# Spatial and temporal non‐stationarity in long‐term population dynamics of over‐wintering birds of North America

**DOI:** 10.1002/ece3.9781

**Published:** 2023-03-16

**Authors:** Stephen J. Murphy, Marta A. Jarzyna

**Affiliations:** ^1^ Department of Evolution, Ecology, and Organismal Biology The Ohio State University Columbus Ohio USA; ^2^ Translational Data Analytics Institute The Ohio State University Columbus Ohio USA

**Keywords:** avian decline, biodiversity, land‐use change, long‐term change, occupancy model, population dynamics, spatial ecology

## Abstract

Understanding population changes across long time scales and at fine spatiotemporal resolutions is important for confronting a broad suite of conservation challenges. However, this task is hampered by a lack of quality long‐term census data for multiple species collected across large geographic regions. Here, we used century‐long (1919–2018) data from the Audubon Christmas Bird Count (CBC) survey to assess population changes in over 300 avian species in North America and evaluate their temporal non‐stationarity. To estimate population sizes across the entire century, we employed a Bayesian hierarchical model that accounts for species detection probabilities, variable sampling effort, and missing data. We evaluated population trends using generalized additive models (GAMs) and assessed temporal non‐stationarity in the rate of population change by extracting the first derivatives from the fitted GAM functions. We then summarized the population dynamics across species, space, and time using a non‐parametric clustering algorithm that categorized individual population trends into four distinct trend clusters. We found that species varied widely in their population trajectories, with over 90% of species showing a considerable degree of spatial and/or temporal non‐stationarity, and many showing strong shifts in the direction and magnitude of population trends throughout the past century. Species were roughly equally distributed across the four clusters of population trajectories, although grassland, forest, and desert specialists more commonly showed declining trends. Interestingly, for many species, region‐wide population trends often differed from those observed at individual sites, suggesting that conservation decisions need to be tailored to fine spatial scales. Together, our results highlight the importance of considering spatial and temporal non‐stationarity when assessing long‐term population changes. More generally, we demonstrate the promise of novel statistical techniques for improving the utility and extending the temporal scope of existing citizen science datasets.

## Graphical abstract

We present 100 years of population dynamics for the birds of North America, observing complex and shifting trends that were previously unavailable from shorter‐term studies. Our research places an emphasis on non‐stationarity in both the spatial and temporal dynamics of species populations, with significant implications for species conservation.

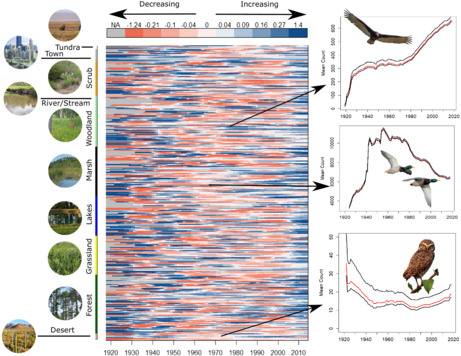

## 1 INTRODUCTION

Accurately documenting long‐term population changes is essential for understanding species extinctions (O'Grady et al., 2004), community composition (Jarzyna & Jetz, 2017), and ecosystem functioning (Sol et al., 2020), as well as for devising relevant conservation/management strategies (Jetz et al., 2019; Lindenmayer et al., 2012). Many of the most important conservation challenges center on determining how species populations have changed through time and use this information to predict future outcomes (Franklin, 1989; Lindenmayer et al., 2012; Robertson et al., 2012). For that reason, species populations, including their change over space and time, have been recognized as one of the essential biodiversity variables (EBVs) necessary for enabling a more focused, integrated, and effective biodiversity monitoring effort (Jetz et al., 2019). Despite this recognition, inherent challenges with tracking long‐term change, including a general lack of quality long‐term monitoring data, leaves our understanding of global population dynamics incomplete (Ho et al., 2019; Jacobson et al., 2018; WWF, 2020).

For avian populations in North America, long‐term systematic monitoring programs such as the USGS Breeding Bird Survey (BBS) serve as a gold standard for tracking the population size of multiple species and for linking observations to relevant environmental and anthropogenic drivers (Hudson et al., 2017; Pardieck et al., 2020). For example, using the BBS and other North American avian monitoring programs, Rosenberg et al. (2019) documented an estimated 30% reduction in the total number of individuals from 1970 to the present. Declines have also been reported for avian communities in Europe (Inger et al., 2015; Reif, 2013) and Asia (Kamp et al., 2015), and for global vertebrates generally (WWF, 2020). An important limitation of existing efforts, however, is that nearly all long‐term studies that track population size are restricted to the last ~50 years. Understanding population dynamics over longer time scales is important for developing a more complete understanding of ecological change and its underlying drivers. For example, the shifting baseline syndrome is a widely acknowledged phenomenon in conservation science, wherein the perceived magnitude of population change is underestimated due to a lack of appropriate reference data (Jones et al., 2020; Papworth et al., 2009; Pauly, 1995; Soga & Gaston, 2018). Assessing the true impacts of anthropogenic drivers like land cover and climate change on populations is also difficult in the absence of adequate data on long‐term species abundances. For birds of North America, reference conditions beginning in the latter part of the 20th century coincide with the advent of federal environmental protections (Grier, 1982; Scott et al., 2005; Watts et al., 2008) and may thus mask important population dynamics resulting from anthropogenic drivers occurring earlier on, including land conversion (Stanton et al., 2018), hunting (Bucher, 1992), and pesticide use (Mineau & Whiteside, 2013).

In addition to underestimating the magnitude of population change, short time series can mask trend reversals and other complex dynamics. The importance of such temporally non‐stationary population dynamics has received relatively little attention in the literature (Rollinson et al., 2021), but might be of particular importance for North American bird populations due to the concurrence of targeted conservation actions and federal efforts to restore critical habitats with the onset of systematic monitoring programs like BBS (King et al., 2006; Pardieck et al., 2020). For example, raptors and wetland birds increased across the US after the ban of dichlorodiphenyltrichloroethane (DDT) in 1972 and through increased availability of wetland habitats (Jarzyna & Jetz, 2017), yet experienced dramatic population declines prior to that time period (Shore & Taggart, 2019). Ignoring such complexities may lead to mischaracterization of important species–environment interactions that are critical to understanding long‐term population change and its underlying drivers (e.g., Rollinson et al., 2021).

The Audubon Christmas Bird Count Survey (CBC) offers an opportunity to track avian populations over a period of a century or more across North America (National Audubon Society, 2020). The CBC was initiated in 1900 and, today, over 2000 sites are sampled annually. Despite the unprecedented spatial coverage and temporal extent of the CBC data, there remain considerable challenges associated with using these data for rigorous dynamic population modeling (Bock & Root, 1981; Link & Sauer, 1999; Meehan et al., 2019). These include non‐random site selection, variable sampling effort across sites and years, and missing or lower‐quality information found in the older sampling periods. Consequently, most research utilizing the CBC data has remained restricted to time periods proceeding from the mid to late 1960s when collecting sampling effort became more common (Meehan et al., 2019; Niven et al., 2004). As a result, data collected prior to this period remains underutilized (but see Saunders et al., 2022, for one notable exception).

Recent advancements in dynamic hierarchical spatiotemporal population models that allow for the inclusion of missing data and for incorporating variability in sampling intensity provide an opportunity for extending analyses of the CBC data to years prior to the 1960s. Here, we take advantage of these methodological advancements to document, for the first time, long‐term (100 years) population trends and their temporal non‐stationarity in over‐wintering bird species across the continental United States. Our goal is to document dynamics at a variety of spatial (i.e., continental vs. local) and ecological (i.e., individual species, habitat guilds, and whole community) scales and to summarize the complexity and heterogeneity in these dynamics using a novel clustering algorithm that groups similar trends into a set of most similar types. We expect that our efforts will help to advance the utility of existing citizen science datasets and enhance our understanding of avian population dynamics in North America.

## 2 MATERIALS AND METHODS

### 2.1 Audubon Christmas Bird Count Survey data

The Audubon Christmas Bird Count Survey is conducted within established “circles” of 24.1 km in diameter. The first CBC survey was conducted in 1900 in the northeastern United States of America and, today, over 2000 circles are sampled annually across North America (Bock & Root, 1981; Dunn et al., 2005; Meehan et al., 2019; Saunders et al., 2022). Sampling consists of volunteer observers recording the number of individuals seen or heard over a 24‐h period on or around December 25. Information on sampling effort is consistently recorded from approximately the mid‐1960s onward but was only sporadically recorded in earlier recording events.

Our analysis focuses on the 100‐year period from 1919 to 2018. Prior to running all models, we removed pelagic and nocturnal species that are unlikely to be well represented. Due to poor convergence of parameter estimates for less common species, we focused our final analyses on the 310 most abundant species in terms of total count and site frequency, which constitute more than 50% of the total avifauna and over 95% of individuals in the data. We also removed circles that overlapped with coasts and large lakes and those that were sampled fewer than 30 times between 1919 and 2018, resulting in a total of 1248 circles (Figure S1). For each individual species analysis, we removed circles where the target species was detected fewer than 10 times between 1919 and 2018.

### 2.2 Environmental data

We used data on elevation, land cover, and human population density to model species population size and its change over space and time. We chose these variables because they are expected to vary significantly over the previous century and are known to influence demographic parameters of bird populations (Meehan et al., 2019; Soykan et al., 2016). Elevation data were obtained from NASA's Shuttle Radar Topography Mission (SRTM) at a resolution of 0.5 arc minutes (Fick & Hijmans, 2017). We used the USGS National Land Cover Database (NLCD) to obtain area of preferred habitat and area of developed land within each CBC circle. NLCD data are compiled every 2–3 years starting in 2001 (NLCD, 2020). For older years, we used predicted NLCD raster maps available from 1938 to 1992 from Sohl et al. (2018). For years when NLCD was not available, we used the most recent data preceding that particular year, and for years prior to 1938, data from the 1938 raster were used. We combined some NLCD categories to produce a final set of 10 relevant land cover types, which included Water, Forest, Grassland, Barren, Urban, Shrubland, Agriculture, Herbaceous Wetland, Woody Wetland, and Ice/Snow. For each species in our analysis, we obtained its preferred habitat type from the eBird/Cornell online database (Cornell Lab of Ornithology, 2019) and matched these preferences to the most relevant NLCD land cover category described above. Habitat guilds from eBird/Cornell included Forest, Open Woodland, Scrub, Grassland, Desert, Tundra, Towns, Lakes, and Marsh habitat.

We obtained predicted human population density data from 1900 to 2010 from Fang and Jawitz (2018). These data are available in 10‐year increments from 1790 to 2010 and were obtained by modeling US Census data and other sources of human population density across the United States as a function of multiple relevant predictor variables. Finally, minimum daily temperature data were obtained from current and historic weather stations (Vose et al., 2020). The minimum daily temperature was used to help estimate detection probability under the assumption that lower temperatures make it more difficult to detect birds (see next section for model details). When possible, data were obtained from one or more stations located within the boundary of each CBC circle. If weather data were not available within a particular circle, the closest weather station to the circle centroid was used instead.

### 2.3 Sampling effort data

Effort data are consistently recorded in the CBC survey after 1967 but are frequently missing from older census years. When available, the overall sampling effort was quantified as the total party hours recorded at a specific circle during a particular sampling year (National Audubon Society, 2020). Where effort data were missing, we found that they could be accurately estimated based on predictable temporal trends observed in the existing data. Specifically, we plotted the relationship between total party hours and census years for individual circles. We observed that prior to 1967, the relationship between total party hours and sampling year was well approximated by a gamma distribution (Figure S2), which we fit to each of the 1248 circles. Using these functions, we then interpolated effort data for years when sampling did not occur or when sampling effort was not recorded. For samples occurring after 1967, approximately 96% of CBC circle‐year combinations contained recorded effort data. To predict the small percentage of remaining missing values, we fit flexible smoothing splines using the “loess” function in the “stats” package of R. For all predicted effort data, uncertainty was summarized as 2 standard errors above and below the predicted values. These prediction intervals were then incorporated into the resulting dynamic population model to ensure proper incorporation of error estimates.

### 2.4 Dail–Madsen dynamic model for open populations

We used a “single‐visit” version of the dynamic *N*‐mixture model described by Dail and Madsen (2011), hereafter, the Dail–Madsen model (DMM), to estimate long‐term species‐specific population change while accounting for imperfect detection and variable sampling. Unlike traditional *N*‐mixture models that assume population closure to processes such as immigration/birth and emigration/death (Royle, 2004), the DMM explicitly incorporates survival and recruitment (Kery & Royle, 2020). Likewise, the DMM allows for the estimation of detection probability when secondary sampling events are not conducted. In this case, the estimation of detection probability is aided using relevant covariate data. These so‐called “single‐visit” *N*‐mixture models have been successfully used to model the abundance of other avian (Dénes et al., 2015, 2017; Sólymos et al., 2012) and vertebrate (Tingley et al., 2016) populations across the globe.

In the DMM, the initial (i.e., at time *t* = 1) size of the population is estimated as:

$$N_{i,1}\sim \text{Pois}\left(\Lambda_{i,1}\right)$$

where $\Lambda_{i,1}\;$ can be modeled as a function of spatially varying predictor variables. Here, predictor variables included elevation, area of preferred habitat, area of developed habitat, and human population density. Temporal population dynamics are then modeled by fitting parameters for both survival (*S*) and recruitment (*R*) as:

Si,t∣Ni,t−1~BinNi,t−1ωRi,t∣Ni,t−1~PoisγNi,t−1

where *ω* is the species' probability of survival between times *t*−1 and *t* and *γ* indicates recruitment between times *t*−1 and *t*. With state variables S and R estimated, abundance in time *t*, $N_{i,t}$, is calculated as $S_{i,t}+R_{i,t}$. As for Λ, we fit S and R using elevation, area of preferred habitat, area of developed habitat, and human population density.

Next, the observation process is modeled as:

$$X_{i,t}\sim \mathrm{Bin}\left(N_{i,t},p\right)$$

where $X_{i,t}$ is the observed count at site *i* and time *t* given the true population size $N_{i,t}$ and detection probability *p*. In our case, *p* was estimated as a function of sampling effort (i.e., total party hours) and the minimum temperature recorded on a particular sampling event. We included minimum temperature as a covariate in the observation process because we assumed that the number of observers and the duration of the survey will be tightly linked to minimum daily temperature. Because sampling effort was imputed for some years, particularly prior to 1967, we included a random error term in the estimation of *p* that was equal to the width of the prediction interval at each site‐by‐year instance. For cases where recorded sampling data were available, this term was set to zero.

All models were fit using the “rjags” package in R (Plummer, 2021). All covariate data were standardized to a mean of zero and a standard deviation of 1. We ran a total of 100,000 MCMC iterations and a burn‐in period of 80,000. Uninformative priors on regression slope and intercept parameters were used except for the relationship between *p* and sampling effort and minimum temperature, for which we forced positive and negative slopes, respectively. We assessed the convergence of parameter estimates via visual inspection of trace plots and posterior distributions for 15 randomly selected species via the Gelman and Rubin (1992) convergence diagnostic statistic for all 310 species, with values <1.1 indicating adequate convergence. Due to large computational requirements, all models were run using parallel processing on the Ohio Supercomputer Center using a total of eight computational nodes, with each node containing a 40‐core 2x Xeon Platinum 8286 processor with 192 total GB of memory. All models ran to completion in under 3 days using this setup. The raw CBC data can be requested using Audubon's online data request portal (https://form.jotform.us/81195823581159).

### 2.5 Assessing long‐term species trends

To document species‐specific population trends and their spatiotemporal non‐stationarity, we conducted analyses at two levels. To explore continent‐wide population trends, we conducted an “all‐circles” analysis, wherein we obtained species‐specific mean estimates of population size (*N*) across all sites for each year. Because our model estimates total population size for each circle, regional summaries were created using simple means and summations across circles. For each species, we removed estimates of *N* occurring outside the range of years where a species was detected in the CBC data to avoid extrapolating population size beyond the sampling timeframe (Figures S1 and S2). While the number of CBC circles varied across years, this variation had minimal effects on the mean estimates of *N* (Figure S3).

We next evaluated temporal trends in mean population size by fitting generalized additive models (GAMs) to each species trend. GAMs were fit using cubic splines and default parameters from the “gam” function in the car package in R (Fox & Weisberg, 2019). GAMs were used instead of the raw output from the DMM to help dampen the influence of occasional large changes in population size occurring between short time intervals (Figure S3). To evaluate the temporal non‐stationarity in the rate of population change, we extracted the first derivatives from the fitted GAM functions for each 3‐year interval between the beginning and end of each time series. We then conducted a “circle‐by‐species” analysis, where a total of 114,352 individual GAMs were fit to all possible circle and species combinations. Occasionally, GAM models fit using cubic splines resulted in an error, in which cases a thin‐plate regression spline was used instead. The “circle‐by‐species” analysis allowed us to determine whether there was a spatial signature in the distribution of specific temporal trend types for each species.

### 2.6 Clustering of spatial and temporal population trends

We next sought to identify commonalities in temporal population trends across species and circles. To do this, we first used a *k*‐medoids clustering algorithm that utilizes partitioning around medoids (PAM) to cluster the 310 species‐specific fitted standardized GAM functions from the all‐circles analysis into distinct groups. The *k*‐medoids algorithm searches for *k* representative patterns in the species‐by‐year matrix and then assigns individual species' trends to the specific medoid that minimizes the sum of dissimilarities between the data and each medoid. We conducted analyses using a total of *k* = 4 medoids. Next, to determine whether clustering was dependent on species' preferred habitat type, we summarized the number of species associated with each medoid according to the number of species belonging to each habitat category.

To determine whether there were spatial signatures in the distribution of specific temporal trends, we next conducted the same clustering procedure for the “circle‐by‐species” analysis. To accommodate the much larger number of trends analyzed in this case, the trend‐by‐year matrix was split into subsamples of 1000 rows for a total of 1000 times (with random sampling) using the “clara” function in the cluster package in R. Once individual trends were assigned to one of the four cluster types, we summarized trends for individual CBC circles at both the community and the individual species scales. At the community level, we quantified the proportion of each cluster type observed across all species present within a specific CBC circle. To visualize this analysis, we color‐coded circles according to the dominant medoid present at that location. Circles were only color coded when the distribution of cluster types was significantly different from expected, as determined using a chi‐square proportionality test. At the individual species scale, circles were color coded according to the associated medoid for a given species at that location.

## 3 RESULTS

**FIGURE 1:**
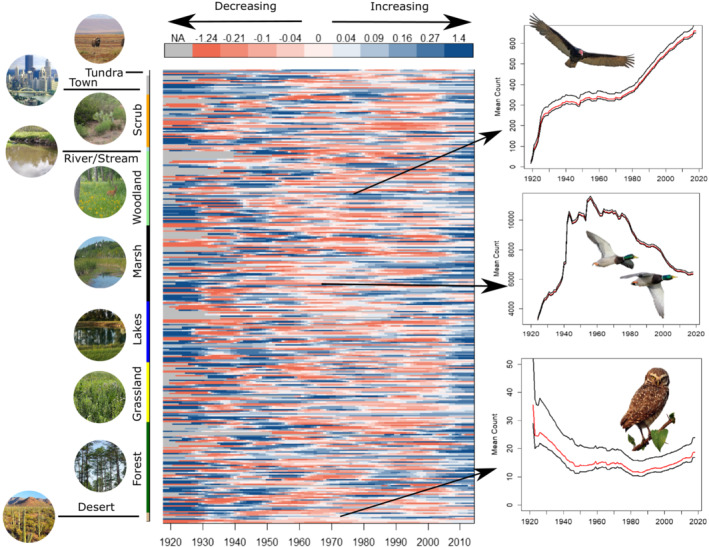
Century‐long temporal trends in avian abundance estimates for 310 species sampled in the conterminous United States of America. Species abundance estimates are mean values across individual circles and are derived from a spatiotemporally dynamic Dail–Madsen model. Abundance estimates were smoothed using a generalized additive model and a cubic regression spline, and the first derivatives from the fitted function are shown in the red‐to‐blue color scale. The order of species is grouped according to each species' habitat guild and color coded along the *y*‐axis. The right‐hand panel shows examples of individual population trends for Turkey Vulture (*Cathartes aura*; top), Mallard (*Anas platyrhynchos*; middle), and Burrowing Owl (*Athene cunicularia*; bottom).

By examining population dynamics at a fine temporal resolution, complex and variable trends were revealed for many species across the century spanning 1919–2018 (Figure 1). Most species showed some level of temporal non‐stationarity in their population dynamics between 1919 and 2018. Specifically, 283 species (91%) showed periods of both decreasing and increasing population trajectories that spanned a minimum of five consecutive years. In contrast, only a small number of species (*n* = 27) showed a constant rate of change between 1919 and 2018, which included species like the Turkey Vulture (*Cathartes aura*) and Loggerhead Shrike (*Lanius ludovicianus*). In about 80% of cases, covariate data (i.e., habitat, population density, and elevation) were significantly correlated with population size and demographic parameters (Appendix S1).

Despite considerable variability in the dynamics among species and significant temporal non‐stationarity in population trends, we observed a period of widespread population decline throughout the latter half of the 20th century. Specifically, 165 species (53%) declined for at least 15 years or more over this period, compared to only 40% of species showing a similar increasing trend. Interestingly, this period of decline in the second half of the 20th century was followed by largely consistent increases in population size over the most recent observed decade. In this case, 196 species (appr. 62% of species) increased over eight consecutive years or more.

**FIGURE 2:**
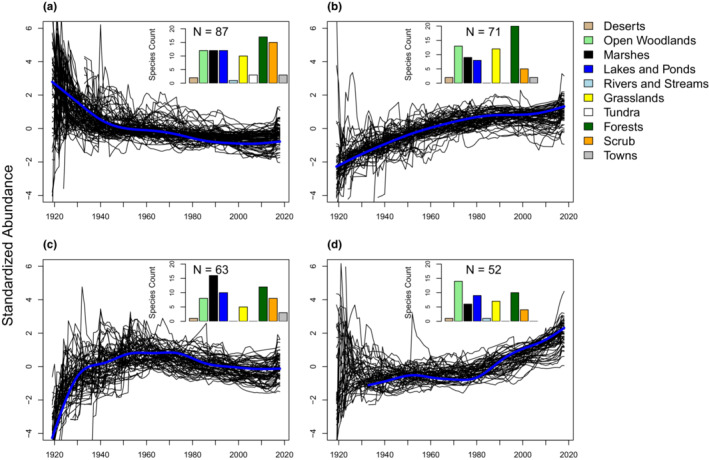
Results from partitioning around medoids (PAM; *k*‐medoids) procedure analyzing continent wide population trends for individual species. Trends were standardized prior to analysis and a total of four medoids, representing the four most common trend patterns across species, was chosen a priori to summarize trends across species. The shape of the four medoids returned from PAM are shown as thick blue lines in panels a, b, c, and d, and the individual species trends associated with each medoid are shown using thinner black lines. Inset color‐coded histograms show the number of species belonging to each cluster, summarized according to the total numbers of species belonging to each habitat guild.

The first cluster grouped species whose populations showed a steady decline since 1919, with the most rapid declines appearing in the first half of the 20th century (Figure 2a). A total of 87 species (32%) were included in this cluster and included species like Vesper Sparrow (*L. ludovicianus*), Canyon Wren (*Catherpes mexicanus*), and American Tree Sparrow (*Spizelloides arborea*). The second medoid revealed steadily increasing population trends, and a total of 71 species (26%) were matched to this medoid (Figure 2b), including species like Carolina Wren (*Thryothorus ludovicianus*), Northern Mockingbird (*Mimus polyglottos*), and Great Horned Owl (*Bubo virginianus*). The third medoid indicates species showing rapidly increasing population trends throughout the first half of the century, followed by a steady decline. Sixty‐three species (23%) were most closely matched to this medoid, including Song Sparrow (*Melospiza melodia*), Mexican Jay (*Aphelocoma wollweberi*), and Snowy Egret (*Egretta thula*). Finally, 53 species (19%) were matched to medoid 4 (Figure 2d), which describes a period of relatively stable dynamics up until the 1970s, followed by a steadily increasing trend. Species associated with this medoid included Bald Eagle, Common Loon (*Gavia immer*), and Turkey Vulture. When examining groupings according to habitat affinities, grassland, forest, and desert specialist species were more commonly associated with declining population trends (i.e., medoids 1 and 3), than medoids describing increasing trends (2 and 4). For other habitat groups, we did not observe any notable affinities toward one cluster group over another.

**FIGURE 3:**
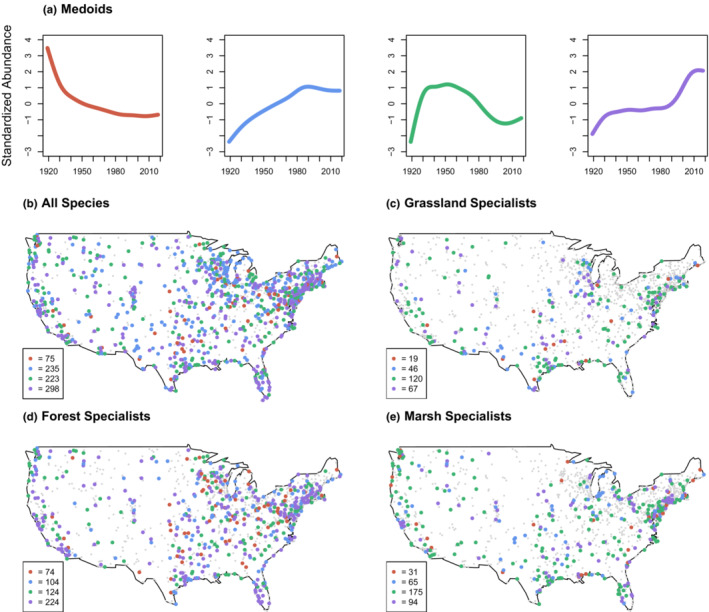
Four medoids representing the most common trends observed across all circle‐by‐species combinations, obtained from a partitioning around medoids (PAM) clustering algorithm; partitioning was conducted on standardized trends for individual circle and species combinations using a subsampling approach (details in the main text; a). Spatial variation in the prevalence of a given cluster type at individual CBC circles was observed across the study region for all species (b) as well as for specific habitat guilds: grassland specialists (c), forest specialists (d), and marsh specialists (e). In (b–e), colored CBC circles indicate locations where a non‐uniform distribution of cluster types was observed based on a chi‐square proportionality test. For these circles, the most dominant cluster type is indicated (colors match those shown in figure [a]). All other sites are shown using smaller gray dots. Figures for additional habitat guilds are found in Figure S5.

When performing the clustering procedure for the “circle‐by‐species” analysis, the PAM algorithm returned relatively similar shapes for the four representative medoids (Figure 3a). Despite declining patterns being most common when examining mean trends across all circles, these were relatively underrepresented when examining site‐level trends (Figure 3a). Among circles where the distribution of medoid types was significantly non‐uniform, cluster 1 was dominant at only 75 sites (9%). Clusters 2, 3, and 4 were dominant at 28%, 27%, and 36% of circles, respectively. Medoid 1 appeared to be overrepresented in the Eastern United States, while medoid 2 was overrepresented in the Northeast. In contrast, medoids 3 and 4 showed little spatial patterning across the conterminous United States. Examining subsets of communities according to specific habitat guilds revealed more noticeable differences across trend types and space (Figure 3c–e; Figures S4 and S5). While declining trends (i.e., medoids 1 and 3) were overrepresented for grassland and forest specialists compared to other guilds, increasing trends (i.e., medoids 2 and 4) were commonly observed at specific circles for these groups as well, suggesting important variability in population trajectories when examining individual circles.

**FIGURE 4:**
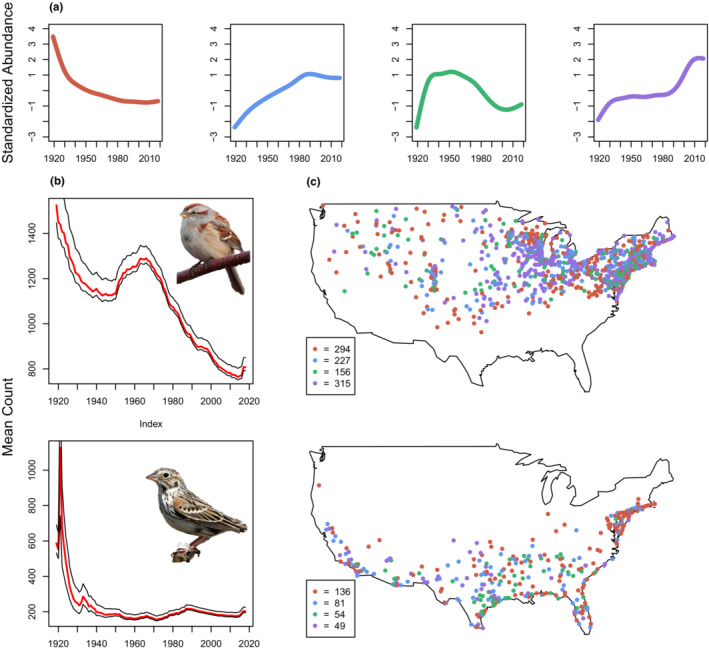
Four medoids obtained from partitioning around medoids (PAM) were used to explore spatial variation in trend types among individual species (a). Two example species (American Tree Sparrow [*Spizelloides arborea*] and Vesper Sparrow [*Pooecetes gramineus*]) are shown in steady decline over the entire study region (b), yet show significant variability in trends when examining individual circles, as indicated by the most‐associated medoid found at each circle (c). Additional plots for all 310 species can be found in Figure S6.

At the individual species level, population trends associated with specific circles were significantly different from region‐wide mean trends (Figure 4; Figure S6). For example, our analysis revealed that Vesper Sparrow (*Pooecetes gramineus*), a grassland specialist species that has been well documented as showing significant declines in population size (Rosenberg et al., 2019; Soykan et al., 2016), showed a significant mean decline across our study region (Figure S3). However, the “circle‐by‐species” analysis revealed that Vesper Sparrow showed declining population trends at only approximately 40% of CBC circles (Figure 4b, lower panel).

## 4 DISCUSSION

We present a century‐long evaluation of trends in avian abundances for a large group of common and widely distributed species in North America. Expectedly, we find high variability in population dynamics over the previous century. For more recent census years (i.e., post‐1966), where comparisons to other work were possible, these species abundance trends were largely in‐line with those reported from both the BBS‐ and CBC‐based studies (National Audubon Society, 2020; Sauer et al., 2017; Soykan et al., 2016). Despite strong among‐species variability, however, some common temporal patterns emerged. Roughly 55% of all species showed significant declines in mean population size between 1960s and early 2000s, which corroborates recent findings on extensive bird population losses (Rosenberg et al., 2019). These widespread losses were most pronounced for grassland, forest, and desert specialists. Grassland specialists, as well as some forest and understory specialists, have undergone precipitous declines across both North America and Europe (Herkert, 1994; King & Degraaf, 2000; Reif, 2013; Rosenberg et al., 2019; Zuckerberg et al., 2009), mostly as a result of habitat loss (Brennan & Kuvlesky, 2005; Jarzyna & Jetz, 2017), forest habitat degradation (Virkkala, 2016), and intensification of agricultural practices (Askins et al., 2007). Our models indicated that the area of preferred habitat of a species was an important predictor of population size and demographic change, with parameter estimates being non‐zero in 80% of cases (Figure S7; Appendix S1). Altogether, these results add to a growing body of literature suggesting the importance of habitat availability and its change over time as a key conservation factor for birds of North America and across the globe.

The 1960s–2000s period of widespread population decline is followed by pervasive trend reversals that conclude in population increases over the most recent decade (i.e., ca. 2008–2018). Rosenberg et al. (2019) found similar trends for most species' groups, wherein their mean abundance trends either taper off or increase toward the end of the time series. Understanding the root causes of these recent increases requires further research, although recent changes in temperature and precipitation are plausible drivers. For example, increased temperature and precipitation due to climate change can increase resource availability and ultimately lead to increases in bird population size (Pearce‐Higgins et al., 2015), although evidence for the negative impacts of climate change on species reproduction and population size also abound (Leech & Crick, 2007; Saunders et al., 2022; Senapathi et al., 2011 and many others). Over‐wintering birds, however, might benefit from winter warming (Princé & Zuckerberg, 2015; Rodenhouse et al., 2009; Soykan et al., 2016) as their demography and population sizes are limited by climatic constraints during the winter season (Brittingham & Temple, 1988; Zuckerberg et al., 2011), and their phenological traits are thought to be more plastic compared to long‐distance migrants (Both et al., 2010).

Importantly, extending the temporal extent to a century revealed unique trends in species population sizes that would have been obscured or lost if observations were constrained solely to the past five decades. Specifically, many species showed highly variable population dynamics pre‐1960s, with shifts in the direction and magnitude of population trends throughout that period. For example, many species characterized by strong declines in recent decades (e.g., Horned Grebe [*Podiceps auratus*], Lark Sparrow [*Chondestes grammacus*], and Ruffed Grouse [*Bonasa umbellus*]) showed either neutral or increasing population sizes pre‐1960s. Our findings emphasize that non‐stationarity in temporal population dynamics is commonplace for North American birds and should be incorporated in analyses seeking to link population changes to natural or anthropogenic drivers (Rollinson et al., 2021).

Likewise, our results highlight the importance of biases inherent to the shifting baseline syndrome (Soga & Gaston, 2018), where population changes are underestimated or misinterpreted due to a lack of appropriate reference data. While examining such long temporal extents is admittedly difficult or impossible for most regions and taxonomic groups, our work highlights the potential opportunity to extend the period of investigation by utilizing citizen science databases in novel ways through the use of statistical techniques that can account for non‐systematic sampling designs and missing or incomplete data.

We found strong spatial variation in how populations changed through time. That is, for many species, population trajectories measured at the scale of a CBC circle differed from region‐wide mean population trends. For example, while populations of Turkey Vulture have seen significant increases over the last 50 years across continental United States, our data indicate that 38% of locations have undergone population declines (Figure S3). Such spatial non‐stationarity in population change was common among species included in our study, underscoring the importance of carefully considering site‐specific population trajectories in addition to region‐wide trends.

Despite strong spatial variation in population trajectories for individual species, we observed few consistent spatial signatures in assemblage‐wide change. One notable exception was that of steady declining population trends being more prevalent across the eastern United States (Figure 3), which is consistent with observations of bird biomass reductions along the eastern flyway (Rosenberg et al., 2019). Both these studies, however, measured population change across much shorter timeframes—35 and 50 years, respectively—and neither accounted for potential temporal non‐stationarity in population trends.

While our study advances knowledge of avian population dynamics in North America, we acknowledge several limitations of our work. First is the use of mean population size across circles, which was necessary to allow for the number of sites to vary across years. Restricting our analysis to just a subset of consistently sampled locations, akin to Saunders et al. (2022), would have allowed assessing total population size, but would have also greatly reduced the generality of our findings. We did find that mean estimates were relatively comparable when analyzing different time periods (i.e., years from 1930, 1960, and 1990 onward; Figure S2), but there were still situations where they did not match well. Ultimately, the accuracy and impact of using mean estimates across a variable number of circles for each year cannot be measured but may have an important impact on our results.

Second, in contrast to many previous studies (Leung et al., 2020; Meehan et al., 2019; Rosenberg et al., 2019; Saunders et al., 2022; Soykan et al., 2016), the principal objective of this work was to evaluate temporal non‐stationarity of population trajectories at a fine spatiotemporal resolution. Summarizing such complex temporal dynamics across >1000 sites and for hundreds of bird species is not without its challenges. Our selected method—a clustering algorithm that utilizes partitioning around medoids—relies on an a priori selection of the number of clusters (medoids), which unsurprisingly results in some population trajectories not being well matched to one of the four medoids obtained from the clustering procedure. This problem was more pronounced when analyzing all pairwise circle and species combinations. While adding additional medoids would likely increase the concordance between species' population trends and medoids, this would come at the expense of interpretability and generalization, which was our goal.

## 5 CONCLUSION

By considering a century‐long record of over‐wintering bird abundances, our findings provide new insights into long‐term avian population dynamics across the continental United States, with significant practical implications for the study of population dynamics and species conservation. We highlight the importance of considering the temporal non‐stationarity of population trajectories and their spatial variation to pinpoint time periods and locations of most drastic change. This can be used to support timely management and conservation actions. Our efforts represent a first step in documenting truly long‐term changes in bird populations in North America, and we expect that this work will be used to help better understand how climate change, land use change, and other environmental and anthropogenic drivers influence species populations in North America and across the globe. This work also provides evidence that the utility of previously underutilized data, including large‐scale citizen science efforts, can be enhanced through the deployment of novel statistical models that can account for variability in sampling effort and missing data. We are confident that the strategies developed here for use with CBC survey data could be applied to other biodiversity datasets in the future.

## AUTHOR CONTRIBUTIONS

**Stephen J. Murphy:** Conceptualization (equal); formal analysis (lead); methodology (equal); writing – original draft (lead). **Marta A. Jarzyna:** Conceptualization (equal); formal analysis (supporting); methodology (equal); writing – original draft (supporting).

## Supporting information


Appendix S1

Figure S1

Figure S2

Figure S3

Figure S4

Figure S5

Figure S6

Figure S7

## Data Availability

The data that support the findings of this study are openly available at https://netapp.audubon.org/cbcobservation/.
