# Supplementary material for: Spatial and temporal non‐stationarity in long‐term population dynamics of over‐wintering birds of North America
Source: Ecol Evol. 2023 Mar 16;13(3):e9781. doi: 10.1002/ece3.9781 (PMC10019912; doi:10.1002/ece3.9781)
Supplement: Supplementary file 1 — Appendix S1 [file ECE3-13-e9781-s007.pdf]

Table 1: American Black Duck

	mean	sd	2.5%	25%	50%	75%	97.5%	n.eff	overlap0	f
alpha.lambda	8.84	0.34	8.45	8.46	8.80	9.23	9.35	3	0	1
alpha.phi	2.31	0.13	2.14	2.16	2.33	2.45	2.47	3	0	1
alpha.p	-2.43	0.39	-2.99	-2.87	-2.40	-1.99	-1.97	3	0	1
beta.hab.lambda	0.39	0.03	0.34	0.36	0.40	0.42	0.43	3	0	1
beta.hab.gamma	-0.49	0.26	-0.90	-0.76	-0.44	-0.20	-0.19	3	0	1
beta.hab.phi	0.75	0.10	0.65	0.66	0.72	0.86	0.92	3	0	1
beta.elev.lambda	-0.12	0.00	-0.12	-0.12	-0.12	-0.11	-0.11	3	0	1
beta.ele.gamma2	-0.18	0.05	-0.23	-0.23	-0.19	-0.11	-0.10	3	0	1
beta.elev.phi	-0.31	0.04	-0.37	-0.36	-0.31	-0.26	-0.26	3	0	1
beta.develop.lambda	0.13	0.01	0.12	0.13	0.13	0.14	0.14	3	0	1
beta.develop.gamma	-0.68	0.14	-0.90	-0.87	-0.60	-0.55	-0.54	3	0	1
beta.develop.phi	-0.13	0.05	-0.18	-0.17	-0.15	-0.06	-0.05	3	0	1
beta.effort	0.13	0.01	0.12	0.12	0.13	0.14	0.15	3	0	1
beta.min_temp	-0.08	0.00	-0.08	-0.08	-0.08	-0.08	-0.07	3	0	1

Table 2: American Coot

	mean	sd	2.5%	25%	50%	75%	97.5%	n.eff	overlap0	f
alpha.lambda	8.00	0	7.99	7.99	8.00	8.00	8.00	27	0	1
alpha.phi	2.11	0	2.10	2.11	2.11	2.11	2.11	9	0	1
alpha.p	-1.55	0	-1.56	-1.55	-1.55	-1.55	-1.55	7	0	1
beta.hab.lambda	0.33	0	0.33	0.33	0.33	0.33	0.34	109	0	1
beta.hab.gamma	-0.11	0	-0.11	-0.11	-0.11	-0.11	-0.10	155	0	1
beta.hab.phi	0.14	0	0.14	0.14	0.14	0.15	0.15	132	0	1
beta.elev.lambda	-0.52	0	-0.52	-0.52	-0.52	-0.52	-0.52	145	0	1
beta.ele.gamma2	-0.62	0	-0.62	-0.62	-0.62	-0.62	-0.62	448	0	1
beta.elev.phi	0.30	0	0.30	0.30	0.30	0.30	0.30	144	0	1
beta.develop.lambda	0.22	0	0.22	0.22	0.22	0.23	0.23	100	0	1
beta.develop.gamma	-0.55	0	-0.55	-0.55	-0.55	-0.55	-0.55	8	0	1
beta.develop.phi	0.20	0	0.20	0.20	0.20	0.20	0.20	8	0	1
beta.effort	0.14	0	0.14	0.14	0.14	0.14	0.14	19	0	1
beta.min_temp	0.00	0	0.00	0.00	0.00	0.00	0.00	1	0	1

Table 3: American Crow

	mean	sd	2.5%	25%	50%	75%	97.5%	n.eff	overlap0	f
alpha.lambda	8.06	0	8.06	8.06	8.06	8.06	8.06	6000	0	1
alpha.phi	1.65	0	1.64	1.64	1.65	1.65	1.65	437	0	1
alpha.p	-1.21	0	-1.22	-1.21	-1.21	-1.21	-1.21	657	0	1
beta.hab.lambda	-0.01	0	-0.02	-0.02	-0.01	-0.01	-0.01	6000	0	1
beta.hab.gamma	-0.06	0	-0.06	-0.06	-0.06	-0.05	-0.05	921	0	1
beta.hab.phi	-0.06	0	-0.06	-0.06	-0.06	-0.06	-0.06	962	0	1
beta.elev.lambda	0.25	0	0.25	0.25	0.25	0.25	0.25	623	0	1
beta.ele.gamma2	-0.09	0	-0.09	-0.09	-0.09	-0.09	-0.08	1592	0	1
beta.elev.phi	0.06	0	0.05	0.06	0.06	0.06	0.06	2851	0	1
beta.develop.lambda	-0.09	0	-0.10	-0.10	-0.09	-0.09	-0.09	1260	0	1
beta.develop.gamma	0.22	0	0.22	0.22	0.22	0.23	0.23	181	0	1

	mean	sd	2.5%	25%	50%	75%	97.5%	n.eff	overlap0	f
beta.develop.phi	0.31	0	0.31	0.31	0.31	0.31	0.32	168	0	1
beta.effort	0.00	0	0.00	0.00	0.00	0.00	0.00	1	0	1
beta.min_temp	-0.05	0	-0.05	-0.05	-0.05	-0.05	-0.05	954	0	1

Table 4: American Goldfinch

	mean	sd	2.5%	25%	50%	75%	97.5%	n.eff	overlap0	f
alpha.lambda	6.57	0.01	6.56	6.57	6.57	6.57	6.59	13	0	1.00
alpha.phi	2.58	0.01	2.57	2.57	2.58	2.58	2.61	9	0	1.00
alpha.p	-1.51	0.01	-1.53	-1.51	-1.51	-1.51	-1.51	12	0	1.00
beta.hab.lambda	0.03	0.00	0.02	0.02	0.03	0.03	0.03	2068	0	1.00
beta.hab.gamma	-0.14	0.00	-0.14	-0.14	-0.14	-0.13	-0.13	12	0	1.00
beta.hab.phi	0.23	0.00	0.22	0.23	0.23	0.23	0.24	10	0	1.00
beta.elev.lambda	-0.33	0.00	-0.34	-0.34	-0.33	-0.33	-0.33	1369	0	1.00
beta.ele.gamma2	-0.23	0.00	-0.23	-0.23	-0.23	-0.22	-0.22	47	0	1.00
beta.elev.phi	0.14	0.00	0.13	0.14	0.14	0.14	0.15	27	0	1.00
beta.develop.lambda	0.46	0.00	0.45	0.46	0.46	0.46	0.47	57	0	1.00
beta.develop.gamma	-0.01	0.00	-0.01	-0.01	-0.01	-0.01	0.00	184	0	0.99
beta.develop.phi	0.20	0.00	0.19	0.20	0.20	0.20	0.21	66	0	1.00
beta.effort	0.02	0.00	0.02	0.02	0.02	0.02	0.02	360	0	1.00
beta.min_temp	-0.02	0.00	-0.02	-0.02	-0.02	-0.02	-0.02	55	0	1.00

Table 5: American Robin

	mean	sd	2.5%	25%	50%	75%	97.5%	n.eff	overlap0	f
alpha.lambda	11.85	0.84	10.55	10.73	12.38	12.48	12.56	3	0	1.00
alpha.phi	0.03	1.16	-1.04	-1.03	-0.52	1.64	1.66	3	1	0.33
alpha.p	-5.55	1.25	-6.61	-6.45	-6.36	-3.83	-3.70	3	0	1.00
beta.hab.lambda	0.15	0.21	-0.17	-0.14	0.28	0.31	0.32	3	1	0.67
beta.hab.gamma	0.01	0.22	-0.33	-0.28	0.14	0.18	0.19	3	1	0.67
beta.hab.phi	0.29	0.31	-0.02	0.02	0.15	0.68	0.77	3	1	0.88
beta.elev.lambda	-0.15	0.10	-0.23	-0.23	-0.20	-0.02	0.01	3	1	0.88
beta.ele.gamma2	-0.16	0.37	-0.43	-0.43	-0.41	0.32	0.42	3	1	0.67
beta.elev.phi	-0.40	0.56	-1.31	-1.11	-0.04	0.02	0.02	3	1	0.67
beta.develop.lambda	0.82	0.33	0.33	0.37	1.02	1.07	1.08	3	0	1.00
beta.develop.gamma	0.39	0.17	0.21	0.22	0.34	0.62	0.63	3	0	1.00
beta.develop.phi	0.03	0.31	-0.41	-0.39	0.20	0.29	0.31	3	1	0.67
beta.effort	0.00	0.00	0.00	0.00	0.00	0.00	0.00	1	0	1.00
beta.min_temp	0.00	0.00	0.00	0.00	0.00	0.00	0.00	1	0	1.00

Table 6: American Wigeon

	mean	sd	2.5%	25%	50%	75%	97.5%	n.eff	overlap0	f
alpha.lambda	8.59	0.03	8.58	8.58	8.58	8.58	8.68	20	0	1
alpha.phi	2.10	0.01	2.09	2.09	2.09	2.10	2.14	21	0	1
alpha.p	-1.35	0.02	-1.40	-1.35	-1.34	-1.34	-1.34	22	0	1
beta.hab.lambda	-0.30	0.01	-0.30	-0.30	-0.30	-0.30	-0.26	23	0	1

	mean	sd	2.5%	25%	50%	75%	97.5%	n.eff	overlap0	f
beta.hab.gamma	0.19	0.00	0.18	0.19	0.19	0.19	0.19	24	0	1
beta.hab.phi	0.28	0.01	0.28	0.28	0.28	0.28	0.31	21	0	1
beta.elev.lambda	-0.48	0.01	-0.48	-0.48	-0.48	-0.48	-0.43	19	0	1
beta.ele.gamma2	-0.14	0.01	-0.16	-0.13	-0.13	-0.13	-0.13	21	0	1
beta.elev.phi	0.16	0.01	0.16	0.16	0.16	0.16	0.18	21	0	1
beta.develop.lambda	-0.08	0.01	-0.10	-0.08	-0.08	-0.08	-0.07	22	0	1
beta.develop.gamma	-0.27	0.01	-0.28	-0.27	-0.27	-0.27	-0.25	17	0	1
beta.develop.phi	0.24	0.00	0.24	0.24	0.24	0.24	0.25	113	0	1
beta.effort	0.00	0.00	0.00	0.00	0.00	0.00	0.00	1	0	1
beta.min_temp	0.00	0.00	0.00	0.00	0.00	0.00	0.00	1	0	1

Table 7: Black-capped Chickadee

	mean	sd	2.5%	25%	50%	75%	97.5%	n.eff	overlap0	f
alpha.lambda	5.95	0.00	5.94	5.95	5.95	5.95	5.96	60	0	1
alpha.phi	2.63	0.00	2.62	2.63	2.63	2.63	2.64	13	0	1
alpha.p	-0.60	0.00	-0.60	-0.60	-0.60	-0.60	-0.59	15	0	1
beta.hab.lambda	0.40	0.00	0.39	0.39	0.40	0.40	0.40	5191	0	1
beta.hab.gamma	0.52	0.00	0.52	0.52	0.52	0.52	0.52	3661	0	1
beta.hab.phi	-0.19	0.00	-0.19	-0.19	-0.19	-0.19	-0.18	1040	0	1
beta.elev.lambda	-0.27	0.00	-0.28	-0.28	-0.27	-0.27	-0.27	3975	0	1
beta.ele.gamma2	-0.30	0.00	-0.30	-0.30	-0.30	-0.30	-0.29	71	0	1
beta.elev.phi	0.03	0.00	0.03	0.03	0.03	0.04	0.04	51	0	1
beta.develop.lambda	0.56	0.01	0.55	0.56	0.56	0.56	0.57	1795	0	1
beta.develop.gamma	0.19	0.00	0.19	0.19	0.19	0.19	0.20	396	0	1
beta.develop.phi	0.10	0.00	0.09	0.10	0.10	0.10	0.11	346	0	1
beta.effort	0.02	0.00	0.02	0.02	0.02	0.02	0.02	158	0	1
beta.min_temp	-0.01	0.00	-0.01	-0.01	-0.01	-0.01	-0.01	4756	0	1

Table 8: Blue Jay

	mean	sd	2.5%	25%	50%	75%	97.5%	n.eff	overlap0	f
alpha.lambda	6.90	0.07	6.83	6.85	6.85	7.00	7.01	3	0	1.00
alpha.phi	3.63	0.08	3.55	3.57	3.58	3.74	3.75	3	0	1.00
alpha.p	-1.63	0.08	-1.75	-1.75	-1.58	-1.58	-1.56	3	0	1.00
beta.hab.lambda	0.40	0.00	0.40	0.40	0.40	0.41	0.41	5	0	1.00
beta.hab.gamma	0.55	0.01	0.54	0.55	0.55	0.56	0.57	3	0	1.00
beta.hab.phi	-0.35	0.02	-0.38	-0.37	-0.35	-0.34	-0.33	3	0	1.00
beta.elev.lambda	-0.30	0.00	-0.31	-0.30	-0.30	-0.30	-0.29	4	0	1.00
beta.ele.gamma2	-0.42	0.01	-0.43	-0.42	-0.41	-0.41	-0.40	3	0	1.00
beta.elev.phi	0.38	0.01	0.36	0.37	0.37	0.39	0.40	3	0	1.00
beta.develop.lambda	0.34	0.00	0.33	0.33	0.33	0.34	0.34	5	0	1.00
beta.develop.gamma	0.06	0.06	-0.03	-0.02	0.10	0.10	0.11	3	1	0.67
beta.develop.phi	-0.16	0.04	-0.21	-0.19	-0.18	-0.10	-0.09	3	0	1.00
beta.effort	0.00	0.00	0.00	0.00	0.00	0.00	0.00	6	0	1.00
beta.min_temp	-0.07	0.00	-0.07	-0.07	-0.07	-0.07	-0.07	4	0	1.00

Table 9: Brant

	mean	sd	2.5%	25%	50%	75%	97.5%	n.eff	overlap0	f
alpha.lambda	9.98	0.87	9.30	9.31	9.42	11.19	11.25	3	0	1.00
alpha.phi	0.17	1.20	-1.38	-1.30	0.25	1.59	1.60	3	1	0.67
alpha.p	-2.21	0.50	-2.65	-2.62	-2.49	-1.51	-1.51	3	0	1.00
beta.hab.lambda	0.52	0.26	0.21	0.22	0.51	0.84	0.84	3	0	1.00
beta.hab.gamma	0.28	1.02	-0.85	-0.84	0.06	1.62	1.62	3	1	0.67
beta.hab.phi	1.14	2.92	-2.38	-2.35	0.98	4.74	4.88	3	1	0.67
beta.elev.lambda	0.26	0.15	0.08	0.10	0.21	0.46	0.46	3	0	1.00
beta.ele.gamma2	0.30	1.13	-0.59	-0.59	-0.40	1.90	1.90	3	1	0.33
beta.elev.phi	-0.70	2.85	-4.61	-4.56	0.27	2.17	2.25	3	1	0.33
beta.develop.lambda	0.70	0.74	0.06	0.07	0.30	1.73	1.76	3	0	1.00
beta.develop.gamma	0.12	0.35	-0.19	-0.19	-0.06	0.60	0.61	3	1	0.33
beta.develop.phi	-0.39	0.82	-1.57	-1.55	0.13	0.25	0.25	3	1	0.33
beta.effort	0.19	0.06	0.10	0.11	0.22	0.25	0.25	3	0	1.00
beta.min__temp	-0.08	0.02	-0.12	-0.12	-0.07	-0.06	-0.06	3	0	1.00

Table 10: Brewer's Blackbird

	mean	sd	2.5%	25%	50%	75%	97.5%	n.eff	overlap0	f
alpha.lambda	9.98	0.00	9.98	9.98	9.98	9.99	9.99	11	0	1.00
alpha.phi	2.01	0.00	2.01	2.01	2.01	2.01	2.02	34	0	1.00
alpha.p	-1.96	0.00	-1.96	-1.96	-1.96	-1.96	-1.96	678	0	1.00
beta.hab.lambda	0.11	2.26	-4.27	-1.41	0.12	1.65	4.47	6000	1	0.52
beta.hab.gamma	-0.53	2.21	-4.86	-2.02	-0.53	0.95	3.87	6000	1	0.59
beta.hab.phi	0.29	2.21	-3.97	-1.21	0.25	1.84	4.57	5915	1	0.54
beta.elev.lambda	-0.13	0.00	-0.14	-0.13	-0.13	-0.13	-0.13	1241	0	1.00
beta.ele.gamma2	-0.13	0.00	-0.13	-0.13	-0.13	-0.13	-0.13	26	0	1.00
beta.elev.phi	-0.26	0.00	-0.26	-0.26	-0.26	-0.25	-0.25	26	0	1.00
beta.develop.lambda	0.13	2.26	-4.23	-1.41	0.12	1.65	4.51	6000	1	0.52
beta.develop.gamma	-0.43	2.21	-4.83	-1.91	-0.43	1.06	3.90	6000	1	0.58
beta.develop.phi	0.25	2.21	-4.02	-1.30	0.29	1.76	4.51	5873	1	0.55
beta.effort	0.01	0.00	0.01	0.01	0.01	0.01	0.01	3	0	1.00
beta.min__temp	0.00	0.00	0.00	0.00	0.00	0.00	0.00	1	0	1.00

Table 11: Brown-headed Cowbird

	mean	sd	2.5%	25%	50%	75%	97.5%	n.eff	overlap0	f
alpha.lambda	13.21	0.24	12.88	13.03	13.14	13.47	13.62	3	0	1.00
alpha.phi	-0.76	0.86	-1.96	-1.96	-0.34	-0.11	0.21	3	1	0.84
alpha.p	-5.95	1.07	-7.56	-7.40	-5.25	-5.18	-5.07	3	0	1.00
beta.hab.lambda	-1.89	0.99	-3.33	-3.26	-1.20	-1.19	-1.16	3	0	1.00
beta.hab.gamma	0.03	0.21	-0.27	-0.27	0.17	0.19	0.21	3	1	0.67
beta.hab.phi	-0.33	0.53	-0.85	-0.82	-0.56	0.40	0.41	3	1	0.67
beta.elev.lambda	0.22	0.65	-0.70	-0.69	0.66	0.70	0.74	3	1	0.67
beta.ele.gamma2	0.47	0.59	-0.36	-0.36	0.87	0.91	0.93	3	1	0.67
beta.elev.phi	-1.51	1.34	-2.73	-2.52	-2.34	0.37	0.38	3	1	0.67
beta.develop.lambda	0.47	0.45	0.06	0.09	0.23	1.09	1.10	3	0	1.00
beta.develop.gamma	0.25	0.58	-0.58	-0.57	0.66	0.67	0.67	3	1	0.67

	mean	sd	2.5%	25%	50%	75%	97.5%	n.eff	overlap0	f
beta.develop.phi	-0.82	1.68	-2.37	-2.10	-1.81	1.53	1.56	3	1	0.67
beta.effort	0.32	0.01	0.31	0.31	0.32	0.32	0.32	4	0	1.00
beta.min_temp	0.00	0.00	0.00	0.00	0.00	0.00	0.00	1	0	1.00

Table 12: Canada Goose

	mean	sd	2.5%	25%	50%	75%	97.5%	n.eff	overlap0	f
alpha.lambda	8.49	0	8.48	8.48	8.49	8.49	8.49	280	0	1
alpha.phi	2.07	0	2.07	2.07	2.07	2.07	2.07	59	0	1
alpha.p	-1.40	0	-1.40	-1.40	-1.40	-1.40	-1.40	52	0	1
beta.hab.lambda	-0.05	0	-0.05	-0.05	-0.05	-0.05	-0.05	106	0	1
beta.hab.gamma	0.11	0	0.11	0.11	0.11	0.11	0.11	1652	0	1
beta.hab.phi	0.00	0	-0.01	-0.01	0.00	0.00	0.00	213	0	1
beta.elev.lambda	-0.36	0	-0.36	-0.36	-0.36	-0.36	-0.36	542	0	1
beta.ele.gamma2	0.12	0	0.12	0.12	0.12	0.12	0.12	2501	0	1
beta.elev.phi	-0.18	0	-0.18	-0.18	-0.18	-0.18	-0.18	570	0	1
beta.develop.lambda	-0.94	0	-0.95	-0.94	-0.94	-0.94	-0.94	246	0	1
beta.develop.gamma	0.03	0	0.03	0.03	0.03	0.03	0.03	70	0	1
beta.develop.phi	0.12	0	0.12	0.12	0.12	0.12	0.12	41	0	1
beta.effort	0.00	0	0.00	0.00	0.00	0.00	0.00	1	0	1
beta.min_temp	-0.02	0	-0.02	-0.02	-0.02	-0.02	-0.02	962	0	1

Table 13: Cedar Waxwing

	mean	sd	2.5%	25%	50%	75%	97.5%	n.eff	overlap0	f
alpha.lambda	8.22	1.03	6.90	6.94	8.28	9.35	9.56	3	0	1.00
alpha.phi	0.33	2.35	-2.86	-2.80	0.91	2.67	2.71	3	1	0.67
alpha.p	-4.04	1.24	-5.52	-5.30	-4.37	-2.42	-2.39	3	0	1.00
beta.hab.lambda	0.36	0.07	0.28	0.29	0.39	0.39	0.49	4	0	1.00
beta.hab.gamma	0.20	0.04	0.11	0.15	0.22	0.23	0.23	3	0	1.00
beta.hab.phi	-0.07	0.12	-0.20	-0.19	-0.10	0.09	0.11	3	1	0.70
beta.elev.lambda	-0.15	0.04	-0.20	-0.20	-0.13	-0.12	-0.09	4	0	1.00
beta.ele.gamma2	-0.21	0.03	-0.25	-0.24	-0.21	-0.18	-0.17	3	0	1.00
beta.elev.phi	0.02	0.04	-0.05	-0.04	0.04	0.05	0.05	3	1	0.68
beta.develop.lambda	0.70	0.20	0.43	0.45	0.73	0.88	1.01	3	0	1.00
beta.develop.gamma	0.10	0.19	-0.17	-0.11	0.13	0.32	0.32	3	1	0.63
beta.develop.phi	0.01	0.32	-0.50	-0.41	0.20	0.26	0.30	3	1	0.67
beta.effort	0.00	0.00	0.00	0.00	0.00	0.00	0.00	1	0	1.00
beta.min_temp	0.00	0.00	0.00	0.00	0.00	0.00	0.00	1	0	1.00

Table 14: Common Grackle

	mean	sd	2.5%	25%	50%	75%	97.5%	n.eff	overlap0	f
alpha.lambda	16.38	0.26	16.02	16.04	16.49	16.58	16.73	3	0	1.00
alpha.phi	0.07	0.77	-1.06	-0.93	0.38	0.74	0.93	3	1	0.67
alpha.p	-6.32	0.56	-7.07	-6.96	-6.32	-5.65	-5.61	3	0	1.00
beta.hab.lambda	0.33	0.46	-0.24	-0.20	0.30	0.90	0.91	3	1	0.67

	mean	sd	2.5%	25%	50%	75%	97.5%	n.eff	overlap0	f
beta.hab.gamma	0.27	0.33	-0.19	-0.15	0.36	0.58	0.68	3	1	0.67
beta.hab.phi	-0.41	0.36	-0.79	-0.69	-0.59	0.05	0.14	3	1	0.67
beta.elev.lambda	-0.02	0.07	-0.12	-0.12	0.01	0.05	0.06	3	1	0.34
beta.ele.gamma2	-0.18	0.11	-0.33	-0.31	-0.16	-0.06	-0.03	3	0	1.00
beta.elev.phi	-0.13	0.19	-0.38	-0.31	-0.17	0.12	0.13	3	1	0.67
beta.develop.lambda	0.64	0.11	0.47	0.52	0.64	0.75	0.82	3	0	1.00
beta.develop.gamma	0.03	1.55	-2.15	-2.11	0.77	1.40	1.52	3	1	0.67
beta.develop.phi	-1.42	2.01	-3.06	-2.89	-2.74	1.37	1.47	3	1	0.67
beta.effort	0.00	0.00	0.00	0.00	0.00	0.00	0.00	1	0	1.00
beta.min_temp	0.00	0.00	0.00	0.00	0.00	0.00	0.00	1	0	1.00

Table 15: Dark-eyed Junco

	mean	sd	2.5%	25%	50%	75%	97.5%	n.eff	overlap0	f
alpha.lambda	7.57	0	7.56	7.57	7.57	7.57	7.57	16	0	1
alpha.phi	2.77	0	2.77	2.77	2.77	2.77	2.77	10	0	1
alpha.p	-1.20	0	-1.21	-1.20	-1.20	-1.20	-1.20	9	0	1
beta.hab.lambda	0.08	0	0.08	0.08	0.08	0.08	0.09	196	0	1
beta.hab.gamma	0.20	0	0.20	0.20	0.20	0.20	0.20	131	0	1
beta.hab.phi	-0.11	0	-0.12	-0.11	-0.11	-0.11	-0.11	82	0	1
beta.elev.lambda	-0.06	0	-0.06	-0.06	-0.06	-0.06	-0.06	326	0	1
beta.ele.gamma2	0.05	0	0.05	0.05	0.05	0.05	0.05	219	0	1
beta.elev.phi	-0.12	0	-0.12	-0.12	-0.12	-0.12	-0.12	154	0	1
beta.develop.lambda	0.31	0	0.31	0.31	0.31	0.31	0.32	125	0	1
beta.develop.gamma	-0.01	0	-0.02	-0.02	-0.01	-0.01	-0.01	133	0	1
beta.develop.phi	0.21	0	0.20	0.21	0.21	0.21	0.21	97	0	1
beta.effort	0.00	0	0.00	0.00	0.00	0.00	0.01	738	0	1
beta.min_temp	-0.15	0	-0.15	-0.15	-0.15	-0.15	-0.15	561	0	1

Table 16: Double-crested Cormorant

	mean	sd	2.5%	25%	50%	75%	97.5%	n.eff	overlap0	f
alpha.lambda	5.67	0.01	5.66	5.67	5.67	5.68	5.69	37	0	1
alpha.phi	1.86	0.00	1.86	1.86	1.86	1.87	1.87	16	0	1
alpha.p	-1.69	0.00	-1.69	-1.69	-1.69	-1.69	-1.69	15	0	1
beta.hab.lambda	0.58	0.00	0.57	0.57	0.58	0.58	0.58	117	0	1
beta.hab.gamma	0.27	0.00	0.27	0.27	0.27	0.27	0.27	44	0	1
beta.hab.phi	0.22	0.00	0.22	0.22	0.22	0.22	0.22	40	0	1
beta.elev.lambda	-0.27	0.00	-0.28	-0.27	-0.27	-0.27	-0.26	297	0	1
beta.ele.gamma2	-0.18	0.00	-0.18	-0.18	-0.18	-0.18	-0.18	220	0	1
beta.elev.phi	-0.11	0.00	-0.12	-0.12	-0.11	-0.11	-0.11	276	0	1
beta.develop.lambda	1.21	0.01	1.20	1.20	1.21	1.21	1.22	40	0	1
beta.develop.gamma	-0.55	0.00	-0.56	-0.55	-0.55	-0.55	-0.55	20	0	1
beta.develop.phi	0.68	0.00	0.68	0.68	0.68	0.68	0.69	44	0	1
beta.effort	0.00	0.00	0.00	0.00	0.00	0.00	0.00	1	0	1
beta.min_temp	0.00	0.00	0.00	0.00	0.00	0.00	0.00	1	0	1

Table 17: European Starling

	mean	sd	2.5%	25%	50%	75%	97.5%	n.eff	overlap0	f
alpha.lambda	13.86	0.15	13.75	13.75	13.76	14.00	14.18	3	0	1.00
alpha.phi	1.90	0.06	1.86	1.86	1.86	1.96	2.03	3	0	1.00
alpha.p	-3.89	0.18	-4.26	-4.05	-3.78	-3.76	-3.76	3	0	1.00
beta.hab.lambda	0.22	2.25	-4.16	-1.34	0.25	1.74	4.66	6000	1	0.54
beta.hab.gamma	-0.08	2.20	-4.33	-1.61	-0.09	1.39	4.24	6000	1	0.52
beta.hab.phi	0.24	2.23	-4.18	-1.30	0.27	1.78	4.53	2445	1	0.55
beta.elev.lambda	0.17	0.00	0.17	0.17	0.17	0.17	0.18	3	0	1.00
beta.ele.gamma2	0.02	0.03	0.00	0.00	0.00	0.05	0.09	3	1	0.67
beta.elev.phi	-0.27	0.03	-0.32	-0.29	-0.25	-0.25	-0.25	3	0	1.00
beta.develop.lambda	0.23	2.25	-4.20	-1.29	0.20	1.79	4.60	3940	1	0.54
beta.develop.gamma	-0.12	2.20	-4.44	-1.60	-0.11	1.40	4.12	6000	1	0.52
beta.develop.phi	0.23	2.23	-4.07	-1.32	0.19	1.77	4.64	2742	1	0.53
beta.effort	0.06	0.00	0.06	0.06	0.06	0.06	0.06	1	0	1.00
beta.min_temp	0.00	0.00	0.00	0.00	0.00	0.00	0.00	1	0	1.00

Table 18: Great-tailed Grackle

	mean	sd	2.5%	25%	50%	75%	97.5%	n.eff	overlap0	f
alpha.lambda	-7.41	1.37	-9.93	-8.44	-7.48	-6.31	-4.98	883	0	1.00
alpha.phi	3.31	1.99	1.91	1.91	1.91	5.78	6.65	3	0	1.00
alpha.p	-3.34	0.51	-4.12	-4.04	-2.98	-2.98	-2.98	3	0	1.00
beta.hab.lambda	-0.11	2.45	-5.03	-1.72	-0.04	1.51	4.63	496	1	0.51
beta.hab.gamma	0.68	2.23	-3.82	-0.76	0.66	2.18	5.03	674	1	0.63
beta.hab.phi	-0.47	2.33	-5.11	-1.99	-0.42	1.13	3.99	38	1	0.57
beta.elev.lambda	0.10	1.17	-1.68	-0.82	0.00	0.82	2.61	40	1	0.50
beta.ele.gamma2	-0.64	0.28	-1.05	-1.01	-0.44	-0.44	-0.44	3	0	1.00
beta.elev.phi	0.93	1.20	0.08	0.09	0.09	2.42	2.93	3	0	1.00
beta.develop.lambda	-0.03	2.42	-4.80	-1.63	-0.02	1.61	4.64	1377	1	0.50
beta.develop.gamma	0.73	2.24	-3.66	-0.79	0.73	2.18	5.23	450	1	0.63
beta.develop.phi	-0.45	2.32	-4.89	-2.06	-0.45	1.11	4.14	41	1	0.58
beta.effort	0.24	0.03	0.18	0.20	0.26	0.26	0.26	3	0	1.00
beta.min_temp	0.00	0.00	0.00	0.00	0.00	0.00	0.00	1	0	1.00

Table 19: Greater Scaup

	mean	sd	2.5%	25%	50%	75%	97.5%	n.eff	overlap0	f
alpha.lambda	10.86	1.38	8.91	8.91	11.76	11.87	11.97	3	0	1.00
alpha.phi	-1.07	1.74	-2.63	-2.58	-1.98	1.36	1.36	3	1	0.67
alpha.p	-4.70	1.76	-6.06	-5.98	-5.87	-2.21	-2.21	3	0	1.00
beta.hab.lambda	0.49	0.08	0.36	0.37	0.54	0.55	0.56	3	0	1.00
beta.hab.gamma	0.36	0.18	0.11	0.11	0.48	0.49	0.49	3	0	1.00
beta.hab.phi	-0.03	0.41	-0.50	-0.44	-0.15	0.53	0.53	3	1	0.67
beta.elev.lambda	-0.11	0.02	-0.14	-0.14	-0.11	-0.10	-0.10	3	0	1.00
beta.ele.gamma2	-0.18	0.04	-0.24	-0.23	-0.16	-0.14	-0.13	3	0	1.00
beta.elev.phi	-0.25	0.21	-0.45	-0.43	-0.35	0.05	0.06	3	1	0.67
beta.develop.lambda	0.69	0.04	0.63	0.63	0.70	0.73	0.74	3	0	1.00
beta.develop.gamma	0.41	0.11	0.25	0.25	0.49	0.49	0.49	3	0	1.00

	mean	sd	2.5%	25%	50%	75%	97.5%	n.eff	overlap0	f
beta.develop.phi	-0.02	0.13	-0.16	-0.14	-0.08	0.16	0.16	3	1	0.67
beta.effort	0.02	0.02	0.00	0.00	0.00	0.05	0.05	3	0	1.00
beta.min_temp	-0.18	0.04	-0.21	-0.21	-0.21	-0.12	-0.12	3	0	1.00

Table 20: Green-winged Teal

	mean	sd	2.5%	25%	50%	75%	97.5%	n.eff	overlap0	f
alpha.lambda	10.19	1.60	7.93	7.93	11.25	11.36	11.43	3	0	1.00
alpha.phi	-1.50	2.25	-3.36	-3.28	-2.86	1.67	1.67	3	1	0.67
alpha.p	-4.94	2.12	-6.55	-6.47	-6.37	-1.95	-1.95	3	0	1.00
beta.hab.lambda	0.04	0.12	-0.05	-0.05	-0.04	0.22	0.22	3	1	0.33
beta.hab.gamma	0.19	0.01	0.18	0.18	0.18	0.21	0.21	3	0	1.00
beta.hab.phi	-0.70	0.57	-1.42	-1.42	-0.65	-0.02	-0.02	3	0	1.00
beta.elev.lambda	-0.34	0.36	-0.60	-0.59	-0.59	0.17	0.17	3	1	0.67
beta.ele.gamma2	-0.33	0.01	-0.34	-0.34	-0.33	-0.31	-0.31	3	0	1.00
beta.elev.phi	0.23	0.29	-0.10	-0.10	0.18	0.60	0.64	3	1	0.67
beta.develop.lambda	0.15	0.15	-0.08	-0.06	0.25	0.26	0.27	3	1	0.67
beta.develop.gamma	-0.33	0.19	-0.49	-0.49	-0.45	-0.07	-0.06	3	0	1.00
beta.develop.phi	0.38	0.71	-0.62	-0.61	0.74	1.01	1.04	3	1	0.67
beta.effort	0.00	0.00	0.00	0.00	0.00	0.00	0.01	3	0	1.00
beta.min_temp	0.00	0.00	0.00	0.00	0.00	0.00	0.00	1	0	1.00

Table 21: House Finch

	mean	sd	2.5%	25%	50%	75%	97.5%	n.eff	overlap0	f
alpha.lambda	7.29	0.00	7.29	7.29	7.29	7.29	7.30	118	0	1.00
alpha.phi	1.81	0.00	1.81	1.81	1.81	1.81	1.82	400	0	1.00
alpha.p	-0.75	0.00	-0.75	-0.75	-0.75	-0.75	-0.75	82	0	1.00
beta.hab.lambda	0.29	2.26	-4.14	-1.20	0.29	1.78	4.66	6000	1	0.55
beta.hab.gamma	0.01	2.25	-4.44	-1.51	0.02	1.53	4.41	3451	1	0.50
beta.hab.phi	0.08	2.22	-4.30	-1.40	0.10	1.61	4.37	2821	1	0.52
beta.elev.lambda	-0.38	0.00	-0.38	-0.38	-0.38	-0.38	-0.38	111	0	1.00
beta.ele.gamma2	-0.03	0.00	-0.04	-0.03	-0.03	-0.03	-0.03	873	0	1.00
beta.elev.phi	-0.01	0.00	-0.02	-0.01	-0.01	-0.01	-0.01	813	0	1.00
beta.develop.lambda	0.24	2.26	-4.13	-1.25	0.24	1.73	4.66	6000	1	0.54
beta.develop.gamma	0.00	2.25	-4.40	-1.52	-0.01	1.52	4.45	3456	1	0.50
beta.develop.phi	0.05	2.22	-4.24	-1.47	0.04	1.54	4.43	2828	1	0.51
beta.effort	0.00	0.00	0.00	0.00	0.00	0.00	0.00	1	0	1.00
beta.min_temp	0.00	0.00	0.00	0.00	0.00	0.00	0.00	1	0	1.00

Table 22: House Sparrow

	mean	sd	2.5%	25%	50%	75%	97.5%	n.eff	overlap0	f
alpha.lambda	8.26	0.00	8.26	8.26	8.26	8.26	8.27	17	0	1.00
alpha.phi	2.41	0.00	2.41	2.41	2.41	2.41	2.42	10	0	1.00
alpha.p	-1.12	0.00	-1.13	-1.12	-1.12	-1.12	-1.12	11	0	1.00
beta.hab.lambda	0.17	2.21	-4.19	-1.31	0.17	1.67	4.41	5533	1	0.53

	mean	sd	2.5%	25%	50%	75%	97.5%	n.eff	overlap0	f
beta.hab.gamma	0.07	2.23	-4.24	-1.42	0.09	1.54	4.47	3104	1	0.52
beta.hab.phi	0.07	2.23	-4.19	-1.45	0.06	1.56	4.50	6000	1	0.51
beta.elev.lambda	0.12	0.00	0.12	0.12	0.12	0.13	0.13	147	0	1.00
beta.ele.gamma2	0.27	0.00	0.27	0.27	0.27	0.27	0.27	47	0	1.00
beta.elev.phi	-0.04	0.00	-0.04	-0.04	-0.04	-0.03	-0.03	42	0	1.00
beta.develop.lambda	0.05	2.21	-4.20	-1.46	0.04	1.52	4.41	5532	1	0.51
beta.develop.gamma	0.03	2.23	-4.37	-1.44	0.01	1.52	4.33	3098	1	0.50
beta.develop.phi	0.05	2.23	-4.39	-1.44	0.06	1.56	4.30	6000	1	0.51
beta.effort	0.03	0.00	0.03	0.03	0.03	0.03	0.03	599	0	1.00
beta.min_temp	-0.10	0.00	-0.10	-0.10	-0.10	-0.10	-0.10	503	0	1.00

Table 23: Lesser Scaup

	mean	sd	2.5%	25%	50%	75%	97.5%	n.eff	overlap0	f
alpha.lambda	8.69	0.03	8.63	8.66	8.69	8.71	8.75	5	0	1
alpha.phi	0.65	0.01	0.64	0.64	0.65	0.65	0.66	5	0	1
alpha.p	-3.14	0.03	-3.19	-3.16	-3.14	-3.12	-3.09	4	0	1
beta.hab.lambda	0.35	0.01	0.34	0.34	0.35	0.35	0.36	5	0	1
beta.hab.gamma	-0.45	0.01	-0.47	-0.46	-0.45	-0.45	-0.44	8	0	1
beta.hab.phi	1.55	0.02	1.51	1.54	1.55	1.56	1.58	8	0	1
beta.elev.lambda	-0.82	0.00	-0.82	-0.82	-0.82	-0.81	-0.81	4	0	1
beta.ele.gamma2	-0.63	0.01	-0.64	-0.63	-0.63	-0.63	-0.62	9	0	1
beta.elev.phi	0.45	0.01	0.43	0.44	0.45	0.46	0.47	7	0	1
beta.develop.lambda	0.88	0.01	0.87	0.88	0.88	0.89	0.90	5	0	1
beta.develop.gamma	-0.70	0.01	-0.71	-0.70	-0.70	-0.69	-0.68	6	0	1
beta.develop.phi	1.25	0.01	1.23	1.24	1.25	1.26	1.27	8	0	1
beta.effort	0.12	0.00	0.11	0.11	0.12	0.12	0.12	7	0	1
beta.min_temp	0.00	0.00	0.00	0.00	0.00	0.00	0.00	1	0	1

Table 24: Long-tailed Duck

	mean	sd	2.5%	25%	50%	75%	97.5%	n.eff	overlap0	f
alpha.lambda	9.76	1.03	8.26	8.46	10.11	10.81	10.82	3	0	1.00
alpha.phi	0.89	0.37	0.36	0.49	0.89	1.30	1.42	3	0	1.00
alpha.p	-4.48	1.27	-5.44	-5.38	-5.35	-2.69	-2.67	3	0	1.00
beta.hab.lambda	0.46	0.39	-0.11	-0.03	0.58	0.86	0.88	3	1	0.67
beta.hab.gamma	0.41	0.44	-0.23	-0.21	0.70	0.74	0.74	3	1	0.67
beta.hab.phi	1.02	1.50	-0.16	-0.12	0.06	2.91	3.49	3	1	0.67
beta.elev.lambda	0.43	0.20	0.12	0.16	0.56	0.58	0.59	3	0	1.00
beta.ele.gamma2	1.49	0.09	1.35	1.38	1.55	1.56	1.63	3	0	1.00
beta.elev.phi	-0.73	0.35	-1.16	-1.10	-0.80	-0.34	-0.21	3	0	1.00
beta.develop.lambda	0.06	0.40	-0.41	-0.36	-0.04	0.58	0.61	3	1	0.33
beta.develop.gamma	-0.54	0.68	-1.34	-1.08	-0.84	0.36	0.41	3	1	0.67
beta.develop.phi	0.52	1.21	-1.67	-0.04	0.26	1.91	2.23	3	1	0.74
beta.effort	0.00	0.00	0.00	0.00	0.00	0.00	0.00	1	0	1.00
beta.min_temp	-0.03	0.02	-0.05	-0.04	-0.04	0.00	0.00	3	0	1.00

Table 25: Mallard

	mean	sd	2.5%	25%	50%	75%	97.5%	n.eff	overlap0	f
alpha.lambda	9.66	0	9.66	9.66	9.66	9.67	9.67	16	0	1
alpha.phi	2.57	0	2.56	2.57	2.57	2.57	2.57	14	0	1
alpha.p	-1.88	0	-1.88	-1.88	-1.88	-1.88	-1.88	5	0	1
beta.hab.lambda	-0.33	0	-0.33	-0.33	-0.33	-0.33	-0.33	243	0	1
beta.hab.gamma	-0.56	0	-0.56	-0.56	-0.56	-0.55	-0.55	48	0	1
beta.hab.phi	1.63	0	1.62	1.63	1.63	1.64	1.64	27	0	1
beta.elev.lambda	0.20	0	0.20	0.20	0.20	0.20	0.20	9	0	1
beta.ele.gamma2	-0.05	0	-0.06	-0.05	-0.05	-0.05	-0.05	16	0	1
beta.elev.phi	0.20	0	0.20	0.20	0.20	0.20	0.21	51	0	1
beta.develop.lambda	-0.83	0	-0.83	-0.83	-0.83	-0.83	-0.82	9	0	1
beta.develop.gamma	-0.20	0	-0.20	-0.20	-0.20	-0.20	-0.20	12	0	1
beta.develop.phi	0.34	0	0.34	0.34	0.34	0.34	0.34	620	0	1
beta.effort	0.03	0	0.03	0.03	0.03	0.03	0.03	18	0	1
beta.min_temp	0.00	0	0.00	0.00	0.00	0.00	0.00	1	0	1

Table 26: Mourning Dove

	mean	sd	2.5%	25%	50%	75%	97.5%	n.eff	overlap0	f
alpha.lambda	6.83	0	6.82	6.83	6.83	6.83	6.83	96	0	1
alpha.phi	2.36	0	2.36	2.36	2.36	2.37	2.37	14	0	1
alpha.p	-1.18	0	-1.18	-1.18	-1.18	-1.18	-1.18	40	0	1
beta.hab.lambda	0.03	0	0.03	0.03	0.03	0.03	0.04	662	0	1
beta.hab.gamma	0.02	0	0.02	0.02	0.02	0.02	0.02	59	0	1
beta.hab.phi	0.03	0	0.02	0.02	0.03	0.03	0.03	45	0	1
beta.elev.lambda	-0.22	0	-0.23	-0.23	-0.22	-0.22	-0.22	1020	0	1
beta.ele.gamma2	-0.30	0	-0.30	-0.30	-0.30	-0.30	-0.30	94	0	1
beta.elev.phi	0.11	0	0.11	0.11	0.11	0.11	0.12	67	0	1
beta.develop.lambda	0.42	0	0.41	0.42	0.42	0.42	0.42	585	0	1
beta.develop.gamma	0.19	0	0.19	0.19	0.19	0.19	0.19	138	0	1
beta.develop.phi	0.06	0	0.05	0.06	0.06	0.06	0.06	116	0	1
beta.effort	0.01	0	0.01	0.01	0.01	0.01	0.02	4286	0	1
beta.min_temp	0.00	0	0.00	0.00	0.00	0.00	0.00	1	0	1

Table 27: Northern Cardinal

	mean	sd	2.5%	25%	50%	75%	97.5%	n.eff	overlap0	f
alpha.lambda	6.45	0.00	6.45	6.45	6.45	6.46	6.46	13	0	1
alpha.phi	3.22	0.00	3.21	3.22	3.22	3.22	3.23	10	0	1
alpha.p	-0.84	0.00	-0.84	-0.84	-0.84	-0.83	-0.83	10	0	1
beta.hab.lambda	0.04	0.00	0.04	0.04	0.04	0.05	0.05	311	0	1
beta.hab.gamma	-0.08	0.00	-0.08	-0.08	-0.08	-0.08	-0.07	82	0	1
beta.hab.phi	0.12	0.01	0.11	0.11	0.12	0.12	0.13	60	0	1
beta.elev.lambda	-0.17	0.00	-0.18	-0.17	-0.17	-0.17	-0.17	730	0	1
beta.ele.gamma2	-0.28	0.00	-0.28	-0.28	-0.28	-0.28	-0.27	87	0	1
beta.elev.phi	0.27	0.00	0.26	0.27	0.27	0.27	0.28	50	0	1
beta.develop.lambda	0.29	0.00	0.29	0.29	0.29	0.30	0.30	1231	0	1
beta.develop.gamma	0.23	0.00	0.22	0.23	0.23	0.23	0.24	223	0	1

	mean	sd	2.5%	25%	50%	75%	97.5%	n.eff	overlap0	f
beta.develop.phi	-0.18	0.01	-0.19	-0.19	-0.18	-0.18	-0.17	98	0	1
beta.effort	0.00	0.00	0.00	0.00	0.00	0.00	0.00	122	0	1
beta.min_temp	-0.07	0.00	-0.07	-0.07	-0.07	-0.07	-0.07	101	0	1

Table 28: Northern Pintail

	mean	sd	2.5%	25%	50%	75%	97.5%	n.eff	overlap0	f
alpha.lambda	10.57	0.77	9.78	9.79	10.32	11.60	11.66	3	0	1.00
alpha.phi	1.21	1.22	-0.61	-0.46	2.07	2.07	2.07	3	1	0.67
alpha.p	-2.76	1.00	-4.19	-4.14	-2.24	-1.87	-1.87	3	0	1.00
beta.hab.lambda	-0.11	0.12	-0.29	-0.27	-0.07	0.02	0.02	3	1	0.67
beta.hab.gamma	-0.51	0.23	-0.77	-0.75	-0.56	-0.21	-0.21	3	0	1.00
beta.hab.phi	1.34	0.84	0.15	0.15	1.84	1.97	2.12	3	0	1.00
beta.elev.lambda	-0.11	0.08	-0.18	-0.18	-0.15	0.01	0.01	3	1	0.67
beta.ele.gamma2	-0.76	0.25	-1.10	-1.09	-0.68	-0.50	-0.49	3	0	1.00
beta.elev.phi	0.60	1.02	-0.28	-0.27	0.04	1.97	2.10	3	1	0.67
beta.develop.lambda	-0.10	0.53	-0.86	-0.85	0.21	0.35	0.35	3	1	0.33
beta.develop.gamma	-0.62	0.78	-1.20	-1.20	-1.13	0.48	0.48	3	1	0.67
beta.develop.phi	-0.57	1.28	-2.47	-2.33	0.30	0.36	0.36	3	1	0.33
beta.effort	0.07	0.03	0.02	0.03	0.08	0.09	0.09	3	0	1.00
beta.min_temp	0.00	0.00	0.00	0.00	0.00	0.00	0.00	1	0	1.00

Table 29: Northern Shoveler

	mean	sd	2.5%	25%	50%	75%	97.5%	n.eff	overlap0	f
alpha.lambda	9.20	0.11	9.06	9.10	9.19	9.34	9.35	3	0	1.00
alpha.phi	1.41	1.55	-0.80	-0.76	2.45	2.55	2.57	3	1	0.67
alpha.p	-3.01	0.84	-4.22	-4.15	-2.57	-2.28	-2.26	3	0	1.00
beta.hab.lambda	-0.41	0.35	-0.92	-0.89	-0.21	-0.12	-0.11	3	0	1.00
beta.hab.gamma	0.12	0.12	0.00	0.00	0.07	0.29	0.29	3	0	1.00
beta.hab.phi	0.88	1.83	-1.74	-1.67	2.04	2.28	2.35	3	1	0.67
beta.elev.lambda	-0.20	0.41	-0.78	-0.77	0.06	0.12	0.12	3	1	0.33
beta.ele.gamma2	-0.05	0.21	-0.25	-0.24	-0.15	0.24	0.24	3	1	0.67
beta.elev.phi	-0.79	0.47	-1.46	-1.45	-0.50	-0.42	-0.39	3	0	1.00
beta.develop.lambda	-0.13	0.15	-0.35	-0.34	-0.05	0.00	0.02	3	1	0.80
beta.develop.gamma	-0.78	0.28	-1.17	-1.17	-0.62	-0.55	-0.54	3	0	1.00
beta.develop.phi	1.44	0.65	0.90	0.95	1.04	2.36	2.36	3	0	1.00
beta.effort	0.00	0.00	0.00	0.00	0.00	0.00	0.00	1	0	1.00
beta.min_temp	0.00	0.00	0.00	0.00	0.00	0.00	0.00	1	0	1.00

Table 30: Red-winged Blackbird

	mean	sd	2.5%	25%	50%	75%	97.5%	n.eff	overlap0	f
alpha.lambda	16.08	0.95	14.74	14.74	16.60	16.86	16.93	3	0	1.00
alpha.phi	-0.04	1.06	-1.12	-1.07	-0.44	1.41	1.41	3	1	0.67
alpha.p	-5.33	1.10	-6.25	-6.16	-6.02	-3.79	-3.79	3	0	1.00
beta.hab.lambda	-0.79	0.57	-1.33	-1.29	-1.08	-0.01	-0.01	3	0	1.00

	mean	sd	2.5%	25%	50%	75%	97.5%	n.eff	overlap0	f
beta.hab.gamma	0.04	0.07	-0.01	-0.01	-0.01	0.14	0.14	3	1	0.33
beta.hab.phi	-0.76	0.52	-1.47	-1.46	-0.60	-0.23	-0.23	3	0	1.00
beta.elev.lambda	-0.16	0.21	-0.32	-0.32	-0.30	0.13	0.13	3	1	0.67
beta.ele.gamma2	-0.28	0.08	-0.34	-0.33	-0.33	-0.17	-0.17	3	0	1.00
beta.elev.phi	-0.31	0.04	-0.35	-0.34	-0.32	-0.26	-0.24	3	0	1.00
beta.develop.lambda	0.08	0.38	-0.22	-0.21	-0.17	0.61	0.61	3	1	0.33
beta.develop.gamma	-1.31	0.59	-1.87	-1.81	-1.61	-0.49	-0.49	3	0	1.00
beta.develop.phi	1.84	0.95	0.51	0.51	2.39	2.58	2.69	3	0	1.00
beta.effort	0.11	0.01	0.10	0.10	0.10	0.13	0.13	3	0	1.00
beta.min_temp	0.00	0.00	0.00	0.00	0.00	0.00	0.00	1	0	1.00

Table 31: Ring-billed Gull

	mean	sd	2.5%	25%	50%	75%	97.5%	n.eff	overlap0	f
alpha.lambda	8.65	0	8.64	8.65	8.65	8.65	8.65	382	0	1
alpha.phi	1.93	0	1.93	1.93	1.93	1.93	1.93	38	0	1
alpha.p	-1.75	0	-1.75	-1.75	-1.75	-1.75	-1.75	144	0	1
beta.hab.lambda	0.16	0	0.16	0.16	0.16	0.16	0.16	496	0	1
beta.hab.gamma	0.10	0	0.09	0.10	0.10	0.10	0.10	553	0	1
beta.hab.phi	0.04	0	0.04	0.04	0.04	0.04	0.04	1033	0	1
beta.elev.lambda	0.11	0	0.11	0.11	0.11	0.11	0.11	569	0	1
beta.ele.gamma2	-0.34	0	-0.34	-0.34	-0.34	-0.33	-0.33	515	0	1
beta.elev.phi	0.02	0	0.02	0.02	0.02	0.02	0.02	398	0	1
beta.develop.lambda	0.43	0	0.43	0.43	0.43	0.43	0.43	416	0	1
beta.develop.gamma	-0.01	0	-0.01	-0.01	-0.01	-0.01	-0.01	498	0	1
beta.develop.phi	0.30	0	0.30	0.30	0.30	0.30	0.30	505	0	1
beta.effort	0.00	0	0.00	0.00	0.00	0.00	0.00	1	0	1
beta.min_temp	0.00	0	0.00	0.00	0.00	0.00	0.00	1	0	1

Table 32: Rock Pigeon

	mean	sd	2.5%	25%	50%	75%	97.5%	n.eff	overlap0	f
alpha.lambda	-7.96	0.77	-9.72	-8.39	-7.97	-7.55	-6.44	10	0	1.00
alpha.phi	2.31	0.00	2.30	2.31	2.31	2.31	2.31	268	0	1.00
alpha.p	-1.16	0.00	-1.16	-1.16	-1.16	-1.16	-1.16	6000	0	1.00
beta.hab.lambda	0.49	2.28	-4.16	-1.02	0.54	2.03	4.95	361	1	0.59
beta.hab.gamma	0.25	2.21	-4.08	-1.25	0.28	1.74	4.60	6000	1	0.55
beta.hab.phi	-0.09	2.20	-4.42	-1.60	-0.08	1.41	4.22	6000	1	0.52
beta.elev.lambda	0.81	1.11	-1.04	-0.16	0.99	1.67	2.80	4	1	0.70
beta.ele.gamma2	-0.01	0.00	-0.01	-0.01	-0.01	-0.01	-0.01	505	0	1.00
beta.elev.phi	0.01	0.00	0.01	0.01	0.01	0.01	0.02	264	0	1.00
beta.develop.lambda	0.47	2.27	-4.08	-1.03	0.45	1.98	4.92	203	1	0.59
beta.develop.gamma	0.28	2.21	-4.07	-1.21	0.25	1.79	4.61	6000	1	0.55
beta.develop.phi	-0.15	2.20	-4.46	-1.64	-0.16	1.36	4.18	6000	1	0.53
beta.effort	0.00	0.00	0.00	0.00	0.00	0.00	0.00	1	0	1.00
beta.min_temp	0.00	0.00	0.00	0.00	0.00	0.00	0.00	1	0	1.00

Table 33: Ruddy Duck

	mean	sd	2.5%	25%	50%	75%	97.5%	n.eff	overlap0	f
alpha.lambda	7.37	0	7.37	7.37	7.37	7.38	7.38	113	0	1
alpha.phi	1.94	0	1.94	1.94	1.94	1.94	1.94	33	0	1
alpha.p	-1.47	0	-1.48	-1.47	-1.47	-1.47	-1.47	538	0	1
beta.hab.lambda	-0.84	0	-0.85	-0.84	-0.84	-0.84	-0.84	678	0	1
beta.hab.gamma	-0.12	0	-0.12	-0.12	-0.12	-0.12	-0.12	783	0	1
beta.hab.phi	0.06	0	0.06	0.06	0.06	0.06	0.07	1697	0	1
beta.elev.lambda	-0.84	0	-0.85	-0.85	-0.84	-0.84	-0.84	91	0	1
beta.ele.gamma2	-0.44	0	-0.45	-0.44	-0.44	-0.44	-0.44	60	0	1
beta.elev.phi	-0.11	0	-0.12	-0.11	-0.11	-0.11	-0.11	51	0	1
beta.develop.lambda	0.22	0	0.22	0.22	0.22	0.22	0.23	6000	0	1
beta.develop.gamma	-0.45	0	-0.45	-0.45	-0.45	-0.45	-0.45	237	0	1
beta.develop.phi	0.46	0	0.46	0.46	0.46	0.47	0.47	84	0	1
beta.effort	0.00	0	0.00	0.00	0.00	0.00	0.00	6000	0	1
beta.min__temp	0.00	0	0.00	0.00	0.00	0.00	0.00	1	0	1

Table 34: Snow Goose

	mean	sd	2.5%	25%	50%	75%	97.5%	n.eff	overlap0	f
alpha.lambda	7.75	0.01	7.74	7.75	7.75	7.75	7.76	54	0	1
alpha.phi	1.72	0.00	1.72	1.72	1.72	1.72	1.72	14	0	1
alpha.p	-2.30	0.00	-2.30	-2.30	-2.30	-2.30	-2.30	11	0	1
beta.hab.lambda	-0.80	0.00	-0.80	-0.80	-0.80	-0.80	-0.80	25	0	1
beta.hab.gamma	-0.56	0.00	-0.56	-0.56	-0.56	-0.56	-0.56	55	0	1
beta.hab.phi	0.53	0.00	0.53	0.53	0.53	0.53	0.53	51	0	1
beta.elev.lambda	-0.60	0.00	-0.60	-0.60	-0.60	-0.60	-0.59	65	0	1
beta.ele.gamma2	-0.16	0.00	-0.16	-0.16	-0.16	-0.16	-0.16	188	0	1
beta.elev.phi	-0.21	0.00	-0.21	-0.21	-0.21	-0.21	-0.21	56	0	1
beta.develop.lambda	1.28	0.00	1.28	1.28	1.28	1.28	1.29	192	0	1
beta.develop.gamma	-0.59	0.00	-0.59	-0.59	-0.59	-0.59	-0.59	122	0	1
beta.develop.phi	-0.05	0.00	-0.05	-0.05	-0.05	-0.04	-0.04	63	0	1
beta.effort	0.08	0.00	0.08	0.08	0.08	0.08	0.08	405	0	1
beta.min__temp	0.00	0.00	0.00	0.00	0.00	0.00	0.00	1	0	1

Table 35: Tree Swallow

	mean	sd	2.5%	25%	50%	75%	97.5%	n.eff	overlap0	f
alpha.lambda	12.15	0.13	11.88	12.06	12.15	12.26	12.36	4	0	1.00
alpha.phi	-1.65	0.48	-2.29	-2.28	-1.55	-1.13	-1.12	3	0	1.00
alpha.p	-6.87	0.09	-7.04	-6.94	-6.87	-6.81	-6.70	8	0	1.00
beta.hab.lambda	-0.96	0.16	-1.20	-1.18	-0.86	-0.85	-0.84	3	0	1.00
beta.hab.gamma	0.01	0.05	-0.06	-0.05	0.04	0.06	0.06	3	1	0.67
beta.hab.phi	-0.22	0.25	-0.45	-0.44	-0.35	0.12	0.14	3	1	0.67
beta.elev.lambda	-1.23	0.12	-1.40	-1.39	-1.17	-1.12	-1.11	3	0	1.00
beta.ele.gamma2	-0.53	0.01	-0.54	-0.54	-0.54	-0.51	-0.51	3	0	1.00
beta.elev.phi	0.02	0.04	-0.03	-0.03	0.05	0.06	0.06	3	1	0.67
beta.develop.lambda	2.26	0.03	2.22	2.24	2.25	2.28	2.30	3	0	1.00
beta.develop.gamma	1.53	0.04	1.48	1.48	1.54	1.58	1.58	3	0	1.00

	mean	sd	2.5%	25%	50%	75%	97.5%	n.eff	overlap0	f
beta.develop.phi	-0.05	0.21	-0.22	-0.21	-0.19	0.23	0.25	3	1	0.67
beta.effort	0.10	0.00	0.09	0.10	0.10	0.10	0.10	3	0	1.00
beta.min_temp	0.00	0.00	0.00	0.00	0.00	0.00	0.00	1	0	1.00

Table 36: White-crowned Sparrow

	mean	sd	2.5%	25%	50%	75%	97.5%	n.eff	overlap0	f
alpha.lambda	7.23	0.00	7.22	7.22	7.23	7.23	7.23	918	0	1.00
alpha.phi	3.03	0.00	3.02	3.02	3.03	3.03	3.03	58	0	1.00
alpha.p	-1.05	0.00	-1.05	-1.05	-1.05	-1.04	-1.04	548	0	1.00
beta.hab.lambda	0.54	0.00	0.54	0.54	0.54	0.54	0.54	435	0	1.00
beta.hab.gamma	0.67	0.00	0.67	0.67	0.67	0.68	0.68	216	0	1.00
beta.hab.phi	-0.09	0.00	-0.10	-0.09	-0.09	-0.09	-0.09	250	0	1.00
beta.elev.lambda	-0.41	0.00	-0.42	-0.41	-0.41	-0.41	-0.41	1439	0	1.00
beta.ele.gamma2	-0.17	0.00	-0.18	-0.17	-0.17	-0.17	-0.17	4423	0	1.00
beta.elev.phi	0.00	0.00	0.00	0.00	0.00	0.00	0.00	440	1	0.58
beta.develop.lambda	0.51	0.00	0.50	0.51	0.51	0.51	0.52	11	0	1.00
beta.develop.gamma	-0.45	0.01	-0.46	-0.45	-0.45	-0.45	-0.44	36	0	1.00
beta.develop.phi	0.26	0.00	0.26	0.26	0.26	0.27	0.27	91	0	1.00
beta.effort	0.07	0.00	0.07	0.07	0.07	0.07	0.07	196	0	1.00
beta.min_temp	0.00	0.00	0.00	0.00	0.00	0.00	0.00	1	0	1.00

Table 37: White-throated Sparrow

	mean	sd	2.5%	25%	50%	75%	97.5%	n.eff	overlap0	f
alpha.lambda	6.63	0.05	6.56	6.57	6.63	6.69	6.70	3	0	1
alpha.phi	3.24	0.04	3.19	3.19	3.24	3.28	3.29	3	0	1
alpha.p	-0.93	0.06	-1.02	-1.00	-0.93	-0.86	-0.85	3	0	1
beta.hab.lambda	0.25	0.01	0.24	0.25	0.25	0.26	0.26	4	0	1
beta.hab.gamma	0.66	0.01	0.64	0.65	0.66	0.66	0.67	3	0	1
beta.hab.phi	-0.35	0.01	-0.37	-0.36	-0.35	-0.33	-0.33	3	0	1
beta.elev.lambda	-0.56	0.00	-0.57	-0.56	-0.56	-0.56	-0.56	4	0	1
beta.ele.gamma2	-0.84	0.01	-0.85	-0.85	-0.84	-0.84	-0.83	3	0	1
beta.elev.phi	0.35	0.01	0.33	0.33	0.35	0.36	0.37	3	0	1
beta.develop.lambda	0.24	0.01	0.22	0.23	0.24	0.25	0.25	3	0	1
beta.develop.gamma	0.02	0.02	0.00	0.01	0.02	0.04	0.05	3	0	1
beta.develop.phi	-0.14	0.01	-0.16	-0.15	-0.14	-0.14	-0.13	4	0	1
beta.effort	0.03	0.00	0.02	0.03	0.03	0.04	0.04	3	0	1
beta.min_temp	-0.05	0.00	-0.05	-0.05	-0.05	-0.05	-0.05	3	0	1

Table 38: Yellow-rumped Warbler

	mean	sd	2.5%	25%	50%	75%	97.5%	n.eff	overlap0	f
alpha.lambda	6.86	0	6.86	6.86	6.86	6.87	6.87	208	0	1
alpha.phi	2.93	0	2.92	2.93	2.93	2.93	2.93	273	0	1
alpha.p	-1.12	0	-1.12	-1.12	-1.12	-1.12	-1.11	417	0	1
beta.hab.lambda	-0.95	0	-0.96	-0.96	-0.95	-0.95	-0.95	494	0	1

	mean	sd	2.5%	25%	50%	75%	97.5%	n.eff	overlap0	f
beta.hab.gamma	-0.52	0	-0.53	-0.52	-0.52	-0.52	-0.52	1294	0	1
beta.hab.phi	0.38	0	0.37	0.37	0.38	0.38	0.38	236	0	1
beta.elev.lambda	-0.48	0	-0.48	-0.48	-0.48	-0.48	-0.47	76	0	1
beta.ele.gamma2	-0.78	0	-0.79	-0.78	-0.78	-0.78	-0.78	87	0	1
beta.elev.phi	0.26	0	0.26	0.26	0.26	0.26	0.26	74	0	1
beta.develop.lambda	0.09	0	0.08	0.09	0.09	0.09	0.09	97	0	1
beta.develop.gamma	-0.28	0	-0.28	-0.28	-0.28	-0.28	-0.28	45	0	1
beta.develop.phi	0.41	0	0.40	0.41	0.41	0.41	0.42	82	0	1
beta.effort	0.00	0	0.00	0.00	0.00	0.00	0.00	1	0	1
beta.min_temp	0.00	0	0.00	0.00	0.00	0.00	0.00	1	0	1

Table 39: American Tree Sparrow

	mean	sd	2.5%	25%	50%	75%	97.5%	n.eff	overlap0	f
alpha.lambda	7.63	0.00	7.62	7.63	7.63	7.63	7.63	8	0	1
alpha.phi	3.00	0.00	3.00	3.00	3.00	3.01	3.01	8	0	1
alpha.p	-1.73	0.00	-1.74	-1.73	-1.73	-1.73	-1.73	7	0	1
beta.hab.lambda	-0.23	0.00	-0.23	-0.23	-0.23	-0.23	-0.22	1463	0	1
beta.hab.gamma	-0.43	0.00	-0.44	-0.43	-0.43	-0.43	-0.42	59	0	1
beta.hab.phi	-0.16	0.00	-0.16	-0.16	-0.16	-0.16	-0.15	27	0	1
beta.elev.lambda	0.07	0.00	0.07	0.07	0.07	0.07	0.08	80	0	1
beta.ele.gamma2	0.35	0.00	0.34	0.35	0.35	0.35	0.36	29	0	1
beta.elev.phi	-0.56	0.01	-0.57	-0.56	-0.56	-0.55	-0.55	24	0	1
beta.develop.lambda	0.12	0.00	0.11	0.11	0.12	0.12	0.12	63	0	1
beta.develop.gamma	-0.18	0.01	-0.19	-0.18	-0.18	-0.18	-0.17	59	0	1
beta.develop.phi	0.14	0.00	0.13	0.14	0.14	0.15	0.15	46	0	1
beta.effort	0.05	0.00	0.05	0.05	0.05	0.05	0.05	418	0	1
beta.min_temp	-0.17	0.00	-0.17	-0.17	-0.17	-0.17	-0.17	223	0	1

Table 40: Boat-tailed Grackle

	mean	sd	2.5%	25%	50%	75%	97.5%	n.eff	overlap0	f
alpha.lambda	-7.10	1.72	-10.00	-8.51	-7.16	-5.68	-4.08	8	0	1.00
alpha.phi	1.38	0.08	1.31	1.32	1.33	1.48	1.51	3	0	1.00
alpha.p	-3.07	0.02	-3.13	-3.08	-3.06	-3.04	-3.04	4	0	1.00
beta.hab.lambda	0.14	1.49	-2.87	-0.81	0.17	1.19	2.97	6	1	0.54
beta.hab.gamma	0.30	0.03	0.25	0.25	0.31	0.33	0.33	3	0	1.00
beta.hab.phi	0.08	0.04	0.03	0.04	0.06	0.13	0.14	3	0	1.00
beta.elev.lambda	1.14	1.73	-2.02	-0.29	1.05	2.69	3.96	5	1	0.70
beta.ele.gamma2	0.15	0.05	0.08	0.09	0.19	0.19	0.19	3	0	1.00
beta.elev.phi	-0.04	0.06	-0.09	-0.09	-0.08	0.03	0.05	3	1	0.67
beta.develop.lambda	1.41	3.45	-3.65	-1.46	0.13	5.55	6.62	3	1	0.52
beta.develop.gamma	0.45	0.21	0.20	0.20	0.43	0.71	0.72	3	0	1.00
beta.develop.phi	0.36	0.32	-0.06	-0.04	0.42	0.72	0.73	3	1	0.67
beta.effort	0.23	0.00	0.23	0.23	0.23	0.24	0.24	3	0	1.00
beta.min_temp	0.00	0.00	0.00	0.00	0.00	0.00	0.00	1	0	1.00

Table 41: Bonaparte's Gull

	mean	sd	2.5%	25%	50%	75%	97.5%	n.eff	overlap0	f
alpha.lambda	10.44	1.51	9.35	9.37	9.38	12.50	12.68	3	0	1.00
alpha.phi	0.69	2.01	-2.16	-2.16	2.09	2.10	2.16	3	1	0.67
alpha.p	-3.76	1.65	-6.19	-6.03	-2.61	-2.59	-2.57	3	0	1.00
beta.hab.lambda	-0.56	0.08	-0.68	-0.67	-0.51	-0.49	-0.49	3	0	1.00
beta.hab.gamma	-0.19	0.16	-0.37	-0.28	-0.28	0.03	0.04	3	1	0.67
beta.hab.phi	0.53	0.23	0.22	0.23	0.64	0.67	0.85	3	0	1.00
beta.elev.lambda	0.00	0.04	-0.03	-0.03	-0.02	0.05	0.06	3	1	0.33
beta.ele.gamma2	-0.07	0.13	-0.19	-0.16	-0.15	0.11	0.11	3	1	0.67
beta.elev.phi	0.50	0.07	0.43	0.43	0.48	0.59	0.62	3	0	1.00
beta.develop.lambda	0.10	0.11	-0.06	-0.05	0.15	0.20	0.20	3	1	0.67
beta.develop.gamma	0.04	0.18	-0.21	-0.20	0.10	0.23	0.23	3	1	0.67
beta.develop.phi	-0.12	0.23	-0.51	-0.34	-0.12	0.14	0.15	3	1	0.67
beta.effort	0.08	0.00	0.08	0.08	0.08	0.08	0.08	3	0	1.00
beta.min_temp	-0.14	0.05	-0.22	-0.22	-0.11	-0.11	-0.10	3	0	1.00

Table 42: Bufflehead

	mean	sd	2.5%	25%	50%	75%	97.5%	n.eff	overlap0	f
alpha.lambda	5.59	0.03	5.57	5.58	5.59	5.59	5.70	25	0	1.00
alpha.phi	2.39	0.02	2.37	2.38	2.38	2.39	2.46	17	0	1.00
alpha.p	-1.12	0.03	-1.23	-1.12	-1.12	-1.11	-1.11	21	0	1.00
beta.hab.lambda	0.74	0.01	0.73	0.74	0.74	0.74	0.75	85	0	1.00
beta.hab.gamma	0.63	0.01	0.62	0.63	0.63	0.63	0.64	44	0	1.00
beta.hab.phi	-0.03	0.01	-0.03	-0.03	-0.03	-0.03	0.00	50	0	0.98
beta.elev.lambda	-0.13	0.00	-0.14	-0.14	-0.13	-0.13	-0.13	26	0	1.00
beta.ele.gamma2	-0.03	0.00	-0.05	-0.04	-0.03	-0.03	-0.03	22	0	1.00
beta.elev.phi	-0.16	0.00	-0.16	-0.16	-0.16	-0.16	-0.14	18	0	1.00
beta.develop.lambda	-0.02	0.01	-0.03	-0.02	-0.02	-0.01	0.01	26	1	0.95
beta.develop.gamma	-0.30	0.01	-0.32	-0.31	-0.31	-0.30	-0.30	121	0	1.00
beta.develop.phi	0.80	0.04	0.79	0.80	0.81	0.81	0.85	121	0	1.00
beta.effort	0.09	0.00	0.09	0.09	0.09	0.09	0.09	124	0	1.00
beta.min_temp	0.00	0.00	0.00	0.00	0.00	0.00	0.00	6000	0	1.00

Table 43: Bushtit

	mean	sd	2.5%	25%	50%	75%	97.5%	n.eff	overlap0	f
alpha.lambda	7.50	0.80	6.40	6.42	7.80	8.28	8.30	3	0	1.00
alpha.phi	3.04	0.42	2.45	2.46	3.22	3.44	3.46	3	0	1.00
alpha.p	-1.94	0.78	-2.76	-2.73	-2.20	-0.89	-0.88	3	0	1.00
beta.hab.lambda	-0.01	0.06	-0.11	-0.09	0.01	0.05	0.06	3	1	0.43
beta.hab.gamma	0.14	0.09	0.01	0.02	0.19	0.22	0.24	3	0	1.00
beta.hab.phi	-0.12	0.06	-0.20	-0.19	-0.11	-0.06	-0.04	3	0	1.00
beta.elev.lambda	-0.17	0.04	-0.21	-0.21	-0.18	-0.12	-0.10	3	0	1.00
beta.ele.gamma2	-0.11	0.07	-0.20	-0.18	-0.13	-0.01	0.00	3	0	1.00
beta.elev.phi	-1.06	0.40	-1.37	-1.35	-1.33	-0.51	-0.47	3	0	1.00
beta.develop.lambda	0.38	0.04	0.32	0.34	0.37	0.43	0.43	3	0	1.00
beta.develop.gamma	0.29	0.04	0.22	0.24	0.31	0.32	0.34	3	0	1.00

	mean	sd	2.5%	25%	50%	75%	97.5%	n.eff	overlap0	f
beta.develop.phi	0.15	0.08	0.05	0.09	0.11	0.25	0.27	3	0	1.00
beta.effort	0.10	0.01	0.09	0.09	0.11	0.11	0.11	3	0	1.00
beta.min_temp	0.00	0.00	0.00	0.00	0.00	0.00	0.00	1	0	1.00

Table 44: Cackling Goose

	mean	sd	2.5%	25%	50%	75%	97.5%	n.eff	overlap0	f
alpha.lambda	-7.82	1.35	-10.55	-8.87	-7.47	-6.77	-5.78	5	0	1.00
alpha.phi	5.40	5.17	1.44	1.45	2.06	12.17	13.56	3	0	1.00
alpha.p	-2.16	0.61	-3.02	-3.01	-1.82	-1.65	-1.65	3	0	1.00
beta.hab.lambda	-0.95	1.16	-4.12	-1.64	-0.83	-0.10	0.82	6	1	0.78
beta.hab.gamma	-0.69	0.41	-1.15	-1.15	-0.79	-0.15	-0.15	3	0	1.00
beta.hab.phi	1.86	3.39	-1.12	-1.11	0.10	6.22	7.18	3	1	0.67
beta.elev.lambda	0.65	1.26	-1.20	-0.26	0.47	1.31	3.60	15	1	0.64
beta.ele.gamma2	-0.55	0.09	-0.67	-0.67	-0.53	-0.45	-0.45	3	0	1.00
beta.elev.phi	0.10	0.47	-0.29	-0.29	-0.17	0.70	0.86	3	1	0.33
beta.develop.lambda	-0.24	1.28	-3.04	-1.02	-0.09	0.61	2.13	37	1	0.54
beta.develop.gamma	1.72	0.35	1.43	1.43	1.51	2.21	2.22	3	0	1.00
beta.develop.phi	-6.54	7.35	-18.22	-16.11	-1.55	-1.17	-1.16	3	0	1.00
beta.effort	0.26	0.01	0.24	0.24	0.26	0.27	0.27	3	0	1.00
beta.min_temp	0.00	0.00	0.00	0.00	0.00	0.00	0.00	1	0	1.00

Table 45: California Gull

	mean	sd	2.5%	25%	50%	75%	97.5%	n.eff	overlap0	f
alpha.lambda	8.91	0.02	8.86	8.89	8.91	8.93	8.95	51	0	1.00
alpha.phi	2.65	0.07	2.51	2.60	2.66	2.72	2.78	16	0	1.00
alpha.p	-2.59	0.02	-2.62	-2.61	-2.59	-2.58	-2.55	17	0	1.00
beta.hab.lambda	-0.28	0.01	-0.29	-0.29	-0.28	-0.27	-0.26	151	0	1.00
beta.hab.gamma	-1.18	0.02	-1.21	-1.20	-1.18	-1.16	-1.13	16	0	1.00
beta.hab.phi	2.81	0.13	2.57	2.71	2.83	2.92	3.02	20	0	1.00
beta.elev.lambda	-0.15	0.01	-0.17	-0.16	-0.15	-0.15	-0.14	4	0	1.00
beta.ele.gamma2	-0.42	0.00	-0.43	-0.43	-0.42	-0.42	-0.42	4	0	1.00
beta.elev.phi	-0.07	0.00	-0.08	-0.08	-0.07	-0.07	-0.06	5	0	1.00
beta.develop.lambda	0.43	0.01	0.42	0.43	0.43	0.44	0.46	150	0	1.00
beta.develop.gamma	-0.06	0.03	-0.11	-0.09	-0.06	-0.03	0.00	30	1	0.94
beta.develop.phi	0.74	0.06	0.63	0.68	0.74	0.79	0.83	27	0	1.00
beta.effort	0.09	0.00	0.08	0.08	0.09	0.09	0.09	94	0	1.00
beta.min_temp	0.00	0.00	0.00	0.00	0.00	0.00	0.00	1	0	1.00

Table 46: Canvasback

	mean	sd	2.5%	25%	50%	75%	97.5%	n.eff	overlap0	f
alpha.lambda	7.48	0	7.47	7.48	7.48	7.49	7.49	57	0	1
alpha.phi	1.80	0	1.80	1.80	1.80	1.80	1.81	35	0	1
alpha.p	-1.86	0	-1.86	-1.86	-1.86	-1.86	-1.86	40	0	1
beta.hab.lambda	0.36	0	0.35	0.36	0.36	0.36	0.36	134	0	1

	mean	sd	2.5%	25%	50%	75%	97.5%	n.eff	overlap0	f
beta.hab.gamma	-0.11	0	-0.11	-0.11	-0.11	-0.11	-0.11	61	0	1
beta.hab.phi	0.66	0	0.66	0.66	0.66	0.67	0.67	44	0	1
beta.elev.lambda	-0.24	0	-0.25	-0.24	-0.24	-0.24	-0.24	341	0	1
beta.ele.gamma2	-0.50	0	-0.50	-0.50	-0.50	-0.50	-0.50	257	0	1
beta.elev.phi	0.33	0	0.32	0.33	0.33	0.33	0.33	101	0	1
beta.develop.lambda	0.36	0	0.36	0.36	0.36	0.36	0.37	76	0	1
beta.develop.gamma	-0.52	0	-0.52	-0.52	-0.52	-0.52	-0.51	45	0	1
beta.develop.phi	0.61	0	0.60	0.61	0.61	0.61	0.61	227	0	1
beta.effort	0.19	0	0.19	0.19	0.19	0.19	0.19	2344	0	1
beta.min_temp	-0.05	0	-0.06	-0.06	-0.05	-0.05	-0.05	899	0	1

Table 47: Carolina Chickadee

	mean	sd	2.5%	25%	50%	75%	97.5%	n.eff	overlap0	f
alpha.lambda	7.02	0.86	5.80	5.81	7.54	7.69	7.71	3	0	1.00
alpha.phi	3.46	0.60	2.61	2.63	3.81	3.95	3.97	3	0	1.00
alpha.p	-1.85	0.88	-2.55	-2.54	-2.39	-0.60	-0.60	3	0	1.00
beta.hab.lambda	0.26	0.02	0.24	0.25	0.25	0.29	0.30	3	0	1.00
beta.hab.gamma	0.43	0.14	0.23	0.24	0.52	0.55	0.56	3	0	1.00
beta.hab.phi	-0.39	0.20	-0.56	-0.55	-0.52	-0.11	-0.09	3	0	1.00
beta.elev.lambda	-0.12	0.00	-0.13	-0.12	-0.12	-0.11	-0.11	9	0	1.00
beta.ele.gamma2	-0.11	0.11	-0.26	-0.25	-0.05	-0.01	0.00	3	1	0.97
beta.elev.phi	0.05	0.09	-0.03	-0.02	0.00	0.17	0.18	3	1	0.53
beta.develop.lambda	0.31	0.07	0.25	0.26	0.27	0.40	0.42	3	0	1.00
beta.develop.gamma	-0.36	0.07	-0.45	-0.43	-0.37	-0.28	-0.25	3	0	1.00
beta.develop.phi	-0.12	0.32	-0.40	-0.37	-0.30	0.32	0.35	3	1	0.67
beta.effort	0.01	0.01	0.00	0.00	0.00	0.03	0.03	3	0	1.00
beta.min_temp	0.00	0.00	0.00	0.00	0.00	0.00	0.00	1	0	1.00

Table 48: Common Goldeneye

	mean	sd	2.5%	25%	50%	75%	97.5%	n.eff	overlap0	f
alpha.lambda	6.72	0.00	6.71	6.71	6.71	6.72	6.72	172	0	1
alpha.phi	1.98	0.00	1.98	1.98	1.98	1.98	1.99	11	0	1
alpha.p	-1.69	0.00	-1.70	-1.69	-1.69	-1.69	-1.69	20	0	1
beta.hab.lambda	0.45	0.00	0.45	0.45	0.45	0.46	0.46	156	0	1
beta.hab.gamma	0.25	0.00	0.25	0.25	0.25	0.26	0.26	45	0	1
beta.hab.phi	0.29	0.00	0.28	0.29	0.29	0.29	0.29	38	0	1
beta.elev.lambda	-0.06	0.00	-0.06	-0.06	-0.06	-0.06	-0.06	125	0	1
beta.ele.gamma2	0.11	0.00	0.11	0.11	0.11	0.11	0.11	132	0	1
beta.elev.phi	0.07	0.00	0.07	0.07	0.07	0.08	0.08	116	0	1
beta.develop.lambda	0.48	0.00	0.47	0.47	0.48	0.48	0.48	6000	0	1
beta.develop.gamma	-0.20	0.00	-0.21	-0.21	-0.20	-0.20	-0.20	30	0	1
beta.develop.phi	0.72	0.01	0.71	0.72	0.72	0.73	0.73	30	0	1
beta.effort	0.00	0.00	0.00	0.00	0.00	0.00	0.00	309	0	1
beta.min_temp	-0.08	0.00	-0.08	-0.08	-0.08	-0.08	-0.08	333	0	1

Table 49: Common Merganser

	mean	sd	2.5%	25%	50%	75%	97.5%	n.eff	overlap0	f
alpha.lambda	7.84	0.32	7.59	7.61	7.63	8.23	8.41	3	0	1.00
alpha.phi	3.38	0.31	2.86	3.00	3.54	3.62	3.70	3	0	1.00
alpha.p	-2.75	0.37	-3.40	-3.20	-2.51	-2.49	-2.46	3	0	1.00
beta.hab.lambda	-0.21	0.23	-0.39	-0.38	-0.36	0.10	0.12	3	1	0.67
beta.hab.gamma	-0.37	0.05	-0.42	-0.42	-0.39	-0.31	-0.27	3	0	1.00
beta.hab.phi	3.01	1.54	0.81	0.84	4.01	4.15	4.27	3	0	1.00
beta.elev.lambda	0.17	0.24	-0.20	-0.16	0.33	0.34	0.35	3	1	0.67
beta.ele.gamma2	-0.17	0.39	-0.74	-0.71	0.10	0.11	0.11	3	1	0.33
beta.elev.phi	0.32	0.67	-0.17	-0.16	-0.14	1.25	1.33	3	1	0.33
beta.develop.lambda	0.11	0.08	0.04	0.05	0.07	0.21	0.24	3	0	1.00
beta.develop.gamma	0.01	0.06	-0.04	-0.03	-0.02	0.08	0.10	3	1	0.33
beta.develop.phi	0.25	0.28	-0.18	-0.14	0.43	0.46	0.48	3	1	0.67
beta.effort	0.00	0.00	0.00	0.00	0.00	0.00	0.00	1	0	1.00
beta.min_temp	-0.04	0.02	-0.08	-0.08	-0.03	-0.03	-0.02	3	0	1.00

Table 50: Downy Woodpecker

	mean	sd	2.5%	25%	50%	75%	97.5%	n.eff	overlap0	f
alpha.lambda	5.75	0.60	4.89	4.90	6.13	6.22	6.23	3	0	1.00
alpha.phi	4.81	0.85	3.60	3.61	5.35	5.43	5.57	3	0	1.00
alpha.p	-1.70	0.68	-2.24	-2.23	-2.13	-0.74	-0.74	3	0	1.00
beta.hab.lambda	0.19	0.03	0.16	0.16	0.18	0.23	0.24	3	0	1.00
beta.hab.gamma	0.20	0.12	0.05	0.06	0.20	0.34	0.36	3	0	1.00
beta.hab.phi	-0.11	0.14	-0.31	-0.29	-0.08	0.04	0.05	3	1	0.67
beta.elev.lambda	-0.02	0.08	-0.14	-0.13	0.02	0.06	0.06	3	1	0.33
beta.ele.gamma2	0.01	0.25	-0.32	-0.31	0.06	0.28	0.30	3	1	0.67
beta.elev.phi	-1.34	1.19	-2.27	-2.24	-2.11	0.33	0.35	3	1	0.67
beta.develop.lambda	0.25	0.05	0.18	0.19	0.24	0.31	0.32	3	0	1.00
beta.develop.gamma	0.15	0.07	0.05	0.05	0.19	0.20	0.22	3	0	1.00
beta.develop.phi	0.33	0.67	-0.55	-0.54	0.49	1.05	1.10	3	1	0.67
beta.effort	0.02	0.00	0.02	0.02	0.03	0.03	0.03	3	0	1.00
beta.min_temp	-0.08	0.02	-0.10	-0.10	-0.09	-0.05	-0.05	3	0	1.00

Table 51: Eared Grebe

	mean	sd	2.5%	25%	50%	75%	97.5%	n.eff	overlap0	f
alpha.lambda	8.59	0.69	7.93	8.00	8.24	9.47	9.67	3	0	1.00
alpha.phi	-0.30	1.49	-2.59	-2.30	0.74	0.75	0.79	3	1	0.33
alpha.p	-3.23	0.43	-3.91	-3.78	-2.99	-2.89	-2.82	3	0	1.00
beta.hab.lambda	0.34	0.21	0.10	0.12	0.27	0.61	0.65	3	0	1.00
beta.hab.gamma	0.53	0.05	0.46	0.47	0.54	0.59	0.59	3	0	1.00
beta.hab.phi	-0.72	0.30	-1.16	-1.09	-0.61	-0.42	-0.41	3	0	1.00
beta.elev.lambda	-0.83	0.14	-1.01	-1.00	-0.81	-0.68	-0.65	3	0	1.00
beta.ele.gamma2	2.04	0.41	1.43	1.48	2.31	2.32	2.36	3	0	1.00
beta.elev.phi	-2.07	0.51	-2.87	-2.72	-1.81	-1.62	-1.60	3	0	1.00
beta.develop.lambda	-0.87	0.32	-1.32	-1.31	-0.71	-0.59	-0.58	3	0	1.00
beta.develop.gamma	-0.76	0.56	-1.49	-1.47	-0.69	-0.10	-0.09	3	0	1.00

	mean	sd	2.5%	25%	50%	75%	97.5%	n.eff	overlap0	f
beta.develop.phi	1.18	1.36	-0.74	-0.63	1.60	2.58	2.60	3	1	0.67
beta.effort	0.00	0.00	0.00	0.00	0.00	0.00	0.00	1	0	1.00
beta.min_temp	-0.04	0.04	-0.10	-0.08	-0.03	0.00	0.00	3	0	1.00

Table 52: Eastern Bluebird

	mean	sd	2.5%	25%	50%	75%	97.5%	n.eff	overlap0	f
alpha.lambda	3.90	0.01	3.88	3.90	3.90	3.91	3.93	216	0	1
alpha.phi	2.88	0.01	2.87	2.88	2.88	2.89	2.89	36	0	1
alpha.p	-0.85	0.00	-0.85	-0.85	-0.85	-0.84	-0.84	14	0	1
beta.hab.lambda	0.14	0.01	0.12	0.13	0.14	0.14	0.15	1320	0	1
beta.hab.gamma	-0.02	0.00	-0.03	-0.02	-0.02	-0.01	-0.01	302	0	1
beta.hab.phi	-0.07	0.01	-0.08	-0.07	-0.07	-0.07	-0.06	169	0	1
beta.elev.lambda	0.03	0.01	0.01	0.03	0.03	0.04	0.05	144	0	1
beta.ele.gamma2	-0.15	0.00	-0.15	-0.15	-0.15	-0.14	-0.14	40	0	1
beta.elev.phi	-0.03	0.01	-0.04	-0.03	-0.03	-0.02	-0.01	33	0	1
beta.develop.lambda	0.88	0.02	0.84	0.87	0.88	0.90	0.93	284	0	1
beta.develop.gamma	-0.34	0.01	-0.35	-0.34	-0.34	-0.33	-0.33	111	0	1
beta.develop.phi	0.57	0.01	0.55	0.56	0.57	0.57	0.59	120	0	1
beta.effort	0.00	0.00	0.00	0.00	0.00	0.00	0.00	1	0	1
beta.min_temp	0.00	0.00	0.00	0.00	0.00	0.00	0.00	1	0	1

Table 53: Gadwall

	mean	sd	2.5%	25%	50%	75%	97.5%	n.eff	overlap0	f
alpha.lambda	6.17	0.72	5.65	5.66	5.67	7.19	7.21	3	0	1.00
alpha.phi	2.47	0.36	2.21	2.22	2.22	2.96	3.00	3	0	1.00
alpha.p	-2.12	0.49	-2.81	-2.81	-1.77	-1.77	-1.77	3	0	1.00
beta.hab.lambda	0.18	0.07	0.08	0.09	0.23	0.23	0.23	3	0	1.00
beta.hab.gamma	0.23	0.04	0.20	0.20	0.21	0.29	0.29	3	0	1.00
beta.hab.phi	0.43	0.57	0.02	0.03	0.03	1.20	1.28	3	0	1.00
beta.elev.lambda	0.14	0.04	0.10	0.11	0.11	0.19	0.20	3	0	1.00
beta.ele.gamma2	-0.22	0.20	-0.36	-0.36	-0.36	0.06	0.07	3	1	0.67
beta.elev.phi	0.03	0.33	-0.44	-0.42	0.26	0.27	0.27	3	1	0.67
beta.develop.lambda	-0.69	0.80	-1.29	-1.26	-1.25	0.45	0.46	3	1	0.67
beta.develop.gamma	-0.54	0.16	-0.79	-0.77	-0.43	-0.43	-0.42	3	0	1.00
beta.develop.phi	0.54	0.27	0.35	0.35	0.35	0.91	0.94	3	0	1.00
beta.effort	0.01	0.01	0.00	0.00	0.02	0.02	0.02	3	0	1.00
beta.min_temp	0.00	0.00	0.00	0.00	0.00	0.00	0.00	1	0	1.00

Table 54: Golden-crowned Sparrow

	mean	sd	2.5%	25%	50%	75%	97.5%	n.eff	overlap0	f
alpha.lambda	7.21	0.44	6.61	6.65	7.33	7.68	7.69	3	0	1
alpha.phi	6.88	0.93	5.51	5.66	7.42	7.61	7.73	3	0	1
alpha.p	-1.80	0.47	-2.32	-2.31	-1.90	-1.19	-1.16	3	0	1
beta.hab.lambda	-0.94	0.11	-1.10	-1.08	-0.87	-0.86	-0.85	3	0	1

	mean	sd	2.5%	25%	50%	75%	97.5%	n.eff	overlap0	f
beta.hab.gamma	-2.14	0.14	-2.32	-2.28	-2.17	-1.98	-1.94	3	0	1
beta.hab.phi	4.31	0.74	3.22	3.34	4.71	4.87	5.11	3	0	1
beta.elev.lambda	-0.20	0.02	-0.22	-0.22	-0.20	-0.18	-0.17	3	0	1
beta.ele.gamma2	-0.40	0.03	-0.44	-0.43	-0.39	-0.38	-0.36	3	0	1
beta.elev.phi	0.26	0.05	0.19	0.21	0.27	0.31	0.32	3	0	1
beta.develop.lambda	0.07	0.01	0.04	0.05	0.07	0.08	0.09	3	0	1
beta.develop.gamma	-0.76	0.08	-0.84	-0.82	-0.79	-0.66	-0.64	3	0	1
beta.develop.phi	1.02	0.06	0.92	0.97	1.02	1.09	1.12	3	0	1
beta.effort	0.09	0.01	0.07	0.07	0.09	0.10	0.11	3	0	1
beta.min_temp	-0.04	0.03	-0.08	-0.07	-0.03	-0.01	-0.01	3	0	1

Table 55: Greater White-fronted Goose

	mean	sd	2.5%	25%	50%	75%	97.5%	n.eff	overlap0	f
alpha.lambda	5.00	0.44	4.66	4.69	4.71	5.50	5.82	3	0	1
alpha.phi	1.68	0.10	1.60	1.60	1.61	1.80	1.84	3	0	1
alpha.p	-2.89	0.29	-3.35	-3.26	-2.70	-2.68	-2.67	3	0	1
beta.hab.lambda	-4.71	0.42	-5.03	-5.00	-4.97	-4.39	-3.79	3	0	1
beta.hab.gamma	-0.21	0.02	-0.23	-0.23	-0.20	-0.19	-0.19	3	0	1
beta.hab.phi	-0.60	0.05	-0.64	-0.64	-0.63	-0.53	-0.52	3	0	1
beta.elev.lambda	-1.33	0.09	-1.48	-1.44	-1.28	-1.27	-1.25	3	0	1
beta.ele.gamma2	0.35	0.12	0.25	0.26	0.27	0.51	0.52	3	0	1
beta.elev.phi	-0.76	0.15	-0.98	-0.97	-0.67	-0.66	-0.65	3	0	1
beta.develop.lambda	1.34	0.05	1.27	1.29	1.37	1.38	1.39	3	0	1
beta.develop.gamma	-2.26	0.07	-2.32	-2.31	-2.31	-2.16	-2.14	3	0	1
beta.develop.phi	0.52	0.23	0.18	0.21	0.67	0.69	0.71	3	0	1
beta.effort	0.00	0.00	0.00	0.00	0.00	0.00	0.00	1	0	1
beta.min_temp	0.00	0.00	0.00	0.00	0.00	0.00	0.00	1	0	1

Table 56: Killdeer

	mean	sd	2.5%	25%	50%	75%	97.5%	n.eff	overlap0	f
alpha.lambda	7.62	1.40	6.60	6.61	6.62	9.54	9.69	3	0	1.00
alpha.phi	1.64	1.83	-1.01	-0.92	2.93	2.93	2.97	3	1	0.67
alpha.p	-2.92	1.88	-5.67	-5.53	-1.59	-1.58	-1.58	3	0	1.00
beta.hab.lambda	0.18	0.08	0.12	0.12	0.13	0.30	0.31	3	0	1.00
beta.hab.gamma	-0.17	0.25	-0.36	-0.35	-0.34	0.18	0.18	3	1	0.67
beta.hab.phi	0.33	0.29	-0.09	-0.08	0.52	0.54	0.56	3	1	0.67
beta.elev.lambda	-0.61	0.03	-0.63	-0.63	-0.63	-0.58	-0.57	3	0	1.00
beta.ele.gamma2	-0.56	0.02	-0.59	-0.58	-0.55	-0.54	-0.53	3	0	1.00
beta.elev.phi	-0.04	0.05	-0.09	-0.08	-0.07	0.04	0.04	3	1	0.67
beta.develop.lambda	0.42	0.15	0.29	0.31	0.31	0.63	0.65	3	0	1.00
beta.develop.gamma	0.14	0.05	0.07	0.07	0.16	0.17	0.19	3	0	1.00
beta.develop.phi	0.19	0.13	0.09	0.10	0.10	0.36	0.39	3	0	1.00
beta.effort	0.00	0.00	0.00	0.00	0.00	0.00	0.00	1	0	1.00
beta.min_temp	0.00	0.00	0.00	0.00	0.00	0.00	0.00	1	0	1.00

Table 57: Least Sandpiper

	mean	sd	2.5%	25%	50%	75%	97.5%	n.eff	overlap0	f
alpha.lambda	7.62	0.10	7.54	7.55	7.56	7.76	7.77	3	0	1.00
alpha.phi	3.76	0.11	3.57	3.64	3.79	3.85	3.89	3	0	1.00
alpha.p	-2.21	0.17	-2.45	-2.44	-2.10	-2.08	-2.06	3	0	1.00
beta.hab.lambda	-0.51	0.52	-1.25	-1.24	-0.14	-0.14	-0.13	3	0	1.00
beta.hab.gamma	-1.27	0.87	-1.98	-1.92	-1.84	-0.05	-0.03	3	0	1.00
beta.hab.phi	1.10	0.53	0.34	0.35	1.41	1.52	1.59	3	0	1.00
beta.elev.lambda	-0.19	0.28	-0.59	-0.58	0.01	0.02	0.02	3	1	0.33
beta.ele.gamma2	-0.64	0.75	-1.19	-1.18	-1.17	0.40	0.45	3	1	0.67
beta.elev.phi	-0.36	0.60	-1.24	-1.18	0.06	0.07	0.08	3	1	0.33
beta.develop.lambda	-0.45	0.02	-0.49	-0.48	-0.44	-0.43	-0.43	3	0	1.00
beta.develop.gamma	-2.28	0.33	-2.81	-2.71	-2.05	-2.04	-2.03	3	0	1.00
beta.develop.phi	3.17	0.22	2.97	3.01	3.03	3.42	3.57	3	0	1.00
beta.effort	0.03	0.01	0.02	0.02	0.02	0.04	0.04	3	0	1.00
beta.min__temp	0.00	0.00	0.00	0.00	0.00	0.00	0.00	1	0	1.00

Table 58: Northern Flicker

	mean	sd	2.5%	25%	50%	75%	97.5%	n.eff	overlap0	f
alpha.lambda	5.68	0.49	5.33	5.33	5.34	6.34	6.40	3	0	1.00
alpha.phi	3.84	0.45	3.52	3.52	3.53	4.46	4.50	3	0	1.00
alpha.p	-1.32	0.52	-2.10	-2.02	-0.95	-0.95	-0.94	3	0	1.00
beta.hab.lambda	-0.09	0.01	-0.11	-0.10	-0.10	-0.07	-0.06	3	0	1.00
beta.hab.gamma	-0.07	0.15	-0.30	-0.28	0.03	0.03	0.04	3	1	0.33
beta.hab.phi	0.10	0.13	0.00	0.01	0.01	0.27	0.30	3	1	0.96
beta.elev.lambda	-0.25	0.01	-0.26	-0.26	-0.25	-0.24	-0.24	3	0	1.00
beta.ele.gamma2	0.68	0.96	-0.01	0.01	0.01	2.00	2.08	3	1	0.90
beta.elev.phi	0.07	0.01	0.05	0.06	0.06	0.08	0.09	4	0	1.00
beta.develop.lambda	0.26	0.03	0.22	0.23	0.28	0.29	0.30	3	0	1.00
beta.develop.gamma	0.17	0.06	0.07	0.09	0.21	0.22	0.23	3	0	1.00
beta.develop.phi	-0.12	0.02	-0.15	-0.14	-0.13	-0.11	-0.07	4	0	1.00
beta.effort	0.03	0.01	0.02	0.02	0.02	0.03	0.04	3	0	1.00
beta.min__temp	0.00	0.00	0.00	0.00	0.00	0.00	0.00	1	0	1.00

Table 59: Northern Mockingbird

	mean	sd	2.5%	25%	50%	75%	97.5%	n.eff	overlap0	f
alpha.lambda	5.37	0.01	5.36	5.37	5.37	5.38	5.39	7	0	1.00
alpha.phi	3.43	0.01	3.42	3.43	3.43	3.44	3.45	16	0	1.00
alpha.p	-0.83	0.00	-0.84	-0.83	-0.83	-0.83	-0.82	7	0	1.00
beta.hab.lambda	0.25	2.24	-4.20	-1.25	0.27	1.76	4.60	6000	1	0.55
beta.hab.gamma	0.16	2.21	-4.17	-1.33	0.15	1.63	4.46	5435	1	0.53
beta.hab.phi	-0.02	2.24	-4.43	-1.50	-0.03	1.44	4.45	5857	1	0.51
beta.elev.lambda	-0.41	0.00	-0.41	-0.41	-0.41	-0.40	-0.40	57	0	1.00
beta.ele.gamma2	-0.64	0.00	-0.65	-0.64	-0.64	-0.64	-0.63	48	0	1.00
beta.elev.phi	0.35	0.01	0.34	0.35	0.35	0.36	0.36	57	0	1.00
beta.develop.lambda	0.30	2.24	-4.04	-1.20	0.29	1.81	4.76	6000	1	0.56
beta.develop.gamma	0.15	2.21	-4.16	-1.32	0.16	1.64	4.49	5754	1	0.53

	mean	sd	2.5%	25%	50%	75%	97.5%	n.eff	overlap0	f
beta.develop.phi	-0.07	2.24	-4.54	-1.53	-0.05	1.41	4.34	5662	1	0.51
beta.effort	0.00	0.00	0.00	0.00	0.00	0.01	0.01	567	0	1.00
beta.min_temp	0.00	0.00	0.00	0.00	0.00	0.00	0.00	1	0	1.00

Table 60: Pine Siskin

	mean	sd	2.5%	25%	50%	75%	97.5%	n.eff	overlap0	f
alpha.lambda	7.07	0.12	6.99	7.00	7.00	7.09	7.42	4	0	1
alpha.phi	2.35	0.14	2.26	2.26	2.26	2.39	2.71	4	0	1
alpha.p	-2.49	0.09	-2.76	-2.50	-2.44	-2.44	-2.44	4	0	1
beta.hab.lambda	0.11	0.02	0.06	0.10	0.12	0.13	0.13	4	0	1
beta.hab.gamma	0.32	0.02	0.28	0.31	0.33	0.33	0.35	5	0	1
beta.hab.phi	-0.20	0.03	-0.24	-0.23	-0.22	-0.17	-0.14	3	0	1
beta.elev.lambda	-0.07	0.01	-0.08	-0.08	-0.08	-0.07	-0.04	6	0	1
beta.ele.gamma2	0.33	0.08	0.11	0.30	0.37	0.38	0.39	4	0	1
beta.elev.phi	-0.36	0.08	-0.42	-0.41	-0.40	-0.32	-0.15	4	0	1
beta.develop.lambda	0.83	0.02	0.77	0.82	0.84	0.84	0.85	4	0	1
beta.develop.gamma	-0.63	0.03	-0.65	-0.65	-0.64	-0.63	-0.55	5	0	1
beta.develop.phi	0.60	0.04	0.49	0.60	0.62	0.63	0.63	4	0	1
beta.effort	0.00	0.00	0.00	0.00	0.00	0.00	0.00	1	0	1
beta.min_temp	0.00	0.00	0.00	0.00	0.00	0.00	0.00	1	0	1

Table 61: Red-breasted Merganser

	mean	sd	2.5%	25%	50%	75%	97.5%	n.eff	overlap0	f
alpha.lambda	8.70	1.28	7.70	7.73	7.88	10.44	10.63	3	0	1.00
alpha.phi	-0.58	2.59	-4.28	-4.22	1.19	1.31	1.32	3	1	0.33
alpha.p	-3.96	1.59	-6.31	-6.14	-2.88	-2.79	-2.77	3	0	1.00
beta.hab.lambda	0.26	0.20	0.11	0.11	0.12	0.53	0.54	3	0	1.00
beta.hab.gamma	0.04	0.30	-0.23	-0.18	-0.15	0.47	0.48	3	1	0.33
beta.hab.phi	0.95	0.35	0.42	0.48	1.12	1.21	1.35	3	0	1.00
beta.elev.lambda	-0.14	0.10	-0.22	-0.21	-0.19	0.00	0.01	3	1	0.78
beta.ele.gamma2	0.21	0.21	-0.09	-0.08	0.34	0.38	0.42	3	1	0.67
beta.elev.phi	0.38	0.93	-0.31	-0.28	-0.26	1.68	1.74	3	1	0.33
beta.develop.lambda	-0.27	0.17	-0.44	-0.43	-0.35	-0.05	-0.02	3	0	1.00
beta.develop.gamma	0.04	0.14	-0.14	-0.14	0.05	0.20	0.22	3	1	0.67
beta.develop.phi	1.04	0.56	0.42	0.44	0.93	1.73	1.85	3	0	1.00
beta.effort	0.02	0.01	0.00	0.00	0.02	0.03	0.04	3	0	1.00
beta.min_temp	-0.16	0.04	-0.23	-0.23	-0.14	-0.13	-0.12	3	0	1.00

Table 62: Red-tailed Hawk

	mean	sd	2.5%	25%	50%	75%	97.5%	n.eff	overlap0	f
alpha.lambda	4.04	0.01	4.02	4.04	4.04	4.05	4.07	38	0	1.00
alpha.phi	3.66	0.01	3.65	3.66	3.66	3.67	3.69	66	0	1.00
alpha.p	-0.81	0.01	-0.82	-0.81	-0.80	-0.80	-0.79	21	0	1.00
beta.hab.lambda	-0.24	0.01	-0.26	-0.25	-0.24	-0.24	-0.23	65	0	1.00

	mean	sd	2.5%	25%	50%	75%	97.5%	n.eff	overlap0	f
beta.hab.gamma	-0.13	0.01	-0.14	-0.13	-0.13	-0.12	-0.11	101	0	1.00
beta.hab.phi	-0.03	0.01	-0.06	-0.04	-0.03	-0.02	0.00	129	0	0.99
beta.elev.lambda	-0.23	0.01	-0.24	-0.23	-0.23	-0.22	-0.21	51	0	1.00
beta.ele.gamma2	-0.12	0.01	-0.13	-0.13	-0.12	-0.12	-0.11	146	0	1.00
beta.elev.phi	0.21	0.01	0.20	0.21	0.21	0.22	0.23	682	0	1.00
beta.develop.lambda	0.14	0.02	0.11	0.13	0.14	0.15	0.17	8	0	1.00
beta.develop.gamma	0.14	0.01	0.12	0.13	0.14	0.15	0.17	4	0	1.00
beta.develop.phi	-0.45	0.02	-0.49	-0.47	-0.45	-0.44	-0.42	4	0	1.00
beta.effort	0.04	0.00	0.03	0.04	0.04	0.04	0.04	112	0	1.00
beta.min_temp	-0.03	0.00	-0.03	-0.03	-0.03	-0.03	-0.02	157	0	1.00

Table 63: Redhead

	mean	sd	2.5%	25%	50%	75%	97.5%	n.eff	overlap0	f
alpha.lambda	5.77	0.34	5.25	5.42	5.77	6.15	6.22	3	0	1.00
alpha.phi	4.54	2.31	1.30	1.31	5.85	6.35	6.77	3	0	1.00
alpha.p	-2.60	0.45	-3.20	-3.12	-2.60	-2.07	-2.06	3	0	1.00
beta.hab.lambda	1.38	0.18	1.12	1.13	1.50	1.51	1.54	3	0	1.00
beta.hab.gamma	0.66	0.19	0.39	0.40	0.78	0.80	0.82	3	0	1.00
beta.hab.phi	0.08	0.42	-0.31	-0.25	-0.17	0.67	0.67	3	1	0.33
beta.elev.lambda	-0.09	0.19	-0.25	-0.24	-0.20	0.17	0.18	3	1	0.67
beta.ele.gamma2	-0.37	0.17	-0.55	-0.53	-0.44	-0.14	-0.14	3	0	1.00
beta.elev.phi	0.86	0.50	0.16	0.17	1.12	1.23	1.39	3	0	1.00
beta.develop.lambda	-0.42	0.14	-0.55	-0.52	-0.49	-0.23	-0.20	3	0	1.00
beta.develop.gamma	-0.39	0.07	-0.49	-0.48	-0.35	-0.33	-0.32	3	0	1.00
beta.develop.phi	-3.52	2.78	-6.24	-5.67	-5.11	0.36	0.37	3	1	0.67
beta.effort	0.02	0.02	0.00	0.00	0.00	0.04	0.05	3	0	1.00
beta.min_temp	0.00	0.00	0.00	0.00	0.00	0.00	0.00	1	0	1.00

Table 64: Ring-necked Duck

	mean	sd	2.5%	25%	50%	75%	97.5%	n.eff	overlap0	f
alpha.lambda	6.39	0.01	6.38	6.39	6.39	6.40	6.41	13	0	1.00
alpha.phi	2.12	0.00	2.11	2.12	2.12	2.12	2.13	7	0	1.00
alpha.p	-2.00	0.00	-2.01	-2.00	-2.00	-2.00	-2.00	8	0	1.00
beta.hab.lambda	-0.32	0.01	-0.34	-0.33	-0.32	-0.32	-0.31	36	0	1.00
beta.hab.gamma	-0.11	0.00	-0.11	-0.11	-0.11	-0.10	-0.10	92	0	1.00
beta.hab.phi	0.02	0.00	0.01	0.01	0.02	0.02	0.02	67	0	1.00
beta.elev.lambda	-0.42	0.00	-0.43	-0.42	-0.42	-0.42	-0.41	91	0	1.00
beta.ele.gamma2	-0.49	0.00	-0.49	-0.49	-0.49	-0.49	-0.48	22	0	1.00
beta.elev.phi	0.21	0.00	0.20	0.21	0.21	0.21	0.21	19	0	1.00
beta.develop.lambda	0.01	0.01	0.00	0.01	0.01	0.02	0.02	25	1	0.96
beta.develop.gamma	0.11	0.00	0.10	0.11	0.11	0.12	0.12	128	0	1.00
beta.develop.phi	0.08	0.00	0.08	0.08	0.09	0.09	0.09	117	0	1.00
beta.effort	0.00	0.00	0.00	0.00	0.00	0.00	0.00	1	0	1.00
beta.min_temp	0.00	0.00	0.00	0.00	0.00	0.00	0.00	1	0	1.00

Table 65: Ruby-crowned Kinglet

	mean	sd	2.5%	25%	50%	75%	97.5%	n.eff	overlap0	f
alpha.lambda	5.54	0.03	5.50	5.51	5.52	5.57	5.59	3	0	1
alpha.phi	3.43	0.02	3.40	3.41	3.42	3.45	3.47	3	0	1
alpha.p	-0.98	0.02	-1.02	-1.01	-0.97	-0.96	-0.95	3	0	1
beta.hab.lambda	-0.02	0.01	-0.03	-0.03	-0.02	-0.02	-0.01	12	0	1
beta.hab.gamma	-0.18	0.01	-0.19	-0.18	-0.18	-0.17	-0.16	64	0	1
beta.hab.phi	0.10	0.01	0.08	0.09	0.10	0.10	0.11	53	0	1
beta.elev.lambda	-0.35	0.00	-0.36	-0.35	-0.35	-0.35	-0.35	372	0	1
beta.ele.gamma2	-0.35	0.00	-0.36	-0.35	-0.35	-0.35	-0.34	900	0	1
beta.elev.phi	0.03	0.00	0.02	0.03	0.03	0.03	0.04	161	0	1
beta.develop.lambda	0.15	0.01	0.14	0.15	0.15	0.16	0.17	19	0	1
beta.develop.gamma	-0.27	0.02	-0.31	-0.30	-0.27	-0.25	-0.24	3	0	1
beta.develop.phi	0.04	0.02	0.01	0.02	0.03	0.05	0.07	4	0	1
beta.effort	0.04	0.00	0.04	0.04	0.04	0.04	0.05	7	0	1
beta.min__temp	0.00	0.00	0.00	0.00	0.00	0.00	0.00	1	0	1

Table 66: Rusty Blackbird

	mean	sd	2.5%	25%	50%	75%	97.5%	n.eff	overlap0	f
alpha.lambda	13.42	0.10	13.21	13.34	13.42	13.50	13.59	8	0	1.00
alpha.phi	-1.51	0.49	-2.20	-2.18	-1.28	-1.09	-1.01	3	0	1.00
alpha.p	-6.92	0.11	-7.14	-6.99	-6.91	-6.83	-6.71	4	0	1.00
beta.hab.lambda	0.13	0.10	0.01	0.02	0.12	0.25	0.26	3	0	1.00
beta.hab.gamma	-0.07	0.12	-0.24	-0.22	-0.03	0.04	0.06	3	1	0.67
beta.hab.phi	0.23	0.36	-0.22	-0.16	0.17	0.69	0.70	3	1	0.67
beta.elev.lambda	-0.10	0.14	-0.30	-0.28	-0.07	0.04	0.06	3	1	0.67
beta.ele.gamma2	0.49	0.09	0.36	0.37	0.52	0.56	0.58	3	0	1.00
beta.elev.phi	-0.33	0.22	-0.59	-0.59	-0.35	-0.07	-0.03	3	0	1.00
beta.develop.lambda	0.04	0.70	-0.47	-0.46	-0.44	1.01	1.06	3	1	0.33
beta.develop.gamma	0.29	0.59	-0.58	-0.52	0.63	0.77	0.80	3	1	0.67
beta.develop.phi	-0.06	1.18	-1.14	-0.97	-0.75	1.60	1.61	3	1	0.67
beta.effort	0.00	0.00	0.00	0.00	0.00	0.00	0.00	1	0	1.00
beta.min__temp	0.00	0.00	0.00	0.00	0.00	0.00	0.00	1	0	1.00

Table 67: Sandhill Crane

	mean	sd	2.5%	25%	50%	75%	97.5%	n.eff	overlap0	f
alpha.lambda	5.39	0.85	4.17	4.20	5.98	6.00	6.02	3	0	1.00
alpha.phi	0.92	0.30	0.70	0.71	0.71	1.33	1.34	3	0	1.00
alpha.p	-1.70	0.24	-1.87	-1.87	-1.87	-1.37	-1.37	3	0	1.00
beta.hab.lambda	-2.41	0.99	-3.12	-3.11	-3.10	-1.01	-1.00	3	0	1.00
beta.hab.gamma	-0.33	0.46	-0.66	-0.66	-0.65	0.32	0.33	3	1	0.67
beta.hab.phi	0.81	0.88	-0.43	-0.43	1.43	1.43	1.44	3	1	0.67
beta.elev.lambda	0.65	0.98	-0.06	-0.05	-0.05	2.03	2.04	3	1	0.33
beta.ele.gamma2	-0.53	0.65	-0.99	-0.99	-0.99	0.39	0.40	3	1	0.67
beta.elev.phi	1.27	1.09	-0.26	-0.26	2.04	2.04	2.05	3	1	0.67
beta.develop.lambda	-1.87	1.54	-2.99	-2.96	-2.94	0.28	0.34	3	1	0.67
beta.develop.gamma	-0.77	0.39	-1.06	-1.05	-1.05	-0.22	-0.21	3	0	1.00

	mean	sd	2.5%	25%	50%	75%	97.5%	n.eff	overlap0	f
beta.develop.phi	0.55	0.87	-0.69	-0.68	1.16	1.17	1.18	3	1	0.67
beta.effort	0.00	0.00	0.00	0.00	0.00	0.00	0.00	1	0	1.00
beta.min_temp	-0.16	0.02	-0.18	-0.18	-0.18	-0.13	-0.13	3	0	1.00

Table 68: Savannah Sparrow

	mean	sd	2.5%	25%	50%	75%	97.5%	n.eff	overlap0	f
alpha.lambda	6.07	0.01	6.06	6.07	6.07	6.08	6.09	32	0	1
alpha.phi	2.49	0.00	2.48	2.49	2.49	2.50	2.50	94	0	1
alpha.p	-1.42	0.00	-1.43	-1.42	-1.42	-1.42	-1.42	21	0	1
beta.hab.lambda	0.23	0.00	0.22	0.23	0.23	0.23	0.24	183	0	1
beta.hab.gamma	0.42	0.00	0.41	0.42	0.42	0.42	0.42	213	0	1
beta.hab.phi	-0.12	0.00	-0.12	-0.12	-0.12	-0.11	-0.11	192	0	1
beta.elev.lambda	-0.29	0.00	-0.30	-0.30	-0.29	-0.29	-0.28	50	0	1
beta.ele.gamma2	-0.46	0.00	-0.47	-0.47	-0.46	-0.46	-0.46	247	0	1
beta.elev.phi	-0.05	0.00	-0.05	-0.05	-0.05	-0.05	-0.04	822	0	1
beta.develop.lambda	0.73	0.00	0.72	0.73	0.73	0.74	0.74	1693	0	1
beta.develop.gamma	-0.67	0.01	-0.69	-0.68	-0.67	-0.67	-0.66	1814	0	1
beta.develop.phi	0.38	0.01	0.37	0.37	0.38	0.38	0.39	923	0	1
beta.effort	0.00	0.00	0.00	0.00	0.00	0.00	0.00	1044	0	1
beta.min_temp	0.00	0.00	0.00	0.00	0.00	0.00	0.00	1	0	1

Table 69: Song Sparrow

	mean	sd	2.5%	25%	50%	75%	97.5%	n.eff	overlap0	f
alpha.lambda	6.25	0.01	6.24	6.24	6.25	6.25	6.30	11	0	1
alpha.phi	3.15	0.01	3.14	3.14	3.14	3.15	3.19	11	0	1
alpha.p	-1.02	0.01	-1.07	-1.02	-1.02	-1.02	-1.02	11	0	1
beta.hab.lambda	0.07	0.00	0.06	0.07	0.07	0.07	0.07	46	0	1
beta.hab.gamma	0.08	0.00	0.07	0.07	0.08	0.08	0.08	32	0	1
beta.hab.phi	0.06	0.00	0.06	0.06	0.06	0.07	0.07	57	0	1
beta.elev.lambda	-0.36	0.00	-0.36	-0.36	-0.36	-0.36	-0.35	427	0	1
beta.ele.gamma2	-0.41	0.00	-0.41	-0.41	-0.41	-0.41	-0.40	129	0	1
beta.elev.phi	0.05	0.00	0.04	0.05	0.05	0.05	0.05	196	0	1
beta.develop.lambda	0.26	0.00	0.25	0.26	0.26	0.26	0.27	353	0	1
beta.develop.gamma	-0.03	0.00	-0.04	-0.03	-0.03	-0.03	-0.02	28	0	1
beta.develop.phi	0.28	0.00	0.27	0.28	0.28	0.28	0.29	37	0	1
beta.effort	0.03	0.00	0.03	0.03	0.03	0.03	0.03	23	0	1
beta.min_temp	-0.05	0.00	-0.05	-0.05	-0.05	-0.05	-0.04	31	0	1

Table 70: Tufted Titmouse

	mean	sd	2.5%	25%	50%	75%	97.5%	n.eff	overlap0	f
alpha.lambda	5.28	0.01	5.27	5.27	5.28	5.29	5.30	5	0	1
alpha.phi	3.08	0.01	3.07	3.08	3.08	3.09	3.10	4	0	1
alpha.p	-0.74	0.01	-0.75	-0.75	-0.74	-0.73	-0.73	3	0	1
beta.hab.lambda	0.31	0.00	0.30	0.31	0.31	0.32	0.32	69	0	1

	mean	sd	2.5%	25%	50%	75%	97.5%	n.eff	overlap0	f
beta.hab.gamma	0.37	0.01	0.36	0.37	0.37	0.38	0.39	9	0	1
beta.hab.phi	0.11	0.01	0.08	0.10	0.11	0.12	0.14	9	0	1
beta.elev.lambda	-0.06	0.00	-0.07	-0.06	-0.06	-0.06	-0.05	124	0	1
beta.ele.gamma2	-0.24	0.01	-0.25	-0.24	-0.24	-0.23	-0.23	13	0	1
beta.elev.phi	0.08	0.01	0.06	0.07	0.08	0.08	0.09	13	0	1
beta.develop.lambda	0.46	0.01	0.44	0.45	0.46	0.46	0.47	1063	0	1
beta.develop.gamma	0.26	0.01	0.25	0.25	0.26	0.26	0.27	137	0	1
beta.develop.phi	-0.33	0.01	-0.35	-0.34	-0.33	-0.32	-0.31	6	0	1
beta.effort	0.03	0.00	0.03	0.03	0.03	0.03	0.03	33	0	1
beta.min_temp	-0.01	0.00	-0.01	-0.01	-0.01	-0.01	-0.01	801	0	1

Table 71: Tundra Swan

	mean	sd	2.5%	25%	50%	75%	97.5%	n.eff	overlap0	f
alpha.lambda	4.13	2.54	2.26	2.31	2.38	7.68	7.80	3	0	1.00
alpha.phi	0.91	1.57	-1.31	-1.30	1.98	2.06	2.07	3	1	0.67
alpha.p	-3.00	1.70	-5.49	-5.34	-1.80	-1.79	-1.79	3	0	1.00
beta.hab.lambda	0.72	0.10	0.58	0.59	0.79	0.79	0.79	3	0	1.00
beta.hab.gamma	-0.58	0.22	-0.74	-0.73	-0.73	-0.26	-0.26	3	0	1.00
beta.hab.phi	0.47	0.32	0.24	0.25	0.25	0.92	0.92	3	0	1.00
beta.elev.lambda	-0.19	0.29	-0.40	-0.39	-0.39	0.22	0.22	3	1	0.67
beta.ele.gamma2	-1.07	0.02	-1.09	-1.09	-1.08	-1.05	-1.05	3	0	1.00
beta.elev.phi	0.89	1.05	0.14	0.14	0.16	2.36	2.39	3	0	1.00
beta.develop.lambda	0.07	1.28	-1.87	-1.66	0.94	0.98	1.04	3	1	0.67
beta.develop.gamma	0.98	1.11	-0.59	-0.59	1.71	1.80	1.81	3	1	0.67
beta.develop.phi	-2.40	2.16	-4.10	-4.08	-3.76	0.65	0.65	3	1	0.67
beta.effort	0.20	0.03	0.15	0.15	0.22	0.22	0.22	3	0	1.00
beta.min_temp	0.00	0.00	0.00	0.00	0.00	0.00	0.00	1	0	1.00

Table 72: Turkey Vulture

	mean	sd	2.5%	25%	50%	75%	97.5%	n.eff	overlap0	f
alpha.lambda	5.57	0.13	5.39	5.46	5.51	5.74	5.76	3	0	1
alpha.phi	4.99	0.25	4.65	4.79	4.89	5.27	5.41	3	0	1
alpha.p	-1.55	0.07	-1.65	-1.64	-1.51	-1.49	-1.46	3	0	1
beta.hab.lambda	-0.26	0.03	-0.31	-0.29	-0.26	-0.25	-0.21	4	0	1
beta.hab.gamma	-0.08	0.01	-0.11	-0.09	-0.08	-0.07	-0.06	4	0	1
beta.hab.phi	-0.60	0.02	-0.62	-0.61	-0.60	-0.58	-0.56	4	0	1
beta.elev.lambda	-0.46	0.04	-0.50	-0.49	-0.48	-0.41	-0.40	3	0	1
beta.ele.gamma2	-0.99	0.01	-1.02	-1.01	-0.99	-0.98	-0.97	4	0	1
beta.elev.phi	1.59	0.09	1.47	1.52	1.56	1.68	1.74	3	0	1
beta.develop.lambda	0.30	0.04	0.24	0.26	0.29	0.35	0.37	3	0	1
beta.develop.gamma	1.07	0.03	1.02	1.04	1.06	1.10	1.11	3	0	1
beta.develop.phi	-0.36	0.04	-0.42	-0.40	-0.35	-0.33	-0.29	3	0	1
beta.effort	0.06	0.00	0.05	0.05	0.05	0.06	0.06	4	0	1
beta.min_temp	0.00	0.00	0.00	0.00	0.00	0.00	0.00	1	0	1

Table 73: Western Grebe

	mean	sd	2.5%	25%	50%	75%	97.5%	n.eff	overlap0	f
alpha.lambda	-6.67	1.68	-10.78	-7.25	-6.22	-5.54	-4.39	10	0	1.00
alpha.phi	1.97	0.24	1.74	1.75	1.88	2.26	2.34	3	0	1.00
alpha.p	-2.88	0.84	-4.34	-3.80	-2.61	-2.02	-2.01	3	0	1.00
beta.hab.lambda	-0.24	1.41	-3.15	-1.40	0.02	0.88	1.91	7	1	0.50
beta.hab.gamma	0.31	0.41	-0.26	-0.25	0.48	0.69	0.72	3	1	0.67
beta.hab.phi	0.60	1.17	-0.43	-0.38	-0.03	2.23	2.28	3	1	0.33
beta.elev.lambda	1.29	1.32	-1.20	0.35	1.28	2.12	4.02	7	1	0.84
beta.ele.gamma2	-0.26	0.07	-0.37	-0.36	-0.22	-0.21	-0.19	3	0	1.00
beta.elev.phi	0.29	0.24	0.09	0.10	0.14	0.63	0.65	3	0	1.00
beta.develop.lambda	-0.01	1.70	-3.58	-1.08	0.11	1.25	2.84	42	1	0.47
beta.develop.gamma	0.50	0.22	0.20	0.24	0.51	0.77	0.77	3	0	1.00
beta.develop.phi	-0.27	0.68	-1.19	-1.18	-0.08	0.43	0.46	3	1	0.67
beta.effort	0.02	0.02	0.00	0.01	0.01	0.04	0.05	3	0	1.00
beta.min_temp	0.00	0.00	0.00	0.00	0.00	0.00	0.00	1	0	1.00

Table 74: Western Meadowlark

	mean	sd	2.5%	25%	50%	75%	97.5%	n.eff	overlap0	f
alpha.lambda	7.34	0.27	7.14	7.15	7.16	7.67	7.78	3	0	1.00
alpha.phi	3.25	0.29	3.03	3.04	3.05	3.65	3.69	3	0	1.00
alpha.p	-1.61	0.33	-2.17	-2.03	-1.39	-1.38	-1.37	3	0	1.00
beta.hab.lambda	0.43	0.04	0.40	0.40	0.41	0.48	0.50	3	0	1.00
beta.hab.gamma	1.85	1.36	0.86	0.88	0.90	3.75	3.79	3	0	1.00
beta.hab.phi	-0.20	0.09	-0.35	-0.31	-0.14	-0.14	-0.13	3	0	1.00
beta.elev.lambda	-0.48	0.01	-0.49	-0.49	-0.47	-0.47	-0.47	3	0	1.00
beta.ele.gamma2	-1.88	1.38	-3.88	-3.82	-0.92	-0.90	-0.88	3	0	1.00
beta.elev.phi	-0.06	0.08	-0.21	-0.15	-0.01	-0.01	0.00	3	1	0.97
beta.develop.lambda	0.35	0.01	0.33	0.35	0.35	0.36	0.36	4	0	1.00
beta.develop.gamma	1.07	1.61	-0.10	-0.07	-0.05	3.33	3.38	3	1	0.33
beta.develop.phi	-0.08	0.19	-0.40	-0.32	0.04	0.05	0.07	3	1	0.33
beta.effort	0.08	0.01	0.06	0.07	0.08	0.09	0.09	3	0	1.00
beta.min_temp	0.00	0.00	0.00	0.00	0.00	0.00	0.00	1	0	1.00

Table 75: White-breasted Nuthatch

	mean	sd	2.5%	25%	50%	75%	97.5%	n.eff	overlap0	f
alpha.lambda	5.74	0.55	4.95	4.97	6.04	6.20	6.22	3	0	1.00
alpha.phi	5.17	0.69	4.15	4.23	5.55	5.69	5.82	3	0	1.00
alpha.p	-1.85	0.58	-2.35	-2.34	-2.18	-1.05	-1.03	3	0	1.00
beta.hab.lambda	0.23	0.00	0.22	0.23	0.23	0.23	0.24	5	0	1.00
beta.hab.gamma	0.44	0.16	0.20	0.21	0.54	0.55	0.56	3	0	1.00
beta.hab.phi	-0.41	0.32	-0.66	-0.64	-0.63	0.04	0.06	3	1	0.67
beta.elev.lambda	-0.05	0.03	-0.08	-0.08	-0.08	-0.01	0.00	3	1	0.93
beta.ele.gamma2	-0.07	0.23	-0.26	-0.24	-0.23	0.25	0.26	3	1	0.67
beta.elev.phi	0.05	0.80	-1.13	-1.07	0.60	0.63	0.64	3	1	0.67
beta.develop.lambda	0.23	0.06	0.17	0.18	0.20	0.32	0.33	3	0	1.00
beta.develop.gamma	0.18	0.10	0.04	0.05	0.25	0.25	0.26	3	0	1.00

	mean	sd	2.5%	25%	50%	75%	97.5%	n.eff	overlap0	f
beta.develop.phi	0.73	0.78	-0.38	-0.34	1.13	1.36	1.54	3	1	0.67
beta.effort	0.03	0.00	0.03	0.03	0.03	0.03	0.03	5	0	1.00
beta.min_temp	-0.02	0.01	-0.03	-0.03	-0.03	0.00	0.00	3	0	1.00

Table 76: White Ibis

	mean	sd	2.5%	25%	50%	75%	97.5%	n.eff	overlap0	f
alpha.lambda	7.87	0.43	7.51	7.55	7.60	8.45	8.52	3	0	1
alpha.phi	3.28	0.10	3.16	3.21	3.23	3.35	3.50	4	0	1
alpha.p	-2.56	0.43	-3.18	-3.16	-2.28	-2.24	-2.20	3	0	1
beta.hab.lambda	-1.53	0.02	-1.57	-1.54	-1.53	-1.52	-1.49	23	0	1
beta.hab.gamma	0.44	0.03	0.42	0.42	0.43	0.47	0.48	3	0	1
beta.hab.phi	-0.07	0.02	-0.11	-0.10	-0.06	-0.06	-0.06	3	0	1
beta.elev.lambda	-1.63	0.10	-1.72	-1.70	-1.69	-1.52	-1.41	3	0	1
beta.ele.gamma2	-0.68	0.05	-0.73	-0.72	-0.71	-0.65	-0.57	3	0	1
beta.elev.phi	0.95	0.05	0.80	0.96	0.97	0.98	1.00	5	0	1
beta.develop.lambda	-1.13	0.02	-1.18	-1.14	-1.12	-1.11	-1.09	5	0	1
beta.develop.gamma	1.16	0.04	1.10	1.13	1.15	1.17	1.26	4	0	1
beta.develop.phi	-0.85	0.05	-0.94	-0.88	-0.86	-0.83	-0.72	20	0	1
beta.effort	0.12	0.01	0.11	0.11	0.12	0.12	0.12	3	0	1
beta.min_temp	0.00	0.00	0.00	0.00	0.00	0.00	0.00	1	0	1

Table 77: American Avocet

	mean	sd	2.5%	25%	50%	75%	97.5%	n.eff	overlap0	f
alpha.lambda	7.92	0.49	7.38	7.39	7.81	8.56	8.58	3	0	1.00
alpha.phi	3.75	0.71	2.91	2.94	3.67	4.55	4.83	3	0	1.00
alpha.p	-2.10	0.33	-2.46	-2.45	-2.19	-1.66	-1.64	3	0	1.00
beta.hab.lambda	-0.53	0.66	-1.41	-1.38	-0.43	0.23	0.23	3	1	0.67
beta.hab.gamma	0.02	0.40	-0.33	-0.31	-0.21	0.58	0.59	3	1	0.33
beta.hab.phi	0.39	0.46	-0.27	-0.26	0.62	0.80	0.81	3	1	0.67
beta.elev.lambda	-0.65	0.28	-1.00	-0.97	-0.68	-0.29	-0.28	3	0	1.00
beta.ele.gamma2	-1.63	1.77	-3.54	-3.30	-2.28	0.77	0.82	3	1	0.67
beta.elev.phi	2.66	2.77	-1.31	-1.21	4.61	4.63	4.65	3	1	0.67
beta.develop.lambda	0.20	0.47	-0.48	-0.47	0.53	0.54	0.55	3	1	0.67
beta.develop.gamma	-0.64	1.20	-2.39	-2.28	0.05	0.33	0.39	3	1	0.33
beta.develop.phi	1.78	2.76	-0.62	-0.49	0.25	5.48	5.97	3	1	0.67
beta.effort	0.00	0.00	0.00	0.00	0.00	0.00	0.00	1	0	1.00
beta.min_temp	0.00	0.00	0.00	0.00	0.00	0.00	0.00	7	0	1.00

Table 78: American Kestrel

	mean	sd	2.5%	25%	50%	75%	97.5%	n.eff	overlap0	f
alpha.lambda	4.38	0.01	4.36	4.37	4.38	4.39	4.41	6	0	1.00
alpha.phi	3.35	0.01	3.33	3.34	3.35	3.36	3.37	7	0	1.00
alpha.p	-0.72	0.01	-0.74	-0.73	-0.72	-0.72	-0.71	5	0	1.00
beta.hab.lambda	0.23	0.00	0.22	0.23	0.23	0.23	0.24	38	0	1.00

	mean	sd	2.5%	25%	50%	75%	97.5%	n.eff	overlap0	f
beta.hab.gamma	-0.11	0.01	-0.13	-0.12	-0.11	-0.10	-0.09	8	0	1.00
beta.hab.phi	0.75	0.02	0.71	0.73	0.76	0.77	0.79	5	0	1.00
beta.elev.lambda	-0.51	0.00	-0.52	-0.52	-0.51	-0.51	-0.50	29	0	1.00
beta.ele.gamma2	-0.18	0.01	-0.19	-0.18	-0.18	-0.17	-0.16	19	0	1.00
beta.elev.phi	0.01	0.01	0.00	0.01	0.01	0.01	0.02	46	1	0.97
beta.develop.lambda	0.35	0.01	0.33	0.34	0.35	0.35	0.36	66	0	1.00
beta.develop.gamma	-0.11	0.01	-0.13	-0.12	-0.11	-0.10	-0.09	95	0	1.00
beta.develop.phi	0.01	0.01	-0.01	0.00	0.01	0.02	0.03	32	1	0.86
beta.effort	0.01	0.00	0.01	0.01	0.01	0.01	0.01	822	0	1.00
beta.min_temp	0.00	0.00	0.00	0.00	0.00	0.00	0.00	1	0	1.00

Table 79: American White Pelican

	mean	sd	2.5%	25%	50%	75%	97.5%	n.eff	overlap0	f
alpha.lambda	6.29	0.04	6.23	6.26	6.29	6.33	6.38	4	0	1.00
alpha.phi	1.43	0.03	1.38	1.40	1.44	1.45	1.47	3	0	1.00
alpha.p	-2.00	0.01	-2.01	-2.00	-2.00	-1.99	-1.99	5	0	1.00
beta.hab.lambda	0.48	0.01	0.47	0.48	0.48	0.48	0.49	8	0	1.00
beta.hab.gamma	-0.13	0.01	-0.15	-0.14	-0.13	-0.13	-0.12	3	0	1.00
beta.hab.phi	0.50	0.01	0.48	0.49	0.50	0.51	0.52	3	0	1.00
beta.elev.lambda	-0.34	0.02	-0.36	-0.35	-0.34	-0.32	-0.30	4	0	1.00
beta.ele.gamma2	-0.59	0.01	-0.60	-0.59	-0.59	-0.58	-0.58	4	0	1.00
beta.elev.phi	0.01	0.01	0.00	0.00	0.01	0.02	0.03	3	1	0.87
beta.develop.lambda	-0.69	0.11	-0.84	-0.81	-0.71	-0.58	-0.51	3	0	1.00
beta.develop.gamma	-0.40	0.07	-0.50	-0.48	-0.40	-0.35	-0.24	3	0	1.00
beta.develop.phi	0.39	0.09	0.20	0.33	0.40	0.49	0.51	3	0	1.00
beta.effort	0.00	0.00	0.00	0.00	0.00	0.00	0.00	1	0	1.00
beta.min_temp	0.00	0.00	0.00	0.00	0.00	0.00	0.00	1	0	1.00

Table 80: Black-billed Magpie

	mean	sd	2.5%	25%	50%	75%	97.5%	n.eff	overlap0	f
alpha.lambda	5.53	0.01	5.50	5.52	5.53	5.54	5.55	69	0	1.00
alpha.phi	2.34	0.01	2.32	2.34	2.34	2.35	2.37	24	0	1.00
alpha.p	-0.46	0.01	-0.47	-0.46	-0.46	-0.46	-0.45	41	0	1.00
beta.hab.lambda	-0.33	0.01	-0.35	-0.33	-0.33	-0.32	-0.31	348	0	1.00
beta.hab.gamma	0.27	0.01	0.26	0.27	0.27	0.28	0.29	27	0	1.00
beta.hab.phi	-0.40	0.01	-0.42	-0.40	-0.40	-0.39	-0.37	22	0	1.00
beta.elev.lambda	1.08	0.02	1.04	1.06	1.08	1.09	1.11	47	0	1.00
beta.ele.gamma2	0.31	0.01	0.29	0.30	0.31	0.32	0.33	273	0	1.00
beta.elev.phi	0.02	0.01	-0.01	0.01	0.02	0.03	0.05	650	1	0.89
beta.develop.lambda	0.44	0.02	0.40	0.42	0.44	0.45	0.47	151	0	1.00
beta.develop.gamma	0.63	0.02	0.60	0.62	0.63	0.64	0.67	464	0	1.00
beta.develop.phi	0.30	0.03	0.25	0.28	0.30	0.31	0.34	311	0	1.00
beta.effort	0.06	0.00	0.06	0.06	0.06	0.07	0.07	4503	0	1.00
beta.min_temp	0.00	0.00	0.00	0.00	0.00	0.00	0.00	1	0	1.00

Table 81: Black Vulture

	mean	sd	2.5%	25%	50%	75%	97.5%	n.eff	overlap0	f
alpha.lambda	3.49	0.06	3.40	3.44	3.47	3.55	3.60	3	0	1.00
alpha.phi	2.63	0.12	2.53	2.54	2.55	2.80	2.81	3	0	1.00
alpha.p	-1.17	0.02	-1.21	-1.20	-1.16	-1.16	-1.15	3	0	1.00
beta.hab.lambda	0.76	0.01	0.73	0.75	0.76	0.77	0.78	12	0	1.00
beta.hab.gamma	0.31	0.00	0.30	0.31	0.31	0.31	0.31	7	0	1.00
beta.hab.phi	-0.20	0.00	-0.21	-0.21	-0.20	-0.20	-0.19	250	0	1.00
beta.elev.lambda	-0.55	0.04	-0.61	-0.58	-0.57	-0.50	-0.47	3	0	1.00
beta.ele.gamma2	-0.22	0.12	-0.31	-0.30	-0.30	-0.05	-0.04	3	0	1.00
beta.elev.phi	-0.08	0.26	-0.46	-0.44	0.09	0.10	0.11	3	1	0.33
beta.develop.lambda	-0.08	0.03	-0.14	-0.10	-0.08	-0.06	-0.02	14	0	1.00
beta.develop.gamma	0.25	0.39	-0.04	-0.03	-0.02	0.79	0.81	3	1	0.33
beta.develop.phi	1.00	0.85	-0.23	-0.19	1.60	1.61	1.64	3	1	0.67
beta.effort	0.00	0.00	0.00	0.00	0.00	0.00	0.00	1	0	1.00
beta.min_temp	0.00	0.00	0.00	0.00	0.00	0.00	0.00	1	0	1.00

Table 82: Bohemian Waxwing

	mean	sd	2.5%	25%	50%	75%	97.5%	n.eff	overlap0	f
alpha.lambda	8.28	0.28	7.88	7.94	8.34	8.57	8.68	3	0	1.00
alpha.phi	1.79	2.42	-1.68	-1.57	3.15	3.76	3.92	3	1	0.67
alpha.p	-3.88	1.10	-5.50	-5.35	-3.39	-2.87	-2.84	3	0	1.00
beta.hab.lambda	0.03	0.43	-0.54	-0.54	0.16	0.49	0.50	3	1	0.67
beta.hab.gamma	-0.09	0.32	-0.52	-0.49	-0.05	0.25	0.30	3	1	0.67
beta.hab.phi	0.61	1.14	-0.53	-0.48	0.16	2.13	2.24	3	1	0.67
beta.elev.lambda	1.06	0.19	0.80	0.83	1.07	1.29	1.29	3	0	1.00
beta.ele.gamma2	0.66	0.39	0.10	0.12	0.86	0.98	1.03	3	0	1.00
beta.elev.phi	-0.26	0.94	-1.52	-1.36	-0.24	0.88	0.90	3	1	0.67
beta.develop.lambda	-0.77	0.93	-2.20	-1.77	-0.65	0.27	0.30	3	1	0.67
beta.develop.gamma	-0.32	1.38	-2.15	-2.13	-0.02	1.20	1.22	3	1	0.67
beta.develop.phi	1.18	2.21	-1.12	-1.11	0.48	4.11	4.26	3	1	0.67
beta.effort	0.00	0.00	0.00	0.00	0.00	0.00	0.00	1	0	1.00
beta.min_temp	0.00	0.00	-0.01	0.00	0.00	0.00	0.00	3	0	1.00

Table 83: California Quail

	mean	sd	2.5%	25%	50%	75%	97.5%	n.eff	overlap0	f
alpha.lambda	6.78	0.01	6.76	6.77	6.78	6.78	6.79	194	0	1
alpha.phi	2.17	0.01	2.16	2.17	2.17	2.18	2.19	28	0	1
alpha.p	-0.93	0.00	-0.94	-0.93	-0.93	-0.92	-0.92	120	0	1
beta.hab.lambda	0.11	0.01	0.10	0.11	0.12	0.12	0.13	373	0	1
beta.hab.gamma	0.19	0.01	0.18	0.19	0.19	0.19	0.20	349	0	1
beta.hab.phi	-0.17	0.01	-0.18	-0.17	-0.17	-0.16	-0.16	191	0	1
beta.elev.lambda	-0.08	0.01	-0.10	-0.09	-0.08	-0.08	-0.07	2138	0	1
beta.ele.gamma2	0.26	0.01	0.25	0.26	0.26	0.27	0.28	50	0	1
beta.elev.phi	-0.26	0.01	-0.28	-0.27	-0.26	-0.26	-0.25	58	0	1
beta.develop.lambda	0.07	0.01	0.05	0.06	0.06	0.07	0.09	830	0	1
beta.develop.gamma	0.12	0.01	0.10	0.12	0.12	0.13	0.14	310	0	1

	mean	sd	2.5%	25%	50%	75%	97.5%	n.eff	overlap0	f
beta.develop.phi	0.29	0.01	0.27	0.28	0.29	0.30	0.32	733	0	1
beta.effort	0.00	0.00	0.00	0.00	0.00	0.00	0.00	2624	0	1
beta.min_temp	-0.10	0.00	-0.10	-0.10	-0.10	-0.10	-0.10	465	0	1

Table 84: Carolina Wren

	mean	sd	2.5%	25%	50%	75%	97.5%	n.eff	overlap0	f
alpha.lambda	4.59	0.29	4.37	4.38	4.39	4.98	5.06	3	0	1.00
alpha.phi	3.53	0.36	3.26	3.27	3.28	4.02	4.10	3	0	1.00
alpha.p	-0.96	0.29	-1.42	-1.35	-0.76	-0.76	-0.75	3	0	1.00
beta.hab.lambda	0.35	0.06	0.26	0.28	0.39	0.40	0.41	3	0	1.00
beta.hab.gamma	0.35	0.10	0.27	0.28	0.28	0.48	0.50	3	0	1.00
beta.hab.phi	-0.25	0.18	-0.53	-0.49	-0.13	-0.13	-0.11	3	0	1.00
beta.elev.lambda	-0.36	0.01	-0.38	-0.37	-0.36	-0.35	-0.34	5	0	1.00
beta.ele.gamma2	-0.41	0.02	-0.45	-0.44	-0.40	-0.40	-0.39	3	0	1.00
beta.elev.phi	0.11	0.07	0.04	0.06	0.06	0.20	0.22	3	0	1.00
beta.develop.lambda	0.44	0.08	0.30	0.33	0.49	0.50	0.52	3	0	1.00
beta.develop.gamma	-0.07	0.14	-0.19	-0.18	-0.17	0.12	0.14	3	1	0.67
beta.develop.phi	0.60	0.43	-0.05	0.01	0.88	0.91	0.93	3	1	0.81
beta.effort	0.05	0.02	0.03	0.03	0.04	0.08	0.08	3	0	1.00
beta.min_temp	0.00	0.00	0.00	0.00	0.00	0.00	0.00	1	0	1.00

Table 85: Cattle Egret

	mean	sd	2.5%	25%	50%	75%	97.5%	n.eff	overlap0	f
alpha.lambda	7.22	0.33	6.91	6.97	7.03	7.69	7.70	3	0	1.00
alpha.phi	3.81	1.20	2.10	2.12	4.58	4.72	4.76	3	0	1.00
alpha.p	-1.88	0.21	-2.06	-2.04	-2.01	-1.59	-1.58	3	0	1.00
beta.hab.lambda	-0.42	0.19	-0.57	-0.56	-0.55	-0.16	-0.15	3	0	1.00
beta.hab.gamma	-1.69	0.13	-1.83	-1.81	-1.73	-1.52	-1.50	3	0	1.00
beta.hab.phi	2.00	0.22	1.68	1.72	2.05	2.24	2.26	3	0	1.00
beta.elev.lambda	-1.20	0.77	-1.81	-1.79	-1.70	-0.12	-0.10	3	0	1.00
beta.ele.gamma2	-1.75	0.88	-2.44	-2.41	-2.33	-0.51	-0.51	3	0	1.00
beta.elev.phi	2.45	2.09	-0.52	-0.51	3.92	3.94	3.96	3	1	0.67
beta.develop.lambda	1.08	0.01	1.06	1.07	1.08	1.09	1.10	6	0	1.00
beta.develop.gamma	0.57	0.11	0.42	0.43	0.61	0.68	0.69	3	0	1.00
beta.develop.phi	0.98	0.33	0.50	0.52	1.18	1.23	1.24	3	0	1.00
beta.effort	0.23	0.03	0.19	0.19	0.26	0.26	0.26	3	0	1.00
beta.min_temp	0.00	0.00	0.00	0.00	0.00	0.00	0.00	1	0	1.00

Table 86: Chipping Sparrow

	mean	sd	2.5%	25%	50%	75%	97.5%	n.eff	overlap0	f
alpha.lambda	4.64	0.03	4.60	4.62	4.63	4.67	4.70	3	0	1.00
alpha.phi	3.15	0.40	2.57	2.60	3.41	3.44	3.46	3	0	1.00
alpha.p	-1.44	0.02	-1.47	-1.46	-1.45	-1.42	-1.41	3	0	1.00
beta.hab.lambda	1.24	0.04	1.17	1.19	1.26	1.27	1.28	3	0	1.00

	mean	sd	2.5%	25%	50%	75%	97.5%	n.eff	overlap0	f
beta.hab.gamma	0.65	0.09	0.50	0.52	0.70	0.72	0.73	3	0	1.00
beta.hab.phi	-0.26	0.18	-0.41	-0.39	-0.38	-0.02	0.01	3	1	0.94
beta.elev.lambda	-0.01	0.02	-0.03	-0.02	-0.01	0.01	0.03	3	1	0.66
beta.ele.gamma2	-0.49	0.09	-0.57	-0.56	-0.55	-0.37	-0.36	3	0	1.00
beta.elev.phi	0.44	0.15	0.22	0.24	0.53	0.56	0.57	3	0	1.00
beta.develop.lambda	0.48	0.04	0.41	0.43	0.50	0.52	0.54	3	0	1.00
beta.develop.gamma	0.25	0.27	-0.02	-0.01	0.15	0.62	0.64	3	1	0.67
beta.develop.phi	0.59	0.38	0.25	0.28	0.39	1.12	1.16	3	0	1.00
beta.effort	0.02	0.00	0.02	0.02	0.02	0.03	0.03	5	0	1.00
beta.min_temp	0.00	0.00	0.00	0.00	0.00	0.00	0.00	1	0	1.00

Table 87: Common Raven

	mean	sd	2.5%	25%	50%	75%	97.5%	n.eff	overlap0	f
alpha.lambda	4.06	0.02	4.03	4.05	4.06	4.07	4.09	12	0	1
alpha.phi	3.10	0.02	3.07	3.09	3.10	3.11	3.13	9	0	1
alpha.p	-0.78	0.00	-0.79	-0.78	-0.78	-0.78	-0.77	10	0	1
beta.hab.lambda	-0.59	0.02	-0.62	-0.60	-0.59	-0.58	-0.56	13	0	1
beta.hab.gamma	-0.40	0.01	-0.41	-0.40	-0.40	-0.39	-0.38	9	0	1
beta.hab.phi	0.29	0.01	0.27	0.28	0.29	0.30	0.31	7	0	1
beta.elev.lambda	-0.33	0.01	-0.34	-0.33	-0.33	-0.32	-0.31	64	0	1
beta.ele.gamma2	0.65	0.01	0.64	0.65	0.65	0.65	0.66	60	0	1
beta.elev.phi	-0.16	0.01	-0.17	-0.16	-0.16	-0.16	-0.15	23	0	1
beta.develop.lambda	-0.42	0.03	-0.47	-0.44	-0.42	-0.40	-0.36	81	0	1
beta.develop.gamma	-0.51	0.01	-0.53	-0.52	-0.51	-0.51	-0.49	8	0	1
beta.develop.phi	0.95	0.03	0.87	0.93	0.94	0.97	1.00	8	0	1
beta.effort	0.13	0.00	0.13	0.13	0.13	0.13	0.13	647	0	1
beta.min_temp	0.00	0.00	0.00	0.00	0.00	0.00	0.00	1	0	1

Table 88: Common Redpoll

	mean	sd	2.5%	25%	50%	75%	97.5%	n.eff	overlap0	f
alpha.lambda	9.01	0.21	8.58	8.84	9.06	9.15	9.36	3	0	1.00
alpha.phi	-1.11	1.41	-2.18	-2.16	-2.04	0.81	1.01	3	1	0.67
alpha.p	-4.80	0.37	-5.15	-5.09	-5.01	-4.32	-4.23	3	0	1.00
beta.hab.lambda	-0.51	0.21	-0.73	-0.68	-0.62	-0.23	-0.19	3	0	1.00
beta.hab.gamma	-0.19	0.20	-0.50	-0.44	-0.05	-0.05	-0.04	3	0	1.00
beta.hab.phi	-0.03	0.47	-0.52	-0.49	-0.20	0.59	0.65	3	1	0.67
beta.elev.lambda	-0.04	0.22	-0.35	-0.33	0.06	0.16	0.17	3	1	0.33
beta.ele.gamma2	0.03	0.09	-0.04	-0.04	-0.03	0.14	0.19	3	1	0.33
beta.elev.phi	-0.01	0.19	-0.31	-0.26	0.10	0.13	0.14	3	1	0.33
beta.develop.lambda	1.31	0.71	0.34	0.37	1.50	2.06	2.09	3	0	1.00
beta.develop.gamma	-0.29	0.17	-0.53	-0.51	-0.23	-0.11	-0.11	3	0	1.00
beta.develop.phi	0.29	0.23	-0.09	0.00	0.40	0.48	0.52	3	1	0.69
beta.effort	0.00	0.00	0.00	0.00	0.00	0.00	0.00	1	0	1.00
beta.min_temp	-0.15	0.01	-0.16	-0.15	-0.15	-0.14	-0.14	3	0	1.00

Table 89: Eastern Meadowlark

	mean	sd	2.5%	25%	50%	75%	97.5%	n.eff	overlap0	f
alpha.lambda	6.51	0.00	6.50	6.51	6.51	6.51	6.52	205	0	1
alpha.phi	2.58	0.01	2.57	2.58	2.58	2.58	2.59	33	0	1
alpha.p	-1.35	0.00	-1.36	-1.35	-1.35	-1.35	-1.34	43	0	1
beta.hab.lambda	0.23	0.00	0.23	0.23	0.23	0.24	0.24	142	0	1
beta.hab.gamma	0.28	0.00	0.28	0.28	0.28	0.29	0.29	175	0	1
beta.hab.phi	-0.13	0.00	-0.14	-0.13	-0.13	-0.13	-0.13	140	0	1
beta.elev.lambda	-0.31	0.00	-0.32	-0.31	-0.31	-0.31	-0.31	92	0	1
beta.ele.gamma2	-0.34	0.00	-0.34	-0.34	-0.34	-0.33	-0.33	121	0	1
beta.elev.phi	0.08	0.00	0.07	0.08	0.08	0.08	0.09	92	0	1
beta.develop.lambda	0.54	0.01	0.53	0.53	0.54	0.54	0.55	681	0	1
beta.develop.gamma	-0.65	0.01	-0.68	-0.66	-0.65	-0.65	-0.63	129	0	1
beta.develop.phi	0.07	0.01	0.05	0.06	0.07	0.07	0.09	51	0	1
beta.effort	0.07	0.00	0.07	0.07	0.07	0.07	0.08	234	0	1
beta.min_temp	0.00	0.00	0.00	0.00	0.00	0.00	0.00	1	0	1

Table 90: Eurasian Collared-Dove

	mean	sd	2.5%	25%	50%	75%	97.5%	n.eff	overlap0	f
alpha.lambda	-9.98	2.26	-13.79	-12.50	-9.34	-8.23	-6.73	3	0	1.00
alpha.phi	3.00	0.02	2.97	2.99	3.00	3.01	3.04	17	0	1.00
alpha.p	-0.66	0.01	-0.68	-0.67	-0.66	-0.66	-0.65	22	0	1.00
beta.hab.lambda	-0.03	2.48	-4.90	-1.69	-0.03	1.60	4.80	192	1	0.50
beta.hab.gamma	0.62	2.27	-3.74	-0.94	0.60	2.15	5.03	6000	1	0.61
beta.hab.phi	-0.81	2.23	-5.23	-2.31	-0.80	0.72	3.59	5339	1	0.64
beta.elev.lambda	-0.69	1.20	-3.26	-1.50	-0.57	0.12	1.32	5	1	0.71
beta.ele.gamma2	0.34	0.00	0.33	0.34	0.34	0.34	0.34	112	0	1.00
beta.elev.phi	-0.12	0.00	-0.12	-0.12	-0.12	-0.11	-0.11	51	0	1.00
beta.develop.lambda	0.05	2.50	-4.94	-1.62	0.06	1.79	4.87	206	1	0.51
beta.develop.gamma	0.68	2.27	-3.72	-0.85	0.70	2.24	5.04	6000	1	0.62
beta.develop.phi	-0.80	2.23	-5.19	-2.34	-0.80	0.71	3.63	6000	1	0.64
beta.effort	0.08	0.00	0.07	0.08	0.08	0.08	0.08	68	0	1.00
beta.min_temp	0.00	0.00	0.00	0.00	0.00	0.00	0.00	1	0	1.00

Table 91: Evening Grosbeak

	mean	sd	2.5%	25%	50%	75%	97.5%	n.eff	overlap0	f
alpha.lambda	7.84	0.21	7.65	7.68	7.72	8.10	8.21	3	0	1
alpha.phi	2.71	0.10	2.59	2.63	2.66	2.84	2.85	3	0	1
alpha.p	-2.87	0.24	-3.28	-3.18	-2.73	-2.69	-2.64	3	0	1
beta.hab.lambda	0.48	0.01	0.47	0.47	0.48	0.48	0.49	4	0	1
beta.hab.gamma	-0.46	0.09	-0.60	-0.57	-0.42	-0.39	-0.36	3	0	1
beta.hab.phi	0.07	0.04	0.01	0.02	0.09	0.10	0.12	3	0	1
beta.elev.lambda	-0.17	0.00	-0.17	-0.17	-0.16	-0.16	-0.16	4	0	1
beta.ele.gamma2	1.32	0.38	0.93	1.02	1.12	1.83	1.92	3	0	1
beta.elev.phi	0.13	0.03	0.10	0.11	0.12	0.17	0.18	3	0	1
beta.develop.lambda	0.25	0.01	0.24	0.25	0.25	0.26	0.27	35	0	1
beta.develop.gamma	0.19	0.04	0.12	0.15	0.21	0.22	0.24	3	0	1

	mean	sd	2.5%	25%	50%	75%	97.5%	n.eff	overlap0	f
beta.develop.phi	-0.17	0.01	-0.19	-0.18	-0.17	-0.16	-0.16	8	0	1
beta.effort	0.00	0.00	0.00	0.00	0.00	0.00	0.00	1	0	1
beta.min_temp	-0.17	0.01	-0.18	-0.18	-0.16	-0.16	-0.16	3	0	1

Table 92: Field Sparrow

	mean	sd	2.5%	25%	50%	75%	97.5%	n.eff	overlap0	f
alpha.lambda	6.23	0.01	6.22	6.22	6.23	6.24	6.24	6	0	1
alpha.phi	3.07	0.01	3.06	3.06	3.07	3.08	3.09	5	0	1
alpha.p	-1.60	0.01	-1.61	-1.61	-1.60	-1.59	-1.59	5	0	1
beta.hab.lambda	-0.19	0.01	-0.20	-0.20	-0.19	-0.19	-0.18	20	0	1
beta.hab.gamma	-0.13	0.01	-0.16	-0.14	-0.13	-0.12	-0.11	25	0	1
beta.hab.phi	0.08	0.01	0.06	0.07	0.08	0.09	0.10	16	0	1
beta.elev.lambda	-0.14	0.00	-0.15	-0.14	-0.14	-0.14	-0.13	166	0	1
beta.ele.gamma2	-0.25	0.01	-0.26	-0.25	-0.25	-0.24	-0.23	75	0	1
beta.elev.phi	0.11	0.00	0.11	0.11	0.11	0.12	0.12	66	0	1
beta.develop.lambda	0.23	0.01	0.22	0.23	0.23	0.24	0.25	1166	0	1
beta.develop.gamma	-1.09	0.03	-1.16	-1.11	-1.09	-1.07	-1.03	16	0	1
beta.develop.phi	0.14	0.01	0.13	0.14	0.14	0.15	0.16	31	0	1
beta.effort	0.00	0.00	0.00	0.00	0.00	0.00	0.00	1	0	1
beta.min_temp	0.00	0.00	0.00	0.00	0.00	0.00	0.00	1	0	1

Table 93: Forster's Tern

	mean	sd	2.5%	25%	50%	75%	97.5%	n.eff	overlap0	f
alpha.lambda	7.08	1.36	6.09	6.11	6.12	8.96	9.08	3	0	1.00
alpha.phi	1.19	1.94	-1.60	-1.51	2.55	2.56	2.58	3	1	0.67
alpha.p	-2.91	1.54	-5.17	-5.05	-1.83	-1.82	-1.81	3	0	1.00
beta.hab.lambda	0.15	0.03	0.12	0.13	0.13	0.19	0.22	3	0	1.00
beta.hab.gamma	0.44	0.04	0.38	0.39	0.46	0.47	0.47	3	0	1.00
beta.hab.phi	-0.16	0.04	-0.23	-0.22	-0.13	-0.13	-0.12	3	0	1.00
beta.elev.lambda	-0.41	0.04	-0.45	-0.44	-0.43	-0.37	-0.33	3	0	1.00
beta.ele.gamma2	0.08	0.06	0.02	0.03	0.04	0.15	0.20	3	0	1.00
beta.elev.phi	-0.34	0.40	-0.96	-0.85	-0.06	-0.06	-0.05	3	0	1.00
beta.develop.lambda	0.53	0.10	0.44	0.46	0.47	0.65	0.71	3	0	1.00
beta.develop.gamma	-0.13	0.35	-0.40	-0.38	-0.36	0.35	0.36	3	1	0.67
beta.develop.phi	0.26	0.45	-0.42	-0.35	0.57	0.58	0.60	3	1	0.67
beta.effort	0.00	0.00	0.00	0.00	0.00	0.00	0.00	1	0	1.00
beta.min_temp	0.00	0.00	0.00	0.00	0.00	0.00	0.00	1	0	1.00

Table 94: Golden-crowned Kinglet

	mean	sd	2.5%	25%	50%	75%	97.5%	n.eff	overlap0	f
alpha.lambda	4.72	0.01	4.70	4.72	4.72	4.73	4.74	32	0	1
alpha.phi	2.74	0.01	2.72	2.73	2.74	2.74	2.75	16	0	1
alpha.p	-1.10	0.00	-1.11	-1.10	-1.10	-1.10	-1.09	17	0	1
beta.hab.lambda	0.24	0.01	0.23	0.23	0.24	0.24	0.25	201	0	1

	mean	sd	2.5%	25%	50%	75%	97.5%	n.eff	overlap0	f
beta.hab.gamma	0.49	0.01	0.48	0.49	0.49	0.49	0.50	61	0	1
beta.hab.phi	-0.16	0.01	-0.17	-0.16	-0.16	-0.15	-0.15	37	0	1
beta.elev.lambda	-0.34	0.01	-0.35	-0.34	-0.34	-0.33	-0.33	35	0	1
beta.ele.gamma2	-0.31	0.00	-0.32	-0.31	-0.31	-0.31	-0.30	183	0	1
beta.elev.phi	-0.07	0.01	-0.08	-0.07	-0.07	-0.07	-0.06	695	0	1
beta.develop.lambda	0.68	0.03	0.64	0.67	0.68	0.70	0.74	13	0	1
beta.develop.gamma	0.17	0.01	0.13	0.16	0.17	0.18	0.19	10	0	1
beta.develop.phi	0.22	0.01	0.20	0.21	0.22	0.23	0.26	10	0	1
beta.effort	0.03	0.00	0.03	0.03	0.03	0.03	0.03	108	0	1
beta.min_temp	0.00	0.00	0.00	0.00	0.00	0.00	0.00	1	0	1

Table 95: Great Blue Heron

	mean	sd	2.5%	25%	50%	75%	97.5%	n.eff	overlap0	f
alpha.lambda	5.35	1.54	4.09	4.12	4.37	7.46	7.60	3	0	1.00
alpha.phi	0.22	4.26	-5.81	-5.79	3.14	3.22	3.51	3	1	0.67
alpha.p	-2.16	1.78	-4.74	-4.62	-0.97	-0.80	-0.78	3	0	1.00
beta.hab.lambda	0.10	0.07	0.00	0.01	0.14	0.15	0.15	3	1	0.95
beta.hab.gamma	-0.02	0.10	-0.28	-0.02	-0.01	0.07	0.07	4	1	0.67
beta.hab.phi	0.08	0.12	-0.01	0.00	0.05	0.06	0.41	6	1	0.81
beta.elev.lambda	-0.50	0.05	-0.57	-0.55	-0.49	-0.47	-0.42	3	0	1.00
beta.ele.gamma2	-0.62	0.11	-0.72	-0.71	-0.69	-0.46	-0.46	3	0	1.00
beta.elev.phi	-0.05	0.37	-0.58	-0.56	0.21	0.22	0.23	3	1	0.33
beta.develop.lambda	0.38	0.09	0.22	0.26	0.43	0.45	0.47	3	0	1.00
beta.develop.gamma	0.02	0.13	-0.12	-0.07	-0.06	0.20	0.20	3	1	0.33
beta.develop.phi	0.55	0.34	0.28	0.30	0.33	1.02	1.05	3	0	1.00
beta.effort	0.01	0.00	0.00	0.00	0.01	0.01	0.02	4	0	1.00
beta.min_temp	0.00	0.00	0.00	0.00	0.00	0.00	0.00	1	0	1.00

Table 96: Great Egret

	mean	sd	2.5%	25%	50%	75%	97.5%	n.eff	overlap0	f
alpha.lambda	4.68	0.01	4.65	4.67	4.68	4.68	4.70	44	0	1
alpha.phi	2.85	0.01	2.82	2.84	2.85	2.86	2.87	53	0	1
alpha.p	-1.30	0.01	-1.31	-1.30	-1.30	-1.30	-1.29	23	0	1
beta.hab.lambda	-0.09	0.01	-0.10	-0.09	-0.09	-0.09	-0.08	110	0	1
beta.hab.gamma	0.21	0.00	0.20	0.21	0.21	0.21	0.21	694	0	1
beta.hab.phi	-0.06	0.00	-0.06	-0.06	-0.06	-0.05	-0.05	672	0	1
beta.elev.lambda	-1.05	0.01	-1.07	-1.06	-1.05	-1.05	-1.04	51	0	1
beta.ele.gamma2	-0.71	0.01	-0.73	-0.72	-0.71	-0.71	-0.70	74	0	1
beta.elev.phi	0.29	0.01	0.27	0.29	0.29	0.30	0.31	62	0	1
beta.develop.lambda	0.46	0.01	0.43	0.45	0.46	0.47	0.49	480	0	1
beta.develop.gamma	0.19	0.01	0.17	0.18	0.19	0.19	0.21	66	0	1
beta.develop.phi	0.35	0.01	0.33	0.35	0.35	0.36	0.38	30	0	1
beta.effort	0.13	0.00	0.12	0.13	0.13	0.13	0.13	661	0	1
beta.min_temp	0.00	0.00	0.00	0.00	0.00	0.00	0.00	1	0	1

Table 97: Hooded Merganser

	mean	sd	2.5%	25%	50%	75%	97.5%	n.eff	overlap0	f
alpha.lambda	3.63	0.12	3.51	3.54	3.55	3.78	3.85	3	0	1.00
alpha.phi	2.60	0.16	2.47	2.48	2.50	2.81	2.87	3	0	1.00
alpha.p	-1.55	0.14	-1.79	-1.73	-1.46	-1.44	-1.43	3	0	1.00
beta.hab.lambda	0.15	0.03	0.10	0.12	0.13	0.18	0.20	3	0	1.00
beta.hab.gamma	-0.01	0.04	-0.05	-0.04	-0.04	0.04	0.06	3	1	0.67
beta.hab.phi	1.24	0.05	1.19	1.20	1.21	1.31	1.32	3	0	1.00
beta.elev.lambda	-0.48	0.01	-0.51	-0.49	-0.48	-0.48	-0.46	7	0	1.00
beta.ele.gamma2	-0.20	0.02	-0.22	-0.22	-0.22	-0.18	-0.17	3	0	1.00
beta.elev.phi	0.16	0.02	0.13	0.14	0.17	0.17	0.18	3	0	1.00
beta.develop.lambda	0.04	0.05	-0.03	0.00	0.03	0.08	0.14	4	1	0.80
beta.develop.gamma	0.02	0.01	0.00	0.01	0.01	0.04	0.05	3	0	1.00
beta.develop.phi	0.49	0.01	0.47	0.48	0.48	0.49	0.51	6	0	1.00
beta.effort	0.00	0.00	0.00	0.00	0.00	0.00	0.00	22	0	1.00
beta.min_temp	-0.07	0.00	-0.08	-0.07	-0.07	-0.07	-0.07	4	0	1.00

Table 98: Lapland Longspur

	mean	sd	2.5%	25%	50%	75%	97.5%	n.eff	overlap0	f
alpha.lambda	10.04	0.20	9.73	9.88	9.99	10.24	10.39	3	0	1
alpha.phi	-1.10	0.23	-1.38	-1.32	-1.16	-0.81	-0.76	3	0	1
alpha.p	-5.73	0.11	-5.95	-5.82	-5.74	-5.65	-5.52	5	0	1
beta.hab.lambda	-0.19	0.05	-0.28	-0.25	-0.17	-0.16	-0.14	3	0	1
beta.hab.gamma	0.26	0.01	0.24	0.25	0.25	0.27	0.28	3	0	1
beta.hab.phi	0.05	0.02	0.02	0.03	0.06	0.07	0.08	3	0	1
beta.elev.lambda	0.73	0.09	0.59	0.64	0.74	0.83	0.87	3	0	1
beta.ele.gamma2	0.16	0.01	0.14	0.15	0.17	0.17	0.18	3	0	1
beta.elev.phi	-0.09	0.03	-0.12	-0.12	-0.10	-0.05	-0.04	3	0	1
beta.develop.lambda	1.35	0.34	0.86	0.91	1.45	1.68	1.74	3	0	1
beta.develop.gamma	0.18	0.01	0.16	0.16	0.18	0.19	0.20	3	0	1
beta.develop.phi	0.64	0.26	0.30	0.38	0.59	0.97	1.02	3	0	1
beta.effort	0.15	0.00	0.15	0.15	0.15	0.15	0.15	31	0	1
beta.min_temp	-0.13	0.00	-0.13	-0.13	-0.13	-0.12	-0.12	3	0	1

Table 99: Lesser Goldfinch

	mean	sd	2.5%	25%	50%	75%	97.5%	n.eff	overlap0	f
alpha.lambda	6.92	0.01	6.91	6.92	6.92	6.93	6.93	12	0	1
alpha.phi	2.92	0.01	2.90	2.91	2.92	2.92	2.94	51	0	1
alpha.p	-1.38	0.00	-1.39	-1.38	-1.38	-1.38	-1.37	11	0	1
beta.hab.lambda	-0.53	0.01	-0.54	-0.53	-0.53	-0.53	-0.52	21	0	1
beta.hab.gamma	0.05	0.01	0.04	0.05	0.05	0.06	0.07	257	0	1
beta.hab.phi	0.31	0.01	0.30	0.31	0.31	0.32	0.33	46	0	1
beta.elev.lambda	-0.22	0.00	-0.23	-0.22	-0.22	-0.21	-0.21	34	0	1
beta.ele.gamma2	-0.12	0.01	-0.13	-0.12	-0.12	-0.11	-0.11	28	0	1
beta.elev.phi	-0.17	0.01	-0.19	-0.18	-0.17	-0.16	-0.15	19	0	1
beta.develop.lambda	0.16	0.01	0.15	0.16	0.16	0.17	0.17	140	0	1
beta.develop.gamma	0.76	0.01	0.75	0.76	0.76	0.77	0.78	26	0	1

	mean	sd	2.5%	25%	50%	75%	97.5%	n.eff	overlap0	f
beta.develop.phi	-0.12	0.01	-0.14	-0.13	-0.12	-0.12	-0.11	21	0	1
beta.effort	0.21	0.00	0.21	0.21	0.21	0.21	0.22	2443	0	1
beta.min_temp	0.00	0.00	0.00	0.00	0.00	0.00	0.00	6000	0	1

Table 100: Long-billed Dowitcher

	mean	sd	2.5%	25%	50%	75%	97.5%	n.eff	overlap0	f
alpha.lambda	6.83	0.45	6.47	6.49	6.51	7.40	7.57	3	0	1.00
alpha.phi	1.63	1.12	-0.14	0.16	2.40	2.42	2.43	3	1	0.87
alpha.p	-3.04	0.90	-4.33	-4.28	-2.40	-2.40	-2.39	3	0	1.00
beta.hab.lambda	0.11	0.32	-0.38	-0.33	0.32	0.33	0.35	3	1	0.67
beta.hab.gamma	-0.14	0.49	-0.84	-0.84	0.21	0.21	0.21	3	1	0.33
beta.hab.phi	0.20	1.00	-0.52	-0.51	-0.50	1.58	1.68	3	1	0.33
beta.elev.lambda	-0.50	0.29	-1.03	-0.81	-0.32	-0.30	-0.25	3	0	1.00
beta.ele.gamma2	-0.89	0.05	-1.00	-0.93	-0.86	-0.86	-0.85	3	0	1.00
beta.elev.phi	0.70	0.43	0.38	0.39	0.40	1.29	1.33	3	0	1.00
beta.develop.lambda	0.18	0.12	-0.06	0.08	0.25	0.26	0.28	3	1	0.87
beta.develop.gamma	0.02	0.03	-0.04	0.00	0.01	0.05	0.06	3	1	0.76
beta.develop.phi	-0.31	0.05	-0.43	-0.33	-0.30	-0.29	-0.24	4	0	1.00
beta.effort	0.02	0.01	0.00	0.00	0.03	0.03	0.03	3	0	1.00
beta.min_temp	-0.02	0.01	-0.03	-0.03	-0.03	0.00	0.00	3	0	1.00

Table 101: Marbled Godwit

	mean	sd	2.5%	25%	50%	75%	97.5%	n.eff	overlap0	f
alpha.lambda	5.66	0.14	5.47	5.51	5.63	5.82	5.86	3	0	1
alpha.phi	4.84	0.15	4.65	4.72	4.79	4.95	5.15	3	0	1
alpha.p	-1.51	0.13	-1.69	-1.69	-1.42	-1.41	-1.40	3	0	1
beta.hab.lambda	-4.74	0.10	-4.92	-4.85	-4.70	-4.66	-4.61	3	0	1
beta.hab.gamma	-1.86	0.03	-1.92	-1.89	-1.87	-1.84	-1.81	4	0	1
beta.hab.phi	0.61	0.05	0.54	0.57	0.60	0.64	0.71	4	0	1
beta.elev.lambda	-0.38	0.01	-0.39	-0.38	-0.38	-0.37	-0.37	4	0	1
beta.ele.gamma2	-0.48	0.01	-0.50	-0.49	-0.48	-0.47	-0.46	6	0	1
beta.elev.phi	0.27	0.01	0.24	0.26	0.27	0.28	0.29	5	0	1
beta.develop.lambda	0.71	0.07	0.62	0.63	0.73	0.78	0.79	3	0	1
beta.develop.gamma	-1.39	0.03	-1.45	-1.42	-1.40	-1.37	-1.33	4	0	1
beta.develop.phi	-1.72	0.05	-1.83	-1.74	-1.71	-1.69	-1.64	5	0	1
beta.effort	0.00	0.00	0.00	0.00	0.00	0.00	0.00	1	0	1
beta.min_temp	-0.02	0.01	-0.04	-0.03	-0.02	-0.02	-0.01	3	0	1

Table 102: Pied-billed Grebe

	mean	sd	2.5%	25%	50%	75%	97.5%	n.eff	overlap0	f
alpha.lambda	4.05	0.01	4.02	4.04	4.05	4.06	4.07	8	0	1
alpha.phi	2.58	0.01	2.56	2.57	2.58	2.59	2.60	5	0	1
alpha.p	-1.05	0.01	-1.06	-1.05	-1.05	-1.04	-1.03	5	0	1
beta.hab.lambda	0.40	0.01	0.39	0.40	0.40	0.41	0.42	161	0	1

	mean	sd	2.5%	25%	50%	75%	97.5%	n.eff	overlap0	f
beta.hab.gamma	-0.11	0.00	-0.12	-0.12	-0.11	-0.11	-0.10	266	0	1
beta.hab.phi	0.27	0.01	0.26	0.27	0.27	0.28	0.28	278	0	1
beta.elev.lambda	-0.43	0.01	-0.44	-0.43	-0.43	-0.42	-0.41	28	0	1
beta.ele.gamma2	-0.37	0.01	-0.38	-0.37	-0.37	-0.37	-0.36	61	0	1
beta.elev.phi	0.03	0.01	0.01	0.02	0.02	0.03	0.04	58	0	1
beta.develop.lambda	0.21	0.01	0.19	0.20	0.21	0.22	0.23	304	0	1
beta.develop.gamma	-0.18	0.01	-0.20	-0.19	-0.18	-0.17	-0.15	17	0	1
beta.develop.phi	0.54	0.02	0.51	0.53	0.54	0.55	0.57	9	0	1
beta.effort	0.00	0.00	0.00	0.00	0.00	0.00	0.00	1	0	1
beta.min_temp	0.00	0.00	0.00	0.00	0.00	0.00	0.00	1	0	1

Table 103: Purple Finch

	mean	sd	2.5%	25%	50%	75%	97.5%	n.eff	overlap0	f
alpha.lambda	6.53	0.04	6.47	6.50	6.54	6.56	6.59	103	0	1
alpha.phi	3.33	0.03	3.28	3.30	3.34	3.35	3.37	60	0	1
alpha.p	-2.71	0.04	-2.77	-2.74	-2.71	-2.68	-2.64	131	0	1
beta.hab.lambda	0.48	0.00	0.47	0.47	0.48	0.48	0.48	14	0	1
beta.hab.gamma	0.34	0.02	0.31	0.33	0.34	0.35	0.38	121	0	1
beta.hab.phi	-0.28	0.01	-0.29	-0.29	-0.28	-0.28	-0.27	209	0	1
beta.elev.lambda	-0.08	0.00	-0.09	-0.09	-0.08	-0.08	-0.08	204	0	1
beta.ele.gamma2	-0.24	0.03	-0.30	-0.26	-0.24	-0.22	-0.18	62	0	1
beta.elev.phi	0.22	0.01	0.21	0.22	0.22	0.22	0.23	59	0	1
beta.develop.lambda	0.58	0.01	0.57	0.57	0.58	0.58	0.59	1017	0	1
beta.develop.gamma	0.28	0.05	0.19	0.23	0.28	0.31	0.38	70	0	1
beta.develop.phi	-0.42	0.01	-0.44	-0.43	-0.42	-0.41	-0.40	86	0	1
beta.effort	0.03	0.00	0.03	0.03	0.03	0.04	0.04	621	0	1
beta.min_temp	0.00	0.00	0.00	0.00	0.00	0.00	0.00	471	0	1

Table 104: Red-bellied Woodpecker

	mean	sd	2.5%	25%	50%	75%	97.5%	n.eff	overlap0	f
alpha.lambda	4.10	0.01	4.08	4.09	4.10	4.11	4.12	10	0	1.00
alpha.phi	3.64	0.01	3.61	3.64	3.65	3.65	3.67	5	0	1.00
alpha.p	-0.63	0.01	-0.64	-0.64	-0.63	-0.62	-0.61	5	0	1.00
beta.hab.lambda	0.00	0.01	-0.02	-0.01	0.00	0.01	0.02	6	1	0.57
beta.hab.gamma	0.05	0.01	0.04	0.04	0.05	0.06	0.08	4	0	1.00
beta.hab.phi	0.05	0.03	-0.01	0.03	0.06	0.07	0.09	4	1	0.92
beta.elev.lambda	-0.33	0.01	-0.34	-0.33	-0.33	-0.32	-0.31	89	0	1.00
beta.ele.gamma2	-0.33	0.00	-0.34	-0.33	-0.33	-0.32	-0.32	1019	0	1.00
beta.elev.phi	0.34	0.01	0.32	0.33	0.34	0.34	0.35	121	0	1.00
beta.develop.lambda	0.41	0.01	0.39	0.40	0.41	0.42	0.44	67	0	1.00
beta.develop.gamma	0.34	0.01	0.32	0.33	0.34	0.34	0.35	24	0	1.00
beta.develop.phi	-0.12	0.01	-0.15	-0.13	-0.12	-0.11	-0.09	212	0	1.00
beta.effort	0.01	0.00	0.01	0.01	0.01	0.01	0.01	103	0	1.00
beta.min_temp	0.00	0.00	0.00	0.00	0.00	0.00	0.00	3123	0	1.00

Table 105: Short-billed Dowitcher

	mean	sd	2.5%	25%	50%	75%	97.5%	n.eff	overlap0	f
alpha.lambda	7.44	0.58	7.01	7.03	7.04	8.26	8.29	3	0	1.00
alpha.phi	1.52	0.77	0.41	0.45	2.05	2.07	2.09	3	0	1.00
alpha.p	-2.43	0.71	-3.45	-3.43	-1.94	-1.93	-1.92	3	0	1.00
beta.hab.lambda	-1.78	0.08	-1.92	-1.87	-1.74	-1.72	-1.69	3	0	1.00
beta.hab.gamma	0.25	0.51	-0.48	-0.48	0.61	0.61	0.62	3	1	0.67
beta.hab.phi	-0.15	0.84	-0.75	-0.75	-0.74	1.04	1.05	3	1	0.67
beta.elev.lambda	-0.34	0.01	-0.36	-0.35	-0.34	-0.34	-0.33	16	0	1.00
beta.ele.gamma2	-0.18	0.57	-0.98	-0.98	0.22	0.23	0.24	3	1	0.33
beta.elev.phi	0.28	1.02	-0.46	-0.45	-0.44	1.71	1.72	3	1	0.33
beta.develop.lambda	1.16	0.25	0.96	0.98	0.98	1.51	1.53	3	0	1.00
beta.develop.gamma	-0.75	0.11	-0.91	-0.90	-0.68	-0.68	-0.66	3	0	1.00
beta.develop.phi	0.57	0.34	0.31	0.33	0.33	1.05	1.07	3	0	1.00
beta.effort	0.00	0.00	0.00	0.00	0.00	0.00	0.00	1	0	1.00
beta.min_temp	0.00	0.00	0.00	0.00	0.00	0.00	0.00	1	0	1.00

Table 106: Snow Bunting

	mean	sd	2.5%	25%	50%	75%	97.5%	n.eff	overlap0	f
alpha.lambda	9.27	0.49	8.62	8.71	9.25	9.82	9.96	3	0	1.00
alpha.phi	-0.44	1.24	-1.52	-1.52	-1.09	1.07	1.70	3	1	0.67
alpha.p	-4.79	0.53	-5.44	-5.30	-4.93	-4.15	-4.01	3	0	1.00
beta.hab.lambda	-0.62	0.32	-1.07	-1.06	-0.46	-0.37	-0.26	3	0	1.00
beta.hab.gamma	-0.33	0.58	-1.15	-1.14	-0.01	0.16	0.18	3	1	0.67
beta.hab.phi	0.31	1.37	-0.90	-0.90	-0.40	2.20	2.28	3	1	0.33
beta.elev.lambda	0.23	0.10	0.13	0.15	0.17	0.34	0.40	3	0	1.00
beta.ele.gamma2	0.10	0.11	-0.04	-0.04	0.11	0.22	0.23	3	1	0.67
beta.elev.phi	-0.05	0.26	-0.42	-0.41	0.09	0.17	0.20	3	1	0.33
beta.develop.lambda	-0.27	0.09	-0.42	-0.37	-0.21	-0.20	-0.18	3	0	1.00
beta.develop.gamma	-0.49	0.37	-1.05	-0.99	-0.26	-0.20	-0.19	3	0	1.00
beta.develop.phi	-0.16	0.64	-0.94	-0.90	-0.20	0.64	0.68	3	1	0.67
beta.effort	0.00	0.00	0.00	0.00	0.00	0.00	0.00	1	0	1.00
beta.min_temp	-0.28	0.00	-0.28	-0.28	-0.28	-0.28	-0.28	3	0	1.00

Table 107: Snowy Egret

	mean	sd	2.5%	25%	50%	75%	97.5%	n.eff	overlap0	f
alpha.lambda	5.57	0.02	5.54	5.56	5.57	5.58	5.59	137	0	1.00
alpha.phi	2.21	0.01	2.19	2.21	2.21	2.22	2.23	45	0	1.00
alpha.p	-1.32	0.01	-1.33	-1.32	-1.32	-1.31	-1.31	13	0	1.00
beta.hab.lambda	-0.69	0.01	-0.71	-0.70	-0.69	-0.68	-0.67	569	0	1.00
beta.hab.gamma	0.48	0.00	0.48	0.48	0.48	0.49	0.49	449	0	1.00
beta.hab.phi	-0.25	0.00	-0.26	-0.25	-0.25	-0.25	-0.24	629	0	1.00
beta.elev.lambda	-0.73	0.01	-0.75	-0.73	-0.73	-0.72	-0.71	128	0	1.00
beta.ele.gamma2	0.05	0.01	0.04	0.05	0.05	0.06	0.07	220	0	1.00
beta.elev.phi	-0.56	0.01	-0.58	-0.57	-0.56	-0.56	-0.54	97	0	1.00
beta.develop.lambda	-0.01	0.02	-0.04	-0.02	-0.01	0.00	0.03	26	1	0.68
beta.develop.gamma	0.16	0.02	0.12	0.15	0.17	0.18	0.19	4	0	1.00

	mean	sd	2.5%	25%	50%	75%	97.5%	n.eff	overlap0	f
beta.develop.phi	0.23	0.02	0.19	0.21	0.22	0.24	0.28	4	0	1.00
beta.effort	0.00	0.00	0.00	0.00	0.00	0.00	0.00	1	0	1.00
beta.min_temp	0.00	0.00	0.00	0.00	0.00	0.00	0.00	1	0	1.00

Table 108: Swamp Sparrow

	mean	sd	2.5%	25%	50%	75%	97.5%	n.eff	overlap0	f
alpha.lambda	4.93	0.01	4.91	4.92	4.93	4.93	4.95	7	0	1
alpha.phi	2.60	0.01	2.59	2.60	2.60	2.61	2.62	10	0	1
alpha.p	-1.24	0.01	-1.25	-1.25	-1.24	-1.24	-1.23	5	0	1
beta.hab.lambda	0.18	0.00	0.18	0.18	0.18	0.18	0.19	231	0	1
beta.hab.gamma	0.21	0.00	0.20	0.21	0.21	0.21	0.21	466	0	1
beta.hab.phi	-0.04	0.00	-0.05	-0.05	-0.04	-0.04	-0.04	928	0	1
beta.elev.lambda	-0.48	0.01	-0.49	-0.48	-0.48	-0.47	-0.47	100	0	1
beta.ele.gamma2	-0.57	0.01	-0.58	-0.57	-0.57	-0.57	-0.56	558	0	1
beta.elev.phi	0.11	0.01	0.10	0.11	0.11	0.11	0.12	6000	0	1
beta.develop.lambda	0.11	0.01	0.09	0.11	0.11	0.12	0.13	177	0	1
beta.develop.gamma	-0.47	0.01	-0.49	-0.47	-0.47	-0.46	-0.45	193	0	1
beta.develop.phi	0.27	0.01	0.25	0.26	0.27	0.28	0.29	139	0	1
beta.effort	0.12	0.00	0.11	0.12	0.12	0.12	0.12	369	0	1
beta.min_temp	0.00	0.00	0.00	0.00	0.00	0.00	0.00	1	0	1

Table 109: Tricolored Blackbird

	mean	sd	2.5%	25%	50%	75%	97.5%	n.eff	overlap0	f
alpha.lambda	11.42	0.47	10.60	10.87	11.69	11.73	11.94	3	0	1.00
alpha.phi	-0.46	0.79	-1.58	-1.44	-0.29	0.32	0.54	3	1	0.67
alpha.p	-4.65	0.21	-4.96	-4.90	-4.58	-4.45	-4.39	3	0	1.00
beta.hab.lambda	-0.07	0.08	-0.21	-0.16	-0.03	-0.01	0.01	3	1	0.93
beta.hab.gamma	0.15	0.67	-0.92	-0.73	0.58	0.65	0.68	3	1	0.67
beta.hab.phi	0.38	1.38	-0.61	-0.60	-0.58	2.23	2.52	3	1	0.33
beta.elev.lambda	-0.48	0.25	-0.89	-0.69	-0.46	-0.25	-0.10	3	0	1.00
beta.ele.gamma2	-0.48	0.29	-0.84	-0.73	-0.58	-0.11	-0.05	3	0	1.00
beta.elev.phi	0.78	0.57	-0.15	0.06	1.15	1.19	1.26	3	1	0.83
beta.develop.lambda	-0.66	0.36	-1.56	-0.77	-0.55	-0.42	-0.28	4	0	1.00
beta.develop.gamma	-0.55	0.92	-1.37	-1.32	-1.06	0.72	0.78	3	1	0.67
beta.develop.phi	-0.83	4.08	-7.21	-6.20	2.01	2.07	2.10	3	1	0.33
beta.effort	0.00	0.00	0.00	0.00	0.00	0.00	0.00	1	0	1.00
beta.min_temp	0.00	0.00	0.00	0.00	0.00	0.00	0.00	8	0	1.00

Table 110: Western Bluebird

	mean	sd	2.5%	25%	50%	75%	97.5%	n.eff	overlap0	f
alpha.lambda	6.27	0.38	5.99	6.00	6.01	6.80	6.81	3	0	1.00
alpha.phi	4.34	0.94	3.33	3.36	4.08	5.54	5.68	3	0	1.00
alpha.p	-1.60	0.44	-2.22	-2.21	-1.36	-1.23	-1.21	3	0	1.00
beta.hab.lambda	0.31	0.25	-0.06	-0.04	0.43	0.53	0.54	3	1	0.67

	mean	sd	2.5%	25%	50%	75%	97.5%	n.eff	overlap0	f
beta.hab.gamma	0.03	0.43	-0.52	-0.50	0.07	0.53	0.54	3	1	0.67
beta.hab.phi	-0.27	1.20	-1.16	-1.13	-1.10	1.43	1.46	3	1	0.67
beta.elev.lambda	-0.27	0.35	-0.56	-0.54	-0.48	0.22	0.24	3	1	0.67
beta.ele.gamma2	-0.33	0.71	-0.87	-0.85	-0.80	0.68	0.69	3	1	0.67
beta.elev.phi	0.50	2.05	-2.40	-2.35	1.57	2.27	2.34	3	1	0.67
beta.develop.lambda	0.42	0.07	0.35	0.37	0.38	0.50	0.53	3	0	1.00
beta.develop.gamma	0.05	0.30	-0.38	-0.35	0.14	0.35	0.38	3	1	0.67
beta.develop.phi	-0.15	0.62	-0.71	-0.63	-0.51	0.70	0.76	3	1	0.67
beta.effort	0.04	0.02	0.02	0.02	0.05	0.06	0.07	3	0	1.00
beta.min_temp	0.00	0.00	0.00	0.00	0.00	0.00	0.00	1	0	1.00

Table 111: White-faced Ibis

	mean	sd	2.5%	25%	50%	75%	97.5%	n.eff	overlap0	f
alpha.lambda	0.93	2.49	-1.21	-0.88	-0.68	4.37	4.60	3	1	0.33
alpha.phi	1.57	0.59	0.71	0.74	1.98	1.99	2.02	3	0	1.00
alpha.p	-2.39	0.26	-2.77	-2.76	-2.21	-2.20	-2.19	3	0	1.00
beta.hab.lambda	2.77	0.21	2.42	2.52	2.89	2.92	2.97	3	0	1.00
beta.hab.gamma	0.53	0.13	0.44	0.44	0.44	0.70	0.71	3	0	1.00
beta.hab.phi	-0.71	0.22	-1.04	-1.03	-0.56	-0.56	-0.55	3	0	1.00
beta.elev.lambda	2.01	0.15	1.70	1.90	2.03	2.13	2.27	5	0	1.00
beta.ele.gamma2	-0.21	0.27	-0.42	-0.41	-0.40	0.16	0.17	3	1	0.67
beta.elev.phi	-0.36	0.48	-1.04	-1.03	-0.03	-0.02	-0.01	3	0	1.00
beta.develop.lambda	3.50	0.26	3.10	3.28	3.44	3.77	3.94	3	0	1.00
beta.develop.gamma	-0.67	1.00	-1.41	-1.38	-1.37	0.73	0.75	3	1	0.67
beta.develop.phi	1.67	1.43	-0.38	-0.32	2.65	2.70	2.79	3	1	0.67
beta.effort	0.08	0.01	0.06	0.07	0.09	0.09	0.10	3	0	1.00
beta.min_temp	-0.19	0.00	-0.19	-0.19	-0.19	-0.18	-0.18	4	0	1.00

Table 112: White-winged Dove

	mean	sd	2.5%	25%	50%	75%	97.5%	n.eff	overlap0	f
alpha.lambda	-0.01	0.70	-1.55	-0.44	0.07	0.49	1.13	10	1	0.46
alpha.phi	0.27	0.01	0.25	0.26	0.27	0.27	0.29	48	0	1.00
alpha.p	-0.92	0.00	-0.92	-0.92	-0.92	-0.91	-0.91	3160	0	1.00
beta.hab.lambda	-1.40	2.26	-5.78	-2.94	-1.41	0.15	2.95	944	1	0.73
beta.hab.gamma	-0.35	2.22	-4.61	-1.84	-0.40	1.16	4.01	4136	1	0.57
beta.hab.phi	2.26	2.25	-2.17	0.73	2.25	3.81	6.69	1975	1	0.84
beta.elev.lambda	2.81	0.66	1.74	2.34	2.74	3.21	4.28	12	0	1.00
beta.ele.gamma2	0.34	0.00	0.33	0.33	0.34	0.34	0.35	14	0	1.00
beta.elev.phi	1.03	0.01	1.01	1.03	1.03	1.04	1.05	22	0	1.00
beta.develop.lambda	-1.42	2.27	-5.75	-2.96	-1.43	0.11	2.99	4837	1	0.73
beta.develop.gamma	-0.42	2.22	-4.78	-1.93	-0.37	1.07	3.84	4803	1	0.57
beta.develop.phi	2.30	2.25	-2.11	0.76	2.30	3.81	6.72	3815	1	0.84
beta.effort	0.00	0.00	0.00	0.00	0.00	0.00	0.00	1	0	1.00
beta.min_temp	-0.05	0.00	-0.06	-0.05	-0.05	-0.05	-0.05	482	0	1.00

Table 113: White-winged Scoter

	mean	sd	2.5%	25%	50%	75%	97.5%	n.eff	overlap0	f
alpha.lambda	8.70	0.61	8.25	8.27	8.27	9.57	9.58	3	0	1.00
alpha.phi	0.90	0.80	-0.23	-0.22	1.46	1.47	1.47	3	1	0.67
alpha.p	-2.96	0.69	-3.94	-3.92	-2.48	-2.47	-2.44	3	0	1.00
beta.hab.lambda	0.22	0.07	0.17	0.18	0.18	0.32	0.33	3	0	1.00
beta.hab.gamma	0.10	0.07	-0.01	-0.01	0.15	0.15	0.16	3	1	0.67
beta.hab.phi	0.66	0.17	0.54	0.54	0.54	0.91	0.91	3	0	1.00
beta.elev.lambda	-0.20	0.01	-0.21	-0.21	-0.20	-0.19	-0.19	3	0	1.00
beta.ele.gamma2	0.08	0.58	-0.74	-0.73	0.48	0.49	0.50	3	1	0.67
beta.elev.phi	0.32	0.97	-0.37	-0.36	-0.36	1.69	1.70	3	1	0.33
beta.develop.lambda	-0.14	0.16	-0.26	-0.26	-0.25	0.08	0.10	3	1	0.67
beta.develop.gamma	-0.93	1.20	-1.79	-1.78	-1.77	0.76	0.77	3	1	0.67
beta.develop.phi	-0.25	2.11	-3.27	-3.20	1.24	1.24	1.26	3	1	0.33
beta.effort	0.05	0.01	0.03	0.03	0.06	0.06	0.06	3	0	1.00
beta.min_temp	-0.20	0.03	-0.25	-0.24	-0.18	-0.18	-0.18	3	0	1.00

Table 114: Wild Turkey

	mean	sd	2.5%	25%	50%	75%	97.5%	n.eff	overlap0	f
alpha.lambda	0.03	0.13	-0.22	-0.06	0.03	0.12	0.27	50	1	0.58
alpha.phi	2.29	0.01	2.27	2.28	2.29	2.29	2.31	7	0	1.00
alpha.p	-1.26	0.00	-1.27	-1.26	-1.26	-1.25	-1.25	14	0	1.00
beta.hab.lambda	-1.65	0.04	-1.74	-1.68	-1.65	-1.62	-1.57	188	0	1.00
beta.hab.gamma	-0.38	0.01	-0.39	-0.39	-0.38	-0.38	-0.37	13	0	1.00
beta.hab.phi	0.51	0.01	0.50	0.51	0.51	0.52	0.53	10	0	1.00
beta.elev.lambda	-0.78	0.03	-0.84	-0.79	-0.78	-0.76	-0.73	12	0	1.00
beta.ele.gamma2	0.10	0.01	0.09	0.10	0.10	0.11	0.11	34	0	1.00
beta.elev.phi	0.08	0.01	0.07	0.08	0.08	0.09	0.10	37	0	1.00
beta.develop.lambda	-2.92	0.24	-3.41	-3.08	-2.92	-2.75	-2.46	132	0	1.00
beta.develop.gamma	0.06	0.01	0.05	0.06	0.06	0.07	0.08	21	0	1.00
beta.develop.phi	0.03	0.01	0.01	0.02	0.03	0.04	0.06	16	0	1.00
beta.effort	0.00	0.00	0.00	0.00	0.00	0.00	0.00	1	0	1.00
beta.min_temp	-0.10	0.00	-0.10	-0.10	-0.10	-0.10	-0.10	100	0	1.00

Table 115: Acorn Woodpecker

	mean	sd	2.5%	25%	50%	75%	97.5%	n.eff	overlap0	f
alpha.lambda	5.96	0.25	5.64	5.67	5.96	6.26	6.28	3	0	1.00
alpha.phi	5.87	0.47	5.16	5.25	6.07	6.29	6.35	3	0	1.00
alpha.p	-1.39	0.36	-1.85	-1.84	-1.38	-0.97	-0.95	3	0	1.00
beta.hab.lambda	-0.05	0.18	-0.21	-0.18	-0.17	0.20	0.22	3	1	0.67
beta.hab.gamma	0.29	0.11	0.15	0.18	0.28	0.43	0.46	3	0	1.00
beta.hab.phi	0.49	0.97	-0.92	-0.85	1.11	1.21	1.26	3	1	0.67
beta.elev.lambda	-0.07	0.49	-0.79	-0.76	0.26	0.28	0.30	3	1	0.33
beta.ele.gamma2	-0.39	0.71	-1.39	-1.38	-0.05	0.24	0.26	3	1	0.67
beta.elev.phi	-1.40	4.14	-4.67	-4.54	-4.07	4.42	4.50	3	1	0.67
beta.develop.lambda	-0.22	0.15	-0.45	-0.41	-0.15	-0.11	-0.05	3	0	1.00
beta.develop.gamma	-0.16	0.14	-0.37	-0.34	-0.09	-0.04	0.00	3	0	0.98

	mean	sd	2.5%	25%	50%	75%	97.5%	n.eff	overlap0	f
beta.develop.phi	2.01	0.85	1.11	1.21	1.66	3.13	3.26	3	0	1.00
beta.effort	0.08	0.01	0.06	0.07	0.09	0.10	0.10	3	0	1.00
beta.min_temp	-0.02	0.01	-0.03	-0.03	-0.02	0.00	0.00	3	0	1.00

Table 116: Anna's Hummingbird

	mean	sd	2.5%	25%	50%	75%	97.5%	n.eff	overlap0	f
alpha.lambda	5.58	0.30	5.27	5.31	5.46	5.99	6.01	3	0	1.00
alpha.phi	4.41	1.22	2.70	2.72	4.98	5.52	5.56	3	0	1.00
alpha.p	-0.80	0.39	-1.37	-1.34	-0.60	-0.45	-0.44	3	0	1.00
beta.hab.lambda	0.16	0.36	-0.38	-0.33	0.36	0.46	0.50	3	1	0.67
beta.hab.gamma	0.09	0.73	-0.95	-0.93	0.55	0.65	0.67	3	1	0.67
beta.hab.phi	-1.46	2.15	-3.16	-3.11	-2.83	1.57	1.61	3	1	0.67
beta.elev.lambda	-0.02	0.21	-0.24	-0.21	-0.10	0.25	0.28	3	1	0.67
beta.ele.gamma2	-0.05	0.28	-0.26	-0.24	-0.24	0.34	0.37	3	1	0.67
beta.elev.phi	-0.18	0.71	-1.23	-1.16	0.28	0.34	0.38	3	1	0.33
beta.develop.lambda	0.68	0.23	0.32	0.37	0.80	0.87	0.91	3	0	1.00
beta.develop.gamma	-0.07	0.80	-1.20	-1.19	0.45	0.53	0.55	3	1	0.33
beta.develop.phi	0.61	1.87	-0.75	-0.72	-0.70	3.24	3.29	3	1	0.33
beta.effort	0.08	0.07	0.03	0.04	0.04	0.18	0.18	3	0	1.00
beta.min_temp	0.00	0.00	0.00	0.00	0.00	0.00	0.00	11	0	1.00

Table 117: Bald Eagle

	mean	sd	2.5%	25%	50%	75%	97.5%	n.eff	overlap0	f
alpha.lambda	1.80	0.04	1.72	1.77	1.80	1.82	1.86	10	0	1.00
alpha.phi	3.27	0.01	3.24	3.26	3.27	3.28	3.30	8	0	1.00
alpha.p	-0.76	0.01	-0.78	-0.77	-0.76	-0.75	-0.74	3	0	1.00
beta.hab.lambda	-0.24	0.04	-0.31	-0.27	-0.24	-0.21	-0.16	5	0	1.00
beta.hab.gamma	-0.08	0.01	-0.10	-0.08	-0.08	-0.07	-0.05	7	0	1.00
beta.hab.phi	-0.31	0.02	-0.36	-0.33	-0.31	-0.29	-0.27	7	0	1.00
beta.elev.lambda	0.17	0.02	0.13	0.15	0.17	0.18	0.20	28	0	1.00
beta.ele.gamma2	0.00	0.01	-0.01	-0.01	0.00	0.00	0.01	27	1	0.73
beta.elev.phi	-0.43	0.01	-0.45	-0.44	-0.43	-0.42	-0.40	511	0	1.00
beta.develop.lambda	-0.08	0.06	-0.20	-0.12	-0.08	-0.03	0.04	26	1	0.89
beta.develop.gamma	0.24	0.02	0.21	0.23	0.24	0.25	0.27	10	0	1.00
beta.develop.phi	0.24	0.04	0.17	0.21	0.23	0.26	0.32	6	0	1.00
beta.effort	0.00	0.00	0.00	0.00	0.00	0.00	0.00	1330	0	1.00
beta.min_temp	-0.04	0.00	-0.05	-0.04	-0.04	-0.04	-0.04	132	0	1.00

Table 118: Band-tailed Pigeon

	mean	sd	2.5%	25%	50%	75%	97.5%	n.eff	overlap0	f
alpha.lambda	8.21	0.06	8.11	8.15	8.23	8.25	8.28	3	0	1
alpha.phi	3.05	0.08	2.92	2.97	3.07	3.10	3.17	3	0	1
alpha.p	-2.55	0.08	-2.65	-2.61	-2.58	-2.46	-2.43	3	0	1
beta.hab.lambda	-0.16	0.01	-0.17	-0.16	-0.16	-0.15	-0.14	8	0	1

	mean	sd	2.5%	25%	50%	75%	97.5%	n.eff	overlap0	f
beta.hab.gamma	-0.29	0.03	-0.33	-0.32	-0.30	-0.27	-0.24	4	0	1
beta.hab.phi	0.64	0.02	0.59	0.62	0.64	0.66	0.68	5	0	1
beta.elev.lambda	0.70	0.01	0.69	0.69	0.70	0.70	0.71	8	0	1
beta.ele.gamma2	1.13	0.05	1.04	1.09	1.13	1.18	1.23	4	0	1
beta.elev.phi	-1.80	0.09	-1.96	-1.87	-1.80	-1.73	-1.64	4	0	1
beta.develop.lambda	0.29	0.01	0.27	0.28	0.29	0.29	0.30	76	0	1
beta.develop.gamma	-1.20	0.04	-1.27	-1.24	-1.21	-1.17	-1.11	4	0	1
beta.develop.phi	0.86	0.02	0.82	0.84	0.86	0.87	0.89	11	0	1
beta.effort	0.06	0.00	0.05	0.05	0.06	0.06	0.06	4	0	1
beta.min_temp	0.00	0.00	0.00	0.00	0.00	0.00	0.00	1	0	1

Table 119: Belted Kingfisher

	mean	sd	2.5%	25%	50%	75%	97.5%	n.eff	overlap0	f
alpha.lambda	3.88	0.54	3.47	3.49	3.51	4.60	4.72	3	0	1.00
alpha.phi	3.73	0.45	3.39	3.41	3.43	4.31	4.45	3	0	1.00
alpha.p	-1.39	0.52	-2.21	-2.08	-1.03	-1.02	-1.01	3	0	1.00
beta.hab.lambda	0.35	0.03	0.30	0.31	0.36	0.37	0.37	3	0	1.00
beta.hab.gamma	-0.17	0.05	-0.27	-0.22	-0.14	-0.13	-0.11	3	0	1.00
beta.hab.phi	0.15	0.04	0.07	0.10	0.18	0.19	0.21	3	0	1.00
beta.elev.lambda	-0.36	0.02	-0.38	-0.37	-0.37	-0.34	-0.33	3	0	1.00
beta.ele.gamma2	-0.51	0.02	-0.55	-0.52	-0.51	-0.50	-0.48	8	0	1.00
beta.elev.phi	0.09	0.03	0.03	0.06	0.10	0.12	0.13	3	0	1.00
beta.develop.lambda	0.52	0.05	0.44	0.46	0.55	0.56	0.58	3	0	1.00
beta.develop.gamma	-0.01	0.18	-0.28	-0.25	0.10	0.12	0.14	3	1	0.33
beta.develop.phi	0.42	0.36	0.13	0.16	0.18	0.87	1.03	3	0	1.00
beta.effort	0.00	0.00	0.00	0.00	0.00	0.00	0.01	7	0	1.00
beta.min_temp	0.00	0.00	0.00	0.00	0.00	0.00	0.00	1	0	1.00

Table 120: Black-crowned Night-Heron

	mean	sd	2.5%	25%	50%	75%	97.5%	n.eff	overlap0	f
alpha.lambda	5.21	0.22	5.05	5.06	5.07	5.51	5.55	3	0	1.00
alpha.phi	3.02	0.22	2.84	2.86	2.87	3.29	3.38	3	0	1.00
alpha.p	-1.45	0.23	-1.80	-1.75	-1.29	-1.28	-1.28	3	0	1.00
beta.hab.lambda	0.27	0.05	0.19	0.20	0.30	0.30	0.31	3	0	1.00
beta.hab.gamma	-2.24	2.53	-6.82	-5.04	-0.55	-0.53	-0.52	3	0	1.00
beta.hab.phi	0.47	0.37	0.21	0.22	0.22	0.77	1.22	3	0	1.00
beta.elev.lambda	0.08	0.06	-0.02	0.00	0.12	0.13	0.14	3	1	0.77
beta.ele.gamma2	-1.17	0.14	-1.40	-1.36	-1.07	-1.07	-1.05	3	0	1.00
beta.elev.phi	0.20	0.02	0.17	0.19	0.21	0.22	0.23	4	0	1.00
beta.develop.lambda	-0.03	0.03	-0.10	-0.06	-0.02	-0.01	0.01	4	1	0.91
beta.develop.gamma	-0.23	0.15	-0.35	-0.33	-0.32	-0.02	0.04	3	1	0.82
beta.develop.phi	0.17	0.07	0.06	0.14	0.15	0.17	0.37	9	0	1.00
beta.effort	0.00	0.00	0.00	0.00	0.00	0.00	0.00	28	0	1.00
beta.min_temp	0.00	0.00	0.00	0.00	0.00	0.00	0.00	132	0	1.00

Table 121: Black-necked Stilt

	mean	sd	2.5%	25%	50%	75%	97.5%	n.eff	overlap0	f
alpha.lambda	4.97	2.48	1.30	1.53	6.68	6.74	6.79	3	0	1.00
alpha.phi	2.81	0.57	2.00	2.01	3.14	3.27	3.30	3	0	1.00
alpha.p	-2.05	0.58	-2.52	-2.50	-2.41	-1.24	-1.22	3	0	1.00
beta.hab.lambda	-3.36	4.07	-9.44	-8.98	-0.53	-0.45	-0.39	3	0	1.00
beta.hab.gamma	-0.09	0.12	-0.18	-0.18	-0.18	0.07	0.08	3	1	0.67
beta.hab.phi	0.50	0.47	-0.17	-0.15	0.83	0.84	0.85	3	1	0.67
beta.elev.lambda	-0.81	0.23	-1.01	-0.98	-0.95	-0.51	-0.46	3	0	1.00
beta.ele.gamma2	-1.70	0.76	-2.28	-2.27	-2.22	-0.63	-0.62	3	0	1.00
beta.elev.phi	3.34	2.37	-0.03	-0.01	4.95	5.08	5.16	3	1	0.67
beta.develop.lambda	1.68	0.27	1.45	1.48	1.52	2.05	2.12	3	0	1.00
beta.develop.gamma	0.39	0.16	0.14	0.16	0.50	0.51	0.52	3	0	1.00
beta.develop.phi	-1.27	0.72	-1.84	-1.81	-1.76	-0.26	-0.23	3	0	1.00
beta.effort	0.20	0.03	0.15	0.16	0.22	0.23	0.23	3	0	1.00
beta.min_temp	-0.20	0.01	-0.22	-0.22	-0.19	-0.19	-0.19	3	0	1.00

Table 122: Black Phoebe

	mean	sd	2.5%	25%	50%	75%	97.5%	n.eff	overlap0	f
alpha.lambda	4.72	0.28	4.38	4.43	4.68	5.05	5.12	3	0	1.00
alpha.phi	5.12	0.47	3.86	4.96	5.32	5.46	5.58	4	0	1.00
alpha.p	-1.18	0.31	-1.58	-1.57	-1.14	-0.83	-0.82	3	0	1.00
beta.hab.lambda	-0.01	0.06	-0.09	-0.05	-0.03	0.05	0.11	3	1	0.66
beta.hab.gamma	-0.17	0.01	-0.20	-0.18	-0.17	-0.16	-0.15	24	0	1.00
beta.hab.phi	0.72	0.13	0.40	0.65	0.78	0.82	0.86	4	0	1.00
beta.elev.lambda	-0.16	0.06	-0.30	-0.22	-0.14	-0.12	-0.09	3	0	1.00
beta.ele.gamma2	-0.11	0.03	-0.15	-0.13	-0.12	-0.08	-0.07	3	0	1.00
beta.elev.phi	-3.53	0.78	-4.20	-4.07	-3.88	-3.18	-1.46	4	0	1.00
beta.develop.lambda	0.23	0.12	0.07	0.14	0.18	0.36	0.43	3	0	1.00
beta.develop.gamma	0.13	0.03	0.09	0.11	0.14	0.15	0.17	4	0	1.00
beta.develop.phi	0.86	0.14	0.65	0.74	0.84	0.96	1.12	3	0	1.00
beta.effort	0.02	0.01	0.00	0.00	0.02	0.02	0.03	3	0	1.00
beta.min_temp	0.00	0.00	0.00	0.00	0.00	0.00	0.00	16	0	1.00

Table 123: Blue-gray Gnatcatcher

	mean	sd	2.5%	25%	50%	75%	97.5%	n.eff	overlap0	f
alpha.lambda	4.07	0.04	4.01	4.05	4.07	4.11	4.14	3	0	1
alpha.phi	6.88	0.04	6.80	6.85	6.88	6.91	6.98	17	0	1
alpha.p	-1.13	0.02	-1.16	-1.15	-1.12	-1.11	-1.09	3	0	1
beta.hab.lambda	-0.78	0.01	-0.80	-0.79	-0.78	-0.77	-0.75	60	0	1
beta.hab.gamma	-0.59	0.02	-0.63	-0.60	-0.59	-0.58	-0.56	16	0	1
beta.hab.phi	-0.59	0.02	-0.63	-0.60	-0.59	-0.57	-0.54	18	0	1
beta.elev.lambda	-1.07	0.02	-1.10	-1.08	-1.07	-1.06	-1.04	4	0	1
beta.ele.gamma2	-1.44	0.01	-1.47	-1.45	-1.44	-1.44	-1.43	36	0	1
beta.elev.phi	3.11	0.03	3.05	3.09	3.11	3.13	3.17	26	0	1
beta.develop.lambda	0.44	0.01	0.41	0.43	0.44	0.45	0.47	26	0	1
beta.develop.gamma	0.83	0.01	0.80	0.82	0.83	0.84	0.86	13	0	1

	mean	sd	2.5%	25%	50%	75%	97.5%	n.eff	overlap0	f
beta.develop.phi	-0.72	0.02	-0.76	-0.73	-0.72	-0.70	-0.67	42	0	1
beta.effort	0.00	0.00	0.00	0.00	0.00	0.00	0.00	6000	0	1
beta.min_temp	0.00	0.00	0.00	0.00	0.00	0.00	0.00	228	0	1

Table 124: Blue-winged Teal

	mean	sd	2.5%	25%	50%	75%	97.5%	n.eff	overlap0	f
alpha.lambda	4.30	0.03	4.25	4.28	4.31	4.33	4.35	14	0	1
alpha.phi	6.88	0.43	5.66	6.71	7.04	7.19	7.28	26	0	1
alpha.p	-2.09	0.05	-2.15	-2.13	-2.11	-2.06	-1.95	25	0	1
beta.hab.lambda	-0.05	0.02	-0.10	-0.05	-0.04	-0.03	-0.03	23	0	1
beta.hab.gamma	0.37	0.00	0.37	0.37	0.37	0.38	0.38	34	0	1
beta.hab.phi	-0.13	0.00	-0.14	-0.13	-0.13	-0.13	-0.12	52	0	1
beta.elev.lambda	-1.41	0.01	-1.43	-1.42	-1.42	-1.41	-1.39	67	0	1
beta.ele.gamma2	-1.22	0.04	-1.26	-1.25	-1.23	-1.19	-1.10	32	0	1
beta.elev.phi	2.16	0.20	1.61	2.09	2.23	2.30	2.34	27	0	1
beta.develop.lambda	-0.57	0.03	-0.63	-0.58	-0.56	-0.55	-0.53	29	0	1
beta.develop.gamma	0.50	0.02	0.46	0.49	0.51	0.52	0.53	30	0	1
beta.develop.phi	-1.16	0.11	-1.26	-1.23	-1.20	-1.12	-0.86	23	0	1
beta.effort	0.00	0.00	0.00	0.00	0.00	0.00	0.00	1	0	1
beta.min_temp	0.00	0.00	0.00	0.00	0.00	0.00	0.00	1	0	1

Table 125: Brown Creeper

	mean	sd	2.5%	25%	50%	75%	97.5%	n.eff	overlap0	f
alpha.lambda	3.66	0.02	3.62	3.64	3.65	3.68	3.70	4	0	1.00
alpha.phi	2.99	0.03	2.95	2.98	2.99	3.02	3.04	4	0	1.00
alpha.p	-1.25	0.02	-1.28	-1.27	-1.25	-1.23	-1.22	3	0	1.00
beta.hab.lambda	0.08	0.01	0.06	0.07	0.08	0.08	0.10	31	0	1.00
beta.hab.gamma	0.14	0.01	0.12	0.13	0.14	0.15	0.17	63	0	1.00
beta.hab.phi	-0.09	0.02	-0.12	-0.10	-0.09	-0.08	-0.06	49	0	1.00
beta.elev.lambda	-0.09	0.01	-0.11	-0.09	-0.09	-0.08	-0.07	23	0	1.00
beta.ele.gamma2	-0.05	0.01	-0.07	-0.06	-0.05	-0.04	-0.03	52	0	1.00
beta.elev.phi	-0.03	0.01	-0.05	-0.04	-0.03	-0.02	-0.01	32	0	0.99
beta.develop.lambda	0.31	0.01	0.28	0.30	0.31	0.32	0.33	42	0	1.00
beta.develop.gamma	0.42	0.02	0.39	0.41	0.42	0.43	0.46	109	0	1.00
beta.develop.phi	-0.50	0.02	-0.55	-0.51	-0.50	-0.48	-0.46	55	0	1.00
beta.effort	0.02	0.00	0.01	0.02	0.02	0.02	0.03	26	0	1.00
beta.min_temp	0.00	0.00	0.00	0.00	0.00	0.00	0.00	1	0	1.00

Table 126: California Towhee

	mean	sd	2.5%	25%	50%	75%	97.5%	n.eff	overlap0	f
alpha.lambda	-7.69	1.74	-11.25	-8.95	-7.72	-6.26	-4.57	9	0	1.00
alpha.phi	2.59	0.01	2.56	2.58	2.59	2.60	2.62	6000	0	1.00
alpha.p	-0.02	0.01	-0.04	-0.02	-0.02	-0.01	0.01	559	1	0.94
beta.hab.lambda	0.00	1.47	-3.01	-0.93	0.07	1.06	2.77	67	1	0.48

	mean	sd	2.5%	25%	50%	75%	97.5%	n.eff	overlap0	f
beta.hab.gamma	0.21	0.01	0.20	0.21	0.21	0.22	0.23	495	0	1.00
beta.hab.phi	-0.30	0.01	-0.33	-0.31	-0.30	-0.29	-0.27	322	0	1.00
beta.elev.lambda	0.44	1.84	-2.80	-0.71	0.22	1.46	4.92	24	1	0.56
beta.ele.gamma2	-0.27	0.01	-0.28	-0.27	-0.27	-0.26	-0.25	1545	0	1.00
beta.elev.phi	0.29	0.02	0.26	0.28	0.29	0.30	0.32	693	0	1.00
beta.develop.lambda	0.33	1.94	-3.58	-0.97	0.47	1.71	3.80	157	1	0.59
beta.develop.gamma	0.79	0.01	0.76	0.78	0.79	0.80	0.82	167	0	1.00
beta.develop.phi	-0.41	0.02	-0.45	-0.43	-0.41	-0.40	-0.37	194	0	1.00
beta.effort	0.00	0.00	0.00	0.00	0.00	0.01	0.01	319	0	1.00
beta.min_temp	-0.05	0.00	-0.05	-0.05	-0.05	-0.05	-0.04	6000	0	1.00

Table 127: Chestnut-backed Chickadee

	mean	sd	2.5%	25%	50%	75%	97.5%	n.eff	overlap0	f
alpha.lambda	6.77	0.27	6.48	6.54	6.66	7.13	7.15	3	0	1.00
alpha.phi	3.70	0.17	3.50	3.56	3.64	3.93	3.95	3	0	1.00
alpha.p	-1.32	0.26	-1.70	-1.68	-1.21	-1.10	-1.04	3	0	1.00
beta.hab.lambda	-0.26	0.02	-0.29	-0.28	-0.26	-0.25	-0.23	4	0	1.00
beta.hab.gamma	-0.65	0.02	-0.69	-0.66	-0.64	-0.63	-0.61	4	0	1.00
beta.hab.phi	2.63	0.12	2.43	2.51	2.61	2.77	2.81	3	0	1.00
beta.elev.lambda	0.05	0.02	0.02	0.03	0.05	0.06	0.09	4	0	1.00
beta.ele.gamma2	-0.06	0.08	-0.16	-0.13	-0.09	0.03	0.06	3	1	0.67
beta.elev.phi	-0.66	0.15	-0.89	-0.86	-0.60	-0.52	-0.48	3	0	1.00
beta.develop.lambda	0.02	0.02	-0.01	0.01	0.03	0.04	0.06	4	1	0.86
beta.develop.gamma	-0.14	0.04	-0.22	-0.18	-0.13	-0.11	-0.09	3	0	1.00
beta.develop.phi	0.74	0.16	0.57	0.62	0.66	0.93	1.00	3	0	1.00
beta.effort	0.05	0.00	0.04	0.05	0.05	0.05	0.06	4	0	1.00
beta.min_temp	0.00	0.00	0.00	0.00	0.00	0.00	0.00	32	0	1.00

Table 128: Common Gallinule

	mean	sd	2.5%	25%	50%	75%	97.5%	n.eff	overlap0	f
alpha.lambda	4.83	0.62	4.30	4.34	4.47	5.69	5.71	3	0	1.00
alpha.phi	4.77	0.74	4.16	4.22	4.32	5.78	5.88	3	0	1.00
alpha.p	-1.79	0.52	-2.53	-2.52	-1.45	-1.40	-1.38	3	0	1.00
beta.hab.lambda	0.15	0.09	0.06	0.08	0.09	0.28	0.29	3	0	1.00
beta.hab.gamma	0.16	0.19	-0.13	-0.10	0.29	0.30	0.31	3	1	0.67
beta.hab.phi	0.39	0.34	0.13	0.14	0.16	0.86	0.90	3	0	1.00
beta.elev.lambda	-0.86	0.03	-0.92	-0.89	-0.85	-0.84	-0.81	4	0	1.00
beta.ele.gamma2	-1.26	0.11	-1.43	-1.39	-1.19	-1.17	-1.16	3	0	1.00
beta.elev.phi	2.34	0.58	1.87	1.91	1.95	3.11	3.23	3	0	1.00
beta.develop.lambda	1.29	0.02	1.23	1.27	1.29	1.30	1.33	4	0	1.00
beta.develop.gamma	1.29	0.03	1.24	1.27	1.30	1.31	1.33	4	0	1.00
beta.develop.phi	-0.47	0.06	-0.56	-0.52	-0.47	-0.41	-0.38	3	0	1.00
beta.effort	0.00	0.00	0.00	0.00	0.00	0.00	0.00	1	0	1.00
beta.min_temp	0.00	0.00	0.00	0.00	0.00	0.00	0.00	1	0	1.00

Table 129: Common Loon

	mean	sd	2.5%	25%	50%	75%	97.5%	n.eff	overlap0	f
alpha.lambda	5.04	0.62	4.55	4.59	4.62	5.91	5.95	3	0	1.00
alpha.phi	1.83	0.29	1.56	1.60	1.67	2.24	2.26	3	0	1.00
alpha.p	-2.01	0.55	-2.79	-2.78	-1.63	-1.62	-1.60	3	0	1.00
beta.hab.lambda	0.08	0.03	0.03	0.04	0.10	0.11	0.12	3	0	1.00
beta.hab.gamma	-0.04	0.07	-0.10	-0.09	-0.09	0.05	0.06	3	1	0.67
beta.hab.phi	2.07	0.03	2.02	2.04	2.07	2.09	2.12	3	0	1.00
beta.elev.lambda	0.11	0.06	0.05	0.07	0.08	0.18	0.22	3	0	1.00
beta.ele.gamma2	0.07	0.12	-0.12	-0.09	0.15	0.16	0.17	3	1	0.67
beta.elev.phi	-0.72	0.10	-0.82	-0.80	-0.79	-0.58	-0.57	3	0	1.00
beta.develop.lambda	-0.23	0.04	-0.29	-0.26	-0.23	-0.21	-0.16	5	0	1.00
beta.develop.gamma	-0.30	0.04	-0.36	-0.34	-0.30	-0.25	-0.23	3	0	1.00
beta.develop.phi	0.66	0.14	0.45	0.48	0.73	0.77	0.80	3	0	1.00
beta.effort	0.06	0.02	0.03	0.04	0.07	0.07	0.08	3	0	1.00
beta.min_temp	-0.02	0.01	-0.04	-0.03	-0.02	-0.02	-0.01	3	0	1.00

Table 130: Eastern Phoebe

	mean	sd	2.5%	25%	50%	75%	97.5%	n.eff	overlap0	f
alpha.lambda	2.64	0.26	2.42	2.45	2.47	2.99	3.06	3	0	1
alpha.phi	5.65	0.09	5.53	5.57	5.61	5.76	5.80	3	0	1
alpha.p	-0.73	0.12	-0.95	-0.86	-0.65	-0.64	-0.64	3	0	1
beta.hab.lambda	-0.45	0.06	-0.53	-0.50	-0.47	-0.39	-0.35	3	0	1
beta.hab.gamma	-0.09	0.01	-0.11	-0.10	-0.09	-0.08	-0.07	5	0	1
beta.hab.phi	-0.47	0.04	-0.53	-0.50	-0.47	-0.44	-0.40	4	0	1
beta.elev.lambda	-0.79	0.09	-0.87	-0.85	-0.84	-0.68	-0.65	3	0	1
beta.ele.gamma2	-0.90	0.02	-0.92	-0.91	-0.91	-0.88	-0.86	3	0	1
beta.elev.phi	1.65	0.02	1.62	1.64	1.65	1.66	1.68	14	0	1
beta.develop.lambda	0.43	0.14	0.23	0.28	0.41	0.59	0.64	3	0	1
beta.develop.gamma	0.66	0.06	0.56	0.58	0.68	0.71	0.74	3	0	1
beta.develop.phi	-1.15	0.08	-1.27	-1.22	-1.16	-1.08	-1.02	4	0	1
beta.effort	0.04	0.01	0.03	0.04	0.04	0.04	0.05	5	0	1
beta.min_temp	0.00	0.00	0.00	0.00	0.00	0.00	0.00	44	0	1

Table 131: Eastern Towhee

	mean	sd	2.5%	25%	50%	75%	97.5%	n.eff	overlap0	f
alpha.lambda	-8.89	0.96	-11.05	-9.30	-8.70	-8.33	-7.34	14	0	1.00
alpha.phi	3.25	0.01	3.22	3.24	3.25	3.26	3.28	993	0	1.00
alpha.p	-0.56	0.01	-0.57	-0.56	-0.56	-0.56	-0.55	13	0	1.00
beta.hab.lambda	0.11	0.71	-1.89	-0.27	0.41	0.59	0.86	5	1	0.68
beta.hab.gamma	-0.47	0.03	-0.52	-0.49	-0.47	-0.45	-0.42	6	0	1.00
beta.hab.phi	0.22	0.05	0.13	0.19	0.22	0.25	0.31	12	0	1.00
beta.elev.lambda	0.51	0.72	-0.92	0.01	0.44	1.07	1.95	5	1	0.76
beta.ele.gamma2	-0.40	0.00	-0.41	-0.40	-0.40	-0.39	-0.39	255	0	1.00
beta.elev.phi	0.31	0.01	0.30	0.31	0.31	0.32	0.33	1747	0	1.00
beta.develop.lambda	0.39	1.37	-2.38	-0.54	0.59	1.43	2.76	73	1	0.64
beta.develop.gamma	0.23	0.01	0.21	0.22	0.22	0.23	0.24	226	0	1.00

	mean	sd	2.5%	25%	50%	75%	97.5%	n.eff	overlap0	f
beta.develop.phi	-0.09	0.02	-0.13	-0.10	-0.09	-0.08	-0.06	166	0	1.00
beta.effort	0.02	0.00	0.02	0.02	0.02	0.03	0.03	758	0	1.00
beta.min_temp	0.00	0.00	0.00	0.00	0.00	0.00	0.00	6000	0	1.00

Table 132: Fox Sparrow

	mean	sd	2.5%	25%	50%	75%	97.5%	n.eff	overlap0	f
alpha.lambda	4.25	0.02	4.20	4.23	4.25	4.26	4.28	4	0	1.00
alpha.phi	3.05	0.01	3.02	3.04	3.05	3.06	3.08	5	0	1.00
alpha.p	-1.04	0.02	-1.07	-1.06	-1.04	-1.03	-1.02	3	0	1.00
beta.hab.lambda	-0.05	0.01	-0.07	-0.05	-0.05	-0.04	-0.03	31	0	1.00
beta.hab.gamma	0.04	0.01	0.02	0.03	0.04	0.05	0.06	23	0	1.00
beta.hab.phi	0.03	0.02	0.00	0.02	0.03	0.05	0.06	23	1	0.97
beta.elev.lambda	-0.26	0.01	-0.27	-0.26	-0.26	-0.25	-0.24	12	0	1.00
beta.ele.gamma2	-0.31	0.01	-0.32	-0.31	-0.31	-0.30	-0.29	90	0	1.00
beta.elev.phi	-0.02	0.01	-0.04	-0.03	-0.02	-0.01	0.00	65	0	0.98
beta.develop.lambda	0.23	0.01	0.21	0.22	0.23	0.23	0.25	22	0	1.00
beta.develop.gamma	-0.12	0.01	-0.14	-0.13	-0.12	-0.11	-0.10	533	0	1.00
beta.develop.phi	0.05	0.01	0.03	0.04	0.05	0.06	0.08	118	0	1.00
beta.effort	0.00	0.00	0.00	0.00	0.00	0.00	0.00	995	0	1.00
beta.min_temp	-0.02	0.00	-0.03	-0.02	-0.02	-0.02	-0.01	5	0	1.00

Table 133: Hairy Woodpecker

	mean	sd	2.5%	25%	50%	75%	97.5%	n.eff	overlap0	f
alpha.lambda	3.61	0.01	3.59	3.60	3.61	3.62	3.63	7	0	1
alpha.phi	3.23	0.01	3.21	3.22	3.23	3.24	3.26	26	0	1
alpha.p	-0.82	0.01	-0.84	-0.83	-0.82	-0.81	-0.81	5	0	1
beta.hab.lambda	0.32	0.01	0.30	0.31	0.32	0.33	0.34	12	0	1
beta.hab.gamma	0.26	0.01	0.24	0.25	0.26	0.26	0.28	226	0	1
beta.hab.phi	-0.10	0.01	-0.13	-0.11	-0.10	-0.09	-0.08	135	0	1
beta.elev.lambda	-0.11	0.01	-0.13	-0.12	-0.11	-0.11	-0.10	76	0	1
beta.ele.gamma2	-0.12	0.01	-0.14	-0.12	-0.12	-0.11	-0.10	32	0	1
beta.elev.phi	0.21	0.01	0.18	0.20	0.21	0.22	0.24	21	0	1
beta.develop.lambda	0.37	0.02	0.34	0.36	0.37	0.38	0.40	7	0	1
beta.develop.gamma	0.08	0.01	0.05	0.07	0.08	0.09	0.11	10	0	1
beta.develop.phi	-0.09	0.04	-0.16	-0.12	-0.10	-0.07	-0.01	4	0	1
beta.effort	0.01	0.00	0.00	0.00	0.01	0.01	0.01	12	0	1
beta.min_temp	-0.08	0.00	-0.08	-0.08	-0.08	-0.07	-0.07	20	0	1

Table 134: Harris's Sparrow

	mean	sd	2.5%	25%	50%	75%	97.5%	n.eff	overlap0	f
alpha.lambda	6.80	0.60	6.34	6.36	6.41	7.64	7.66	3	0	1.00
alpha.phi	3.74	0.25	3.49	3.52	3.67	3.92	4.28	4	0	1.00
alpha.p	-2.00	0.66	-3.02	-2.92	-1.54	-1.53	-1.51	3	0	1.00
beta.hab.lambda	-0.70	0.05	-0.78	-0.76	-0.68	-0.66	-0.65	3	0	1.00

	mean	sd	2.5%	25%	50%	75%	97.5%	n.eff	overlap0	f
beta.hab.gamma	-0.82	0.57	-1.76	-1.55	-0.65	-0.28	-0.25	3	0	1.00
beta.hab.phi	-0.24	0.19	-0.44	-0.42	-0.31	-0.01	0.11	3	1	0.79
beta.elev.lambda	0.50	0.23	0.13	0.19	0.63	0.70	0.71	3	0	1.00
beta.ele.gamma2	0.25	0.08	0.14	0.18	0.23	0.34	0.36	3	0	1.00
beta.elev.phi	-3.20	0.66	-3.67	-3.63	-3.41	-3.21	-1.02	5	0	1.00
beta.develop.lambda	0.20	0.06	0.14	0.16	0.17	0.22	0.34	4	0	1.00
beta.develop.gamma	-2.63	0.61	-3.56	-3.18	-2.72	-1.90	-1.84	3	0	1.00
beta.develop.phi	0.86	0.28	0.29	0.61	1.00	1.08	1.12	3	0	1.00
beta.effort	0.08	0.02	0.06	0.06	0.07	0.10	0.12	3	0	1.00
beta.min_temp	0.00	0.00	0.00	0.00	0.00	0.00	0.00	1	0	1.00

Table 135: Hermit Thrush

	mean	sd	2.5%	25%	50%	75%	97.5%	n.eff	overlap0	f
alpha.lambda	4.28	0.03	4.23	4.26	4.29	4.30	4.32	4	0	1.00
alpha.phi	3.29	0.02	3.25	3.27	3.29	3.30	3.33	5	0	1.00
alpha.p	-1.24	0.02	-1.28	-1.26	-1.24	-1.22	-1.20	3	0	1.00
beta.hab.lambda	-0.02	0.01	-0.04	-0.03	-0.02	-0.01	0.00	10	1	0.95
beta.hab.gamma	0.02	0.01	0.00	0.01	0.02	0.03	0.04	77	0	0.98
beta.hab.phi	0.32	0.01	0.29	0.31	0.31	0.32	0.34	27	0	1.00
beta.elev.lambda	-0.07	0.01	-0.08	-0.07	-0.07	-0.06	-0.05	35	0	1.00
beta.ele.gamma2	-0.26	0.01	-0.28	-0.27	-0.26	-0.25	-0.24	103	0	1.00
beta.elev.phi	-0.25	0.01	-0.27	-0.26	-0.25	-0.24	-0.22	53	0	1.00
beta.develop.lambda	0.22	0.02	0.17	0.20	0.22	0.24	0.26	4	0	1.00
beta.develop.gamma	-0.40	0.01	-0.43	-0.41	-0.40	-0.39	-0.38	11	0	1.00
beta.develop.phi	-0.02	0.02	-0.05	-0.03	-0.02	-0.01	0.02	26	1	0.83
beta.effort	0.01	0.00	0.00	0.01	0.01	0.01	0.02	5	0	1.00
beta.min_temp	-0.07	0.00	-0.08	-0.08	-0.07	-0.07	-0.07	6	0	1.00

Table 136: Horned Grebe

	mean	sd	2.5%	25%	50%	75%	97.5%	n.eff	overlap0	f
alpha.lambda	5.24	0.03	5.18	5.20	5.24	5.26	5.30	4	0	1
alpha.phi	3.57	0.29	3.16	3.31	3.50	3.84	4.02	3	0	1
alpha.p	-1.94	0.06	-2.03	-1.99	-1.93	-1.87	-1.84	3	0	1
beta.hab.lambda	0.77	0.02	0.74	0.75	0.77	0.80	0.81	3	0	1
beta.hab.gamma	0.53	0.08	0.42	0.46	0.52	0.62	0.66	3	0	1
beta.hab.phi	-0.25	0.13	-0.45	-0.38	-0.22	-0.13	-0.07	3	0	1
beta.elev.lambda	-0.26	0.00	-0.27	-0.26	-0.26	-0.26	-0.25	27	0	1
beta.ele.gamma2	-0.35	0.01	-0.37	-0.35	-0.35	-0.34	-0.33	13	0	1
beta.elev.phi	0.36	0.02	0.33	0.34	0.36	0.38	0.40	4	0	1
beta.develop.lambda	0.25	0.01	0.23	0.24	0.25	0.25	0.26	26	0	1
beta.develop.gamma	-0.42	0.04	-0.47	-0.44	-0.43	-0.38	-0.36	3	0	1
beta.develop.phi	0.60	0.03	0.54	0.57	0.61	0.63	0.66	3	0	1
beta.effort	0.02	0.00	0.01	0.02	0.02	0.02	0.03	4	0	1
beta.min_temp	0.00	0.00	0.00	0.00	0.00	0.00	0.00	1	0	1

Table 137: Lark Sparrow

	mean	sd	2.5%	25%	50%	75%	97.5%	n.eff	overlap0	f
alpha.lambda	6.10	0.09	6.03	6.04	6.05	6.17	6.33	3	0	1.0
alpha.phi	2.49	0.12	2.39	2.40	2.42	2.57	2.77	3	0	1.0
alpha.p	-1.83	0.10	-2.07	-1.89	-1.77	-1.76	-1.76	3	0	1.0
beta.hab.lambda	0.52	0.01	0.51	0.52	0.52	0.52	0.53	8	0	1.0
beta.hab.gamma	0.06	0.01	0.04	0.06	0.06	0.07	0.08	5	0	1.0
beta.hab.phi	0.27	0.02	0.23	0.27	0.28	0.28	0.29	4	0	1.0
beta.elev.lambda	0.01	0.03	-0.02	-0.01	0.00	0.04	0.06	3	1	0.4
beta.ele.gamma2	1.05	0.14	0.93	0.95	0.97	1.18	1.35	3	0	1.0
beta.elev.phi	-0.61	0.06	-0.73	-0.67	-0.58	-0.57	-0.55	3	0	1.0
beta.develop.lambda	-0.18	0.12	-0.39	-0.33	-0.12	-0.09	-0.06	3	0	1.0
beta.develop.gamma	0.14	0.06	0.02	0.09	0.16	0.19	0.23	4	0	1.0
beta.develop.phi	-0.21	0.13	-0.35	-0.31	-0.28	-0.05	-0.01	3	0	1.0
beta.effort	0.17	0.00	0.16	0.17	0.17	0.17	0.18	95	0	1.0
beta.min_temp	0.00	0.00	0.00	0.00	0.00	0.00	0.00	1	0	1.0

Table 138: Loggerhead Shrike

	mean	sd	2.5%	25%	50%	75%	97.5%	n.eff	overlap0	f
alpha.lambda	4.44	0.02	4.41	4.42	4.44	4.45	4.47	4	0	1
alpha.phi	3.09	0.02	3.05	3.07	3.09	3.10	3.12	5	0	1
alpha.p	-0.72	0.02	-0.74	-0.73	-0.72	-0.70	-0.69	4	0	1
beta.hab.lambda	-0.16	0.01	-0.17	-0.16	-0.16	-0.15	-0.14	64	0	1
beta.hab.gamma	-0.64	0.03	-0.69	-0.66	-0.64	-0.62	-0.58	7	0	1
beta.hab.phi	0.07	0.01	0.05	0.07	0.07	0.08	0.10	55	0	1
beta.elev.lambda	-0.40	0.01	-0.41	-0.41	-0.40	-0.40	-0.39	30	0	1
beta.ele.gamma2	-0.55	0.01	-0.57	-0.55	-0.55	-0.54	-0.52	16	0	1
beta.elev.phi	0.05	0.01	0.02	0.04	0.05	0.06	0.07	26	0	1
beta.develop.lambda	0.53	0.01	0.50	0.52	0.53	0.54	0.56	24	0	1
beta.develop.gamma	0.59	0.02	0.55	0.57	0.59	0.60	0.62	9	0	1
beta.develop.phi	-0.32	0.01	-0.34	-0.33	-0.32	-0.31	-0.29	15	0	1
beta.effort	0.05	0.00	0.05	0.05	0.05	0.06	0.06	110	0	1
beta.min_temp	0.00	0.00	0.00	0.00	0.00	0.00	0.00	1	0	1

Table 139: Long-billed Curlew

	mean	sd	2.5%	25%	50%	75%	97.5%	n.eff	overlap0	f
alpha.lambda	5.40	0.02	5.36	5.39	5.40	5.42	5.45	8	0	1.00
alpha.phi	2.94	0.03	2.88	2.92	2.94	2.97	3.00	25	0	1.00
alpha.p	-1.78	0.01	-1.80	-1.79	-1.78	-1.77	-1.76	4	0	1.00
beta.hab.lambda	0.66	0.02	0.62	0.65	0.66	0.68	0.71	8	0	1.00
beta.hab.gamma	0.47	0.00	0.46	0.47	0.47	0.47	0.48	168	0	1.00
beta.hab.phi	-0.39	0.01	-0.41	-0.40	-0.39	-0.38	-0.37	517	0	1.00
beta.elev.lambda	-1.00	0.01	-1.03	-1.01	-1.00	-0.99	-0.97	133	0	1.00
beta.ele.gamma2	-0.02	0.01	-0.04	-0.03	-0.02	-0.02	0.00	31	0	0.99
beta.elev.phi	-0.92	0.02	-0.95	-0.93	-0.92	-0.91	-0.89	91	0	1.00
beta.develop.lambda	1.00	0.02	0.97	0.99	1.00	1.01	1.03	15	0	1.00
beta.develop.gamma	-3.12	0.05	-3.20	-3.15	-3.13	-3.08	-3.03	51	0	1.00

	mean	sd	2.5%	25%	50%	75%	97.5%	n.eff	overlap0	f
beta.develop.phi	5.93	0.11	5.69	5.84	5.95	6.01	6.12	27	0	1.00
beta.effort	0.00	0.00	0.00	0.00	0.00	0.00	0.00	1	0	1.00
beta.min_temp	0.00	0.00	0.00	0.00	0.00	0.00	0.00	6000	0	1.00

Table 140: Mute Swan

	mean	sd	2.5%	25%	50%	75%	97.5%	n.eff	overlap0	f
alpha.lambda	5.72	1.93	4.32	4.35	4.37	8.37	8.55	3	0	1.00
alpha.phi	0.07	2.42	-3.38	-3.33	1.78	1.79	1.80	3	1	0.67
alpha.p	-2.02	2.15	-5.14	-5.01	-0.50	-0.50	-0.49	3	0	1.00
beta.hab.lambda	0.02	0.02	-0.03	0.01	0.02	0.03	0.05	9	1	0.87
beta.hab.gamma	0.35	0.02	0.33	0.34	0.34	0.38	0.39	3	0	1.00
beta.hab.phi	-0.12	0.39	-0.70	-0.67	0.15	0.16	0.17	3	1	0.33
beta.elev.lambda	-0.31	0.09	-0.39	-0.38	-0.36	-0.19	-0.16	3	0	1.00
beta.ele.gamma2	-0.04	0.04	-0.10	-0.10	-0.01	0.00	0.00	3	1	0.91
beta.elev.phi	-0.31	0.30	-0.75	-0.71	-0.10	-0.09	-0.08	3	0	1.00
beta.develop.lambda	-0.66	0.12	-0.79	-0.75	-0.72	-0.57	-0.39	3	0	1.00
beta.develop.gamma	0.06	0.14	-0.05	-0.04	-0.03	0.26	0.26	3	1	0.33
beta.develop.phi	0.33	0.23	-0.03	0.01	0.48	0.49	0.51	3	1	0.86
beta.effort	0.00	0.00	0.00	0.00	0.00	0.00	0.00	11	0	1.00
beta.min_temp	-0.08	0.05	-0.15	-0.15	-0.05	-0.05	-0.04	3	0	1.00

Table 141: Northern Bobwhite

	mean	sd	2.5%	25%	50%	75%	97.5%	n.eff	overlap0	f
alpha.lambda	6.38	0.12	6.27	6.28	6.30	6.51	6.59	3	0	1.00
alpha.phi	2.74	0.05	2.69	2.70	2.71	2.80	2.84	3	0	1.00
alpha.p	-2.11	0.14	-2.35	-2.26	-2.03	-2.01	-1.99	3	0	1.00
beta.hab.lambda	0.15	0.01	0.14	0.15	0.15	0.16	0.16	5	0	1.00
beta.hab.gamma	0.07	0.05	-0.03	0.03	0.09	0.10	0.12	3	1	0.88
beta.hab.phi	0.24	0.02	0.21	0.22	0.23	0.26	0.28	3	0	1.00
beta.elev.lambda	-0.23	0.00	-0.24	-0.23	-0.23	-0.23	-0.22	8	0	1.00
beta.ele.gamma2	-0.23	0.04	-0.30	-0.26	-0.21	-0.20	-0.18	3	0	1.00
beta.elev.phi	0.06	0.01	0.04	0.05	0.06	0.07	0.08	4	0	1.00
beta.develop.lambda	0.17	0.01	0.15	0.16	0.16	0.17	0.18	537	0	1.00
beta.develop.gamma	-1.04	0.35	-1.97	-1.22	-0.85	-0.81	-0.75	4	0	1.00
beta.develop.phi	-0.03	0.02	-0.06	-0.04	-0.03	-0.02	-0.01	4	0	0.99
beta.effort	0.00	0.00	0.00	0.00	0.00	0.00	0.00	903	0	1.00
beta.min_temp	-0.01	0.00	-0.01	-0.01	-0.01	-0.01	0.00	6	0	1.00

Table 142: Northern Harrier

	mean	sd	2.5%	25%	50%	75%	97.5%	n.eff	overlap0	f
alpha.lambda	3.73	0.22	3.51	3.54	3.61	4.03	4.05	3	0	1.00
alpha.phi	3.65	0.24	3.44	3.46	3.51	3.95	4.04	3	0	1.00
alpha.p	-1.17	0.19	-1.45	-1.43	-1.07	-1.01	-0.99	3	0	1.00
beta.hab.lambda	0.21	0.01	0.18	0.19	0.21	0.22	0.23	4	0	1.00

	mean	sd	2.5%	25%	50%	75%	97.5%	n.eff	overlap0	f
beta.hab.gamma	0.20	0.22	-0.13	-0.10	0.35	0.36	0.37	3	1	0.67
beta.hab.phi	0.49	0.86	-0.15	-0.13	-0.11	1.54	1.92	3	1	0.33
beta.elev.lambda	-0.35	0.01	-0.38	-0.36	-0.35	-0.34	-0.33	4	0	1.00
beta.ele.gamma2	-0.59	0.01	-0.61	-0.60	-0.59	-0.58	-0.55	7	0	1.00
beta.elev.phi	0.11	0.06	0.01	0.04	0.14	0.15	0.16	3	0	0.99
beta.develop.lambda	-0.11	0.02	-0.15	-0.12	-0.11	-0.10	-0.08	35	0	1.00
beta.develop.gamma	-0.38	0.02	-0.42	-0.39	-0.38	-0.37	-0.34	47	0	1.00
beta.develop.phi	0.04	0.03	0.00	0.03	0.05	0.06	0.09	5	1	0.96
beta.effort	0.02	0.01	0.01	0.02	0.02	0.03	0.03	4	0	1.00
beta.min_temp	0.00	0.00	0.00	0.00	0.00	0.00	0.00	47	0	1.00

Table 143: Palm Warbler

	mean	sd	2.5%	25%	50%	75%	97.5%	n.eff	overlap0	f
alpha.lambda	3.51	0.29	3.22	3.27	3.36	3.90	3.95	3	0	1
alpha.phi	5.04	1.45	2.97	3.00	6.03	6.07	6.13	3	0	1
alpha.p	-1.02	0.04	-1.08	-1.07	-1.02	-0.97	-0.96	3	0	1
beta.hab.lambda	-1.62	0.02	-1.67	-1.64	-1.62	-1.61	-1.58	38	0	1
beta.hab.gamma	-0.62	0.07	-0.73	-0.71	-0.59	-0.56	-0.55	3	0	1
beta.hab.phi	-0.23	0.11	-0.34	-0.31	-0.29	-0.09	-0.07	3	0	1
beta.elev.lambda	-0.75	0.17	-0.90	-0.88	-0.85	-0.51	-0.50	3	0	1
beta.ele.gamma2	-0.82	0.32	-1.07	-1.05	-1.03	-0.37	-0.36	3	0	1
beta.elev.phi	1.71	1.09	0.16	0.19	2.46	2.49	2.53	3	0	1
beta.develop.lambda	0.53	0.25	0.16	0.19	0.67	0.72	0.75	3	0	1
beta.develop.gamma	0.73	0.47	0.06	0.08	1.05	1.06	1.08	3	0	1
beta.develop.phi	1.78	2.35	0.08	0.10	0.13	5.06	5.18	3	0	1
beta.effort	0.00	0.00	0.00	0.00	0.00	0.00	0.00	1	0	1
beta.min_temp	0.00	0.00	0.00	0.00	0.00	0.00	0.00	1	0	1

Table 144: Pine Warbler

	mean	sd	2.5%	25%	50%	75%	97.5%	n.eff	overlap0	f
alpha.lambda	3.80	0.03	3.75	3.78	3.80	3.83	3.86	5	0	1.00
alpha.phi	2.95	0.01	2.91	2.94	2.95	2.96	2.97	15	0	1.00
alpha.p	-1.15	0.01	-1.16	-1.15	-1.15	-1.14	-1.13	18	0	1.00
beta.hab.lambda	0.21	0.02	0.17	0.19	0.21	0.22	0.25	71	0	1.00
beta.hab.gamma	0.35	0.01	0.34	0.35	0.35	0.36	0.37	63	0	1.00
beta.hab.phi	-0.08	0.01	-0.10	-0.09	-0.08	-0.07	-0.06	98	0	1.00
beta.elev.lambda	-0.17	0.02	-0.21	-0.18	-0.17	-0.16	-0.13	22	0	1.00
beta.ele.gamma2	-0.34	0.01	-0.36	-0.35	-0.34	-0.33	-0.31	9	0	1.00
beta.elev.phi	0.00	0.02	-0.05	-0.02	0.00	0.01	0.04	9	1	0.50
beta.develop.lambda	-0.09	0.04	-0.18	-0.12	-0.09	-0.06	-0.01	27	0	0.99
beta.develop.gamma	0.54	0.02	0.51	0.53	0.54	0.55	0.58	138	0	1.00
beta.develop.phi	0.40	0.02	0.35	0.38	0.40	0.41	0.44	104	0	1.00
beta.effort	0.00	0.00	0.00	0.00	0.00	0.00	0.00	998	0	1.00
beta.min_temp	0.00	0.00	0.00	0.00	0.00	0.00	0.00	1	0	1.00

Table 145: Red-breasted Nuthatch

	mean	sd	2.5%	25%	50%	75%	97.5%	n.eff	overlap0	f
alpha.lambda	4.15	0.02	4.13	4.14	4.15	4.17	4.19	8	0	1
alpha.phi	3.45	0.02	3.40	3.43	3.45	3.47	3.49	9	0	1
alpha.p	-1.99	0.01	-2.01	-2.00	-1.99	-1.99	-1.98	11	0	1
beta.hab.lambda	0.37	0.01	0.36	0.37	0.37	0.38	0.39	60	0	1
beta.hab.gamma	0.38	0.01	0.35	0.38	0.38	0.39	0.40	7	0	1
beta.hab.phi	-0.34	0.02	-0.37	-0.35	-0.34	-0.33	-0.30	7	0	1
beta.elev.lambda	0.40	0.02	0.36	0.38	0.40	0.41	0.44	8	0	1
beta.ele.gamma2	-0.32	0.01	-0.36	-0.33	-0.32	-0.31	-0.30	10	0	1
beta.elev.phi	0.40	0.02	0.37	0.38	0.39	0.41	0.43	8	0	1
beta.develop.lambda	0.37	0.01	0.34	0.36	0.37	0.38	0.39	14	0	1
beta.develop.gamma	0.12	0.01	0.09	0.11	0.12	0.13	0.14	297	0	1
beta.develop.phi	-0.08	0.02	-0.11	-0.09	-0.08	-0.07	-0.04	41	0	1
beta.effort	0.00	0.00	0.00	0.00	0.00	0.00	0.00	6000	0	1
beta.min_temp	-0.04	0.00	-0.04	-0.04	-0.04	-0.04	-0.04	102	0	1

Table 146: Red-throated Loon

	mean	sd	2.5%	25%	50%	75%	97.5%	n.eff	overlap0	f
alpha.lambda	4.87	0.13	4.63	4.72	4.93	4.98	5.04	3	0	1.00
alpha.phi	1.94	0.15	1.77	1.81	1.88	2.14	2.16	3	0	1.00
alpha.p	-2.55	0.09	-2.70	-2.65	-2.51	-2.48	-2.46	3	0	1.00
beta.hab.lambda	-0.60	0.29	-1.04	-0.99	-0.49	-0.33	-0.29	3	0	1.00
beta.hab.gamma	0.08	0.26	-0.23	-0.21	0.04	0.41	0.43	3	1	0.67
beta.hab.phi	1.63	0.94	0.33	0.36	1.95	2.53	2.65	3	0	1.00
beta.elev.lambda	-0.33	0.13	-0.46	-0.43	-0.41	-0.17	-0.14	3	0	1.00
beta.ele.gamma2	-0.40	0.31	-0.65	-0.64	-0.60	0.03	0.05	3	1	0.67
beta.elev.phi	0.53	0.80	-0.61	-0.58	1.01	1.17	1.19	3	1	0.67
beta.develop.lambda	-1.24	0.71	-1.90	-1.78	-1.67	-0.28	-0.18	3	0	1.00
beta.develop.gamma	-0.02	0.54	-0.50	-0.46	-0.33	0.74	0.75	3	1	0.67
beta.develop.phi	-0.53	1.26	-2.32	-2.28	0.12	0.57	0.63	3	1	0.33
beta.effort	0.00	0.00	0.00	0.00	0.00	0.00	0.00	1	0	1.00
beta.min_temp	0.00	0.00	0.00	0.00	0.00	0.00	0.00	1	0	1.00

Table 147: Ring-necked Pheasant

	mean	sd	2.5%	25%	50%	75%	97.5%	n.eff	overlap0	f
alpha.lambda	5.85	0.01	5.84	5.85	5.85	5.86	5.87	46	0	1
alpha.phi	2.32	0.01	2.31	2.31	2.32	2.32	2.33	46	0	1
alpha.p	-1.56	0.01	-1.57	-1.56	-1.56	-1.56	-1.55	28	0	1
beta.hab.lambda	-0.30	0.01	-0.31	-0.30	-0.30	-0.30	-0.29	56	0	1
beta.hab.gamma	-0.21	0.01	-0.22	-0.21	-0.21	-0.21	-0.20	45	0	1
beta.hab.phi	0.37	0.01	0.35	0.37	0.37	0.38	0.38	54	0	1
beta.elev.lambda	0.11	0.00	0.10	0.11	0.11	0.12	0.12	31	0	1
beta.ele.gamma2	0.77	0.01	0.76	0.77	0.77	0.78	0.79	52	0	1
beta.elev.phi	-0.06	0.00	-0.07	-0.07	-0.06	-0.06	-0.05	42	0	1
beta.develop.lambda	0.18	0.01	0.17	0.17	0.18	0.18	0.19	93	0	1
beta.develop.gamma	0.12	0.02	0.08	0.11	0.12	0.14	0.16	128	0	1

	mean	sd	2.5%	25%	50%	75%	97.5%	n.eff	overlap0	f
beta.develop.phi	0.11	0.01	0.10	0.11	0.11	0.12	0.12	120	0	1
beta.effort	0.01	0.00	0.01	0.01	0.01	0.01	0.01	577	0	1
beta.min_temp	-0.22	0.00	-0.22	-0.22	-0.22	-0.22	-0.22	352	0	1

Table 148: Spotted Towhee

	mean	sd	2.5%	25%	50%	75%	97.5%	n.eff	overlap0	f
alpha.lambda	-7.97	1.19	-10.35	-8.88	-7.93	-7.06	-5.92	18	0	1.00
alpha.phi	4.53	0.21	4.29	4.38	4.45	4.76	4.91	3	0	1.00
alpha.p	-0.91	0.12	-1.07	-1.07	-0.83	-0.82	-0.81	3	0	1.00
beta.hab.lambda	-1.33	1.70	-4.93	-2.72	-1.07	0.06	1.39	5	1	0.73
beta.hab.gamma	0.20	0.02	0.17	0.18	0.21	0.22	0.23	3	0	1.00
beta.hab.phi	-0.88	0.03	-0.94	-0.90	-0.88	-0.86	-0.84	4	0	1.00
beta.elev.lambda	0.95	1.02	-0.75	0.11	0.84	1.74	2.98	22	1	0.79
beta.ele.gamma2	-0.49	0.01	-0.51	-0.50	-0.48	-0.48	-0.47	4	0	1.00
beta.elev.phi	0.79	0.07	0.72	0.74	0.76	0.87	0.91	3	0	1.00
beta.develop.lambda	-0.70	1.86	-4.44	-2.04	-0.61	0.67	2.64	8	1	0.64
beta.develop.gamma	0.08	0.02	0.05	0.06	0.07	0.09	0.10	4	0	1.00
beta.develop.phi	0.29	0.07	0.16	0.23	0.28	0.34	0.41	4	0	1.00
beta.effort	0.09	0.01	0.07	0.08	0.08	0.10	0.10	3	0	1.00
beta.min_temp	-0.08	0.00	-0.09	-0.08	-0.08	-0.08	-0.07	34	0	1.00

Table 149: Steller's Jay

	mean	sd	2.5%	25%	50%	75%	97.5%	n.eff	overlap0	f
alpha.lambda	6.34	0.87	5.70	5.72	5.74	7.52	7.66	3	0	1.00
alpha.phi	1.24	2.73	-2.82	-2.49	3.14	3.19	3.22	3	1	0.67
alpha.p	-2.24	1.15	-3.94	-3.81	-1.43	-1.42	-1.40	3	0	1.00
beta.hab.lambda	0.24	0.03	0.20	0.22	0.22	0.26	0.31	4	0	1.00
beta.hab.gamma	-0.06	0.26	-0.26	-0.24	-0.23	0.30	0.31	3	1	0.67
beta.hab.phi	1.13	0.52	0.38	0.40	1.47	1.52	1.57	3	0	1.00
beta.elev.lambda	0.16	0.10	-0.01	0.04	0.22	0.23	0.24	3	1	0.94
beta.ele.gamma2	-0.29	0.01	-0.31	-0.30	-0.29	-0.28	-0.26	4	0	1.00
beta.elev.phi	0.49	1.07	-0.30	-0.27	-0.25	1.87	2.18	3	1	0.33
beta.develop.lambda	0.44	0.06	0.33	0.38	0.47	0.48	0.50	3	0	1.00
beta.develop.gamma	0.33	0.05	0.26	0.29	0.31	0.40	0.41	3	0	1.00
beta.develop.phi	0.35	0.08	0.24	0.28	0.31	0.45	0.48	3	0	1.00
beta.effort	0.02	0.01	0.01	0.01	0.03	0.03	0.04	3	0	1.00
beta.min_temp	0.00	0.00	-0.01	0.00	0.00	0.00	0.00	6	0	1.00

Table 150: Vesper Sparrow

	mean	sd	2.5%	25%	50%	75%	97.5%	n.eff	overlap0	f
alpha.lambda	5.18	0.02	5.15	5.16	5.18	5.19	5.21	13	0	1
alpha.phi	5.32	0.37	4.66	5.00	5.35	5.64	5.91	177	0	1
alpha.p	-1.82	0.01	-1.83	-1.82	-1.82	-1.81	-1.80	13	0	1
beta.hab.lambda	-0.11	0.01	-0.13	-0.12	-0.11	-0.09	-0.08	201	0	1

	mean	sd	2.5%	25%	50%	75%	97.5%	n.eff	overlap0	f
beta.hab.gamma	-0.96	0.01	-0.98	-0.97	-0.96	-0.95	-0.94	6	0	1
beta.hab.phi	5.42	0.77	4.05	4.78	5.49	6.10	6.57	250	0	1
beta.elev.lambda	0.09	0.01	0.07	0.08	0.09	0.09	0.10	256	0	1
beta.ele.gamma2	0.05	0.02	0.02	0.03	0.05	0.06	0.08	72	0	1
beta.elev.phi	0.26	0.01	0.24	0.26	0.26	0.27	0.29	31	0	1
beta.develop.lambda	0.42	0.03	0.37	0.40	0.42	0.45	0.48	15	0	1
beta.develop.gamma	-7.65	0.11	-7.85	-7.74	-7.66	-7.56	-7.47	415	0	1
beta.develop.phi	2.94	0.17	2.60	2.80	2.94	3.08	3.23	175	0	1
beta.effort	0.05	0.00	0.04	0.04	0.05	0.05	0.05	14	0	1
beta.min_temp	0.00	0.00	0.00	0.00	0.00	0.00	0.00	1	0	1

Table 151: Western Scrub-Jay

	mean	sd	2.5%	25%	50%	75%	97.5%	n.eff	overlap0	f
alpha.lambda	-8.68	1.79	-12.62	-9.85	-8.53	-7.30	-5.81	40	0	1.00
alpha.phi	5.13	2.78	3.14	3.16	3.17	8.99	9.26	3	0	1.00
alpha.p	-0.71	0.71	-1.72	-1.72	-0.22	-0.21	-0.20	3	0	1.00
beta.hab.lambda	-0.62	1.38	-3.23	-1.52	-0.58	0.45	1.71	45	1	0.63
beta.hab.gamma	-0.22	0.05	-0.31	-0.30	-0.19	-0.18	-0.18	3	0	1.00
beta.hab.phi	2.07	2.68	0.14	0.17	0.18	5.75	5.99	3	0	1.00
beta.elev.lambda	0.55	1.42	-2.13	-0.56	0.55	1.75	3.08	551	1	0.60
beta.ele.gamma2	-0.55	0.07	-0.61	-0.61	-0.60	-0.45	-0.44	3	0	1.00
beta.elev.phi	0.38	0.25	-0.02	0.06	0.55	0.56	0.57	3	1	0.92
beta.develop.lambda	-0.48	1.74	-3.64	-1.84	-0.48	0.84	2.83	25	1	0.59
beta.develop.gamma	0.42	0.06	0.31	0.35	0.46	0.47	0.48	3	0	1.00
beta.develop.phi	-0.98	1.34	-3.52	-2.37	-0.08	-0.06	-0.04	3	0	1.00
beta.effort	0.02	0.00	0.01	0.01	0.02	0.02	0.02	11	0	1.00
beta.min_temp	0.00	0.00	0.00	0.00	0.00	0.00	0.00	10	0	1.00

Table 152: Wood Duck

	mean	sd	2.5%	25%	50%	75%	97.5%	n.eff	overlap0	f
alpha.lambda	4.25	0.02	4.21	4.23	4.25	4.26	4.29	35	0	1
alpha.phi	1.97	0.01	1.96	1.96	1.97	1.97	1.98	33	0	1
alpha.p	-1.72	0.00	-1.73	-1.73	-1.73	-1.72	-1.72	21	0	1
beta.hab.lambda	-0.83	0.02	-0.86	-0.84	-0.83	-0.82	-0.80	144	0	1
beta.hab.gamma	-0.31	0.01	-0.32	-0.31	-0.31	-0.30	-0.29	210	0	1
beta.hab.phi	0.07	0.01	0.06	0.07	0.07	0.08	0.09	215	0	1
beta.elev.lambda	-0.67	0.01	-0.69	-0.68	-0.67	-0.67	-0.65	159	0	1
beta.ele.gamma2	-0.37	0.01	-0.38	-0.38	-0.37	-0.37	-0.36	104	0	1
beta.elev.phi	-0.11	0.01	-0.12	-0.12	-0.11	-0.11	-0.10	71	0	1
beta.develop.lambda	-0.63	0.04	-0.71	-0.66	-0.63	-0.61	-0.55	510	0	1
beta.develop.gamma	-0.63	0.01	-0.64	-0.63	-0.63	-0.62	-0.61	45	0	1
beta.develop.phi	0.89	0.01	0.87	0.89	0.89	0.90	0.91	39	0	1
beta.effort	0.01	0.00	0.01	0.01	0.01	0.01	0.01	70	0	1
beta.min_temp	0.00	0.00	0.00	0.00	0.00	0.00	0.00	1	0	1

Table 153: Yellow-headed Blackbird

	mean	sd	2.5%	25%	50%	75%	97.5%	n.eff	overlap0	f
alpha.lambda	4.91	0.30	4.31	4.71	4.91	5.15	5.41	4	0	1.00
alpha.phi	-13.66	1.37	-16.34	-14.89	-13.23	-12.57	-11.90	3	0	1.00
alpha.p	-4.26	0.39	-4.75	-4.66	-4.33	-3.78	-3.72	3	0	1.00
beta.hab.lambda	-0.82	1.08	-2.37	-1.61	-1.25	0.49	0.82	3	1	0.67
beta.hab.gamma	-2.69	0.05	-2.78	-2.74	-2.69	-2.64	-2.62	3	0	1.00
beta.hab.phi	-22.34	3.19	-28.22	-25.81	-20.66	-19.86	-18.98	3	0	1.00
beta.elev.lambda	0.93	0.88	0.02	0.27	0.40	2.10	2.31	3	0	0.98
beta.ele.gamma2	-0.13	0.01	-0.15	-0.15	-0.12	-0.12	-0.11	3	0	1.00
beta.elev.phi	2.91	0.41	2.26	2.46	2.99	3.23	3.59	3	0	1.00
beta.develop.lambda	-0.13	0.55	-0.98	-0.83	0.09	0.31	0.58	3	1	0.35
beta.develop.gamma	-0.24	0.13	-0.40	-0.38	-0.28	-0.07	-0.06	3	0	1.00
beta.develop.phi	0.22	2.33	-3.28	-2.94	1.86	1.88	1.90	3	1	0.67
beta.effort	0.36	0.02	0.33	0.33	0.35	0.38	0.38	3	0	1.00
beta.min_temp	0.00	0.00	0.00	0.00	0.00	0.00	0.00	1	0	1.00

Table 154: Anhinga

	mean	sd	2.5%	25%	50%	75%	97.5%	n.eff	overlap0	f
alpha.lambda	5.49	0.38	5.17	5.20	5.23	6.02	6.04	3	0	1
alpha.phi	3.42	0.40	3.07	3.12	3.16	3.97	4.01	3	0	1
alpha.p	-1.21	0.44	-1.83	-1.82	-0.92	-0.88	-0.85	3	0	1
beta.hab.lambda	0.43	0.03	0.38	0.41	0.43	0.45	0.48	5	0	1
beta.hab.gamma	0.22	0.06	0.11	0.15	0.25	0.27	0.29	3	0	1
beta.hab.phi	-1.00	0.10	-1.19	-1.11	-0.96	-0.93	-0.88	3	0	1
beta.elev.lambda	0.75	0.04	0.69	0.72	0.75	0.78	0.81	5	0	1
beta.ele.gamma2	0.17	0.06	0.05	0.09	0.20	0.22	0.24	3	0	1
beta.elev.phi	-2.13	0.44	-2.85	-2.70	-1.85	-1.81	-1.72	3	0	1
beta.develop.lambda	0.91	0.03	0.84	0.89	0.91	0.93	0.96	8	0	1
beta.develop.gamma	0.62	0.26	0.39	0.43	0.46	0.97	1.00	3	0	1
beta.develop.phi	2.48	0.12	2.22	2.35	2.53	2.57	2.64	3	0	1
beta.effort	0.00	0.00	0.00	0.00	0.00	0.00	0.00	21	0	1
beta.min_temp	0.00	0.00	0.00	0.00	0.00	0.00	0.00	298	0	1

Table 155: Bewick's Wren

	mean	sd	2.5%	25%	50%	75%	97.5%	n.eff	overlap0	f
alpha.lambda	4.47	0.02	4.44	4.46	4.47	4.48	4.50	9	0	1.00
alpha.phi	3.20	0.02	3.16	3.19	3.20	3.21	3.25	5	0	1.00
alpha.p	-1.03	0.01	-1.05	-1.04	-1.03	-1.02	-1.01	4	0	1.00
beta.hab.lambda	0.01	0.02	-0.02	0.00	0.01	0.02	0.04	25	1	0.74
beta.hab.gamma	0.23	0.02	0.19	0.21	0.23	0.24	0.26	404	0	1.00
beta.hab.phi	0.31	0.02	0.27	0.30	0.31	0.32	0.34	478	0	1.00
beta.elev.lambda	-0.07	0.01	-0.09	-0.08	-0.07	-0.06	-0.05	26	0	1.00
beta.ele.gamma2	-0.04	0.02	-0.08	-0.06	-0.04	-0.03	-0.01	7	0	0.99
beta.elev.phi	-0.22	0.03	-0.27	-0.24	-0.22	-0.19	-0.16	6	0	1.00
beta.develop.lambda	0.11	0.02	0.07	0.10	0.11	0.12	0.14	430	0	1.00
beta.develop.gamma	0.68	0.02	0.64	0.66	0.68	0.69	0.72	154	0	1.00

	mean	sd	2.5%	25%	50%	75%	97.5%	n.eff	overlap0	f
beta.develop.phi	-0.11	0.03	-0.18	-0.13	-0.11	-0.09	-0.05	22	0	1.00
beta.effort	0.04	0.00	0.04	0.04	0.04	0.05	0.05	18	0	1.00
beta.min_temp	0.00	0.00	0.00	0.00	0.00	0.00	0.00	6000	0	1.00

Table 156: Brewer's Sparrow

	mean	sd	2.5%	25%	50%	75%	97.5%	n.eff	overlap0	f
alpha.lambda	-6.32	9.36	-19.22	-18.15	-4.57	3.87	4.81	3	1	0.67
alpha.phi	0.73	0.81	-0.05	0.10	0.25	1.85	1.89	3	1	0.95
alpha.p	-4.24	0.88	-4.91	-4.89	-4.83	-3.00	-2.96	3	0	1.00
beta.hab.lambda	12.51	7.67	3.22	4.03	11.37	22.09	22.96	3	0	1.00
beta.hab.gamma	0.19	0.77	-0.53	-0.50	-0.18	1.19	1.36	3	1	0.33
beta.hab.phi	0.56	1.45	-1.58	-1.35	1.17	1.91	1.97	3	1	0.67
beta.elev.lambda	-4.54	3.24	-9.32	-8.95	-2.64	-2.04	-1.54	3	0	1.00
beta.ele.gamma2	0.22	0.75	-0.48	-0.38	-0.19	1.26	1.29	3	1	0.33
beta.elev.phi	0.12	1.47	-1.99	-1.92	0.88	1.35	1.49	3	1	0.67
beta.develop.lambda	8.69	4.73	2.66	3.13	8.59	14.22	15.06	3	0	1.00
beta.develop.gamma	-0.54	1.48	-2.35	-2.29	-0.62	1.30	1.33	3	1	0.67
beta.develop.phi	1.53	1.79	-1.00	-0.94	2.43	3.06	3.22	3	1	0.67
beta.effort	0.36	0.02	0.33	0.34	0.35	0.38	0.39	3	0	1.00
beta.min_temp	0.00	0.00	0.00	0.00	0.00	0.00	0.00	6	0	1.00

Table 157: Brown-headed Nuthatch

	mean	sd	2.5%	25%	50%	75%	97.5%	n.eff	overlap0	f
alpha.lambda	4.43	0.02	4.39	4.42	4.43	4.45	4.48	57	0	1.00
alpha.phi	2.78	0.02	2.74	2.77	2.78	2.80	2.83	12	0	1.00
alpha.p	-0.88	0.02	-0.91	-0.89	-0.88	-0.87	-0.85	9	0	1.00
beta.hab.lambda	0.16	0.02	0.12	0.15	0.16	0.18	0.20	68	0	1.00
beta.hab.gamma	-0.06	0.02	-0.09	-0.07	-0.06	-0.05	-0.03	95	0	1.00
beta.hab.phi	-0.10	0.02	-0.13	-0.11	-0.10	-0.09	-0.06	109	0	1.00
beta.elev.lambda	-0.09	0.02	-0.13	-0.10	-0.09	-0.08	-0.05	76	0	1.00
beta.ele.gamma2	0.13	0.02	0.09	0.11	0.13	0.14	0.16	47	0	1.00
beta.elev.phi	-0.04	0.02	-0.08	-0.05	-0.04	-0.03	-0.01	51	0	0.99
beta.develop.lambda	-0.46	0.04	-0.53	-0.49	-0.46	-0.44	-0.39	471	0	1.00
beta.develop.gamma	-1.31	0.06	-1.43	-1.35	-1.31	-1.26	-1.18	36	0	1.00
beta.develop.phi	0.45	0.06	0.34	0.41	0.46	0.50	0.56	30	0	1.00
beta.effort	0.01	0.00	0.00	0.00	0.00	0.01	0.01	6000	0	1.00
beta.min_temp	0.00	0.00	0.00	0.00	0.00	0.00	0.00	6000	0	1.00

Table 158: Brown Thrasher

	mean	sd	2.5%	25%	50%	75%	97.5%	n.eff	overlap0	f
alpha.lambda	3.49	0.03	3.44	3.47	3.49	3.51	3.54	5	0	1
alpha.phi	2.97	0.04	2.88	2.93	2.98	3.00	3.04	4	0	1
alpha.p	-1.06	0.03	-1.10	-1.08	-1.06	-1.03	-1.01	3	0	1
beta.hab.lambda	-0.18	0.04	-0.27	-0.21	-0.18	-0.15	-0.10	11	0	1

	mean	sd	2.5%	25%	50%	75%	97.5%	n.eff	overlap0	f
beta.hab.gamma	0.20	0.06	0.10	0.16	0.19	0.24	0.32	4	0	1
beta.hab.phi	-0.52	0.08	-0.67	-0.58	-0.50	-0.46	-0.41	4	0	1
beta.elev.lambda	-0.50	0.01	-0.52	-0.51	-0.50	-0.50	-0.49	24	0	1
beta.ele.gamma2	-0.46	0.01	-0.50	-0.47	-0.46	-0.45	-0.44	25	0	1
beta.elev.phi	0.20	0.02	0.17	0.19	0.19	0.21	0.23	10	0	1
beta.develop.lambda	0.64	0.02	0.60	0.63	0.64	0.66	0.69	64	0	1
beta.develop.gamma	-0.28	0.04	-0.36	-0.31	-0.29	-0.26	-0.21	42	0	1
beta.develop.phi	0.19	0.03	0.13	0.17	0.19	0.21	0.26	20	0	1
beta.effort	0.03	0.00	0.02	0.02	0.03	0.03	0.04	88	0	1
beta.min_temp	0.00	0.00	0.00	0.00	0.00	0.00	0.00	234	0	1

Table 159: Common Yellowthroat

	mean	sd	2.5%	25%	50%	75%	97.5%	n.eff	overlap0	f
alpha.lambda	4.44	0.40	4.13	4.16	4.17	4.99	5.02	3	0	1
alpha.phi	3.51	0.56	3.02	3.08	3.17	4.20	4.45	3	0	1
alpha.p	-1.18	0.41	-1.77	-1.76	-0.90	-0.88	-0.87	3	0	1
beta.hab.lambda	-0.16	0.03	-0.21	-0.18	-0.15	-0.14	-0.12	4	0	1
beta.hab.gamma	0.18	0.10	0.10	0.11	0.12	0.31	0.33	3	0	1
beta.hab.phi	2.98	1.27	1.88	2.00	2.22	4.47	5.17	3	0	1
beta.elev.lambda	-0.75	0.05	-0.80	-0.79	-0.78	-0.69	-0.67	3	0	1
beta.ele.gamma2	-0.44	0.04	-0.48	-0.47	-0.46	-0.39	-0.38	3	0	1
beta.elev.phi	-0.24	0.03	-0.30	-0.28	-0.23	-0.22	-0.21	3	0	1
beta.develop.lambda	0.99	0.03	0.94	0.96	1.00	1.01	1.03	3	0	1
beta.develop.gamma	0.36	0.01	0.33	0.35	0.36	0.37	0.38	9	0	1
beta.develop.phi	-0.30	0.02	-0.33	-0.31	-0.29	-0.28	-0.26	4	0	1
beta.effort	0.01	0.01	0.00	0.00	0.01	0.02	0.02	3	0	1
beta.min_temp	0.00	0.00	0.00	0.00	0.00	0.00	0.00	1	0	1

Table 160: Cooper's Hawk

	mean	sd	2.5%	25%	50%	75%	97.5%	n.eff	overlap0	f
alpha.lambda	1.42	0.03	1.36	1.40	1.42	1.44	1.49	37	0	1.00
alpha.phi	3.55	0.05	3.45	3.51	3.55	3.59	3.66	4	0	1.00
alpha.p	-0.95	0.03	-1.01	-0.98	-0.94	-0.93	-0.91	5	0	1.00
beta.hab.lambda	-0.33	0.03	-0.39	-0.35	-0.33	-0.30	-0.26	53	0	1.00
beta.hab.gamma	-0.16	0.02	-0.20	-0.17	-0.16	-0.15	-0.13	13	0	1.00
beta.hab.phi	0.09	0.03	0.03	0.07	0.09	0.11	0.16	9	0	1.00
beta.elev.lambda	0.00	0.03	-0.05	-0.02	0.00	0.02	0.06	24	1	0.53
beta.ele.gamma2	-0.04	0.01	-0.06	-0.05	-0.04	-0.03	-0.01	58	0	1.00
beta.elev.phi	-0.11	0.02	-0.16	-0.13	-0.11	-0.10	-0.07	888	0	1.00
beta.develop.lambda	0.13	0.05	0.04	0.10	0.13	0.16	0.22	100	0	1.00
beta.develop.gamma	0.12	0.02	0.09	0.11	0.12	0.13	0.15	9	0	1.00
beta.develop.phi	0.39	0.09	0.20	0.34	0.40	0.45	0.54	5	0	1.00
beta.effort	0.02	0.00	0.01	0.02	0.02	0.03	0.03	6000	0	1.00
beta.min_temp	-0.02	0.01	-0.03	-0.02	-0.02	-0.02	-0.01	7	0	1.00

Table 161: Eastern Screech-Owl

	mean	sd	2.5%	25%	50%	75%	97.5%	n.eff	overlap0	f
alpha.lambda	-7.20	0.97	-9.34	-7.91	-7.20	-6.41	-5.54	5	0	1.00
alpha.phi	2.67	0.02	2.63	2.66	2.67	2.68	2.71	7	0	1.00
alpha.p	-0.67	0.01	-0.69	-0.68	-0.67	-0.66	-0.64	7	0	1.00
beta.hab.lambda	-0.87	0.70	-2.26	-1.36	-0.82	-0.38	0.36	6	1	0.88
beta.hab.gamma	0.08	0.01	0.05	0.07	0.08	0.08	0.10	54	0	1.00
beta.hab.phi	0.09	0.02	0.05	0.07	0.09	0.10	0.12	54	0	1.00
beta.elev.lambda	0.53	0.61	-0.63	0.12	0.53	0.95	1.69	10	1	0.80
beta.ele.gamma2	-0.22	0.01	-0.24	-0.23	-0.22	-0.21	-0.20	45	0	1.00
beta.elev.phi	0.02	0.02	-0.01	0.01	0.02	0.04	0.06	75	1	0.93
beta.develop.lambda	-0.28	1.54	-3.43	-1.32	-0.38	0.81	2.72	31	1	0.58
beta.develop.gamma	0.14	0.02	0.09	0.12	0.14	0.15	0.17	5	0	1.00
beta.develop.phi	-0.46	0.04	-0.53	-0.49	-0.47	-0.43	-0.39	4	0	1.00
beta.effort	0.01	0.00	0.00	0.01	0.01	0.01	0.02	74	0	1.00
beta.min_temp	-0.04	0.00	-0.04	-0.04	-0.04	-0.03	-0.03	93	0	1.00

Table 162: Gambel's Quail

	mean	sd	2.5%	25%	50%	75%	97.5%	n.eff	overlap0	f
alpha.lambda	5.96	0.02	5.91	5.95	5.96	5.98	6.01	11	0	1
alpha.phi	2.52	0.02	2.49	2.51	2.52	2.54	2.55	14	0	1
alpha.p	-0.98	0.01	-1.00	-0.99	-0.98	-0.97	-0.95	8	0	1
beta.hab.lambda	0.50	0.02	0.45	0.49	0.50	0.51	0.54	17	0	1
beta.hab.gamma	0.98	0.01	0.95	0.97	0.98	0.99	1.00	83	0	1
beta.hab.phi	-1.29	0.02	-1.34	-1.31	-1.29	-1.27	-1.25	41	0	1
beta.elev.lambda	-0.31	0.02	-0.34	-0.32	-0.31	-0.29	-0.27	15	0	1
beta.ele.gamma2	-0.20	0.01	-0.22	-0.21	-0.20	-0.19	-0.18	24	0	1
beta.elev.phi	0.23	0.02	0.19	0.22	0.23	0.24	0.26	21	0	1
beta.develop.lambda	0.40	0.05	0.31	0.37	0.40	0.44	0.50	168	0	1
beta.develop.gamma	1.30	0.02	1.25	1.28	1.30	1.31	1.34	459	0	1
beta.develop.phi	-1.48	0.04	-1.56	-1.50	-1.48	-1.45	-1.39	185	0	1
beta.effort	0.00	0.00	0.00	0.00	0.00	0.00	0.00	6000	0	1
beta.min_temp	0.00	0.00	0.00	0.00	0.00	0.00	0.00	6000	0	1

Table 163: Glossy Ibis

	mean	sd	2.5%	25%	50%	75%	97.5%	n.eff	overlap0	f
alpha.lambda	5.44	0.07	5.31	5.39	5.45	5.49	5.55	4	0	1.0
alpha.phi	2.49	0.06	2.42	2.45	2.46	2.56	2.61	3	0	1.0
alpha.p	-1.53	0.03	-1.57	-1.56	-1.52	-1.50	-1.49	3	0	1.0
beta.hab.lambda	-0.54	0.09	-0.71	-0.62	-0.54	-0.45	-0.40	3	0	1.0
beta.hab.gamma	-1.04	0.09	-1.13	-1.11	-1.09	-0.91	-0.89	3	0	1.0
beta.hab.phi	4.49	0.29	4.01	4.12	4.65	4.70	4.80	3	0	1.0
beta.elev.lambda	-0.15	0.06	-0.27	-0.21	-0.15	-0.11	-0.04	4	0	1.0
beta.ele.gamma2	0.20	0.02	0.16	0.17	0.19	0.22	0.24	4	0	1.0
beta.elev.phi	0.32	0.05	0.24	0.27	0.32	0.35	0.39	4	0	1.0
beta.develop.lambda	-0.38	0.04	-0.46	-0.41	-0.39	-0.36	-0.30	5	0	1.0
beta.develop.gamma	0.05	0.07	-0.03	-0.01	0.02	0.14	0.18	3	1	0.7

	mean	sd	2.5%	25%	50%	75%	97.5%	n.eff	overlap0	f
beta.develop.phi	2.41	0.30	1.90	2.05	2.54	2.67	2.73	3	0	1.0
beta.effort	0.00	0.00	0.00	0.00	0.00	0.00	0.00	1394	0	1.0
beta.min_temp	0.00	0.00	0.00	0.00	0.00	0.00	0.00	931	0	1.0

Table 164: Gray-crowned Rosy-Finch

	mean	sd	2.5%	25%	50%	75%	97.5%	n.eff	overlap0	f
alpha.lambda	9.65	0.18	9.35	9.45	9.69	9.80	9.89	3	0	1.00
alpha.phi	-1.10	1.00	-2.33	-2.29	-1.14	0.09	0.23	3	1	0.67
alpha.p	-4.56	0.24	-4.82	-4.76	-4.66	-4.26	-4.15	3	0	1.00
beta.hab.lambda	-1.57	0.11	-1.80	-1.66	-1.57	-1.49	-1.38	6	0	1.00
beta.hab.gamma	0.01	0.10	-0.14	-0.13	0.07	0.10	0.10	3	1	0.67
beta.hab.phi	-0.32	0.55	-1.09	-0.89	-0.38	0.37	0.38	3	1	0.67
beta.elev.lambda	0.90	0.19	0.61	0.67	0.94	1.08	1.14	3	0	1.00
beta.ele.gamma2	-0.29	0.06	-0.35	-0.34	-0.31	-0.22	-0.20	3	0	1.00
beta.elev.phi	0.54	0.15	0.31	0.35	0.61	0.66	0.69	3	0	1.00
beta.develop.lambda	1.13	0.40	0.54	0.64	1.24	1.48	1.66	3	0	1.00
beta.develop.gamma	-0.95	0.39	-1.47	-1.40	-0.98	-0.47	-0.44	3	0	1.00
beta.develop.phi	0.52	0.99	-0.87	-0.79	0.86	1.51	1.56	3	1	0.67
beta.effort	0.00	0.00	0.00	0.00	0.00	0.00	0.00	1	0	1.00
beta.min_temp	-0.01	0.00	-0.02	-0.01	-0.01	-0.01	-0.01	4	0	1.00

Table 165: Gray Catbird

	mean	sd	2.5%	25%	50%	75%	97.5%	n.eff	overlap0	f
alpha.lambda	3.70	0.89	2.93	2.98	3.18	4.94	4.99	3	0	1.00
alpha.phi	1.51	0.79	0.33	0.43	1.99	2.11	2.18	3	0	1.00
alpha.p	-1.82	0.63	-2.71	-2.70	-1.45	-1.30	-1.27	3	0	1.00
beta.hab.lambda	-1.33	0.29	-1.59	-1.54	-1.51	-0.94	-0.91	3	0	1.00
beta.hab.gamma	-0.64	0.64	-1.57	-1.53	-0.25	-0.14	-0.09	3	0	1.00
beta.hab.phi	-0.20	1.02	-1.07	-1.00	-0.82	1.21	1.26	3	1	0.67
beta.elev.lambda	-0.23	0.07	-0.30	-0.28	-0.27	-0.13	-0.11	3	0	1.00
beta.ele.gamma2	0.43	0.57	-0.01	0.02	0.05	1.23	1.25	3	1	0.93
beta.elev.phi	-2.21	1.19	-3.95	-3.84	-1.44	-1.30	-1.26	3	0	1.00
beta.develop.lambda	0.45	0.11	0.33	0.36	0.38	0.60	0.63	3	0	1.00
beta.develop.gamma	0.30	0.60	-0.57	-0.55	0.71	0.73	0.75	3	1	0.67
beta.develop.phi	-0.23	0.91	-0.93	-0.90	-0.84	1.04	1.07	3	1	0.67
beta.effort	0.03	0.01	0.01	0.02	0.02	0.03	0.04	4	0	1.00
beta.min_temp	0.00	0.00	0.00	0.00	0.00	0.00	0.00	1	0	1.00

Table 166: Great Horned Owl

	mean	sd	2.5%	25%	50%	75%	97.5%	n.eff	overlap0	f
alpha.lambda	2.89	0.09	2.77	2.81	2.88	2.97	3.05	4	0	1.00
alpha.phi	2.96	0.05	2.88	2.91	2.97	3.01	3.05	4	0	1.00
alpha.p	-1.32	0.09	-1.47	-1.39	-1.32	-1.22	-1.19	4	0	1.00
beta.hab.lambda	-0.16	0.02	-0.20	-0.18	-0.16	-0.15	-0.13	235	0	1.00

	mean	sd	2.5%	25%	50%	75%	97.5%	n.eff	overlap0	f
beta.hab.gamma	-0.01	0.02	-0.05	-0.02	-0.01	0.01	0.04	5	1	0.60
beta.hab.phi	-0.17	0.03	-0.23	-0.19	-0.17	-0.15	-0.11	5	0	1.00
beta.elev.lambda	-0.11	0.01	-0.14	-0.12	-0.11	-0.11	-0.09	12	0	1.00
beta.ele.gamma2	-0.07	0.01	-0.09	-0.08	-0.07	-0.06	-0.05	17	0	1.00
beta.elev.phi	-0.13	0.01	-0.16	-0.14	-0.13	-0.12	-0.10	276	0	1.00
beta.develop.lambda	0.05	0.02	0.01	0.04	0.05	0.07	0.10	6	0	0.99
beta.develop.gamma	-0.10	0.04	-0.17	-0.14	-0.12	-0.06	-0.02	3	0	1.00
beta.develop.phi	-0.45	0.03	-0.50	-0.47	-0.45	-0.43	-0.40	12	0	1.00
beta.effort	0.02	0.00	0.01	0.01	0.02	0.02	0.03	11	0	1.00
beta.min_temp	-0.01	0.00	-0.02	-0.01	-0.01	0.00	0.00	6	0	1.00

Table 167: Greater Yellowlegs

	mean	sd	2.5%	25%	50%	75%	97.5%	n.eff	overlap0	f
alpha.lambda	4.43	0.17	4.26	4.30	4.33	4.66	4.69	3	0	1.00
alpha.phi	3.12	0.14	2.99	3.02	3.04	3.31	3.36	3	0	1.00
alpha.p	-1.81	0.14	-2.03	-1.99	-1.73	-1.71	-1.70	3	0	1.00
beta.hab.lambda	0.10	0.01	0.08	0.09	0.10	0.12	0.13	3	0	1.00
beta.hab.gamma	-0.17	0.02	-0.22	-0.19	-0.17	-0.16	-0.15	4	0	1.00
beta.hab.phi	0.59	0.07	0.50	0.53	0.55	0.67	0.71	3	0	1.00
beta.elev.lambda	-0.37	0.04	-0.42	-0.40	-0.39	-0.33	-0.31	3	0	1.00
beta.ele.gamma2	-0.47	0.01	-0.50	-0.48	-0.47	-0.46	-0.45	328	0	1.00
beta.elev.phi	0.15	0.01	0.13	0.14	0.15	0.16	0.18	86	0	1.00
beta.develop.lambda	0.05	0.06	-0.06	-0.02	0.07	0.10	0.13	3	1	0.68
beta.develop.gamma	-0.23	0.03	-0.28	-0.26	-0.22	-0.20	-0.17	4	0	1.00
beta.develop.phi	-0.37	0.03	-0.44	-0.39	-0.37	-0.35	-0.31	8	0	1.00
beta.effort	0.04	0.00	0.04	0.04	0.04	0.05	0.05	6	0	1.00
beta.min_temp	0.00	0.00	0.00	0.00	0.00	0.00	0.00	778	0	1.00

Table 168: House Wren

	mean	sd	2.5%	25%	50%	75%	97.5%	n.eff	overlap0	f
alpha.lambda	2.97	0.61	2.53	2.59	2.63	3.18	4.33	4	0	1.00
alpha.phi	2.90	0.31	2.67	2.70	2.72	3.04	3.60	4	0	1.00
alpha.p	-1.17	0.41	-2.14	-1.25	-0.94	-0.93	-0.92	4	0	1.00
beta.hab.lambda	-0.92	0.17	-1.09	-1.04	-1.00	-0.77	-0.61	3	0	1.00
beta.hab.gamma	-0.03	0.09	-0.22	-0.02	0.01	0.03	0.06	5	1	0.37
beta.hab.phi	-0.20	0.14	-0.32	-0.28	-0.26	-0.22	0.13	5	1	0.83
beta.elev.lambda	-0.47	0.08	-0.54	-0.52	-0.51	-0.46	-0.29	4	0	1.00
beta.ele.gamma2	-0.27	0.07	-0.36	-0.30	-0.28	-0.27	-0.08	12	0	1.00
beta.elev.phi	-0.07	0.12	-0.39	-0.06	-0.03	-0.01	0.08	11	1	0.84
beta.develop.lambda	0.36	0.06	0.22	0.34	0.37	0.40	0.43	6	0	1.00
beta.develop.gamma	0.06	0.18	-0.43	0.11	0.14	0.16	0.19	6	1	0.81
beta.develop.phi	0.12	0.35	-0.13	-0.06	-0.03	0.02	1.13	6	1	0.35
beta.effort	0.01	0.00	0.00	0.00	0.01	0.01	0.02	35	0	1.00
beta.min_temp	0.00	0.00	0.00	0.00	0.00	0.00	0.00	1	0	1.00

Table 169: Lark Bunting

	mean	sd	2.5%	25%	50%	75%	97.5%	n.eff	overlap0	f
alpha.lambda	9.84	0.13	9.74	9.75	9.76	10.01	10.04	3	0	1
alpha.phi	1.23	0.36	0.72	0.74	1.34	1.58	1.62	3	0	1
alpha.p	-3.18	0.21	-3.46	-3.45	-3.15	-2.96	-2.93	3	0	1
beta.hab.lambda	0.69	0.03	0.63	0.66	0.69	0.72	0.73	4	0	1
beta.hab.gamma	-0.15	0.04	-0.19	-0.18	-0.17	-0.12	-0.07	3	0	1
beta.hab.phi	-0.39	0.03	-0.46	-0.43	-0.38	-0.37	-0.35	3	0	1
beta.elev.lambda	0.94	0.02	0.91	0.92	0.94	0.97	0.98	5	0	1
beta.ele.gamma2	0.23	0.06	0.08	0.19	0.24	0.28	0.29	4	0	1
beta.elev.phi	0.67	0.09	0.55	0.60	0.64	0.79	0.81	3	0	1
beta.develop.lambda	-0.50	0.37	-1.04	-1.01	-0.25	-0.24	-0.22	3	0	1
beta.develop.gamma	-4.79	0.33	-5.19	-5.04	-4.94	-4.36	-4.31	3	0	1
beta.develop.phi	5.85	0.66	4.96	5.31	5.66	6.70	6.74	3	0	1
beta.effort	0.00	0.00	0.00	0.00	0.00	0.00	0.00	11	0	1
beta.min_temp	-0.13	0.02	-0.15	-0.14	-0.13	-0.11	-0.10	3	0	1

Table 170: Lincoln's Sparrow

	mean	sd	2.5%	25%	50%	75%	97.5%	n.eff	overlap0	f
alpha.lambda	4.84	0.09	4.76	4.78	4.79	4.94	5.02	3	0	1.00
alpha.phi	2.96	0.06	2.90	2.91	2.92	3.00	3.08	3	0	1.00
alpha.p	-1.64	0.07	-1.79	-1.72	-1.60	-1.59	-1.58	3	0	1.00
beta.hab.lambda	0.07	0.01	0.05	0.06	0.07	0.08	0.10	5	0	1.00
beta.hab.gamma	0.14	0.06	0.01	0.08	0.17	0.19	0.21	3	0	0.98
beta.hab.phi	0.44	0.11	0.33	0.36	0.37	0.55	0.66	3	0	1.00
beta.elev.lambda	-0.22	0.01	-0.24	-0.23	-0.22	-0.21	-0.20	6	0	1.00
beta.ele.gamma2	-0.12	0.01	-0.14	-0.13	-0.12	-0.11	-0.09	14	0	1.00
beta.elev.phi	-0.37	0.02	-0.40	-0.38	-0.37	-0.36	-0.34	5	0	1.00
beta.develop.lambda	0.33	0.02	0.30	0.32	0.33	0.35	0.37	20	0	1.00
beta.develop.gamma	-0.32	0.05	-0.39	-0.36	-0.34	-0.27	-0.23	3	0	1.00
beta.develop.phi	0.53	0.06	0.41	0.46	0.55	0.58	0.61	3	0	1.00
beta.effort	0.02	0.01	0.00	0.01	0.02	0.02	0.03	4	0	1.00
beta.min_temp	0.00	0.00	0.00	0.00	0.00	0.00	0.00	1	0	1.00

Table 171: Little Blue Heron

	mean	sd	2.5%	25%	50%	75%	97.5%	n.eff	overlap0	f
alpha.lambda	5.33	0.47	4.95	4.99	5.02	5.99	6.01	3	0	1.00
alpha.phi	4.30	1.08	3.49	3.53	3.56	5.66	6.01	3	0	1.00
alpha.p	-1.50	0.37	-2.03	-2.02	-1.25	-1.24	-1.23	3	0	1.00
beta.hab.lambda	-0.56	0.31	-0.82	-0.79	-0.77	-0.13	-0.10	3	0	1.00
beta.hab.gamma	0.37	0.02	0.34	0.35	0.38	0.38	0.39	3	0	1.00
beta.hab.phi	-0.24	0.01	-0.25	-0.25	-0.24	-0.23	-0.21	4	0	1.00
beta.elev.lambda	-1.17	0.16	-1.31	-1.28	-1.26	-0.95	-0.94	3	0	1.00
beta.ele.gamma2	-1.24	0.52	-2.09	-1.90	-0.88	-0.87	-0.85	3	0	1.00
beta.elev.phi	1.47	0.72	0.93	0.96	0.98	2.37	2.63	3	0	1.00
beta.develop.lambda	0.09	0.11	-0.01	0.01	0.03	0.23	0.26	3	1	0.88
beta.develop.gamma	1.01	0.38	0.71	0.74	0.76	1.50	1.61	3	0	1.00

	mean	sd	2.5%	25%	50%	75%	97.5%	n.eff	overlap0	f
beta.develop.phi	-0.13	0.41	-0.79	-0.65	0.14	0.17	0.21	3	1	0.33
beta.effort	0.00	0.00	0.00	0.00	0.00	0.01	0.01	126	0	1.00
beta.min_temp	0.00	0.00	0.00	0.00	0.00	0.00	0.00	1	0	1.00

Table 172: Marsh Wren

	mean	sd	2.5%	25%	50%	75%	97.5%	n.eff	overlap0	f
alpha.lambda	3.67	0.02	3.63	3.66	3.67	3.69	3.72	20	0	1
alpha.phi	2.53	0.02	2.50	2.51	2.52	2.54	2.56	14	0	1
alpha.p	-1.18	0.01	-1.20	-1.19	-1.18	-1.17	-1.16	10	0	1
beta.hab.lambda	-0.18	0.02	-0.22	-0.19	-0.18	-0.17	-0.14	691	0	1
beta.hab.gamma	0.27	0.01	0.26	0.27	0.27	0.27	0.28	103	0	1
beta.hab.phi	-0.08	0.01	-0.09	-0.09	-0.08	-0.08	-0.07	78	0	1
beta.elev.lambda	-0.27	0.02	-0.29	-0.28	-0.27	-0.26	-0.23	55	0	1
beta.ele.gamma2	0.09	0.01	0.06	0.08	0.09	0.09	0.11	48	0	1
beta.elev.phi	-0.27	0.01	-0.29	-0.27	-0.27	-0.26	-0.24	30	0	1
beta.develop.lambda	-0.27	0.02	-0.32	-0.29	-0.27	-0.26	-0.23	353	0	1
beta.develop.gamma	-0.35	0.02	-0.38	-0.36	-0.35	-0.33	-0.31	33	0	1
beta.develop.phi	0.20	0.03	0.15	0.18	0.20	0.22	0.26	18	0	1
beta.effort	0.08	0.00	0.07	0.07	0.08	0.08	0.08	283	0	1
beta.min_temp	0.00	0.00	0.00	0.00	0.00	0.00	0.00	1257	0	1

Table 173: Mottled Duck

	mean	sd	2.5%	25%	50%	75%	97.5%	n.eff	overlap0	f
alpha.lambda	5.08	0.03	5.02	5.06	5.08	5.11	5.15	249	0	1.00
alpha.phi	0.97	0.02	0.93	0.96	0.97	0.99	1.02	216	0	1.00
alpha.p	-1.00	0.01	-1.02	-1.00	-1.00	-0.99	-0.97	25	0	1.00
beta.hab.lambda	-0.03	0.03	-0.09	-0.05	-0.03	-0.01	0.03	282	1	0.84
beta.hab.gamma	0.23	0.01	0.20	0.22	0.23	0.23	0.25	381	0	1.00
beta.hab.phi	-0.02	0.02	-0.05	-0.03	-0.02	-0.01	0.01	565	1	0.93
beta.elev.lambda	-0.64	0.04	-0.73	-0.67	-0.64	-0.61	-0.55	187	0	1.00
beta.ele.gamma2	0.49	0.02	0.46	0.48	0.49	0.51	0.53	210	0	1.00
beta.elev.phi	-0.71	0.03	-0.76	-0.73	-0.71	-0.69	-0.66	194	0	1.00
beta.develop.lambda	-0.70	0.05	-0.79	-0.73	-0.70	-0.67	-0.61	672	0	1.00
beta.develop.gamma	-0.74	0.03	-0.79	-0.76	-0.74	-0.72	-0.69	92	0	1.00
beta.develop.phi	1.26	0.04	1.19	1.23	1.26	1.29	1.33	97	0	1.00
beta.effort	0.02	0.00	0.01	0.01	0.02	0.02	0.02	263	0	1.00
beta.min_temp	0.00	0.00	0.00	0.00	0.00	0.00	0.00	1843	0	1.00

Table 174: Mountain Bluebird

	mean	sd	2.5%	25%	50%	75%	97.5%	n.eff	overlap0	f
alpha.lambda	5.65	0.15	5.35	5.55	5.67	5.78	5.86	3	0	1.00
alpha.phi	1.87	0.22	1.58	1.75	1.77	1.94	2.39	8	0	1.00
alpha.p	-2.85	0.22	-3.32	-2.98	-2.83	-2.61	-2.59	3	0	1.00
beta.hab.lambda	-0.52	0.30	-1.14	-0.66	-0.61	-0.18	-0.13	4	0	1.00

	mean	sd	2.5%	25%	50%	75%	97.5%	n.eff	overlap0	f
beta.hab.gamma	-0.42	0.25	-0.76	-0.70	-0.25	-0.19	-0.12	4	0	1.00
beta.hab.phi	0.17	0.23	-0.12	-0.02	0.03	0.38	0.64	5	1	0.56
beta.elev.lambda	0.59	0.54	-0.08	0.01	0.51	1.23	1.34	3	1	0.76
beta.ele.gamma2	0.18	1.05	-0.59	-0.58	-0.54	1.53	1.94	3	1	0.33
beta.elev.phi	1.31	1.28	-0.98	-0.22	2.17	2.24	2.26	3	1	0.67
beta.develop.lambda	-0.07	0.82	-1.09	-1.03	0.01	0.85	0.91	3	1	0.50
beta.develop.gamma	-0.15	0.72	-1.19	-1.08	0.16	0.57	0.60	3	1	0.43
beta.develop.phi	0.81	0.61	0.07	0.11	0.68	1.43	1.71	3	0	1.00
beta.effort	0.00	0.00	0.00	0.00	0.00	0.00	0.00	1	0	1.00
beta.min_temp	-0.02	0.02	-0.05	-0.04	0.00	0.00	0.00	3	0	1.00

Table 175: Mountain Chickadee

	mean	sd	2.5%	25%	50%	75%	97.5%	n.eff	overlap0	f
alpha.lambda	5.04	0.17	4.83	4.89	4.97	5.27	5.29	3	0	1.00
alpha.phi	2.78	0.57	2.28	2.35	2.42	3.57	3.63	3	0	1.00
alpha.p	-1.58	0.03	-1.61	-1.61	-1.60	-1.55	-1.52	3	0	1.00
beta.hab.lambda	0.47	0.04	0.40	0.41	0.49	0.50	0.51	3	0	1.00
beta.hab.gamma	0.33	0.06	0.27	0.28	0.29	0.41	0.44	3	0	1.00
beta.hab.phi	0.18	0.02	0.15	0.17	0.18	0.19	0.22	6	0	1.00
beta.elev.lambda	1.55	0.22	1.22	1.25	1.67	1.72	1.78	3	0	1.00
beta.ele.gamma2	0.54	1.13	-0.30	-0.27	-0.24	2.12	2.19	3	1	0.33
beta.elev.phi	1.03	0.98	-0.39	-0.34	1.71	1.74	1.76	3	1	0.67
beta.develop.lambda	0.52	0.08	0.39	0.42	0.57	0.59	0.61	3	0	1.00
beta.develop.gamma	-0.38	0.35	-0.71	-0.64	-0.58	0.07	0.20	3	1	0.69
beta.develop.phi	1.98	0.11	1.80	1.90	1.98	2.09	2.16	4	0	1.00
beta.effort	0.09	0.01	0.07	0.08	0.09	0.09	0.09	4	0	1.00
beta.min_temp	0.00	0.00	0.00	0.00	0.00	0.00	0.00	57	0	1.00

Table 176: Neotropic Cormorant

	mean	sd	2.5%	25%	50%	75%	97.5%	n.eff	overlap0	f
alpha.lambda	-20.90	26.74	-59.28	-58.29	-4.78	0.11	1.54	3	1	0.73
alpha.phi	1.47	1.44	0.06	0.32	0.69	3.48	3.53	3	0	1.00
alpha.p	-3.09	0.85	-4.22	-4.19	-2.95	-2.13	-2.09	3	0	1.00
beta.hab.lambda	-0.93	0.66	-1.78	-1.63	-0.95	-0.28	0.10	3	1	0.92
beta.hab.gamma	0.37	0.46	-0.01	0.00	0.09	1.01	1.02	3	1	0.77
beta.hab.phi	-0.51	1.16	-2.20	-2.13	0.20	0.42	0.46	3	1	0.33
beta.elev.lambda	-0.42	1.31	-2.60	-1.43	-0.69	1.23	1.37	3	1	0.67
beta.ele.gamma2	-0.63	0.53	-1.42	-1.36	-0.27	-0.26	-0.23	3	0	1.00
beta.elev.phi	0.08	1.62	-1.71	-1.64	-0.36	2.19	2.34	3	1	0.33
beta.develop.lambda	-17.36	16.32	-41.37	-39.39	-9.43	-3.49	-0.62	3	0	0.99
beta.develop.gamma	1.00	1.28	-0.65	-0.33	0.76	2.65	2.72	3	1	0.67
beta.develop.phi	-1.02	2.65	-4.61	-4.40	-0.56	1.72	2.57	3	1	0.67
beta.effort	0.28	0.07	0.22	0.23	0.23	0.37	0.38	3	0	1.00
beta.min_temp	0.00	0.00	-0.01	-0.01	0.00	0.00	0.00	4	0	1.00

Table 177: Orange-crowned Warbler

	mean	sd	2.5%	25%	50%	75%	97.5%	n.eff	overlap0	f
alpha.lambda	2.87	0.04	2.79	2.84	2.87	2.91	2.95	4	0	1.00
alpha.phi	3.31	0.04	3.24	3.28	3.30	3.33	3.38	5	0	1.00
alpha.p	-0.70	0.04	-0.77	-0.75	-0.68	-0.68	-0.67	3	0	1.00
beta.hab.lambda	-0.69	0.03	-0.74	-0.71	-0.69	-0.67	-0.62	7	0	1.00
beta.hab.gamma	-1.13	0.02	-1.17	-1.14	-1.13	-1.12	-1.09	16	0	1.00
beta.hab.phi	0.76	0.02	0.72	0.74	0.76	0.77	0.80	114	0	1.00
beta.elev.lambda	-0.47	0.02	-0.50	-0.48	-0.47	-0.46	-0.44	656	0	1.00
beta.ele.gamma2	-0.10	0.01	-0.12	-0.10	-0.10	-0.09	-0.08	120	0	1.00
beta.elev.phi	-0.41	0.01	-0.43	-0.42	-0.41	-0.40	-0.39	105	0	1.00
beta.develop.lambda	-0.01	0.04	-0.09	-0.04	-0.01	0.01	0.06	10	1	0.62
beta.develop.gamma	-0.22	0.01	-0.24	-0.23	-0.22	-0.21	-0.20	28	0	1.00
beta.develop.phi	-0.16	0.03	-0.22	-0.18	-0.16	-0.13	-0.09	11	0	1.00
beta.effort	0.00	0.00	0.00	0.00	0.00	0.00	0.01	21	0	1.00
beta.min_temp	0.00	0.00	0.00	0.00	0.00	0.00	0.00	933	0	1.00

Table 178: Pacific Loon

	mean	sd	2.5%	25%	50%	75%	97.5%	n.eff	overlap0	f
alpha.lambda	8.44	0.08	8.29	8.38	8.43	8.51	8.59	16	0	1.00
alpha.phi	-1.75	0.14	-1.96	-1.94	-1.67	-1.63	-1.61	3	0	1.00
alpha.p	-5.82	0.07	-5.94	-5.88	-5.82	-5.76	-5.69	229	0	1.00
beta.hab.lambda	0.91	0.04	0.83	0.88	0.90	0.94	1.00	9	0	1.00
beta.hab.gamma	0.09	0.02	0.06	0.07	0.09	0.10	0.10	3	0	1.00
beta.hab.phi	-0.09	0.10	-0.19	-0.17	-0.15	0.05	0.06	3	1	0.67
beta.elev.lambda	1.36	0.09	1.18	1.28	1.38	1.43	1.50	4	0	1.00
beta.ele.gamma2	0.08	0.03	0.03	0.03	0.09	0.10	0.11	3	0	1.00
beta.elev.phi	-0.12	0.20	-0.34	-0.31	-0.20	0.16	0.16	3	1	0.67
beta.develop.lambda	-0.20	0.13	-0.45	-0.28	-0.19	-0.11	0.04	5	1	0.94
beta.develop.gamma	-0.04	0.02	-0.06	-0.06	-0.03	-0.03	-0.02	3	0	1.00
beta.develop.phi	-0.07	0.11	-0.16	-0.15	-0.13	0.08	0.10	3	1	0.67
beta.effort	0.04	0.00	0.04	0.04	0.04	0.04	0.05	303	0	1.00
beta.min_temp	0.00	0.00	0.00	0.00	0.00	0.00	0.00	1	0	1.00

Table 179: Pileated Woodpecker

	mean	sd	2.5%	25%	50%	75%	97.5%	n.eff	overlap0	f
alpha.lambda	2.55	0.05	2.45	2.52	2.55	2.58	2.63	4	0	1.00
alpha.phi	3.52	0.03	3.46	3.50	3.52	3.54	3.57	8	0	1.00
alpha.p	-0.89	0.04	-0.95	-0.91	-0.90	-0.86	-0.82	4	0	1.00
beta.hab.lambda	0.14	0.02	0.10	0.12	0.14	0.15	0.18	7	0	1.00
beta.hab.gamma	0.19	0.01	0.17	0.18	0.19	0.20	0.22	22	0	1.00
beta.hab.phi	0.18	0.02	0.12	0.16	0.17	0.19	0.22	9	0	1.00
beta.elev.lambda	-0.39	0.01	-0.42	-0.40	-0.39	-0.38	-0.37	11	0	1.00
beta.ele.gamma2	-0.22	0.02	-0.26	-0.24	-0.22	-0.21	-0.19	7	0	1.00
beta.elev.phi	-0.05	0.03	-0.10	-0.07	-0.05	-0.03	0.00	6	1	0.96
beta.develop.lambda	-0.07	0.03	-0.14	-0.09	-0.07	-0.05	-0.01	124	0	0.98
beta.develop.gamma	0.30	0.02	0.25	0.28	0.30	0.32	0.35	15	0	1.00

	mean	sd	2.5%	25%	50%	75%	97.5%	n.eff	overlap0	f
beta.develop.phi	-0.29	0.04	-0.38	-0.32	-0.29	-0.26	-0.21	12	0	1.00
beta.effort	0.05	0.00	0.05	0.05	0.05	0.06	0.06	104	0	1.00
beta.min_temp	0.00	0.00	0.00	0.00	0.00	0.00	0.00	6000	0	1.00

Table 180: Pinyon Jay

	mean	sd	2.5%	25%	50%	75%	97.5%	n.eff	overlap0	f
alpha.lambda	7.73	0.06	7.60	7.69	7.73	7.77	7.82	4	0	1.00
alpha.phi	2.86	0.11	2.54	2.84	2.89	2.94	2.97	7	0	1.00
alpha.p	-2.75	0.25	-3.12	-3.09	-2.61	-2.57	-2.53	3	0	1.00
beta.hab.lambda	-0.41	0.04	-0.47	-0.44	-0.42	-0.38	-0.35	4	0	1.00
beta.hab.gamma	-0.71	0.31	-1.23	-1.07	-0.57	-0.48	-0.39	3	0	1.00
beta.hab.phi	1.15	0.31	0.80	0.91	1.00	1.55	1.63	3	0	1.00
beta.elev.lambda	0.18	0.11	0.07	0.10	0.11	0.32	0.34	3	0	1.00
beta.ele.gamma2	0.21	1.66	-0.99	-0.97	-0.95	2.52	2.64	3	1	0.33
beta.elev.phi	-0.04	1.76	-2.57	-2.52	1.19	1.21	1.23	3	1	0.33
beta.develop.lambda	-1.49	1.21	-2.44	-2.37	-2.30	0.16	0.37	3	1	0.67
beta.develop.gamma	-1.10	0.48	-1.83	-1.75	-0.83	-0.71	-0.68	3	0	1.00
beta.develop.phi	2.93	0.47	2.15	2.33	3.18	3.30	3.42	3	0	1.00
beta.effort	0.00	0.00	0.00	0.00	0.00	0.00	0.00	15	0	1.00
beta.min_temp	-0.01	0.01	-0.02	-0.01	-0.01	0.00	0.00	3	0	1.00

Table 181: Pygmy Nuthatch

	mean	sd	2.5%	25%	50%	75%	97.5%	n.eff	overlap0	f
alpha.lambda	6.04	0.25	5.81	5.84	5.89	6.37	6.40	3	0	1.00
alpha.phi	3.17	0.13	3.01	3.06	3.10	3.32	3.39	3	0	1.00
alpha.p	-1.40	0.29	-1.81	-1.80	-1.24	-1.17	-1.16	3	0	1.00
beta.hab.lambda	-0.03	0.03	-0.07	-0.05	-0.04	-0.01	0.02	4	1	0.80
beta.hab.gamma	0.10	0.03	0.05	0.07	0.09	0.14	0.16	3	0	1.00
beta.hab.phi	0.64	0.04	0.57	0.60	0.65	0.67	0.71	3	0	1.00
beta.elev.lambda	-0.06	0.02	-0.10	-0.08	-0.06	-0.04	-0.01	4	0	1.00
beta.ele.gamma2	0.02	0.08	-0.10	-0.08	0.06	0.08	0.11	3	1	0.67
beta.elev.phi	0.21	0.07	0.13	0.16	0.17	0.29	0.33	3	0	1.00
beta.develop.lambda	-0.21	0.03	-0.27	-0.23	-0.21	-0.18	-0.15	5	0	1.00
beta.develop.gamma	-0.96	0.09	-1.10	-1.07	-0.94	-0.86	-0.83	3	0	1.00
beta.develop.phi	0.47	0.18	0.20	0.30	0.44	0.62	0.78	3	0	1.00
beta.effort	0.03	0.02	0.00	0.00	0.04	0.05	0.05	3	0	1.00
beta.min_temp	0.00	0.00	0.00	0.00	0.00	0.00	0.00	66	0	1.00

Table 182: Red-headed Woodpecker

	mean	sd	2.5%	25%	50%	75%	97.5%	n.eff	overlap0	f
alpha.lambda	4.55	0.03	4.50	4.52	4.56	4.57	4.60	3	0	1.00
alpha.phi	3.56	0.06	3.46	3.50	3.56	3.63	3.66	3	0	1.00
alpha.p	-1.89	0.02	-1.92	-1.90	-1.89	-1.87	-1.86	4	0	1.00
beta.hab.lambda	-0.06	0.01	-0.09	-0.07	-0.06	-0.05	-0.04	5	0	1.00

	mean	sd	2.5%	25%	50%	75%	97.5%	n.eff	overlap0	f
beta.hab.gamma	0.21	0.02	0.17	0.19	0.21	0.22	0.25	5	0	1.00
beta.hab.phi	-0.15	0.01	-0.18	-0.16	-0.15	-0.15	-0.13	12	0	1.00
beta.elev.lambda	0.40	0.02	0.37	0.39	0.40	0.41	0.43	4	0	1.00
beta.ele.gamma2	0.65	0.07	0.55	0.58	0.65	0.71	0.76	3	0	1.00
beta.elev.phi	-1.59	0.05	-1.68	-1.62	-1.59	-1.56	-1.50	5	0	1.00
beta.develop.lambda	-0.27	0.07	-0.39	-0.35	-0.23	-0.21	-0.17	3	0	1.00
beta.develop.gamma	0.16	0.23	-0.03	0.00	0.02	0.46	0.53	3	1	0.75
beta.develop.phi	0.25	0.12	0.04	0.10	0.31	0.34	0.37	3	0	1.00
beta.effort	0.01	0.00	0.00	0.00	0.01	0.01	0.01	10	0	1.00
beta.min_temp	0.00	0.00	0.00	0.00	0.00	0.00	0.00	5000	0	1.00

Table 183: Red-shouldered Hawk

	mean	sd	2.5%	25%	50%	75%	97.5%	n.eff	overlap0	f
alpha.lambda	2.23	0.47	1.83	1.88	1.92	2.86	2.95	3	0	1.00
alpha.phi	3.95	0.50	3.53	3.57	3.62	4.64	4.69	3	0	1.00
alpha.p	-0.81	0.46	-1.51	-1.44	-0.50	-0.47	-0.44	3	0	1.00
beta.hab.lambda	-0.56	0.04	-0.63	-0.59	-0.57	-0.53	-0.49	4	0	1.00
beta.hab.gamma	-0.09	0.02	-0.14	-0.11	-0.09	-0.08	-0.05	4	0	1.00
beta.hab.phi	0.21	0.12	0.07	0.11	0.15	0.35	0.39	3	0	1.00
beta.elev.lambda	-0.46	0.03	-0.51	-0.49	-0.47	-0.43	-0.40	3	0	1.00
beta.ele.gamma2	-0.30	0.03	-0.34	-0.33	-0.31	-0.28	-0.26	3	0	1.00
beta.elev.phi	-0.02	0.03	-0.08	-0.05	-0.02	0.01	0.03	5	1	0.66
beta.develop.lambda	0.34	0.10	0.21	0.26	0.30	0.46	0.52	3	0	1.00
beta.develop.gamma	0.08	0.02	0.03	0.06	0.08	0.09	0.11	5	0	1.00
beta.develop.phi	1.20	0.48	0.75	0.81	0.92	1.82	1.93	3	0	1.00
beta.effort	0.01	0.01	0.00	0.01	0.01	0.02	0.02	5	0	1.00
beta.min_temp	0.00	0.00	0.00	0.00	0.00	0.00	0.00	27	0	1.00

Table 184: Red Crossbill

	mean	sd	2.5%	25%	50%	75%	97.5%	n.eff	overlap0	f
alpha.lambda	6.44	0.15	6.21	6.26	6.51	6.57	6.62	3	0	1.00
alpha.phi	1.41	0.32	0.99	1.16	1.31	1.65	2.10	4	0	1.00
alpha.p	-3.27	0.11	-3.41	-3.37	-3.30	-3.12	-3.10	3	0	1.00
beta.hab.lambda	-0.02	0.02	-0.05	-0.03	-0.01	0.00	0.01	18	1	0.85
beta.hab.gamma	0.14	0.04	0.04	0.12	0.16	0.16	0.17	4	0	1.00
beta.hab.phi	-0.12	0.05	-0.17	-0.16	-0.15	-0.09	0.00	3	1	0.97
beta.elev.lambda	0.76	0.04	0.71	0.73	0.74	0.80	0.85	3	0	1.00
beta.ele.gamma2	-0.55	0.04	-0.61	-0.58	-0.54	-0.52	-0.50	3	0	1.00
beta.elev.phi	0.85	0.08	0.67	0.79	0.89	0.92	0.95	3	0	1.00
beta.develop.lambda	-0.38	0.04	-0.44	-0.42	-0.36	-0.34	-0.32	3	0	1.00
beta.develop.gamma	-1.13	0.06	-1.20	-1.18	-1.16	-1.06	-1.04	3	0	1.00
beta.develop.phi	1.64	0.14	1.42	1.47	1.72	1.74	1.77	3	0	1.00
beta.effort	0.00	0.00	0.00	0.00	0.00	0.01	0.01	13	0	1.00
beta.min_temp	0.00	0.00	0.00	0.00	0.00	0.00	0.00	1	0	1.00

Table 185: Sharp-shinned Hawk

	mean	sd	2.5%	25%	50%	75%	97.5%	n.eff	overlap0	f
alpha.lambda	1.62	0.06	1.54	1.58	1.61	1.68	1.74	4	0	1.00
alpha.phi	2.97	0.05	2.90	2.93	2.96	3.00	3.06	4	0	1.00
alpha.p	-0.85	0.04	-0.92	-0.89	-0.83	-0.81	-0.79	3	0	1.00
beta.hab.lambda	-0.25	0.04	-0.32	-0.27	-0.24	-0.22	-0.18	6	0	1.00
beta.hab.gamma	-0.07	0.02	-0.12	-0.09	-0.07	-0.05	-0.03	10	0	1.00
beta.hab.phi	0.05	0.04	-0.01	0.03	0.05	0.07	0.14	8	1	0.93
beta.elev.lambda	-0.15	0.03	-0.20	-0.17	-0.15	-0.13	-0.09	7	0	1.00
beta.ele.gamma2	-0.33	0.01	-0.36	-0.34	-0.33	-0.32	-0.30	14	0	1.00
beta.elev.phi	0.18	0.02	0.15	0.17	0.18	0.19	0.22	12	0	1.00
beta.develop.lambda	0.12	0.05	0.02	0.09	0.12	0.16	0.22	15	0	0.99
beta.develop.gamma	0.24	0.02	0.20	0.22	0.24	0.25	0.27	35	0	1.00
beta.develop.phi	-0.23	0.03	-0.29	-0.25	-0.23	-0.21	-0.18	37	0	1.00
beta.effort	0.02	0.01	0.01	0.01	0.02	0.02	0.03	90	0	1.00
beta.min_temp	0.00	0.00	-0.01	-0.01	0.00	0.00	0.00	27	0	1.00

Table 186: Tricolored Heron

	mean	sd	2.5%	25%	50%	75%	97.5%	n.eff	overlap0	f
alpha.lambda	5.98	1.64	4.77	4.81	4.83	8.26	8.38	3	0	1.00
alpha.phi	0.99	2.96	-3.28	-3.15	3.04	3.10	3.18	3	1	0.67
alpha.p	-2.35	1.75	-4.90	-4.79	-1.13	-1.11	-1.10	3	0	1.00
beta.hab.lambda	-0.90	0.40	-1.51	-1.43	-0.63	-0.61	-0.58	3	0	1.00
beta.hab.gamma	0.35	0.13	0.16	0.17	0.44	0.44	0.45	3	0	1.00
beta.hab.phi	-1.05	1.07	-2.62	-2.52	-0.30	-0.29	-0.28	3	0	1.00
beta.elev.lambda	-1.01	0.03	-1.06	-1.03	-1.02	-1.00	-0.94	7	0	1.00
beta.ele.gamma2	-0.56	0.12	-0.68	-0.65	-0.63	-0.39	-0.38	3	0	1.00
beta.elev.phi	0.11	0.42	-0.50	-0.47	0.39	0.42	0.47	3	1	0.67
beta.develop.lambda	0.30	0.09	0.13	0.21	0.34	0.36	0.40	3	0	1.00
beta.develop.gamma	0.75	0.15	0.53	0.54	0.84	0.86	0.90	3	0	1.00
beta.develop.phi	-0.81	0.45	-1.55	-1.38	-0.51	-0.48	-0.44	3	0	1.00
beta.effort	0.01	0.02	0.00	0.00	0.00	0.03	0.04	3	0	1.00
beta.min_temp	0.00	0.00	0.00	0.00	0.00	0.00	0.00	11	0	1.00

Table 187: Trumpeter Swan

	mean	sd	2.5%	25%	50%	75%	97.5%	n.eff	overlap0	f
alpha.lambda	-5.48	1.46	-8.52	-6.47	-5.30	-4.56	-2.50	23	0	1.00
alpha.phi	0.89	0.72	-0.10	-0.03	1.05	1.68	1.70	3	1	0.70
alpha.p	-1.39	0.52	-2.11	-2.07	-1.24	-0.85	-0.83	3	0	1.00
beta.hab.lambda	-1.37	1.65	-5.21	-2.75	-0.90	0.11	0.51	4	1	0.69
beta.hab.gamma	0.36	0.14	0.20	0.20	0.34	0.53	0.55	3	0	1.00
beta.hab.phi	0.02	0.51	-0.67	-0.58	0.05	0.62	0.64	3	1	0.67
beta.elev.lambda	3.98	0.95	1.70	3.45	4.15	4.66	5.52	7	0	1.00
beta.ele.gamma2	-0.47	0.43	-1.05	-1.05	-0.34	-0.04	-0.02	3	0	1.00
beta.elev.phi	1.25	1.70	-0.39	-0.37	0.54	3.58	3.63	3	1	0.67
beta.develop.lambda	-3.30	1.43	-6.09	-4.26	-3.47	-2.18	-0.56	6	0	1.00
beta.develop.gamma	0.33	1.35	-1.23	-1.21	0.13	2.07	2.09	3	1	0.67

	mean	sd	2.5%	25%	50%	75%	97.5%	n.eff	overlap0	f
beta.develop.phi	3.06	4.45	-3.30	-3.19	6.10	6.35	6.44	3	1	0.67
beta.effort	0.00	0.00	0.00	0.00	0.00	0.00	0.00	7	0	1.00
beta.min_temp	0.00	0.00	0.00	0.00	0.00	0.00	0.00	1	0	1.00

Table 188: Varied Thrush

	mean	sd	2.5%	25%	50%	75%	97.5%	n.eff	overlap0	f
alpha.lambda	7.69	1.03	6.62	6.73	7.28	9.06	9.19	3	0	1.00
alpha.phi	1.53	3.47	-3.40	-3.31	3.53	4.42	4.44	3	1	0.67
alpha.p	-3.56	1.20	-5.35	-5.20	-2.74	-2.70	-2.67	3	0	1.00
beta.hab.lambda	0.62	0.44	0.21	0.28	0.37	1.24	1.26	3	0	1.00
beta.hab.gamma	0.86	0.66	0.32	0.34	0.45	1.72	1.91	3	0	1.00
beta.hab.phi	-0.69	0.72	-1.69	-1.64	-0.49	0.07	0.10	3	1	0.67
beta.elev.lambda	-0.09	0.22	-0.38	-0.34	-0.12	0.19	0.19	3	1	0.67
beta.ele.gamma2	-0.06	0.34	-0.50	-0.32	-0.21	0.40	0.42	3	1	0.67
beta.elev.phi	-0.50	0.86	-1.78	-1.65	0.04	0.08	0.29	3	1	0.38
beta.develop.lambda	0.15	0.18	-0.05	-0.03	0.10	0.36	0.47	3	1	0.67
beta.develop.gamma	-0.21	1.08	-2.18	-1.27	0.06	0.63	1.47	3	1	0.33
beta.develop.phi	0.47	0.76	-1.03	-0.36	0.96	1.01	1.13	3	1	0.67
beta.effort	0.00	0.00	0.00	0.00	0.00	0.00	0.00	10	0	1.00
beta.min_temp	0.00	0.00	0.00	0.00	0.00	0.00	0.00	1	0	1.00

Table 189: White-throated Swift

	mean	sd	2.5%	25%	50%	75%	97.5%	n.eff	overlap0	f
alpha.lambda	7.90	0.59	6.85	7.25	8.28	8.32	8.36	3	0	1.00
alpha.phi	0.35	1.58	-1.62	-1.51	0.29	2.06	2.71	3	1	0.67
alpha.p	-3.91	0.74	-4.72	-4.64	-4.15	-3.01	-2.77	3	0	1.00
beta.hab.lambda	0.62	0.25	0.26	0.38	0.65	0.88	0.98	3	0	1.00
beta.hab.gamma	0.61	0.23	0.33	0.35	0.62	0.77	1.03	3	0	1.00
beta.hab.phi	-0.62	0.12	-0.90	-0.67	-0.64	-0.50	-0.47	5	0	1.00
beta.elev.lambda	-0.70	0.28	-1.12	-1.06	-0.61	-0.46	-0.36	3	0	1.00
beta.ele.gamma2	0.06	0.12	-0.34	0.06	0.07	0.14	0.17	7	1	0.83
beta.elev.phi	-0.30	0.28	-0.59	-0.57	-0.35	-0.13	0.36	3	1	0.83
beta.develop.lambda	0.21	0.44	-0.23	-0.14	-0.04	0.77	0.89	3	1	0.44
beta.develop.gamma	0.94	0.24	0.66	0.68	0.91	1.12	1.44	3	0	1.00
beta.develop.phi	-0.84	0.19	-1.13	-1.06	-0.76	-0.70	-0.55	4	0	1.00
beta.effort	0.01	0.01	0.00	0.00	0.00	0.02	0.02	3	0	1.00
beta.min_temp	0.00	0.00	0.00	0.00	0.00	0.00	0.00	86	0	1.00

Table 190: Wrentit

	mean	sd	2.5%	25%	50%	75%	97.5%	n.eff	overlap0	f
alpha.lambda	5.65	0.02	5.61	5.64	5.66	5.67	5.69	5	0	1.00
alpha.phi	3.34	0.03	3.28	3.32	3.34	3.36	3.38	6	0	1.00
alpha.p	-0.93	0.02	-0.97	-0.95	-0.94	-0.92	-0.90	4	0	1.00
beta.hab.lambda	0.06	0.02	0.03	0.05	0.06	0.07	0.09	823	0	1.00

	mean	sd	2.5%	25%	50%	75%	97.5%	n.eff	overlap0	f
beta.hab.gamma	0.56	0.02	0.52	0.55	0.56	0.57	0.60	16	0	1.00
beta.hab.phi	-0.23	0.02	-0.27	-0.24	-0.23	-0.22	-0.20	41	0	1.00
beta.elev.lambda	-0.06	0.01	-0.09	-0.07	-0.06	-0.05	-0.03	157	0	1.00
beta.ele.gamma2	-0.09	0.03	-0.16	-0.11	-0.09	-0.07	-0.04	8	0	1.00
beta.elev.phi	-0.20	0.04	-0.29	-0.23	-0.21	-0.17	-0.11	8	0	1.00
beta.develop.lambda	0.29	0.02	0.24	0.27	0.29	0.30	0.33	145	0	1.00
beta.develop.gamma	0.04	0.04	-0.03	0.02	0.04	0.07	0.12	14	1	0.87
beta.develop.phi	0.44	0.04	0.37	0.41	0.44	0.47	0.52	18	0	1.00
beta.effort	0.01	0.01	0.00	0.01	0.01	0.01	0.02	54	0	1.00
beta.min_temp	0.00	0.00	0.00	0.00	0.00	0.00	0.00	475	0	1.00

Table 191: Yellow-bellied Sapsucker

	mean	sd	2.5%	25%	50%	75%	97.5%	n.eff	overlap0	f
alpha.lambda	3.13	0.02	3.09	3.11	3.13	3.14	3.17	6	0	1.00
alpha.phi	3.36	0.02	3.32	3.34	3.36	3.38	3.40	23	0	1.00
alpha.p	-0.98	0.02	-1.02	-0.99	-0.98	-0.97	-0.95	4	0	1.00
beta.hab.lambda	0.15	0.01	0.12	0.14	0.15	0.16	0.17	29	0	1.00
beta.hab.gamma	0.25	0.01	0.22	0.24	0.25	0.26	0.28	446	0	1.00
beta.hab.phi	0.19	0.02	0.15	0.17	0.18	0.20	0.24	107	0	1.00
beta.elev.lambda	-0.42	0.01	-0.44	-0.42	-0.42	-0.41	-0.40	6000	0	1.00
beta.ele.gamma2	-0.39	0.01	-0.42	-0.40	-0.39	-0.38	-0.37	44	0	1.00
beta.elev.phi	0.02	0.02	-0.03	0.00	0.02	0.03	0.05	31	1	0.75
beta.develop.lambda	0.65	0.02	0.61	0.64	0.65	0.67	0.69	35	0	1.00
beta.develop.gamma	-0.04	0.02	-0.08	-0.05	-0.04	-0.02	0.00	146	1	0.97
beta.develop.phi	0.24	0.02	0.20	0.22	0.24	0.25	0.28	160	0	1.00
beta.effort	0.00	0.00	0.00	0.00	0.00	0.00	0.01	1332	0	1.00
beta.min_temp	-0.07	0.00	-0.08	-0.07	-0.07	-0.07	-0.06	17	0	1.00

Table 192: Barred Owl

	mean	sd	2.5%	25%	50%	75%	97.5%	n.eff	overlap0	f
alpha.lambda	2.14	0.11	1.98	2.04	2.13	2.25	2.30	3	0	1.00
alpha.phi	3.31	0.12	3.13	3.19	3.30	3.42	3.51	3	0	1.00
alpha.p	-1.21	0.10	-1.35	-1.32	-1.22	-1.11	-1.07	3	0	1.00
beta.hab.lambda	-0.10	0.03	-0.15	-0.12	-0.10	-0.08	-0.04	11	0	1.00
beta.hab.gamma	-0.07	0.03	-0.13	-0.10	-0.08	-0.05	-0.01	16	0	0.98
beta.hab.phi	-0.04	0.04	-0.13	-0.07	-0.04	-0.01	0.03	8	1	0.84
beta.elev.lambda	-0.42	0.02	-0.46	-0.43	-0.42	-0.41	-0.39	30	0	1.00
beta.ele.gamma2	-0.26	0.03	-0.31	-0.28	-0.26	-0.24	-0.22	5	0	1.00
beta.elev.phi	0.18	0.05	0.10	0.14	0.17	0.22	0.27	4	0	1.00
beta.develop.lambda	-0.06	0.05	-0.16	-0.10	-0.06	-0.02	0.04	28	1	0.87
beta.develop.gamma	-0.10	0.10	-0.31	-0.16	-0.08	-0.03	0.04	4	1	0.89
beta.develop.phi	0.21	0.13	0.02	0.12	0.17	0.28	0.49	4	0	0.99
beta.effort	0.00	0.00	0.00	0.00	0.00	0.00	0.00	957	0	1.00
beta.min_temp	0.00	0.00	0.00	0.00	0.00	0.00	0.00	600	0	1.00

Table 193: Barrow's Goldeneye

	mean	sd	2.5%	25%	50%	75%	97.5%	n.eff	overlap0	f
alpha.lambda	5.15	0.14	4.92	4.96	5.24	5.26	5.28	3	0	1.00
alpha.phi	3.02	0.20	2.72	2.75	3.14	3.16	3.19	3	0	1.00
alpha.p	-1.16	0.10	-1.24	-1.23	-1.22	-1.01	-1.00	3	0	1.00
beta.hab.lambda	-0.41	0.23	-0.77	-0.71	-0.34	-0.18	-0.16	3	0	1.00
beta.hab.gamma	-0.72	0.25	-1.12	-1.05	-0.55	-0.53	-0.50	3	0	1.00
beta.hab.phi	2.52	0.48	1.82	1.90	2.66	2.99	3.09	3	0	1.00
beta.elev.lambda	-0.37	0.05	-0.45	-0.42	-0.37	-0.33	-0.30	3	0	1.00
beta.ele.gamma2	-0.24	0.05	-0.34	-0.29	-0.22	-0.20	-0.16	3	0	1.00
beta.elev.phi	1.31	0.11	1.14	1.20	1.33	1.41	1.47	3	0	1.00
beta.develop.lambda	0.43	0.24	0.16	0.20	0.34	0.75	0.78	3	0	1.00
beta.develop.gamma	0.71	0.82	-0.48	-0.43	1.27	1.30	1.33	3	1	0.67
beta.develop.phi	0.00	0.70	-0.64	-0.57	-0.39	0.95	1.01	3	1	0.67
beta.effort	0.01	0.01	0.00	0.00	0.00	0.01	0.02	3	0	1.00
beta.min__temp	0.00	0.00	0.00	0.00	0.00	0.00	0.00	881	0	1.00

Table 194: Black-throated Sparrow

	mean	sd	2.5%	25%	50%	75%	97.5%	n.eff	overlap0	f
alpha.lambda	5.78	0.03	5.71	5.76	5.78	5.81	5.84	33	0	1.00
alpha.phi	4.74	0.15	4.43	4.61	4.75	4.87	4.98	56	0	1.00
alpha.p	-2.04	0.04	-2.10	-2.07	-2.04	-2.01	-1.95	31	0	1.00
beta.hab.lambda	0.92	0.02	0.88	0.91	0.92	0.93	0.95	4	0	1.00
beta.hab.gamma	0.86	0.04	0.78	0.83	0.87	0.90	0.94	8	0	1.00
beta.hab.phi	-0.60	0.05	-0.69	-0.64	-0.60	-0.55	-0.49	197	0	1.00
beta.elev.lambda	0.37	0.03	0.31	0.35	0.37	0.38	0.41	6	0	1.00
beta.ele.gamma2	-0.01	0.04	-0.10	-0.04	-0.01	0.02	0.07	8	1	0.56
beta.elev.phi	-0.58	0.14	-0.82	-0.70	-0.58	-0.46	-0.33	27	0	1.00
beta.develop.lambda	-1.61	0.09	-1.77	-1.68	-1.61	-1.56	-1.41	5	0	1.00
beta.develop.gamma	-11.79	1.00	-13.74	-12.56	-11.50	-11.05	-10.36	10	0	1.00
beta.develop.phi	2.09	0.05	2.00	2.06	2.09	2.12	2.18	19	0	1.00
beta.effort	0.00	0.00	0.00	0.00	0.00	0.00	0.00	6000	0	1.00
beta.min__temp	-0.14	0.01	-0.15	-0.14	-0.14	-0.14	-0.13	12	0	1.00

Table 195: Cactus Wren

	mean	sd	2.5%	25%	50%	75%	97.5%	n.eff	overlap0	f
alpha.lambda	5.42	0.56	4.70	4.78	5.40	6.09	6.18	3	0	1.00
alpha.phi	3.90	0.93	2.55	2.63	4.37	4.71	4.78	3	0	1.00
alpha.p	-1.56	0.58	-2.34	-2.26	-1.50	-0.90	-0.86	3	0	1.00
beta.hab.lambda	-0.74	0.23	-1.17	-0.98	-0.65	-0.53	-0.48	3	0	1.00
beta.hab.gamma	0.03	0.05	-0.06	0.00	0.03	0.08	0.12	4	1	0.72
beta.hab.phi	0.35	0.22	0.04	0.09	0.37	0.57	0.64	3	0	1.00
beta.elev.lambda	0.44	0.03	0.36	0.42	0.44	0.46	0.50	7	0	1.00
beta.ele.gamma2	-0.43	0.24	-0.78	-0.74	-0.31	-0.23	-0.18	3	0	1.00
beta.elev.phi	0.75	0.48	0.36	0.40	0.43	1.41	1.47	3	0	1.00
beta.develop.lambda	0.91	0.06	0.79	0.86	0.90	0.96	1.02	4	0	1.00
beta.develop.gamma	0.24	0.31	-0.26	-0.14	0.40	0.48	0.58	3	1	0.67

	mean	sd	2.5%	25%	50%	75%	97.5%	n.eff	overlap0	f
beta.develop.phi	-2.01	1.59	-3.62	-3.45	-2.74	0.15	0.30	3	1	0.67
beta.effort	0.08	0.02	0.05	0.07	0.09	0.09	0.11	4	0	1.00
beta.min_temp	0.00	0.00	-0.01	0.00	0.00	0.00	0.00	18	0	1.00

Table 196: California Thrasher

	mean	sd	2.5%	25%	50%	75%	97.5%	n.eff	overlap0	f
alpha.lambda	5.36	0.30	4.91	4.96	5.47	5.66	5.68	3	0	1.00
alpha.phi	4.33	0.89	3.05	3.12	4.75	5.08	5.25	3	0	1.00
alpha.p	-1.88	0.41	-2.31	-2.30	-2.00	-1.34	-1.31	3	0	1.00
beta.hab.lambda	0.40	0.04	0.32	0.37	0.41	0.43	0.46	4	0	1.00
beta.hab.gamma	0.37	0.27	0.09	0.17	0.22	0.73	0.81	3	0	1.00
beta.hab.phi	1.23	0.49	0.49	0.57	1.54	1.59	1.67	3	0	1.00
beta.elev.lambda	-0.40	0.11	-0.52	-0.49	-0.47	-0.27	-0.22	3	0	1.00
beta.ele.gamma2	-0.51	0.39	-0.86	-0.80	-0.74	-0.01	0.12	3	1	0.76
beta.elev.phi	0.39	0.45	-0.34	-0.21	0.65	0.74	0.81	3	1	0.67
beta.develop.lambda	0.49	0.05	0.39	0.46	0.50	0.53	0.57	4	0	1.00
beta.develop.gamma	0.96	0.23	0.56	0.71	1.03	1.13	1.30	3	0	1.00
beta.develop.phi	0.27	0.17	0.05	0.14	0.20	0.44	0.58	3	0	1.00
beta.effort	0.04	0.01	0.02	0.03	0.04	0.05	0.06	4	0	1.00
beta.min_temp	0.00	0.00	0.00	0.00	0.00	0.00	0.00	194	0	1.00

Table 197: Canyon Towhee

	mean	sd	2.5%	25%	50%	75%	97.5%	n.eff	overlap0	f
alpha.lambda	-6.90	1.64	-10.19	-8.00	-6.78	-5.74	-4.07	41	0	1.00
alpha.phi	2.69	0.14	2.44	2.52	2.76	2.80	2.86	3	0	1.00
alpha.p	-0.70	0.03	-0.76	-0.72	-0.69	-0.67	-0.65	5	0	1.00
beta.hab.lambda	0.31	1.23	-2.23	-0.53	0.39	1.25	2.38	102	1	0.61
beta.hab.gamma	0.61	0.04	0.56	0.58	0.59	0.65	0.68	3	0	1.00
beta.hab.phi	-0.60	0.12	-0.81	-0.75	-0.53	-0.50	-0.45	3	0	1.00
beta.elev.lambda	0.78	1.49	-1.76	-0.25	0.62	1.62	4.18	22	1	0.68
beta.ele.gamma2	0.76	0.06	0.65	0.70	0.78	0.81	0.84	3	0	1.00
beta.elev.phi	0.06	0.09	-0.09	-0.01	0.04	0.12	0.24	4	1	0.71
beta.develop.lambda	-0.30	2.10	-5.00	-1.56	-0.15	1.17	3.29	70	1	0.53
beta.develop.gamma	-0.01	0.13	-0.18	-0.11	-0.06	0.14	0.22	3	1	0.65
beta.develop.phi	0.09	0.14	-0.17	-0.02	0.09	0.20	0.36	5	1	0.71
beta.effort	0.19	0.01	0.16	0.18	0.19	0.20	0.21	7	0	1.00
beta.min_temp	-0.01	0.01	-0.03	-0.02	-0.01	0.00	0.00	6	0	1.00

Table 198: Cassin's Finch

	mean	sd	2.5%	25%	50%	75%	97.5%	n.eff	overlap0	f
alpha.lambda	5.87	0.49	5.09	5.36	5.97	6.33	6.65	3	0	1.00
alpha.phi	-1.07	1.68	-2.56	-2.48	-2.02	1.24	1.44	3	1	0.67
alpha.p	-4.29	0.66	-4.95	-4.83	-4.63	-3.39	-3.31	3	0	1.00
beta.hab.lambda	0.31	0.08	0.14	0.27	0.29	0.36	0.48	4	0	1.00

	mean	sd	2.5%	25%	50%	75%	97.5%	n.eff	overlap0	f
beta.hab.gamma	-0.19	0.45	-0.86	-0.80	0.11	0.14	0.16	3	1	0.33
beta.hab.phi	0.77	0.33	0.50	0.53	0.55	1.23	1.26	3	0	1.00
beta.elev.lambda	0.94	0.78	0.08	0.30	0.57	1.92	2.12	3	0	1.00
beta.ele.gamma2	0.19	0.13	-0.01	0.05	0.22	0.32	0.34	3	1	0.95
beta.elev.phi	1.44	0.72	0.37	0.47	1.91	1.95	2.04	3	0	1.00
beta.develop.lambda	1.34	0.32	0.88	1.01	1.36	1.62	1.89	4	0	1.00
beta.develop.gamma	-0.04	0.33	-0.56	-0.46	0.10	0.27	0.31	3	1	0.33
beta.develop.phi	-0.19	0.41	-0.81	-0.74	0.00	0.12	0.32	3	1	0.50
beta.effort	0.00	0.00	0.00	0.00	0.00	0.01	0.01	14	0	1.00
beta.min_temp	-0.02	0.01	-0.03	-0.02	-0.02	-0.01	-0.01	12	0	1.00

Table 199: Cinnamon Teal

	mean	sd	2.5%	25%	50%	75%	97.5%	n.eff	overlap0	f
alpha.lambda	4.30	1.19	3.33	3.44	3.50	5.92	6.08	3	0	1.00
alpha.phi	-0.85	2.15	-4.09	-3.77	0.52	0.81	0.86	3	1	0.33
alpha.p	-2.26	1.01	-3.74	-3.64	-1.64	-1.46	-1.43	3	0	1.00
beta.hab.lambda	-0.98	0.35	-1.48	-1.24	-1.09	-0.63	-0.35	3	0	1.00
beta.hab.gamma	0.12	0.27	-0.09	-0.07	-0.06	0.50	0.50	3	1	0.33
beta.hab.phi	-1.01	1.65	-3.57	-3.20	0.15	0.16	0.18	3	1	0.33
beta.elev.lambda	-0.55	0.48	-0.97	-0.91	-0.86	0.11	0.17	3	1	0.67
beta.ele.gamma2	-0.10	0.38	-0.42	-0.40	-0.32	0.43	0.44	3	1	0.67
beta.elev.phi	-1.14	1.44	-3.29	-3.10	-0.18	-0.08	-0.05	3	0	1.00
beta.develop.lambda	0.00	0.06	-0.13	-0.04	0.00	0.04	0.11	7	1	0.50
beta.develop.gamma	0.72	0.02	0.68	0.71	0.72	0.73	0.77	326	0	1.00
beta.develop.phi	0.00	0.26	-0.26	-0.20	-0.14	0.35	0.38	3	1	0.33
beta.effort	0.12	0.00	0.11	0.11	0.11	0.12	0.12	83	0	1.00
beta.min_temp	-0.02	0.02	-0.06	-0.04	-0.01	0.00	0.00	3	0	1.00

Table 200: Clark's Grebe

	mean	sd	2.5%	25%	50%	75%	97.5%	n.eff	overlap0	f
alpha.lambda	-7.41	1.40	-9.89	-8.47	-7.44	-6.40	-4.71	8	0	1.00
alpha.phi	5.51	2.39	2.36	2.40	5.97	7.94	8.52	3	0	1.00
alpha.p	-2.29	0.11	-2.45	-2.44	-2.23	-2.22	-2.20	3	0	1.00
beta.hab.lambda	-0.59	1.72	-4.34	-1.57	-0.33	0.62	2.47	30	1	0.58
beta.hab.gamma	0.09	0.42	-0.50	-0.48	0.27	0.50	0.52	3	1	0.67
beta.hab.phi	-0.66	6.65	-5.98	-5.33	-5.18	8.10	9.84	3	1	0.67
beta.elev.lambda	0.70	1.68	-1.79	-0.63	0.27	2.13	3.91	9	1	0.56
beta.ele.gamma2	0.05	0.64	-0.66	-0.65	-0.09	0.88	0.90	3	1	0.33
beta.elev.phi	-1.12	6.15	-10.26	-9.40	2.19	4.04	4.39	3	1	0.33
beta.develop.lambda	0.42	1.85	-3.24	-0.80	0.48	1.72	3.74	27	1	0.60
beta.develop.gamma	0.55	0.42	-0.05	-0.03	0.79	0.88	0.90	3	1	0.67
beta.develop.phi	-0.98	2.39	-3.15	-2.84	-2.34	2.31	2.49	3	1	0.67
beta.effort	0.16	0.03	0.12	0.13	0.15	0.20	0.21	3	0	1.00
beta.min_temp	-0.13	0.04	-0.19	-0.17	-0.13	-0.09	-0.08	3	0	1.00

Table 201: Common Ground Dove

	mean	sd	2.5%	25%	50%	75%	97.5%	n.eff	overlap0	f
alpha.lambda	5.19	0.02	5.15	5.18	5.19	5.20	5.23	29	0	1.00
alpha.phi	2.39	0.02	2.35	2.37	2.39	2.40	2.43	11	0	1.00
alpha.p	-1.31	0.01	-1.33	-1.32	-1.31	-1.30	-1.29	11	0	1.00
beta.hab.lambda	0.00	0.03	-0.05	-0.02	0.00	0.02	0.05	119	1	0.49
beta.hab.gamma	0.87	0.03	0.82	0.85	0.87	0.89	0.92	126	0	1.00
beta.hab.phi	-0.41	0.02	-0.45	-0.42	-0.41	-0.39	-0.36	6000	0	1.00
beta.elev.lambda	-0.33	0.02	-0.36	-0.34	-0.33	-0.32	-0.30	1896	0	1.00
beta.ele.gamma2	-0.70	0.03	-0.75	-0.72	-0.71	-0.68	-0.64	35	0	1.00
beta.elev.phi	0.02	0.02	-0.03	0.01	0.03	0.04	0.07	45	1	0.86
beta.develop.lambda	0.80	0.02	0.76	0.78	0.80	0.81	0.83	141	0	1.00
beta.develop.gamma	0.38	0.04	0.31	0.36	0.38	0.41	0.46	45	0	1.00
beta.develop.phi	-0.39	0.03	-0.45	-0.41	-0.39	-0.37	-0.33	79	0	1.00
beta.effort	0.00	0.00	0.00	0.00	0.00	0.00	0.01	1015	0	1.00
beta.min_temp	0.00	0.00	0.00	0.00	0.00	0.00	0.00	3598	0	1.00

Table 202: Gray Partridge

	mean	sd	2.5%	25%	50%	75%	97.5%	n.eff	overlap0	f
alpha.lambda	6.30	0.36	5.91	6.04	6.09	6.77	6.90	3	0	1.00
alpha.phi	1.77	1.18	-0.12	0.26	2.56	2.64	2.67	3	1	0.87
alpha.p	-2.98	0.58	-3.83	-3.78	-2.59	-2.56	-2.50	3	0	1.00
beta.hab.lambda	-0.37	0.13	-0.59	-0.44	-0.41	-0.24	-0.12	3	0	1.00
beta.hab.gamma	-0.12	0.67	-1.26	-0.97	0.32	0.36	0.45	3	1	0.33
beta.hab.phi	0.19	0.87	-0.56	-0.43	-0.39	1.33	1.63	3	1	0.33
beta.elev.lambda	0.05	0.04	0.00	0.02	0.05	0.08	0.14	4	1	0.96
beta.ele.gamma2	-0.13	0.34	-0.47	-0.38	-0.34	0.33	0.40	3	1	0.67
beta.elev.phi	0.45	0.49	-0.34	-0.22	0.76	0.81	0.88	3	1	0.67
beta.develop.lambda	0.11	0.45	-0.63	-0.48	0.41	0.44	0.49	3	1	0.67
beta.develop.gamma	1.00	0.83	-0.18	-0.14	1.50	1.68	1.81	3	1	0.67
beta.develop.phi	-0.47	0.79	-1.20	-1.08	-0.95	0.61	0.69	3	1	0.67
beta.effort	0.04	0.01	0.03	0.04	0.04	0.05	0.05	12	0	1.00
beta.min_temp	-0.19	0.01	-0.20	-0.20	-0.19	-0.19	-0.18	5	0	1.00

Table 203: Green Heron

	mean	sd	2.5%	25%	50%	75%	97.5%	n.eff	overlap0	f
alpha.lambda	3.14	0.10	2.96	3.03	3.18	3.22	3.27	3	0	1.00
alpha.phi	3.14	0.07	3.01	3.07	3.16	3.20	3.26	4	0	1.00
alpha.p	-1.18	0.08	-1.28	-1.24	-1.22	-1.08	-1.05	3	0	1.00
beta.hab.lambda	-0.06	0.02	-0.10	-0.07	-0.06	-0.05	-0.03	47	0	1.00
beta.hab.gamma	-0.10	0.03	-0.17	-0.13	-0.10	-0.08	-0.04	28	0	1.00
beta.hab.phi	0.03	0.03	-0.03	0.01	0.03	0.06	0.10	26	1	0.85
beta.elev.lambda	-0.91	0.03	-0.97	-0.94	-0.91	-0.89	-0.86	5	0	1.00
beta.ele.gamma2	-0.58	0.04	-0.66	-0.61	-0.58	-0.55	-0.50	22	0	1.00
beta.elev.phi	0.26	0.04	0.19	0.24	0.26	0.29	0.33	37	0	1.00
beta.develop.lambda	0.56	0.03	0.51	0.54	0.55	0.57	0.61	181	0	1.00
beta.develop.gamma	-0.18	0.04	-0.25	-0.20	-0.17	-0.15	-0.10	35	0	1.00

	mean	sd	2.5%	25%	50%	75%	97.5%	n.eff	overlap0	f
beta.develop.phi	0.57	0.03	0.51	0.55	0.57	0.59	0.64	10	0	1.00
beta.effort	0.00	0.00	0.00	0.00	0.00	0.00	0.00	1009	0	1.00
beta.min_temp	0.00	0.00	0.00	0.00	0.00	0.00	0.00	371	0	1.00

Table 204: Inca Dove

	mean	sd	2.5%	25%	50%	75%	97.5%	n.eff	overlap0	f
alpha.lambda	7.17	1.23	5.55	5.63	7.30	8.55	8.69	3	0	1.00
alpha.phi	-0.21	2.66	-3.69	-3.61	0.16	2.79	2.87	3	1	0.39
alpha.p	-3.39	1.62	-5.48	-5.32	-3.40	-1.43	-1.37	3	0	1.00
beta.hab.lambda	0.17	2.31	-4.34	-1.41	0.14	1.78	4.69	31	1	0.52
beta.hab.gamma	0.06	2.24	-4.38	-1.45	0.07	1.54	4.41	75	1	0.51
beta.hab.phi	-0.95	2.25	-5.33	-2.45	-0.95	0.56	3.46	306	1	0.66
beta.elev.lambda	0.15	0.23	-0.21	-0.15	0.24	0.33	0.43	3	1	0.67
beta.ele.gamma2	0.46	0.79	-0.41	-0.38	0.25	1.51	1.54	3	1	0.67
beta.elev.phi	-0.93	1.22	-2.79	-2.32	-0.79	0.48	0.51	3	1	0.67
beta.develop.lambda	0.24	2.33	-4.30	-1.34	0.27	1.84	4.77	26	1	0.55
beta.develop.gamma	0.11	2.24	-4.20	-1.39	0.09	1.60	4.44	81	1	0.52
beta.develop.phi	-0.89	2.25	-5.29	-2.39	-0.87	0.65	3.45	330	1	0.66
beta.effort	0.16	0.02	0.13	0.14	0.17	0.17	0.18	3	0	1.00
beta.min_temp	0.00	0.00	0.00	0.00	0.00	0.00	0.00	1	0	1.00

Table 205: Ladder-backed Woodpecker

	mean	sd	2.5%	25%	50%	75%	97.5%	n.eff	overlap0	f
alpha.lambda	3.45	0.05	3.35	3.41	3.45	3.49	3.54	4	0	1.00
alpha.phi	3.90	0.07	3.77	3.84	3.90	3.95	4.01	5	0	1.00
alpha.p	-0.88	0.04	-0.94	-0.91	-0.89	-0.85	-0.81	3	0	1.00
beta.hab.lambda	-0.03	0.03	-0.09	-0.05	-0.03	-0.01	0.02	420	1	0.87
beta.hab.gamma	-0.18	0.04	-0.28	-0.21	-0.18	-0.15	-0.11	25	0	1.00
beta.hab.phi	0.14	0.05	0.05	0.10	0.13	0.18	0.25	29	0	1.00
beta.elev.lambda	-0.20	0.03	-0.26	-0.22	-0.20	-0.18	-0.14	11	0	1.00
beta.ele.gamma2	-0.23	0.03	-0.29	-0.25	-0.23	-0.21	-0.17	46	0	1.00
beta.elev.phi	0.45	0.04	0.36	0.42	0.45	0.48	0.53	51	0	1.00
beta.develop.lambda	-0.45	0.09	-0.63	-0.51	-0.45	-0.38	-0.28	5	0	1.00
beta.develop.gamma	-0.69	0.13	-0.90	-0.79	-0.72	-0.57	-0.44	4	0	1.00
beta.develop.phi	2.91	0.20	2.55	2.76	2.93	3.04	3.33	7	0	1.00
beta.effort	0.11	0.01	0.09	0.10	0.11	0.12	0.13	164	0	1.00
beta.min_temp	0.00	0.00	0.00	0.00	0.00	0.00	0.00	6000	0	1.00

Table 206: Lesser Yellowlegs

	mean	sd	2.5%	25%	50%	75%	97.5%	n.eff	overlap0	f
alpha.lambda	5.83	0.06	5.69	5.78	5.84	5.88	5.93	5	0	1.00
alpha.phi	2.40	0.05	2.31	2.35	2.40	2.44	2.49	5	0	1.00
alpha.p	-2.53	0.03	-2.58	-2.55	-2.53	-2.50	-2.46	5	0	1.00
beta.hab.lambda	0.17	0.00	0.17	0.17	0.17	0.18	0.18	46	0	1.00

	mean	sd	2.5%	25%	50%	75%	97.5%	n.eff	overlap0	f
beta.hab.gamma	-1.01	0.29	-1.51	-1.19	-1.05	-0.76	-0.51	5	0	1.00
beta.hab.phi	1.04	0.20	0.67	0.87	1.06	1.18	1.39	4	0	1.00
beta.elev.lambda	-0.03	0.03	-0.09	-0.05	-0.02	0.00	0.03	5	1	0.77
beta.ele.gamma2	-0.02	0.03	-0.07	-0.04	-0.02	0.00	0.05	5	1	0.75
beta.elev.phi	-0.35	0.06	-0.46	-0.38	-0.35	-0.30	-0.26	4	0	1.00
beta.develop.lambda	0.63	0.02	0.60	0.62	0.63	0.64	0.67	16	0	1.00
beta.develop.gamma	-0.68	0.02	-0.72	-0.69	-0.68	-0.66	-0.63	25	0	1.00
beta.develop.phi	0.51	0.03	0.46	0.49	0.51	0.53	0.55	8	0	1.00
beta.effort	0.04	0.00	0.03	0.04	0.04	0.04	0.05	45	0	1.00
beta.min_temp	0.00	0.00	0.00	0.00	0.00	0.00	0.00	783	0	1.00

Table 207: Monk Parakeet

	mean	sd	2.5%	25%	50%	75%	97.5%	n.eff	overlap0	f
alpha.lambda	-7.42	1.59	-10.96	-8.43	-7.20	-6.29	-4.80	7	0	1.00
alpha.phi	1.58	0.02	1.54	1.56	1.58	1.60	1.63	1631	0	1.00
alpha.p	-0.43	0.02	-0.46	-0.44	-0.43	-0.42	-0.39	93	0	1.00
beta.hab.lambda	0.08	2.38	-4.62	-1.51	0.08	1.67	4.71	67	1	0.51
beta.hab.gamma	0.26	2.24	-4.03	-1.25	0.28	1.76	4.65	6000	1	0.55
beta.hab.phi	0.10	2.25	-4.35	-1.38	0.10	1.63	4.52	6000	1	0.52
beta.elev.lambda	0.65	1.15	-1.46	-0.19	0.61	1.39	2.99	98	1	0.70
beta.ele.gamma2	-0.65	0.02	-0.68	-0.66	-0.65	-0.64	-0.62	1533	0	1.00
beta.elev.phi	0.33	0.02	0.29	0.32	0.33	0.34	0.37	6000	0	1.00
beta.develop.lambda	0.03	2.42	-4.81	-1.58	0.11	1.70	4.60	48	1	0.52
beta.develop.gamma	0.21	2.24	-4.19	-1.30	0.18	1.71	4.47	6000	1	0.54
beta.develop.phi	0.18	2.25	-4.25	-1.36	0.18	1.66	4.64	6000	1	0.53
beta.effort	0.13	0.01	0.11	0.13	0.13	0.14	0.15	497	0	1.00
beta.min_temp	0.00	0.00	0.00	0.00	0.00	0.00	0.00	2282	0	1.00

Table 208: Nuttall's Woodpecker

	mean	sd	2.5%	25%	50%	75%	97.5%	n.eff	overlap0	f
alpha.lambda	3.09	0.05	2.99	3.06	3.09	3.13	3.20	72	0	1.00
alpha.phi	3.65	0.05	3.55	3.61	3.64	3.68	3.77	7	0	1.00
alpha.p	-0.59	0.02	-0.63	-0.60	-0.58	-0.57	-0.54	8	0	1.00
beta.hab.lambda	-0.39	0.04	-0.47	-0.42	-0.39	-0.36	-0.30	204	0	1.00
beta.hab.gamma	0.13	0.03	0.06	0.10	0.13	0.15	0.18	101	0	1.00
beta.hab.phi	-0.43	0.07	-0.54	-0.48	-0.43	-0.38	-0.29	98	0	1.00
beta.elev.lambda	-0.08	0.04	-0.16	-0.11	-0.08	-0.05	0.00	88	1	0.97
beta.ele.gamma2	-0.46	0.03	-0.51	-0.48	-0.46	-0.44	-0.39	151	0	1.00
beta.elev.phi	0.51	0.07	0.36	0.47	0.52	0.56	0.62	94	0	1.00
beta.develop.lambda	-0.22	0.08	-0.38	-0.28	-0.22	-0.17	-0.07	1574	0	1.00
beta.develop.gamma	0.49	0.03	0.42	0.47	0.49	0.51	0.55	476	0	1.00
beta.develop.phi	0.16	0.09	-0.03	0.10	0.16	0.22	0.31	51	1	0.95
beta.effort	0.00	0.00	0.00	0.00	0.00	0.00	0.01	828	0	1.00
beta.min_temp	-0.05	0.01	-0.06	-0.06	-0.05	-0.05	-0.04	429	0	1.00

Table 209: Oak Titmouse

	mean	sd	2.5%	25%	50%	75%	97.5%	n.eff	overlap0	f
alpha.lambda	-7.33	1.52	-11.07	-8.13	-7.17	-6.27	-4.86	43	0	1.00
alpha.phi	6.16	1.16	4.96	5.04	5.75	7.66	7.85	3	0	1.00
alpha.p	-0.67	0.45	-1.11	-1.11	-0.84	-0.06	-0.04	3	0	1.00
beta.hab.lambda	0.39	1.60	-2.73	-0.65	0.35	1.39	3.75	1338	1	0.59
beta.hab.gamma	-0.04	0.11	-0.19	-0.17	-0.03	0.08	0.11	3	1	0.67
beta.hab.phi	0.80	1.02	-0.77	-0.51	1.32	1.67	1.79	3	1	0.67
beta.elev.lambda	0.22	1.54	-2.66	-0.84	0.17	1.34	3.22	21	1	0.54
beta.ele.gamma2	-0.14	0.23	-0.47	-0.45	-0.02	0.05	0.08	3	1	0.66
beta.elev.phi	-0.86	2.99	-4.53	-4.28	-1.09	2.85	3.01	3	1	0.67
beta.develop.lambda	0.28	1.87	-3.61	-0.99	0.39	1.60	3.64	606	1	0.57
beta.develop.gamma	0.56	0.26	0.21	0.24	0.60	0.85	0.88	3	0	1.00
beta.develop.phi	-0.93	6.53	-7.15	-6.87	-3.94	8.01	8.53	3	1	0.67
beta.effort	0.00	0.00	0.00	0.00	0.00	0.00	0.00	18	0	1.00
beta.min_temp	-0.07	0.01	-0.09	-0.08	-0.06	-0.05	-0.04	3	0	1.00

Table 210: Osprey

	mean	sd	2.5%	25%	50%	75%	97.5%	n.eff	overlap0	f
alpha.lambda	-0.02	2.47	-2.05	-1.80	-1.66	3.45	3.52	3	1	0.67
alpha.phi	4.60	1.20	2.88	2.96	5.14	5.62	5.92	3	0	1.00
alpha.p	-0.92	1.16	-2.58	-2.55	-0.11	-0.09	-0.06	3	0	1.00
beta.hab.lambda	0.75	0.19	0.46	0.49	0.86	0.89	0.93	3	0	1.00
beta.hab.gamma	0.56	0.15	0.35	0.36	0.66	0.67	0.69	3	0	1.00
beta.hab.phi	0.04	0.64	-0.53	-0.43	-0.35	0.92	0.97	3	1	0.33
beta.elev.lambda	-0.51	0.35	-0.81	-0.76	-0.73	-0.04	0.01	3	1	0.93
beta.ele.gamma2	-0.46	0.24	-0.66	-0.64	-0.63	-0.12	-0.10	3	0	1.00
beta.elev.phi	0.18	1.10	-1.41	-1.36	0.90	0.97	1.09	3	1	0.67
beta.develop.lambda	1.45	0.31	0.96	1.05	1.63	1.69	1.77	3	0	1.00
beta.develop.gamma	0.93	0.04	0.86	0.90	0.94	0.96	0.98	9	0	1.00
beta.develop.phi	-0.16	0.48	-0.82	-0.78	-0.02	0.27	0.44	3	1	0.52
beta.effort	0.03	0.01	0.02	0.02	0.03	0.03	0.04	30	0	1.00
beta.min_temp	0.00	0.00	0.00	0.00	0.00	0.00	0.00	14	0	1.00

Table 211: Phainopepla

	mean	sd	2.5%	25%	50%	75%	97.5%	n.eff	overlap0	f
alpha.lambda	2.24	0.19	1.90	2.09	2.24	2.39	2.56	5	0	1
alpha.phi	3.43	0.34	3.13	3.18	3.21	3.87	3.96	3	0	1
alpha.p	-1.53	0.45	-2.19	-2.14	-1.23	-1.21	-1.19	3	0	1
beta.hab.lambda	1.92	0.13	1.76	1.82	1.87	2.06	2.15	3	0	1
beta.hab.gamma	0.79	0.09	0.67	0.71	0.74	0.90	0.94	3	0	1
beta.hab.phi	-0.33	0.22	-0.68	-0.61	-0.20	-0.17	-0.10	3	0	1
beta.elev.lambda	2.52	0.34	1.89	2.27	2.48	2.83	3.07	4	0	1
beta.ele.gamma2	-0.71	0.02	-0.75	-0.73	-0.71	-0.70	-0.67	1062	0	1
beta.elev.phi	0.56	0.04	0.49	0.54	0.56	0.60	0.64	5	0	1
beta.develop.lambda	2.17	0.19	1.87	2.02	2.11	2.37	2.49	3	0	1
beta.develop.gamma	-1.58	0.13	-1.77	-1.68	-1.62	-1.44	-1.36	3	0	1

	mean	sd	2.5%	25%	50%	75%	97.5%	n.eff	overlap0	f
beta.develop.phi	3.85	0.66	3.21	3.36	3.45	4.68	4.95	3	0	1
beta.effort	0.20	0.01	0.19	0.20	0.20	0.21	0.22	10	0	1
beta.min_temp	0.00	0.00	0.00	0.00	0.00	0.00	0.00	1039	0	1

Table 212: Pine Grosbeak

	mean	sd	2.5%	25%	50%	75%	97.5%	n.eff	overlap0	f
alpha.lambda	6.12	0.08	5.92	6.08	6.15	6.18	6.25	4	0	1.00
alpha.phi	1.90	2.27	-1.34	-1.29	3.43	3.56	3.68	3	1	0.67
alpha.p	-3.38	0.80	-4.57	-4.46	-2.85	-2.81	-2.75	3	0	1.00
beta.hab.lambda	0.79	0.59	0.35	0.37	0.37	1.59	1.68	3	0	1.00
beta.hab.gamma	0.42	0.21	0.14	0.14	0.48	0.61	0.68	3	0	1.00
beta.hab.phi	-0.33	0.25	-0.61	-0.56	-0.41	0.00	0.01	3	1	0.78
beta.elev.lambda	-0.69	0.26	-1.11	-1.03	-0.52	-0.51	-0.46	3	0	1.00
beta.ele.gamma2	-0.86	0.46	-1.29	-1.22	-1.13	-0.22	-0.21	3	0	1.00
beta.elev.phi	0.84	0.58	0.02	0.03	1.17	1.31	1.38	3	0	1.00
beta.develop.lambda	-0.02	0.27	-0.30	-0.26	-0.14	0.31	0.46	3	1	0.67
beta.develop.gamma	0.07	0.20	-0.17	-0.14	0.03	0.26	0.42	3	1	0.56
beta.develop.phi	0.28	0.46	-0.21	-0.14	0.10	0.91	0.95	3	1	0.67
beta.effort	0.04	0.01	0.03	0.03	0.04	0.04	0.05	4	0	1.00
beta.min_temp	-0.13	0.02	-0.17	-0.16	-0.11	-0.11	-0.11	3	0	1.00

Table 213: Pyrrhuloxia

	mean	sd	2.5%	25%	50%	75%	97.5%	n.eff	overlap0	f
alpha.lambda	6.13	0.06	6.06	6.08	6.11	6.19	6.23	3	0	1.00
alpha.phi	3.10	0.03	3.04	3.08	3.10	3.12	3.18	8	0	1.00
alpha.p	-1.76	0.05	-1.85	-1.82	-1.74	-1.72	-1.70	3	0	1.00
beta.hab.lambda	-0.12	0.04	-0.19	-0.16	-0.13	-0.09	-0.03	4	0	1.00
beta.hab.gamma	-0.74	0.05	-0.86	-0.77	-0.73	-0.70	-0.66	20	0	1.00
beta.hab.phi	1.42	0.06	1.33	1.38	1.42	1.46	1.57	32	0	1.00
beta.elev.lambda	-0.04	0.05	-0.13	-0.07	-0.03	0.00	0.04	4	1	0.77
beta.ele.gamma2	0.24	0.05	0.13	0.20	0.24	0.27	0.34	5	0	1.00
beta.elev.phi	-0.68	0.07	-0.80	-0.73	-0.68	-0.64	-0.56	4	0	1.00
beta.develop.lambda	-0.31	0.19	-0.56	-0.49	-0.36	-0.08	0.00	3	0	0.98
beta.develop.gamma	-6.80	0.34	-7.42	-7.02	-6.82	-6.58	-6.11	4	0	1.00
beta.develop.phi	5.95	0.41	5.24	5.64	5.98	6.37	6.53	3	0	1.00
beta.effort	0.09	0.01	0.08	0.08	0.09	0.09	0.10	15	0	1.00
beta.min_temp	-0.11	0.01	-0.12	-0.12	-0.11	-0.11	-0.10	6	0	1.00

Table 214: Red-necked Grebe

	mean	sd	2.5%	25%	50%	75%	97.5%	n.eff	overlap0	f
alpha.lambda	5.26	0.18	5.06	5.11	5.16	5.48	5.57	3	0	1.00
alpha.phi	2.01	0.90	0.68	0.77	2.62	2.66	2.70	3	0	1.00
alpha.p	-1.89	0.03	-1.94	-1.91	-1.89	-1.88	-1.84	11	0	1.00
beta.hab.lambda	-0.15	0.12	-0.32	-0.29	-0.14	-0.01	0.01	3	1	0.86

	mean	sd	2.5%	25%	50%	75%	97.5%	n.eff	overlap0	f
beta.hab.gamma	-0.34	0.03	-0.39	-0.36	-0.34	-0.31	-0.28	4	0	1.00
beta.hab.phi	1.71	0.14	1.54	1.61	1.65	1.85	1.97	3	0	1.00
beta.elev.lambda	0.85	0.07	0.75	0.78	0.83	0.93	0.98	3	0	1.00
beta.ele.gamma2	0.73	0.05	0.66	0.69	0.72	0.78	0.82	3	0	1.00
beta.elev.phi	-1.87	0.07	-2.00	-1.92	-1.89	-1.80	-1.74	7	0	1.00
beta.develop.lambda	0.87	0.14	0.75	0.77	0.79	1.06	1.09	3	0	1.00
beta.develop.gamma	-0.05	0.61	-0.57	-0.51	-0.45	0.80	0.83	3	1	0.67
beta.develop.phi	0.93	0.51	0.43	0.50	0.67	1.59	1.71	3	0	1.00
beta.effort	0.06	0.01	0.04	0.05	0.06	0.06	0.07	4	0	1.00
beta.min_temp	0.00	0.00	0.00	0.00	0.00	0.00	0.00	120	0	1.00

Table 215: Roseate Spoonbill

	mean	sd	2.5%	25%	50%	75%	97.5%	n.eff	overlap0	f
alpha.lambda	-7.73	0.50	-8.74	-8.06	-7.69	-7.37	-6.83	9	0	1
alpha.phi	3.08	0.05	3.00	3.05	3.08	3.11	3.19	32	0	1
alpha.p	-1.48	0.02	-1.51	-1.50	-1.48	-1.47	-1.45	6	0	1
beta.hab.lambda	-1.04	0.03	-1.09	-1.06	-1.04	-1.02	-0.98	20	0	1
beta.hab.gamma	0.61	0.01	0.59	0.60	0.61	0.61	0.62	50	0	1
beta.hab.phi	-0.27	0.01	-0.29	-0.28	-0.27	-0.27	-0.26	97	0	1
beta.elev.lambda	-1.30	0.04	-1.38	-1.33	-1.30	-1.27	-1.23	5	0	1
beta.ele.gamma2	-0.26	0.02	-0.30	-0.28	-0.26	-0.25	-0.21	7	0	1
beta.elev.phi	0.25	0.03	0.19	0.24	0.25	0.27	0.31	7	0	1
beta.develop.lambda	-17.61	0.64	-18.91	-18.05	-17.58	-17.15	-16.46	86	0	1
beta.develop.gamma	0.34	0.05	0.25	0.30	0.34	0.37	0.44	20	0	1
beta.develop.phi	-1.85	0.10	-2.06	-1.91	-1.85	-1.78	-1.66	9	0	1
beta.effort	0.00	0.00	0.00	0.00	0.00	0.00	0.00	2127	0	1
beta.min_temp	0.00	0.00	0.00	0.00	0.00	0.00	0.00	6000	0	1

Table 216: Rough-legged Hawk

	mean	sd	2.5%	25%	50%	75%	97.5%	n.eff	overlap0	f
alpha.lambda	3.40	0.04	3.32	3.36	3.42	3.44	3.46	3	0	1.00
alpha.phi	3.02	0.03	2.97	3.00	3.02	3.04	3.06	4	0	1.00
alpha.p	-1.60	0.04	-1.65	-1.63	-1.61	-1.55	-1.52	3	0	1.00
beta.hab.lambda	-0.03	0.01	-0.05	-0.04	-0.03	-0.03	-0.01	29	0	1.00
beta.hab.gamma	-0.08	0.01	-0.11	-0.09	-0.08	-0.07	-0.05	43	0	1.00
beta.hab.phi	0.20	0.03	0.15	0.18	0.20	0.22	0.27	16	0	1.00
beta.elev.lambda	0.01	0.02	-0.03	0.00	0.01	0.02	0.04	6	1	0.65
beta.ele.gamma2	0.90	0.02	0.85	0.88	0.89	0.91	0.94	78	0	1.00
beta.elev.phi	-0.13	0.01	-0.16	-0.14	-0.13	-0.12	-0.10	78	0	1.00
beta.develop.lambda	-0.22	0.03	-0.27	-0.24	-0.22	-0.20	-0.17	19	0	1.00
beta.develop.gamma	-0.08	0.05	-0.18	-0.11	-0.08	-0.05	0.01	22	1	0.97
beta.develop.phi	-0.18	0.05	-0.25	-0.21	-0.18	-0.14	-0.08	15	0	1.00
beta.effort	0.00	0.00	0.00	0.00	0.00	0.00	0.00	842	0	1.00
beta.min_temp	-0.15	0.00	-0.16	-0.15	-0.15	-0.15	-0.14	17	0	1.00

Table 217: Ruffed Grouse

	mean	sd	2.5%	25%	50%	75%	97.5%	n.eff	overlap0	f
alpha.lambda	3.32	0.04	3.24	3.29	3.32	3.35	3.39	5	0	1.00
alpha.phi	2.28	0.04	2.22	2.26	2.28	2.31	2.35	6	0	1.00
alpha.p	-1.33	0.04	-1.39	-1.36	-1.33	-1.30	-1.26	4	0	1.00
beta.hab.lambda	0.28	0.02	0.24	0.26	0.28	0.30	0.33	48	0	1.00
beta.hab.gamma	0.12	0.03	0.06	0.10	0.12	0.13	0.18	64	0	1.00
beta.hab.phi	-0.04	0.03	-0.10	-0.06	-0.04	-0.02	0.01	58	1	0.92
beta.elev.lambda	-0.10	0.02	-0.14	-0.11	-0.10	-0.08	-0.06	19	0	1.00
beta.ele.gamma2	-0.01	0.03	-0.06	-0.03	-0.01	0.01	0.06	633	1	0.61
beta.elev.phi	0.01	0.03	-0.06	-0.02	0.01	0.03	0.06	209	1	0.61
beta.develop.lambda	0.53	0.04	0.44	0.50	0.53	0.56	0.62	41	0	1.00
beta.develop.gamma	-1.06	0.06	-1.18	-1.10	-1.06	-1.02	-0.94	30	0	1.00
beta.develop.phi	0.06	0.05	-0.04	0.03	0.06	0.09	0.16	92	1	0.90
beta.effort	0.00	0.00	0.00	0.00	0.00	0.00	0.01	1835	0	1.00
beta.min_temp	-0.03	0.01	-0.04	-0.04	-0.03	-0.03	-0.02	208	0	1.00

Table 218: Say's Phoebe

	mean	sd	2.5%	25%	50%	75%	97.5%	n.eff	overlap0	f
alpha.lambda	3.65	0.04	3.57	3.62	3.65	3.68	3.73	4	0	1.00
alpha.phi	4.05	0.06	3.95	4.01	4.05	4.08	4.18	6	0	1.00
alpha.p	-0.71	0.03	-0.76	-0.73	-0.71	-0.68	-0.66	3	0	1.00
beta.hab.lambda	-0.09	0.03	-0.16	-0.11	-0.08	-0.06	-0.03	11	0	1.00
beta.hab.gamma	0.41	0.02	0.37	0.40	0.41	0.43	0.46	8	0	1.00
beta.hab.phi	-0.56	0.03	-0.62	-0.58	-0.56	-0.54	-0.51	8	0	1.00
beta.elev.lambda	0.03	0.02	-0.01	0.01	0.03	0.04	0.07	22	1	0.92
beta.ele.gamma2	-0.05	0.02	-0.08	-0.06	-0.05	-0.03	-0.01	123	0	1.00
beta.elev.phi	-0.91	0.06	-1.04	-0.95	-0.91	-0.87	-0.81	9	0	1.00
beta.develop.lambda	0.19	0.04	0.11	0.16	0.19	0.22	0.27	17	0	1.00
beta.develop.gamma	0.16	0.03	0.10	0.14	0.16	0.18	0.21	380	0	1.00
beta.develop.phi	0.47	0.04	0.39	0.44	0.47	0.50	0.55	46	0	1.00
beta.effort	0.10	0.01	0.08	0.09	0.10	0.10	0.11	17	0	1.00
beta.min_temp	0.00	0.00	0.00	0.00	0.00	0.00	0.00	1109	0	1.00

Table 219: Scaled Quail

	mean	sd	2.5%	25%	50%	75%	97.5%	n.eff	overlap0	f
alpha.lambda	6.39	0.06	6.30	6.34	6.38	6.44	6.50	4	0	1.00
alpha.phi	2.74	0.06	2.66	2.69	2.72	2.78	2.86	4	0	1.00
alpha.p	-2.17	0.06	-2.29	-2.22	-2.15	-2.12	-2.10	3	0	1.00
beta.hab.lambda	-0.15	0.02	-0.18	-0.16	-0.16	-0.14	-0.12	34	0	1.00
beta.hab.gamma	0.56	0.03	0.50	0.54	0.56	0.58	0.62	6	0	1.00
beta.hab.phi	-0.23	0.02	-0.27	-0.25	-0.24	-0.22	-0.19	36	0	1.00
beta.elev.lambda	0.05	0.03	0.00	0.04	0.05	0.07	0.10	218	0	0.99
beta.ele.gamma2	-0.56	0.03	-0.63	-0.58	-0.56	-0.53	-0.49	15	0	1.00
beta.elev.phi	0.88	0.04	0.80	0.85	0.88	0.90	0.95	43	0	1.00
beta.develop.lambda	0.63	0.06	0.51	0.59	0.63	0.67	0.76	99	0	1.00
beta.develop.gamma	-0.79	0.07	-0.93	-0.84	-0.79	-0.74	-0.66	11	0	1.00

	mean	sd	2.5%	25%	50%	75%	97.5%	n.eff	overlap0	f
beta.develop.phi	0.52	0.11	0.30	0.43	0.52	0.60	0.72	6	0	1.00
beta.effort	0.01	0.00	0.00	0.00	0.01	0.01	0.02	76	0	1.00
beta.min_temp	-0.08	0.01	-0.09	-0.09	-0.08	-0.08	-0.07	18	0	1.00

Table 220: Sedge Wren

	mean	sd	2.5%	25%	50%	75%	97.5%	n.eff	overlap0	f
alpha.lambda	3.47	0.05	3.38	3.44	3.47	3.51	3.56	13	0	1
alpha.phi	2.36	0.03	2.31	2.34	2.36	2.38	2.42	12	0	1
alpha.p	-1.28	0.01	-1.31	-1.29	-1.28	-1.27	-1.25	13	0	1
beta.hab.lambda	-0.13	0.04	-0.22	-0.16	-0.13	-0.10	-0.05	622	0	1
beta.hab.gamma	0.22	0.03	0.16	0.20	0.22	0.24	0.28	11	0	1
beta.hab.phi	-0.25	0.04	-0.32	-0.28	-0.25	-0.22	-0.16	12	0	1
beta.elev.lambda	-0.26	0.03	-0.31	-0.28	-0.26	-0.24	-0.21	91	0	1
beta.ele.gamma2	-0.47	0.02	-0.51	-0.49	-0.47	-0.46	-0.43	27	0	1
beta.elev.phi	-0.20	0.02	-0.24	-0.21	-0.20	-0.19	-0.16	53	0	1
beta.develop.lambda	-0.44	0.07	-0.59	-0.49	-0.44	-0.39	-0.30	89	0	1
beta.develop.gamma	-0.70	0.05	-0.80	-0.74	-0.71	-0.67	-0.59	53	0	1
beta.develop.phi	0.33	0.07	0.18	0.29	0.34	0.38	0.46	49	0	1
beta.effort	0.04	0.01	0.03	0.04	0.04	0.04	0.05	414	0	1
beta.min_temp	0.00	0.00	0.00	0.00	0.00	0.00	0.00	6000	0	1

Table 221: Sharp-tailed Grouse

	mean	sd	2.5%	25%	50%	75%	97.5%	n.eff	overlap0	f
alpha.lambda	5.73	1.06	4.59	4.80	5.27	7.14	7.31	3	0	1.00
alpha.phi	1.49	2.65	-2.24	-2.02	2.40	3.77	4.72	3	1	0.67
alpha.p	-3.11	0.88	-4.38	-4.34	-2.54	-2.48	-2.38	3	0	1.00
beta.hab.lambda	0.51	0.38	0.07	0.17	0.35	0.95	1.09	3	0	1.00
beta.hab.gamma	-0.04	0.85	-1.21	-1.17	0.23	0.79	0.91	3	1	0.33
beta.hab.phi	0.52	2.22	-1.87	-1.46	-0.40	3.52	3.67	3	1	0.33
beta.elev.lambda	-0.15	0.49	-0.89	-0.80	0.06	0.24	0.45	3	1	0.41
beta.ele.gamma2	-0.01	0.14	-0.14	-0.10	-0.07	0.02	0.42	4	1	0.71
beta.elev.phi	0.08	0.63	-1.02	-0.72	0.48	0.56	0.60	3	1	0.67
beta.develop.lambda	1.00	0.33	0.17	0.86	1.03	1.24	1.49	5	0	0.99
beta.develop.gamma	0.85	0.27	0.43	0.52	0.94	1.09	1.17	3	0	1.00
beta.develop.phi	-0.07	1.59	-2.24	-1.36	-0.57	2.04	2.14	3	1	0.67
beta.effort	0.00	0.00	0.00	0.00	0.00	0.00	0.00	15	0	1.00
beta.min_temp	-0.22	0.01	-0.24	-0.23	-0.22	-0.20	-0.19	3	0	1.00

Table 222: Sora

	mean	sd	2.5%	25%	50%	75%	97.5%	n.eff	overlap0	f
alpha.lambda	3.30	0.04	3.21	3.27	3.30	3.33	3.38	124	0	1
alpha.phi	1.35	0.06	1.25	1.32	1.35	1.39	1.47	4	0	1
alpha.p	-0.99	0.02	-1.04	-1.01	-0.99	-0.98	-0.95	7	0	1
beta.hab.lambda	0.23	0.02	0.20	0.22	0.23	0.25	0.27	148	0	1

	mean	sd	2.5%	25%	50%	75%	97.5%	n.eff	overlap0	f
beta.hab.gamma	0.43	0.01	0.40	0.42	0.43	0.44	0.45	55	0	1
beta.hab.phi	-0.30	0.02	-0.33	-0.31	-0.30	-0.29	-0.26	29	0	1
beta.elev.lambda	-0.48	0.03	-0.54	-0.50	-0.48	-0.45	-0.41	185	0	1
beta.ele.gamma2	0.25	0.03	0.19	0.24	0.26	0.27	0.31	7	0	1
beta.elev.phi	-0.55	0.04	-0.63	-0.57	-0.55	-0.53	-0.45	6	0	1
beta.develop.lambda	0.41	0.04	0.33	0.38	0.41	0.43	0.48	44	0	1
beta.develop.gamma	0.69	0.03	0.63	0.67	0.69	0.71	0.75	7	0	1
beta.develop.phi	-0.24	0.04	-0.31	-0.27	-0.24	-0.22	-0.17	6	0	1
beta.effort	0.25	0.01	0.23	0.24	0.25	0.26	0.27	66	0	1
beta.min_temp	0.00	0.00	0.00	0.00	0.00	0.00	0.00	2201	0	1

Table 223: Townsend's Solitaire

	mean	sd	2.5%	25%	50%	75%	97.5%	n.eff	overlap0	f
alpha.lambda	3.54	0.14	3.40	3.43	3.46	3.73	3.77	3	0	1.0
alpha.phi	4.28	0.10	4.12	4.19	4.29	4.35	4.46	4	0	1.0
alpha.p	-1.57	0.05	-1.65	-1.64	-1.55	-1.54	-1.51	3	0	1.0
beta.hab.lambda	0.20	0.01	0.17	0.18	0.19	0.21	0.22	16	0	1.0
beta.hab.gamma	-0.23	0.02	-0.28	-0.25	-0.23	-0.22	-0.19	11	0	1.0
beta.hab.phi	0.18	0.03	0.13	0.16	0.19	0.21	0.24	17	0	1.0
beta.elev.lambda	1.51	0.05	1.42	1.47	1.51	1.55	1.60	4	0	1.0
beta.ele.gamma2	2.31	0.10	2.07	2.25	2.33	2.38	2.45	6	0	1.0
beta.elev.phi	-0.27	0.14	-0.47	-0.39	-0.32	-0.13	-0.03	3	0	1.0
beta.develop.lambda	1.04	0.07	0.93	0.99	1.04	1.09	1.16	4	0	1.0
beta.develop.gamma	-0.19	0.20	-0.42	-0.33	-0.27	-0.07	0.31	4	1	0.8
beta.develop.phi	2.48	0.18	2.06	2.33	2.50	2.61	2.80	4	0	1.0
beta.effort	0.00	0.00	0.00	0.00	0.00	0.01	0.01	9	0	1.0
beta.min_temp	0.00	0.00	0.00	0.00	0.00	0.00	0.00	4459	0	1.0

Table 224: Townsend's Warbler

	mean	sd	2.5%	25%	50%	75%	97.5%	n.eff	overlap0	f
alpha.lambda	3.68	1.05	2.83	2.91	2.97	5.14	5.23	3	0	1
alpha.phi	3.84	0.39	3.48	3.56	3.60	4.33	4.50	3	0	1
alpha.p	-1.20	0.82	-2.36	-2.36	-0.64	-0.62	-0.61	3	0	1
beta.hab.lambda	-0.76	0.20	-1.06	-1.02	-0.65	-0.60	-0.53	3	0	1
beta.hab.gamma	-0.38	0.07	-0.49	-0.47	-0.35	-0.33	-0.32	3	0	1
beta.hab.phi	2.64	0.79	1.99	2.06	2.12	3.70	3.86	3	0	1
beta.elev.lambda	0.77	0.26	0.49	0.57	0.63	1.12	1.17	3	0	1
beta.ele.gamma2	0.40	0.19	0.24	0.26	0.28	0.66	0.68	3	0	1
beta.elev.phi	-1.51	0.85	-2.81	-2.67	-0.93	-0.90	-0.85	3	0	1
beta.develop.lambda	-0.43	0.09	-0.59	-0.49	-0.44	-0.38	-0.25	18	0	1
beta.develop.gamma	-0.70	0.02	-0.74	-0.71	-0.70	-0.69	-0.67	9	0	1
beta.develop.phi	-0.88	0.46	-1.34	-1.23	-1.14	-0.28	-0.16	3	0	1
beta.effort	0.02	0.02	0.00	0.00	0.00	0.04	0.05	3	0	1
beta.min_temp	0.00	0.00	0.00	0.00	0.00	0.00	0.00	13	0	1

Table 225: Verdin

	mean	sd	2.5%	25%	50%	75%	97.5%	n.eff	overlap0	f
alpha.lambda	4.97	0.44	4.47	4.54	4.83	5.55	5.59	3	0	1.00
alpha.phi	5.47	0.66	4.57	4.71	5.52	6.15	6.43	3	0	1.00
alpha.p	-1.45	0.45	-2.06	-2.05	-1.30	-0.99	-0.98	3	0	1.00
beta.hab.lambda	1.46	0.05	1.36	1.42	1.45	1.51	1.55	3	0	1.00
beta.hab.gamma	1.44	1.05	0.08	0.11	1.54	2.59	2.83	3	0	1.00
beta.hab.phi	-0.49	2.40	-2.41	-2.24	-2.07	2.86	2.99	3	1	0.67
beta.elev.lambda	-1.16	0.02	-1.21	-1.17	-1.16	-1.15	-1.11	59	0	1.00
beta.ele.gamma2	2.10	3.50	-1.76	-1.73	1.37	6.32	7.13	3	1	0.67
beta.elev.phi	2.13	1.62	0.93	0.99	1.00	4.34	4.55	3	0	1.00
beta.develop.lambda	0.27	0.59	-0.65	-0.36	0.31	0.92	1.06	3	1	0.67
beta.develop.gamma	2.59	1.03	1.10	1.18	3.13	3.47	3.57	3	0	1.00
beta.develop.phi	-0.45	0.54	-0.94	-0.84	-0.78	0.25	0.50	3	1	0.71
beta.effort	0.09	0.03	0.06	0.07	0.07	0.14	0.15	3	0	1.00
beta.min_temp	-0.04	0.04	-0.11	-0.09	-0.01	0.00	0.00	3	0	1.00

Table 226: Virginia Rail

	mean	sd	2.5%	25%	50%	75%	97.5%	n.eff	overlap0	f
alpha.lambda	3.20	0.93	2.47	2.53	2.58	4.51	4.53	3	0	1.00
alpha.phi	2.75	0.48	2.34	2.40	2.43	3.38	3.50	3	0	1.00
alpha.p	-1.75	0.81	-2.91	-2.89	-1.19	-1.17	-1.14	3	0	1.00
beta.hab.lambda	-0.02	0.06	-0.13	-0.09	0.01	0.03	0.06	3	1	0.38
beta.hab.gamma	-0.02	0.07	-0.10	-0.07	-0.05	0.08	0.10	3	1	0.67
beta.hab.phi	0.41	0.26	0.18	0.22	0.24	0.76	0.81	3	0	1.00
beta.elev.lambda	-0.25	0.07	-0.36	-0.31	-0.27	-0.16	-0.13	3	0	1.00
beta.ele.gamma2	-0.18	0.27	-0.42	-0.37	-0.35	0.19	0.24	3	1	0.67
beta.elev.phi	0.08	0.28	-0.35	-0.30	0.26	0.29	0.34	3	1	0.67
beta.develop.lambda	-0.10	0.10	-0.23	-0.18	-0.14	-0.03	0.11	4	1	0.81
beta.develop.gamma	-0.11	0.08	-0.27	-0.19	-0.08	-0.05	0.00	3	1	0.97
beta.develop.phi	0.04	0.15	-0.12	-0.07	-0.03	0.22	0.31	3	1	0.38
beta.effort	0.11	0.01	0.09	0.10	0.11	0.12	0.13	4	0	1.00
beta.min_temp	0.00	0.00	0.00	0.00	0.00	0.00	0.00	88	0	1.00

Table 227: White-tailed Kite

	mean	sd	2.5%	25%	50%	75%	97.5%	n.eff	overlap0	f
alpha.lambda	4.24	0.04	4.18	4.22	4.24	4.27	4.32	8	0	1.00
alpha.phi	2.88	0.04	2.81	2.85	2.87	2.90	2.94	17	0	1.00
alpha.p	-1.28	0.02	-1.31	-1.29	-1.28	-1.26	-1.23	10	0	1.00
beta.hab.lambda	-0.03	0.02	-0.08	-0.05	-0.03	-0.02	0.01	10	1	0.91
beta.hab.gamma	-0.31	0.03	-0.36	-0.33	-0.31	-0.29	-0.26	38	0	1.00
beta.hab.phi	1.07	0.06	0.94	1.03	1.07	1.11	1.17	10	0	1.00
beta.elev.lambda	-0.40	0.02	-0.44	-0.41	-0.40	-0.39	-0.35	16	0	1.00
beta.ele.gamma2	-0.17	0.03	-0.23	-0.19	-0.17	-0.15	-0.11	88	0	1.00
beta.elev.phi	-0.29	0.03	-0.34	-0.31	-0.29	-0.27	-0.23	29	0	1.00
beta.develop.lambda	0.67	0.04	0.60	0.65	0.67	0.70	0.75	35	0	1.00
beta.develop.gamma	-0.11	0.07	-0.23	-0.16	-0.12	-0.07	0.03	13	1	0.94

	mean	sd	2.5%	25%	50%	75%	97.5%	n.eff	overlap0	f
beta.develop.phi	0.47	0.06	0.34	0.42	0.47	0.51	0.57	9	0	1.00
beta.effort	0.04	0.01	0.02	0.03	0.04	0.04	0.06	49	0	1.00
beta.min_temp	0.00	0.00	-0.01	-0.01	0.00	0.00	0.00	6000	0	1.00

Table 228: White-winged Crossbill

	mean	sd	2.5%	25%	50%	75%	97.5%	n.eff	overlap0	f
alpha.lambda	4.49	0.82	2.99	3.69	4.69	5.27	5.49	3	0	1.00
alpha.phi	-0.65	0.35	-1.03	-0.99	-0.77	-0.18	-0.14	3	0	1.00
alpha.p	-4.65	0.10	-4.81	-4.74	-4.65	-4.59	-4.44	5	0	1.00
beta.hab.lambda	0.54	0.33	0.14	0.30	0.46	0.66	1.35	4	0	1.00
beta.hab.gamma	0.26	0.06	0.17	0.18	0.27	0.33	0.33	3	0	1.00
beta.hab.phi	-0.16	0.20	-0.35	-0.34	-0.26	0.12	0.13	3	1	0.67
beta.elev.lambda	-0.10	0.16	-0.48	-0.16	-0.08	-0.02	0.22	8	1	0.80
beta.ele.gamma2	0.07	0.07	-0.03	-0.02	0.08	0.14	0.15	3	1	0.67
beta.elev.phi	-0.12	0.20	-0.29	-0.28	-0.24	0.16	0.18	3	1	0.67
beta.develop.lambda	-0.36	1.25	-3.34	-1.07	0.02	0.63	1.16	4	1	0.50
beta.develop.gamma	-0.40	0.17	-0.61	-0.58	-0.42	-0.18	-0.17	3	0	1.00
beta.develop.phi	0.51	0.31	0.06	0.09	0.69	0.74	0.79	3	0	1.00
beta.effort	0.10	0.00	0.09	0.10	0.10	0.10	0.11	8	0	1.00
beta.min_temp	-0.13	0.01	-0.14	-0.13	-0.13	-0.13	-0.12	5	0	1.00

Table 229: Wilson's Snipe

	mean	sd	2.5%	25%	50%	75%	97.5%	n.eff	overlap0	f
alpha.lambda	-8.42	1.22	-10.28	-9.32	-8.69	-7.72	-5.90	4	0	1.00
alpha.phi	5.67	0.90	4.94	5.01	5.07	6.88	7.10	3	0	1.00
alpha.p	-1.42	0.13	-1.63	-1.60	-1.34	-1.33	-1.32	3	0	1.00
beta.hab.lambda	-0.33	0.77	-2.00	-0.85	-0.19	0.23	0.90	11	1	0.61
beta.hab.gamma	0.04	0.01	0.03	0.03	0.04	0.05	0.06	3	0	1.00
beta.hab.phi	0.07	0.03	0.02	0.05	0.08	0.09	0.11	4	0	1.00
beta.elev.lambda	0.69	1.19	-1.13	-0.21	0.42	1.43	3.33	32	1	0.68
beta.ele.gamma2	-0.67	0.02	-0.70	-0.69	-0.68	-0.64	-0.63	3	0	1.00
beta.elev.phi	1.42	0.25	1.19	1.24	1.27	1.75	1.81	3	0	1.00
beta.develop.lambda	0.52	1.21	-2.22	-0.12	0.59	1.37	2.50	73	1	0.72
beta.develop.gamma	0.77	0.05	0.68	0.70	0.80	0.82	0.84	3	0	1.00
beta.develop.phi	-2.09	0.35	-2.67	-2.54	-1.88	-1.83	-1.73	3	0	1.00
beta.effort	0.10	0.01	0.09	0.09	0.10	0.11	0.11	4	0	1.00
beta.min_temp	0.00	0.00	0.00	0.00	0.00	0.00	0.00	35	0	1.00

Table 230: Wood Stork

	mean	sd	2.5%	25%	50%	75%	97.5%	n.eff	overlap0	f
alpha.lambda	5.15	0.09	5.04	5.08	5.11	5.23	5.35	3	0	1.00
alpha.phi	3.60	0.27	3.34	3.38	3.45	3.96	4.01	3	0	1.00
alpha.p	-1.90	0.05	-1.97	-1.95	-1.91	-1.84	-1.82	3	0	1.00
beta.hab.lambda	-1.61	0.11	-1.76	-1.70	-1.66	-1.48	-1.43	3	0	1.00

	mean	sd	2.5%	25%	50%	75%	97.5%	n.eff	overlap0	f
beta.hab.gamma	-0.06	0.03	-0.12	-0.09	-0.07	-0.04	-0.01	3	0	1.00
beta.hab.phi	0.37	0.03	0.31	0.34	0.38	0.40	0.43	4	0	1.00
beta.elev.lambda	-1.55	0.18	-1.82	-1.78	-1.50	-1.39	-1.31	3	0	1.00
beta.ele.gamma2	-0.13	0.21	-0.44	-0.42	-0.03	0.06	0.09	3	1	0.67
beta.elev.phi	0.30	0.41	-0.10	-0.07	0.11	0.87	0.91	3	1	0.67
beta.develop.lambda	-0.75	0.06	-0.87	-0.80	-0.74	-0.70	-0.65	4	0	1.00
beta.develop.gamma	0.98	0.47	0.49	0.53	0.81	1.61	1.65	3	0	1.00
beta.develop.phi	-0.74	0.73	-1.80	-1.72	-0.41	-0.07	-0.02	3	0	1.00
beta.effort	0.00	0.00	0.00	0.00	0.00	0.00	0.00	57	0	1.00
beta.min_temp	0.00	0.00	0.00	0.00	0.00	0.00	0.00	101	0	1.00

Table 231: American Bittern

	mean	sd	2.5%	25%	50%	75%	97.5%	n.eff	overlap0	f
alpha.lambda	3.88	0.52	3.33	3.40	3.68	4.58	4.62	3	0	1.00
alpha.phi	2.40	0.05	2.32	2.36	2.39	2.42	2.54	34	0	1.00
alpha.p	-1.95	0.51	-2.72	-2.62	-1.74	-1.47	-1.43	3	0	1.00
beta.hab.lambda	-0.02	0.02	-0.06	-0.04	-0.03	-0.01	0.02	5	1	0.87
beta.hab.gamma	-0.40	0.16	-0.74	-0.50	-0.40	-0.27	-0.16	6	0	1.00
beta.hab.phi	0.57	0.19	0.31	0.38	0.56	0.69	0.92	3	0	1.00
beta.elev.lambda	0.03	0.05	-0.06	-0.01	0.03	0.08	0.12	4	1	0.70
beta.ele.gamma2	-0.04	0.14	-0.23	-0.15	-0.09	0.09	0.23	3	1	0.65
beta.elev.phi	-0.08	0.10	-0.25	-0.19	-0.04	0.00	0.06	3	1	0.76
beta.develop.lambda	0.07	0.05	-0.02	0.03	0.07	0.10	0.15	5	1	0.92
beta.develop.gamma	0.11	0.10	-0.08	0.04	0.11	0.17	0.27	11	1	0.85
beta.develop.phi	-0.06	0.05	-0.16	-0.09	-0.06	-0.02	0.04	11	1	0.84
beta.effort	0.07	0.02	0.04	0.06	0.07	0.08	0.10	5	0	1.00
beta.min_temp	0.00	0.00	0.00	0.00	0.00	0.00	0.00	81	0	1.00

Table 232: American Dipper

	mean	sd	2.5%	25%	50%	75%	97.5%	n.eff	overlap0	f
alpha.lambda	2.12	0.12	1.87	2.04	2.12	2.20	2.34	10	0	1.00
alpha.phi	2.95	0.05	2.85	2.91	2.95	2.99	3.04	12	0	1.00
alpha.p	-1.13	0.03	-1.19	-1.15	-1.13	-1.11	-1.07	16	0	1.00
beta.hab.lambda	-0.15	0.06	-0.26	-0.19	-0.15	-0.11	-0.04	5	0	1.00
beta.hab.gamma	-0.69	0.07	-0.83	-0.73	-0.69	-0.65	-0.54	11	0	1.00
beta.hab.phi	1.10	0.10	0.85	1.04	1.10	1.15	1.31	10	0	1.00
beta.elev.lambda	1.78	0.14	1.51	1.69	1.77	1.87	2.06	23	0	1.00
beta.ele.gamma2	-0.64	0.06	-0.76	-0.69	-0.65	-0.61	-0.51	18	0	1.00
beta.elev.phi	1.05	0.08	0.87	1.00	1.06	1.11	1.19	10	0	1.00
beta.develop.lambda	0.10	0.11	-0.12	0.03	0.10	0.18	0.31	81	1	0.83
beta.develop.gamma	-0.08	0.13	-0.35	-0.17	-0.08	0.01	0.18	16	1	0.73
beta.develop.phi	-0.20	0.15	-0.52	-0.30	-0.21	-0.10	0.09	12	1	0.92
beta.effort	0.14	0.01	0.11	0.13	0.14	0.15	0.16	45	0	1.00
beta.min_temp	-0.06	0.01	-0.08	-0.07	-0.06	-0.05	-0.04	36	0	1.00

Table 233: American Woodcock

	mean	sd	2.5%	25%	50%	75%	97.5%	n.eff	overlap0	f
alpha.lambda	2.87	0.12	2.67	2.77	2.86	2.97	3.10	3	0	1.00
alpha.phi	2.52	0.04	2.45	2.50	2.53	2.55	2.59	7	0	1.00
alpha.p	-1.98	0.08	-2.14	-2.07	-1.94	-1.92	-1.87	3	0	1.00
beta.hab.lambda	-0.31	0.05	-0.40	-0.35	-0.31	-0.27	-0.20	4	0	1.00
beta.hab.gamma	0.33	0.11	0.17	0.23	0.31	0.44	0.52	4	0	1.00
beta.hab.phi	-0.32	0.11	-0.52	-0.43	-0.30	-0.23	-0.16	4	0	1.00
beta.elev.lambda	-0.52	0.03	-0.58	-0.54	-0.52	-0.50	-0.45	5	0	1.00
beta.ele.gamma2	-0.01	0.05	-0.10	-0.05	-0.01	0.03	0.09	5	1	0.58
beta.elev.phi	-0.11	0.06	-0.23	-0.16	-0.11	-0.07	-0.01	5	0	0.99
beta.develop.lambda	-0.50	0.07	-0.63	-0.55	-0.50	-0.45	-0.36	9	0	1.00
beta.develop.gamma	0.14	0.06	-0.01	0.10	0.15	0.18	0.23	6	1	0.97
beta.develop.phi	-0.15	0.06	-0.26	-0.19	-0.16	-0.12	-0.02	9	0	0.98
beta.effort	0.07	0.01	0.05	0.06	0.07	0.07	0.08	35	0	1.00
beta.min_temp	-0.01	0.01	-0.03	-0.02	-0.01	-0.01	0.00	27	0	1.00

Table 234: Barn Owl

	mean	sd	2.5%	25%	50%	75%	97.5%	n.eff	overlap0	f
alpha.lambda	2.69	0.12	2.48	2.55	2.72	2.78	2.88	3	0	1.00
alpha.phi	2.83	0.06	2.70	2.80	2.84	2.87	2.94	8	0	1.00
alpha.p	-1.77	0.10	-1.96	-1.86	-1.78	-1.66	-1.63	3	0	1.00
beta.hab.lambda	0.42	0.04	0.34	0.39	0.42	0.45	0.50	38	0	1.00
beta.hab.gamma	0.49	0.04	0.43	0.47	0.49	0.52	0.58	18	0	1.00
beta.hab.phi	-0.44	0.04	-0.55	-0.47	-0.43	-0.41	-0.37	26	0	1.00
beta.elev.lambda	-0.35	0.03	-0.41	-0.37	-0.35	-0.33	-0.28	5	0	1.00
beta.ele.gamma2	0.06	0.04	-0.02	0.02	0.06	0.09	0.14	4	1	0.90
beta.elev.phi	-0.18	0.04	-0.26	-0.22	-0.18	-0.15	-0.11	4	0	1.00
beta.develop.lambda	0.79	0.05	0.68	0.75	0.79	0.82	0.89	21	0	1.00
beta.develop.gamma	0.06	0.06	-0.08	0.03	0.07	0.10	0.18	7	1	0.86
beta.develop.phi	-0.25	0.07	-0.39	-0.30	-0.26	-0.22	-0.07	7	0	1.00
beta.effort	0.02	0.01	0.00	0.01	0.02	0.02	0.04	221	0	1.00
beta.min_temp	0.00	0.00	0.00	0.00	0.00	0.00	0.00	5015	0	1.00

Table 235: Black-and-white Warbler

	mean	sd	2.5%	25%	50%	75%	97.5%	n.eff	overlap0	f
alpha.lambda	3.08	0.78	2.40	2.48	2.61	4.15	4.22	3	0	1.00
alpha.phi	3.73	0.54	3.06	3.21	3.63	4.14	4.92	3	0	1.00
alpha.p	-1.51	0.60	-2.37	-2.35	-1.14	-1.06	-0.99	3	0	1.00
beta.hab.lambda	-0.29	0.08	-0.40	-0.35	-0.32	-0.21	-0.17	3	0	1.00
beta.hab.gamma	0.02	0.33	-0.37	-0.24	-0.16	0.45	0.52	3	1	0.35
beta.hab.phi	-0.09	0.54	-0.93	-0.79	0.25	0.31	0.40	3	1	0.33
beta.elev.lambda	-0.23	0.21	-0.59	-0.45	-0.20	0.01	0.04	3	1	0.71
beta.ele.gamma2	-0.10	0.69	-1.14	-0.69	-0.25	0.76	0.87	3	1	0.67
beta.elev.phi	-0.74	1.35	-2.68	-2.44	-0.34	0.38	1.18	3	1	0.67
beta.develop.lambda	0.61	0.09	0.46	0.53	0.63	0.69	0.77	4	0	1.00
beta.develop.gamma	1.00	0.25	0.60	0.71	1.05	1.22	1.35	3	0	1.00

	mean	sd	2.5%	25%	50%	75%	97.5%	n.eff	overlap0	f
beta.develop.phi	-0.92	0.25	-1.31	-1.13	-0.96	-0.64	-0.51	3	0	1.00
beta.effort	0.00	0.00	0.00	0.00	0.00	0.00	0.01	26	0	1.00
beta.min_temp	0.00	0.00	-0.01	0.00	0.00	0.00	0.00	22	0	1.00

Table 236: Black-tailed Gnatcatcher

	mean	sd	2.5%	25%	50%	75%	97.5%	n.eff	overlap0	f
alpha.lambda	3.42	0.10	3.23	3.34	3.41	3.49	3.63	6	0	1
alpha.phi	4.77	0.10	4.58	4.69	4.78	4.84	4.95	6	0	1
alpha.p	-1.33	0.04	-1.41	-1.36	-1.33	-1.31	-1.23	10	0	1
beta.hab.lambda	1.87	0.09	1.68	1.81	1.89	1.95	2.04	5	0	1
beta.hab.gamma	1.06	0.06	0.94	1.02	1.06	1.10	1.18	12	0	1
beta.hab.phi	-2.35	0.10	-2.54	-2.42	-2.35	-2.28	-2.17	16	0	1
beta.elev.lambda	-1.22	0.06	-1.33	-1.26	-1.22	-1.17	-1.10	6	0	1
beta.ele.gamma2	-0.95	0.04	-1.03	-0.98	-0.95	-0.92	-0.87	40	0	1
beta.elev.phi	1.20	0.07	1.07	1.15	1.20	1.25	1.35	14	0	1
beta.develop.lambda	0.60	0.21	0.19	0.44	0.61	0.76	0.97	8	0	1
beta.develop.gamma	1.89	0.07	1.76	1.84	1.89	1.94	2.03	87	0	1
beta.develop.phi	-0.66	0.15	-0.97	-0.76	-0.65	-0.55	-0.38	94	0	1
beta.effort	0.02	0.01	0.00	0.01	0.02	0.03	0.04	59	0	1
beta.min_temp	0.00	0.00	-0.01	0.00	0.00	0.00	0.00	589	0	1

Table 237: Blue-headed Vireo

	mean	sd	2.5%	25%	50%	75%	97.5%	n.eff	overlap0	f
alpha.lambda	-8.65	1.68	-11.67	-9.93	-8.74	-7.28	-5.63	7	0	1.00
alpha.phi	3.89	0.12	3.66	3.81	3.90	3.98	4.09	5	0	1.00
alpha.p	-0.63	0.02	-0.66	-0.64	-0.63	-0.61	-0.58	10	0	1.00
beta.hab.lambda	-1.42	1.46	-4.96	-2.18	-1.23	-0.42	1.12	6	1	0.86
beta.hab.gamma	-0.21	0.02	-0.25	-0.22	-0.21	-0.20	-0.17	73	0	1.00
beta.hab.phi	0.59	0.06	0.47	0.55	0.59	0.63	0.70	24	0	1.00
beta.elev.lambda	0.03	1.21	-1.77	-0.76	-0.26	0.60	3.20	8	1	0.40
beta.ele.gamma2	-0.49	0.02	-0.52	-0.50	-0.49	-0.48	-0.46	270	0	1.00
beta.elev.phi	0.28	0.03	0.22	0.26	0.28	0.31	0.36	61	0	1.00
beta.develop.lambda	0.20	1.17	-2.21	-0.66	0.25	1.09	2.43	18	1	0.56
beta.develop.gamma	0.37	0.10	0.19	0.26	0.41	0.44	0.50	3	0	1.00
beta.develop.phi	-0.61	0.27	-0.95	-0.82	-0.71	-0.35	-0.05	3	0	0.99
beta.effort	0.01	0.01	0.00	0.00	0.00	0.01	0.02	684	0	1.00
beta.min_temp	-0.02	0.01	-0.04	-0.03	-0.02	-0.02	-0.01	49	0	1.00

Table 238: Burrowing Owl

	mean	sd	2.5%	25%	50%	75%	97.5%	n.eff	overlap0	f
alpha.lambda	3.17	0.10	2.98	3.09	3.17	3.24	3.38	5	0	1.00
alpha.phi	4.42	0.09	4.21	4.37	4.42	4.48	4.61	5	0	1.00
alpha.p	-1.70	0.11	-1.89	-1.77	-1.71	-1.63	-1.47	6	0	1.00
beta.hab.lambda	0.06	0.04	-0.01	0.03	0.05	0.09	0.14	5	1	0.94

	mean	sd	2.5%	25%	50%	75%	97.5%	n.eff	overlap0	f
beta.hab.gamma	-1.31	0.37	-2.16	-1.54	-1.22	-1.03	-0.79	6	0	1.00
beta.hab.phi	0.21	0.12	-0.01	0.12	0.23	0.29	0.44	8	1	0.96
beta.elev.lambda	-0.30	0.03	-0.35	-0.32	-0.30	-0.28	-0.25	8	0	1.00
beta.ele.gamma2	-5.05	1.00	-6.50	-5.76	-5.19	-4.58	-2.60	4	0	1.00
beta.elev.phi	0.40	0.05	0.30	0.36	0.40	0.44	0.49	12	0	1.00
beta.develop.lambda	0.27	0.05	0.17	0.23	0.27	0.30	0.39	7	0	1.00
beta.develop.gamma	3.56	0.39	2.69	3.31	3.59	3.84	4.18	4	0	1.00
beta.develop.phi	-0.25	0.12	-0.46	-0.35	-0.25	-0.16	-0.05	4	0	1.00
beta.effort	0.00	0.00	0.00	0.00	0.00	0.00	0.01	1650	0	1.00
beta.min_temp	-0.01	0.01	-0.03	-0.02	-0.01	-0.01	0.00	120	0	1.00

Table 239: Canyon Wren

	mean	sd	2.5%	25%	50%	75%	97.5%	n.eff	overlap0	f
alpha.lambda	3.22	0.09	3.04	3.16	3.24	3.29	3.35	4	0	1.00
alpha.phi	2.81	0.08	2.66	2.75	2.81	2.88	2.96	4	0	1.00
alpha.p	-1.26	0.05	-1.33	-1.30	-1.28	-1.22	-1.16	3	0	1.00
beta.hab.lambda	-0.77	0.10	-0.98	-0.83	-0.76	-0.70	-0.58	21	0	1.00
beta.hab.gamma	0.18	0.14	-0.06	0.02	0.27	0.28	0.30	3	1	0.83
beta.hab.phi	-0.35	0.21	-0.56	-0.51	-0.46	-0.09	-0.01	3	0	0.98
beta.elev.lambda	0.11	0.04	0.03	0.08	0.11	0.14	0.19	32	0	1.00
beta.ele.gamma2	0.06	0.05	-0.04	0.03	0.06	0.09	0.15	52	1	0.89
beta.elev.phi	0.05	0.05	-0.04	0.02	0.05	0.08	0.15	67	1	0.83
beta.develop.lambda	-0.55	0.11	-0.79	-0.63	-0.54	-0.48	-0.36	15	0	1.00
beta.develop.gamma	-0.14	0.07	-0.29	-0.19	-0.14	-0.09	-0.01	24	0	0.98
beta.develop.phi	-0.08	0.07	-0.21	-0.14	-0.08	-0.03	0.04	20	1	0.88
beta.effort	0.23	0.01	0.21	0.22	0.23	0.24	0.25	308	0	1.00
beta.min_temp	0.00	0.00	0.00	0.00	0.00	0.00	0.00	1622	0	1.00

Table 240: Chihuahuan Raven

	mean	sd	2.5%	25%	50%	75%	97.5%	n.eff	overlap0	f
alpha.lambda	7.55	1.23	6.44	6.55	6.85	9.21	9.40	3	0	1.00
alpha.phi	0.48	1.45	-1.56	-1.40	0.89	1.99	2.07	3	1	0.67
alpha.p	-3.67	1.08	-5.29	-5.13	-3.01	-2.82	-2.80	3	0	1.00
beta.hab.lambda	-0.25	0.53	-1.10	-0.92	0.04	0.16	0.28	3	1	0.44
beta.hab.gamma	-0.22	0.46	-0.88	-0.84	-0.01	0.20	0.22	3	1	0.55
beta.hab.phi	1.50	1.53	0.14	0.21	0.67	3.58	3.77	3	0	1.00
beta.elev.lambda	-1.26	0.22	-1.63	-1.52	-1.14	-1.09	-1.00	3	0	1.00
beta.ele.gamma2	-0.07	0.39	-0.58	-0.57	-0.03	0.37	0.44	3	1	0.66
beta.elev.phi	-0.22	0.86	-1.20	-1.13	-0.43	0.90	0.93	3	1	0.67
beta.develop.lambda	1.11	0.91	0.09	0.26	0.84	2.19	2.70	3	0	1.00
beta.develop.gamma	-0.70	1.32	-2.22	-2.13	-0.97	1.01	1.06	3	1	0.67
beta.develop.phi	3.15	2.00	0.67	0.75	3.11	5.54	5.79	3	0	1.00
beta.effort	0.39	0.04	0.34	0.35	0.38	0.44	0.45	3	0	1.00
beta.min_temp	-0.05	0.05	-0.12	-0.11	-0.05	0.00	0.00	3	0	1.00

Table 241: Clark's Nutcracker

	mean	sd	2.5%	25%	50%	75%	97.5%	n.eff	overlap0	f
alpha.lambda	5.28	0.11	5.15	5.20	5.24	5.40	5.48	3	0	1.00
alpha.phi	3.20	0.29	2.86	2.92	3.09	3.51	3.73	3	0	1.00
alpha.p	-2.70	0.10	-2.87	-2.80	-2.66	-2.62	-2.58	3	0	1.00
beta.hab.lambda	0.09	0.02	0.05	0.07	0.09	0.11	0.13	4	0	1.00
beta.hab.gamma	0.22	0.06	0.10	0.17	0.23	0.28	0.32	4	0	1.00
beta.hab.phi	-0.17	0.09	-0.30	-0.25	-0.18	-0.08	-0.03	3	0	1.00
beta.elev.lambda	0.94	0.09	0.78	0.87	0.96	1.01	1.09	6	0	1.00
beta.ele.gamma2	-0.09	0.18	-0.35	-0.27	-0.07	0.08	0.13	4	1	0.53
beta.elev.phi	0.21	0.44	-0.50	-0.22	0.29	0.64	0.77	4	1	0.57
beta.develop.lambda	-0.65	0.05	-0.74	-0.69	-0.65	-0.61	-0.55	16	0	1.00
beta.develop.gamma	0.14	0.26	-0.23	-0.10	0.06	0.41	0.57	3	1	0.61
beta.develop.phi	0.27	0.21	-0.14	0.10	0.30	0.43	0.60	4	1	0.91
beta.effort	0.00	0.00	0.00	0.00	0.00	0.00	0.01	414	0	1.00
beta.min_temp	-0.02	0.01	-0.03	-0.02	-0.01	-0.01	0.00	122	0	1.00

Table 242: Crested Caracara

	mean	sd	2.5%	25%	50%	75%	97.5%	n.eff	overlap0	f
alpha.lambda	-1.58	1.15	-3.76	-2.44	-1.60	-0.54	0.28	4	1	0.93
alpha.phi	7.03	0.62	5.92	6.55	7.28	7.49	7.85	3	0	1.00
alpha.p	-0.64	0.03	-0.70	-0.66	-0.63	-0.62	-0.60	4	0	1.00
beta.hab.lambda	0.95	0.41	0.10	0.68	0.96	1.22	1.74	10	0	0.99
beta.hab.gamma	-0.31	0.04	-0.38	-0.34	-0.32	-0.28	-0.23	4	0	1.00
beta.hab.phi	7.54	1.11	5.59	6.68	8.03	8.38	8.97	4	0	1.00
beta.elev.lambda	-1.88	0.54	-3.02	-2.24	-1.84	-1.49	-0.98	8	0	1.00
beta.ele.gamma2	-0.68	0.04	-0.75	-0.71	-0.68	-0.65	-0.60	5	0	1.00
beta.elev.phi	1.49	0.10	1.30	1.42	1.50	1.56	1.66	4	0	1.00
beta.develop.lambda	-3.01	1.80	-6.41	-4.45	-2.86	-1.54	0.09	6	1	0.97
beta.develop.gamma	-0.25	0.06	-0.36	-0.28	-0.24	-0.21	-0.14	47	0	1.00
beta.develop.phi	-0.10	0.13	-0.34	-0.19	-0.11	-0.02	0.14	9	1	0.78
beta.effort	0.01	0.01	0.00	0.00	0.01	0.01	0.02	36	0	1.00
beta.min_temp	0.00	0.00	-0.01	-0.01	0.00	0.00	0.00	255	0	1.00

Table 243: Curve-billed Thrasher

	mean	sd	2.5%	25%	50%	75%	97.5%	n.eff	overlap0	f
alpha.lambda	4.53	0.12	4.31	4.39	4.56	4.63	4.70	3	0	1.00
alpha.phi	3.69	0.60	3.01	3.09	3.51	4.45	4.56	3	0	1.00
alpha.p	-1.44	0.16	-1.65	-1.60	-1.45	-1.25	-1.22	3	0	1.00
beta.hab.lambda	1.38	0.15	1.16	1.21	1.38	1.54	1.59	3	0	1.00
beta.hab.gamma	0.13	0.37	-0.47	-0.31	0.33	0.44	0.51	3	1	0.67
beta.hab.phi	0.15	0.76	-0.85	-0.74	0.15	0.98	1.18	3	1	0.58
beta.elev.lambda	-0.67	0.12	-0.87	-0.81	-0.64	-0.57	-0.51	3	0	1.00
beta.ele.gamma2	1.00	0.90	-0.21	-0.02	1.00	2.02	2.29	3	1	0.73
beta.elev.phi	0.17	0.55	-0.63	-0.48	0.26	0.74	0.85	3	1	0.67
beta.develop.lambda	0.61	0.67	-0.10	0.11	0.24	1.51	1.62	3	1	0.93
beta.develop.gamma	1.07	0.63	0.42	0.60	0.71	1.86	2.10	3	0	1.00

	mean	sd	2.5%	25%	50%	75%	97.5%	n.eff	overlap0	f
beta.develop.phi	0.33	1.72	-2.29	-1.87	1.08	1.89	2.11	3	1	0.67
beta.effort	0.14	0.01	0.13	0.14	0.14	0.15	0.16	9	0	1.00
beta.min_temp	-0.02	0.01	-0.04	-0.02	-0.02	-0.01	0.00	7	0	1.00

Table 244: Ferruginous Hawk

	mean	sd	2.5%	25%	50%	75%	97.5%	n.eff	overlap0	f
alpha.lambda	1.53	0.24	1.12	1.32	1.52	1.74	1.94	4	0	1.00
alpha.phi	2.44	0.09	2.28	2.38	2.46	2.51	2.59	4	0	1.00
alpha.p	-0.95	0.10	-1.13	-1.04	-0.93	-0.87	-0.81	3	0	1.00
beta.hab.lambda	-0.02	0.09	-0.19	-0.10	-0.03	0.05	0.16	6	1	0.60
beta.hab.gamma	0.08	0.04	0.01	0.05	0.08	0.10	0.15	18	0	0.99
beta.hab.phi	0.17	0.05	0.09	0.14	0.17	0.21	0.27	60	0	1.00
beta.elev.lambda	1.04	0.14	0.73	0.95	1.04	1.14	1.30	8	0	1.00
beta.ele.gamma2	-0.11	0.04	-0.18	-0.13	-0.11	-0.08	-0.02	21	0	0.99
beta.elev.phi	0.12	0.05	0.02	0.09	0.12	0.16	0.22	11	0	0.99
beta.develop.lambda	-0.24	0.10	-0.44	-0.31	-0.24	-0.17	-0.02	5	0	0.98
beta.develop.gamma	-0.50	0.06	-0.62	-0.55	-0.51	-0.46	-0.38	8	0	1.00
beta.develop.phi	1.01	0.08	0.85	0.95	1.01	1.07	1.16	5	0	1.00
beta.effort	0.10	0.02	0.07	0.09	0.10	0.11	0.13	26	0	1.00
beta.min_temp	-0.02	0.01	-0.05	-0.03	-0.02	-0.01	0.00	24	0	1.00

Table 245: Golden-fronted Woodpecker

	mean	sd	2.5%	25%	50%	75%	97.5%	n.eff	overlap0	f
alpha.lambda	4.93	0.39	4.61	4.65	4.68	5.46	5.50	3	0	1.00
alpha.phi	6.37	0.53	5.68	5.92	6.19	6.97	7.22	3	0	1.00
alpha.p	-1.47	0.45	-2.13	-2.11	-1.16	-1.14	-1.13	3	0	1.00
beta.hab.lambda	-0.44	0.29	-0.90	-0.83	-0.26	-0.22	-0.17	3	0	1.00
beta.hab.gamma	-0.86	0.93	-2.21	-2.16	-0.32	-0.12	0.00	3	1	0.97
beta.hab.phi	3.08	3.78	-0.12	0.16	0.75	8.27	8.65	3	1	0.93
beta.elev.lambda	-0.02	0.20	-0.21	-0.17	-0.14	0.24	0.30	3	1	0.67
beta.ele.gamma2	-0.12	0.84	-0.79	-0.73	-0.69	1.05	1.09	3	1	0.67
beta.elev.phi	-1.09	2.30	-4.50	-4.27	0.47	0.55	0.68	3	1	0.33
beta.develop.lambda	-0.61	0.42	-1.36	-1.10	-0.41	-0.28	-0.09	3	0	1.00
beta.develop.gamma	-0.71	1.07	-2.34	-2.17	-0.05	0.08	0.26	3	1	0.59
beta.develop.phi	6.94	0.92	4.99	6.39	6.90	7.78	8.22	4	0	1.00
beta.effort	0.01	0.01	0.00	0.00	0.00	0.01	0.02	5	0	1.00
beta.min_temp	0.00	0.00	-0.01	0.00	0.00	0.00	0.00	23	0	1.00

Table 246: Golden Eagle

	mean	sd	2.5%	25%	50%	75%	97.5%	n.eff	overlap0	f
alpha.lambda	2.72	0.16	2.50	2.60	2.65	2.87	3.02	3	0	1.00
alpha.phi	3.70	0.19	3.43	3.57	3.63	3.93	4.04	3	0	1.00
alpha.p	-1.59	0.12	-1.80	-1.70	-1.56	-1.49	-1.41	3	0	1.00
beta.hab.lambda	0.24	0.04	0.17	0.21	0.24	0.28	0.31	4	0	1.00

	mean	sd	2.5%	25%	50%	75%	97.5%	n.eff	overlap0	f
beta.hab.gamma	0.07	0.07	-0.05	0.01	0.09	0.12	0.17	3	1	0.79
beta.hab.phi	-0.01	0.09	-0.15	-0.09	-0.03	0.07	0.14	3	1	0.54
beta.elev.lambda	0.05	0.03	-0.01	0.03	0.05	0.07	0.09	6	1	0.95
beta.ele.gamma2	1.41	0.15	1.21	1.29	1.36	1.57	1.70	4	0	1.00
beta.elev.phi	-0.48	0.17	-0.77	-0.66	-0.42	-0.35	-0.27	3	0	1.00
beta.develop.lambda	-0.12	0.07	-0.25	-0.17	-0.13	-0.08	0.01	5	1	0.95
beta.develop.gamma	0.36	0.20	-0.04	0.18	0.41	0.50	0.71	4	1	0.95
beta.develop.phi	-0.31	0.13	-0.50	-0.41	-0.33	-0.21	-0.02	4	0	0.98
beta.effort	0.00	0.00	0.00	0.00	0.00	0.00	0.01	281	0	1.00
beta.min_temp	0.00	0.00	-0.01	0.00	0.00	0.00	0.00	136	0	1.00

Table 247: Gray Jay

	mean	sd	2.5%	25%	50%	75%	97.5%	n.eff	overlap0	f
alpha.lambda	2.55	1.39	1.09	1.50	1.78	4.41	4.66	3	0	1.00
alpha.phi	0.35	2.53	-3.36	-3.13	2.09	2.17	2.25	3	1	0.67
alpha.p	-2.13	0.35	-2.66	-2.56	-2.02	-1.80	-1.72	3	0	1.00
beta.hab.lambda	0.37	0.24	0.12	0.17	0.25	0.64	0.82	3	0	1.00
beta.hab.gamma	-0.22	0.22	-0.53	-0.40	-0.29	0.06	0.08	3	1	0.67
beta.hab.phi	-0.12	0.77	-1.26	-1.16	0.33	0.46	0.62	3	1	0.33
beta.elev.lambda	-0.17	0.29	-0.64	-0.54	0.00	0.05	0.12	3	1	0.48
beta.ele.gamma2	-0.81	0.65	-1.31	-1.28	-1.25	0.09	0.12	3	1	0.67
beta.elev.phi	0.52	1.56	-1.76	-1.66	1.57	1.65	1.74	3	1	0.67
beta.develop.lambda	-1.57	6.42	-6.87	-6.22	-5.81	7.40	7.74	3	1	0.67
beta.develop.gamma	0.60	1.24	-1.23	-1.12	1.42	1.50	1.62	3	1	0.67
beta.develop.phi	-1.71	1.96	-3.31	-3.13	-3.00	1.02	1.19	3	1	0.67
beta.effort	0.07	0.01	0.04	0.06	0.07	0.07	0.09	9	0	1.00
beta.min_temp	-0.06	0.04	-0.13	-0.11	-0.05	-0.03	-0.02	3	0	1.00

Table 248: Greater Roadrunner

	mean	sd	2.5%	25%	50%	75%	97.5%	n.eff	overlap0	f
alpha.lambda	3.72	0.53	2.94	3.06	3.83	4.28	4.34	3	0	1.00
alpha.phi	3.11	0.29	2.81	2.88	2.95	3.44	3.63	3	0	1.00
alpha.p	-2.22	0.47	-2.75	-2.73	-2.32	-1.64	-1.57	3	0	1.00
beta.hab.lambda	0.58	0.10	0.43	0.48	0.56	0.69	0.74	3	0	1.00
beta.hab.gamma	-0.64	0.96	-2.00	-1.71	-0.61	0.44	0.65	3	1	0.67
beta.hab.phi	1.41	1.18	-0.05	0.19	1.16	2.85	3.21	3	1	0.95
beta.elev.lambda	-0.37	0.10	-0.55	-0.48	-0.34	-0.27	-0.24	3	0	1.00
beta.ele.gamma2	-0.11	0.76	-1.15	-1.11	0.07	0.69	0.77	3	1	0.38
beta.elev.phi	0.14	1.02	-1.08	-1.00	-0.01	1.44	1.52	3	1	0.46
beta.develop.lambda	0.29	0.08	0.14	0.22	0.30	0.35	0.44	4	0	1.00
beta.develop.gamma	0.00	0.58	-0.85	-0.69	0.10	0.56	0.81	3	1	0.37
beta.develop.phi	0.31	0.62	-0.47	-0.21	0.10	1.09	1.25	3	1	0.66
beta.effort	0.03	0.01	0.00	0.02	0.03	0.04	0.05	5	0	1.00
beta.min_temp	-0.03	0.02	-0.06	-0.05	-0.03	-0.02	0.00	4	0	1.00

Table 249: Harlequin Duck

	mean	sd	2.5%	25%	50%	75%	97.5%	n.eff	overlap0	f
alpha.lambda	2.18	0.19	1.82	2.05	2.17	2.30	2.55	34	0	1
alpha.phi	-0.29	0.06	-0.38	-0.33	-0.29	-0.25	-0.16	12	0	1
alpha.p	-1.06	0.09	-1.21	-1.18	-1.01	-0.98	-0.96	3	0	1
beta.hab.lambda	0.42	0.09	0.24	0.35	0.41	0.48	0.60	31	0	1
beta.hab.gamma	-0.39	0.02	-0.43	-0.40	-0.39	-0.38	-0.36	17	0	1
beta.hab.phi	2.24	0.09	2.11	2.17	2.23	2.32	2.40	4	0	1
beta.elev.lambda	0.54	0.06	0.43	0.50	0.54	0.58	0.67	25	0	1
beta.ele.gamma2	0.22	0.02	0.18	0.20	0.22	0.23	0.26	5	0	1
beta.elev.phi	-1.62	0.09	-1.75	-1.69	-1.63	-1.55	-1.41	4	0	1
beta.develop.lambda	-0.86	0.26	-1.38	-1.02	-0.85	-0.68	-0.40	49	0	1
beta.develop.gamma	0.37	0.02	0.33	0.36	0.37	0.39	0.41	50	0	1
beta.develop.phi	0.60	0.07	0.44	0.56	0.61	0.65	0.71	19	0	1
beta.effort	0.00	0.00	0.00	0.00	0.00	0.00	0.01	47	0	1
beta.min_temp	-0.01	0.01	-0.02	-0.01	-0.01	0.00	0.00	66	0	1

Table 250: Hutton's Vireo

	mean	sd	2.5%	25%	50%	75%	97.5%	n.eff	overlap0	f
alpha.lambda	3.29	0.05	3.20	3.25	3.29	3.32	3.38	19	0	1.00
alpha.phi	2.95	0.04	2.87	2.92	2.95	2.98	3.04	15	0	1.00
alpha.p	-1.03	0.02	-1.06	-1.04	-1.03	-1.01	-0.99	9	0	1.00
beta.hab.lambda	-0.31	0.04	-0.38	-0.33	-0.31	-0.28	-0.23	51	0	1.00
beta.hab.gamma	-0.43	0.03	-0.51	-0.45	-0.43	-0.41	-0.37	5862	0	1.00
beta.hab.phi	1.03	0.07	0.90	0.98	1.02	1.07	1.19	88	0	1.00
beta.elev.lambda	0.04	0.03	-0.02	0.02	0.04	0.06	0.11	6000	1	0.89
beta.ele.gamma2	0.18	0.04	0.11	0.16	0.19	0.21	0.25	21	0	1.00
beta.elev.phi	-0.34	0.05	-0.44	-0.38	-0.34	-0.30	-0.25	19	0	1.00
beta.develop.lambda	-0.29	0.07	-0.43	-0.33	-0.28	-0.24	-0.15	163	0	1.00
beta.develop.gamma	-0.28	0.06	-0.39	-0.33	-0.28	-0.25	-0.17	57	0	1.00
beta.develop.phi	-0.13	0.10	-0.31	-0.20	-0.13	-0.06	0.05	164	1	0.92
beta.effort	0.01	0.01	0.00	0.01	0.01	0.02	0.03	34	0	1.00
beta.min_temp	-0.01	0.00	-0.02	-0.01	0.00	0.00	0.00	4924	0	1.00

Table 251: King Rail

	mean	sd	2.5%	25%	50%	75%	97.5%	n.eff	overlap0	f
alpha.lambda	3.62	0.06	3.51	3.58	3.62	3.66	3.73	11	0	1.00
alpha.phi	2.36	0.07	2.23	2.31	2.36	2.41	2.52	6	0	1.00
alpha.p	-1.49	0.04	-1.57	-1.52	-1.49	-1.46	-1.42	13	0	1.00
beta.hab.lambda	0.31	0.04	0.24	0.28	0.31	0.33	0.38	13	0	1.00
beta.hab.gamma	0.69	0.04	0.62	0.66	0.69	0.71	0.76	32	0	1.00
beta.hab.phi	-0.26	0.04	-0.33	-0.29	-0.26	-0.23	-0.19	60	0	1.00
beta.elev.lambda	-0.28	0.05	-0.38	-0.31	-0.28	-0.25	-0.19	22	0	1.00
beta.ele.gamma2	0.54	0.09	0.38	0.48	0.55	0.60	0.70	20	0	1.00
beta.elev.phi	-0.53	0.10	-0.73	-0.60	-0.53	-0.47	-0.36	11	0	1.00
beta.develop.lambda	0.31	0.09	0.15	0.25	0.30	0.36	0.51	24	0	1.00
beta.develop.gamma	-0.03	0.10	-0.19	-0.10	-0.03	0.03	0.17	19	1	0.63

	mean	sd	2.5%	25%	50%	75%	97.5%	n.eff	overlap0	f
beta.develop.phi	0.27	0.12	0.02	0.19	0.29	0.37	0.47	10	0	0.98
beta.effort	0.07	0.01	0.05	0.06	0.07	0.08	0.10	83	0	1.00
beta.min_temp	0.00	0.00	0.00	0.00	0.00	0.00	0.00	2996	0	1.00

Table 252: Le Conte's Sparrow

	mean	sd	2.5%	25%	50%	75%	97.5%	n.eff	overlap0	f
alpha.lambda	2.86	0.14	2.59	2.77	2.84	2.94	3.17	7	0	1.00
alpha.phi	1.78	0.09	1.59	1.69	1.80	1.85	1.92	3	0	1.00
alpha.p	-1.73	0.02	-1.77	-1.74	-1.73	-1.71	-1.68	11	0	1.00
beta.hab.lambda	-0.39	0.15	-0.71	-0.49	-0.38	-0.28	-0.13	41	0	1.00
beta.hab.gamma	0.61	0.02	0.56	0.59	0.61	0.62	0.64	25	0	1.00
beta.hab.phi	-0.95	0.05	-1.04	-0.99	-0.96	-0.92	-0.86	9	0	1.00
beta.elev.lambda	0.15	0.08	0.00	0.09	0.14	0.21	0.29	11	1	0.97
beta.ele.gamma2	-0.33	0.03	-0.39	-0.35	-0.33	-0.30	-0.25	7	0	1.00
beta.elev.phi	0.01	0.05	-0.07	-0.02	0.01	0.04	0.10	6	1	0.59
beta.develop.lambda	0.18	0.22	-0.25	0.04	0.19	0.33	0.63	56	1	0.80
beta.develop.gamma	-0.15	0.31	-0.52	-0.40	-0.31	0.24	0.34	3	1	0.67
beta.develop.phi	0.35	0.37	-0.26	-0.08	0.51	0.65	0.86	3	1	0.68
beta.effort	0.00	0.00	0.00	0.00	0.00	0.00	0.01	254	0	1.00
beta.min_temp	0.00	0.00	0.00	0.00	0.00	0.00	0.00	5693	0	1.00

Table 253: Lewis's Woodpecker

	mean	sd	2.5%	25%	50%	75%	97.5%	n.eff	overlap0	f
alpha.lambda	4.64	0.08	4.49	4.58	4.62	4.70	4.79	5	0	1
alpha.phi	3.98	0.07	3.87	3.93	3.97	4.02	4.14	15	0	1
alpha.p	-2.60	0.03	-2.66	-2.63	-2.61	-2.58	-2.53	34	0	1
beta.hab.lambda	-0.78	0.05	-0.87	-0.81	-0.77	-0.73	-0.70	3	0	1
beta.hab.gamma	-1.64	0.13	-1.90	-1.74	-1.62	-1.55	-1.44	5	0	1
beta.hab.phi	1.16	0.12	0.97	1.06	1.15	1.23	1.41	4	0	1
beta.elev.lambda	0.31	0.08	0.21	0.23	0.31	0.38	0.43	3	0	1
beta.ele.gamma2	1.22	0.29	0.76	0.99	1.20	1.43	1.85	4	0	1
beta.elev.phi	-1.50	0.18	-1.82	-1.64	-1.49	-1.31	-1.21	3	0	1
beta.develop.lambda	-1.66	0.10	-1.85	-1.74	-1.64	-1.57	-1.49	4	0	1
beta.develop.gamma	-1.23	0.27	-1.88	-1.38	-1.20	-1.04	-0.74	7	0	1
beta.develop.phi	0.75	0.13	0.51	0.65	0.75	0.85	0.97	11	0	1
beta.effort	0.26	0.01	0.24	0.25	0.26	0.27	0.28	20	0	1
beta.min_temp	0.00	0.00	-0.01	0.00	0.00	0.00	0.00	105	0	1

Table 254: Merlin

	mean	sd	2.5%	25%	50%	75%	97.5%	n.eff	overlap0	f
alpha.lambda	2.11	0.26	1.74	1.87	2.03	2.43	2.51	3	0	1.00
alpha.phi	3.52	0.27	3.10	3.25	3.48	3.76	3.95	3	0	1.00
alpha.p	-1.88	0.26	-2.25	-2.21	-1.80	-1.67	-1.53	3	0	1.00
beta.hab.lambda	-0.09	0.05	-0.17	-0.12	-0.09	-0.06	0.03	6	1	0.91

	mean	sd	2.5%	25%	50%	75%	97.5%	n.eff	overlap0	f
beta.hab.gamma	-0.39	0.11	-0.55	-0.48	-0.39	-0.31	-0.20	4	0	1.00
beta.hab.phi	0.23	0.14	0.00	0.15	0.24	0.32	0.47	4	1	0.97
beta.elev.lambda	-0.10	0.04	-0.18	-0.12	-0.10	-0.08	-0.03	8	0	1.00
beta.ele.gamma2	0.01	0.04	-0.05	-0.02	0.00	0.05	0.08	4	1	0.51
beta.elev.phi	-0.11	0.04	-0.19	-0.15	-0.10	-0.07	-0.04	4	0	1.00
beta.develop.lambda	-0.08	0.07	-0.22	-0.13	-0.08	-0.03	0.07	23	1	0.86
beta.develop.gamma	-0.09	0.04	-0.17	-0.12	-0.10	-0.06	0.00	4	0	0.98
beta.develop.phi	0.17	0.06	0.06	0.11	0.18	0.21	0.27	10	0	1.00
beta.effort	0.04	0.01	0.02	0.03	0.04	0.04	0.06	30	0	1.00
beta.min_temp	0.00	0.00	0.00	0.00	0.00	0.00	0.00	274	0	1.00

Table 255: Muscovy Duck

	mean	sd	2.5%	25%	50%	75%	97.5%	n.eff	overlap0	f
alpha.lambda	-7.51	1.69	-10.83	-8.66	-7.47	-6.39	-4.09	11	0	1.00
alpha.phi	7.82	0.34	7.26	7.54	7.76	8.14	8.46	3	0	1.00
alpha.p	-0.66	0.04	-0.73	-0.71	-0.64	-0.62	-0.59	3	0	1.00
beta.hab.lambda	-1.00	1.82	-5.24	-1.90	-0.61	0.28	1.65	10	1	0.68
beta.hab.gamma	-0.26	0.03	-0.31	-0.28	-0.26	-0.24	-0.20	12	0	1.00
beta.hab.phi	-1.07	0.10	-1.29	-1.14	-1.08	-1.00	-0.89	8	0	1.00
beta.elev.lambda	-0.37	1.53	-3.29	-1.42	-0.50	0.76	2.59	13	1	0.62
beta.ele.gamma2	-0.23	0.02	-0.27	-0.24	-0.23	-0.21	-0.19	9	0	1.00
beta.elev.phi	-0.48	0.06	-0.60	-0.52	-0.48	-0.43	-0.35	35	0	1.00
beta.develop.lambda	0.52	1.76	-2.83	-0.67	0.50	1.69	4.04	333	1	0.61
beta.develop.gamma	1.97	0.06	1.86	1.92	1.98	2.02	2.07	4	0	1.00
beta.develop.phi	-7.15	0.35	-7.78	-7.46	-7.08	-6.91	-6.41	3	0	1.00
beta.effort	0.04	0.01	0.02	0.03	0.04	0.04	0.05	8	0	1.00
beta.min_temp	0.00	0.00	-0.01	0.00	0.00	0.00	0.00	139	0	1.00

Table 256: Northern Rough-winged Swallow

	mean	sd	2.5%	25%	50%	75%	97.5%	n.eff	overlap0	f
alpha.lambda	2.39	0.75	1.47	1.81	2.02	3.18	3.86	3	0	1.00
alpha.phi	-0.59	0.20	-0.87	-0.74	-0.68	-0.34	-0.27	3	0	1.00
alpha.p	-2.55	0.32	-3.04	-2.99	-2.34	-2.31	-2.27	3	0	1.00
beta.hab.lambda	-6.09	1.51	-7.86	-7.26	-6.87	-4.37	-3.26	3	0	1.00
beta.hab.gamma	-1.13	0.41	-1.47	-1.43	-1.40	-0.56	-0.50	3	0	1.00
beta.hab.phi	0.81	1.25	-1.02	-0.91	1.66	1.71	1.77	3	1	0.67
beta.elev.lambda	-1.26	0.41	-1.87	-1.57	-1.39	-0.84	-0.53	3	0	1.00
beta.ele.gamma2	-0.55	0.33	-1.05	-1.01	-0.32	-0.31	-0.29	3	0	1.00
beta.elev.phi	-0.12	0.70	-0.69	-0.62	-0.58	0.85	0.91	3	1	0.67
beta.develop.lambda	2.32	0.74	1.23	1.74	2.11	3.21	3.41	3	0	1.00
beta.develop.gamma	0.33	1.01	-0.45	-0.40	-0.36	1.75	1.78	3	1	0.33
beta.develop.phi	-0.09	2.65	-3.92	-3.78	1.74	1.80	1.88	3	1	0.33
beta.effort	0.00	0.00	0.00	0.00	0.00	0.00	0.00	81	0	1.00
beta.min_temp	-0.07	0.04	-0.12	-0.10	-0.09	-0.03	-0.01	3	0	1.00

Table 257: Northern Shrike

	mean	sd	2.5%	25%	50%	75%	97.5%	n.eff	overlap0	f
alpha.lambda	3.31	0.19	3.03	3.10	3.38	3.46	3.60	3	0	1.00
alpha.phi	2.55	0.42	1.74	2.19	2.76	2.90	2.97	3	0	1.00
alpha.p	-2.21	0.23	-2.51	-2.48	-2.23	-1.96	-1.90	3	0	1.00
beta.hab.lambda	-0.05	0.03	-0.10	-0.07	-0.05	-0.03	0.00	9	0	0.98
beta.hab.gamma	-0.16	0.12	-0.32	-0.25	-0.19	-0.03	0.05	3	1	0.82
beta.hab.phi	0.08	0.12	-0.14	-0.05	0.12	0.17	0.25	4	1	0.72
beta.elev.lambda	0.04	0.18	-0.13	-0.09	-0.07	0.24	0.36	3	1	0.33
beta.ele.gamma2	0.15	0.41	-0.53	-0.36	0.41	0.46	0.52	3	1	0.67
beta.elev.phi	0.04	0.38	-0.27	-0.23	-0.19	0.45	0.73	3	1	0.33
beta.develop.lambda	0.18	0.07	0.07	0.13	0.17	0.22	0.34	6	0	1.00
beta.develop.gamma	-0.27	0.08	-0.45	-0.32	-0.28	-0.22	-0.12	6	0	1.00
beta.develop.phi	0.23	0.08	0.07	0.18	0.24	0.29	0.37	7	0	1.00
beta.effort	0.00	0.00	0.00	0.00	0.00	0.01	0.01	475	0	1.00
beta.min_temp	-0.01	0.00	-0.01	-0.01	0.00	0.00	0.00	89	0	1.00

Table 258: Prairie Falcon

	mean	sd	2.5%	25%	50%	75%	97.5%	n.eff	overlap0	f
alpha.lambda	2.80	1.39	1.66	1.79	1.90	4.53	5.10	3	0	1.00
alpha.phi	3.78	0.28	3.47	3.59	3.67	3.89	4.44	5	0	1.00
alpha.p	-2.02	1.36	-4.29	-3.71	-1.10	-1.04	-0.96	3	0	1.00
beta.hab.lambda	-0.13	0.04	-0.22	-0.15	-0.12	-0.10	-0.06	6	0	1.00
beta.hab.gamma	0.35	0.06	0.23	0.32	0.35	0.38	0.46	94	0	1.00
beta.hab.phi	-0.15	0.08	-0.32	-0.20	-0.13	-0.09	-0.03	9	0	0.99
beta.elev.lambda	0.22	0.11	0.05	0.11	0.25	0.31	0.40	4	0	1.00
beta.ele.gamma2	-0.51	0.13	-0.75	-0.63	-0.47	-0.41	-0.27	3	0	1.00
beta.elev.phi	0.51	0.14	0.27	0.41	0.48	0.64	0.76	3	0	1.00
beta.develop.lambda	0.18	0.10	-0.01	0.11	0.17	0.24	0.41	16	1	0.97
beta.develop.gamma	-0.19	0.23	-0.66	-0.35	-0.16	-0.03	0.23	4	1	0.80
beta.develop.phi	0.16	0.21	-0.27	0.05	0.15	0.31	0.53	4	1	0.82
beta.effort	0.01	0.01	0.00	0.00	0.01	0.02	0.03	8	0	1.00
beta.min_temp	-0.01	0.01	-0.03	-0.01	-0.01	0.00	0.00	8	0	1.00

Table 259: Prairie Warbler

	mean	sd	2.5%	25%	50%	75%	97.5%	n.eff	overlap0	f
alpha.lambda	3.48	0.28	2.96	3.20	3.54	3.70	3.91	3	0	1.00
alpha.phi	2.28	0.15	2.04	2.12	2.31	2.41	2.53	3	0	1.00
alpha.p	-1.21	0.07	-1.31	-1.27	-1.23	-1.14	-1.09	3	0	1.00
beta.hab.lambda	0.38	0.36	-0.36	0.10	0.43	0.69	0.92	3	1	0.82
beta.hab.gamma	0.28	0.13	0.01	0.19	0.28	0.38	0.49	5	0	0.98
beta.hab.phi	-1.41	0.35	-1.92	-1.82	-1.34	-1.14	-0.80	3	0	1.00
beta.elev.lambda	-0.36	0.03	-0.43	-0.38	-0.36	-0.34	-0.29	7	0	1.00
beta.ele.gamma2	-0.63	0.06	-0.74	-0.67	-0.62	-0.58	-0.51	5	0	1.00
beta.elev.phi	0.48	0.04	0.39	0.45	0.48	0.50	0.56	92	0	1.00
beta.develop.lambda	-0.05	0.10	-0.23	-0.12	-0.05	0.02	0.15	6	1	0.68
beta.develop.gamma	1.99	0.17	1.71	1.81	2.03	2.14	2.23	3	0	1.00

	mean	sd	2.5%	25%	50%	75%	97.5%	n.eff	overlap0	f
beta.develop.phi	-1.98	0.29	-2.41	-2.27	-2.00	-1.68	-1.53	3	0	1.00
beta.effort	0.10	0.01	0.08	0.09	0.10	0.11	0.13	1819	0	1.00
beta.min_temp	0.00	0.00	0.00	0.00	0.00	0.00	0.00	193	0	1.00

Table 260: Red-breasted Sapsucker

	mean	sd	2.5%	25%	50%	75%	97.5%	n.eff	overlap0	f
alpha.lambda	-7.18	1.51	-10.53	-8.09	-7.12	-6.06	-4.78	19	0	1.00
alpha.phi	4.19	0.45	3.44	3.63	4.43	4.55	4.67	3	0	1.00
alpha.p	-1.42	0.05	-1.51	-1.47	-1.42	-1.38	-1.34	6	0	1.00
beta.hab.lambda	-0.24	1.26	-3.02	-0.98	0.03	0.70	1.60	44	1	0.48
beta.hab.gamma	0.33	0.19	0.02	0.09	0.44	0.47	0.51	3	0	0.99
beta.hab.phi	-0.55	0.62	-1.12	-1.02	-0.92	0.24	0.45	3	1	0.67
beta.elev.lambda	0.42	1.24	-1.41	-0.50	0.14	1.14	3.24	11	1	0.55
beta.ele.gamma2	-0.44	0.39	-0.76	-0.73	-0.70	0.09	0.13	3	1	0.67
beta.elev.phi	0.50	1.32	-1.48	-1.29	1.39	1.46	1.54	3	1	0.67
beta.develop.lambda	-0.31	1.99	-4.41	-1.75	-0.21	1.30	2.89	16	1	0.54
beta.develop.gamma	0.18	0.11	0.04	0.10	0.13	0.30	0.40	3	0	1.00
beta.develop.phi	0.13	0.50	-0.72	-0.49	0.41	0.51	0.62	3	1	0.67
beta.effort	0.03	0.01	0.01	0.02	0.03	0.04	0.05	12	0	1.00
beta.min_temp	-0.22	0.01	-0.24	-0.22	-0.21	-0.21	-0.19	8	0	1.00

Table 261: Reddish Egret

	mean	sd	2.5%	25%	50%	75%	97.5%	n.eff	overlap0	f
alpha.lambda	3.84	0.13	3.62	3.74	3.82	3.96	4.08	4	0	1.00
alpha.phi	4.27	0.29	3.90	4.00	4.19	4.54	4.79	4	0	1.00
alpha.p	-1.85	0.15	-2.10	-2.03	-1.78	-1.72	-1.68	3	0	1.00
beta.hab.lambda	-0.38	0.06	-0.49	-0.43	-0.37	-0.32	-0.29	4	0	1.00
beta.hab.gamma	-0.40	0.17	-0.64	-0.56	-0.43	-0.23	-0.12	3	0	1.00
beta.hab.phi	1.82	0.20	1.51	1.66	1.78	2.04	2.13	3	0	1.00
beta.elev.lambda	-0.64	0.05	-0.72	-0.68	-0.65	-0.61	-0.53	14	0	1.00
beta.ele.gamma2	-0.68	0.27	-1.11	-1.01	-0.59	-0.47	-0.32	3	0	1.00
beta.elev.phi	1.33	0.83	0.44	0.68	0.93	2.34	2.67	3	0	1.00
beta.develop.lambda	-2.33	0.58	-3.02	-2.78	-2.63	-1.62	-1.37	3	0	1.00
beta.develop.gamma	0.23	0.91	-0.88	-0.54	-0.15	1.44	1.53	3	1	0.42
beta.develop.phi	-1.74	2.22	-5.01	-4.81	-0.35	-0.05	0.13	3	1	0.83
beta.effort	0.01	0.01	0.00	0.00	0.01	0.02	0.03	5	0	1.00
beta.min_temp	0.00	0.00	0.00	0.00	0.00	0.00	0.00	774	0	1.00

Table 262: Rock Wren

	mean	sd	2.5%	25%	50%	75%	97.5%	n.eff	overlap0	f
alpha.lambda	3.83	0.06	3.70	3.78	3.83	3.88	3.94	4	0	1.00
alpha.phi	3.10	0.06	3.00	3.06	3.10	3.13	3.21	4	0	1.00
alpha.p	-1.33	0.05	-1.42	-1.37	-1.34	-1.30	-1.25	4	0	1.00
beta.hab.lambda	-0.38	0.04	-0.46	-0.41	-0.38	-0.36	-0.31	38	0	1.00

	mean	sd	2.5%	25%	50%	75%	97.5%	n.eff	overlap0	f
beta.hab.gamma	0.01	0.02	-0.03	-0.01	0.01	0.02	0.05	69	1	0.64
beta.hab.phi	0.27	0.06	0.15	0.23	0.26	0.31	0.41	8	0	1.00
beta.elev.lambda	0.15	0.03	0.10	0.13	0.15	0.17	0.21	11	0	1.00
beta.ele.gamma2	0.21	0.04	0.12	0.18	0.21	0.24	0.28	18	0	1.00
beta.elev.phi	-0.01	0.05	-0.09	-0.05	-0.02	0.02	0.08	20	1	0.61
beta.develop.lambda	-0.56	0.07	-0.71	-0.61	-0.56	-0.51	-0.43	132	0	1.00
beta.develop.gamma	0.70	0.07	0.58	0.65	0.70	0.74	0.84	28	0	1.00
beta.develop.phi	-0.47	0.06	-0.59	-0.50	-0.47	-0.43	-0.35	94	0	1.00
beta.effort	0.23	0.01	0.21	0.22	0.23	0.23	0.24	204	0	1.00
beta.min_temp	0.00	0.00	0.00	0.00	0.00	0.00	0.00	6000	0	1.00

Table 263: Rufous-crowned Sparrow

	mean	sd	2.5%	25%	50%	75%	97.5%	n.eff	overlap0	f
alpha.lambda	4.70	0.04	4.62	4.67	4.71	4.73	4.78	4	0	1.00
alpha.phi	3.26	0.08	3.11	3.20	3.27	3.32	3.40	4	0	1.00
alpha.p	-1.88	0.04	-1.96	-1.91	-1.89	-1.85	-1.81	4	0	1.00
beta.hab.lambda	0.44	0.02	0.39	0.42	0.44	0.45	0.48	12	0	1.00
beta.hab.gamma	0.08	0.05	-0.04	0.04	0.08	0.11	0.18	41	1	0.93
beta.hab.phi	0.14	0.04	0.07	0.12	0.14	0.17	0.21	28	0	1.00
beta.elev.lambda	0.01	0.03	-0.06	-0.02	0.01	0.03	0.07	10	1	0.58
beta.ele.gamma2	-0.21	0.08	-0.35	-0.26	-0.22	-0.16	-0.04	10	0	0.99
beta.elev.phi	0.15	0.09	-0.03	0.09	0.16	0.22	0.30	7	1	0.93
beta.develop.lambda	-0.26	0.05	-0.37	-0.30	-0.26	-0.23	-0.16	37	0	1.00
beta.develop.gamma	1.21	0.12	0.97	1.12	1.21	1.30	1.42	14	0	1.00
beta.develop.phi	-1.02	0.11	-1.24	-1.09	-1.01	-0.94	-0.83	118	0	1.00
beta.effort	0.16	0.01	0.14	0.15	0.16	0.16	0.17	293	0	1.00
beta.min_temp	0.00	0.00	0.00	0.00	0.00	0.00	0.00	1094	0	1.00

Table 264: Seaside Sparrow

	mean	sd	2.5%	25%	50%	75%	97.5%	n.eff	overlap0	f
alpha.lambda	5.09	0.52	4.34	4.41	5.27	5.60	5.66	3	0	1.00
alpha.phi	4.08	0.84	2.63	3.08	4.45	4.68	5.09	3	0	1.00
alpha.p	-3.26	0.35	-3.69	-3.66	-3.27	-2.83	-2.81	3	0	1.00
beta.hab.lambda	0.69	0.05	0.60	0.63	0.70	0.73	0.77	3	0	1.00
beta.hab.gamma	0.70	0.04	0.64	0.66	0.68	0.75	0.77	3	0	1.00
beta.hab.phi	-0.24	0.04	-0.31	-0.28	-0.22	-0.20	-0.17	4	0	1.00
beta.elev.lambda	-0.19	0.03	-0.24	-0.21	-0.19	-0.17	-0.13	4	0	1.00
beta.ele.gamma2	1.46	0.09	1.33	1.38	1.44	1.56	1.60	3	0	1.00
beta.elev.phi	-2.40	0.06	-2.49	-2.45	-2.40	-2.35	-2.26	6	0	1.00
beta.develop.lambda	-0.23	0.21	-0.55	-0.45	-0.23	0.00	0.06	3	1	0.73
beta.develop.gamma	-4.83	0.56	-5.56	-5.34	-4.94	-4.36	-3.75	3	0	1.00
beta.develop.phi	8.02	1.01	6.25	7.19	7.86	8.98	9.57	3	0	1.00
beta.effort	0.29	0.01	0.27	0.28	0.29	0.30	0.31	5	0	1.00
beta.min_temp	0.00	0.00	0.00	0.00	0.00	0.00	0.00	436	0	1.00

Table 265: Short-eared Owl

	mean	sd	2.5%	25%	50%	75%	97.5%	n.eff	overlap0	f
alpha.lambda	3.50	0.09	3.33	3.44	3.47	3.59	3.66	4	0	1.00
alpha.phi	2.15	0.08	1.99	2.09	2.16	2.21	2.26	4	0	1.00
alpha.p	-2.06	0.08	-2.21	-2.14	-2.04	-2.01	-1.91	3	0	1.00
beta.hab.lambda	-0.01	0.03	-0.09	-0.03	-0.01	0.01	0.04	5	1	0.60
beta.hab.gamma	-0.25	0.08	-0.41	-0.30	-0.24	-0.19	-0.13	4	0	1.00
beta.hab.phi	0.23	0.07	0.12	0.16	0.22	0.29	0.38	4	0	1.00
beta.elev.lambda	-0.13	0.03	-0.19	-0.15	-0.13	-0.11	-0.08	8	0	1.00
beta.ele.gamma2	0.07	0.06	-0.05	0.03	0.09	0.12	0.17	4	1	0.84
beta.elev.phi	-0.14	0.05	-0.23	-0.18	-0.15	-0.09	-0.04	4	0	1.00
beta.develop.lambda	0.09	0.04	0.03	0.07	0.09	0.12	0.16	103	0	1.00
beta.develop.gamma	-0.39	0.08	-0.55	-0.45	-0.39	-0.33	-0.26	6	0	1.00
beta.develop.phi	0.18	0.07	0.07	0.12	0.18	0.23	0.31	7	0	1.00
beta.effort	0.03	0.01	0.01	0.02	0.03	0.03	0.05	49	0	1.00
beta.min_temp	-0.06	0.01	-0.08	-0.06	-0.06	-0.05	-0.04	12	0	1.00

Table 266: Vermilion Flycatcher

	mean	sd	2.5%	25%	50%	75%	97.5%	n.eff	overlap0	f
alpha.lambda	2.48	0.34	2.04	2.22	2.33	2.92	2.99	3	0	1.00
alpha.phi	3.61	1.16	2.58	2.71	2.91	5.15	5.41	3	0	1.00
alpha.p	-1.13	0.57	-1.96	-1.92	-0.81	-0.67	-0.61	3	0	1.00
beta.hab.lambda	0.00	0.06	-0.14	-0.02	0.02	0.04	0.09	5	1	0.63
beta.hab.gamma	-2.27	0.39	-2.90	-2.72	-2.15	-1.94	-1.75	3	0	1.00
beta.hab.phi	1.50	0.42	0.96	1.16	1.33	2.00	2.25	3	0	1.00
beta.elev.lambda	-0.26	0.07	-0.37	-0.31	-0.27	-0.21	-0.11	8	0	1.00
beta.ele.gamma2	0.88	0.28	0.60	0.67	0.73	1.25	1.33	3	0	1.00
beta.elev.phi	-0.58	0.28	-1.02	-0.94	-0.44	-0.37	-0.27	3	0	1.00
beta.develop.lambda	-0.85	0.25	-1.33	-1.04	-0.83	-0.64	-0.45	5	0	1.00
beta.develop.gamma	0.54	0.21	0.33	0.38	0.42	0.80	0.90	3	0	1.00
beta.develop.phi	0.16	1.36	-2.07	-1.61	1.05	1.16	1.27	3	1	0.67
beta.effort	0.24	0.04	0.19	0.21	0.22	0.28	0.31	3	0	1.00
beta.min_temp	0.00	0.00	-0.01	0.00	0.00	0.00	0.00	182	0	1.00

Table 267: White-eyed Vireo

	mean	sd	2.5%	25%	50%	75%	97.5%	n.eff	overlap0	f
alpha.lambda	3.78	0.04	3.72	3.76	3.78	3.81	3.86	15	0	1
alpha.phi	3.06	0.10	2.87	3.01	3.07	3.12	3.26	13	0	1
alpha.p	-1.15	0.02	-1.18	-1.16	-1.15	-1.14	-1.12	7	0	1
beta.hab.lambda	-0.40	0.05	-0.50	-0.44	-0.40	-0.37	-0.31	6	0	1
beta.hab.gamma	-0.24	0.03	-0.30	-0.26	-0.24	-0.22	-0.18	5	0	1
beta.hab.phi	3.22	0.34	2.58	2.97	3.28	3.47	3.77	20	0	1
beta.elev.lambda	-0.29	0.03	-0.35	-0.31	-0.29	-0.27	-0.23	26	0	1
beta.ele.gamma2	0.11	0.03	0.06	0.10	0.11	0.13	0.17	69	0	1
beta.elev.phi	-0.57	0.04	-0.65	-0.59	-0.57	-0.54	-0.49	16	0	1
beta.develop.lambda	0.19	0.03	0.13	0.17	0.19	0.21	0.26	7	0	1
beta.develop.gamma	0.28	0.04	0.20	0.25	0.28	0.30	0.35	13	0	1

	mean	sd	2.5%	25%	50%	75%	97.5%	n.eff	overlap0	f
beta.develop.phi	0.10	0.04	0.02	0.08	0.10	0.13	0.18	11	0	1
beta.effort	0.03	0.01	0.02	0.03	0.03	0.04	0.05	2492	0	1
beta.min_temp	0.00	0.00	0.00	0.00	0.00	0.00	0.00	6000	0	1

Table 268: Yellow-crowned Night-Heron

	mean	sd	2.5%	25%	50%	75%	97.5%	n.eff	overlap0	f
alpha.lambda	3.26	0.12	3.05	3.17	3.24	3.37	3.46	4	0	1.00
alpha.phi	2.79	0.08	2.64	2.72	2.79	2.85	2.96	4	0	1.00
alpha.p	-1.65	0.06	-1.74	-1.72	-1.65	-1.60	-1.56	3	0	1.00
beta.hab.lambda	-2.17	0.09	-2.33	-2.23	-2.17	-2.11	-1.98	14	0	1.00
beta.hab.gamma	0.12	0.03	0.07	0.10	0.12	0.14	0.17	40	0	1.00
beta.hab.phi	-0.04	0.03	-0.09	-0.06	-0.04	-0.02	0.01	97	1	0.92
beta.elev.lambda	-1.33	0.05	-1.43	-1.37	-1.33	-1.29	-1.24	4	0	1.00
beta.ele.gamma2	-0.32	0.03	-0.38	-0.34	-0.32	-0.30	-0.27	7	0	1.00
beta.elev.phi	0.35	0.05	0.26	0.31	0.35	0.39	0.46	5	0	1.00
beta.develop.lambda	0.78	0.06	0.67	0.73	0.79	0.83	0.90	5	0	1.00
beta.develop.gamma	-0.93	0.06	-1.05	-0.98	-0.93	-0.88	-0.81	12	0	1.00
beta.develop.phi	0.70	0.04	0.62	0.68	0.71	0.73	0.78	50	0	1.00
beta.effort	0.00	0.00	0.00	0.00	0.00	0.00	0.00	856	0	1.00
beta.min_temp	0.00	0.00	0.00	0.00	0.00	0.00	0.00	446	0	1.00

Table 269: Yellow-throated Warbler

	mean	sd	2.5%	25%	50%	75%	97.5%	n.eff	overlap0	f
alpha.lambda	3.74	0.16	3.53	3.62	3.71	3.81	4.12	5	0	1.00
alpha.phi	2.93	0.15	2.67	2.83	2.96	3.03	3.20	4	0	1.00
alpha.p	-1.71	0.11	-1.96	-1.80	-1.72	-1.62	-1.53	6	0	1.00
beta.hab.lambda	-0.23	0.06	-0.35	-0.27	-0.22	-0.18	-0.12	5	0	1.00
beta.hab.gamma	-0.95	0.10	-1.15	-1.03	-0.94	-0.87	-0.79	14	0	1.00
beta.hab.phi	0.57	0.07	0.45	0.52	0.56	0.61	0.71	44	0	1.00
beta.elev.lambda	0.01	0.09	-0.14	-0.05	0.00	0.08	0.18	4	1	0.49
beta.ele.gamma2	0.21	0.21	-0.06	0.04	0.17	0.29	0.66	4	1	0.82
beta.elev.phi	-0.34	0.25	-0.86	-0.43	-0.30	-0.14	-0.02	4	0	1.00
beta.develop.lambda	0.21	0.10	0.03	0.14	0.21	0.29	0.41	4	0	0.99
beta.develop.gamma	0.54	0.20	0.15	0.40	0.54	0.69	0.87	4	0	1.00
beta.develop.phi	-0.17	0.21	-0.54	-0.35	-0.15	0.00	0.18	4	1	0.76
beta.effort	0.01	0.01	0.00	0.00	0.01	0.01	0.03	67	0	1.00
beta.min_temp	0.00	0.00	-0.01	0.00	0.00	0.00	0.00	208	0	1.00

Table 270: Baltimore Oriole

	mean	sd	2.5%	25%	50%	75%	97.5%	n.eff	overlap0	f
alpha.lambda	2.96	0.18	2.66	2.77	3.04	3.11	3.18	3	0	1.00
alpha.phi	2.41	0.16	2.14	2.22	2.48	2.54	2.62	3	0	1.00
alpha.p	-1.58	0.21	-1.81	-1.75	-1.69	-1.31	-1.23	3	0	1.00
beta.hab.lambda	0.41	0.05	0.31	0.37	0.41	0.44	0.50	10	0	1.00

	mean	sd	2.5%	25%	50%	75%	97.5%	n.eff	overlap0	f
beta.hab.gamma	0.13	0.10	-0.05	0.06	0.14	0.20	0.31	5	1	0.90
beta.hab.phi	-0.31	0.09	-0.46	-0.39	-0.33	-0.24	-0.14	4	0	1.00
beta.elev.lambda	0.17	0.07	0.04	0.12	0.16	0.22	0.30	4	0	1.00
beta.ele.gamma2	0.23	0.08	0.09	0.17	0.23	0.29	0.39	4	0	1.00
beta.elev.phi	-0.41	0.10	-0.58	-0.48	-0.44	-0.32	-0.22	4	0	1.00
beta.develop.lambda	0.39	0.07	0.27	0.35	0.39	0.44	0.53	12	0	1.00
beta.develop.gamma	0.24	0.06	0.11	0.21	0.25	0.28	0.34	15	0	1.00
beta.develop.phi	0.15	0.06	0.05	0.11	0.15	0.19	0.29	25	0	0.99
beta.effort	0.01	0.01	0.00	0.00	0.01	0.01	0.02	115	0	1.00
beta.min_temp	0.00	0.00	-0.01	0.00	0.00	0.00	0.00	118	0	1.00

Table 271: Barn Swallow

	mean	sd	2.5%	25%	50%	75%	97.5%	n.eff	overlap0	f
alpha.lambda	0.47	1.35	-2.78	0.09	0.96	1.37	1.93	4	1	0.76
alpha.phi	-4.54	5.62	-14.09	-10.42	-3.19	1.41	1.65	3	1	0.67
alpha.p	-2.96	0.66	-3.95	-3.79	-2.77	-2.31	-2.16	3	0	1.00
beta.hab.lambda	-1.17	1.60	-5.68	-1.56	-0.33	-0.22	-0.11	4	0	1.00
beta.hab.gamma	0.06	0.54	-0.71	-0.69	0.35	0.51	0.57	3	1	0.67
beta.hab.phi	2.31	2.35	-0.59	-0.49	2.43	4.84	5.78	3	1	0.67
beta.elev.lambda	-0.15	0.59	-1.03	-0.76	-0.05	0.36	0.81	4	1	0.53
beta.ele.gamma2	-0.46	0.95	-1.78	-1.71	-0.17	0.52	0.56	3	1	0.67
beta.elev.phi	-2.70	3.90	-7.41	-5.70	-4.38	2.64	2.72	3	1	0.67
beta.develop.lambda	-0.55	0.76	-2.22	-0.98	-0.47	-0.15	1.21	16	1	0.83
beta.develop.gamma	0.15	0.09	-0.01	0.06	0.17	0.23	0.30	3	1	0.96
beta.develop.phi	0.80	0.69	0.03	0.33	0.43	1.71	1.97	3	0	0.99
beta.effort	0.23	0.05	0.16	0.19	0.23	0.28	0.31	3	0	1.00
beta.min_temp	0.00	0.00	-0.02	-0.01	0.00	0.00	0.00	13	0	1.00

Table 272: Black-throated Gray Warbler

	mean	sd	2.5%	25%	50%	75%	97.5%	n.eff	overlap0	f
alpha.lambda	2.77	0.18	2.41	2.65	2.76	2.90	3.10	11	0	1.00
alpha.phi	2.05	0.33	1.47	1.69	2.20	2.31	2.46	3	0	1.00
alpha.p	-1.85	0.15	-2.13	-1.97	-1.84	-1.74	-1.58	4	0	1.00
beta.hab.lambda	0.25	0.17	-0.01	0.10	0.28	0.38	0.58	4	1	0.96
beta.hab.gamma	-0.40	0.15	-0.67	-0.51	-0.39	-0.28	-0.13	4	0	1.00
beta.hab.phi	0.10	0.17	-0.23	0.00	0.08	0.23	0.45	4	1	0.75
beta.elev.lambda	0.23	0.14	-0.07	0.14	0.25	0.32	0.46	10	1	0.93
beta.ele.gamma2	-0.23	0.30	-0.73	-0.54	-0.18	0.03	0.20	3	1	0.71
beta.elev.phi	0.46	0.40	-0.06	0.10	0.34	0.89	1.16	3	1	0.89
beta.develop.lambda	-0.30	0.23	-0.76	-0.45	-0.30	-0.15	0.18	12	1	0.91
beta.develop.gamma	-0.22	0.11	-0.41	-0.31	-0.23	-0.14	-0.02	25	0	0.98
beta.develop.phi	0.29	0.14	0.02	0.19	0.30	0.39	0.55	13	0	0.98
beta.effort	0.04	0.02	0.00	0.03	0.04	0.06	0.10	25	0	1.00
beta.min_temp	-0.01	0.01	-0.03	-0.01	-0.01	0.00	0.00	102	0	1.00

Table 273: Costa's Hummingbird

	mean	sd	2.5%	25%	50%	75%	97.5%	n.eff	overlap0	f
alpha.lambda	3.26	0.12	3.02	3.17	3.27	3.35	3.46	4	0	1
alpha.phi	3.48	0.14	3.22	3.38	3.46	3.60	3.72	6	0	1
alpha.p	-1.14	0.12	-1.32	-1.26	-1.15	-1.02	-0.93	3	0	1
beta.hab.lambda	0.33	0.15	0.09	0.21	0.33	0.44	0.63	4	0	1
beta.hab.gamma	1.03	0.07	0.91	0.98	1.03	1.08	1.17	6	0	1
beta.hab.phi	-0.30	0.11	-0.56	-0.38	-0.30	-0.22	-0.11	5	0	1
beta.elev.lambda	0.73	0.21	0.39	0.49	0.80	0.89	1.04	3	0	1
beta.ele.gamma2	2.87	0.14	2.63	2.77	2.87	2.97	3.14	7	0	1
beta.elev.phi	-2.92	0.22	-3.31	-3.13	-2.87	-2.74	-2.56	5	0	1
beta.develop.lambda	-2.54	0.27	-3.09	-2.74	-2.52	-2.34	-2.07	10	0	1
beta.develop.gamma	0.88	0.09	0.70	0.82	0.88	0.95	1.05	31	0	1
beta.develop.phi	0.78	0.15	0.50	0.67	0.78	0.88	1.09	10	0	1
beta.effort	0.49	0.04	0.43	0.47	0.49	0.52	0.56	5	0	1
beta.min_temp	0.00	0.00	-0.01	0.00	0.00	0.00	0.00	2363	0	1

Table 274: Crissal Thrasher

	mean	sd	2.5%	25%	50%	75%	97.5%	n.eff	overlap0	f
alpha.lambda	3.10	0.54	2.53	2.73	2.84	3.44	4.21	3	0	1.00
alpha.phi	2.93	0.32	2.63	2.72	2.77	3.04	3.72	4	0	1.00
alpha.p	-1.22	0.31	-1.89	-1.41	-1.06	-1.01	-0.94	3	0	1.00
beta.hab.lambda	0.35	0.15	0.11	0.20	0.36	0.47	0.61	4	0	1.00
beta.hab.gamma	0.50	0.10	0.24	0.45	0.52	0.57	0.65	5	0	1.00
beta.hab.phi	-1.16	0.12	-1.38	-1.27	-1.16	-1.07	-0.93	5	0	1.00
beta.elev.lambda	-0.11	0.13	-0.31	-0.21	-0.13	0.00	0.13	4	1	0.75
beta.ele.gamma2	0.49	0.09	0.34	0.43	0.48	0.54	0.69	36	0	1.00
beta.elev.phi	-0.25	0.10	-0.49	-0.30	-0.24	-0.17	-0.08	12	0	1.00
beta.develop.lambda	1.19	0.32	0.52	1.00	1.23	1.42	1.73	6	0	1.00
beta.develop.gamma	0.10	0.22	-0.22	-0.05	0.06	0.22	0.65	5	1	0.64
beta.develop.phi	-0.58	0.42	-1.52	-0.87	-0.45	-0.28	-0.02	4	0	0.98
beta.effort	0.01	0.00	0.00	0.00	0.00	0.01	0.02	73	0	1.00
beta.min_temp	-0.03	0.01	-0.06	-0.04	-0.03	-0.02	0.00	25	0	1.00

Table 275: Eurasian Wigeon

	mean	sd	2.5%	25%	50%	75%	97.5%	n.eff	overlap0	f
alpha.lambda	1.15	0.23	0.61	1.03	1.16	1.30	1.56	51	0	1.00
alpha.phi	2.48	0.10	2.29	2.40	2.50	2.56	2.65	6	0	1.00
alpha.p	-1.22	0.04	-1.30	-1.25	-1.22	-1.19	-1.14	5	0	1.00
beta.hab.lambda	0.35	0.17	0.05	0.23	0.34	0.45	0.73	14	0	0.99
beta.hab.gamma	-0.64	0.05	-0.74	-0.68	-0.64	-0.60	-0.54	5	0	1.00
beta.hab.phi	1.28	0.11	1.08	1.20	1.29	1.37	1.49	4	0	1.00
beta.elev.lambda	-0.71	0.10	-0.93	-0.78	-0.70	-0.64	-0.53	12	0	1.00
beta.ele.gamma2	-0.62	0.06	-0.73	-0.66	-0.62	-0.58	-0.51	7	0	1.00
beta.elev.phi	1.13	0.11	0.94	1.06	1.12	1.21	1.35	6	0	1.00
beta.develop.lambda	0.32	0.18	-0.03	0.20	0.33	0.46	0.64	14	1	0.96
beta.develop.gamma	0.25	0.12	0.04	0.16	0.25	0.34	0.46	5	0	1.00

	mean	sd	2.5%	25%	50%	75%	97.5%	n.eff	overlap0	f
beta.develop.phi	-0.25	0.18	-0.55	-0.40	-0.27	-0.12	0.09	4	1	0.88
beta.effort	0.01	0.00	0.00	0.00	0.00	0.01	0.02	112	0	1.00
beta.min_temp	0.00	0.00	-0.01	-0.01	0.00	0.00	0.00	6000	0	1.00

Table 276: Grasshopper Sparrow

	mean	sd	2.5%	25%	50%	75%	97.5%	n.eff	overlap0	f
alpha.lambda	3.65	0.31	3.22	3.44	3.55	3.80	4.32	4	0	1.00
alpha.phi	0.06	1.46	-2.08	-1.94	1.03	1.12	1.24	3	1	0.67
alpha.p	-2.71	0.62	-3.77	-3.39	-2.35	-2.22	-2.17	3	0	1.00
beta.hab.lambda	0.70	0.12	0.45	0.63	0.71	0.76	0.98	23	0	1.00
beta.hab.gamma	-0.12	0.02	-0.17	-0.14	-0.12	-0.10	-0.08	5	0	1.00
beta.hab.phi	0.21	0.18	0.01	0.07	0.13	0.45	0.50	3	0	0.99
beta.elev.lambda	-0.42	0.48	-1.33	-0.96	-0.13	-0.07	0.01	3	1	0.96
beta.ele.gamma2	0.88	0.51	0.14	0.17	1.21	1.26	1.30	3	0	1.00
beta.elev.phi	-1.56	0.47	-2.05	-1.91	-1.82	-0.95	-0.84	3	0	1.00
beta.develop.lambda	0.62	0.19	0.36	0.46	0.56	0.79	0.98	3	0	1.00
beta.develop.gamma	-0.25	0.16	-0.50	-0.44	-0.19	-0.13	-0.04	3	0	0.99
beta.develop.phi	0.21	0.59	-0.35	-0.26	-0.11	1.00	1.08	3	1	0.34
beta.effort	0.01	0.01	0.00	0.00	0.01	0.02	0.04	4	0	1.00
beta.min_temp	0.00	0.00	-0.01	0.00	0.00	0.00	0.00	49	0	1.00

Table 277: Green-tailed Towhee

	mean	sd	2.5%	25%	50%	75%	97.5%	n.eff	overlap0	f
alpha.lambda	2.06	0.51	1.34	1.63	1.91	2.67	2.83	3	0	1.00
alpha.phi	0.92	0.42	0.43	0.58	0.78	1.25	1.90	3	0	1.00
alpha.p	-2.52	0.23	-2.85	-2.78	-2.51	-2.27	-2.19	3	0	1.00
beta.hab.lambda	1.59	0.14	1.37	1.47	1.59	1.72	1.83	3	0	1.00
beta.hab.gamma	-1.11	0.11	-1.31	-1.20	-1.11	-1.02	-0.93	6	0	1.00
beta.hab.phi	2.68	0.33	1.95	2.50	2.58	2.91	3.35	6	0	1.00
beta.elev.lambda	0.55	0.14	0.27	0.46	0.55	0.65	0.80	4	0	1.00
beta.ele.gamma2	-0.10	0.06	-0.21	-0.14	-0.09	-0.05	0.01	5	1	0.97
beta.elev.phi	0.82	0.14	0.47	0.70	0.84	0.94	1.02	5	0	1.00
beta.develop.lambda	-0.61	0.59	-1.53	-1.09	-0.71	-0.13	0.51	3	1	0.83
beta.develop.gamma	-0.31	0.31	-1.07	-0.52	-0.17	-0.06	0.07	4	1	0.90
beta.develop.phi	0.41	0.34	-0.12	0.15	0.33	0.73	0.99	4	1	0.90
beta.effort	0.19	0.02	0.17	0.18	0.19	0.21	0.22	4	0	1.00
beta.min_temp	-0.02	0.01	-0.05	-0.03	-0.02	-0.01	0.00	7	0	1.00

Table 278: Indigo Bunting

	mean	sd	2.5%	25%	50%	75%	97.5%	n.eff	overlap0	f
alpha.lambda	1.08	0.33	0.39	0.88	1.12	1.32	1.61	9	0	1.00
alpha.phi	1.69	0.15	1.43	1.54	1.71	1.82	1.95	4	0	1.00
alpha.p	-1.69	0.18	-2.03	-1.88	-1.61	-1.55	-1.45	3	0	1.00
beta.hab.lambda	0.48	0.30	-0.16	0.29	0.52	0.69	0.97	51	1	0.92

	mean	sd	2.5%	25%	50%	75%	97.5%	n.eff	overlap0	f
beta.hab.gamma	-0.10	0.19	-0.42	-0.25	-0.12	0.07	0.24	4	1	0.67
beta.hab.phi	-0.35	0.30	-0.95	-0.61	-0.29	-0.10	0.12	4	1	0.88
beta.elev.lambda	-2.78	0.19	-3.20	-2.92	-2.75	-2.63	-2.47	18	0	1.00
beta.ele.gamma2	-0.29	0.10	-0.50	-0.37	-0.27	-0.22	-0.12	7	0	1.00
beta.elev.phi	0.40	0.14	0.17	0.30	0.36	0.48	0.70	8	0	1.00
beta.develop.lambda	1.13	0.09	0.99	1.07	1.12	1.19	1.31	21	0	1.00
beta.develop.gamma	0.21	0.05	0.11	0.18	0.21	0.25	0.32	58	0	1.00
beta.develop.phi	0.27	0.05	0.19	0.24	0.26	0.31	0.36	6	0	1.00
beta.effort	0.07	0.02	0.02	0.05	0.07	0.09	0.12	93	0	1.00
beta.min__temp	0.00	0.00	-0.02	-0.01	0.00	0.00	0.00	86	0	1.00

Table 279: Juniper Titmouse

	mean	sd	2.5%	25%	50%	75%	97.5%	n.eff	overlap0	f
alpha.lambda	-6.82	2.19	-12.37	-7.60	-6.41	-5.39	-3.76	14	0	1.00
alpha.phi	2.19	0.92	0.76	0.96	2.74	2.86	3.07	3	0	1.00
alpha.p	-1.03	0.11	-1.21	-1.15	-0.98	-0.94	-0.89	3	0	1.00
beta.hab.lambda	0.79	1.46	-1.38	-0.29	0.57	1.60	4.37	28	1	0.66
beta.hab.gamma	1.20	0.06	1.09	1.16	1.20	1.24	1.32	42	0	1.00
beta.hab.phi	-0.09	0.11	-0.31	-0.16	-0.09	-0.01	0.12	19	1	0.78
beta.elev.lambda	0.57	1.19	-1.60	-0.24	0.51	1.37	3.09	27	1	0.66
beta.ele.gamma2	0.50	0.04	0.43	0.47	0.50	0.53	0.58	6	0	1.00
beta.elev.phi	-0.89	0.09	-1.09	-0.95	-0.89	-0.83	-0.74	26	0	1.00
beta.develop.lambda	-0.30	2.07	-4.71	-1.60	-0.21	1.08	3.67	142	1	0.54
beta.develop.gamma	1.83	0.81	0.57	0.78	2.25	2.46	2.71	3	0	1.00
beta.develop.phi	7.54	2.10	5.29	5.94	6.43	9.94	11.28	3	0	1.00
beta.effort	0.17	0.04	0.11	0.14	0.16	0.20	0.24	4	0	1.00
beta.min__temp	-0.01	0.01	-0.02	-0.01	-0.01	0.00	0.00	60	0	1.00

Table 280: Least Bittern

	mean	sd	2.5%	25%	50%	75%	97.5%	n.eff	overlap0	f
alpha.lambda	3.19	0.38	2.30	2.92	3.33	3.47	3.65	4	0	1.00
alpha.phi	2.17	0.17	1.81	2.03	2.21	2.31	2.43	4	0	1.00
alpha.p	-1.47	0.17	-1.71	-1.61	-1.52	-1.32	-1.13	3	0	1.00
beta.hab.lambda	1.36	0.11	1.12	1.30	1.37	1.43	1.57	125	0	1.00
beta.hab.gamma	0.66	0.07	0.50	0.62	0.66	0.70	0.78	20	0	1.00
beta.hab.phi	-1.30	0.15	-1.57	-1.37	-1.32	-1.26	-0.85	6	0	1.00
beta.elev.lambda	-0.18	0.22	-0.80	-0.28	-0.15	-0.04	0.14	11	1	0.83
beta.ele.gamma2	-0.21	0.12	-0.41	-0.31	-0.23	-0.13	0.06	6	1	0.93
beta.elev.phi	0.22	0.17	-0.16	0.10	0.25	0.35	0.50	6	1	0.89
beta.develop.lambda	-0.32	0.21	-0.72	-0.45	-0.33	-0.18	0.12	8	1	0.94
beta.develop.gamma	-0.21	0.13	-0.42	-0.31	-0.21	-0.12	0.04	13	1	0.94
beta.develop.phi	0.79	0.15	0.50	0.68	0.80	0.91	1.03	10	0	1.00
beta.effort	0.34	0.03	0.28	0.32	0.34	0.37	0.41	27	0	1.00
beta.min__temp	-0.01	0.01	-0.02	-0.01	0.00	0.00	0.00	562	0	1.00

Table 281: Long-eared Owl

	mean	sd	2.5%	25%	50%	75%	97.5%	n.eff	overlap0	f
alpha.lambda	3.28	0.10	3.12	3.20	3.27	3.34	3.48	6	0	1.00
alpha.phi	1.40	0.19	1.03	1.25	1.45	1.54	1.68	5	0	1.00
alpha.p	-2.26	0.06	-2.40	-2.29	-2.26	-2.23	-2.15	8	0	1.00
beta.hab.lambda	-0.03	0.12	-0.23	-0.16	0.00	0.07	0.15	3	1	0.49
beta.hab.gamma	-0.85	0.25	-1.24	-1.15	-0.78	-0.68	-0.45	3	0	1.00
beta.hab.phi	1.01	0.39	0.43	0.73	0.91	1.43	1.65	3	0	1.00
beta.elev.lambda	0.02	0.09	-0.14	-0.05	0.02	0.09	0.21	6	1	0.56
beta.ele.gamma2	-0.11	0.08	-0.25	-0.18	-0.08	-0.05	0.01	3	1	0.96
beta.elev.phi	0.41	0.09	0.22	0.34	0.41	0.49	0.57	4	0	1.00
beta.develop.lambda	-0.13	0.15	-0.42	-0.23	-0.11	-0.02	0.12	4	1	0.79
beta.develop.gamma	-0.48	0.12	-0.65	-0.58	-0.52	-0.39	-0.24	4	0	1.00
beta.develop.phi	0.71	0.11	0.48	0.63	0.72	0.79	0.89	6	0	1.00
beta.effort	0.01	0.00	0.00	0.00	0.00	0.01	0.02	149	0	1.00
beta.min__temp	0.00	0.00	-0.01	0.00	0.00	0.00	0.00	6000	0	1.00

Table 282: Mountain Quail

	mean	sd	2.5%	25%	50%	75%	97.5%	n.eff	overlap0	f
alpha.lambda	5.41	0.24	4.77	5.34	5.44	5.55	5.82	14	0	1.00
alpha.phi	1.32	1.89	-1.54	-1.31	2.62	2.67	2.76	3	1	0.67
alpha.p	-2.63	0.09	-2.79	-2.70	-2.64	-2.57	-2.46	6	0	1.00
beta.hab.lambda	0.20	0.19	-0.02	0.10	0.14	0.20	0.79	6	1	0.97
beta.hab.gamma	-0.17	0.31	-0.55	-0.44	-0.29	0.25	0.28	3	1	0.67
beta.hab.phi	-0.03	0.48	-0.85	-0.61	0.24	0.34	0.40	3	1	0.33
beta.elev.lambda	0.27	0.16	0.08	0.15	0.20	0.43	0.57	3	0	1.00
beta.ele.gamma2	-0.29	0.62	-0.83	-0.75	-0.69	0.56	0.62	3	1	0.67
beta.elev.phi	0.16	0.82	-1.08	-0.94	0.69	0.76	0.85	3	1	0.67
beta.develop.lambda	0.88	0.41	0.32	0.62	0.74	1.00	1.96	6	0	1.00
beta.develop.gamma	-0.23	0.22	-0.73	-0.38	-0.16	-0.06	0.07	8	1	0.89
beta.develop.phi	-0.20	0.21	-0.57	-0.35	-0.22	-0.08	0.26	43	1	0.83
beta.effort	0.11	0.02	0.08	0.10	0.11	0.12	0.14	7	0	1.00
beta.min__temp	-0.12	0.02	-0.15	-0.13	-0.12	-0.11	-0.09	12	0	1.00

Table 283: Nashville Warbler

	mean	sd	2.5%	25%	50%	75%	97.5%	n.eff	overlap0	f
alpha.lambda	1.35	1.08	-0.70	0.50	1.49	2.05	3.24	4	1	0.87
alpha.phi	0.07	2.55	-3.73	-3.30	1.53	2.04	2.65	3	1	0.67
alpha.p	-1.05	1.11	-2.77	-1.95	-1.30	0.16	0.65	3	1	0.67
beta.hab.lambda	-1.19	0.96	-3.88	-1.47	-0.80	-0.59	-0.21	5	0	0.99
beta.hab.gamma	-0.18	0.18	-0.65	-0.21	-0.12	-0.08	0.03	7	1	0.95
beta.hab.phi	0.41	0.32	-0.09	0.13	0.29	0.74	0.92	4	1	0.92
beta.elev.lambda	0.13	0.22	-0.23	-0.04	0.11	0.27	0.61	11	1	0.67
beta.ele.gamma2	0.11	0.16	-0.19	-0.01	0.11	0.23	0.41	7	1	0.70
beta.elev.phi	-0.54	0.38	-1.16	-0.94	-0.44	-0.27	0.17	3	1	0.93
beta.develop.lambda	0.53	0.91	-0.47	-0.17	0.19	1.21	2.55	4	1	0.65
beta.develop.gamma	-0.25	0.31	-0.83	-0.47	-0.13	-0.05	0.24	4	1	0.84

	mean	sd	2.5%	25%	50%	75%	97.5%	n.eff	overlap0	f
beta.develop.phi	-0.04	0.52	-0.85	-0.48	-0.07	0.41	0.80	3	1	0.53
beta.effort	0.09	0.06	0.01	0.05	0.08	0.12	0.24	5	0	1.00
beta.min_temp	-0.01	0.01	-0.04	-0.02	-0.01	0.00	0.00	37	0	1.00

Table 284: Nelson's Sparrow

	mean	sd	2.5%	25%	50%	75%	97.5%	n.eff	overlap0	f
alpha.lambda	-6.49	1.41	-9.16	-7.42	-6.43	-5.70	-3.39	13	0	1.00
alpha.phi	3.81	0.67	2.80	2.91	4.23	4.29	4.41	3	0	1.00
alpha.p	-2.63	0.29	-3.07	-3.02	-2.46	-2.41	-2.35	3	0	1.00
beta.hab.lambda	-0.81	1.52	-3.58	-2.02	-0.78	0.41	1.84	35	1	0.66
beta.hab.gamma	0.51	0.10	0.41	0.44	0.45	0.65	0.68	3	0	1.00
beta.hab.phi	-1.18	0.50	-1.95	-1.86	-0.86	-0.82	-0.75	3	0	1.00
beta.elev.lambda	0.18	1.17	-1.89	-0.63	0.14	0.87	2.67	20	1	0.55
beta.ele.gamma2	-0.57	0.52	-1.34	-1.30	-0.22	-0.20	-0.16	3	0	1.00
beta.elev.phi	0.45	1.41	-0.66	-0.58	-0.50	2.40	2.52	3	1	0.33
beta.develop.lambda	-0.22	1.59	-3.50	-1.21	-0.13	0.82	2.83	16	1	0.54
beta.develop.gamma	0.67	0.35	0.32	0.39	0.47	1.13	1.21	3	0	1.00
beta.develop.phi	-1.76	0.33	-2.26	-2.16	-1.66	-1.45	-1.35	3	0	1.00
beta.effort	0.13	0.03	0.08	0.11	0.13	0.15	0.17	4	0	1.00
beta.min_temp	0.00	0.00	-0.01	0.00	0.00	0.00	0.00	97	0	1.00

Table 285: Northern Goshawk

	mean	sd	2.5%	25%	50%	75%	97.5%	n.eff	overlap0	f
alpha.lambda	1.45	0.74	0.53	0.80	1.51	1.80	3.32	6	0	1.00
alpha.phi	1.74	1.43	-0.82	0.45	2.08	3.07	3.67	3	1	0.85
alpha.p	-0.63	0.98	-2.90	-1.23	-0.77	0.33	0.60	5	1	0.64
beta.hab.lambda	-0.04	0.12	-0.37	-0.08	-0.02	0.02	0.17	29	1	0.61
beta.hab.gamma	-0.23	0.18	-0.49	-0.37	-0.28	-0.11	0.12	4	1	0.81
beta.hab.phi	0.18	0.22	-0.41	0.11	0.27	0.32	0.37	4	1	0.80
beta.elev.lambda	-0.08	0.13	-0.39	-0.15	-0.06	0.02	0.11	8	1	0.66
beta.ele.gamma2	-0.04	0.17	-0.26	-0.19	-0.13	0.14	0.24	3	1	0.61
beta.elev.phi	0.19	0.26	-0.18	-0.06	0.19	0.39	0.68	4	1	0.65
beta.develop.lambda	0.09	0.17	-0.15	0.00	0.04	0.14	0.54	7	1	0.74
beta.develop.gamma	-0.24	0.29	-0.77	-0.53	-0.15	-0.02	0.20	3	1	0.80
beta.develop.phi	0.13	0.21	-0.34	0.01	0.14	0.28	0.50	4	1	0.77
beta.effort	0.02	0.02	0.00	0.01	0.02	0.03	0.07	24	0	1.00
beta.min_temp	-0.08	0.04	-0.17	-0.11	-0.07	-0.06	-0.03	5	0	1.00

Table 286: Northern Pygmy-Owl

	mean	sd	2.5%	25%	50%	75%	97.5%	n.eff	overlap0	f
alpha.lambda	3.24	0.32	2.32	3.19	3.33	3.44	3.62	7	0	1.00
alpha.phi	1.52	0.55	0.22	1.28	1.49	1.96	2.17	7	0	1.00
alpha.p	-2.37	0.23	-2.64	-2.52	-2.46	-2.29	-1.65	8	0	1.00
beta.hab.lambda	0.10	0.12	-0.09	0.03	0.08	0.14	0.46	36	1	0.85

	mean	sd	2.5%	25%	50%	75%	97.5%	n.eff	overlap0	f
beta.hab.gamma	-0.12	0.09	-0.30	-0.19	-0.12	-0.06	0.05	6	1	0.90
beta.hab.phi	0.25	0.12	0.05	0.16	0.24	0.33	0.49	4	0	1.00
beta.elev.lambda	0.12	0.09	-0.06	0.06	0.11	0.17	0.30	13	1	0.93
beta.ele.gamma2	0.39	0.18	0.04	0.25	0.40	0.55	0.68	4	0	1.00
beta.elev.phi	-0.71	0.26	-1.17	-0.96	-0.68	-0.49	-0.29	4	0	1.00
beta.develop.lambda	0.50	0.35	-0.44	0.32	0.51	0.73	1.15	7	1	0.93
beta.develop.gamma	0.39	0.26	-0.16	0.15	0.48	0.61	0.75	4	1	0.93
beta.develop.phi	-0.71	0.41	-1.41	-1.01	-0.74	-0.37	0.10	4	1	0.96
beta.effort	0.11	0.02	0.07	0.09	0.11	0.12	0.14	39	0	1.00
beta.min_temp	-0.01	0.01	-0.03	-0.01	-0.01	0.00	0.00	82	0	1.00

Table 287: Northern Saw-whet Owl

	mean	sd	2.5%	25%	50%	75%	97.5%	n.eff	overlap0	f
alpha.lambda	2.74	0.67	1.72	1.85	3.10	3.21	3.54	3	0	1.00
alpha.phi	2.28	0.64	1.31	1.67	2.29	2.96	3.18	4	0	1.00
alpha.p	-2.29	0.44	-2.76	-2.62	-2.54	-1.73	-1.62	3	0	1.00
beta.hab.lambda	-0.05	0.11	-0.25	-0.15	-0.03	0.03	0.13	4	1	0.63
beta.hab.gamma	-0.17	0.18	-0.56	-0.30	-0.13	-0.01	0.05	5	1	0.81
beta.hab.phi	0.43	0.26	0.13	0.21	0.33	0.61	1.05	6	0	1.00
beta.elev.lambda	0.08	0.13	-0.21	0.01	0.11	0.17	0.30	4	1	0.77
beta.ele.gamma2	-0.01	0.18	-0.28	-0.20	0.03	0.15	0.26	3	1	0.45
beta.elev.phi	-0.10	0.22	-0.46	-0.29	-0.15	0.13	0.21	3	1	0.61
beta.develop.lambda	0.07	0.13	-0.17	-0.03	0.07	0.17	0.29	4	1	0.68
beta.develop.gamma	-0.17	0.12	-0.39	-0.28	-0.17	-0.09	0.08	5	1	0.92
beta.develop.phi	0.07	0.16	-0.24	-0.05	0.05	0.20	0.39	5	1	0.65
beta.effort	0.04	0.01	0.01	0.03	0.04	0.05	0.06	13	0	1.00
beta.min_temp	0.00	0.00	-0.01	0.00	0.00	0.00	0.00	10	0	1.00

Table 288: Ovenbird

	mean	sd	2.5%	25%	50%	75%	97.5%	n.eff	overlap0	f
alpha.lambda	2.55	0.19	2.19	2.40	2.59	2.70	2.85	4	0	1.00
alpha.phi	2.81	0.28	2.35	2.56	2.81	3.00	3.38	4	0	1.00
alpha.p	-1.48	0.16	-1.72	-1.60	-1.53	-1.33	-1.20	3	0	1.00
beta.hab.lambda	-0.49	0.27	-0.99	-0.77	-0.38	-0.28	-0.13	3	0	1.00
beta.hab.gamma	-0.99	0.68	-2.12	-1.78	-0.86	-0.35	-0.09	3	0	0.99
beta.hab.phi	0.72	0.58	-0.08	0.24	0.56	1.32	1.87	3	1	0.95
beta.elev.lambda	-0.28	0.25	-0.68	-0.50	-0.30	-0.03	0.13	3	1	0.80
beta.ele.gamma2	-0.03	0.29	-0.55	-0.27	0.00	0.23	0.42	3	1	0.50
beta.elev.phi	0.03	0.37	-0.52	-0.31	-0.01	0.36	0.71	3	1	0.49
beta.develop.lambda	0.46	0.17	0.22	0.32	0.43	0.60	0.83	5	0	1.00
beta.develop.gamma	-0.28	0.52	-1.10	-0.90	-0.10	0.18	0.35	3	1	0.57
beta.develop.phi	0.34	0.56	-0.24	-0.14	0.08	0.96	1.33	3	1	0.58
beta.effort	0.01	0.01	0.00	0.00	0.01	0.02	0.04	42	0	1.00
beta.min_temp	0.00	0.00	-0.02	-0.01	0.00	0.00	0.00	190	0	1.00

Table 289: Painted Bunting

	mean	sd	2.5%	25%	50%	75%	97.5%	n.eff	overlap0	f
alpha.lambda	0.86	0.24	0.41	0.70	0.86	1.01	1.36	15	0	1.00
alpha.phi	1.90	0.09	1.73	1.83	1.90	1.95	2.09	11	0	1.00
alpha.p	-0.62	0.04	-0.70	-0.64	-0.62	-0.59	-0.54	16	0	1.00
beta.hab.lambda	-10.82	0.78	-12.28	-11.35	-10.85	-10.28	-9.25	19	0	1.00
beta.hab.gamma	-1.01	0.17	-1.34	-1.14	-1.01	-0.88	-0.70	7	0	1.00
beta.hab.phi	1.13	0.26	0.64	0.95	1.16	1.33	1.56	11	0	1.00
beta.elev.lambda	-1.13	0.09	-1.30	-1.19	-1.13	-1.06	-0.96	15	0	1.00
beta.ele.gamma2	-0.29	0.07	-0.41	-0.34	-0.30	-0.25	-0.14	12	0	1.00
beta.elev.phi	0.10	0.08	-0.07	0.04	0.10	0.16	0.23	9	1	0.88
beta.develop.lambda	0.48	0.04	0.41	0.46	0.48	0.51	0.56	9	0	1.00
beta.develop.gamma	0.18	0.08	0.04	0.12	0.17	0.25	0.34	4	0	1.00
beta.develop.phi	0.49	0.09	0.34	0.40	0.50	0.57	0.62	3	0	1.00
beta.effort	0.00	0.00	0.00	0.00	0.00	0.01	0.02	3463	0	1.00
beta.min_temp	0.00	0.00	-0.01	0.00	0.00	0.00	0.00	3939	0	1.00

Table 290: Red-cockaded Woodpecker

	mean	sd	2.5%	25%	50%	75%	97.5%	n.eff	overlap0	f
alpha.lambda	2.55	0.11	2.31	2.48	2.56	2.63	2.75	23	0	1.00
alpha.phi	2.81	0.09	2.65	2.75	2.81	2.87	2.99	14	0	1.00
alpha.p	-1.06	0.06	-1.16	-1.10	-1.05	-1.02	-0.95	11	0	1.00
beta.hab.lambda	-0.14	0.16	-0.48	-0.25	-0.13	-0.03	0.15	47	1	0.82
beta.hab.gamma	-0.27	0.07	-0.41	-0.32	-0.27	-0.22	-0.13	36	0	1.00
beta.hab.phi	1.64	0.14	1.37	1.54	1.64	1.73	1.89	24	0	1.00
beta.elev.lambda	-0.07	0.16	-0.39	-0.18	-0.07	0.03	0.23	22	1	0.67
beta.ele.gamma2	0.15	0.09	-0.02	0.09	0.15	0.20	0.33	16	1	0.96
beta.elev.phi	-1.07	0.12	-1.30	-1.15	-1.08	-1.00	-0.81	12	0	1.00
beta.develop.lambda	-0.99	0.32	-1.67	-1.19	-0.97	-0.76	-0.40	44	0	1.00
beta.develop.gamma	-1.21	0.15	-1.51	-1.31	-1.21	-1.11	-0.92	38	0	1.00
beta.develop.phi	2.38	0.28	1.80	2.21	2.40	2.57	2.88	9	0	1.00
beta.effort	0.01	0.01	0.00	0.00	0.00	0.01	0.02	6000	0	1.00
beta.min_temp	-0.05	0.02	-0.09	-0.07	-0.05	-0.04	-0.02	132	0	1.00

Table 291: Red-naped Sapsucker

	mean	sd	2.5%	25%	50%	75%	97.5%	n.eff	overlap0	f
alpha.lambda	-8.08	1.66	-11.63	-9.12	-7.89	-6.77	-5.46	5	0	1.00
alpha.phi	4.31	0.18	4.03	4.15	4.30	4.42	4.68	7	0	1.00
alpha.p	-1.16	0.05	-1.24	-1.19	-1.16	-1.12	-1.06	4	0	1.00
beta.hab.lambda	-1.30	2.26	-5.37	-3.21	-1.42	0.91	2.03	4	1	0.62
beta.hab.gamma	-0.11	0.03	-0.17	-0.13	-0.11	-0.09	-0.06	6	0	1.00
beta.hab.phi	0.86	0.11	0.67	0.78	0.85	0.93	1.11	28	0	1.00
beta.elev.lambda	1.30	1.26	-0.71	0.36	1.18	2.17	4.00	8	1	0.84
beta.ele.gamma2	0.59	0.06	0.49	0.56	0.59	0.63	0.70	18	0	1.00
beta.elev.phi	-0.43	0.19	-0.87	-0.50	-0.38	-0.30	-0.17	19	0	1.00
beta.develop.lambda	0.36	1.62	-3.89	-0.56	0.52	1.49	2.93	72	1	0.63
beta.develop.gamma	-0.58	0.12	-0.82	-0.66	-0.58	-0.50	-0.33	8	0	1.00

	mean	sd	2.5%	25%	50%	75%	97.5%	n.eff	overlap0	f
beta.develop.phi	-0.80	0.29	-1.36	-1.02	-0.78	-0.58	-0.27	4	0	1.00
beta.effort	0.17	0.02	0.14	0.16	0.17	0.18	0.20	16	0	1.00
beta.min_temp	-0.13	0.02	-0.17	-0.15	-0.13	-0.12	-0.10	21	0	1.00

Table 292: Ruby-throated Hummingbird

	mean	sd	2.5%	25%	50%	75%	97.5%	n.eff	overlap0	f
alpha.lambda	1.39	0.52	0.62	0.98	1.23	1.97	2.29	3	0	1.00
alpha.phi	2.75	0.10	2.52	2.69	2.76	2.82	2.91	36	0	1.00
alpha.p	-1.08	0.17	-1.42	-1.23	-1.02	-0.92	-0.85	3	0	1.00
beta.hab.lambda	-1.74	0.44	-2.48	-2.05	-1.82	-1.40	-0.84	4	0	1.00
beta.hab.gamma	-0.56	0.12	-0.77	-0.65	-0.58	-0.49	-0.27	14	0	1.00
beta.hab.phi	-0.04	0.13	-0.35	-0.12	-0.03	0.06	0.20	20	1	0.58
beta.elev.lambda	-0.62	0.15	-0.91	-0.73	-0.61	-0.51	-0.35	6	0	1.00
beta.ele.gamma2	-0.44	0.13	-0.67	-0.53	-0.46	-0.33	-0.19	5	0	1.00
beta.elev.phi	0.82	0.16	0.53	0.69	0.84	0.95	1.11	4	0	1.00
beta.develop.lambda	0.08	0.26	-0.39	-0.16	0.14	0.29	0.50	4	1	0.65
beta.develop.gamma	0.21	0.19	-0.19	0.08	0.25	0.36	0.51	5	1	0.83
beta.develop.phi	-0.45	0.24	-0.81	-0.63	-0.50	-0.31	0.07	4	1	0.93
beta.effort	0.05	0.02	0.01	0.04	0.05	0.07	0.10	29	0	1.00
beta.min_temp	0.00	0.00	-0.01	0.00	0.00	0.00	0.00	99	0	1.00

Table 293: Sage Thrasher

	mean	sd	2.5%	25%	50%	75%	97.5%	n.eff	overlap0	f
alpha.lambda	4.14	0.52	3.09	3.64	4.37	4.52	4.77	3	0	1.00
alpha.phi	0.42	0.89	-0.97	-0.71	0.78	1.22	1.44	3	1	0.67
alpha.p	-4.54	0.07	-4.66	-4.60	-4.54	-4.48	-4.42	6	0	1.00
beta.hab.lambda	-0.73	0.78	-2.15	-1.41	-0.60	0.10	0.18	3	1	0.67
beta.hab.gamma	0.10	0.03	0.06	0.08	0.10	0.13	0.17	4	0	1.00
beta.hab.phi	-0.02	0.13	-0.26	-0.17	0.04	0.08	0.15	3	1	0.34
beta.elev.lambda	-0.54	0.36	-1.13	-0.94	-0.46	-0.17	-0.08	3	0	1.00
beta.ele.gamma2	1.20	0.44	0.80	0.87	0.92	1.75	1.90	3	0	1.00
beta.elev.phi	-0.15	1.06	-1.70	-1.57	0.32	0.83	0.93	3	1	0.33
beta.develop.lambda	-0.14	0.45	-0.85	-0.64	-0.05	0.21	0.62	3	1	0.58
beta.develop.gamma	-1.97	0.66	-3.12	-2.69	-1.60	-1.46	-1.32	3	0	1.00
beta.develop.phi	1.49	0.87	0.30	0.48	1.49	2.27	2.79	3	0	1.00
beta.effort	0.31	0.01	0.29	0.30	0.31	0.32	0.34	18	0	1.00
beta.min_temp	-0.06	0.02	-0.09	-0.07	-0.06	-0.05	-0.02	7	0	1.00

Table 294: Western Screech-Owl

	mean	sd	2.5%	25%	50%	75%	97.5%	n.eff	overlap0	f
alpha.lambda	-7.34	1.50	-10.48	-8.34	-7.14	-6.20	-4.96	34	0	1.00
alpha.phi	3.25	0.07	3.13	3.21	3.25	3.30	3.39	7	0	1.00
alpha.p	-0.75	0.05	-0.84	-0.79	-0.75	-0.72	-0.68	4	0	1.00
beta.hab.lambda	0.54	1.06	-1.80	-0.15	0.59	1.28	2.35	25	1	0.71

	mean	sd	2.5%	25%	50%	75%	97.5%	n.eff	overlap0	f
beta.hab.gamma	0.28	0.04	0.21	0.26	0.28	0.31	0.35	18	0	1.00
beta.hab.phi	-0.17	0.09	-0.35	-0.23	-0.18	-0.12	0.01	36	1	0.96
beta.elev.lambda	0.20	1.56	-2.22	-0.85	-0.10	0.87	3.92	13	1	0.46
beta.ele.gamma2	-0.45	0.03	-0.50	-0.47	-0.45	-0.43	-0.39	20	0	1.00
beta.elev.phi	0.69	0.05	0.59	0.67	0.70	0.72	0.79	284	0	1.00
beta.develop.lambda	0.23	1.68	-3.33	-0.93	0.45	1.44	3.09	130	1	0.60
beta.develop.gamma	0.70	0.04	0.61	0.67	0.70	0.73	0.79	99	0	1.00
beta.develop.phi	-0.83	0.09	-1.00	-0.90	-0.83	-0.76	-0.66	41	0	1.00
beta.effort	0.14	0.02	0.10	0.12	0.14	0.15	0.17	2093	0	1.00
beta.min_temp	-0.02	0.01	-0.06	-0.03	-0.02	-0.01	0.00	29	0	1.00

Table 295: Wilson's Warbler

	mean	sd	2.5%	25%	50%	75%	97.5%	n.eff	overlap0	f
alpha.lambda	2.84	0.81	1.95	2.15	2.50	3.75	4.24	3	0	1.00
alpha.phi	2.35	0.41	1.40	2.16	2.51	2.68	2.80	4	0	1.00
alpha.p	-2.02	0.48	-2.78	-2.61	-1.85	-1.58	-1.50	3	0	1.00
beta.hab.lambda	-0.37	0.23	-0.70	-0.52	-0.44	-0.30	0.15	5	1	0.86
beta.hab.gamma	0.02	0.33	-0.38	-0.22	-0.14	0.35	0.60	3	1	0.34
beta.hab.phi	0.04	0.41	-0.69	-0.42	0.23	0.34	0.54	3	1	0.67
beta.elev.lambda	0.13	0.17	-0.21	0.00	0.16	0.24	0.41	5	1	0.75
beta.ele.gamma2	0.16	0.52	-0.55	-0.16	-0.01	0.55	1.14	3	1	0.49
beta.elev.phi	-0.52	0.63	-1.81	-0.90	-0.33	-0.17	0.31	4	1	0.82
beta.develop.lambda	-0.58	0.28	-1.03	-0.80	-0.61	-0.38	-0.03	4	0	0.99
beta.develop.gamma	-0.44	0.09	-0.59	-0.50	-0.46	-0.40	-0.22	9	0	1.00
beta.develop.phi	0.81	0.15	0.48	0.73	0.84	0.91	1.05	6	0	1.00
beta.effort	0.01	0.01	0.00	0.00	0.00	0.01	0.02	29	0	1.00
beta.min_temp	-0.01	0.01	-0.02	-0.01	0.00	0.00	0.00	21	0	1.00

Table 296: Yellow-breasted Chat

	mean	sd	2.5%	25%	50%	75%	97.5%	n.eff	overlap0	f
alpha.lambda	2.40	0.87	0.87	1.38	2.76	3.10	3.44	3	0	1.00
alpha.phi	0.41	0.55	-0.60	-0.10	0.46	0.93	1.13	5	1	0.72
alpha.p	-1.92	1.02	-2.89	-2.65	-2.53	-0.62	-0.27	3	0	1.00
beta.hab.lambda	-0.12	0.68	-2.04	-0.45	0.09	0.34	0.77	44	1	0.43
beta.hab.gamma	0.11	0.25	-0.35	-0.15	0.17	0.30	0.46	3	1	0.67
beta.hab.phi	-0.23	0.45	-0.83	-0.57	-0.42	0.29	0.57	3	1	0.67
beta.elev.lambda	-0.26	0.15	-0.61	-0.35	-0.24	-0.16	-0.02	8	0	0.98
beta.ele.gamma2	-0.31	0.14	-0.55	-0.43	-0.31	-0.19	-0.04	6	0	0.99
beta.elev.phi	0.30	0.22	-0.13	0.10	0.33	0.45	0.66	9	1	0.90
beta.develop.lambda	-0.16	0.23	-0.62	-0.31	-0.15	0.00	0.28	104	1	0.75
beta.develop.gamma	0.48	0.23	0.16	0.27	0.45	0.67	0.88	4	0	1.00
beta.develop.phi	0.07	0.31	-0.50	-0.19	0.14	0.23	0.71	4	1	0.67
beta.effort	0.01	0.01	0.00	0.00	0.01	0.02	0.04	71	0	1.00
beta.min_temp	-0.11	0.04	-0.22	-0.14	-0.11	-0.08	-0.04	7	0	1.00
