# Supplementary figures and images for: Spatial and temporal non‐stationarity in long‐term population dynamics of over‐wintering birds of North America
Source: Ecol Evol. 2023 Mar 16;13(3):e9781. doi: 10.1002/ece3.9781 (PMC10019912; doi:10.1002/ece3.9781)

Circles

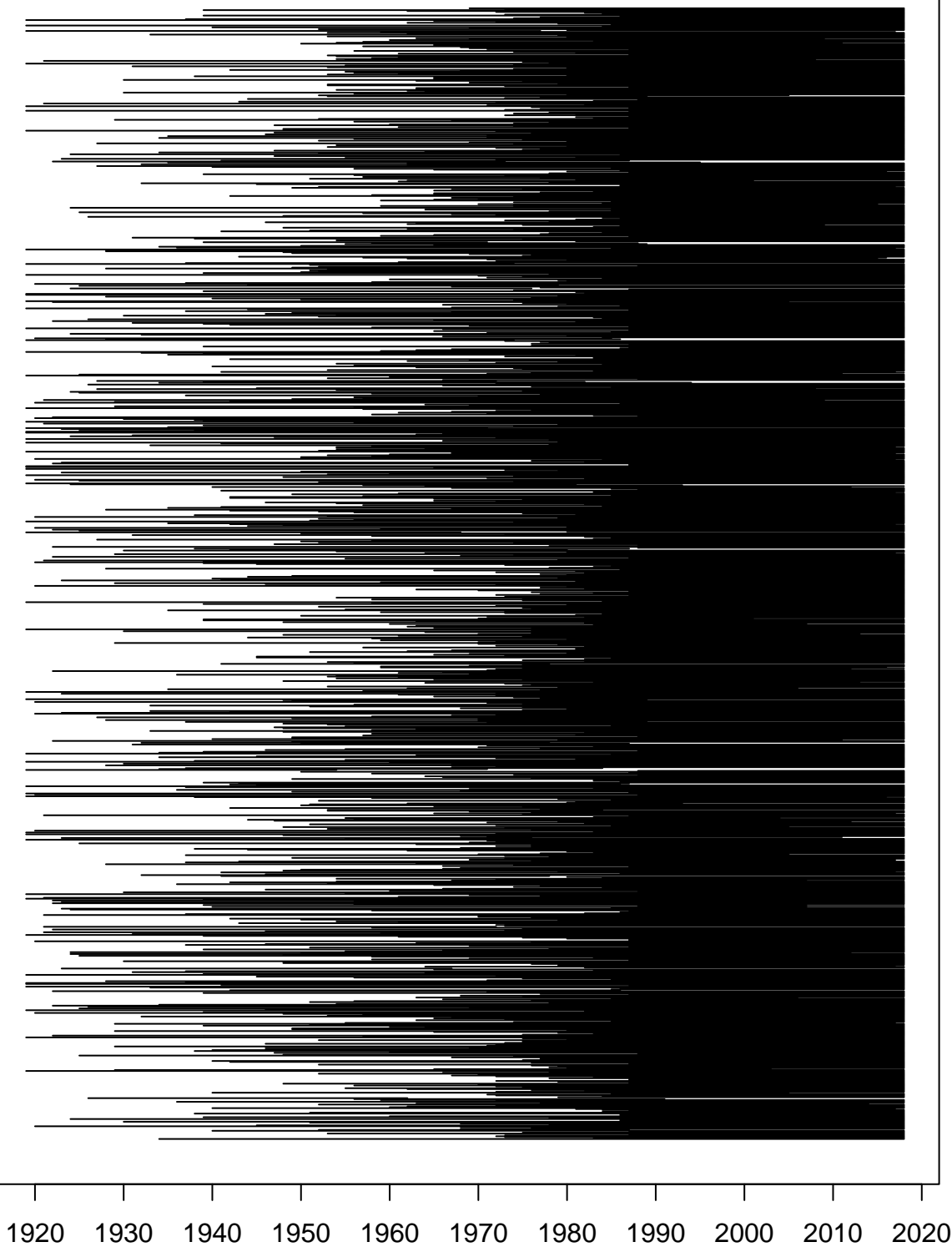

Supplement: Supplementary file 2 — Figure S1 [file ECE3-13-e9781-s006.pdf]

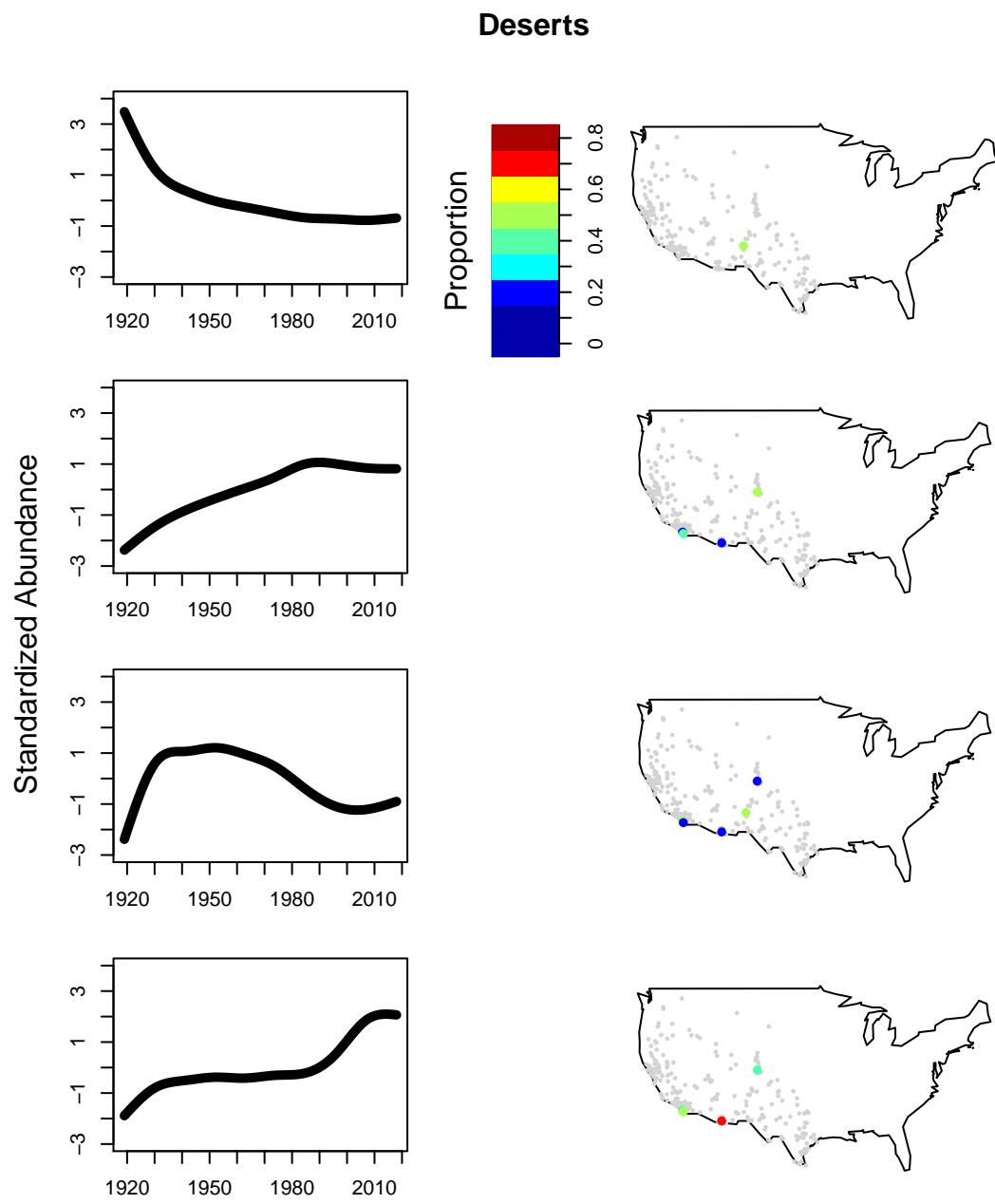

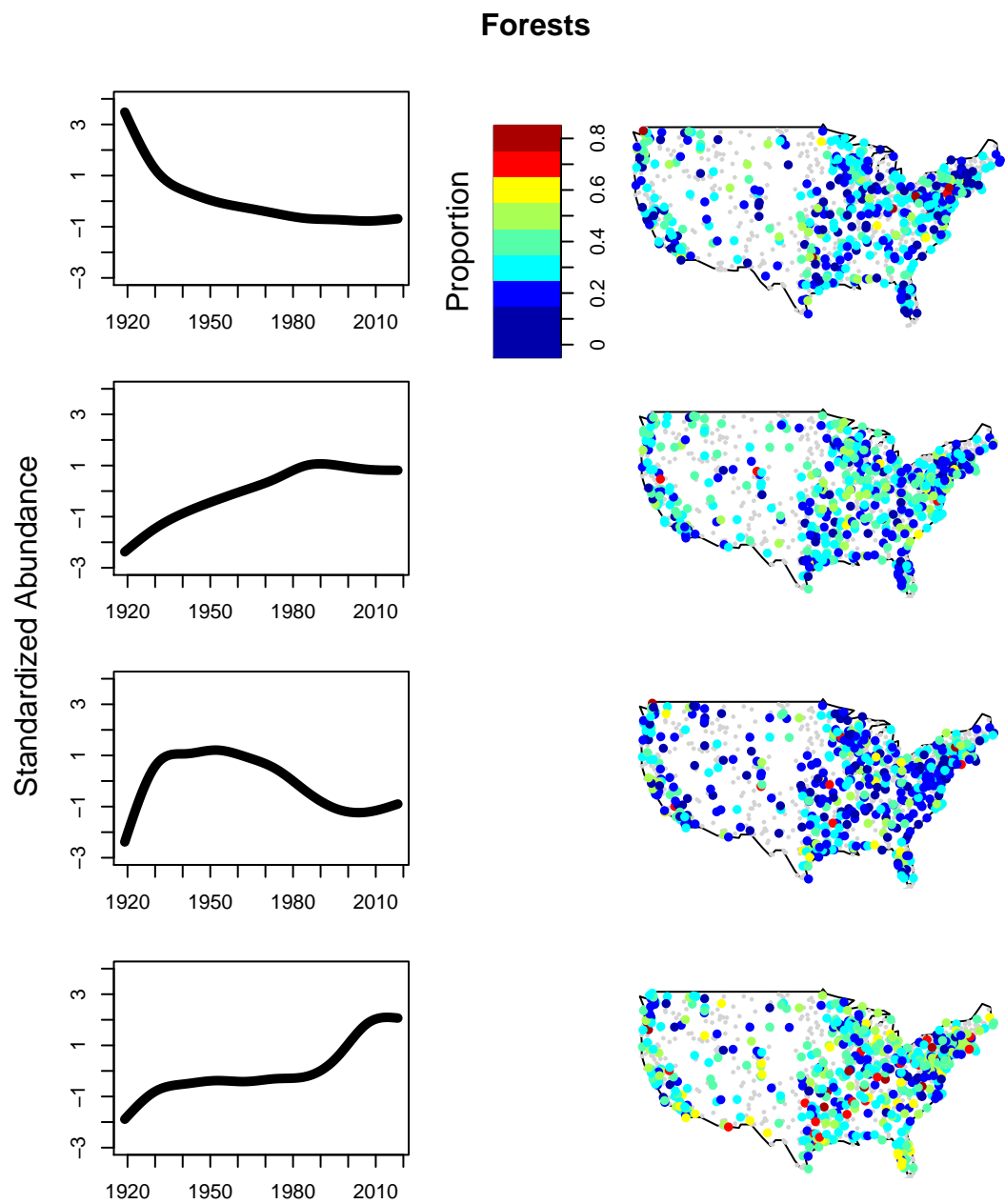

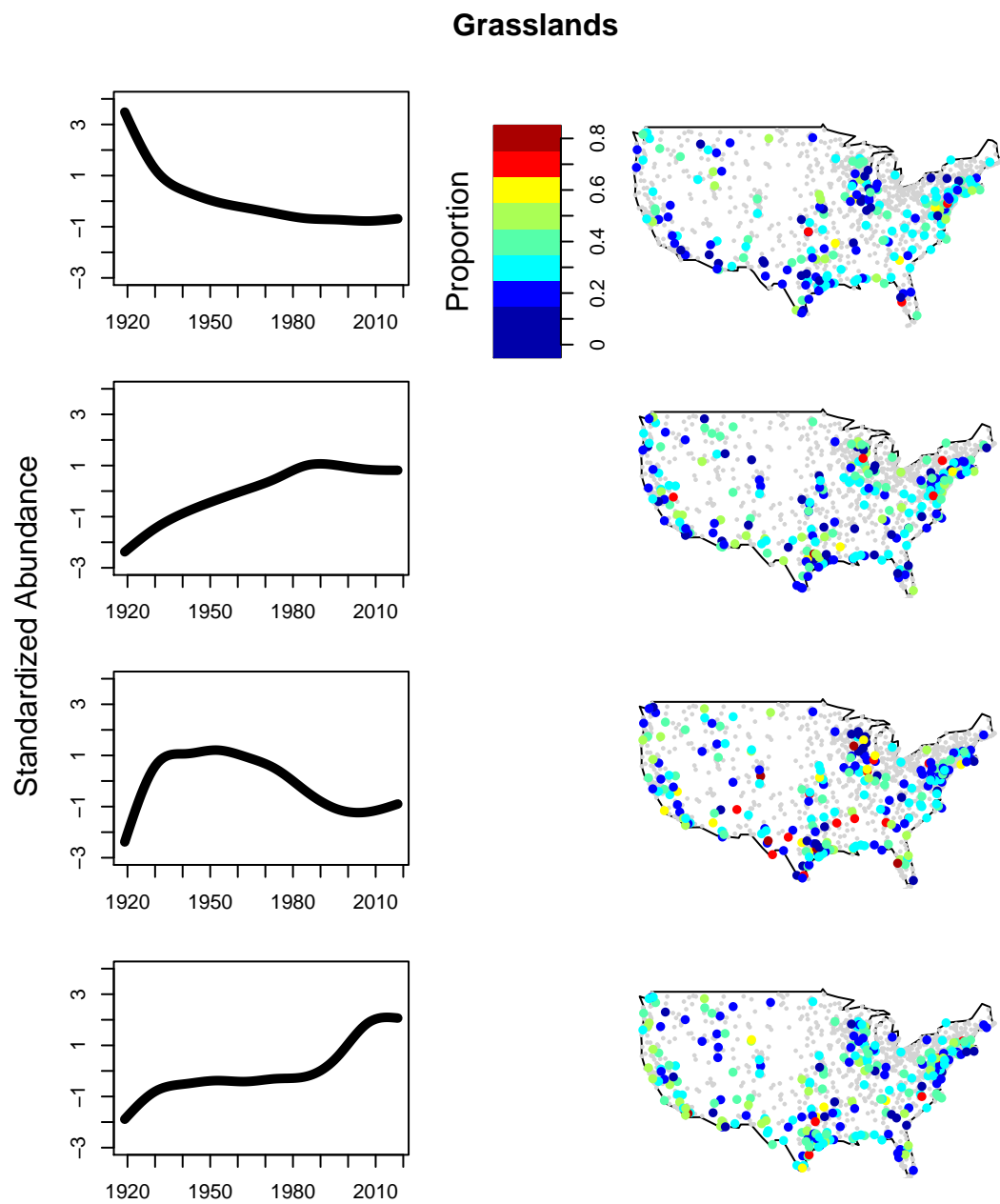

## Lakes and Ponds

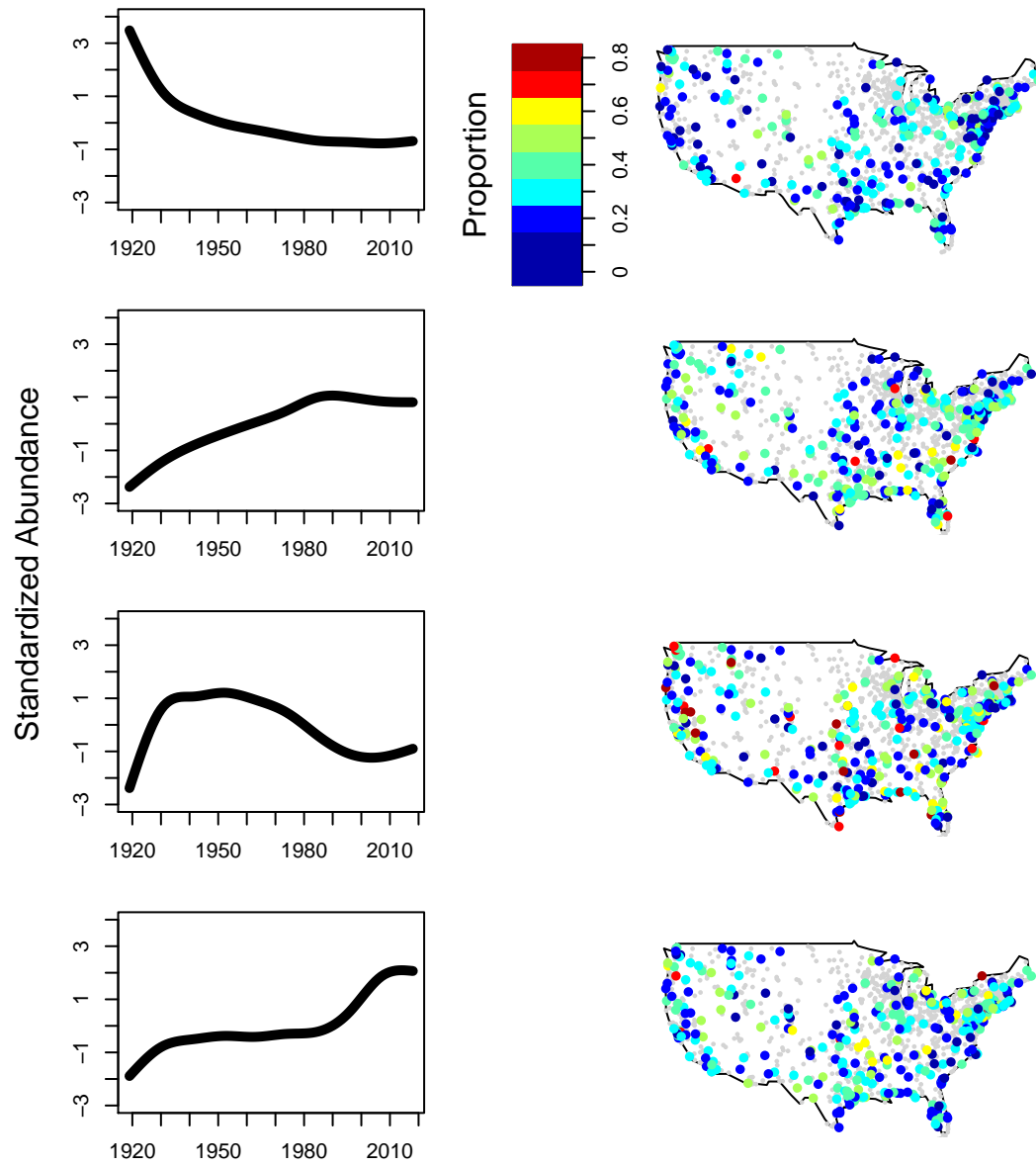

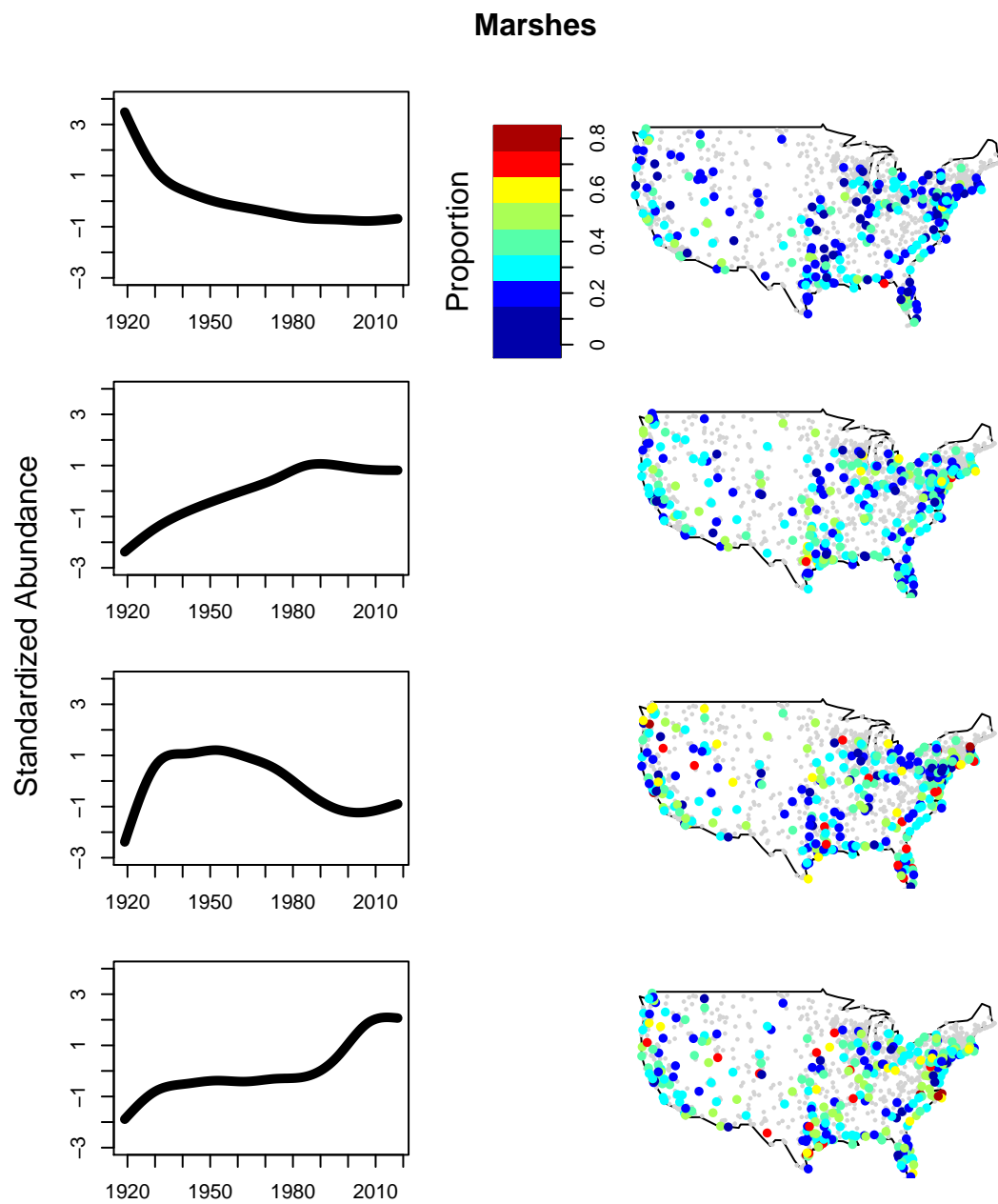

## Open Woodlands

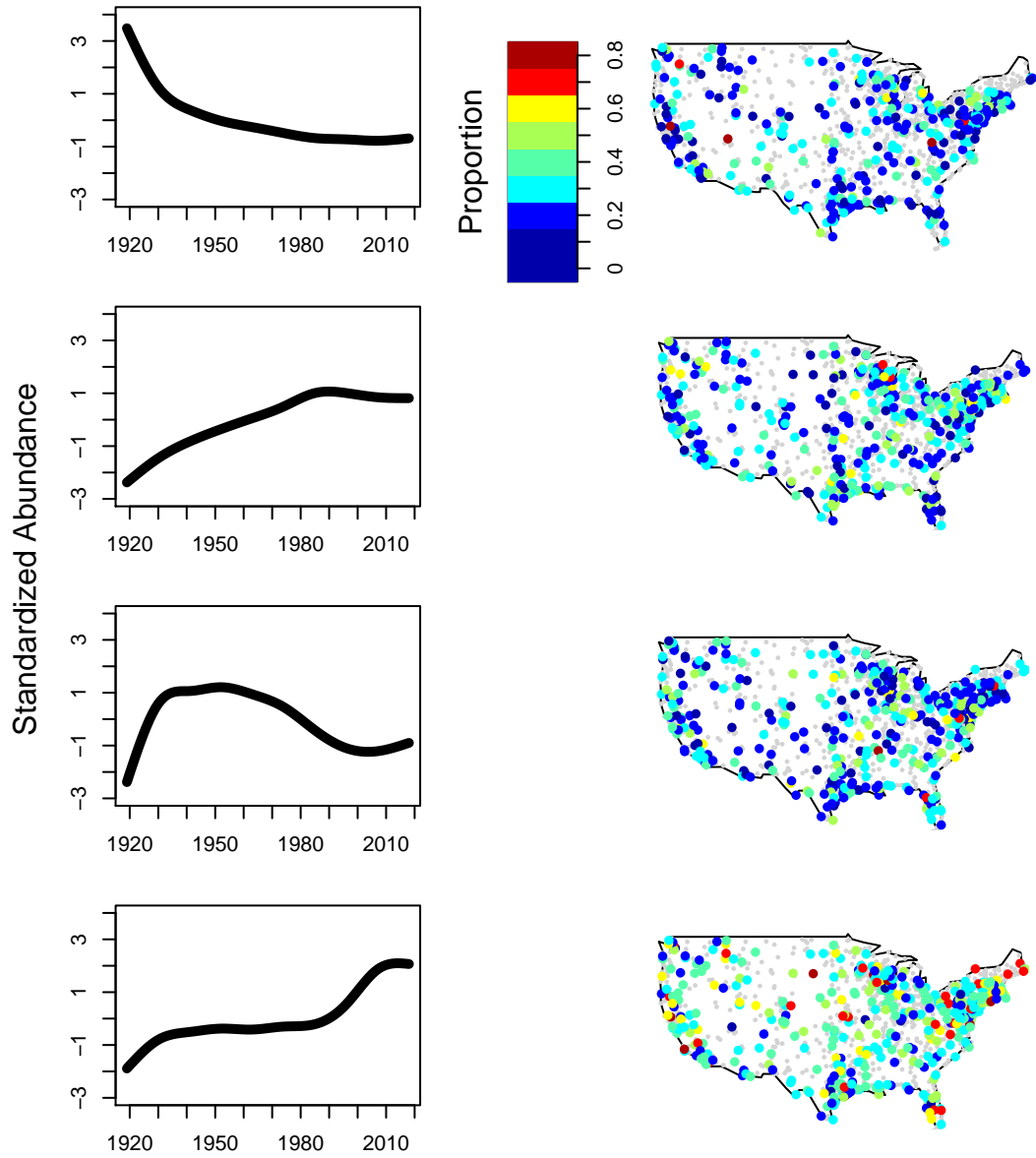

## Rivers and Streams

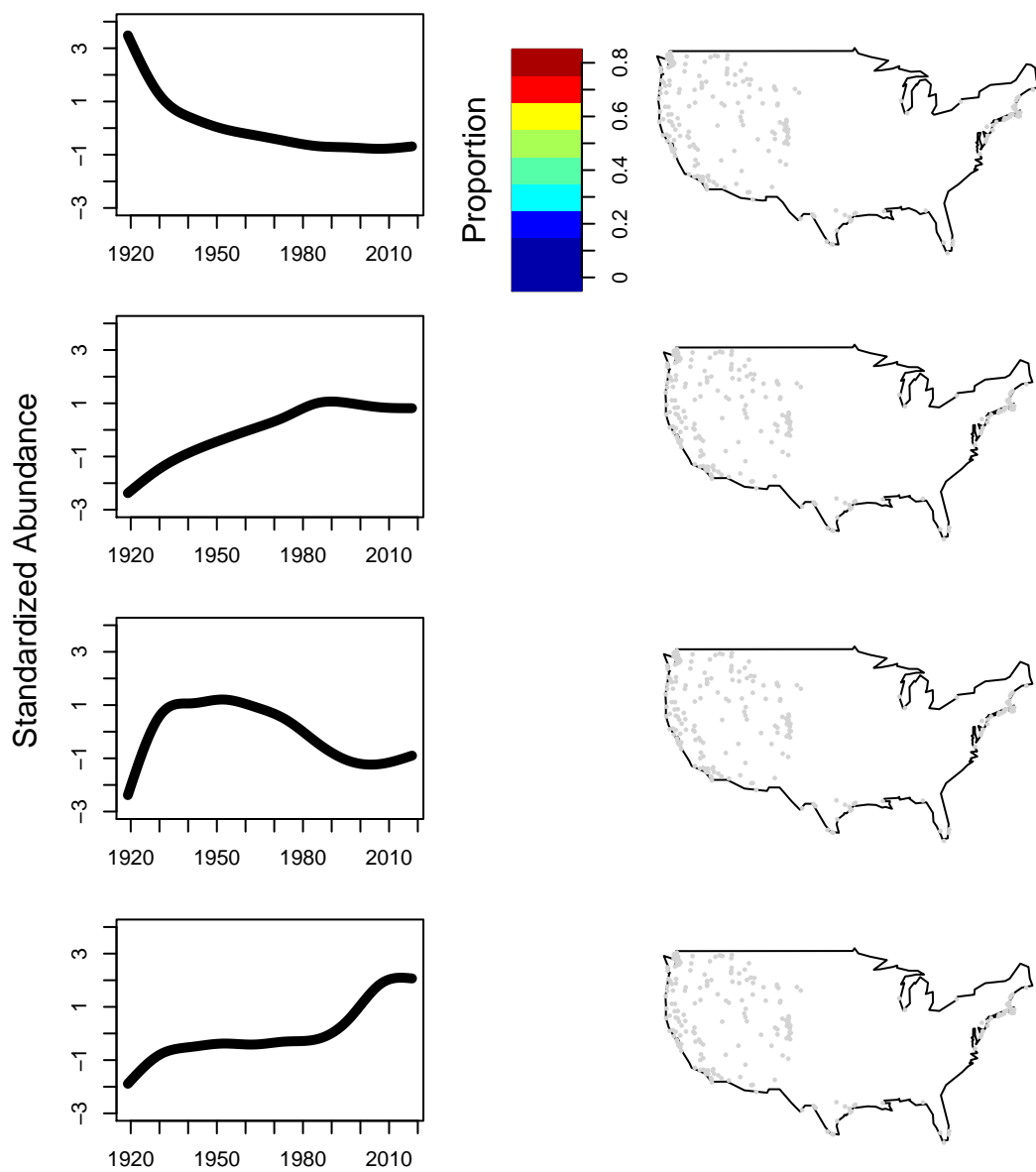

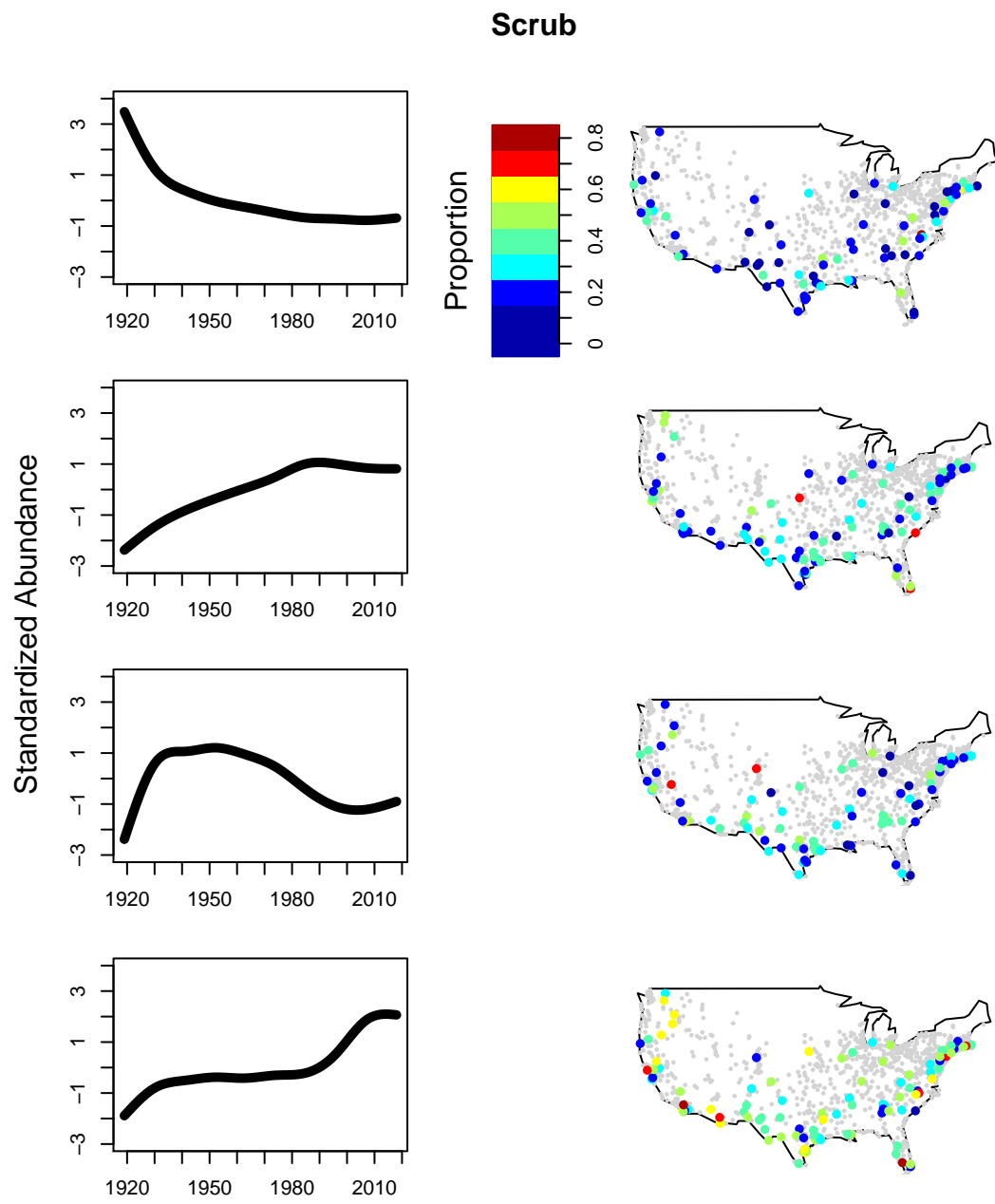

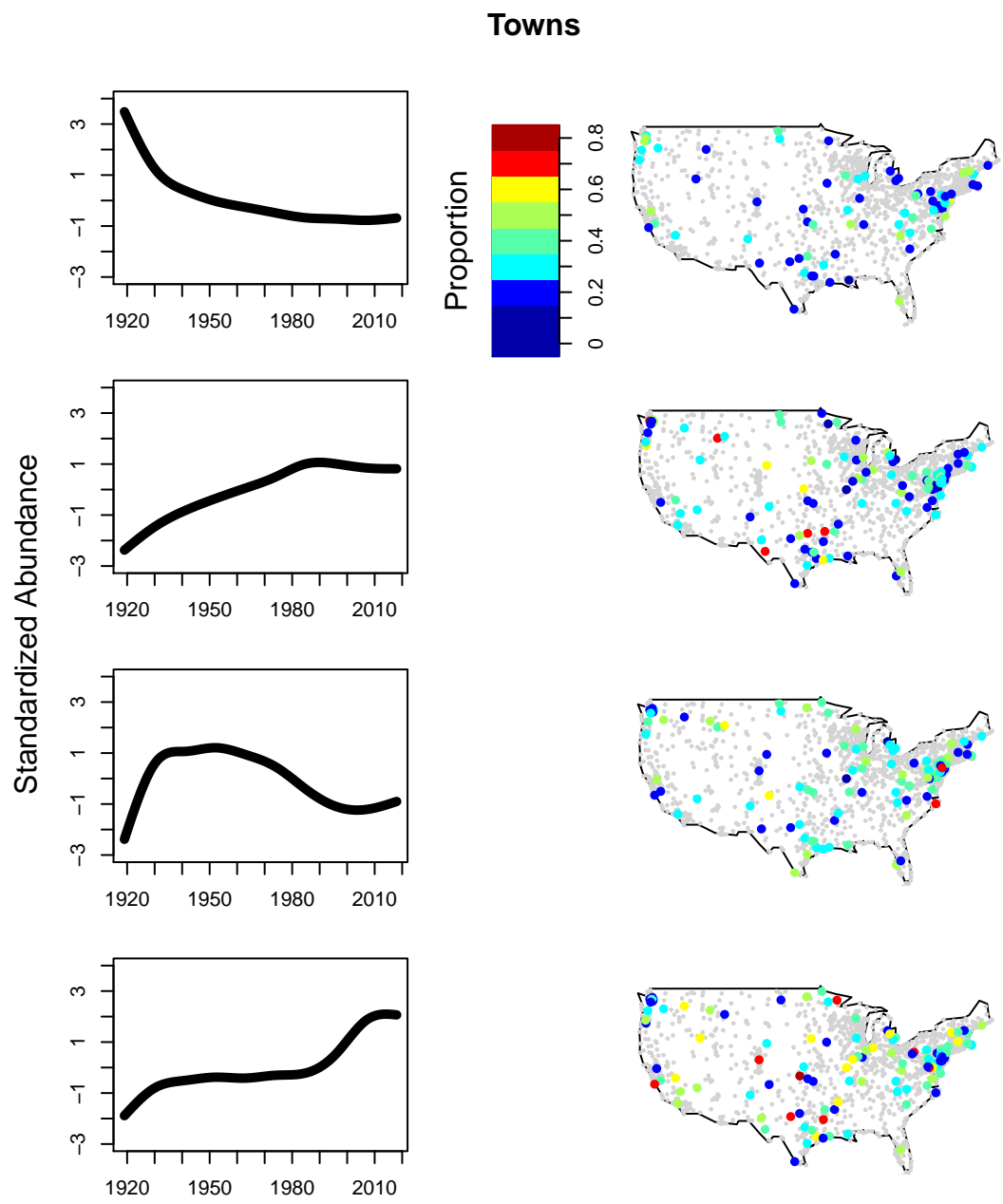

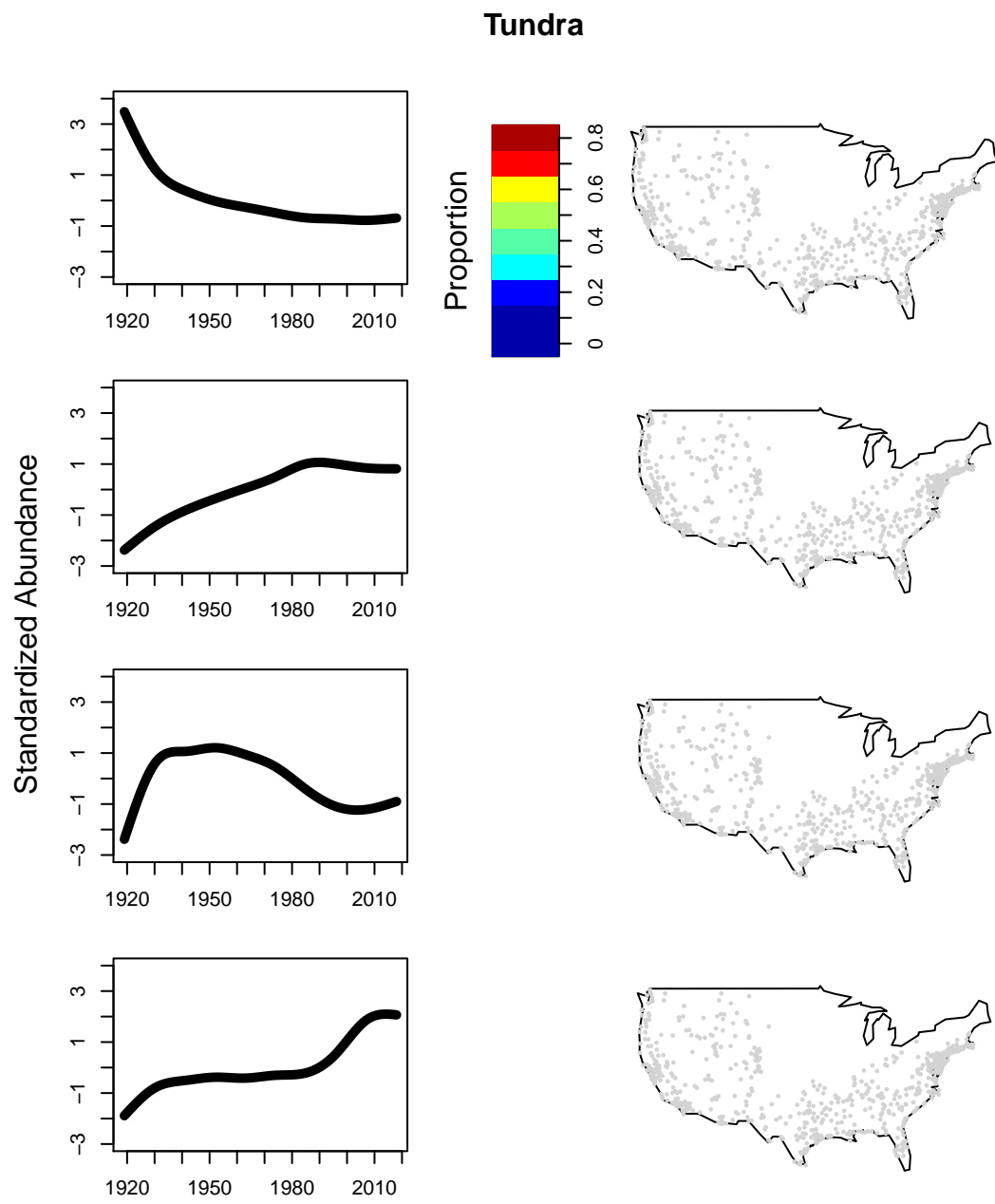

Supplement: Supplementary file 5 — Figure S4 [file ECE3-13-e9781-s003.pdf]
