# Supplementary material for: Spatial and temporal non‐stationarity in long‐term population dynamics of over‐wintering birds of North America
Source: Ecol Evol. 2023 Mar 16;13(3):e9781. doi: 10.1002/ece3.9781 (PMC10019912; doi:10.1002/ece3.9781)

American Black Duck

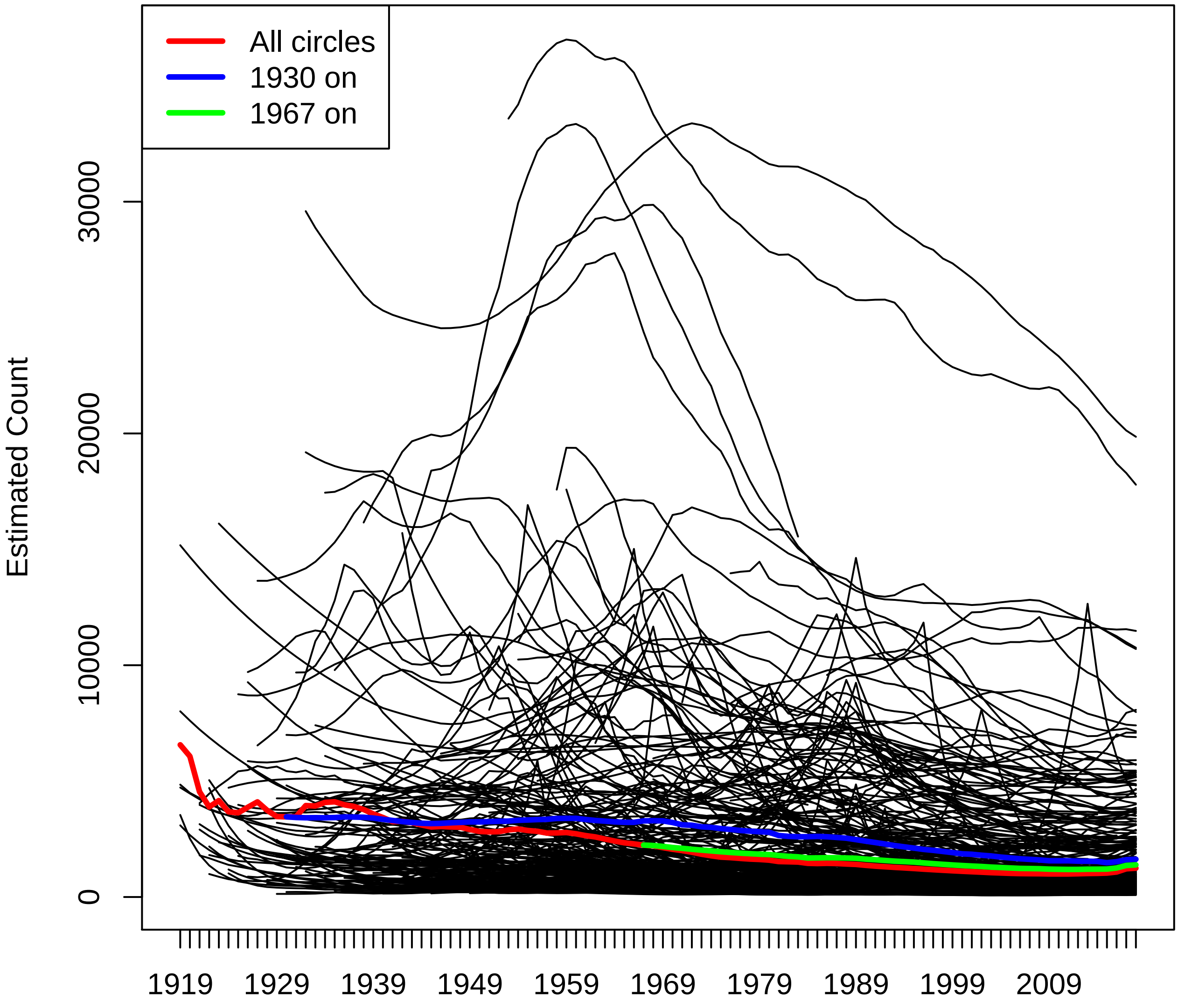

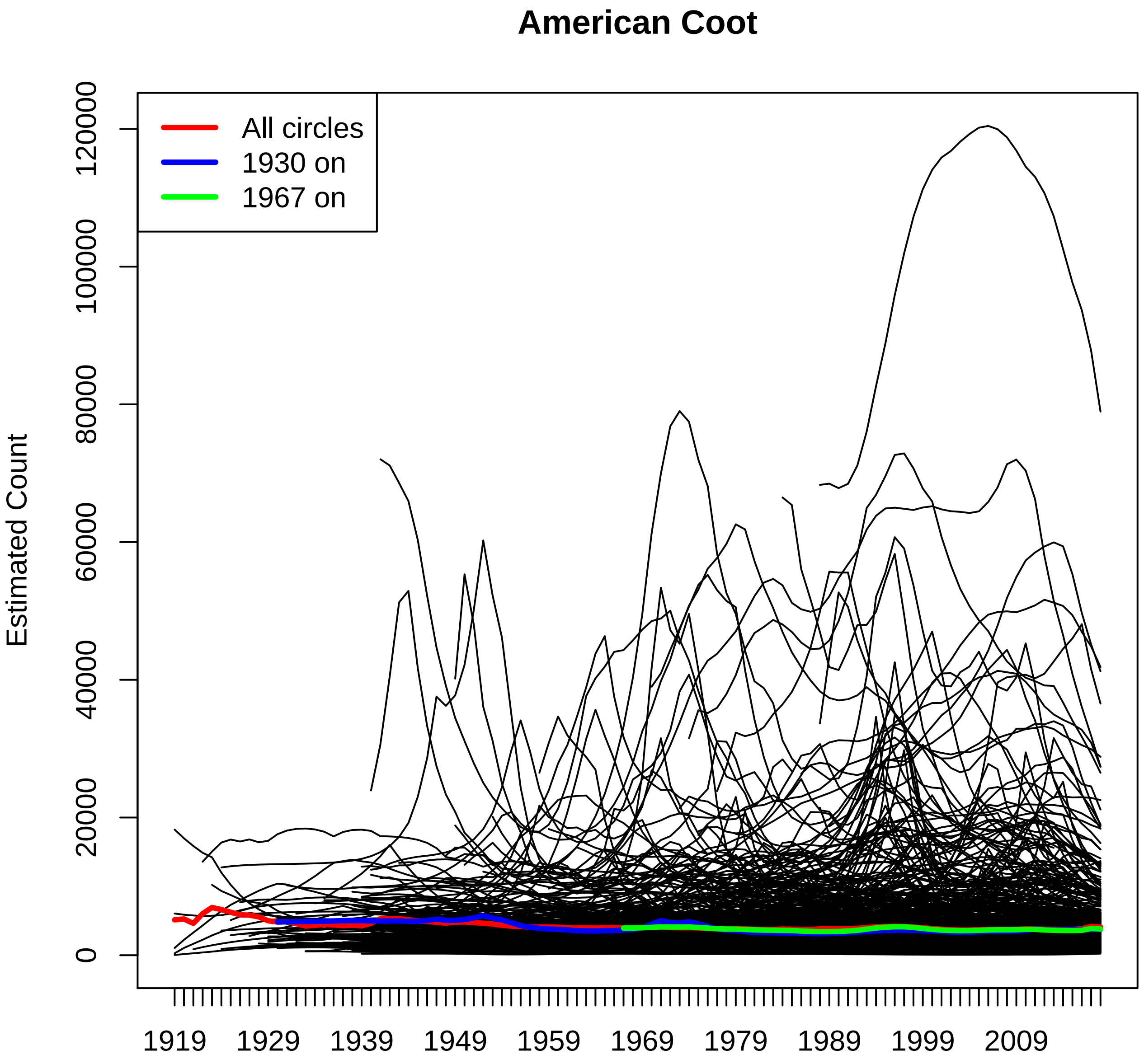

American Coot
Estimated Count
0
20000
40000
60000
80000
100000
120000
1919
1929
1939
1949
1959
1969
1979
1989
1999
2009
All circles
1930 on
1967 on

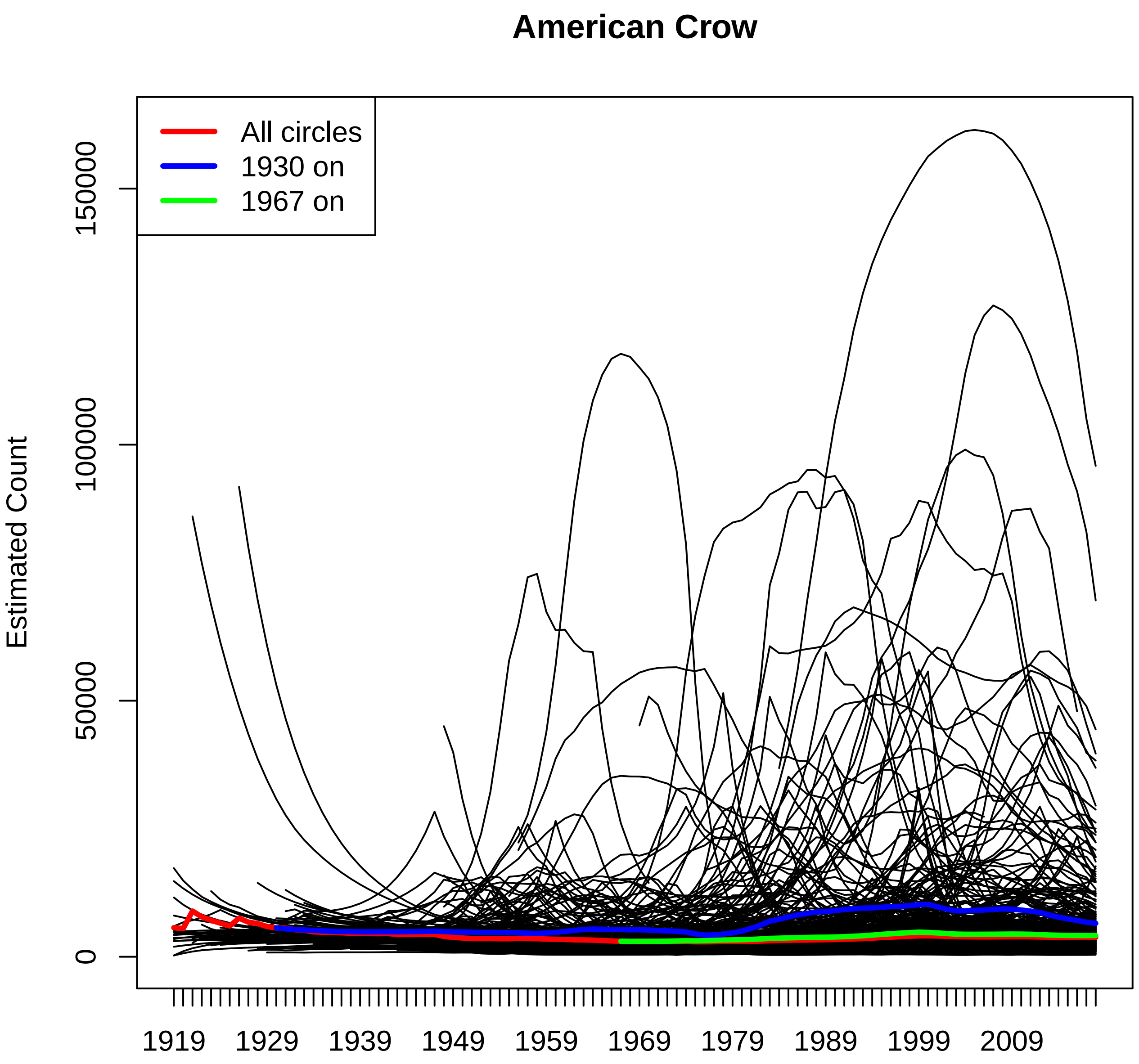
American Crow
All circles
1930 on
1967 on
Estimated Count
0
50000
100000
150000
1919
1929
1939
1949
1959
1969
1979
1989
1999
2009

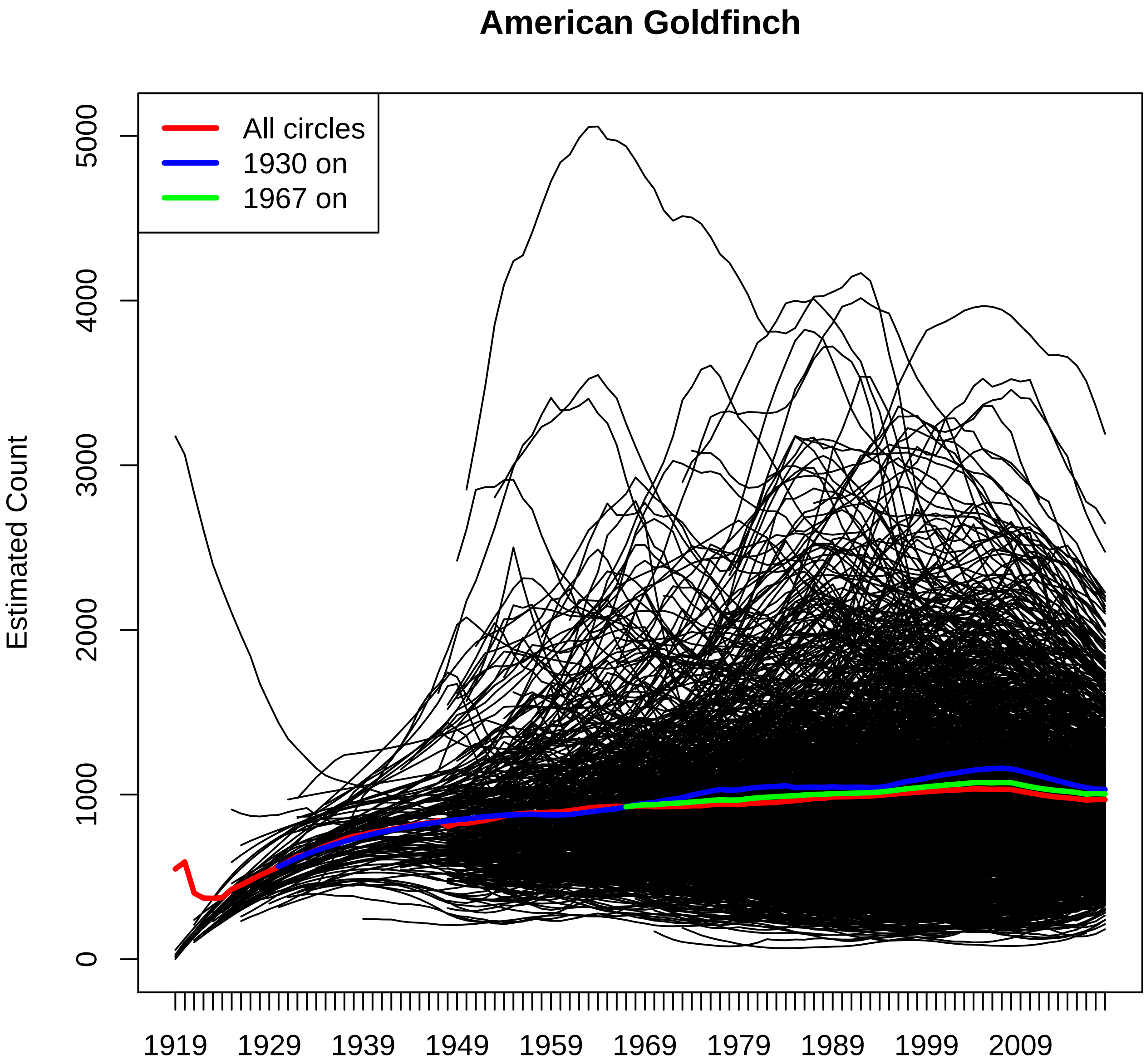
American Goldfinch
All circles
1930 on
1967 on
Estimated Count
0
1000
2000
3000
4000
5000
1919
1929
1939
1949
1959
1969
1979
1989
1999
2009

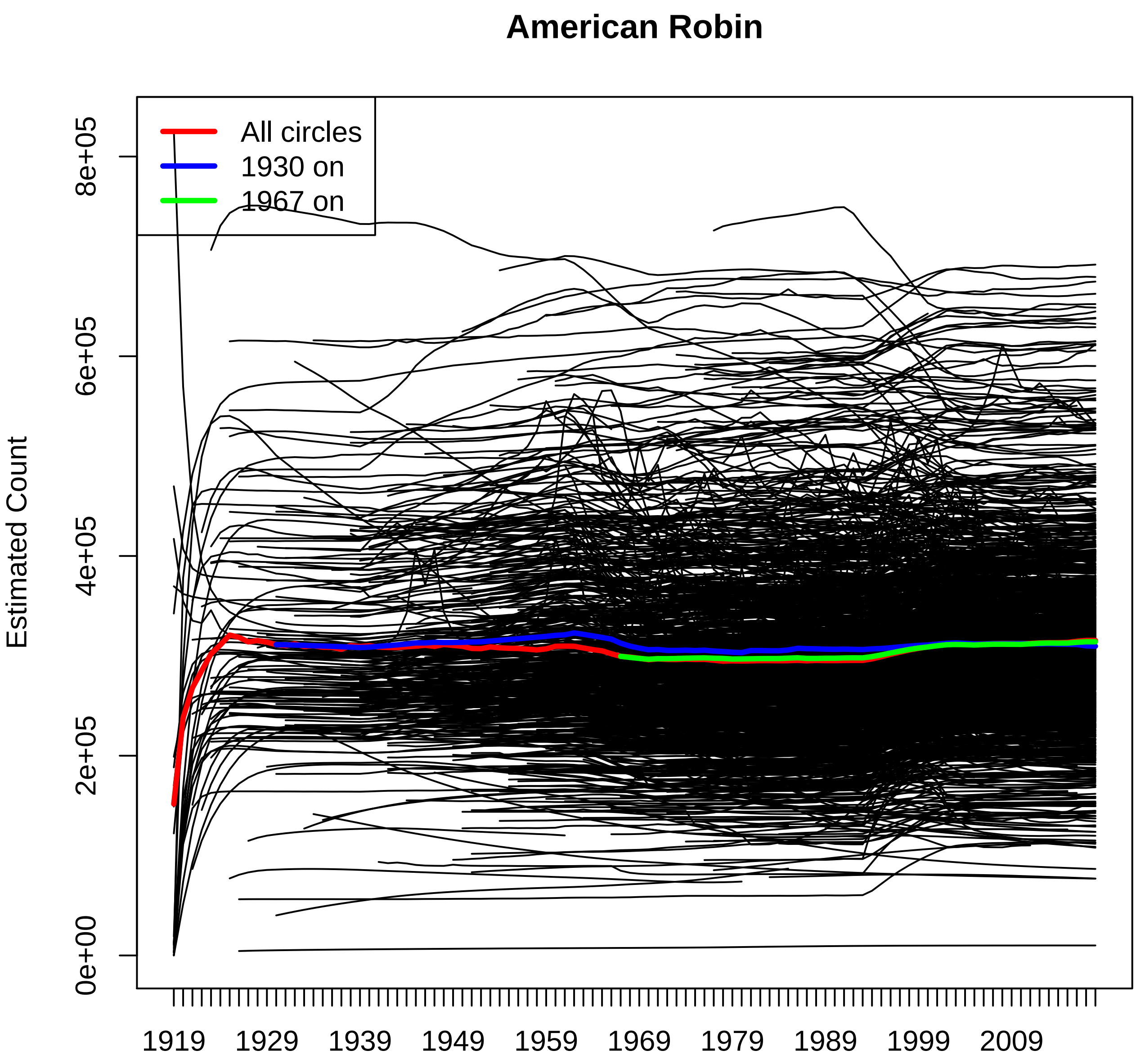
American Robin
All circles
1930 on
1967 on
Estimated Count
0e+00
2e+05
4e+05
6e+05
8e+05
1919
1929
1939
1949
1959
1969
1979
1989
1999
2009

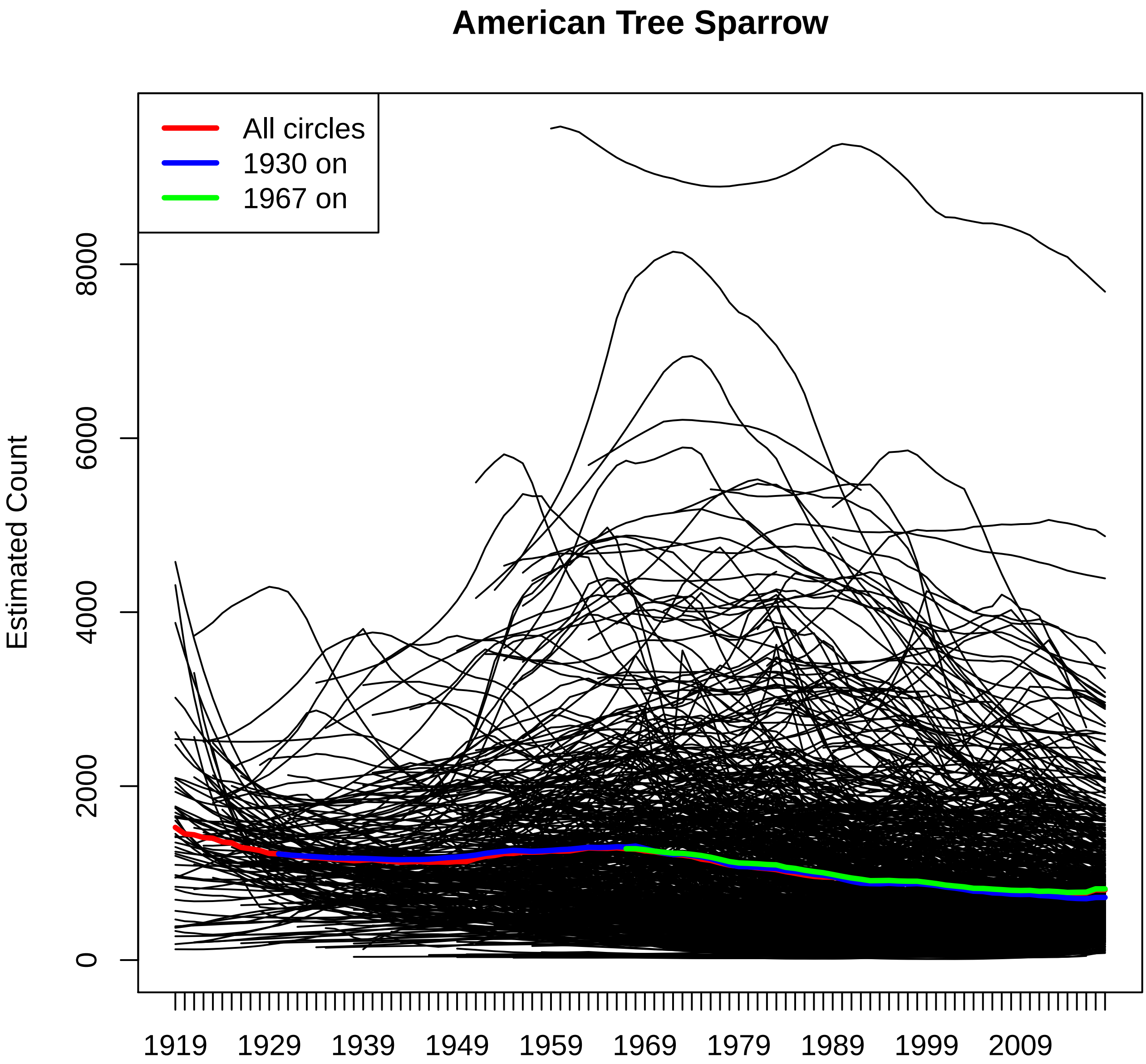

American Tree Sparrow
All circles
1930 on
1967 on
Estimated Count
0
2000
4000
6000
8000
1919
1929
1939
1949
1959
1969
1979
1989
1999
2009

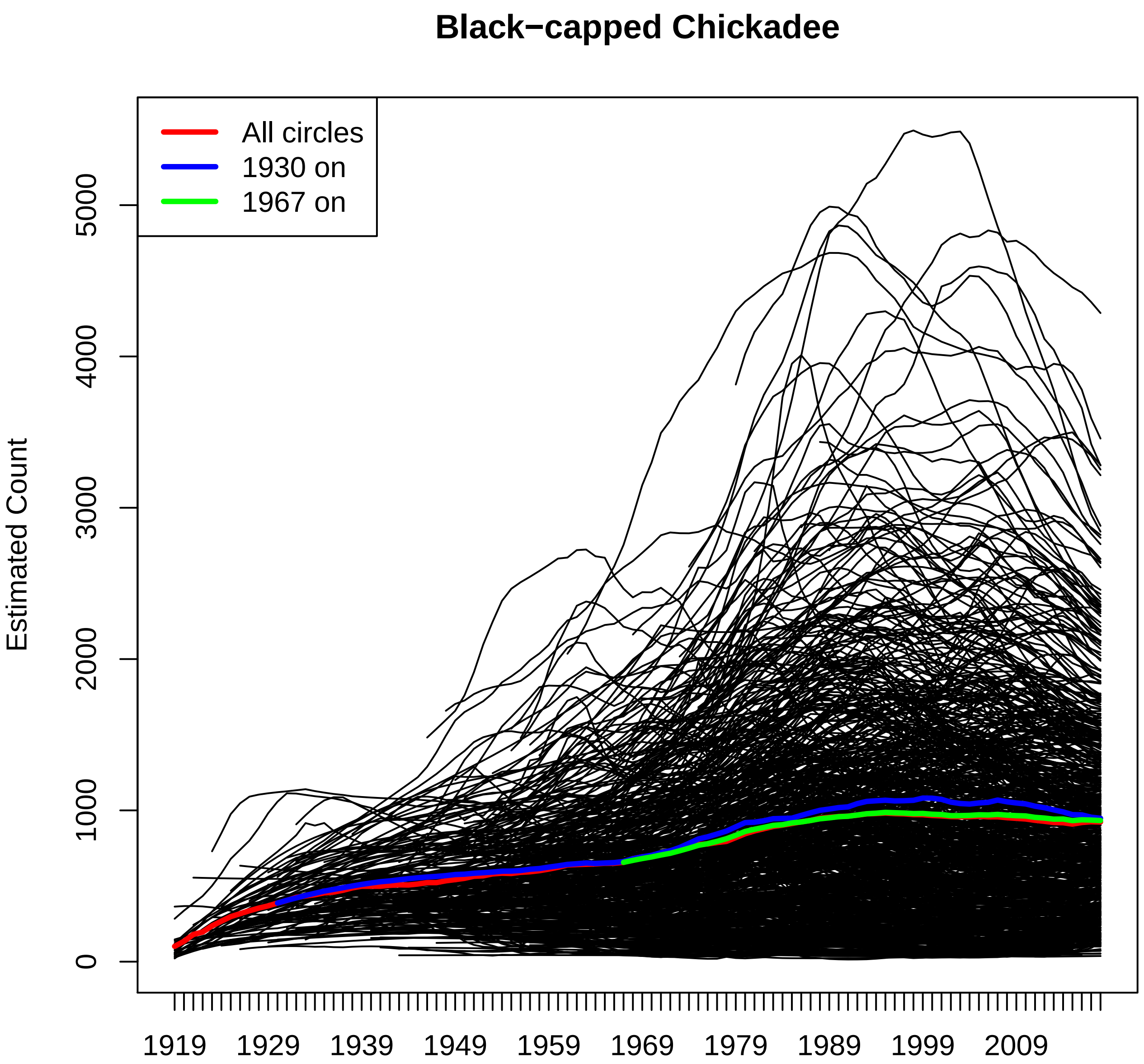
Black−capped Chickadee
All circles
1930 on
1967 on
Estimated Count
0
1000
2000
3000
4000
5000
1919
1929
1939
1949
1959
1969
1979
1989
1999
2009

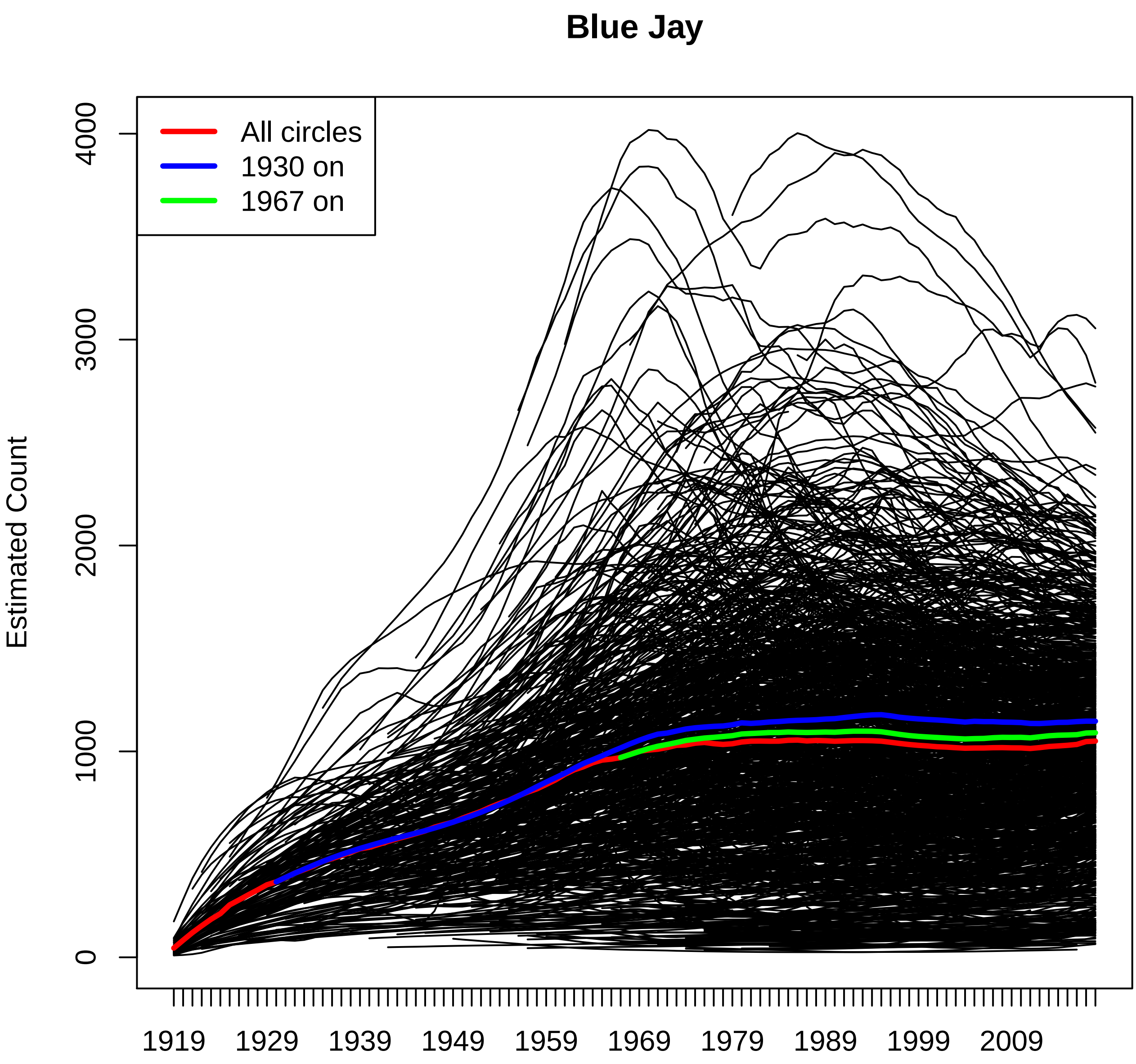

Blue Jay
Estimated Count
0
1000
2000
3000
4000
1919
1929
1939
1949
1959
1969
1979
1989
1999
2009
All circles
1930 on
1967 on

Brant

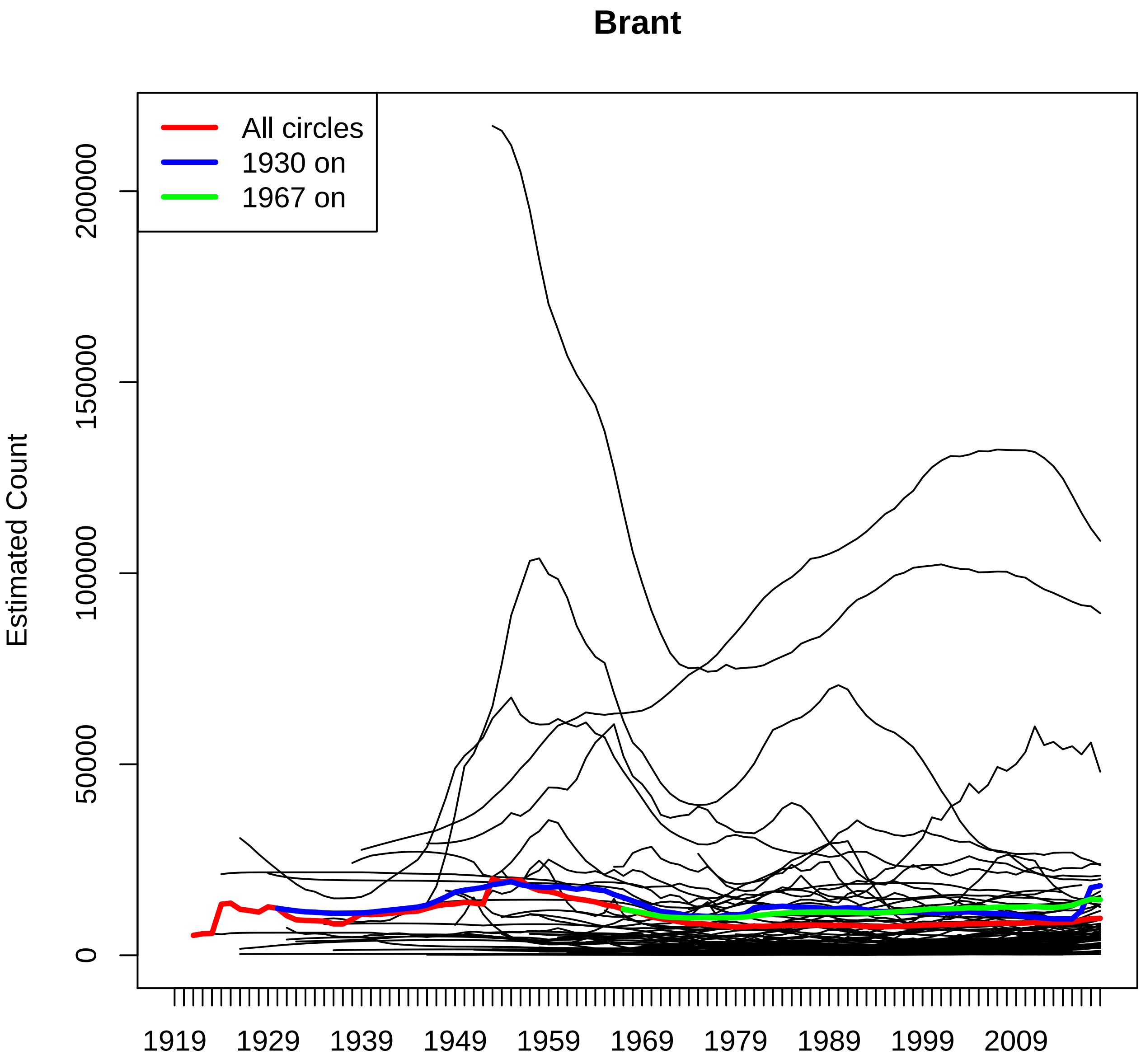

Brant
All circles
1930 on
1967 on
Estimated Count
0
50000
100000
150000
200000
1919
1929
1939
1949
1959
1969
1979
1989
1999
2009

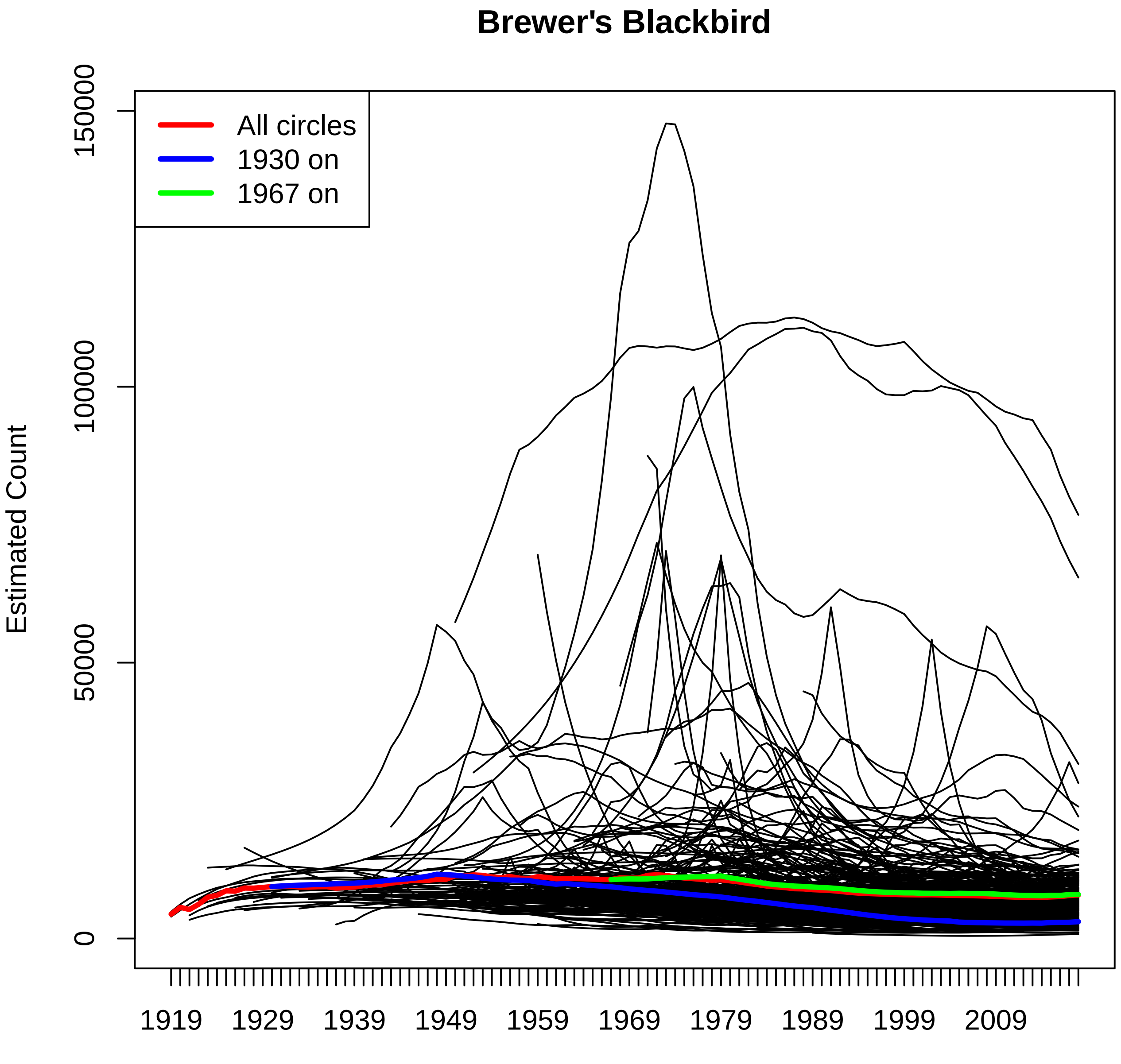
Brewer's Blackbird
Estimated Count
0
50000
100000
150000
1919
1929
1939
1949
1959
1969
1979
1989
1999
2009
All circles
1930 on
1967 on

Brown−headed Cowbird

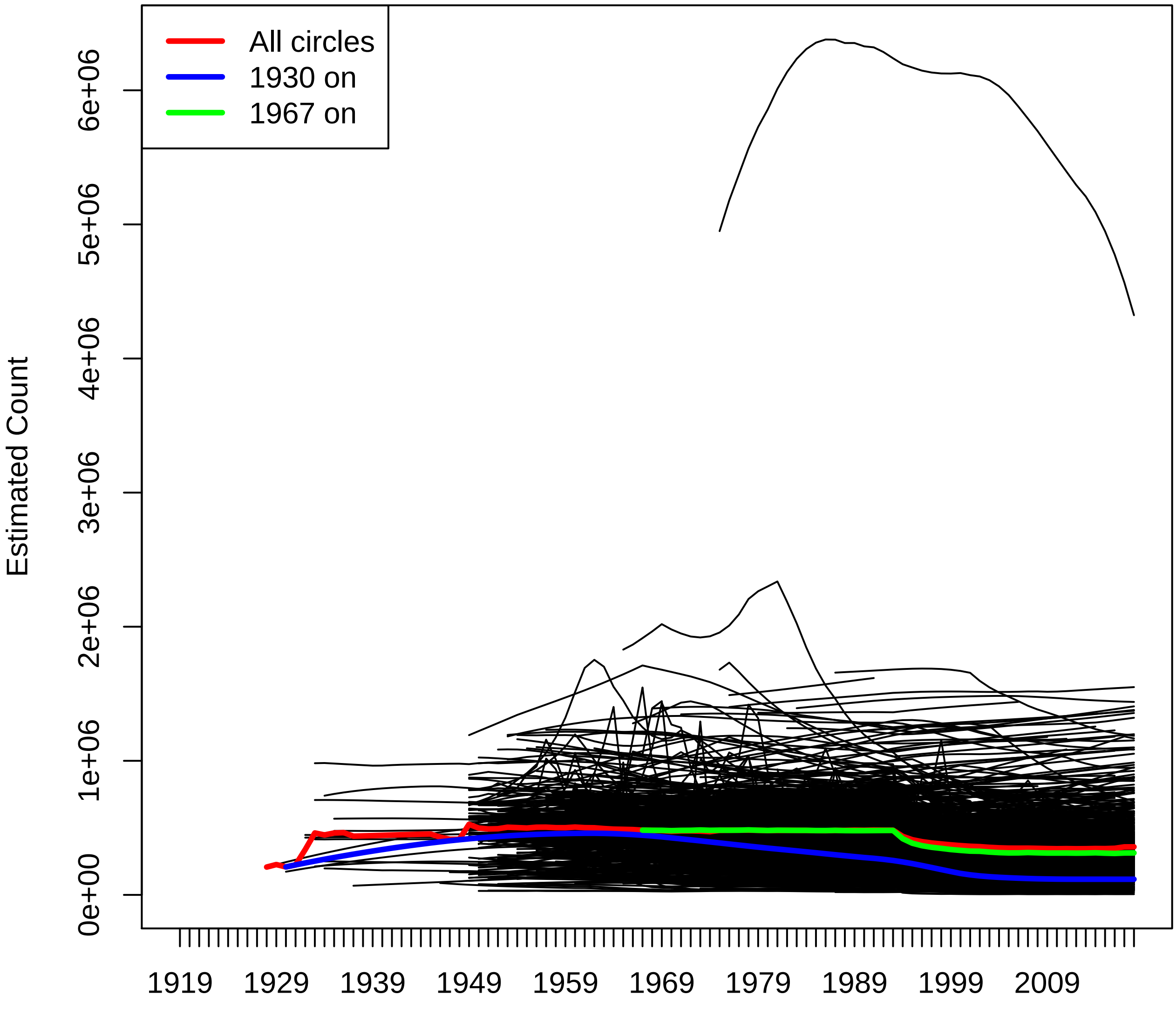

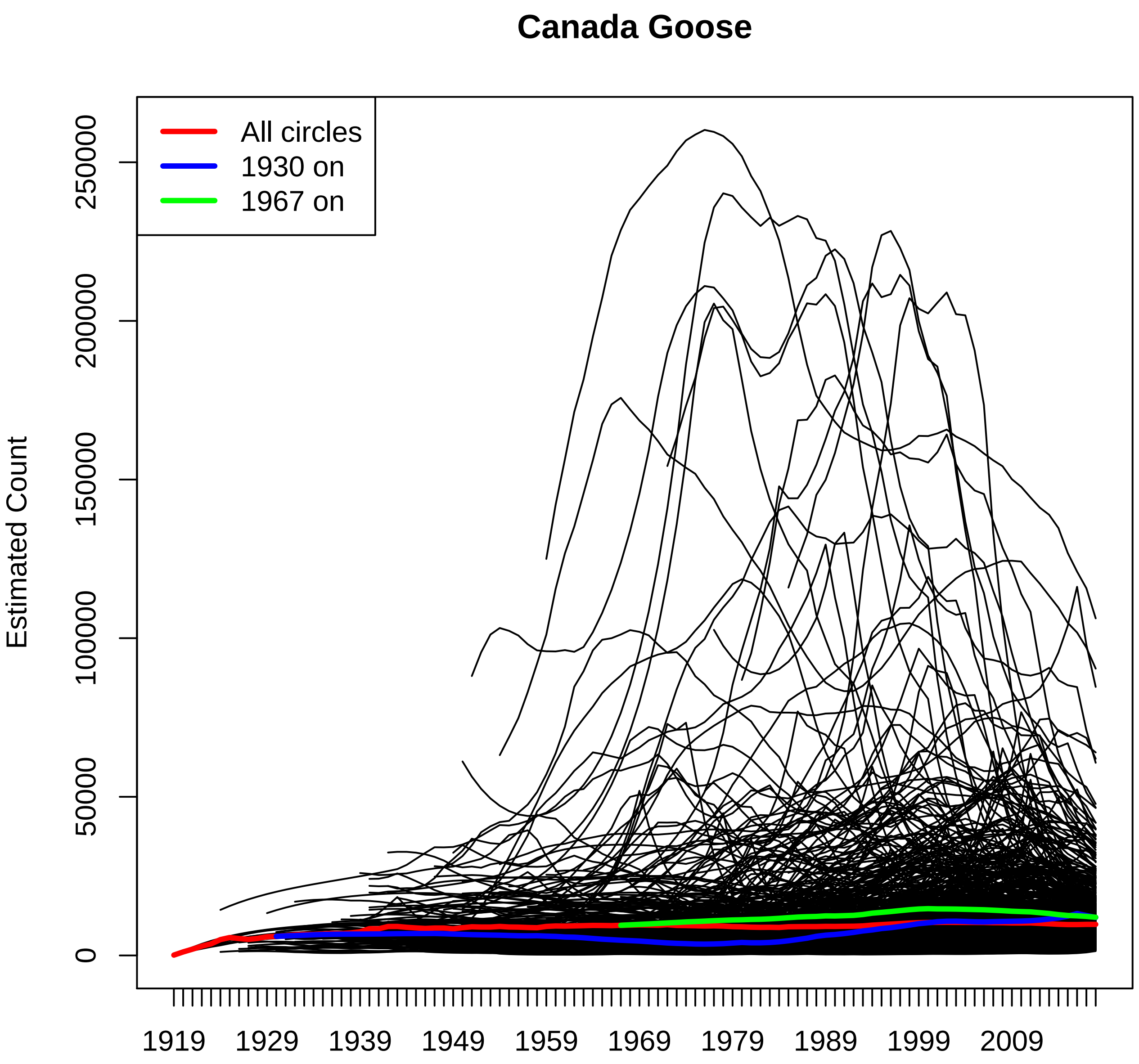

Canada Goose
All circles
1930 on
1967 on
Estimated Count
0
50000
100000
150000
200000
250000
1919
1929
1939
1949
1959
1969
1979
1989
1999
2009

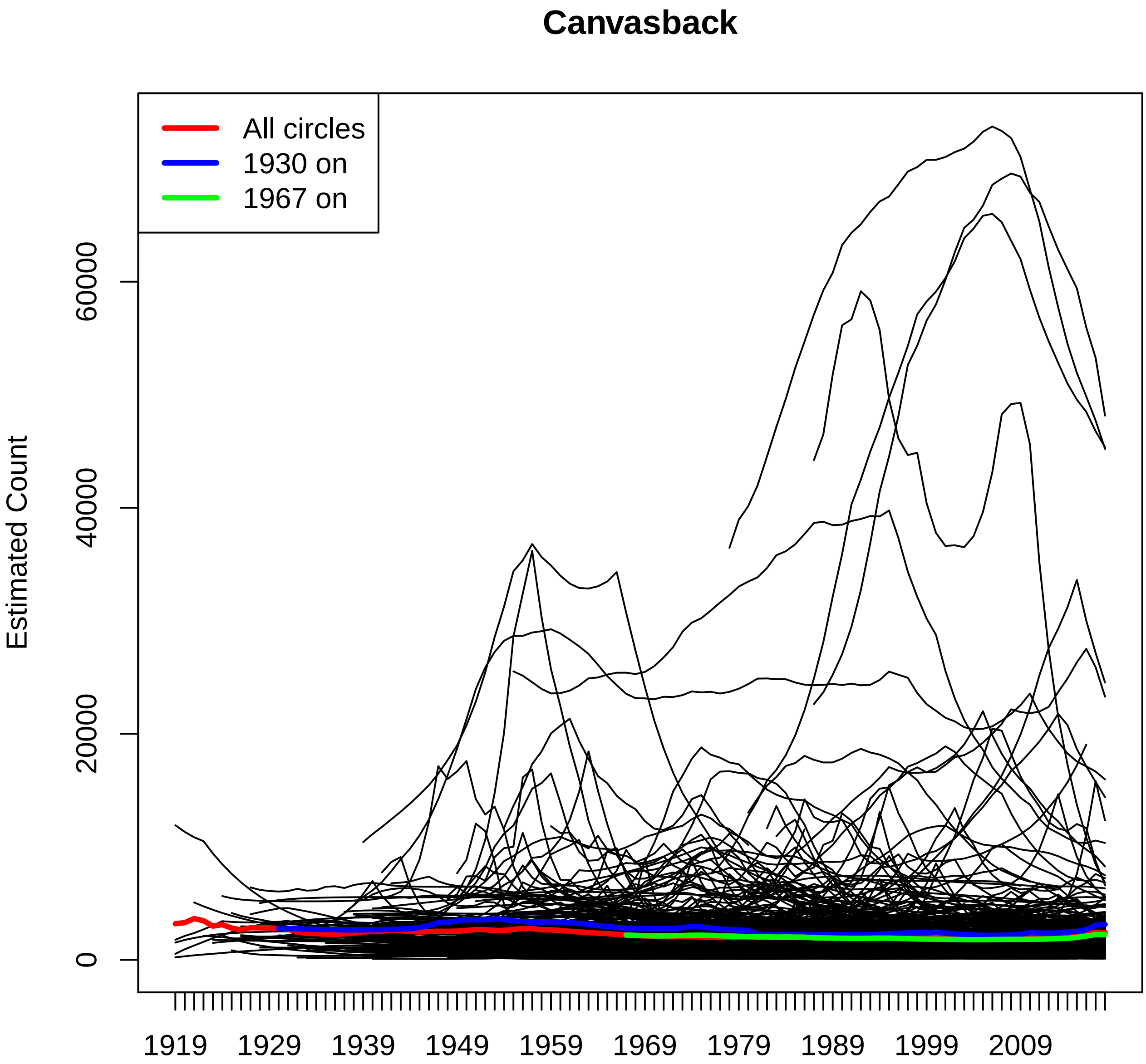

Canvasback
All circles
1930 on
1967 on
Estimated Count
0
20000
40000
60000
1919
1929
1939
1949
1959
1969
1979
1989
1999
2009

Common Grackle

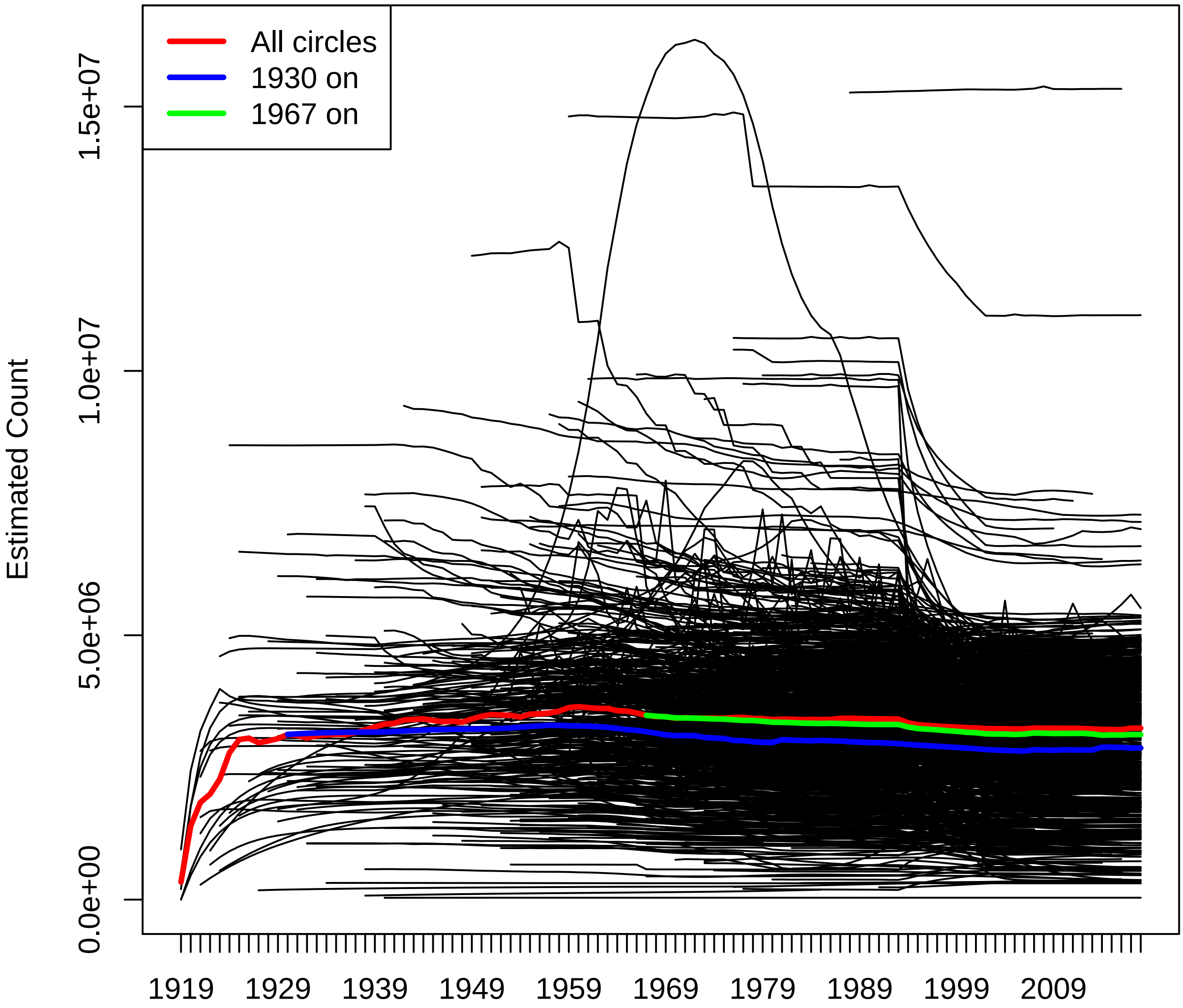

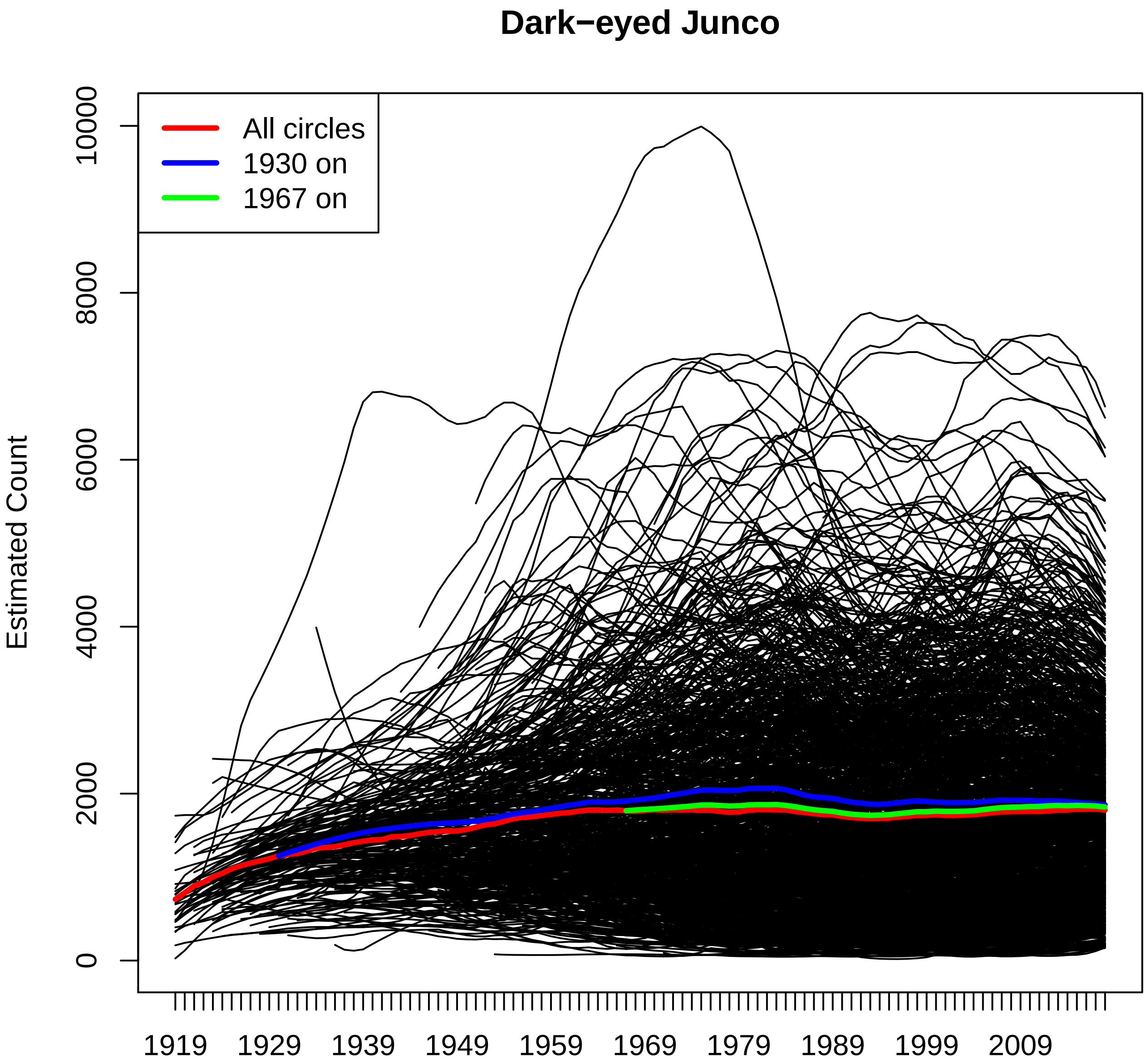

Dark−eyed Junco
All circles
1930 on
1967 on
Estimated Count
0
2000
4000
6000
8000
10000
1919
1929
1939
1949
1959
1969
1979
1989
1999
2009

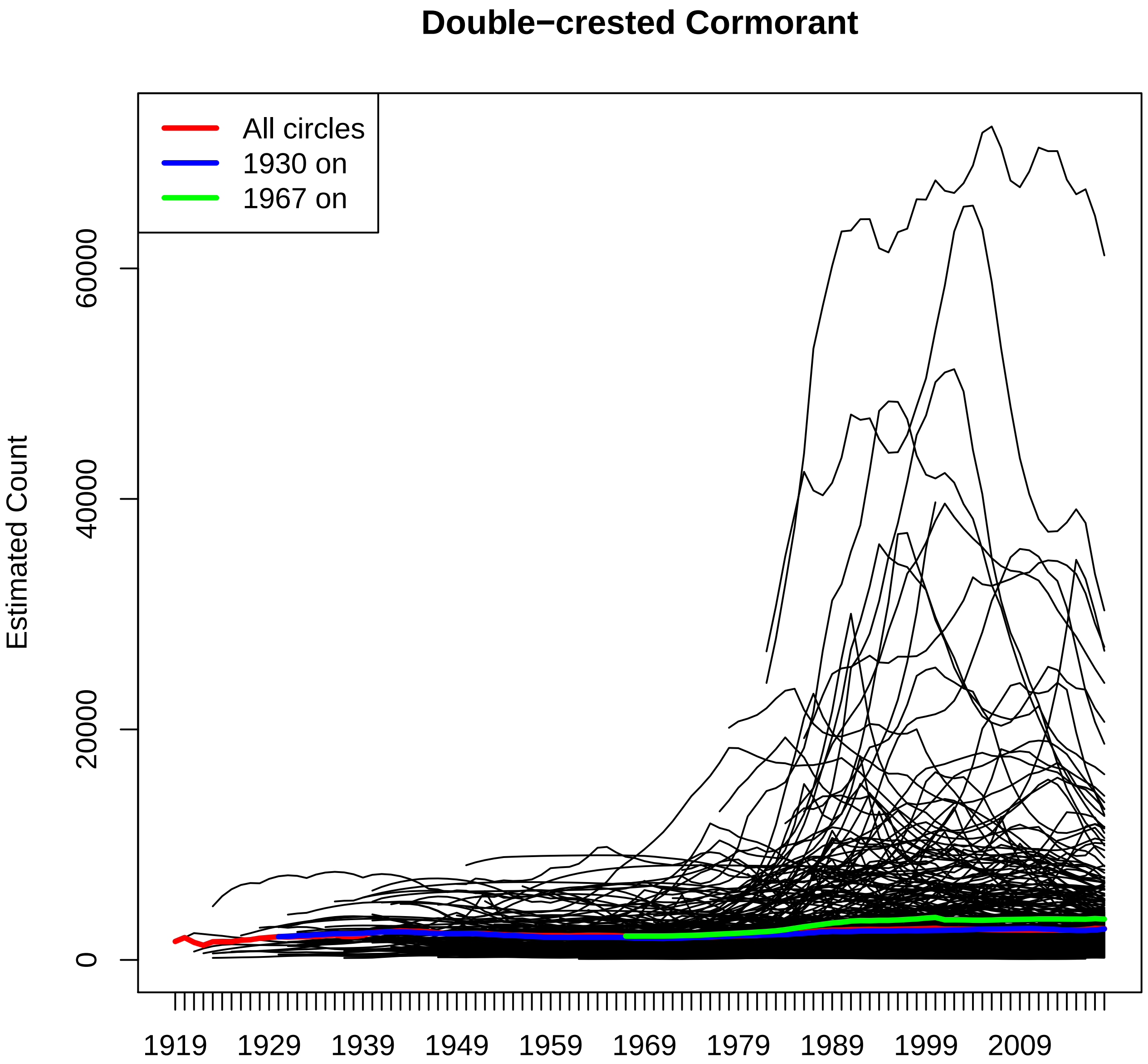
Double−crested Cormorant
All circles
1930 on
1967 on
Estimated Count
0
20000
40000
60000
1919
1929
1939
1949
1959
1969
1979
1989
1999
2009

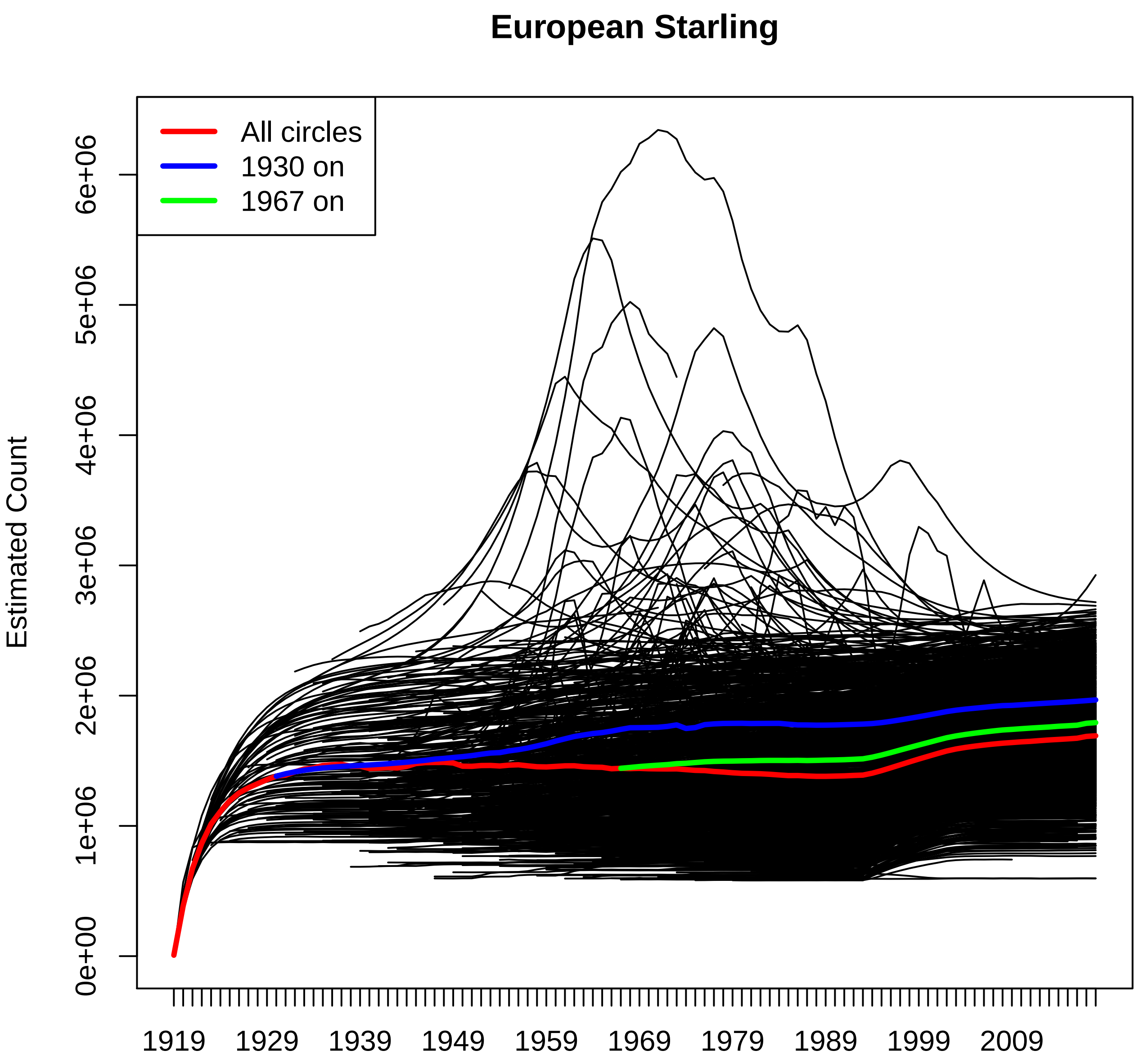
European Starling
All circles
1930 on
1967 on
Estimated Count
0e+00
1e+06
2e+06
3e+06
4e+06
5e+06
6e+06
1919
1929
1939
1949
1959
1969
1979
1989
1999
2009

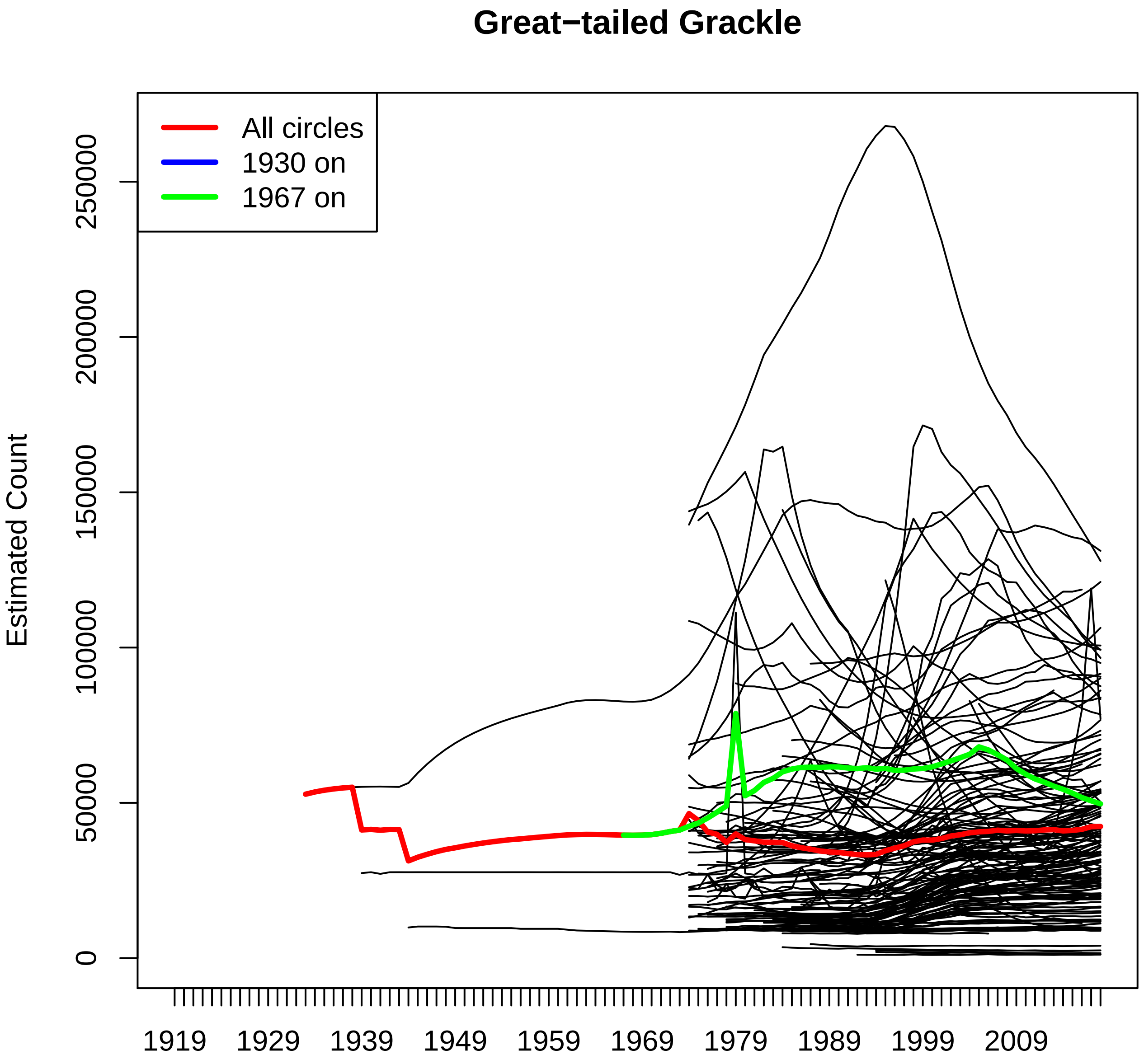

Great−tailed Grackle
Estimated Count
All circles
1930 on
1967 on
0
50000
100000
150000
200000
250000
1919
1929
1939
1949
1959
1969
1979
1989
1999
2009

Greater Scaup

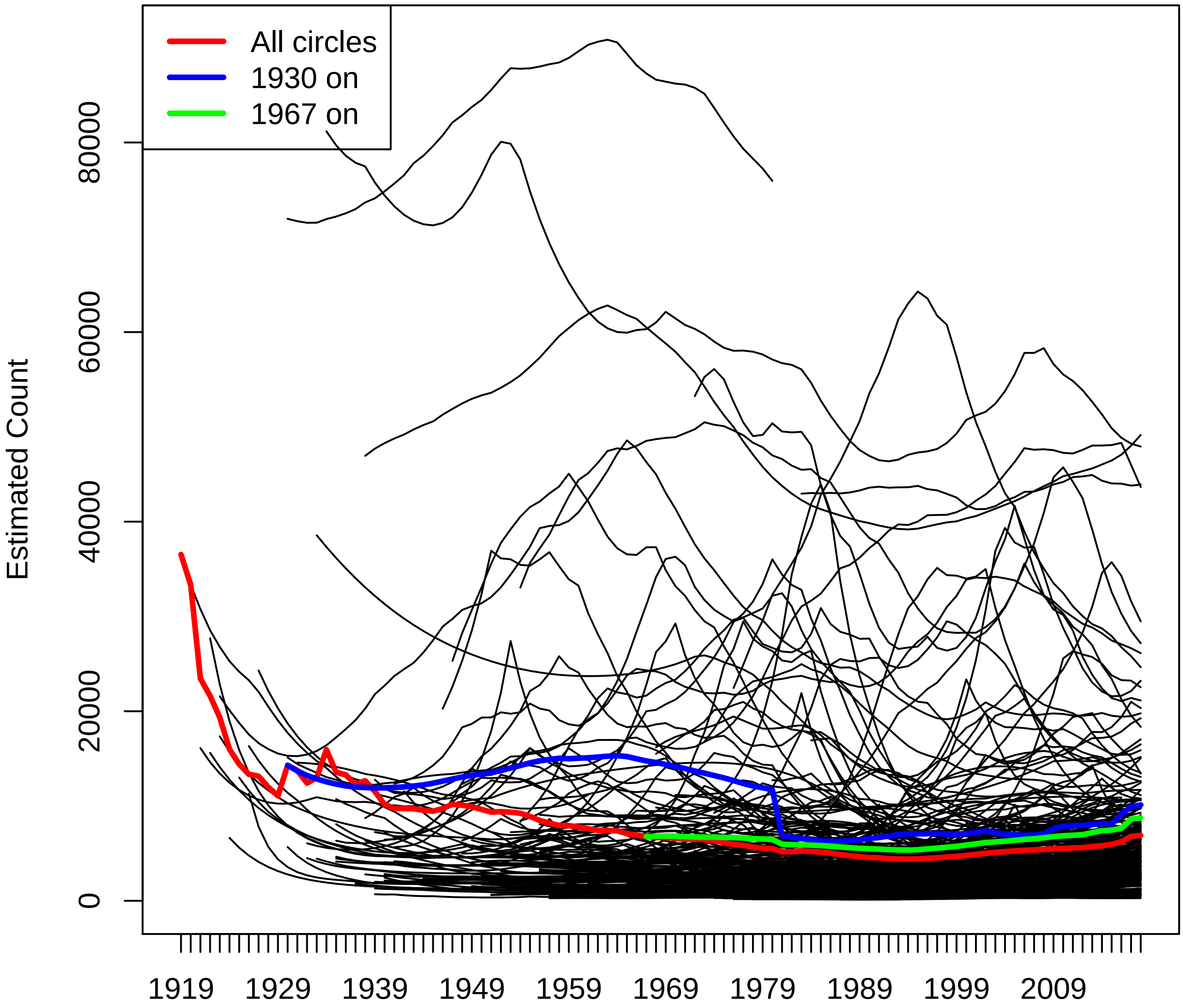

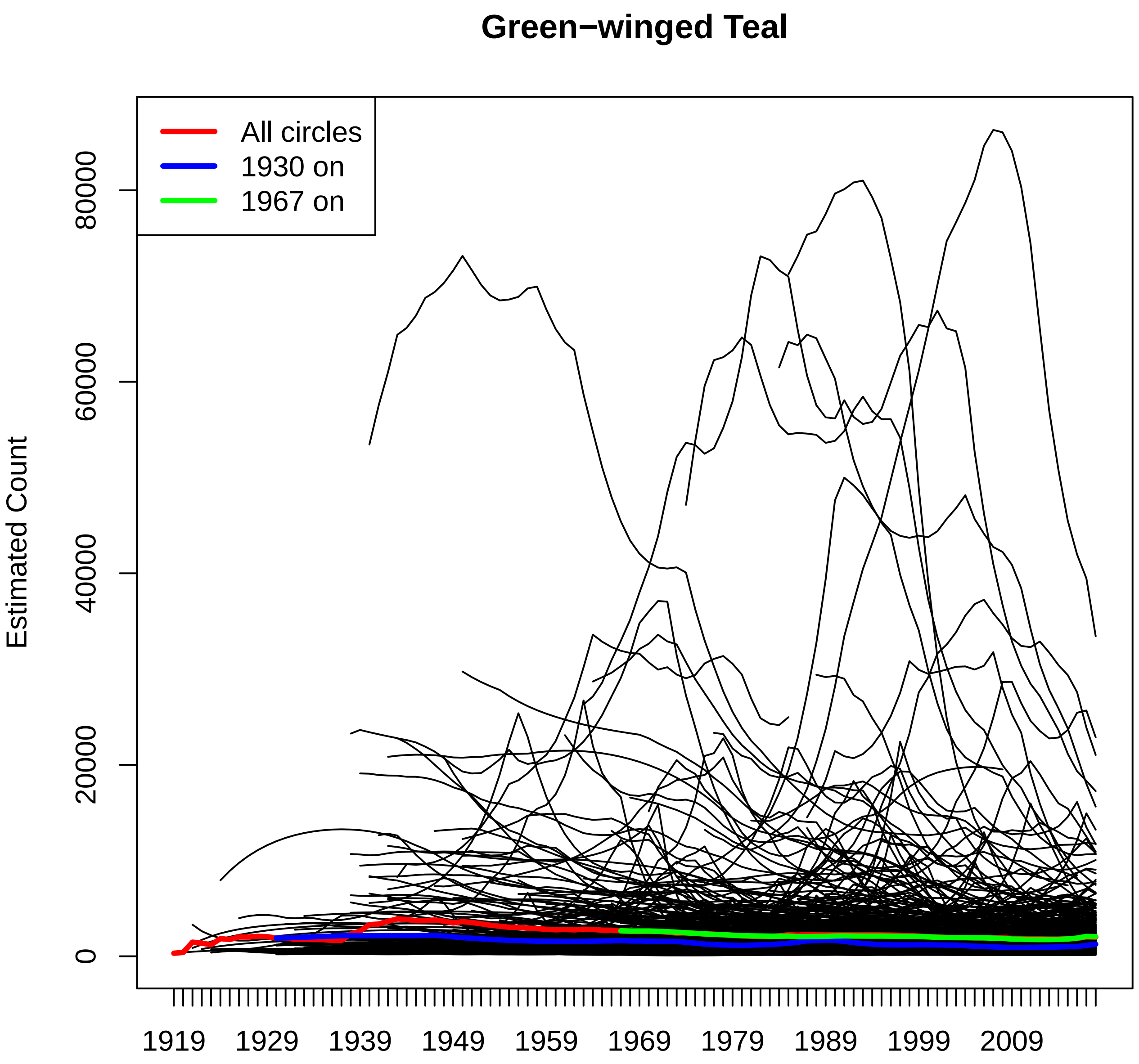
Green−winged Teal
All circles
1930 on
1967 on
Estimated Count
0
20000
40000
60000
80000
1919
1929
1939
1949
1959
1969
1979
1989
1999
2009

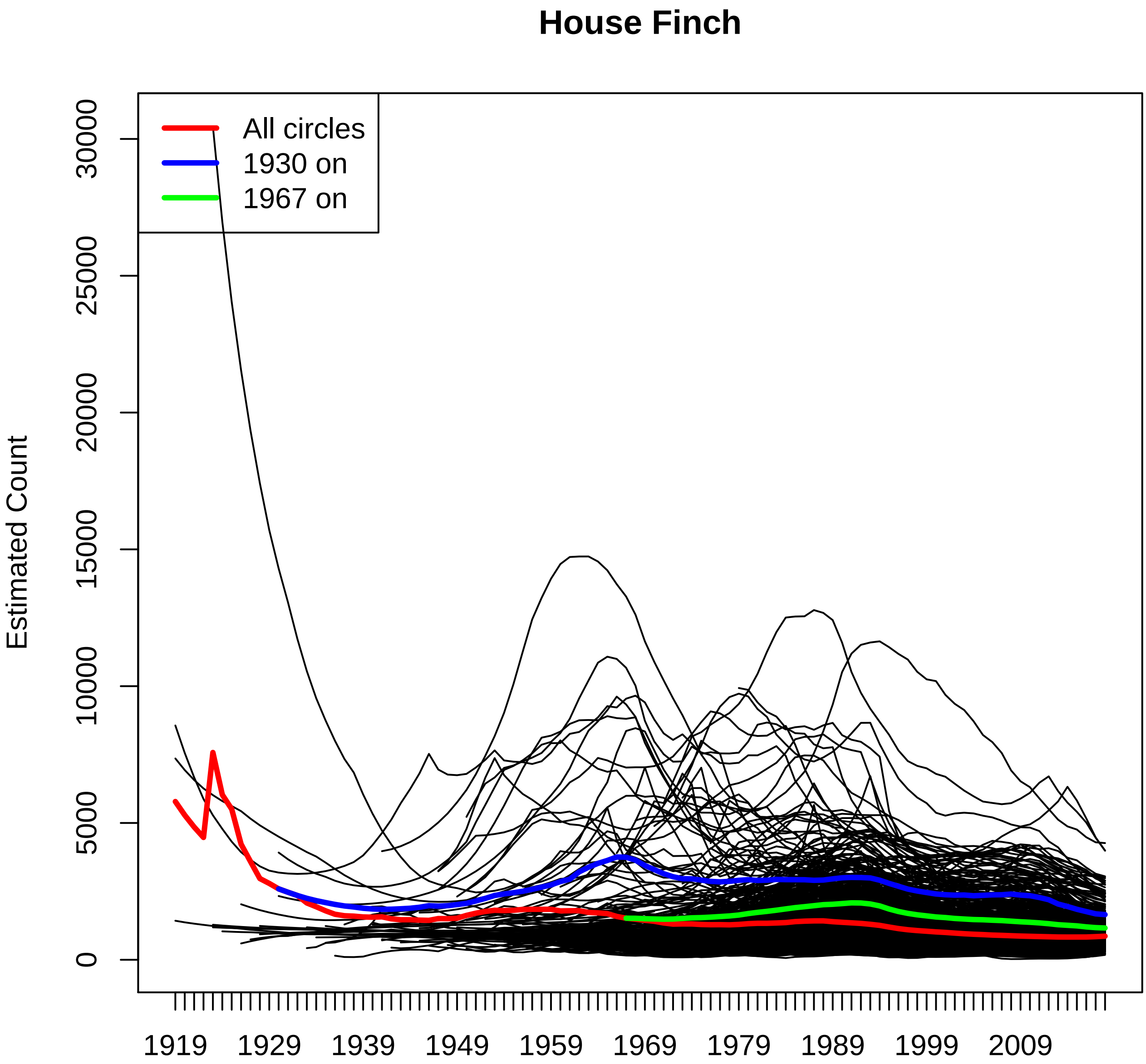
House Finch
All circles
1930 on
1967 on
Estimated Count
0
5000
10000
15000
20000
25000
30000
1919
1929
1939
1949
1959
1969
1979
1989
1999
2009

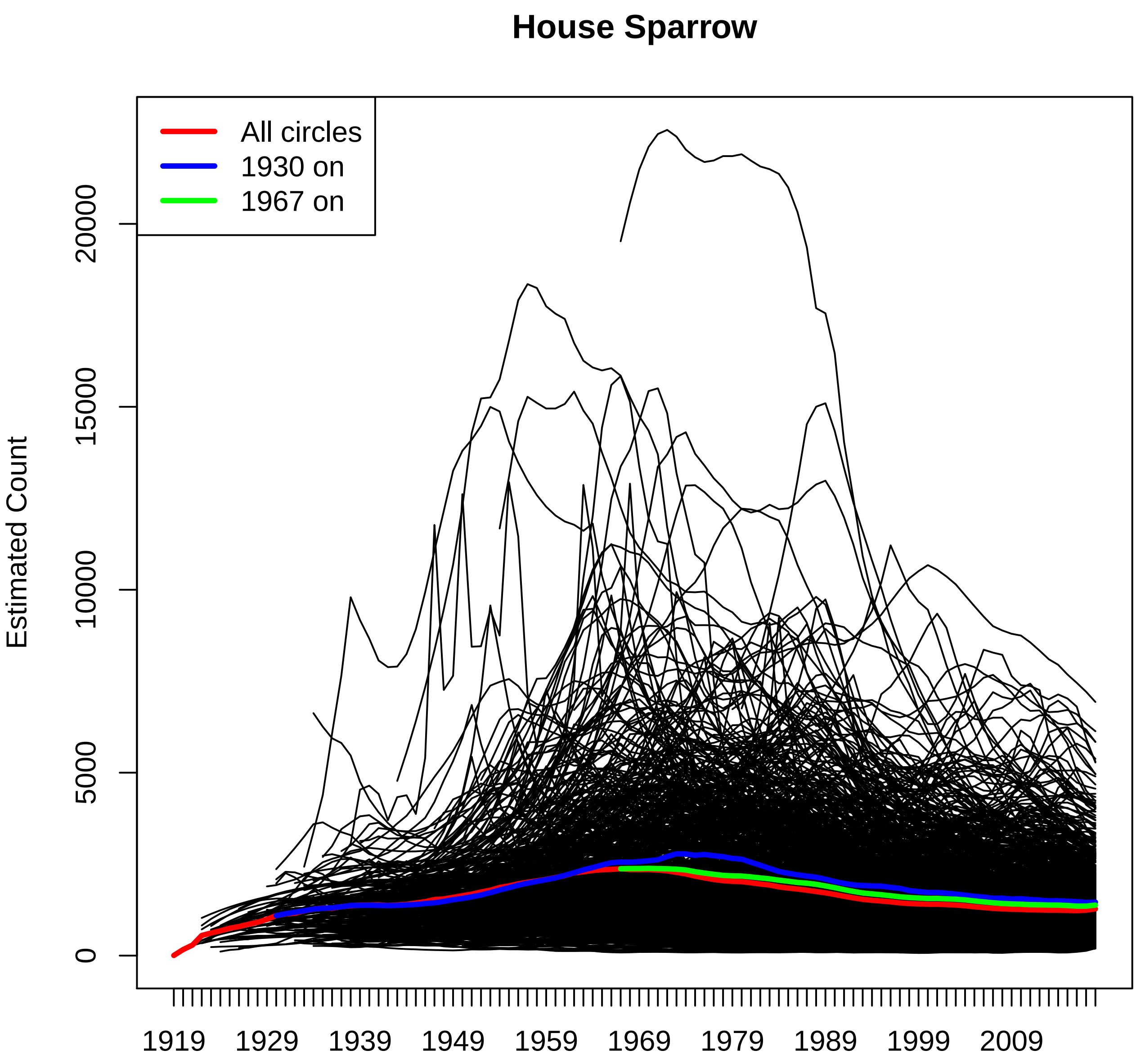
House Sparrow
All circles
1930 on
1967 on
Estimated Count
0
5000
10000
15000
20000
1919
1929
1939
1949
1959
1969
1979
1989
1999
2009

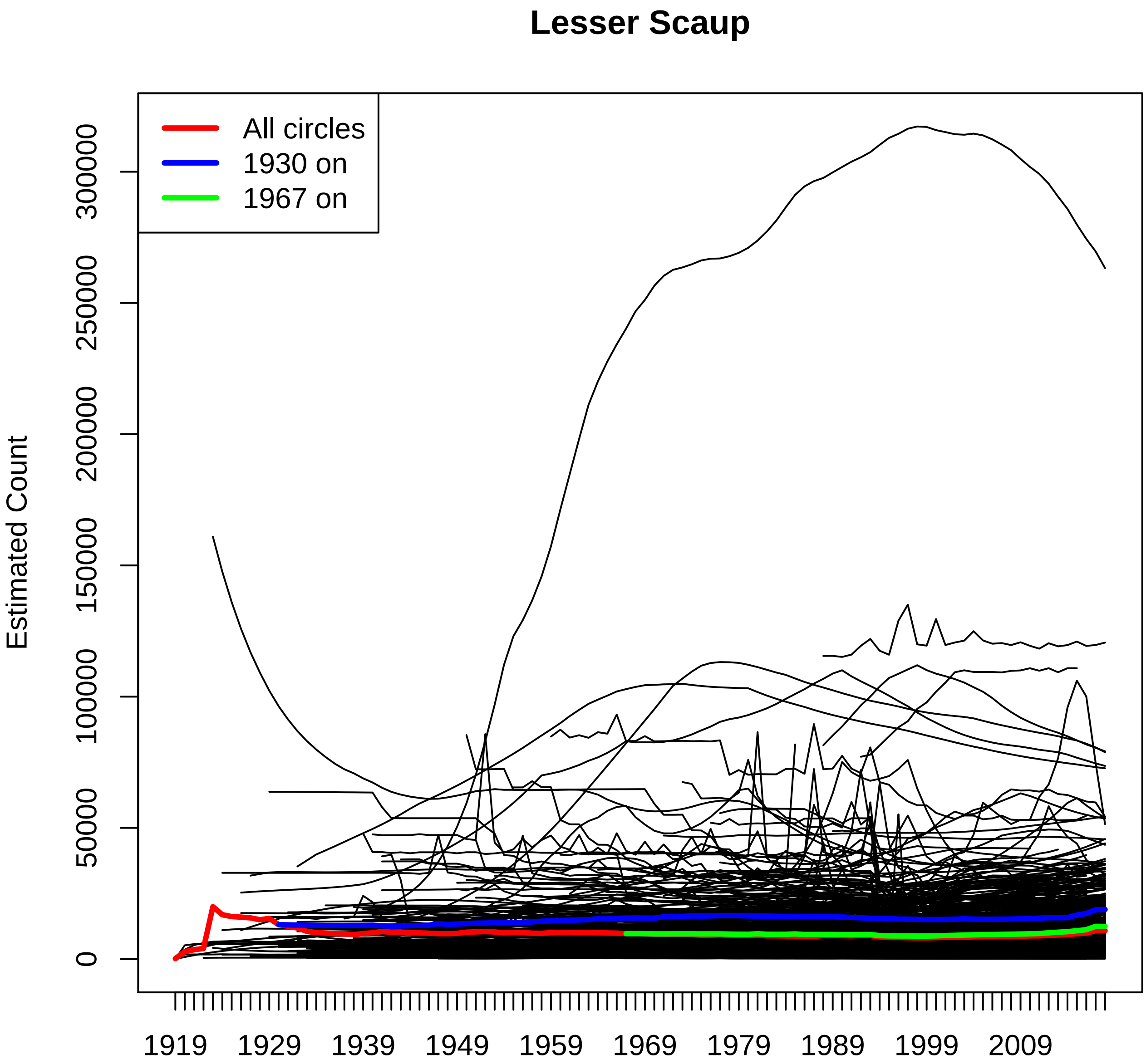
Lesser Scaup
All circles
1930 on
1967 on
Estimated Count
0
50000
100000
150000
200000
250000
300000
1919
1929
1939
1949
1959
1969
1979
1989
1999
2009

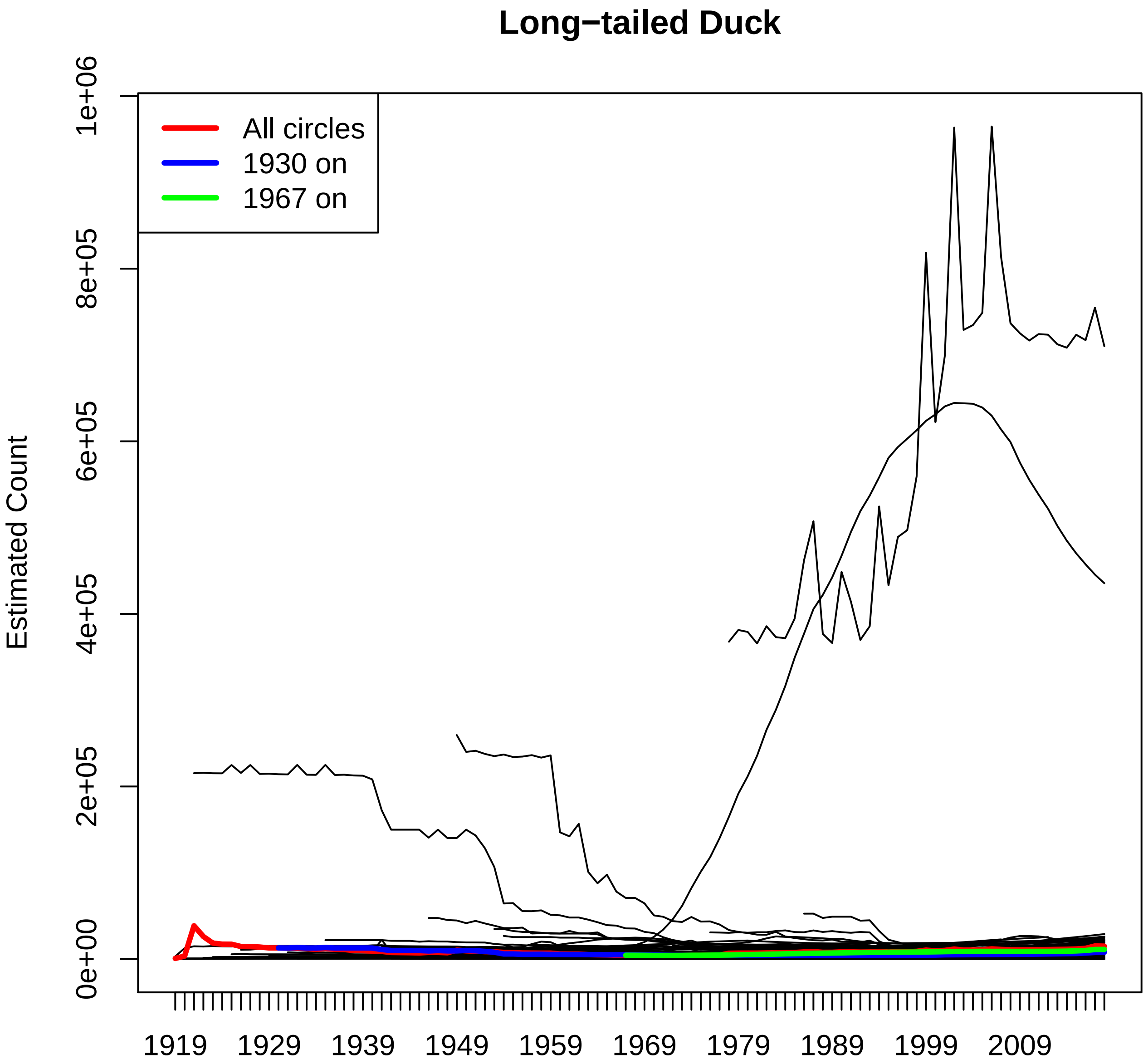
Long−tailed Duck
Estimated Count
0e+00
2e+05
4e+05
6e+05
8e+05
1e+06
1919
1929
1939
1949
1959
1969
1979
1989
1999
2009
All circles
1930 on
1967 on

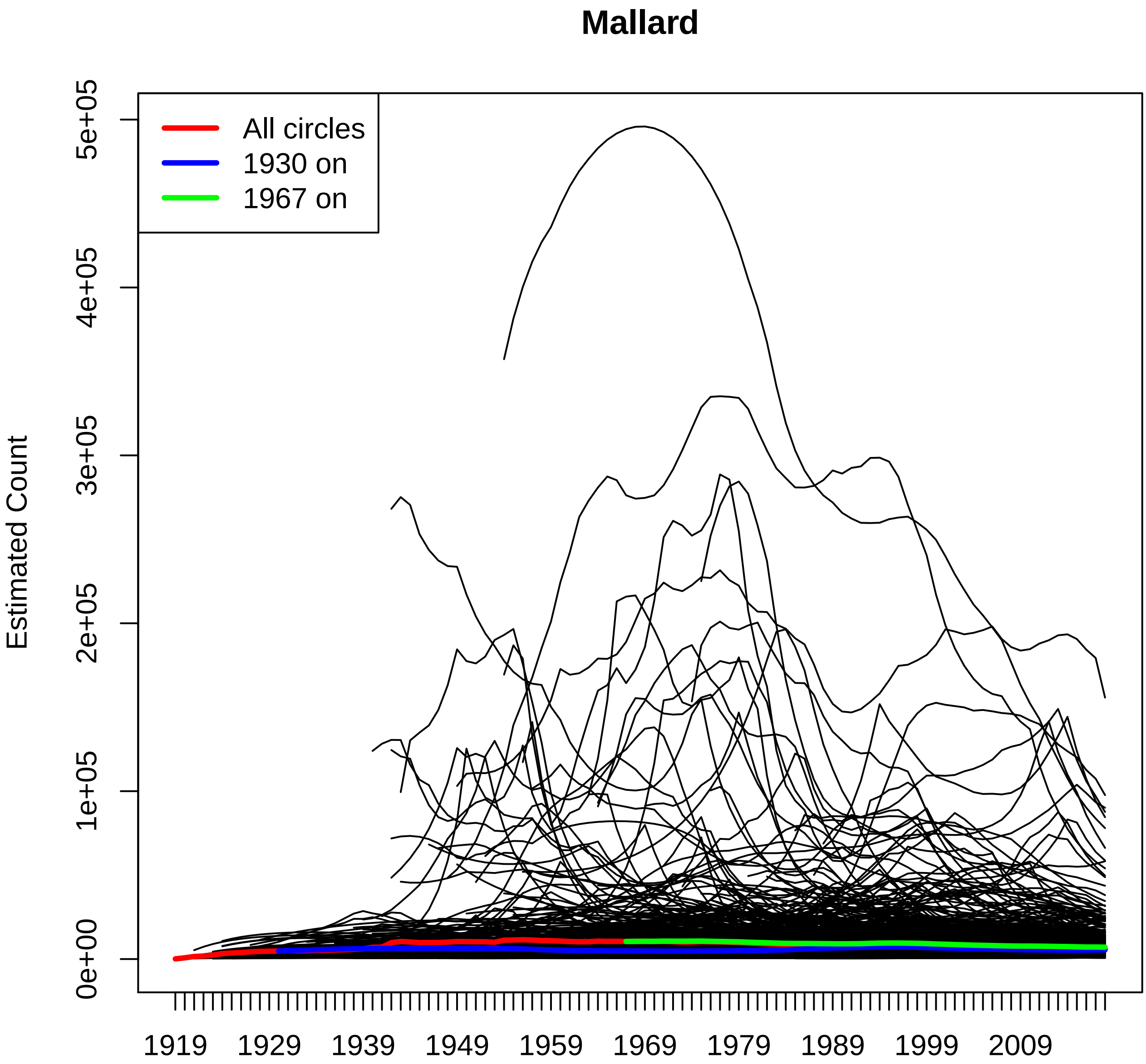

Mallard
All circles
1930 on
1967 on
Estimated Count
0e+00
1e+05
2e+05
3e+05
4e+05
5e+05
1919
1929
1939
1949
1959
1969
1979
1989
1999
2009

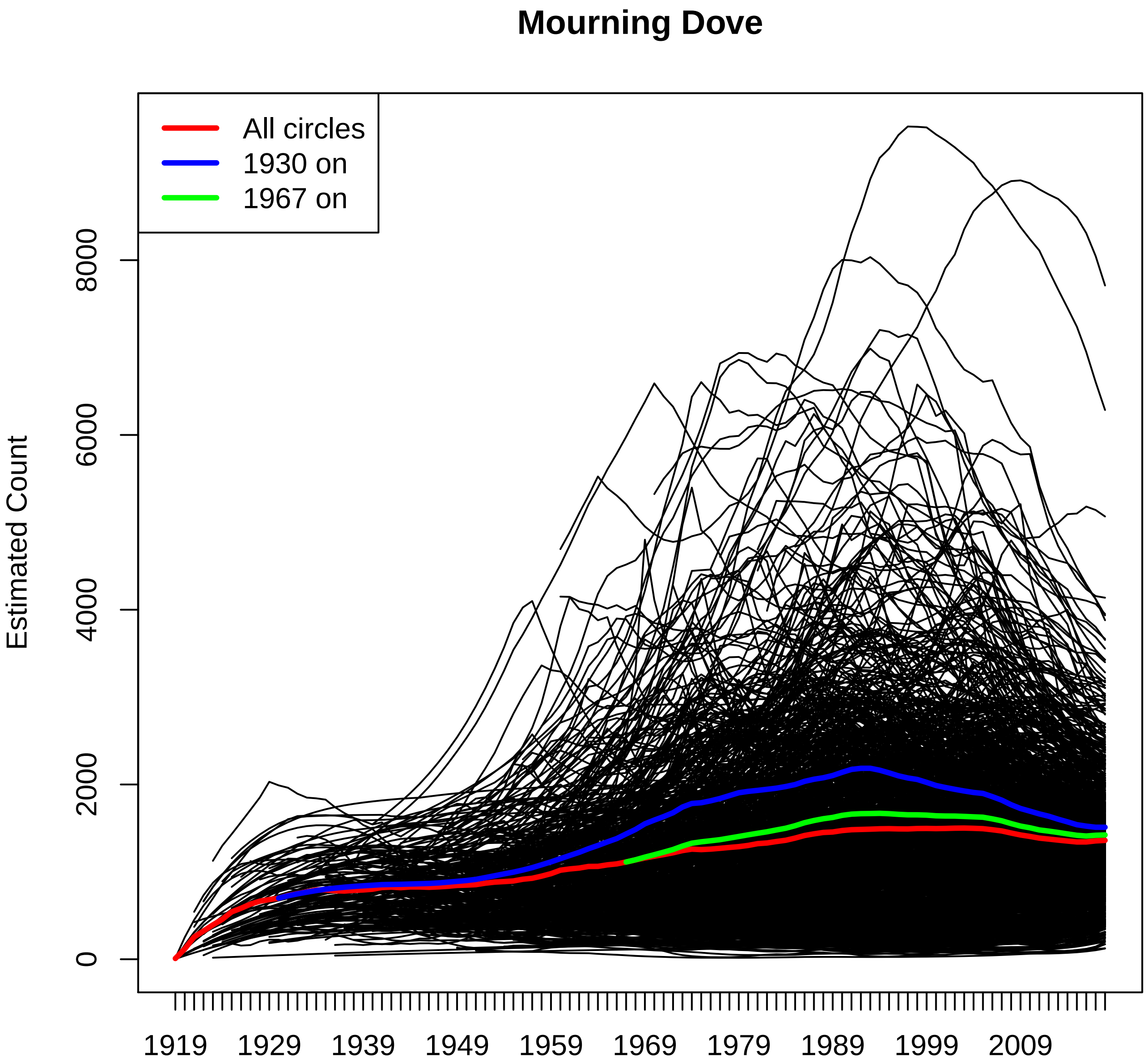
Mourning Dove
All circles
1930 on
1967 on
Estimated Count
0
2000
4000
6000
8000
1919
1929
1939
1949
1959
1969
1979
1989
1999
2009

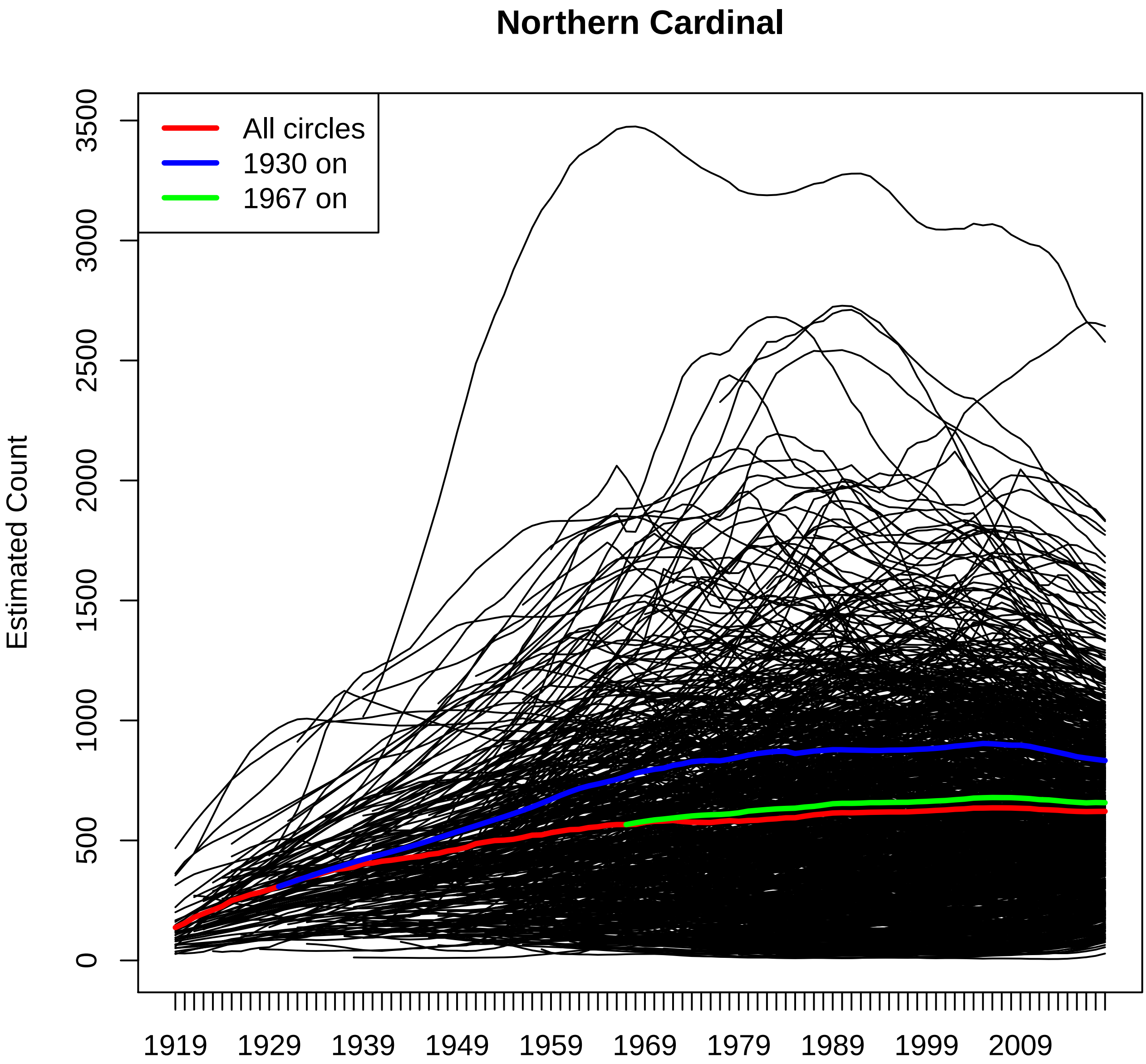

Northern Cardinal
Estimated Count
0
500
1000
1500
2000
2500
3000
3500
1919
1929
1939
1949
1959
1969
1979
1989
1999
2009
All circles
1930 on
1967 on

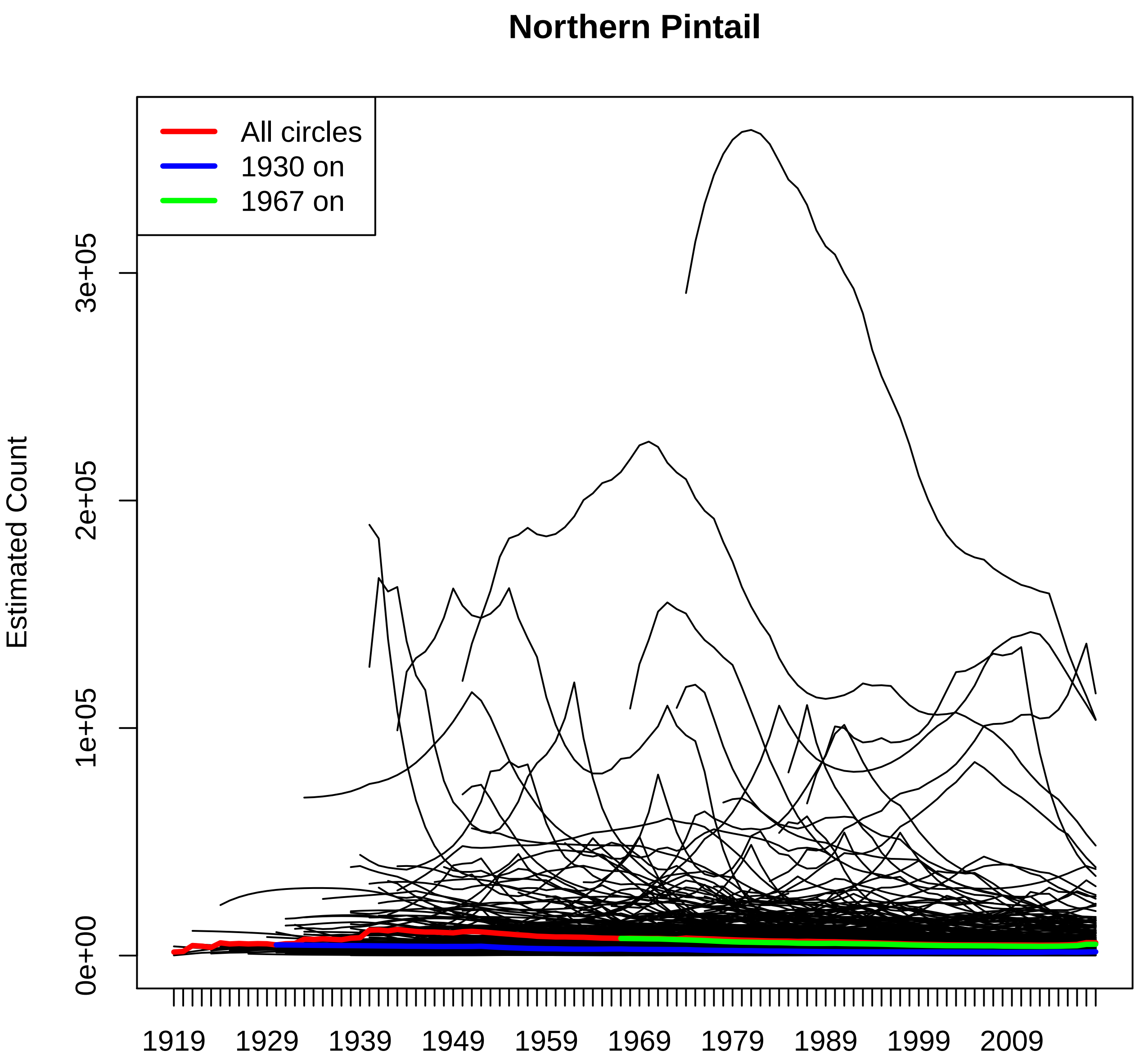
Northern Pintail
All circles
1930 on
1967 on
Estimated Count
0e+00
1e+05
2e+05
3e+05
1919
1929
1939
1949
1959
1969
1979
1989
1999
2009

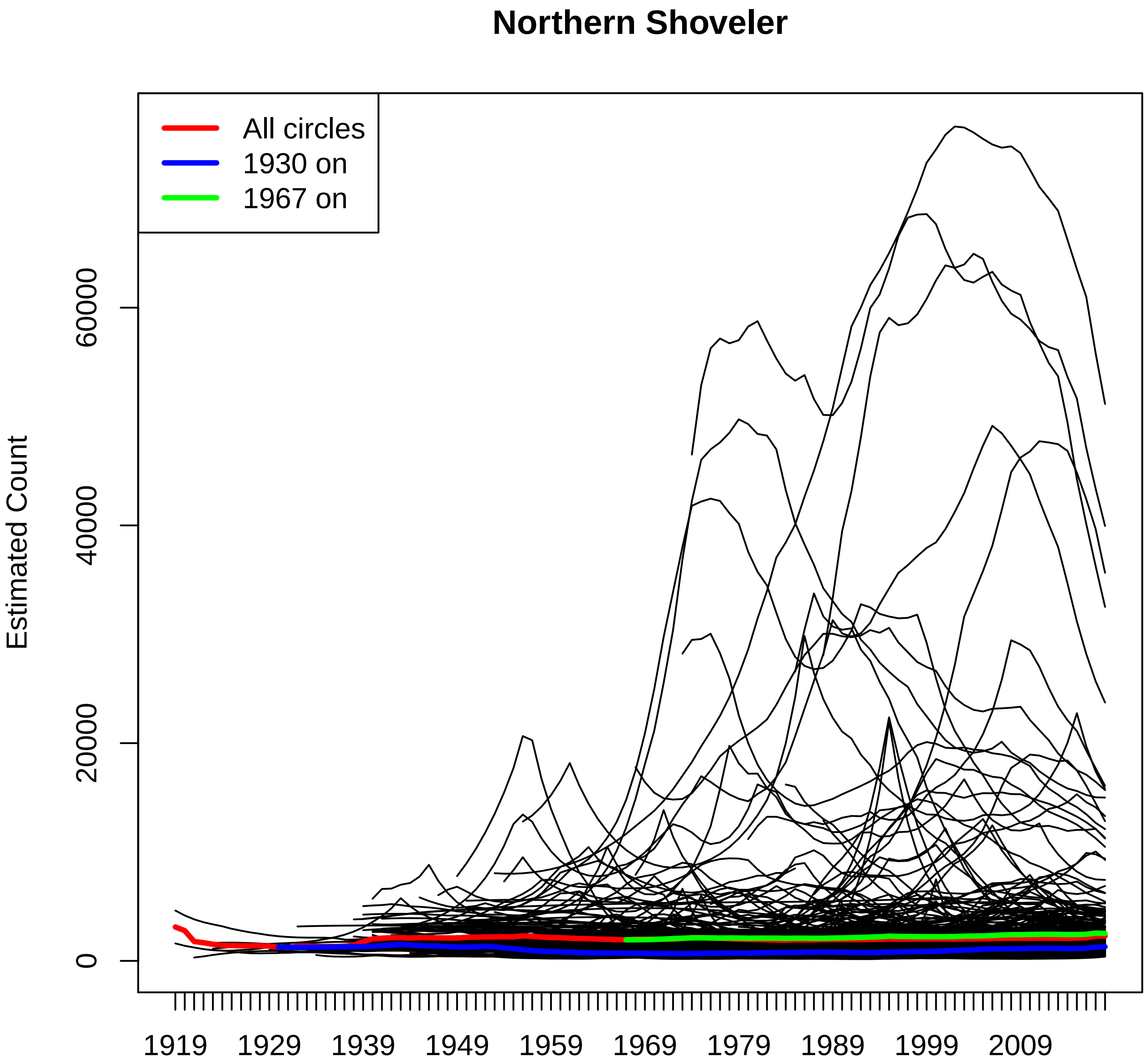
Northern Shoveler
All circles
1930 on
1967 on
Estimated Count
0
20000
40000
60000
1919
1929
1939
1949
1959
1969
1979
1989
1999
2009

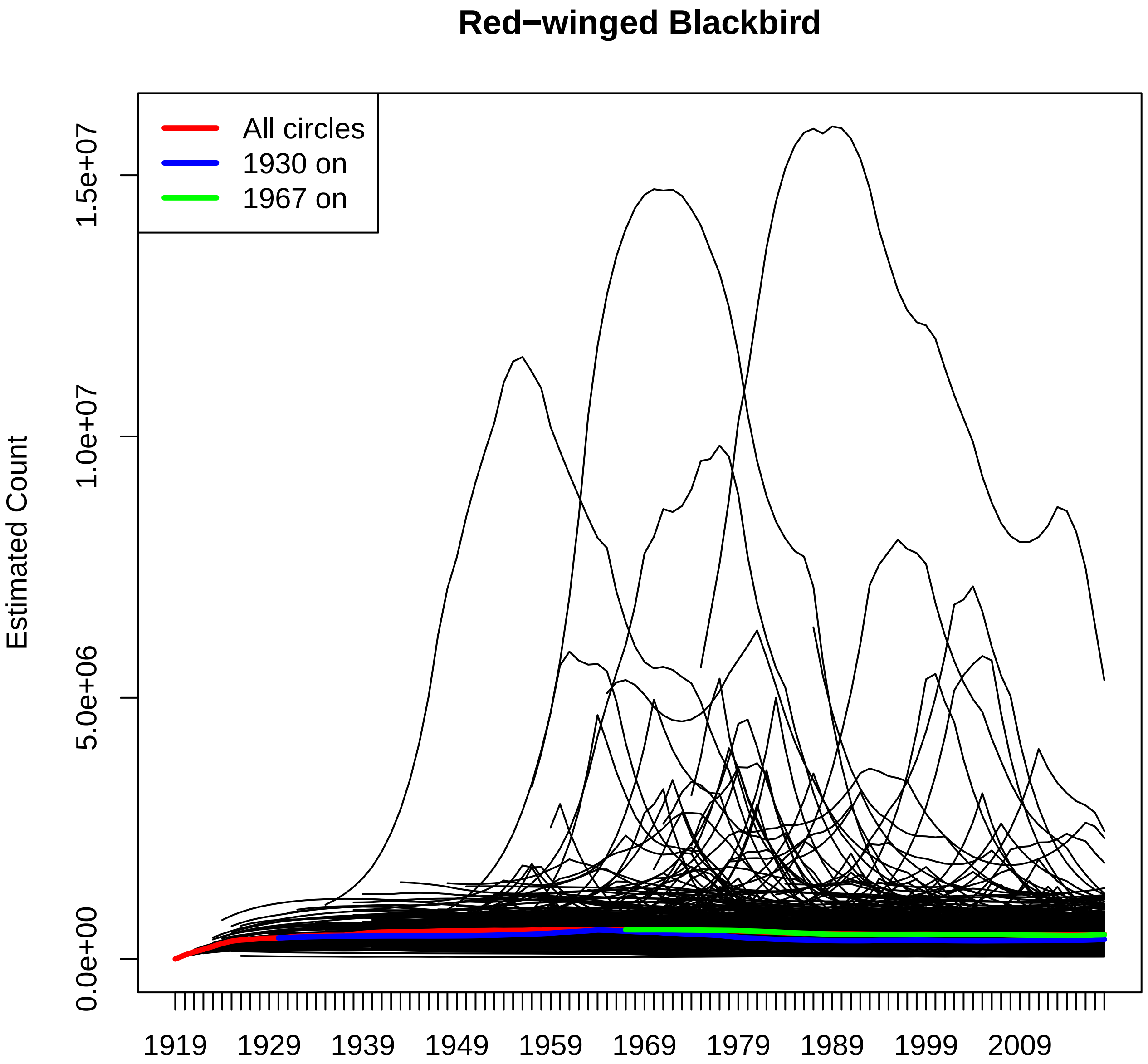
Red−winged Blackbird
Estimated Count
0.0e+00
5.0e+06
1.0e+07
1.5e+07
1919
1929
1939
1949
1959
1969
1979
1989
1999
2009
All circles
1930 on
1967 on

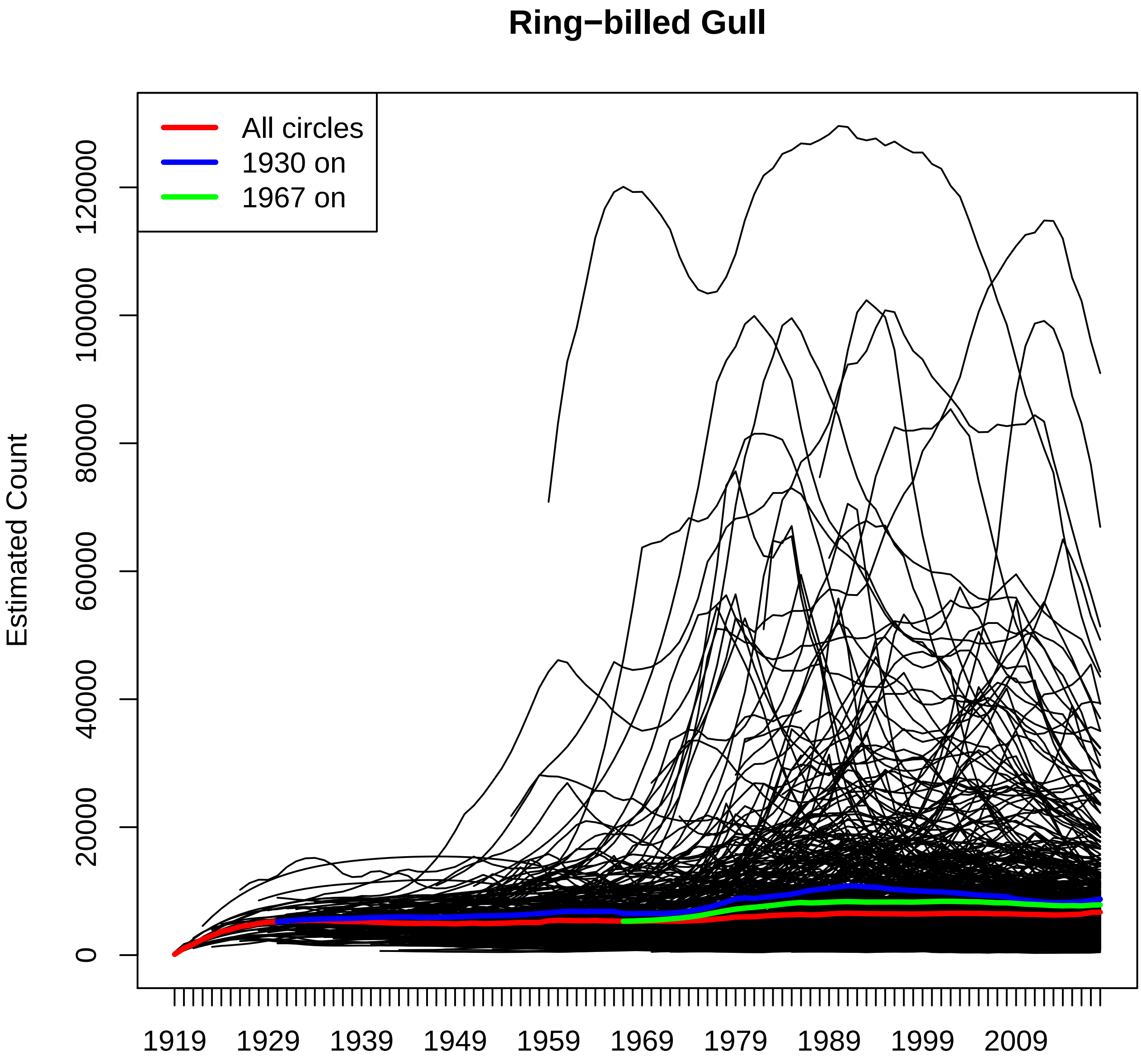
Ring−billed Gull
All circles
1930 on
1967 on
Estimated Count
0
20000
40000
60000
80000
100000
120000
1919
1929
1939
1949
1959
1969
1979
1989
1999
2009

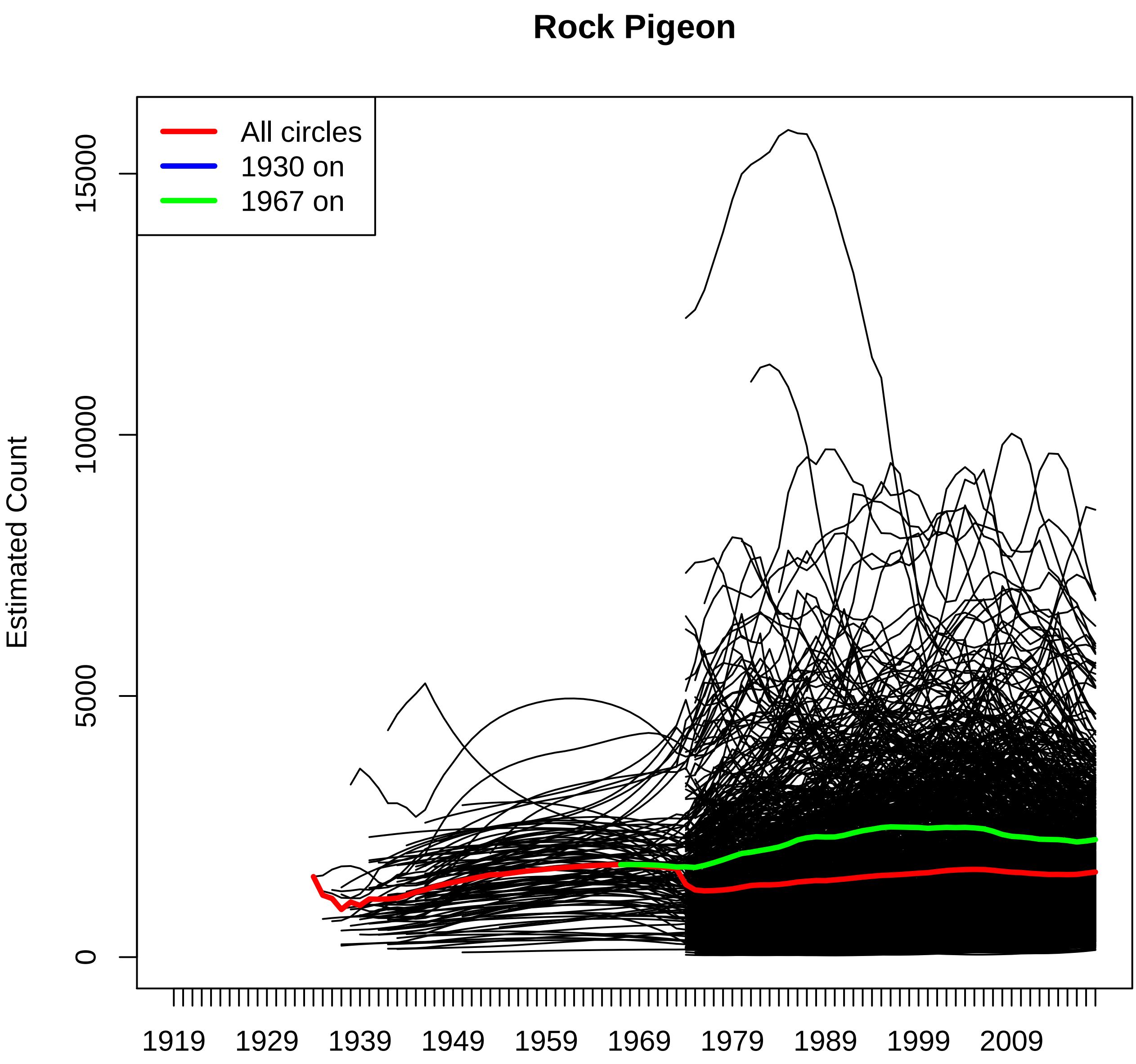

Rock Pigeon
All circles
1930 on
1967 on
Estimated Count
0
5000
10000
15000
1919
1929
1939
1949
1959
1969
1979
1989
1999
2009

Ruddy Duck

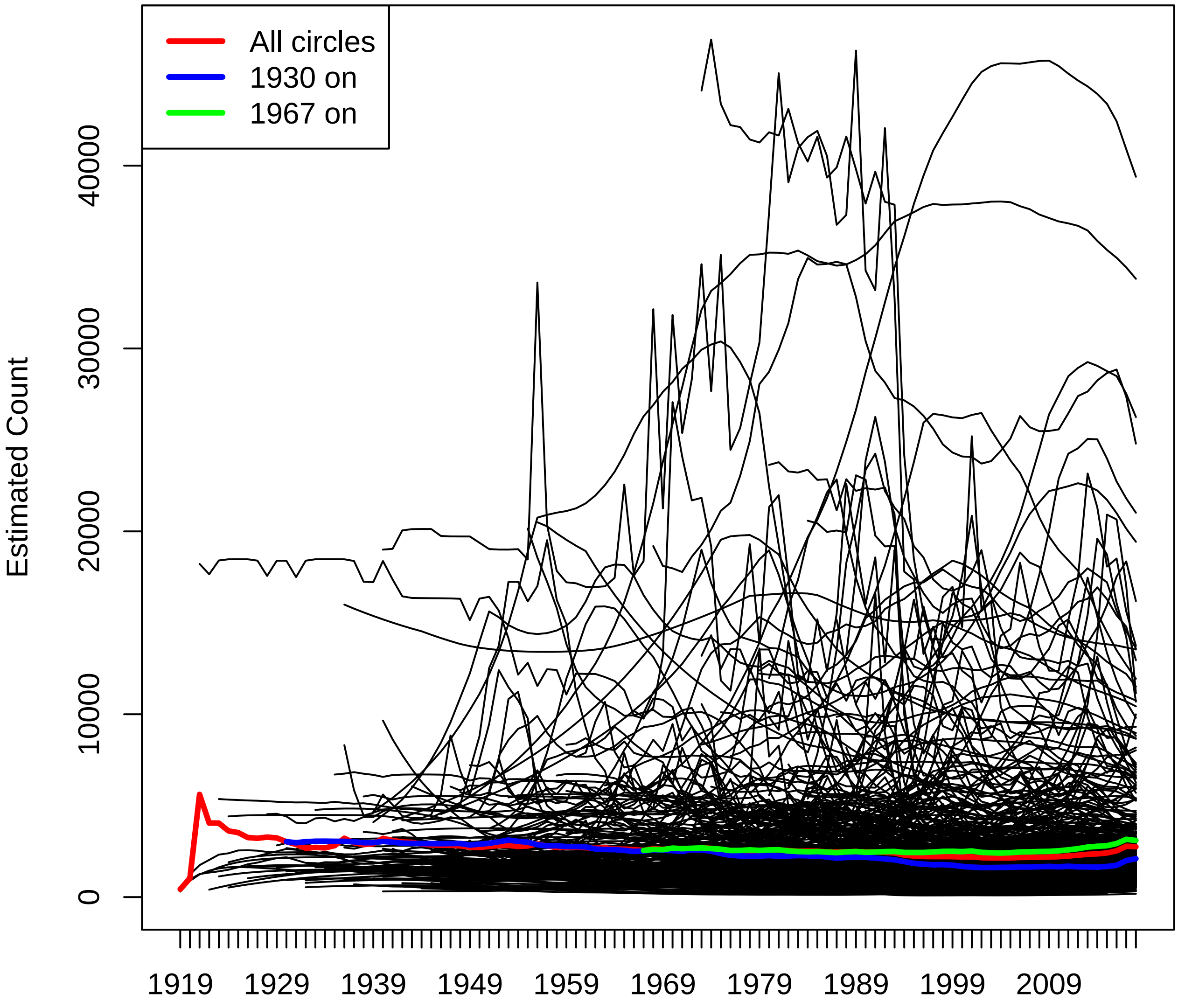

All circles
1930 on
1967 on
Estimated Count
0
10000
20000
30000
40000
1919
1929
1939
1949
1959
1969
1979
1989
1999
2009

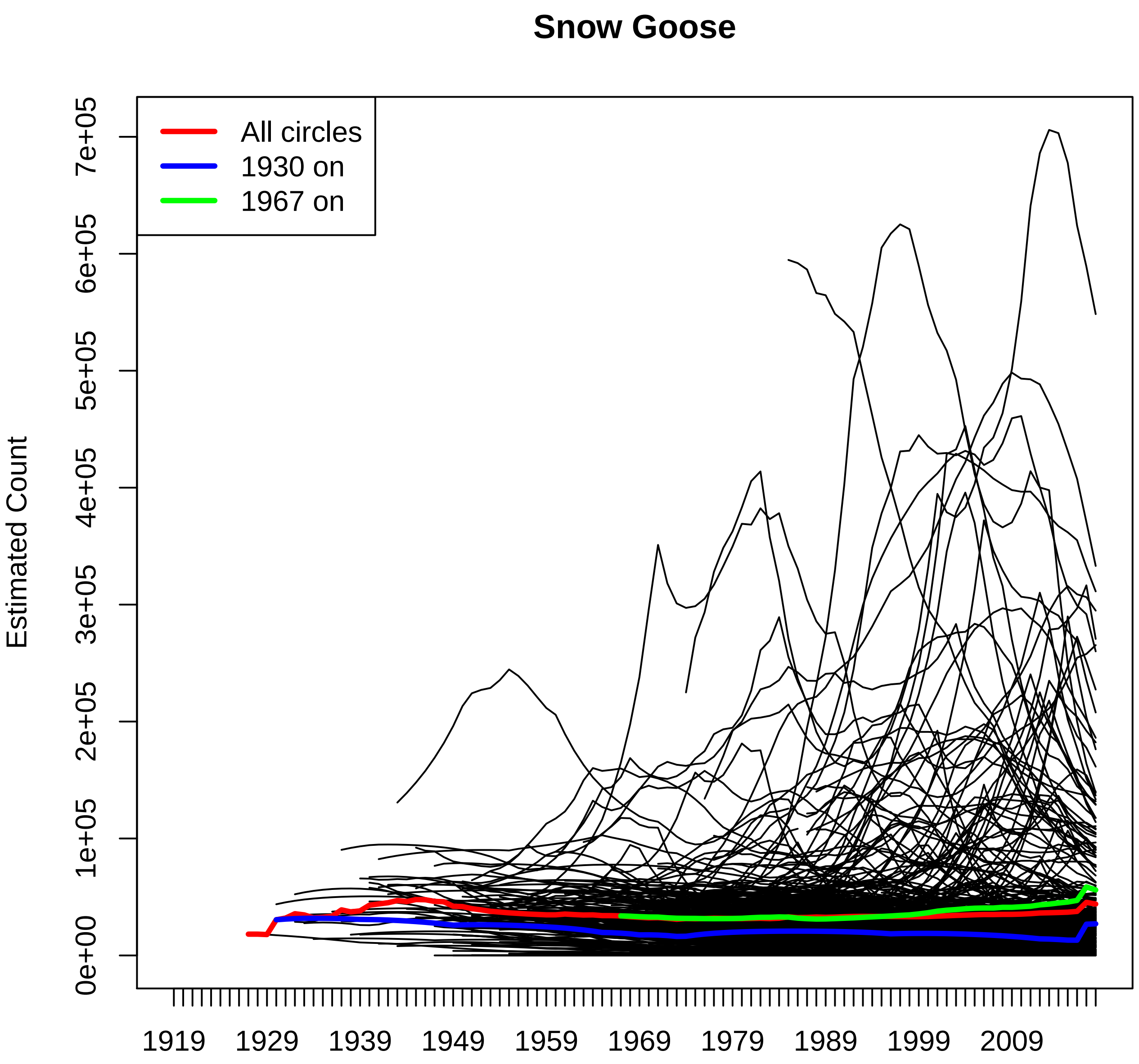
Snow Goose
All circles
1930 on
1967 on
Estimated Count
0e+00
1e+05
2e+05
3e+05
4e+05
5e+05
6e+05
7e+05
1919
1929
1939
1949
1959
1969
1979
1989
1999
2009

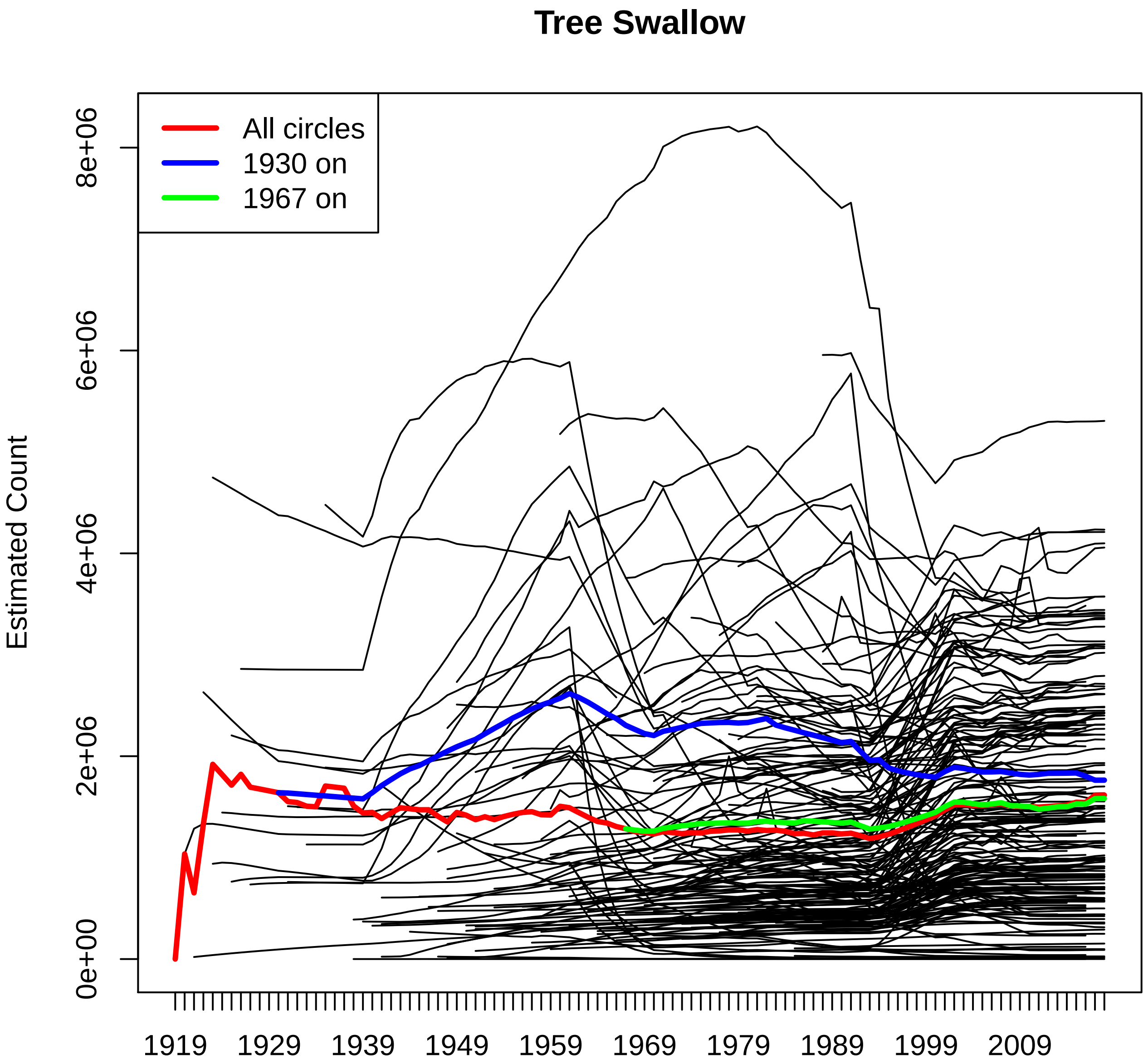
Tree Swallow
Estimated Count
All circles
1930 on
1967 on
0e+00
2e+06
4e+06
6e+06
8e+06
1919
1929
1939
1949
1959
1969
1979
1989
1999
2009

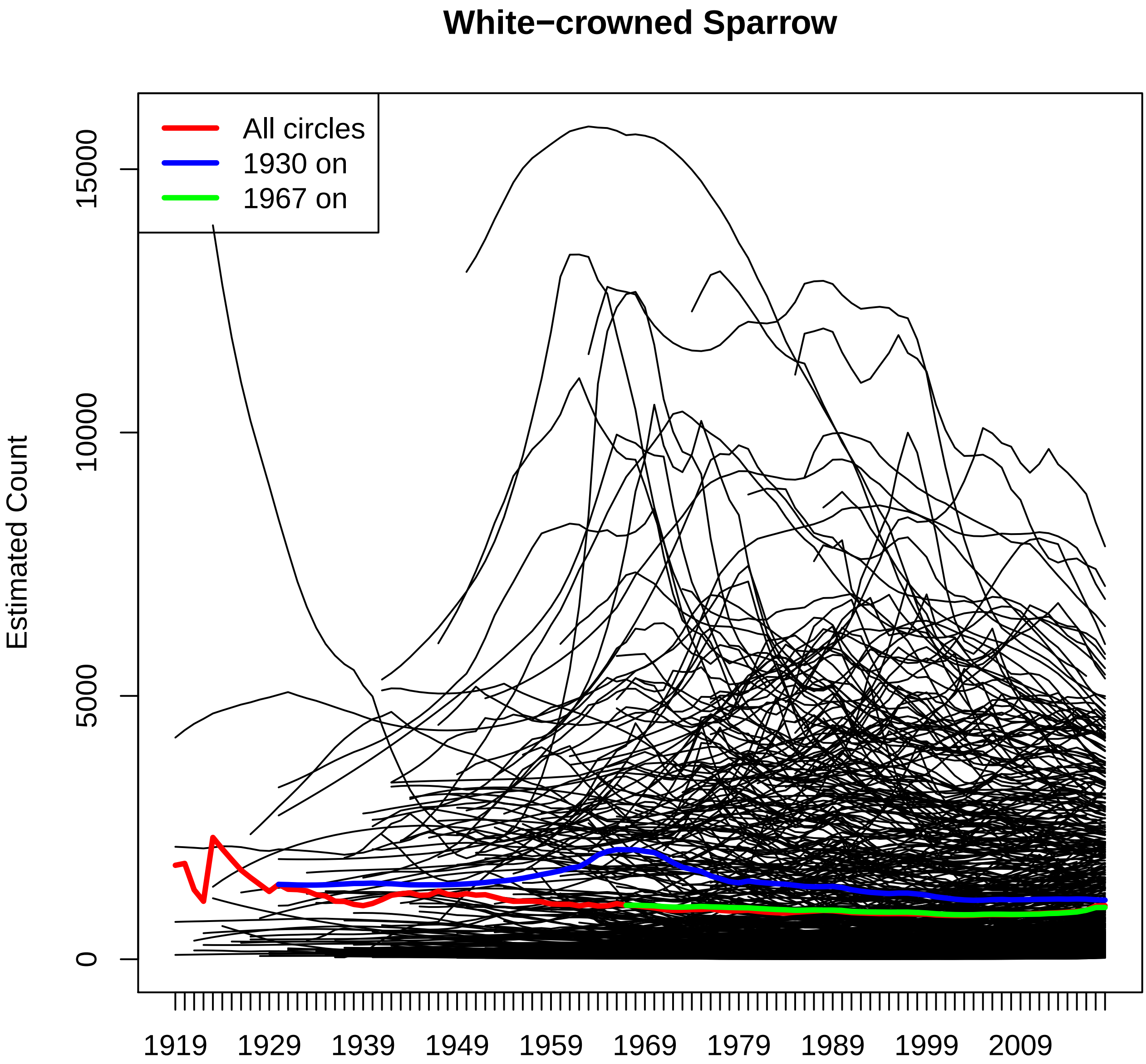

White−crowned Sparrow
Estimated Count
0
5000
10000
15000
1919
1929
1939
1949
1959
1969
1979
1989
1999
2009
All circles
1930 on
1967 on

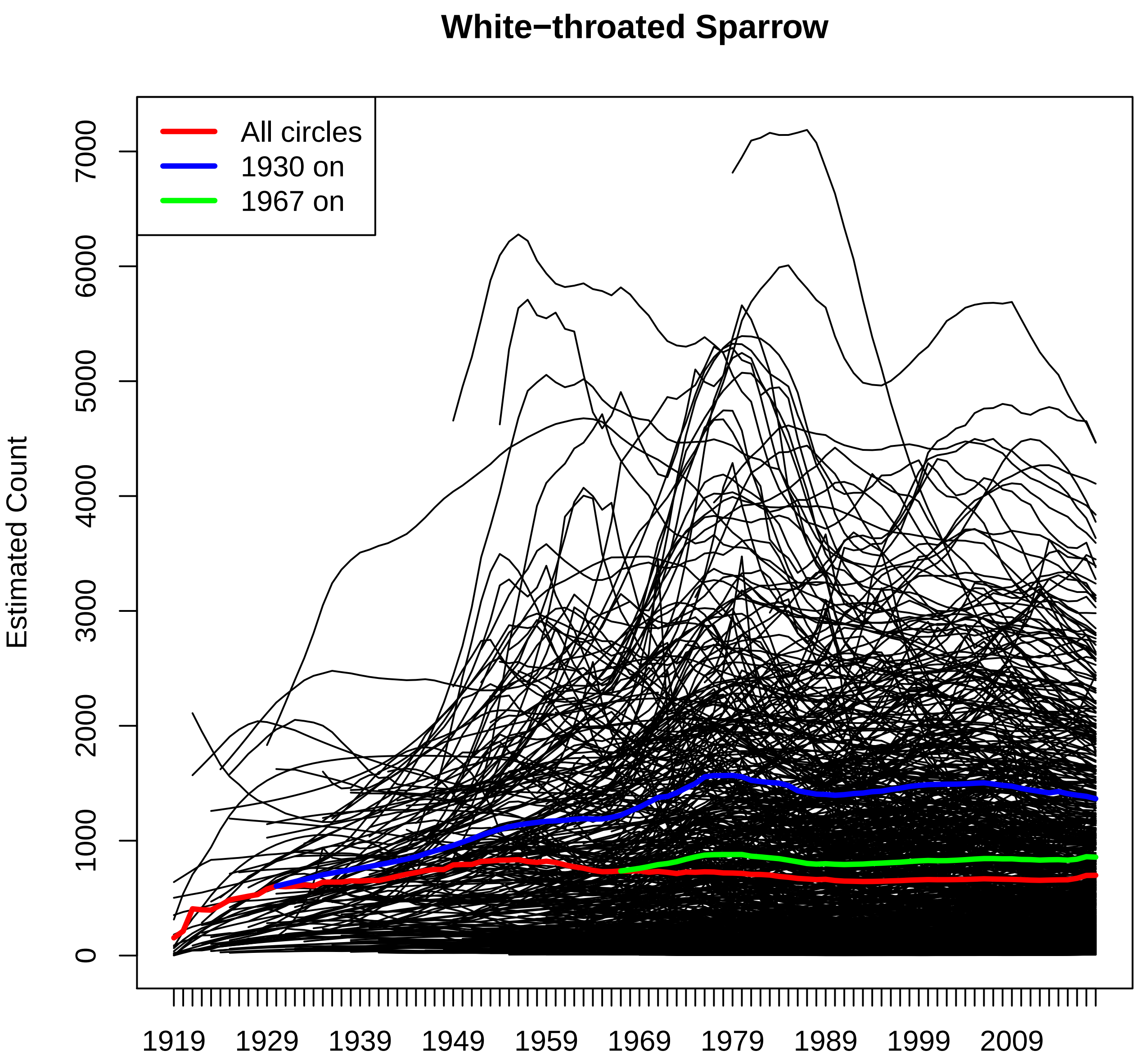

White−throated Sparrow
All circles
1930 on
1967 on
Estimated Count
0
1000
2000
3000
4000
5000
6000
7000
1919
1929
1939
1949
1959
1969
1979
1989
1999
2009

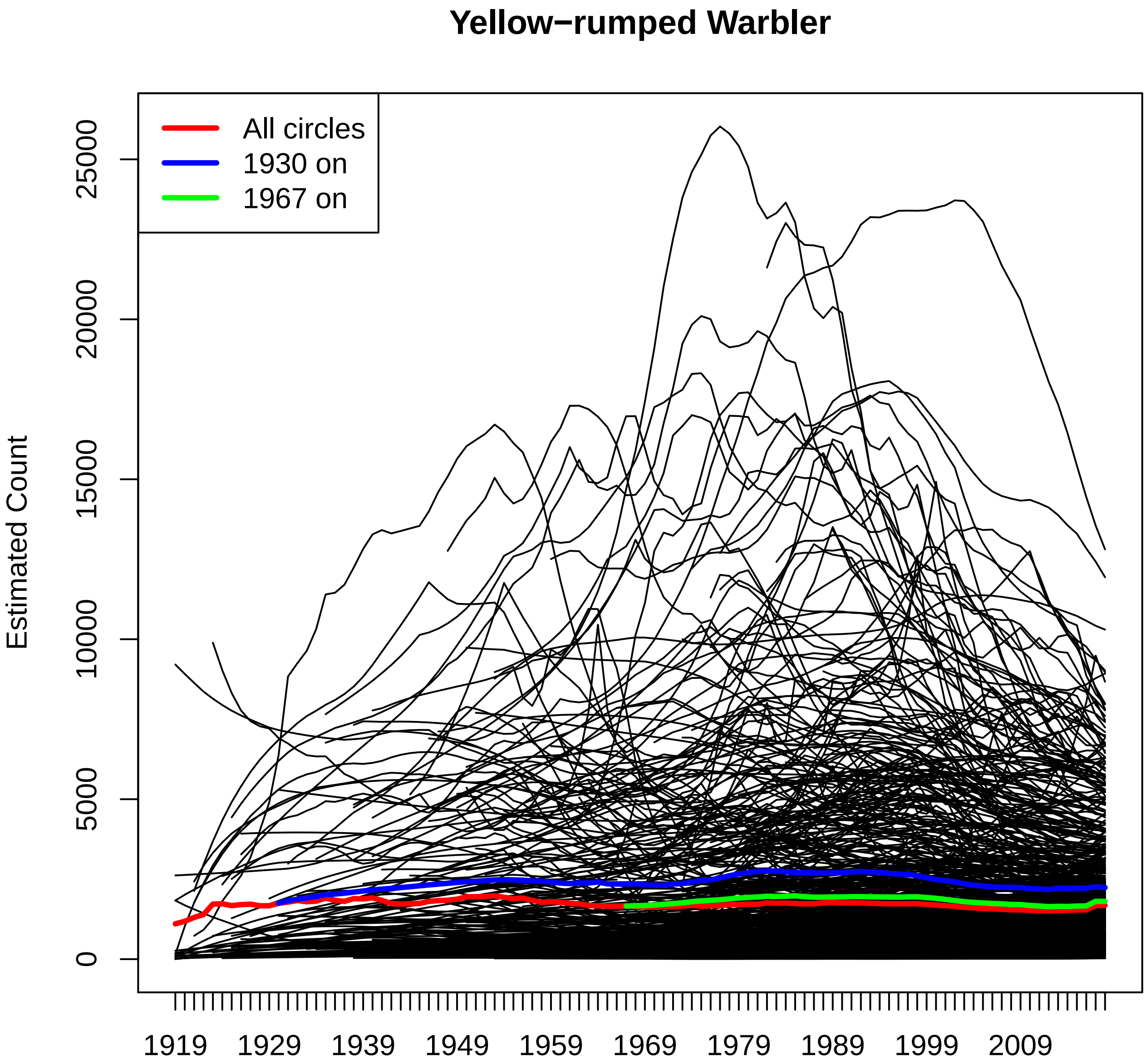
Yellow−rumped Warbler
All circles
1930 on
1967 on
Estimated Count
0
5000
10000
15000
20000
25000
1919
1929
1939
1949
1959
1969
1979
1989
1999
2009

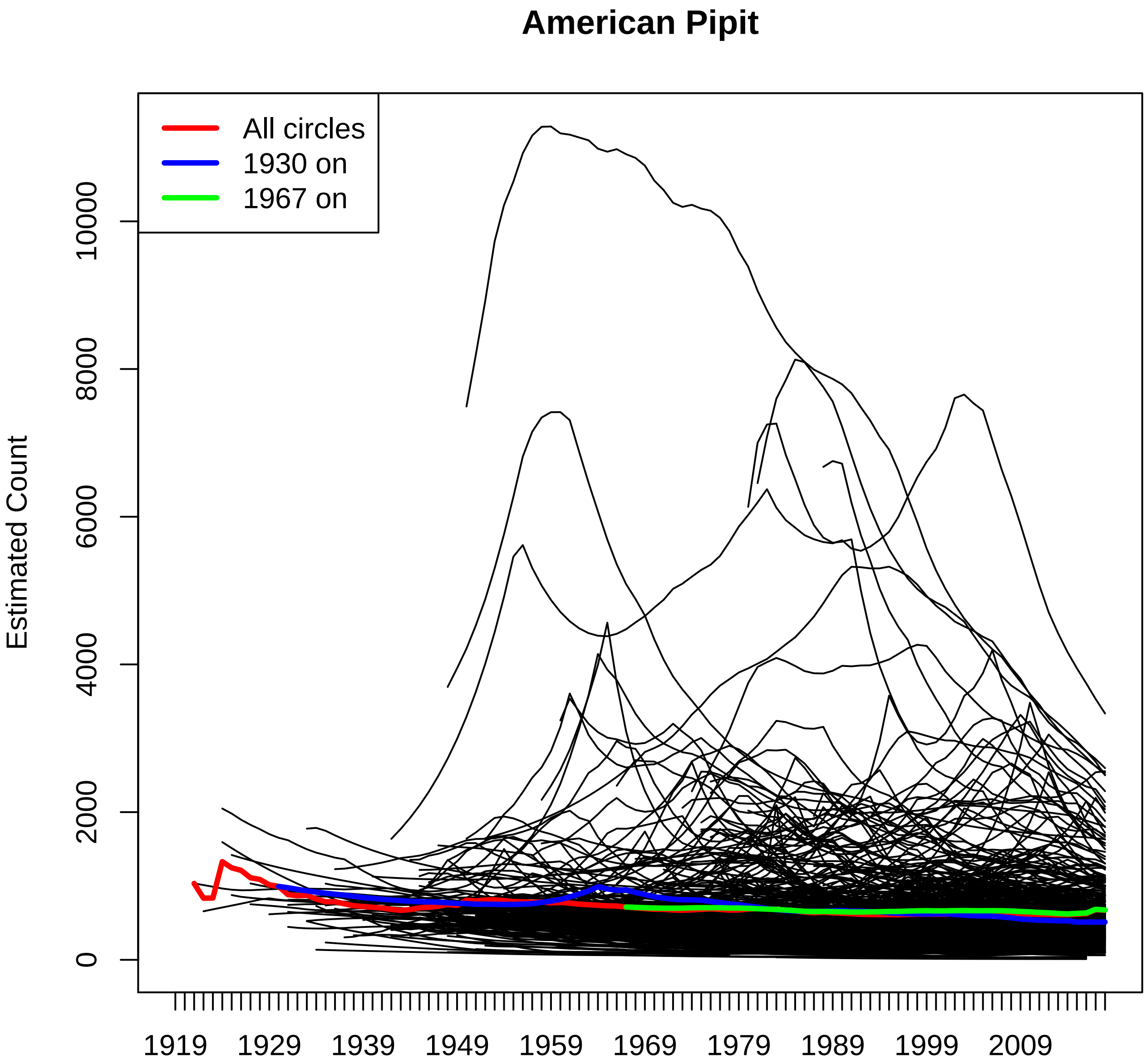
American Pipit
All circles
1930 on
1967 on
Estimated Count
0
2000
4000
6000
8000
10000
1919
1929
1939
1949
1959
1969
1979
1989
1999
2009

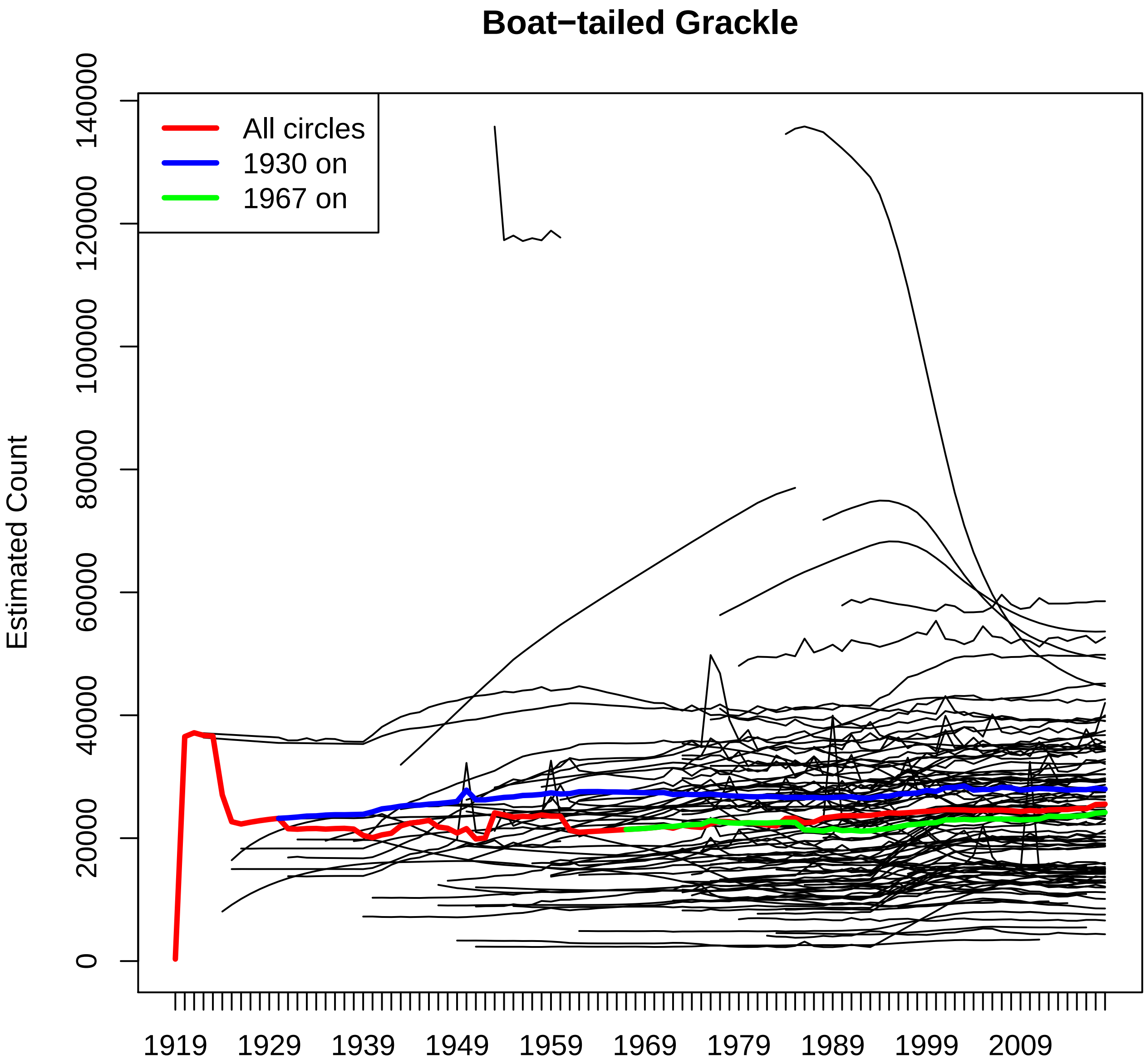

Boat−tailed Grackle
Estimated Count
0
20000
40000
60000
80000
100000
120000
140000
All circles
1930 on
1967 on
1919
1929
1939
1949
1959
1969
1979
1989
1999
2009

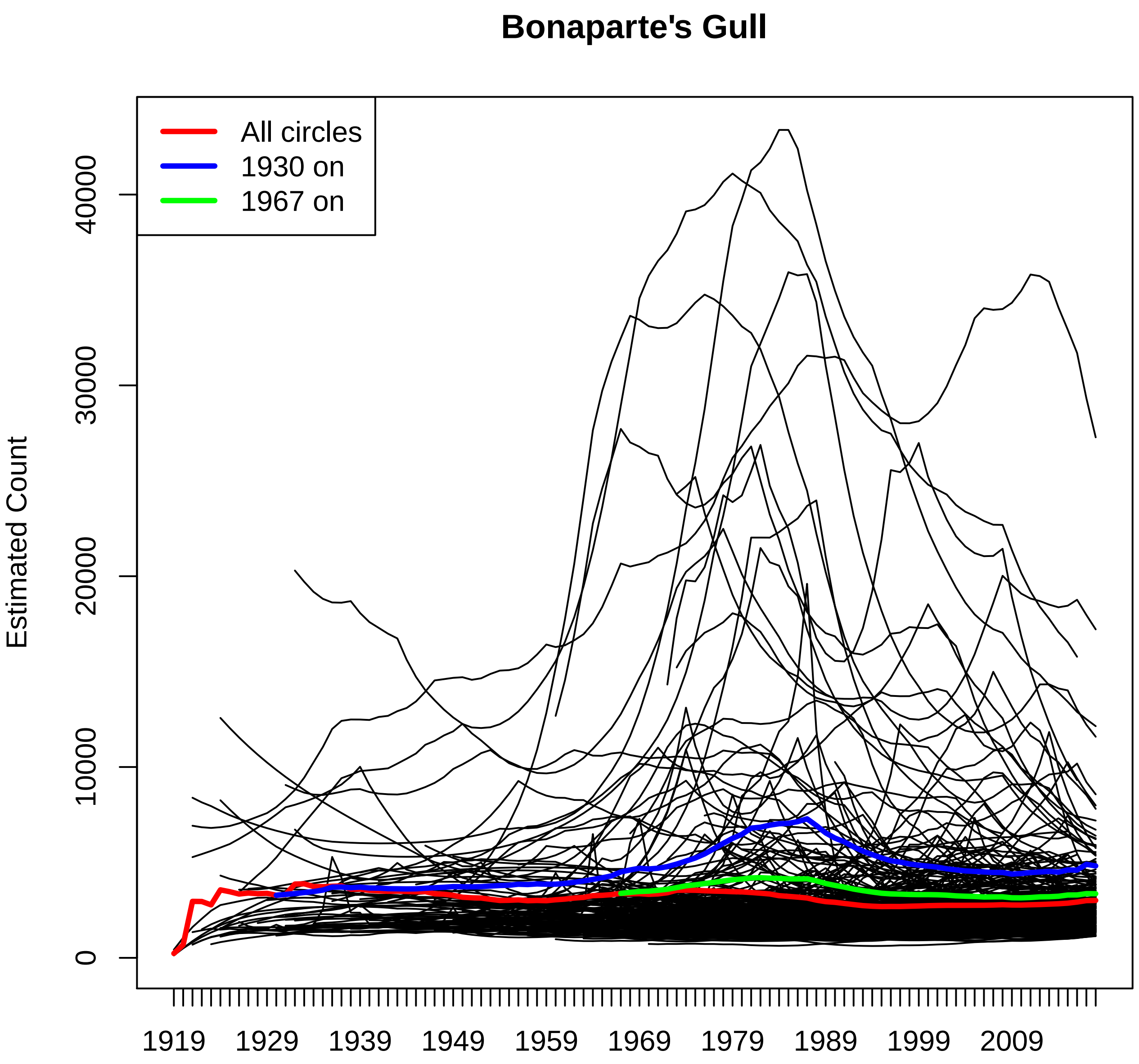

Bonaparte's Gull
All circles
1930 on
1967 on
Estimated Count
0
10000
20000
30000
40000
1919
1929
1939
1949
1959
1969
1979
1989
1999
2009

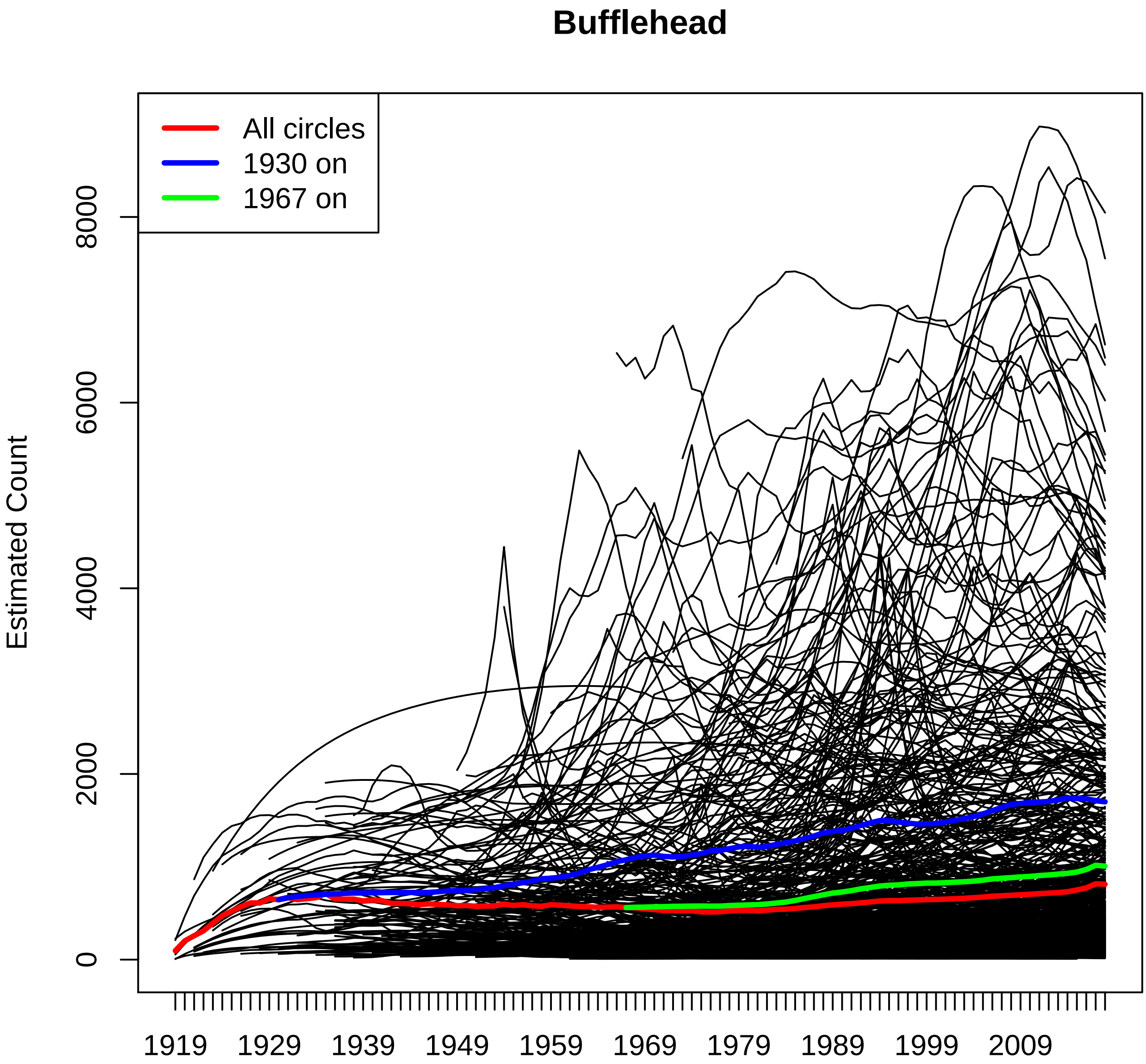

Bufflehead
All circles
1930 on
1967 on
Estimated Count
0
2000
4000
6000
8000
1919
1929
1939
1949
1959
1969
1979
1989
1999
2009

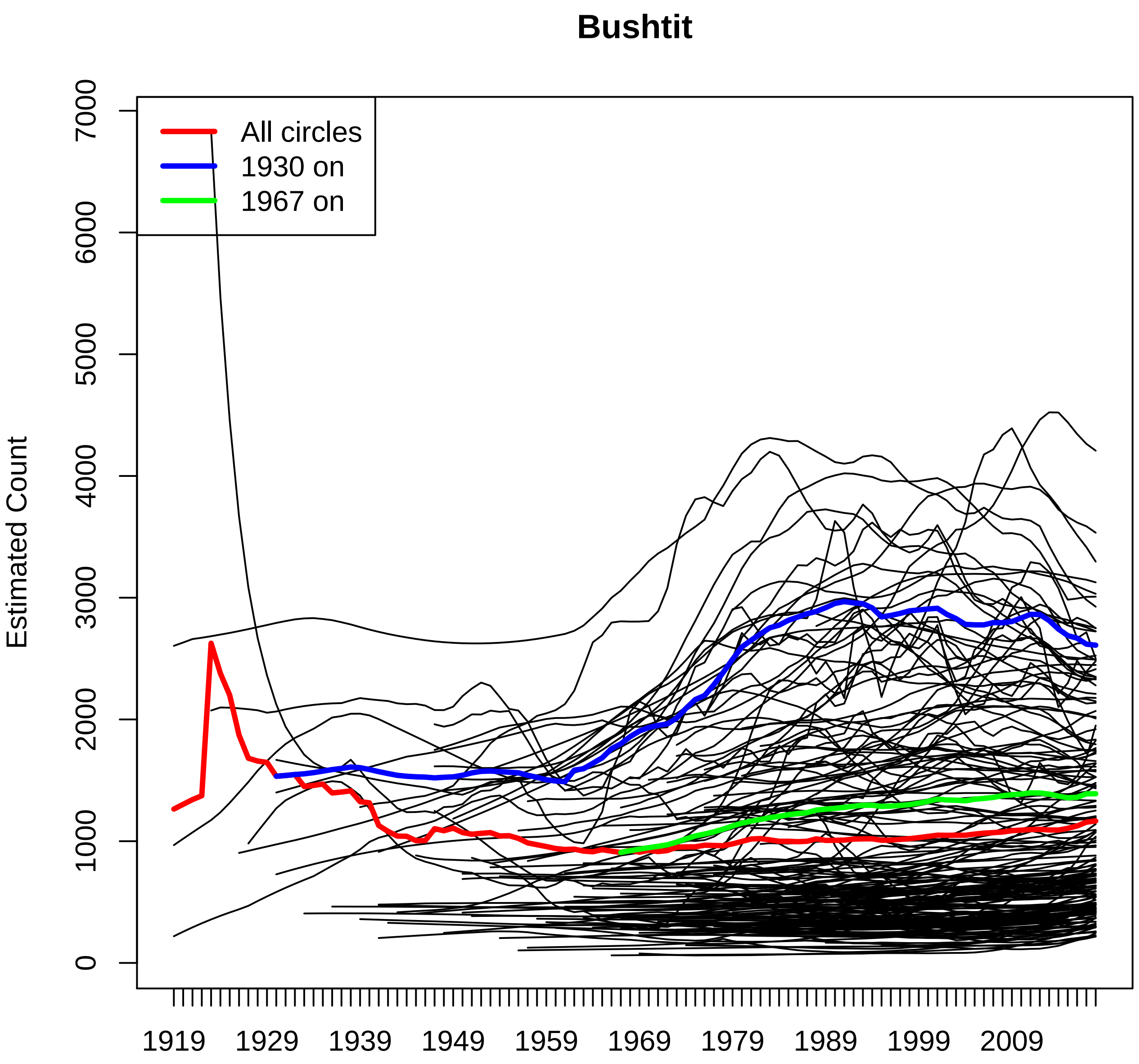
Bushtit
All circles
1930 on
1967 on
Estimated Count
0
1000
2000
3000
4000
5000
6000
7000
1919
1929
1939
1949
1959
1969
1979
1989
1999
2009

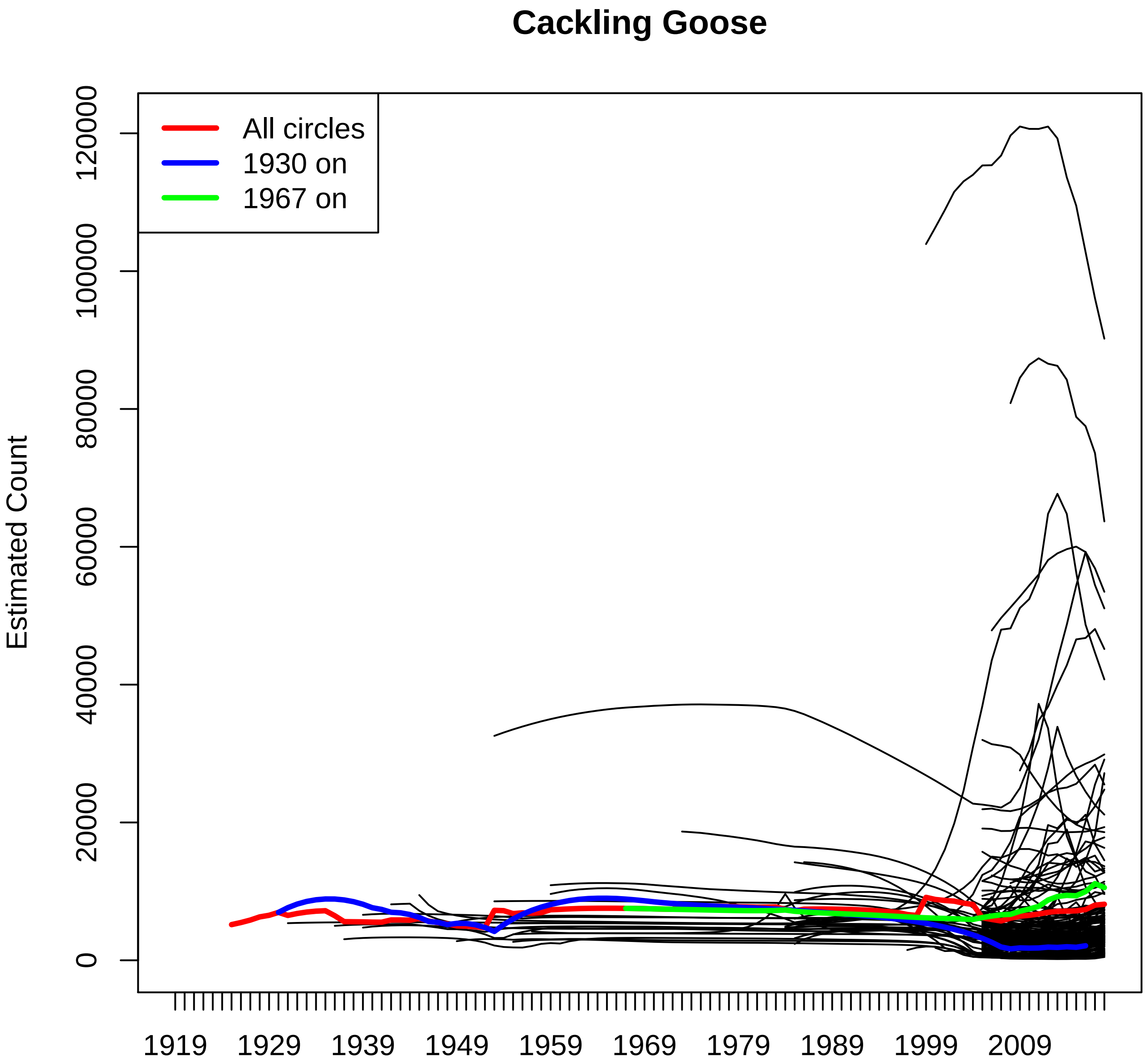
Cackling Goose
All circles
1930 on
1967 on
Estimated Count
0
20000
40000
60000
80000
100000
120000
1919
1929
1939
1949
1959
1969
1979
1989
1999
2009

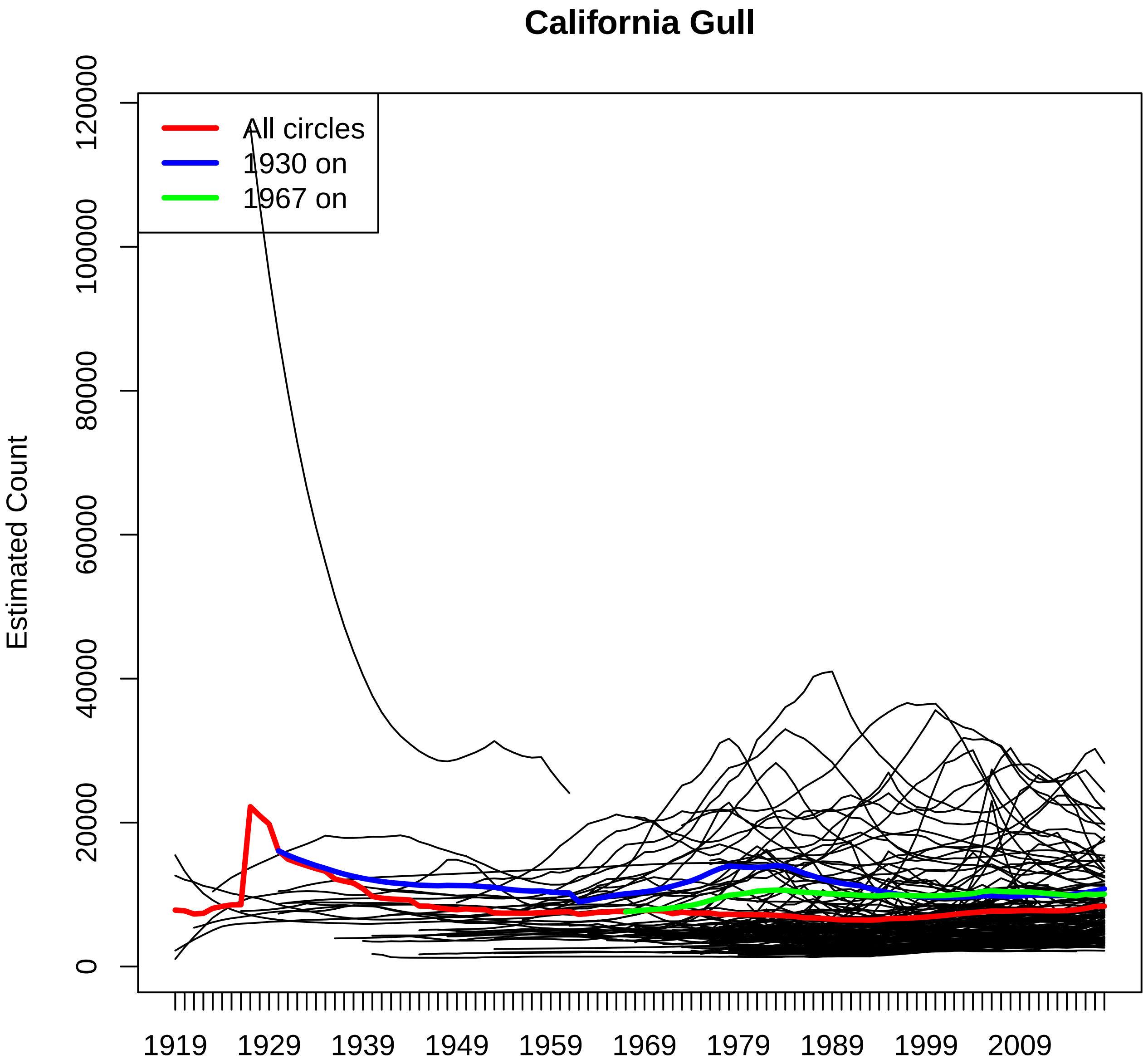
California Gull
Estimated Count
0
20000
40000
60000
80000
100000
120000
All circles
1930 on
1967 on
1919
1929
1939
1949
1959
1969
1979
1989
1999
2009

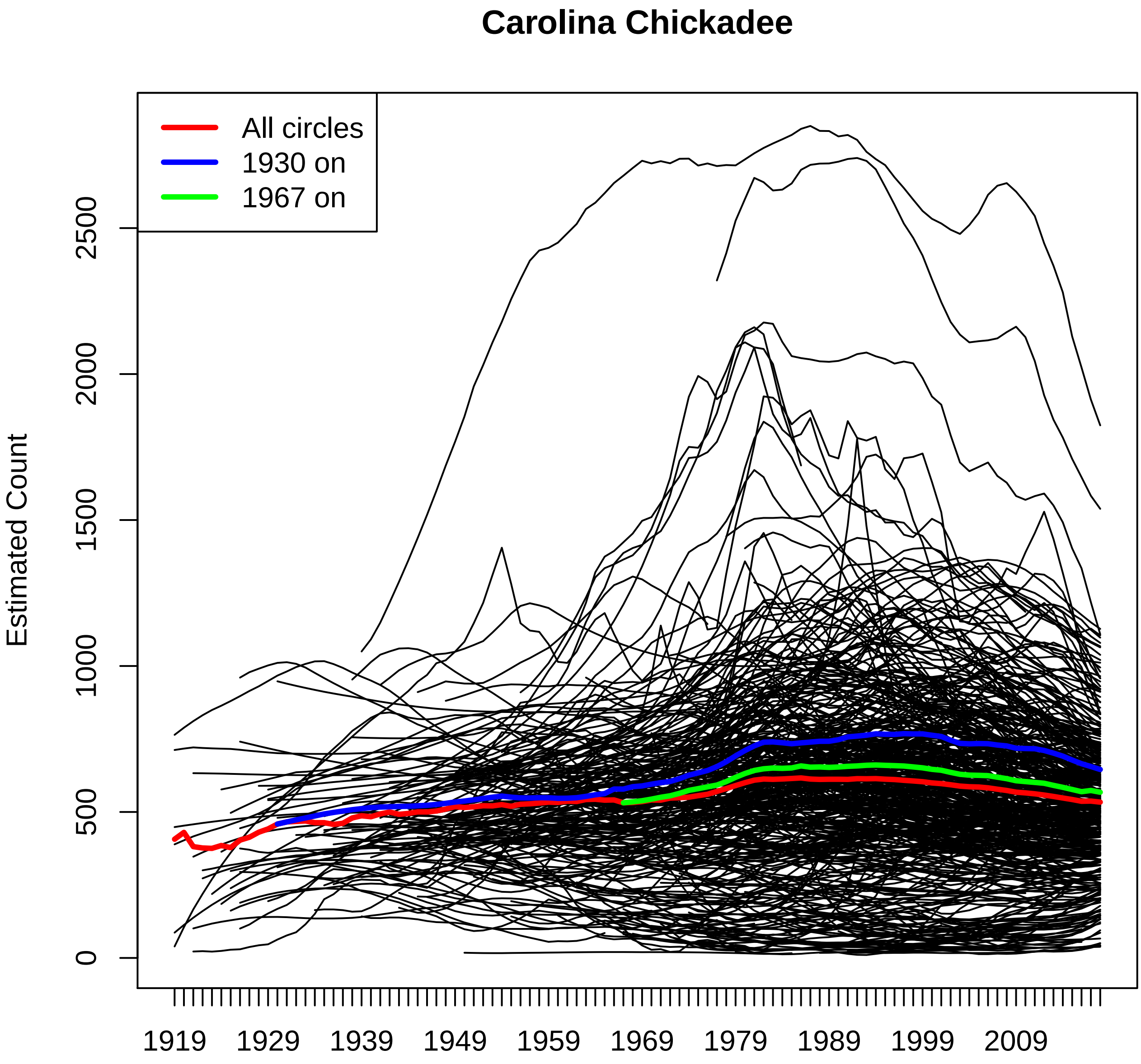
Carolina Chickadee
All circles
1930 on
1967 on
Estimated Count
0
500
1000
1500
2000
2500
1919
1929
1939
1949
1959
1969
1979
1989
1999
2009

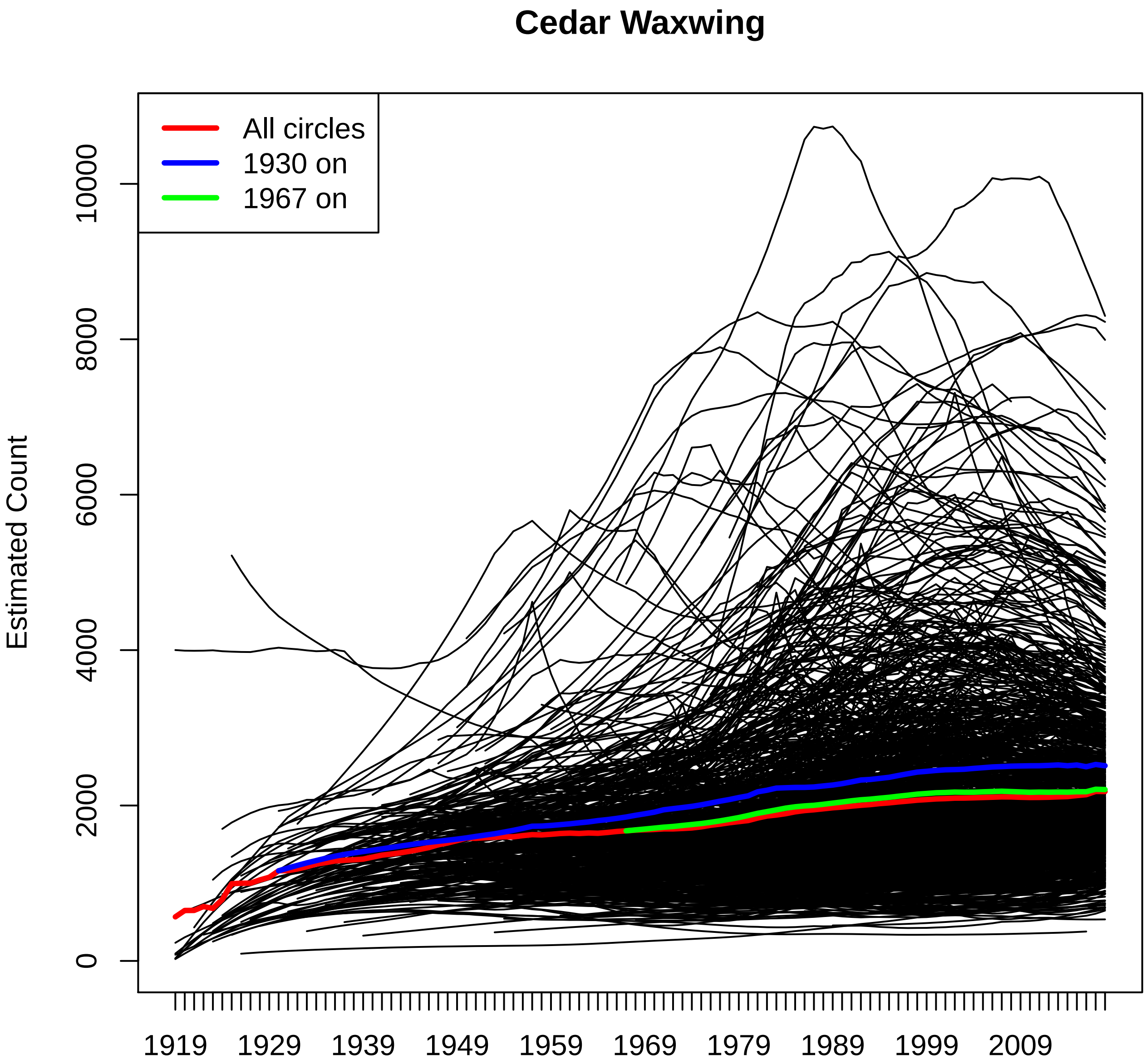
Cedar Waxwing
All circles
1930 on
1967 on
Estimated Count
0
2000
4000
6000
8000
10000
1919
1929
1939
1949
1959
1969
1979
1989
1999
2009

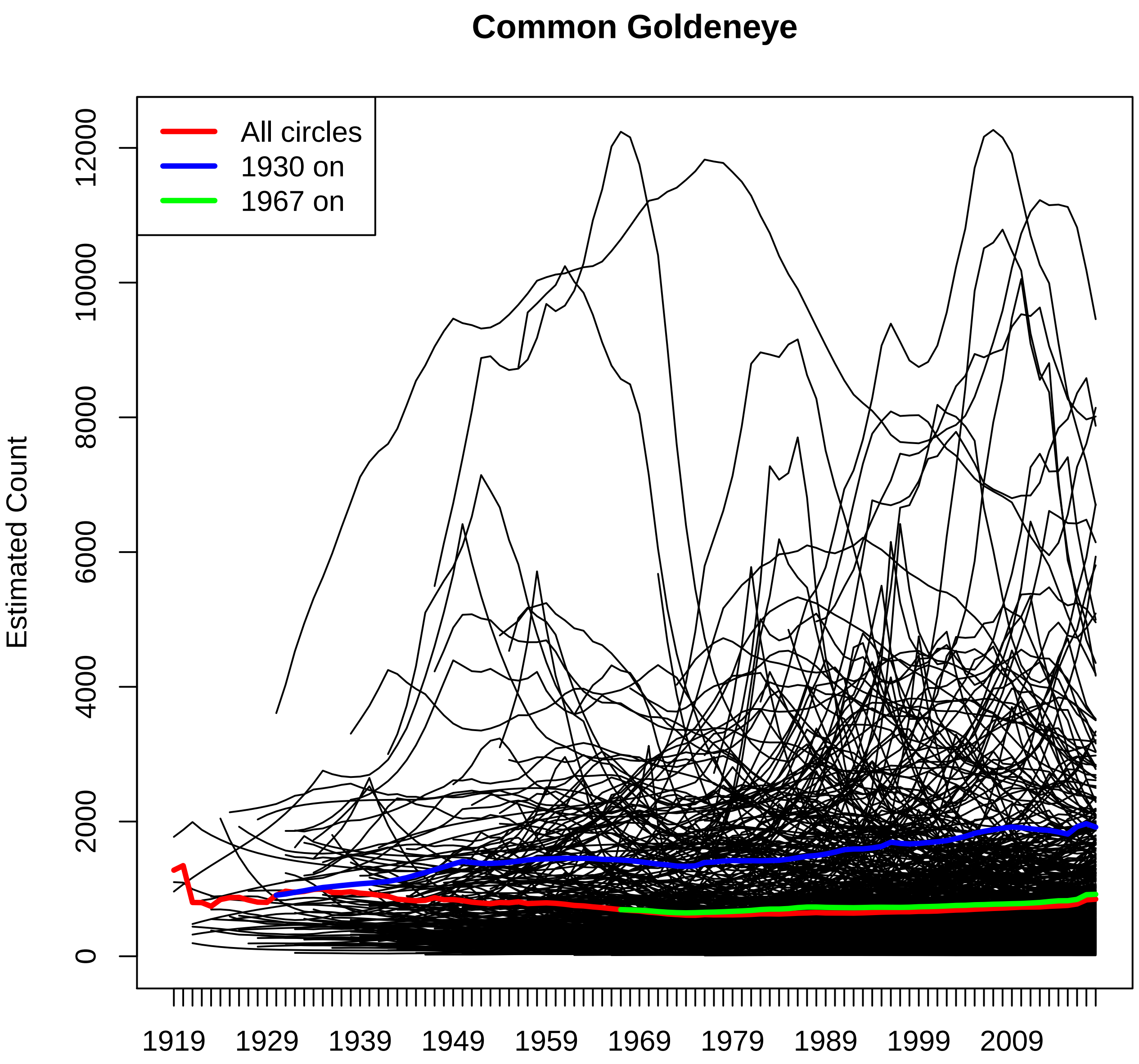

Common Goldeneye
Estimated Count
0
2000
4000
6000
8000
10000
12000
1919
1929
1939
1949
1959
1969
1979
1989
1999
2009
All circles
1930 on
1967 on

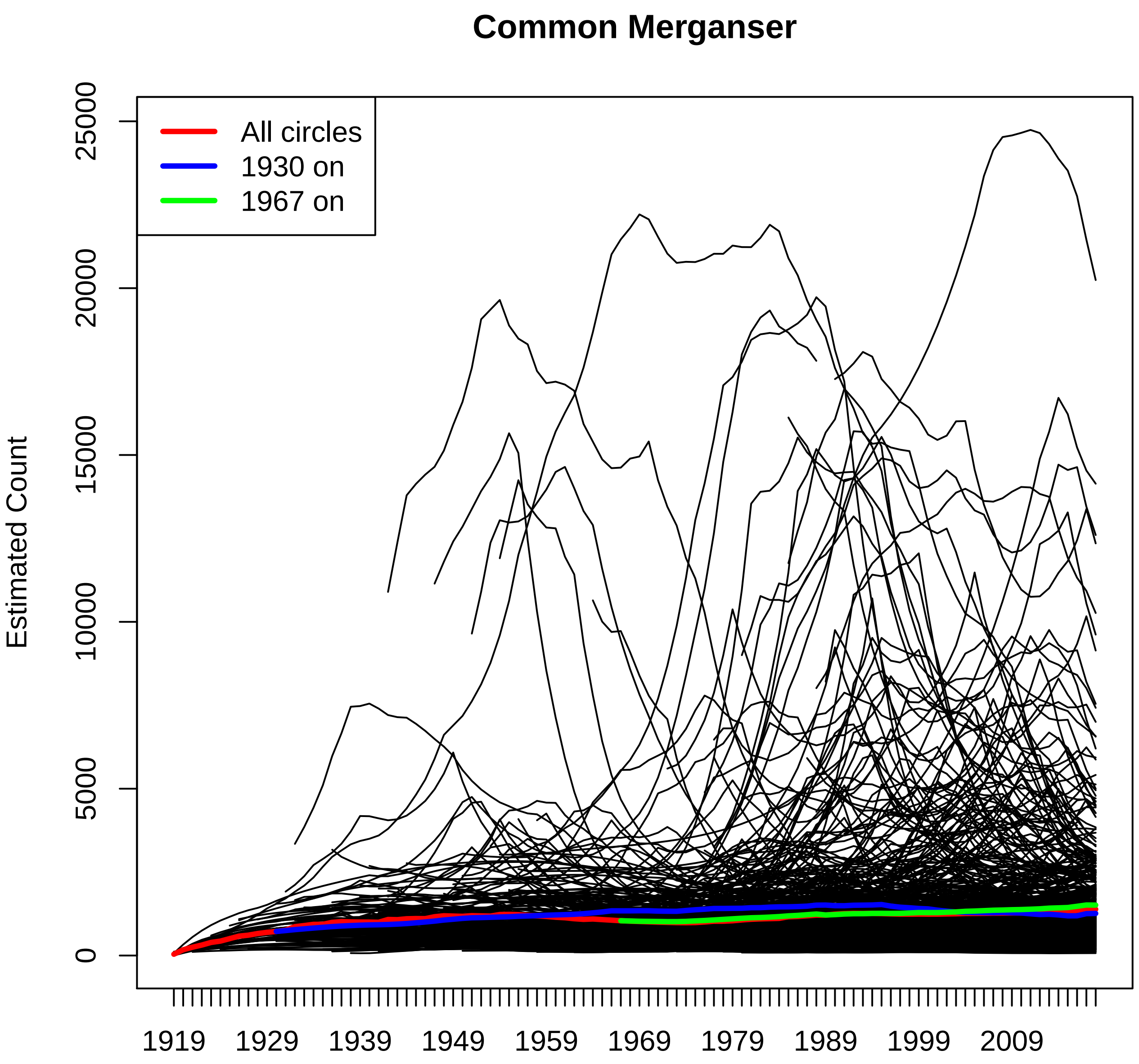
Common Merganser
All circles
1930 on
1967 on
Estimated Count
0
5000
10000
15000
20000
25000
1919
1929
1939
1949
1959
1969
1979
1989
1999
2009

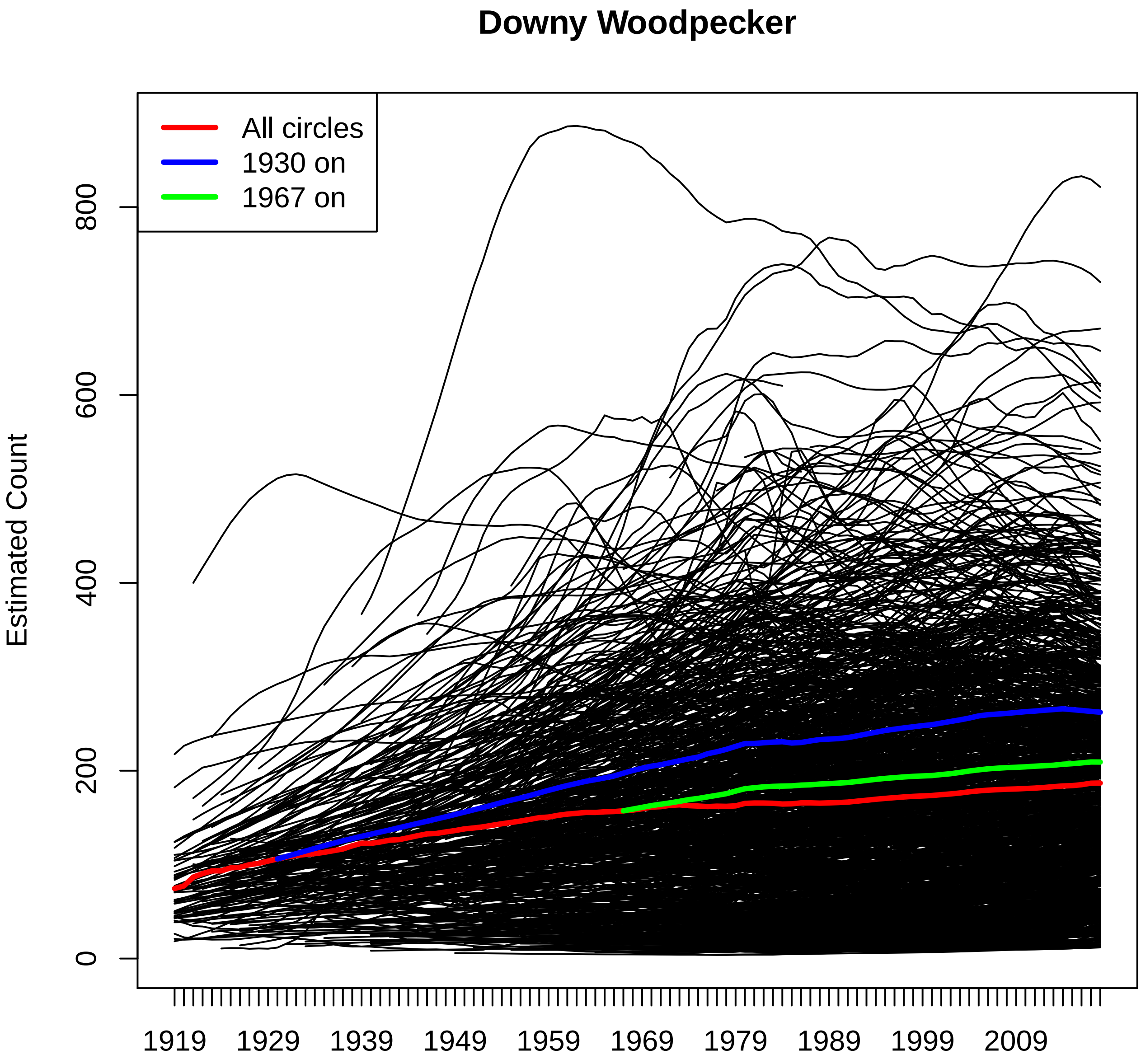
Downy Woodpecker
All circles
1930 on
1967 on
Estimated Count
0
200
400
600
800
1919
1929
1939
1949
1959
1969
1979
1989
1999
2009

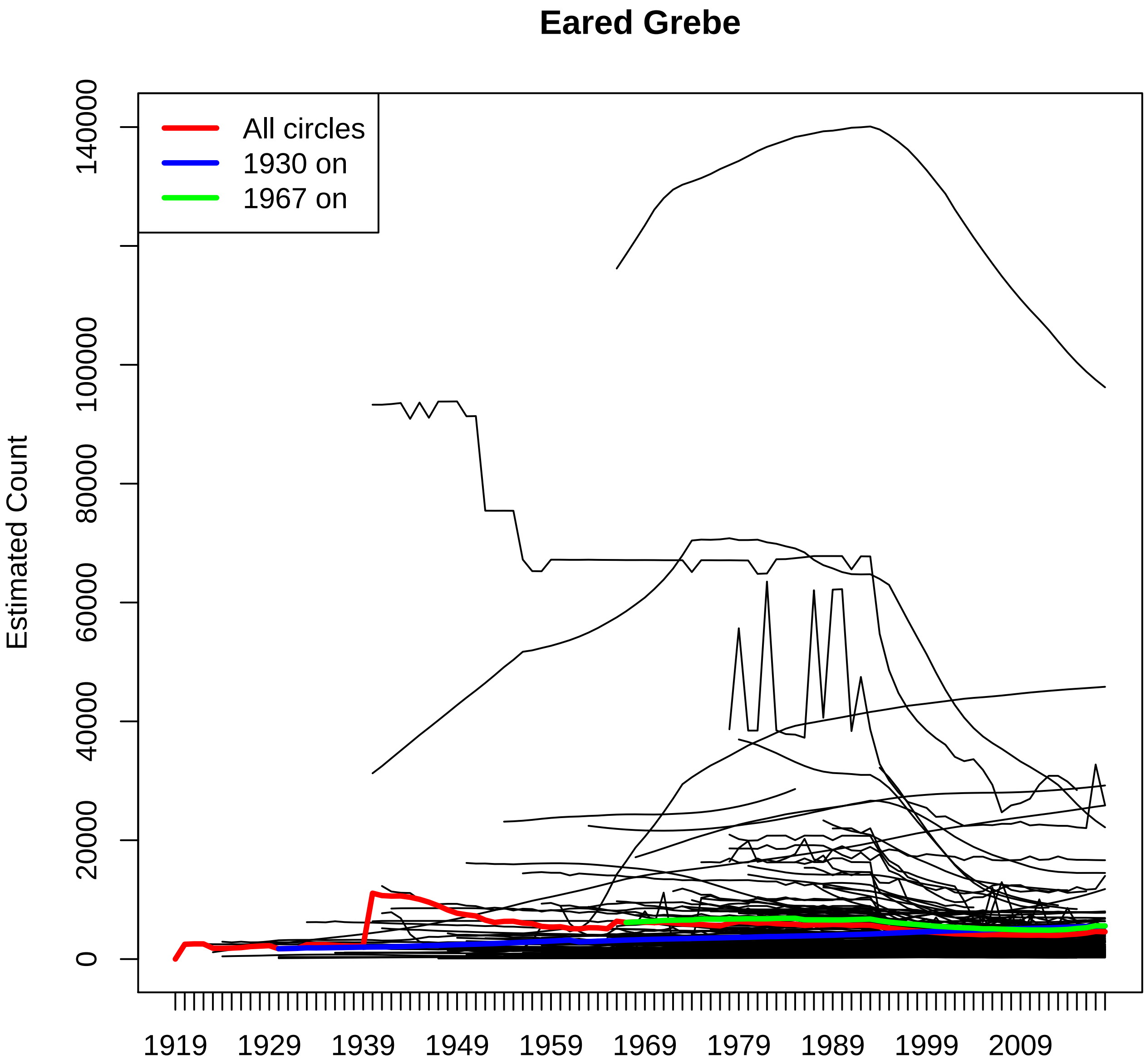
Eared Grebe
All circles
1930 on
1967 on
Estimated Count
0
20000
40000
60000
80000
100000
140000
1919
1929
1939
1949
1959
1969
1979
1989
1999
2009

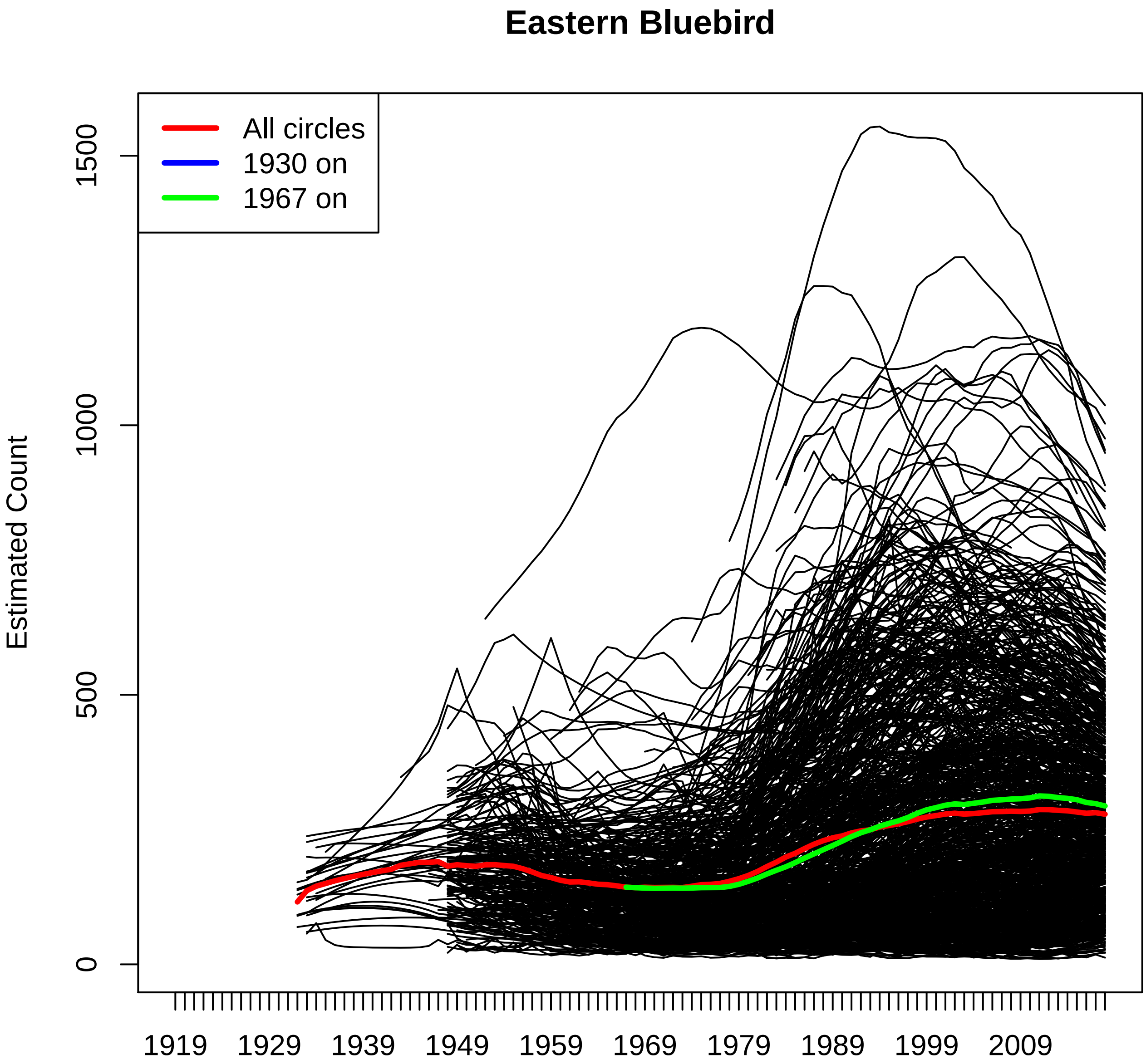

Eastern Bluebird
All circles
1930 on
1967 on
Estimated Count
0
500
1000
1500
1919
1929
1939
1949
1959
1969
1979
1989
1999
2009

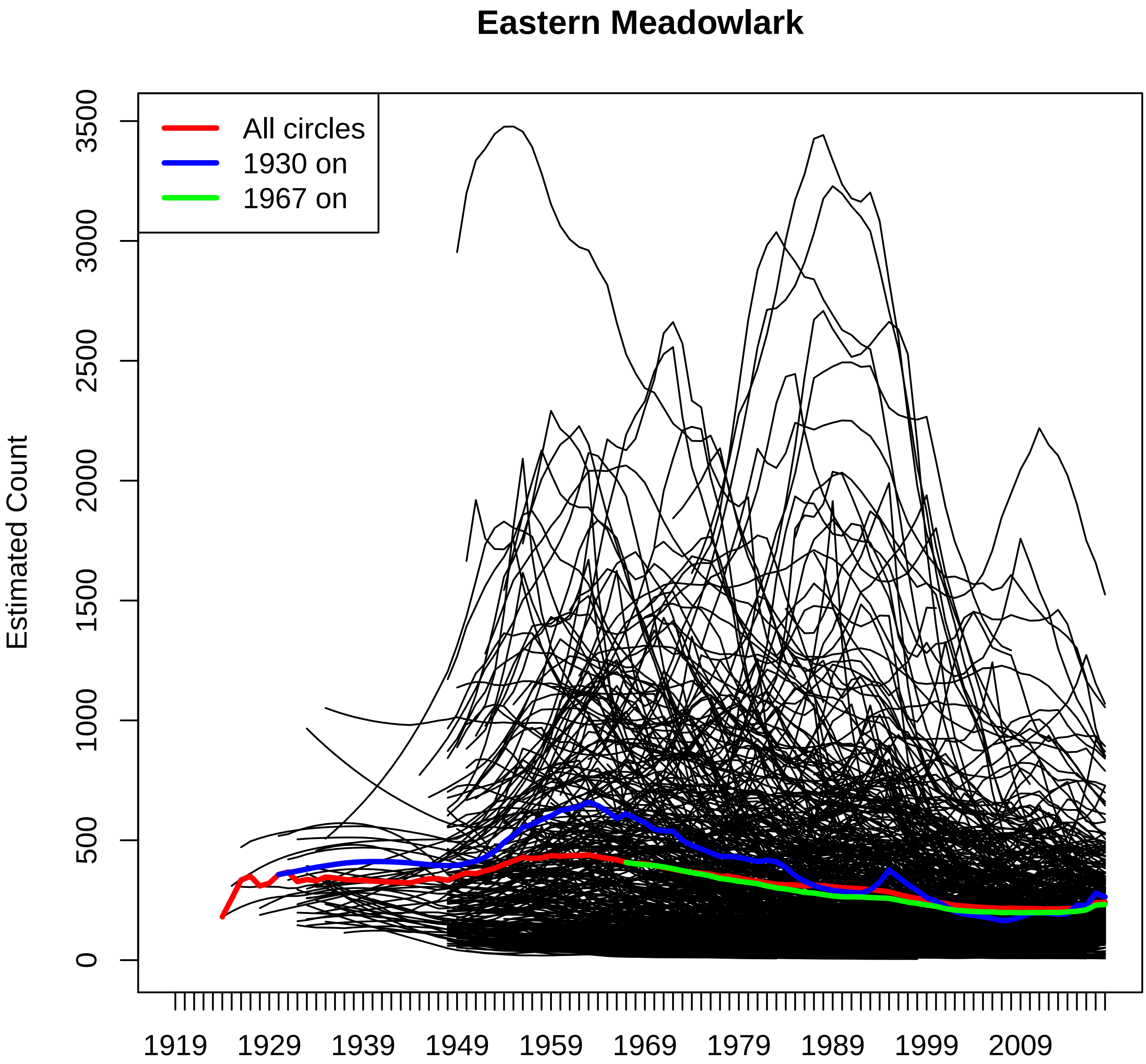

Eastern Meadowlark
All circles
1930 on
1967 on
Estimated Count
0
500
1000
1500
2000
2500
3000
3500
1919
1929
1939
1949
1959
1969
1979
1989
1999
2009

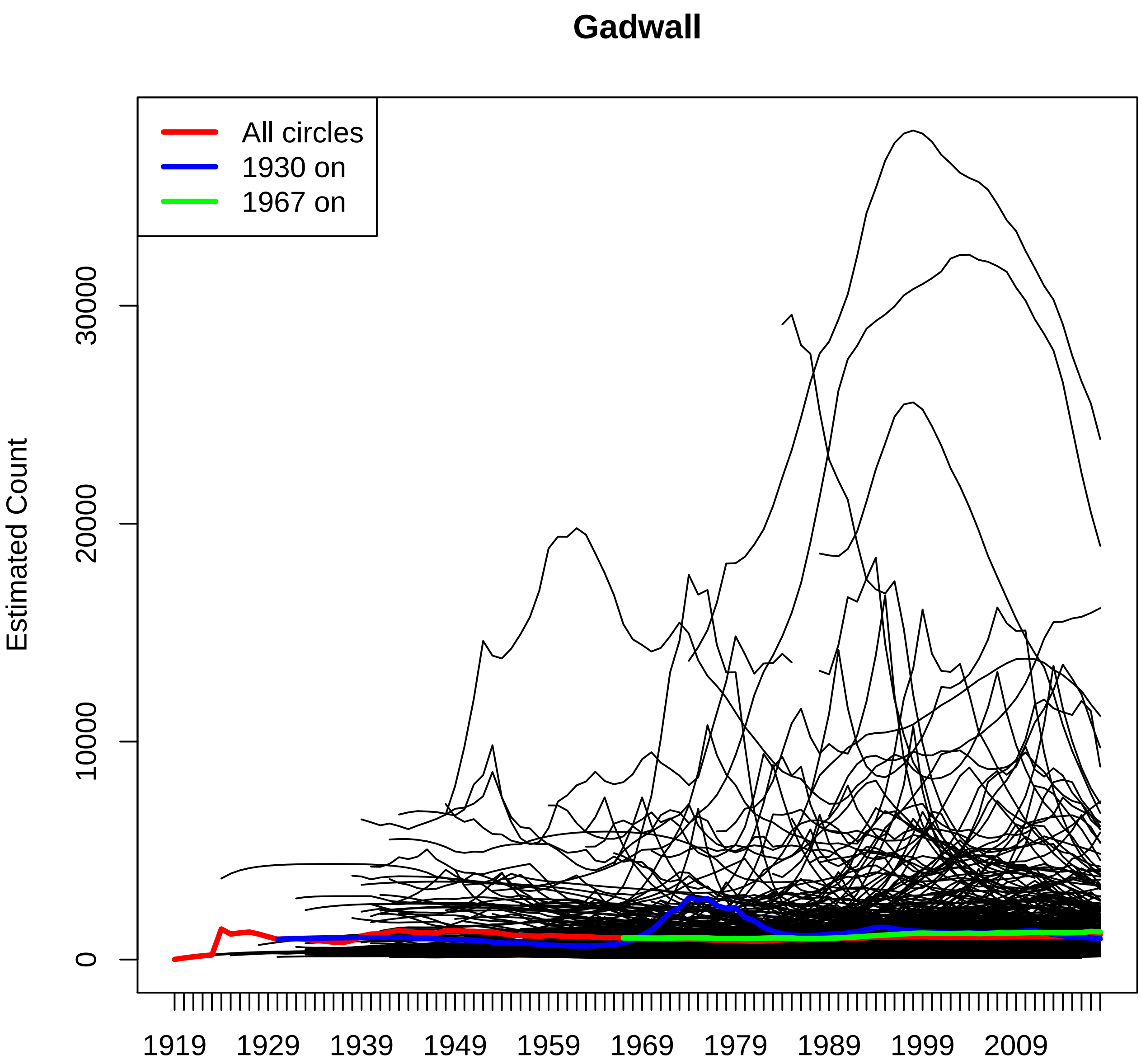

Gadwall
All circles
1930 on
1967 on
Estimated Count
0
10000
20000
30000
1919
1929
1939
1949
1959
1969
1979
1989
1999
2009

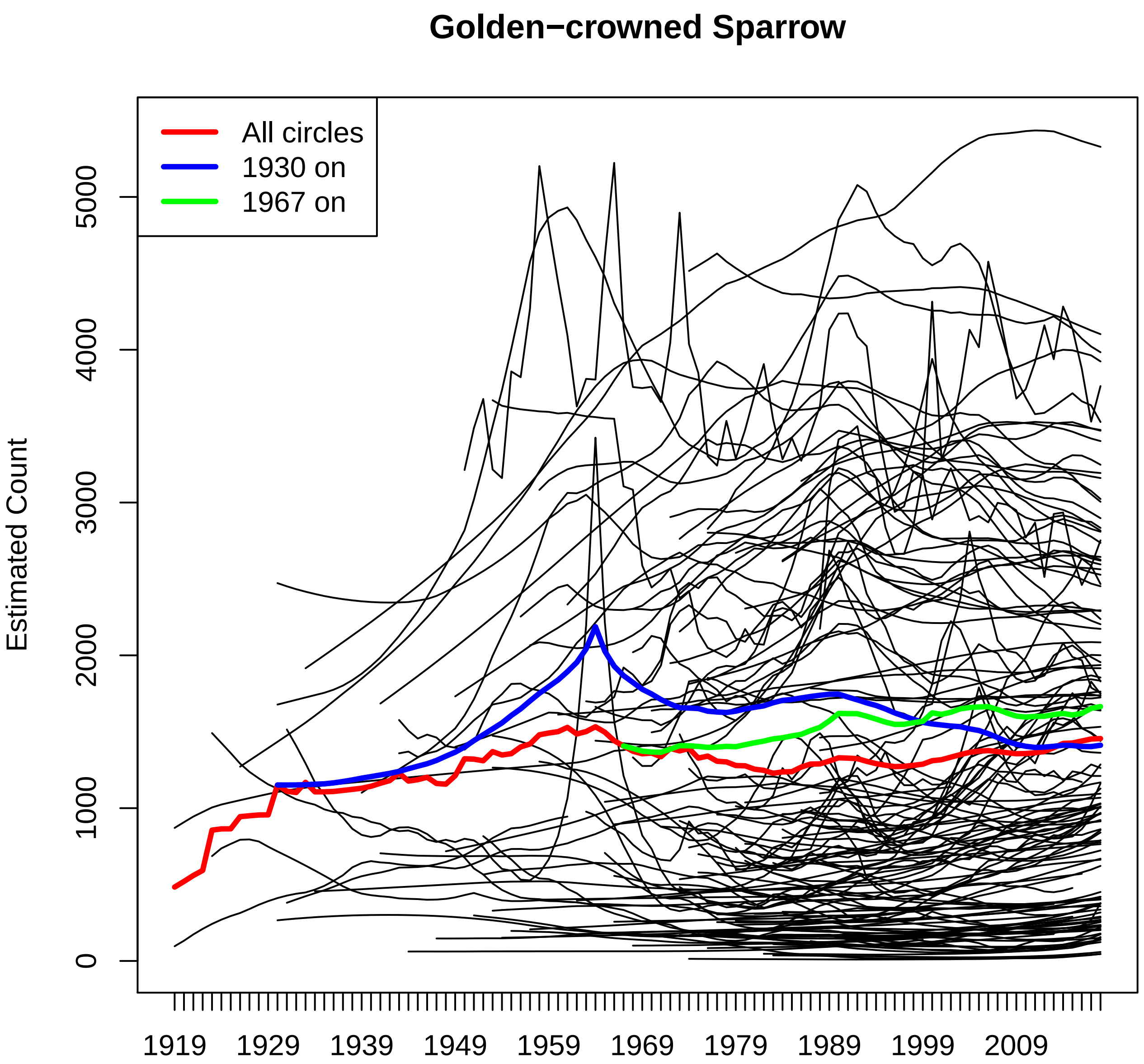

Golden−crowned Sparrow
All circles
1930 on
1967 on
Estimated Count
0
1000
2000
3000
4000
5000
1919
1929
1939
1949
1959
1969
1979
1989
1999
2009

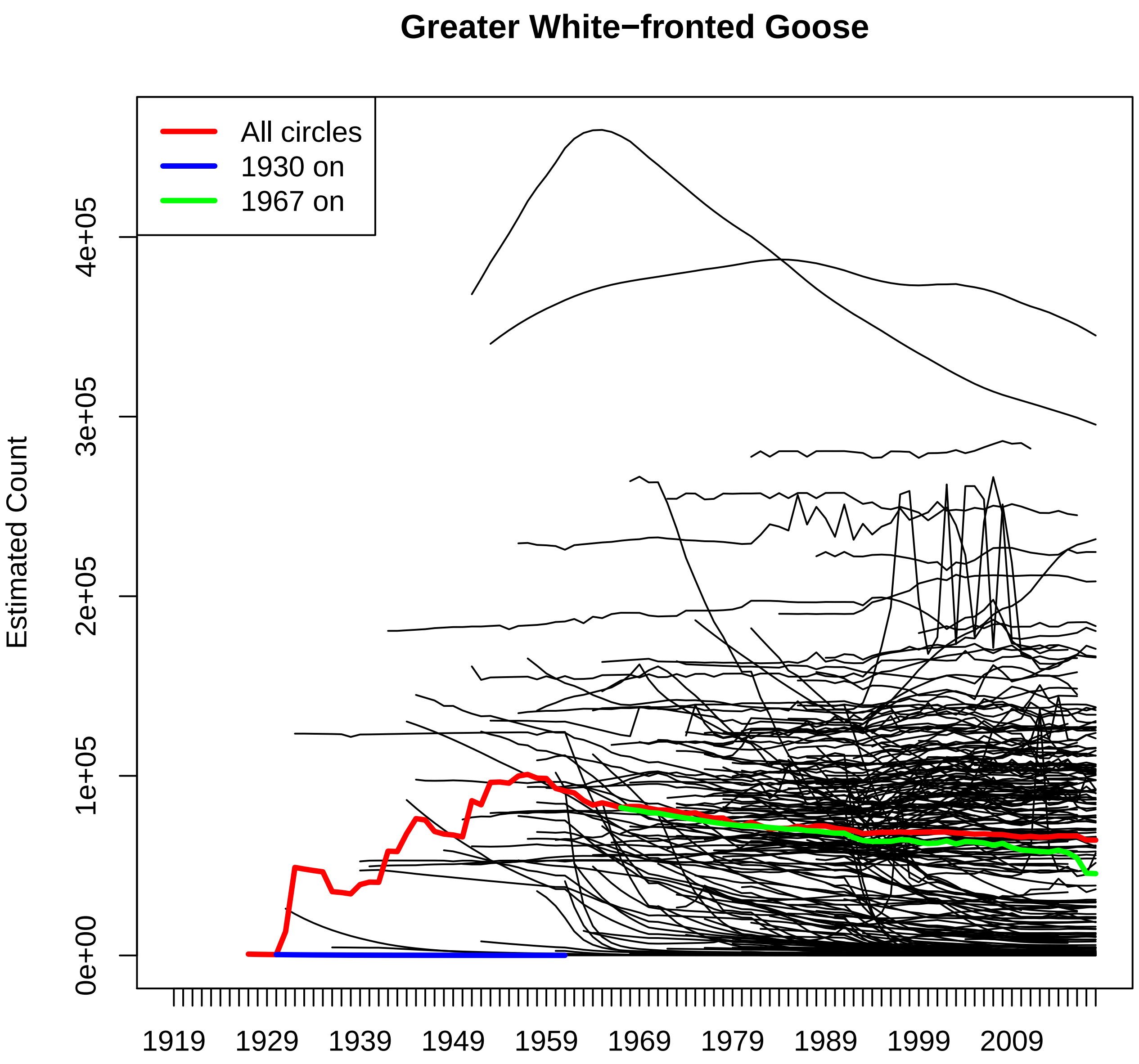
Greater White−fronted Goose
All circles
1930 on
1967 on
Estimated Count
0e+00
1e+05
2e+05
3e+05
4e+05
1919
1929
1939
1949
1959
1969
1979
1989
1999
2009

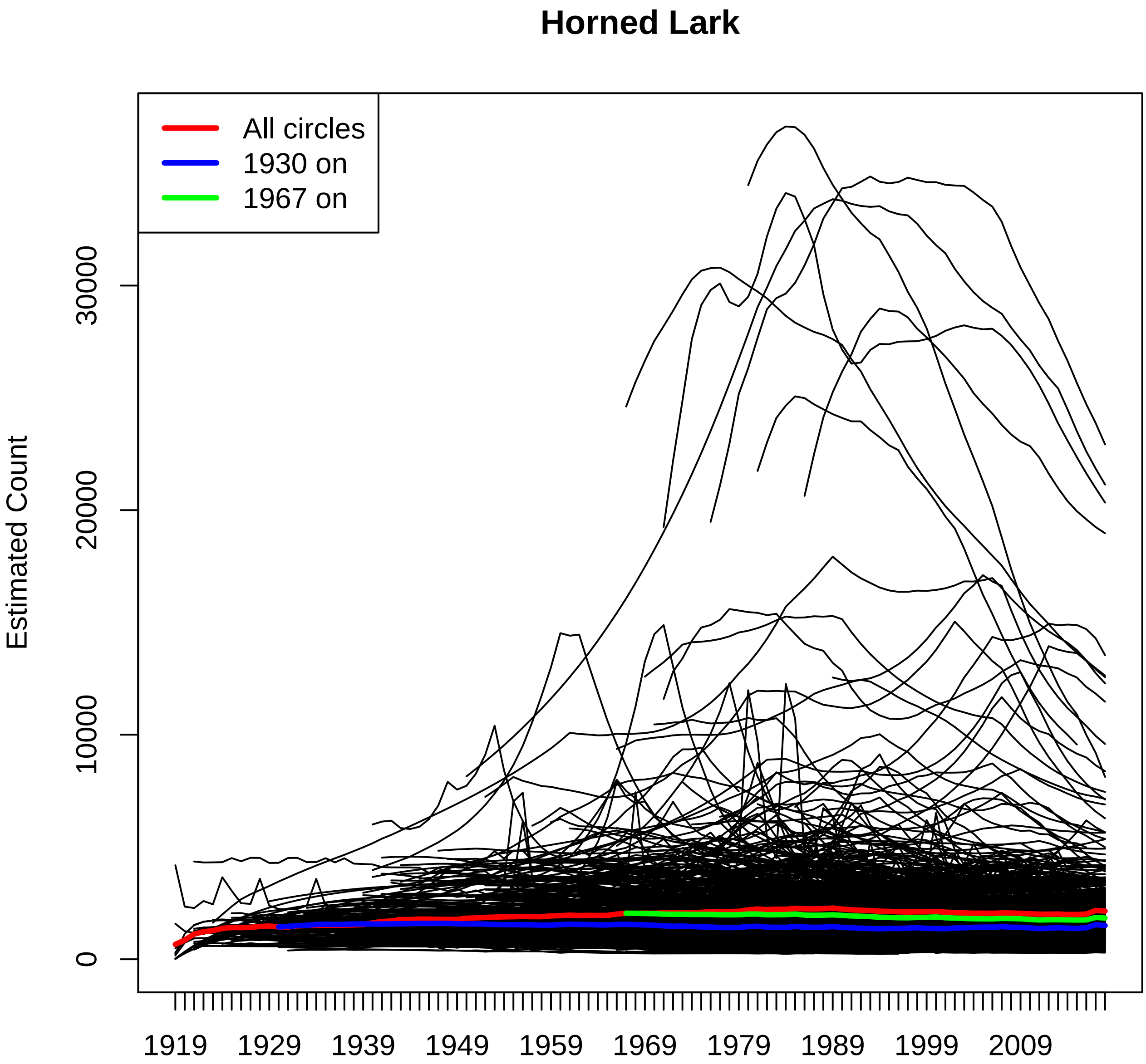
Horned Lark
All circles
1930 on
1967 on
Estimated Count
0
10000
20000
30000
1919
1929
1939
1949
1959
1969
1979
1989
1999
2009

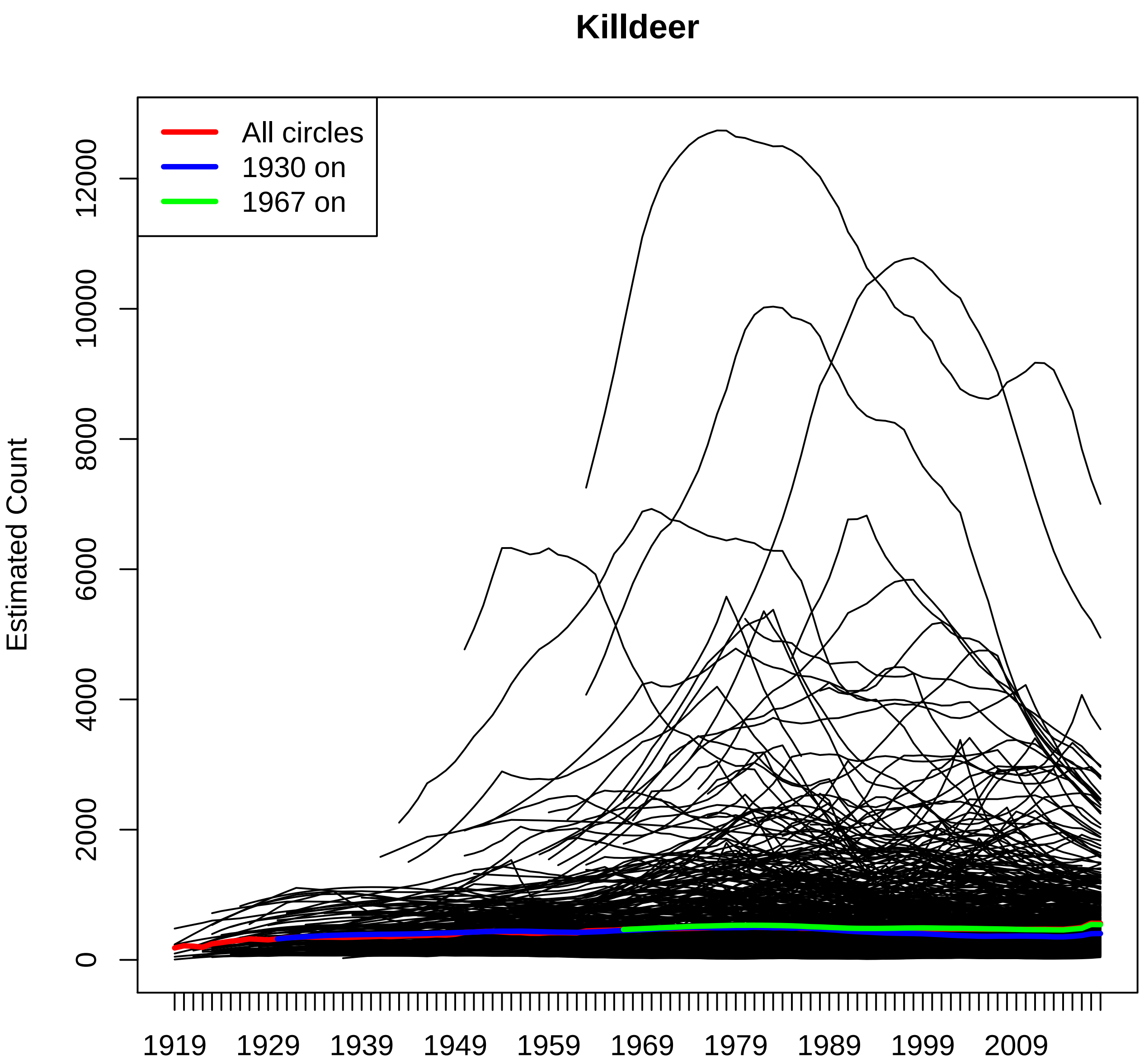

Killdeer
All circles
1930 on
1967 on
Estimated Count
0
2000
4000
6000
8000
10000
12000
1919
1929
1939
1949
1959
1969
1979
1989
1999
2009

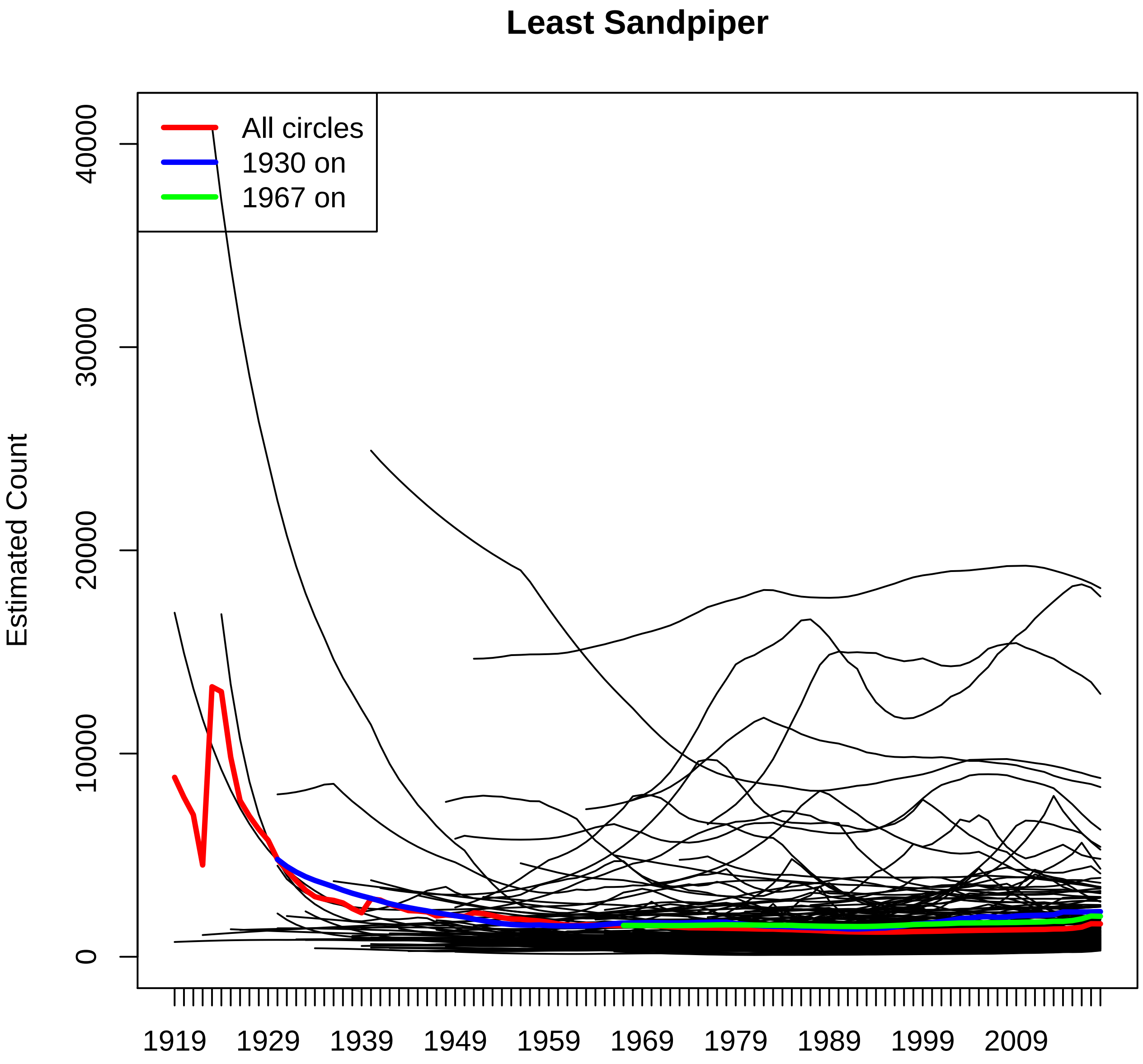

Least Sandpiper
Estimated Count
0
10000
20000
30000
40000
1919
1929
1939
1949
1959
1969
1979
1989
1999
2009
All circles
1930 on
1967 on

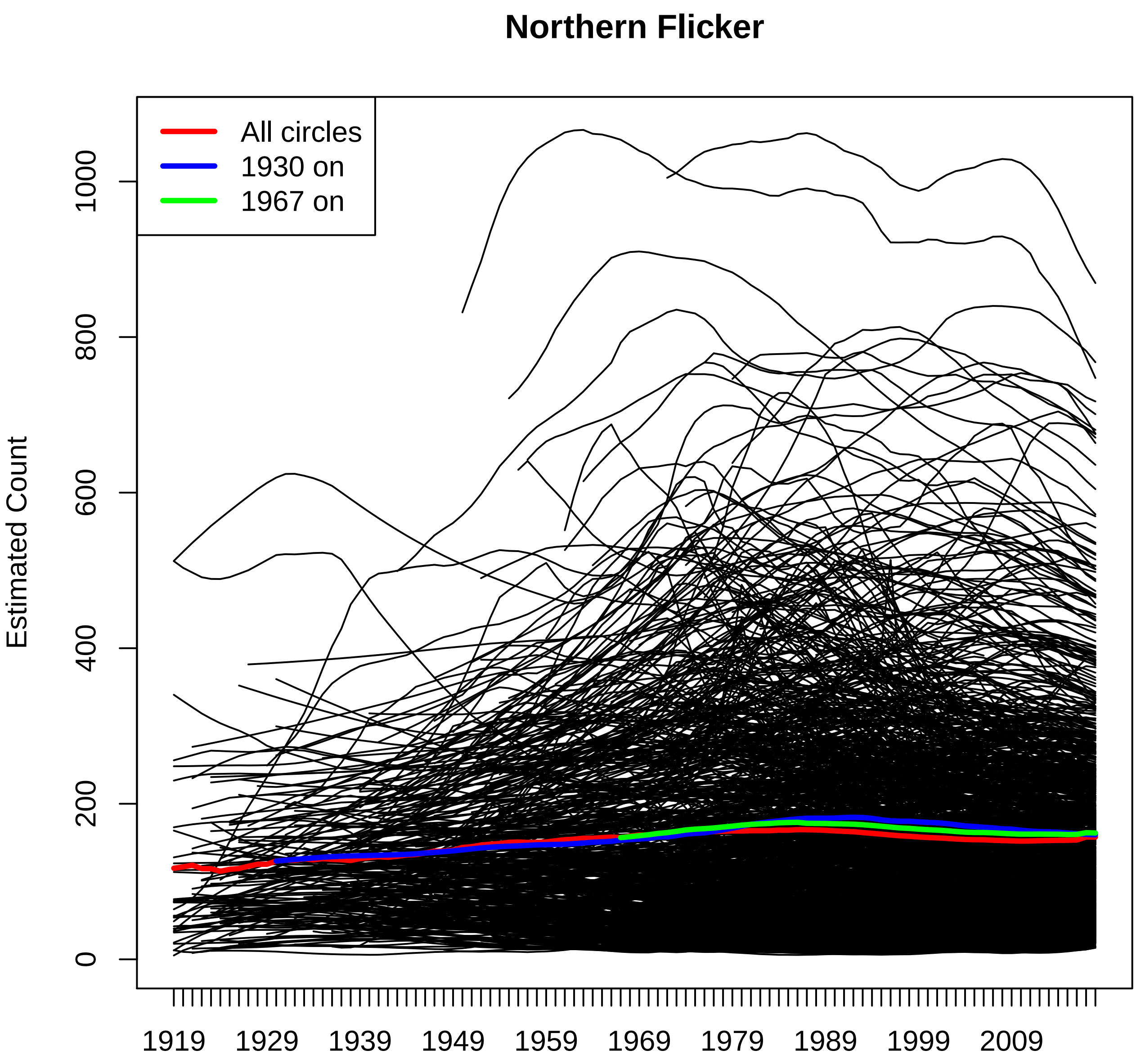

Northern Flicker
All circles
1930 on
1967 on
Estimated Count
0
200
400
600
800
1000
1919
1929
1939
1949
1959
1969
1979
1989
1999
2009

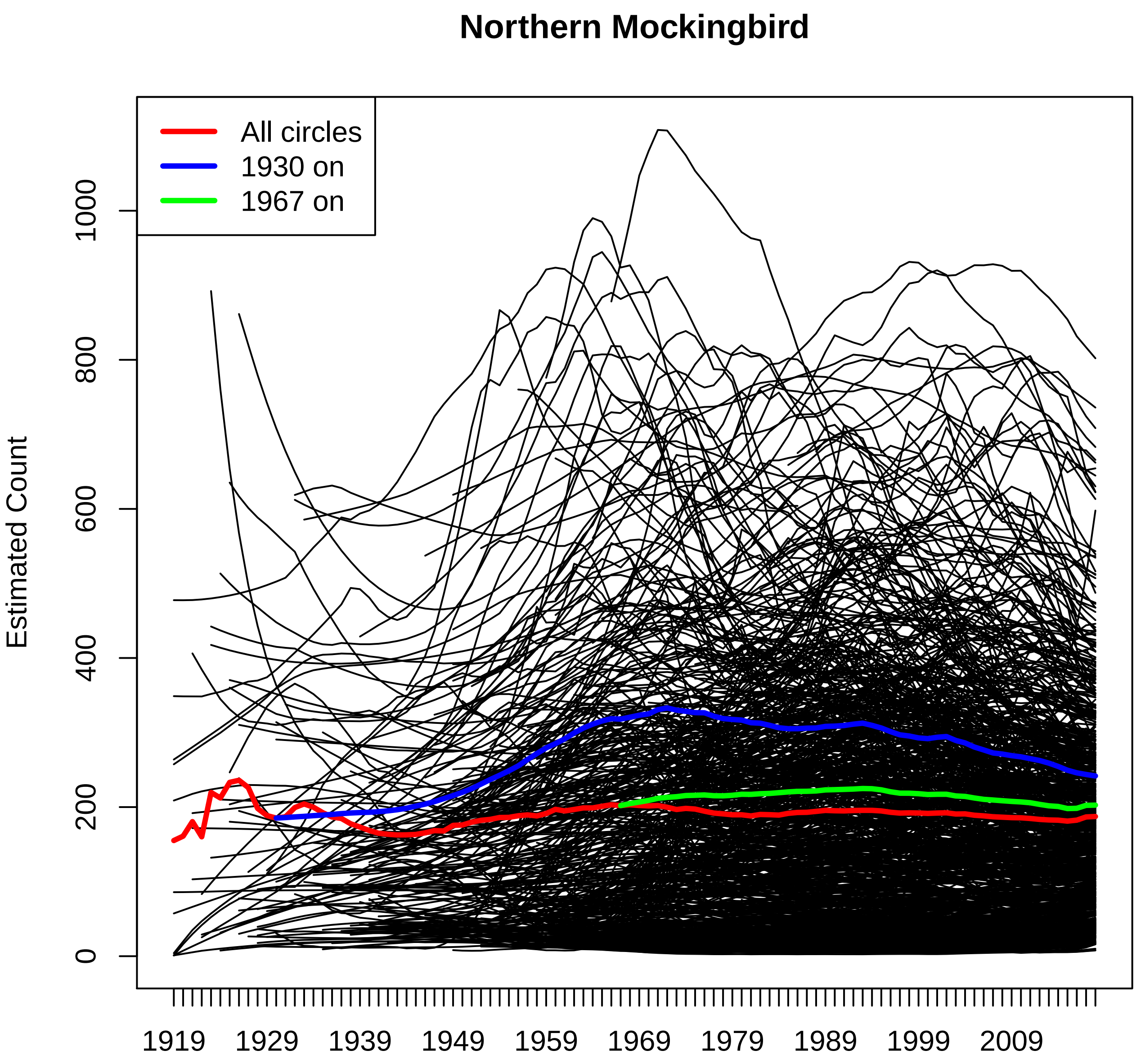

Northern Mockingbird
All circles
1930 on
1967 on
Estimated Count
0
200
400
600
800
1000
1919
1929
1939
1949
1959
1969
1979
1989
1999
2009

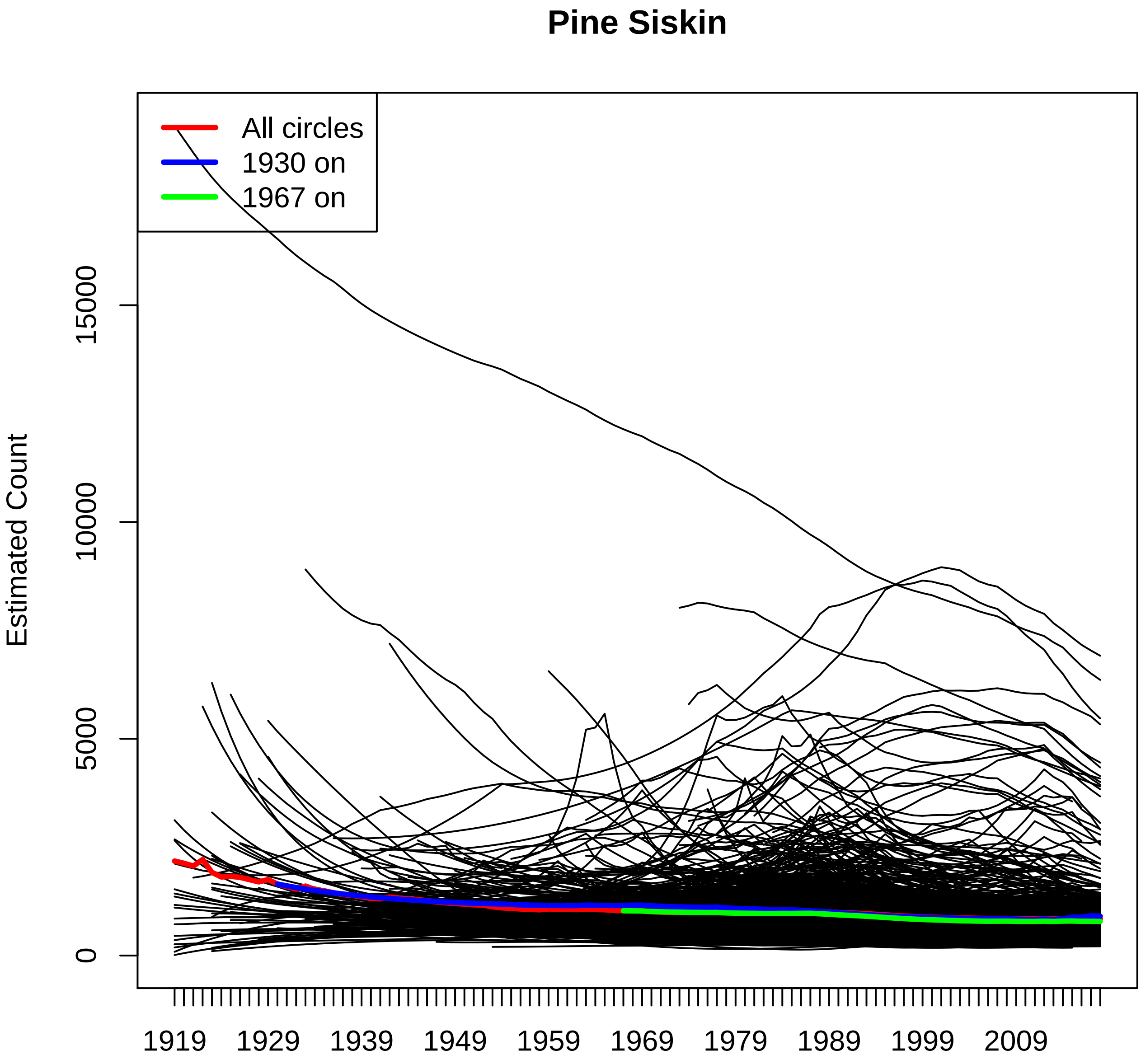

Pine Siskin
All circles
1930 on
1967 on
Estimated Count
0
5000
10000
15000
1919
1929
1939
1949
1959
1969
1979
1989
1999
2009

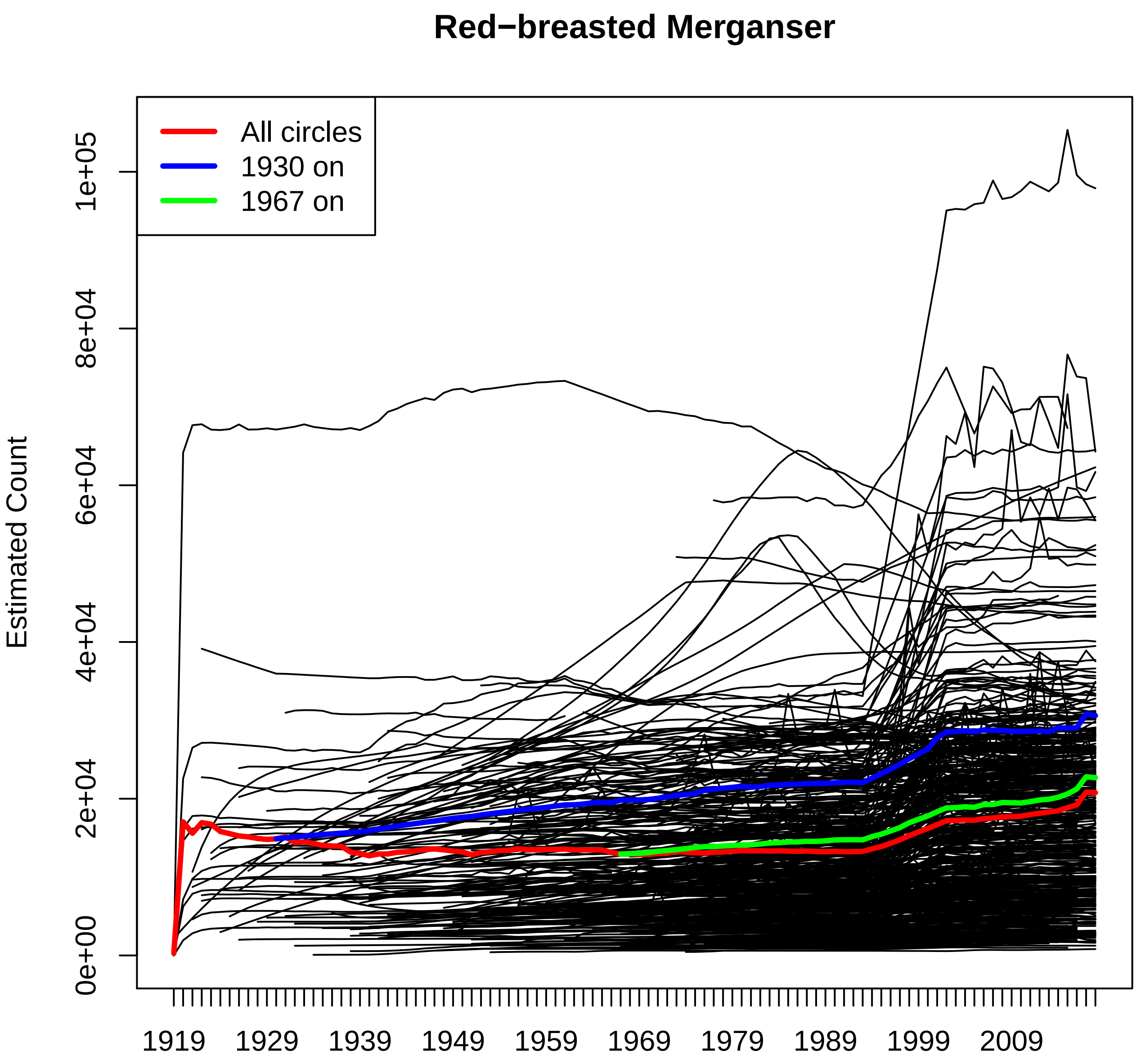
Red−breasted Merganser
All circles
1930 on
1967 on
Estimated Count
0e+00
2e+04
4e+04
6e+04
8e+04
1e+05
1919
1929
1939
1949
1959
1969
1979
1989
1999
2009

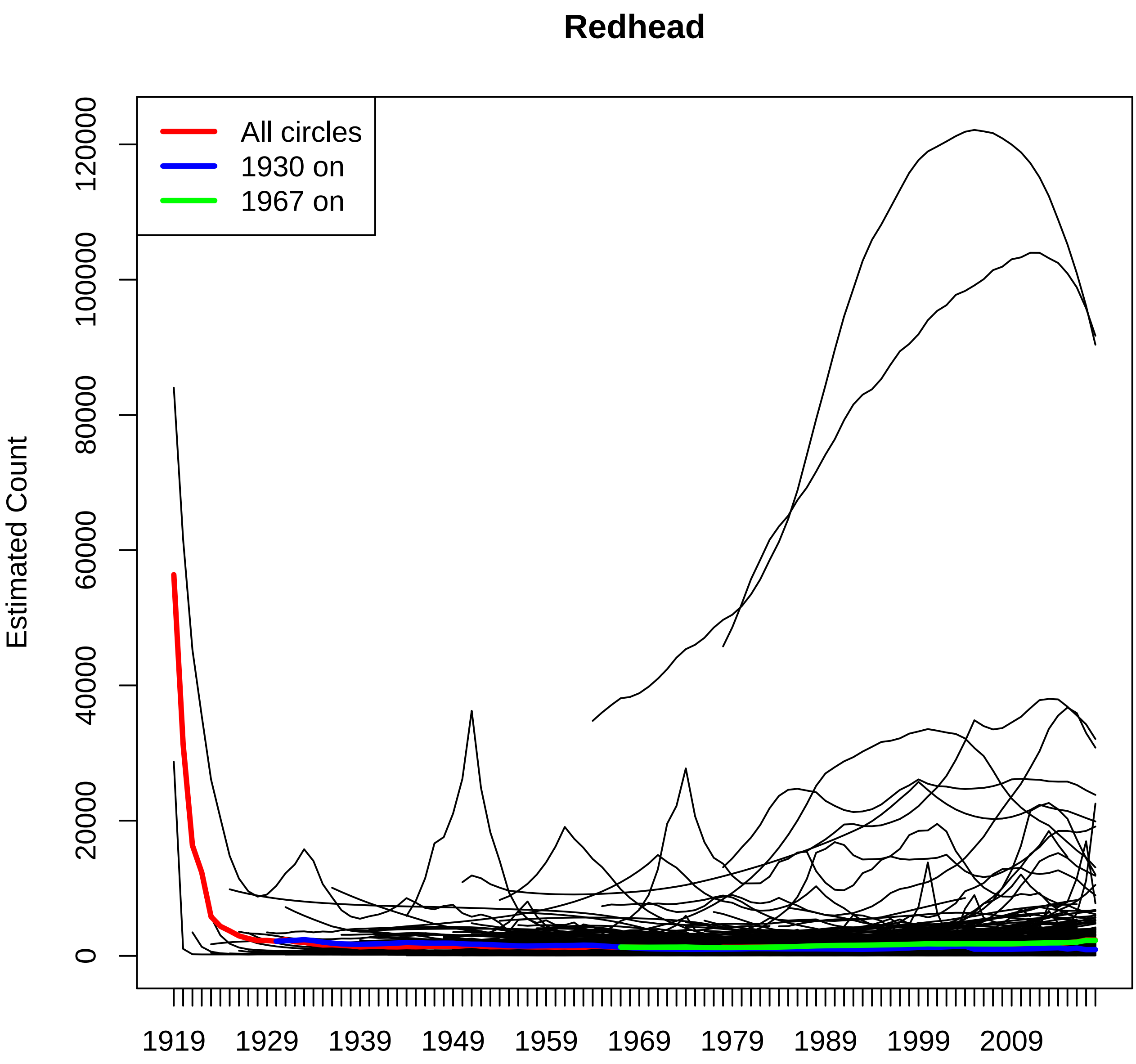
Redhead
Estimated Count
0
20000
40000
60000
80000
100000
120000
All circles
1930 on
1967 on
1919
1929
1939
1949
1959
1969
1979
1989
1999
2009

Ring−necked Duck

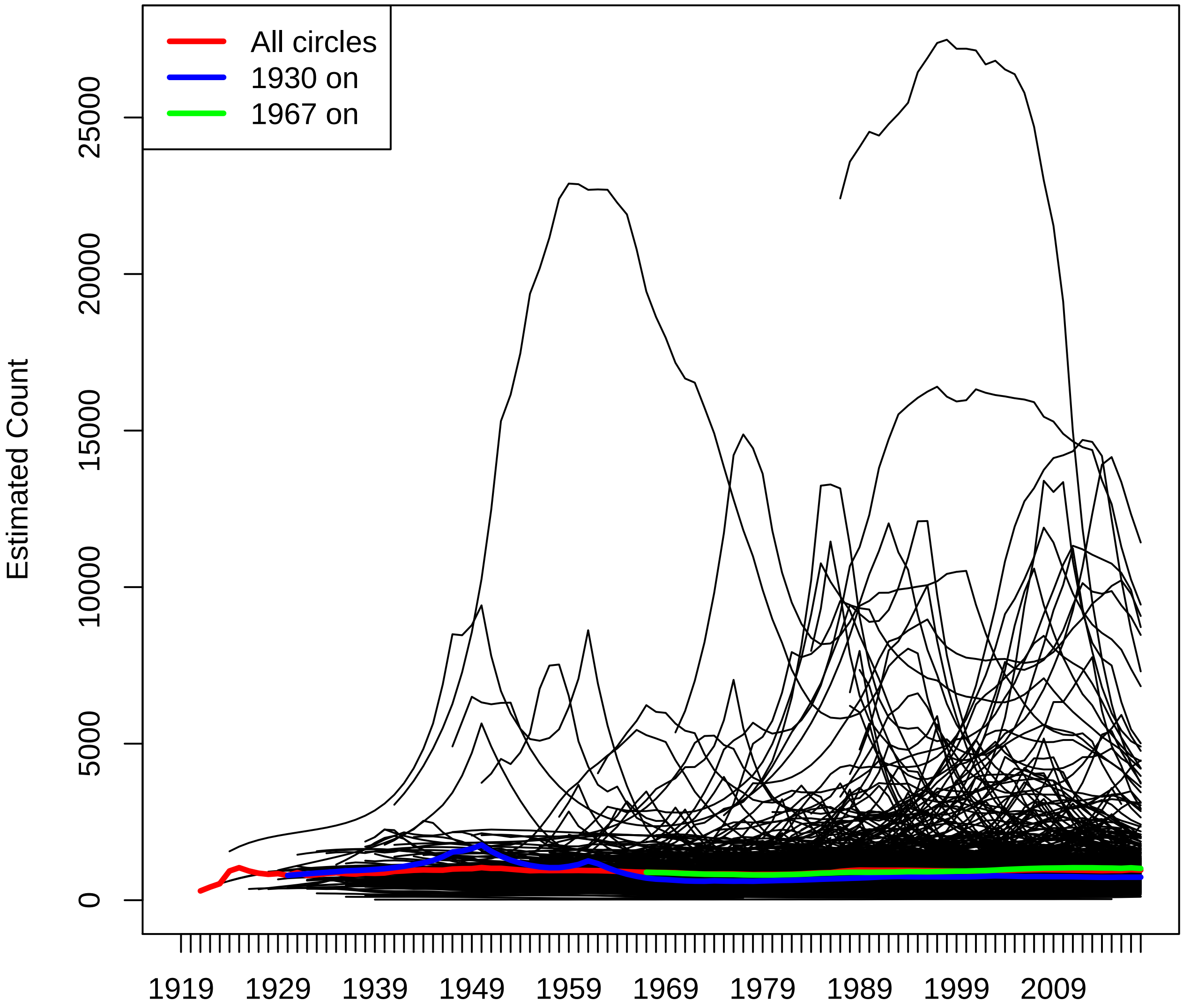

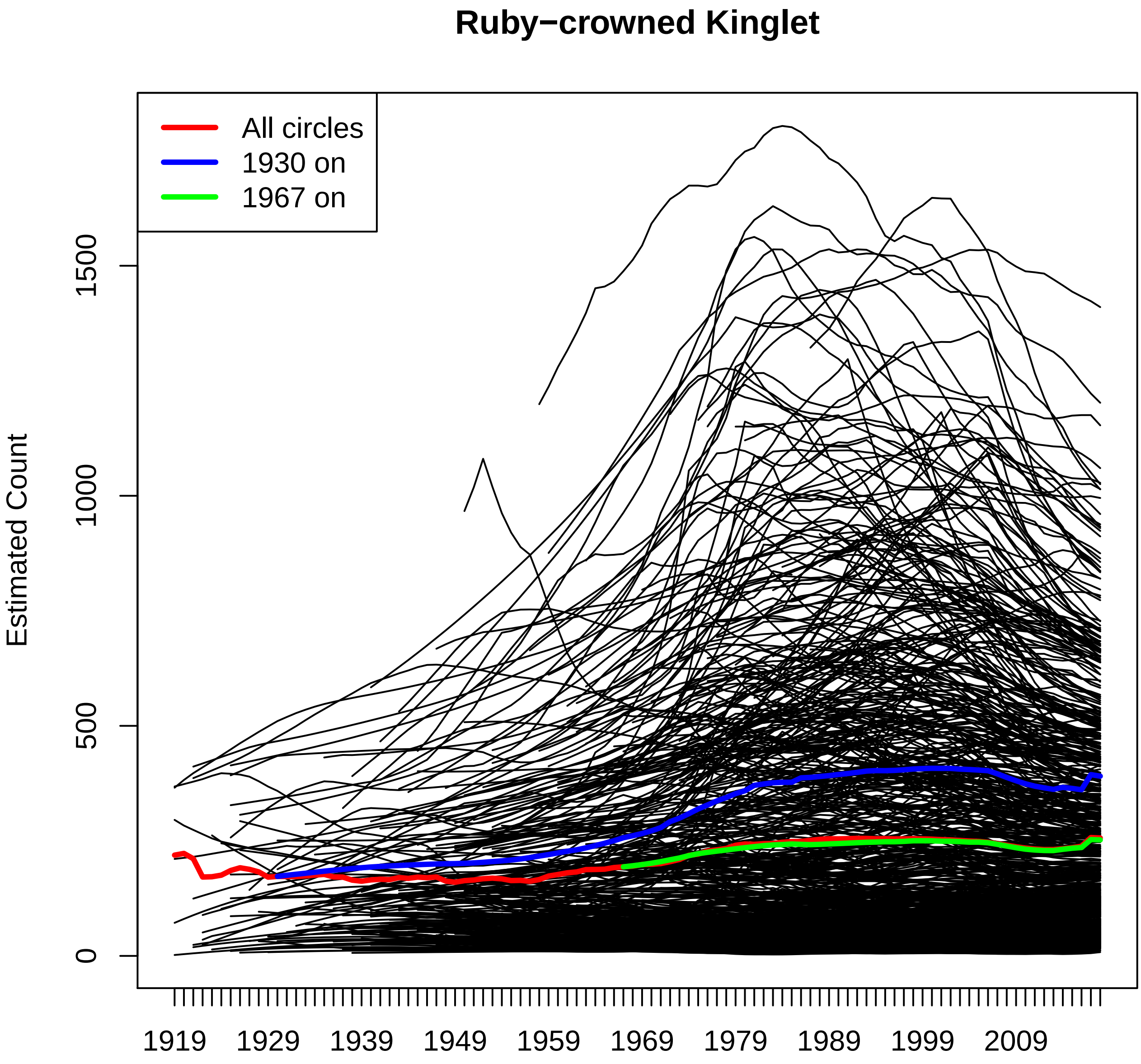

Ruby−crowned Kinglet
All circles
1930 on
1967 on
Estimated Count
0
500
1000
1500
1919
1929
1939
1949
1959
1969
1979
1989
1999
2009

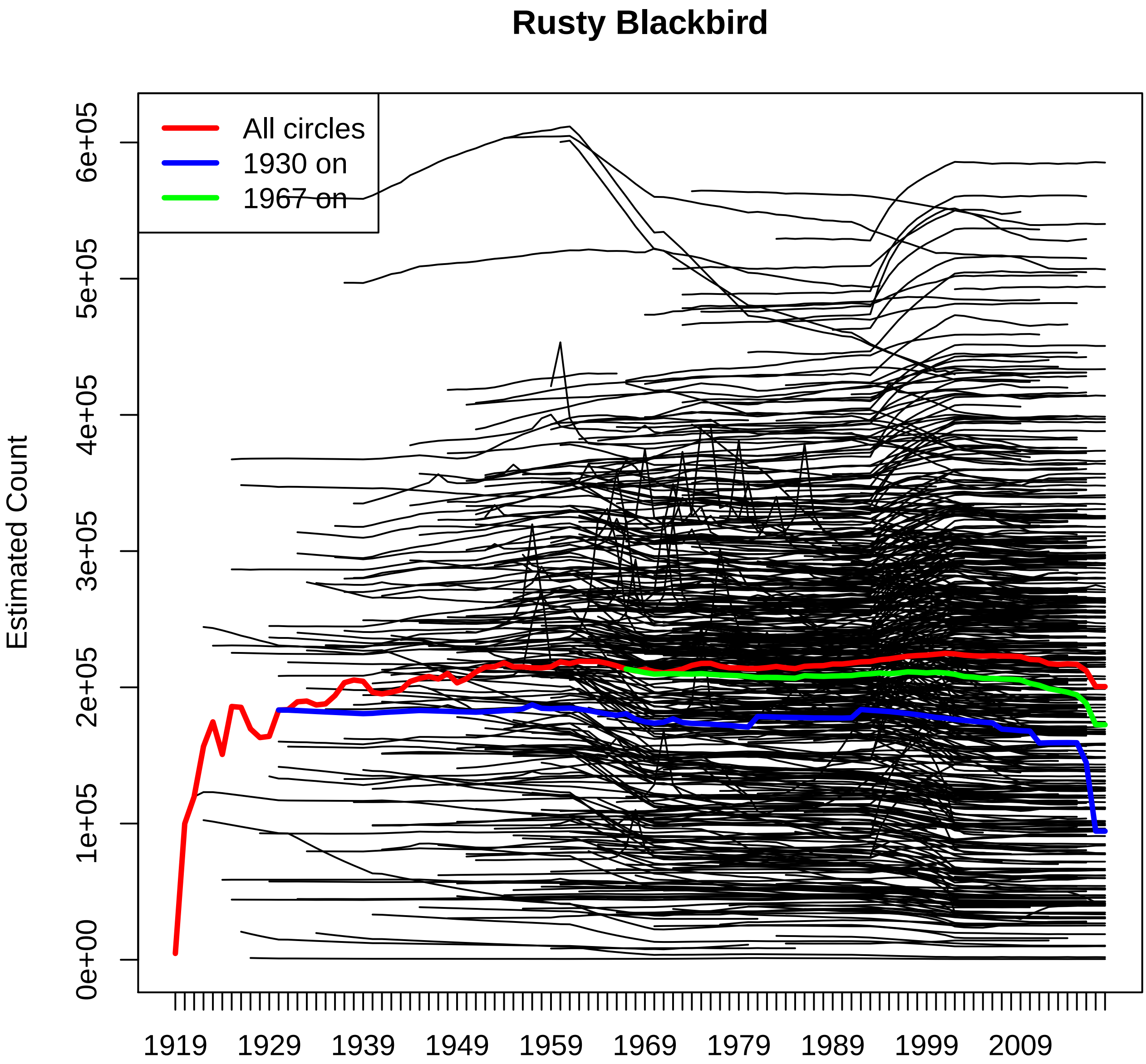
Rusty Blackbird
All circles
1930 on
1967 on
Estimated Count
0e+00
1e+05
2e+05
3e+05
4e+05
5e+05
6e+05
1919
1929
1939
1949
1959
1969
1979
1989
1999
2009

Sandhill Crane

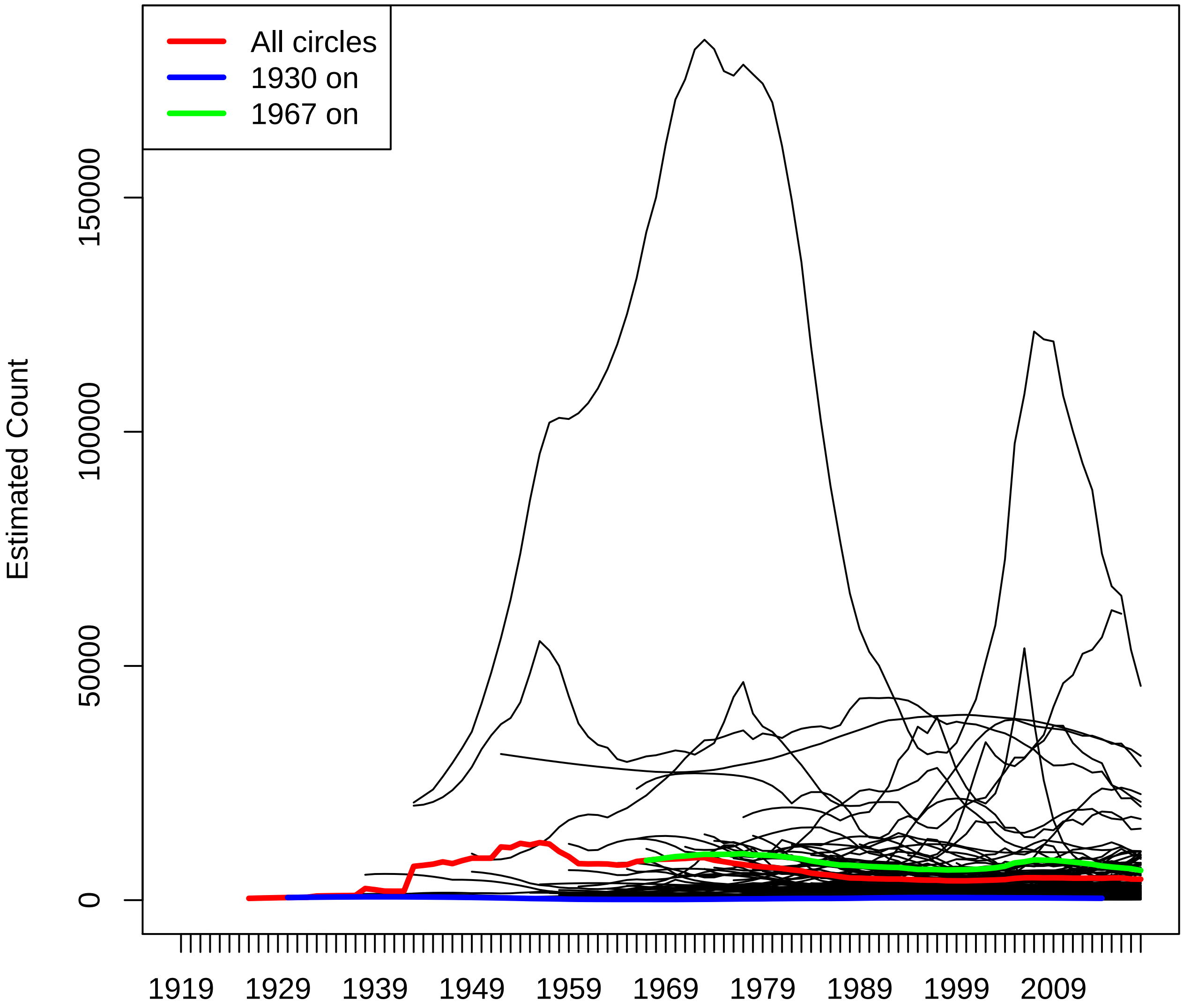

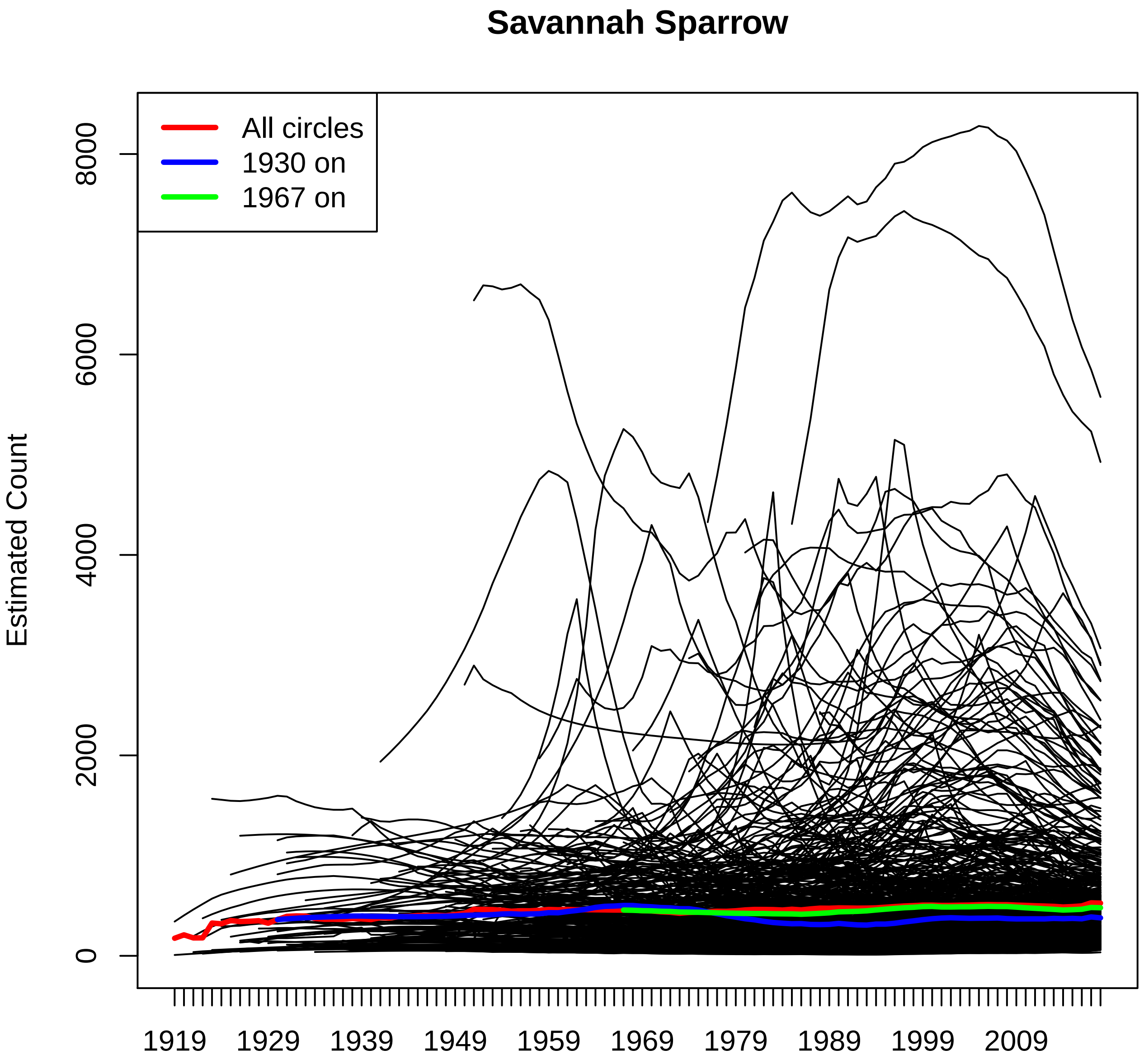

Savannah Sparrow
Estimated Count
All circles
1930 on
1967 on
0
2000
4000
6000
8000
1919
1929
1939
1949
1959
1969
1979
1989
1999
2009

Song Sparrow

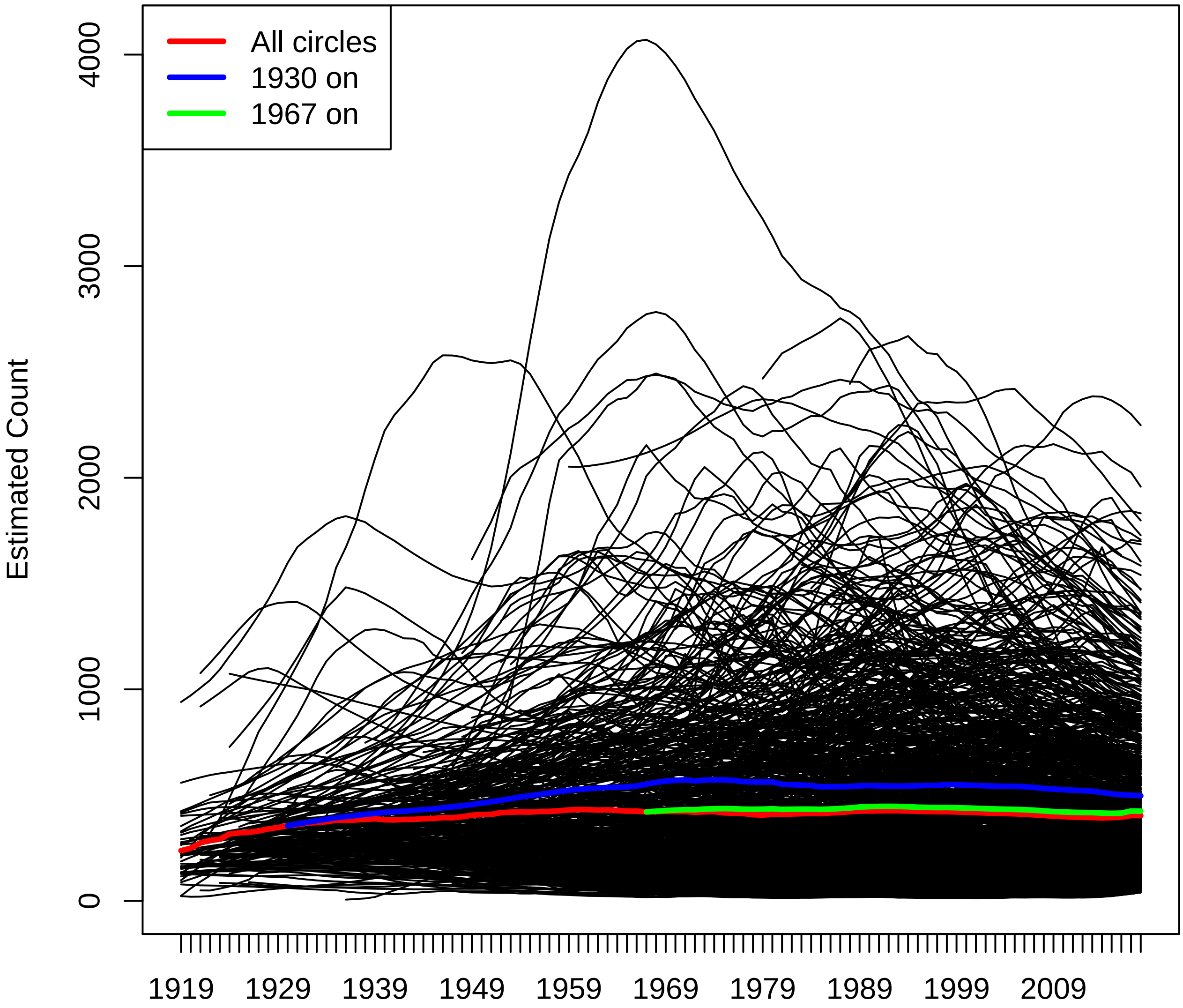

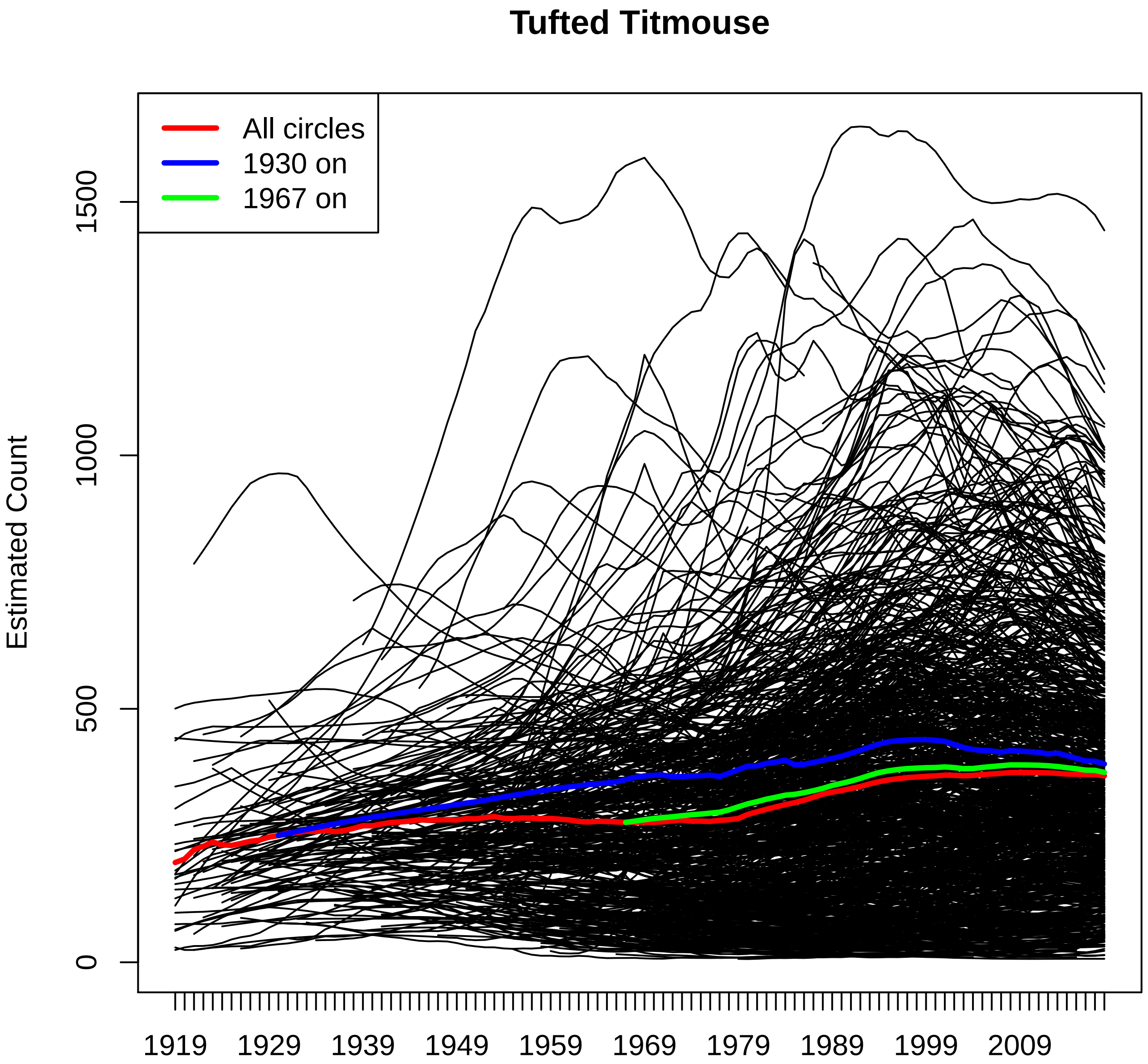
Tufted Titmouse
All circles
1930 on
1967 on
Estimated Count
0
500
1000
1500
1919
1929
1939
1949
1959
1969
1979
1989
1999
2009

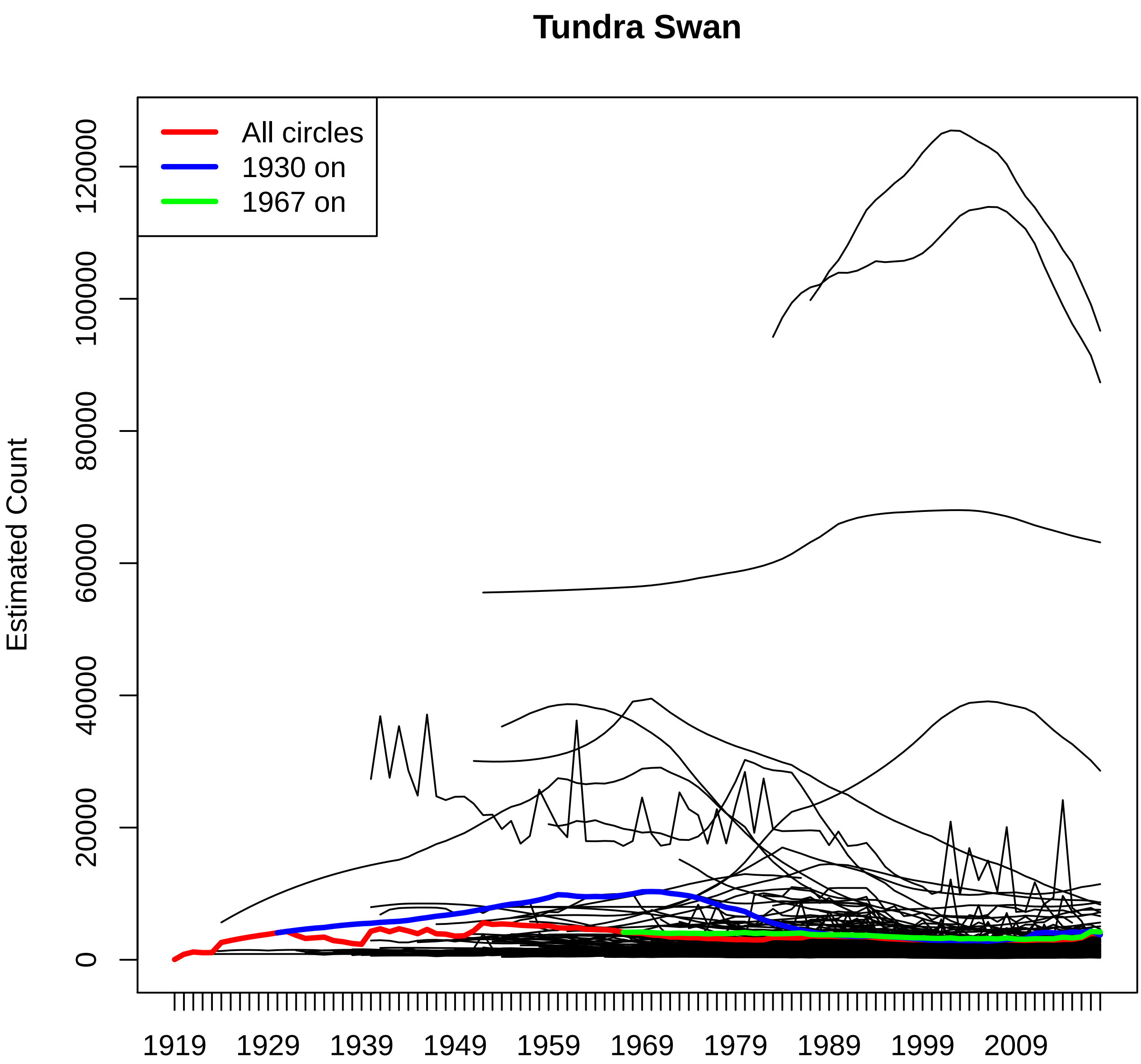
Tundra Swan
All circles
1930 on
1967 on
Estimated Count
0
20000
40000
60000
80000
100000
120000
1919
1929
1939
1949
1959
1969
1979
1989
1999
2009

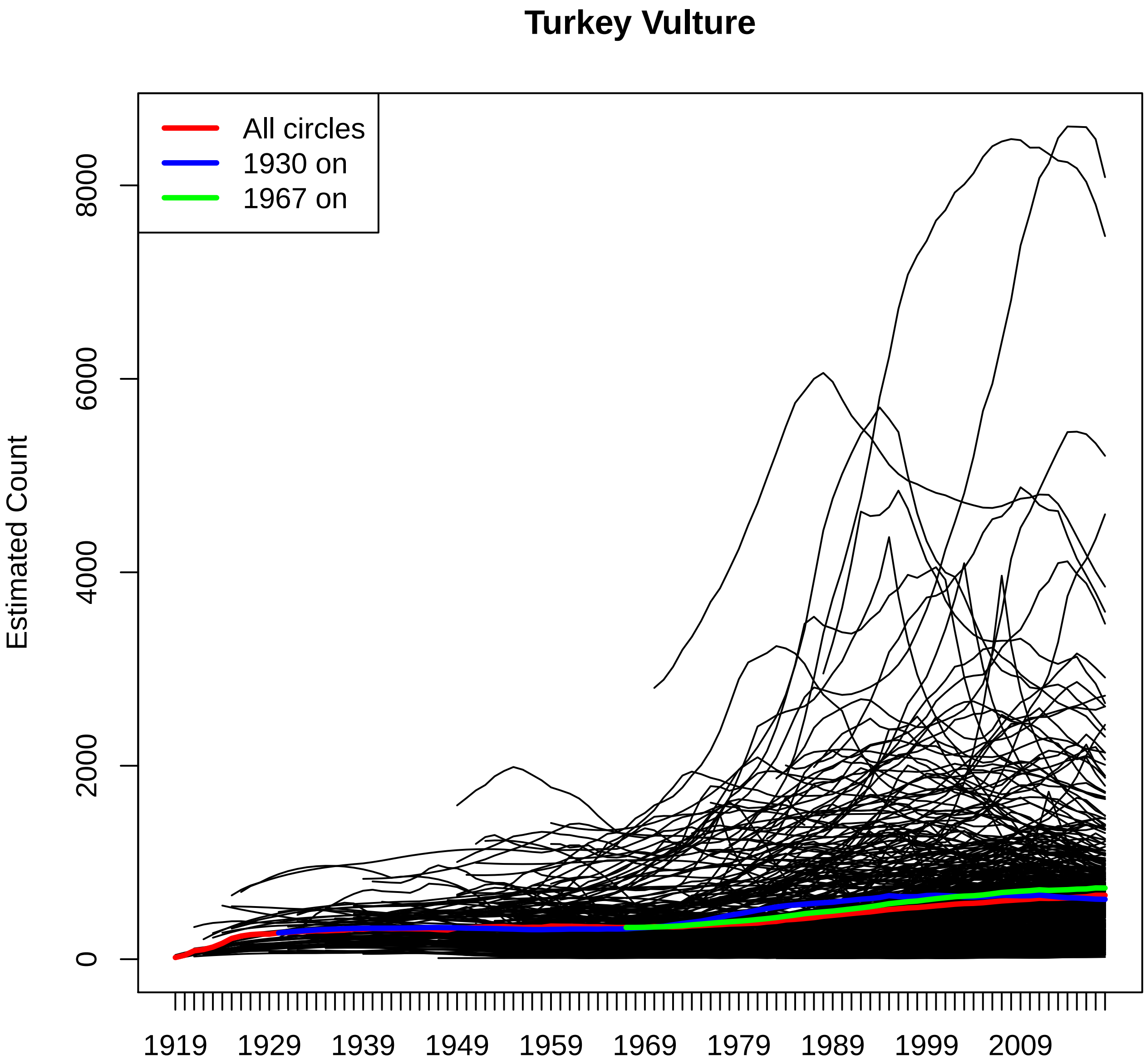

Turkey Vulture
All circles
1930 on
1967 on
Estimated Count
0
2000
4000
6000
8000
1919
1929
1939
1949
1959
1969
1979
1989
1999
2009

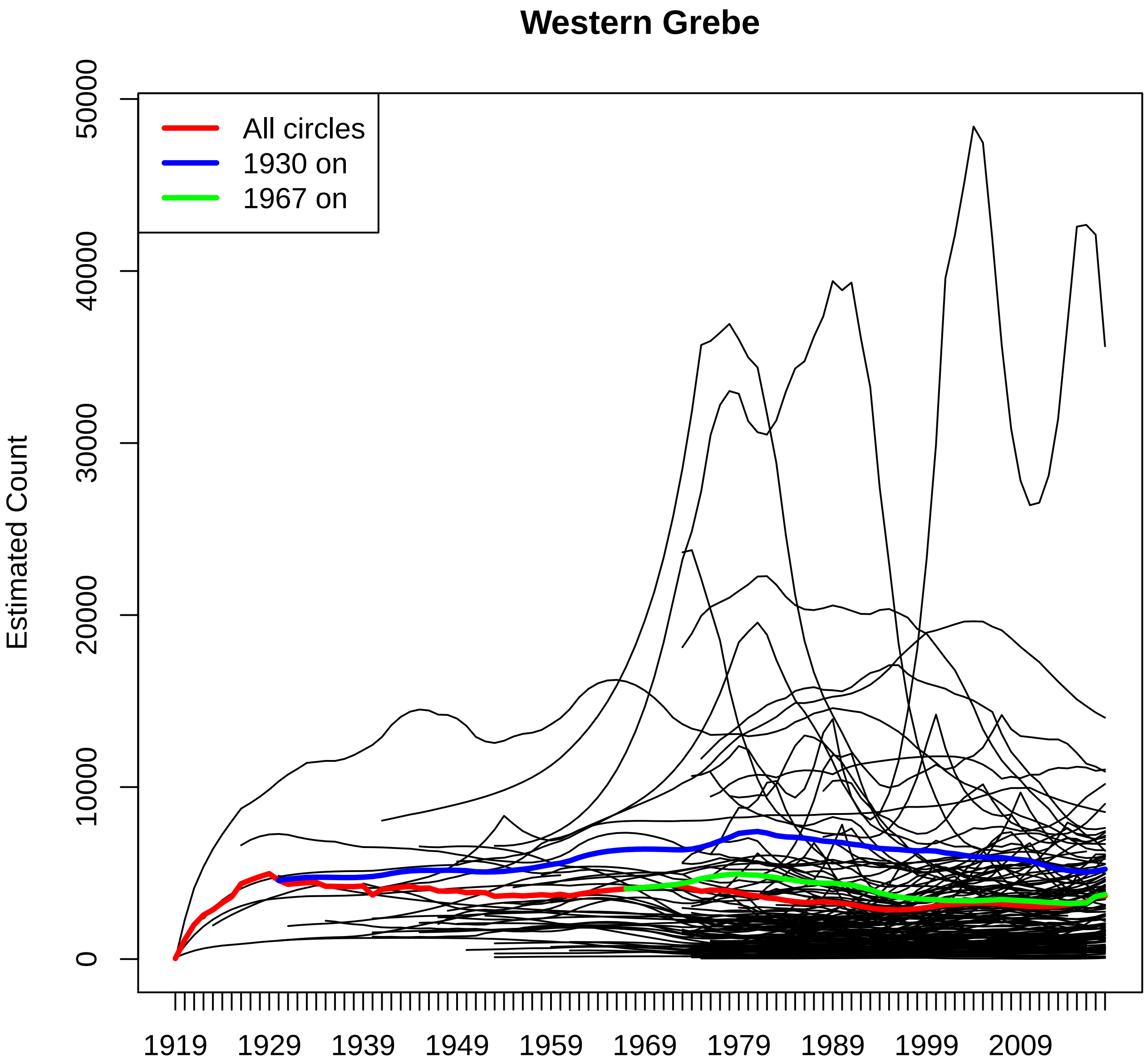

Western Grebe
Estimated Count
50000
40000
30000
20000
10000
0
All circles
1930 on
1967 on
1919
1929
1939
1949
1959
1969
1979
1989
1999
2009

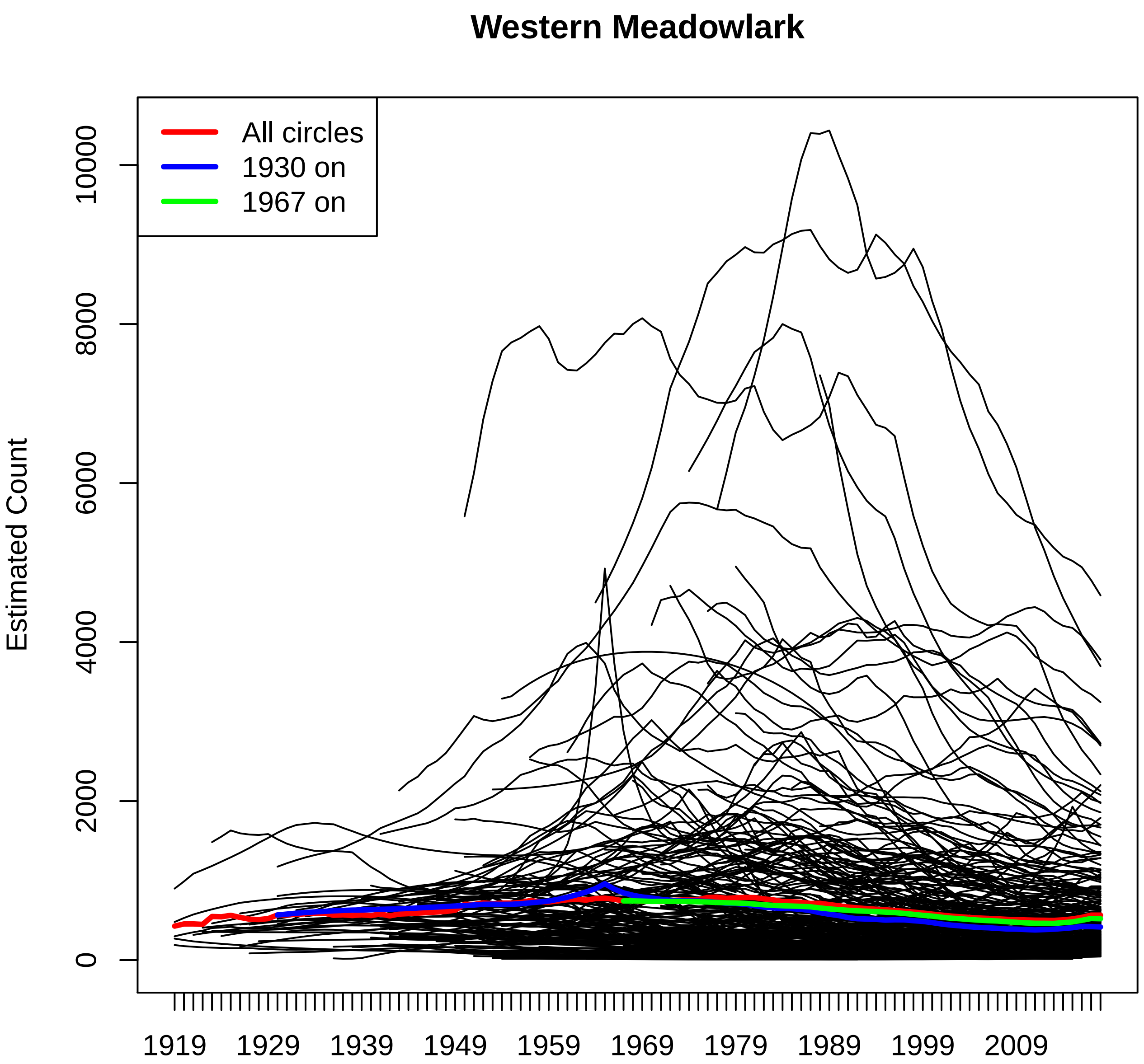

Western Meadowlark
Estimated Count
All circles
1930 on
1967 on
0
2000
4000
6000
8000
10000
1919
1929
1939
1949
1959
1969
1979
1989
1999
2009

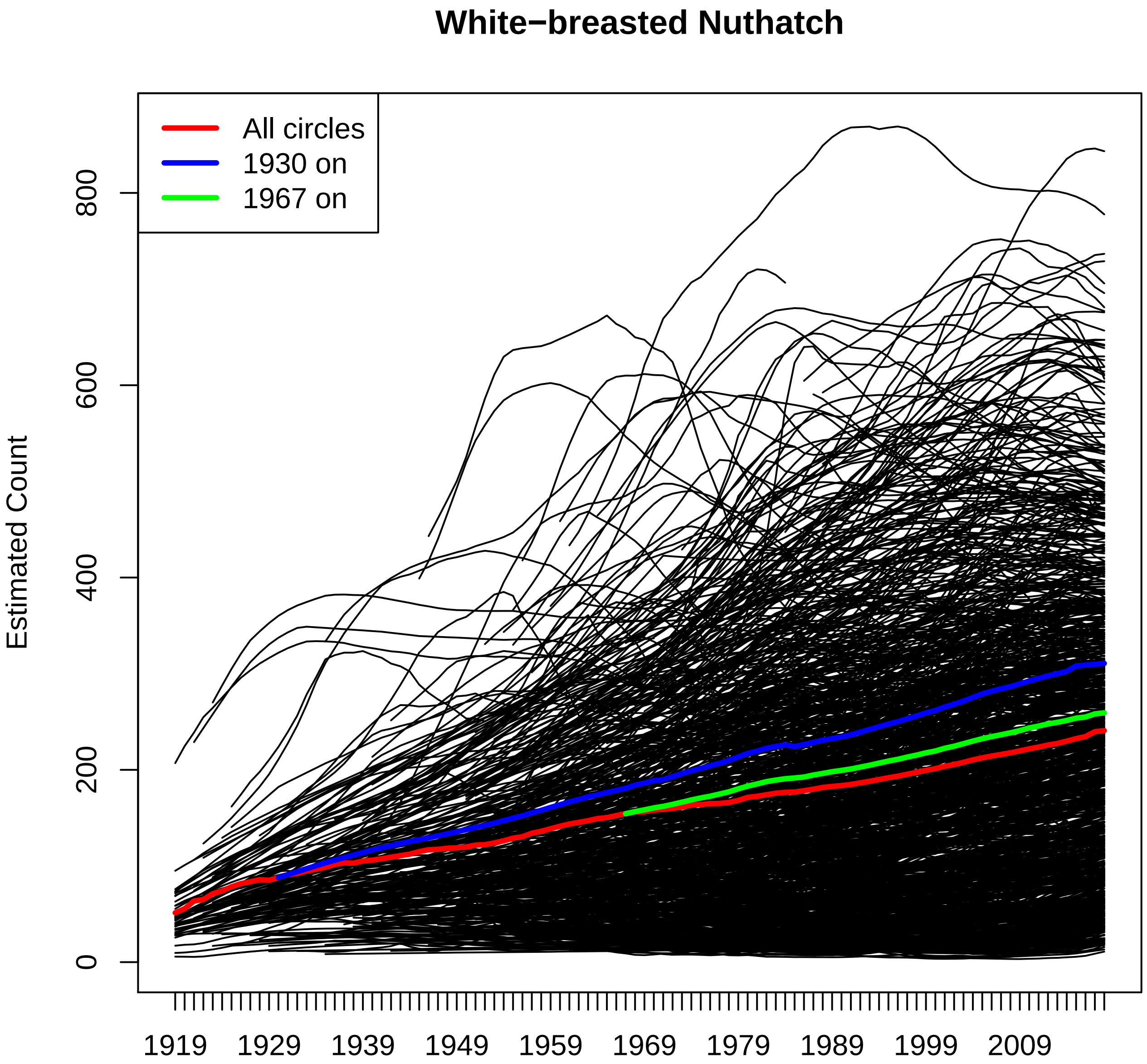

White−breasted Nuthatch
All circles
1930 on
1967 on
Estimated Count
0
200
400
600
800
1919
1929
1939
1949
1959
1969
1979
1989
1999
2009

White Ibis

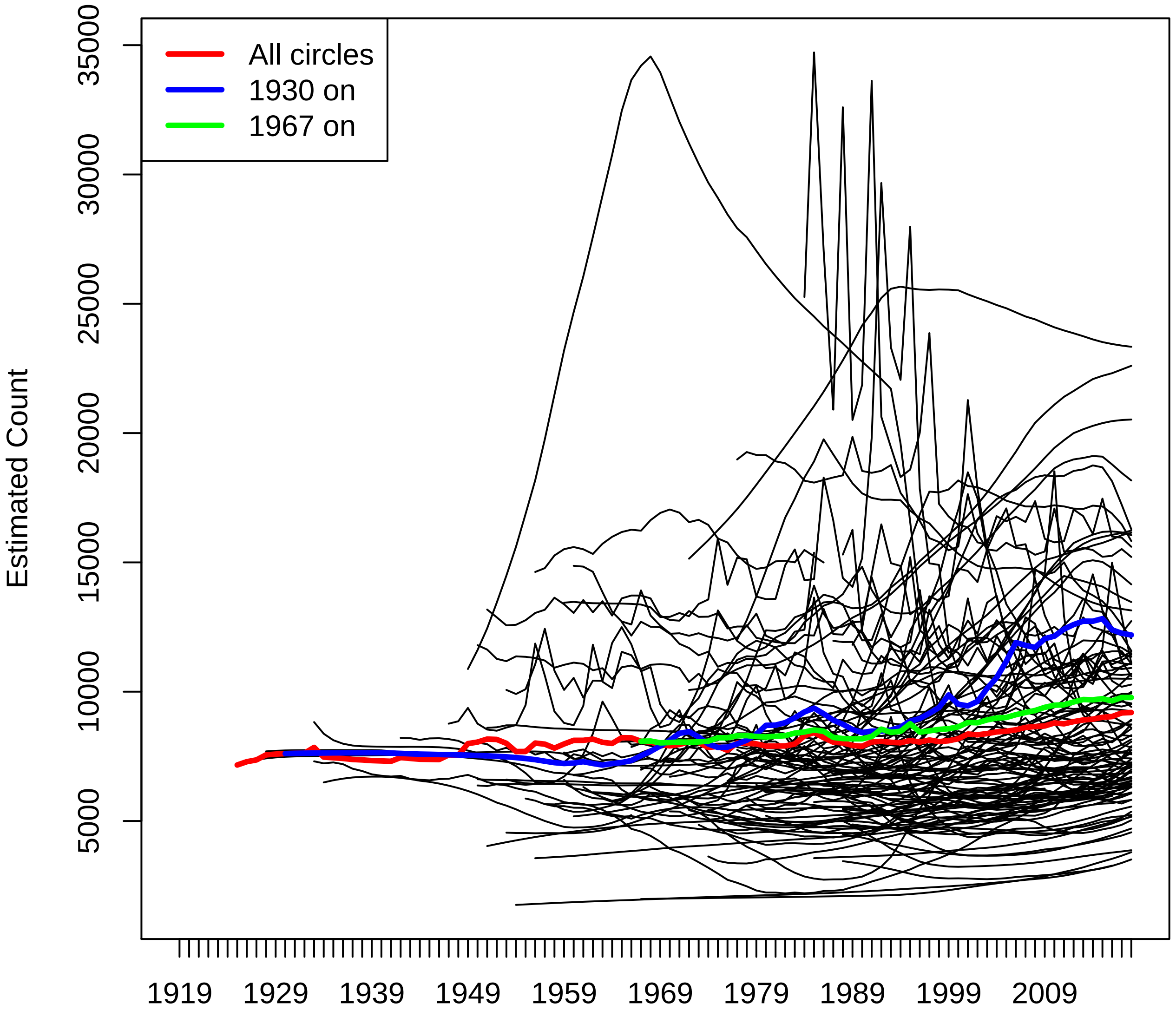

All circles
1930 on
1967 on
Estimated Count
5000
10000
15000
20000
25000
30000
35000
1919
1929
1939
1949
1959
1969
1979
1989
1999
2009

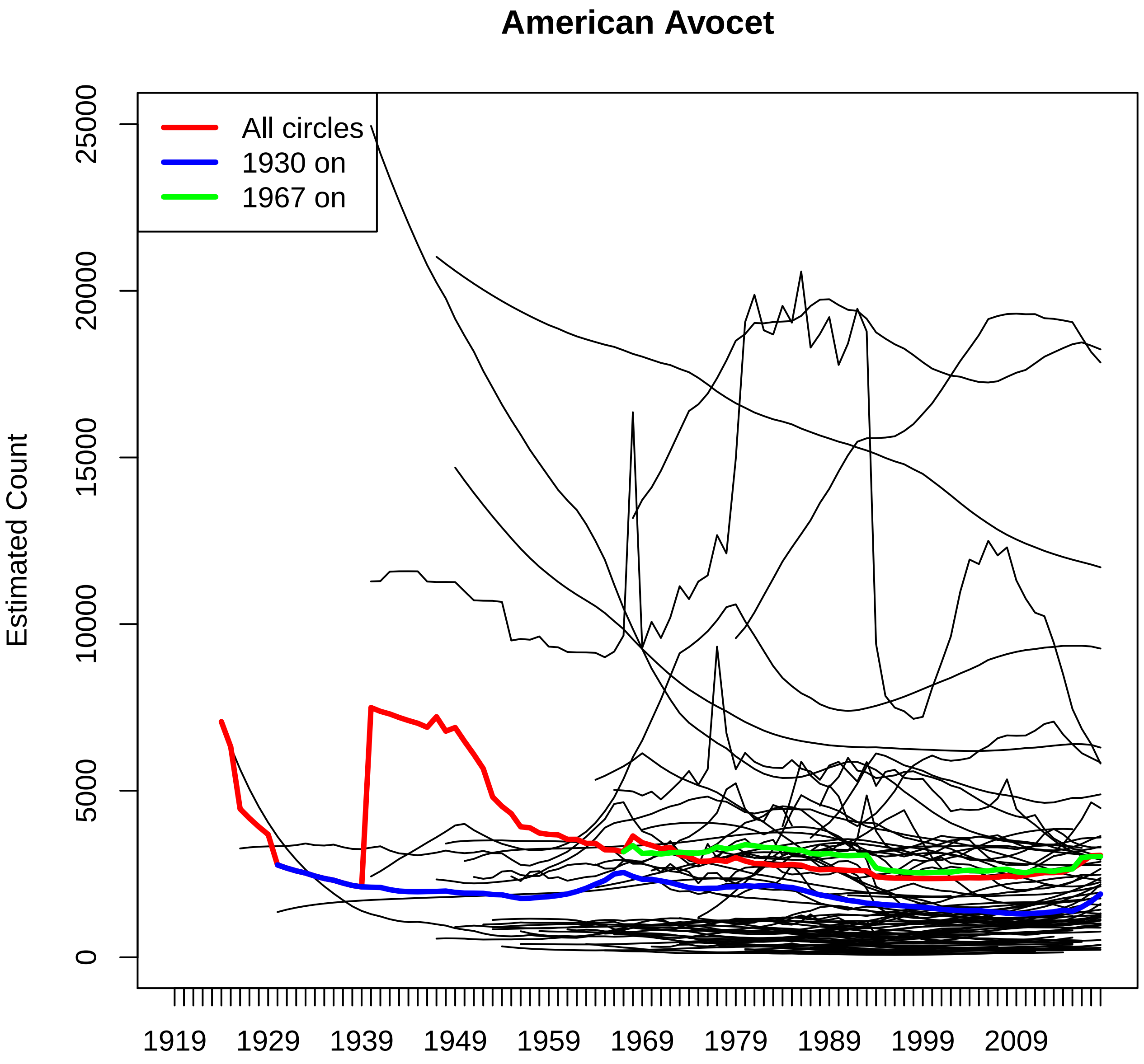

American Avocet
Estimated Count
All circles
1930 on
1967 on
0
5000
10000
15000
20000
25000
1919
1929
1939
1949
1959
1969
1979
1989
1999
2009

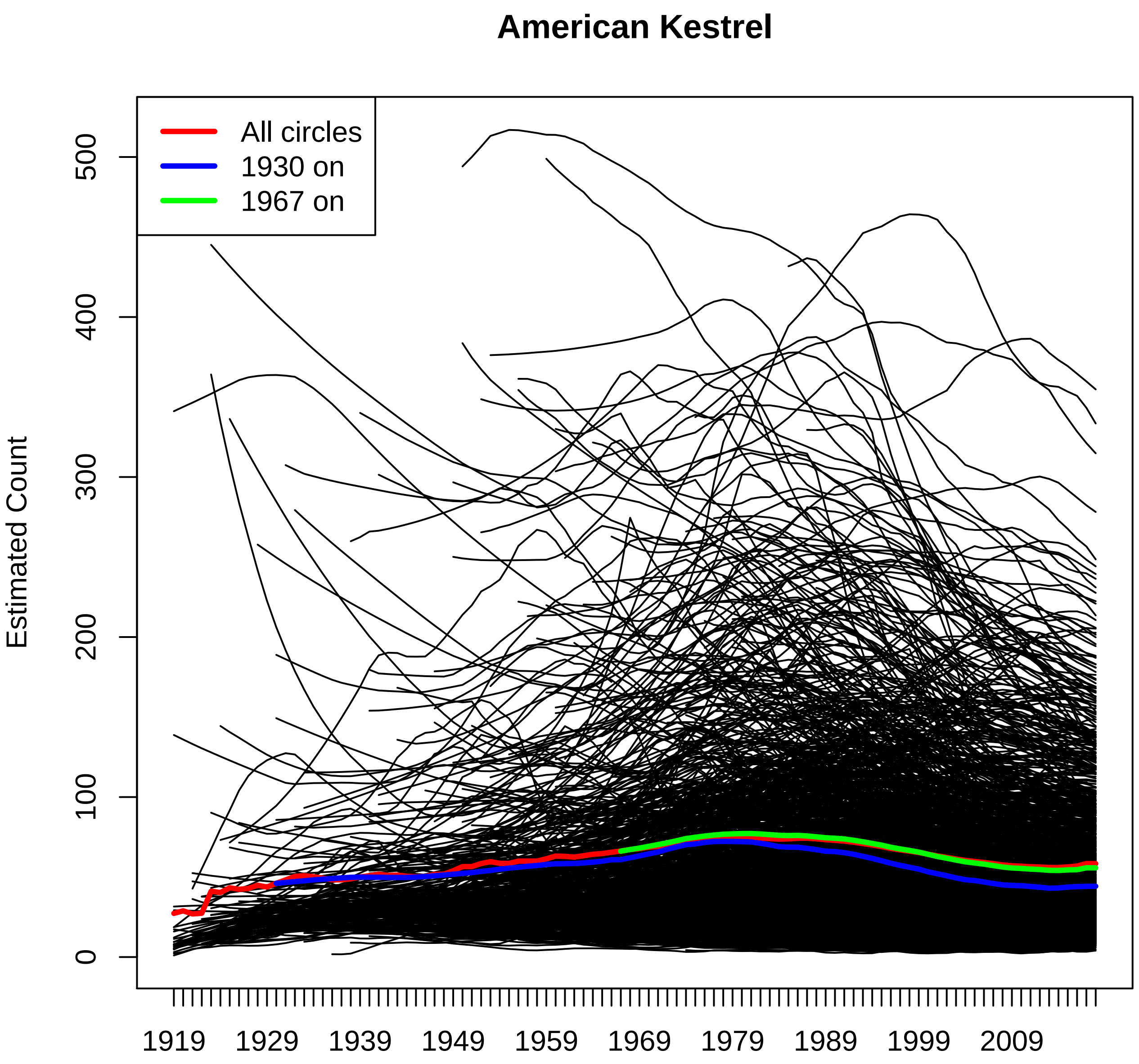
American Kestrel
All circles
1930 on
1967 on
Estimated Count
0
100
200
300
400
500
1919
1929
1939
1949
1959
1969
1979
1989
1999
2009

American White Pelican

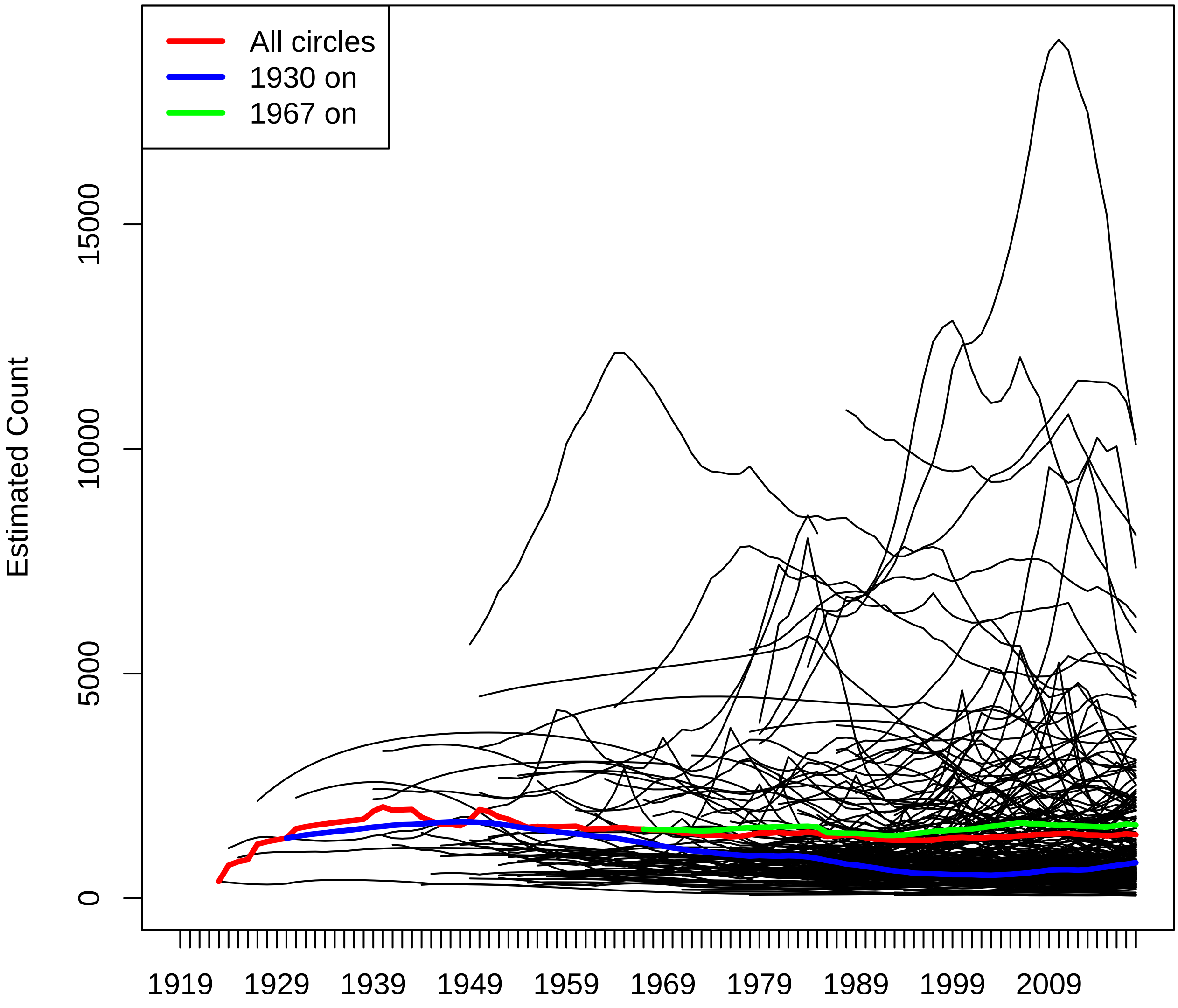

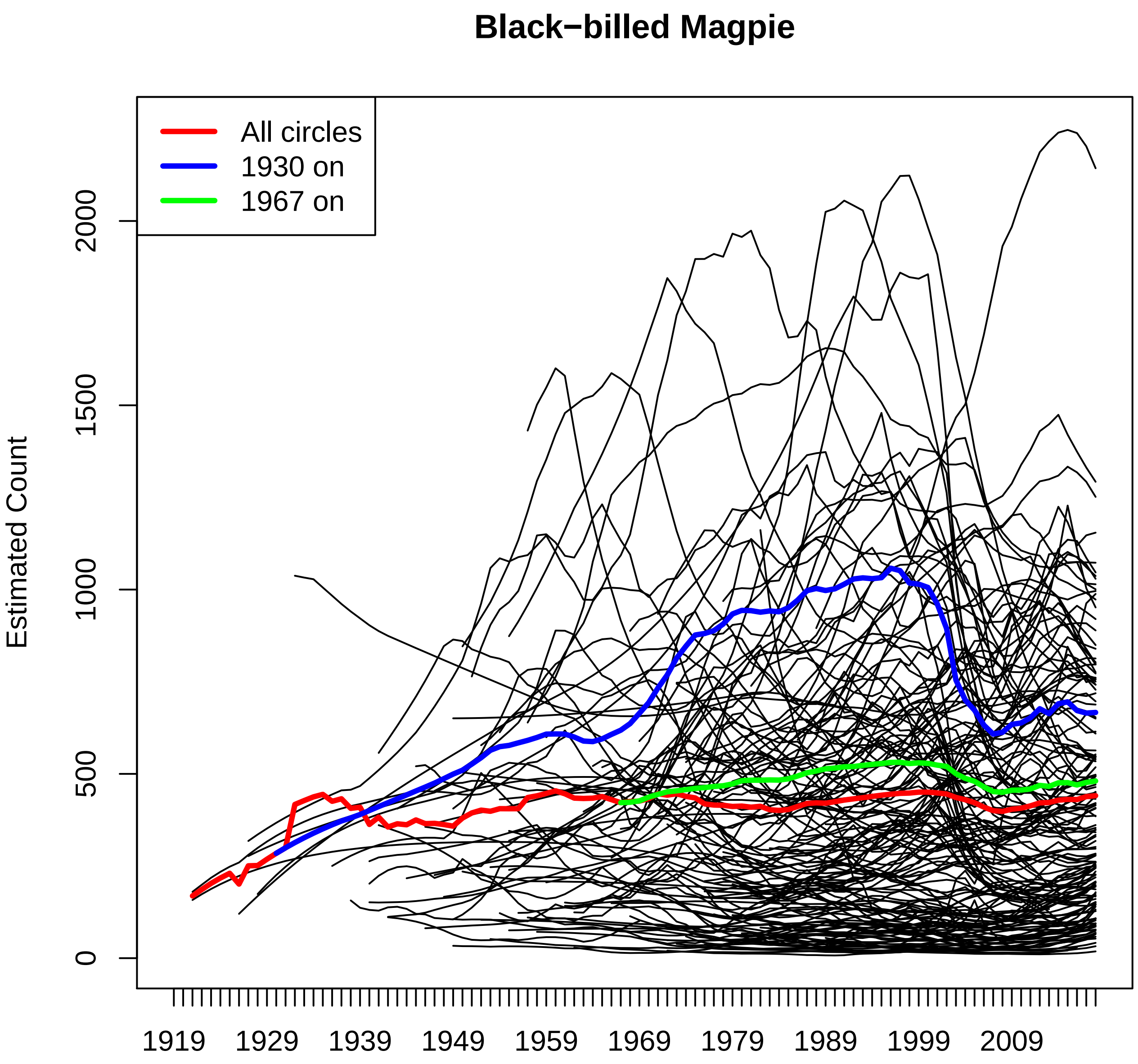

Black−billed Magpie
All circles
1930 on
1967 on
Estimated Count
0
500
1000
1500
2000
1919
1929
1939
1949
1959
1969
1979
1989
1999
2009

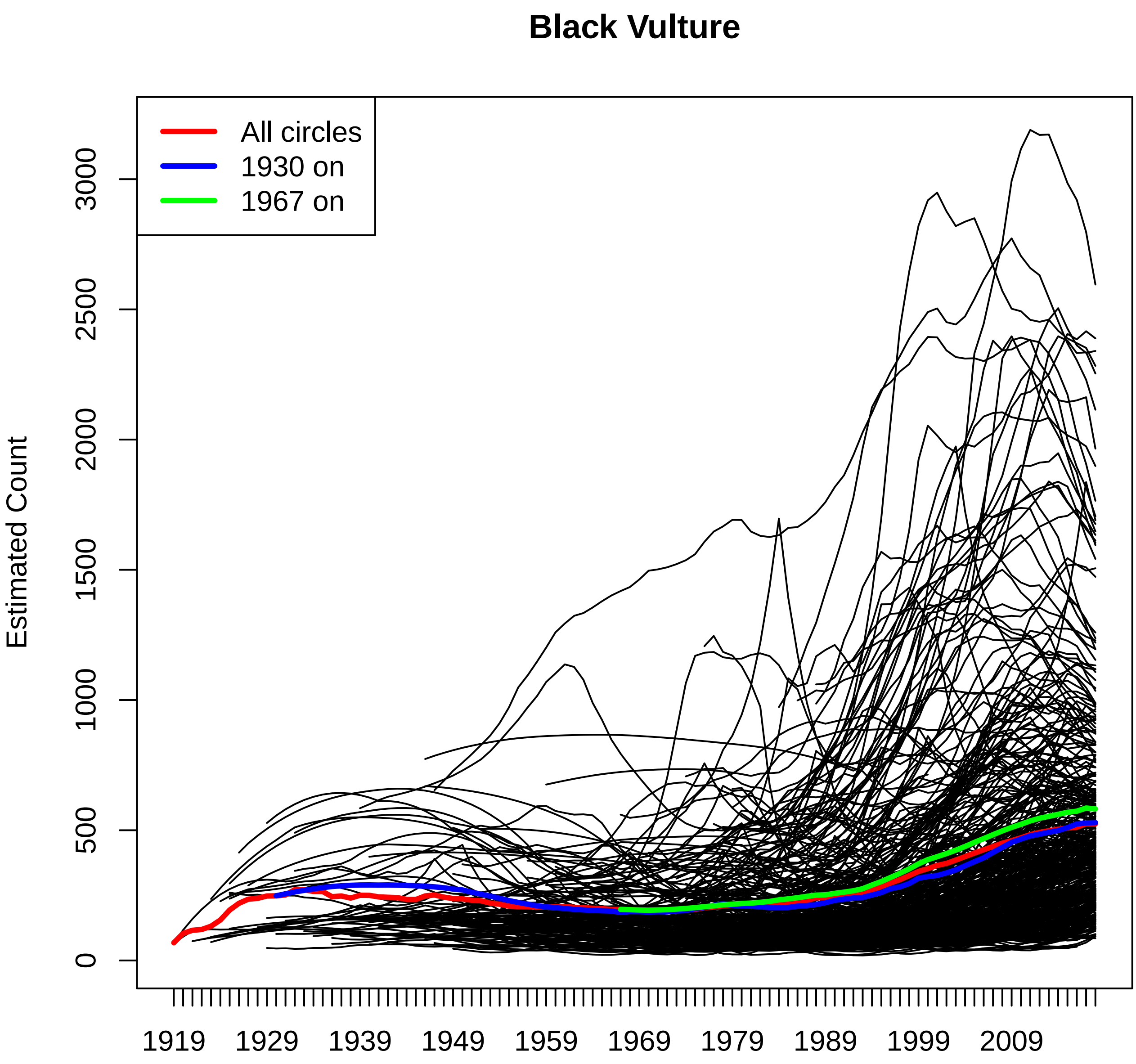

Black Vulture
All circles
1930 on
1967 on
Estimated Count
0
500
1000
1500
2000
2500
3000
1919
1929
1939
1949
1959
1969
1979
1989
1999
2009

Blue−winged Teal

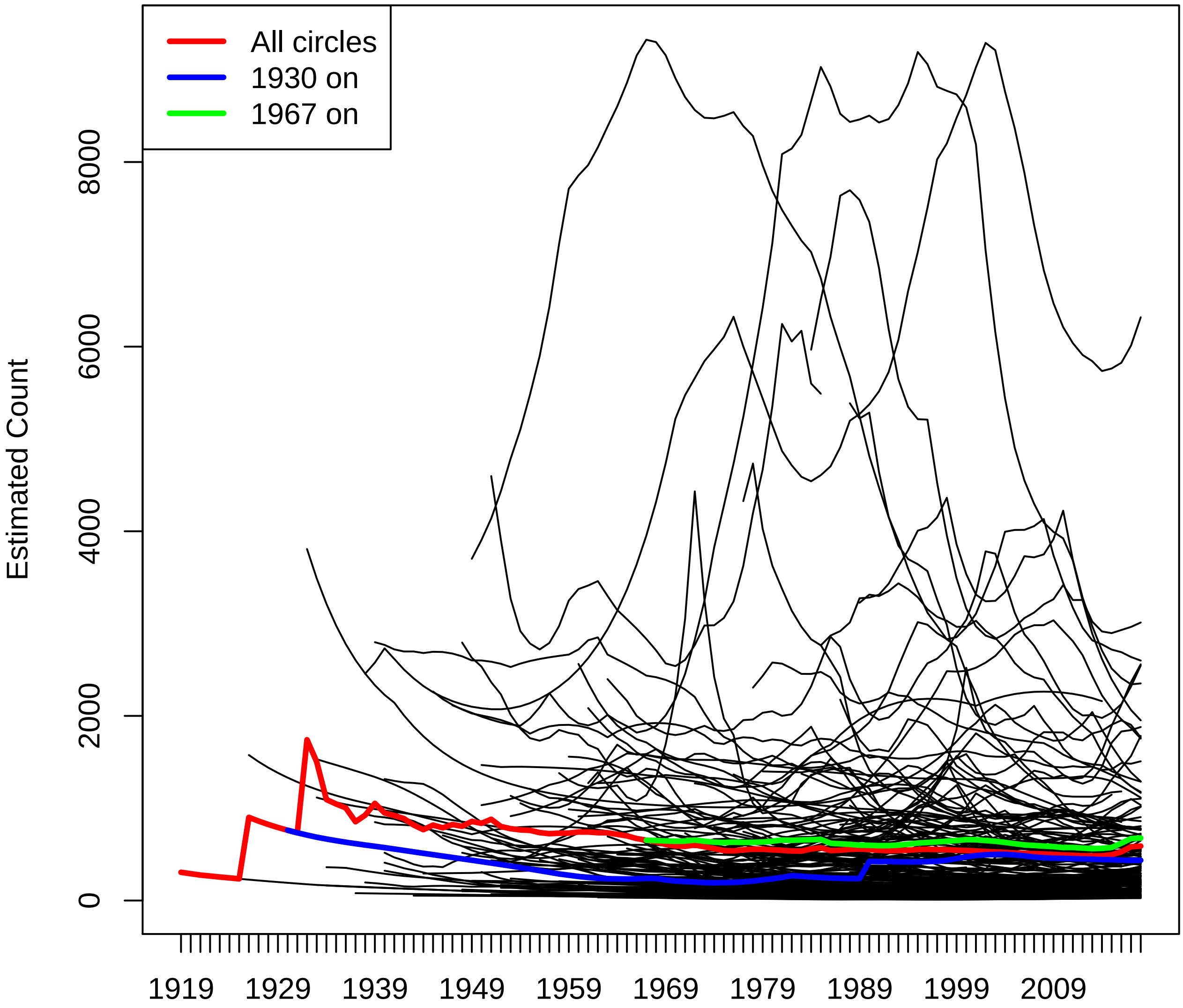

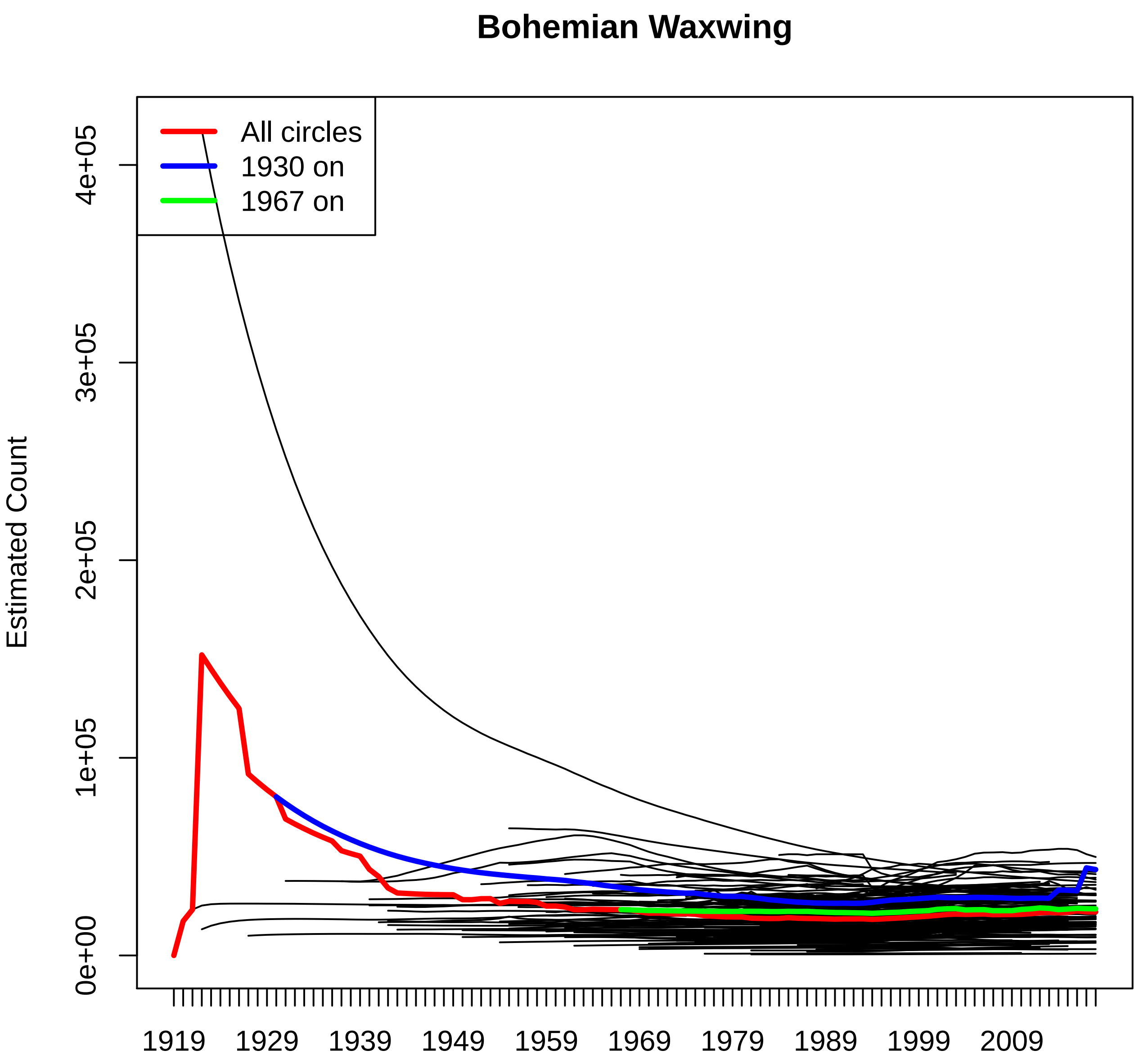
Bohemian Waxwing
All circles
1930 on
1967 on
Estimated Count
0e+00
1e+05
2e+05
3e+05
4e+05
1919
1929
1939
1949
1959
1969
1979
1989
1999
2009

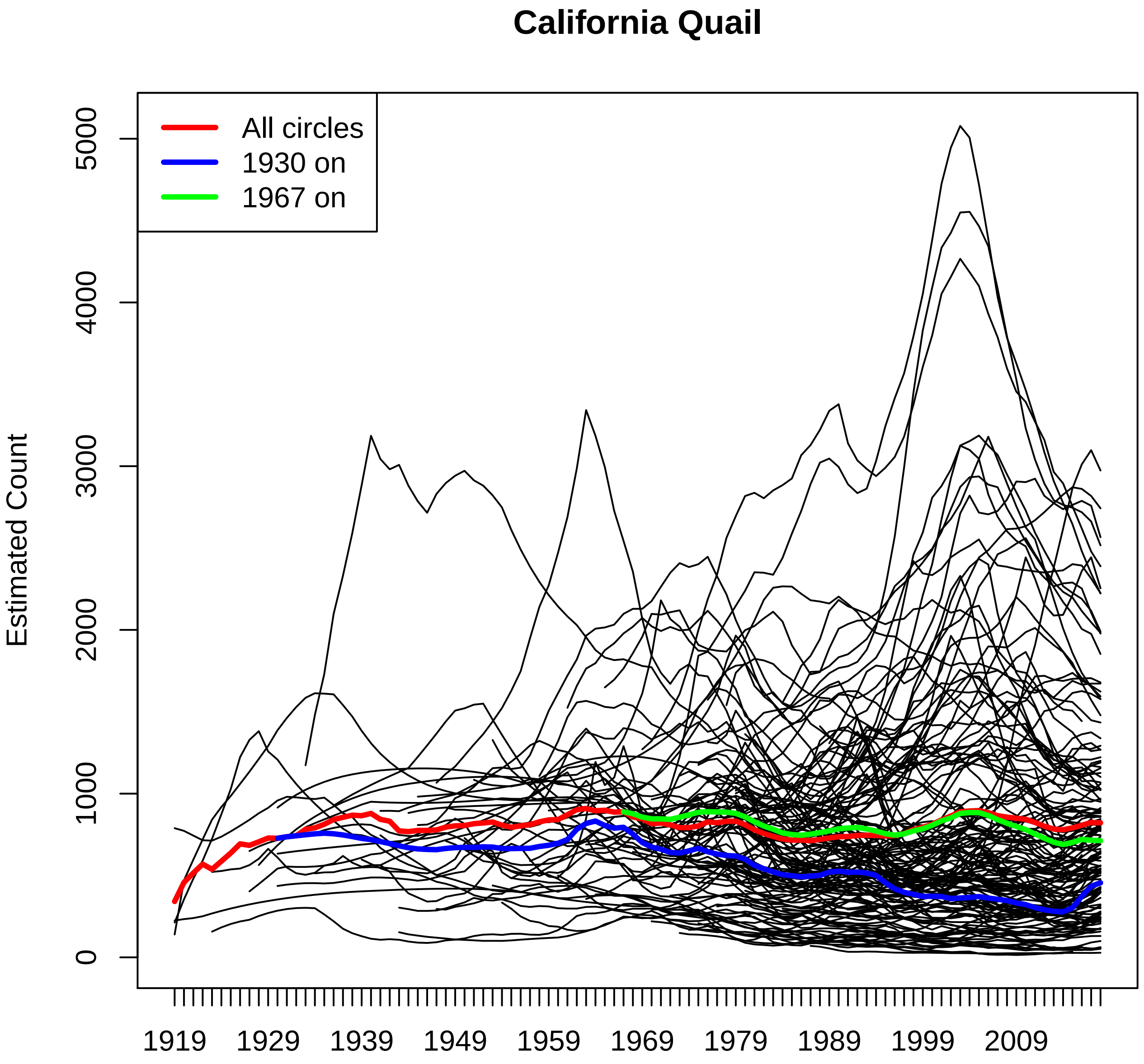
California Quail
Estimated Count
All circles
1930 on
1967 on
0
1000
2000
3000
4000
5000
1919
1929
1939
1949
1959
1969
1979
1989
1999
2009

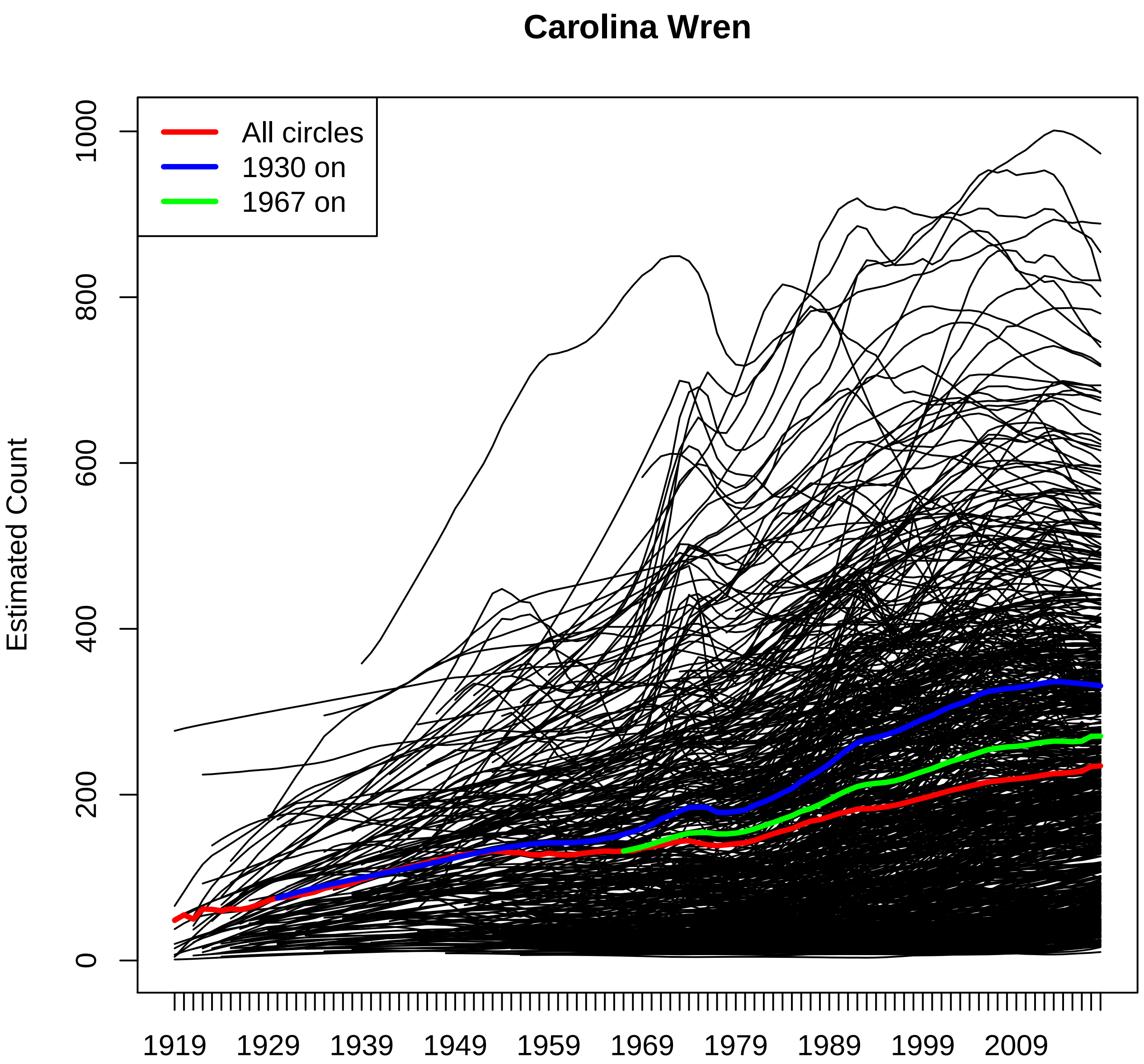

Carolina Wren
All circles
1930 on
1967 on
Estimated Count
0
200
400
600
800
1000
1919
1929
1939
1949
1959
1969
1979
1989
1999
2009

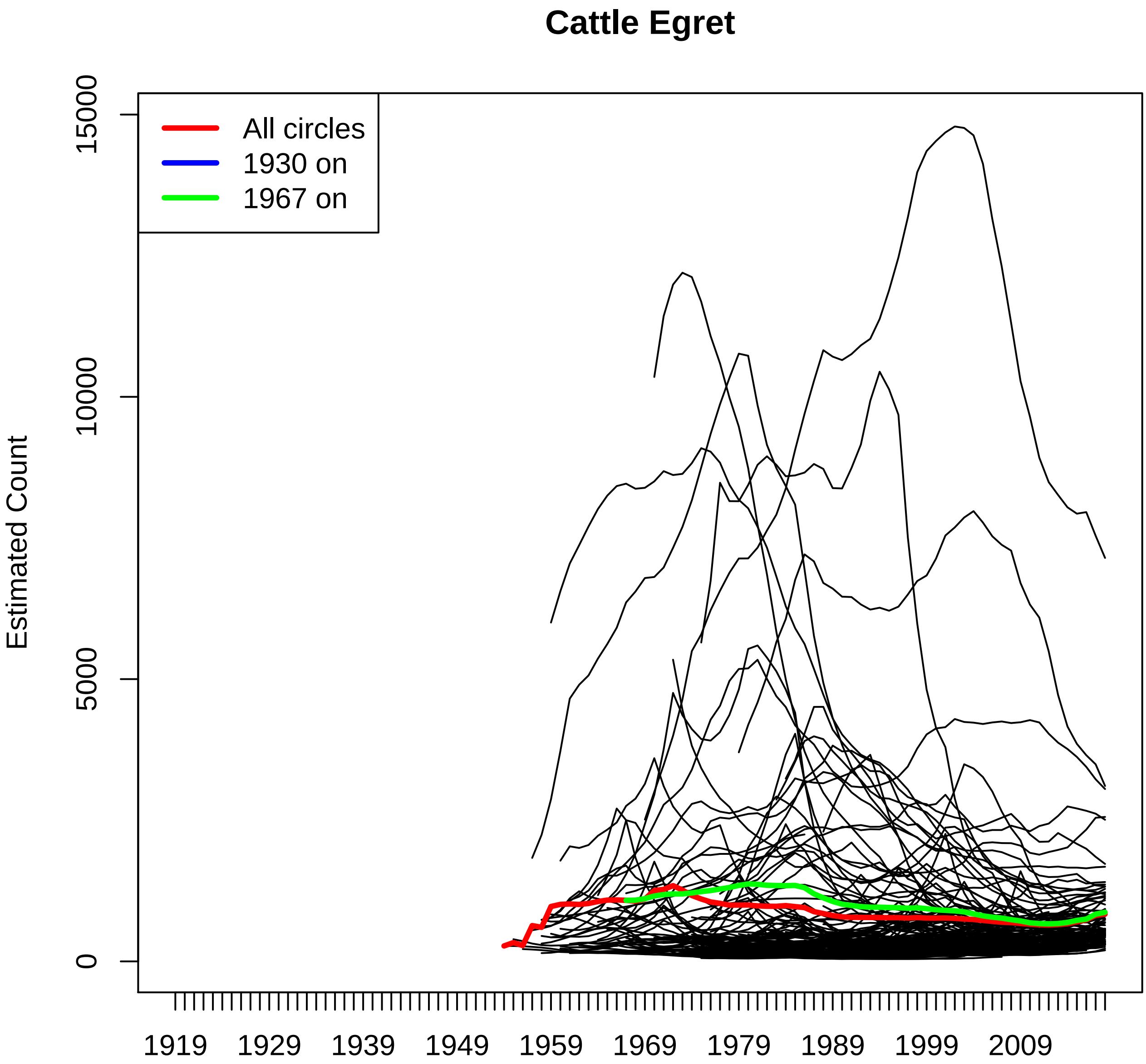
Cattle Egret
All circles
1930 on
1967 on
Estimated Count
0
5000
10000
15000
1919
1929
1939
1949
1959
1969
1979
1989
1999
2009

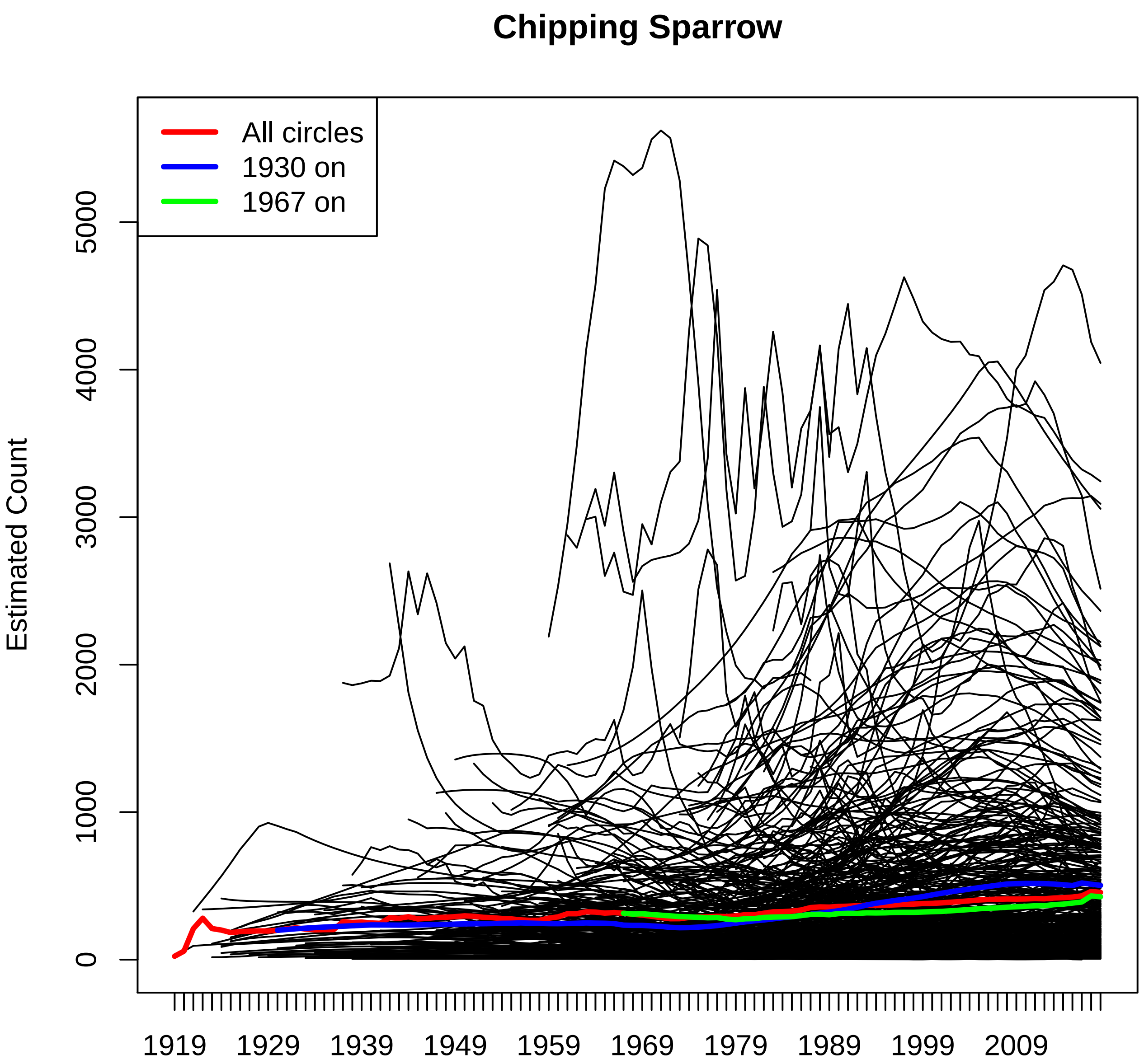
Chipping Sparrow
All circles
1930 on
1967 on
Estimated Count
0
1000
2000
3000
4000
5000
1919
1929
1939
1949
1959
1969
1979
1989
1999
2009

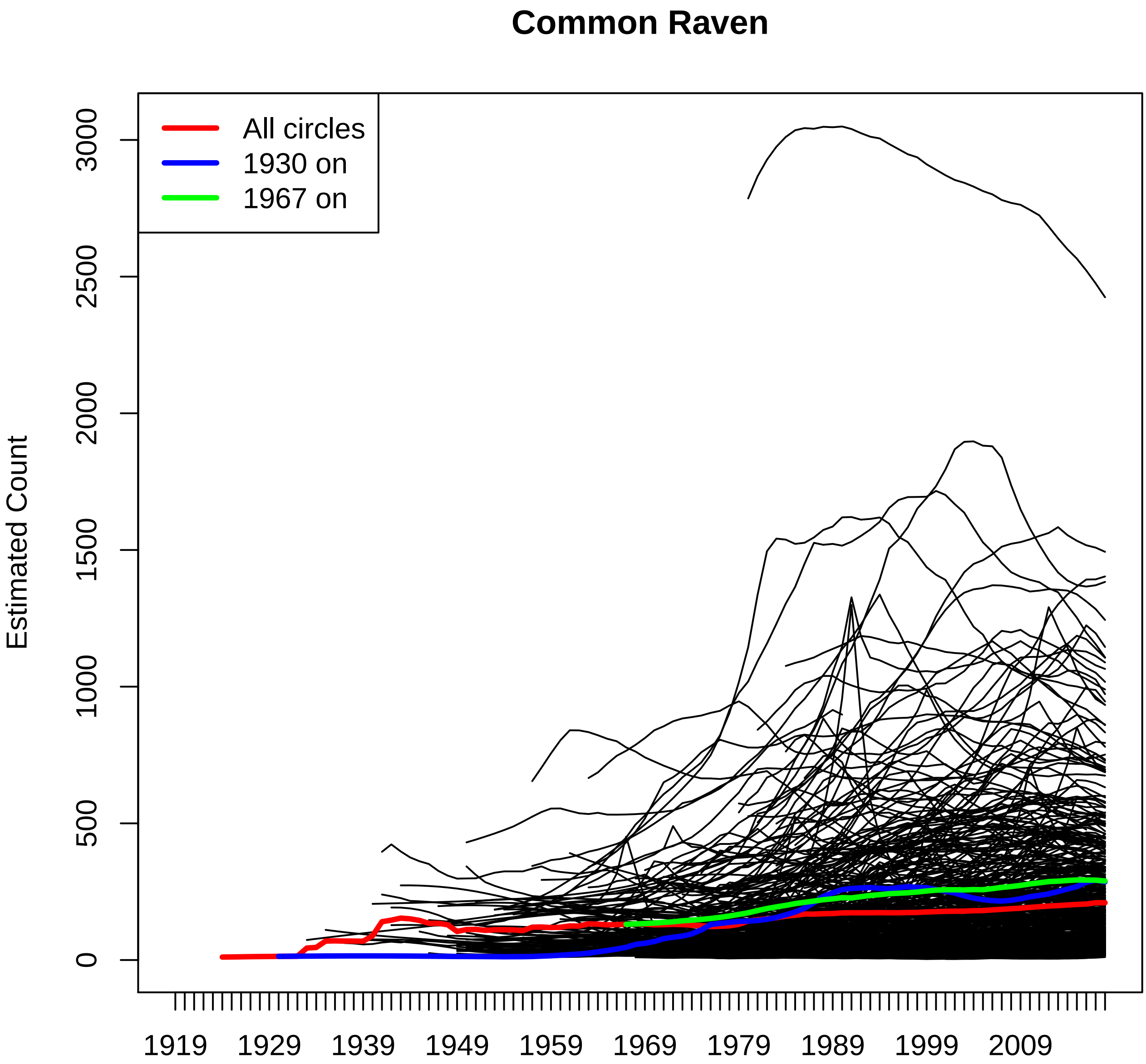

Common Raven
All circles
1930 on
1967 on
Estimated Count
0
500
1000
1500
2000
2500
3000
1919
1929
1939
1949
1959
1969
1979
1989
1999
2009

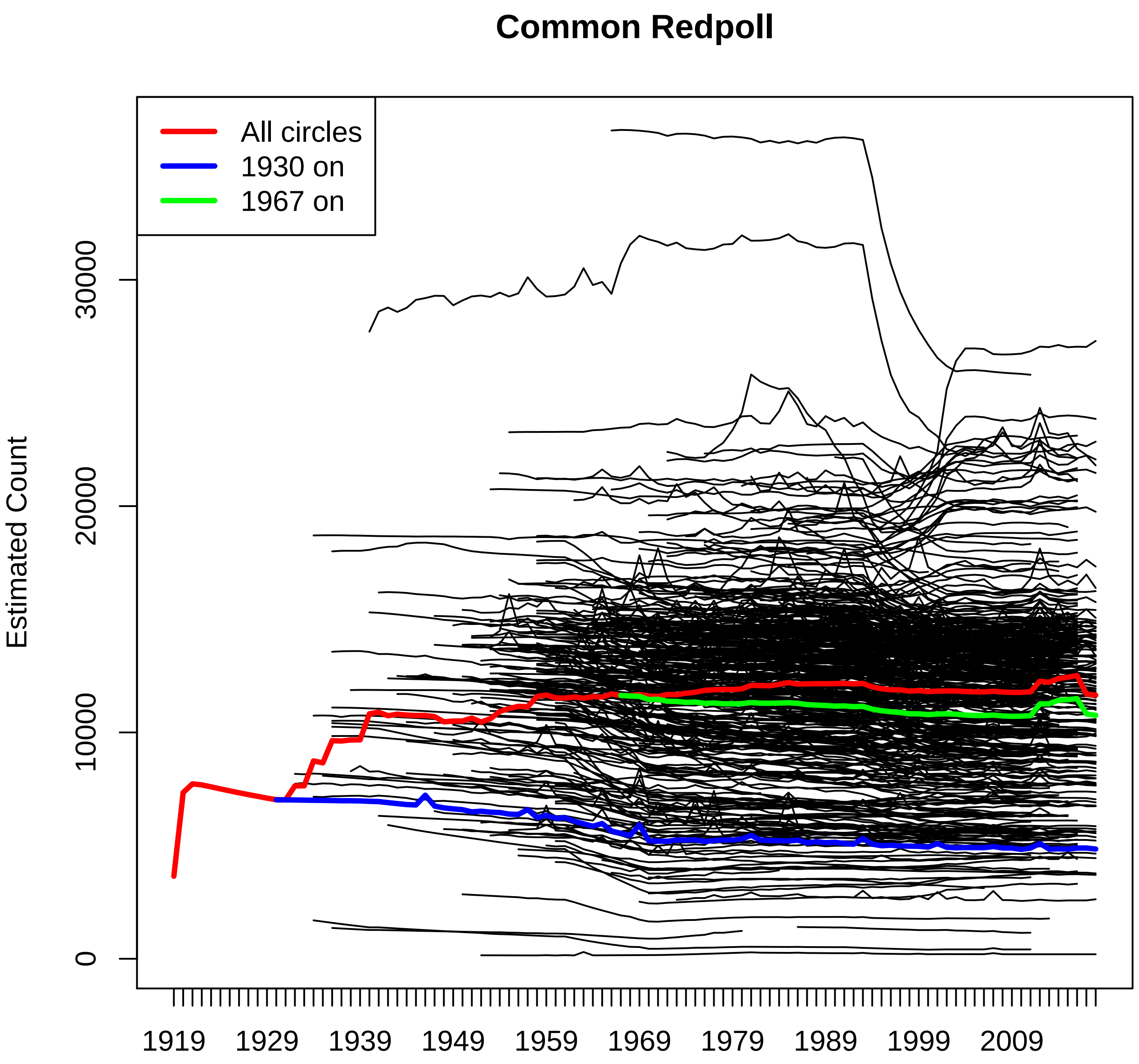
Common Redpoll
All circles
1930 on
1967 on
Estimated Count
0
10000
20000
30000
1919
1929
1939
1949
1959
1969
1979
1989
1999
2009

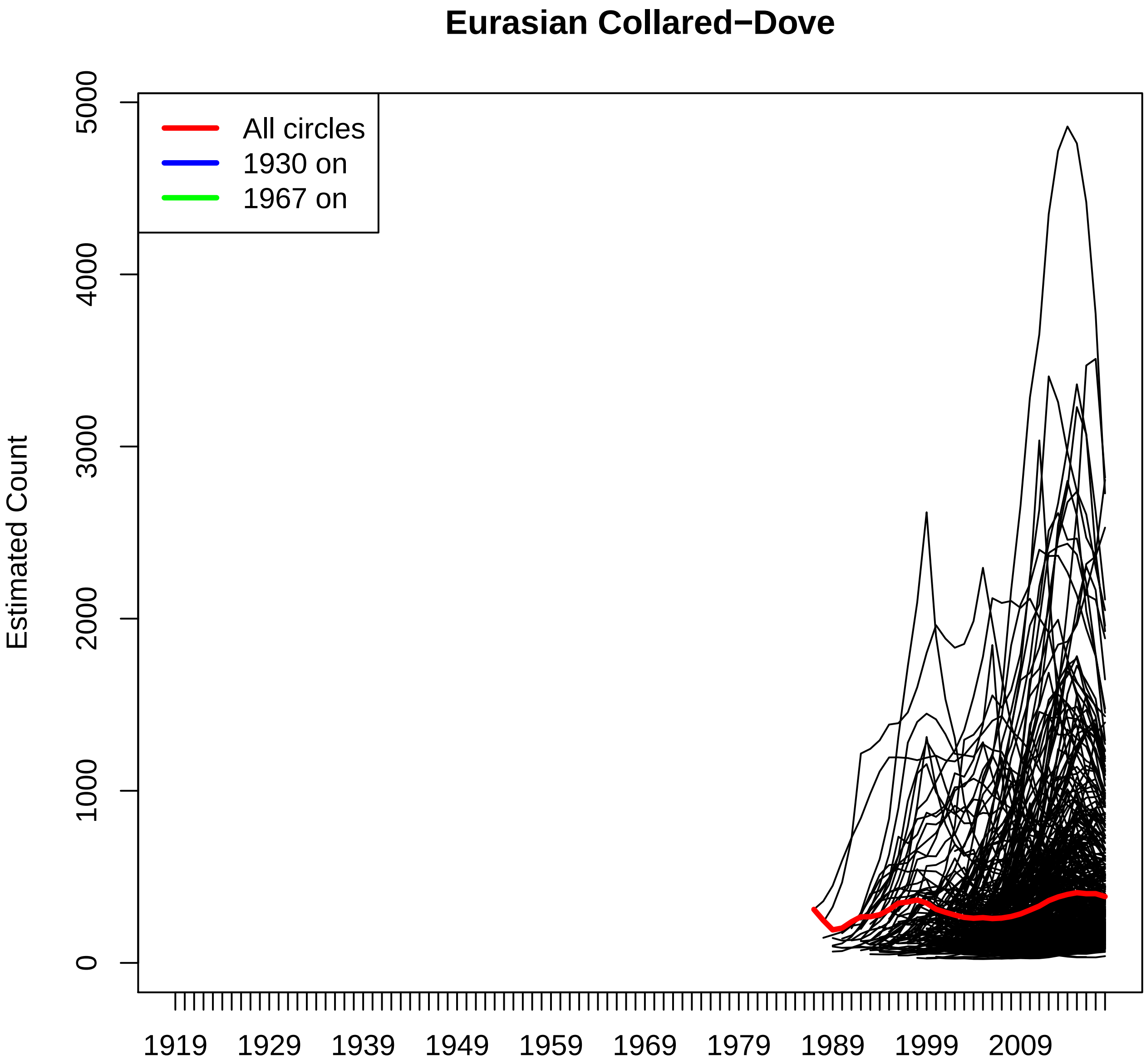
Eurasian Collared−Dove
All circles
1930 on
1967 on
Estimated Count
0
1000
2000
3000
4000
5000
1919
1929
1939
1949
1959
1969
1979
1989
1999
2009

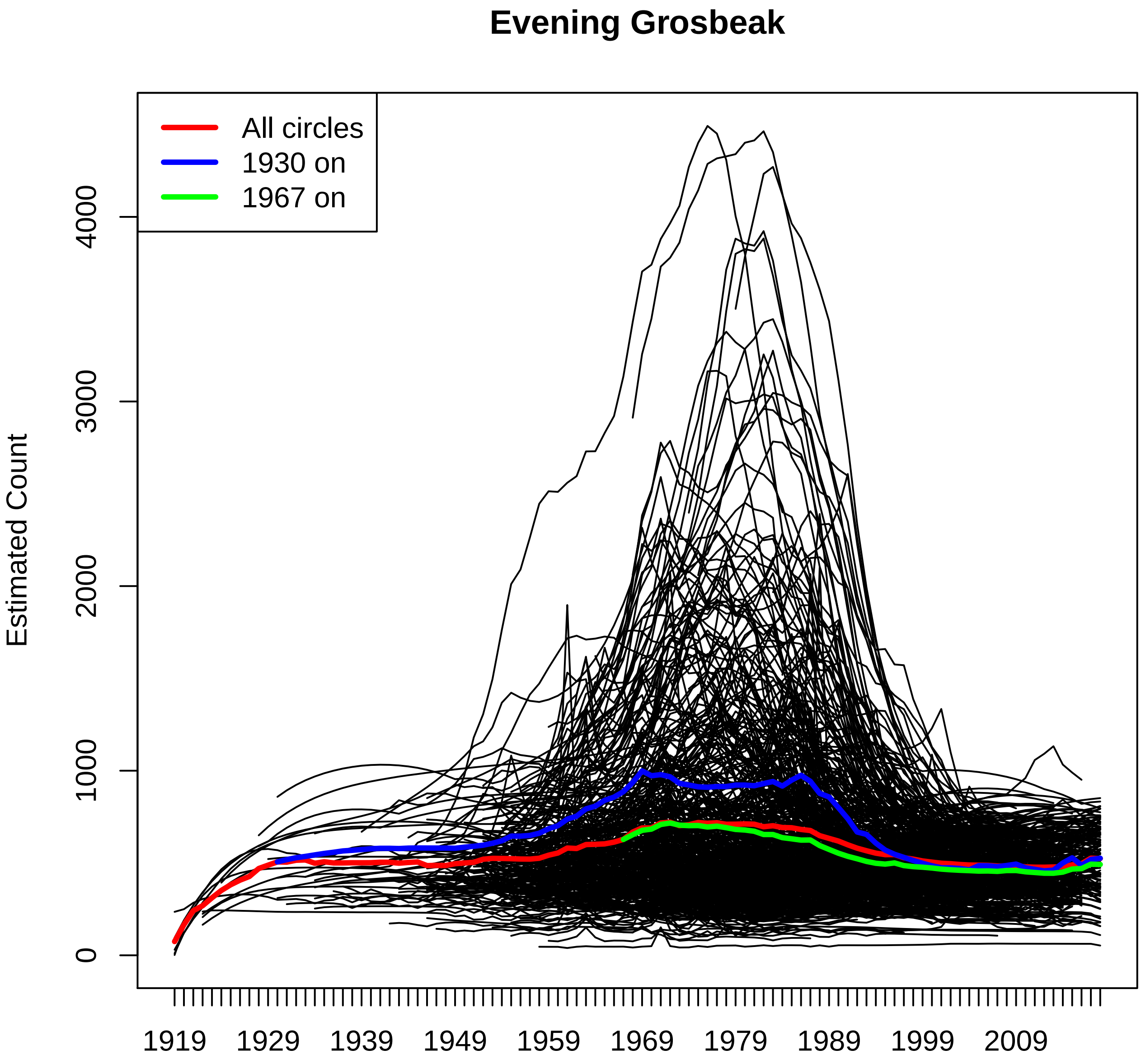

Evening Grosbeak
All circles
1930 on
1967 on
Estimated Count
0
1000
2000
3000
4000
1919
1929
1939
1949
1959
1969
1979
1989
1999
2009

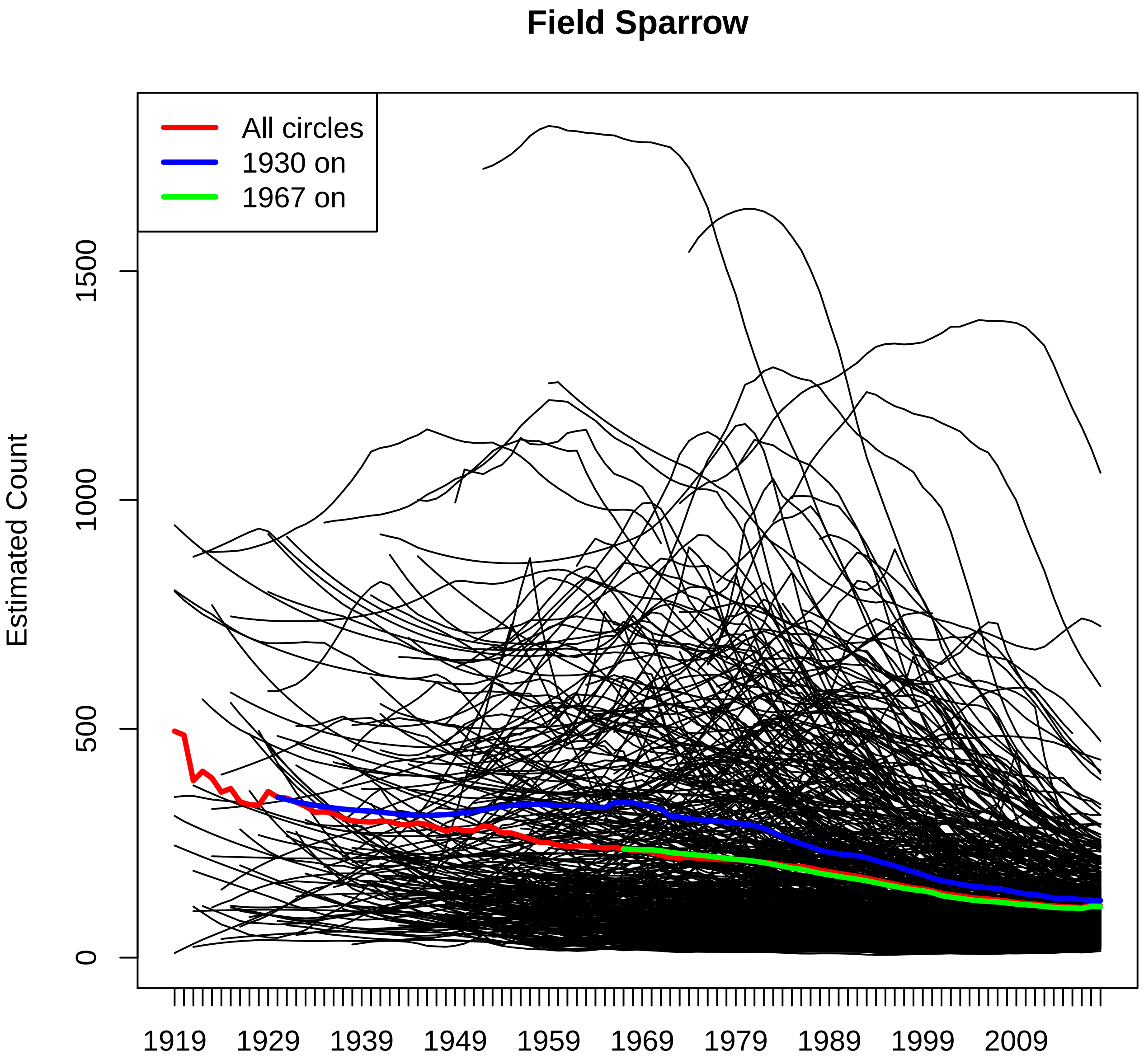

Field Sparrow
All circles
1930 on
1967 on
Estimated Count
0
500
1000
1500
1919
1929
1939
1949
1959
1969
1979
1989
1999
2009

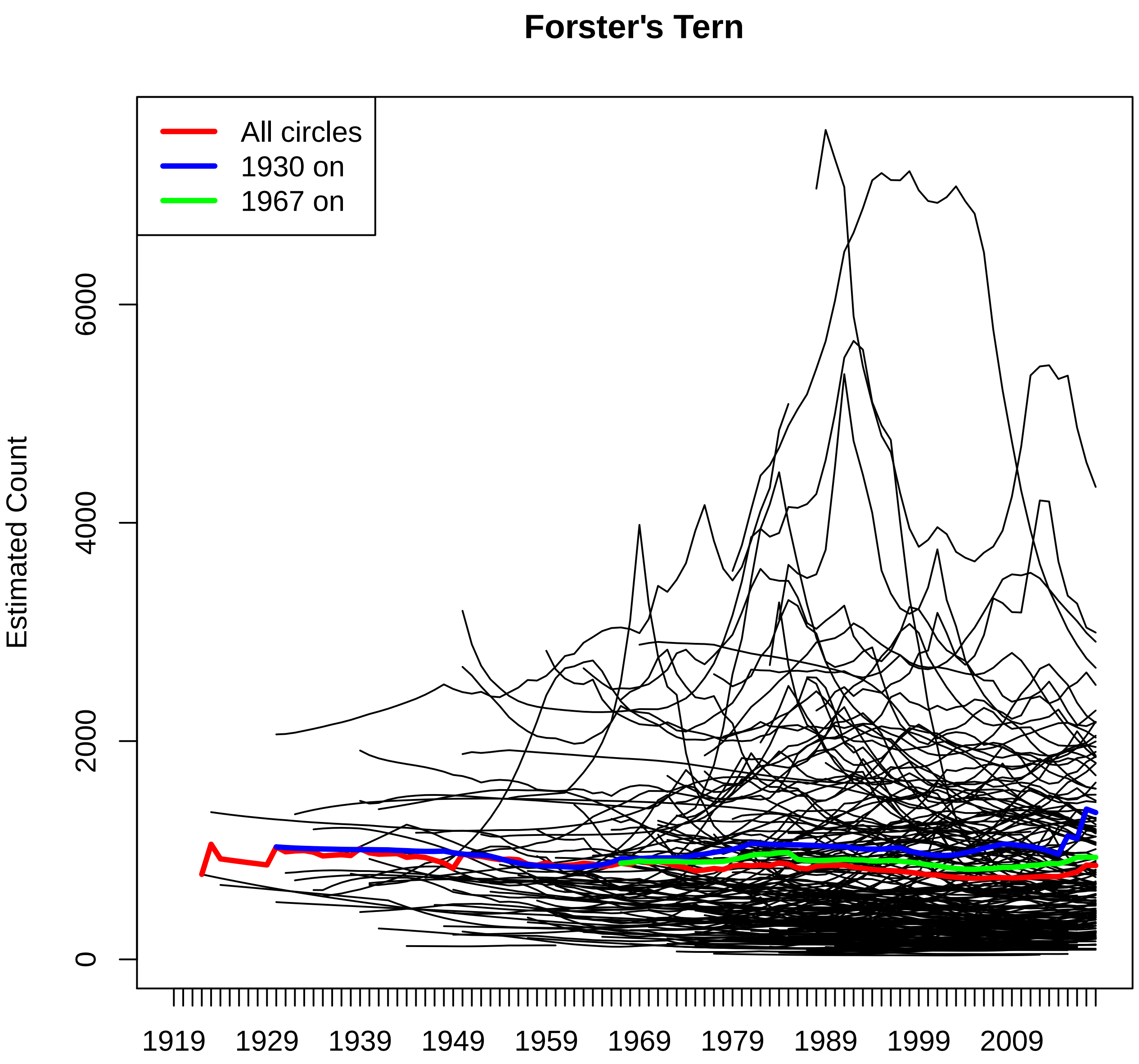

Forster's Tern
All circles
1930 on
1967 on
Estimated Count
0
2000
4000
6000
1919
1929
1939
1949
1959
1969
1979
1989
1999
2009

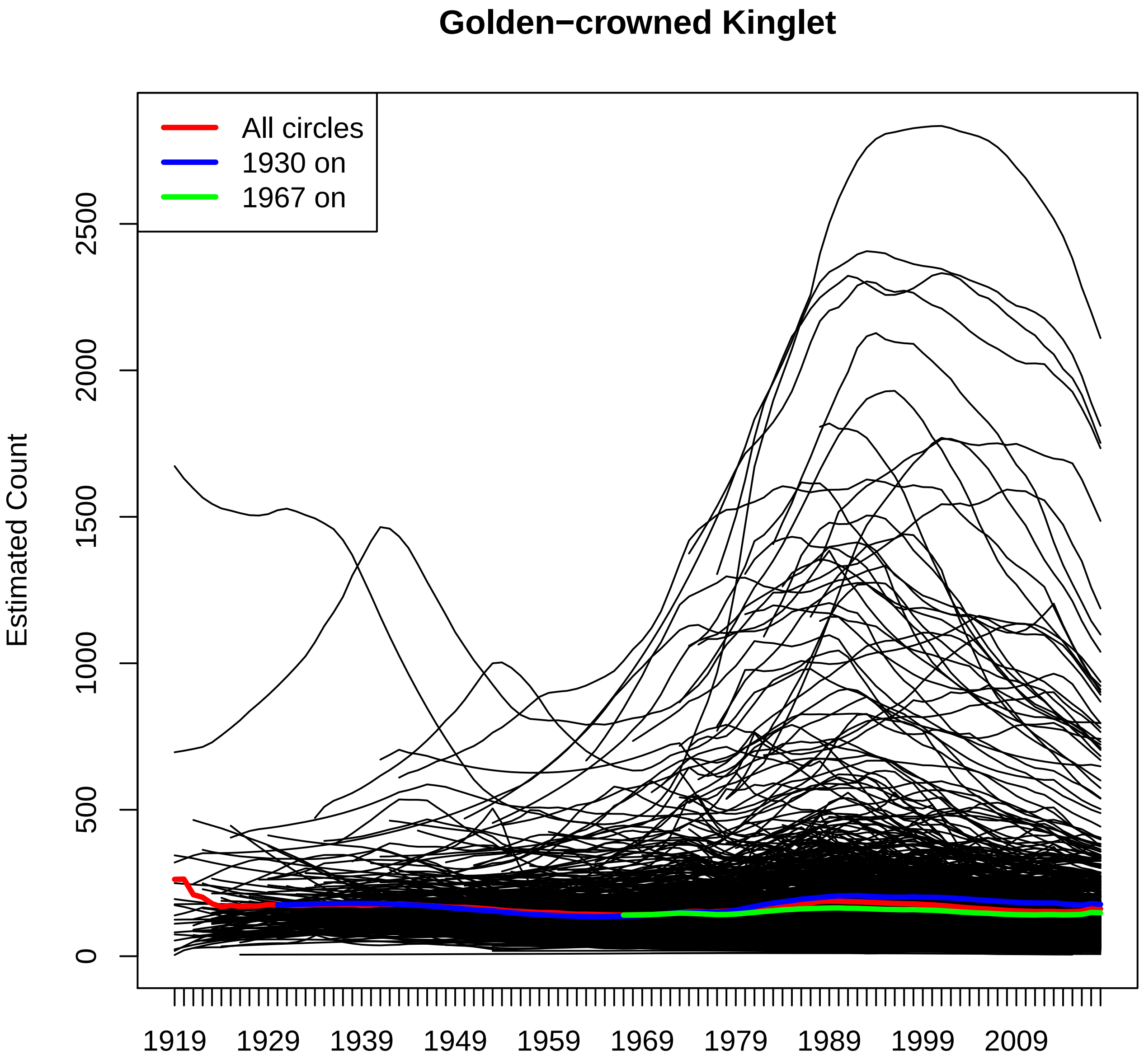

Golden−crowned Kinglet
All circles
1930 on
1967 on
Estimated Count
0
500
1000
1500
2000
2500
1919
1929
1939
1949
1959
1969
1979
1989
1999
2009

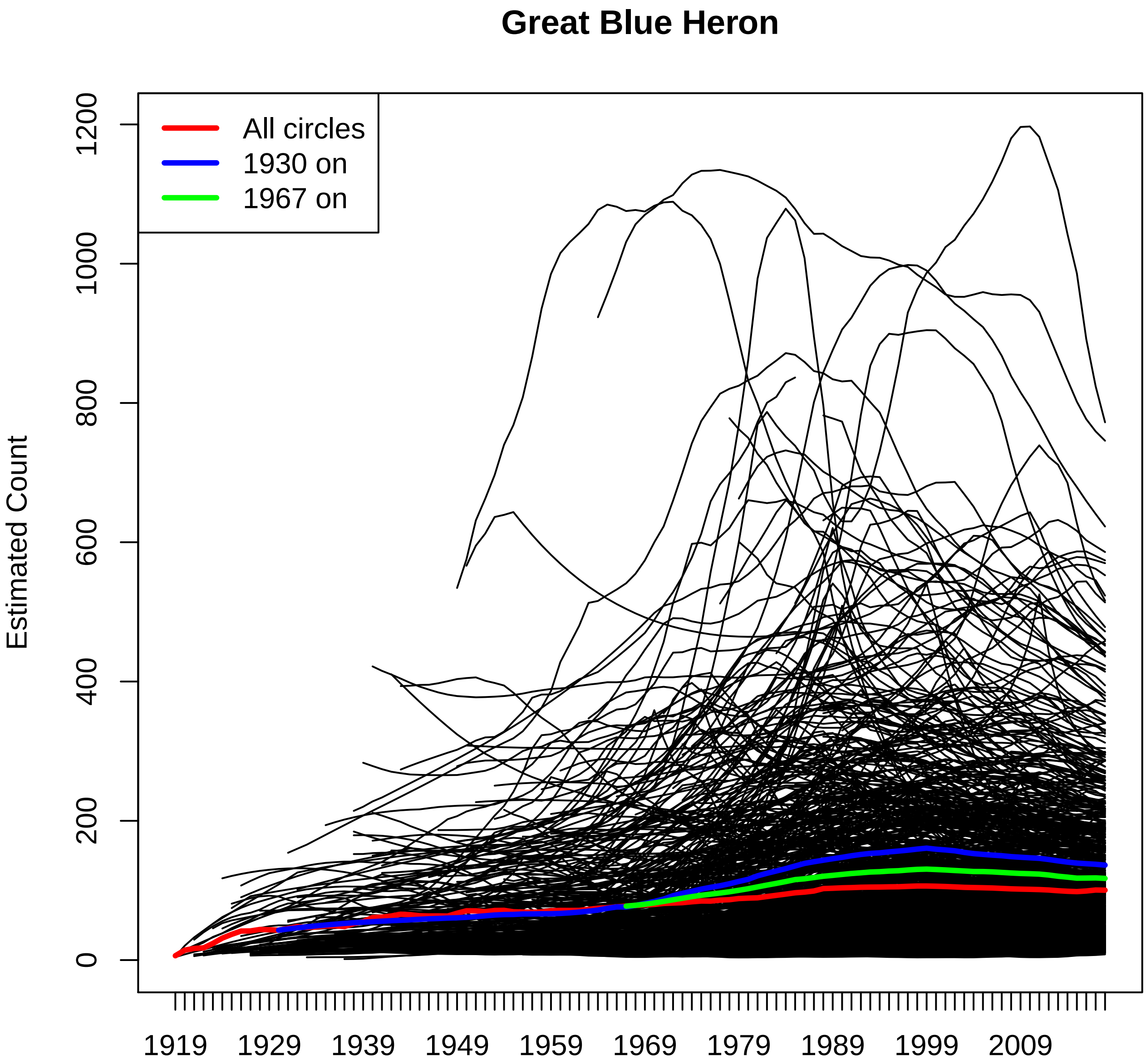
Great Blue Heron
Estimated Count
All circles
1930 on
1967 on
0
200
400
600
800
1000
1200
1919
1929
1939
1949
1959
1969
1979
1989
1999
2009

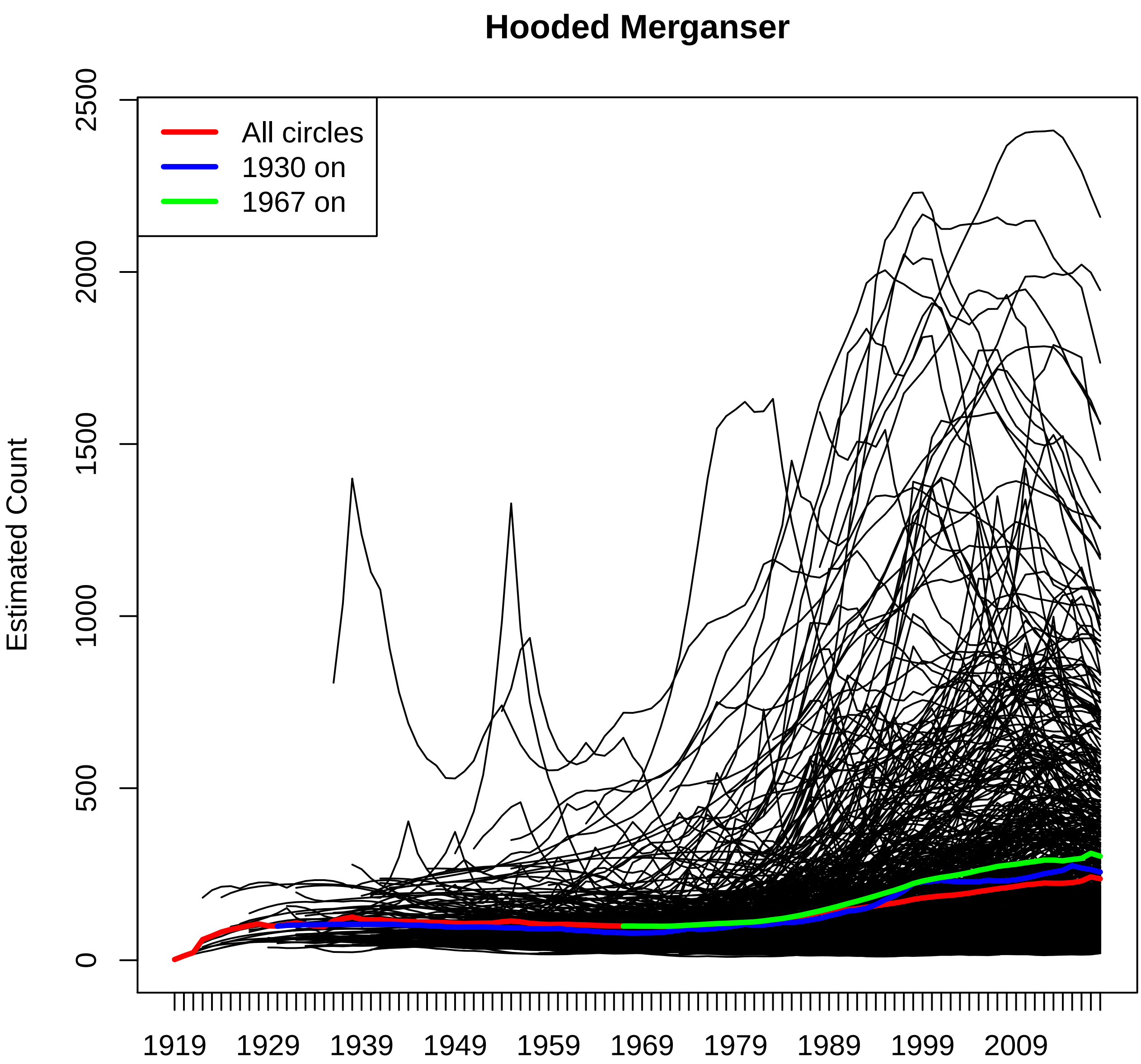
Hooded Merganser
All circles
1930 on
1967 on
Estimated Count
0
500
1000
1500
2000
2500
1919
1929
1939
1949
1959
1969
1979
1989
1999
2009

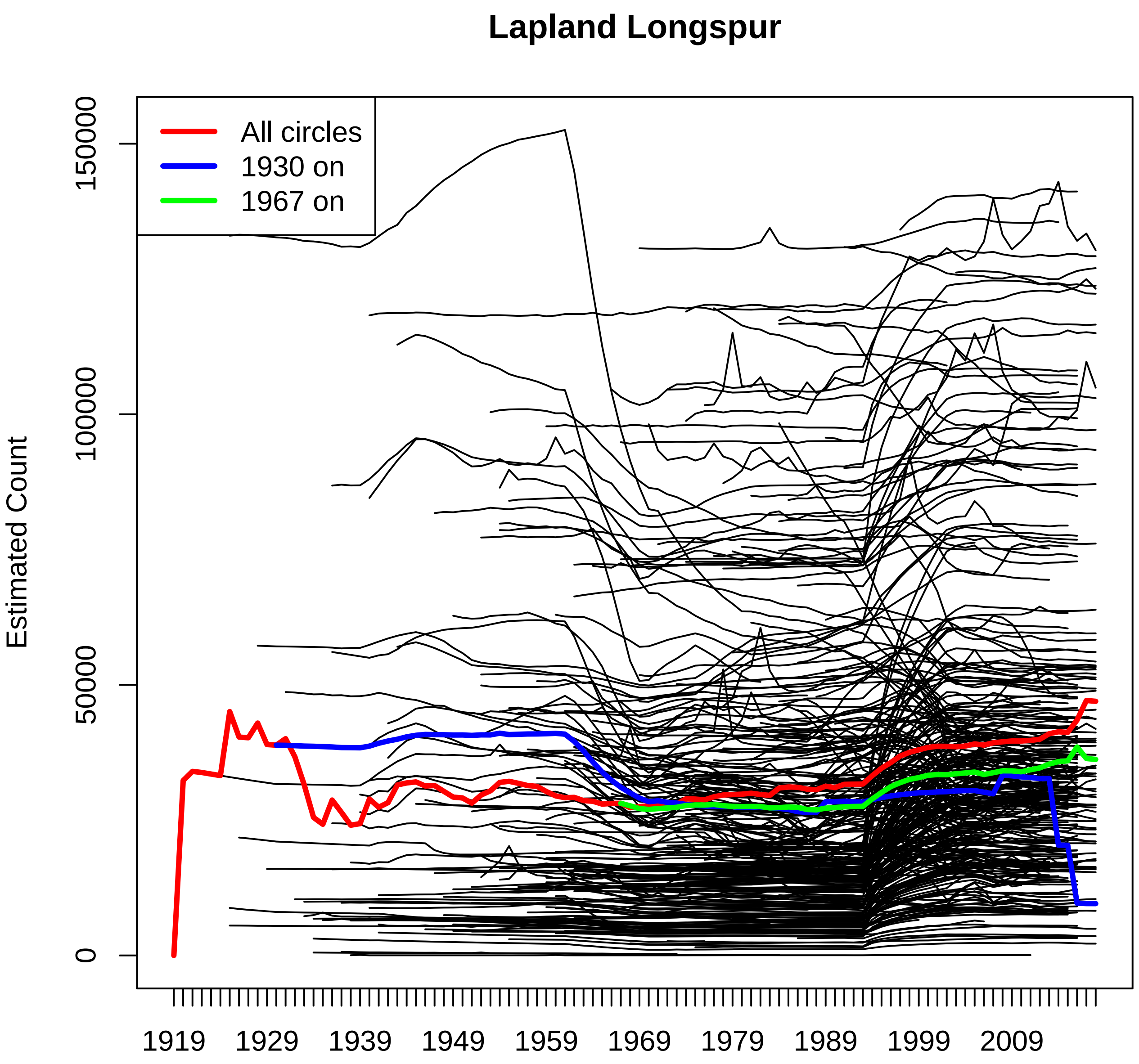

Lapland Longspur
All circles
1930 on
1967 on
Estimated Count
150000
100000
50000
0
1919
1929
1939
1949
1959
1969
1979
1989
1999
2009

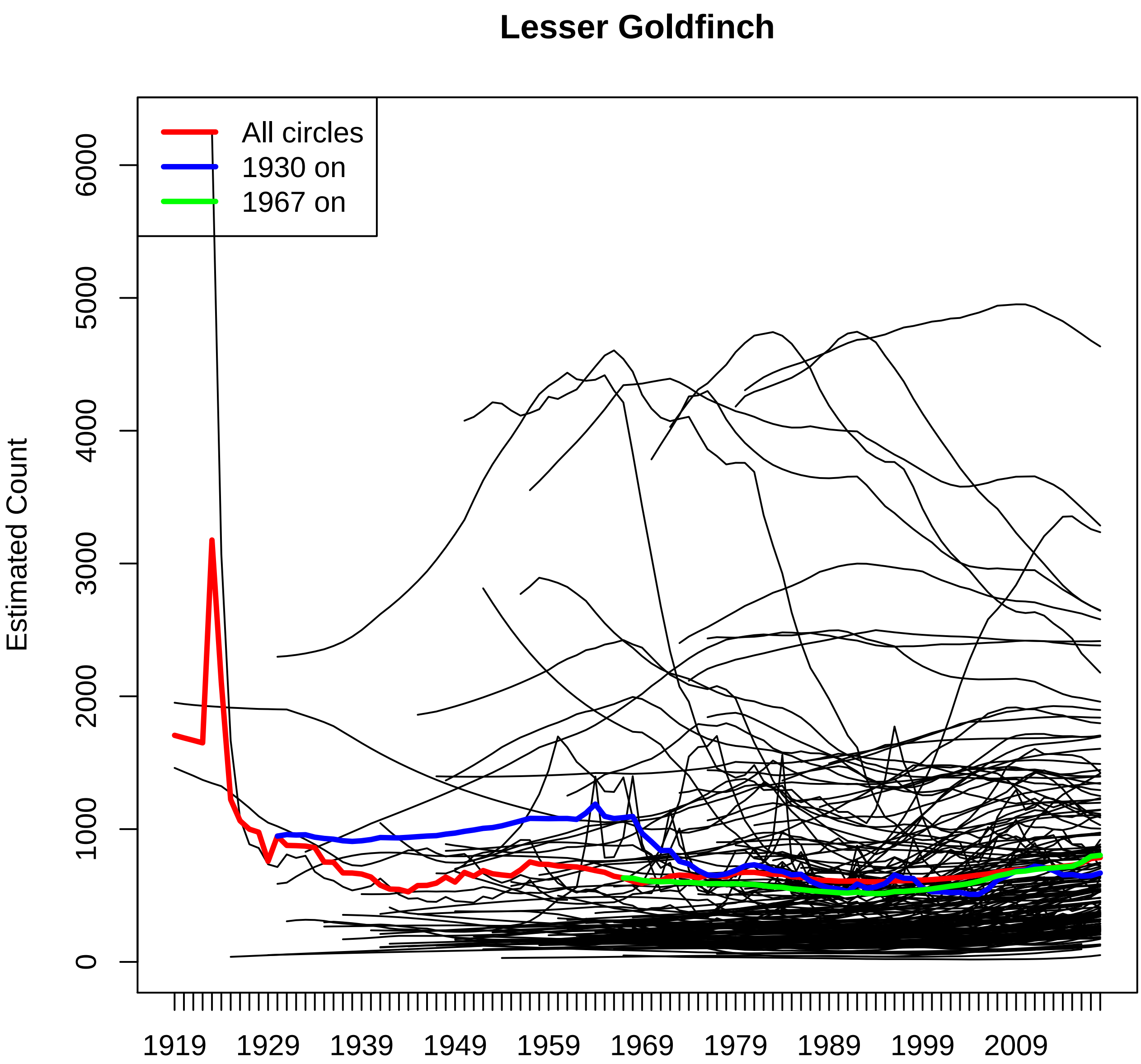

Lesser Goldfinch
All circles
1930 on
1967 on
Estimated Count
0
1000
2000
3000
4000
5000
6000
1919
1929
1939
1949
1959
1969
1979
1989
1999
2009

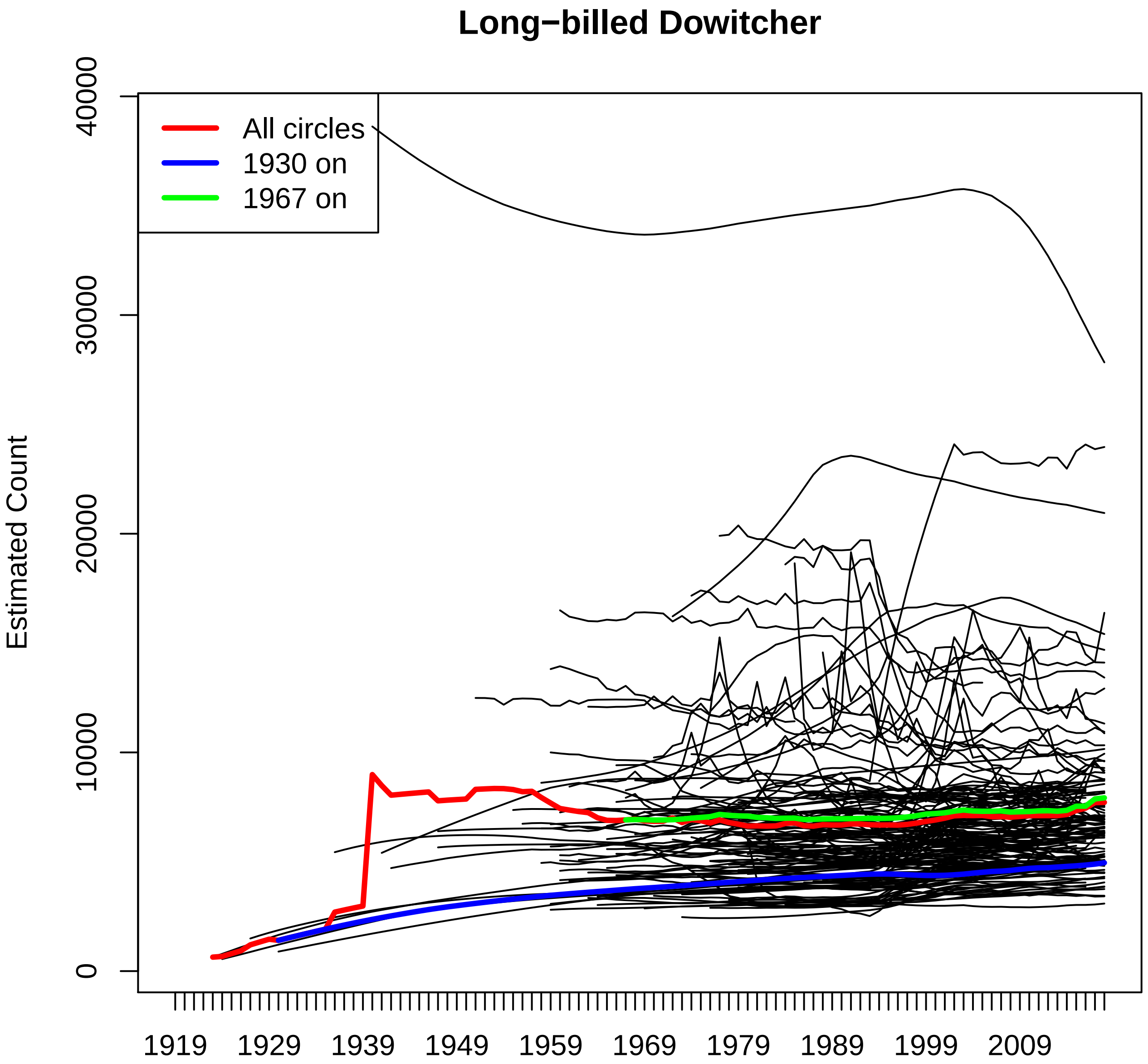
Long−billed Dowitcher
All circles
1930 on
1967 on
Estimated Count
0
10000
20000
30000
40000
1919
1929
1939
1949
1959
1969
1979
1989
1999
2009

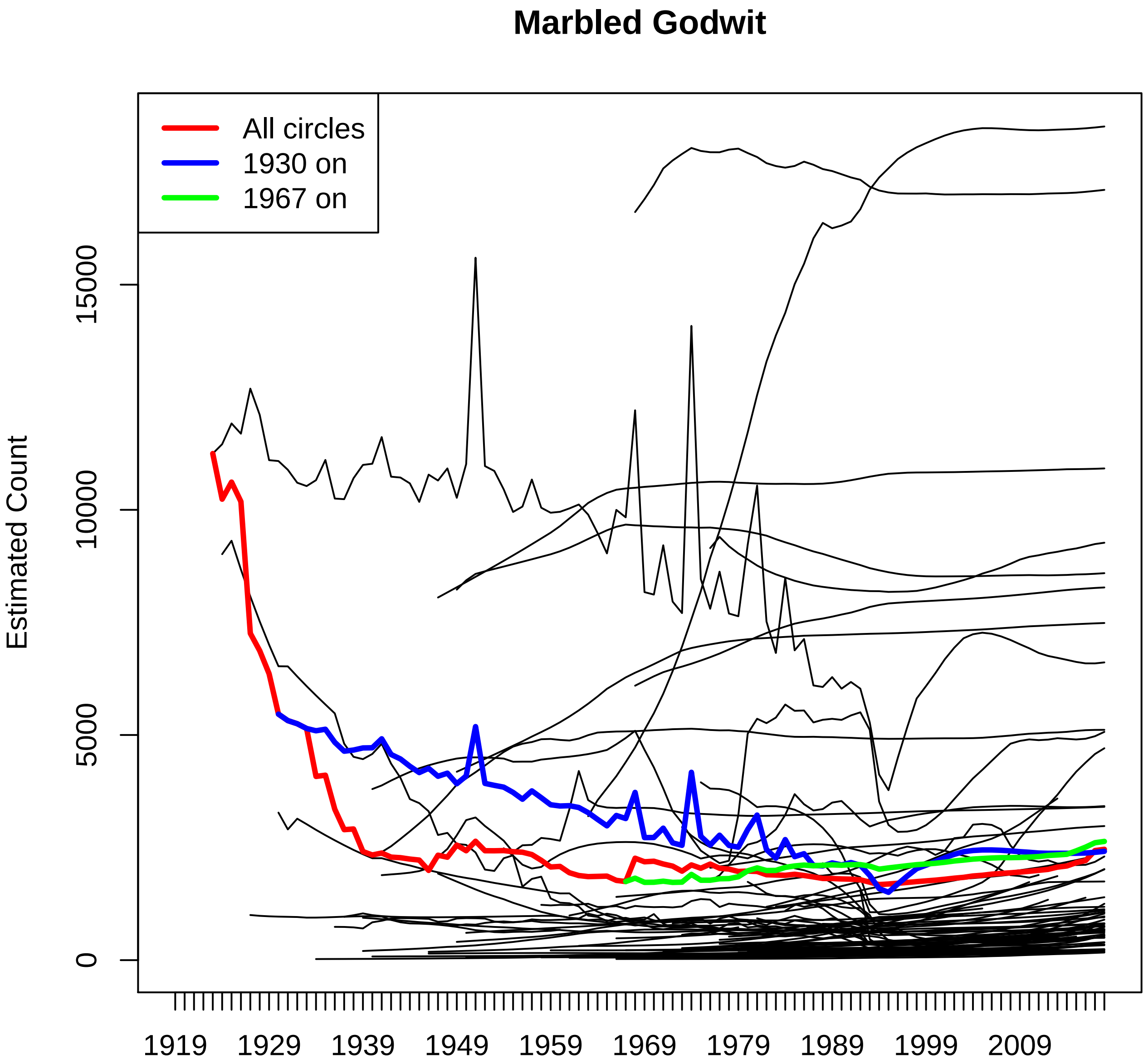

Marbled Godwit
All circles
1930 on
1967 on
Estimated Count
0
5000
10000
15000
1919
1929
1939
1949
1959
1969
1979
1989
1999
2009

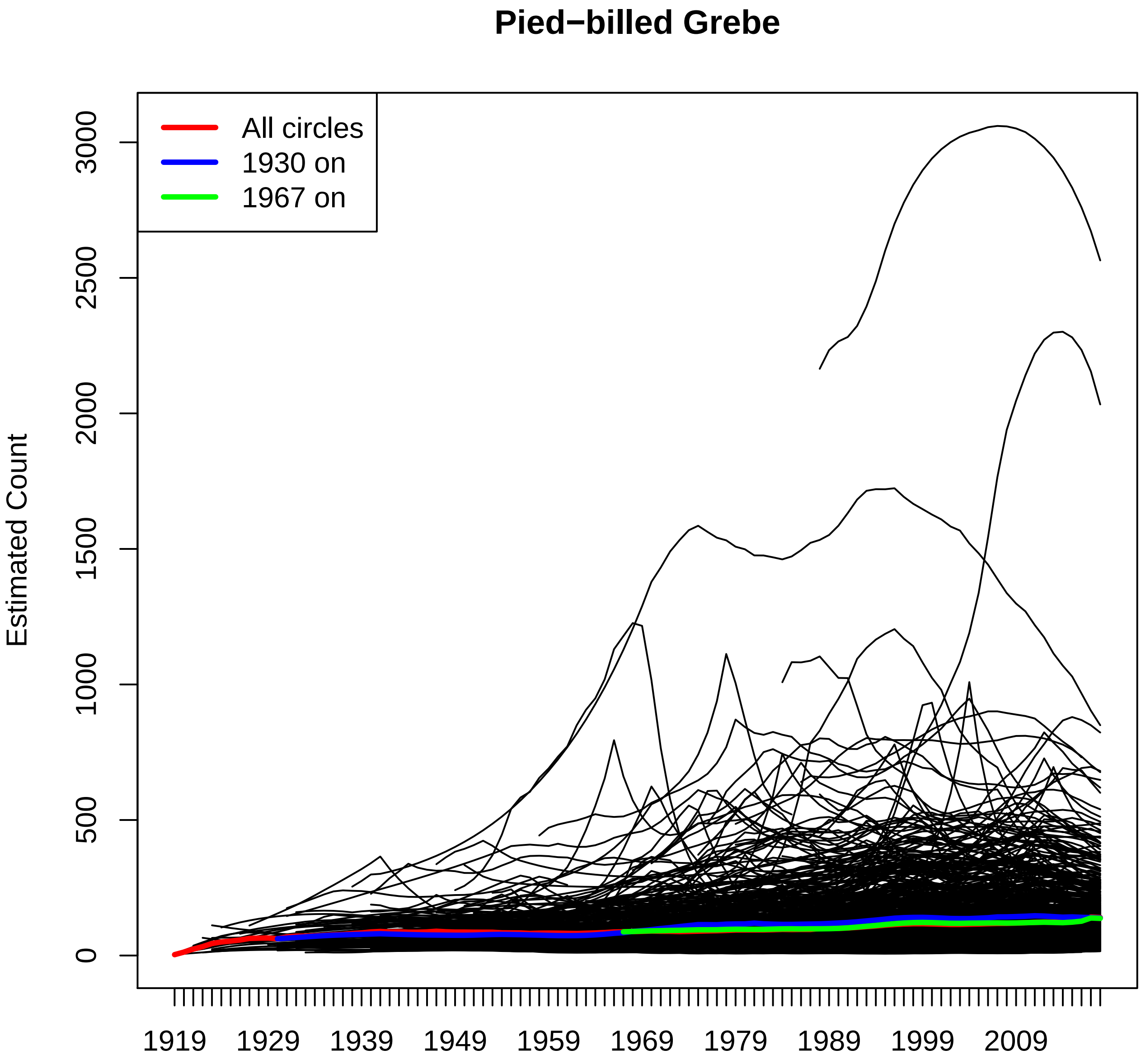

Pied−billed Grebe
Estimated Count
0
500
1000
1500
2000
2500
3000
All circles
1930 on
1967 on
1919
1929
1939
1949
1959
1969
1979
1989
1999
2009

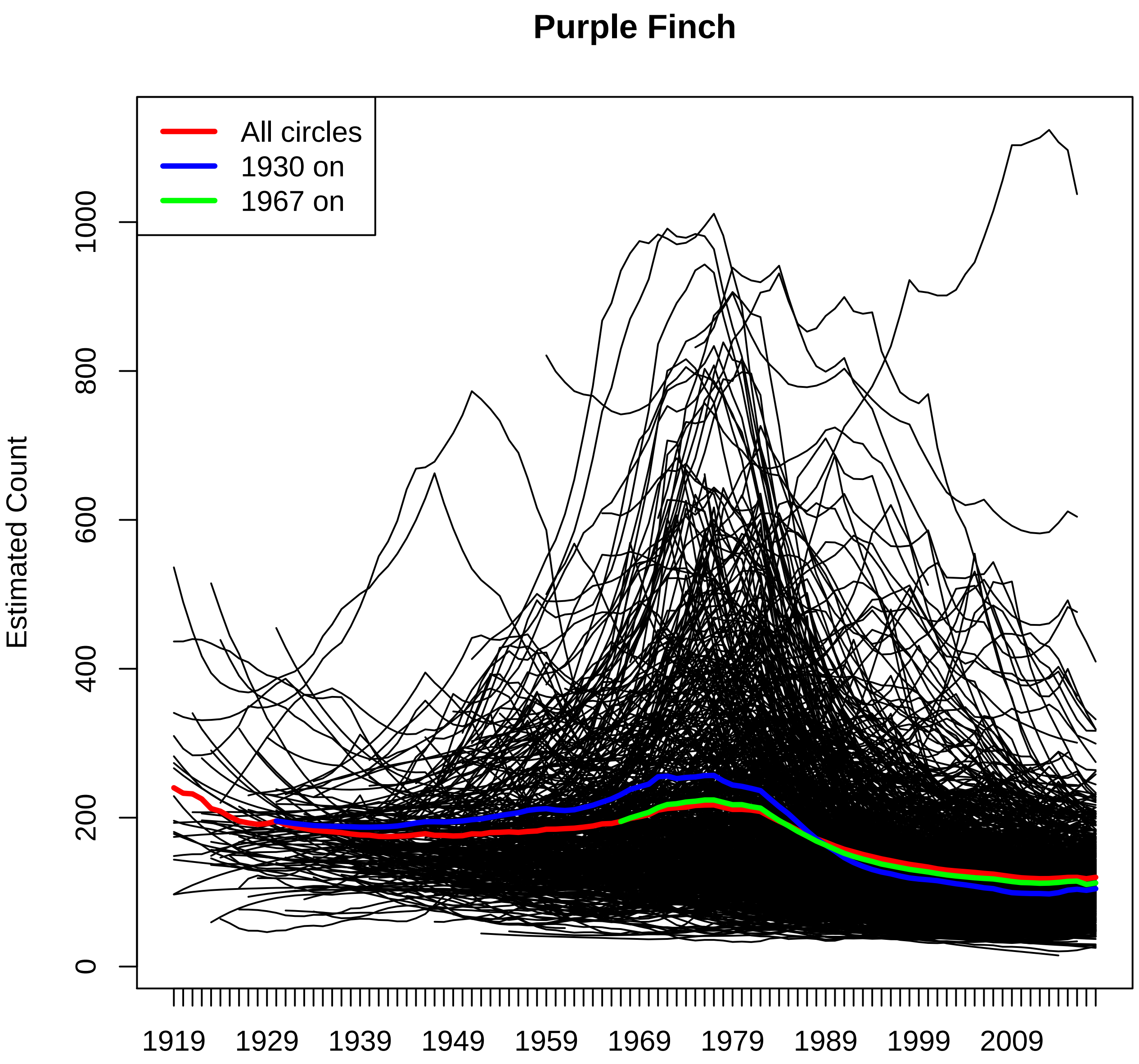

Purple Finch
All circles
1930 on
1967 on
Estimated Count
0
200
400
600
800
1000
1919
1929
1939
1949
1959
1969
1979
1989
1999
2009

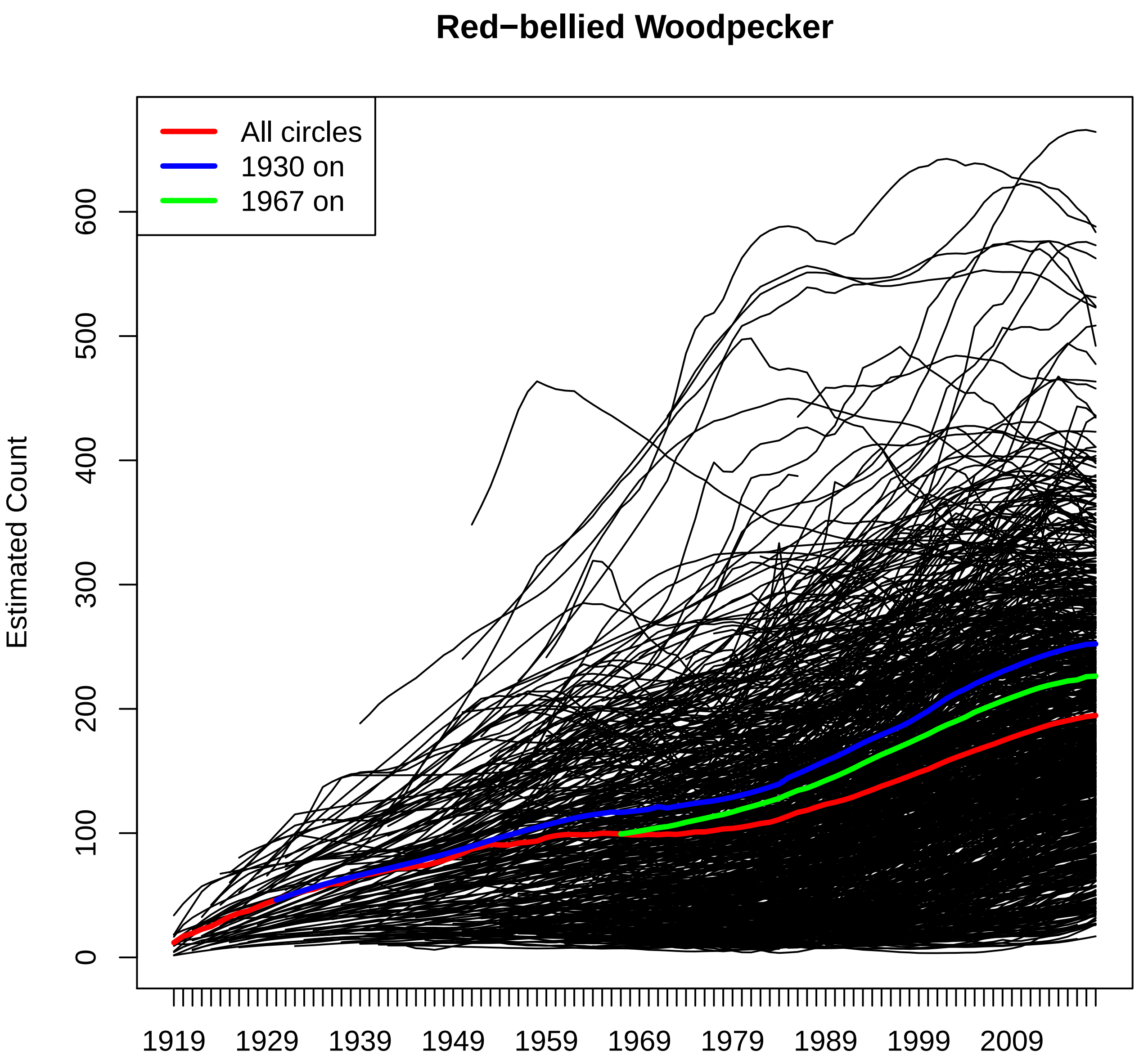
Red−bellied Woodpecker
All circles
1930 on
1967 on
Estimated Count
0
100
200
300
400
500
600
1919
1929
1939
1949
1959
1969
1979
1989
1999
2009

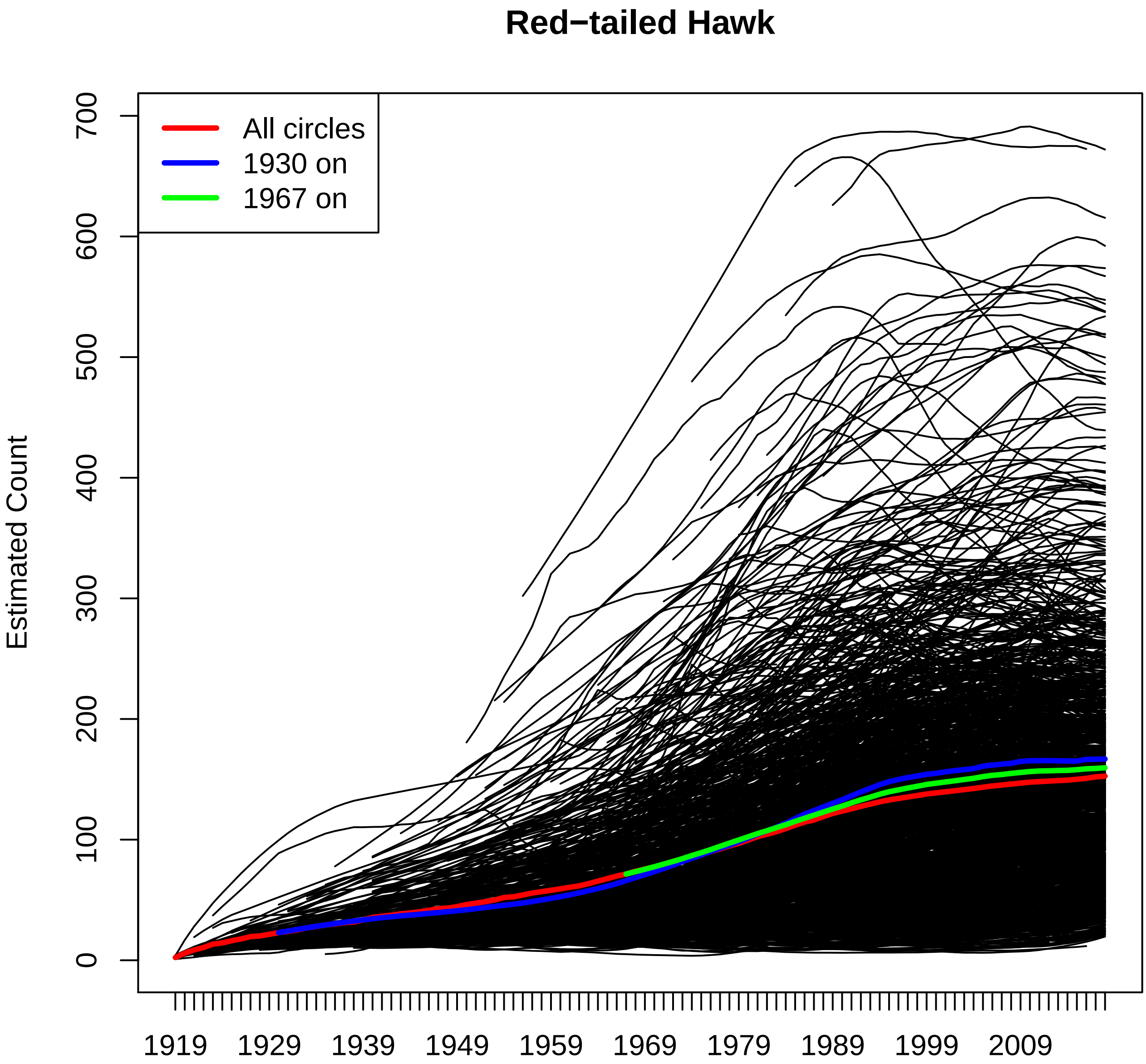

Red−tailed Hawk
All circles
1930 on
1967 on
Estimated Count
0
100
200
300
400
500
600
700
1919
1929
1939
1949
1959
1969
1979
1989
1999
2009

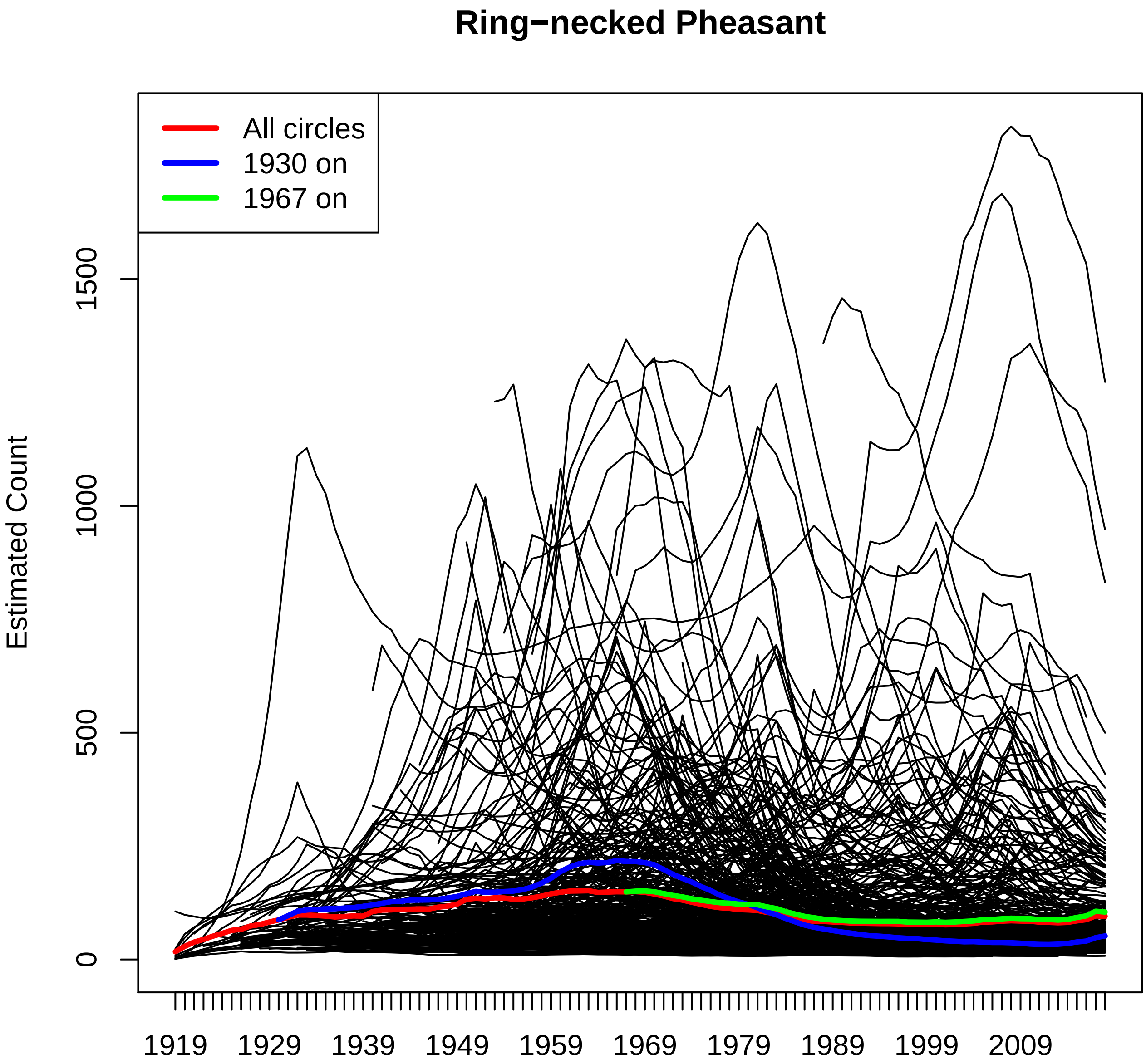

Ring−necked Pheasant
All circles
1930 on
1967 on
Estimated Count
0
500
1000
1500
1919
1929
1939
1949
1959
1969
1979
1989
1999
2009

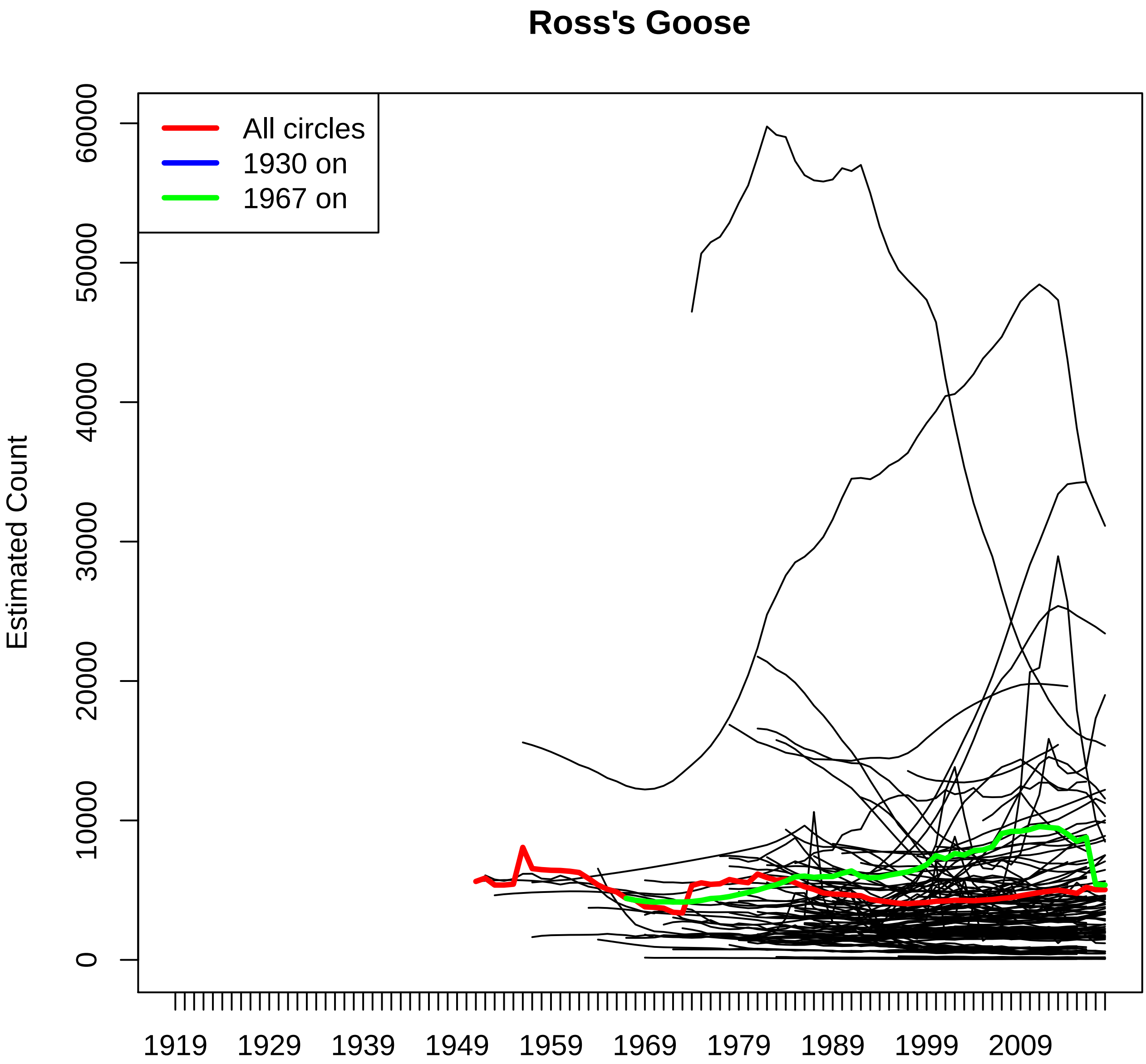

Ross's Goose
All circles
1930 on
1967 on
Estimated Count
0
10000
20000
30000
40000
50000
60000
1919
1929
1939
1949
1959
1969
1979
1989
1999
2009

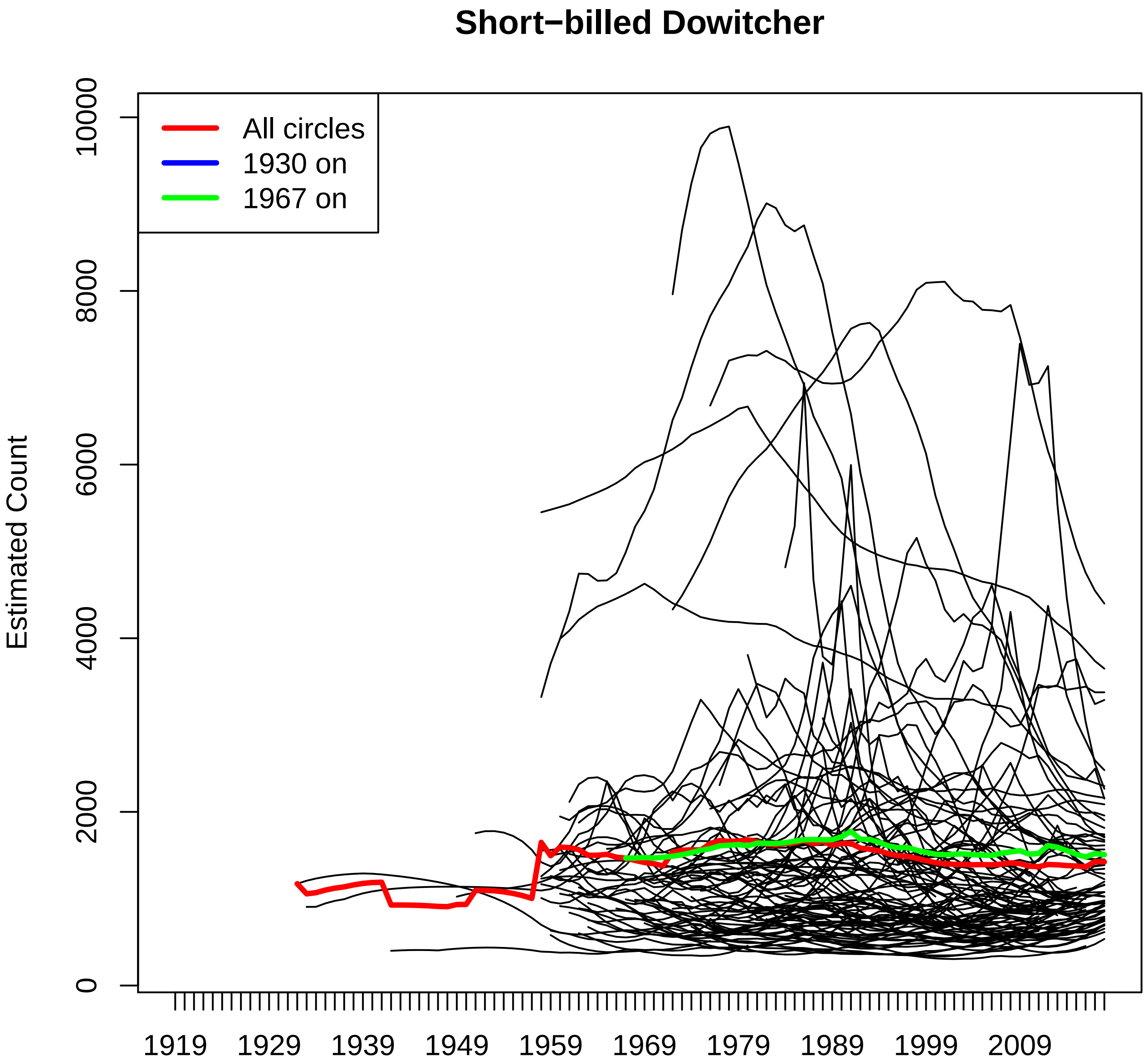
Short−billed Dowitcher
All circles
1930 on
1967 on
Estimated Count
0
2000
4000
6000
8000
10000
1919
1929
1939
1949
1959
1969
1979
1989
1999
2009

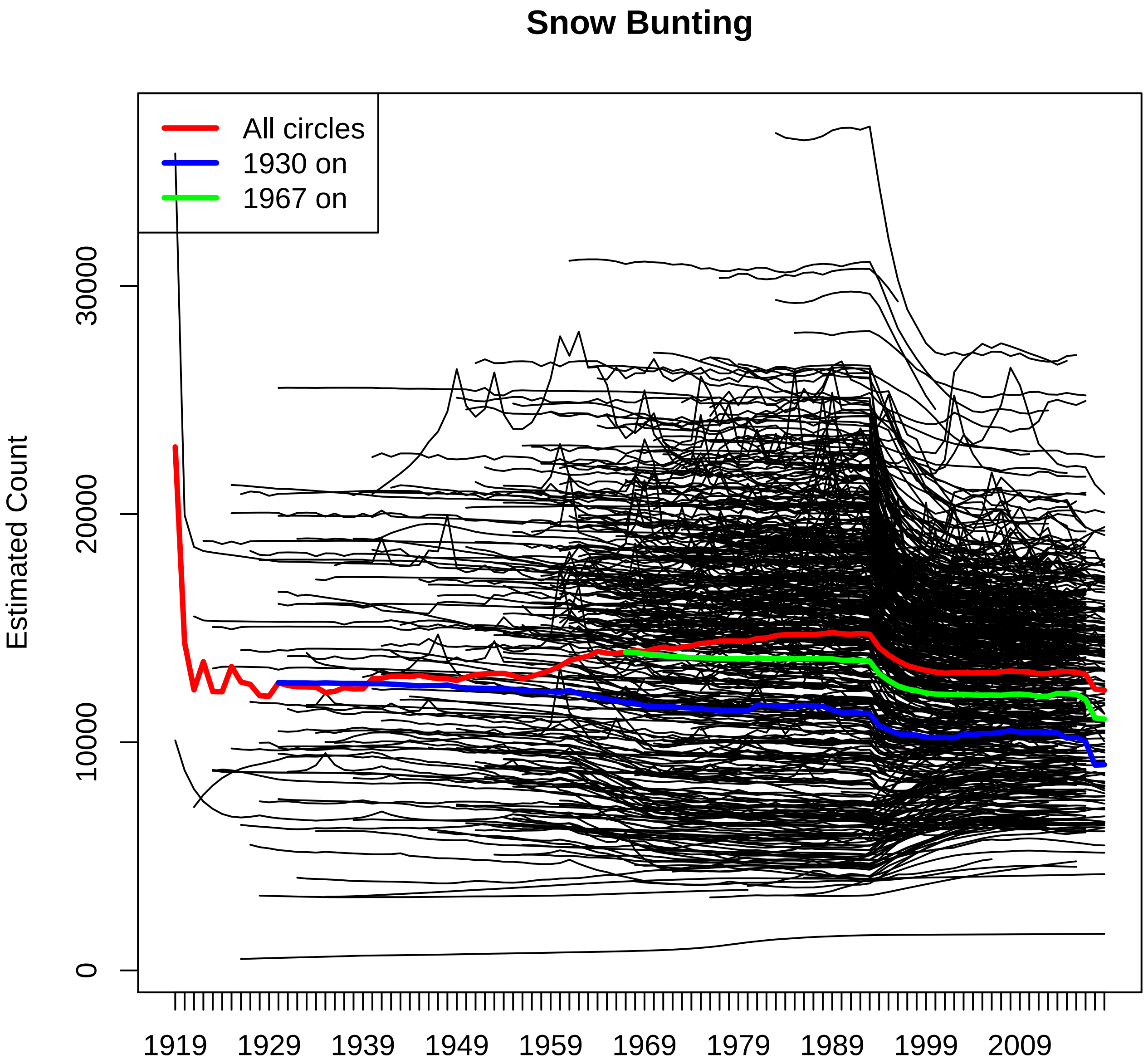

Snow Bunting
All circles
1930 on
1967 on
Estimated Count
0
10000
20000
30000
1919
1929
1939
1949
1959
1969
1979
1989
1999
2009

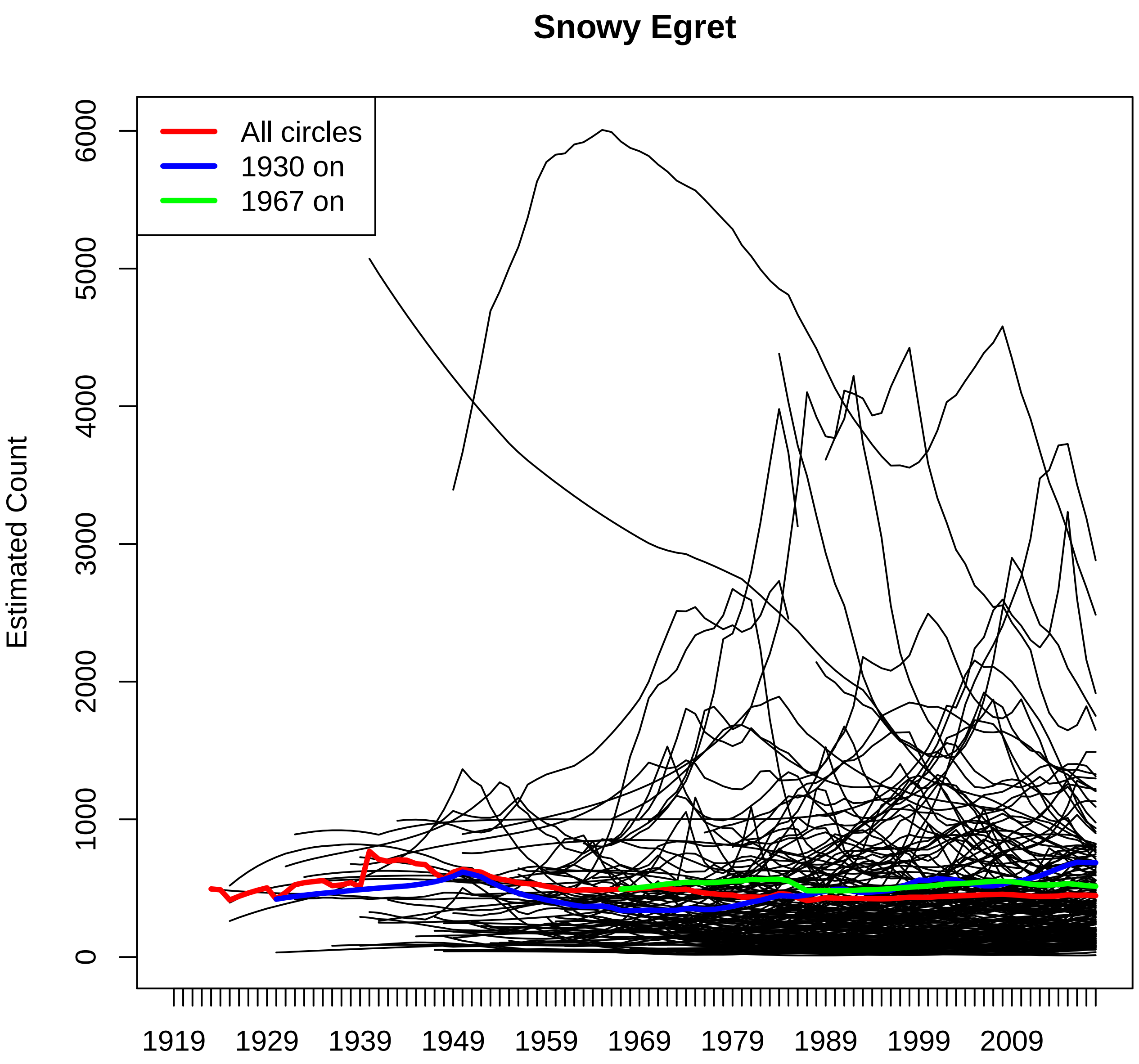
Snowy Egret
All circles
1930 on
1967 on
Estimated Count
0
1000
2000
3000
4000
5000
6000
1919
1929
1939
1949
1959
1969
1979
1989
1999
2009

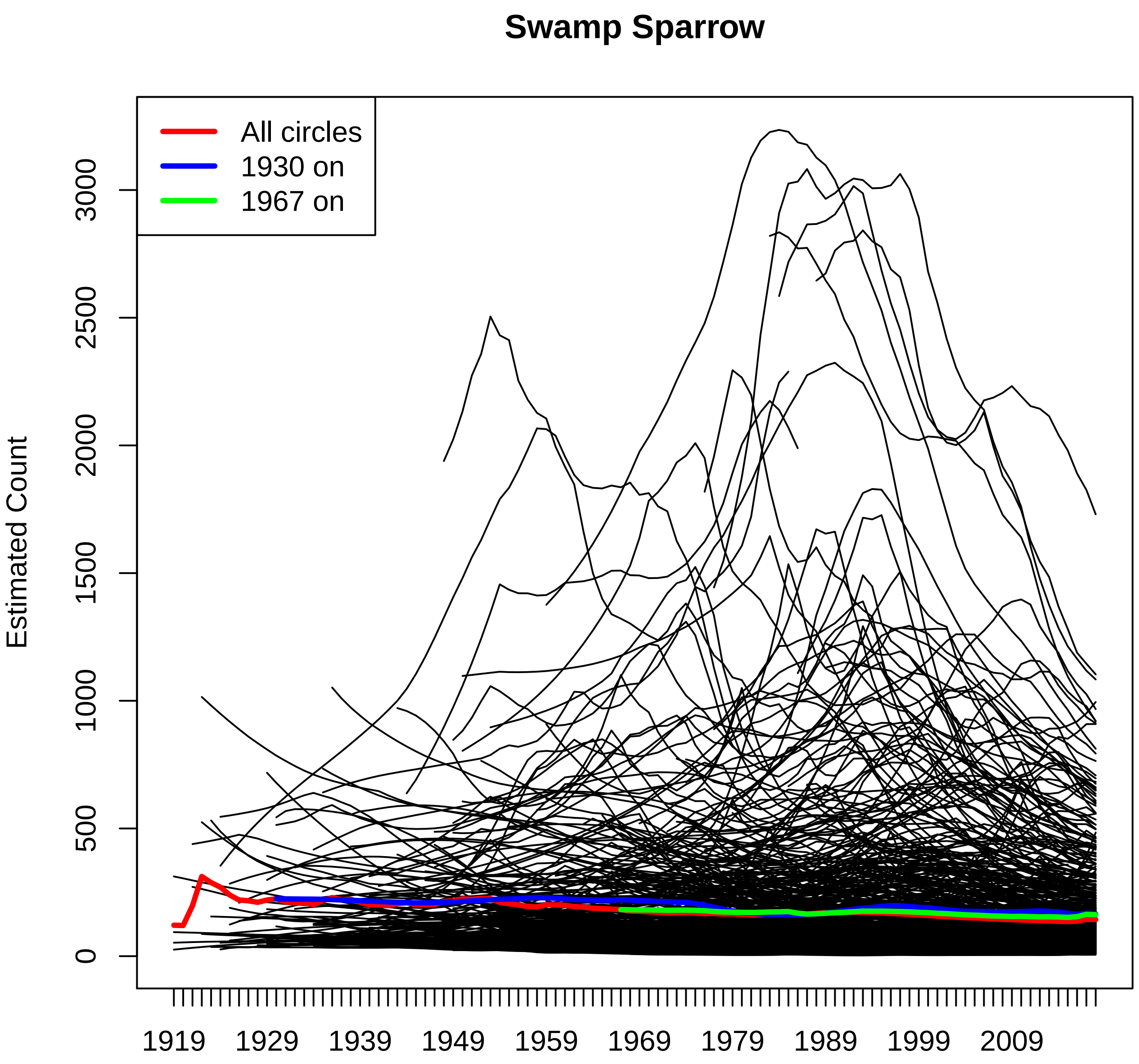
Swamp Sparrow
All circles
1930 on
1967 on
Estimated Count
0
500
1000
1500
2000
2500
3000
1919
1929
1939
1949
1959
1969
1979
1989
1999
2009

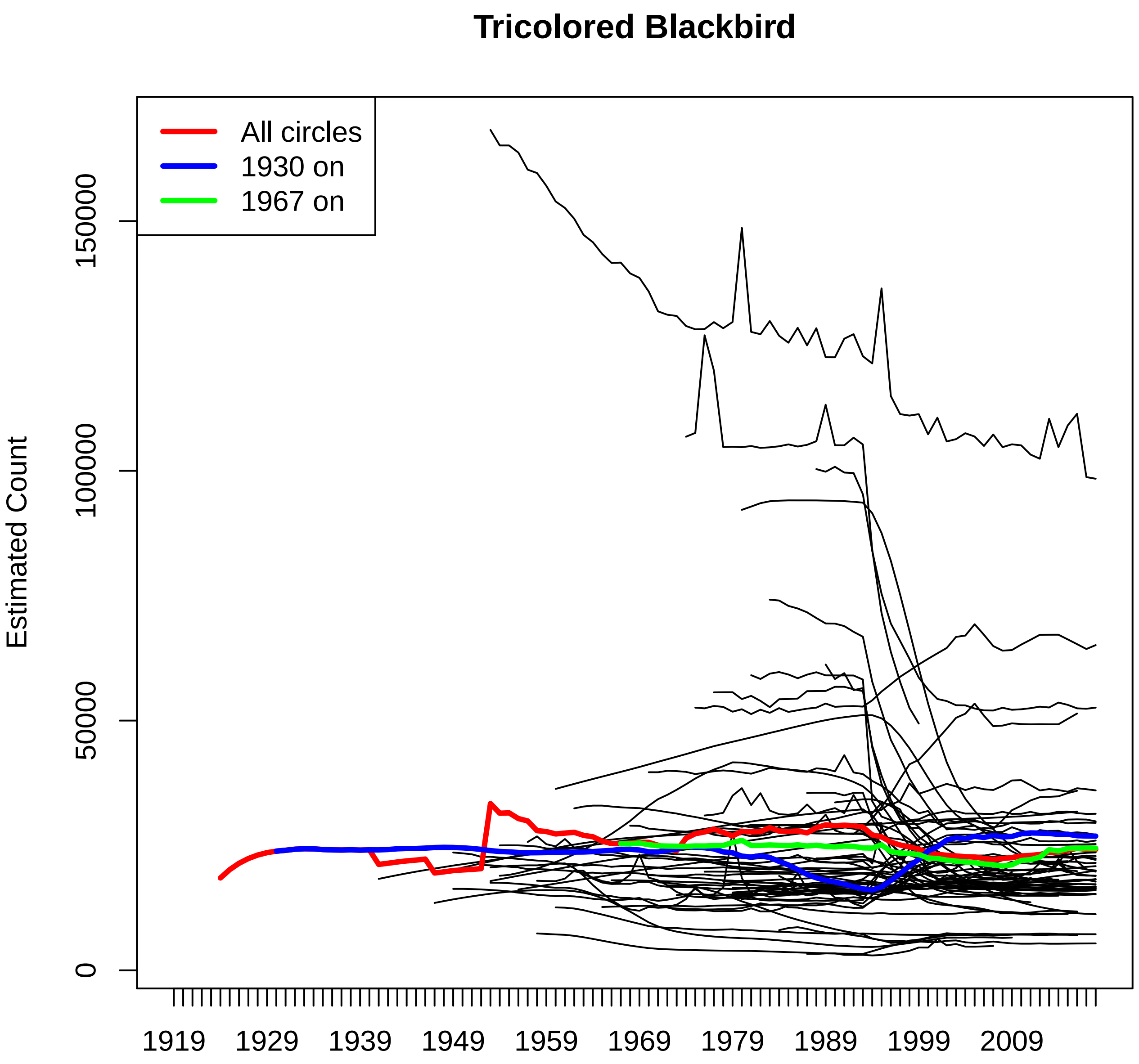

Tricolored Blackbird
All circles
1930 on
1967 on
Estimated Count
0
50000
100000
150000
1919
1929
1939
1949
1959
1969
1979
1989
1999
2009

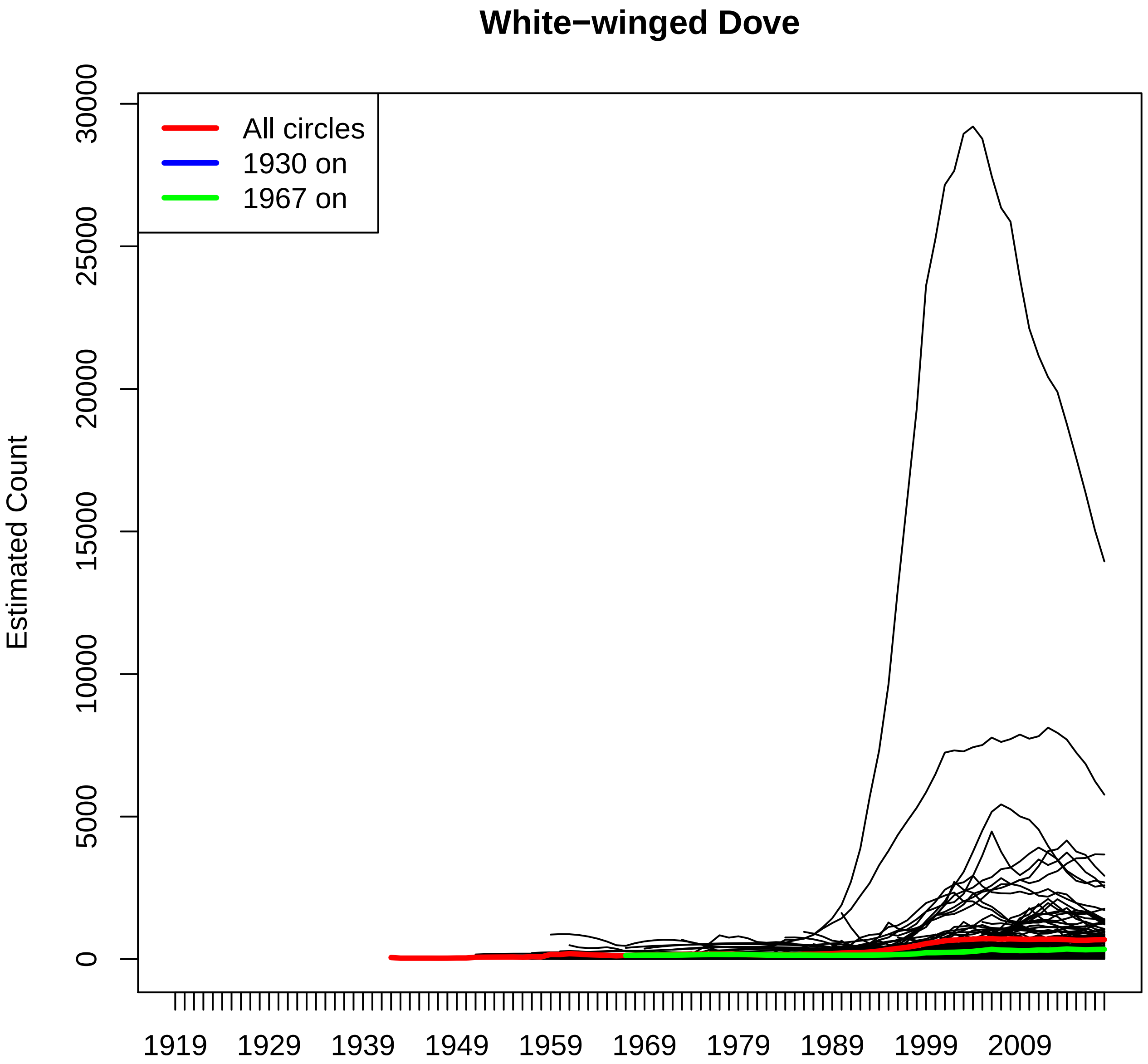
White−winged Dove
Estimated Count
0
5000
10000
15000
20000
25000
30000
1919
1929
1939
1949
1959
1969
1979
1989
1999
2009
All circles
1930 on
1967 on

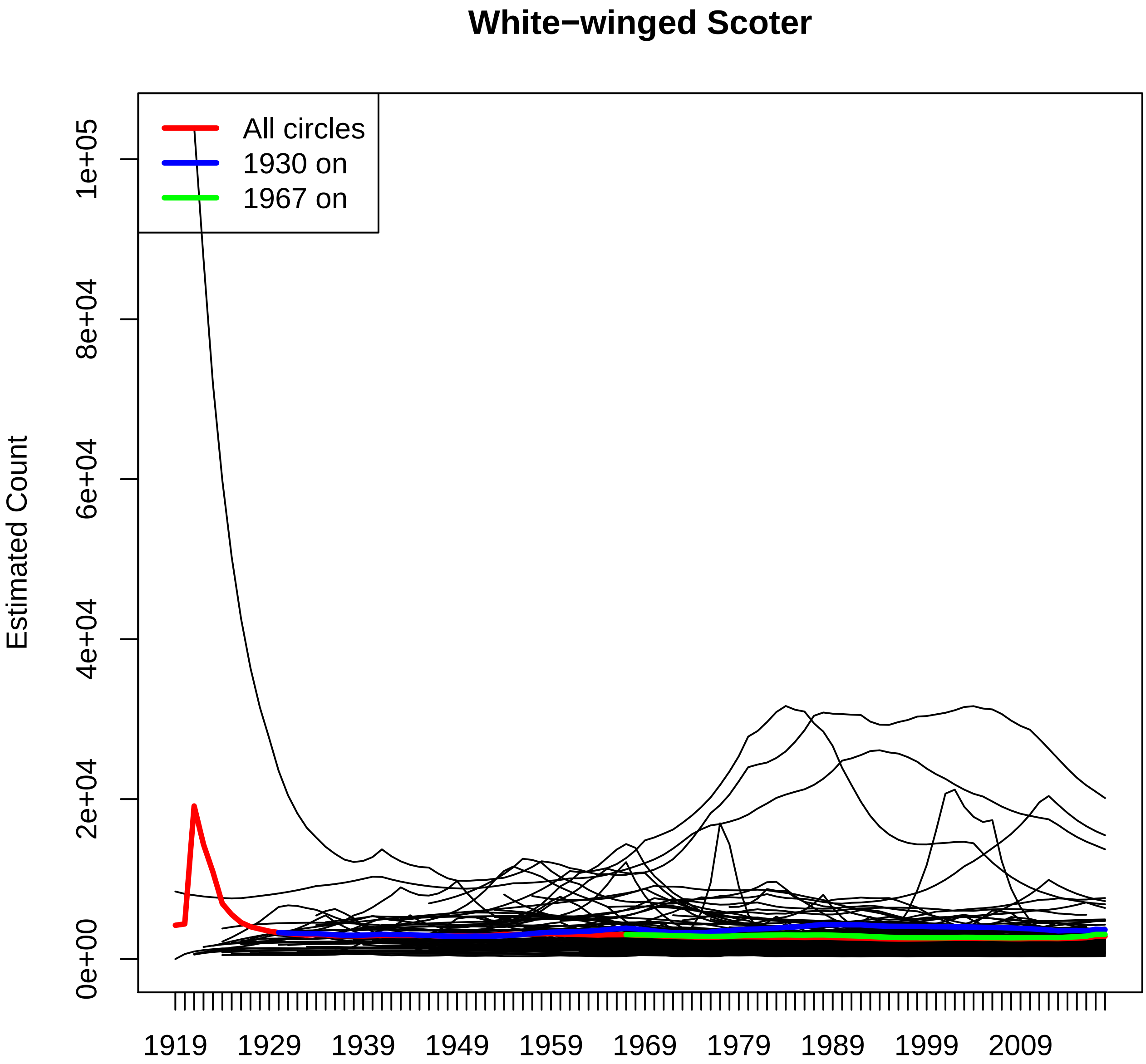
White−winged Scoter
All circles
1930 on
1967 on
Estimated Count
0e+00
2e+04
4e+04
6e+04
8e+04
1e+05
1919
1929
1939
1949
1959
1969
1979
1989
1999
2009

Wild Turkey

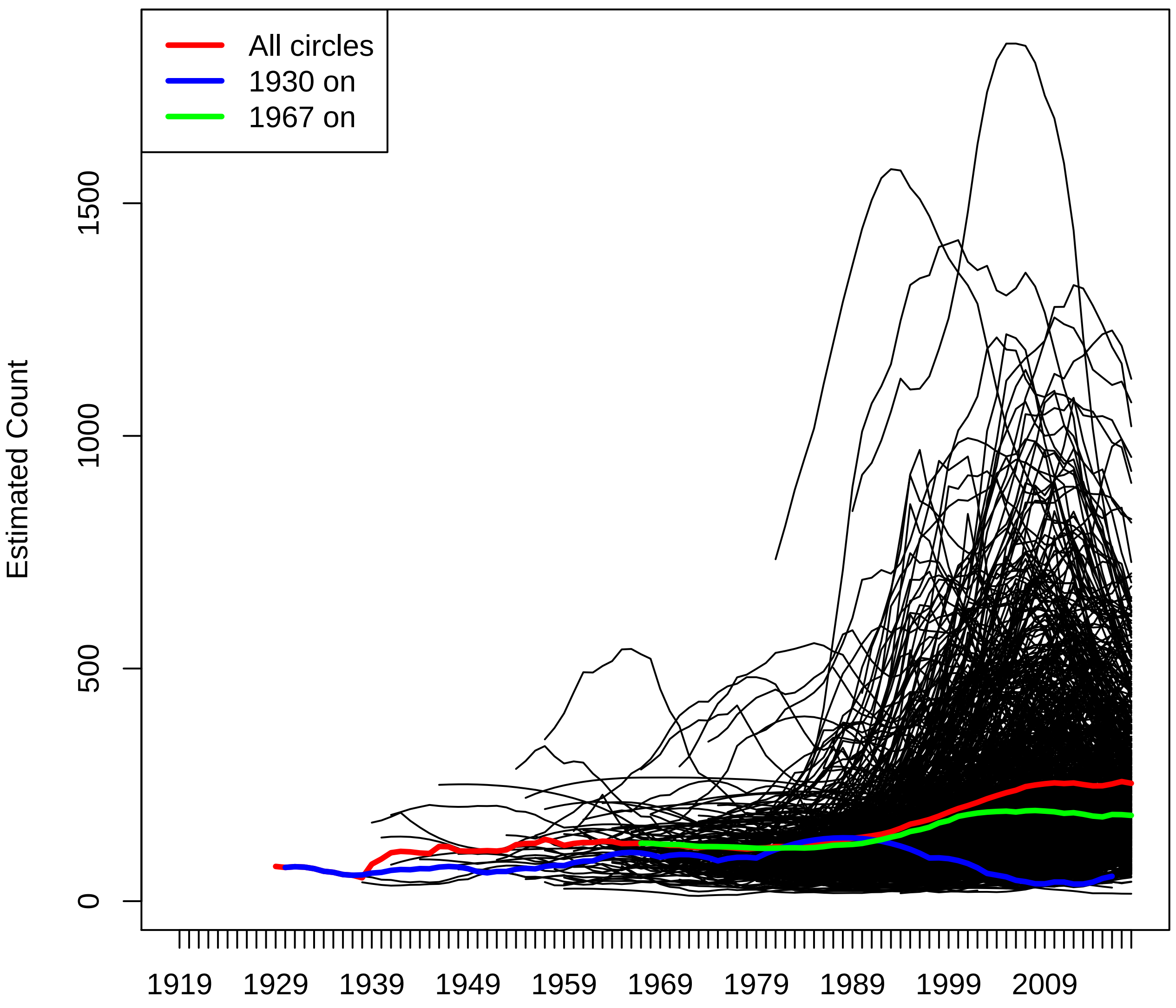

All circles
1930 on
1967 on
Estimated Count
0
500
1000
1500
1919
1929
1939
1949
1959
1969
1979
1989
1999
2009

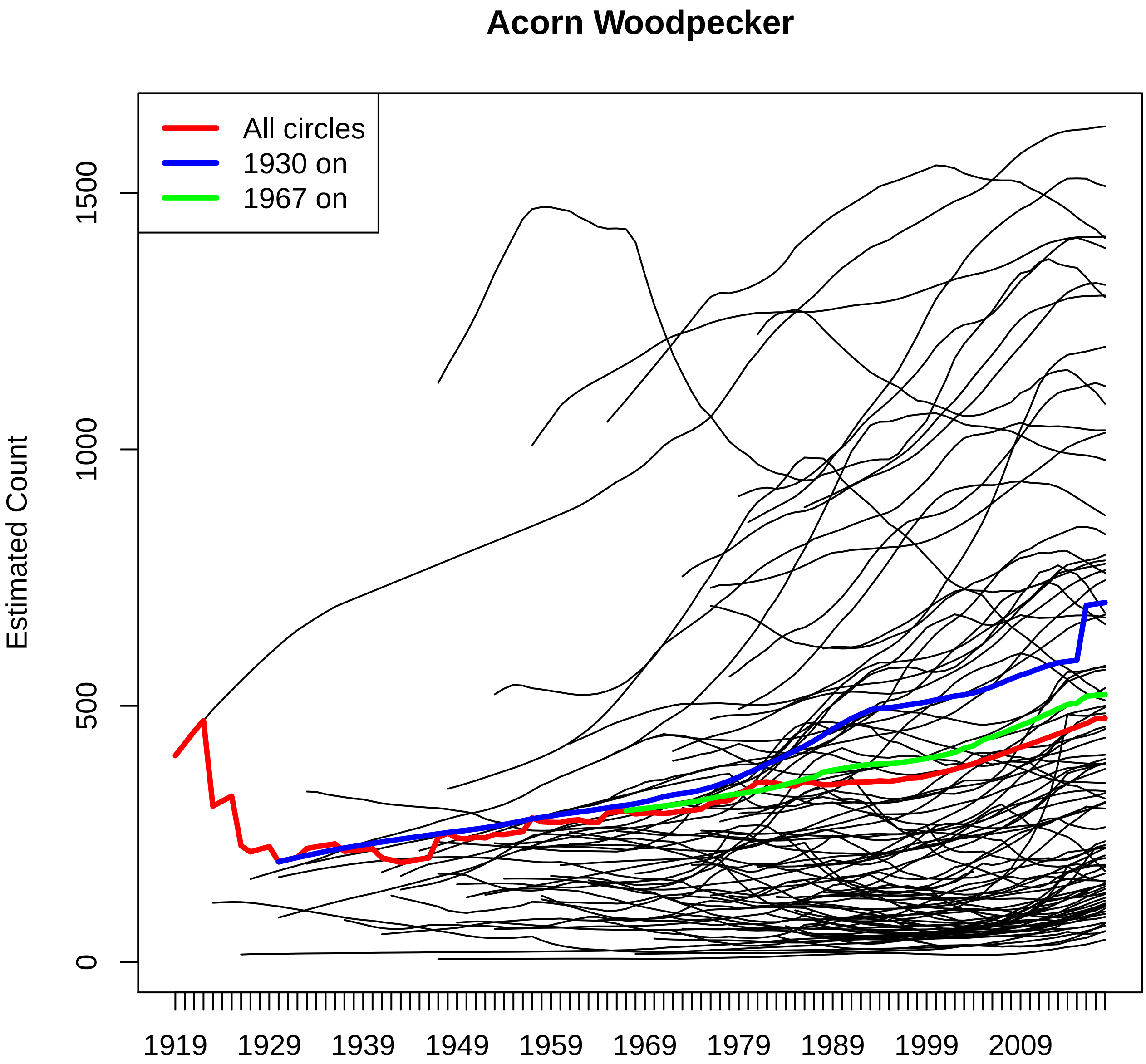

Acorn Woodpecker
All circles
1930 on
1967 on
Estimated Count
0
500
1000
1500
1919
1929
1939
1949
1959
1969
1979
1989
1999
2009

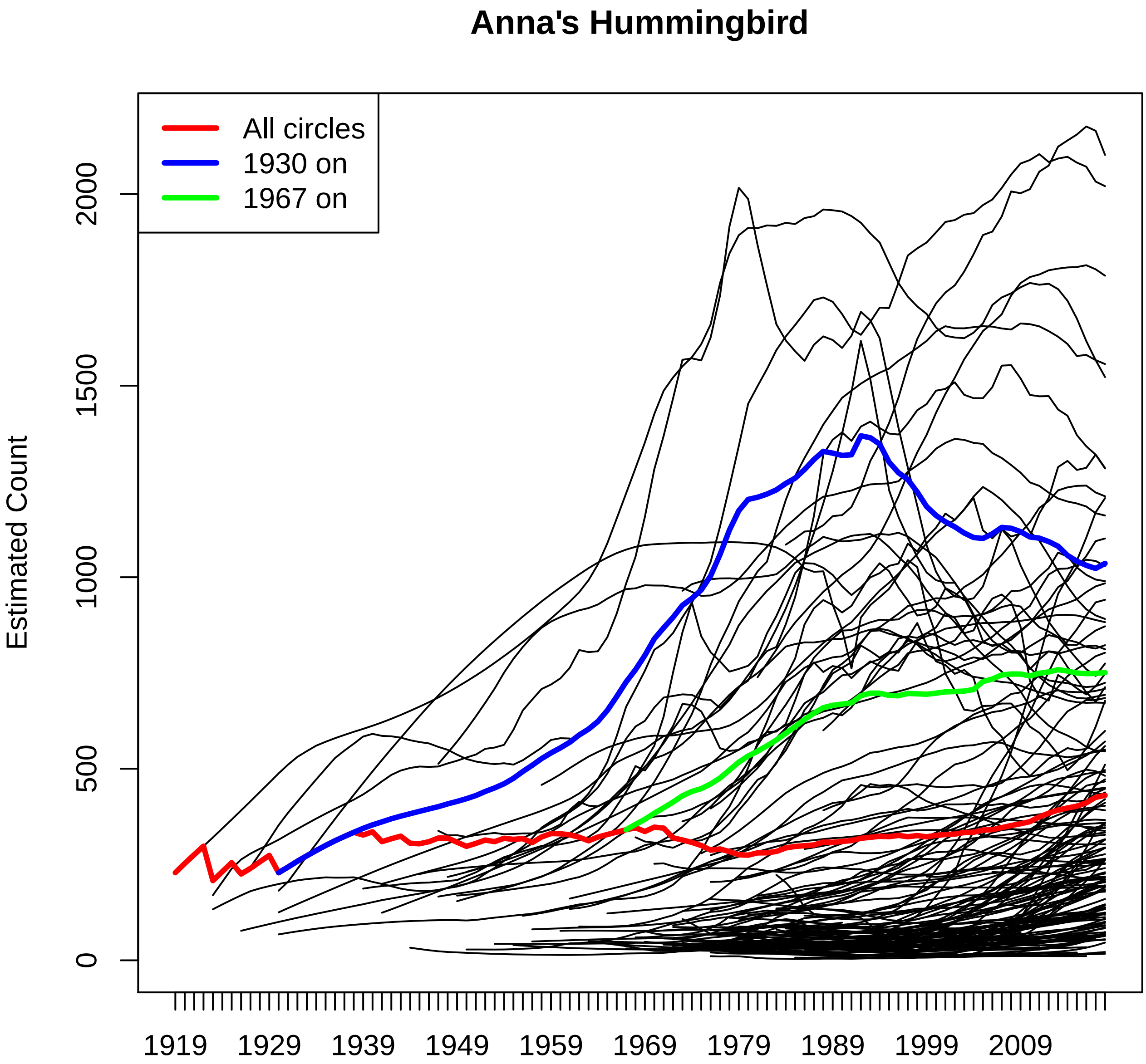

Anna's Hummingbird
All circles
1930 on
1967 on
Estimated Count
0
500
1000
1500
2000
1919
1929
1939
1949
1959
1969
1979
1989
1999
2009

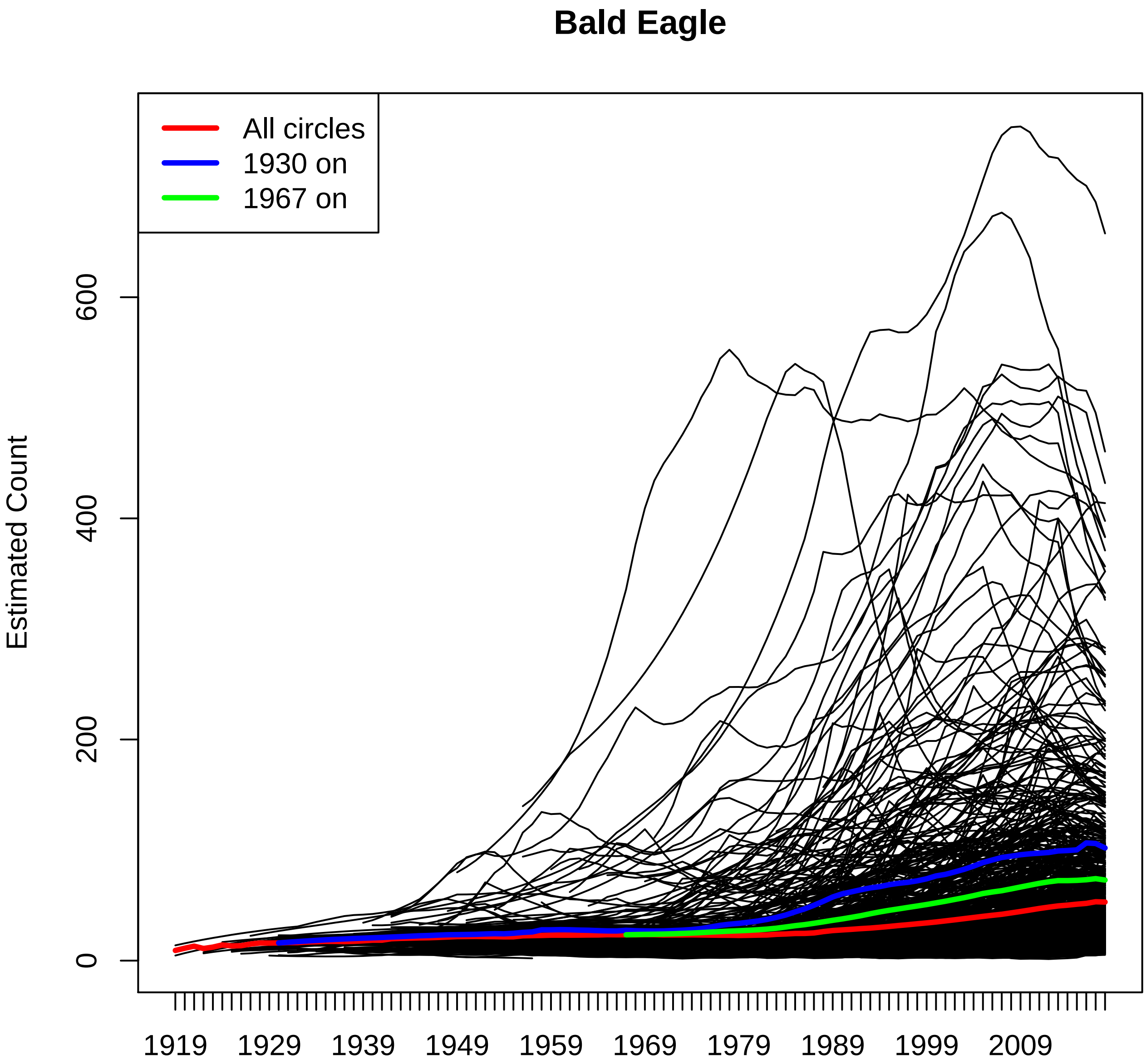

Bald Eagle
All circles
1930 on
1967 on
Estimated Count
0
200
400
600
1919
1929
1939
1949
1959
1969
1979
1989
1999
2009

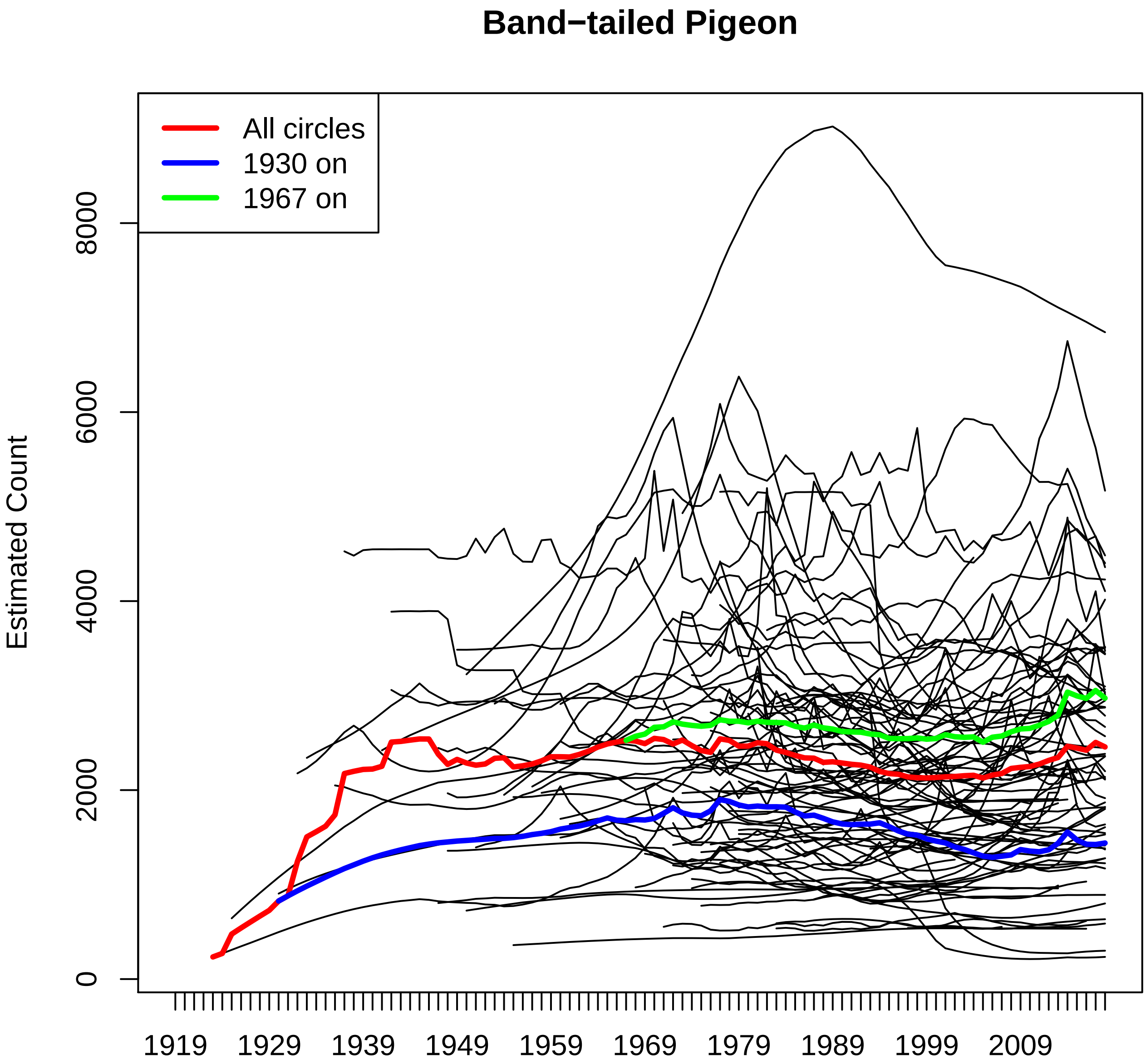

Band−tailed Pigeon
All circles
1930 on
1967 on
Estimated Count
0
2000
4000
6000
8000
1919
1929
1939
1949
1959
1969
1979
1989
1999
2009

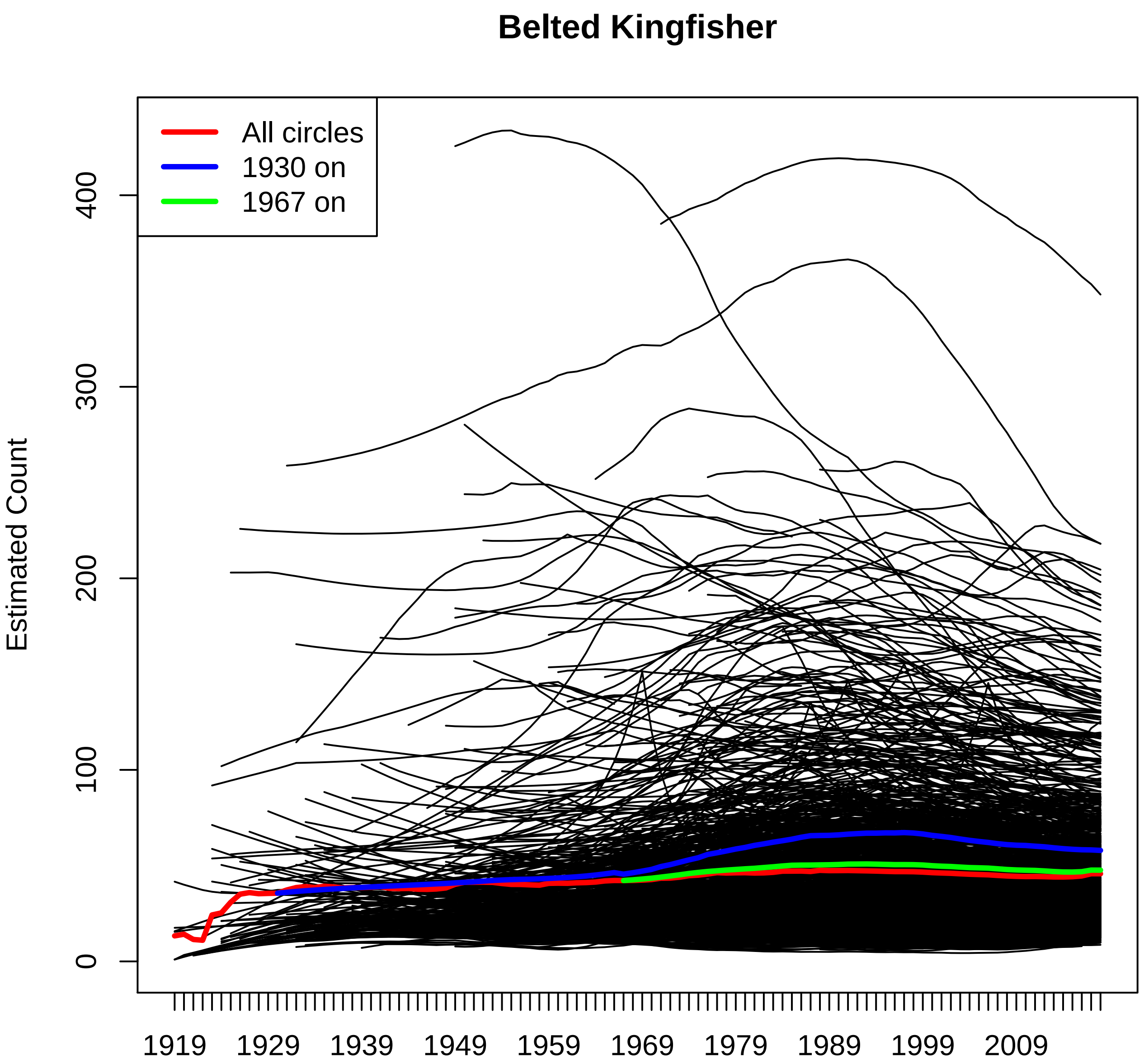

Belted Kingfisher
All circles
1930 on
1967 on
Estimated Count
0
100
200
300
400
1919
1929
1939
1949
1959
1969
1979
1989
1999
2009

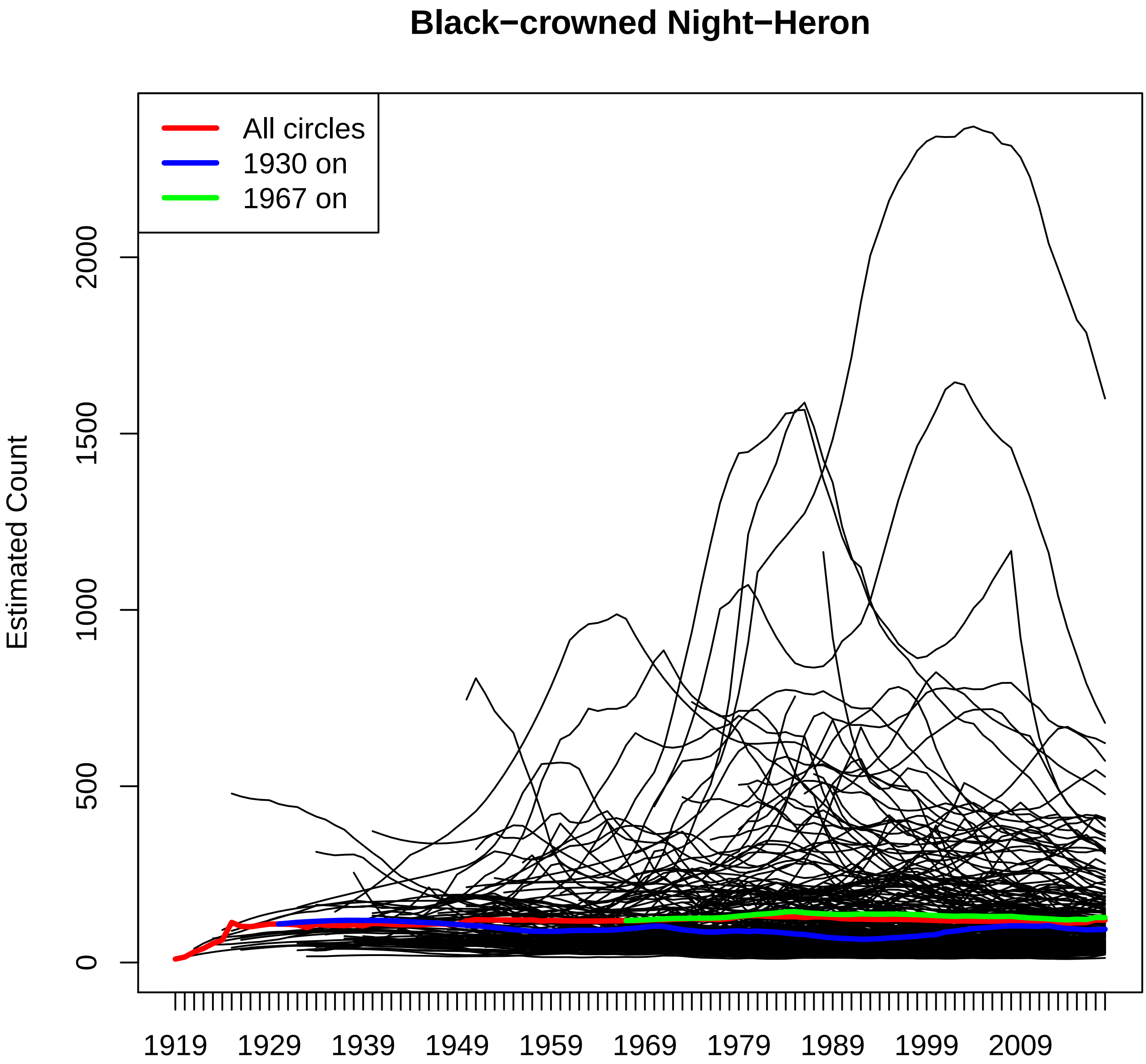

Black−crowned Night−Heron
All circles
1930 on
1967 on
Estimated Count
0
500
1000
1500
2000
1919
1929
1939
1949
1959
1969
1979
1989
1999
2009

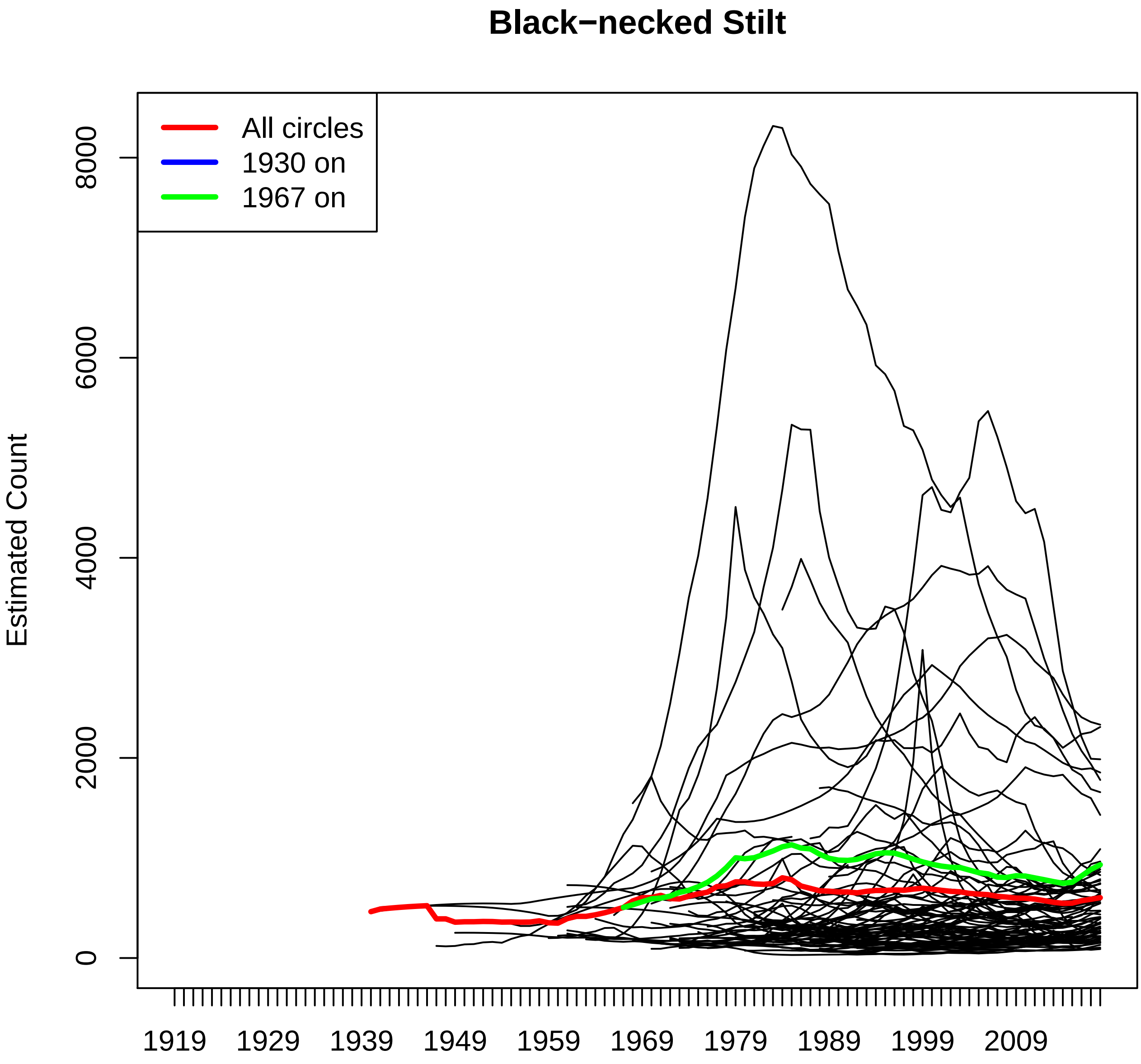

Black−necked Stilt
All circles
1930 on
1967 on
Estimated Count
0
2000
4000
6000
8000
1919
1929
1939
1949
1959
1969
1979
1989
1999
2009

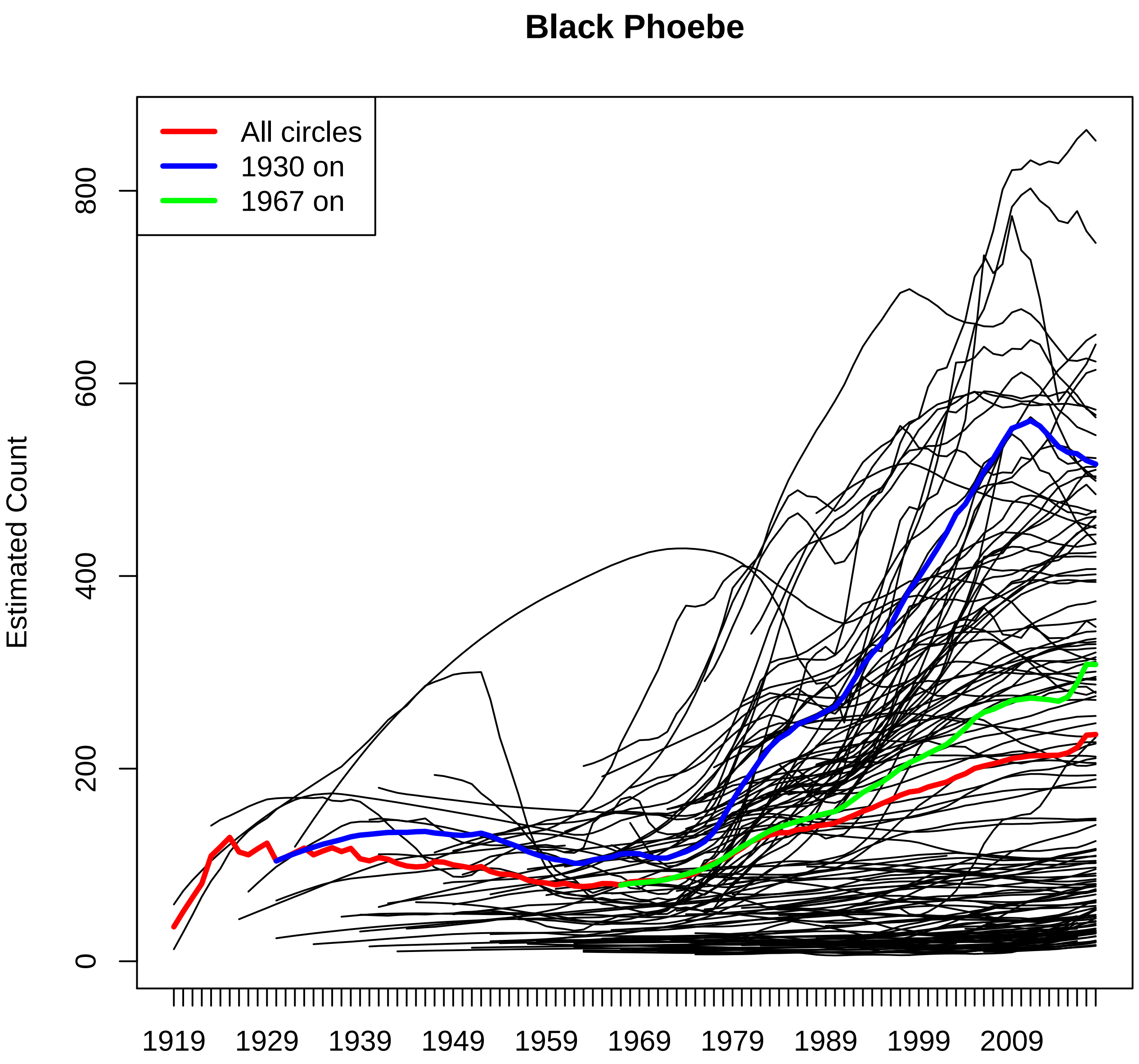
Black Phoebe
All circles
1930 on
1967 on
Estimated Count
0
200
400
600
800
1919
1929
1939
1949
1959
1969
1979
1989
1999
2009

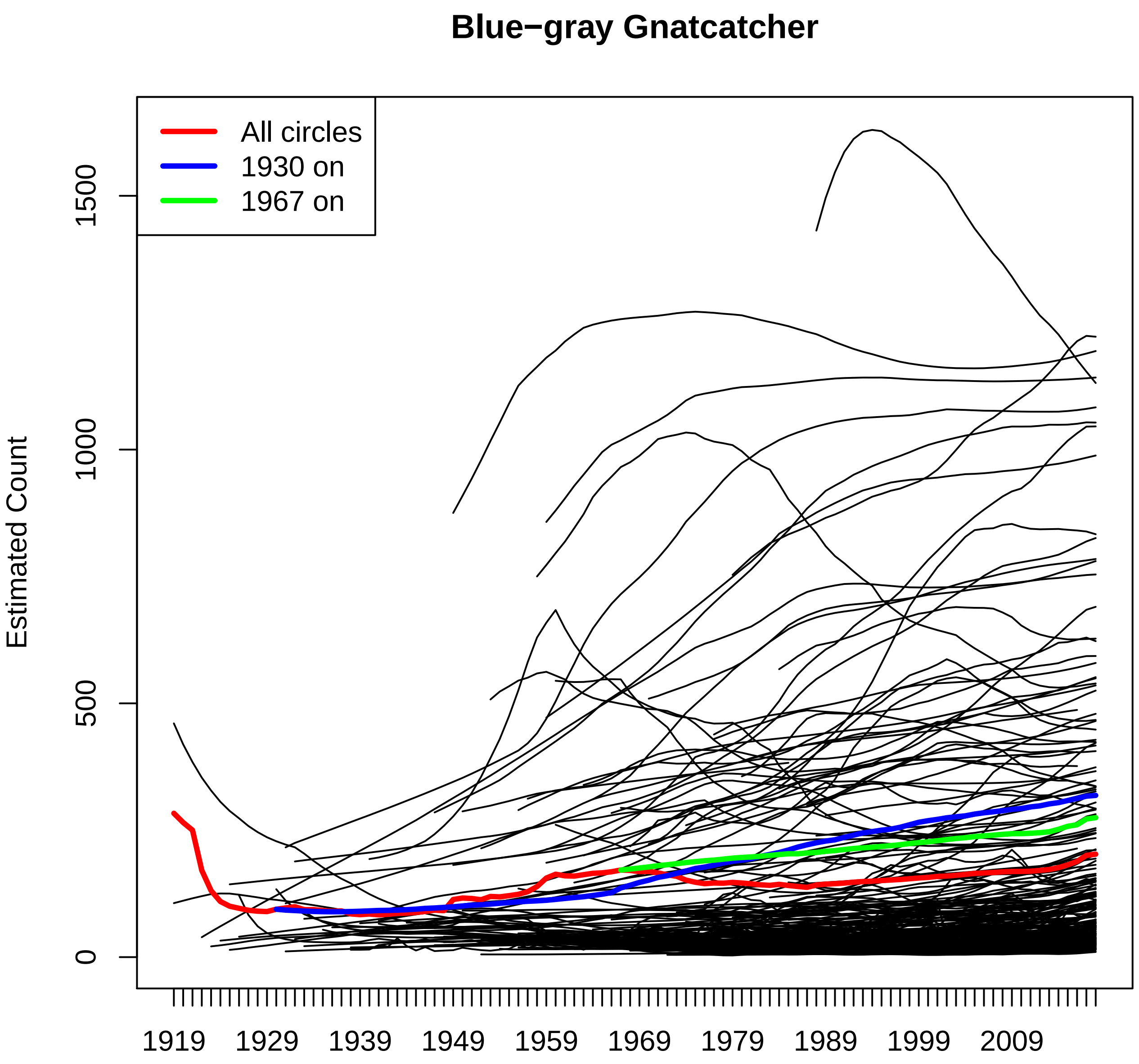

Blue−gray Gnatcatcher
All circles
1930 on
1967 on
Estimated Count
0
500
1000
1500
1919
1929
1939
1949
1959
1969
1979
1989
1999
2009

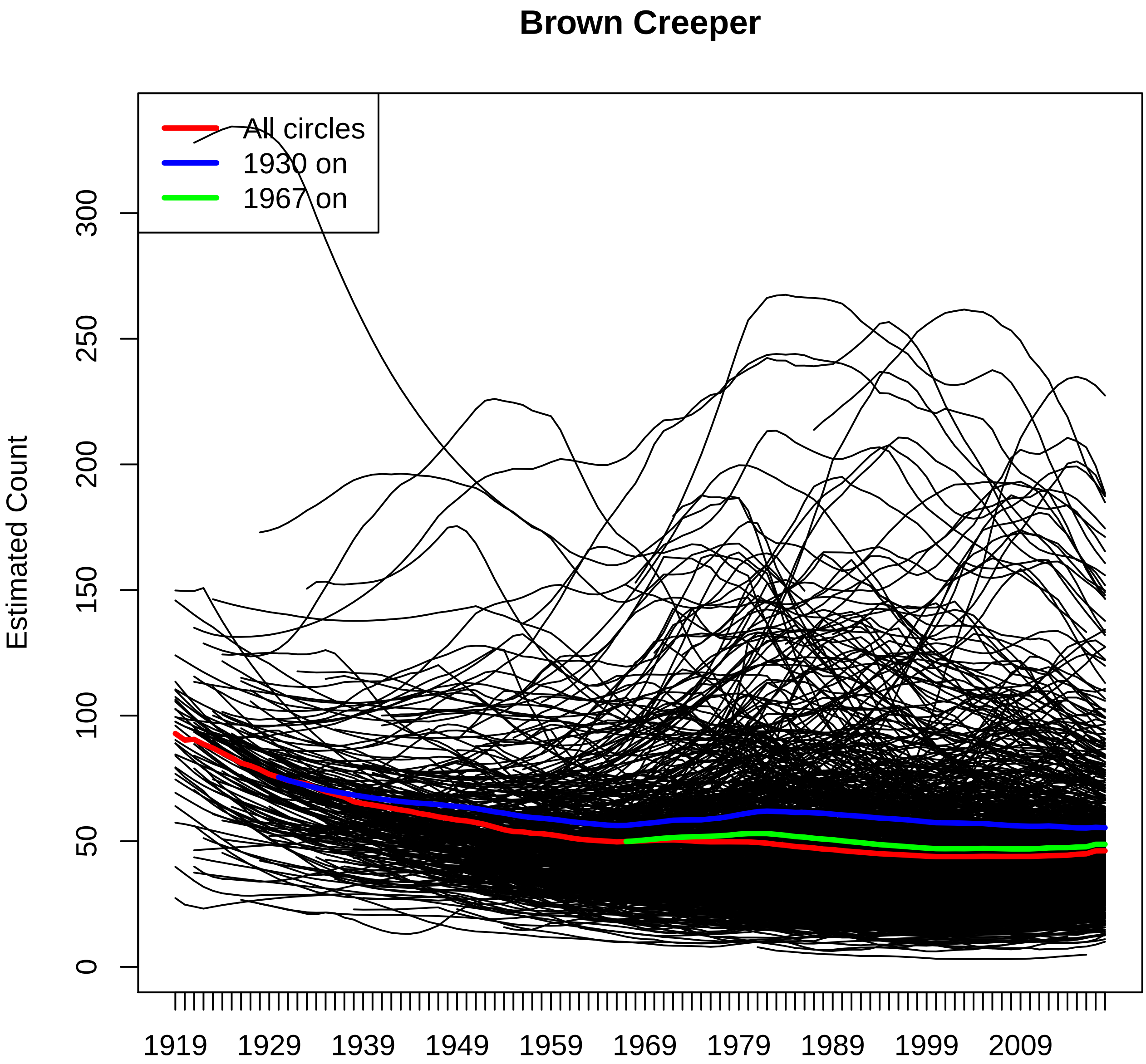

Brown Creeper
All circles
1930 on
1967 on
Estimated Count
0
50
100
150
200
250
300
1919
1929
1939
1949
1959
1969
1979
1989
1999
2009

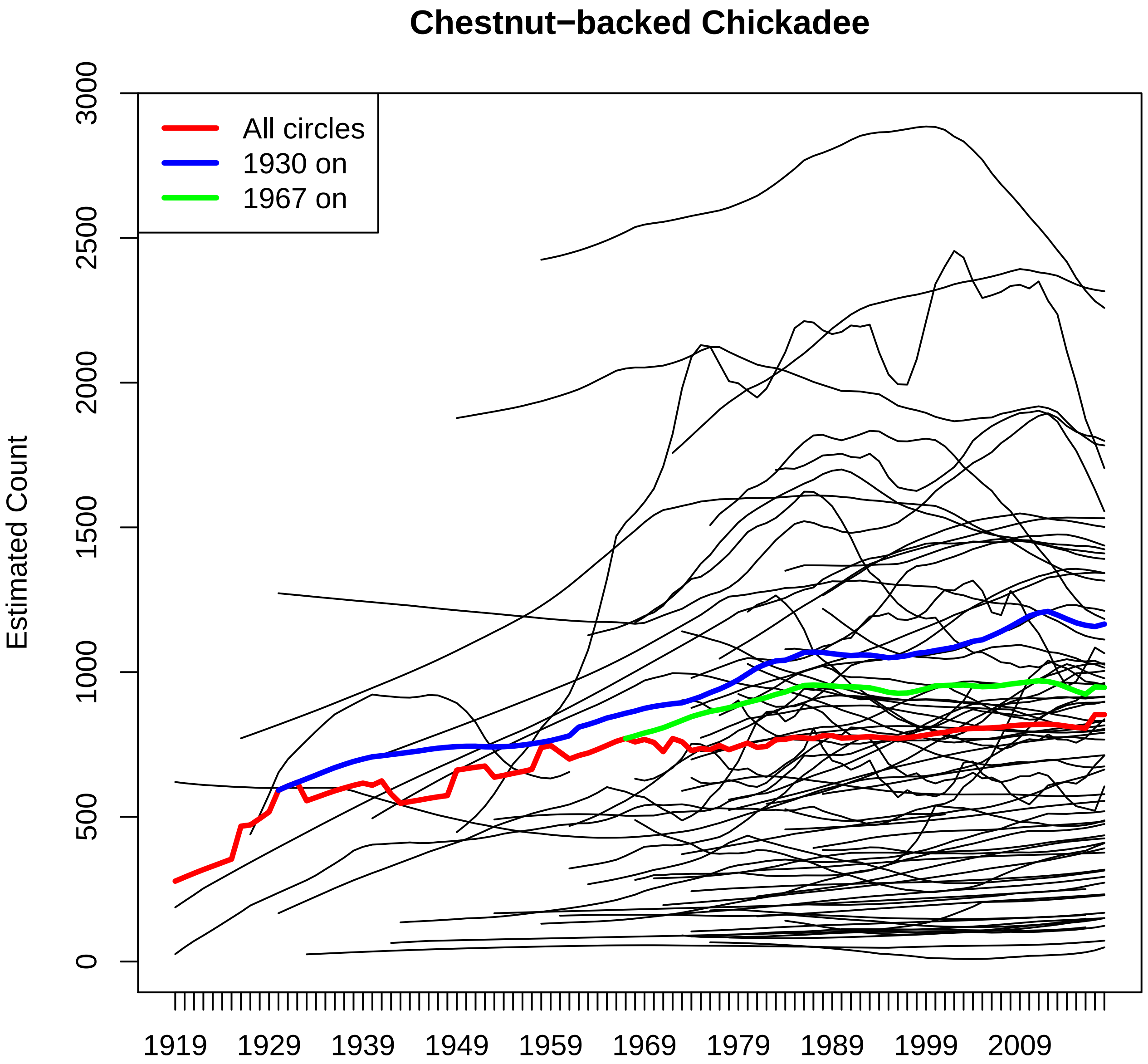
Chestnut−backed Chickadee
Estimated Count
All circles
1930 on
1967 on
0
500
1000
1500
2000
2500
3000
1919
1929
1939
1949
1959
1969
1979
1989
1999
2009

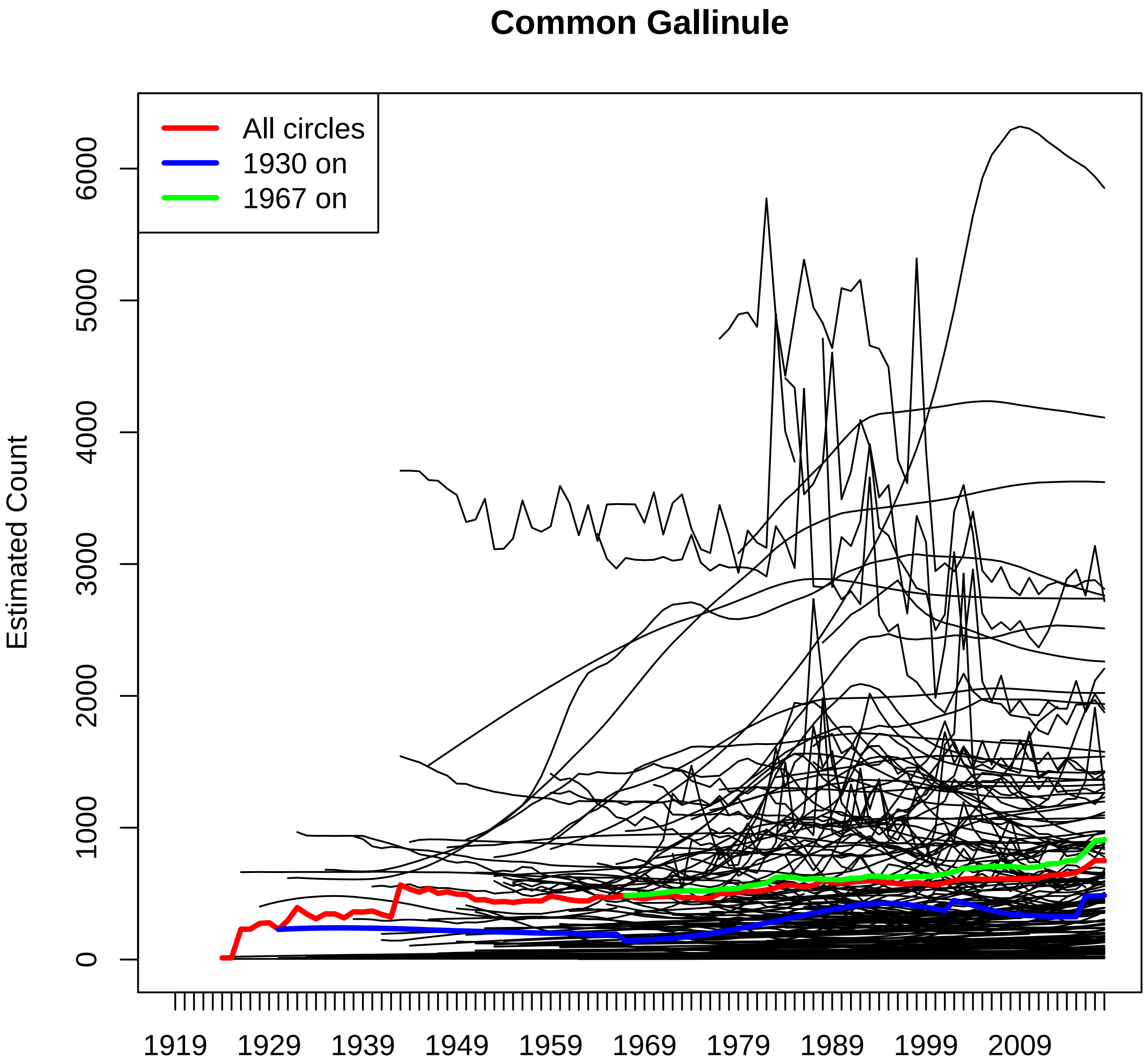
Common Gallinule
All circles
1930 on
1967 on
Estimated Count
0
1000
2000
3000
4000
5000
6000
1919
1929
1939
1949
1959
1969
1979
1989
1999
2009

Common Loon

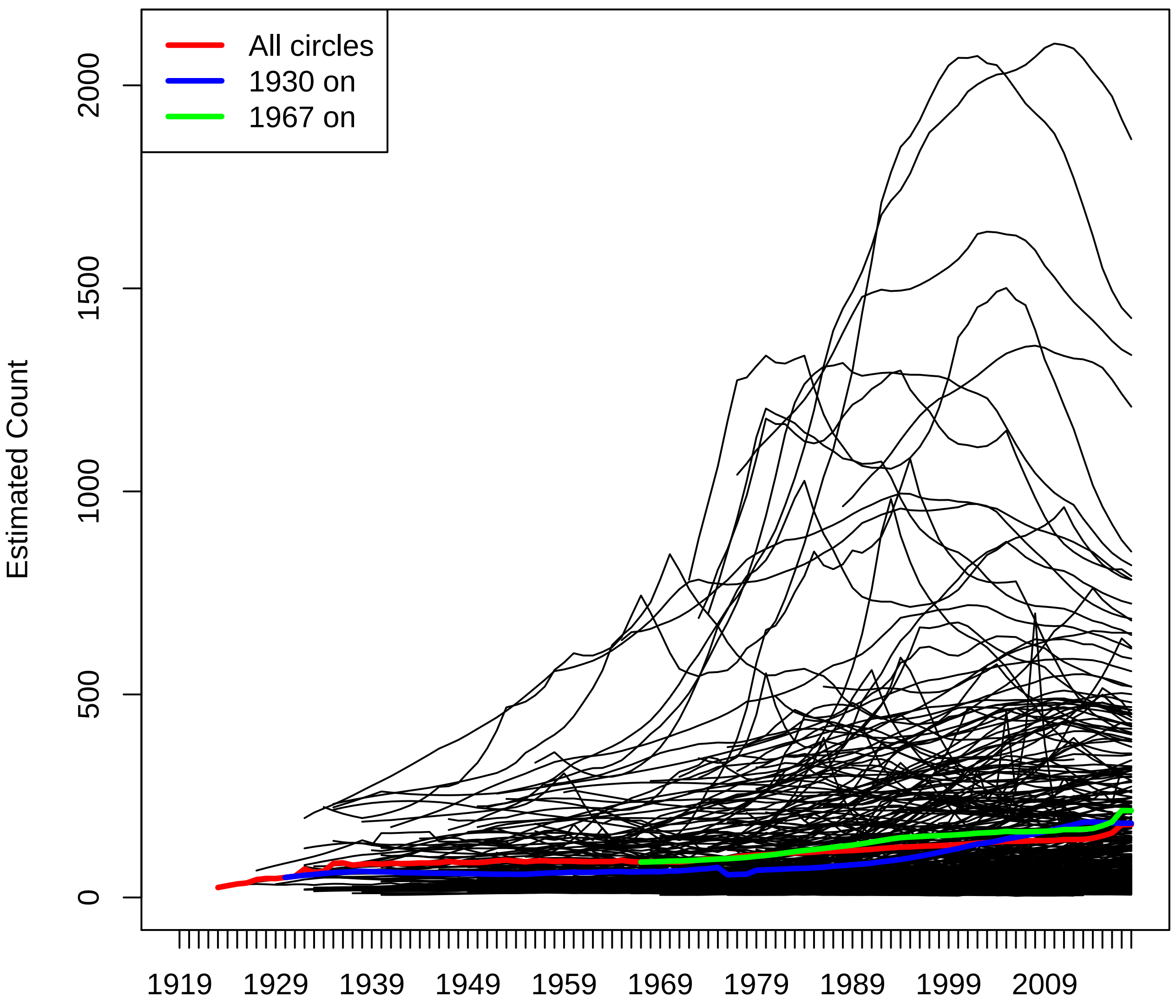
All circles
1930 on
1967 on
Estimated Count
0
500
1000
1500
2000
1919
1929
1939
1949
1959
1969
1979
1989
1999
2009

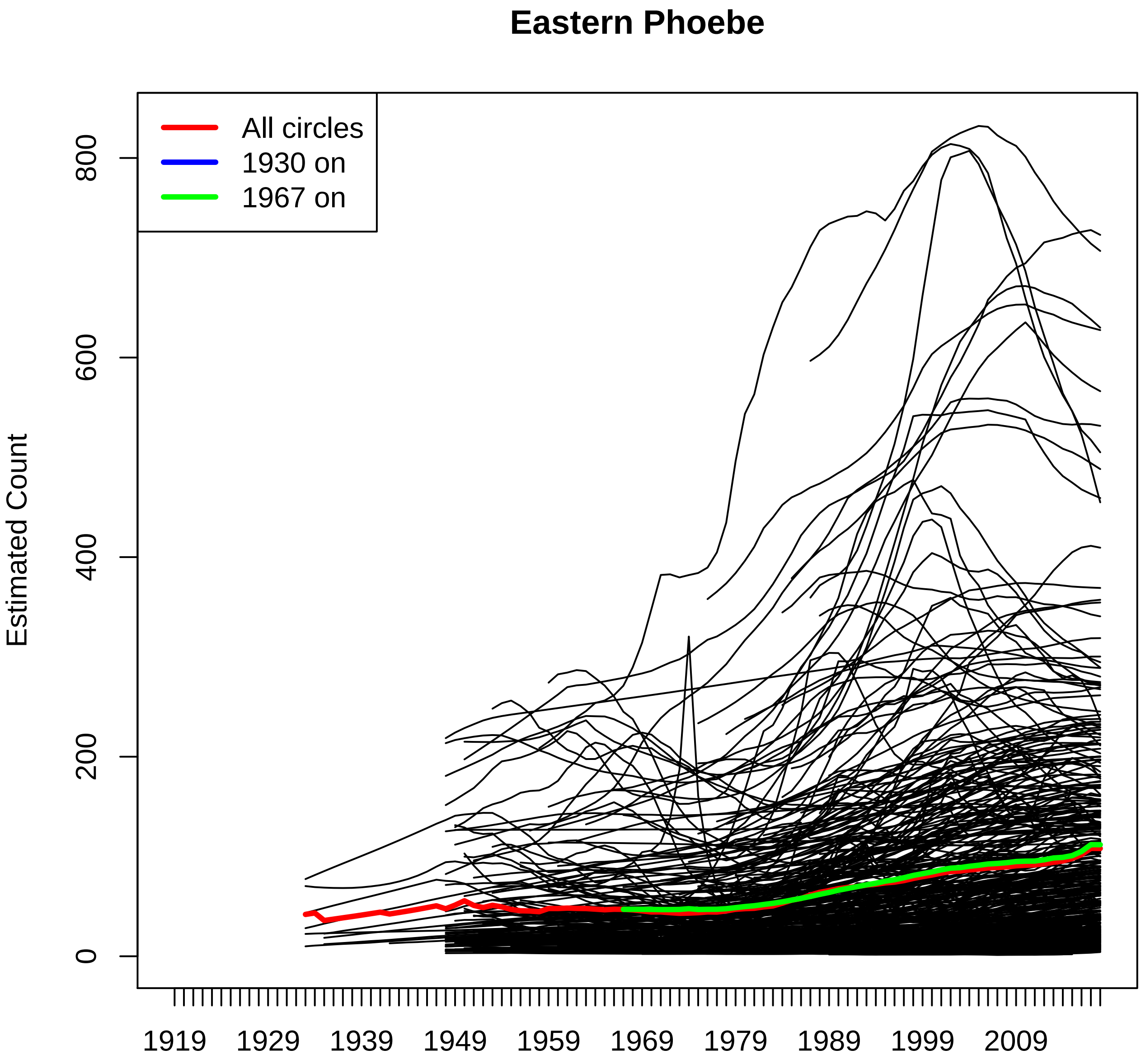
Eastern Phoebe
All circles
1930 on
1967 on
Estimated Count
0
200
400
600
800
1919
1929
1939
1949
1959
1969
1979
1989
1999
2009

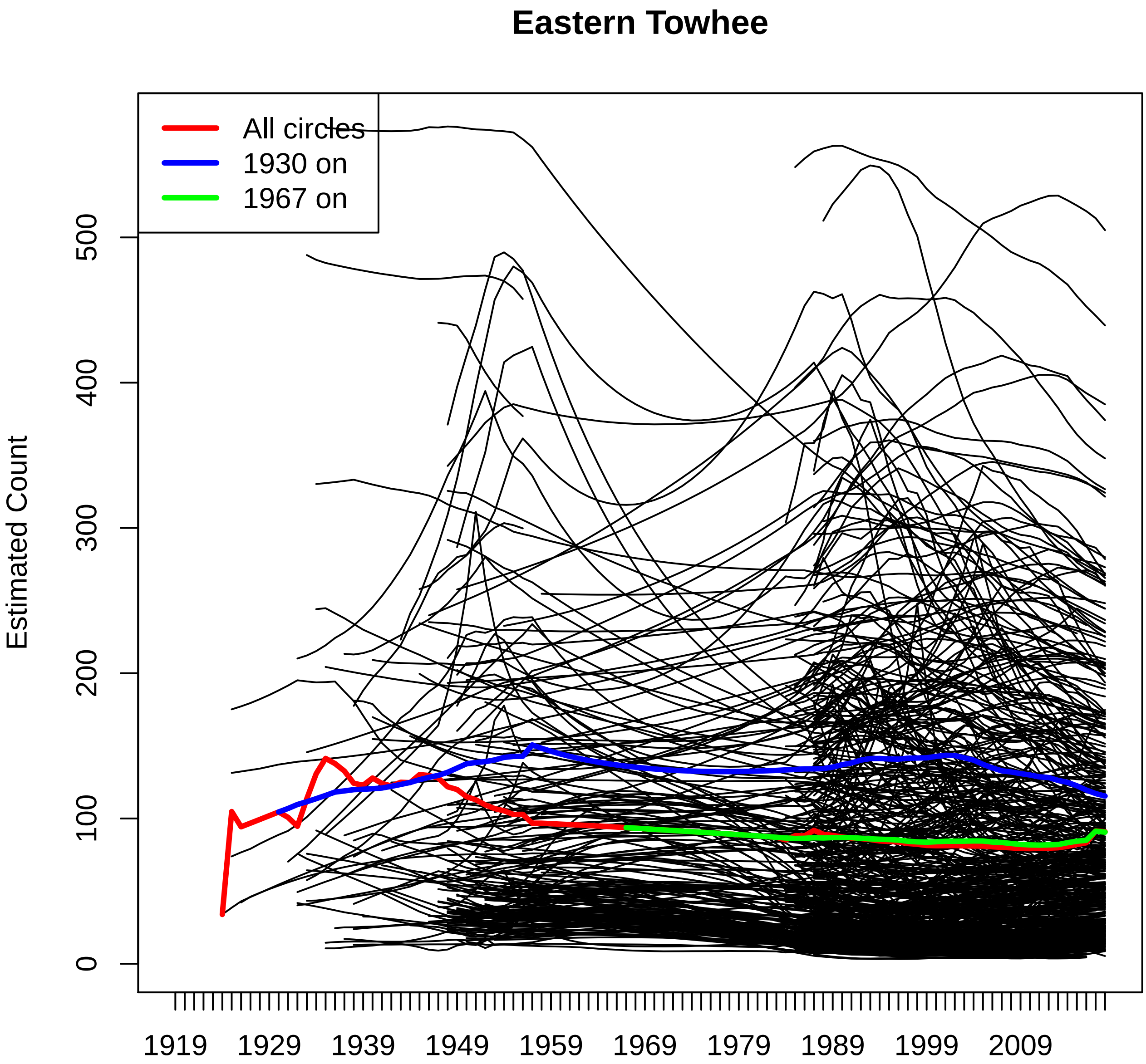

Eastern Towhee
All circles
1930 on
1967 on
Estimated Count
0
100
200
300
400
500
1919
1929
1939
1949
1959
1969
1979
1989
1999
2009

Fox Sparrow

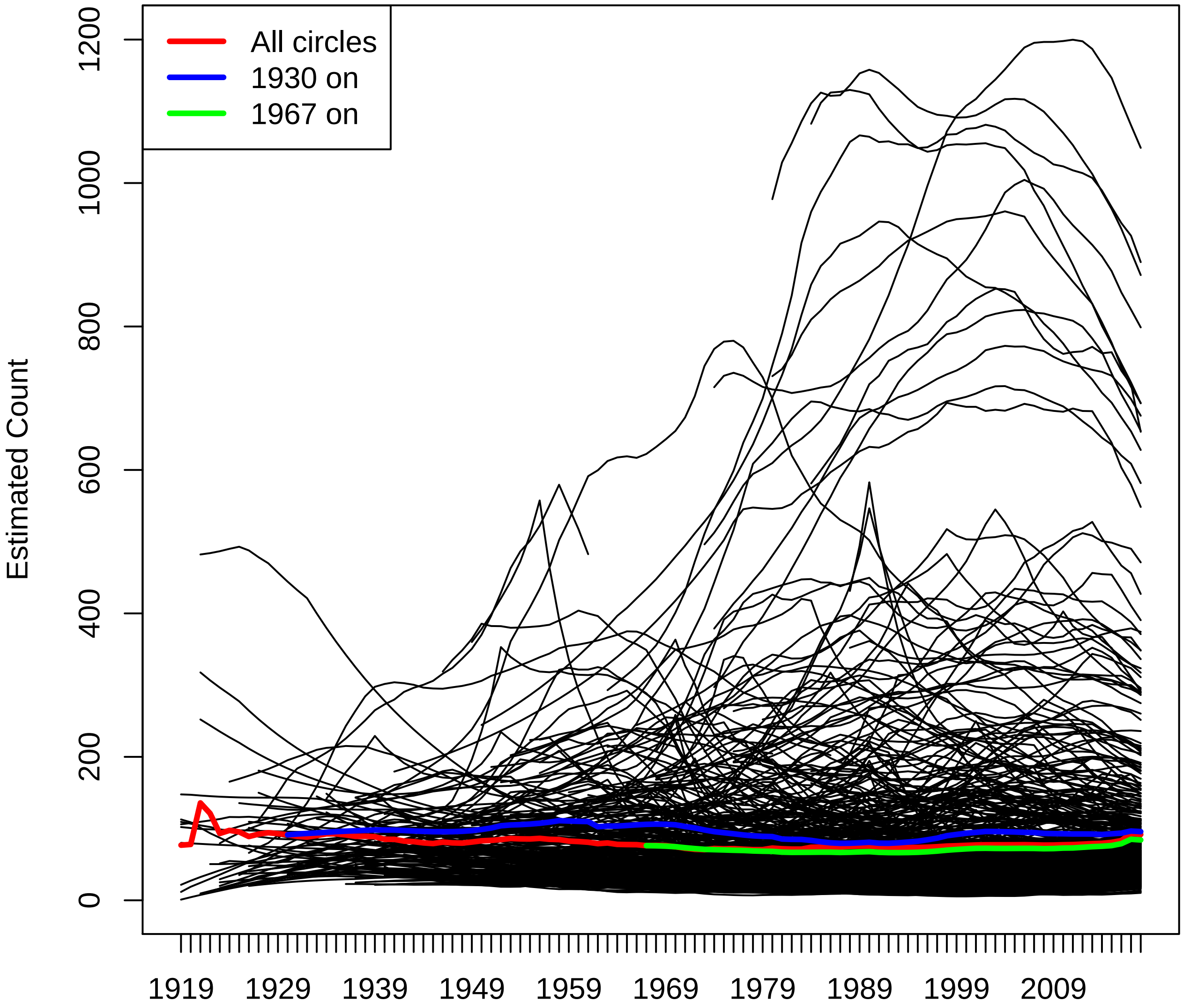

All circles
1930 on
1967 on
Estimated Count
0
200
400
600
800
1000
1200
1919
1929
1939
1949
1959
1969
1979
1989
1999
2009

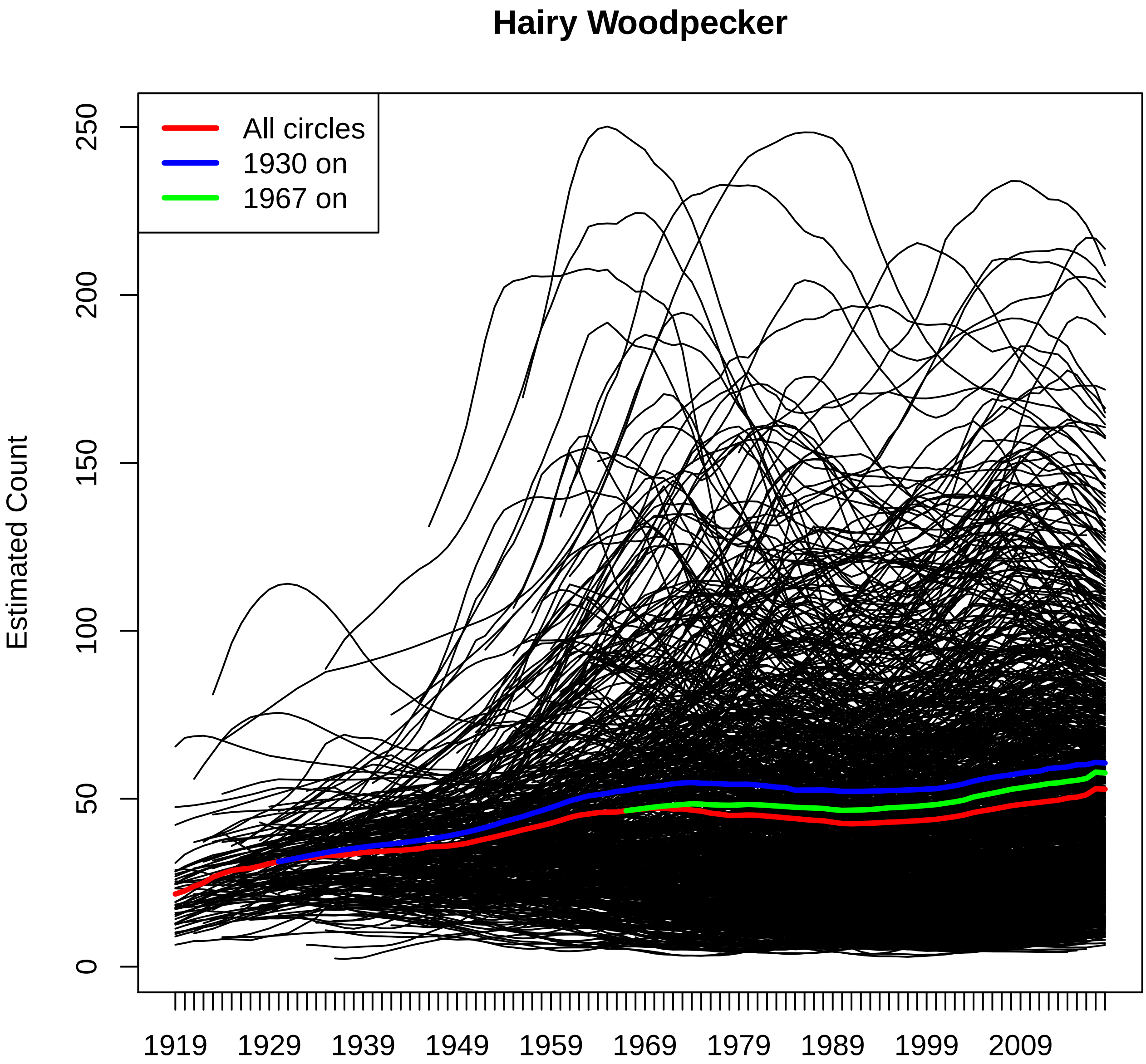

Hairy Woodpecker
All circles
1930 on
1967 on
Estimated Count
0
50
100
150
200
250
1919
1929
1939
1949
1959
1969
1979
1989
1999
2009

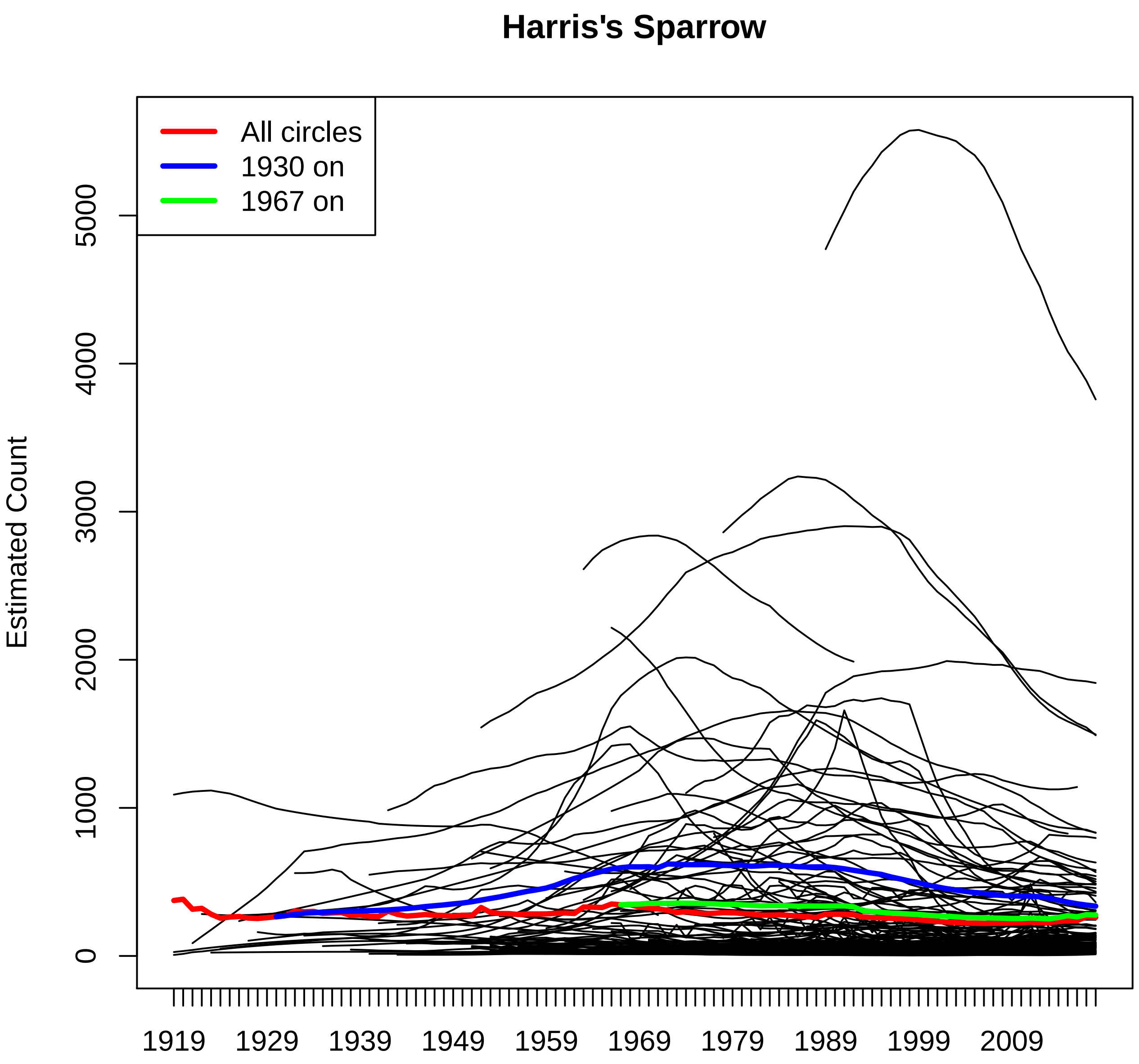
Harris's Sparrow
Estimated Count
0
1000
2000
3000
4000
5000
1919
1929
1939
1949
1959
1969
1979
1989
1999
2009
All circles
1930 on
1967 on

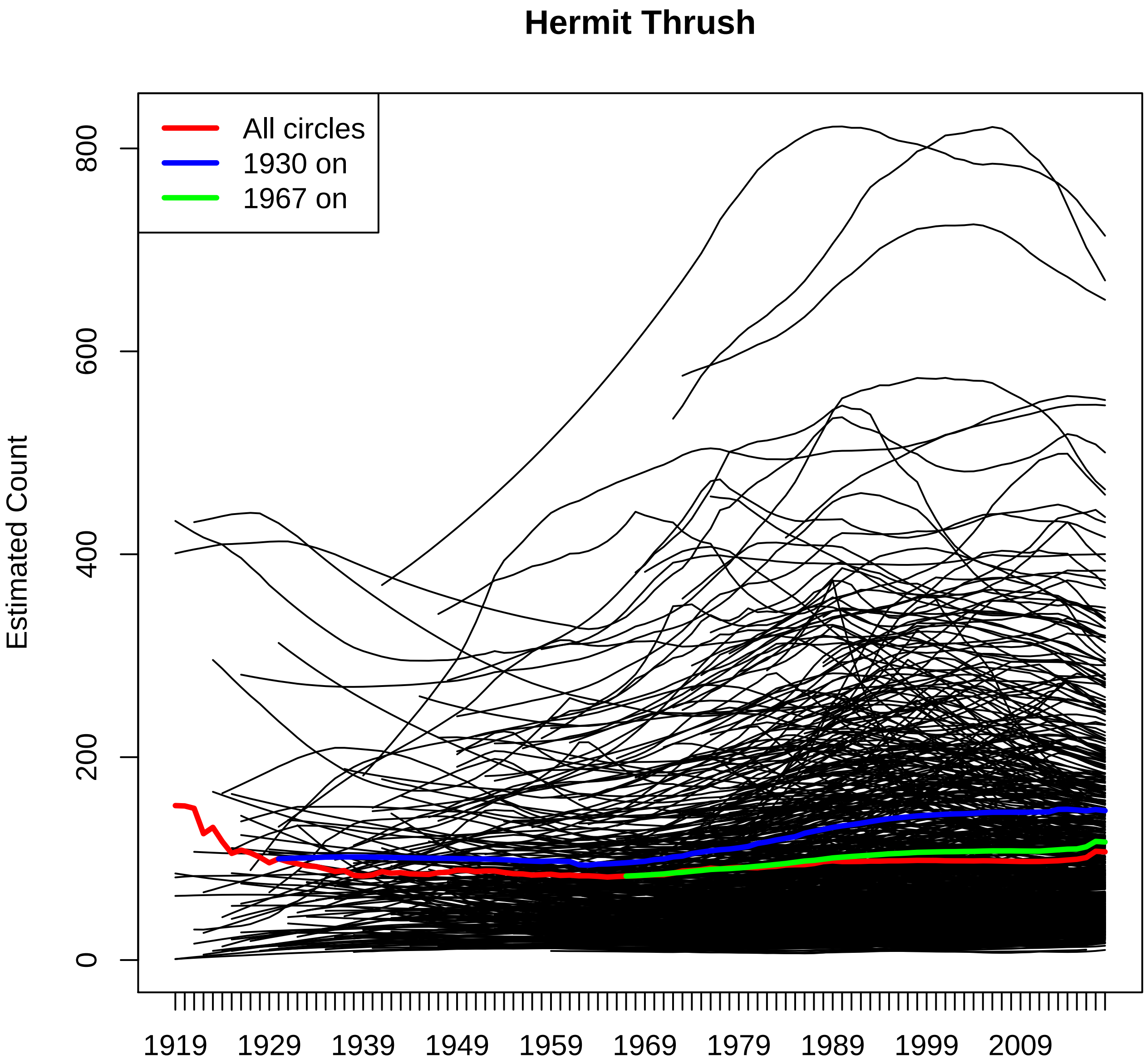

Hermit Thrush
All circles
1930 on
1967 on
Estimated Count
0
200
400
600
800
1919
1929
1939
1949
1959
1969
1979
1989
1999
2009

Horned Grebe

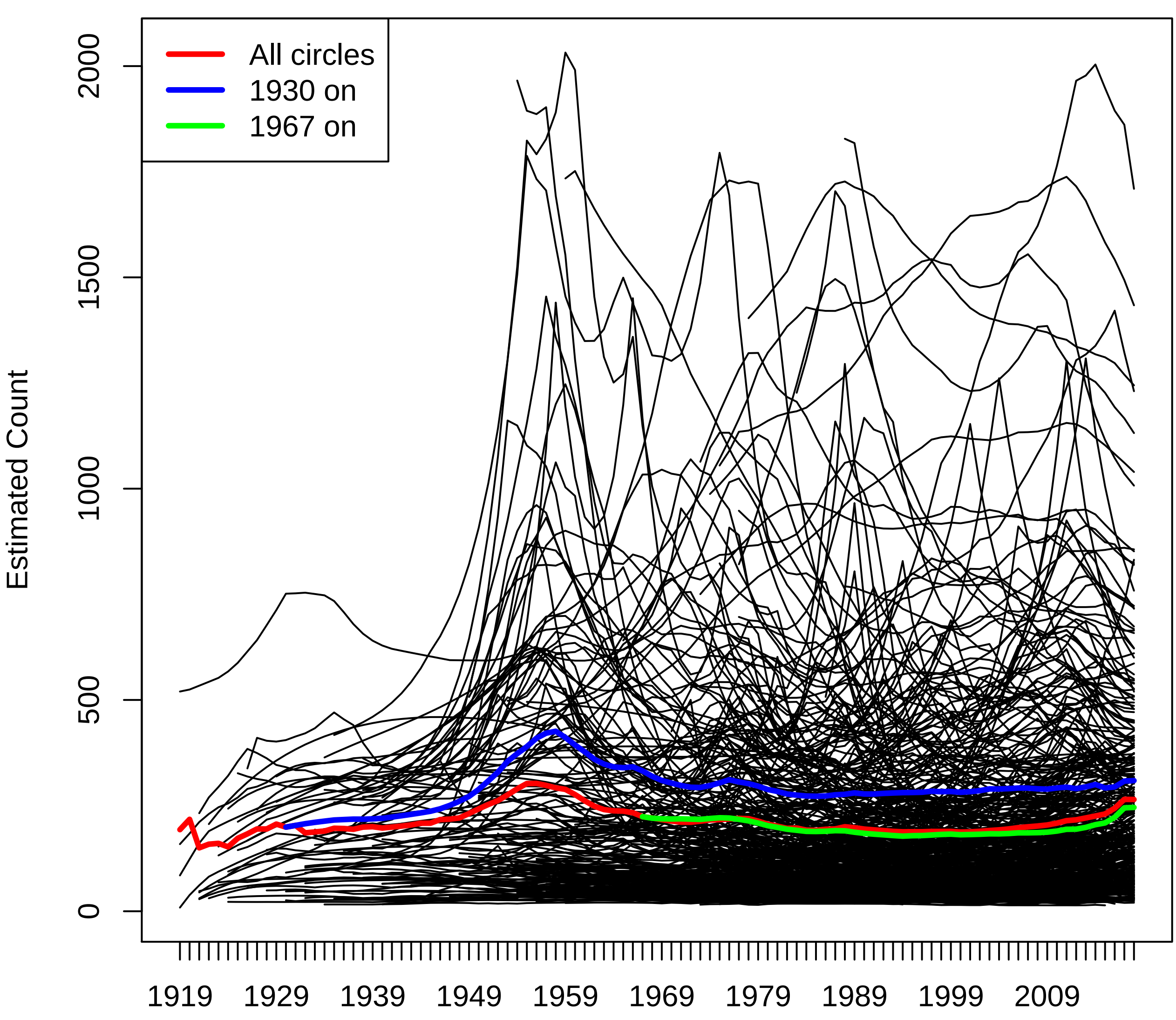

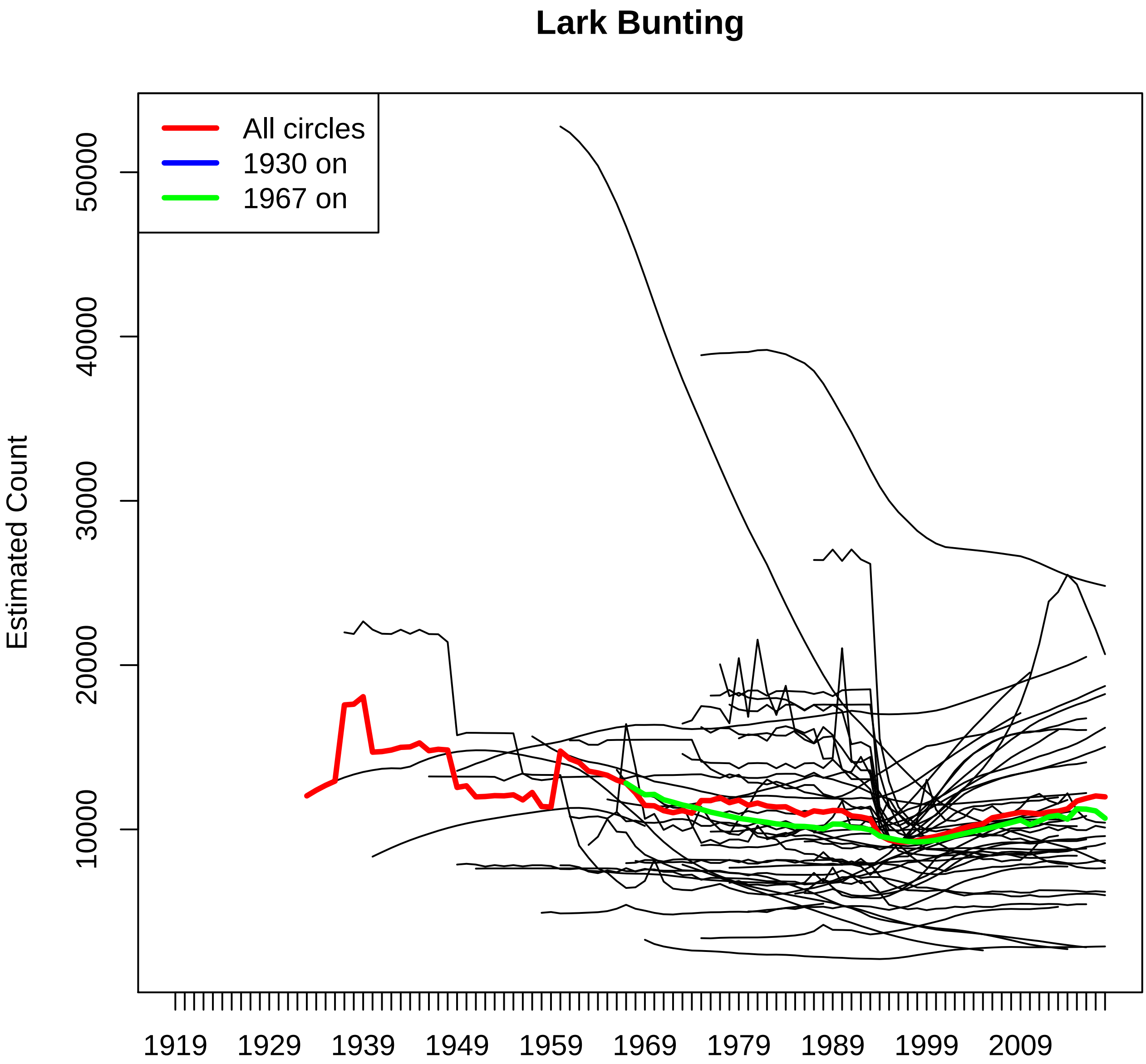

Lark Bunting
All circles
1930 on
1967 on
Estimated Count
50000
40000
30000
20000
10000
1919
1929
1939
1949
1959
1969
1979
1989
1999
2009

Lark Sparrow

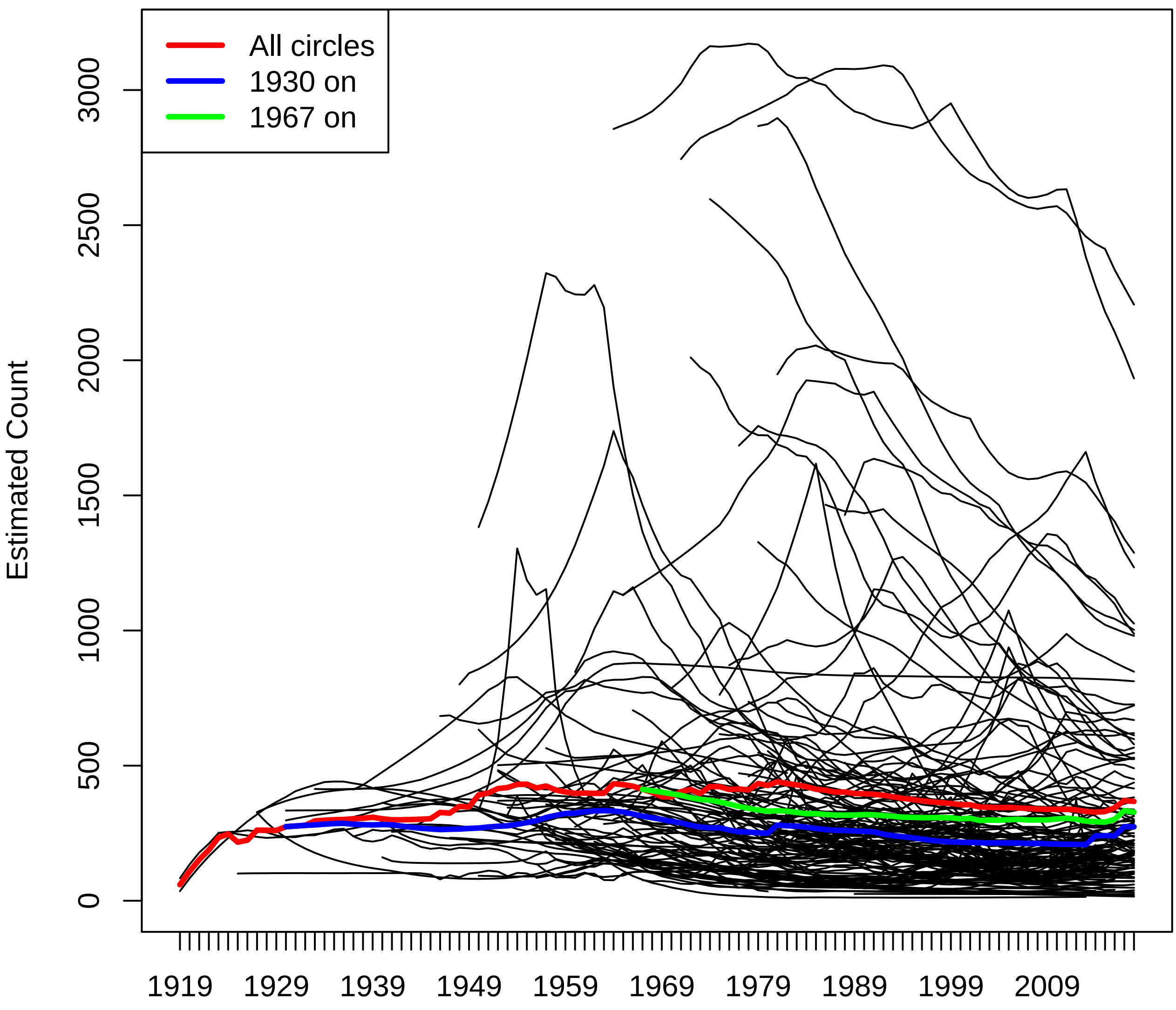

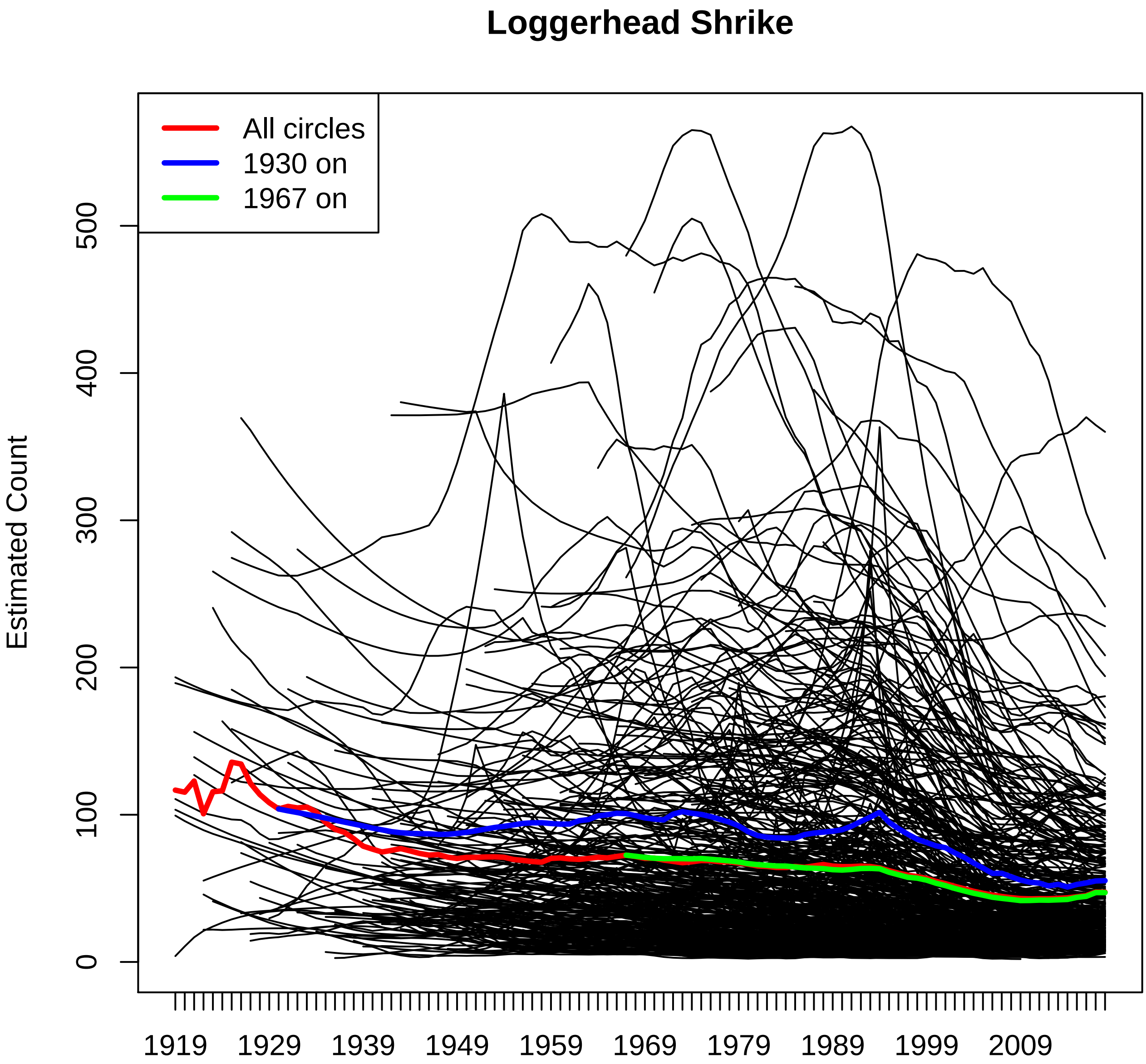

Loggerhead Shrike
All circles
1930 on
1967 on
Estimated Count
0
100
200
300
400
500
1919
1929
1939
1949
1959
1969
1979
1989
1999
2009

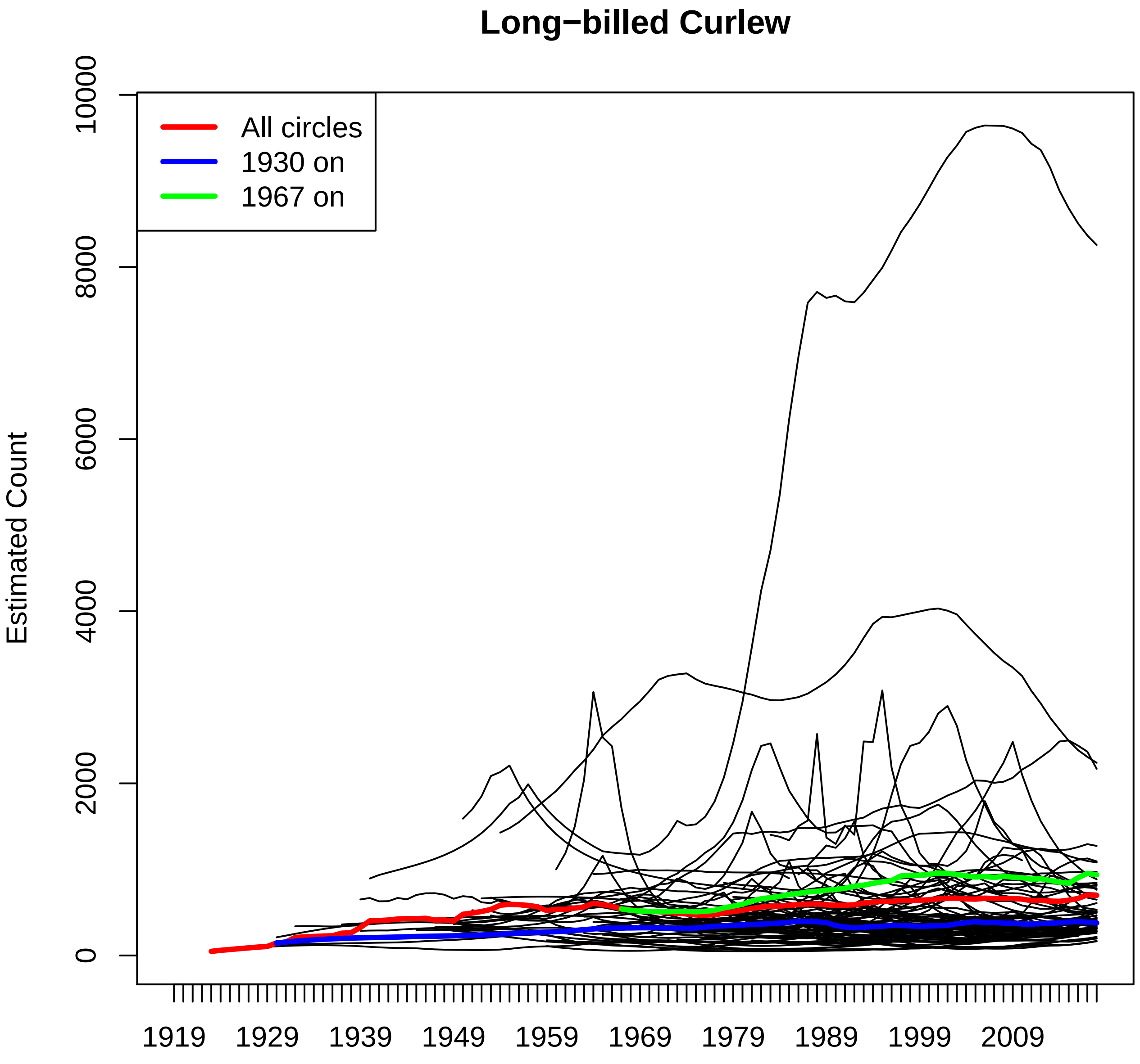

Long−billed Curlew
All circles
1930 on
1967 on
Estimated Count
0
2000
4000
6000
8000
10000
1919
1929
1939
1949
1959
1969
1979
1989
1999
2009

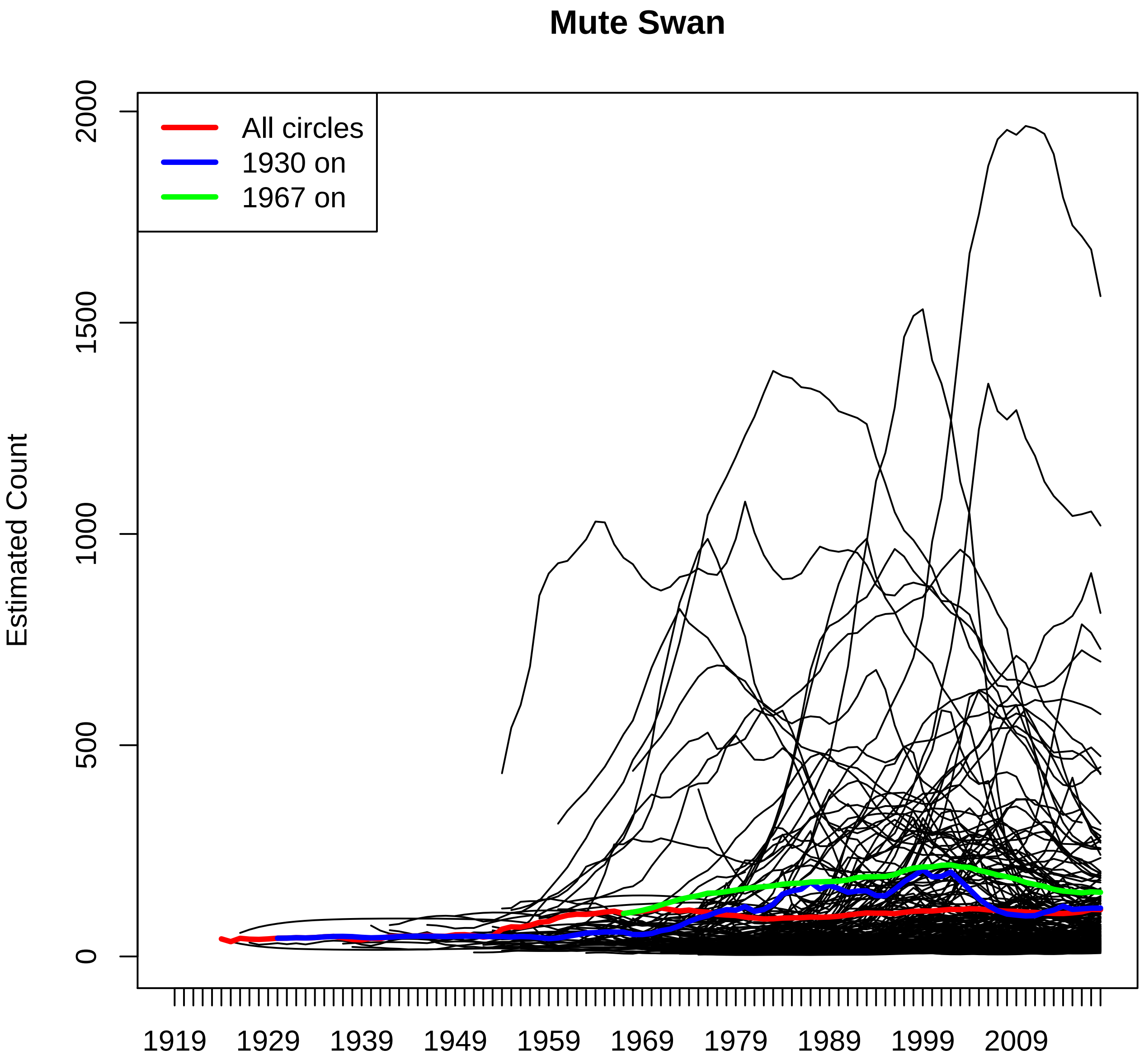

Mute Swan
All circles
1930 on
1967 on
Estimated Count
0
500
1000
1500
2000
1919
1929
1939
1949
1959
1969
1979
1989
1999
2009

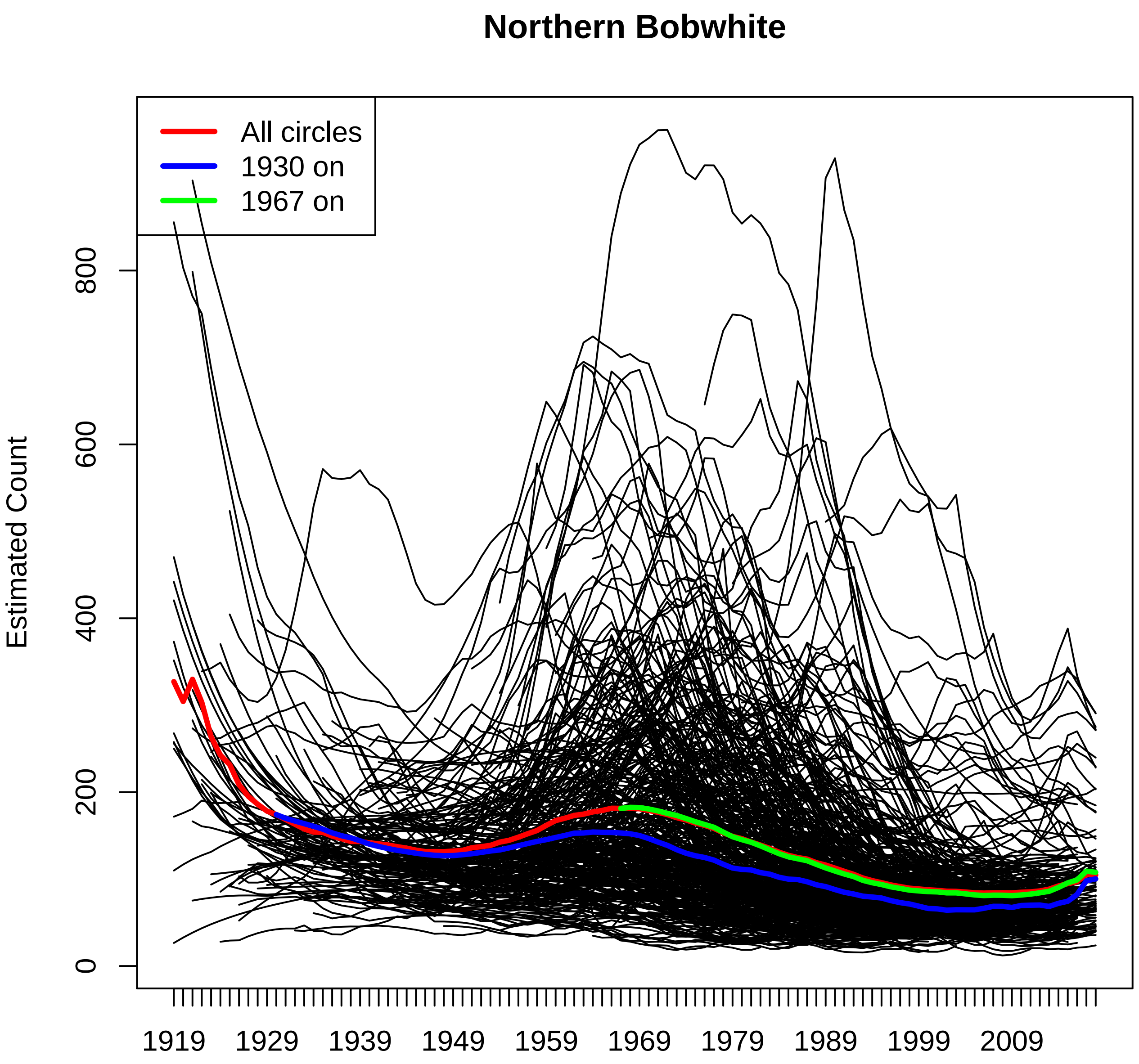

Northern Bobwhite
All circles
1930 on
1967 on
Estimated Count
0
200
400
600
800
1919
1929
1939
1949
1959
1969
1979
1989
1999
2009

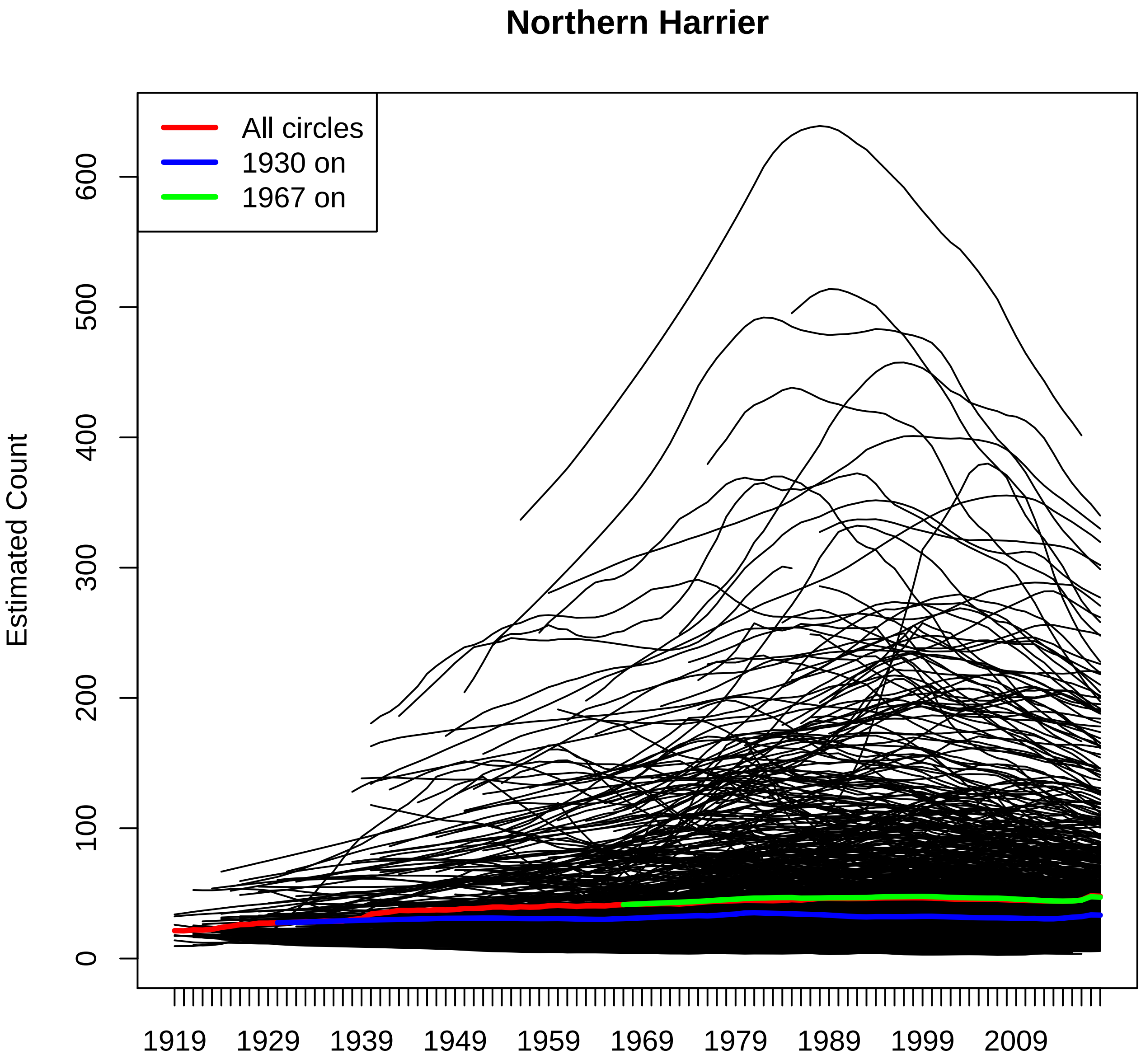
Northern Harrier
All circles
1930 on
1967 on
Estimated Count
0
100
200
300
400
500
600
1919
1929
1939
1949
1959
1969
1979
1989
1999
2009

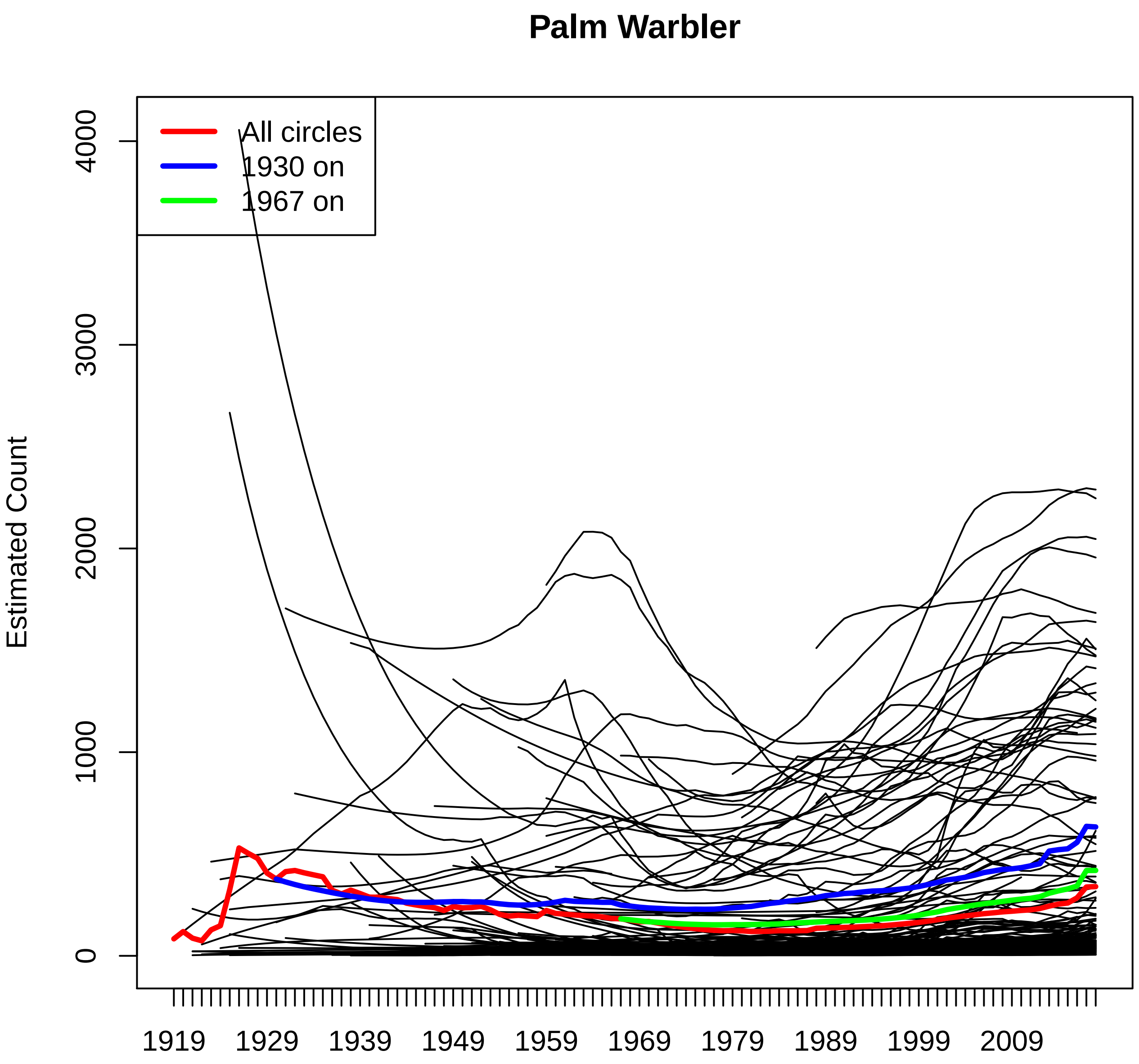

Palm Warbler
All circles
1930 on
1967 on
Estimated Count
0
1000
2000
3000
4000
1919
1929
1939
1949
1959
1969
1979
1989
1999
2009

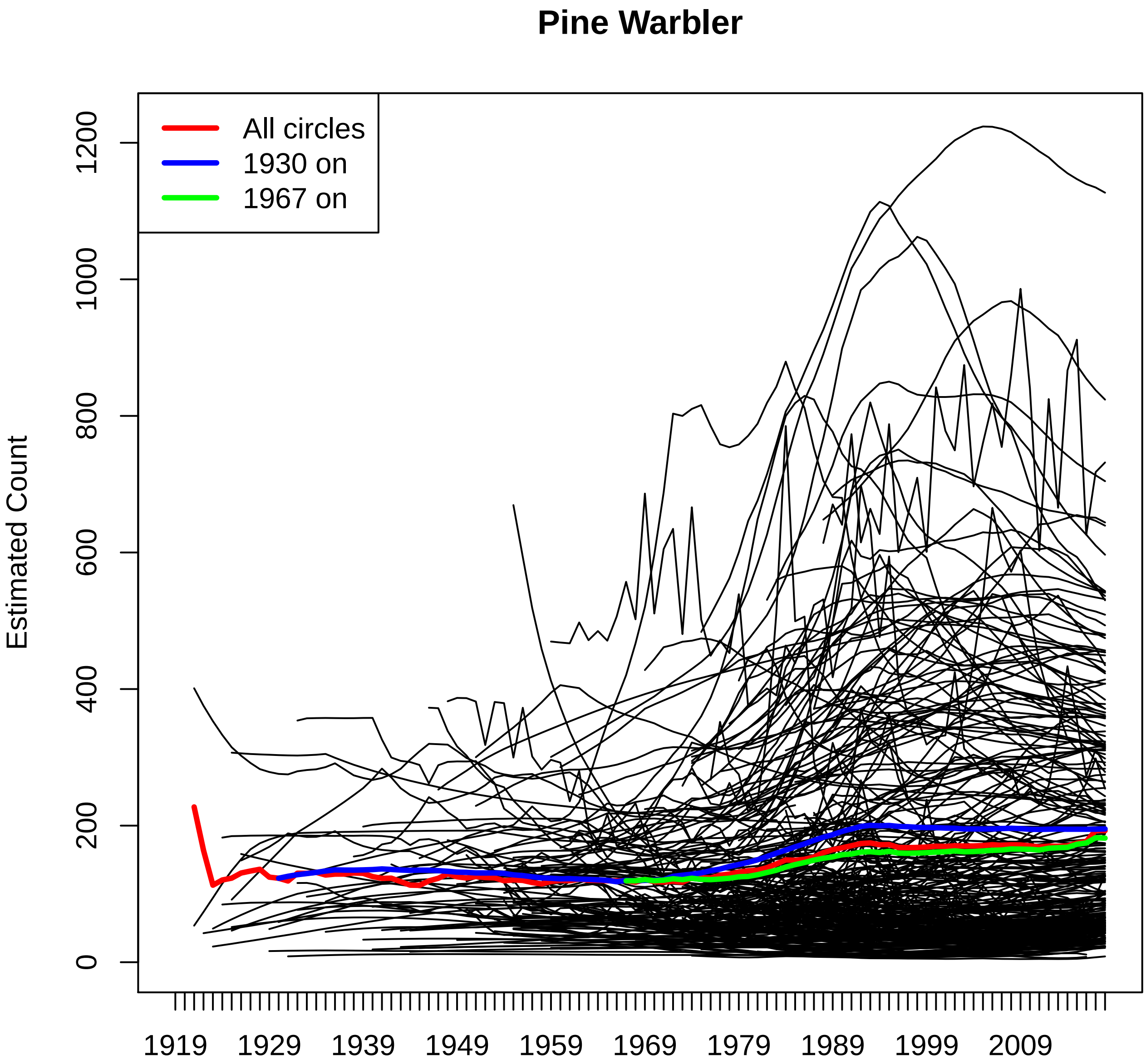

Pine Warbler
Estimated Count
All circles
1930 on
1967 on
0
200
400
600
800
1000
1200
1919
1929
1939
1949
1959
1969
1979
1989
1999
2009

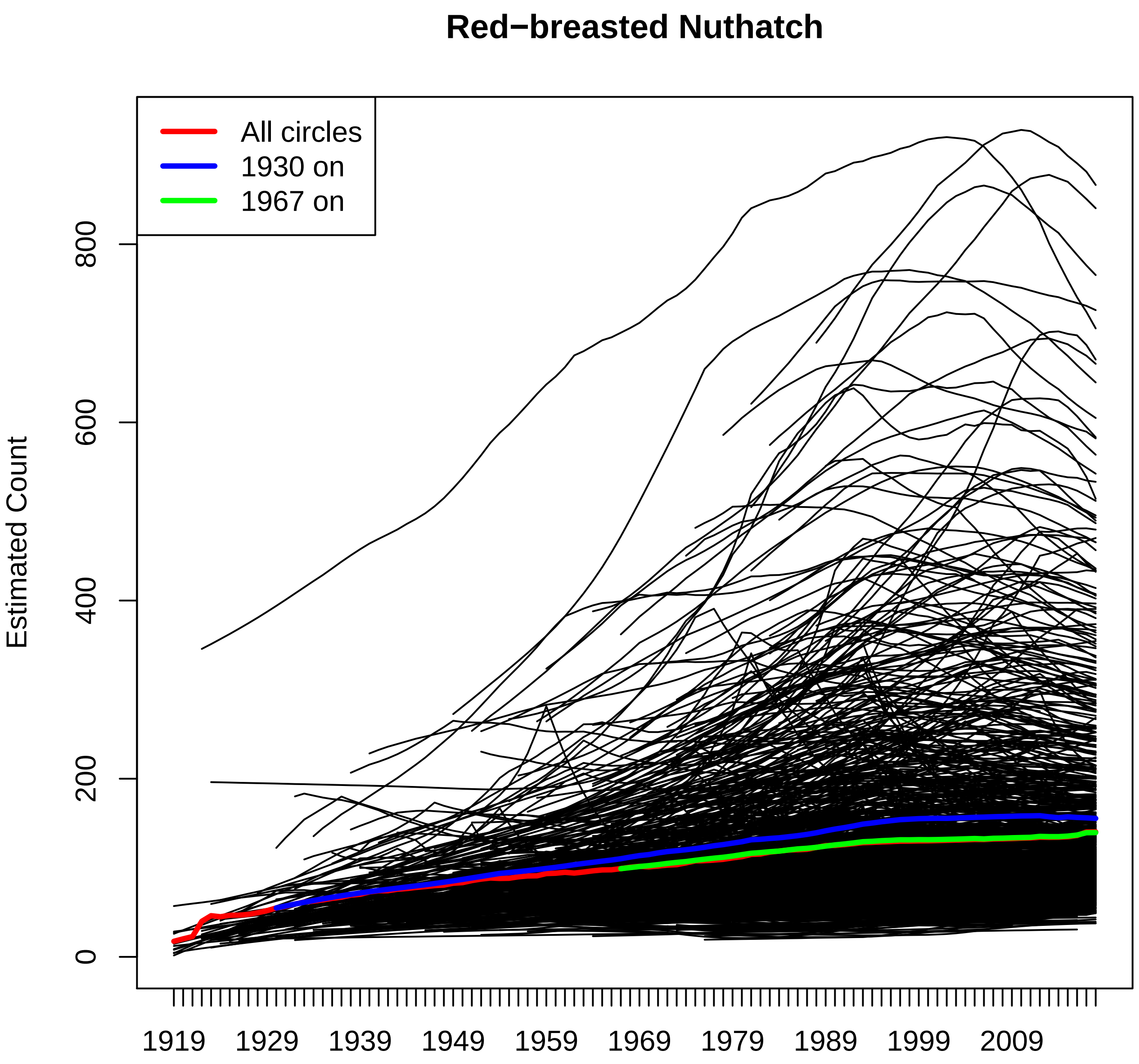

Red−breasted Nuthatch
All circles
1930 on
1967 on
Estimated Count
0
200
400
600
800
1919
1929
1939
1949
1959
1969
1979
1989
1999
2009

Red−throated Loon

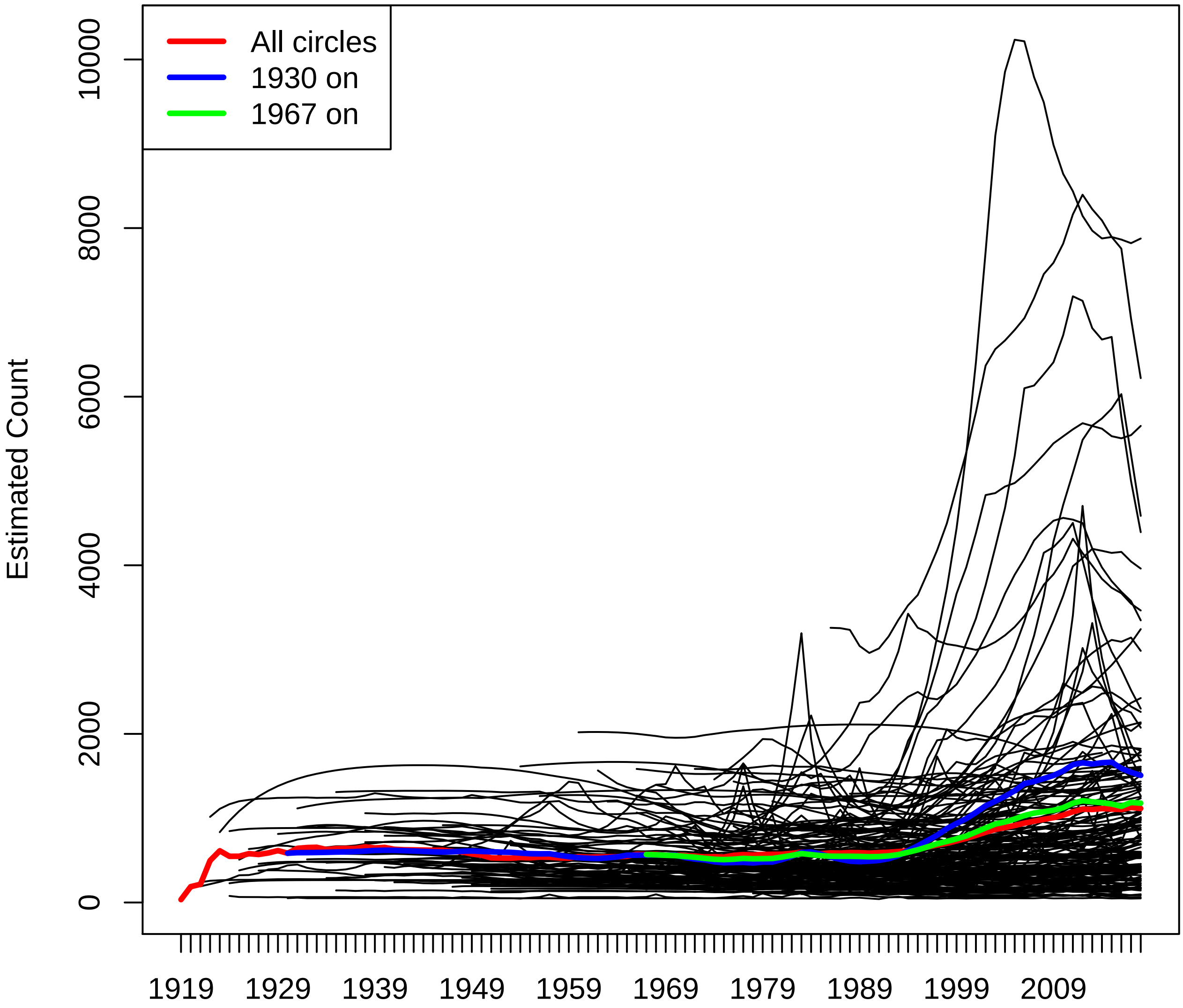

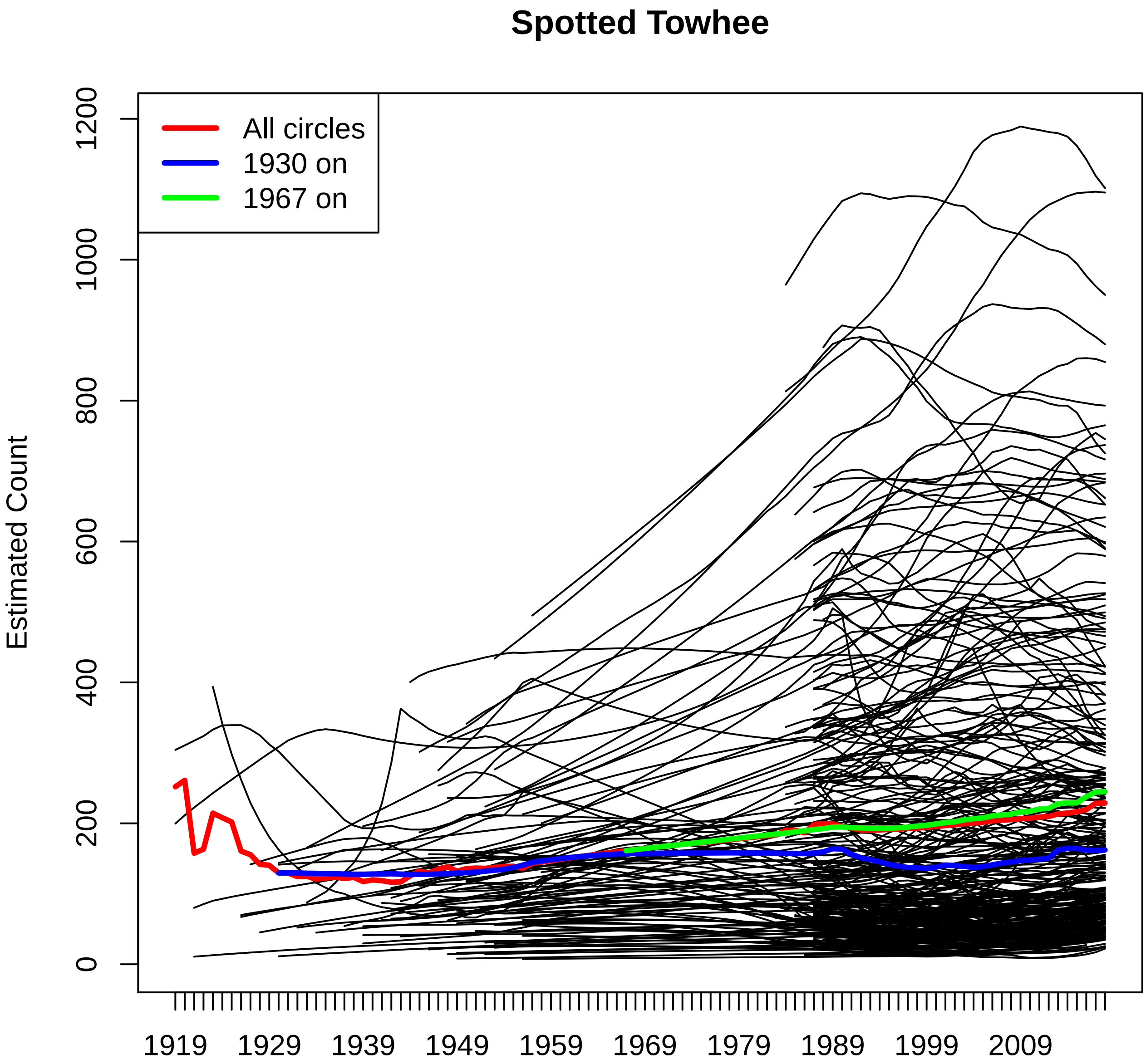

Spotted Towhee
All circles
1930 on
1967 on
Estimated Count
0
200
400
600
800
1000
1200
1919
1929
1939
1949
1959
1969
1979
1989
1999
2009

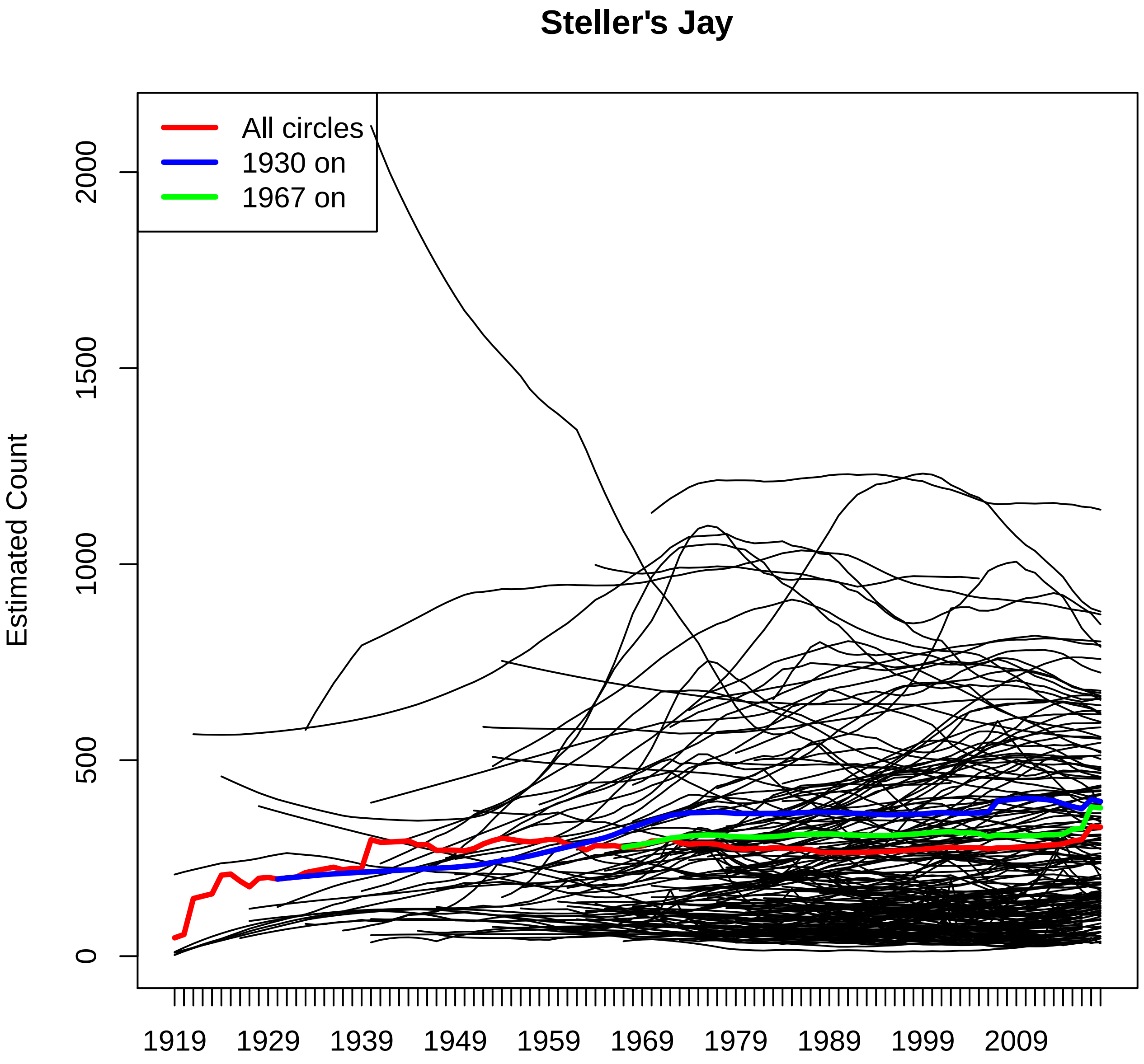

Steller's Jay
Estimated Count
0
500
1000
1500
2000
All circles
1930 on
1967 on
1919
1929
1939
1949
1959
1969
1979
1989
1999
2009

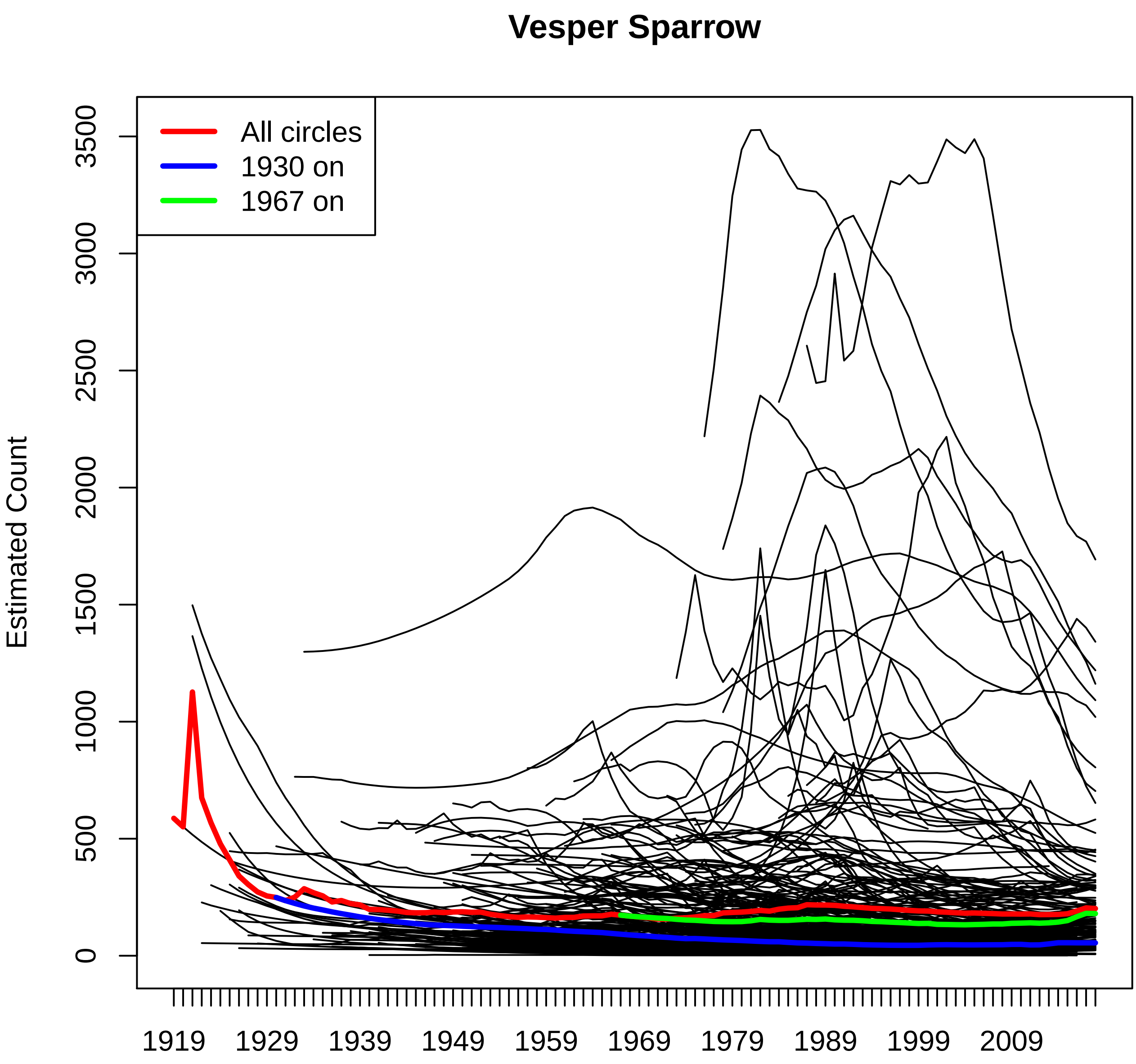
Vesper Sparrow
All circles
1930 on
1967 on
Estimated Count
0
500
1000
1500
2000
2500
3000
3500
1919
1929
1939
1949
1959
1969
1979
1989
1999
2009

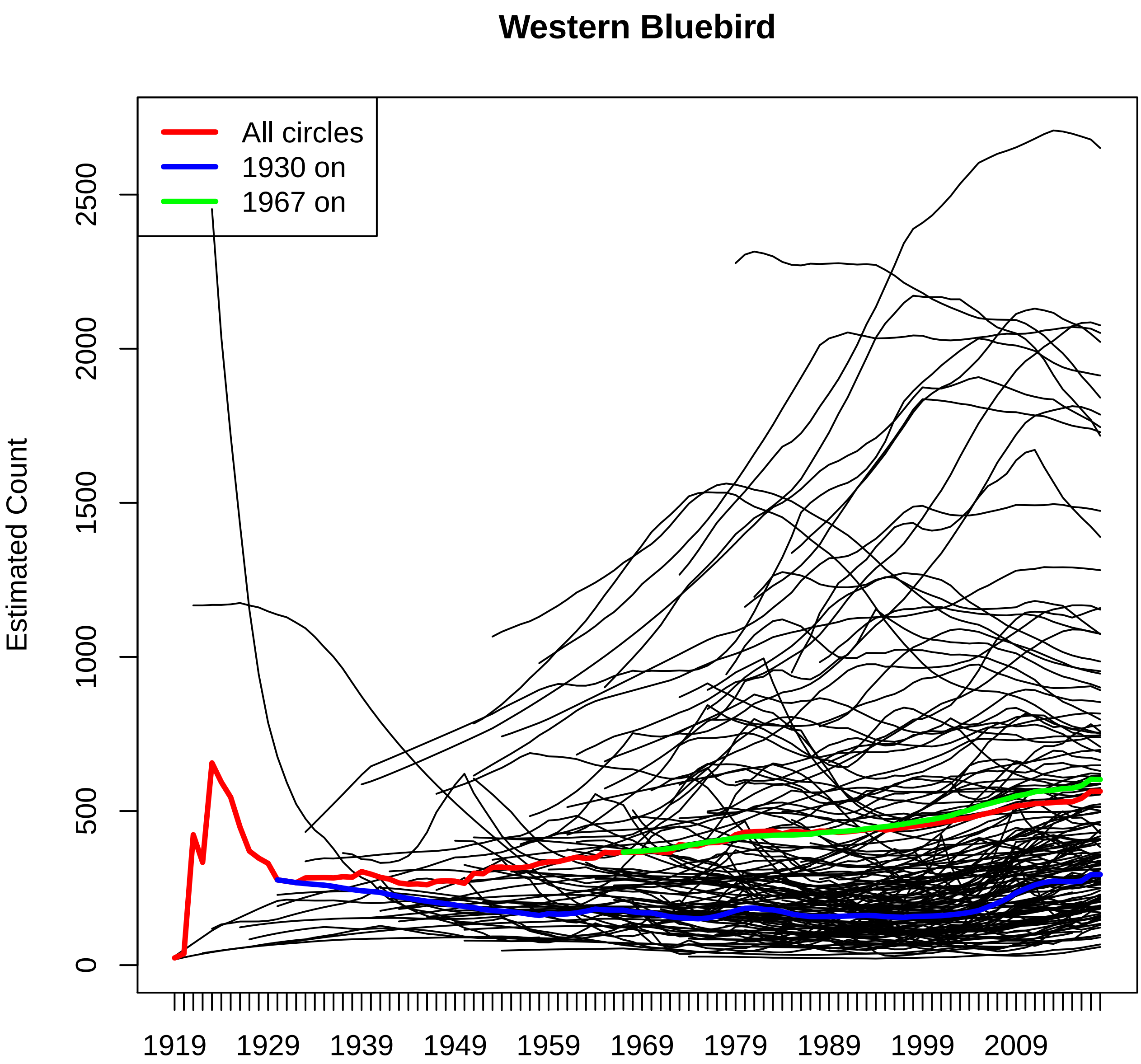

Western Bluebird
All circles
1930 on
1967 on
Estimated Count
0
500
1000
1500
2000
2500
1919
1929
1939
1949
1959
1969
1979
1989
1999
2009

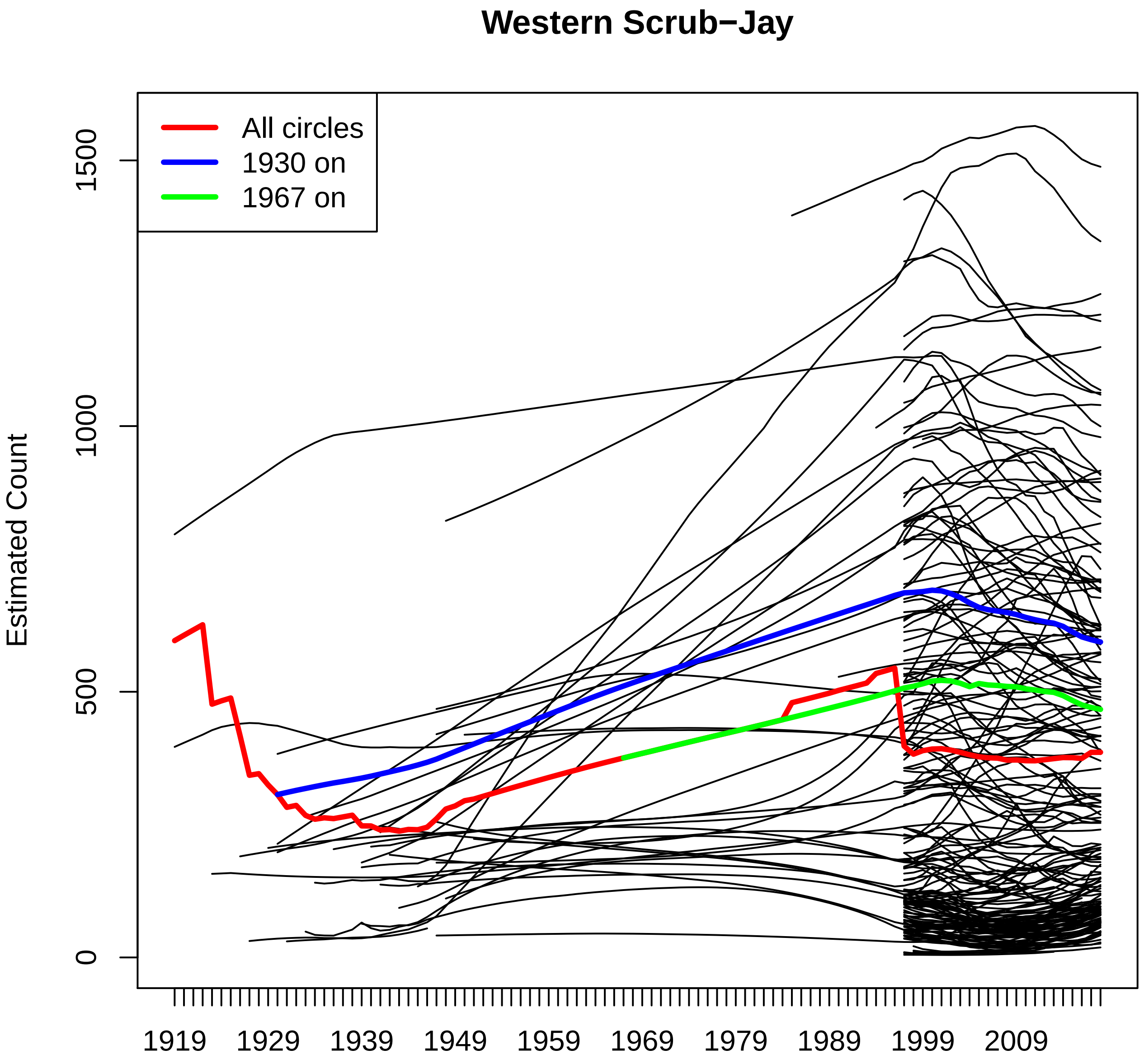
Western Scrub−Jay
All circles
1930 on
1967 on
Estimated Count
0
500
1000
1500
1919
1929
1939
1949
1959
1969
1979
1989
1999
2009

White−faced Ibis

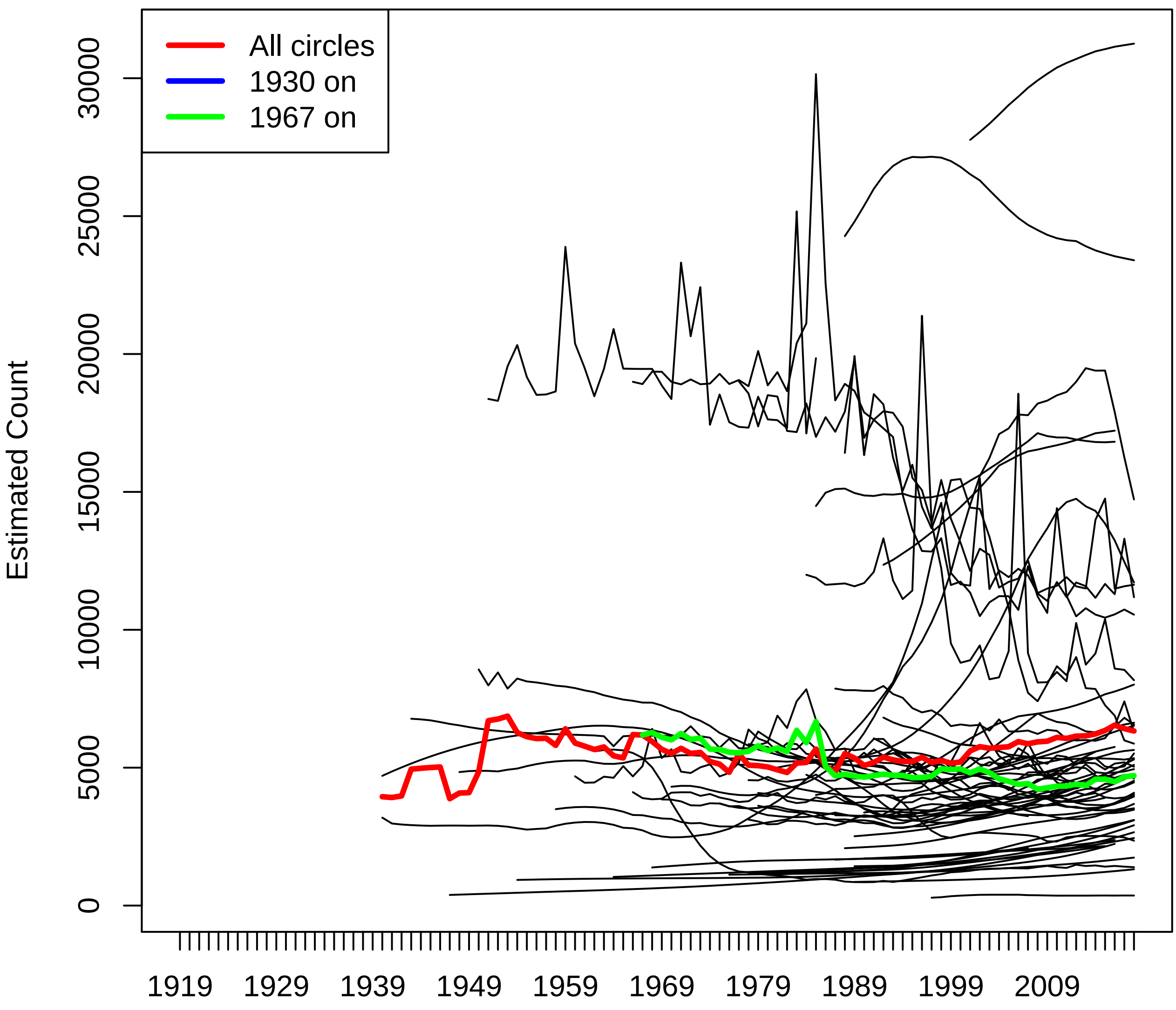

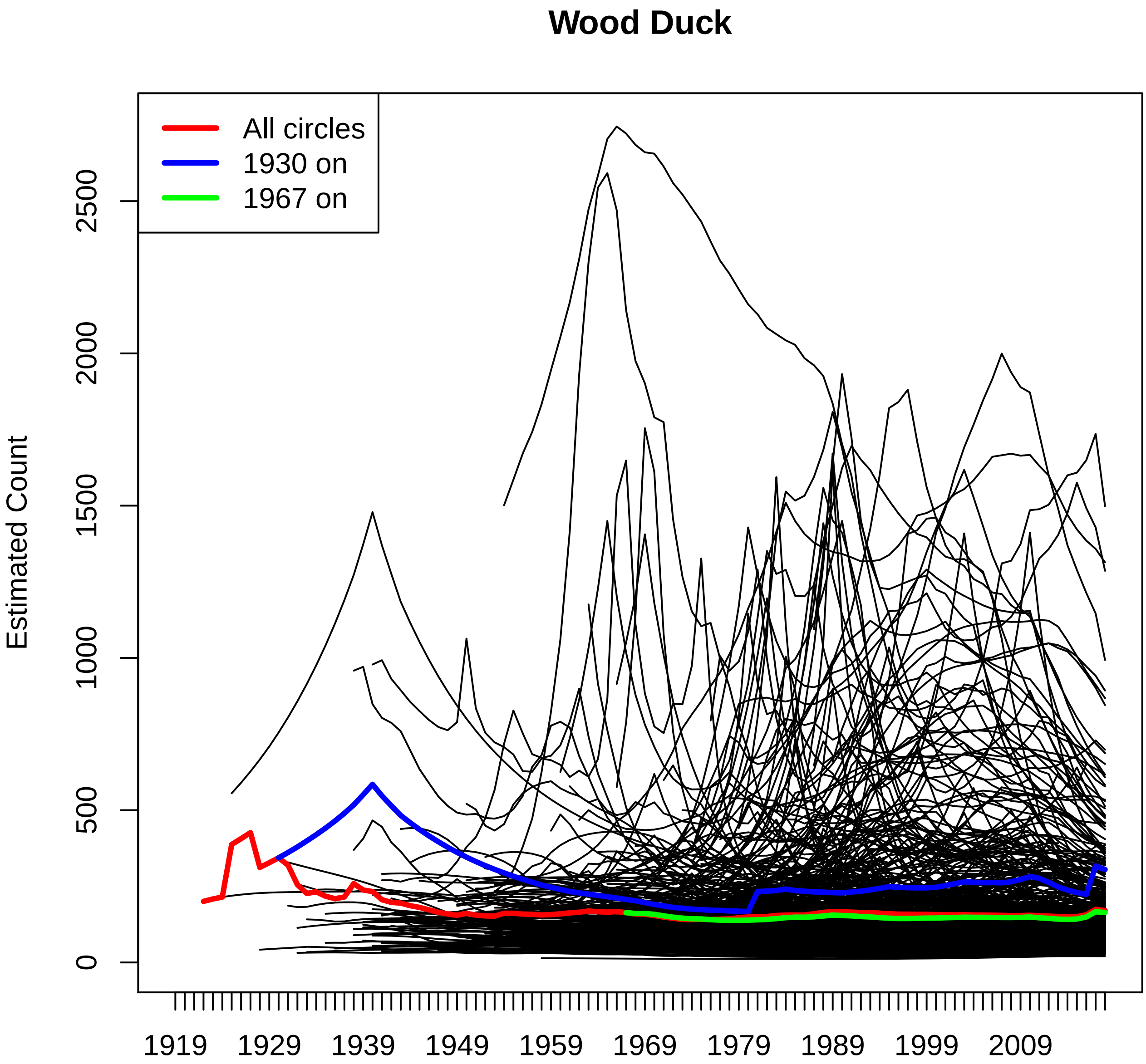
Wood Duck
All circles
1930 on
1967 on
Estimated Count
0
500
1000
1500
2000
2500
1919
1929
1939
1949
1959
1969
1979
1989
1999
2009

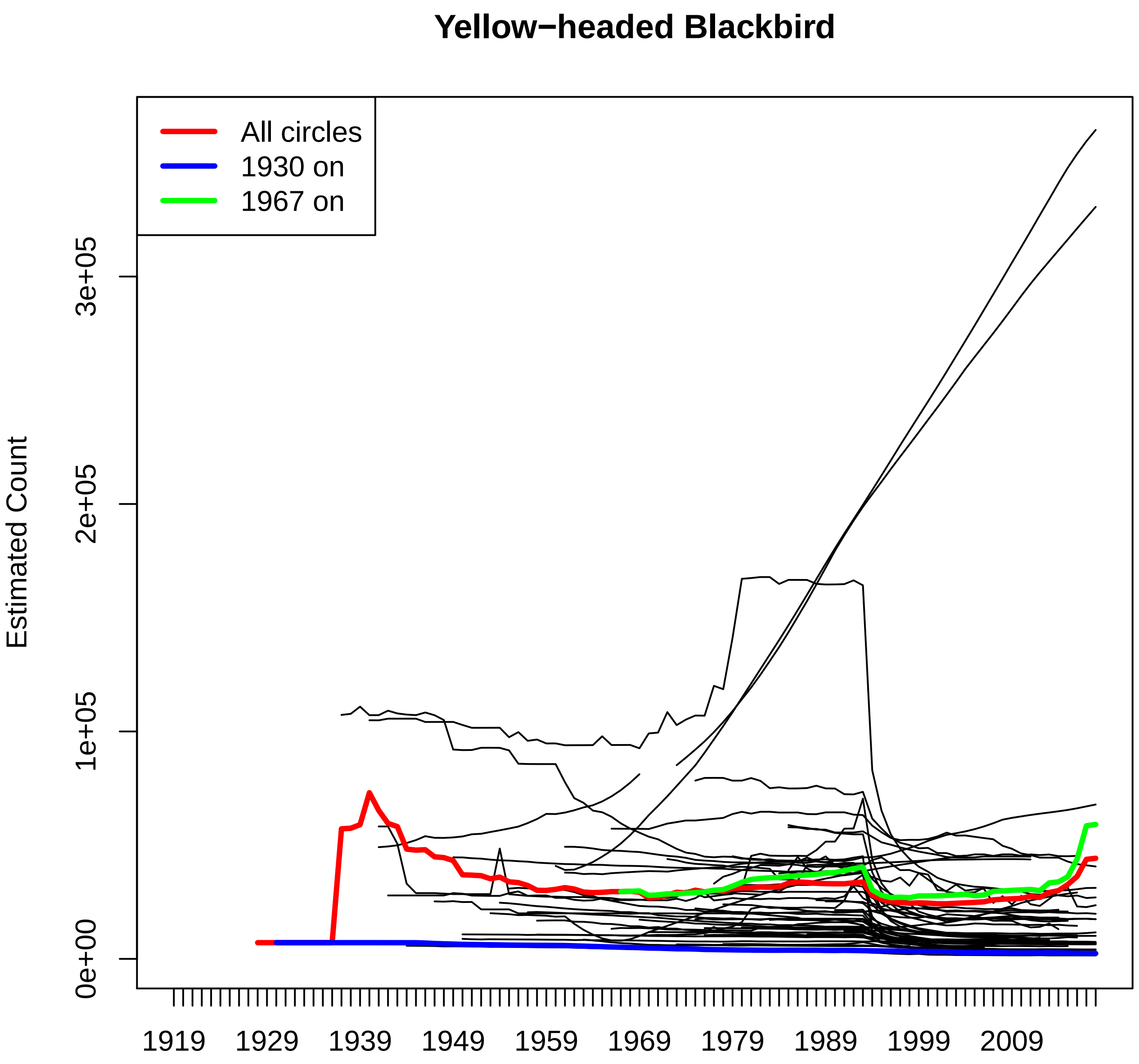
Yellow−headed Blackbird
All circles
1930 on
1967 on
Estimated Count
0e+00
1e+05
2e+05
3e+05
1919
1929
1939
1949
1959
1969
1979
1989
1999
2009

Anhinga

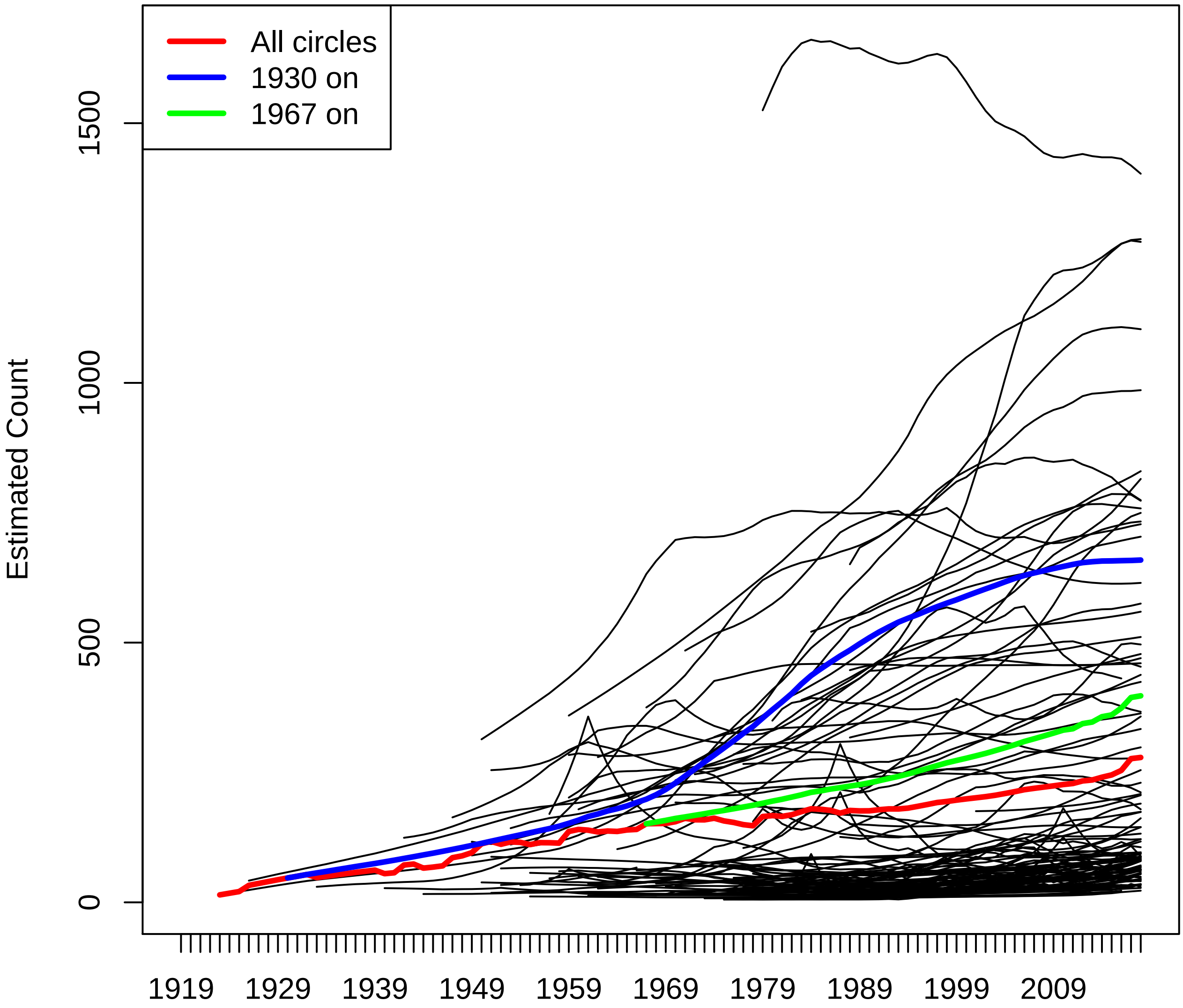

All circles
1930 on
1967 on
Estimated Count
0
500
1000
1500
1919
1929
1939
1949
1959
1969
1979
1989
1999
2009

Bewick's Wren

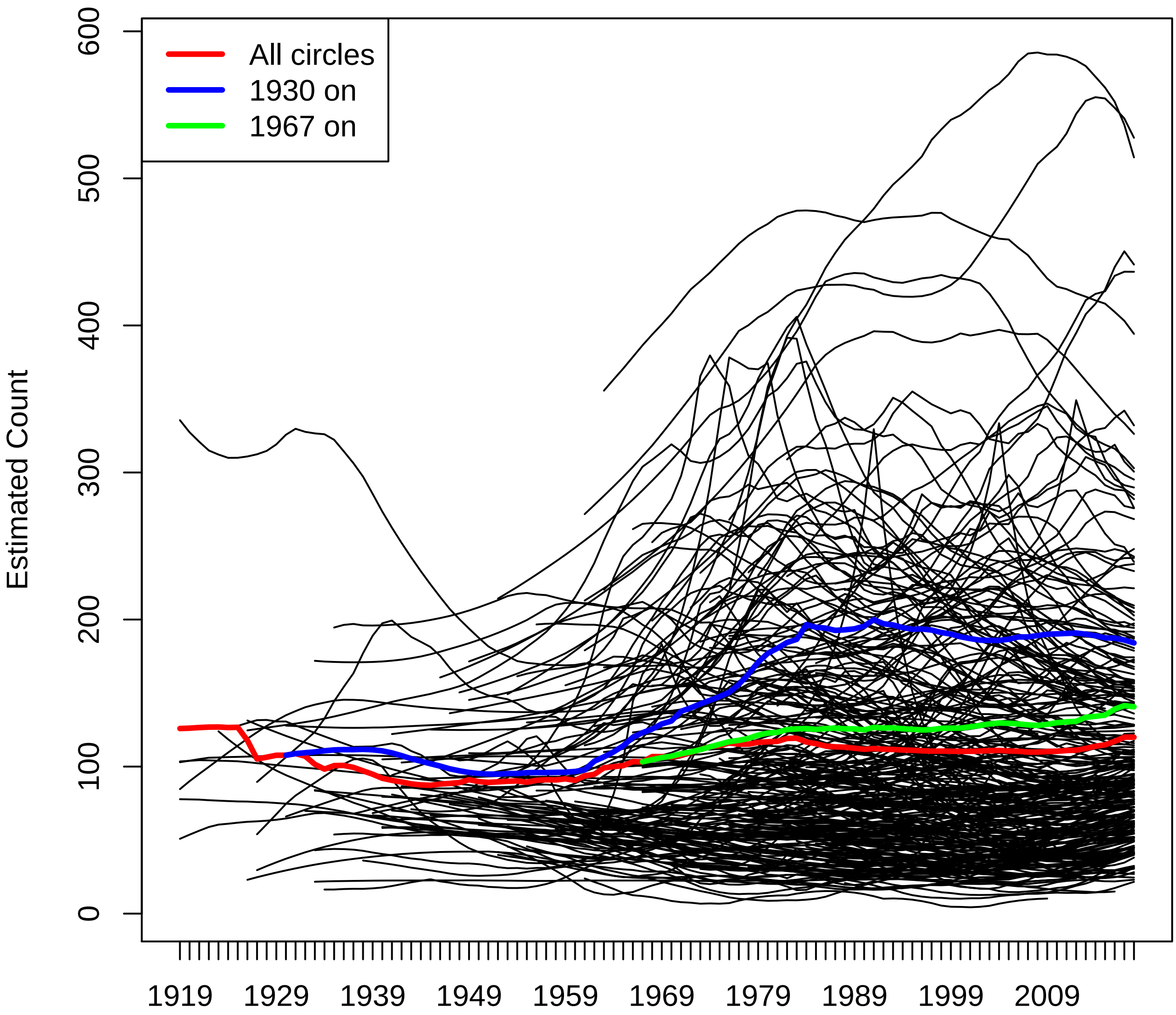

Black−bellied Whistling−Duck

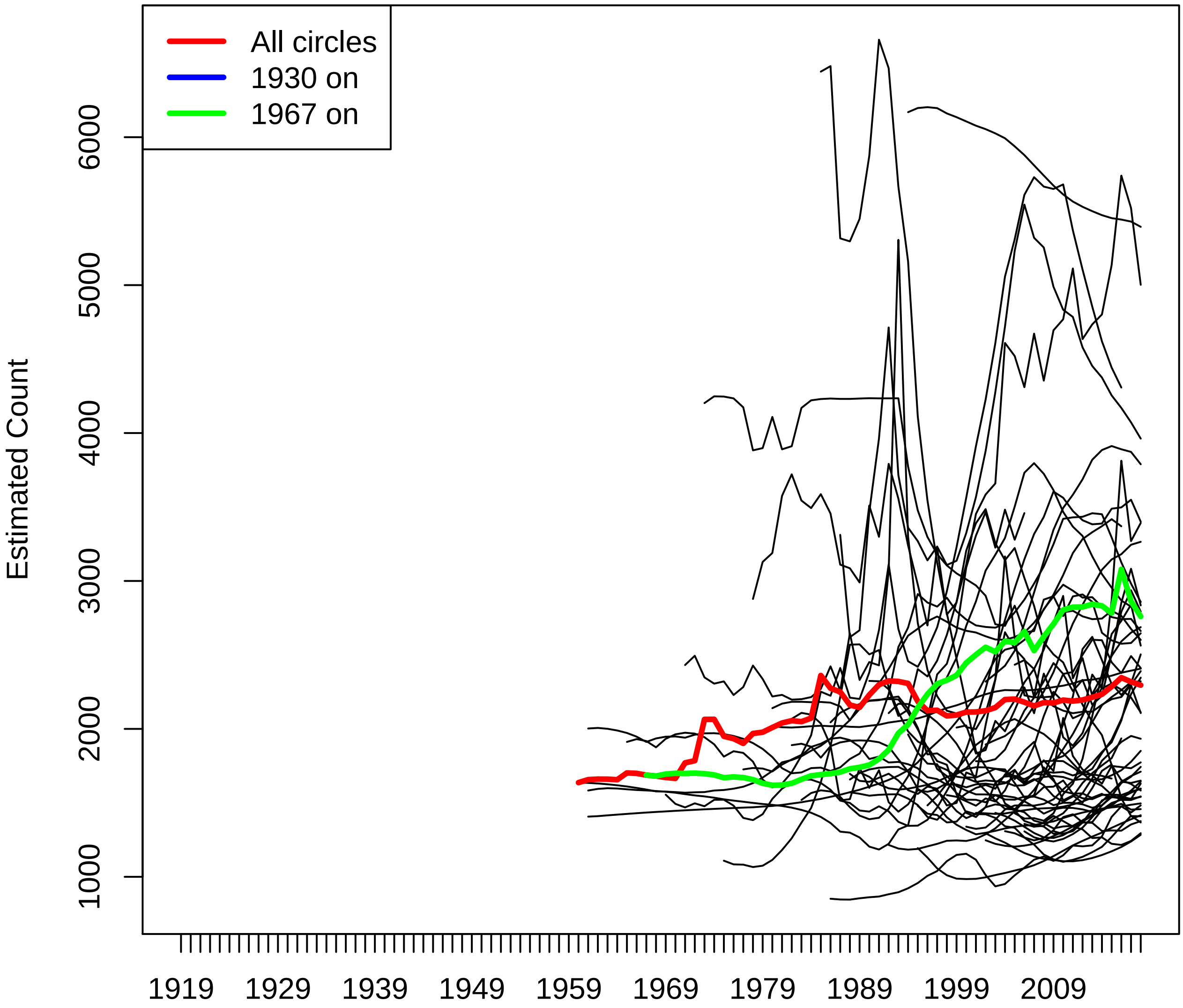

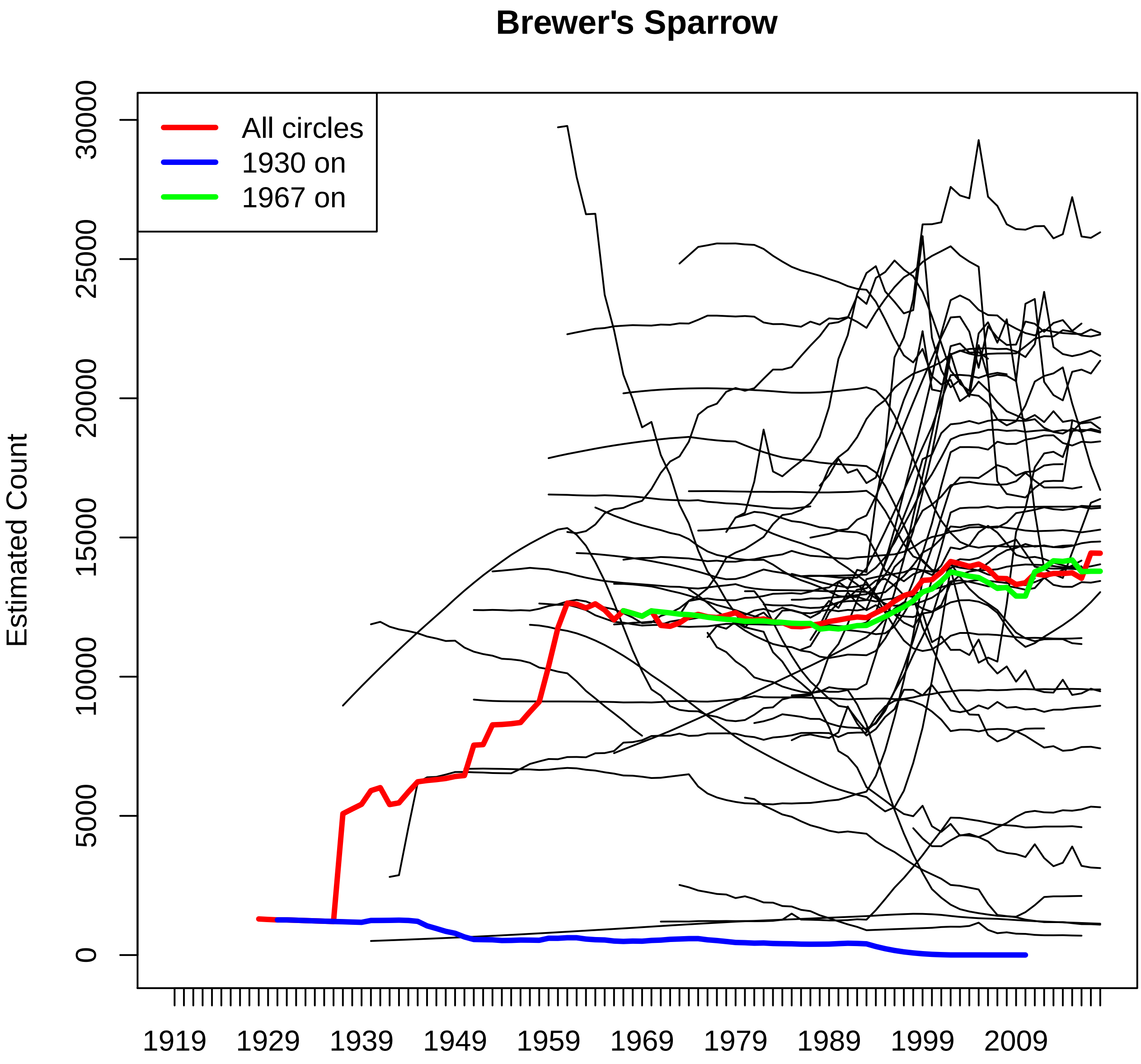
Brewer's Sparrow
Estimated Count
0
5000
10000
15000
20000
25000
30000
1919
1929
1939
1949
1959
1969
1979
1989
1999
2009
All circles
1930 on
1967 on

Bronzed Cowbird

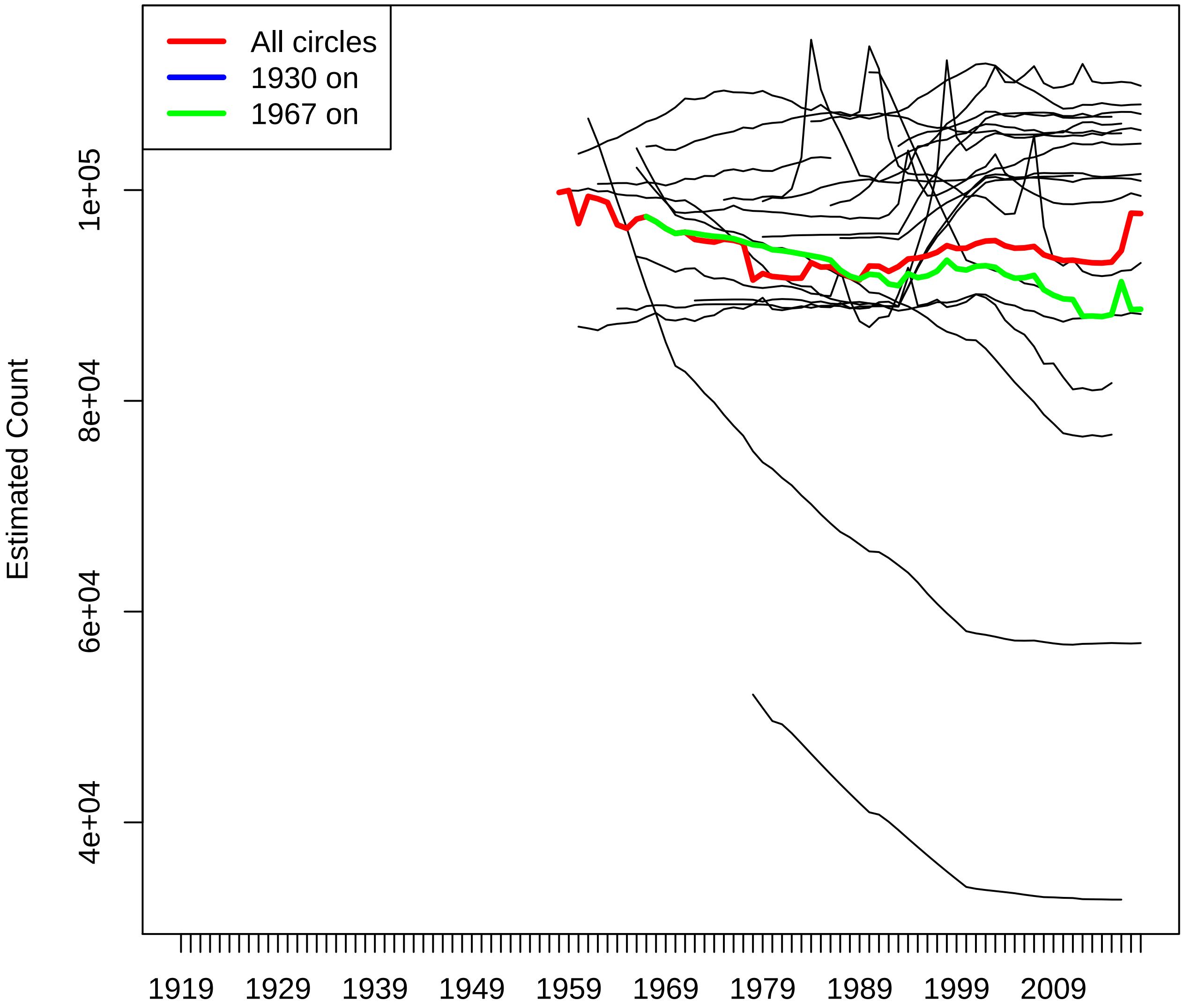

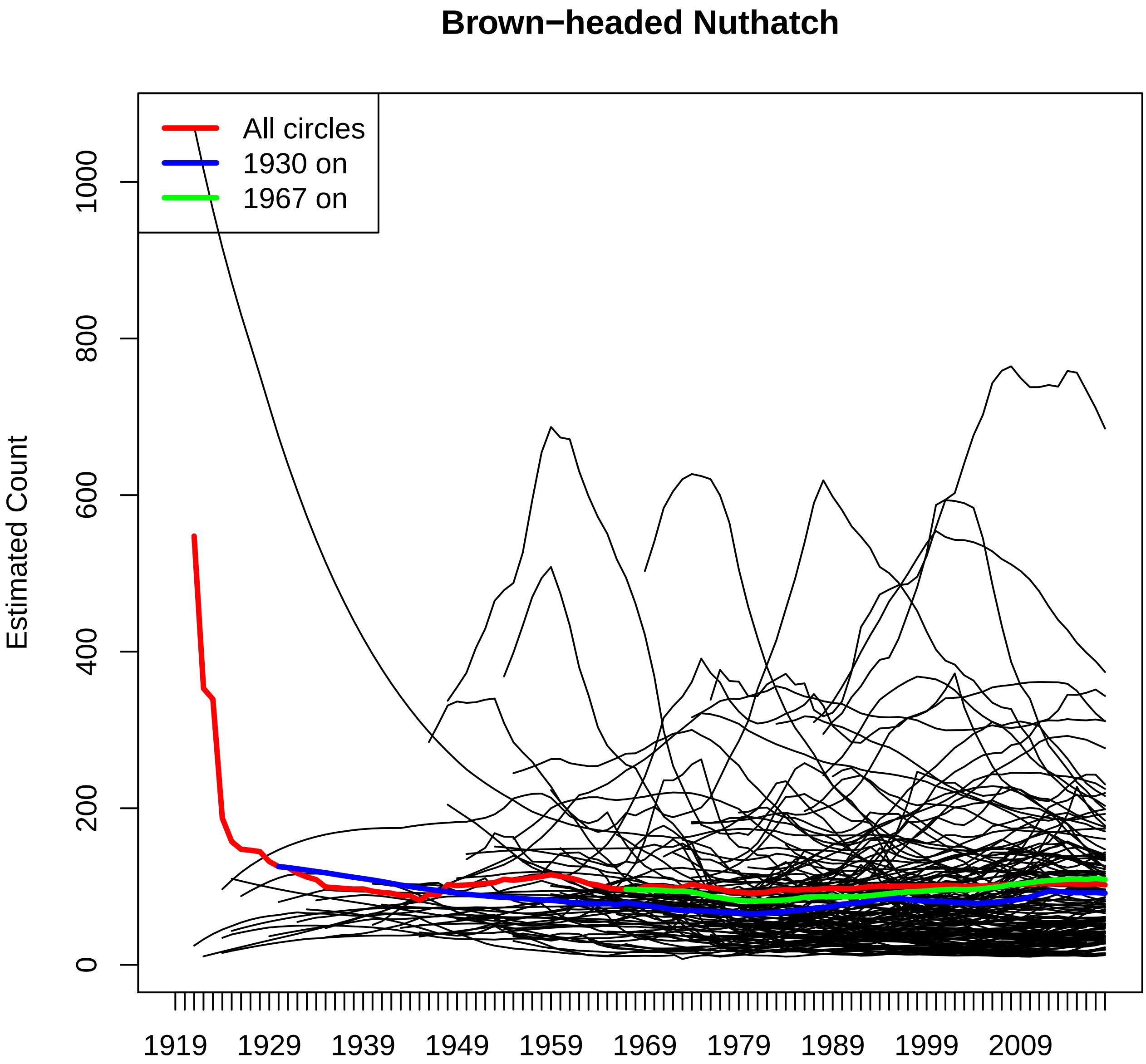

Brown−headed Nuthatch
All circles
1930 on
1967 on
Estimated Count
0
200
400
600
800
1000
1919
1929
1939
1949
1959
1969
1979
1989
1999
2009

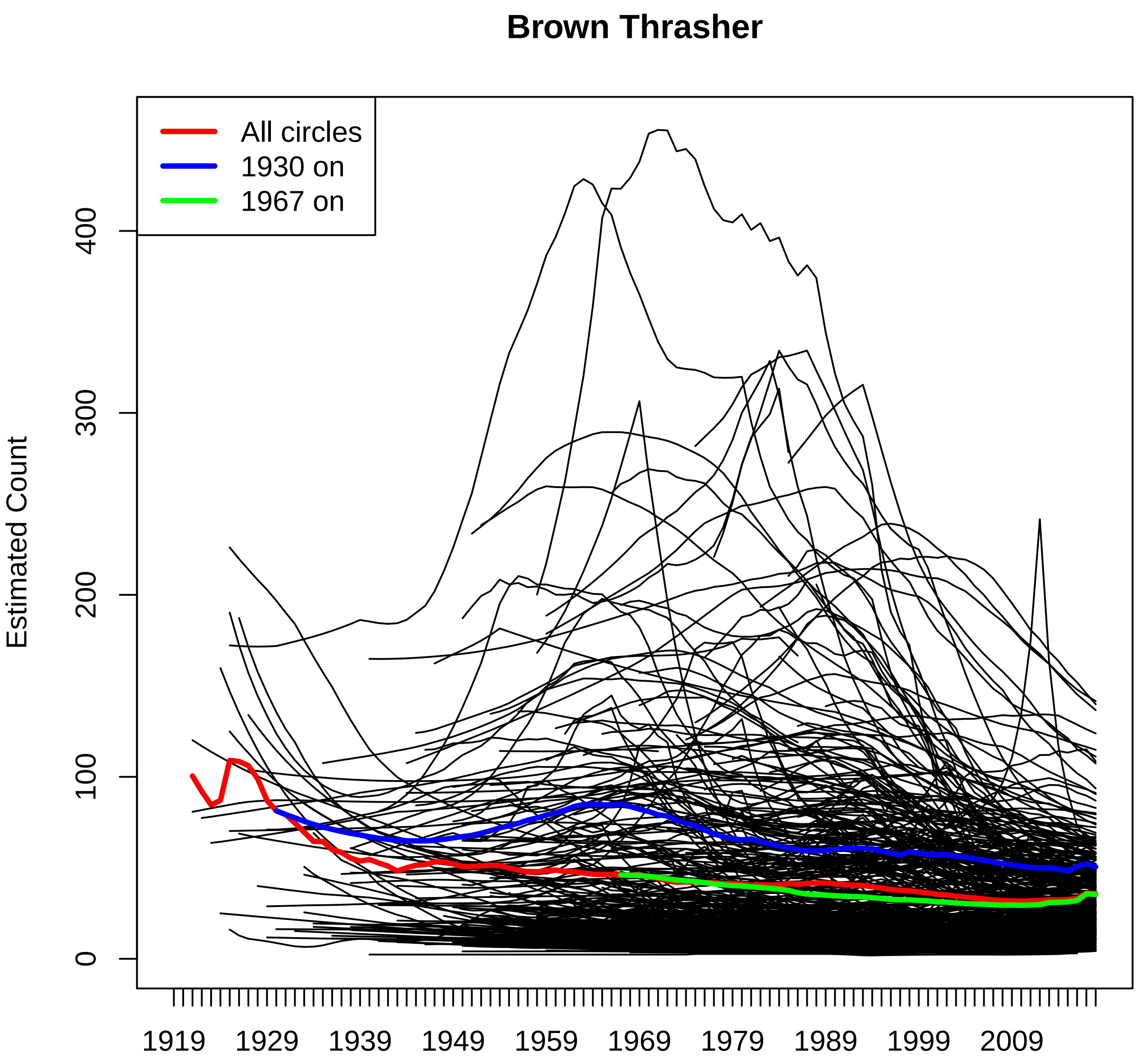

Brown Thrasher
All circles
1930 on
1967 on
Estimated Count
0
100
200
300
400
1919
1929
1939
1949
1959
1969
1979
1989
1999
2009

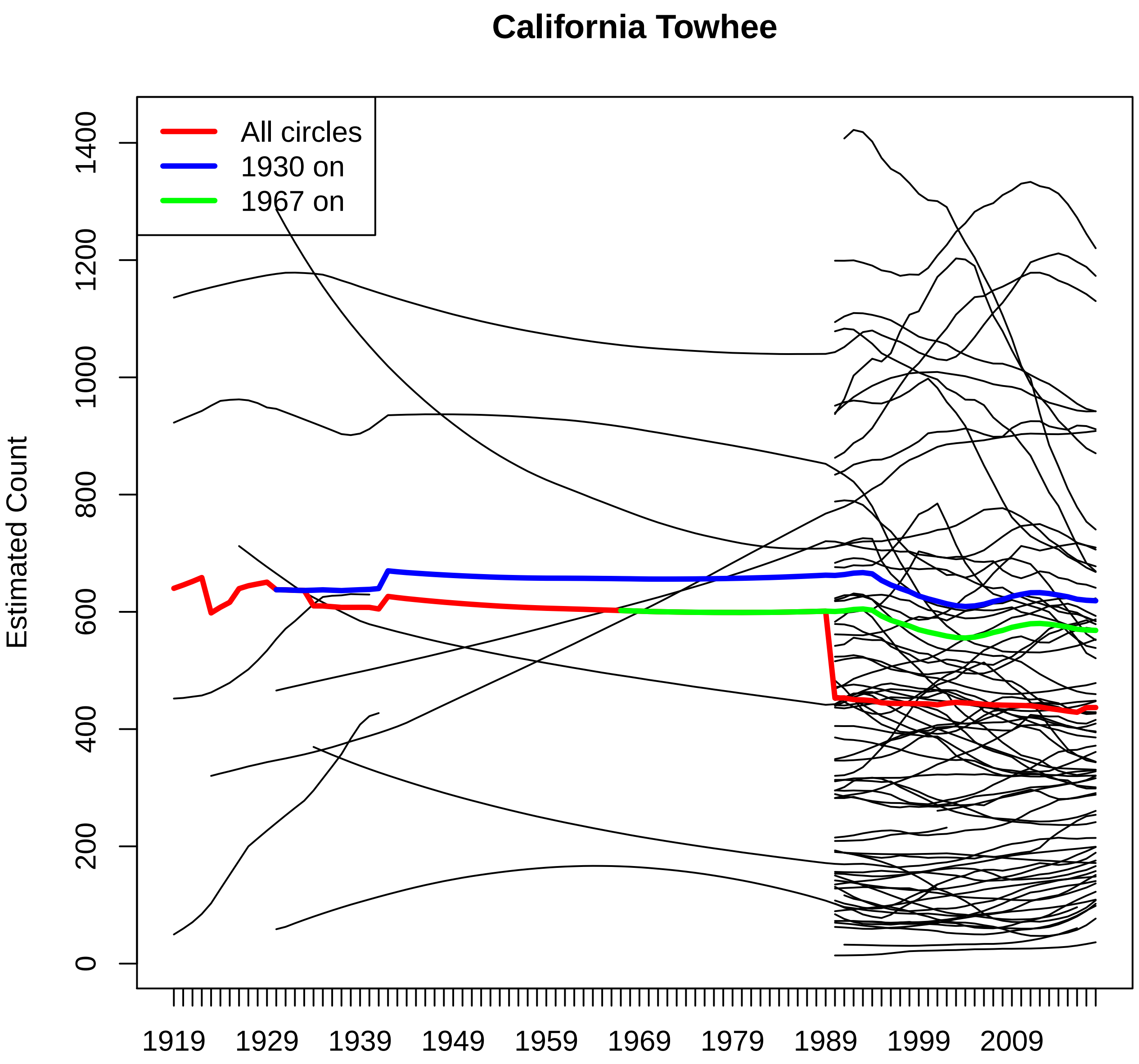

California Towhee
All circles
1930 on
1967 on
Estimated Count
0
200
400
600
800
1000
1200
1400
1919
1929
1939
1949
1959
1969
1979
1989
1999
2009

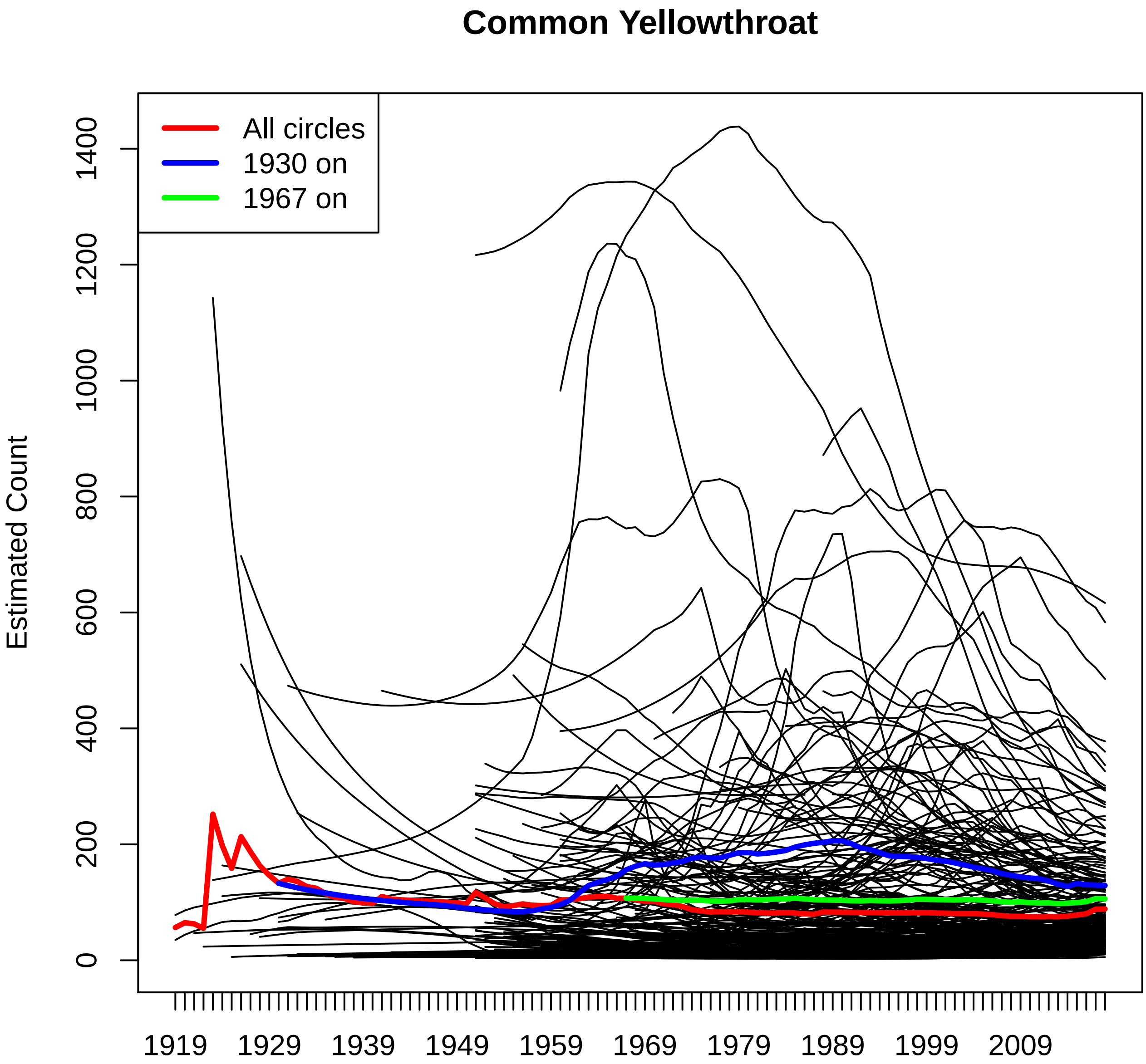

Common Yellowthroat
All circles
1930 on
1967 on
Estimated Count
0
200
400
600
800
1000
1200
1400
1919
1929
1939
1949
1959
1969
1979
1989
1999
2009

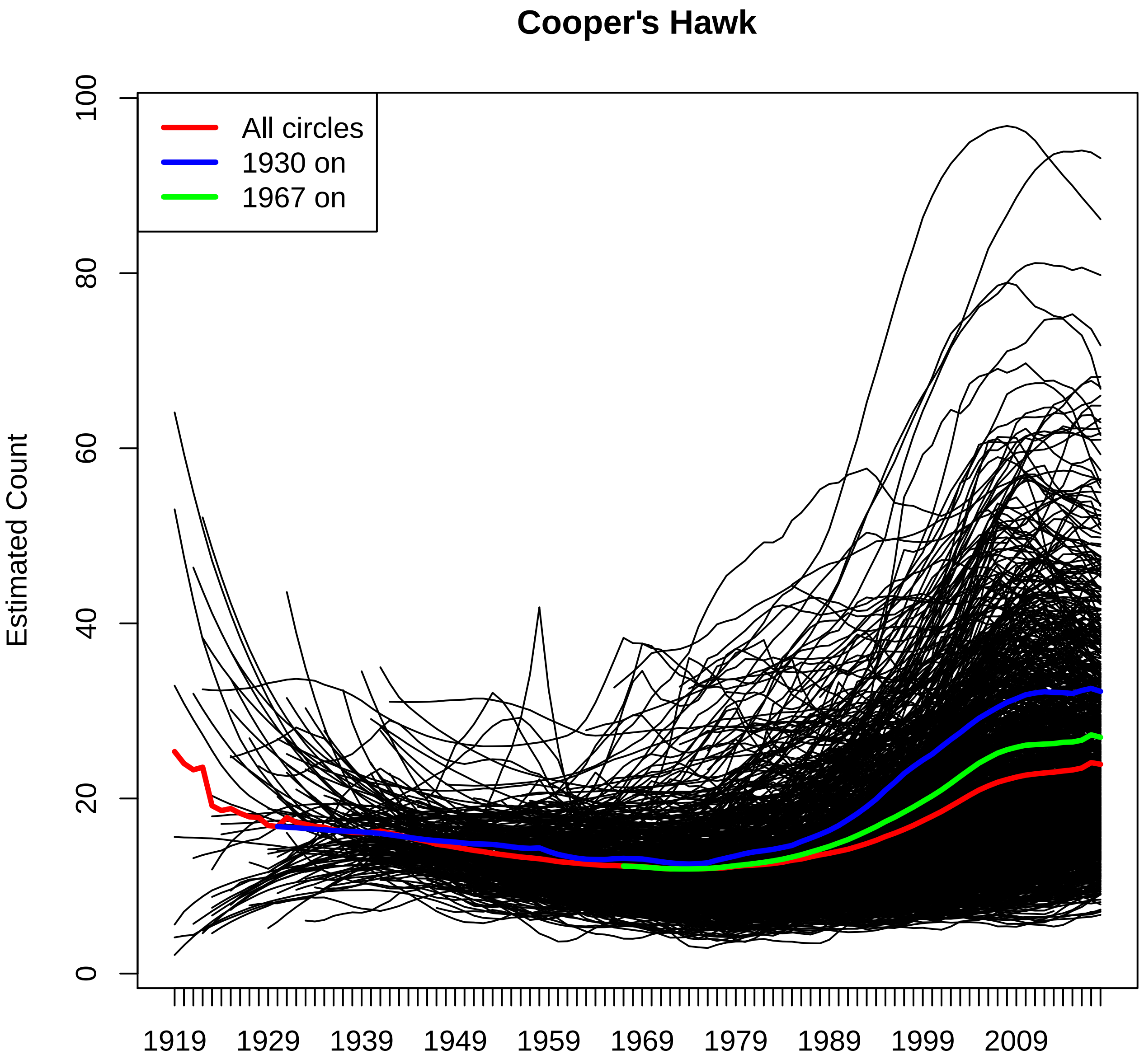

Cooper's Hawk
Estimated Count
0
20
40
60
80
100
1919
1929
1939
1949
1959
1969
1979
1989
1999
2009
All circles
1930 on
1967 on

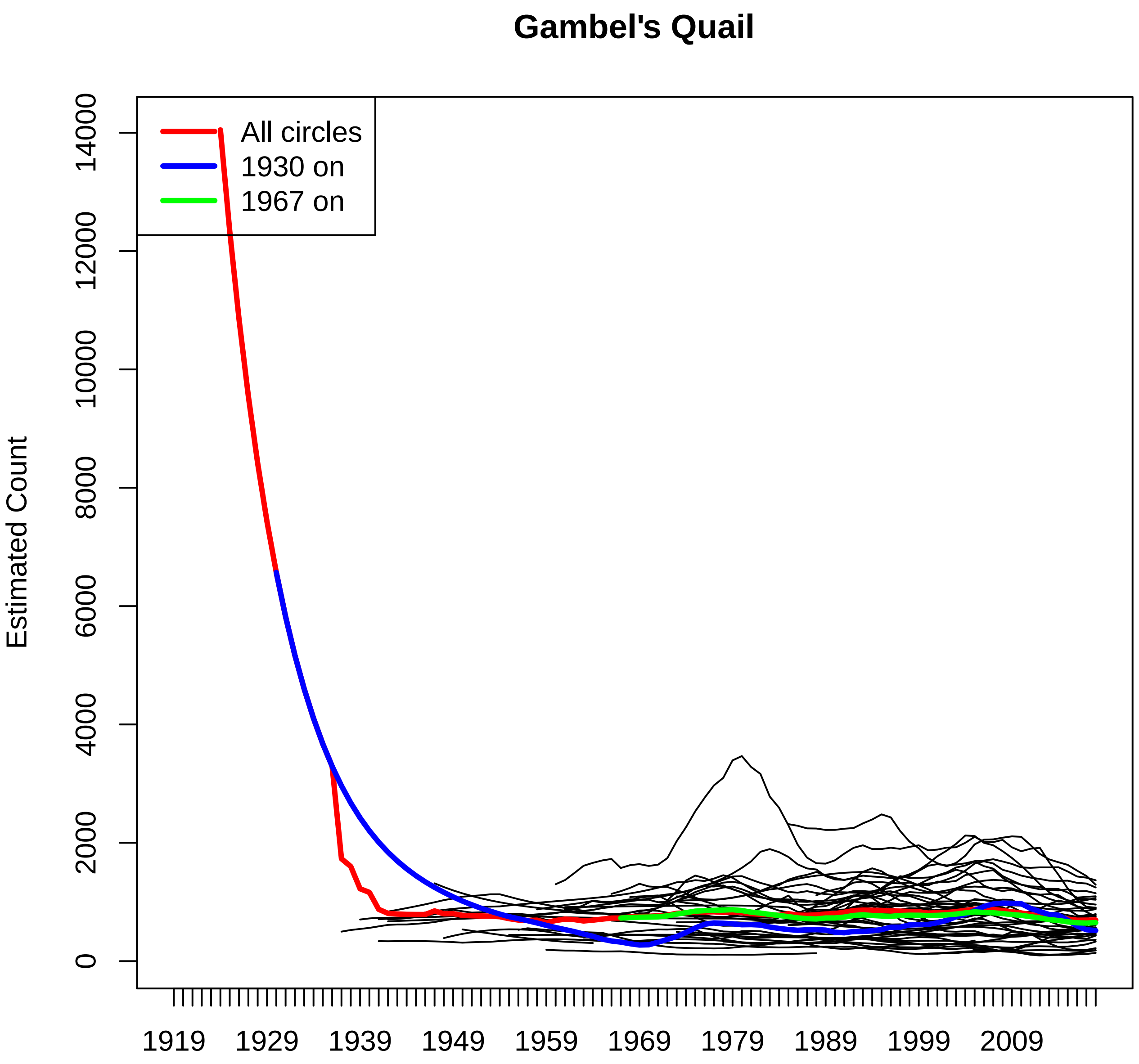
Gambel's Quail
Estimated Count
0
2000
4000
6000
8000
10000
12000
14000
All circles
1930 on
1967 on
1919
1929
1939
1949
1959
1969
1979
1989
1999
2009

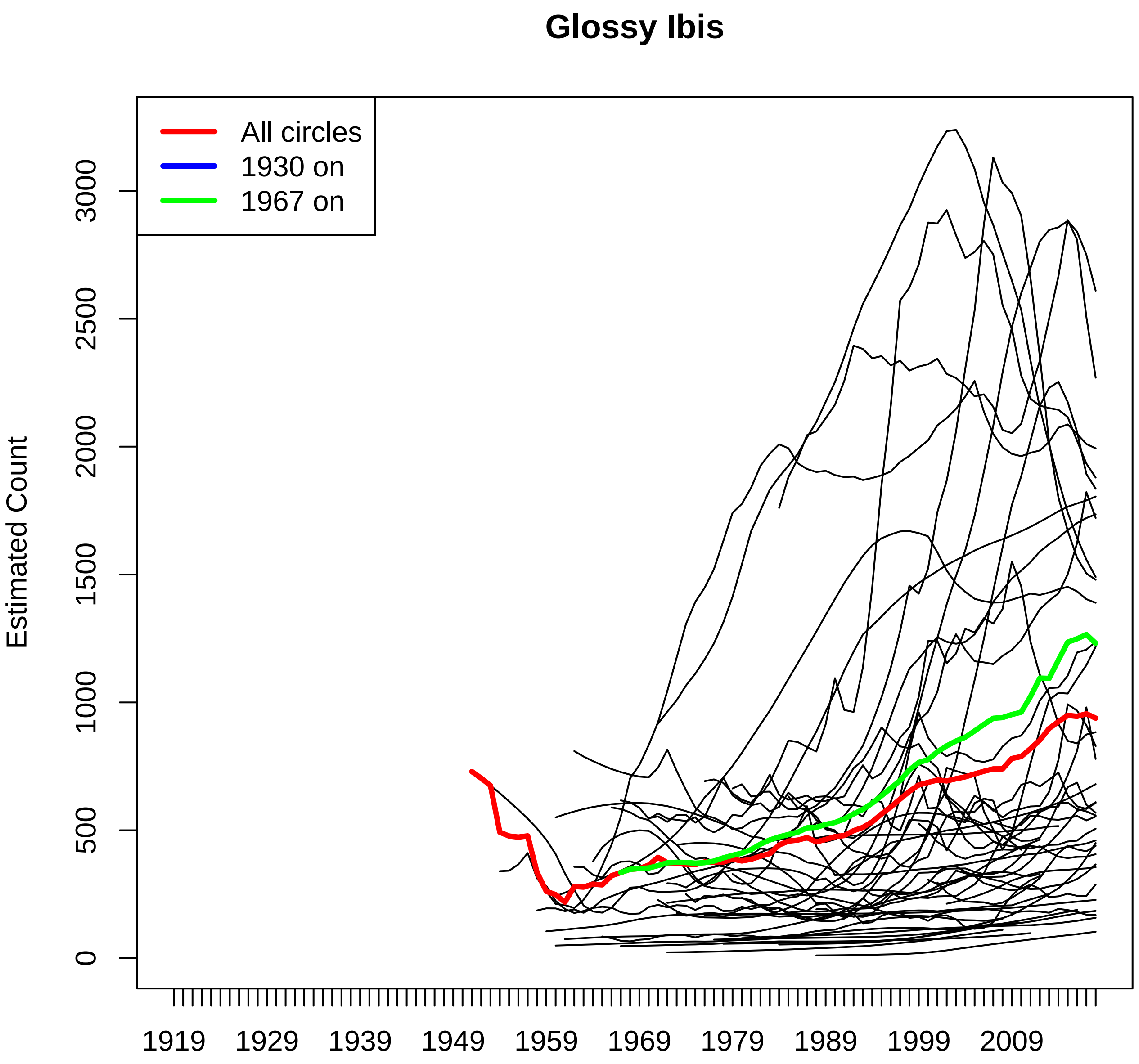

Glossy Ibis
All circles
1930 on
1967 on
Estimated Count
0
500
1000
1500
2000
2500
3000
1919
1929
1939
1949
1959
1969
1979
1989
1999
2009

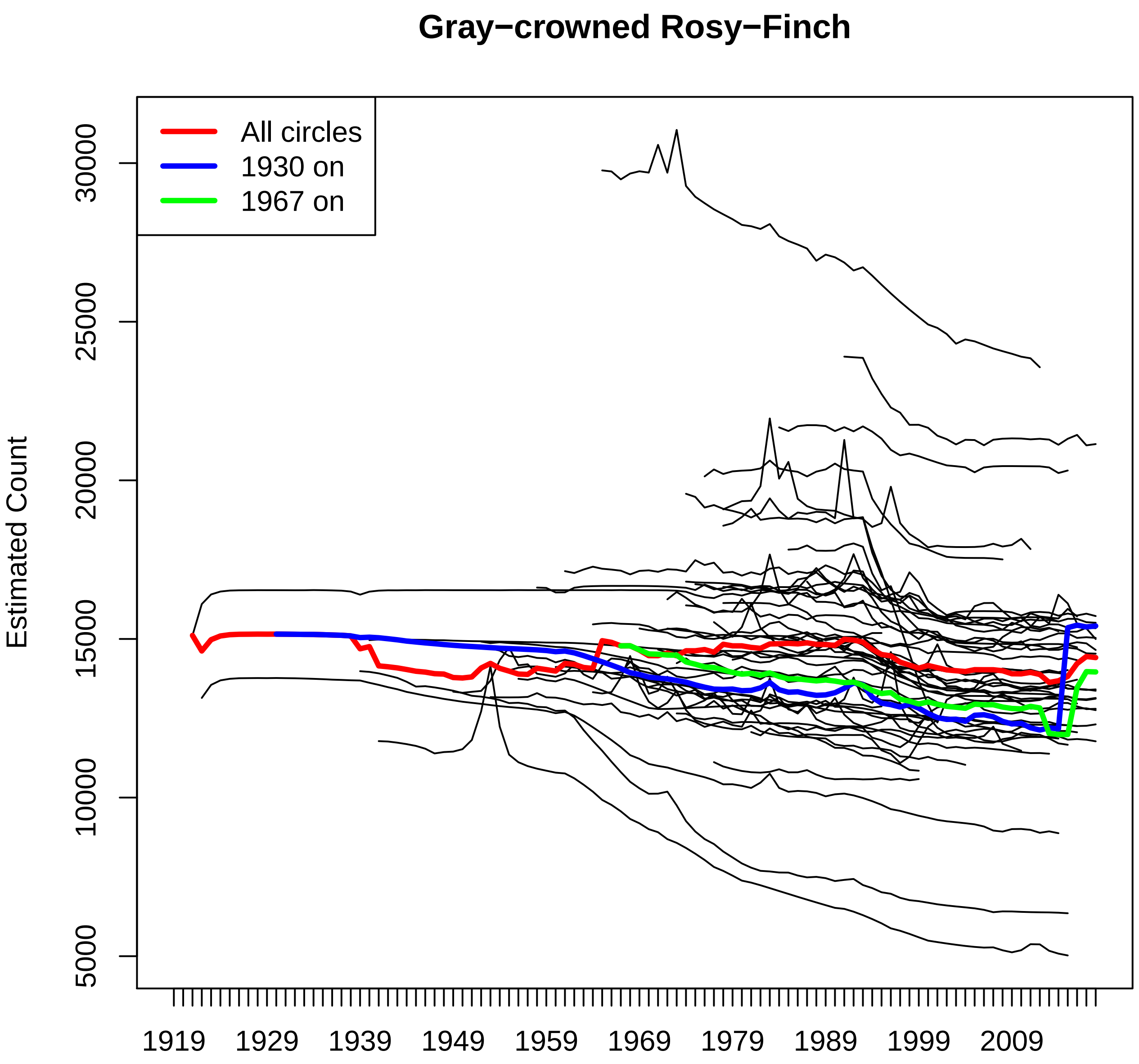

Gray−crowned Rosy−Finch
All circles
1930 on
1967 on
Estimated Count
5000
10000
15000
20000
25000
30000
1919
1929
1939
1949
1959
1969
1979
1989
1999
2009

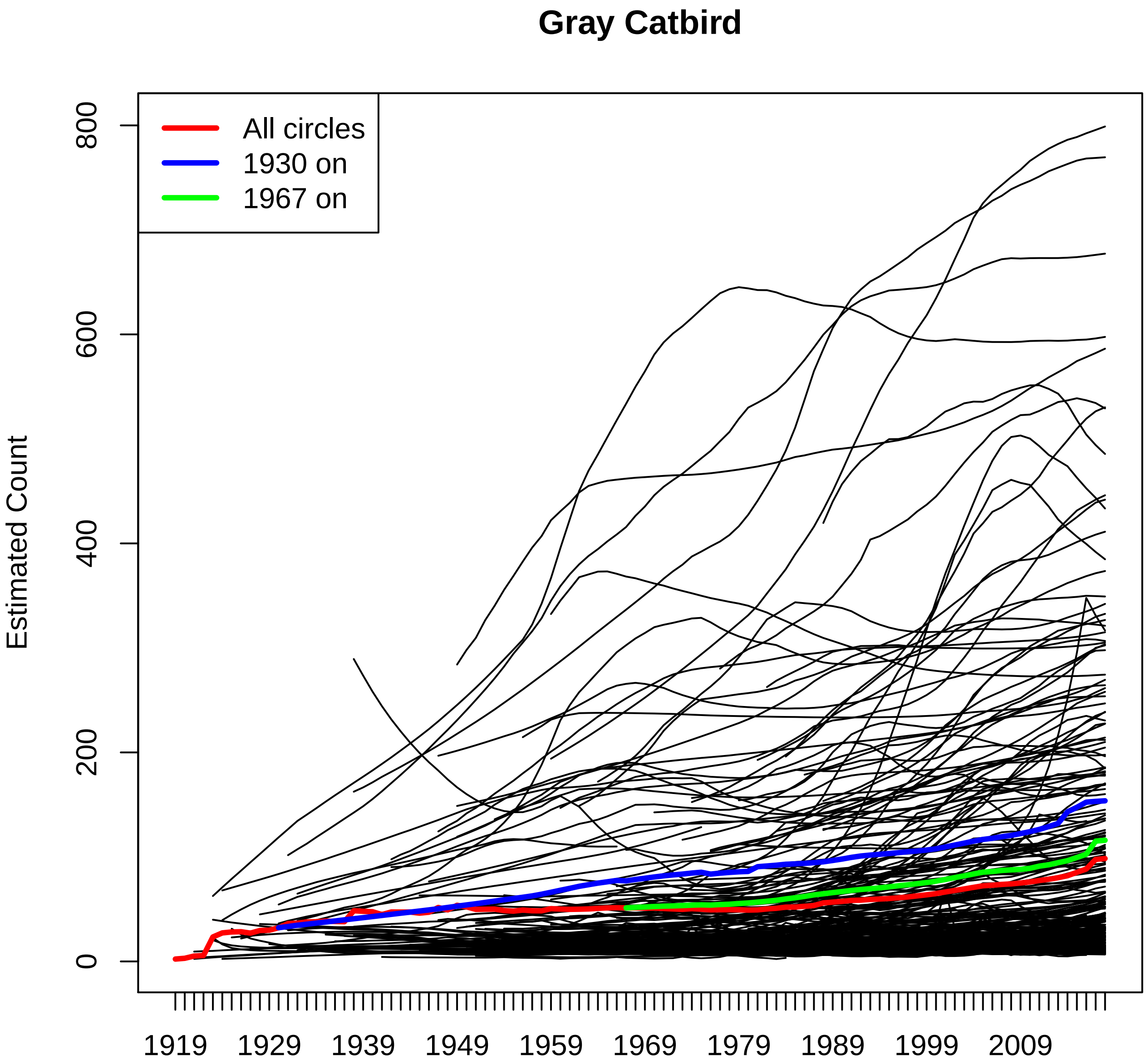

Gray Catbird
All circles
1930 on
1967 on
Estimated Count
0
200
400
600
800
1919
1929
1939
1949
1959
1969
1979
1989
1999
2009

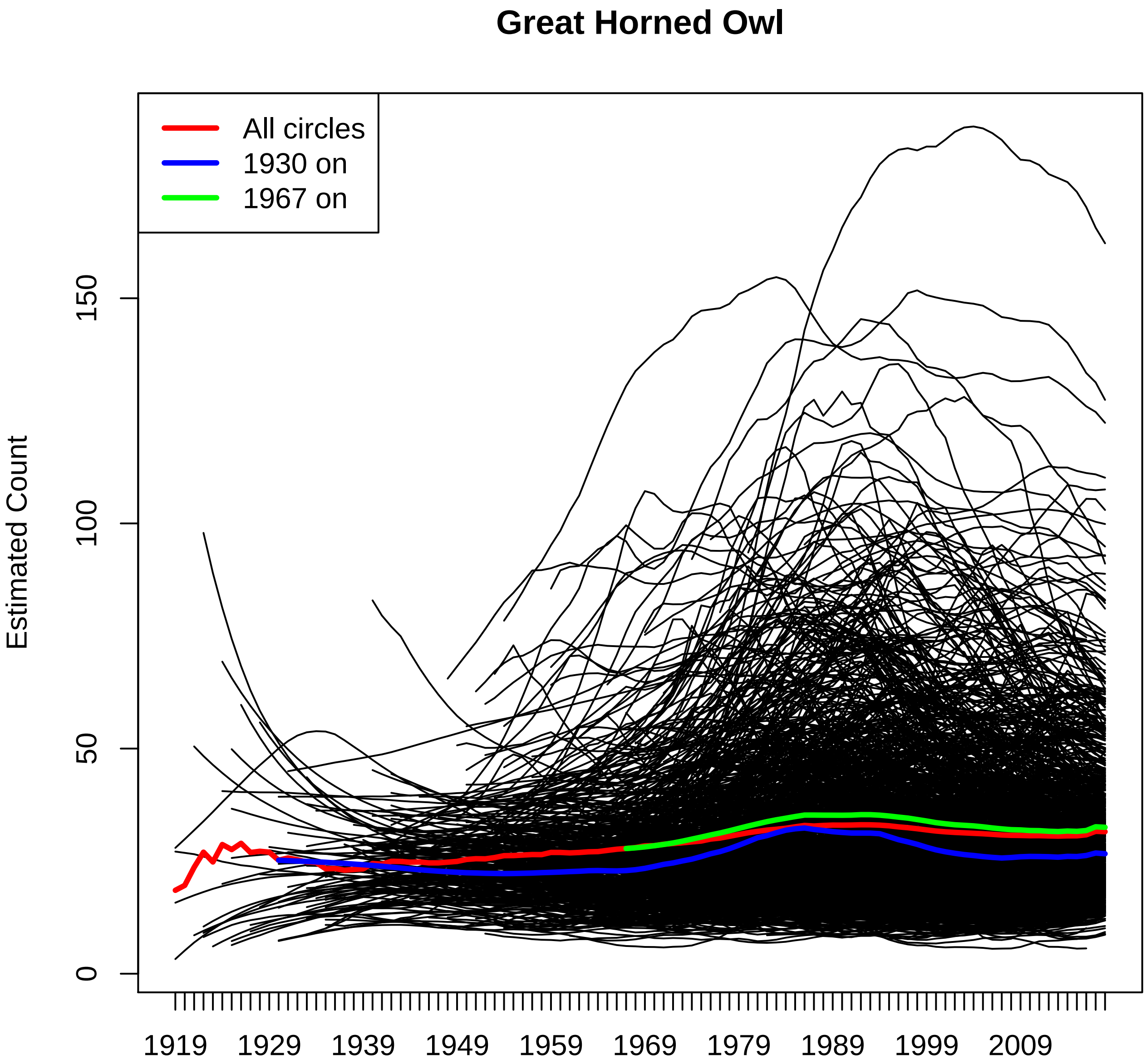
Great Horned Owl
All circles
1930 on
1967 on
Estimated Count
0
50
100
150
1919
1929
1939
1949
1959
1969
1979
1989
1999
2009

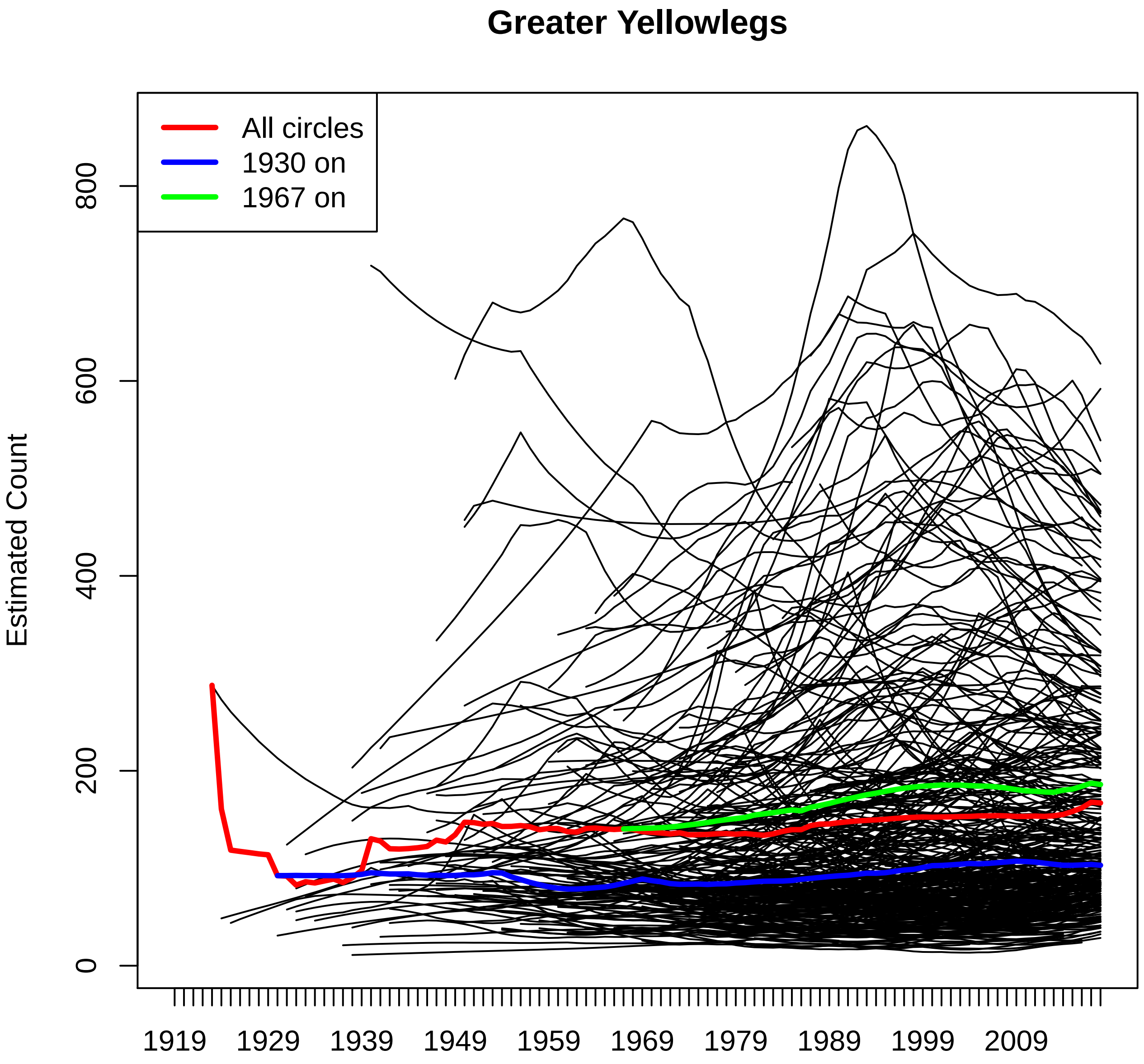
Greater Yellowlegs
All circles
1930 on
1967 on
Estimated Count
0
200
400
600
800
1919
1929
1939
1949
1959
1969
1979
1989
1999
2009

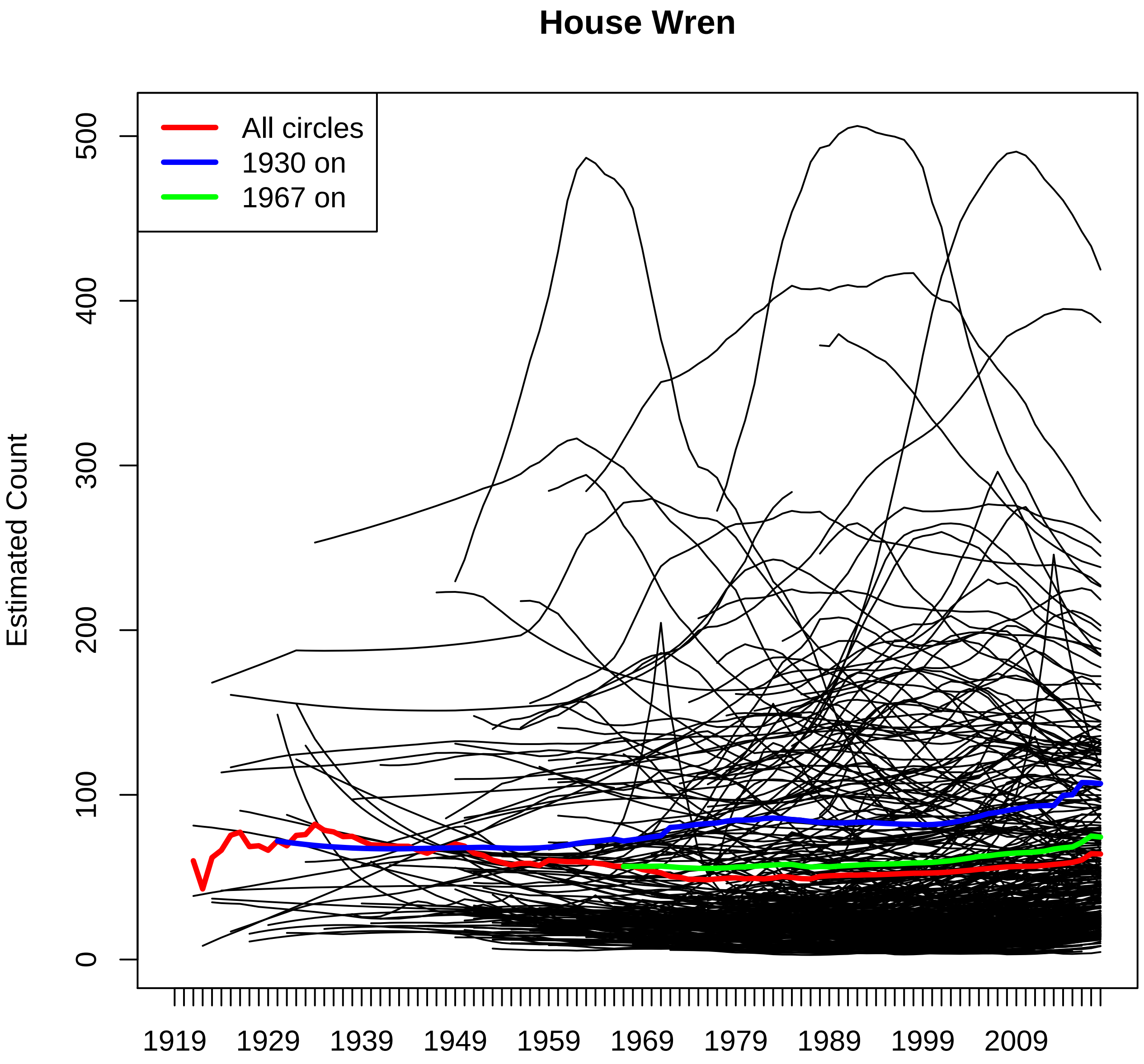
House Wren
All circles
1930 on
1967 on
Estimated Count
0
100
200
300
400
500
1919
1929
1939
1949
1959
1969
1979
1989
1999
2009

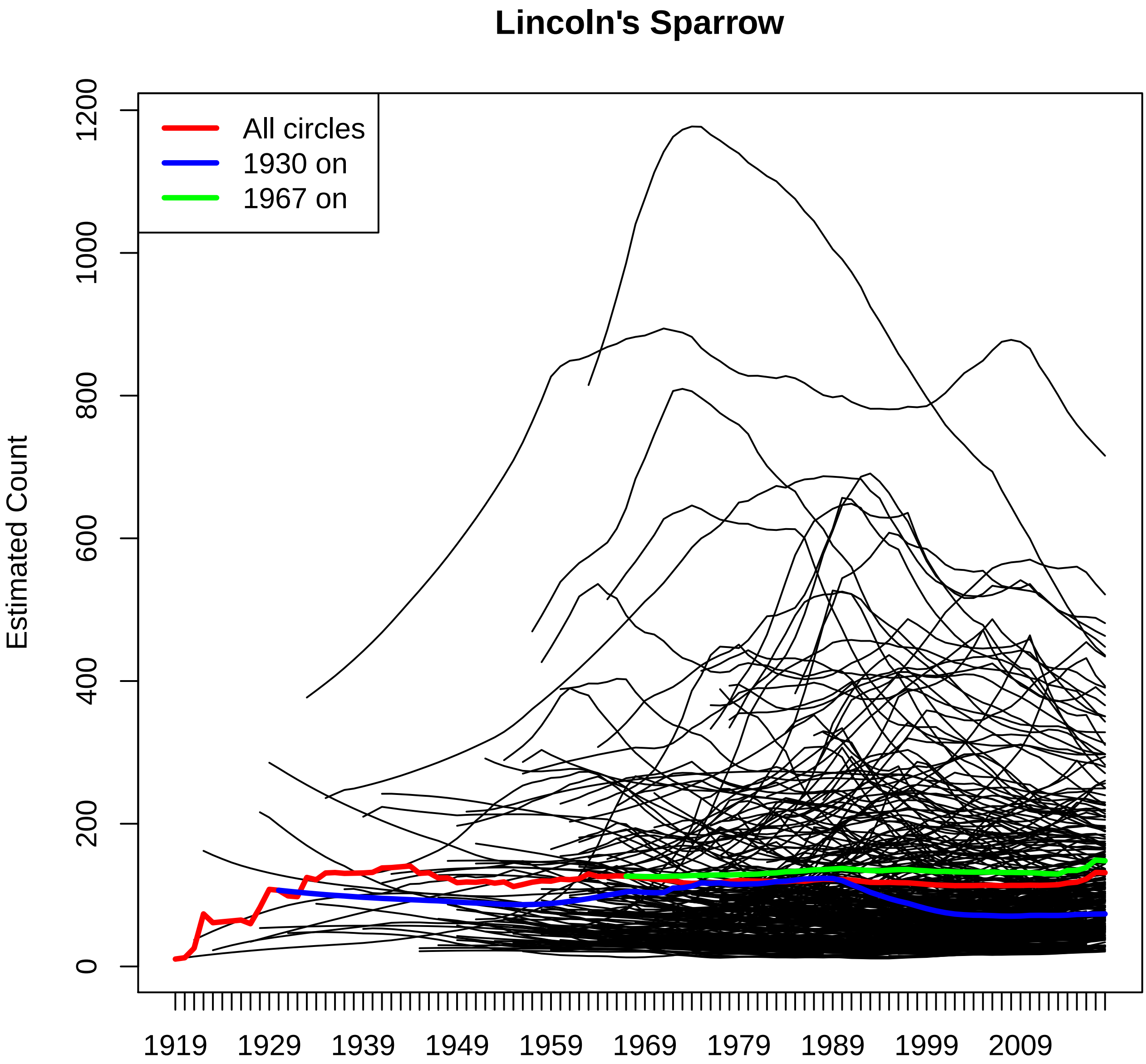

Lincoln's Sparrow
Estimated Count
0
200
400
600
800
1000
1200
All circles
1930 on
1967 on
1919
1929
1939
1949
1959
1969
1979
1989
1999
2009

Little Blue Heron

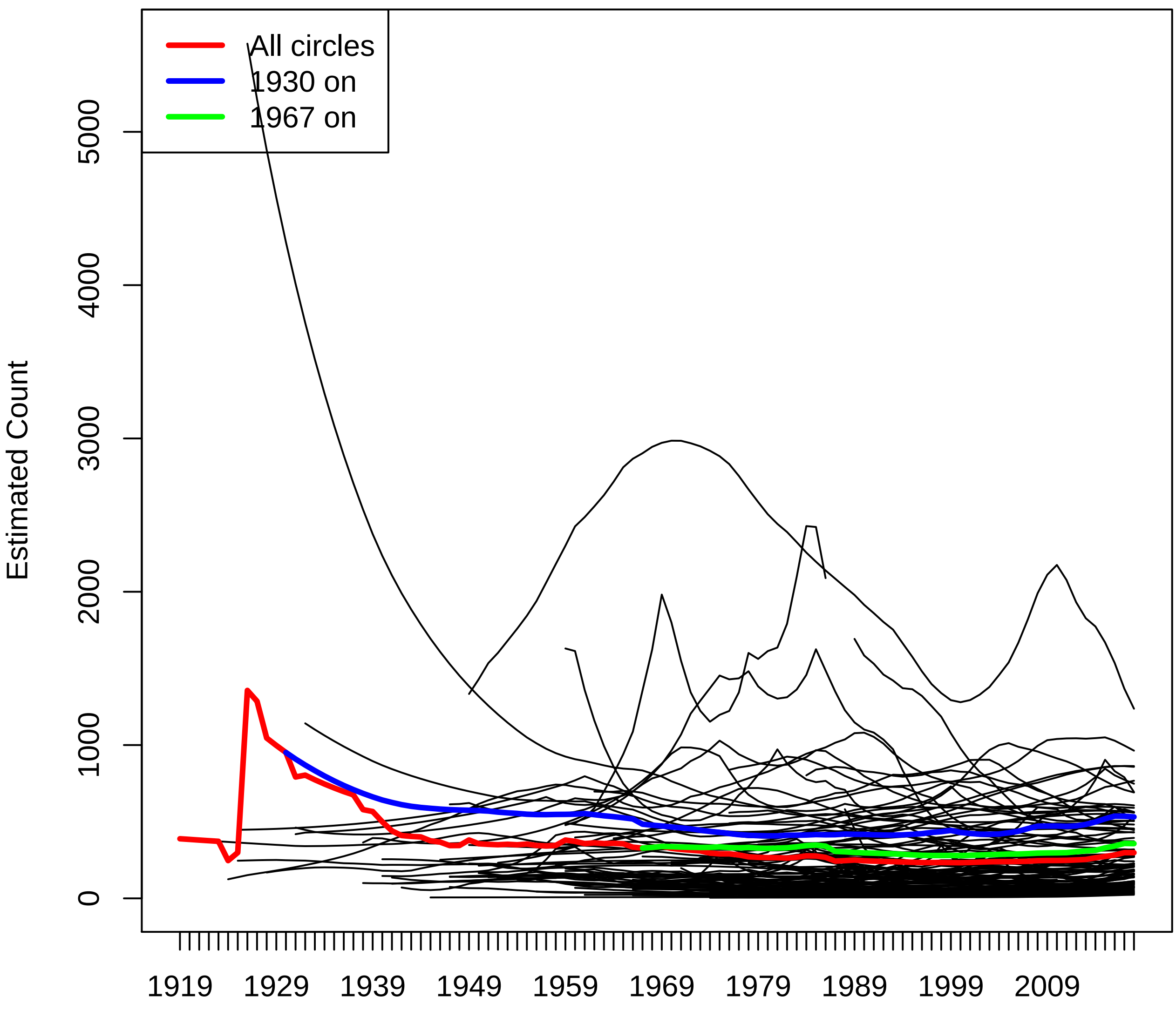

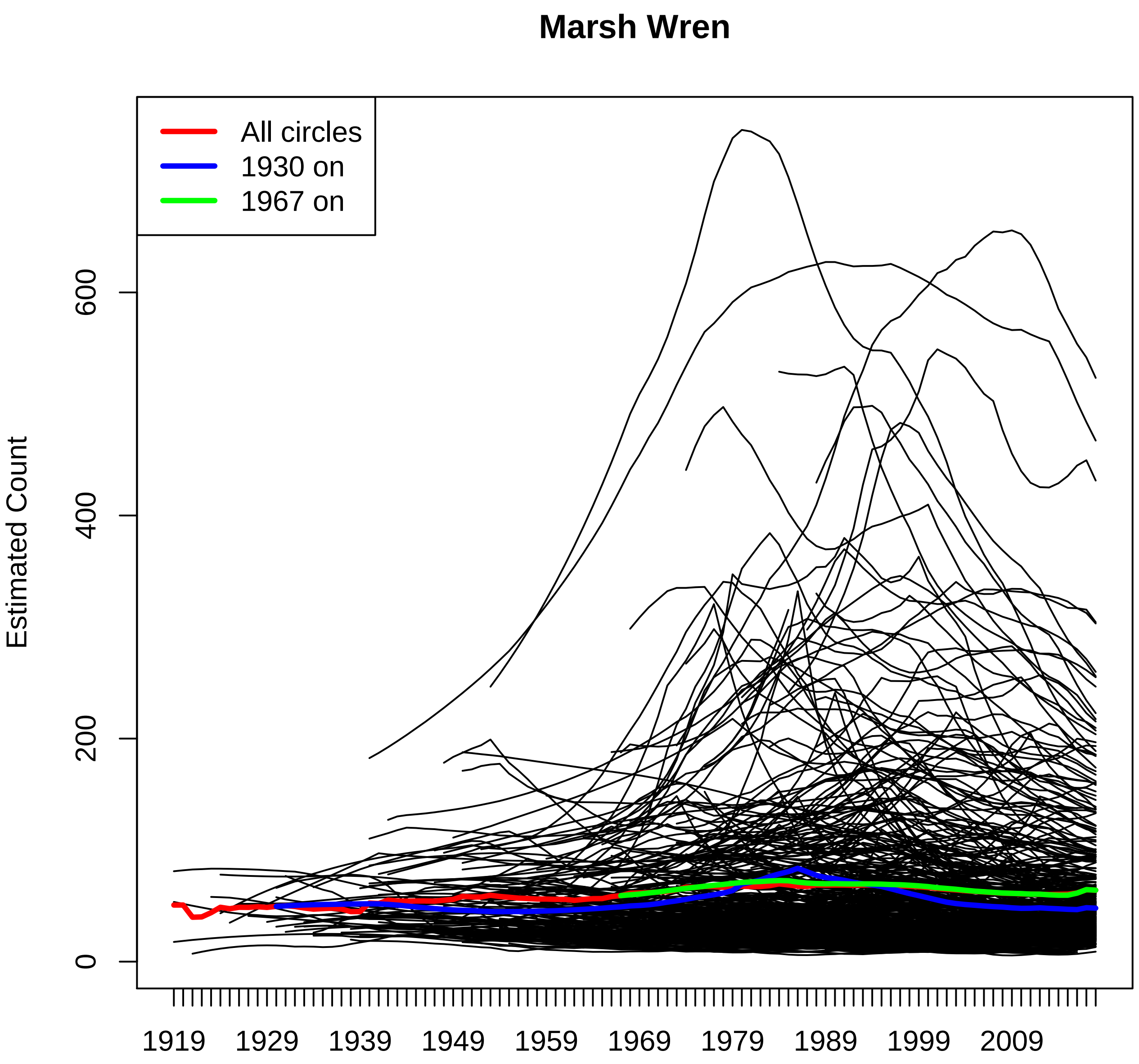
Marsh Wren
All circles
1930 on
1967 on
Estimated Count
0
200
400
600
1919
1929
1939
1949
1959
1969
1979
1989
1999
2009

Mottled Duck

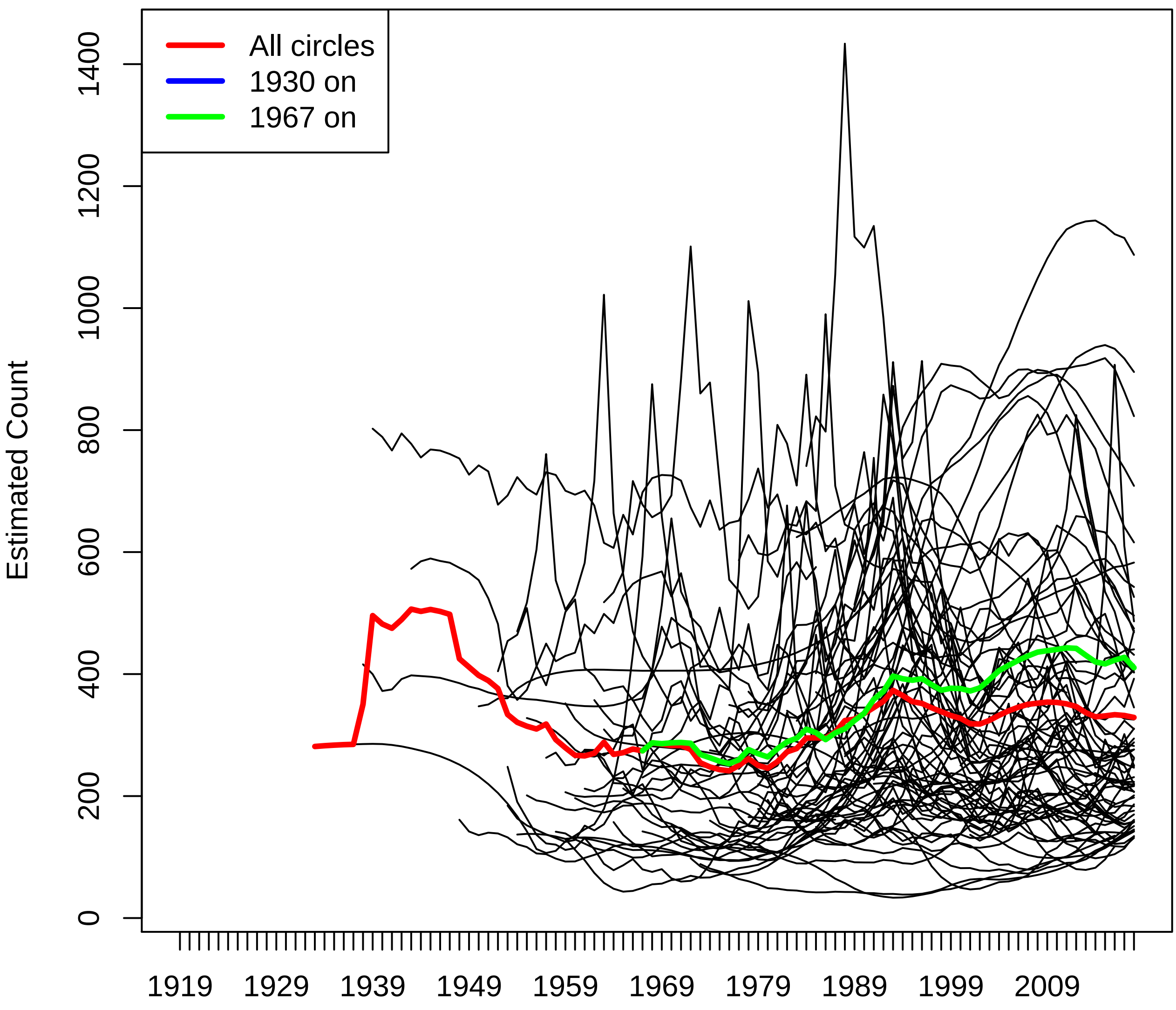

Mountain Bluebird

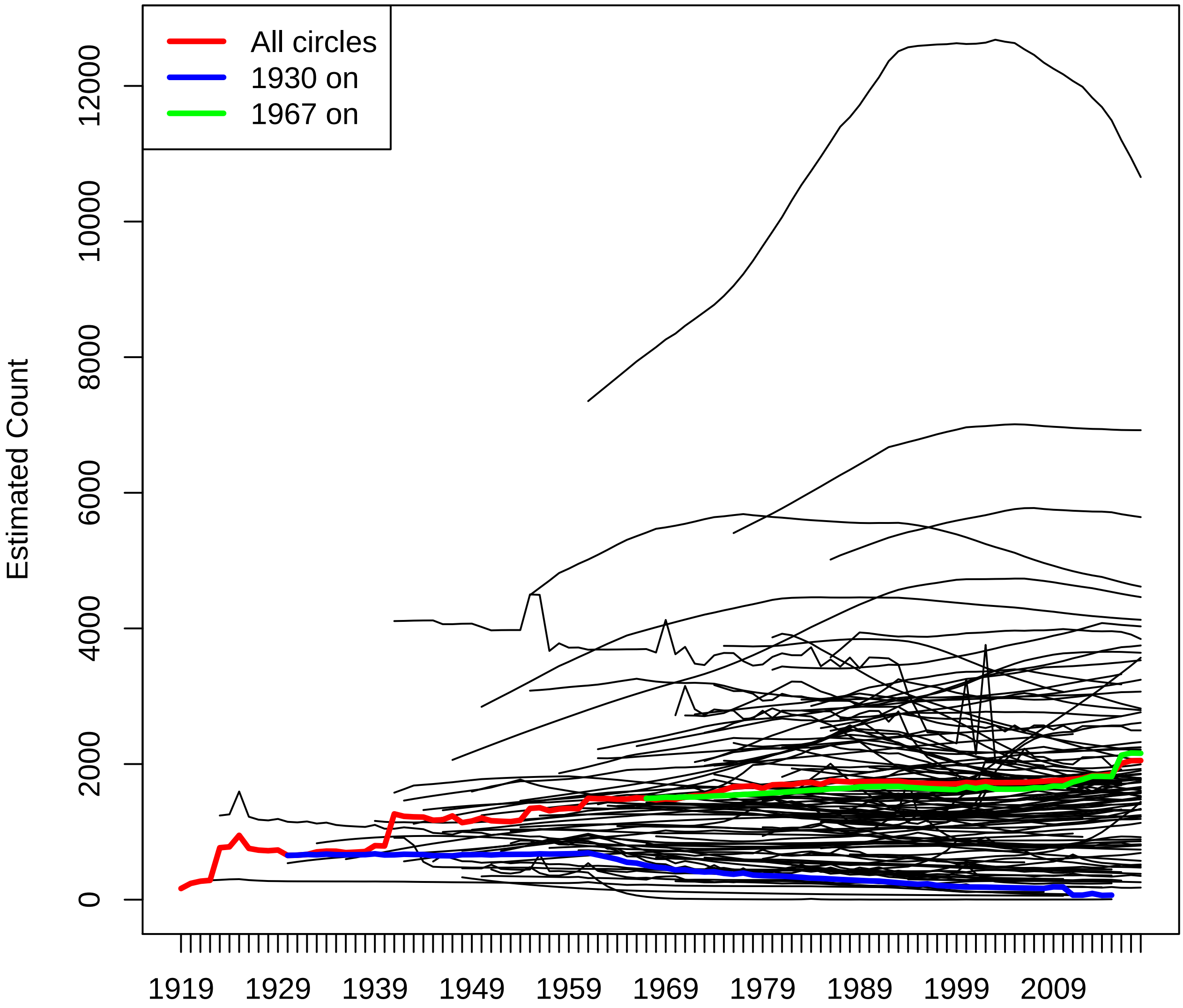

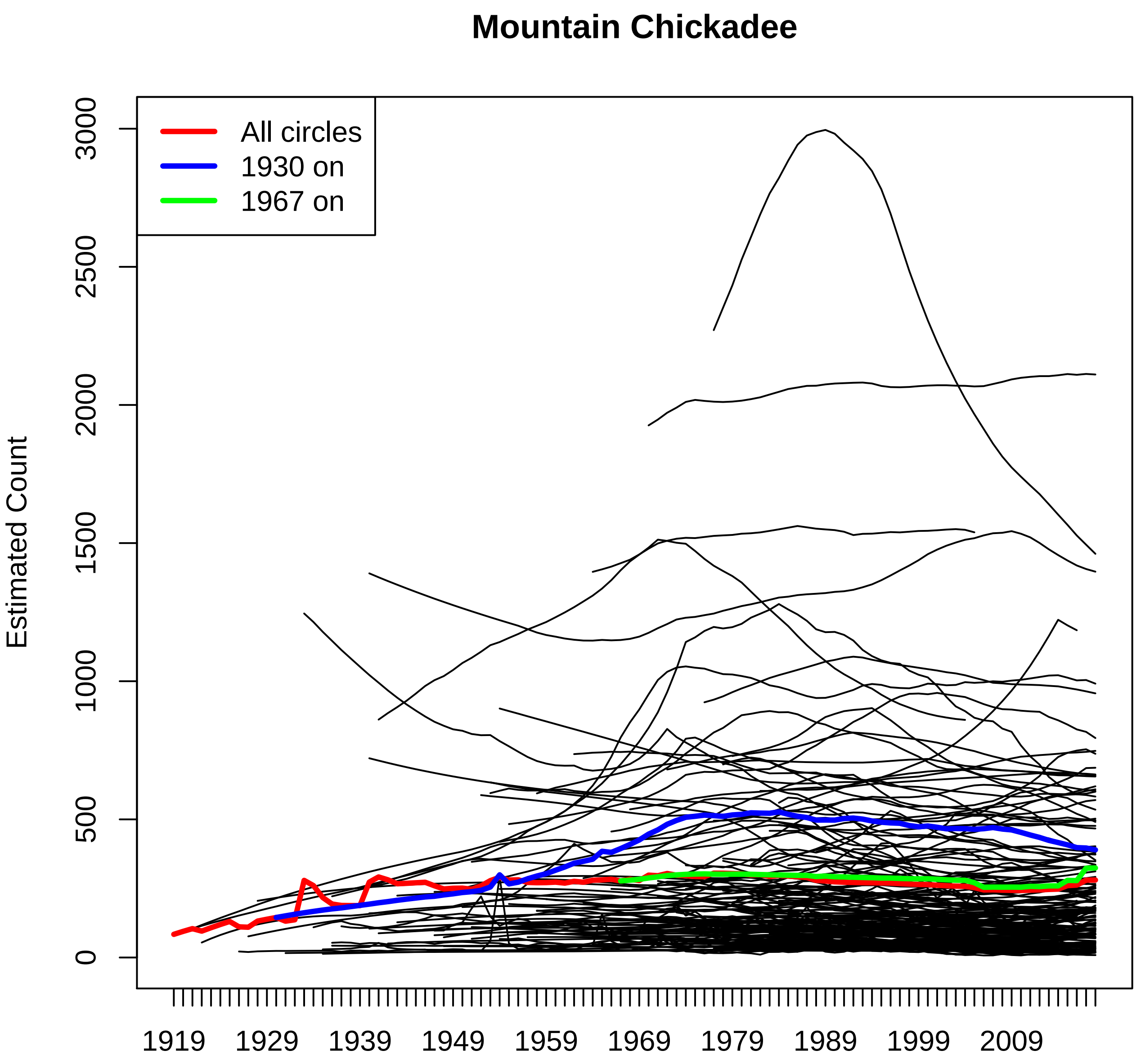

Mountain Chickadee
All circles
1930 on
1967 on
Estimated Count
0
500
1000
1500
2000
2500
3000
1919
1929
1939
1949
1959
1969
1979
1989
1999
2009

Neotropic Cormorant

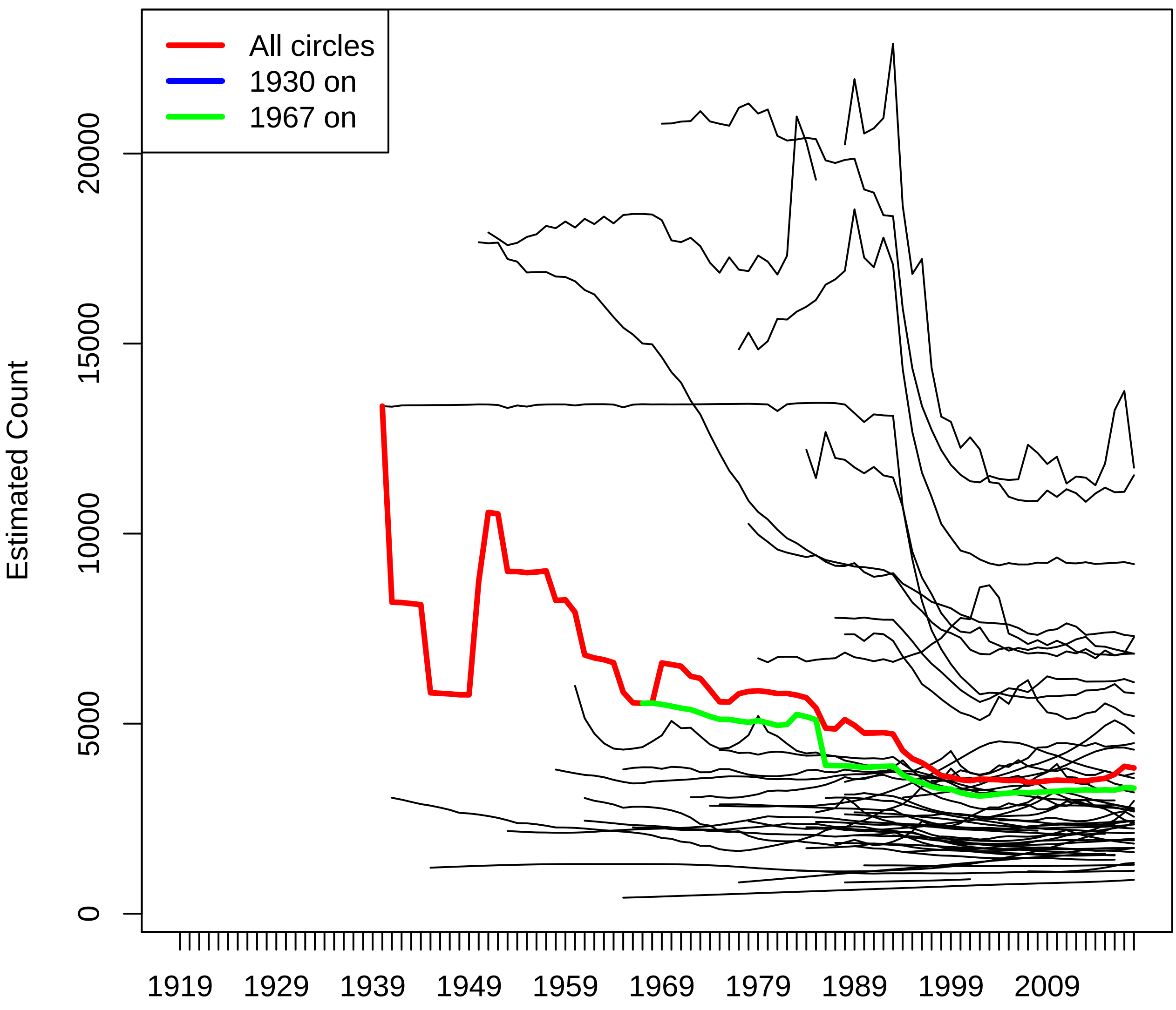

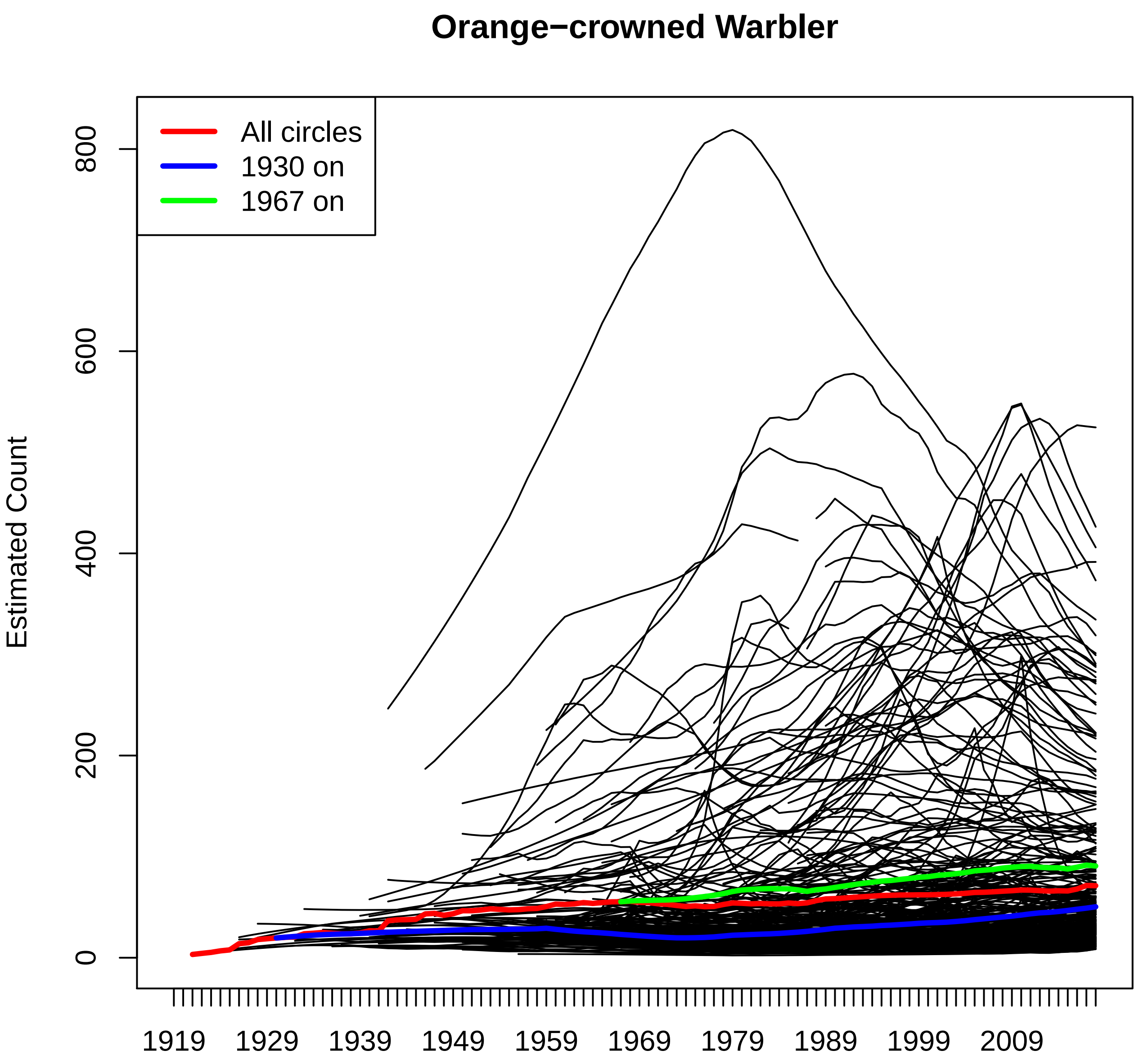
Orange−crowned Warbler
All circles
1930 on
1967 on
Estimated Count
0
200
400
600
800
1919
1929
1939
1949
1959
1969
1979
1989
1999
2009

Pacific Loon

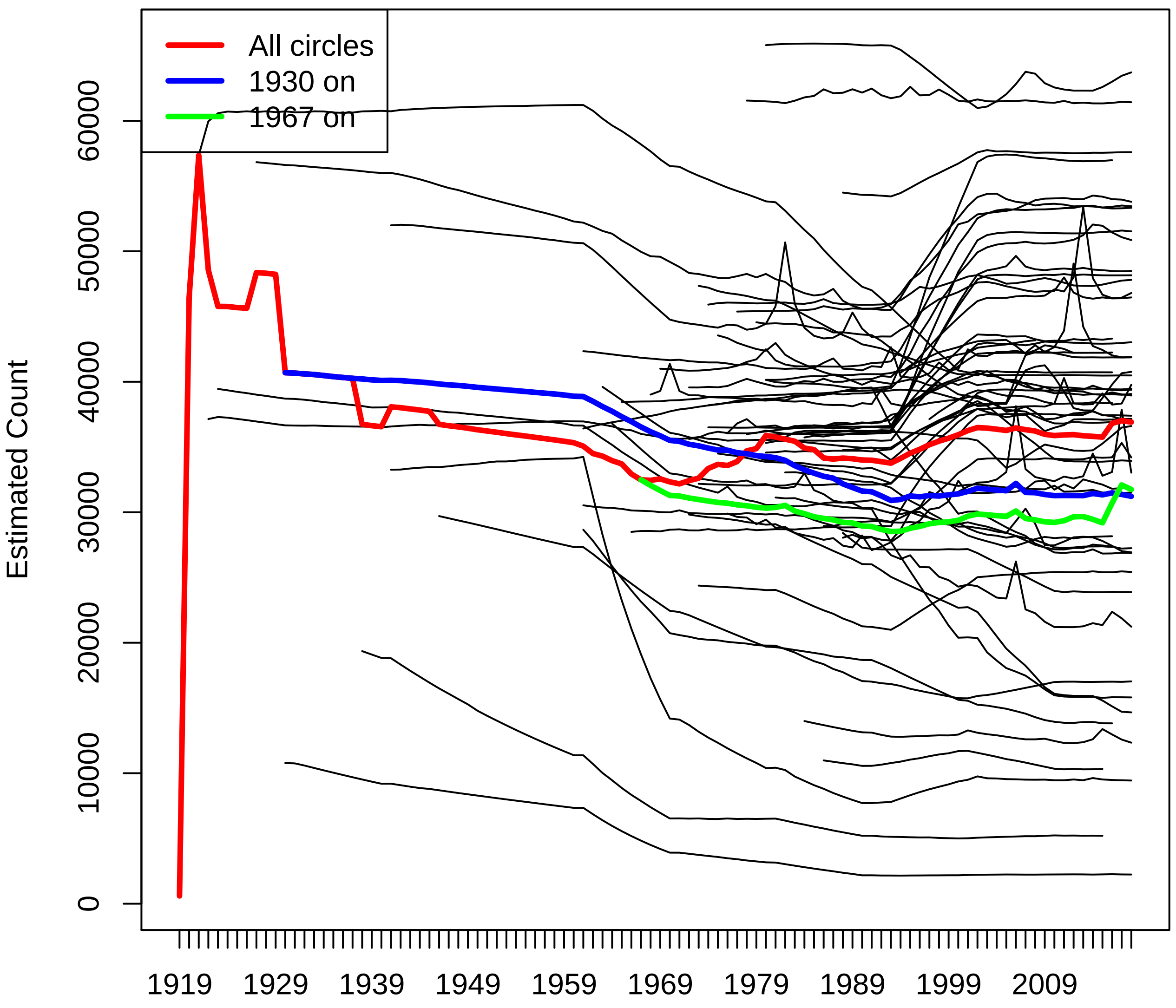

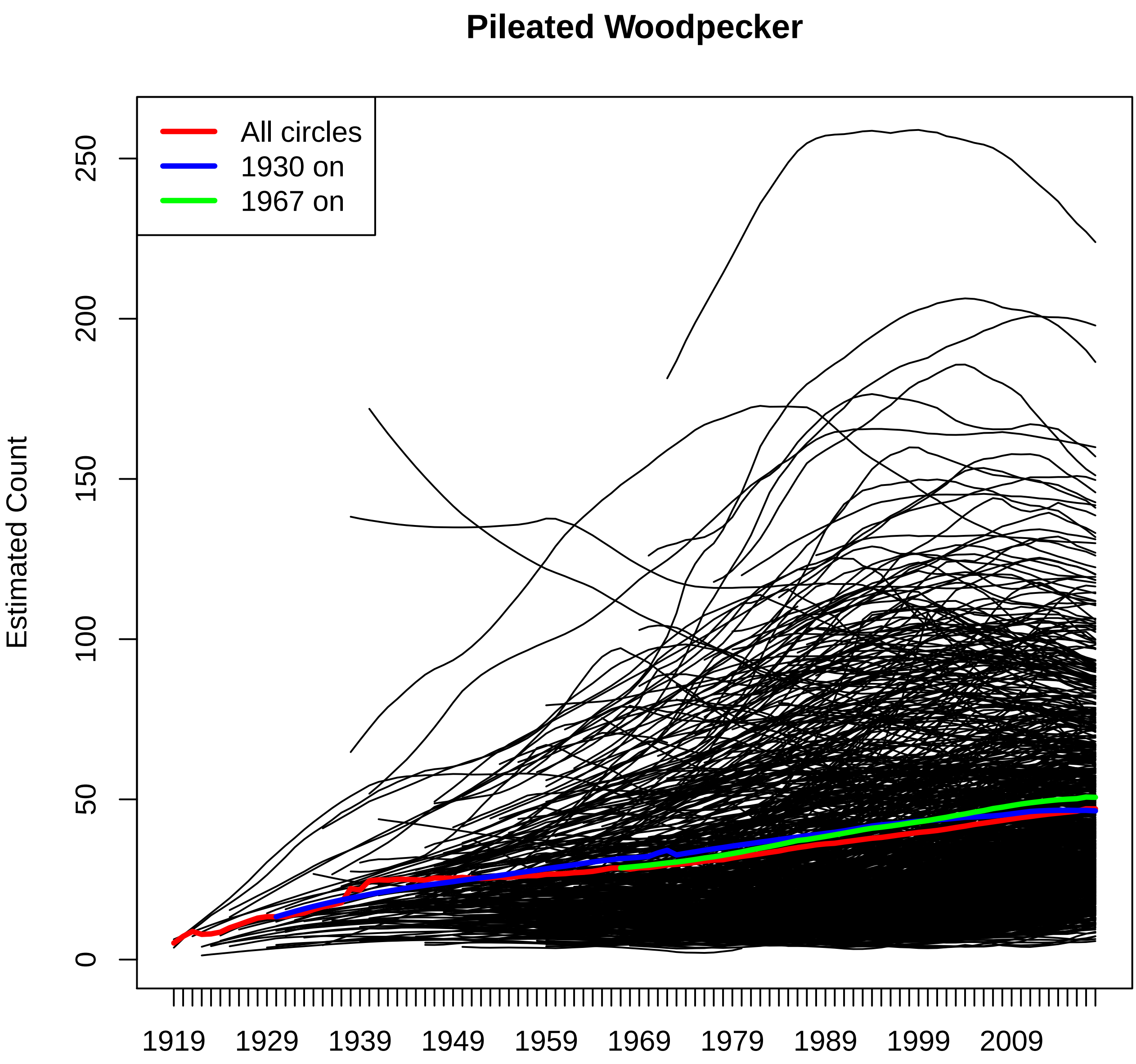

Pileated Woodpecker
All circles
1930 on
1967 on
Estimated Count
0
50
100
150
200
250
1919
1929
1939
1949
1959
1969
1979
1989
1999
2009

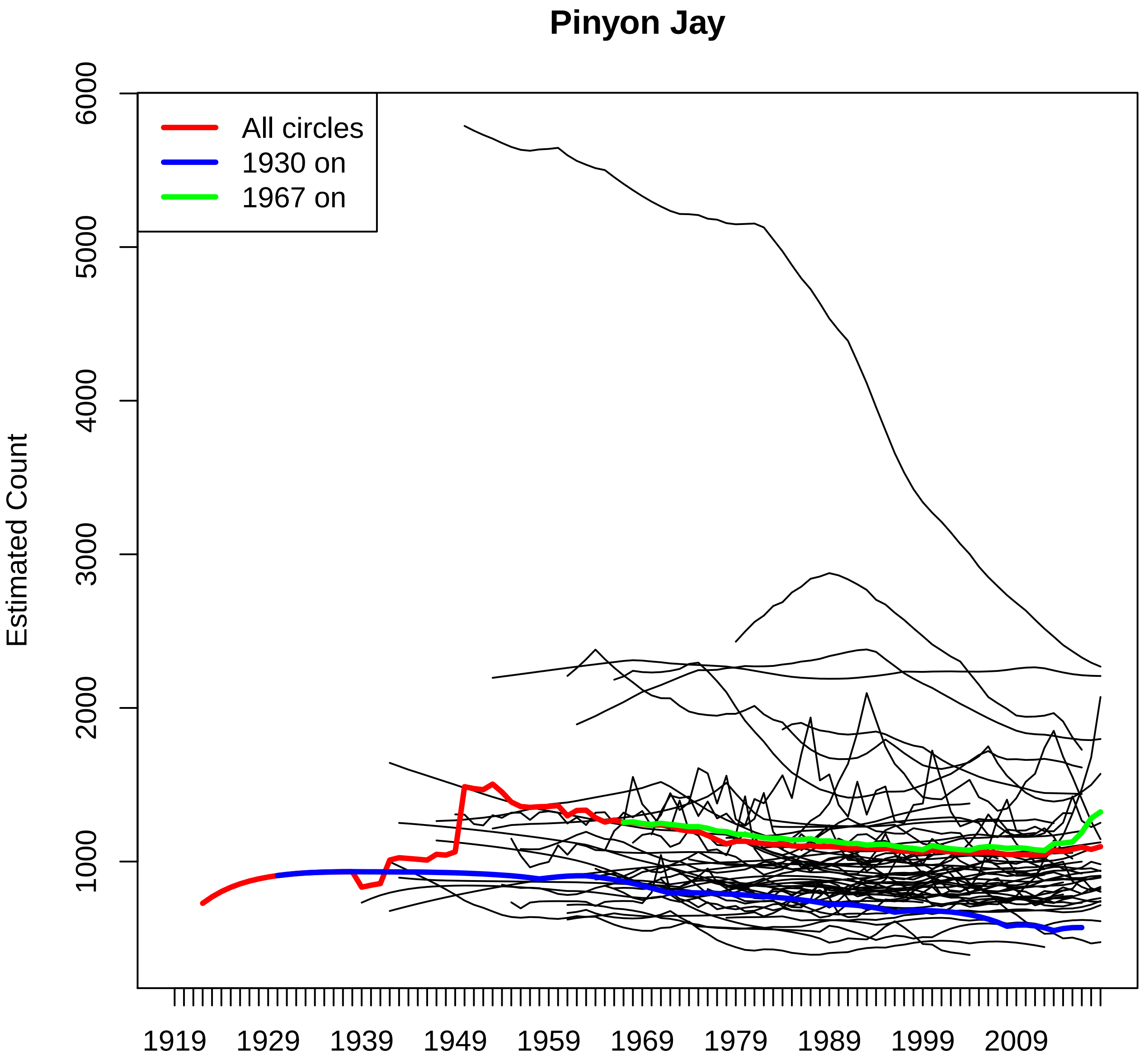

Pinyon Jay
Estimated Count
6000
5000
4000
3000
2000
1000
All circles
1930 on
1967 on
1919
1929
1939
1949
1959
1969
1979
1989
1999
2009

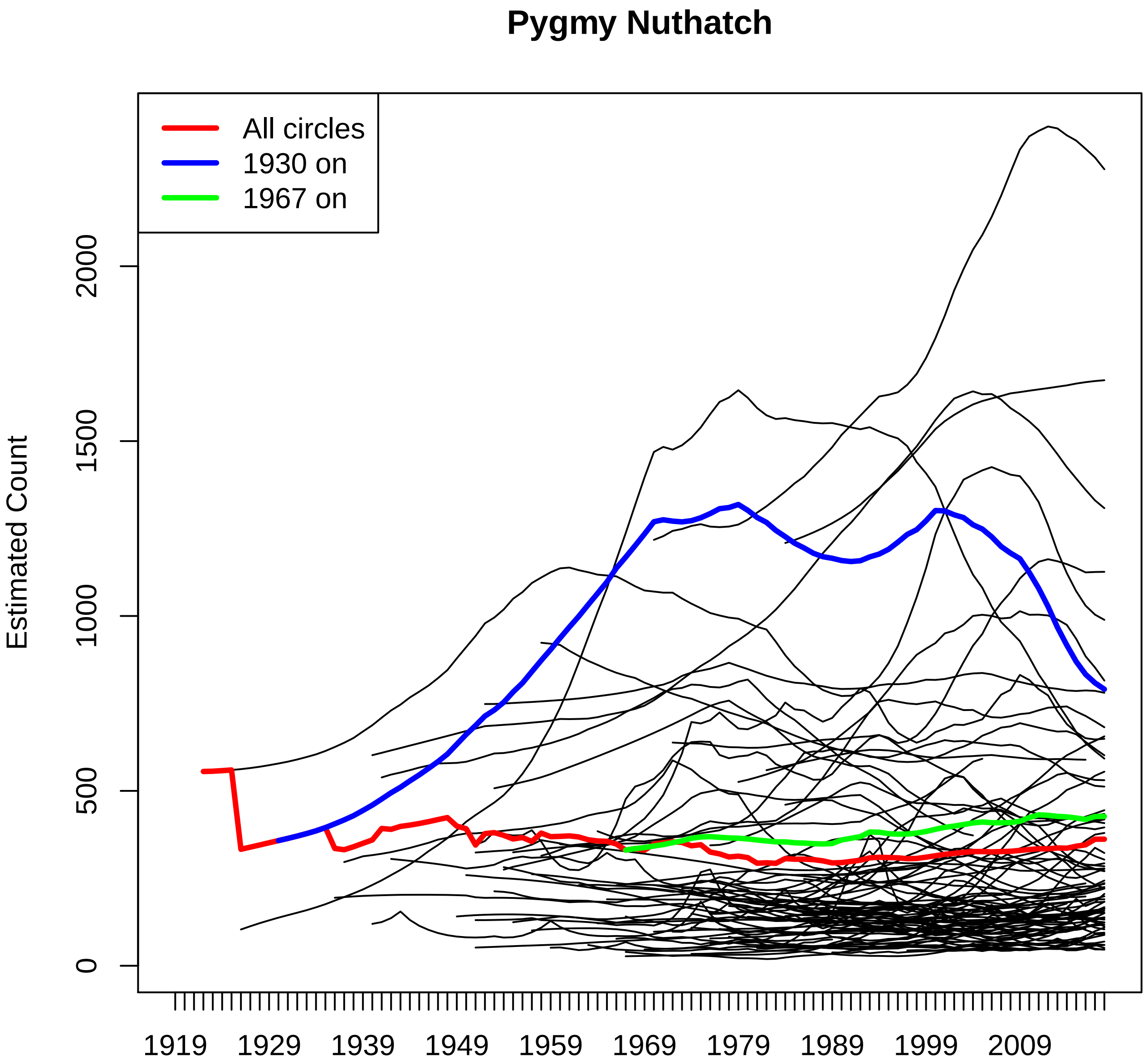

Pygmy Nuthatch
All circles
1930 on
1967 on
Estimated Count
0
500
1000
1500
2000
1919
1929
1939
1949
1959
1969
1979
1989
1999
2009

Red−headed Woodpecker

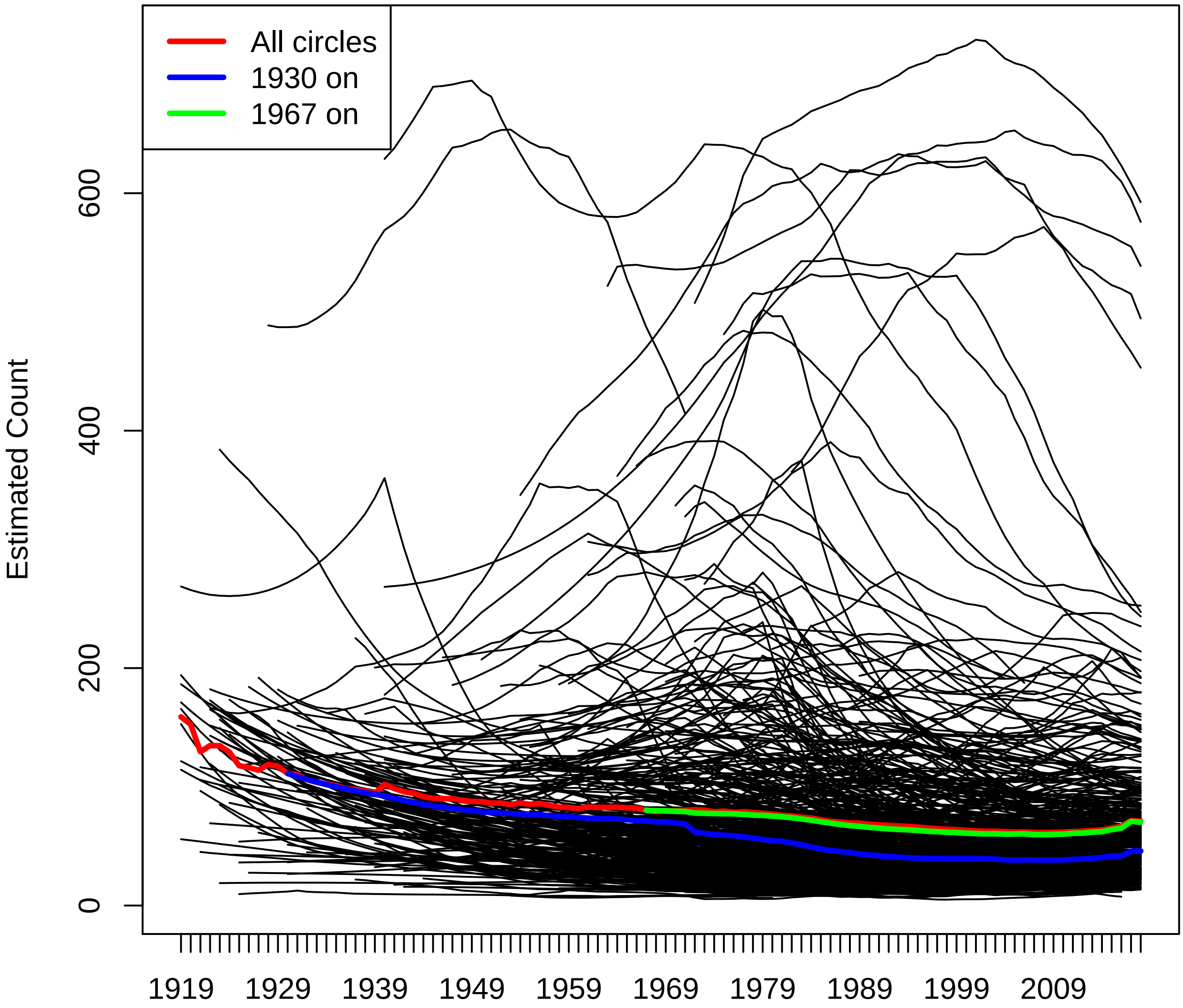

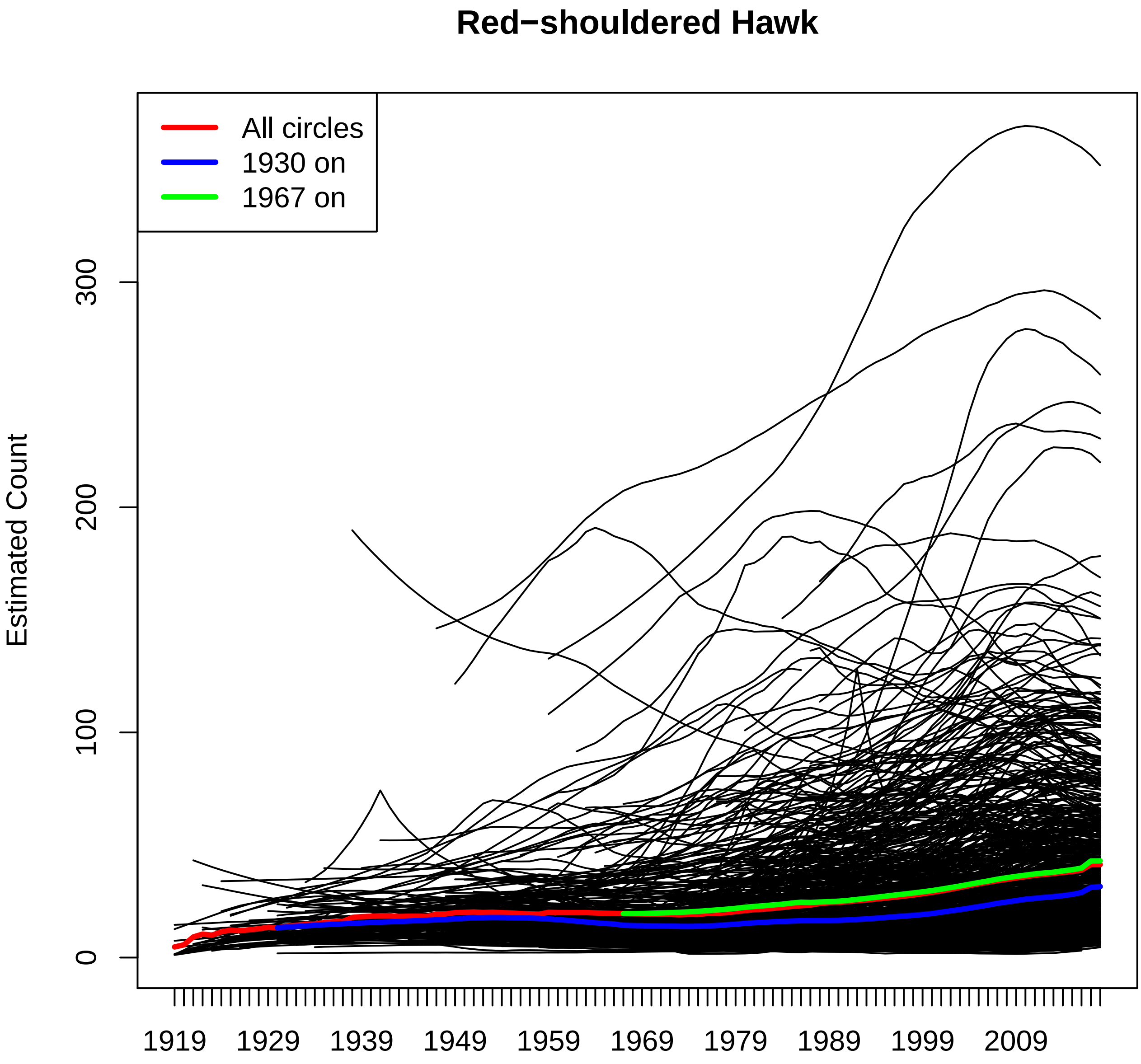

Red−shouldered Hawk
All circles
1930 on
1967 on
Estimated Count
0
100
200
300
1919
1929
1939
1949
1959
1969
1979
1989
1999
2009

Red Crossbill

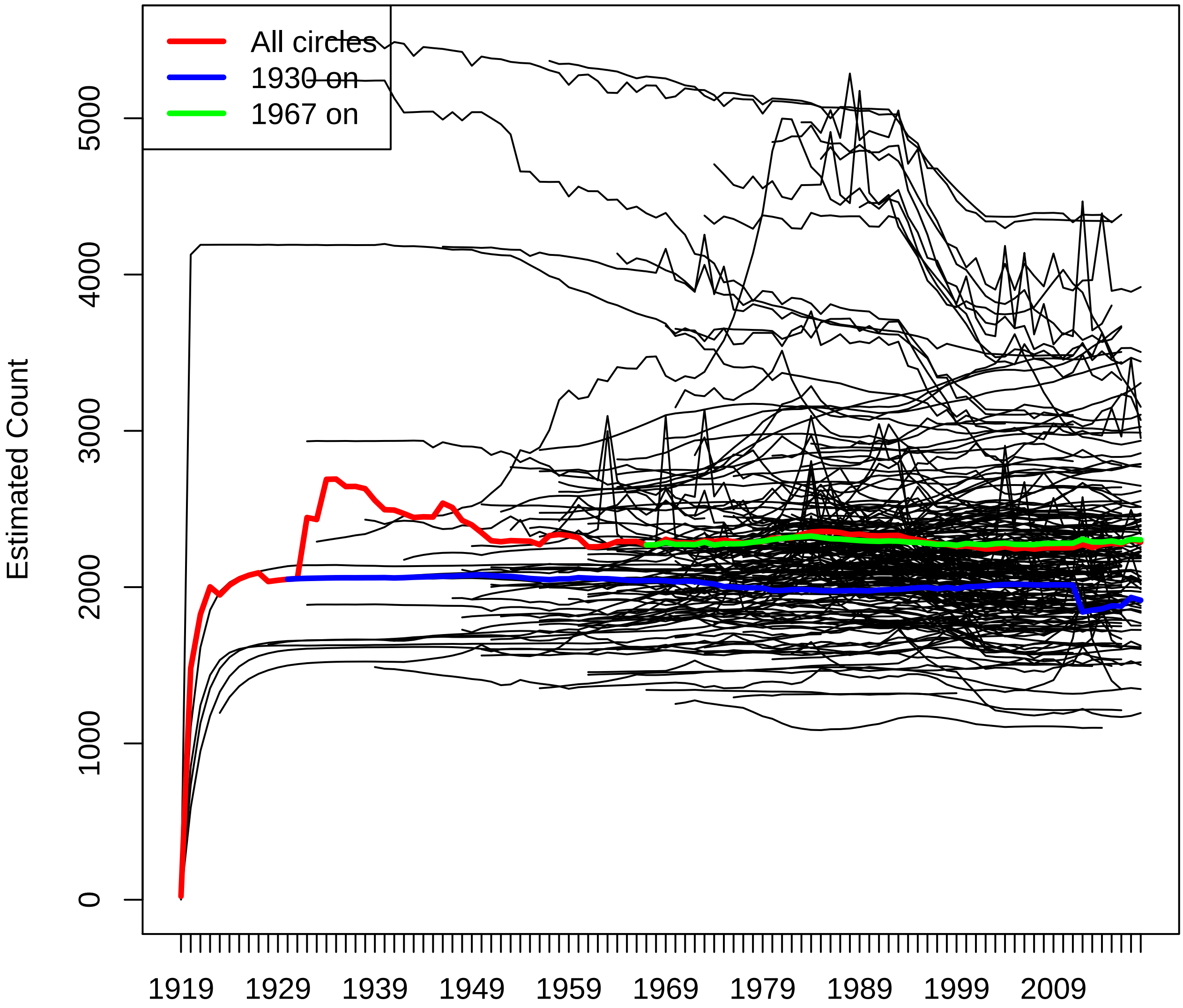

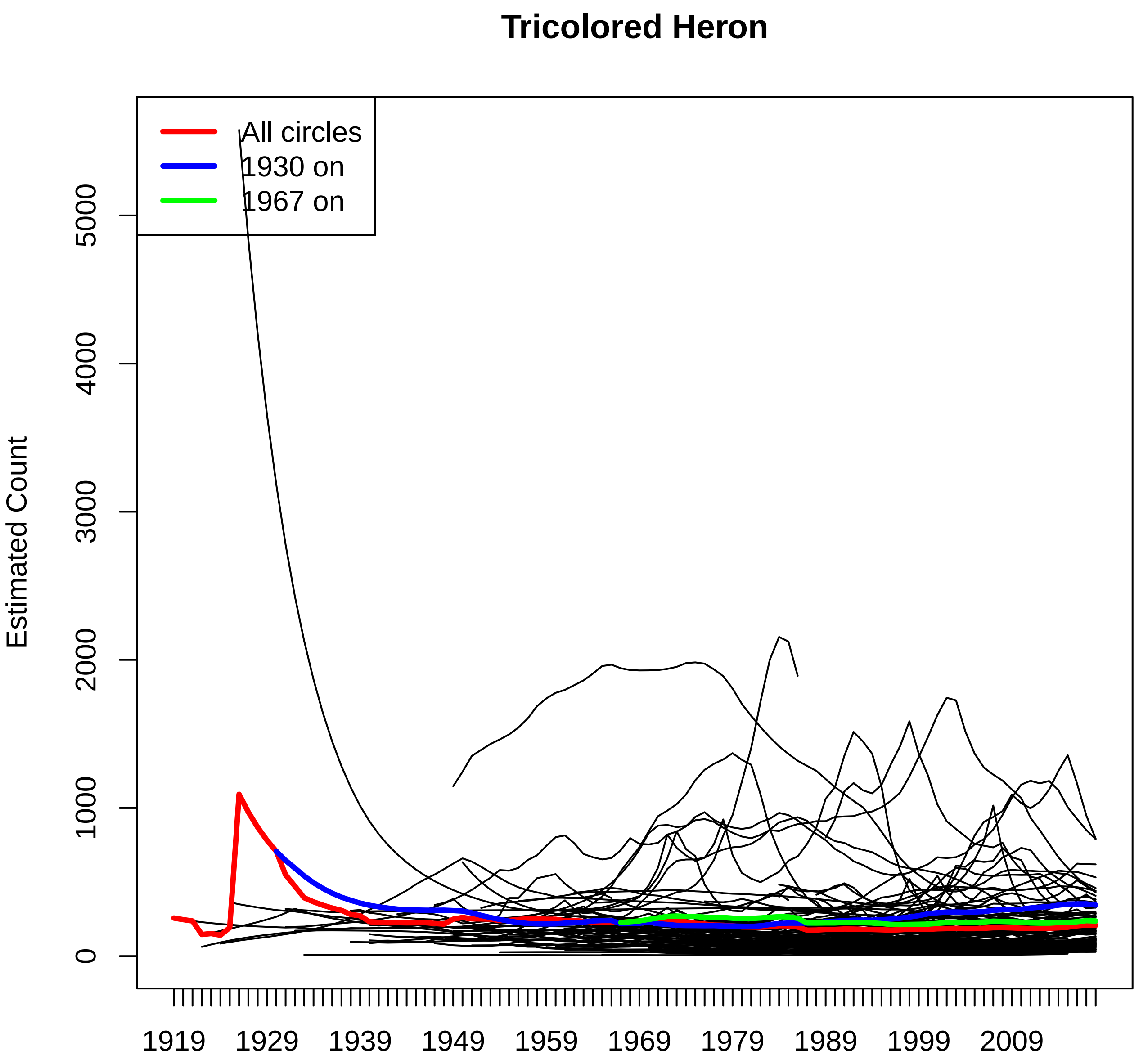

Tricolored Heron
All circles
1930 on
1967 on
Estimated Count
0
1000
2000
3000
4000
5000
1919
1929
1939
1949
1959
1969
1979
1989
1999
2009

Trumpeter Swan

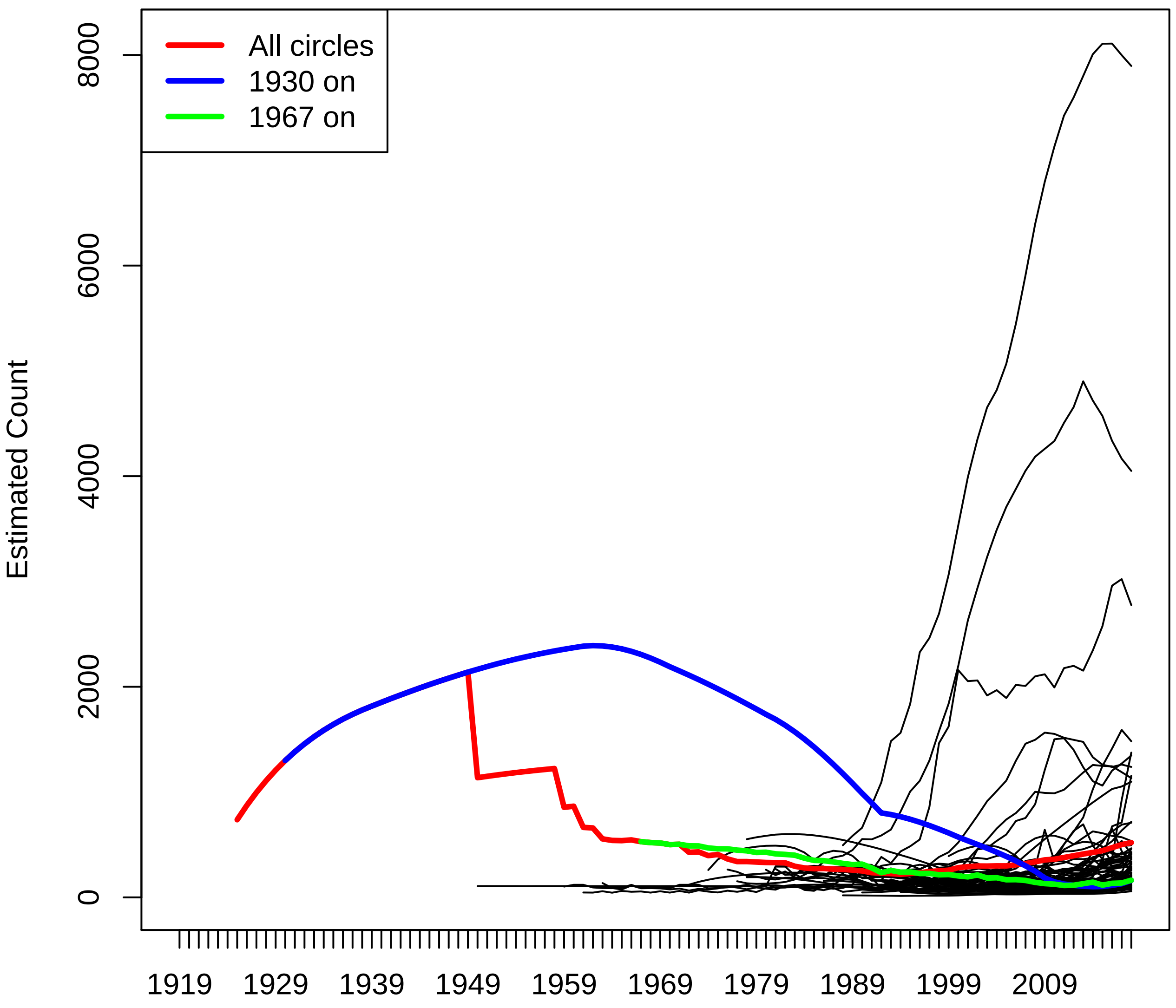

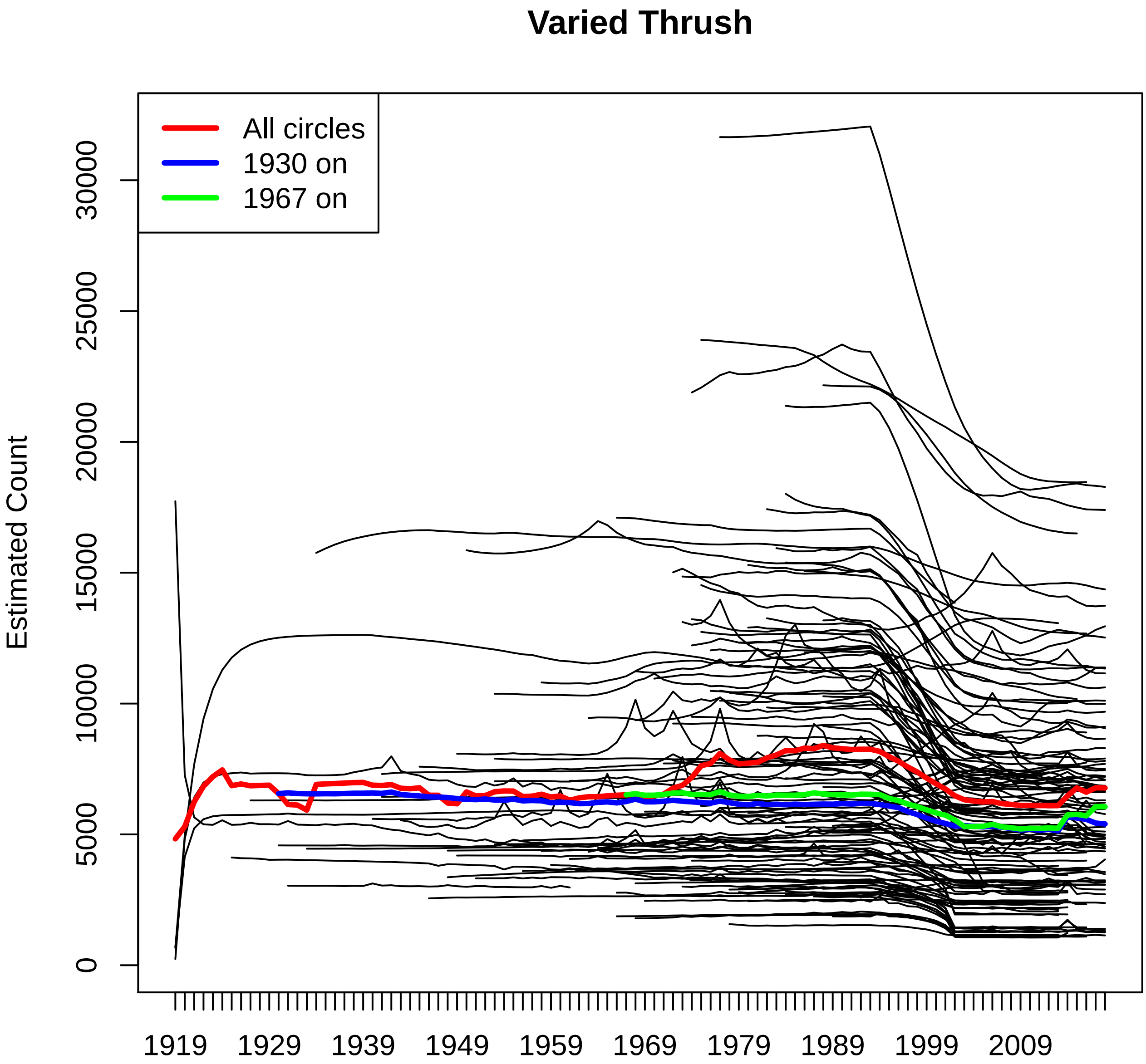

Varied Thrush
All circles
1930 on
1967 on
Estimated Count
0
5000
10000
15000
20000
25000
30000
1919
1929
1939
1949
1959
1969
1979
1989
1999
2009

Wood Stork

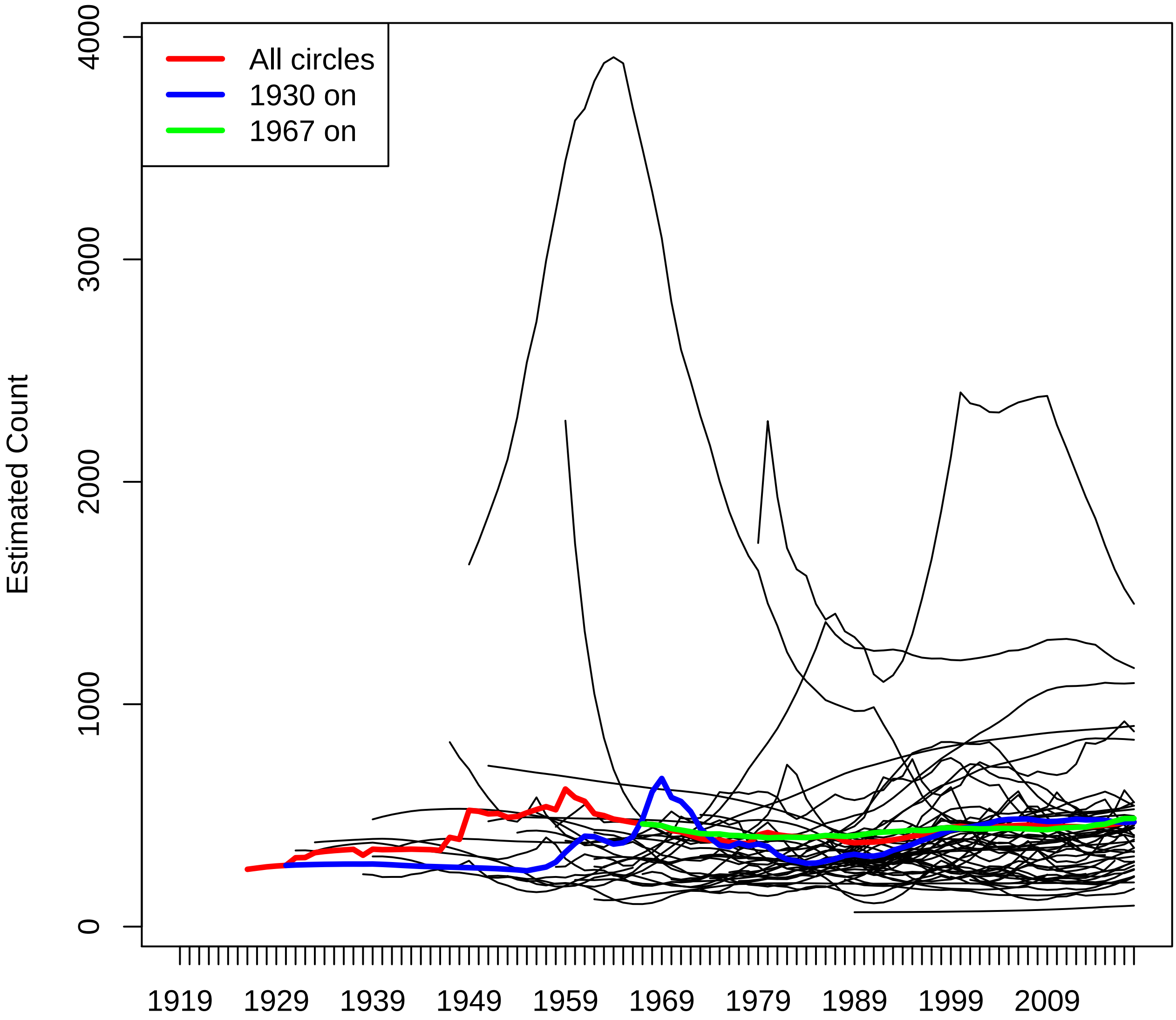

Estimated Count
0
1000
2000
3000
4000
All circles
1930 on
1967 on
1919
1929
1939
1949
1959
1969
1979
1989
1999
2009

Wrentit

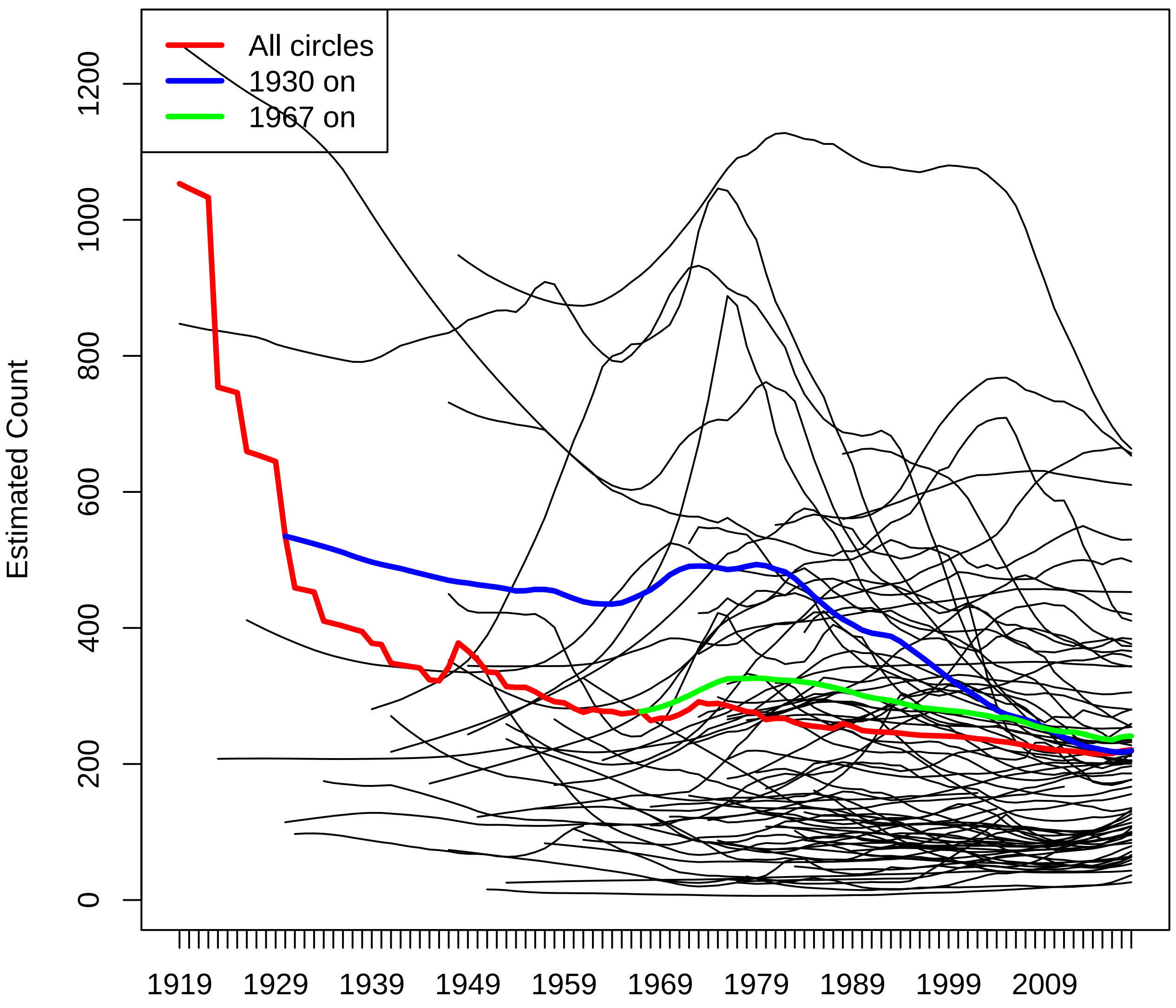

All circles
1930 on
1967 on
Estimated Count
0
200
400
600
800
1000
1200
1919
1929
1939
1949
1959
1969
1979
1989
1999
2009

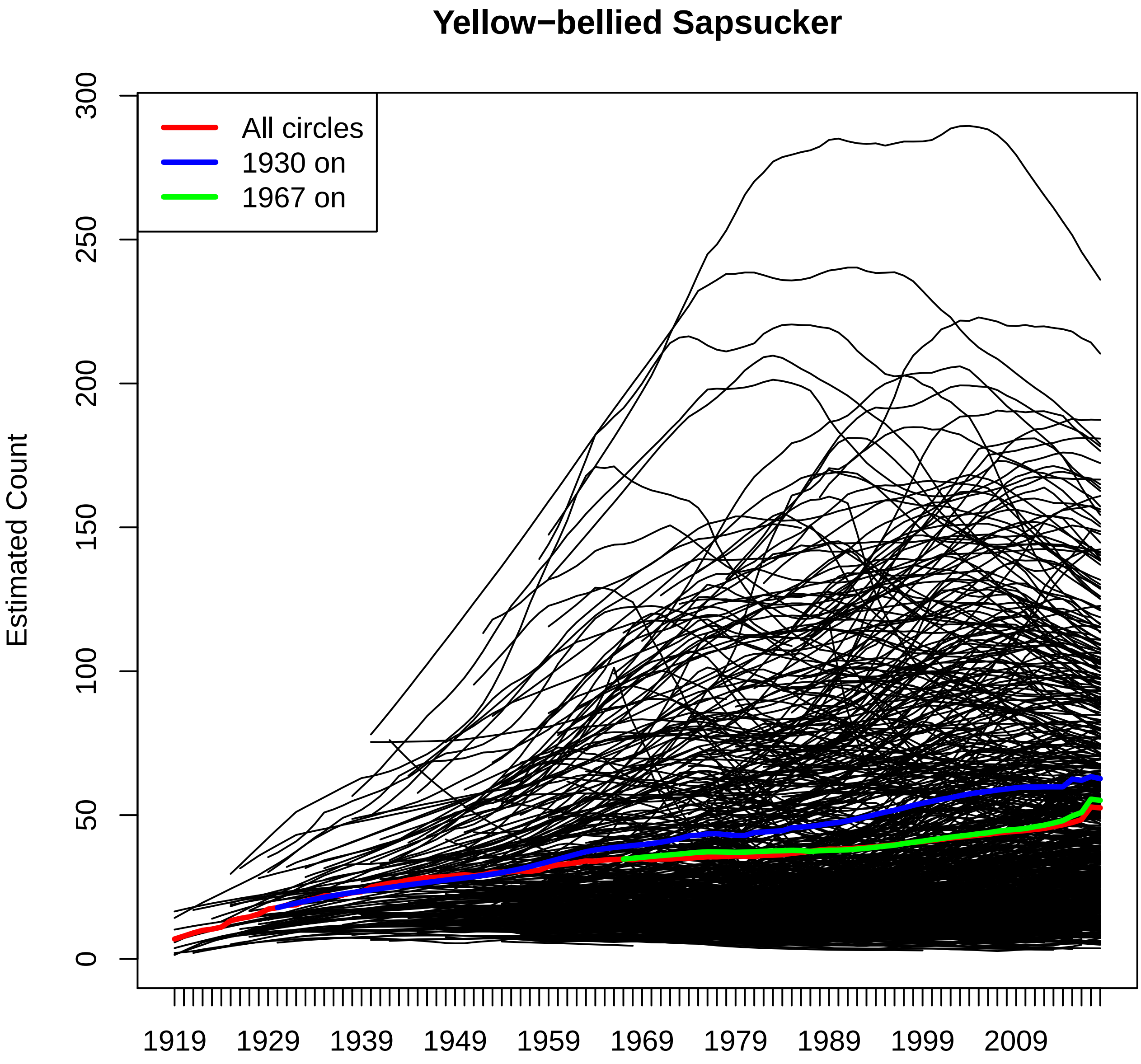

Yellow−bellied Sapsucker
All circles
1930 on
1967 on
Estimated Count
0
50
100
150
200
250
300
1919
1929
1939
1949
1959
1969
1979
1989
1999
2009

Yellow−billed Magpie

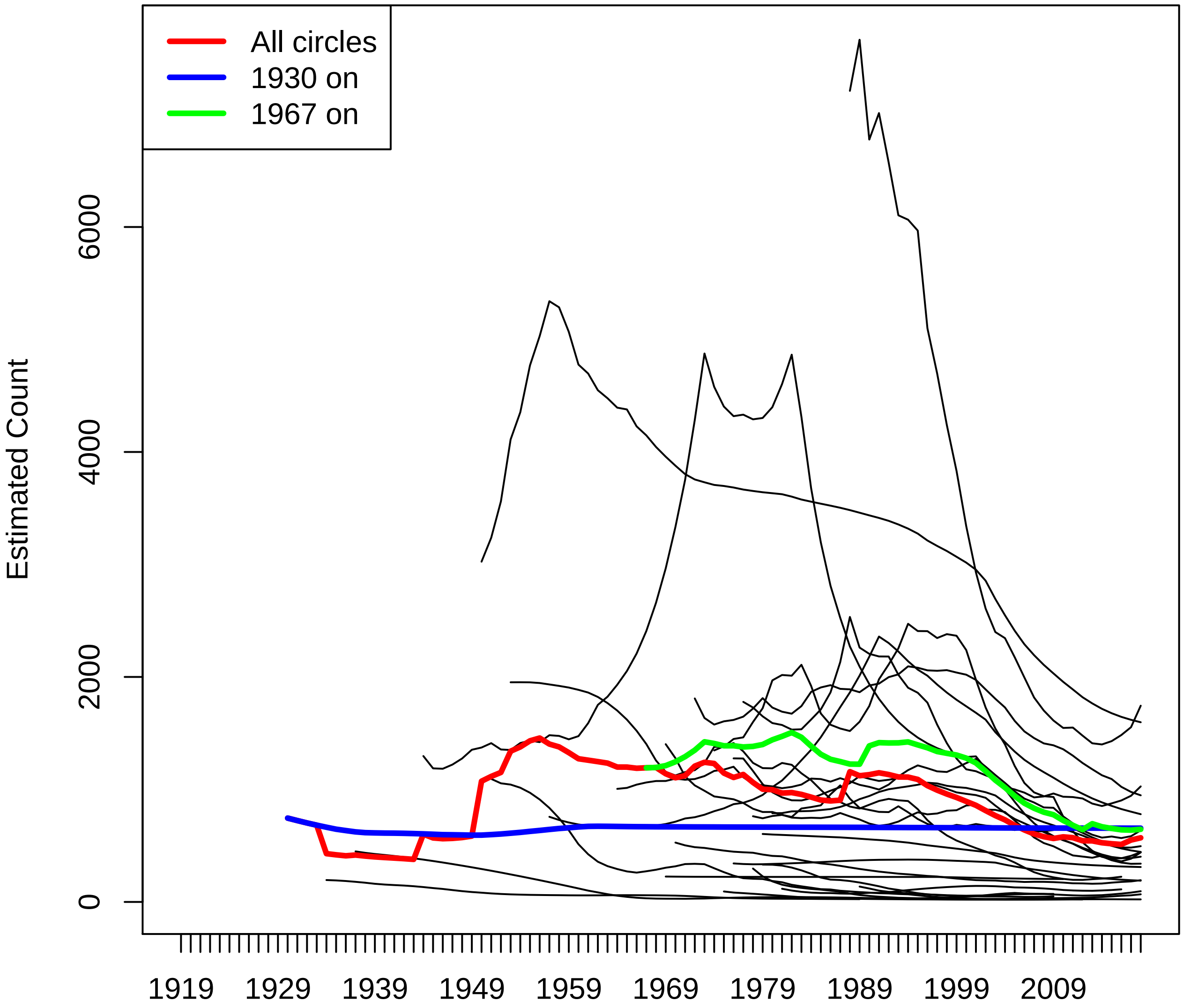

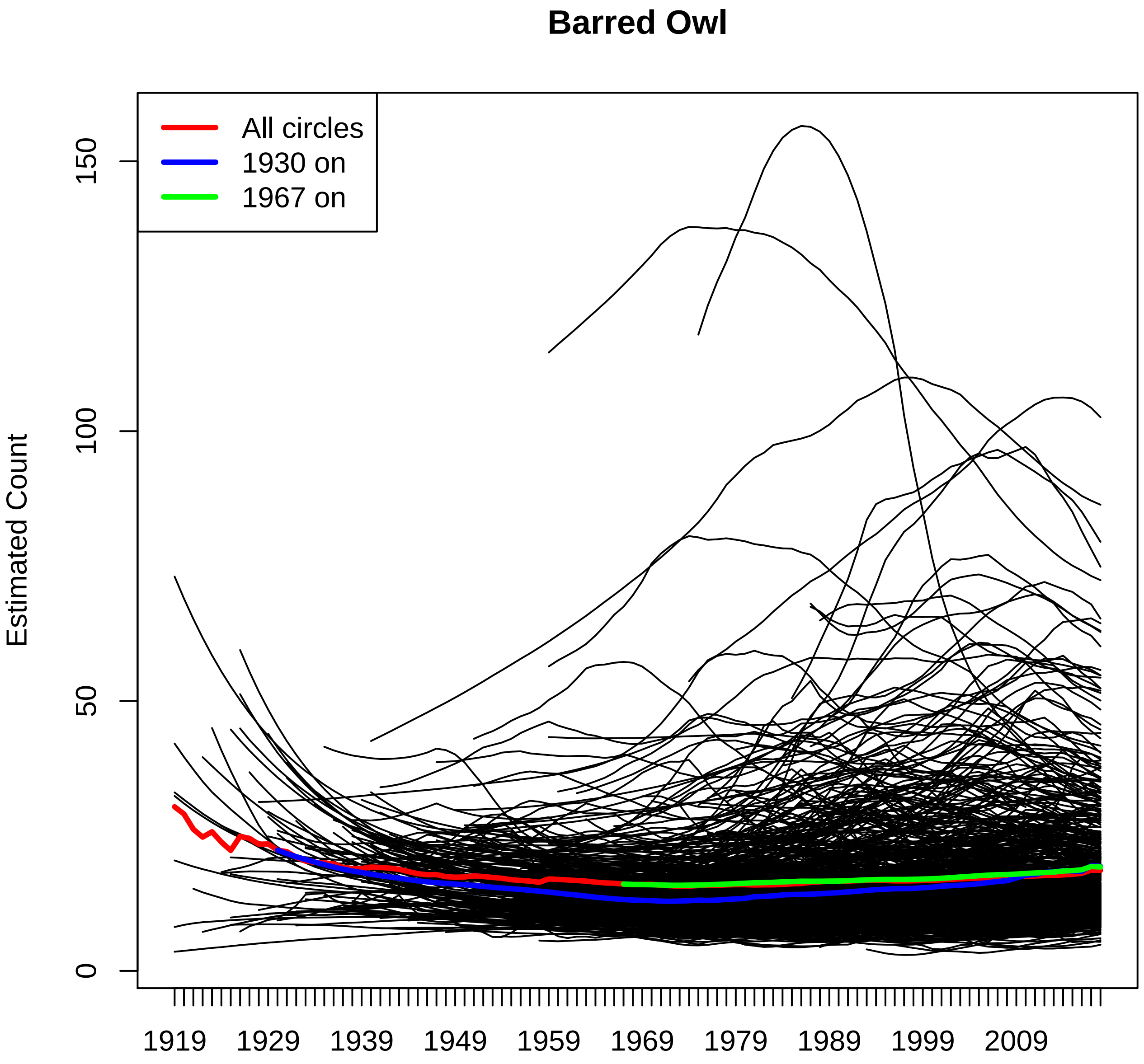
Barred Owl
All circles
1930 on
1967 on
Estimated Count
0
50
100
150
1919
1929
1939
1949
1959
1969
1979
1989
1999
2009

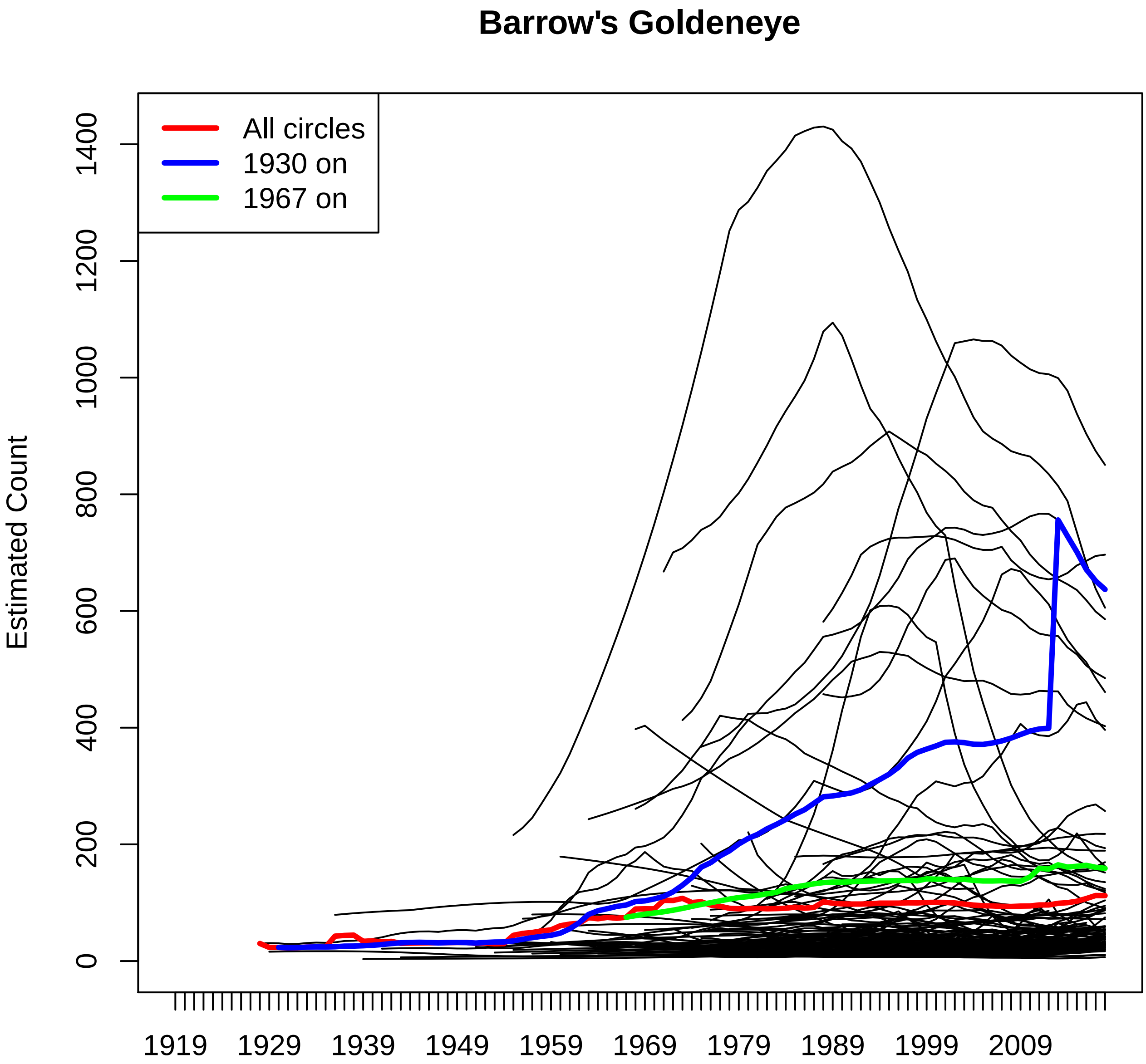
Barrow's Goldeneye
All circles
1930 on
1967 on
Estimated Count
0
200
400
600
800
1000
1200
1400
1919
1929
1939
1949
1959
1969
1979
1989
1999
2009

Black−throated Sparrow

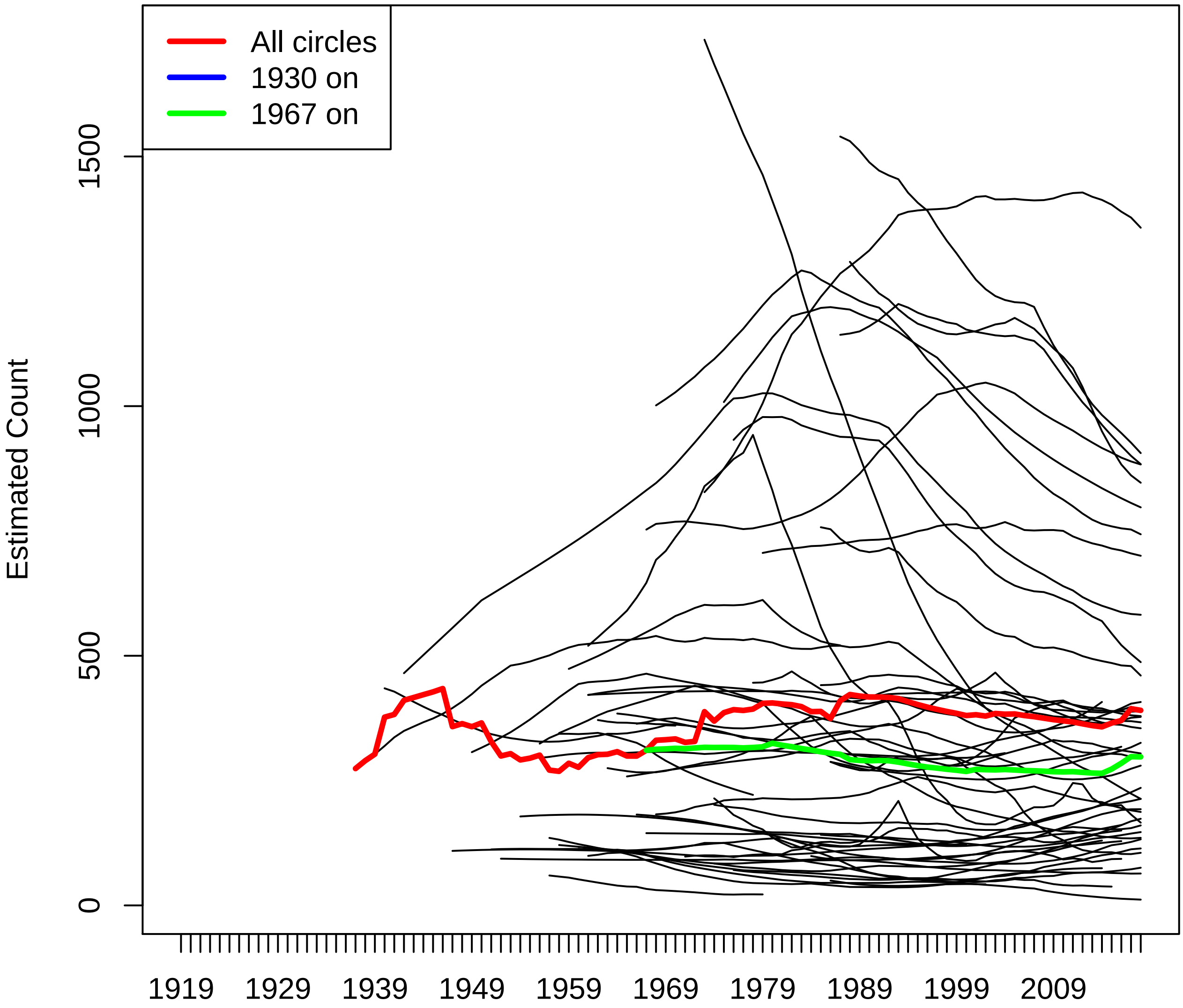

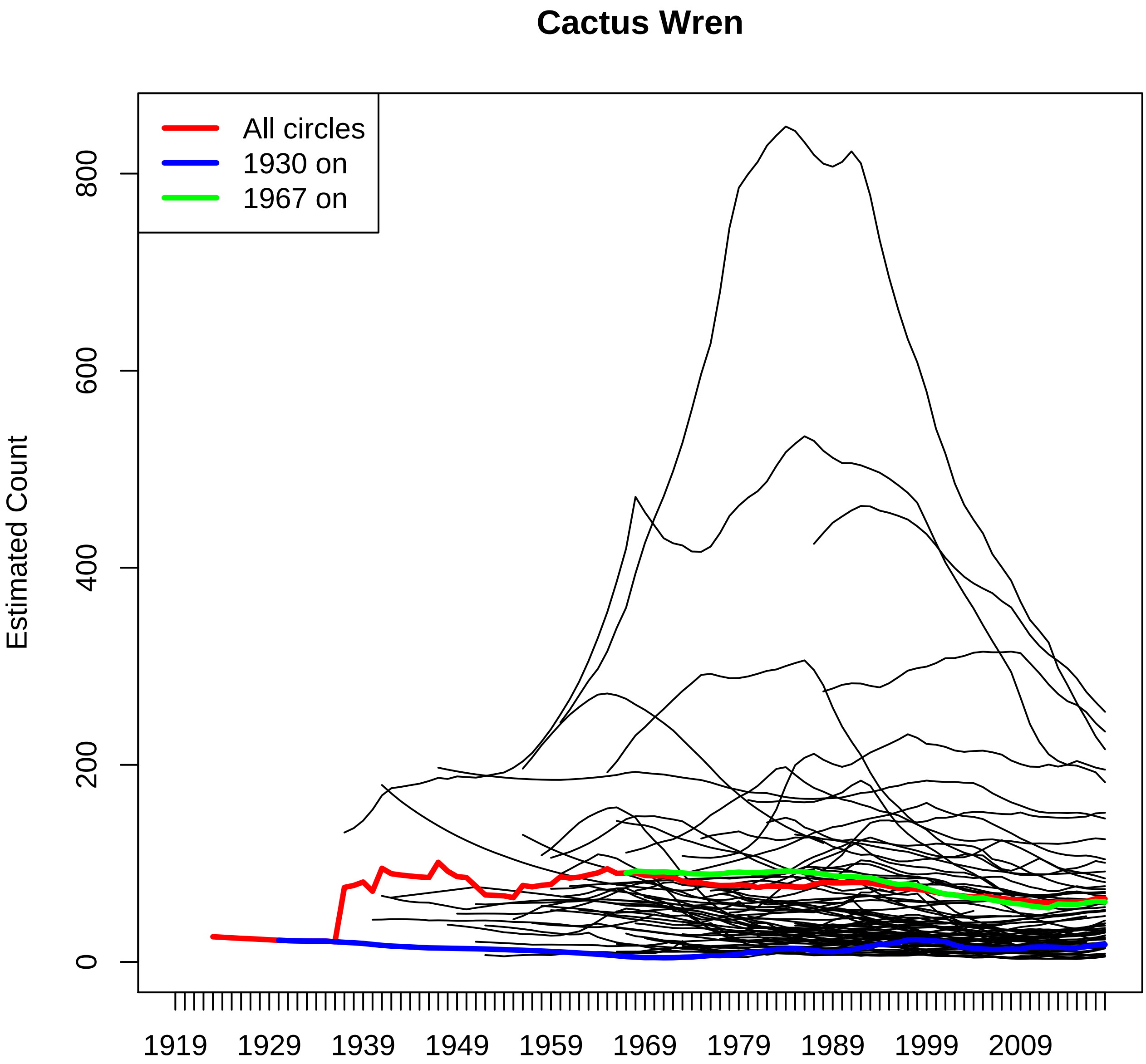
Cactus Wren
All circles
1930 on
1967 on
Estimated Count
0
200
400
600
800
1919
1929
1939
1949
1959
1969
1979
1989
1999
2009

California Thrasher

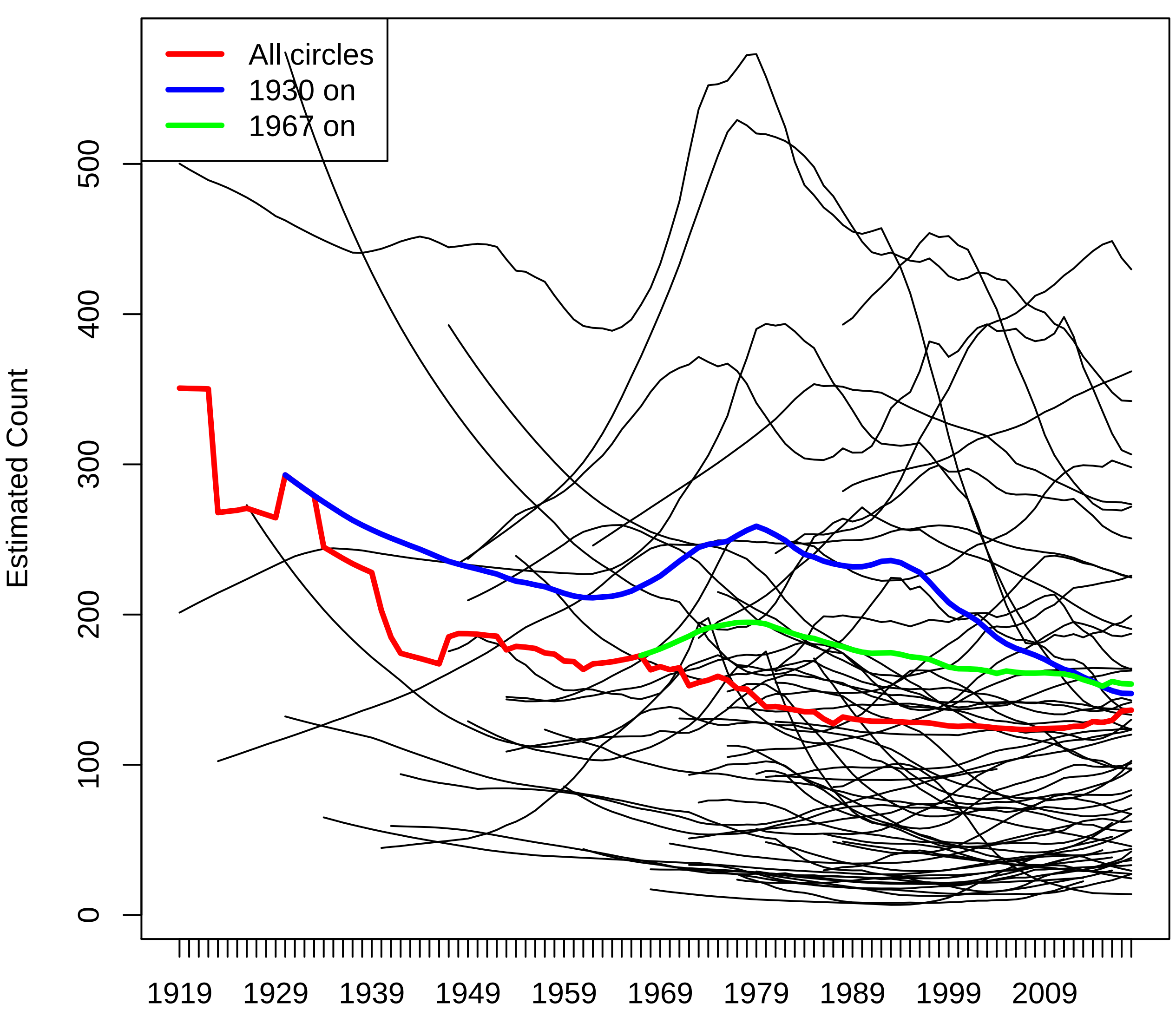

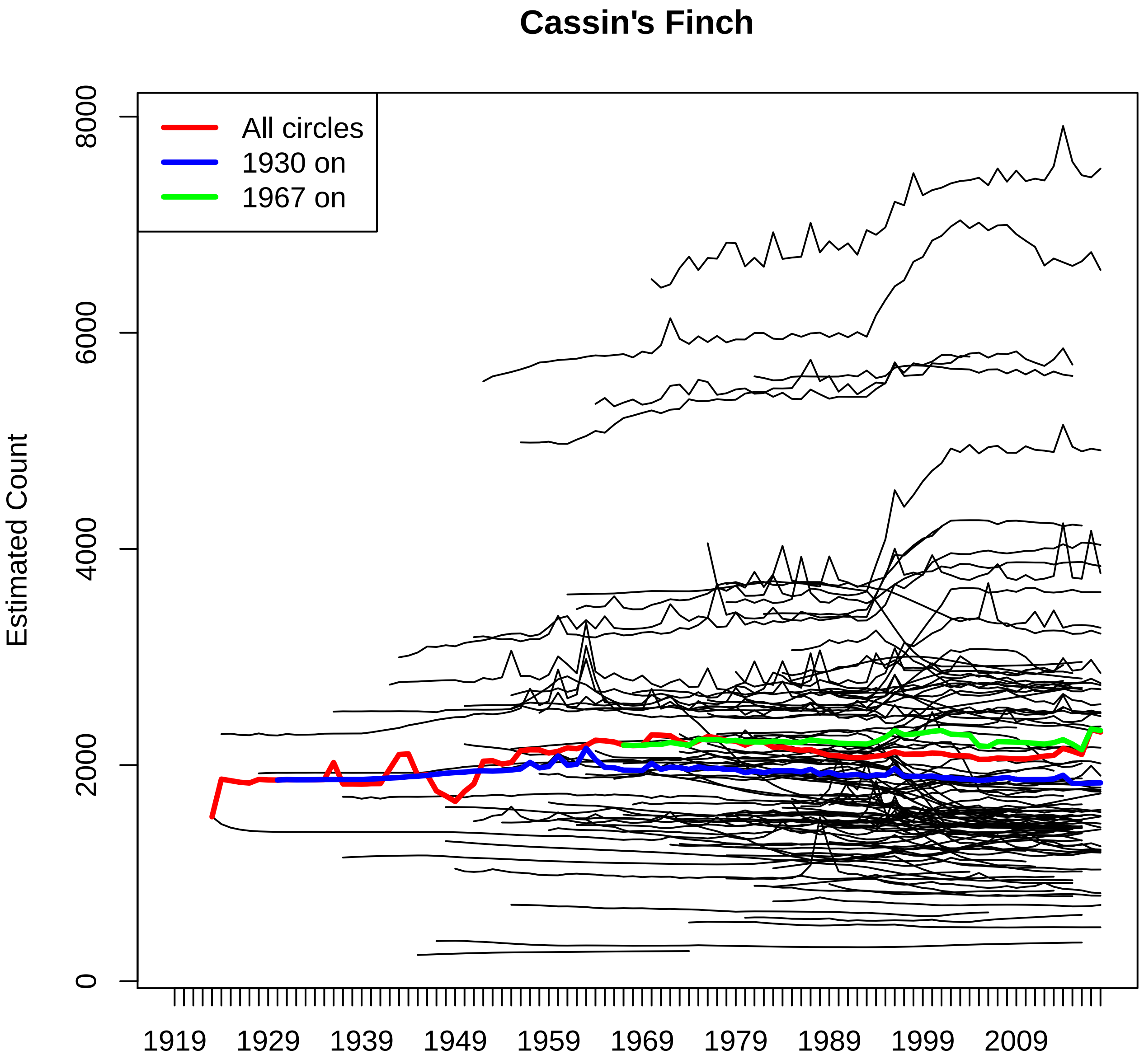

Cassin's Finch
Estimated Count
0
2000
4000
6000
8000
1919
1929
1939
1949
1959
1969
1979
1989
1999
2009
All circles
1930 on
1967 on

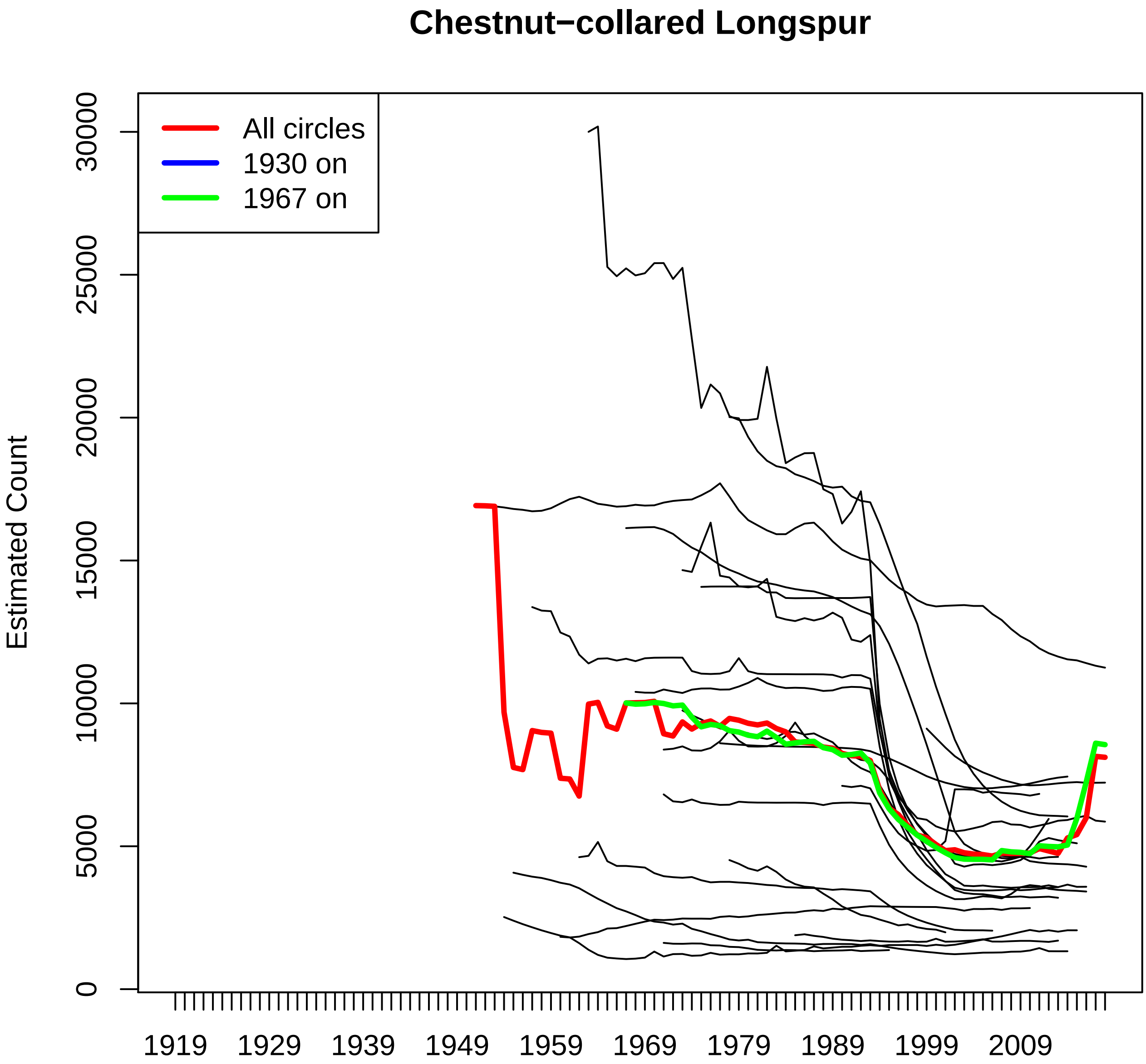

Chestnut−collared Longspur
Estimated Count
0
5000
10000
15000
20000
25000
30000
1919
1929
1939
1949
1959
1969
1979
1989
1999
2009
All circles
1930 on
1967 on

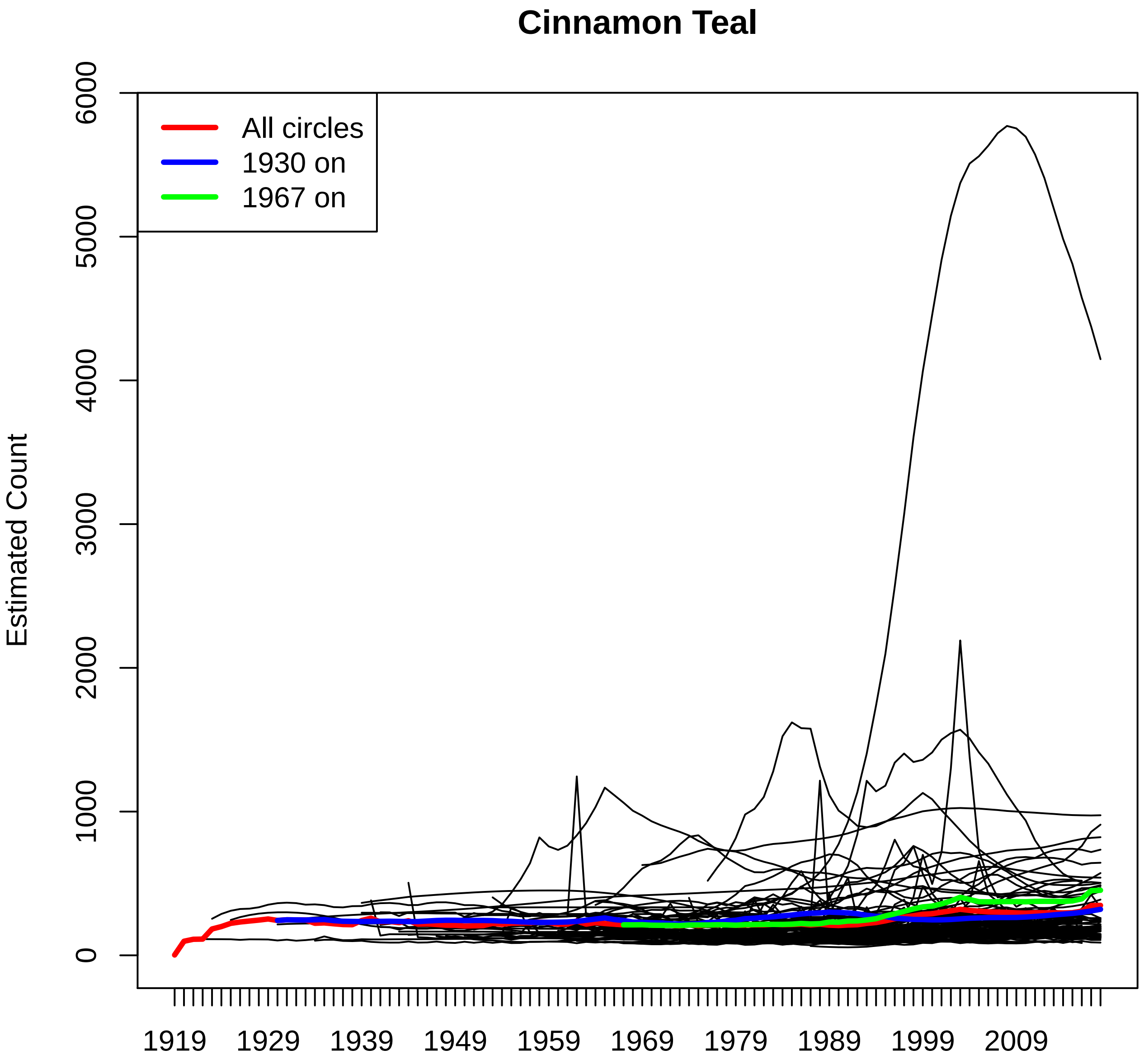

Cinnamon Teal
Estimated Count
0
1000
2000
3000
4000
5000
6000
All circles
1930 on
1967 on
1919
1929
1939
1949
1959
1969
1979
1989
1999
2009

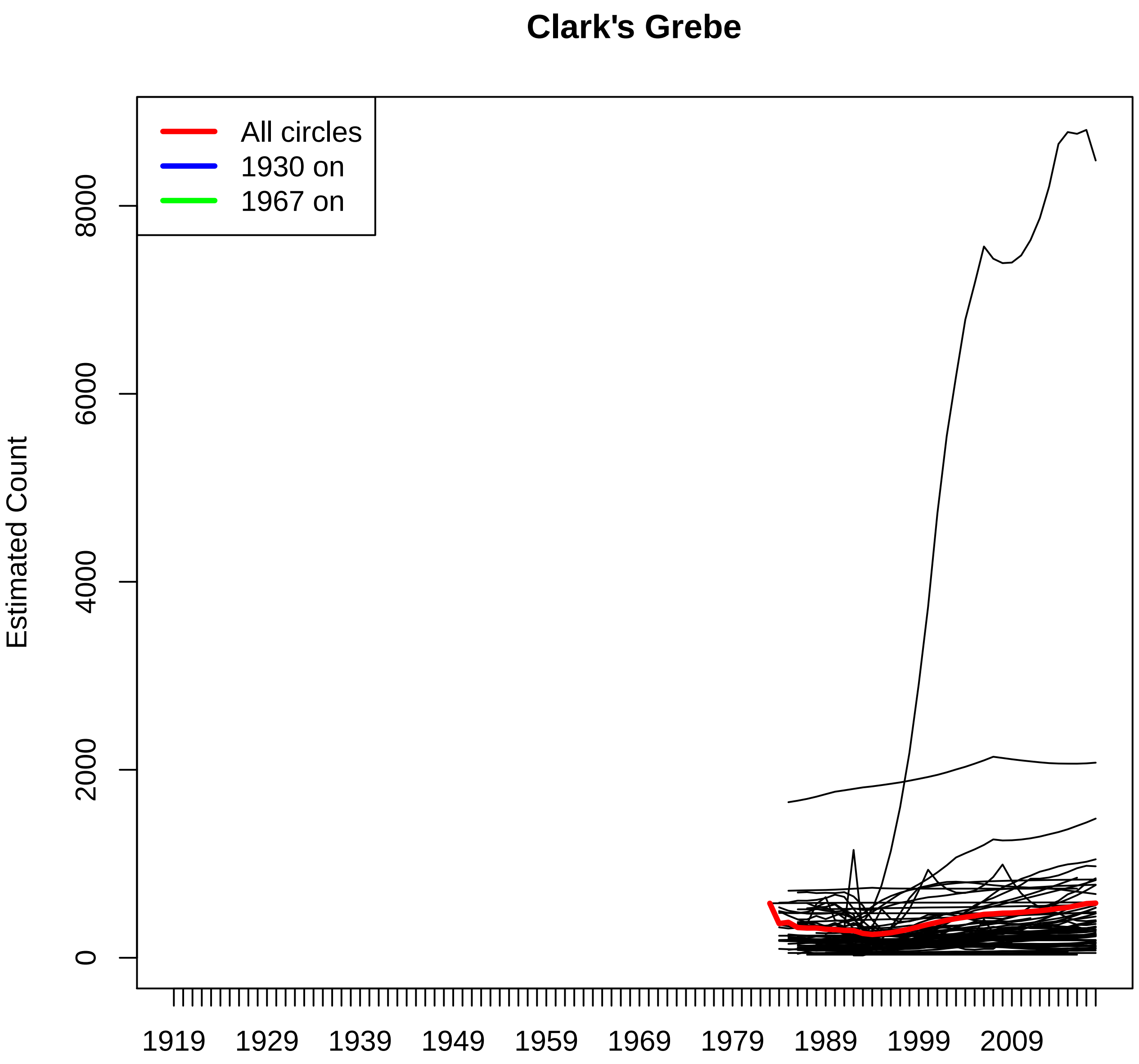
Clark's Grebe
All circles
1930 on
1967 on
Estimated Count
0
2000
4000
6000
8000
1919
1929
1939
1949
1959
1969
1979
1989
1999
2009

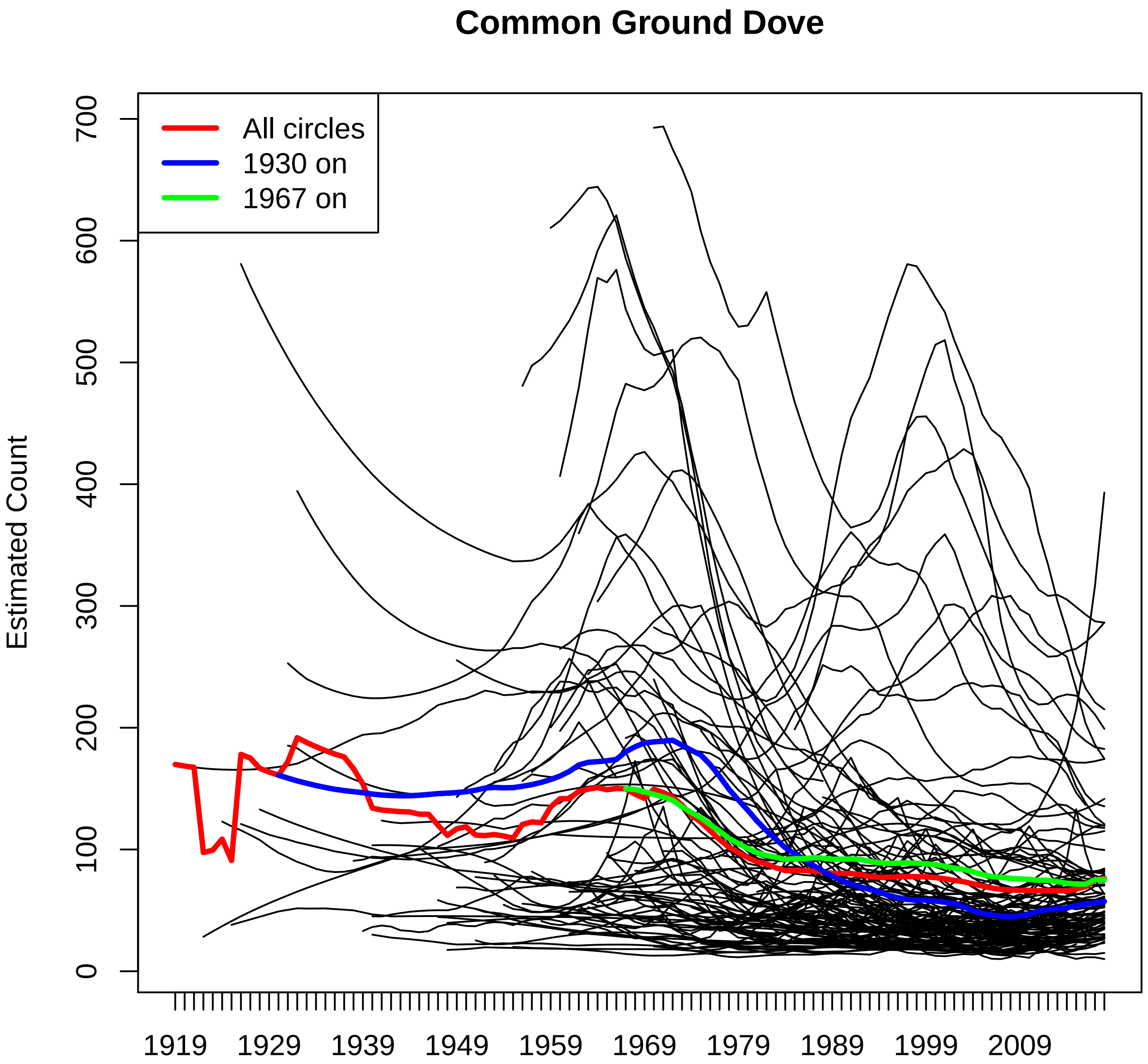

Common Ground Dove
All circles
1930 on
1967 on
Estimated Count
0
100
200
300
400
500
600
700
1919
1929
1939
1949
1959
1969
1979
1989
1999
2009

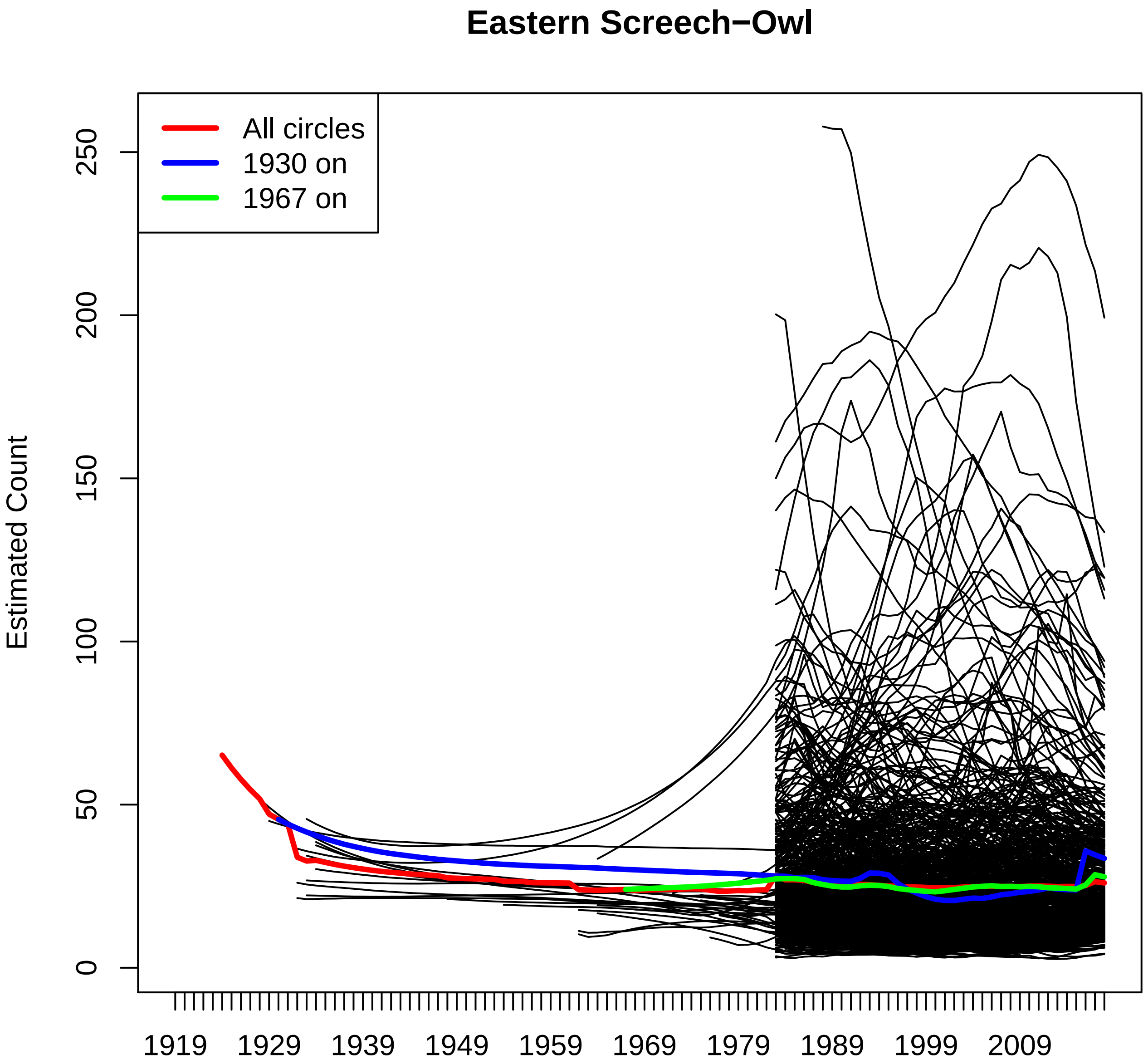

Eastern Screech−Owl
All circles
1930 on
1967 on
Estimated Count
0
50
100
150
200
250
1919
1929
1939
1949
1959
1969
1979
1989
1999
2009

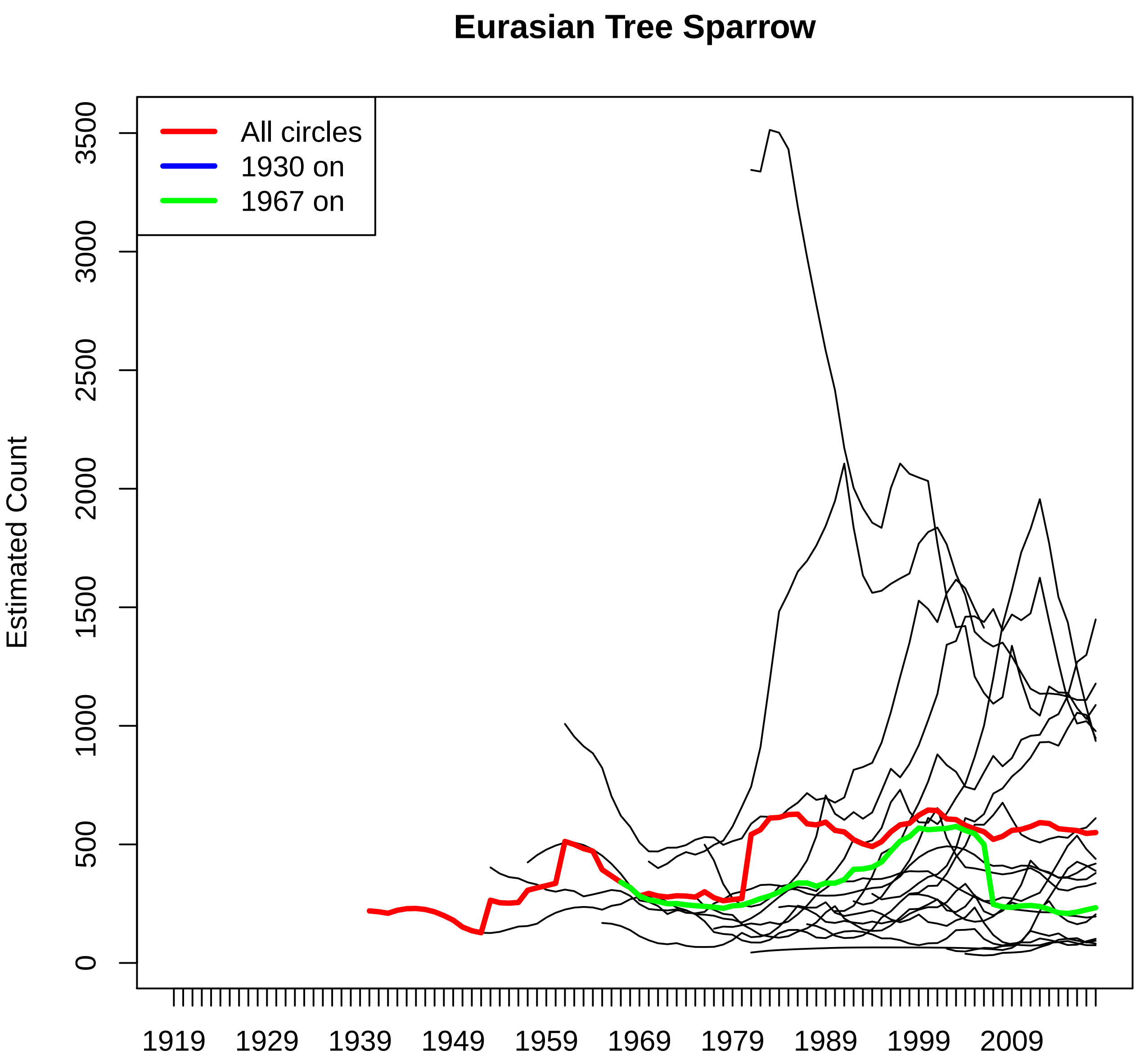
Eurasian Tree Sparrow
All circles
1930 on
1967 on
Estimated Count
0
500
1000
1500
2000
2500
3000
3500
1919
1929
1939
1949
1959
1969
1979
1989
1999
2009

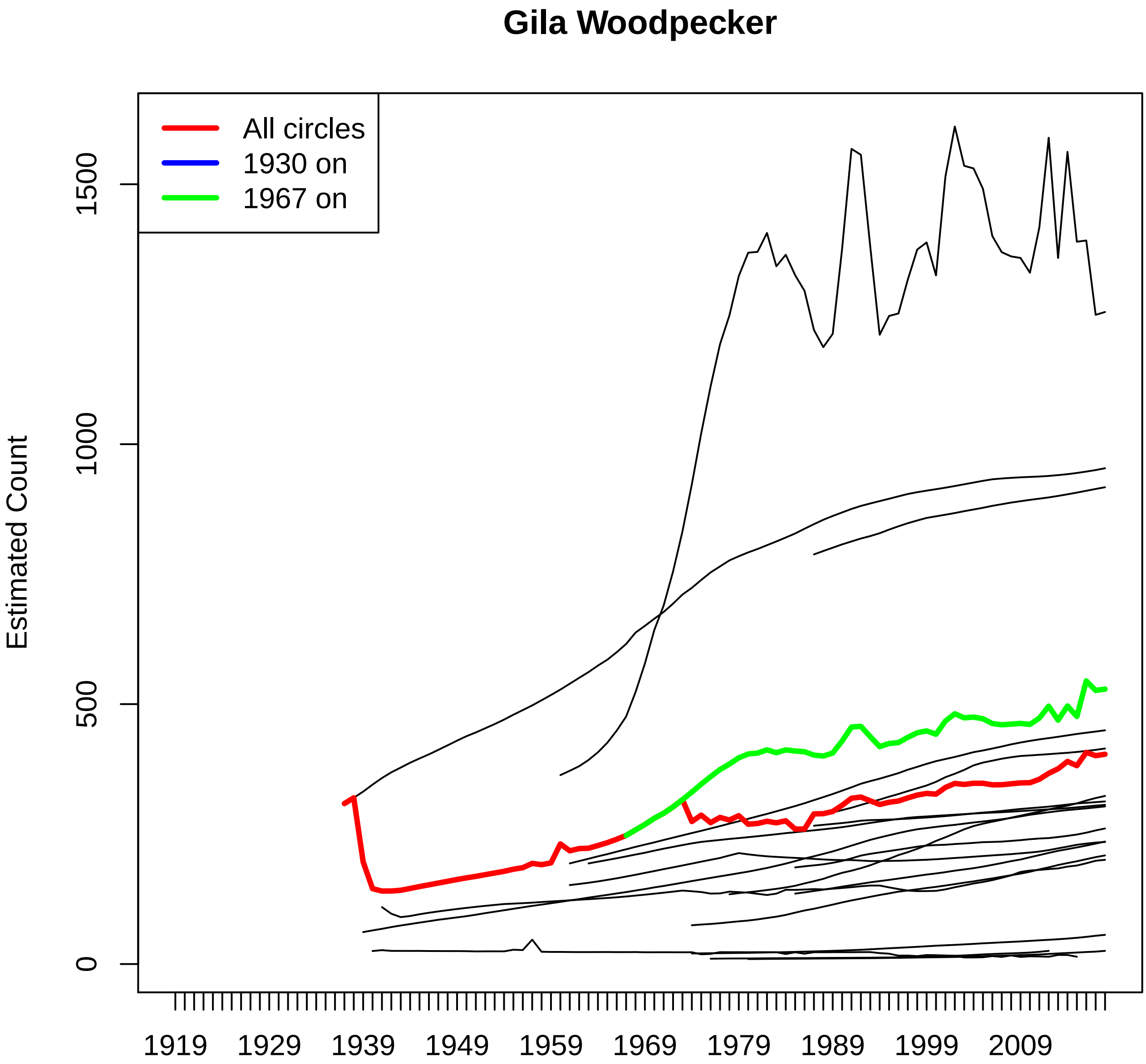

Gila Woodpecker
All circles
1930 on
1967 on
Estimated Count
0
500
1000
1500
1919
1929
1939
1949
1959
1969
1979
1989
1999
2009

Gray Partridge

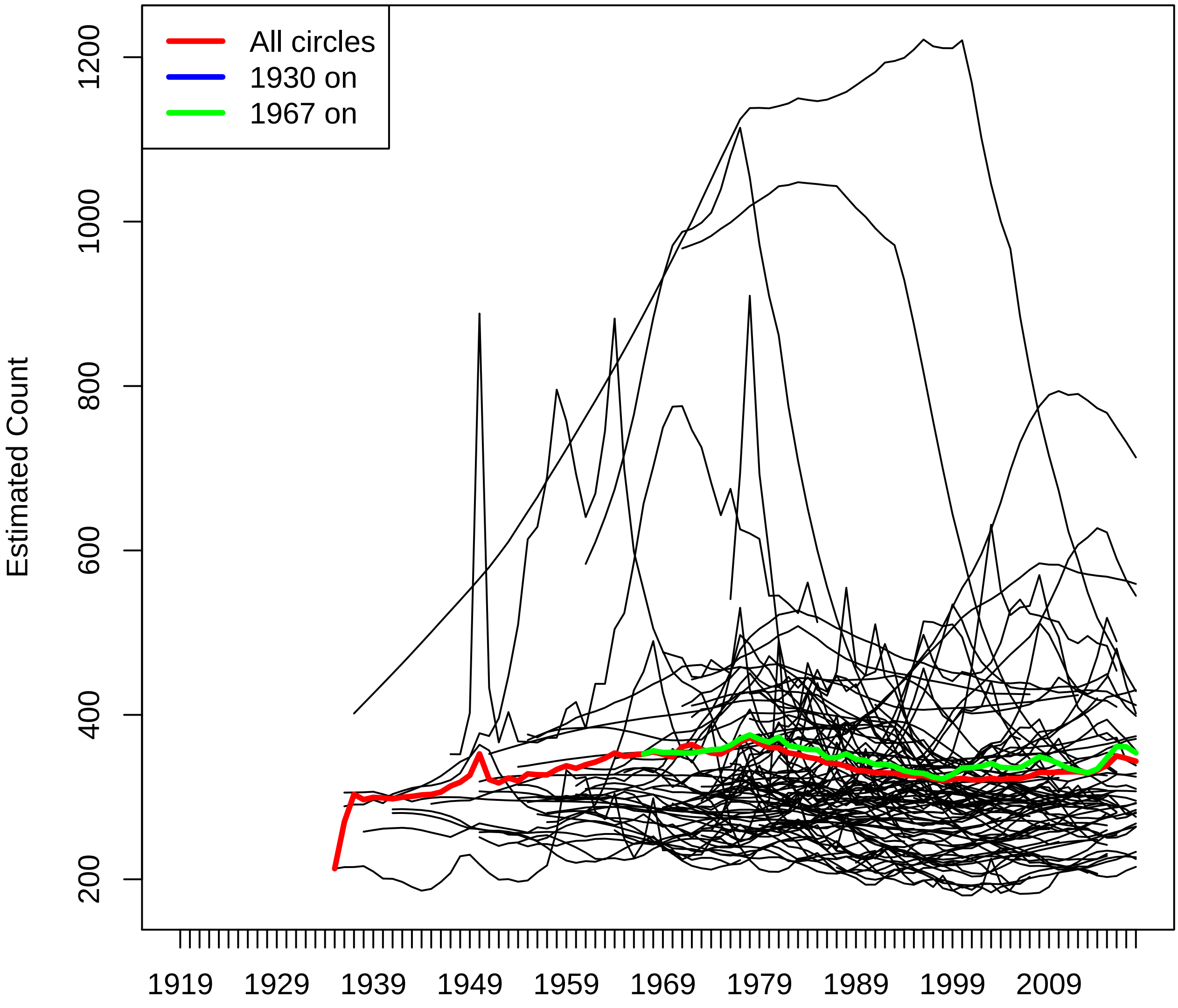

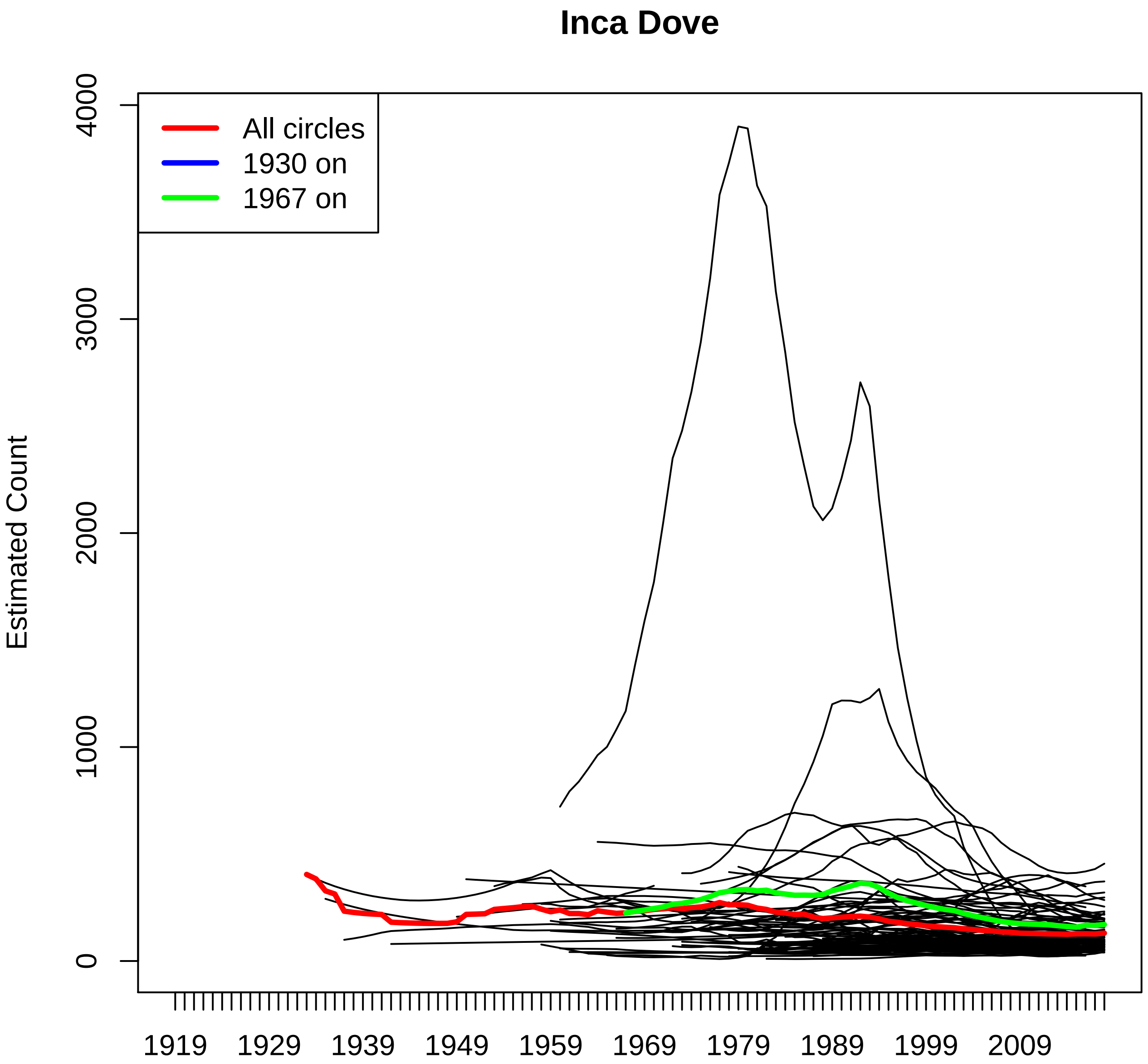
Inca Dove
Estimated Count
0
1000
2000
3000
4000
All circles
1930 on
1967 on
1919
1929
1939
1949
1959
1969
1979
1989
1999
2009

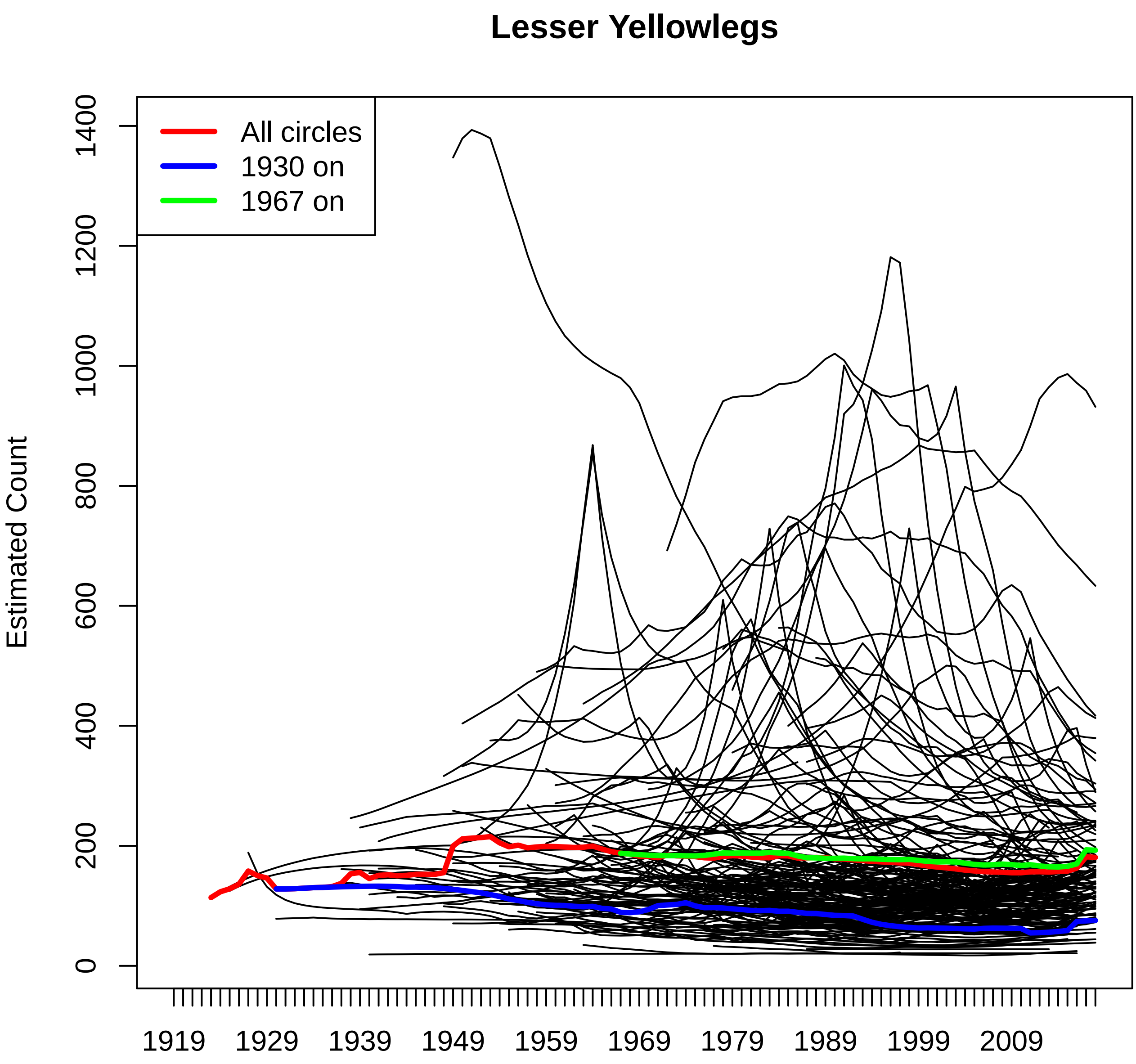

Lesser Yellowlegs
All circles
1930 on
1967 on
Estimated Count
0
200
400
600
800
1000
1200
1400
1919
1929
1939
1949
1959
1969
1979
1989
1999
2009

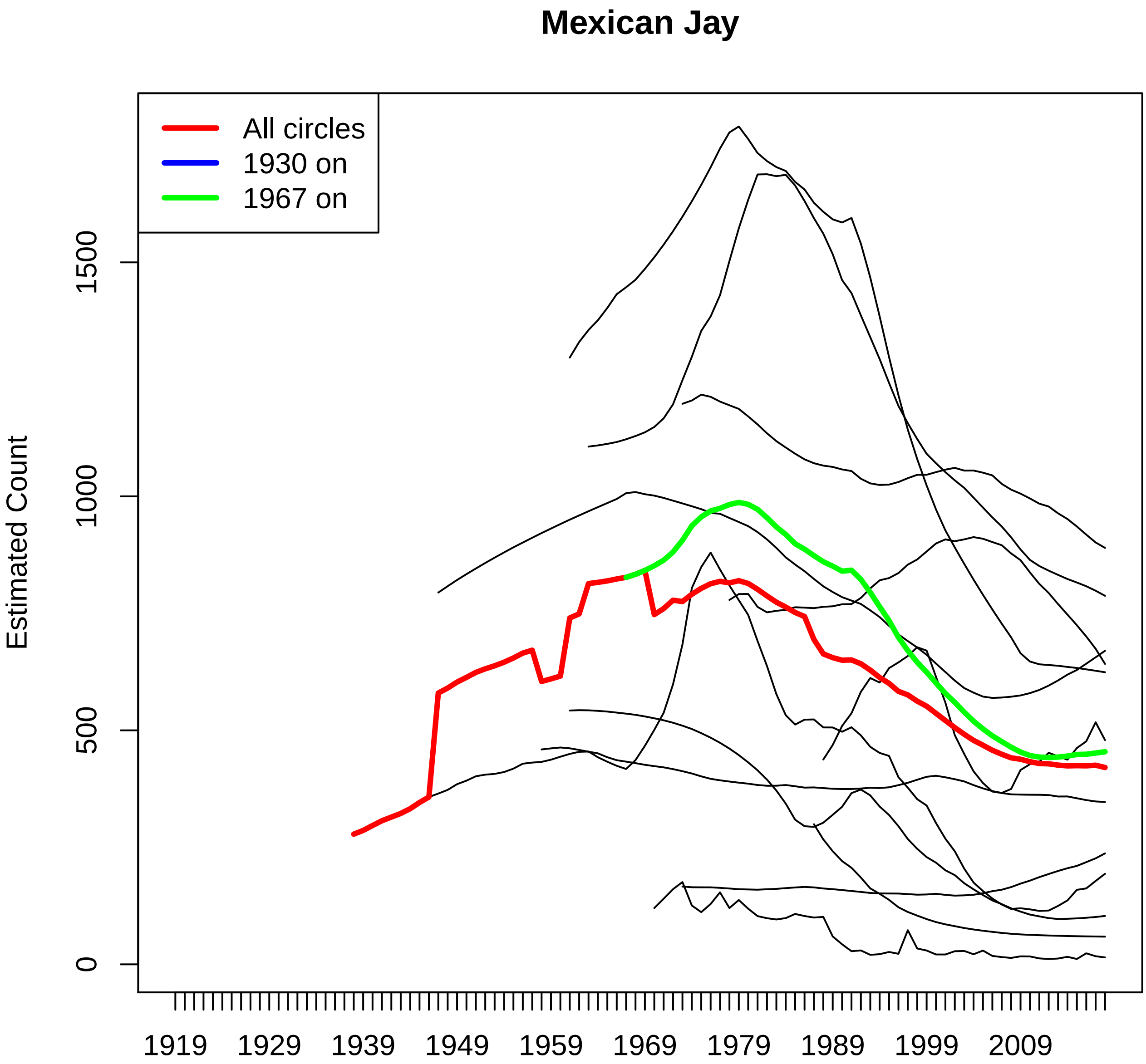

Mexican Jay
All circles
1930 on
1967 on
Estimated Count
0
500
1000
1500
1919
1929
1939
1949
1959
1969
1979
1989
1999
2009

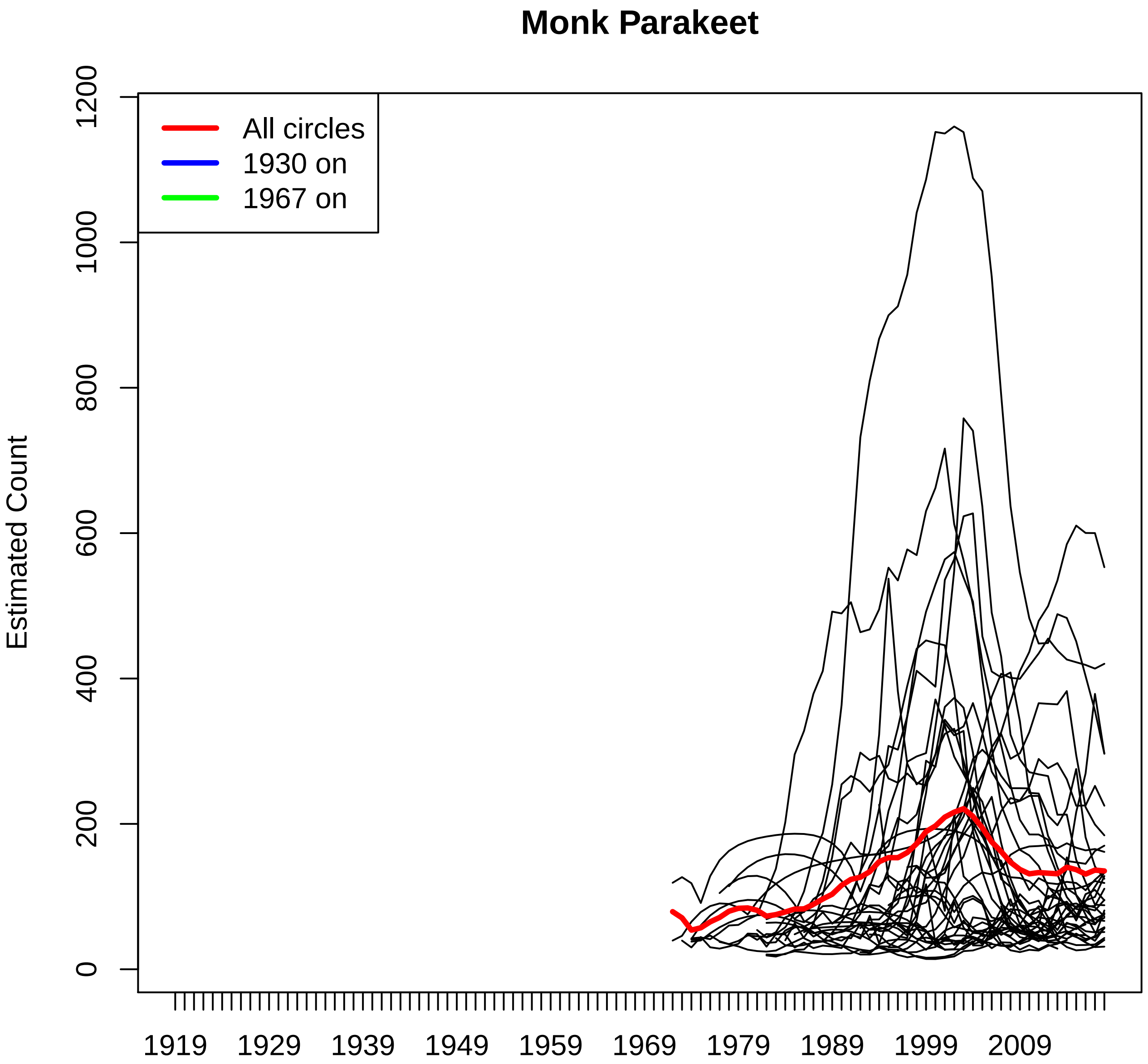
Monk Parakeet
All circles
1930 on
1967 on
Estimated Count
0
200
400
600
800
1000
1200
1919
1929
1939
1949
1959
1969
1979
1989
1999
2009

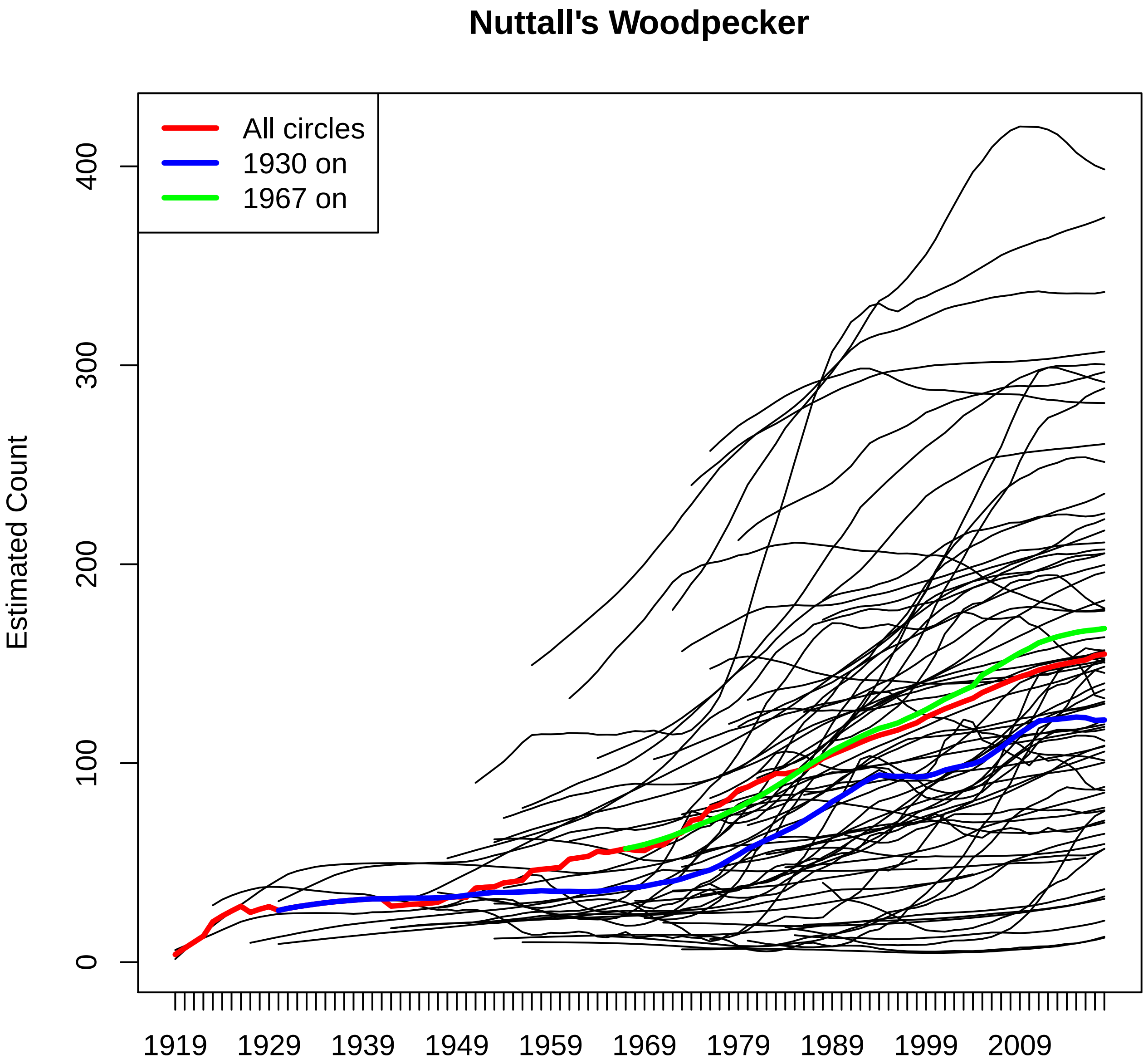

Nuttall's Woodpecker
All circles
1930 on
1967 on
Estimated Count
0
100
200
300
400
1919
1929
1939
1949
1959
1969
1979
1989
1999
2009

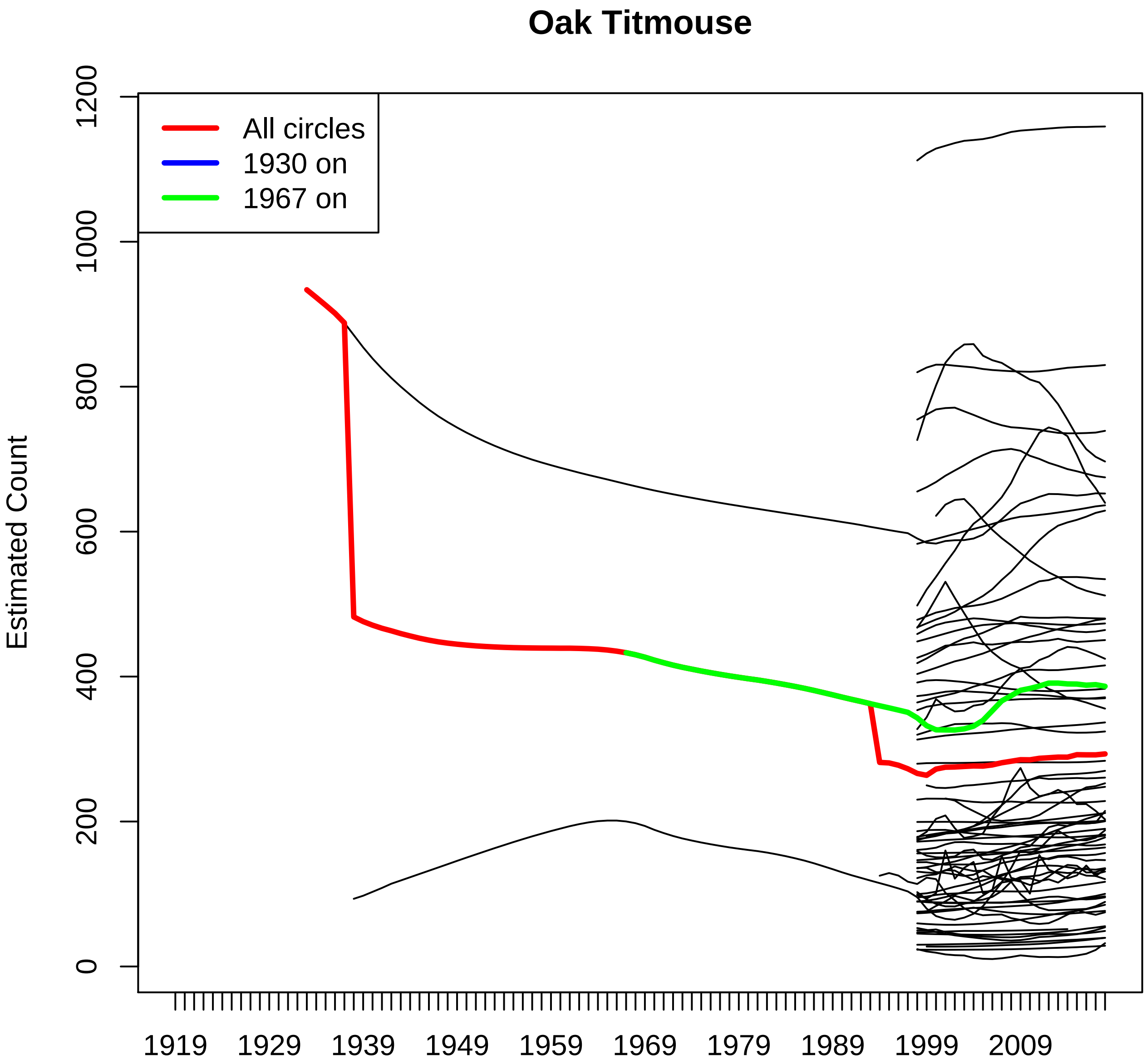

Oak Titmouse
All circles
1930 on
1967 on
Estimated Count
0
200
400
600
800
1000
1200
1919
1929
1939
1949
1959
1969
1979
1989
1999
2009

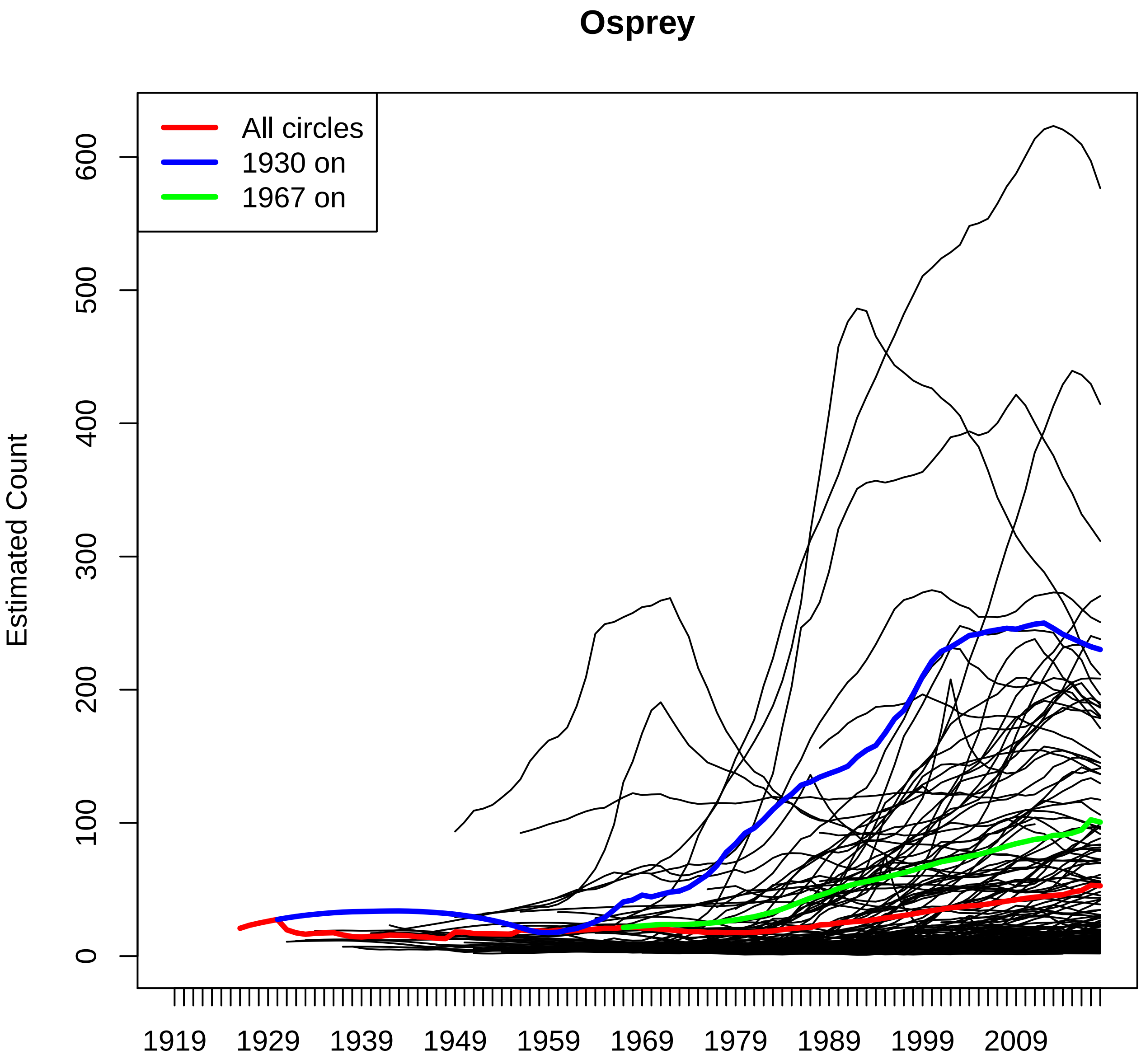
Osprey
All circles
1930 on
1967 on
Estimated Count
0
100
200
300
400
500
600
1919
1929
1939
1949
1959
1969
1979
1989
1999
2009

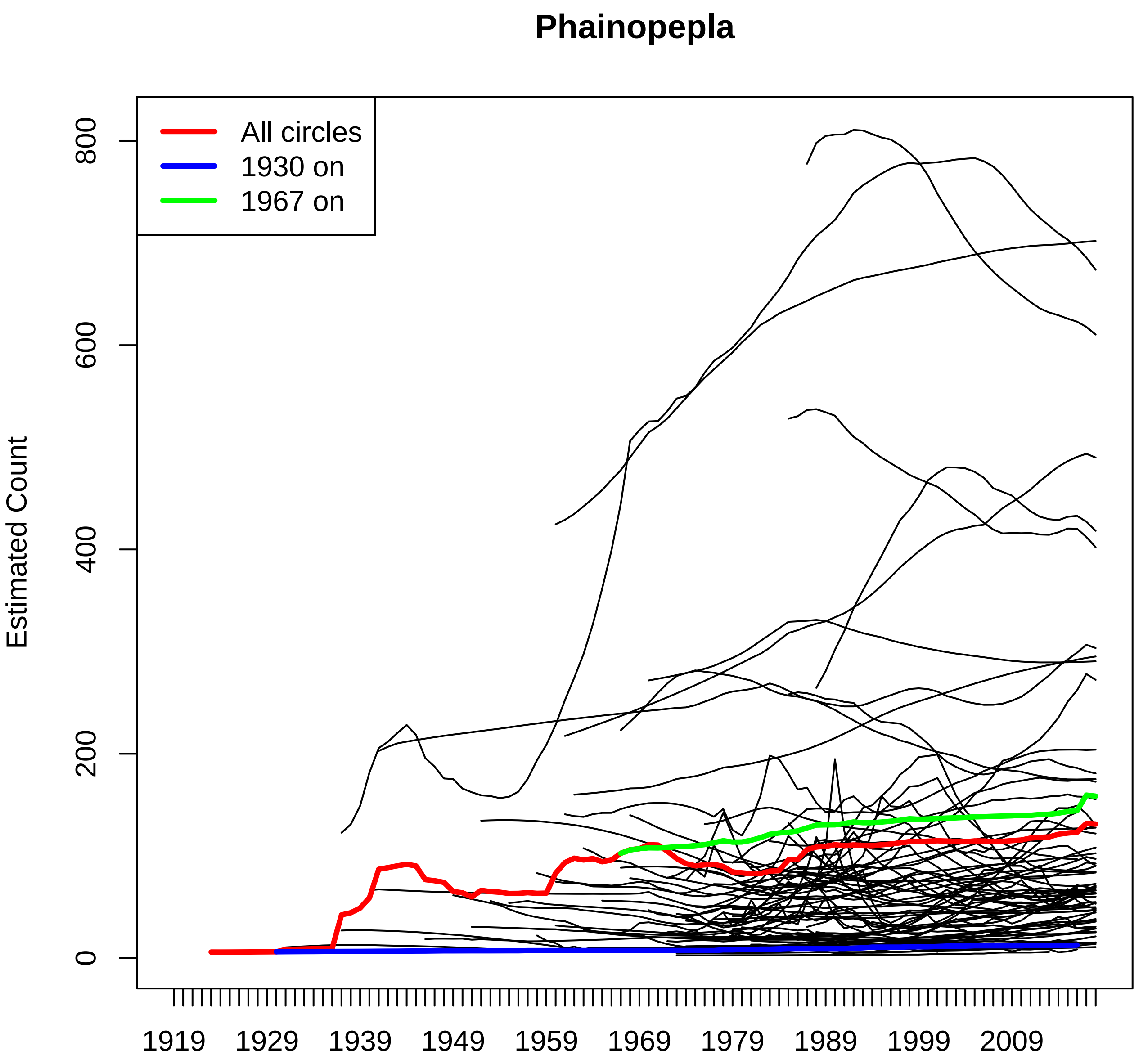

Phainopepla
Estimated Count
All circles
1930 on
1967 on
0
200
400
600
800
1919
1929
1939
1949
1959
1969
1979
1989
1999
2009

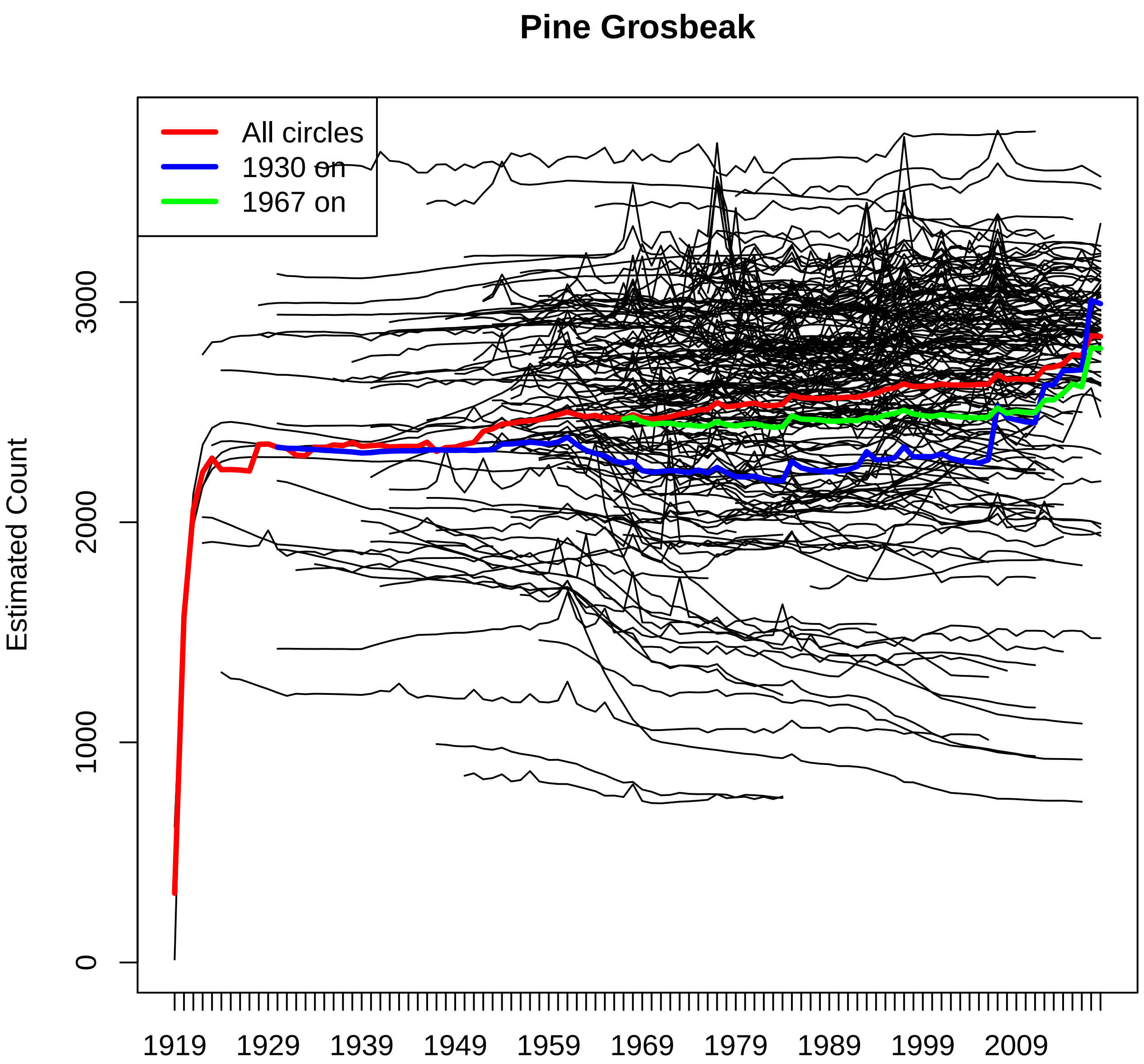

Pine Grosbeak
All circles
1930 on
1967 on
Estimated Count
0
1000
2000
3000
1919
1929
1939
1949
1959
1969
1979
1989
1999
2009

Pyrrhuloxia

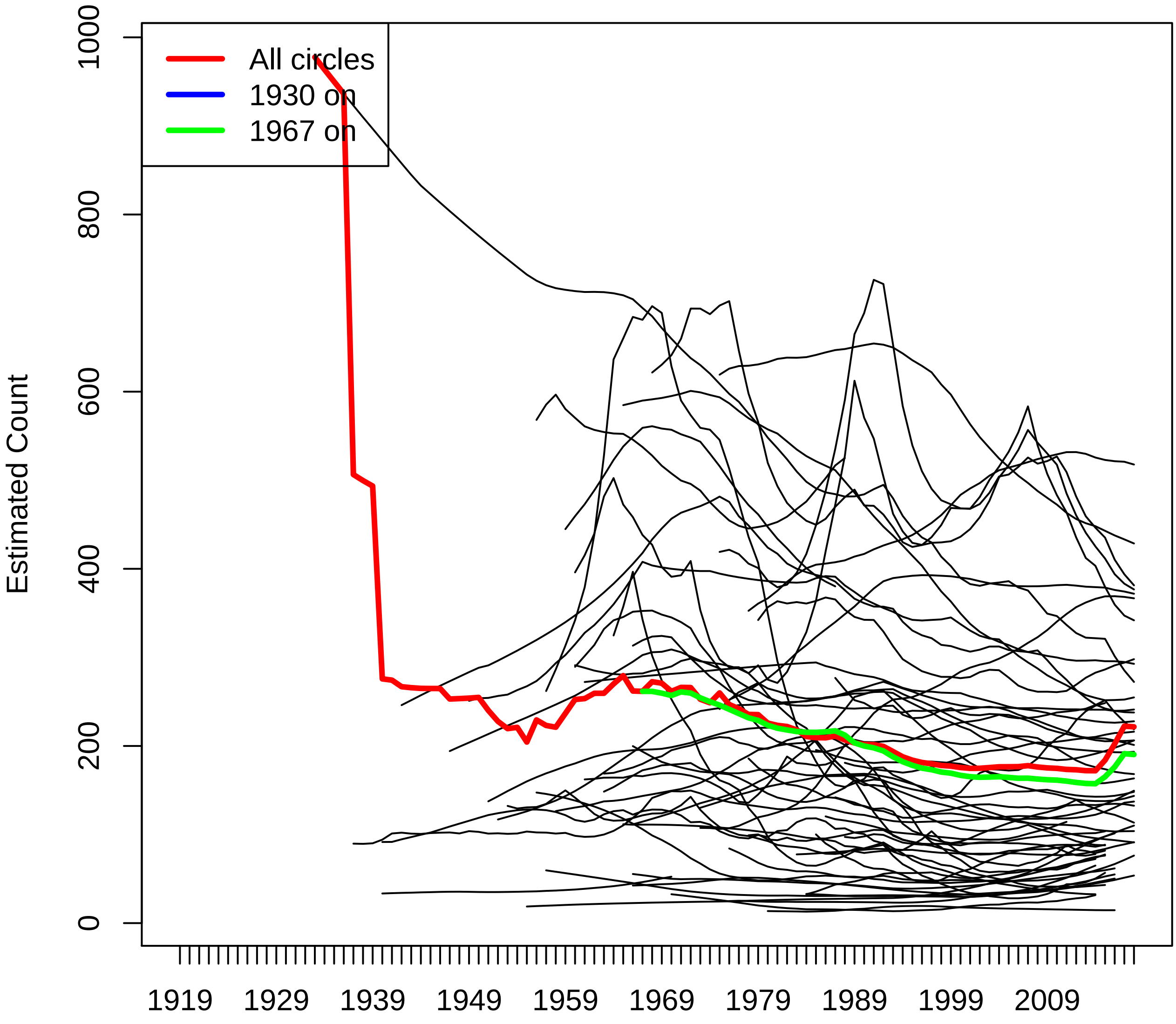

Red−necked Grebe

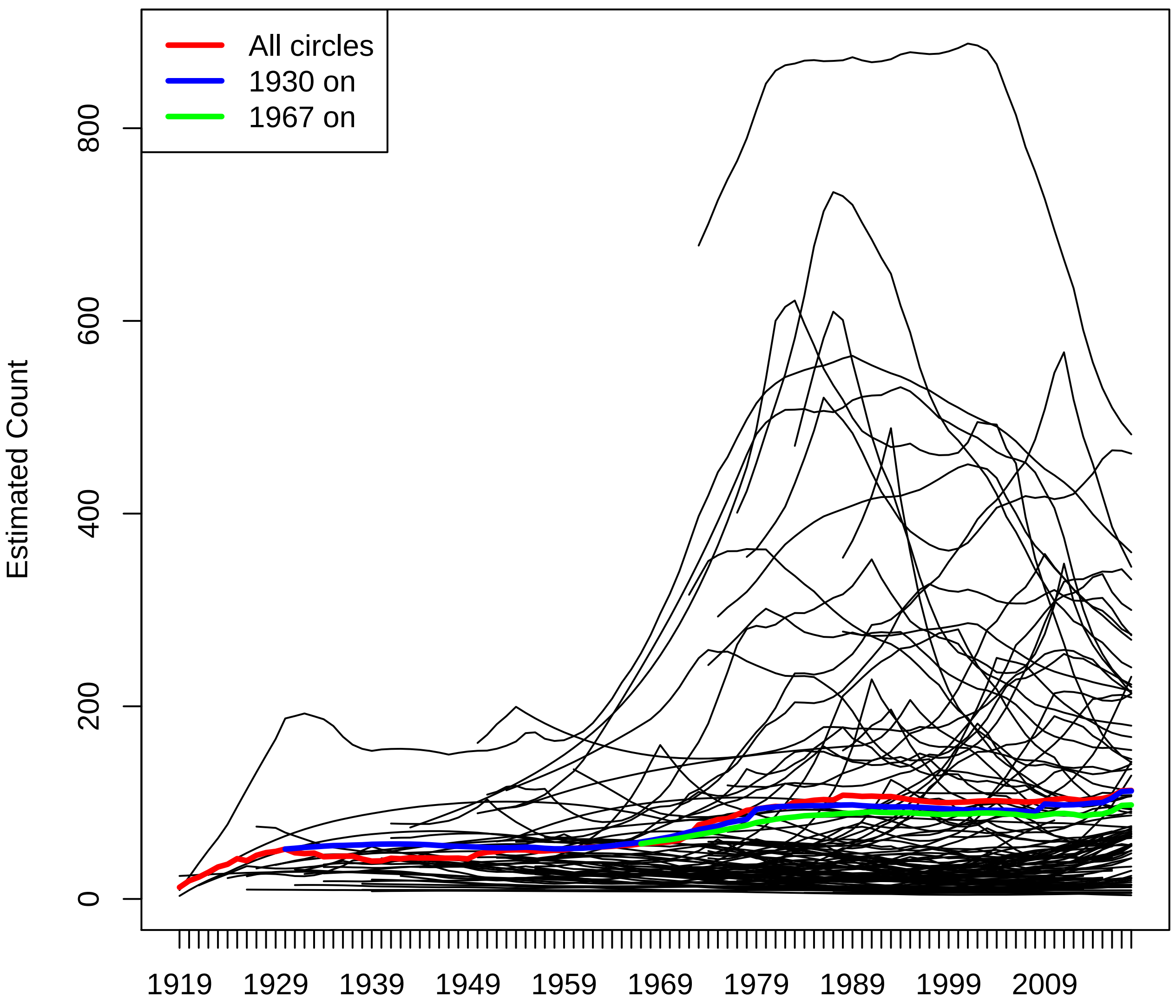

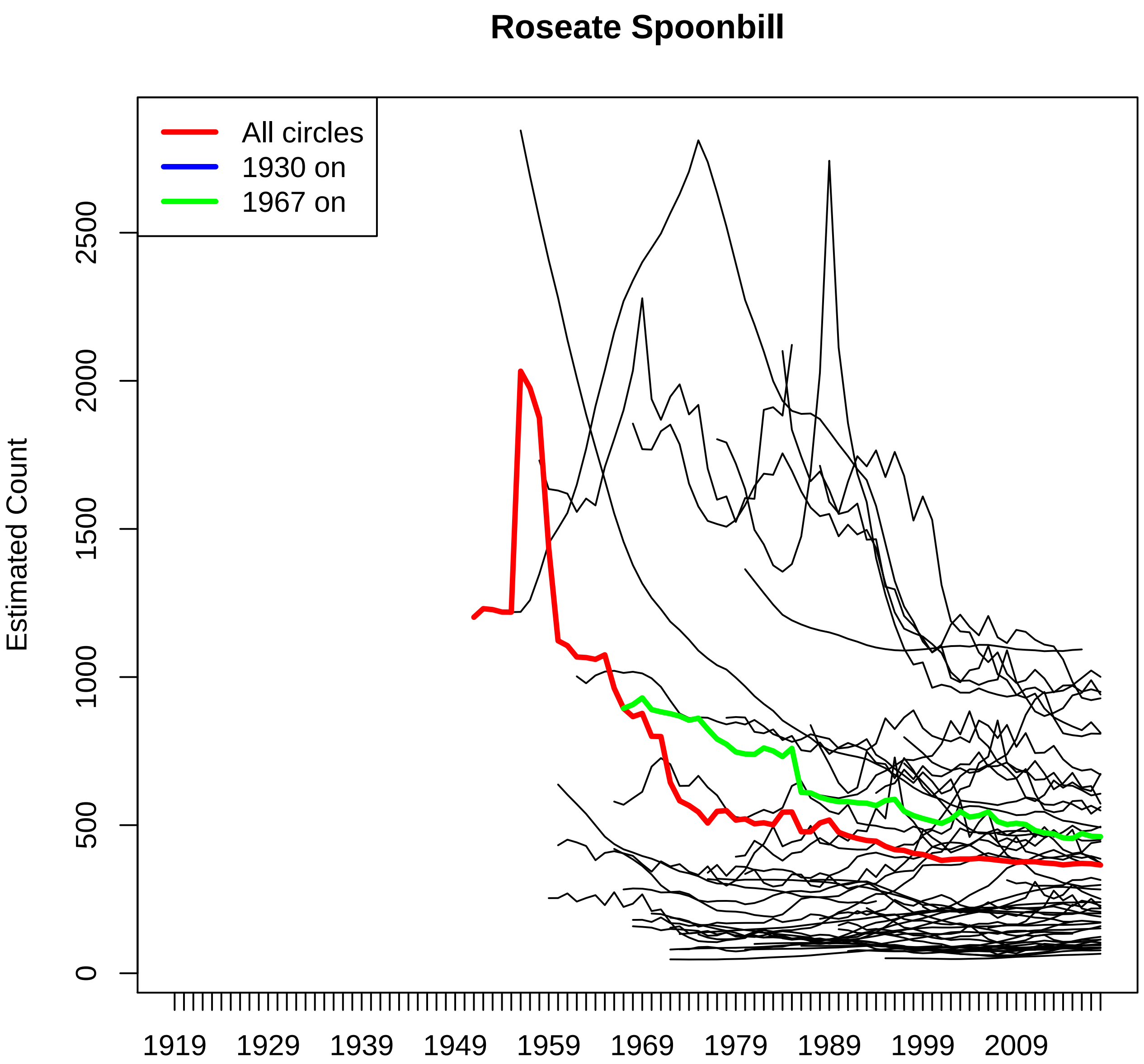
Roseate Spoonbill
All circles
1930 on
1967 on
Estimated Count
0
500
1000
1500
2000
2500
1919
1929
1939
1949
1959
1969
1979
1989
1999
2009

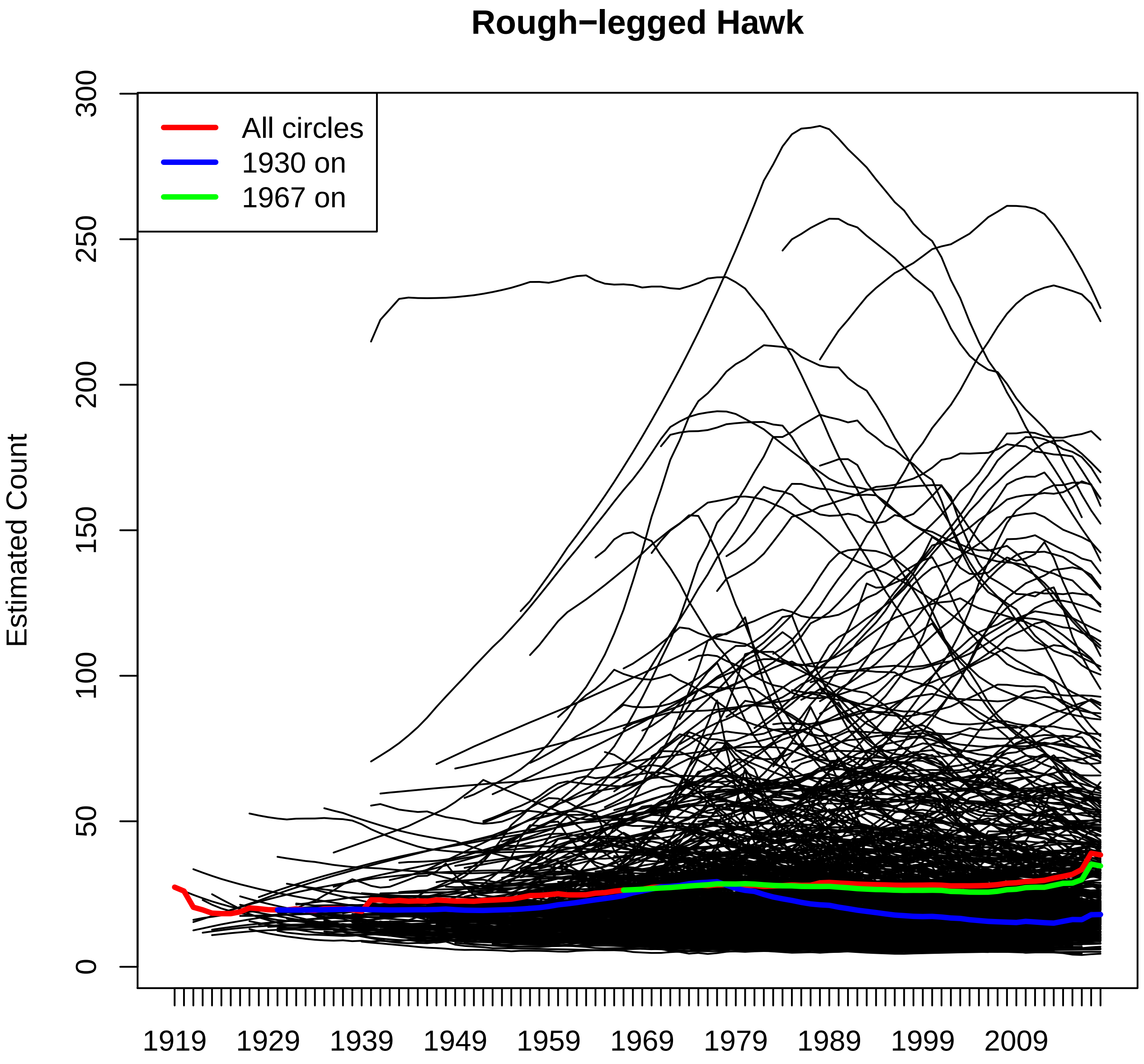

Rough−legged Hawk
Estimated Count
0
50
100
150
200
250
300
All circles
1930 on
1967 on
1919
1929
1939
1949
1959
1969
1979
1989
1999
2009

Ruffed Grouse

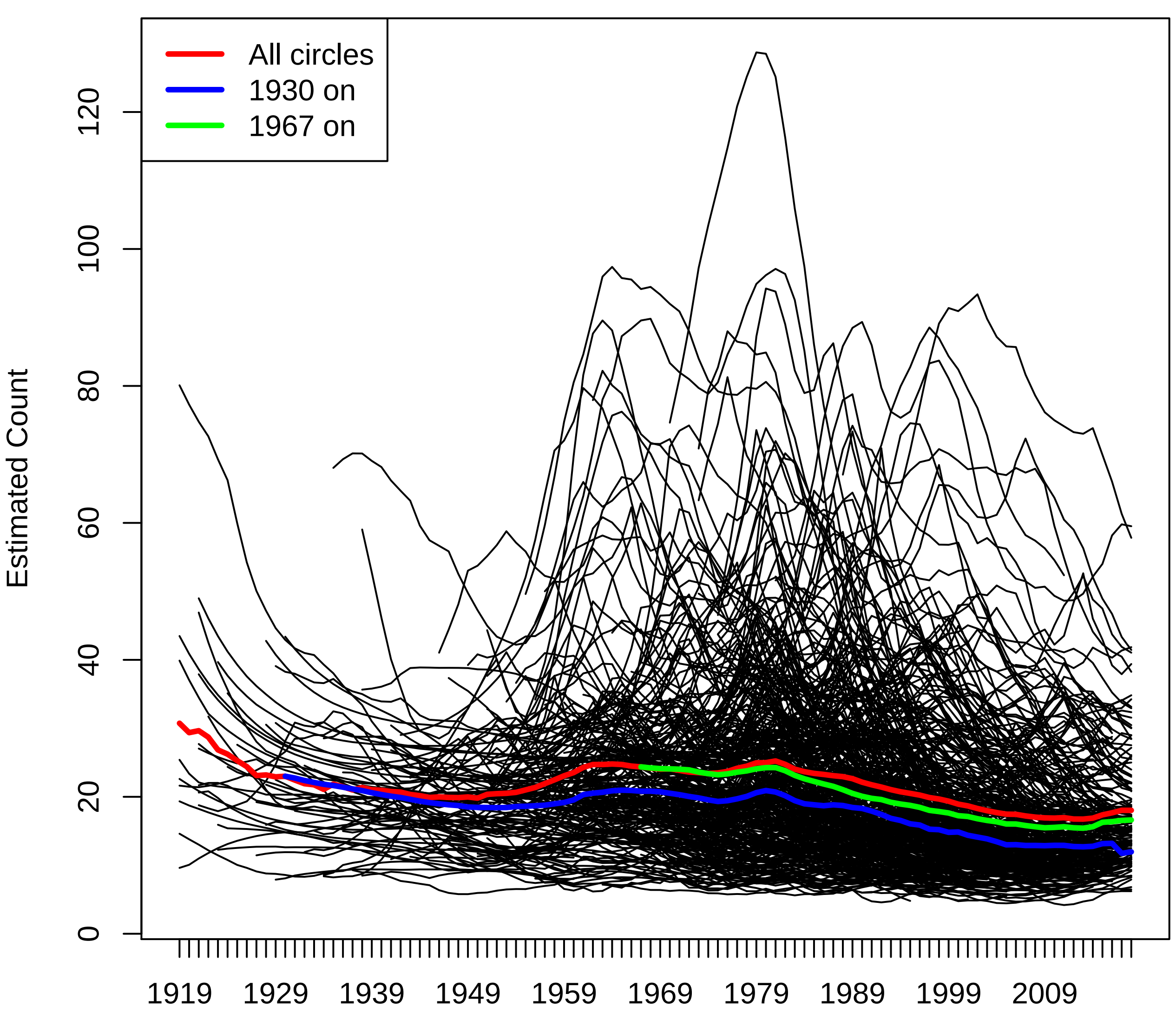

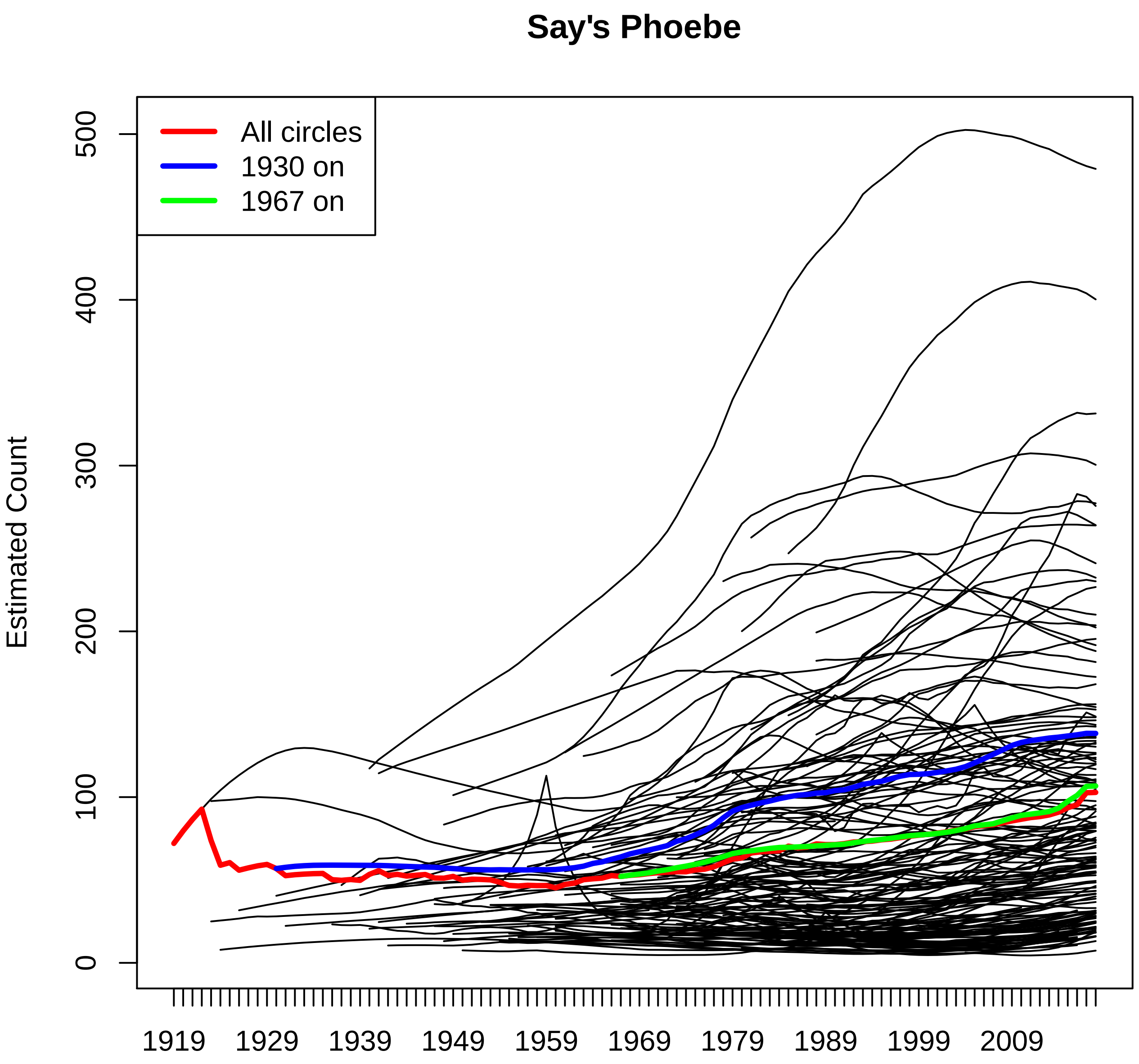

Say's Phoebe
All circles
1930 on
1967 on
Estimated Count
0
100
200
300
400
500
1919
1929
1939
1949
1959
1969
1979
1989
1999
2009

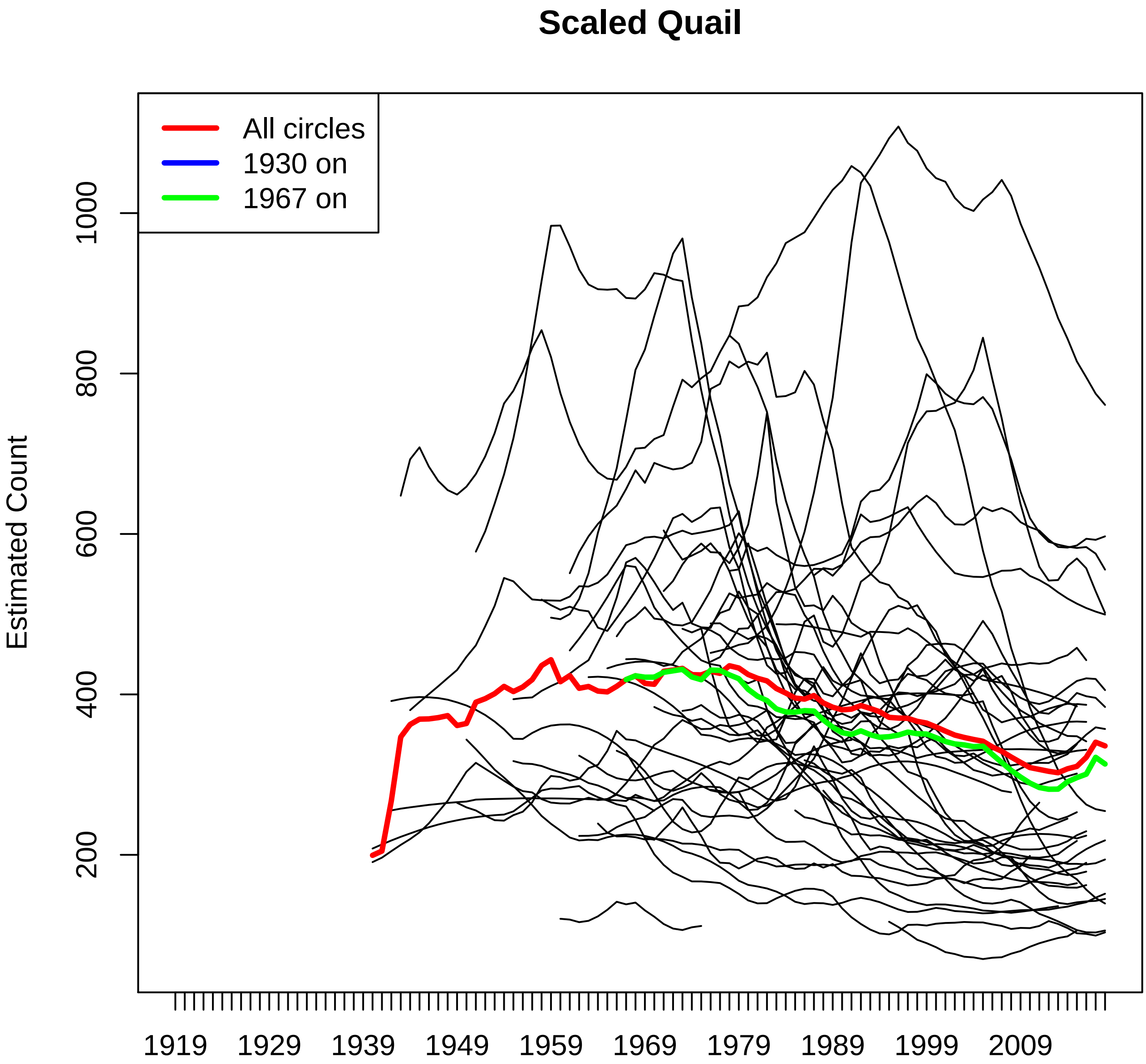

Scaled Quail
All circles
1930 on
1967 on
Estimated Count
200
400
600
800
1000
1919
1929
1939
1949
1959
1969
1979
1989
1999
2009

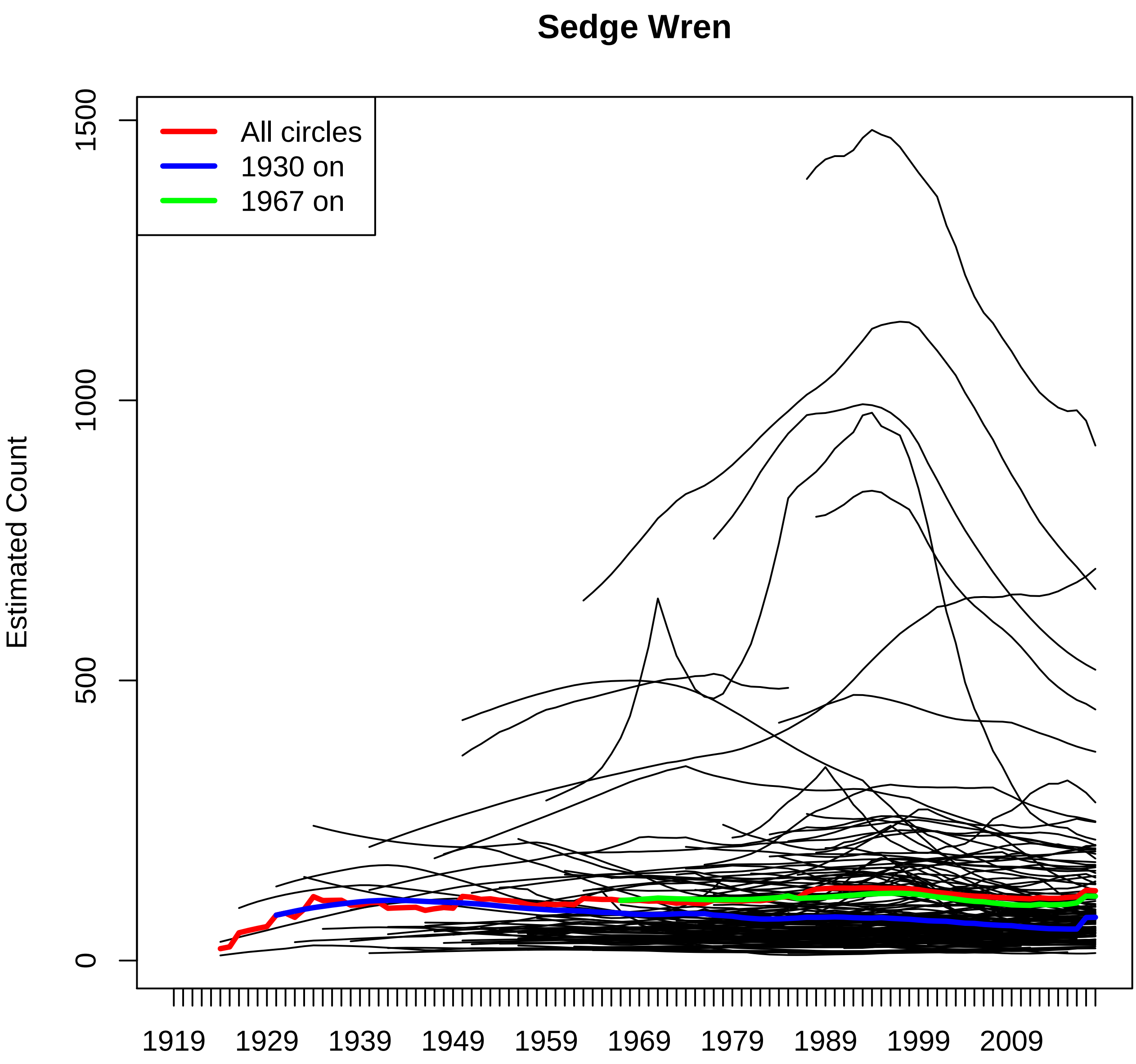
Sedge Wren
Estimated Count
0
500
1000
1500
All circles
1930 on
1967 on
1919
1929
1939
1949
1959
1969
1979
1989
1999
2009

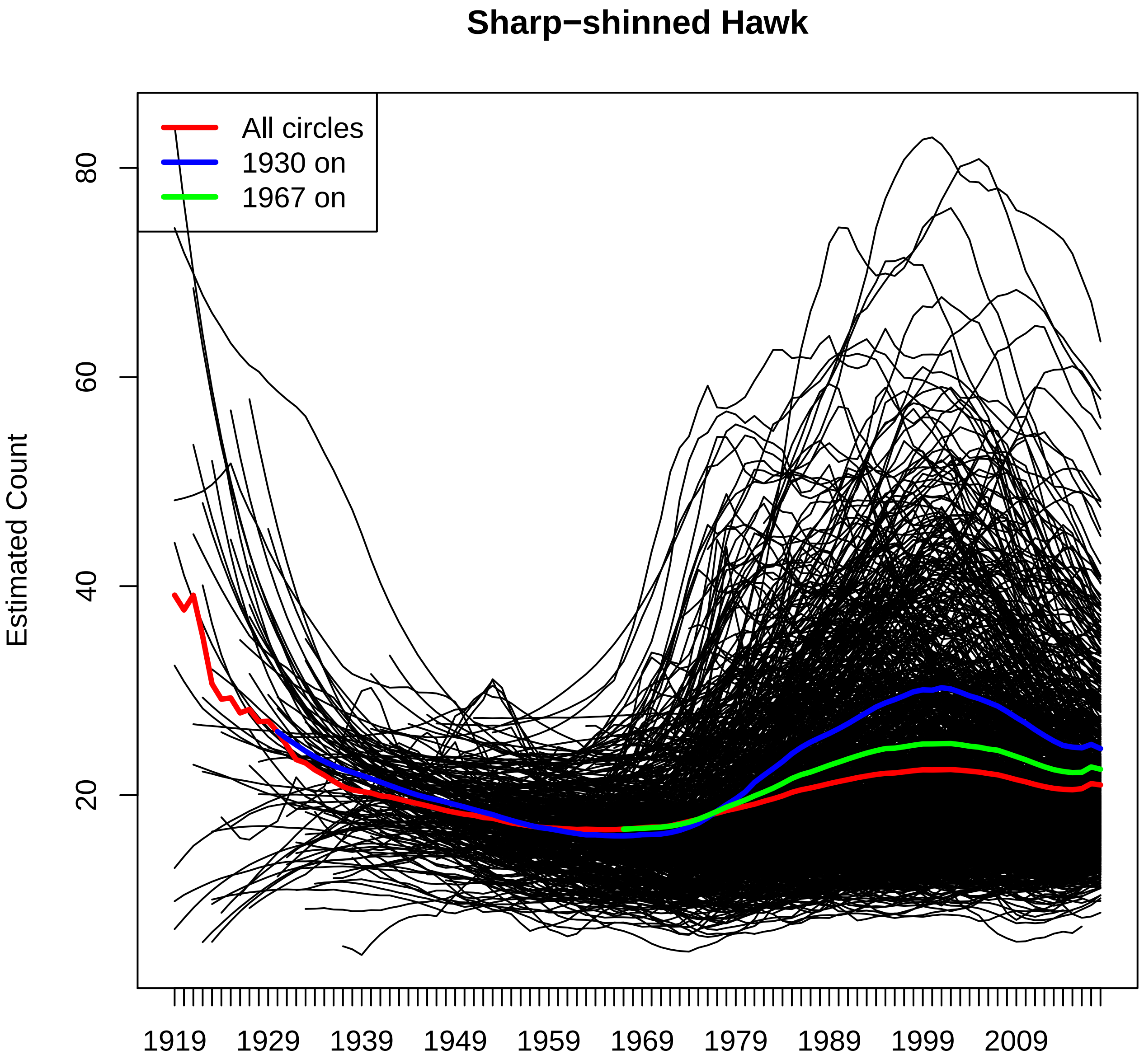
Sharp−shinned Hawk
All circles
1930 on
1967 on
Estimated Count
20
40
60
80
1919
1929
1939
1949
1959
1969
1979
1989
1999
2009

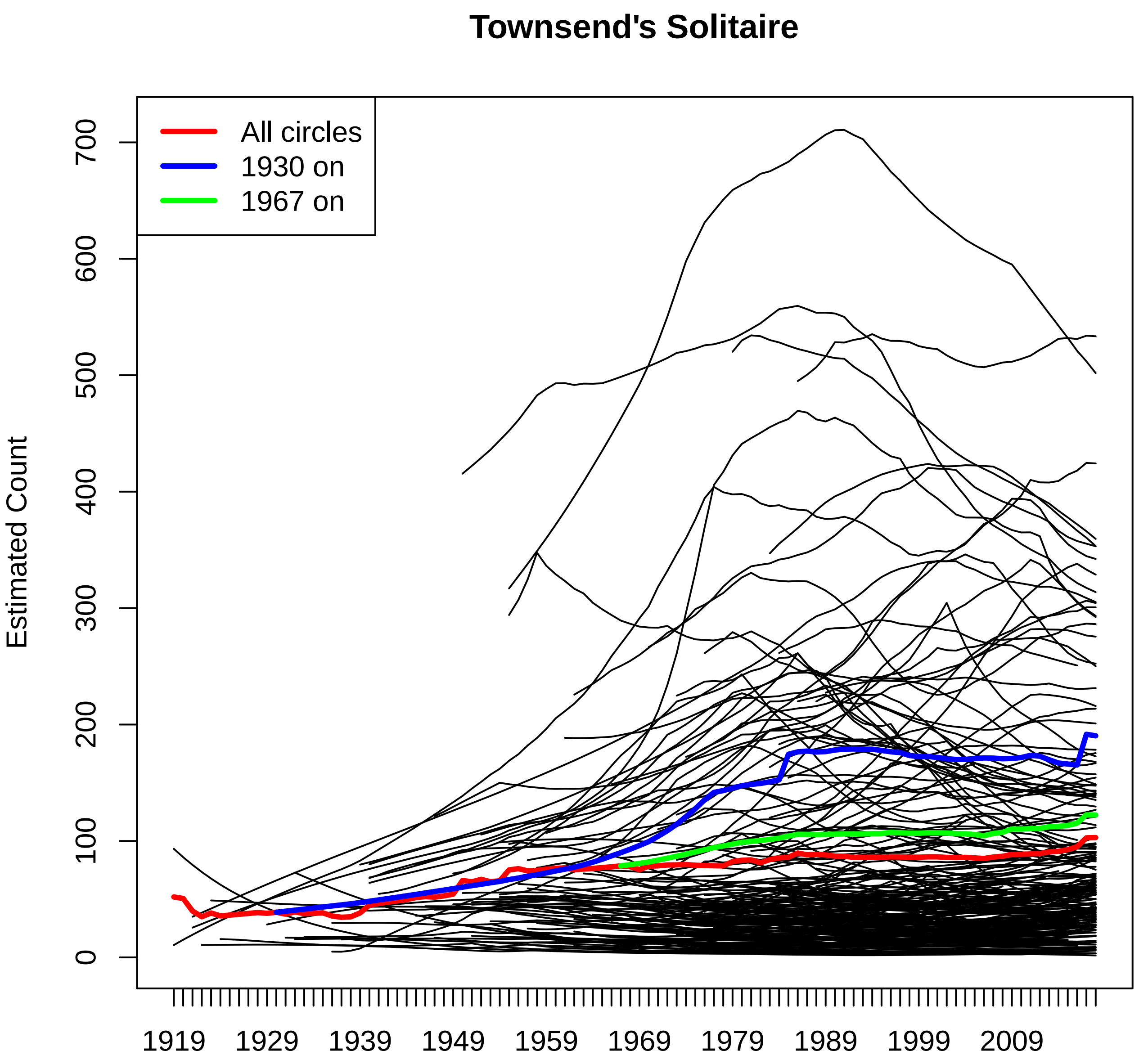
Townsend's Solitaire
All circles
1930 on
1967 on
Estimated Count
0
100
200
300
400
500
600
700
1919
1929
1939
1949
1959
1969
1979
1989
1999
2009

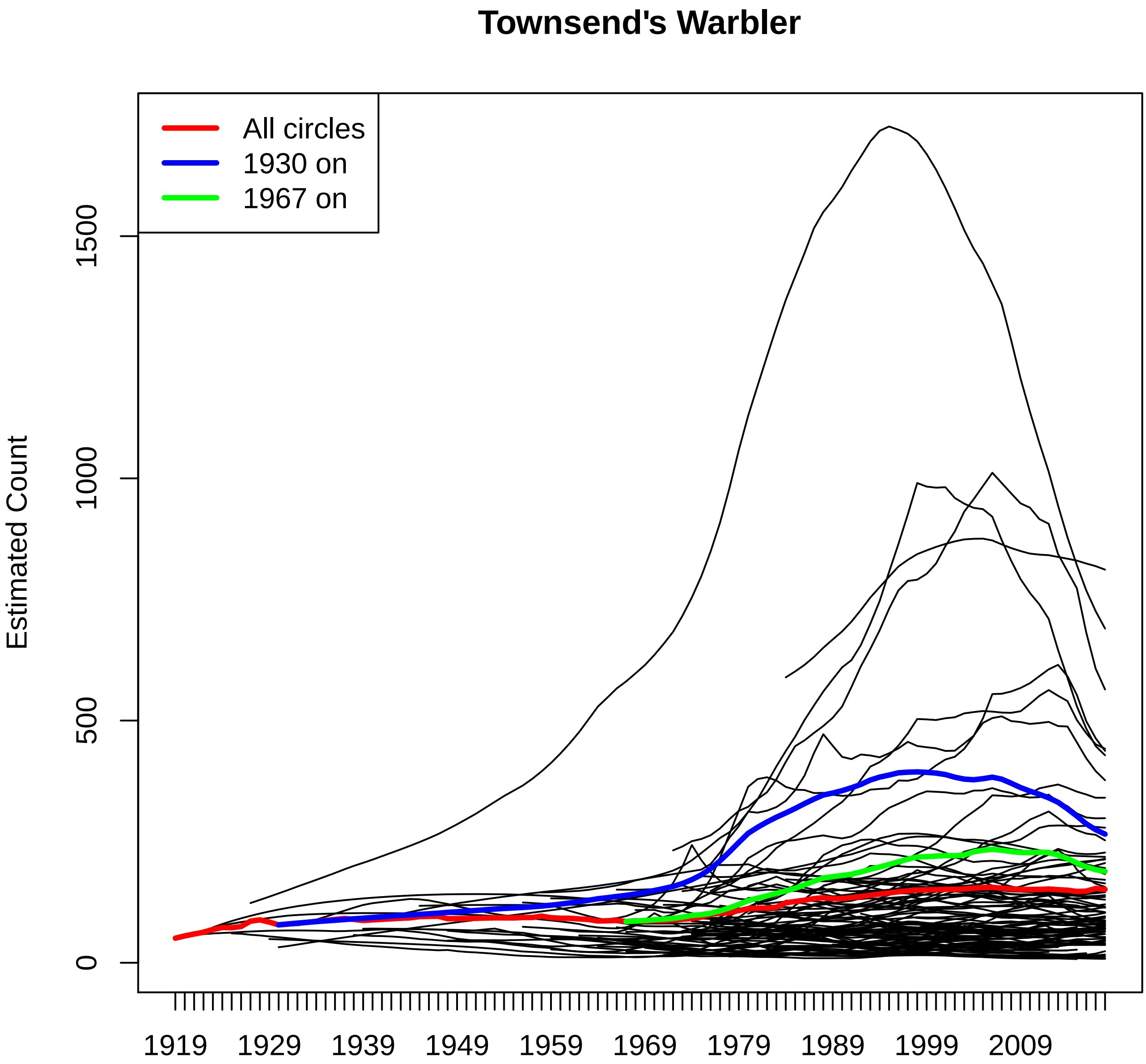

Townsend's Warbler
All circles
1930 on
1967 on
Estimated Count
0
500
1000
1500
1919
1929
1939
1949
1959
1969
1979
1989
1999
2009

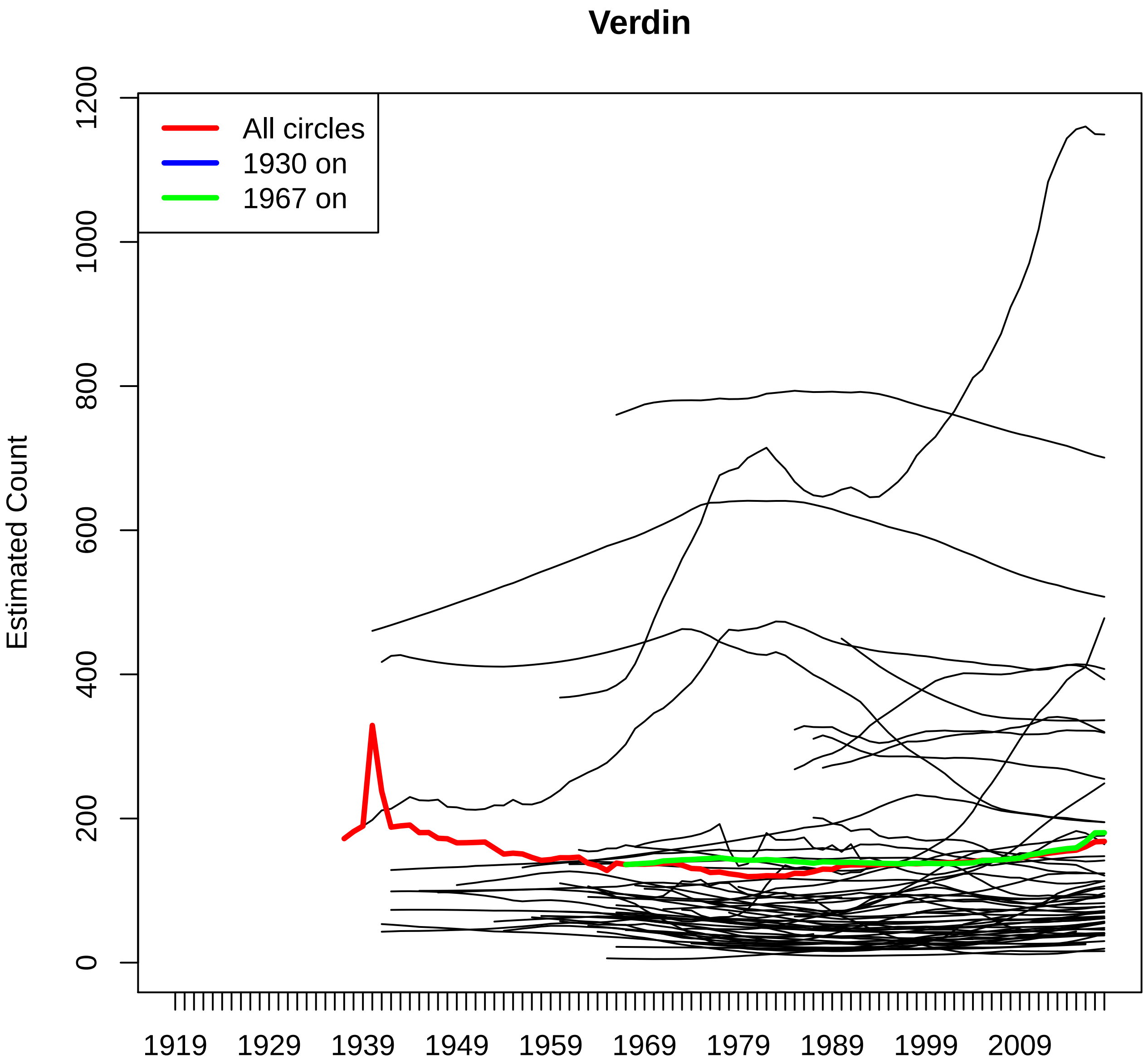

Verdin
All circles
1930 on
1967 on
Estimated Count
0
200
400
600
800
1000
1200
1919
1929
1939
1949
1959
1969
1979
1989
1999
2009

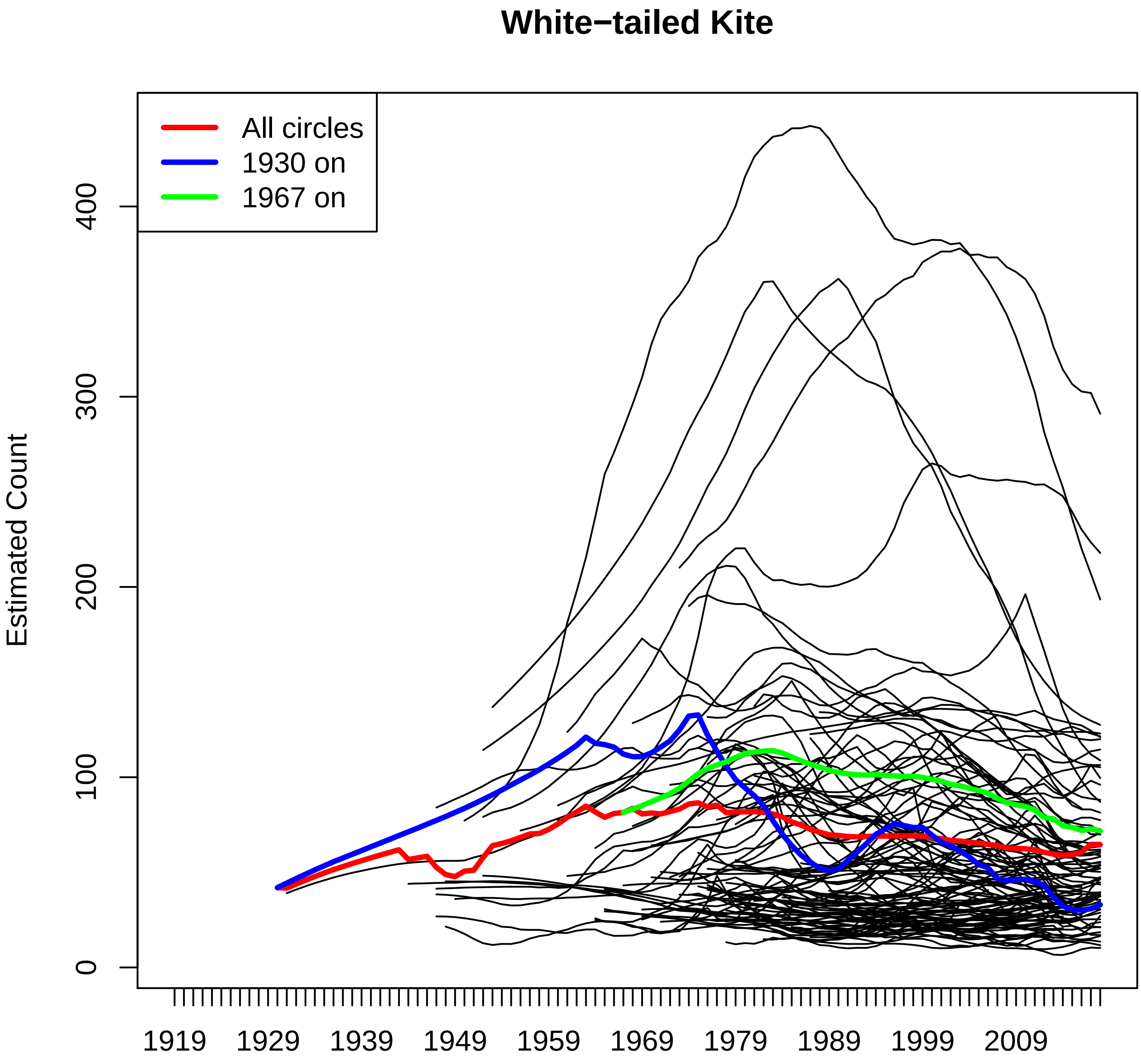

White−tailed Kite
All circles
1930 on
1967 on
Estimated Count
0
100
200
300
400
1919
1929
1939
1949
1959
1969
1979
1989
1999
2009

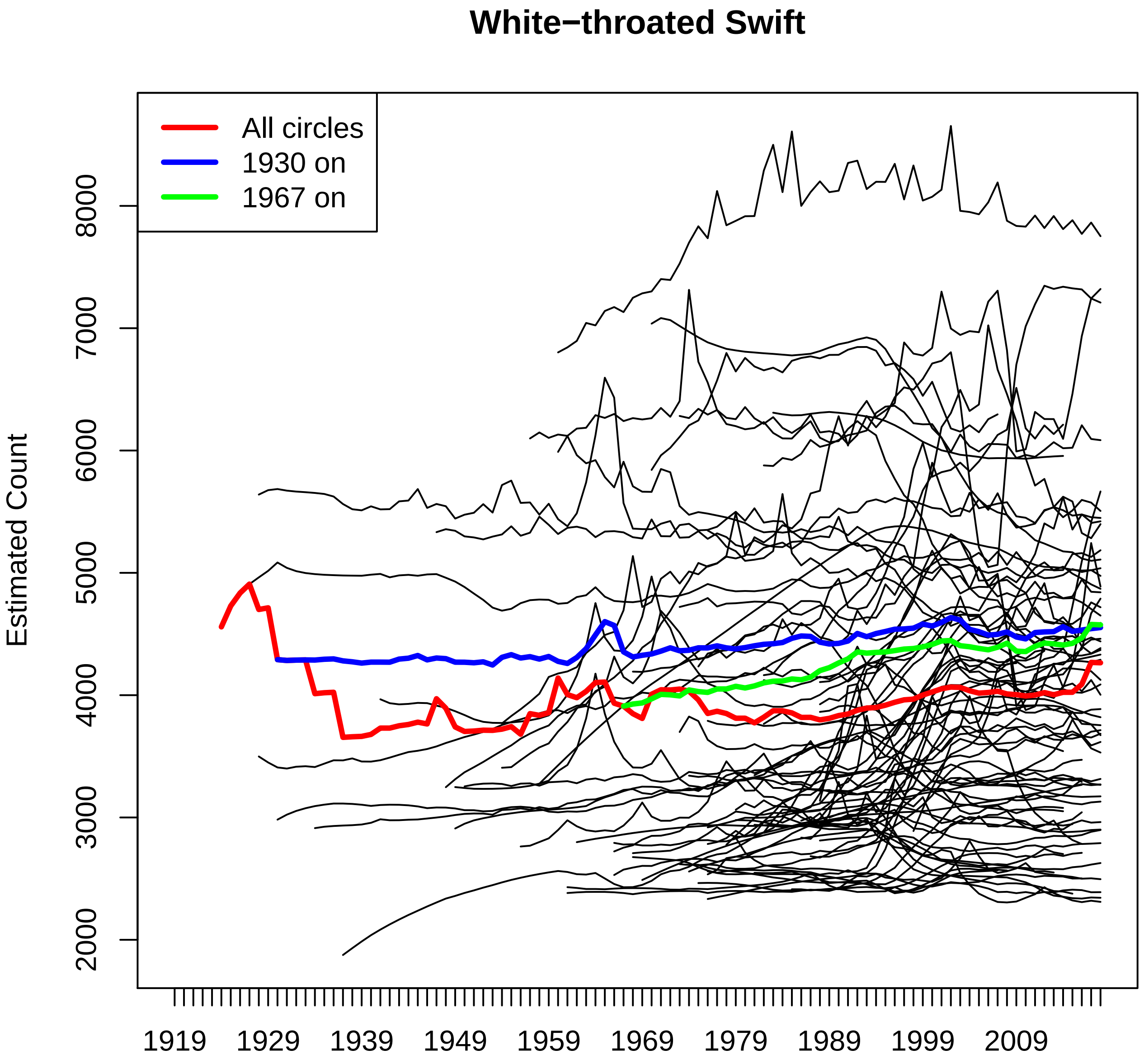
White−throated Swift
All circles
1930 on
1967 on
Estimated Count
2000
3000
4000
5000
6000
7000
8000
1919
1929
1939
1949
1959
1969
1979
1989
1999
2009

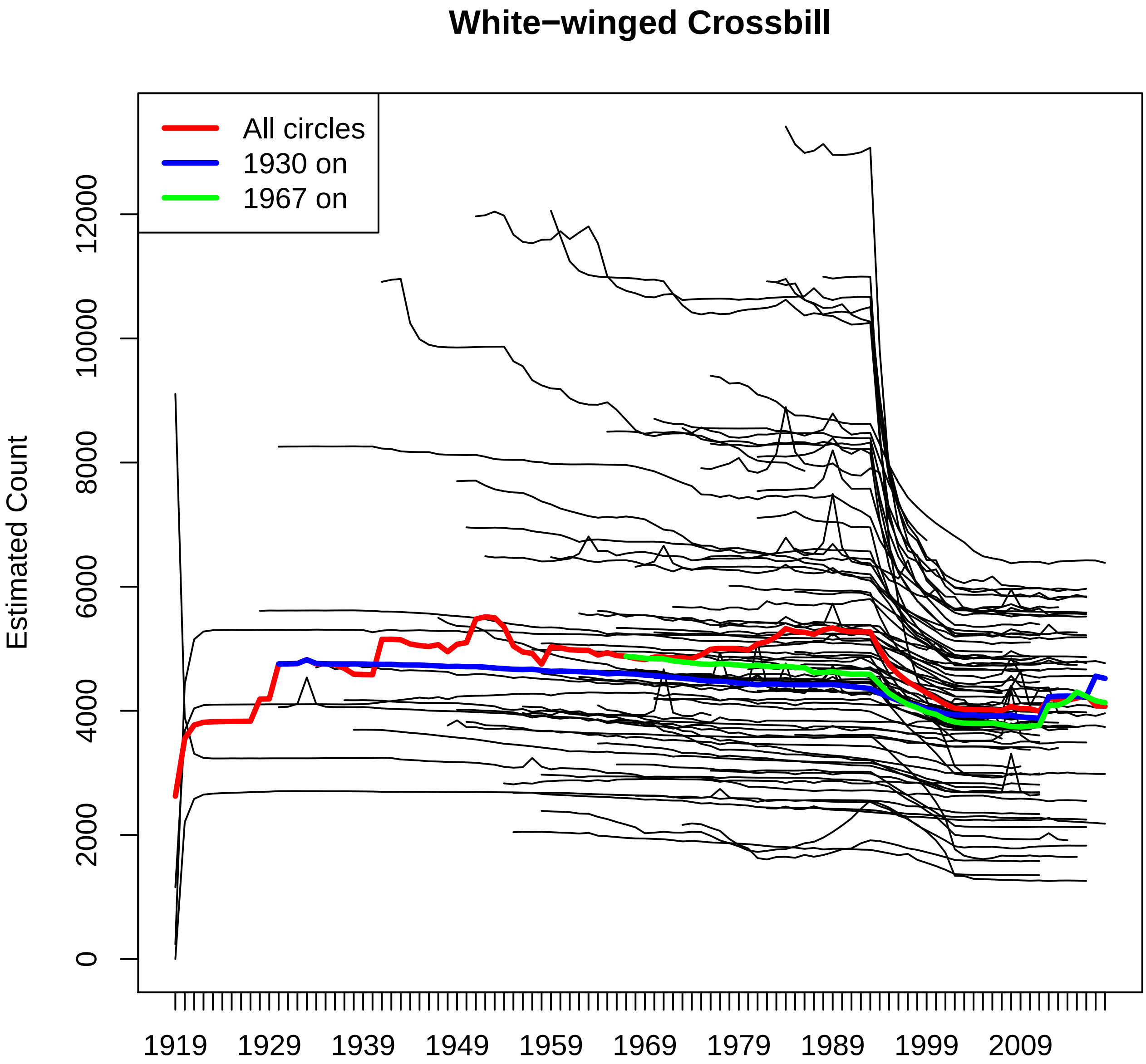
White−winged Crossbill
All circles
1930 on
1967 on
Estimated Count
0
2000
4000
6000
8000
10000
12000
1919
1929
1939
1949
1959
1969
1979
1989
1999
2009

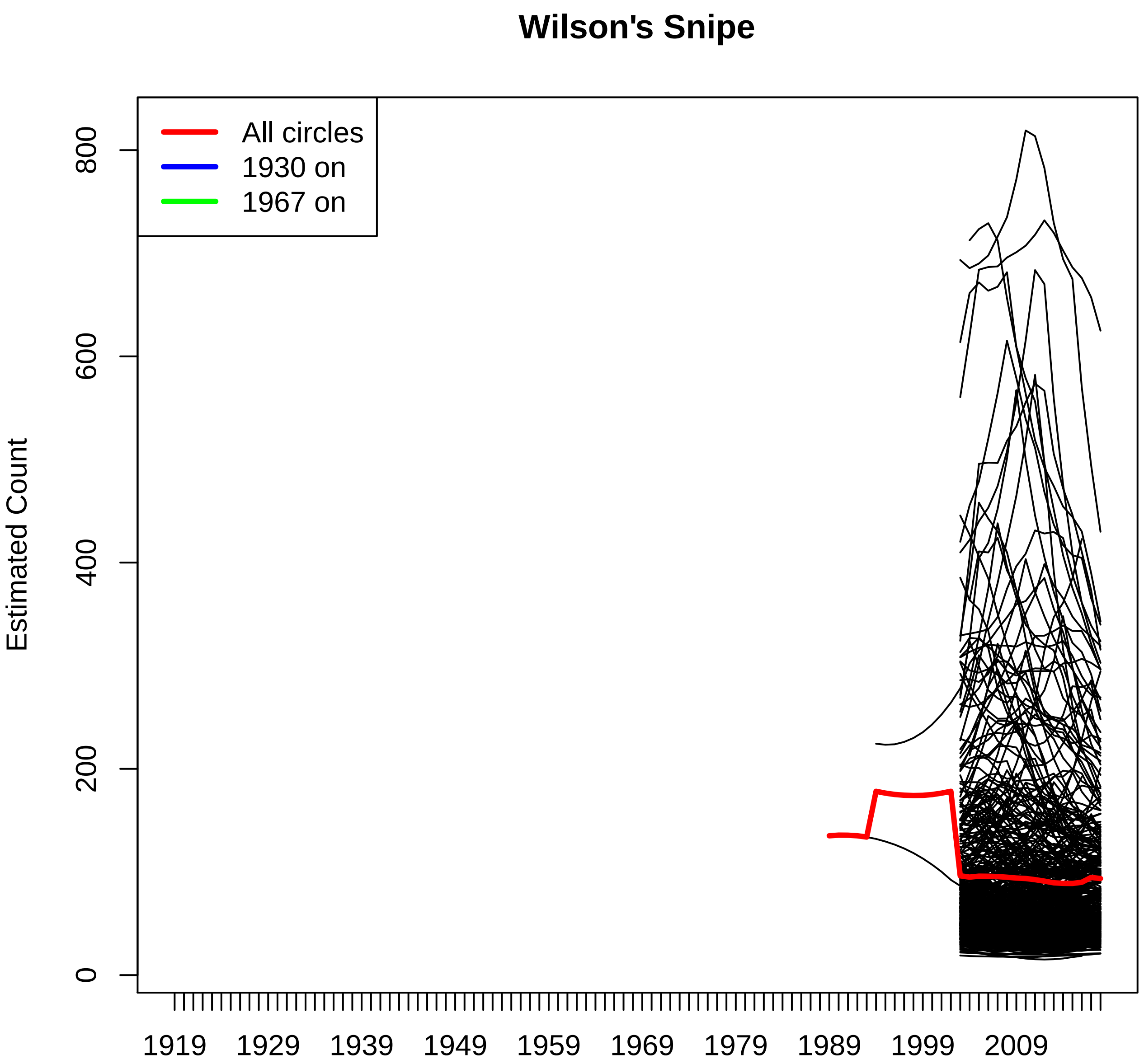
Wilson's Snipe
Estimated Count
All circles
1930 on
1967 on
0
200
400
600
800
1919
1929
1939
1949
1959
1969
1979
1989
1999
2009

Abert's Towhee

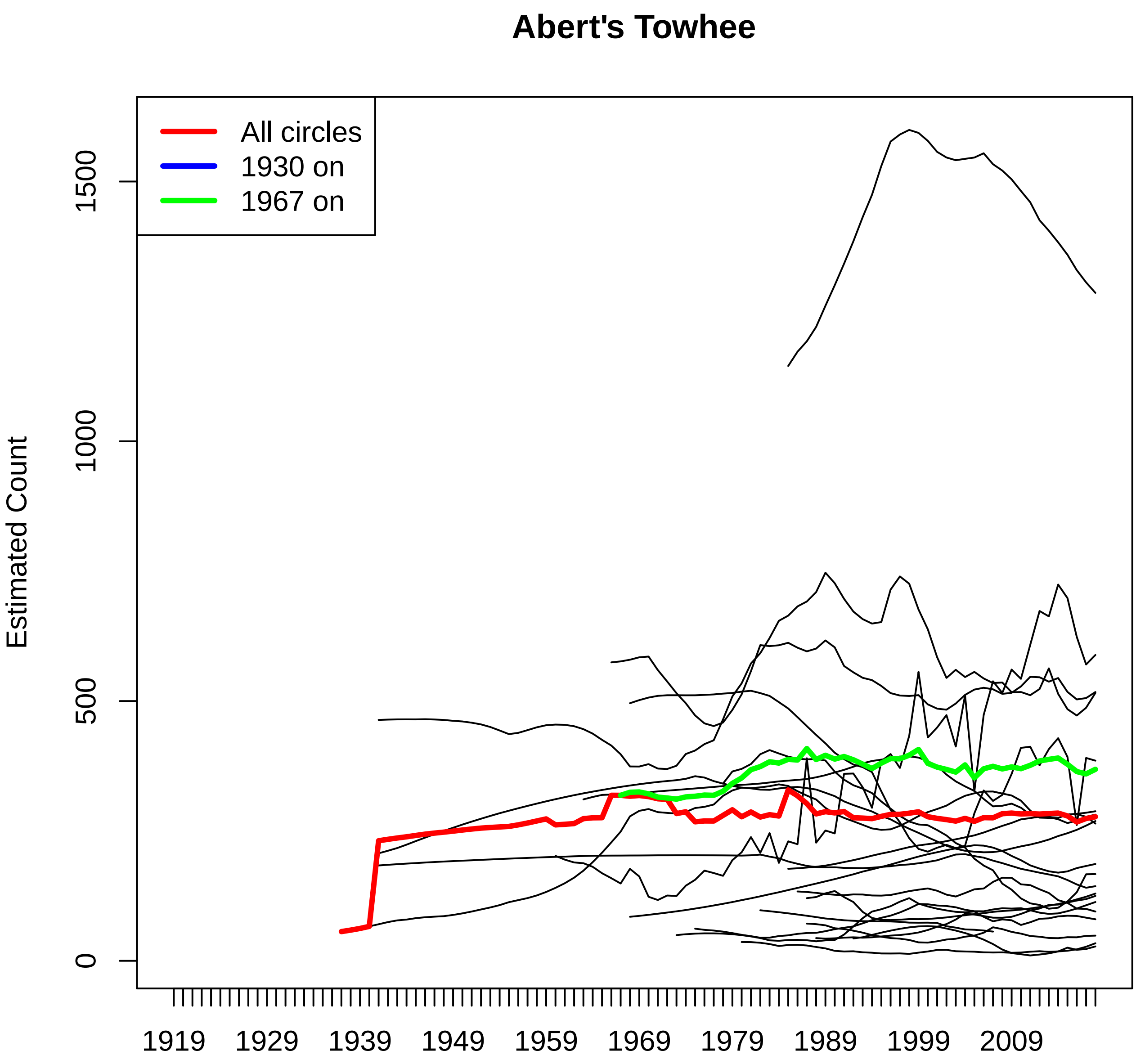

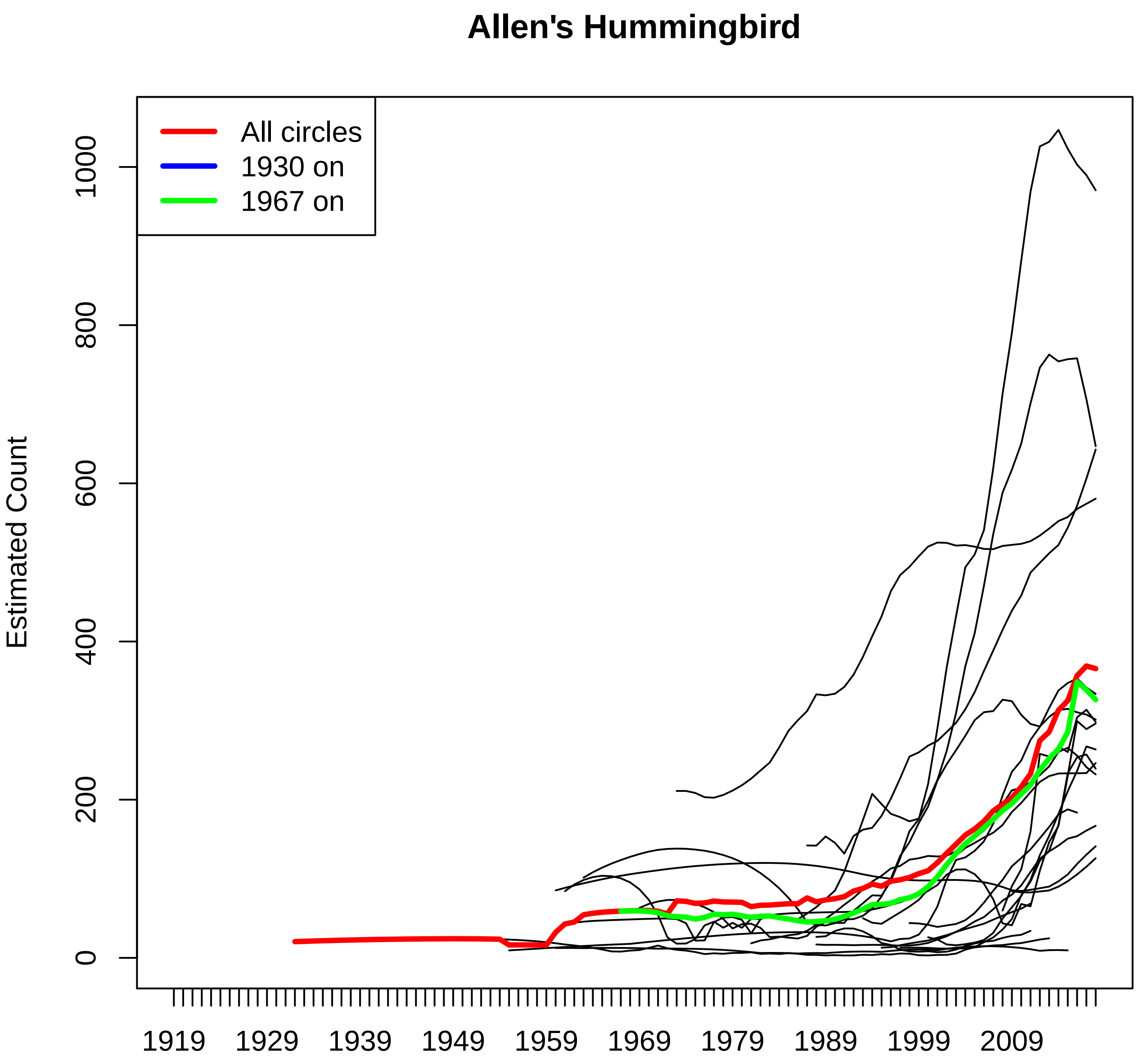

Allen's Hummingbird
All circles
1930 on
1967 on
Estimated Count
0
200
400
600
800
1000
1919
1929
1939
1949
1959
1969
1979
1989
1999
2009

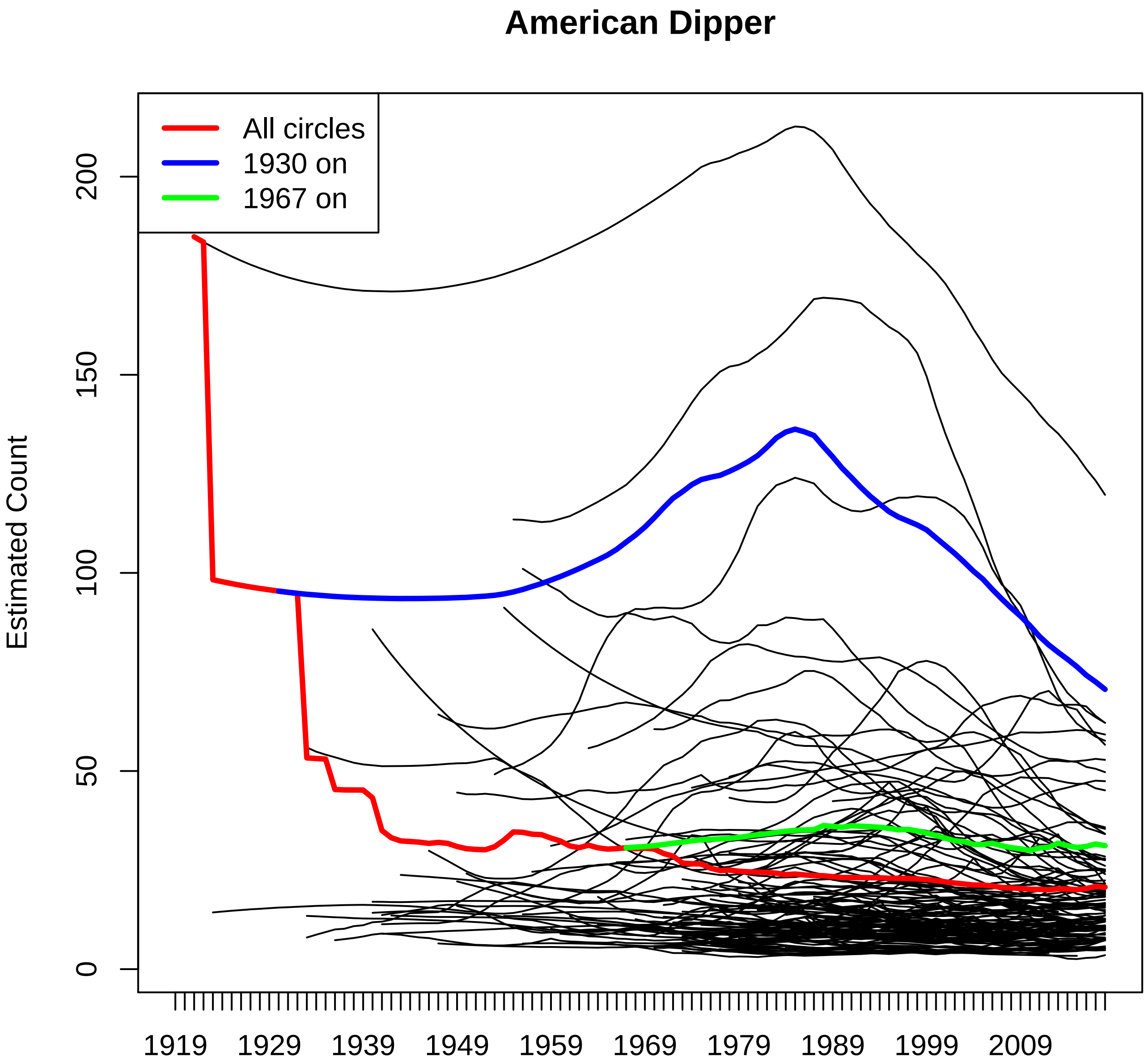

American Dipper
All circles
1930 on
1967 on
Estimated Count
0
50
100
150
200
1919
1929
1939
1949
1959
1969
1979
1989
1999
2009

American Woodcock

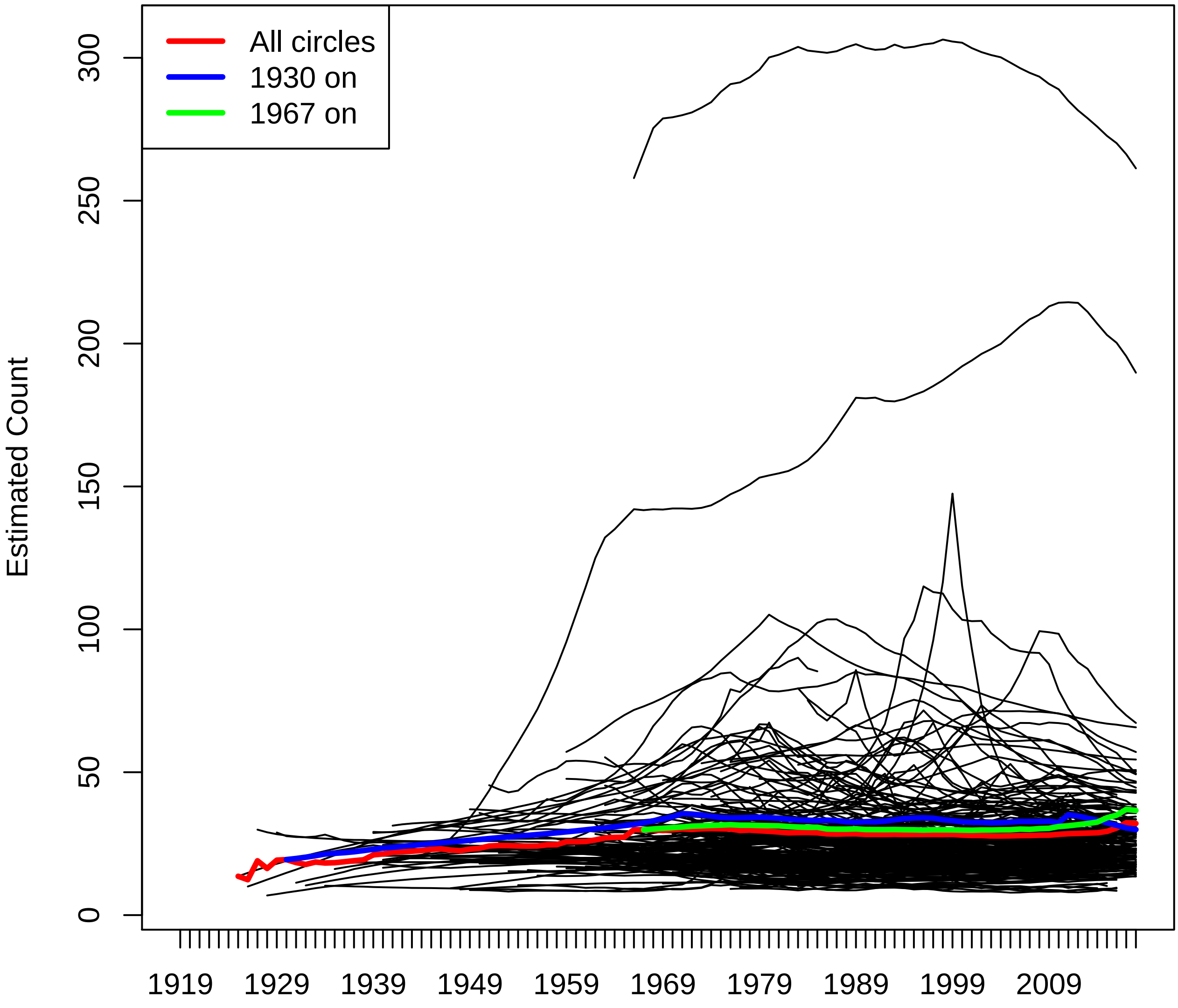

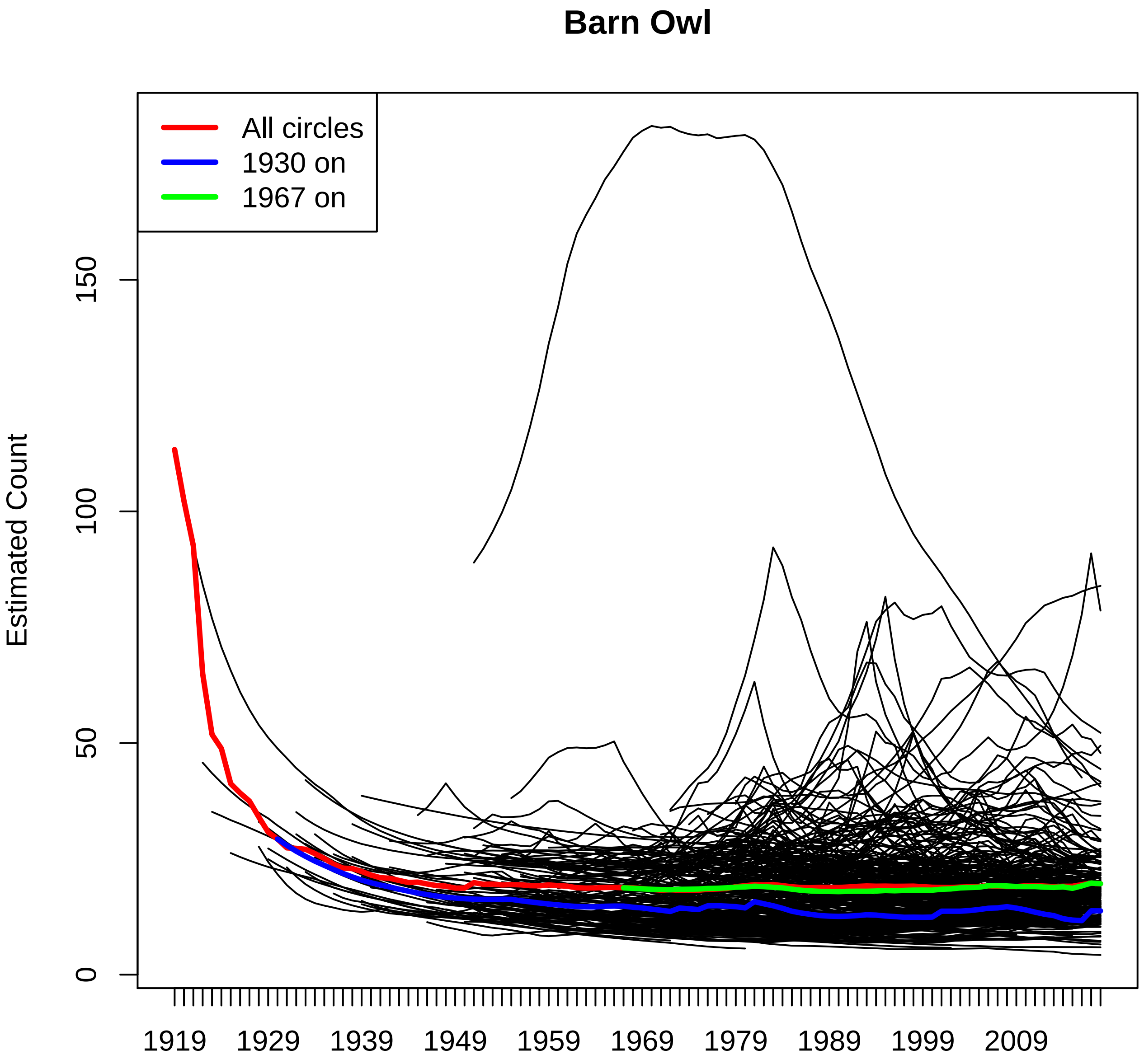
Barn Owl
All circles
1930 on
1967 on
Estimated Count
0
50
100
150
1919
1929
1939
1949
1959
1969
1979
1989
1999
2009

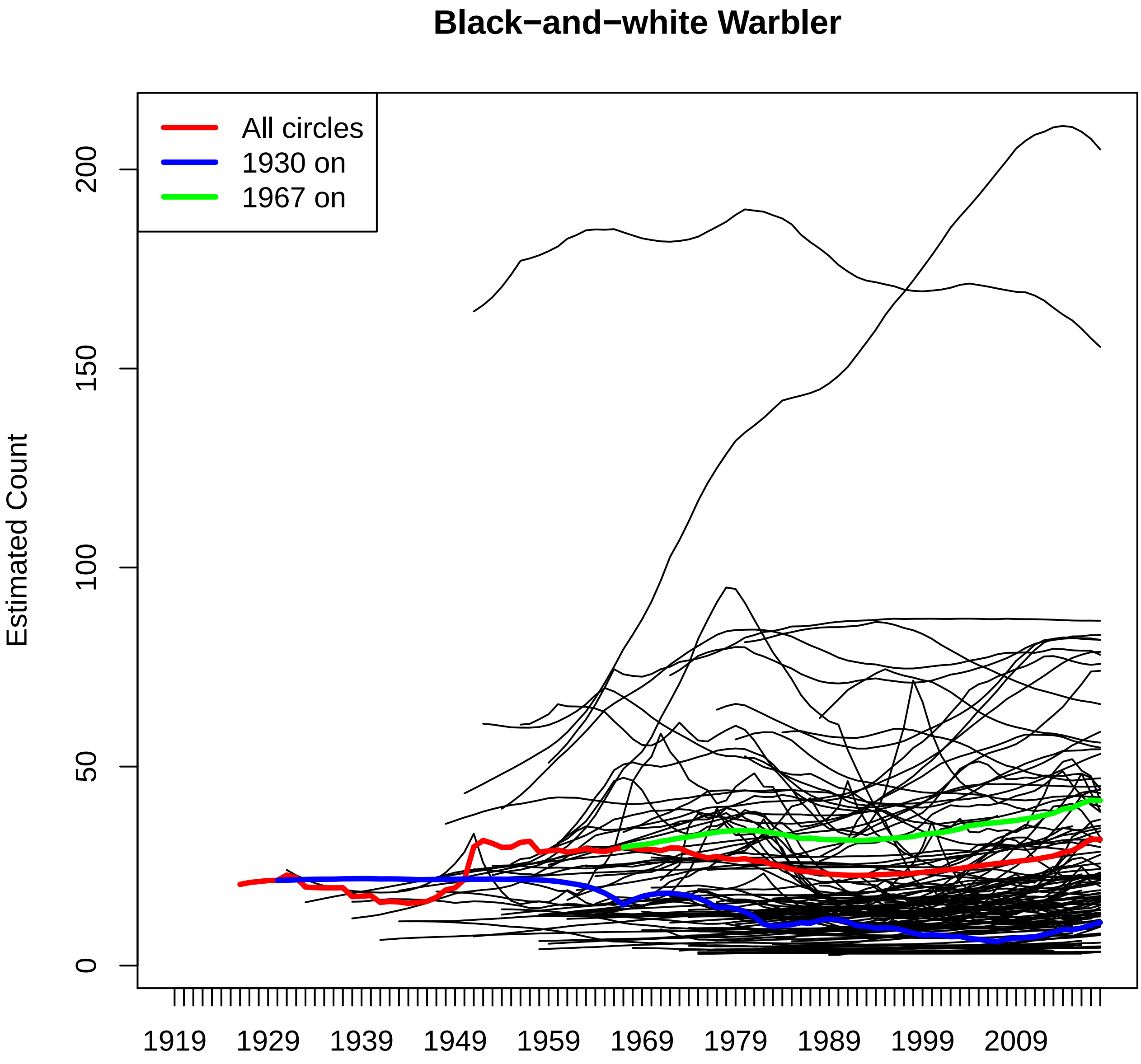

Black−and−white Warbler
All circles
1930 on
1967 on
Estimated Count
0
50
100
150
200
1919
1929
1939
1949
1959
1969
1979
1989
1999
2009

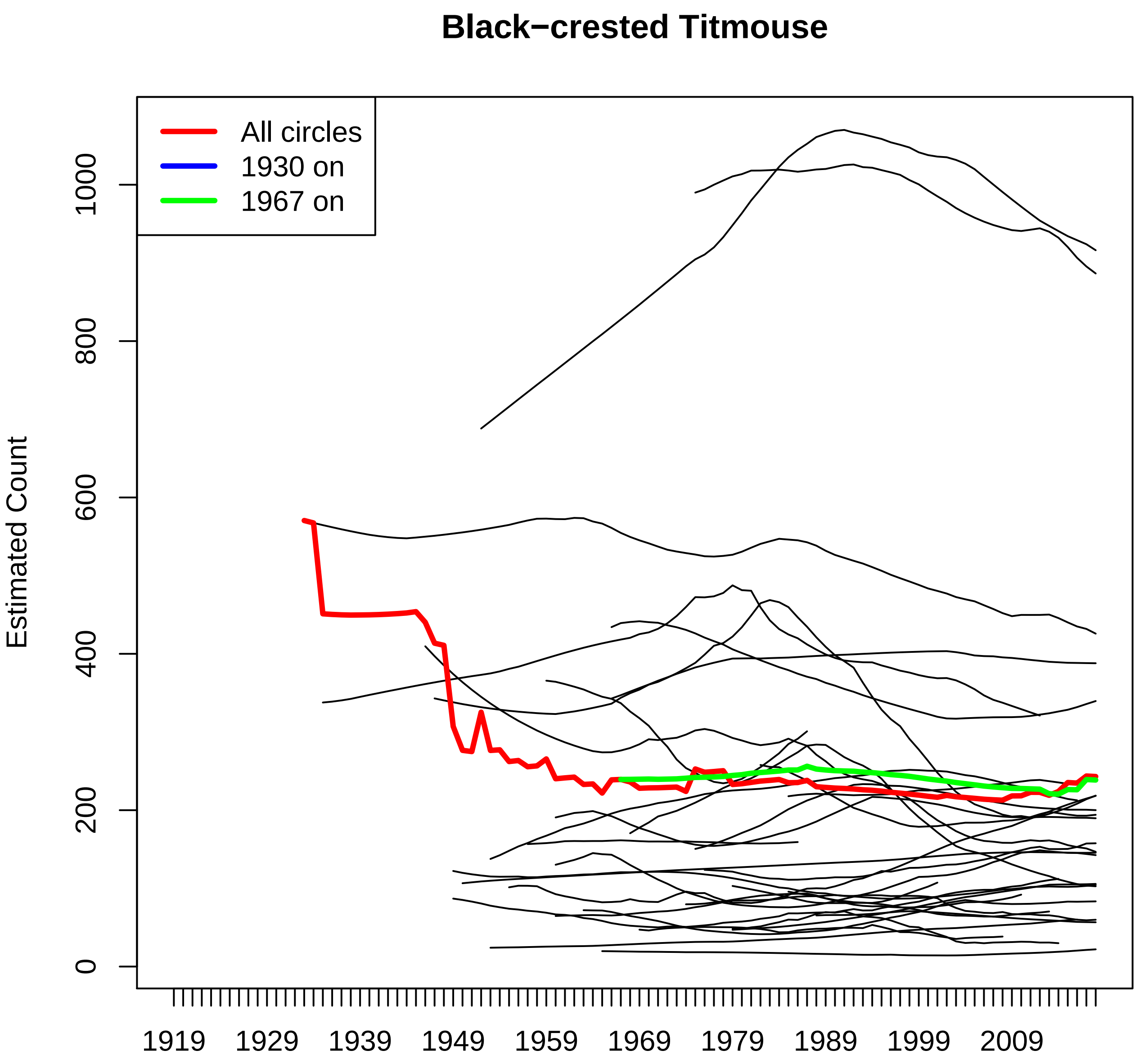

Black−crested Titmouse
All circles
1930 on
1967 on
Estimated Count
0
200
400
600
800
1000
1919
1929
1939
1949
1959
1969
1979
1989
1999
2009

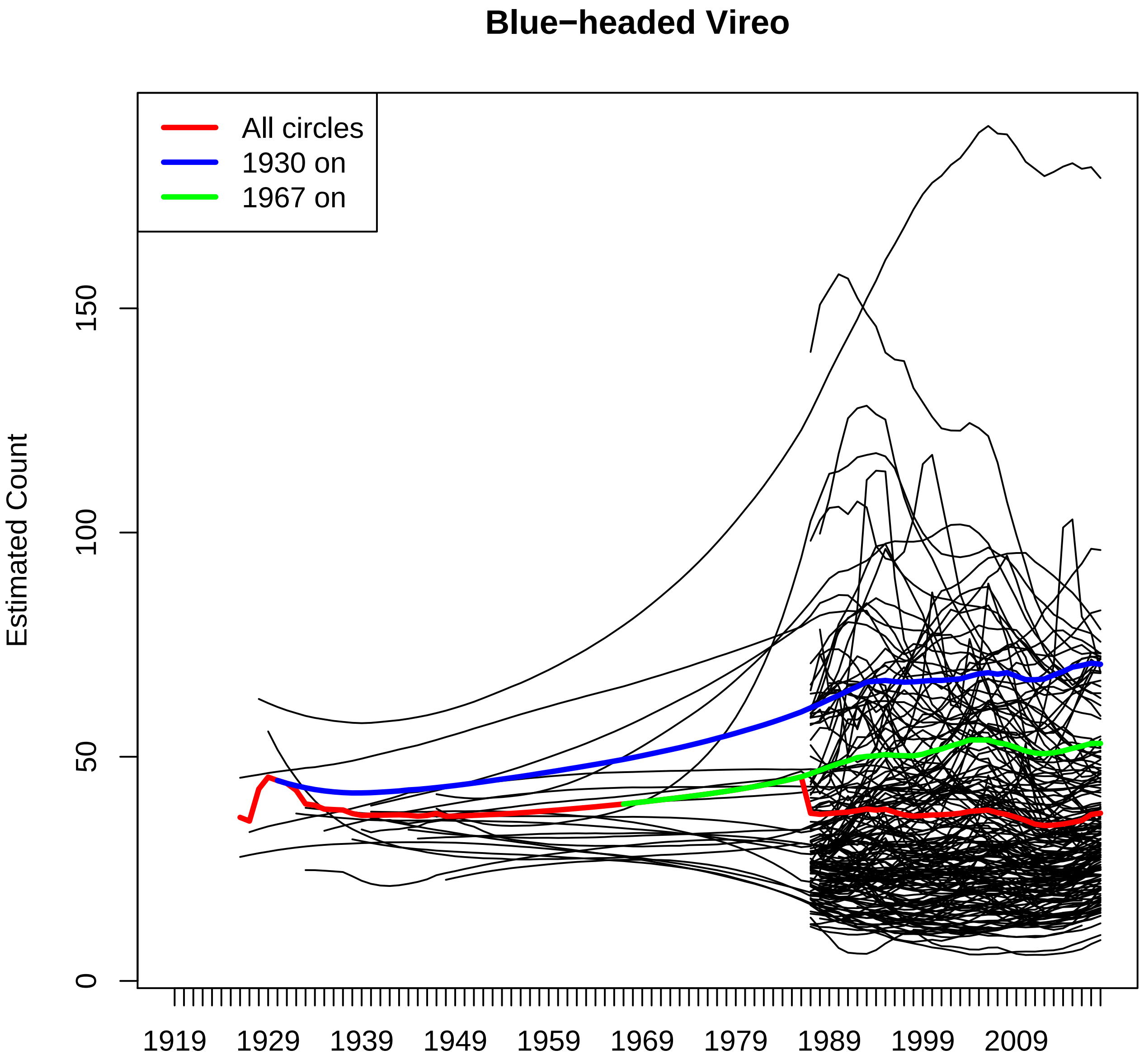
Blue−headed Vireo
All circles
1930 on
1967 on
Estimated Count
0
50
100
150
1919
1929
1939
1949
1959
1969
1979
1989
1999
2009

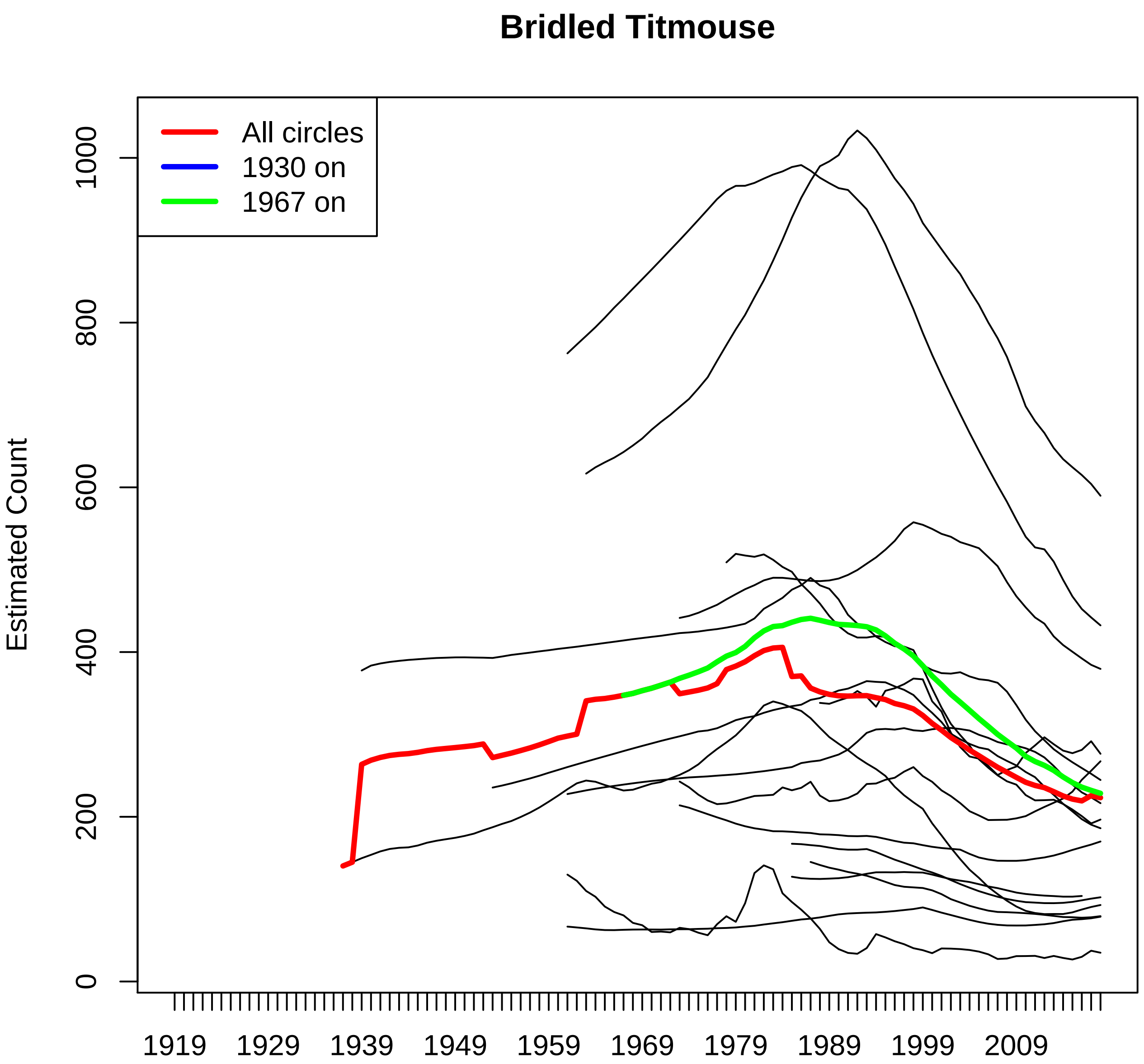

Bridled Titmouse
All circles
1930 on
1967 on
Estimated Count
0
200
400
600
800
1000
1919
1929
1939
1949
1959
1969
1979
1989
1999
2009

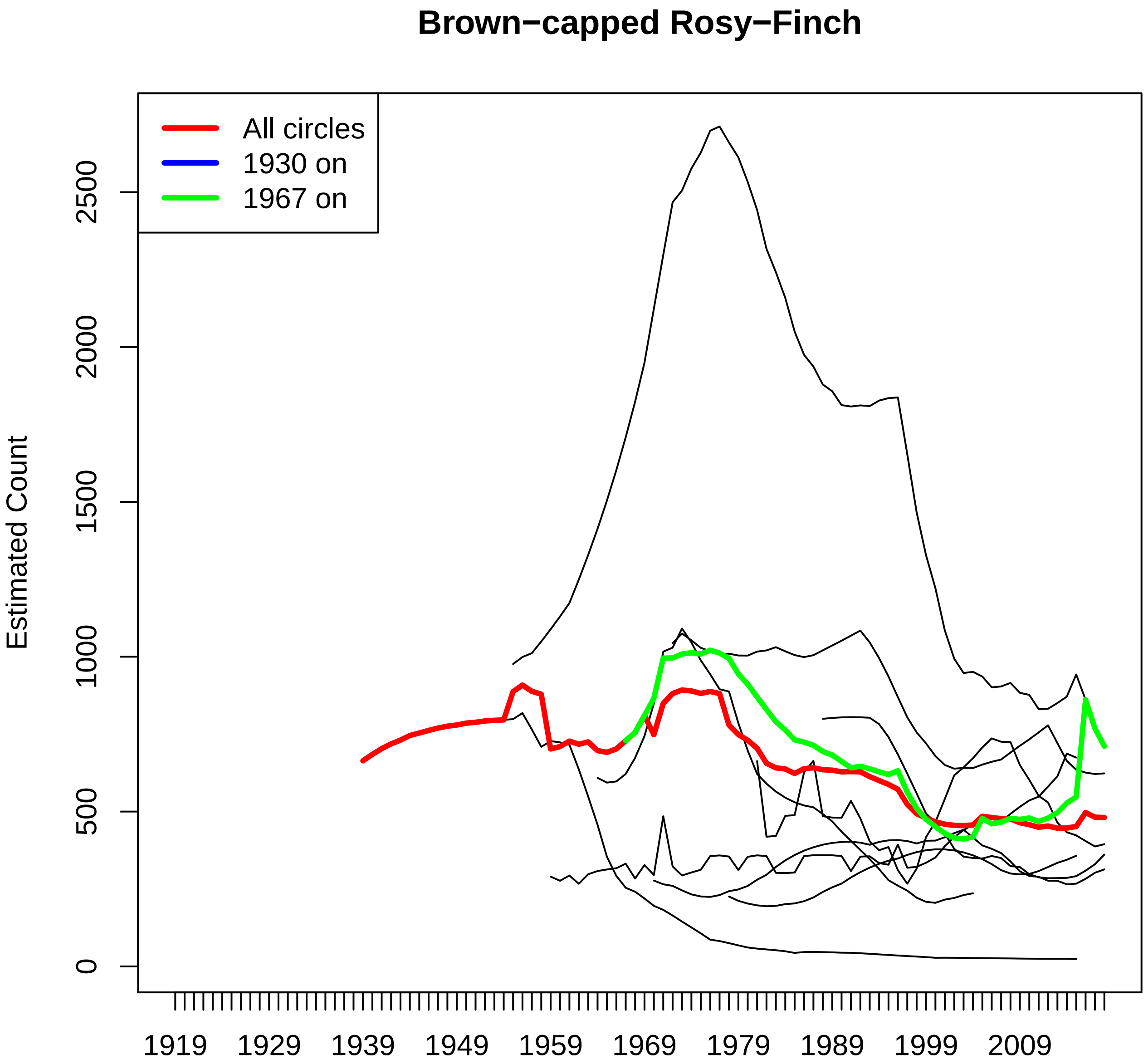

Brown−capped Rosy−Finch
All circles
1930 on
1967 on
Estimated Count
0
500
1000
1500
2000
2500
1919
1929
1939
1949
1959
1969
1979
1989
1999
2009

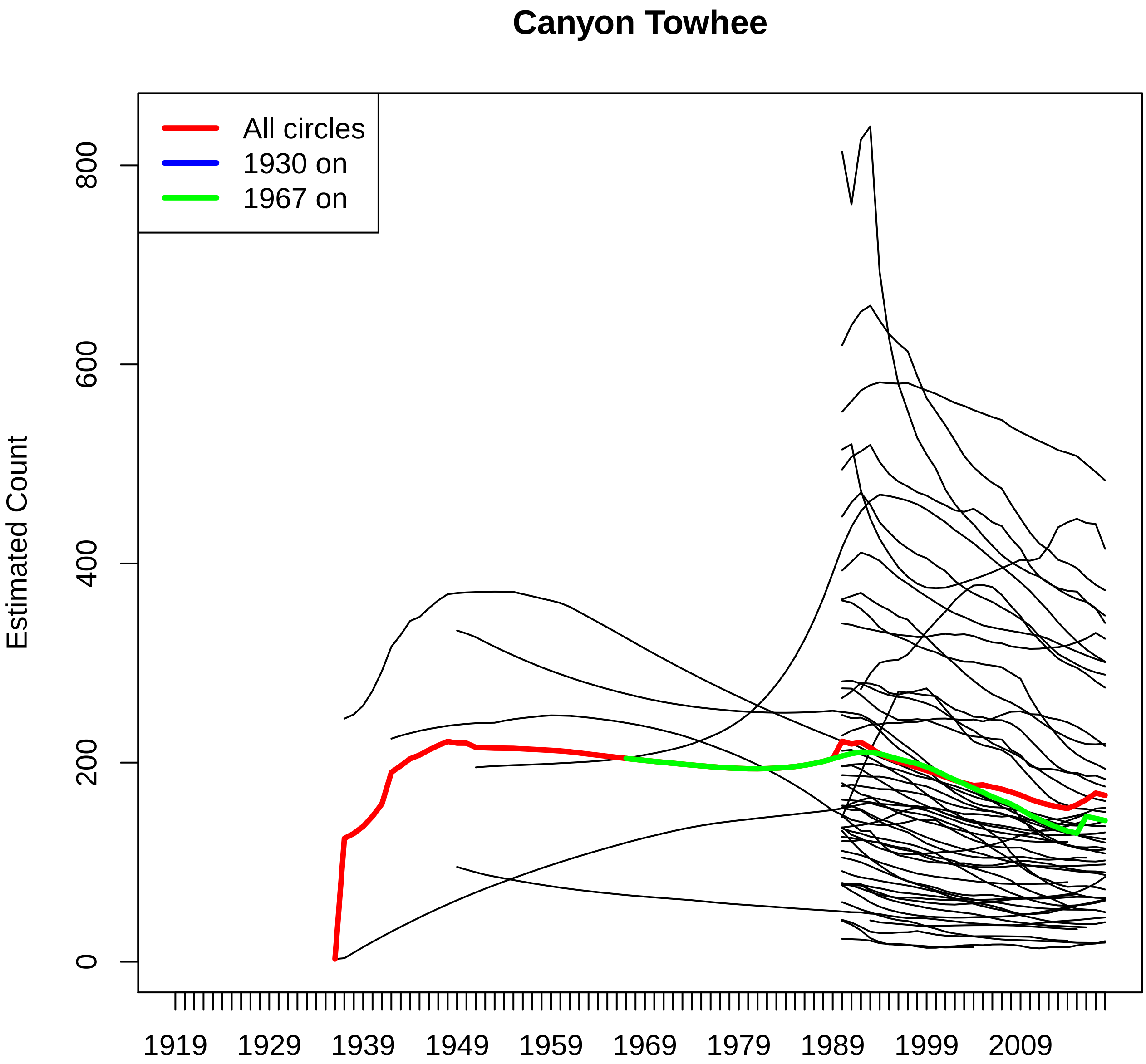

Canyon Towhee
All circles
1930 on
1967 on
Estimated Count
0
200
400
600
800
1919
1929
1939
1949
1959
1969
1979
1989
1999
2009

Canyon Wren

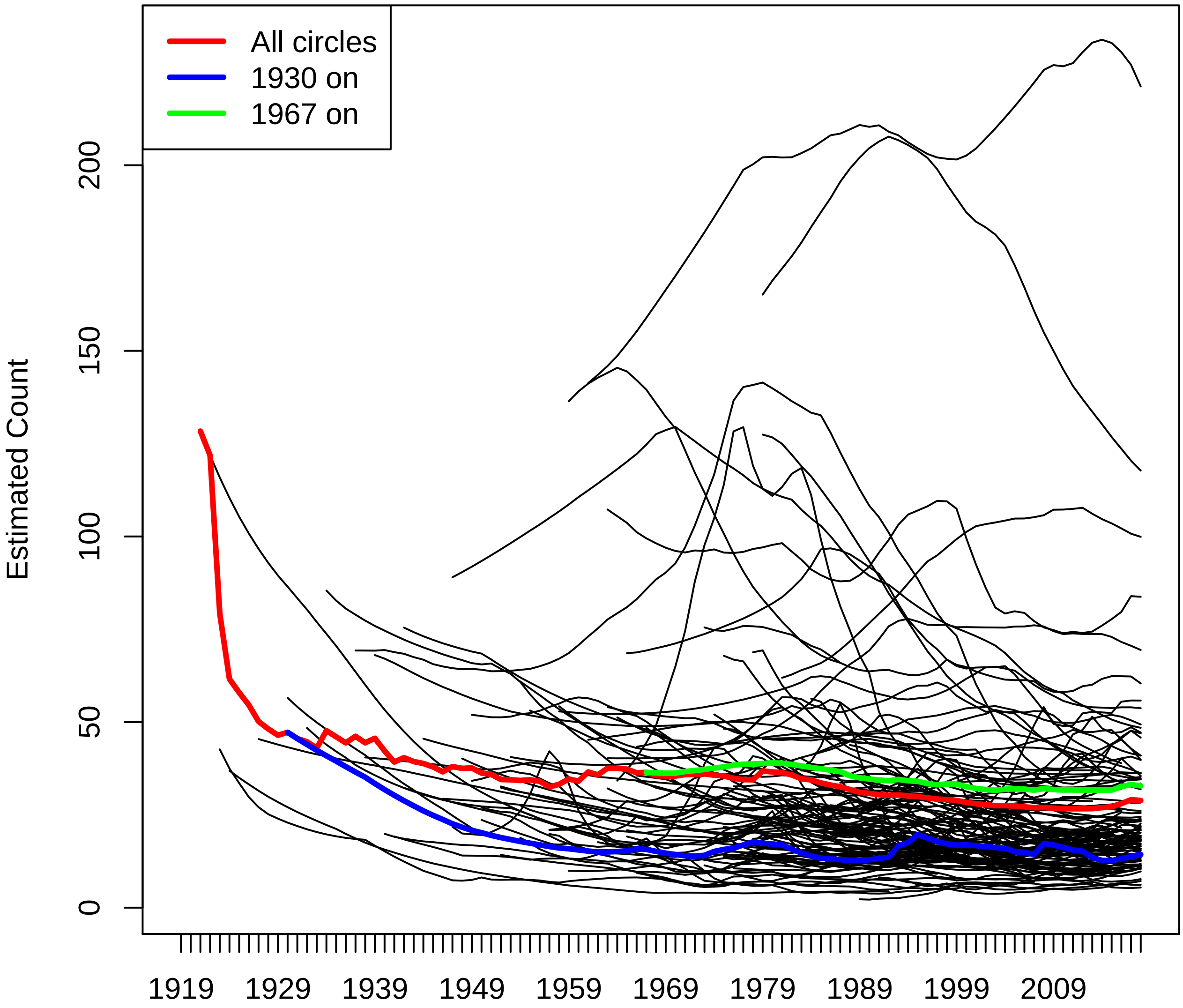

All circles
1930 on
1967 on
Estimated Count
0
50
100
150
200
1919
1929
1939
1949
1959
1969
1979
1989
1999
2009

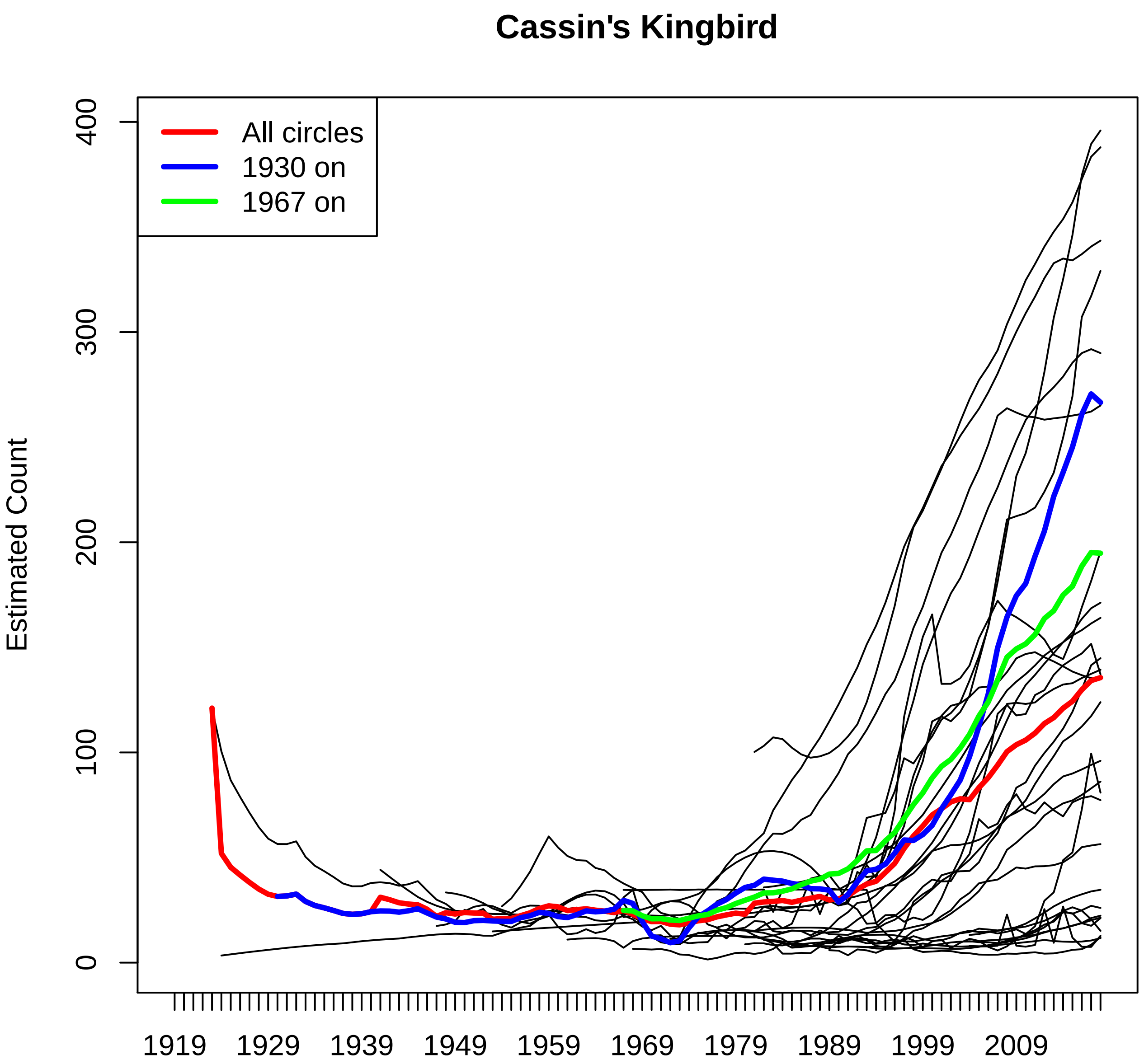

Cassin's Kingbird
All circles
1930 on
1967 on
Estimated Count
0
100
200
300
400
1919
1929
1939
1949
1959
1969
1979
1989
1999
2009

Chihuahuan Raven

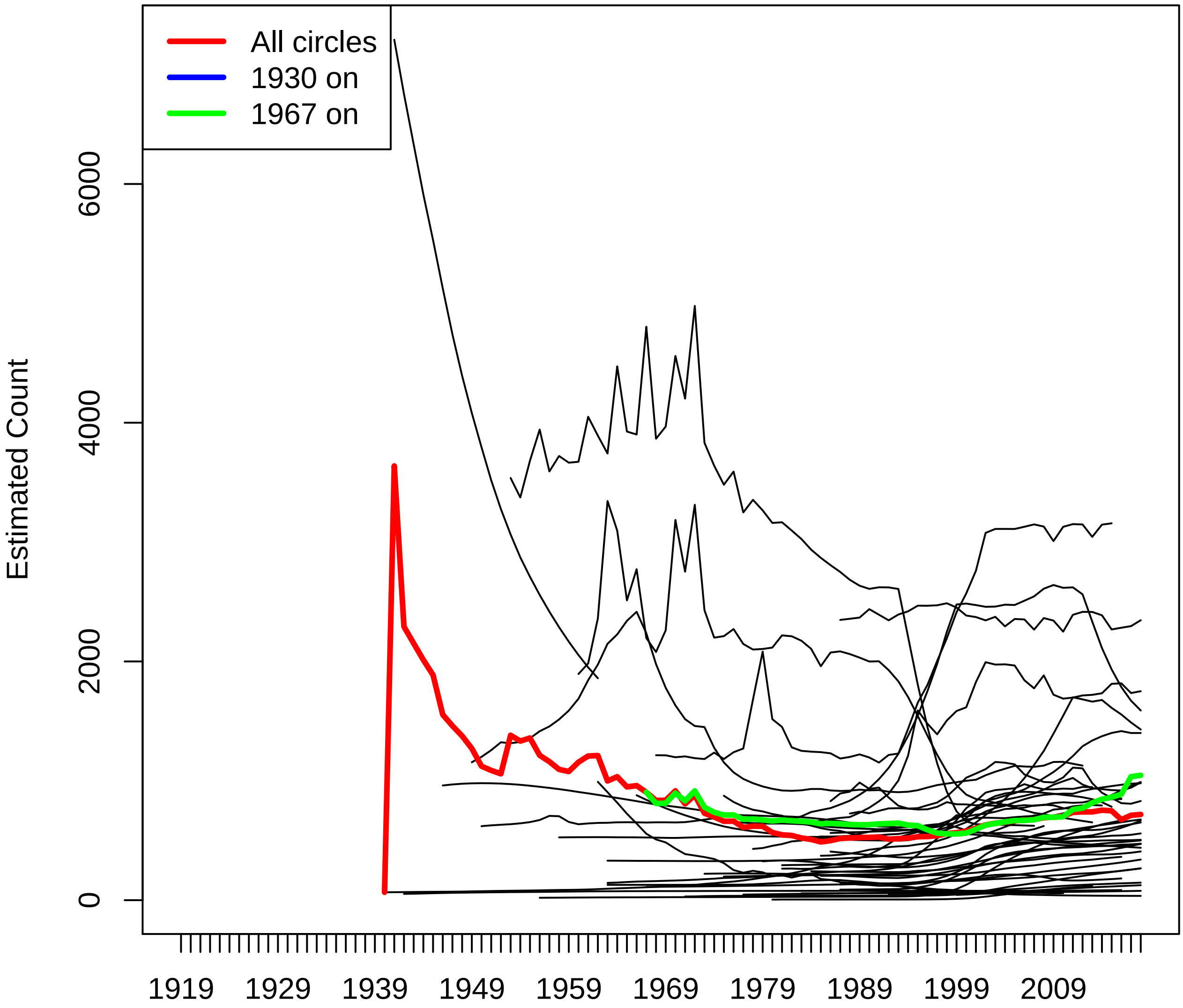

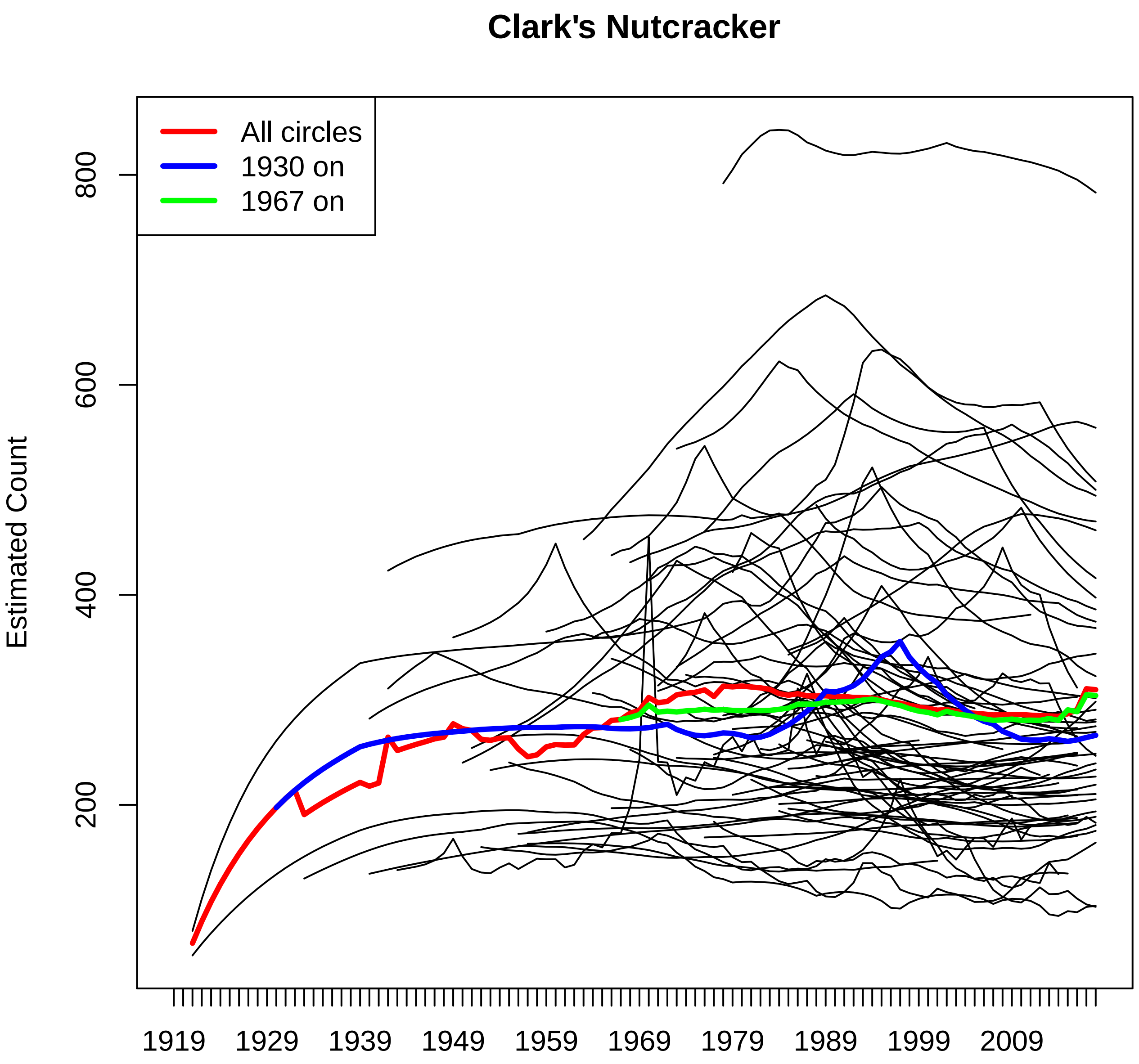

Clark's Nutcracker
All circles
1930 on
1967 on
Estimated Count
200
400
600
800
1919
1929
1939
1949
1959
1969
1979
1989
1999
2009

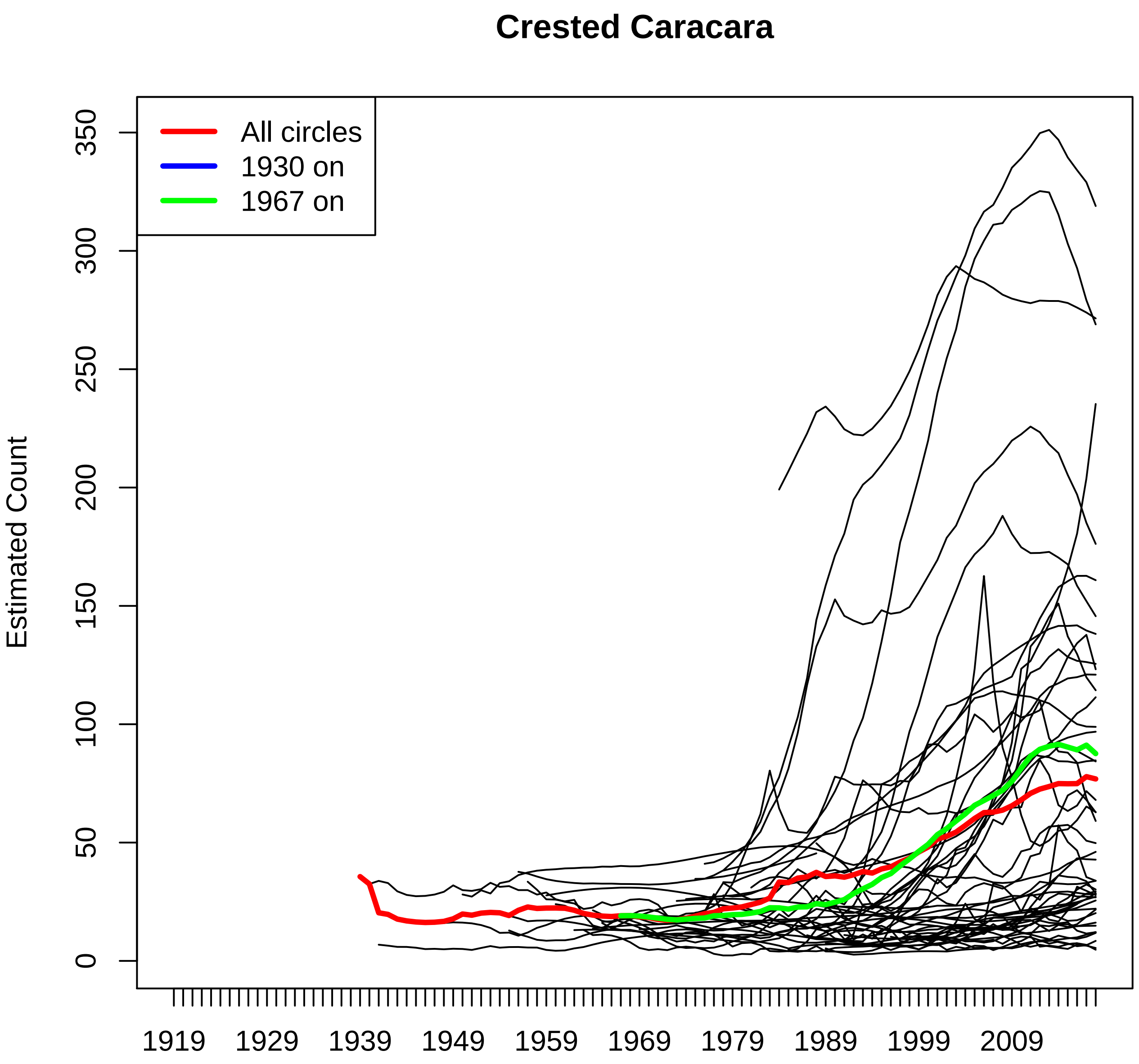
Crested Caracara
All circles
1930 on
1967 on
Estimated Count
0
50
100
150
200
250
300
350
1919
1929
1939
1949
1959
1969
1979
1989
1999
2009

Curve−billed Thrasher

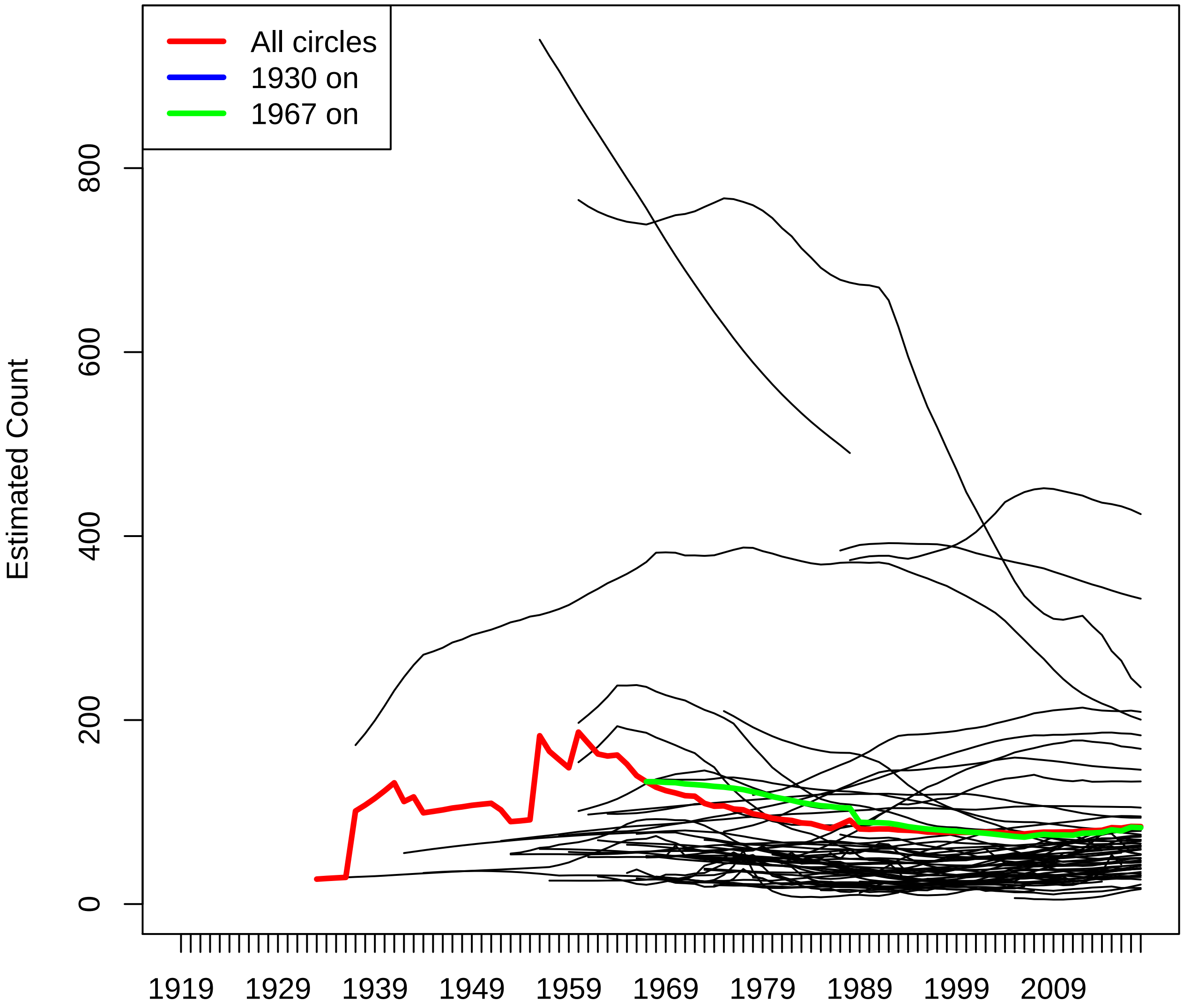

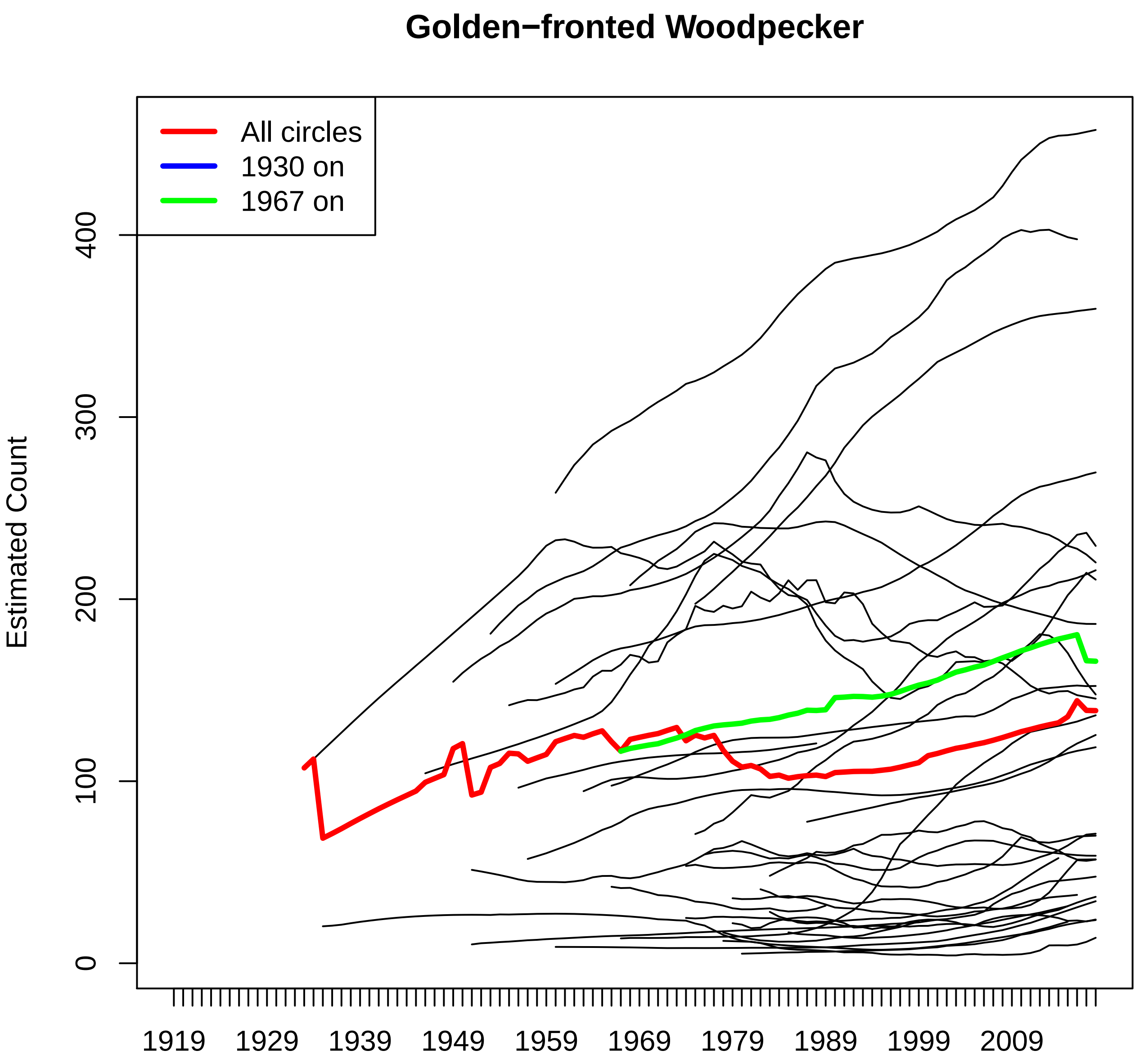

Golden−fronted Woodpecker
All circles
1930 on
1967 on
Estimated Count
0
100
200
300
400
1919
1929
1939
1949
1959
1969
1979
1989
1999
2009

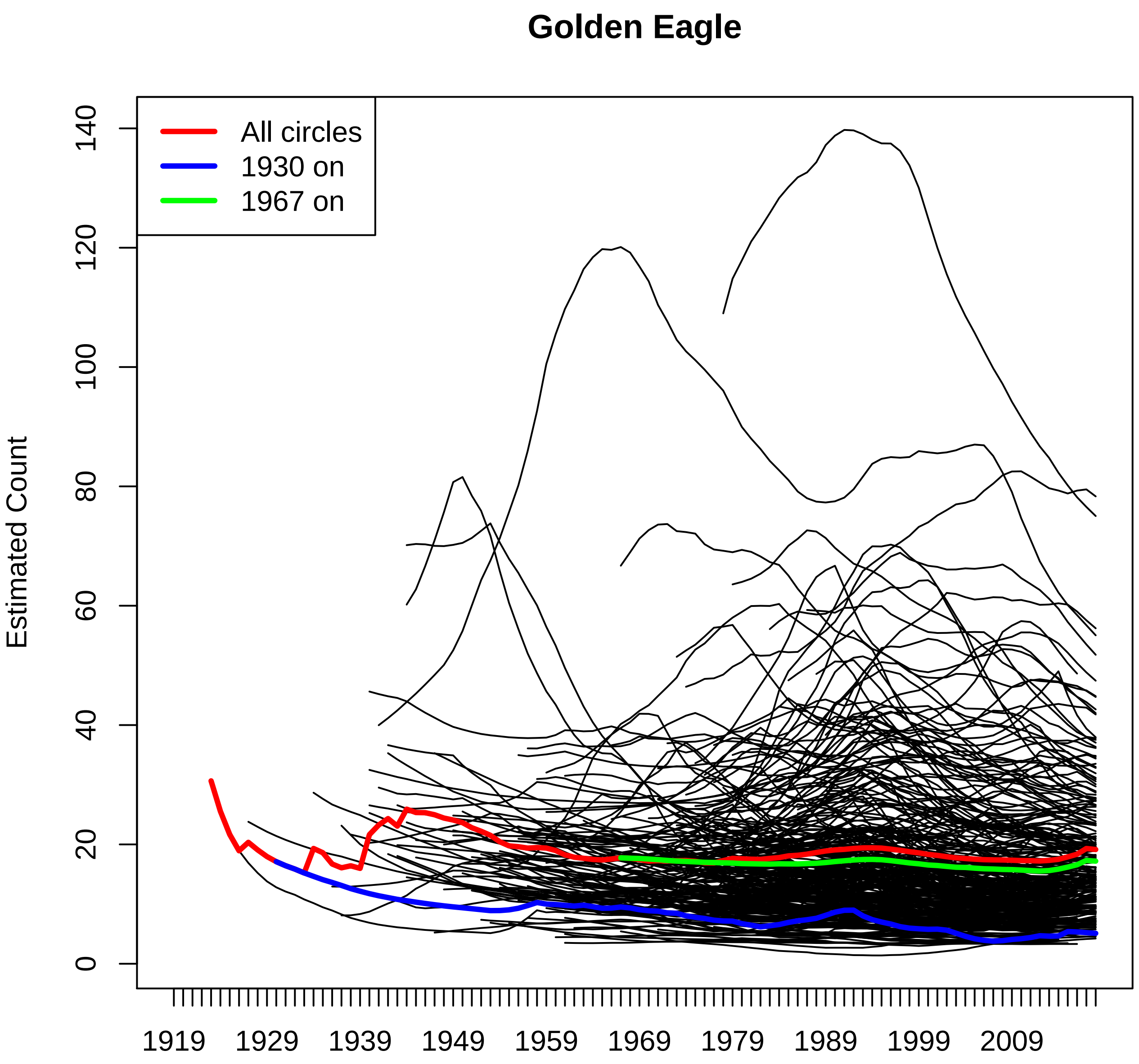
Golden Eagle
All circles
1930 on
1967 on
Estimated Count
0
20
40
60
80
100
120
140
1919
1929
1939
1949
1959
1969
1979
1989
1999
2009

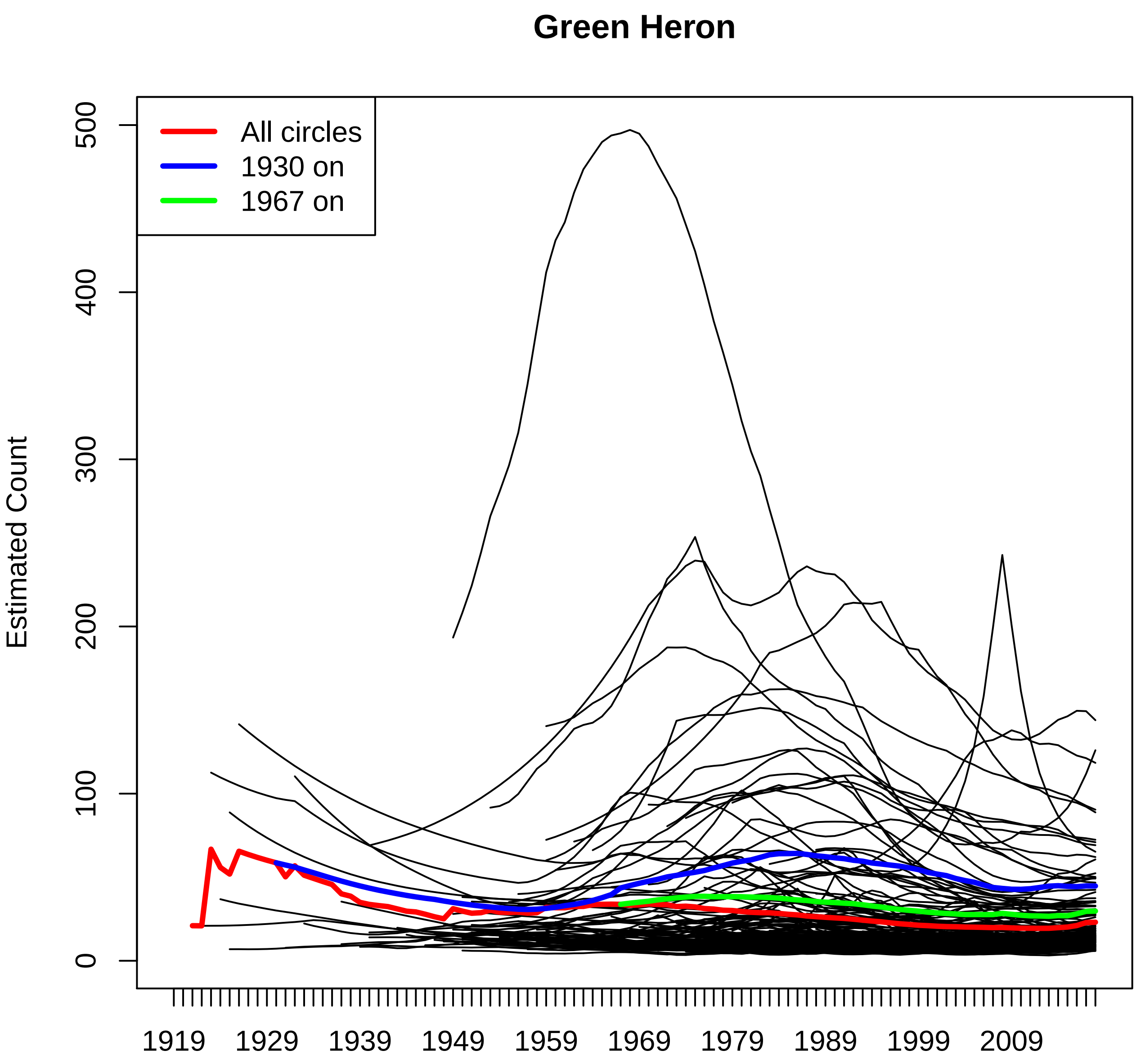
Green Heron
Estimated Count
All circles
1930 on
1967 on
0
100
200
300
400
500
1919
1929
1939
1949
1959
1969
1979
1989
1999
2009

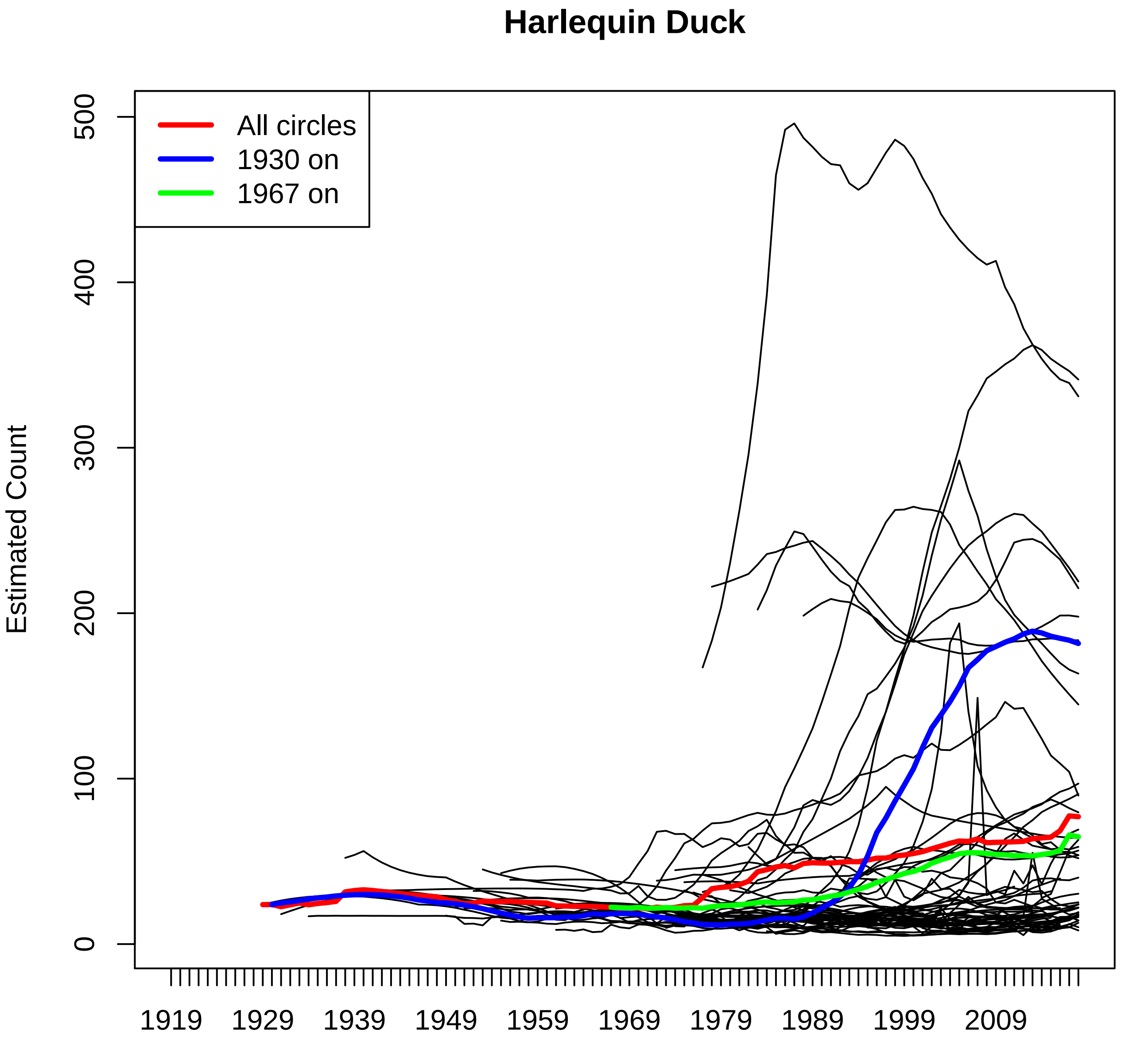

Harlequin Duck
All circles
1930 on
1967 on
Estimated Count
0
100
200
300
400
500
1919
1929
1939
1949
1959
1969
1979
1989
1999
2009

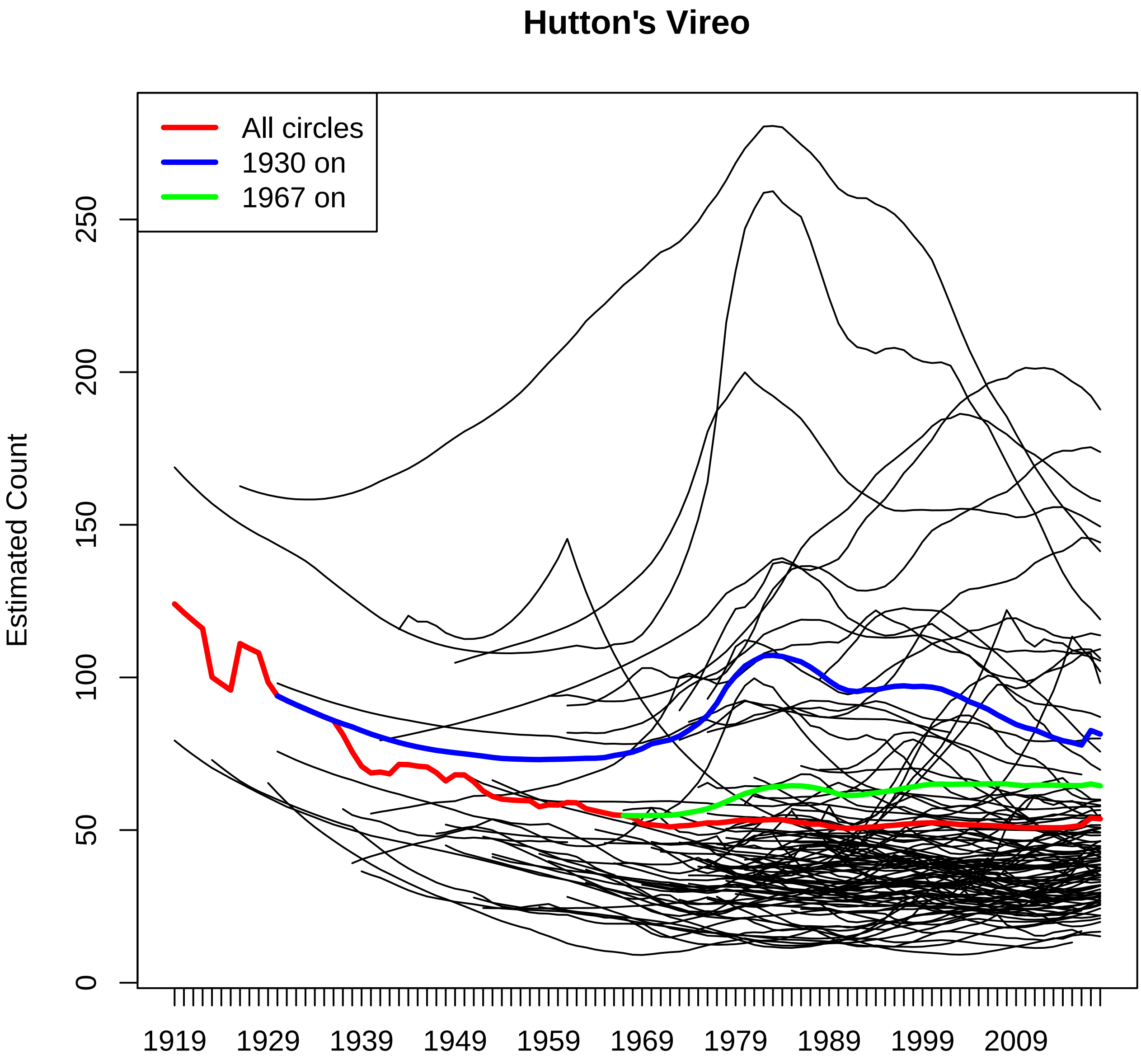
Hutton's Vireo
All circles
1930 on
1967 on
Estimated Count
0
50
100
150
200
250
1919
1929
1939
1949
1959
1969
1979
1989
1999
2009

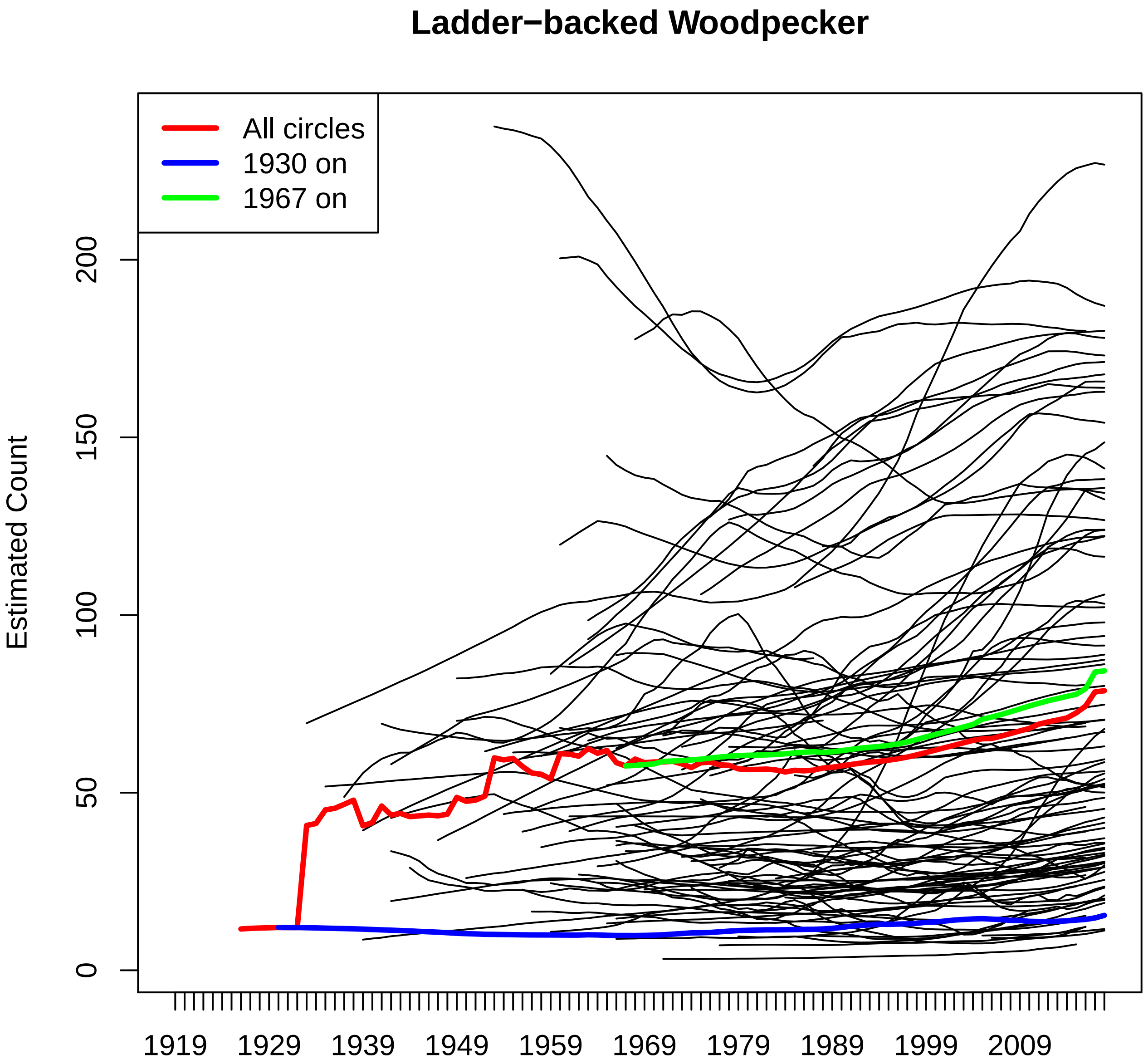

Ladder−backed Woodpecker
All circles
1930 on
1967 on
Estimated Count
0
50
100
150
200
1919
1929
1939
1949
1959
1969
1979
1989
1999
2009

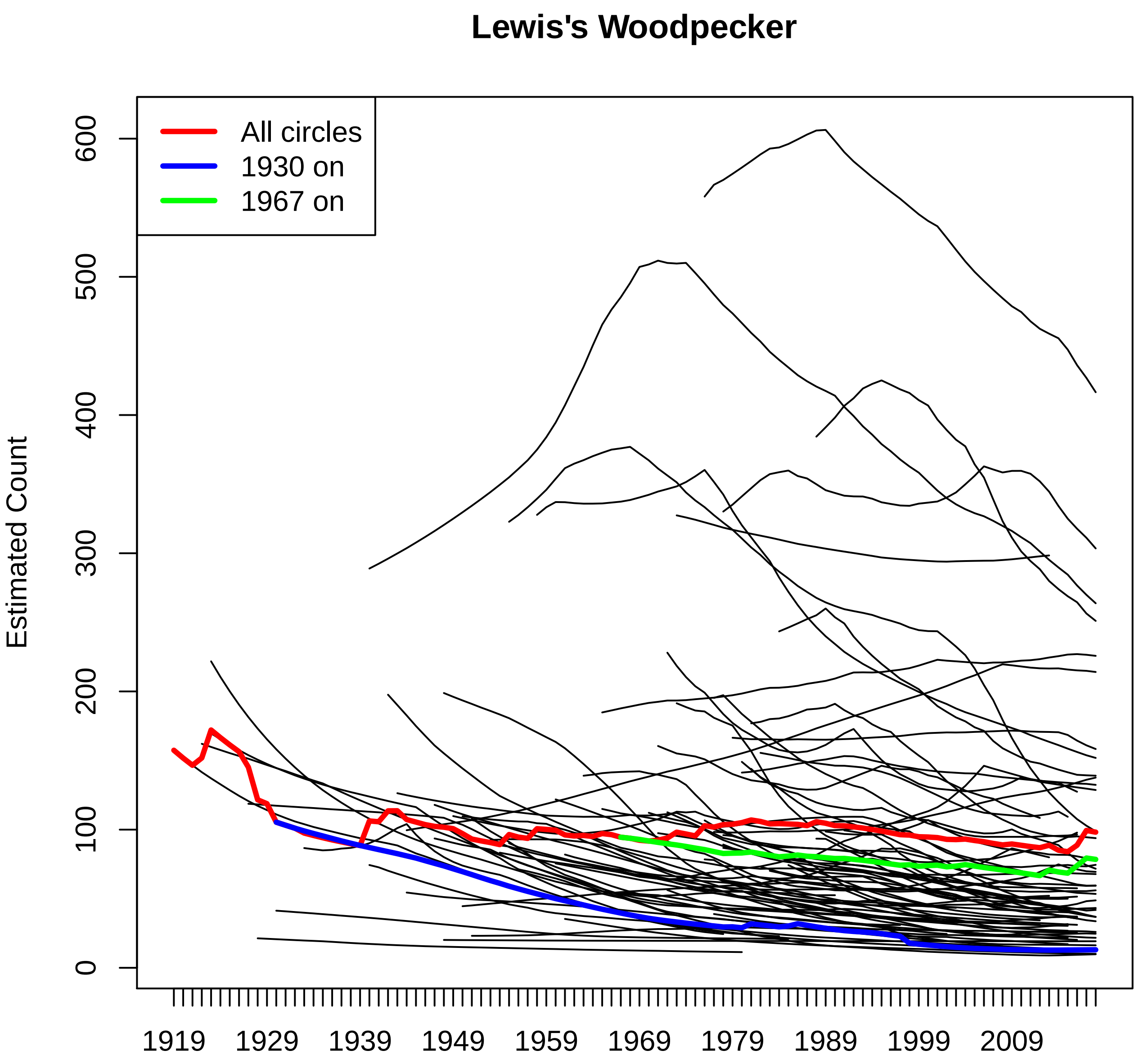
Lewis's Woodpecker
All circles
1930 on
1967 on
Estimated Count
0
100
200
300
400
500
600
1919
1929
1939
1949
1959
1969
1979
1989
1999
2009

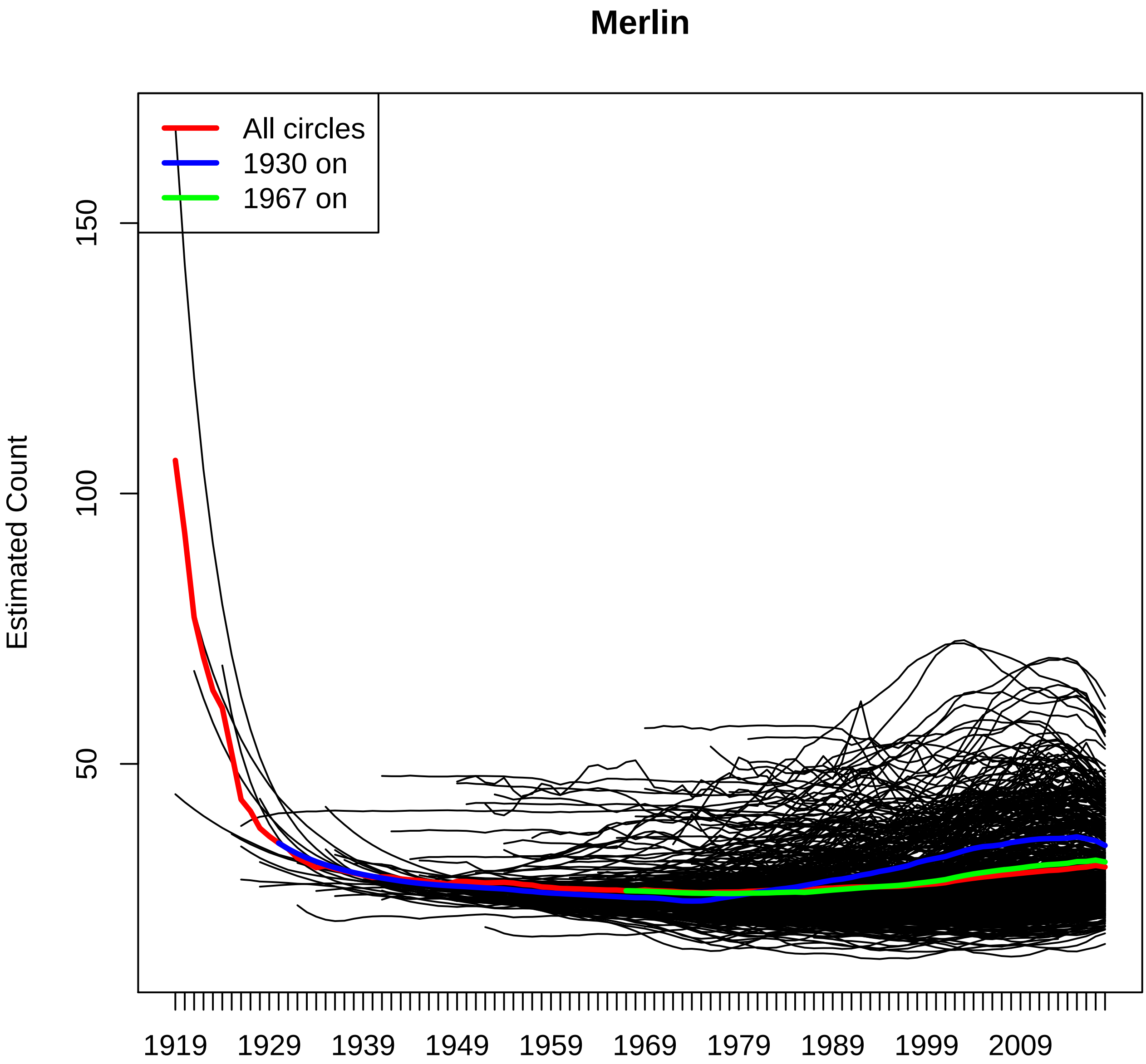
Merlin
All circles
1930 on
1967 on
Estimated Count
50
100
150
1919
1929
1939
1949
1959
1969
1979
1989
1999
2009

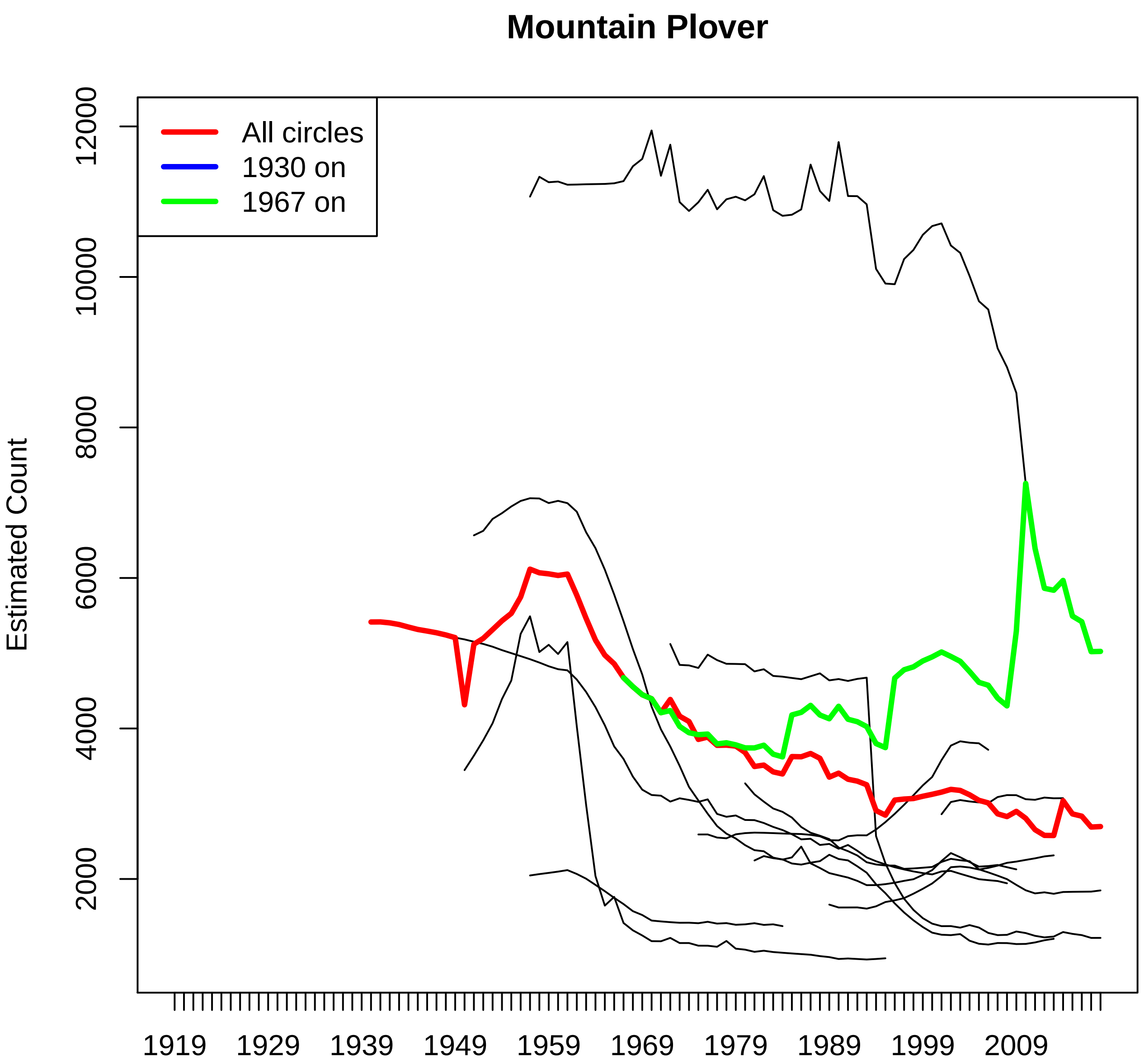

Mountain Plover
All circles
1930 on
1967 on
Estimated Count
2000
4000
6000
8000
10000
12000
1919
1929
1939
1949
1959
1969
1979
1989
1999
2009

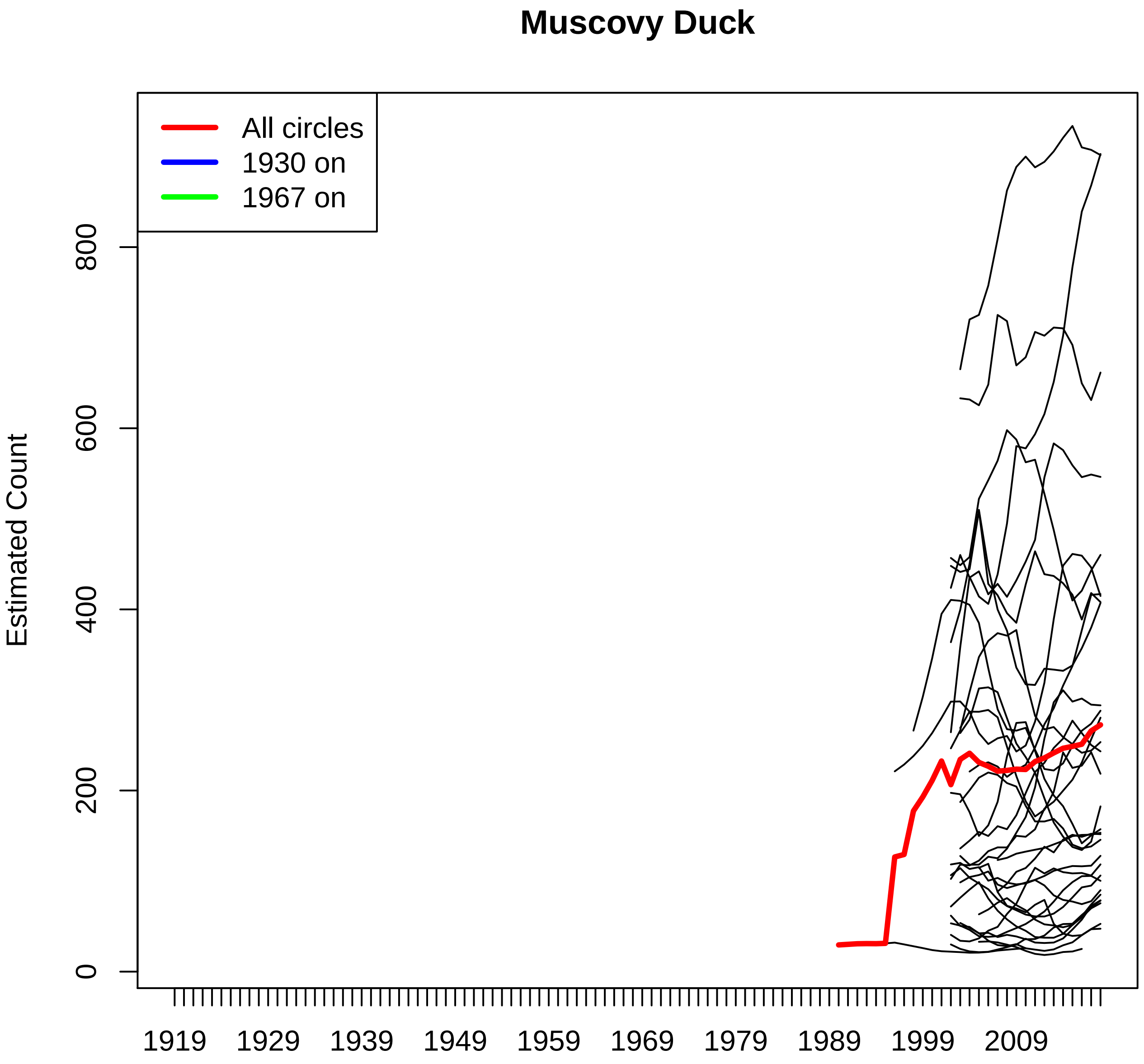

Muscovy Duck
All circles
1930 on
1967 on
Estimated Count
0
200
400
600
800
1919
1929
1939
1949
1959
1969
1979
1989
1999
2009

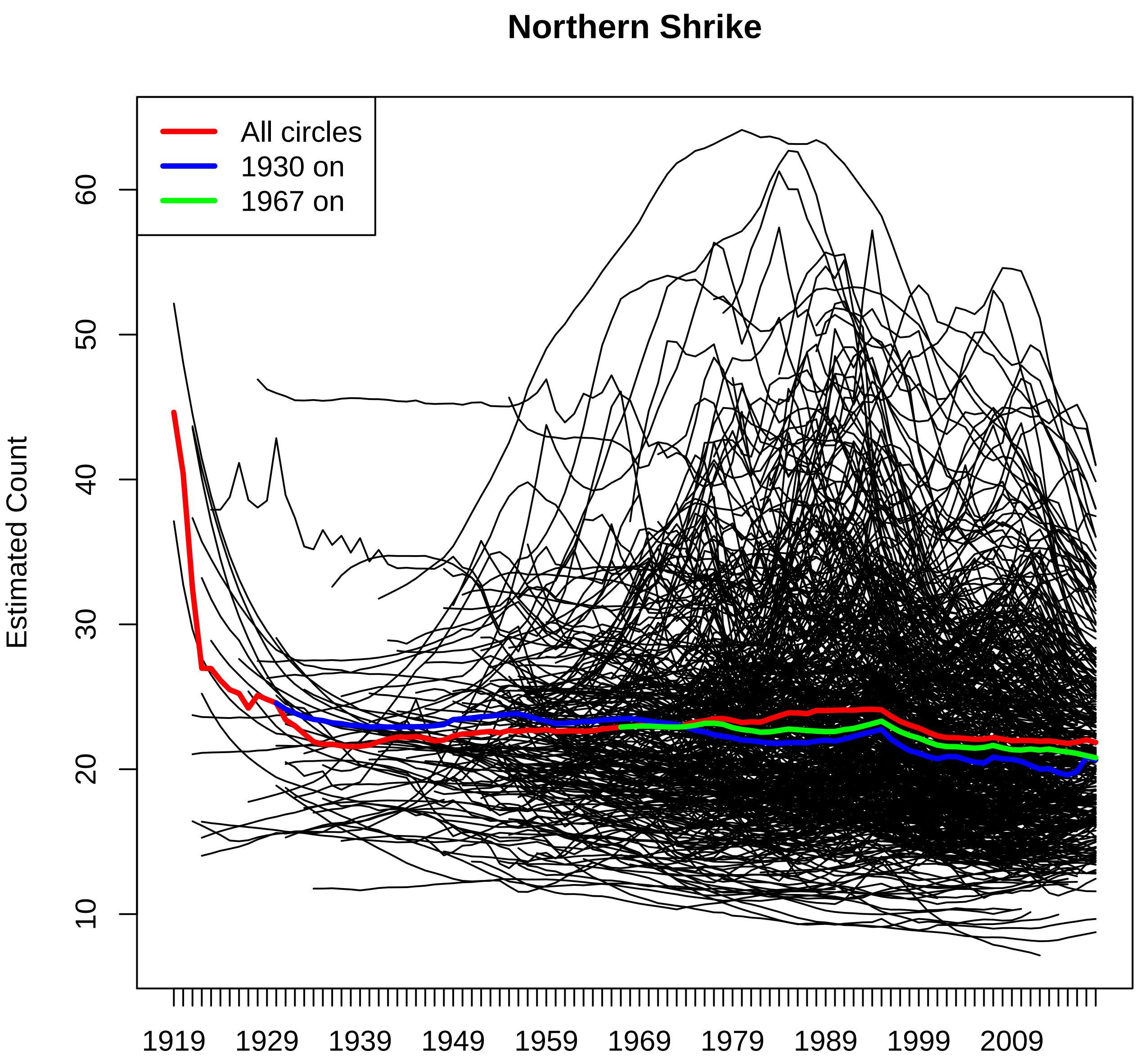

Northern Shrike
All circles
1930 on
1967 on
Estimated Count
10
20
30
40
50
60
1919
1929
1939
1949
1959
1969
1979
1989
1999
2009

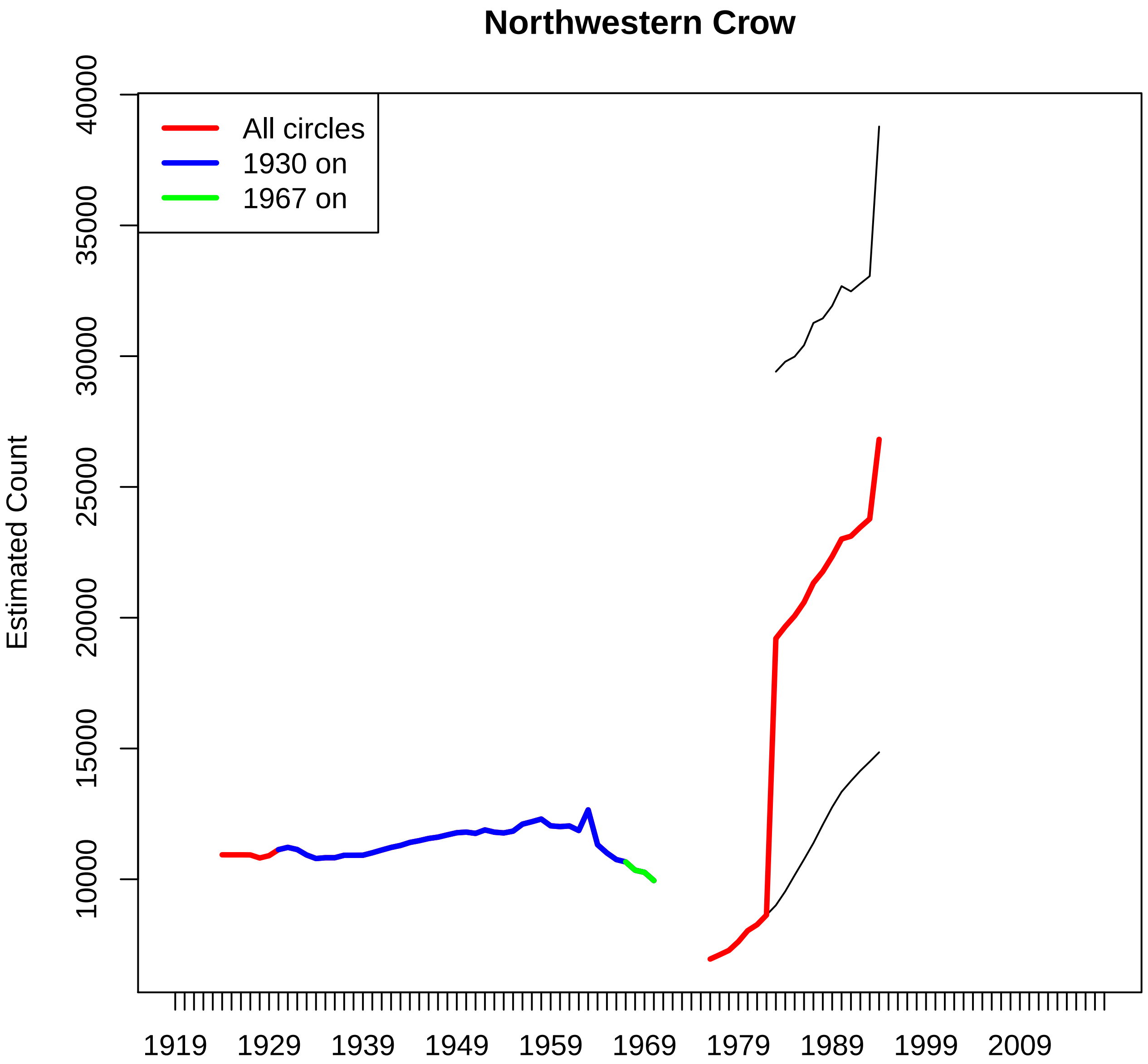
Northwestern Crow
Estimated Count
All circles
1930 on
1967 on
10000
15000
20000
25000
30000
35000
40000
1919
1929
1939
1949
1959
1969
1979
1989
1999
2009

Rock Wren

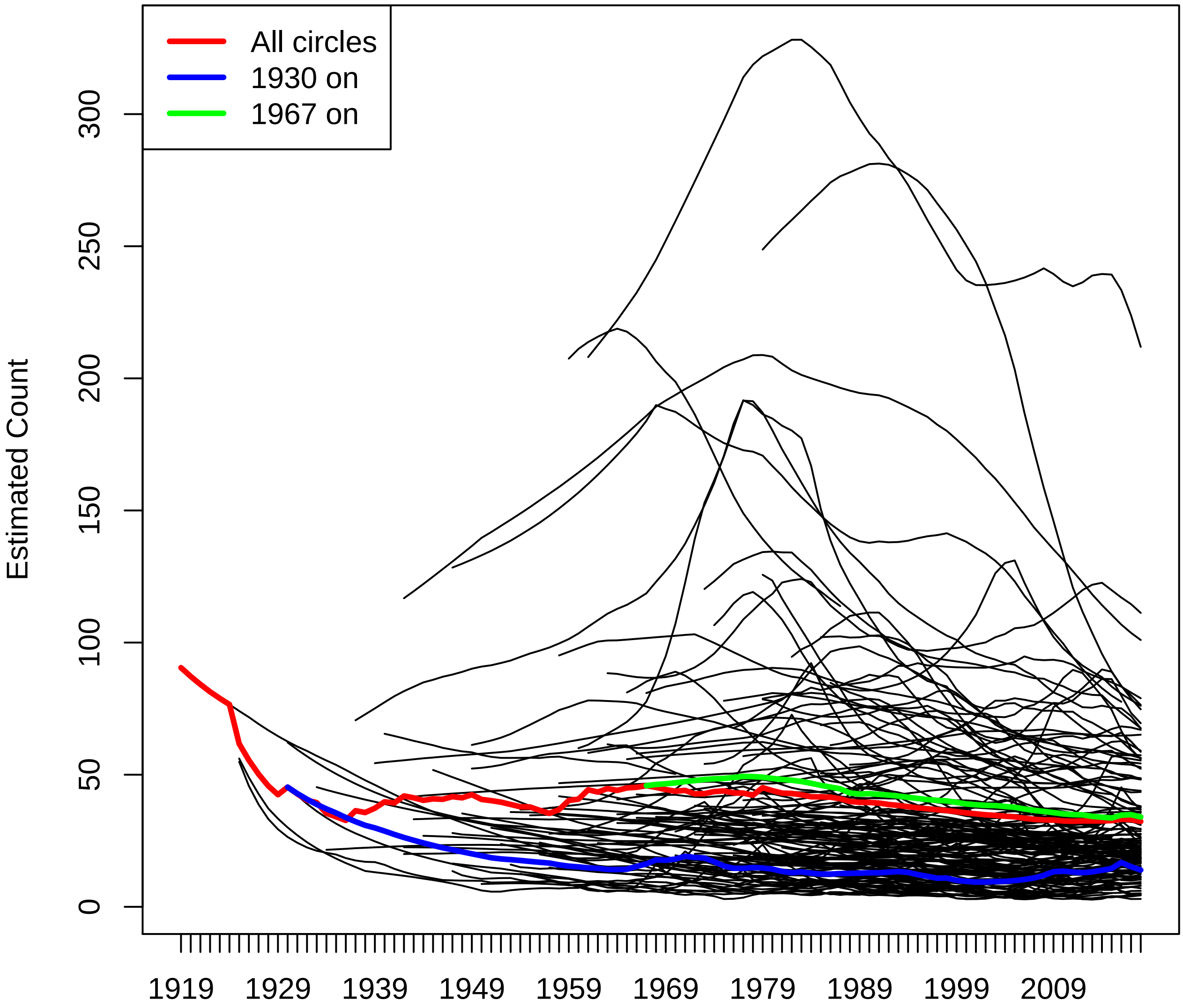

All circles
1930 on
1967 on
Estimated Count
0
50
100
150
200
250
300
1919
1929
1939
1949
1959
1969
1979
1989
1999
2009

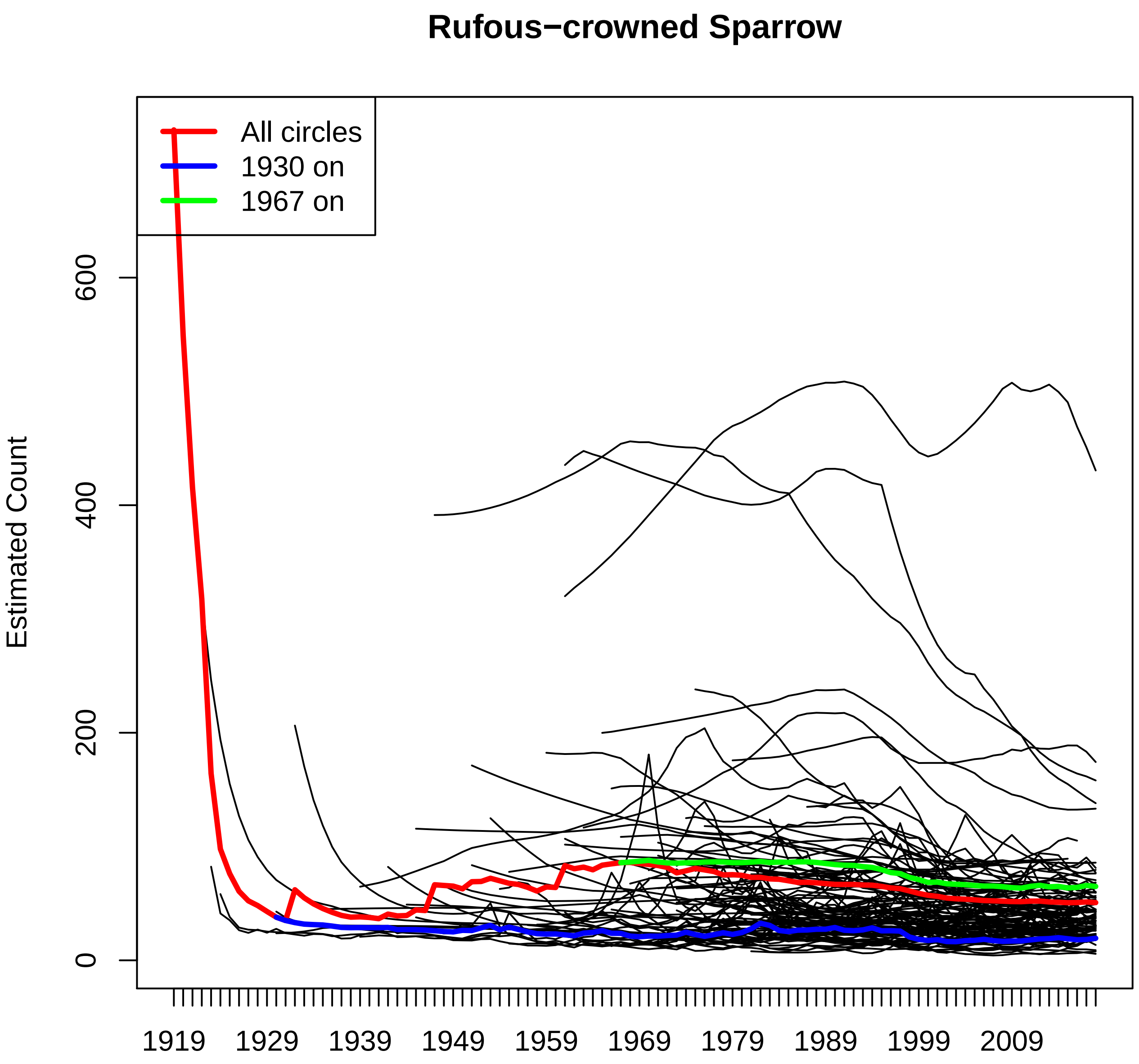

Rufous−crowned Sparrow
All circles
1930 on
1967 on
Estimated Count
0
200
400
600
1919
1929
1939
1949
1959
1969
1979
1989
1999
2009

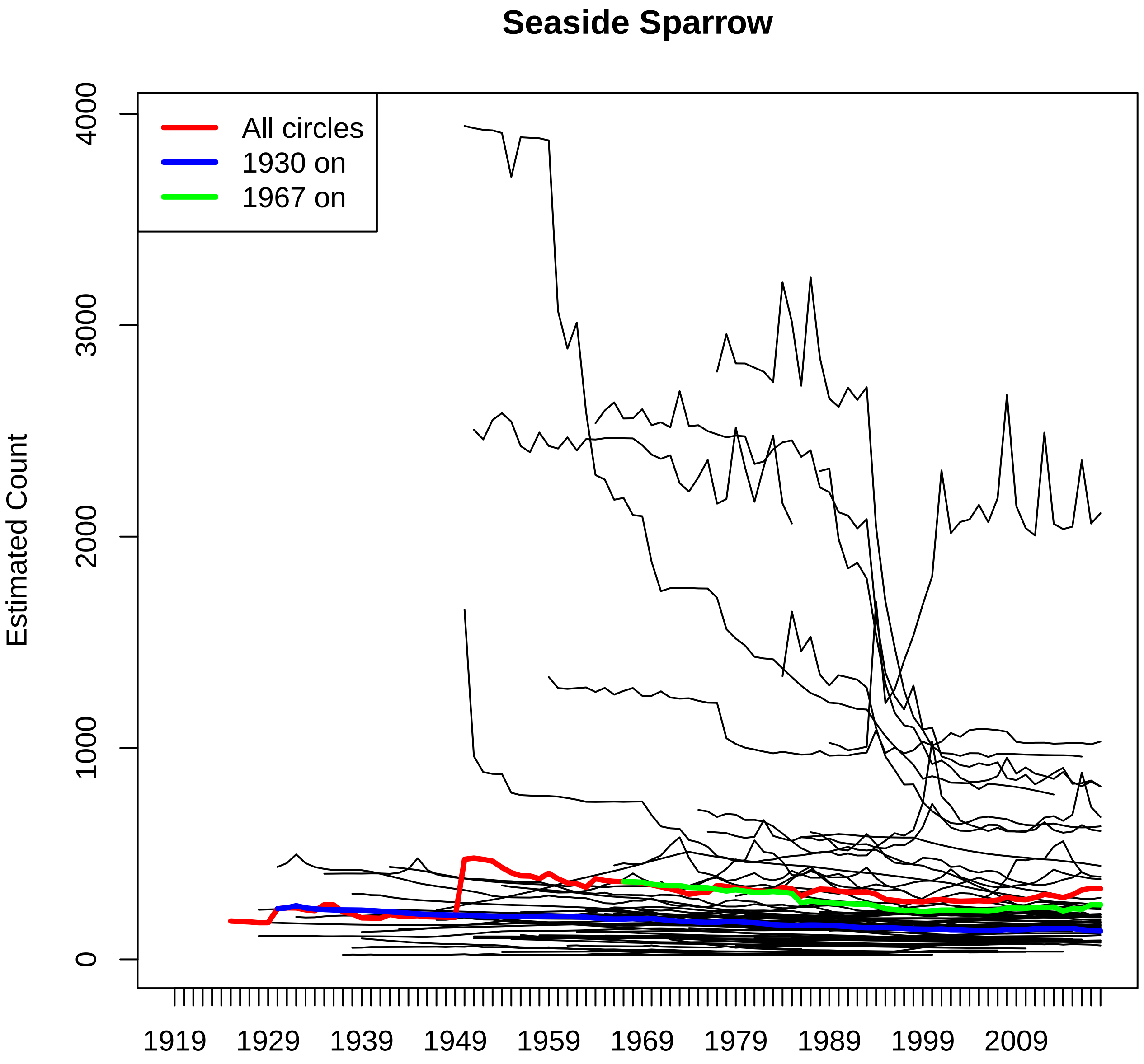

Seaside Sparrow
Estimated Count
0
1000
2000
3000
4000
1919
1929
1939
1949
1959
1969
1979
1989
1999
2009
All circles
1930 on
1967 on

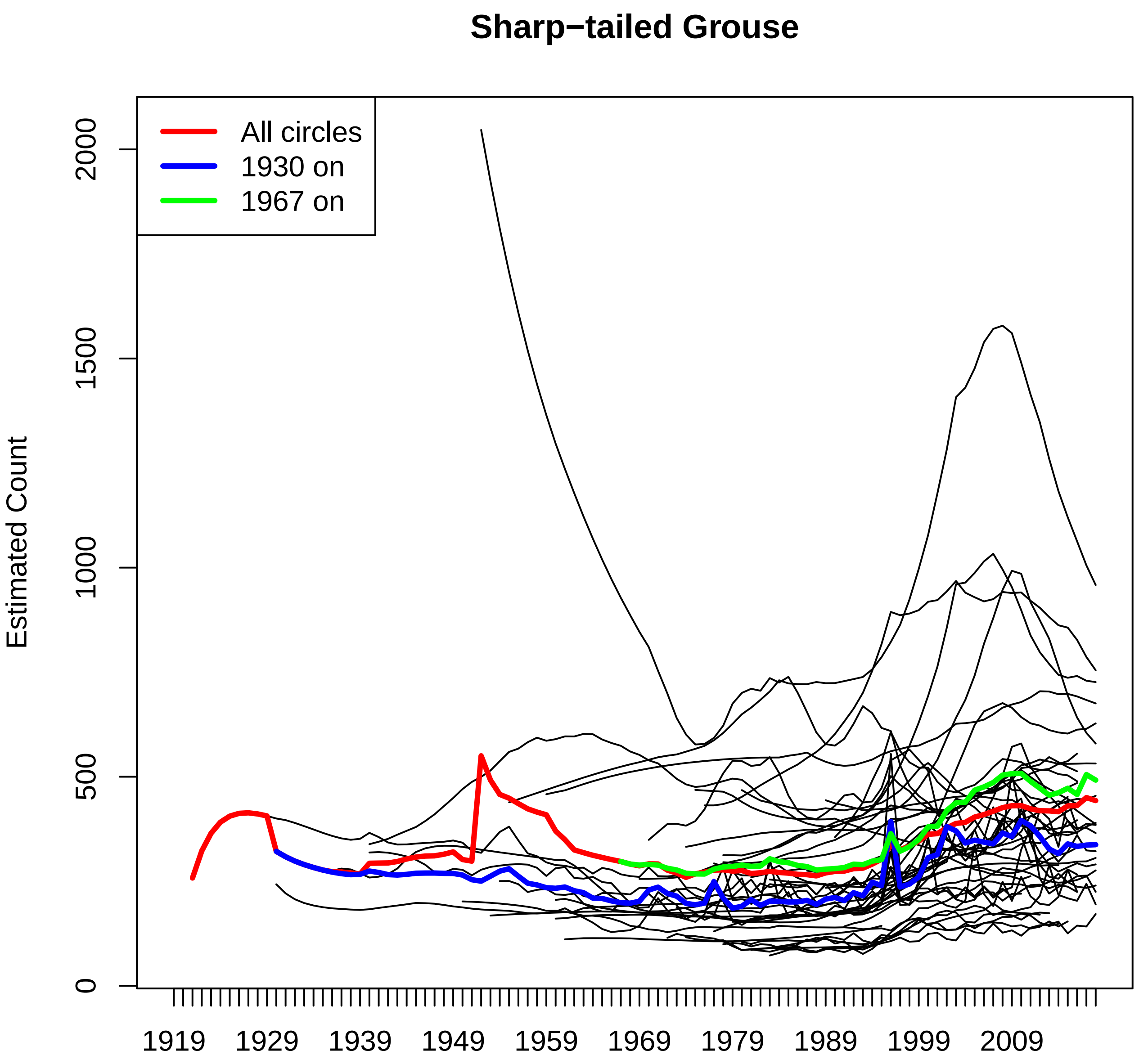

Sharp−tailed Grouse
Estimated Count
0
500
1000
1500
2000
1919
1929
1939
1949
1959
1969
1979
1989
1999
2009
All circles
1930 on
1967 on

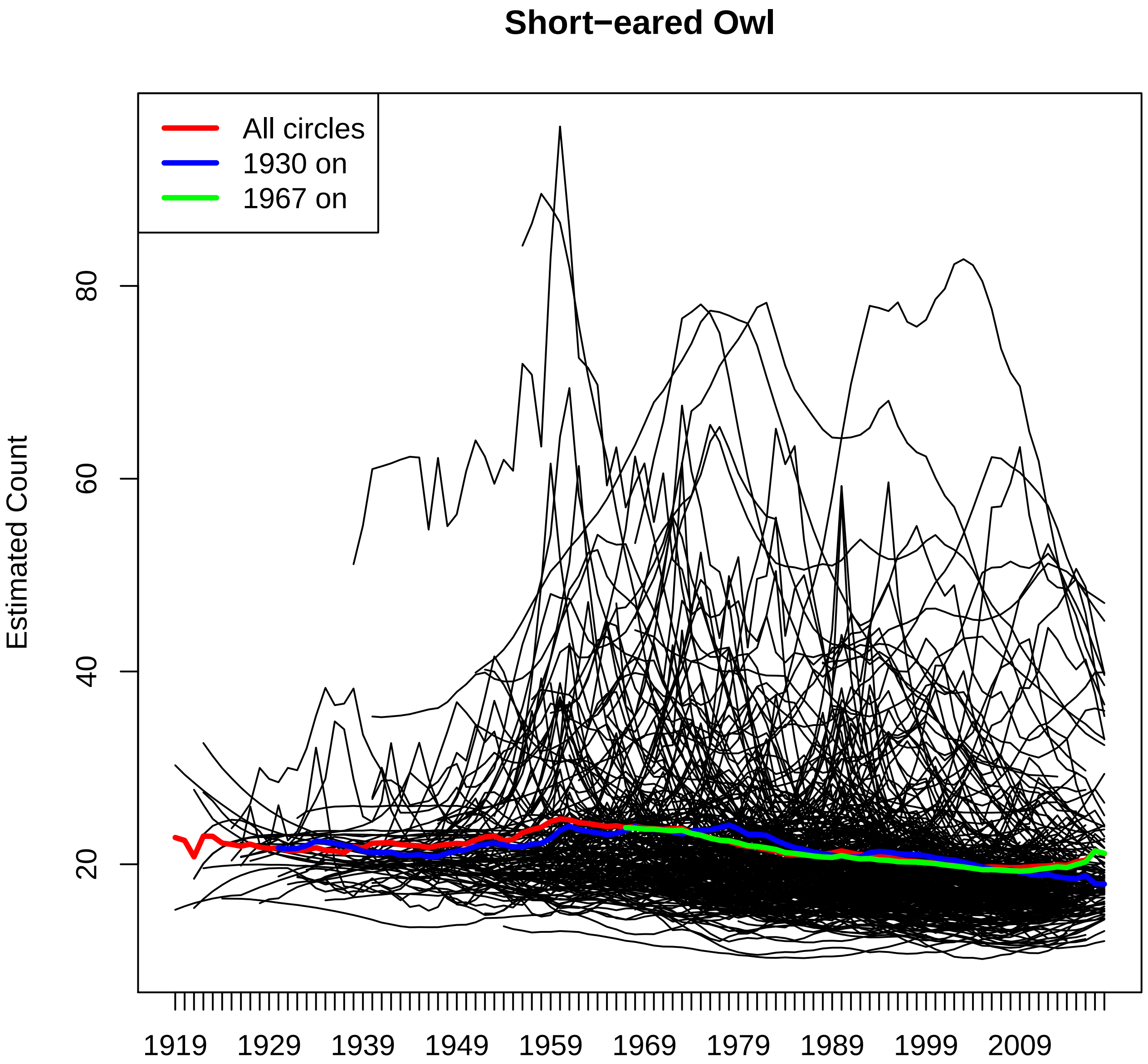
Short−eared Owl
All circles
1930 on
1967 on
Estimated Count
20
40
60
80
1919
1929
1939
1949
1959
1969
1979
1989
1999
2009

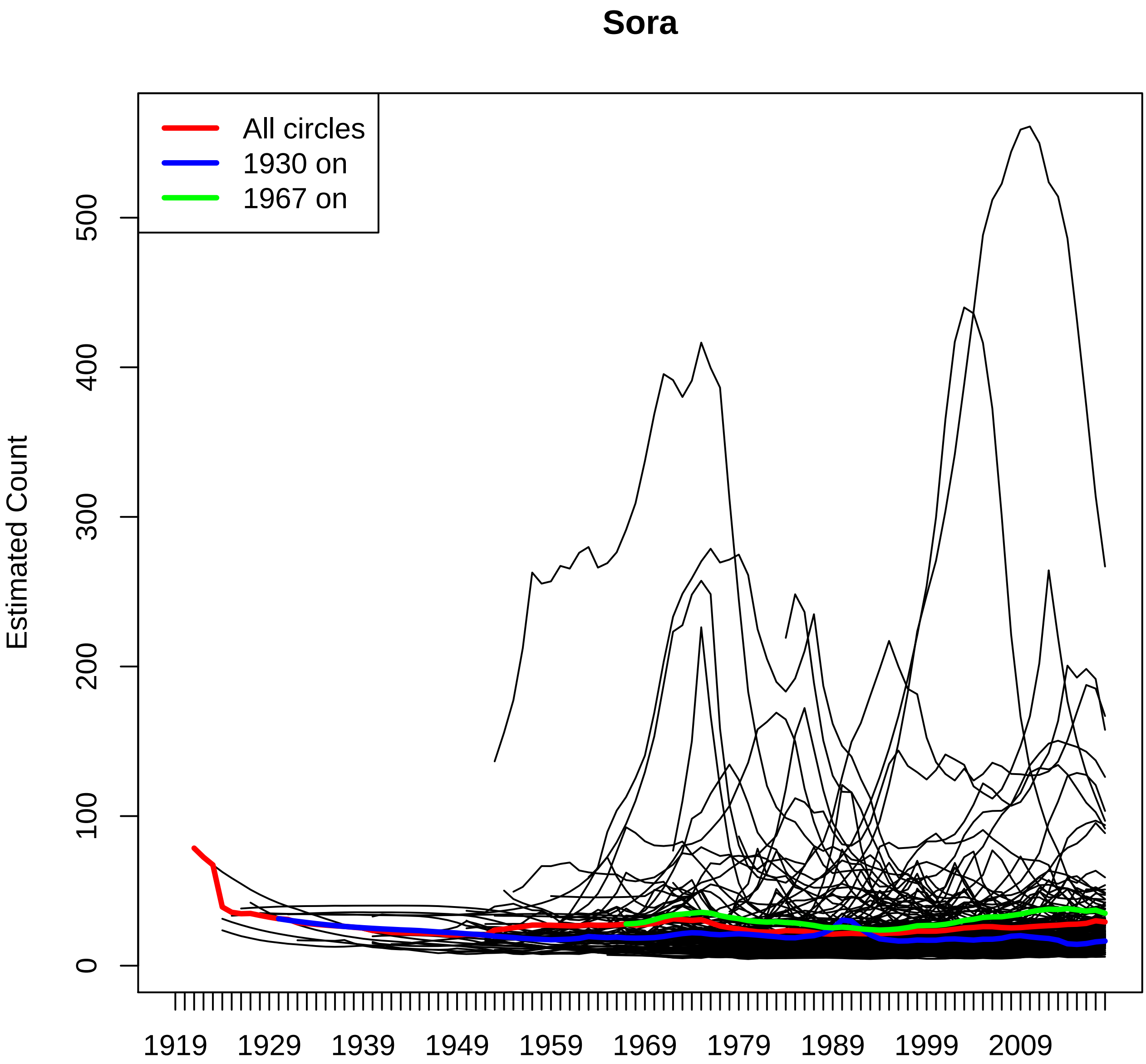

Sora
All circles
1930 on
1967 on
Estimated Count
0
100
200
300
400
500
1919
1929
1939
1949
1959
1969
1979
1989
1999
2009

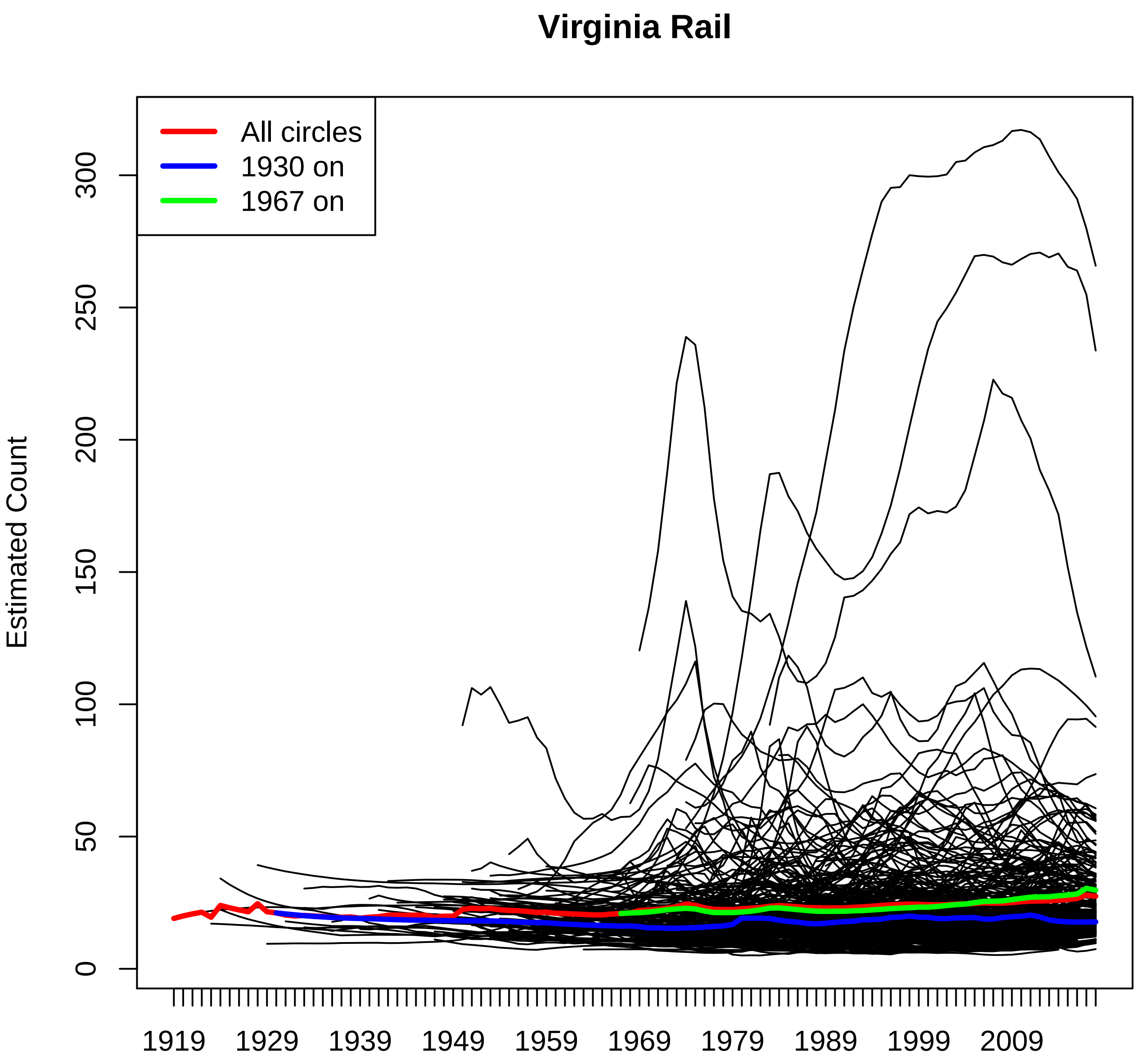

Virginia Rail
All circles
1930 on
1967 on
Estimated Count
0
50
100
150
200
250
300
1919
1929
1939
1949
1959
1969
1979
1989
1999
2009

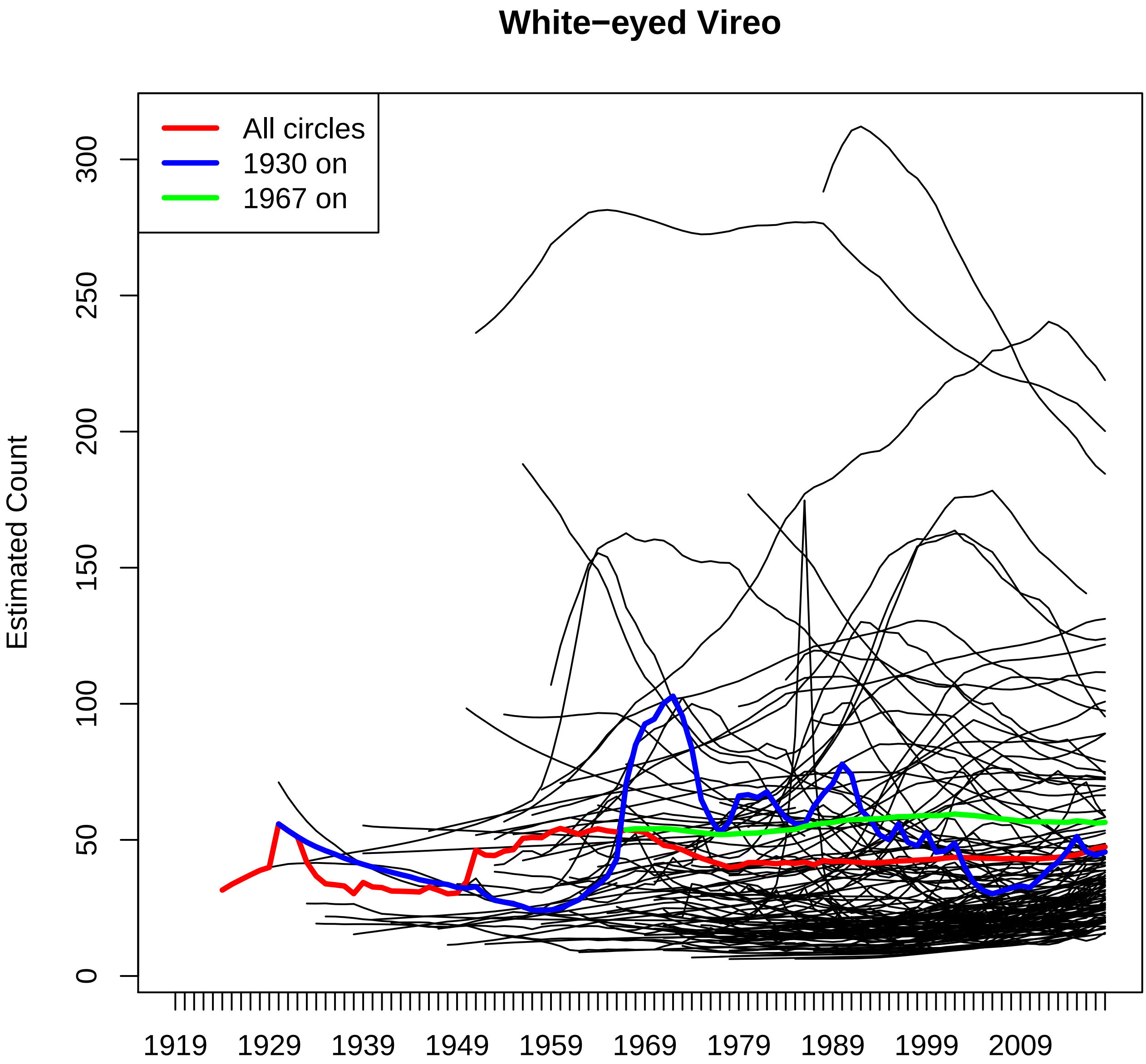

White−eyed Vireo
All circles
1930 on
1967 on
Estimated Count
0
50
100
150
200
250
300
1919
1929
1939
1949
1959
1969
1979
1989
1999
2009

Winter Wren

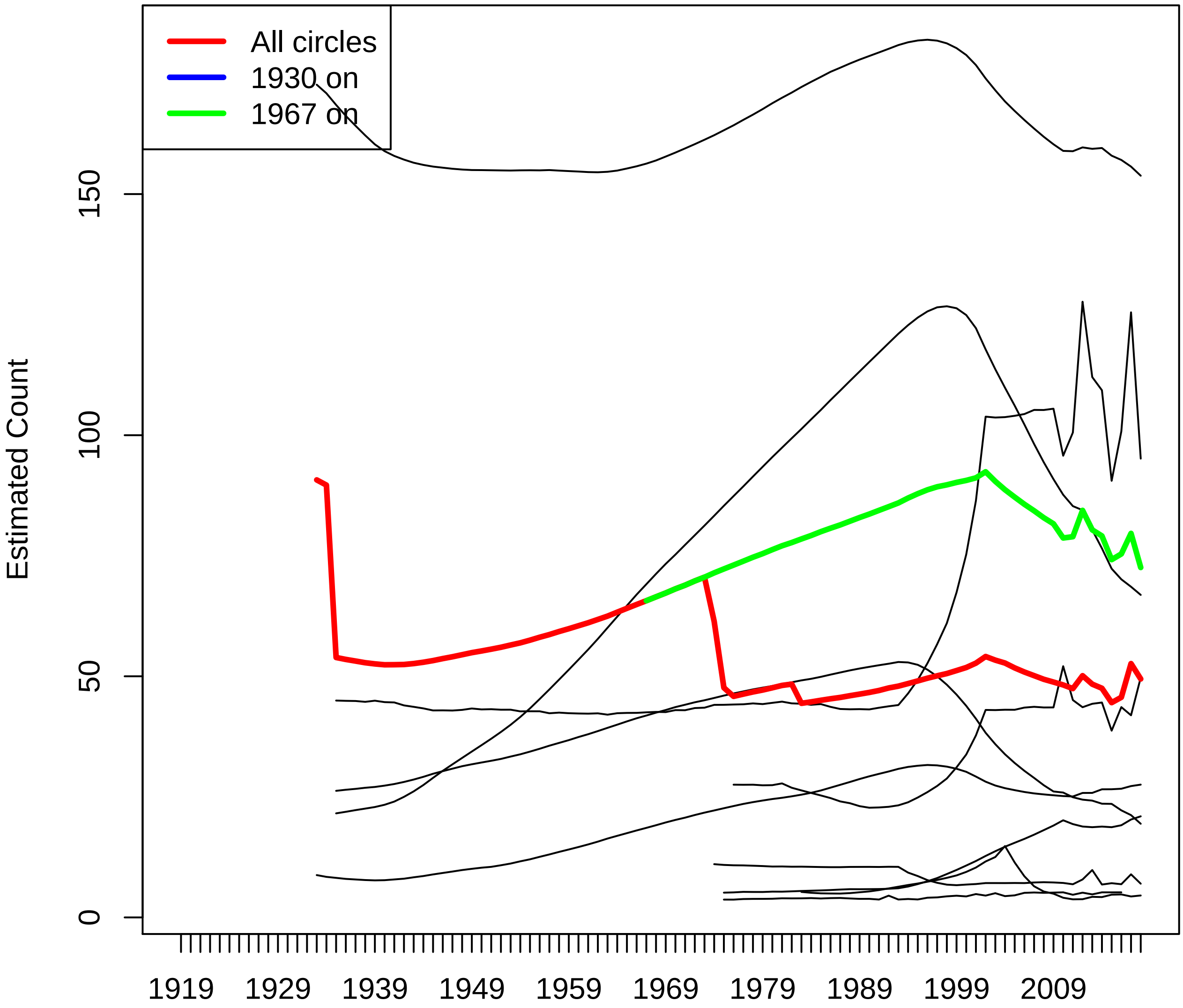

All circles
1930 on
1967 on
Estimated Count
0
50
100
150
1919
1929
1939
1949
1959
1969
1979
1989
1999
2009

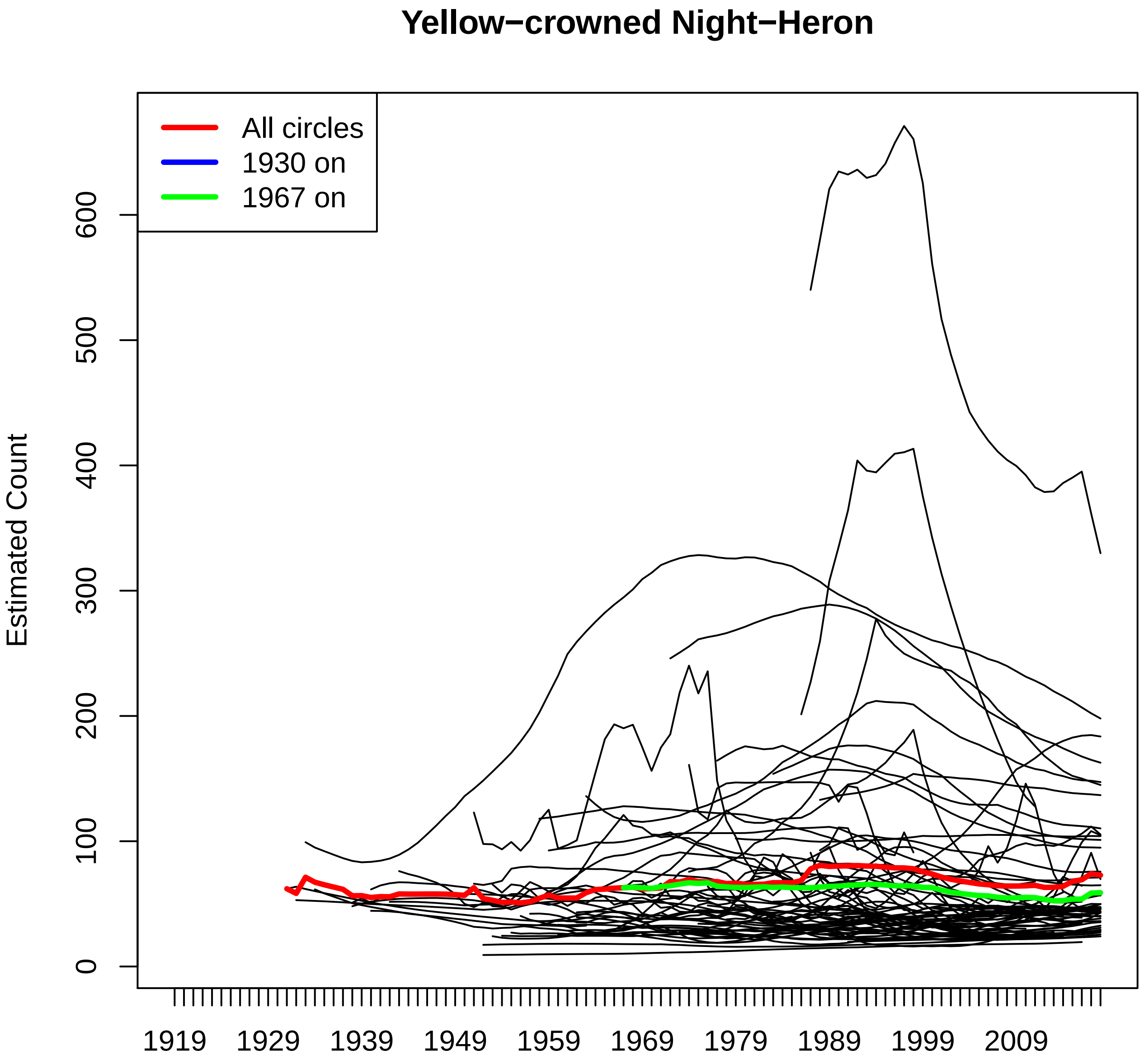

Yellow−crowned Night−Heron
All circles
1930 on
1967 on
Estimated Count
0
100
200
300
400
500
600
1919
1929
1939
1949
1959
1969
1979
1989
1999
2009

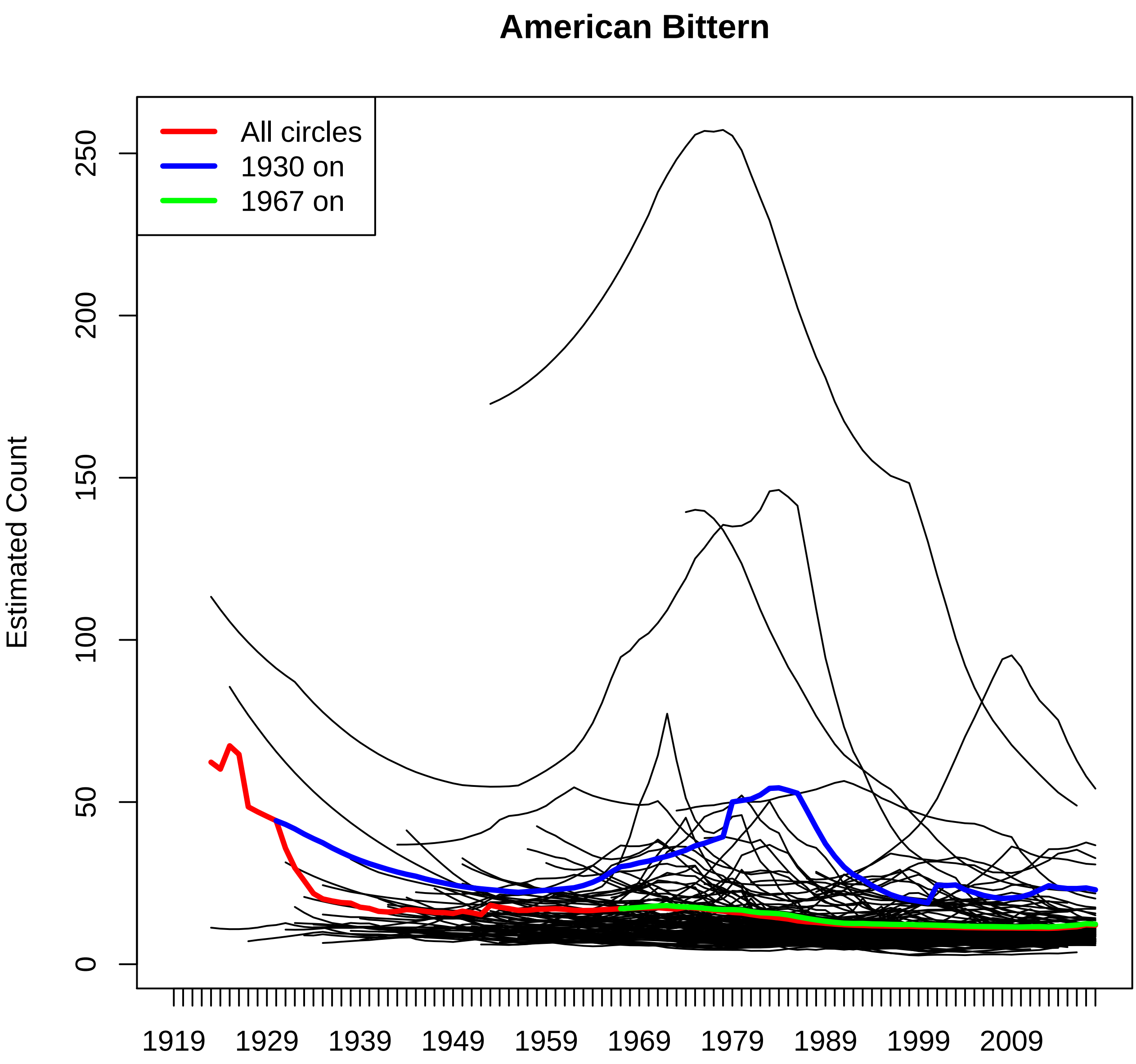
American Bittern
Estimated Count
0
50
100
150
200
250
1919
1929
1939
1949
1959
1969
1979
1989
1999
2009
All circles
1930 on
1967 on

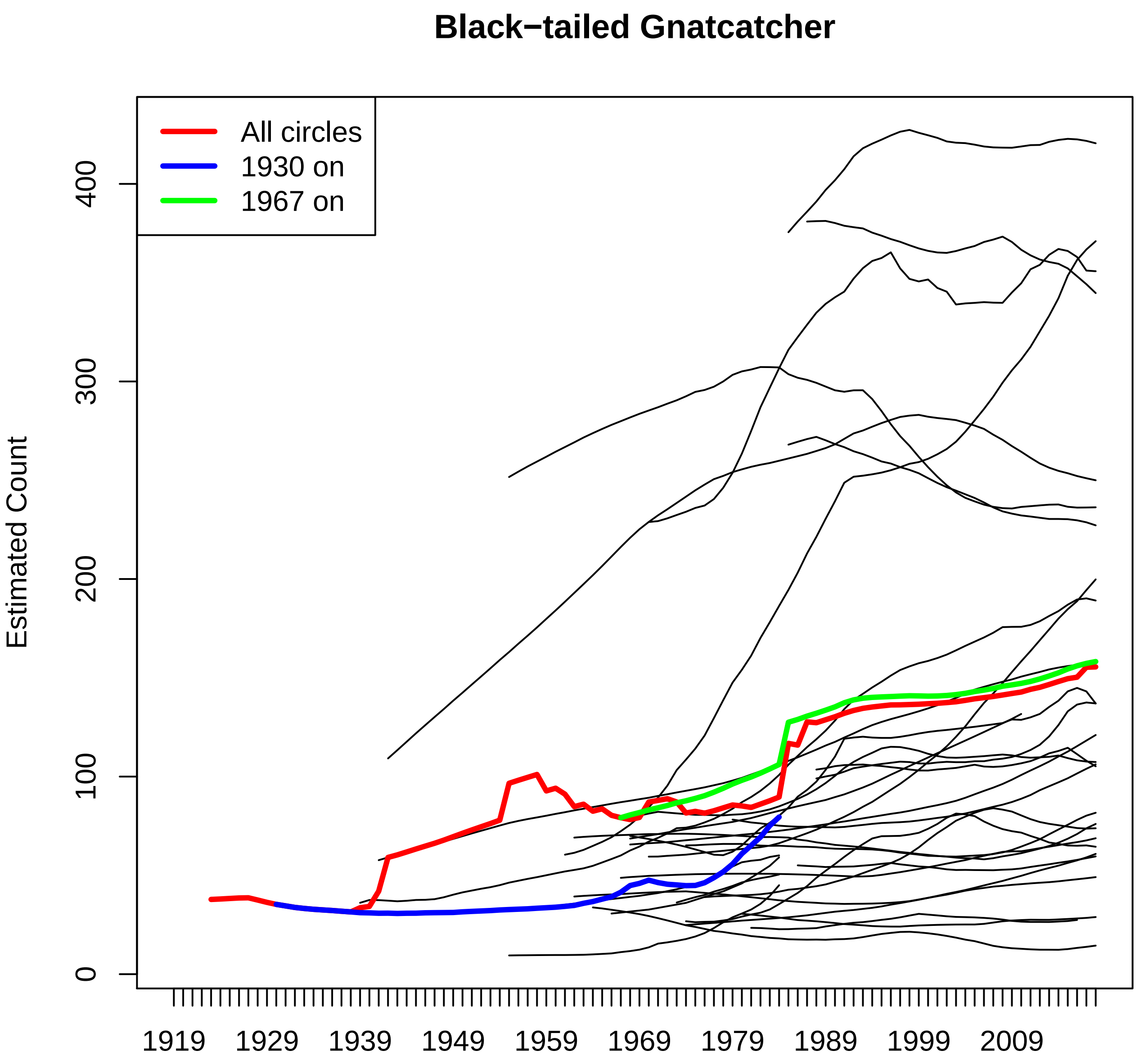
Black−tailed Gnatcatcher
All circles
1930 on
1967 on
Estimated Count
0
100
200
300
400
1919
1929
1939
1949
1959
1969
1979
1989
1999
2009

Black Rosy−Finch

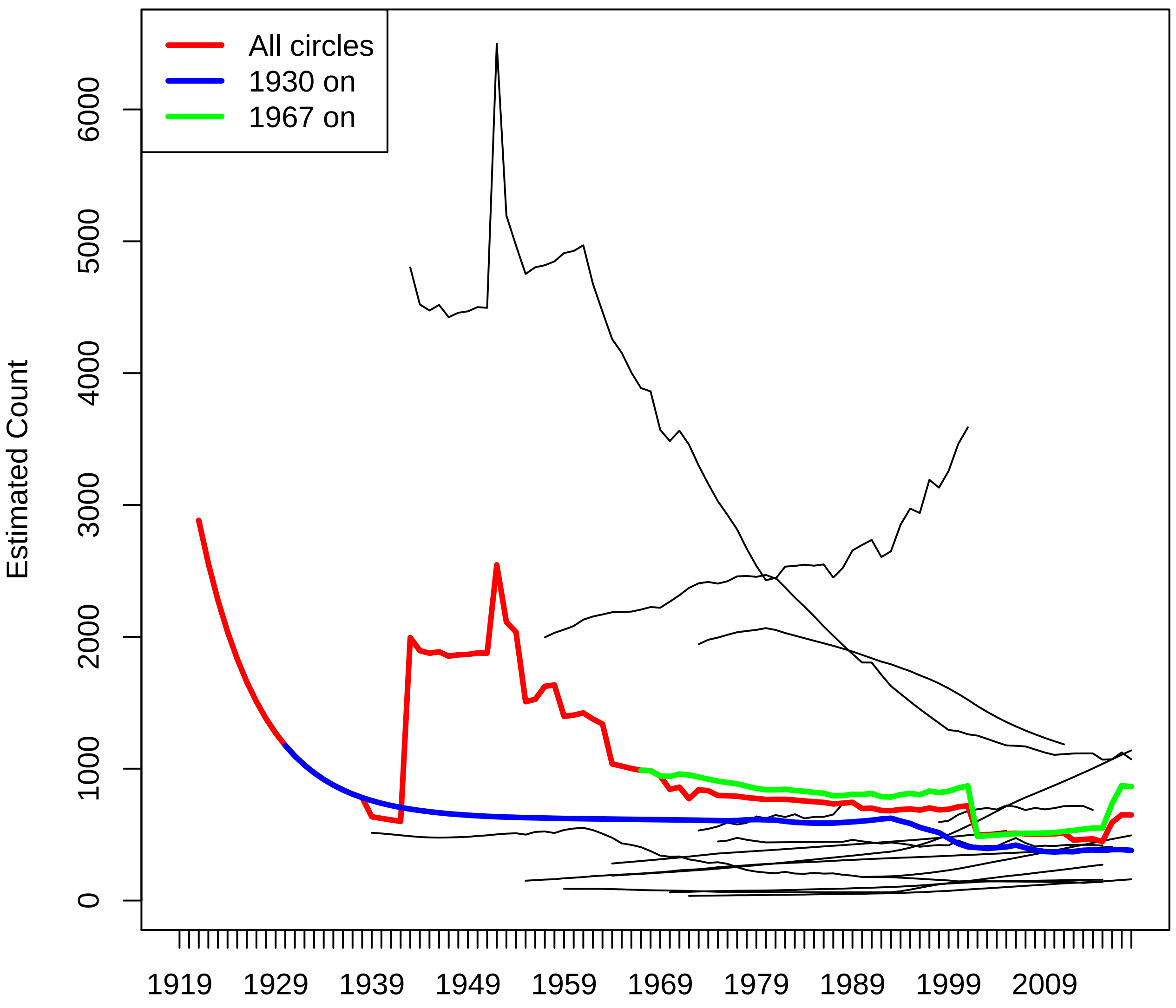

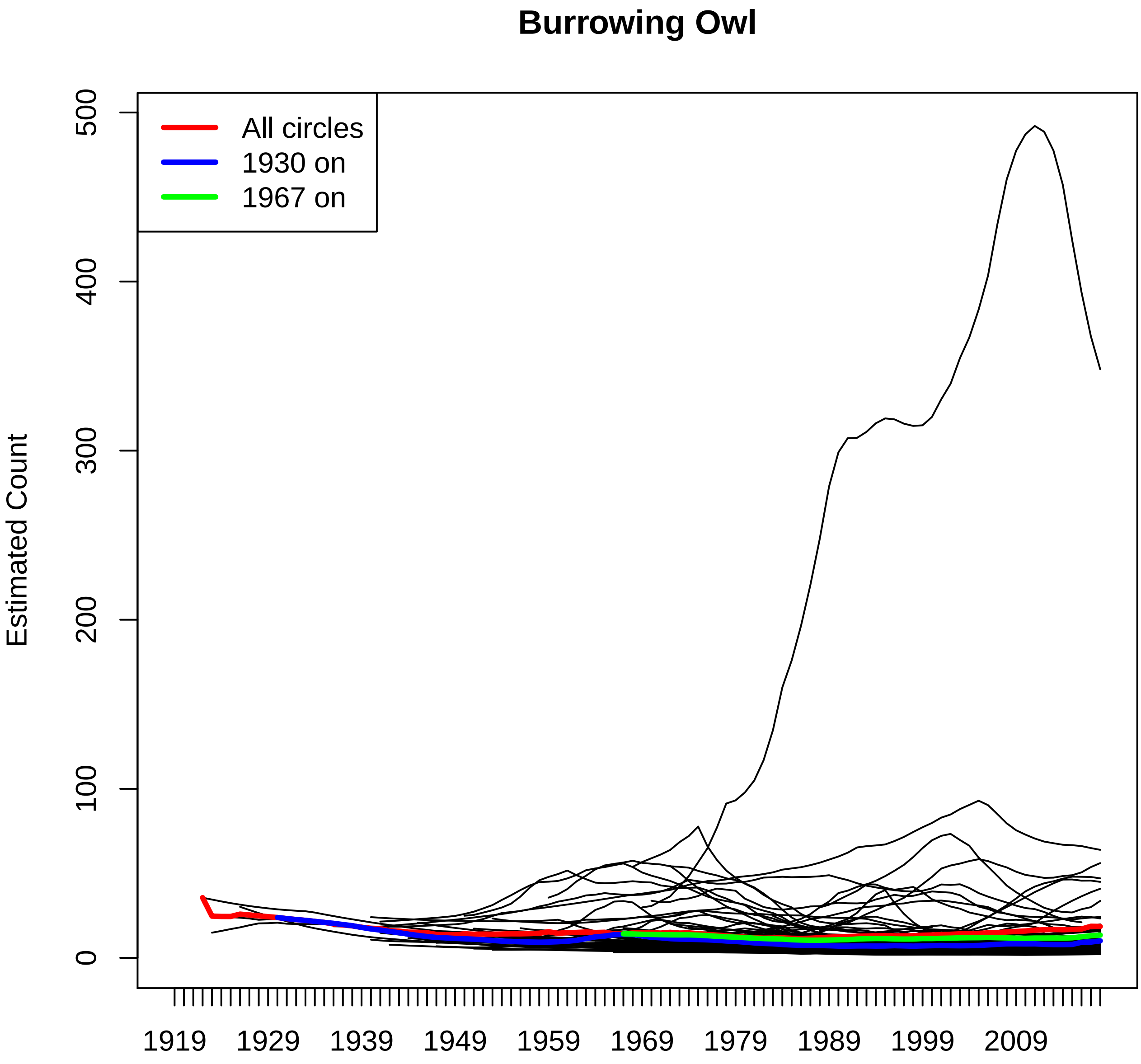

Burrowing Owl
All circles
1930 on
1967 on
Estimated Count
0
100
200
300
400
500
1919
1929
1939
1949
1959
1969
1979
1989
1999
2009

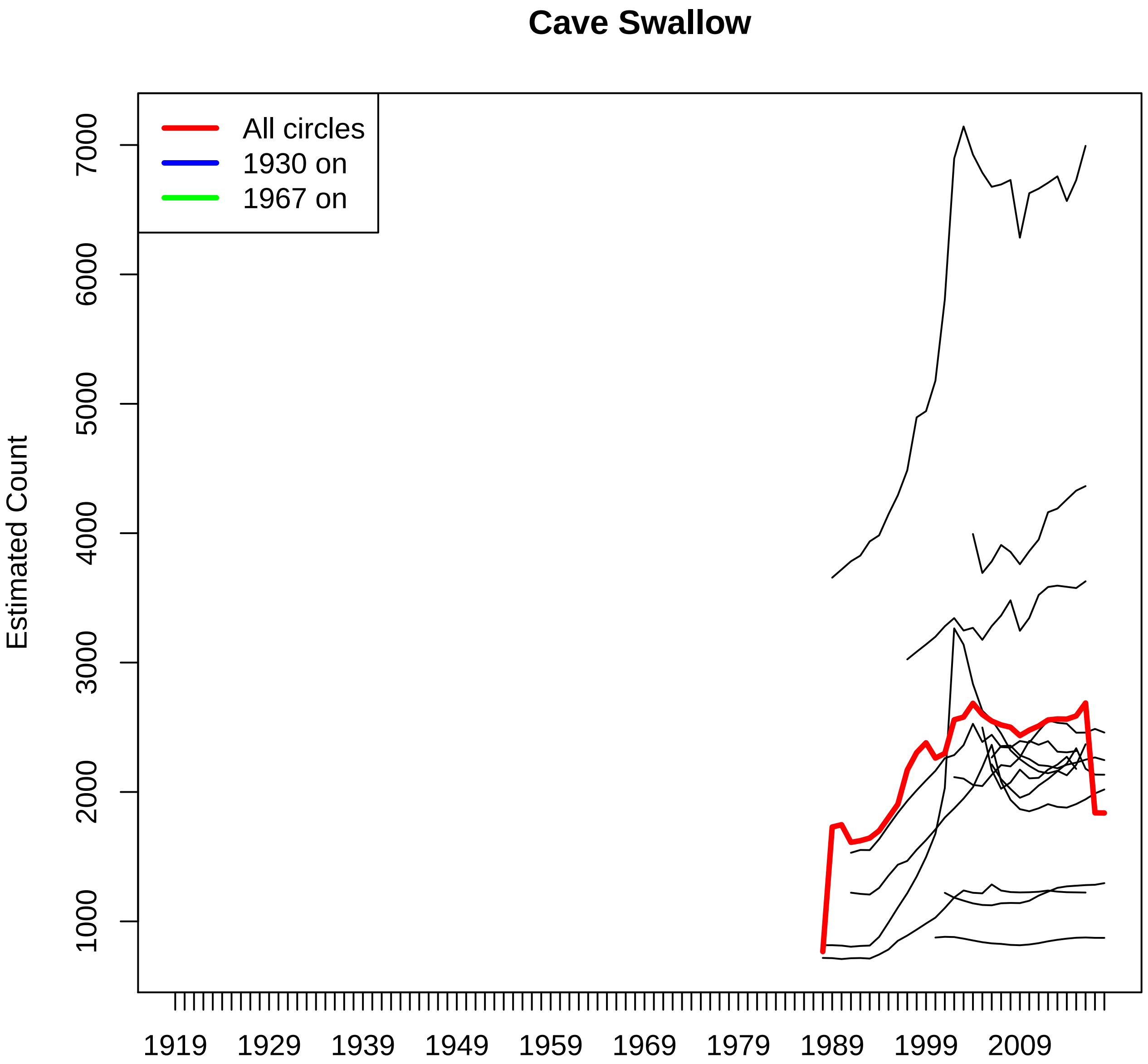
Cave Swallow
All circles
1930 on
1967 on
Estimated Count
1000
2000
3000
4000
5000
6000
7000
1919
1929
1939
1949
1959
1969
1979
1989
1999
2009

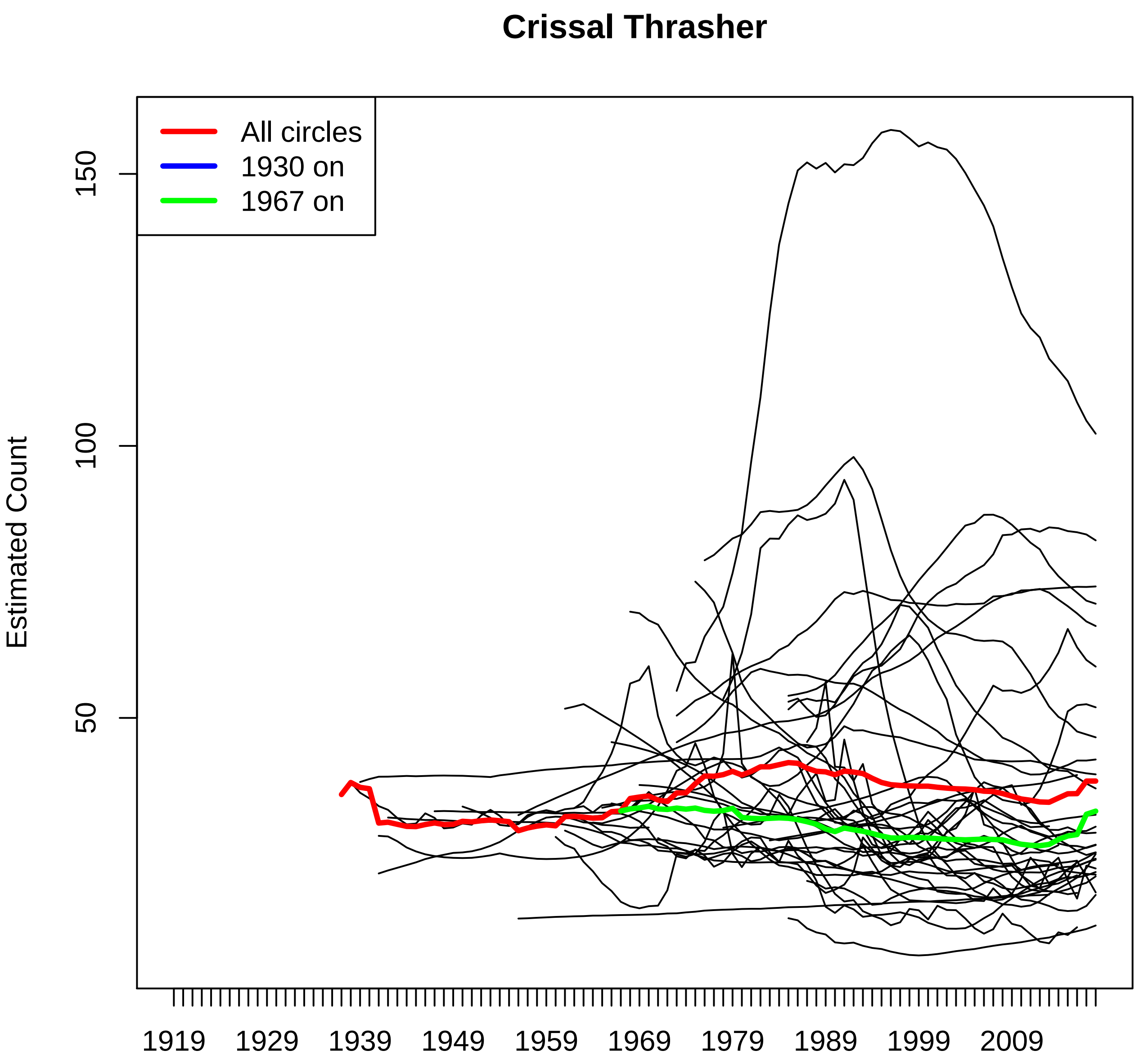
Crissal Thrasher
All circles
1930 on
1967 on
Estimated Count
50
100
150
1919
1929
1939
1949
1959
1969
1979
1989
1999
2009

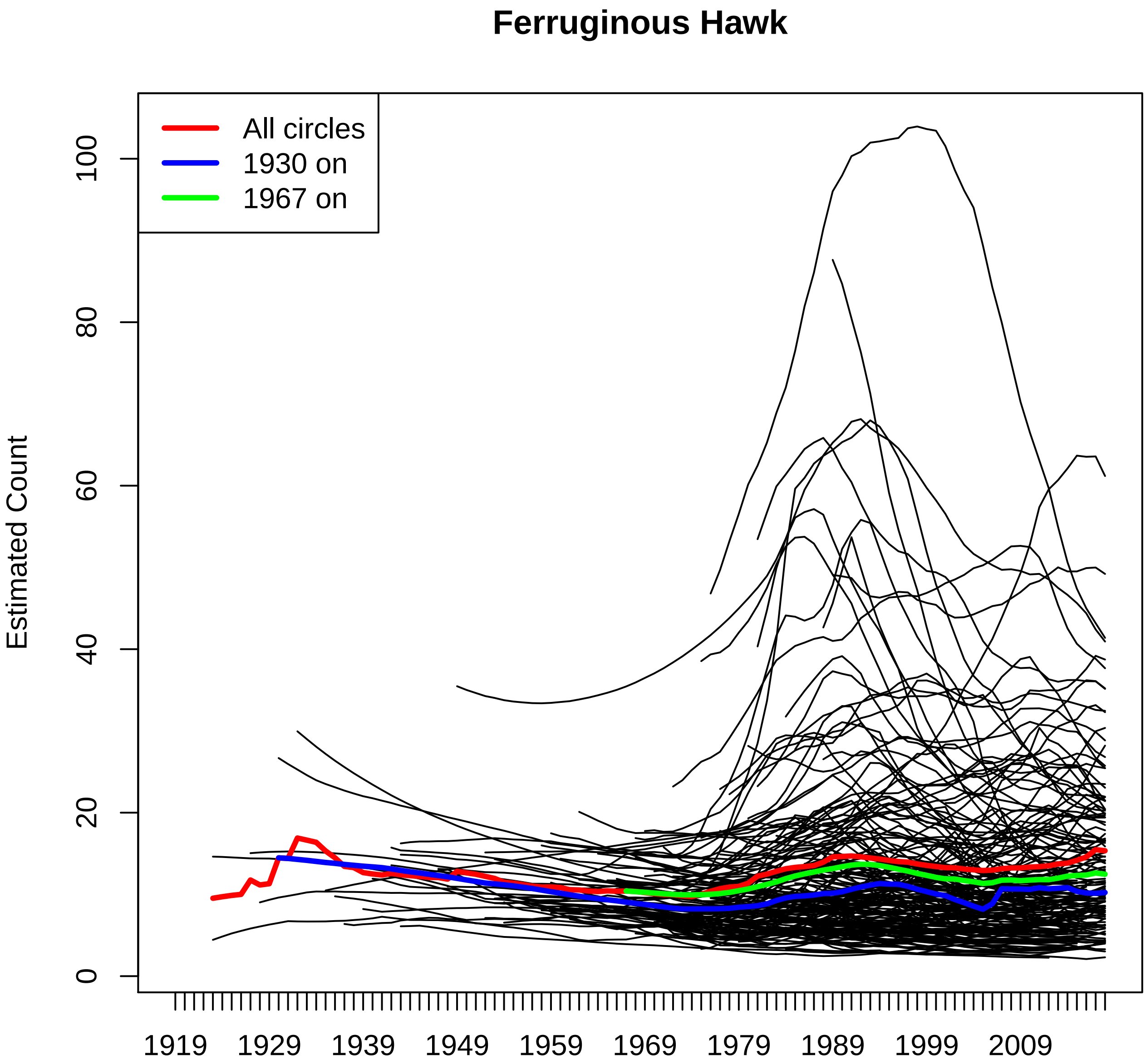

Ferruginous Hawk
All circles
1930 on
1967 on
Estimated Count
0
20
40
60
80
100
1919
1929
1939
1949
1959
1969
1979
1989
1999
2009

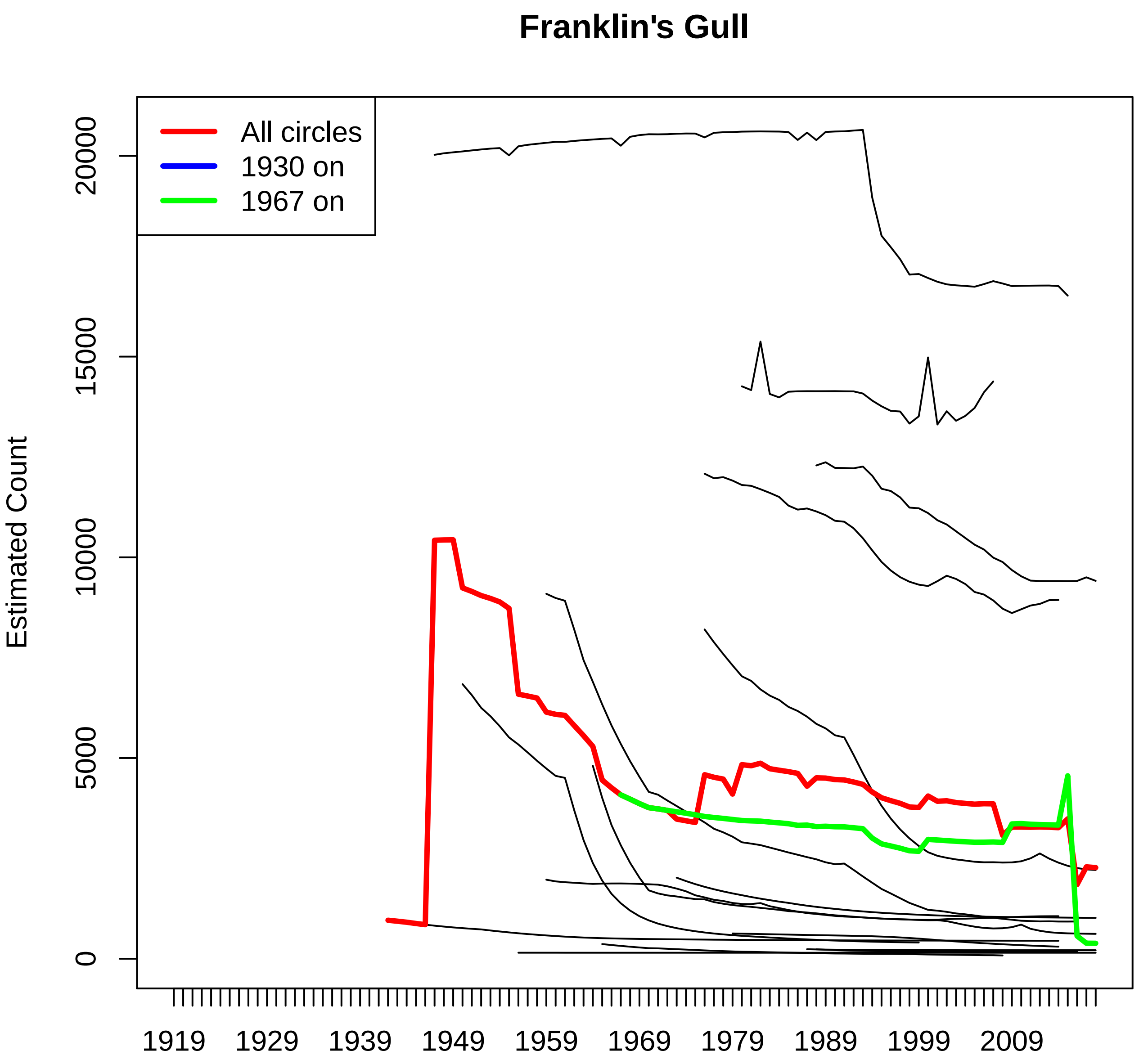
Franklin's Gull
All circles
1930 on
1967 on
Estimated Count
0
5000
10000
15000
20000
1919
1929
1939
1949
1959
1969
1979
1989
1999
2009

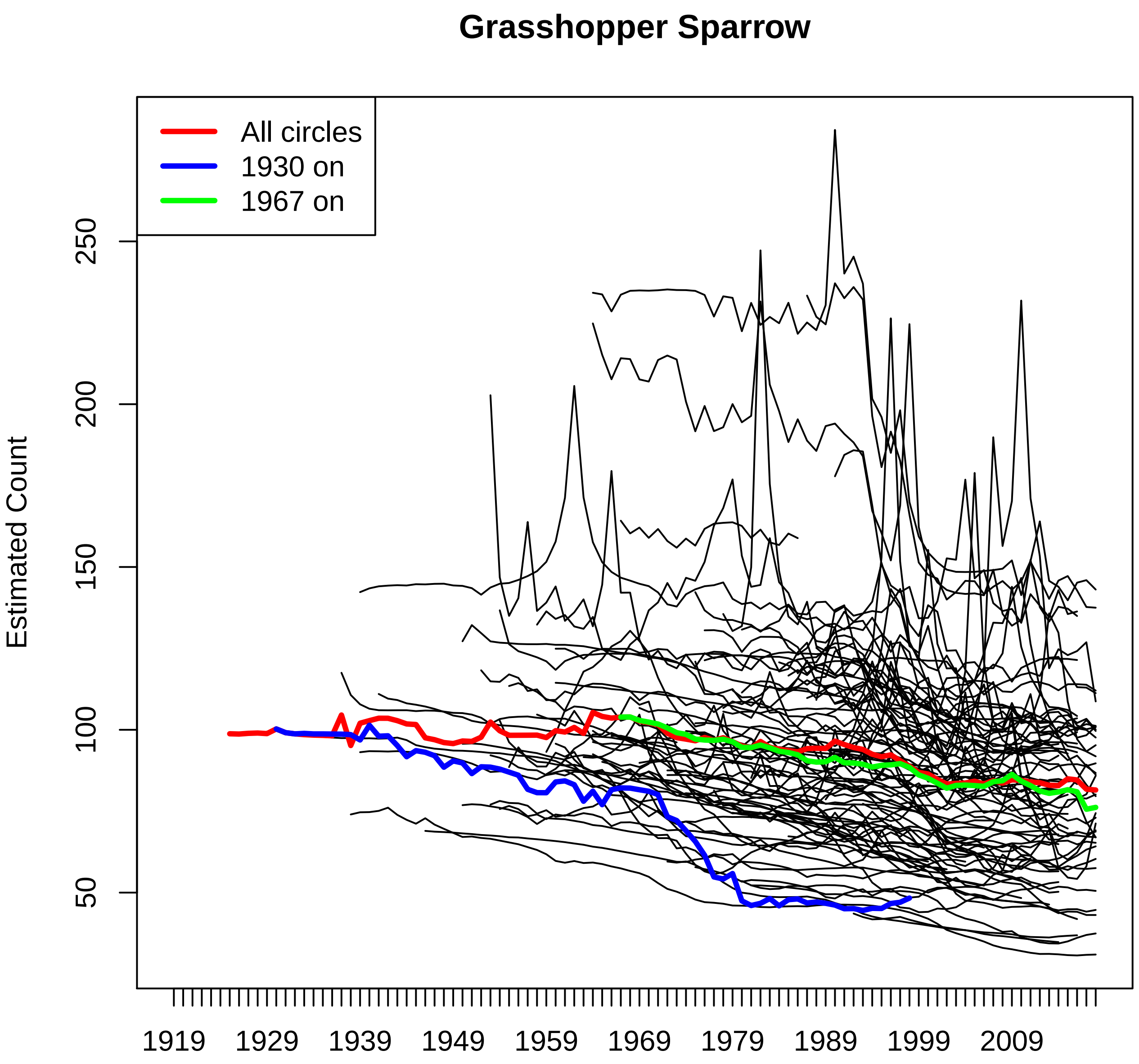

Grasshopper Sparrow
All circles
1930 on
1967 on
Estimated Count
50
100
150
200
250
1919
1929
1939
1949
1959
1969
1979
1989
1999
2009

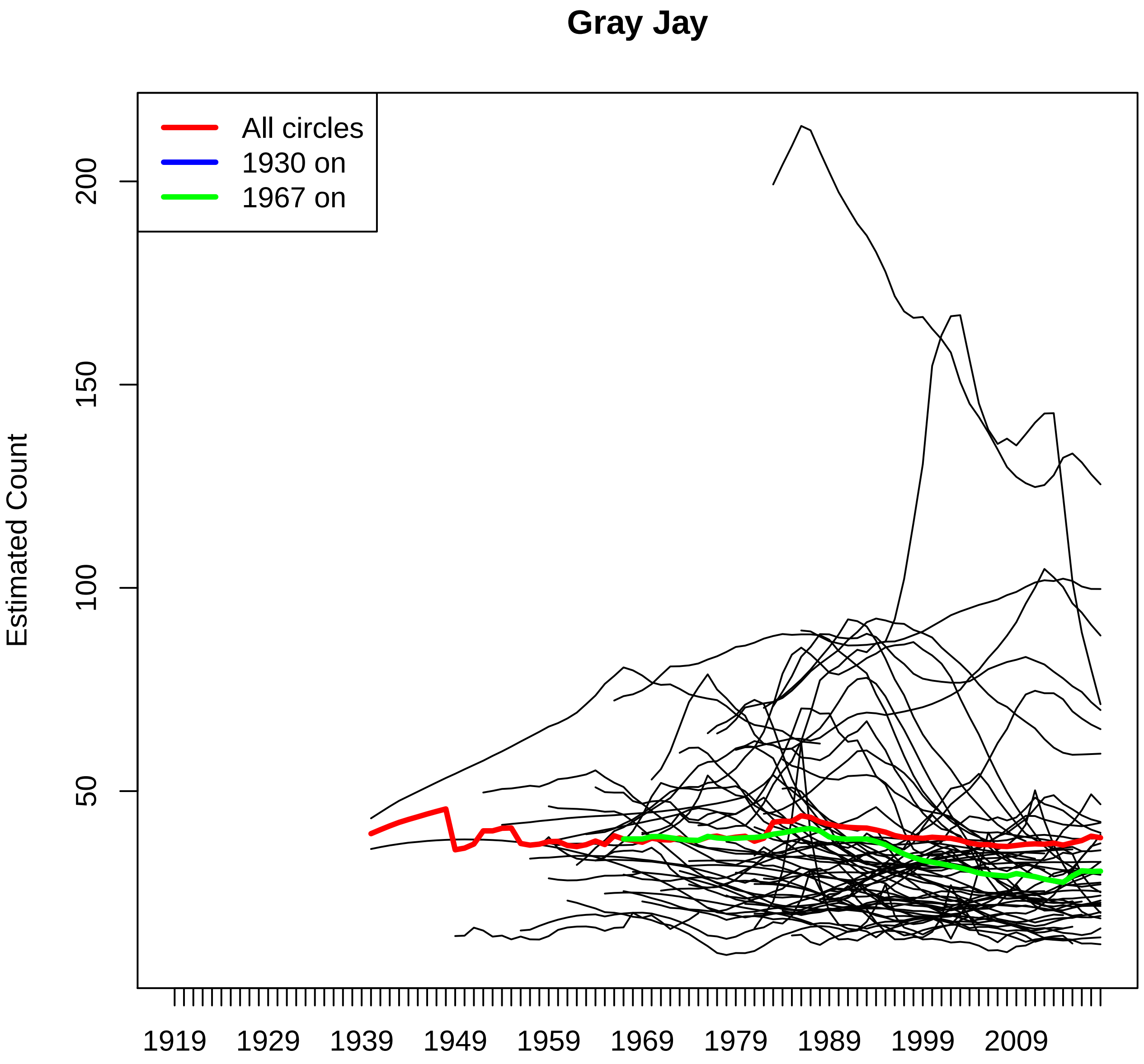
Gray Jay
All circles
1930 on
1967 on
Estimated Count
50
100
150
200
1919
1929
1939
1949
1959
1969
1979
1989
1999
2009

Great Kiskadee

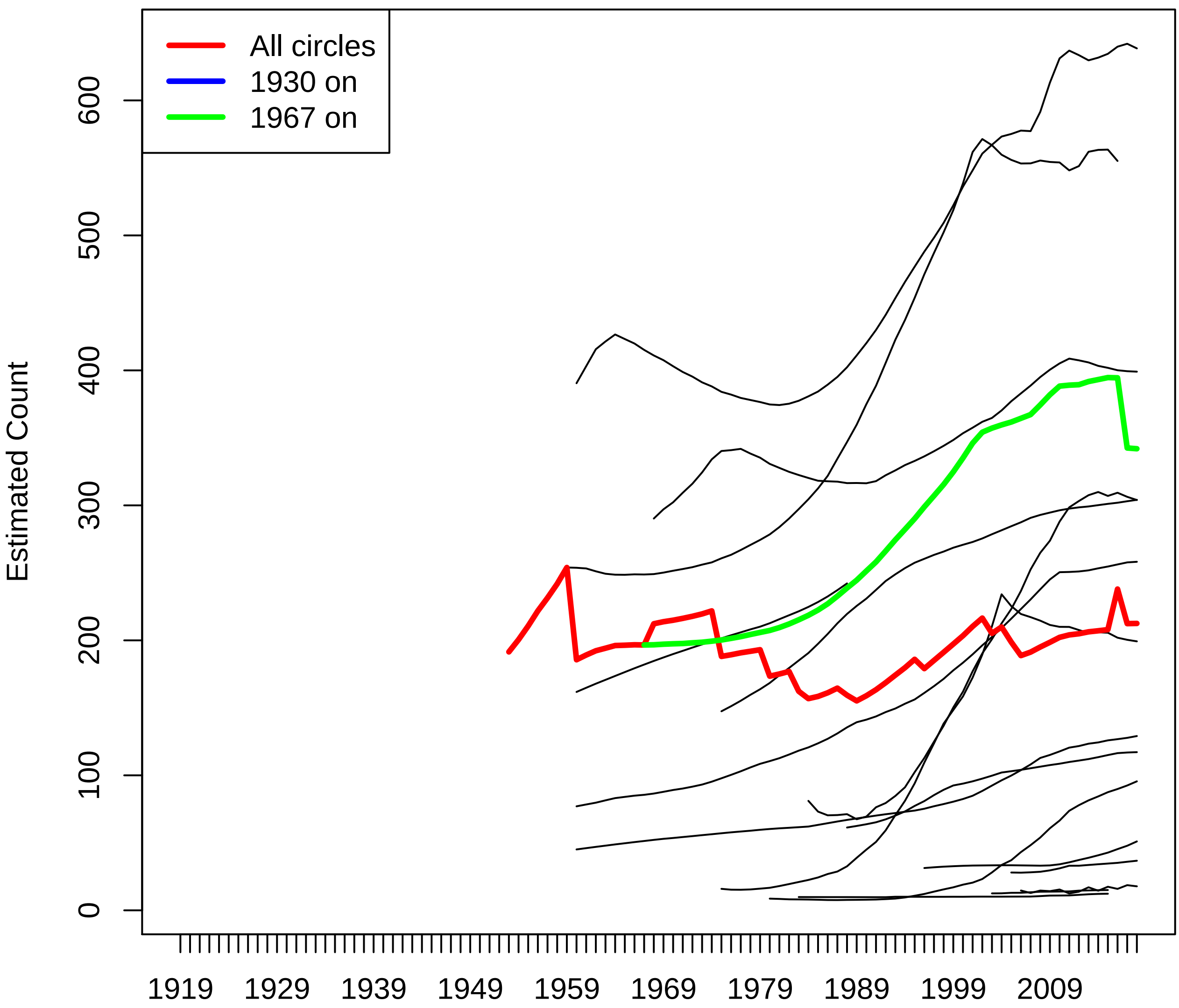

Greater Prairie−Chicken

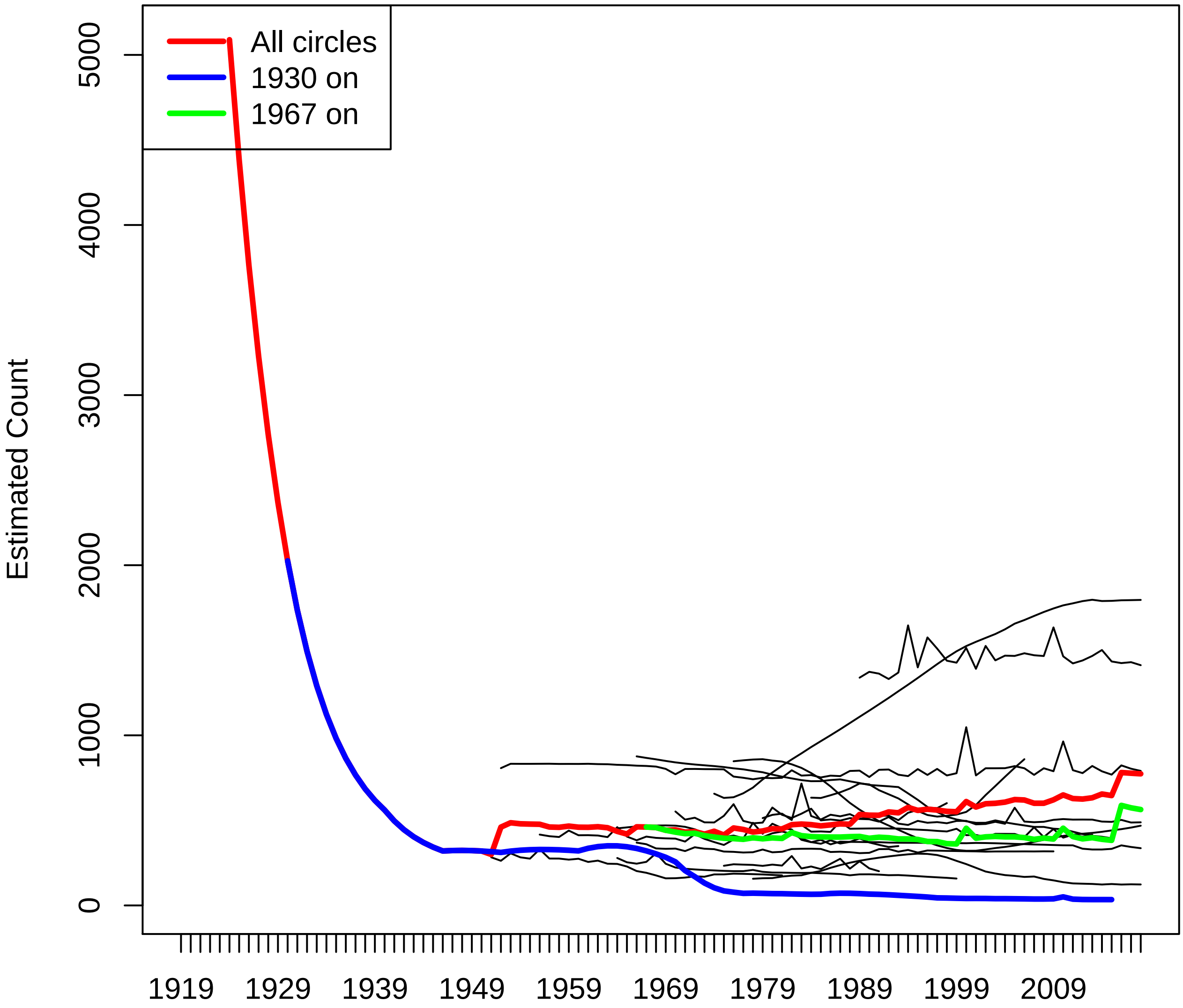

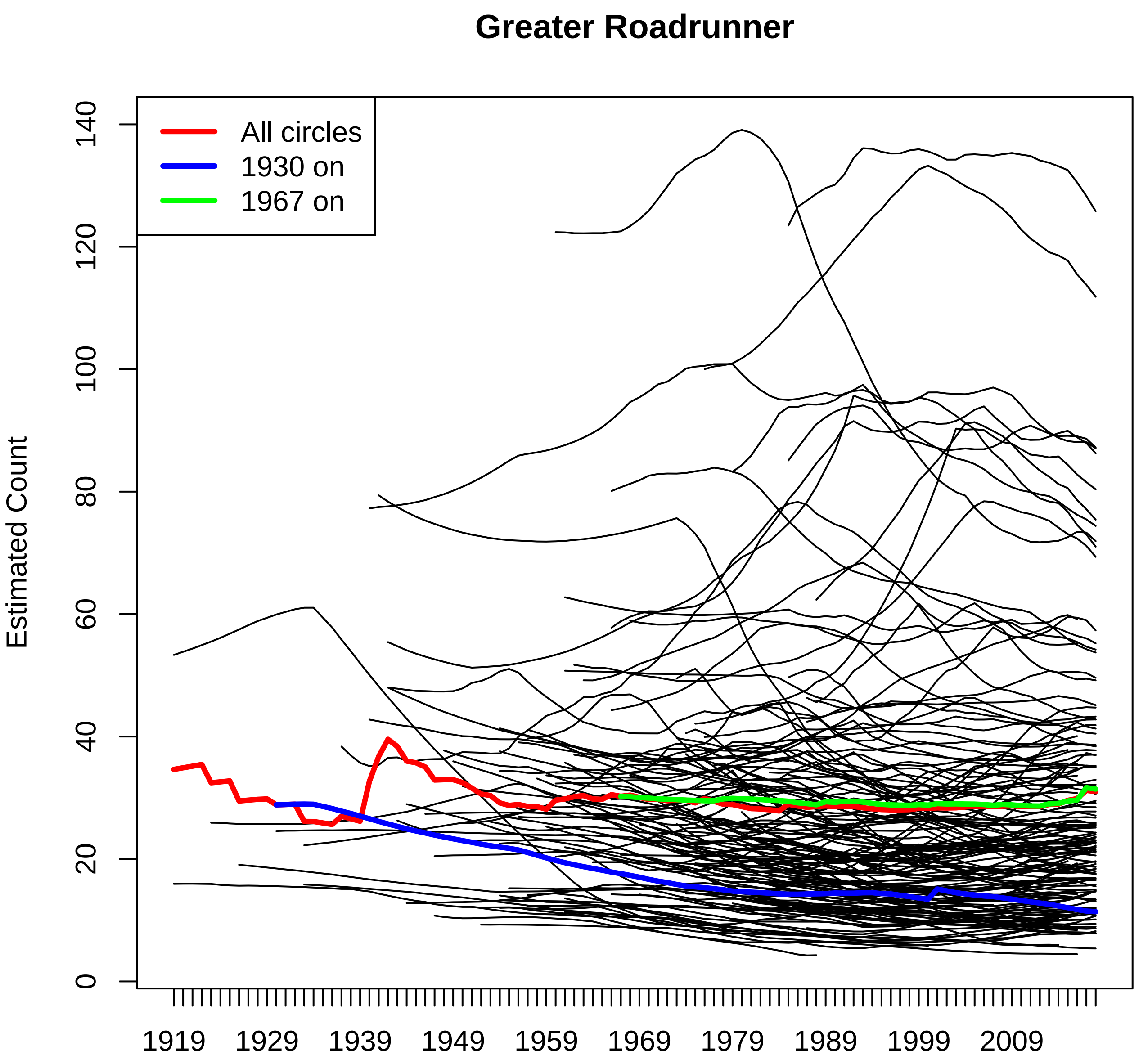

Greater Roadrunner
All circles
1930 on
1967 on
Estimated Count
0
20
40
60
80
100
120
140
1919
1929
1939
1949
1959
1969
1979
1989
1999
2009

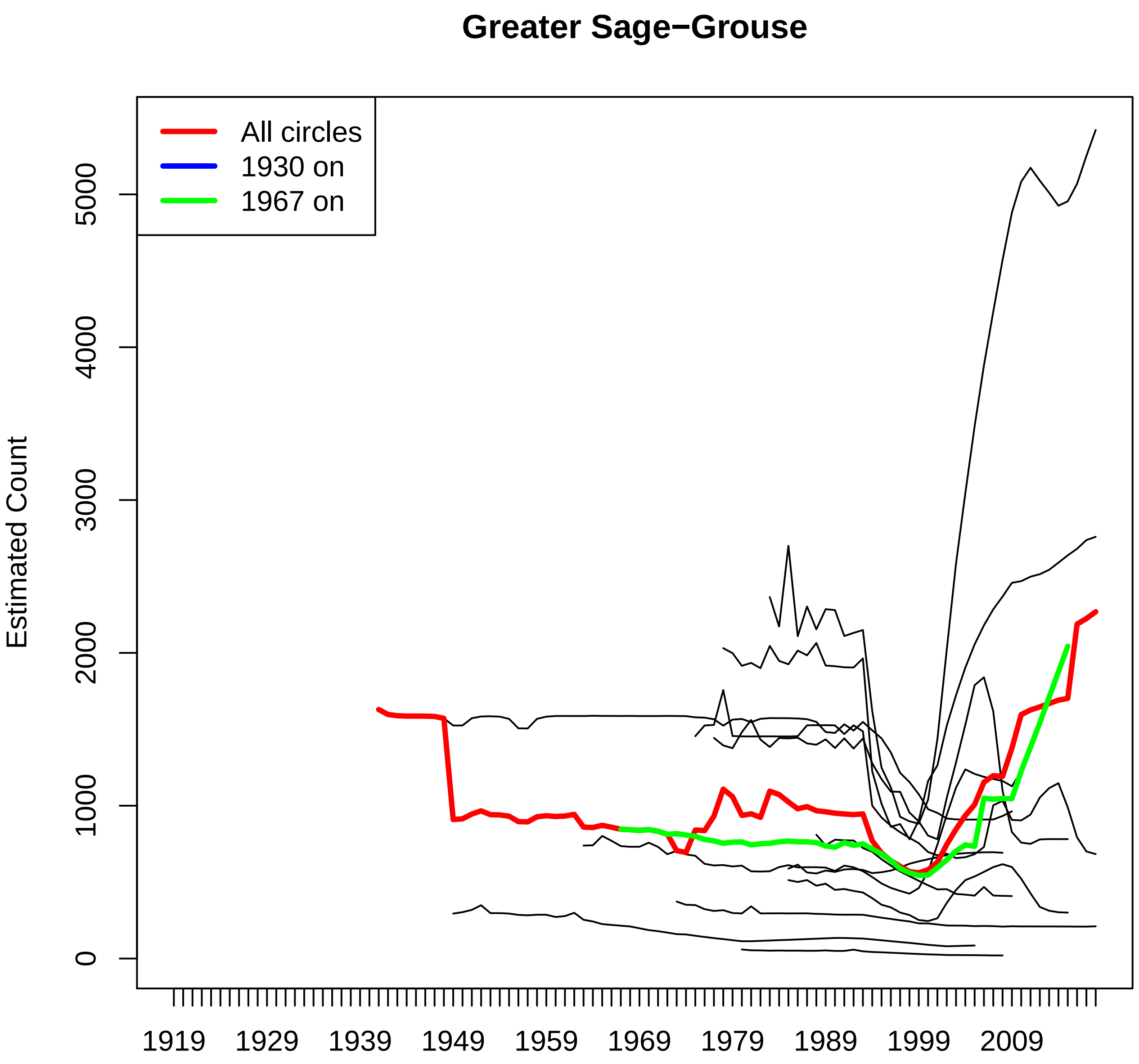

Greater Sage−Grouse
All circles
1930 on
1967 on
Estimated Count
0
1000
2000
3000
4000
5000
1919
1929
1939
1949
1959
1969
1979
1989
1999
2009

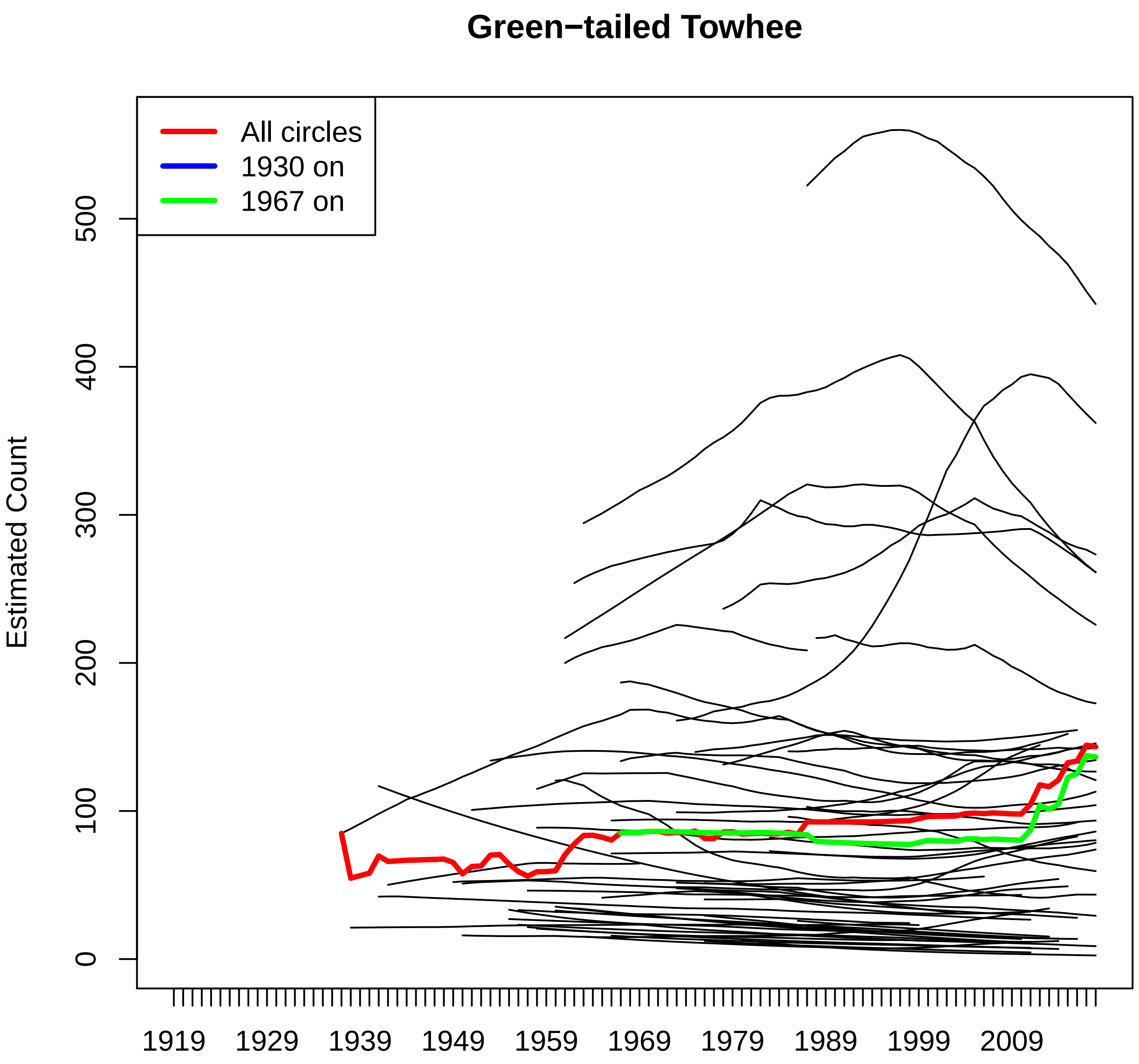
Green−tailed Towhee
All circles
1930 on
1967 on
Estimated Count
0
100
200
300
400
500
1919
1929
1939
1949
1959
1969
1979
1989
1999
2009

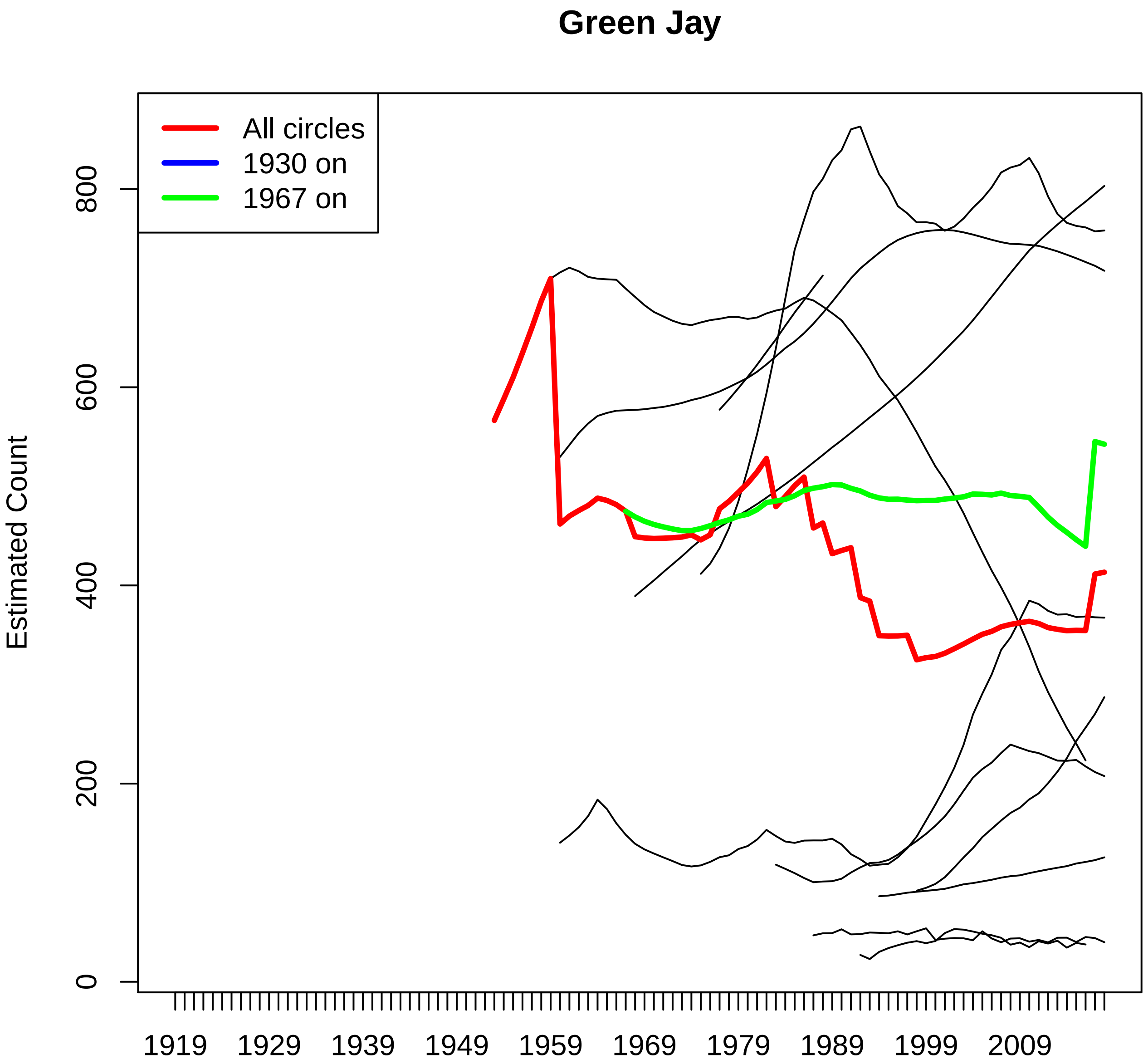

Green Jay
All circles
1930 on
1967 on
Estimated Count
0
200
400
600
800
1919
1929
1939
1949
1959
1969
1979
1989
1999
2009

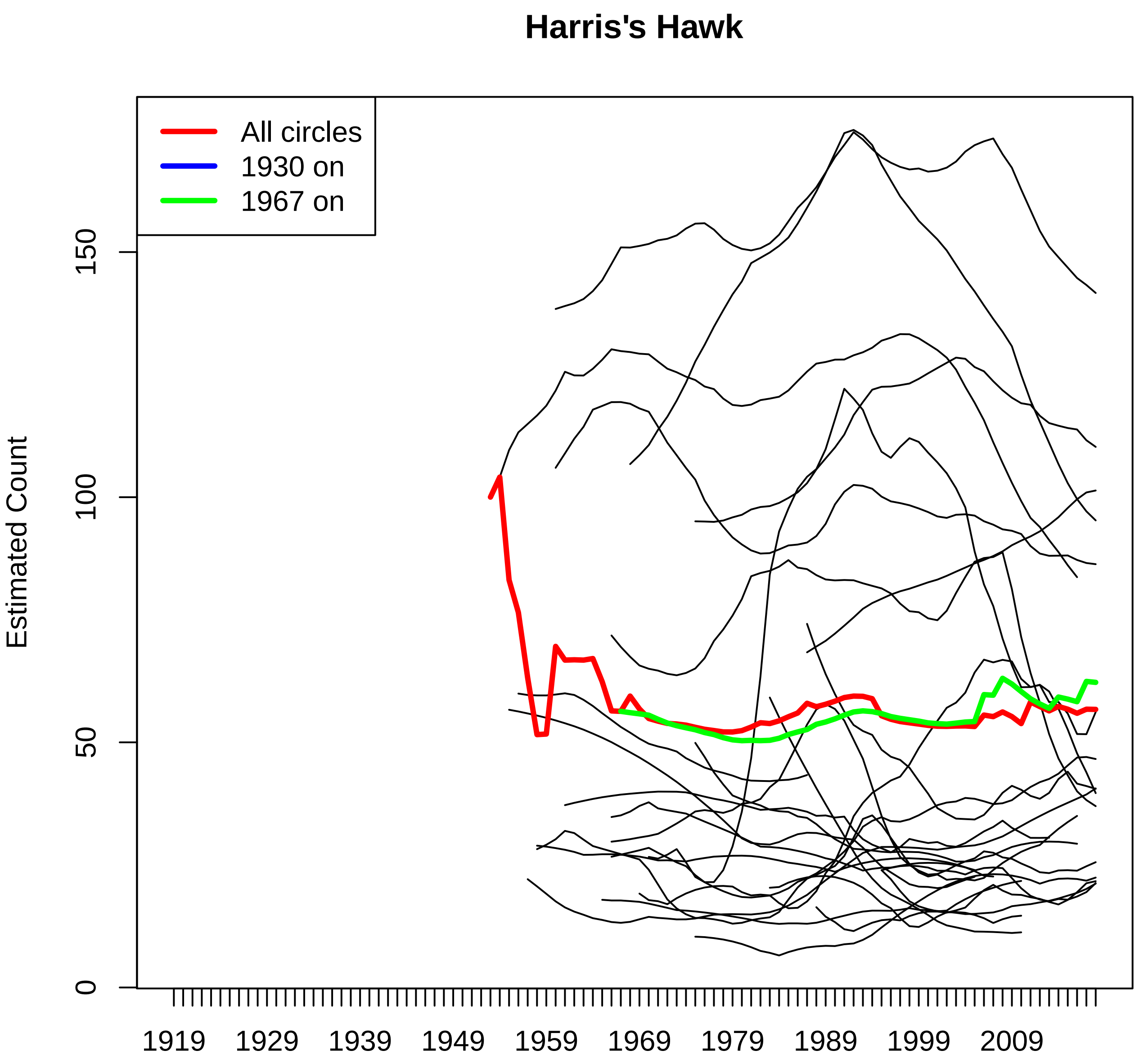
Harris's Hawk
All circles
1930 on
1967 on
Estimated Count
0
50
100
150
1919
1929
1939
1949
1959
1969
1979
1989
1999
2009

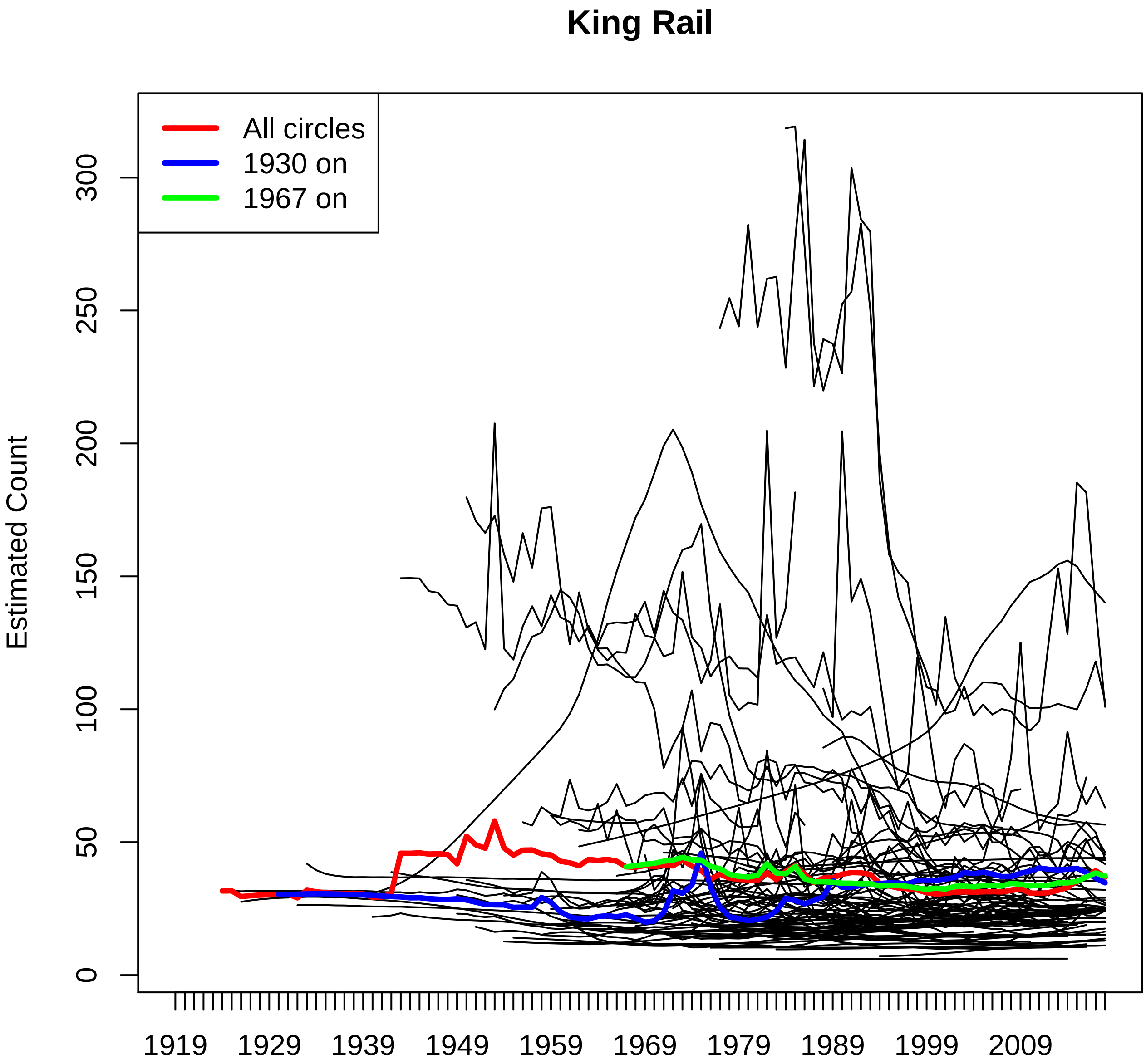
King Rail
All circles
1930 on
1967 on
Estimated Count
0
50
100
150
200
250
300
1919
1929
1939
1949
1959
1969
1979
1989
1999
2009

Lawrence's Goldfinch

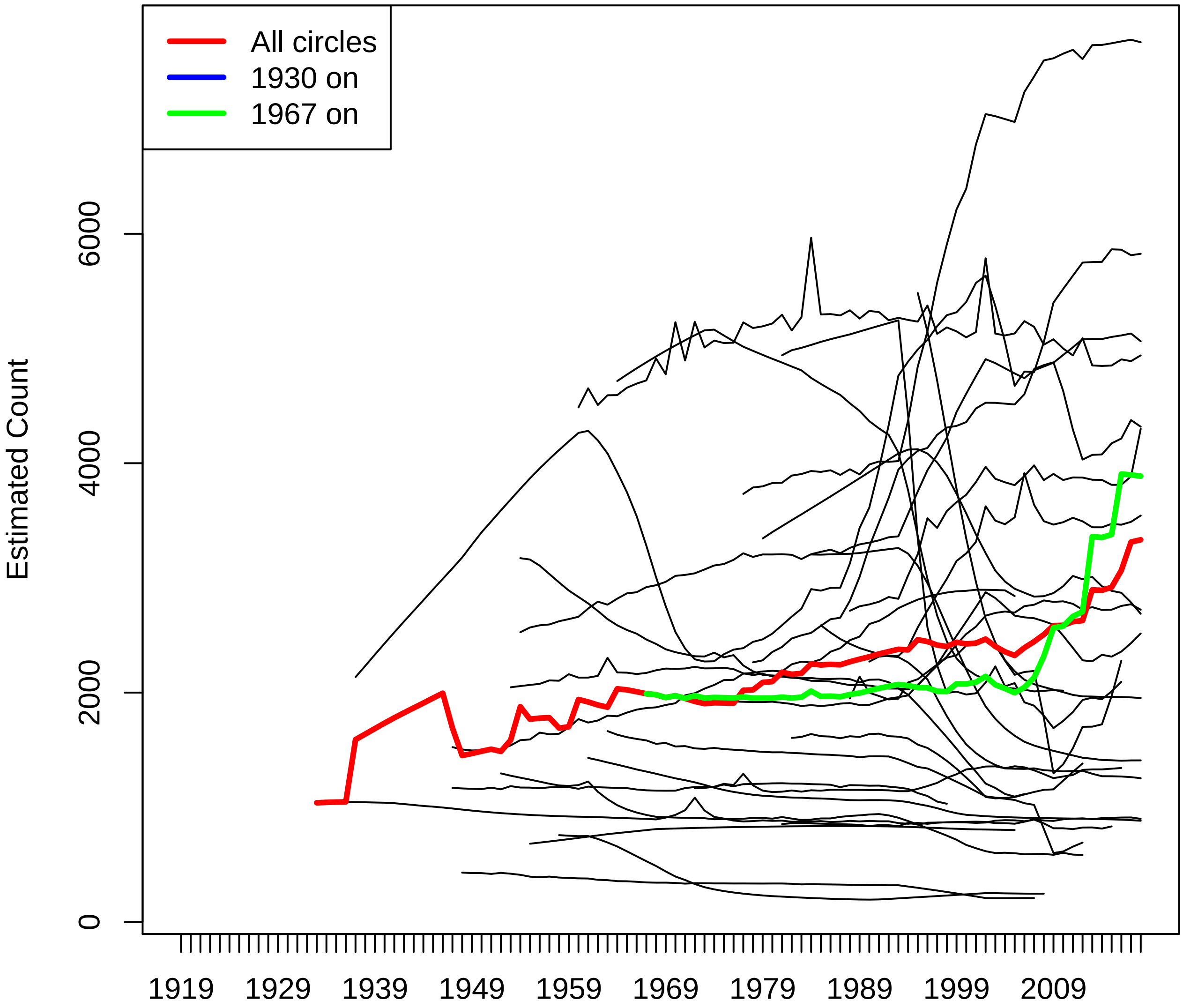

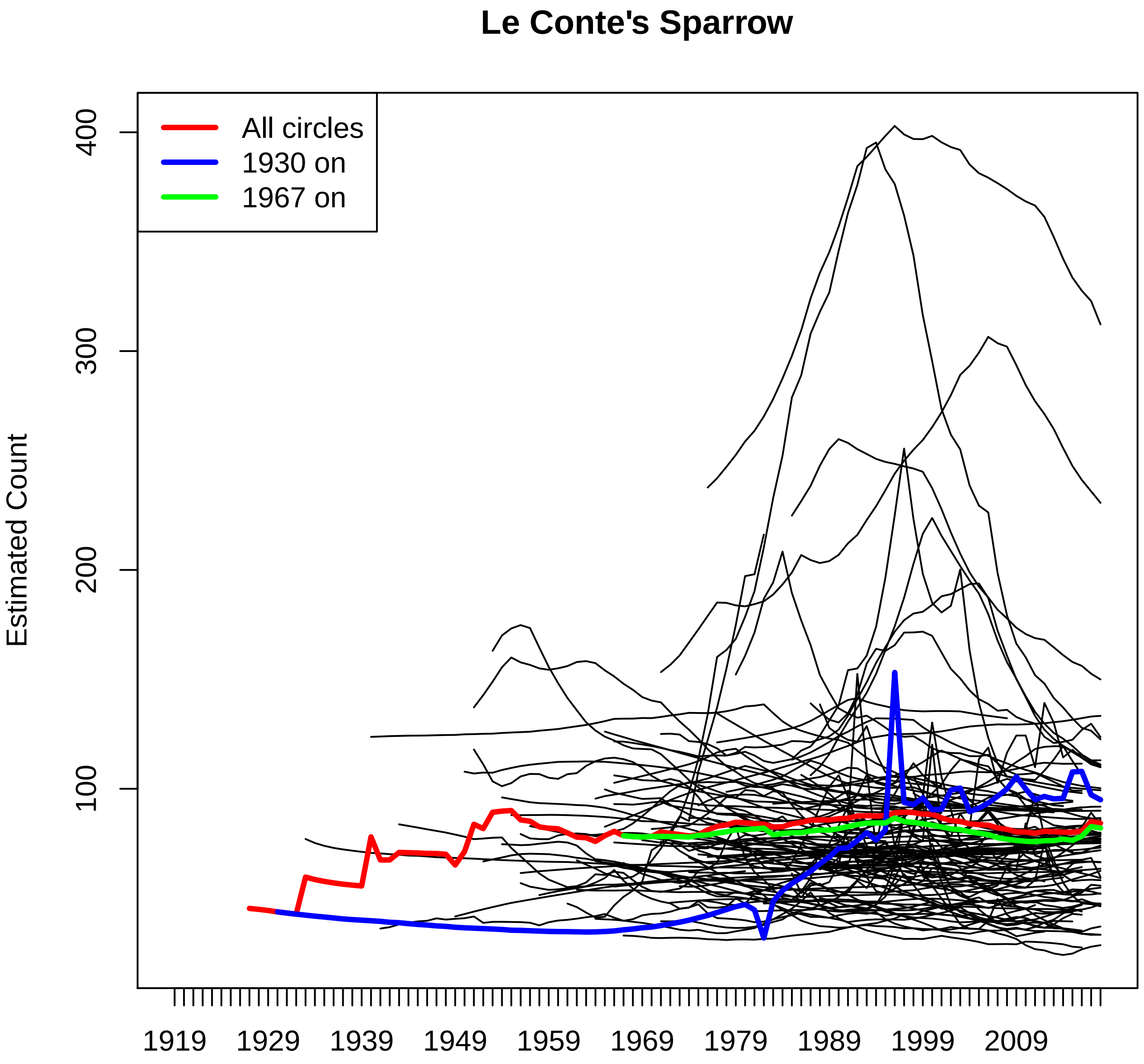

Le Conte's Sparrow
Estimated Count
400
300
200
100
All circles
1930 on
1967 on
1919
1929
1939
1949
1959
1969
1979
1989
1999
2009

Limpkin

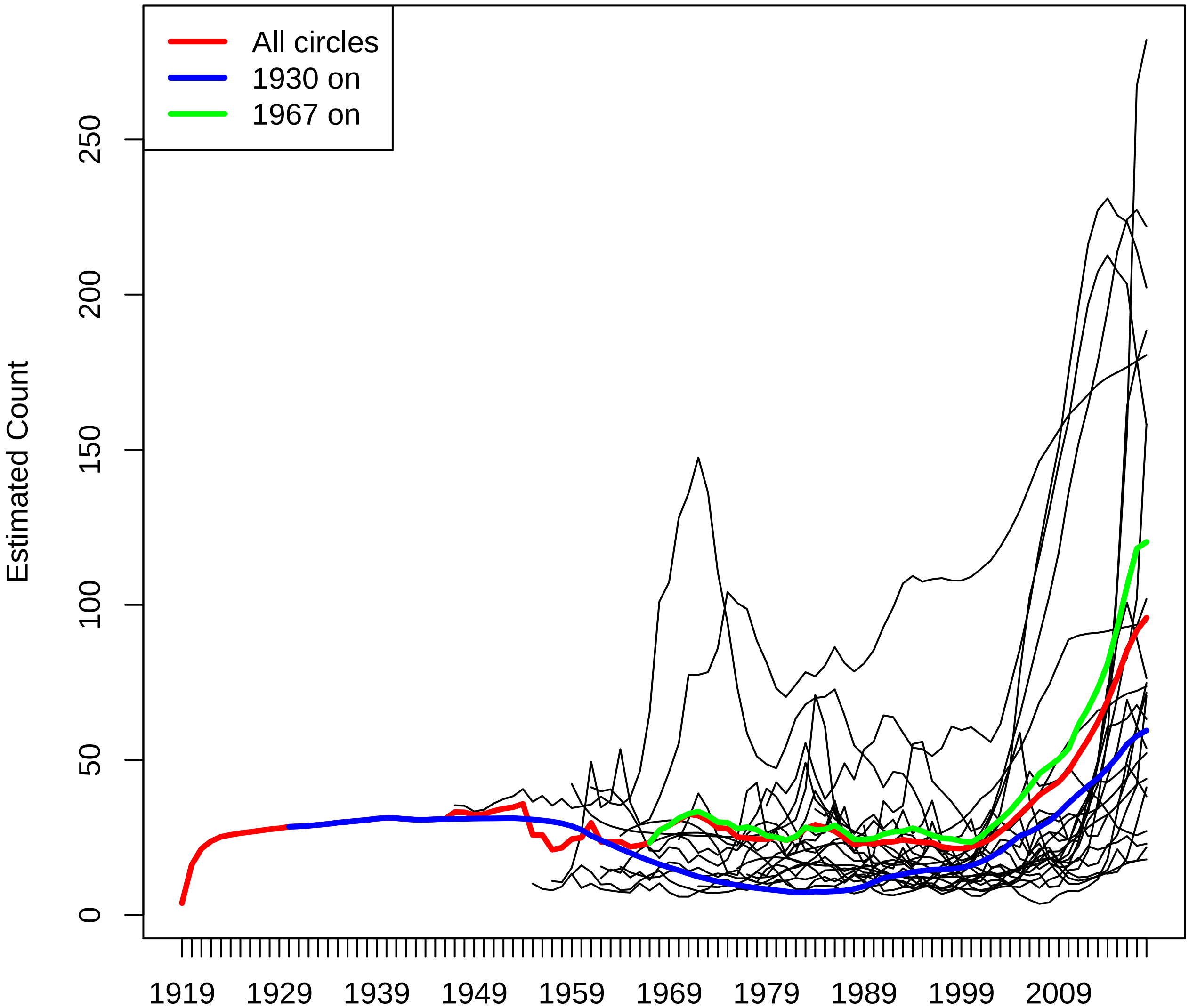

All circles
1930 on
1967 on
Estimated Count
0
50
100
150
200
250
1919
1929
1939
1949
1959
1969
1979
1989
1999
2009

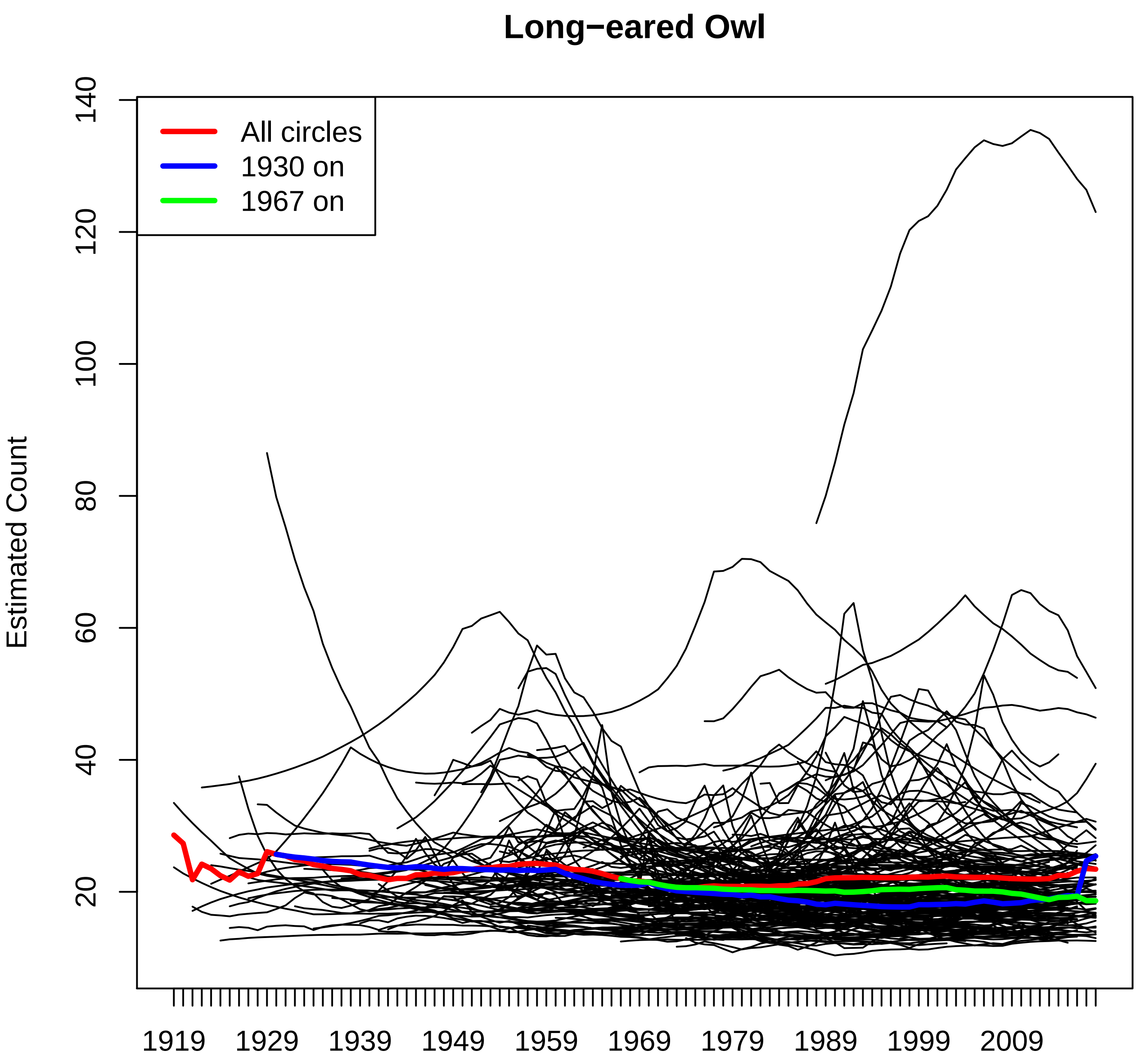

Long−eared Owl
All circles
1930 on
1967 on
Estimated Count
140
120
100
80
60
40
20
1919
1929
1939
1949
1959
1969
1979
1989
1999
2009

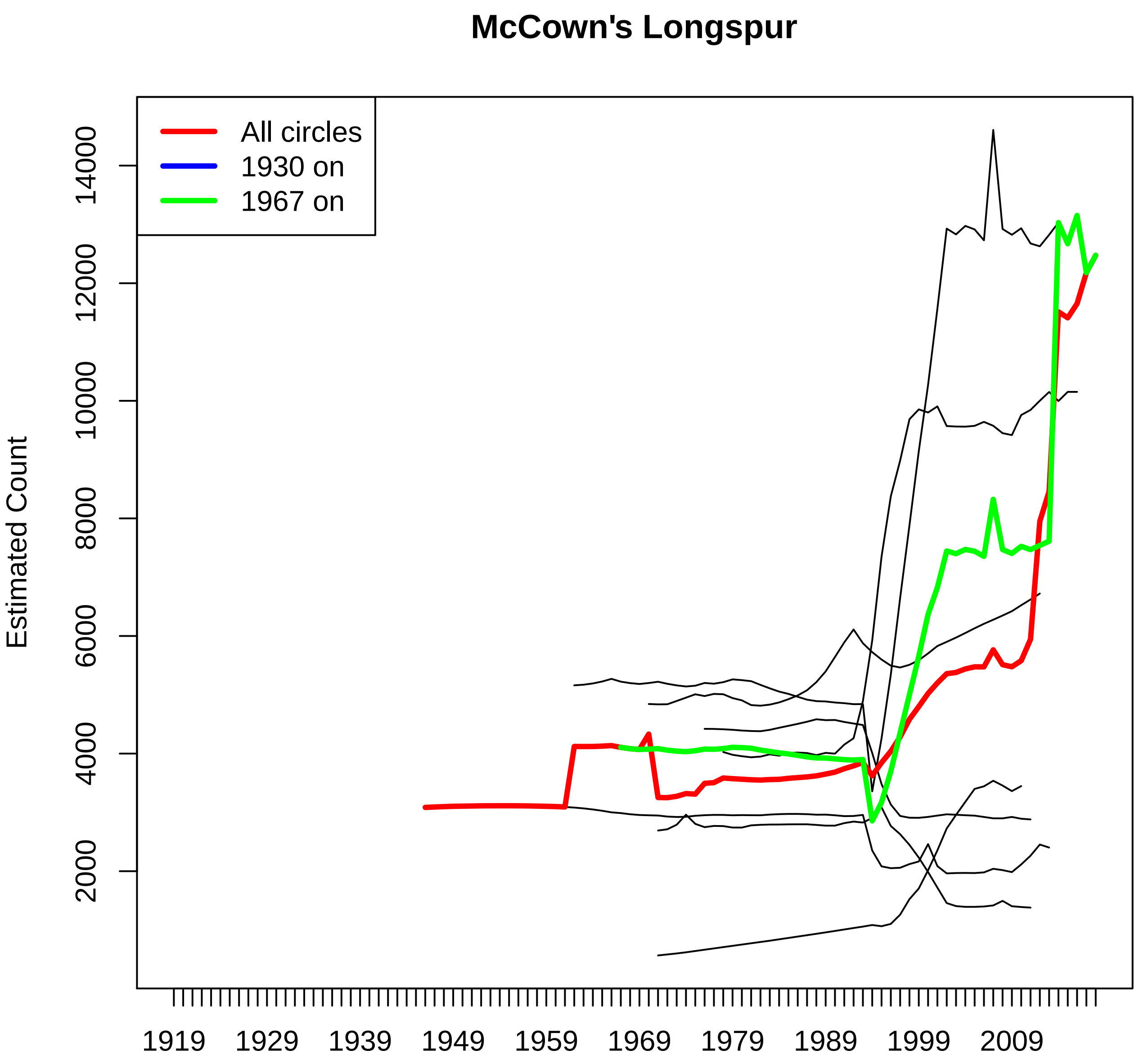
McCown's Longspur
All circles
1930 on
1967 on
Estimated Count
2000
4000
6000
8000
10000
12000
14000
1919
1929
1939
1949
1959
1969
1979
1989
1999
2009

Northern Rough−winged Swallow

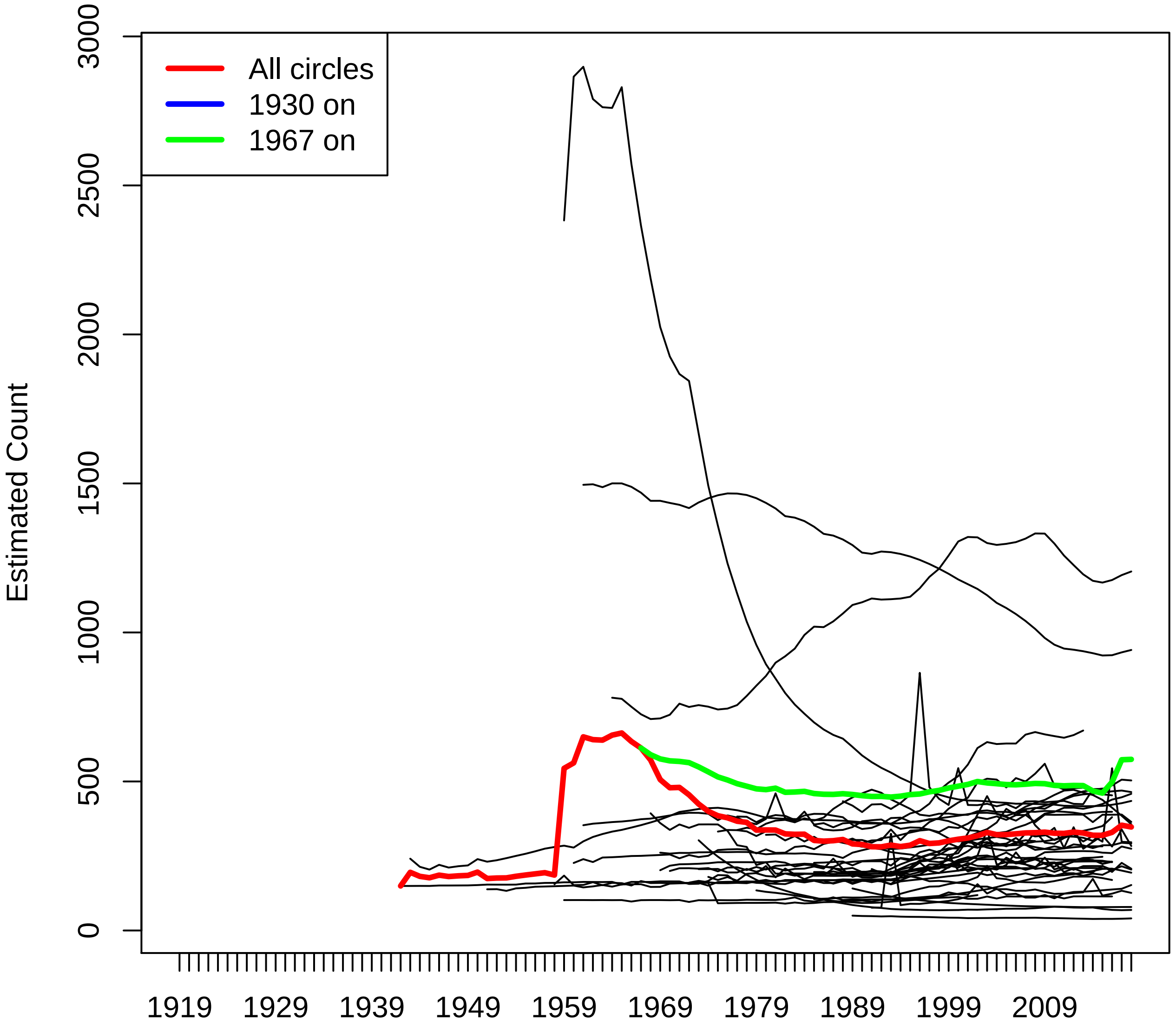

Pacific Wren

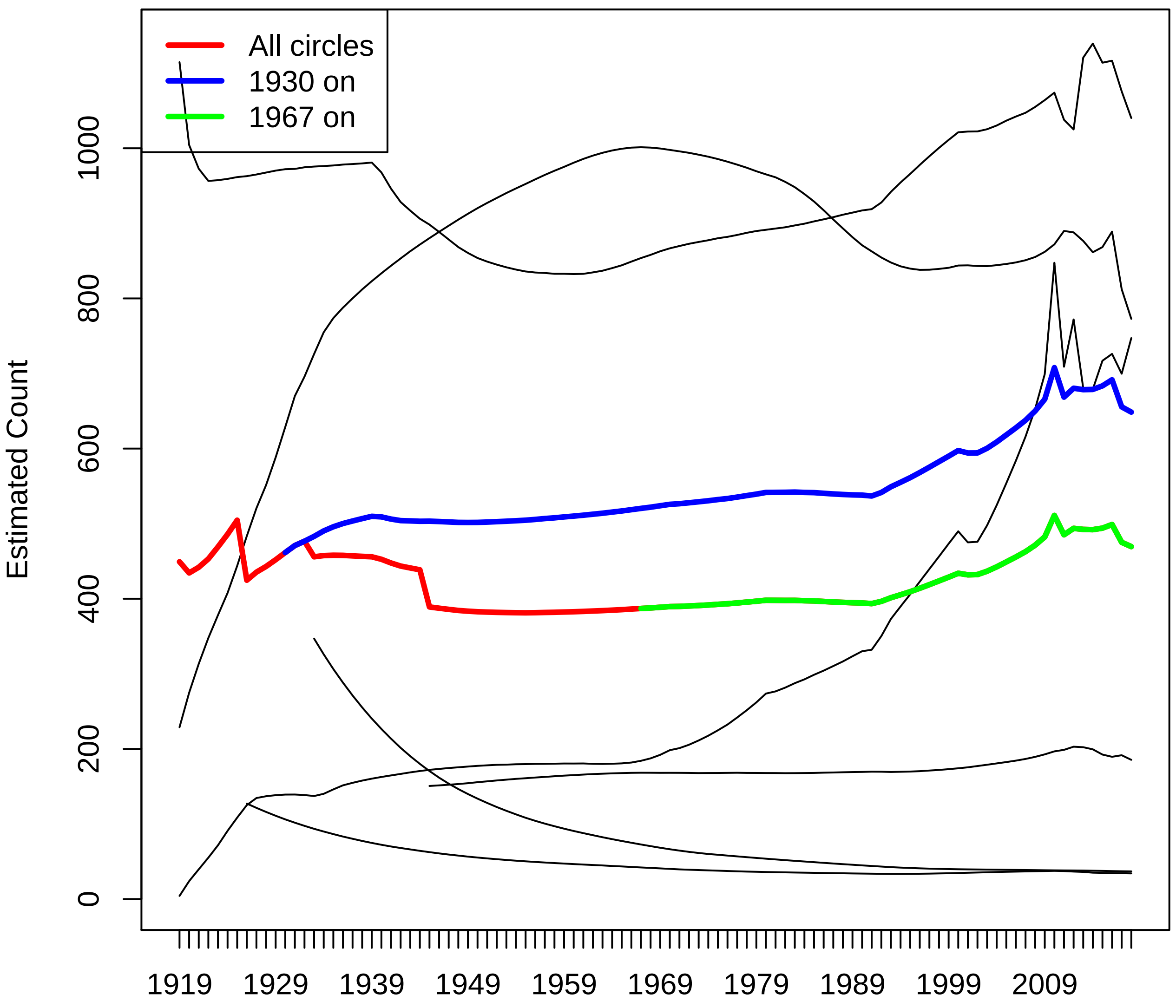

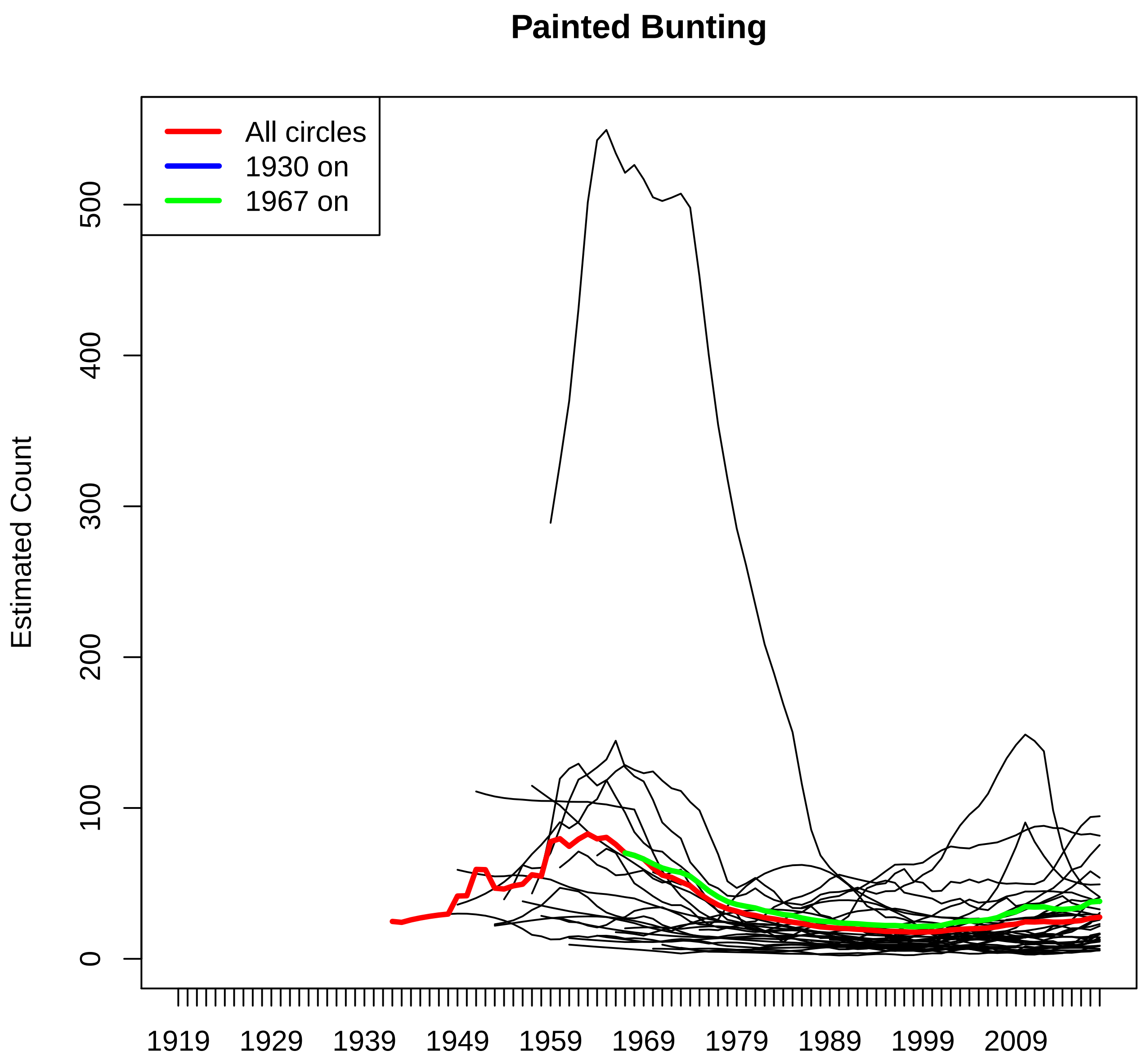

Painted Bunting
All circles
1930 on
1967 on
Estimated Count
0
100
200
300
400
500
1919
1929
1939
1949
1959
1969
1979
1989
1999
2009

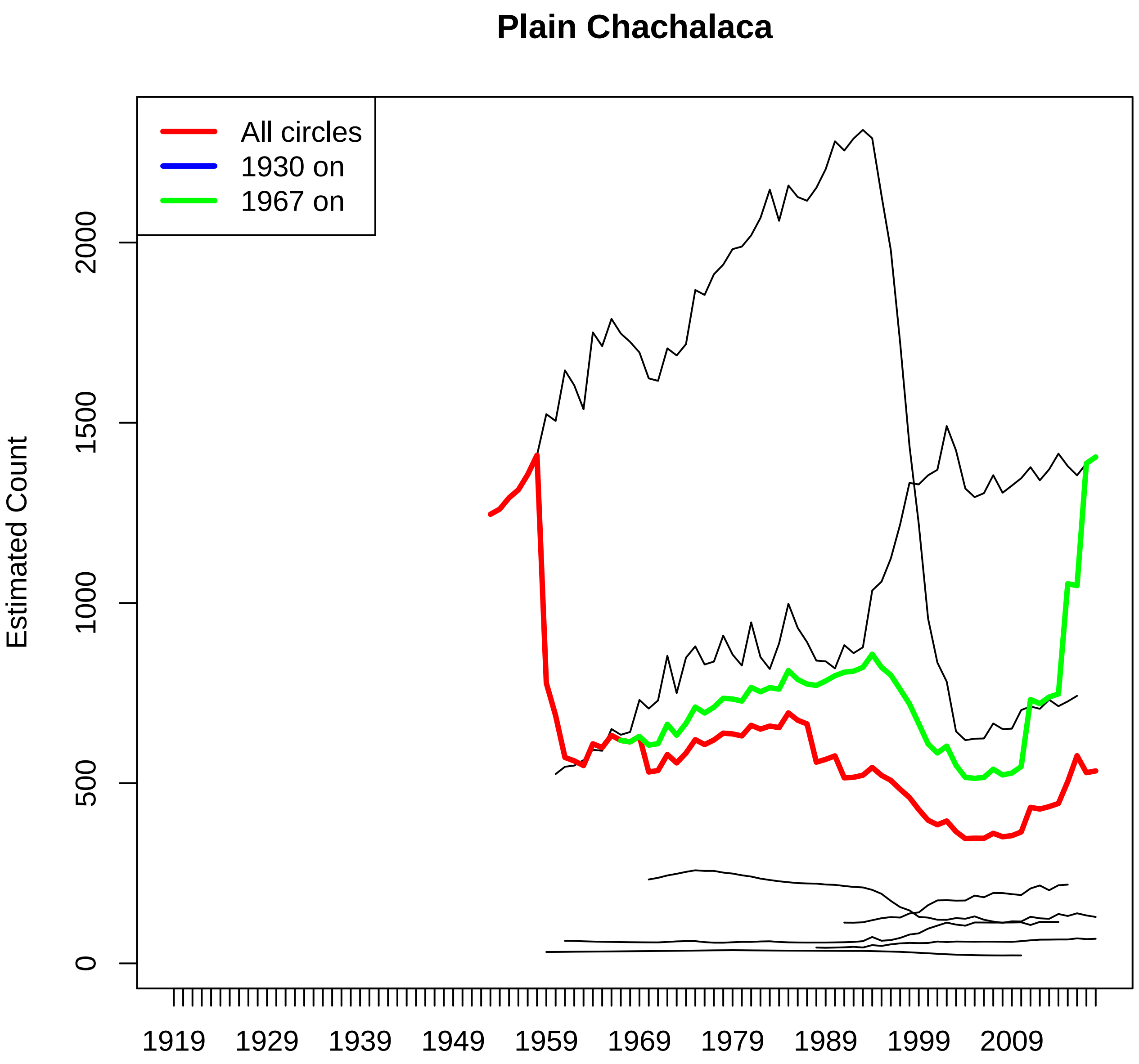

Plain Chachalaca
All circles
1930 on
1967 on
Estimated Count
0
500
1000
1500
2000
1919
1929
1939
1949
1959
1969
1979
1989
1999
2009

Prairie Falcon

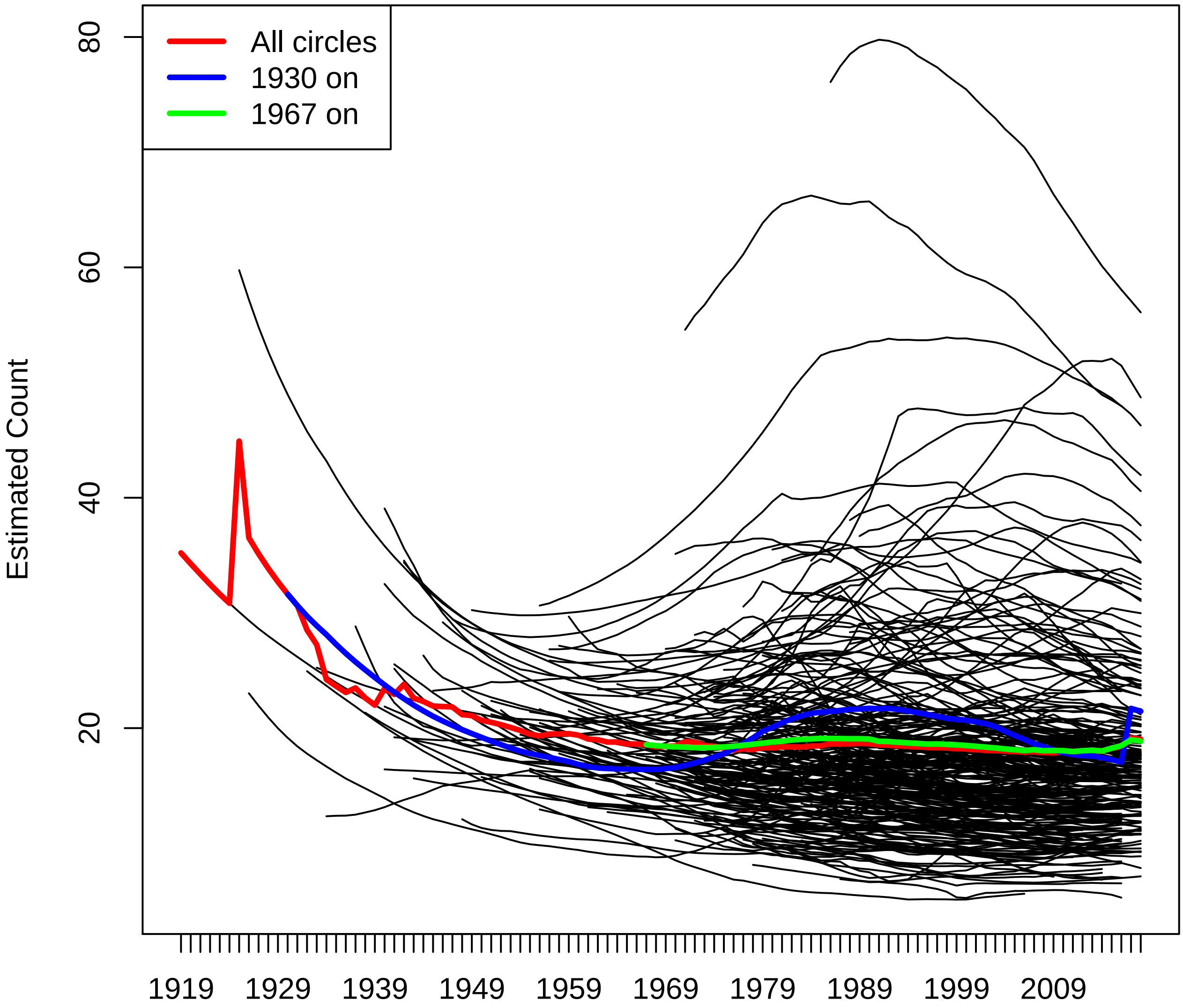

Prairie Warbler

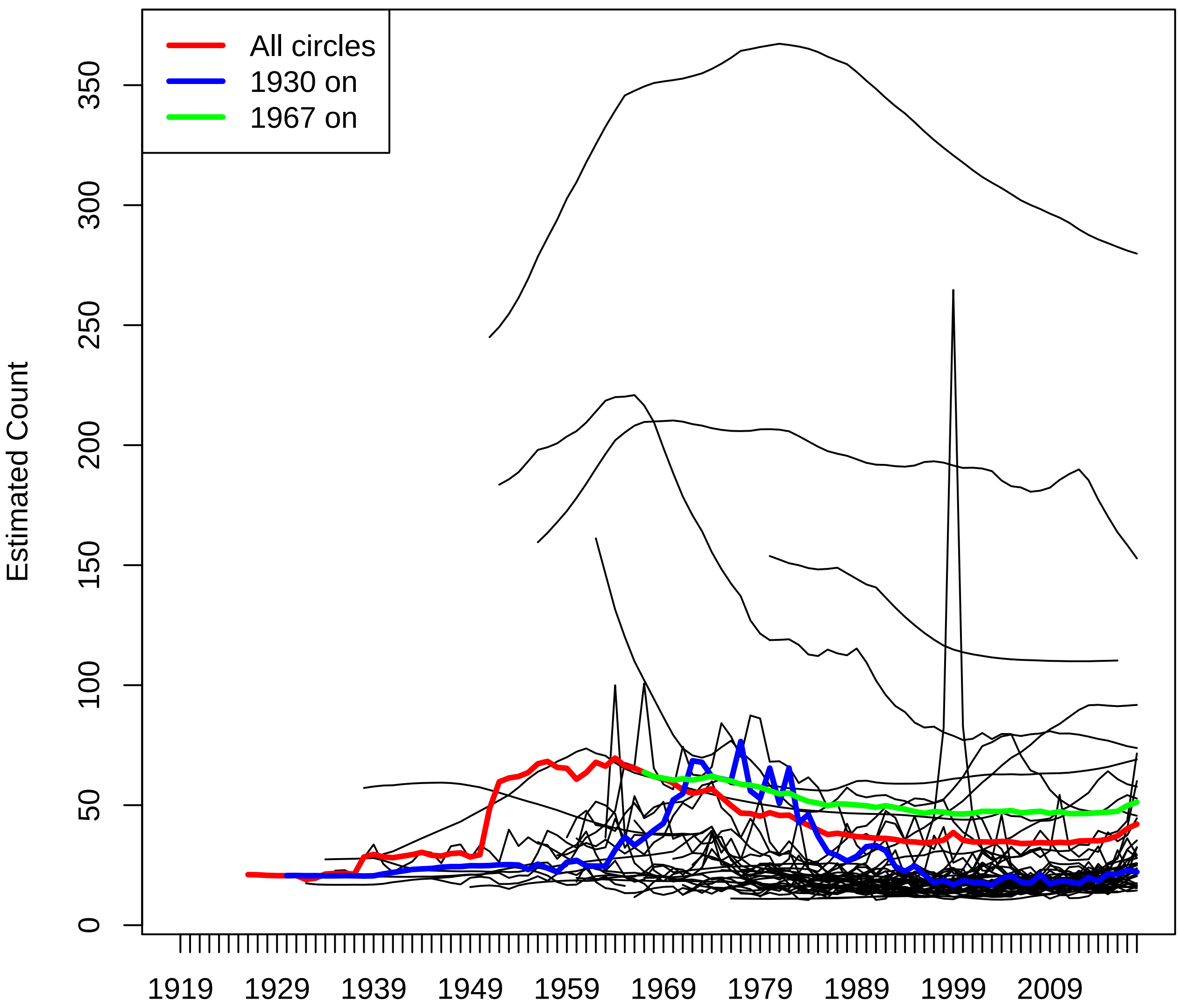

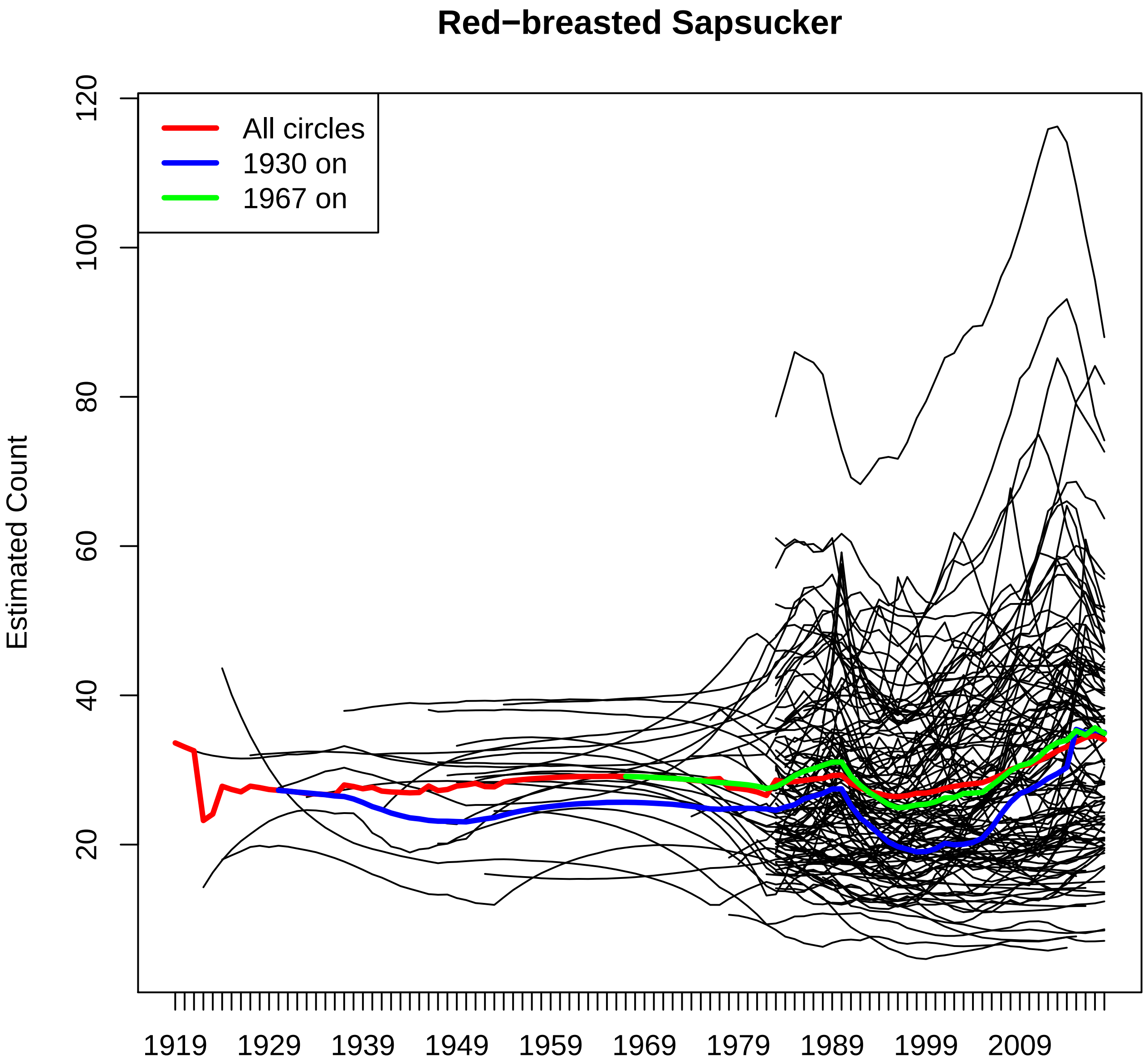

Red−breasted Sapsucker
Estimated Count
120
100
80
60
40
20
All circles
1930 on
1967 on
1919
1929
1939
1949
1959
1969
1979
1989
1999
2009

Reddish Egret

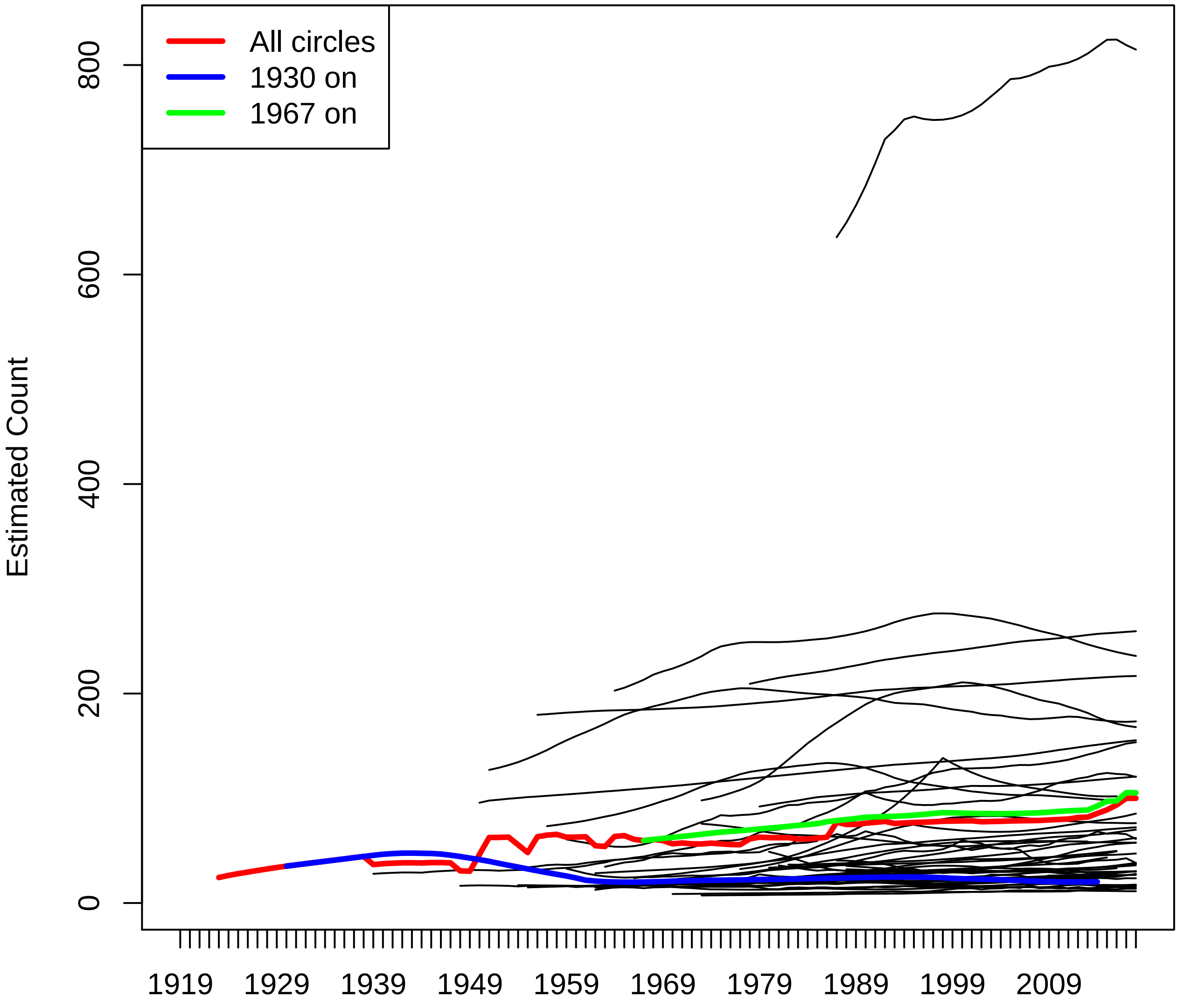

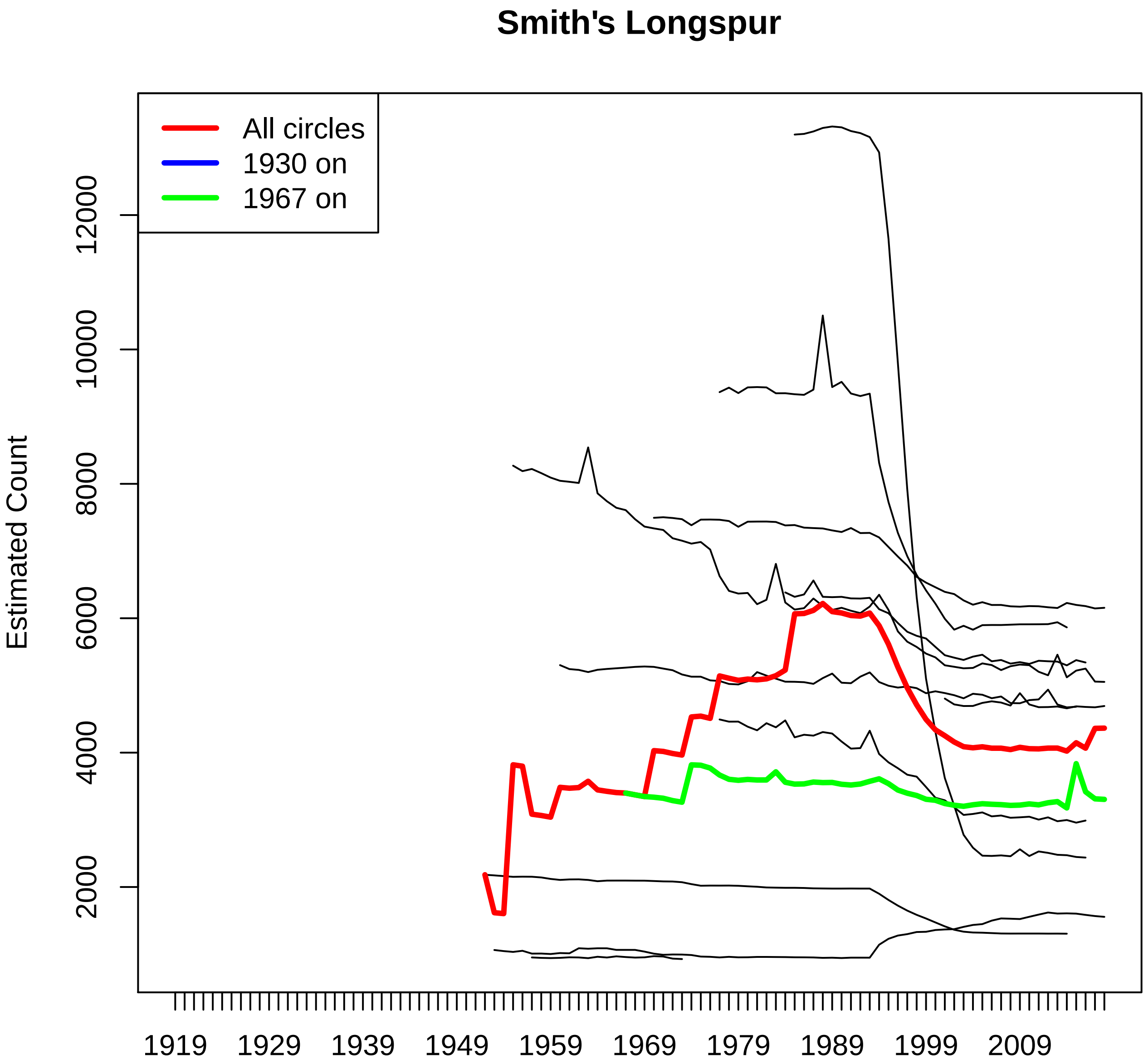
Smith's Longspur
All circles
1930 on
1967 on
Estimated Count
2000
4000
6000
8000
10000
12000
1919
1929
1939
1949
1959
1969
1979
1989
1999
2009

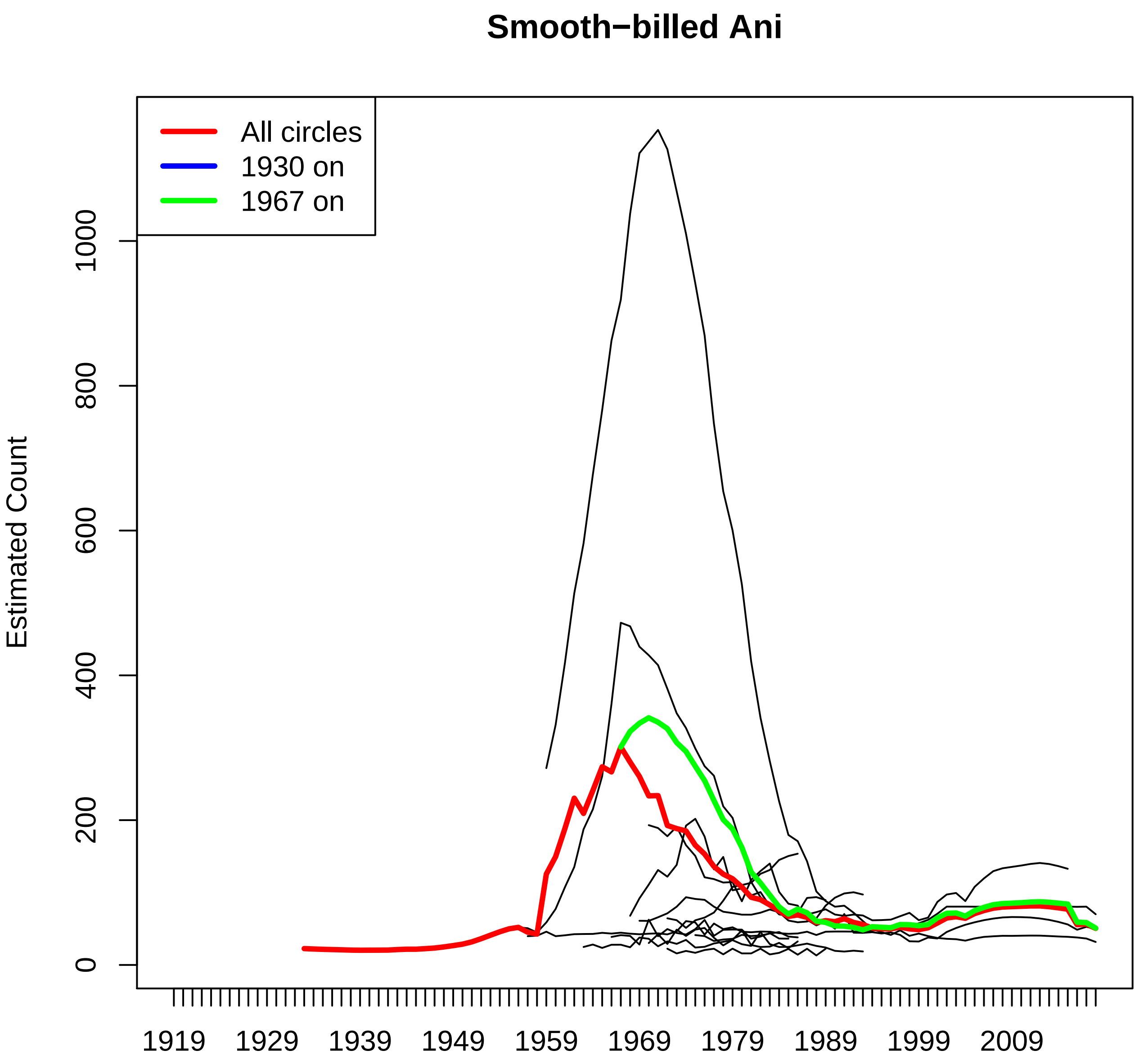

Smooth−billed Ani
All circles
1930 on
1967 on
Estimated Count
0
200
400
600
800
1000
1919
1929
1939
1949
1959
1969
1979
1989
1999
2009

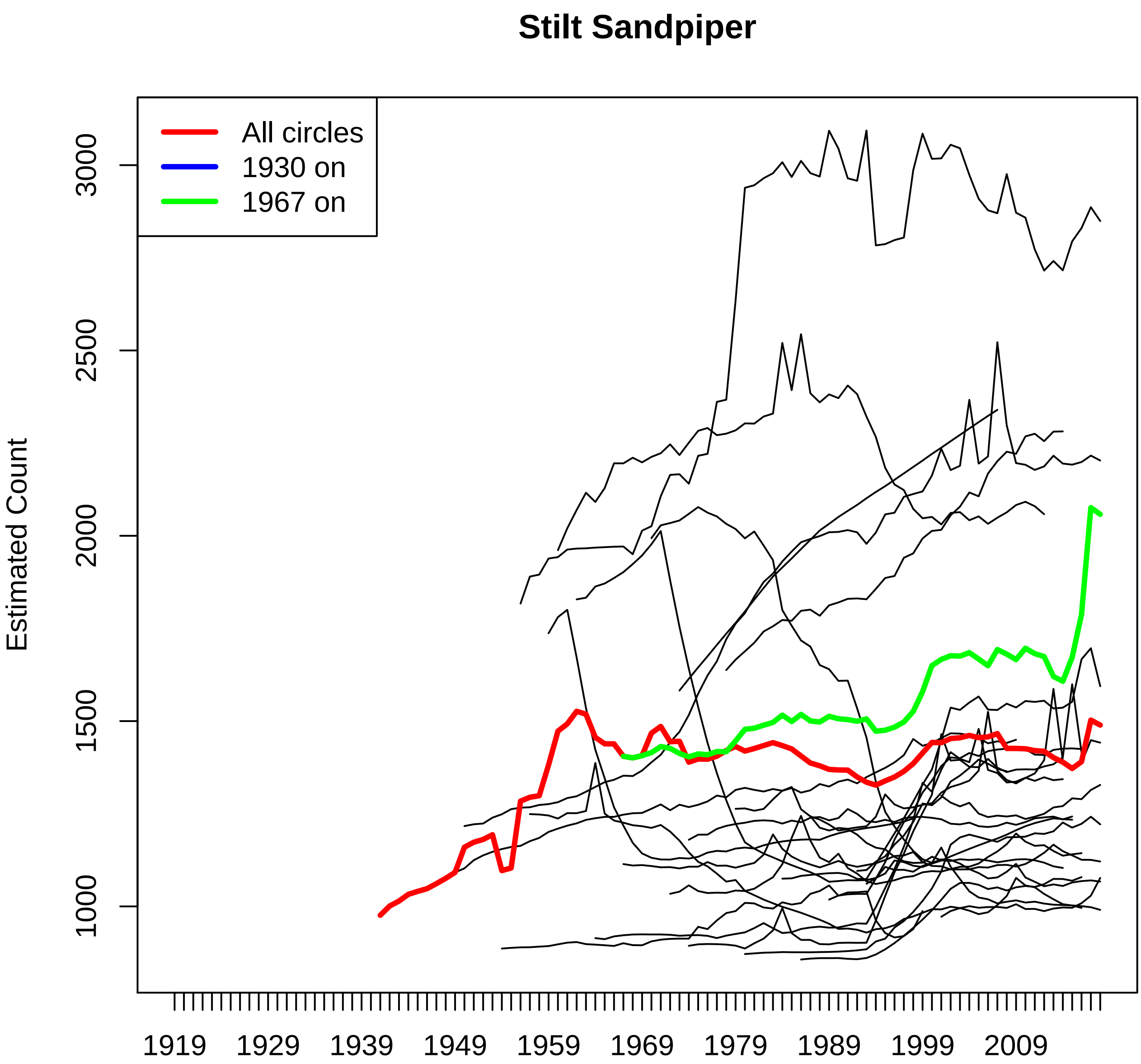
Stilt Sandpiper
All circles
1930 on
1967 on
Estimated Count
1000
1500
2000
2500
3000
1919
1929
1939
1949
1959
1969
1979
1989
1999
2009

Vermilion Flycatcher

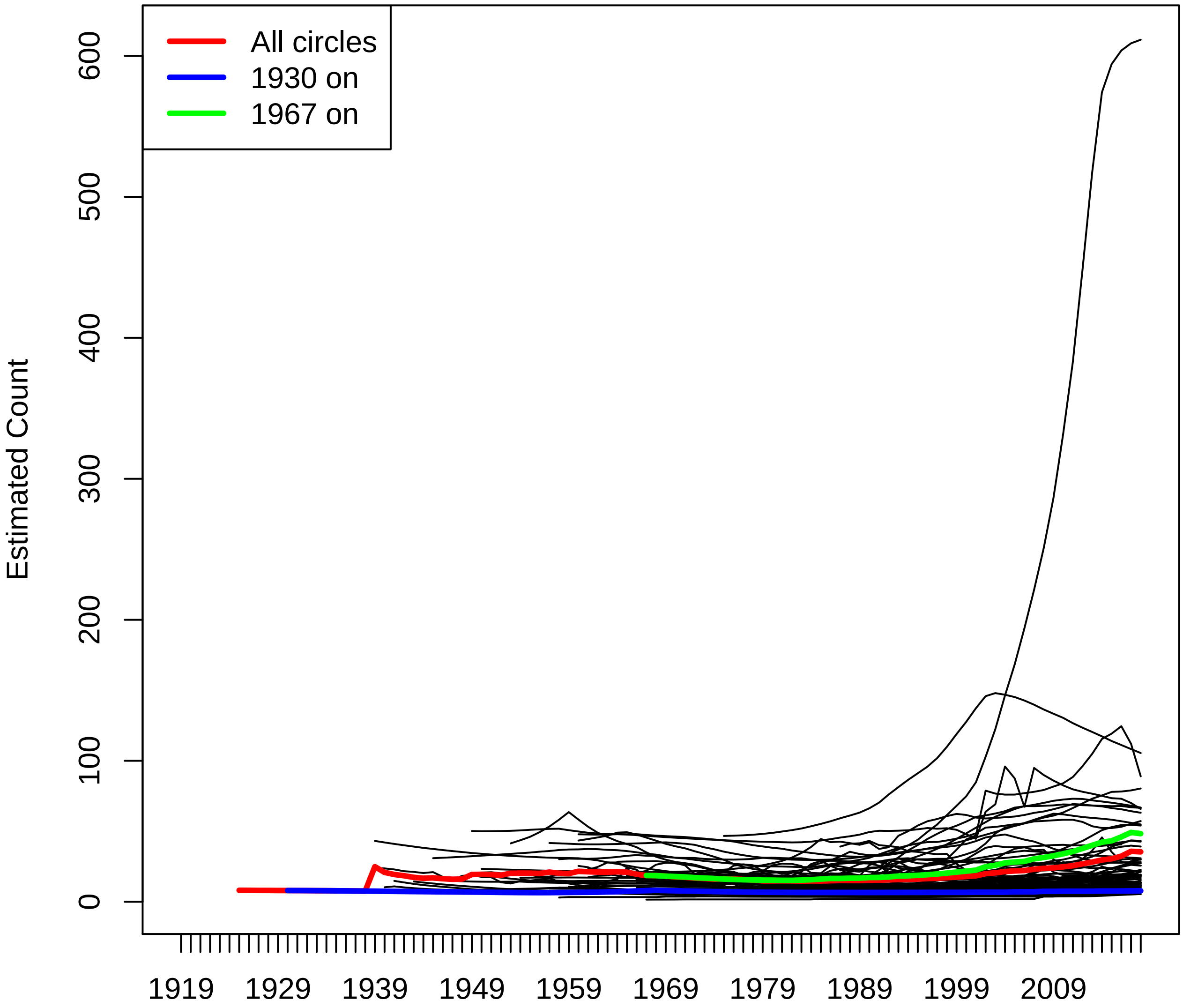

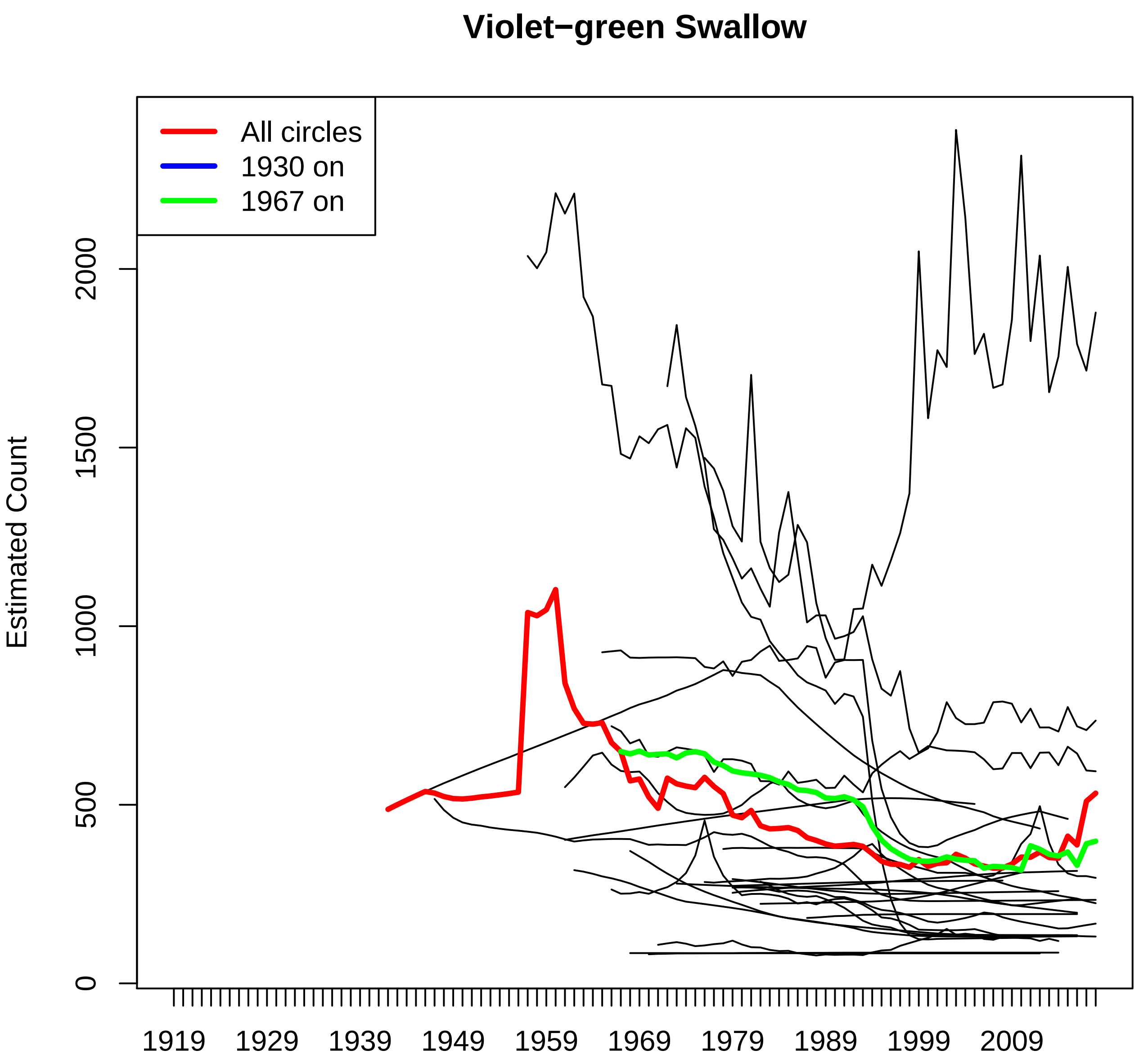

Violet−green Swallow
All circles
1930 on
1967 on
Estimated Count
0
500
1000
1500
2000
1919
1929
1939
1949
1959
1969
1979
1989
1999
2009

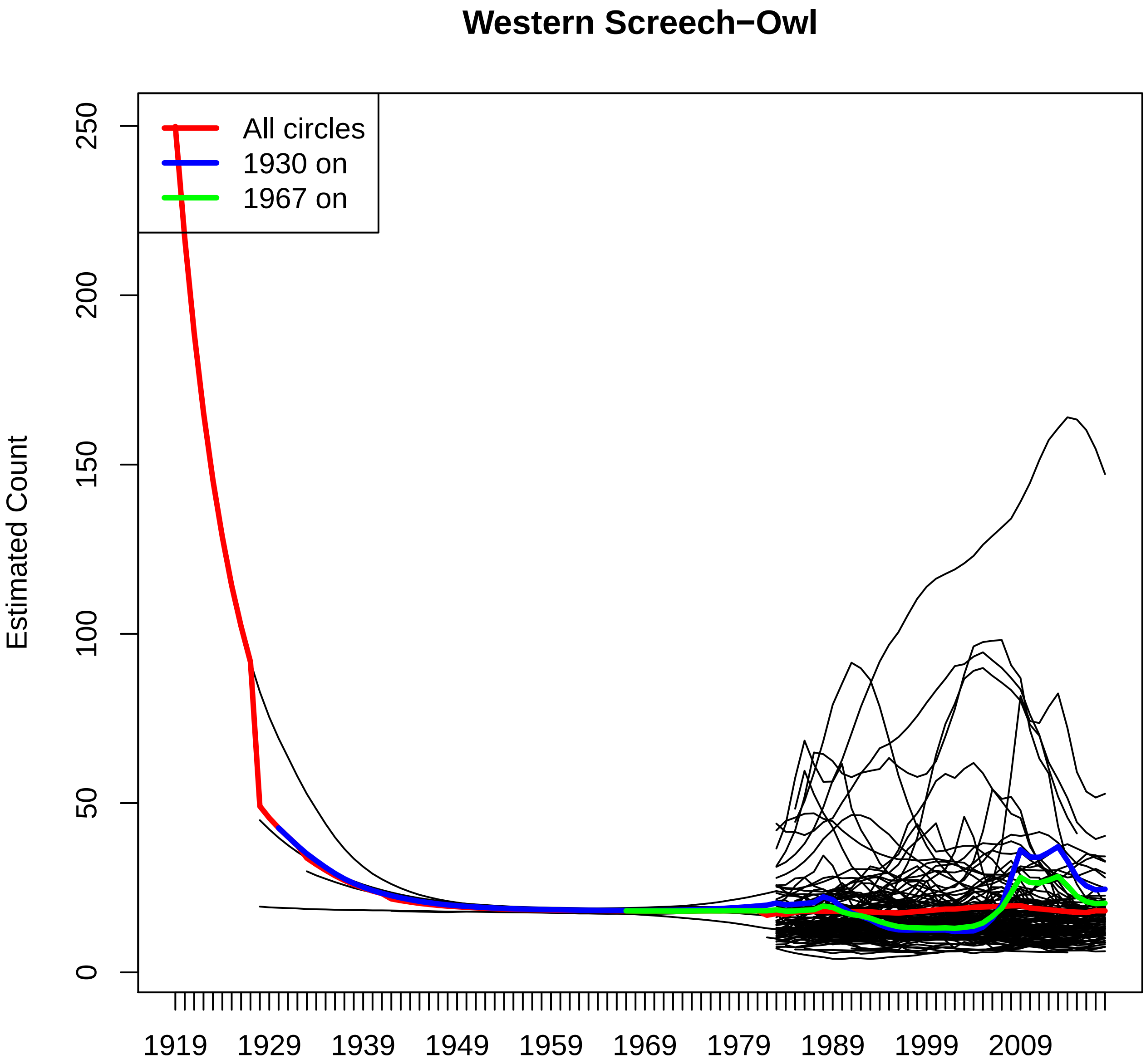
Western Screech−Owl
Estimated Count
0
50
100
150
200
250
All circles
1930 on
1967 on
1919
1929
1939
1949
1959
1969
1979
1989
1999
2009

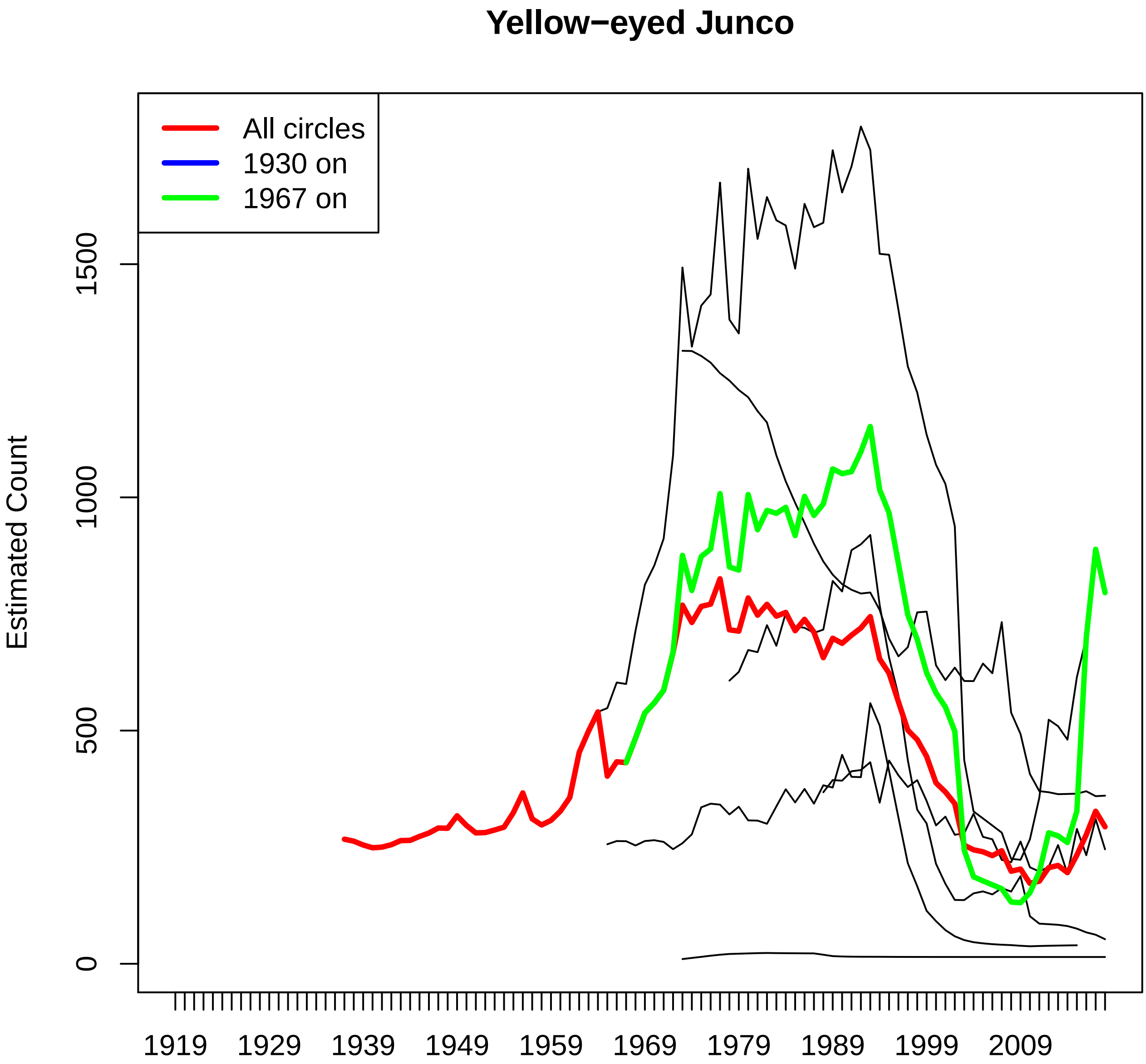
Yellow−eyed Junco
All circles
1930 on
1967 on
Estimated Count
0
500
1000
1500
1919
1929
1939
1949
1959
1969
1979
1989
1999
2009

Yellow−throated Warbler

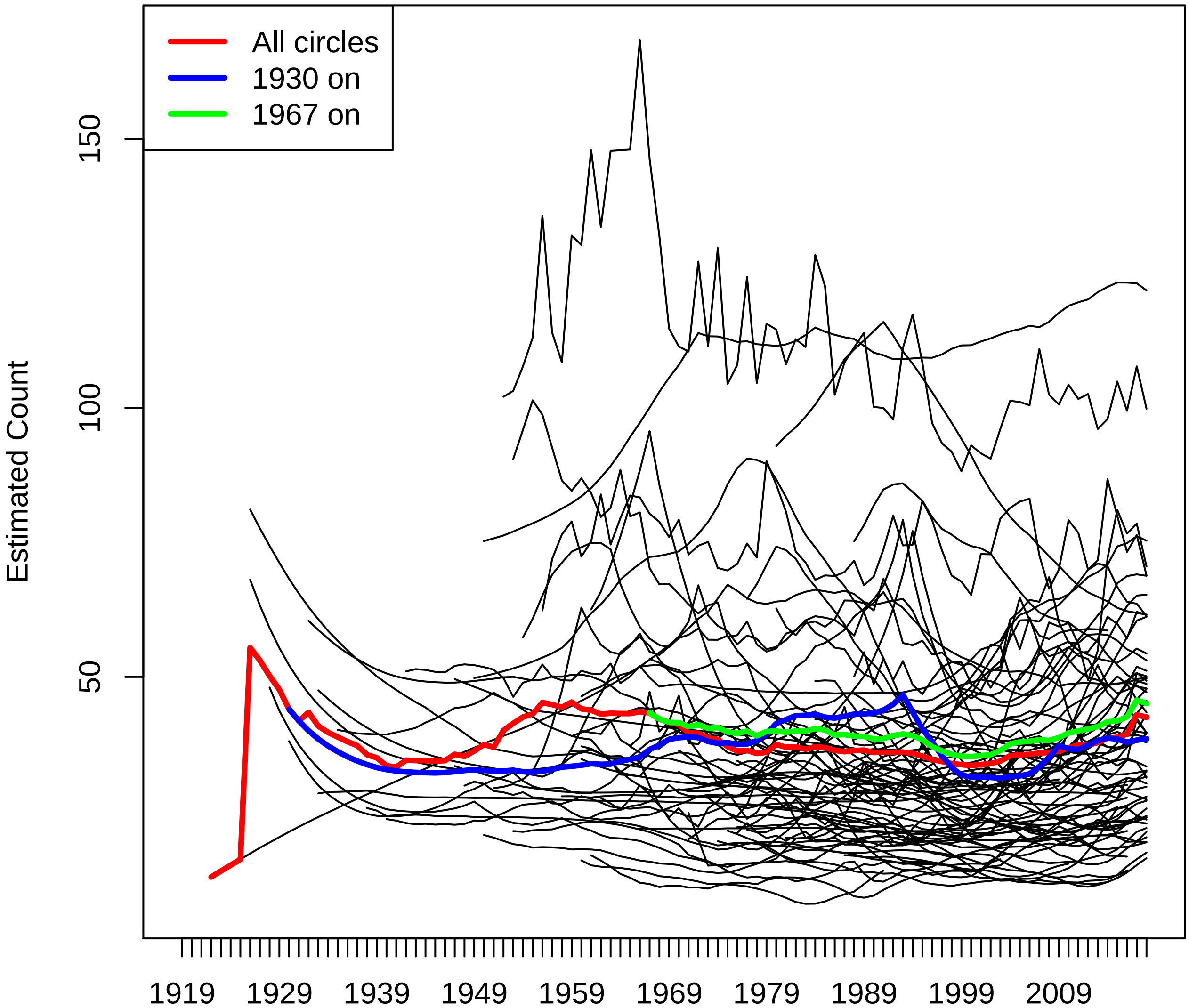

Supplement: Supplementary file 4 — Figure S3 [file ECE3-13-e9781-s008.pdf]
