# Supplementary material for: Spatial and temporal non‐stationarity in long‐term population dynamics of over‐wintering birds of North America
Source: Ecol Evol. 2023 Mar 16;13(3):e9781. doi: 10.1002/ece3.9781 (PMC10019912; doi:10.1002/ece3.9781)

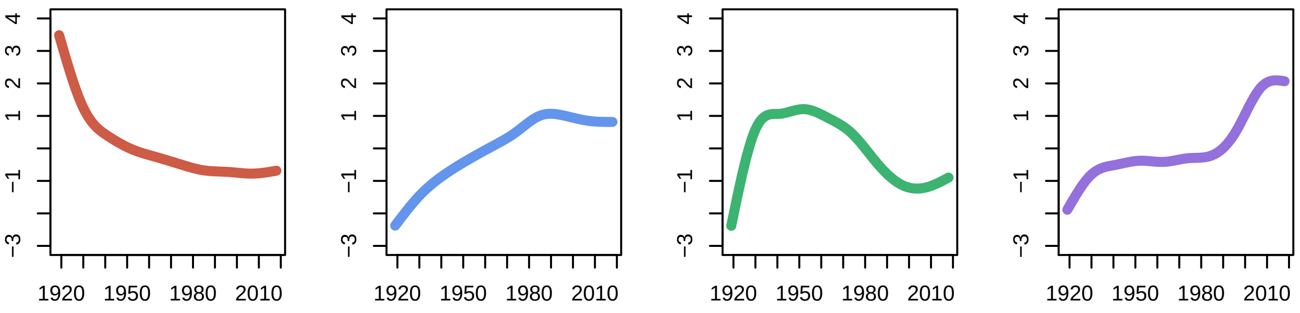

Deserts

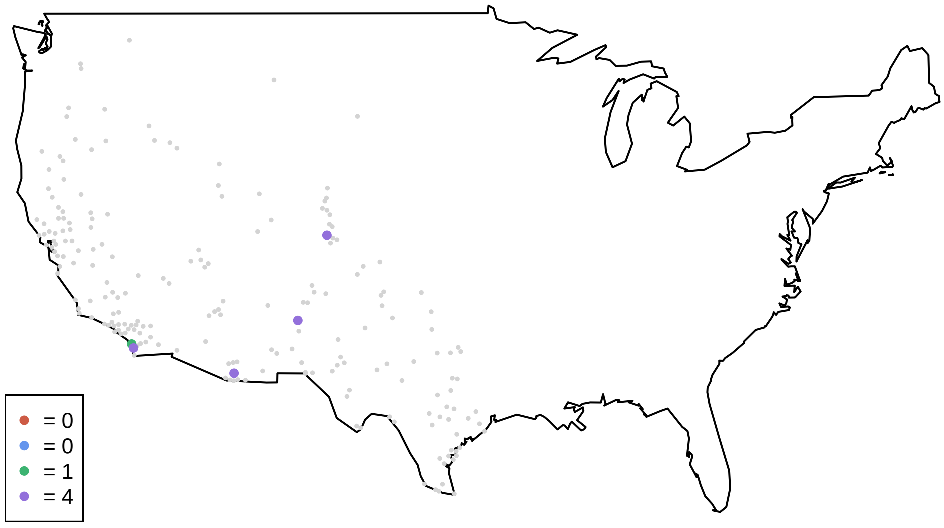

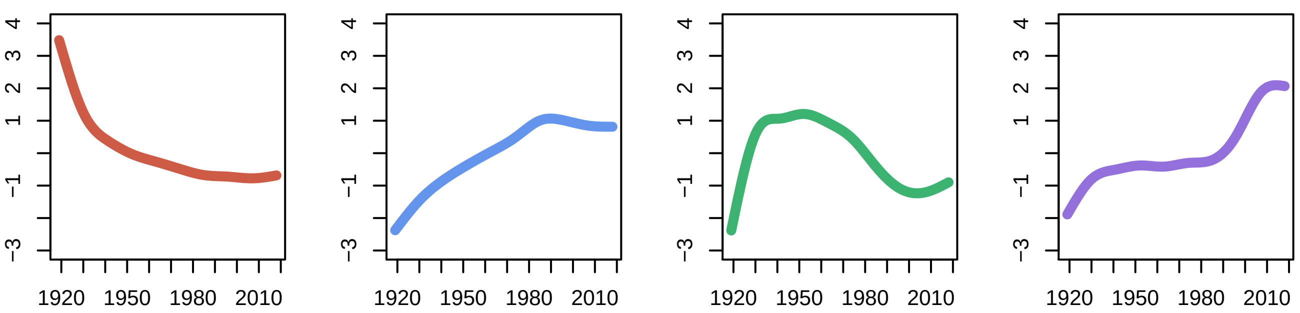
−3 −1 1 2 3 4
1920 1950 1980 2010
−3 −1 1 2 3 4
1920 1950 1980 2010
−3 −1 1 2 3 4
1920 1950 1980 2010
−3 −1 1 2 3 4
1920 1950 1980 2010

Forests

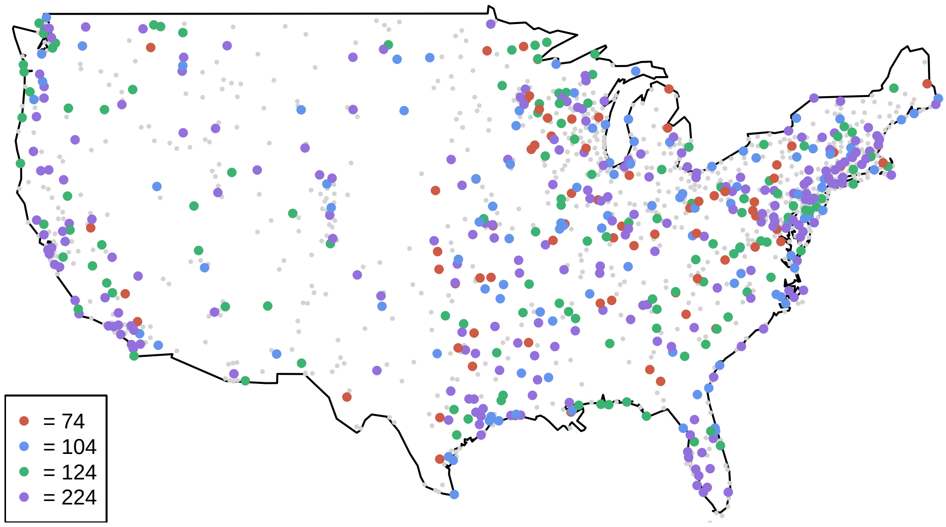
= 74
= 104
= 124
= 224

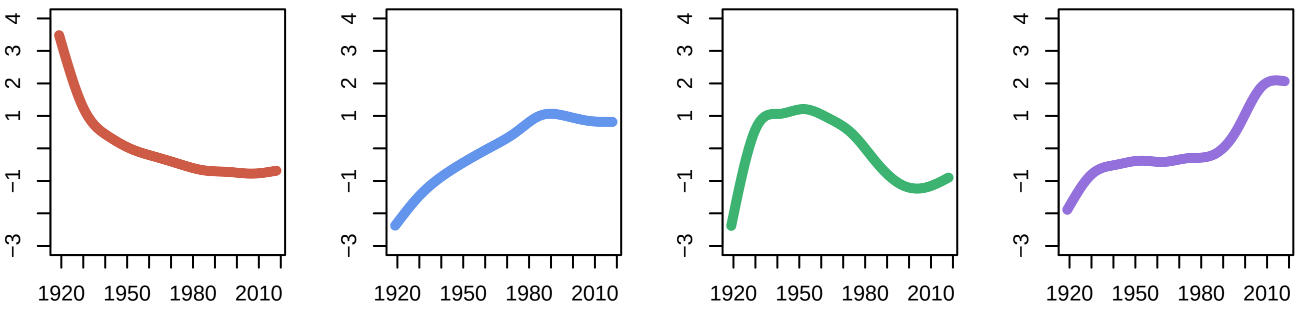

Grasslands

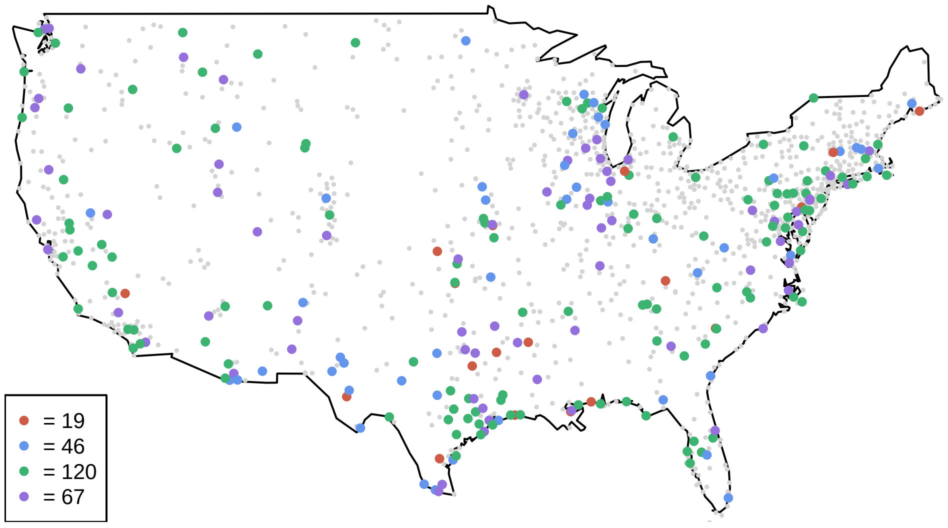

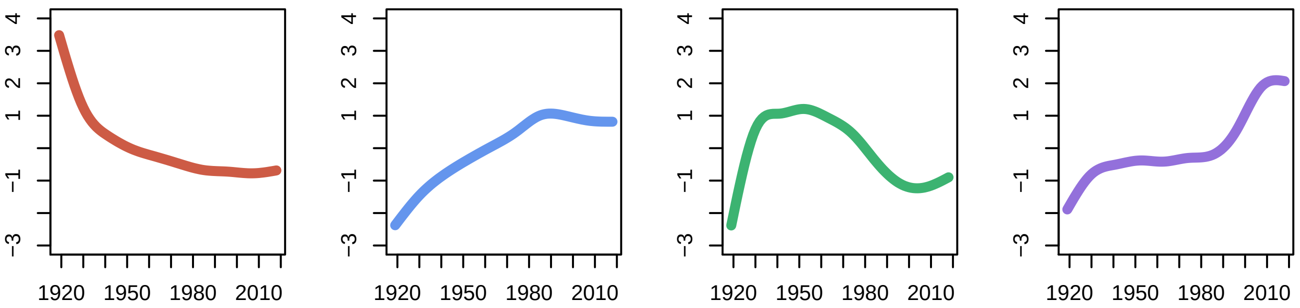

Lakes and Ponds

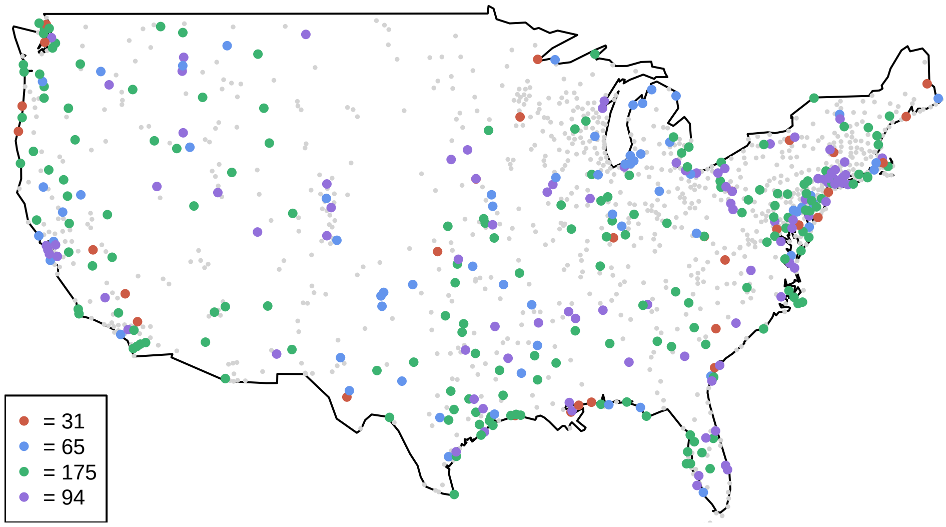

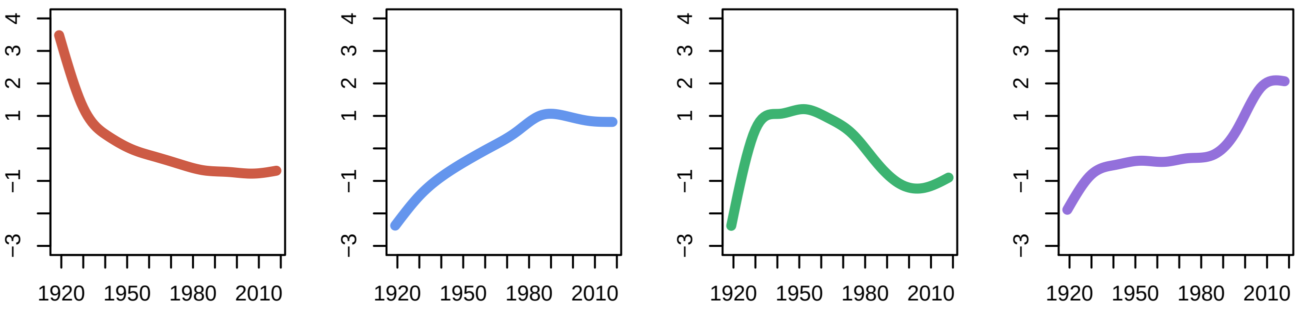

Marshes

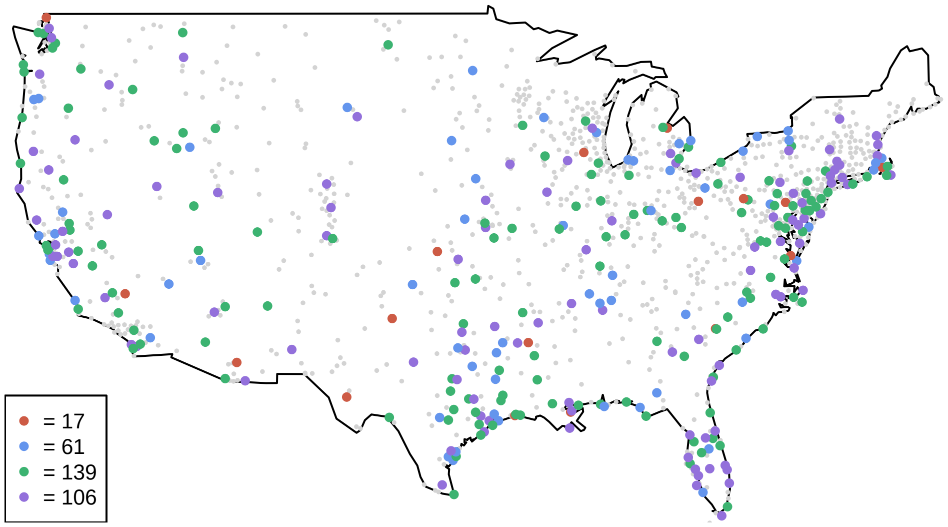

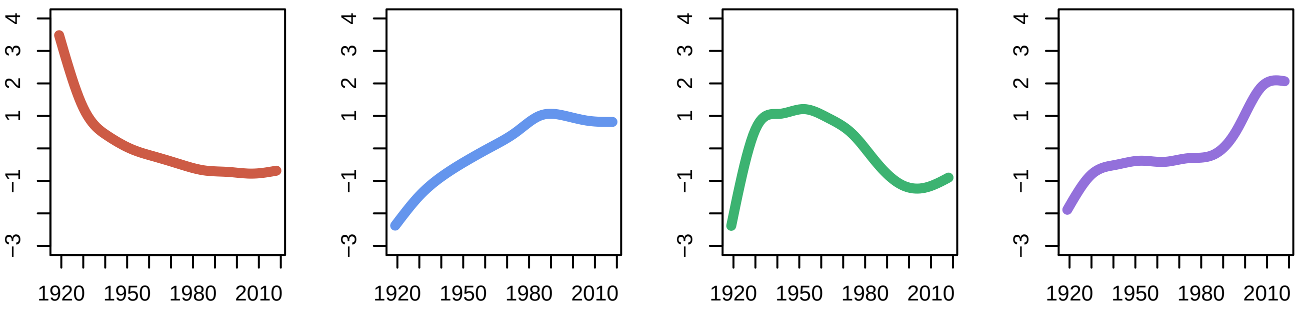

Open Woodlands

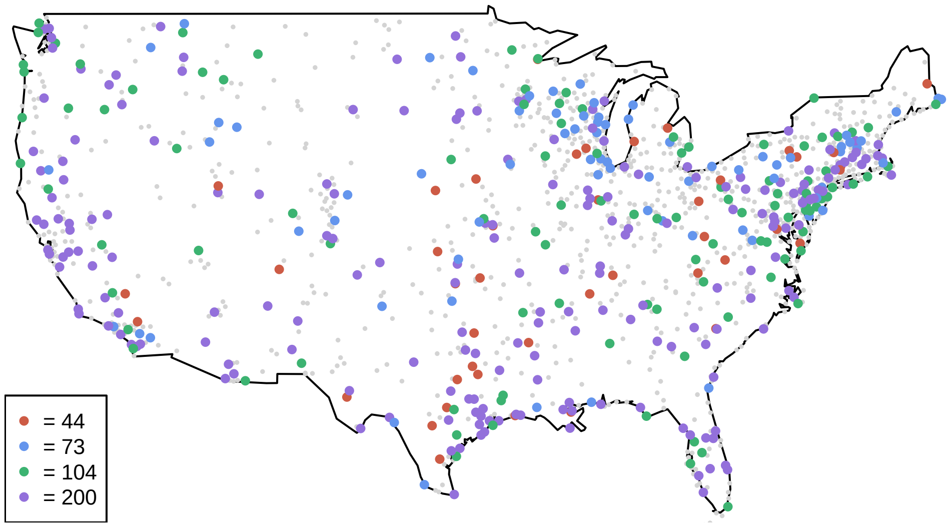

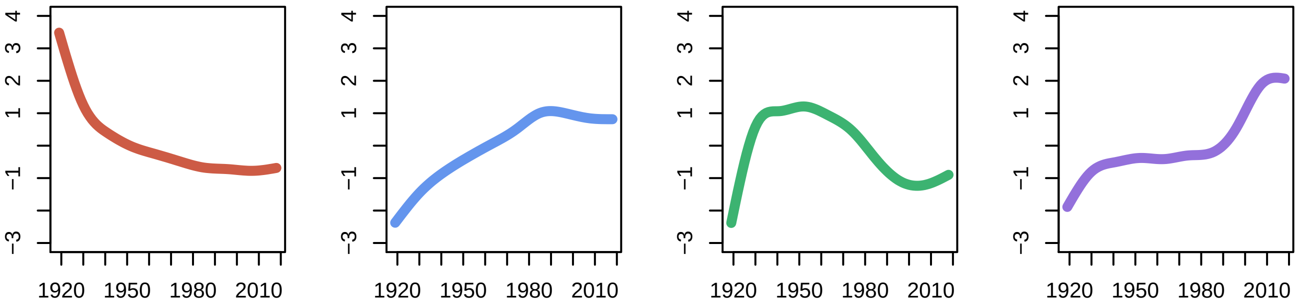

Rivers and Streams

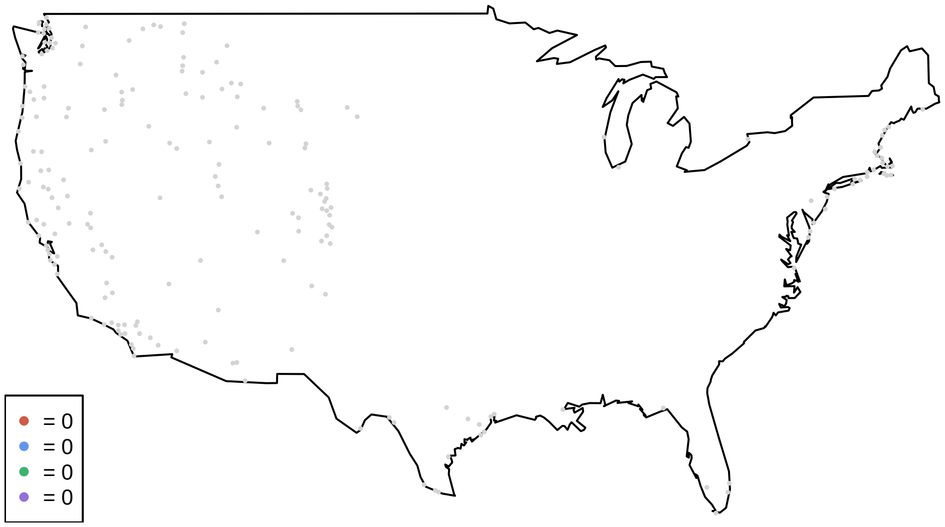

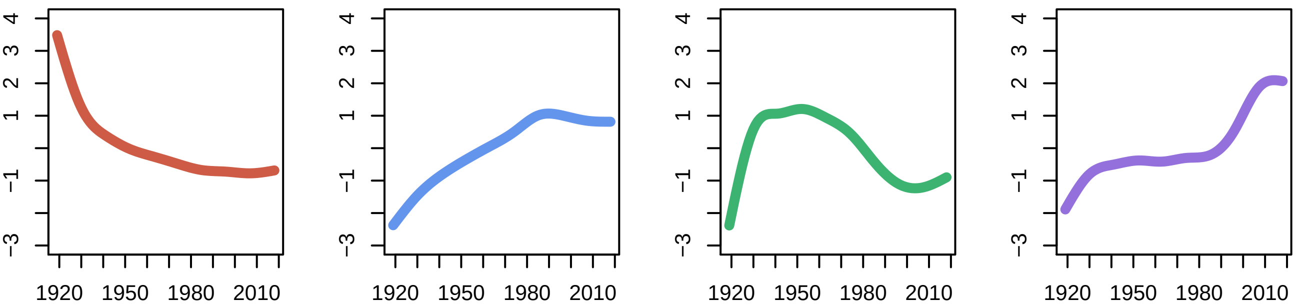

Scrub

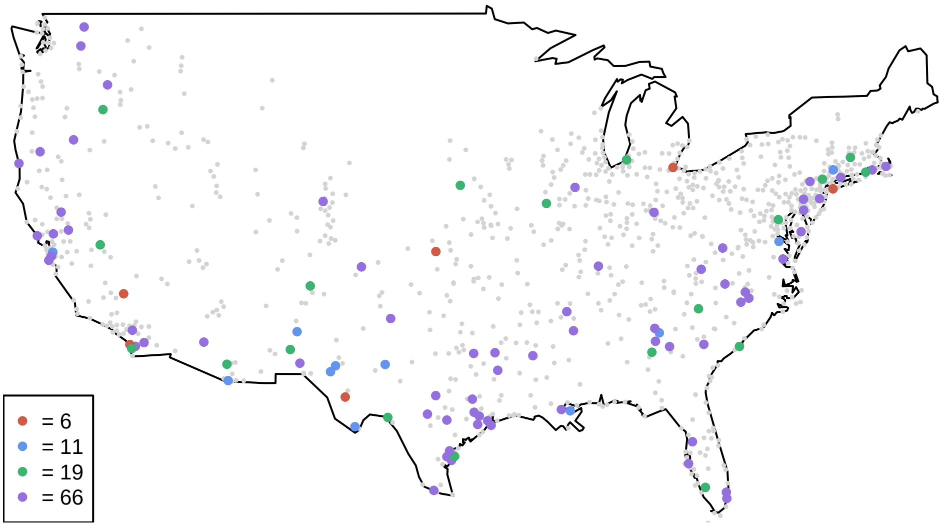

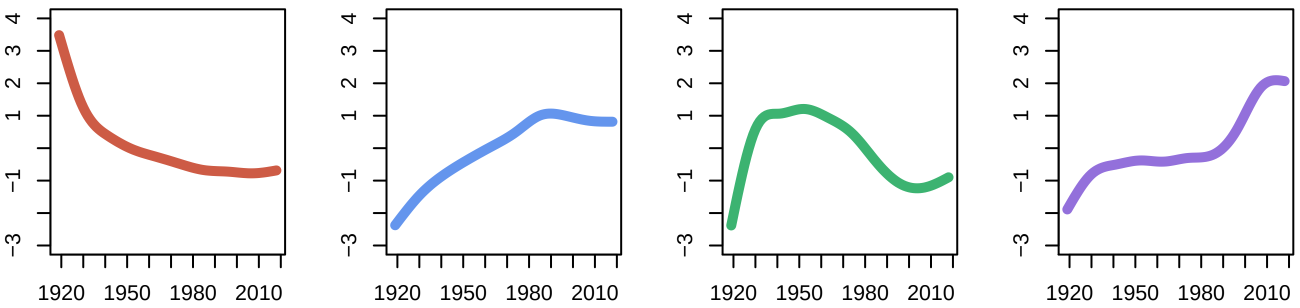

Towns

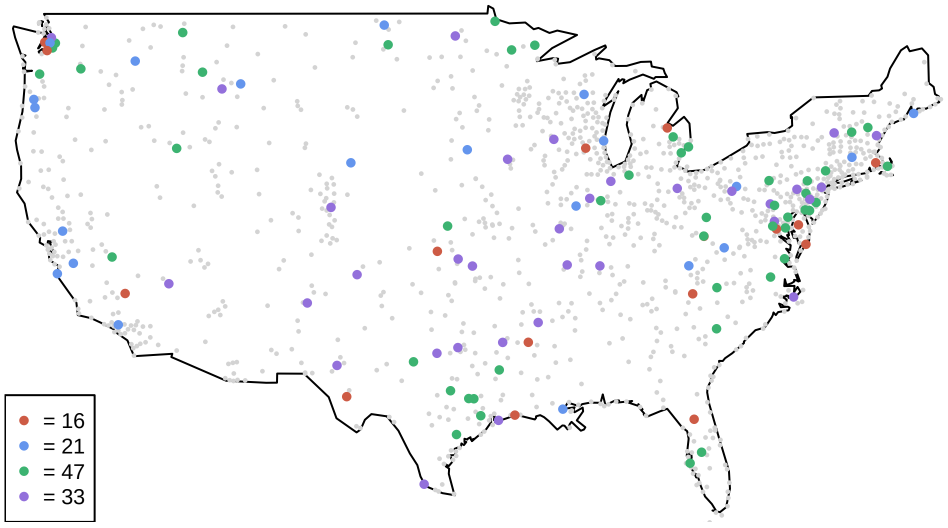

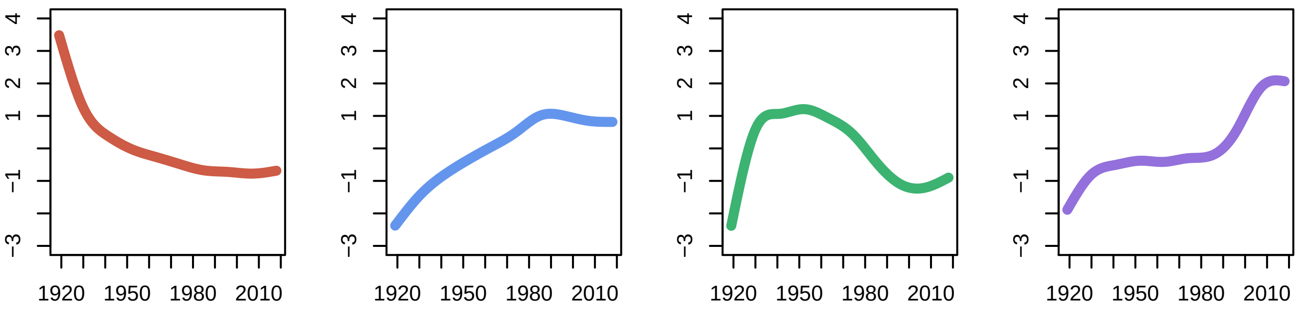

Tundra

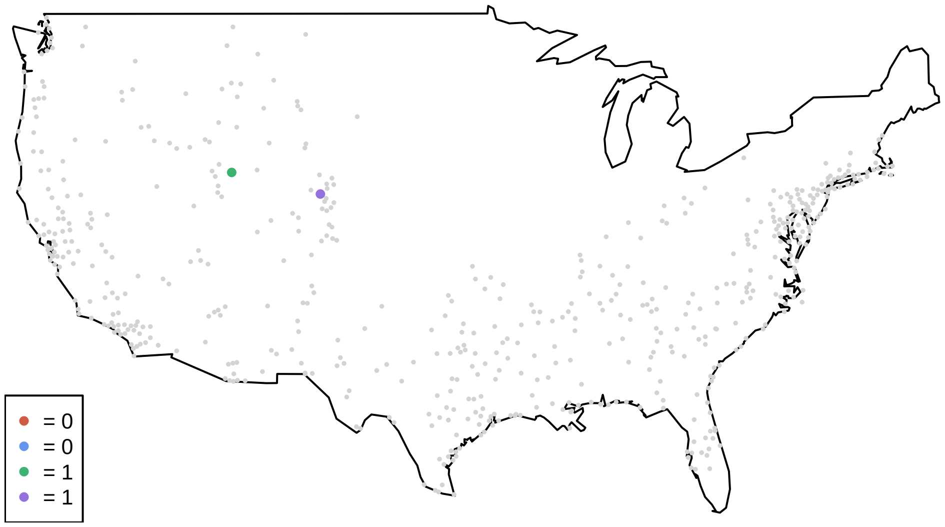

Supplement: Supplementary file 6 — Figure S5 [file ECE3-13-e9781-s001.pdf]
