# Supplementary material for: Spatial and temporal non‐stationarity in long‐term population dynamics of over‐wintering birds of North America
Source: Ecol Evol. 2023 Mar 16;13(3):e9781. doi: 10.1002/ece3.9781 (PMC10019912; doi:10.1002/ece3.9781)

American Black Duck

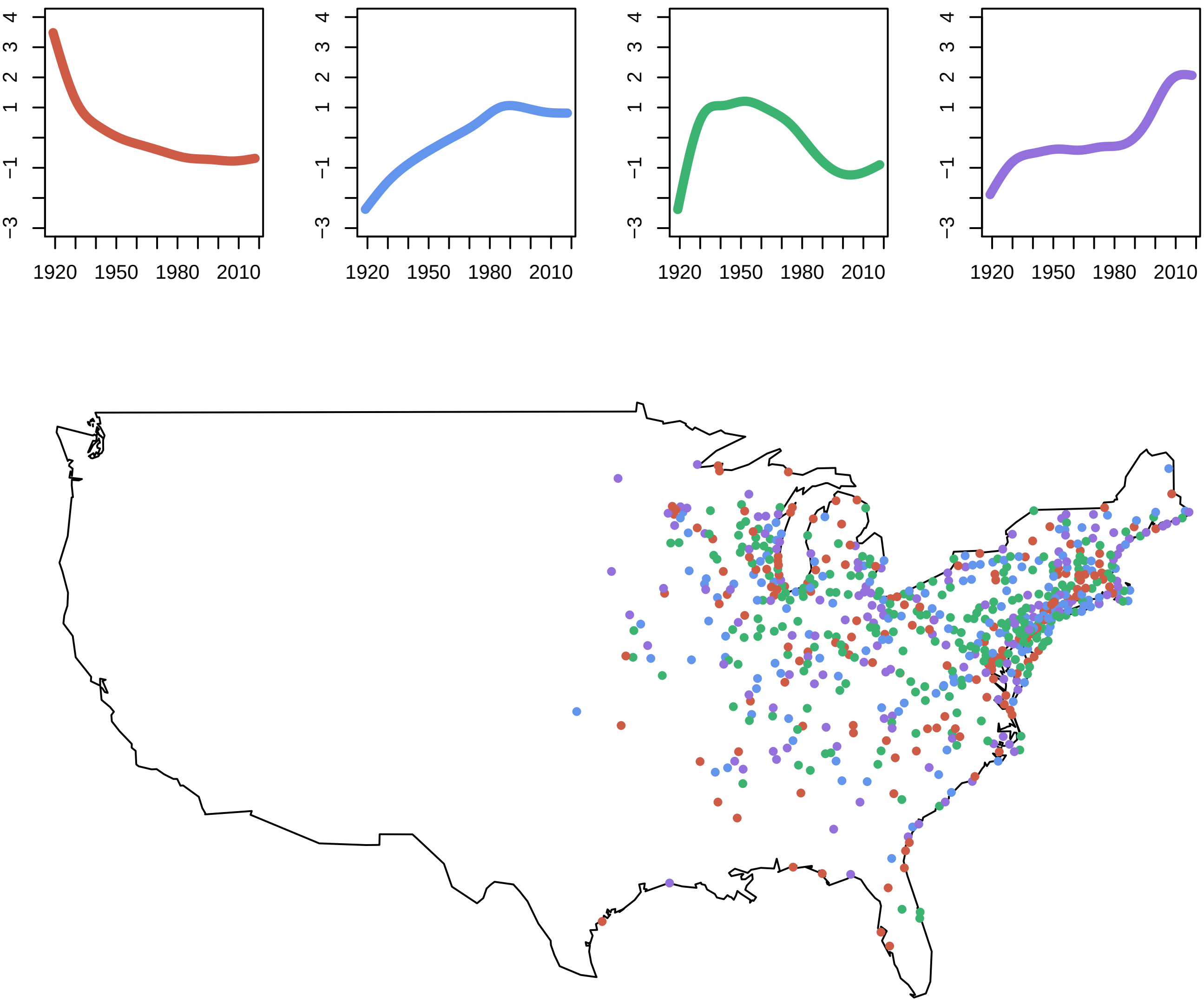

American Coot

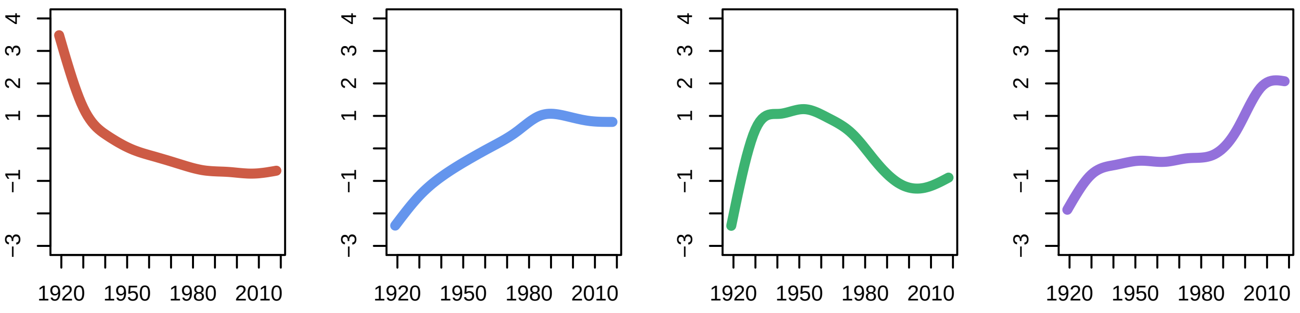

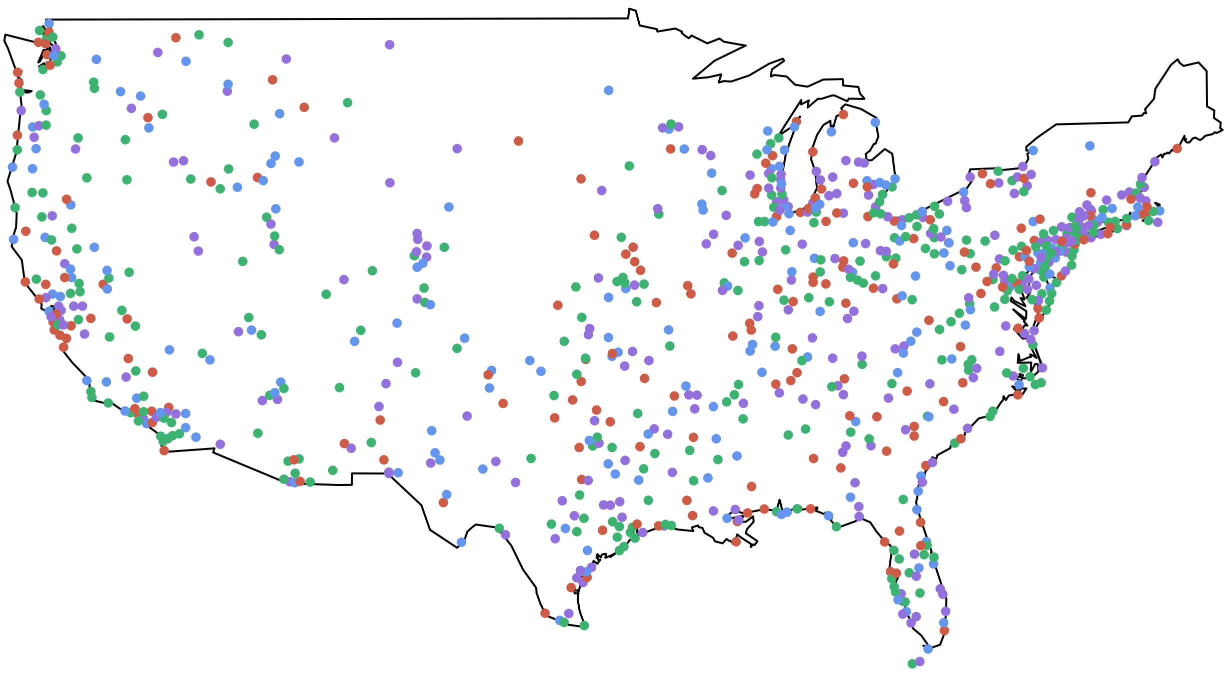

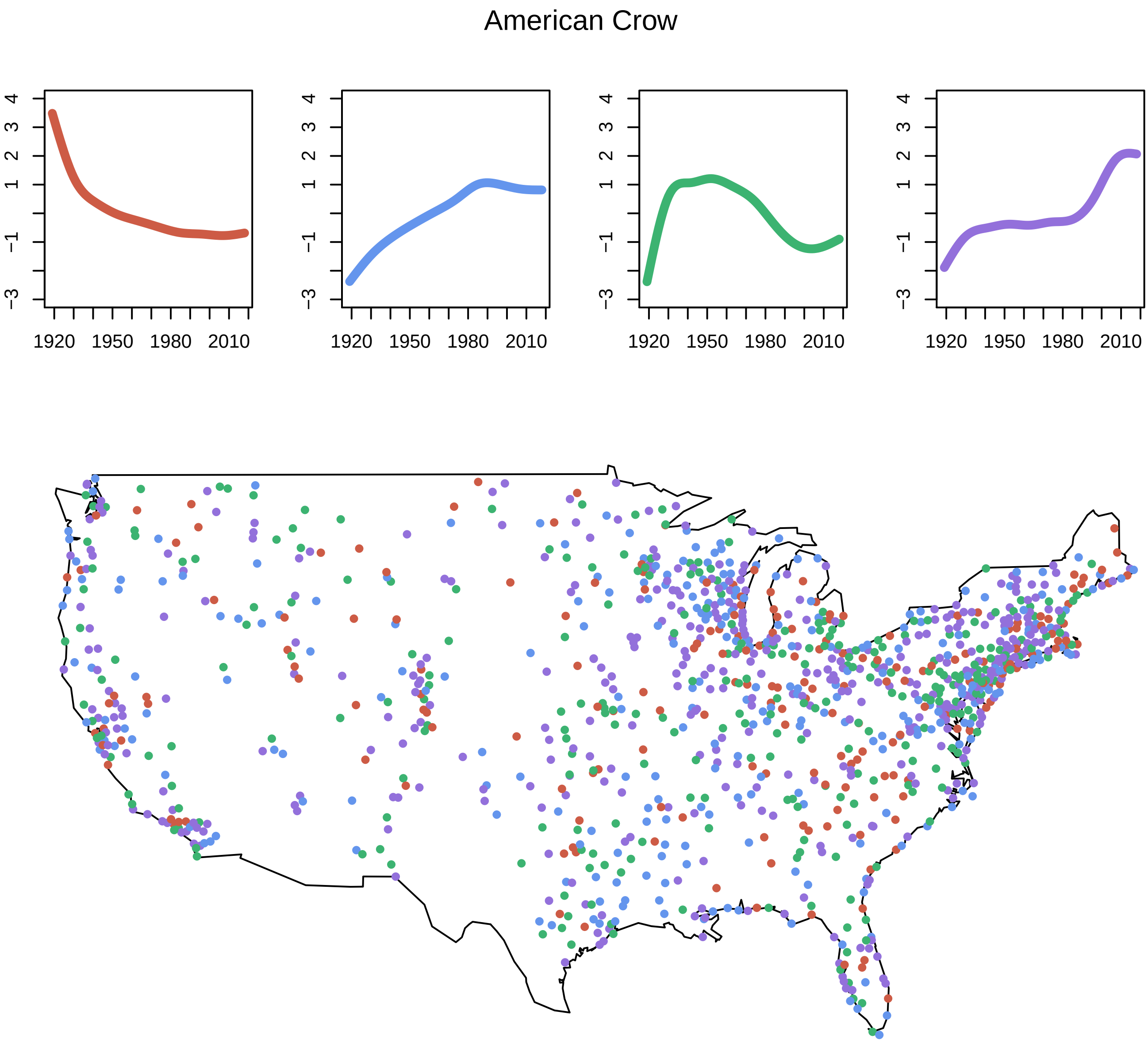
American Crow
4
3
2
1
−1
−3
1920
1950
1980
2010

American Goldfinch

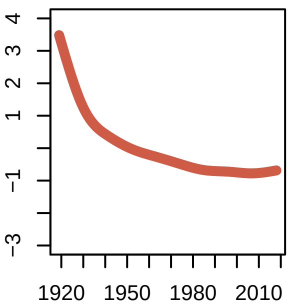

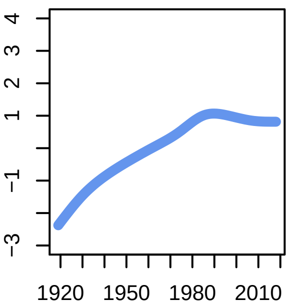

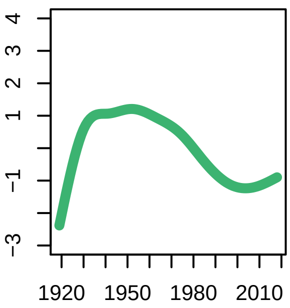

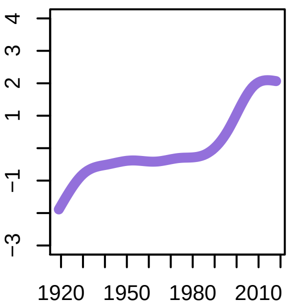

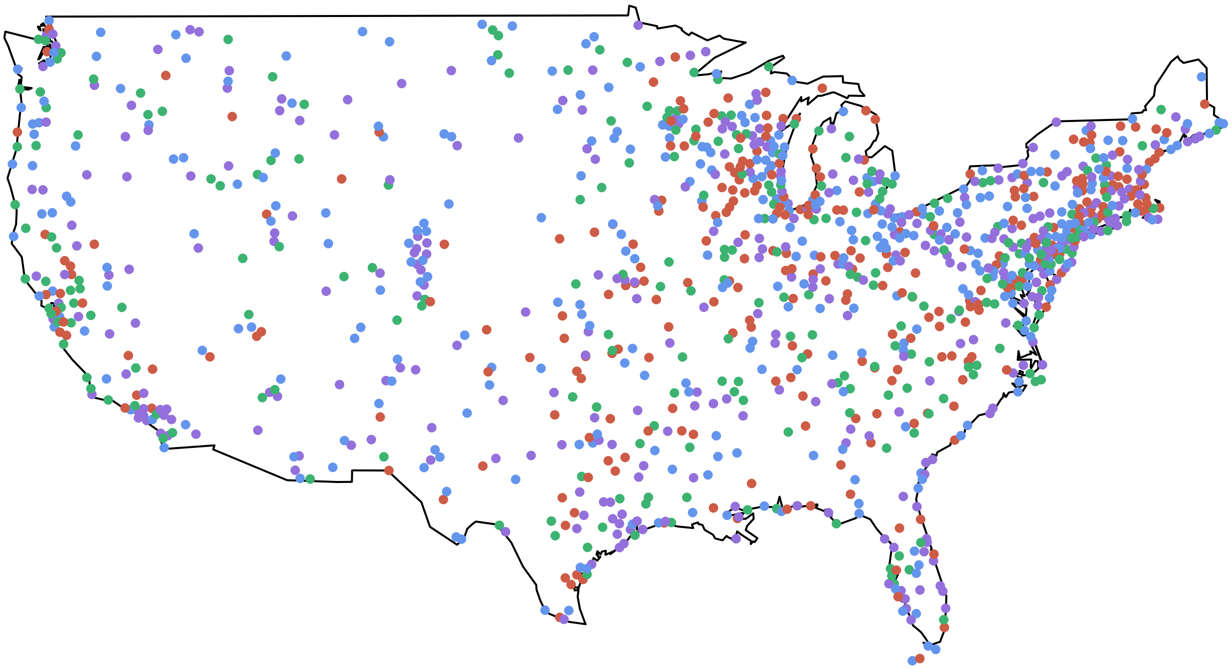

American Robin

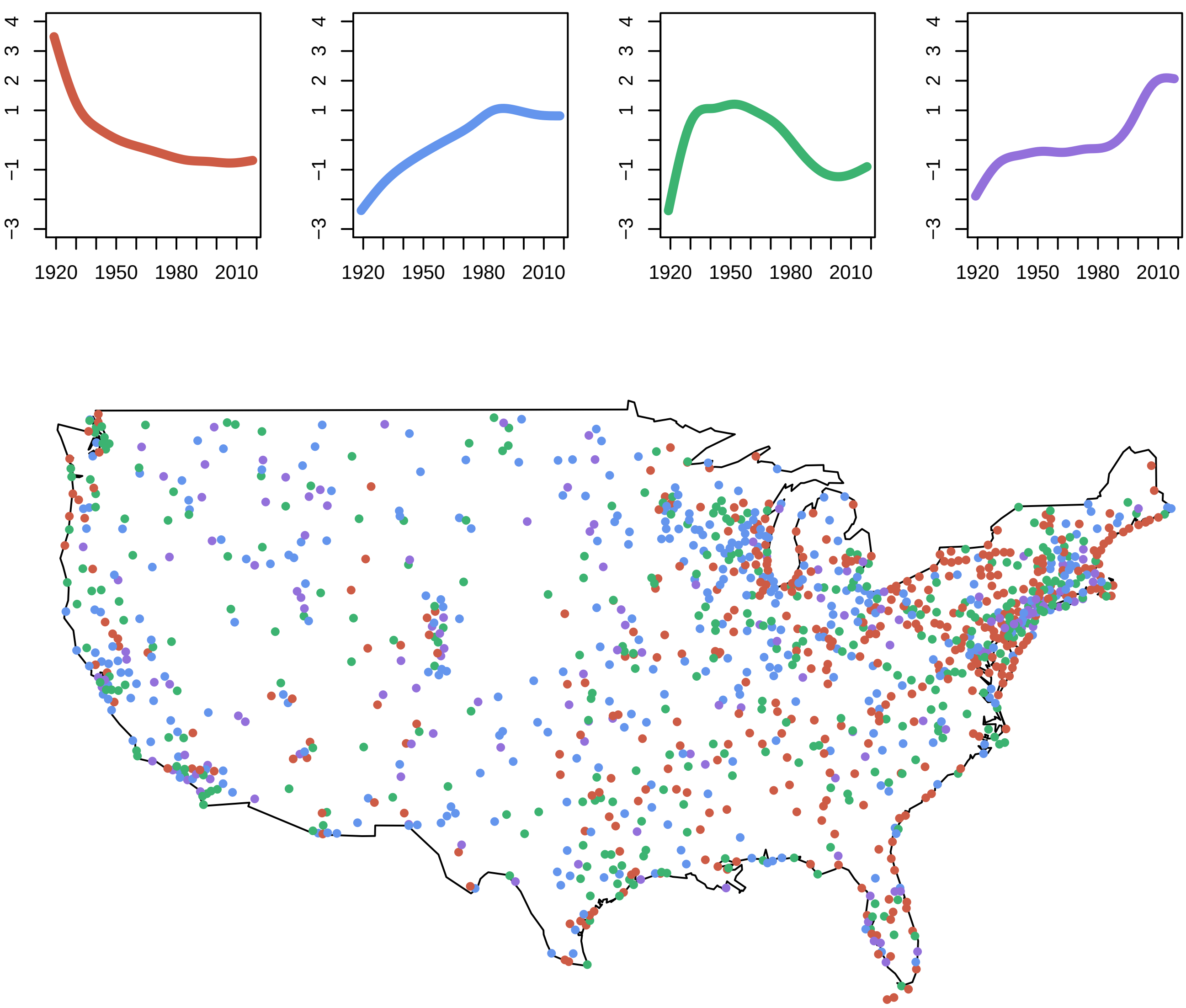

American Tree Sparrow

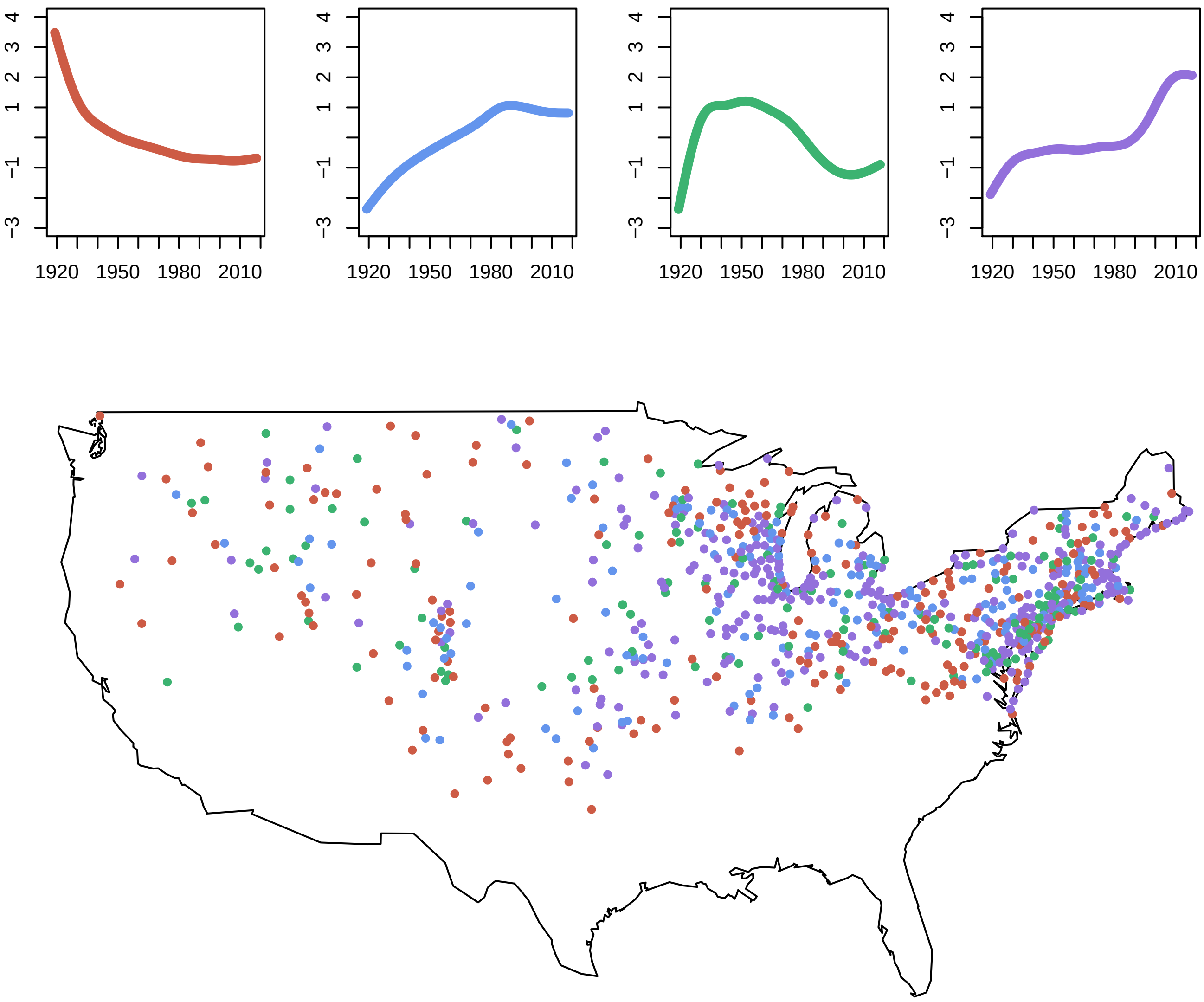

Black−capped Chickadee

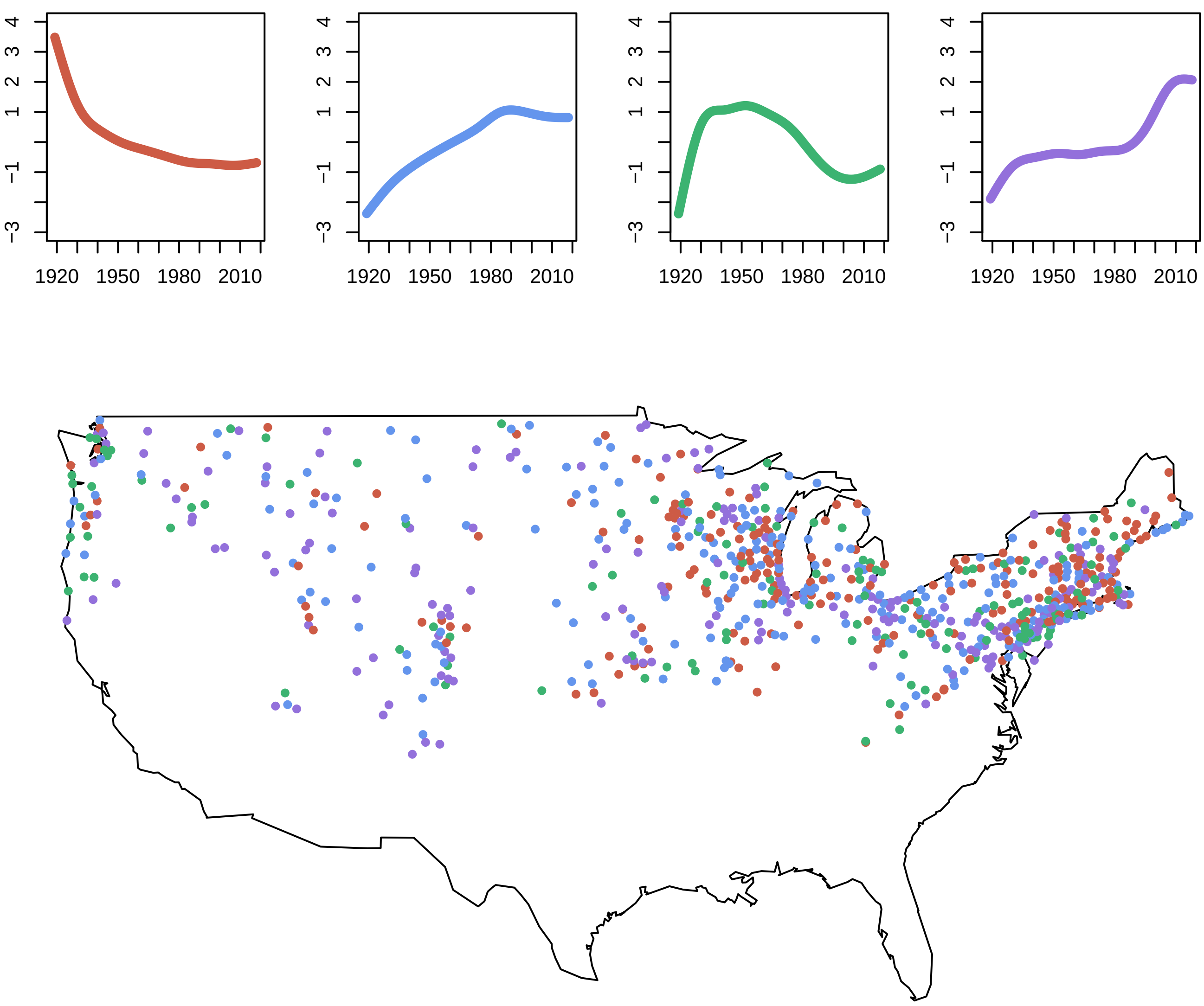

Blue Jay

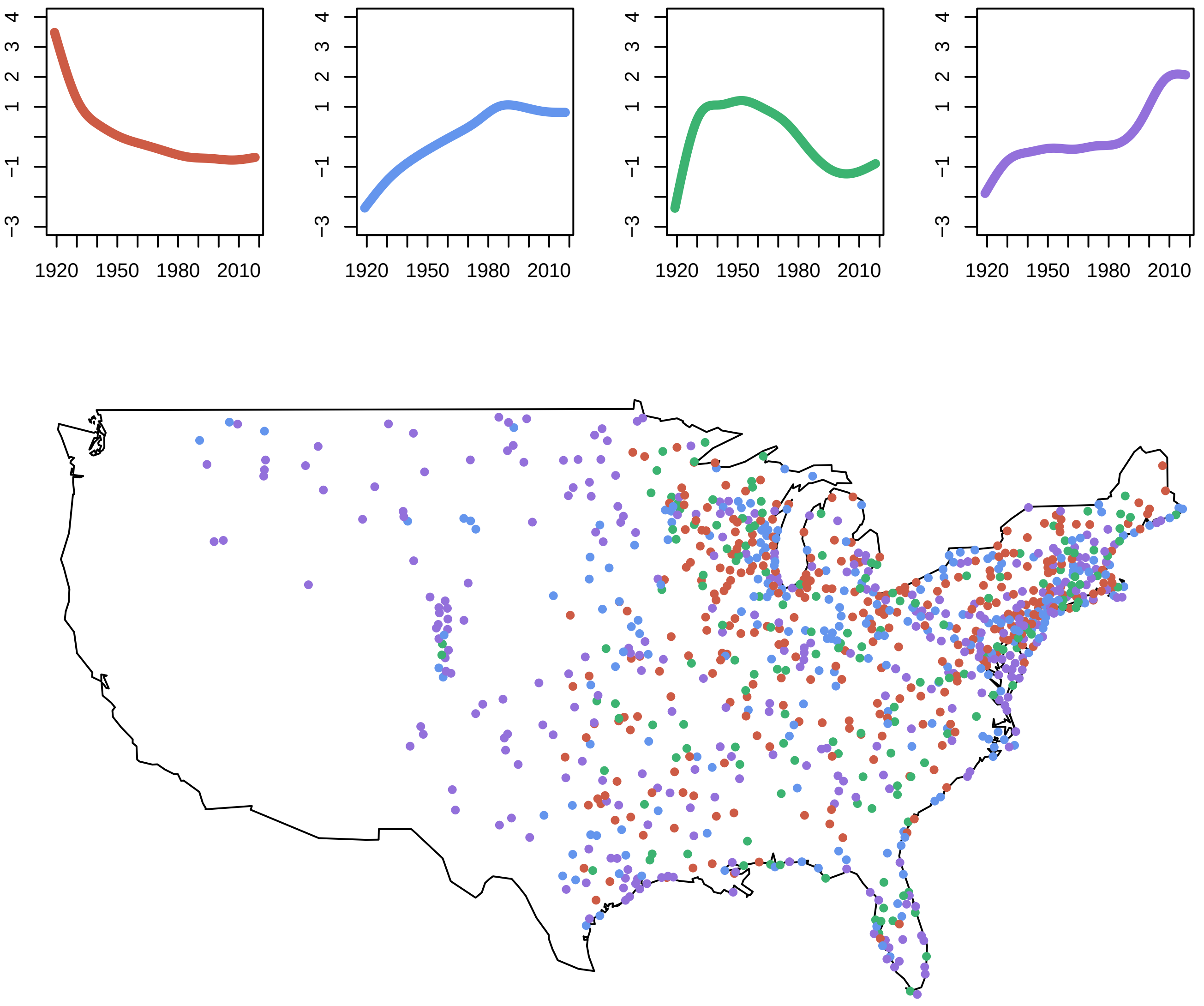

Brant

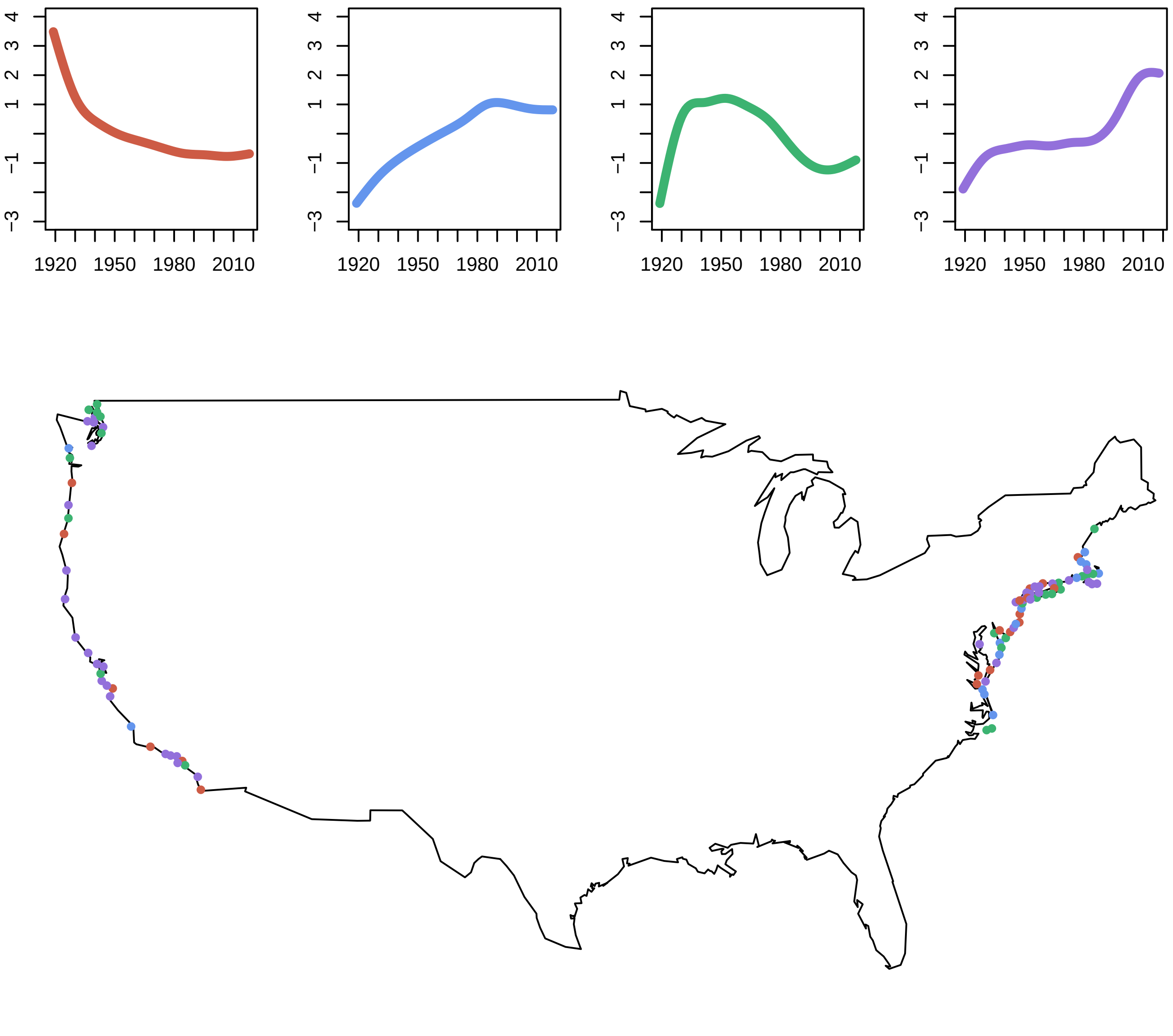

Brewer's Blackbird

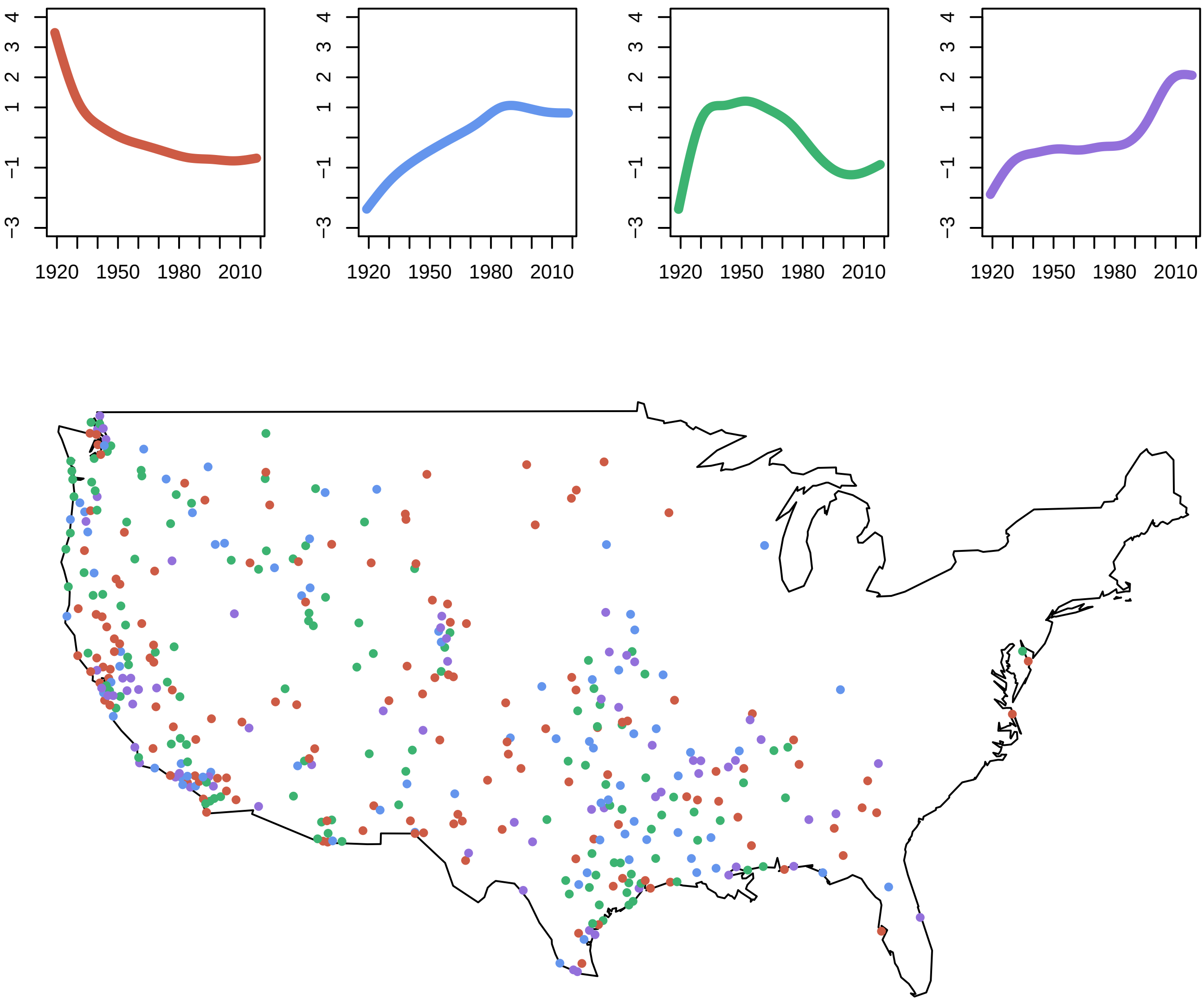

Brown−headed Cowbird

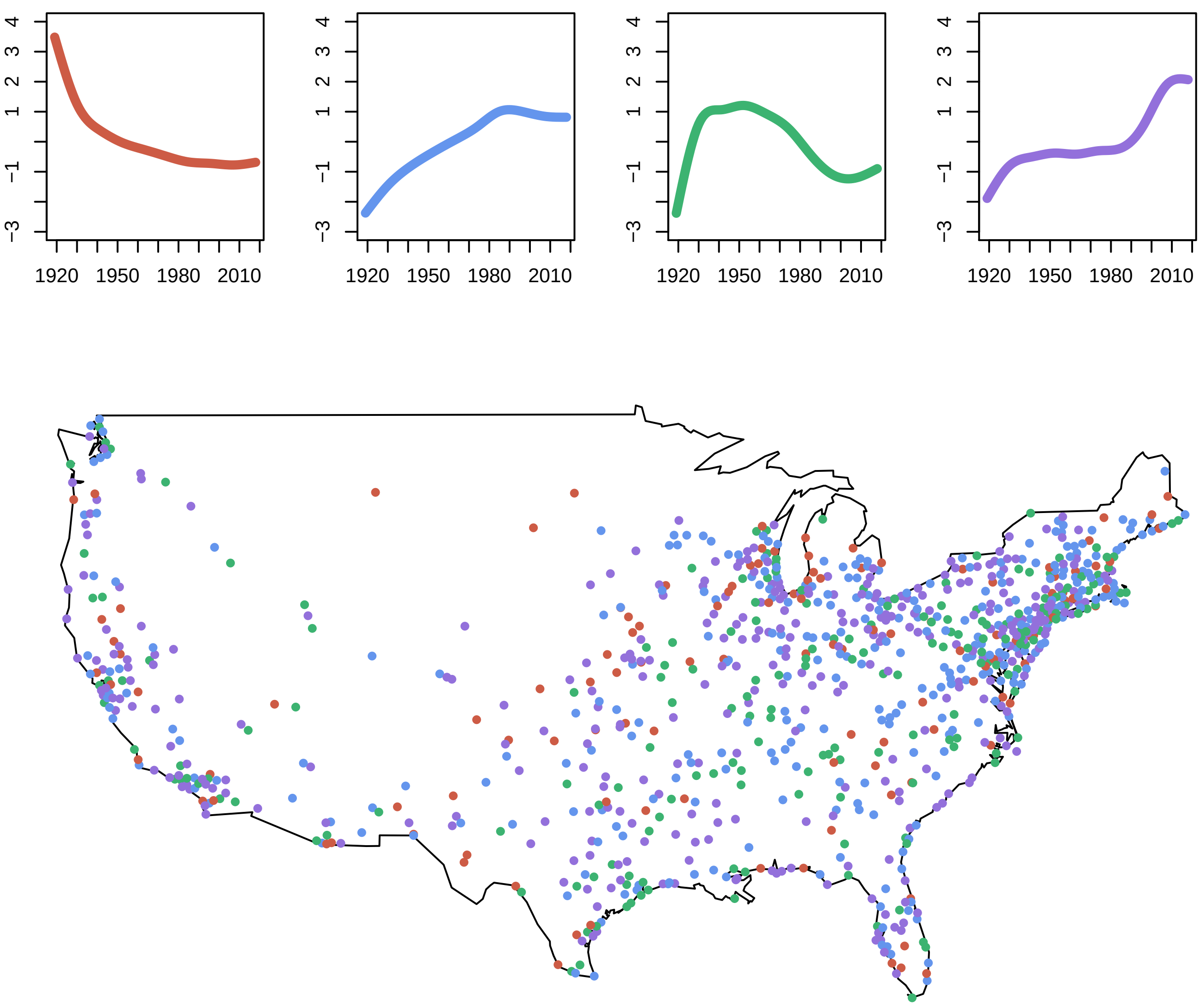

Canada Goose

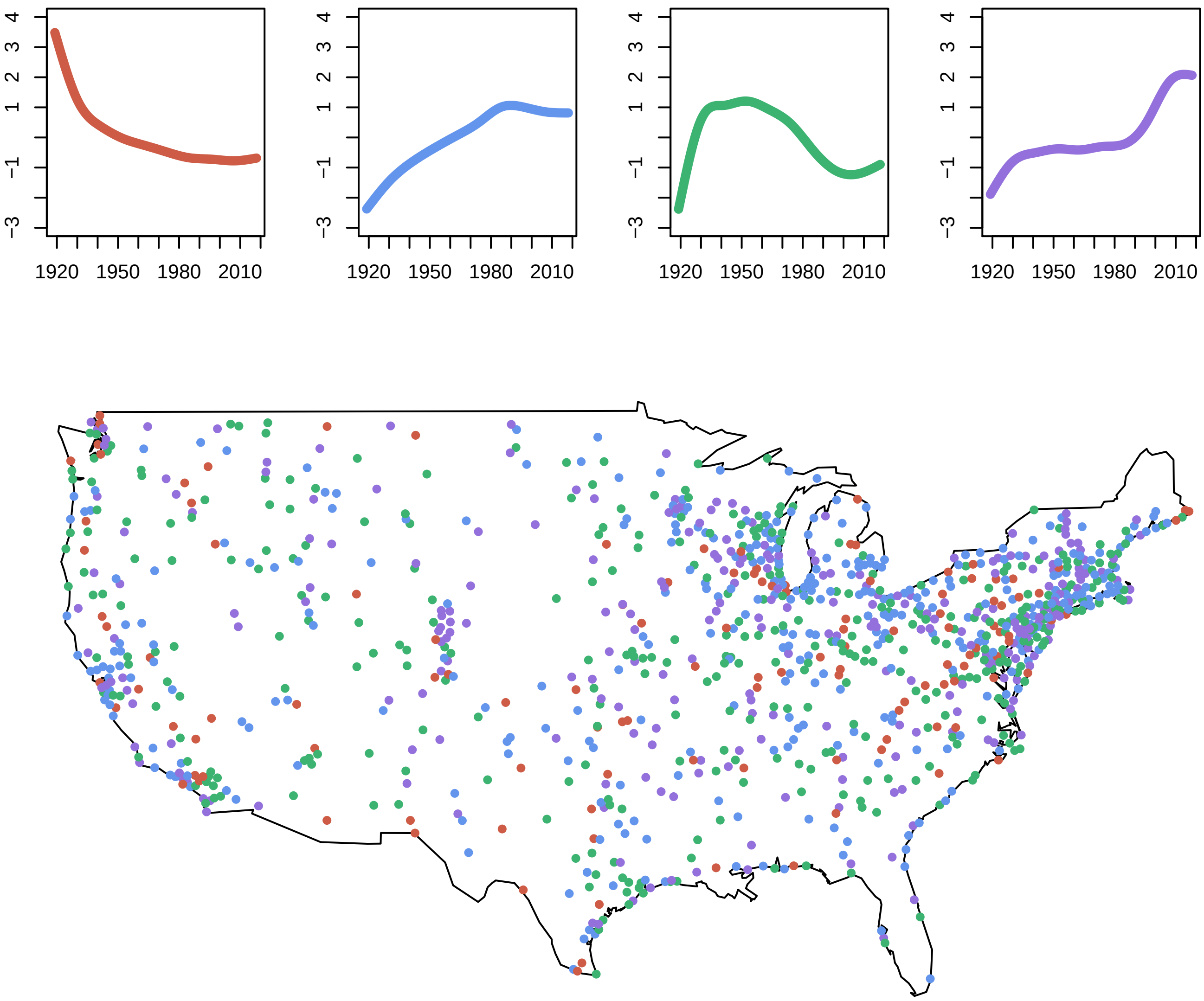

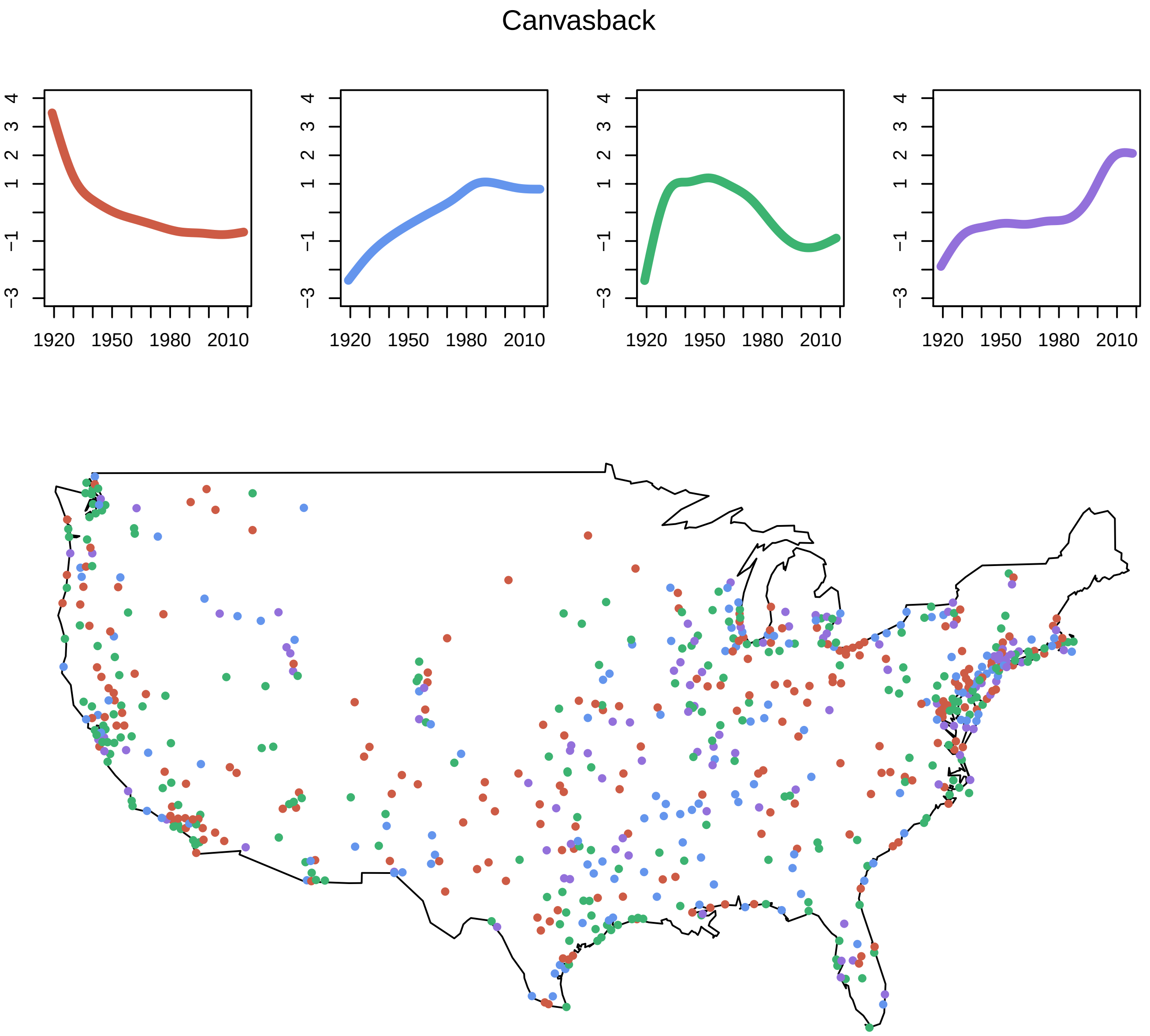
Canvasback
4
3
2
1
−1
−3
1920
1950
1980
2010

Common Grackle

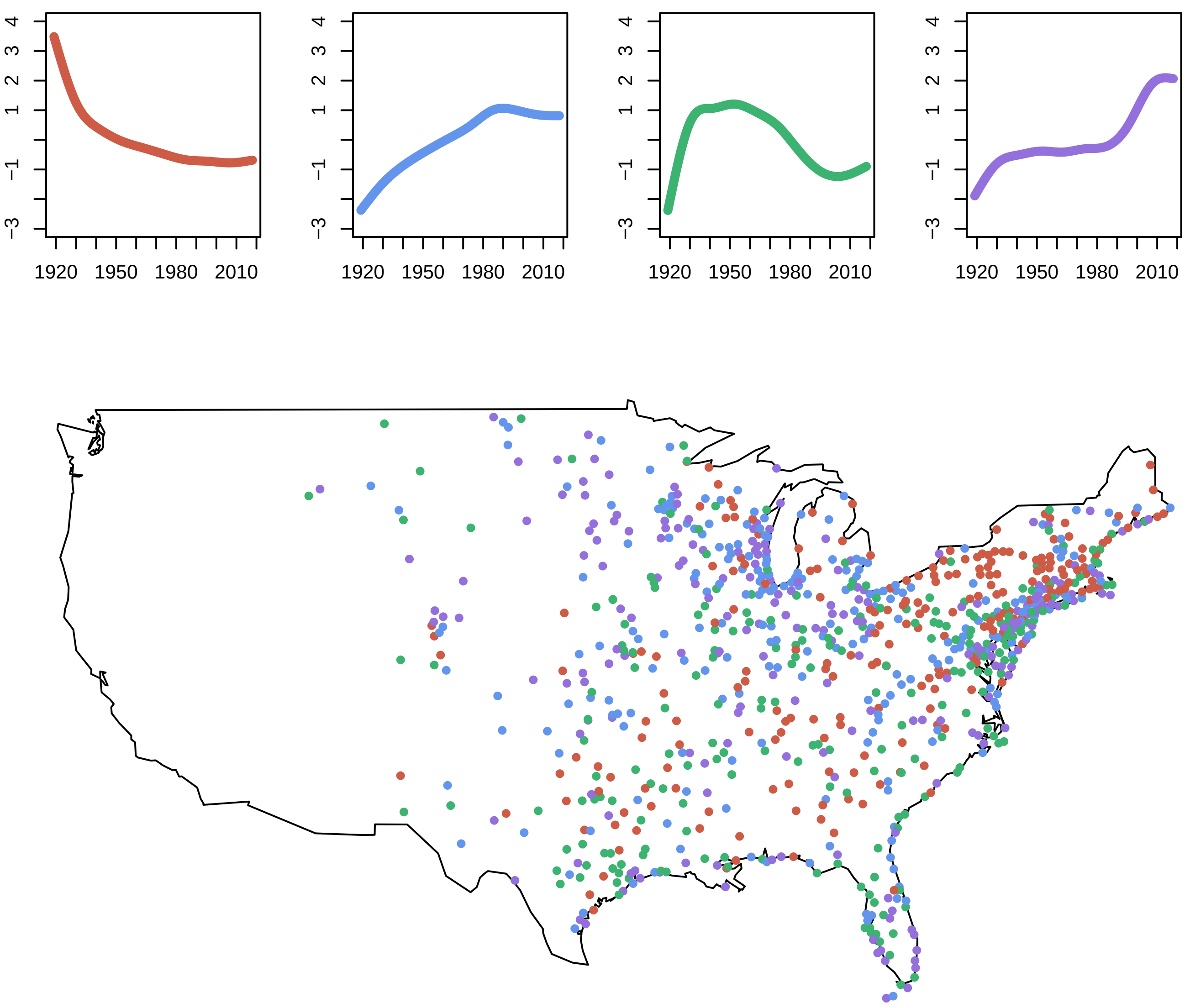

Dark−eyed Junco

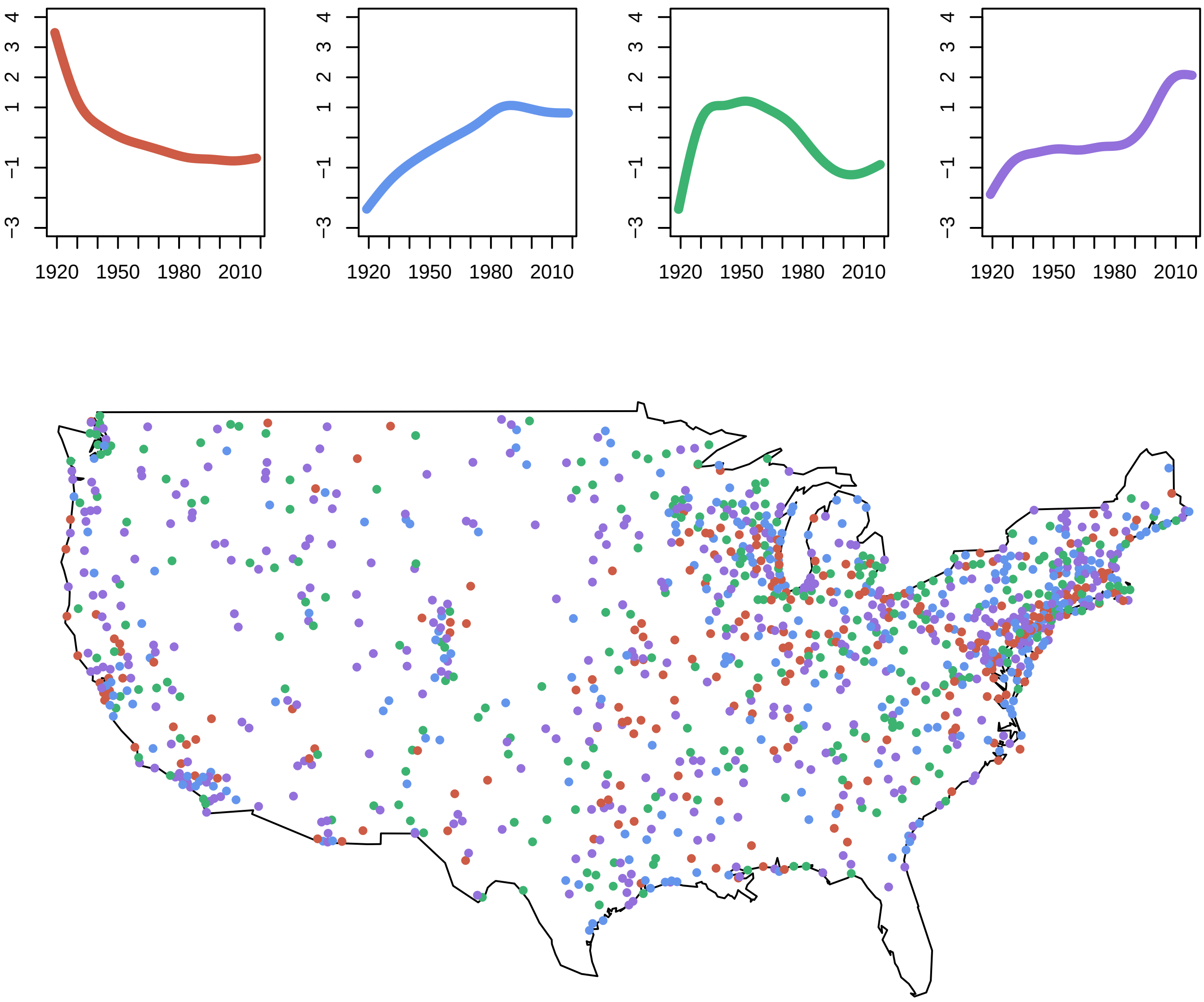

Double−crested Cormorant

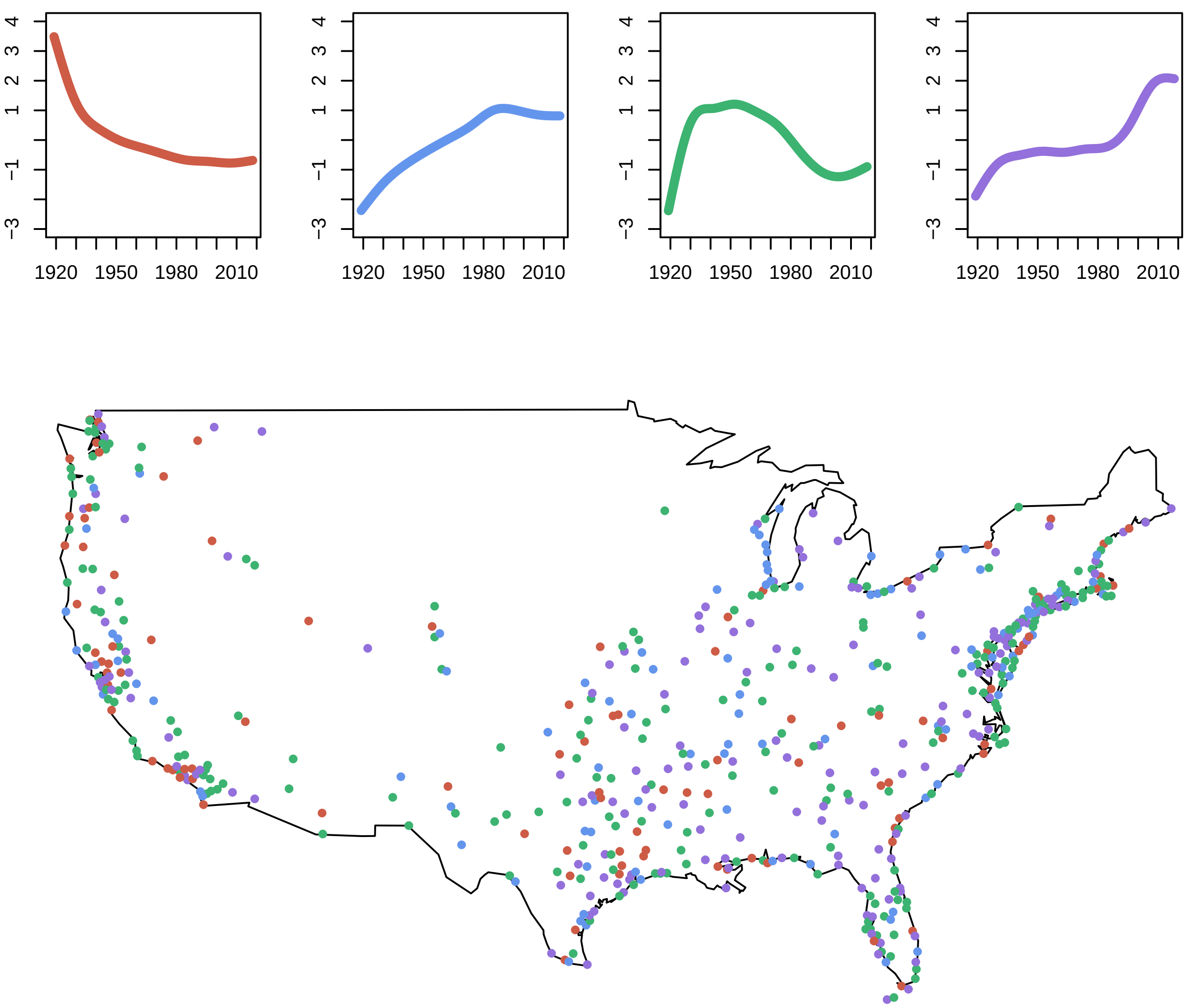

European Starling

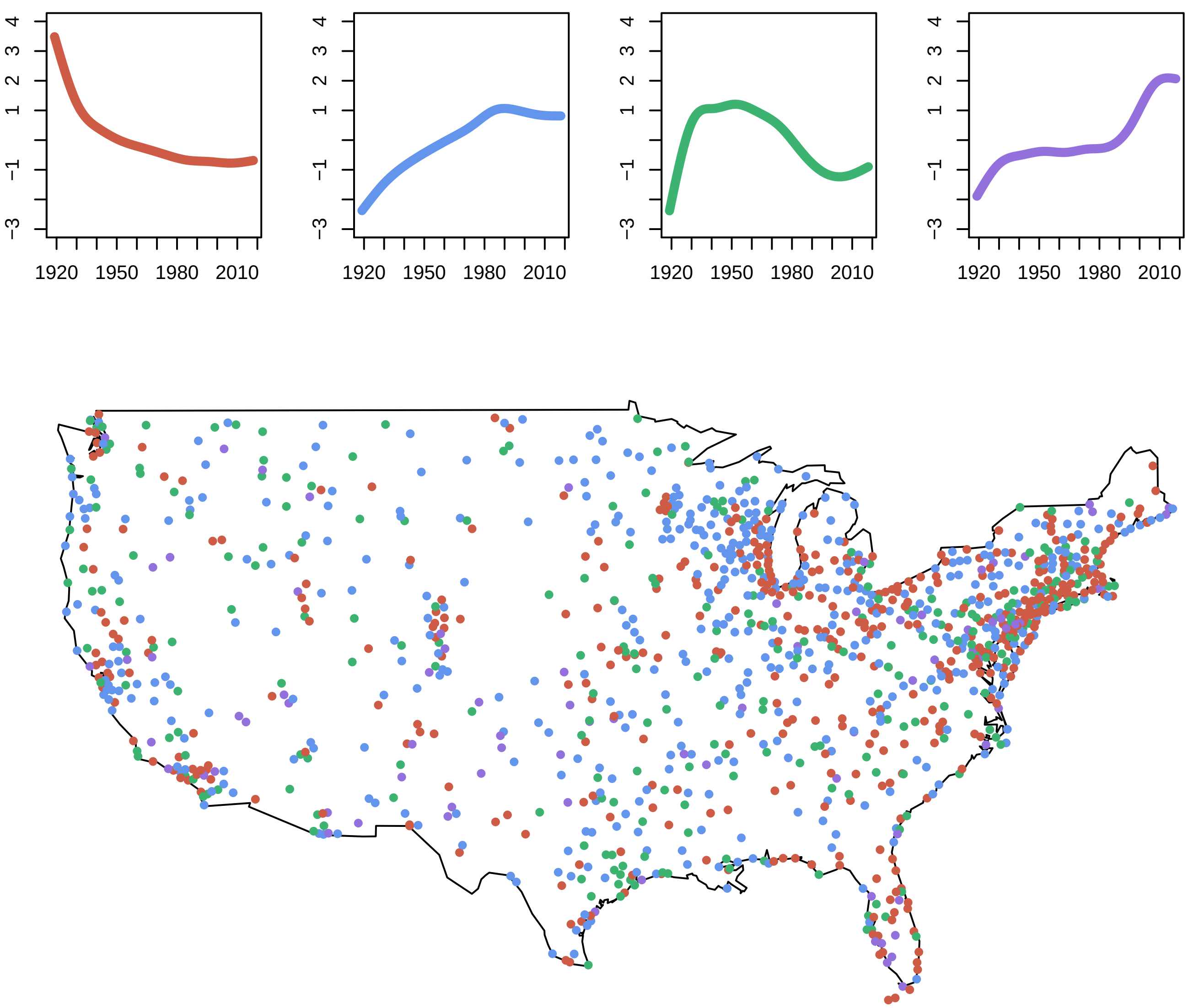

Great−tailed Grackle

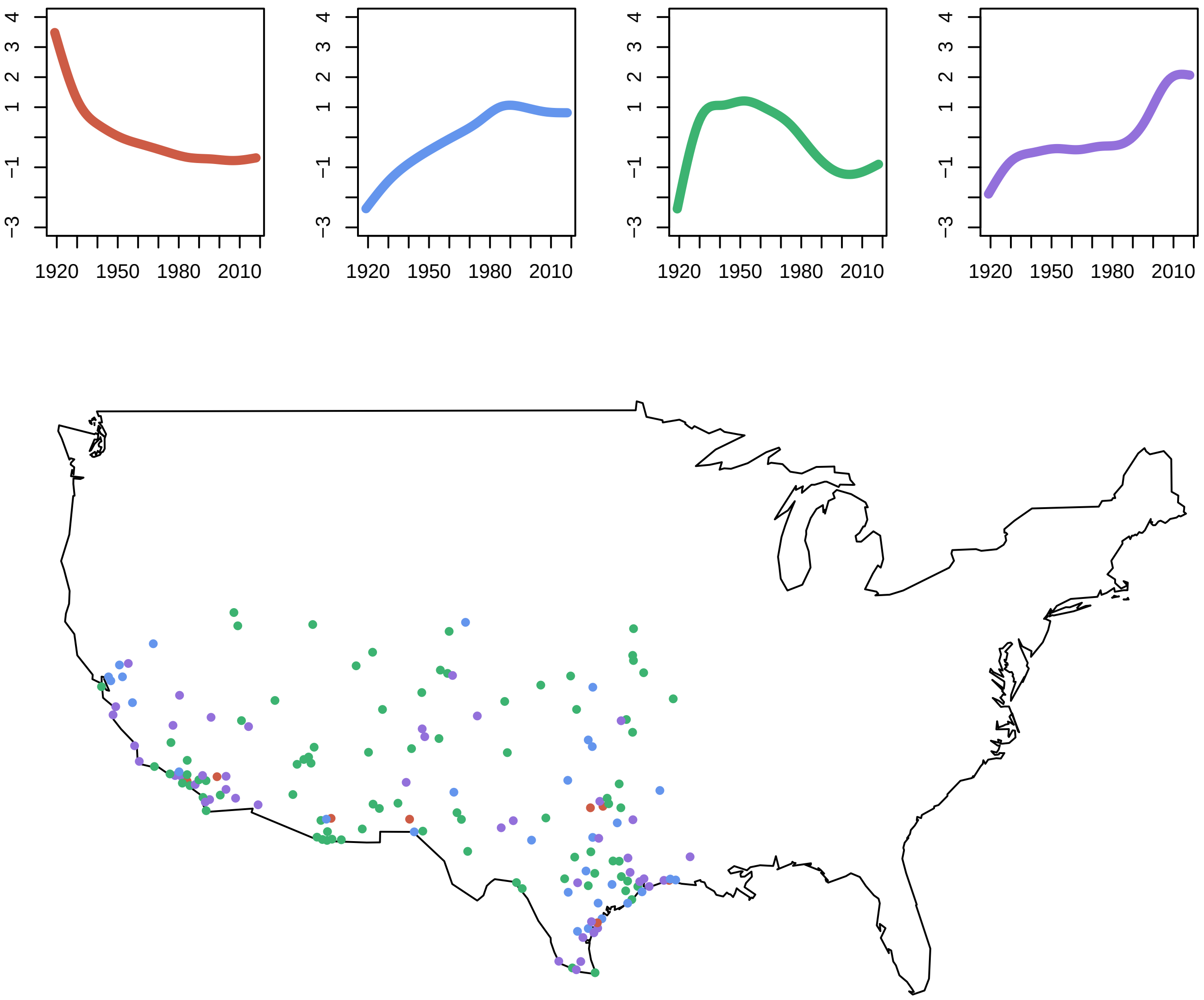

Greater Scaup

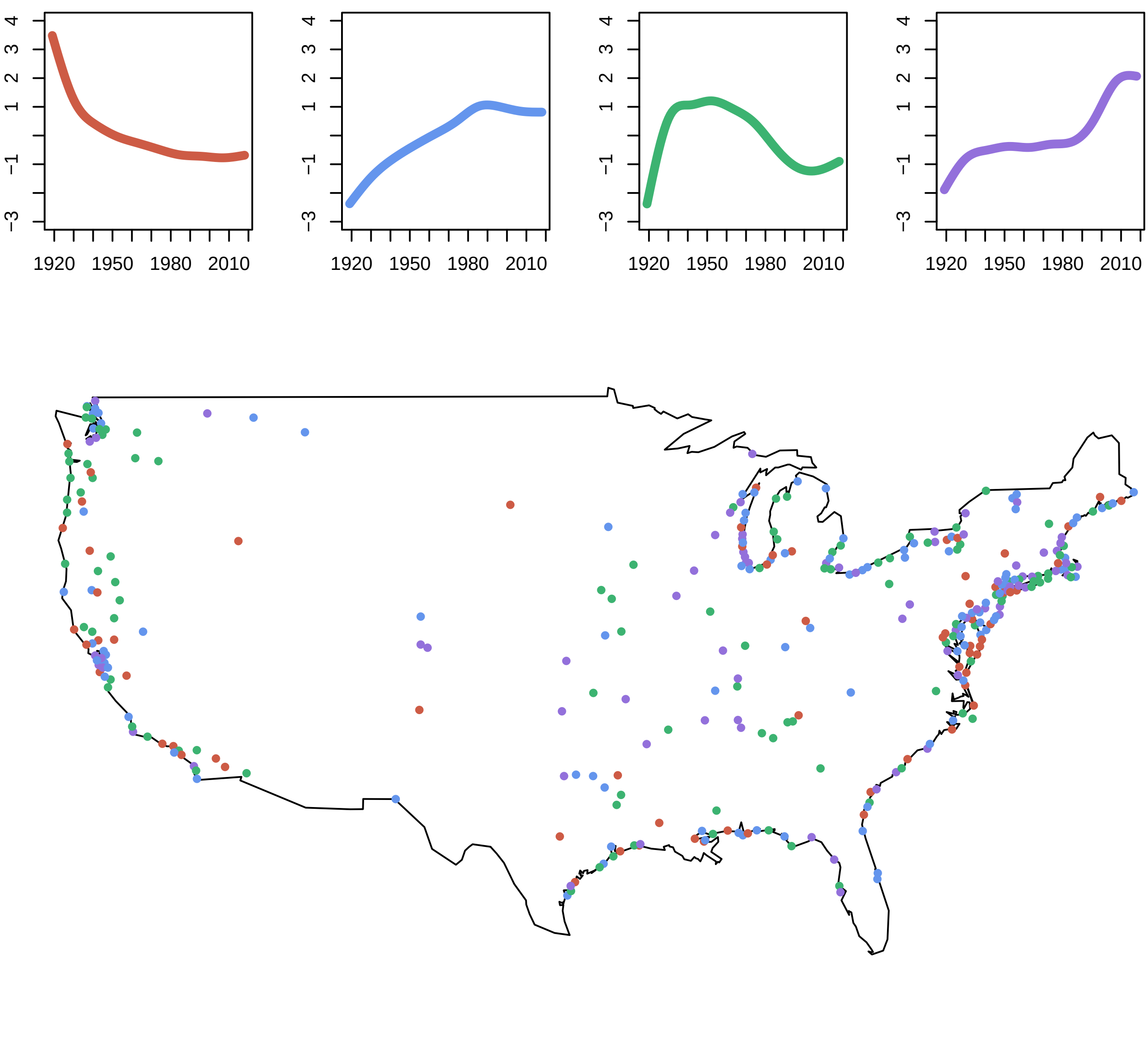

Green−winged Teal

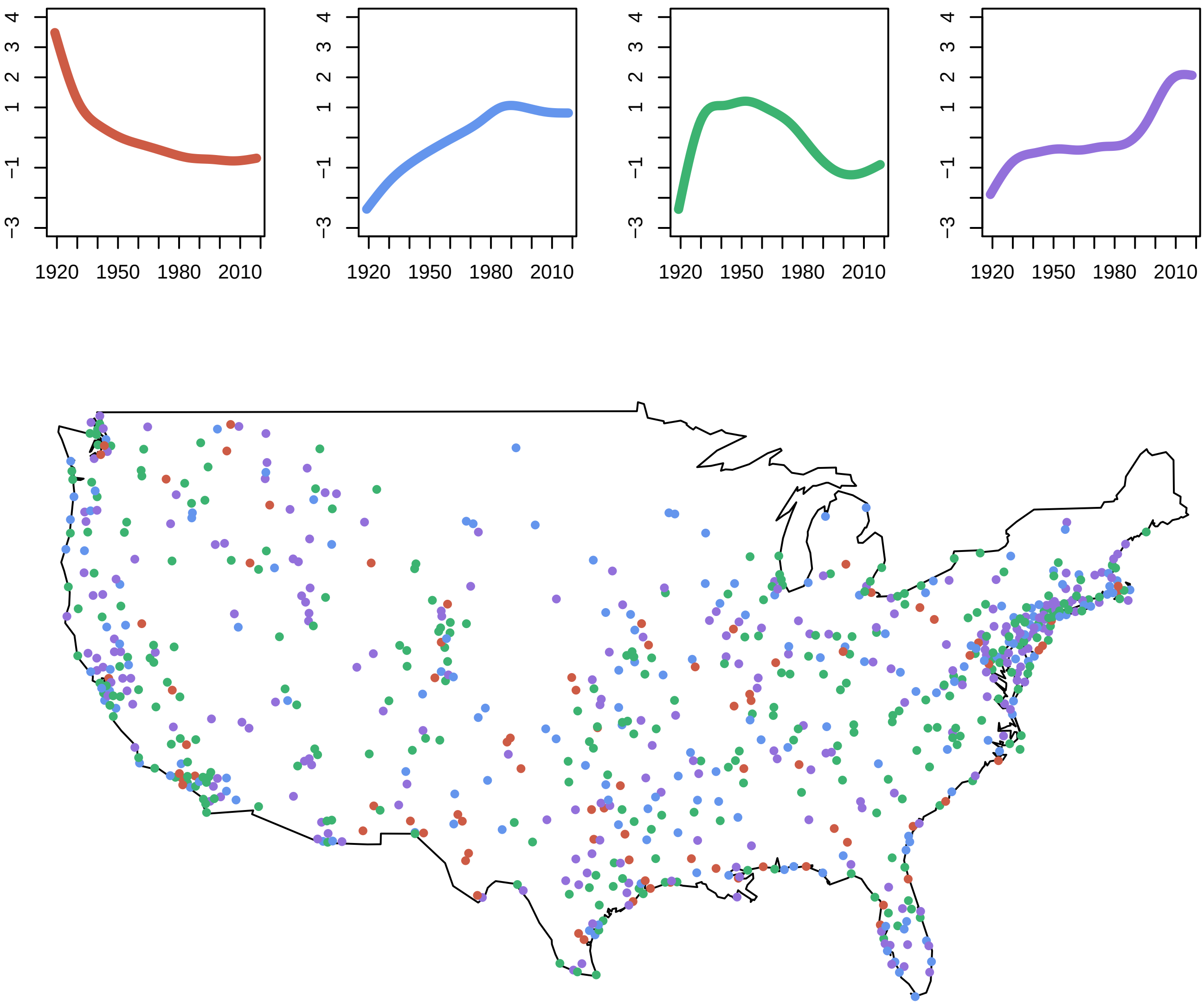

House Finch

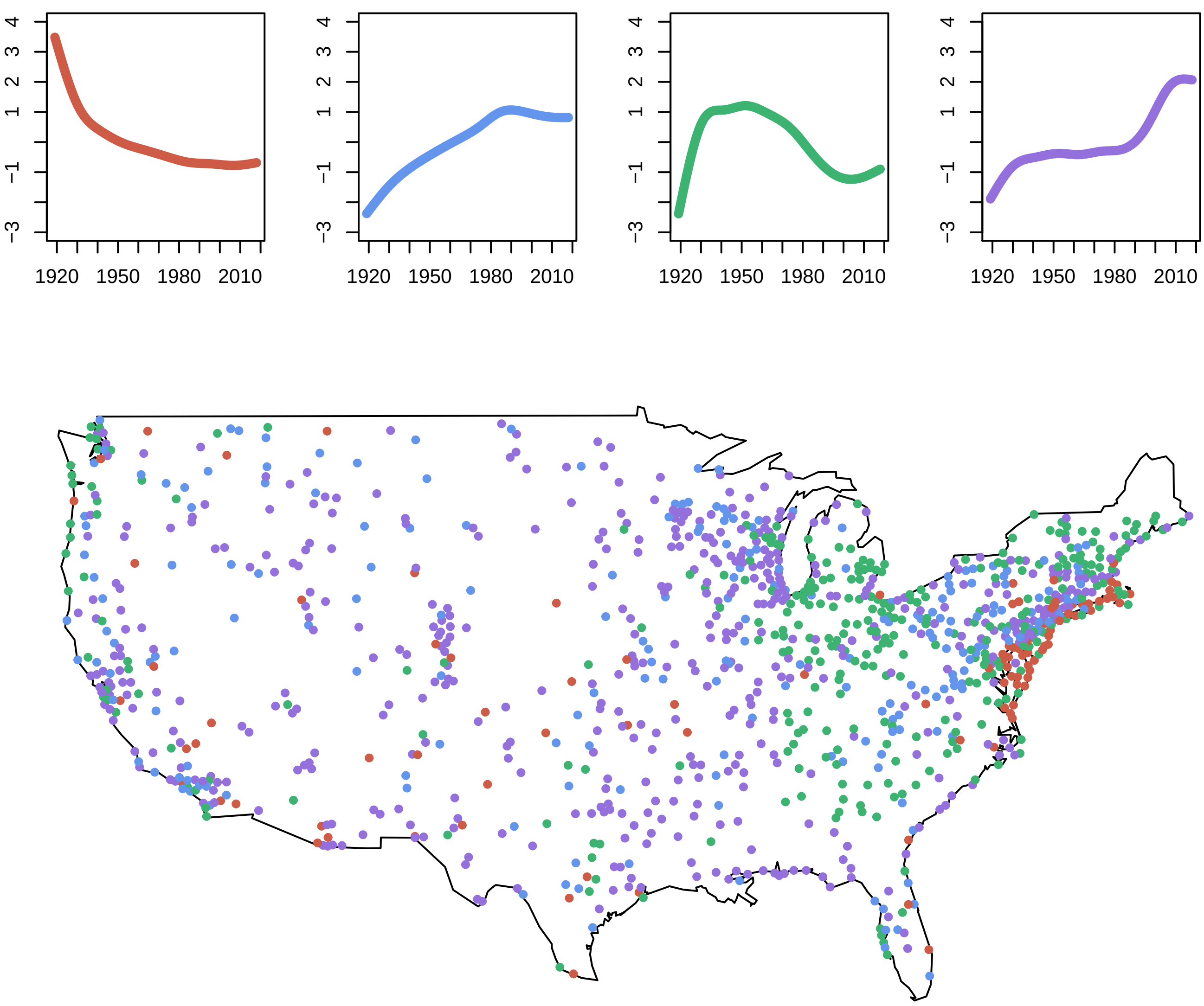

House Sparrow

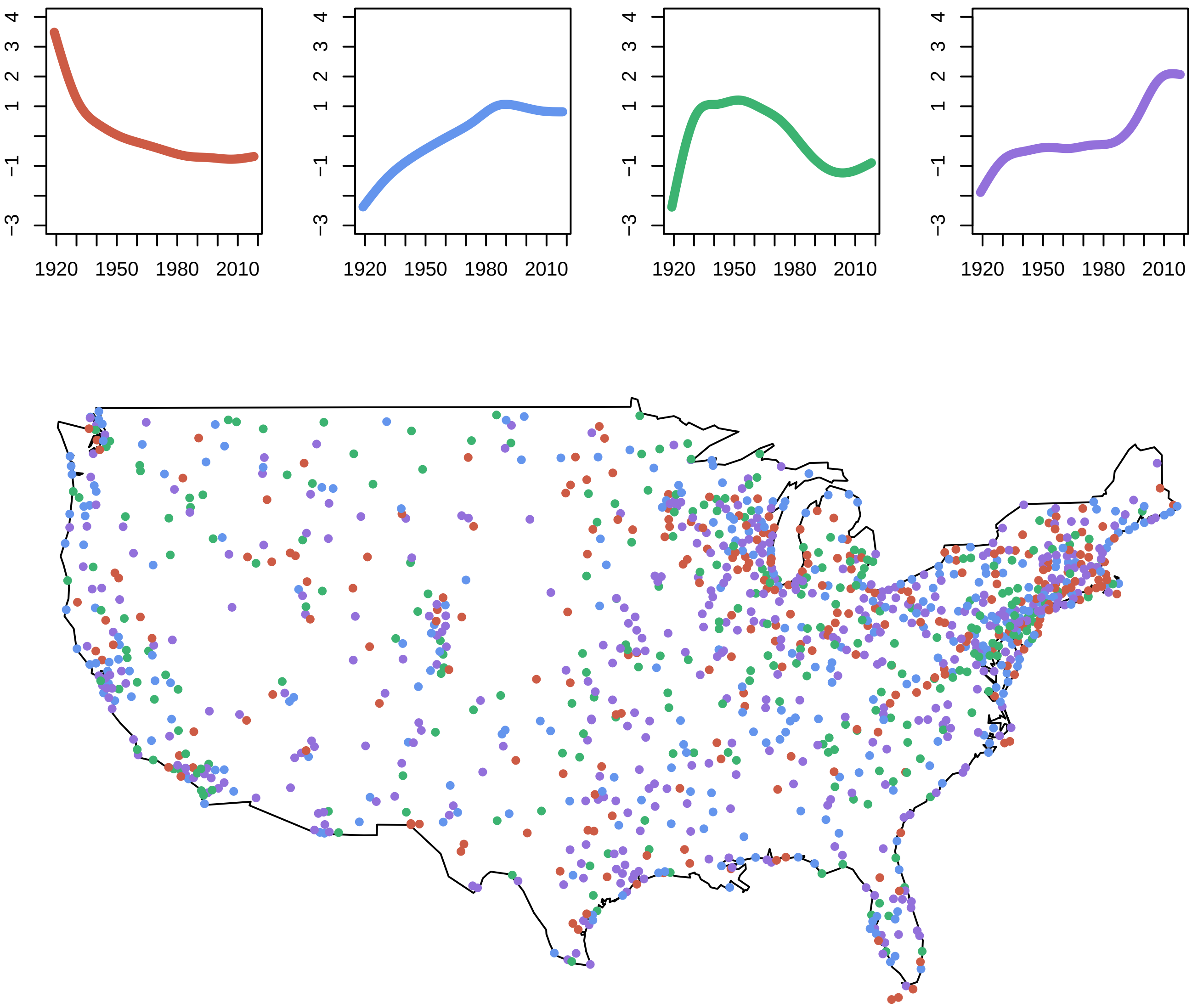

Lesser Scaup

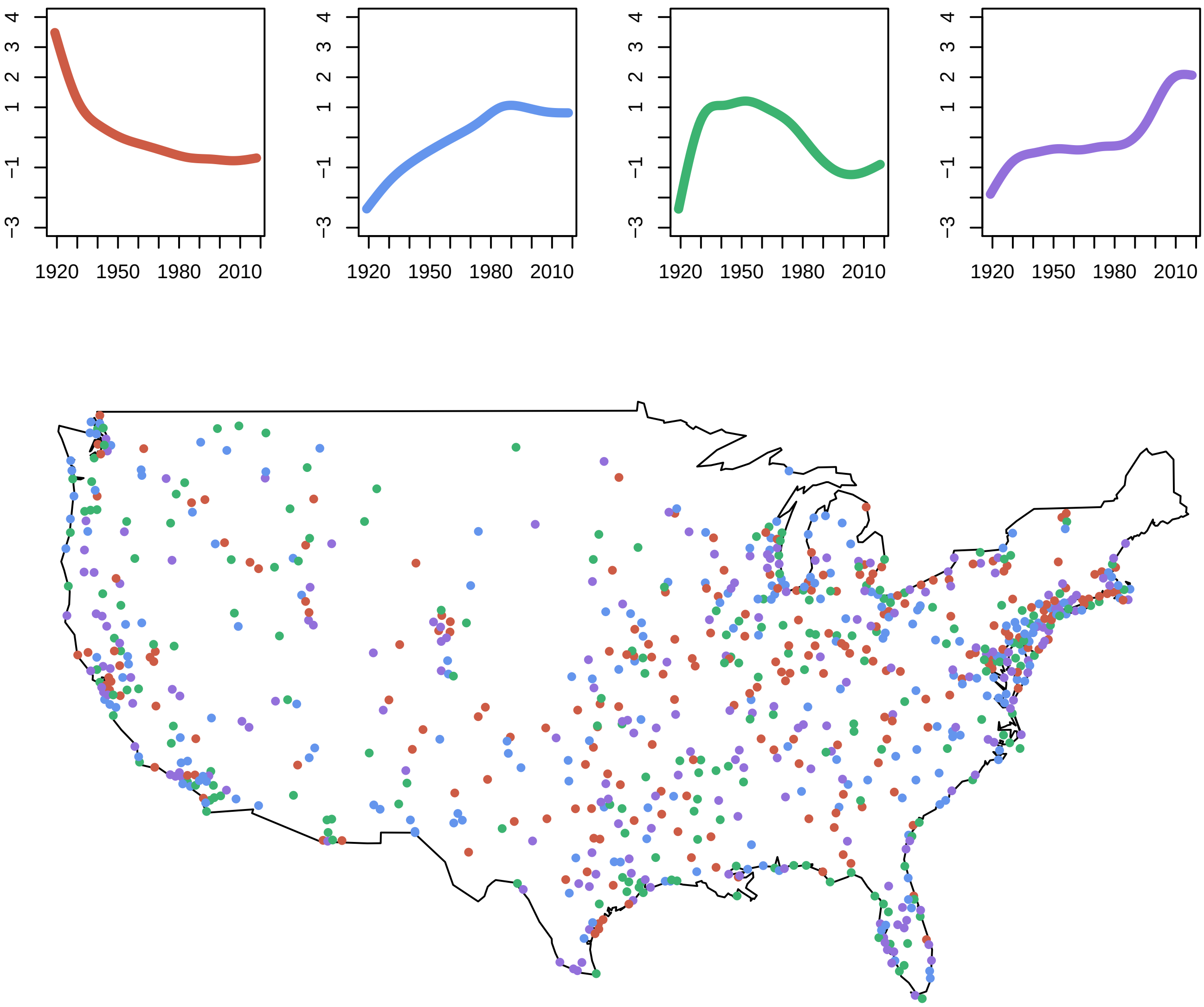

Long−tailed Duck

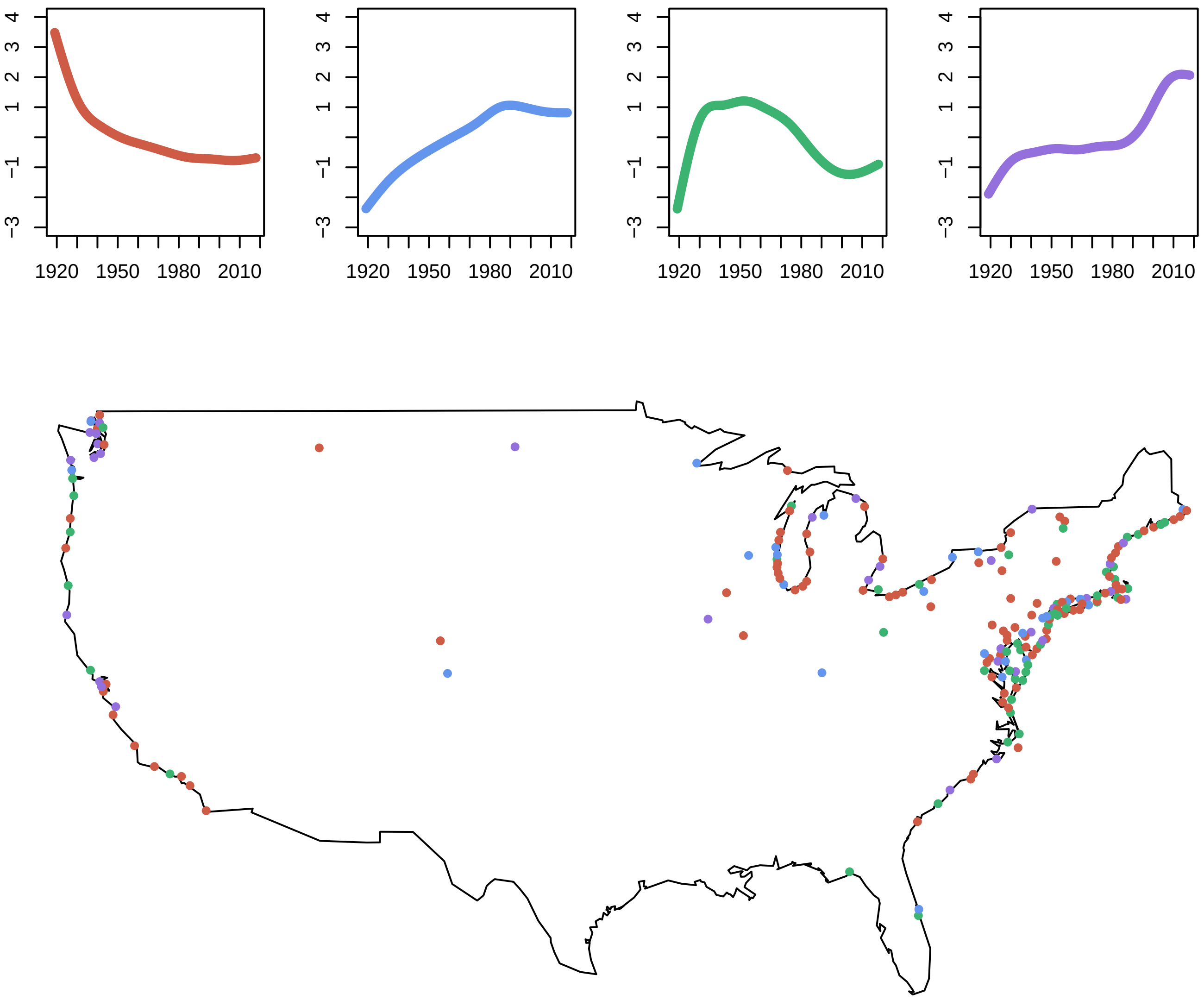

Mallard

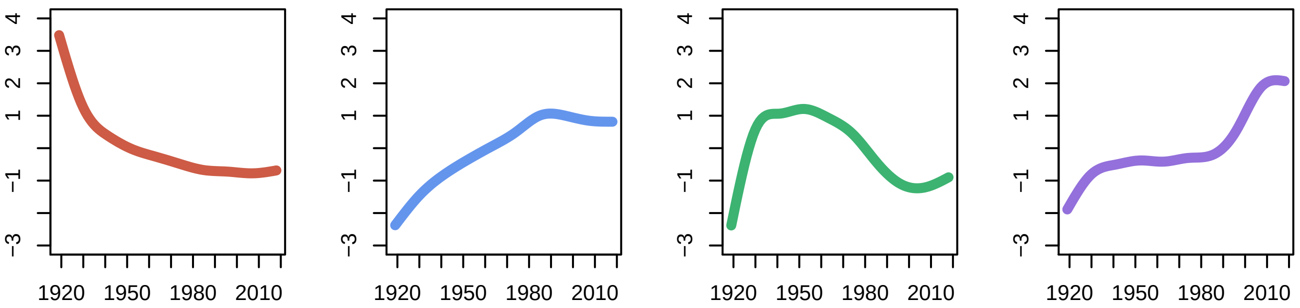

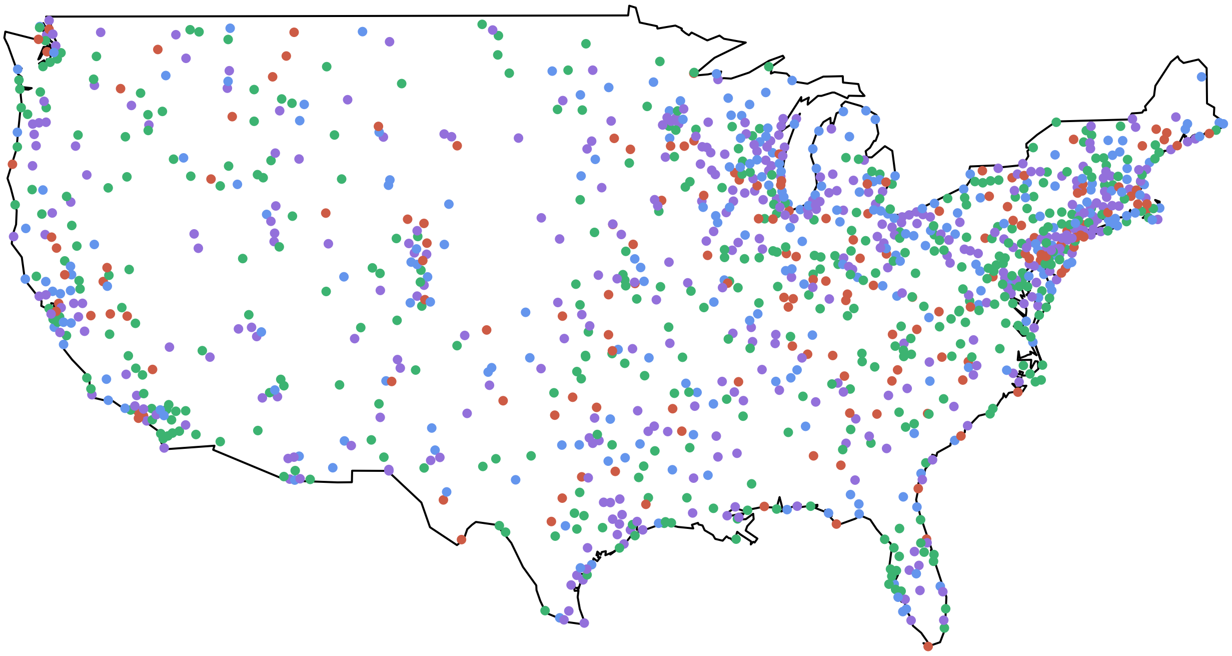

Mourning Dove

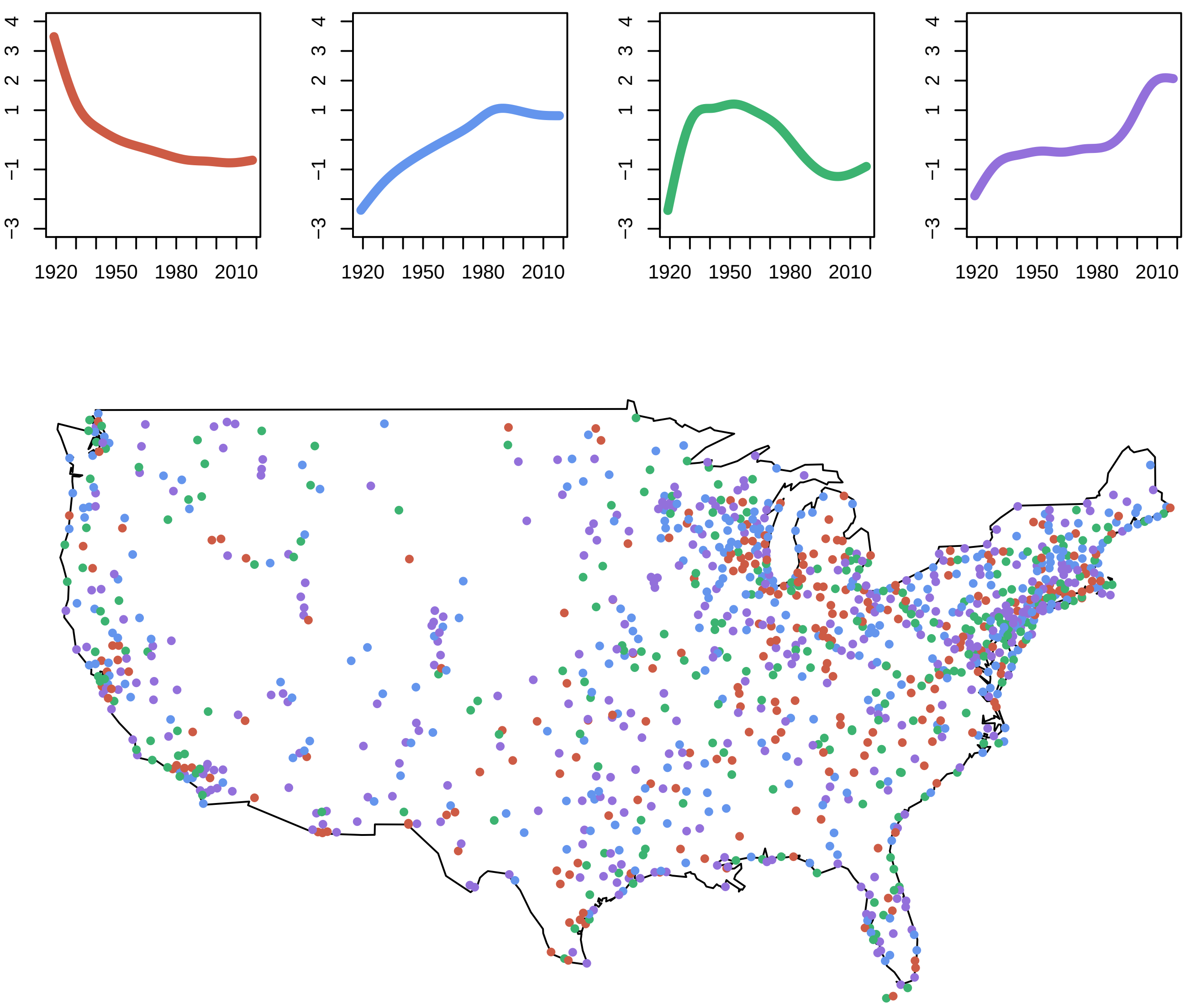

Northern Cardinal

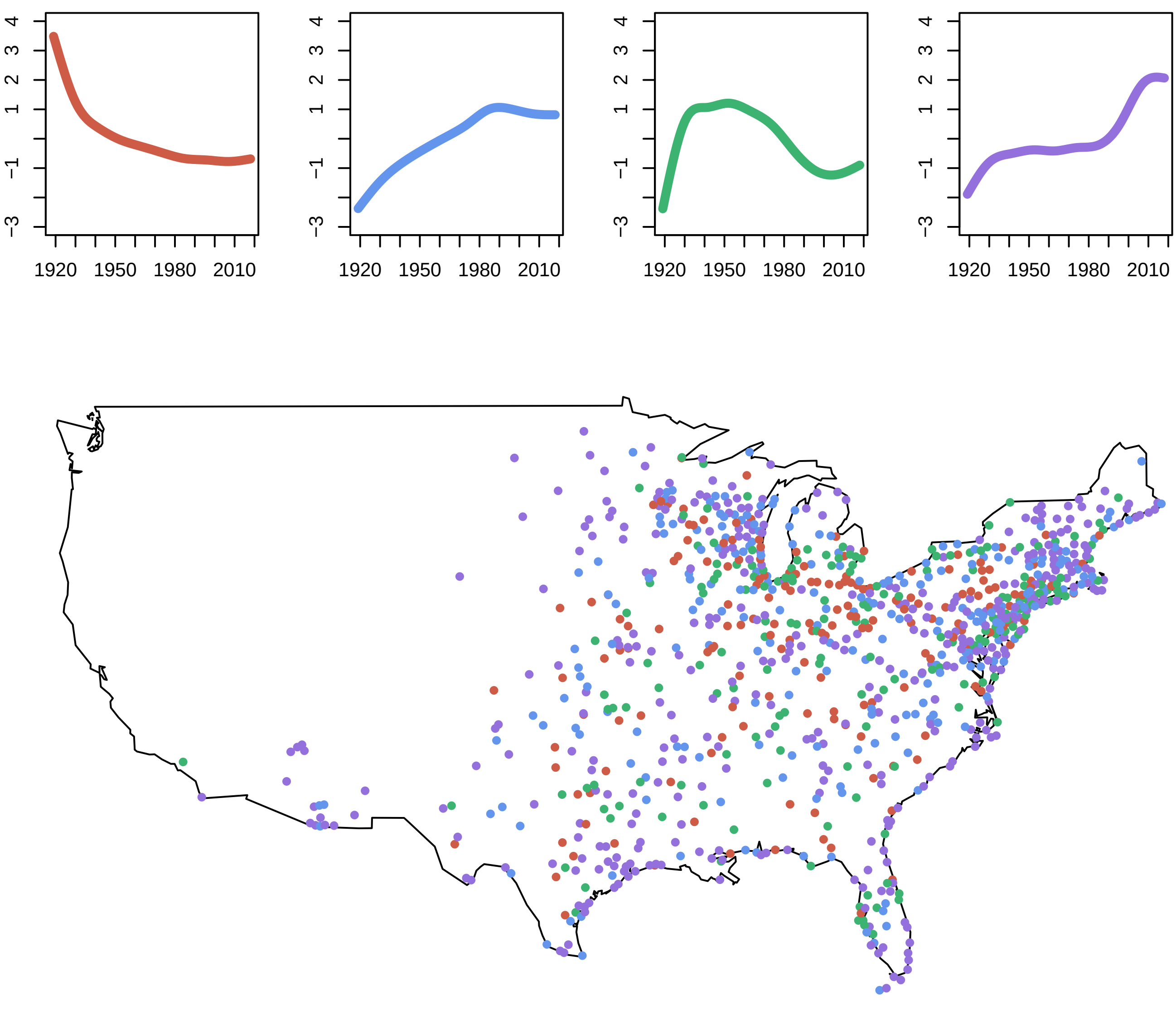

Northern Pintail

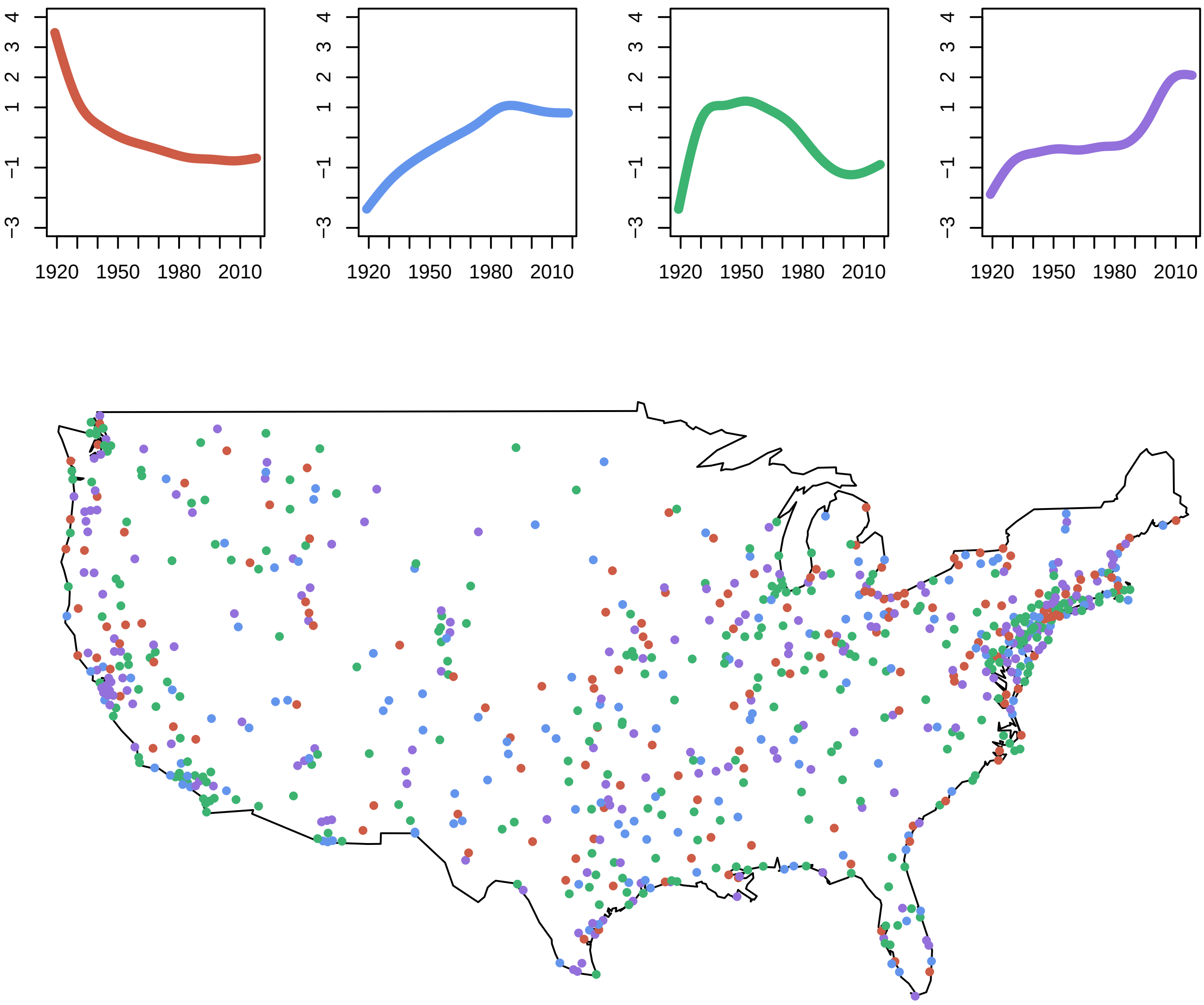

Northern Shoveler

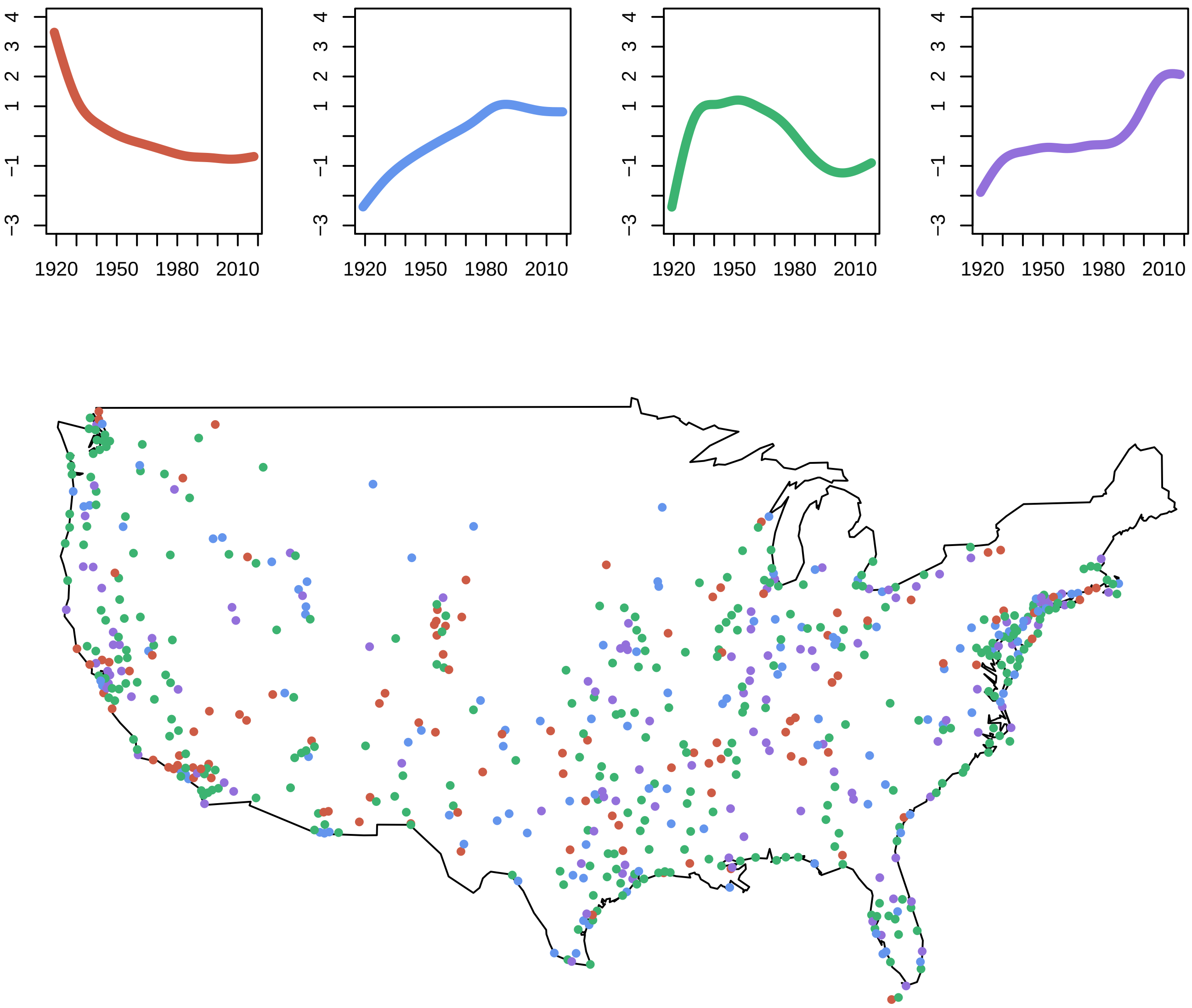

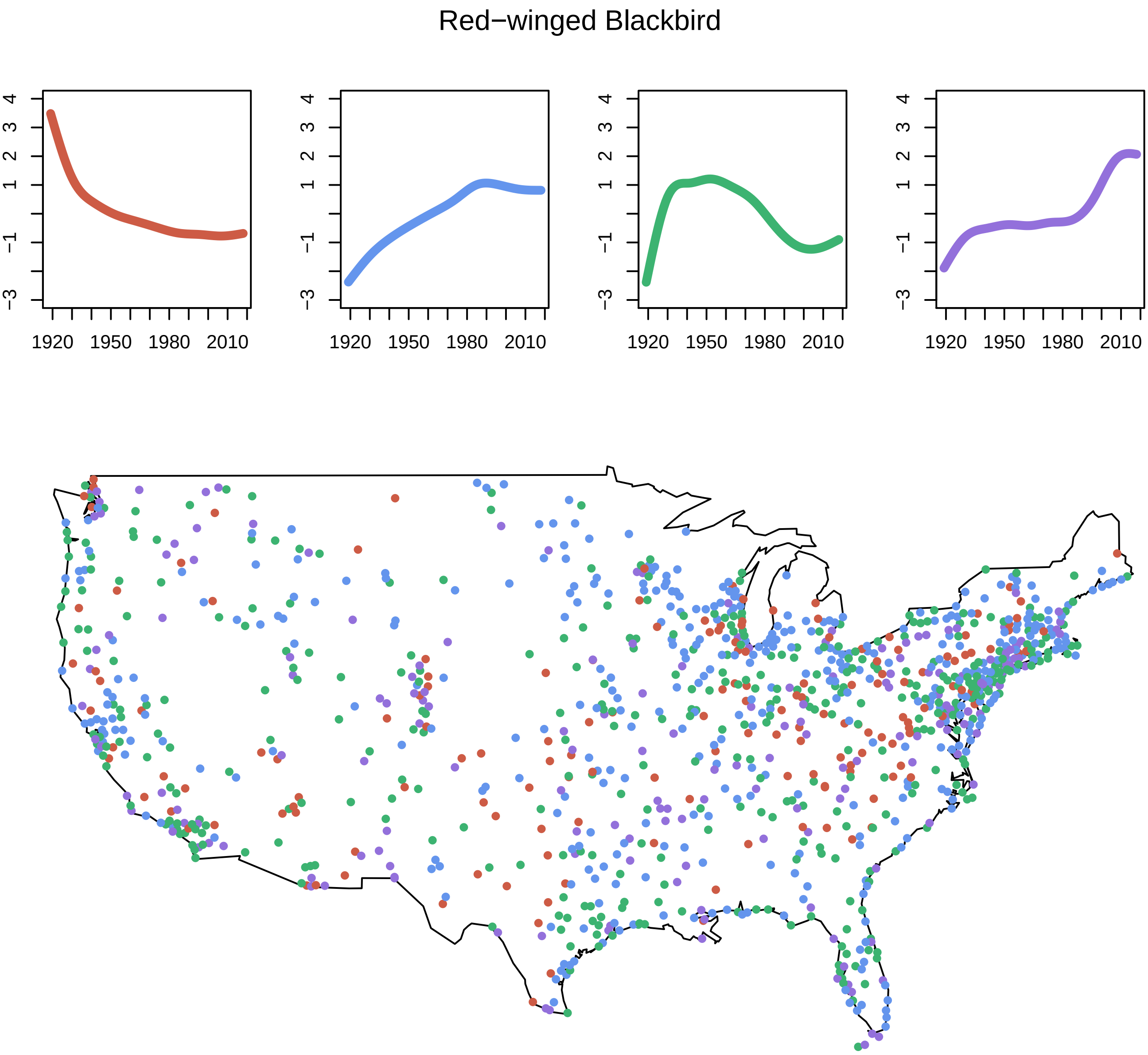
Red−winged Blackbird
4
3
2
1
−1
−3
1920
1950
1980
2010

Ring−billed Gull

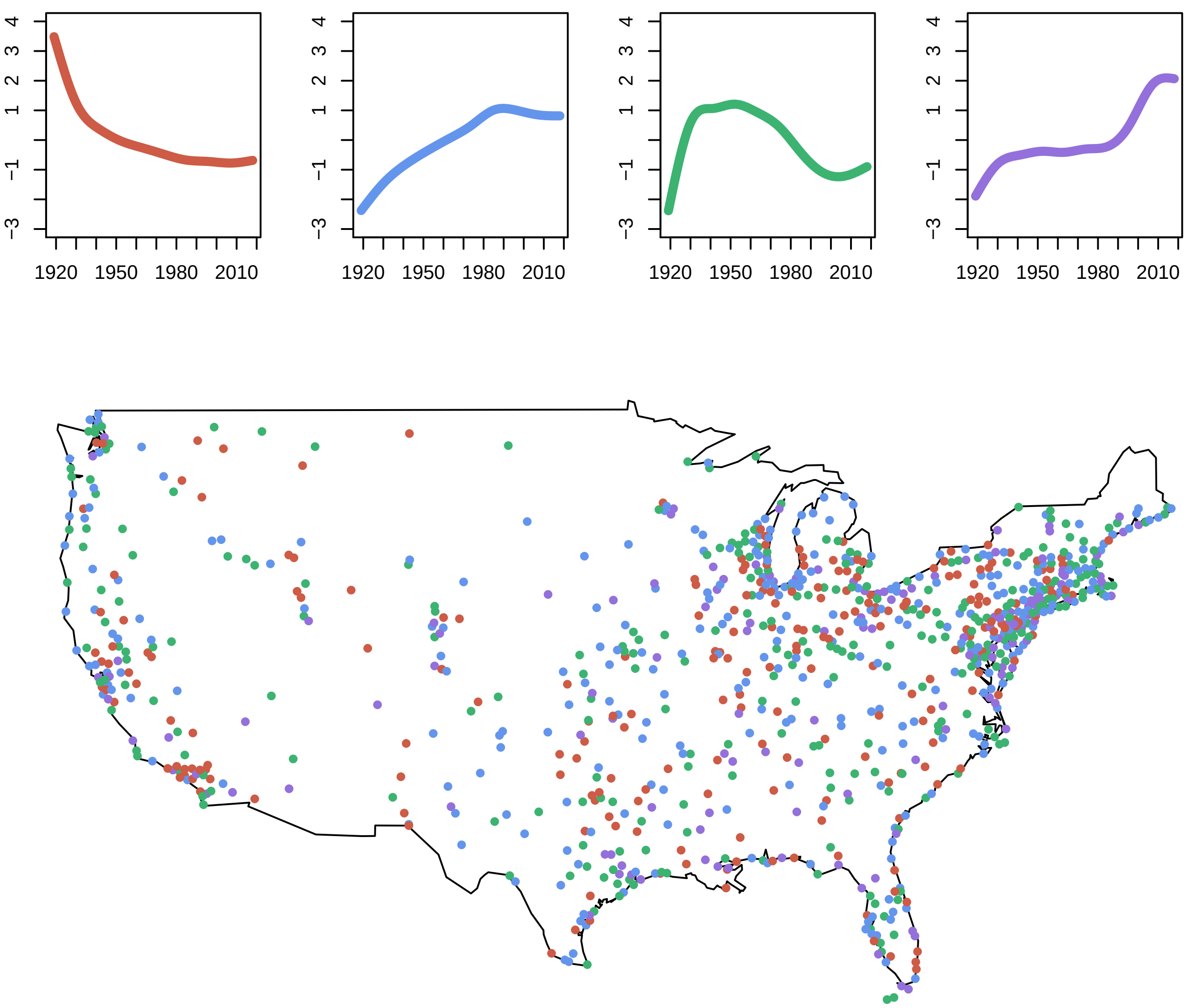

Rock Pigeon

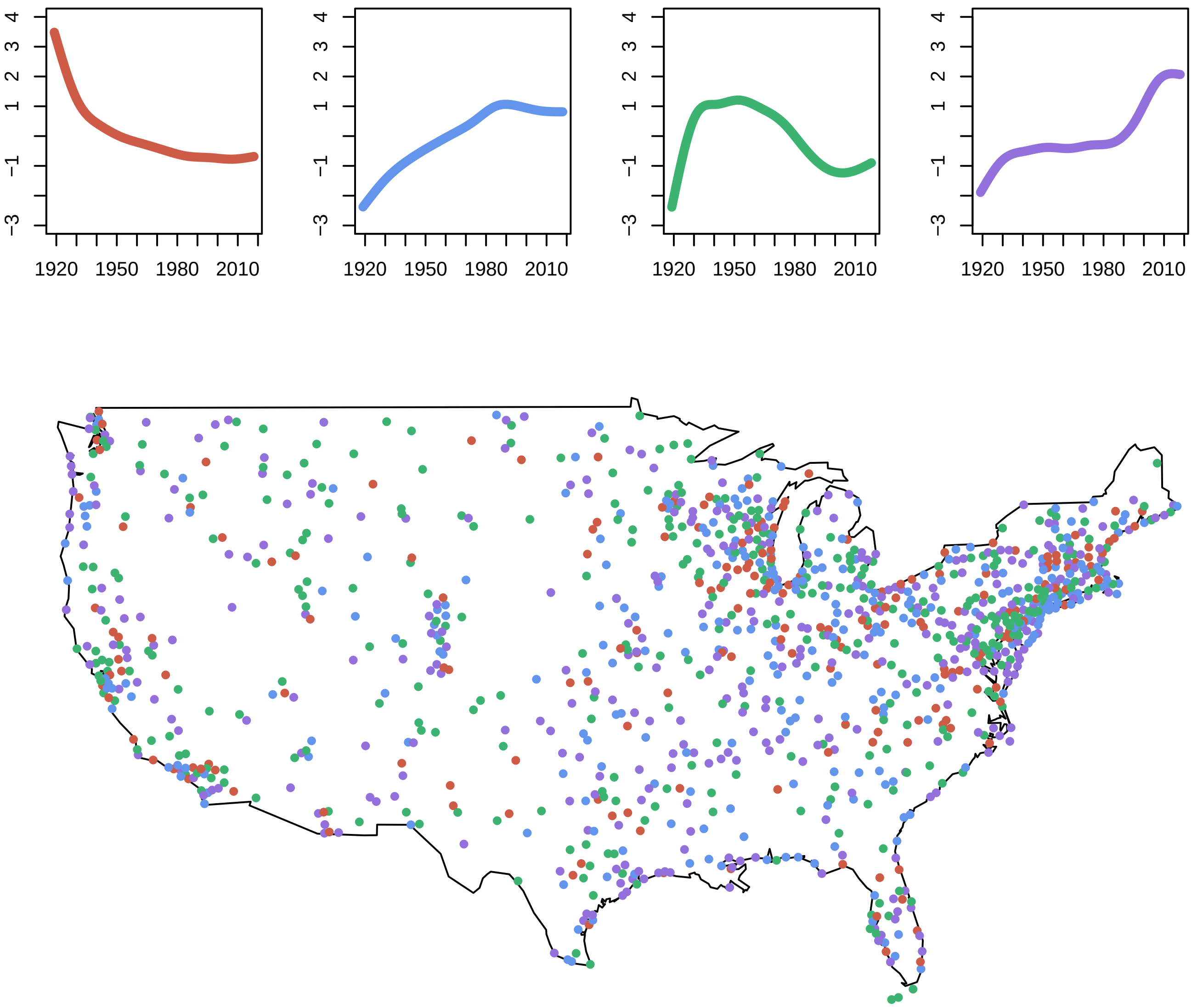

Ruddy Duck

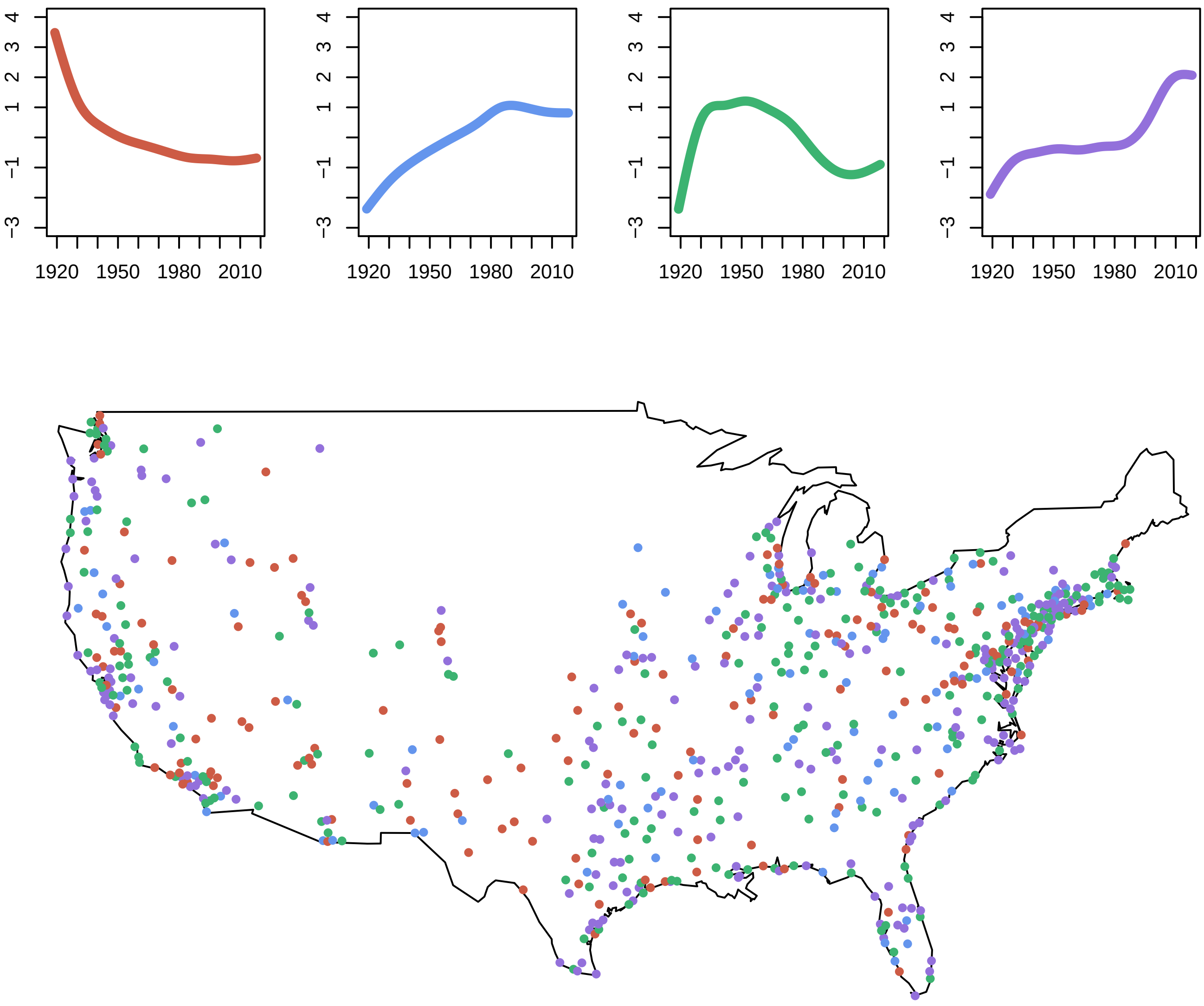

Snow Goose

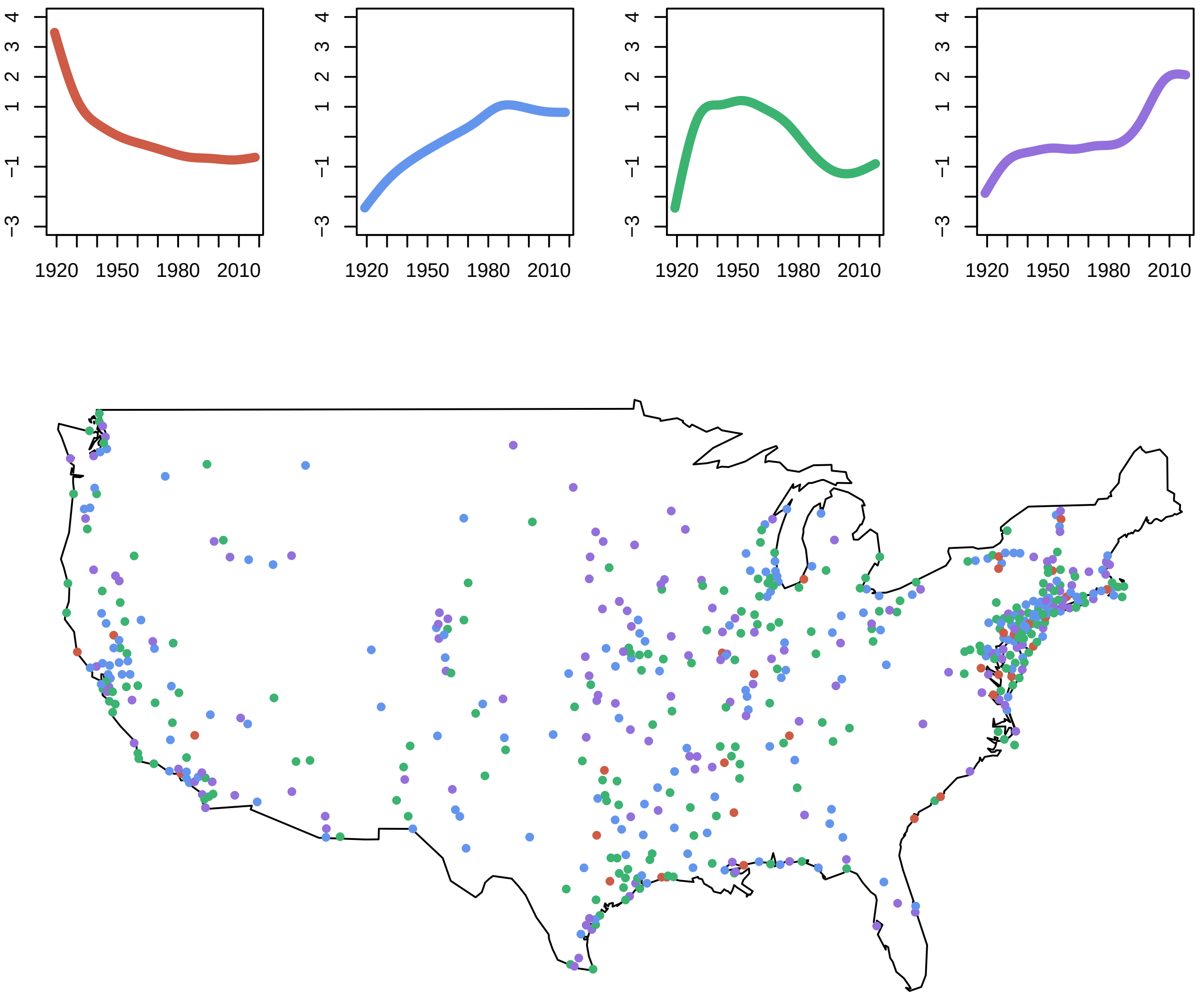

Tree Swallow

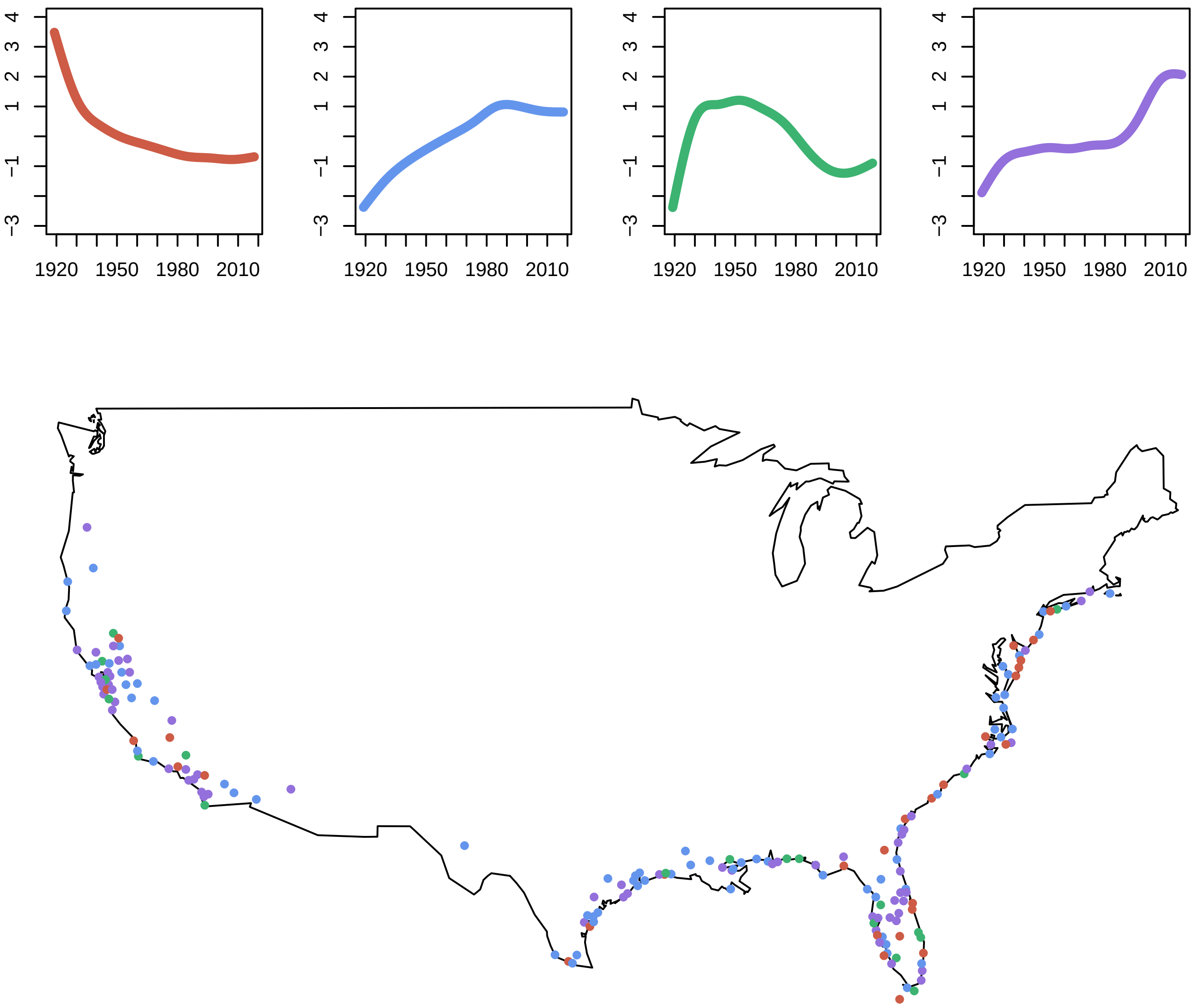

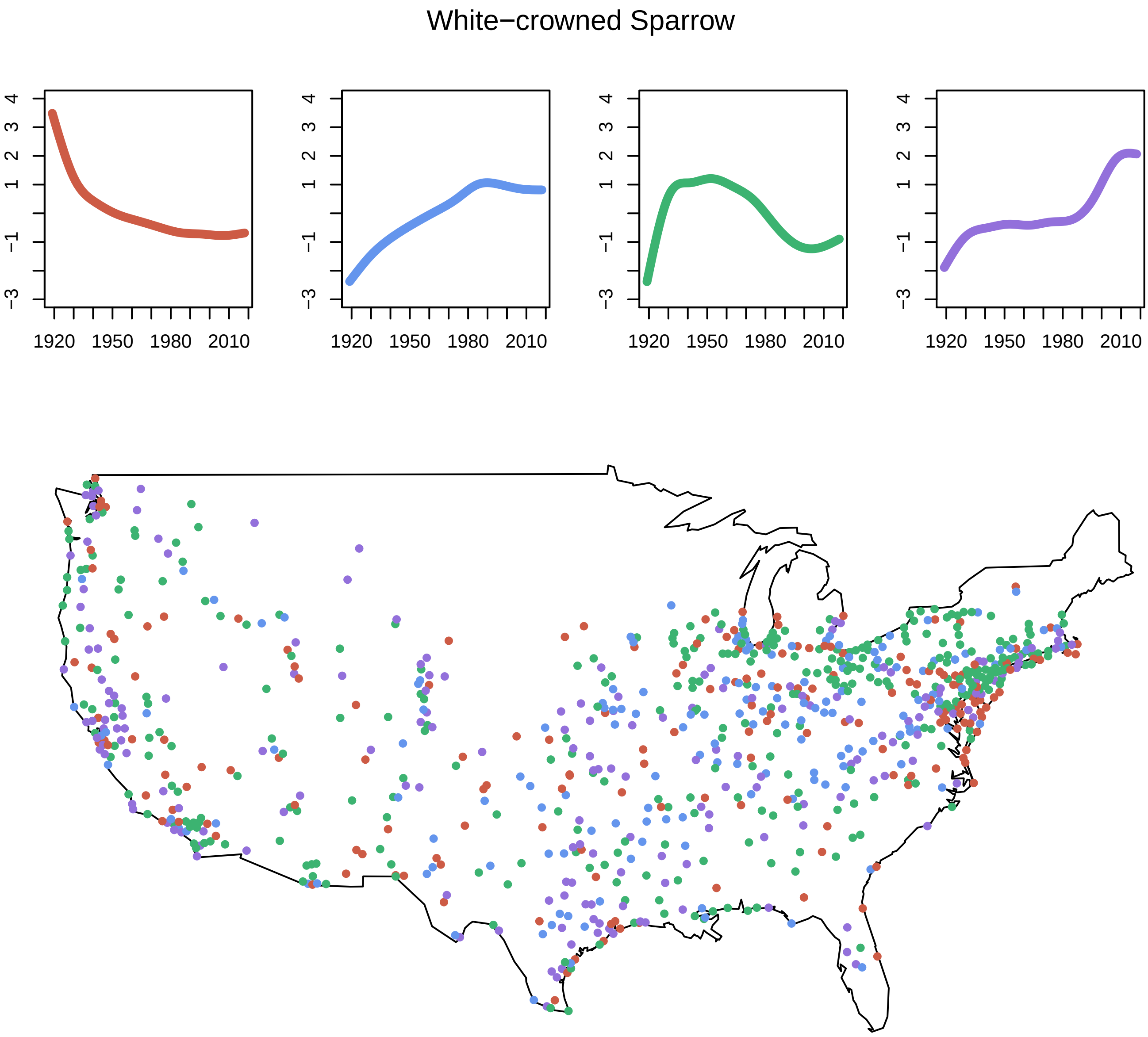
White−crowned Sparrow
4
3
2
1
−1
−3
1920
1950
1980
2010

White−throated Sparrow

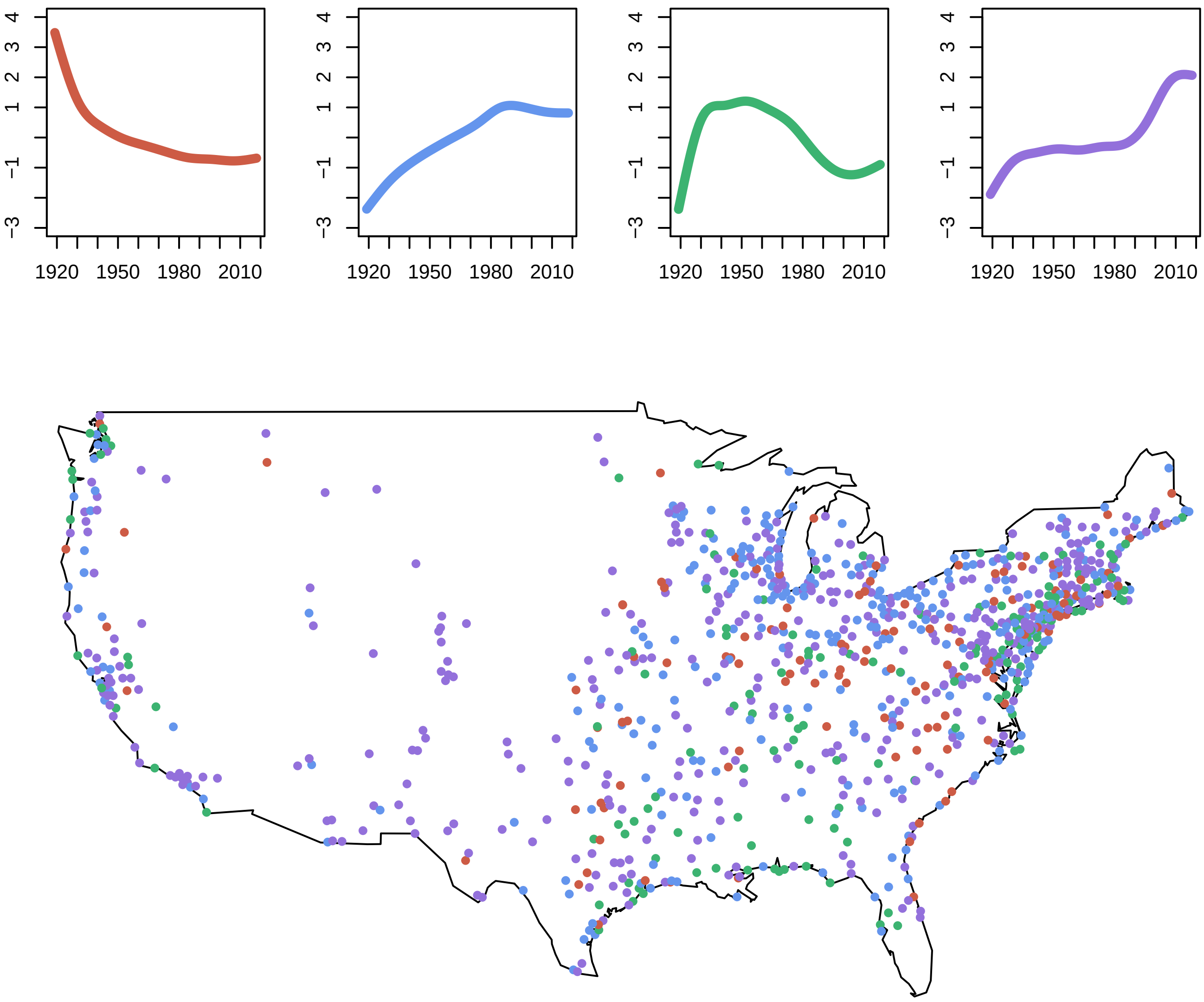

Yellow−rumped Warbler

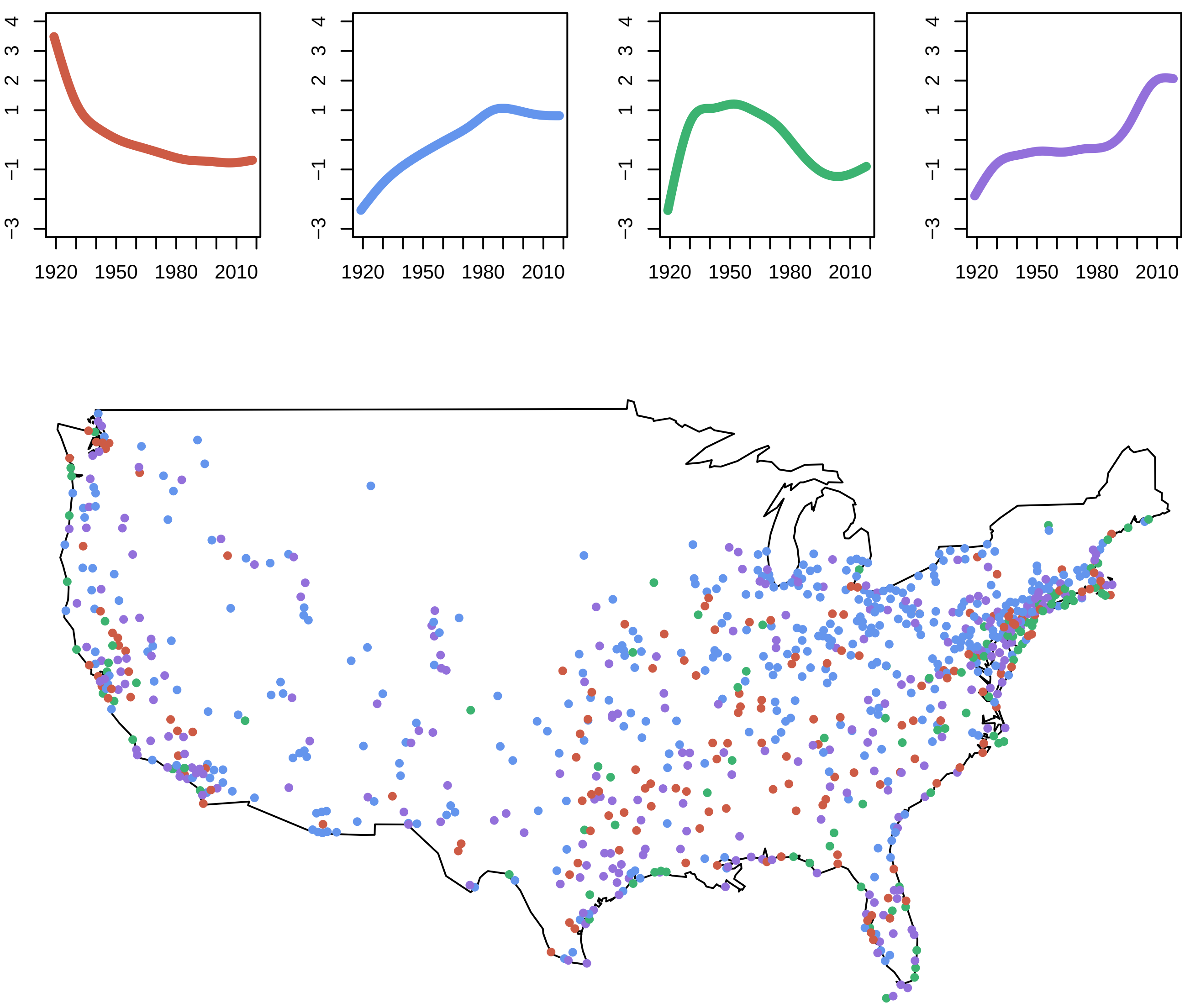

American Pipit

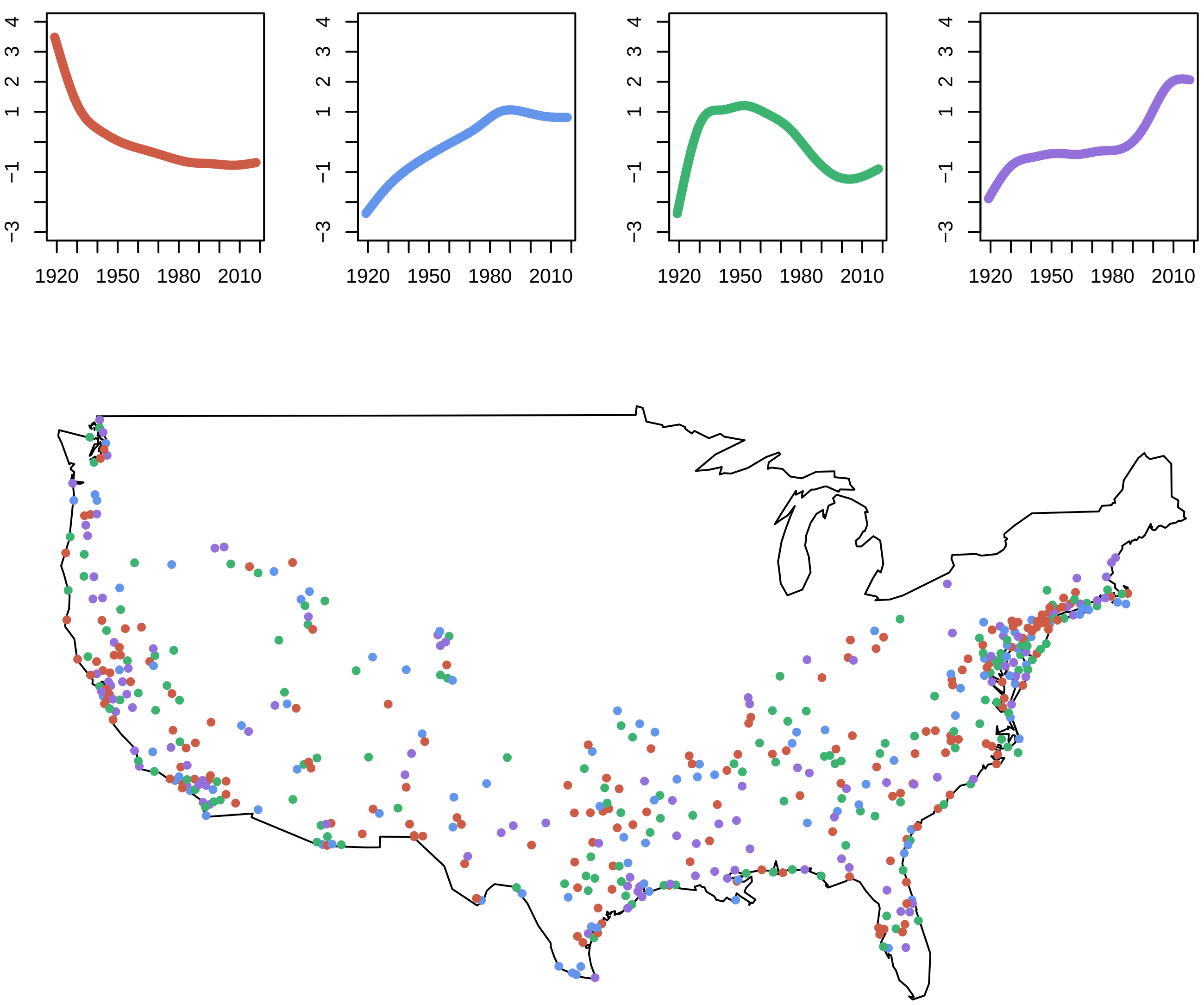

Boat−tailed Grackle

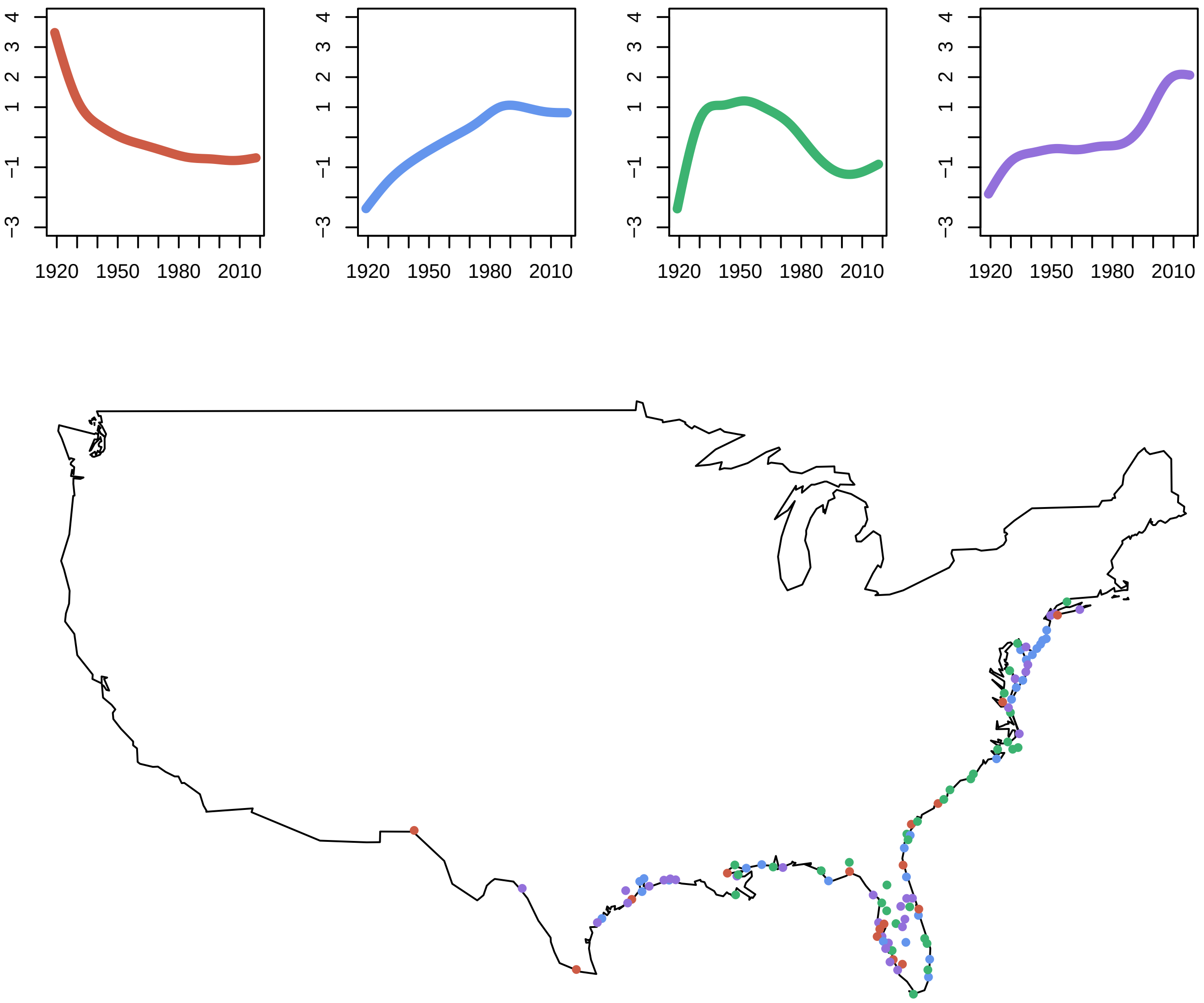

Bonaparte's Gull

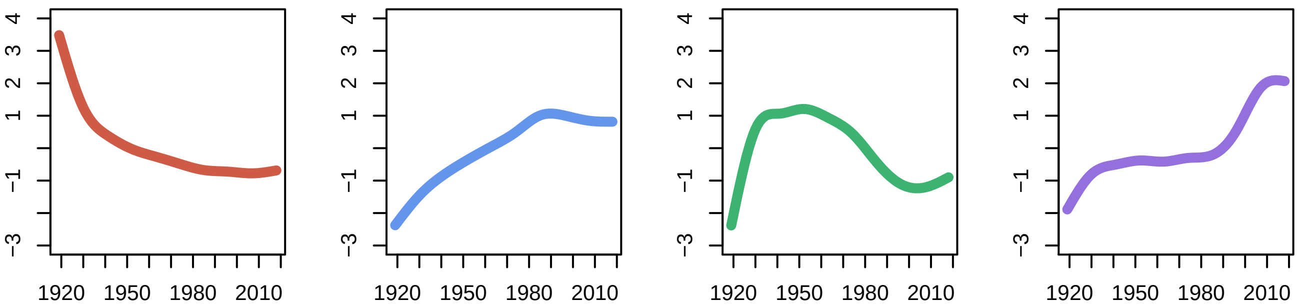

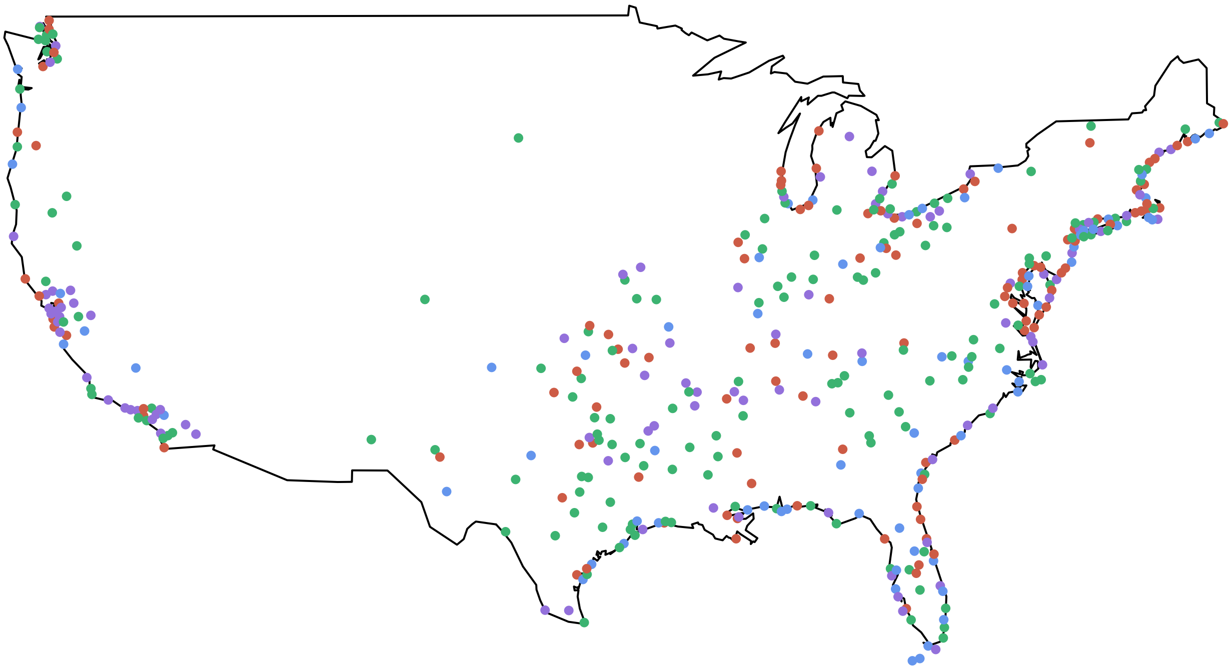

Bufflehead

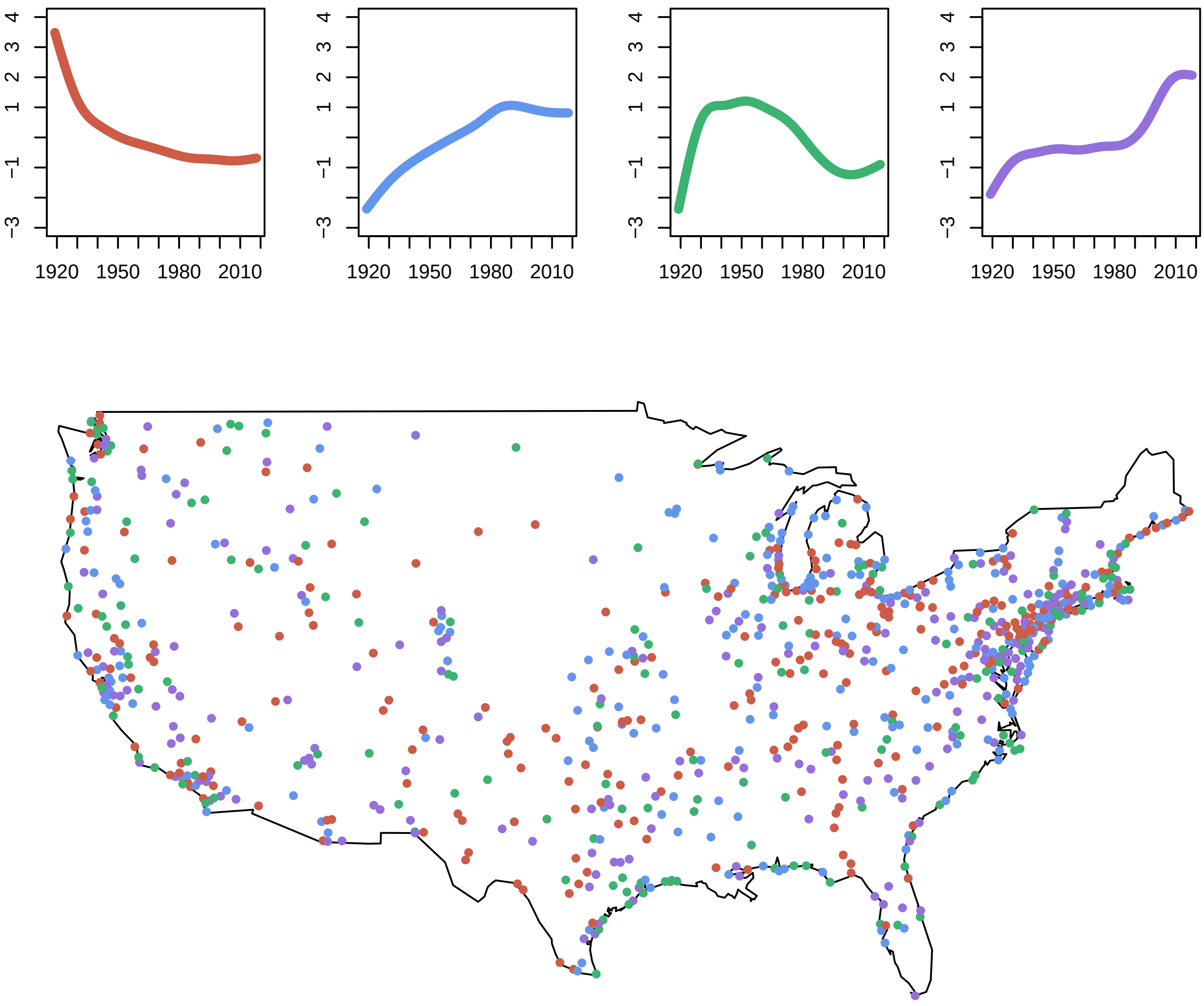

Bushtit

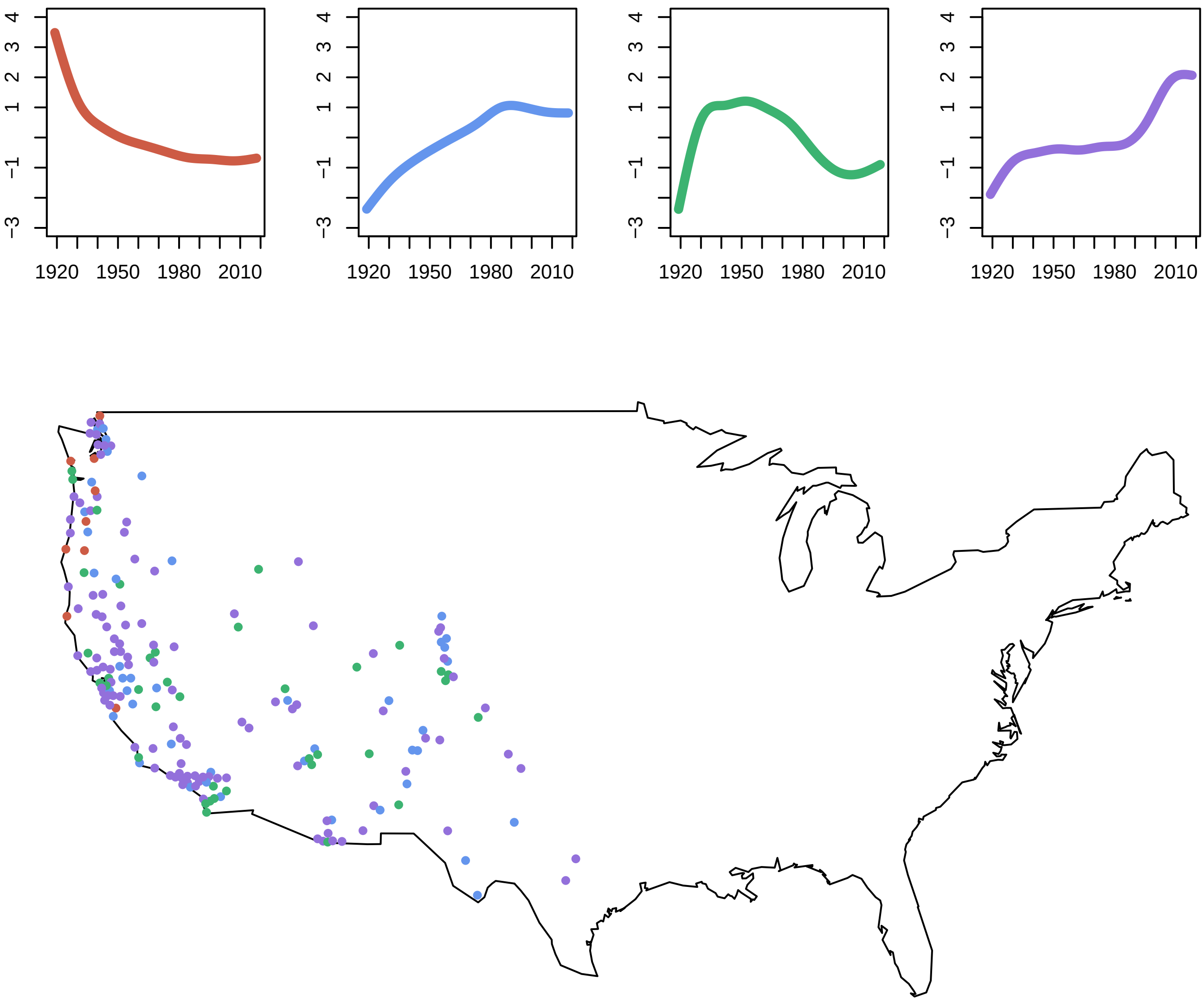

Cackling Goose

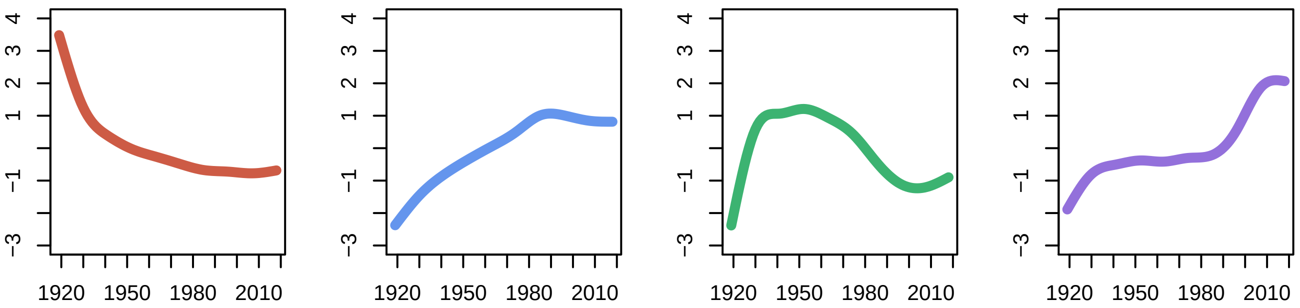

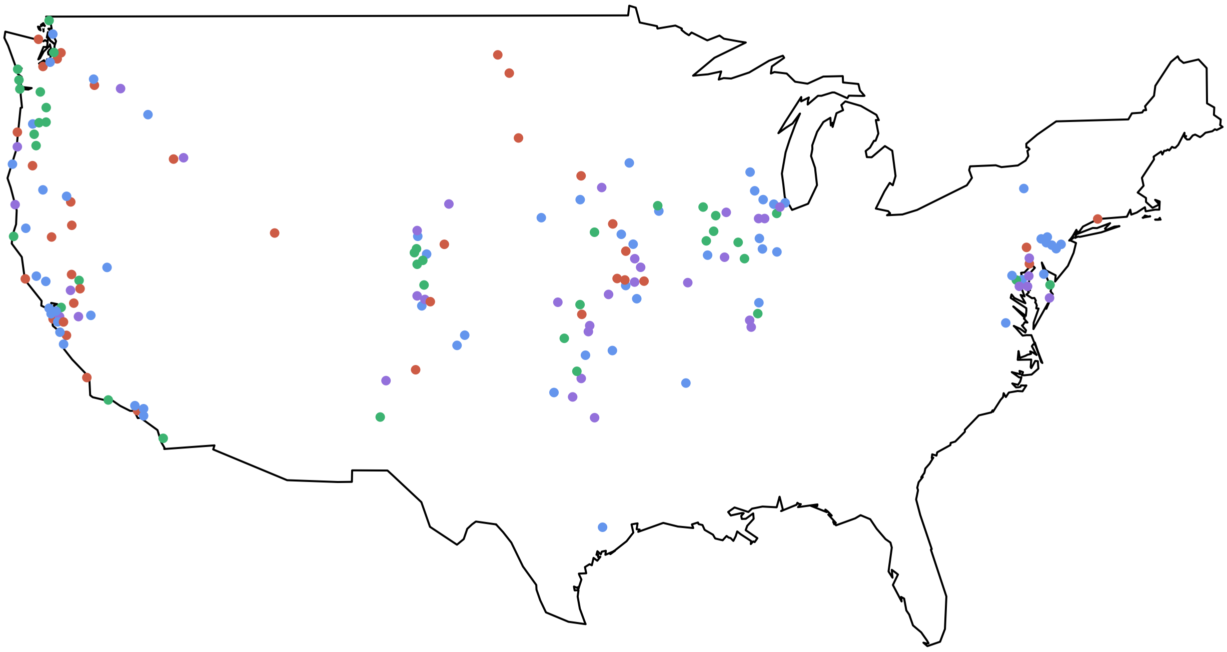

California Gull

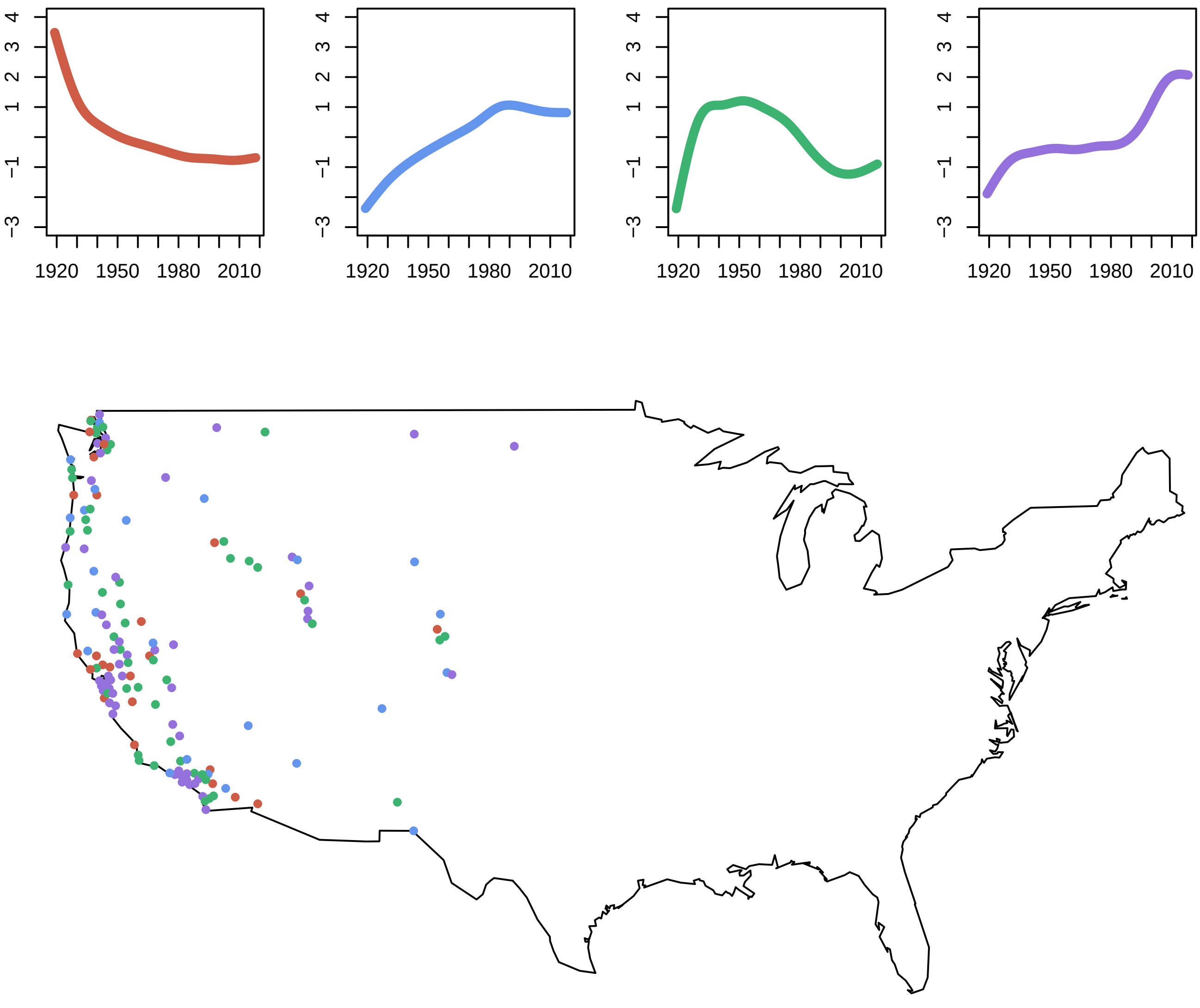

Carolina Chickadee

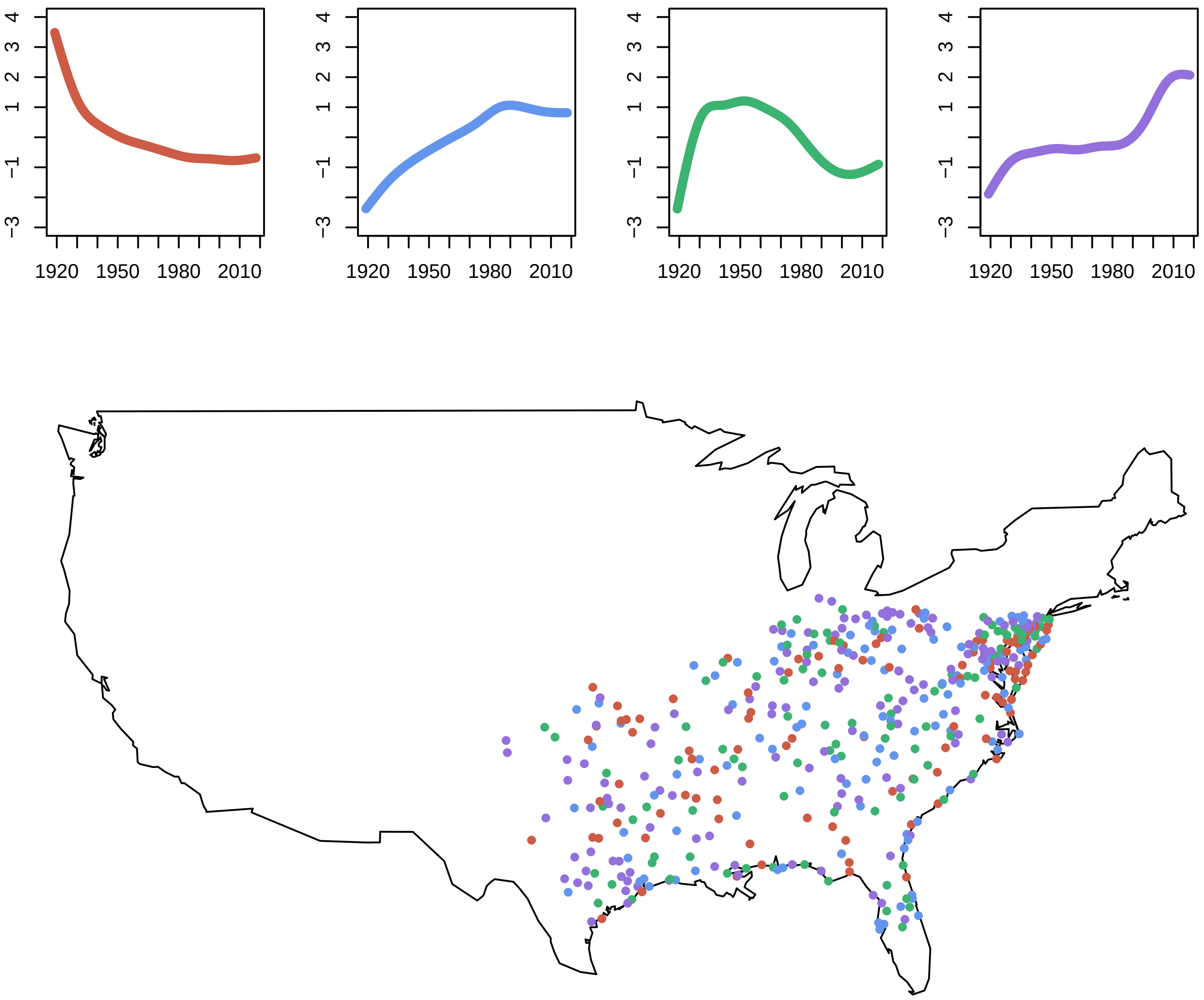

Cedar Waxwing

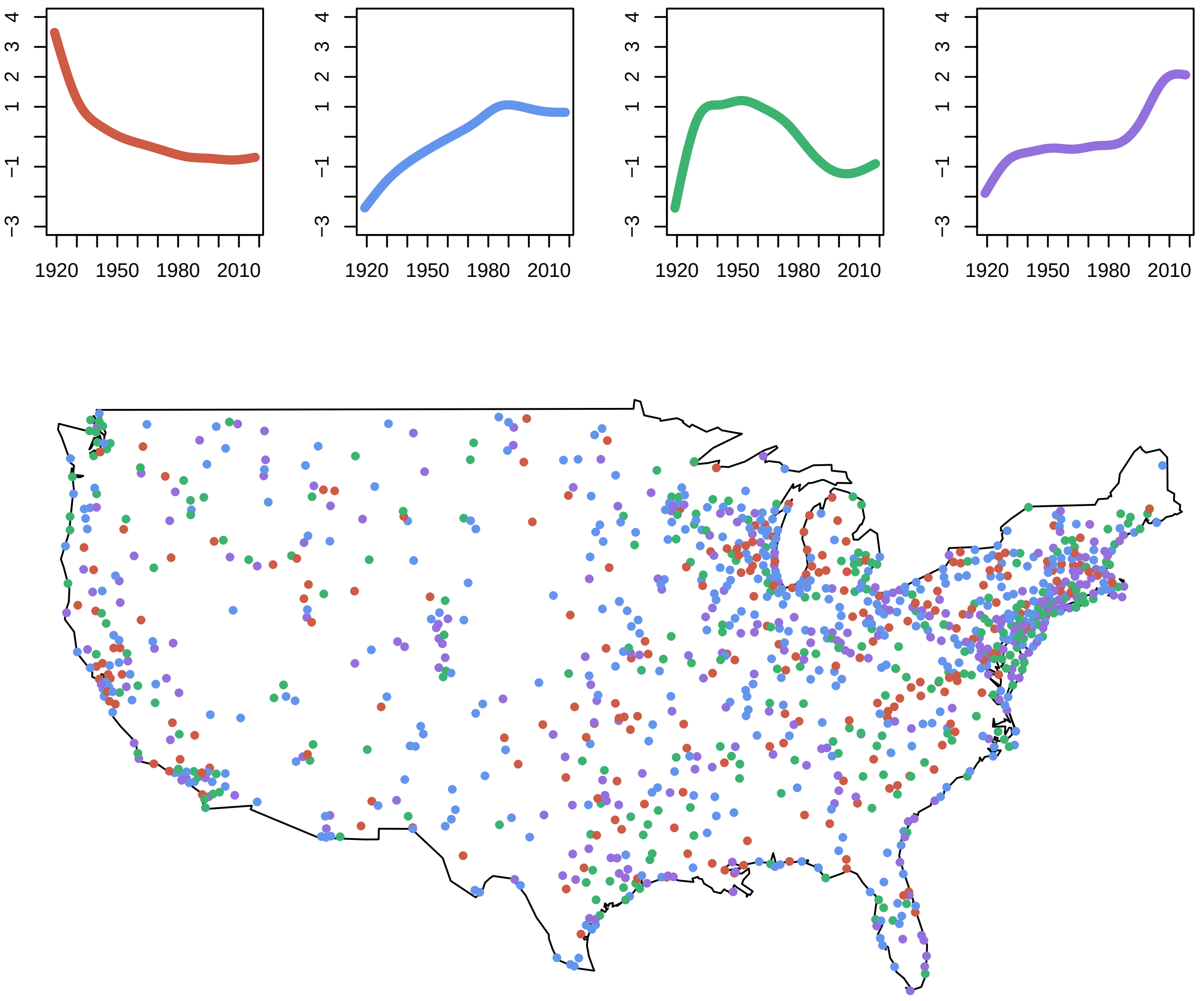

Common Goldeneye

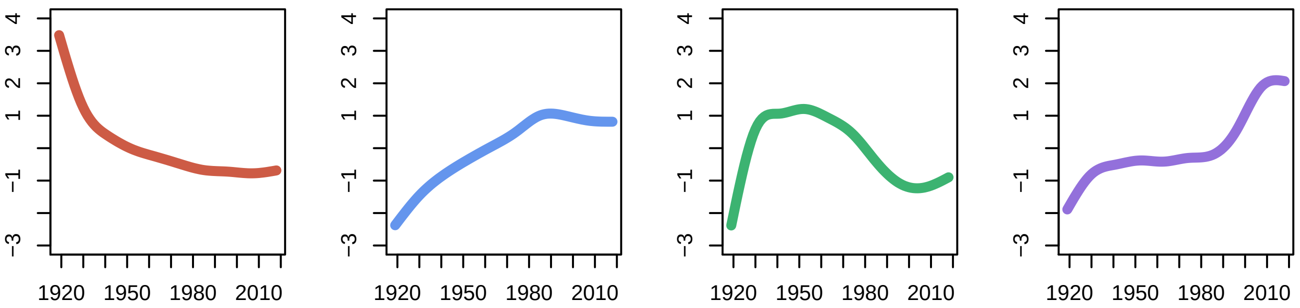

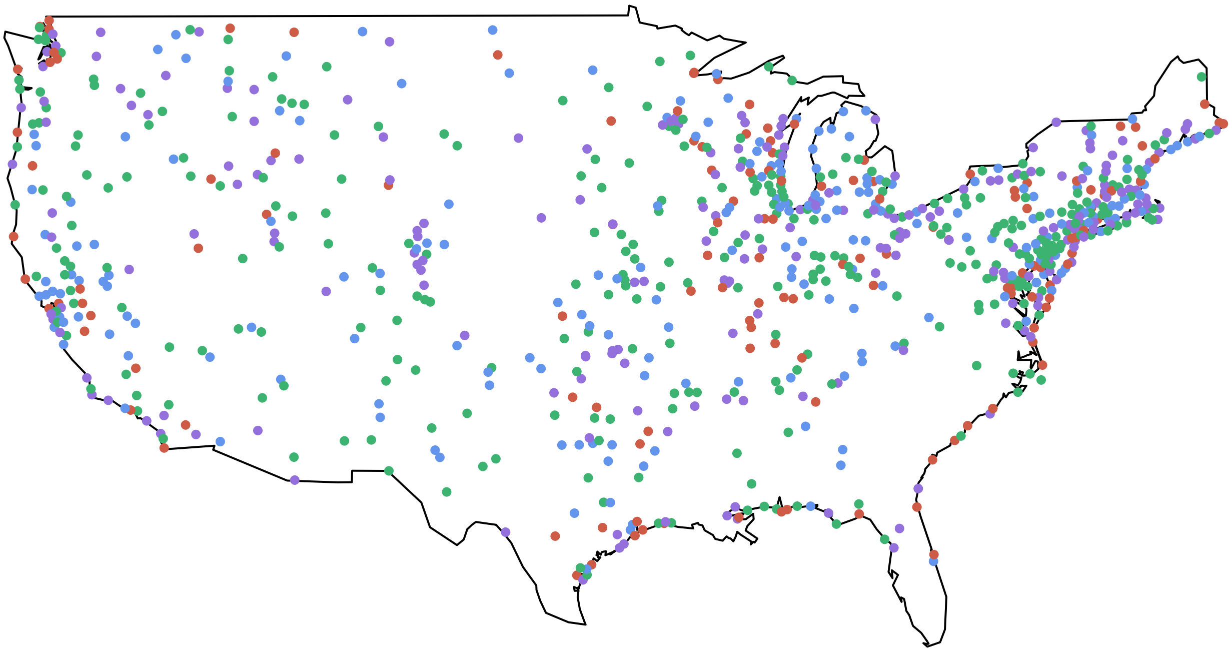

Common Merganser

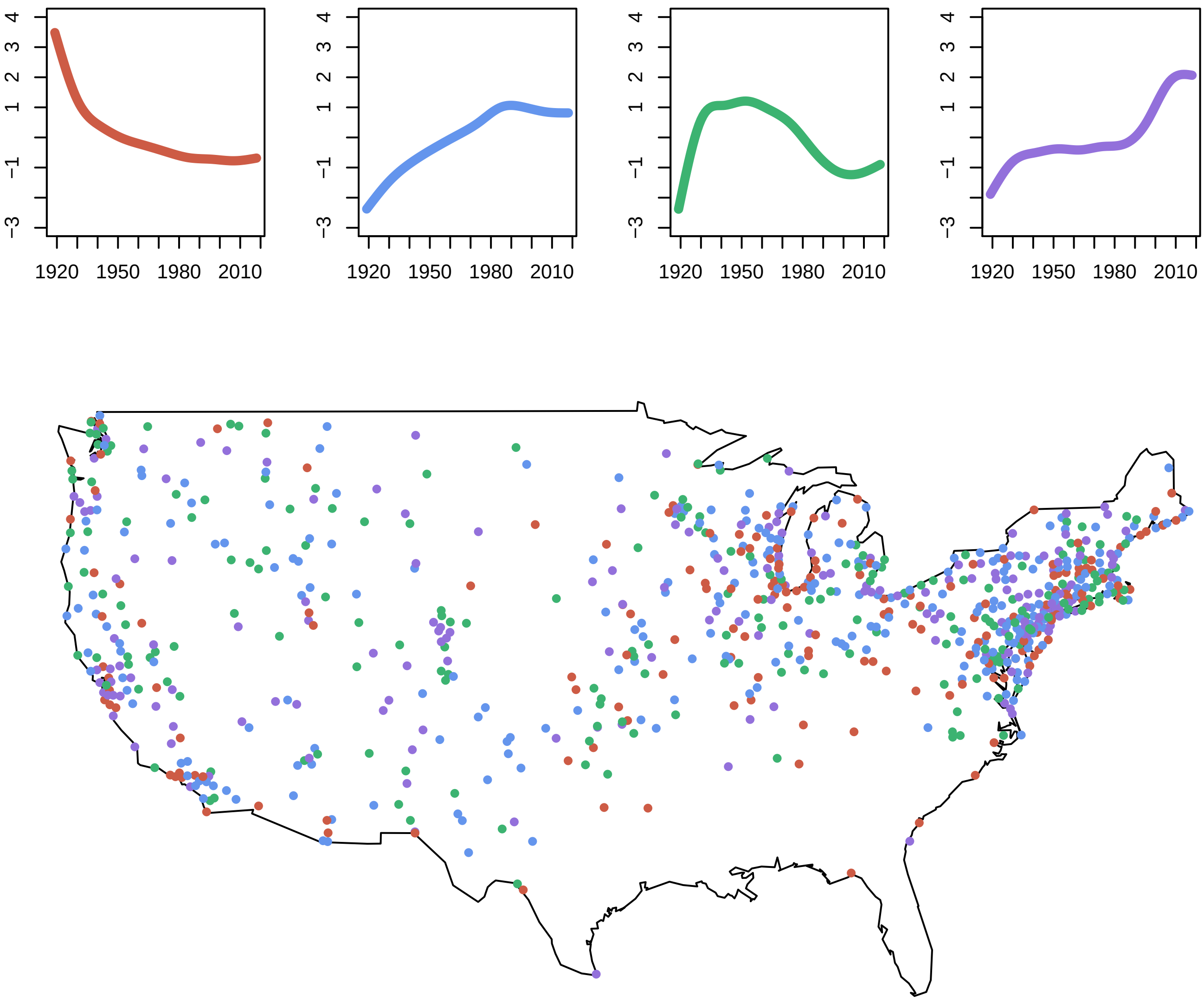

Downy Woodpecker

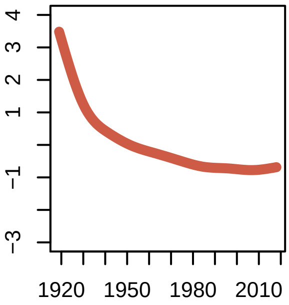

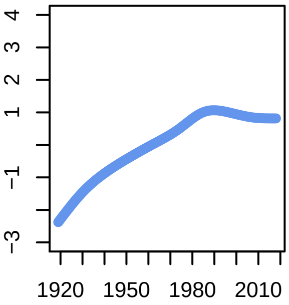

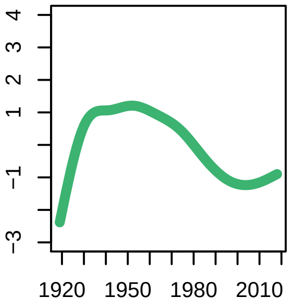

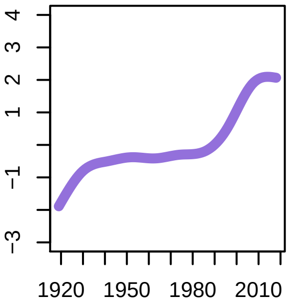

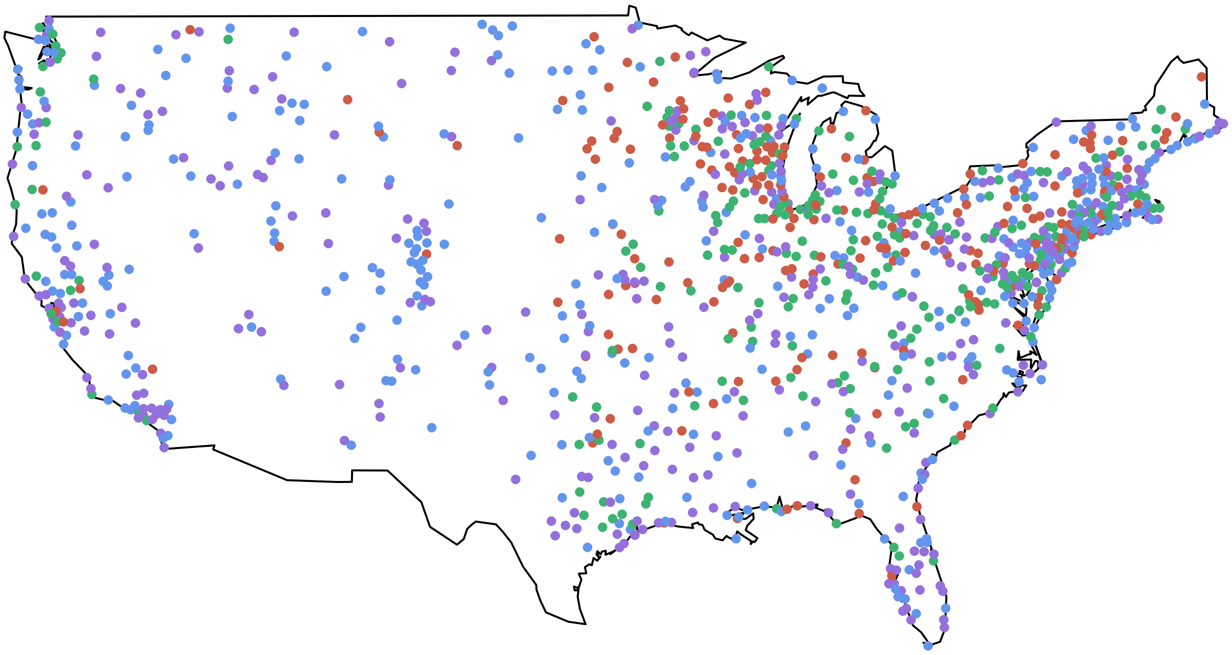

Eared Grebe

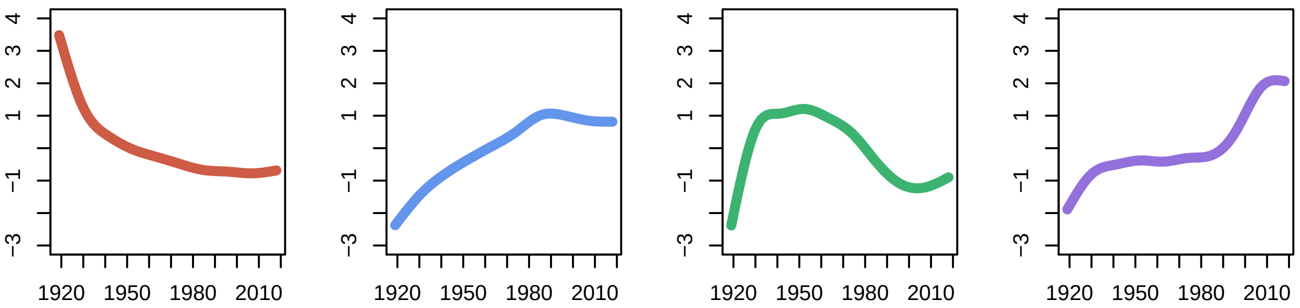

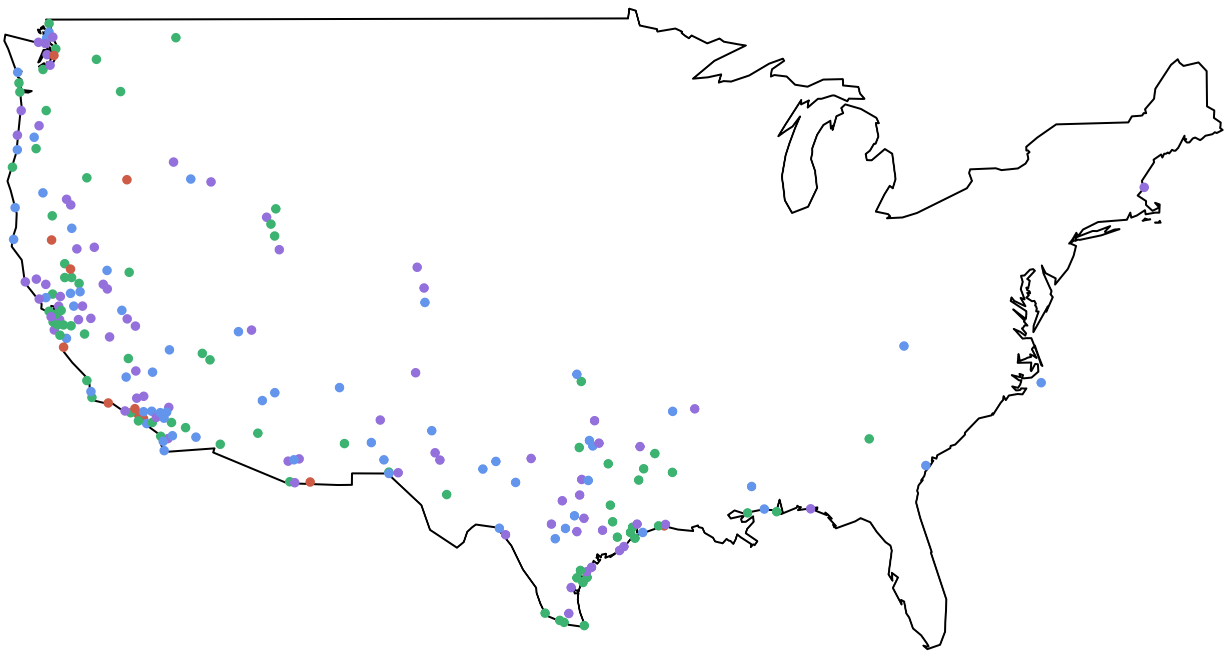

Eastern Bluebird

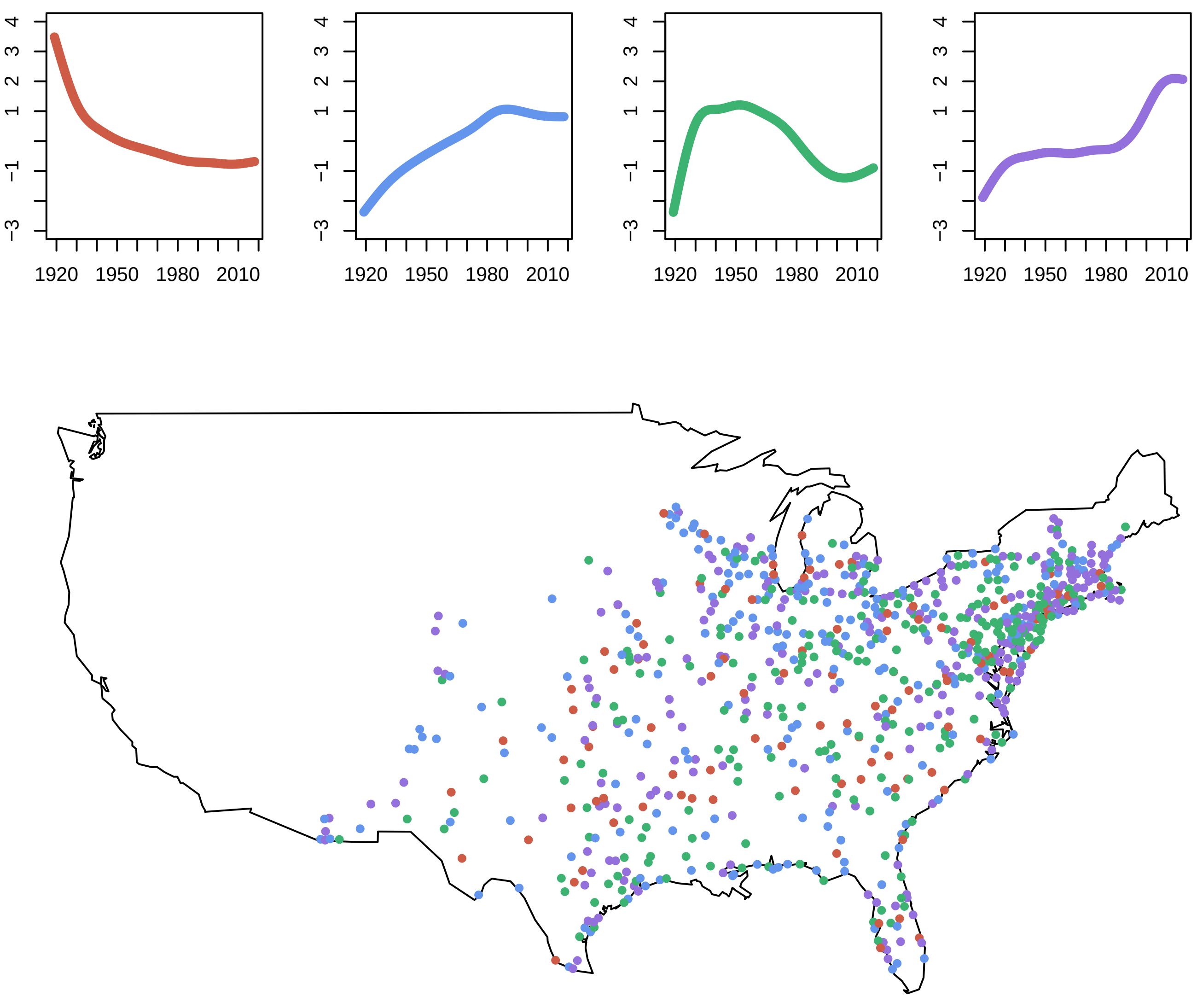

Eastern Meadowlark

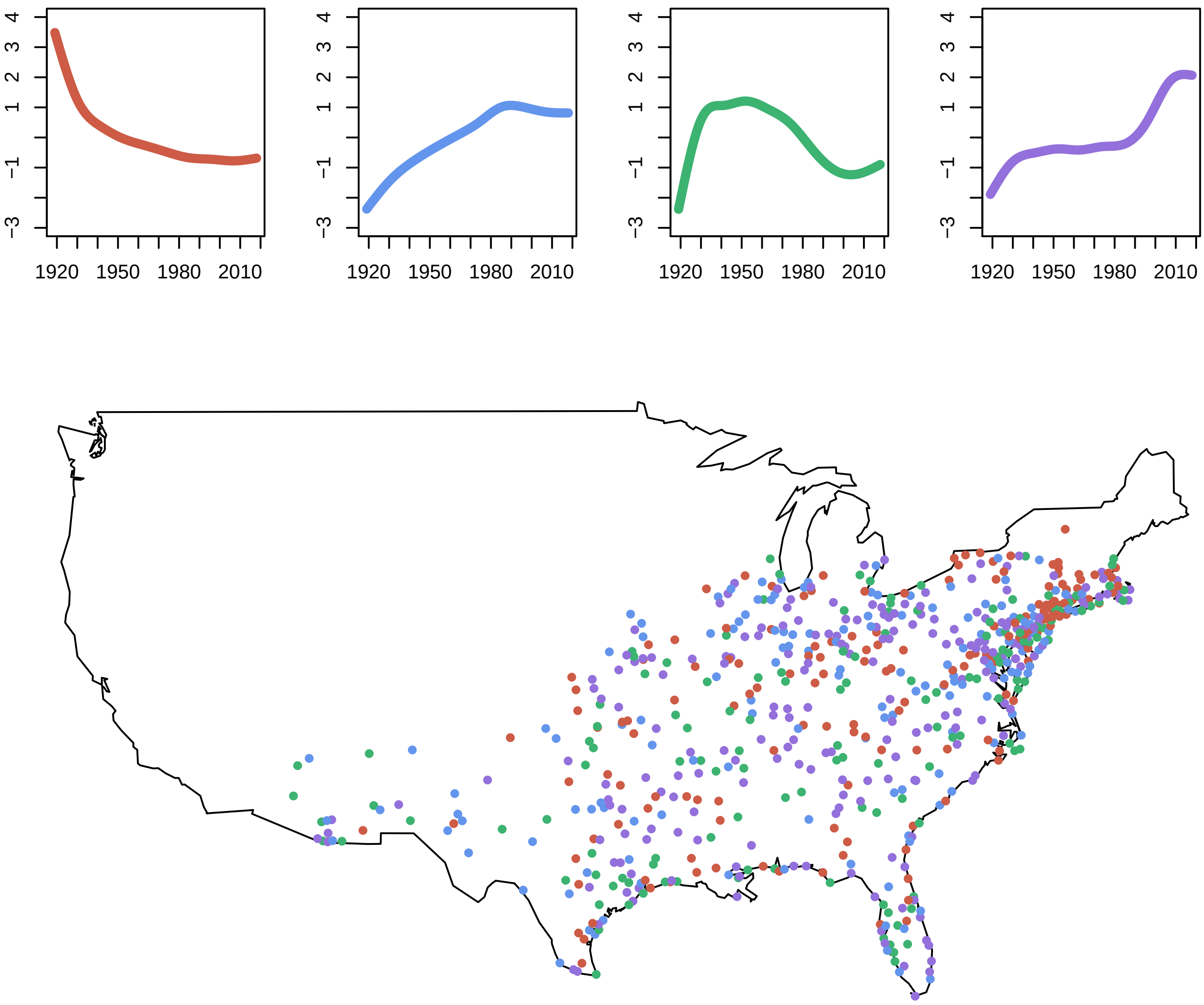

Gadwall

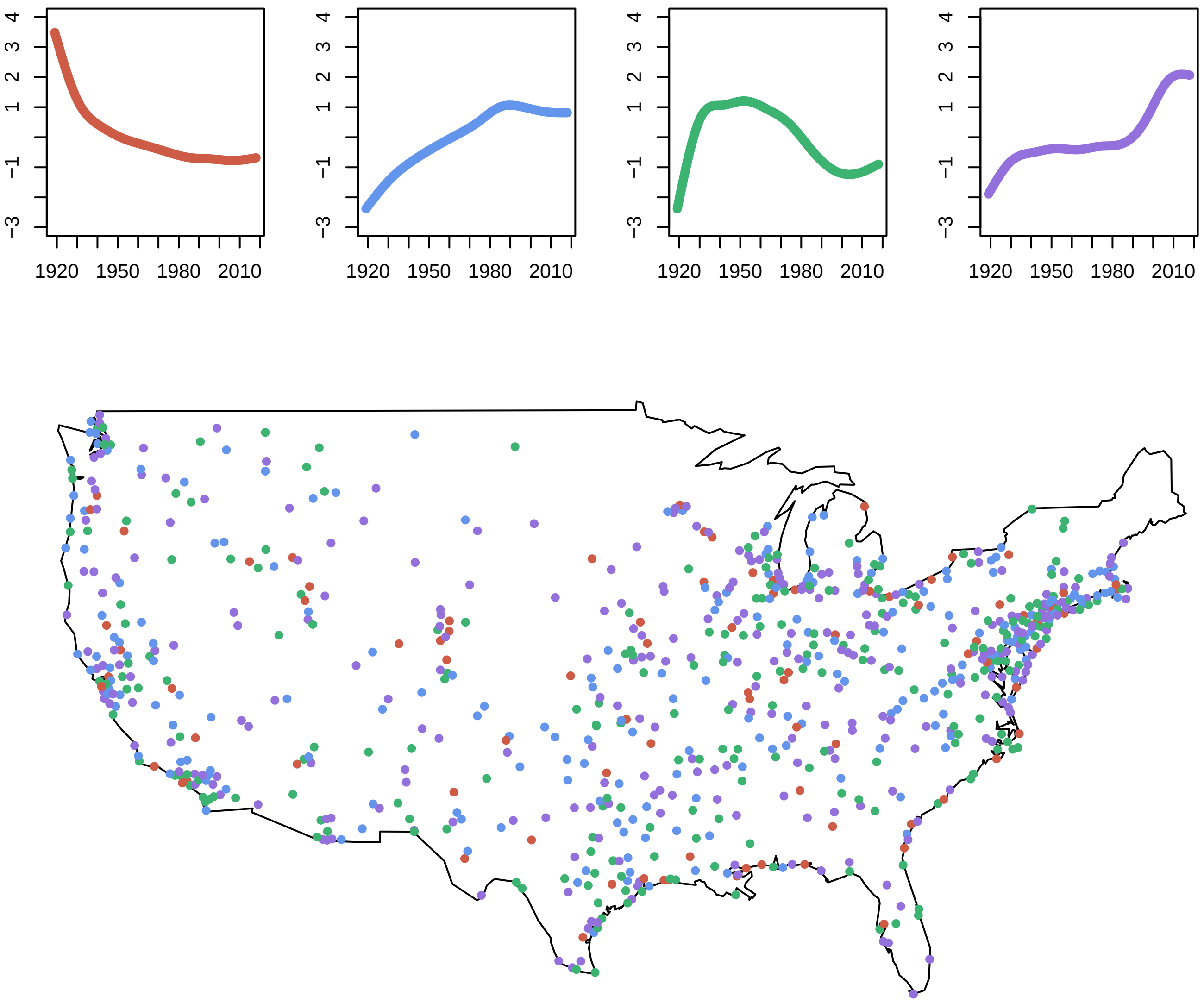

Golden−crowned Sparrow

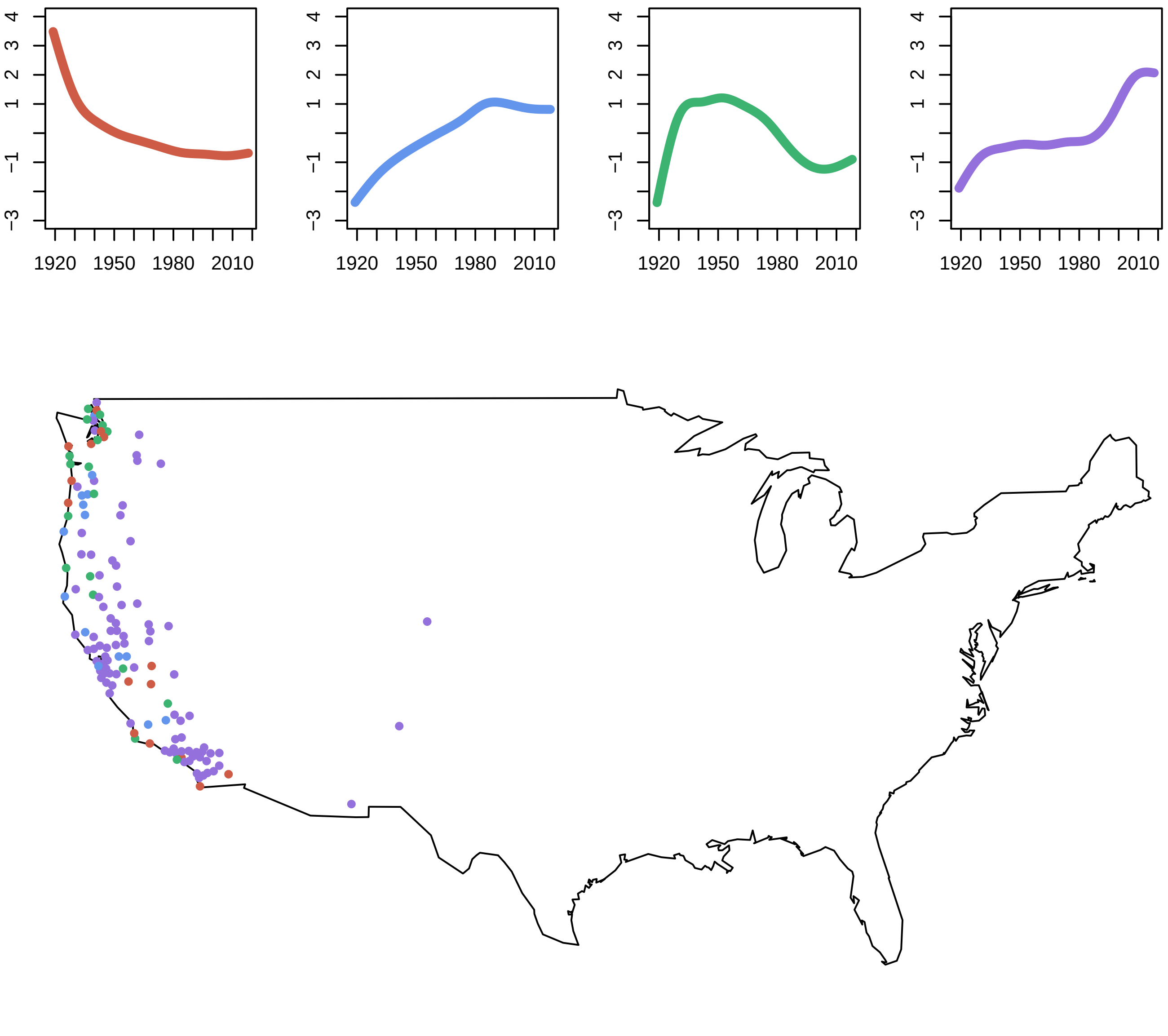

Greater White−fronted Goose

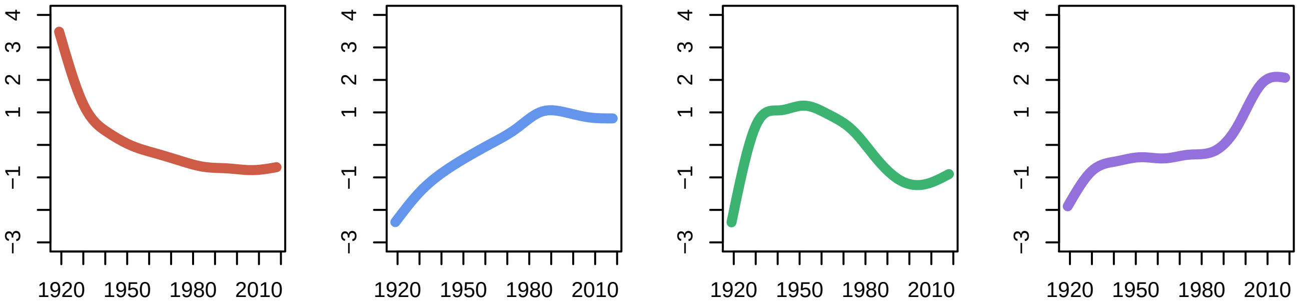

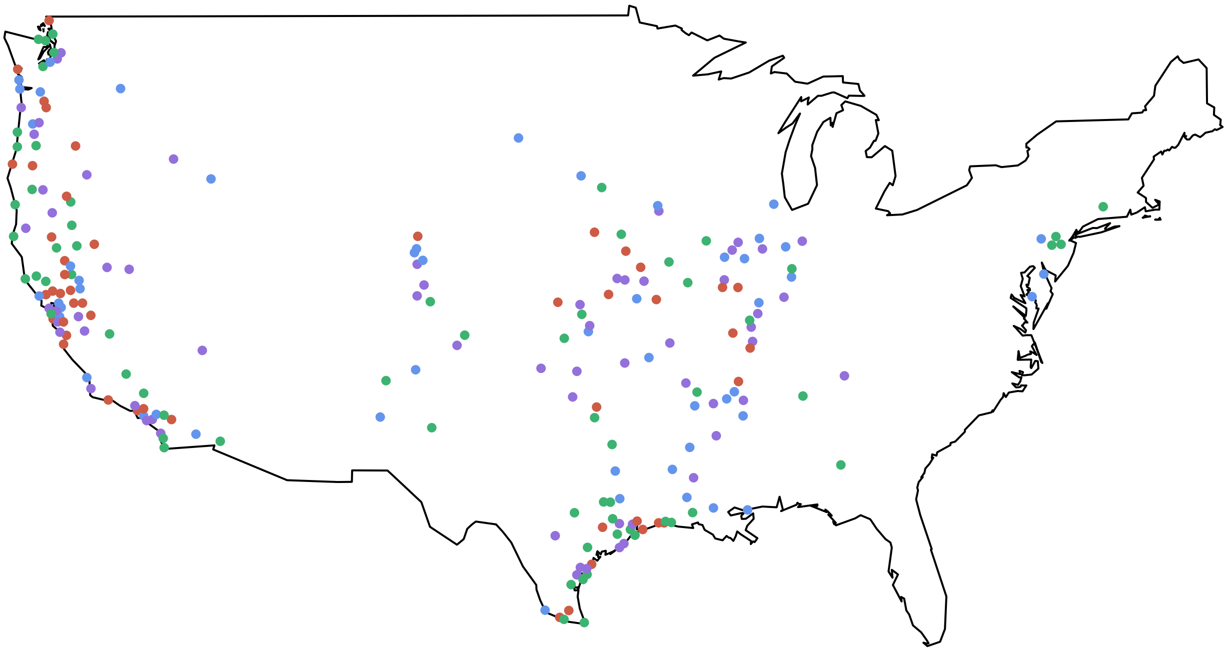

Horned Lark

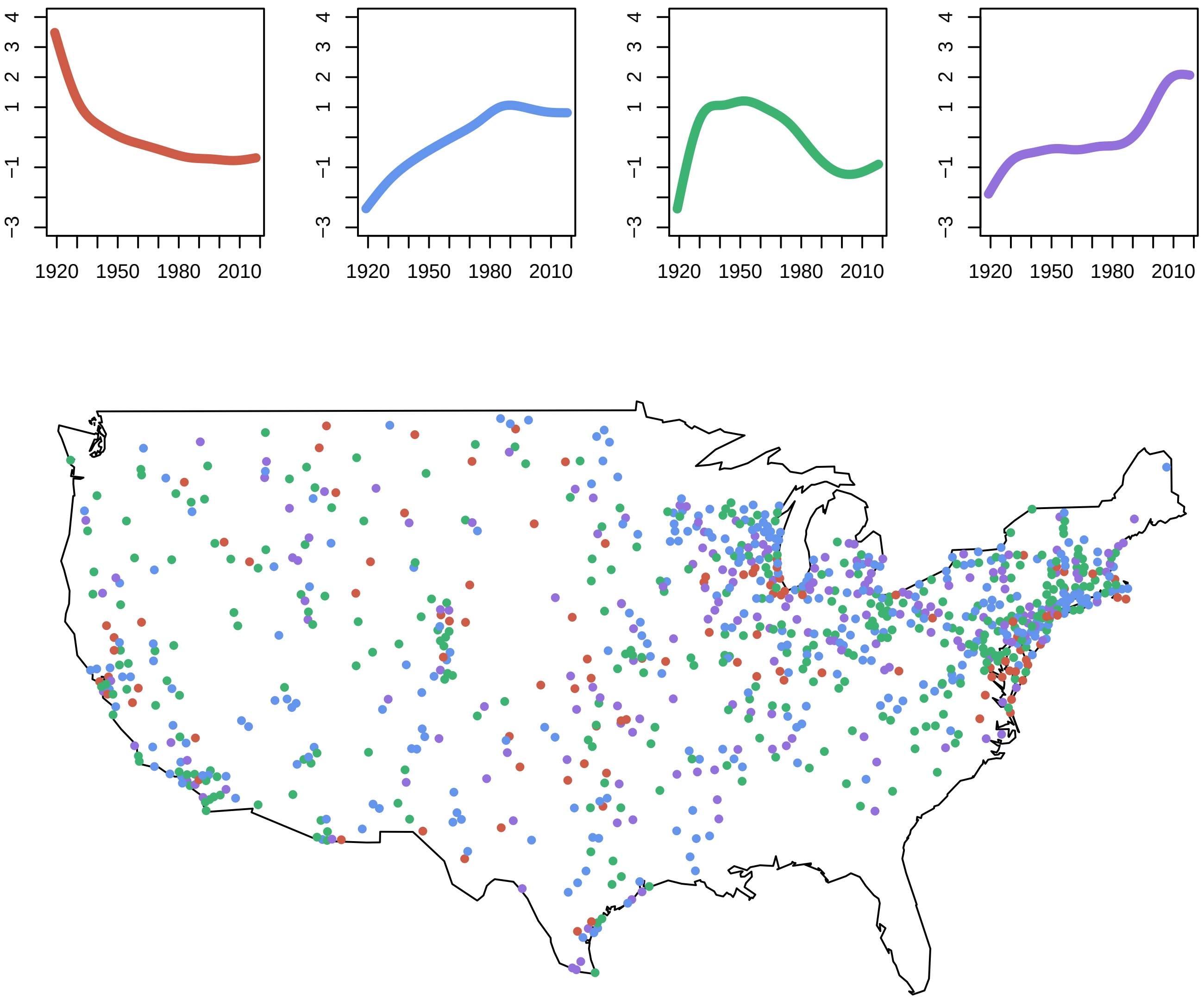

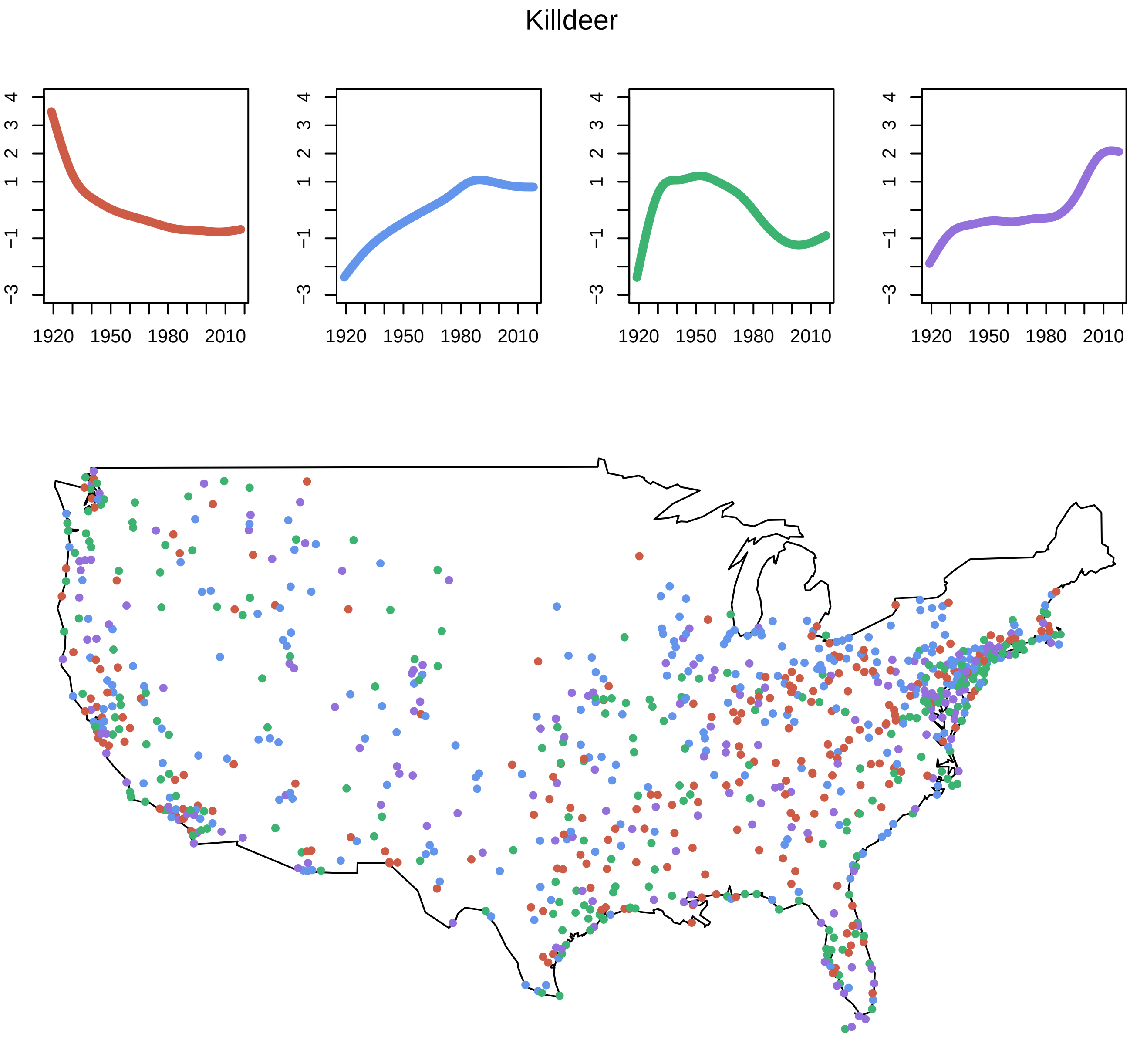
Killdeer
4
3
2
1
−1
−3
1920
1950
1980
2010

Least Sandpiper

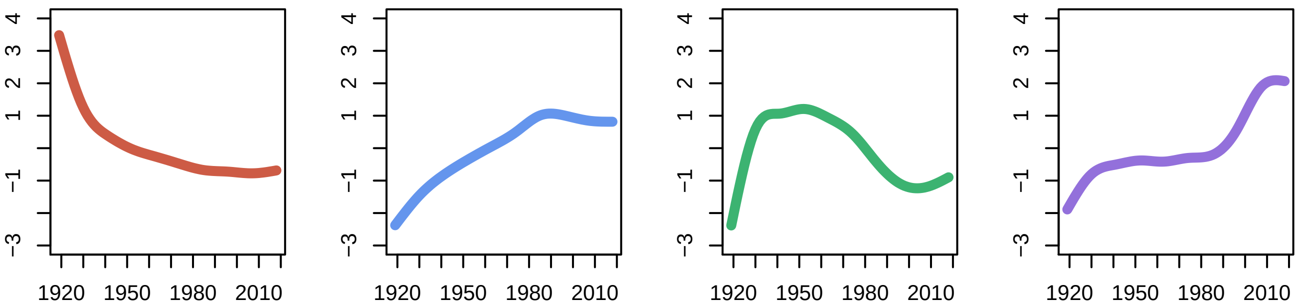

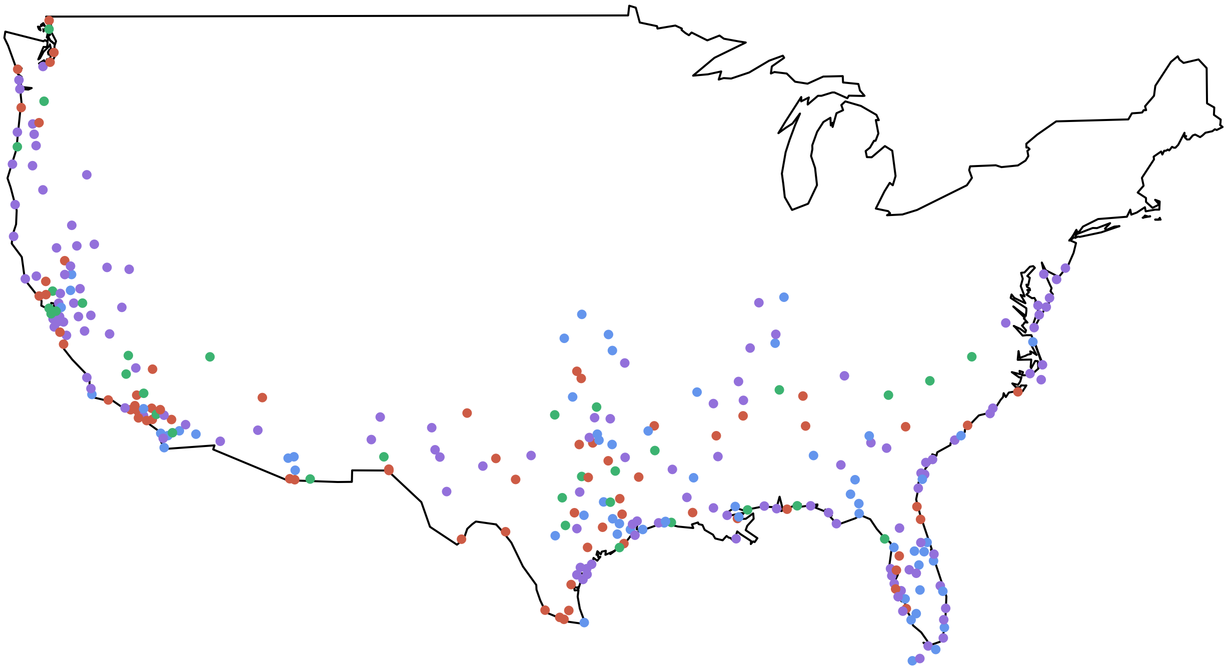

Northern Flicker

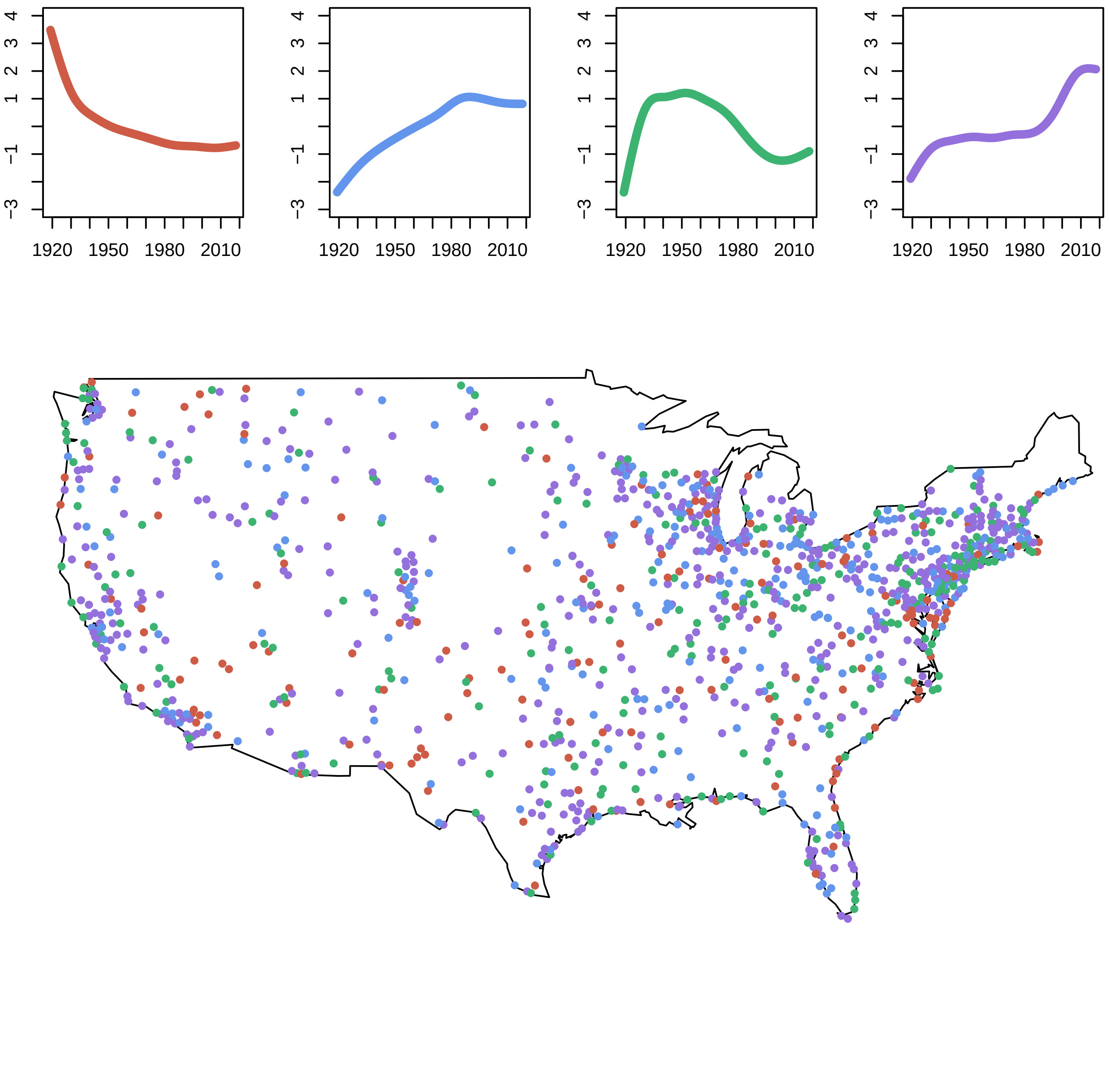

Northern Mockingbird

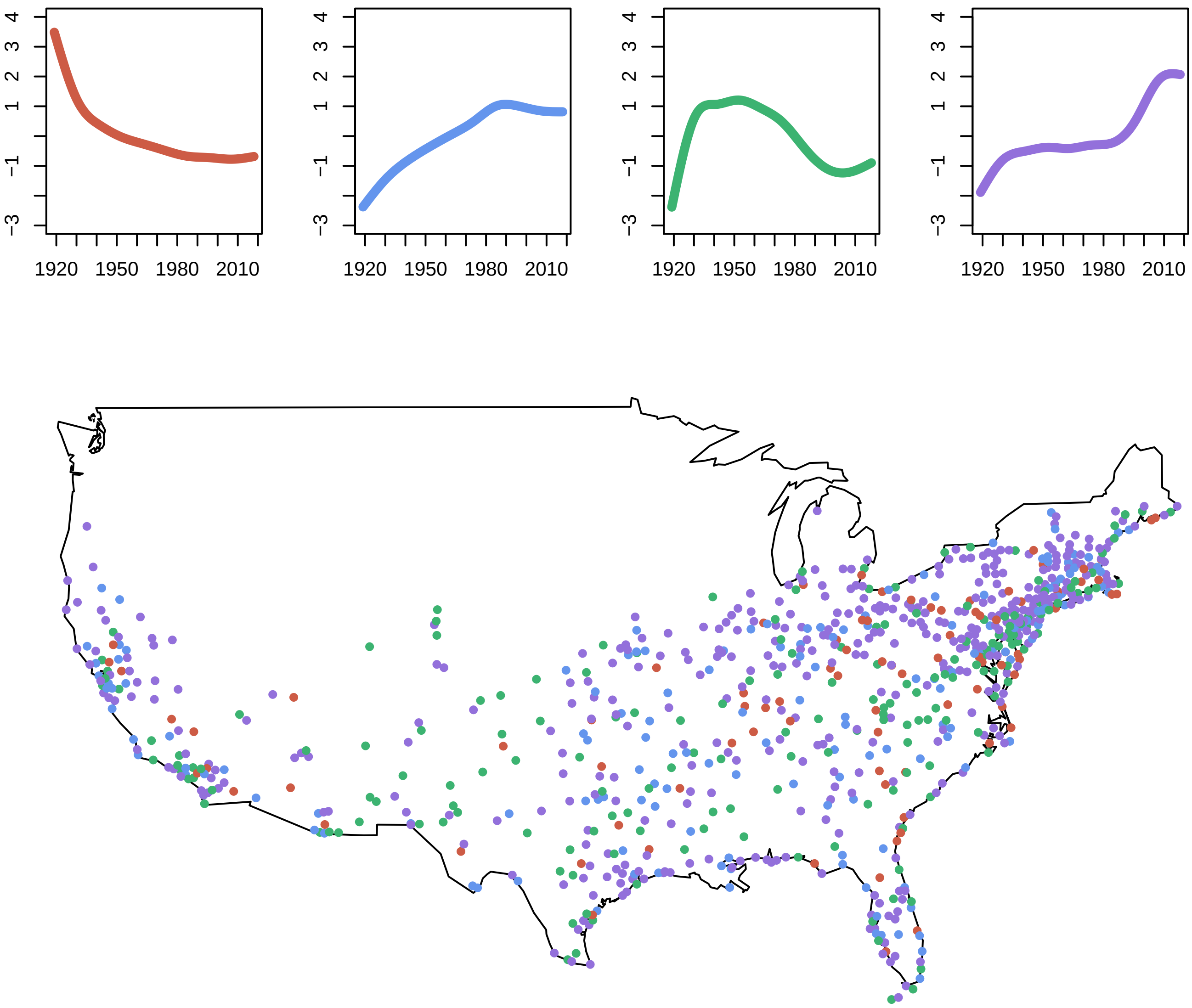

Pine Siskin

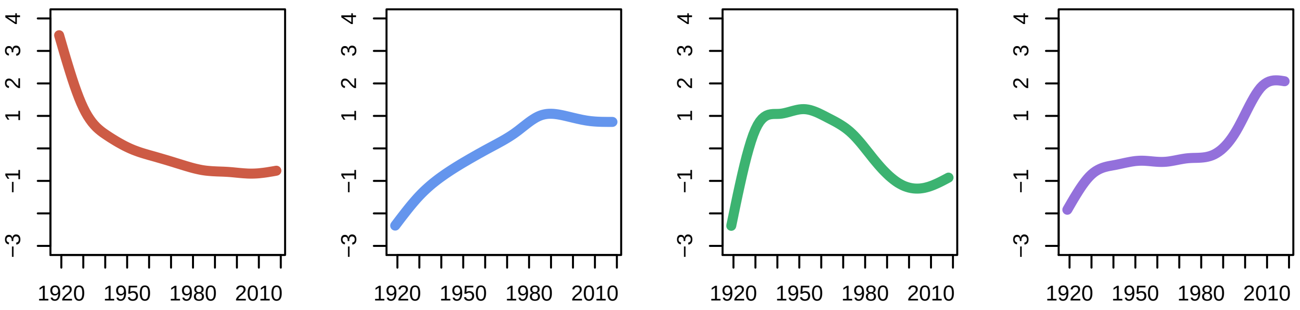

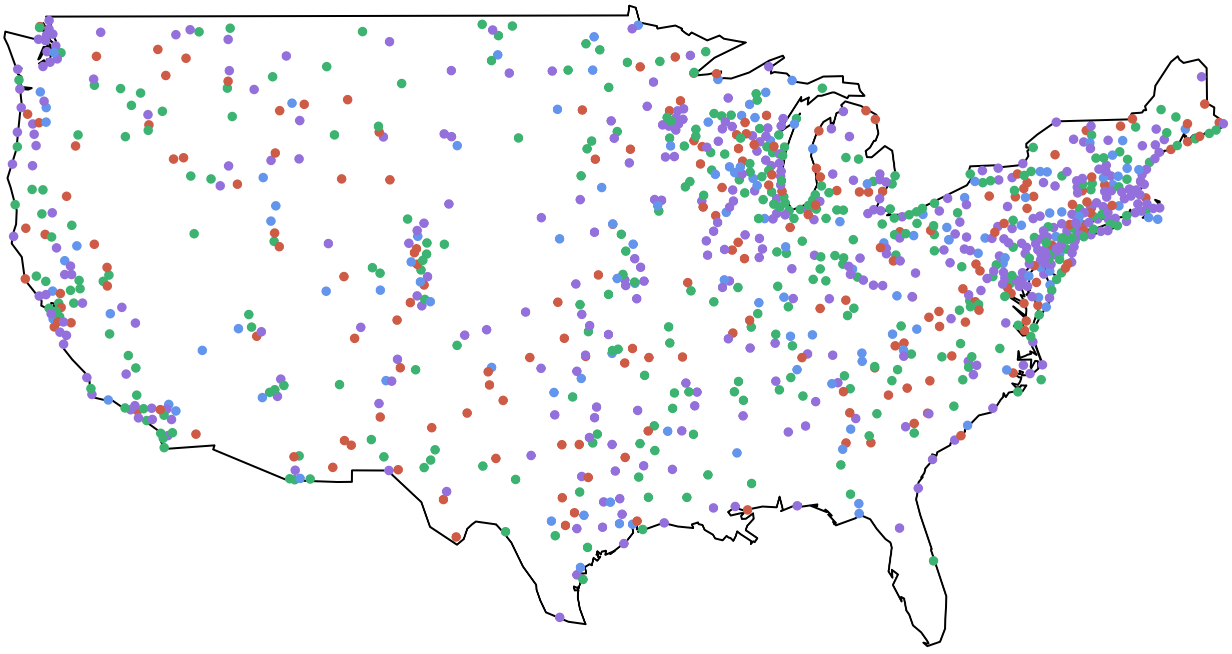

Red−breasted Merganser

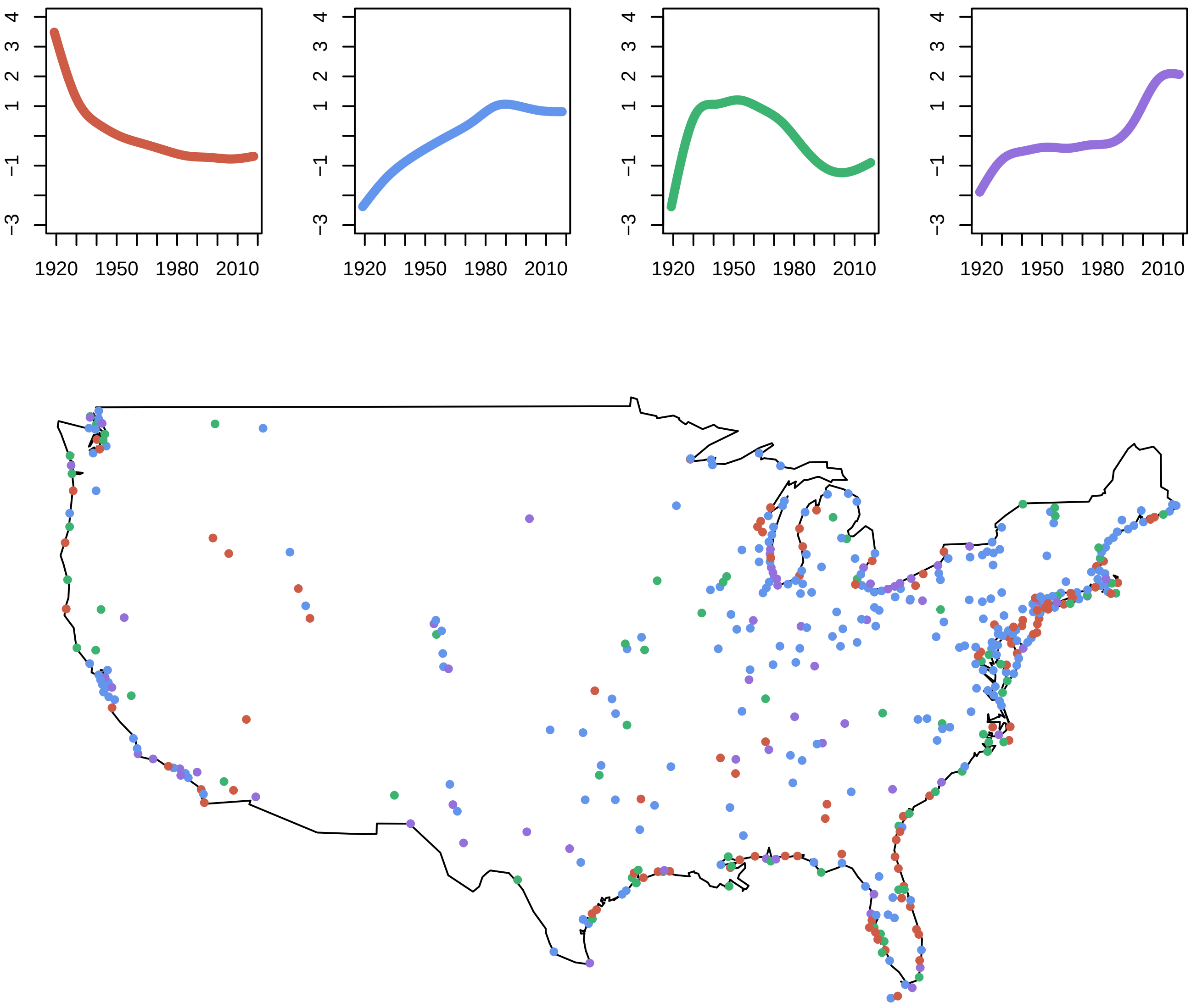

Redhead

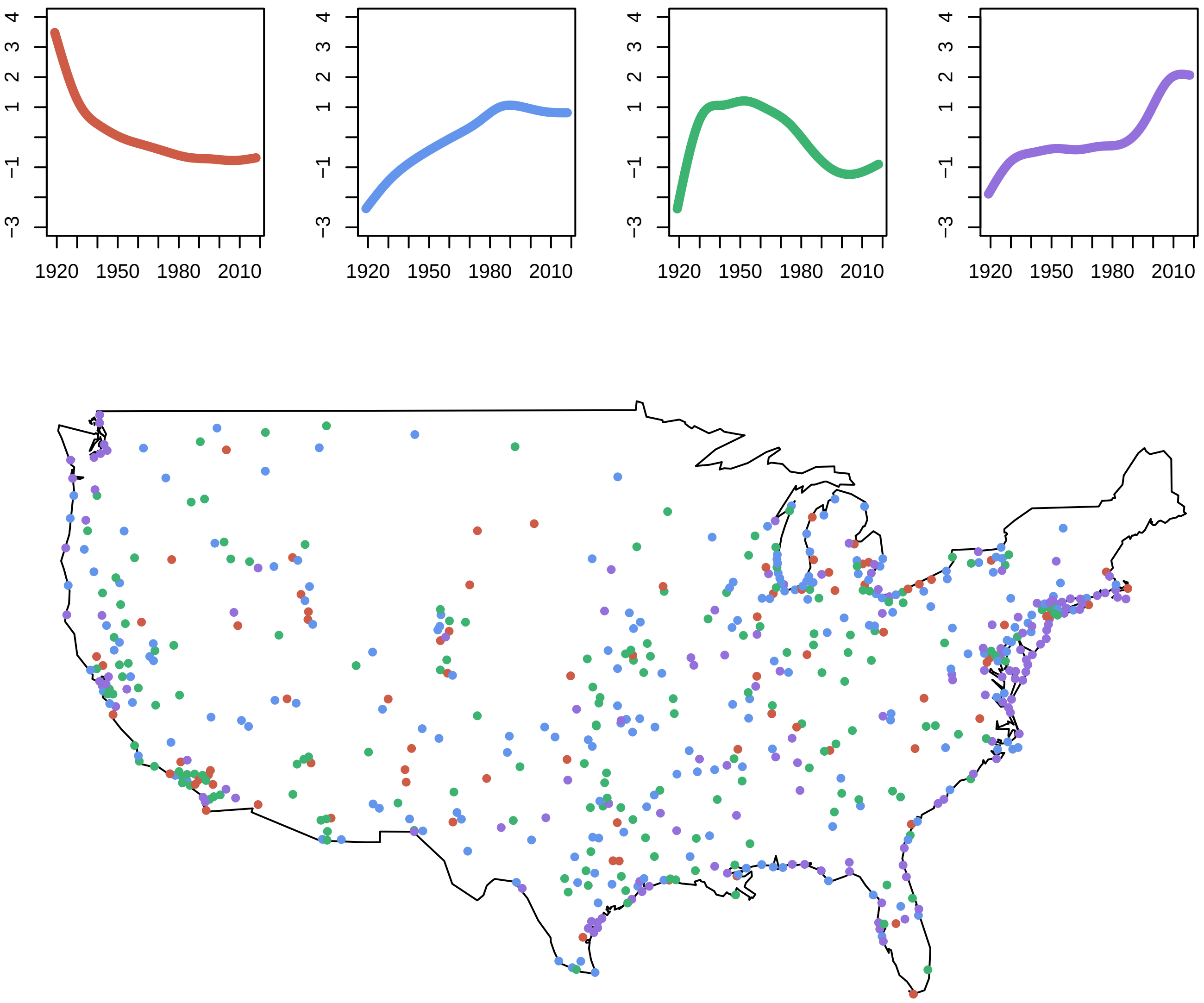

Ring−necked Duck

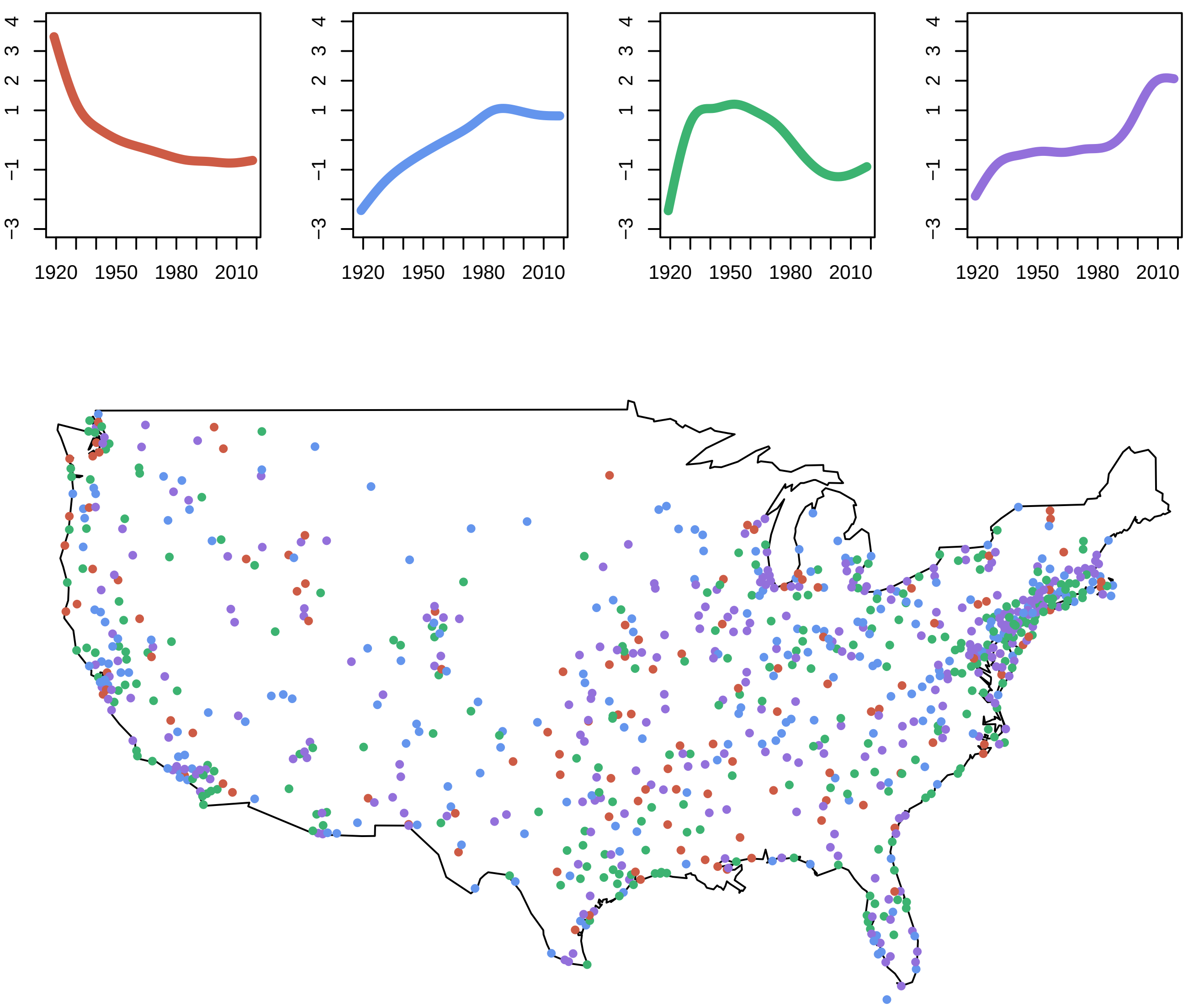

Ruby−crowned Kinglet

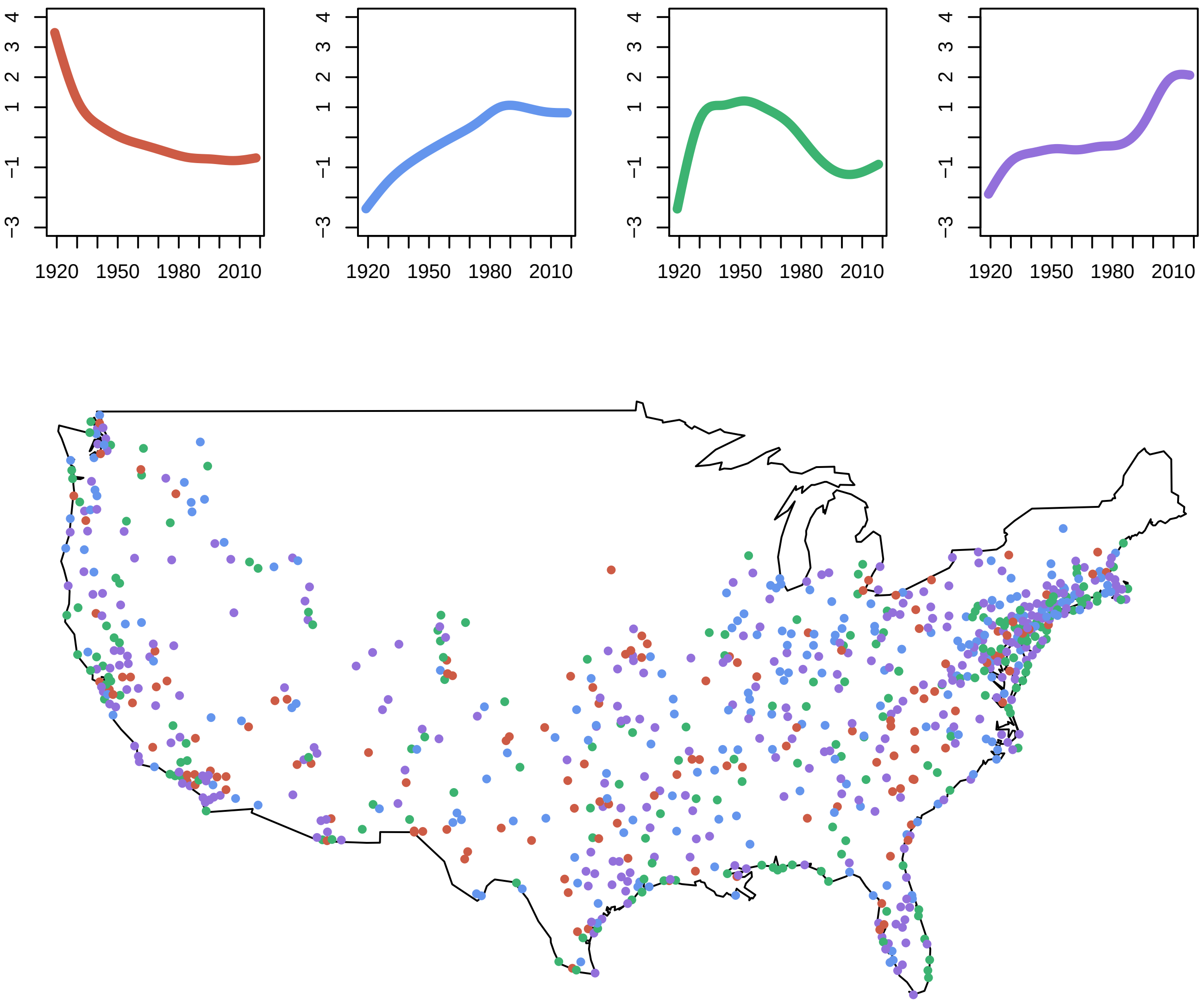

Rusty Blackbird

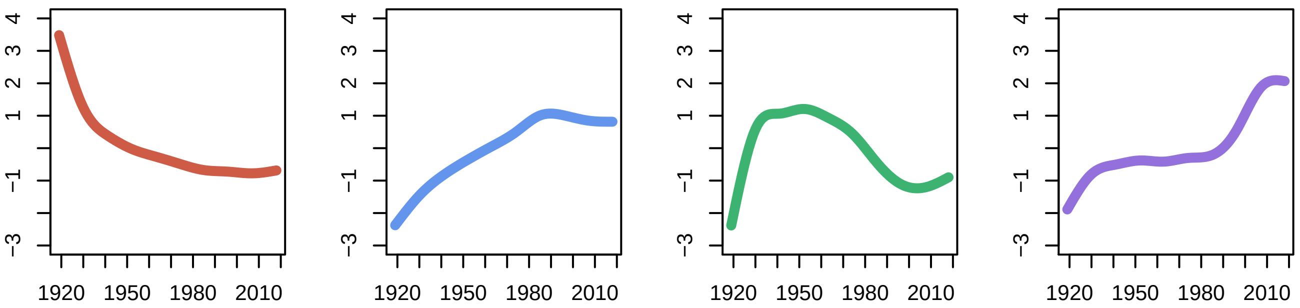

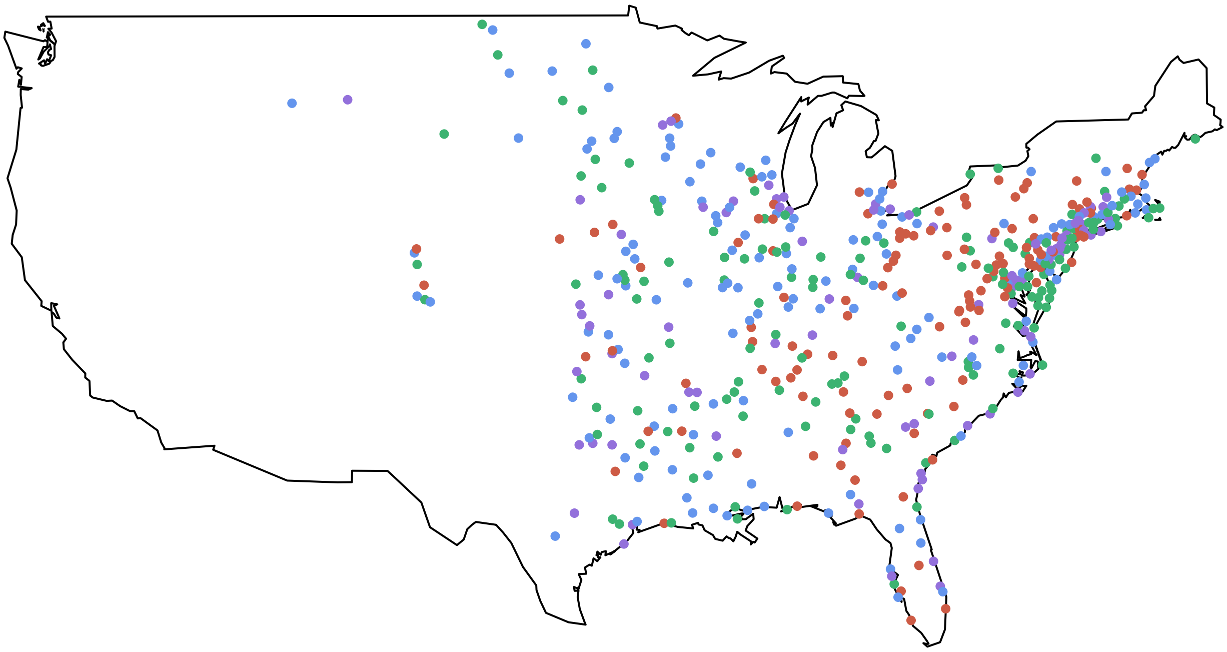

Sandhill Crane

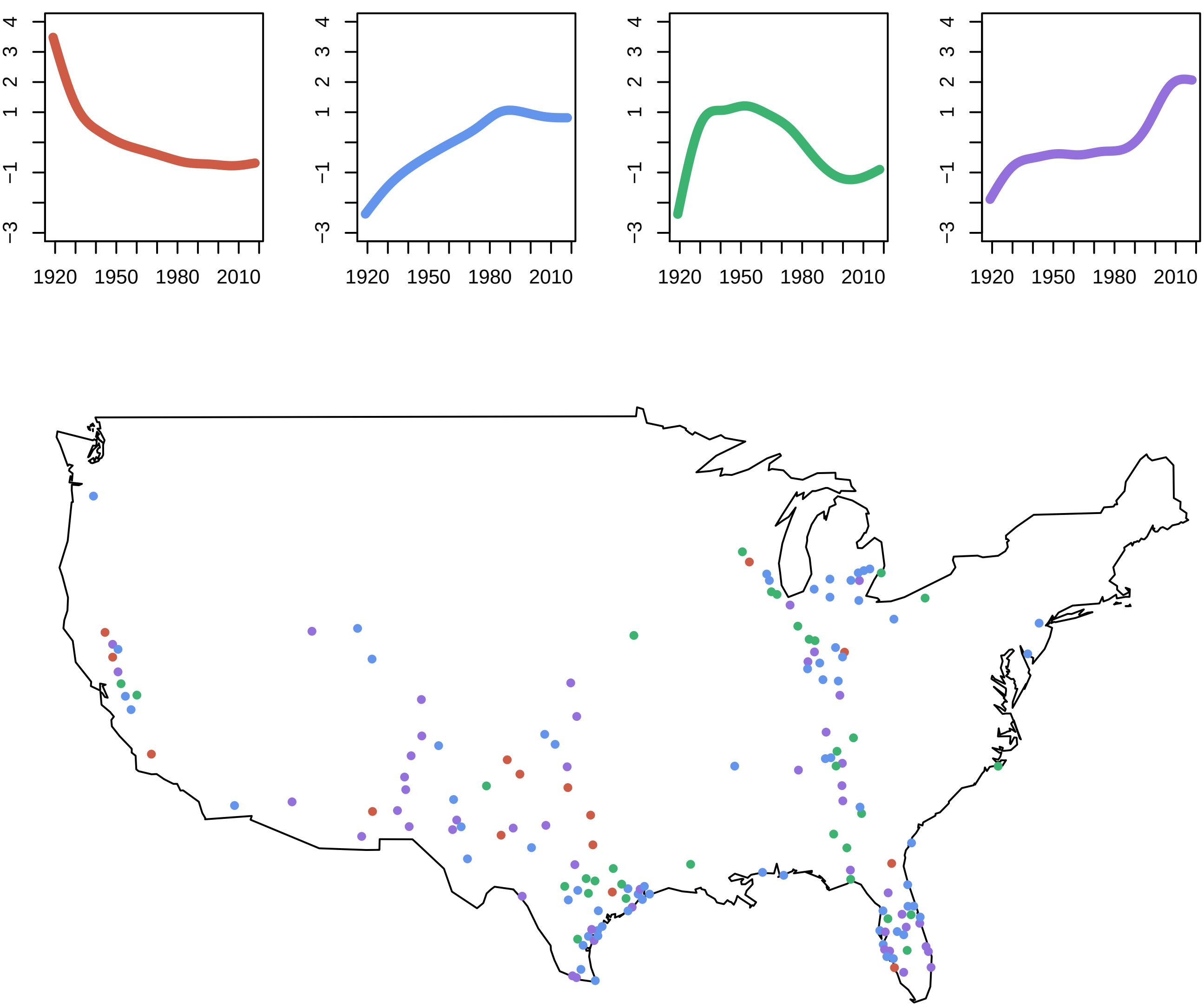

Savannah Sparrow

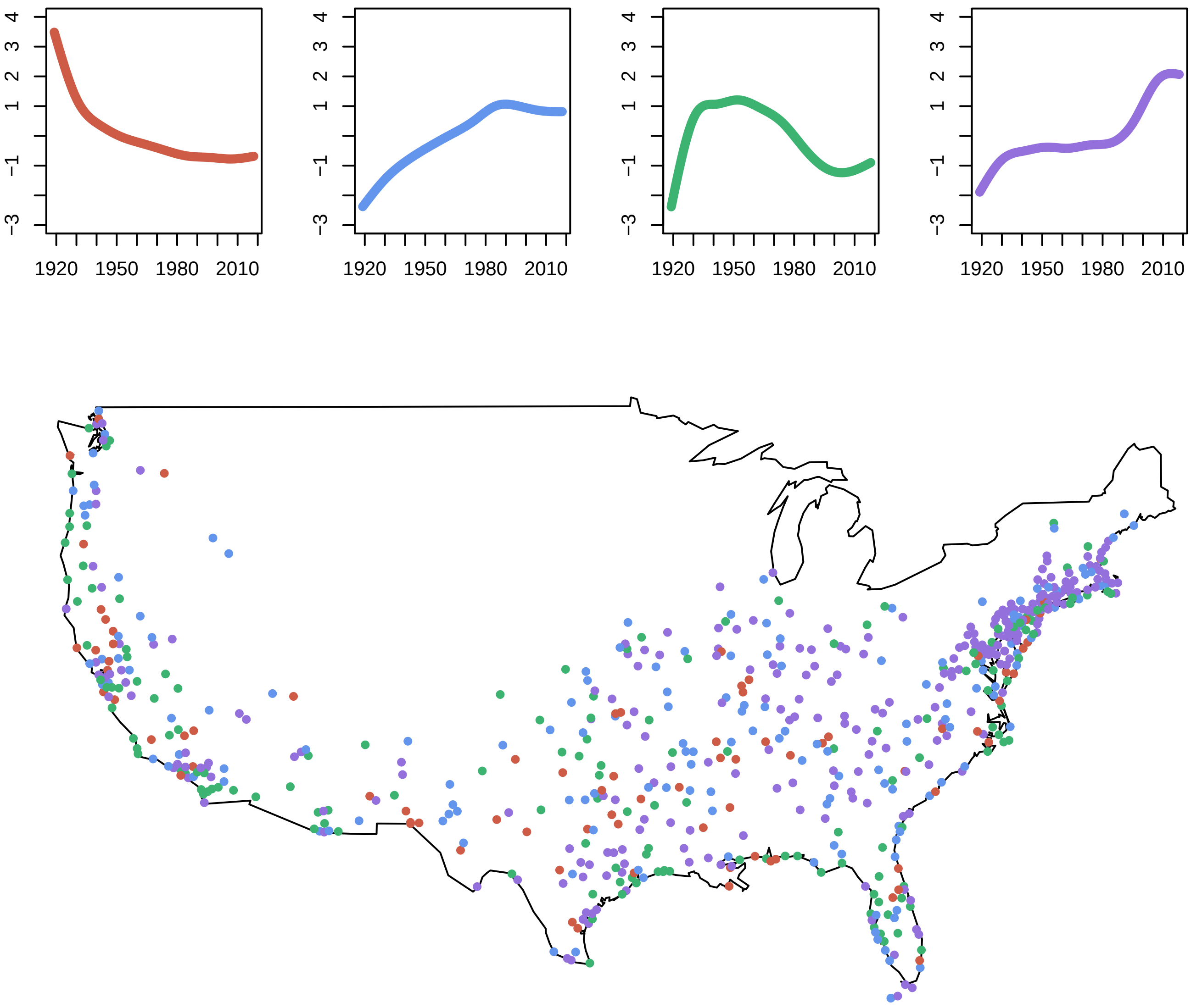

Song Sparrow

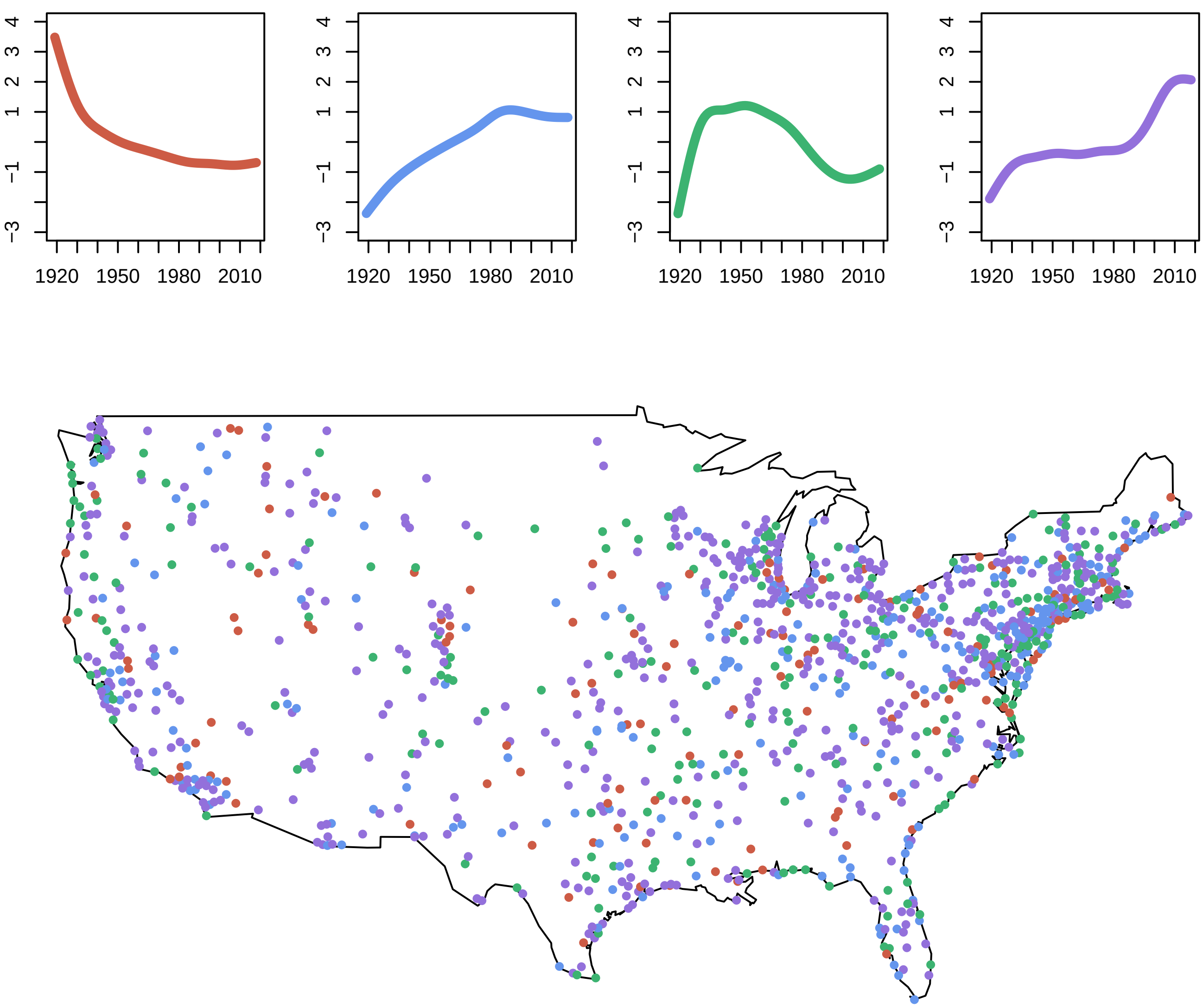

Tufted Titmouse

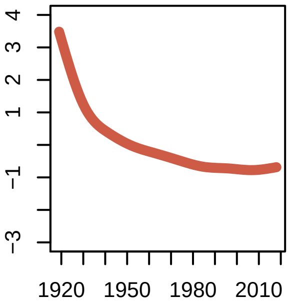

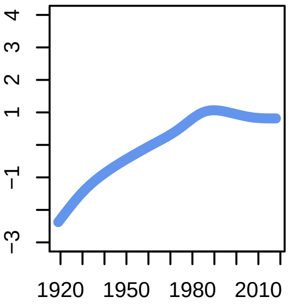

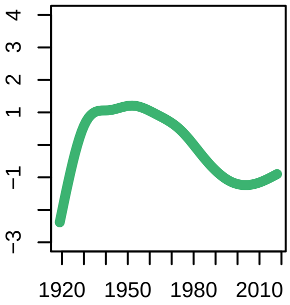

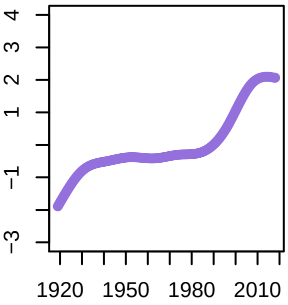

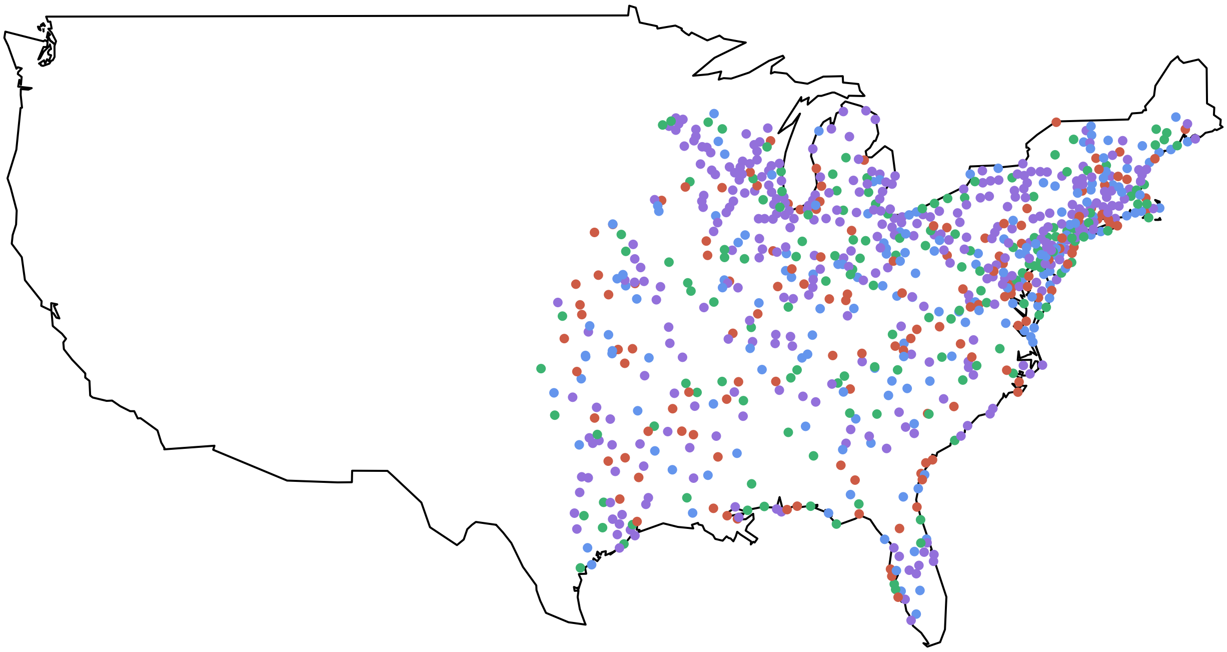

Tundra Swan

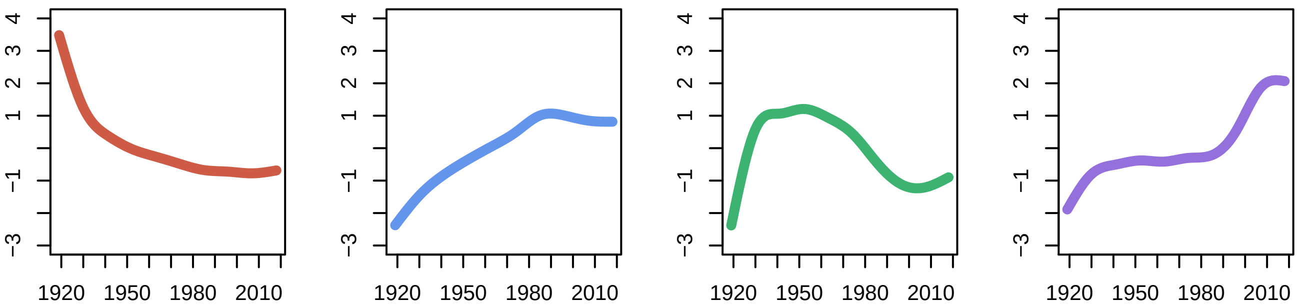

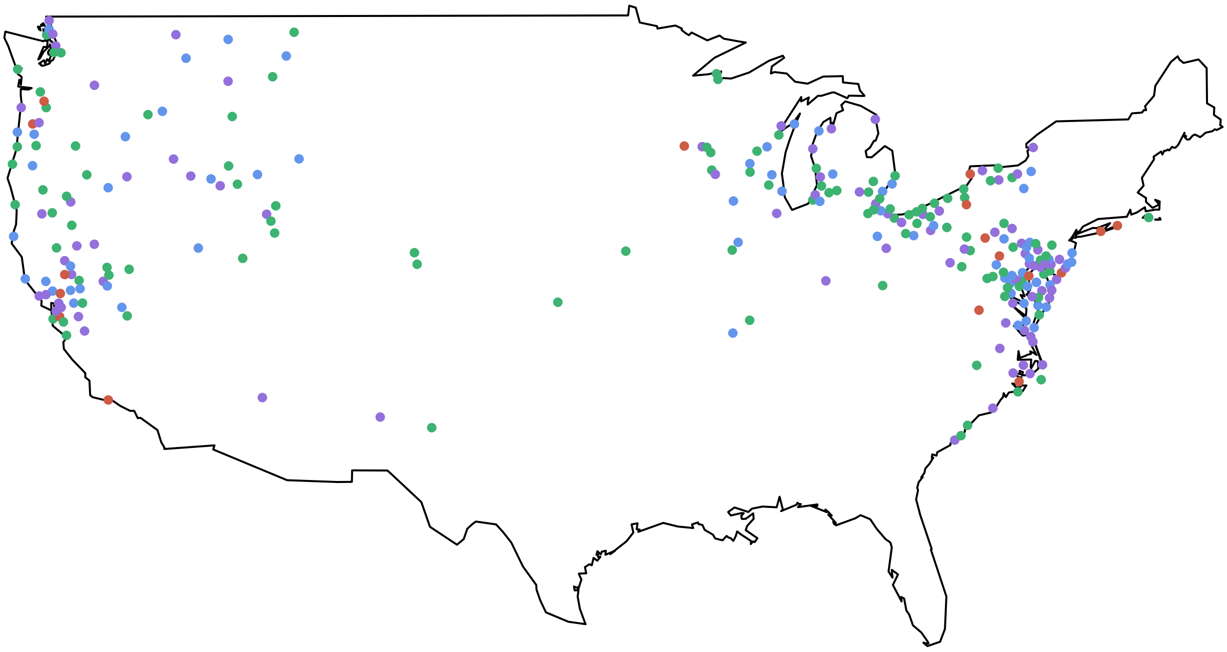

Turkey Vulture

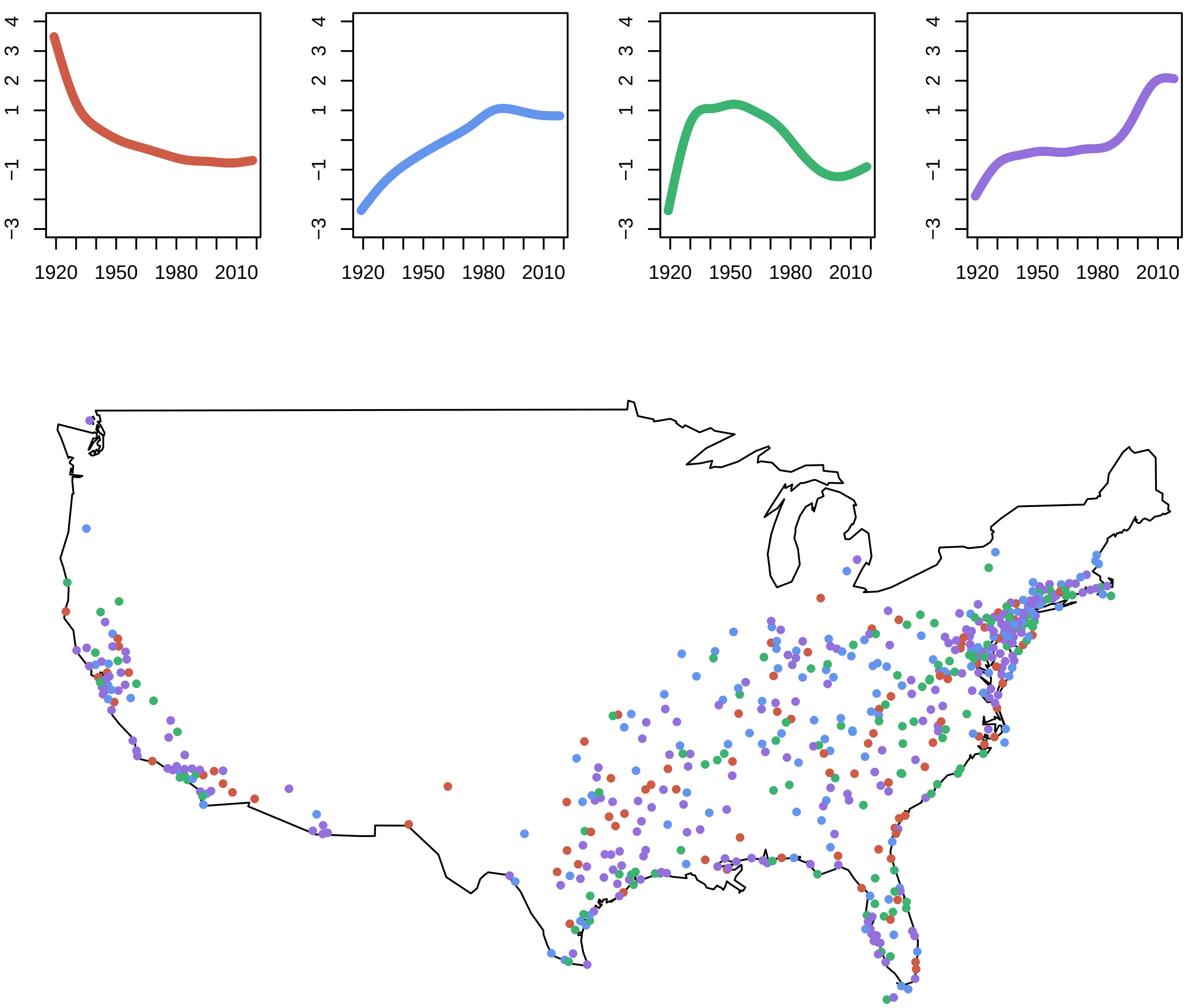

Western Grebe

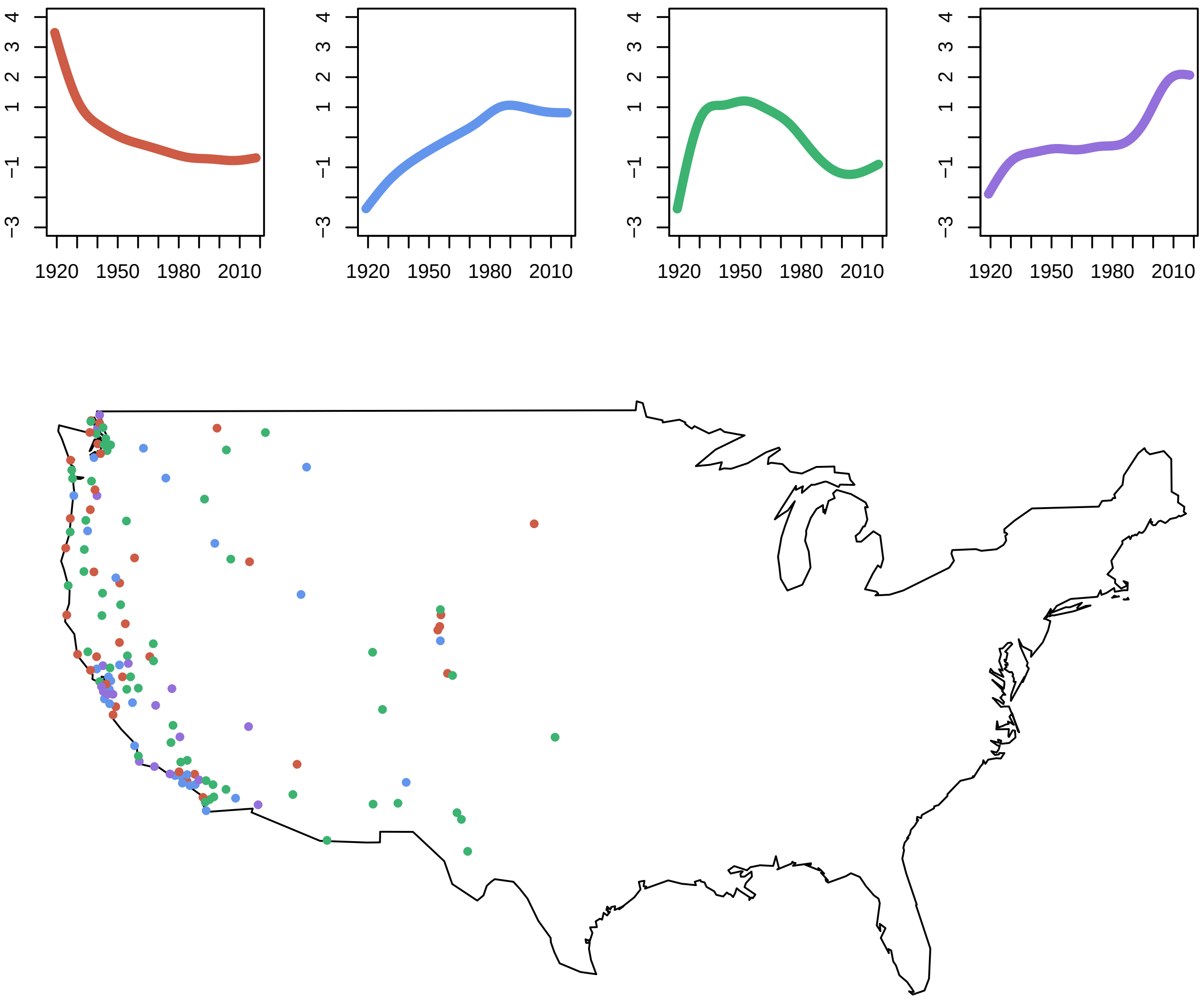

Western Meadowlark

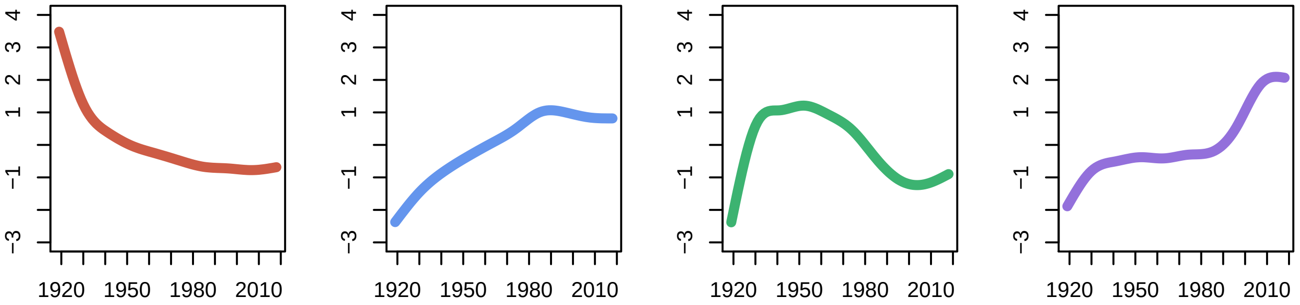

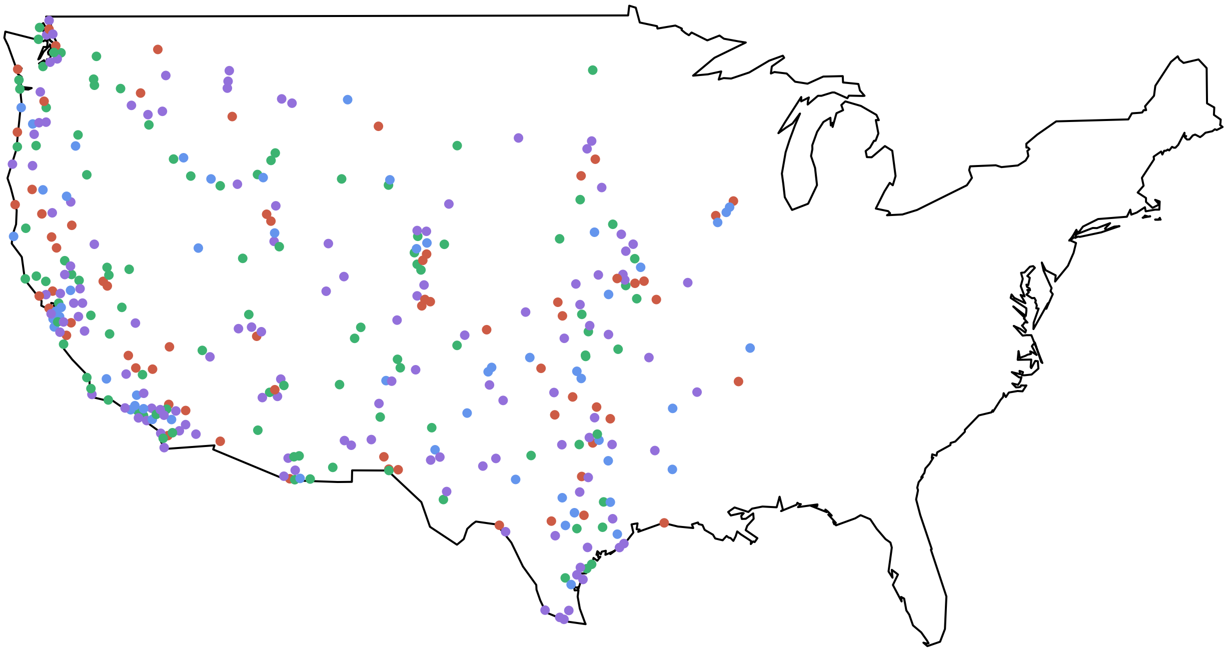

White−breasted Nuthatch

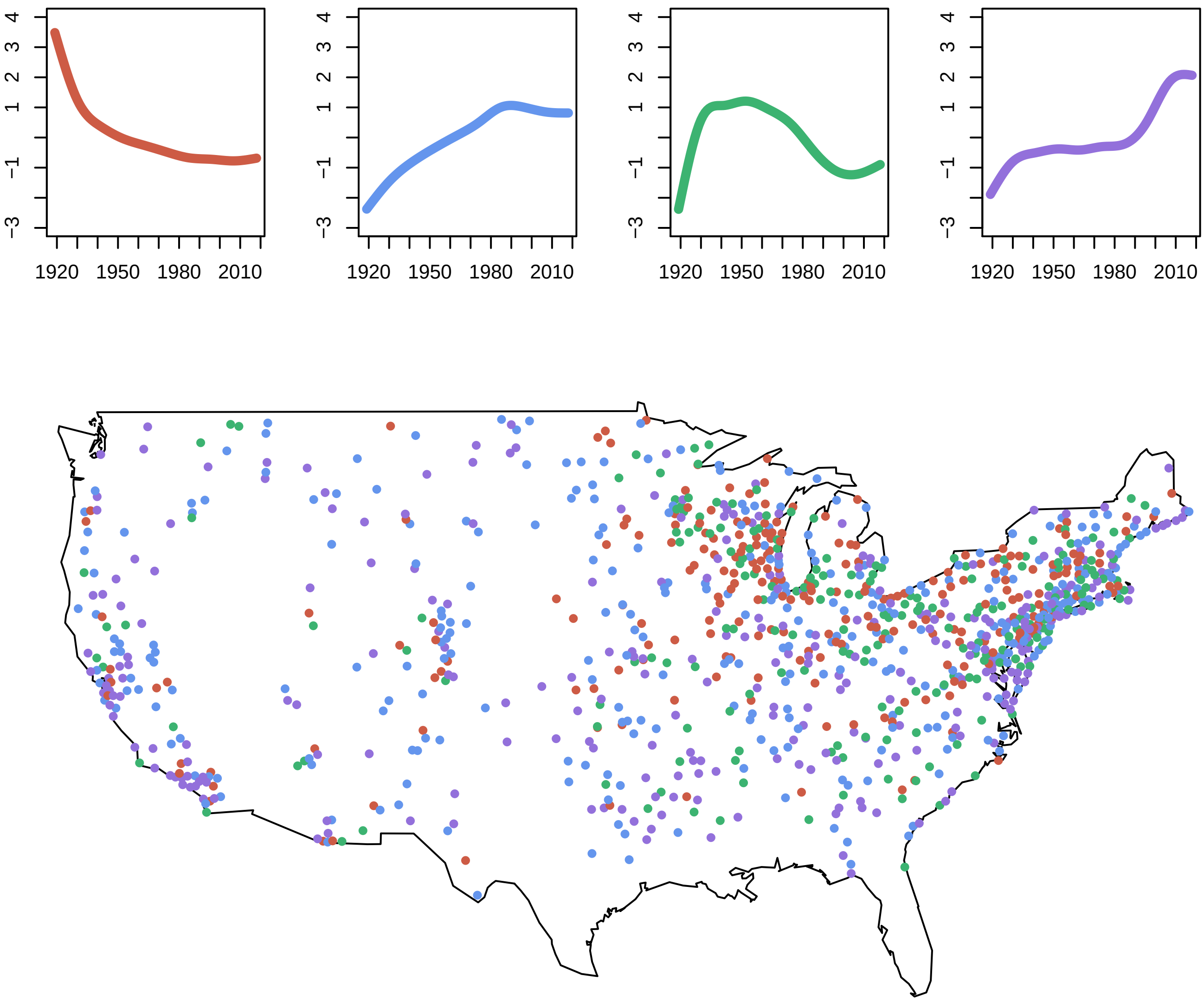

White Ibis

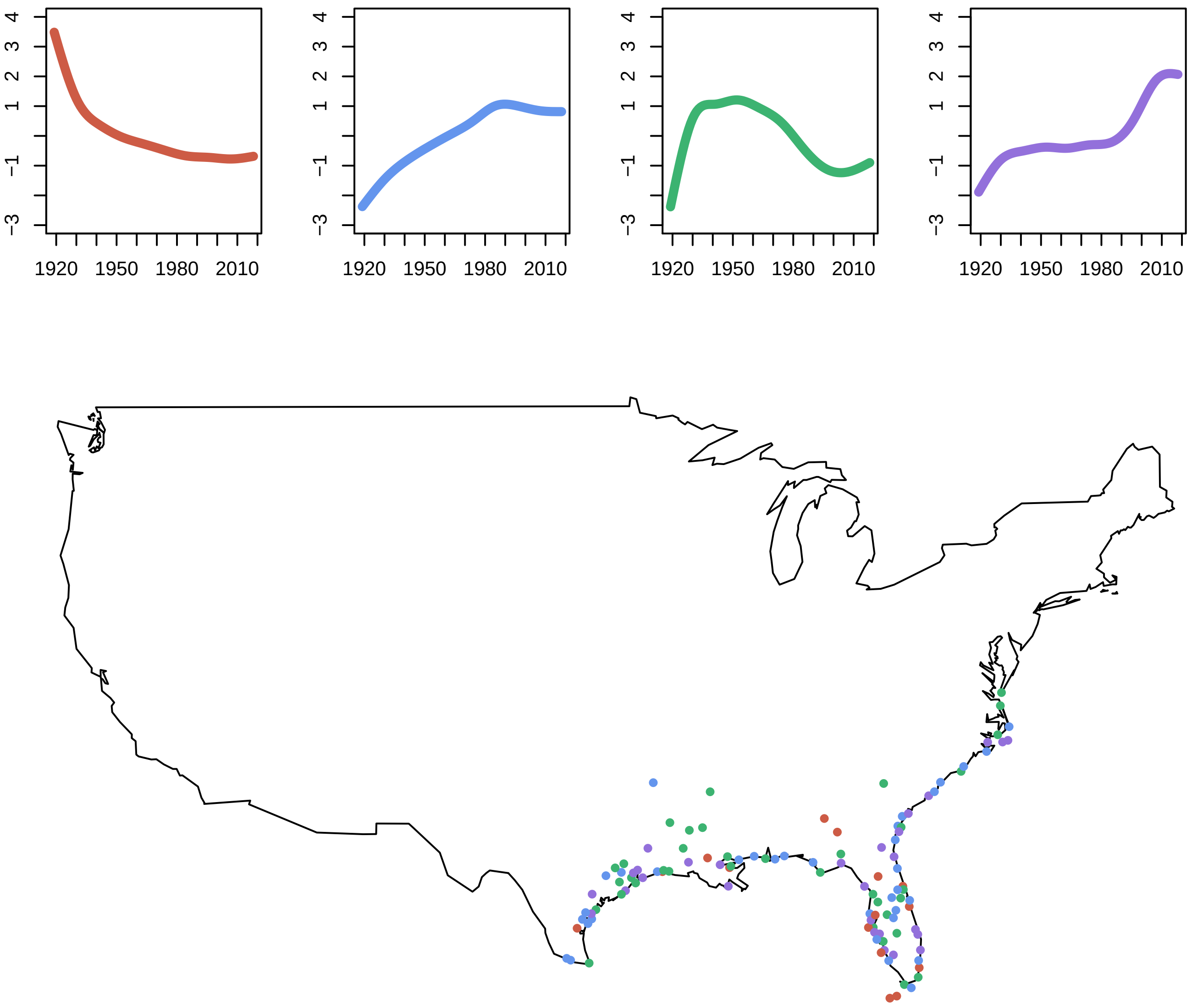

American Avocet

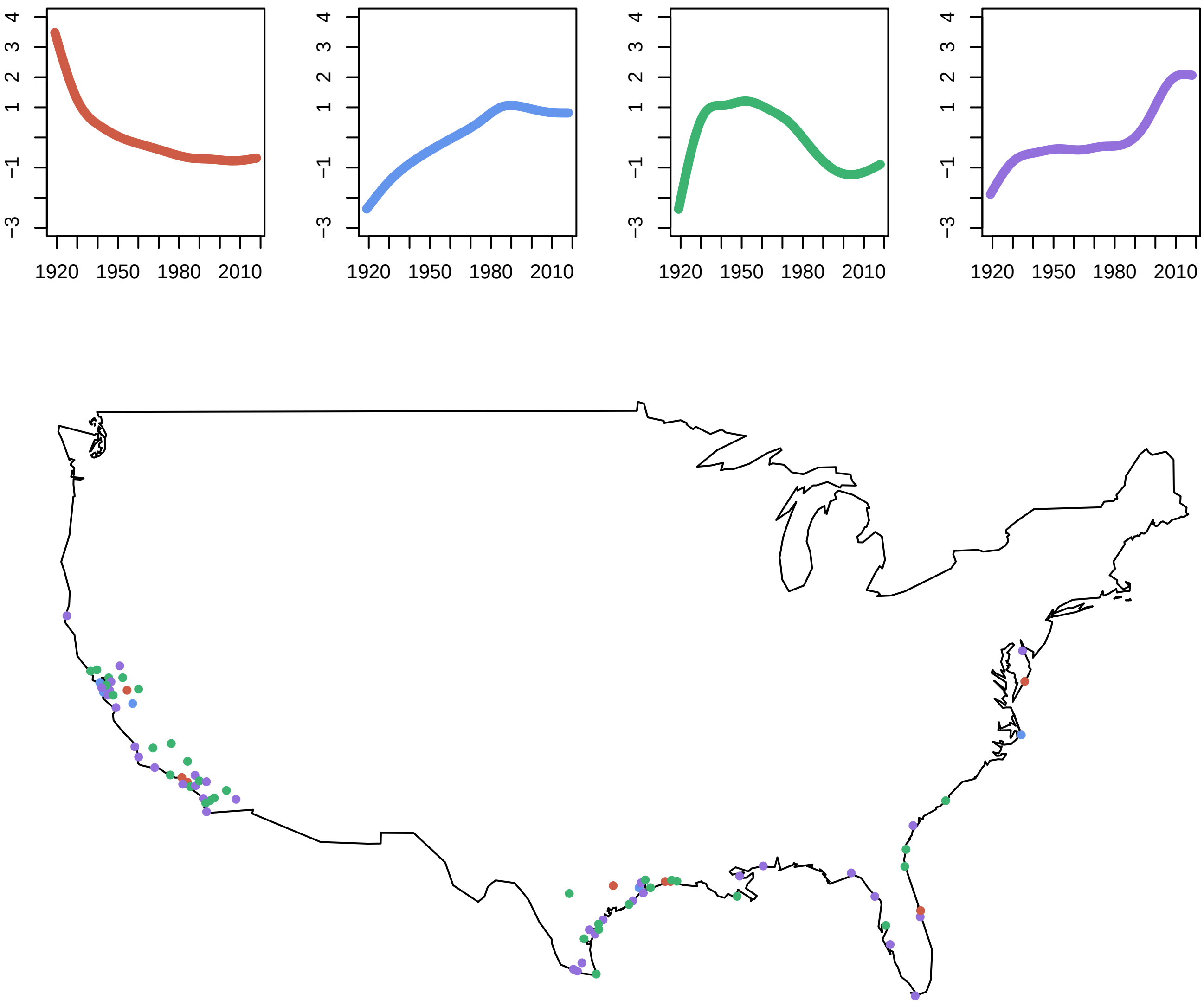

American Kestrel

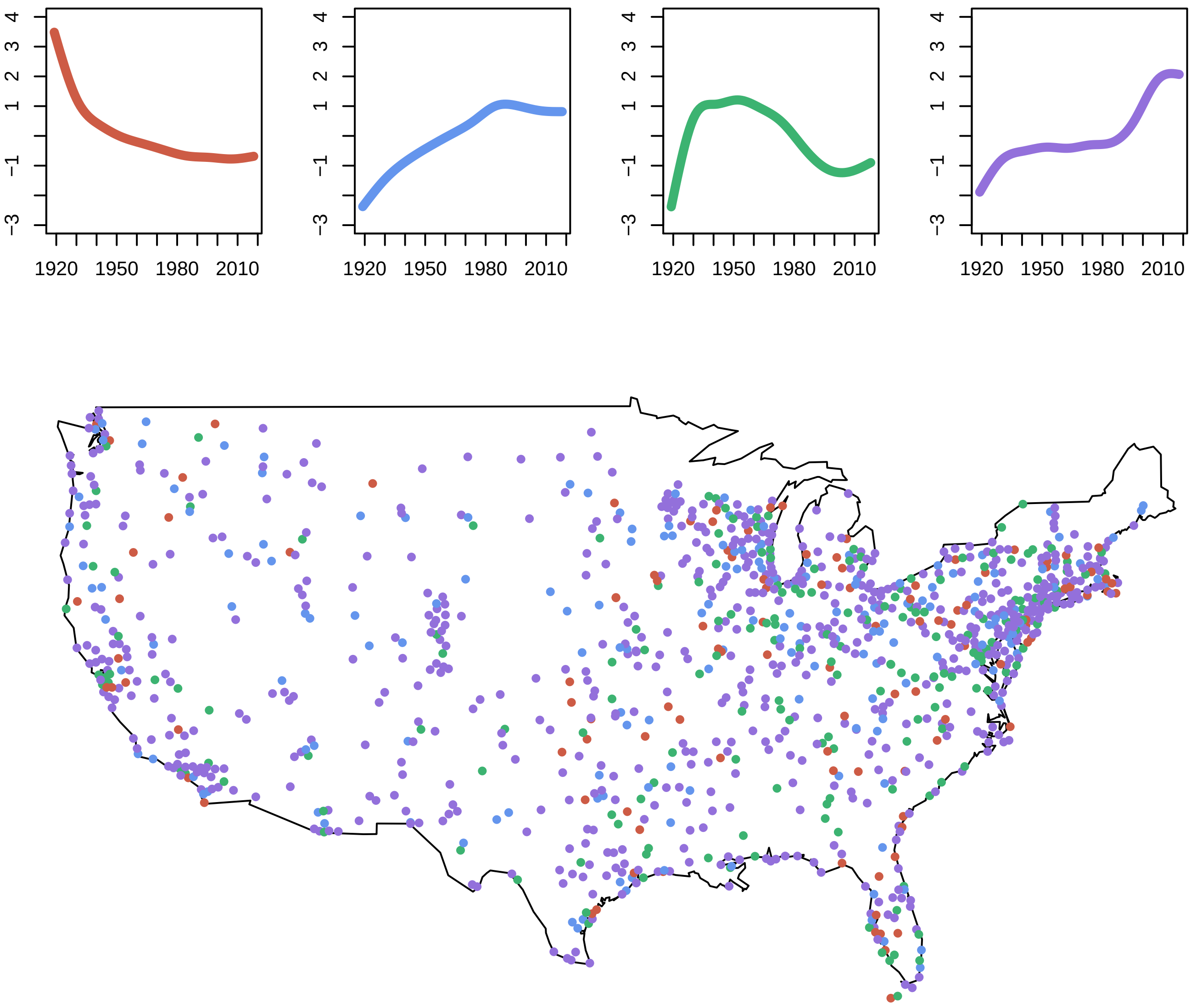

American White Pelican

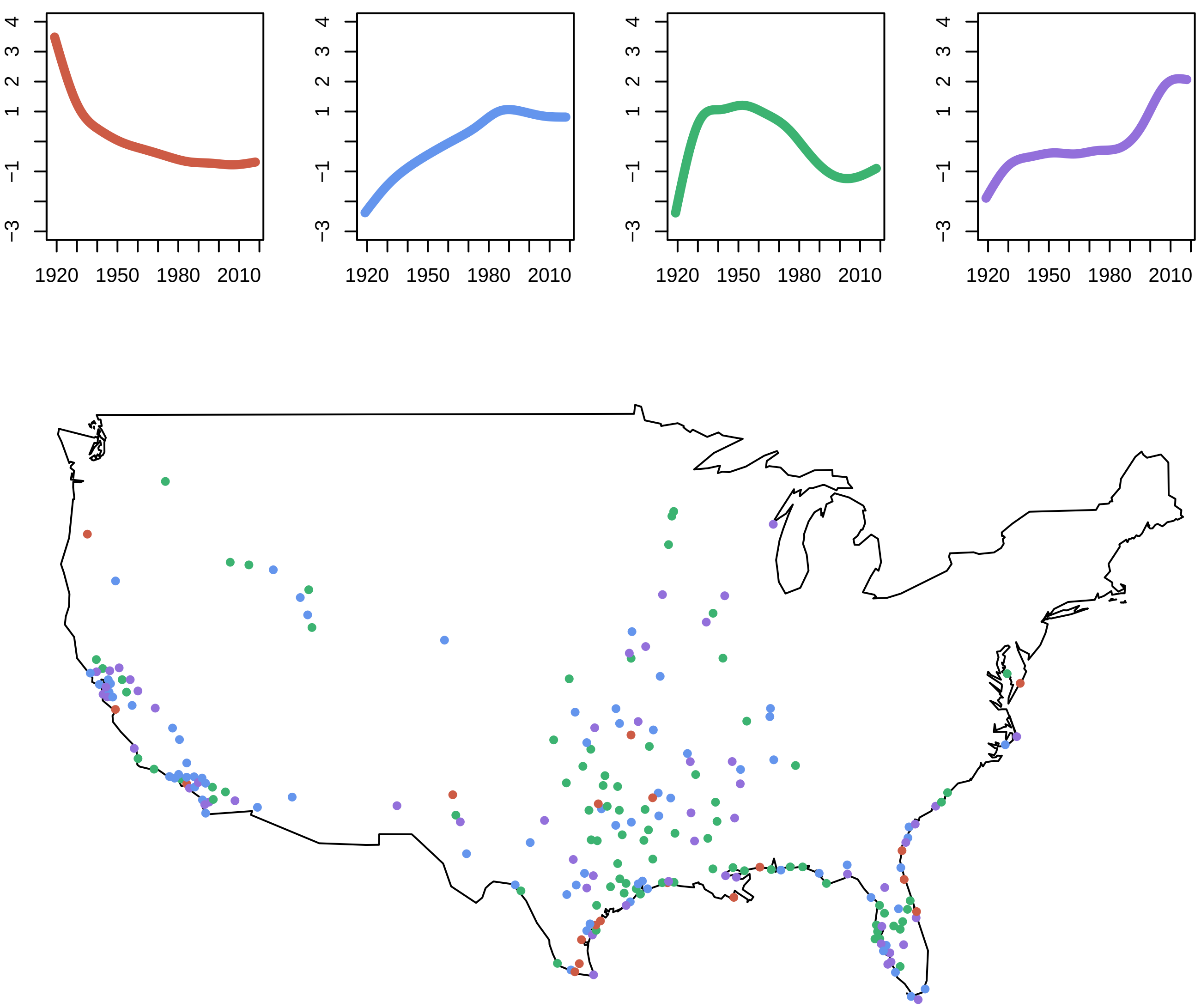

Black−billed Magpie

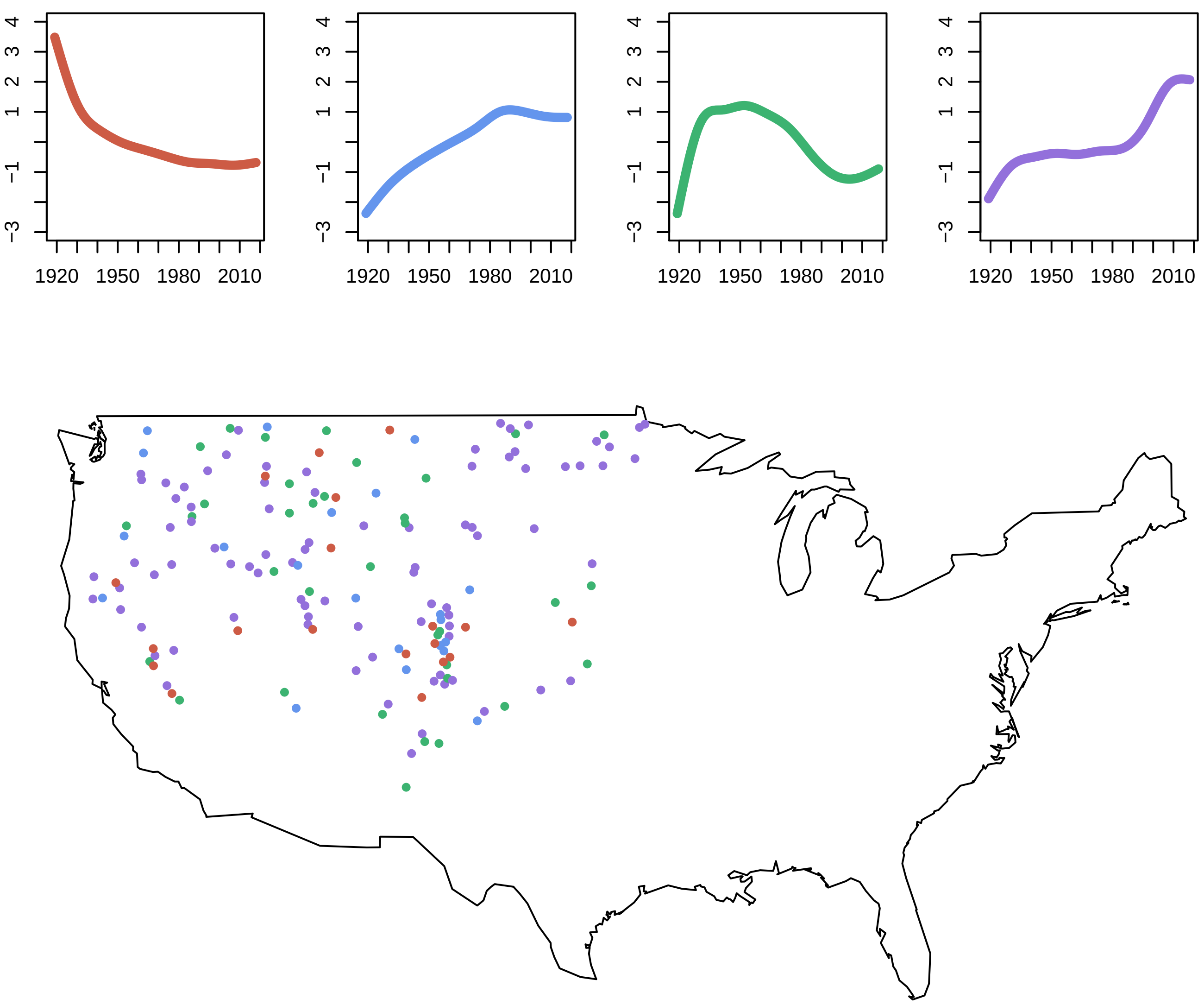

Black Vulture

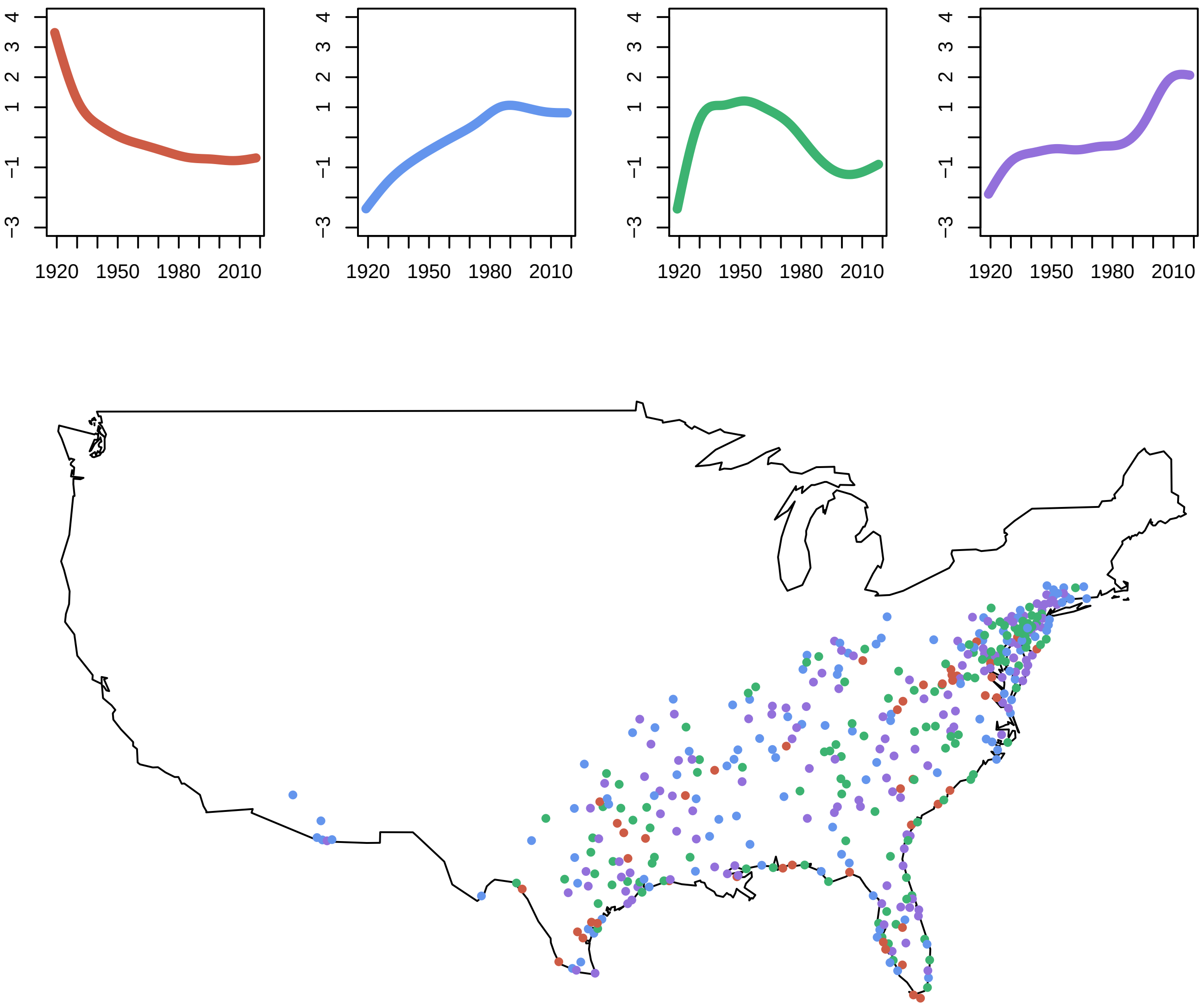

Blue−winged Teal

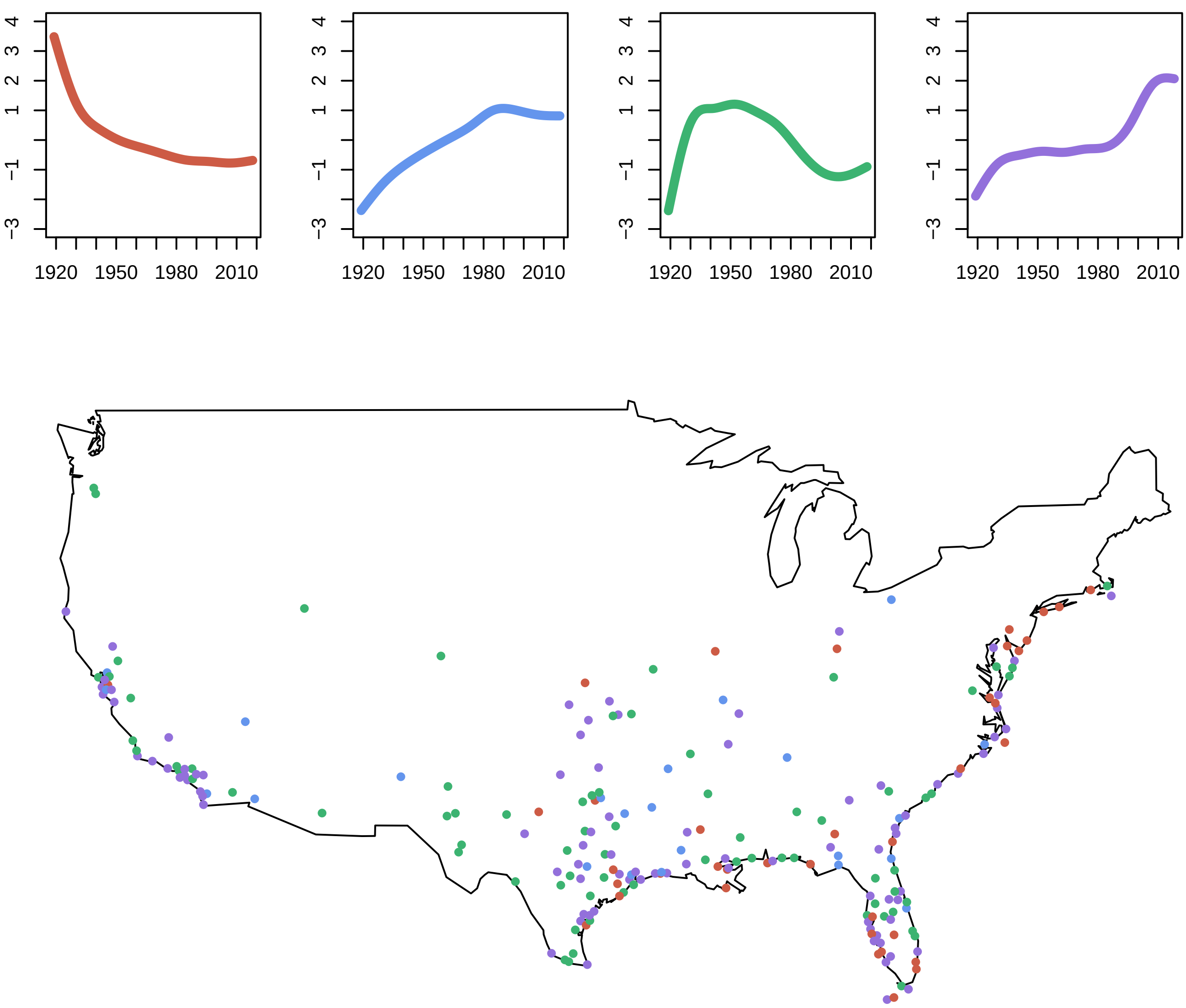

Bohemian Waxwing

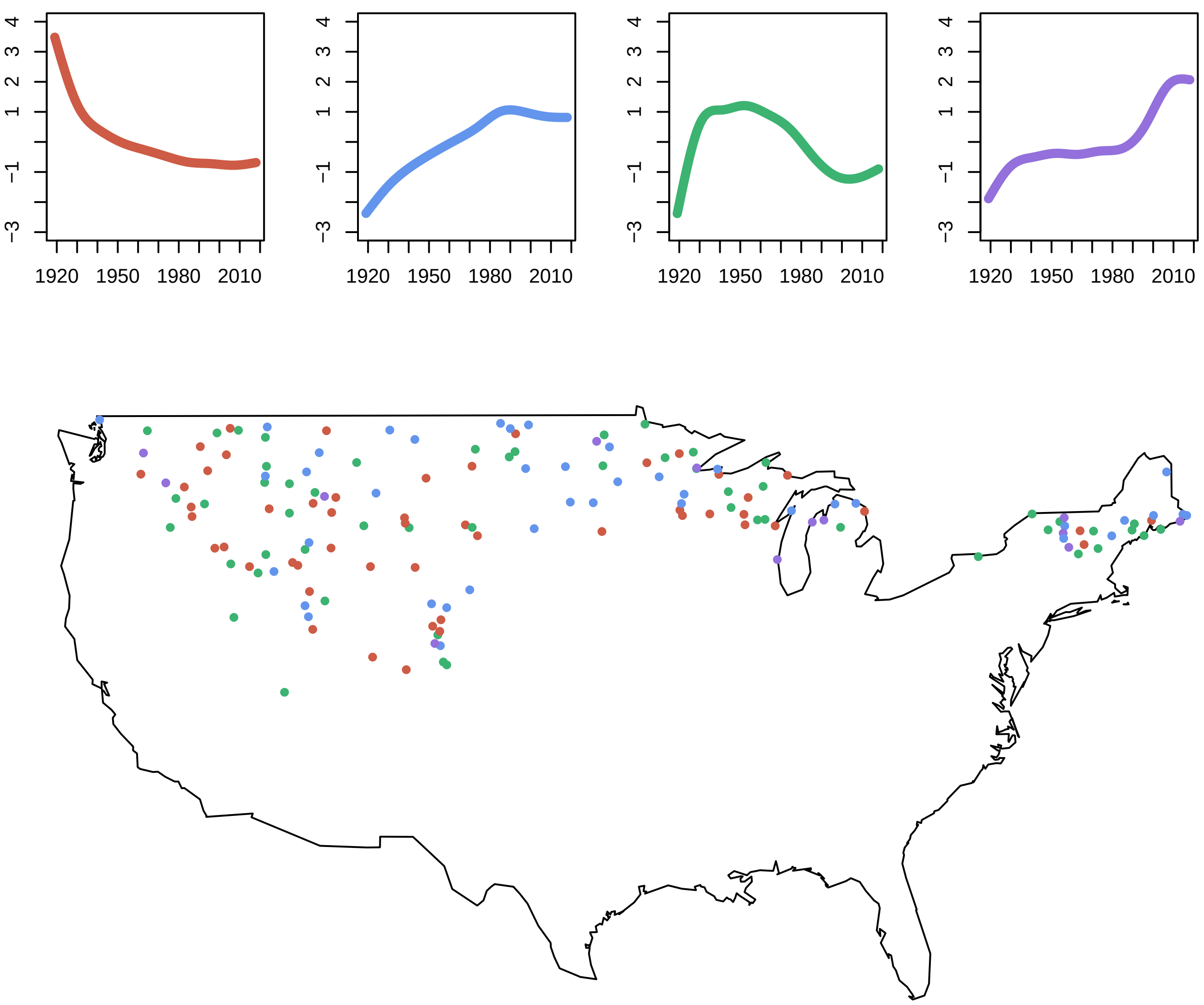

California Quail

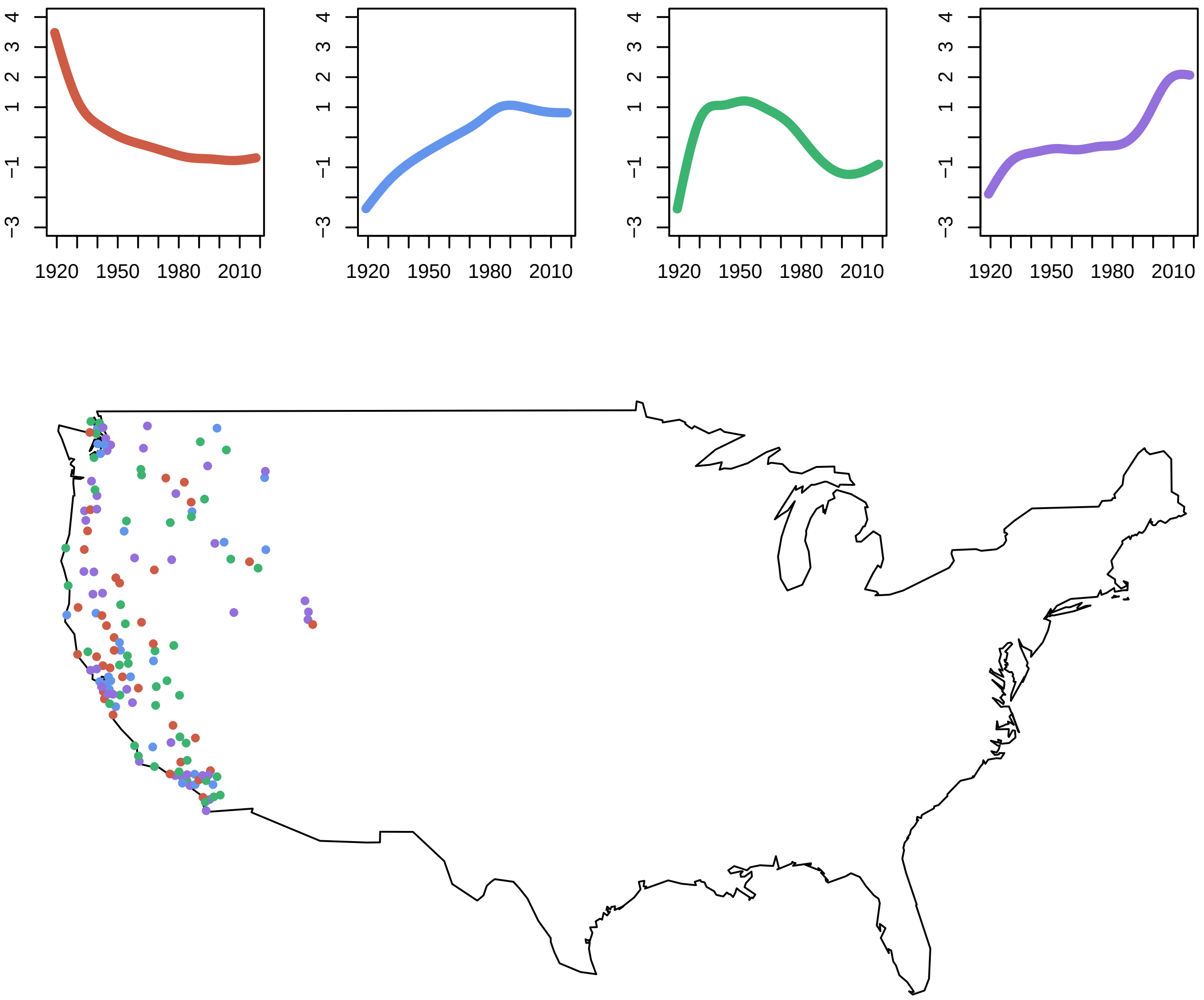

Carolina Wren

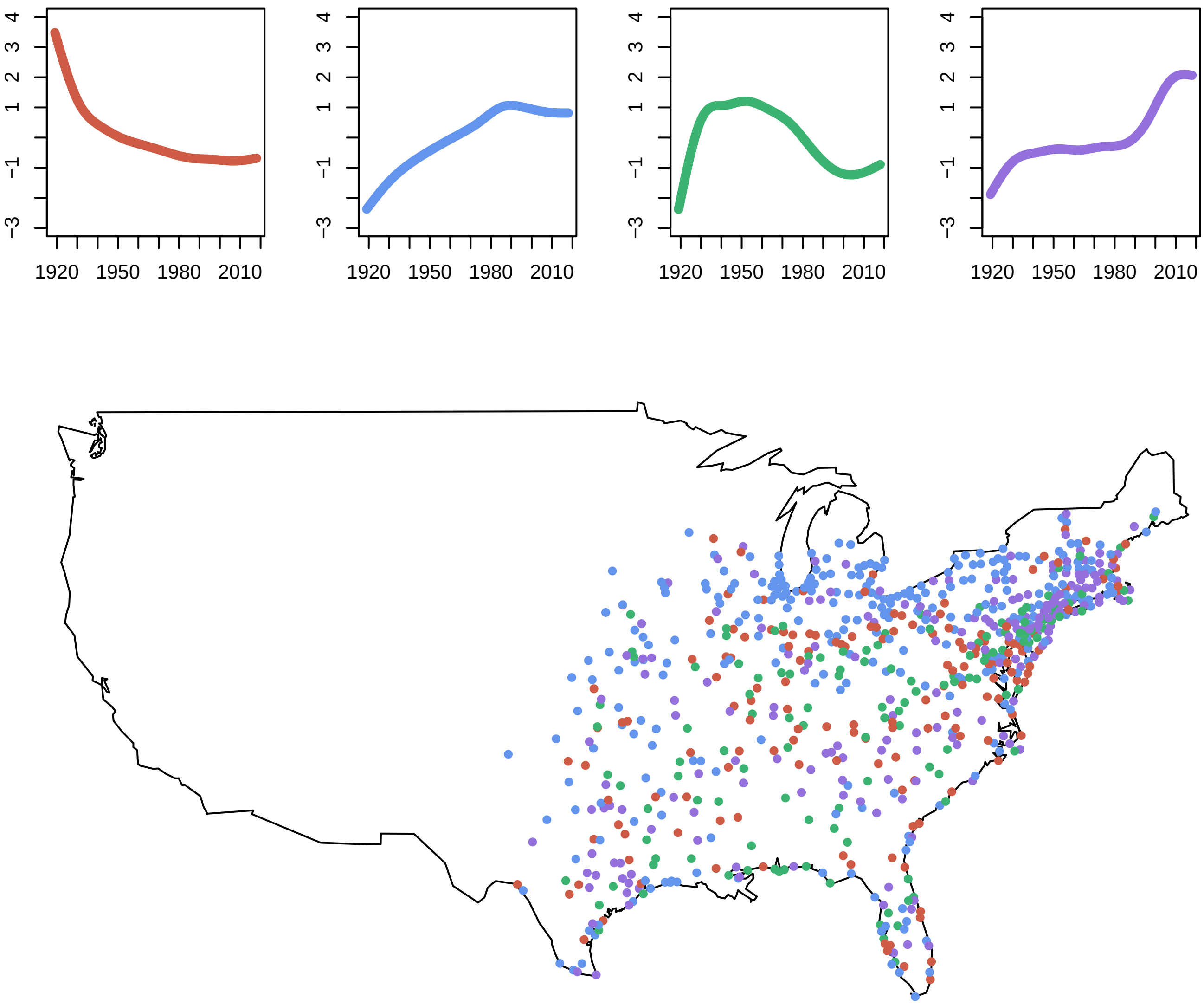

Cattle Egret

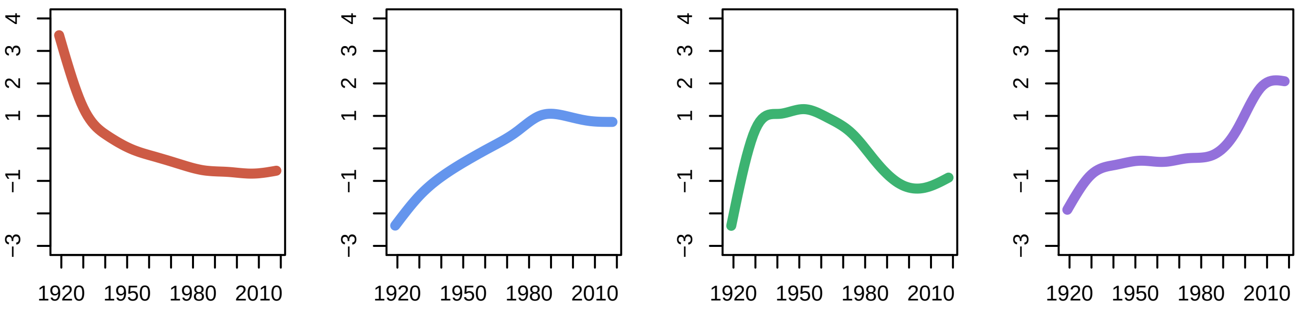

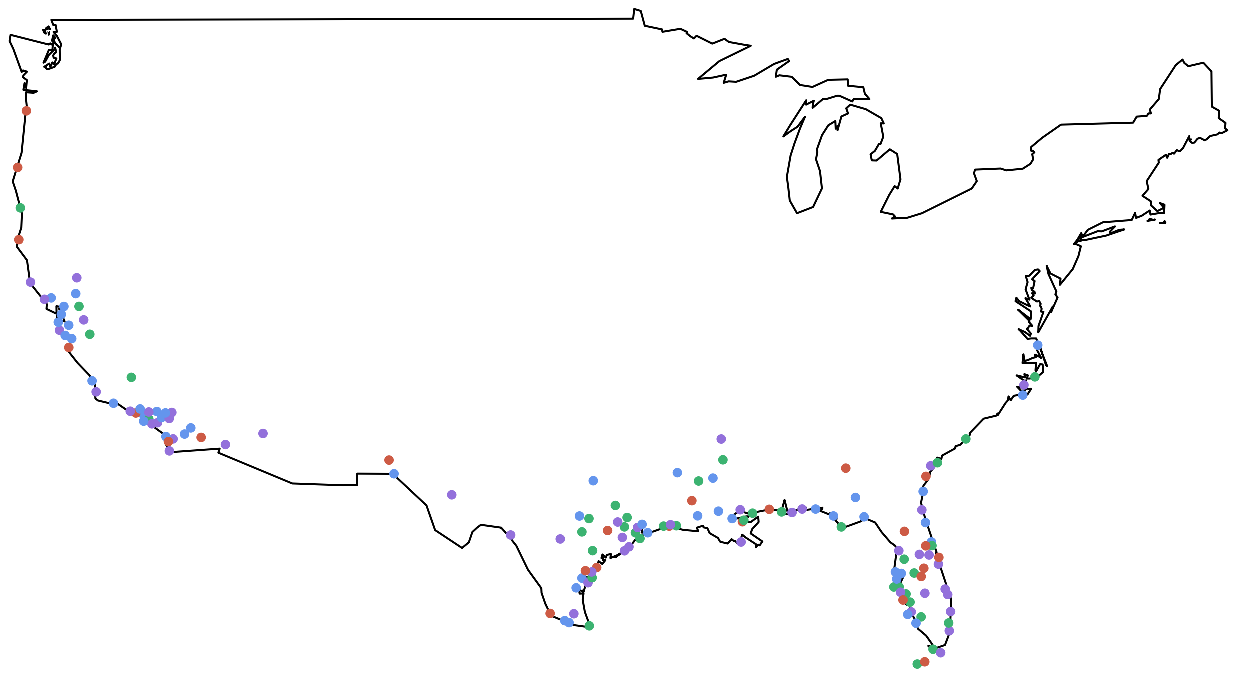

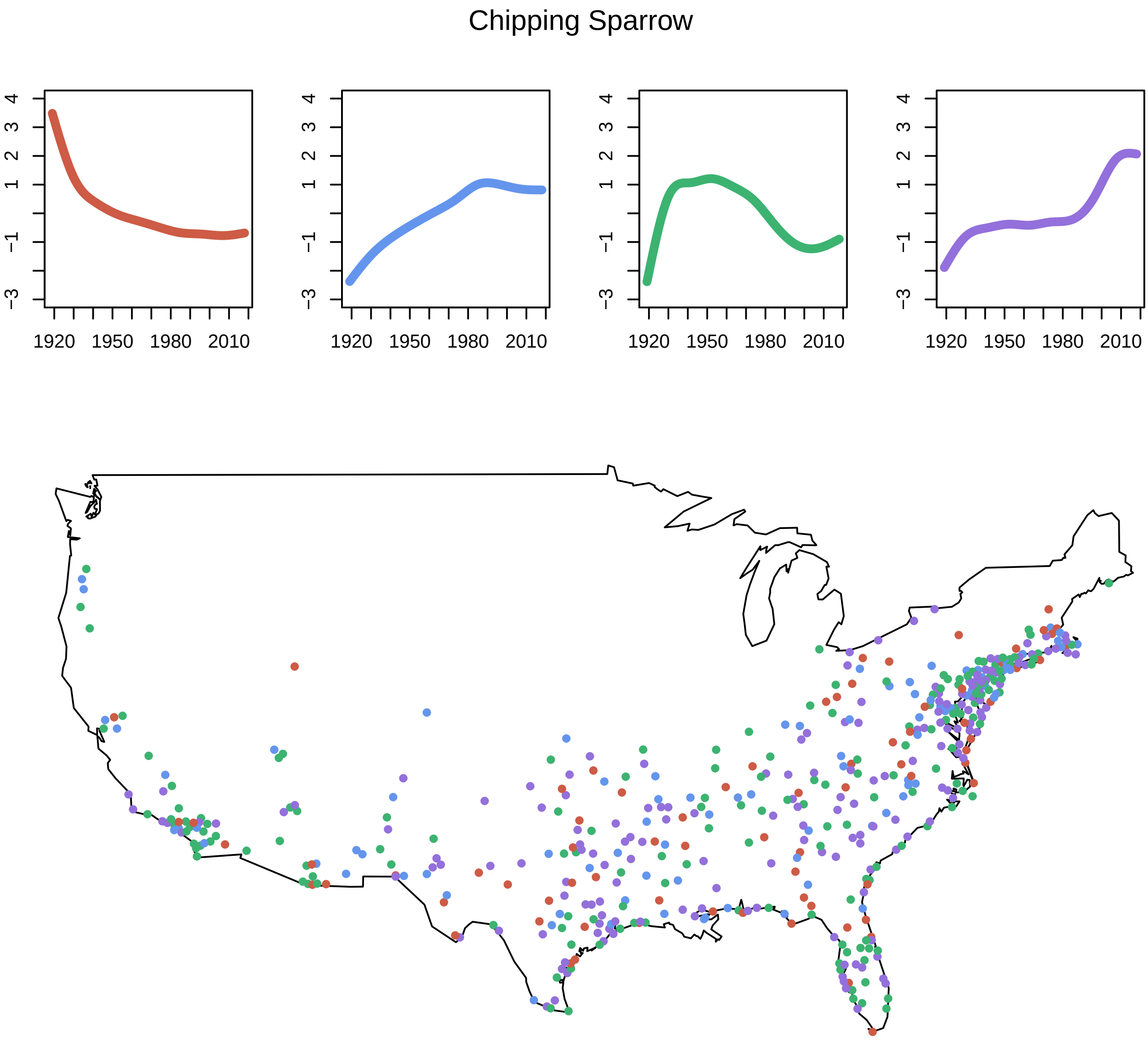

Chipping Sparrow
4
3
2
1
−1
−3
1920
1950
1980
2010

Common Raven

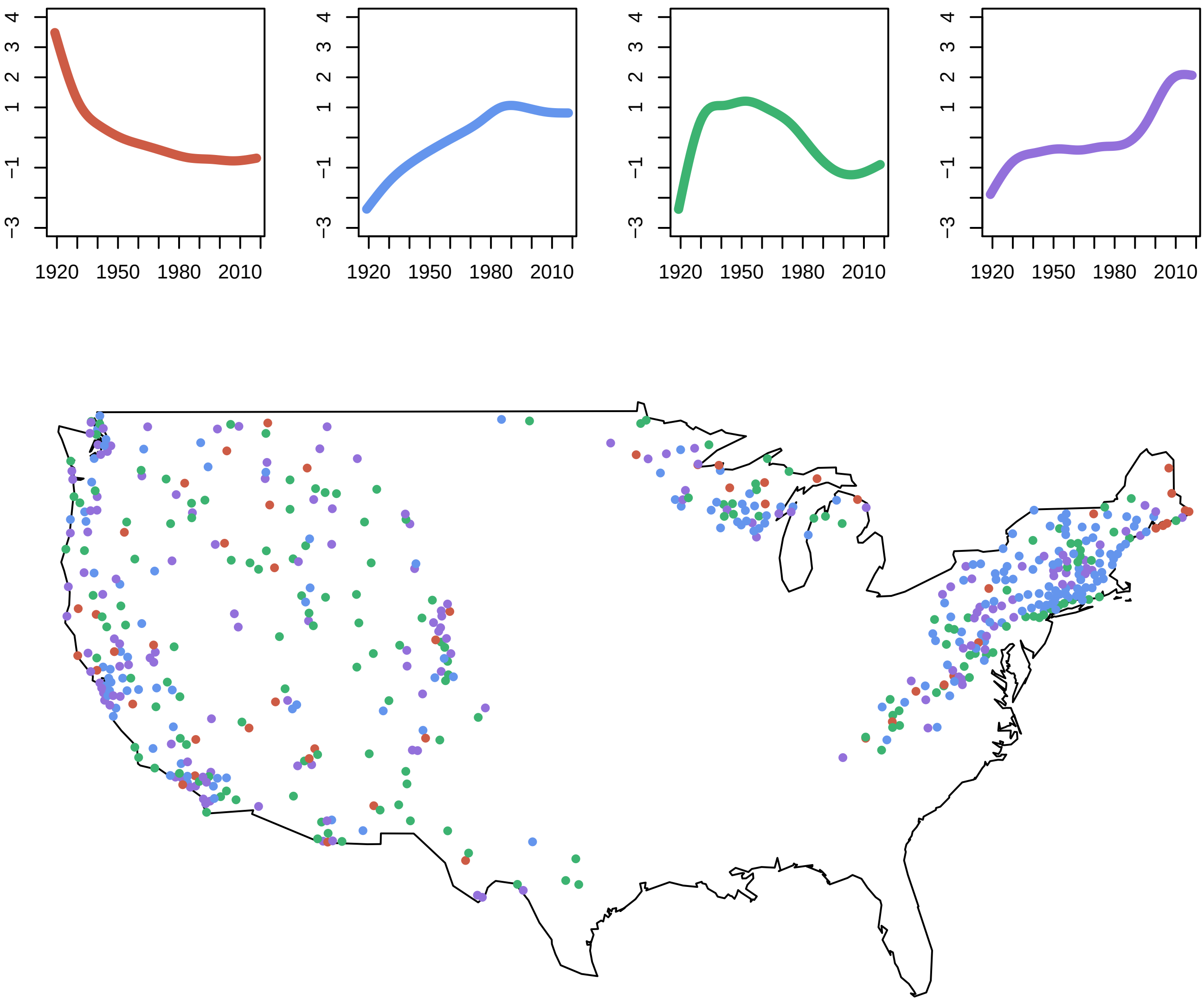

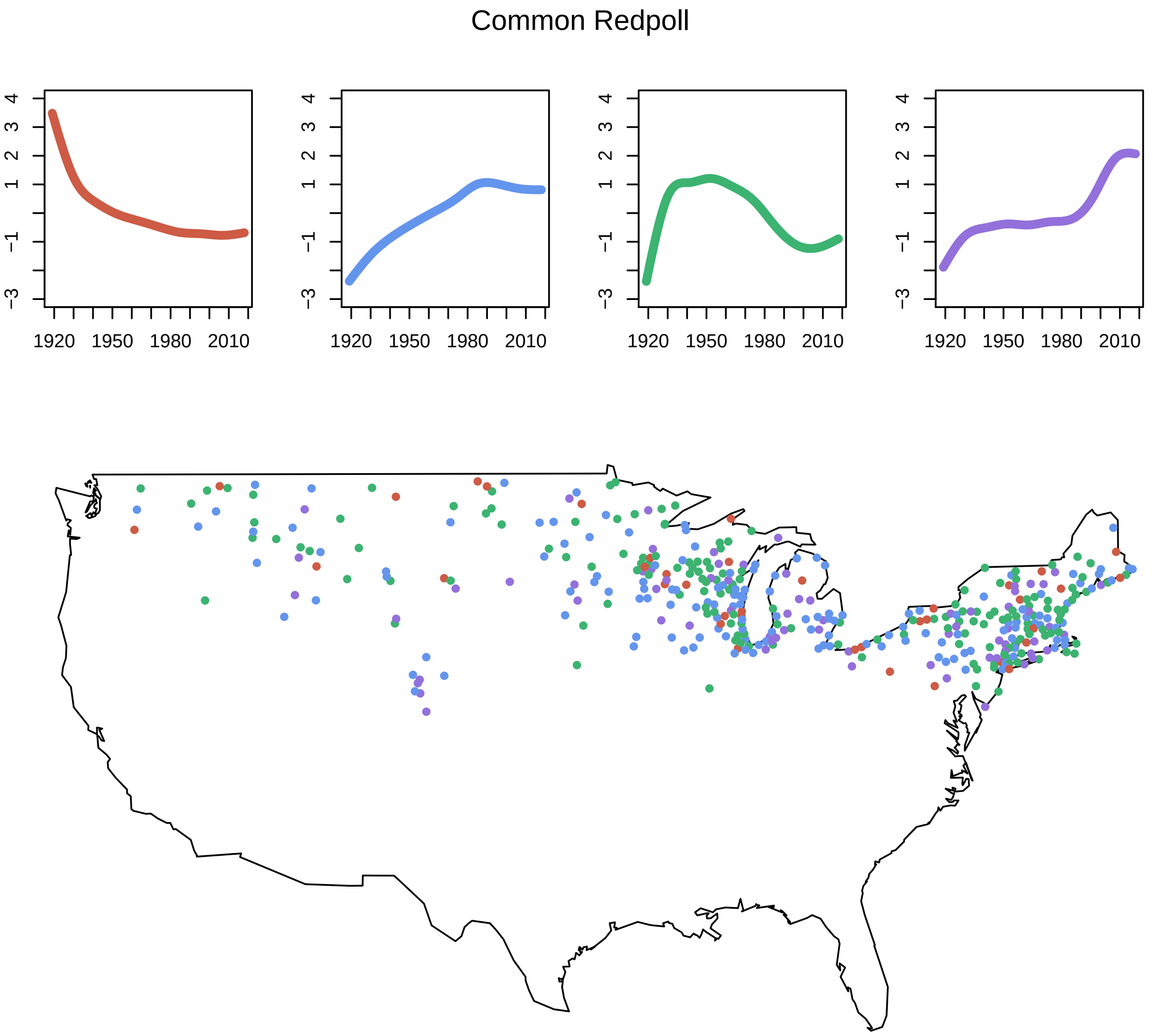
Common Redpoll
4
3
2
1
−1
−3
1920
1950
1980
2010

Eurasian Collared−Dove

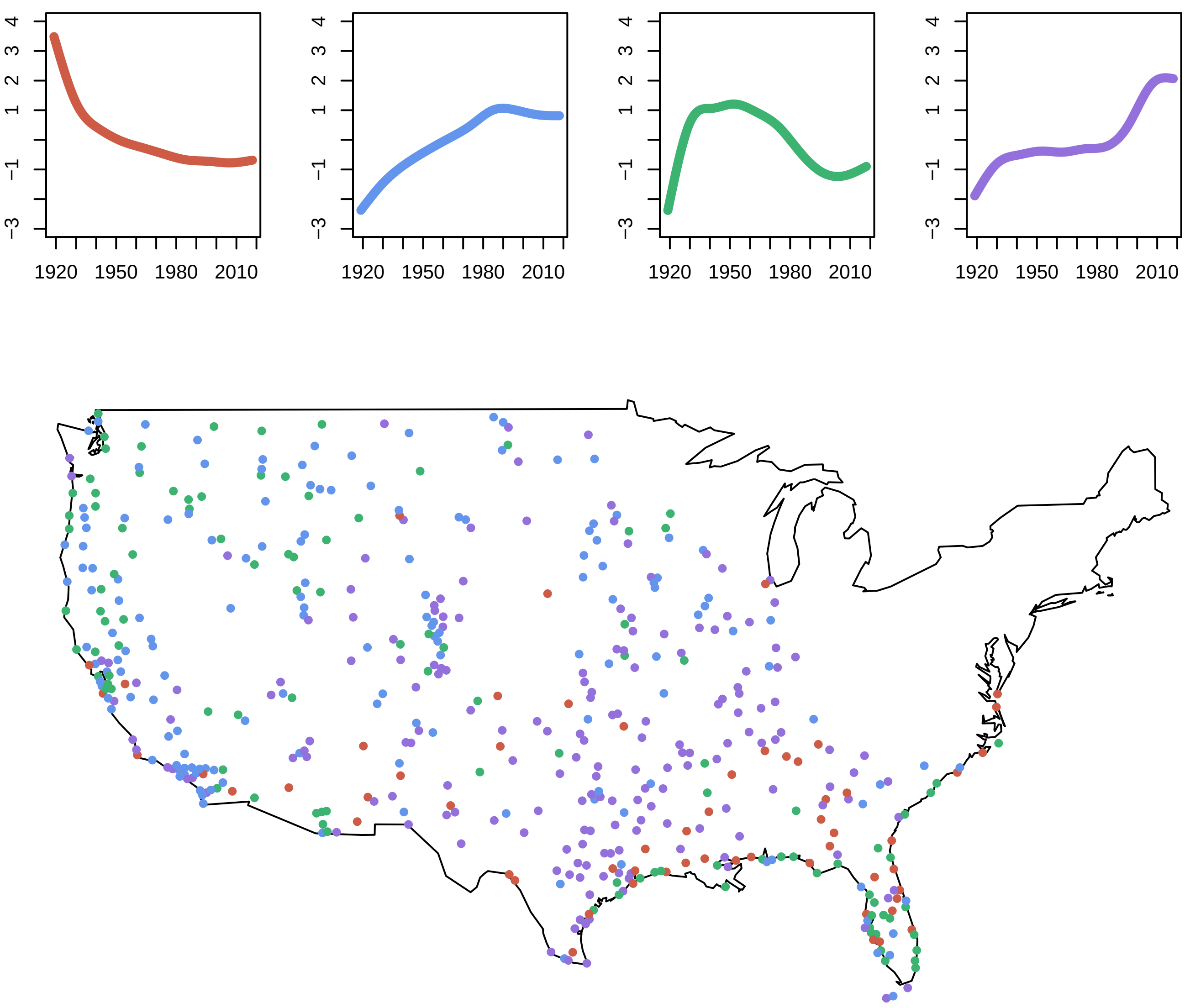

Evening Grosbeak

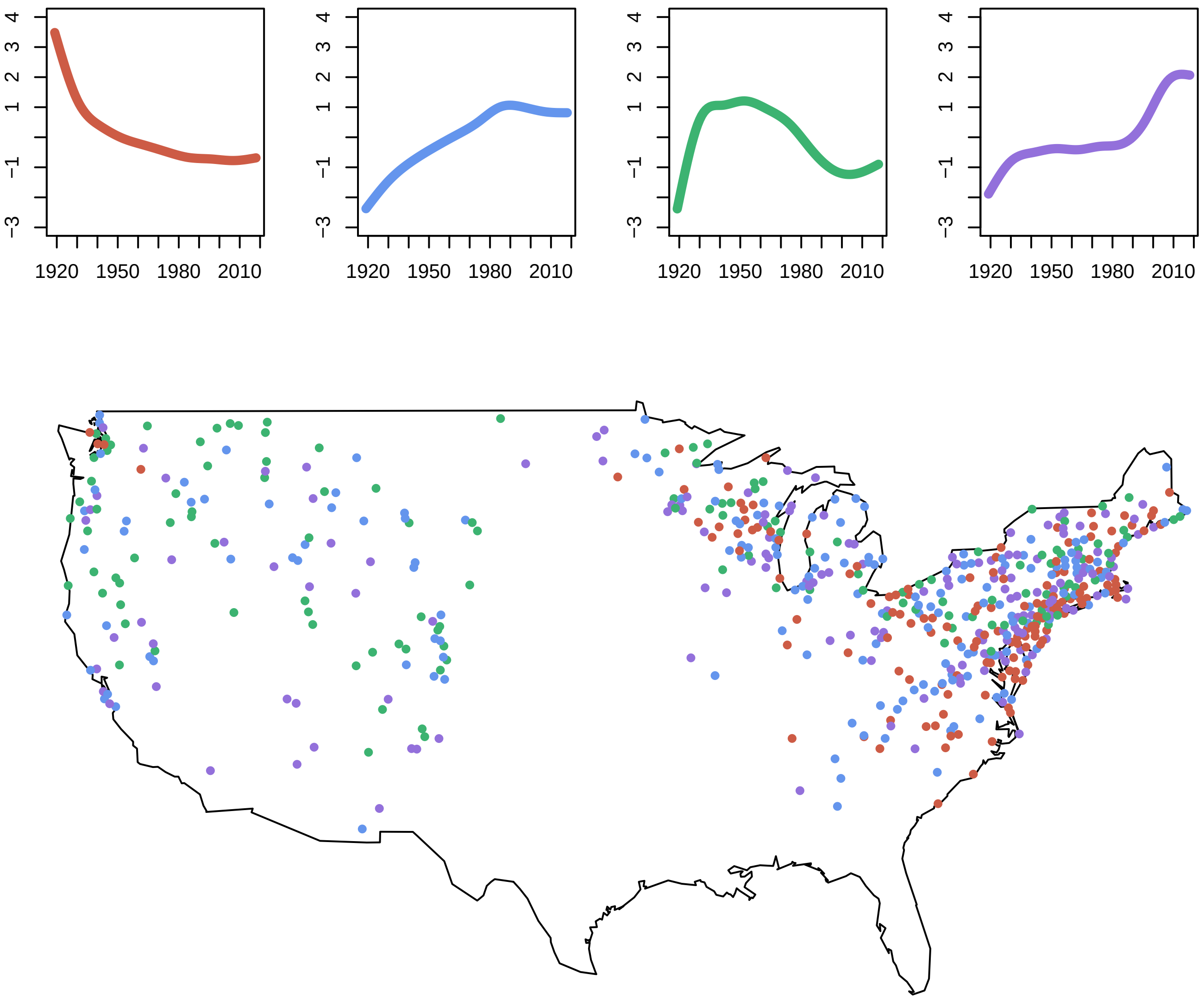

Field Sparrow

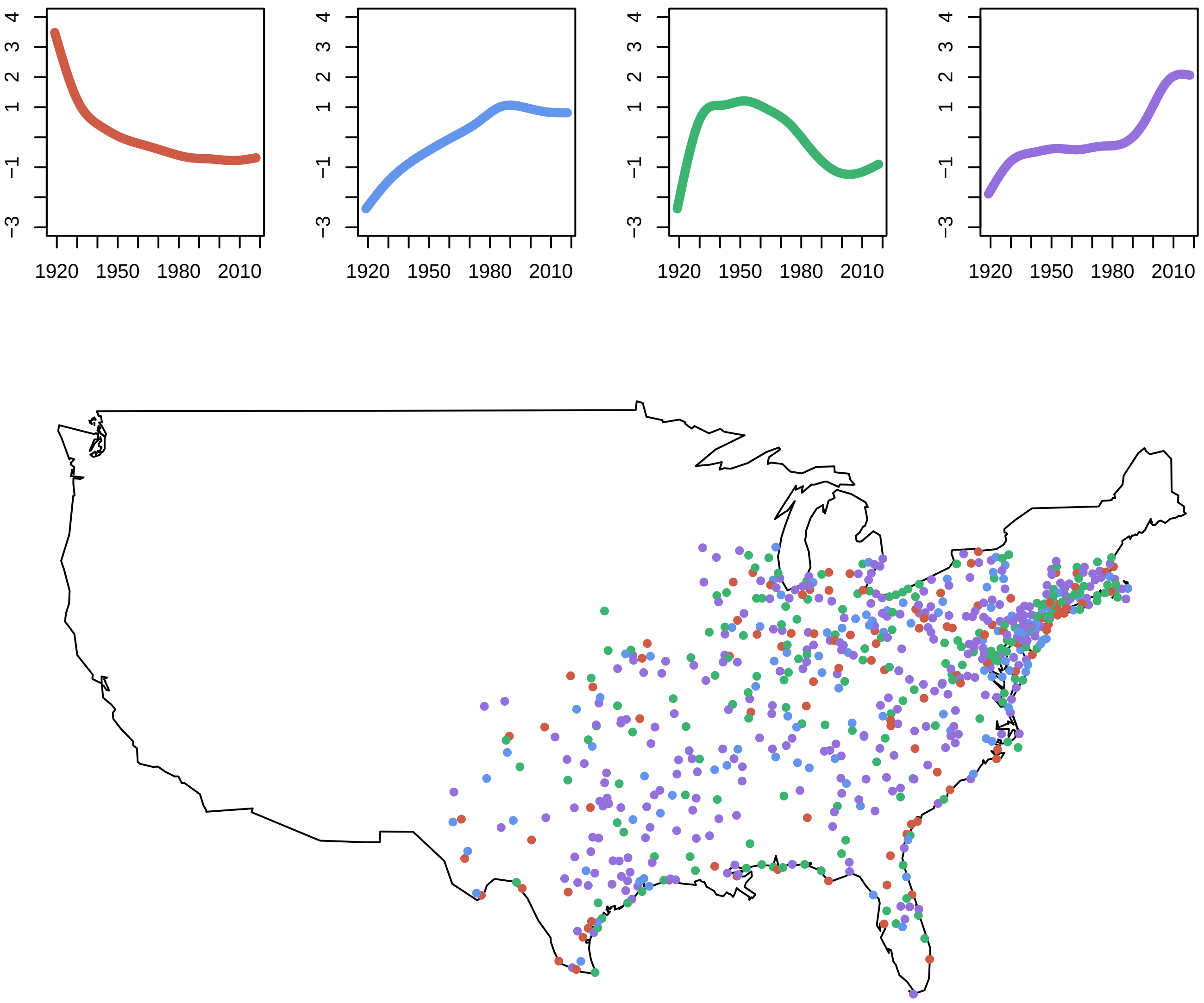

Forster's Tern

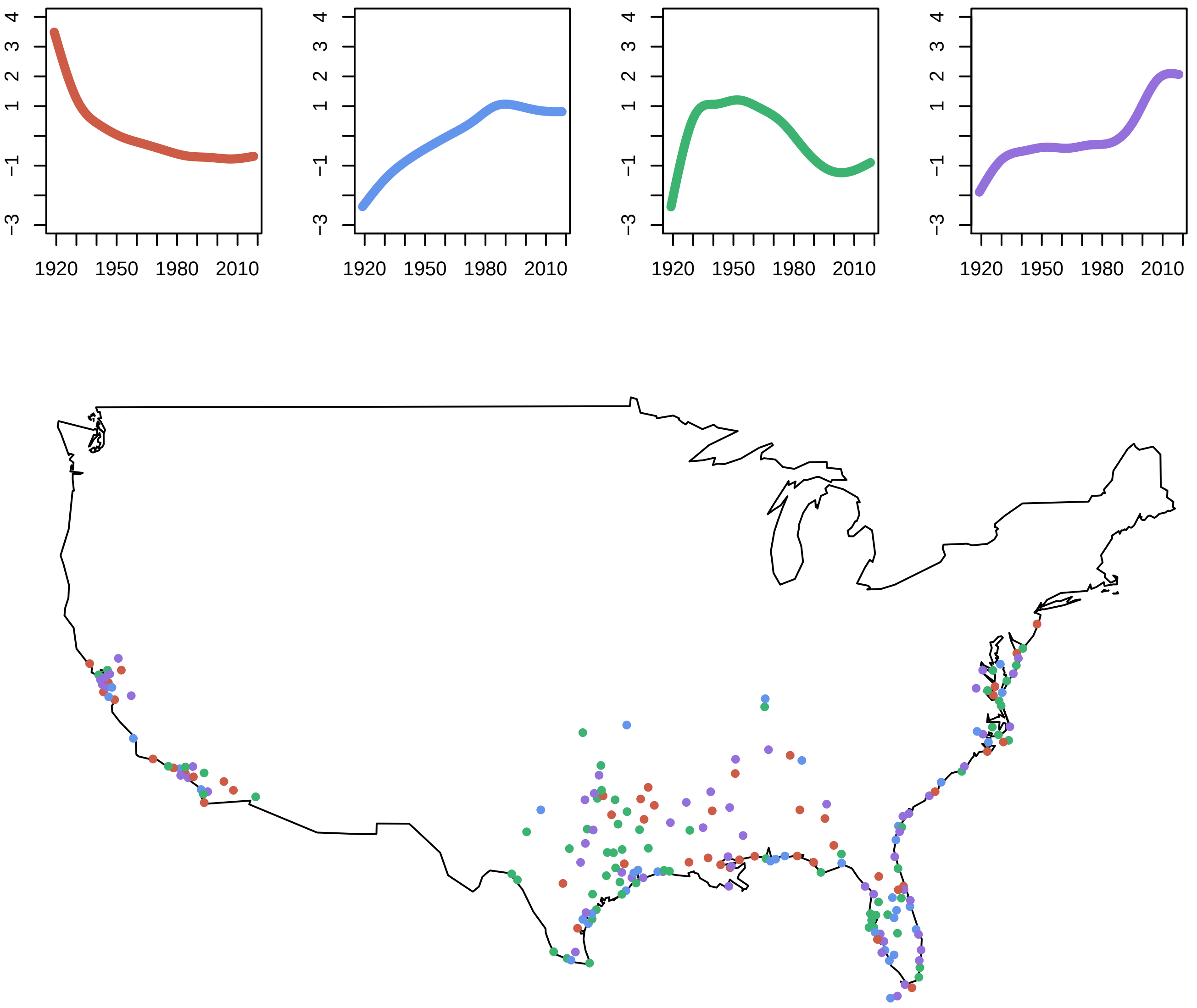

Golden−crowned Kinglet

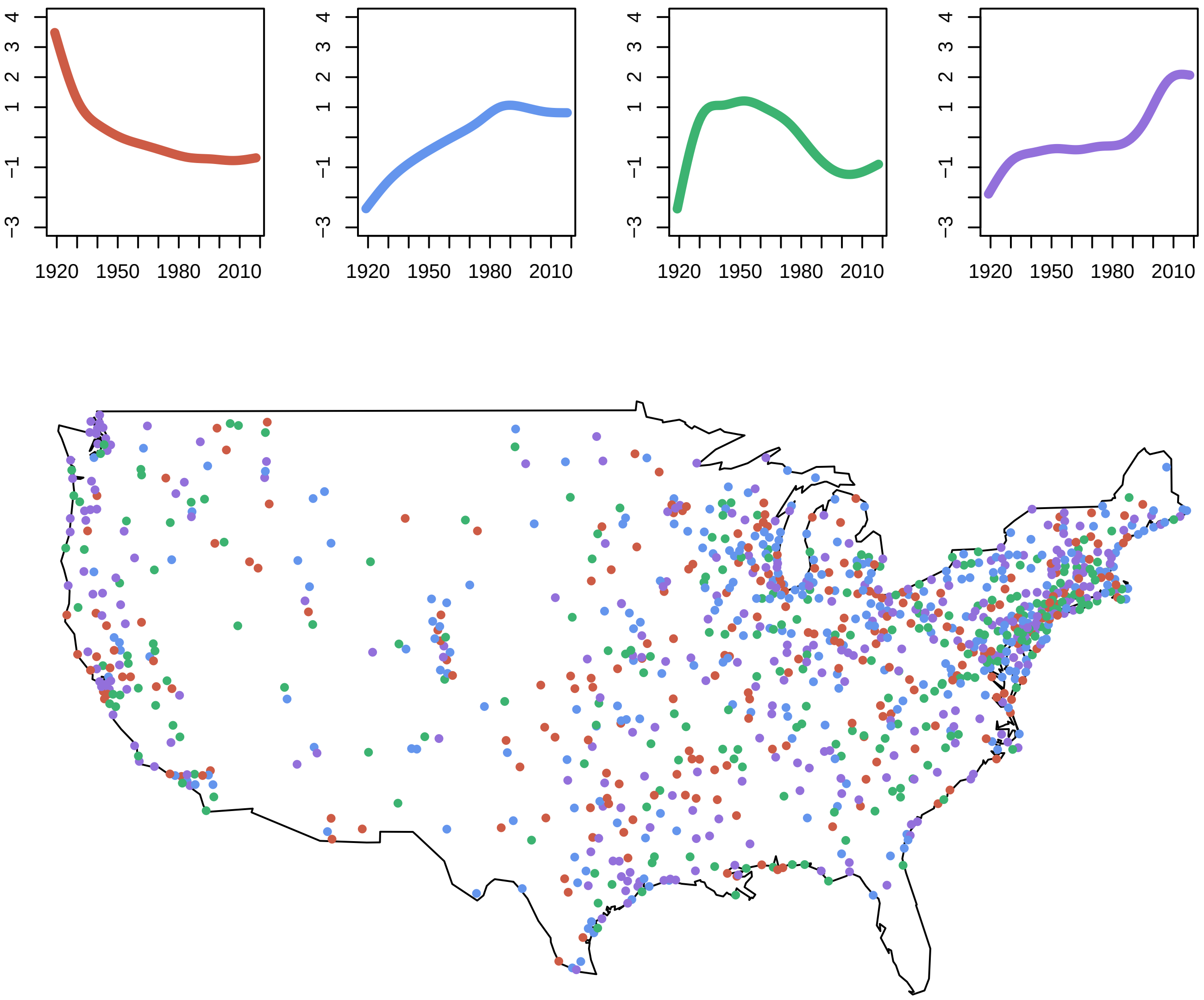

Great Blue Heron

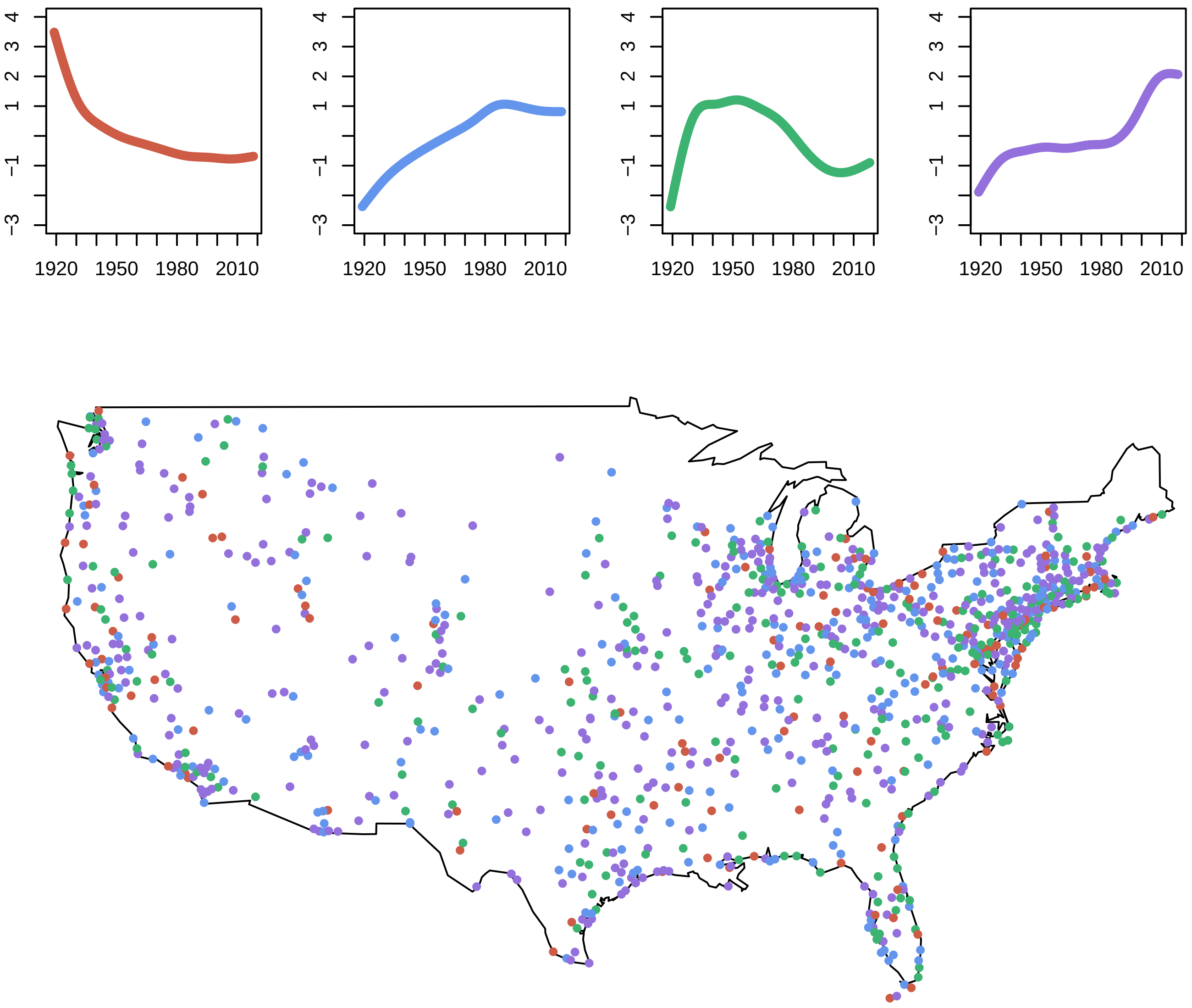

Hooded Merganser

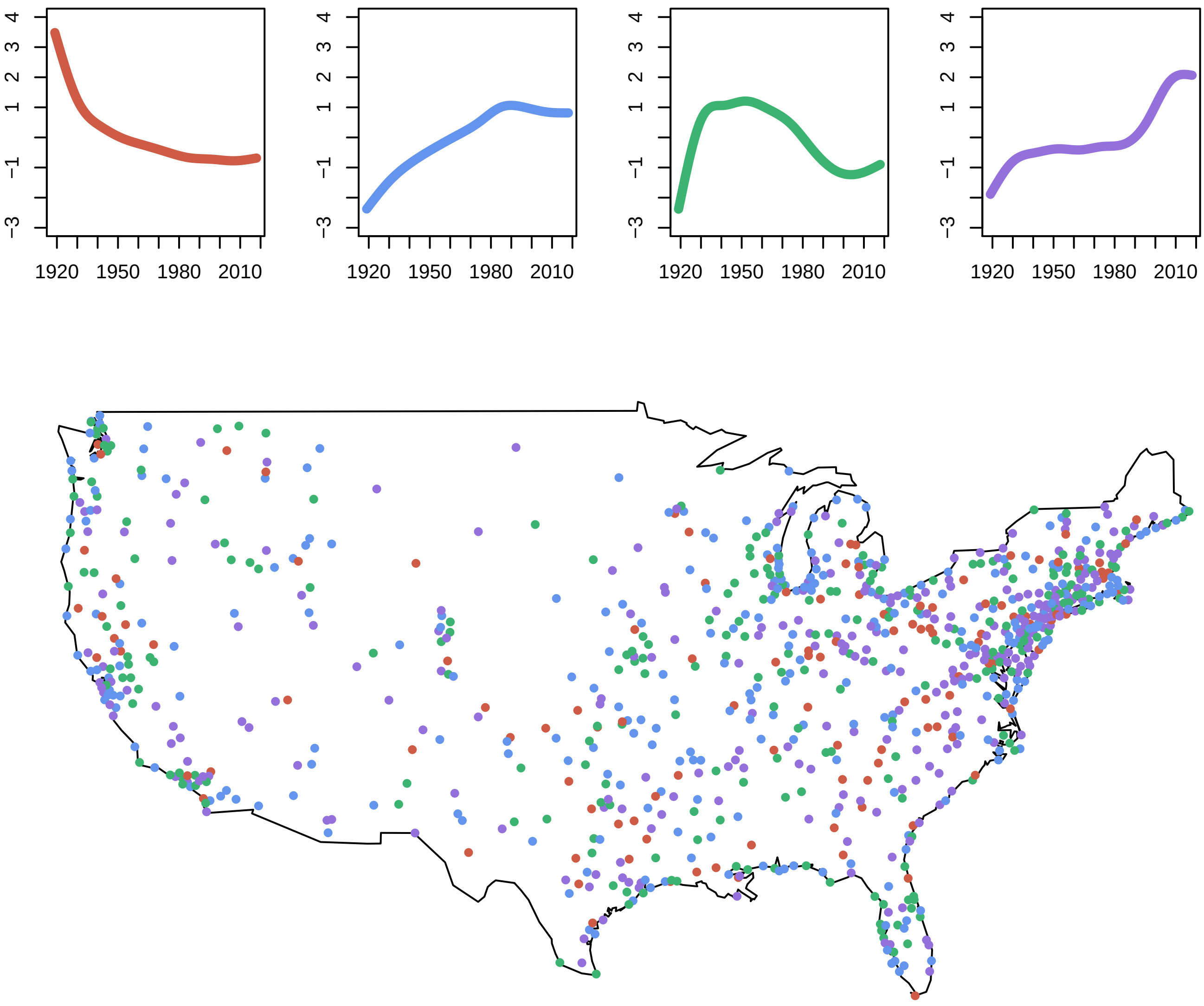

Lapland Longspur

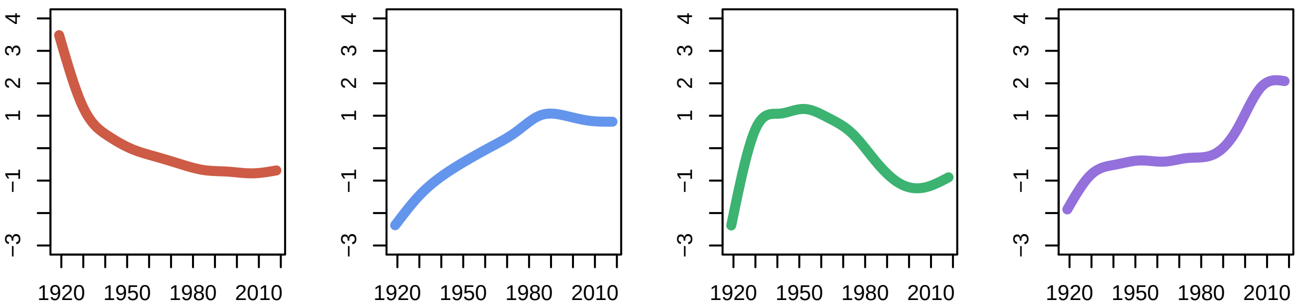

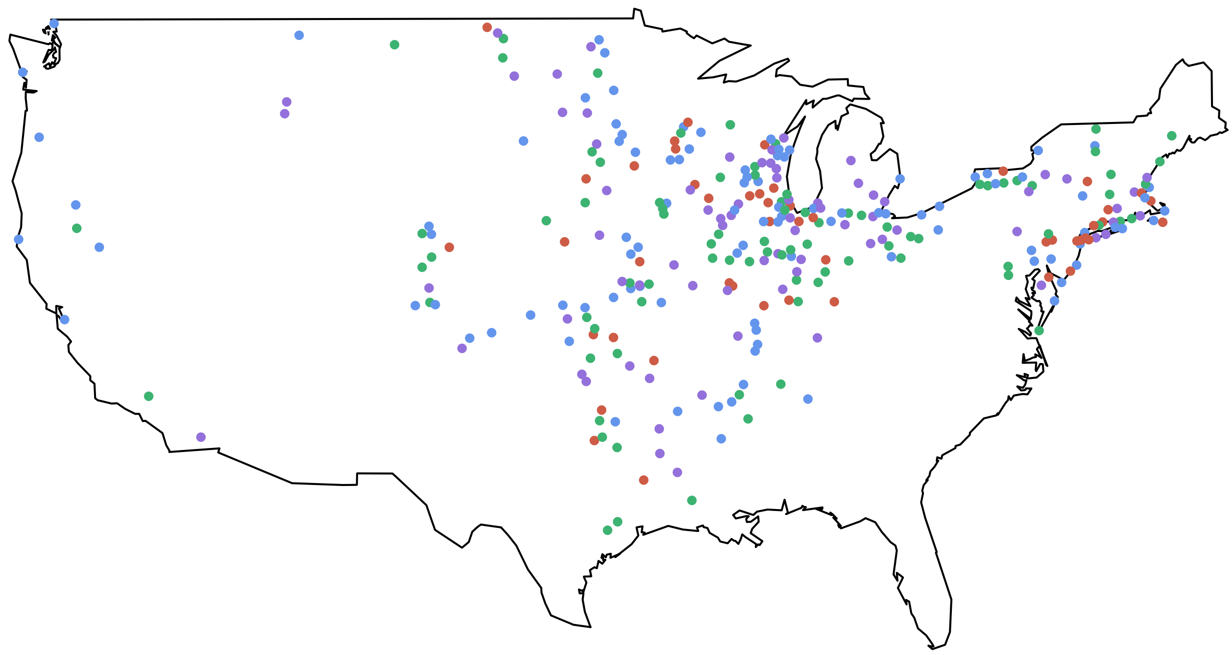

Lesser Goldfinch

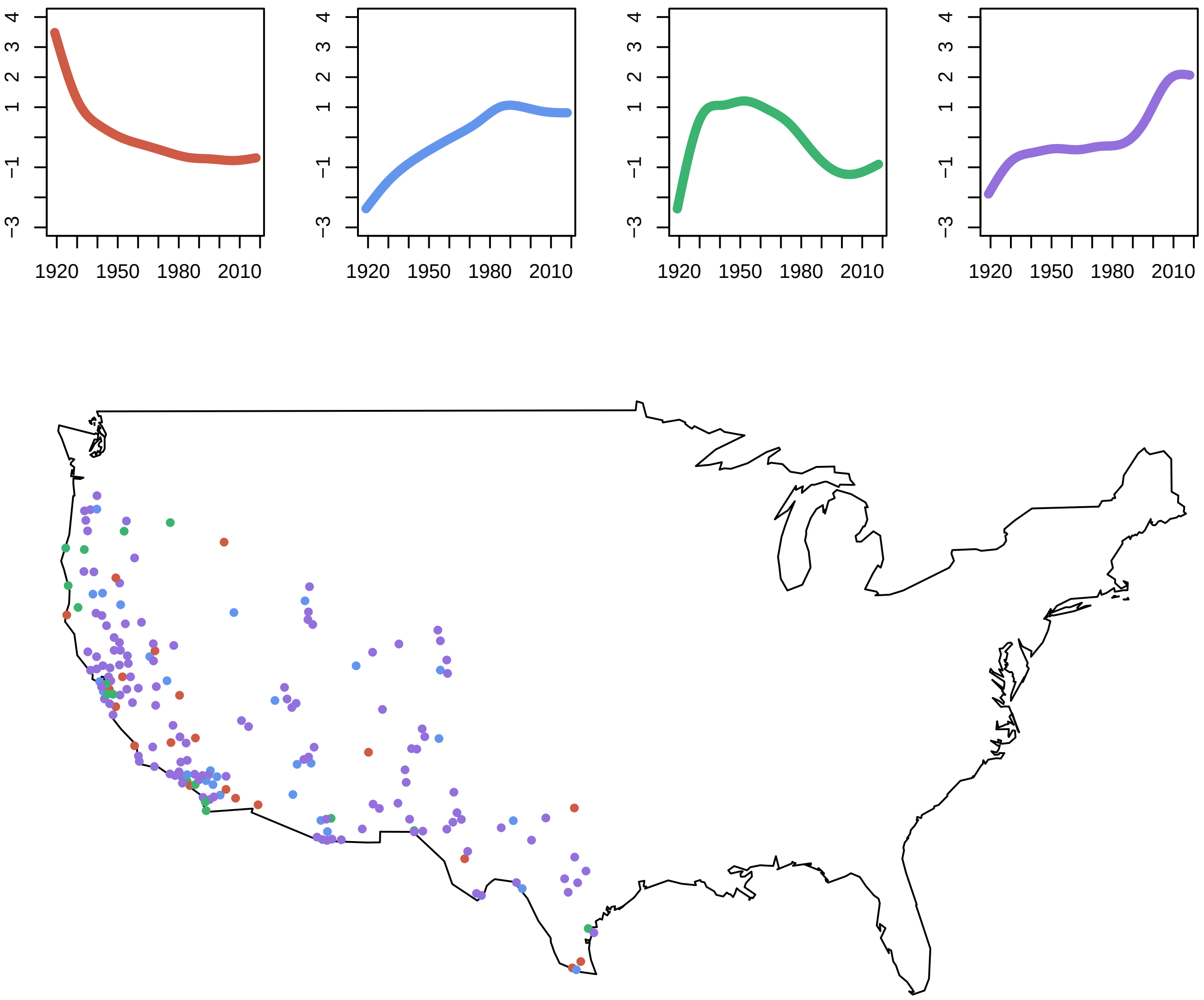

Long−billed Dowitcher

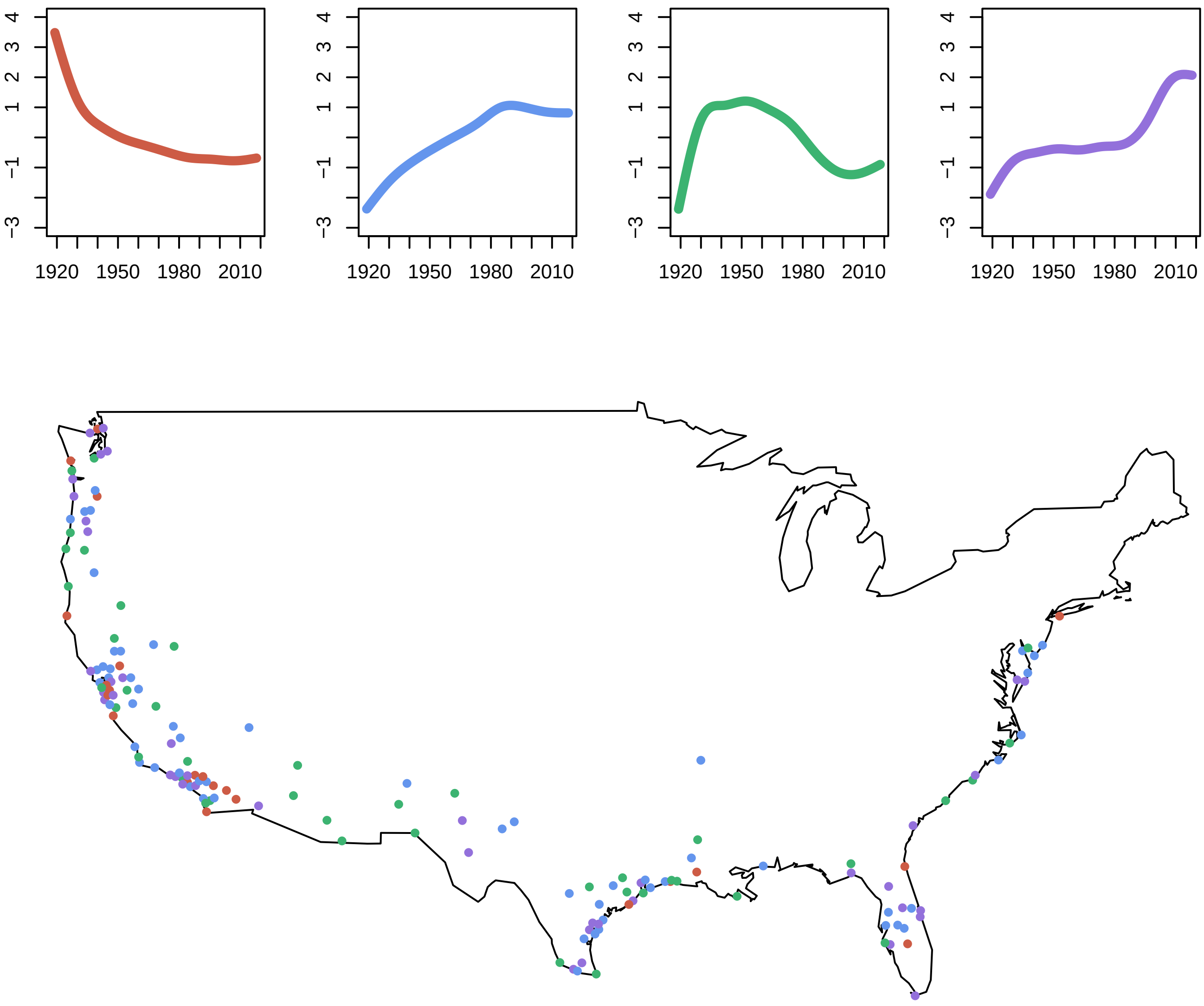

Marbled Godwit

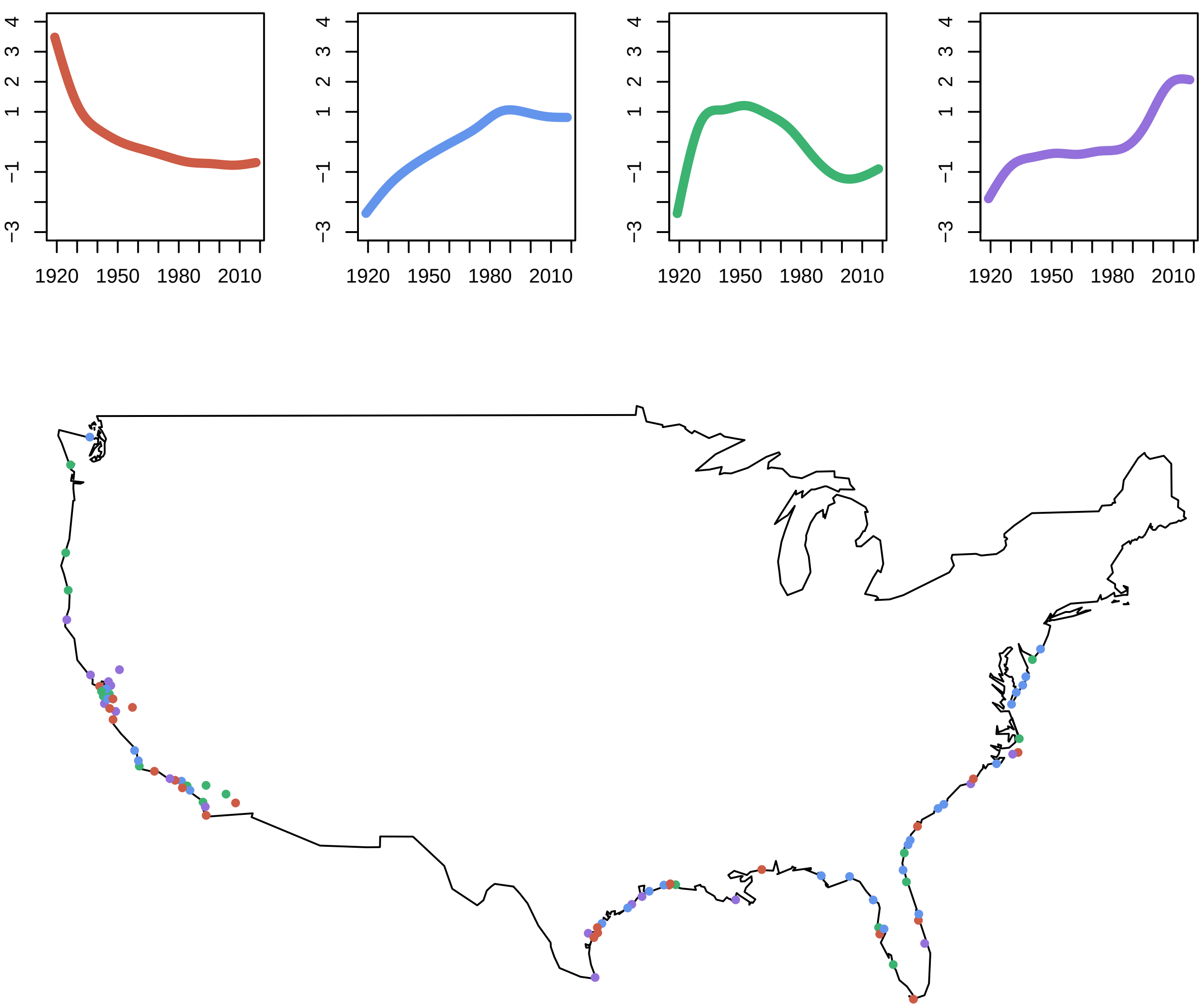

Pied−billed Grebe

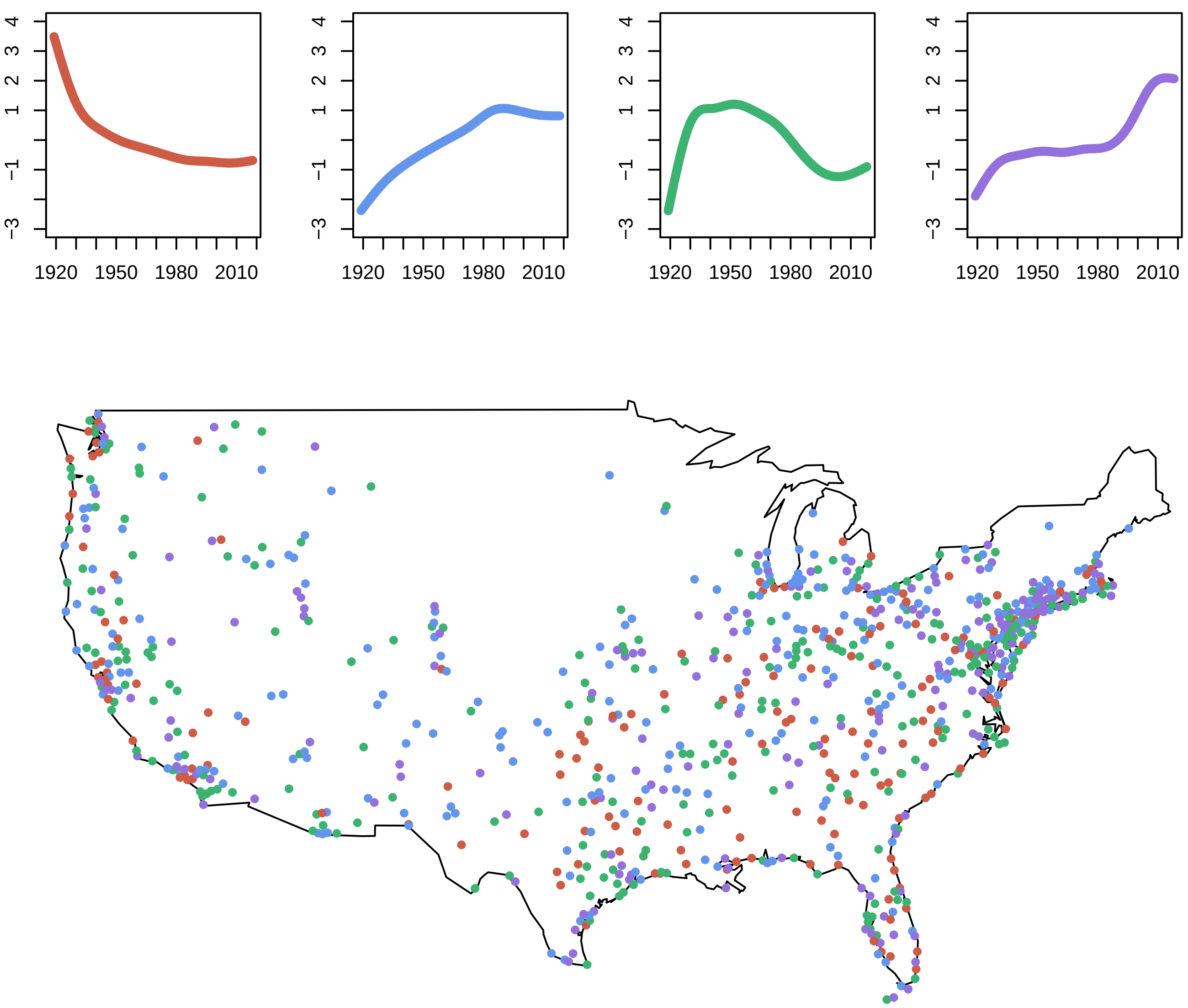

Purple Finch

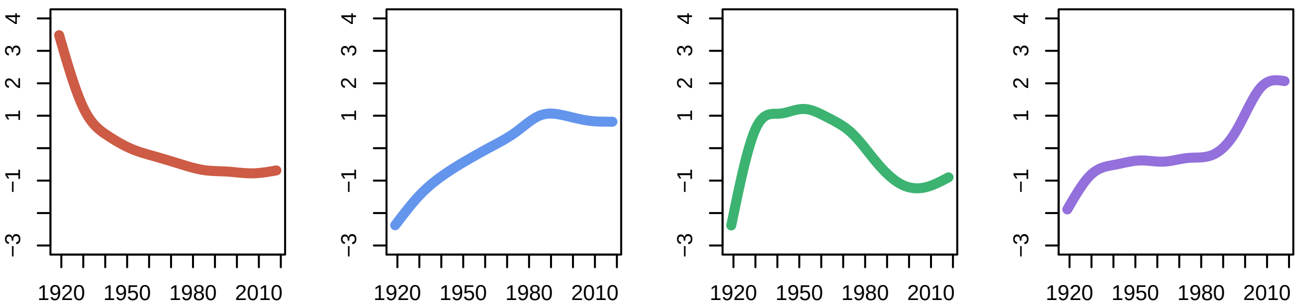

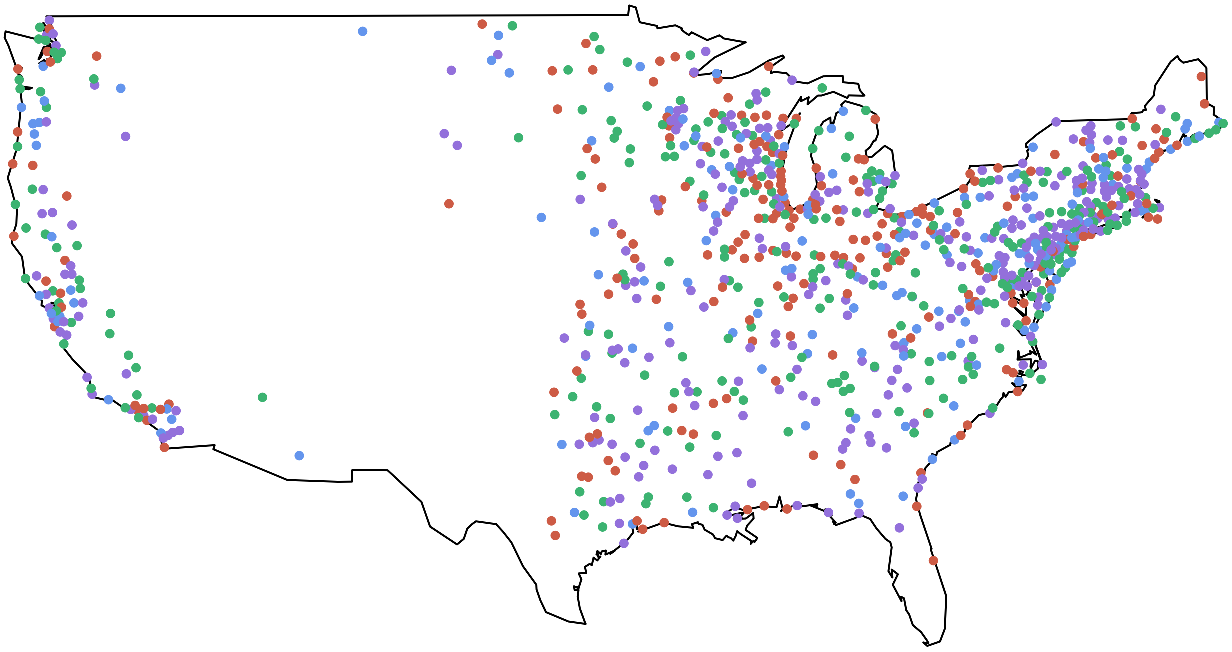

Red−bellied Woodpecker

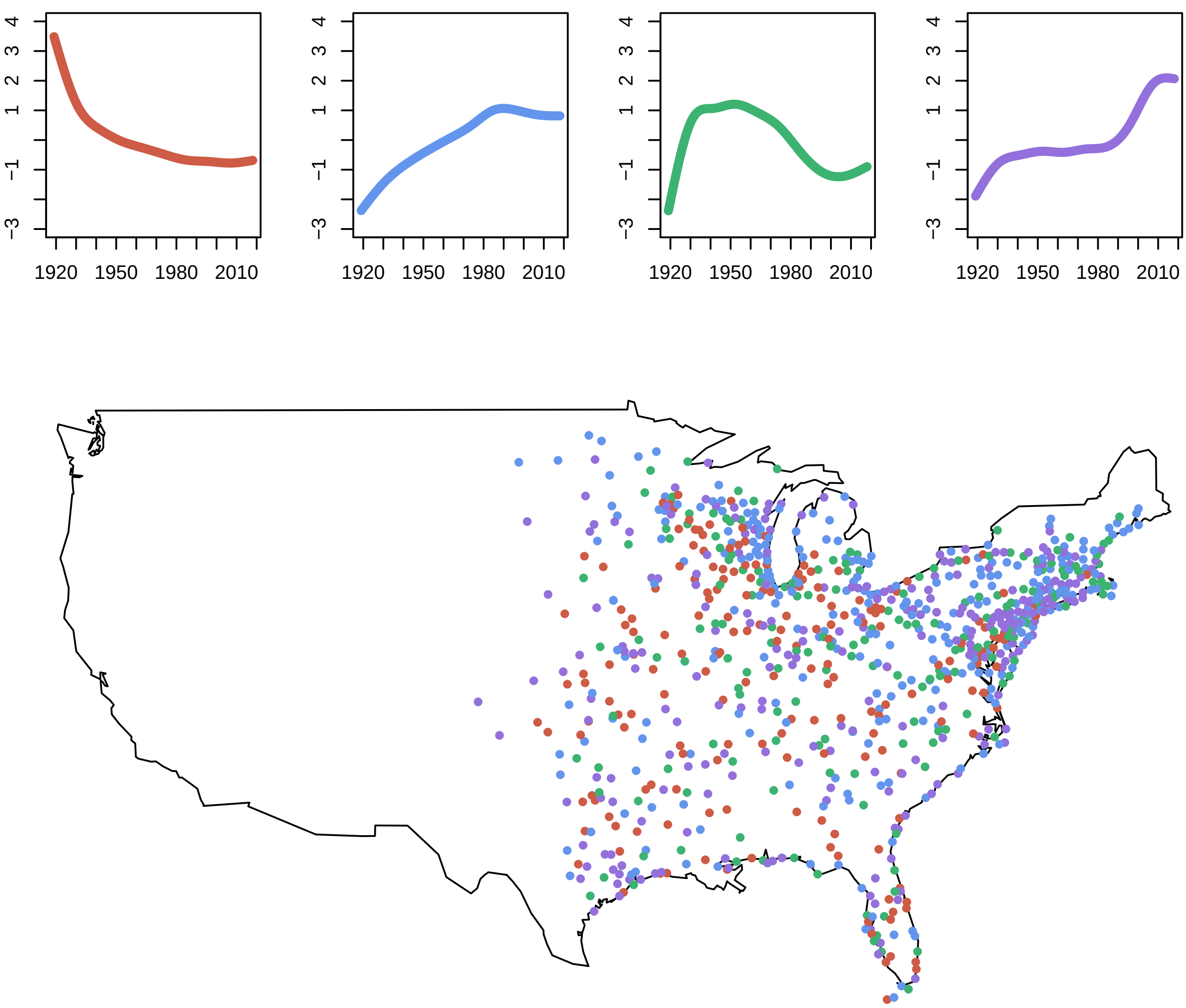

Red−tailed Hawk

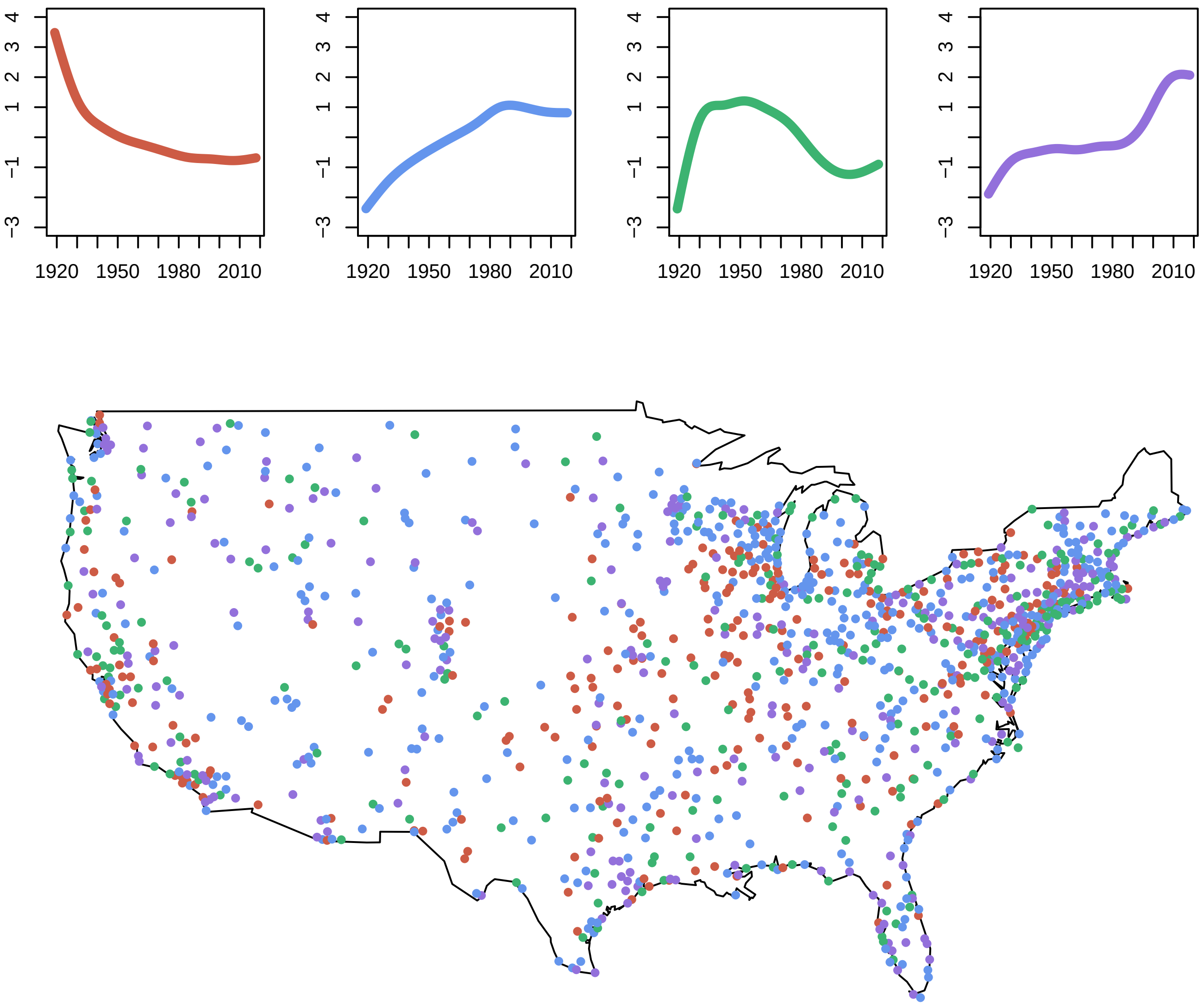

Ring−necked Pheasant

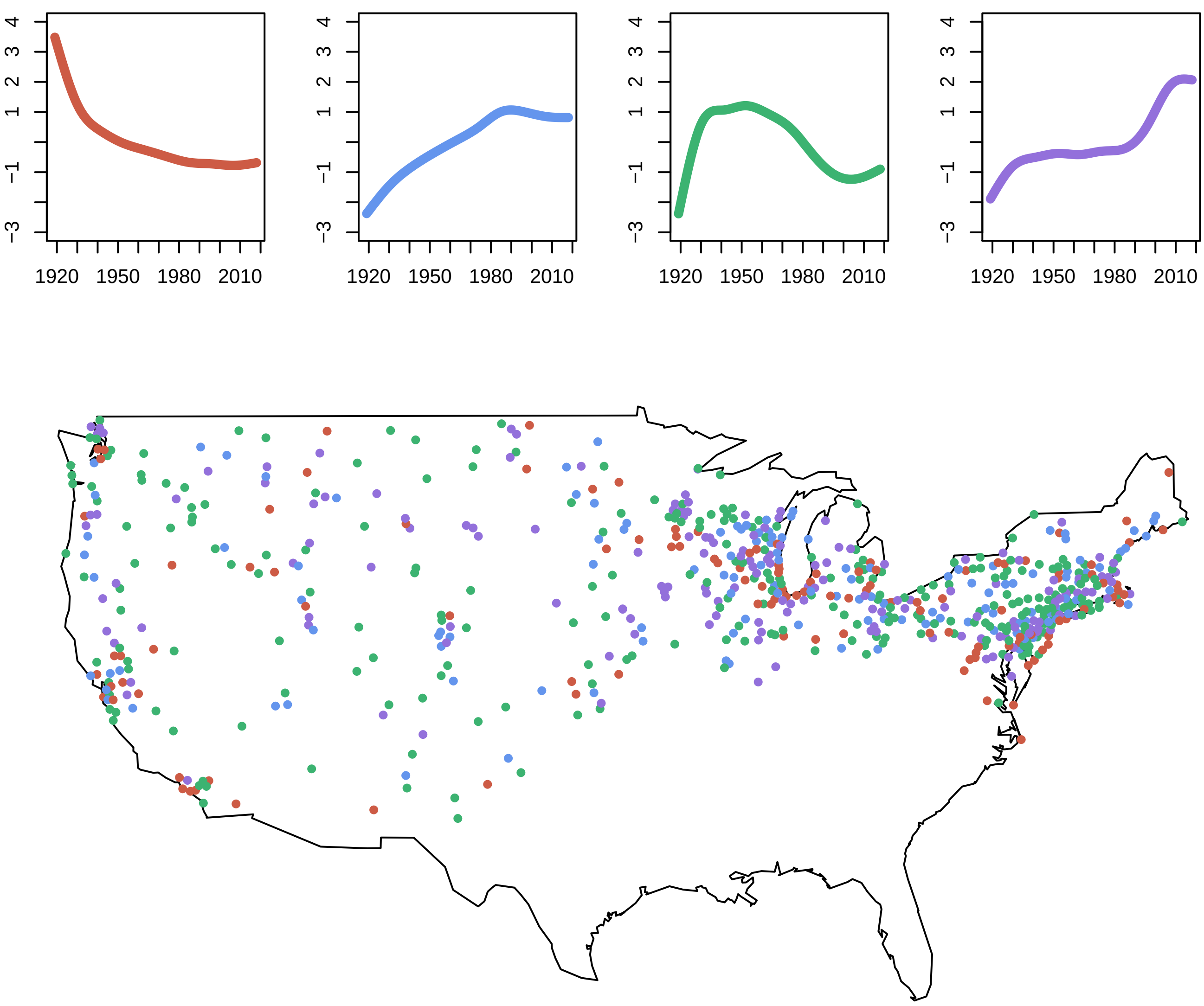

Ross's Goose

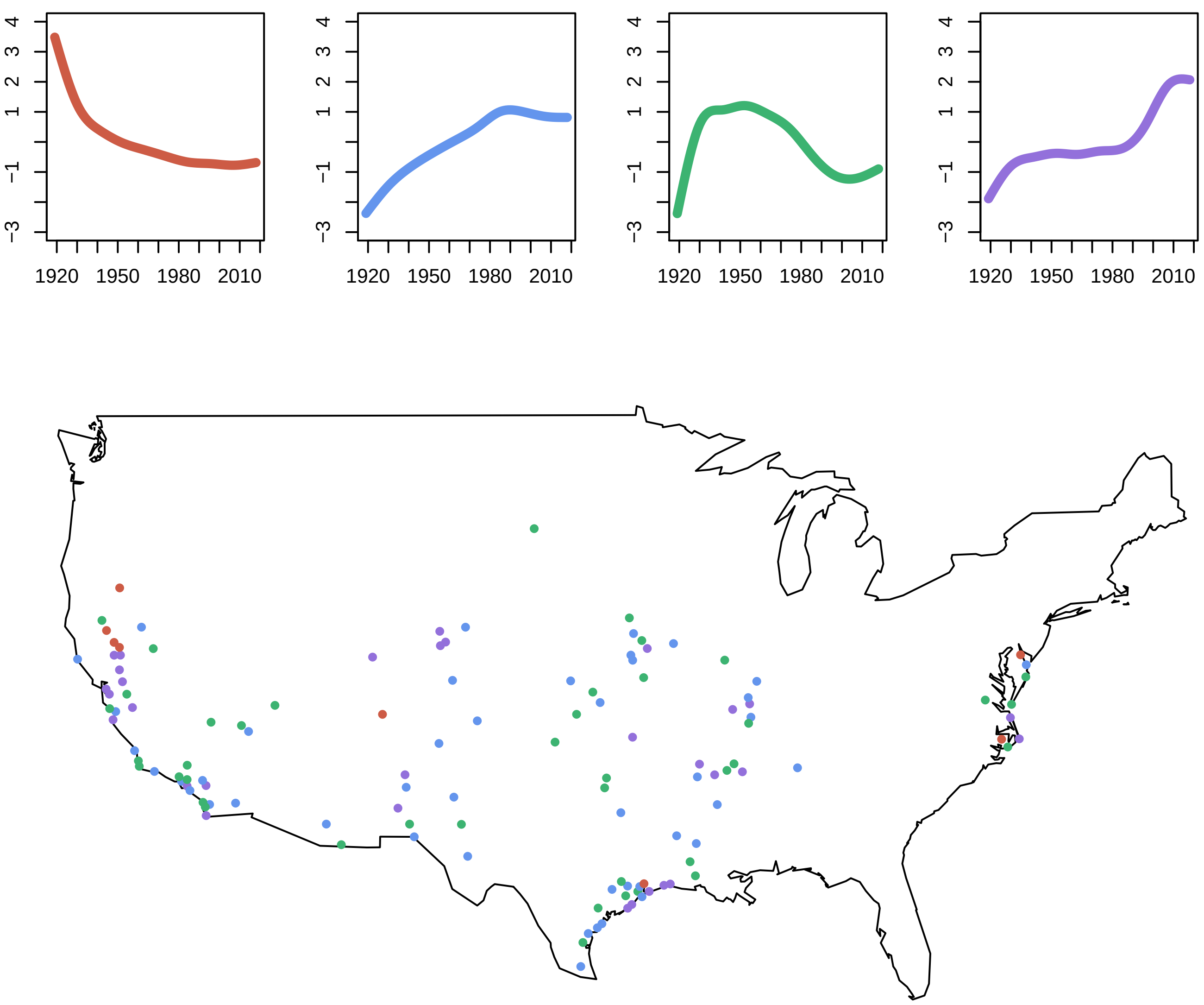

Short−billed Dowitcher

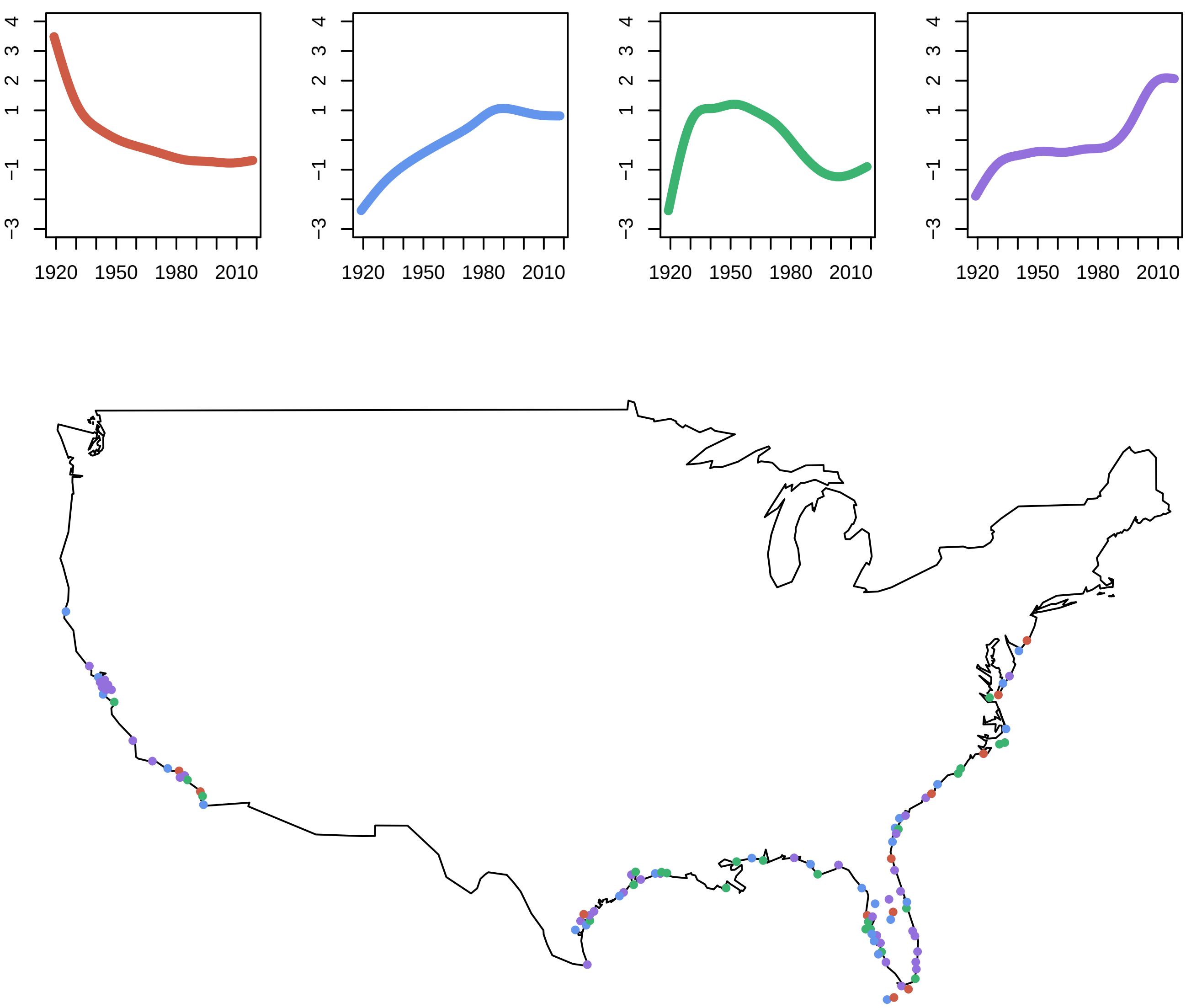

Snow Bunting

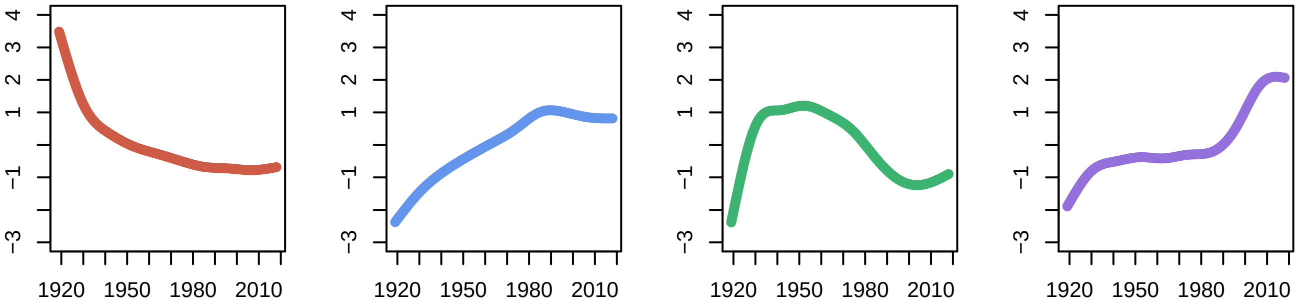

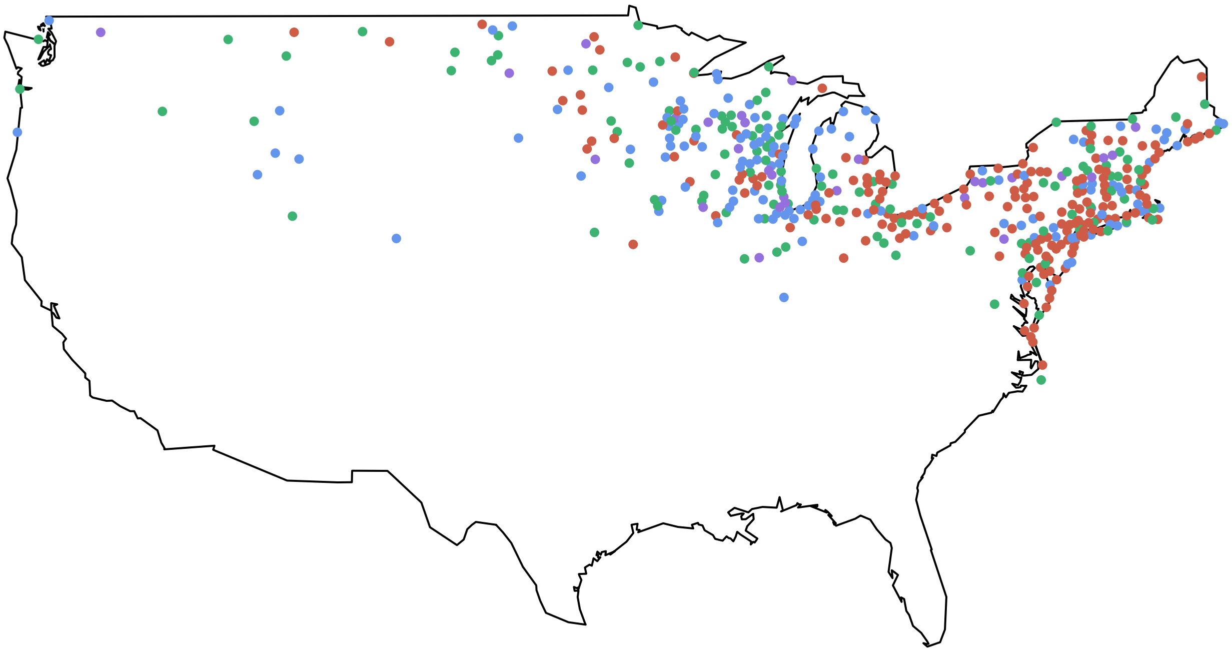

Snowy Egret

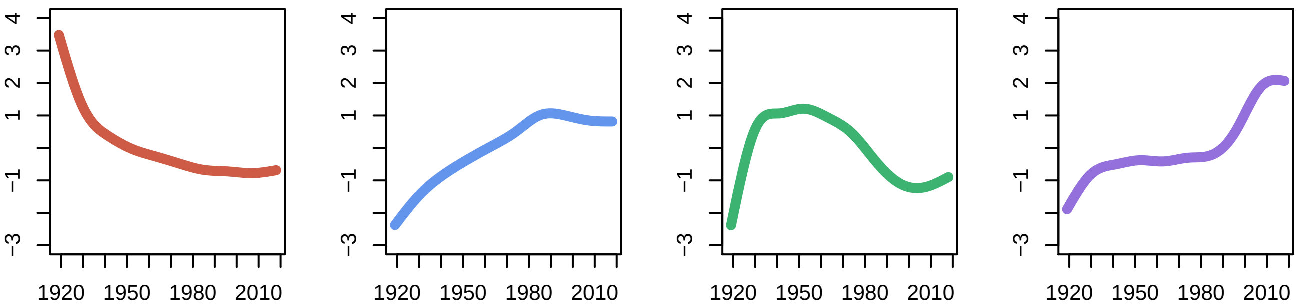

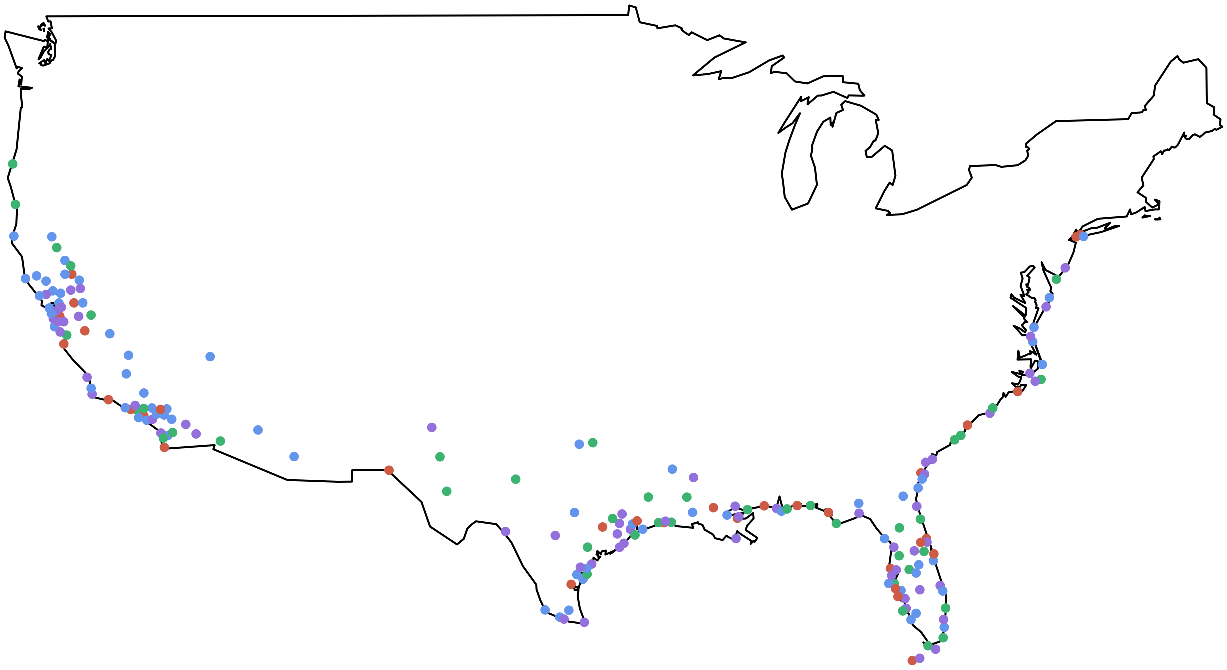

Swamp Sparrow

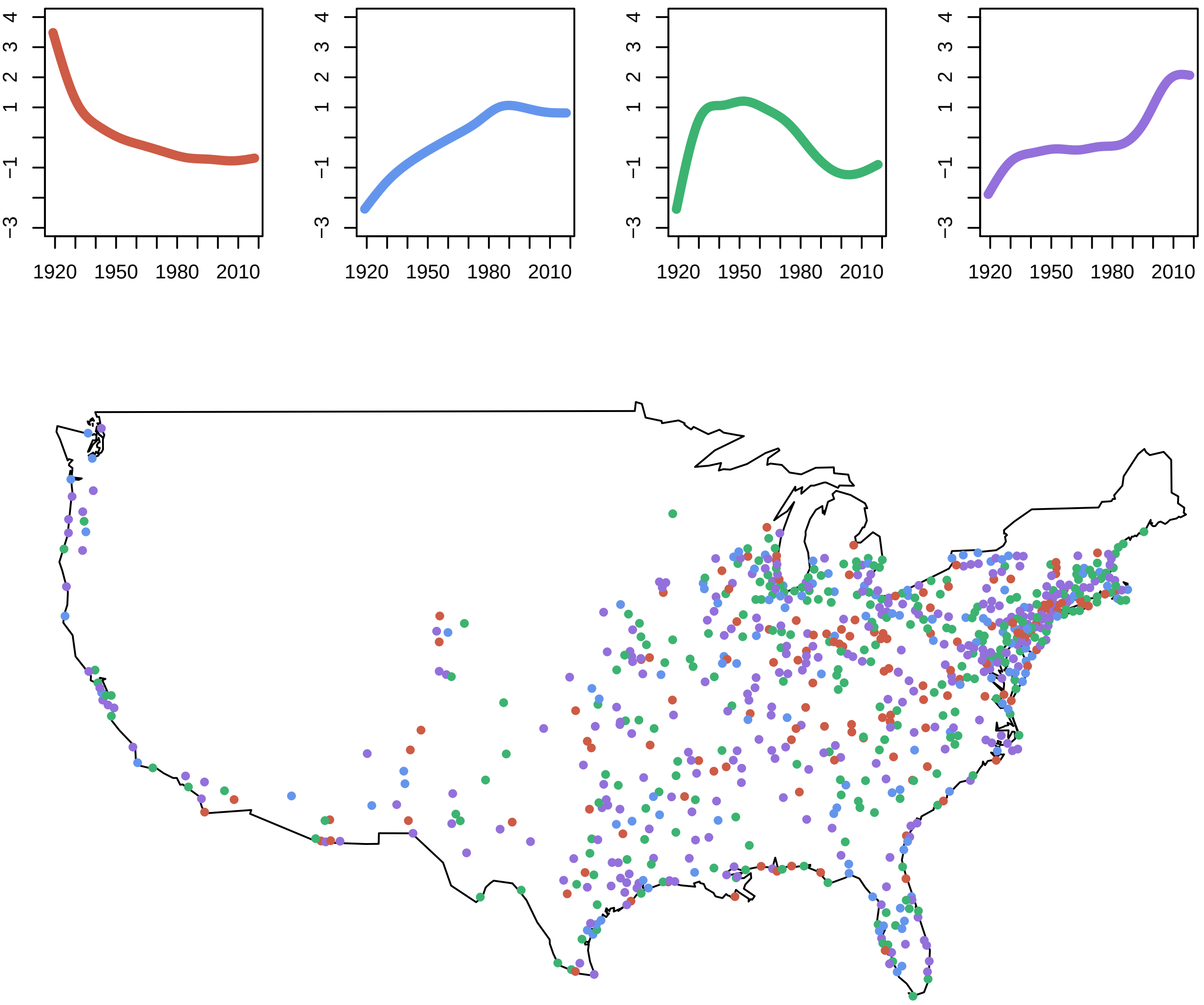

Tricolored Blackbird

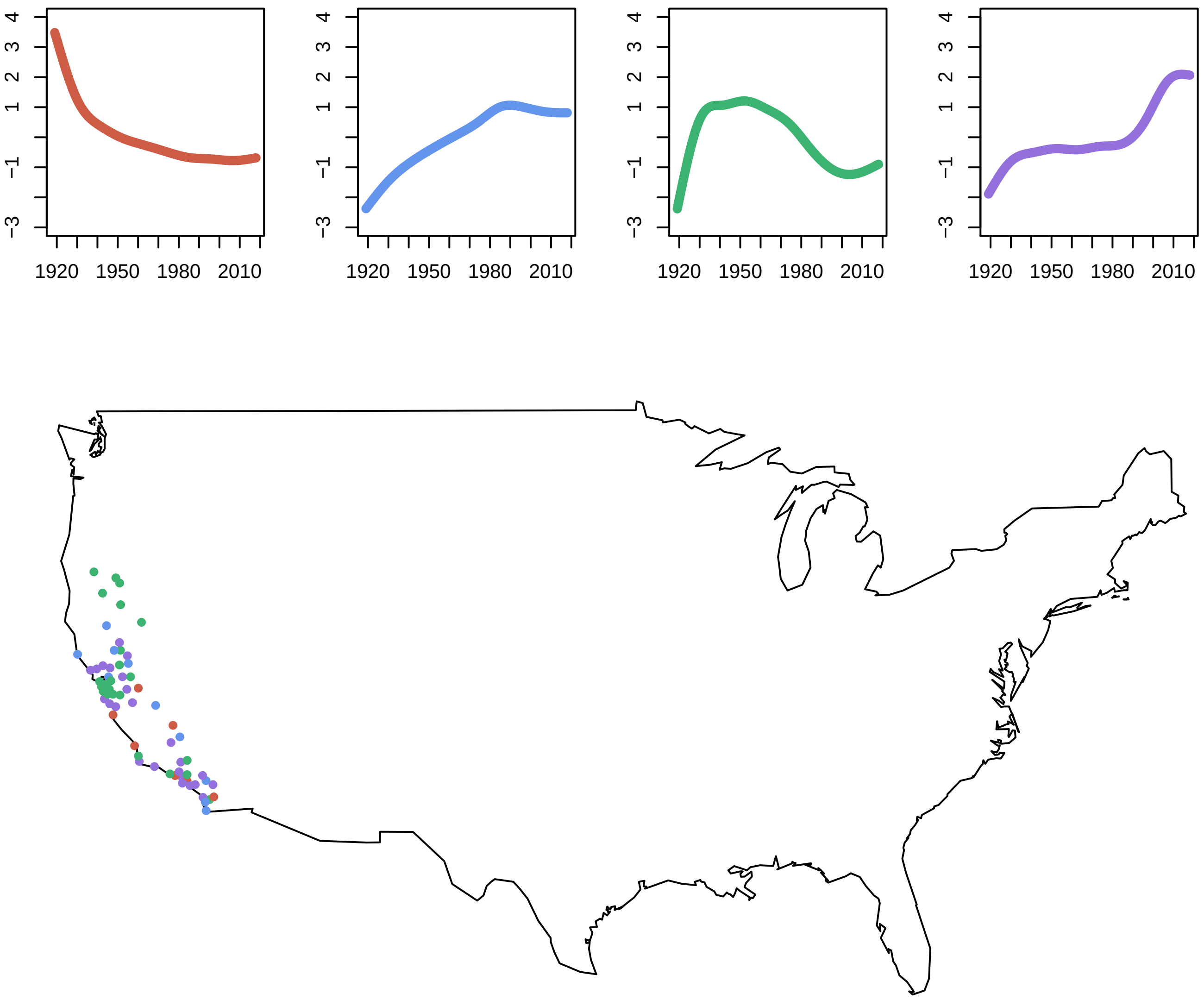

White−winged Dove

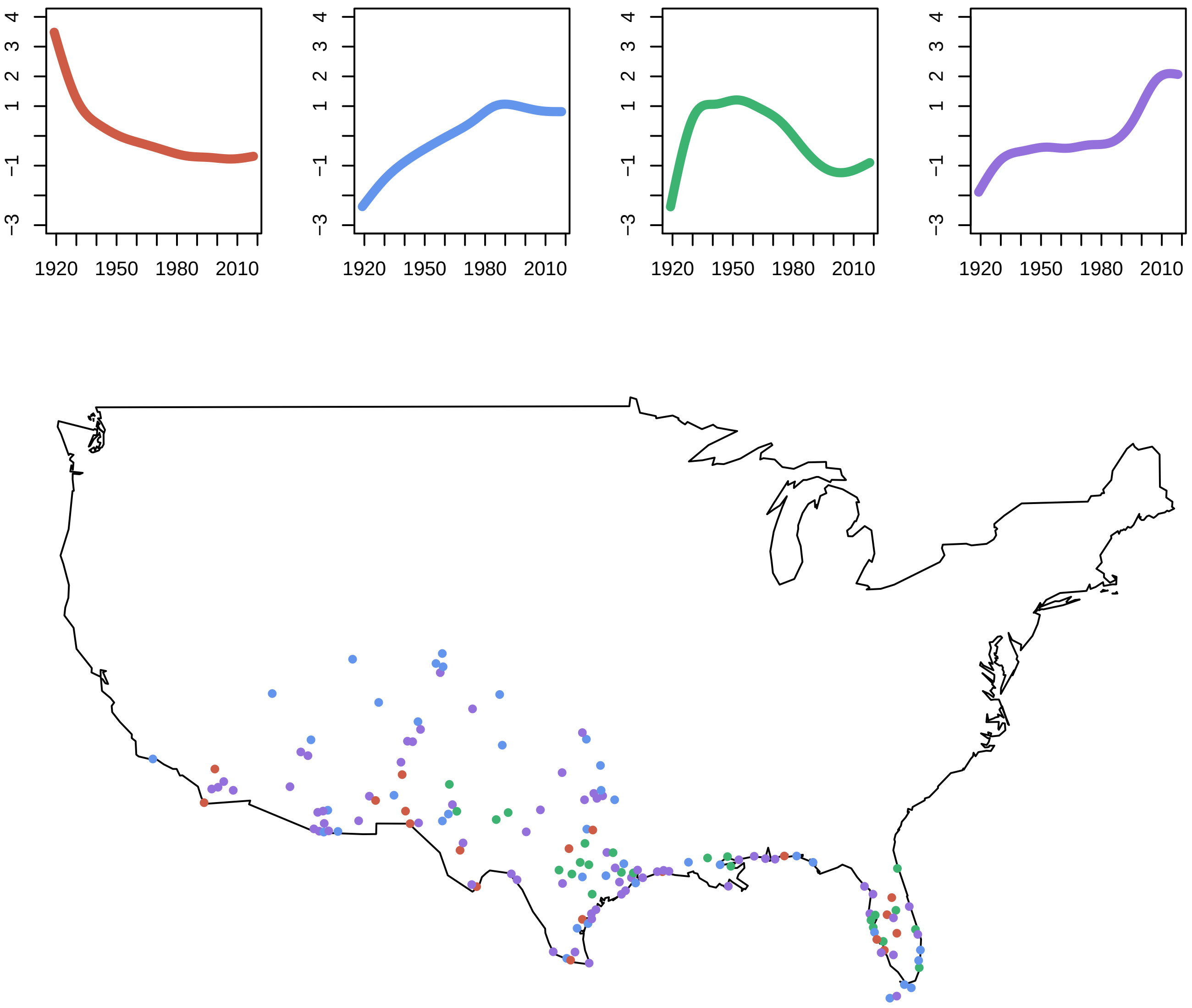

White−winged Scoter

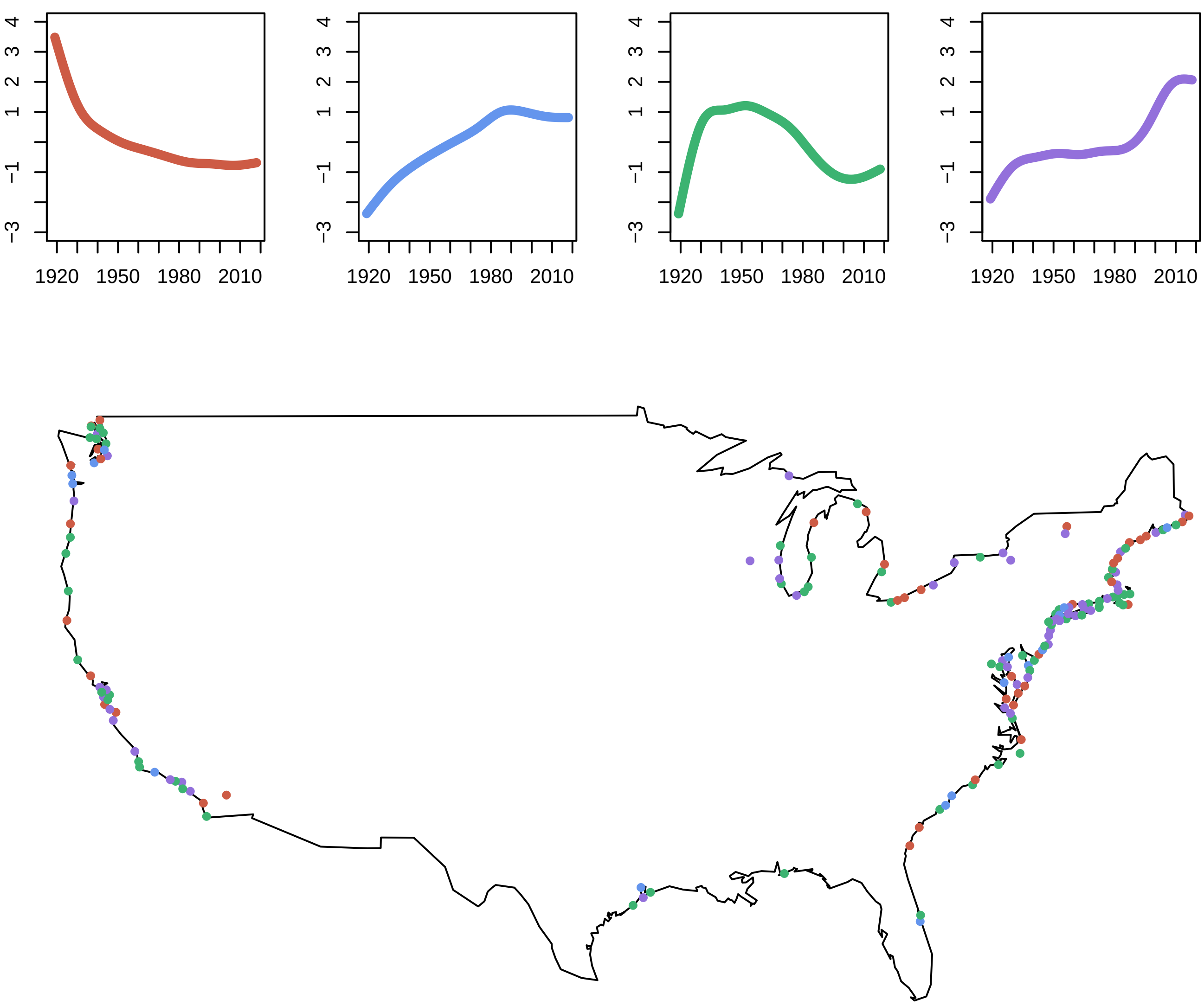

Wild Turkey

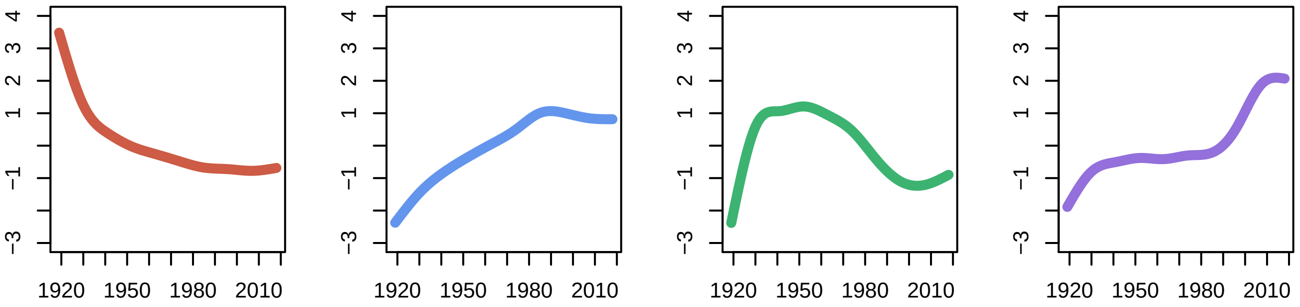

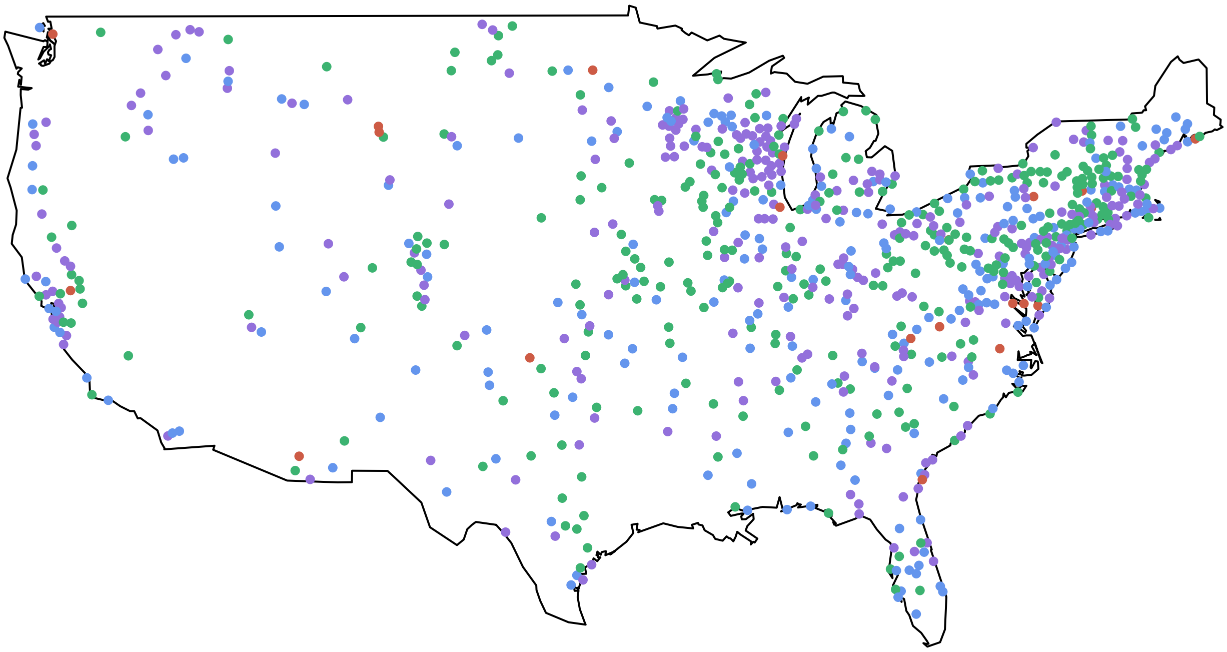

Acorn Woodpecker

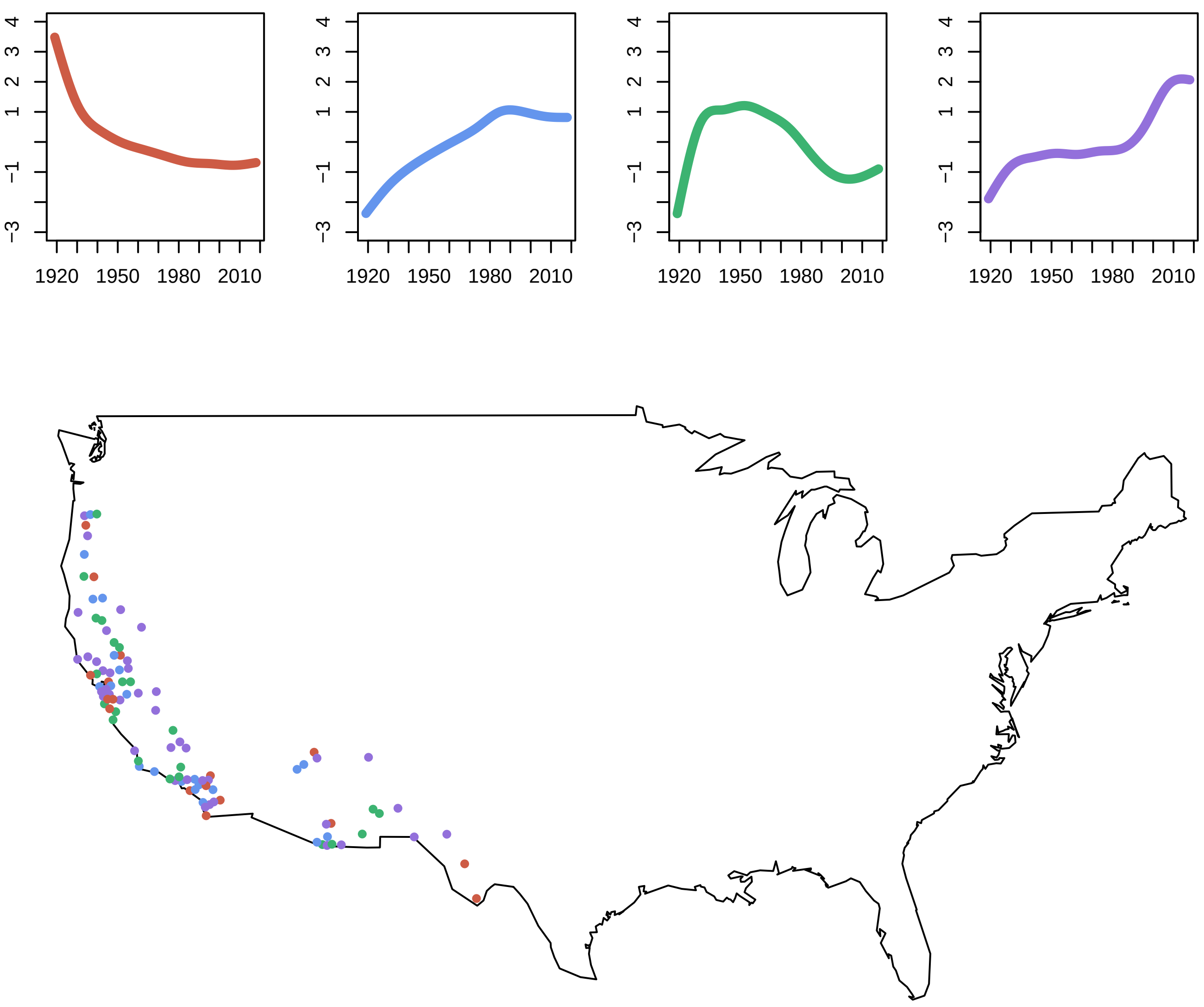

Anna's Hummingbird

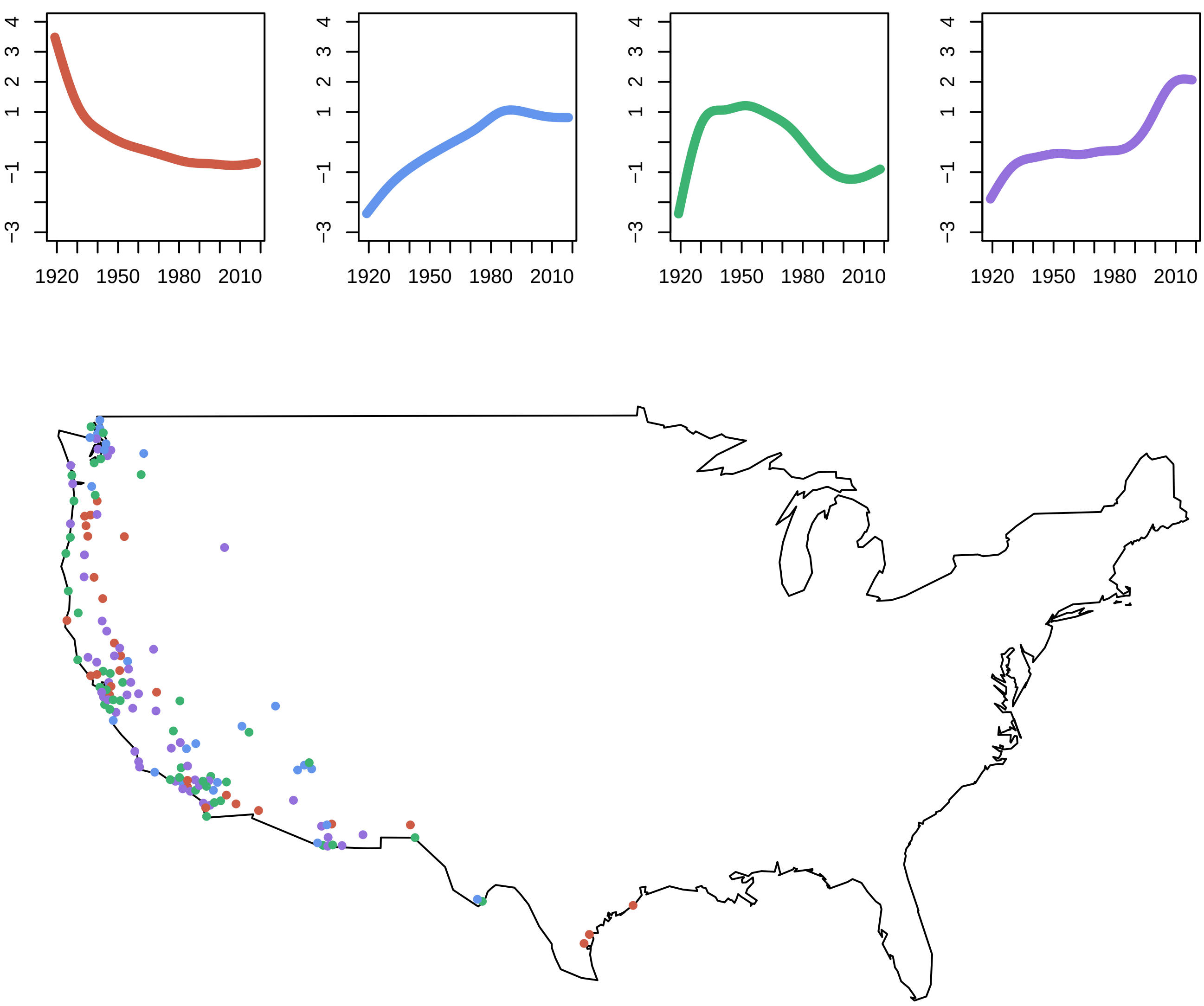

Bald Eagle

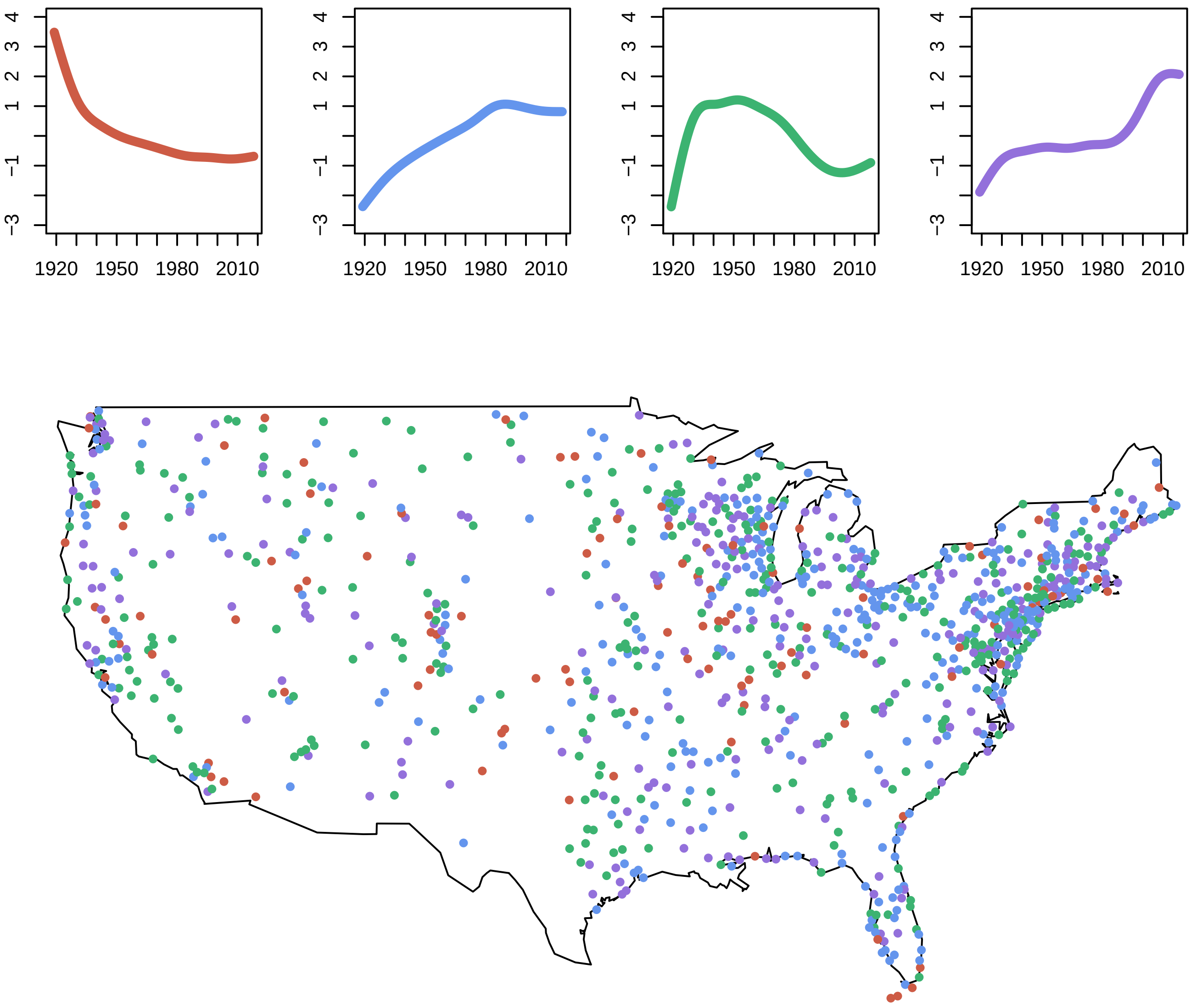

Band−tailed Pigeon

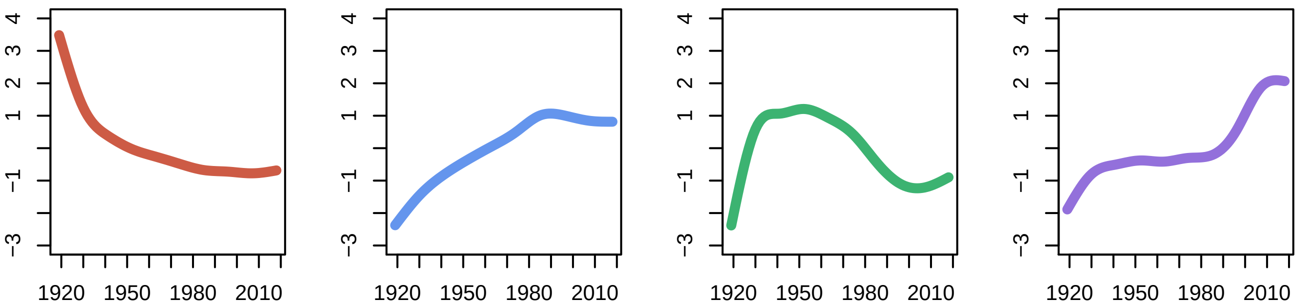

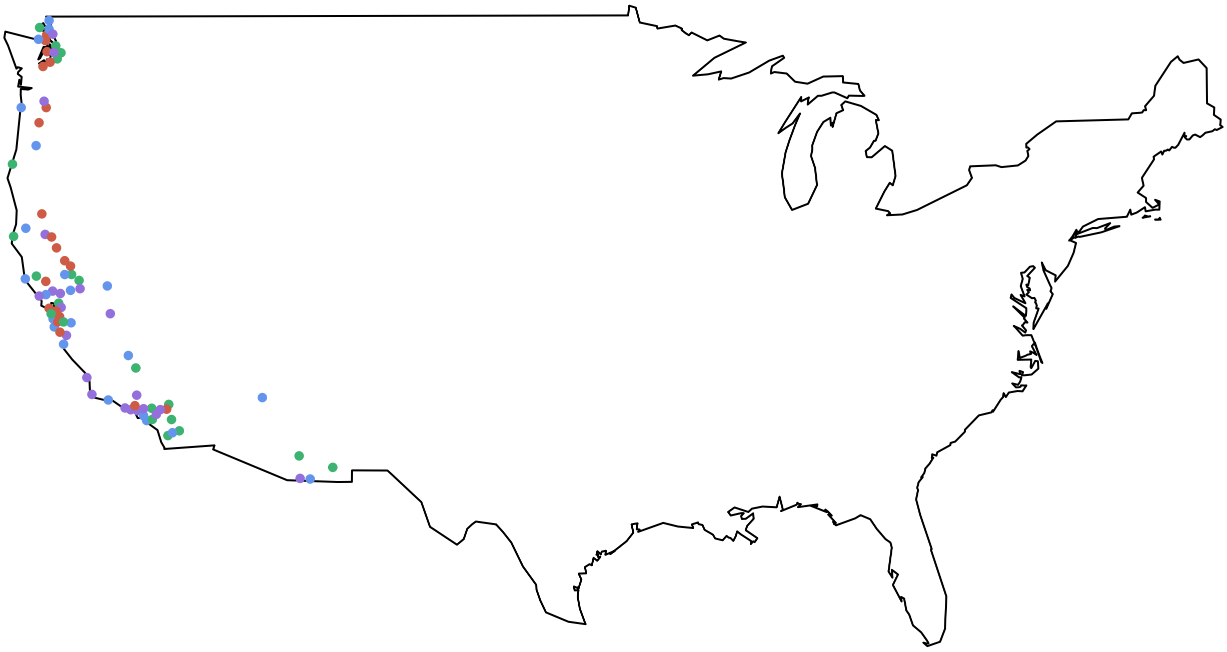

Belted Kingfisher

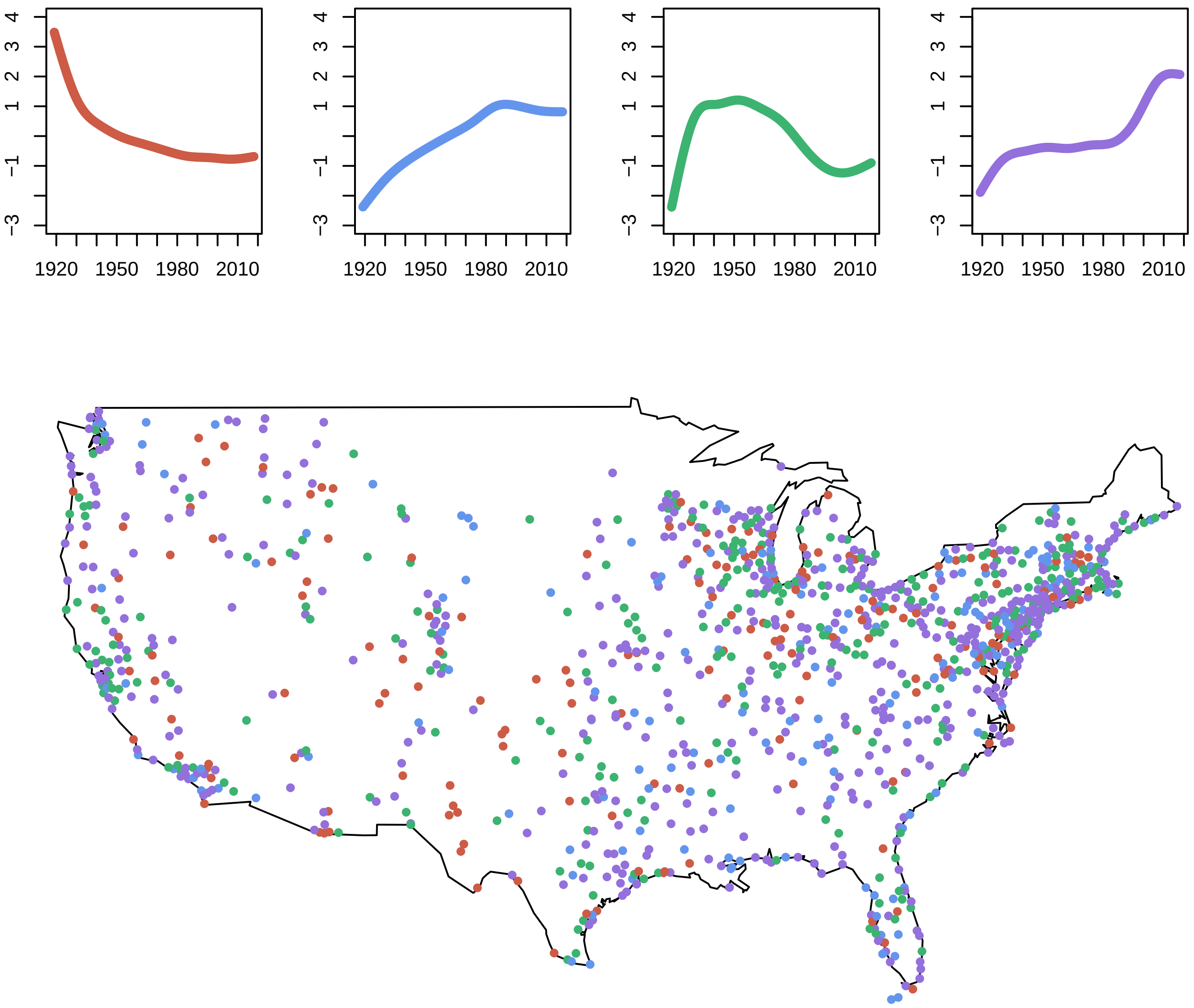

Black−crowned Night−Heron

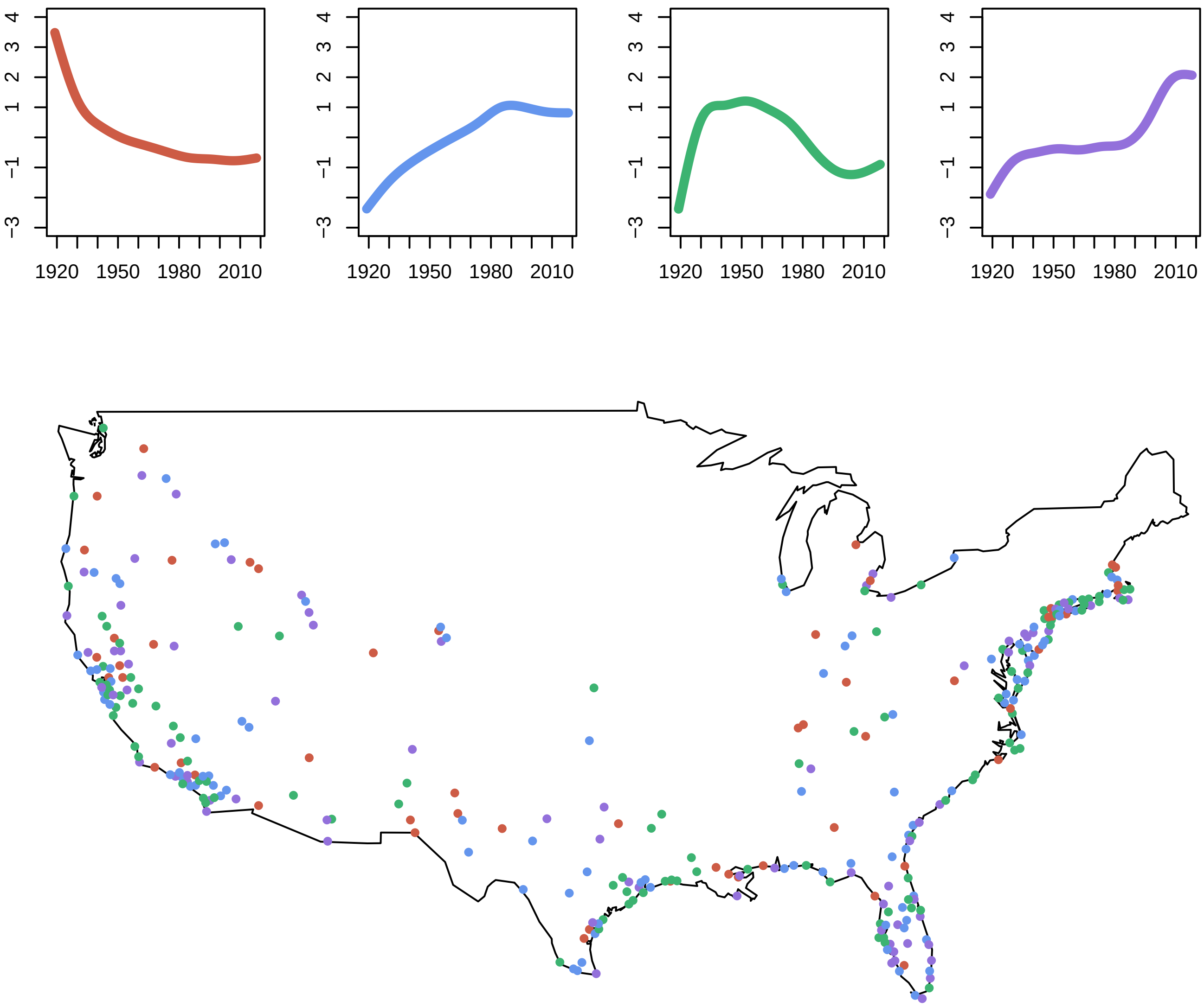

Black−necked Stilt

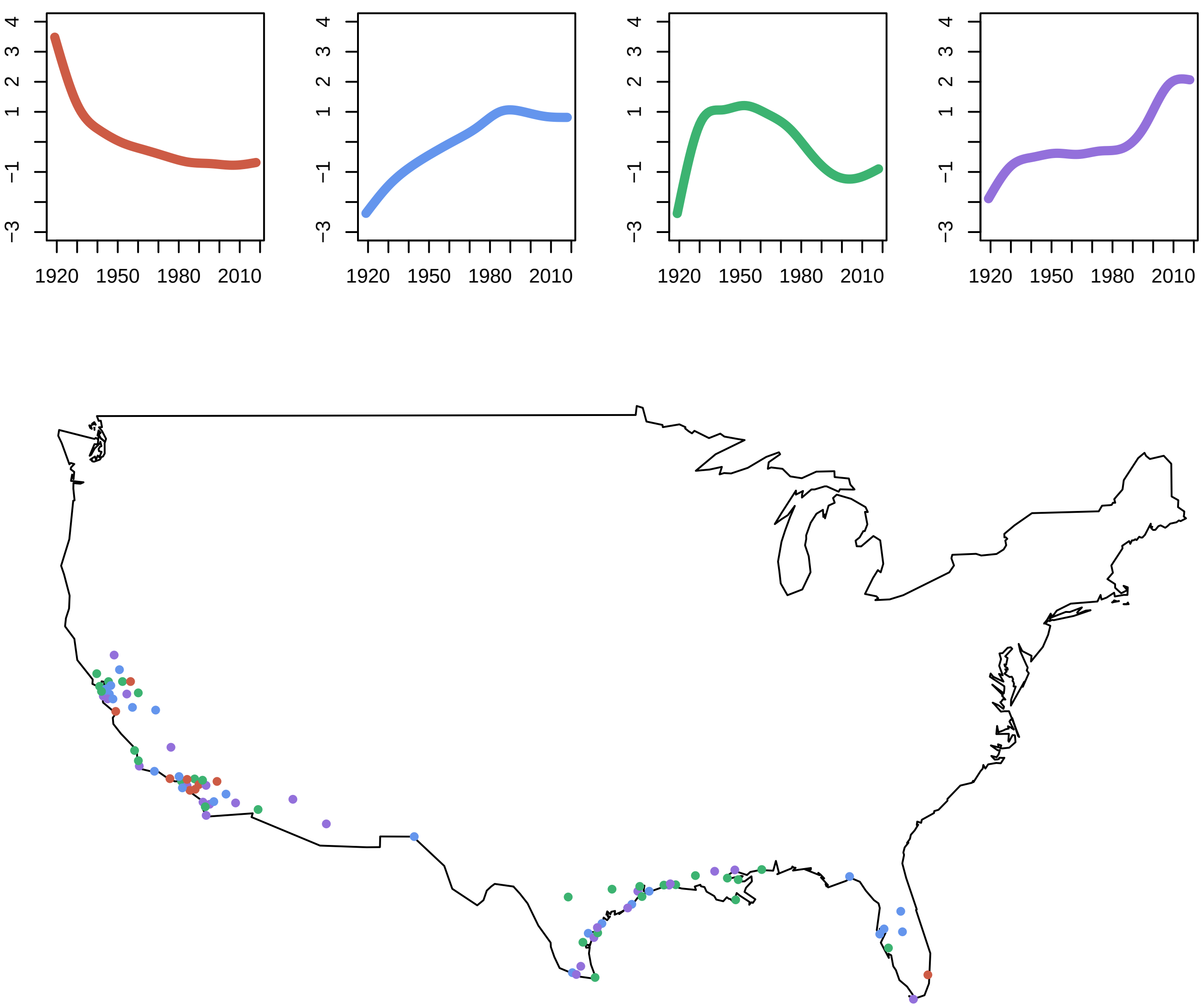

Black Phoebe

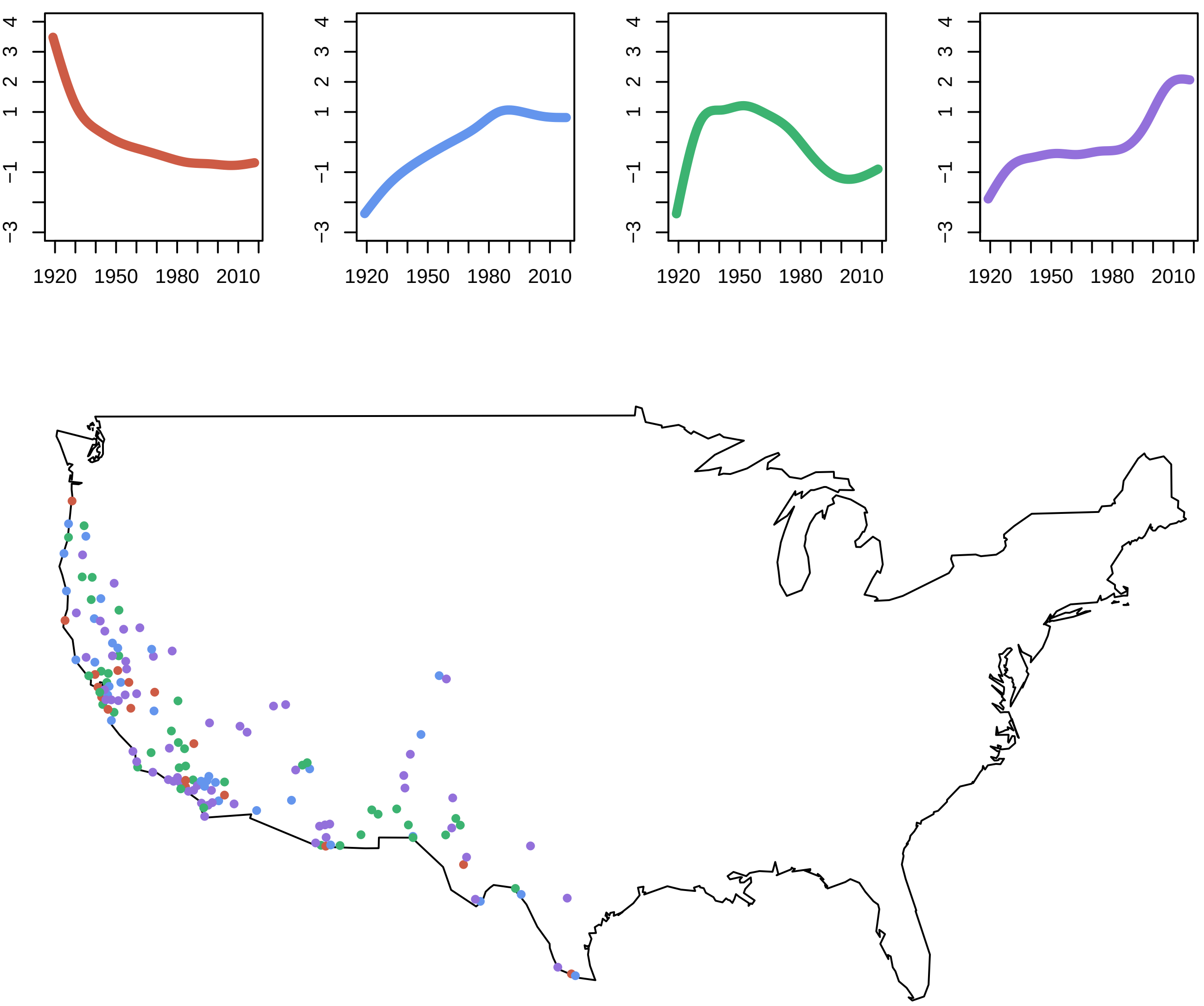

Blue−gray Gnatcatcher

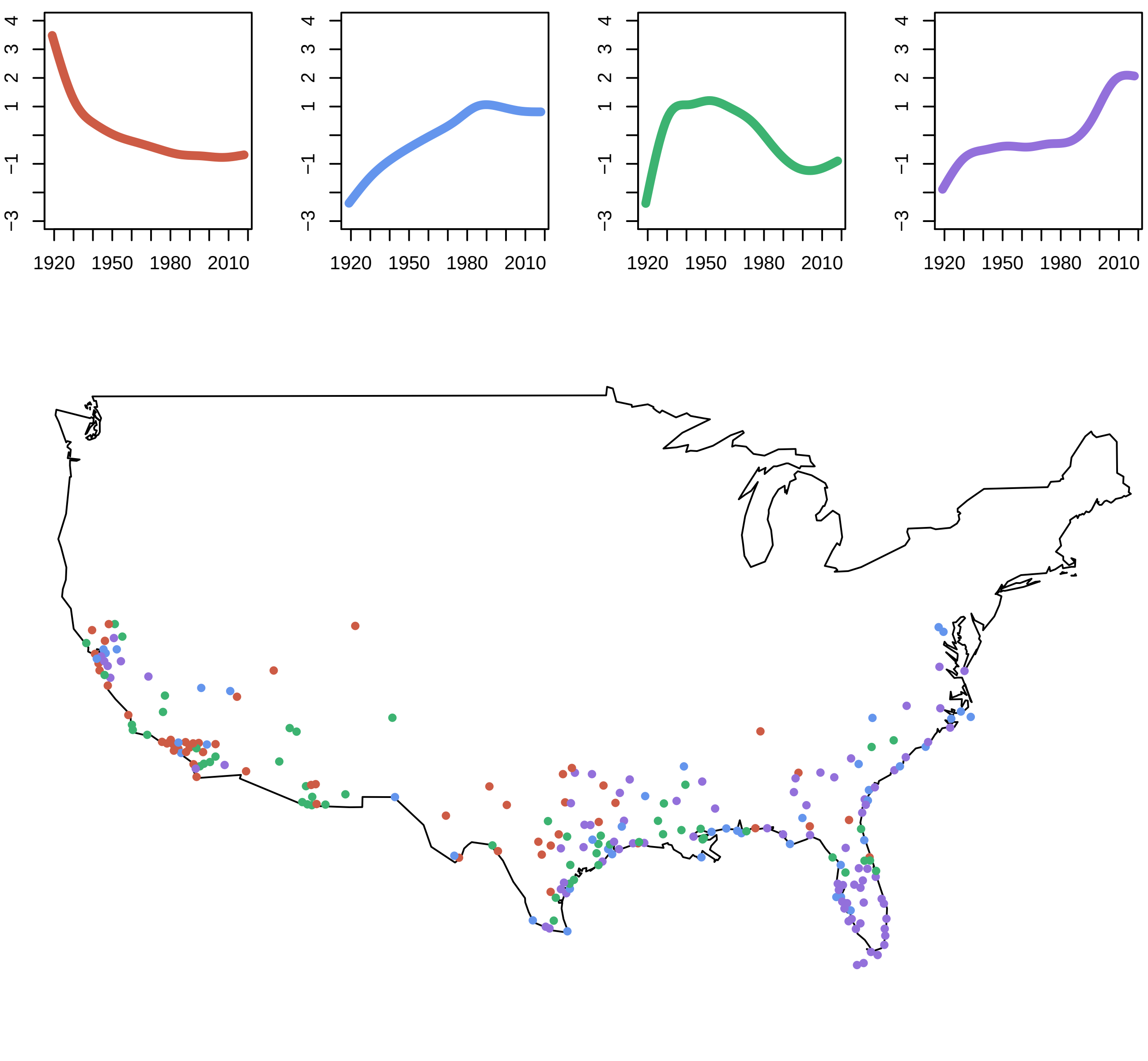

Brown Creeper

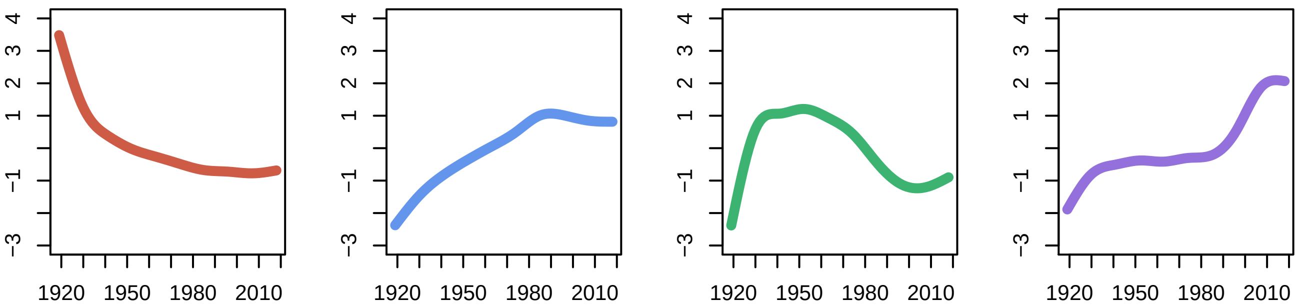

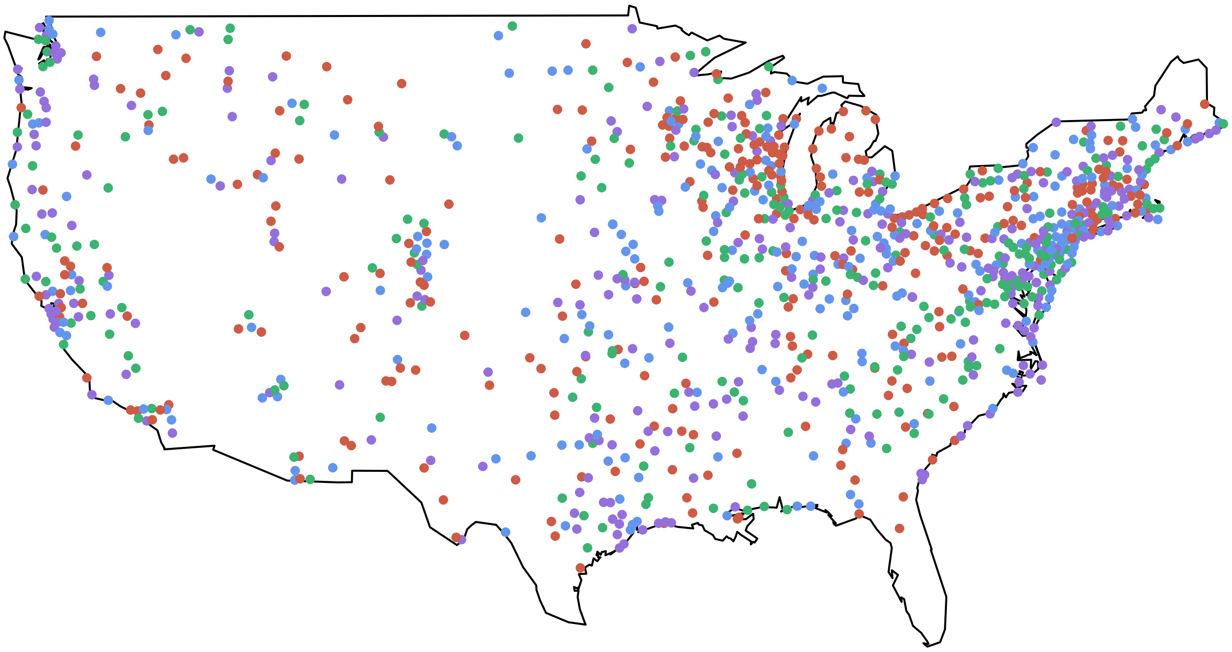

Chestnut−backed Chickadee

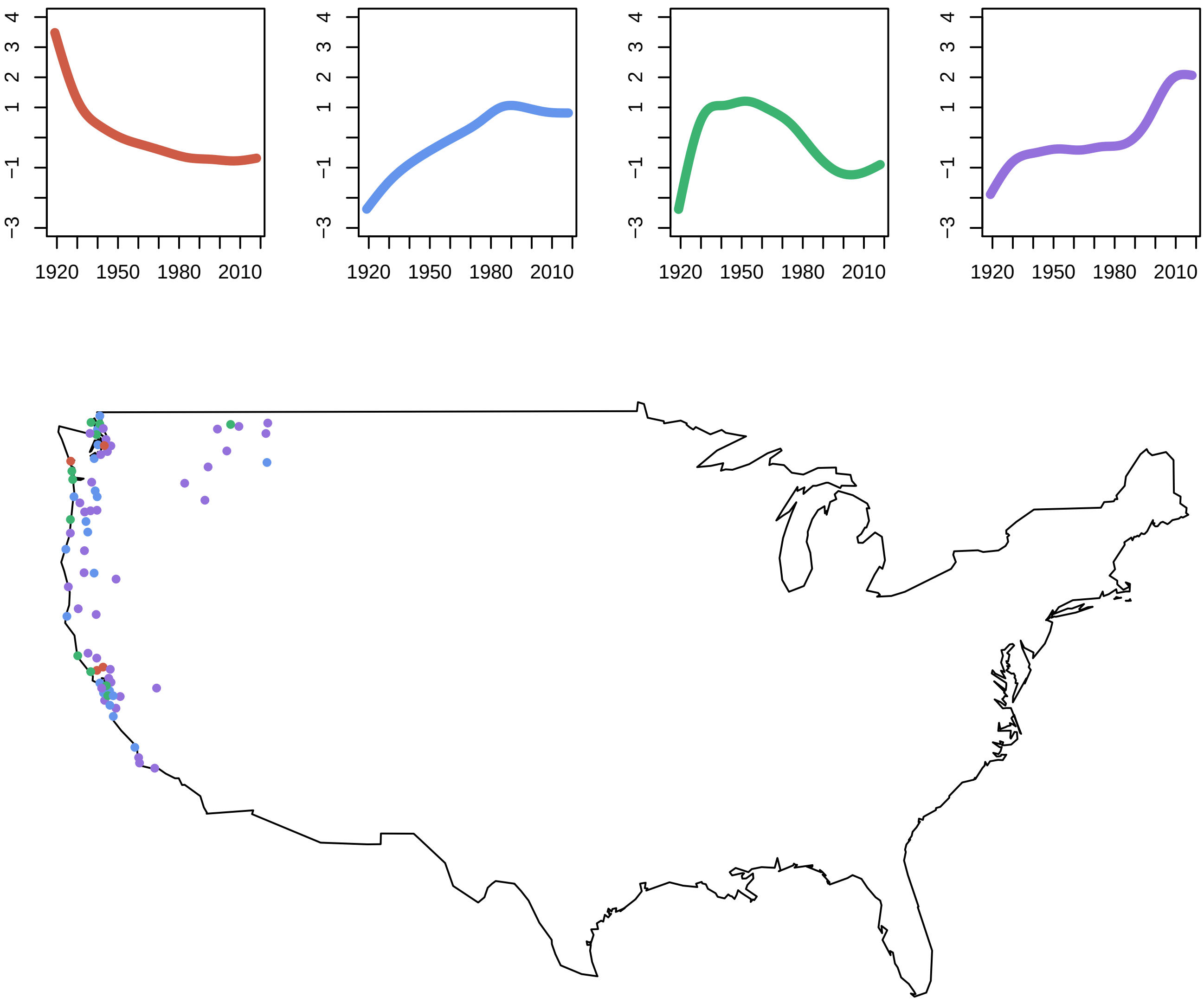

Common Gallinule

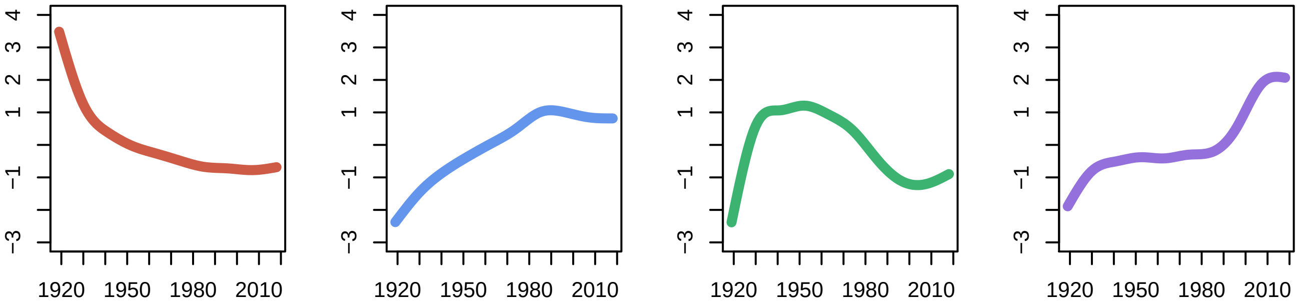

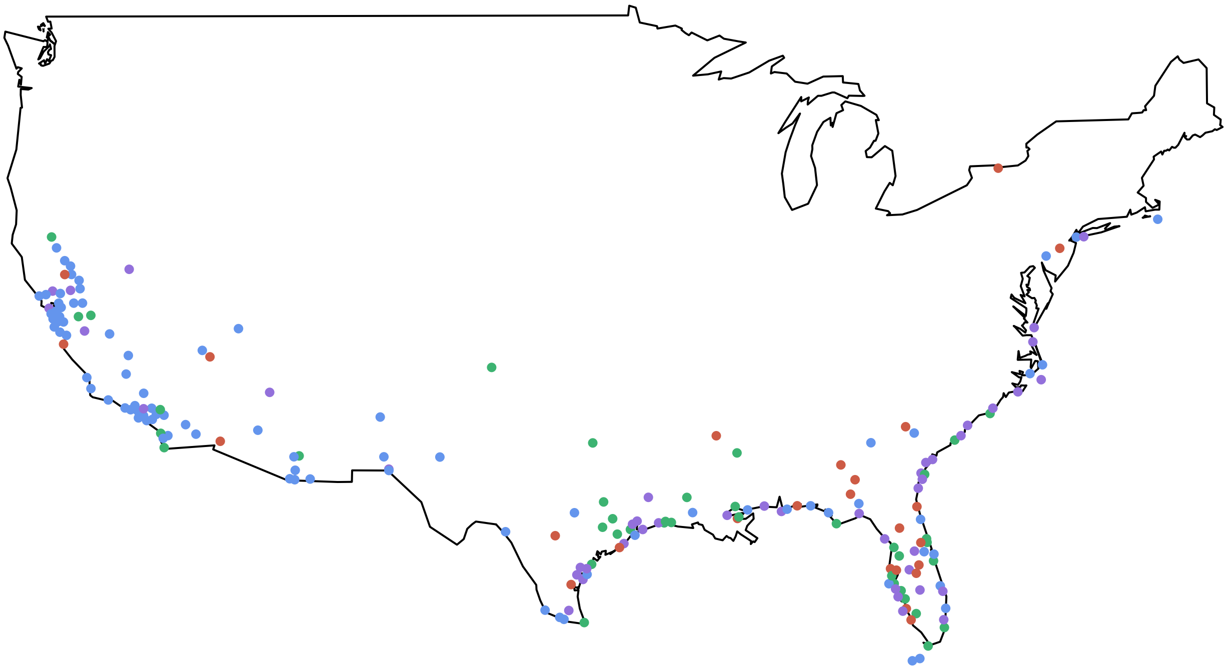

Common Loon

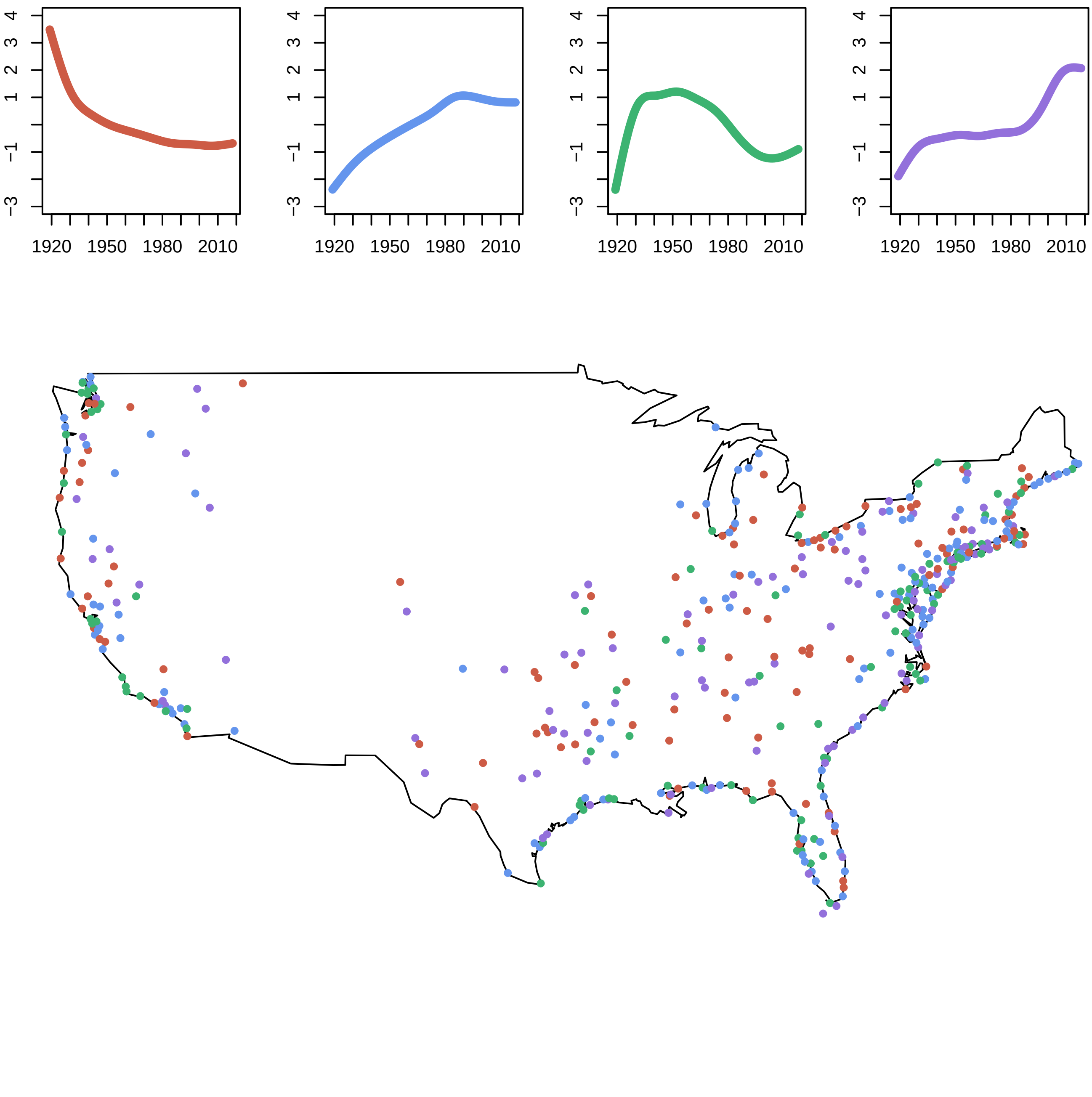

Eastern Phoebe

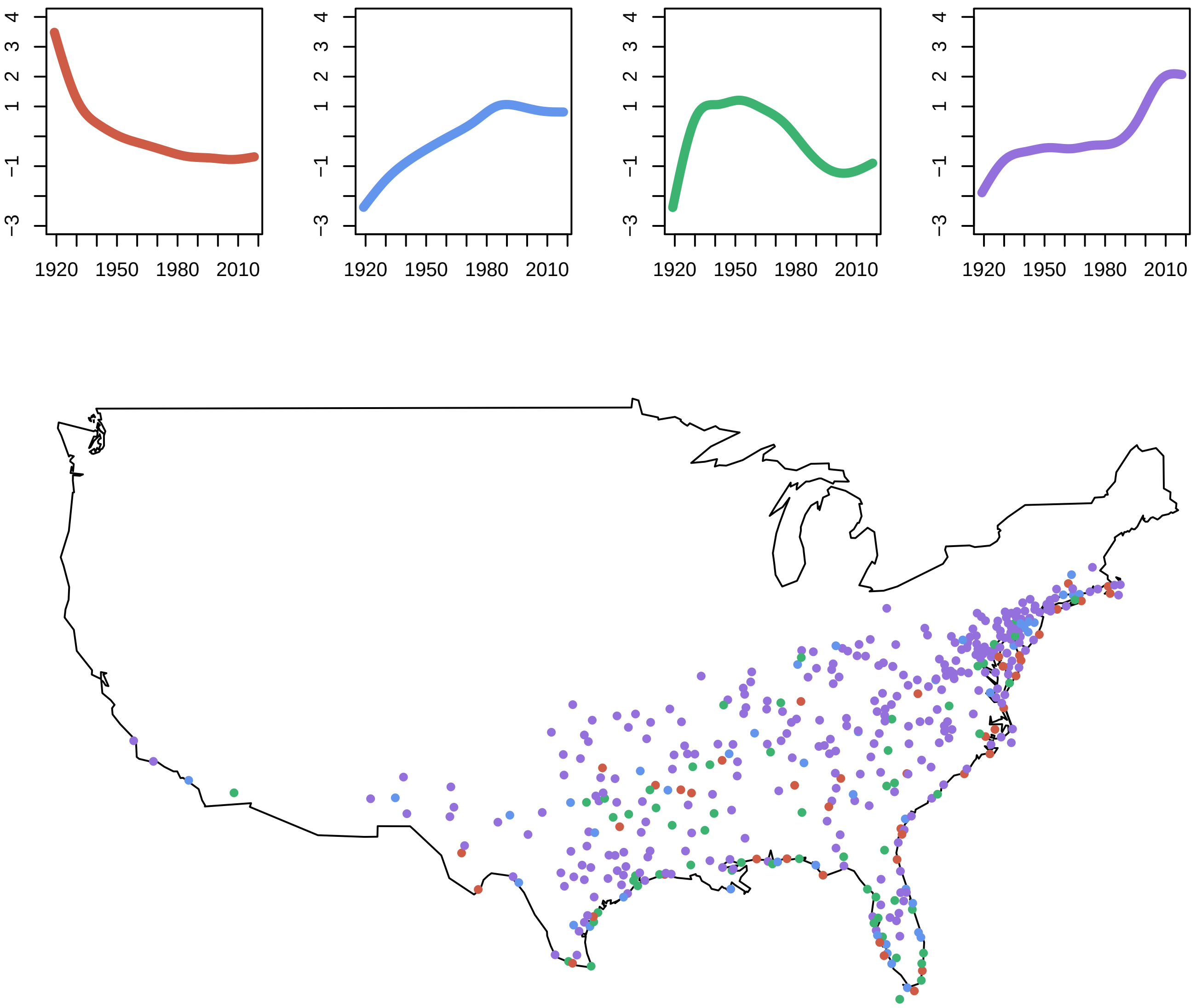

Eastern Towhee

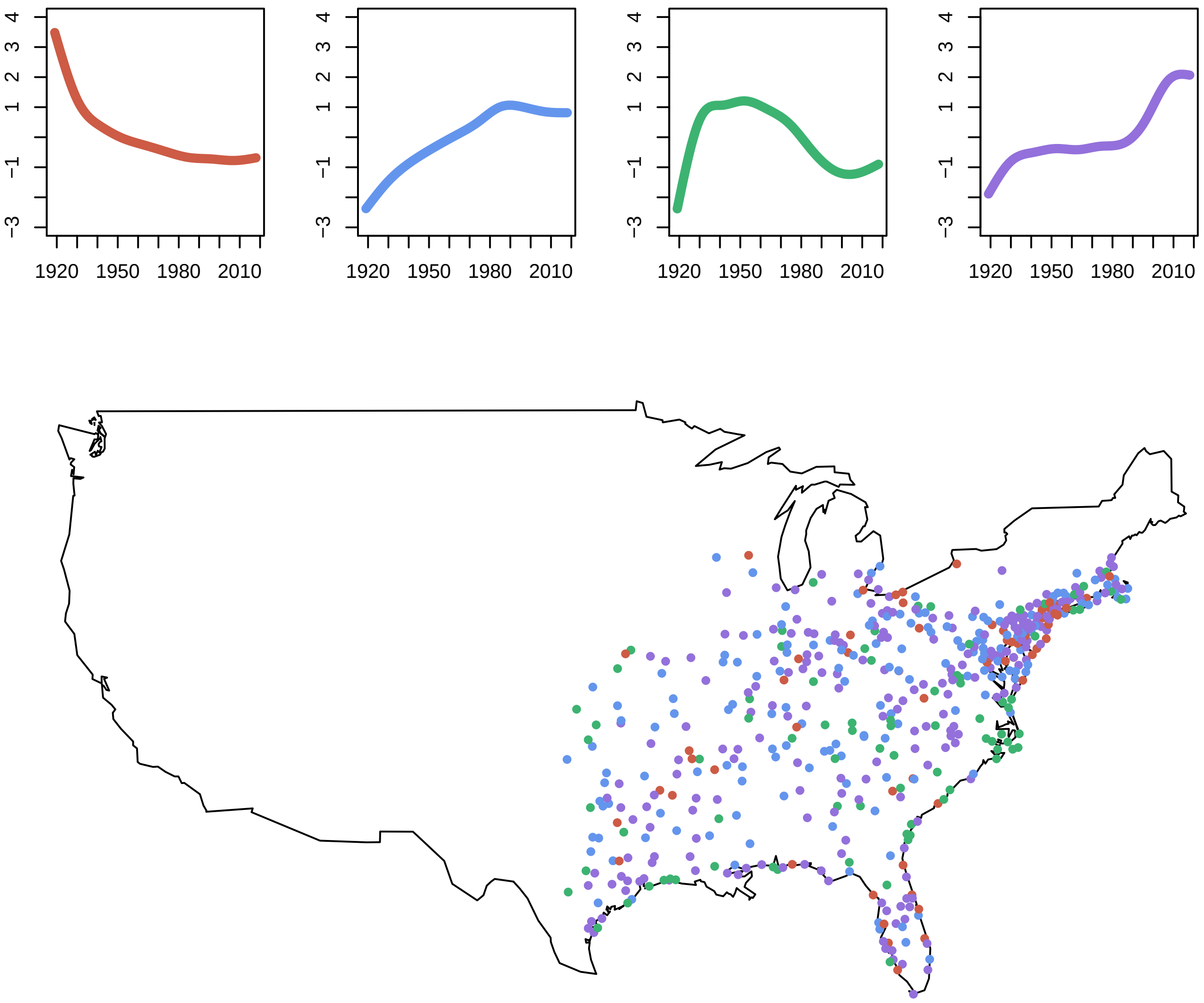

Fox Sparrow

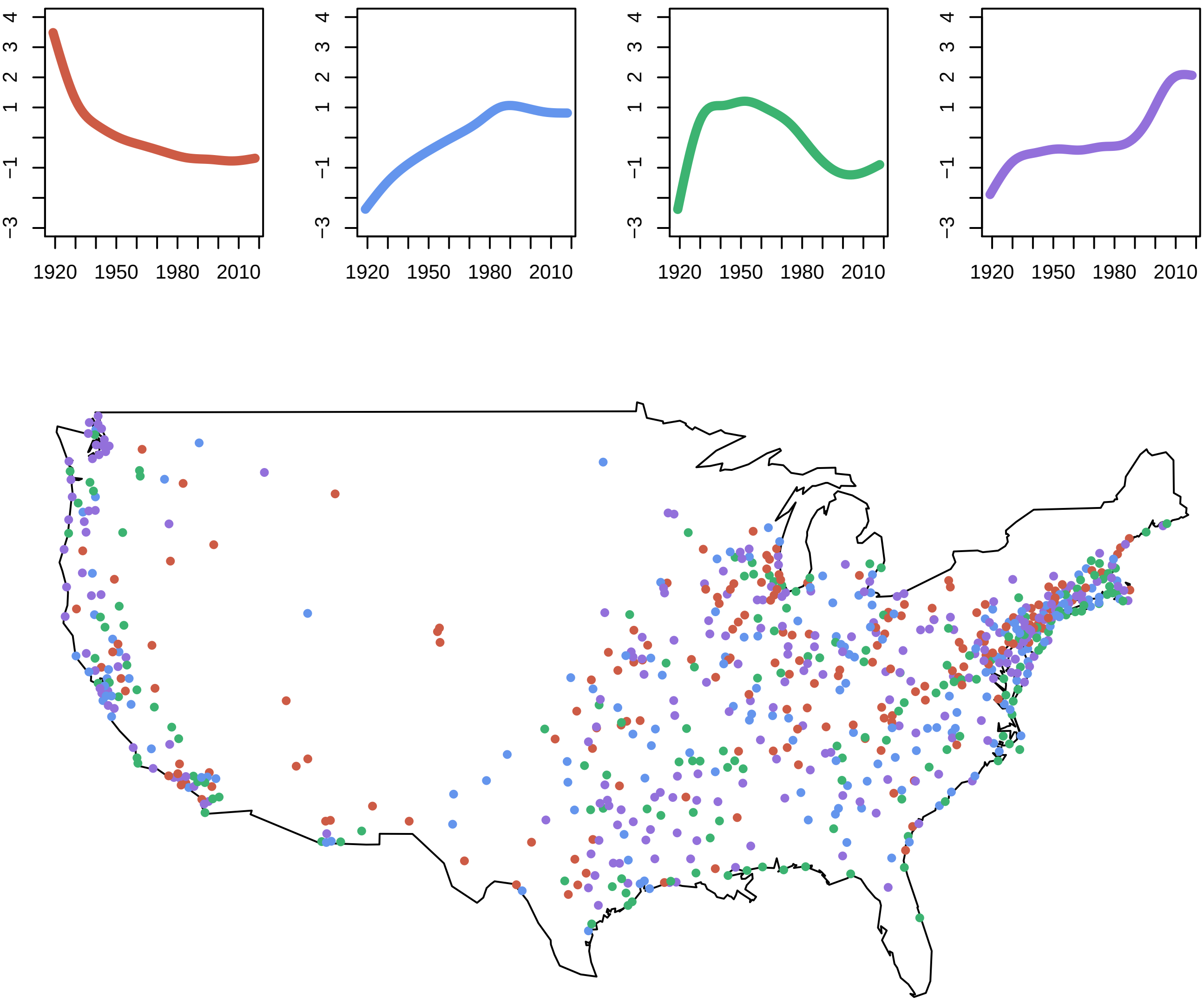

Hairy Woodpecker

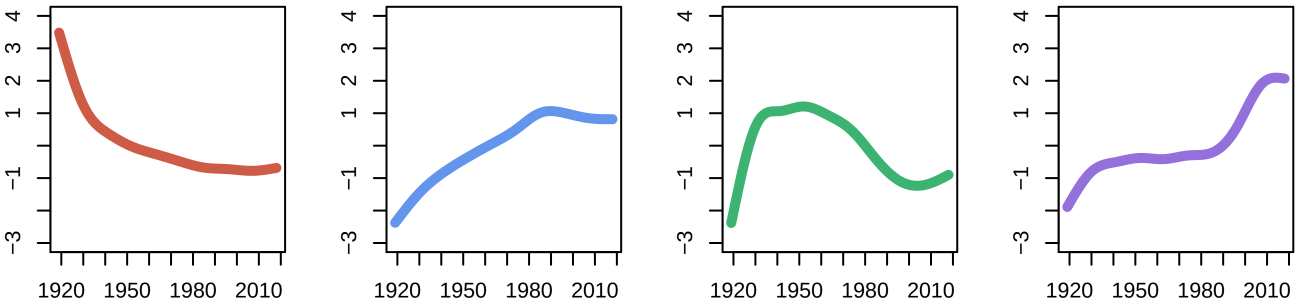

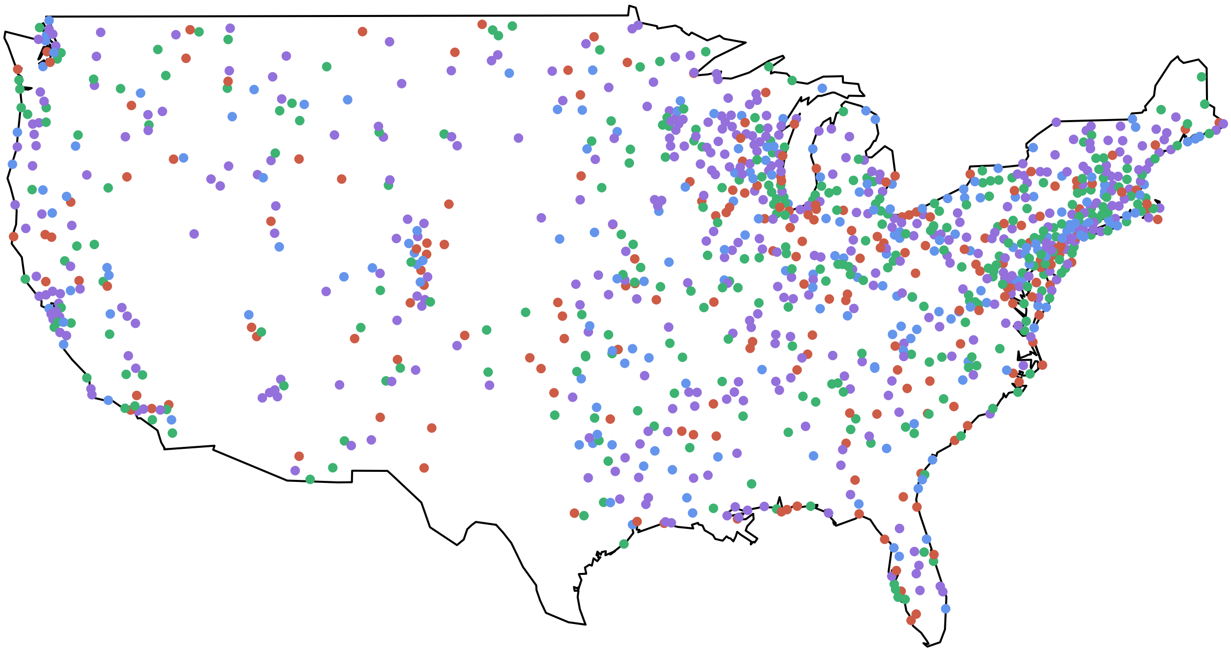

Harris's Sparrow

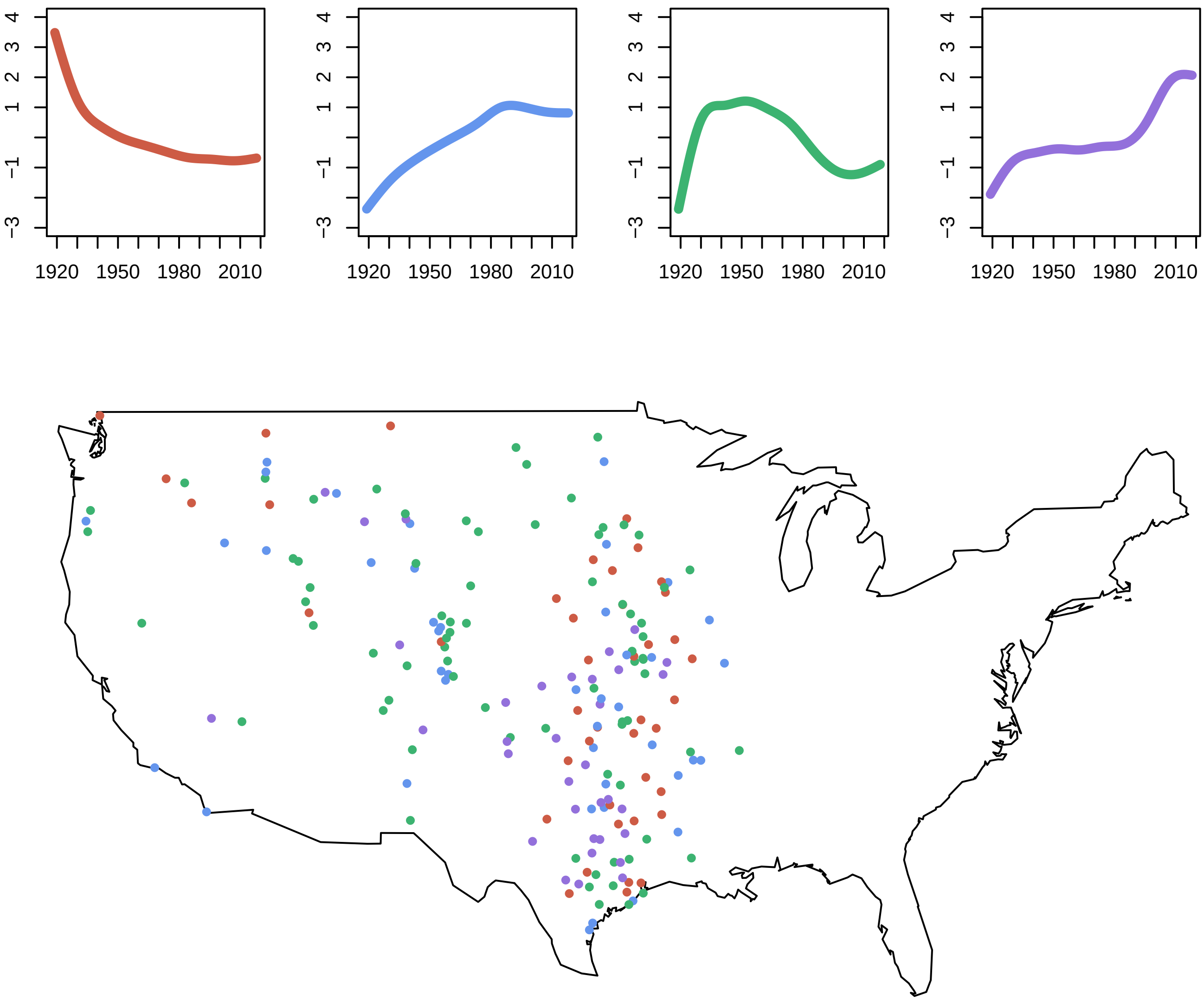

Hermit Thrush

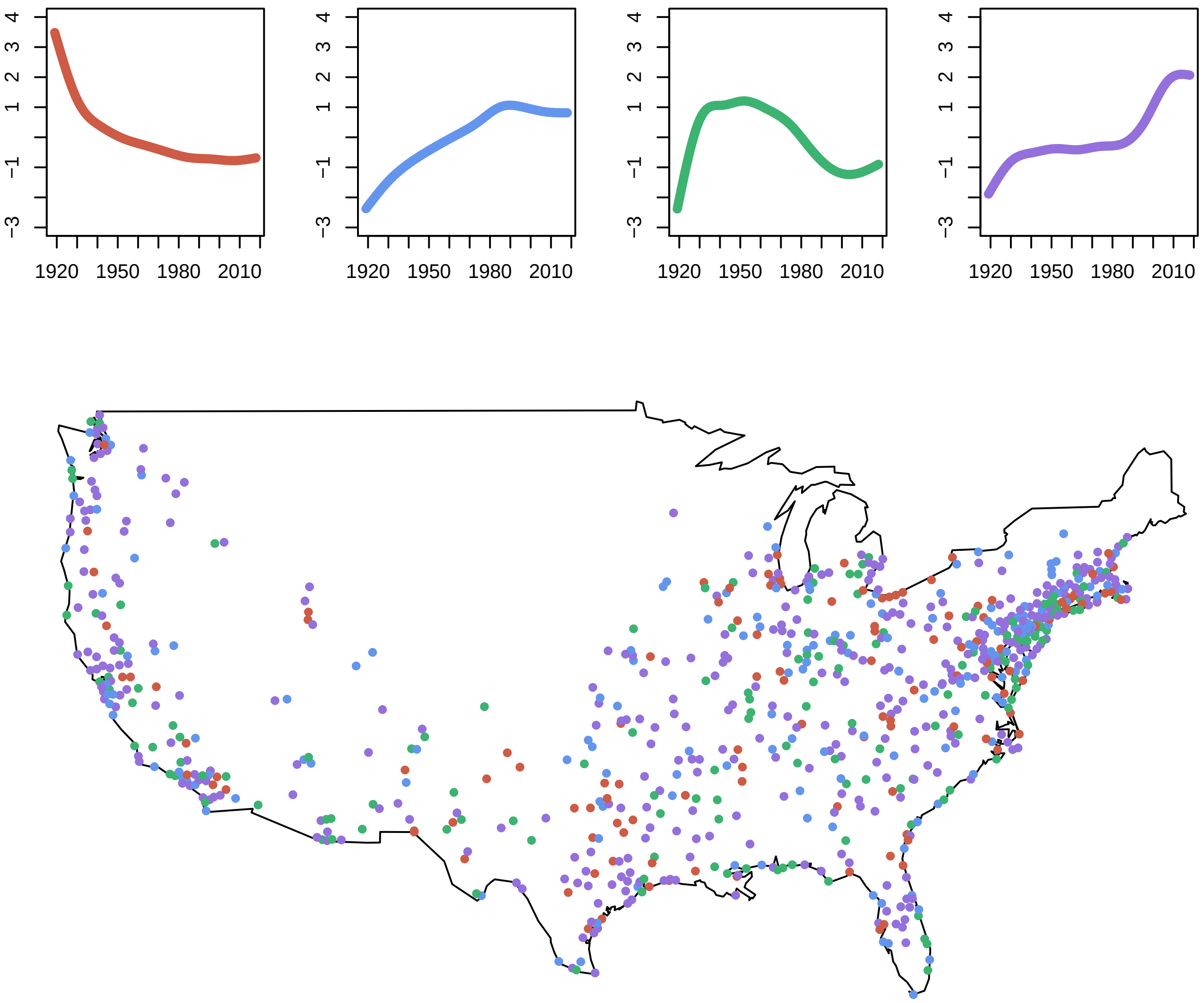

Horned Grebe

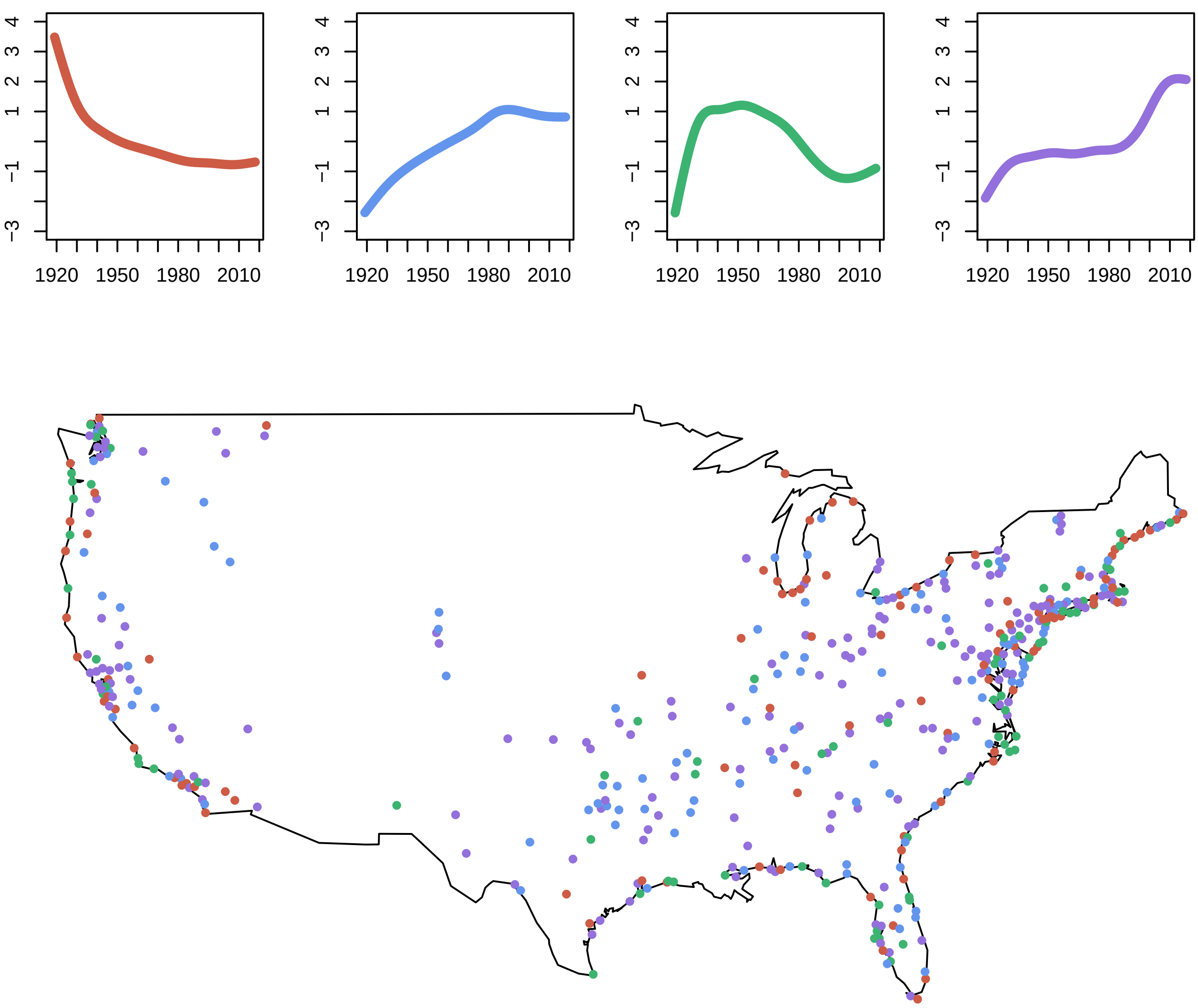

Lark Bunting

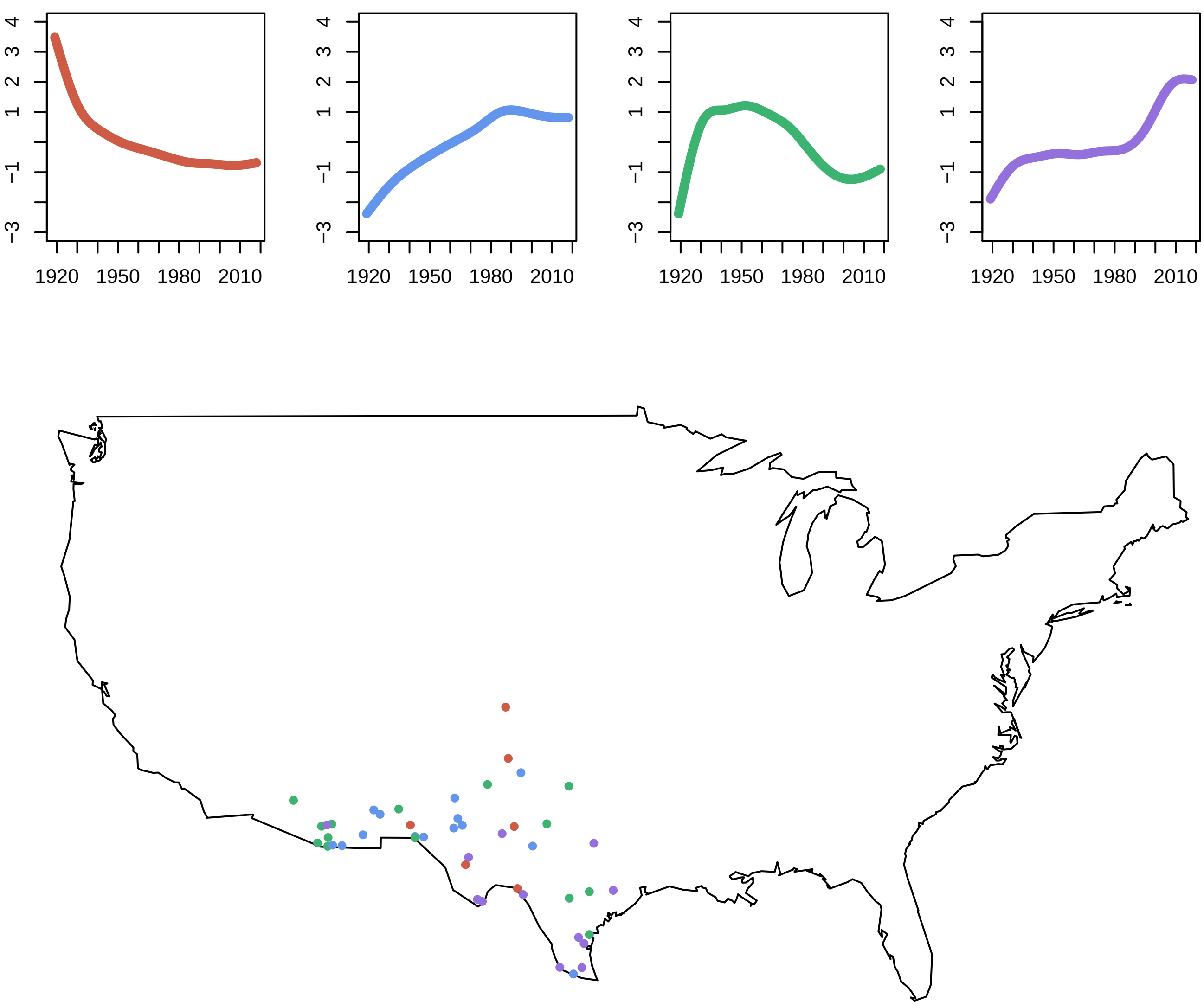

Lark Sparrow

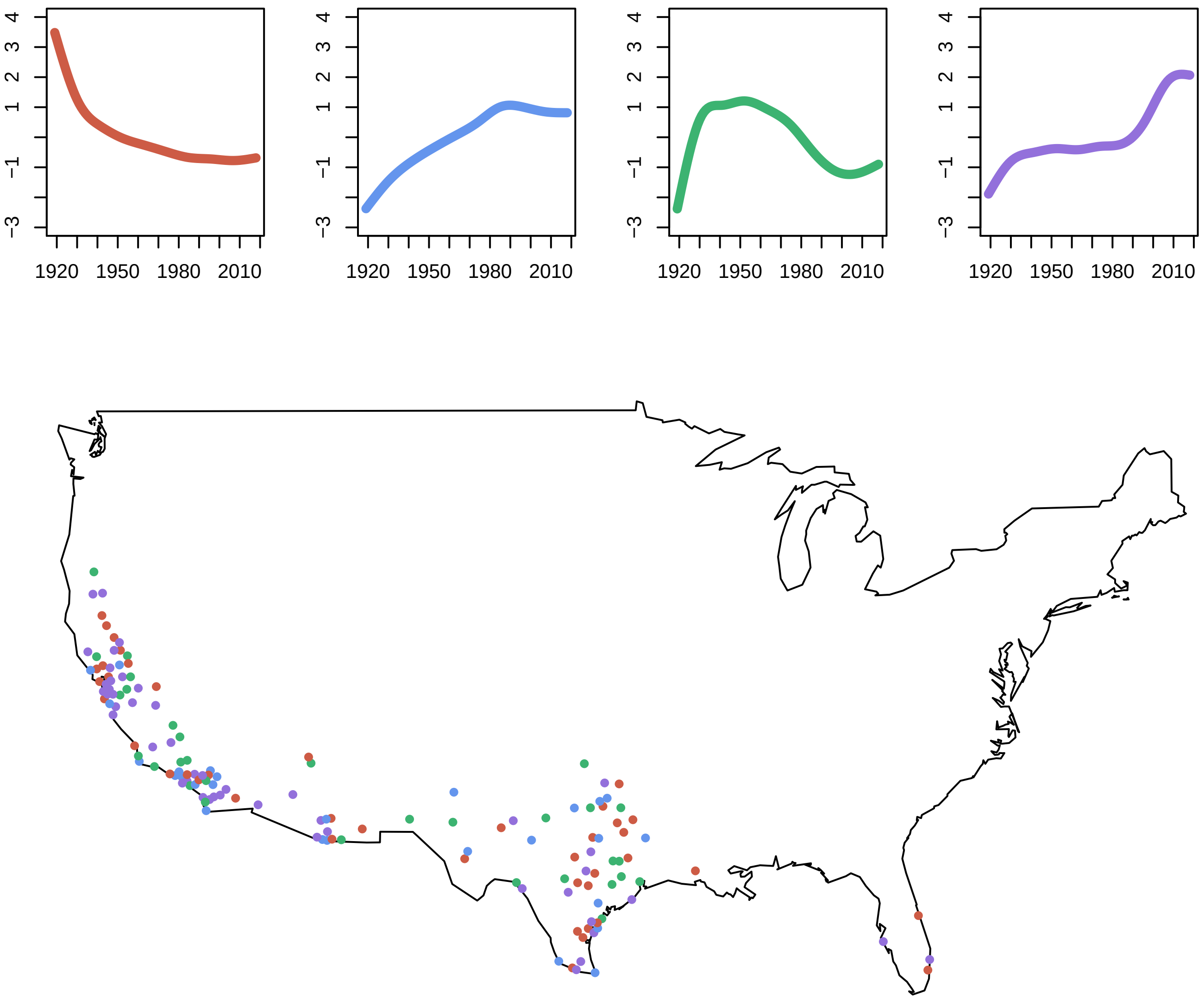

Loggerhead Shrike

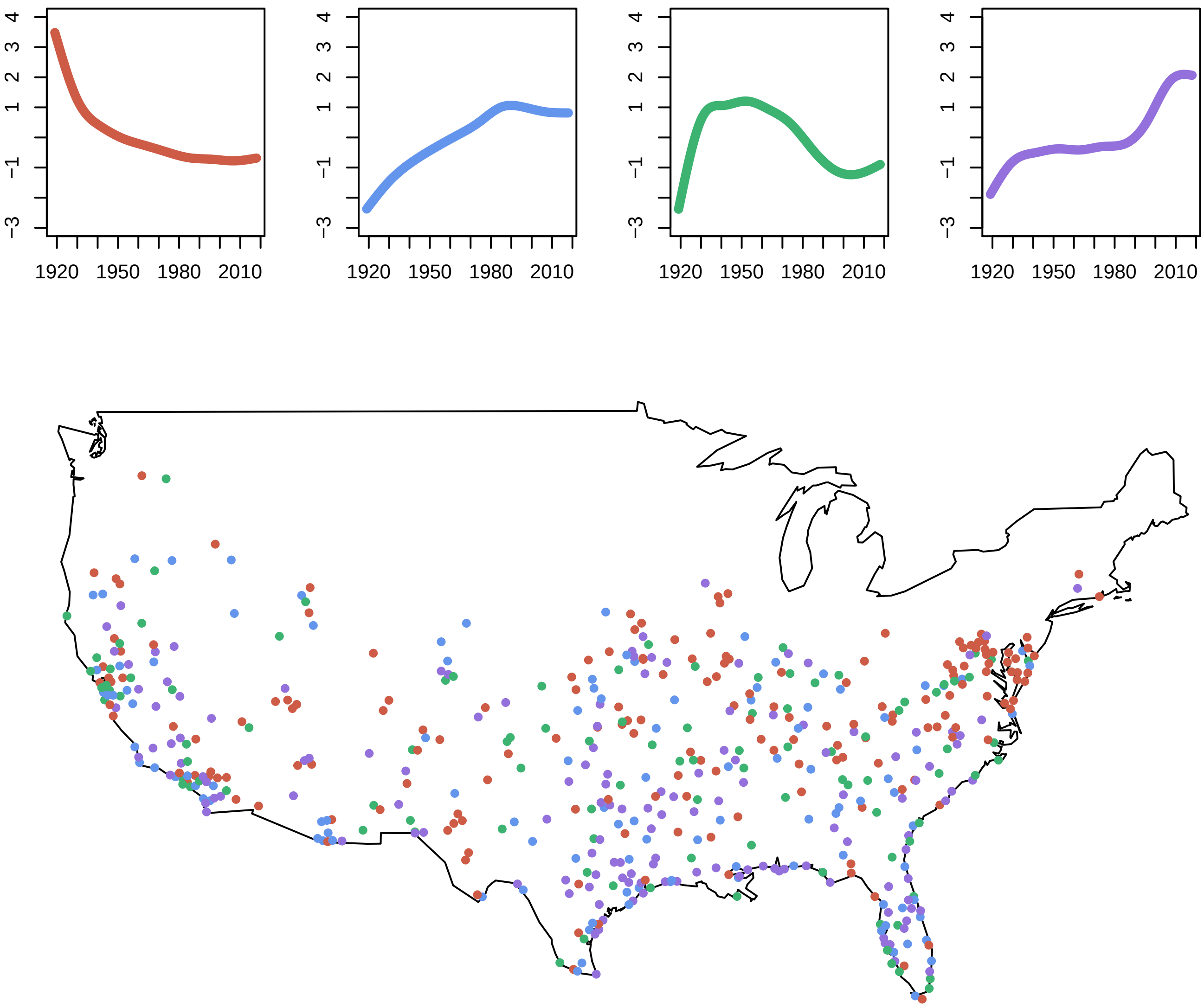

Long−billed Curlew

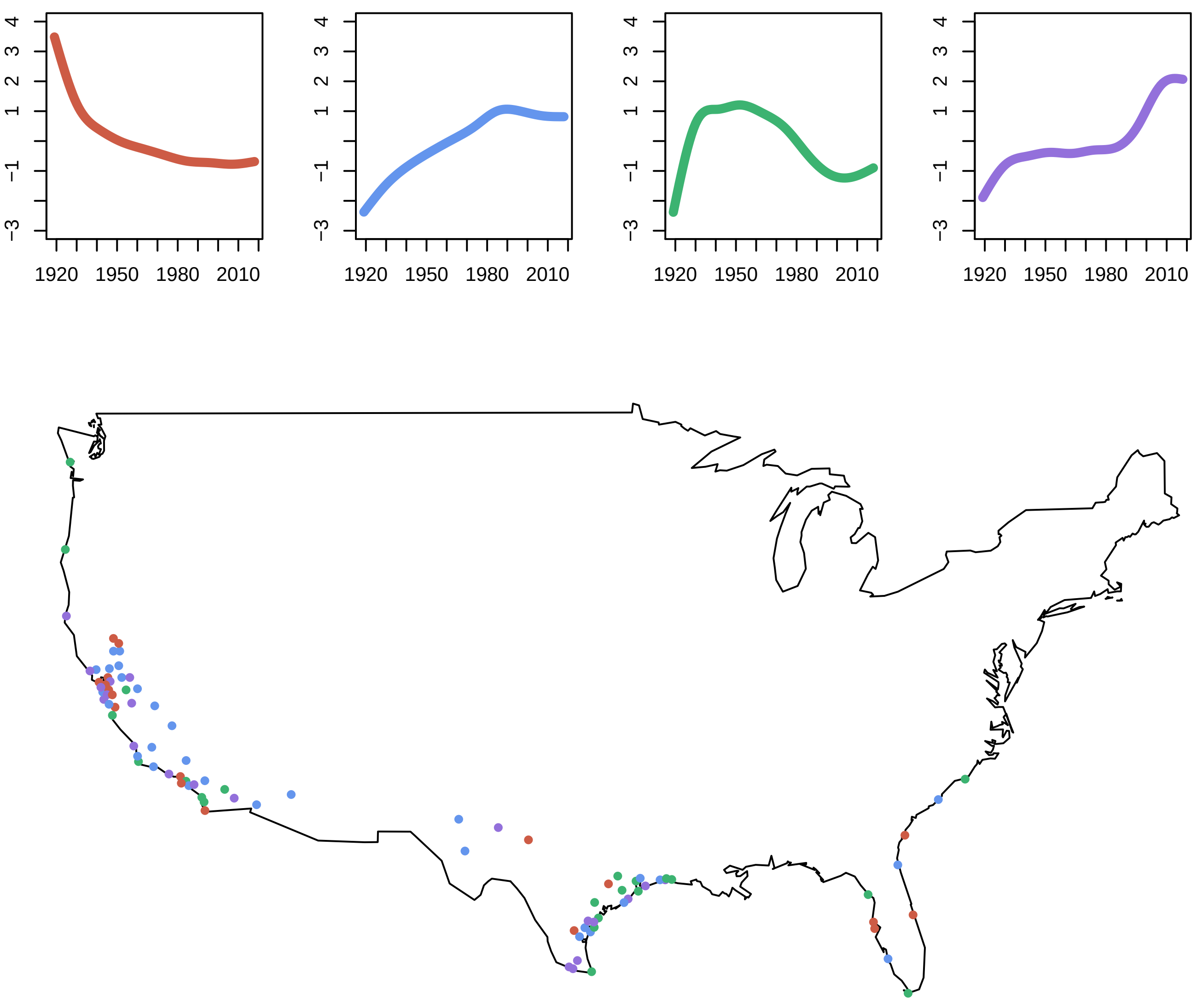

Mute Swan

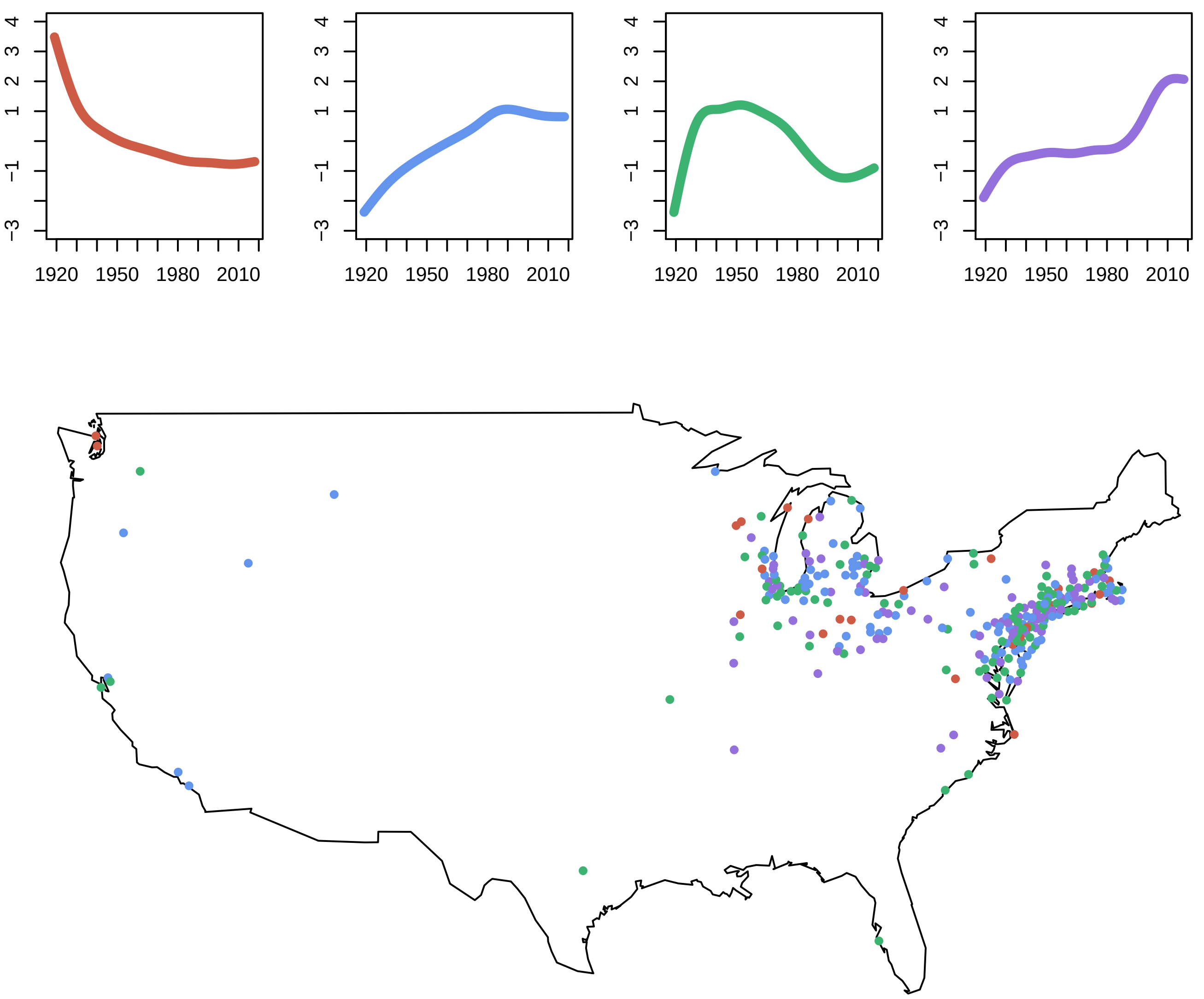

Northern Bobwhite

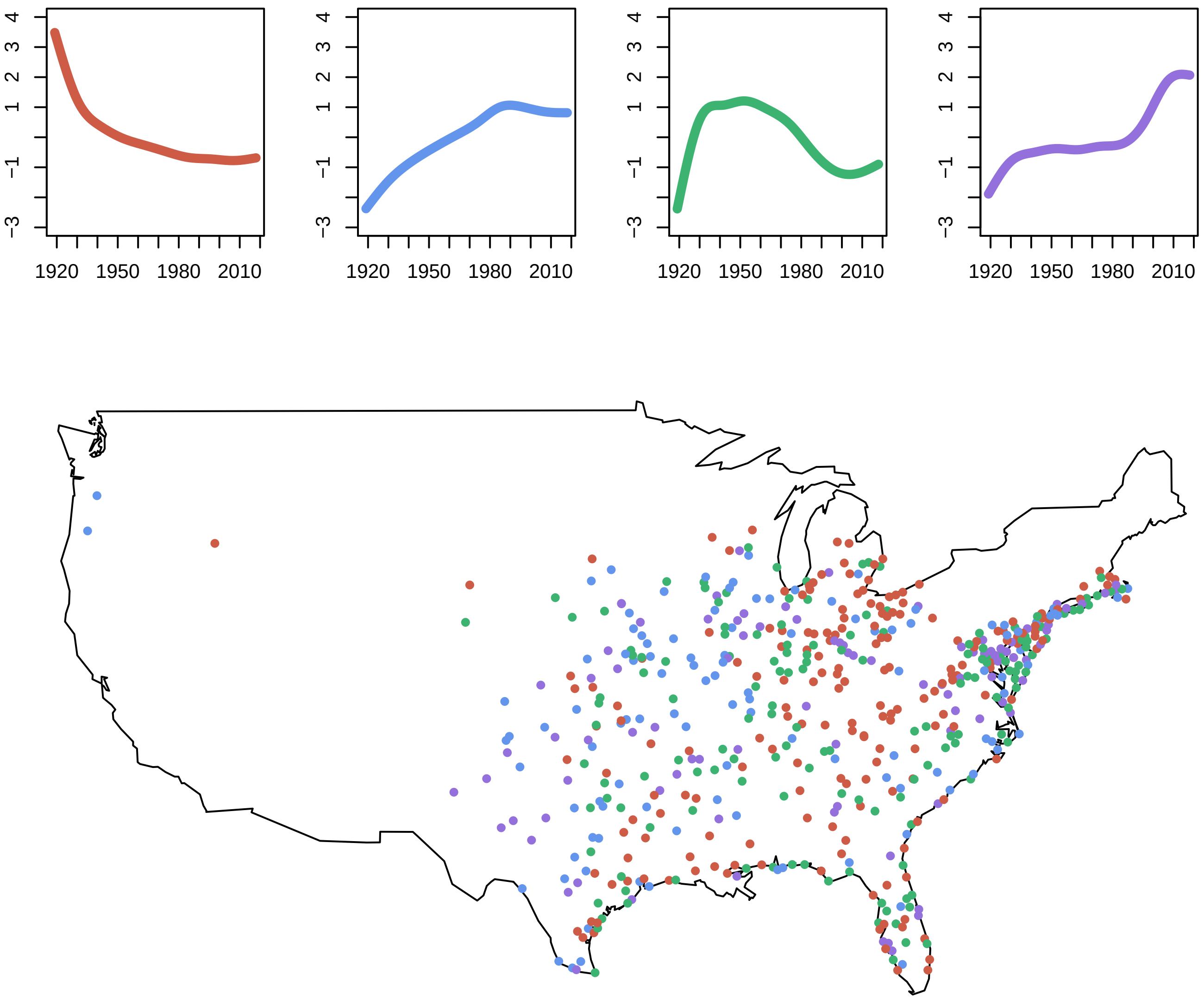

Northern Harrier

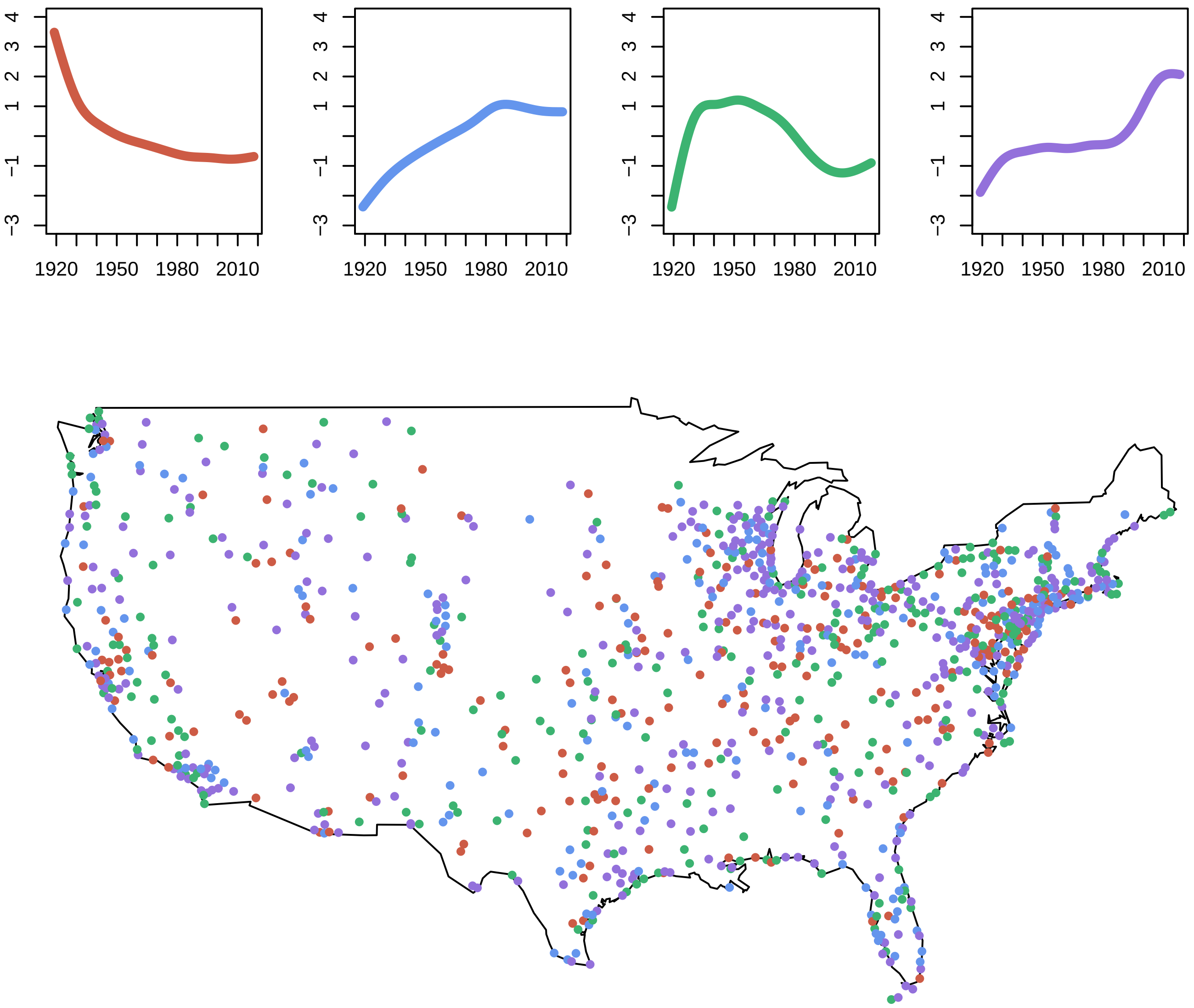

Palm Warbler

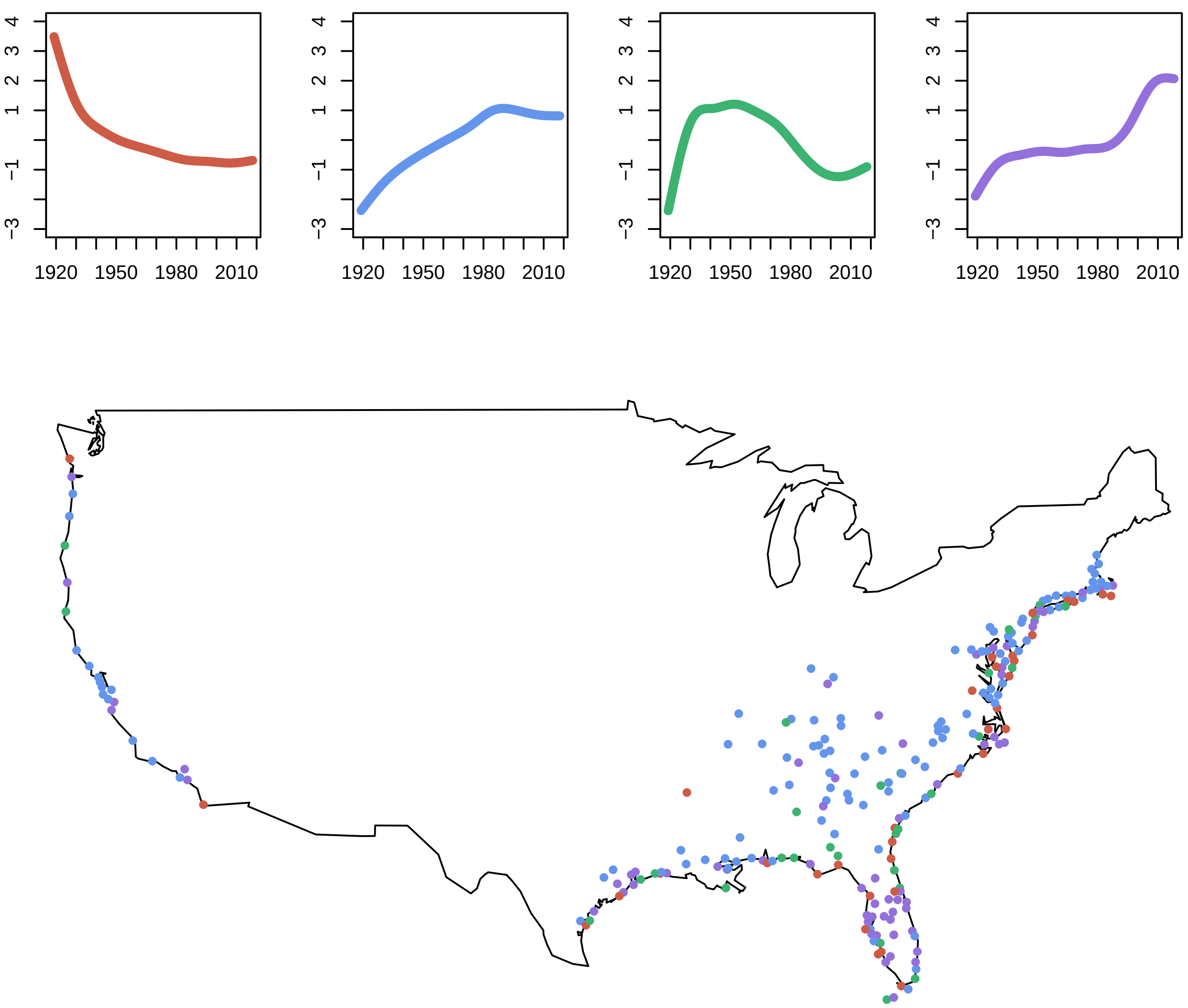

Pine Warbler

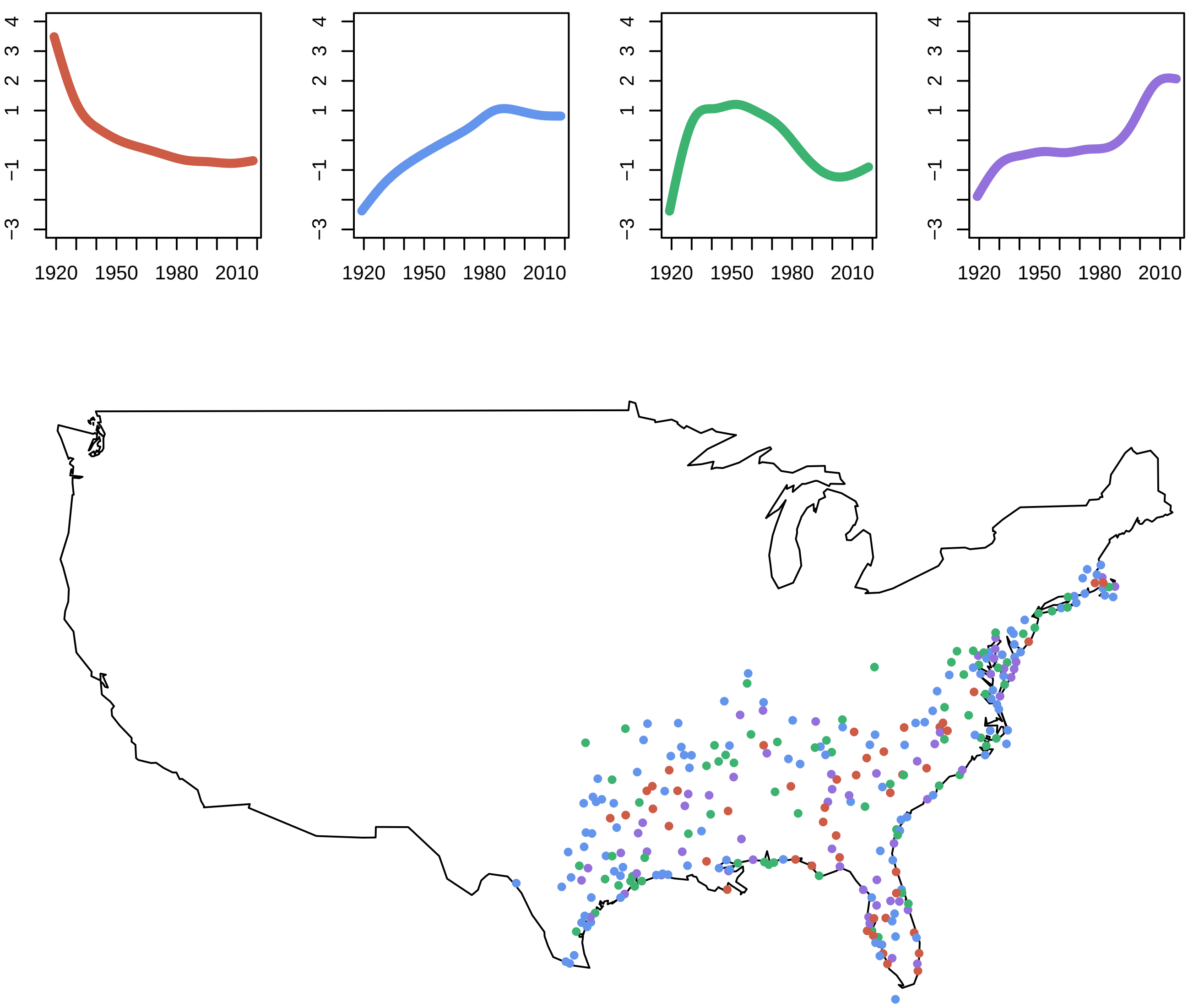

Red−breasted Nuthatch

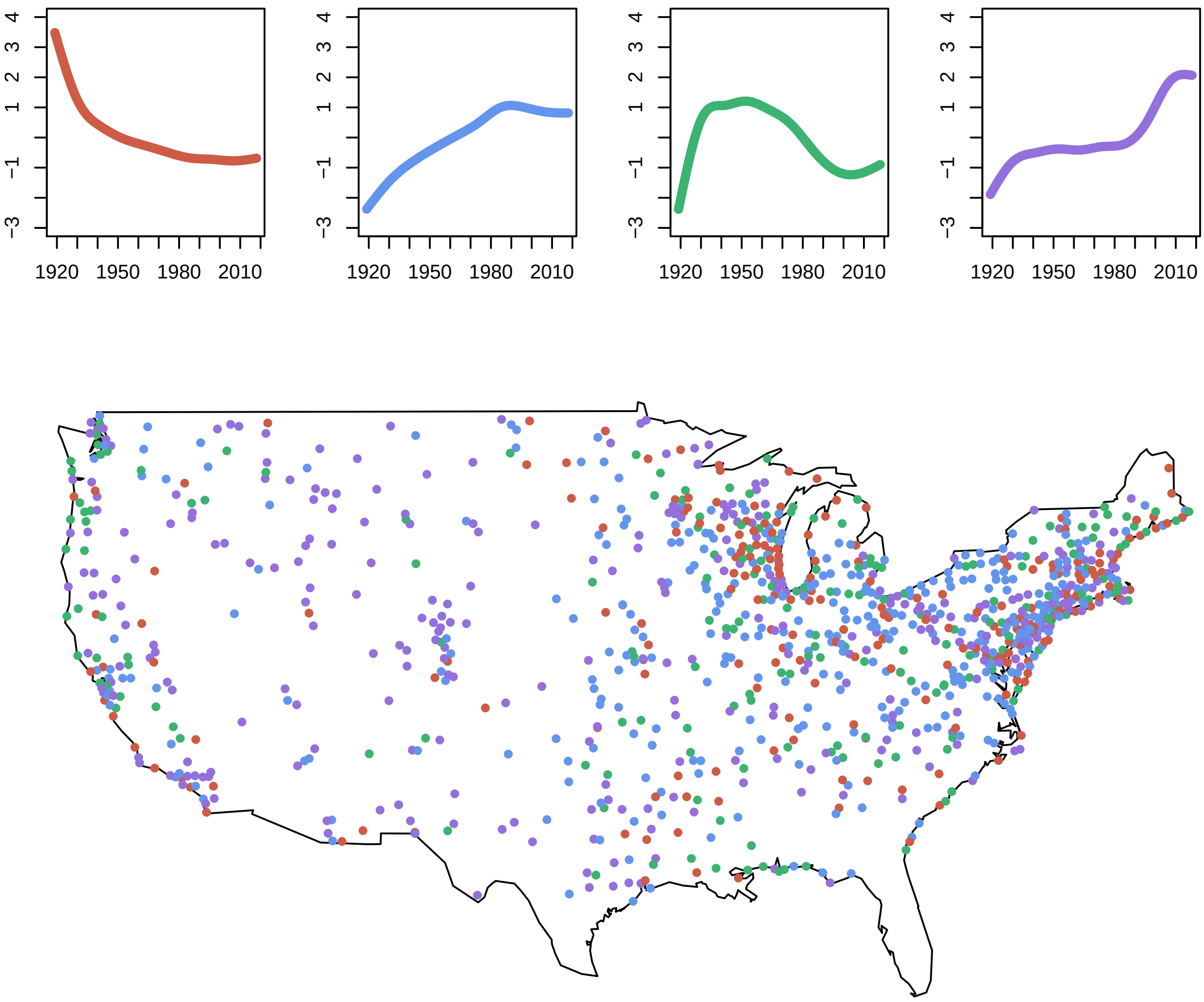

Red−throated Loon

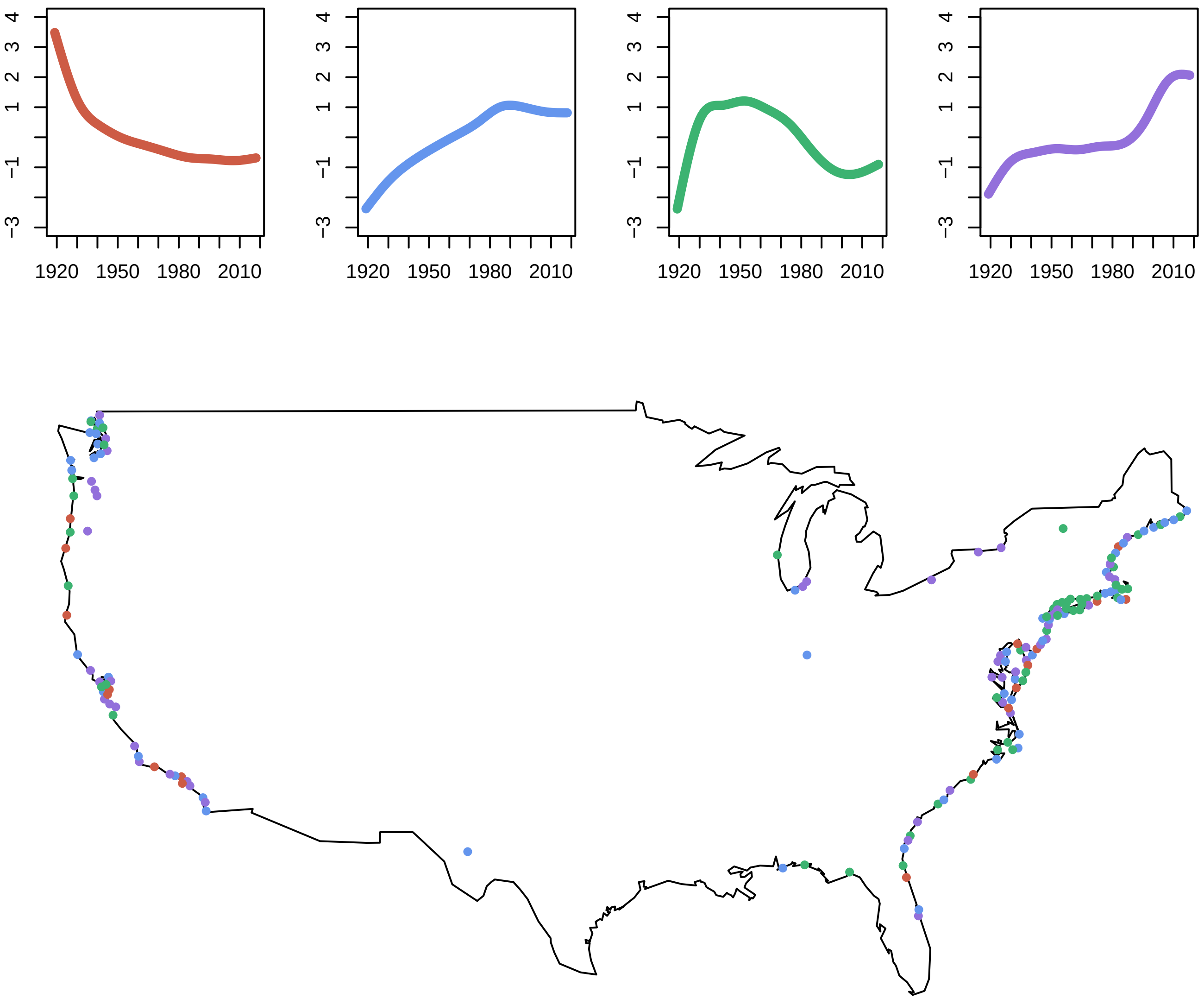

Spotted Towhee

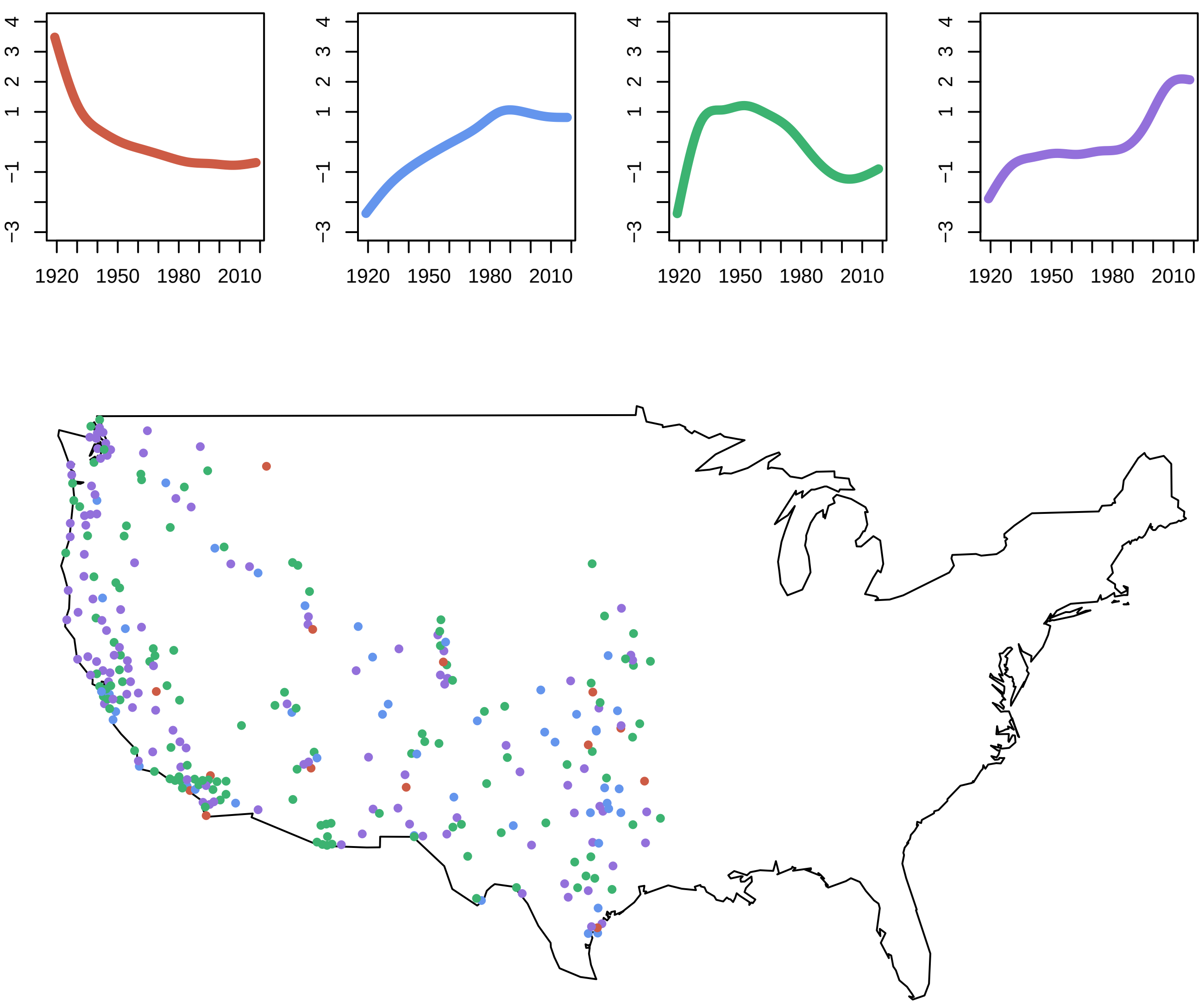

Steller's Jay

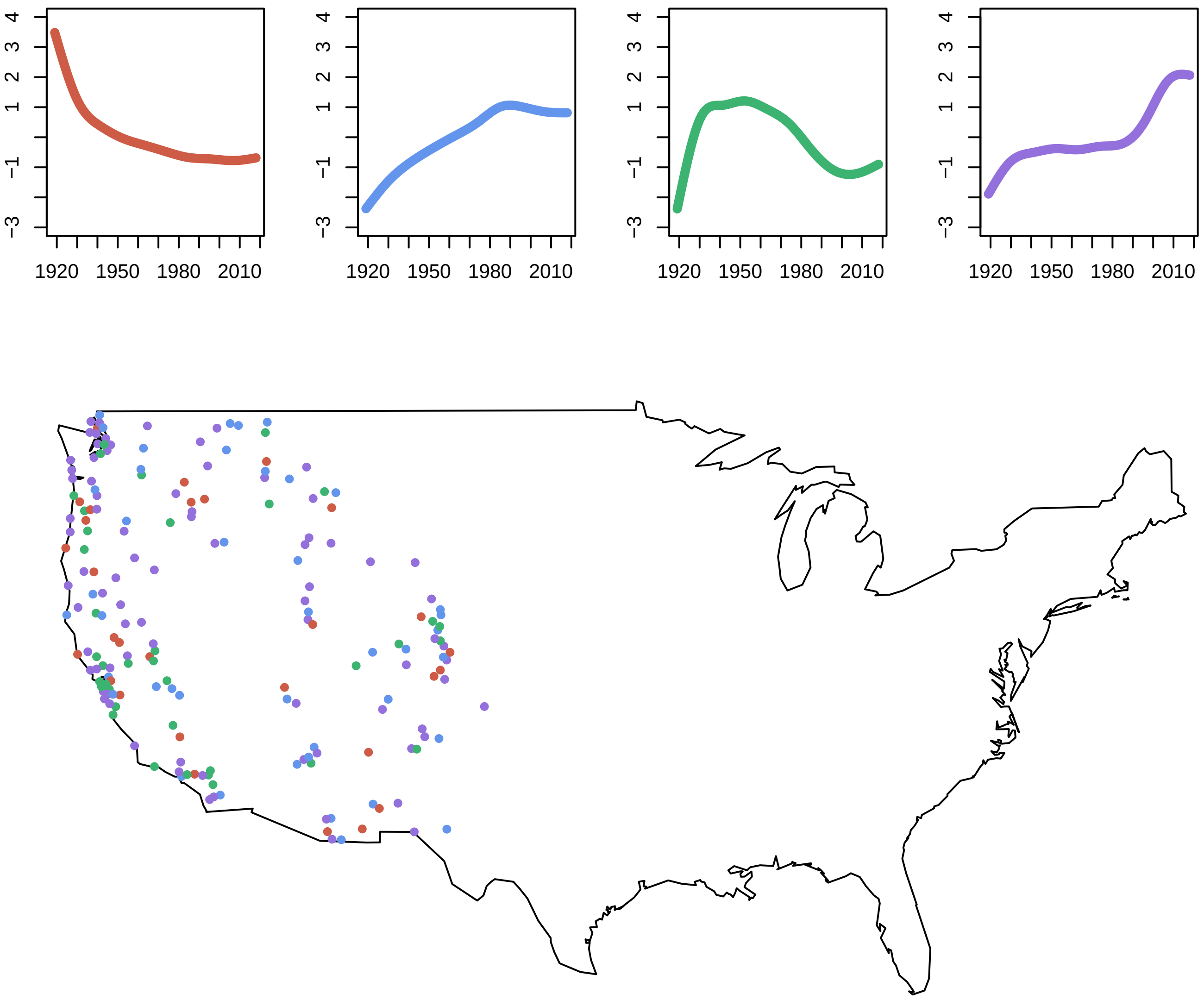

Vesper Sparrow

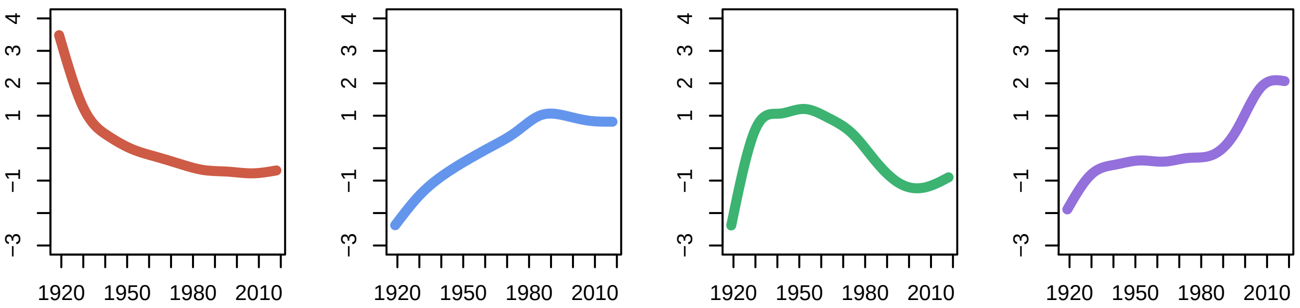

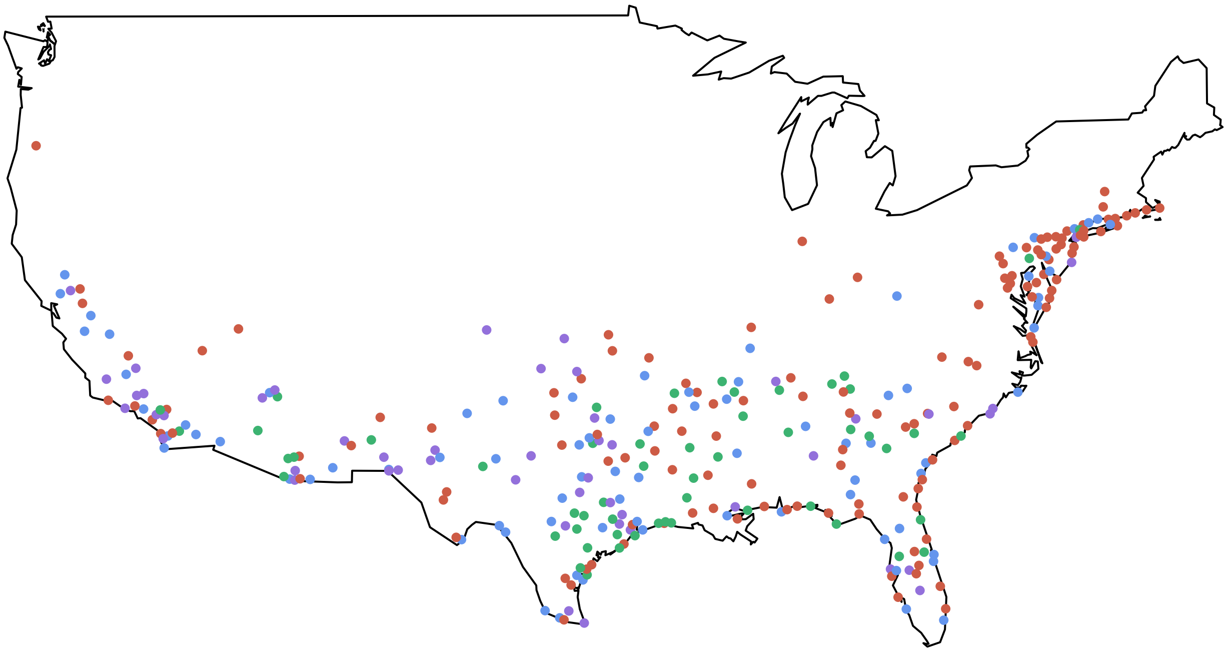

Western Bluebird

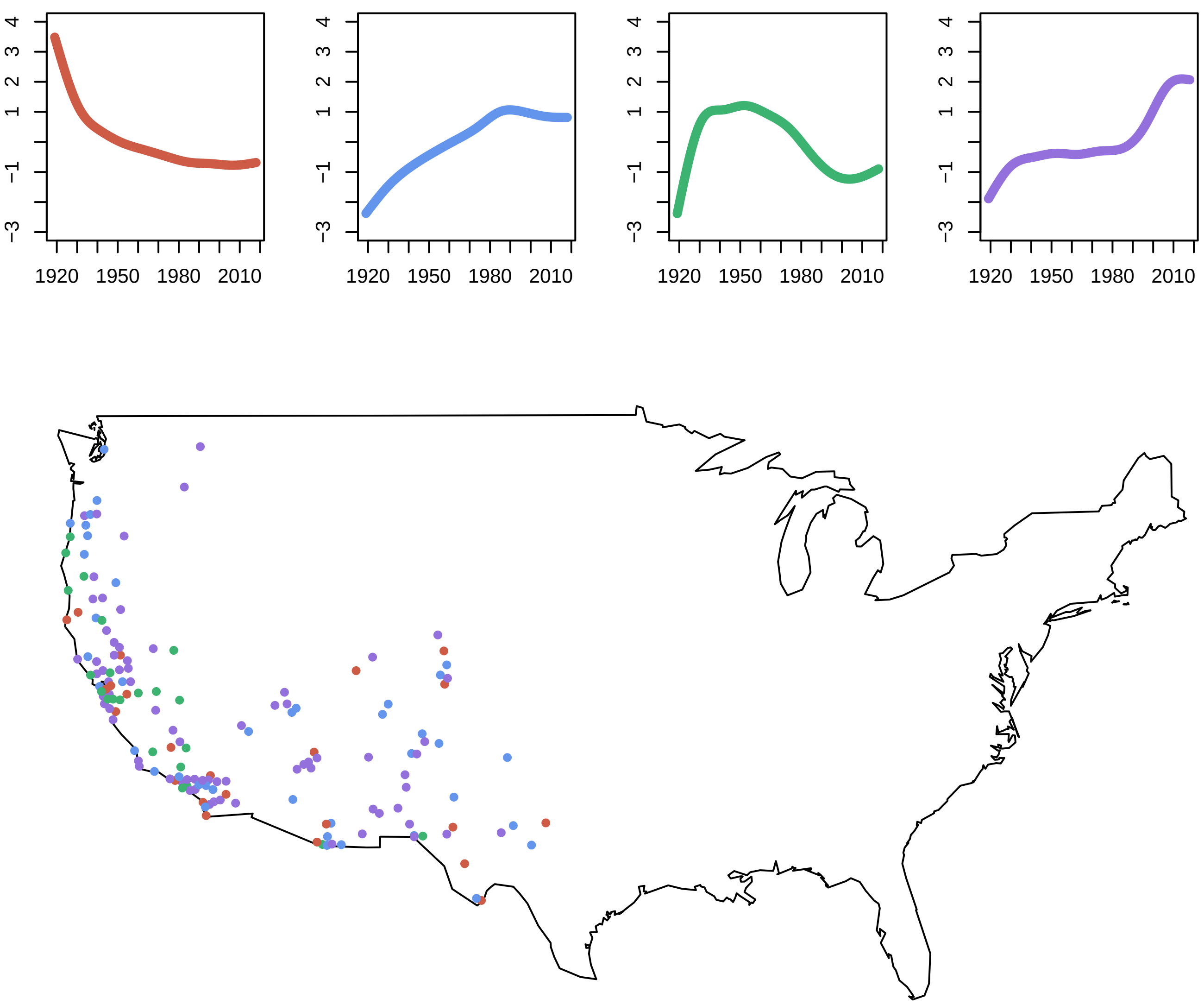

Western Scrub−Jay

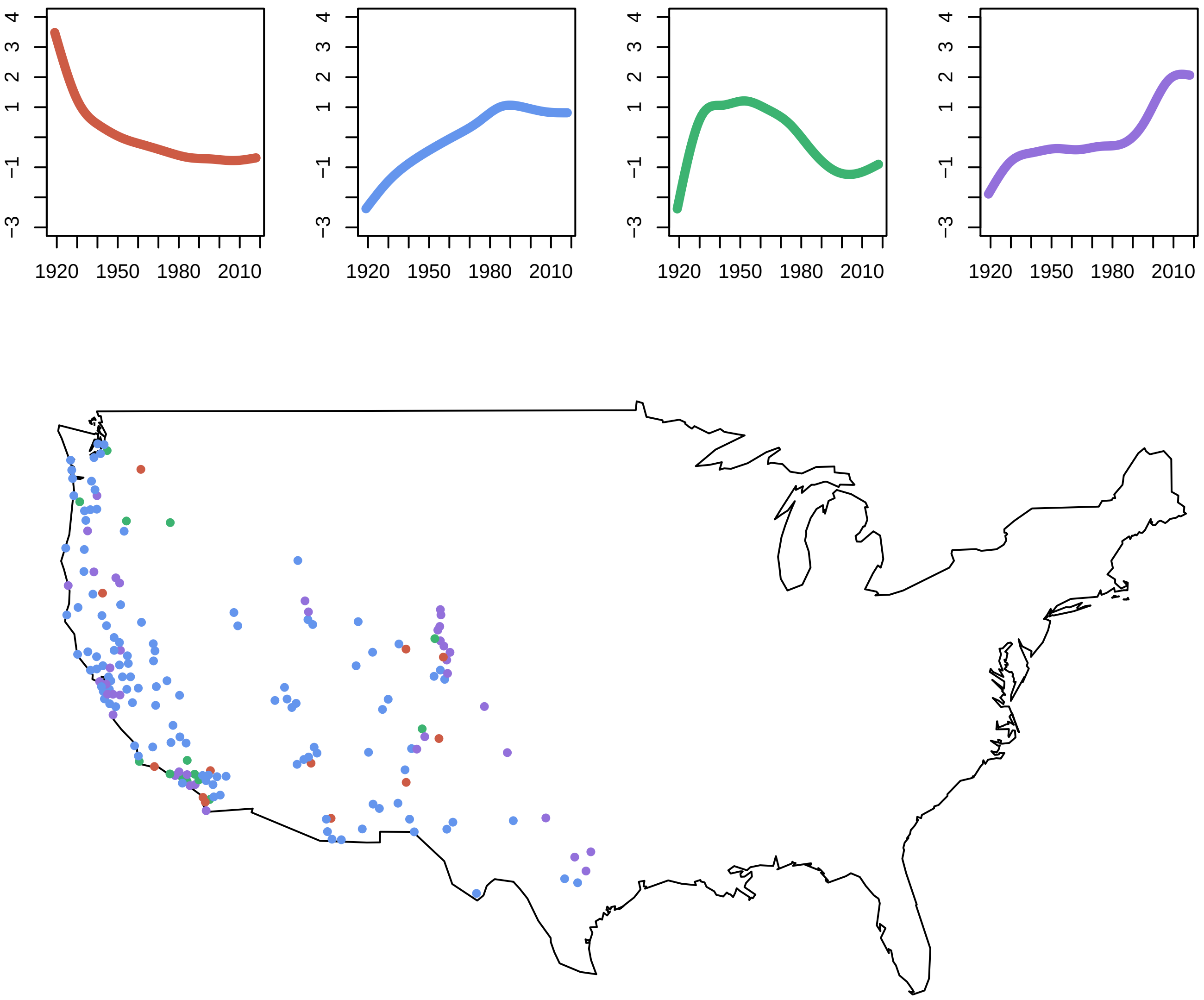

White−faced Ibis

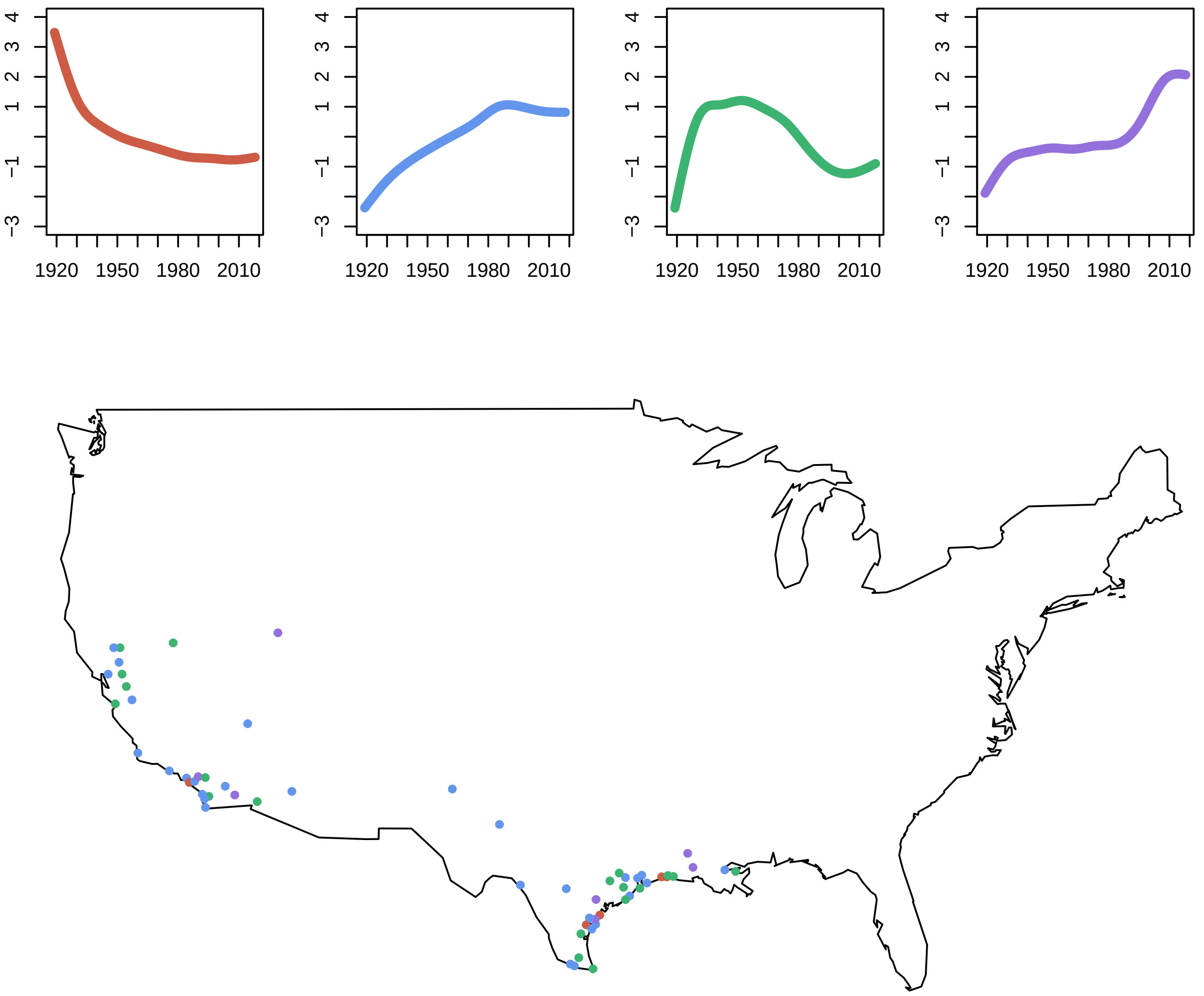

Wood Duck

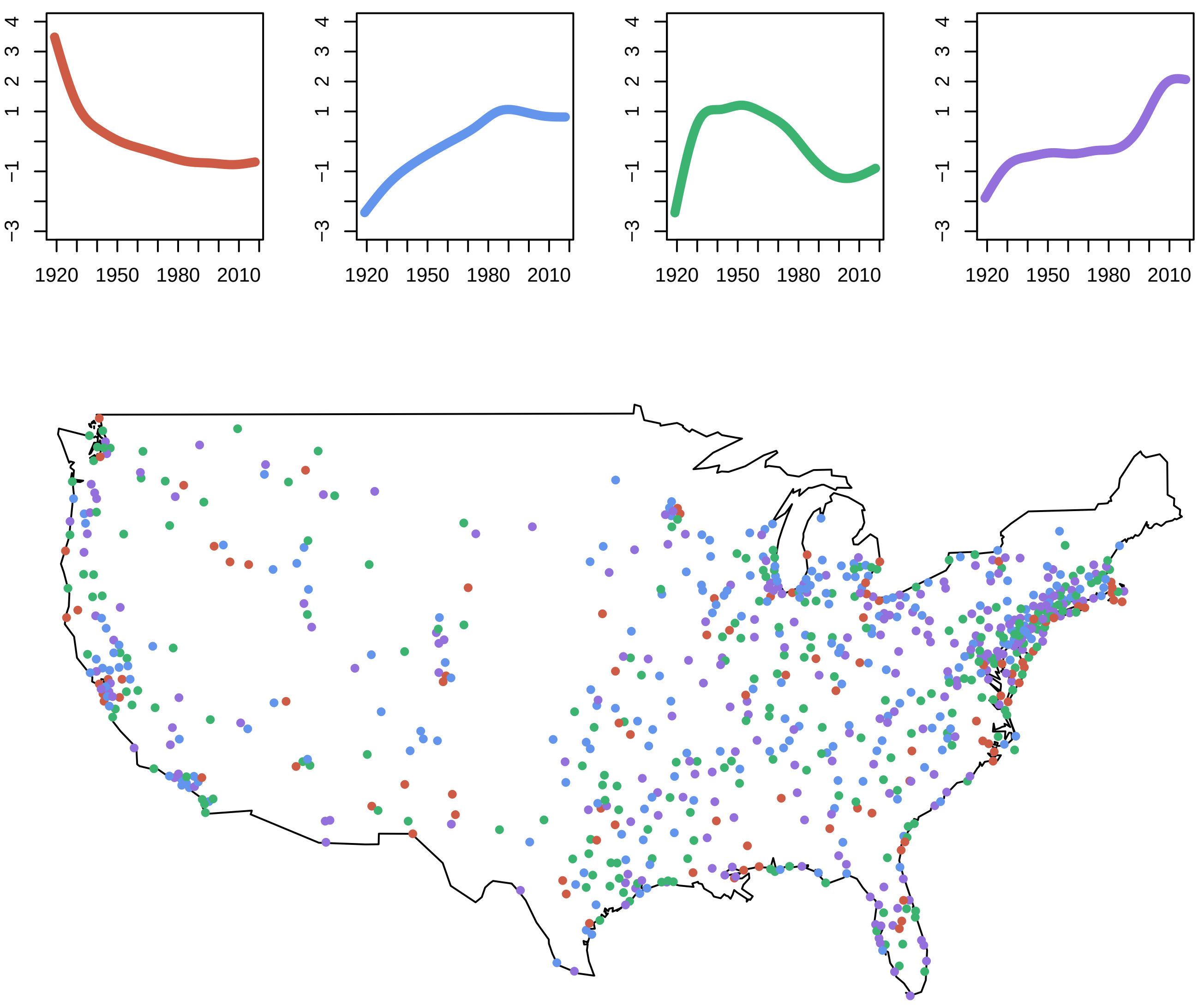

Yellow−headed Blackbird

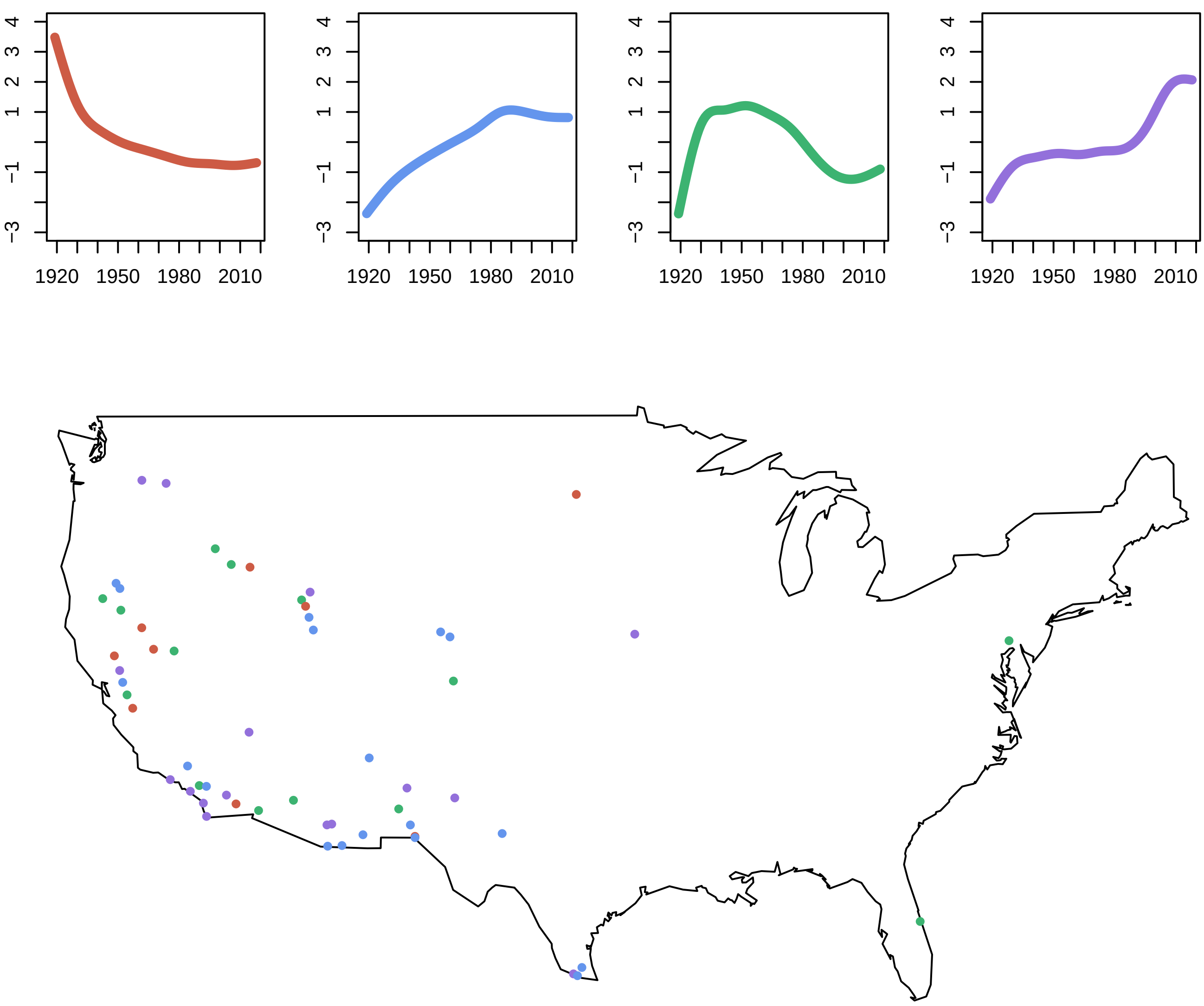

Anhinga

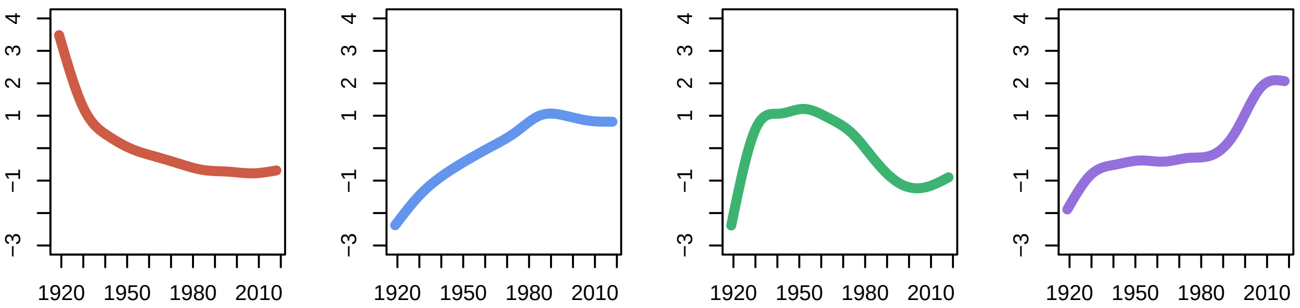

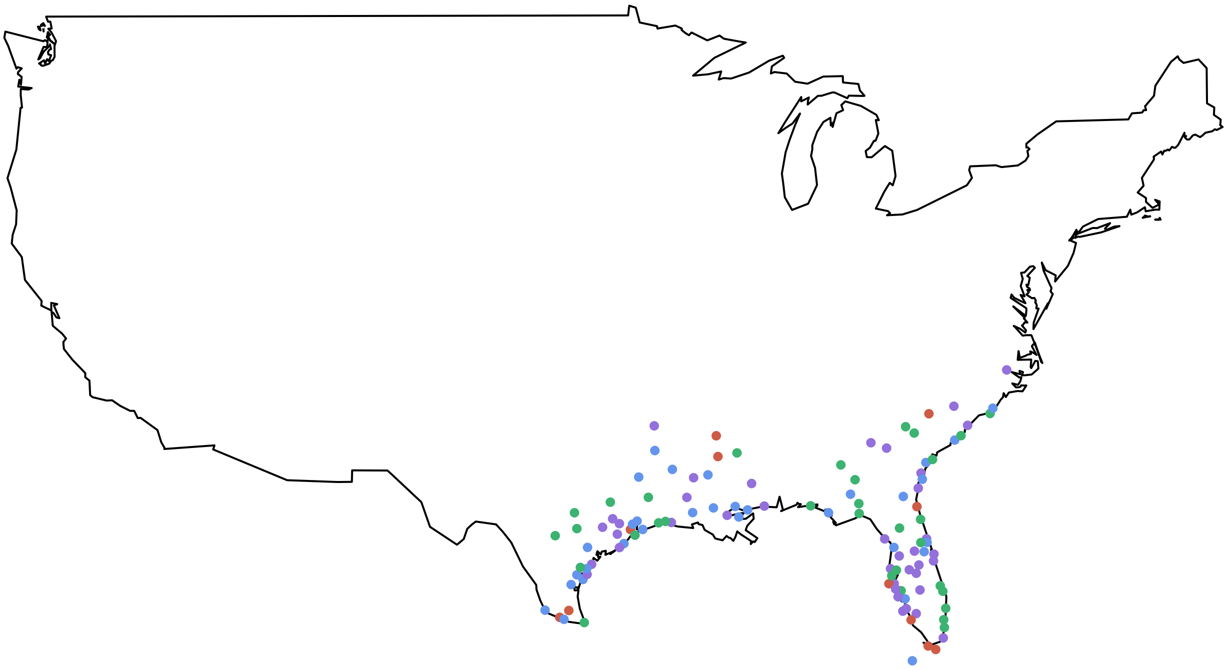

Bewick's Wren

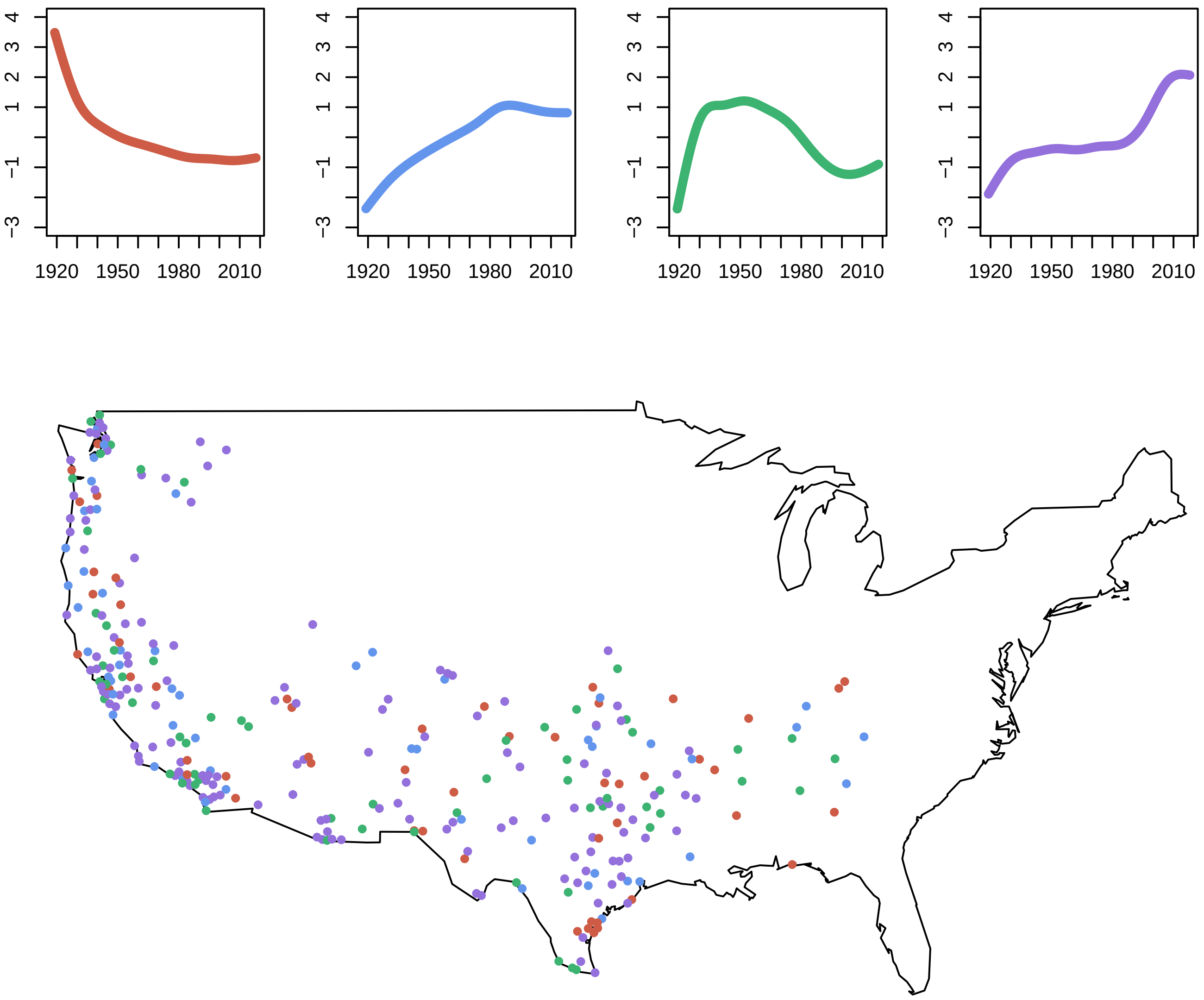

Black−bellied Whistling−Duck

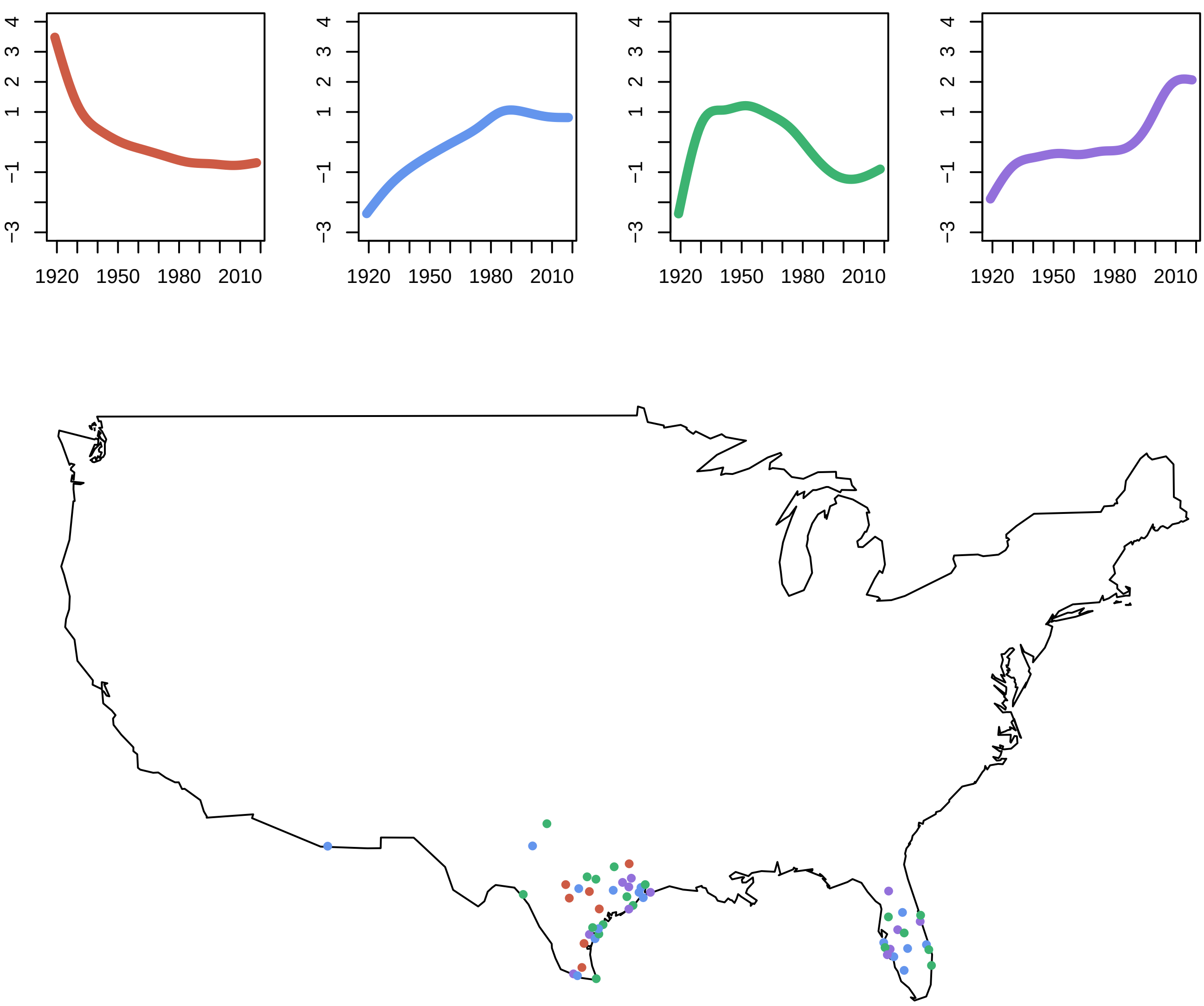

Brewer's Sparrow

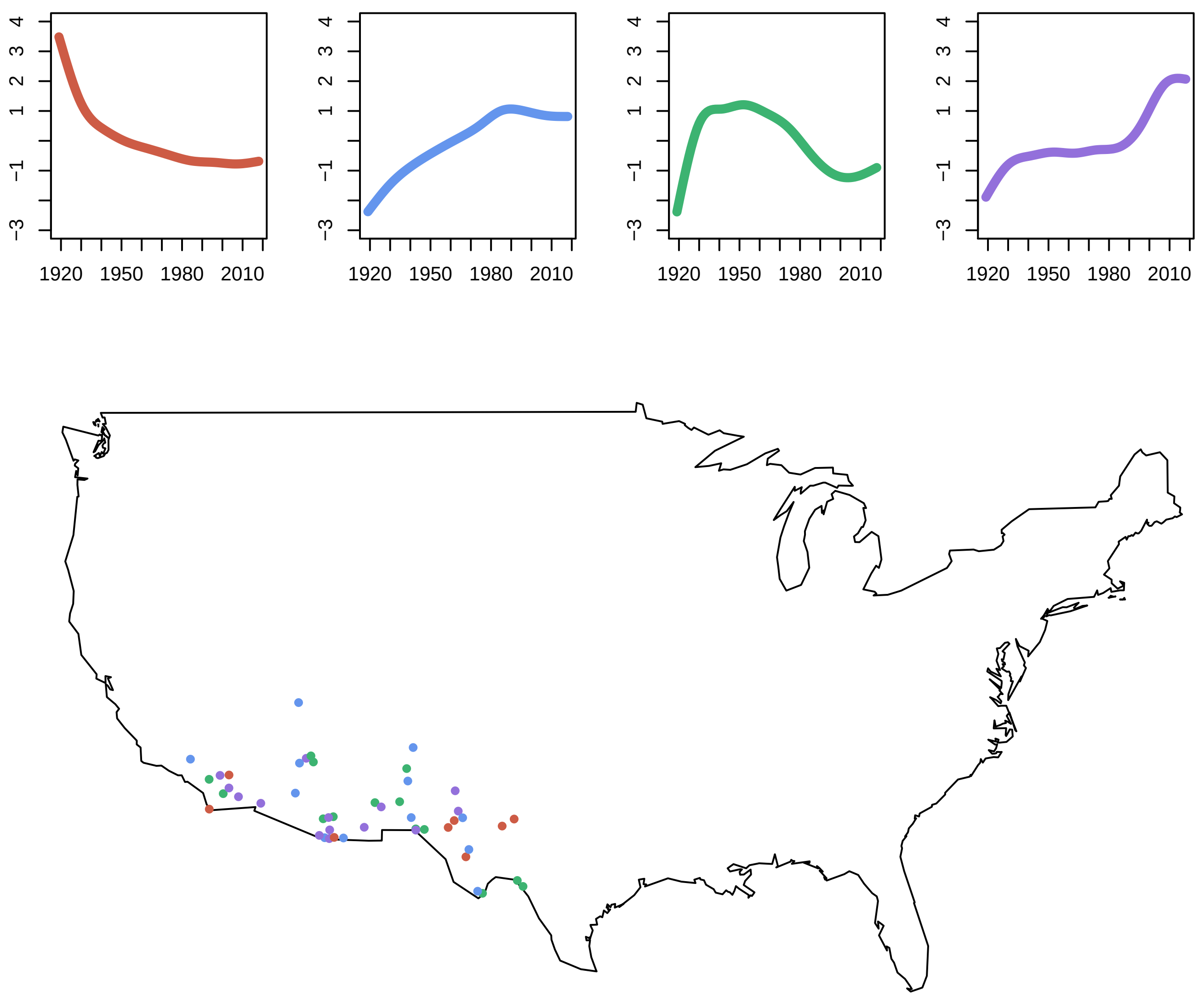

Bronzed Cowbird

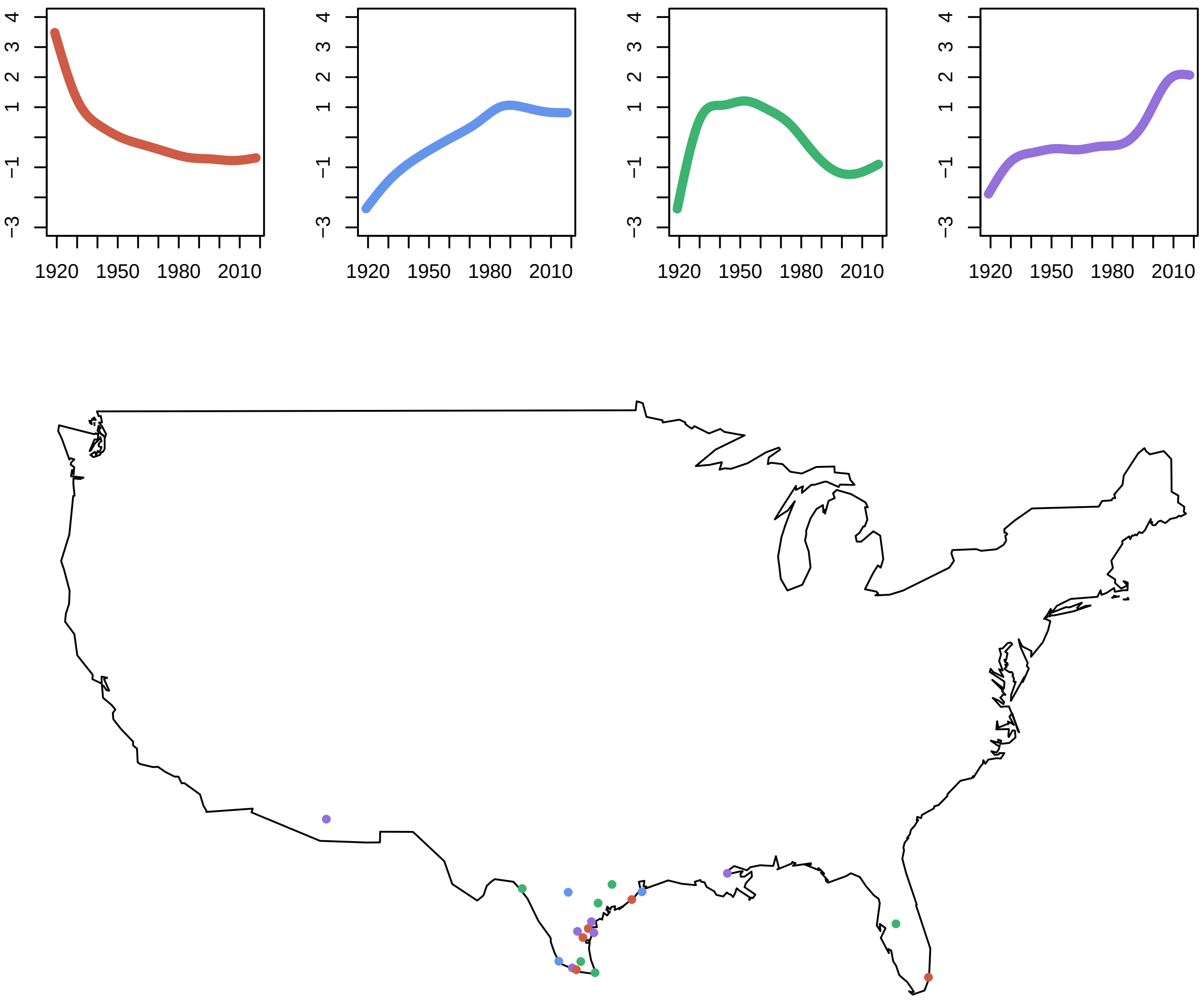

Brown−headed Nuthatch

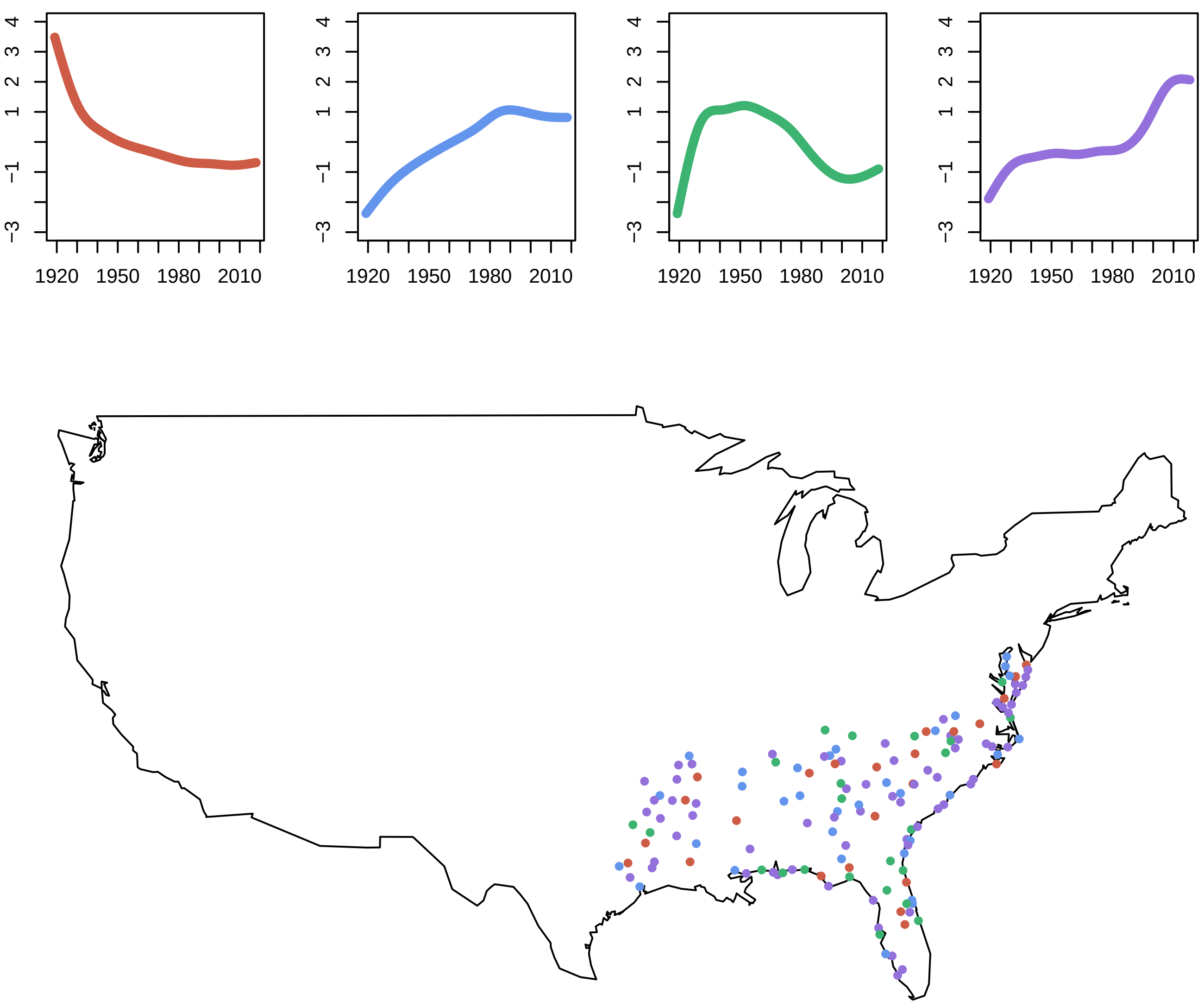

Brown Thrasher

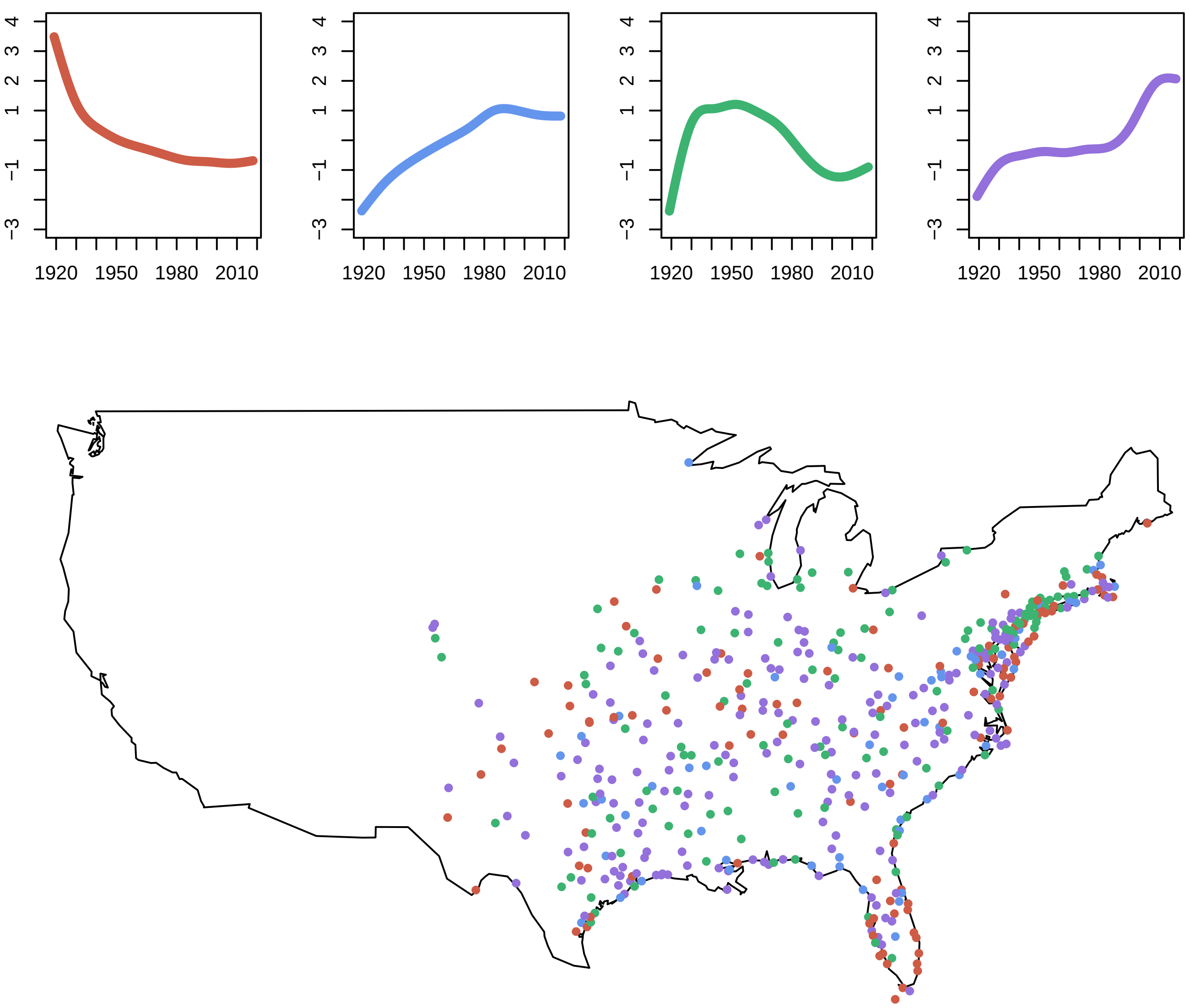

California Towhee

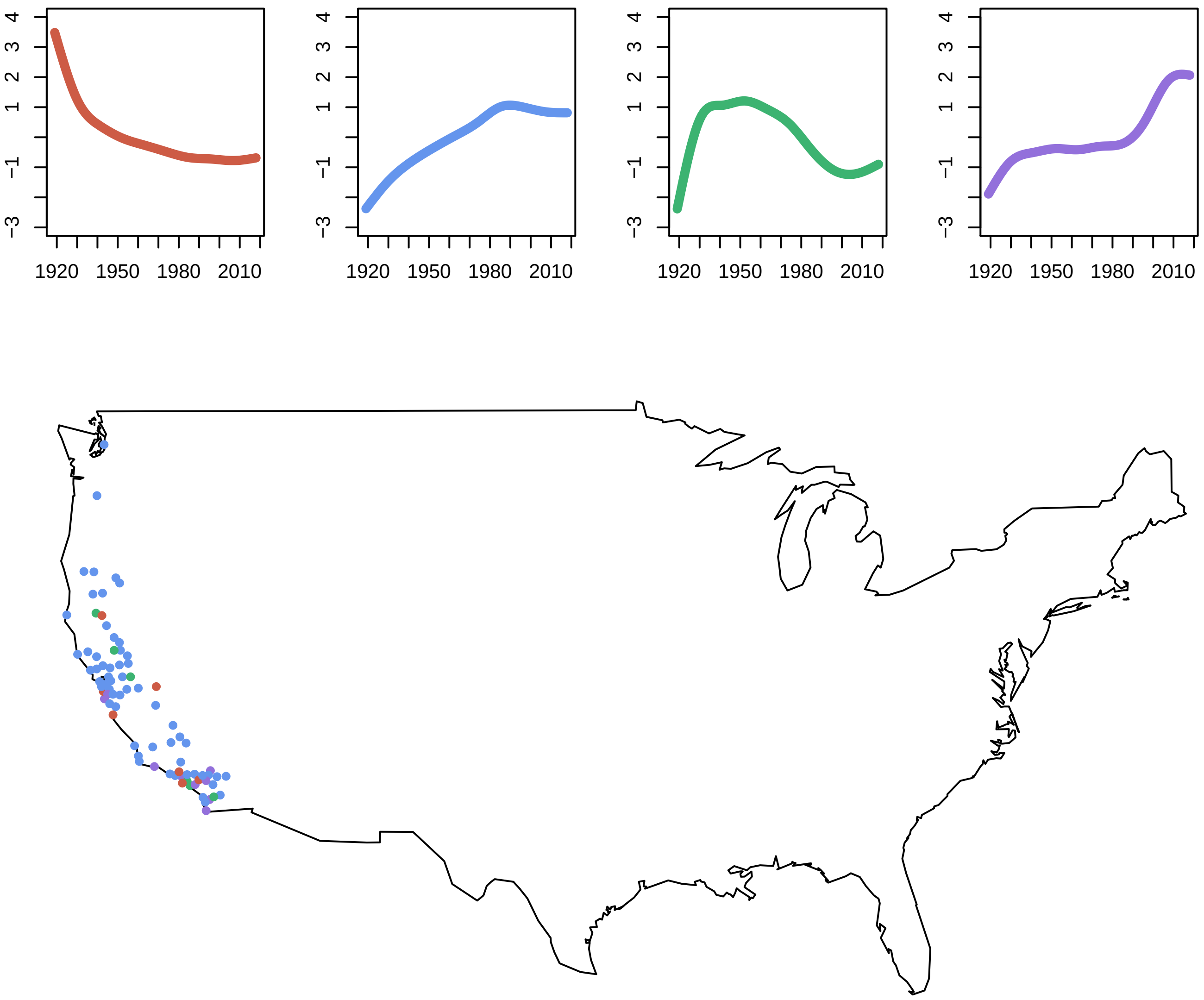

Common Yellowthroat

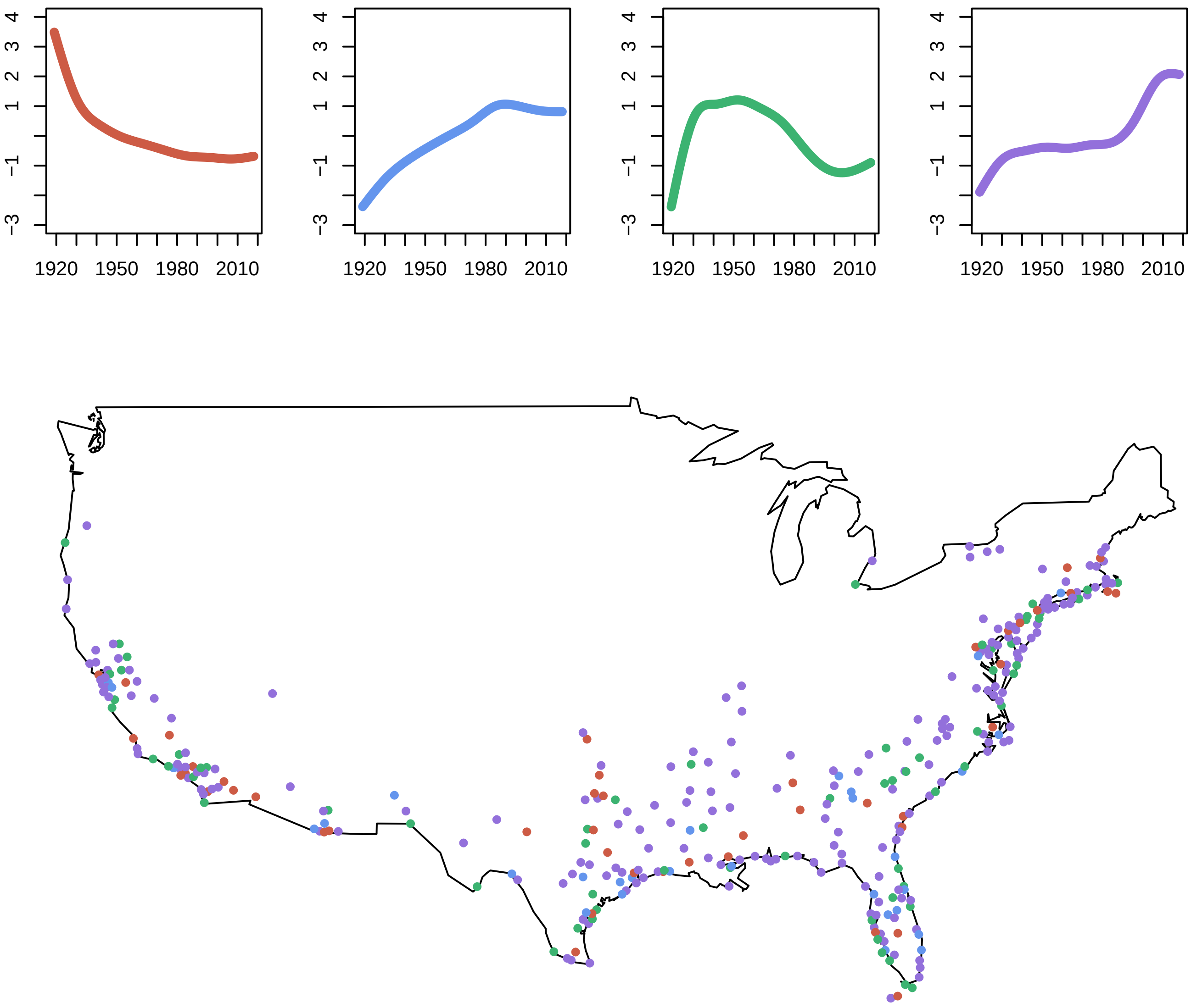

Cooper's Hawk

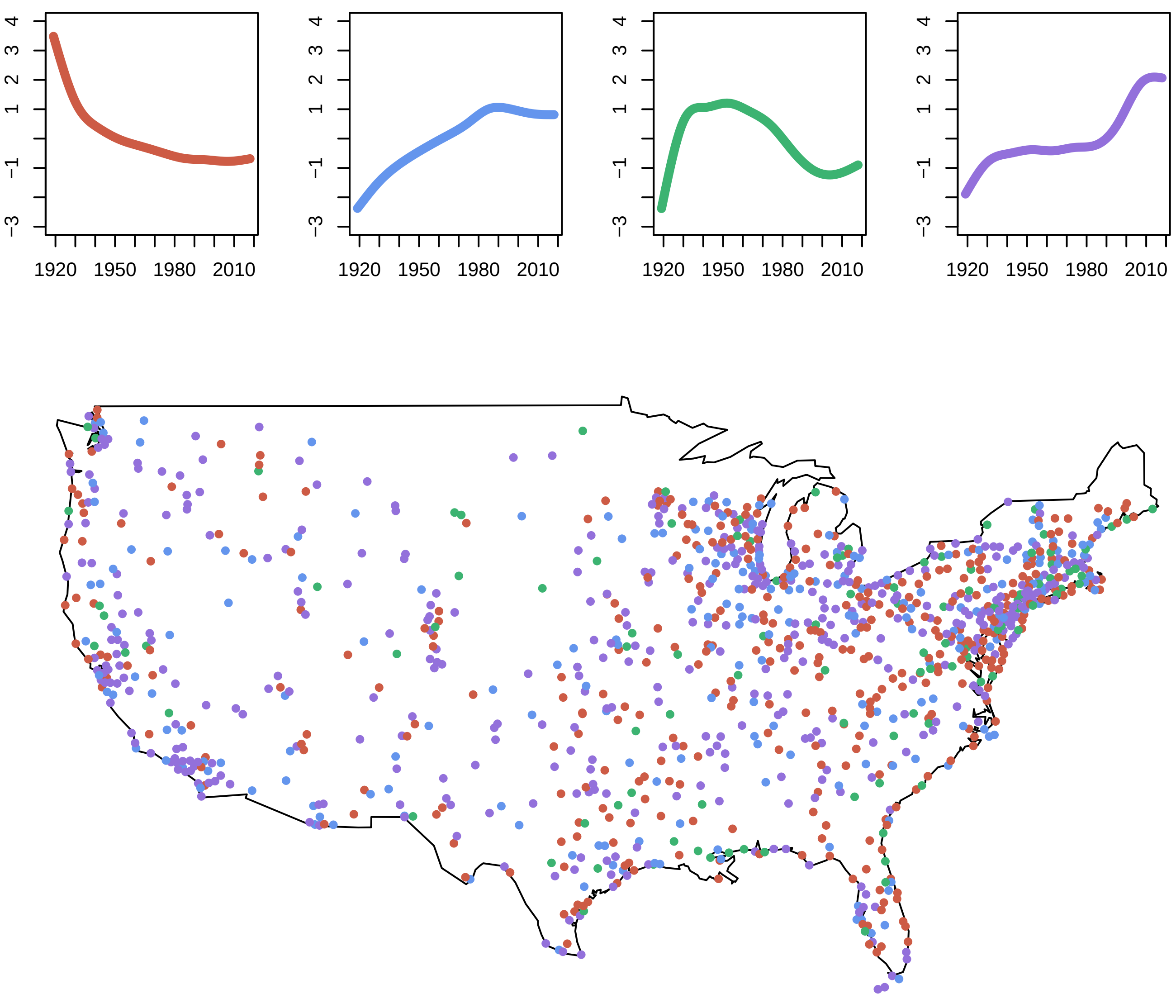

Gambel's Quail

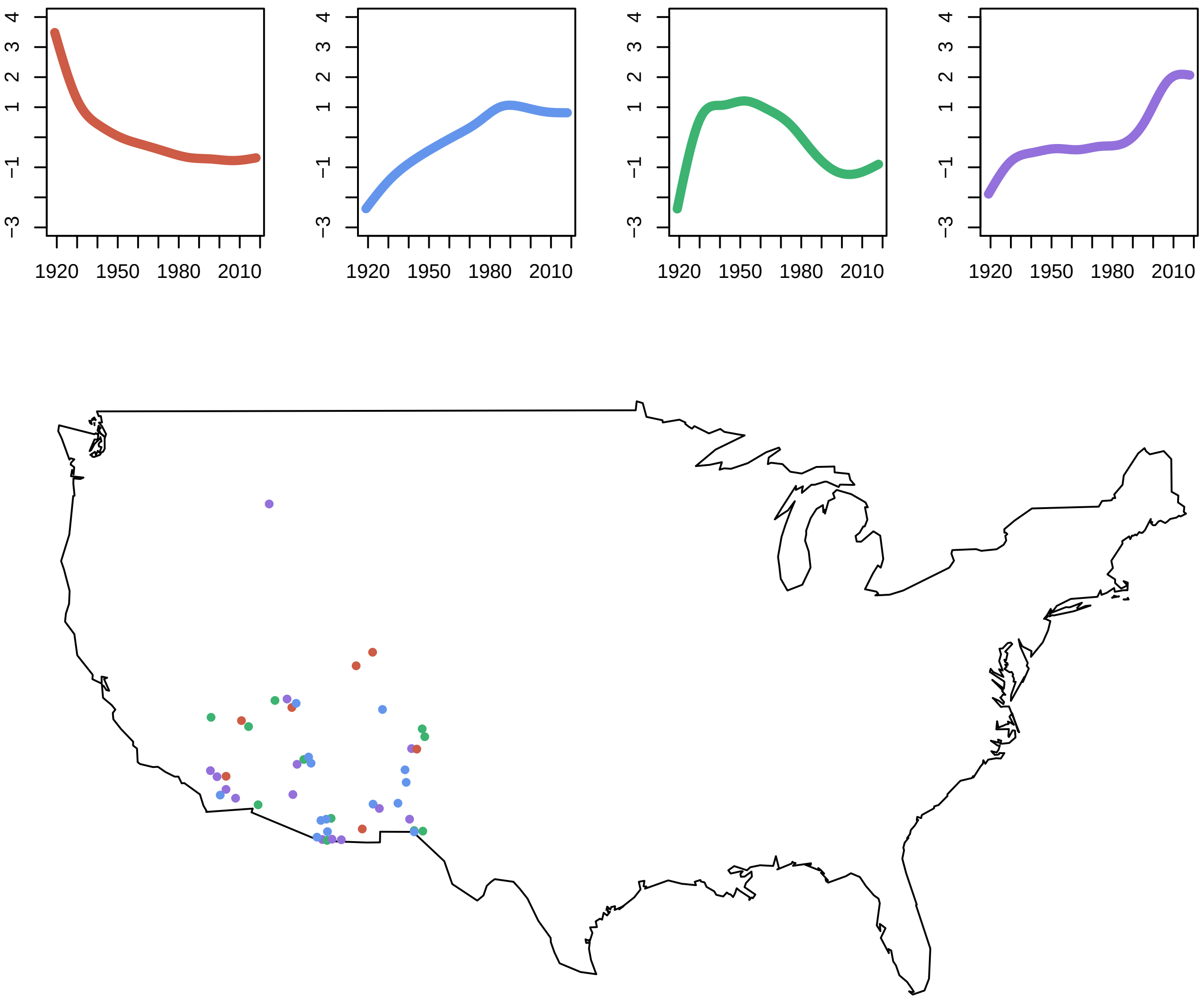

Glossy Ibis

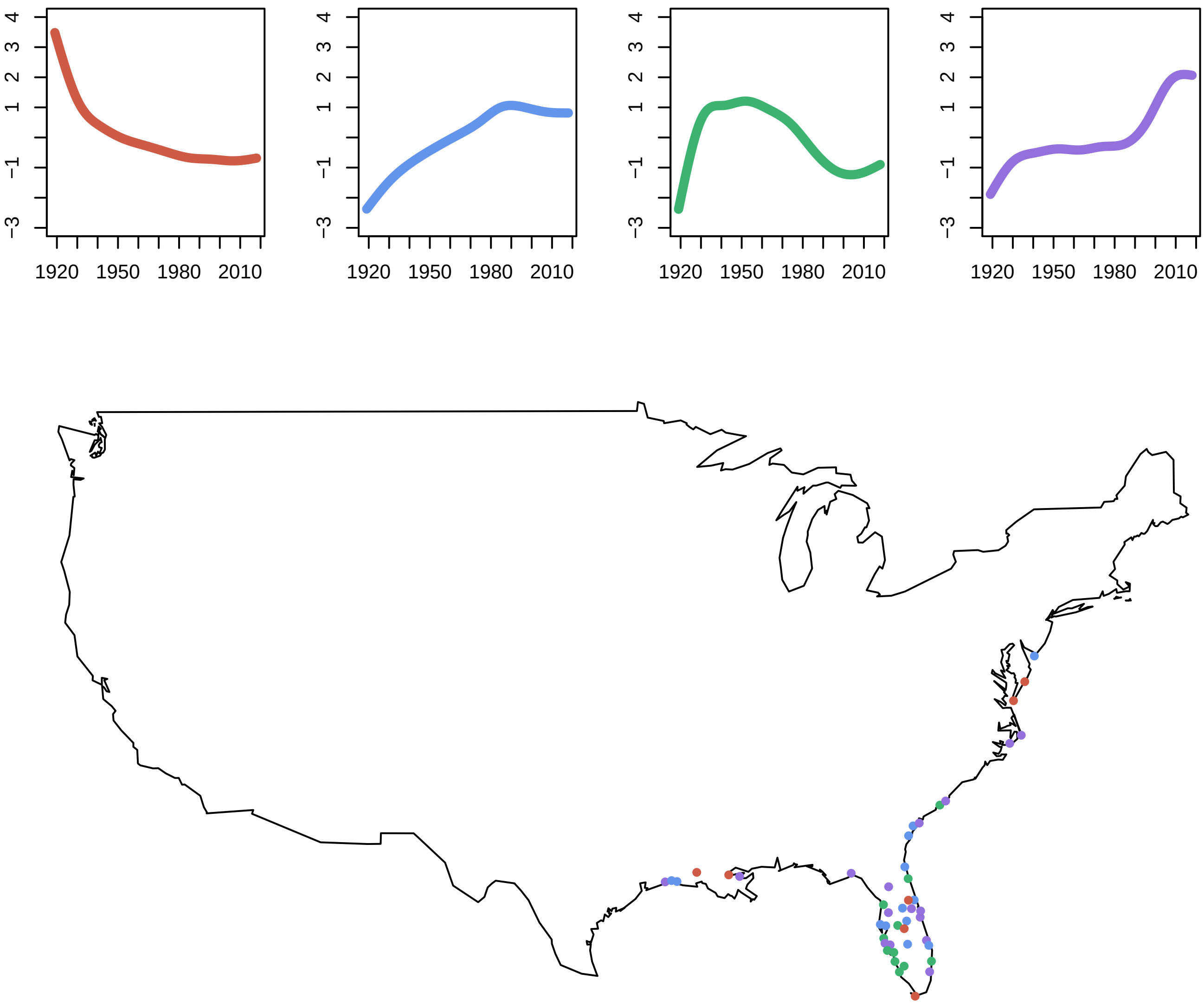

Gray−crowned Rosy−Finch

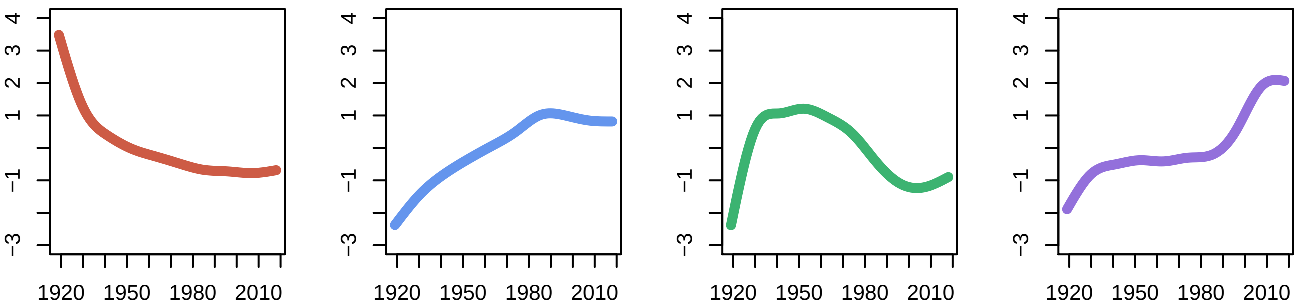

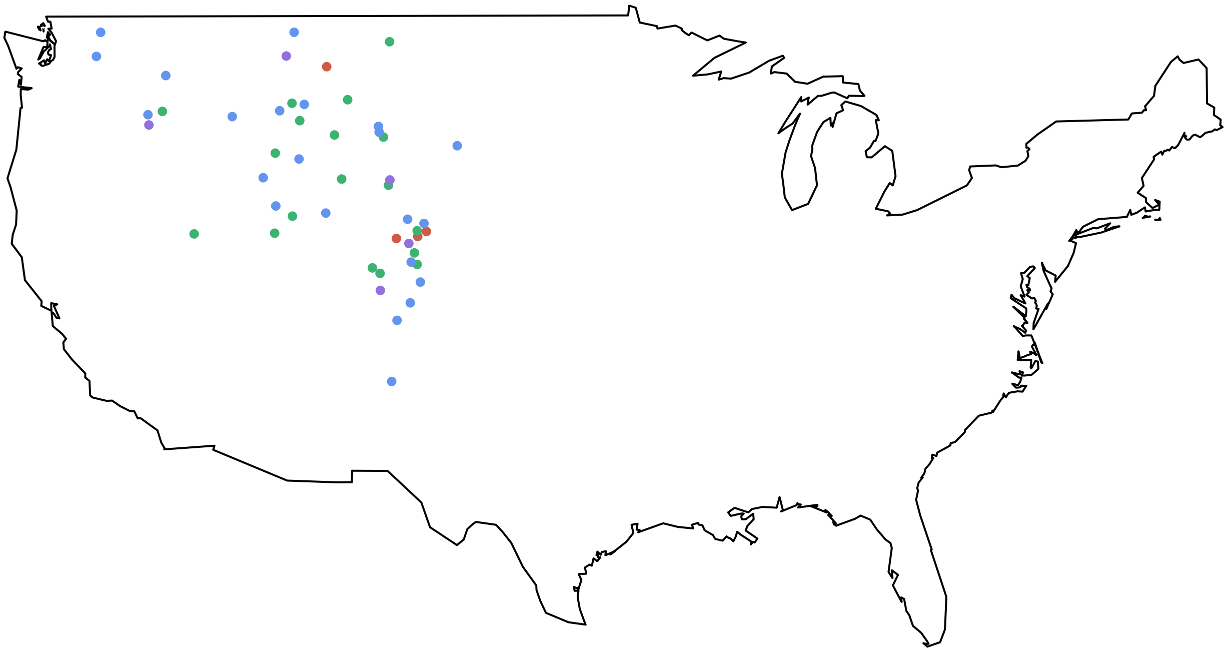

Gray Catbird

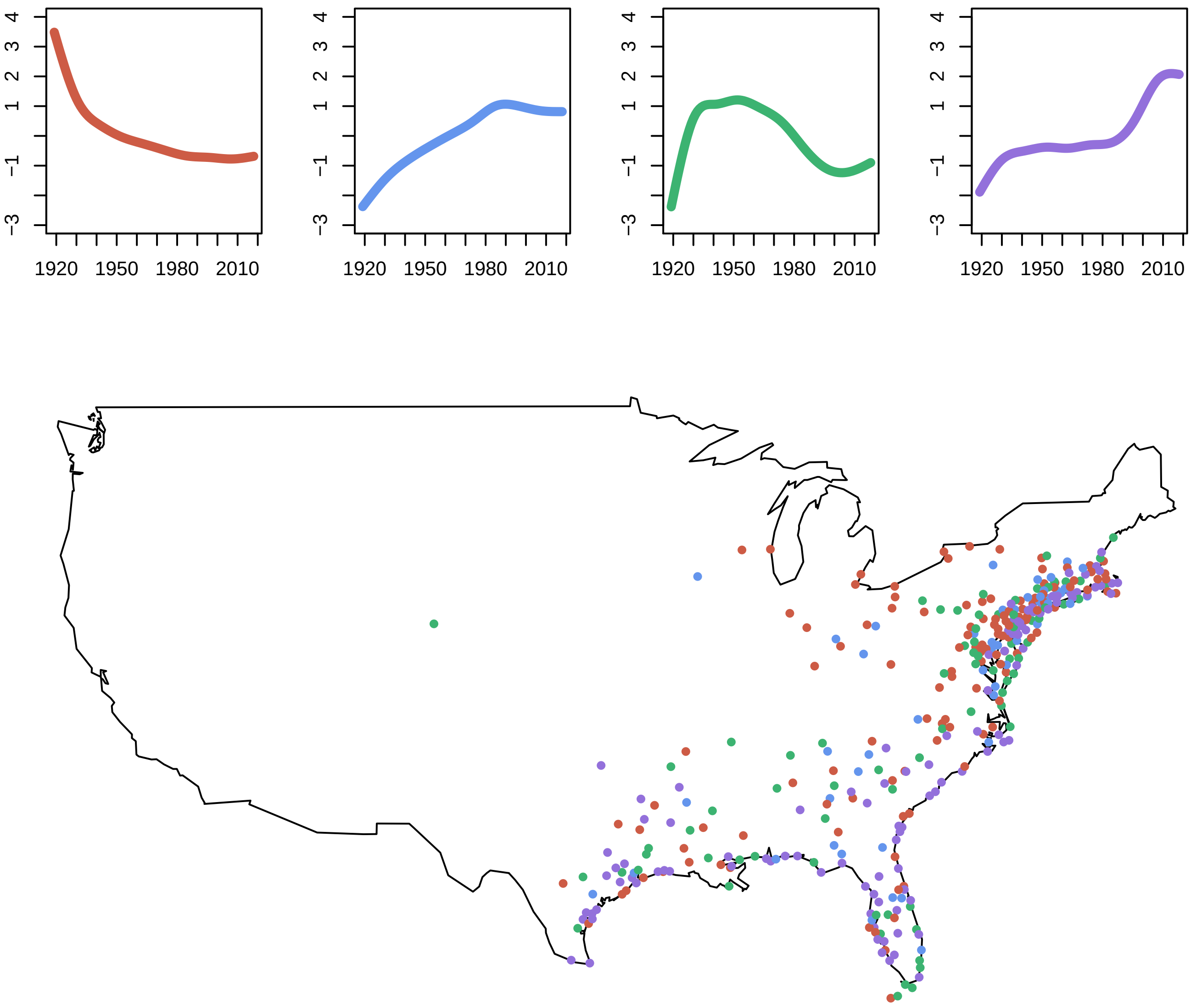

Great Horned Owl

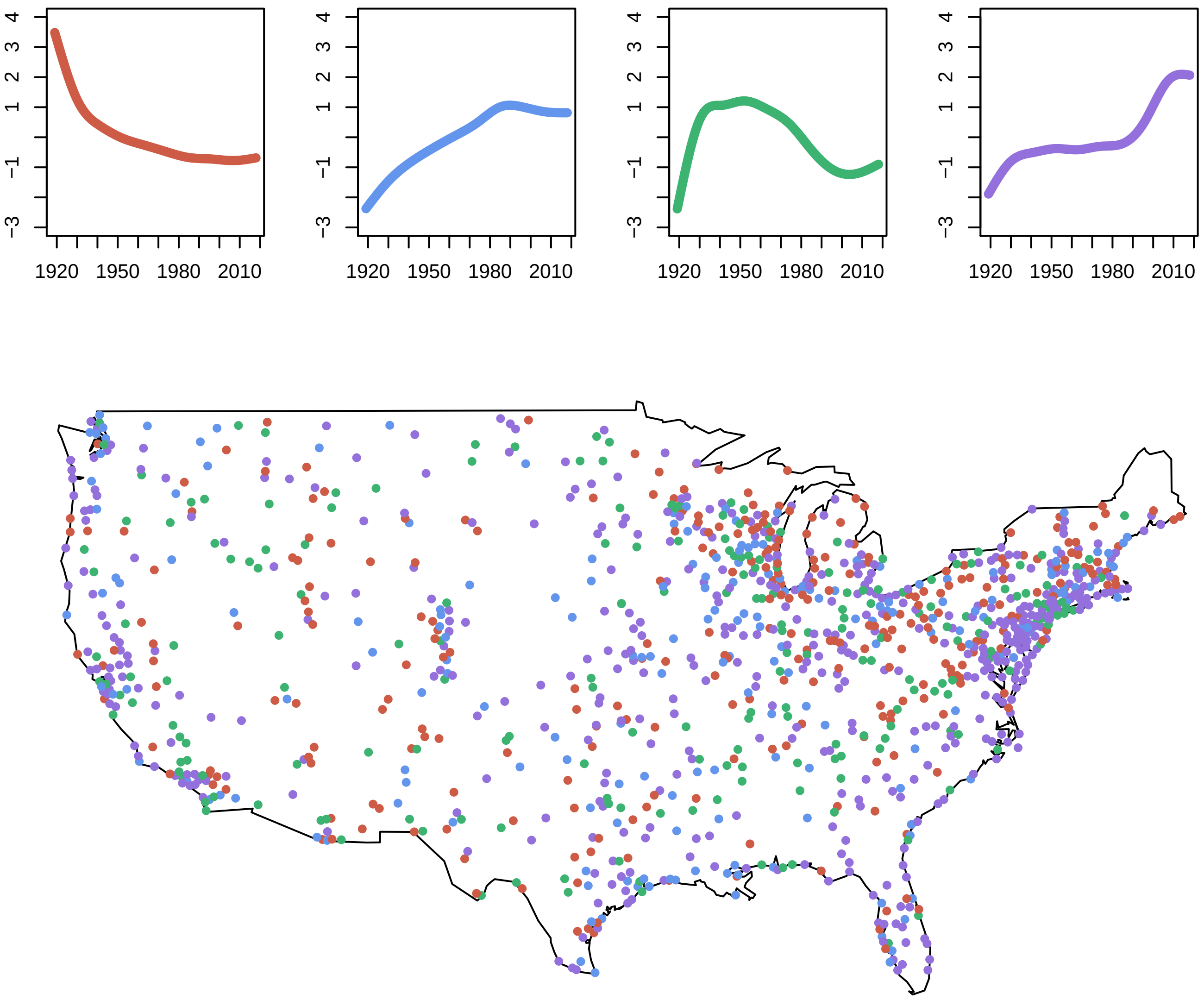

Greater Yellowlegs

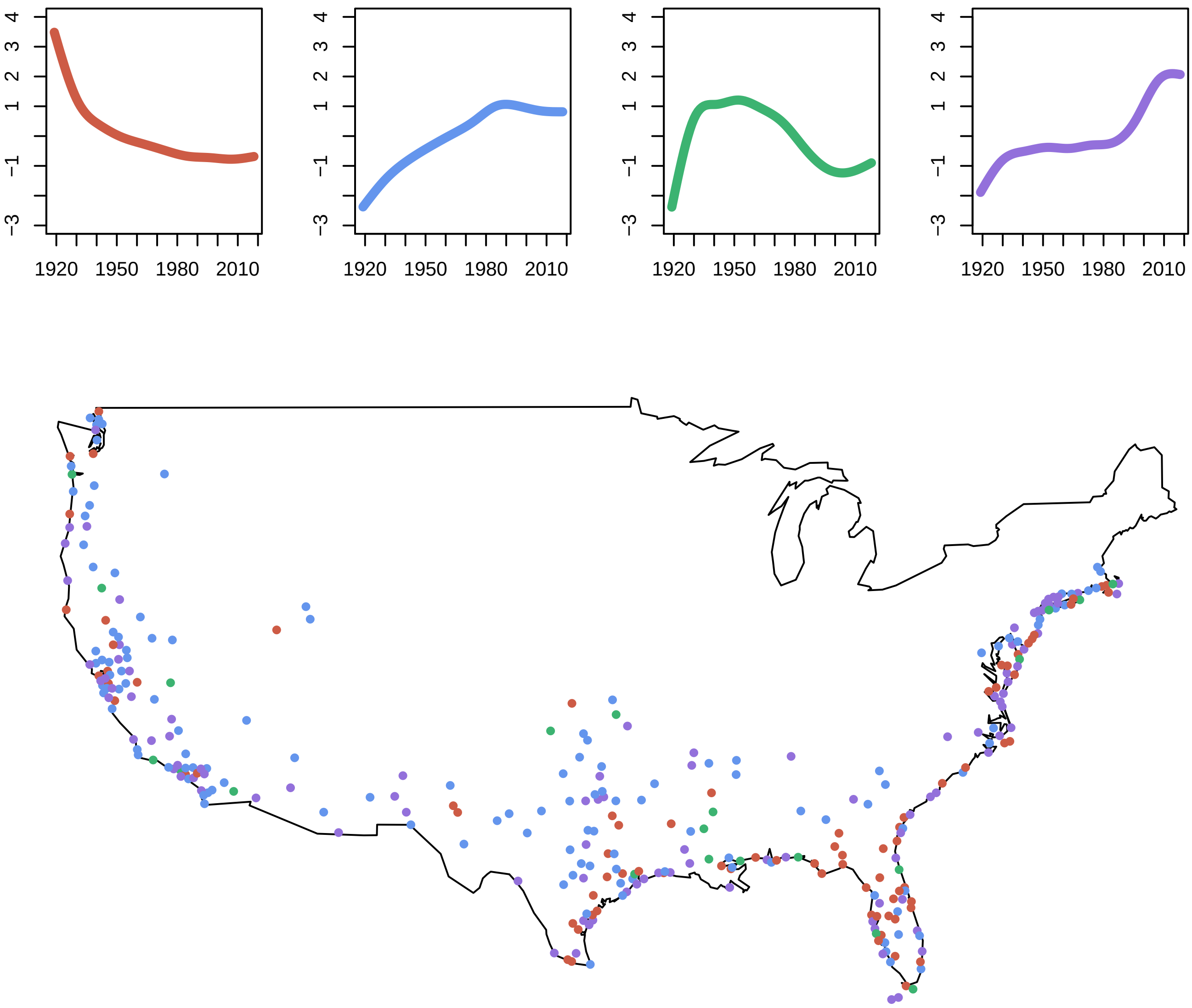

House Wren

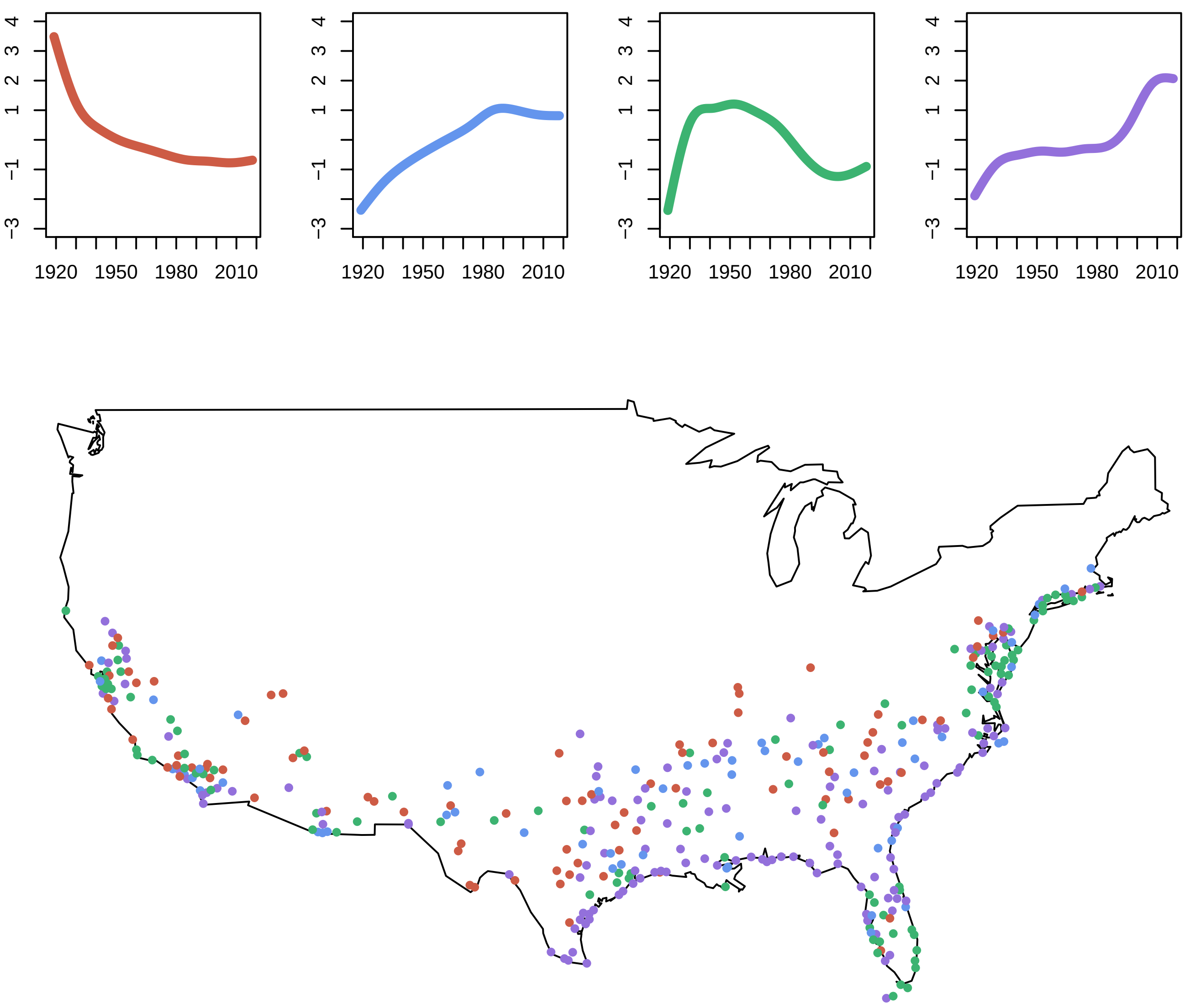

Lincoln's Sparrow

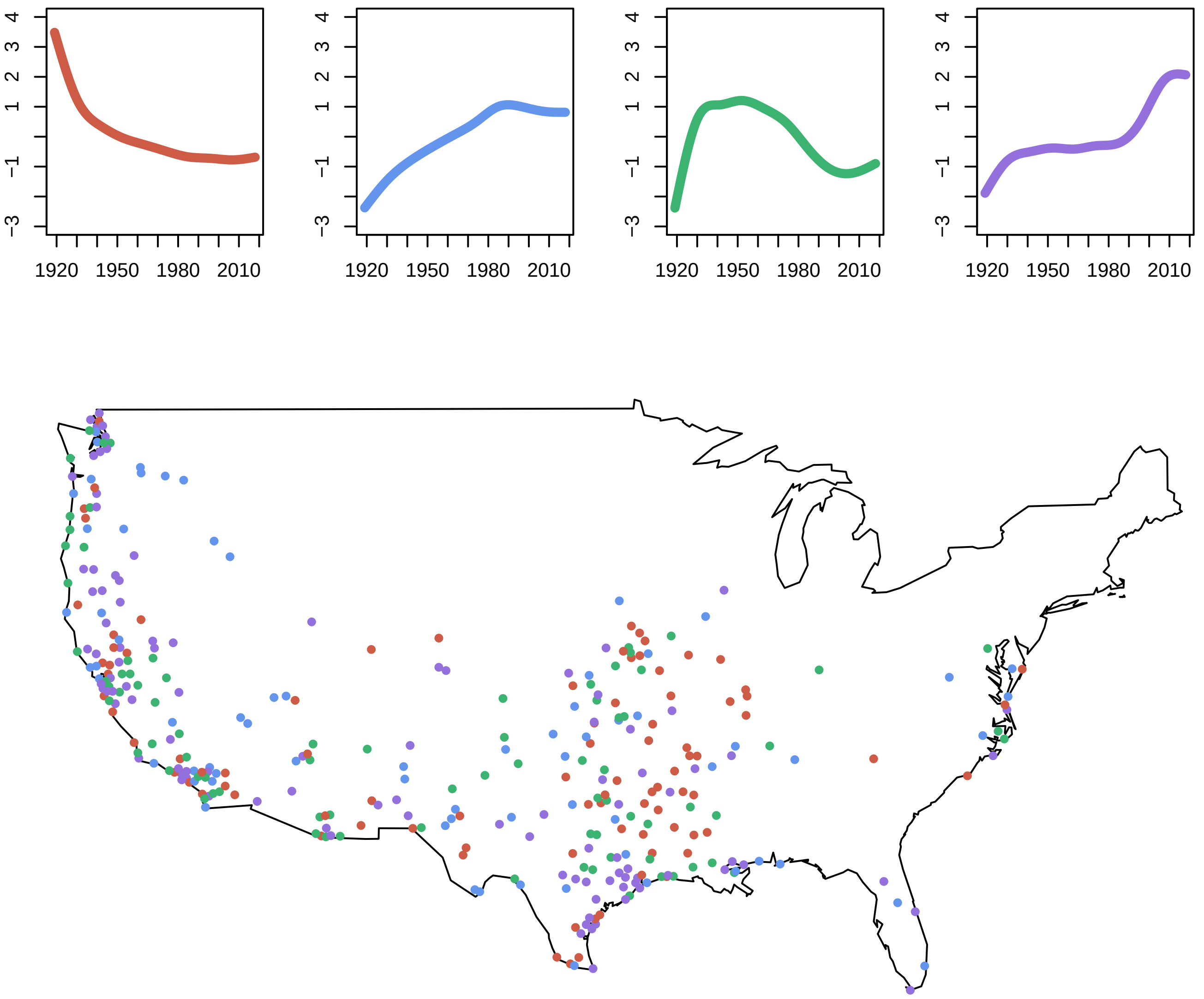

Little Blue Heron

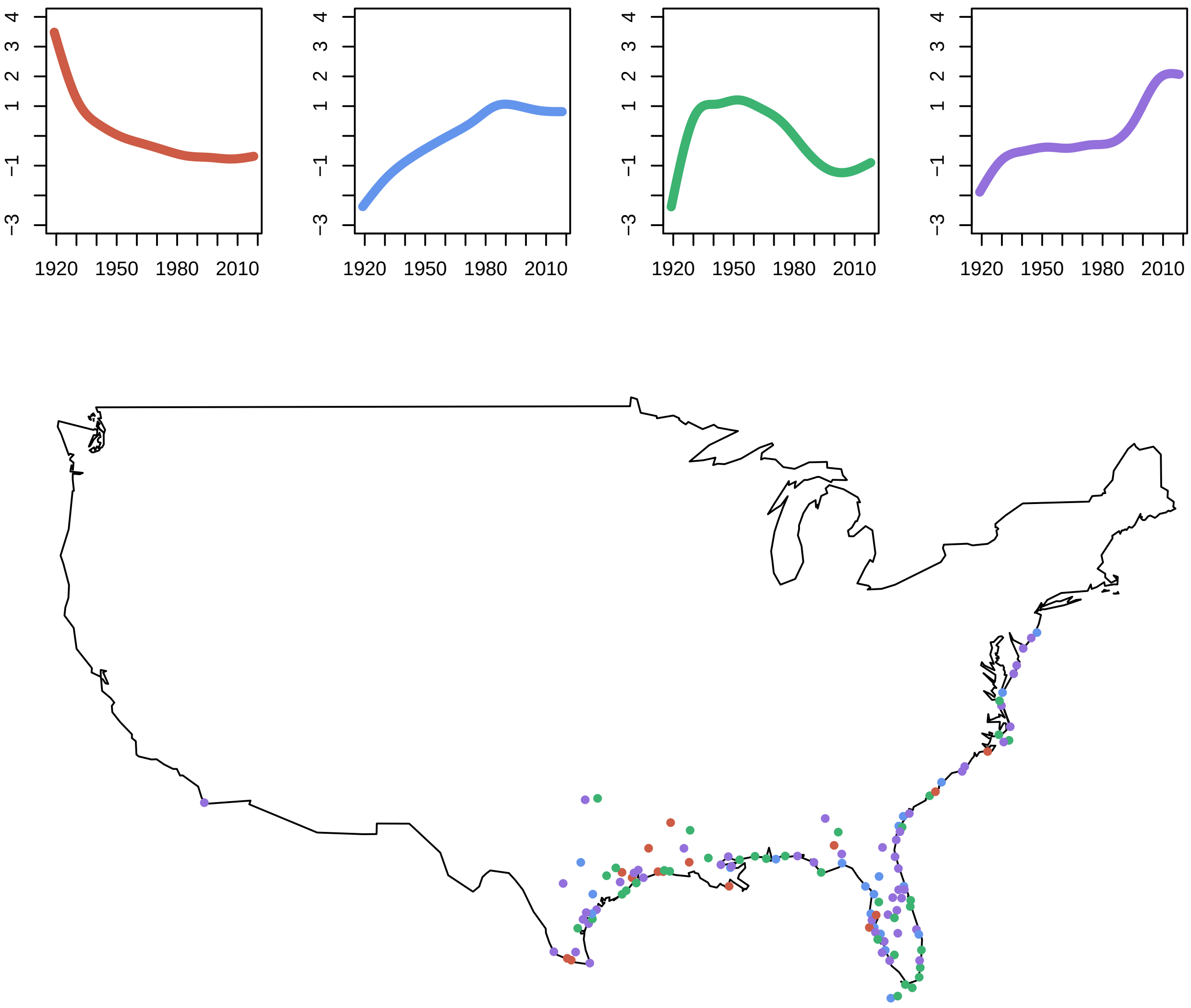

Marsh Wren

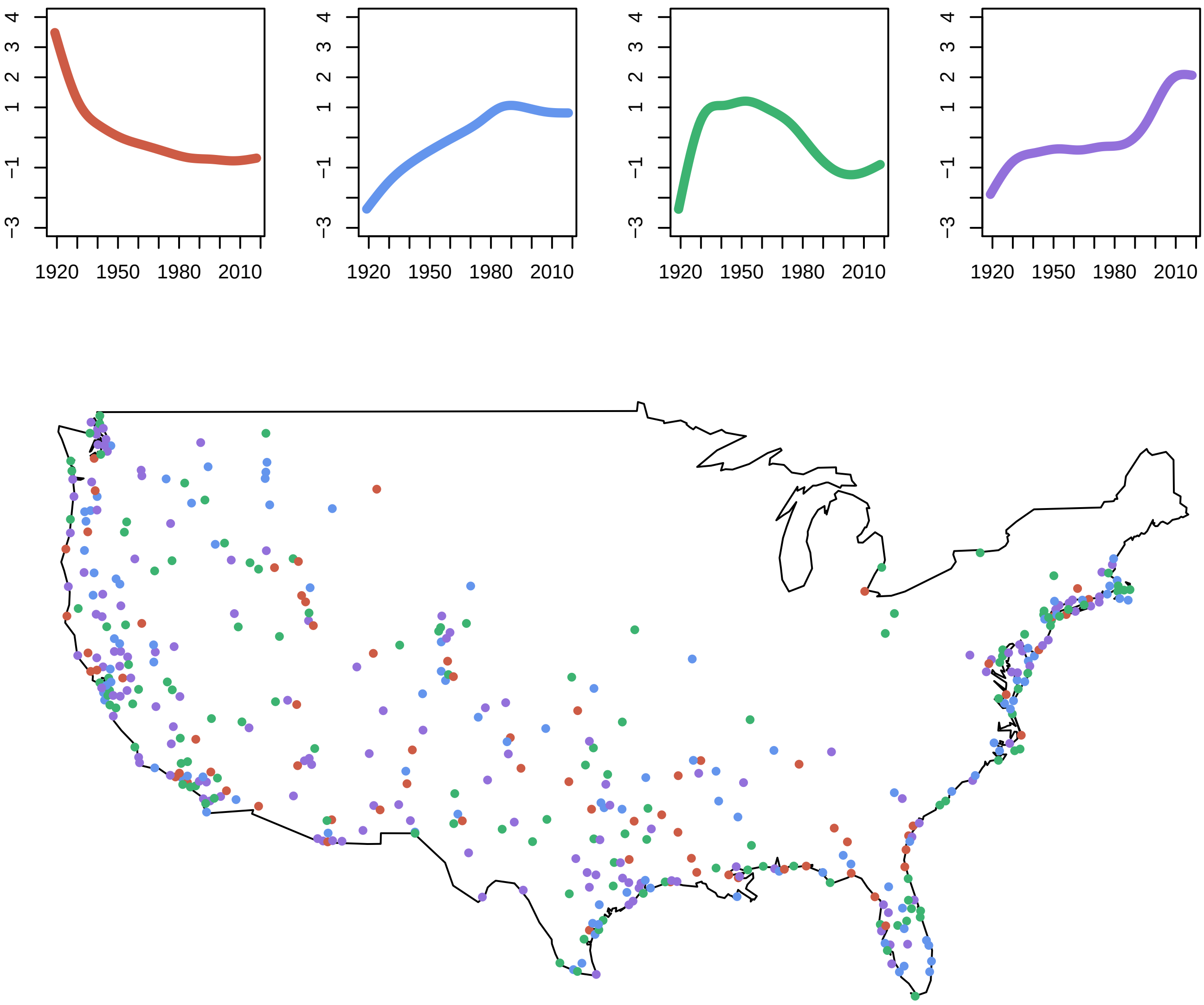

Mottled Duck

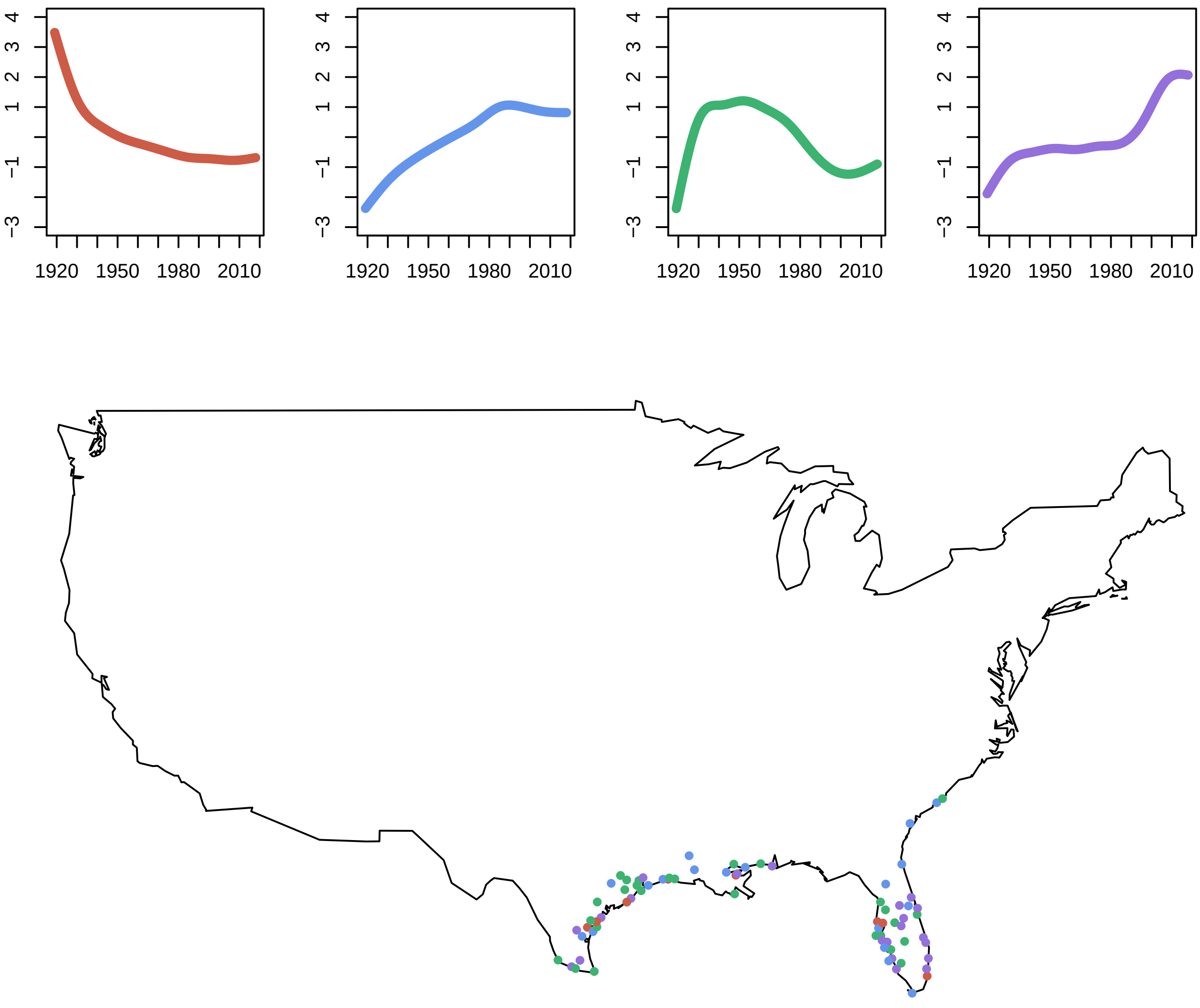

Mountain Bluebird

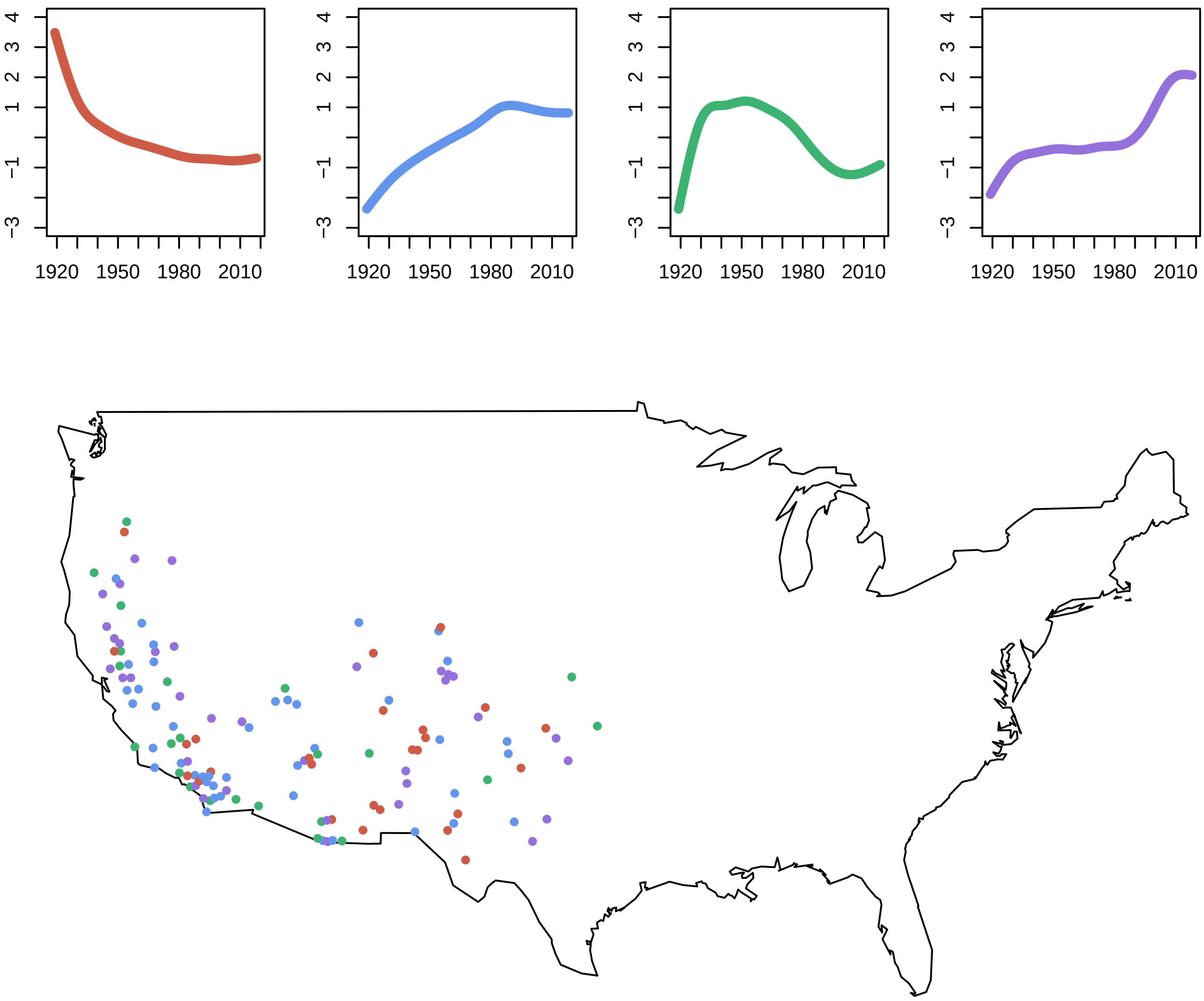

Mountain Chickadee

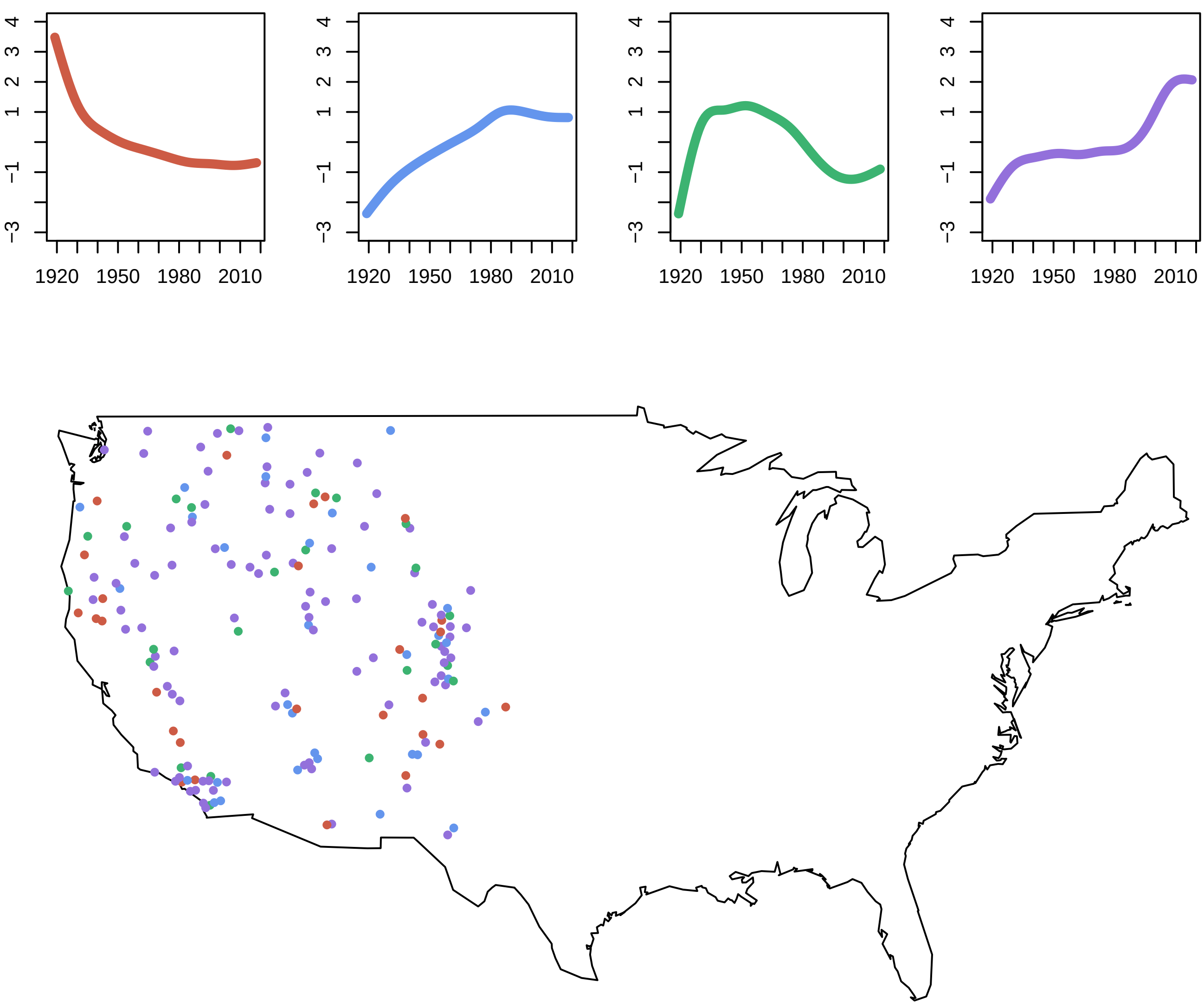

Neotropic Cormorant

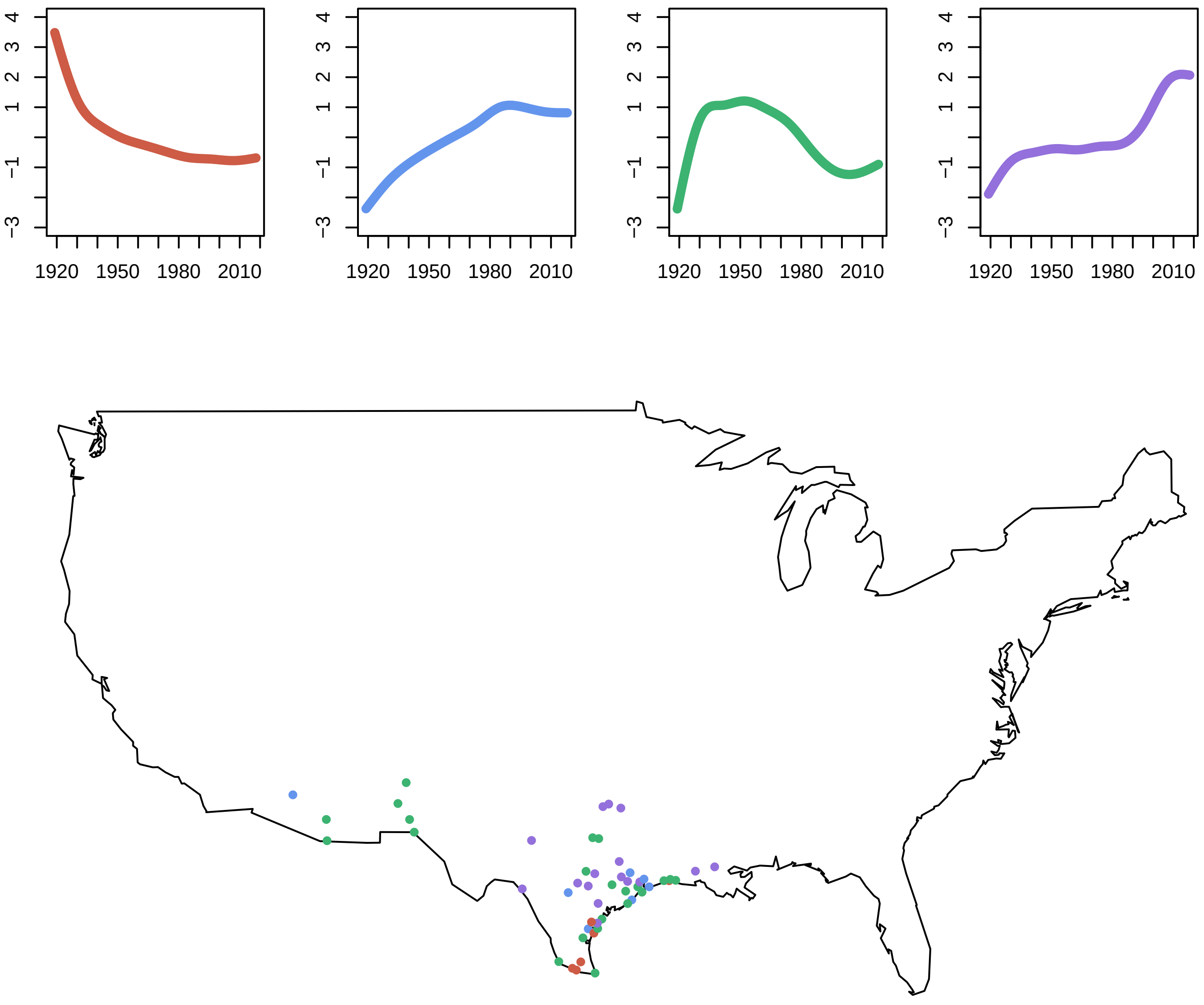

Orange−crowned Warbler

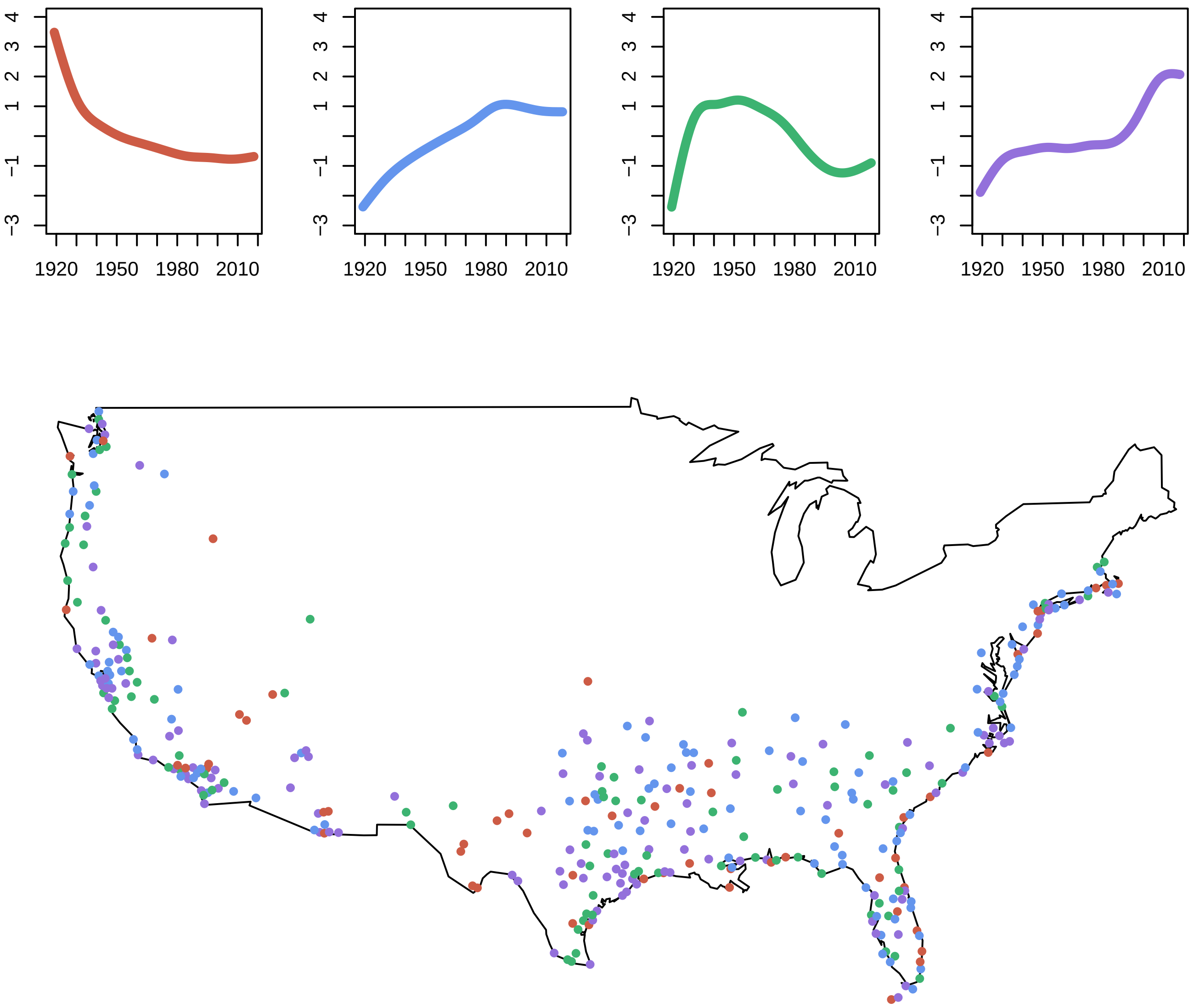

Pacific Loon

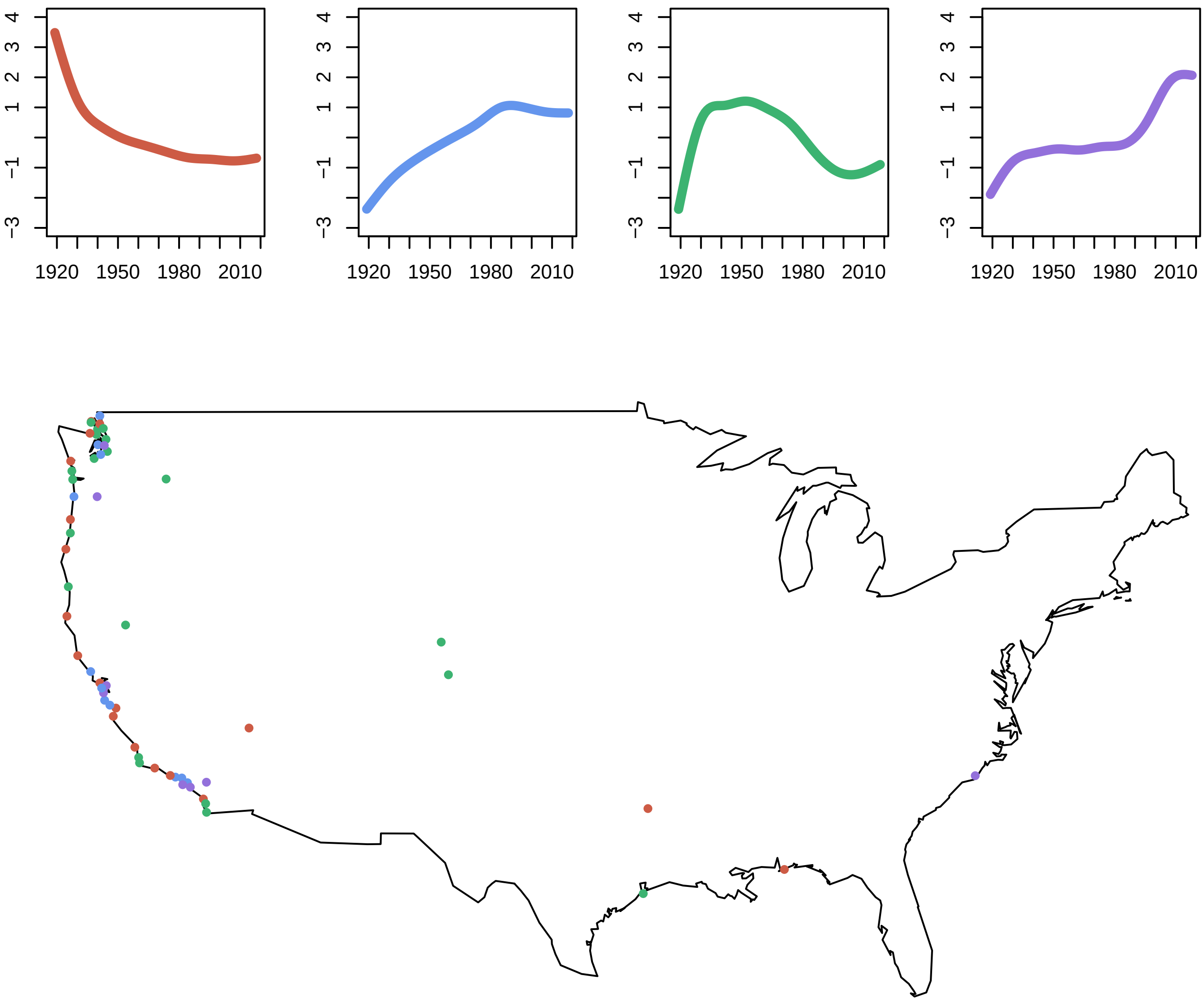

Pileated Woodpecker

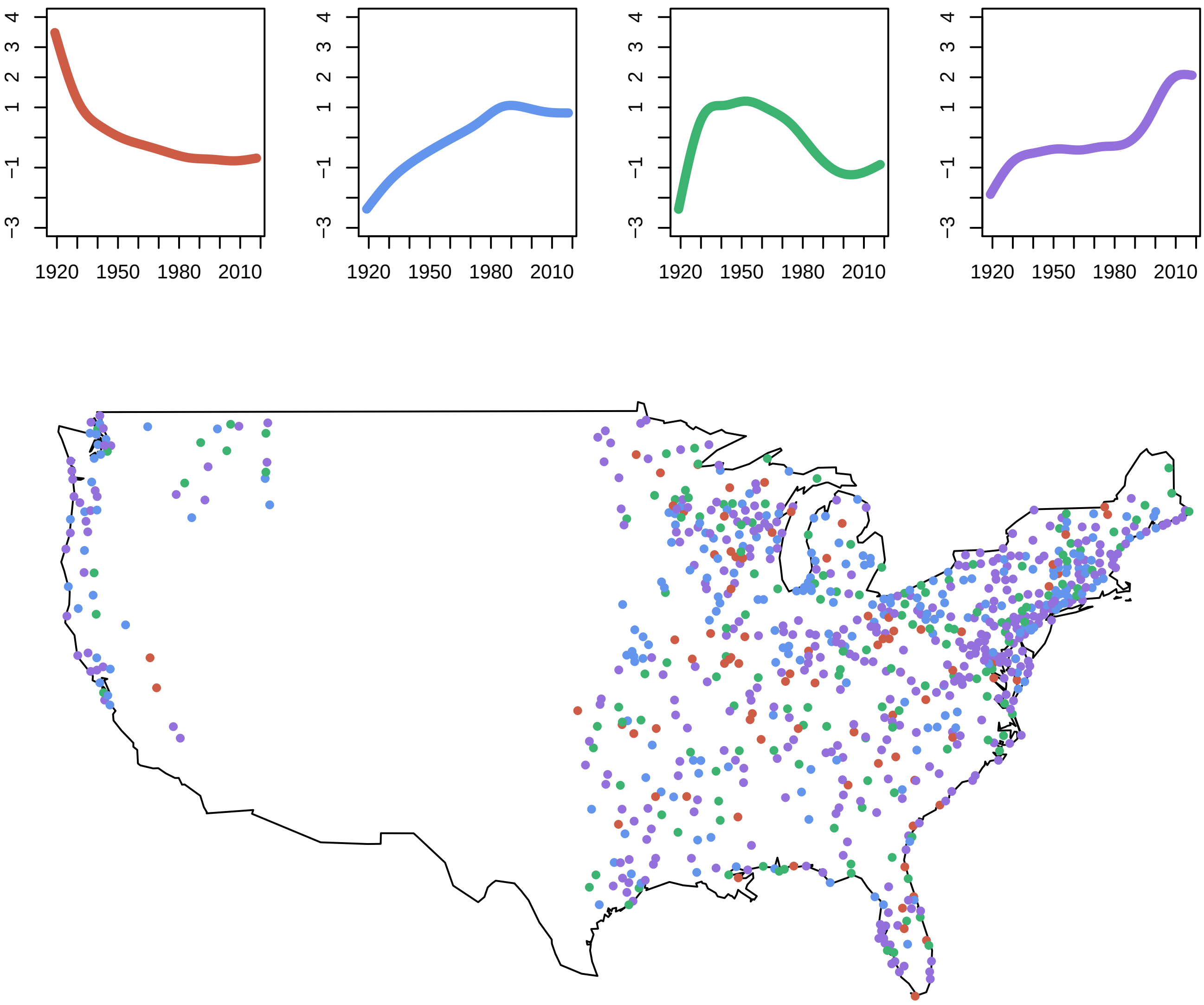

Pinyon Jay

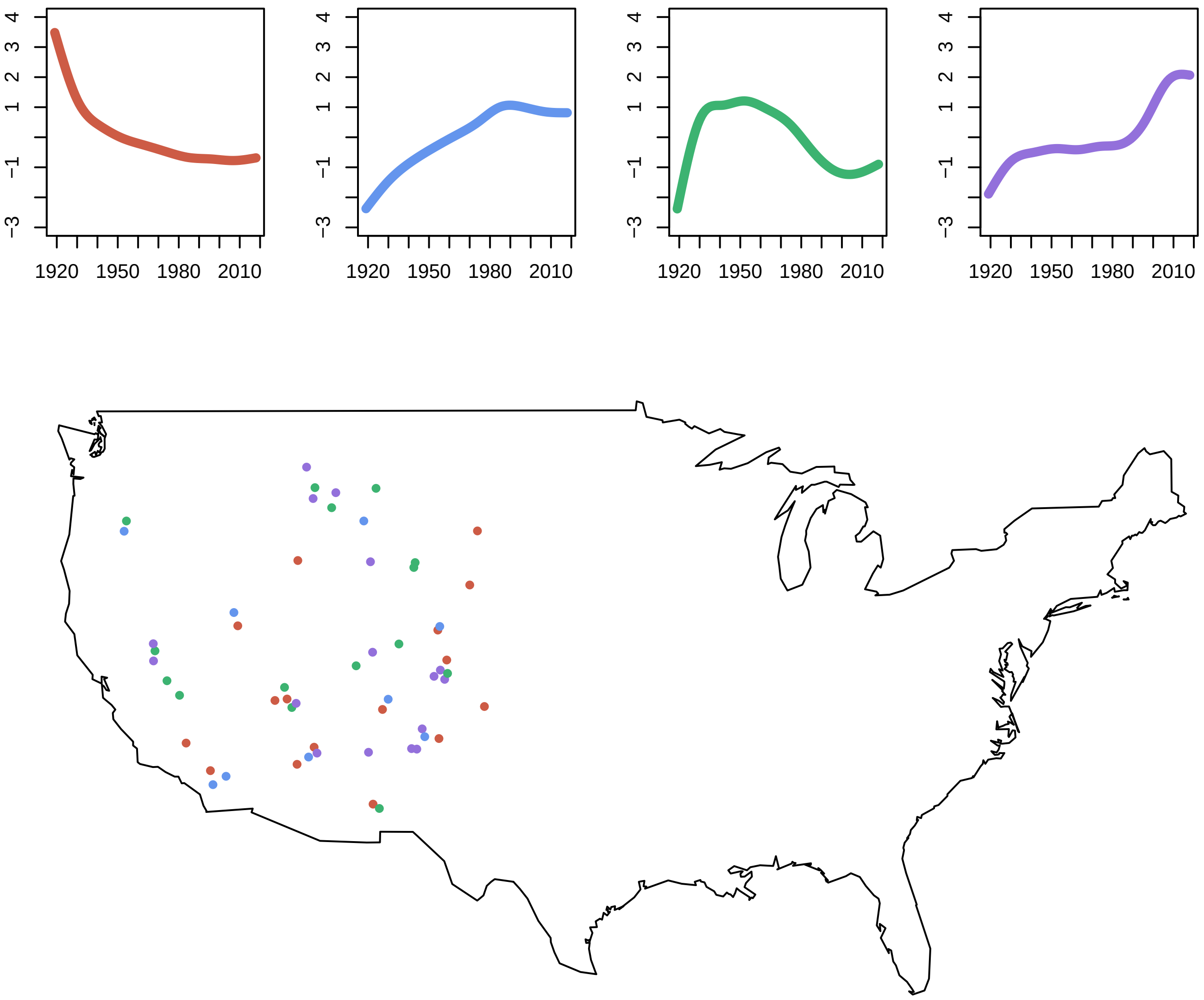

Pygmy Nuthatch

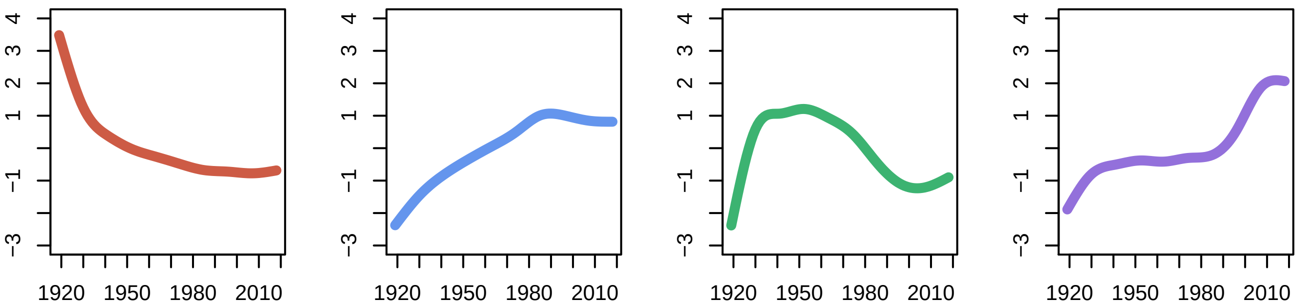

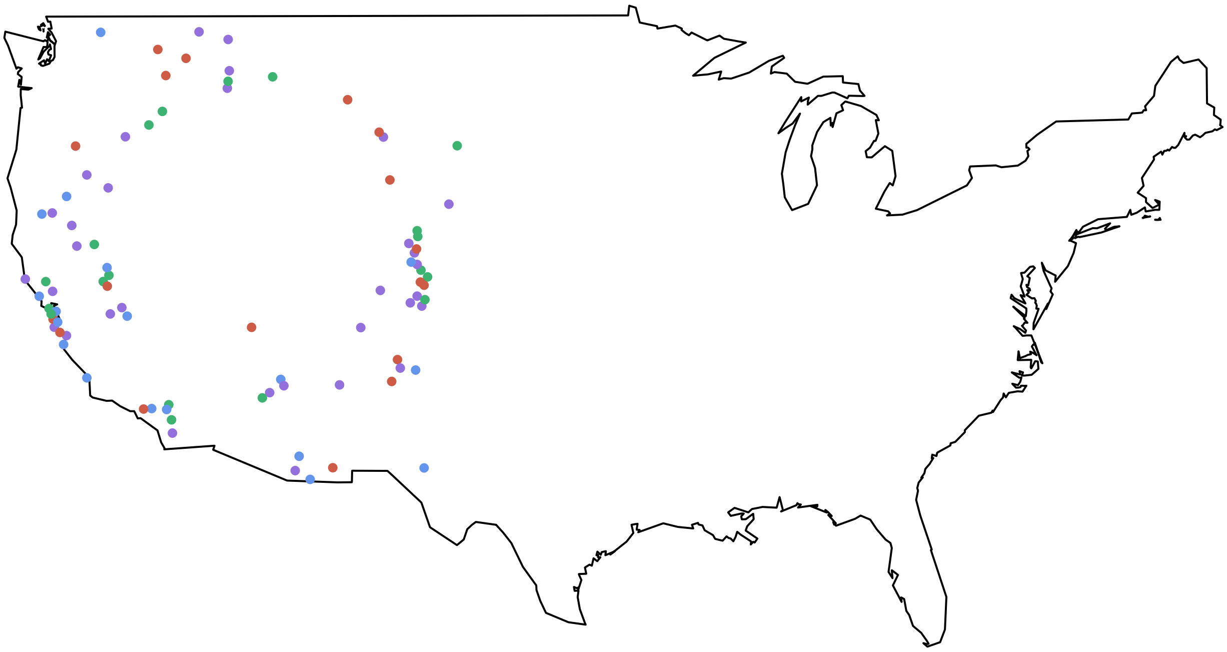

Red−headed Woodpecker

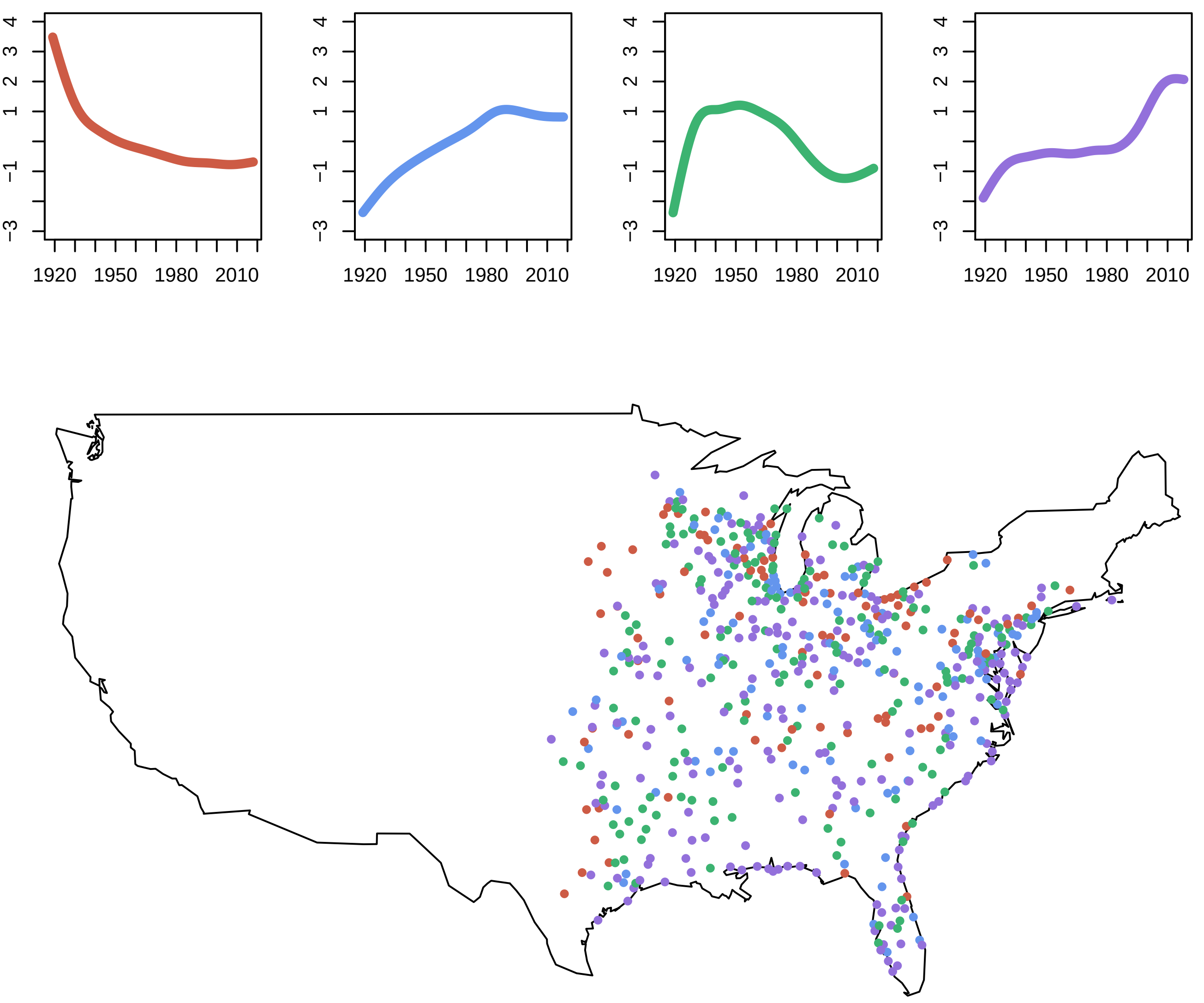

Red−shouldered Hawk

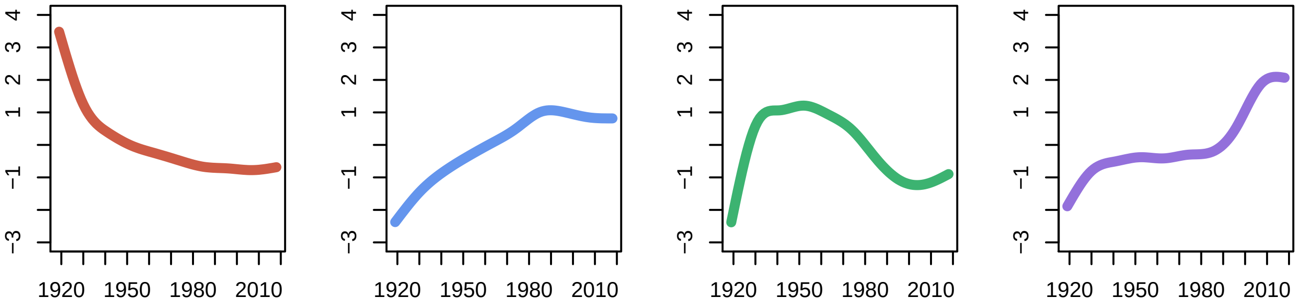

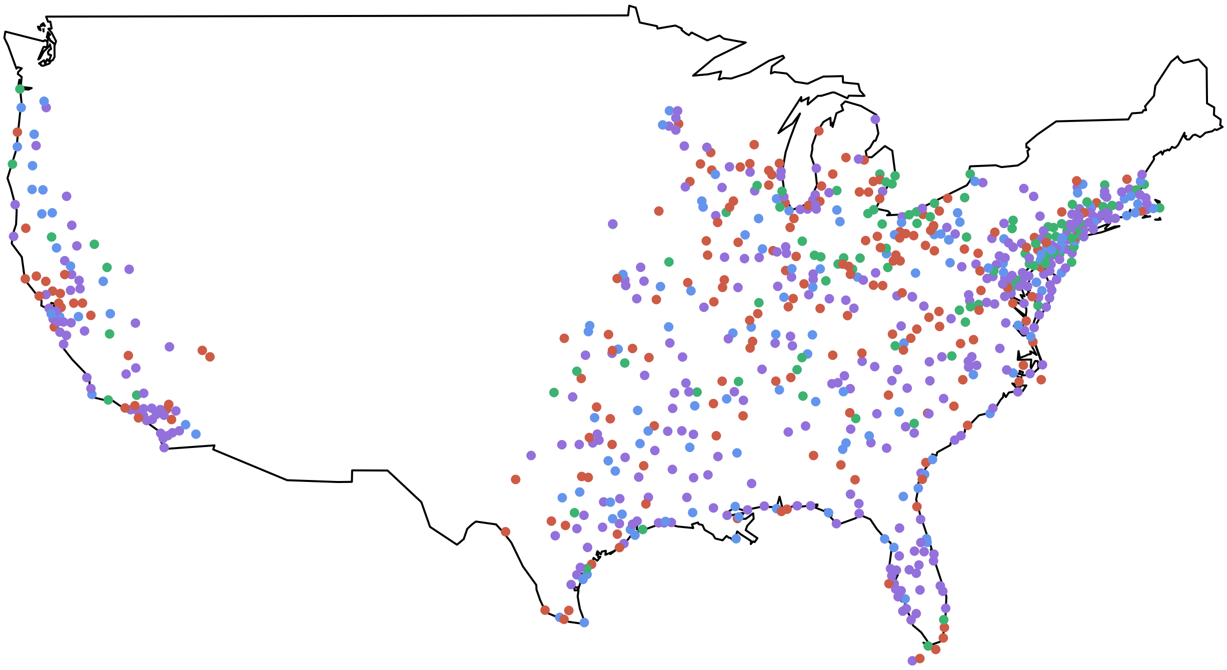

Red Crossbill

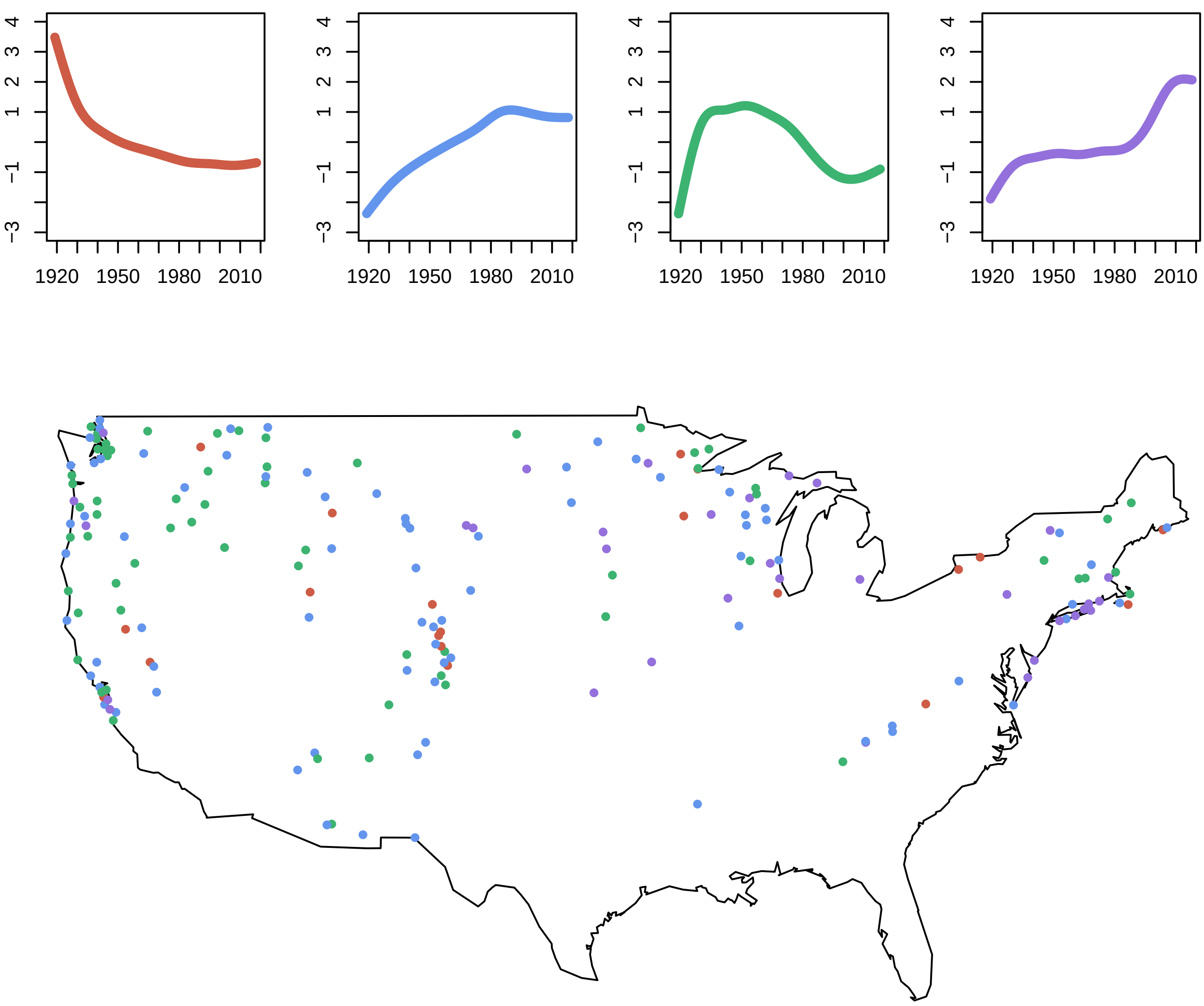

Tricolored Heron

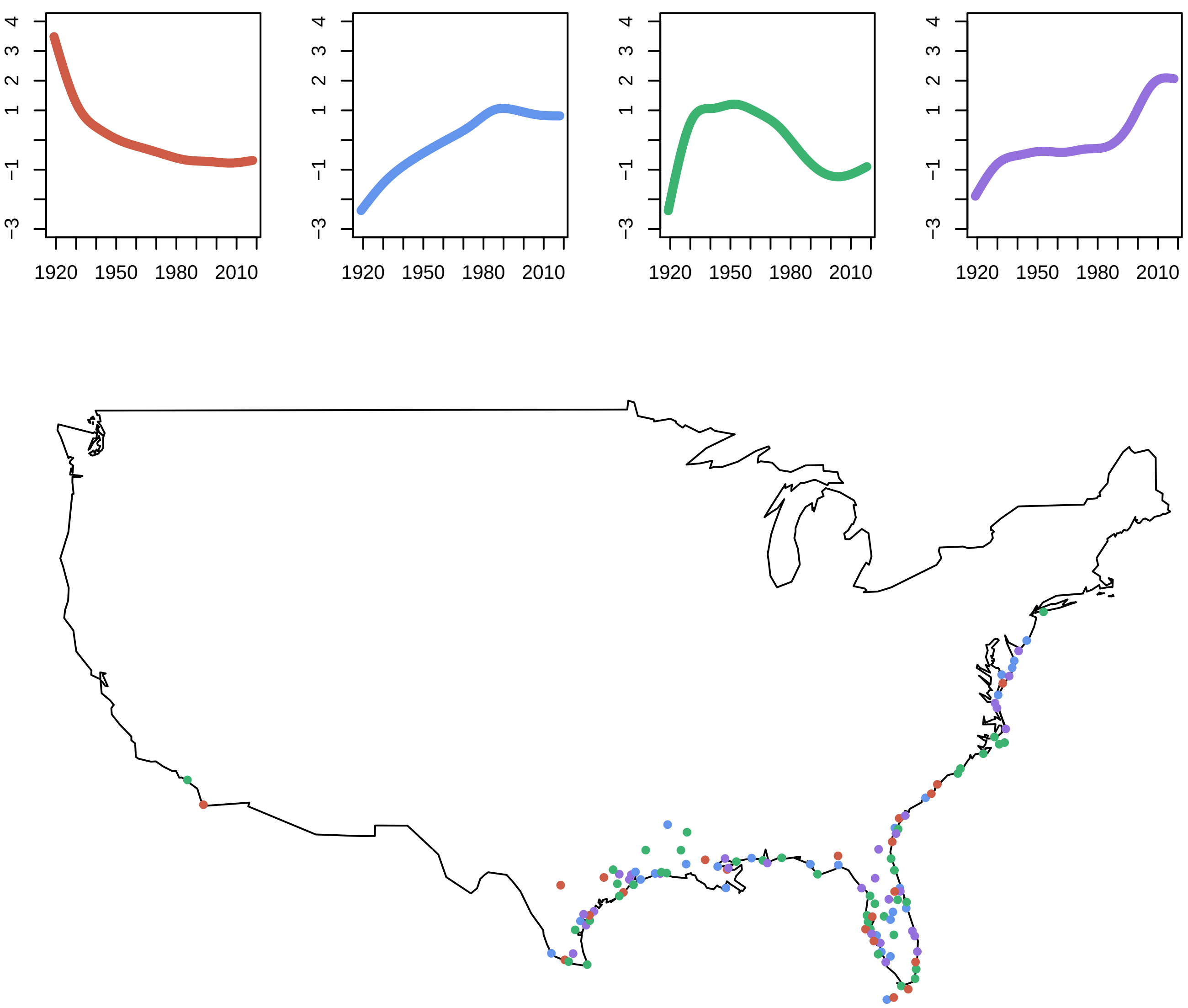

Trumpeter Swan

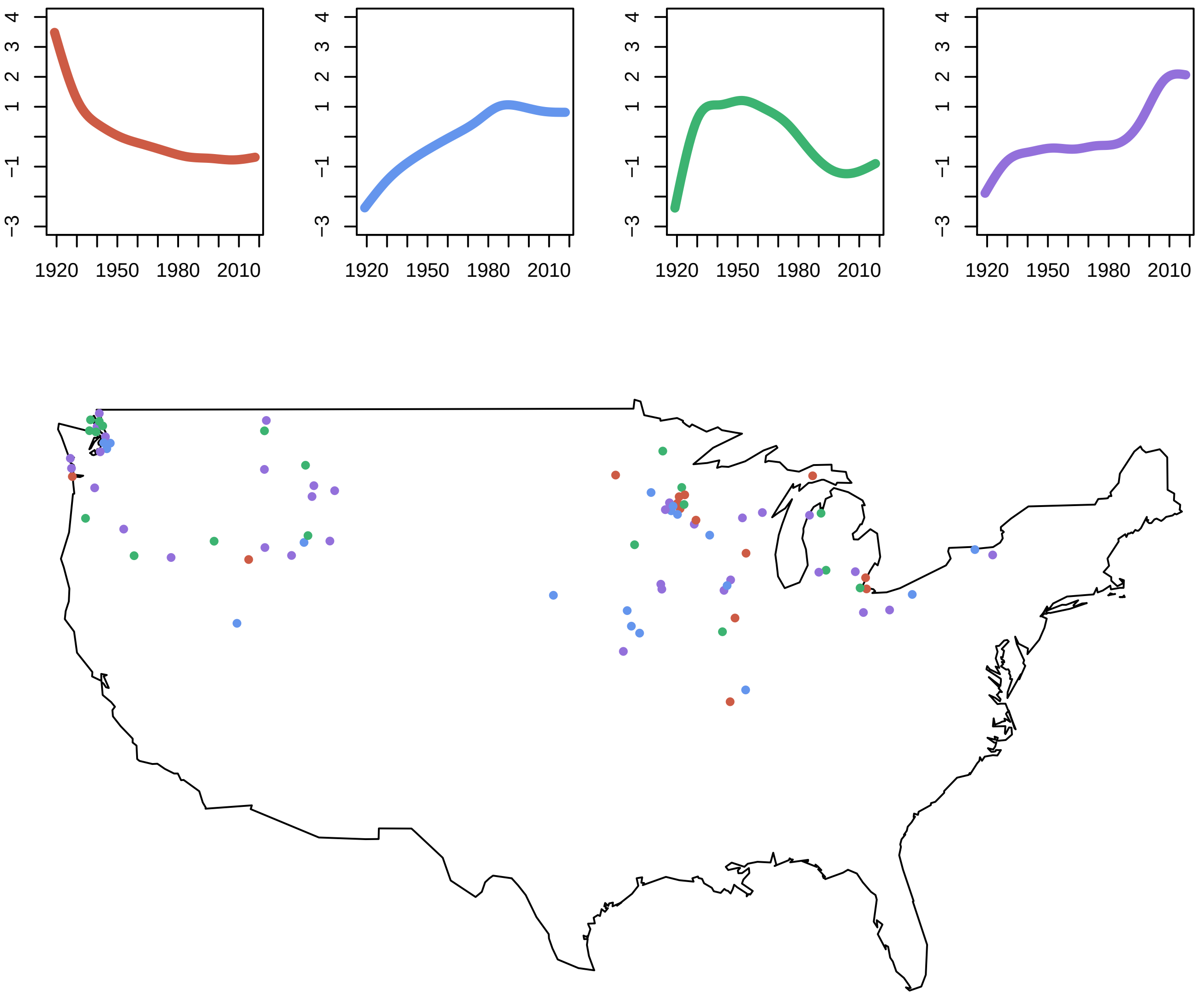

Varied Thrush

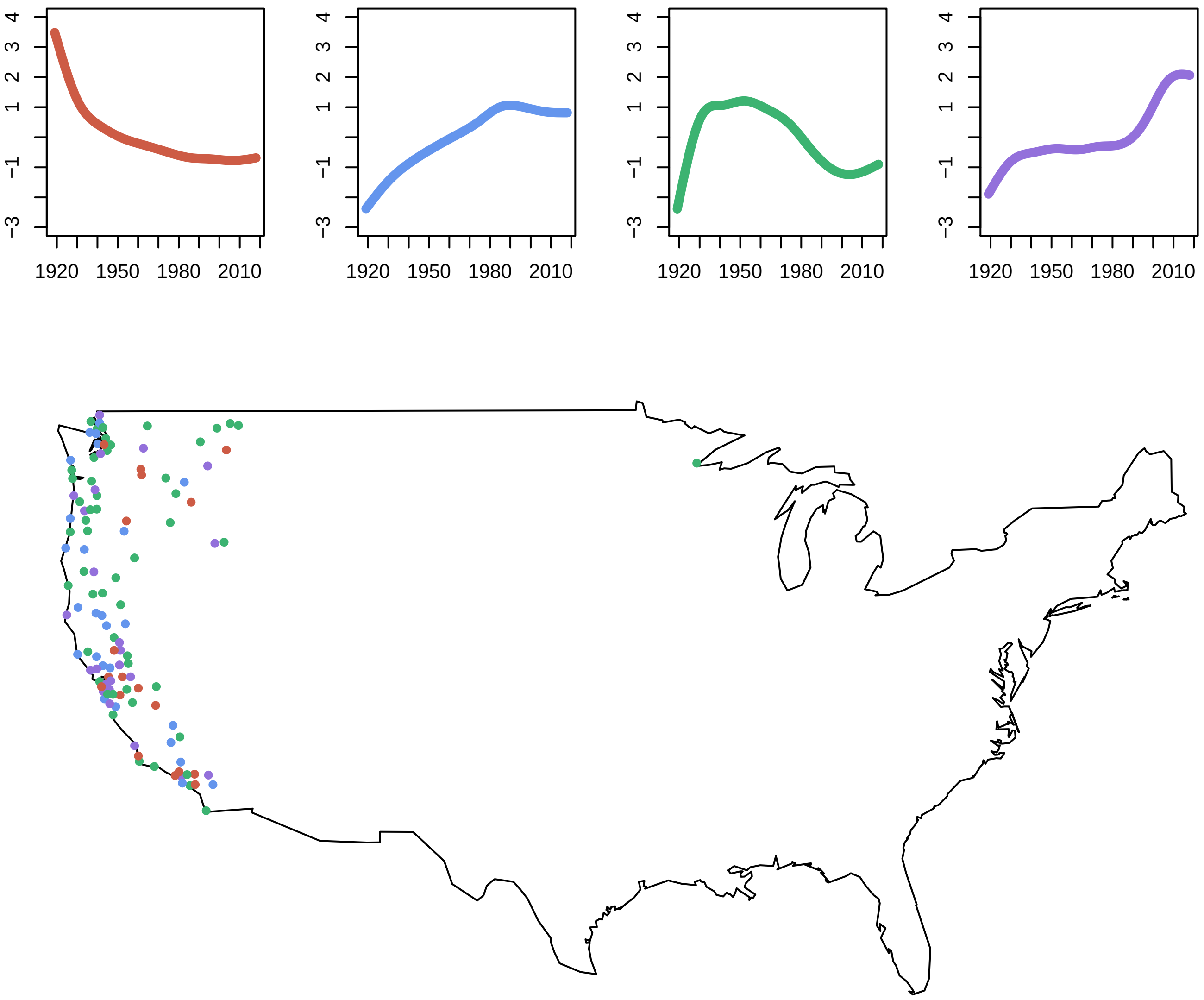

Wood Stork

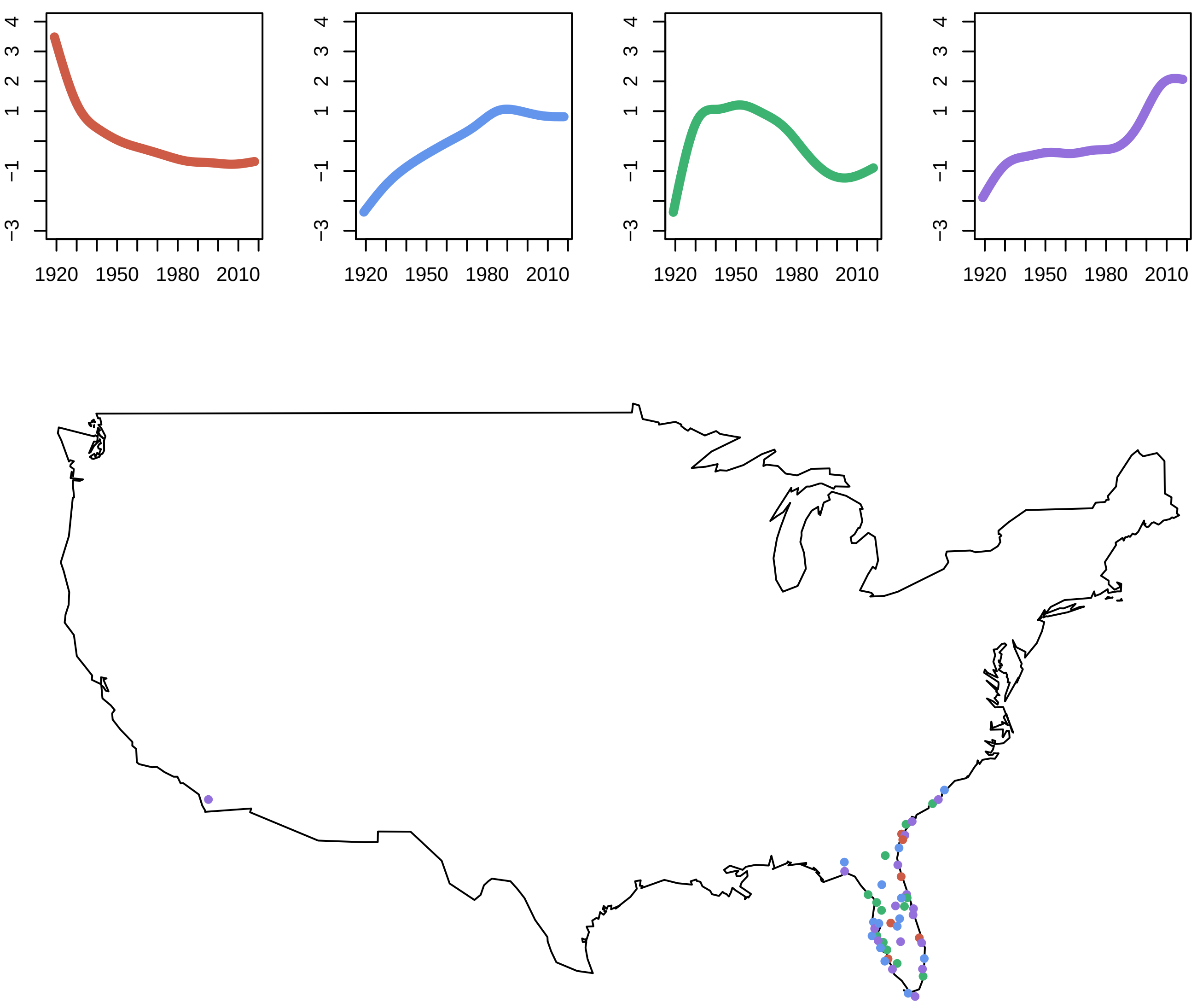

Wrentit

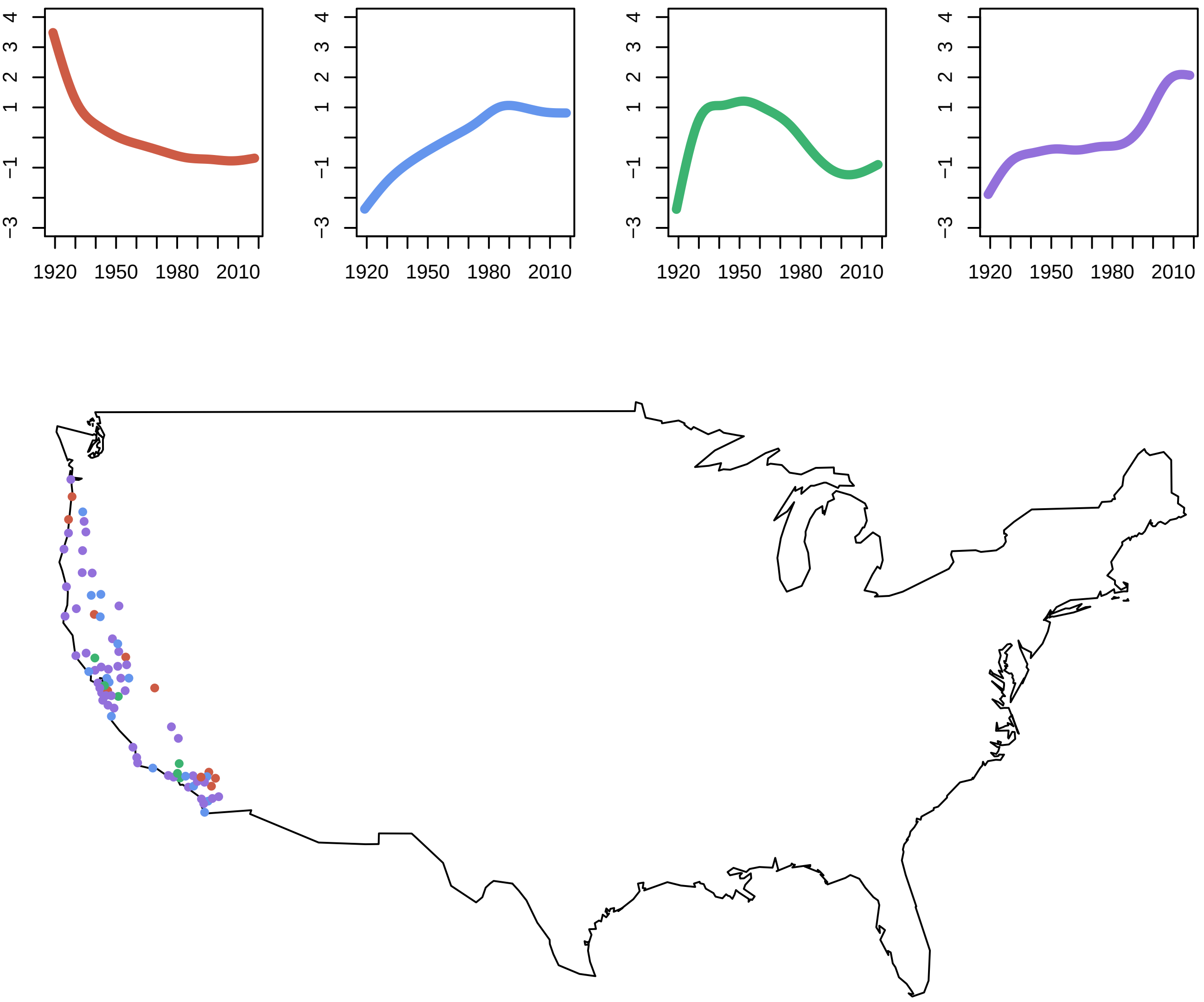

Yellow−bellied Sapsucker

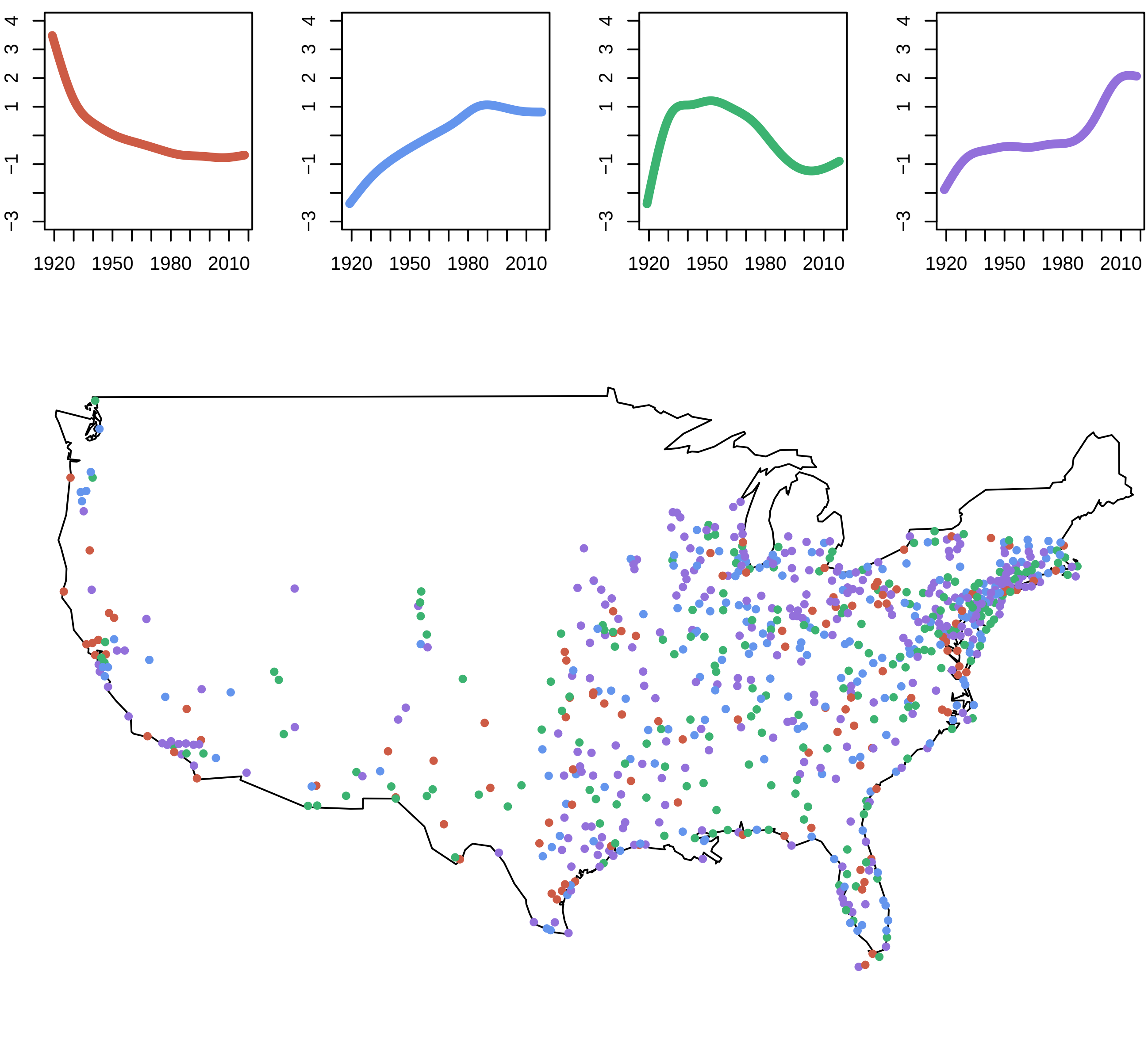

Yellow−billed Magpie

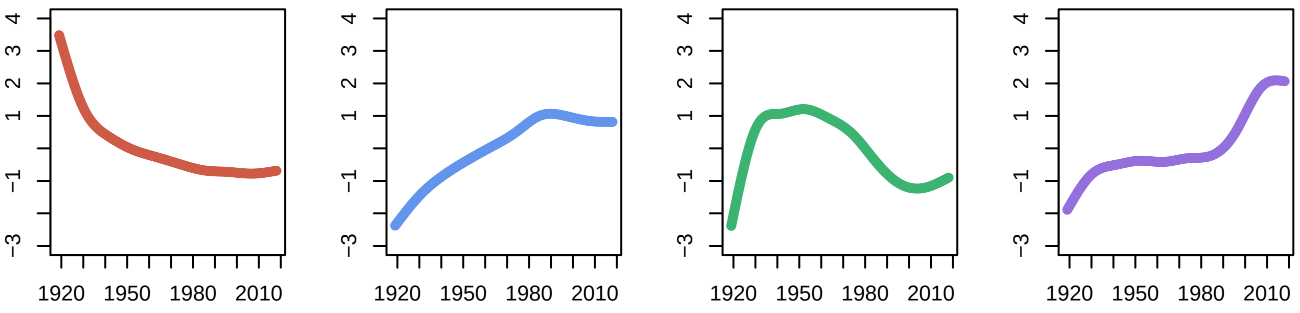

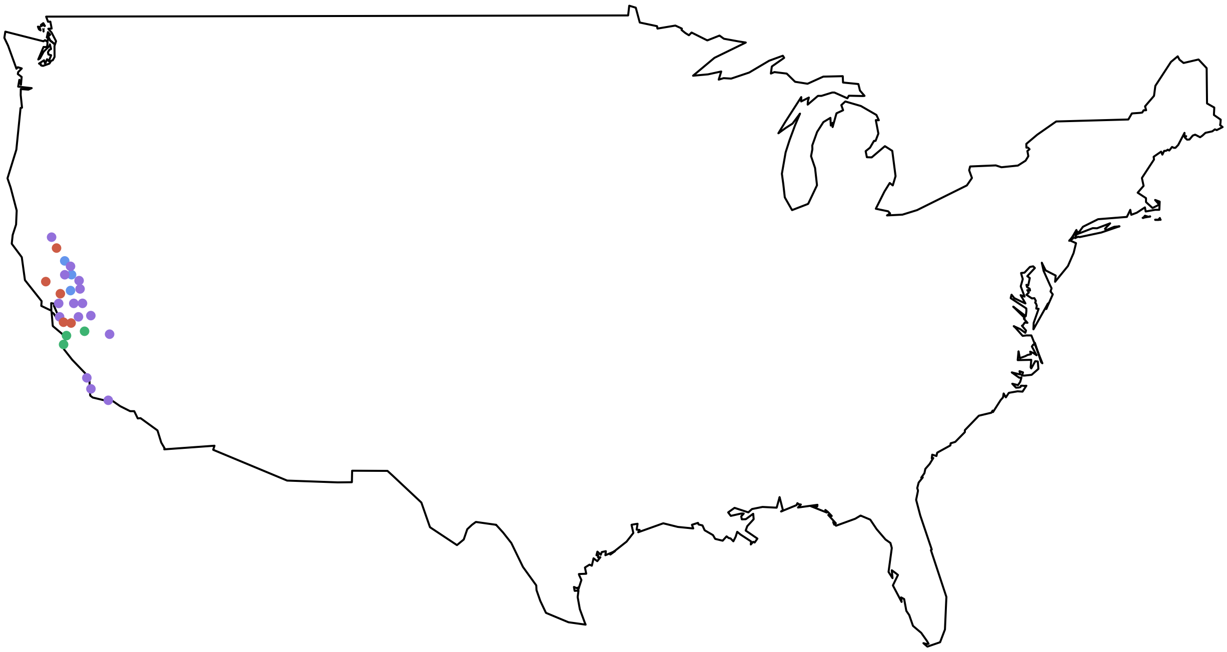

Barred Owl

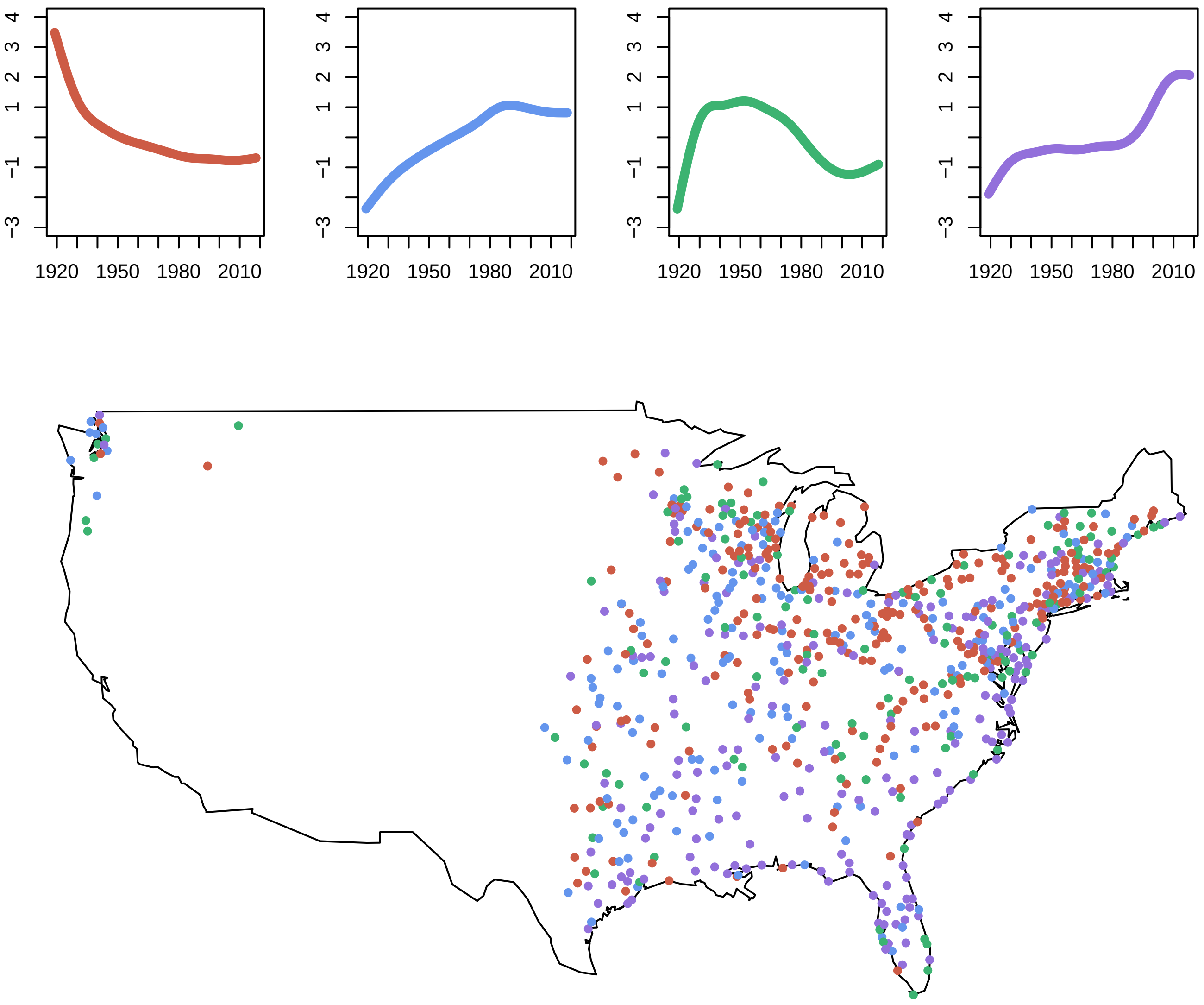

Barrow's Goldeneye

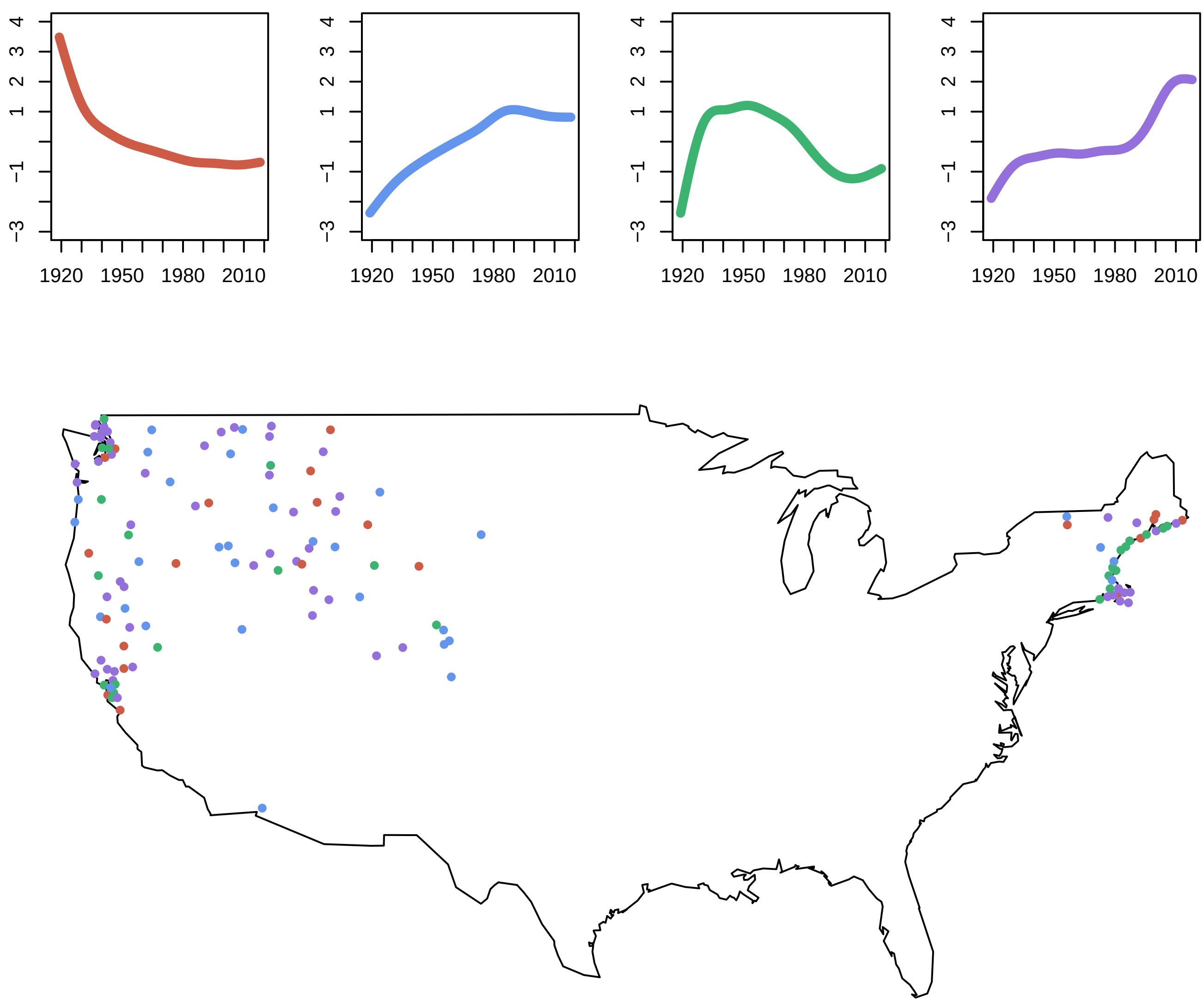

Black−throated Sparrow

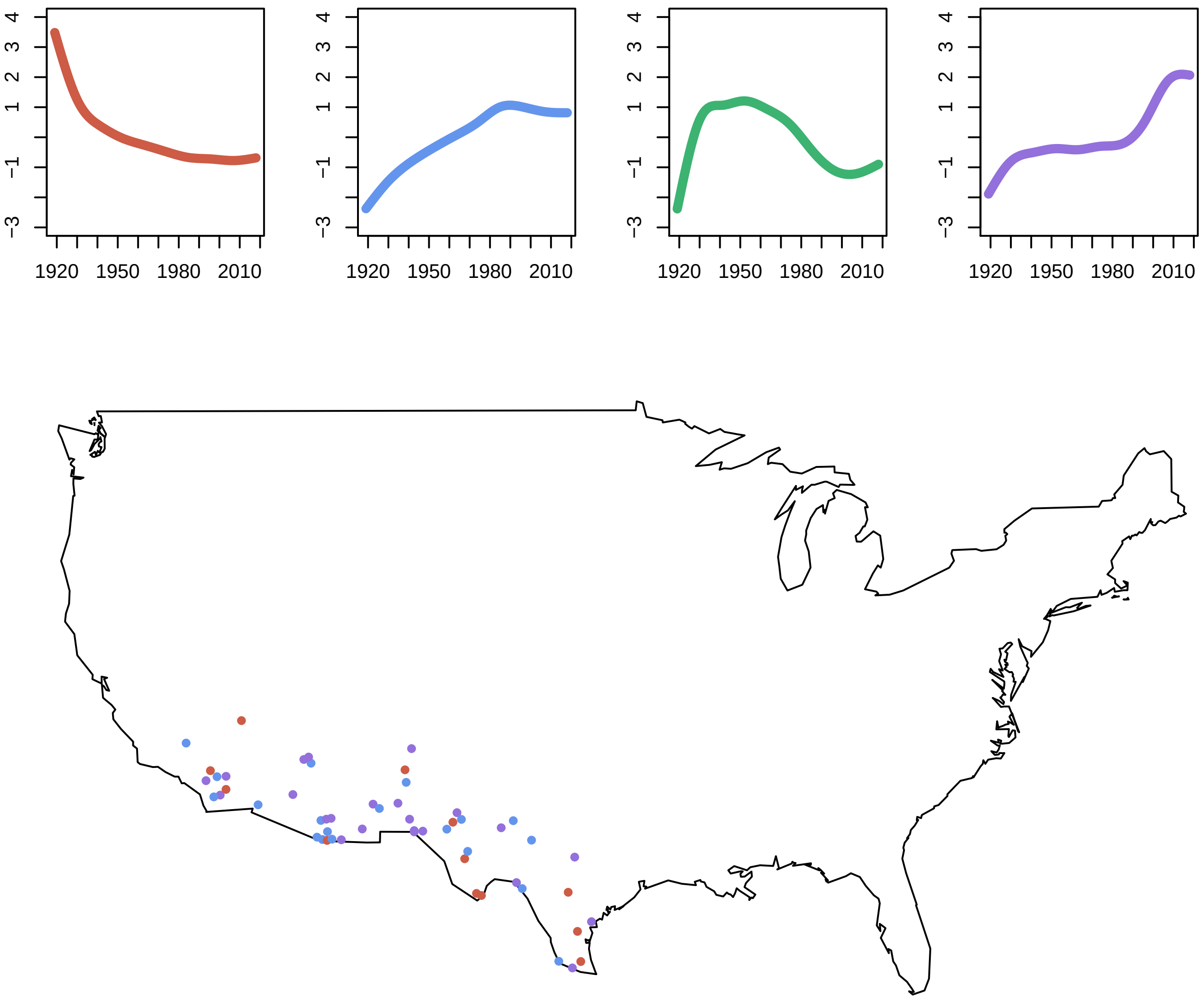

Cactus Wren

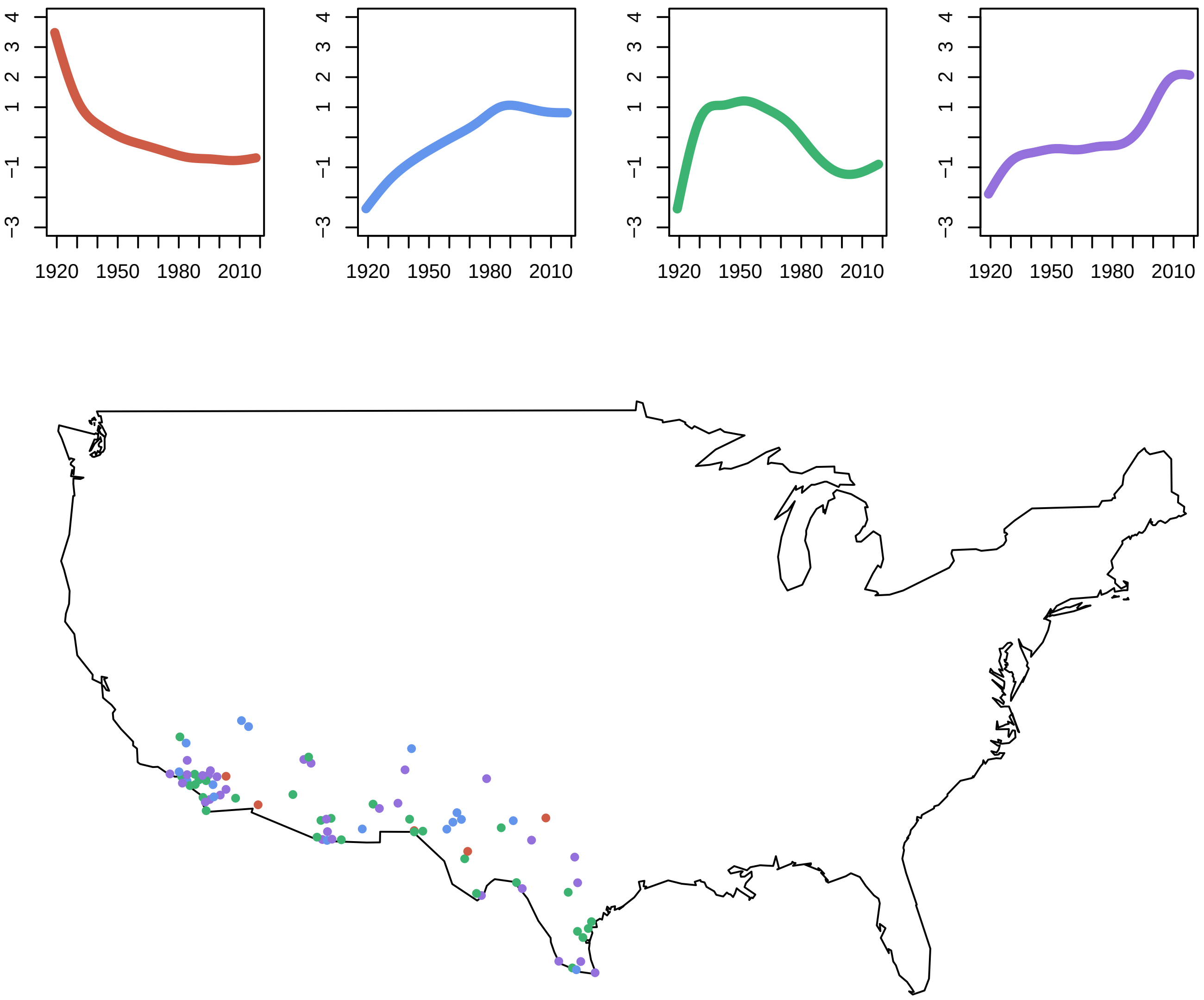

California Thrasher

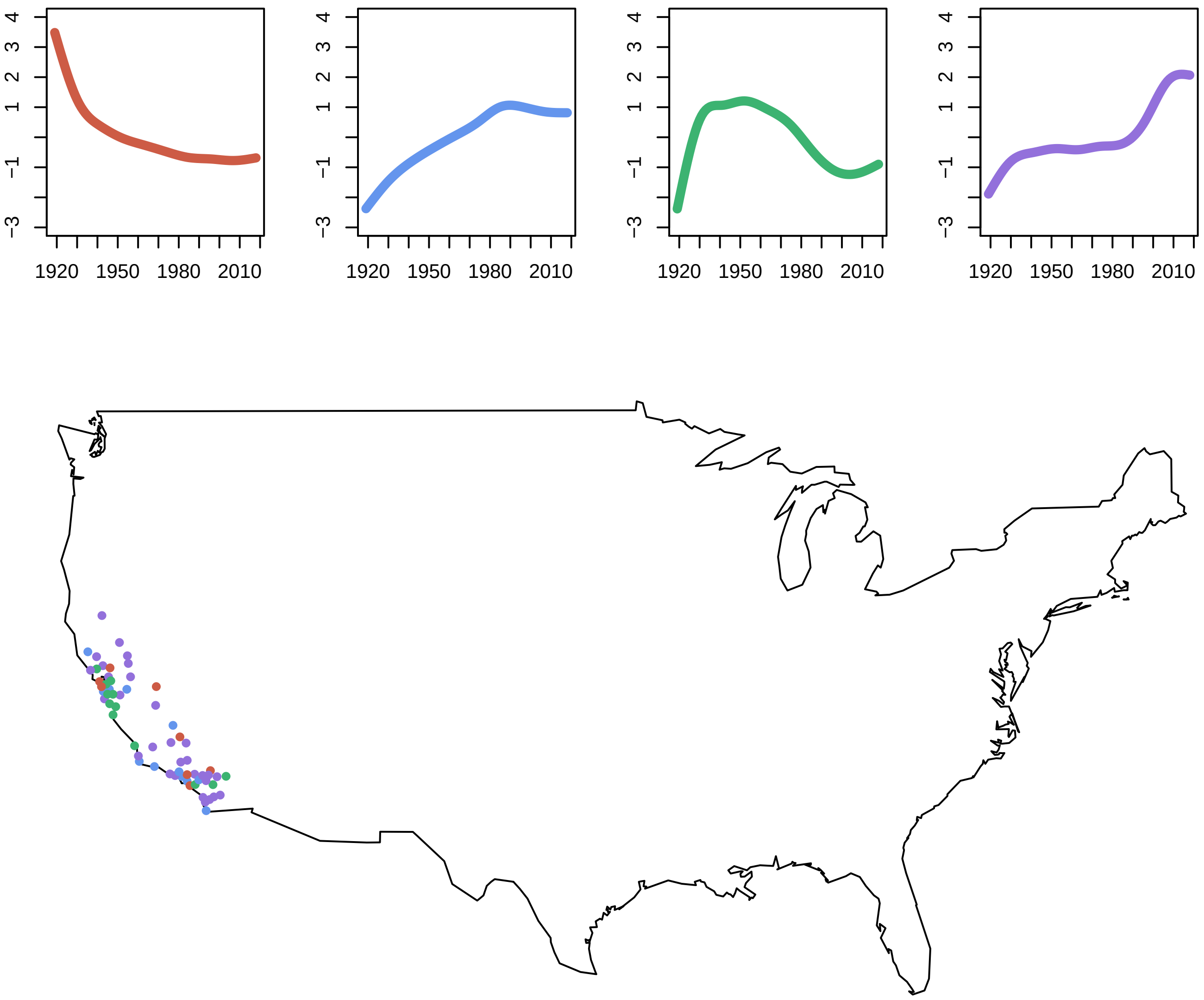

Cassin's Finch

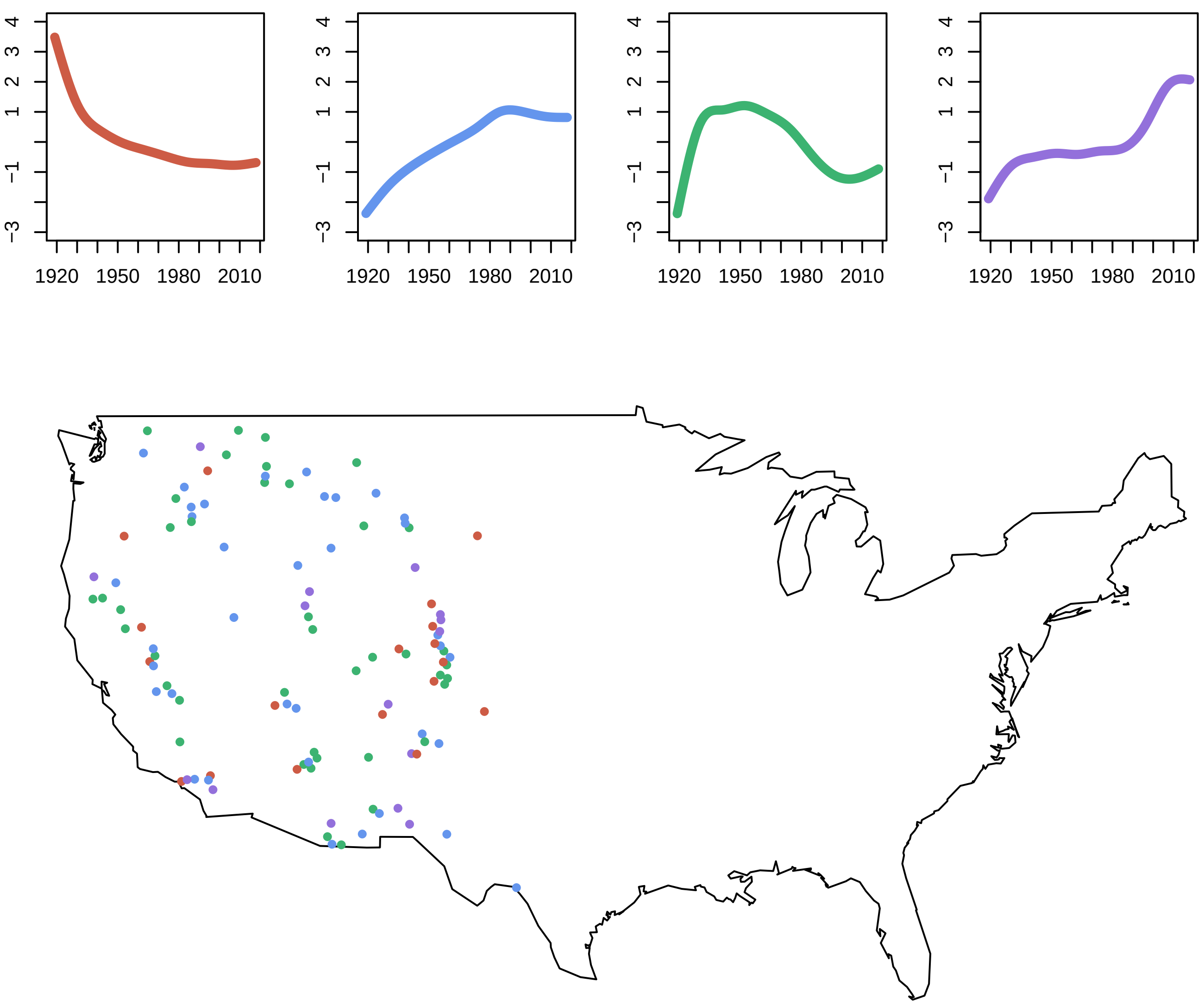

Chestnut−collared Longspur

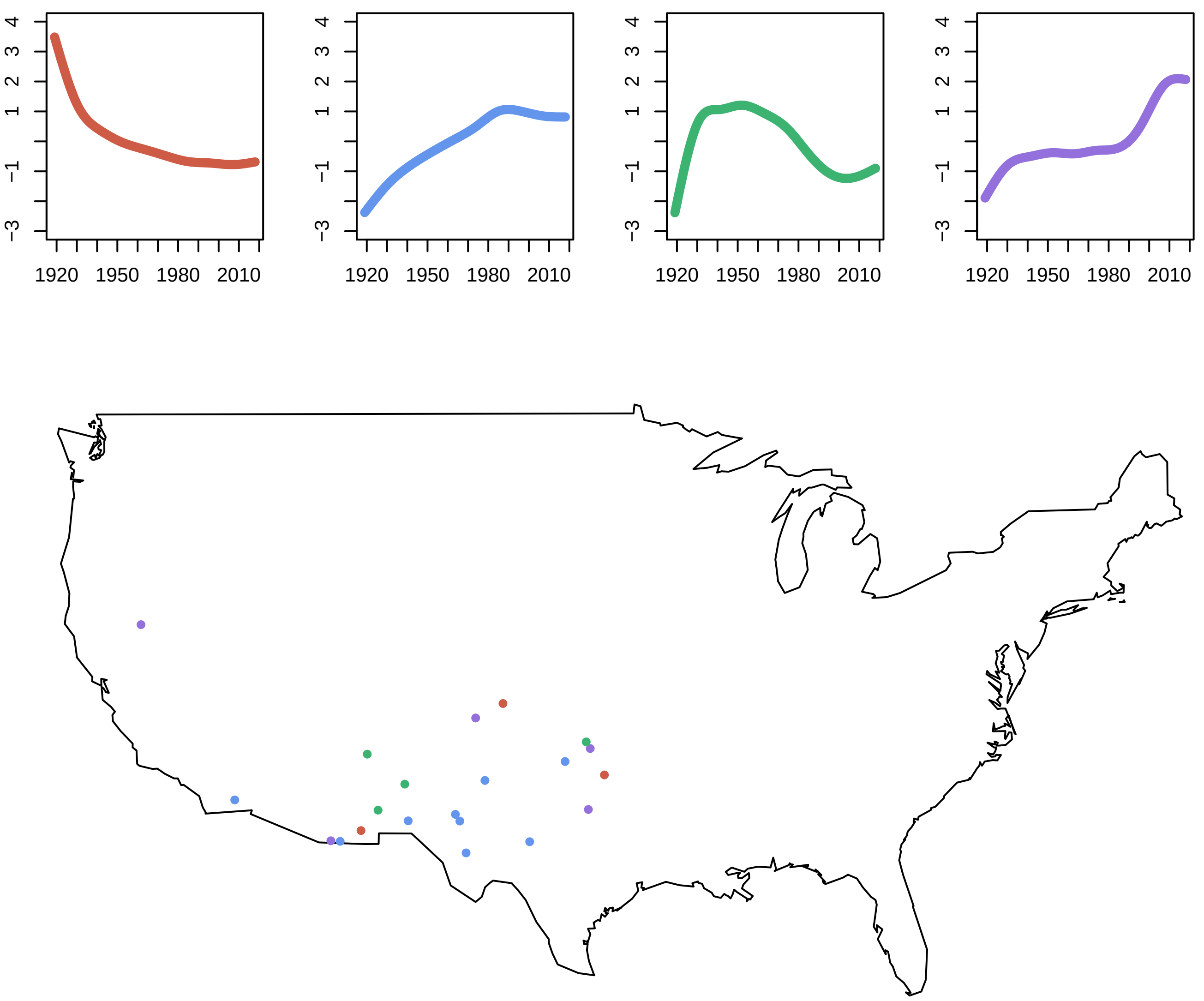

Cinnamon Teal

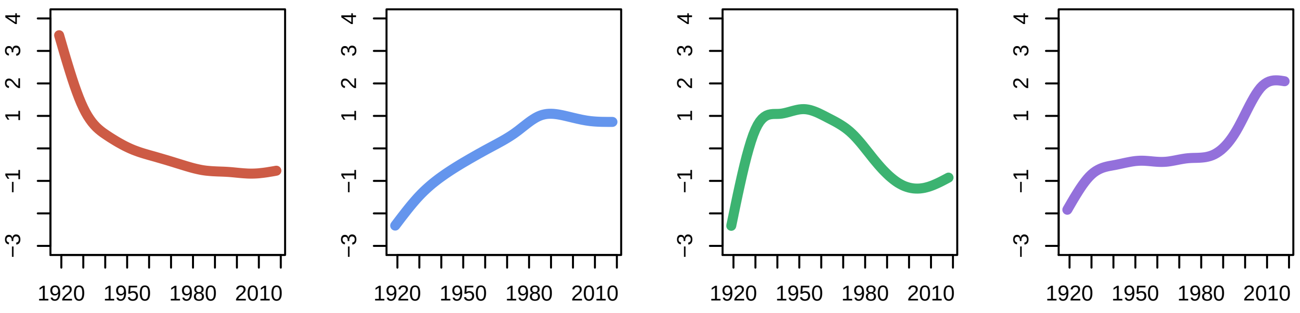

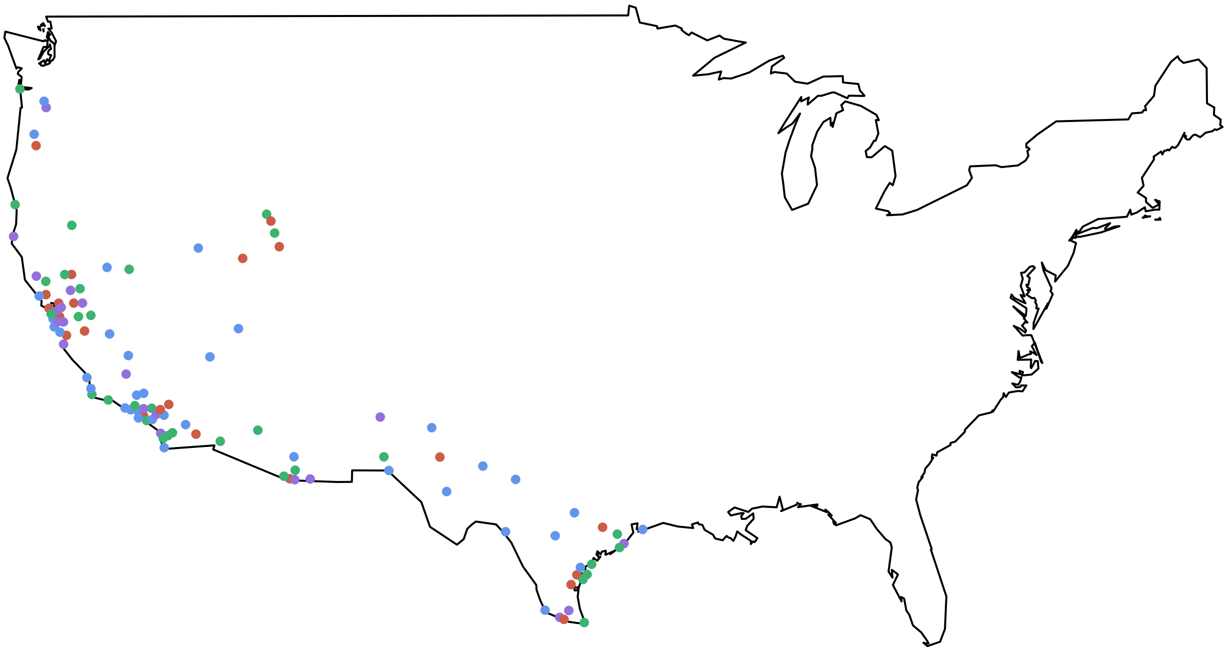

Clark's Grebe

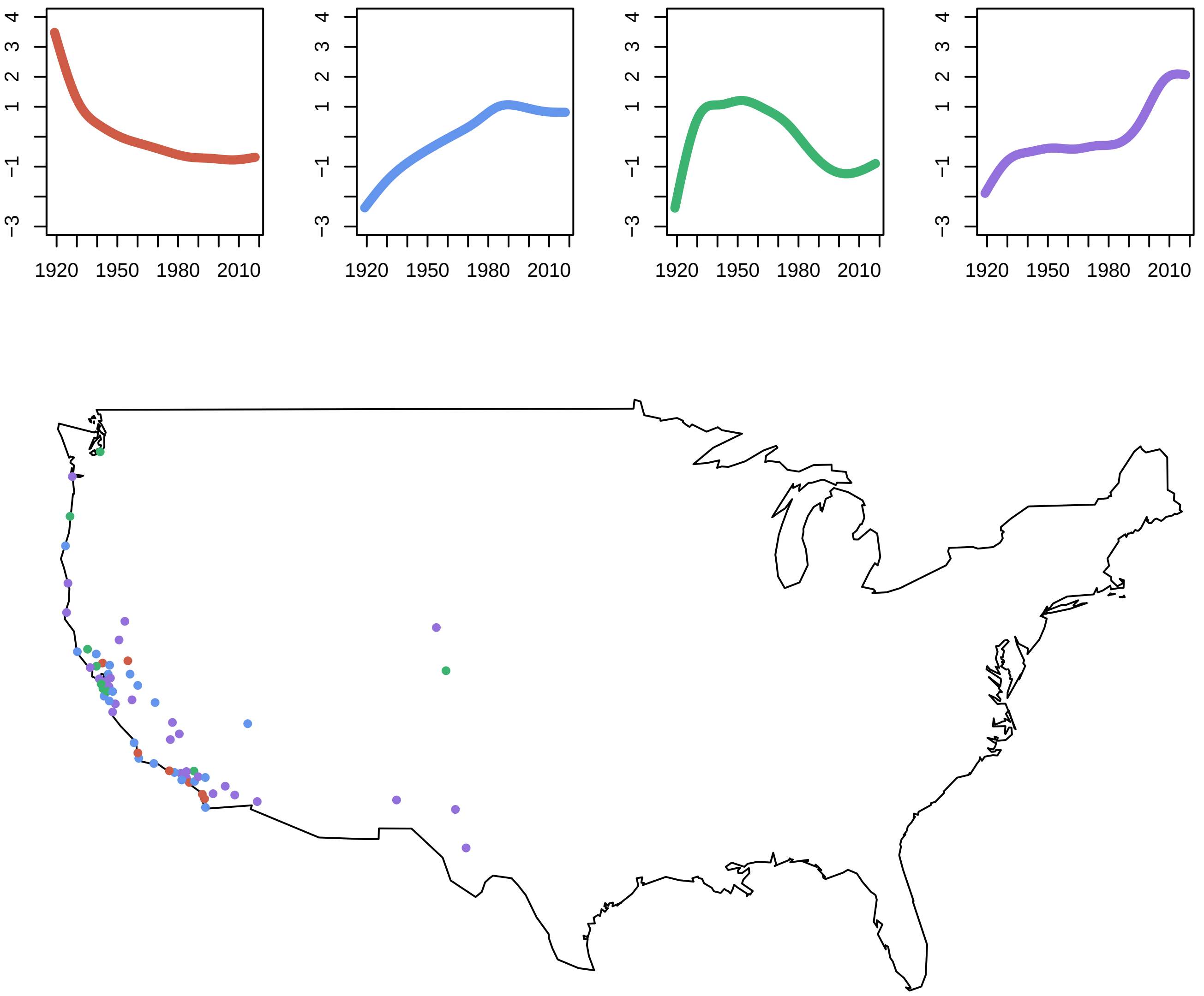

Common Ground Dove

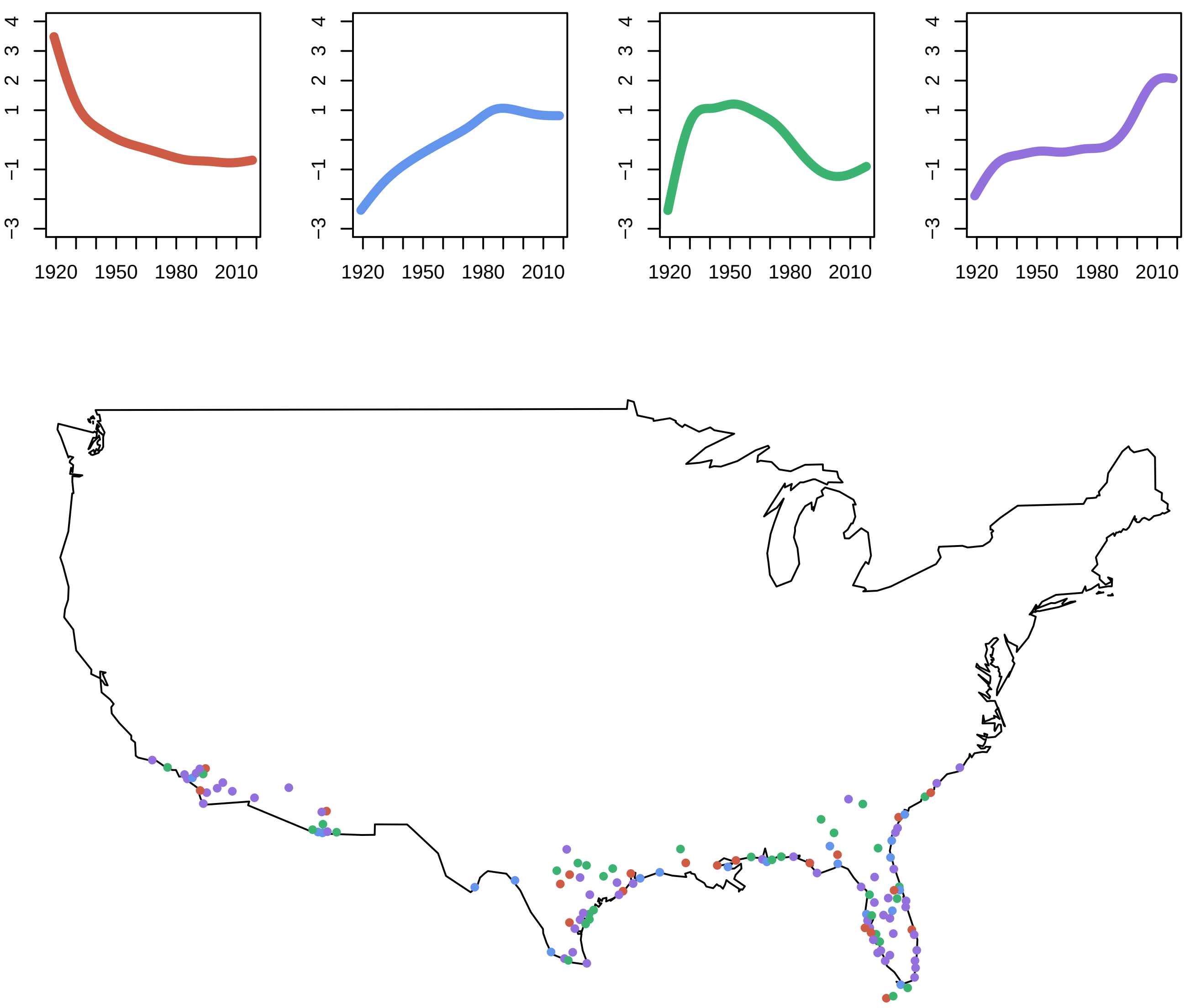

Eastern Screech−Owl

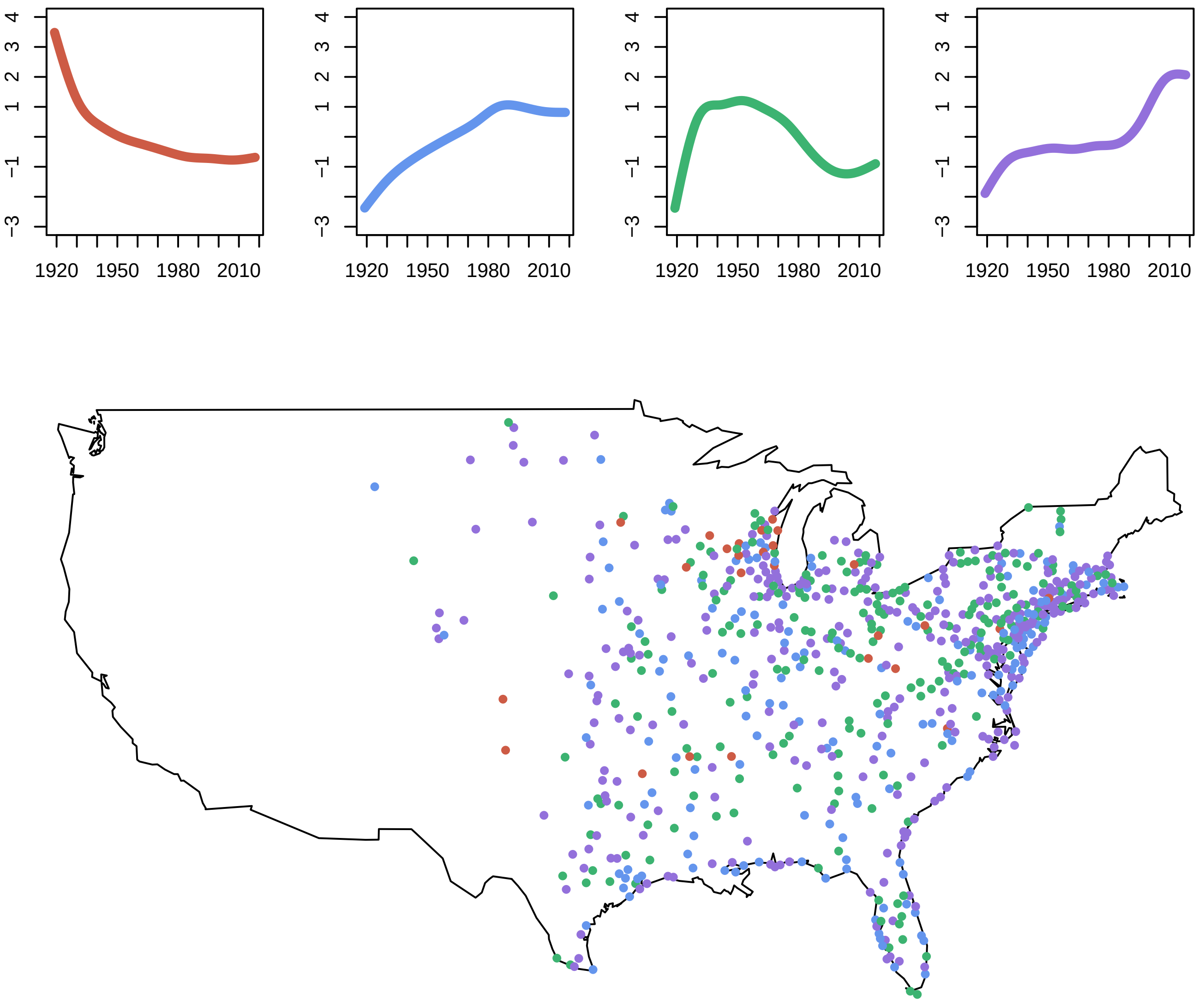

Eurasian Tree Sparrow

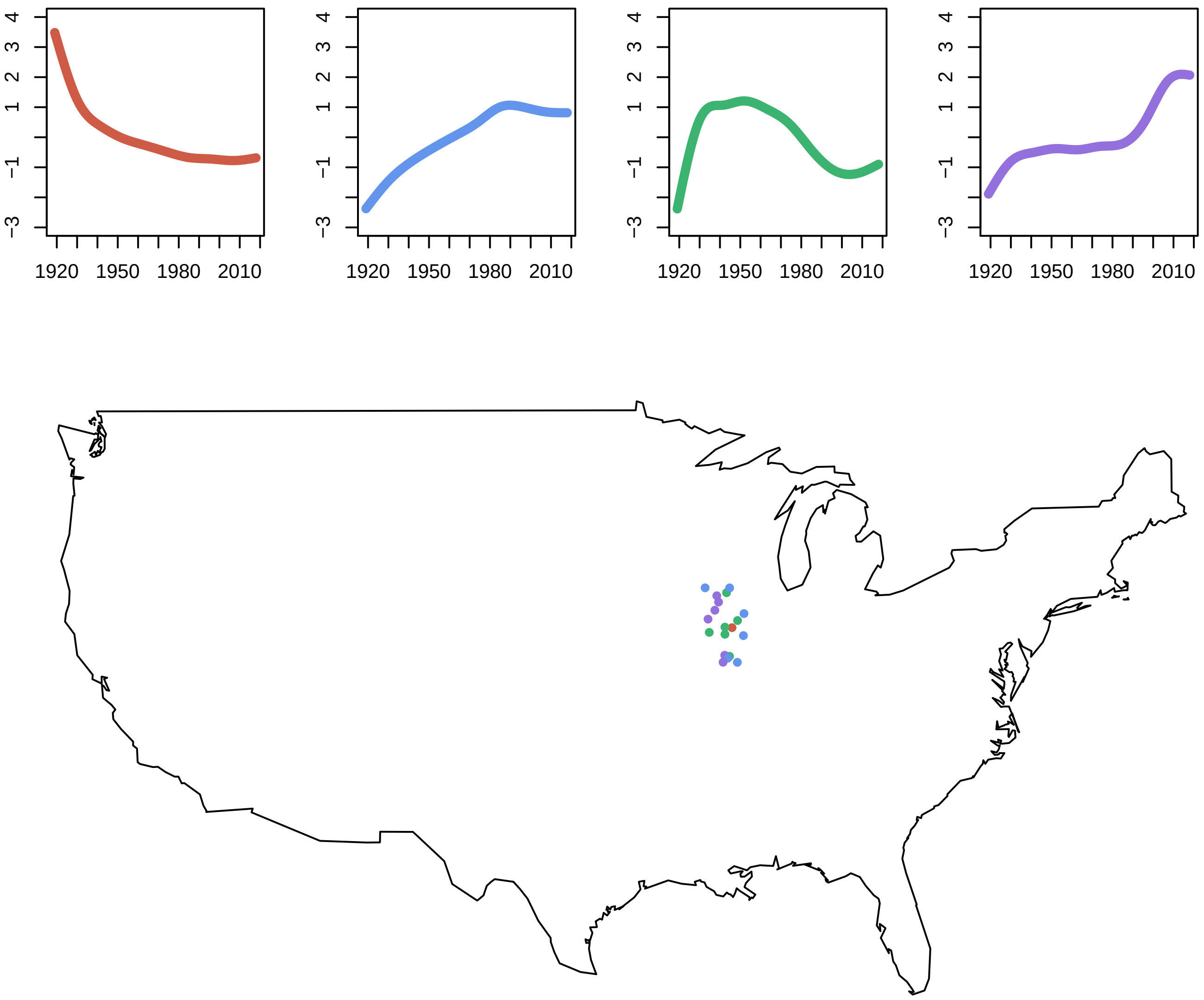

Gila Woodpecker

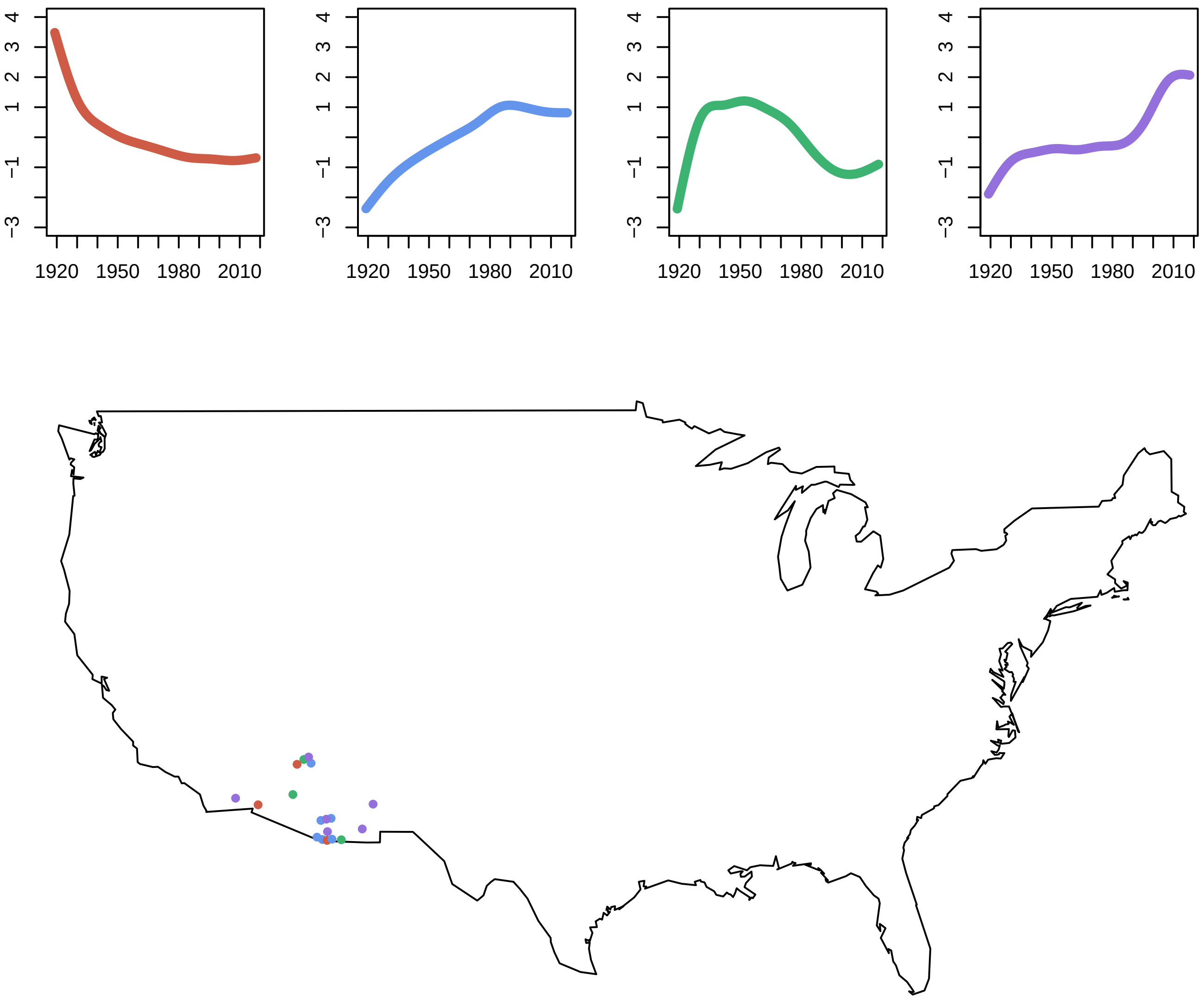

Gray Partridge

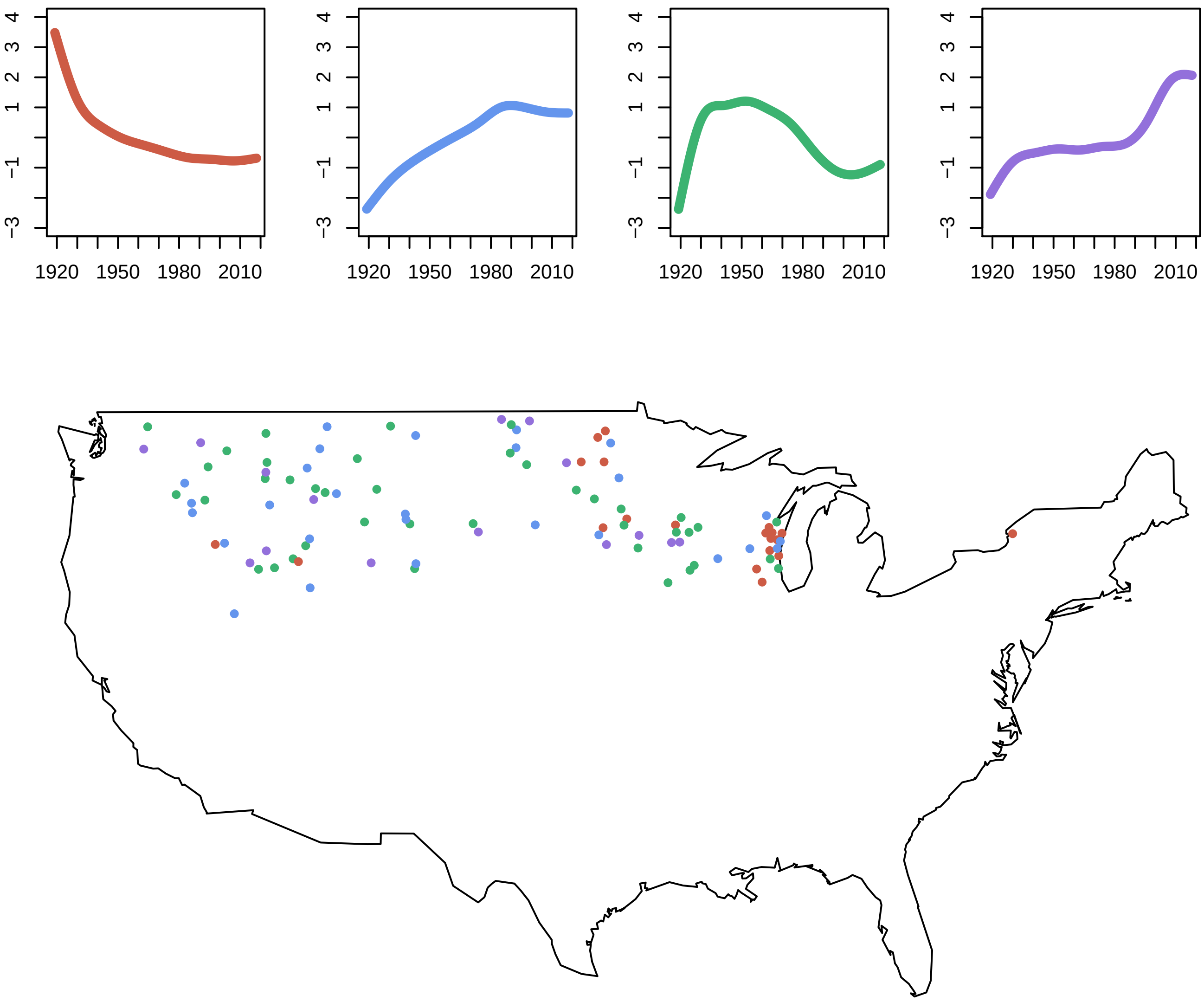

Inca Dove

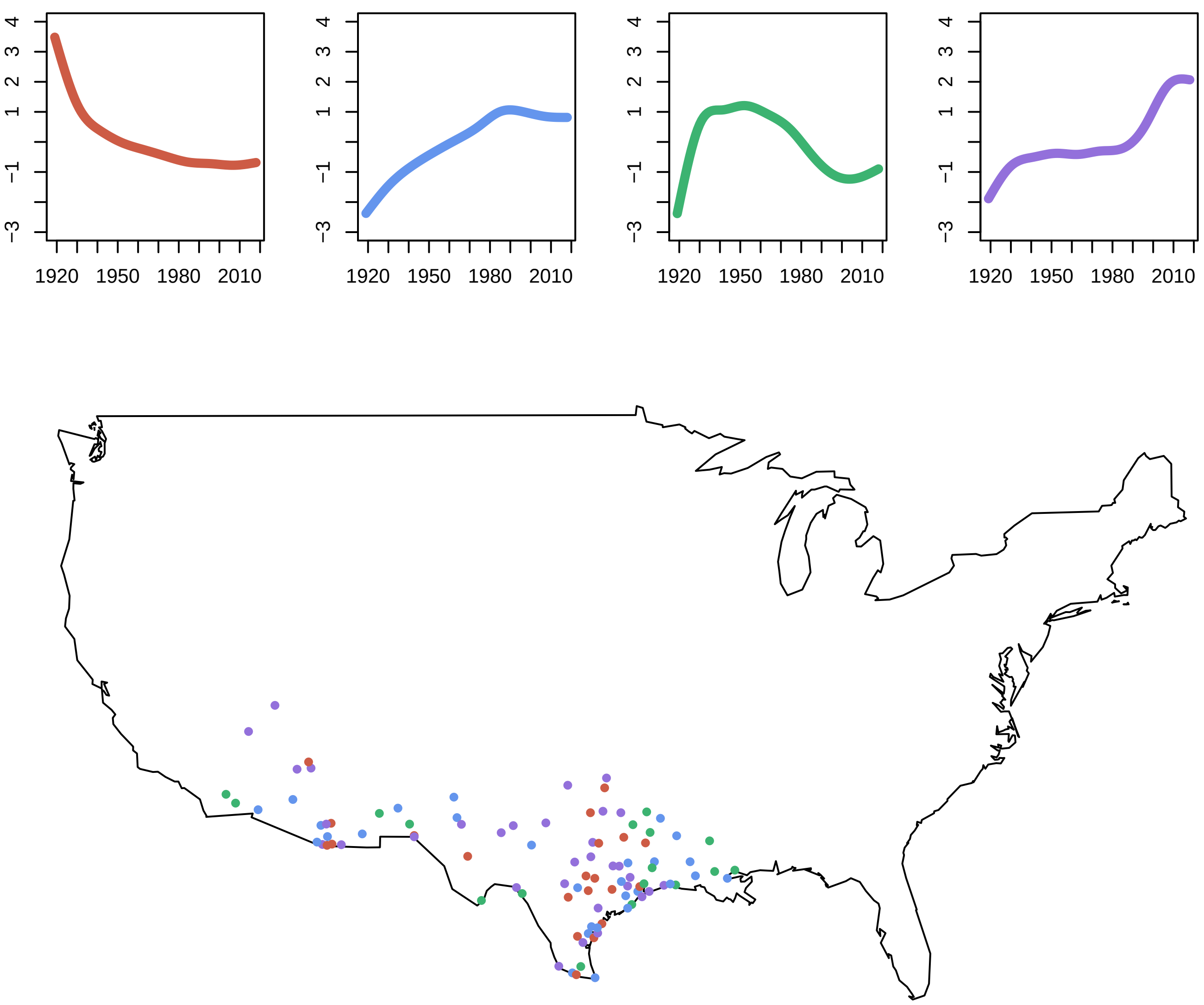

Lesser Yellowlegs

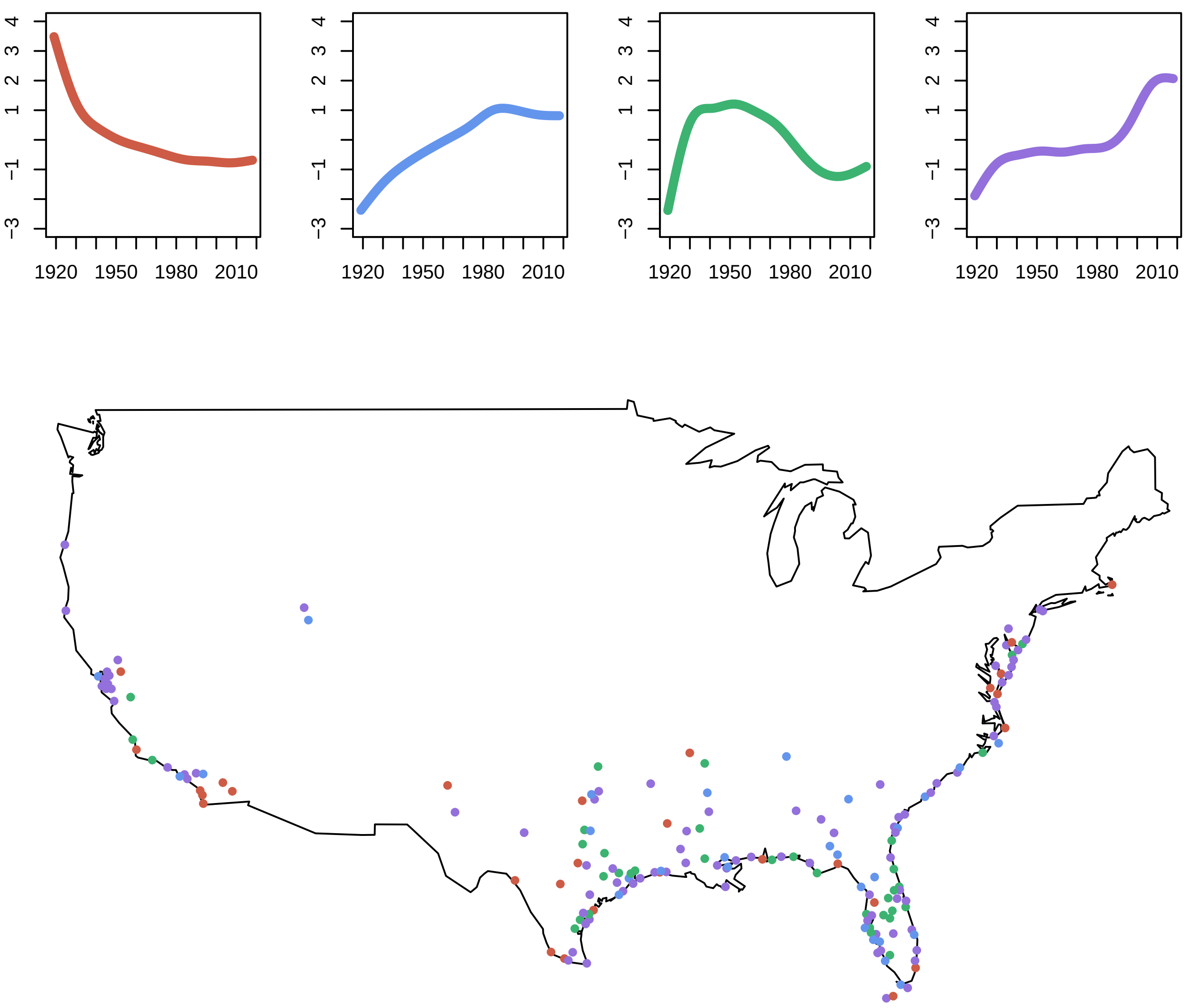

Mexican Jay

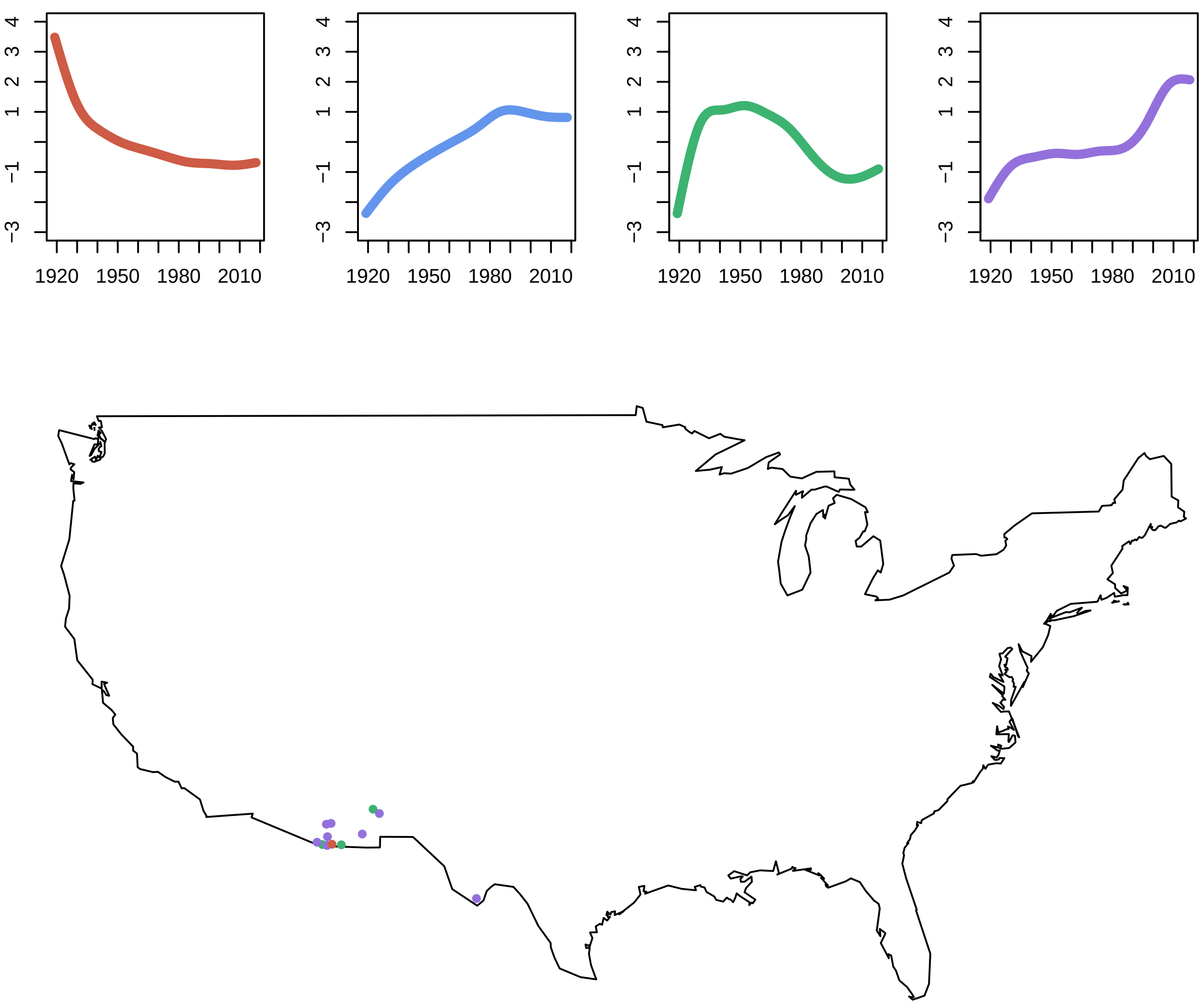

Monk Parakeet

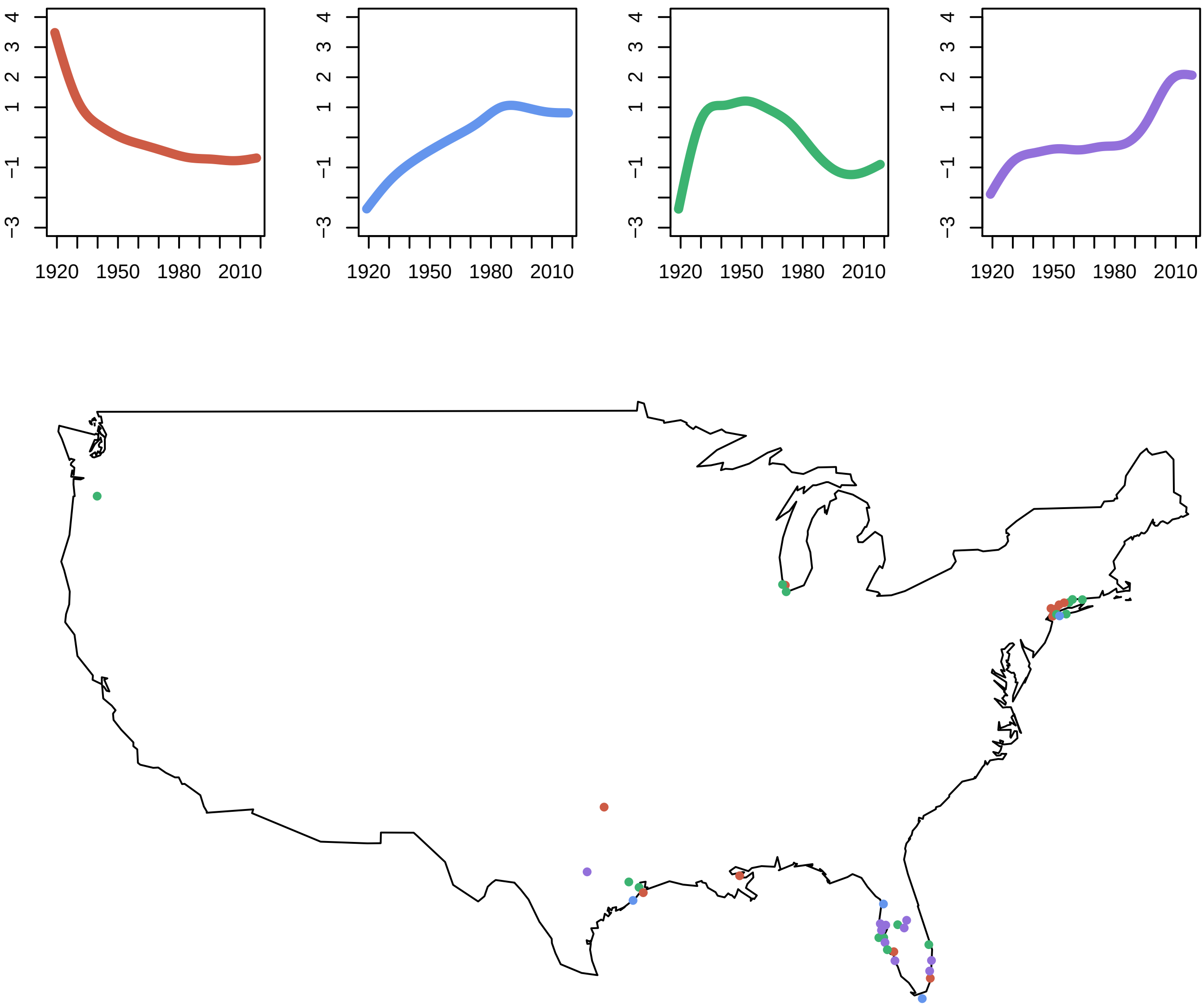

Nuttall's Woodpecker

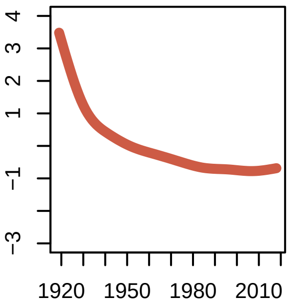

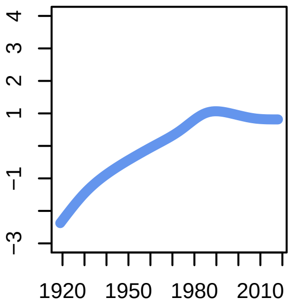

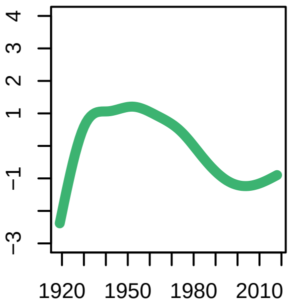

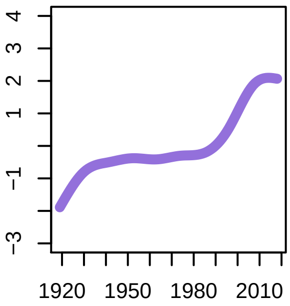

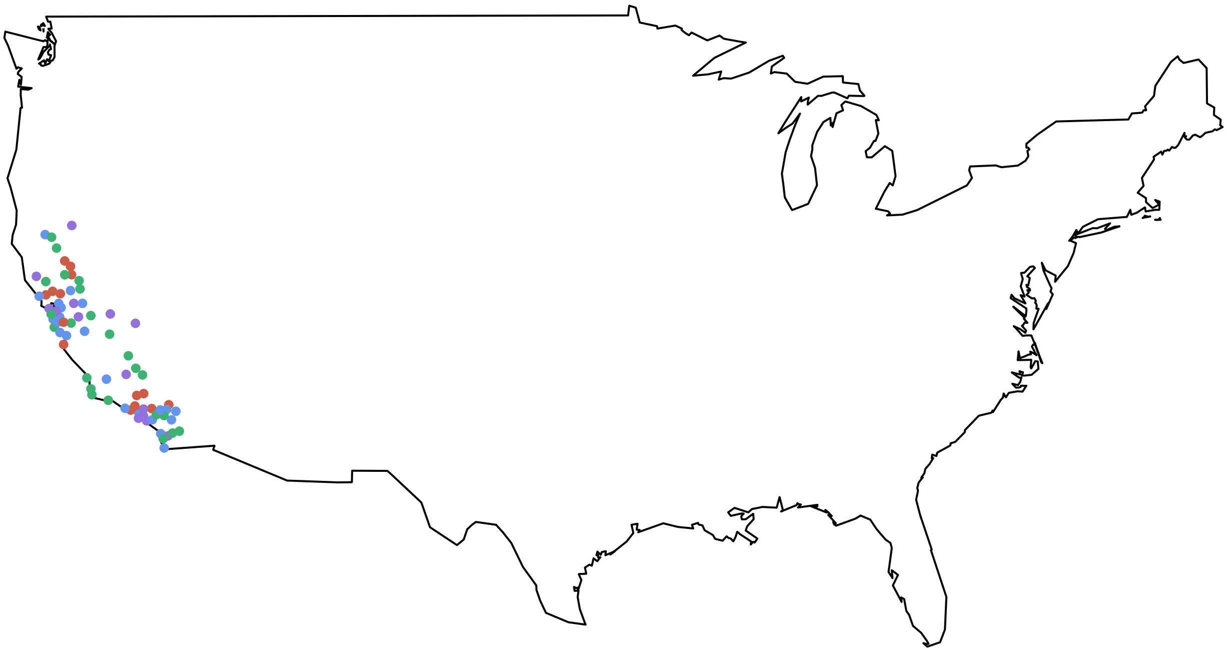

Oak Titmouse

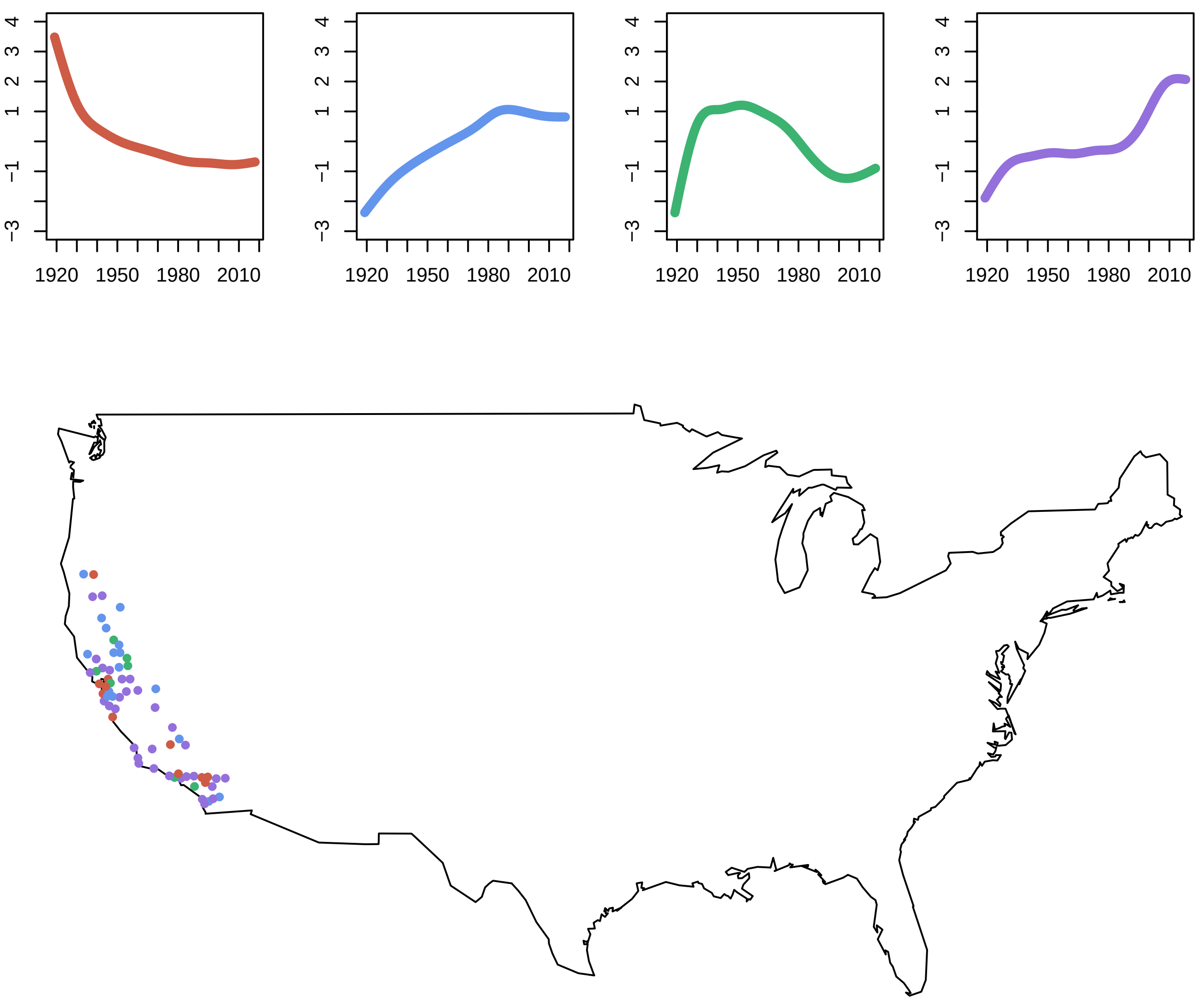

Osprey

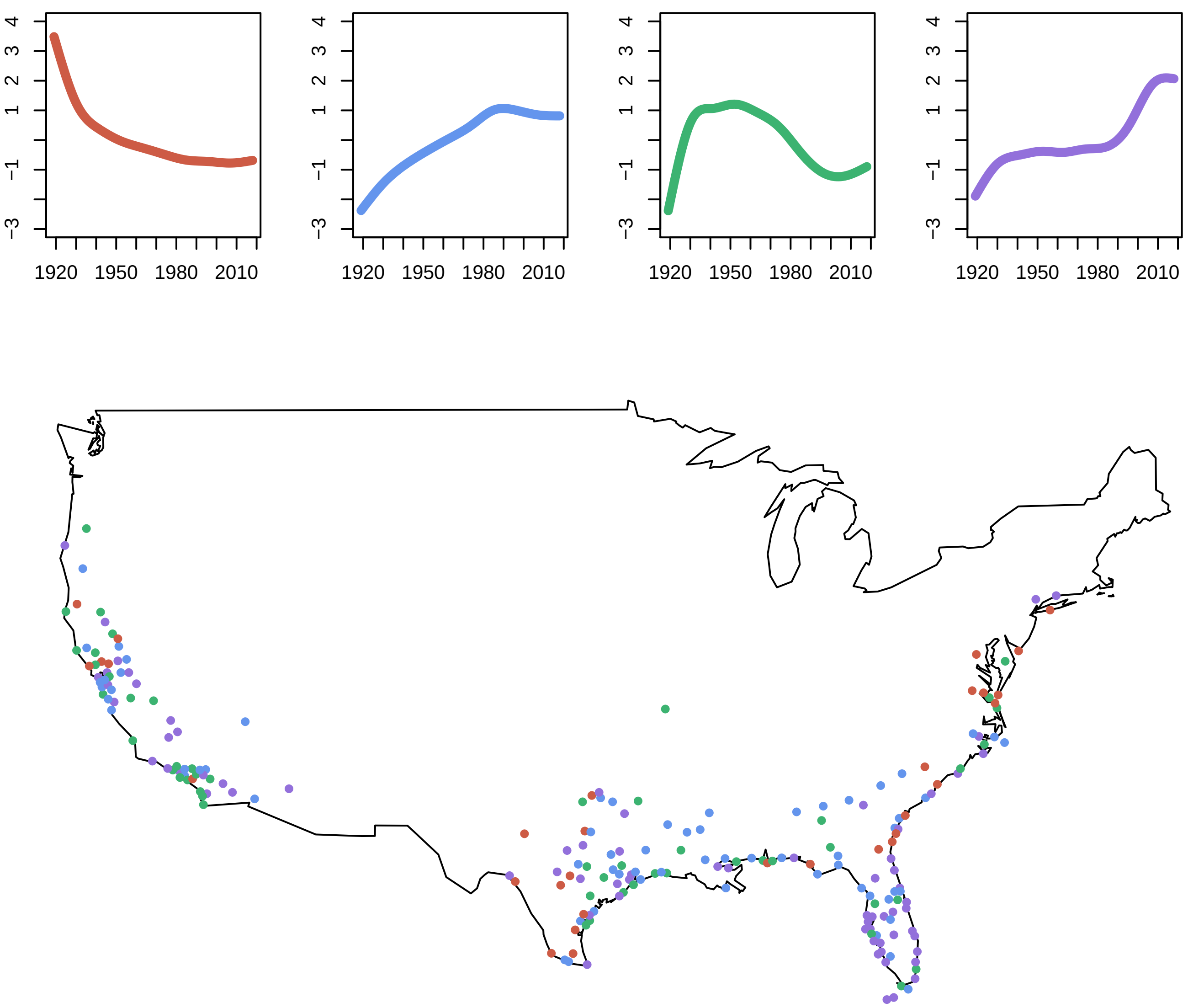

Phainopepla

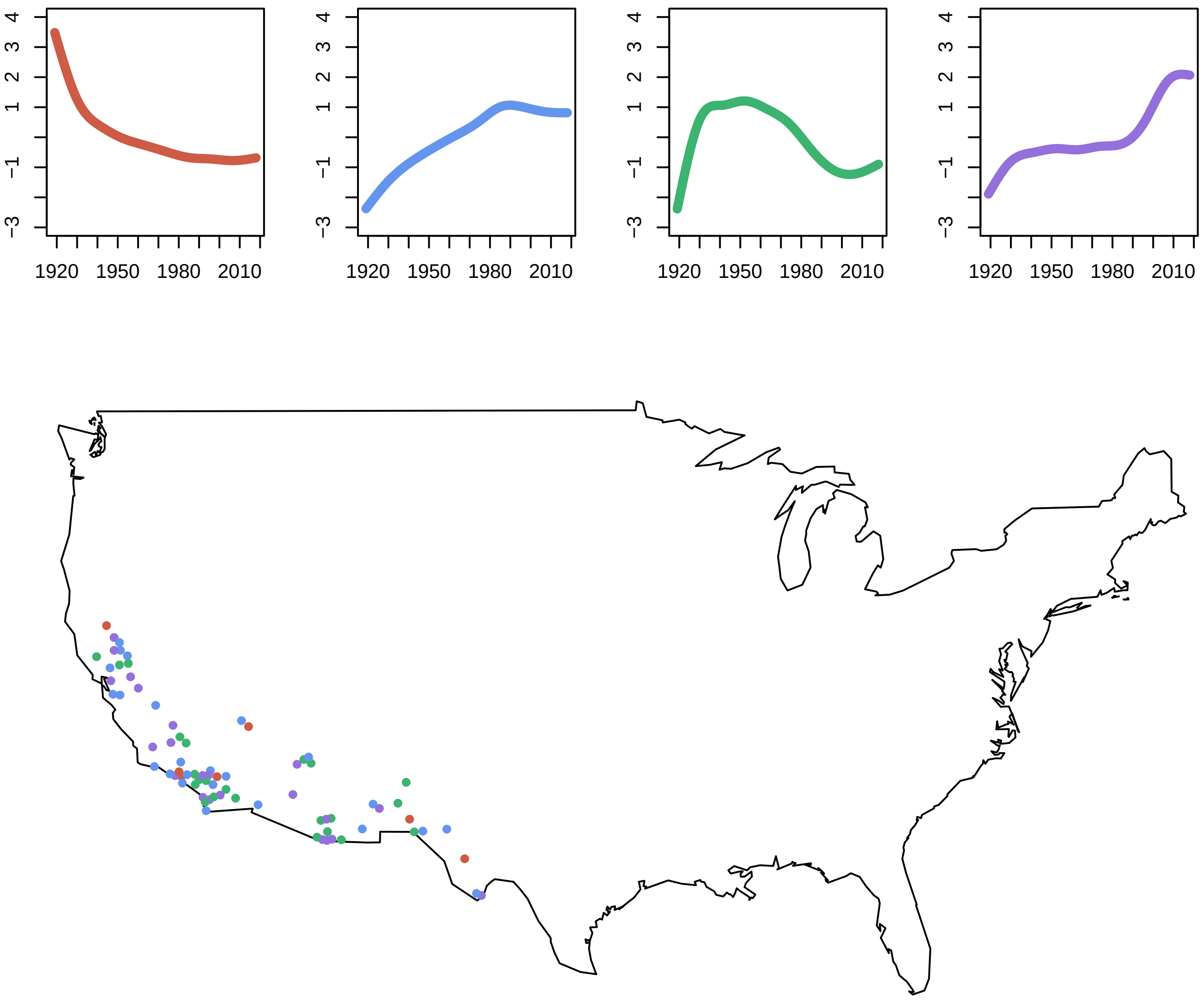

Pine Grosbeak

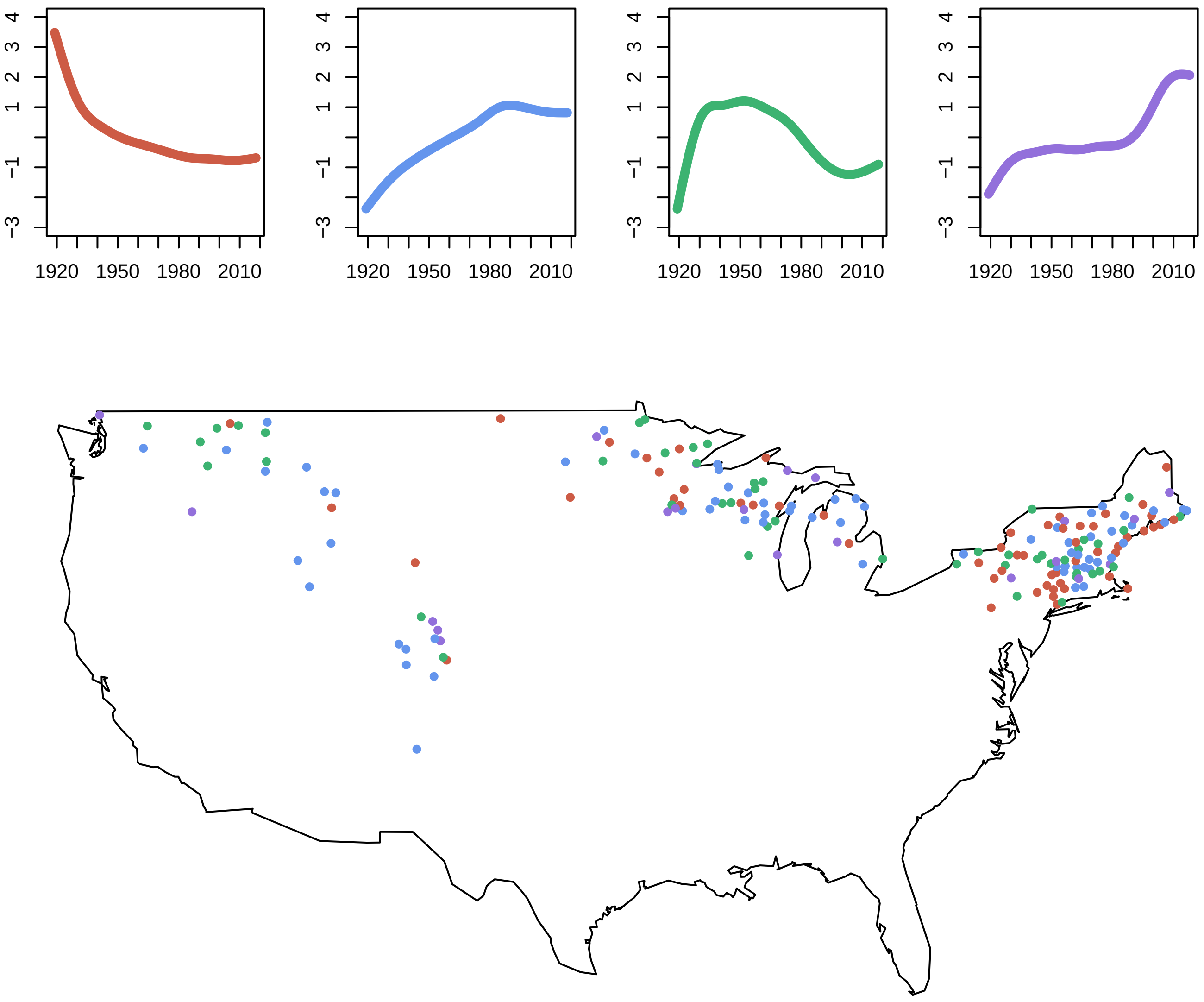

Pyrrhuloxia

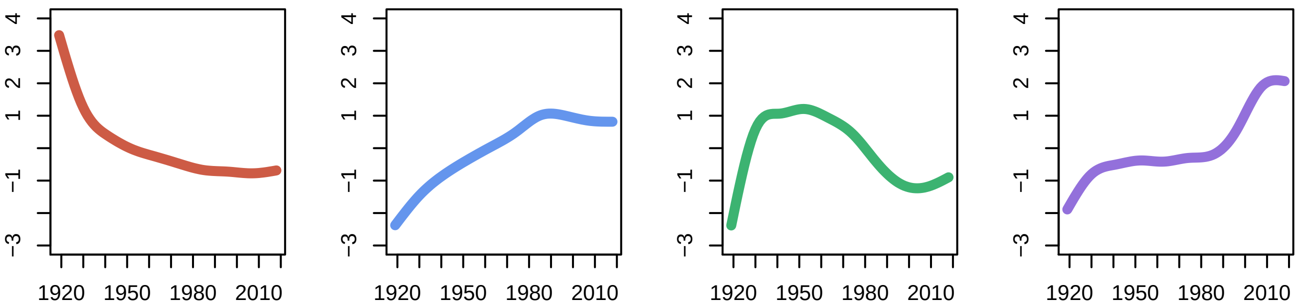

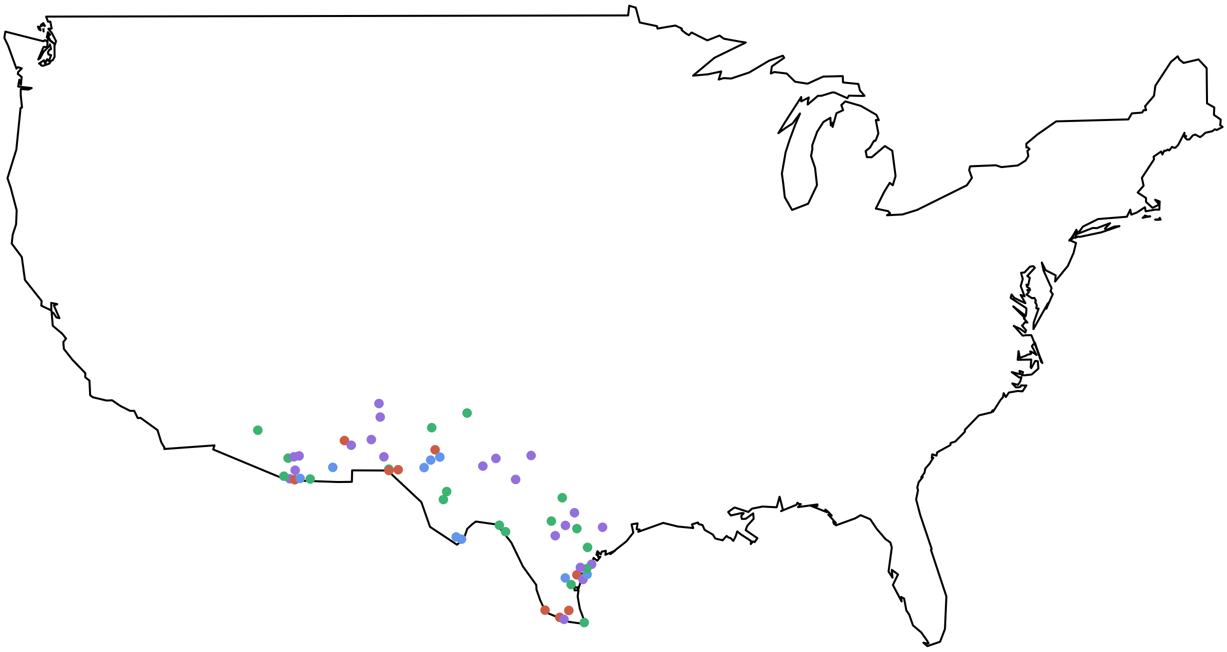

Red−necked Grebe

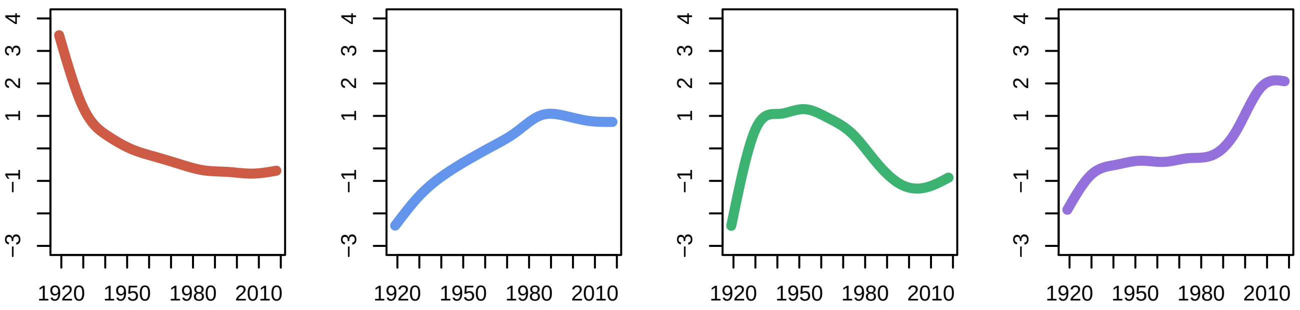

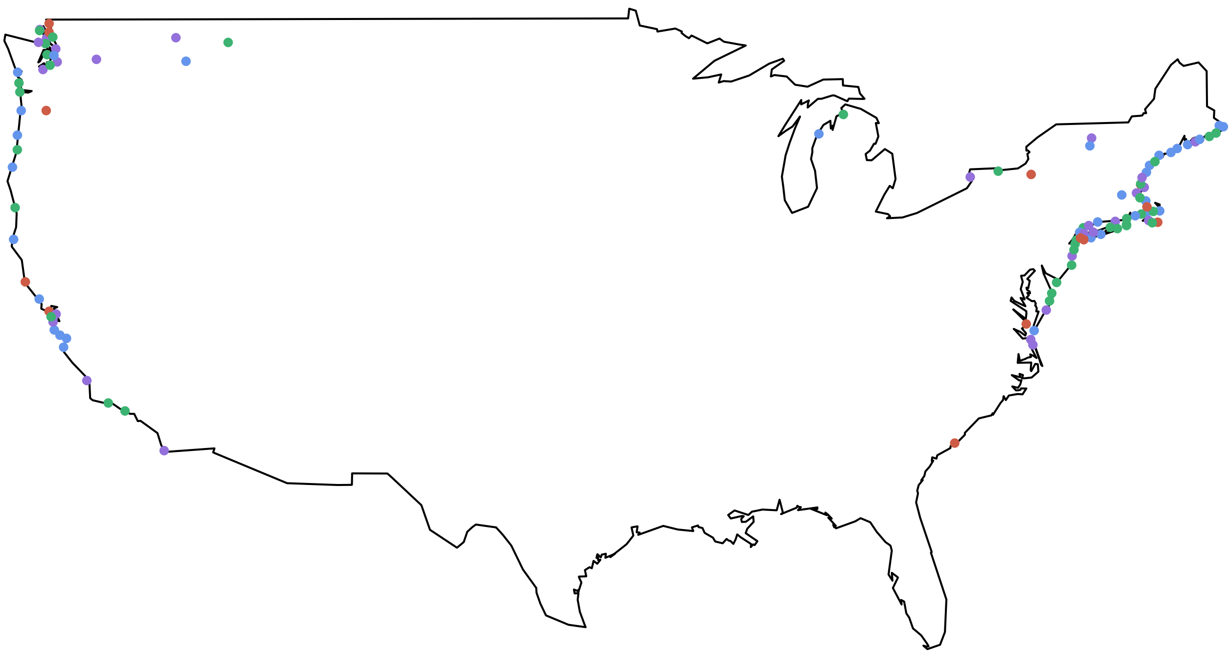

Roseate Spoonbill

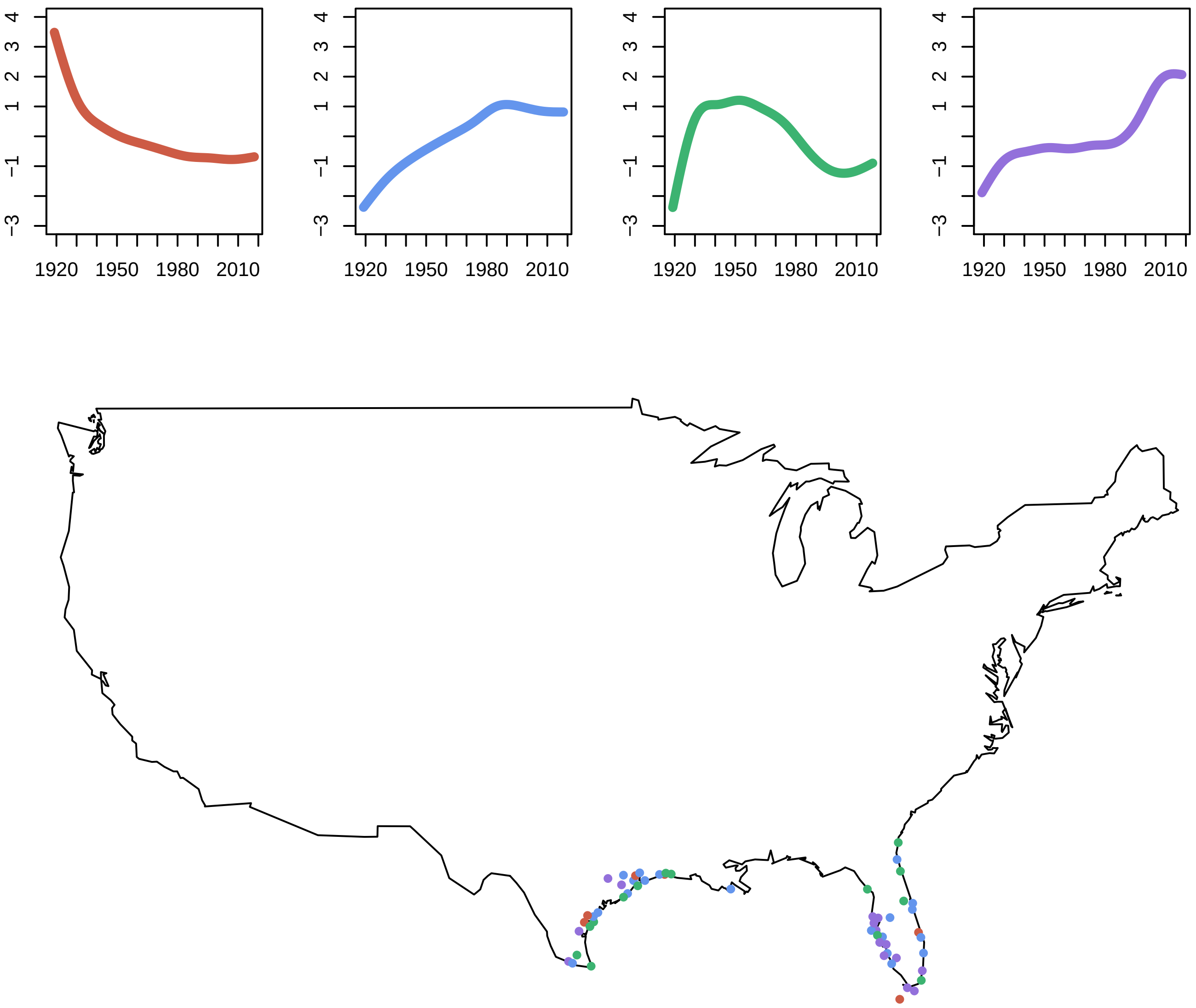

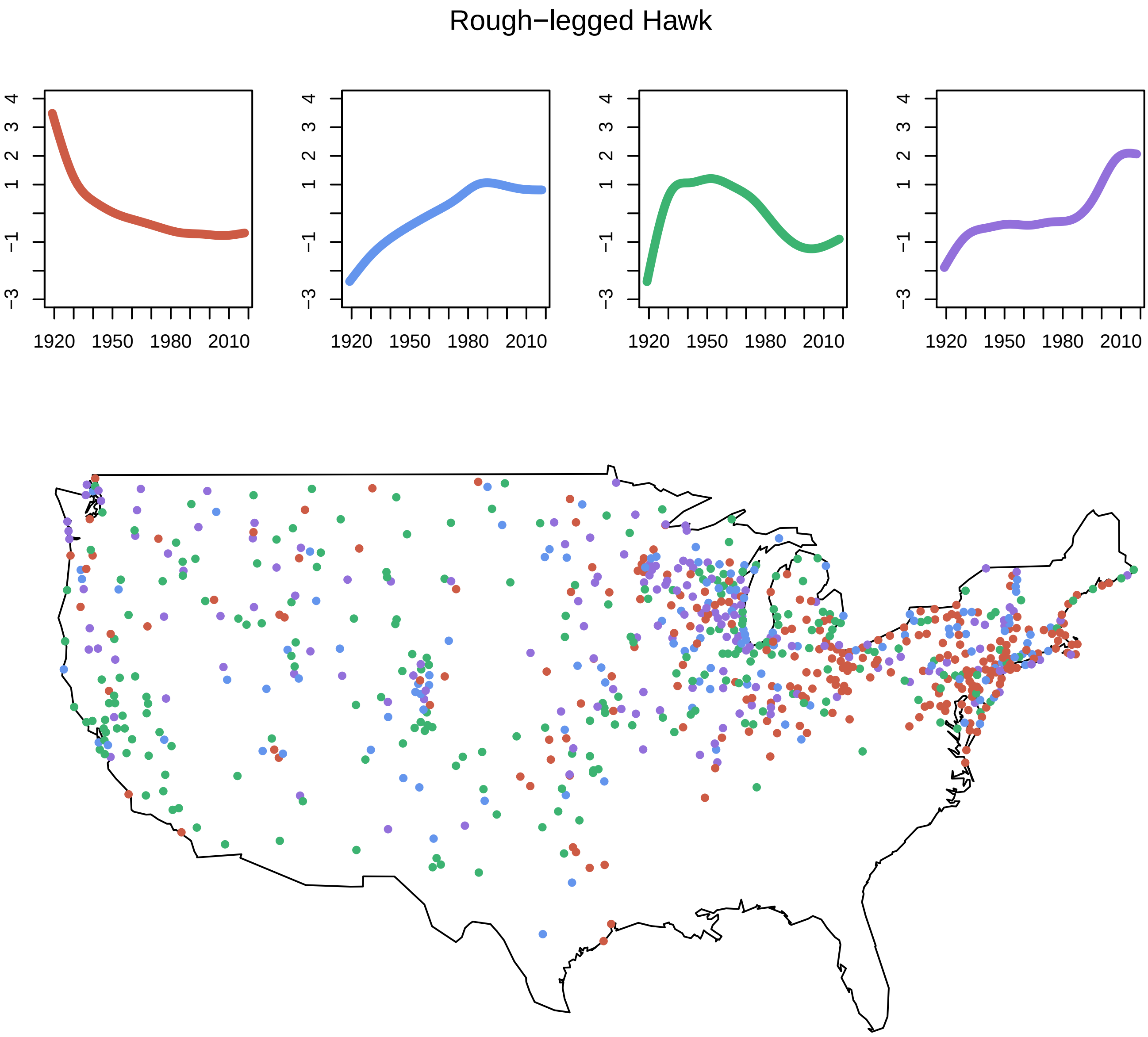
Rough−legged Hawk
4
3
2
1
−1
−3
1920
1950
1980
2010

Ruffed Grouse

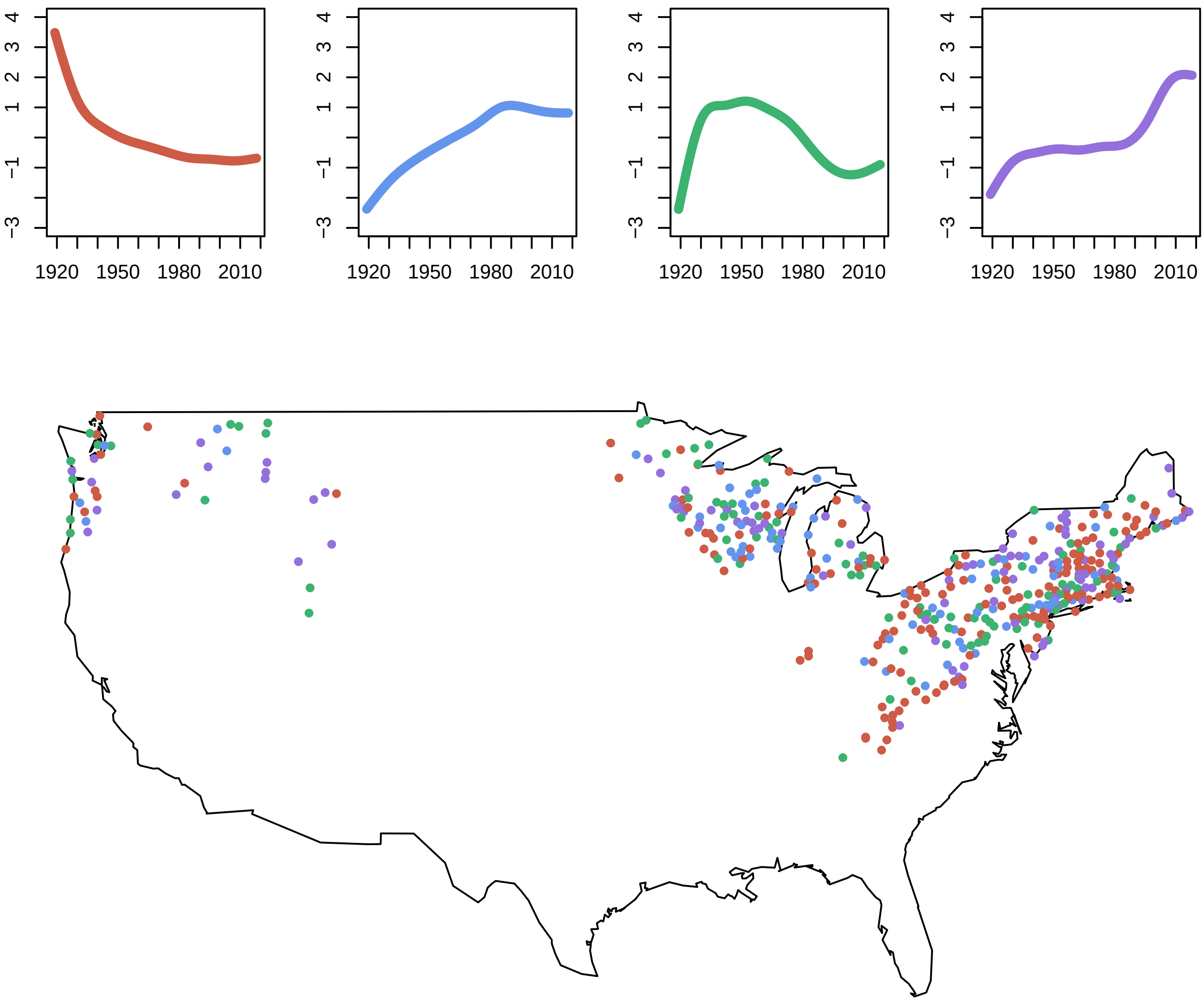

Say's Phoebe

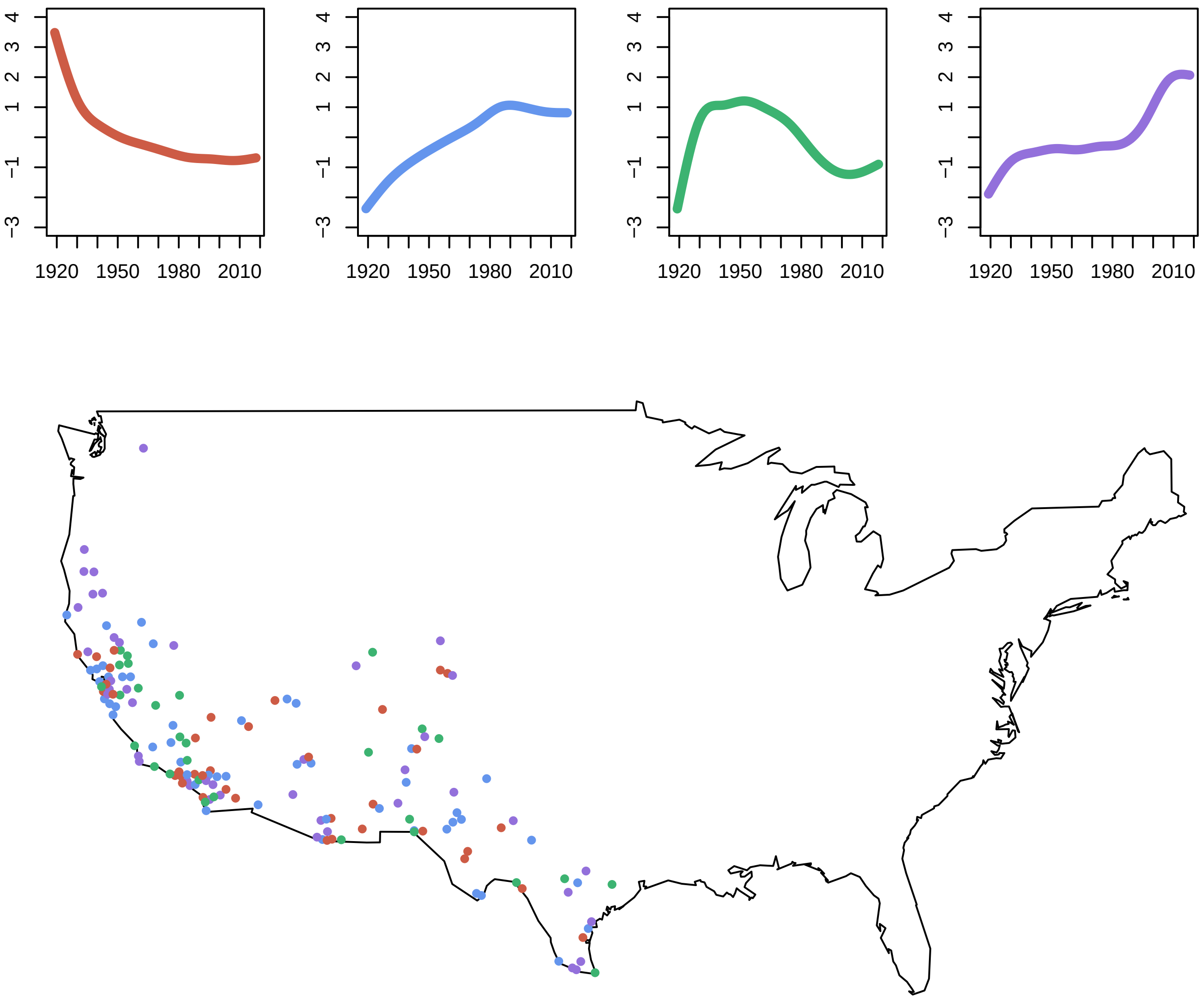

Scaled Quail

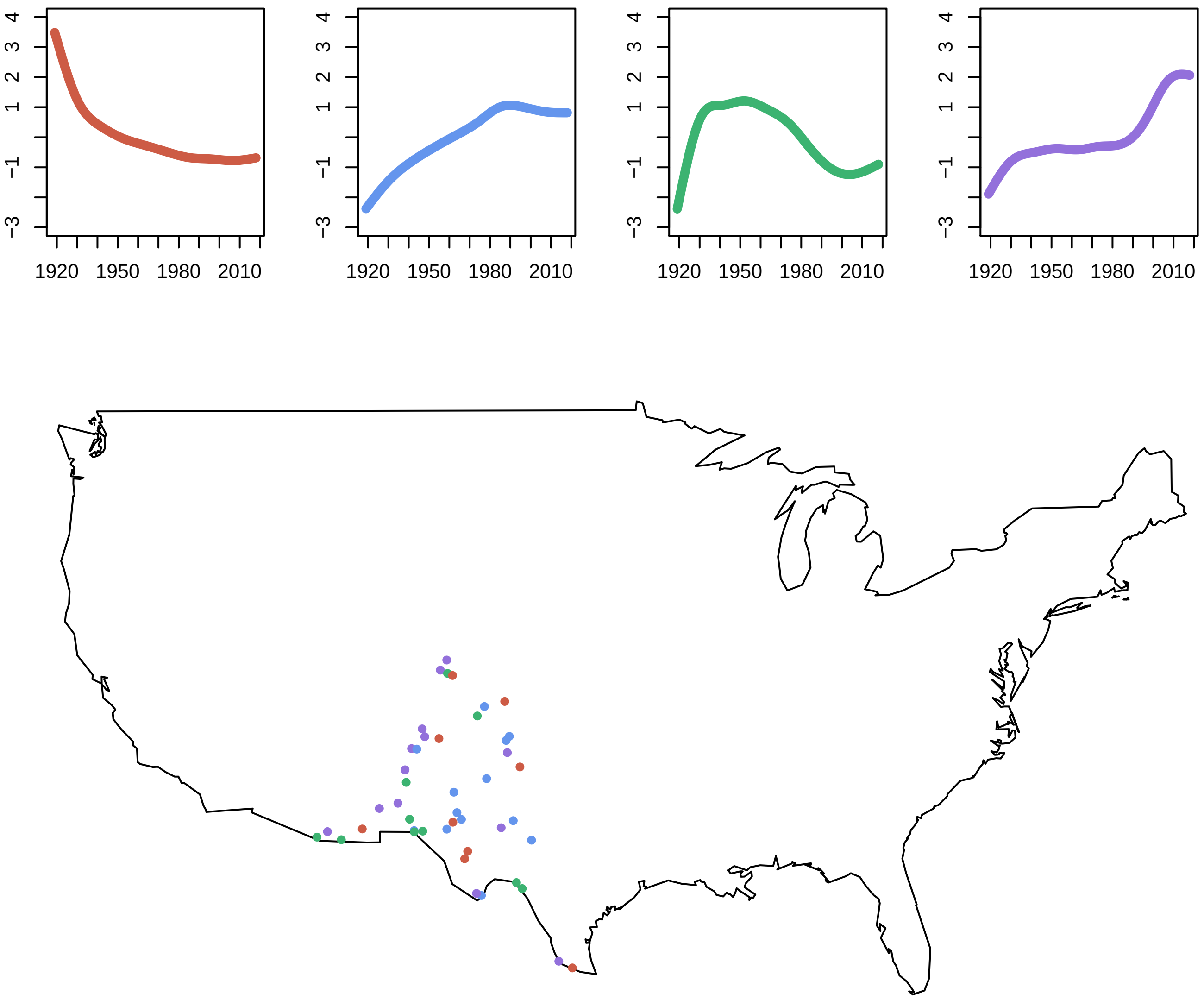

Sedge Wren

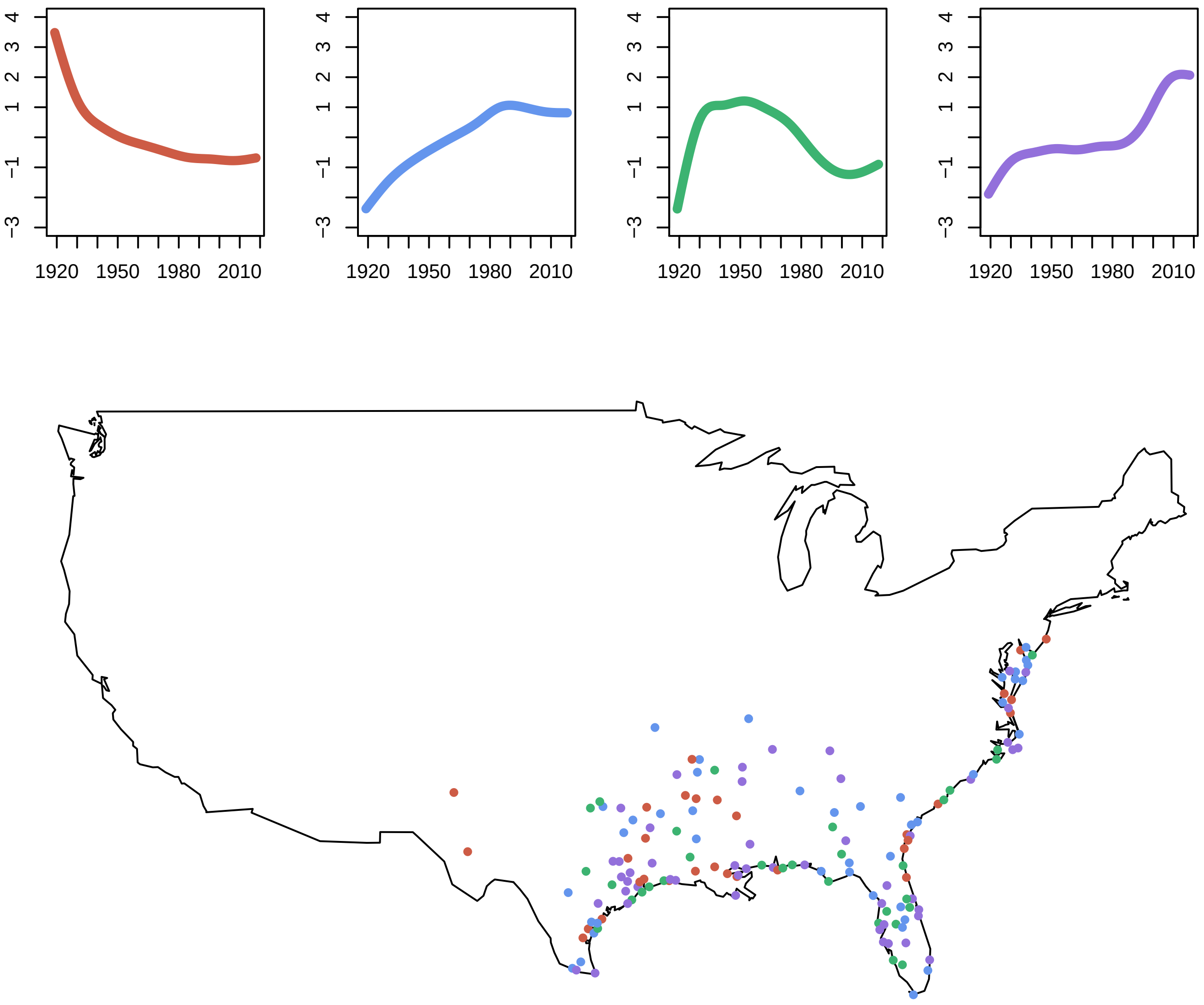

Sharp−shinned Hawk

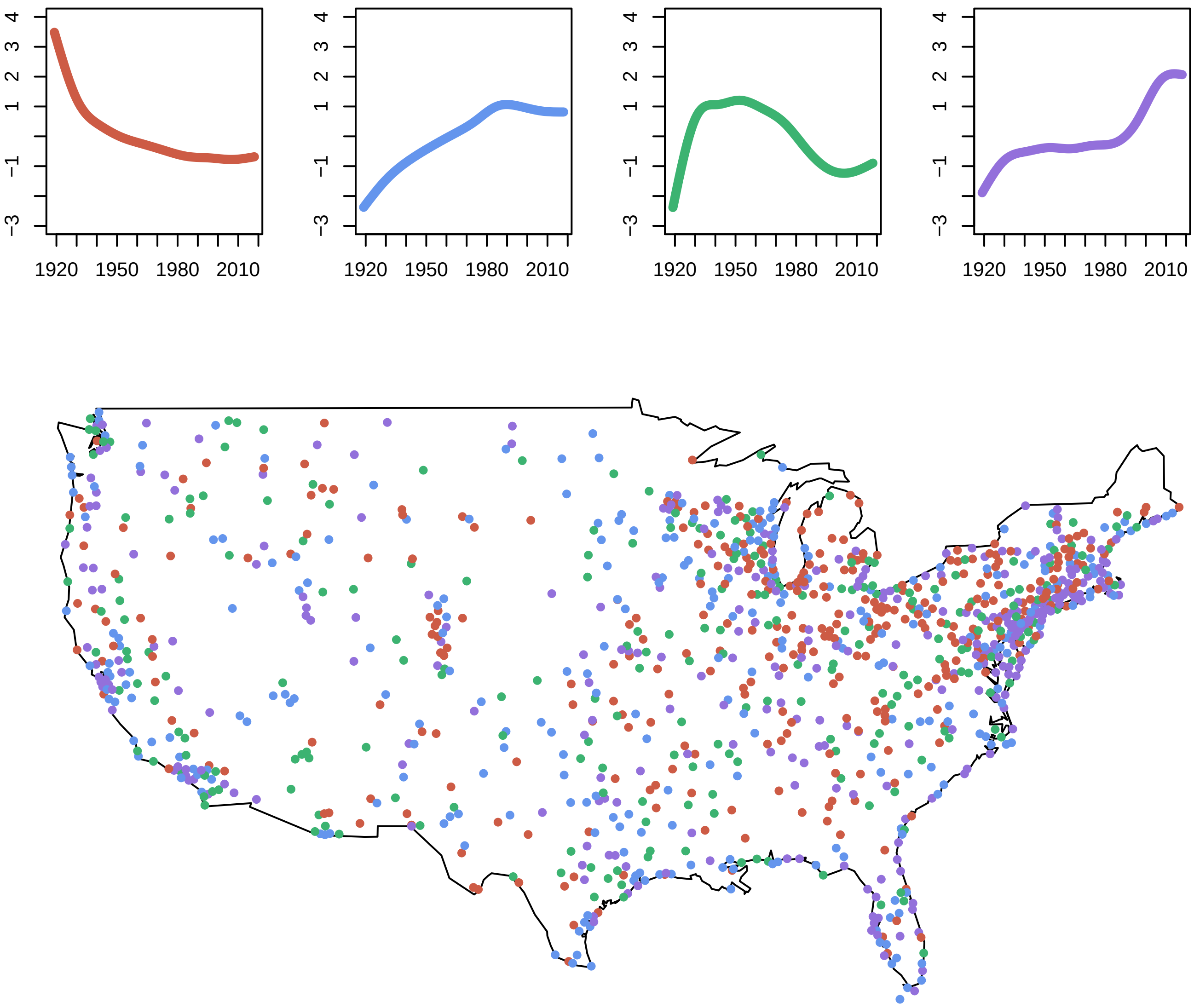

Townsend's Solitaire

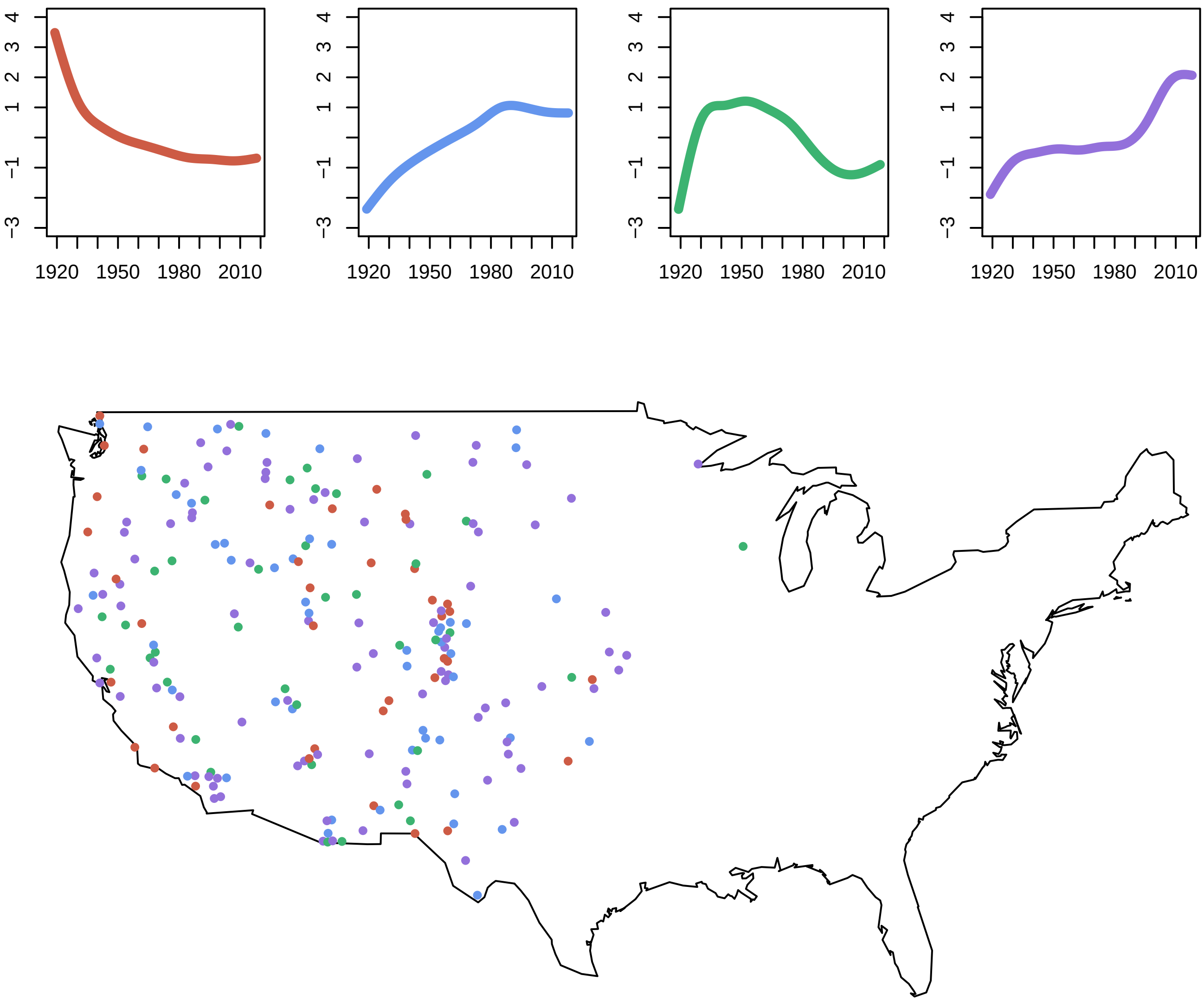

Townsend's Warbler

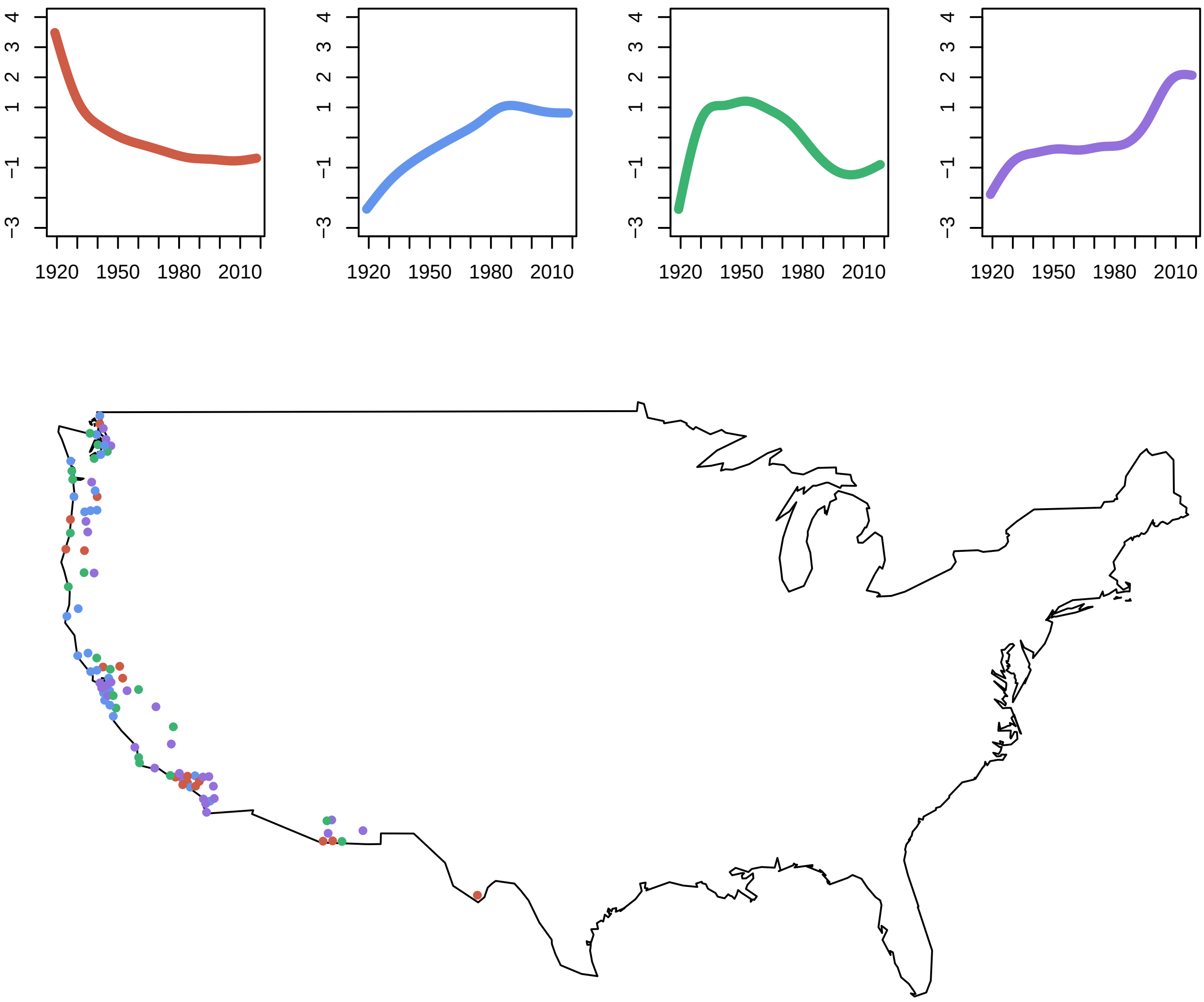

Verdin

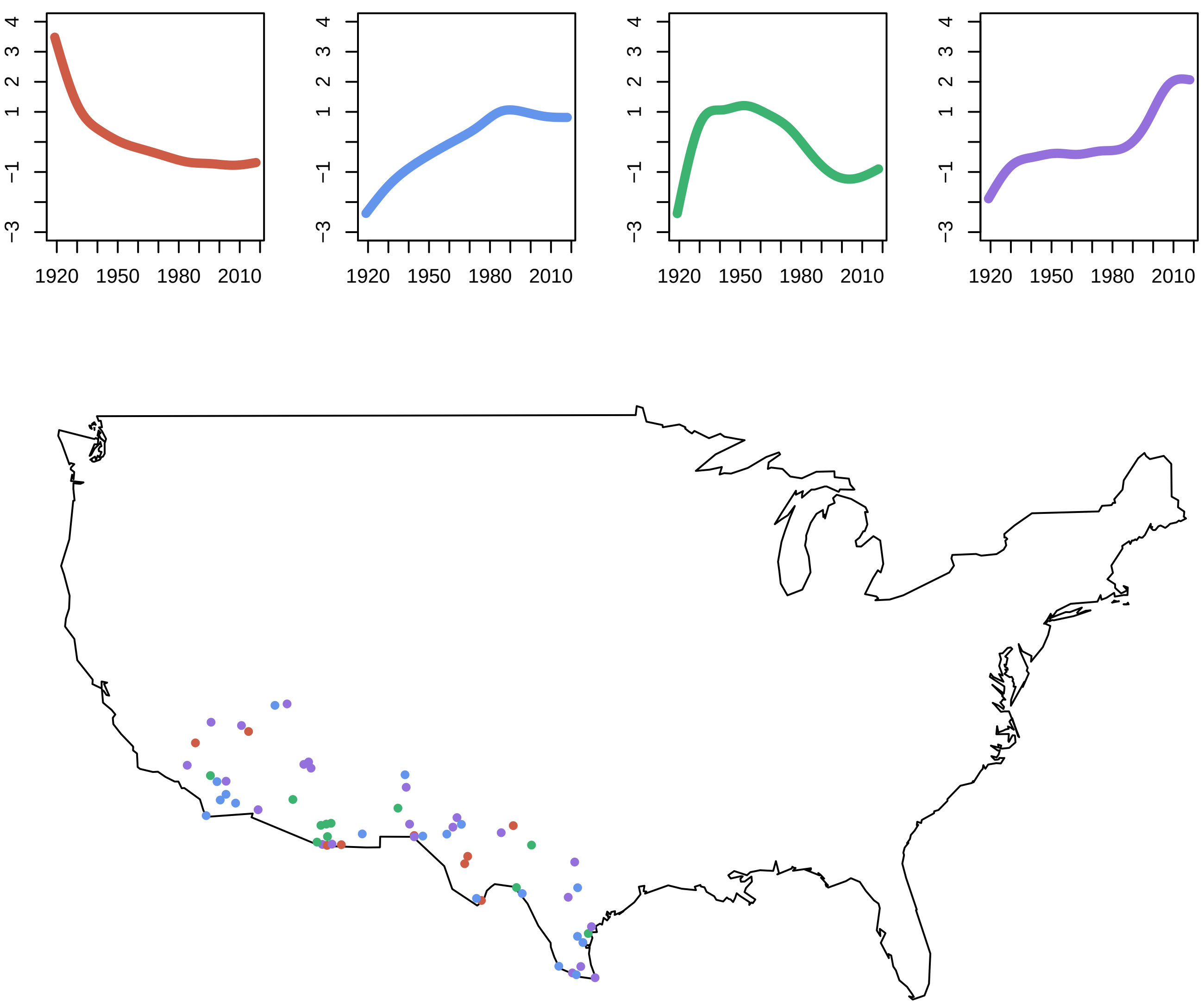

White−tailed Kite

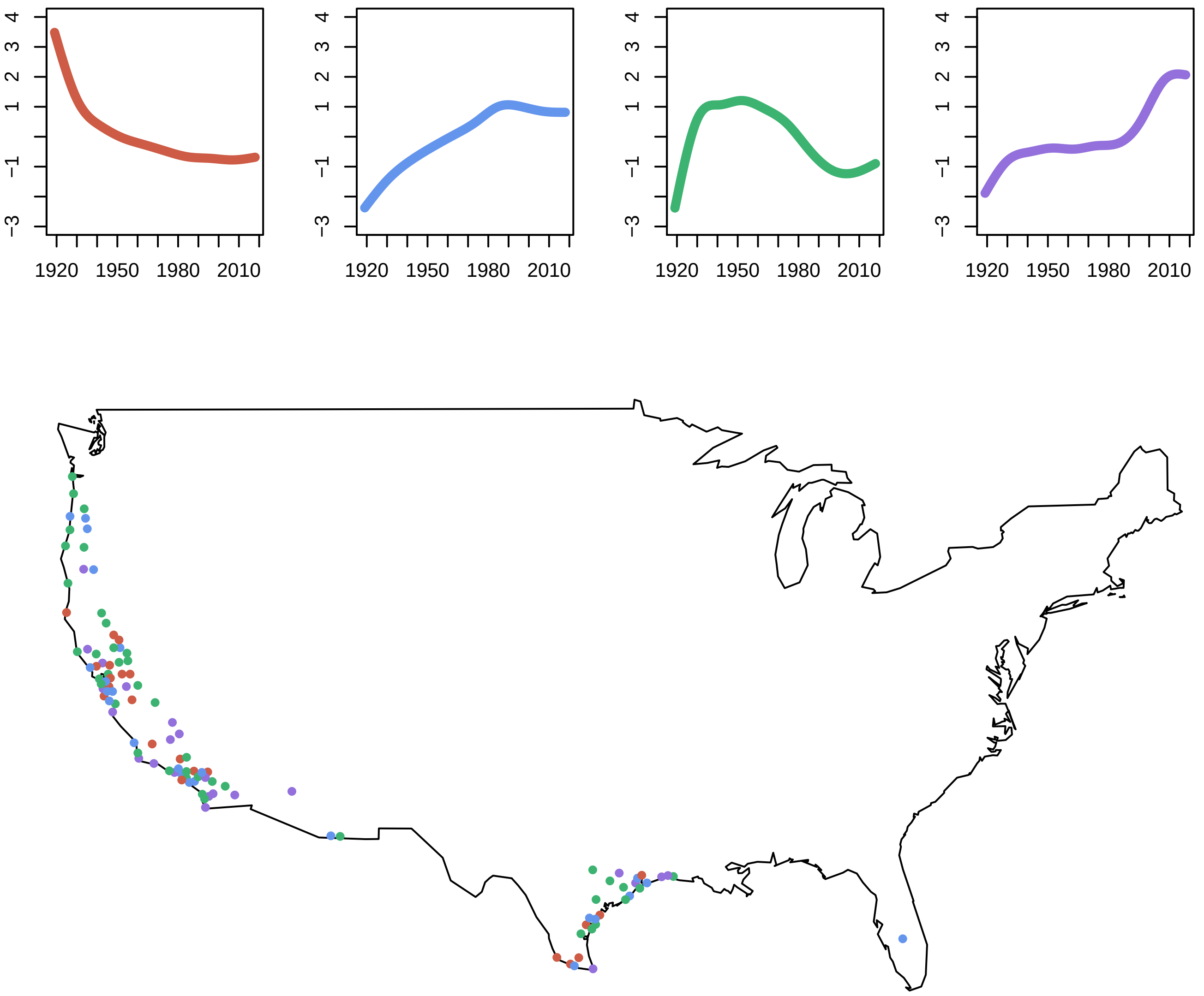

White−throated Swift

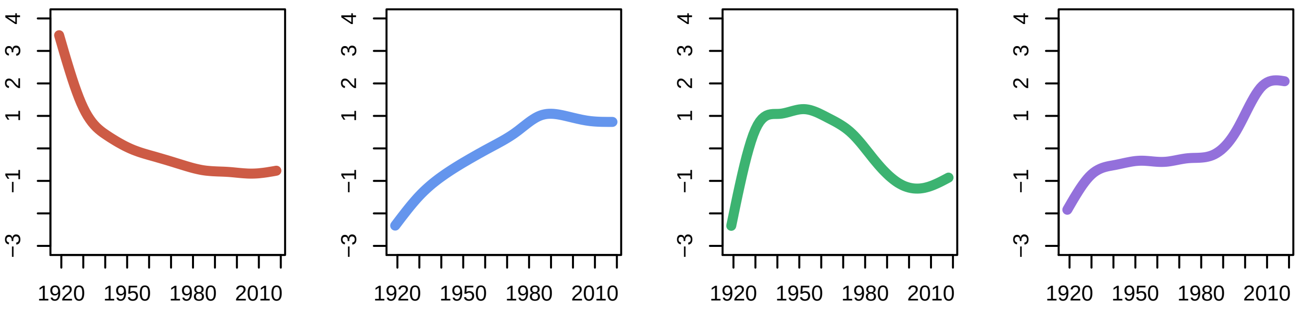

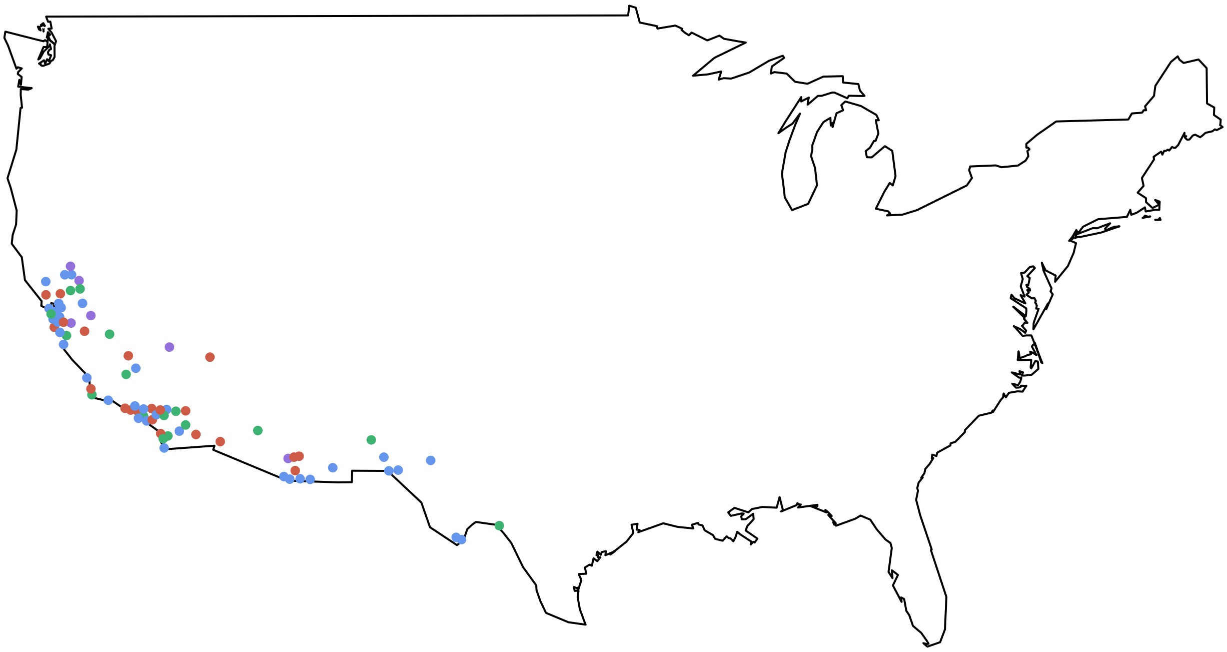

White−winged Crossbill

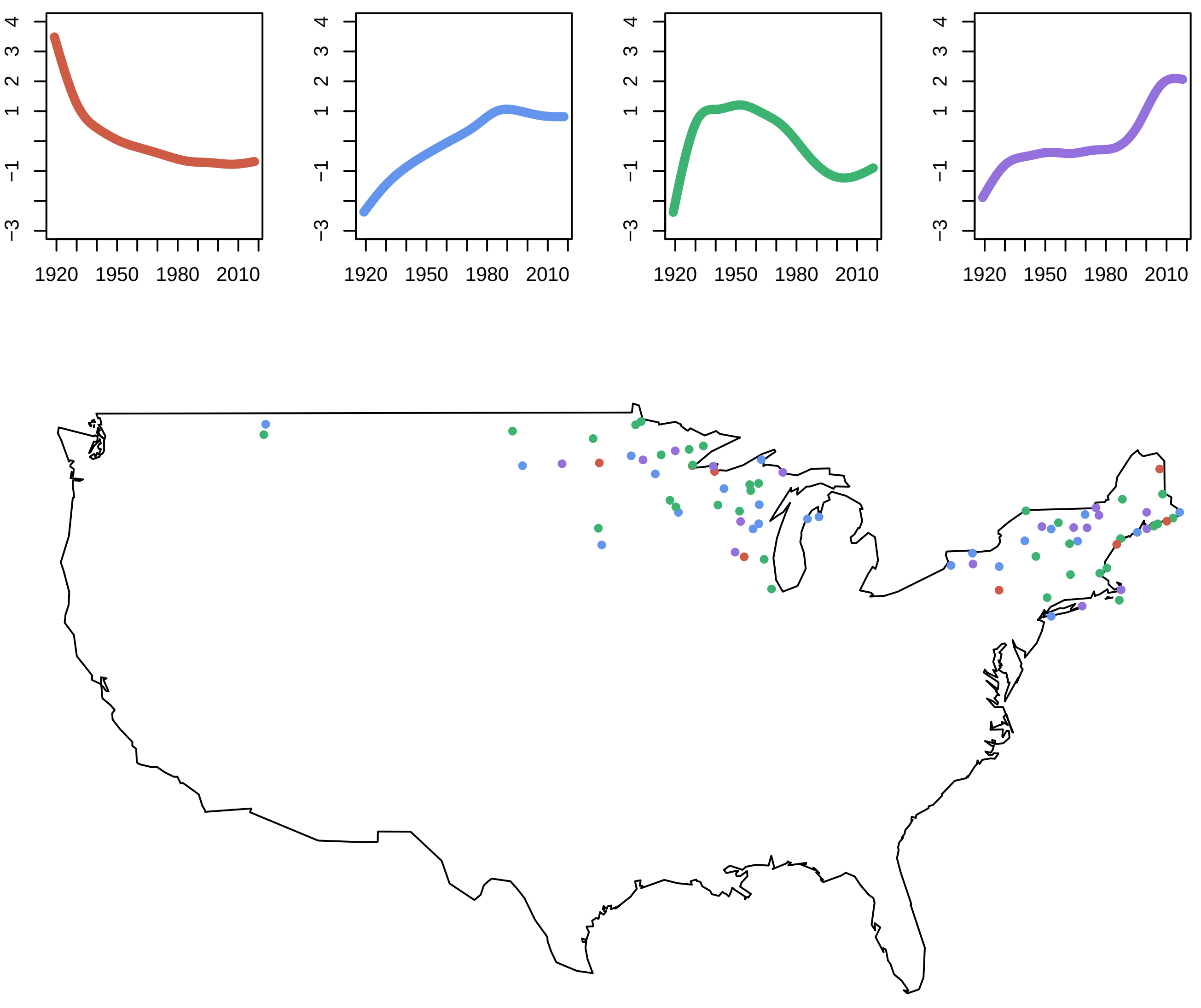

Wilson's Snipe

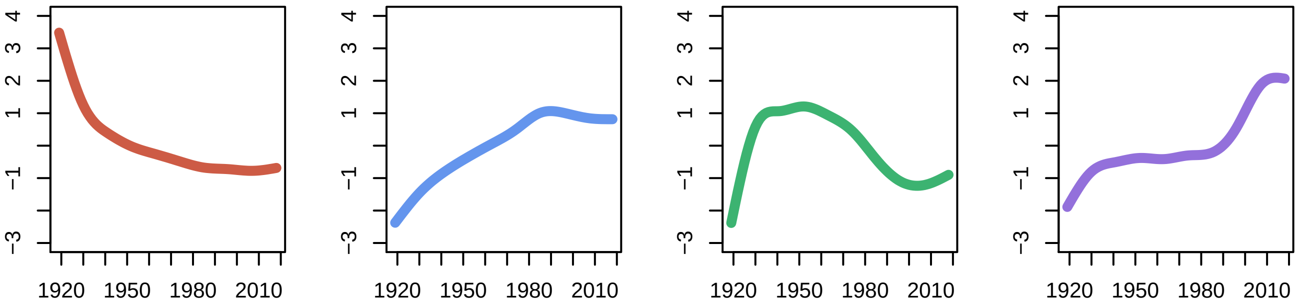

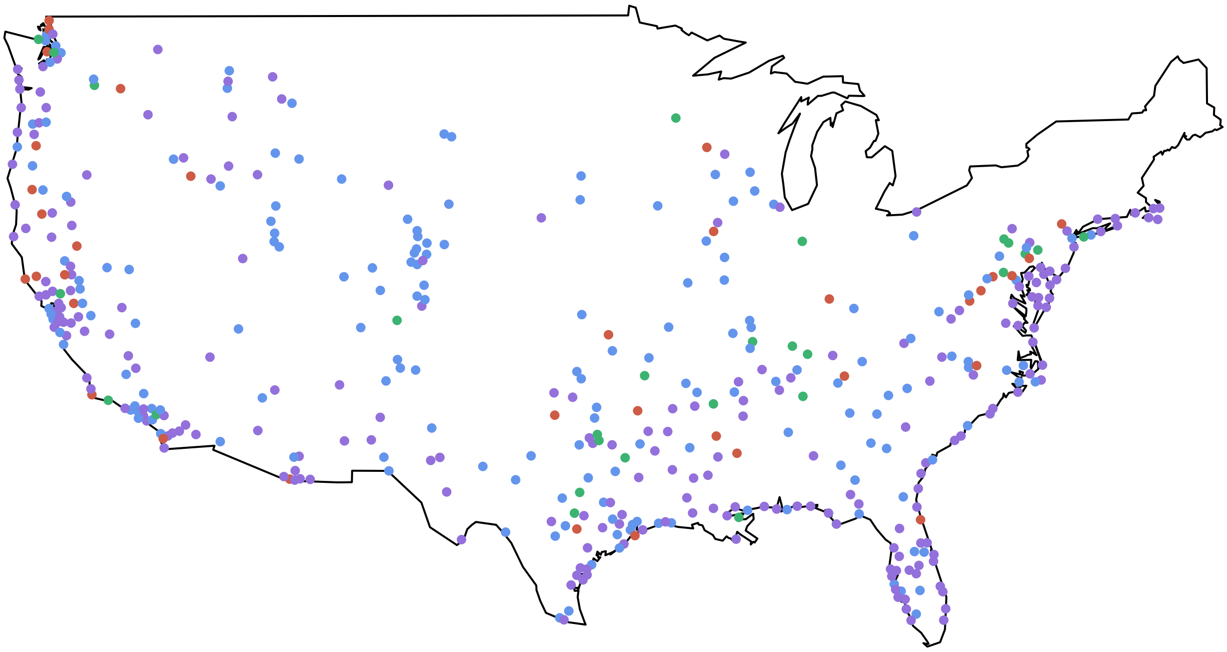

Abert's Towhee

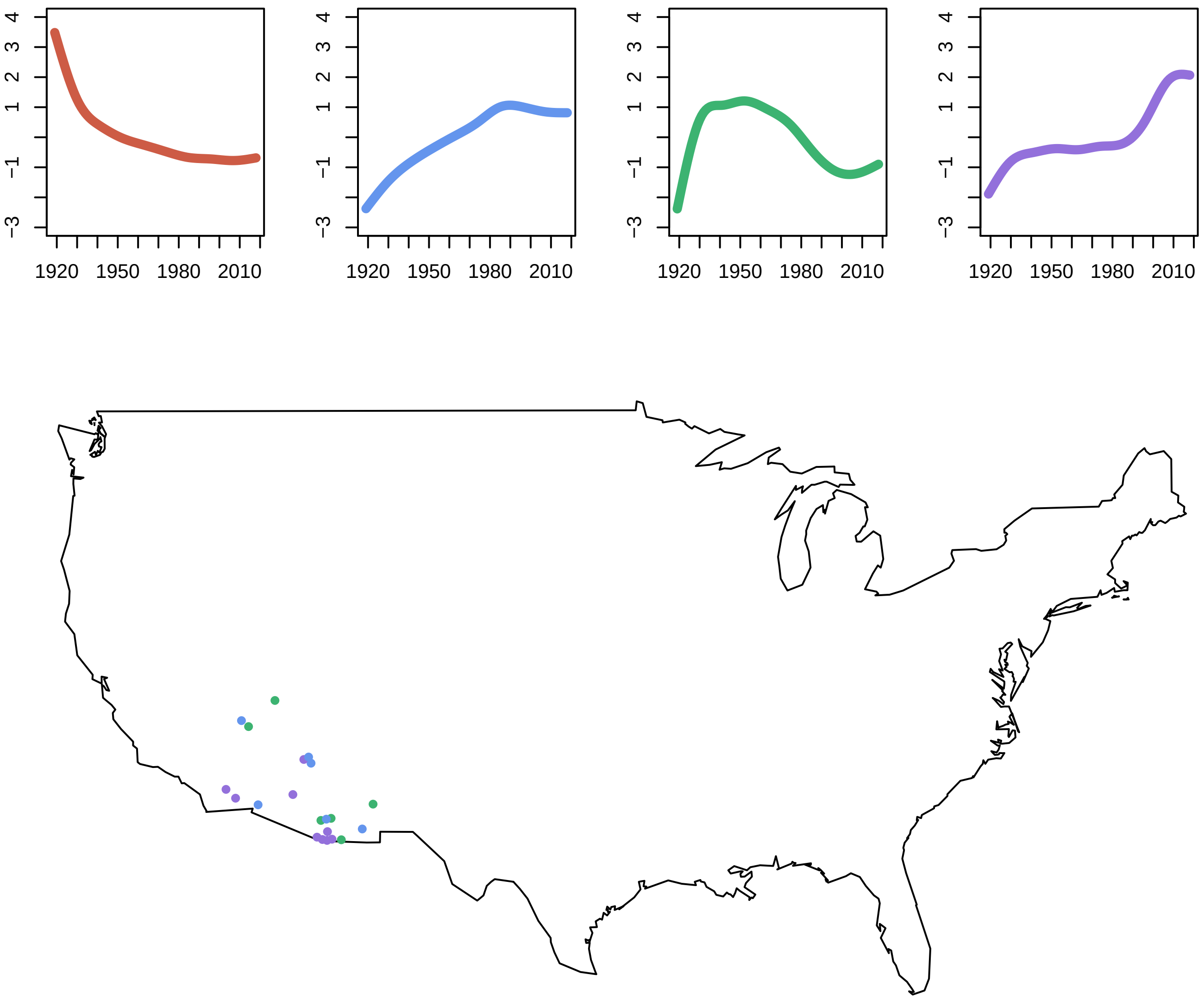

Allen's Hummingbird

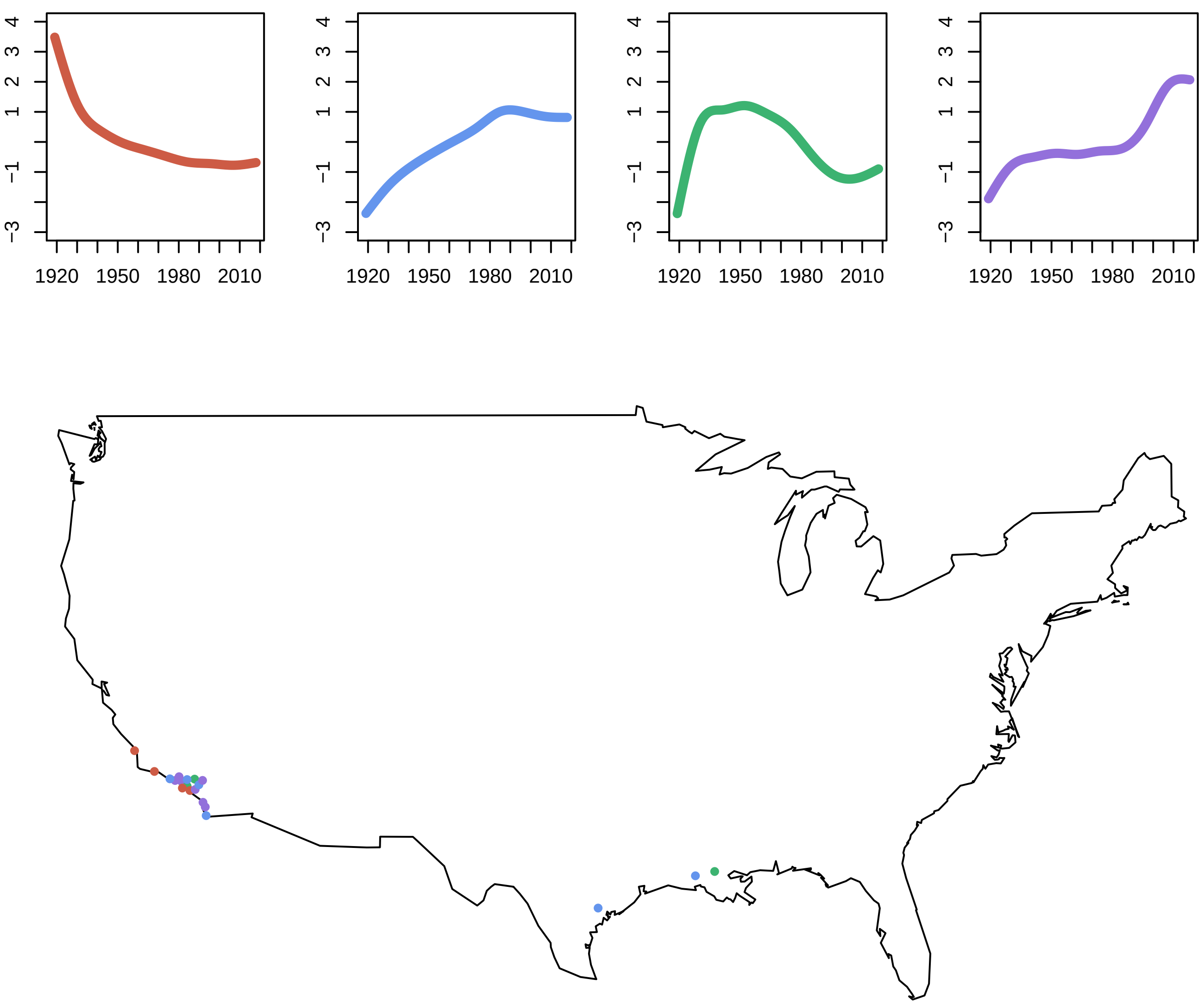

American Dipper

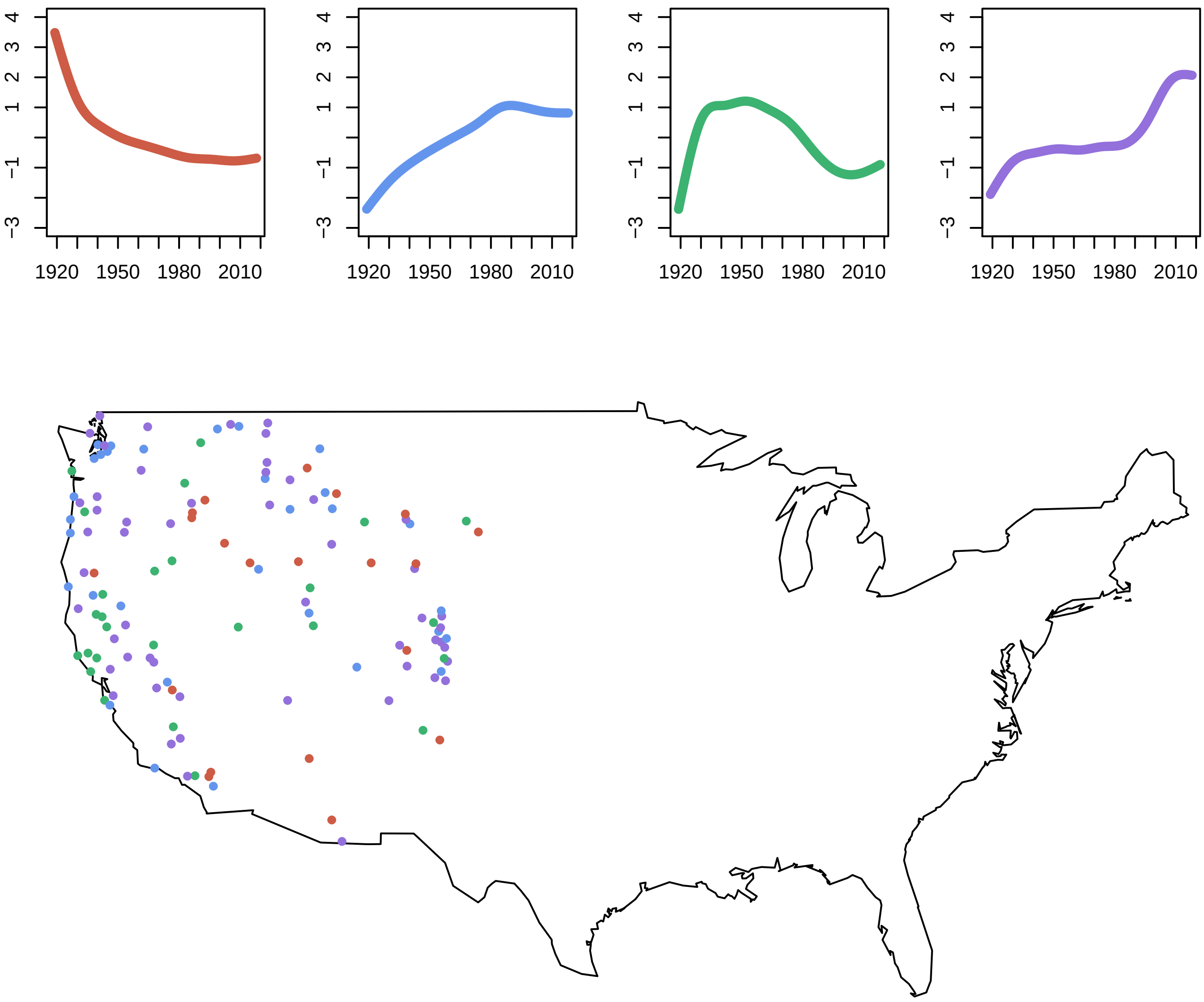

American Woodcock

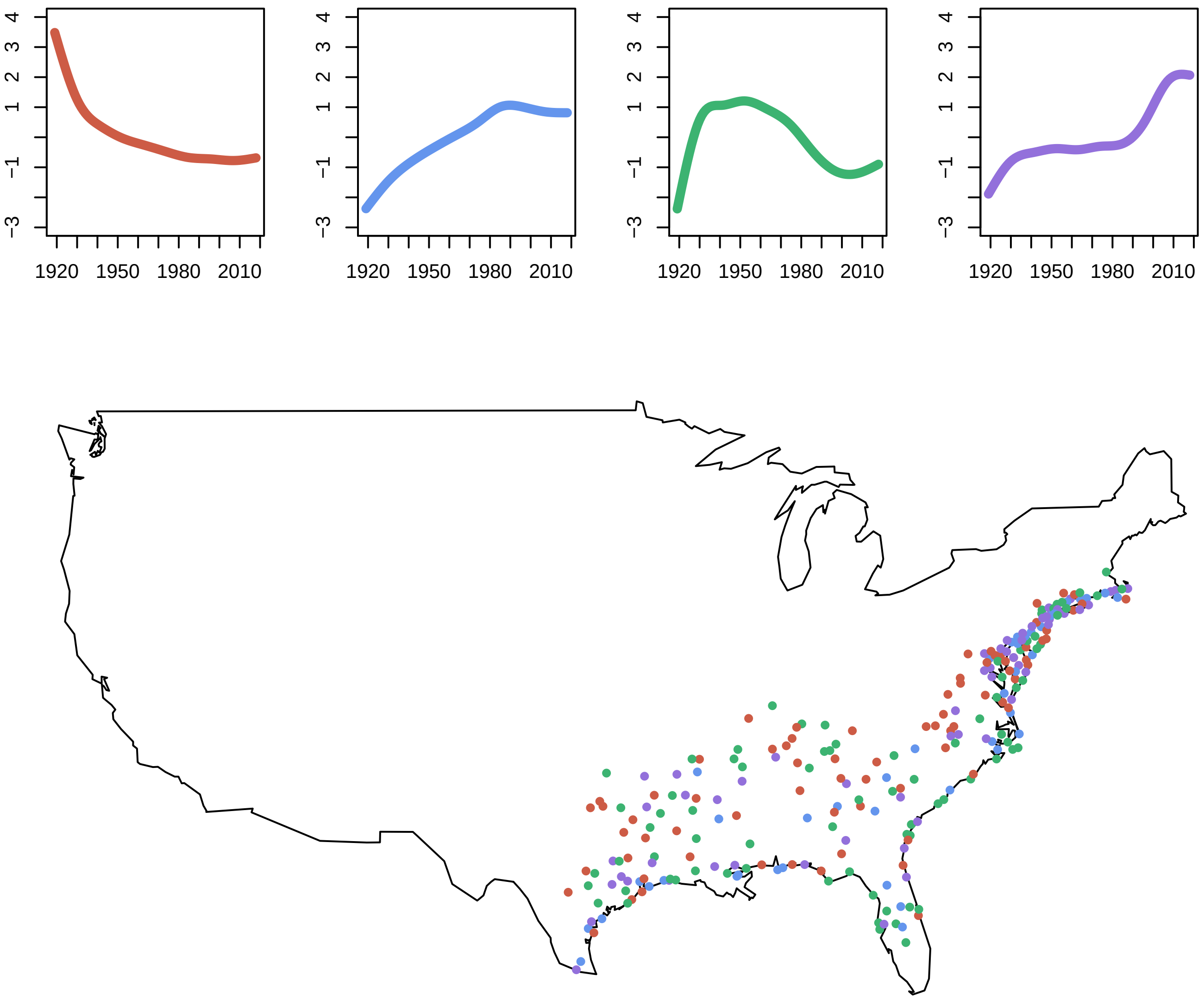

Barn Owl

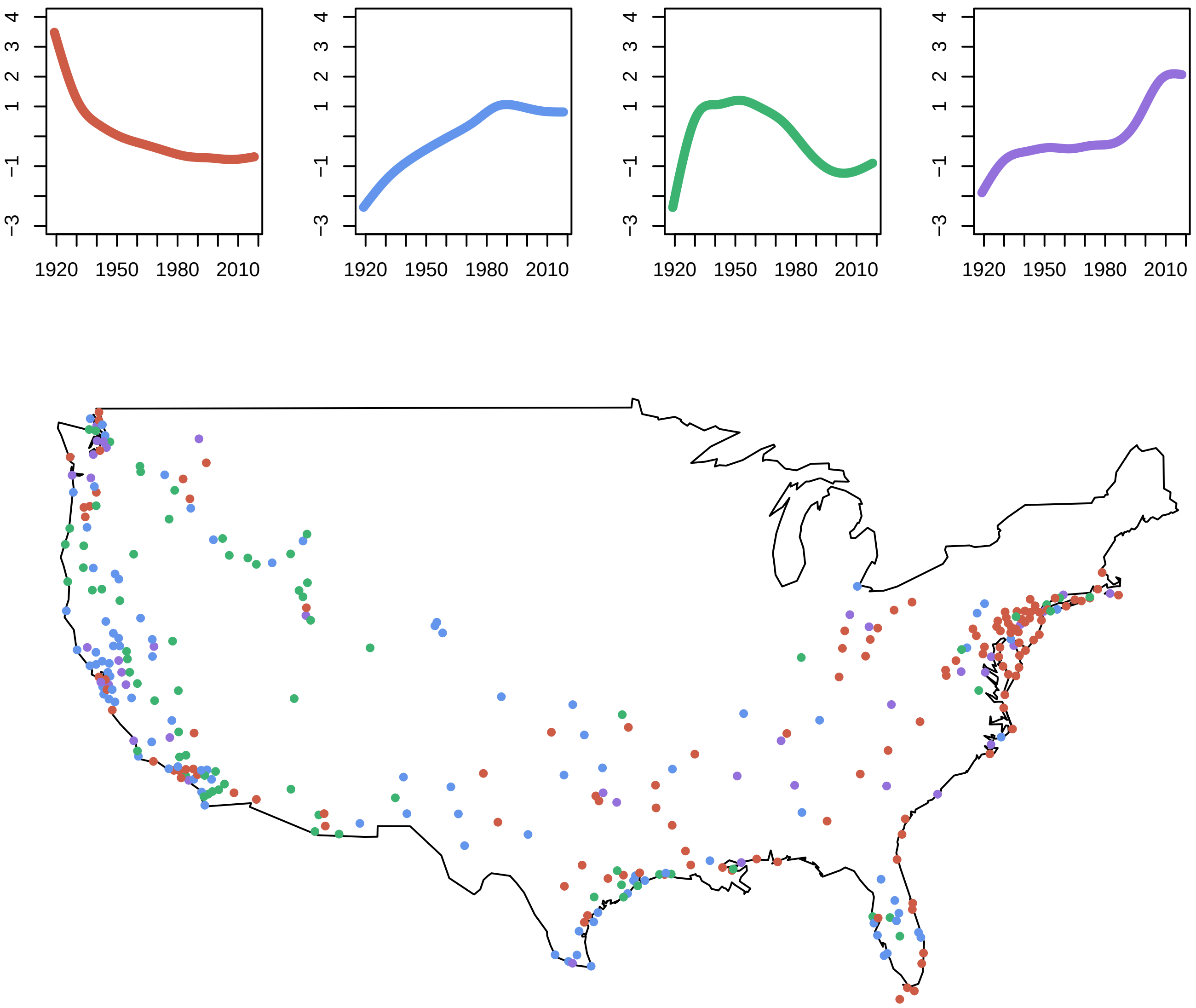

Black−and−white Warbler

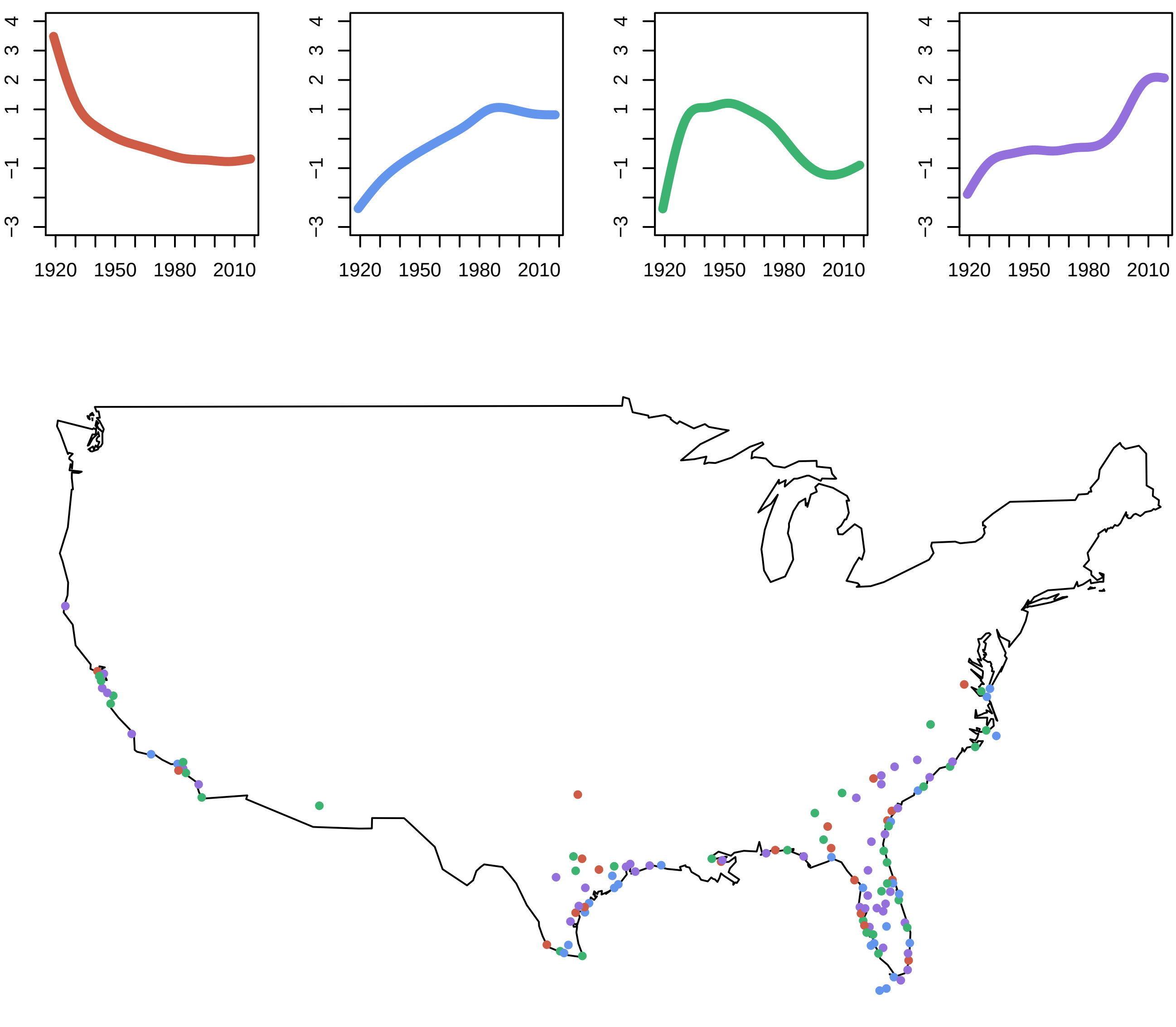

Black−crested Titmouse

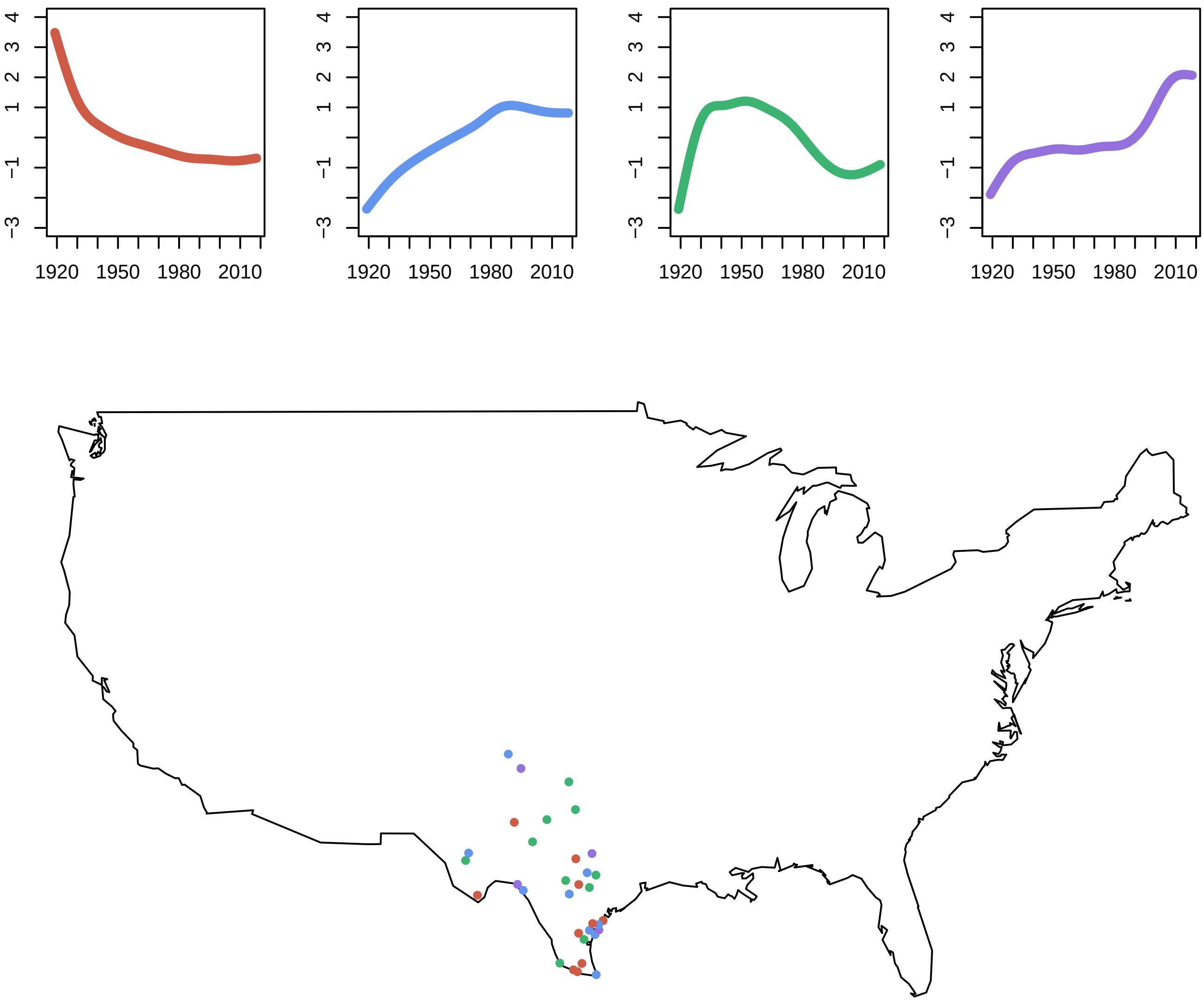

Blue−headed Vireo

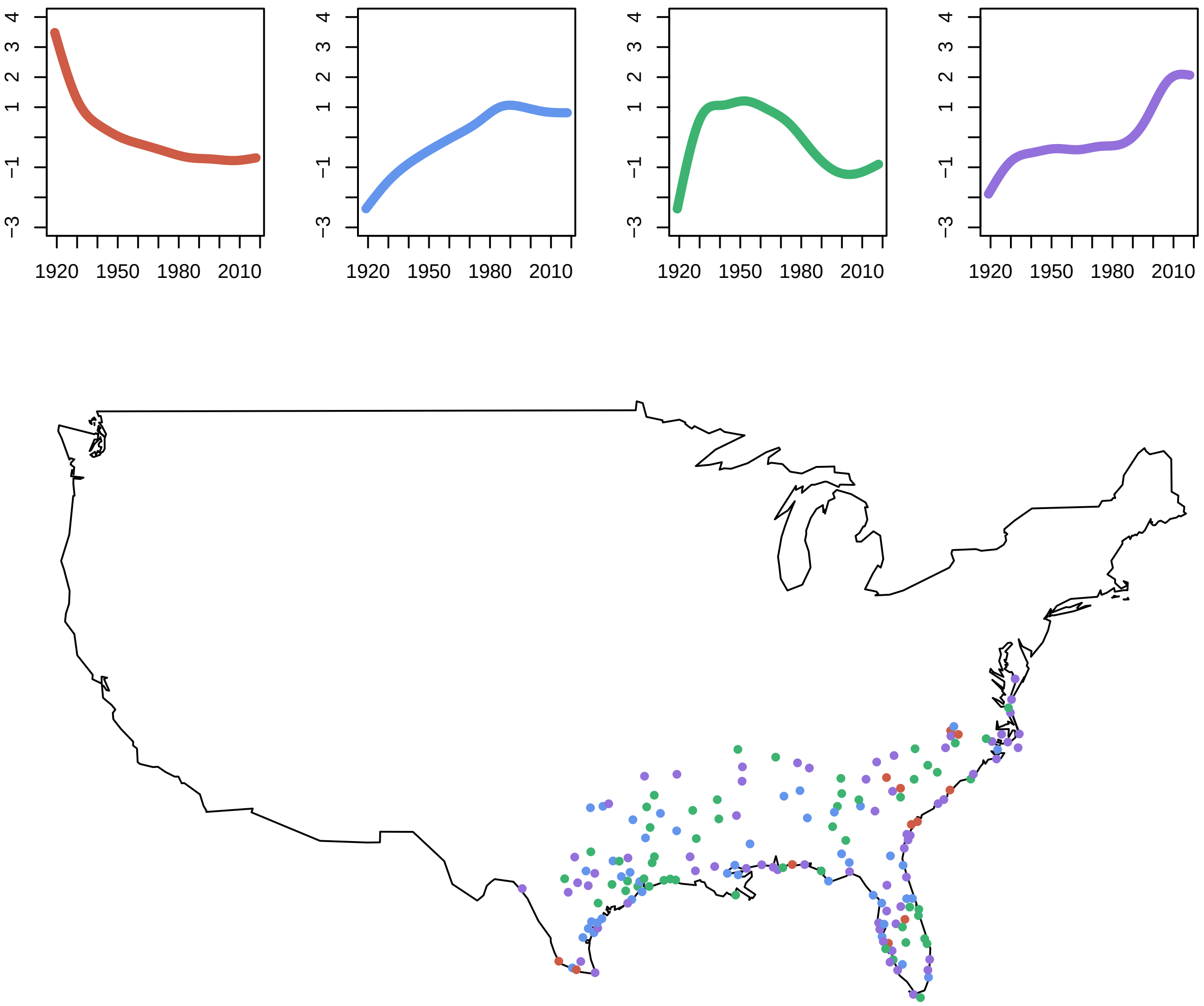

Bridled Titmouse

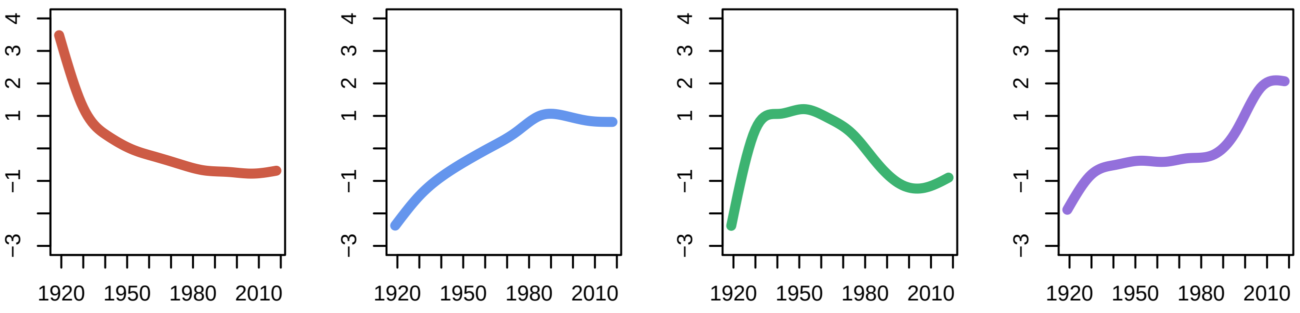

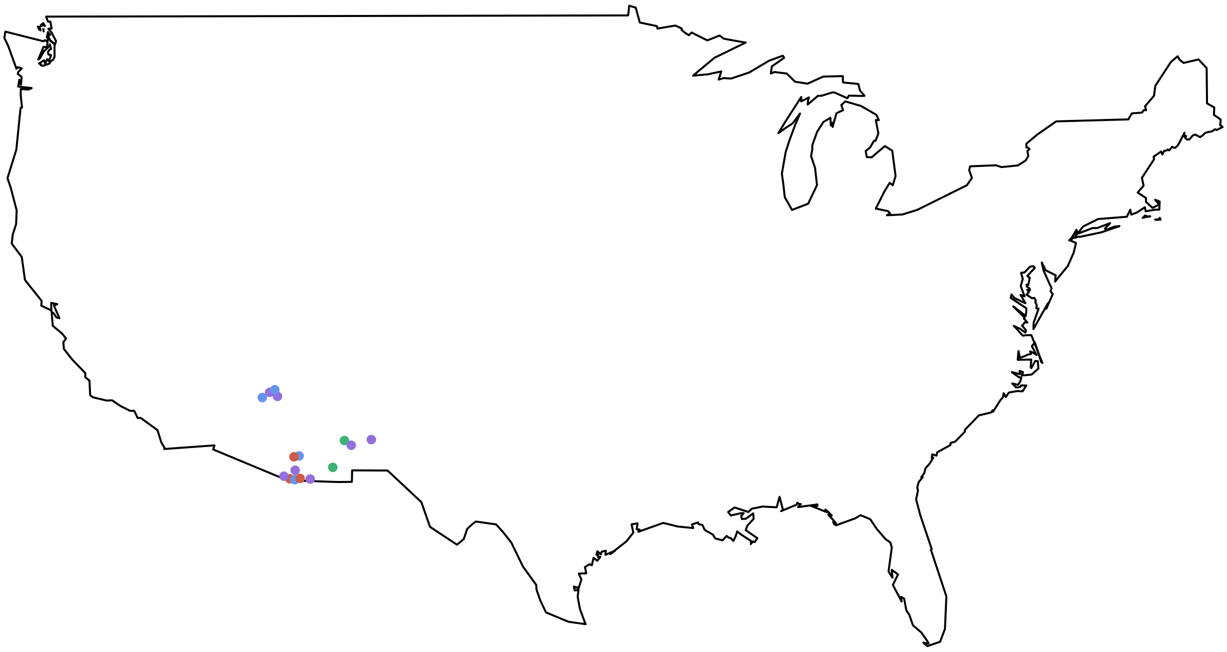

Brown−capped Rosy−Finch

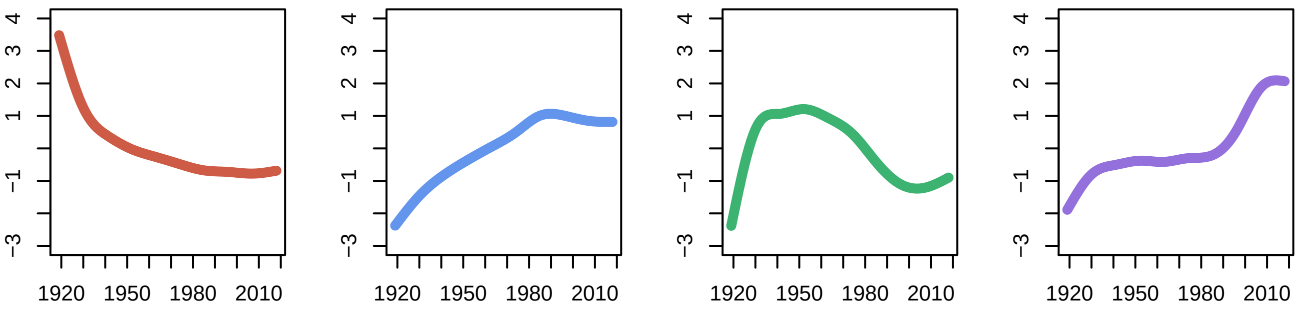

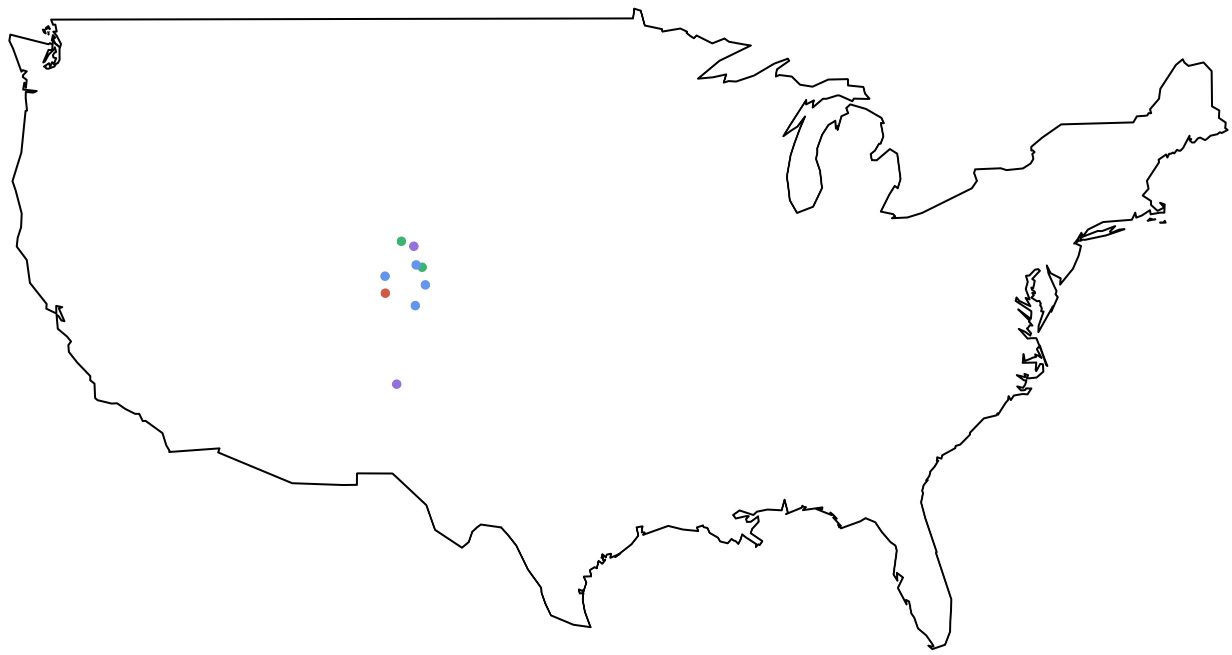

Canyon Towhee

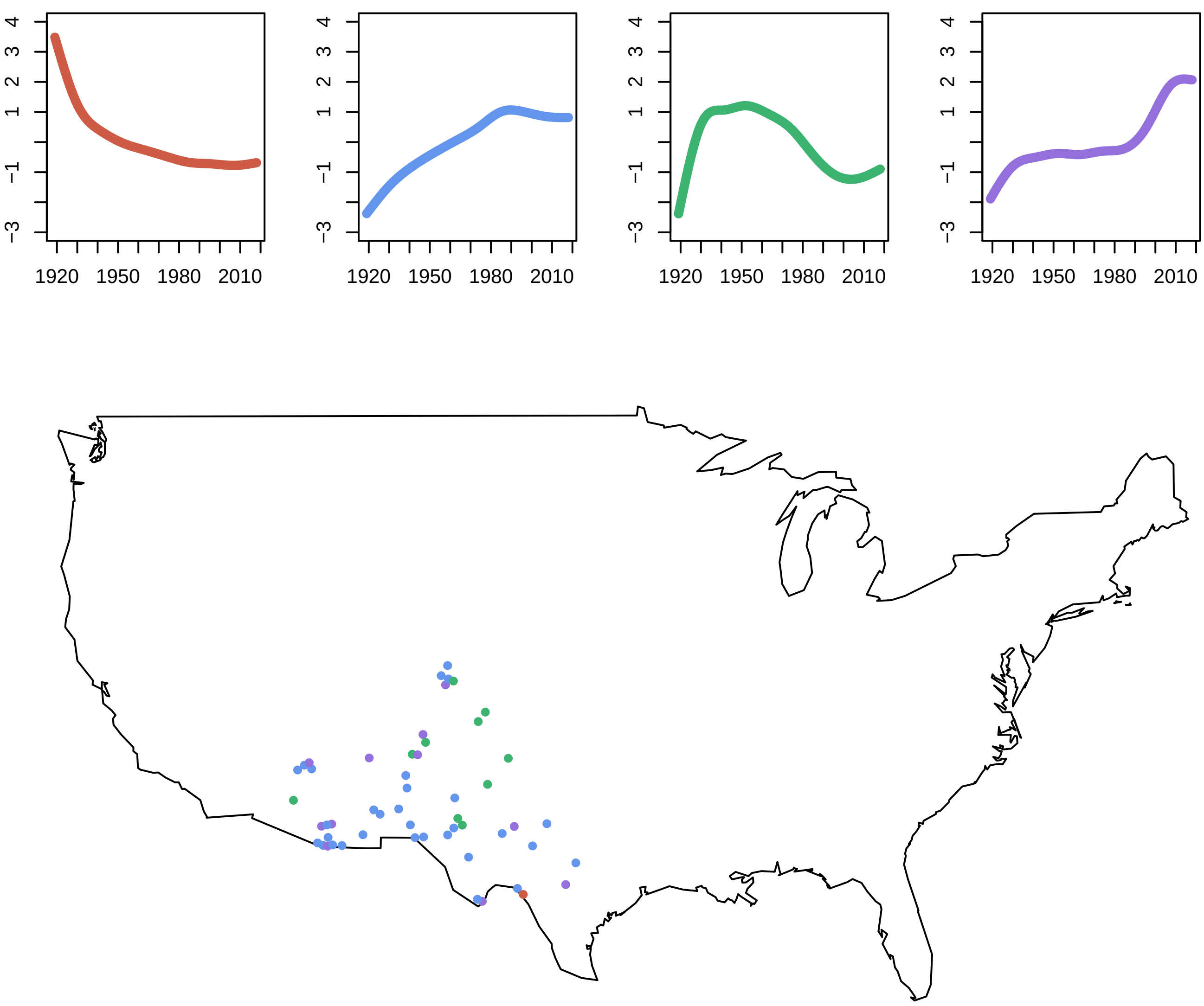

Canyon Wren

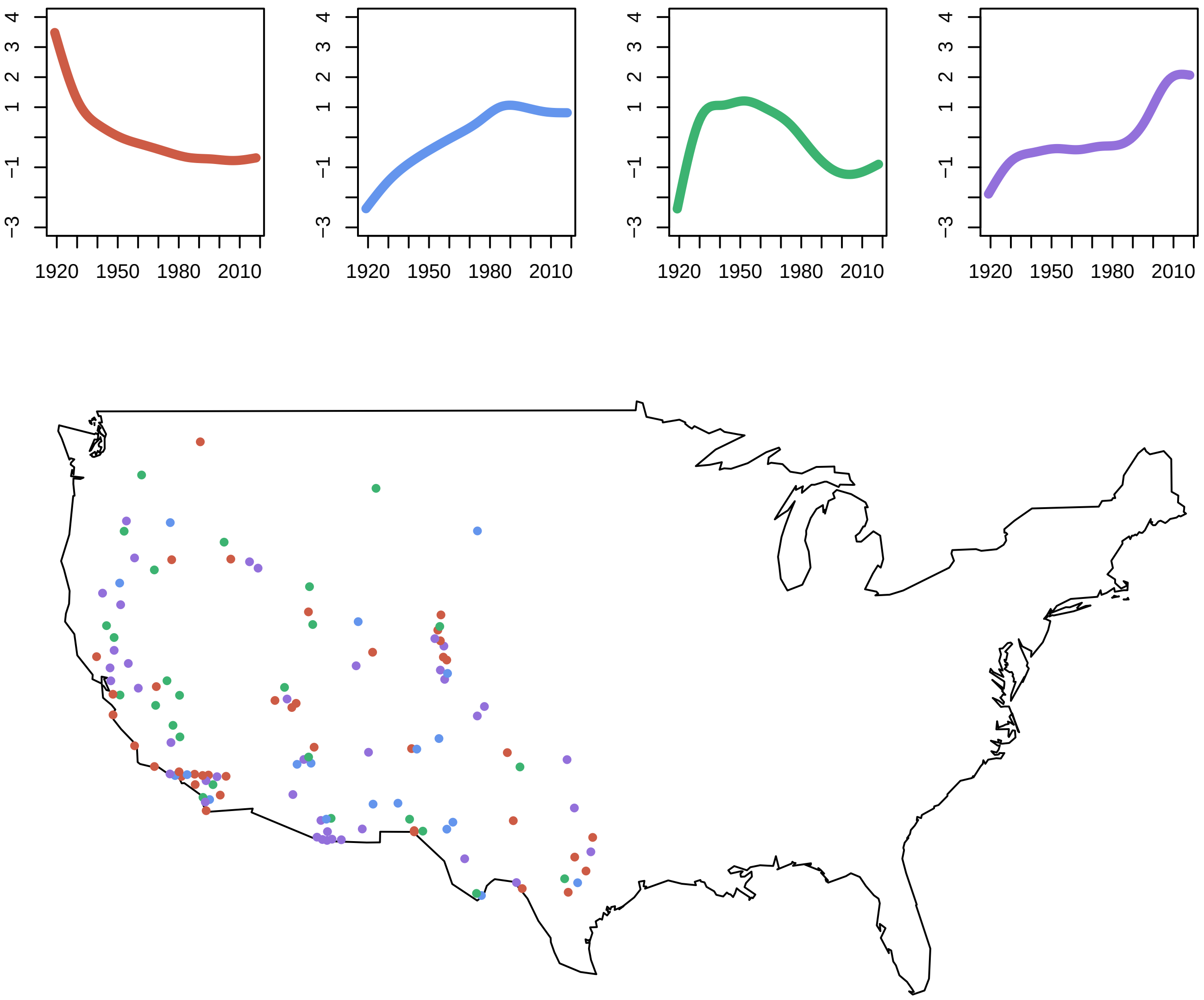

Cassin's Kingbird

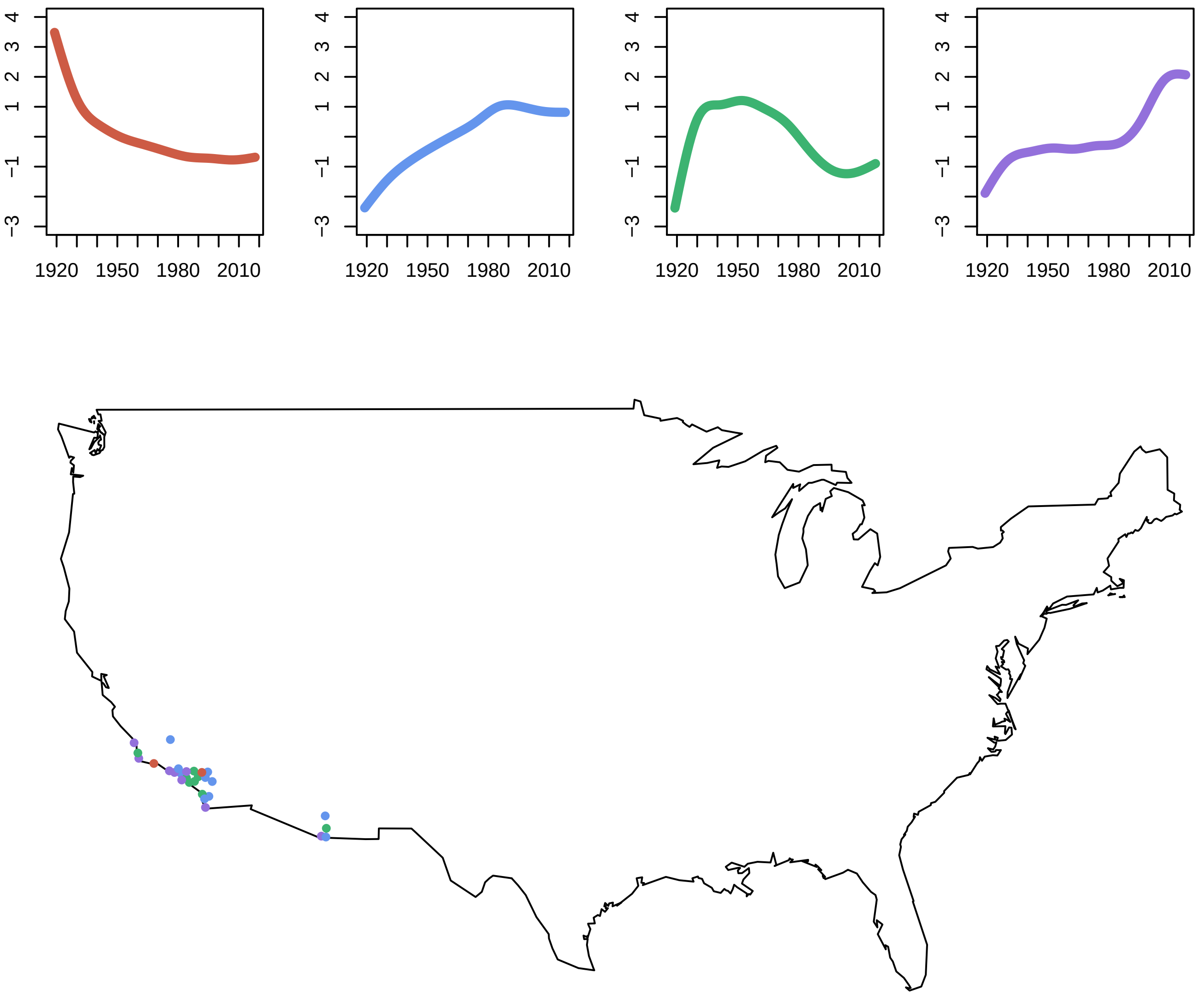

Chihuahuan Raven

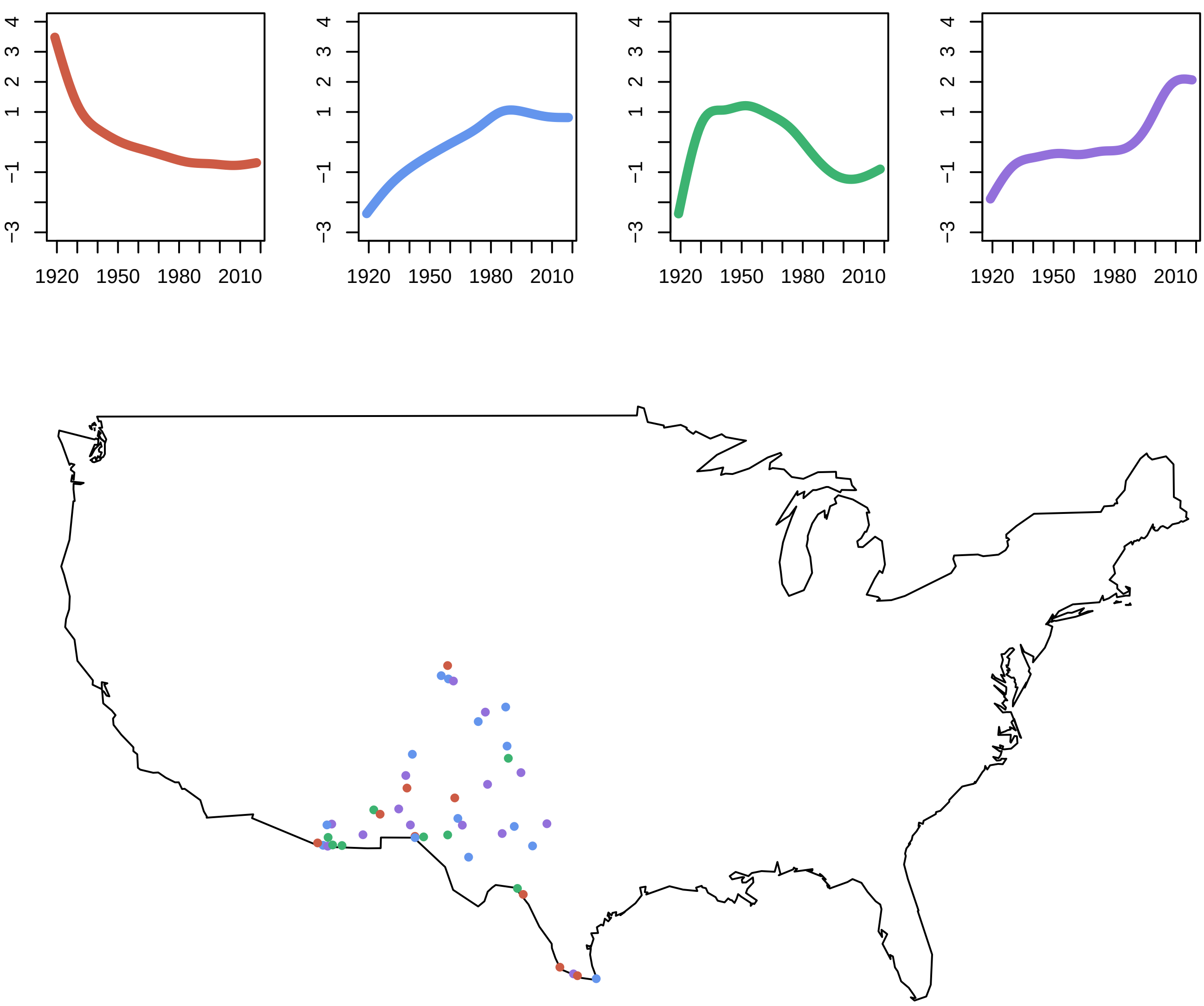

Clark's Nutcracker

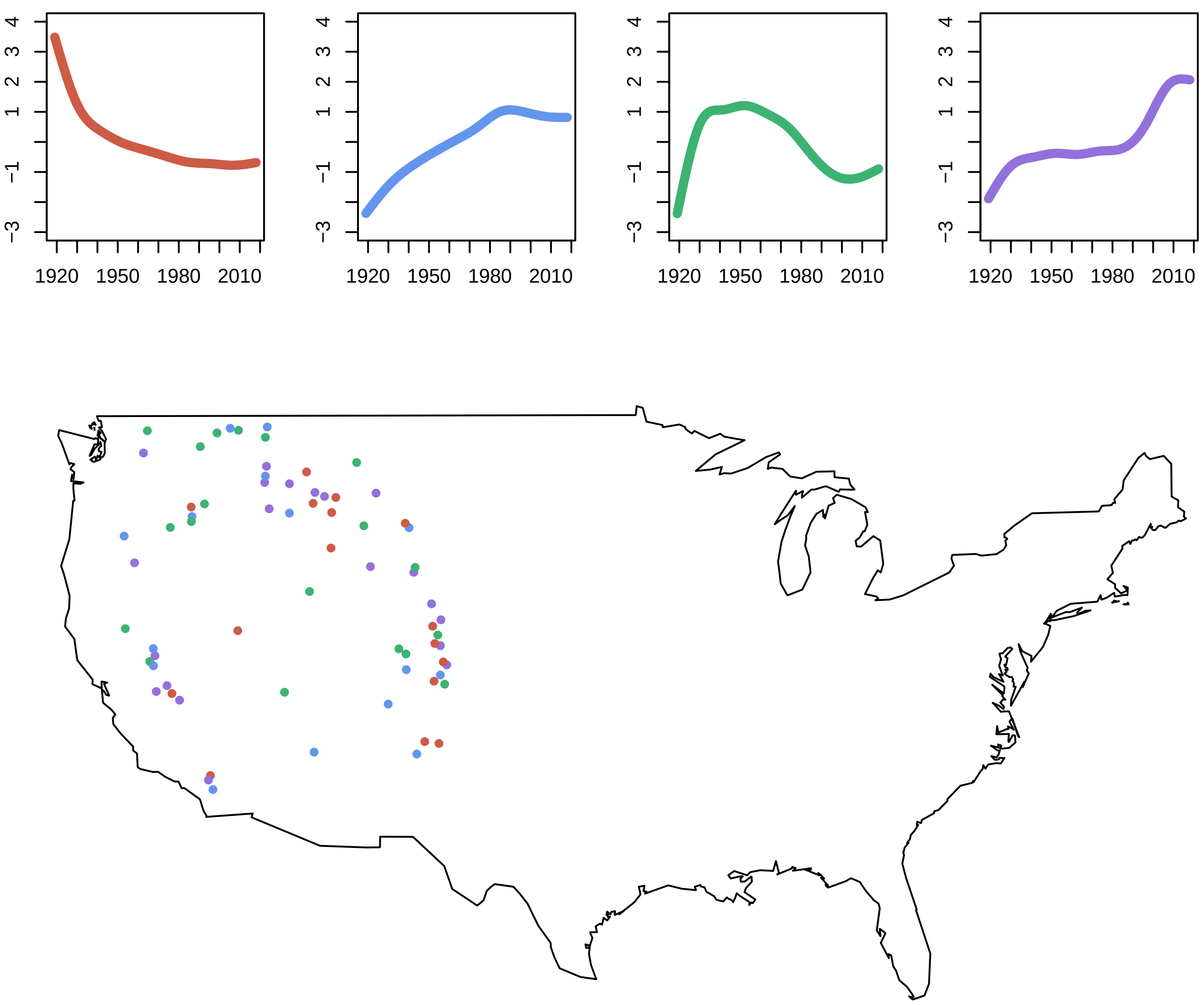

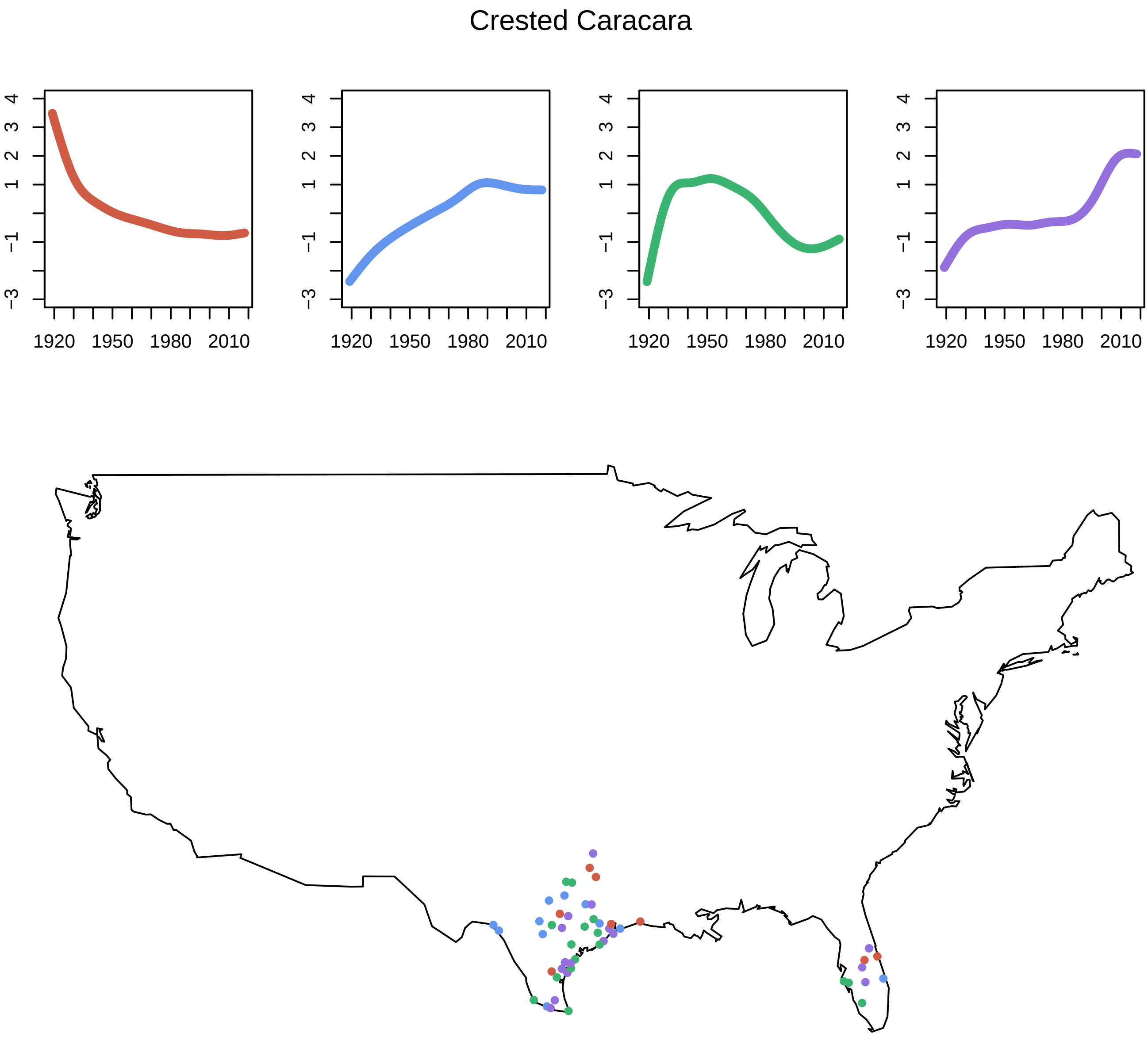
Crested Caracara
4
3
2
1
−1
−3
1920
1950
1980
2010

Curve−billed Thrasher

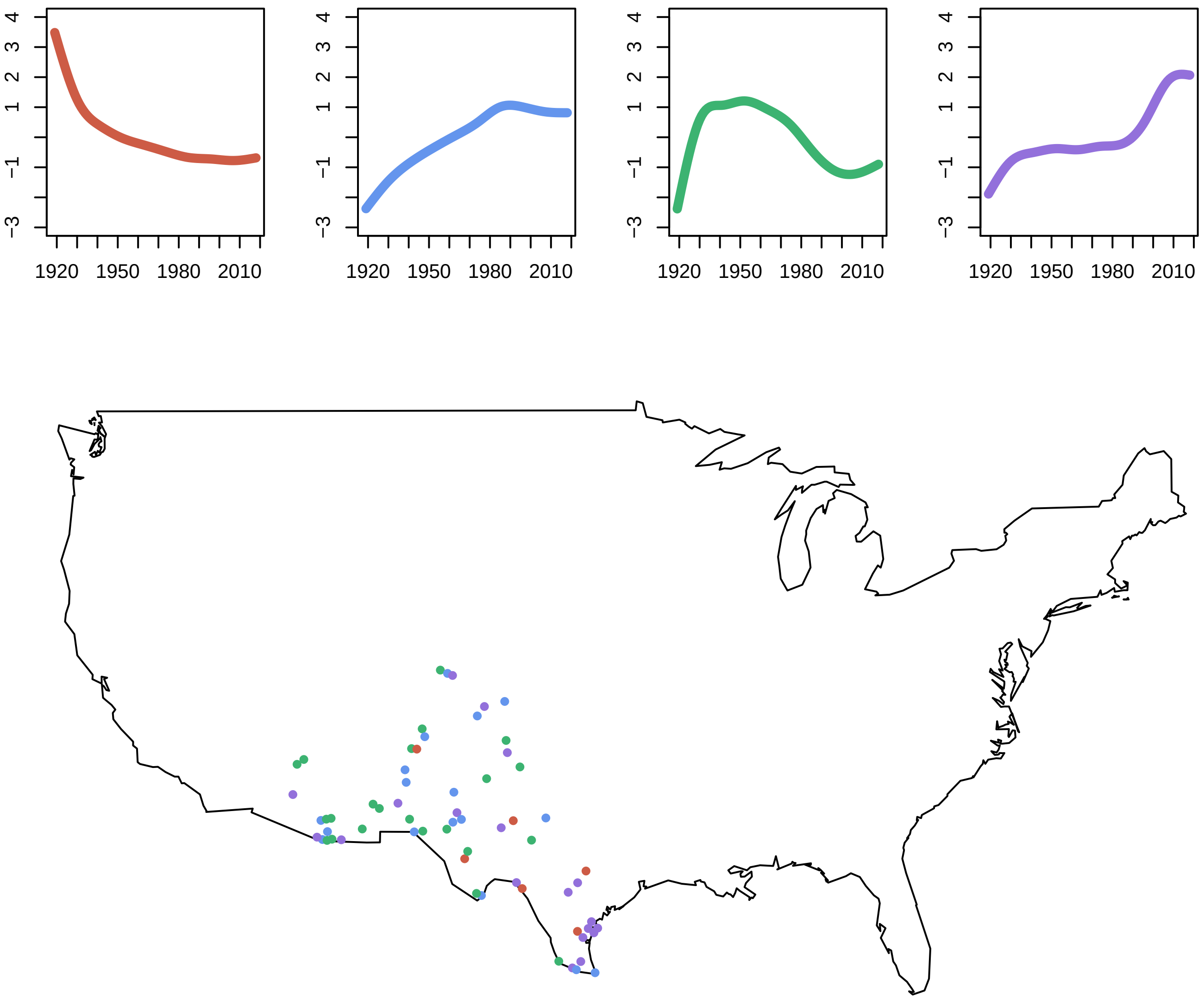

Golden−fronted Woodpecker

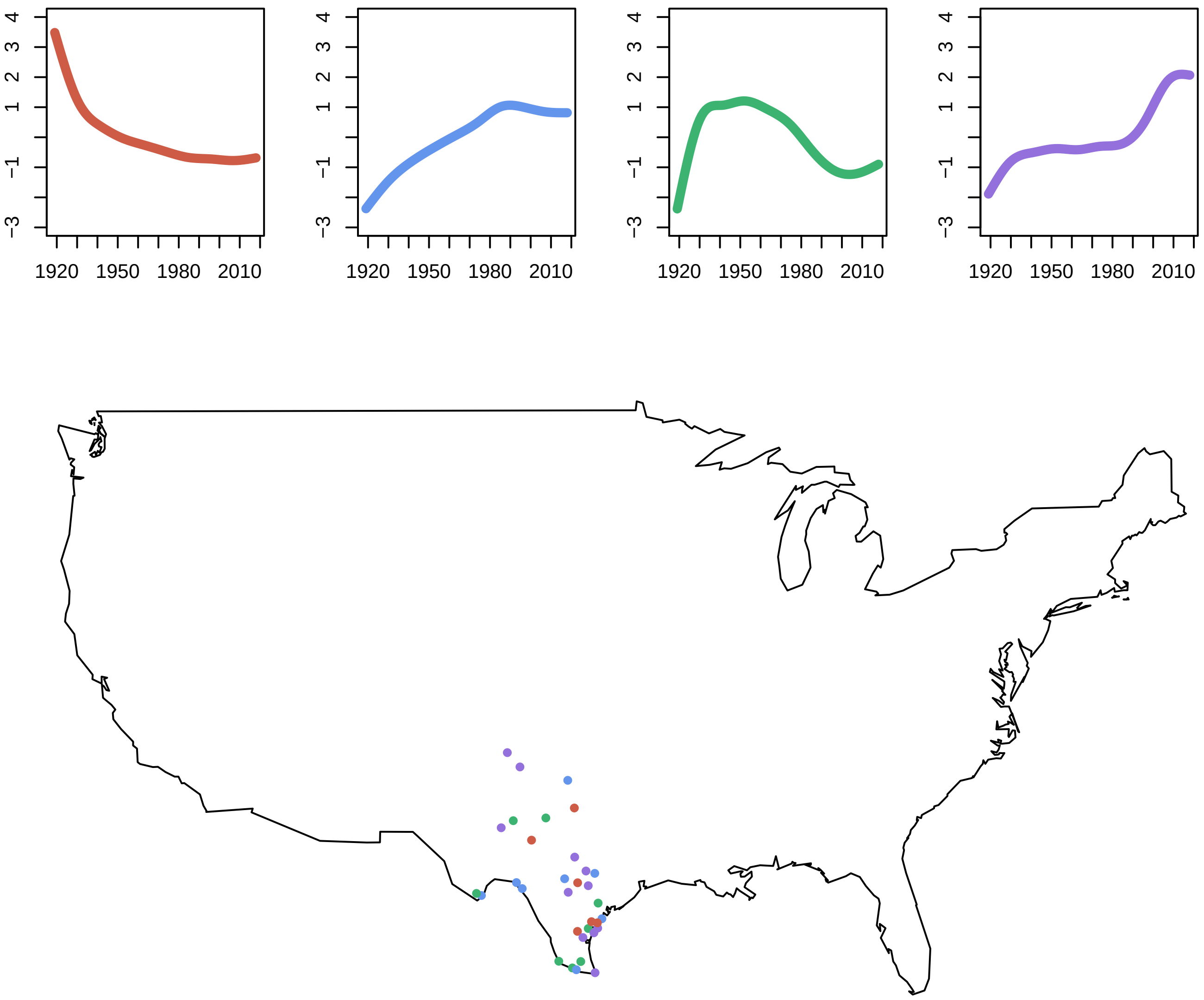

Golden Eagle

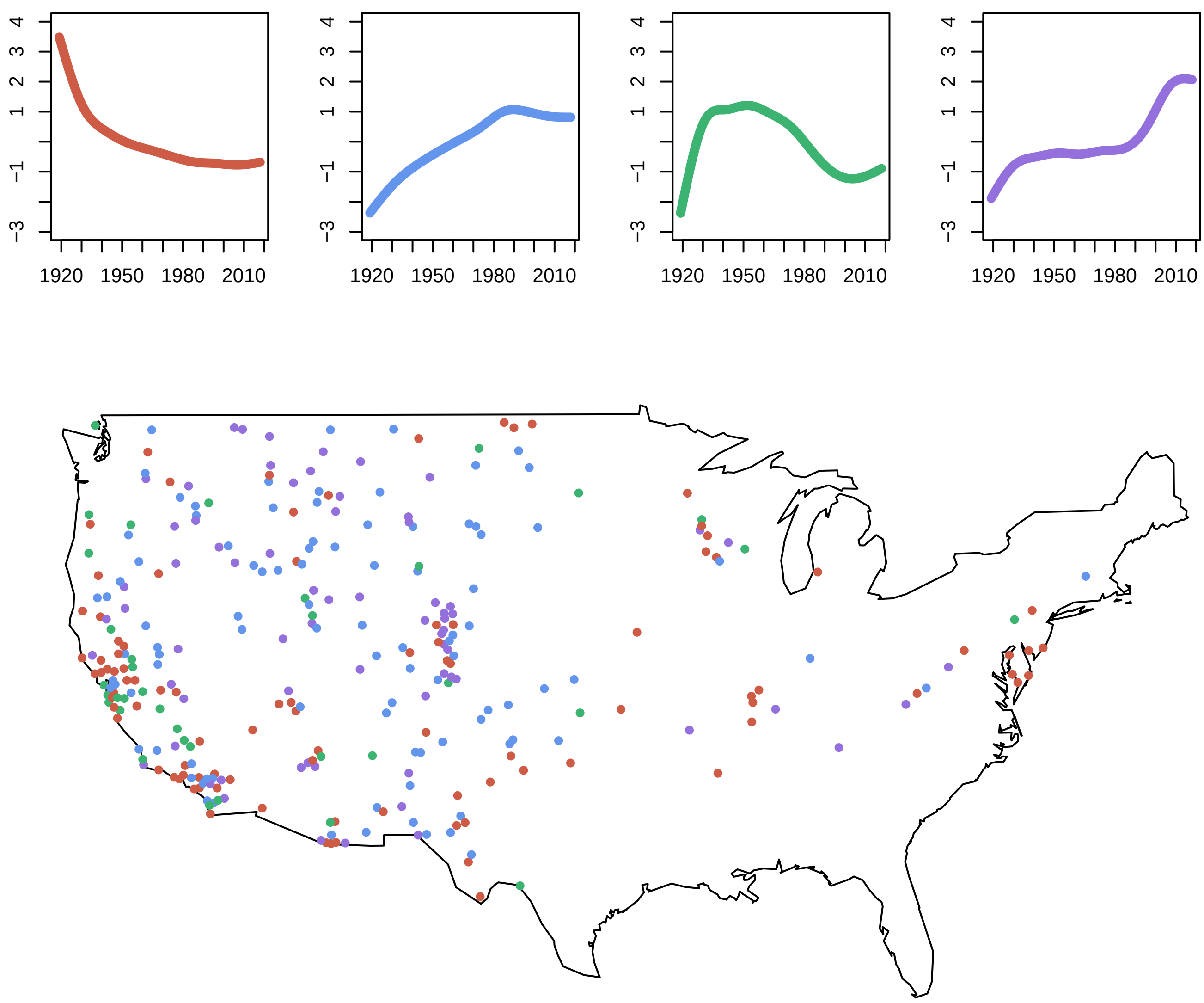

Green Heron

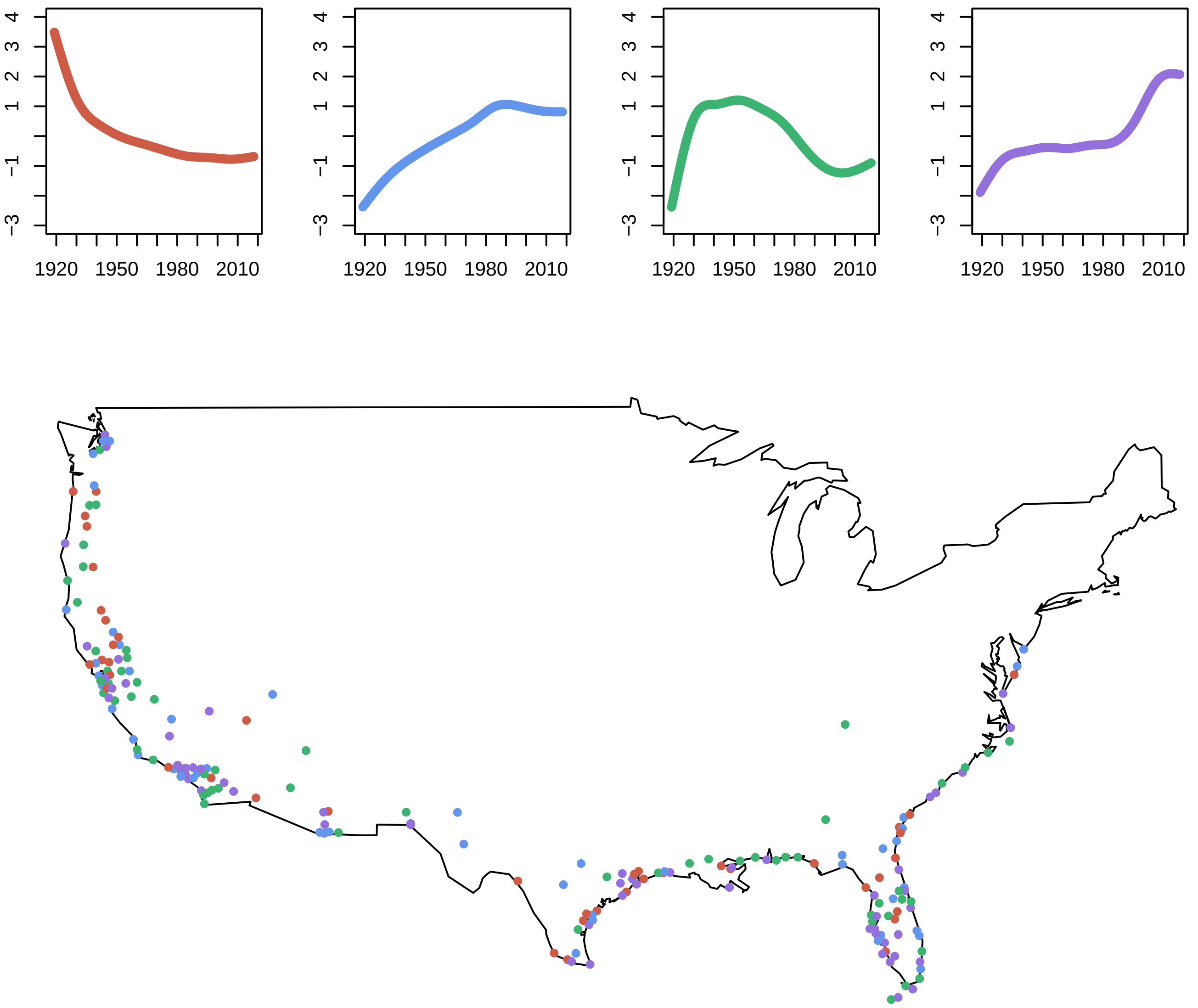

Harlequin Duck

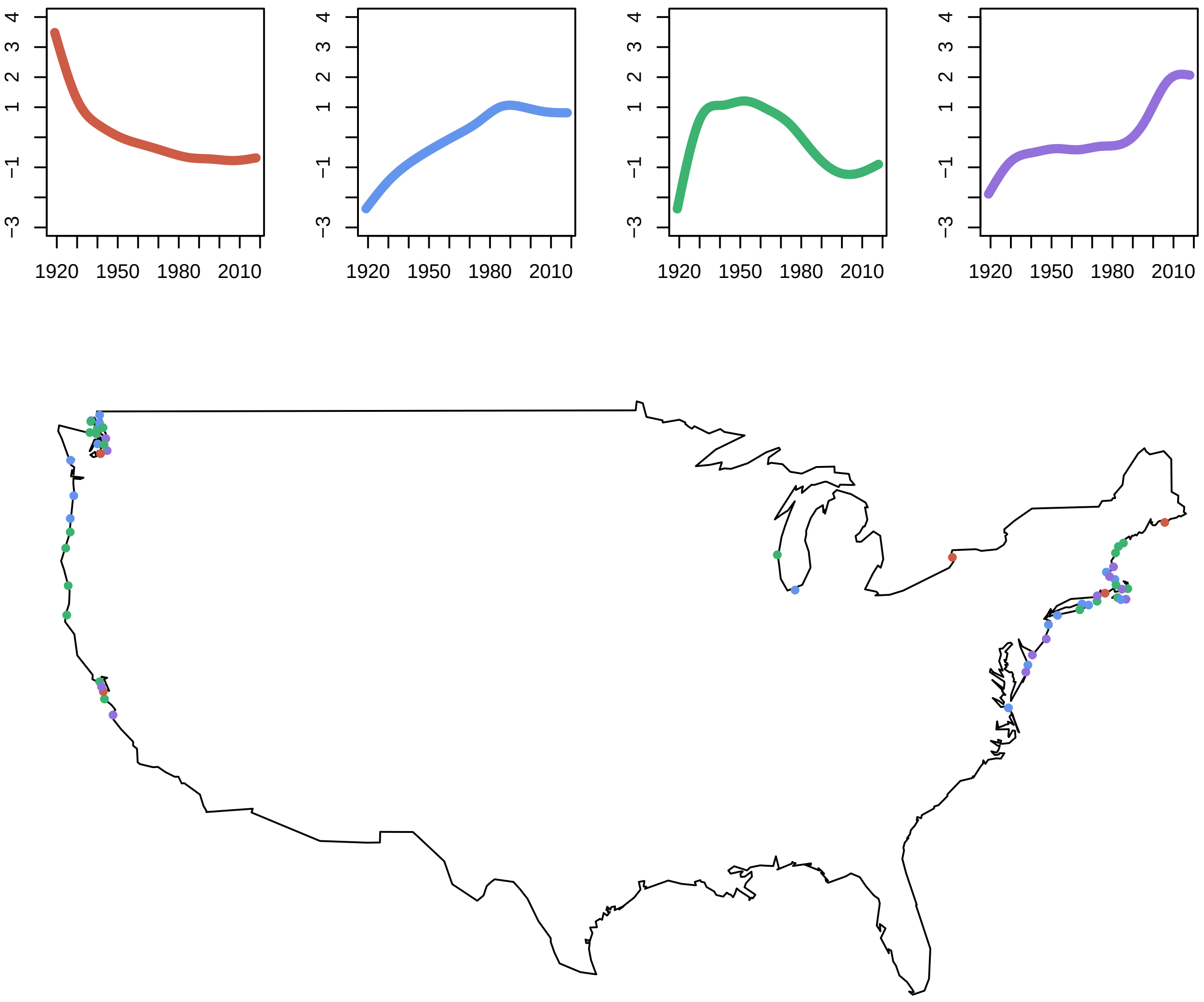

Hutton's Vireo

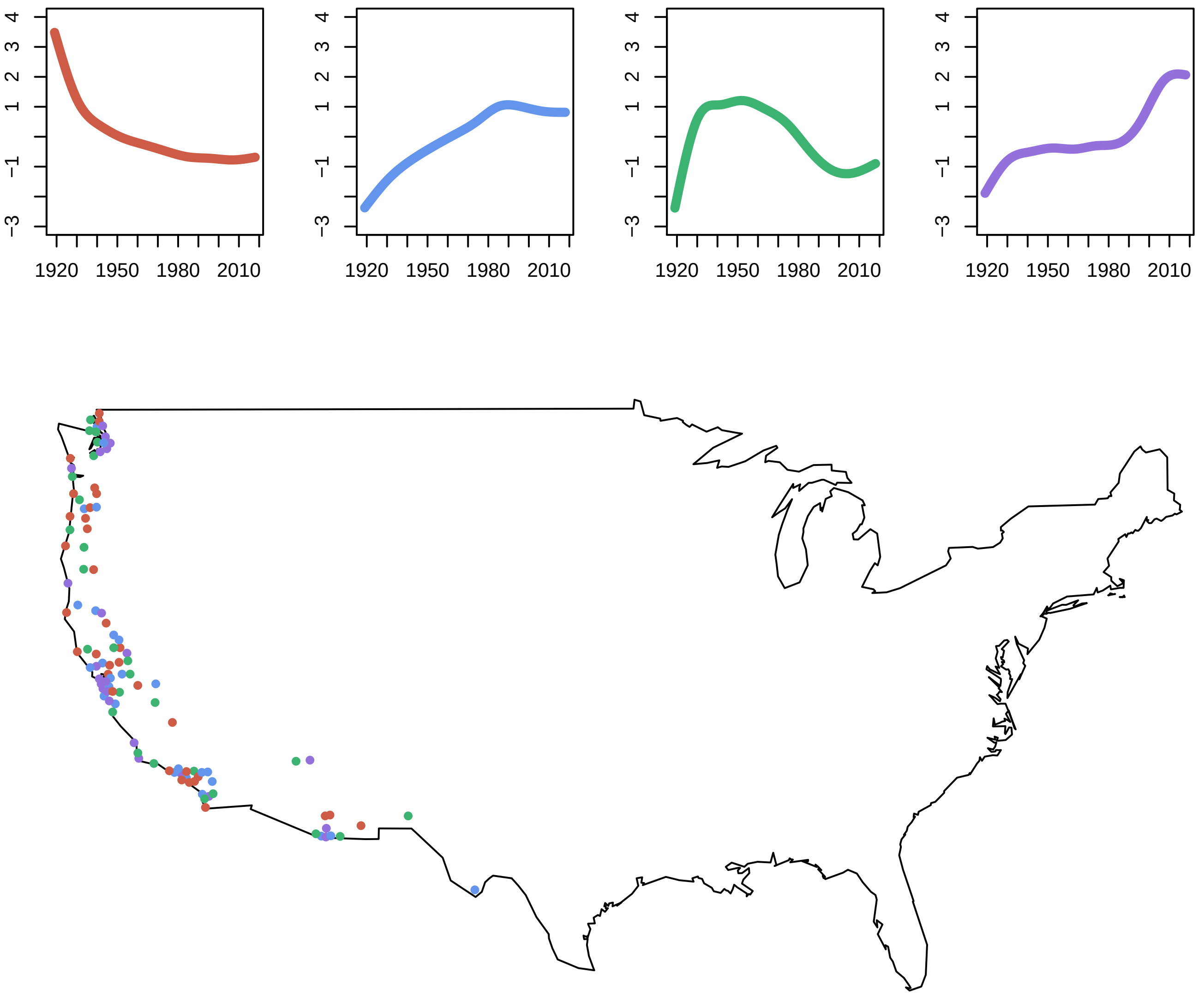

Ladder−backed Woodpecker

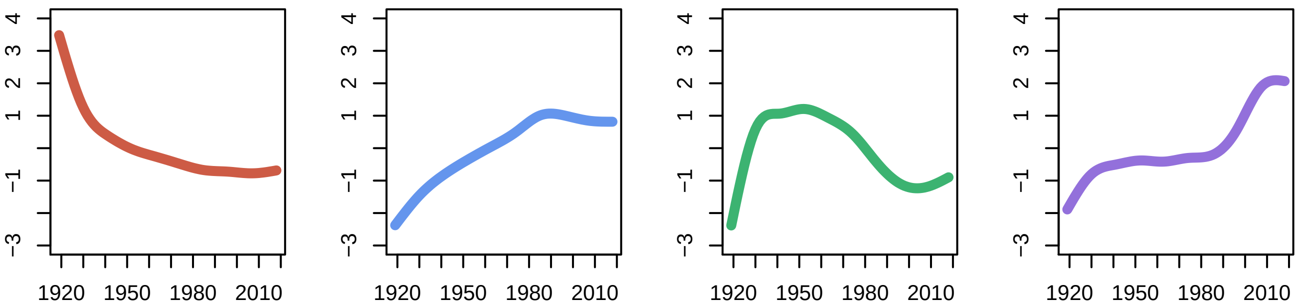

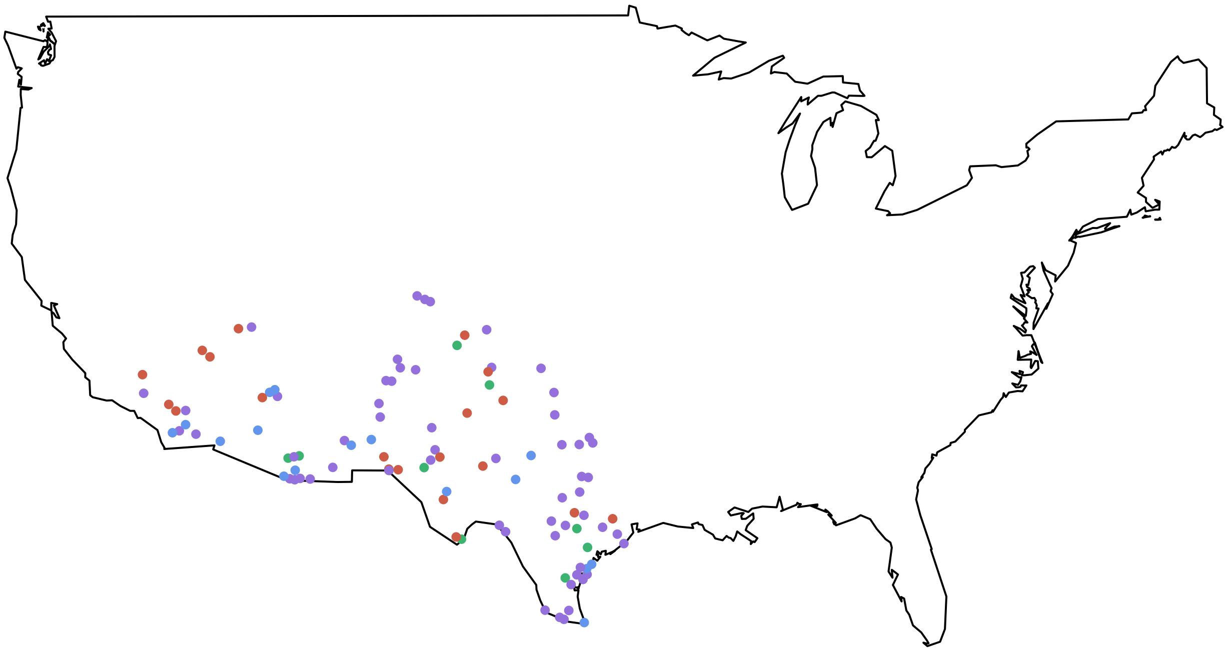

Lewis's Woodpecker

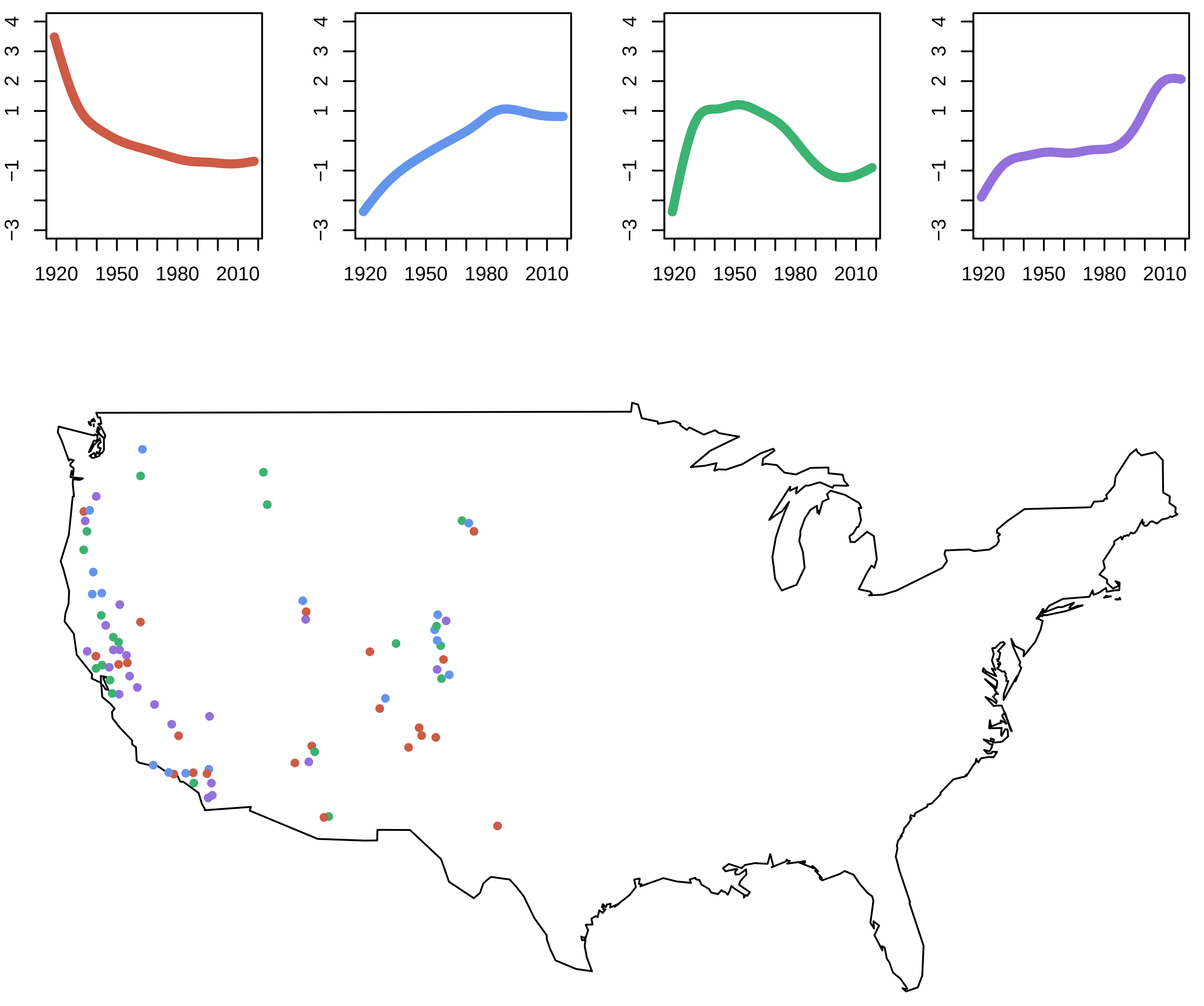

Merlin

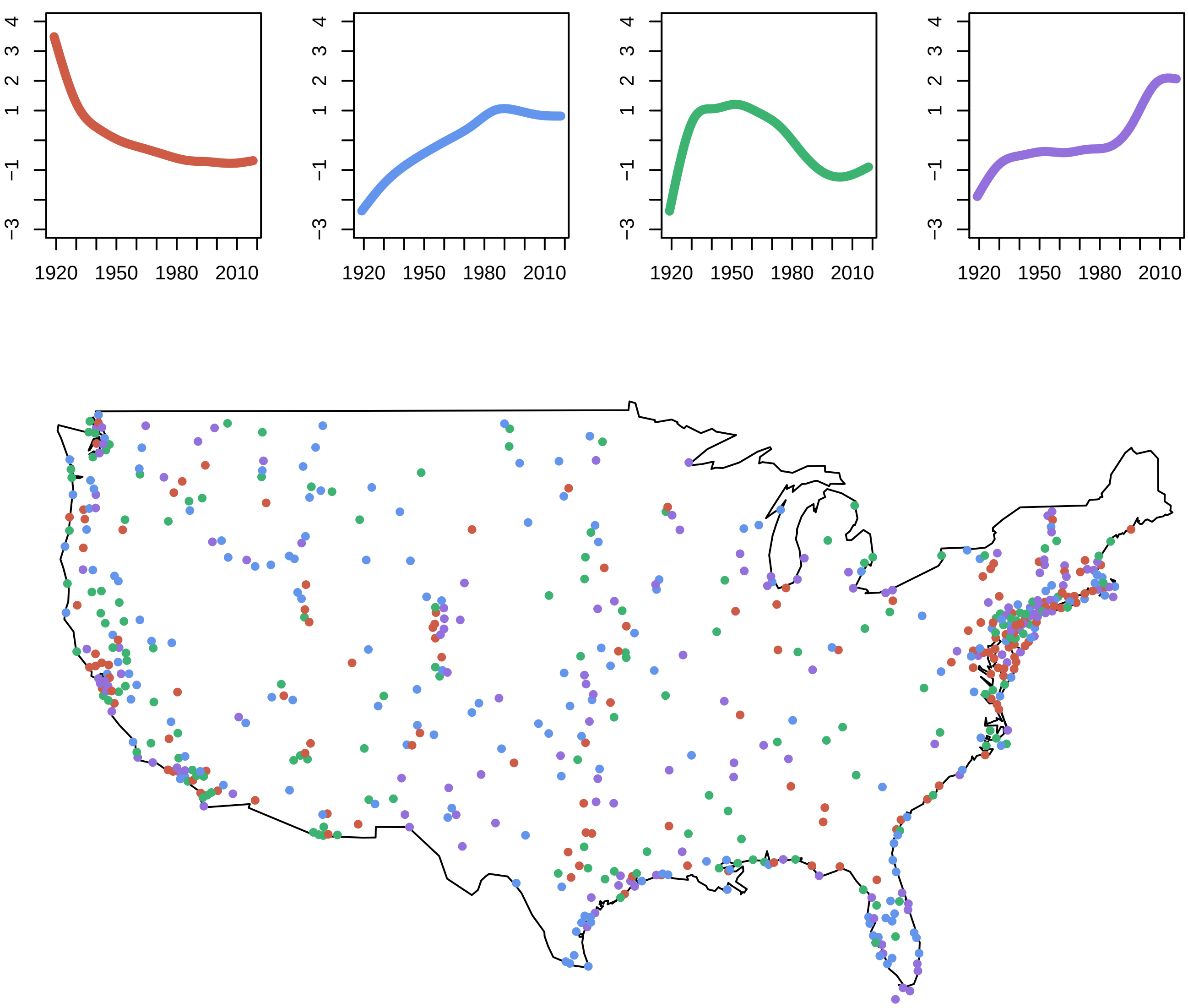

Mountain Plover

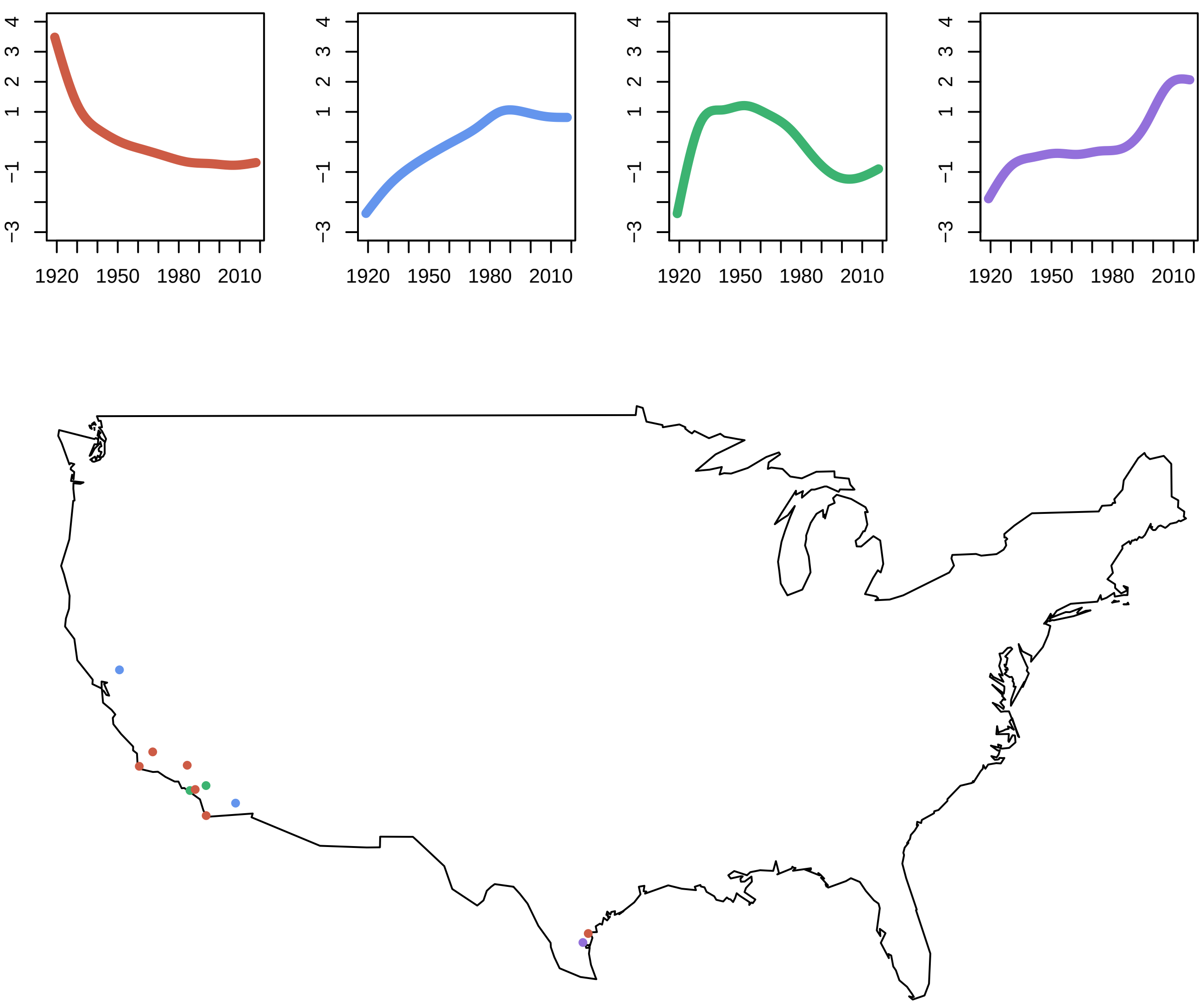

Muscovy Duck

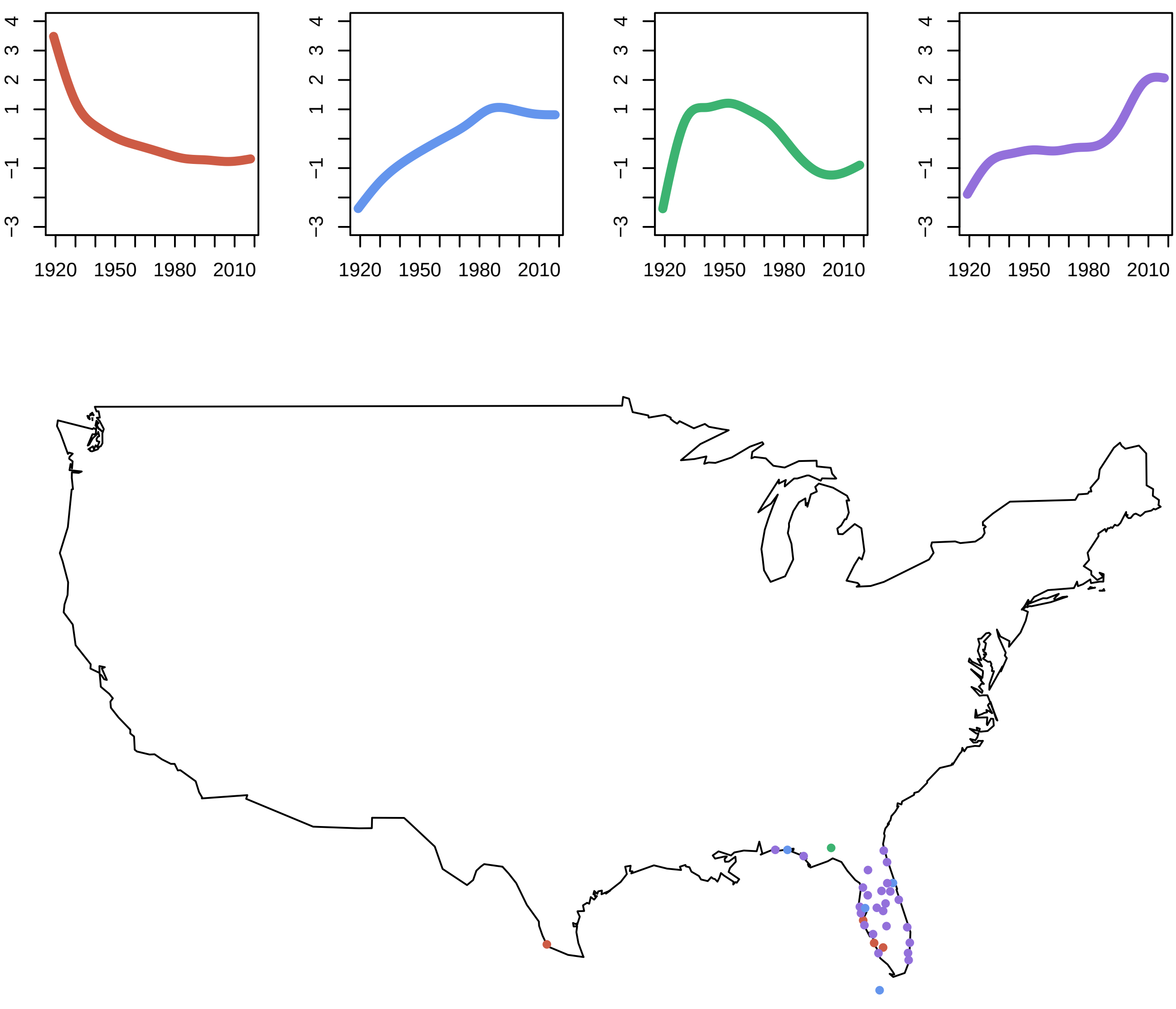

Northern Shrike

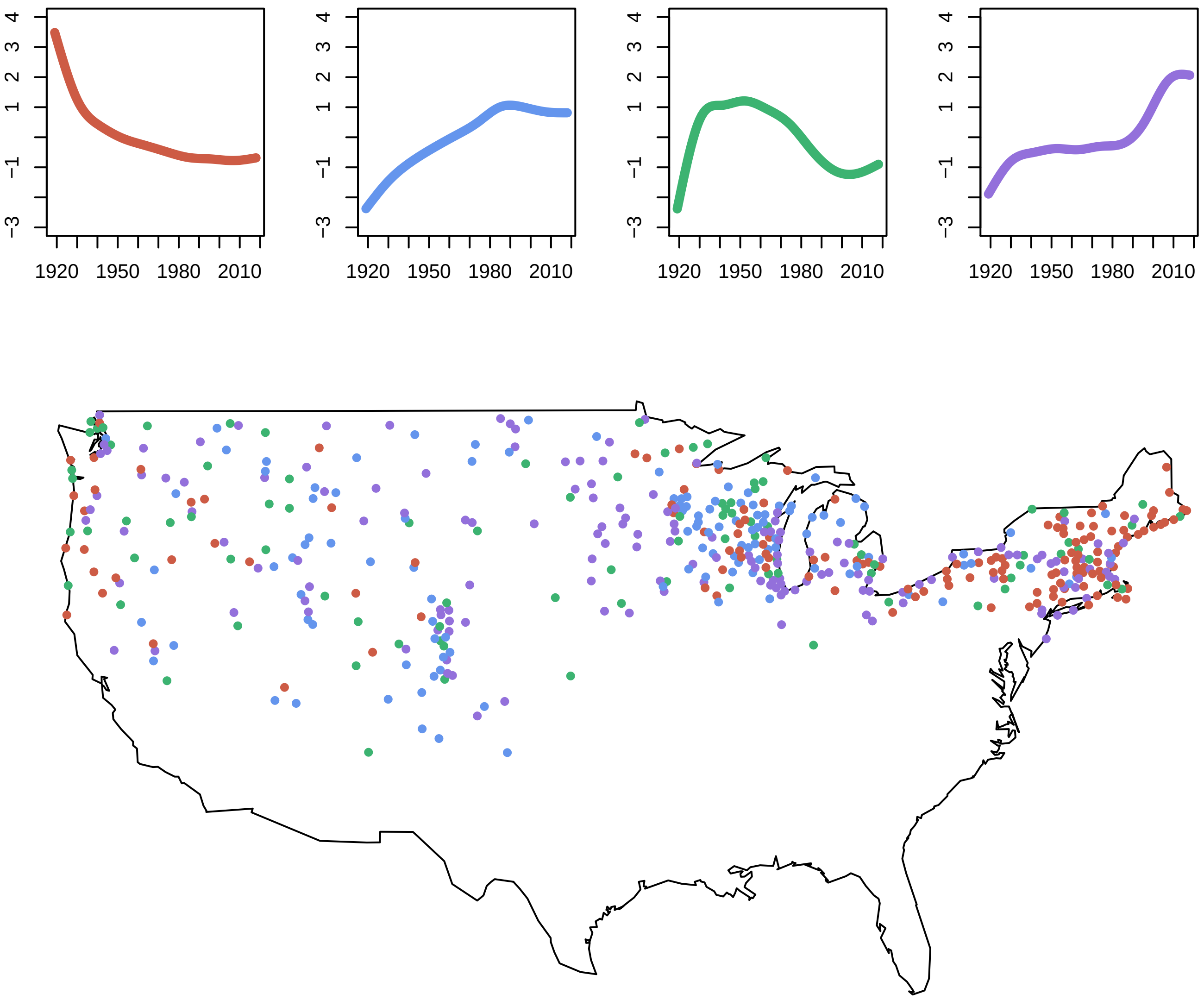

Rock Wren

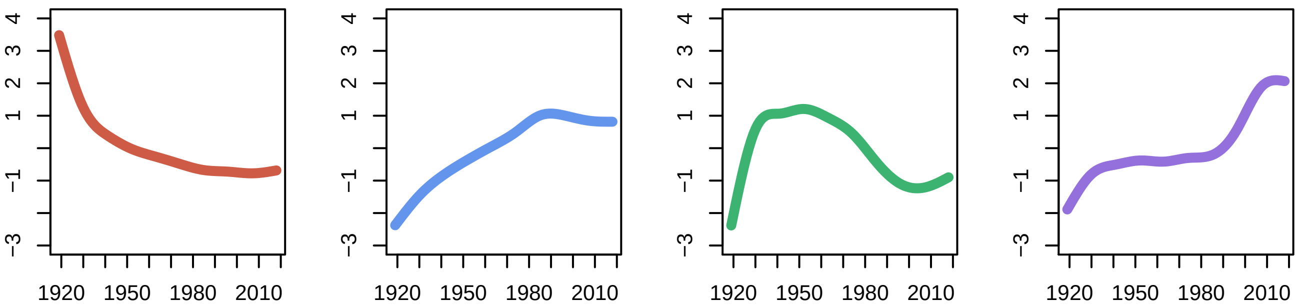

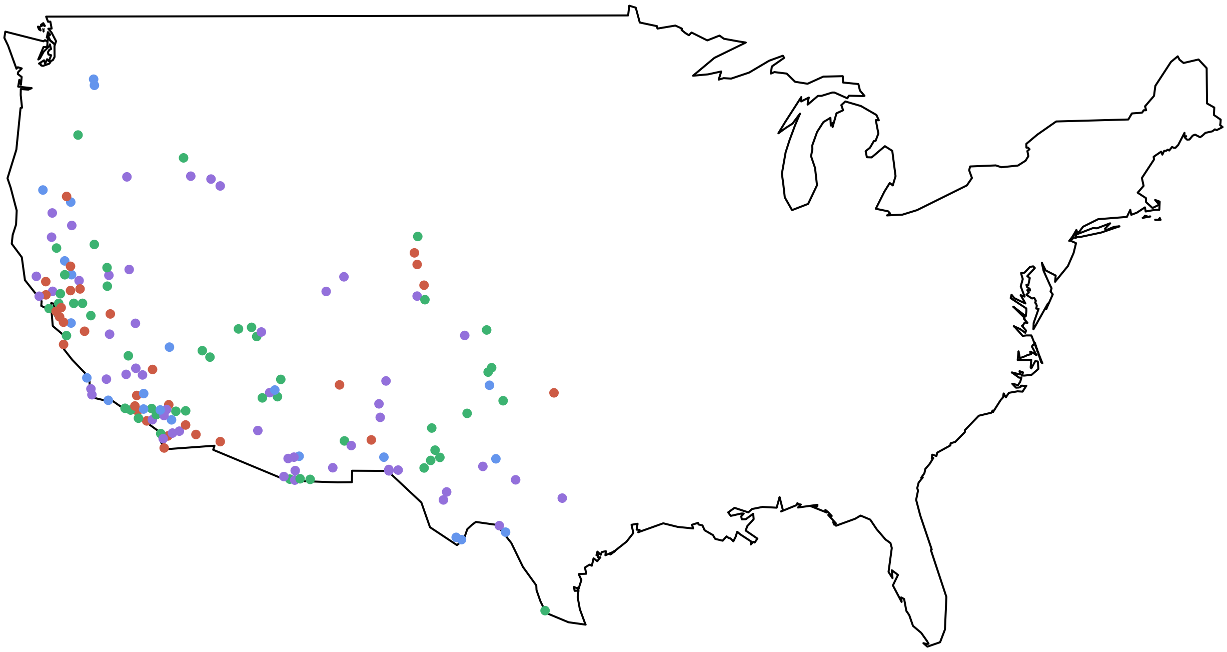

Rufous−crowned Sparrow

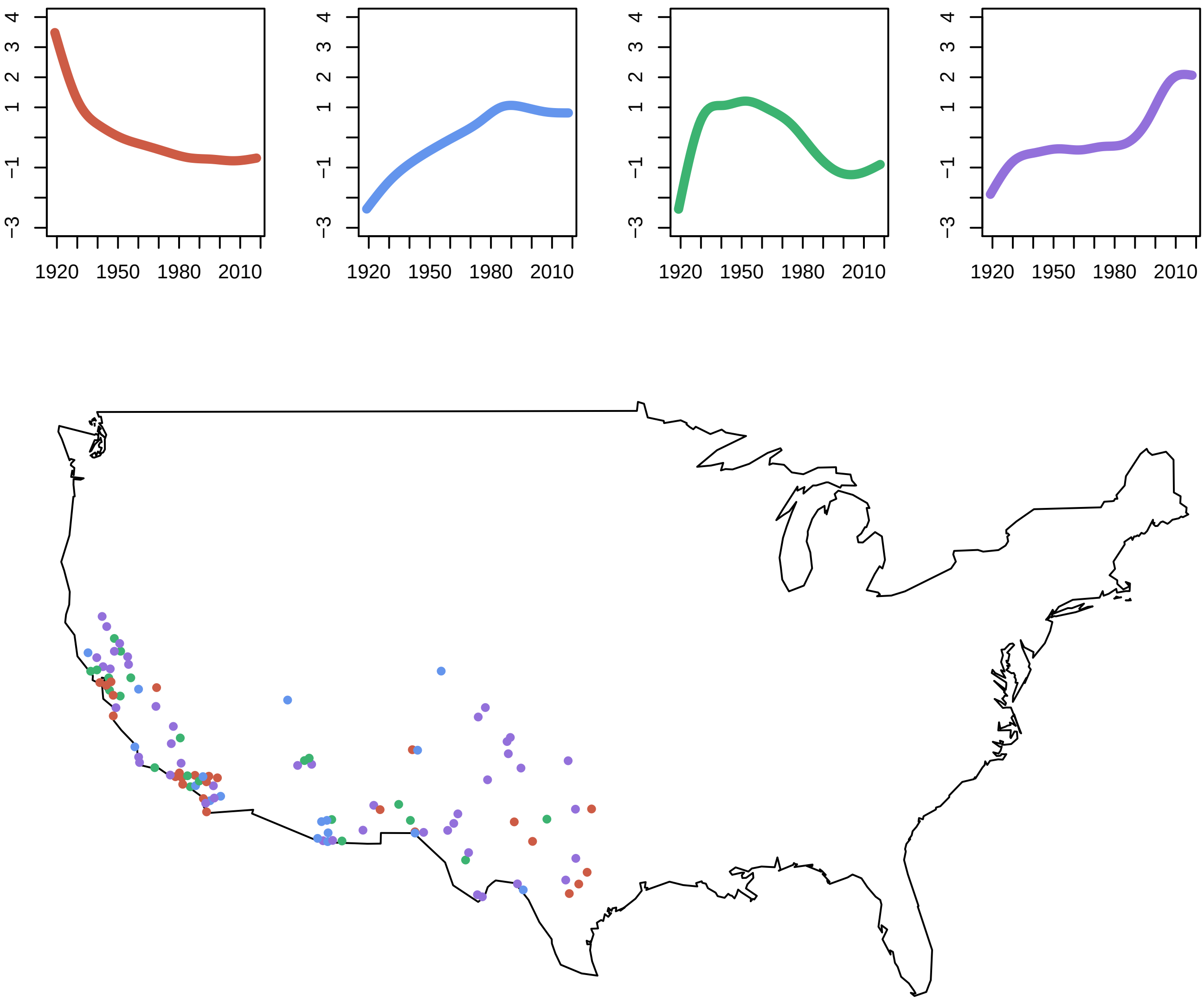

Seaside Sparrow

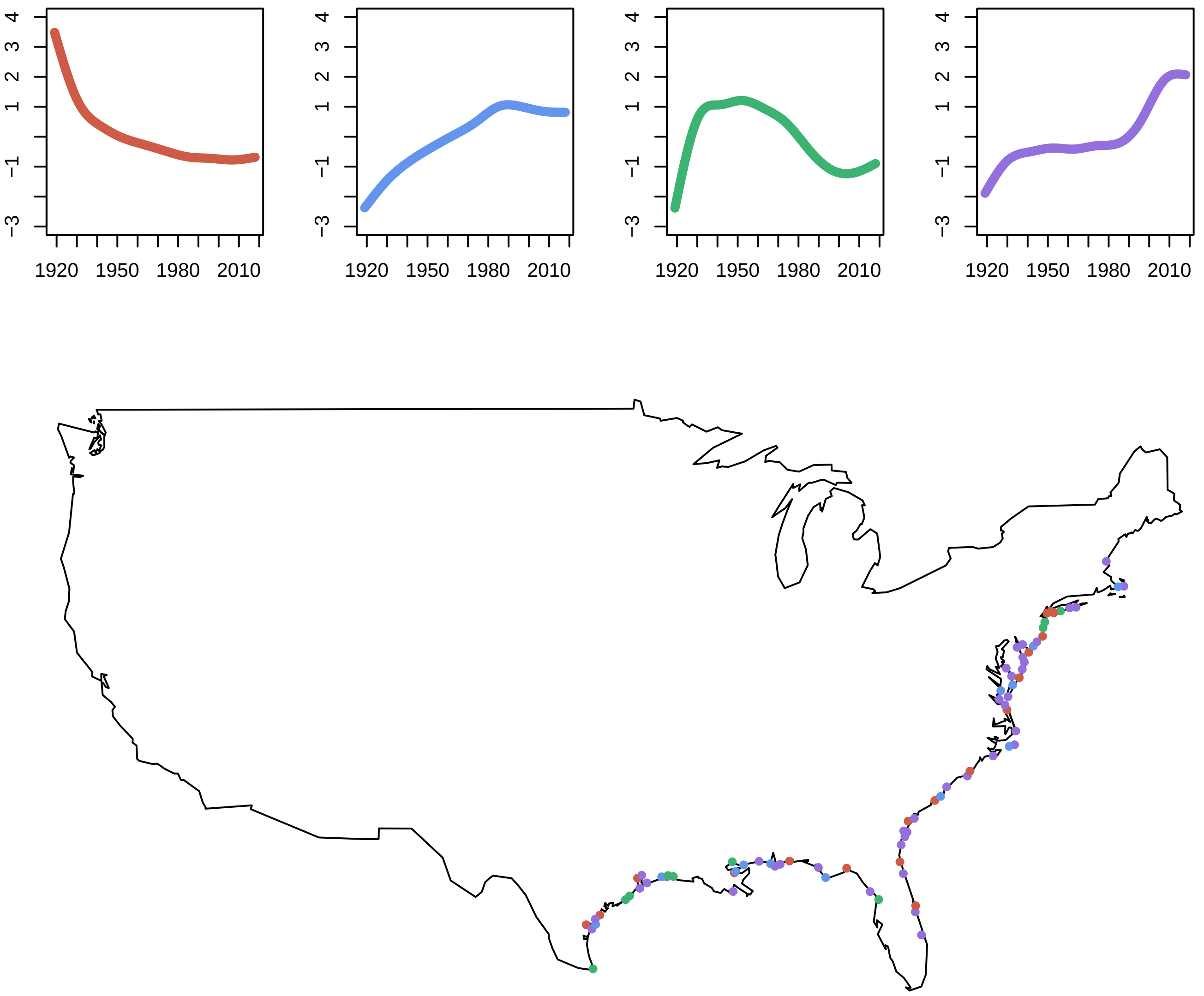

Sharp−tailed Grouse

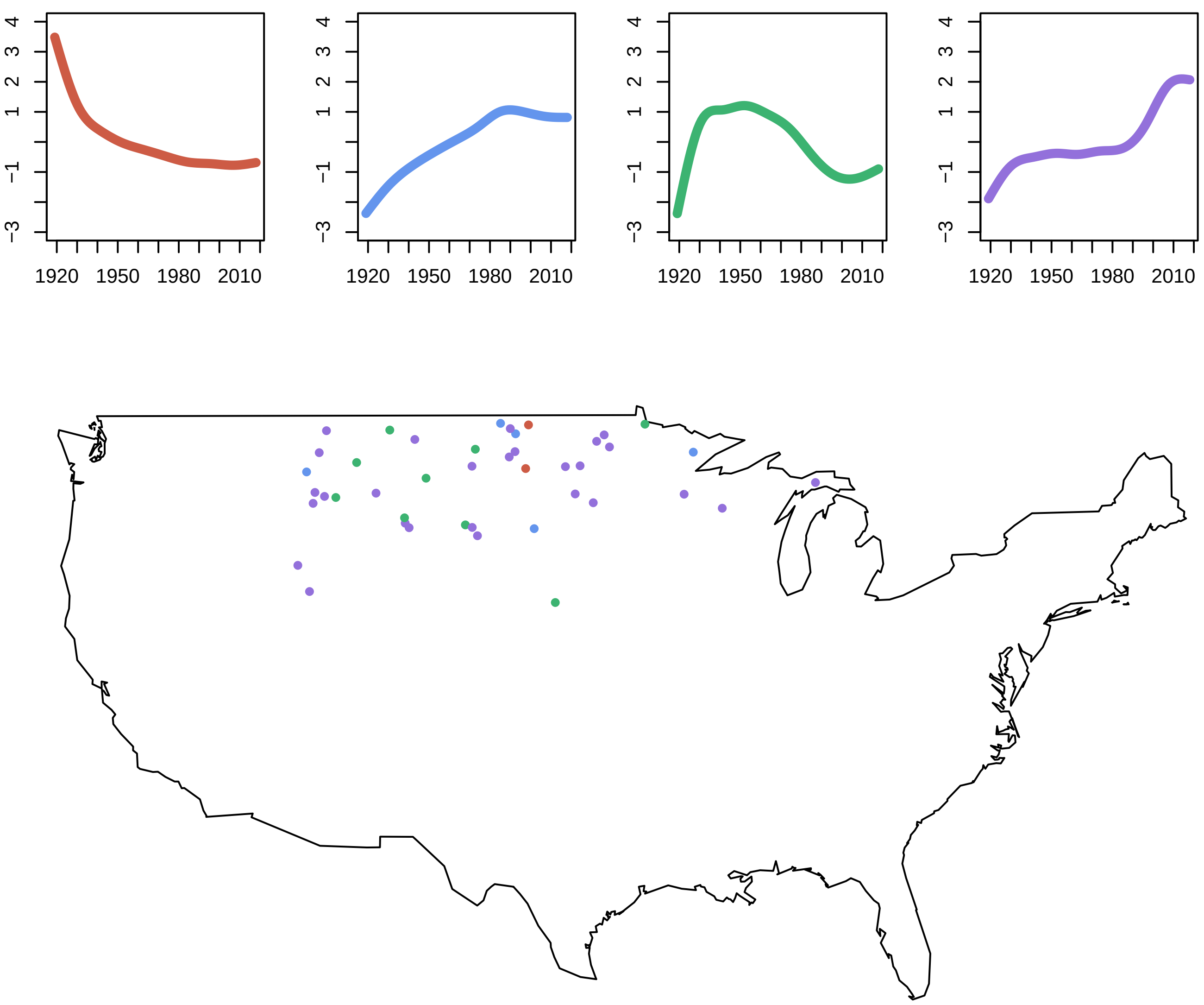

Short−eared Owl

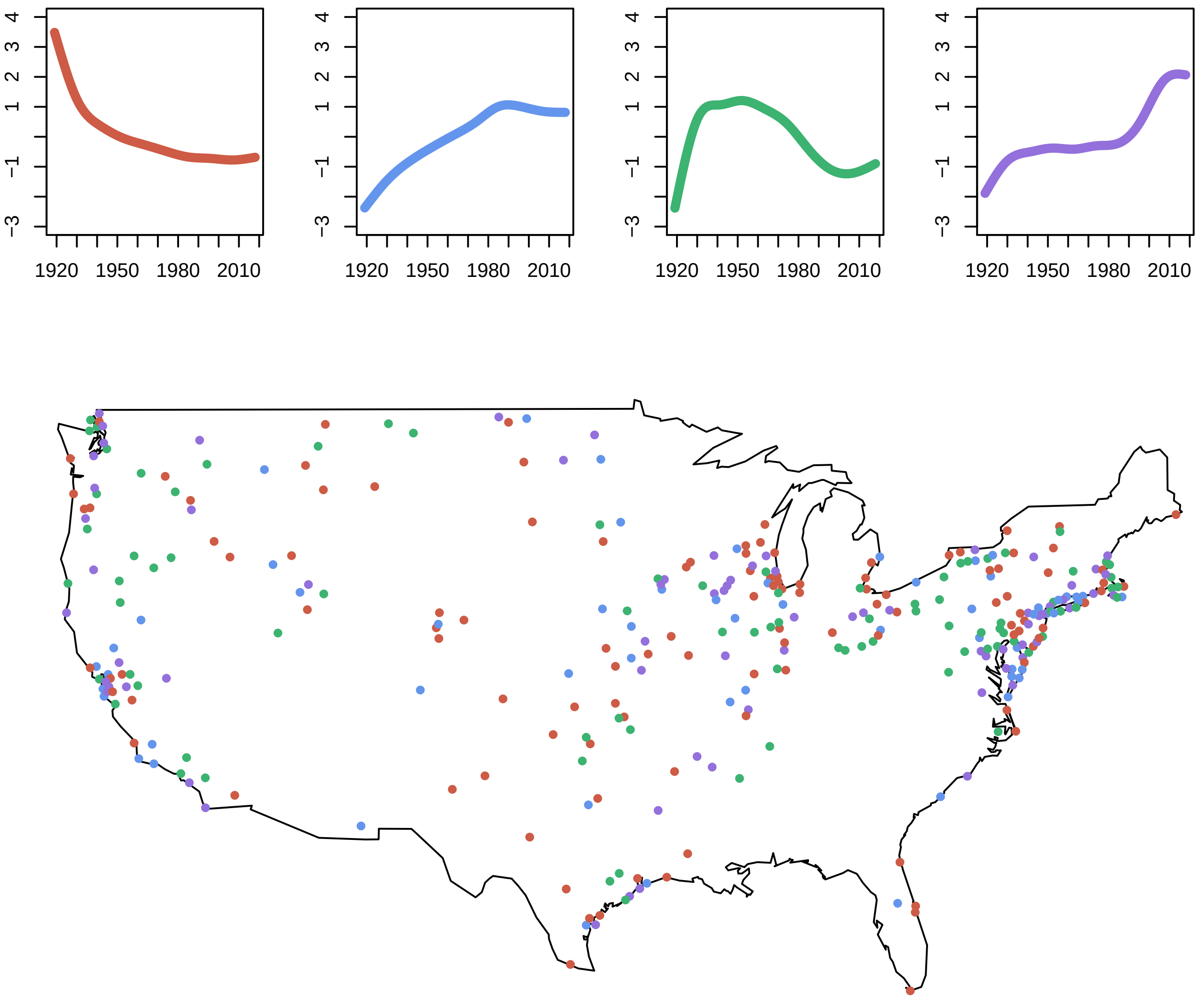

Sora

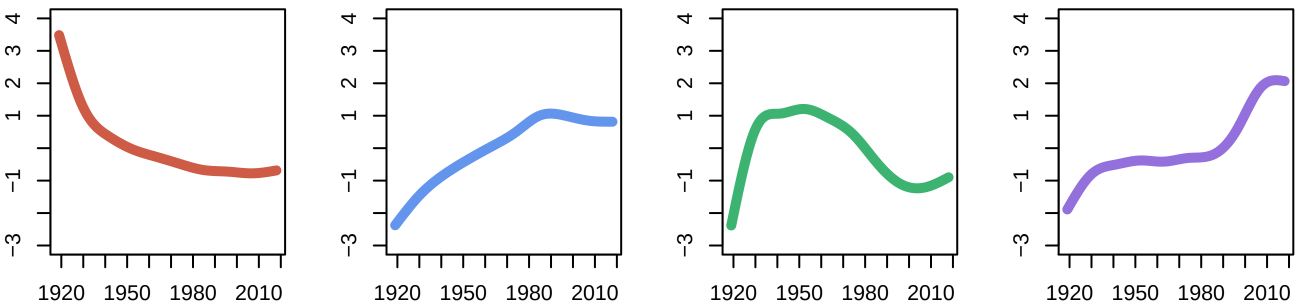

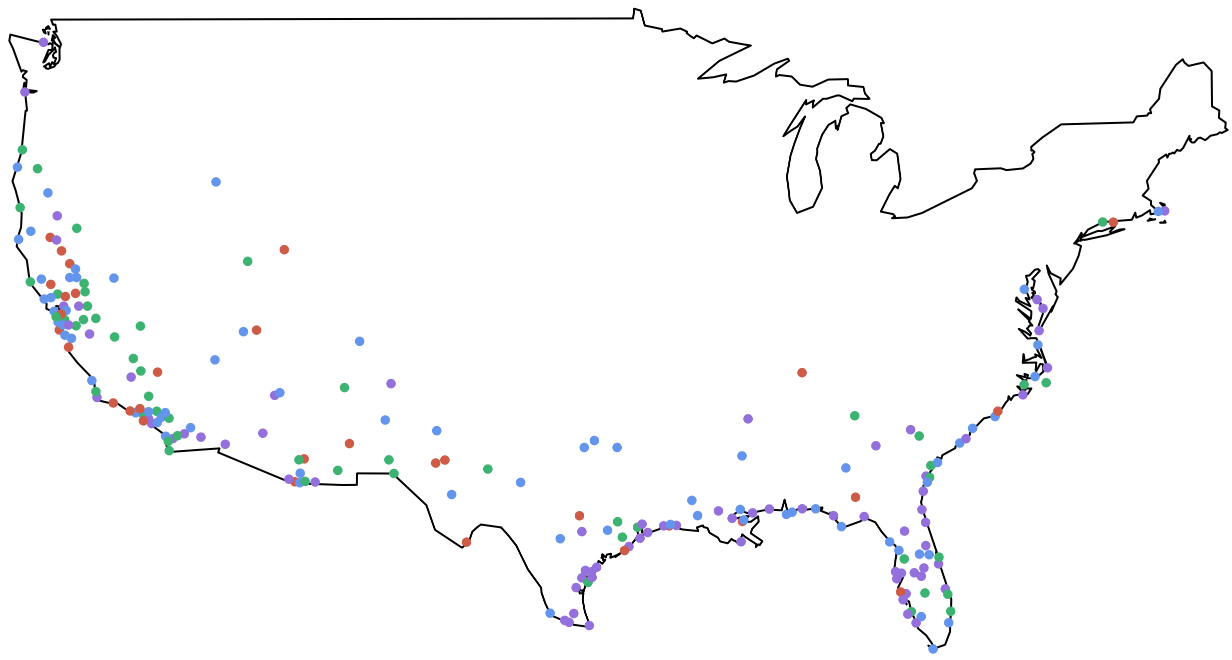

Virginia Rail

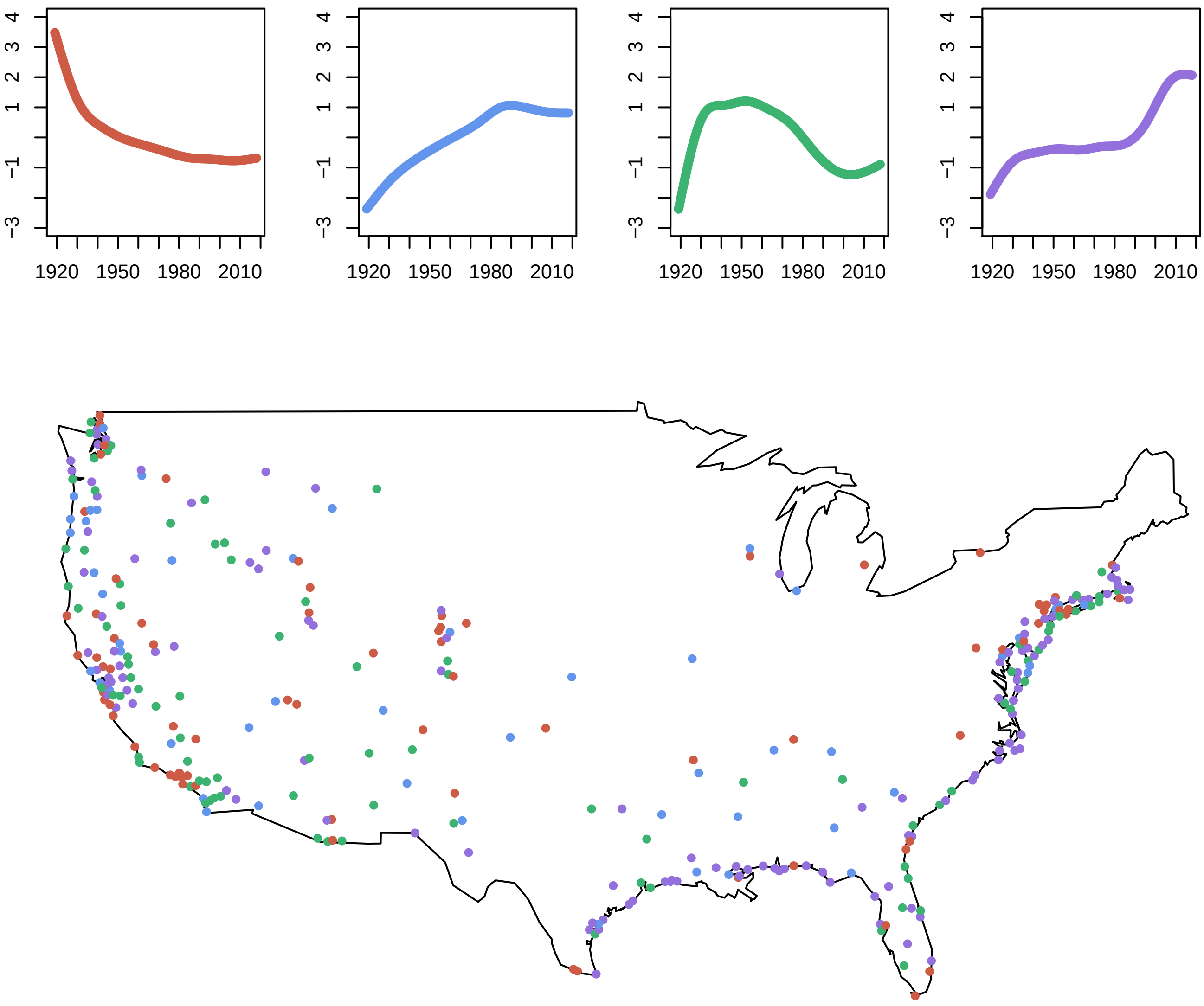

White−eyed Vireo

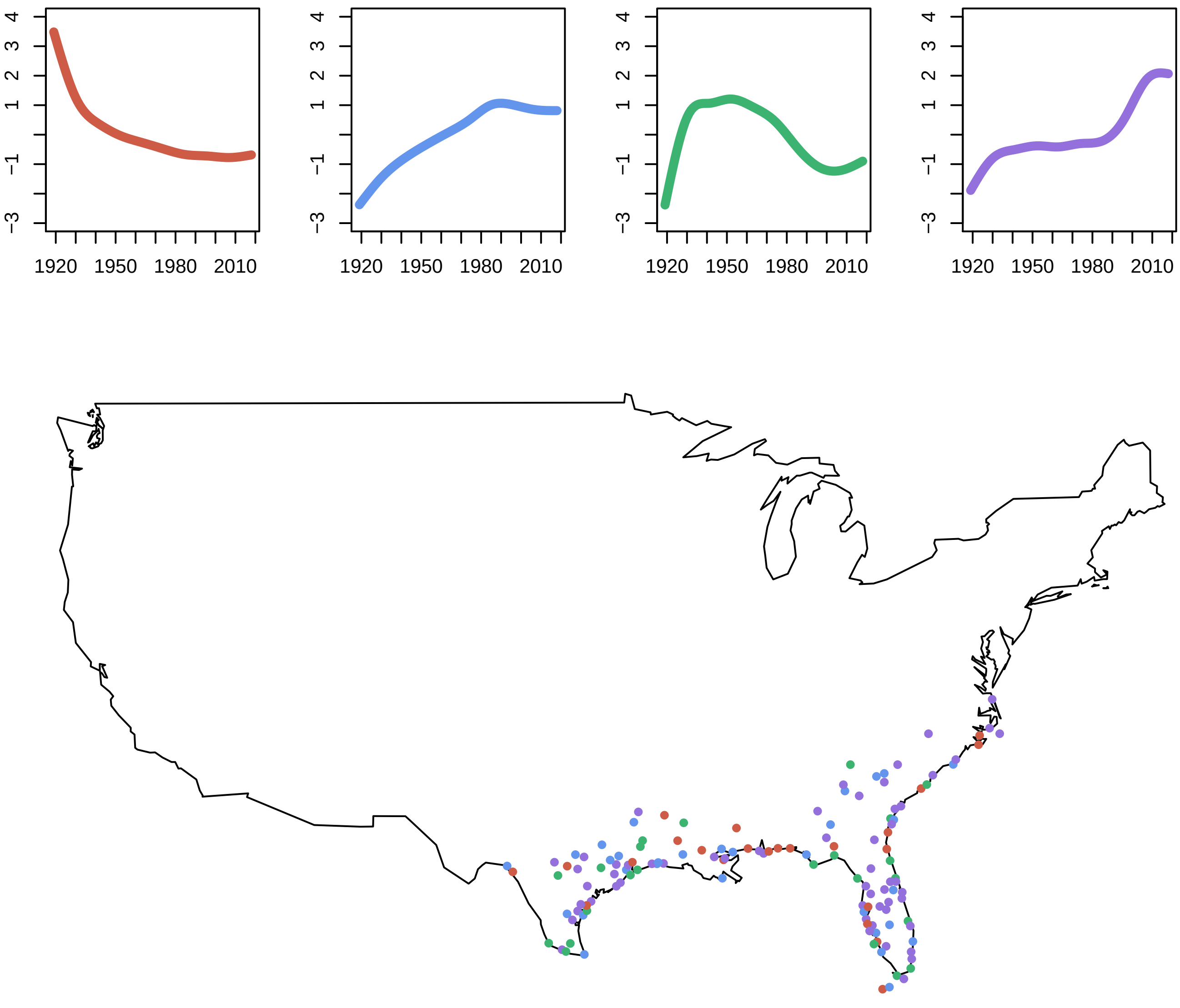

Winter Wren

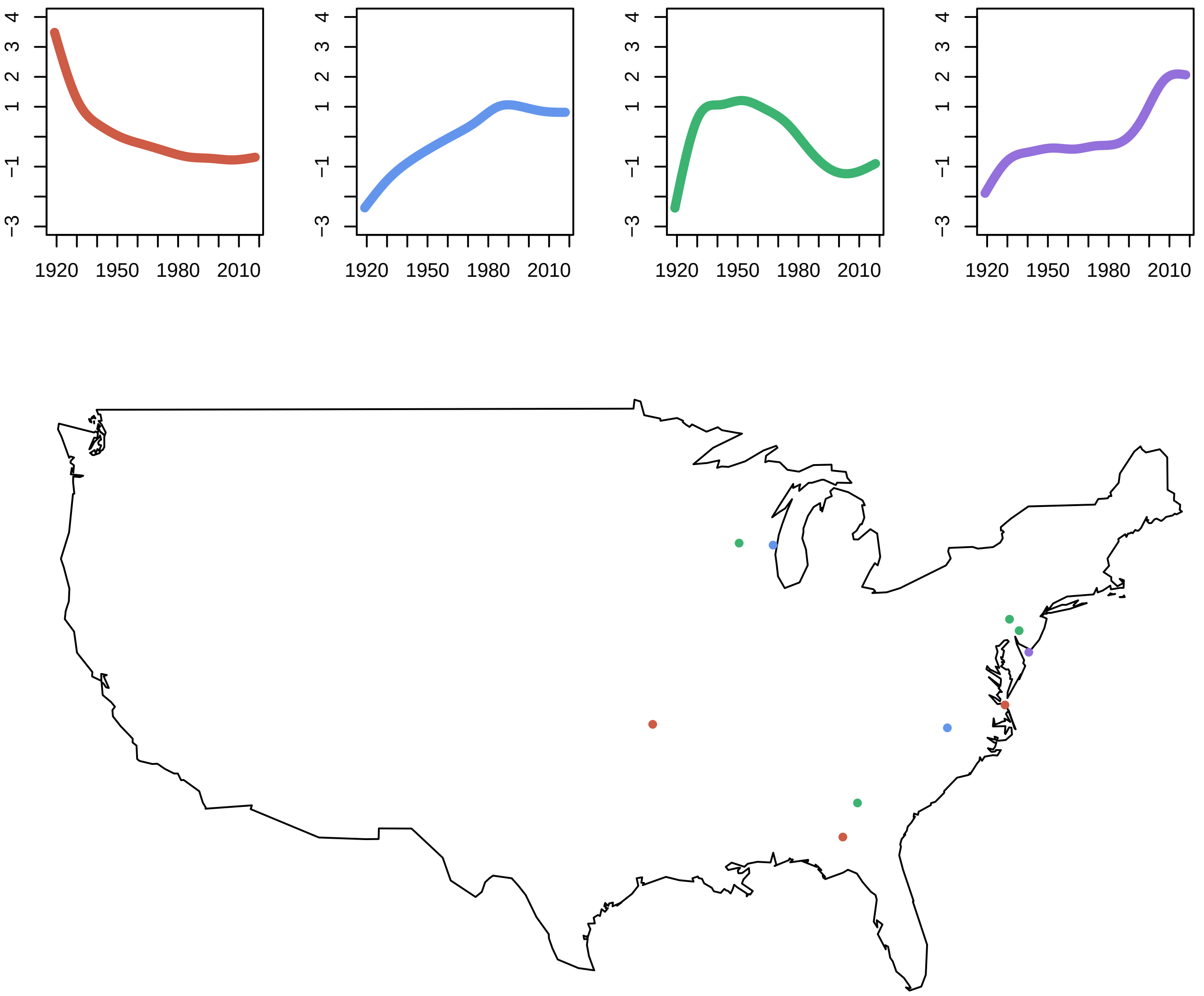

Yellow−crowned Night−Heron

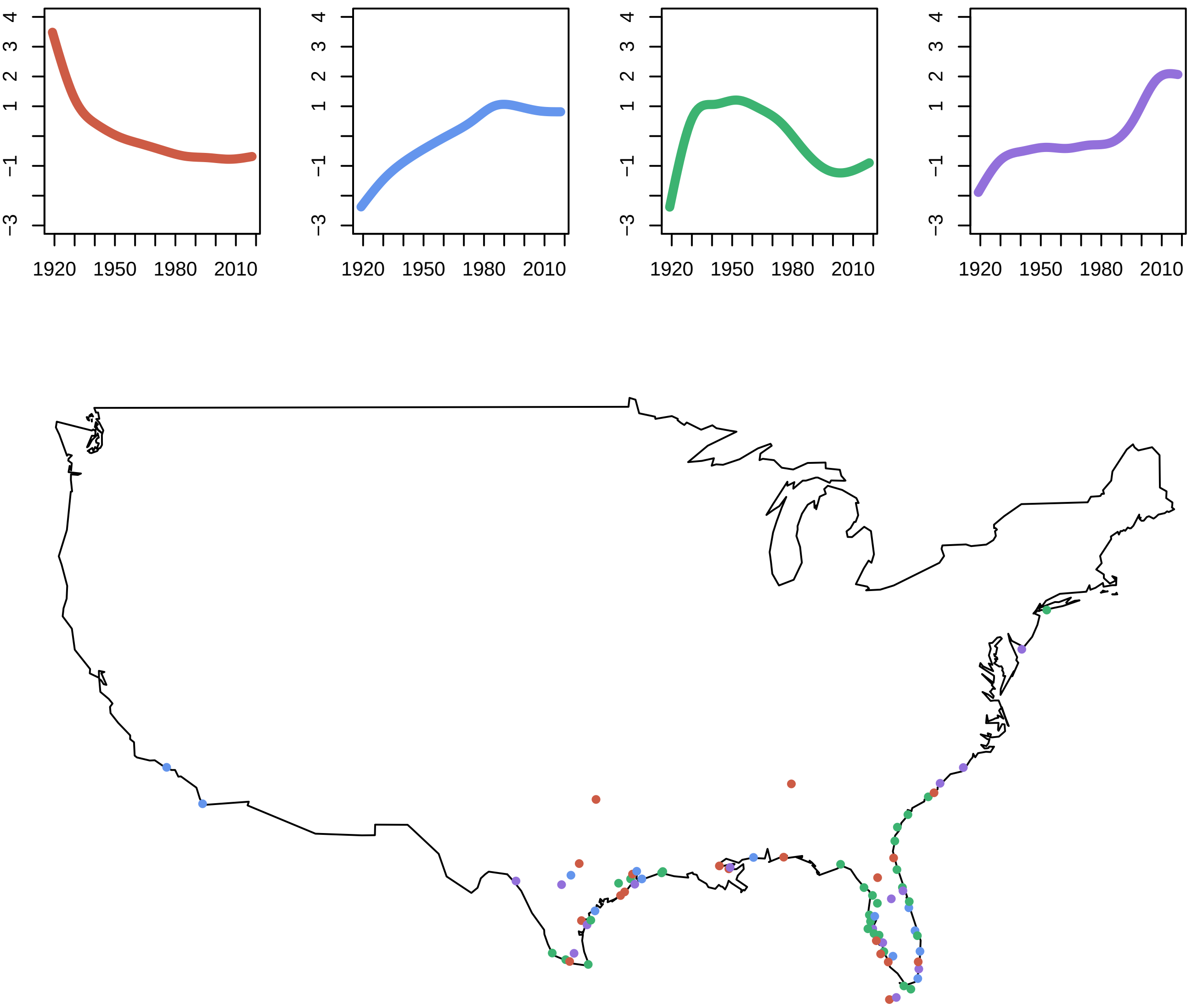

American Bittern

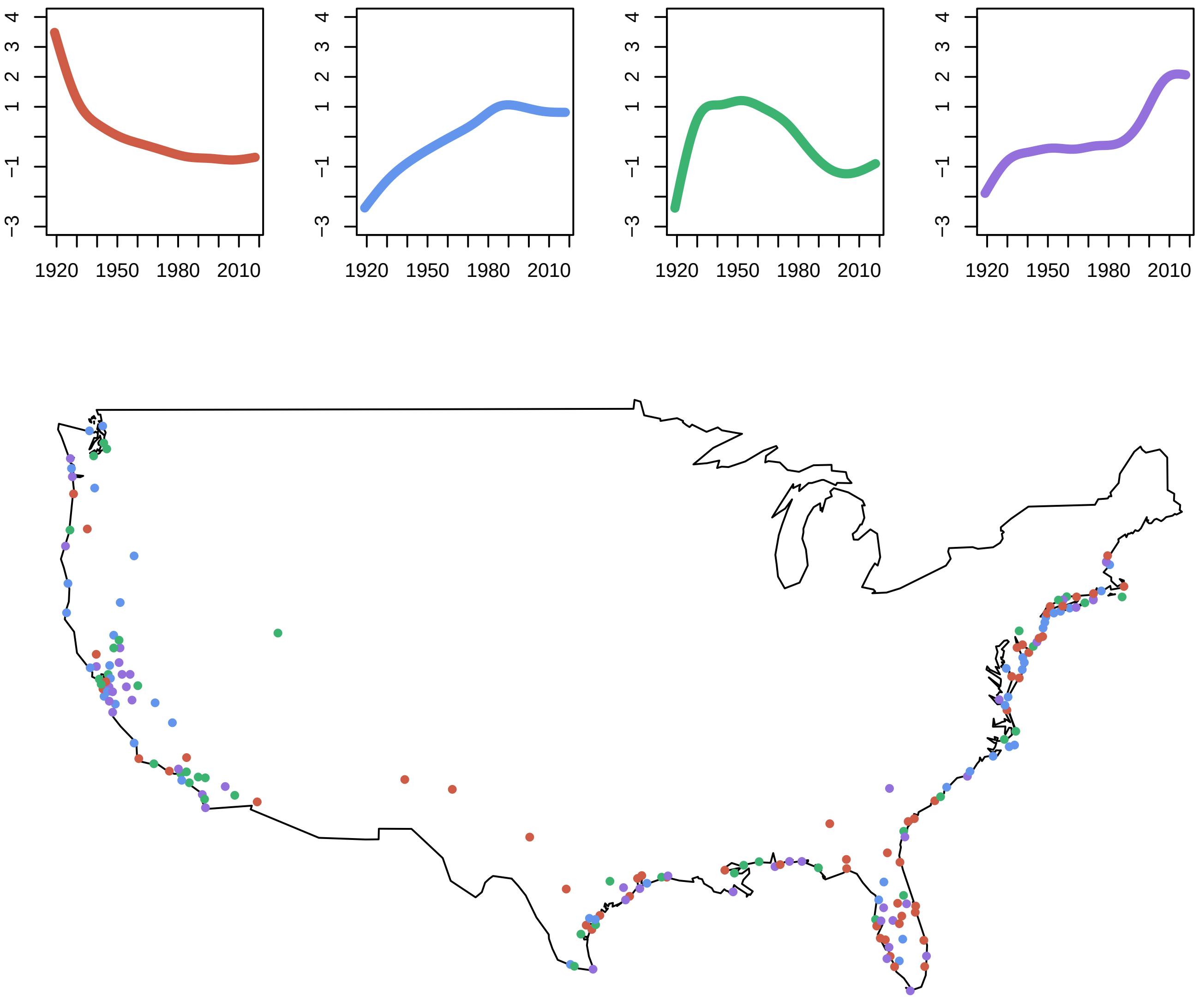

Black−tailed Gnatcatcher

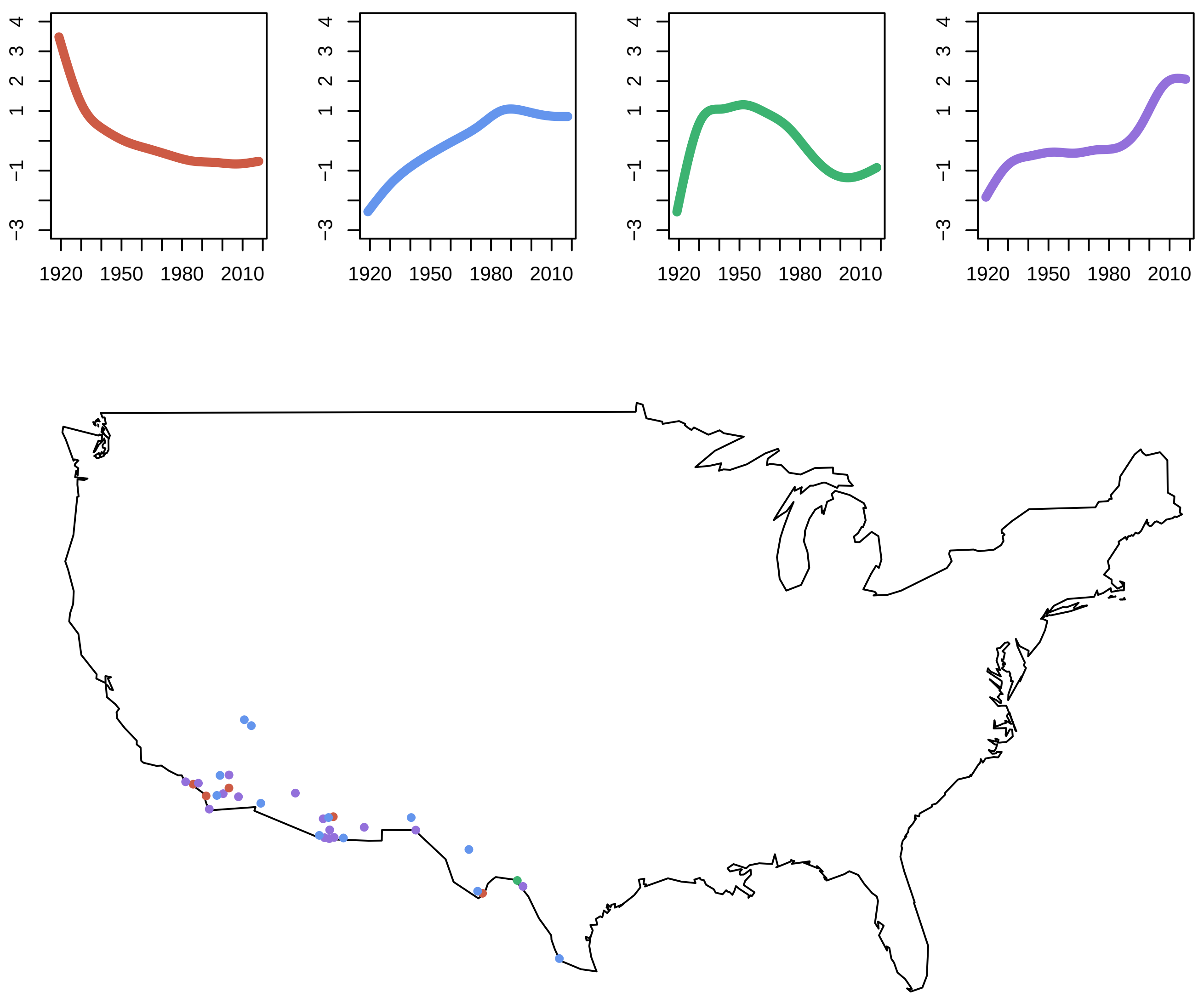

Black Rosy−Finch

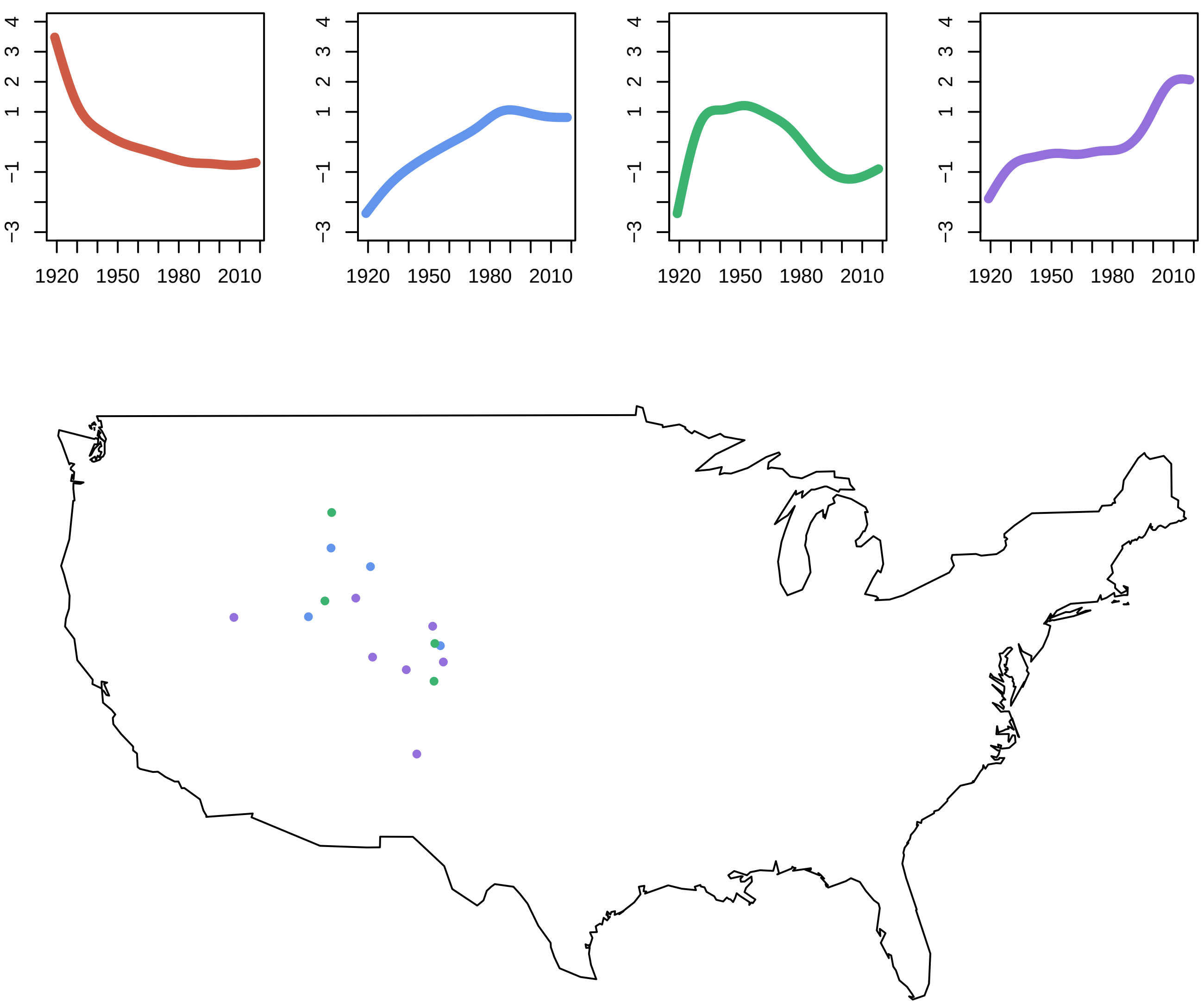

Burrowing Owl

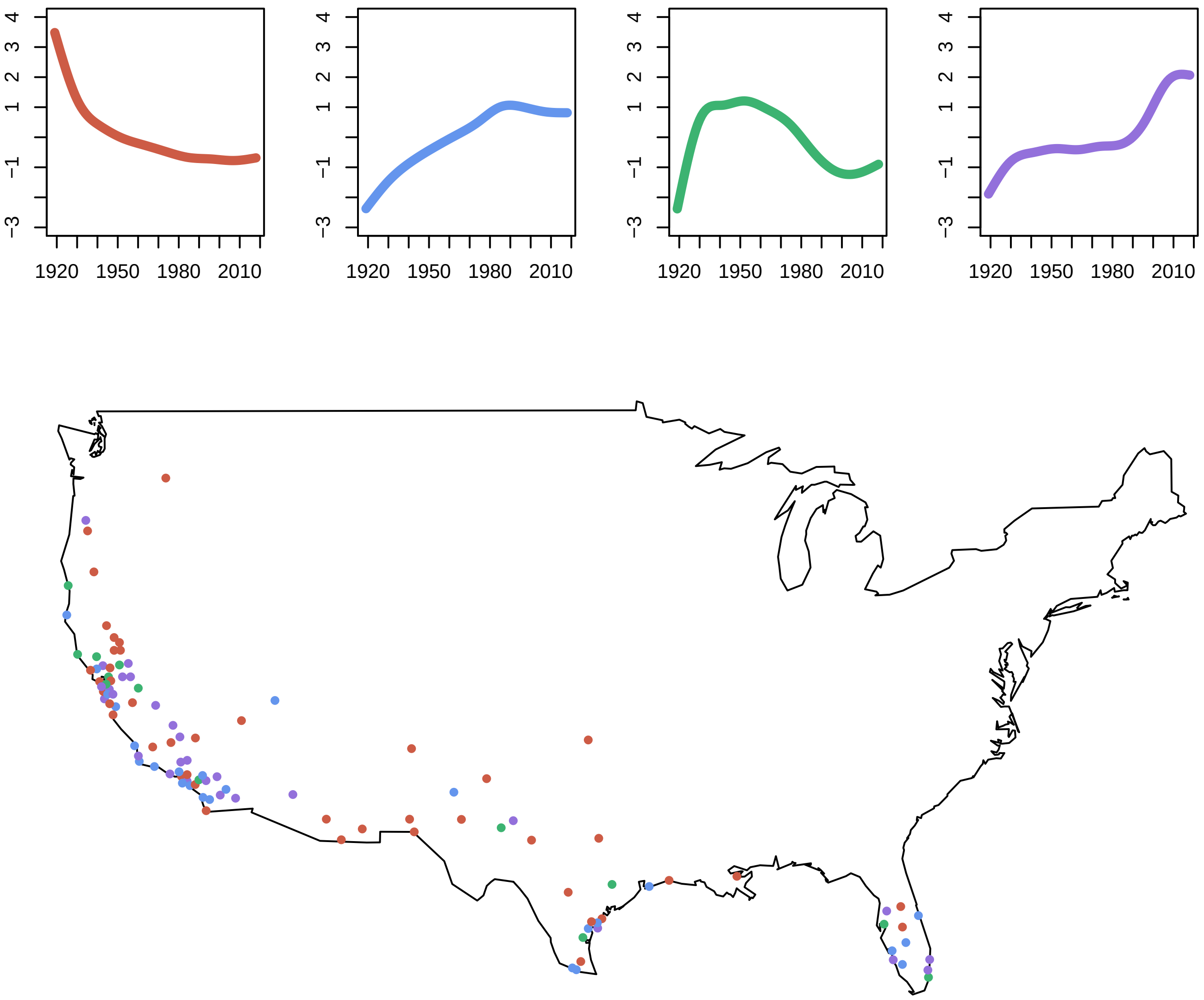

Cave Swallow

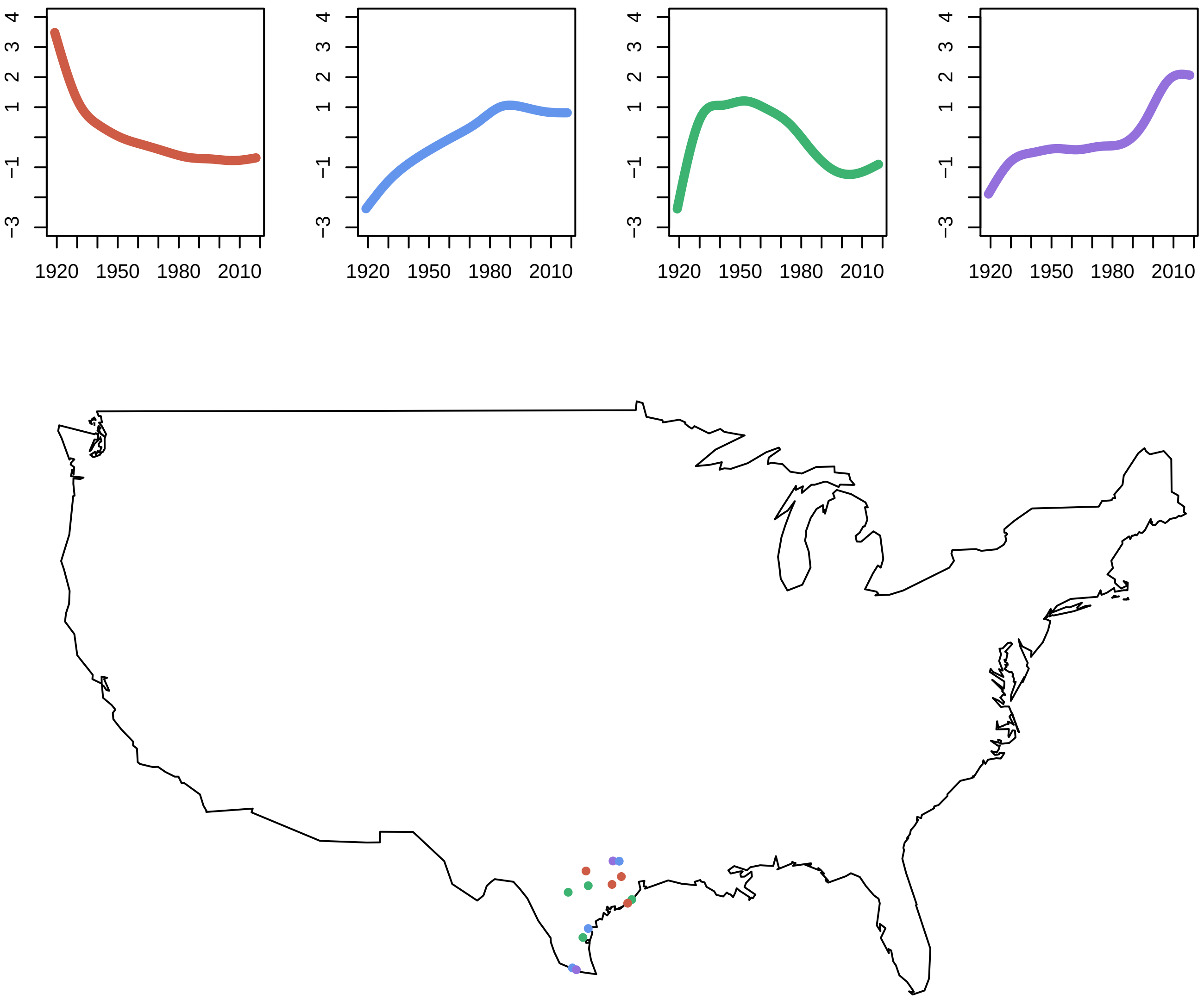

Crissal Thrasher

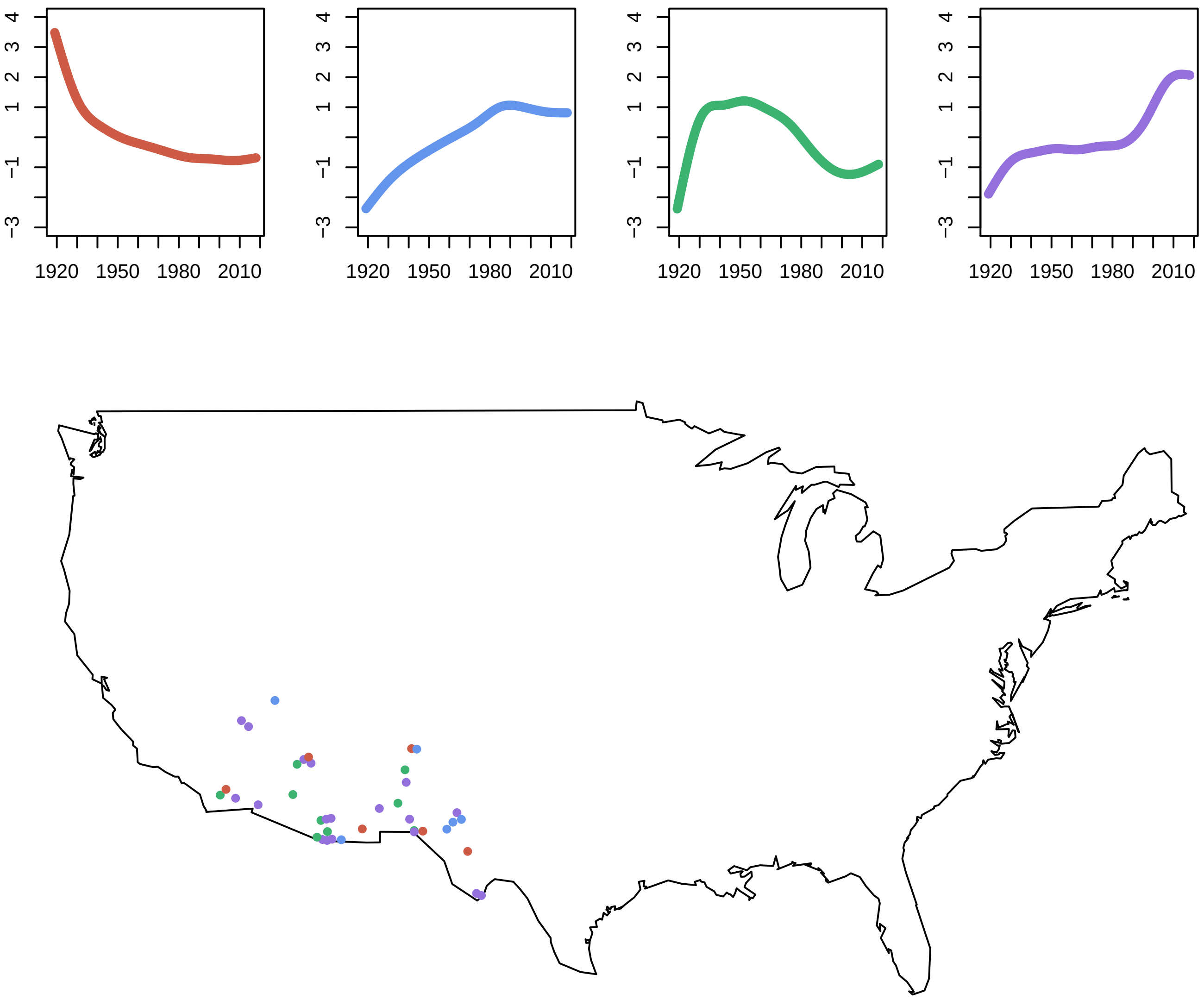

Ferruginous Hawk

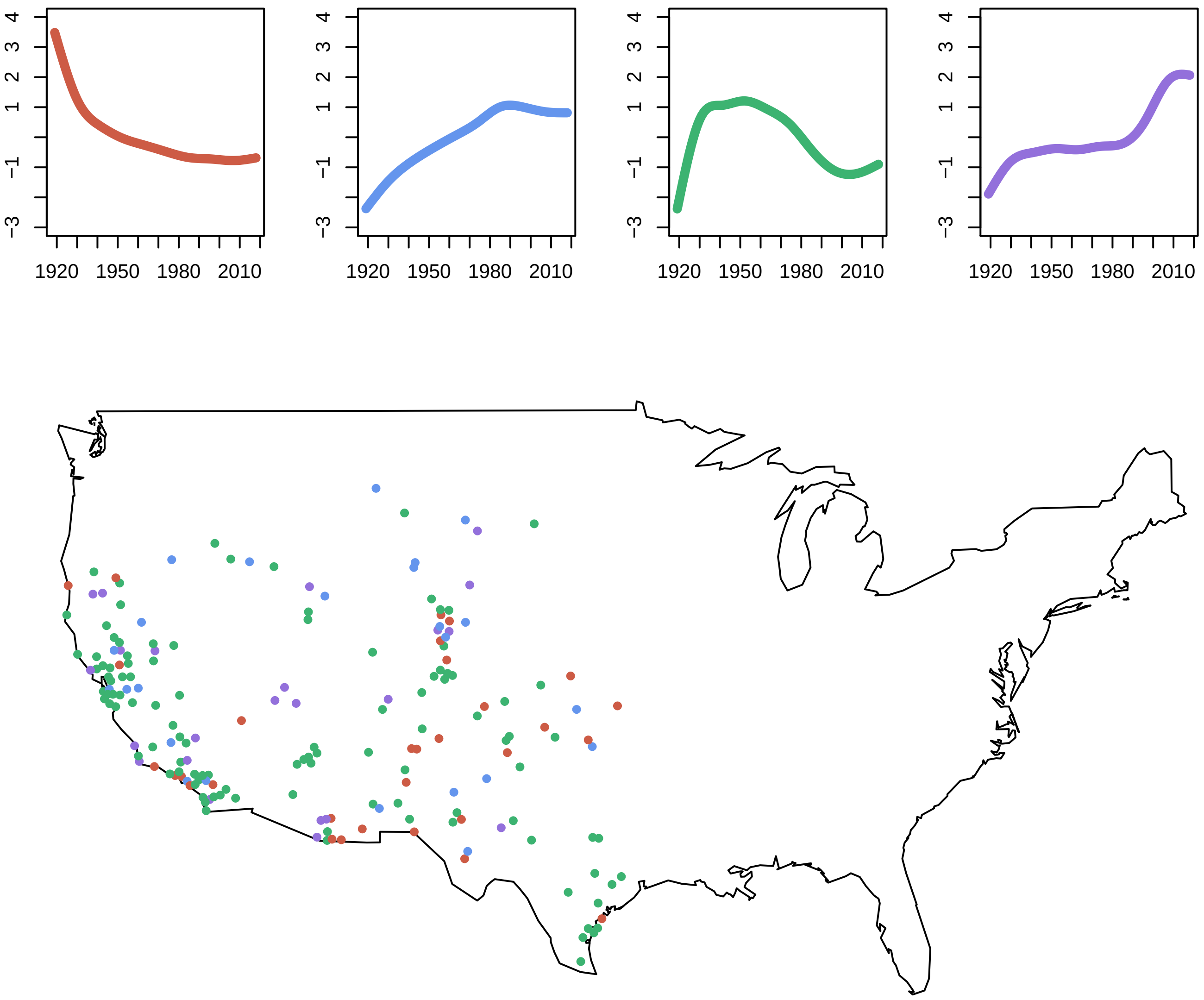

Franklin's Gull

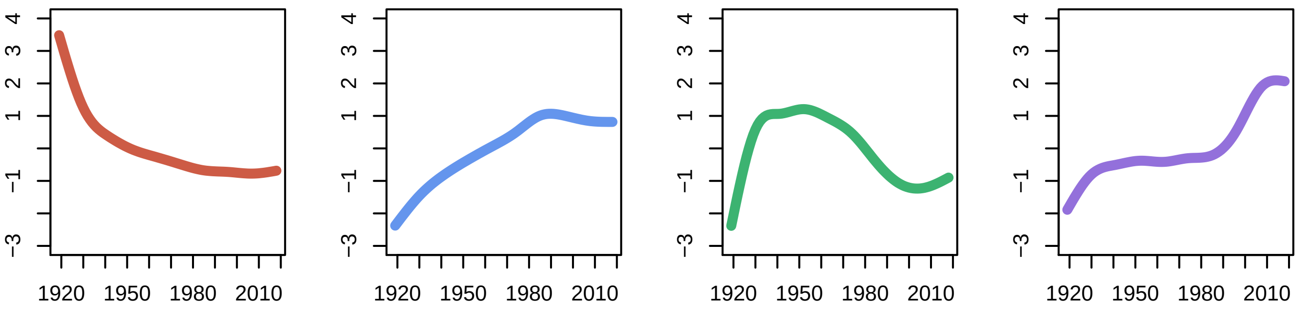

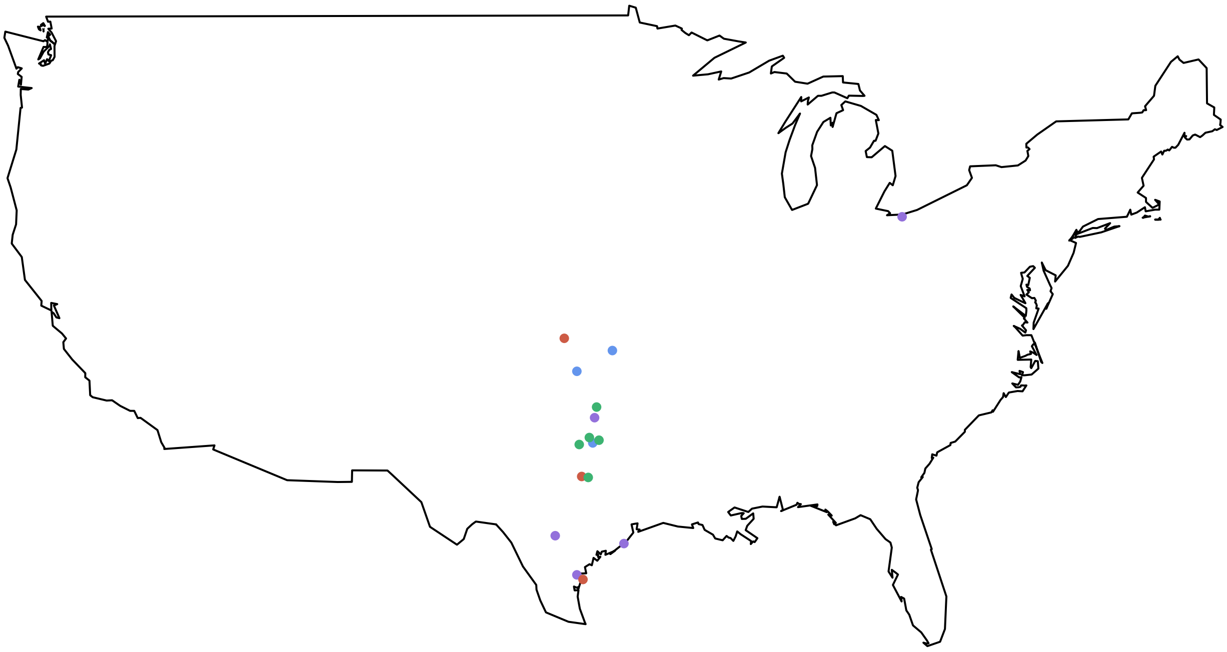

Grasshopper Sparrow

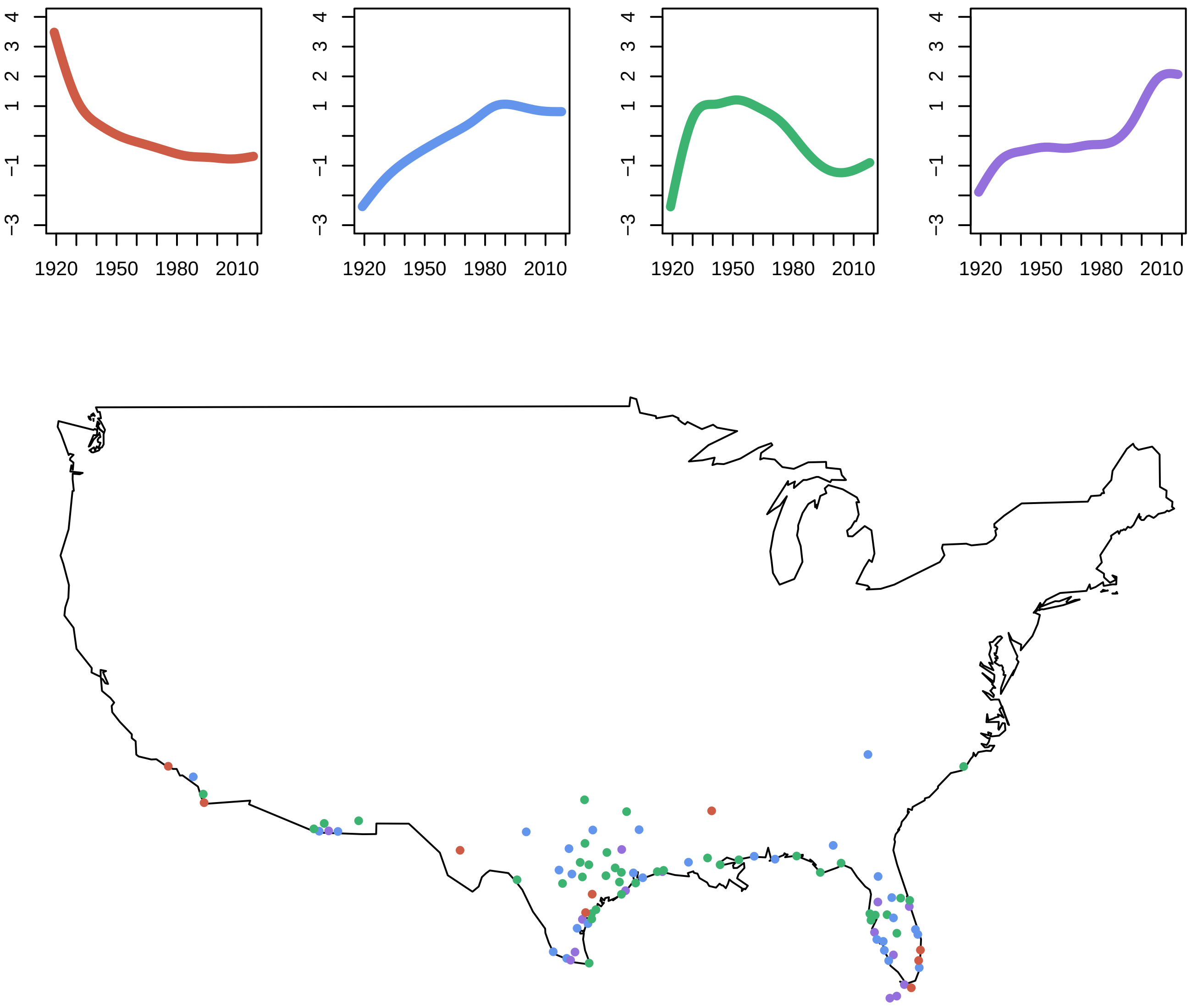

Gray Jay

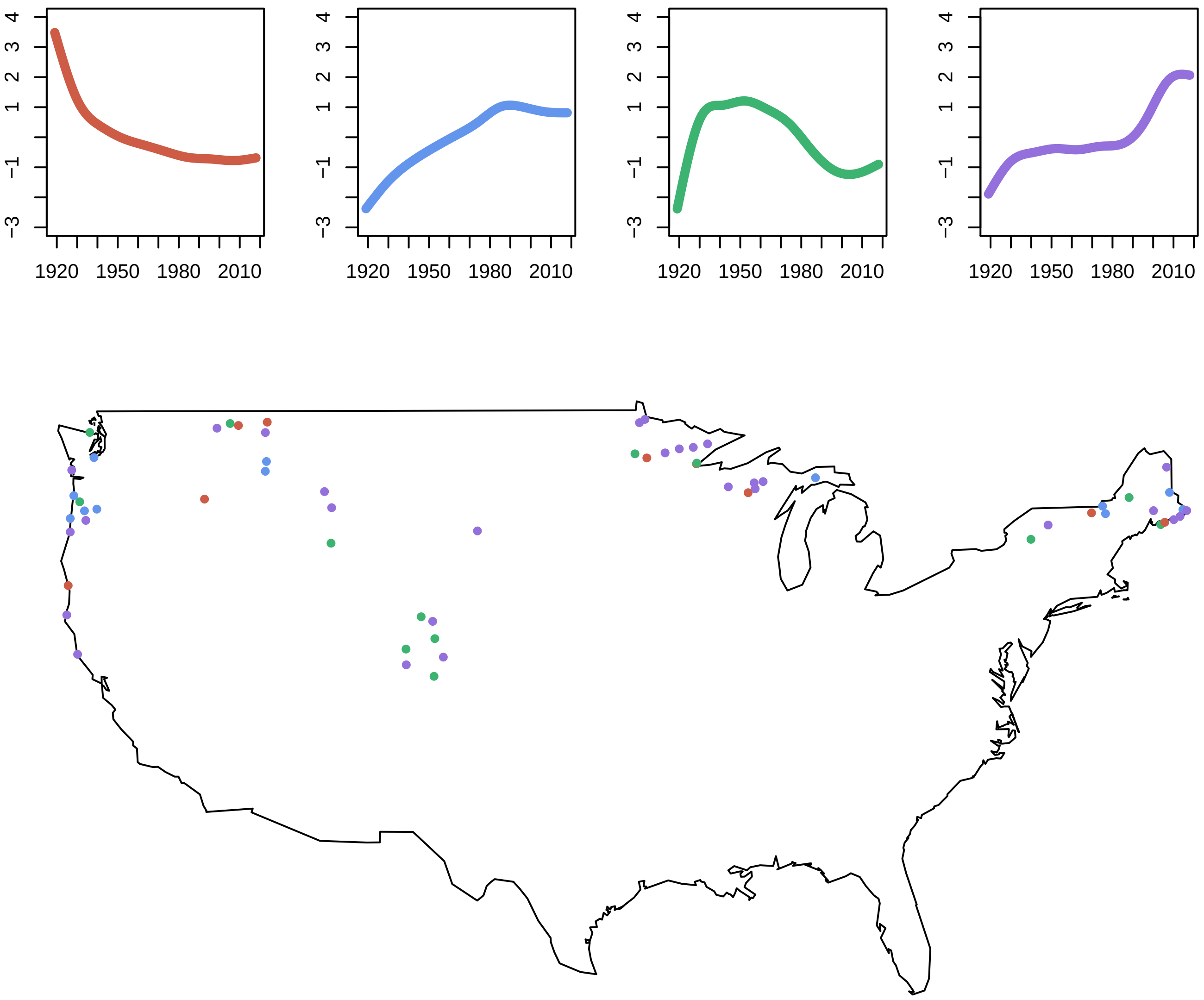

Great Kiskadee

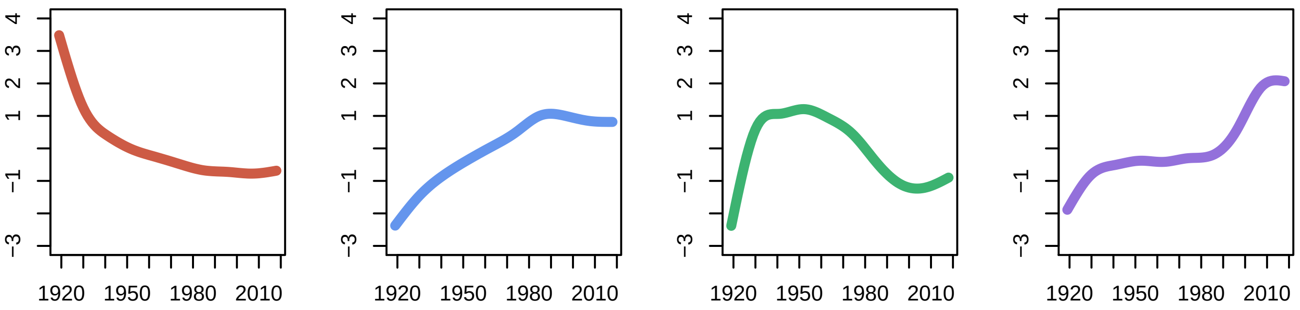

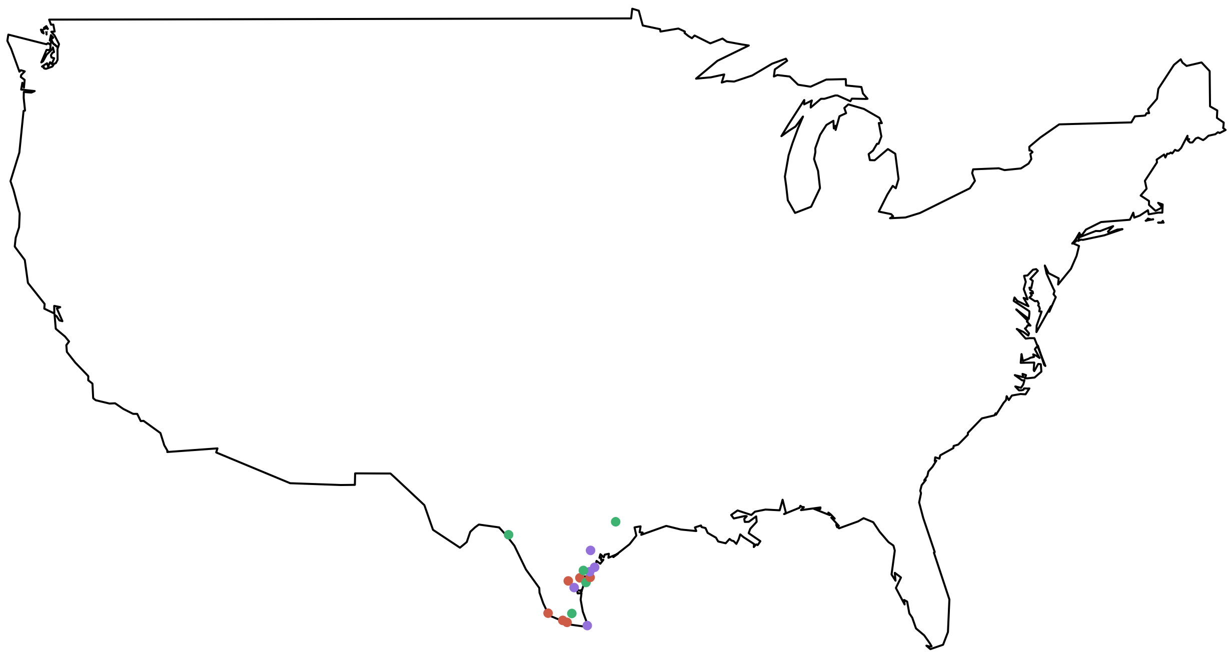

Greater Prairie−Chicken

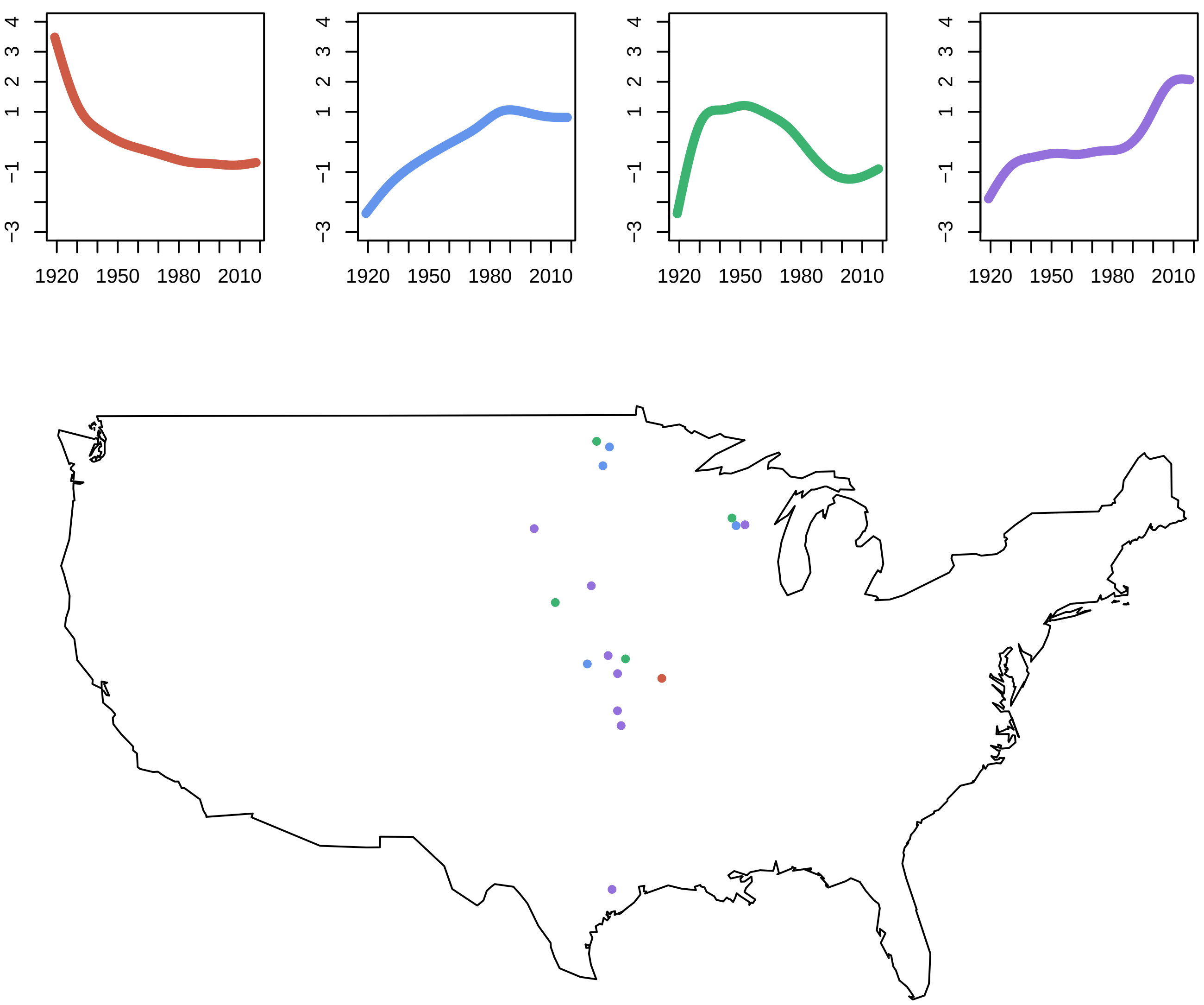

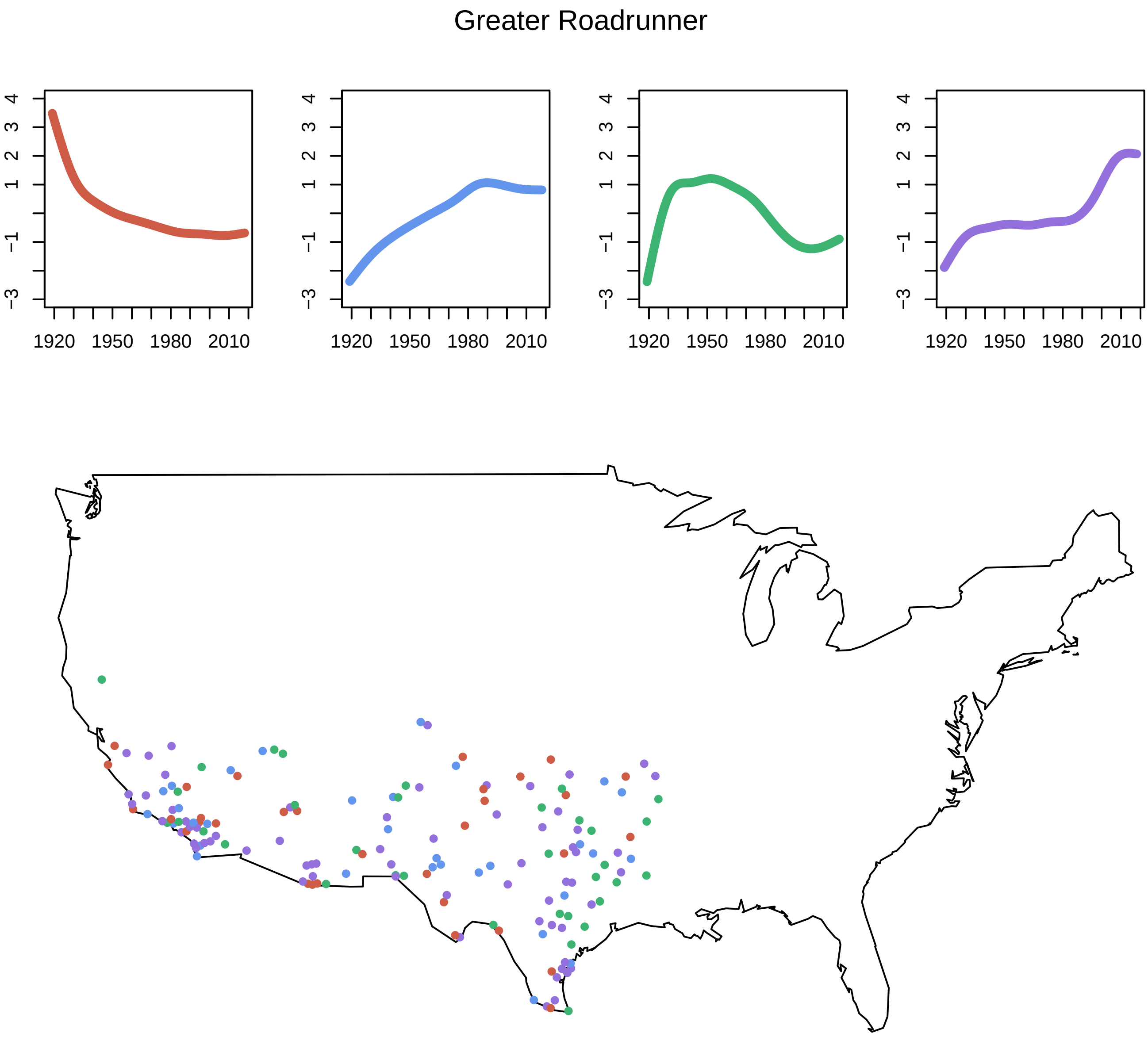
Greater Roadrunner
4
3
2
1
−1
−3
1920
1950
1980
2010

Greater Sage−Grouse

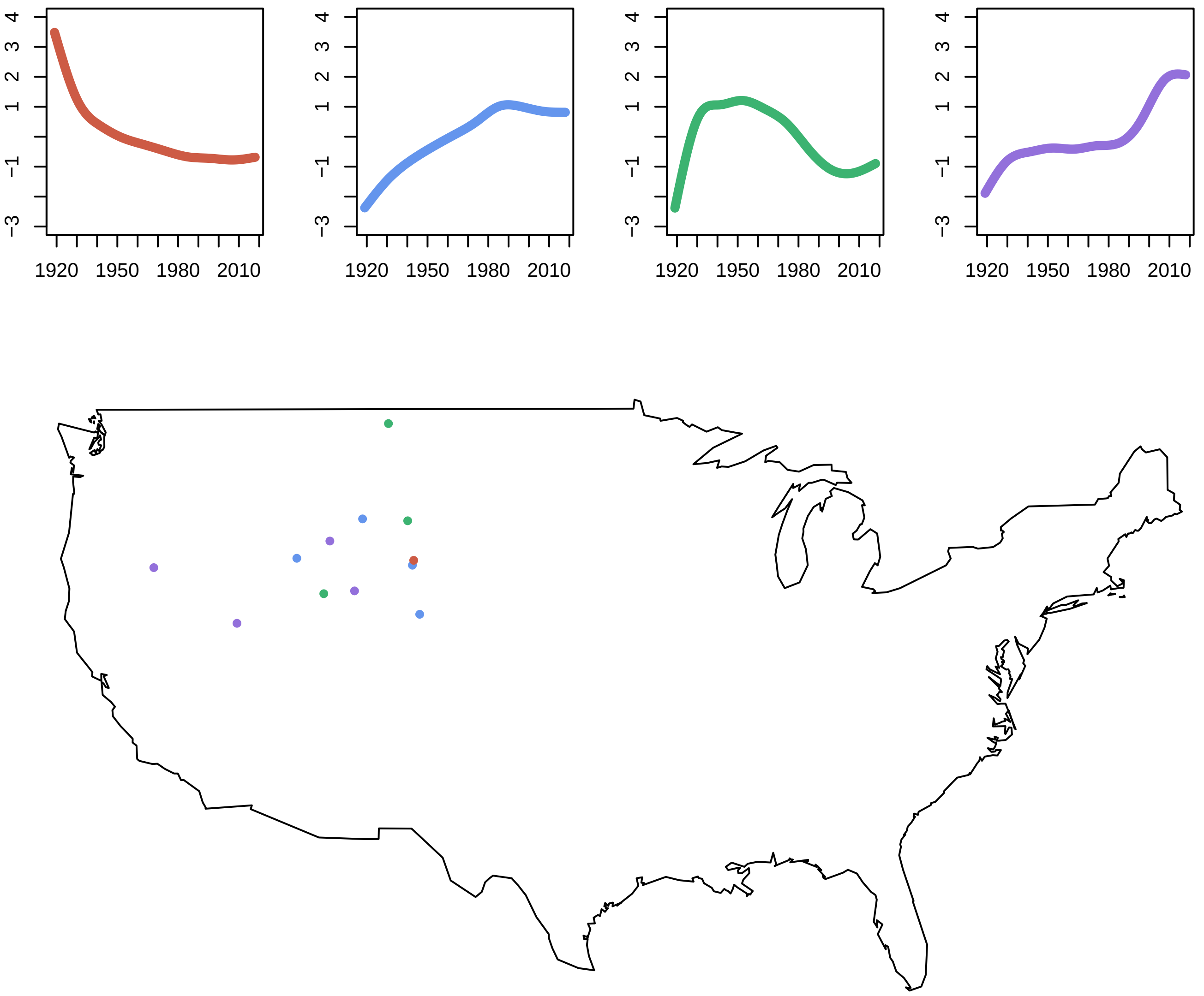

Green−tailed Towhee

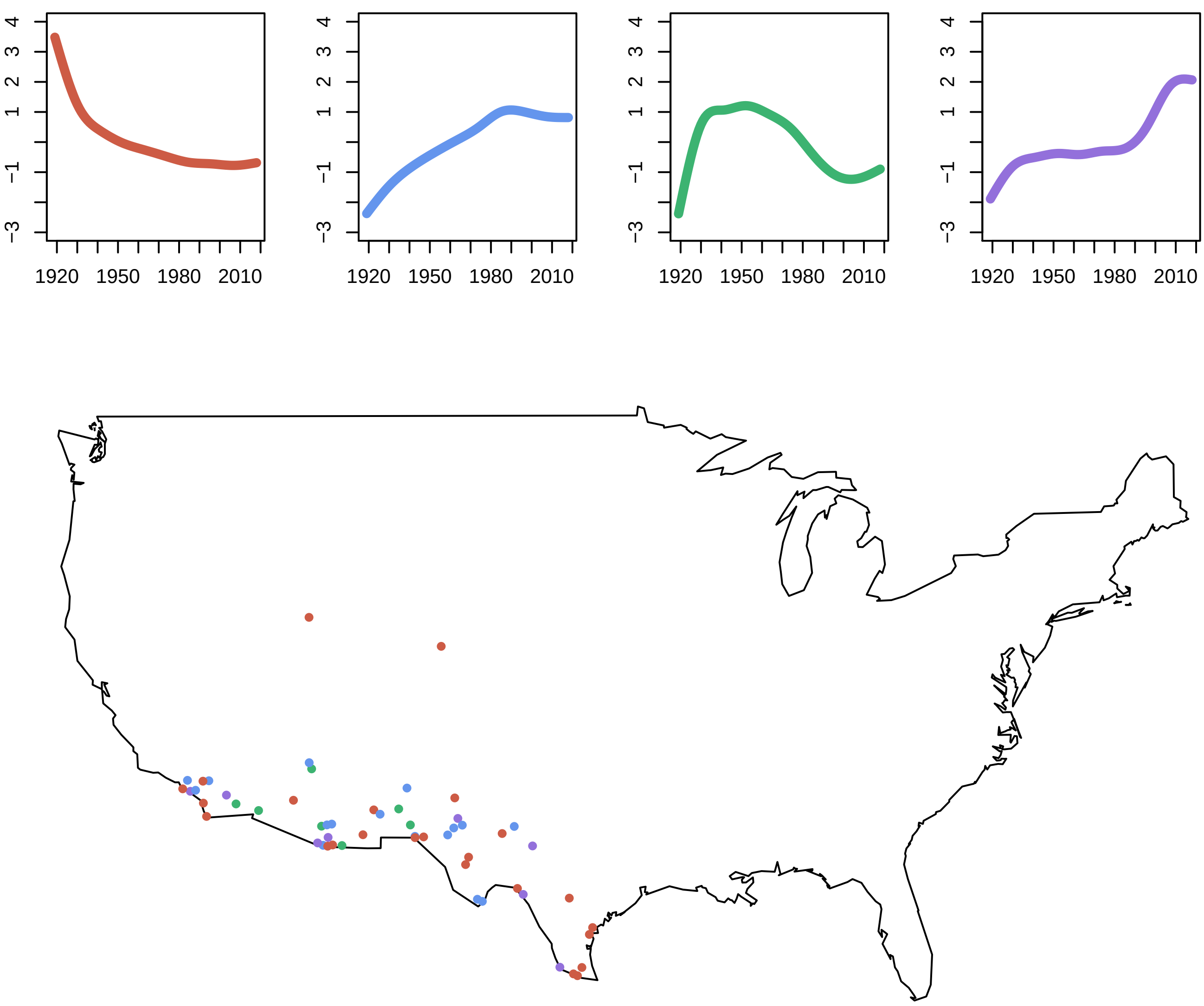

Green Jay

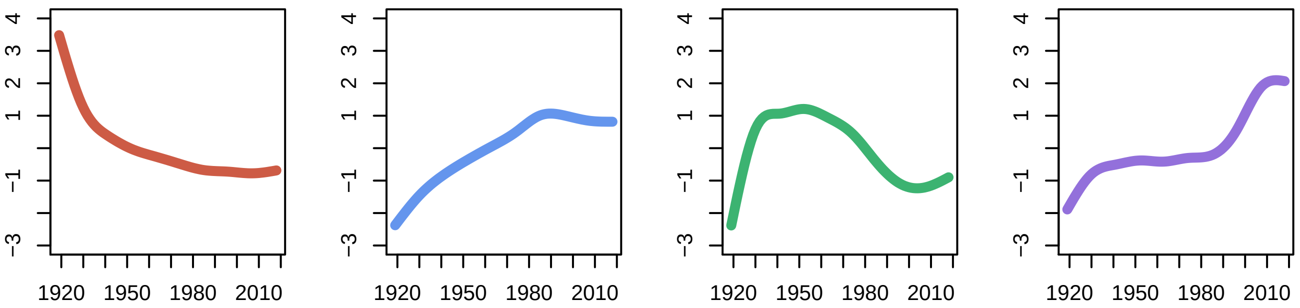

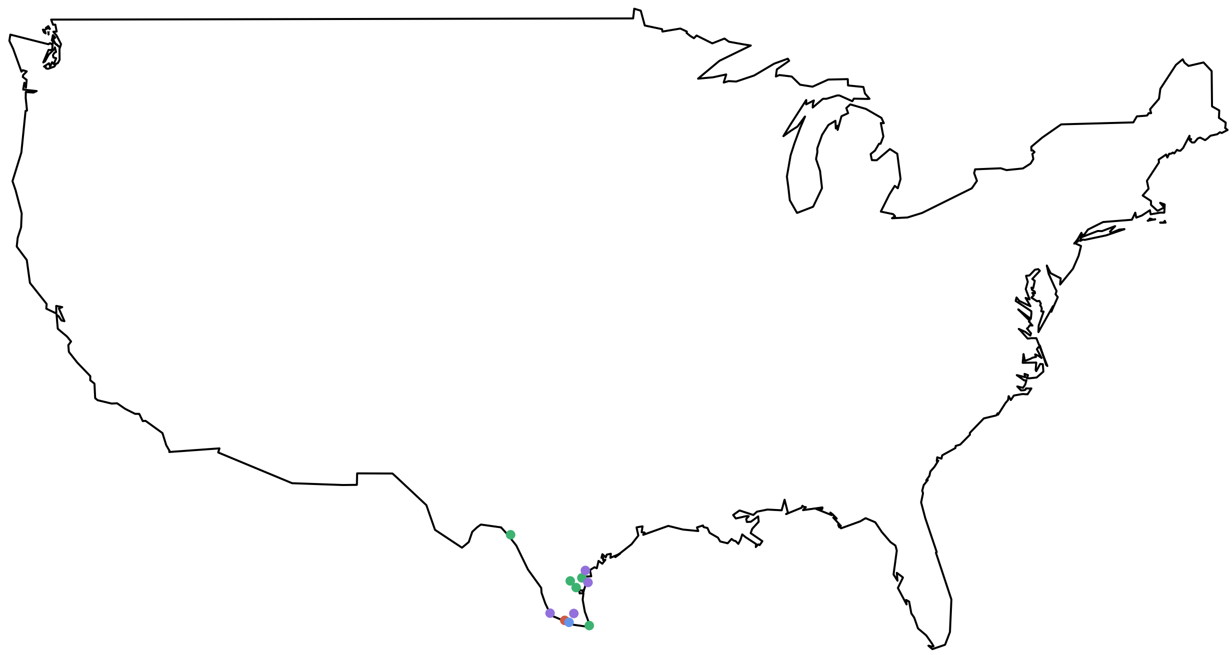

Harris's Hawk

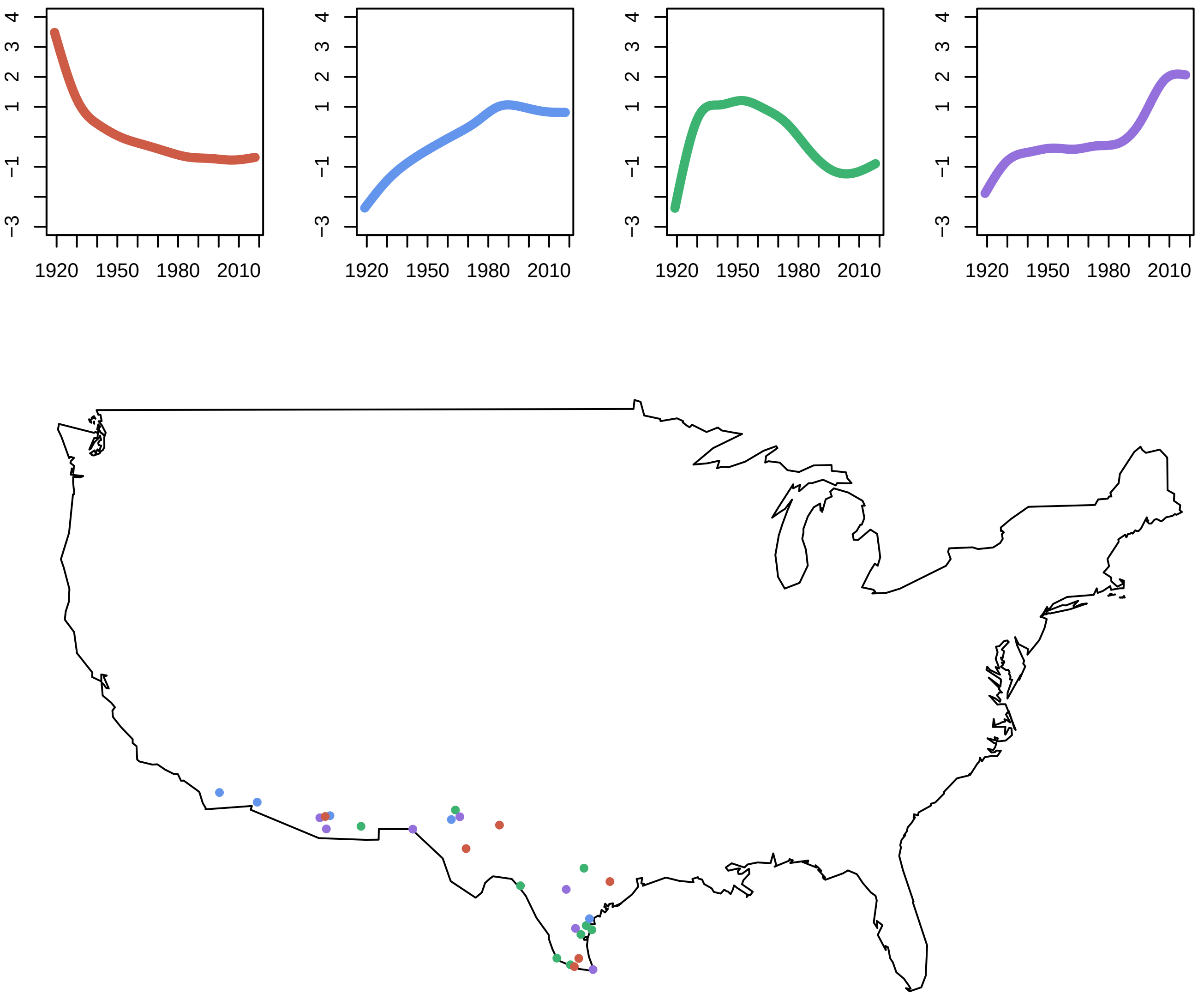

King Rail

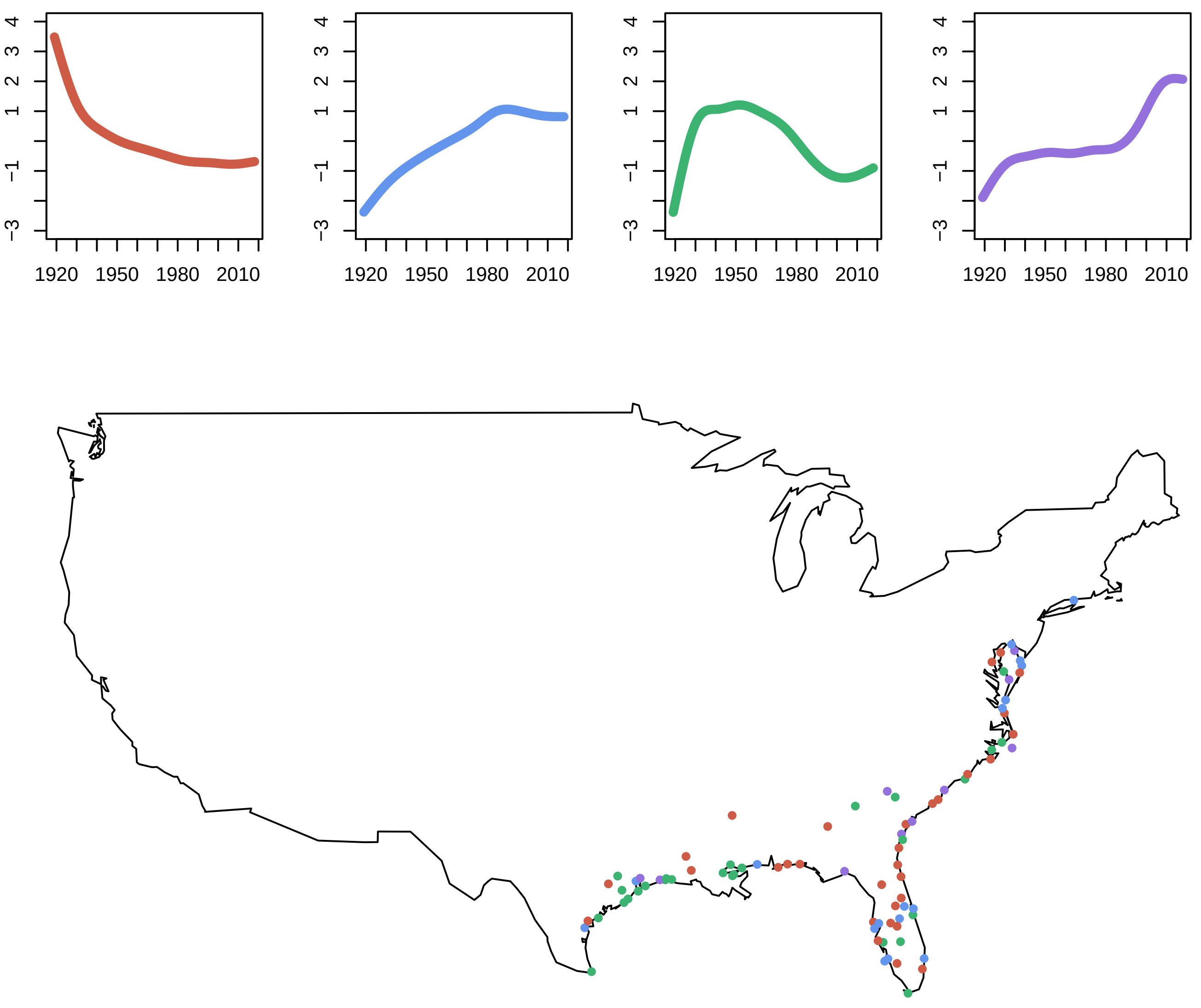

Lawrence's Goldfinch

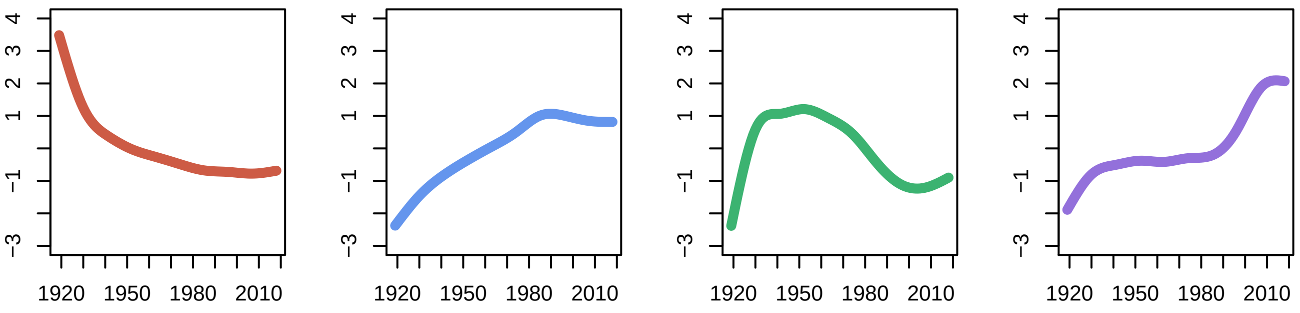

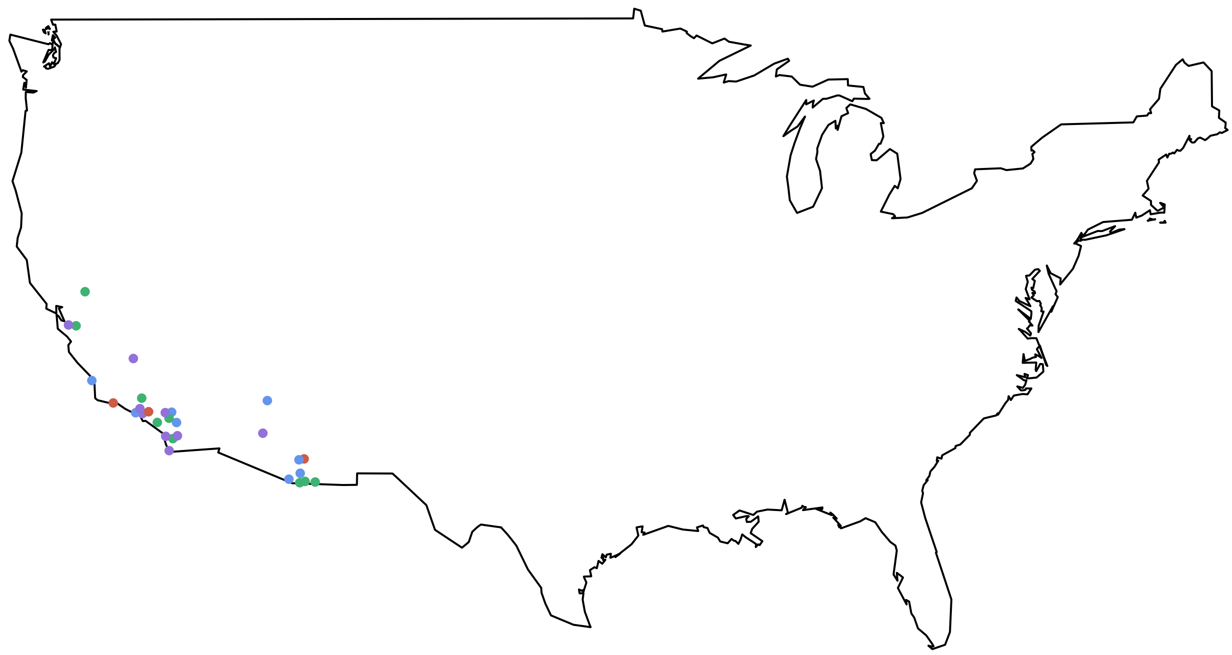

Le Conte's Sparrow

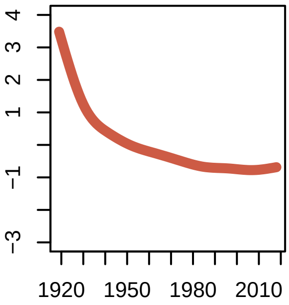

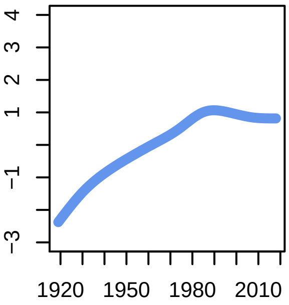

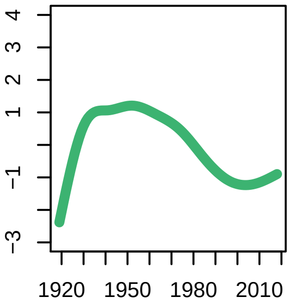

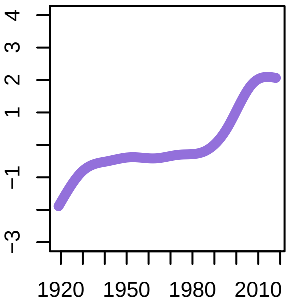

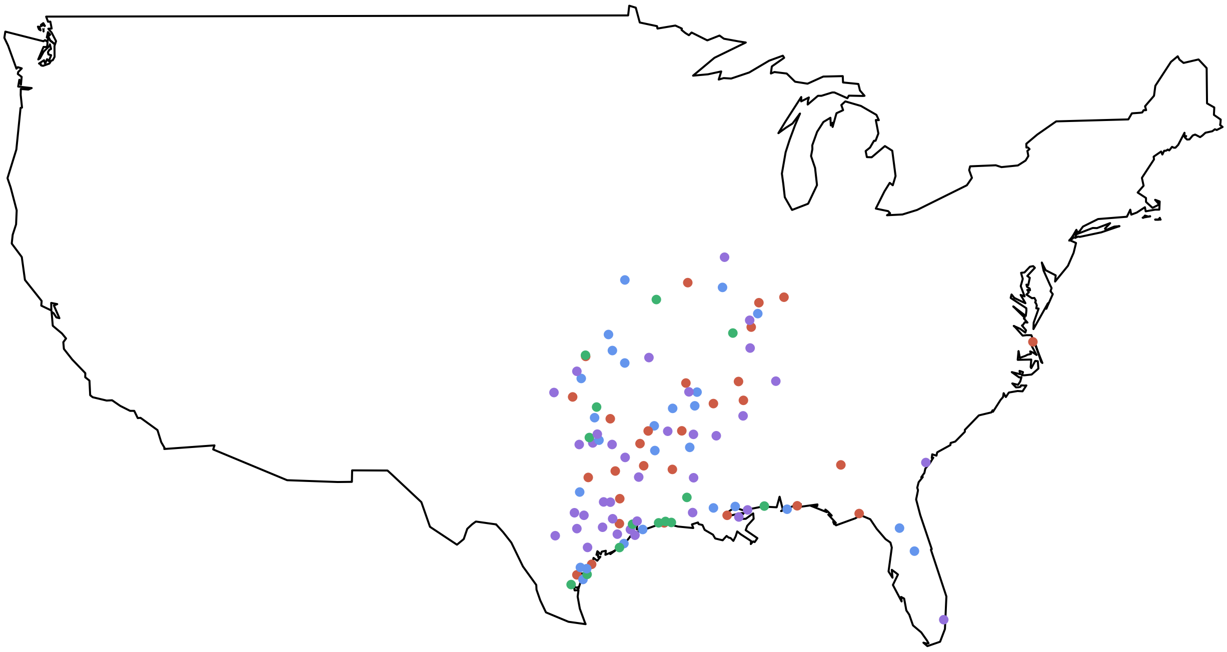

Limpkin

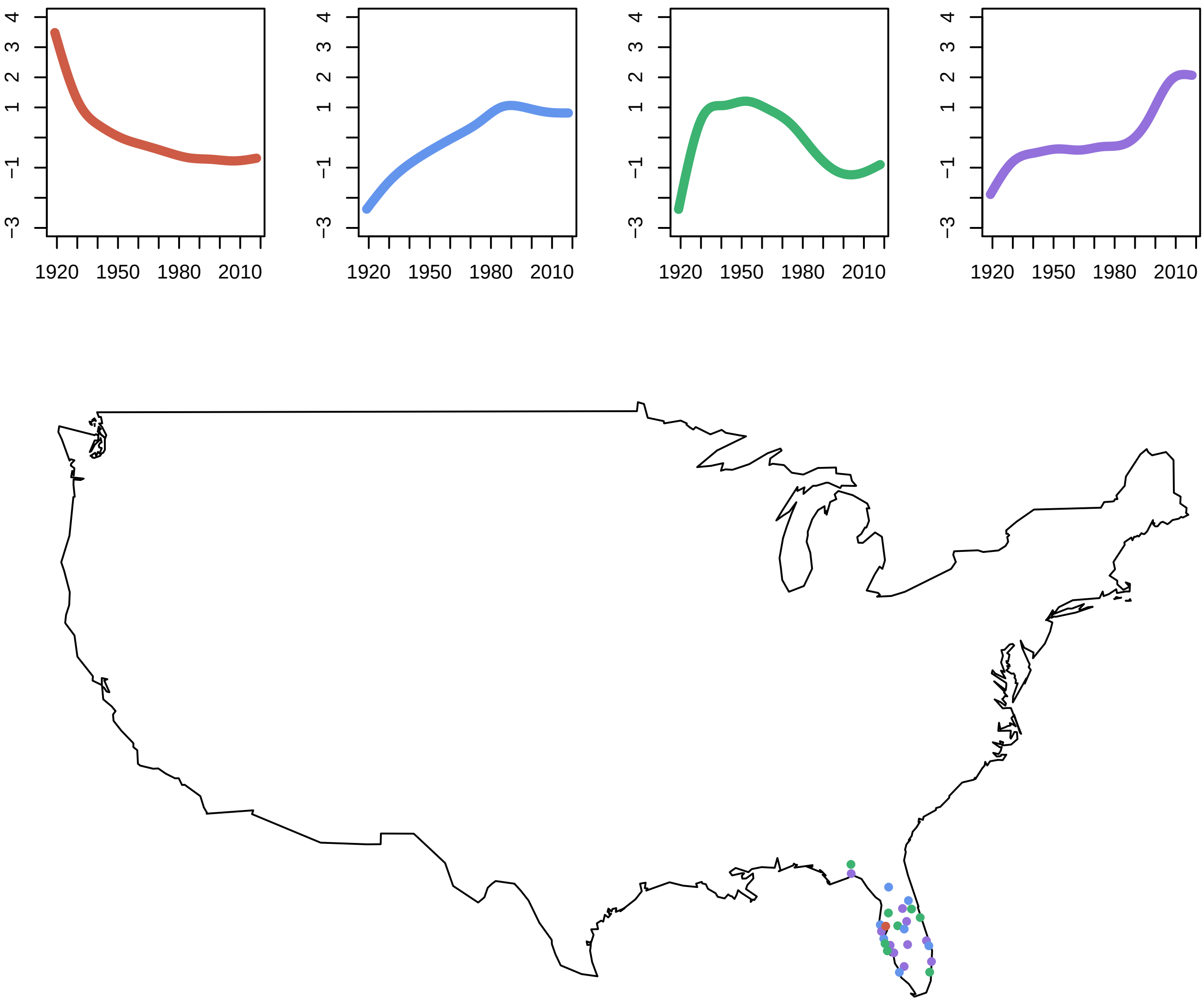

Long−eared Owl

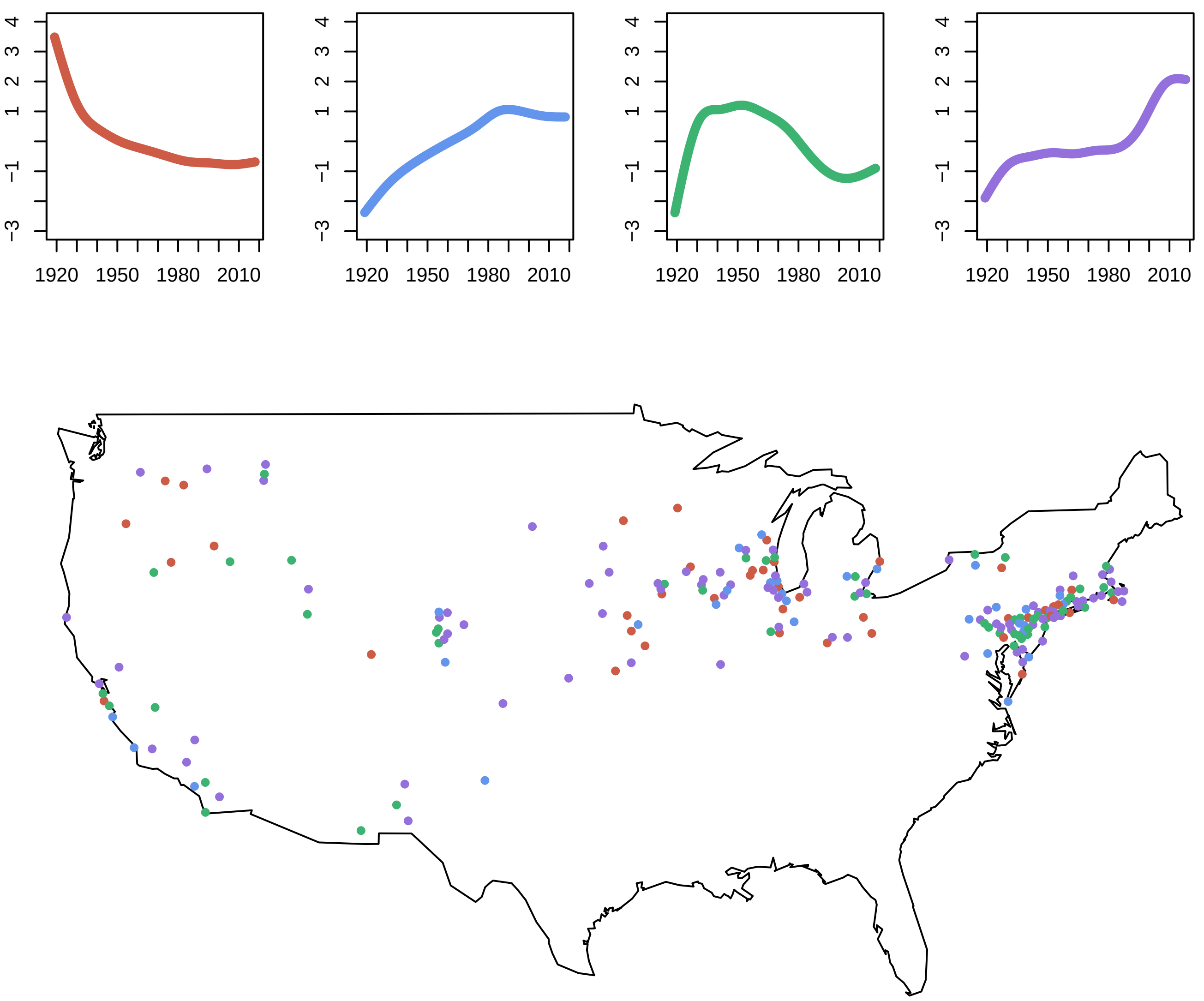

McCown's Longspur

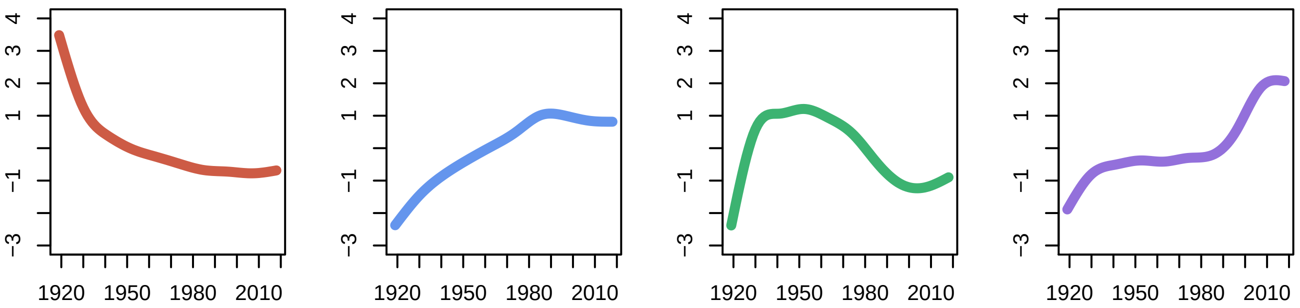

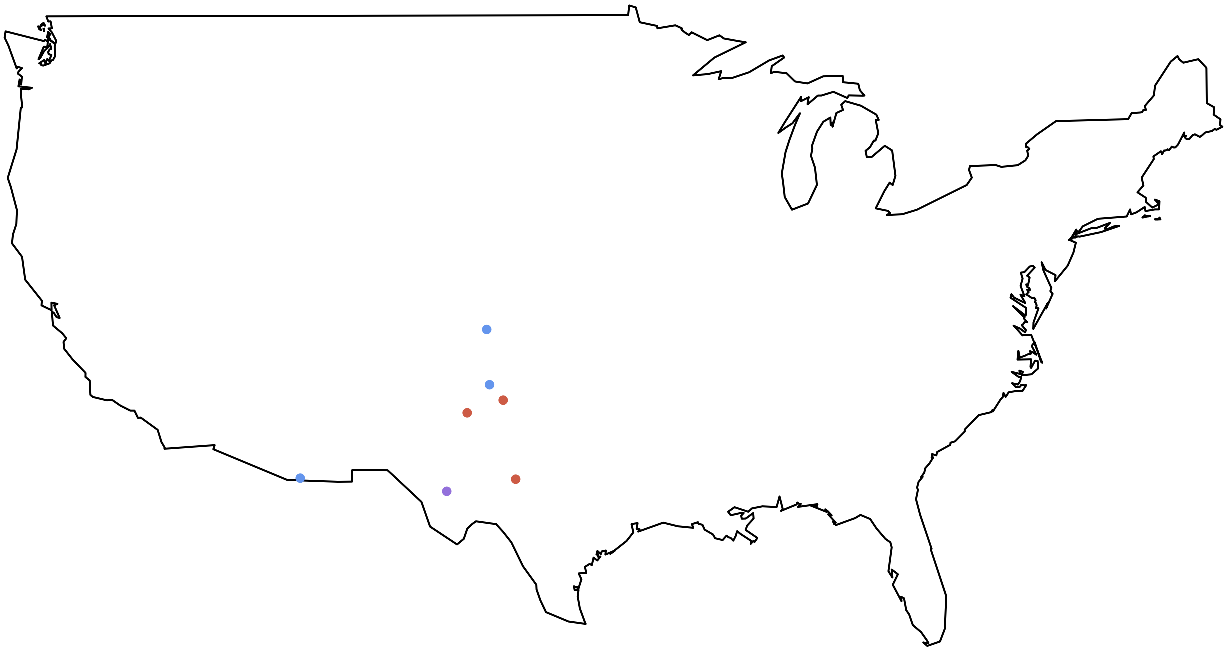

Northern Rough−winged Swallow

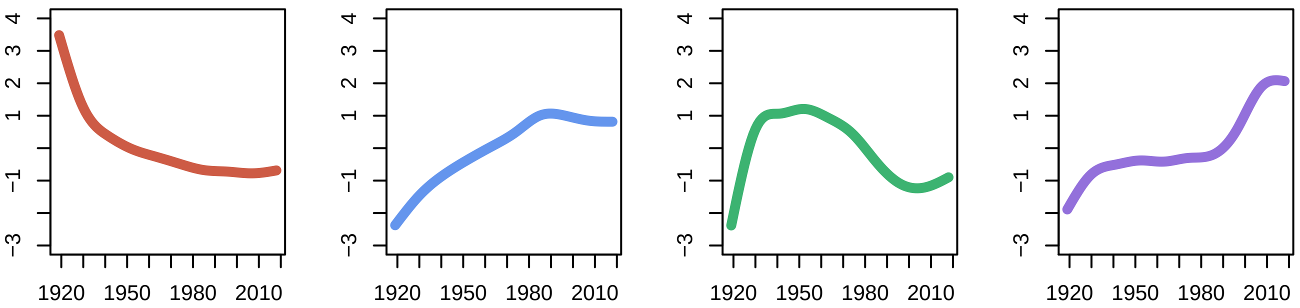

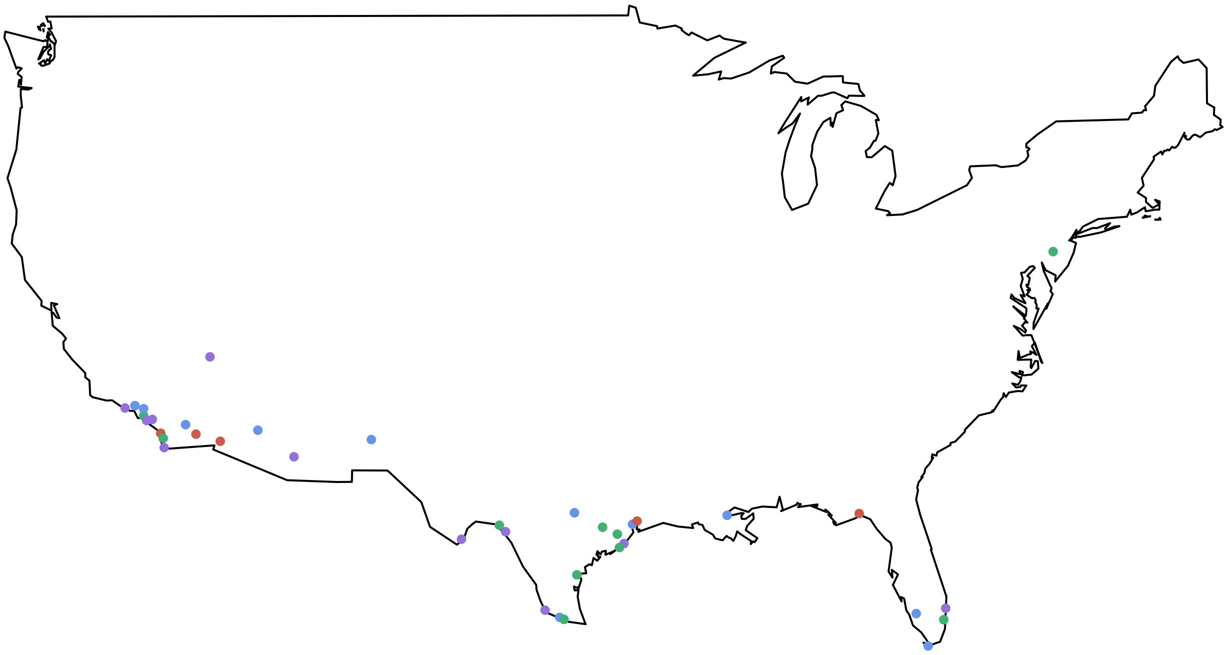

Pacific Wren

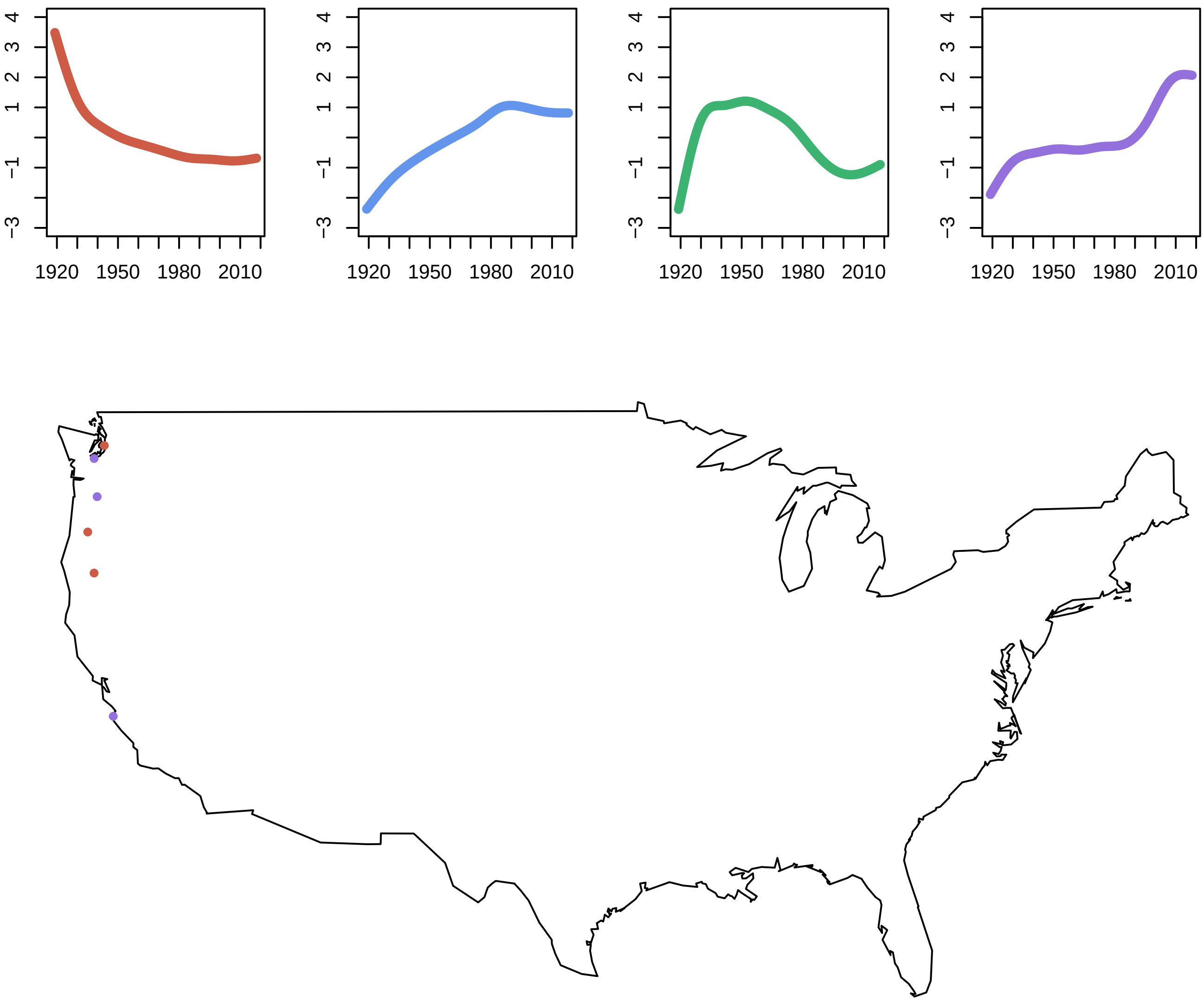

Painted Bunting

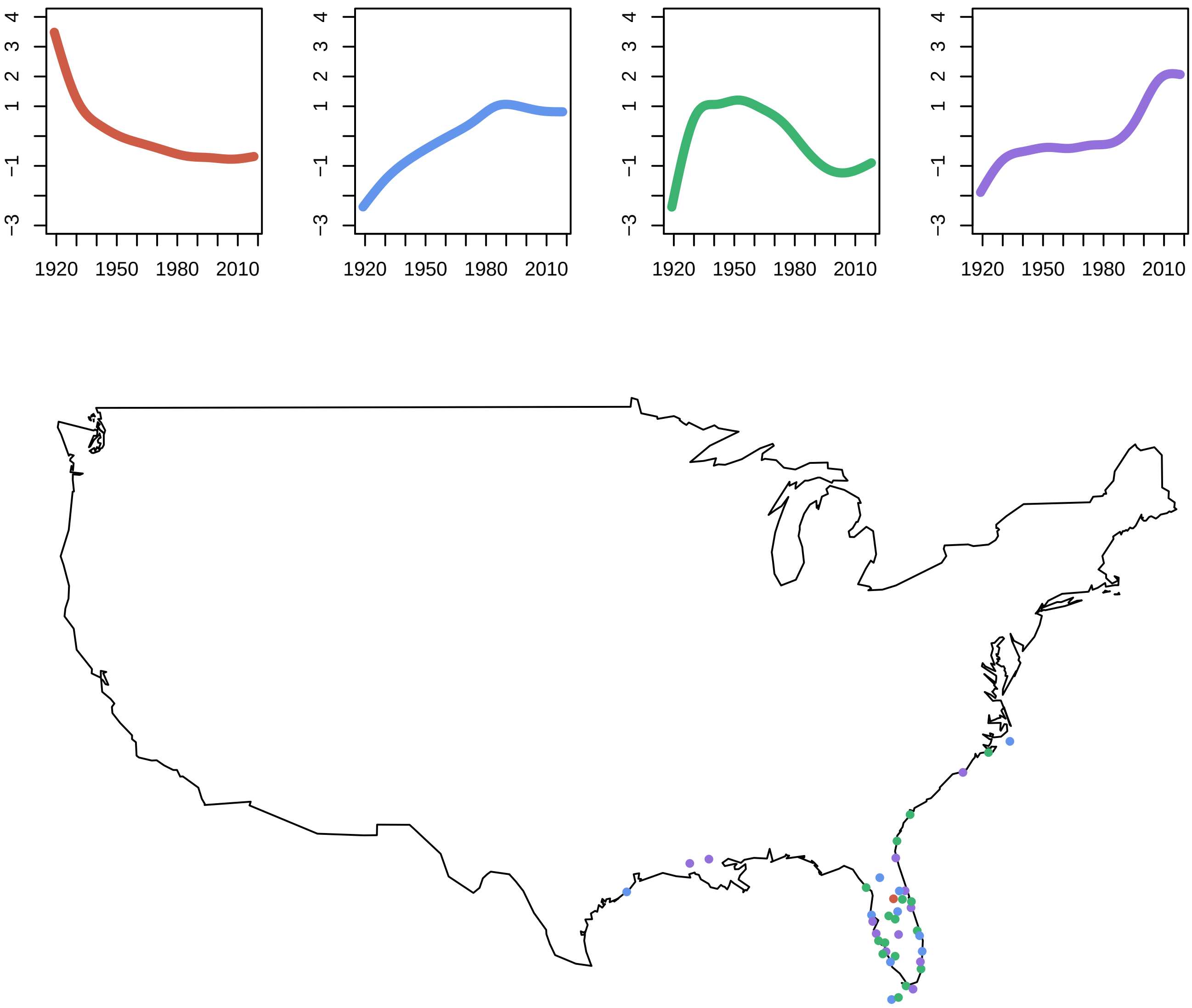

Plain Chachalaca

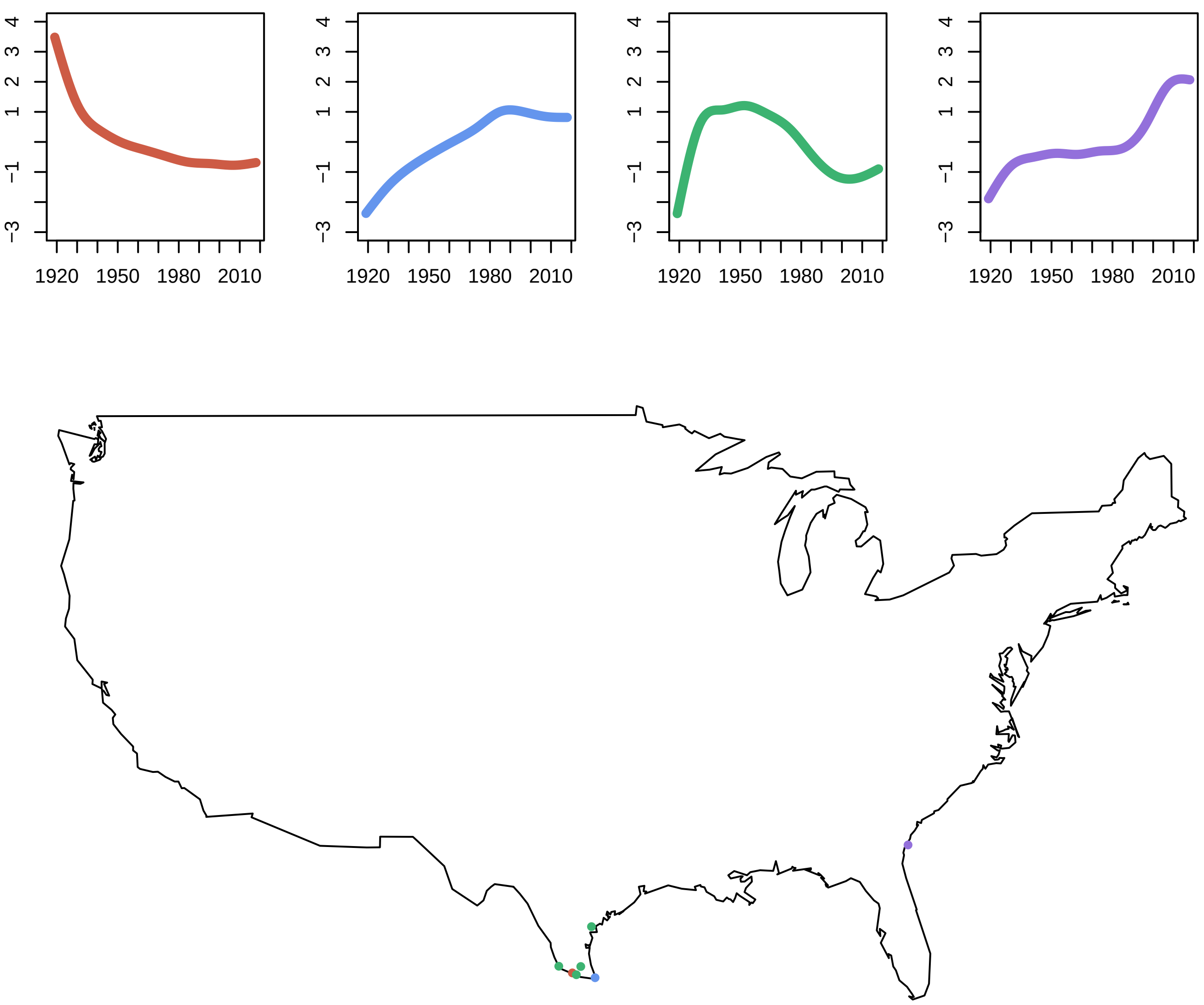

Prairie Falcon

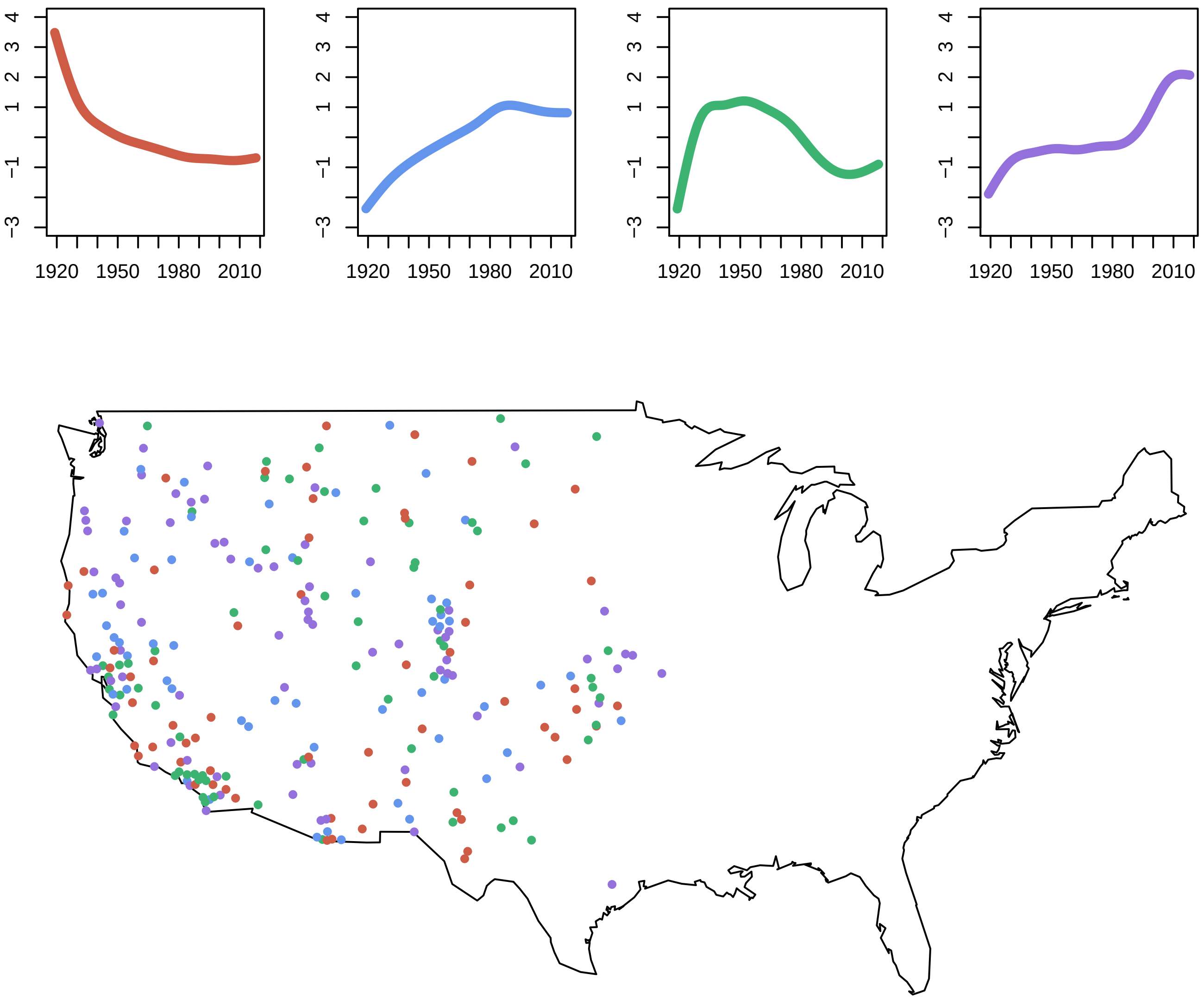

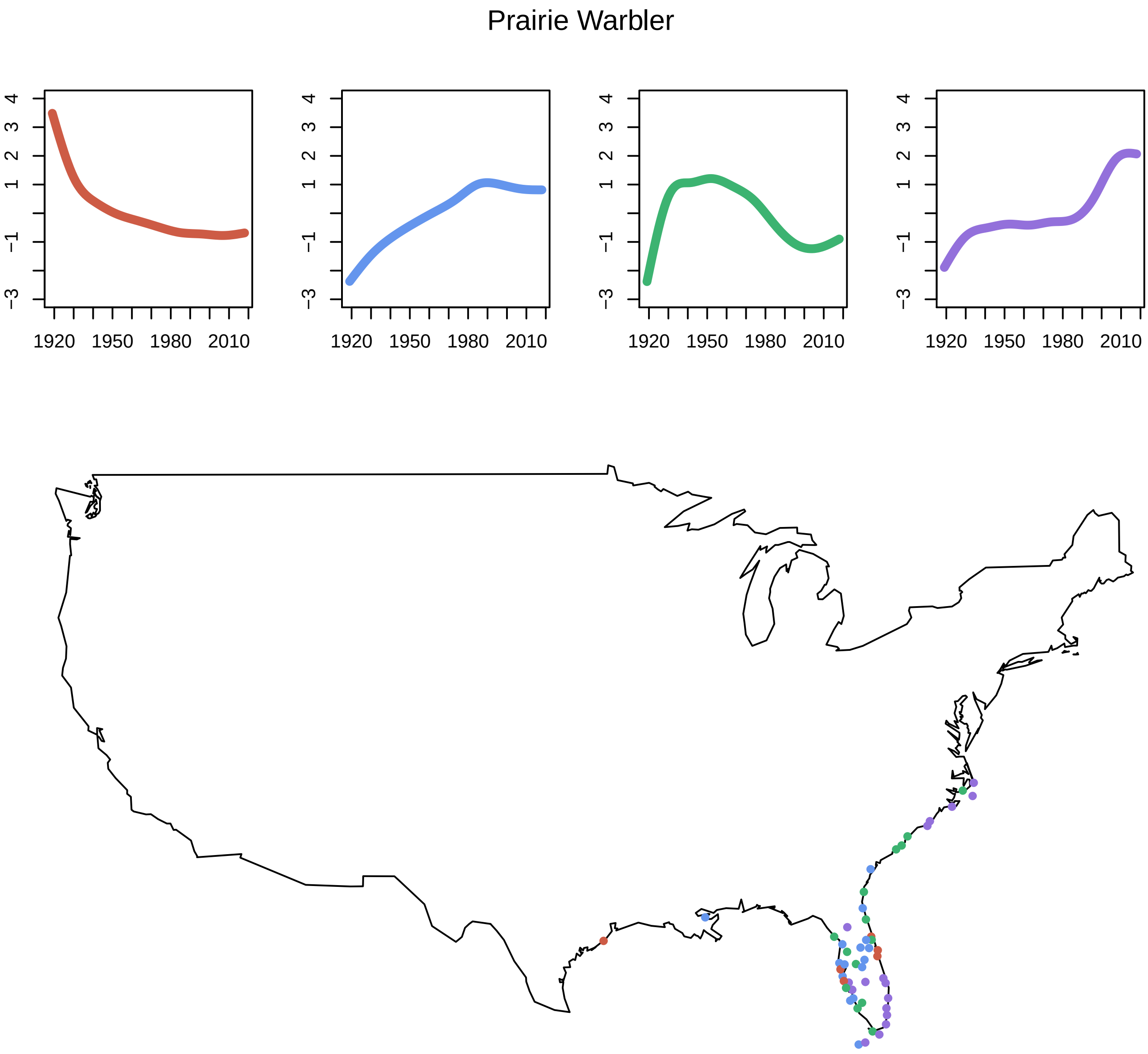

Prairie Warbler
−3
−1
1
2
3
4
1920
1950
1980
2010

Red−breasted Sapsucker

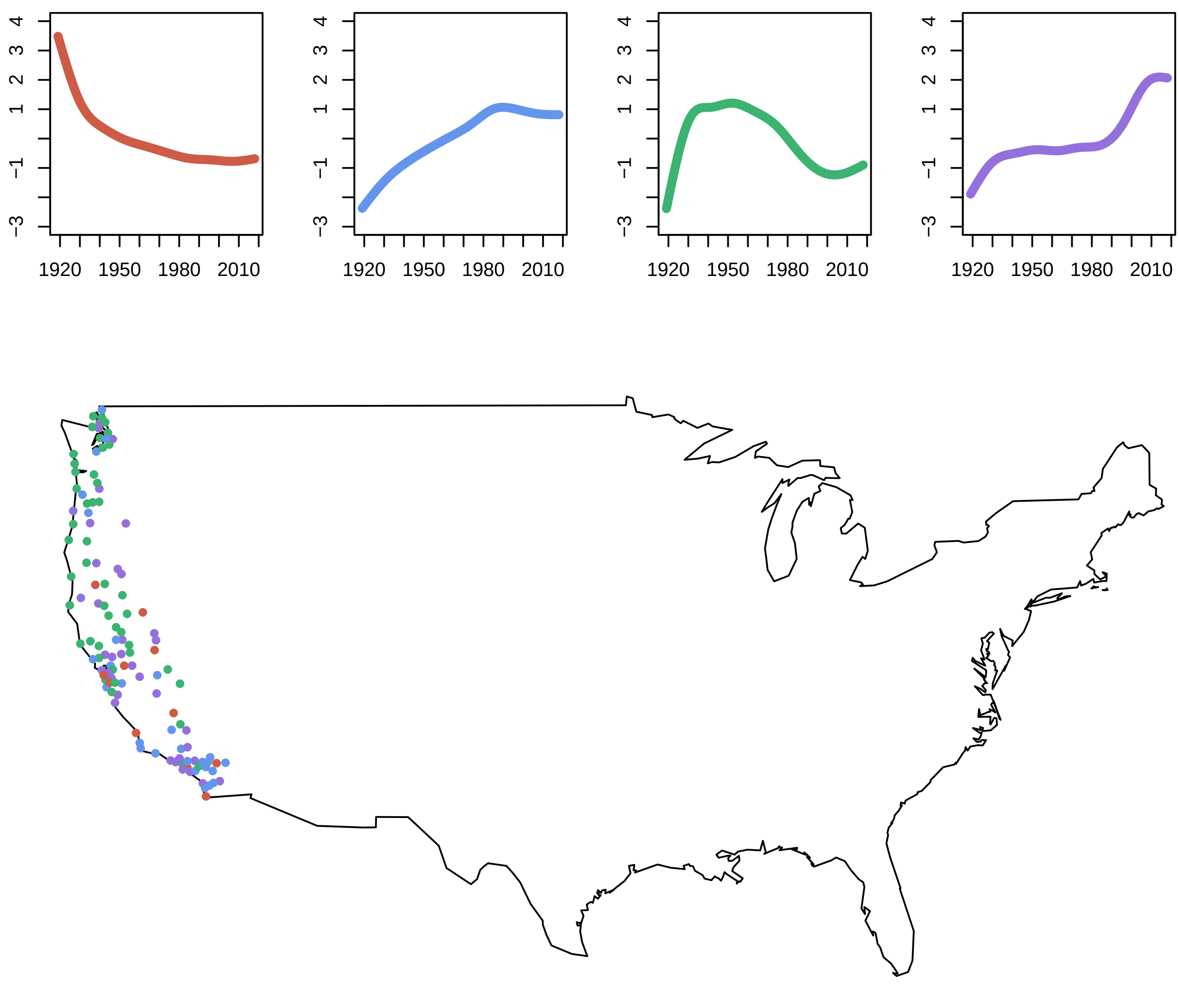

Reddish Egret

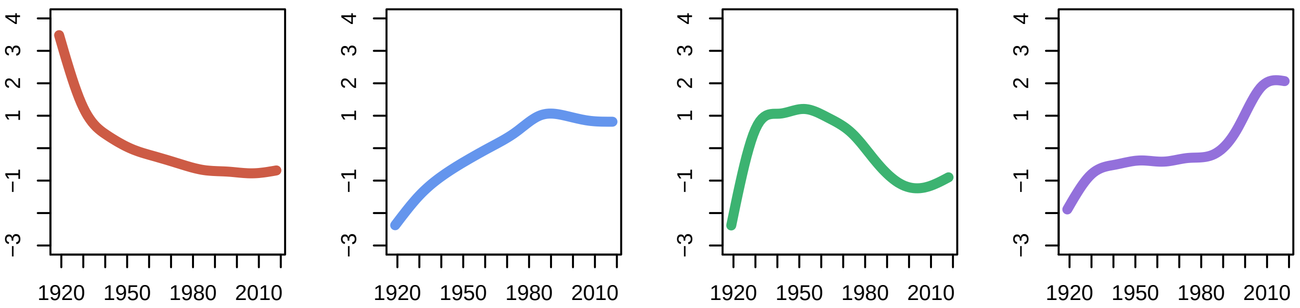

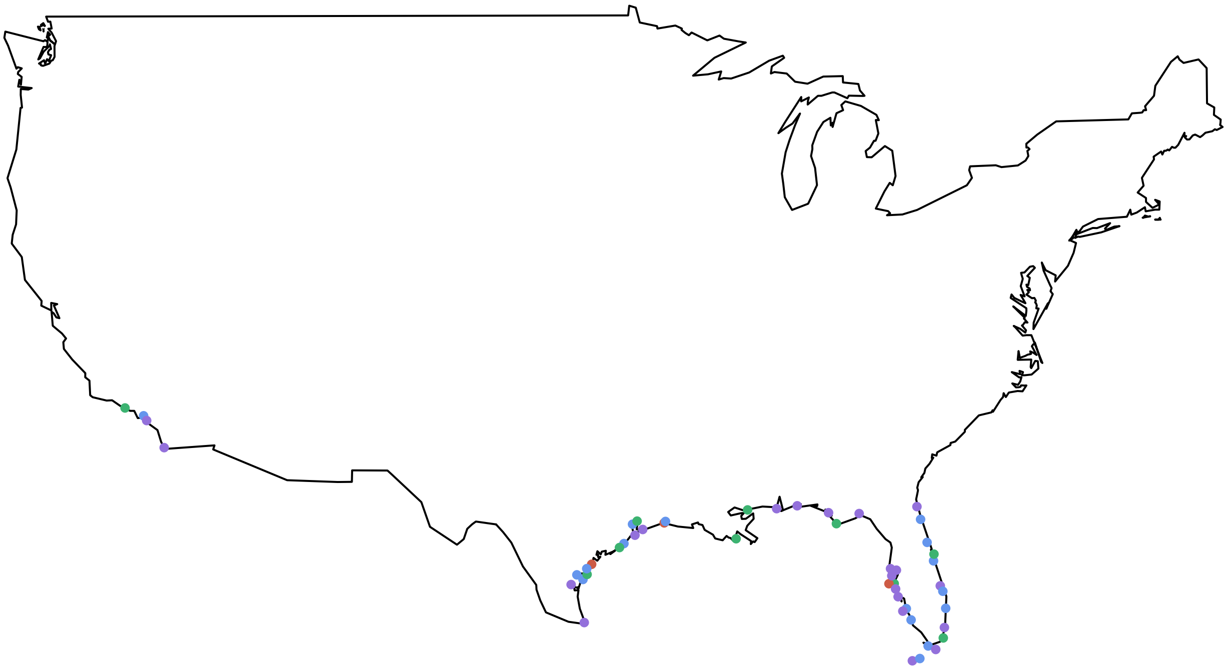

Smith's Longspur

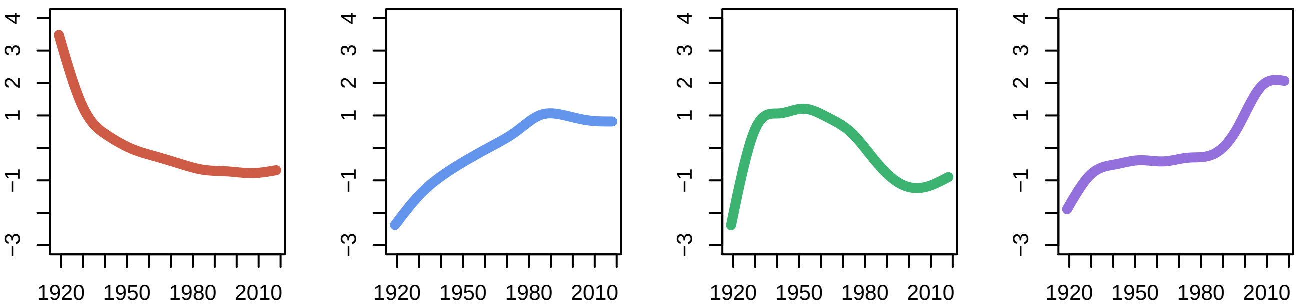

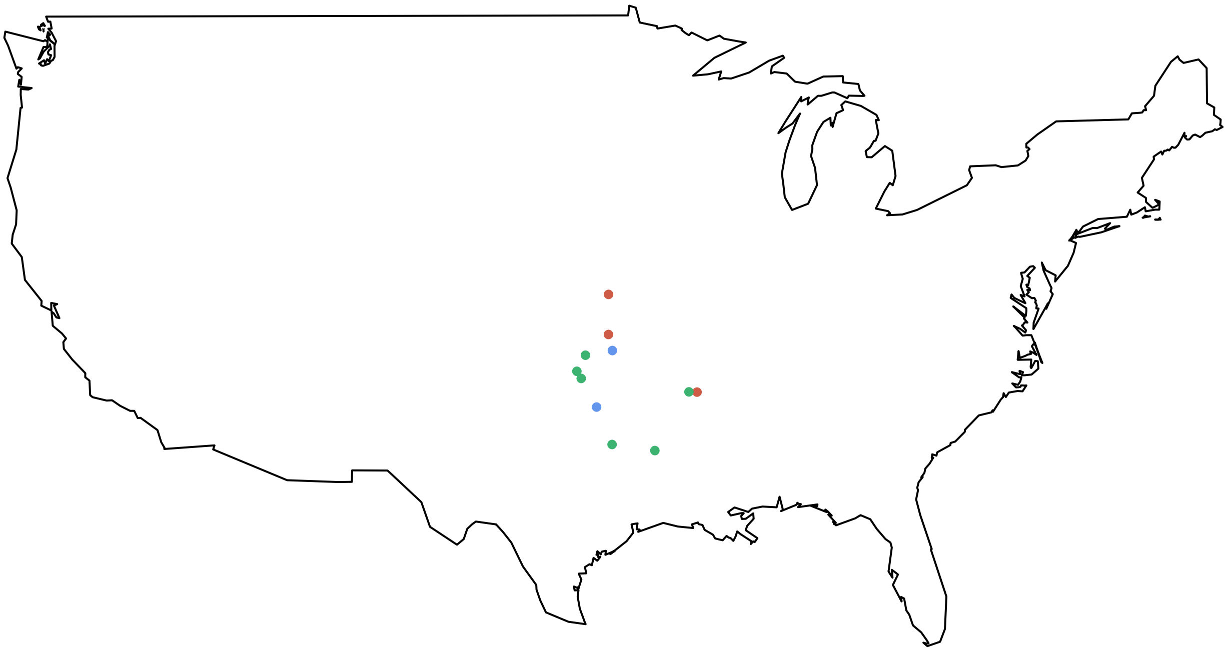

Smooth−billed Ani

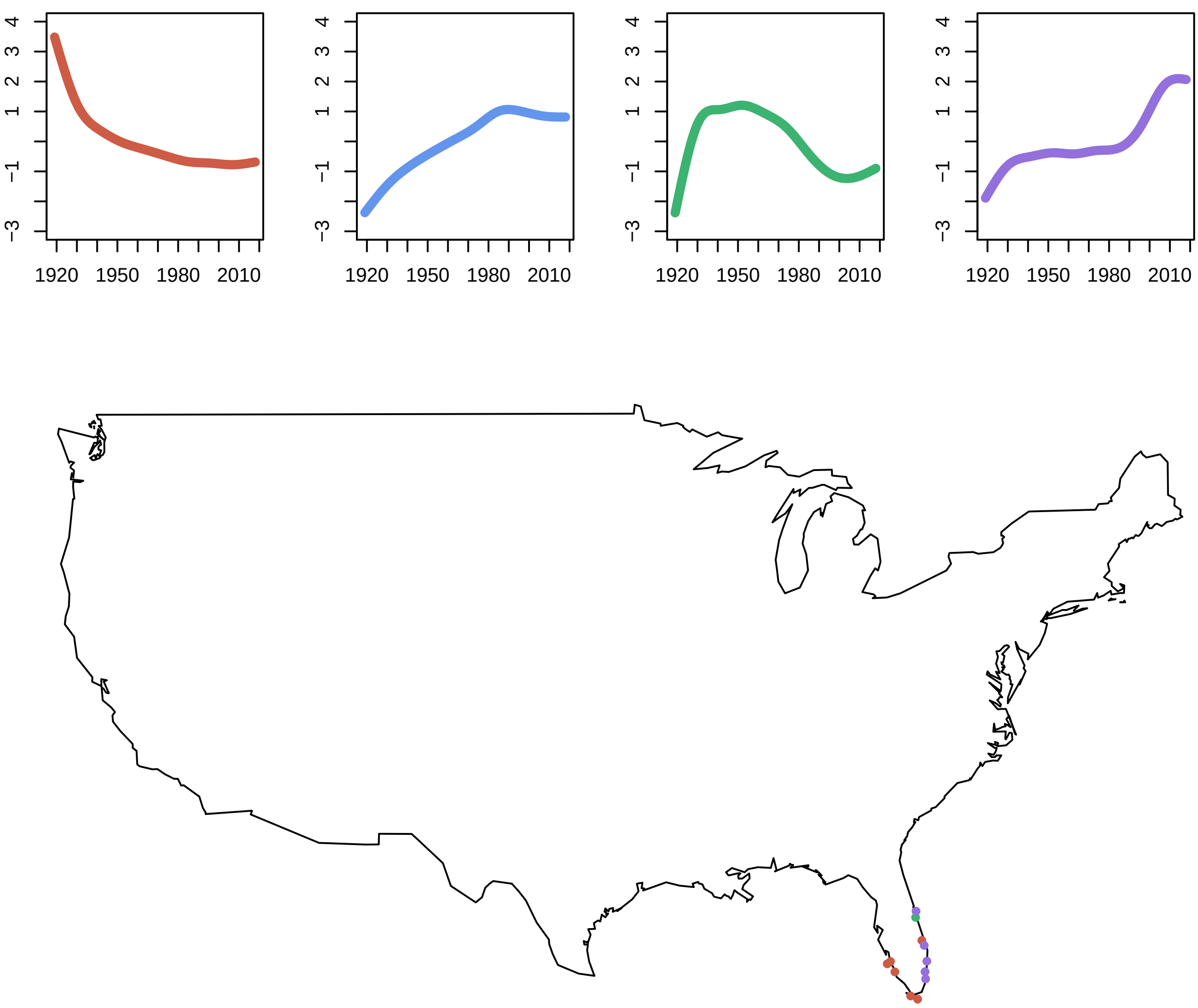

Stilt Sandpiper

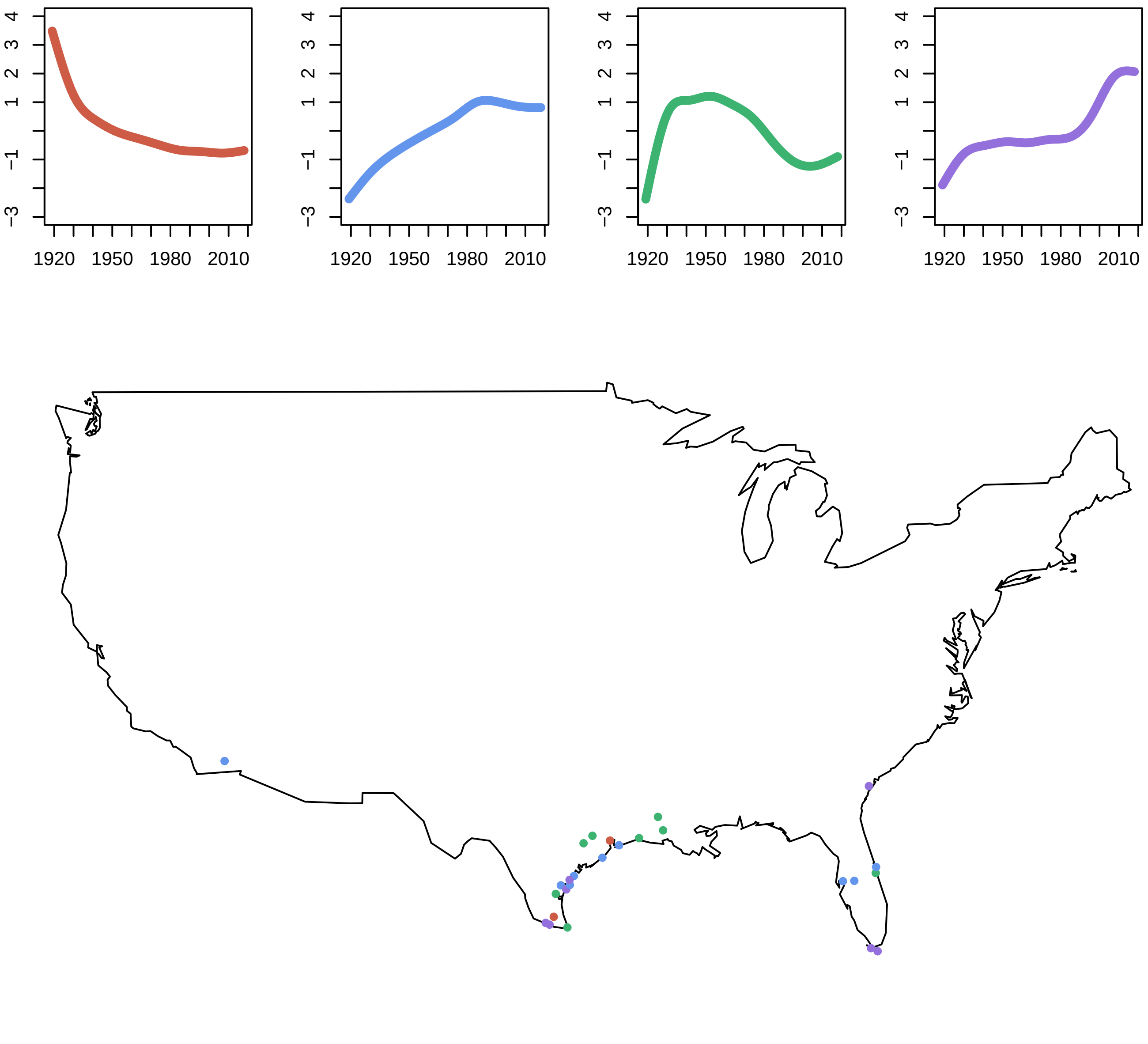

Vermilion Flycatcher

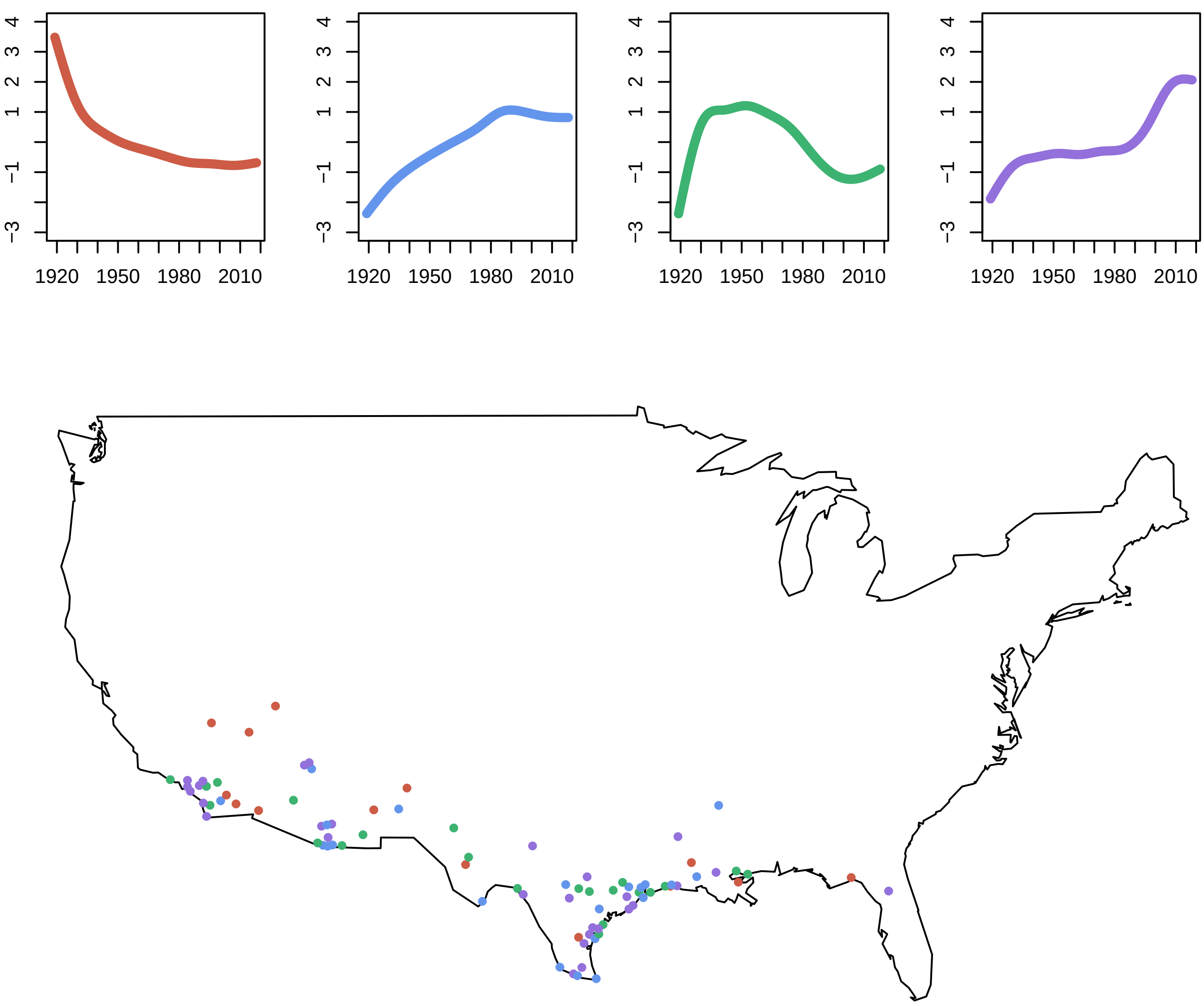

Violet−green Swallow

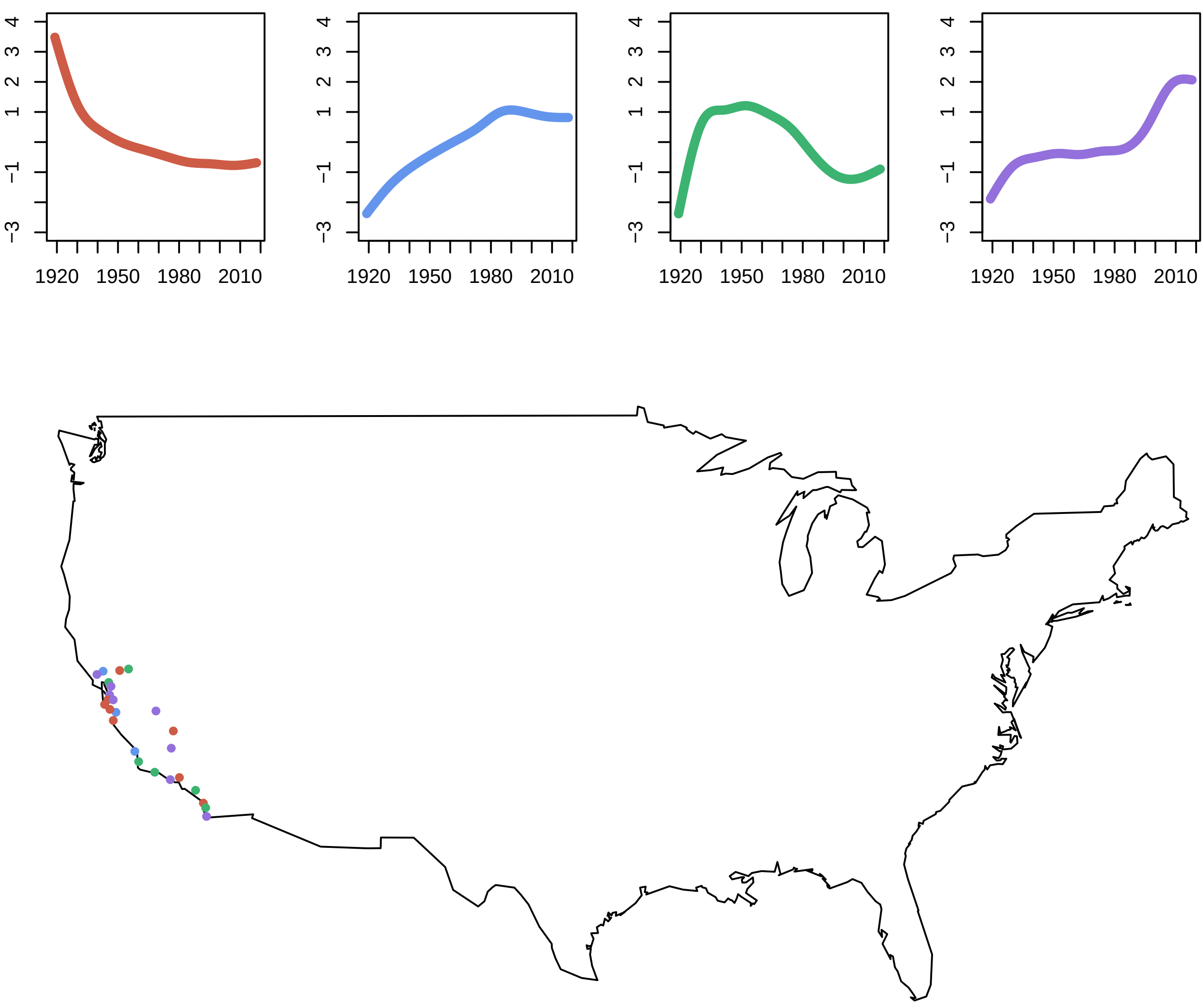

Western Screech−Owl

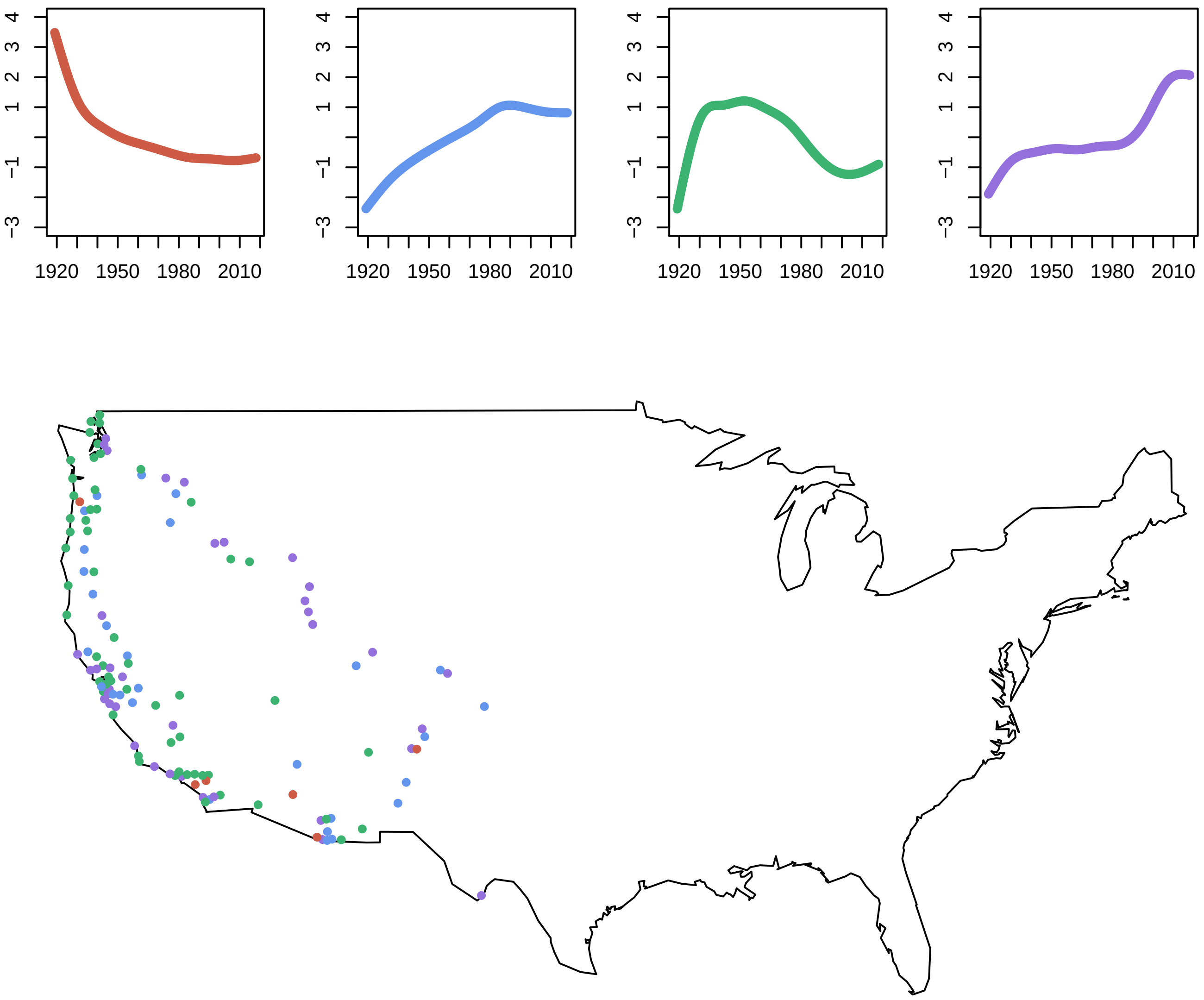

Yellow−eyed Junco

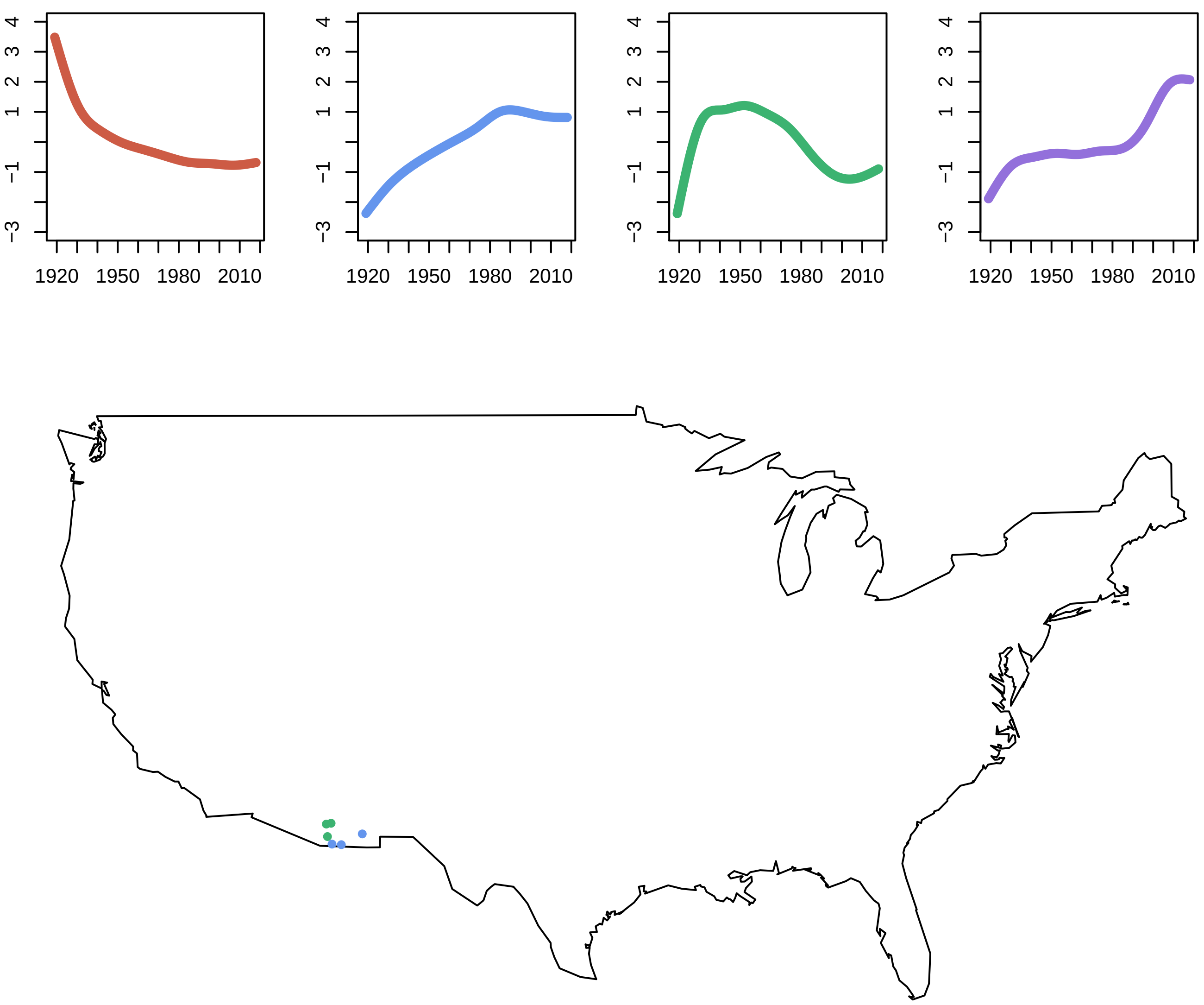

Yellow−throated Warbler

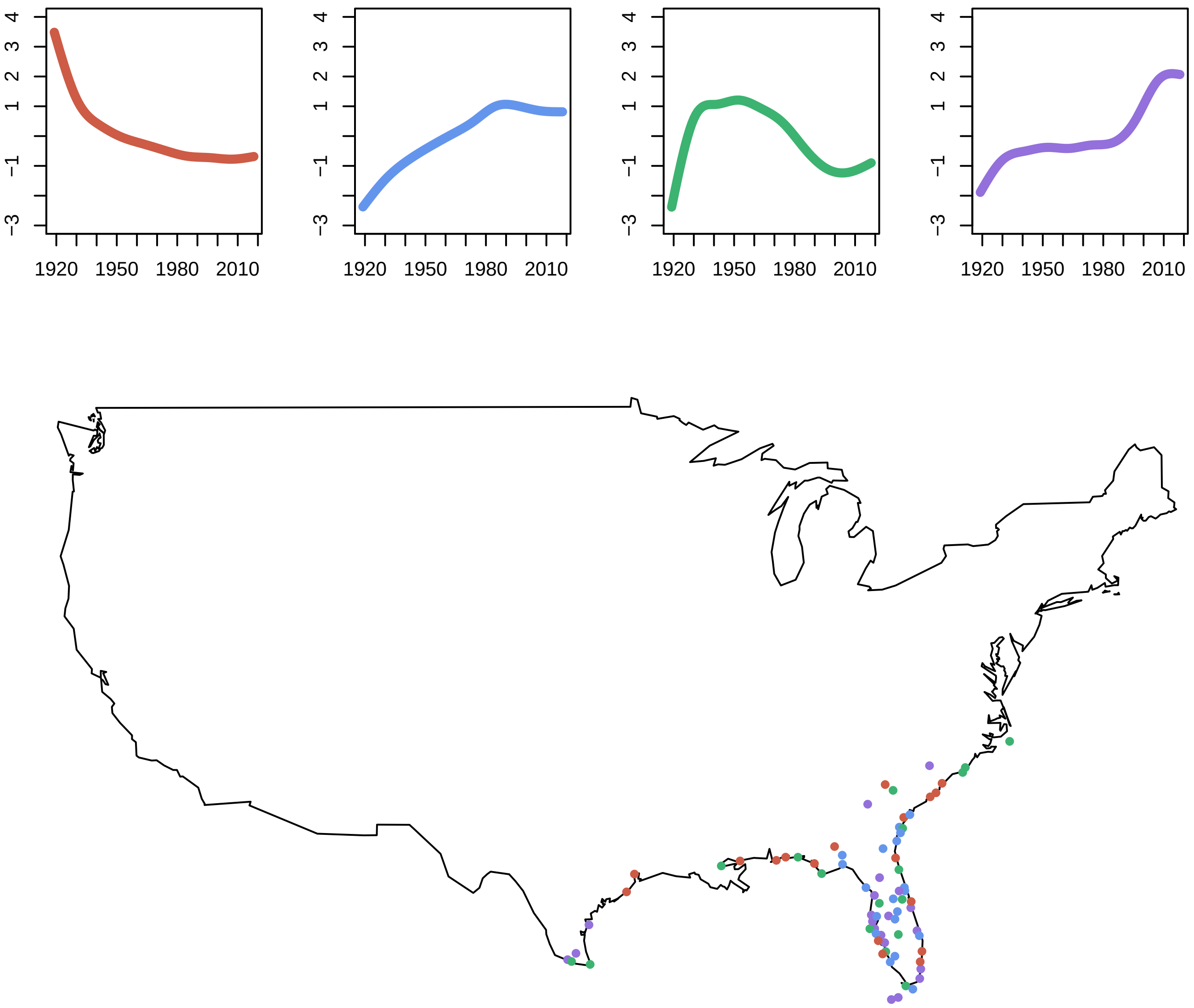

Supplement: Supplementary file 7 — Figure S6 [file ECE3-13-e9781-s005.pdf]
