# Supplementary material for: Spatial and temporal non‐stationarity in long‐term population dynamics of over‐wintering birds of North America
Source: Ecol Evol. 2023 Mar 16;13(3):e9781. doi: 10.1002/ece3.9781 (PMC10019912; doi:10.1002/ece3.9781)
Supplement: Supplementary file 8 — Figure S7 [file ECE3-13-e9781-s004.pdf]

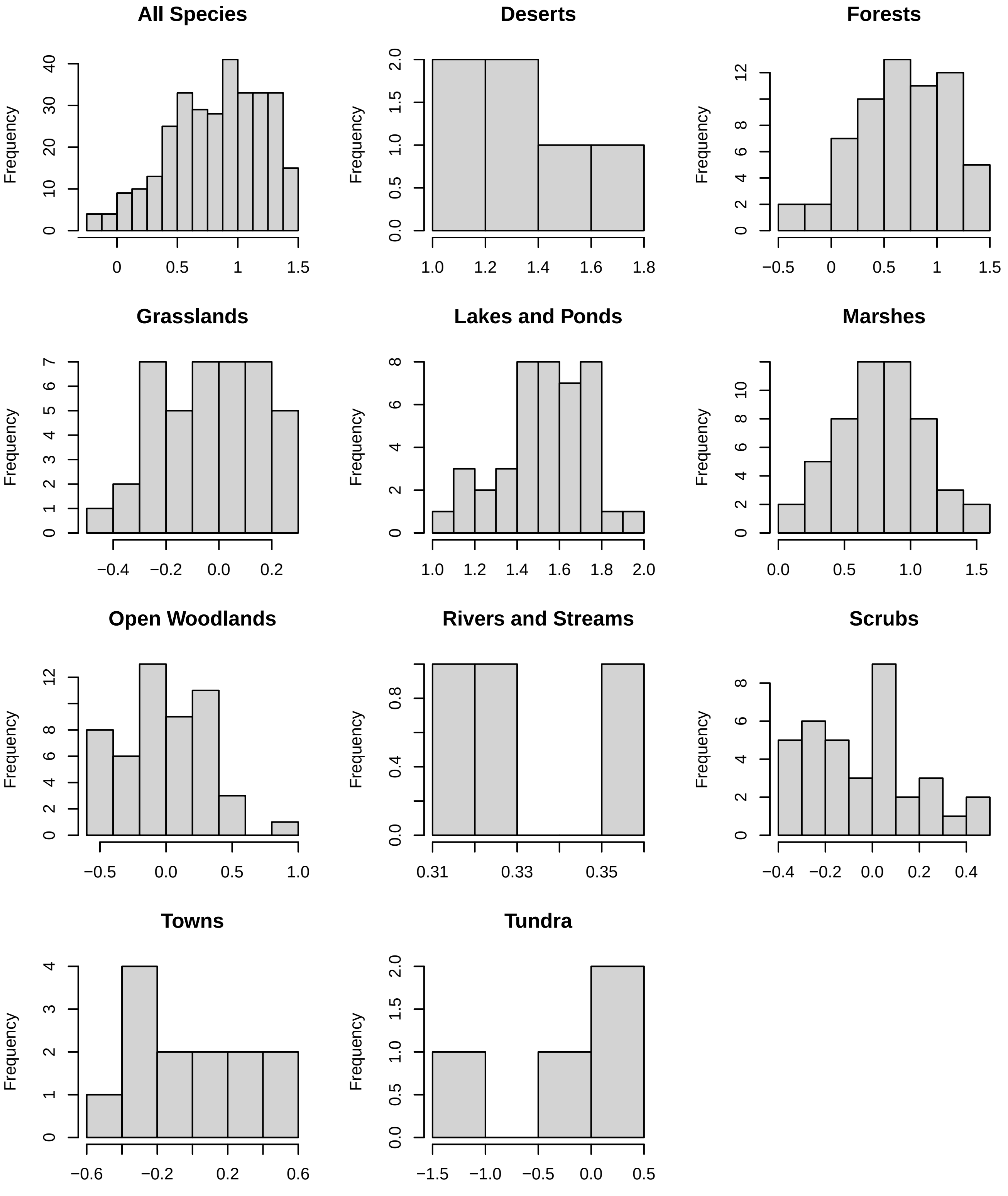
All Species
Frequency
0 10 20 30 40
0 0.5 1 1.5
Deserts
Frequency
0.0 0.5 1.0 1.5 2.0
1.0 1.2 1.4 1.6 1.8
Forests
Frequency
0 2 4 6 8 12
−0.5 0 0.5 1 1.5
Grasslands
Frequency
0 1 2 3 4 5 6 7
−0.4 −0.2 0.0 0.2
Lakes and Ponds
Frequency
0 2 4 6 8
1.0 1.2 1.4 1.6 1.8 2.0
Marshes
Frequency
0 2 4 6 8 10
0.0 0.5 1.0 1.5
Open Woodlands
Frequency
0 2 4 6 8 12
−0.5 0.0 0.5 1.0
Rivers and Streams
Frequency
0.0 0.4 0.8
0.31 0.33 0.35
Scrubs
Frequency
0 2 4 6 8
−0.4 −0.2 0.0 0.2 0.4
Towns
Frequency
0 1 2 3 4
−0.6 −0.2 0.2 0.6
Tundra
Frequency
0.0 0.5 1.0 1.5 2.0
−1.5 −1.0 −0.5 0.0 0.5
