# Supplementary material for: Spatial and temporal non‐stationarity in long‐term population dynamics of over‐wintering birds of North America
Source: Ecol Evol. 2023 Mar 16;13(3):e9781. doi: 10.1002/ece3.9781 (PMC10019912; doi:10.1002/ece3.9781)
Supplement: Supplementary file 3 — Figure S2 [file ECE3-13-e9781-s002.pdf]

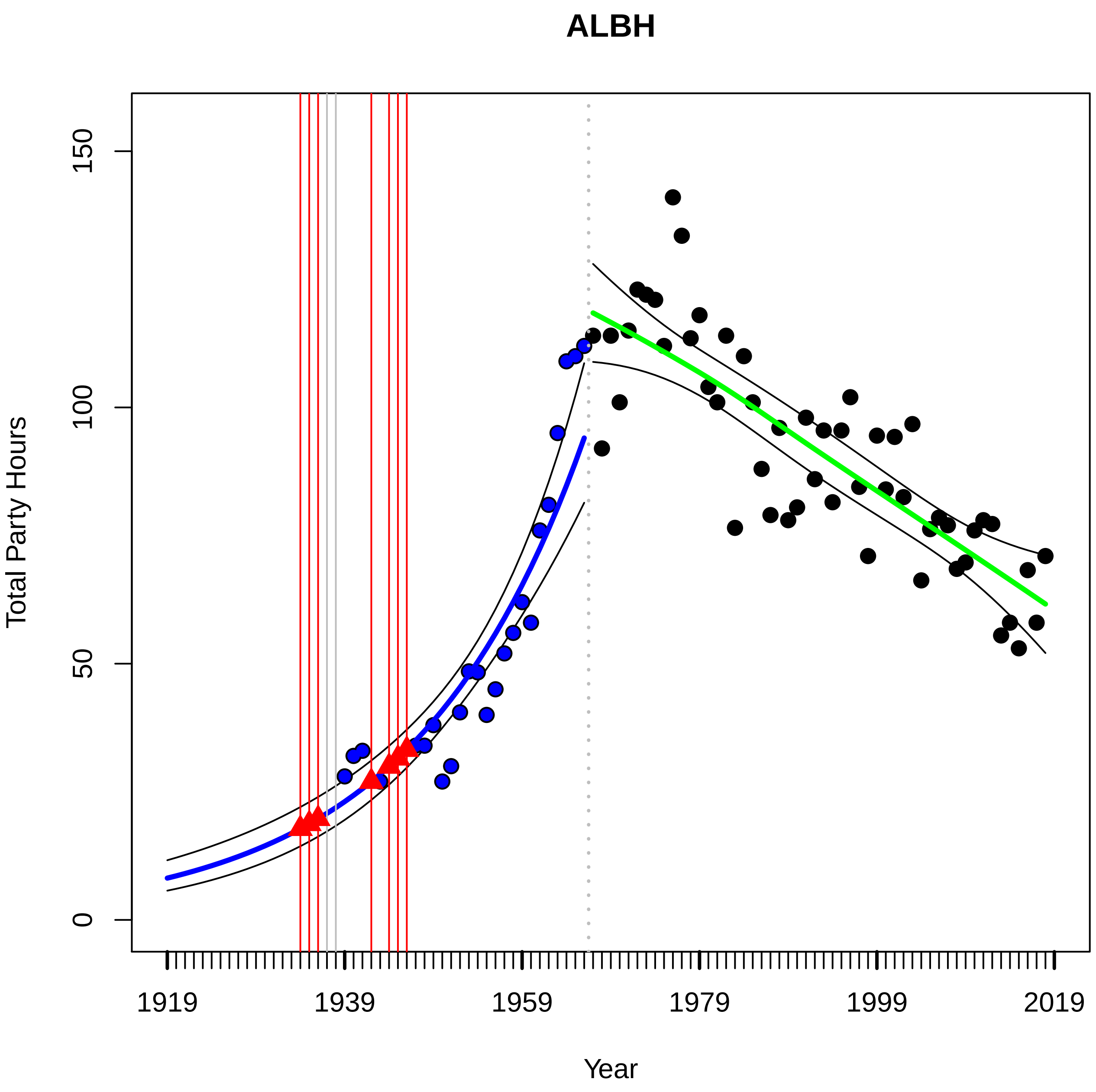

ALBH
Total Party Hours
0
50
100
150
1919
1939
1959
1979
1999
2019
Year

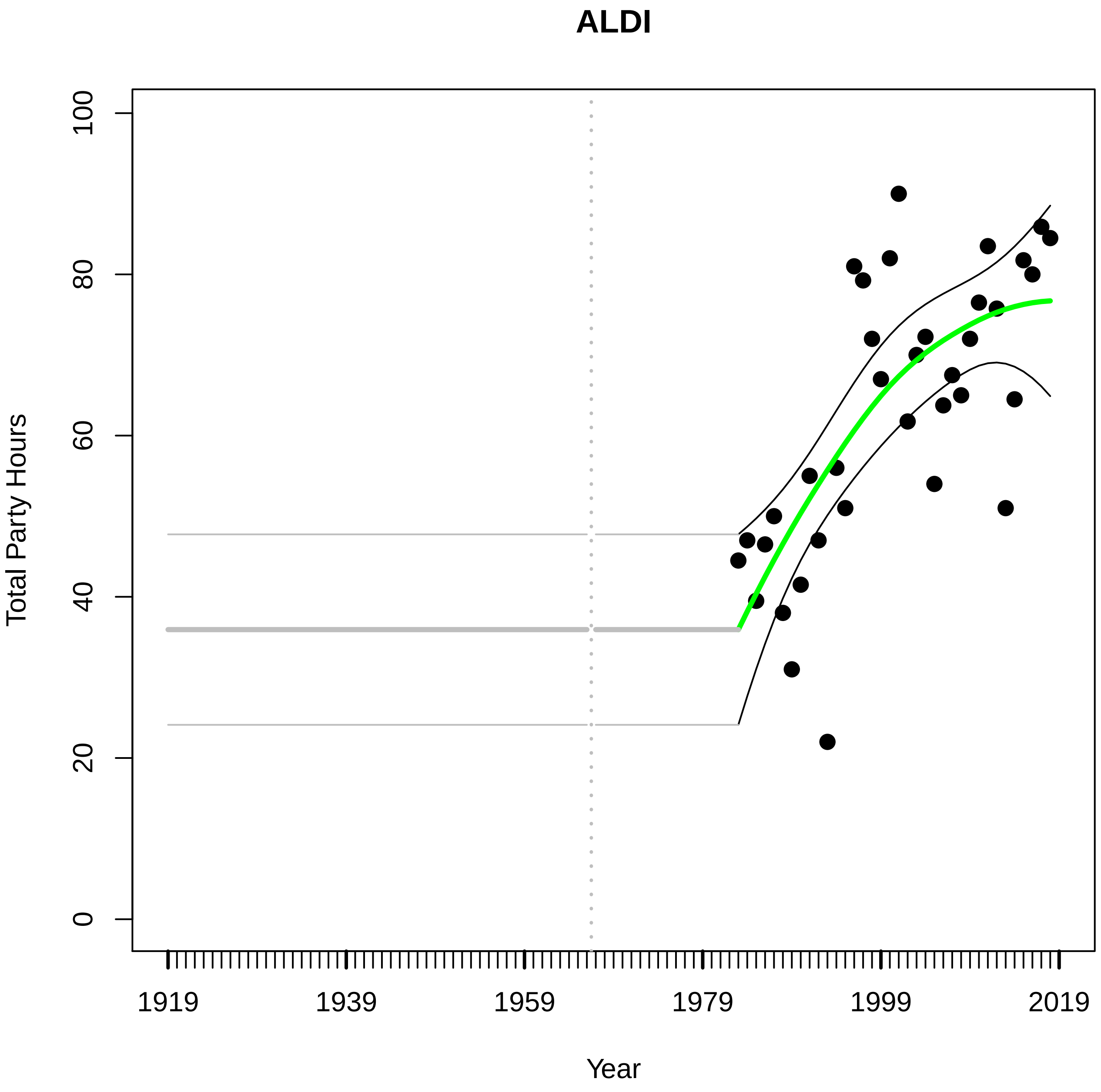

ALDI
Total Party Hours
0
20
40
60
80
100
1919
1939
1959
1979
1999
2019
Year

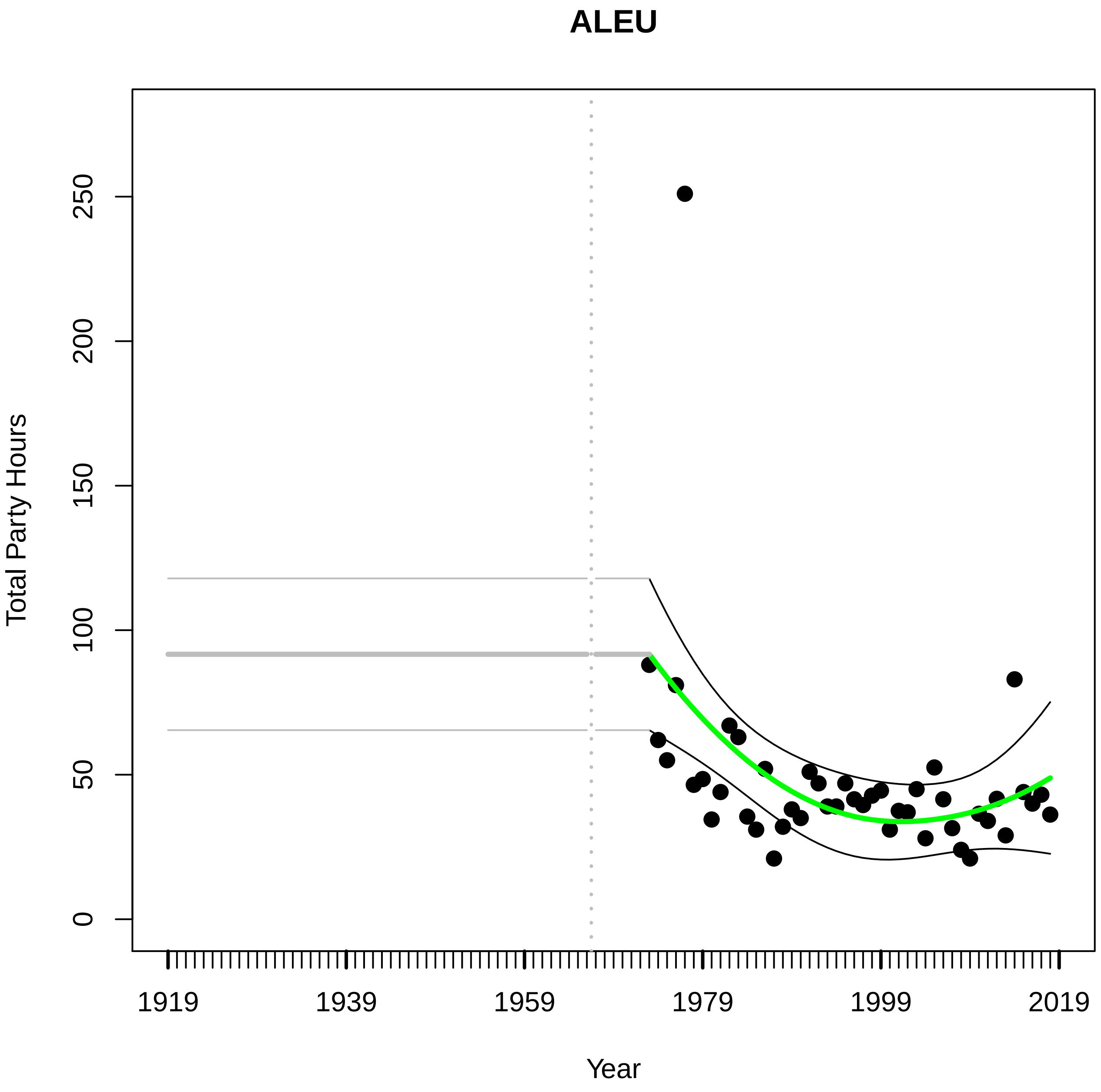

ALEU
Total Party Hours
0
50
100
150
200
250
1919
1939
1959
1979
1999
2019
Year

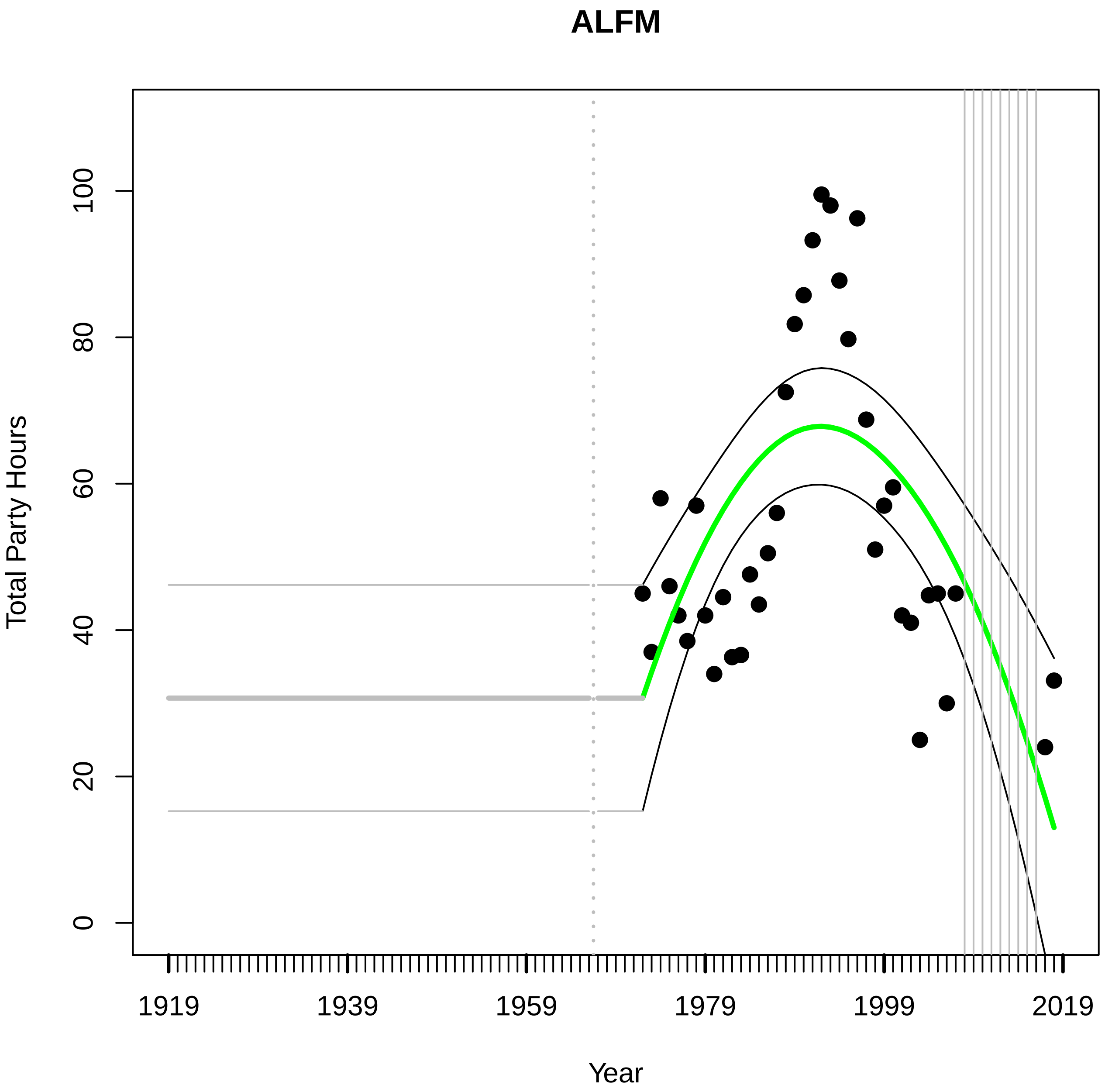

ALFM
Total Party Hours
0
20
40
60
80
100
1919
1939
1959
1979
1999
2019
Year

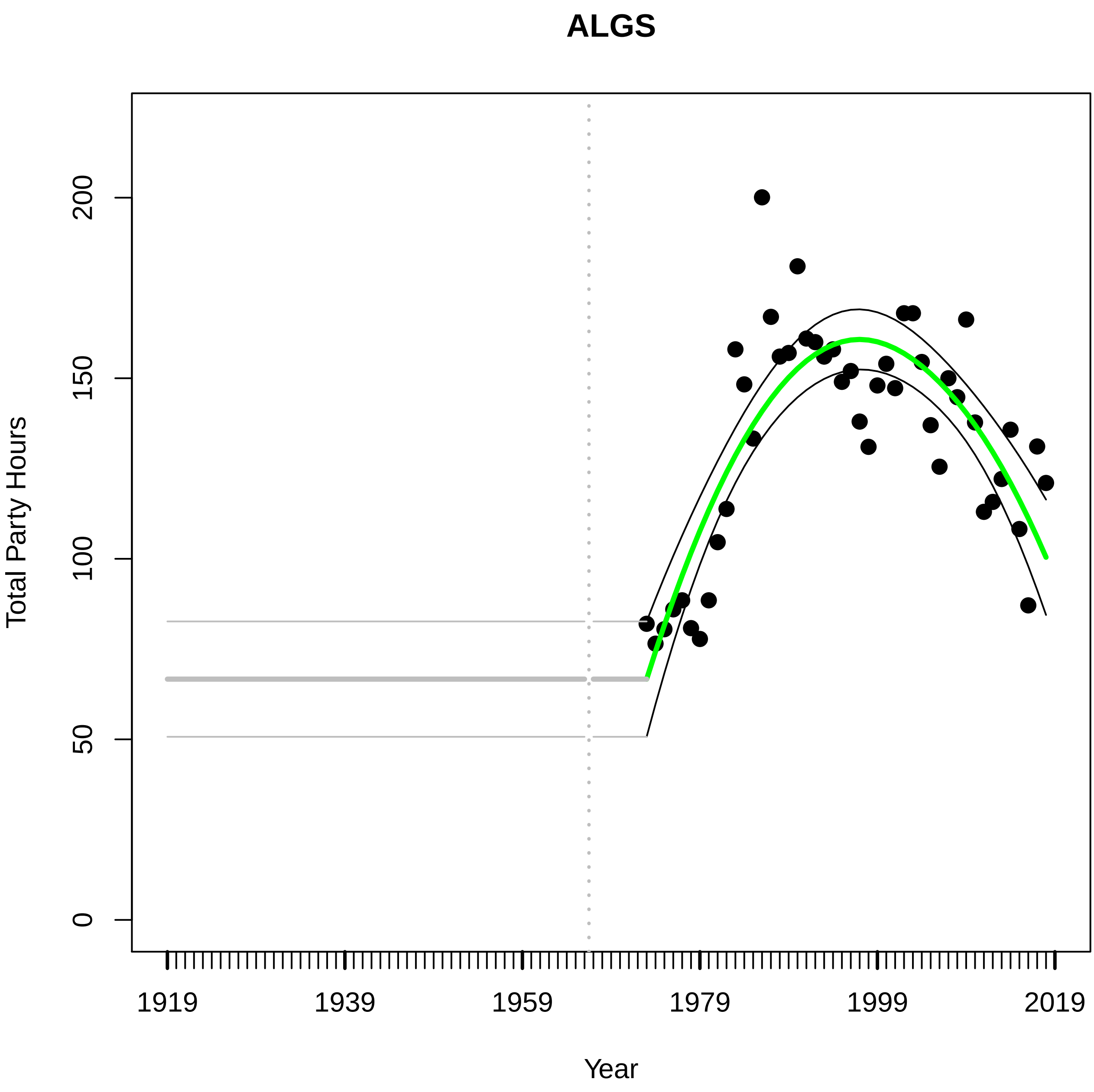

ALGS
Total Party Hours
0
50
100
150
200
1919
1939
1959
1979
1999
2019
Year

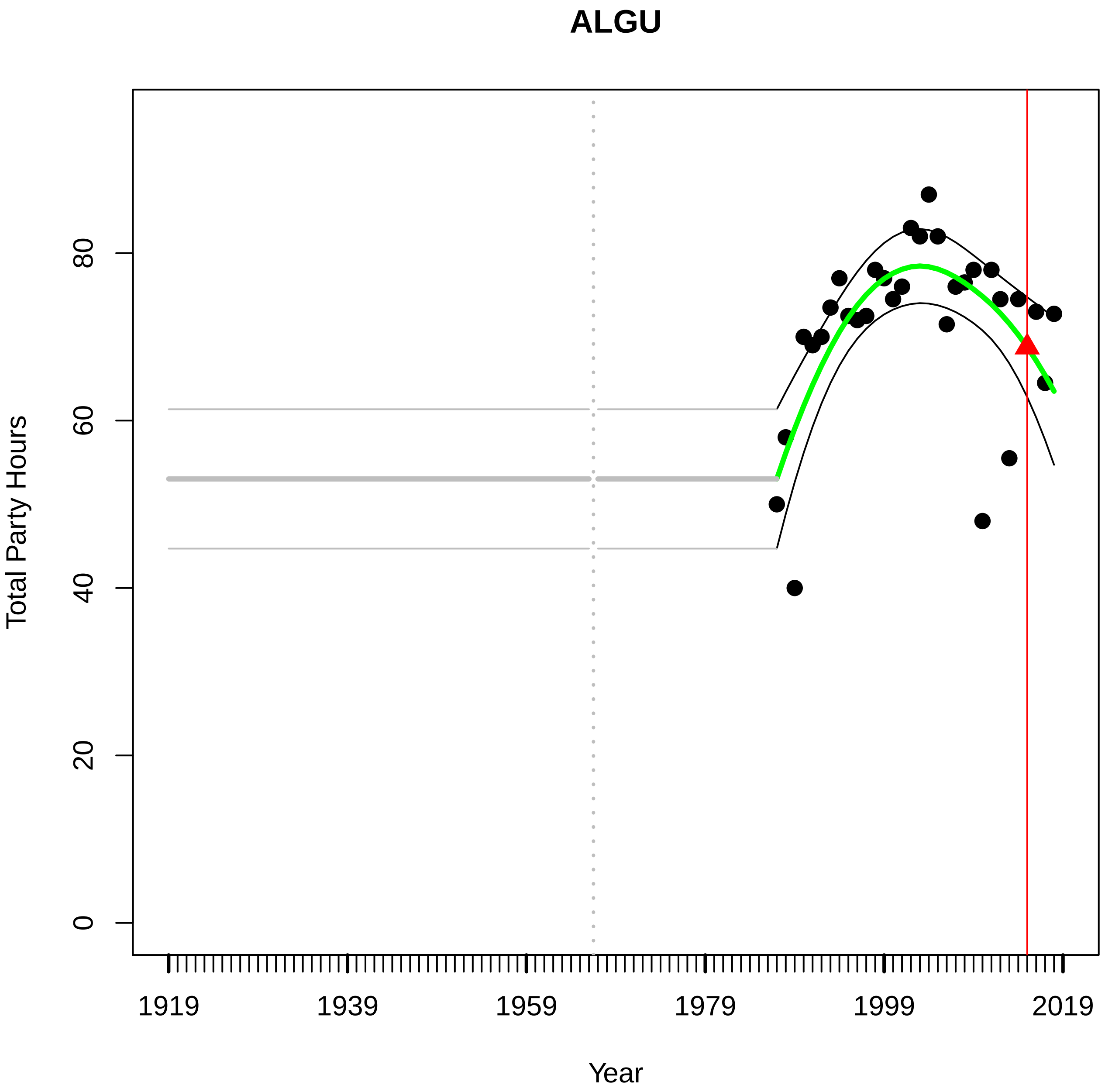

ALGU
Total Party Hours
0
20
40
60
80
1919
1939
1959
1979
1999
2019
Year

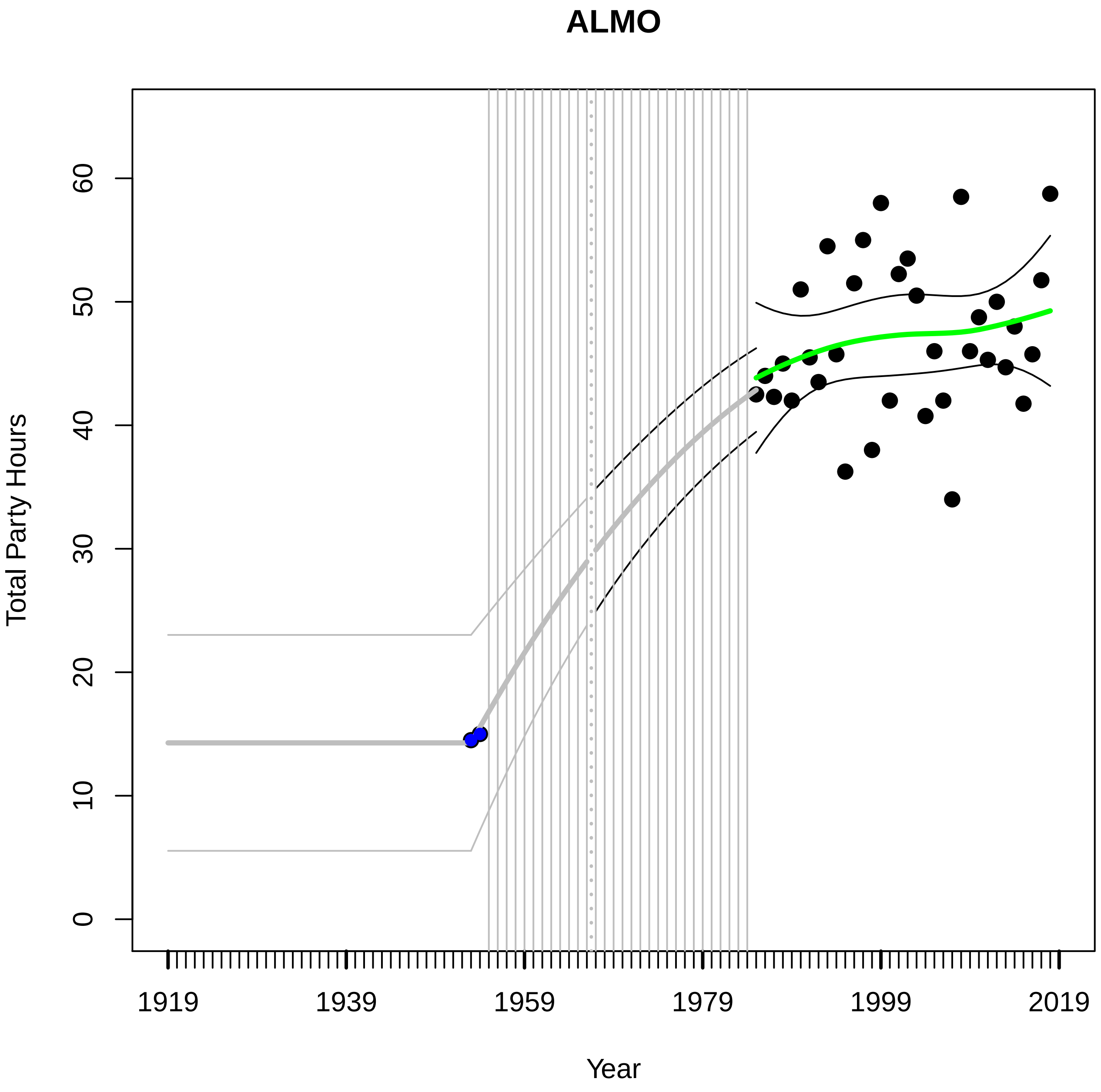
ALMO
Total Party Hours
0
10
20
30
40
50
60
1919
1939
1959
1979
1999
2019
Year

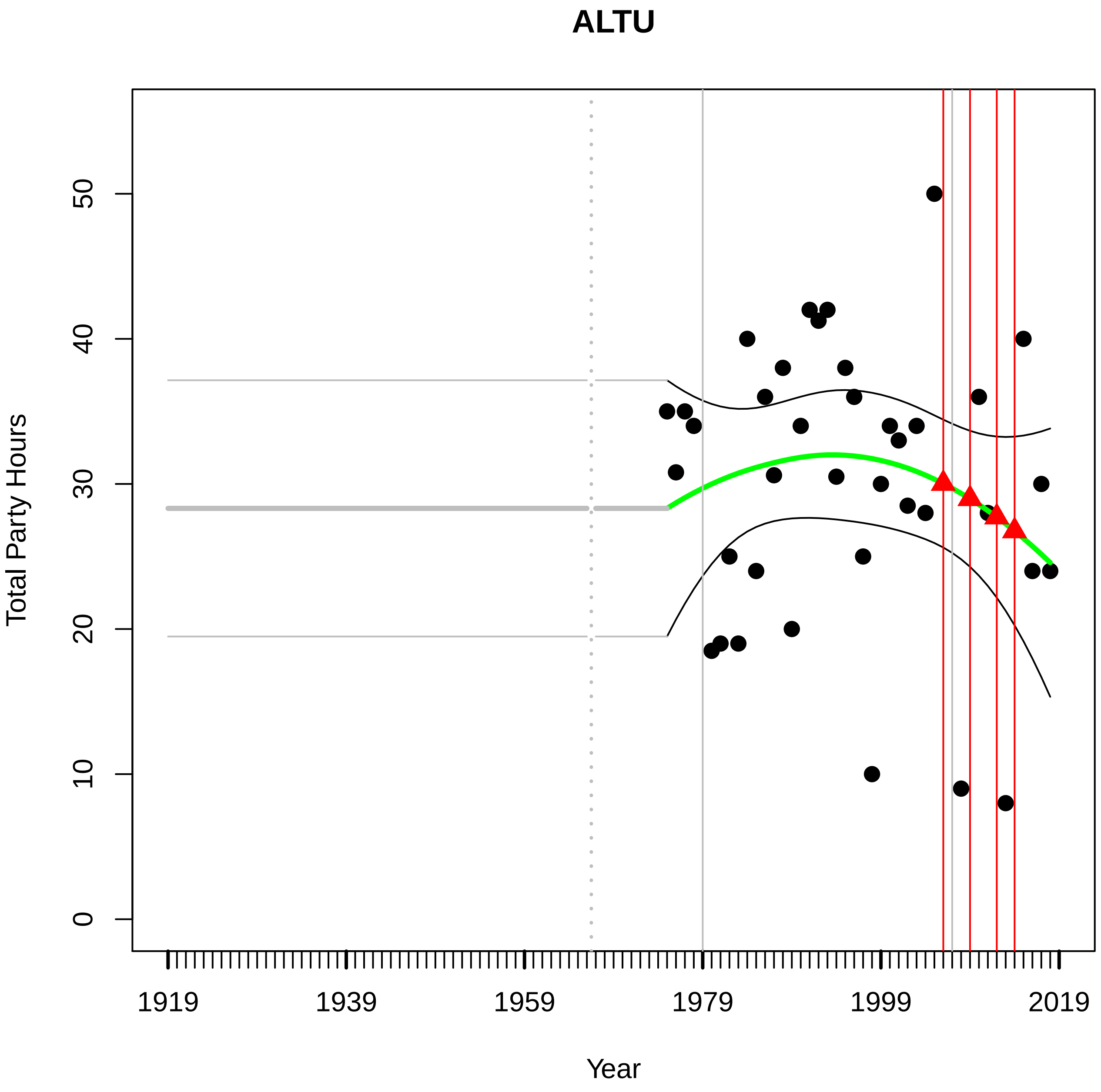
ALTU
Total Party Hours
0
10
20
30
40
50
1919
1939
1959
1979
1999
2019
Year

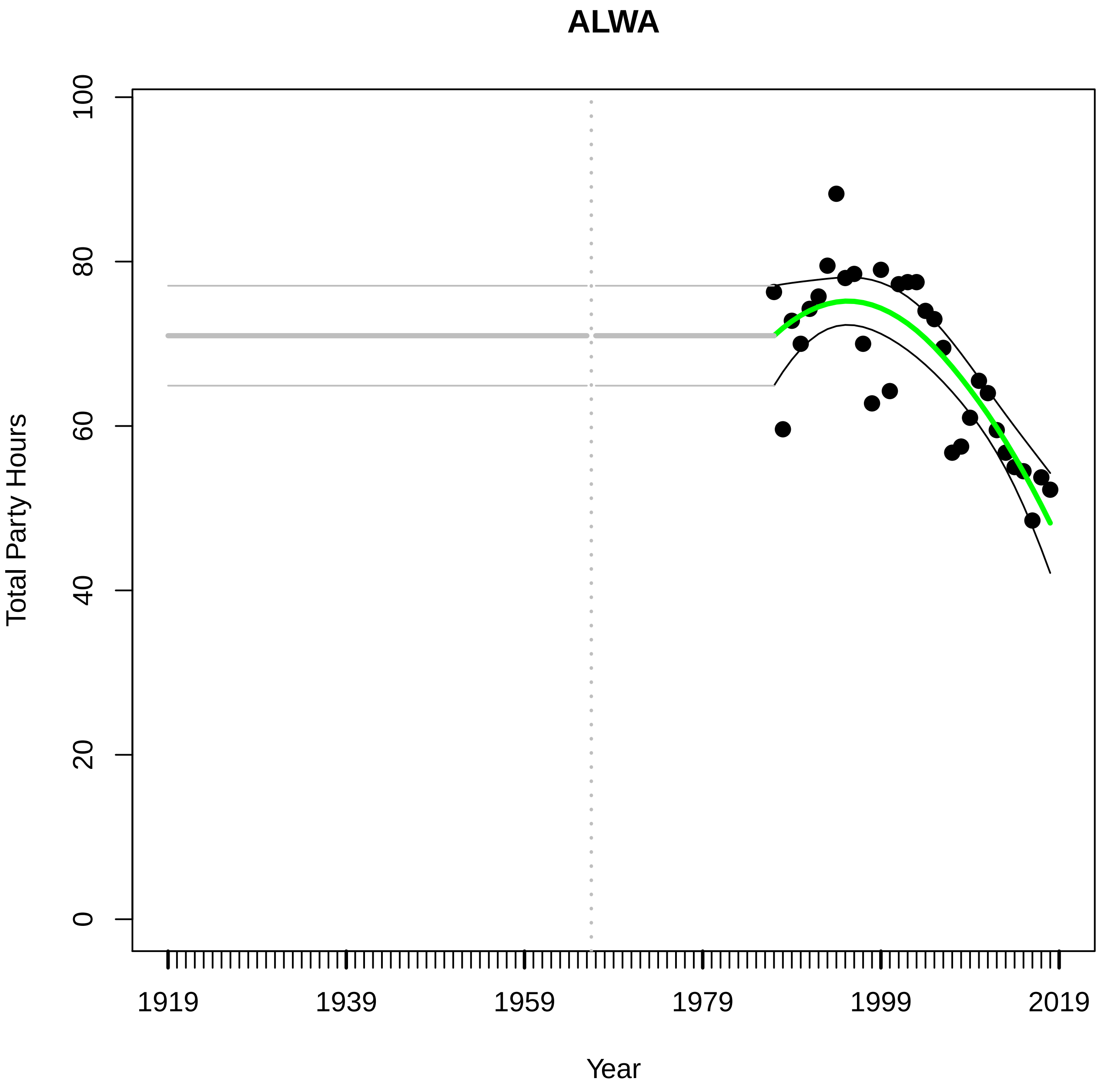
ALWA
Total Party Hours
0
20
40
60
80
100
1919
1939
1959
1979
1999
2019
Year

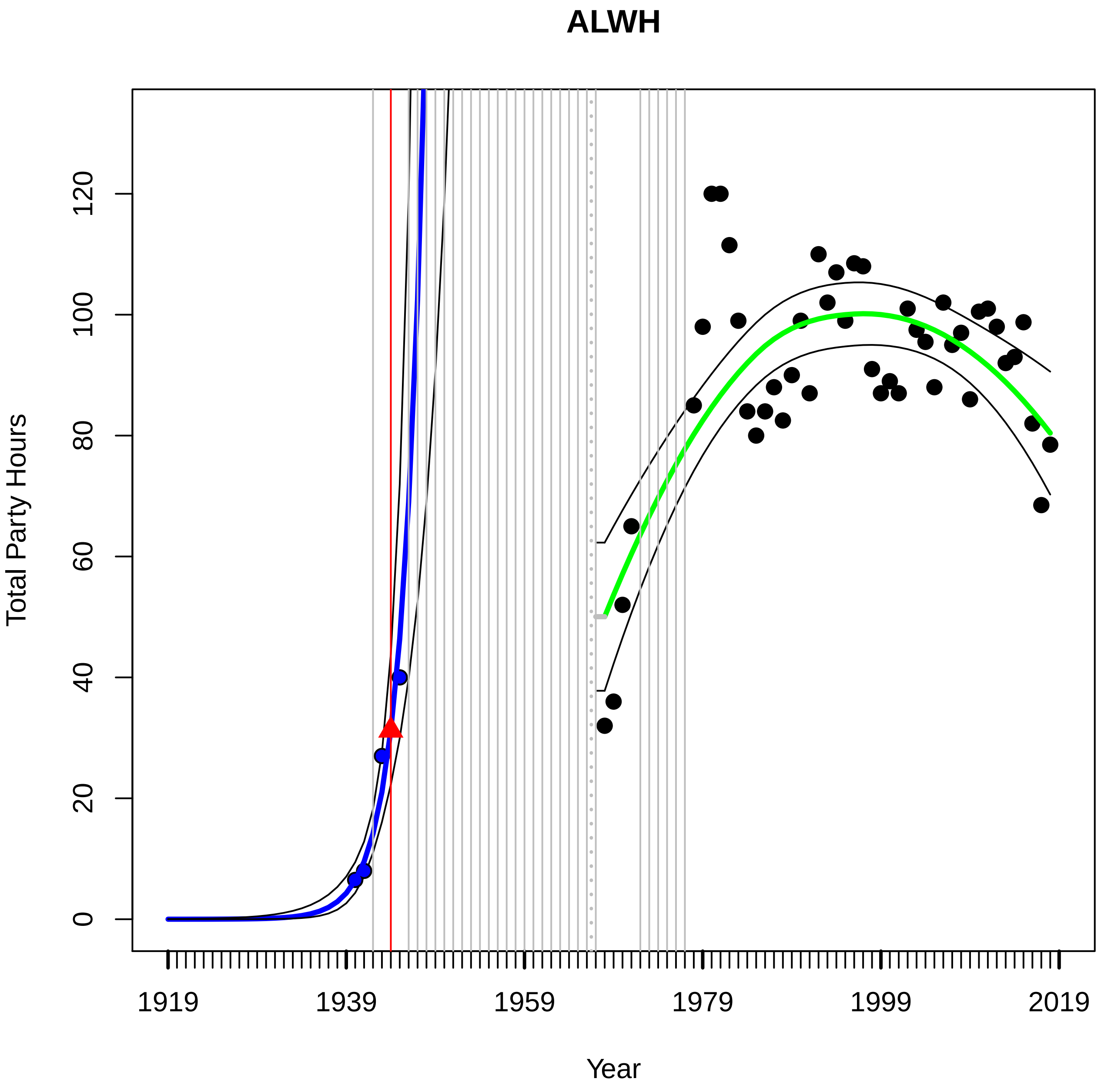
ALWH
Total Party Hours
0
20
40
60
80
100
120
1919
1939
1959
1979
1999
2019
Year

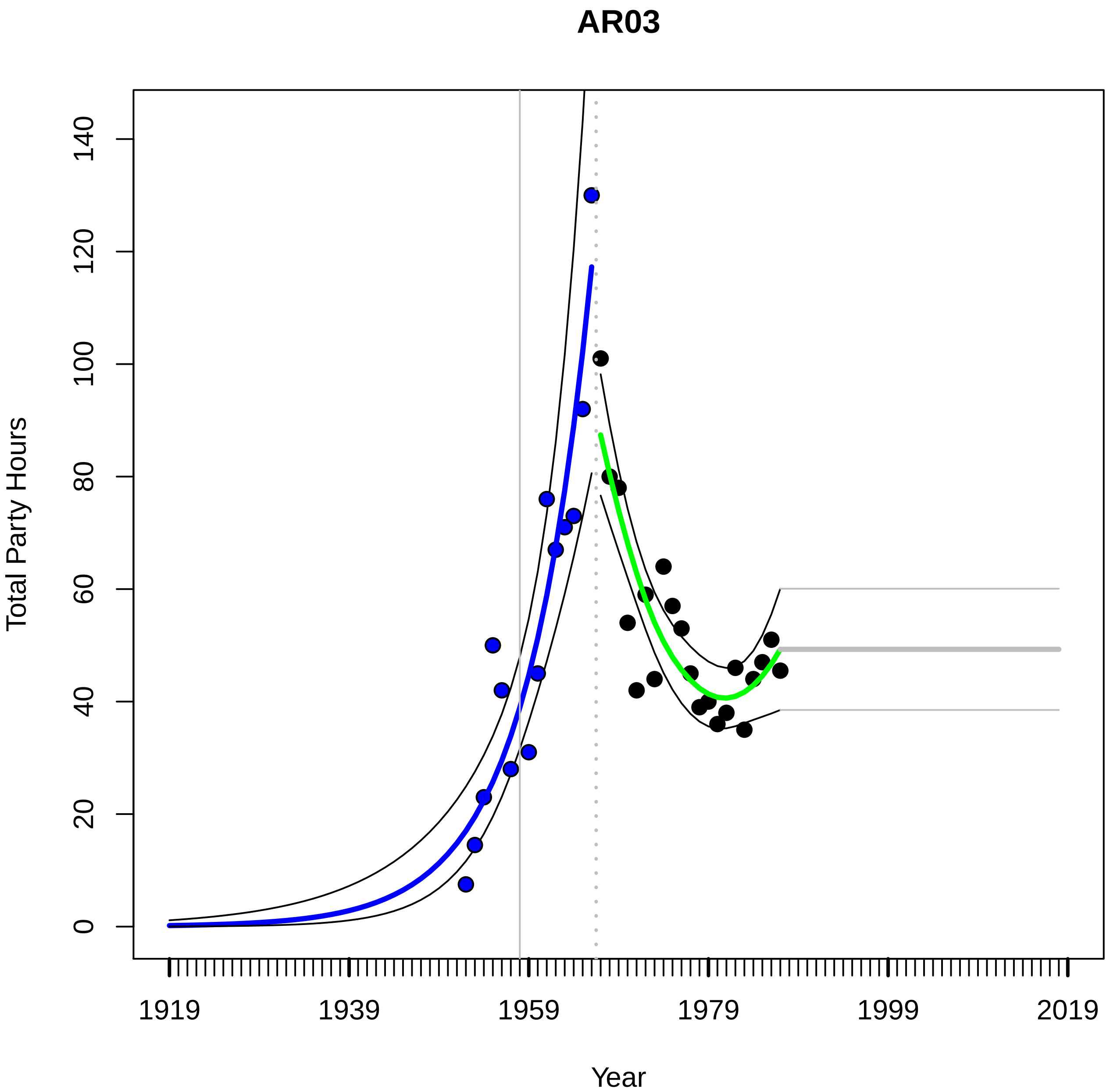

AR03
Total Party Hours
Year
0
20
40
60
80
100
120
140
1919
1939
1959
1979
1999
2019

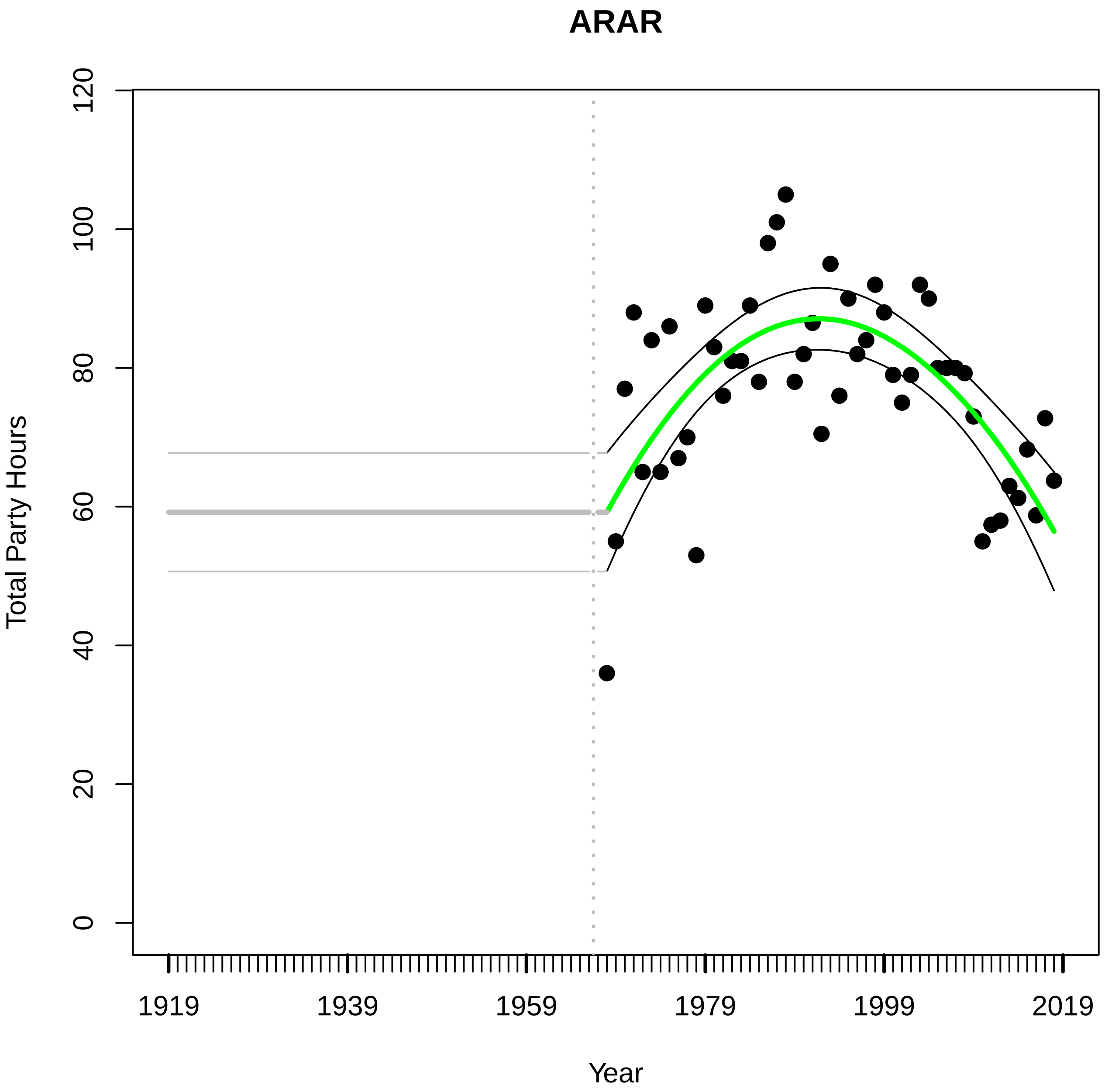
ARAR
Total Party Hours
0
20
40
60
80
100
120
1919
1939
1959
1979
1999
2019
Year

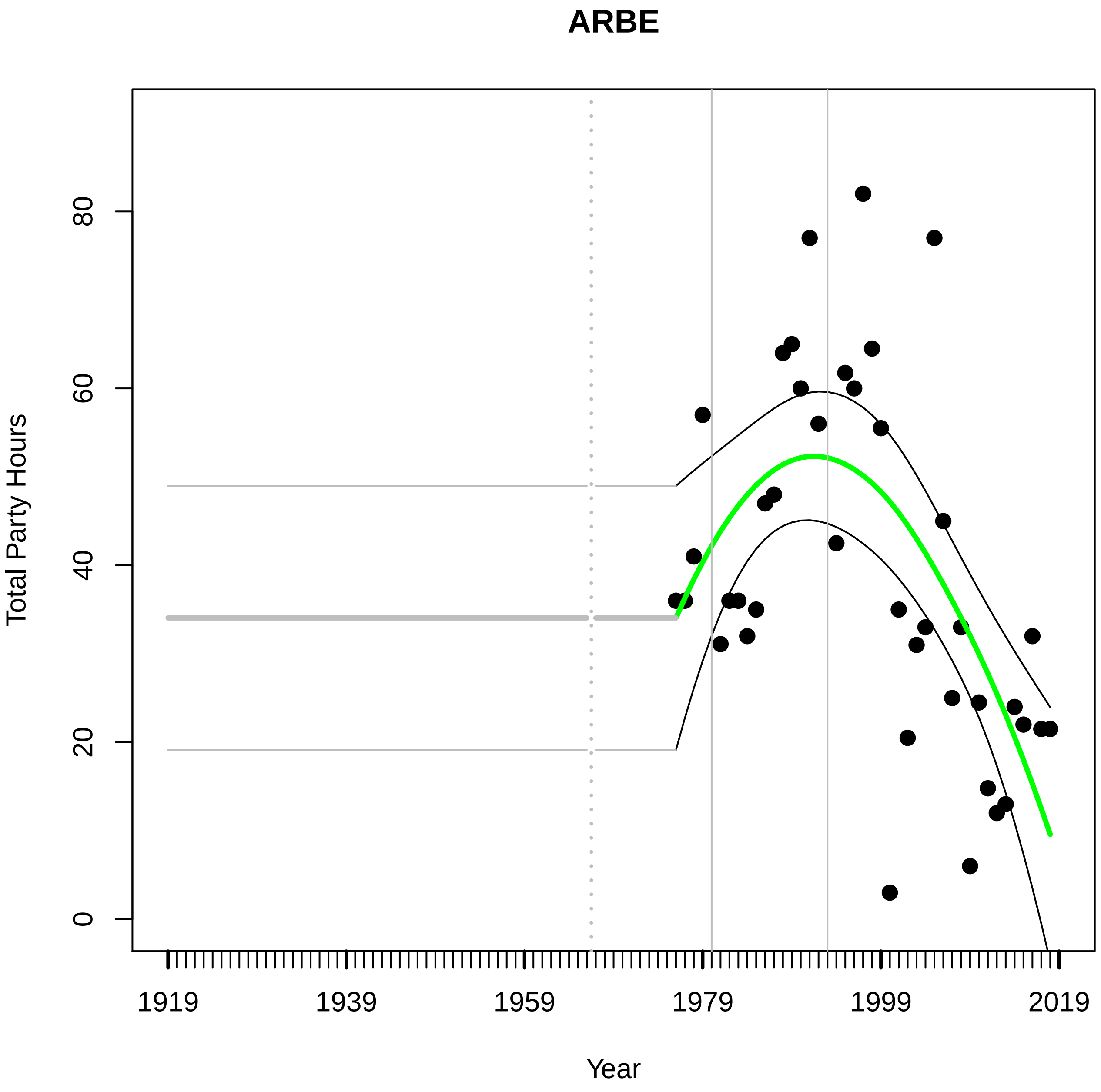
ARBE
Total Party Hours
0
20
40
60
80
1919
1939
1959
1979
1999
2019
Year

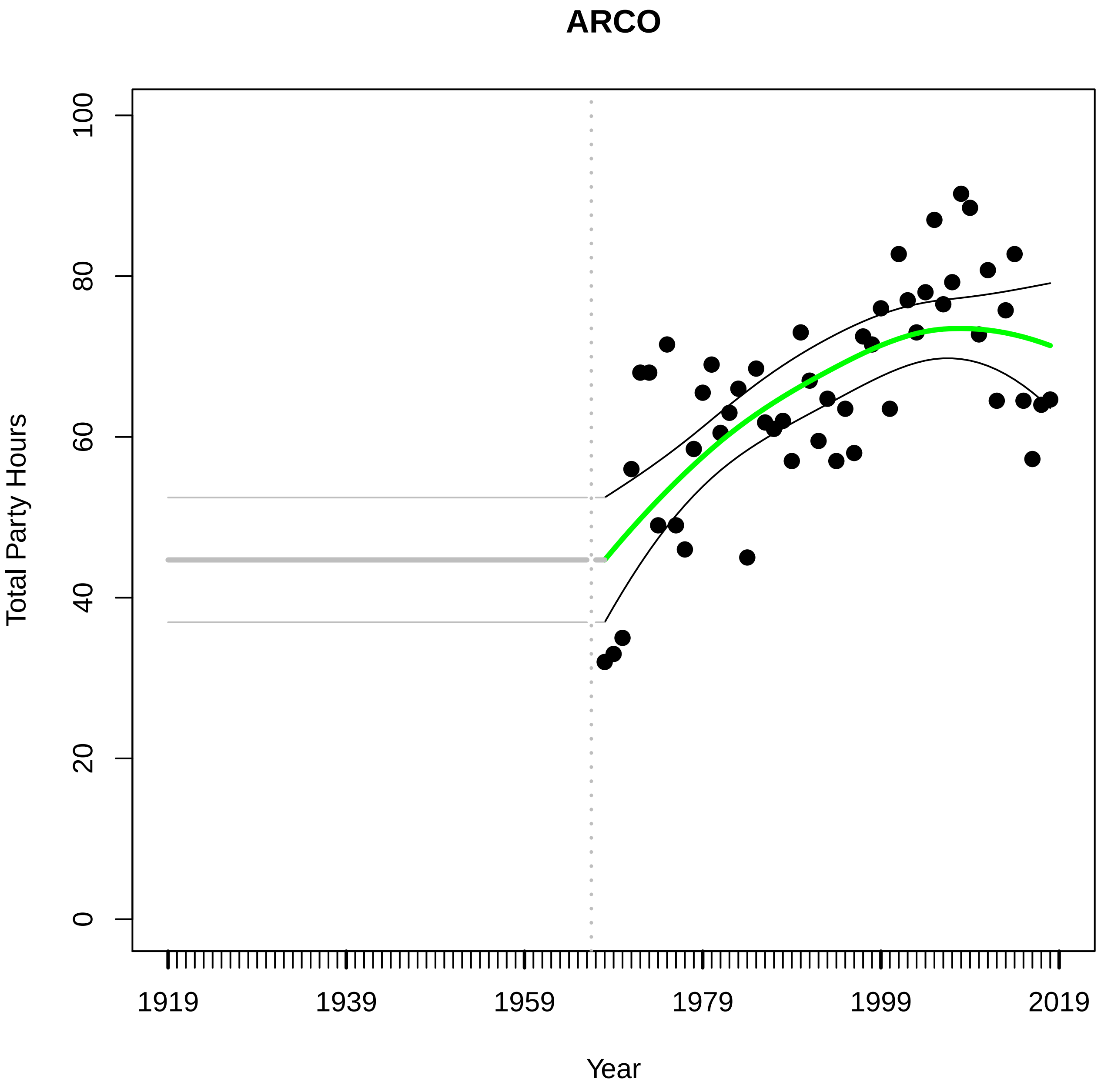

ARCO
Total Party Hours
0
20
40
60
80
100
1919
1939
1959
1979
1999
2019
Year

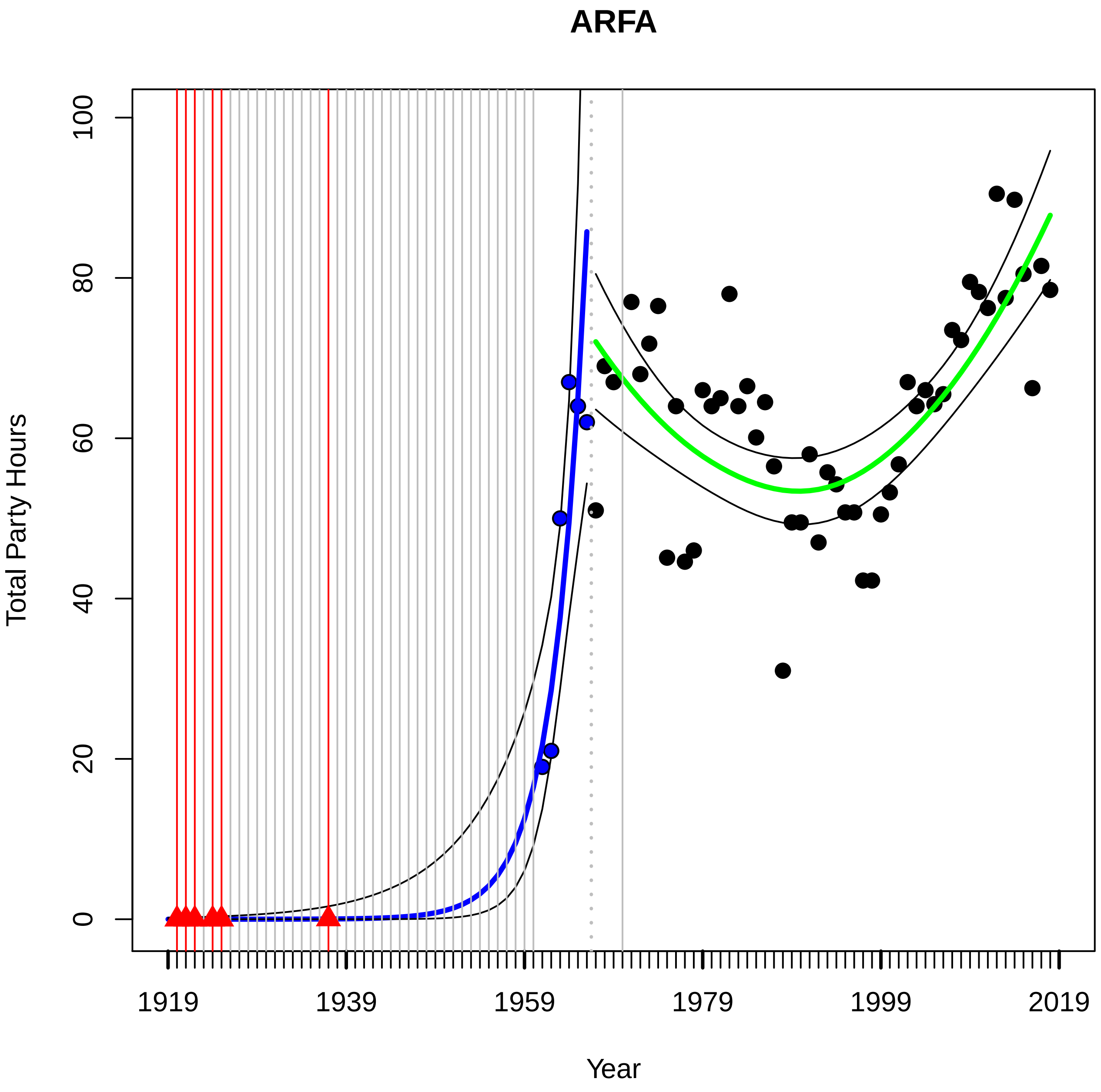

ARFA
Total Party Hours
0
20
40
60
80
100
1919
1939
1959
1979
1999
2019
Year

ARFS

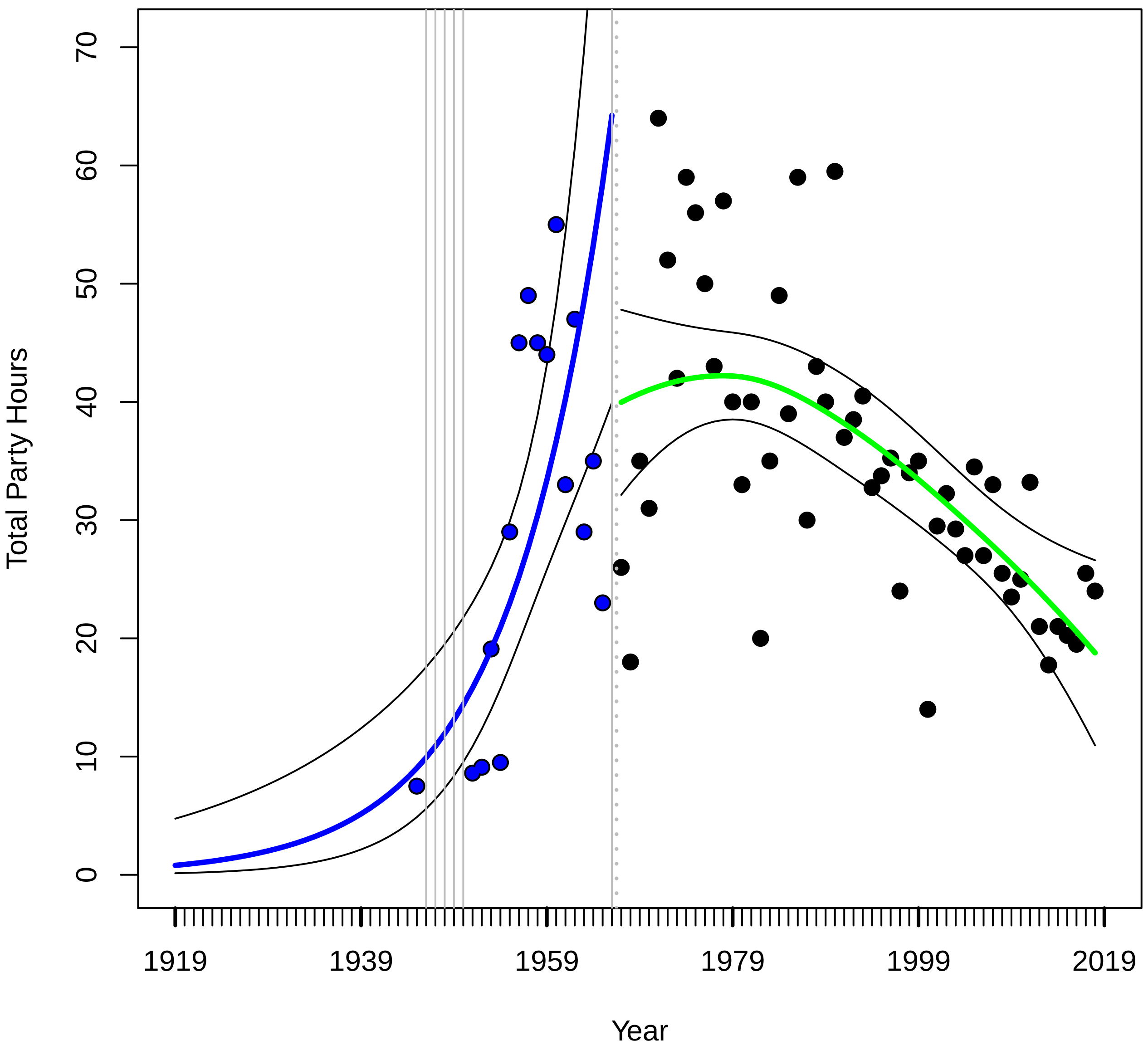

Total Party Hours
0
10
20
30
40
50
60
70
1919
1939
1959
1979
1999
2019
Year

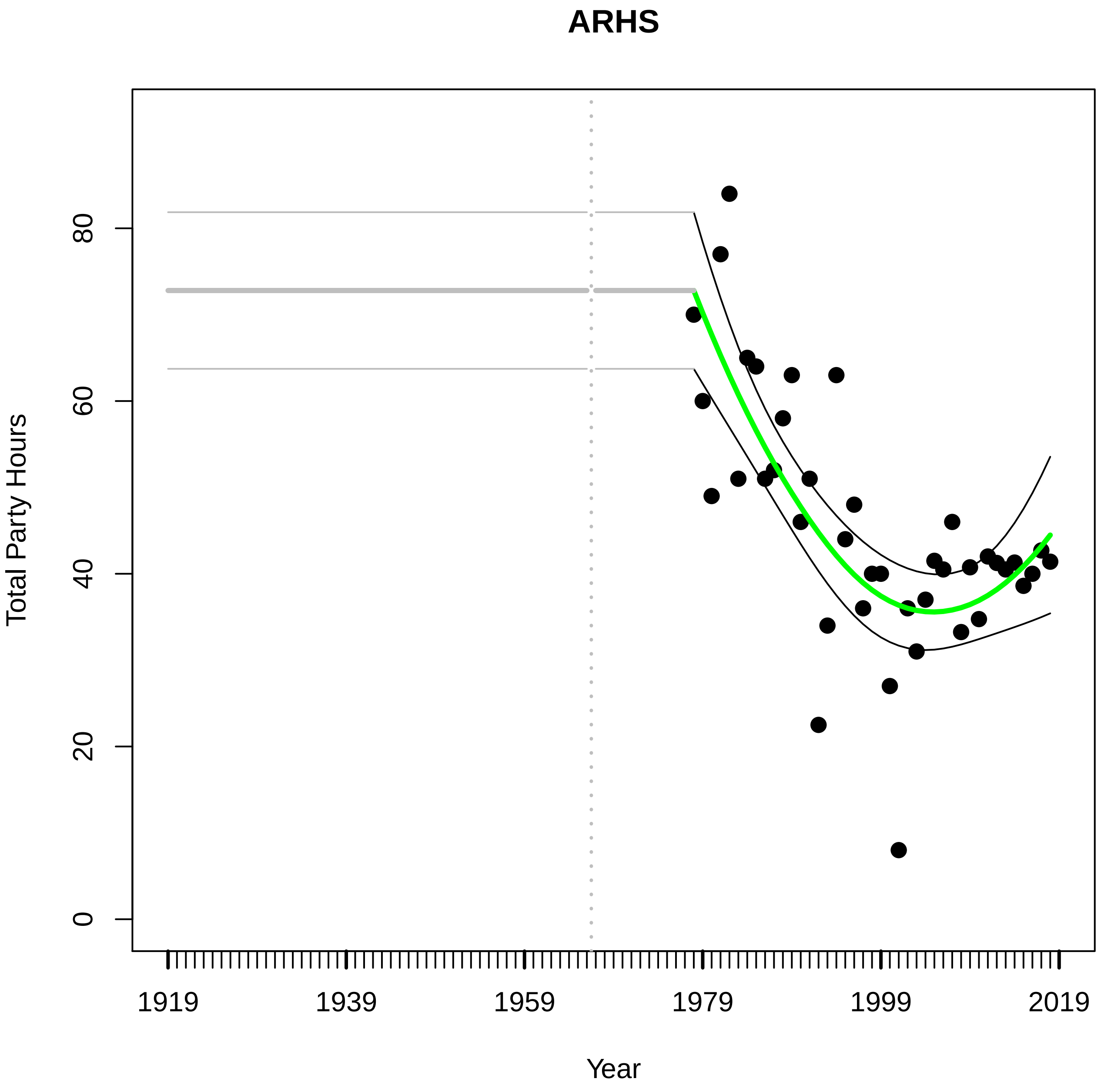

ARHS
Total Party Hours
0
20
40
60
80
1919
1939
1959
1979
1999
2019
Year

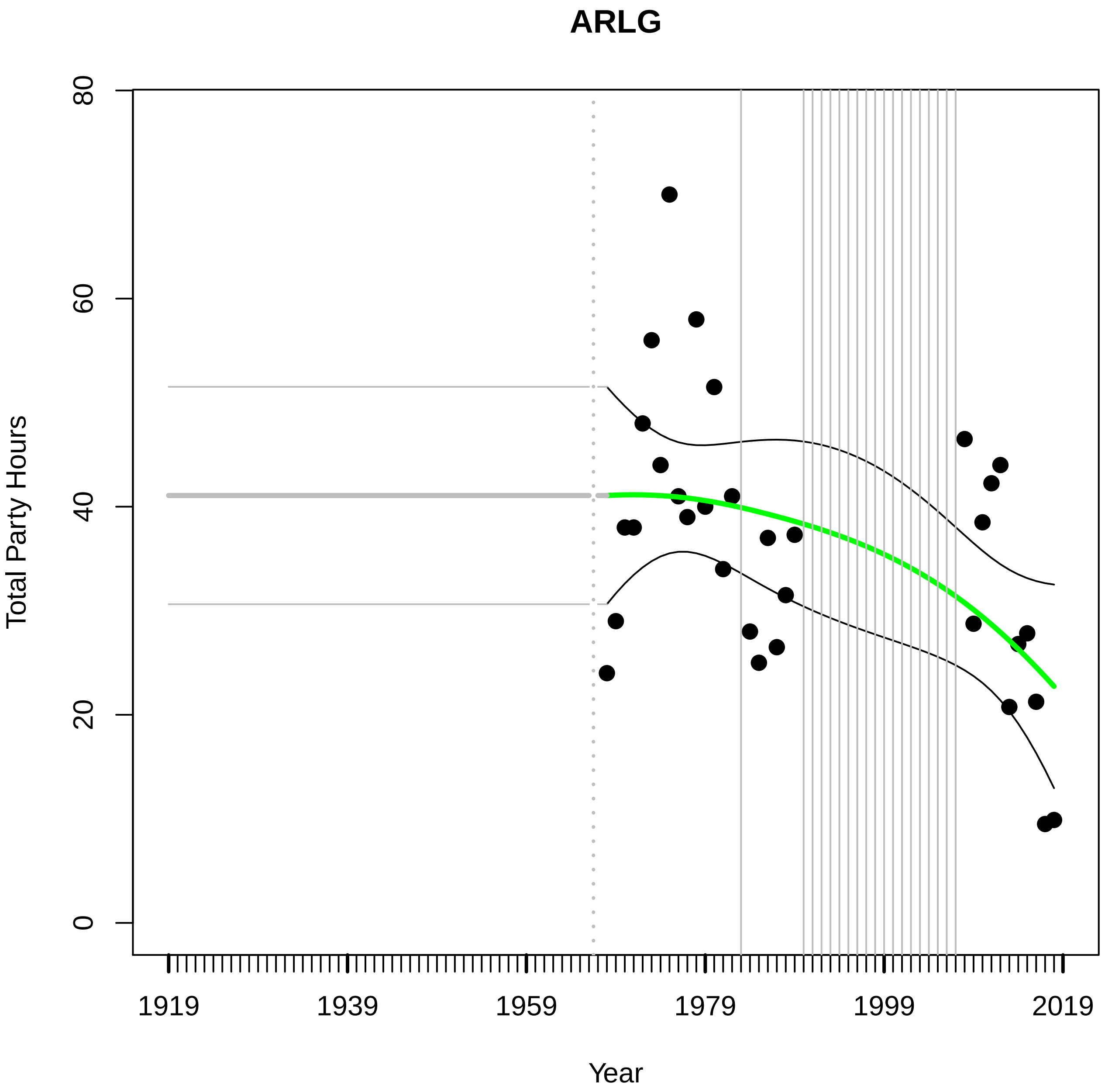

ARLG
Total Party Hours
Year
0
20
40
60
80
1919
1939
1959
1979
1999
2019

ARLO

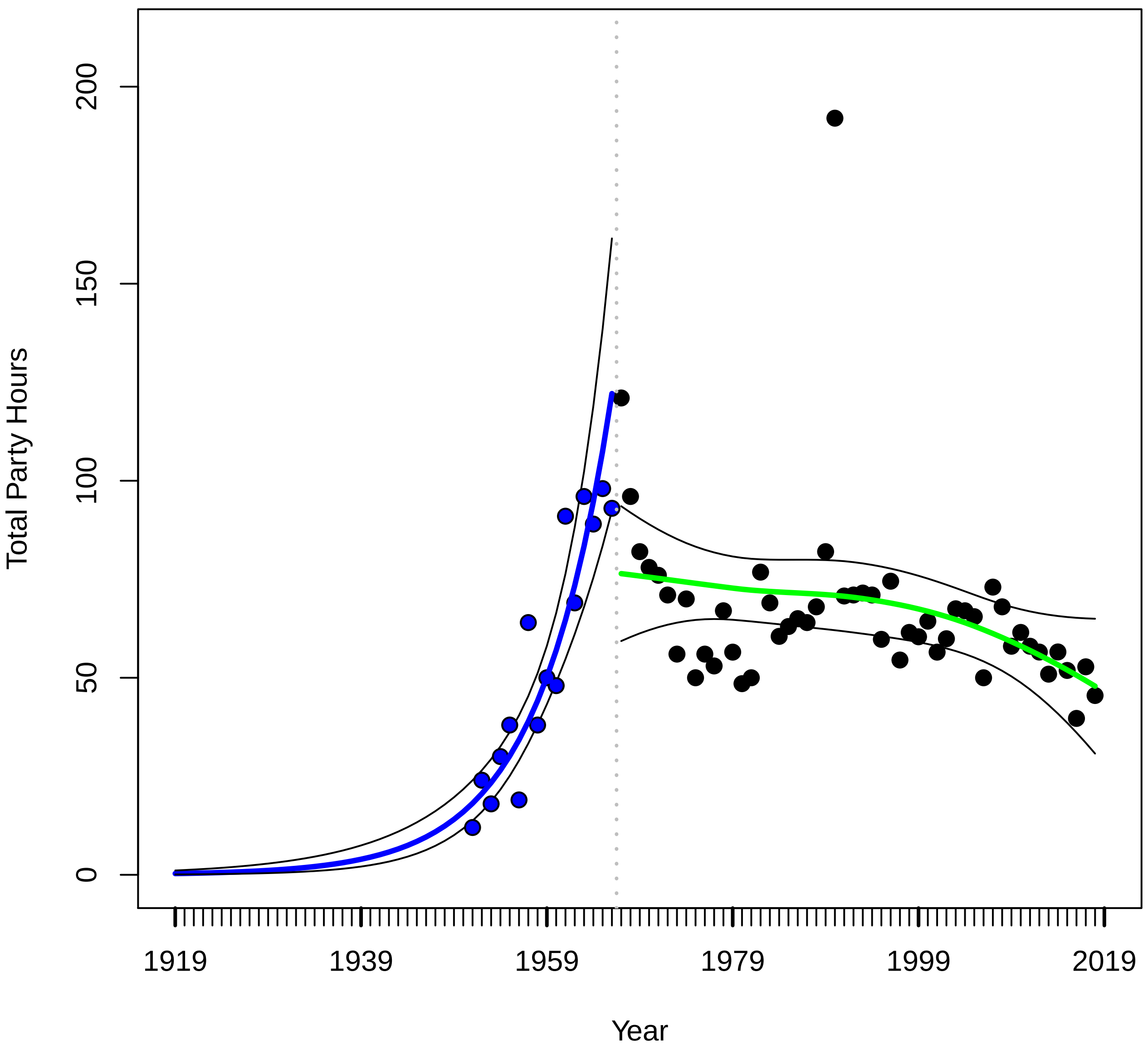

ARLO
Total Party Hours
0
50
100
150
200
1919
1939
1959
1979
1999
2019
Year

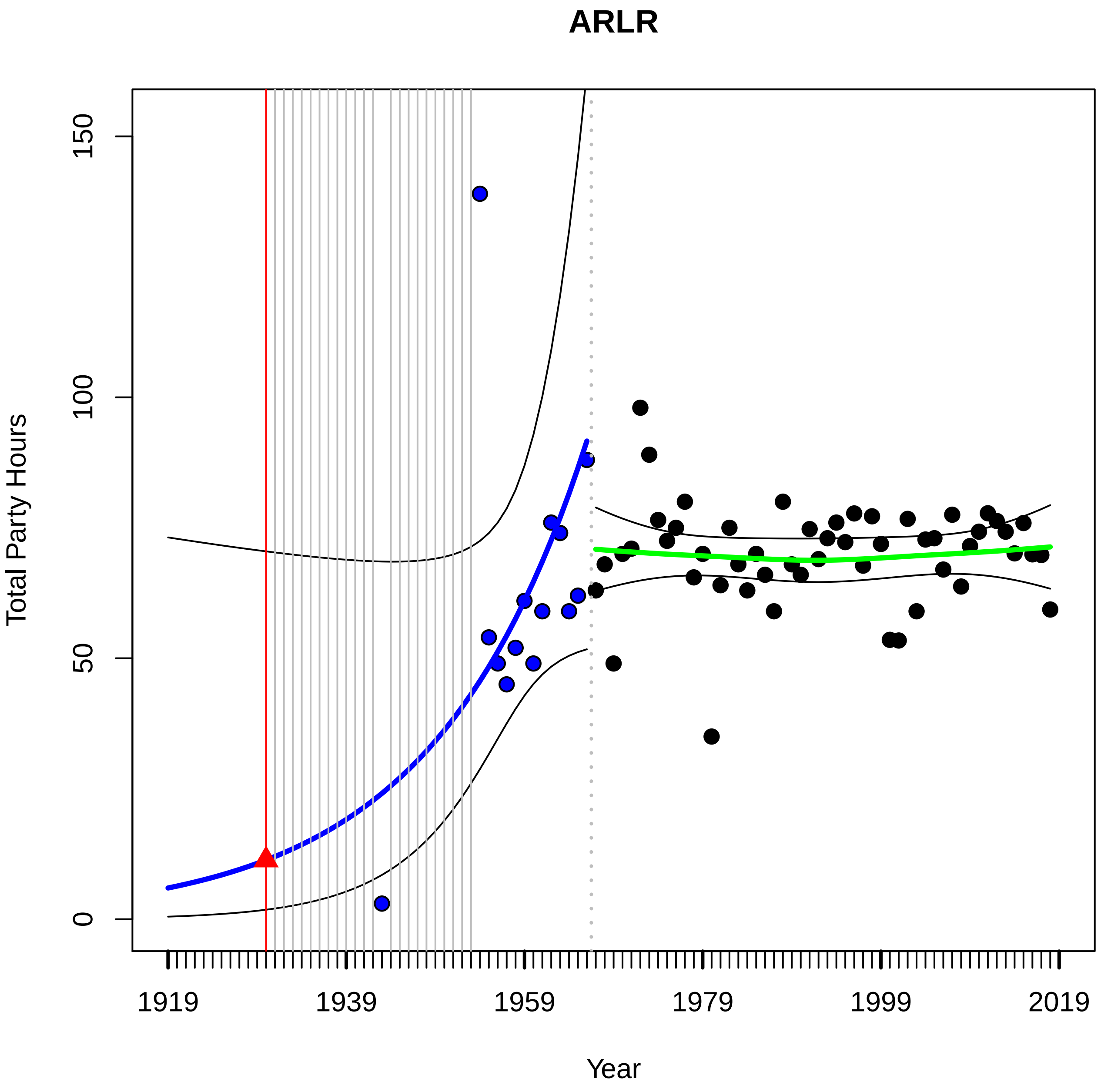

ARLR
Total Party Hours
0
50
100
150
1919
1939
1959
1979
1999
2019
Year

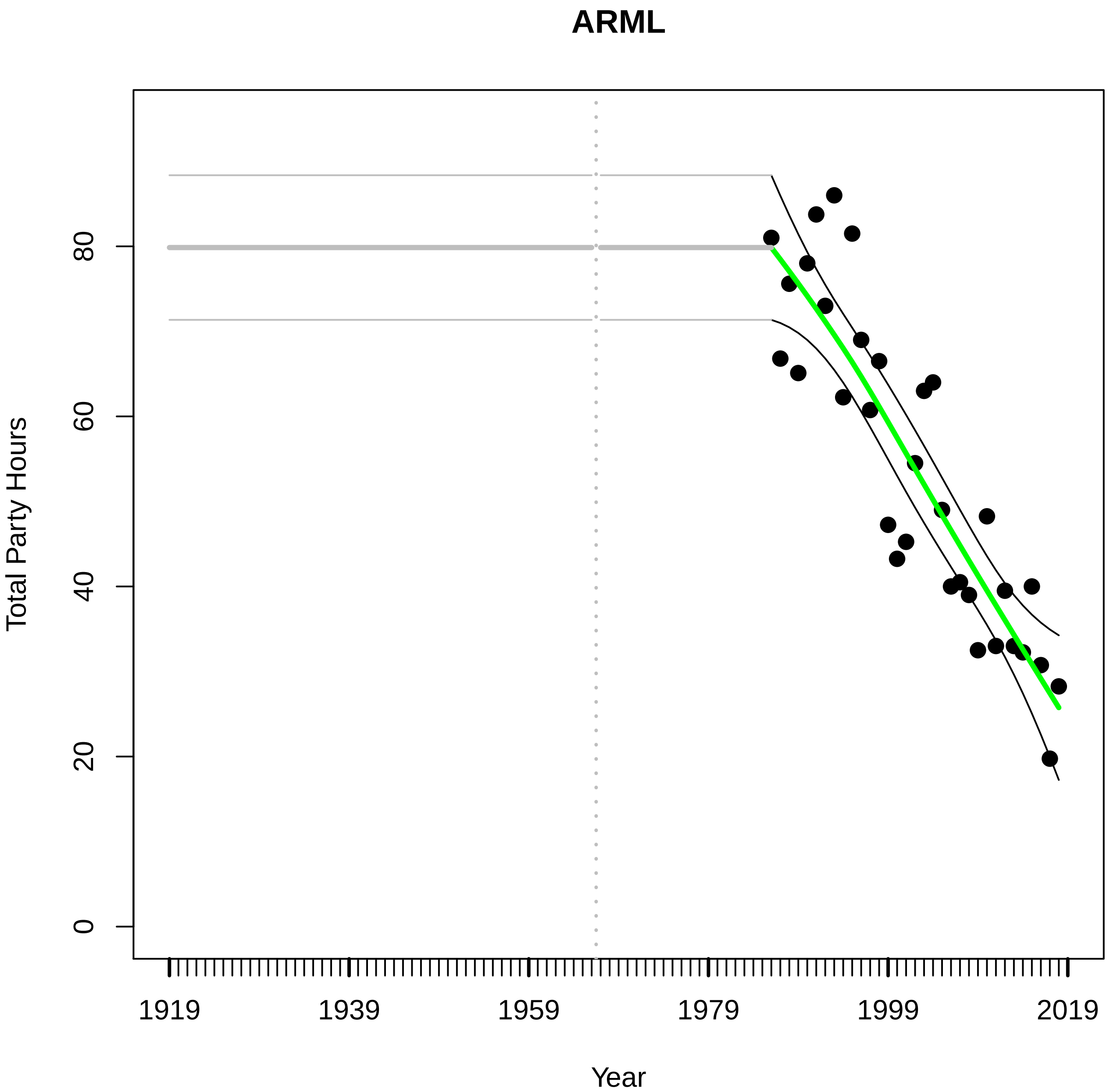

ARML
Total Party Hours
0
20
40
60
80
1919
1939
1959
1979
1999
2019
Year

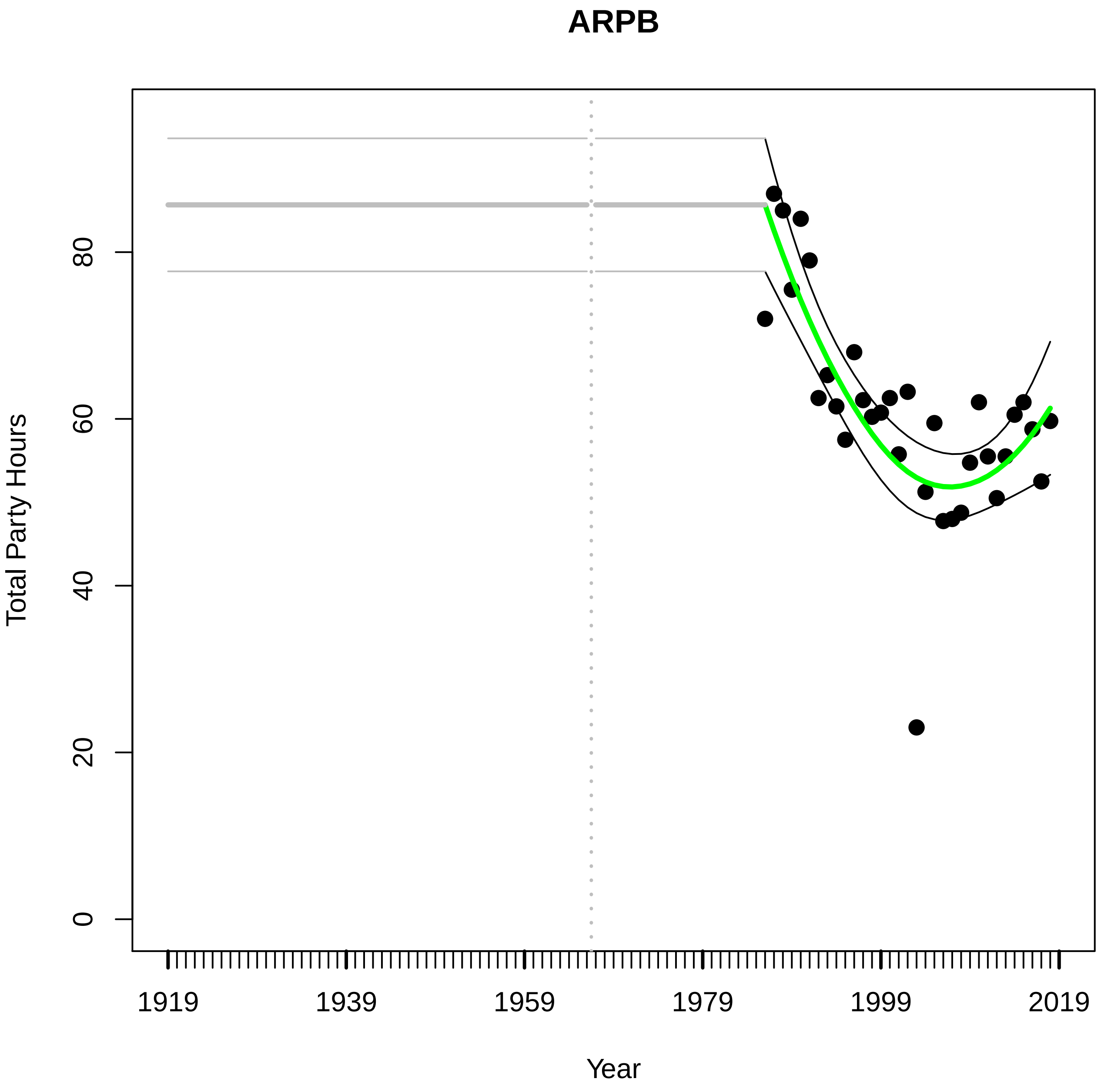
ARPB
Total Party Hours
0
20
40
60
80
1919
1939
1959
1979
1999
2019
Year

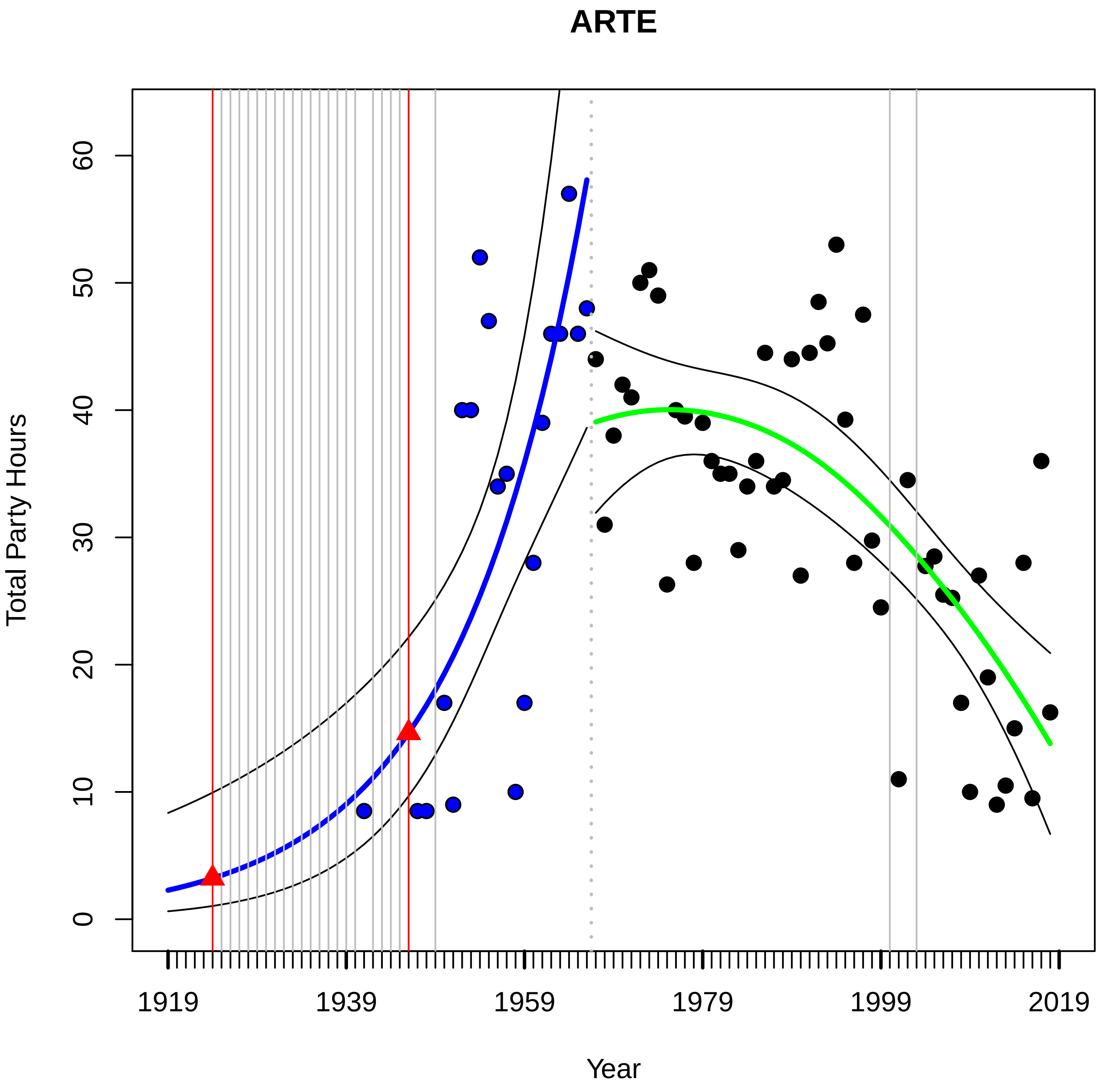

ARTE
Total Party Hours
0
10
20
30
40
50
60
1919
1939
1959
1979
1999
2019
Year

ARVC

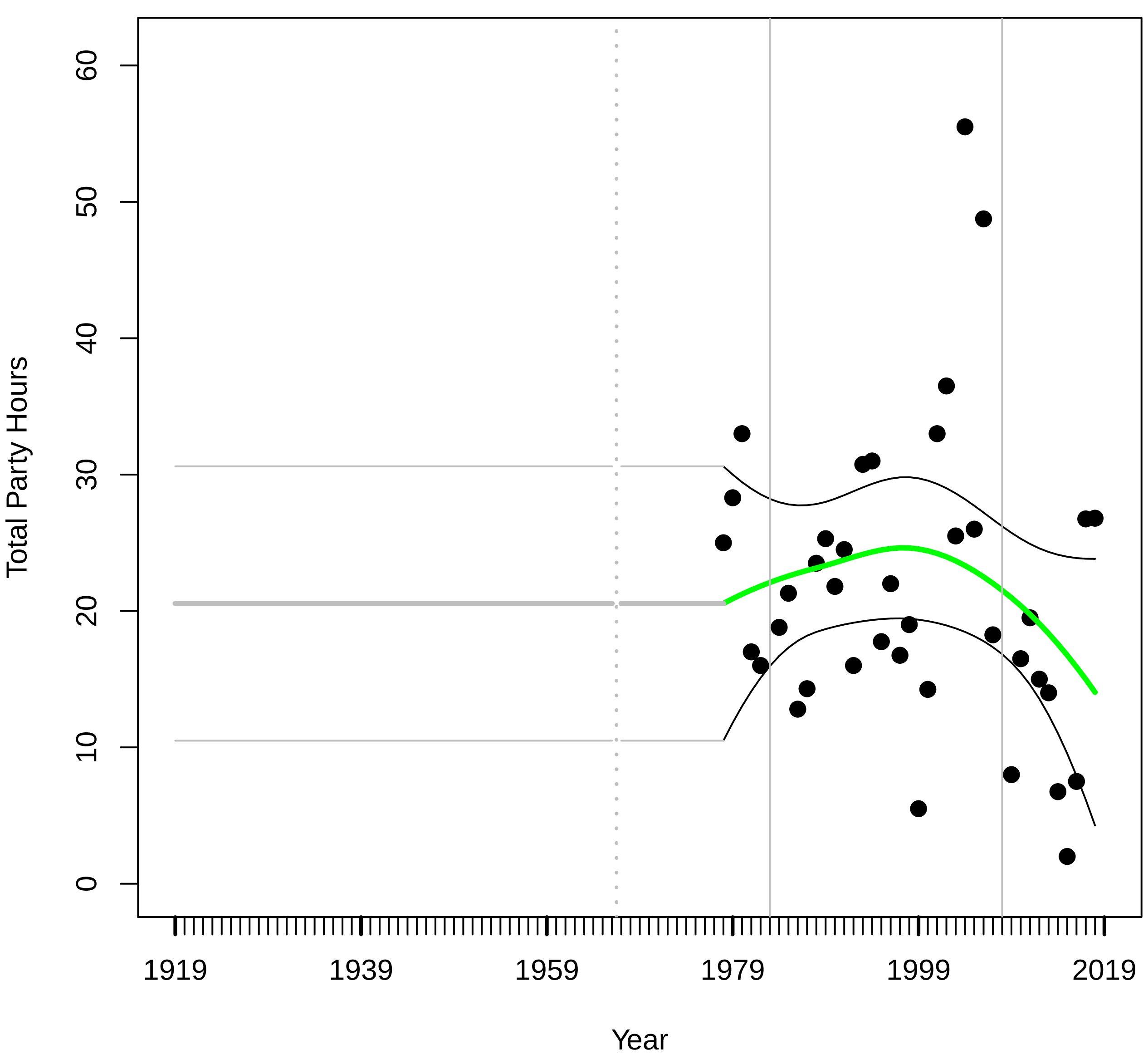

ARVC
Total Party Hours
0
10
20
30
40
50
60
1919
1939
1959
1979
1999
2019
Year

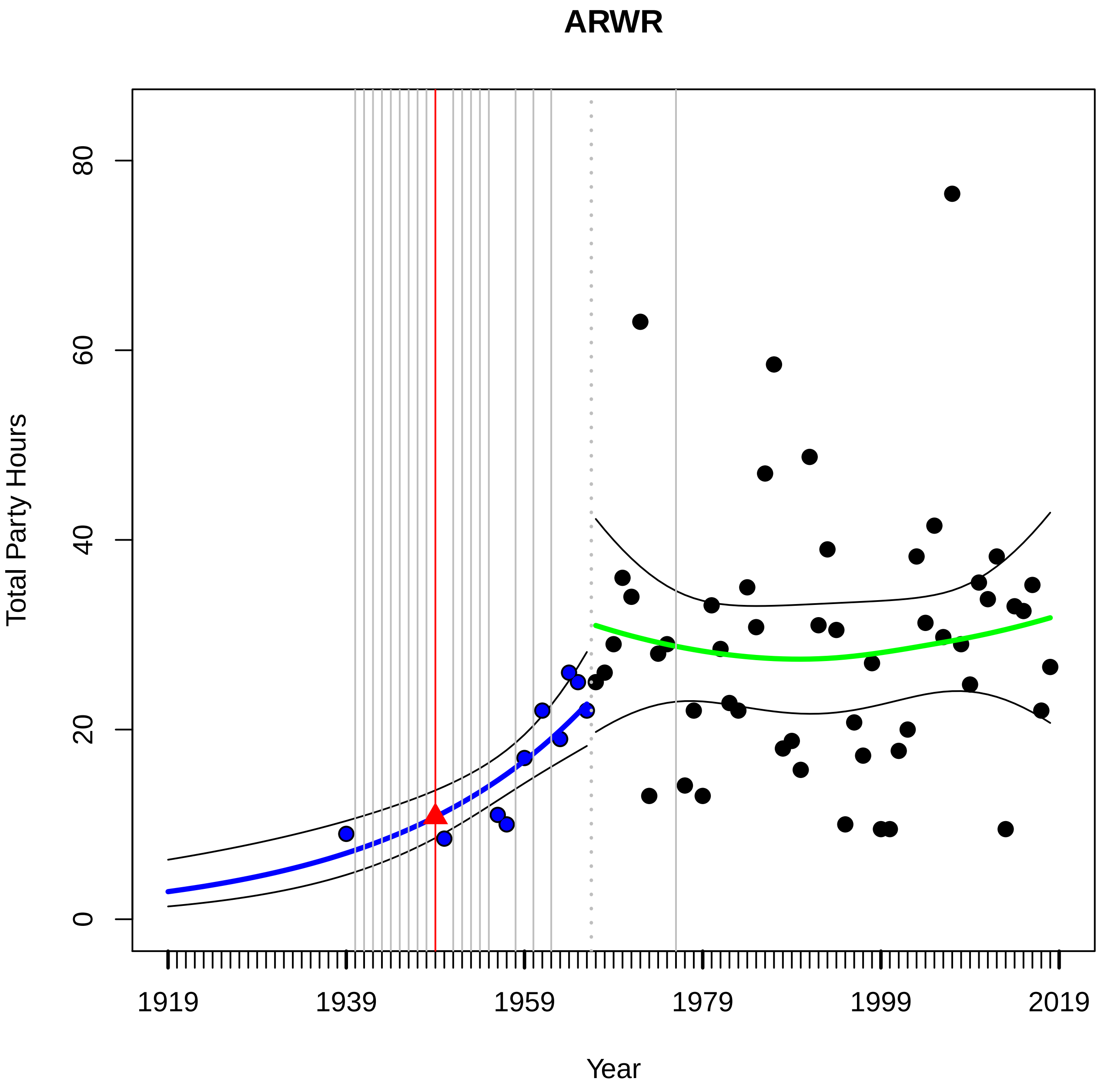
ARWR
Total Party Hours
0
20
40
60
80
1919
1939
1959
1979
1999
2019
Year

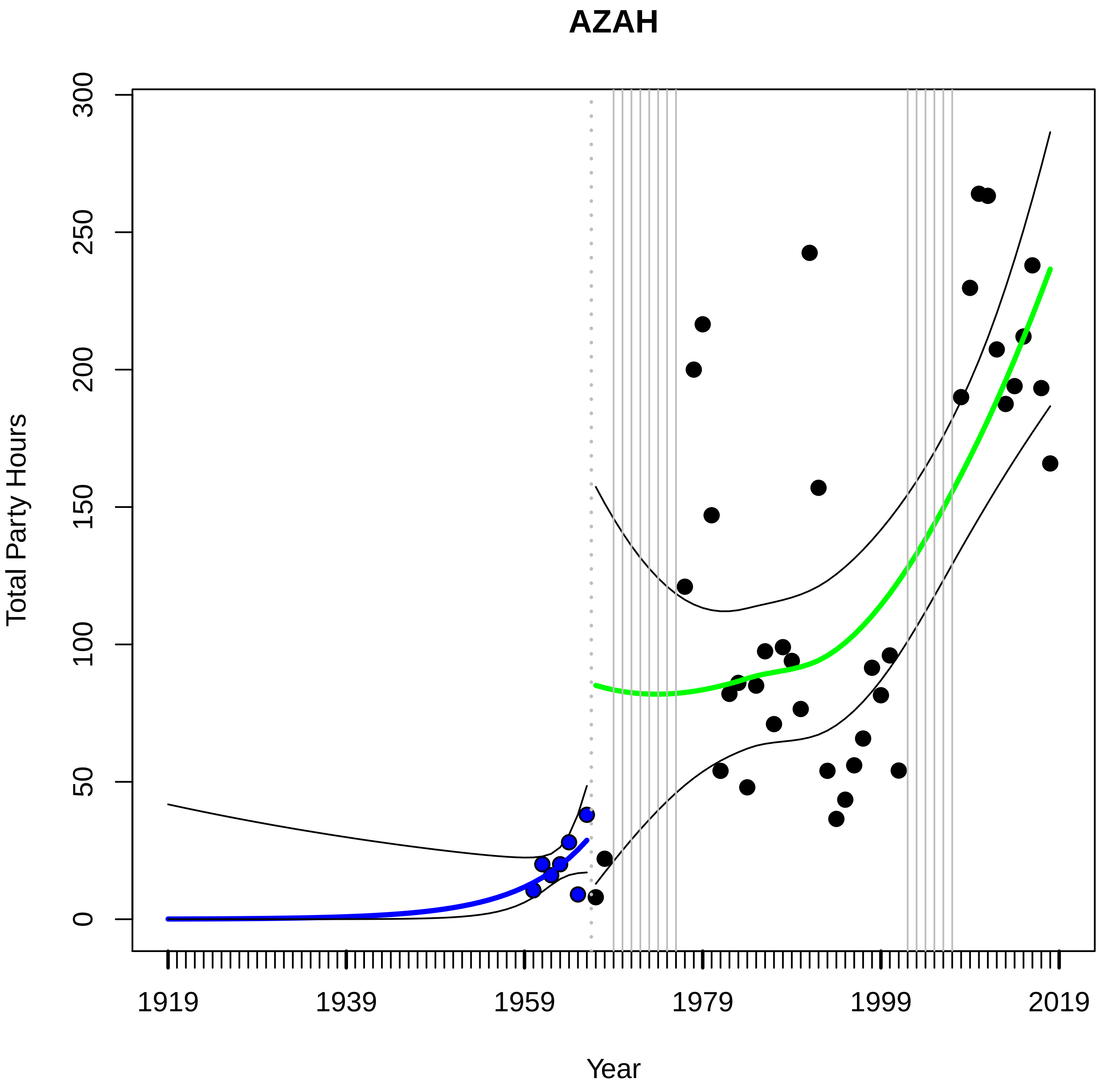
AZAH
Total Party Hours
300
250
200
150
100
50
0
1919
1939
1959
1979
1999
2019
Year

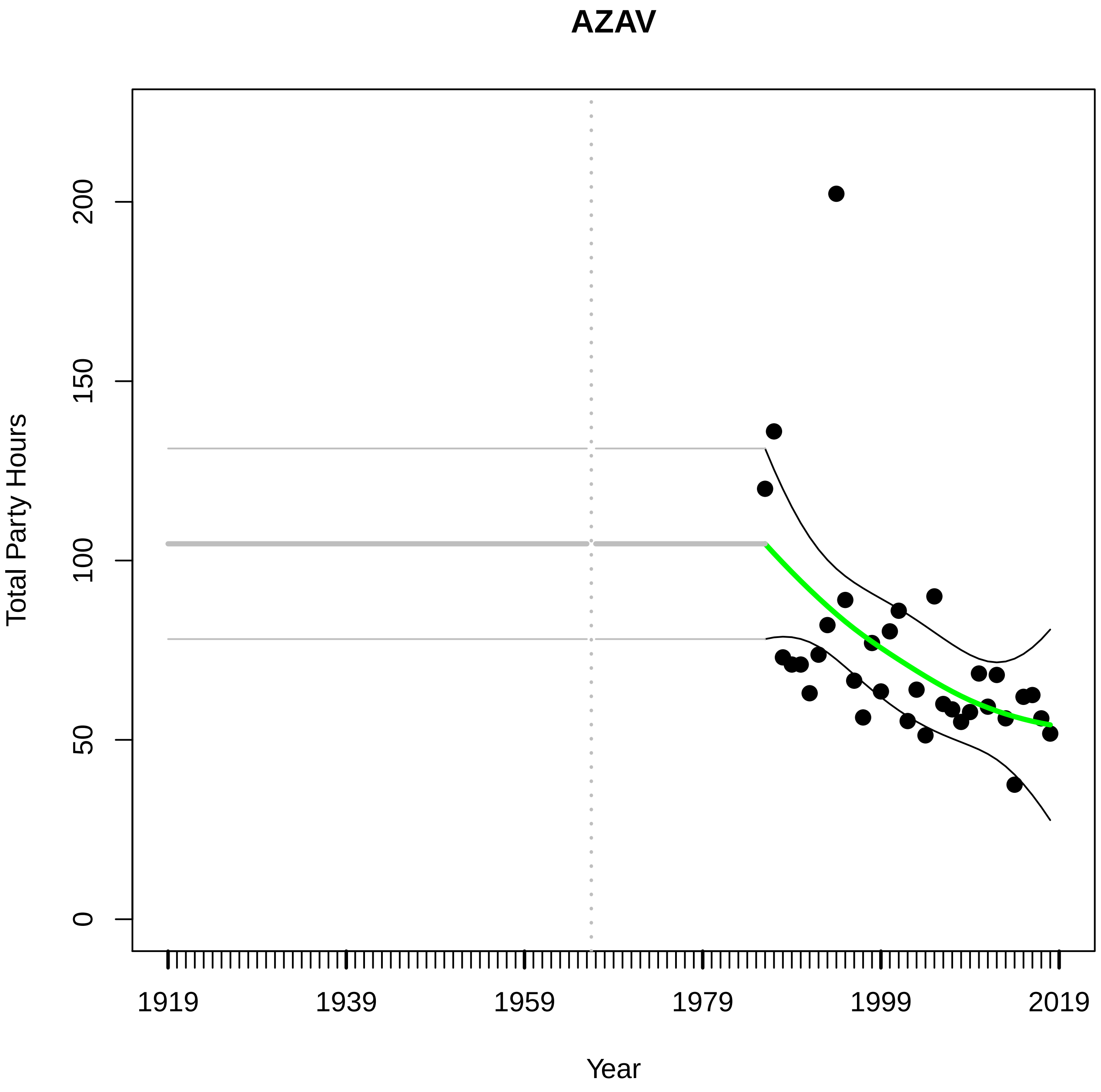

AZAV
Total Party Hours
0
50
100
150
200
1919
1939
1959
1979
1999
2019
Year

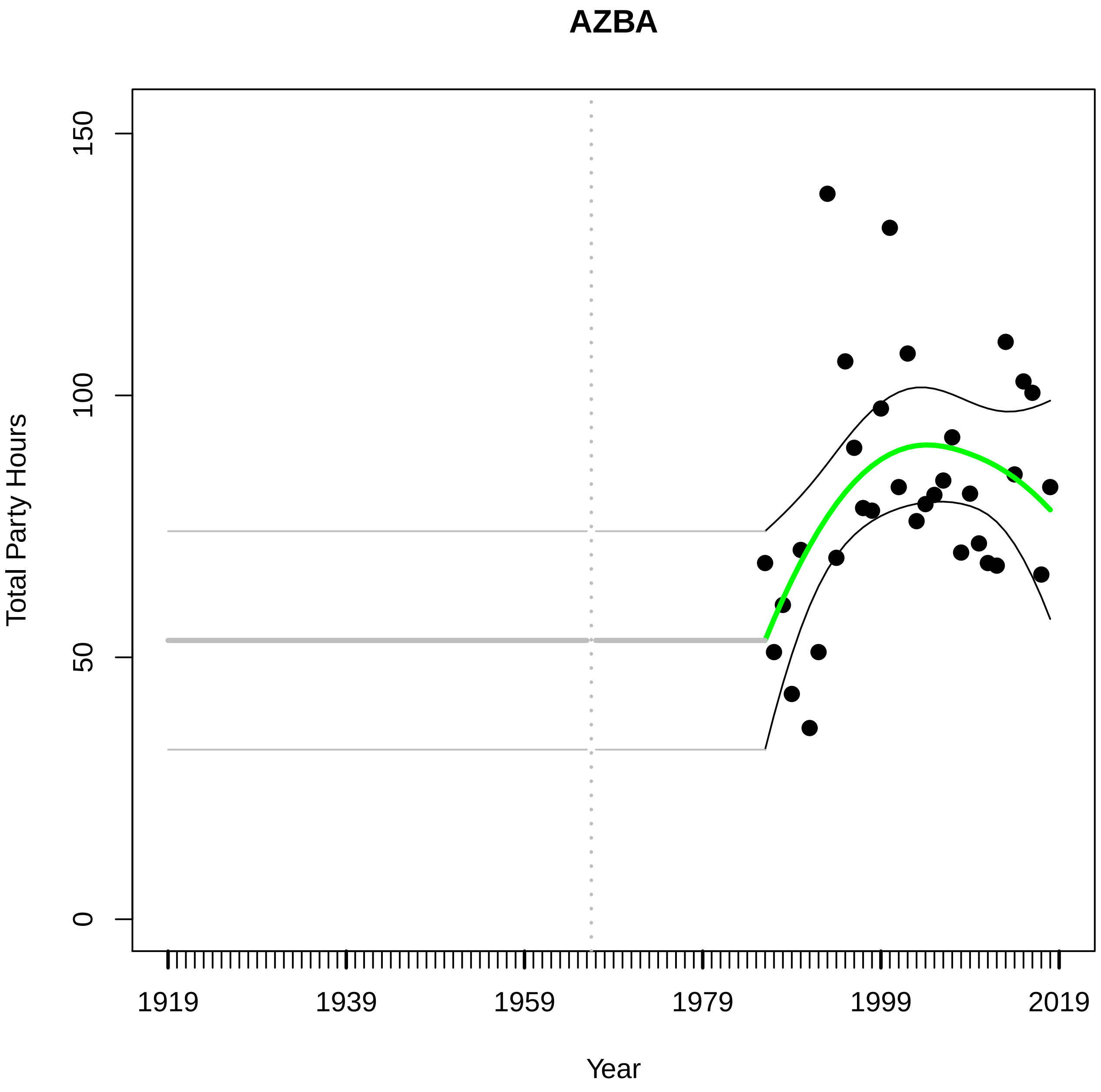

AZBA
Total Party Hours
150
100
50
0
1919
1939
1959
1979
1999
2019
Year

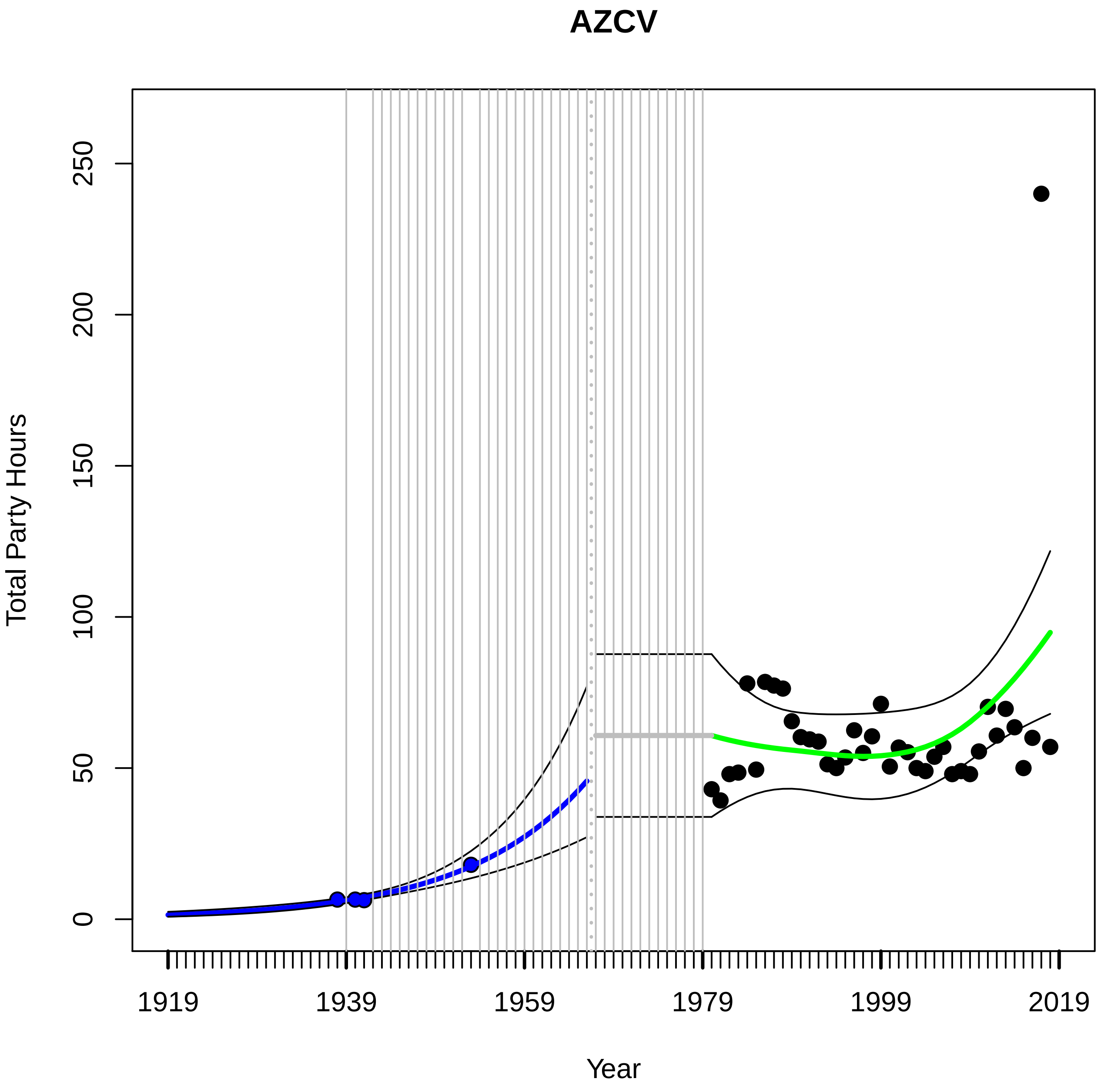

AZCV
Total Party Hours
0
50
100
150
200
250
1919
1939
1959
1979
1999
2019
Year

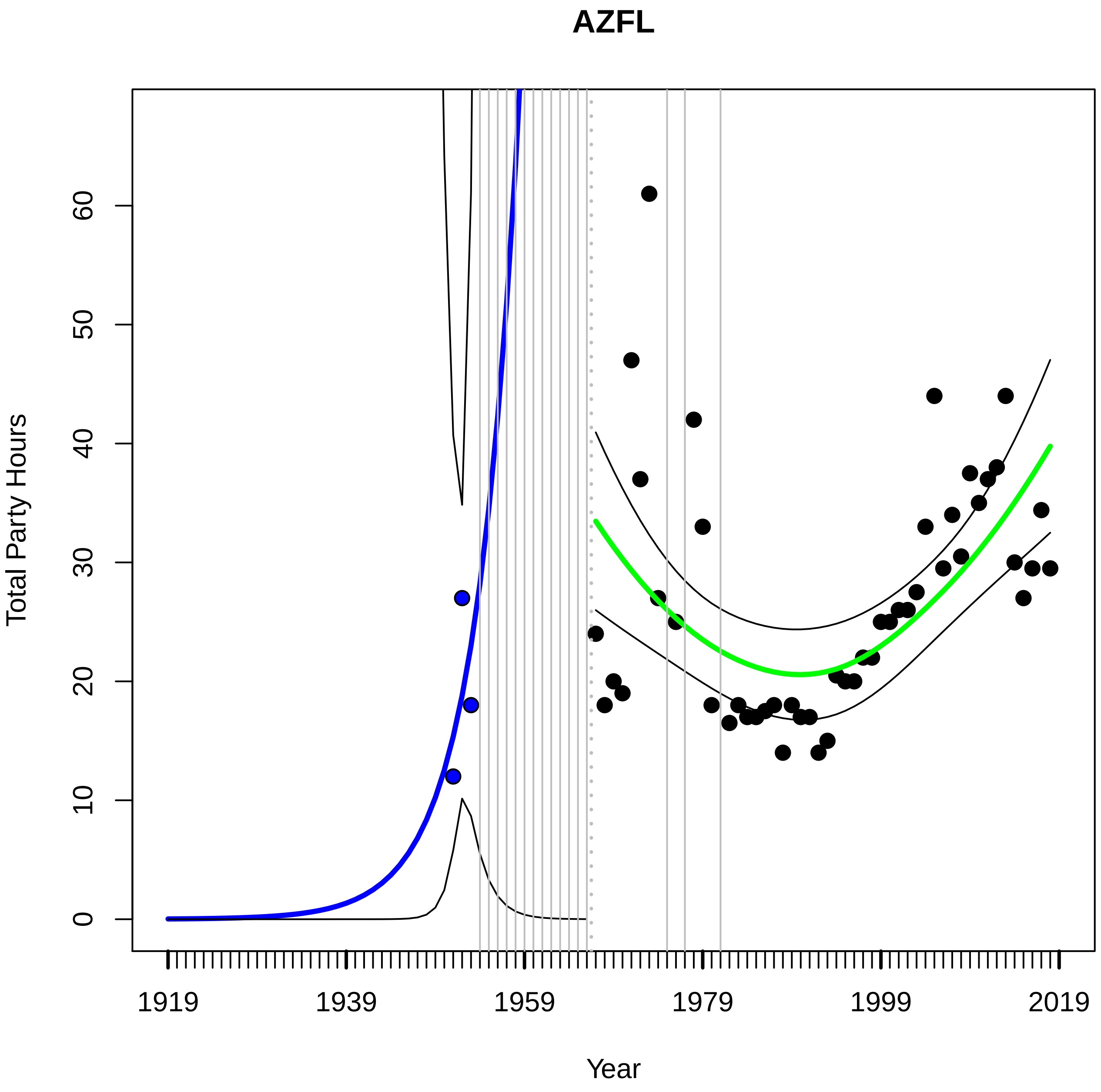
AZFL
Total Party Hours
0
10
20
30
40
50
60
1919
1939
1959
1979
1999
2019
Year

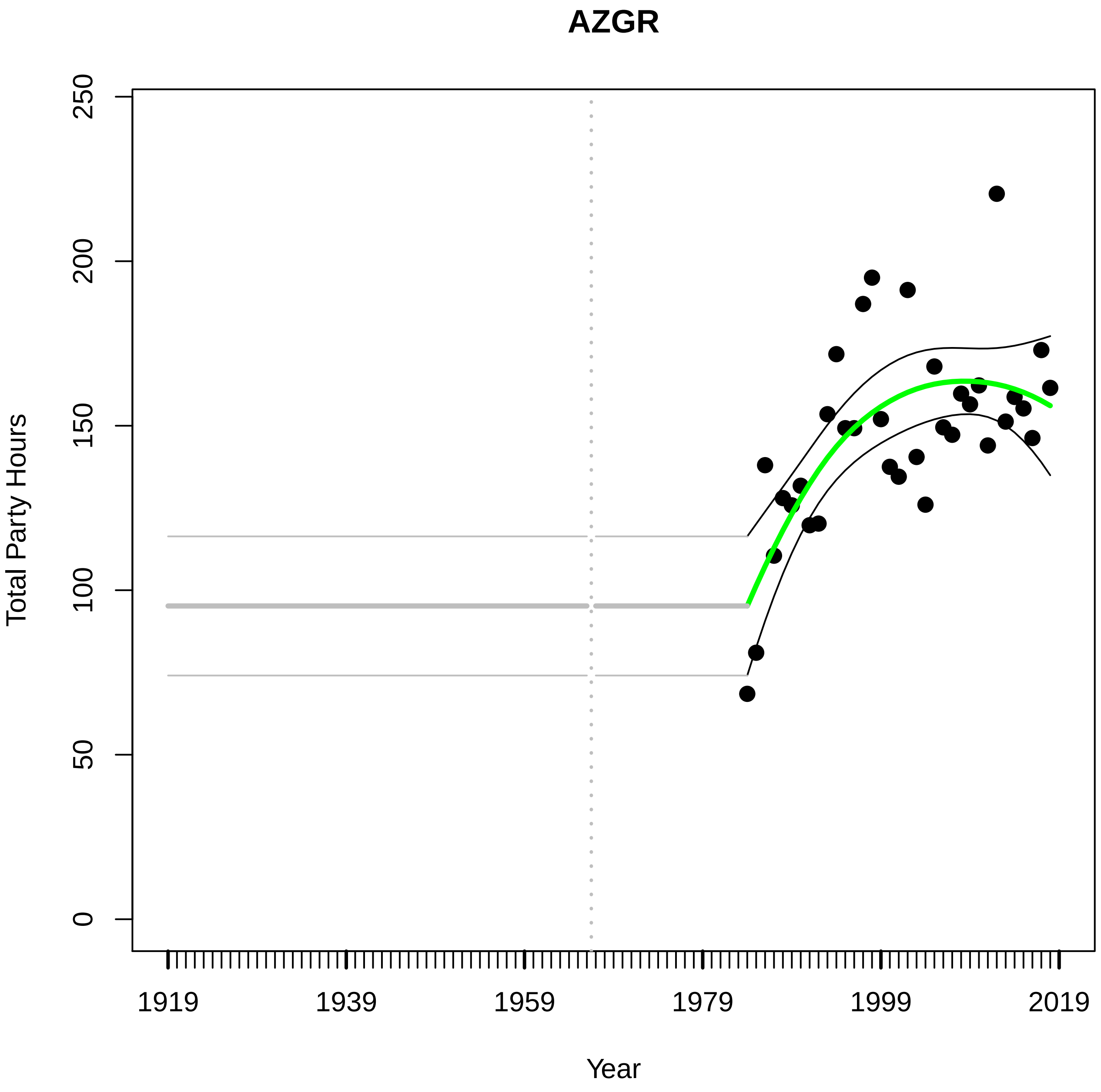

AZGR
Total Party Hours
Year
0
50
100
150
200
250
1919
1939
1959
1979
1999
2019

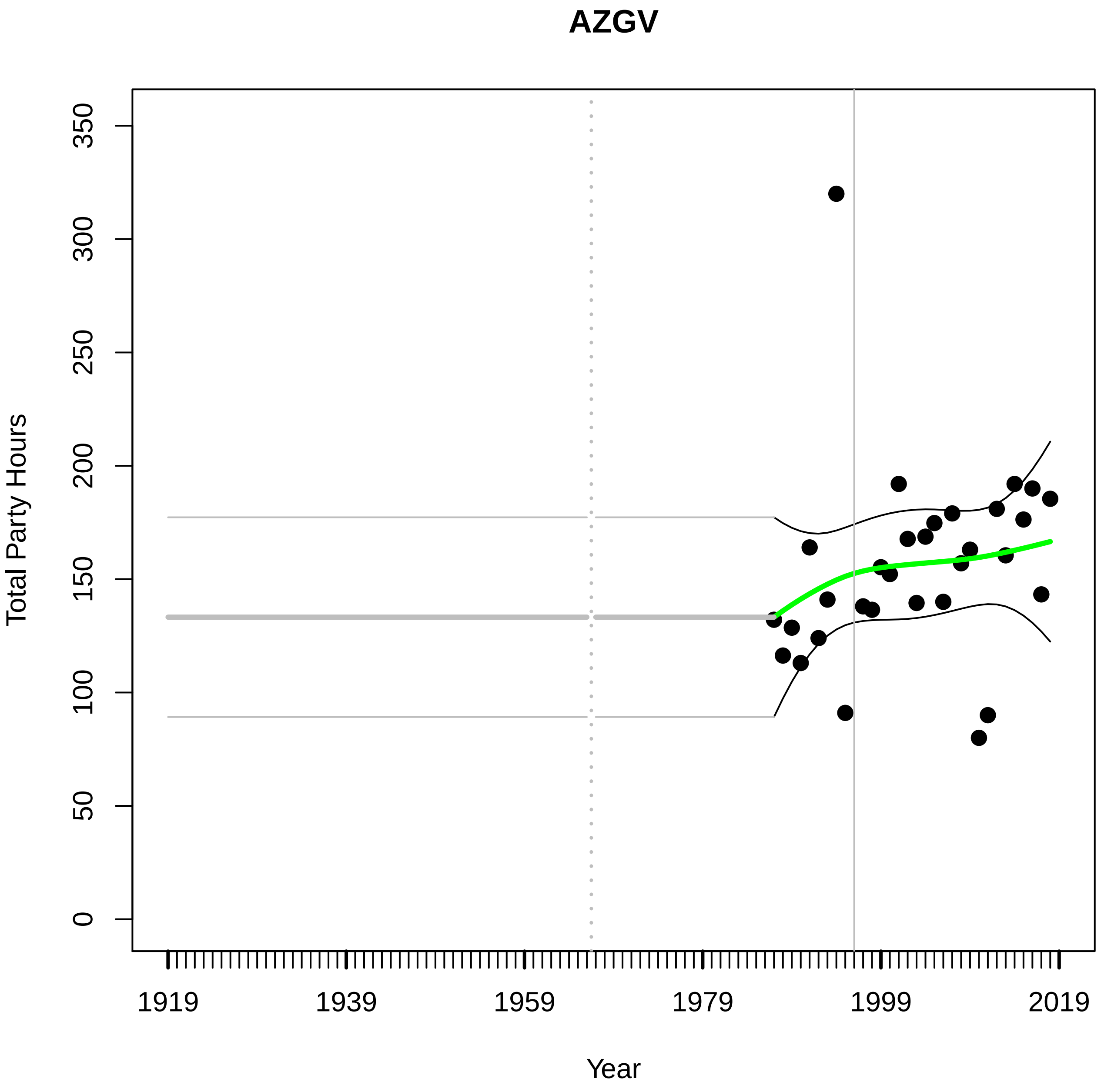

AZGV
Total Party Hours
Year
0
50
100
150
200
250
300
350
1919
1939
1959
1979
1999
2019

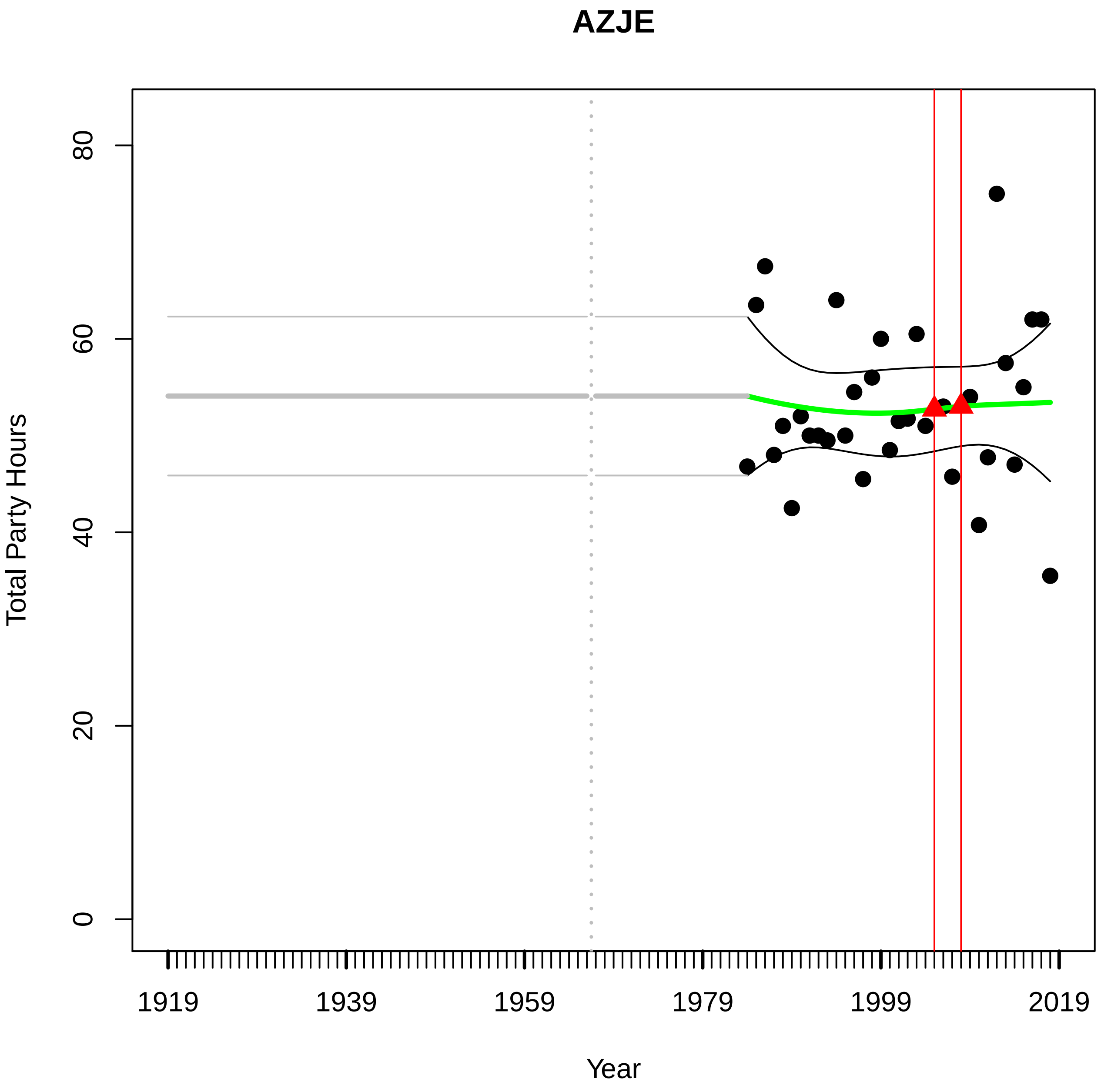

AZJE
Total Party Hours
0
20
40
60
80
1919
1939
1959
1979
1999
2019
Year

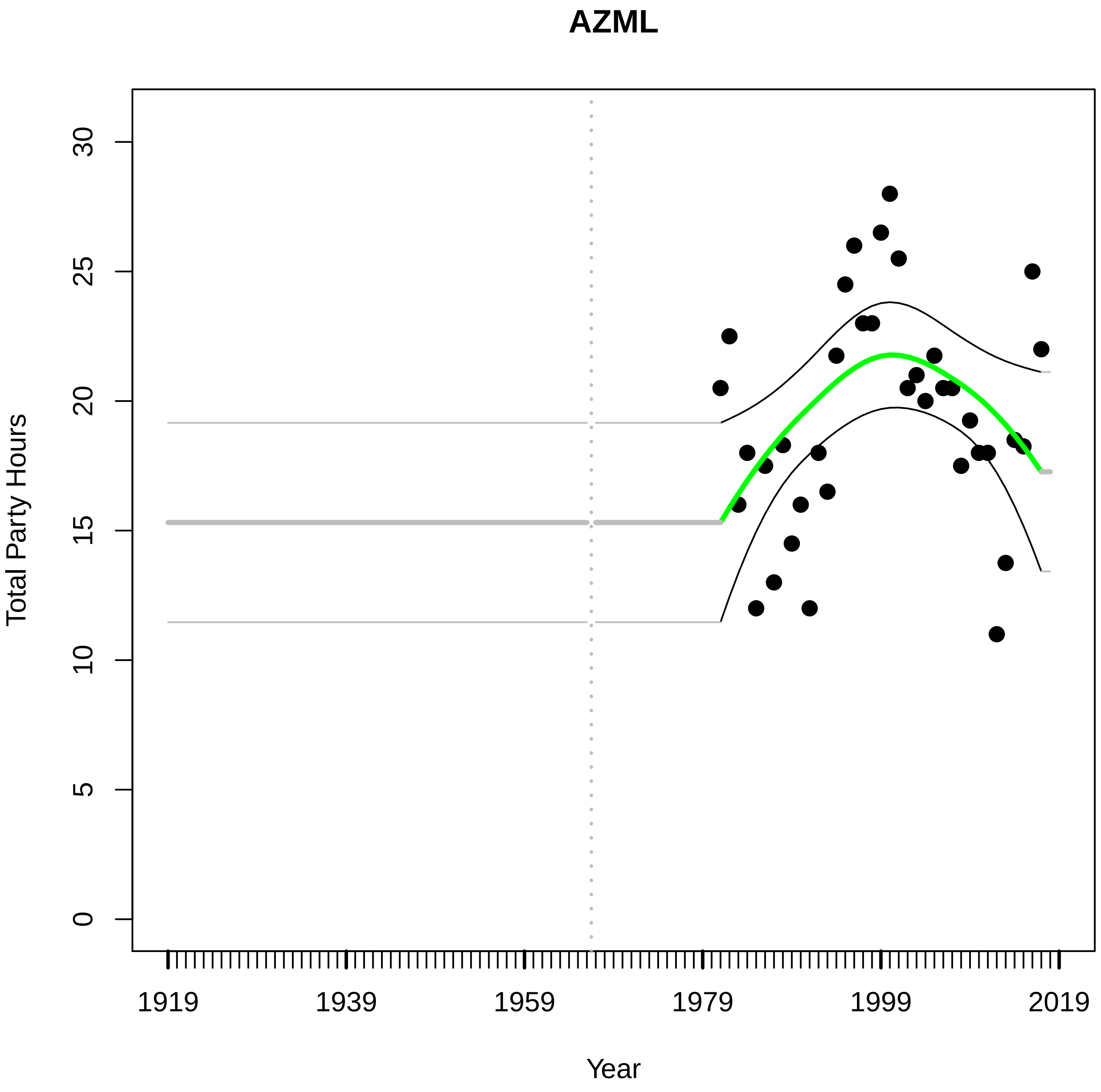

AZML
Total Party Hours
Year
0
5
10
15
20
25
30
1919
1939
1959
1979
1999
2019

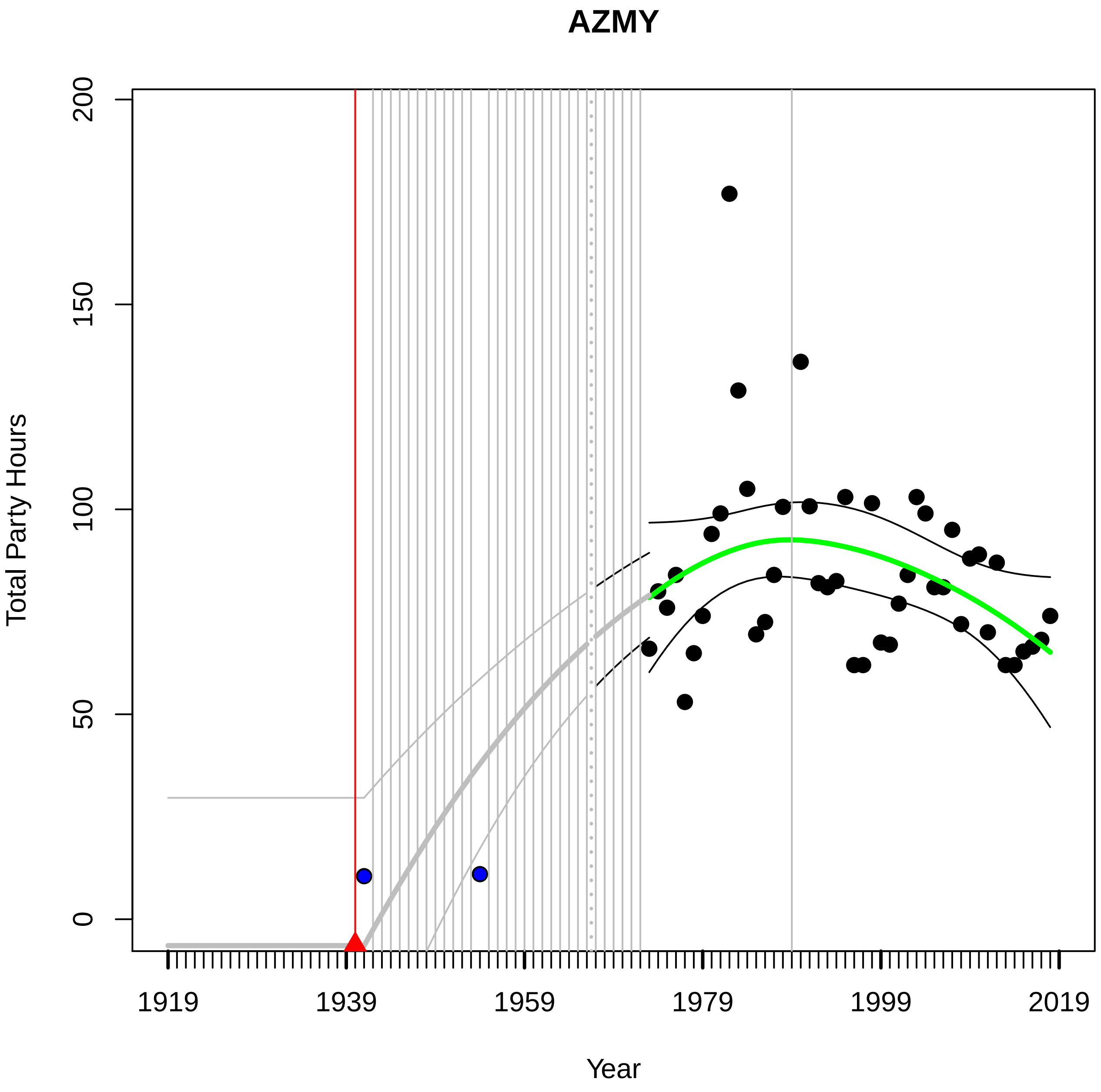

AZMY
Total Party Hours
0
50
100
150
200
Year
1919
1939
1959
1979
1999
2019

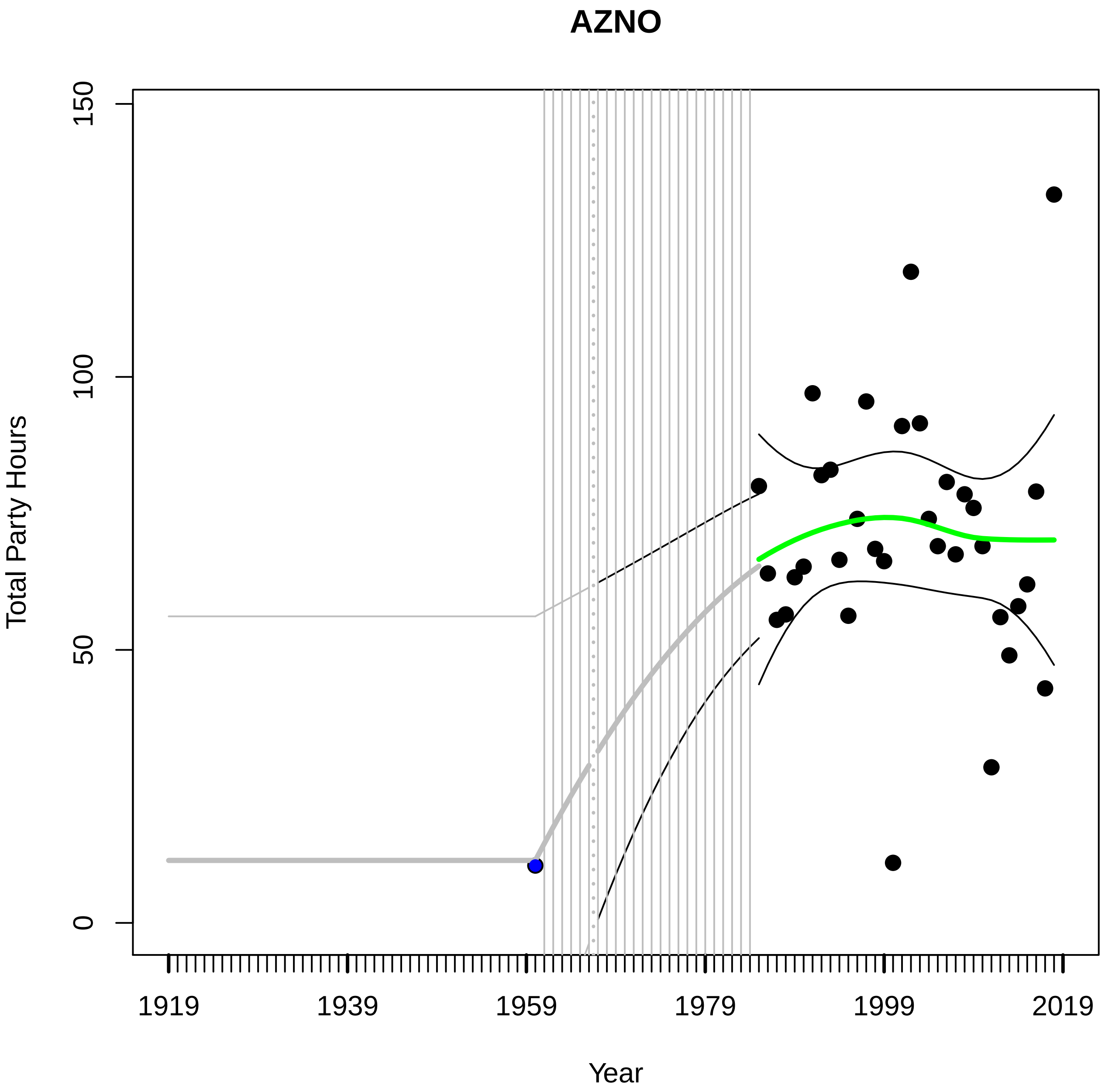

AZNO
Total Party Hours
Year
0
50
100
150
1919
1939
1959
1979
1999
2019

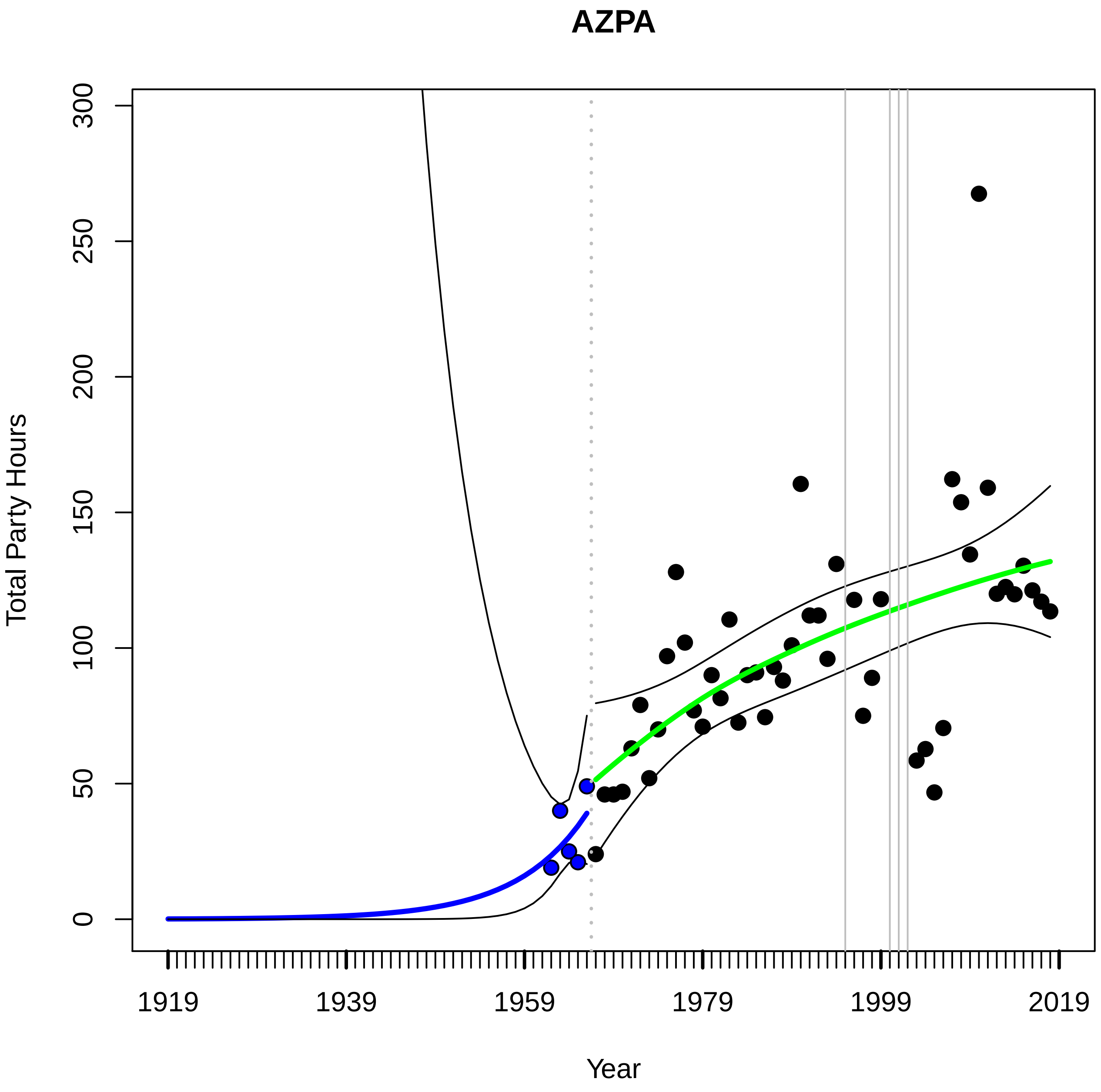
AZPA
Total Party Hours
0
50
100
150
200
250
300
1919
1939
1959
1979
1999
2019
Year

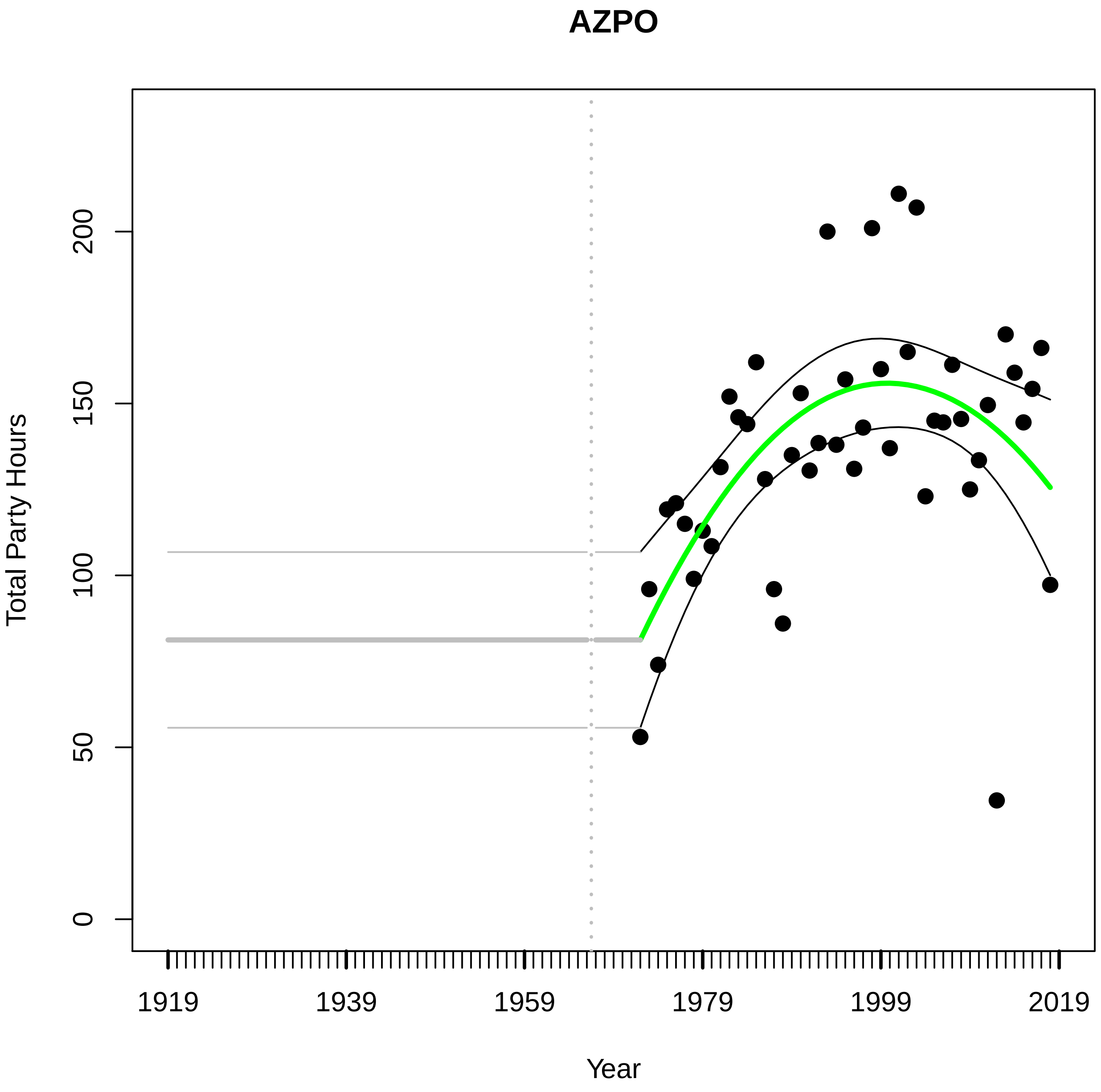

AZPO
Total Party Hours
0
50
100
150
200
1919
1939
1959
1979
1999
2019
Year

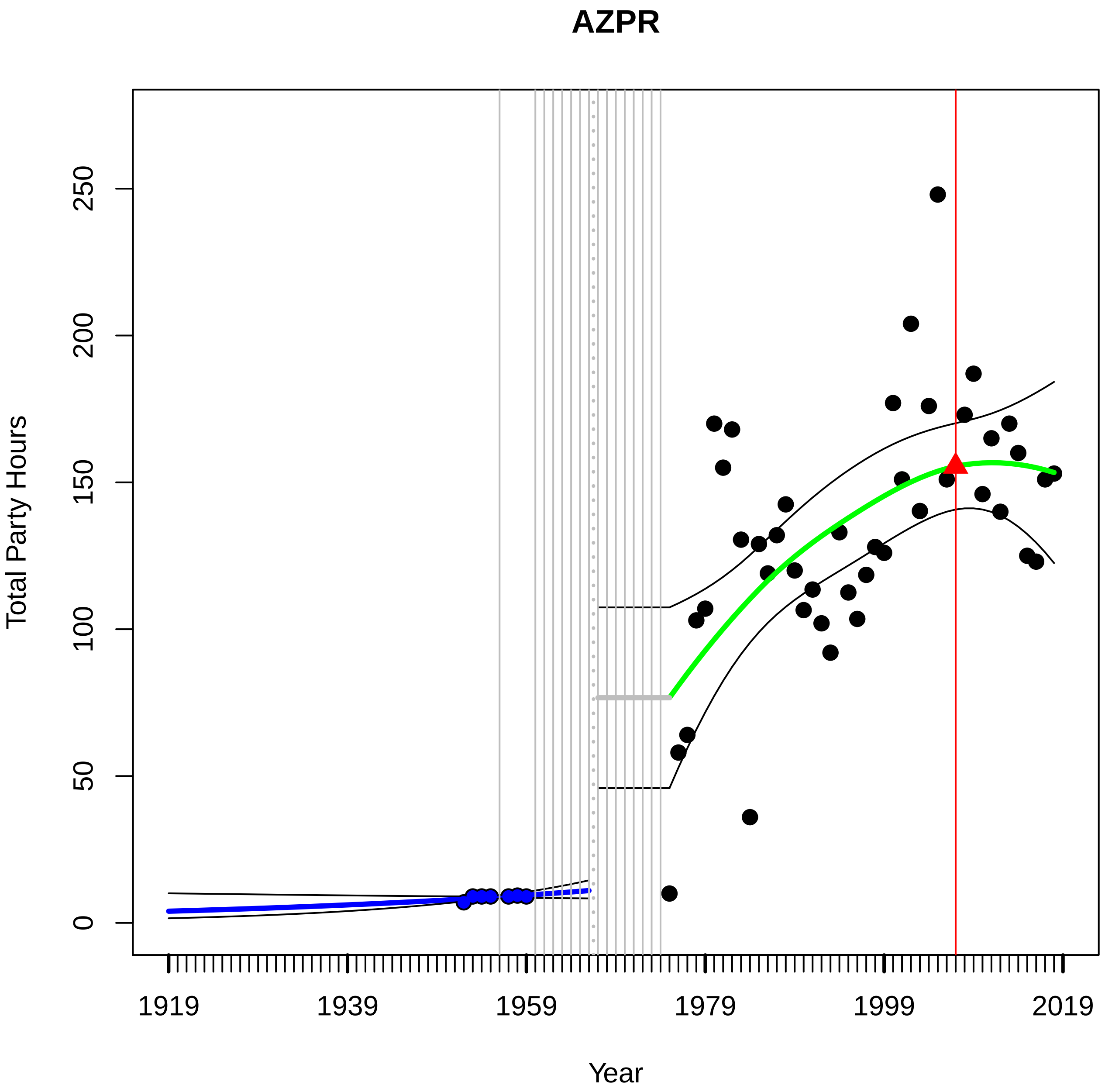

AZPR
Total Party Hours
0
50
100
150
200
250
1919
1939
1959
1979
1999
2019
Year

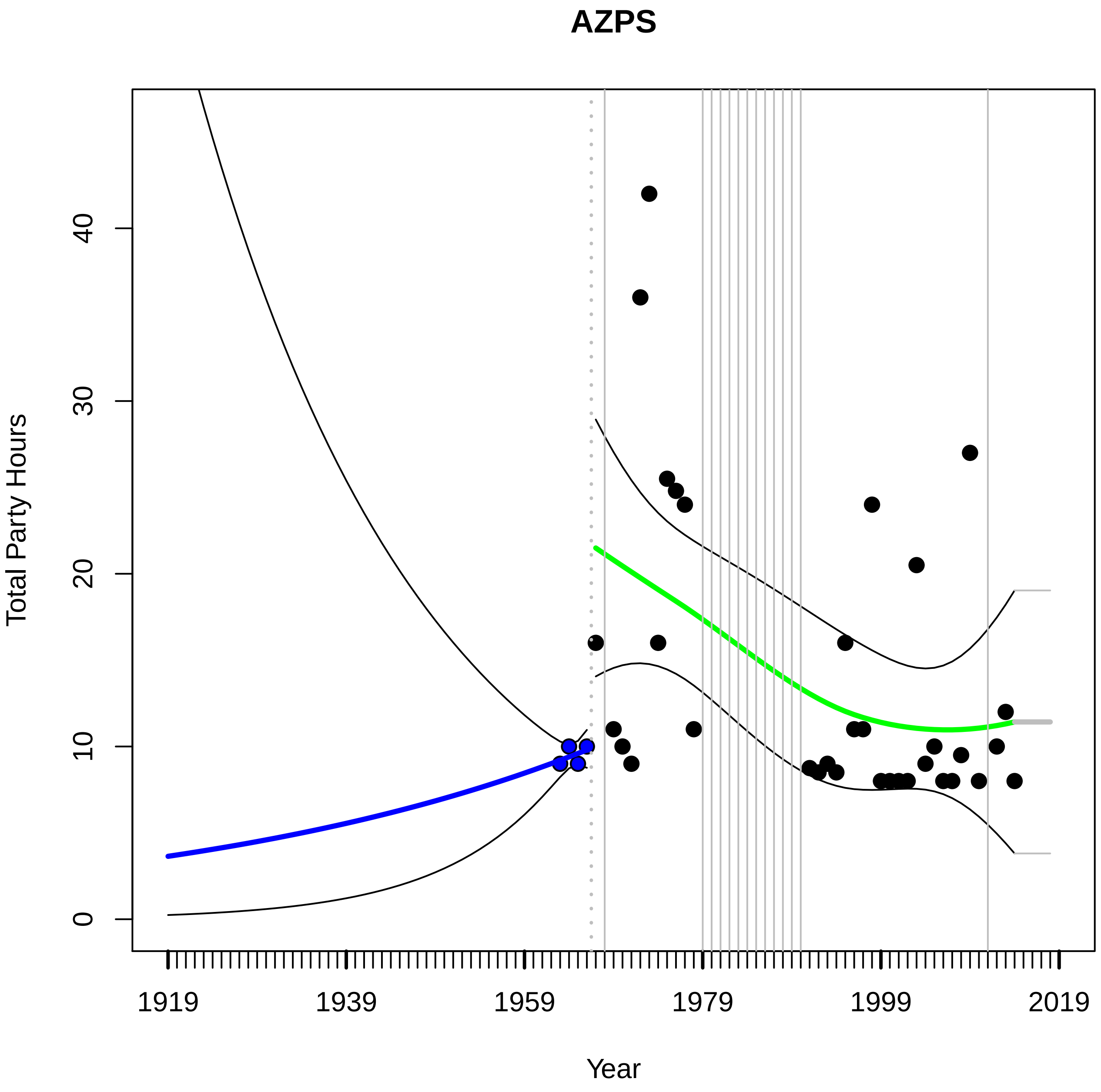
AZPS
Total Party Hours
Year
0
10
20
30
40
1919
1939
1959
1979
1999
2019

AZRC

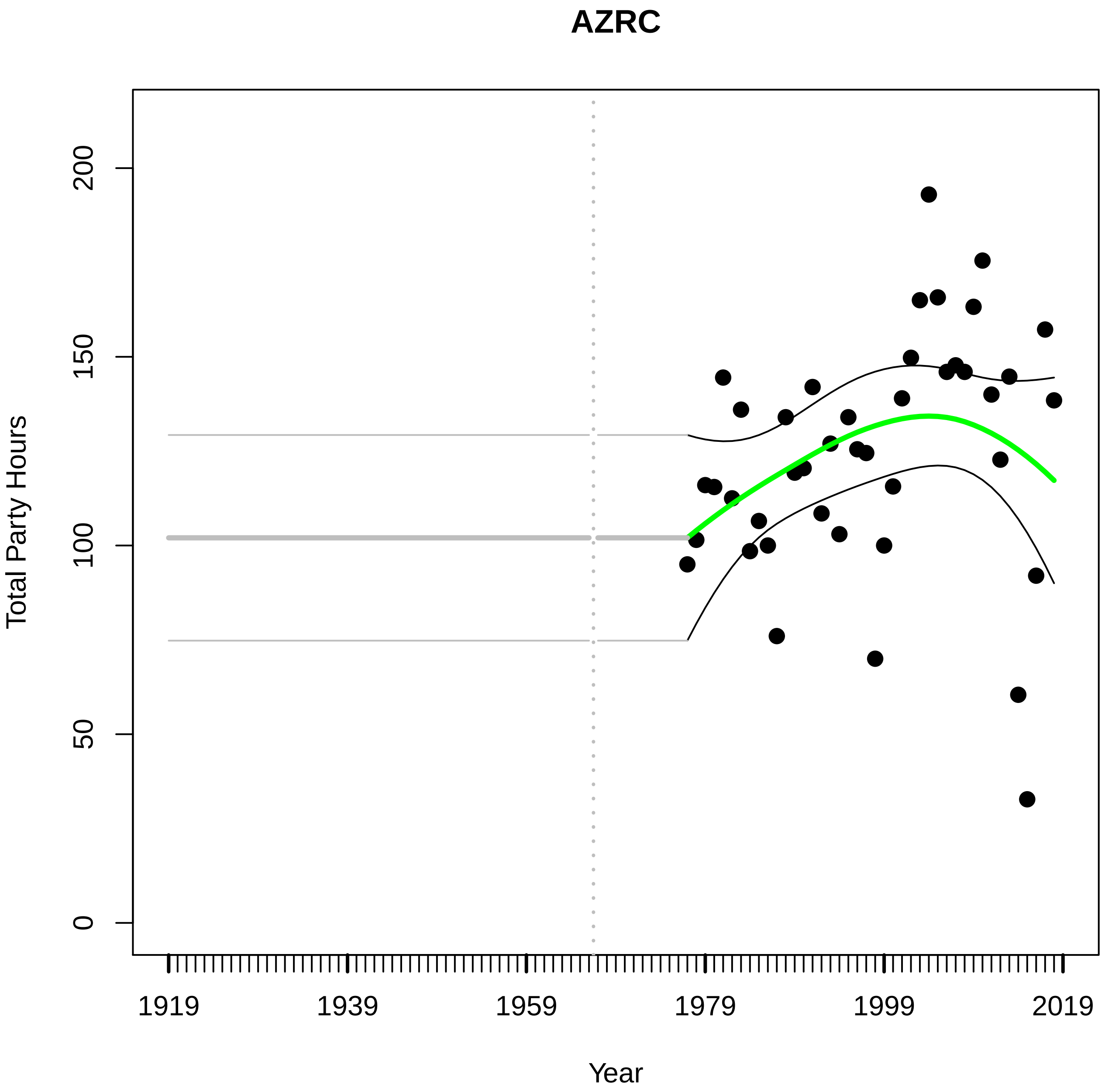

AZRC
Total Party Hours
200
150
100
50
0
1919
1939
1959
1979
1999
2019
Year

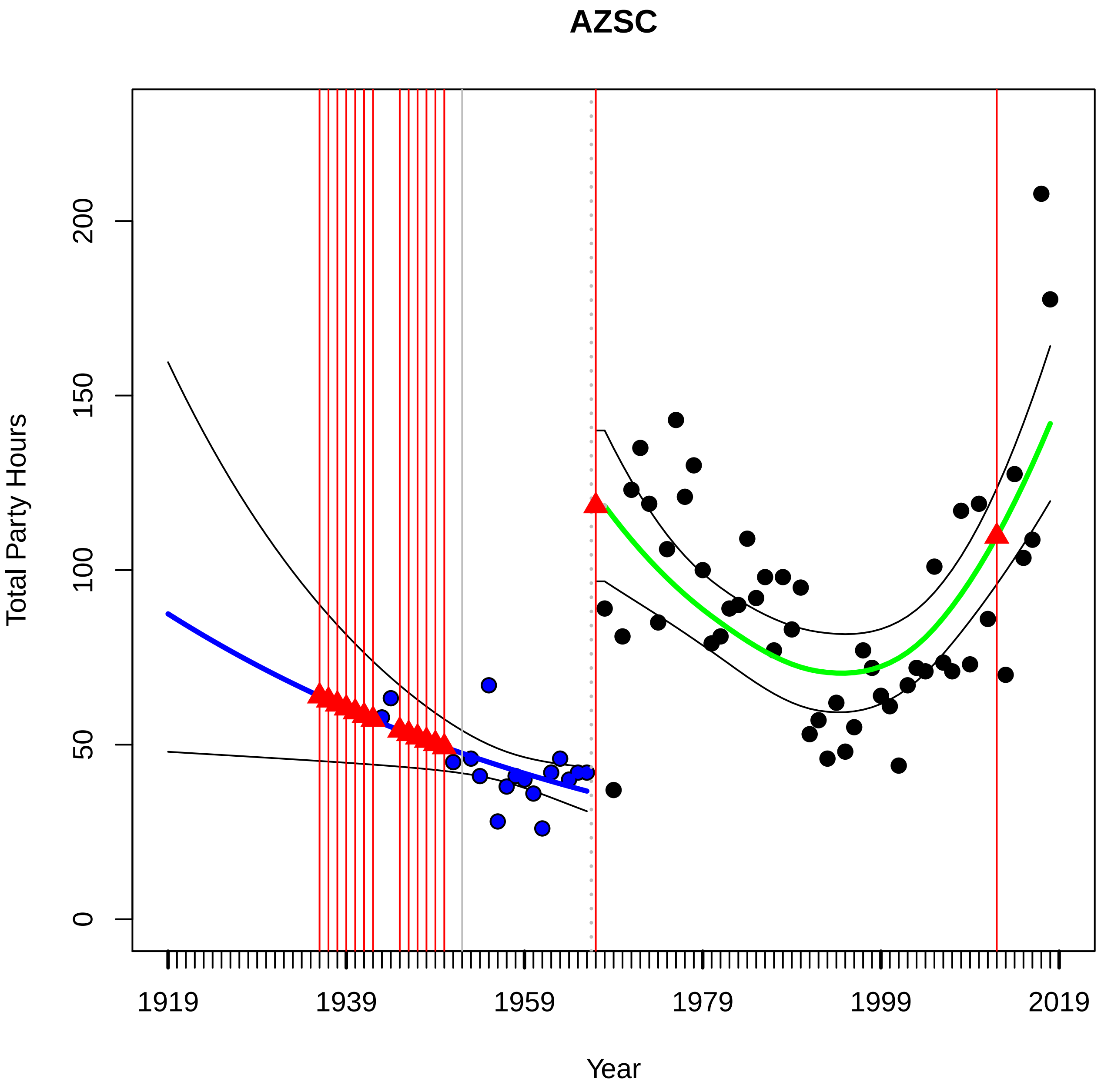
AZSC
Total Party Hours
0
50
100
150
200
1919
1939
1959
1979
1999
2019
Year

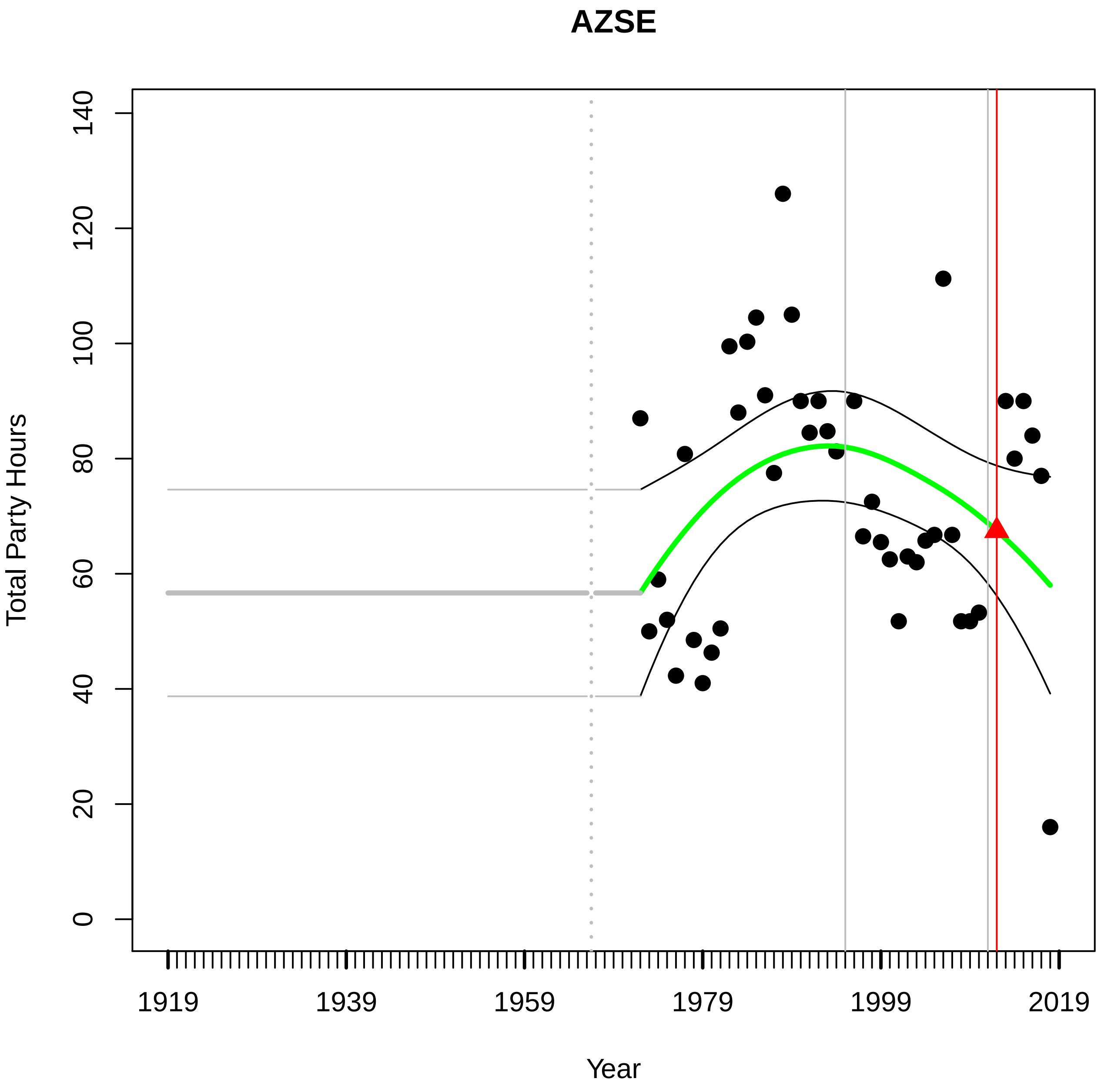

AZSE
Total Party Hours
0
20
40
60
80
100
120
140
1919
1939
1959
1979
1999
2019
Year

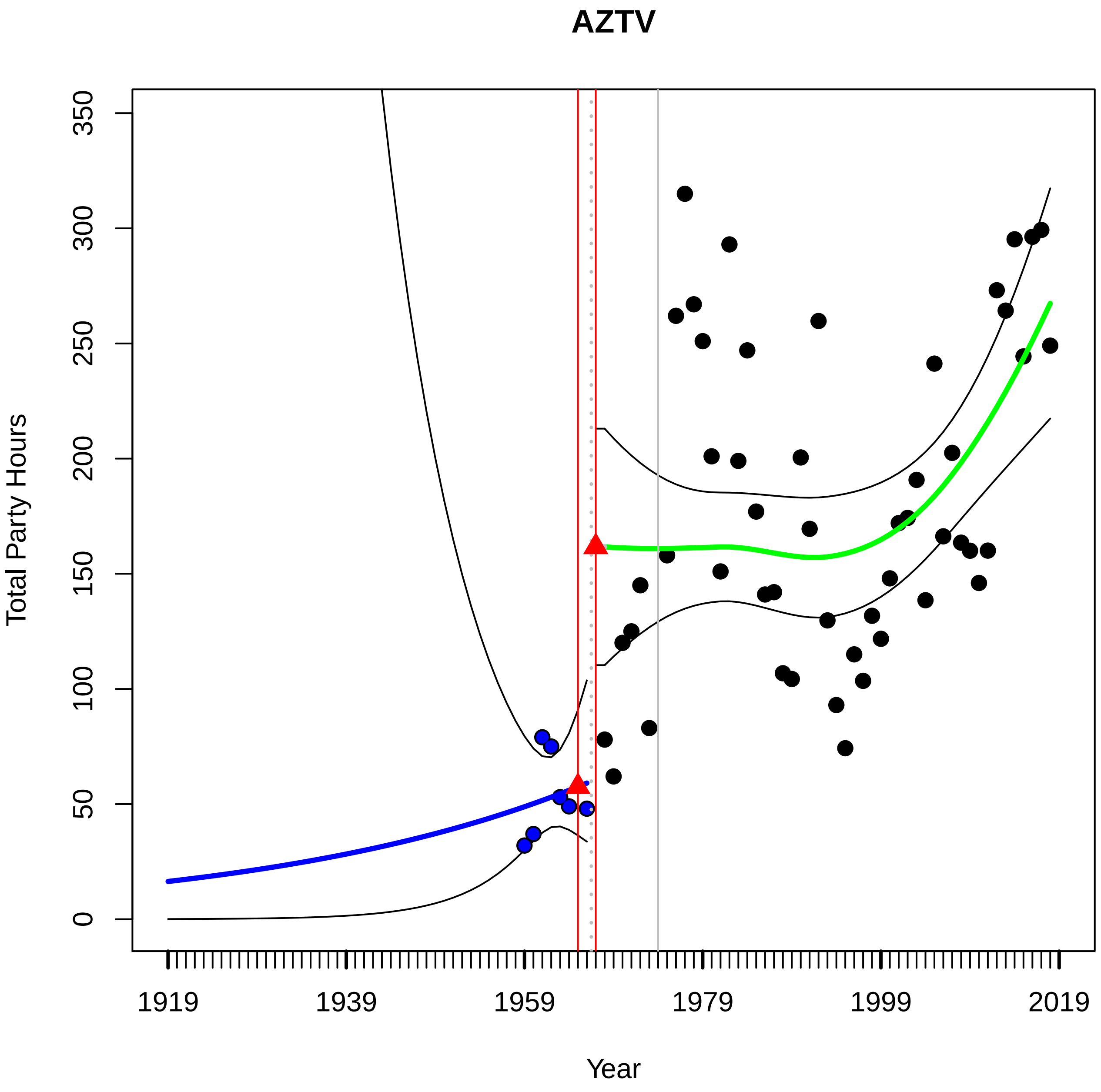
AZTV
Total Party Hours
0
50
100
150
200
250
300
350
1919
1939
1959
1979
1999
2019
Year

CA1B

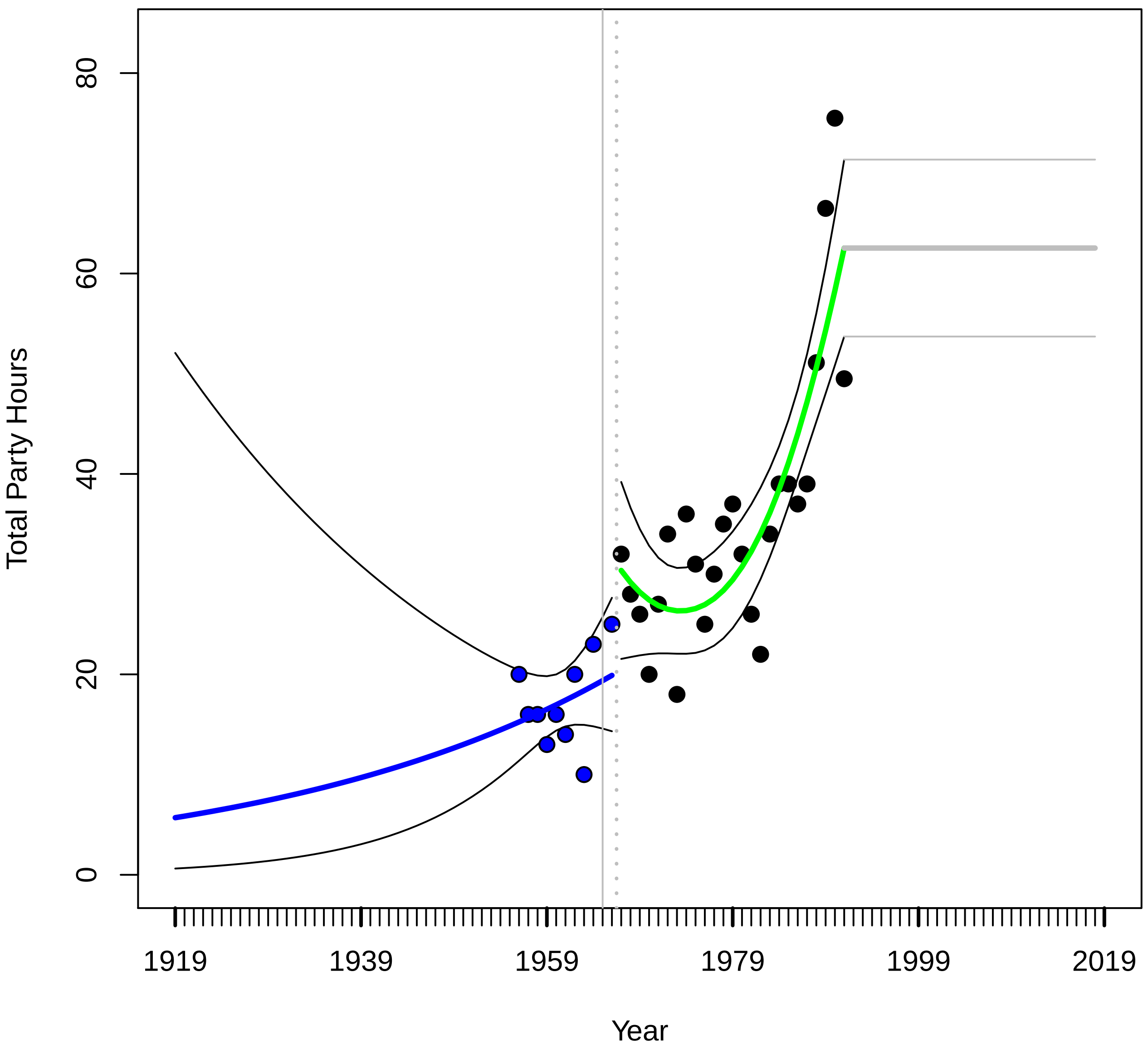

Total Party Hours
Year
0
20
40
60
80
1919
1939
1959
1979
1999
2019

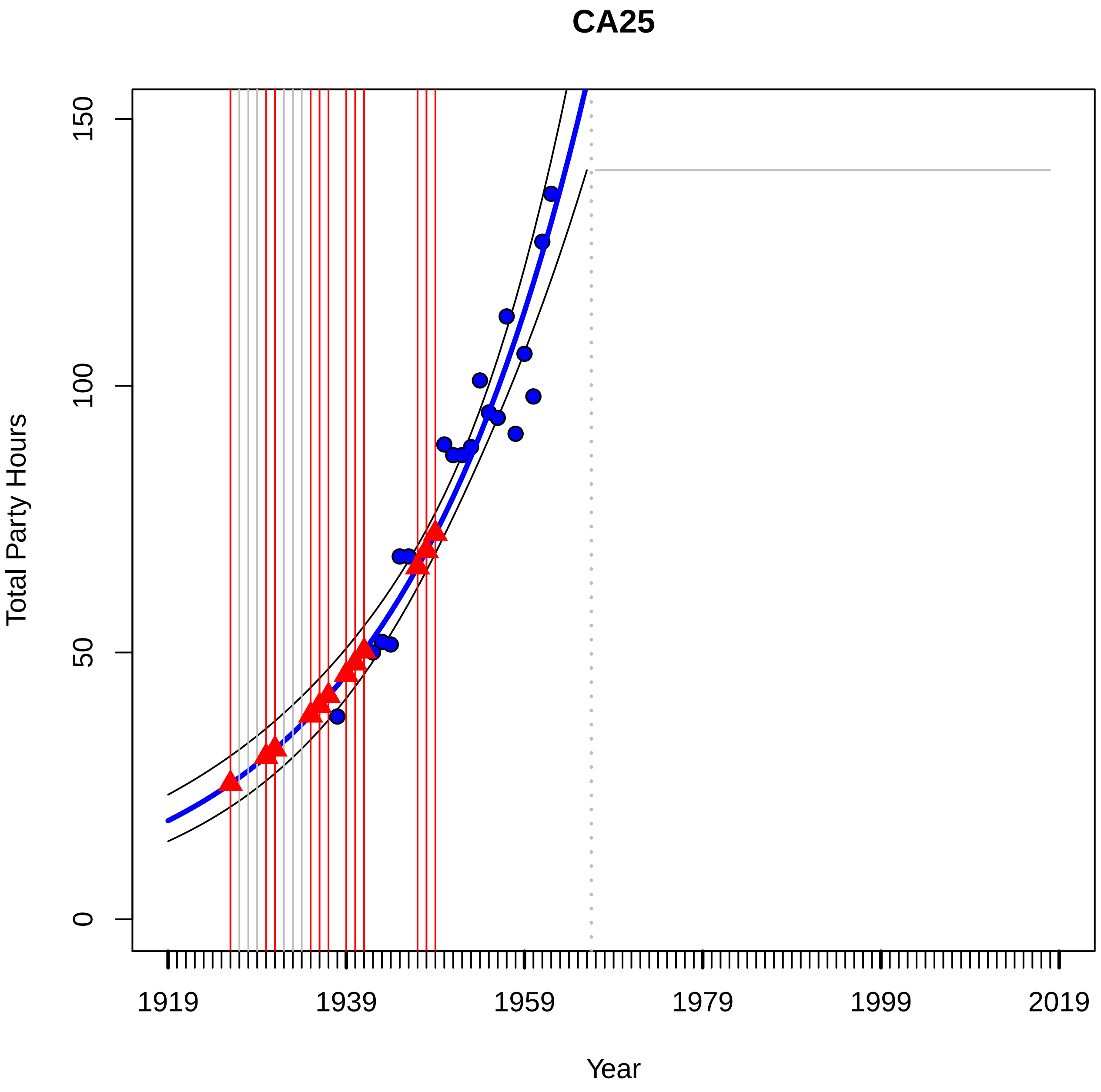

CA25
Total Party Hours
0
50
100
150
1919
1939
1959
1979
1999
2019
Year

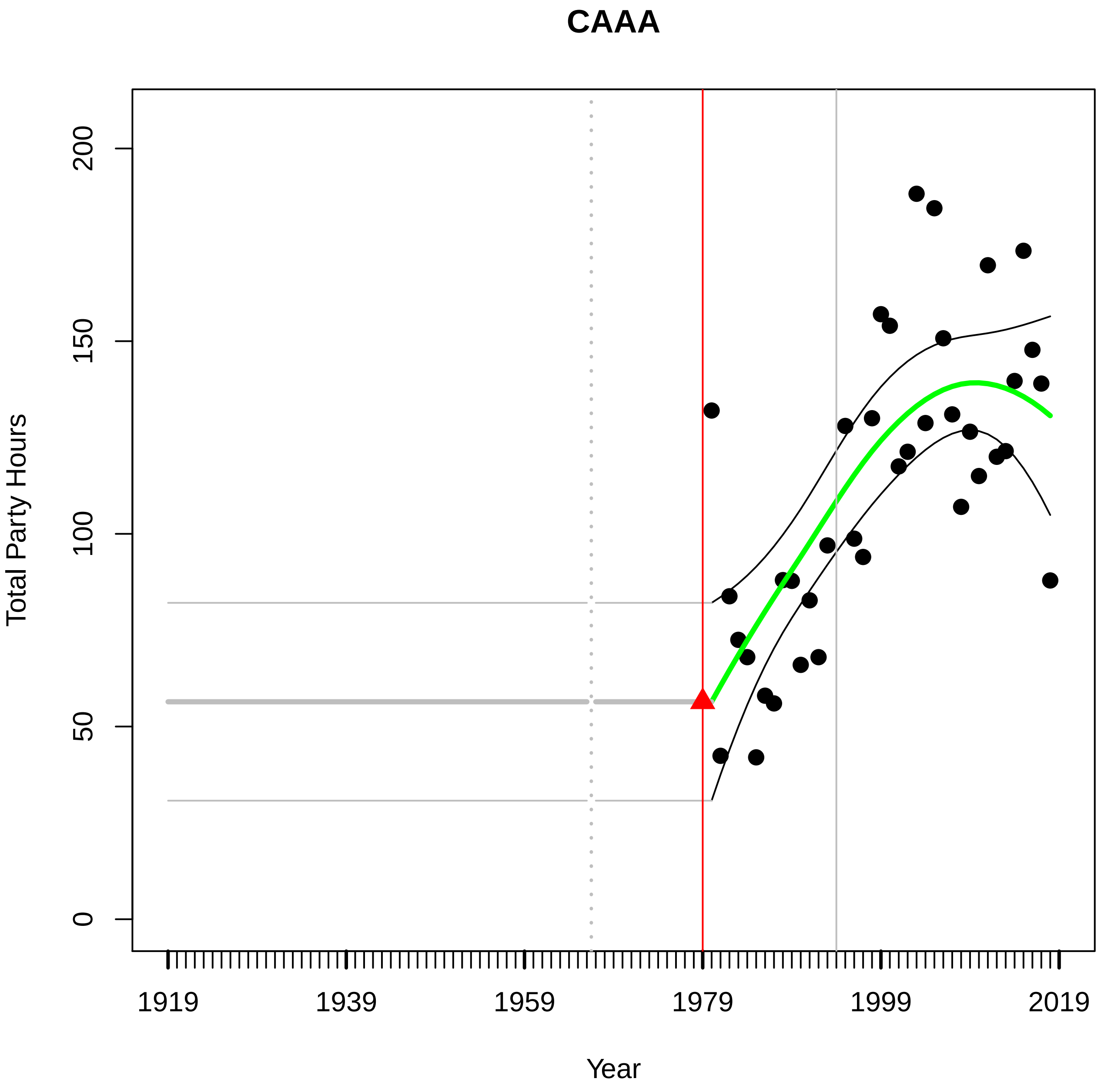
CAAA
Total Party Hours
0
50
100
150
200
1919
1939
1959
1979
1999
2019
Year

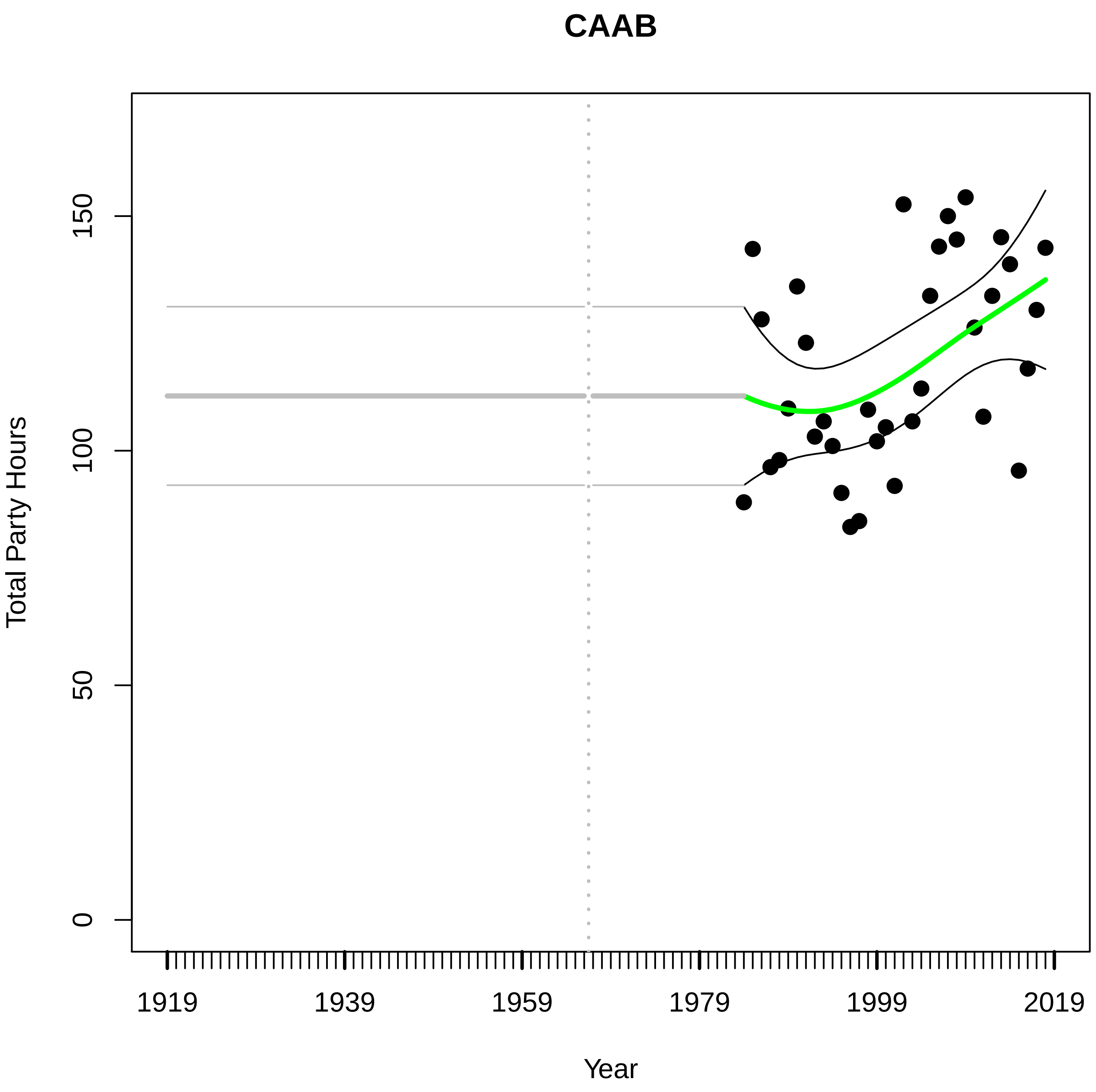

CAAB
Total Party Hours
0
50
100
150
1919
1939
1959
1979
1999
2019
Year

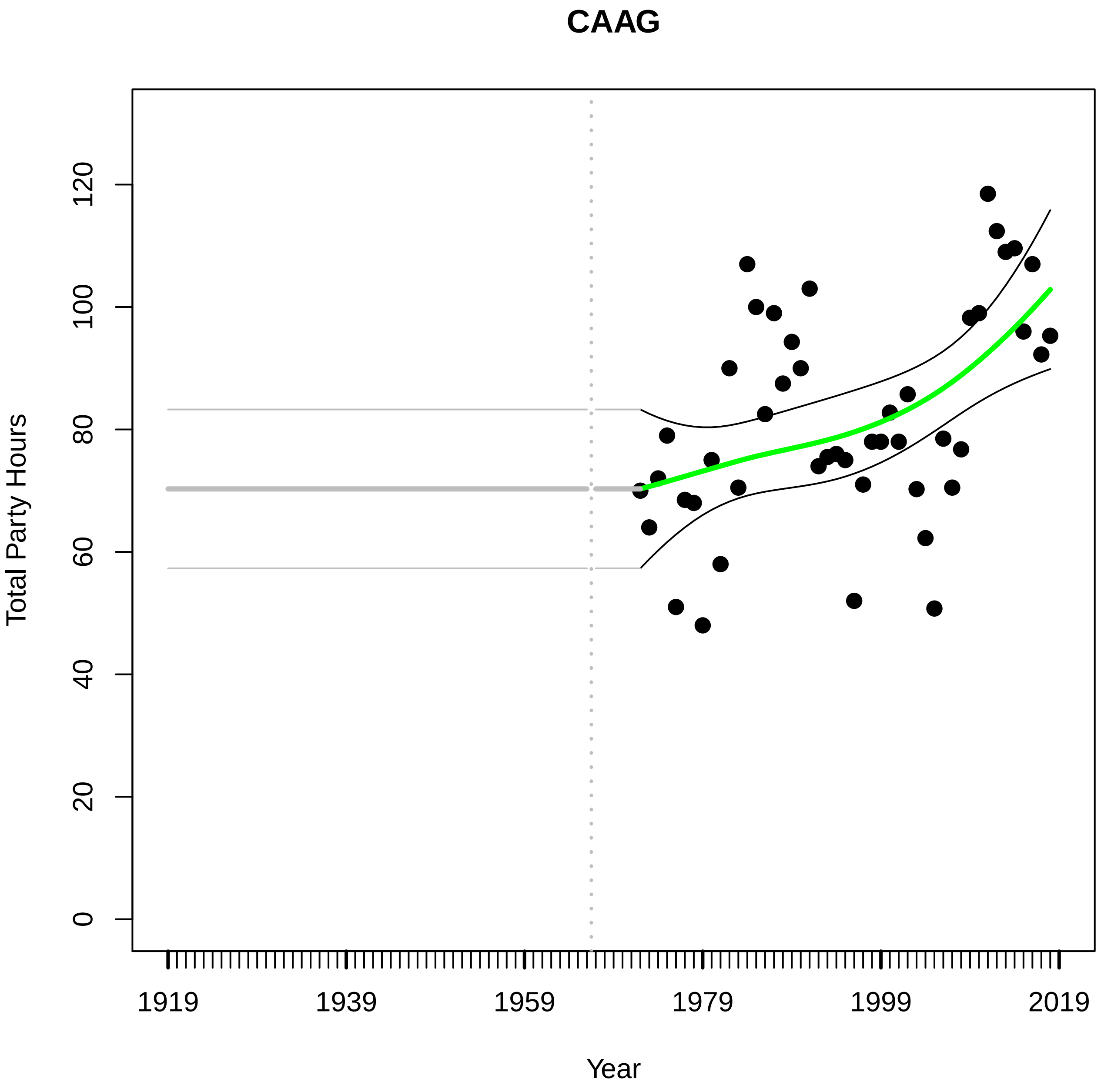

CAAG
Total Party Hours
120
100
80
60
40
20
0
1919
1939
1959
1979
1999
2019
Year

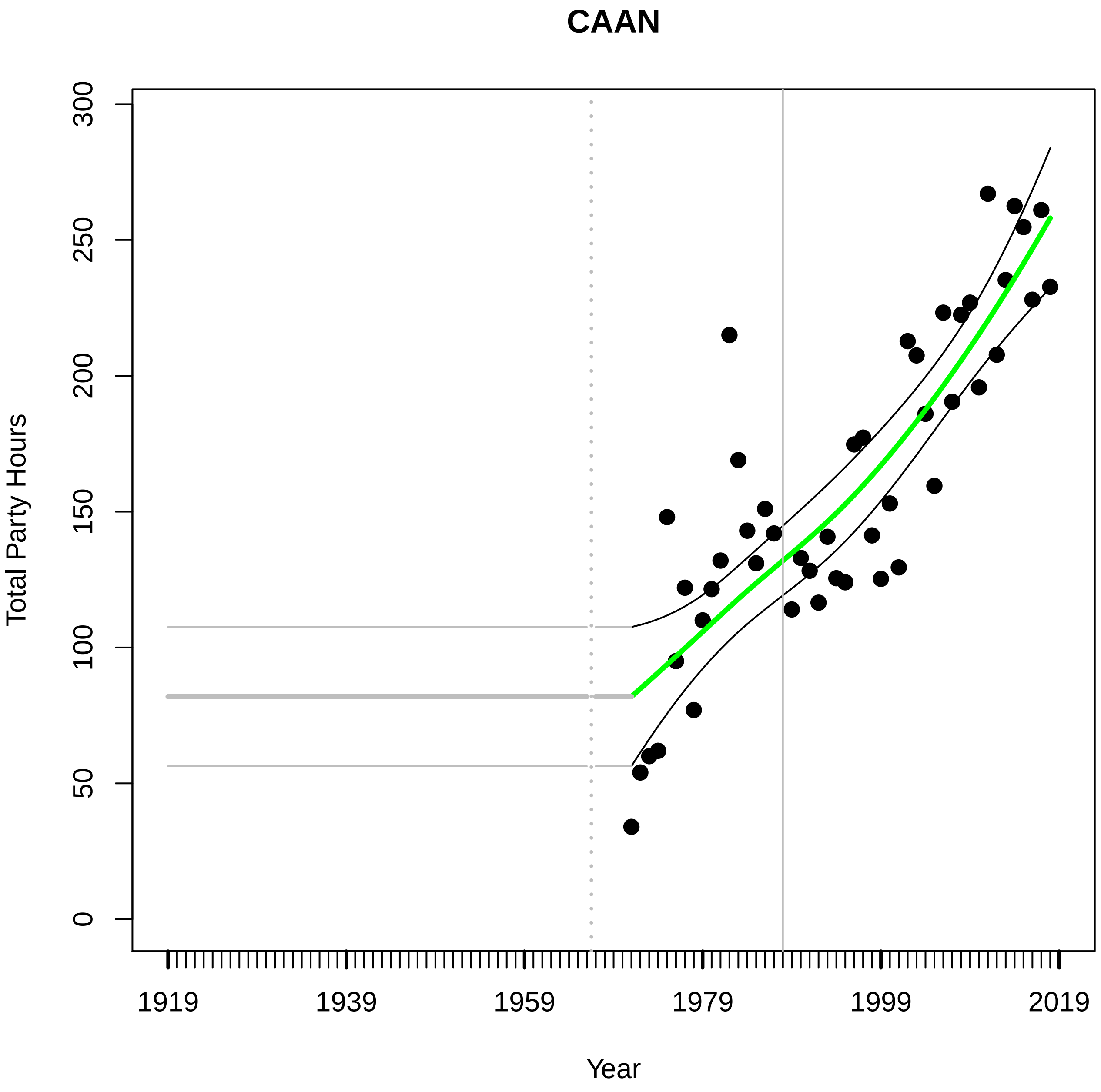
CAAN
Total Party Hours
300
250
200
150
100
50
0
1919
1939
1959
1979
1999
2019
Year

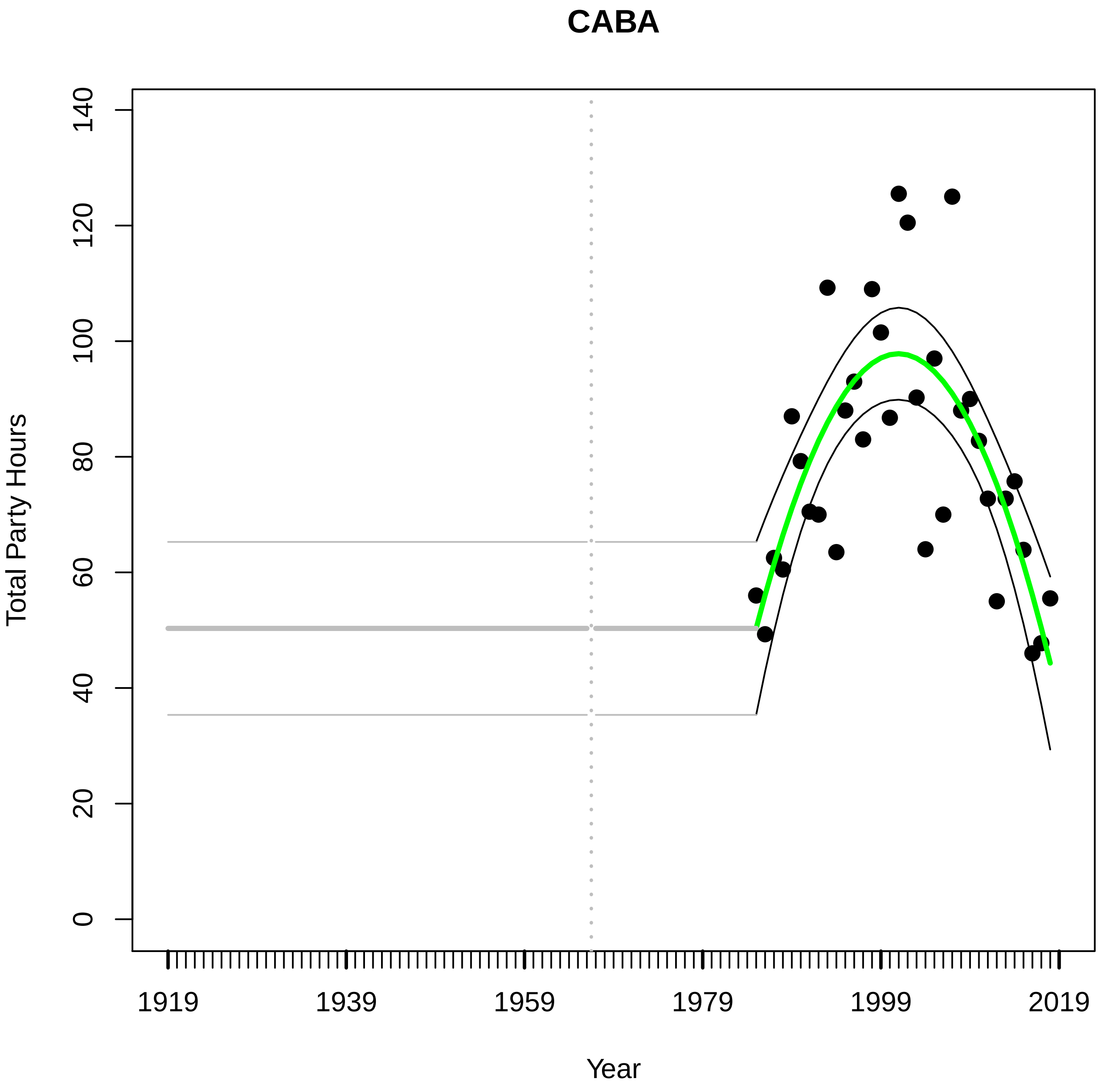

CABA
Total Party Hours
0
20
40
60
80
100
120
140
1919
1939
1959
1979
1999
2019
Year

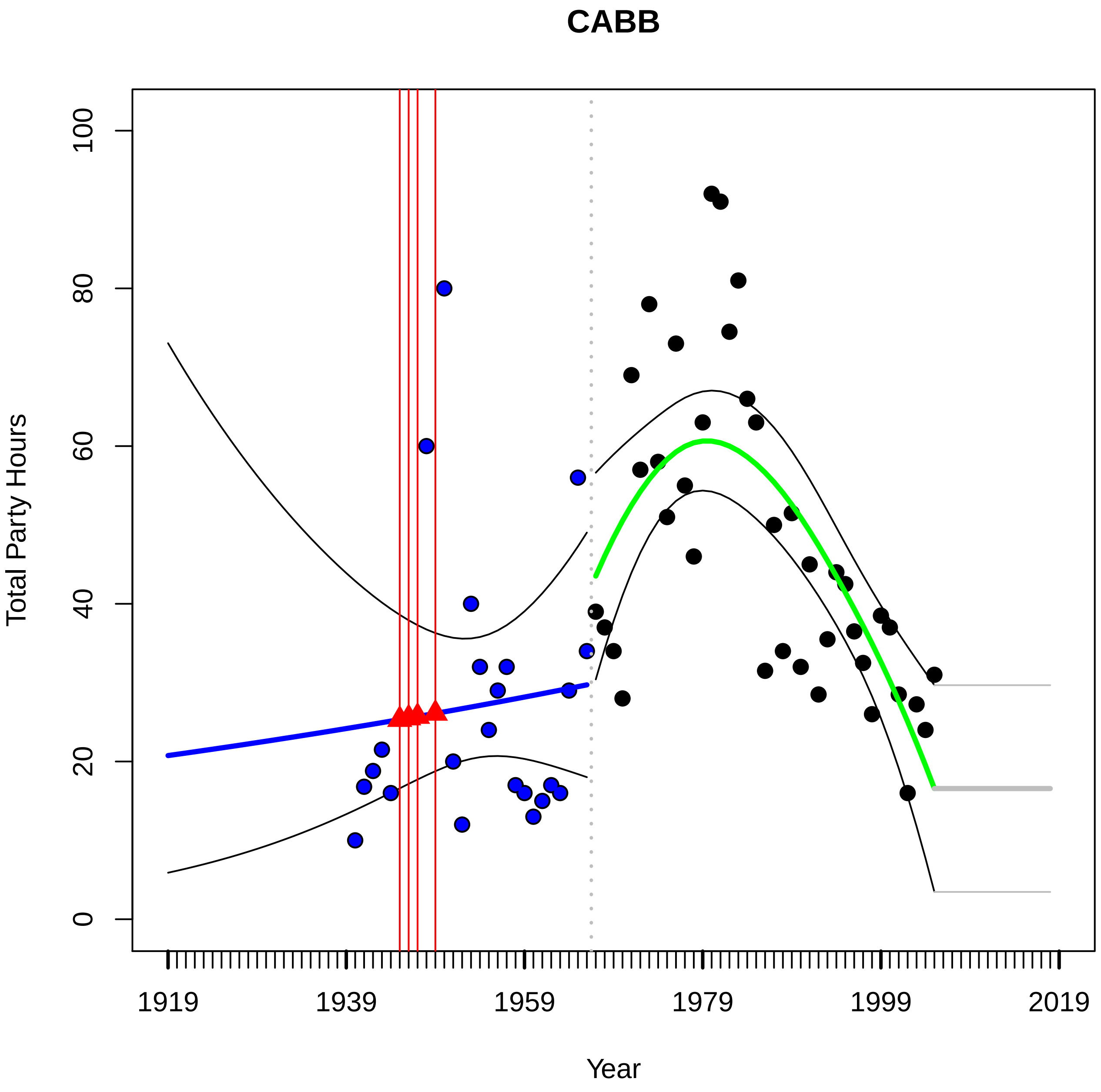
CABB
Total Party Hours
Year
0
20
40
60
80
100
1919
1939
1959
1979
1999
2019

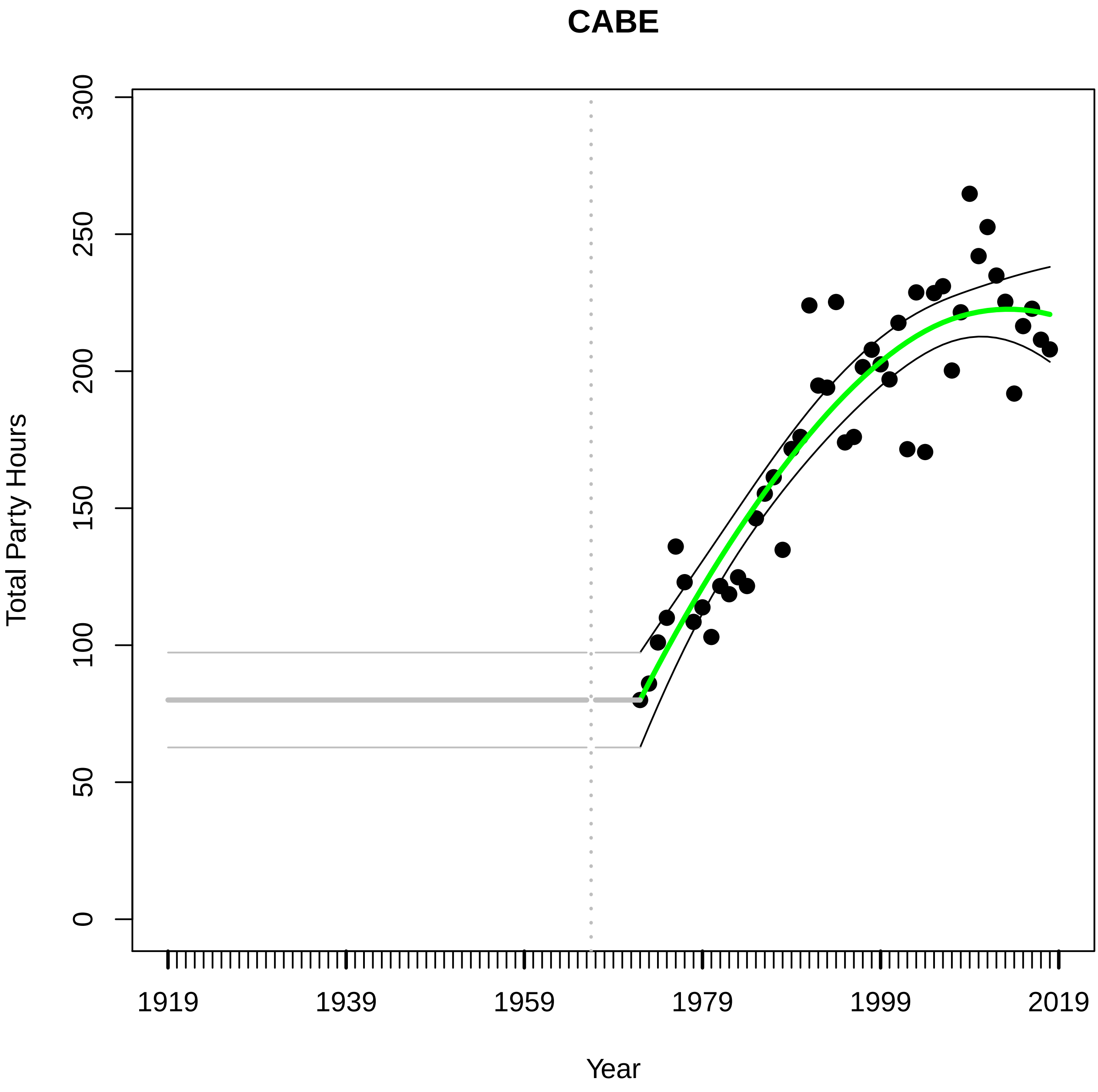

CABE
Total Party Hours
300
250
200
150
100
50
0
1919
1939
1959
1979
1999
2019
Year

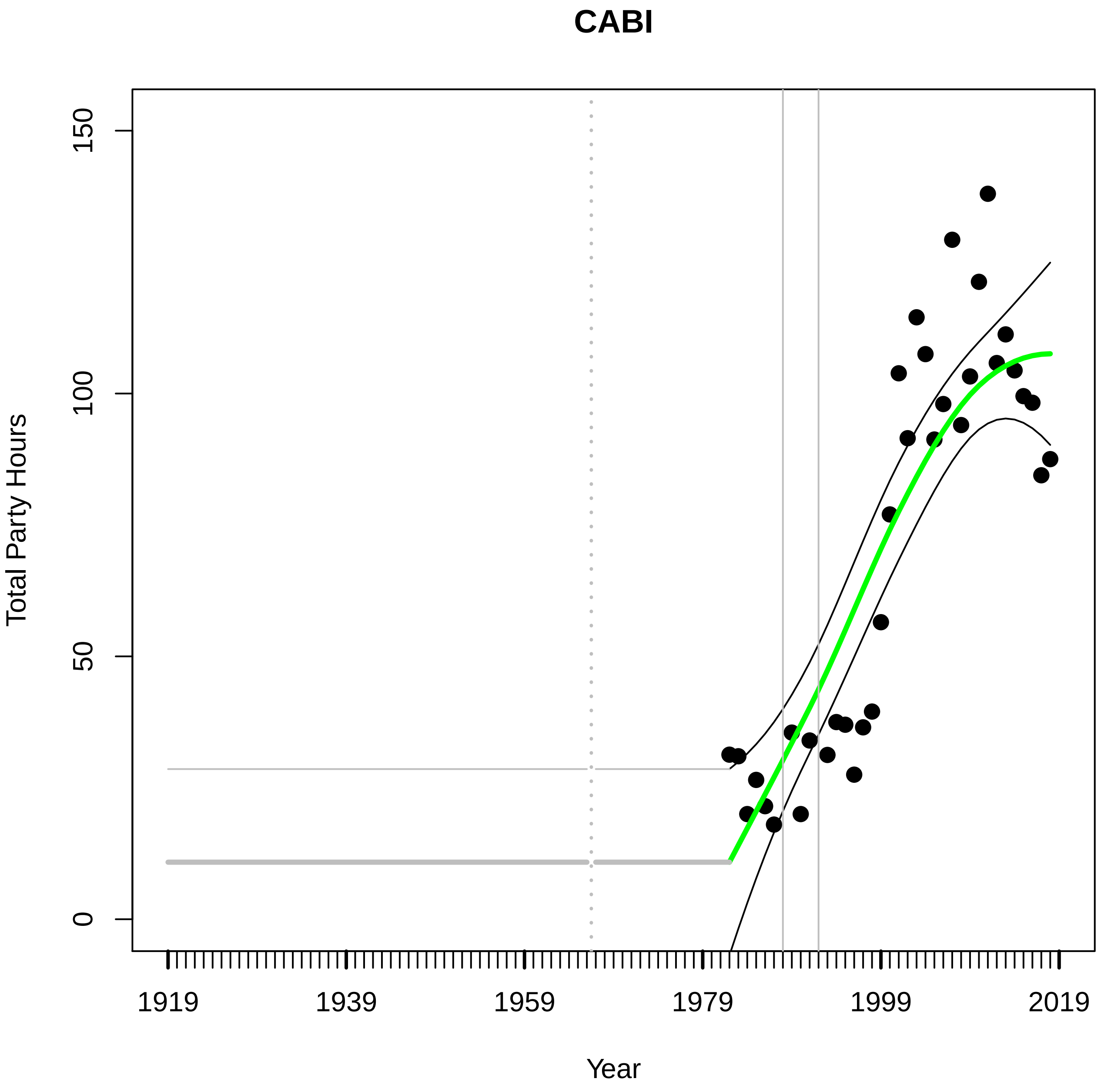

CABI
Total Party Hours
0
50
100
150
1919
1939
1959
1979
1999
2019
Year

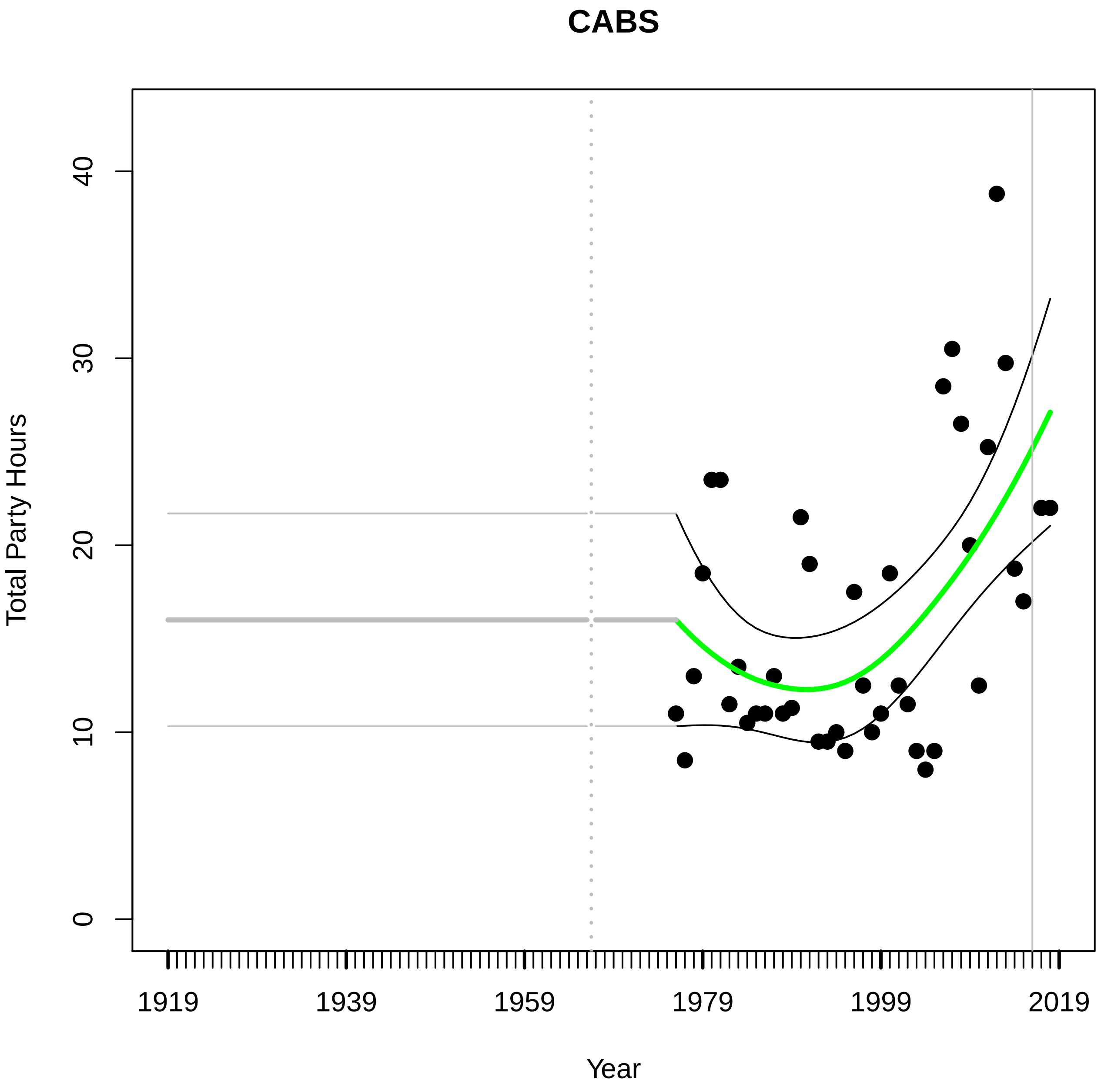

CABS
Total Party Hours
Year
0
10
20
30
40
1919
1939
1959
1979
1999
2019

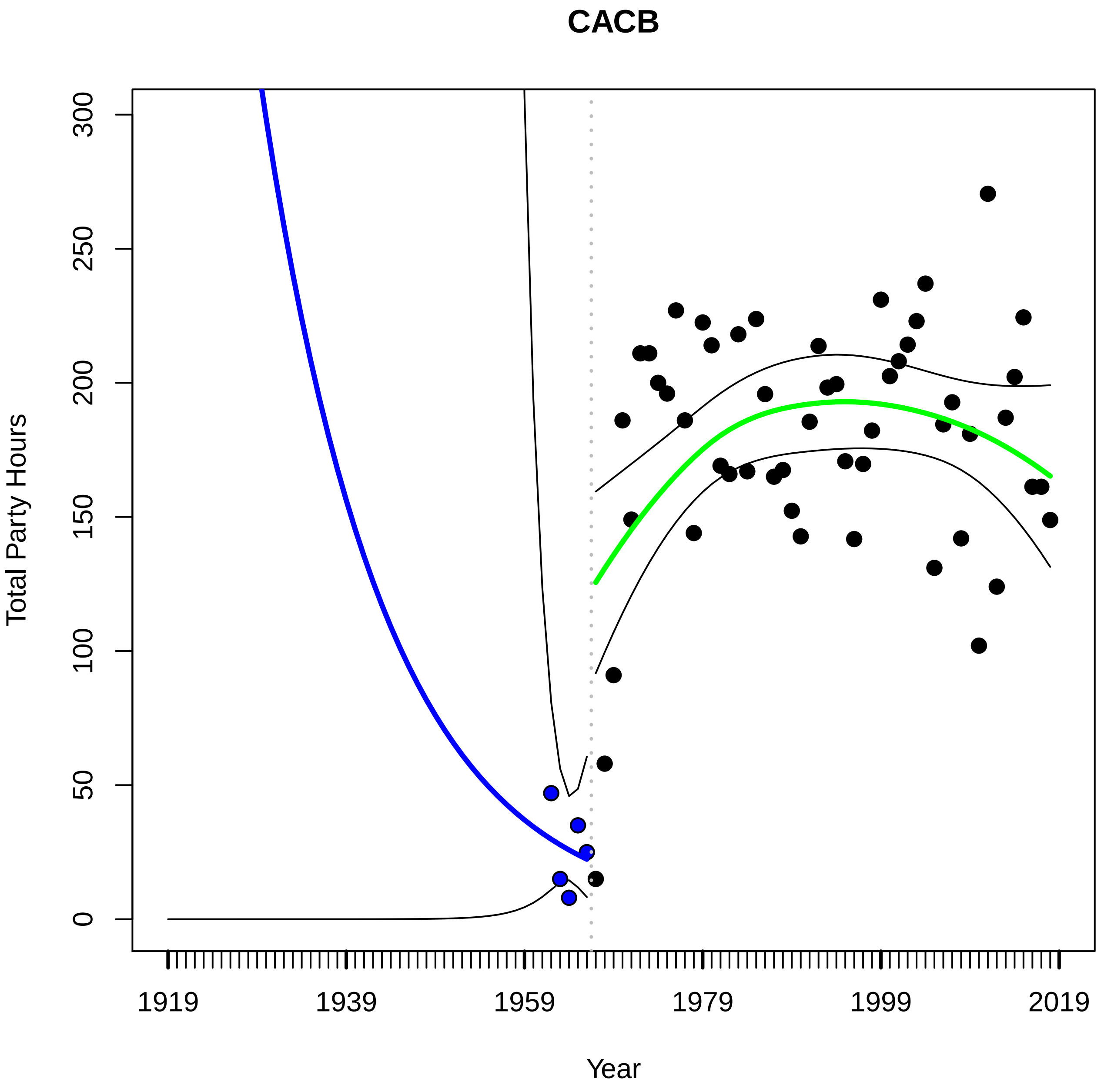
CACB
Total Party Hours
Year
0
50
100
150
200
250
300
1919
1939
1959
1979
1999
2019

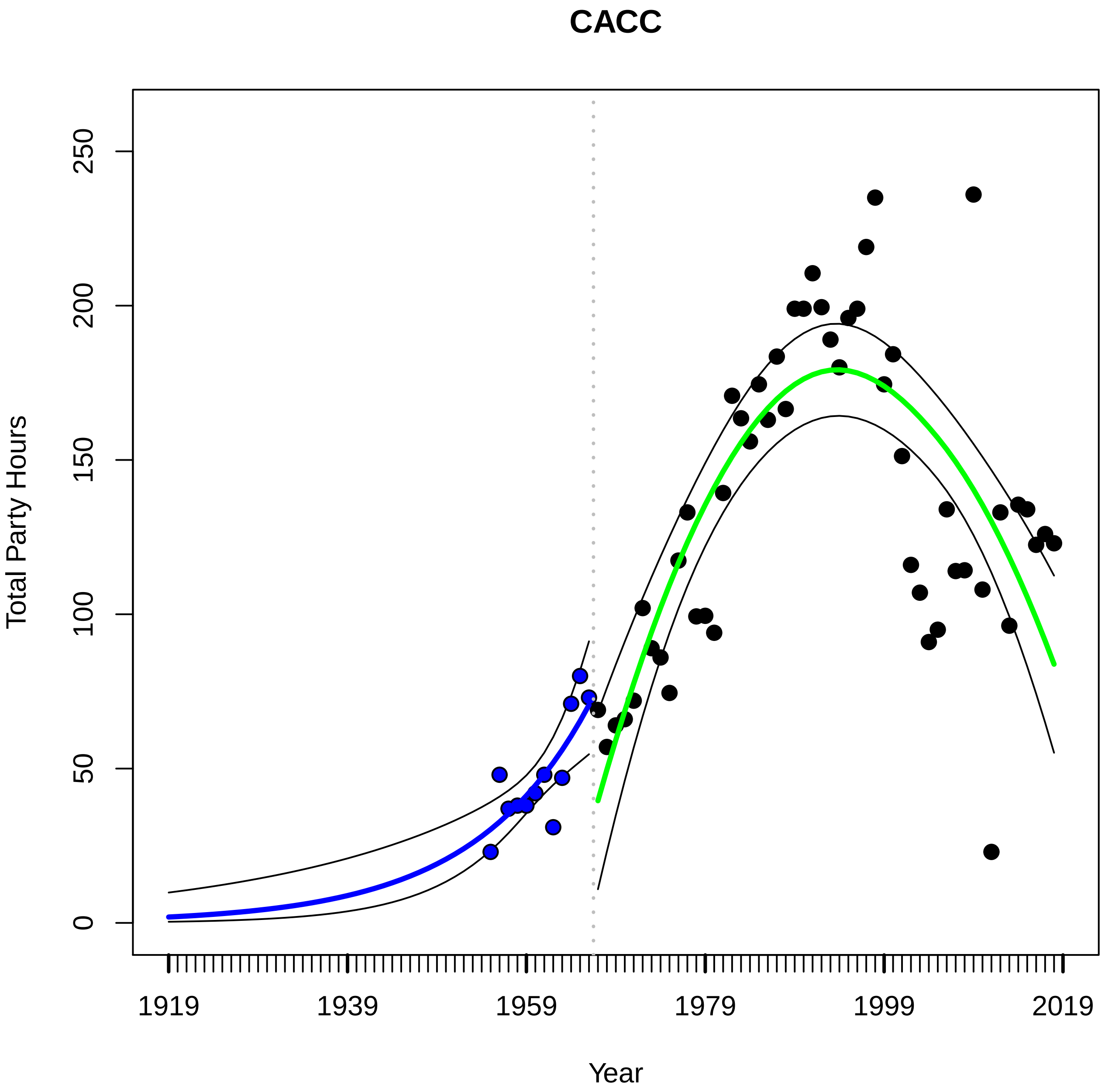

CACC
Total Party Hours
0
50
100
150
200
250
1919
1939
1959
1979
1999
2019
Year

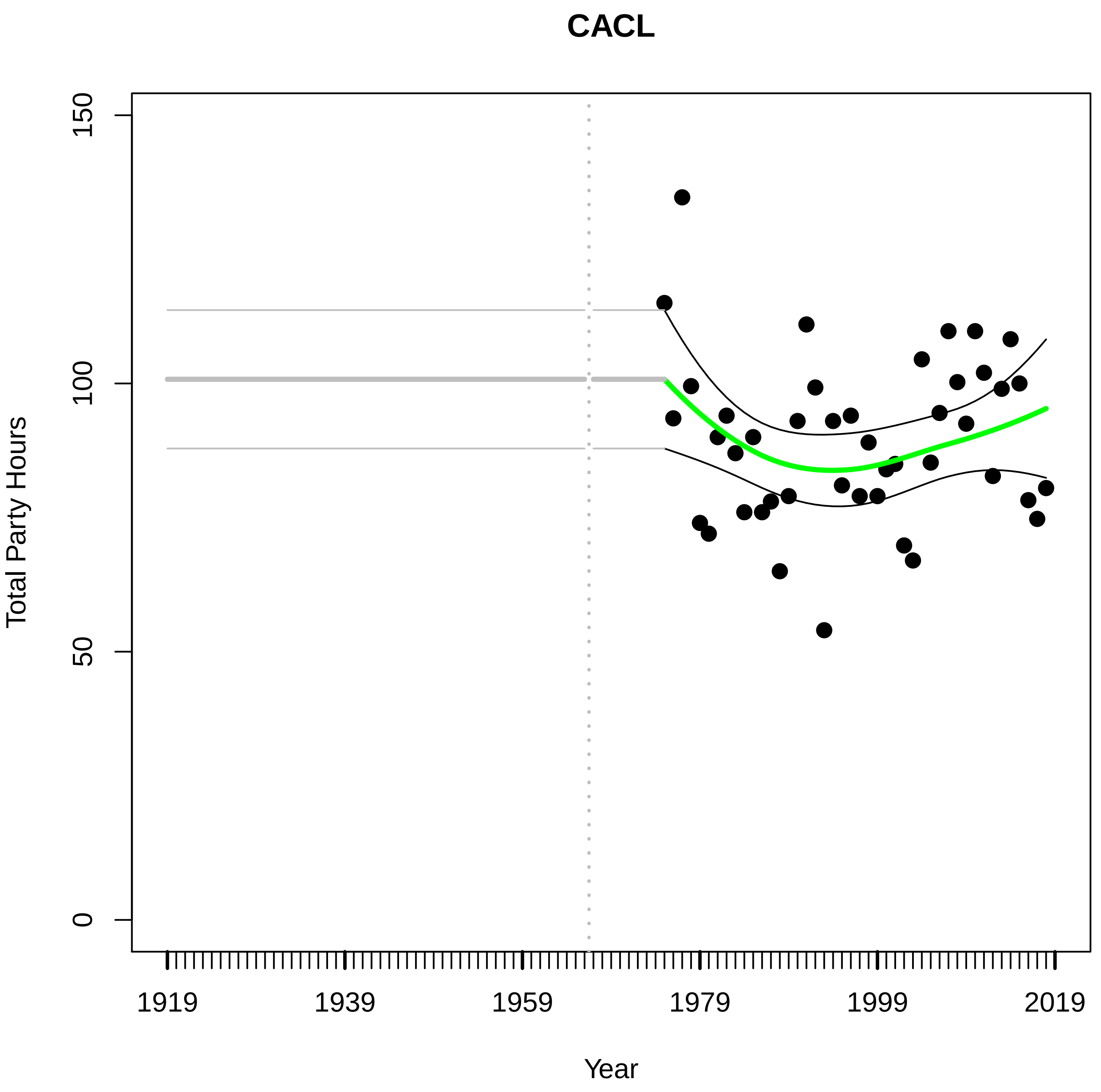

CACL
Total Party Hours
0
50
100
150
1919
1939
1959
1979
1999
2019
Year

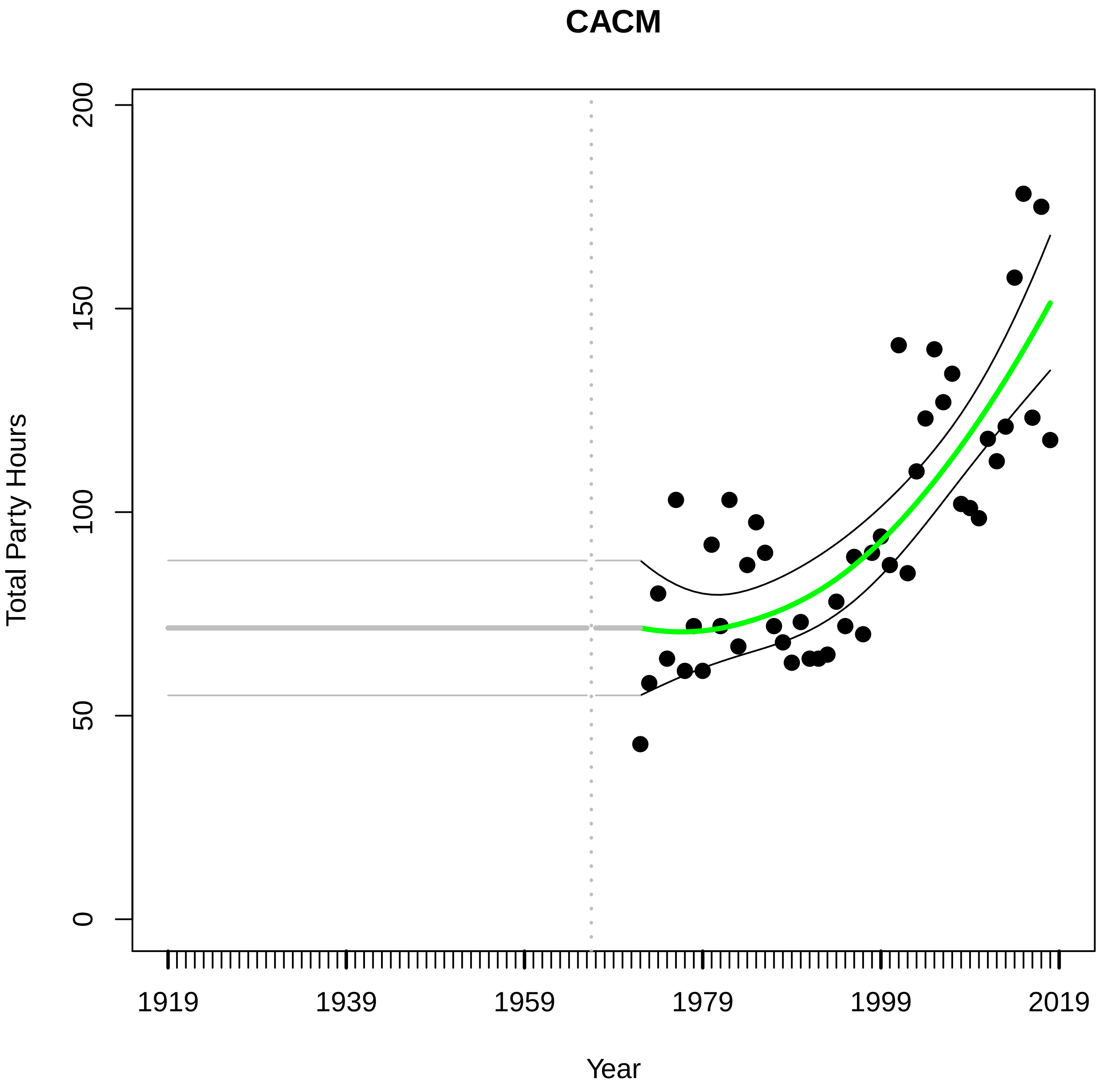

CACM
Total Party Hours
0
50
100
150
200
Year
1919
1939
1959
1979
1999
2019

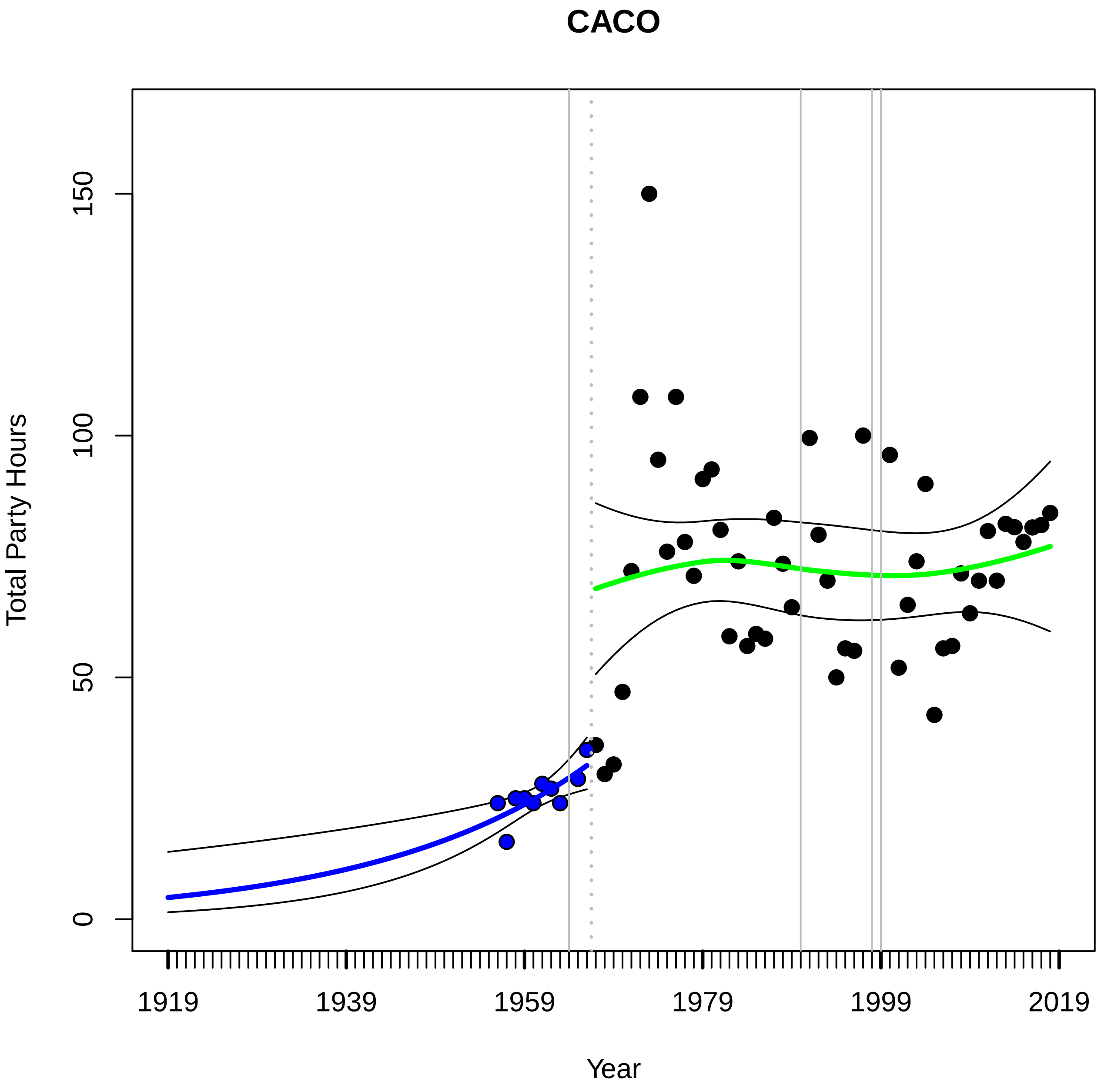

CACO
Total Party Hours
0
50
100
150
1919
1939
1959
1979
1999
2019
Year

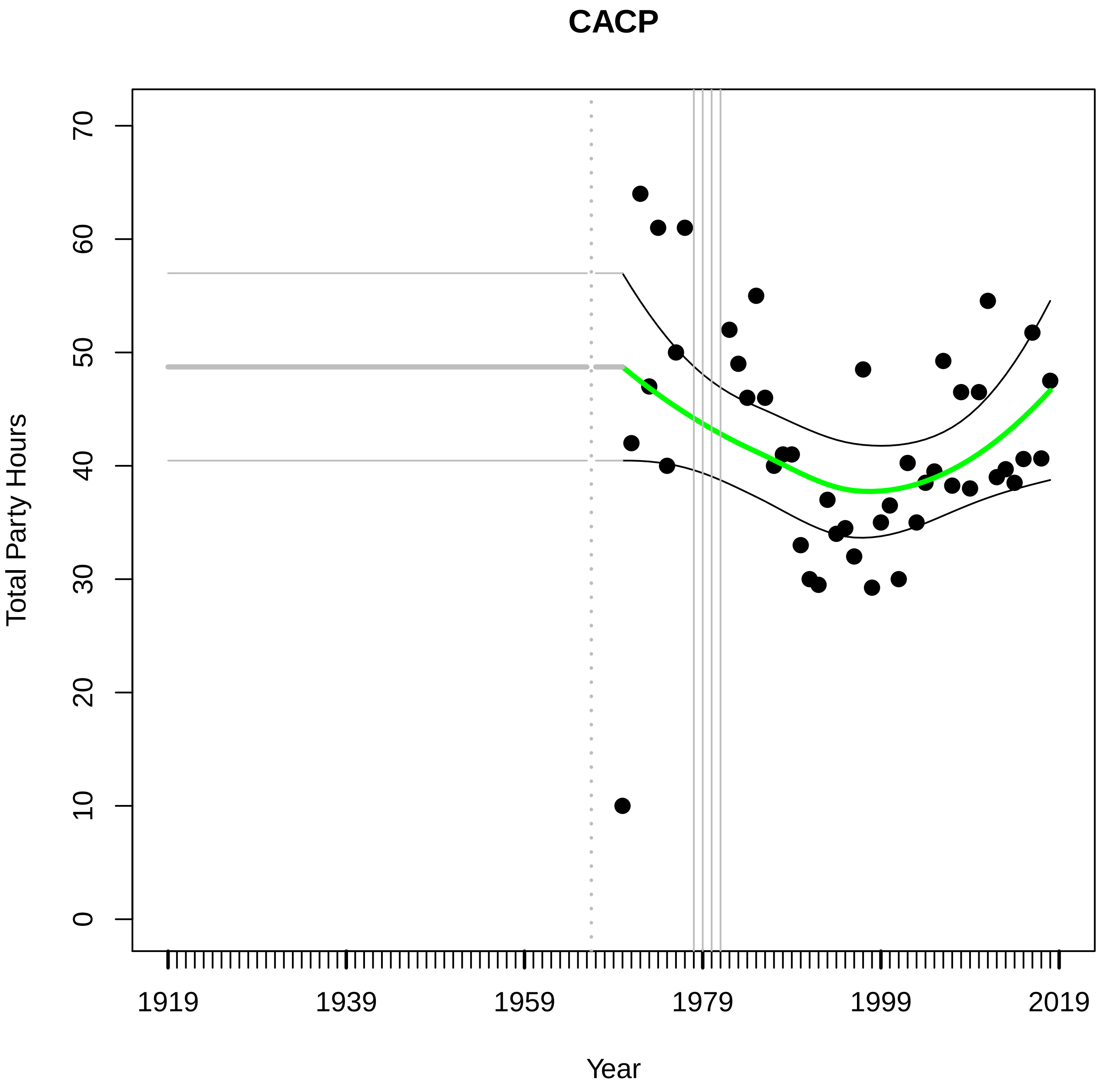
CACP
Total Party Hours
Year
0
10
20
30
40
50
60
70
1919
1939
1959
1979
1999
2019

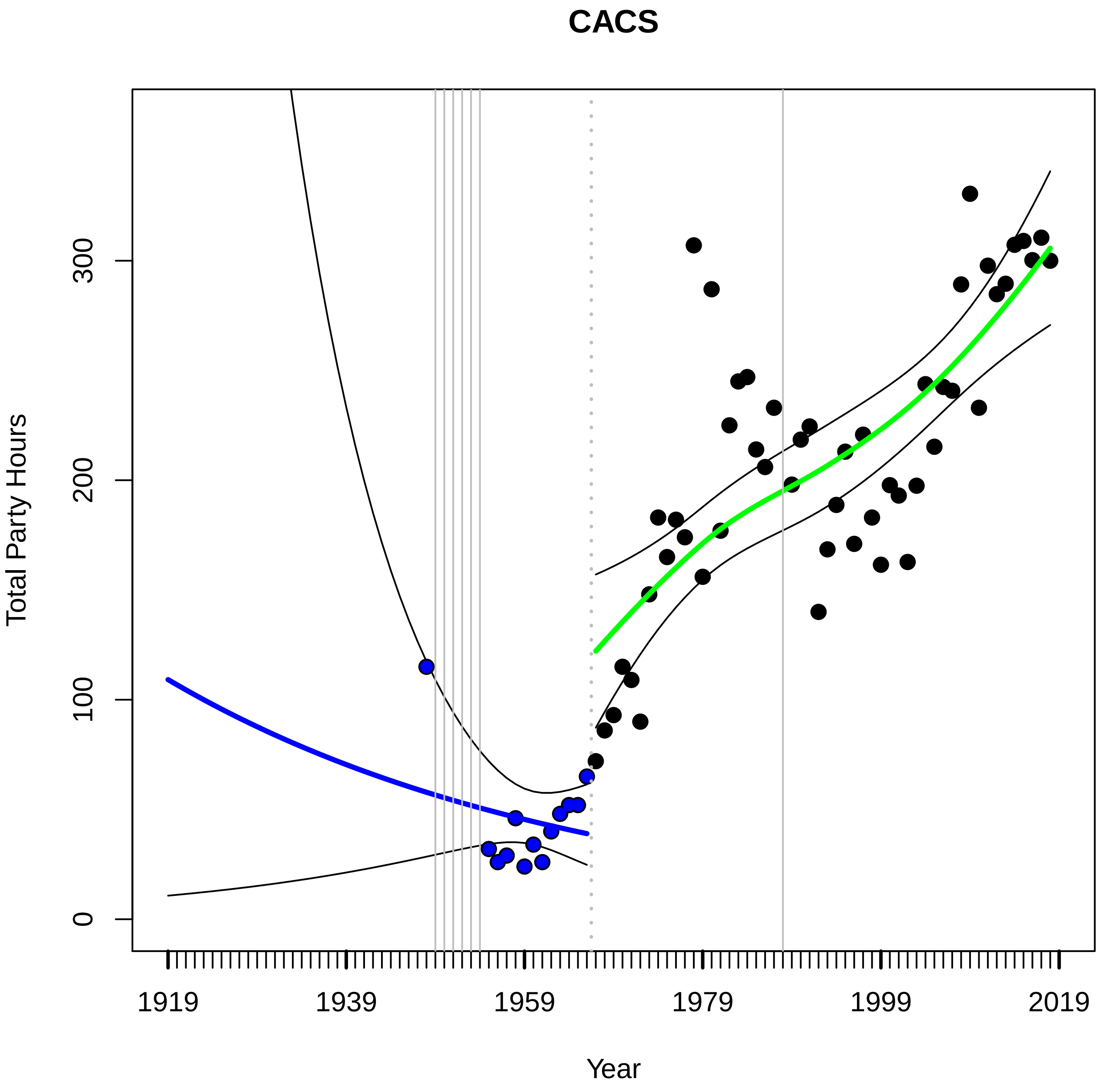
CACS
Total Party Hours
0
100
200
300
1919
1939
1959
1979
1999
2019
Year

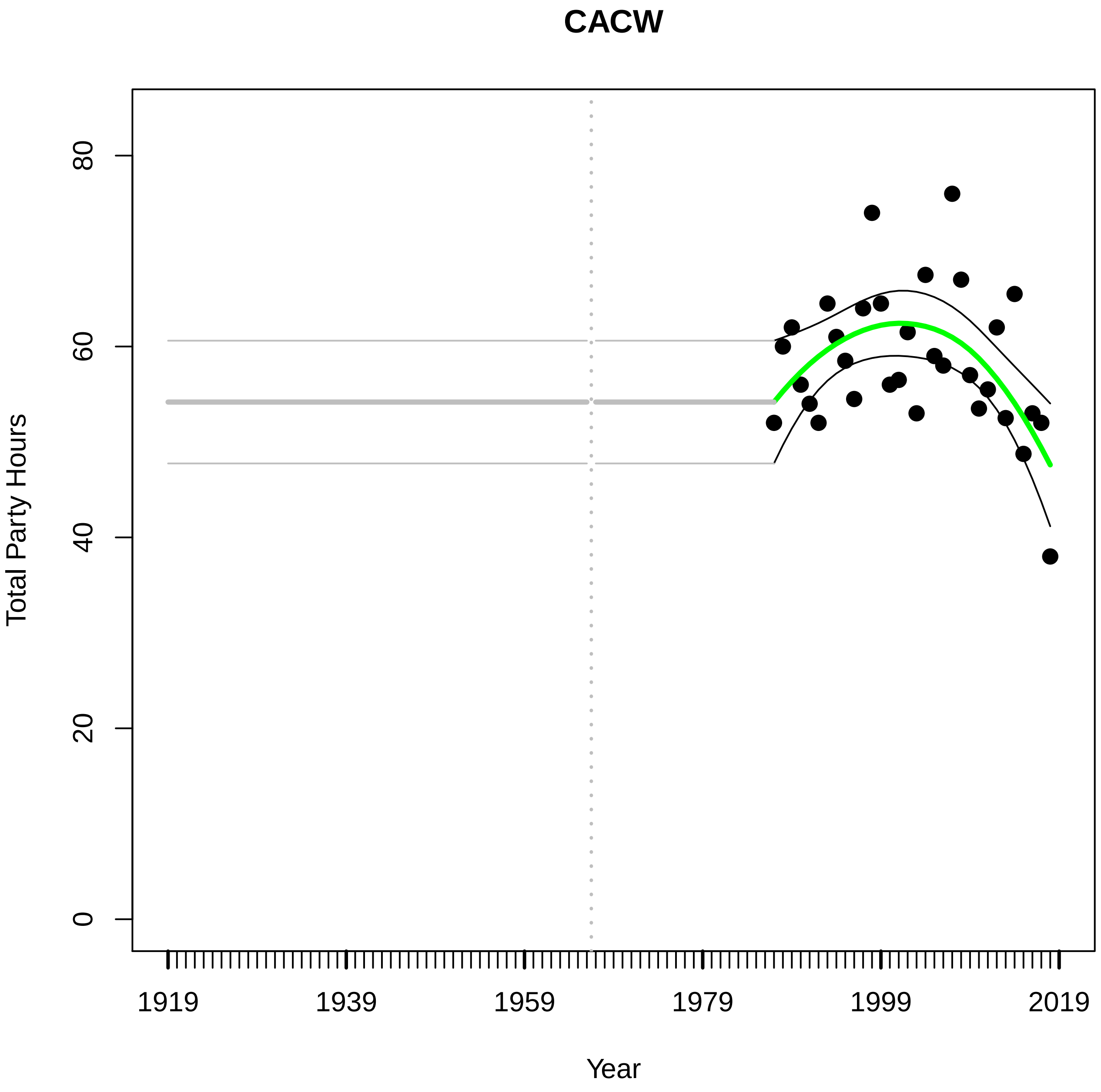
CACW
Total Party Hours
0
20
40
60
80
1919
1939
1959
1979
1999
2019
Year

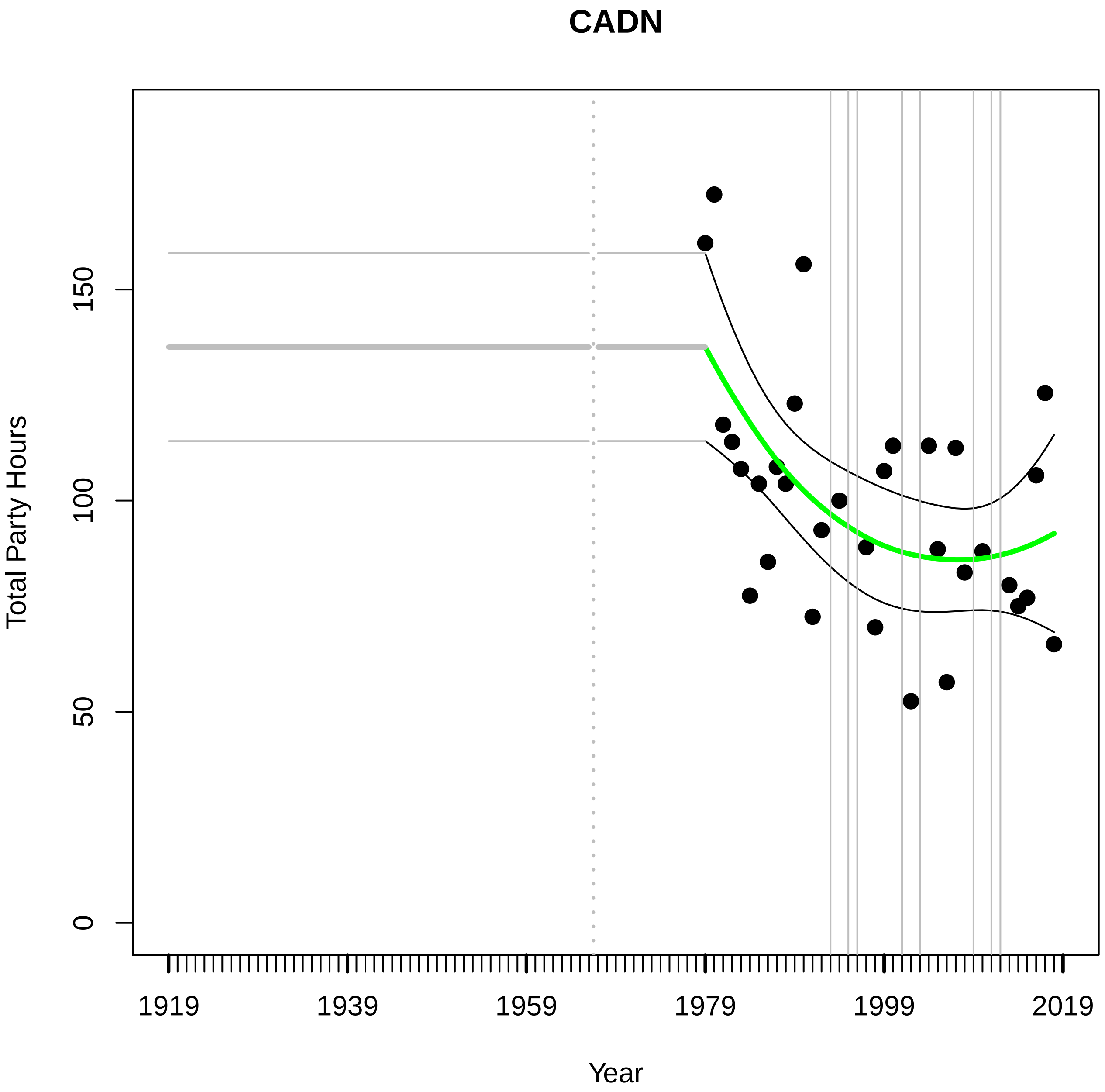
CADN
Total Party Hours
0
50
100
150
1919
1939
1959
1979
1999
2019
Year

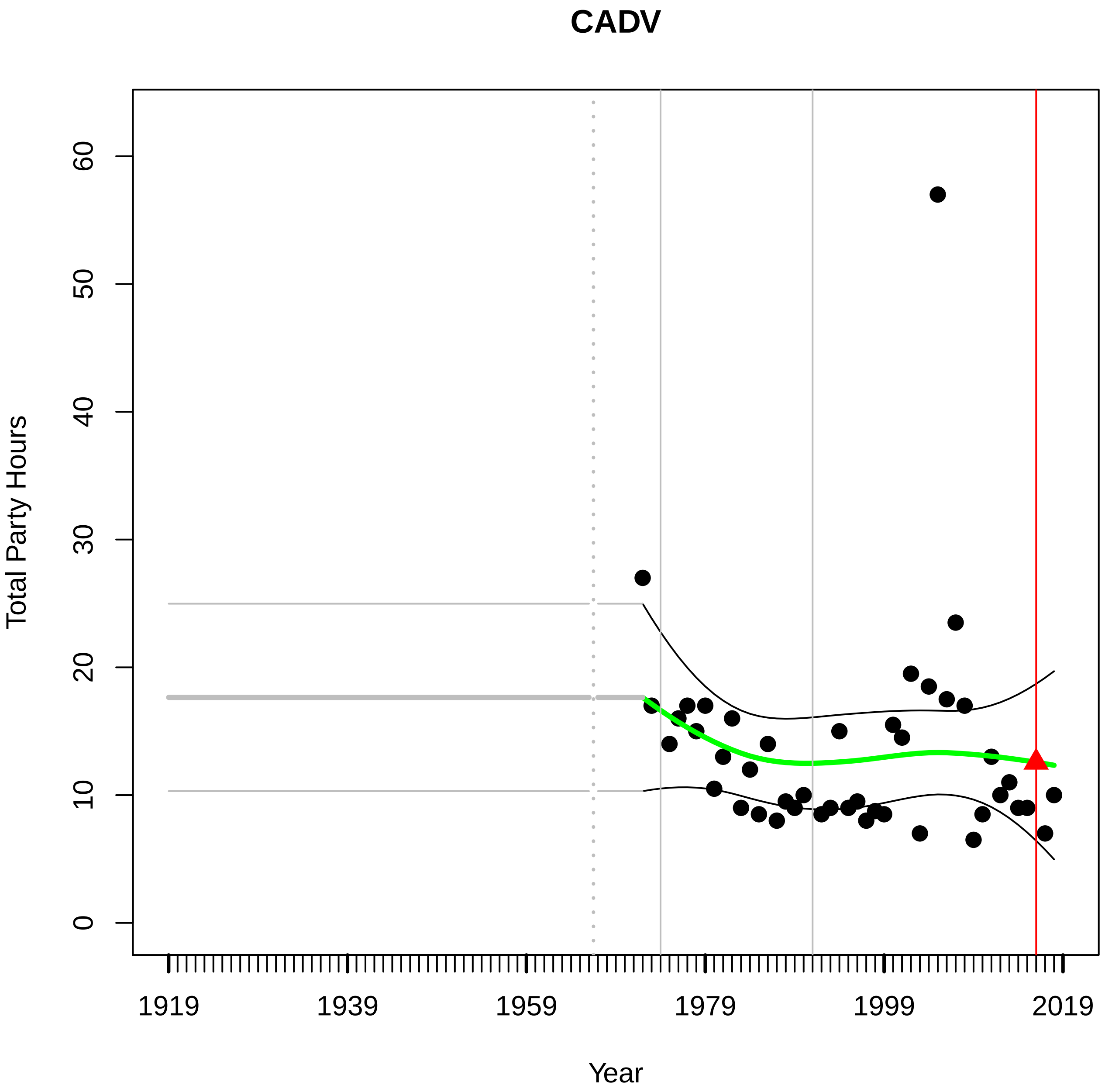

CADV
Total Party Hours
0
10
20
30
40
50
60
1919
1939
1959
1979
1999
2019
Year

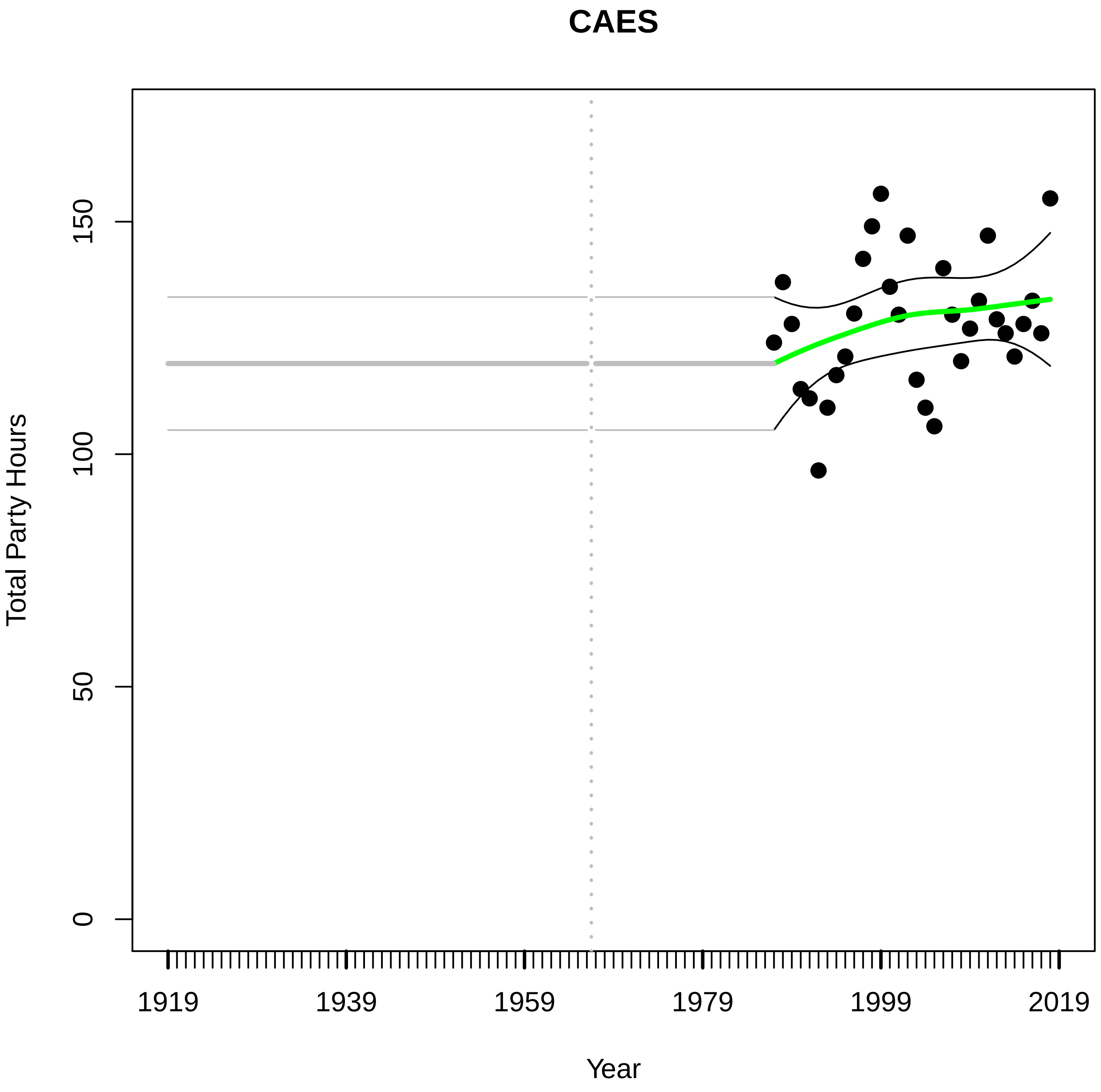

CAES
Total Party Hours
0
50
100
150
1919
1939
1959
1979
1999
2019
Year

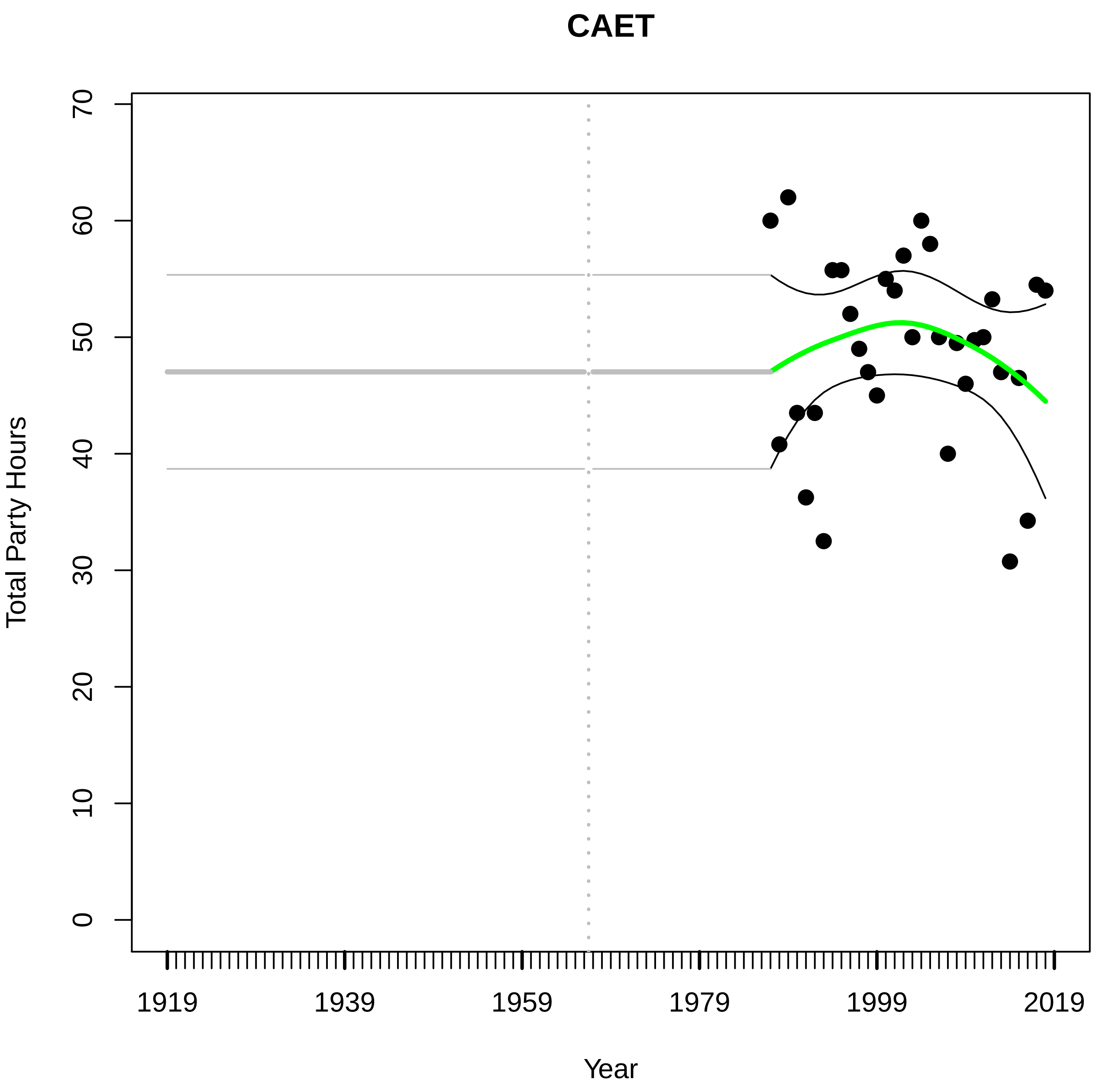

CAET
Total Party Hours
0
10
20
30
40
50
60
70
1919
1939
1959
1979
1999
2019
Year

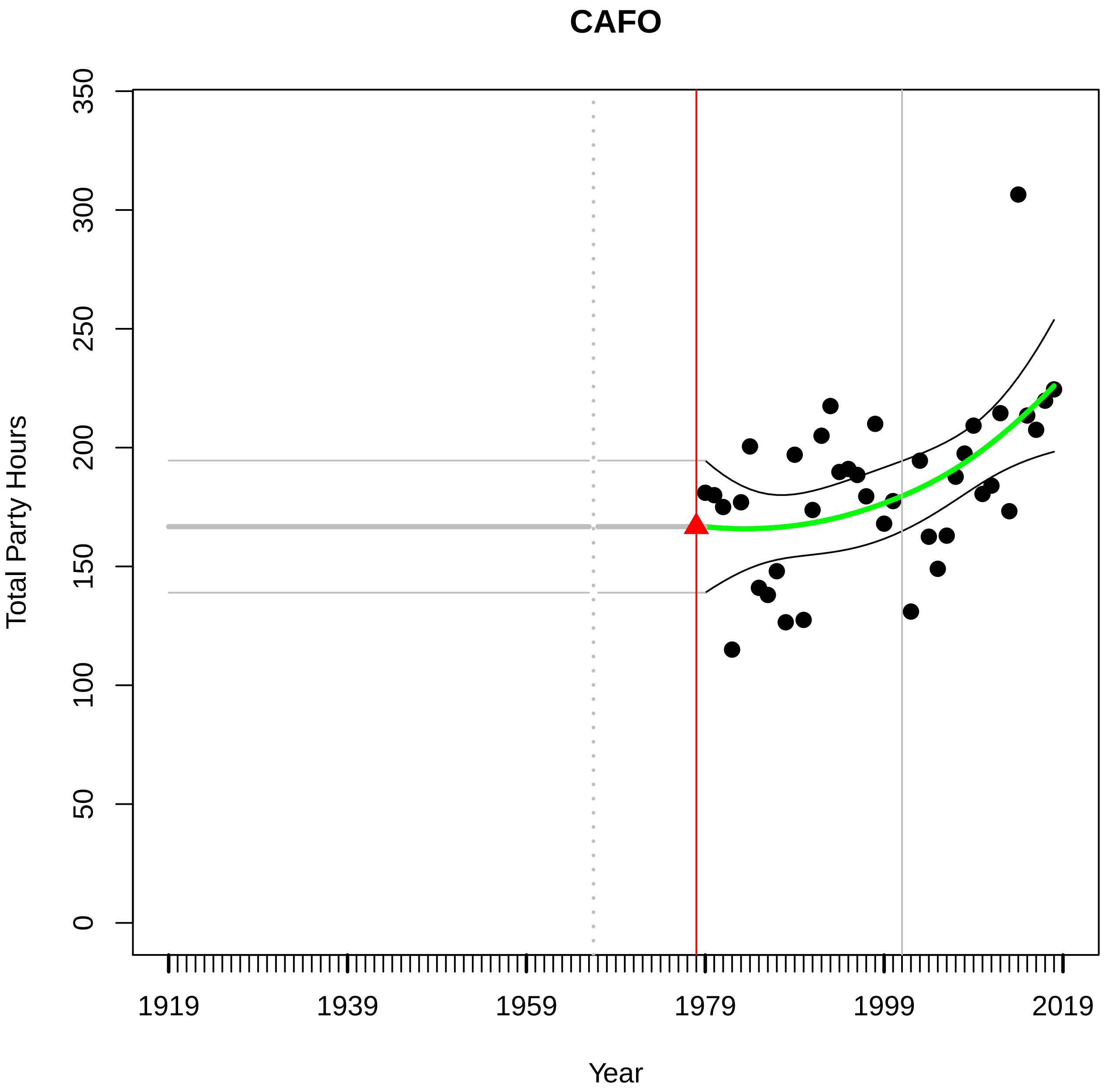
CAFO
Total Party Hours
0
50
100
150
200
250
300
350
1919
1939
1959
1979
1999
2019
Year

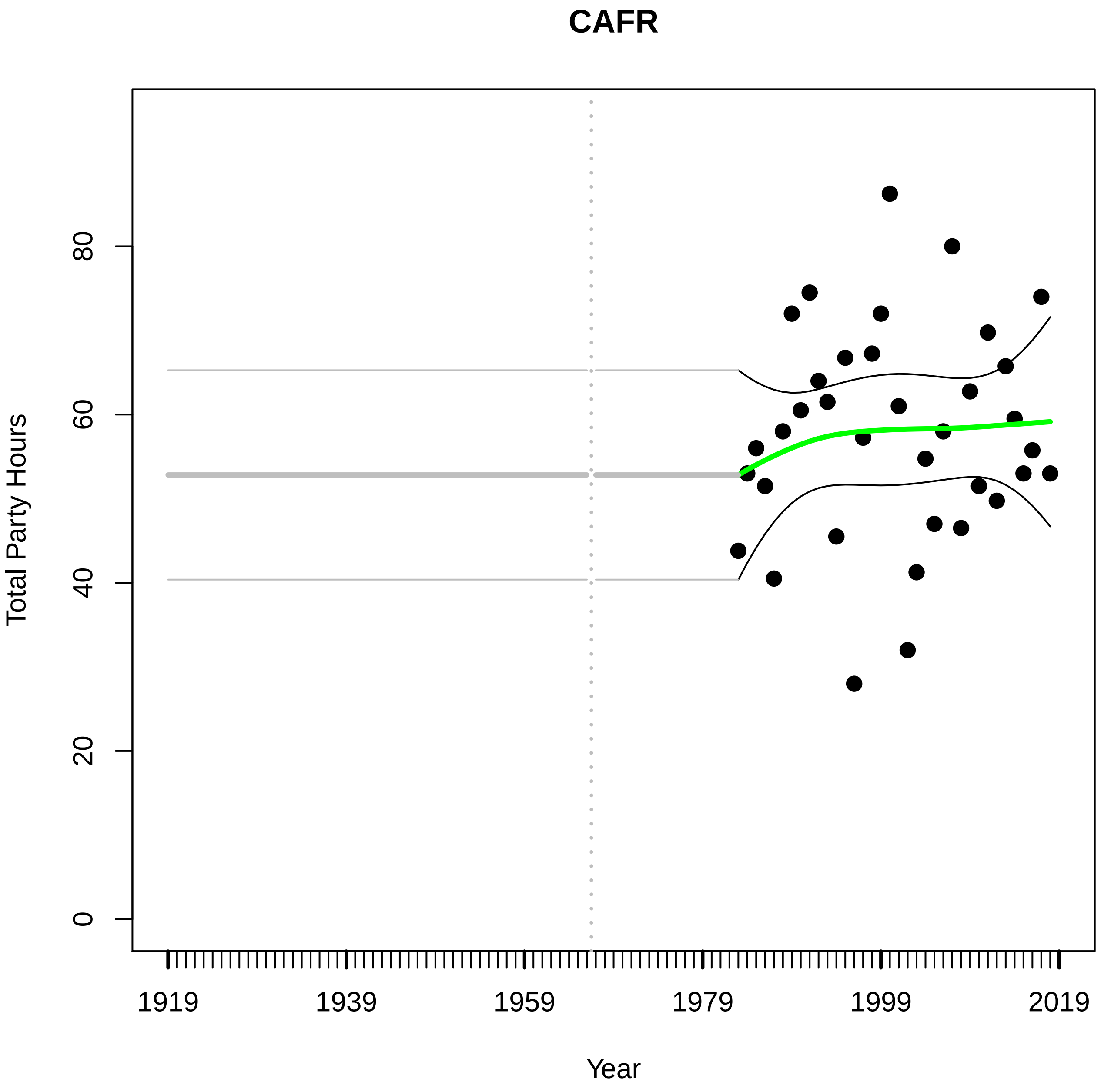

CAFR
Total Party Hours
0
20
40
60
80
1919
1939
1959
1979
1999
2019
Year

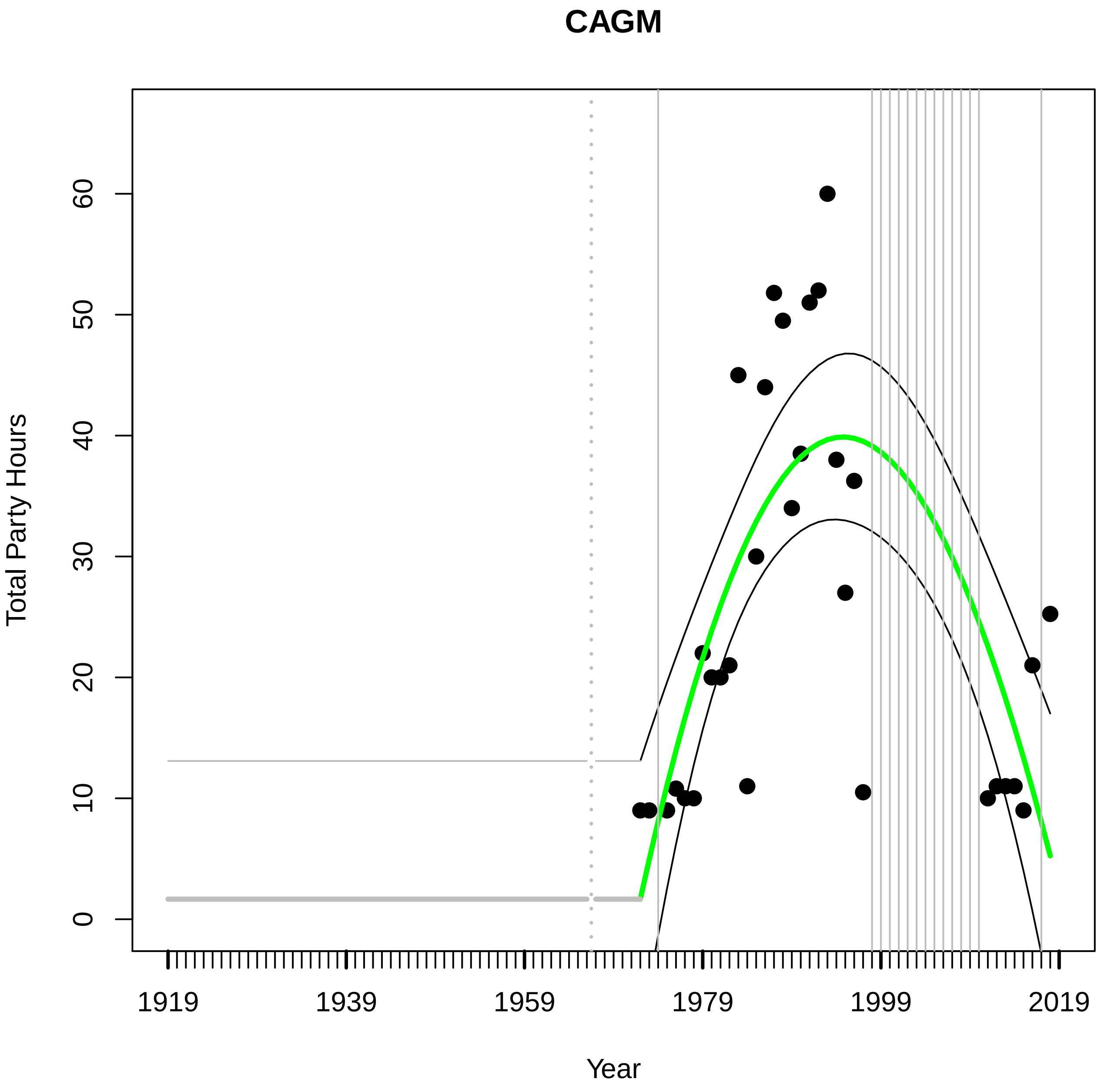
CAGM
Total Party Hours
Year
0
10
20
30
40
50
60
1919
1939
1959
1979
1999
2019

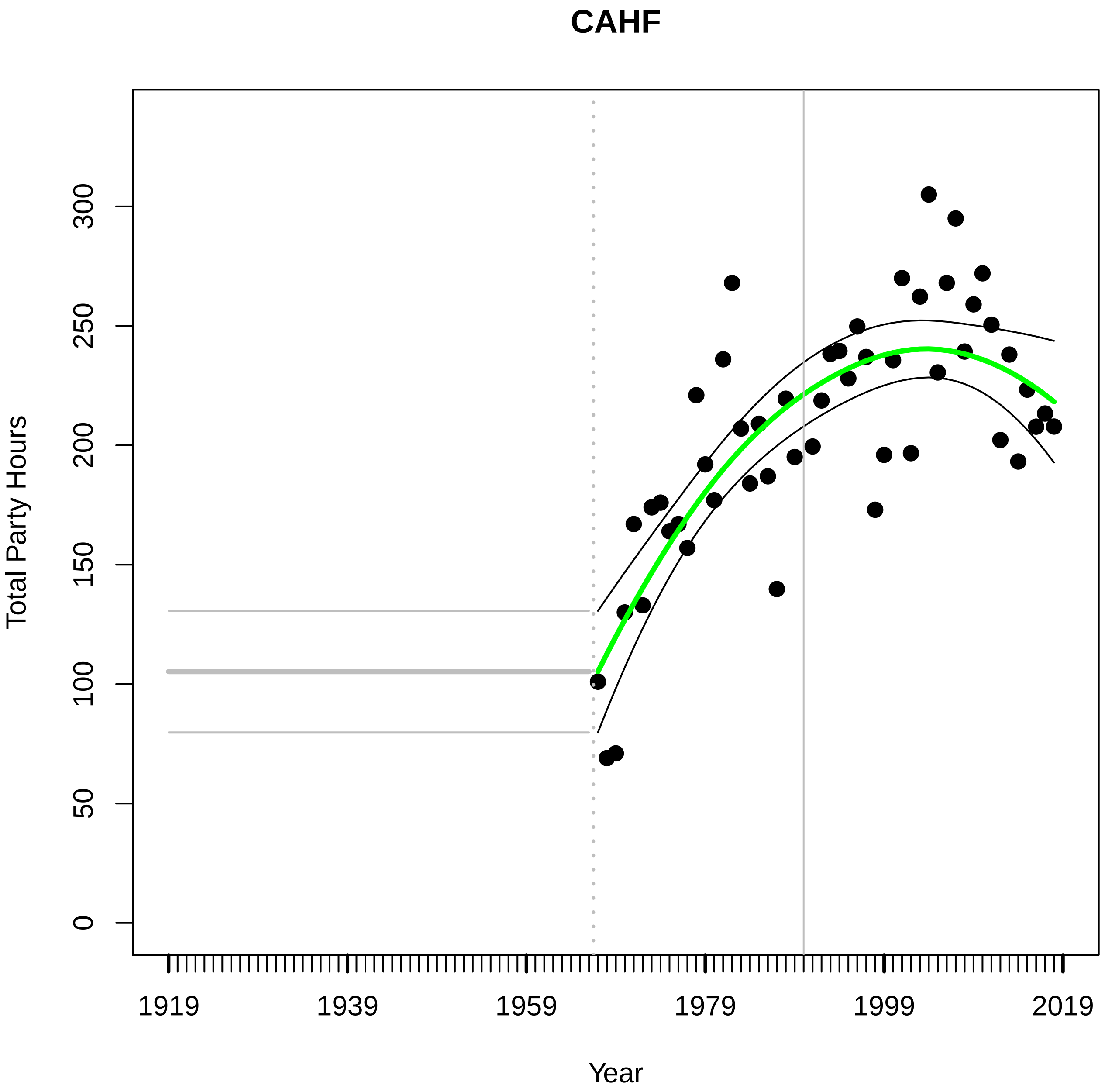

CAHF
Total Party Hours
0
50
100
150
200
250
300
1919
1939
1959
1979
1999
2019
Year

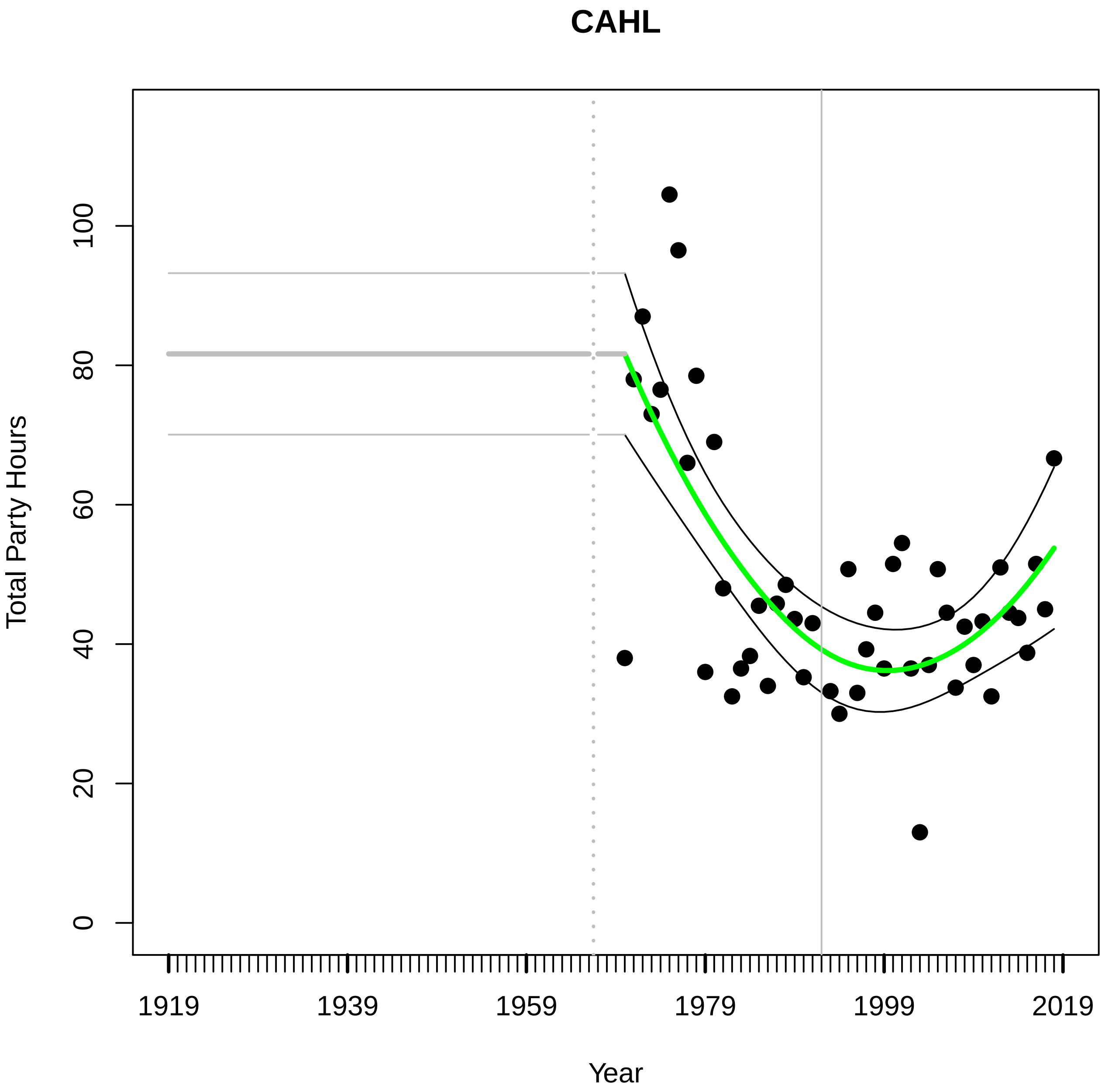

CAHL
Total Party Hours
0
20
40
60
80
100
1919
1939
1959
1979
1999
2019
Year

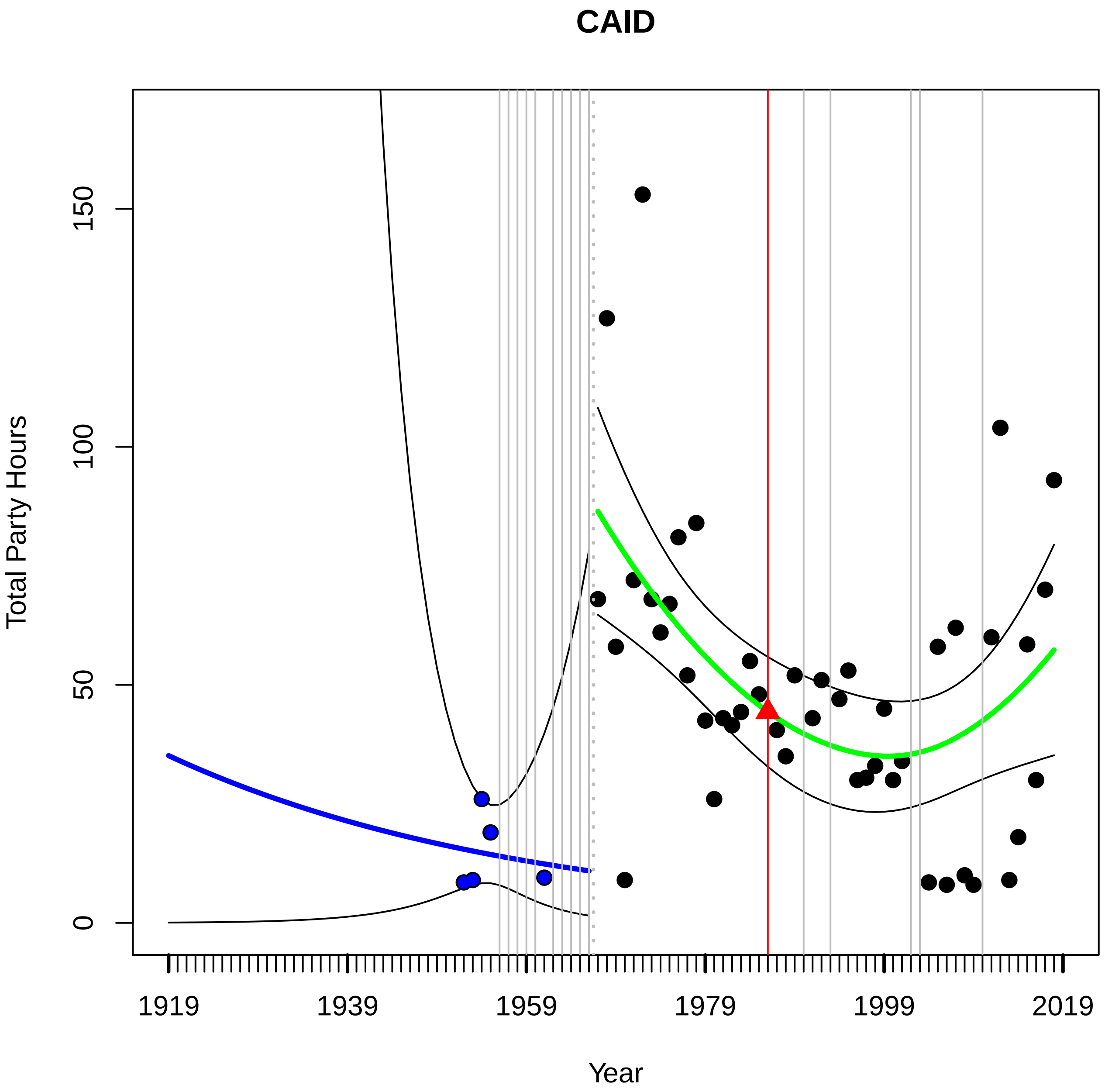
CAID
Total Party Hours
0
50
100
150
1919
1939
1959
1979
1999
2019
Year

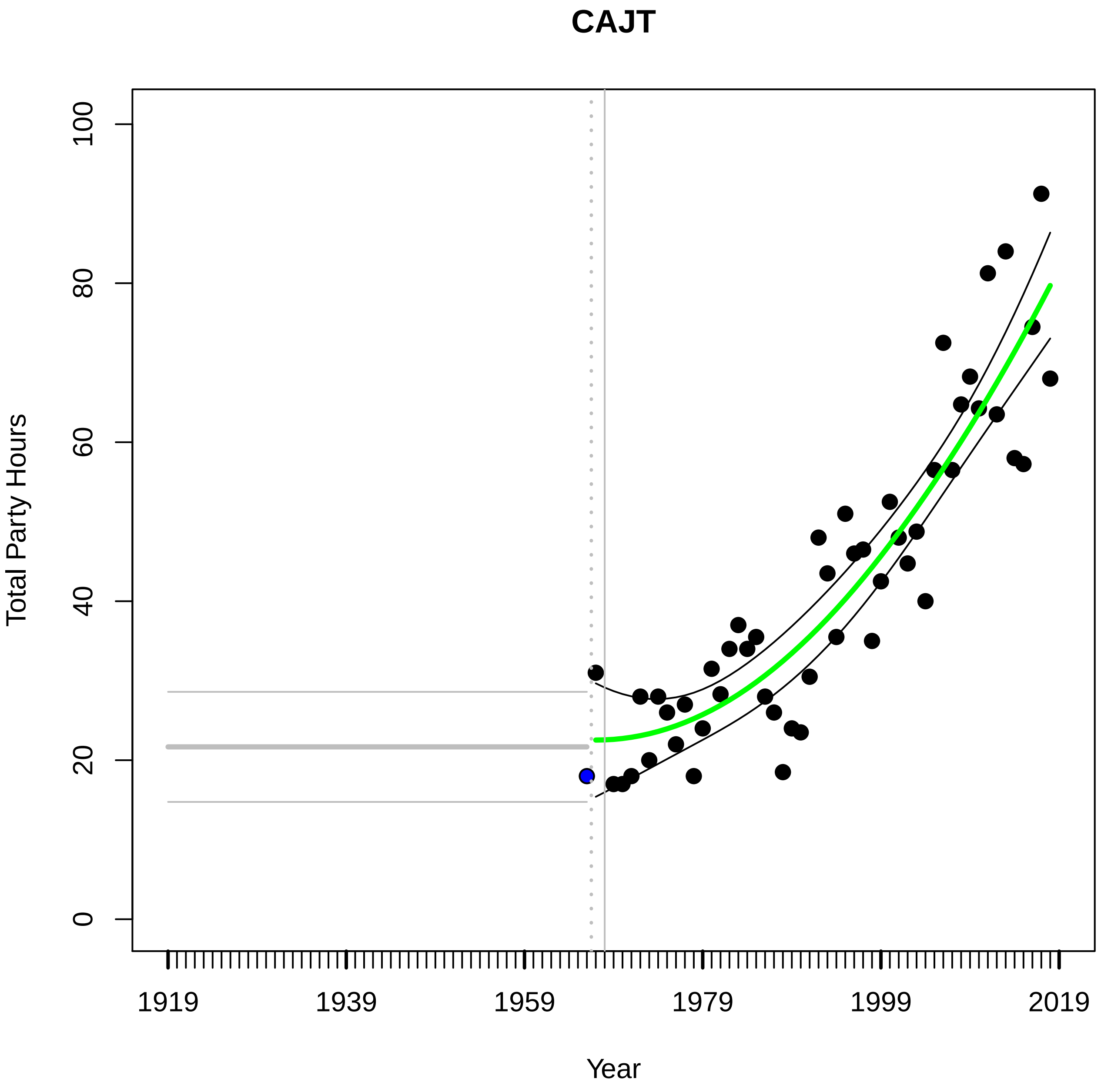

CAJT
Total Party Hours
0
20
40
60
80
100
1919
1939
1959
1979
1999
2019
Year

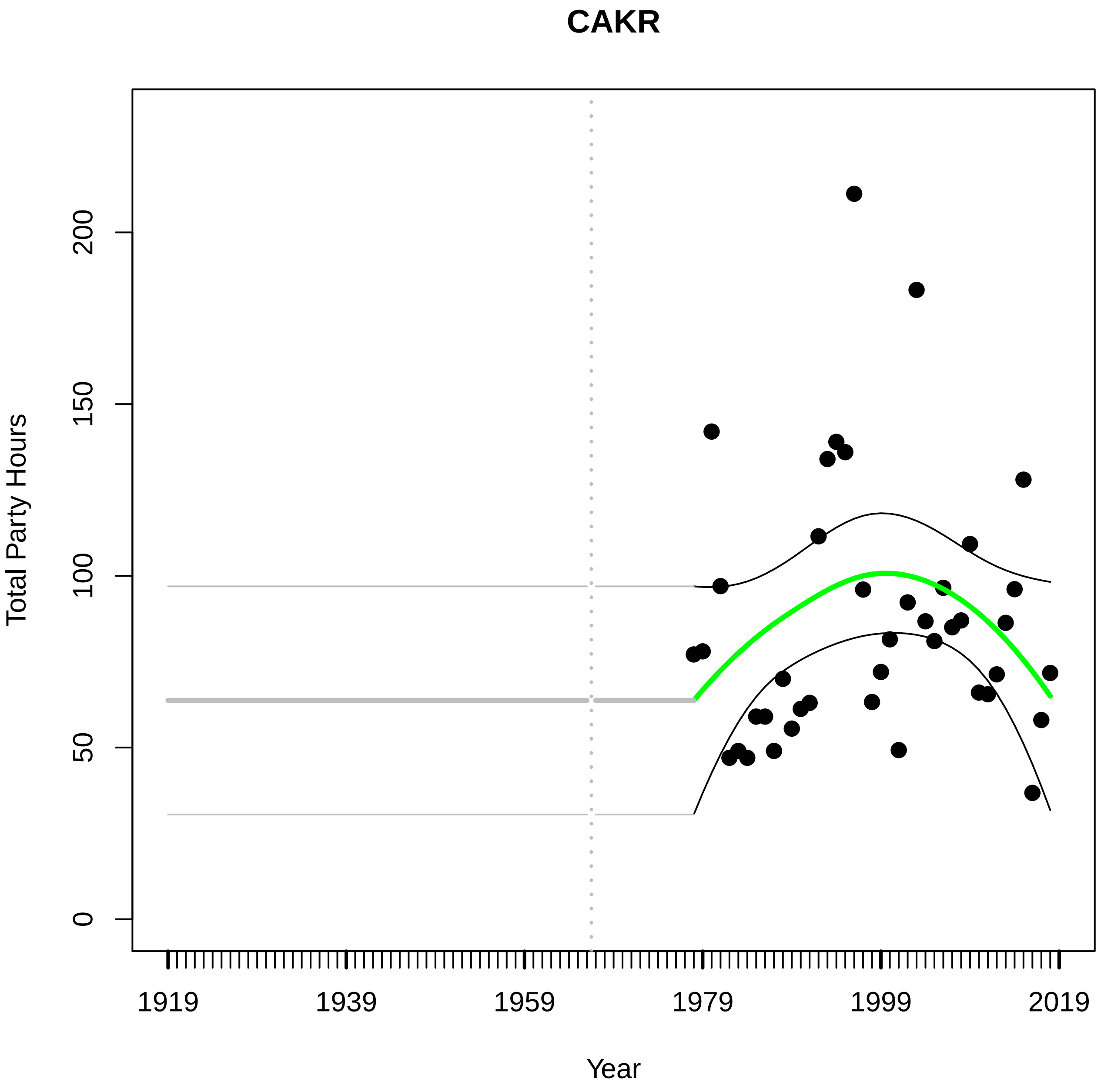

CAKR
Total Party Hours
0
50
100
150
200
1919
1939
1959
1979
1999
2019
Year

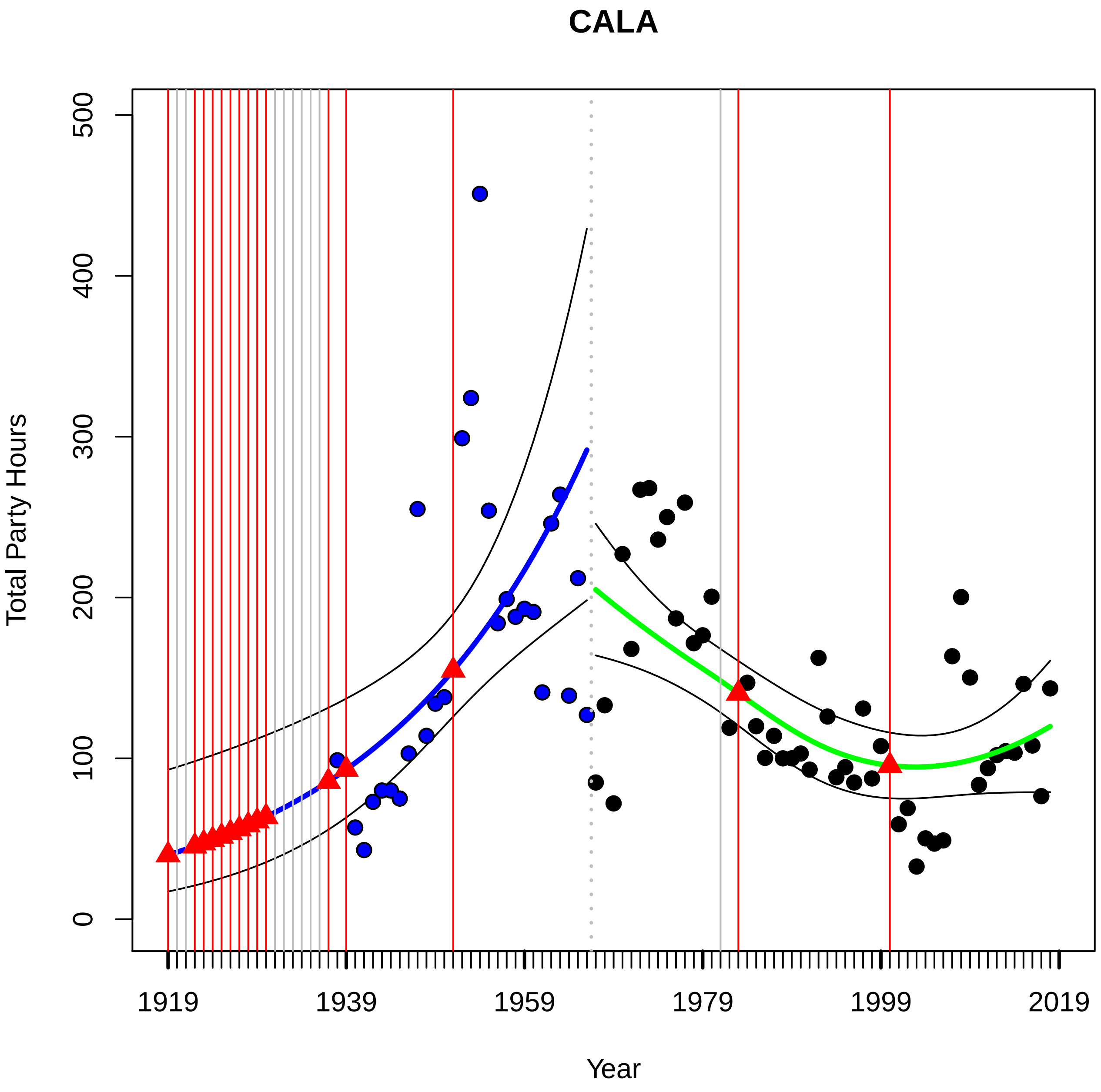

CALA
Total Party Hours
0
100
200
300
400
500
1919
1939
1959
1979
1999
2019
Year

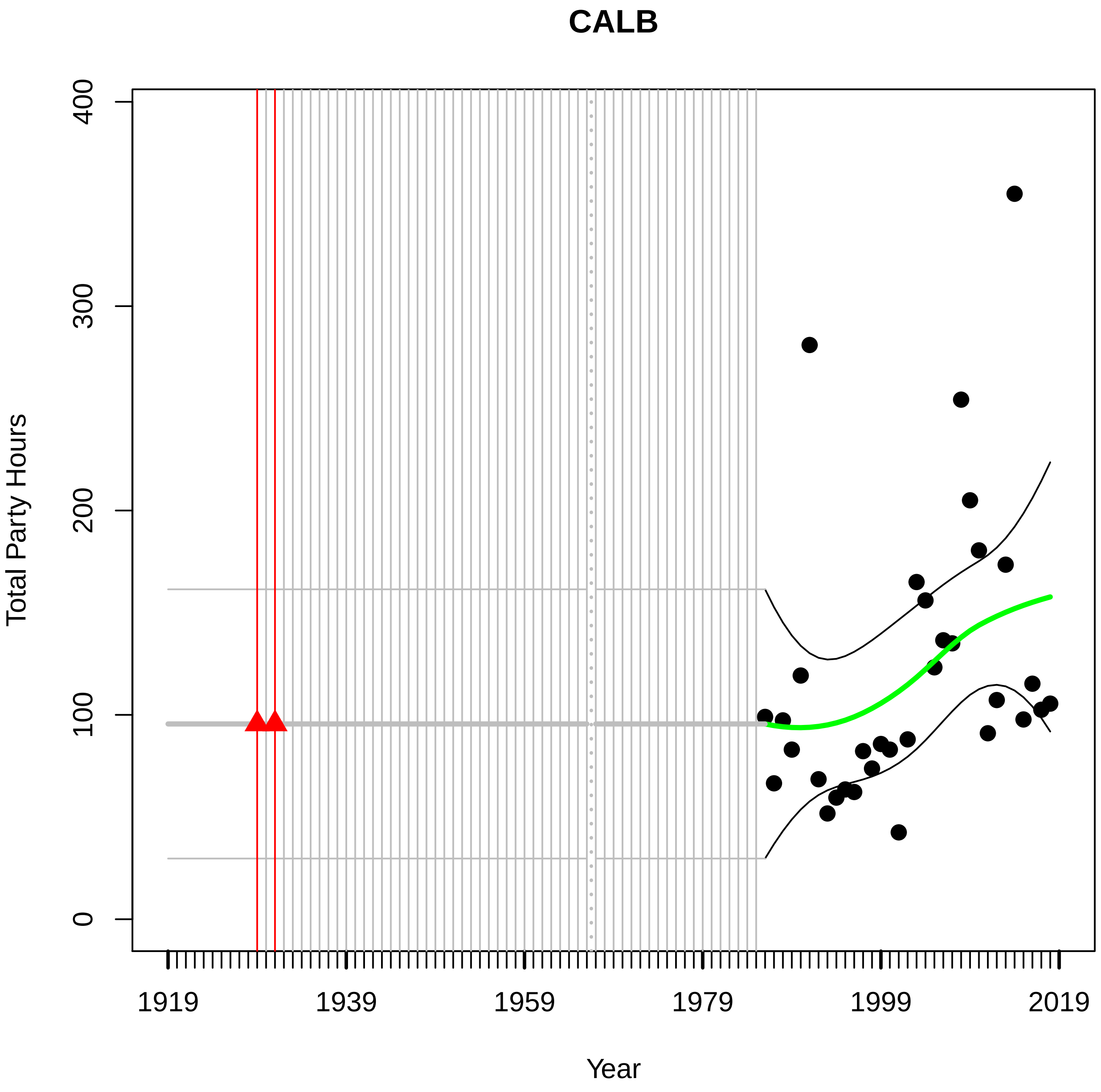
CALB
Total Party Hours
0
100
200
300
400
1919
1939
1959
1979
1999
2019
Year

CALE

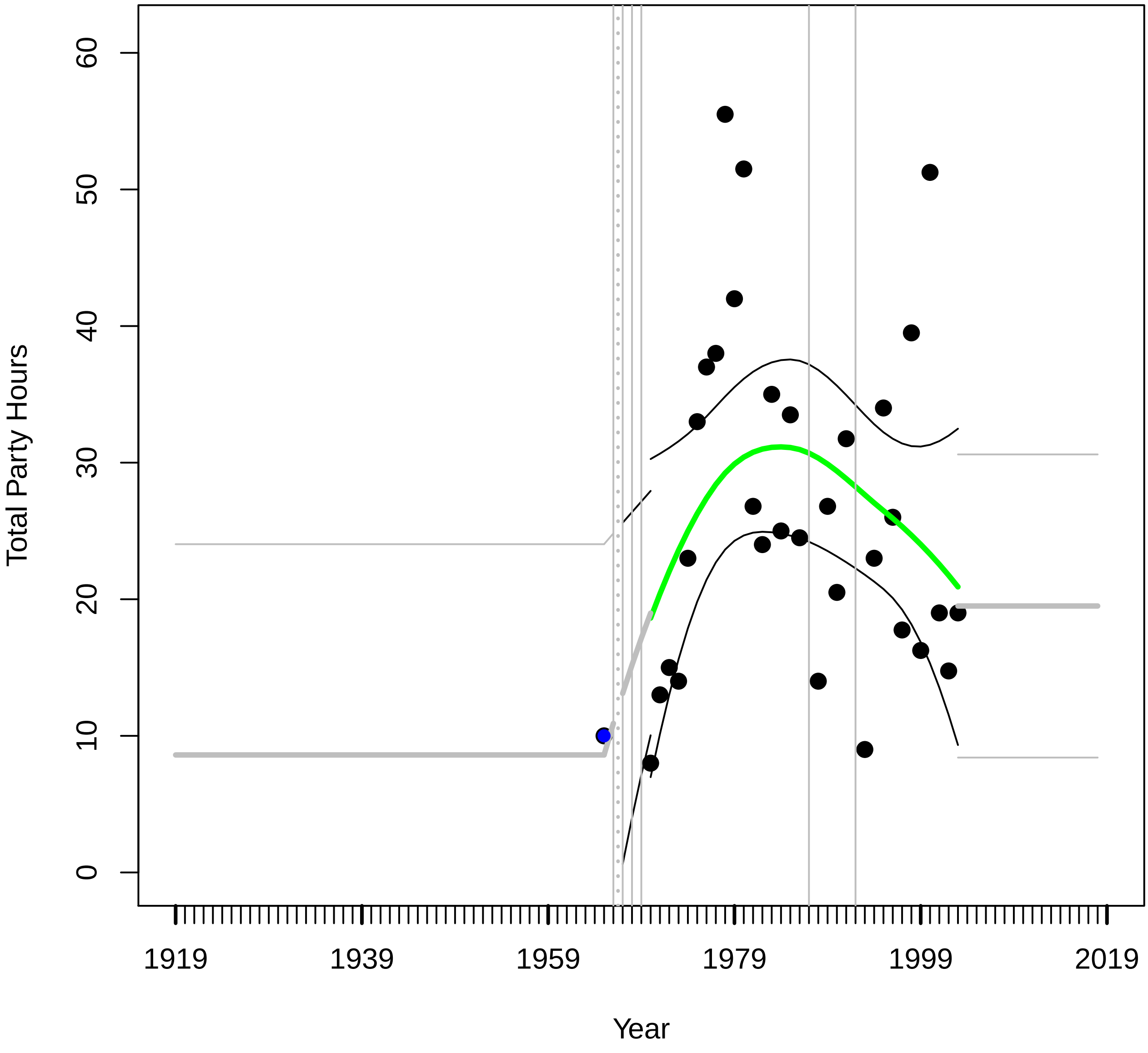

Total Party Hours
0
10
20
30
40
50
60
1919
1939
1959
1979
1999
2019
Year

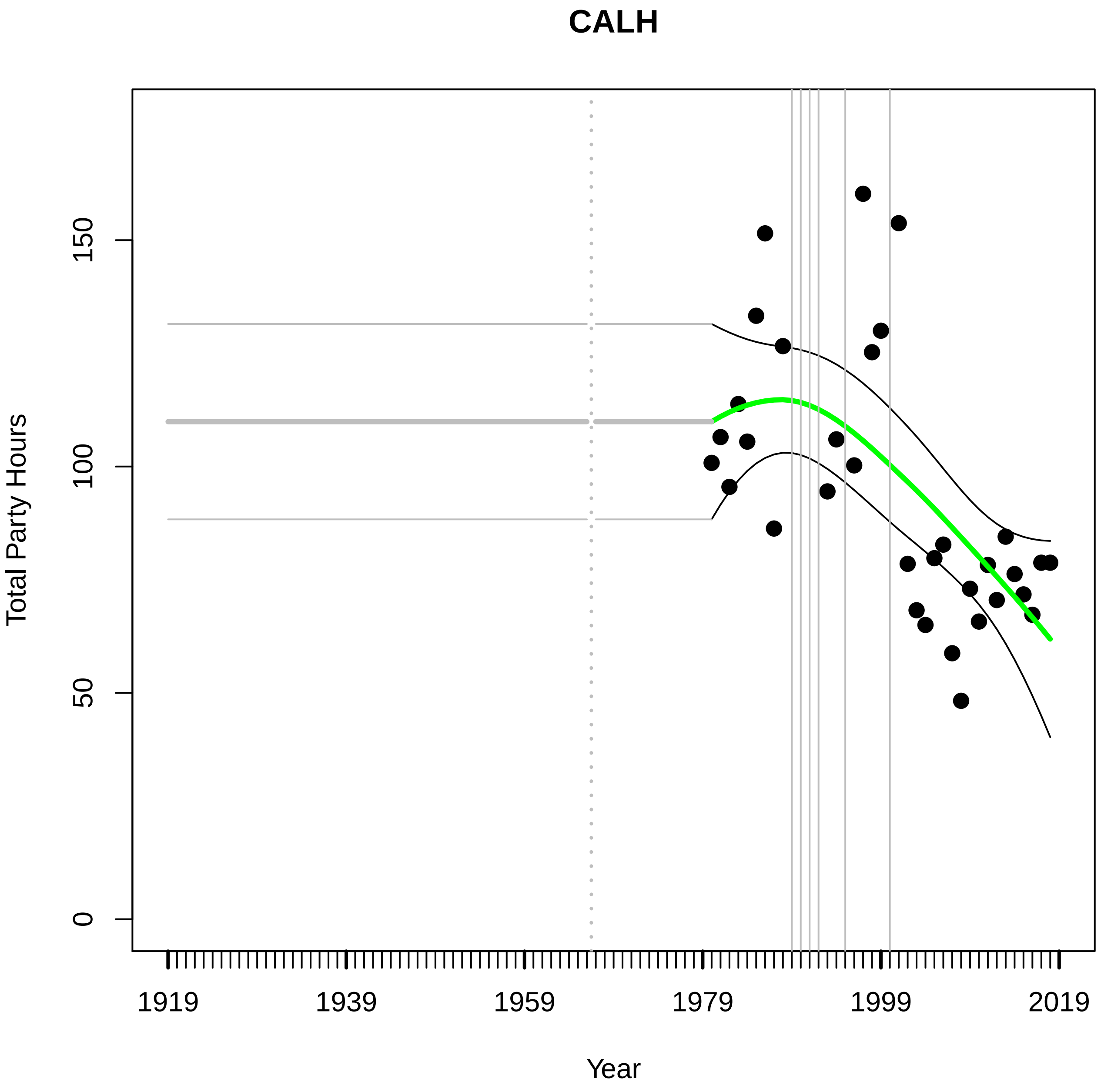

CALH
Total Party Hours
0
50
100
150
1919
1939
1959
1979
1999
2019
Year

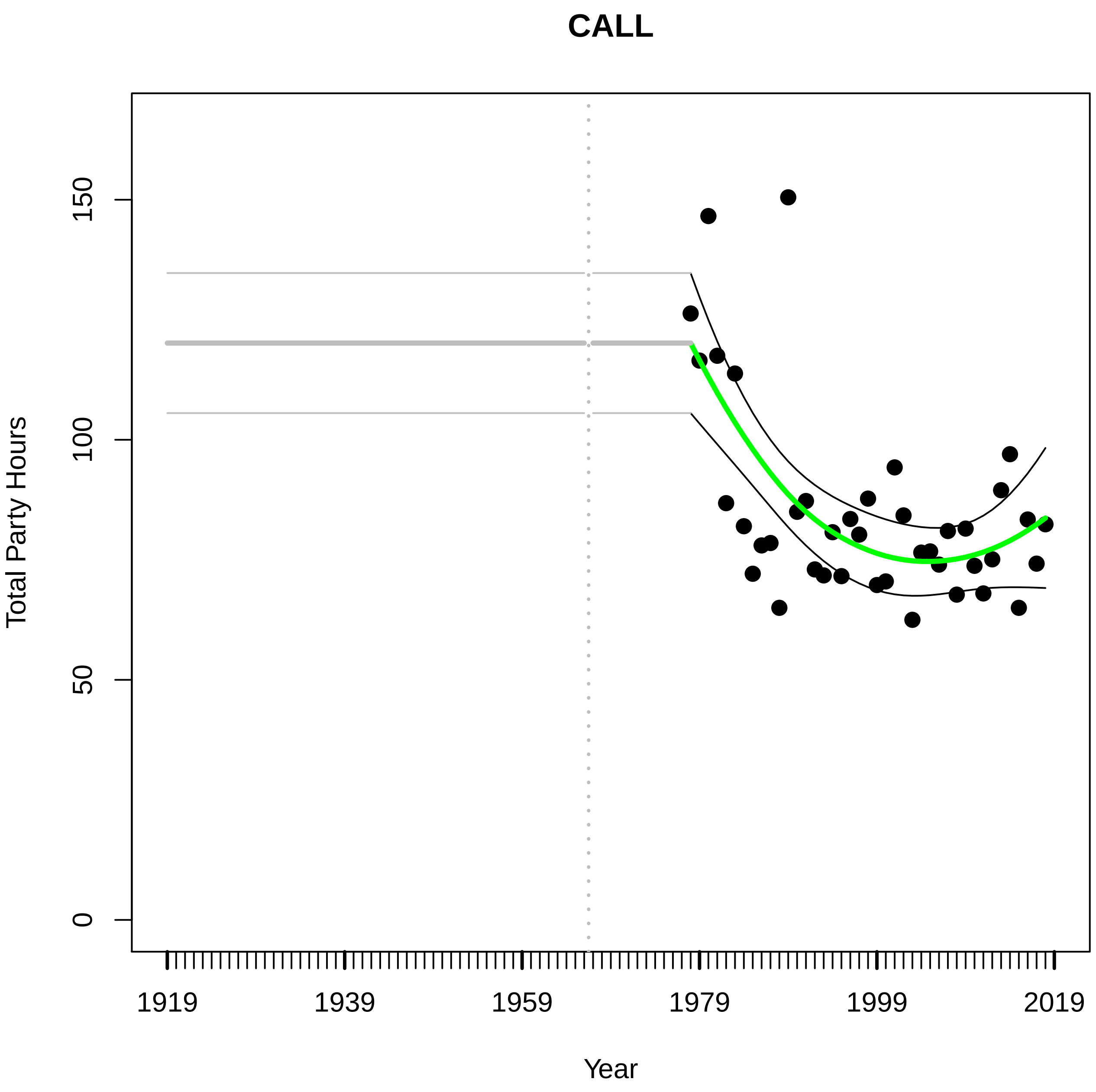

CALL
Total Party Hours
0
50
100
150
1919
1939
1959
1979
1999
2019
Year

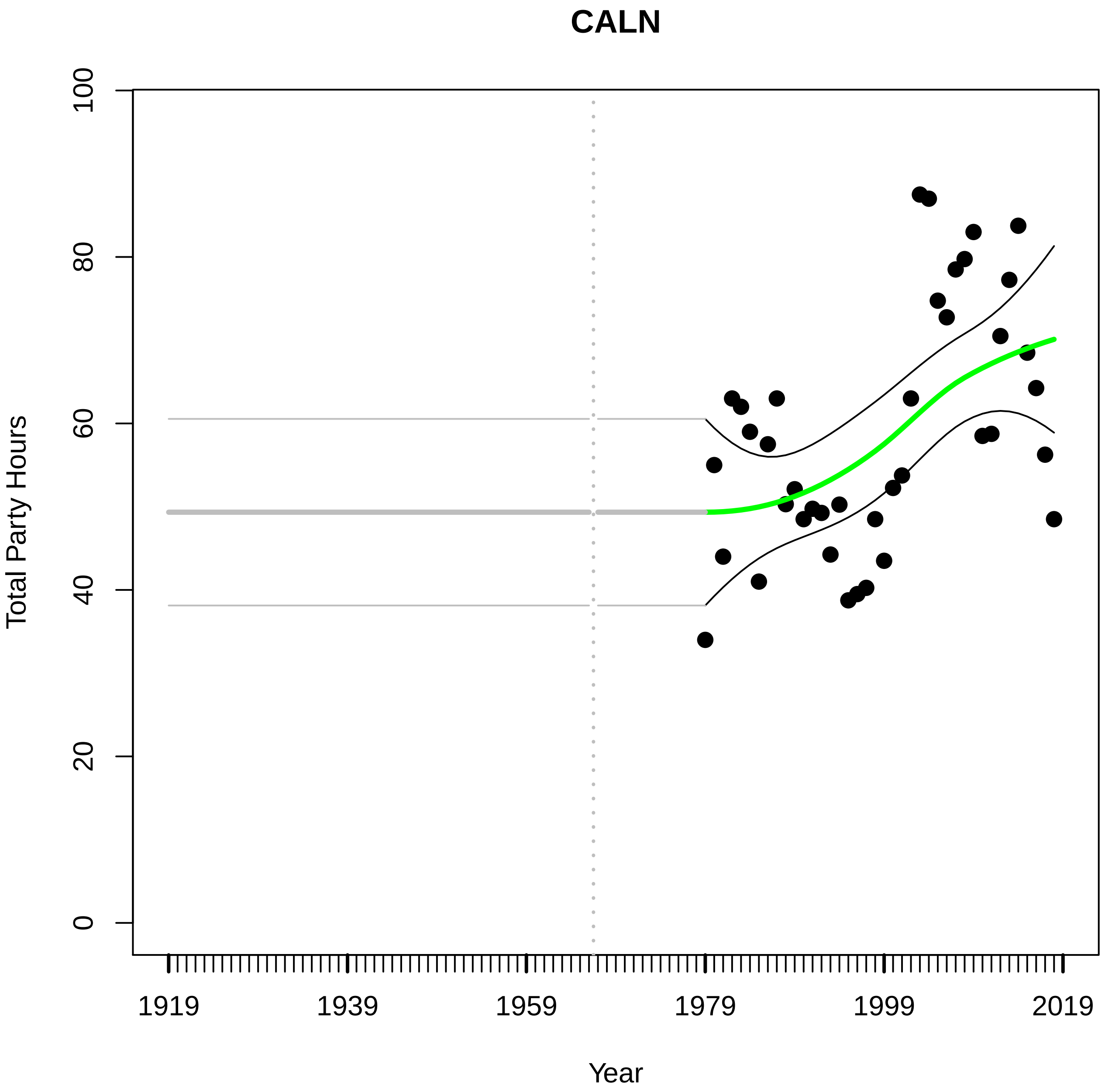

CALN
Total Party Hours
Year
0
20
40
60
80
100
1919
1939
1959
1979
1999
2019

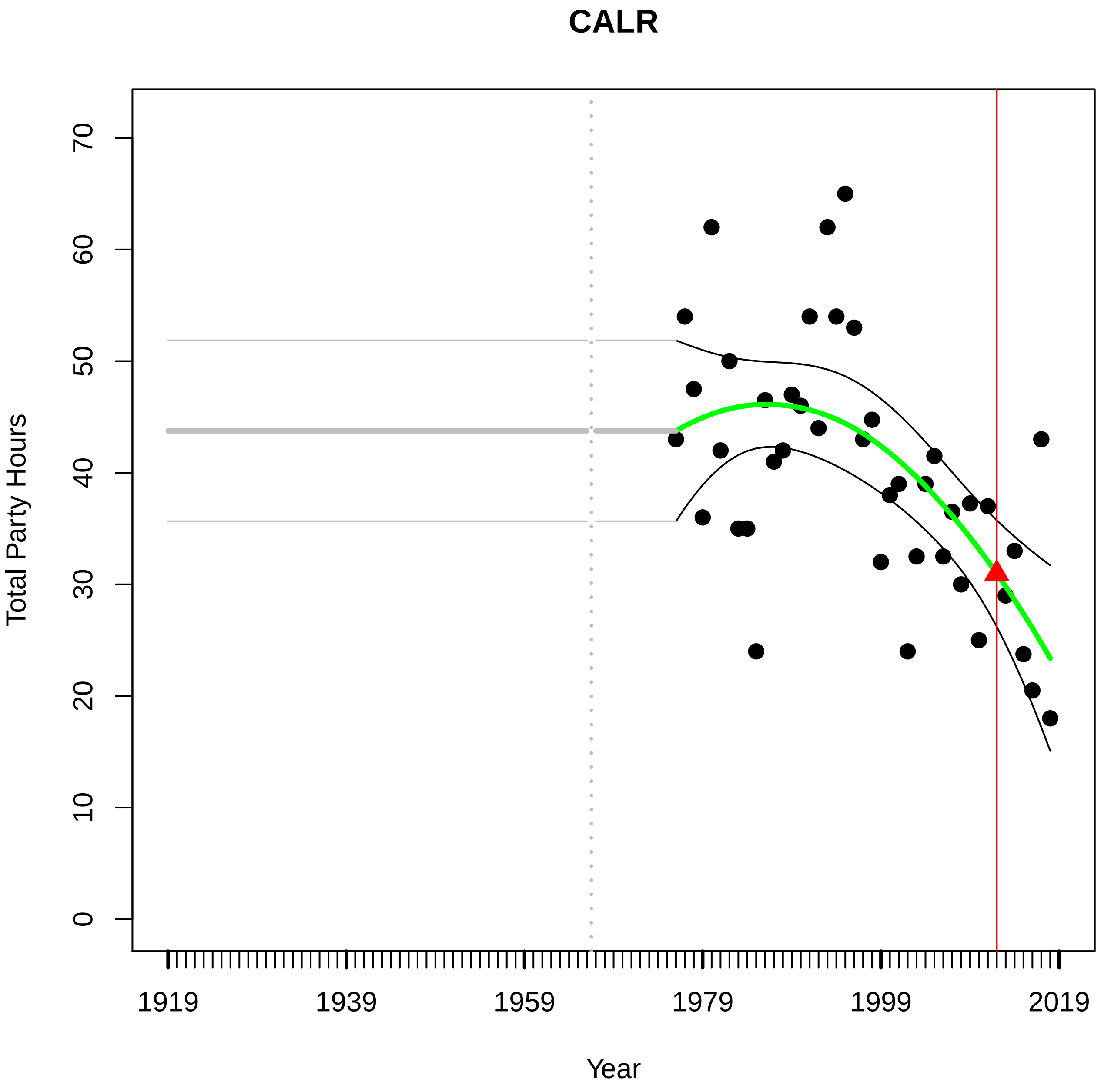

CALR
Total Party Hours
0
10
20
30
40
50
60
70
1919
1939
1959
1979
1999
2019
Year

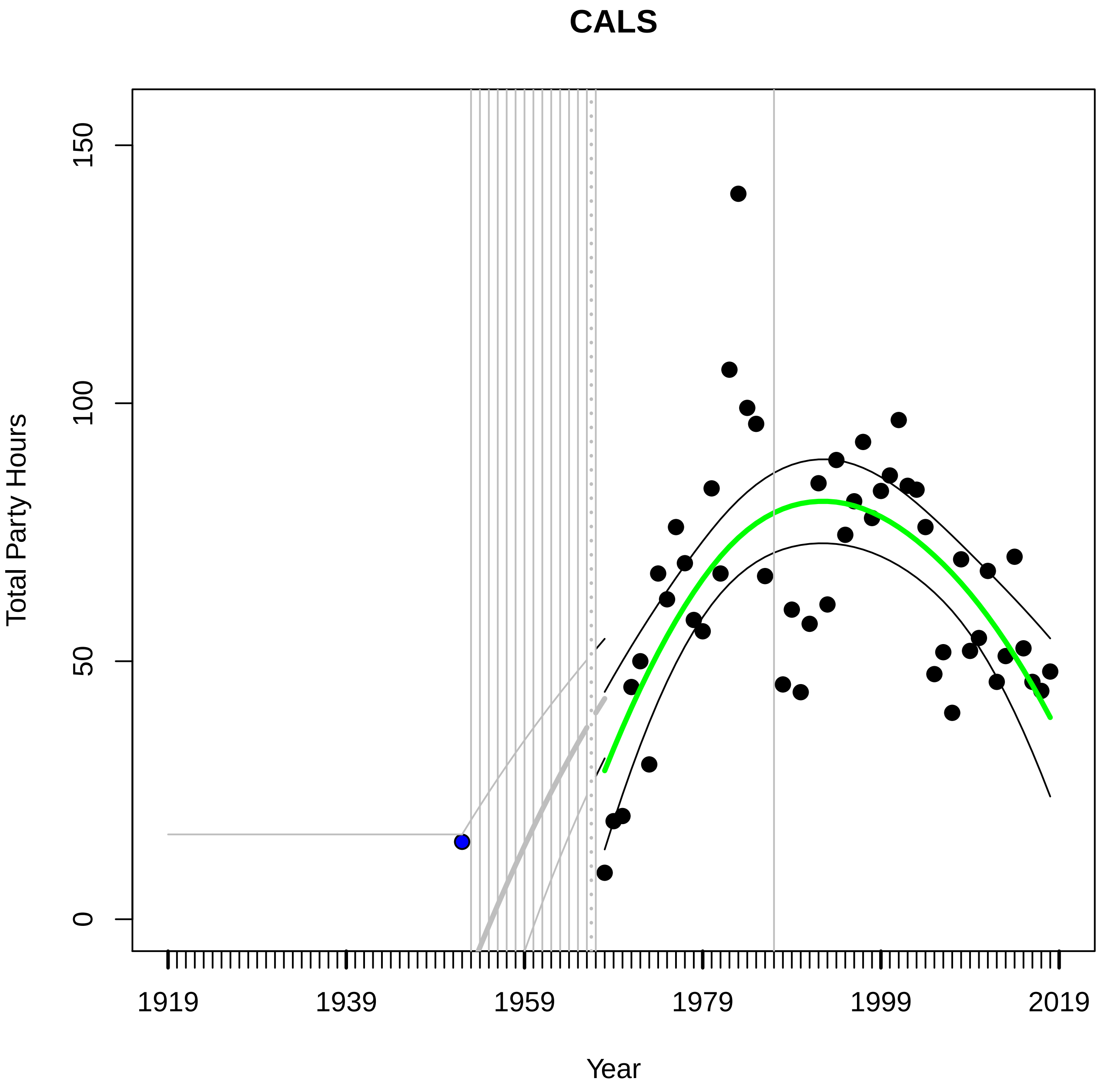

CALS
Total Party Hours
0
50
100
150
1919
1939
1959
1979
1999
2019
Year

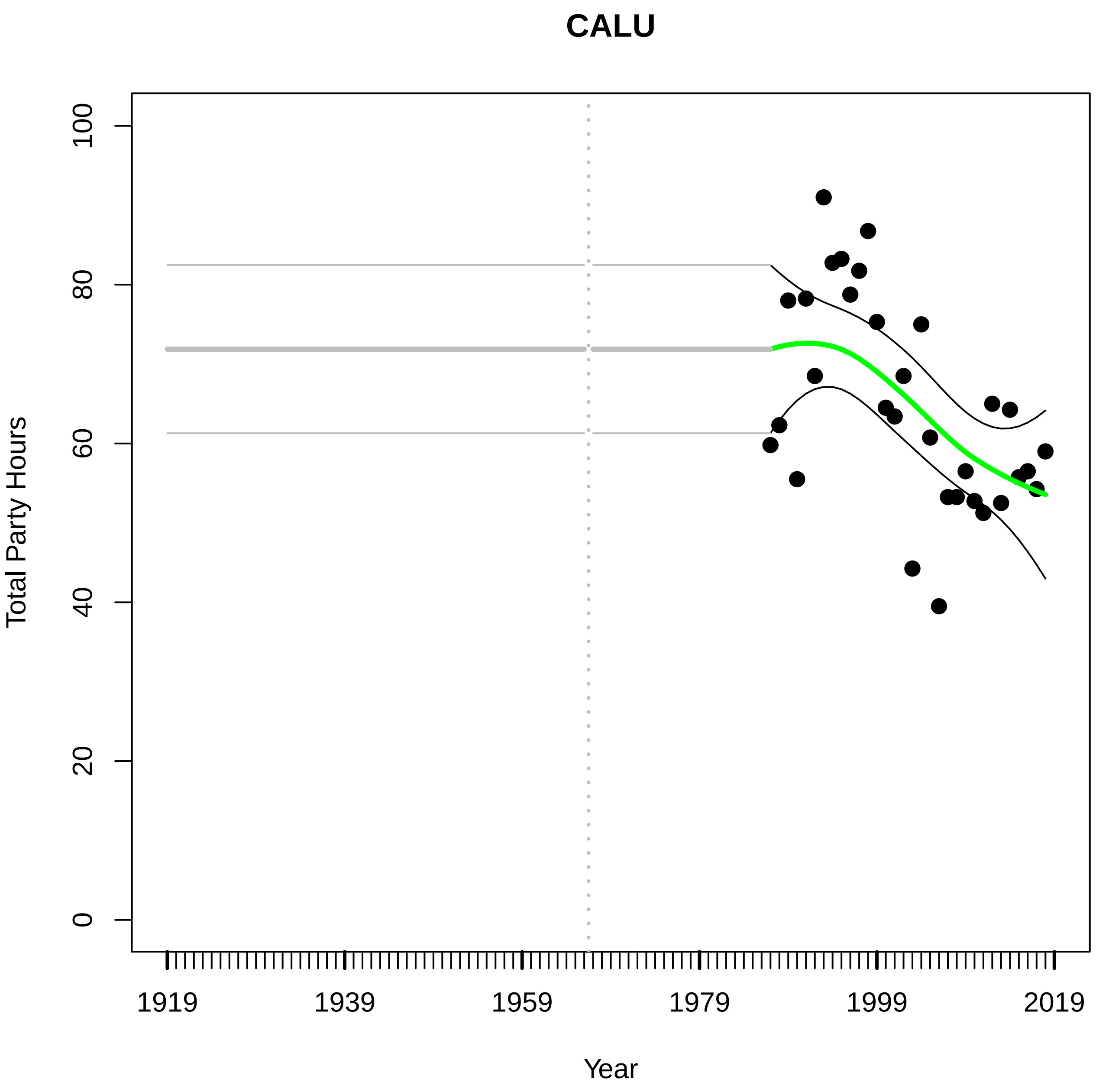
CALU
Total Party Hours
0
20
40
60
80
100
1919
1939
1959
1979
1999
2019
Year

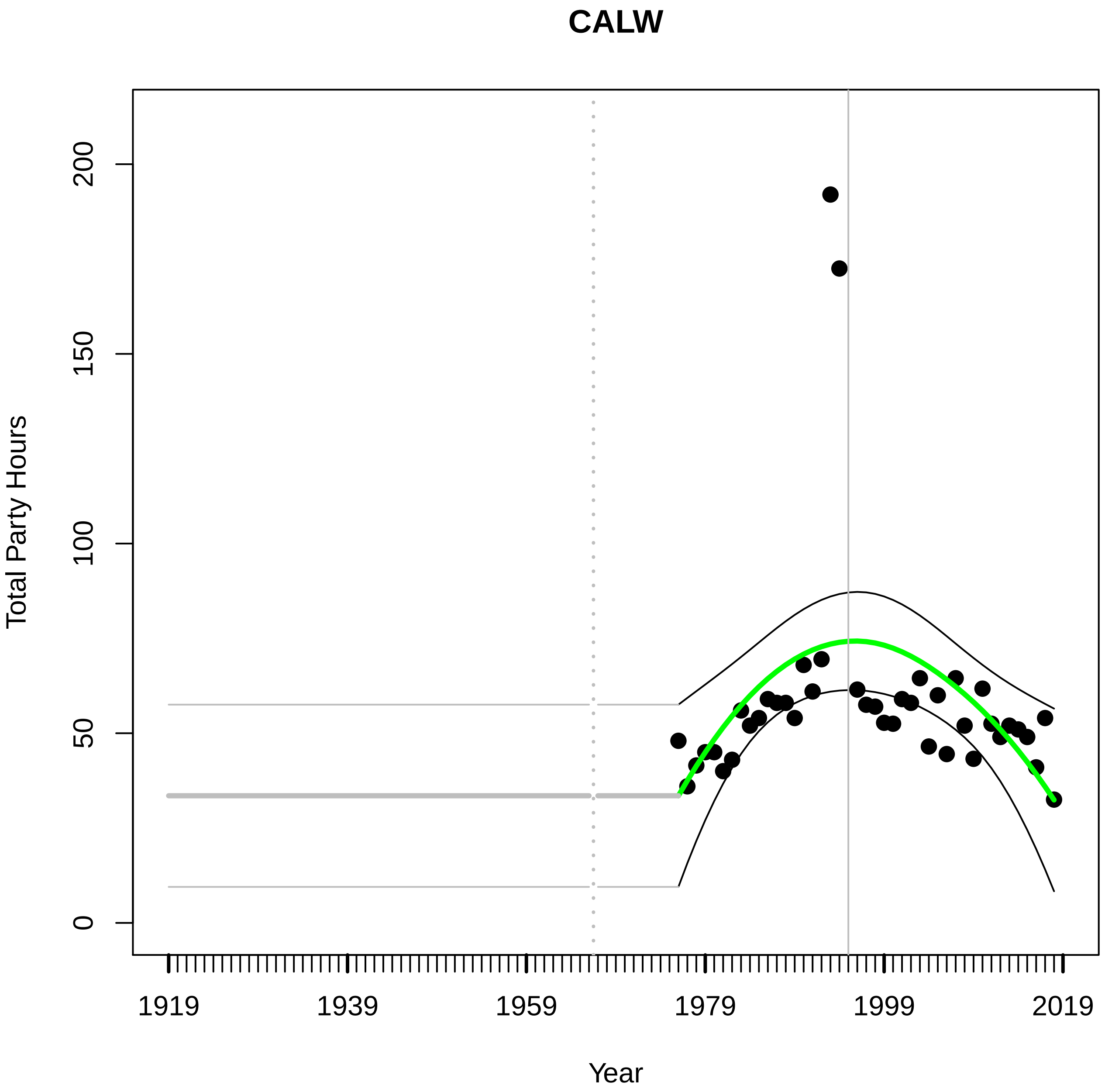

CALW
Total Party Hours
0
50
100
150
200
1919
1939
1959
1979
1999
2019
Year

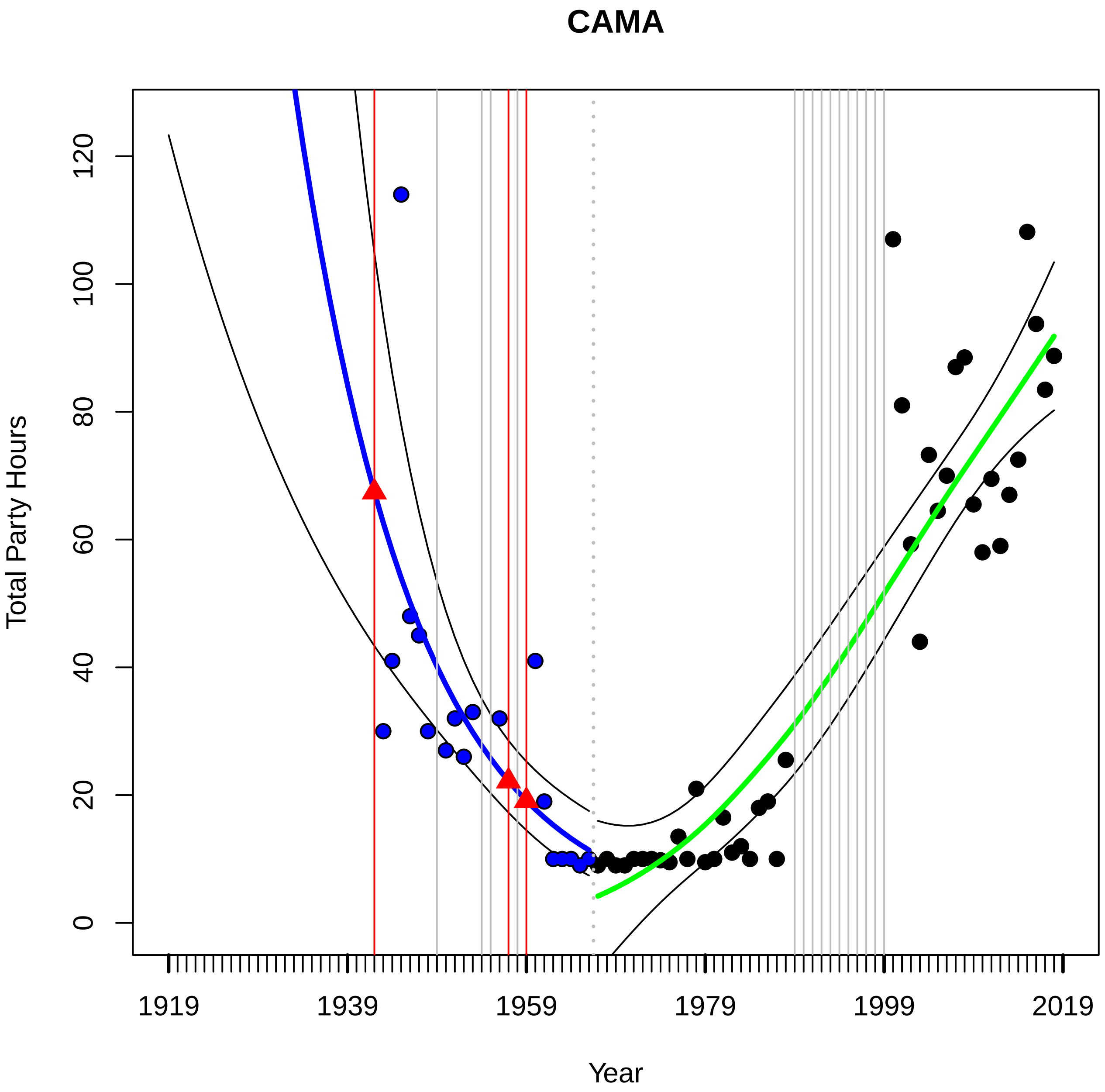
CAMA
Total Party Hours
Year
0
20
40
60
80
100
120
1919
1939
1959
1979
1999
2019

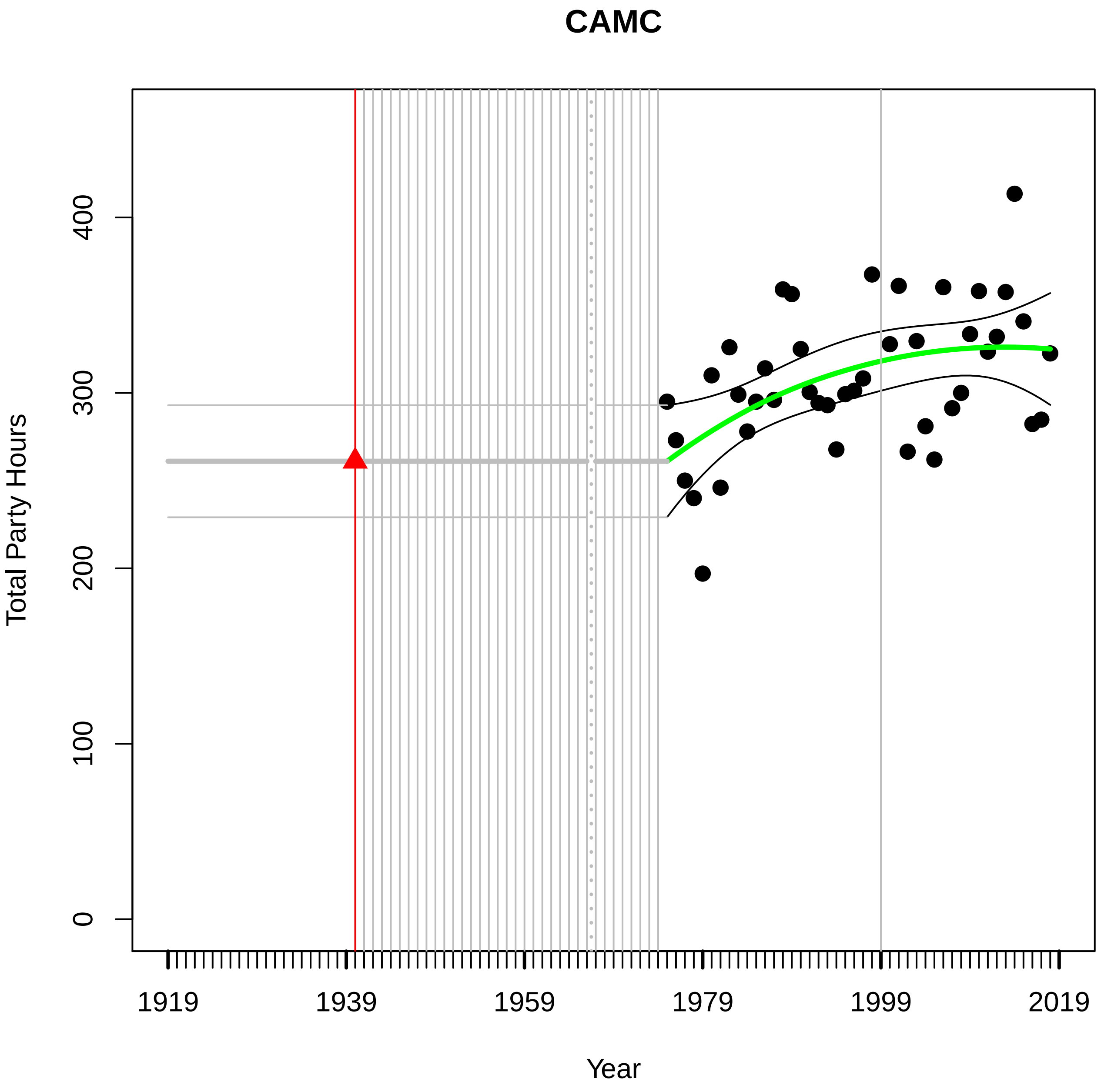

CAMC
Total Party Hours
0
100
200
300
400
Year
1919
1939
1959
1979
1999
2019

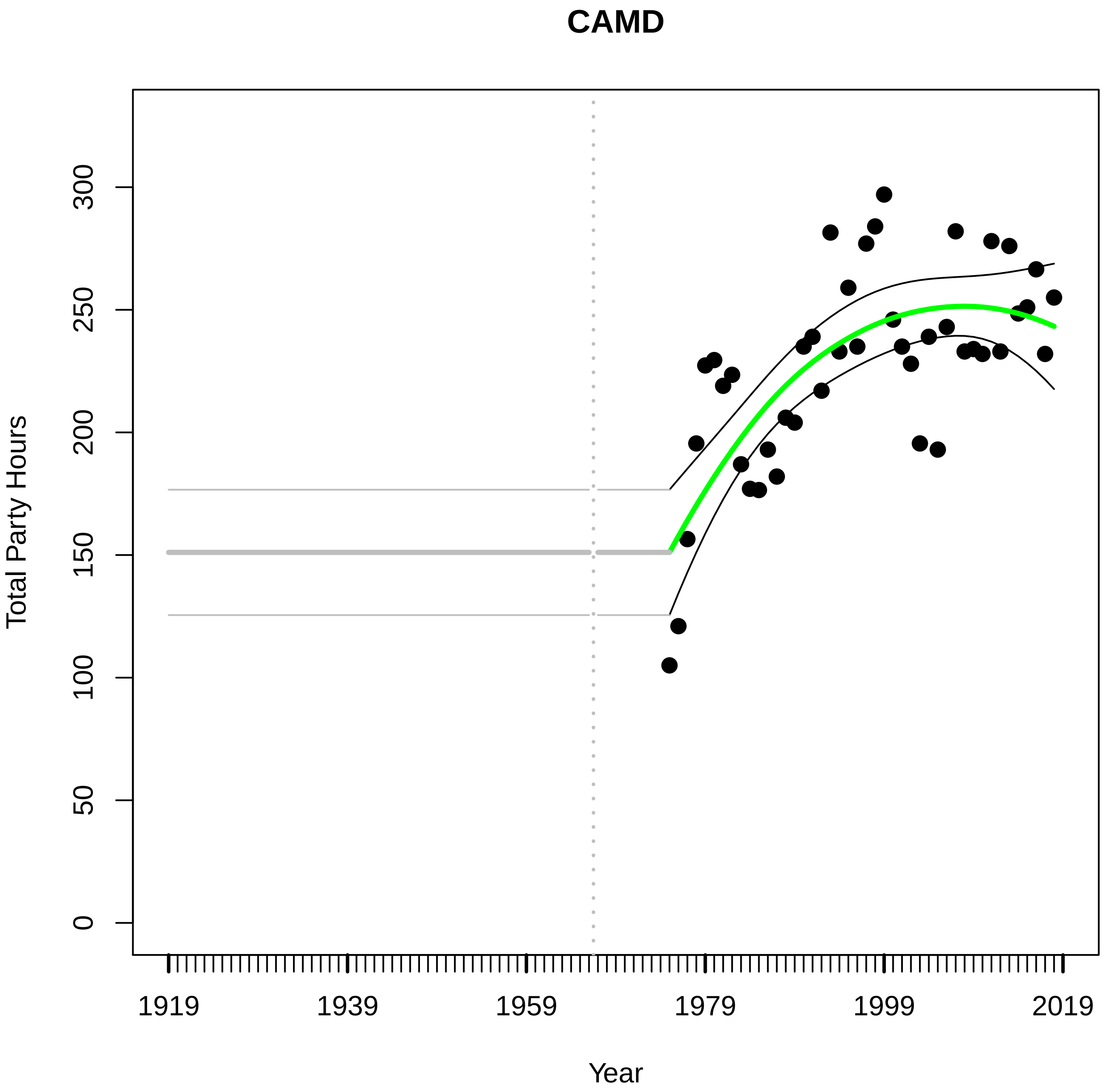
CAMD
Total Party Hours
0
50
100
150
200
250
300
1919
1939
1959
1979
1999
2019
Year

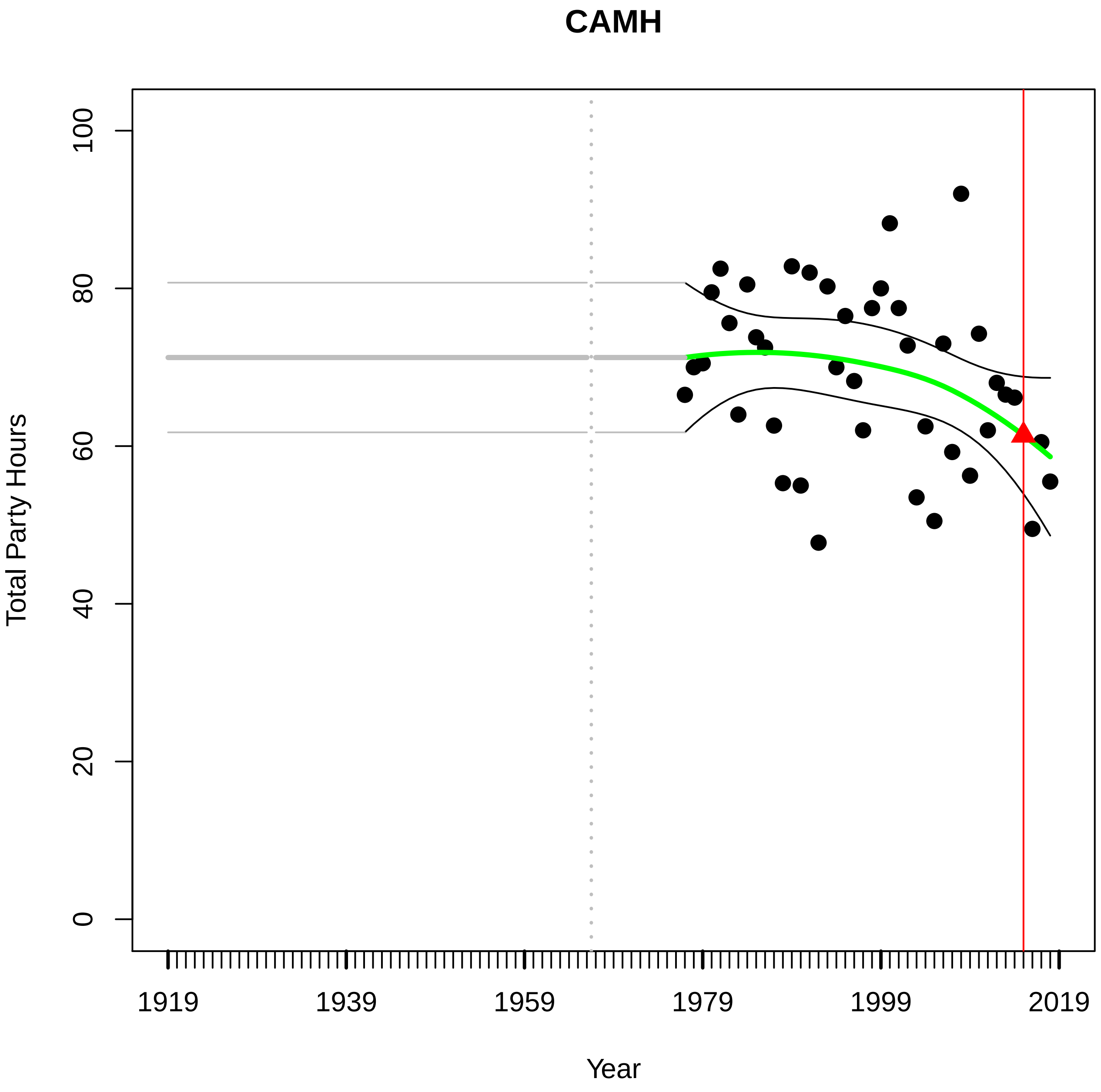

CAMH
Total Party Hours
0
20
40
60
80
100
1919
1939
1959
1979
1999
2019
Year

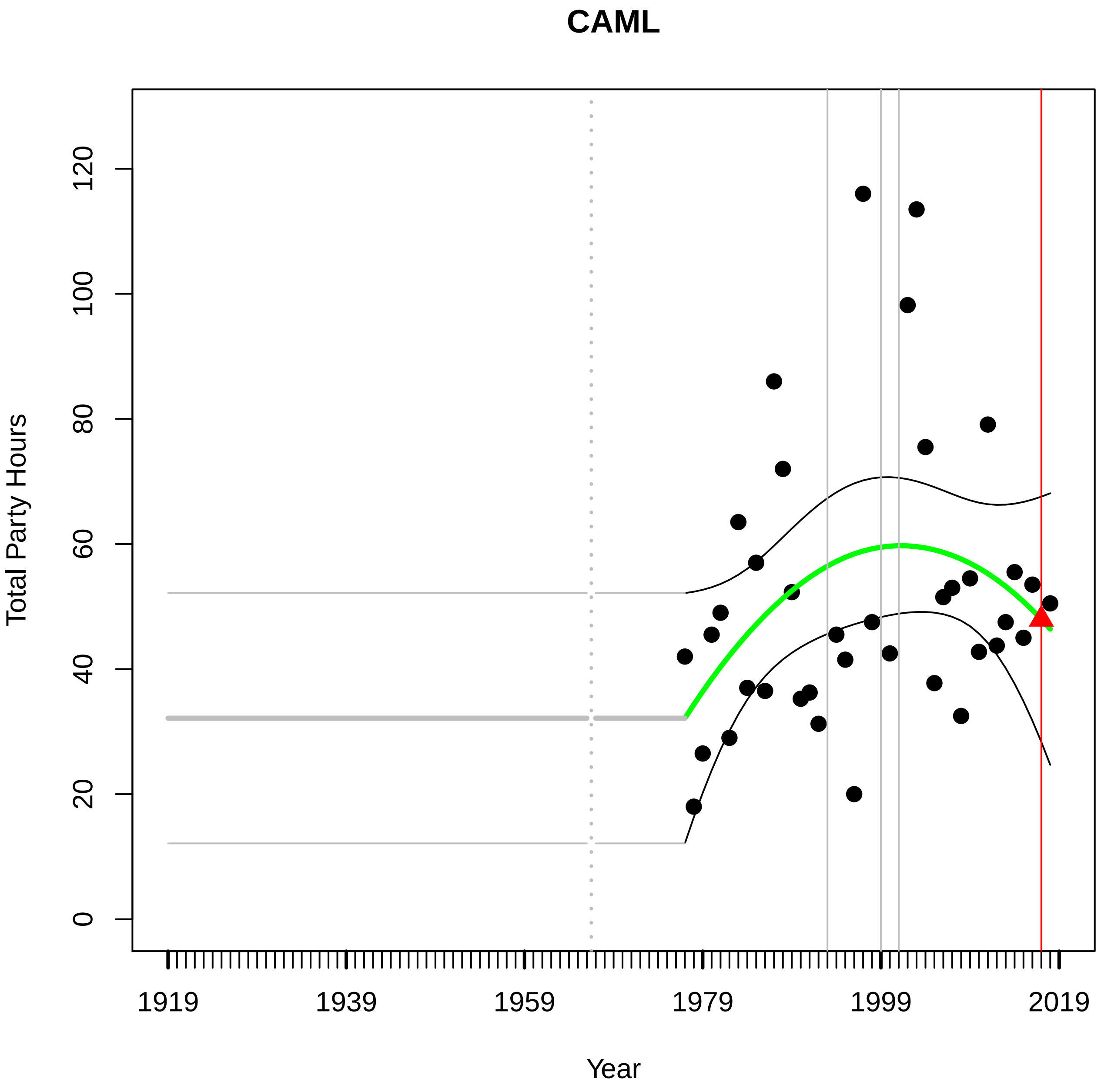

CAML
Total Party Hours
0
20
40
60
80
100
120
1919
1939
1959
1979
1999
2019
Year

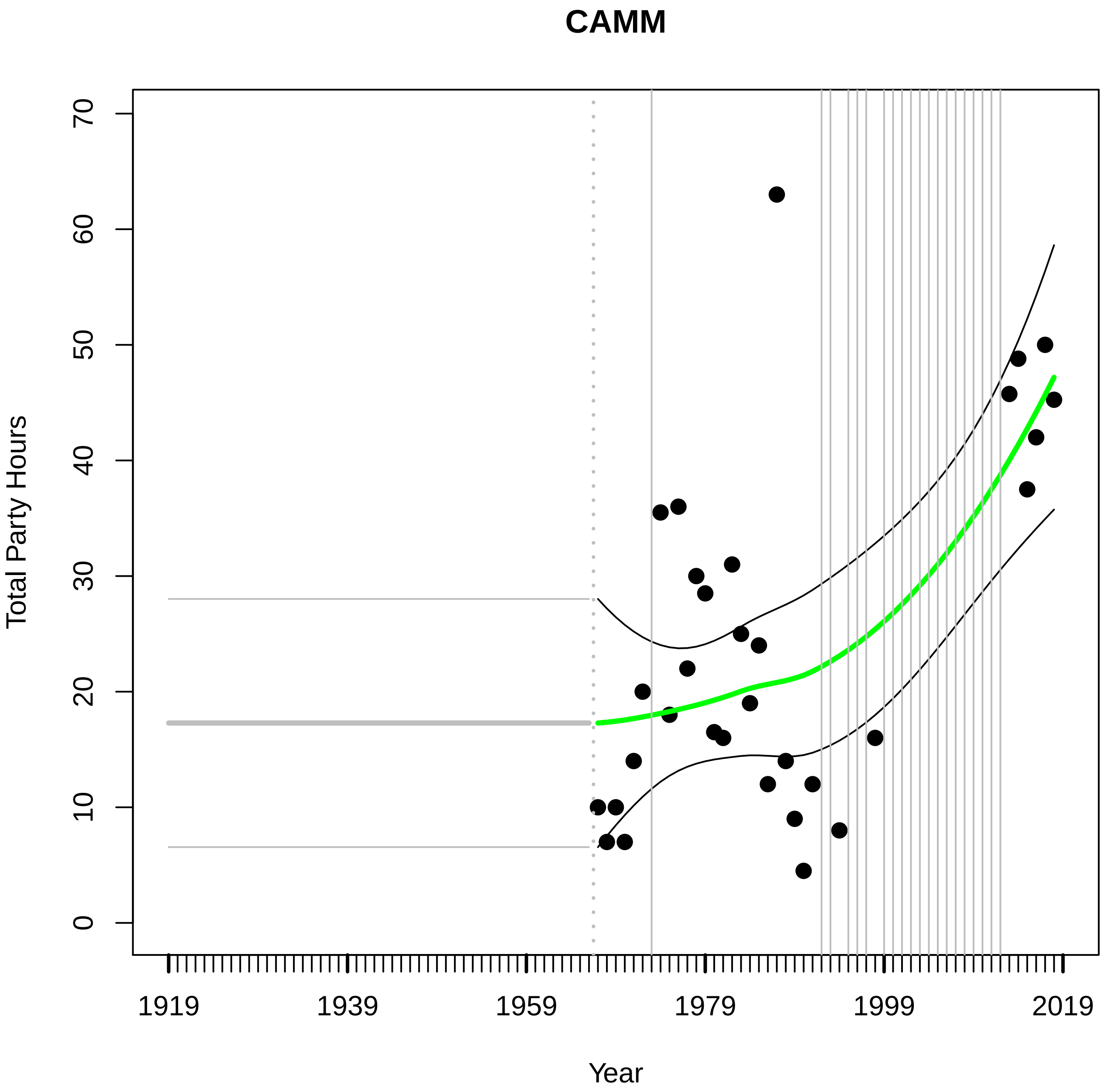
CAMM
Total Party Hours
0
10
20
30
40
50
60
70
1919
1939
1959
1979
1999
2019
Year

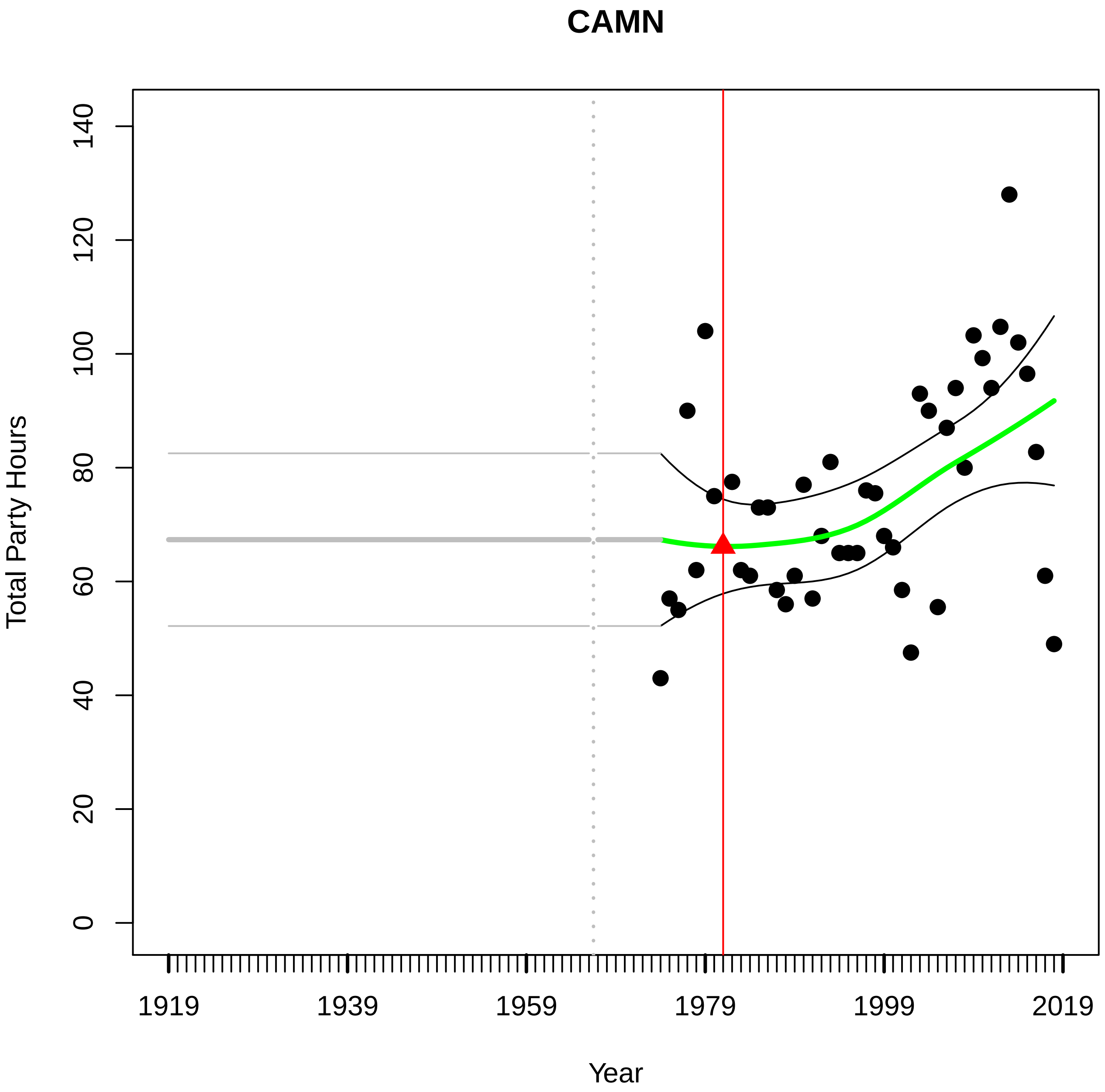

CAMN
Total Party Hours
0
20
40
60
80
100
120
140
1919
1939
1959
1979
1999
2019
Year

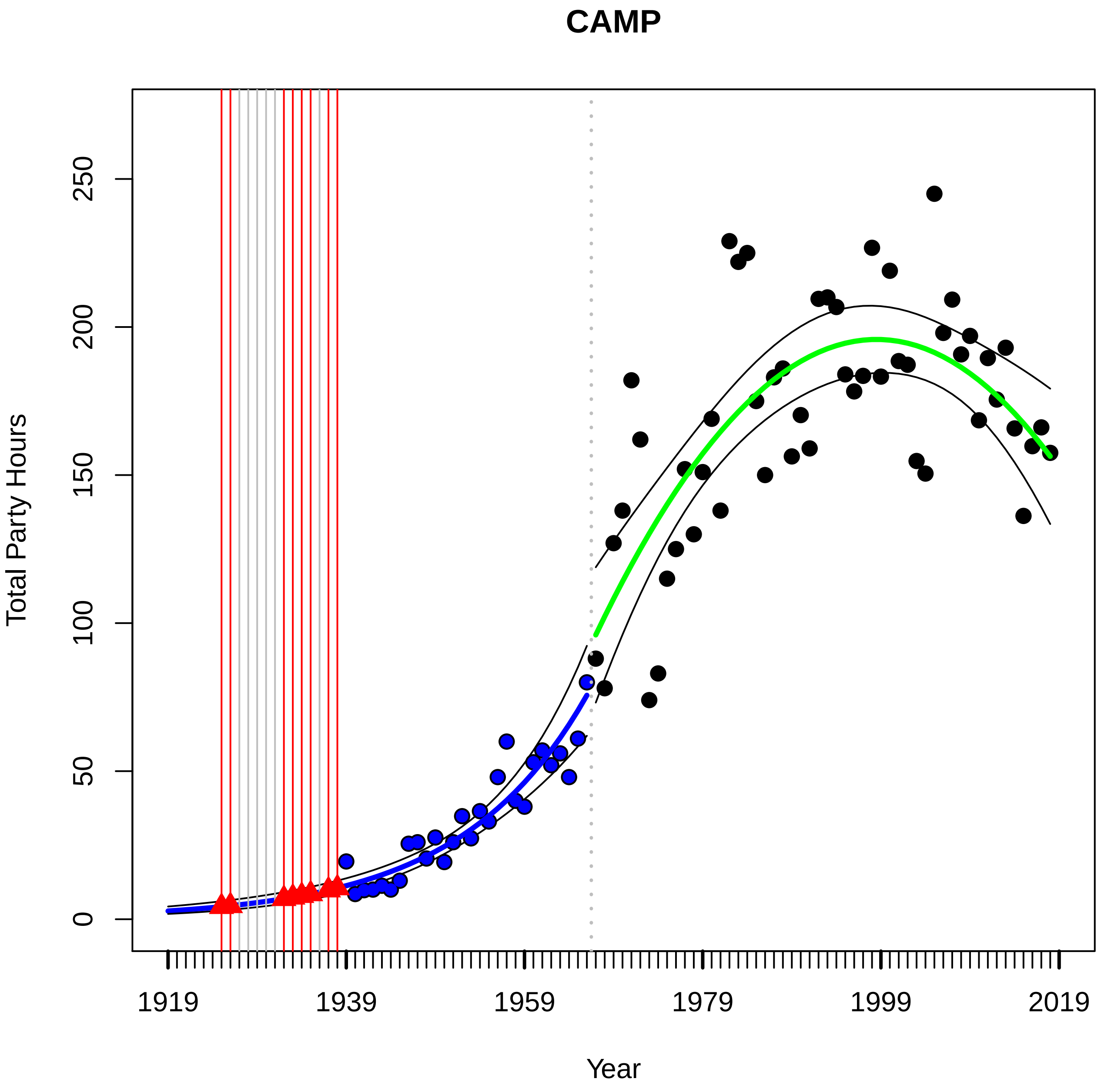

CAMP
Total Party Hours
0
50
100
150
200
250
1919
1939
1959
1979
1999
2019
Year

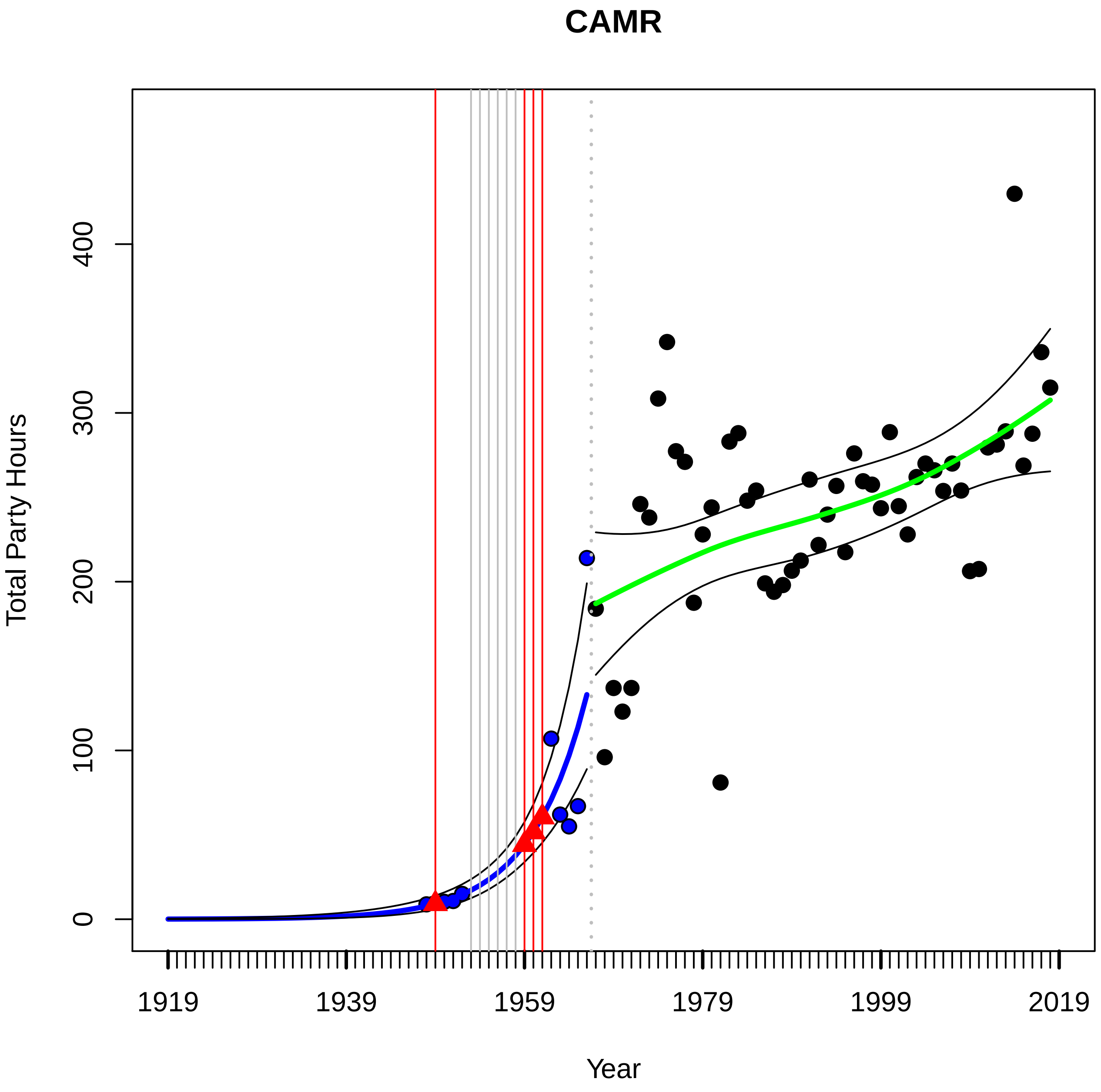
CAMR
Total Party Hours
0
100
200
300
400
Year
1919
1939
1959
1979
1999
2019

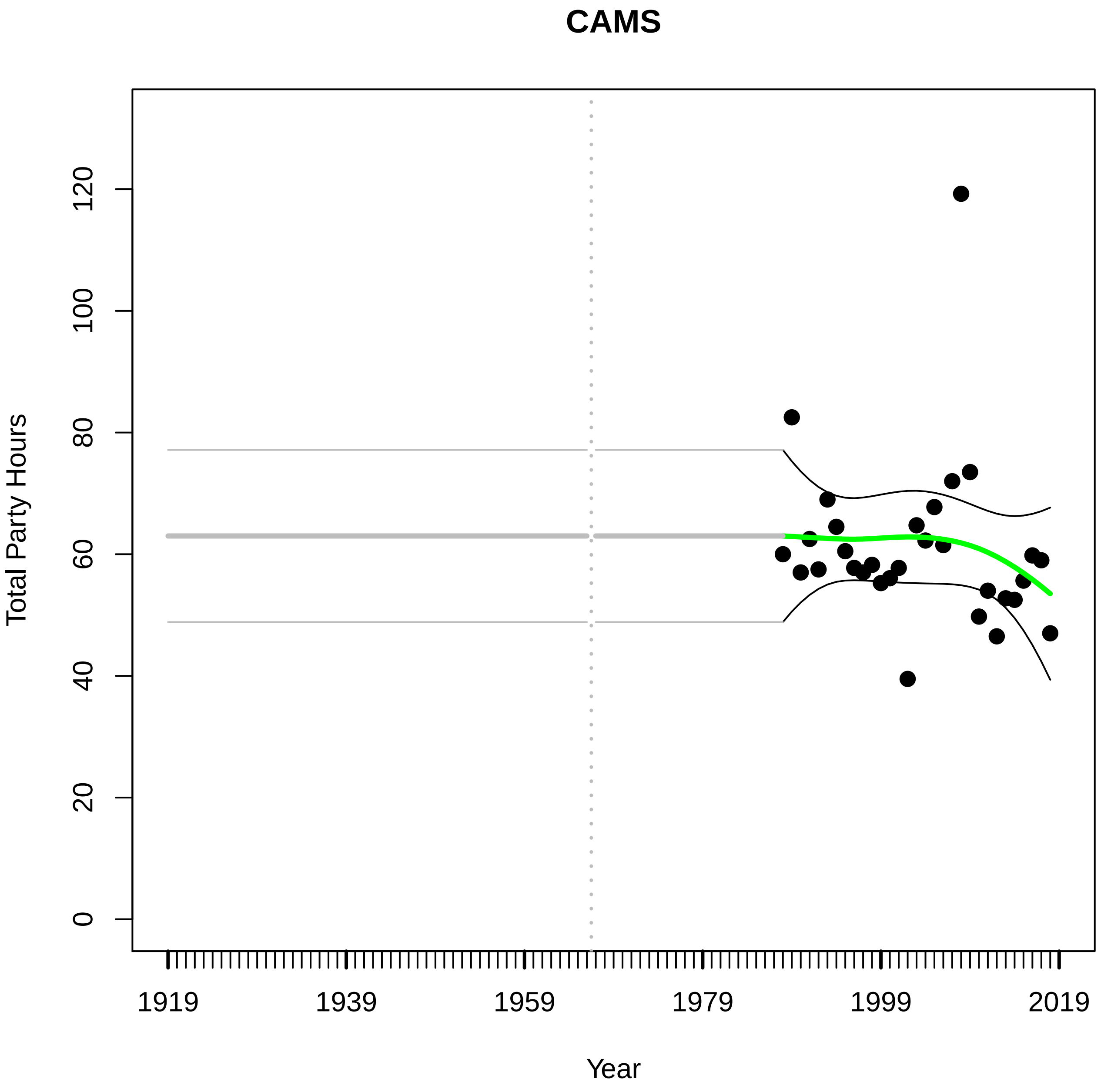
CAMS
Total Party Hours
0
20
40
60
80
100
120
1919
1939
1959
1979
1999
2019
Year

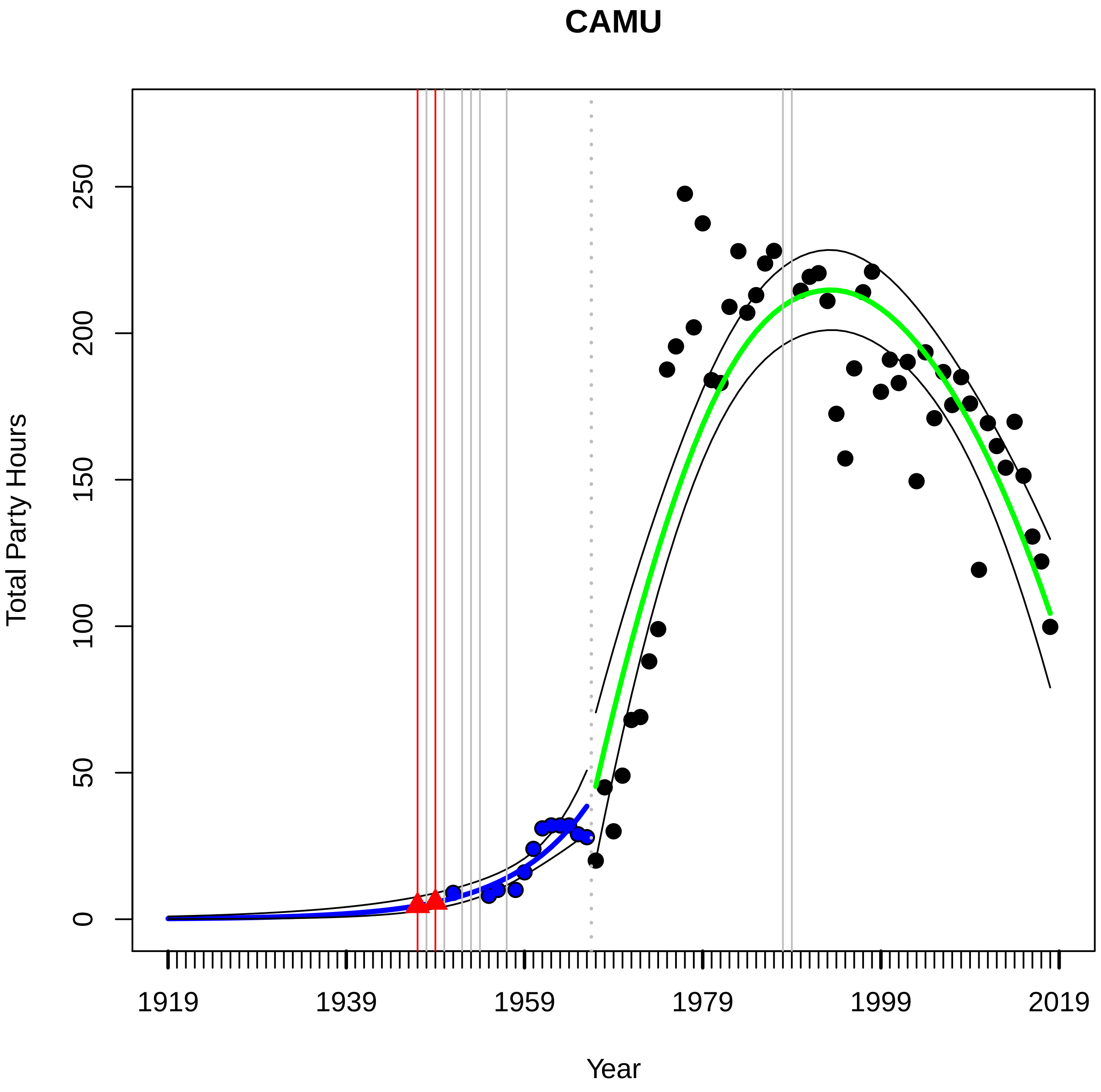

CAMU
Total Party Hours
0
50
100
150
200
250
1919
1939
1959
1979
1999
2019
Year

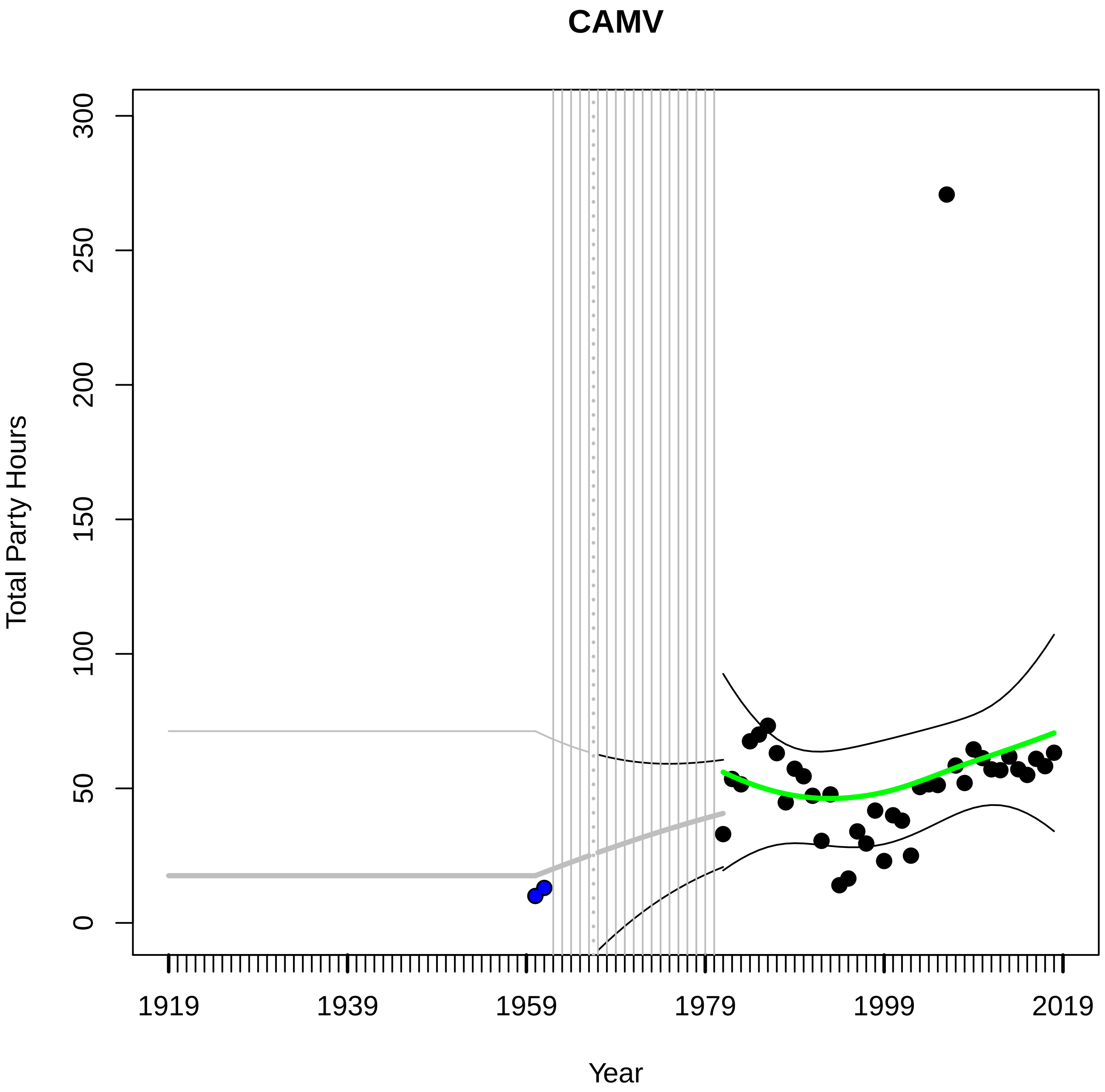

CAMV
Total Party Hours
0
50
100
150
200
250
300
1919
1939
1959
1979
1999
2019
Year

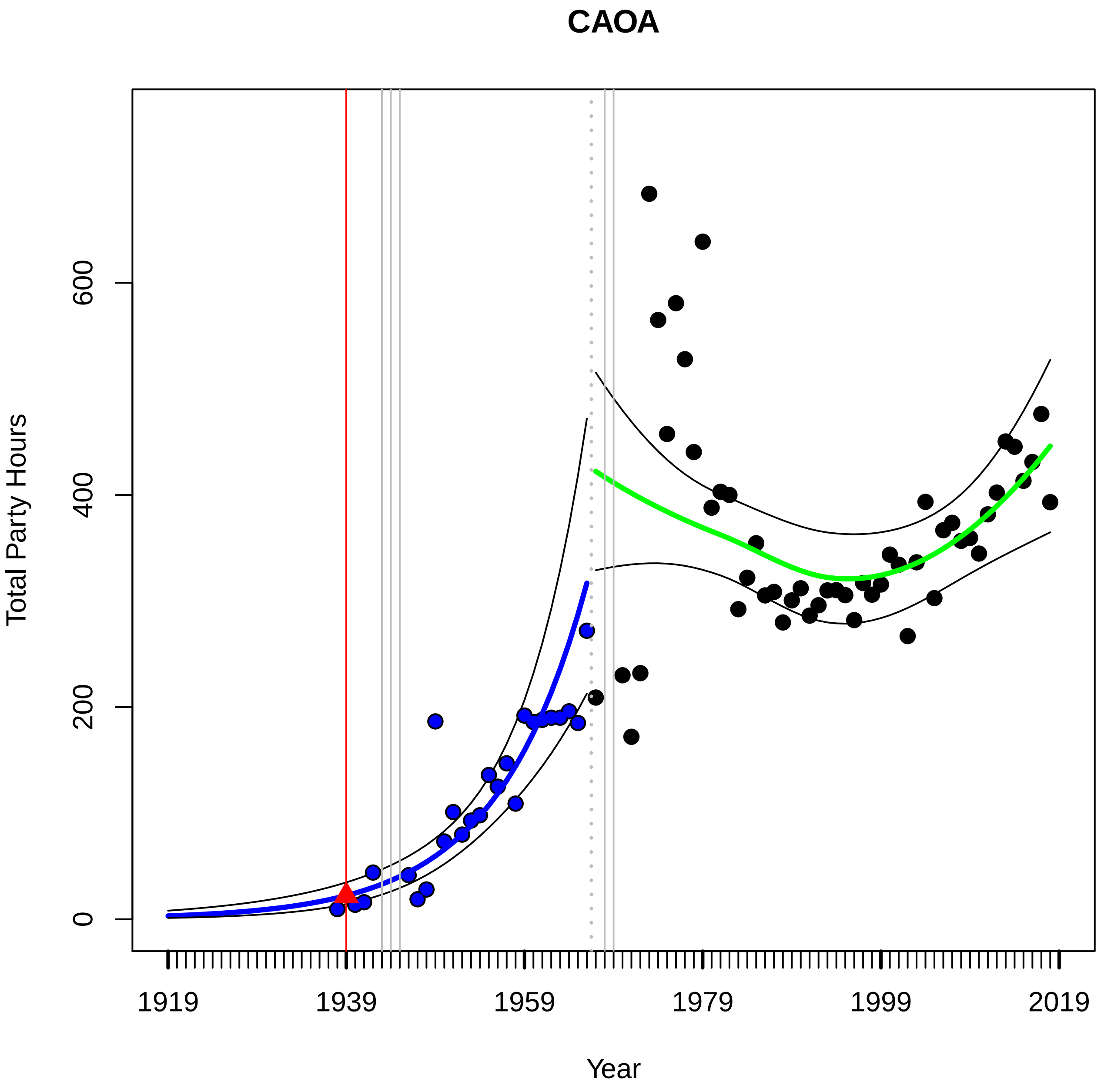

CAOA
Total Party Hours
0
200
400
600
1919
1939
1959
1979
1999
2019
Year

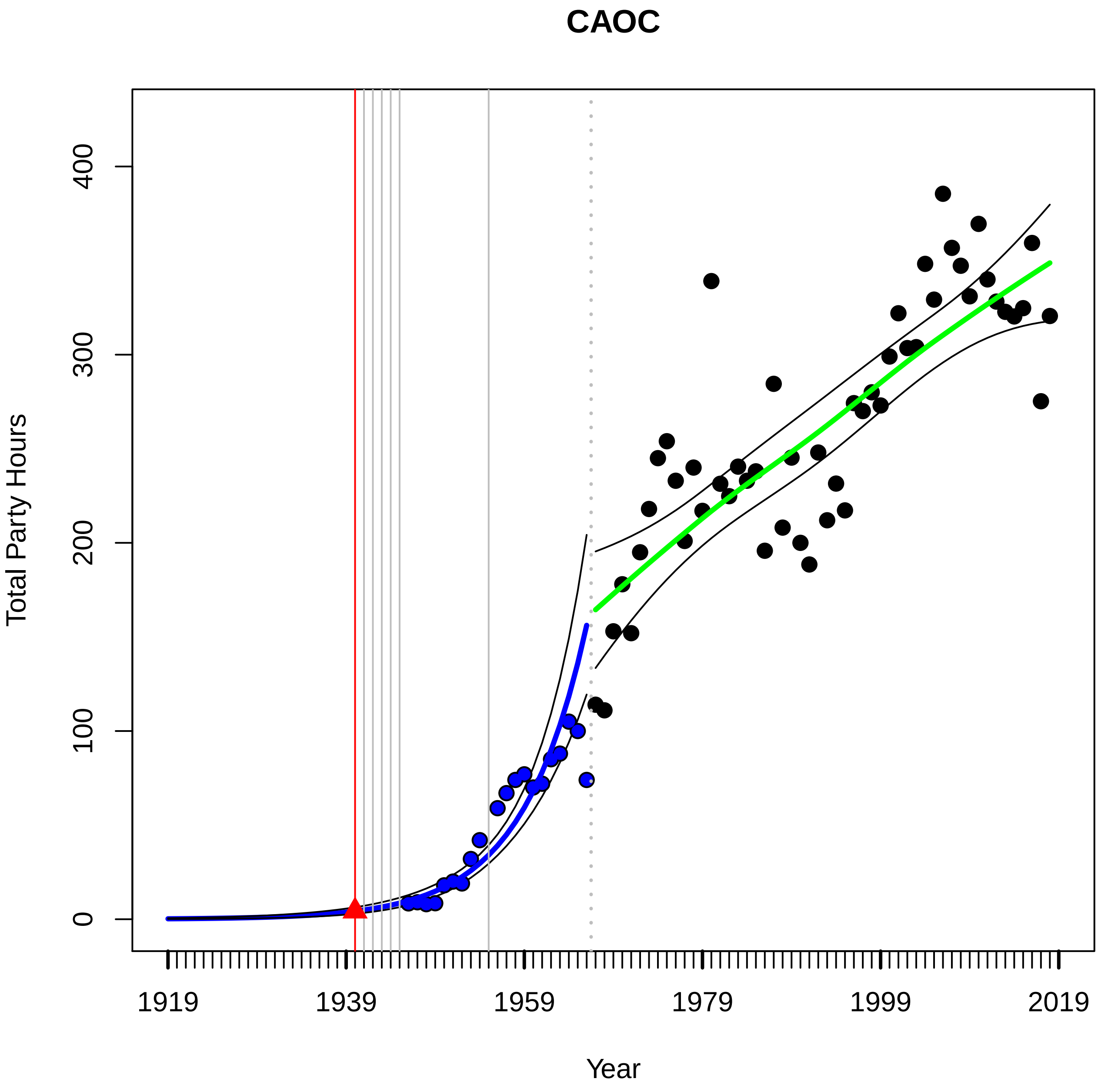
CAOC
Total Party Hours
0
100
200
300
400
1919
1939
1959
1979
1999
2019
Year

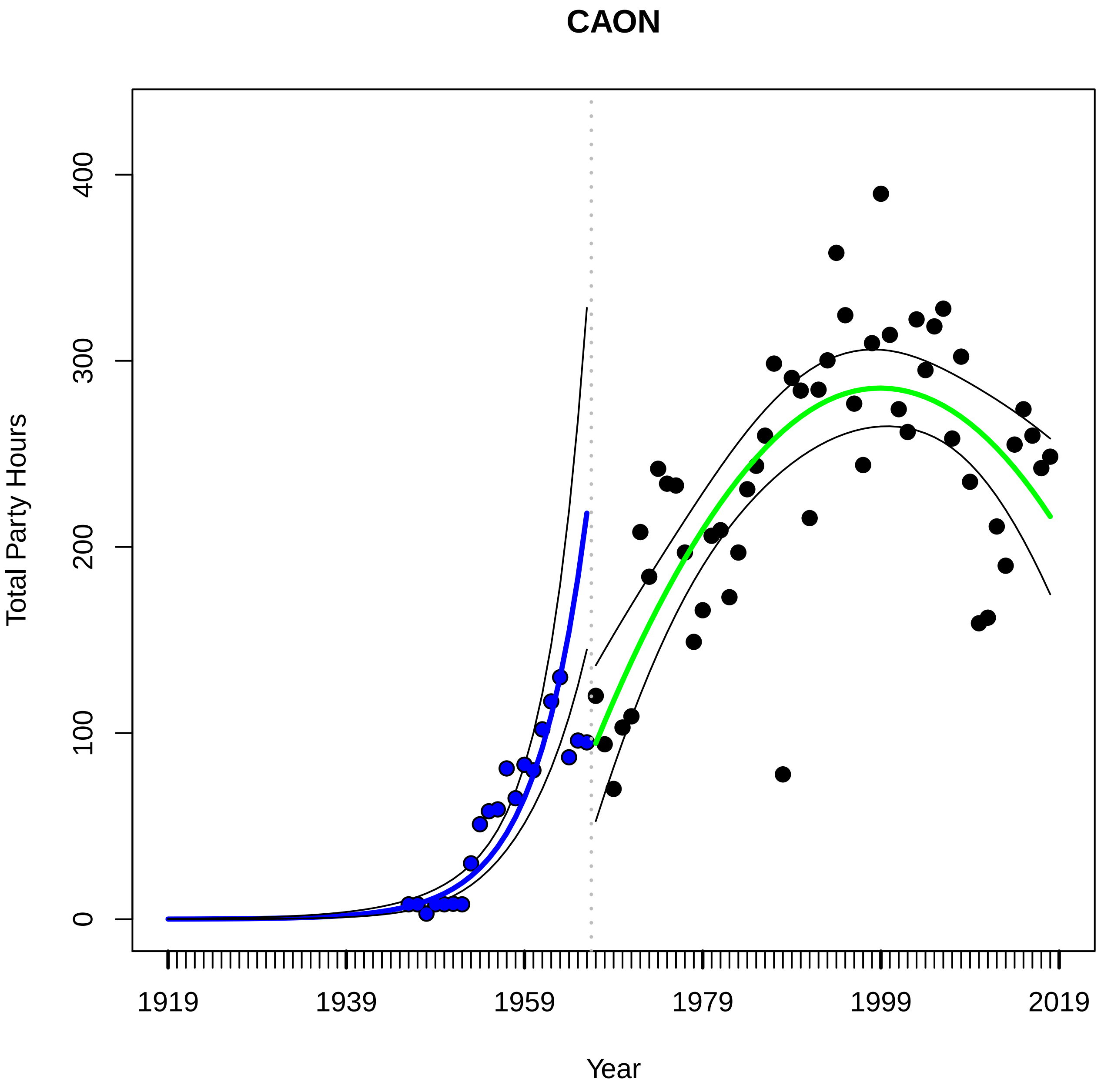
CAON
Total Party Hours
0
100
200
300
400
1919
1939
1959
1979
1999
2019
Year

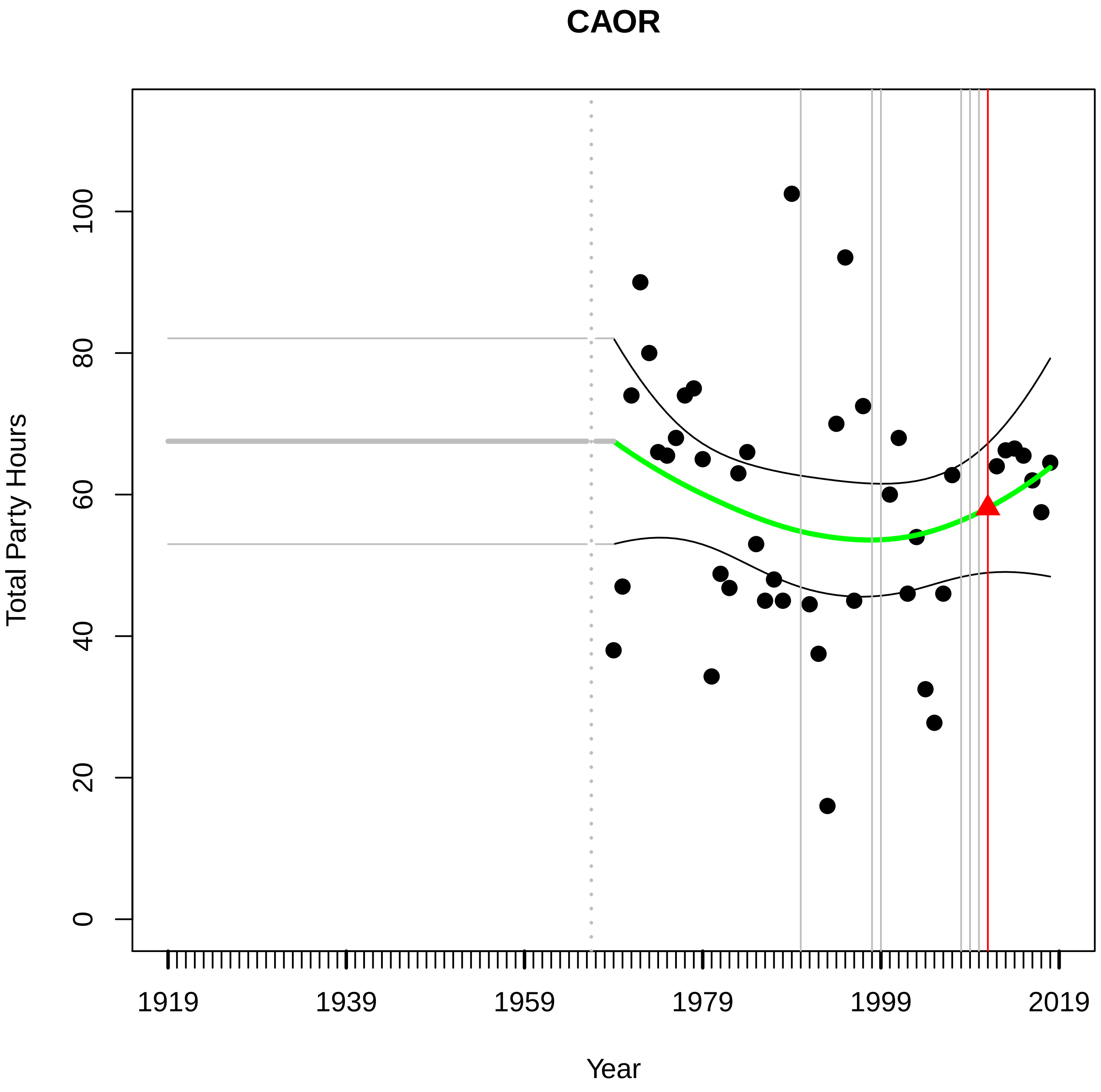

CAOR
Total Party Hours
0
20
40
60
80
100
1919
1939
1959
1979
1999
2019
Year

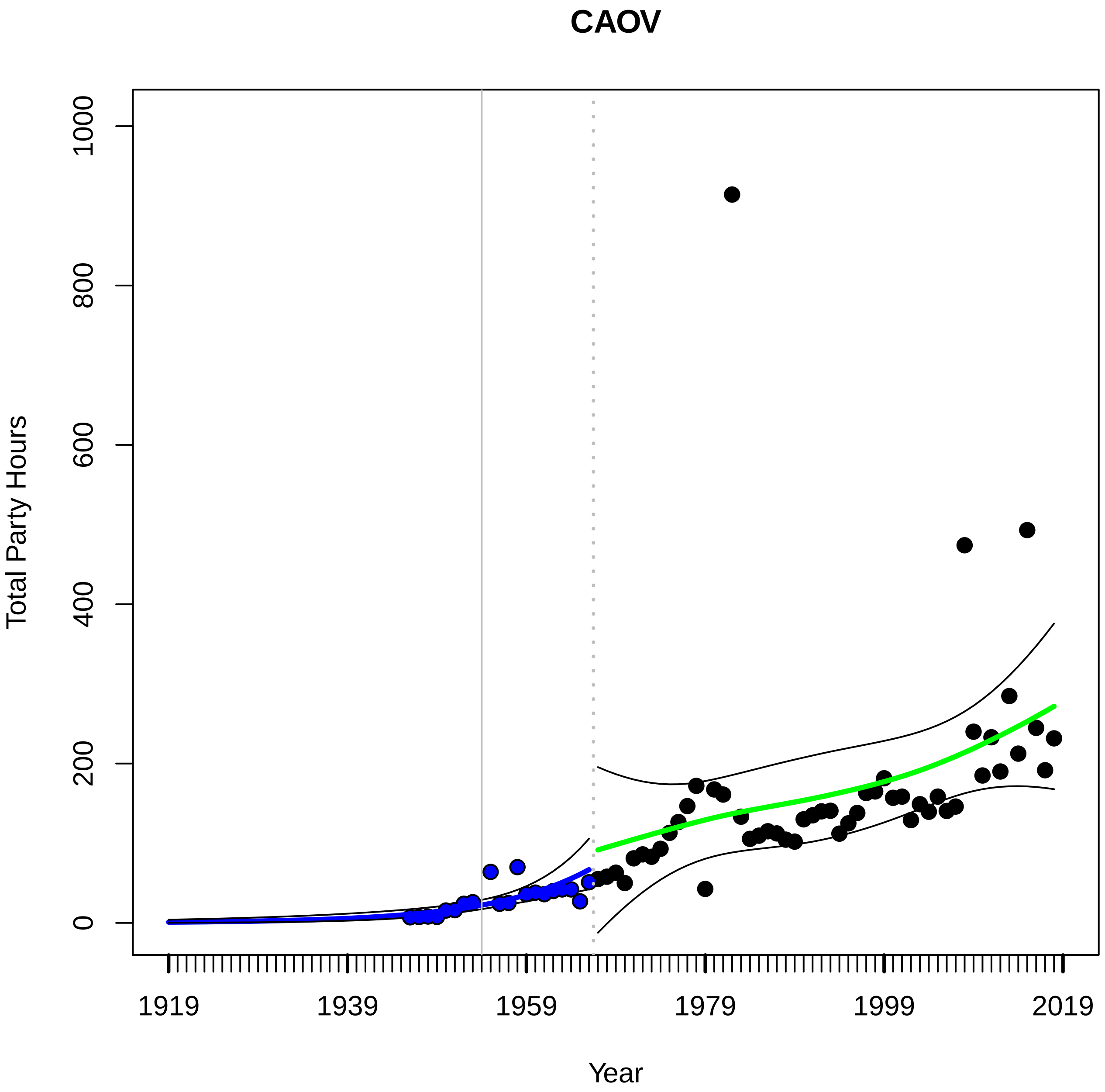
CAOV
Total Party Hours
0
200
400
600
800
1000
1919
1939
1959
1979
1999
2019
Year

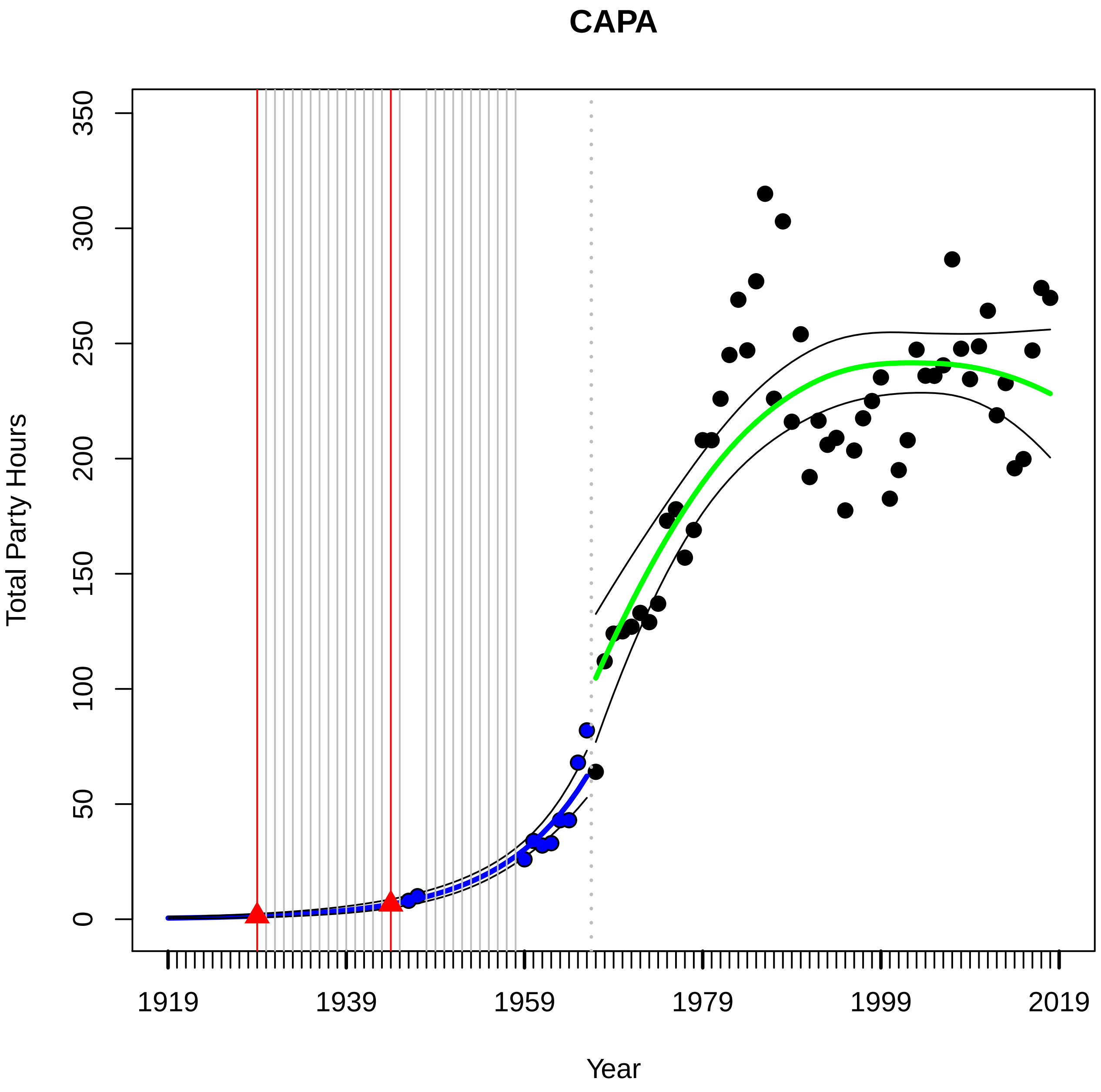

CAPA
Total Party Hours
Year
0
50
100
150
200
250
300
350
1919
1939
1959
1979
1999
2019

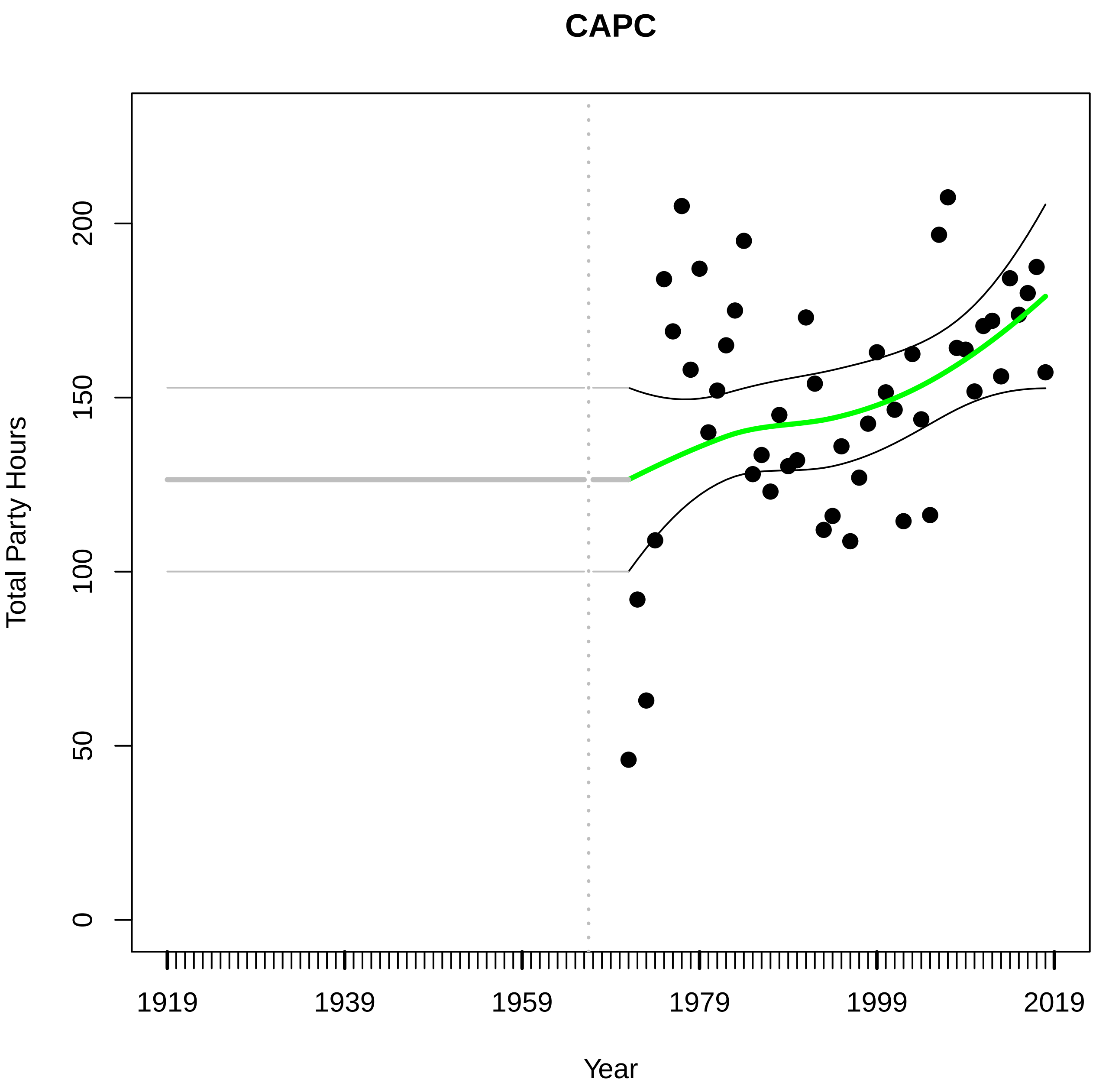
CAPC
Total Party Hours
0
50
100
150
200
1919
1939
1959
1979
1999
2019
Year

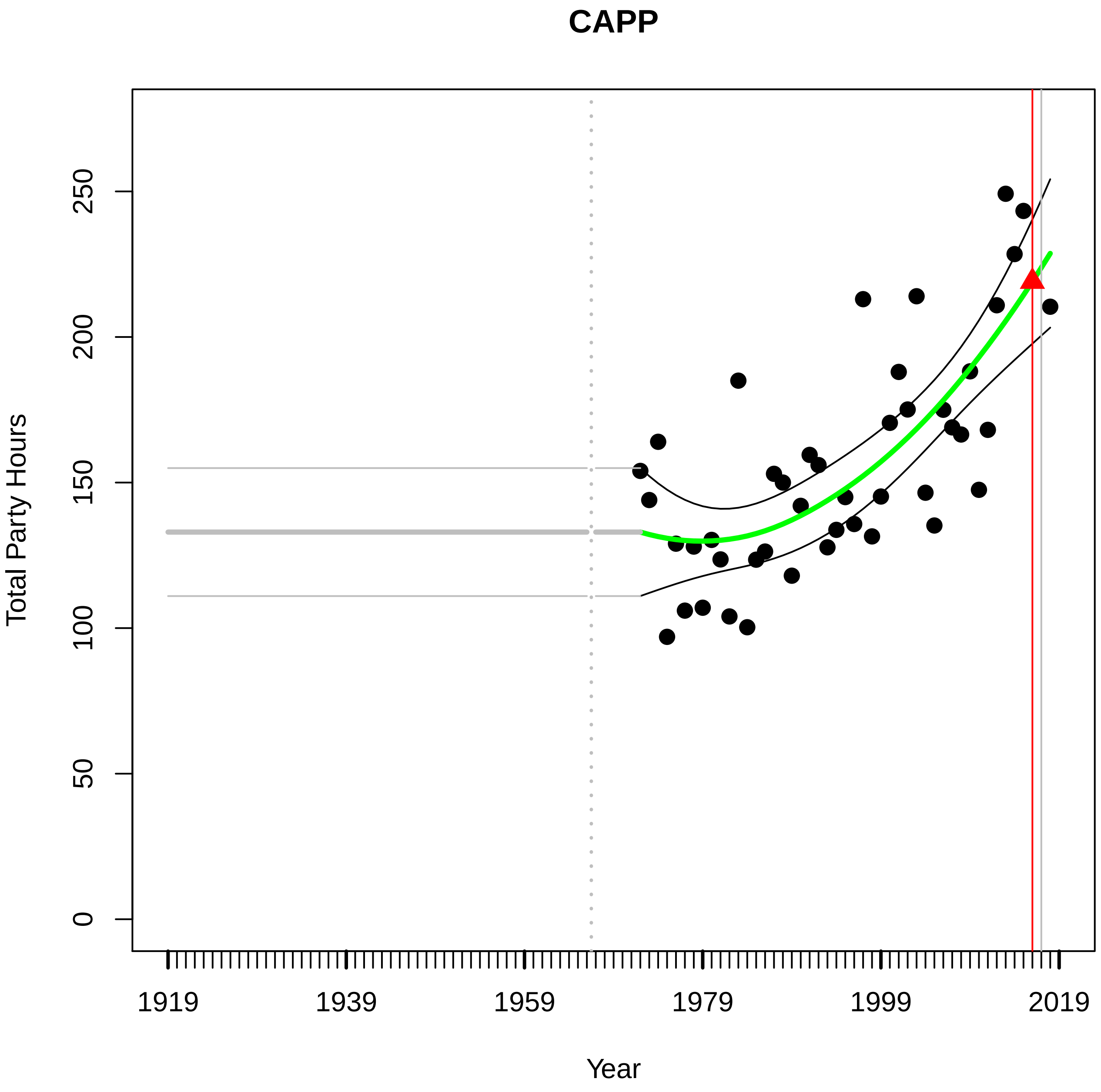

CAPP
Total Party Hours
0
50
100
150
200
250
1919
1939
1959
1979
1999
2019
Year

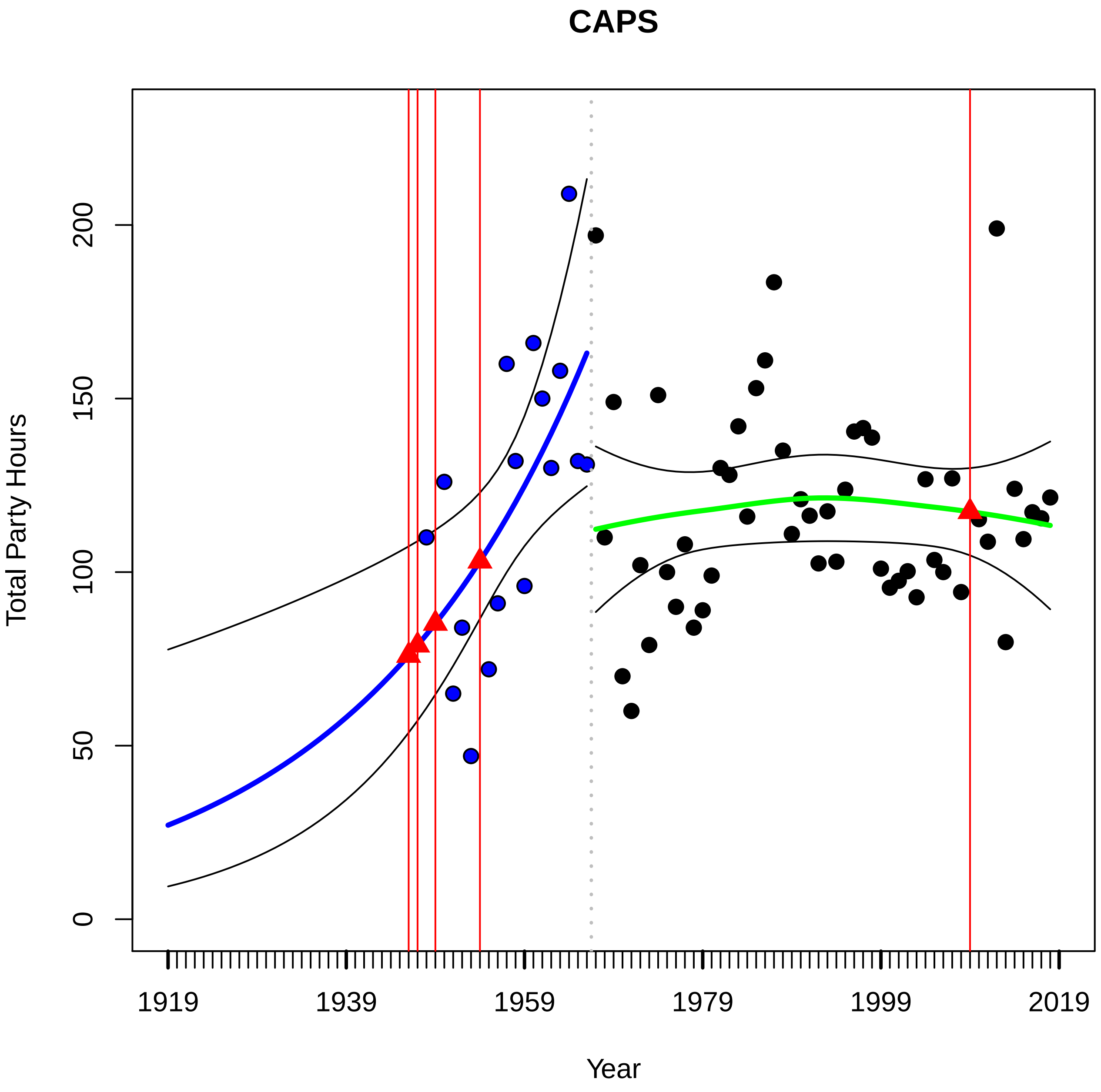

CAPS
Total Party Hours
0
50
100
150
200
Year
1919
1939
1959
1979
1999
2019

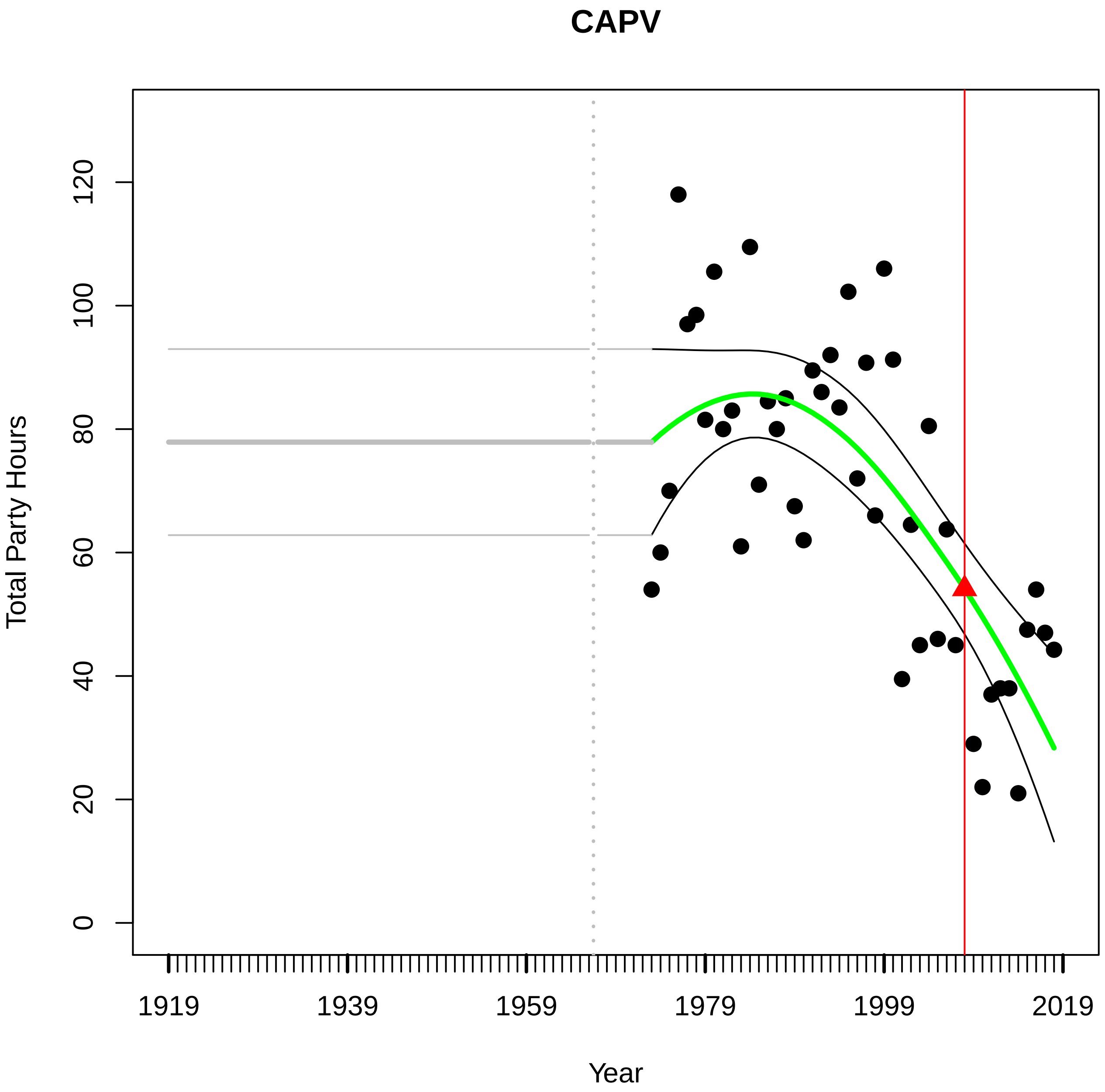

CAPV
Total Party Hours
Year
0
20
40
60
80
100
120
1919
1939
1959
1979
1999
2019

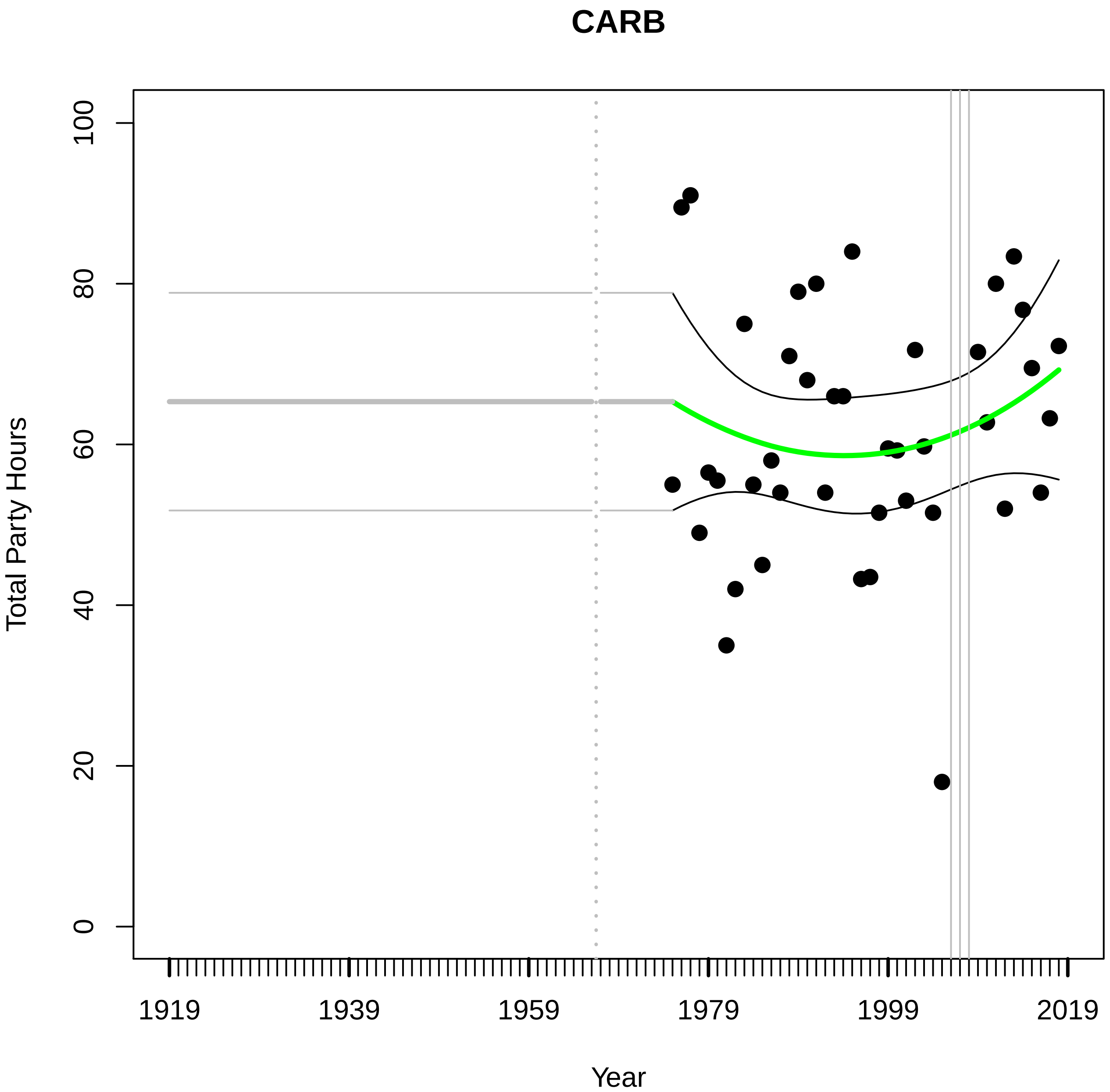
CARB
Total Party Hours
0
20
40
60
80
100
1919
1939
1959
1979
1999
2019
Year

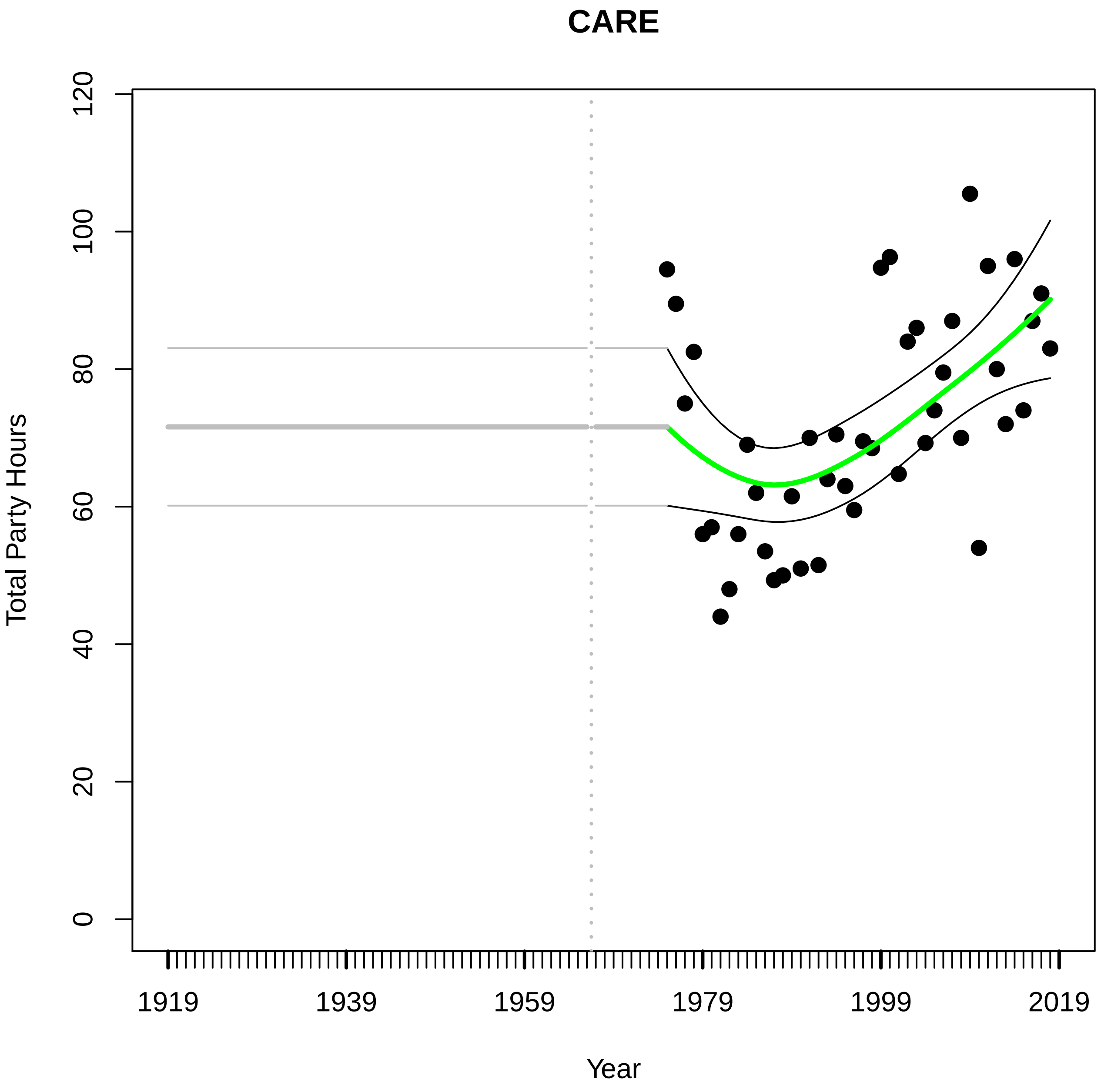

CARE
Total Party Hours
Year
0
20
40
60
80
100
120
1919
1939
1959
1979
1999
2019

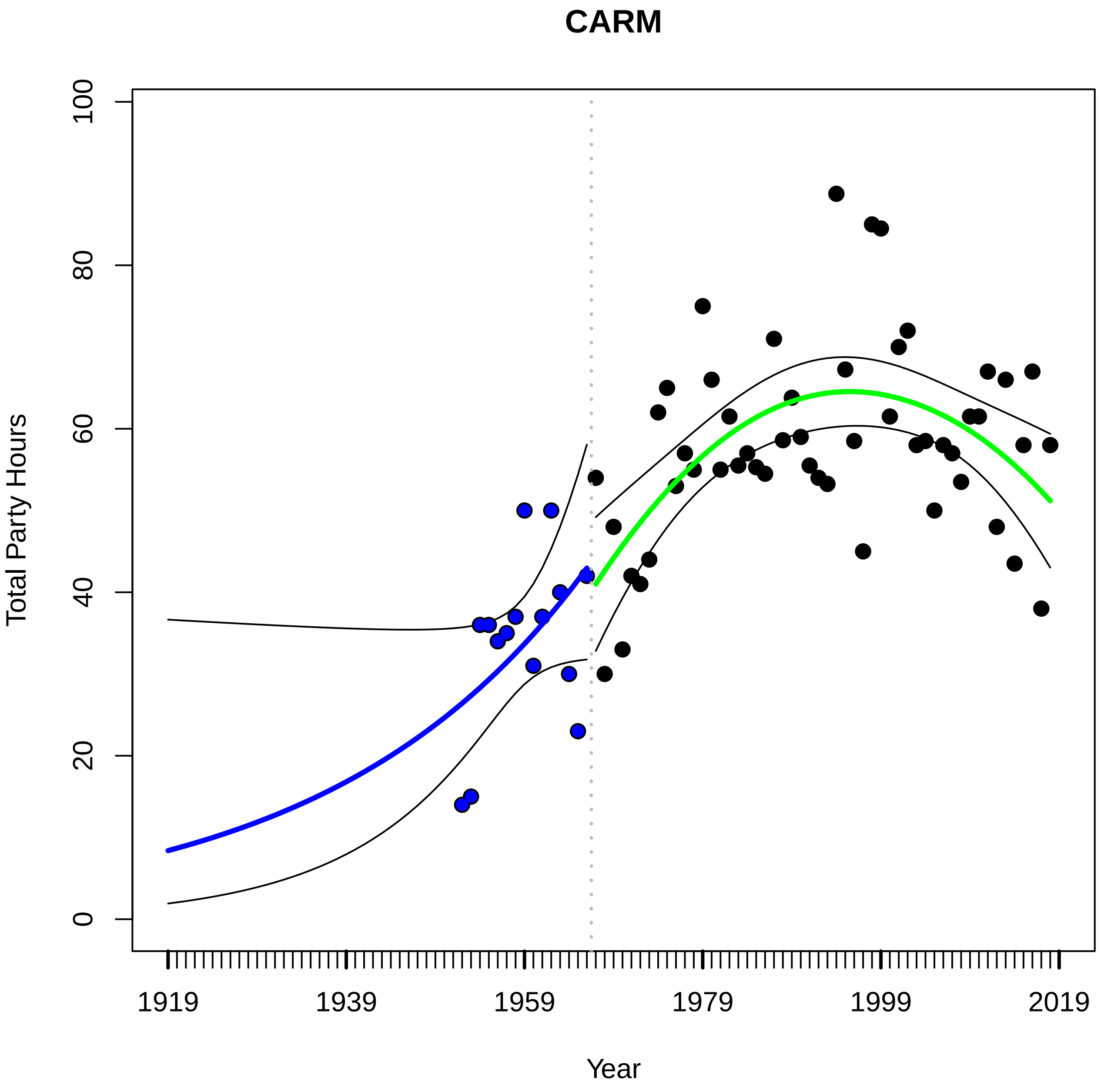

CARM
Total Party Hours
Year
0
20
40
60
80
100
1919
1939
1959
1979
1999
2019

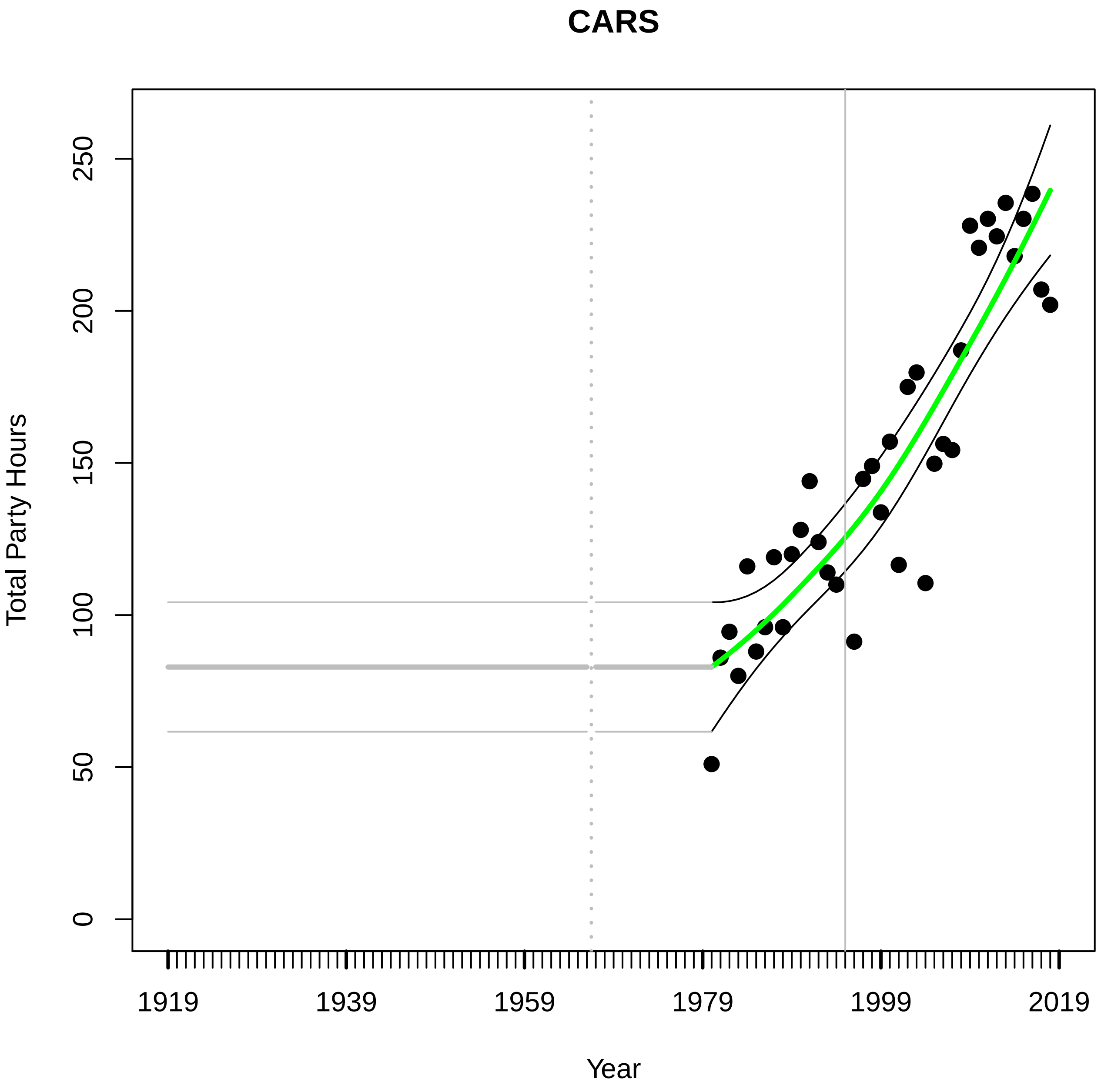
CARS
Total Party Hours
0
50
100
150
200
250
1919
1939
1959
1979
1999
2019
Year

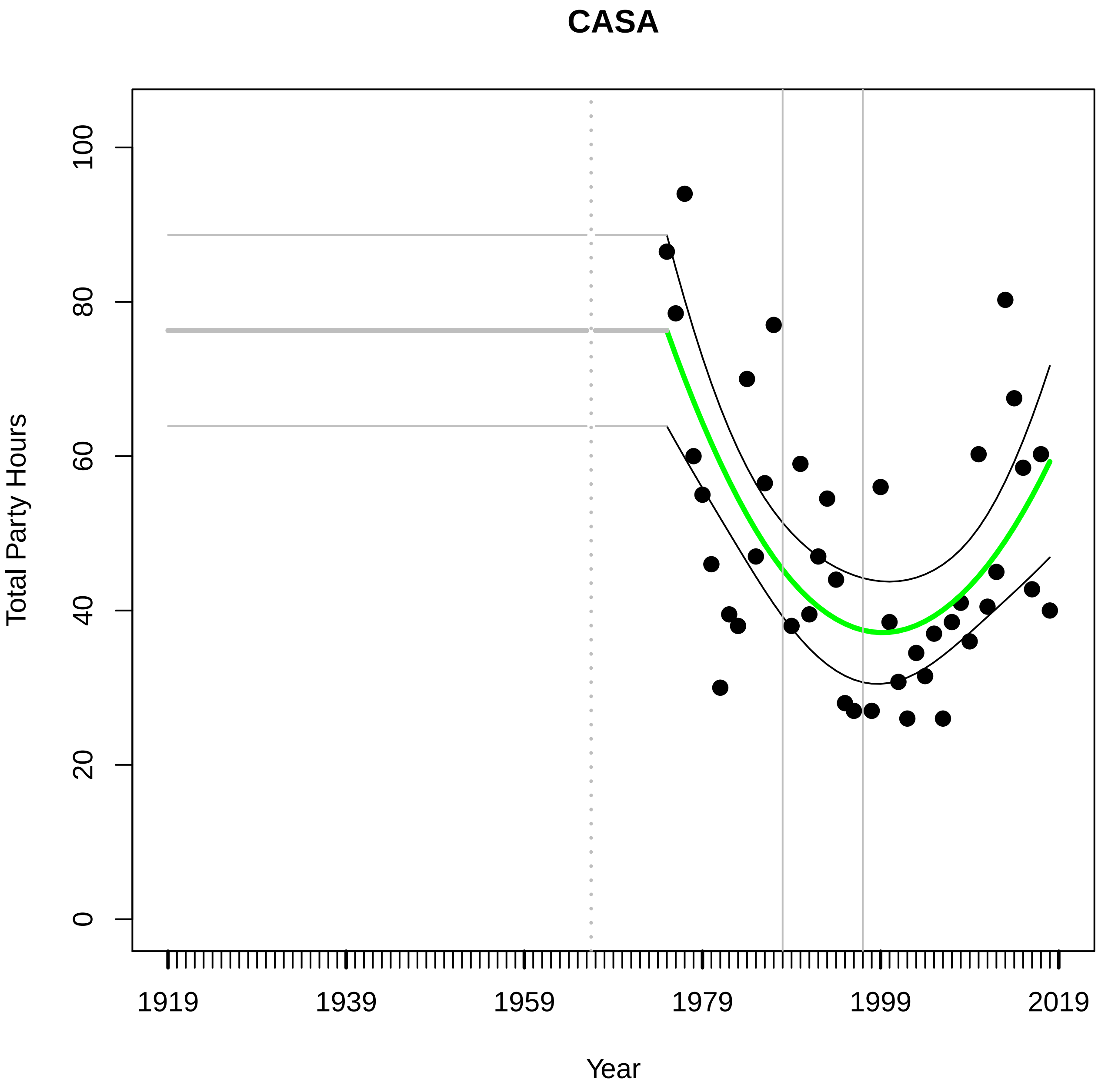

CASA
Total Party Hours
0
20
40
60
80
100
1919
1939
1959
1979
1999
2019
Year

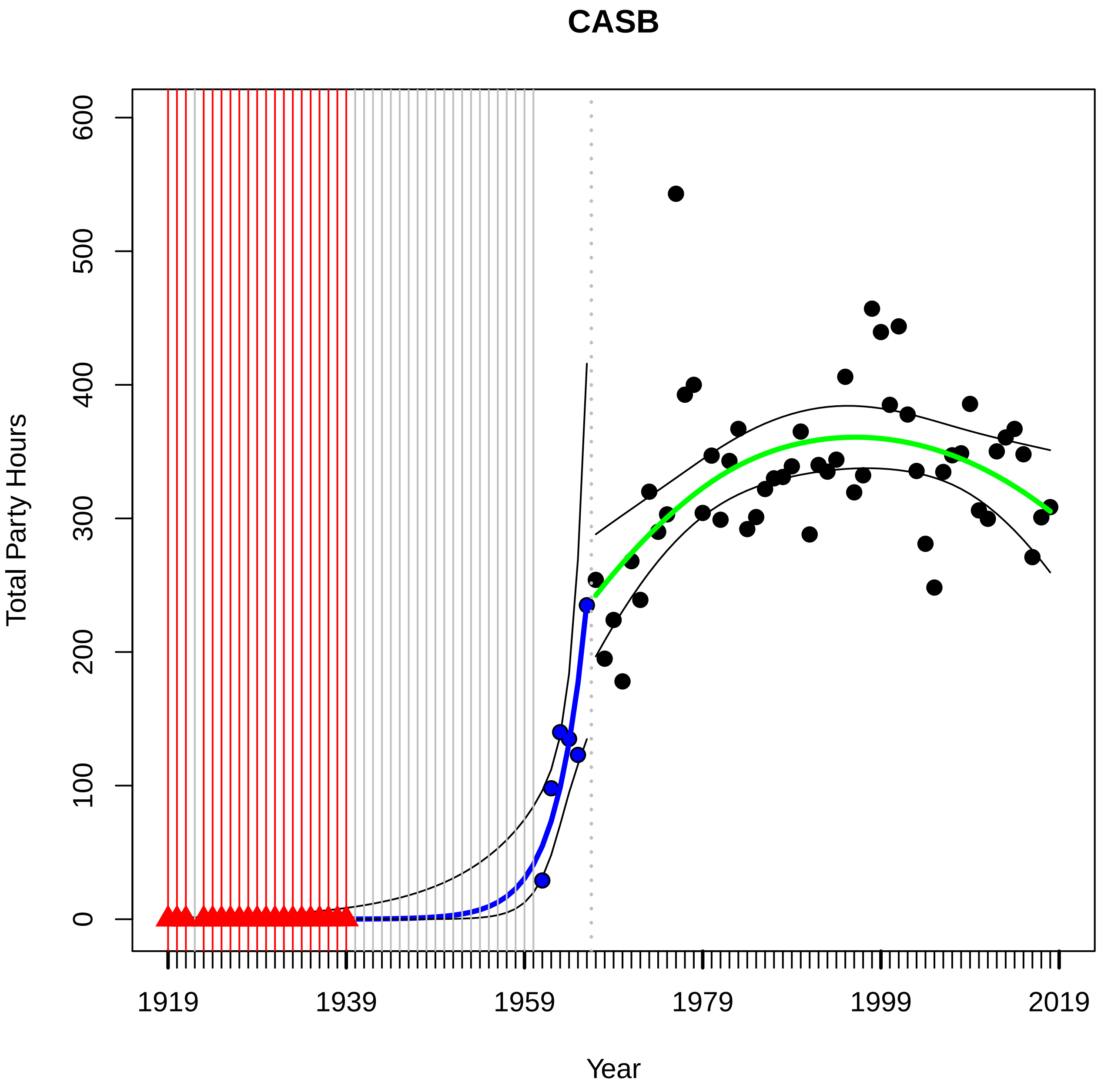

CASB
Total Party Hours
Year
0
100
200
300
400
500
600
1919
1939
1959
1979
1999
2019

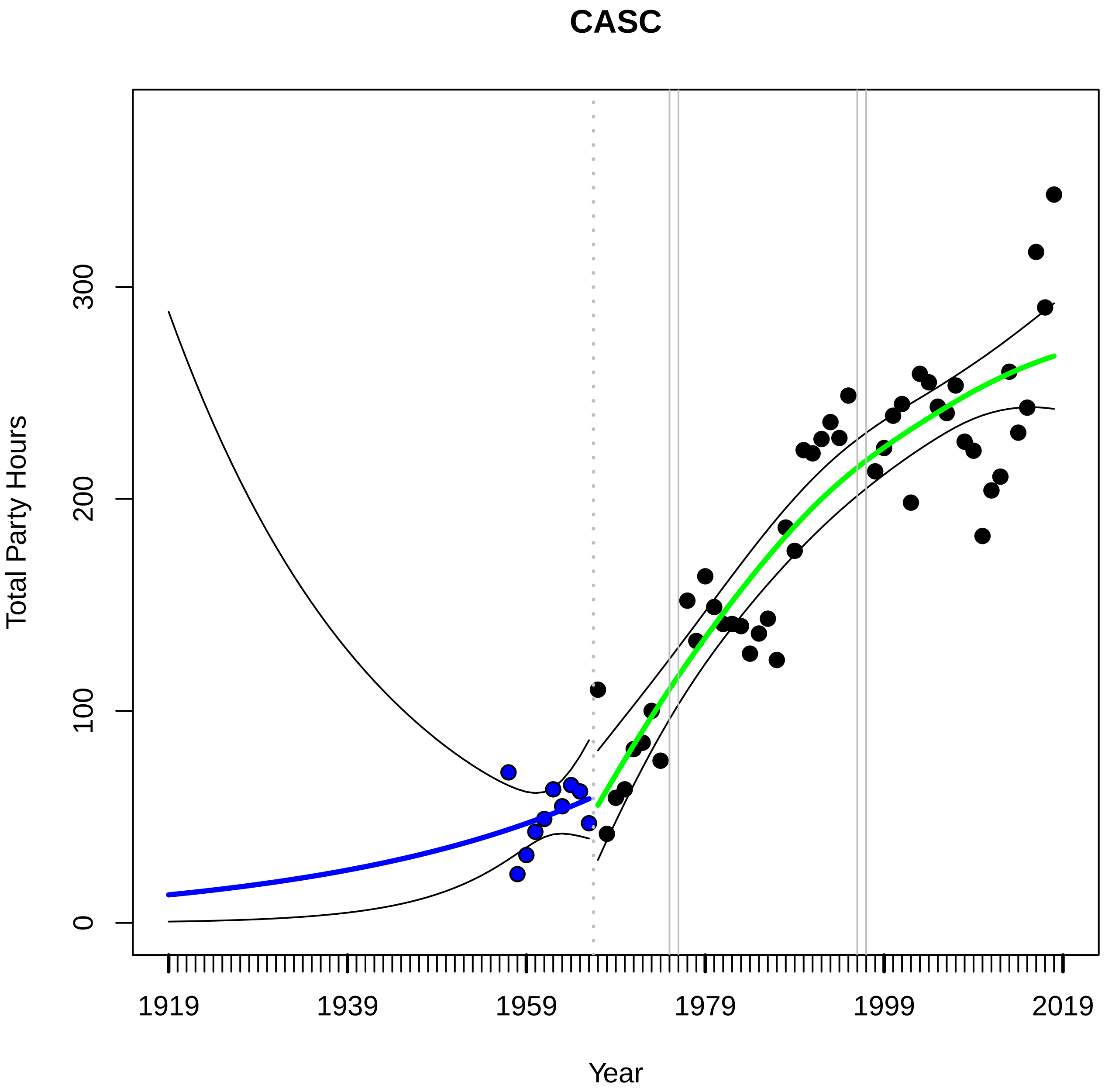

CASC
Total Party Hours
0
100
200
300
1919
1939
1959
1979
1999
2019
Year

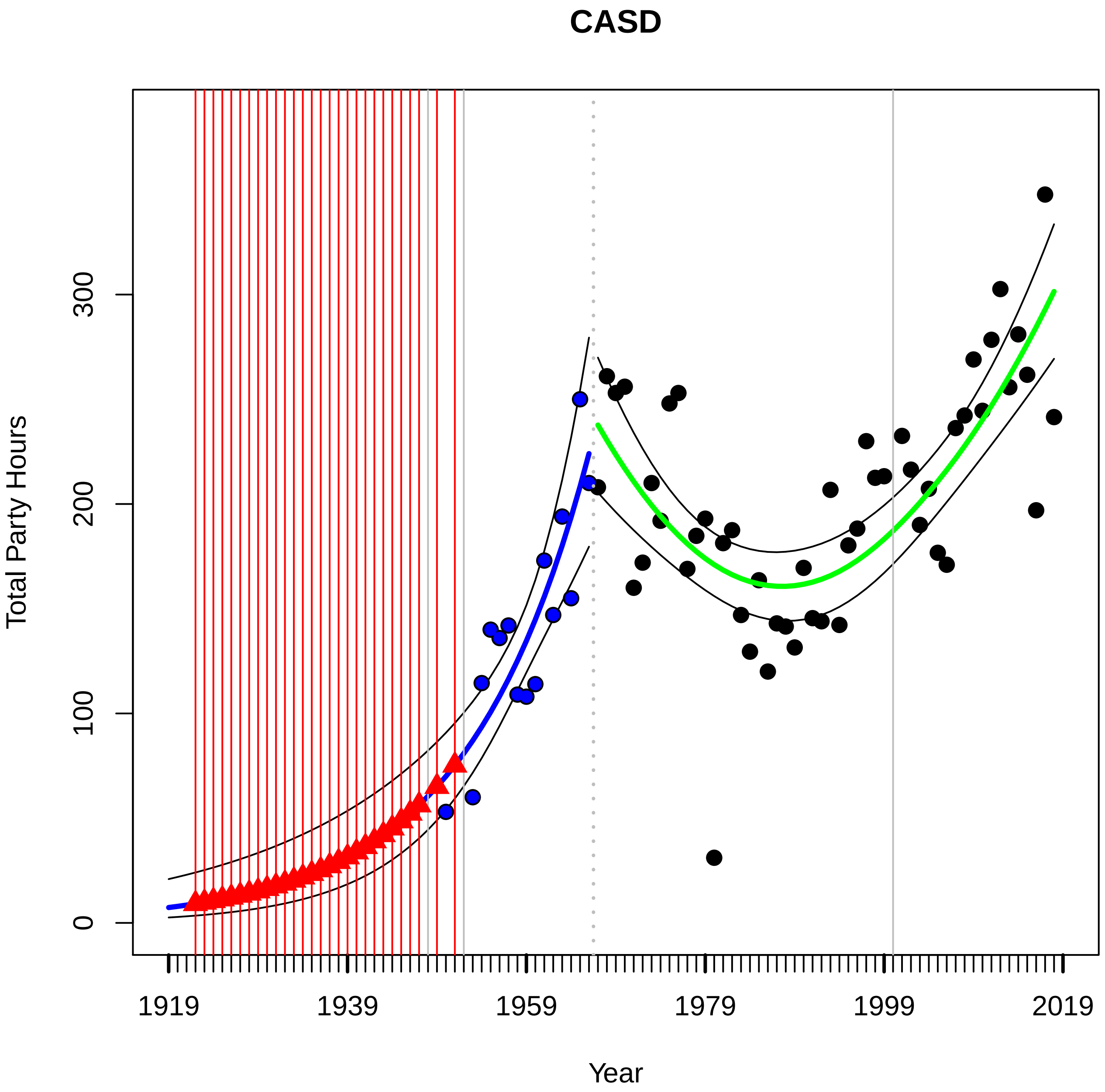
CASD
Total Party Hours
0
100
200
300
1919
1939
1959
1979
1999
2019
Year

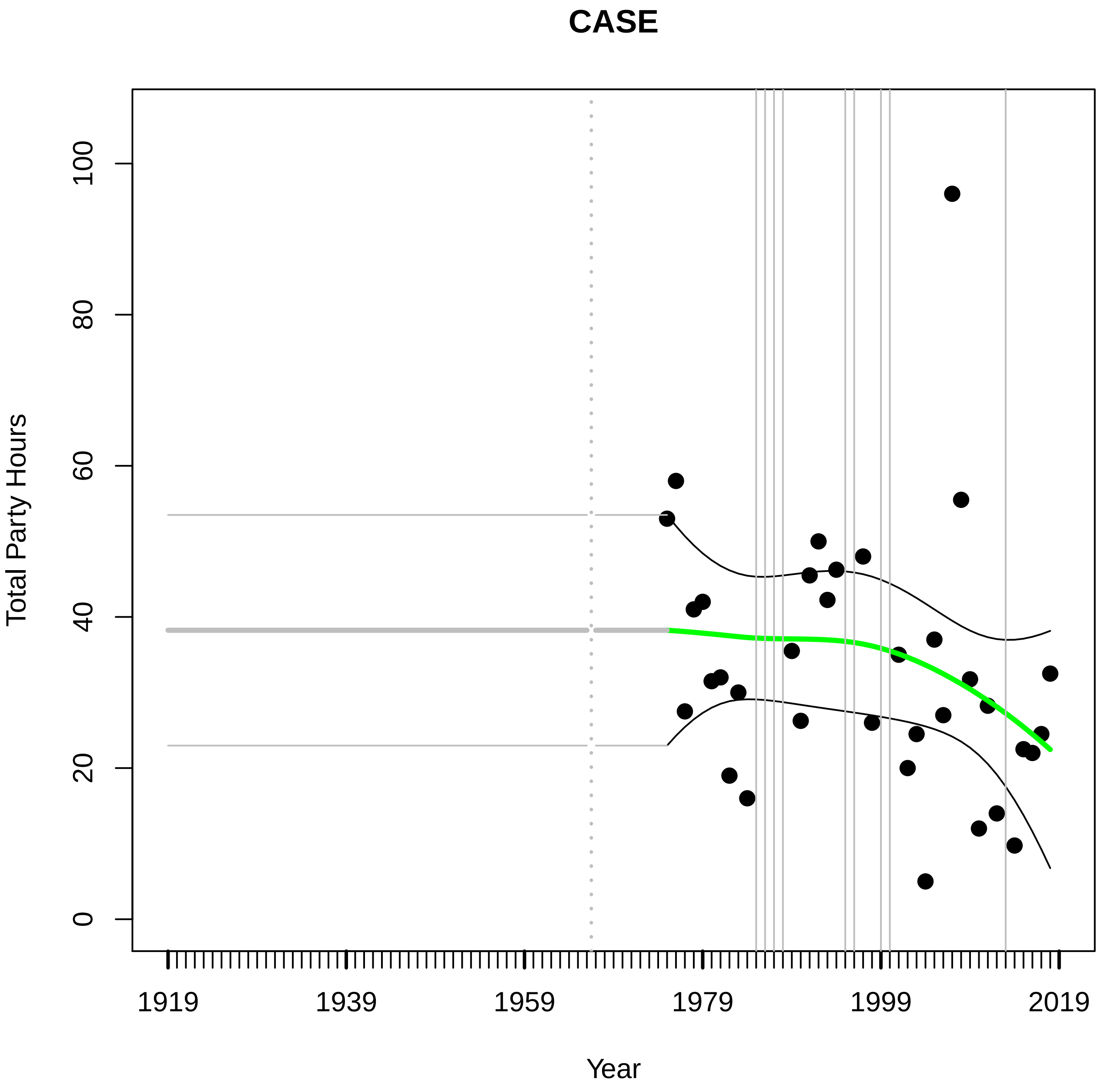

CASE
100
80
60
40
20
0
Total Party Hours
1919
1939
1959
1979
1999
2019
Year

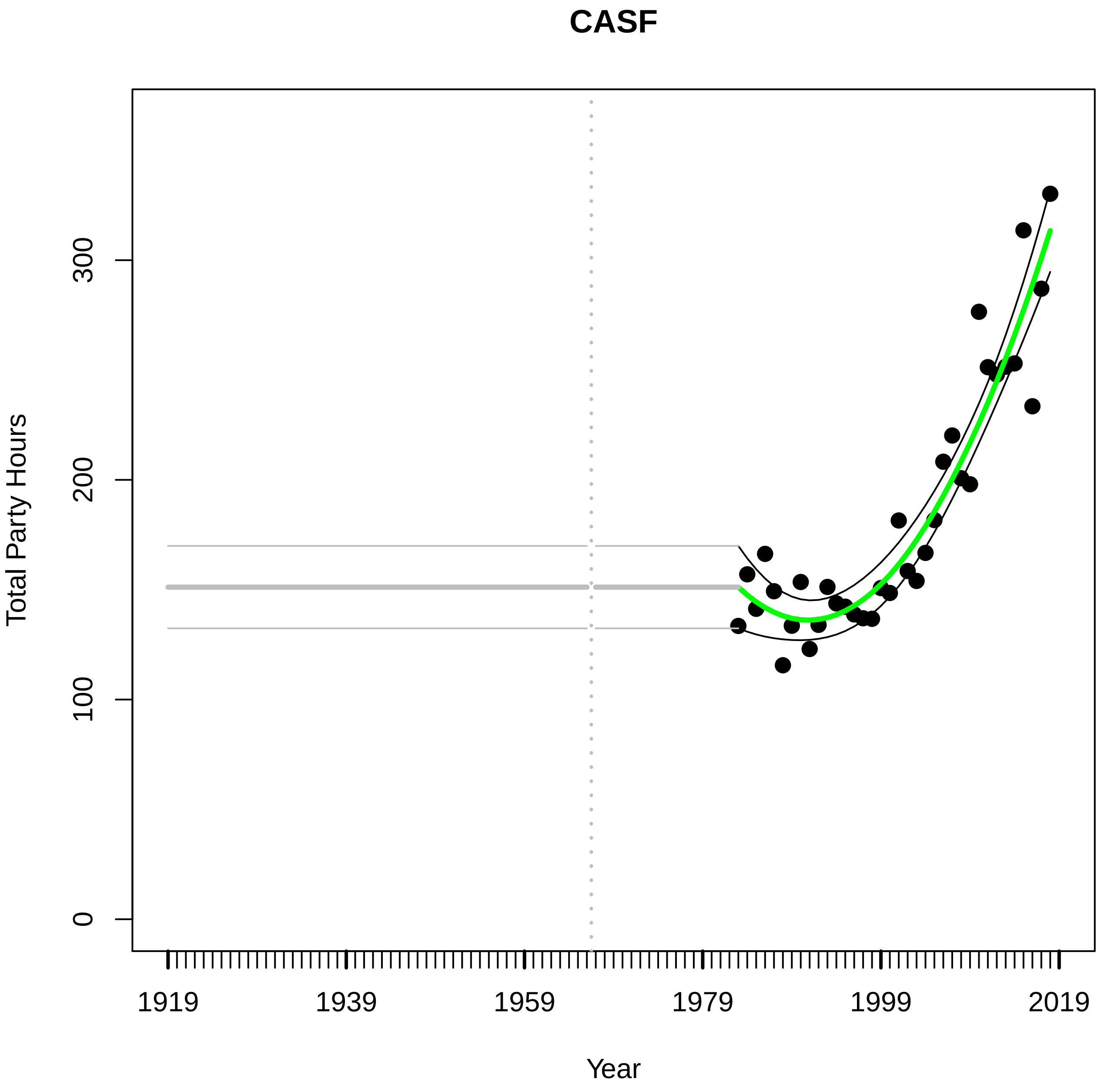
CASF
Total Party Hours
0
100
200
300
1919
1939
1959
1979
1999
2019
Year

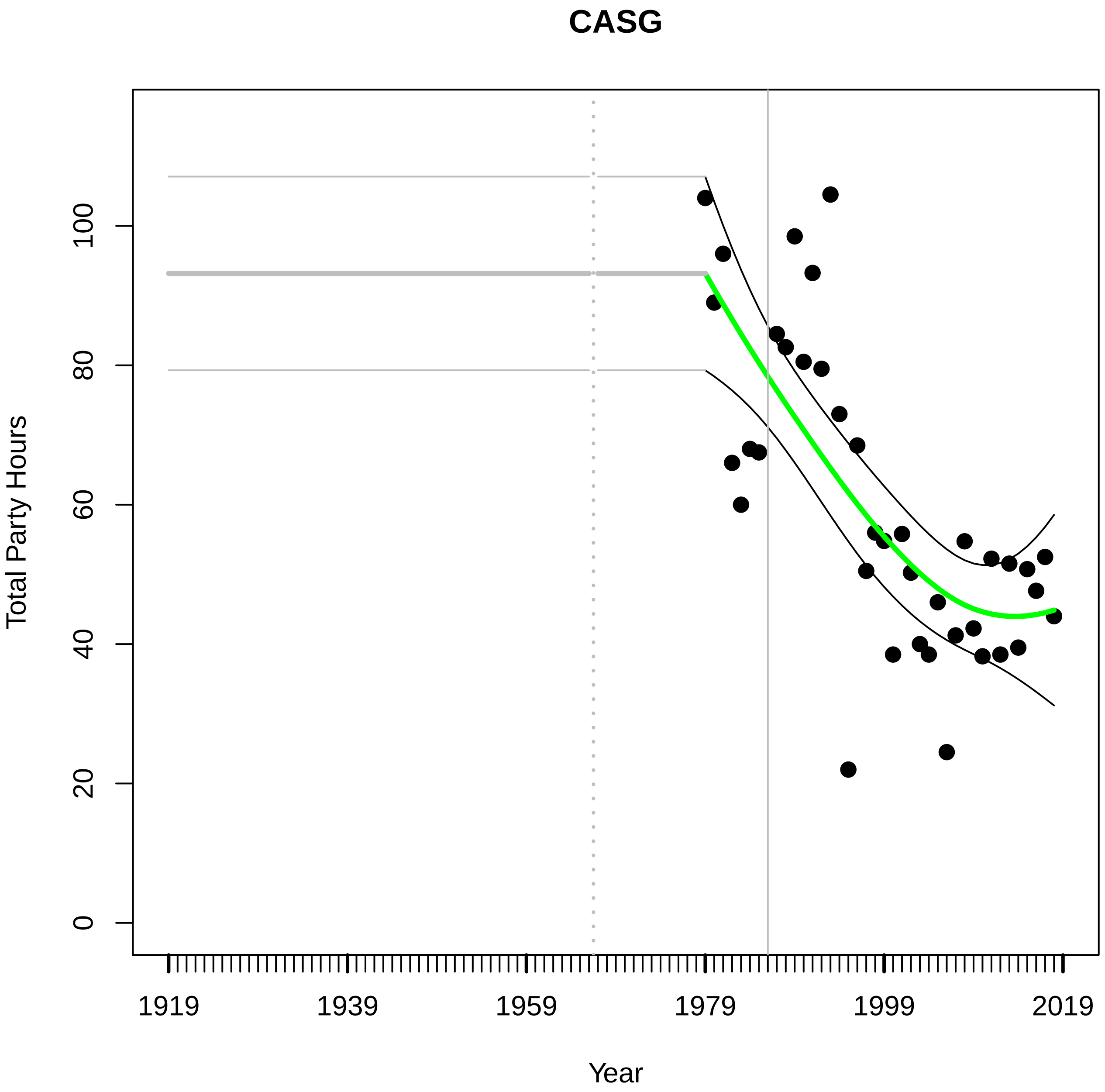

CASG
Total Party Hours
0
20
40
60
80
100
1919
1939
1959
1979
1999
2019
Year

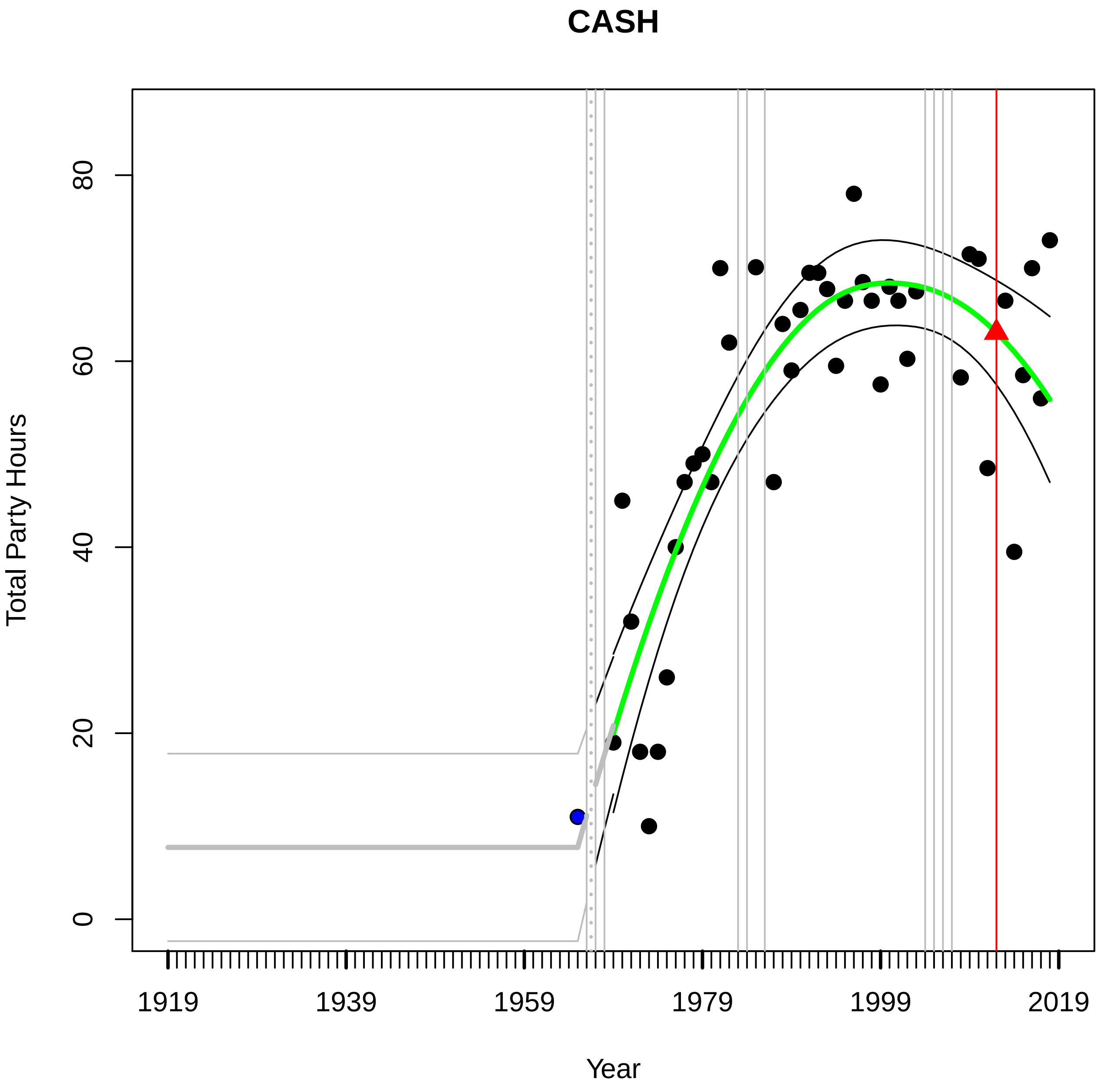

CASH
Total Party Hours
0
20
40
60
80
1919
1939
1959
1979
1999
2019
Year

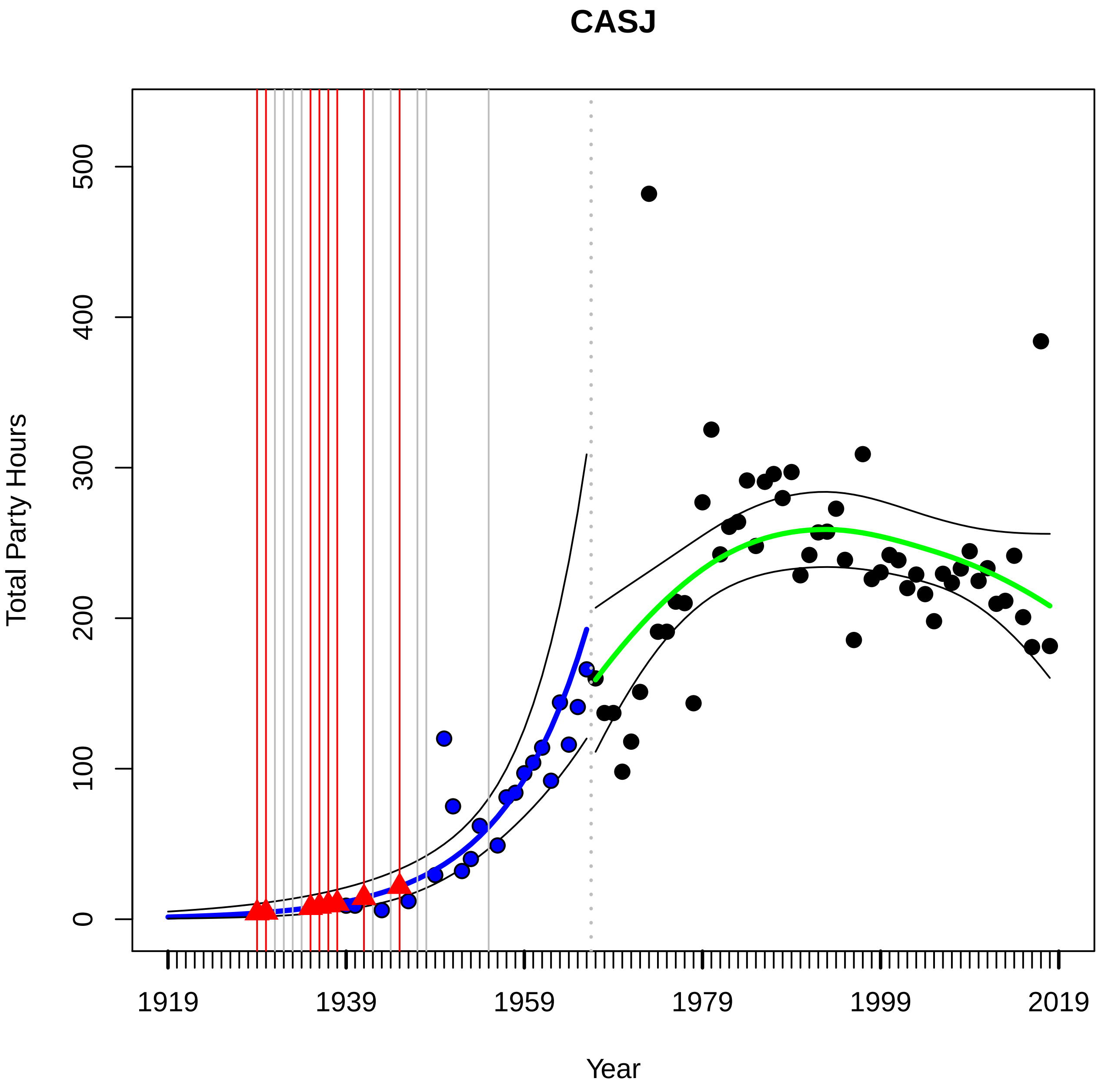

CASJ
Total Party Hours
0
100
200
300
400
500
1919
1939
1959
1979
1999
2019
Year

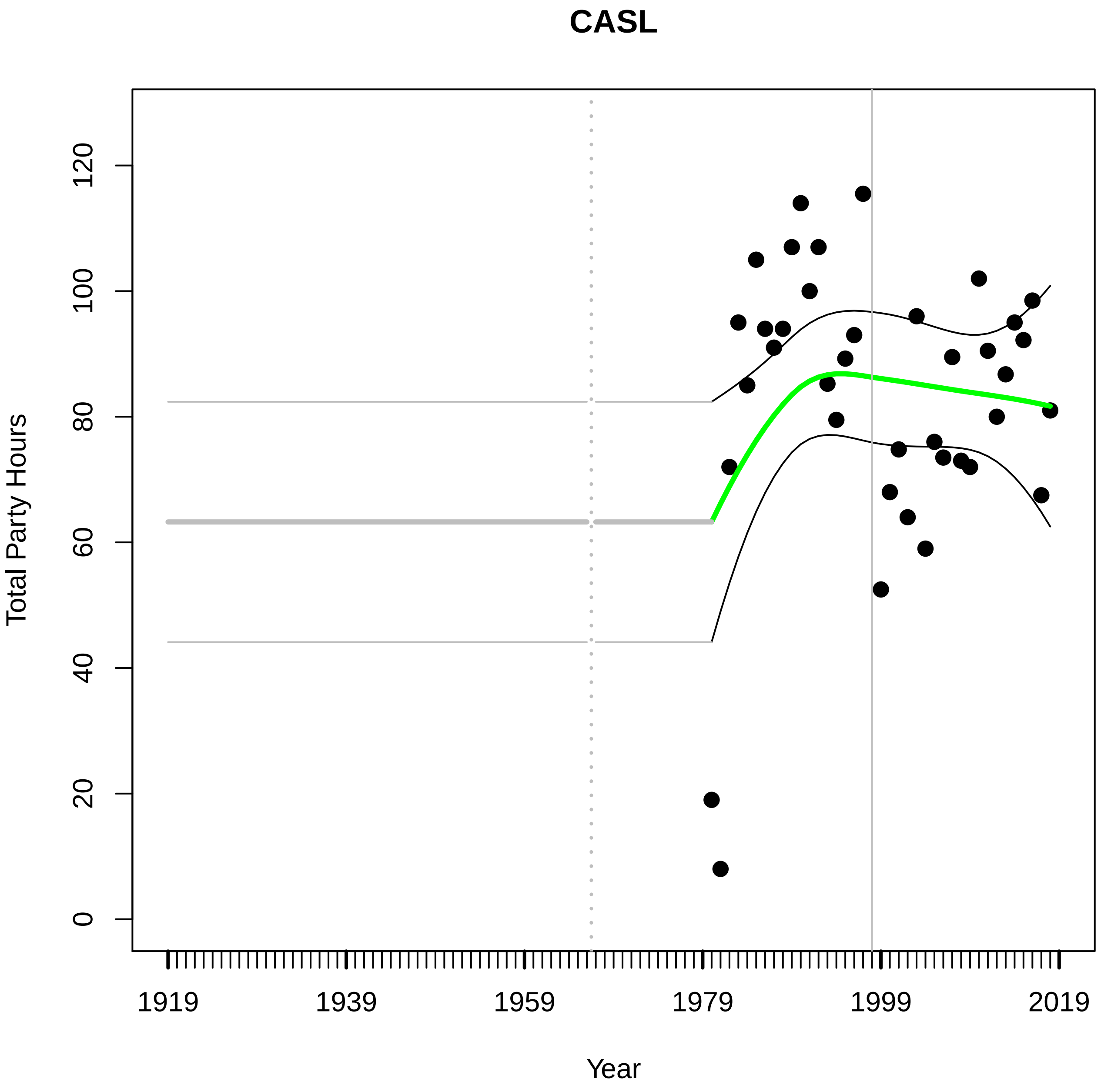

CASL
Total Party Hours
0
20
40
60
80
100
120
1919
1939
1959
1979
1999
2019
Year

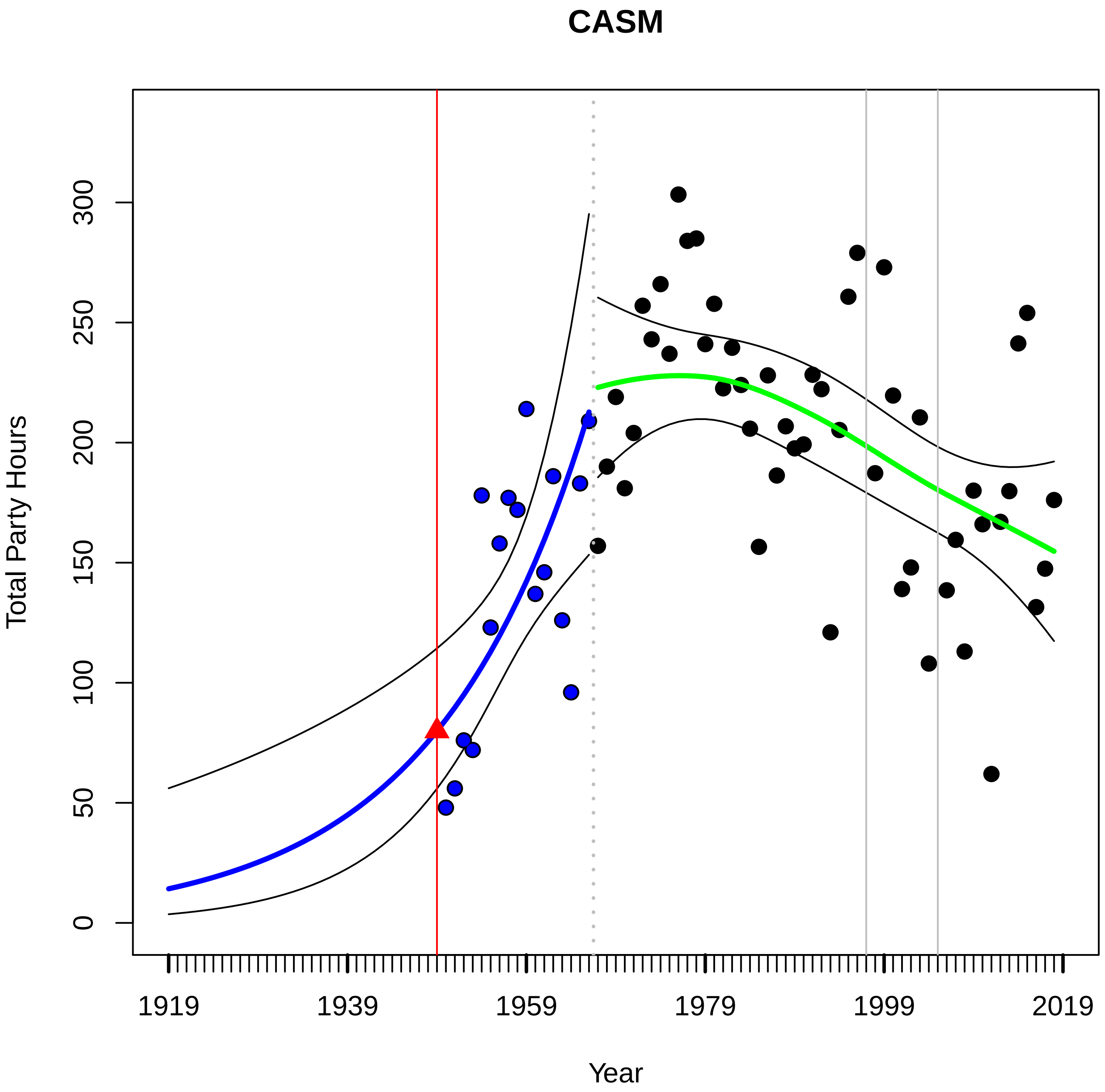

CASM
Total Party Hours
300
250
200
150
100
50
0
1919
1939
1959
1979
1999
2019
Year

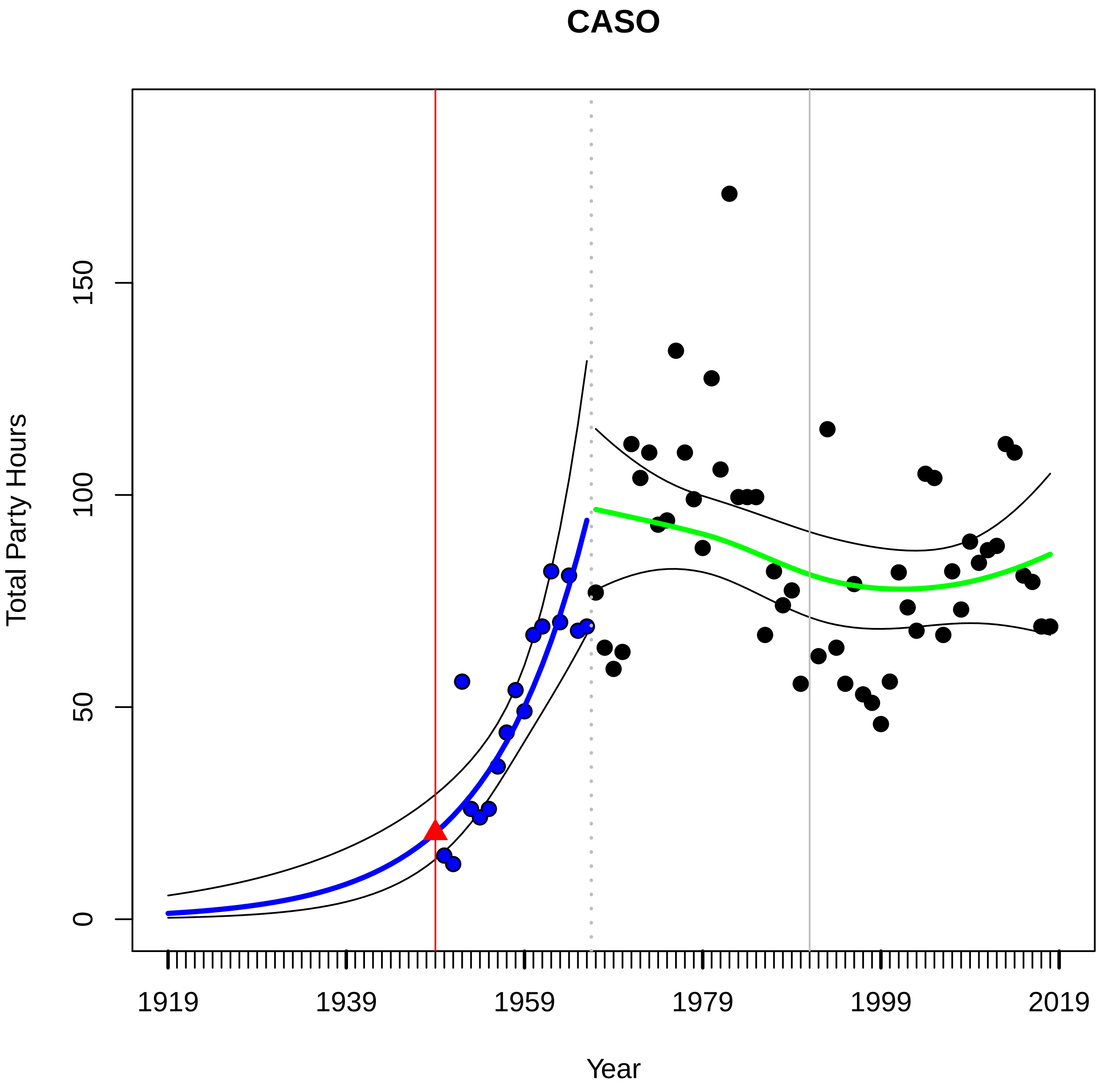

CASO
Total Party Hours
0
50
100
150
1919
1939
1959
1979
1999
2019
Year

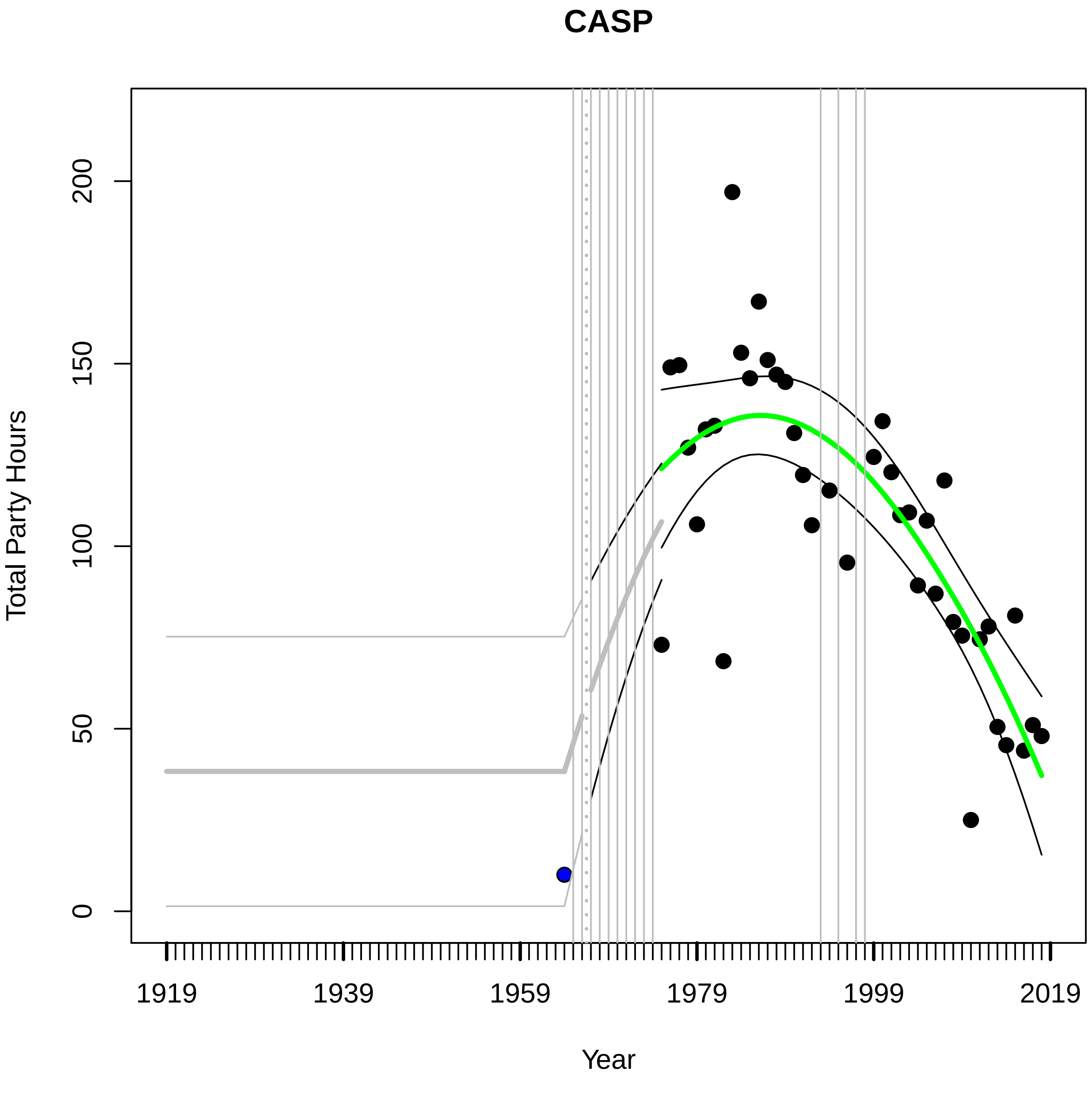

CASP
Total Party Hours
0
50
100
150
200
1919
1939
1959
1979
1999
2019
Year

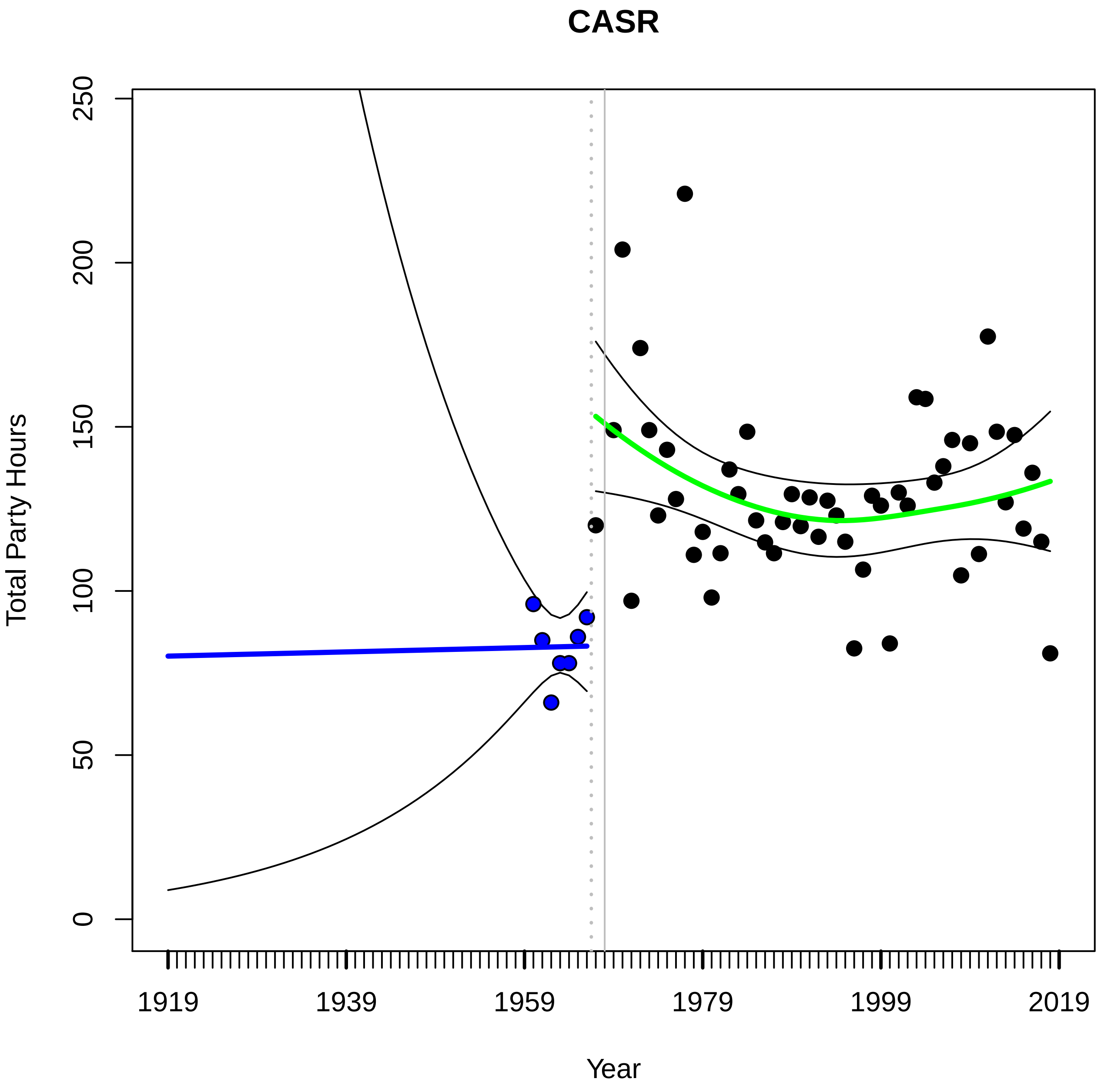

CASR
Total Party Hours
0
50
100
150
200
250
1919
1939
1959
1979
1999
2019
Year

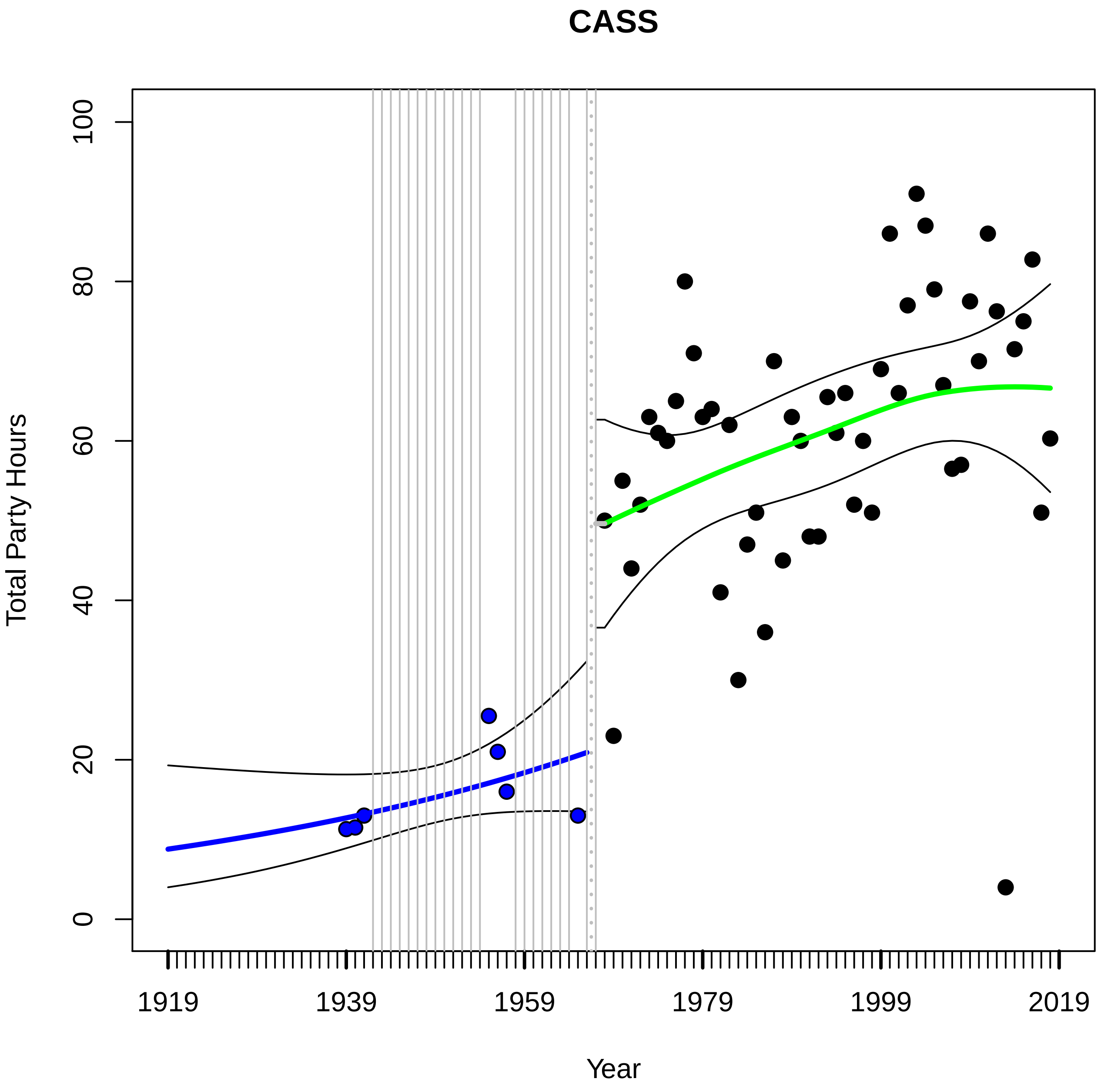

CASS
Total Party Hours
0
20
40
60
80
100
1919
1939
1959
1979
1999
2019
Year

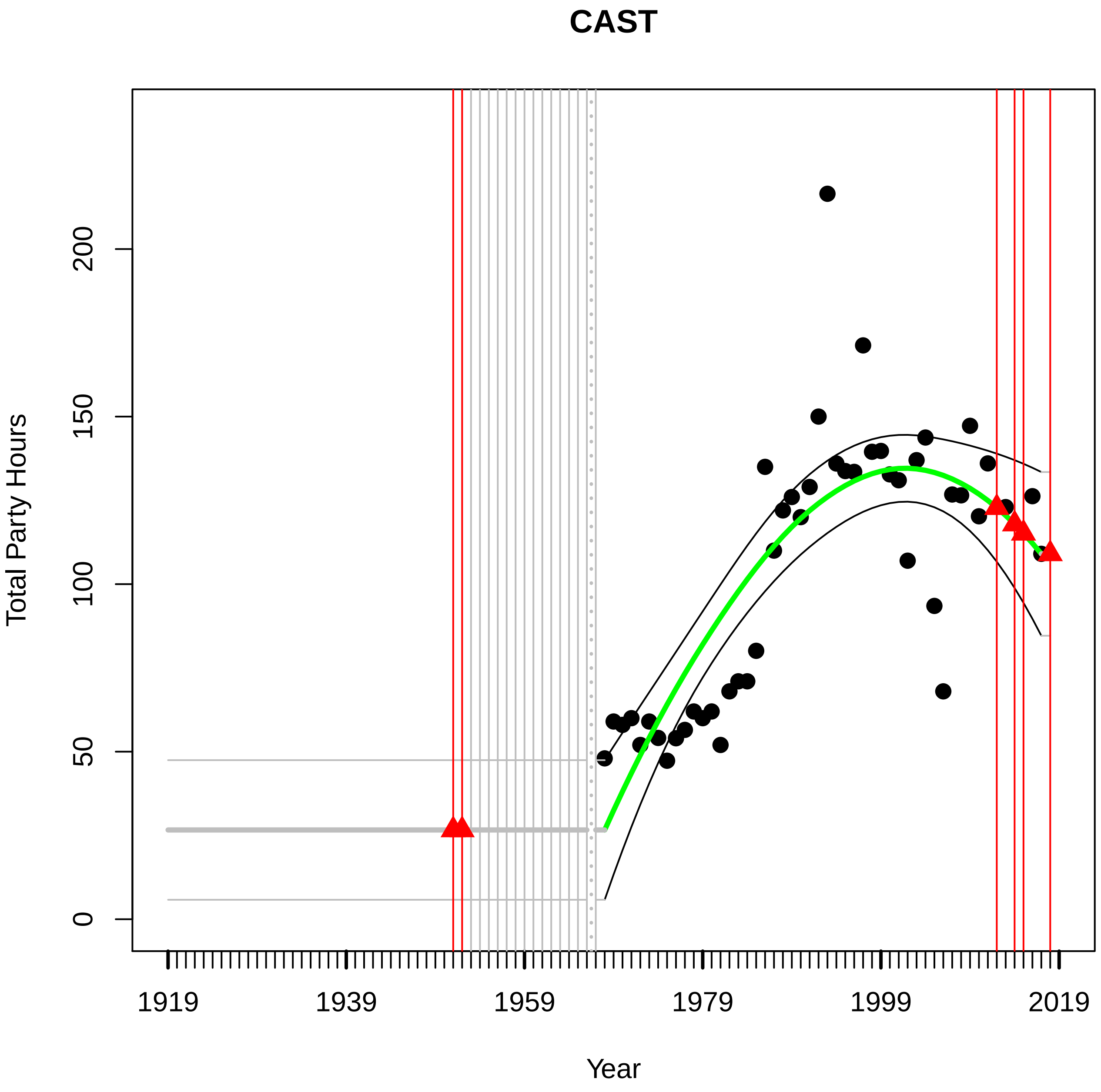

CAST
Total Party Hours
0
50
100
150
200
1919
1939
1959
1979
1999
2019
Year

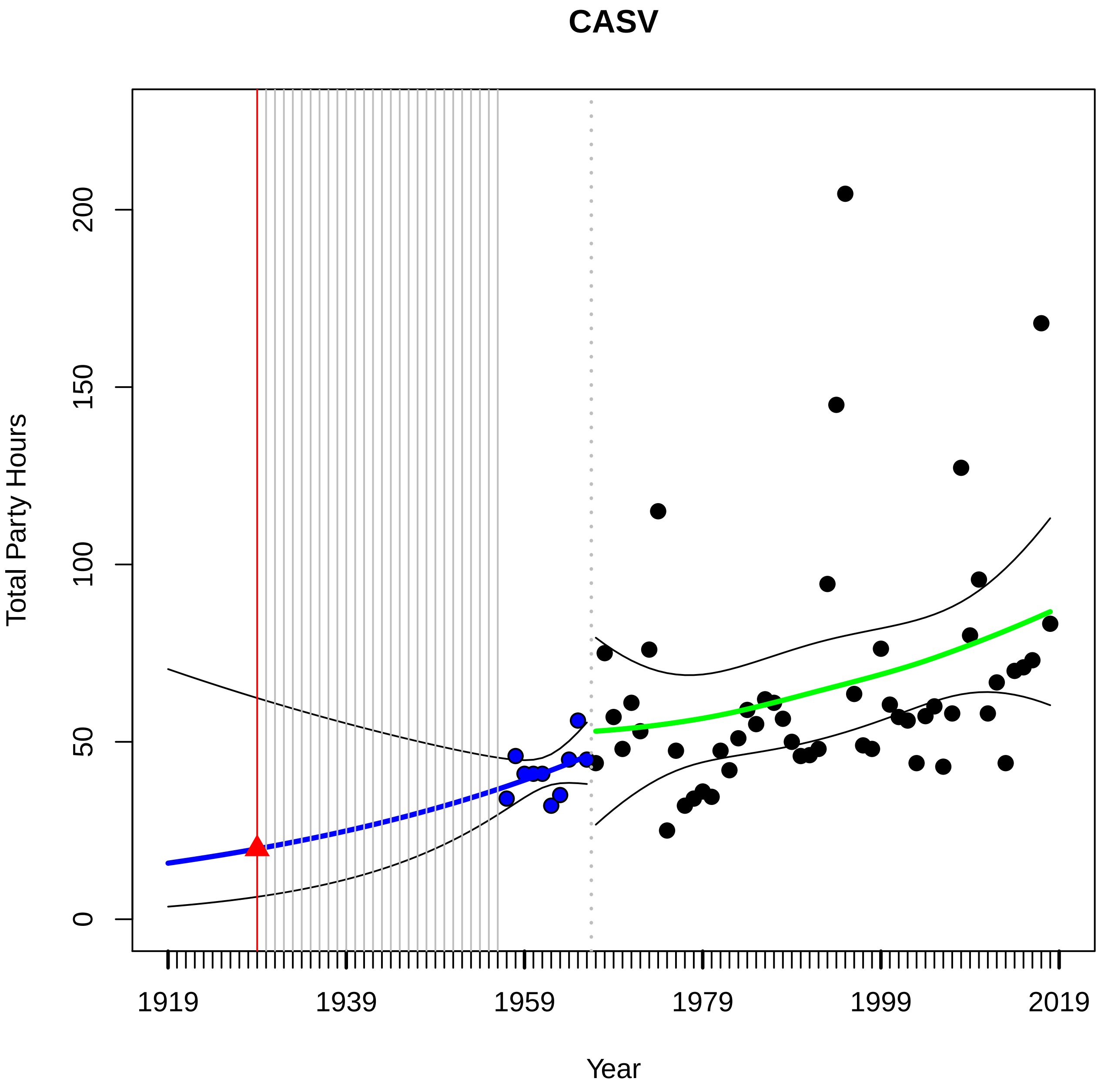

CASV
Total Party Hours
0
50
100
150
200
1919
1939
1959
1979
1999
2019
Year

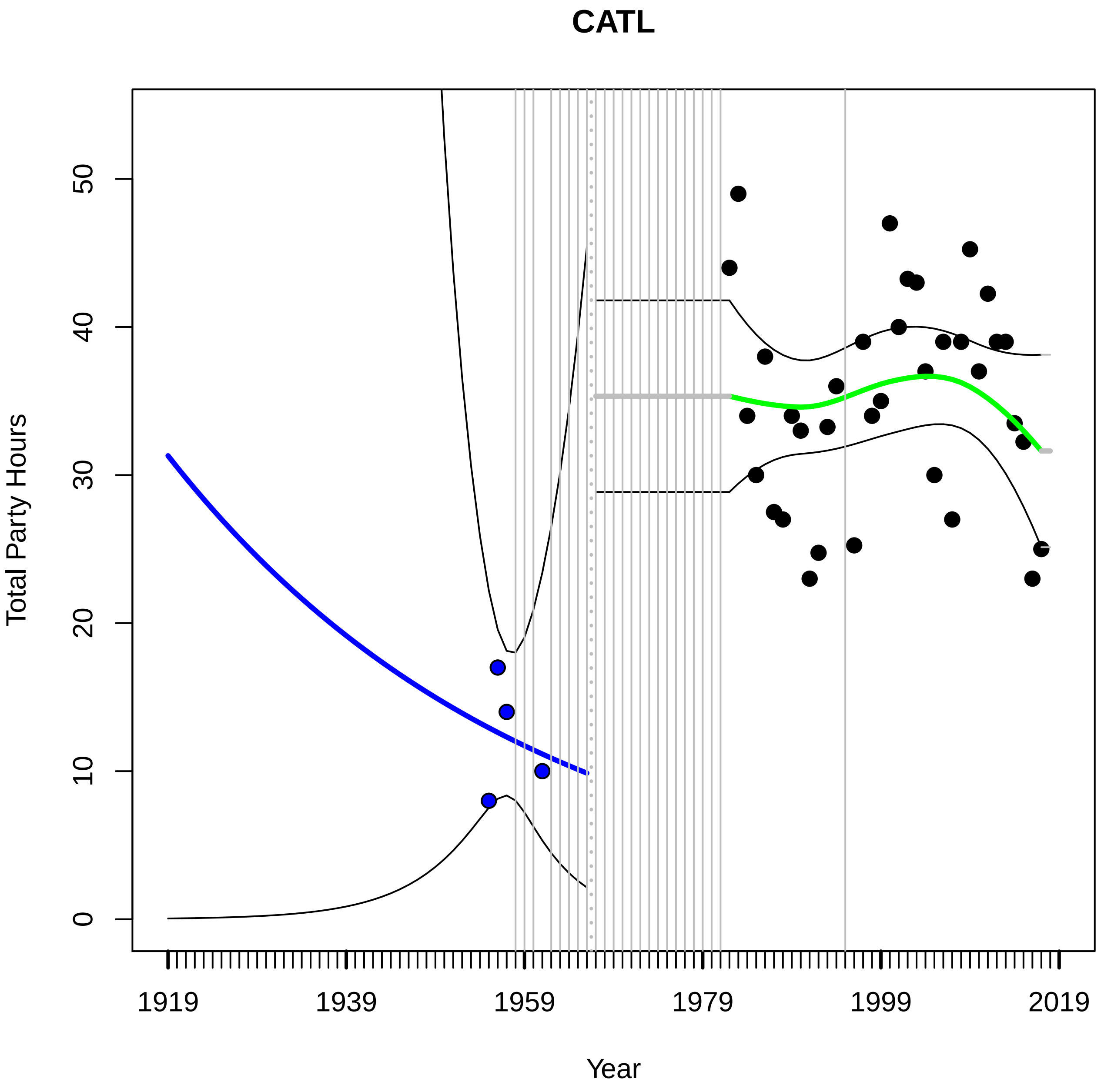

CATL
Total Party Hours
0
10
20
30
40
50
1919
1939
1959
1979
1999
2019
Year

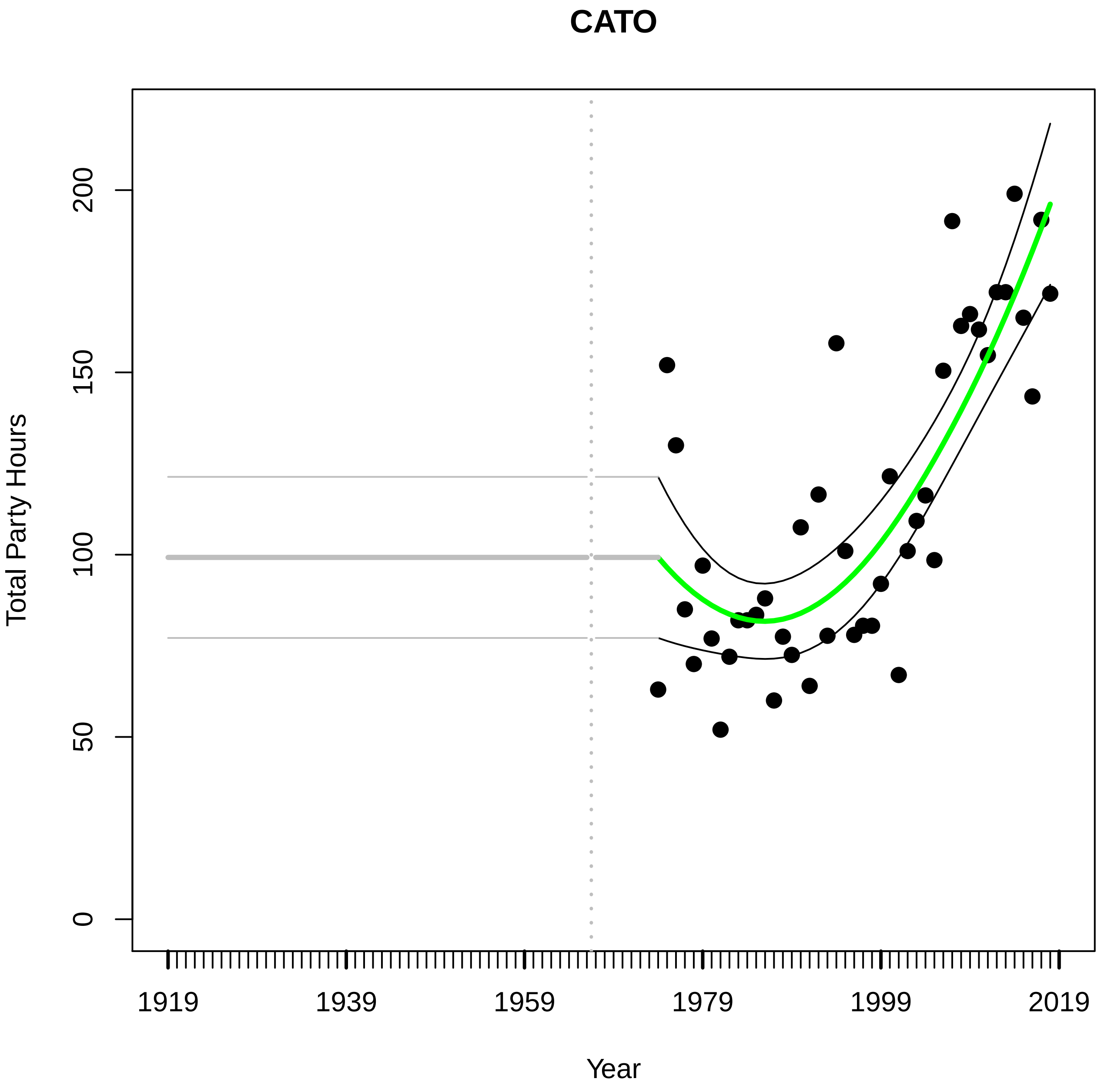
CATO
Total Party Hours
0
50
100
150
200
Year
1919
1939
1959
1979
1999
2019

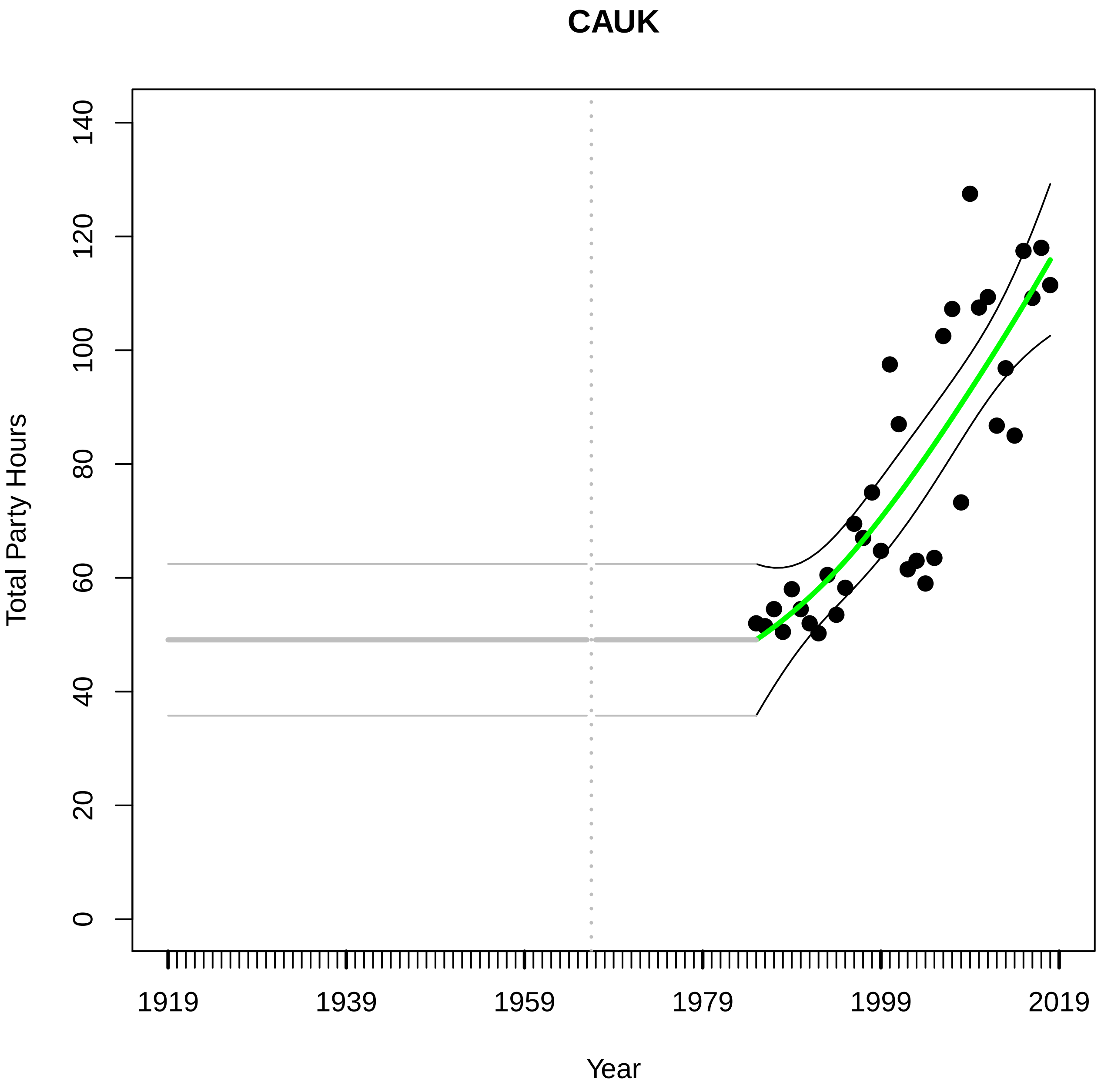
CAUK
Total Party Hours
0
20
40
60
80
100
120
140
1919
1939
1959
1979
1999
2019
Year

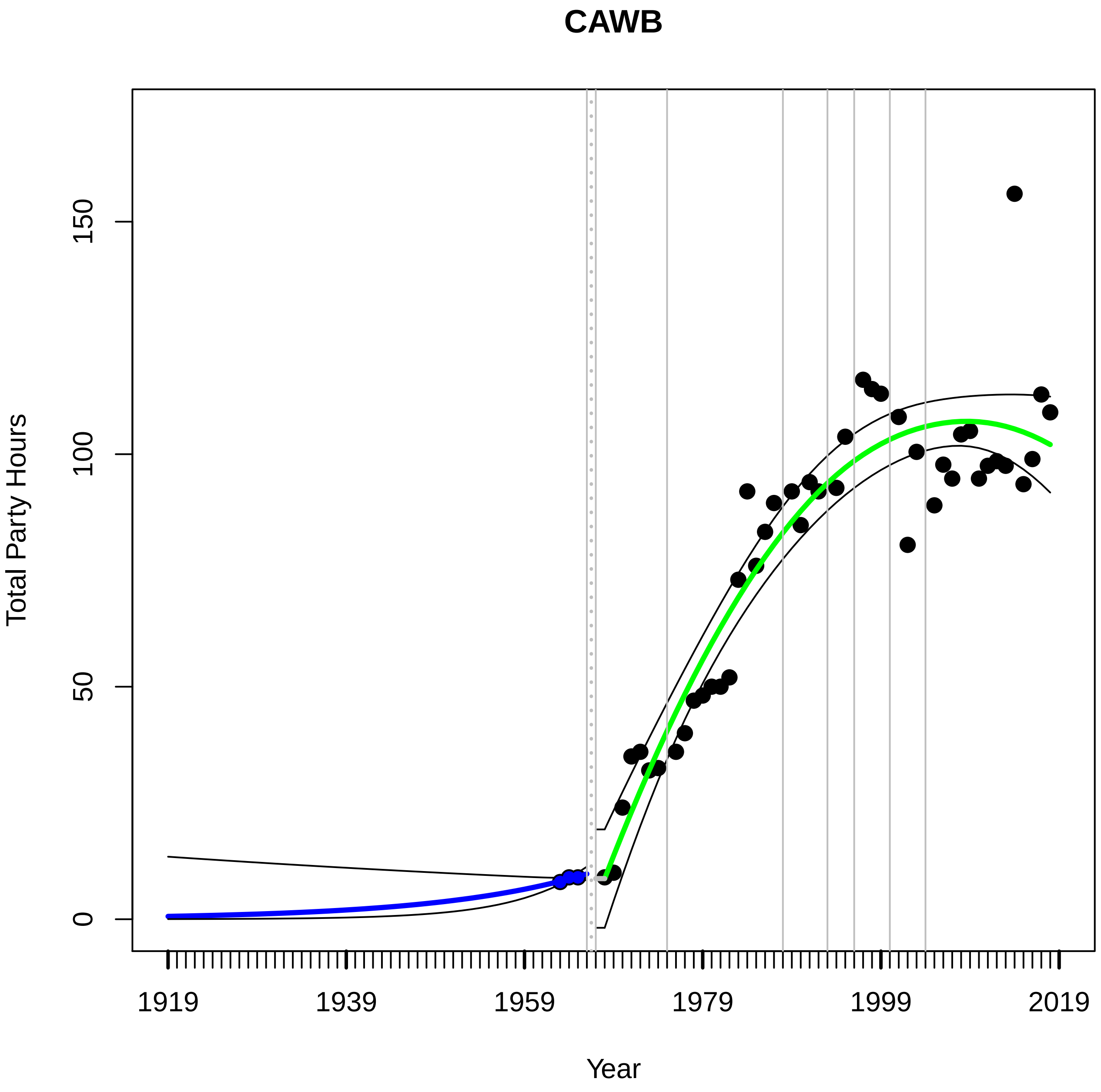
CAWB
Total Party Hours
0
50
100
150
1919
1939
1959
1979
1999
2019
Year

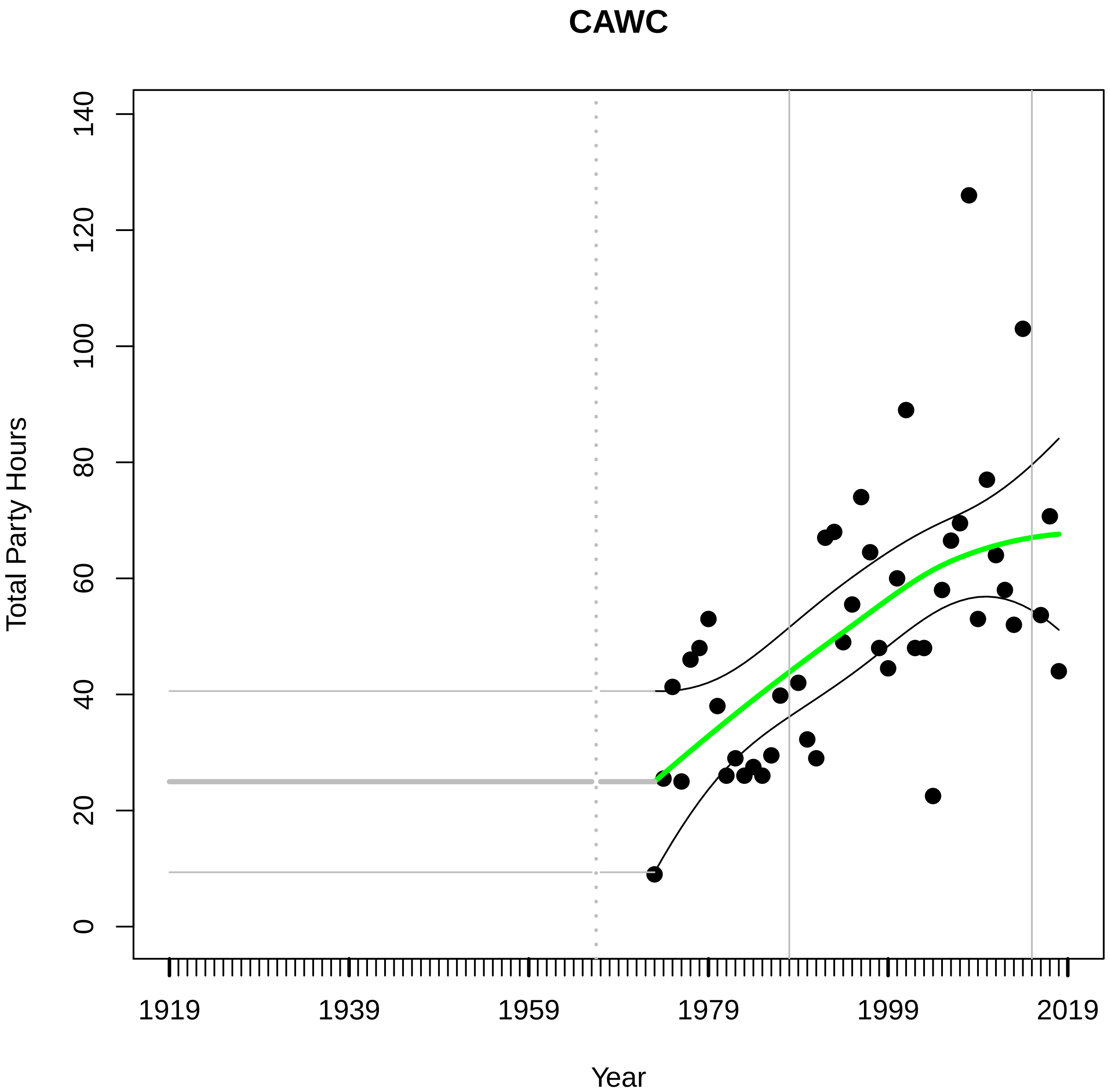

CAWC
Total Party Hours
0
20
40
60
80
100
120
140
1919
1939
1959
1979
1999
2019
Year

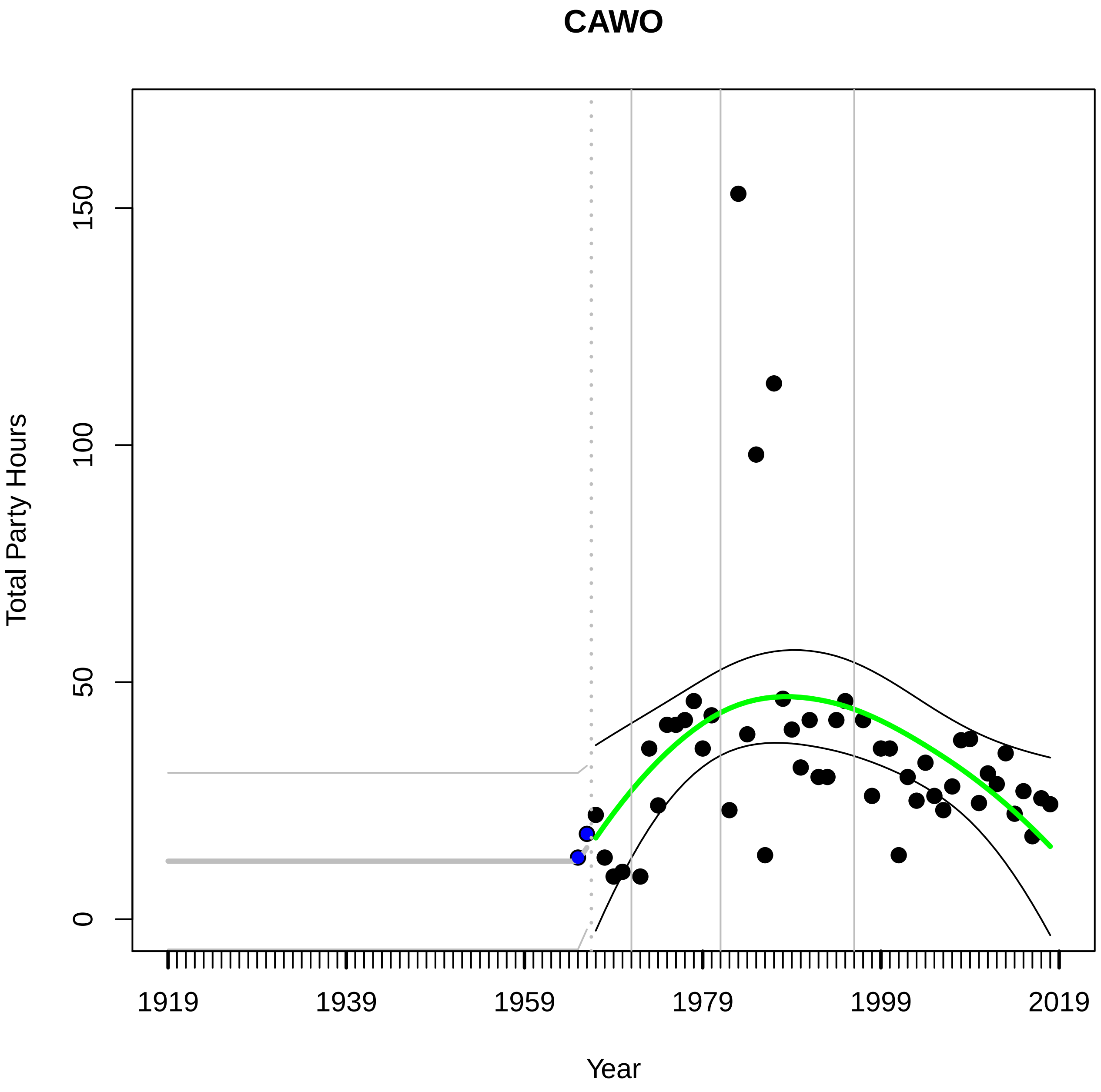
CAWO
Total Party Hours
0
50
100
150
1919
1939
1959
1979
1999
2019
Year

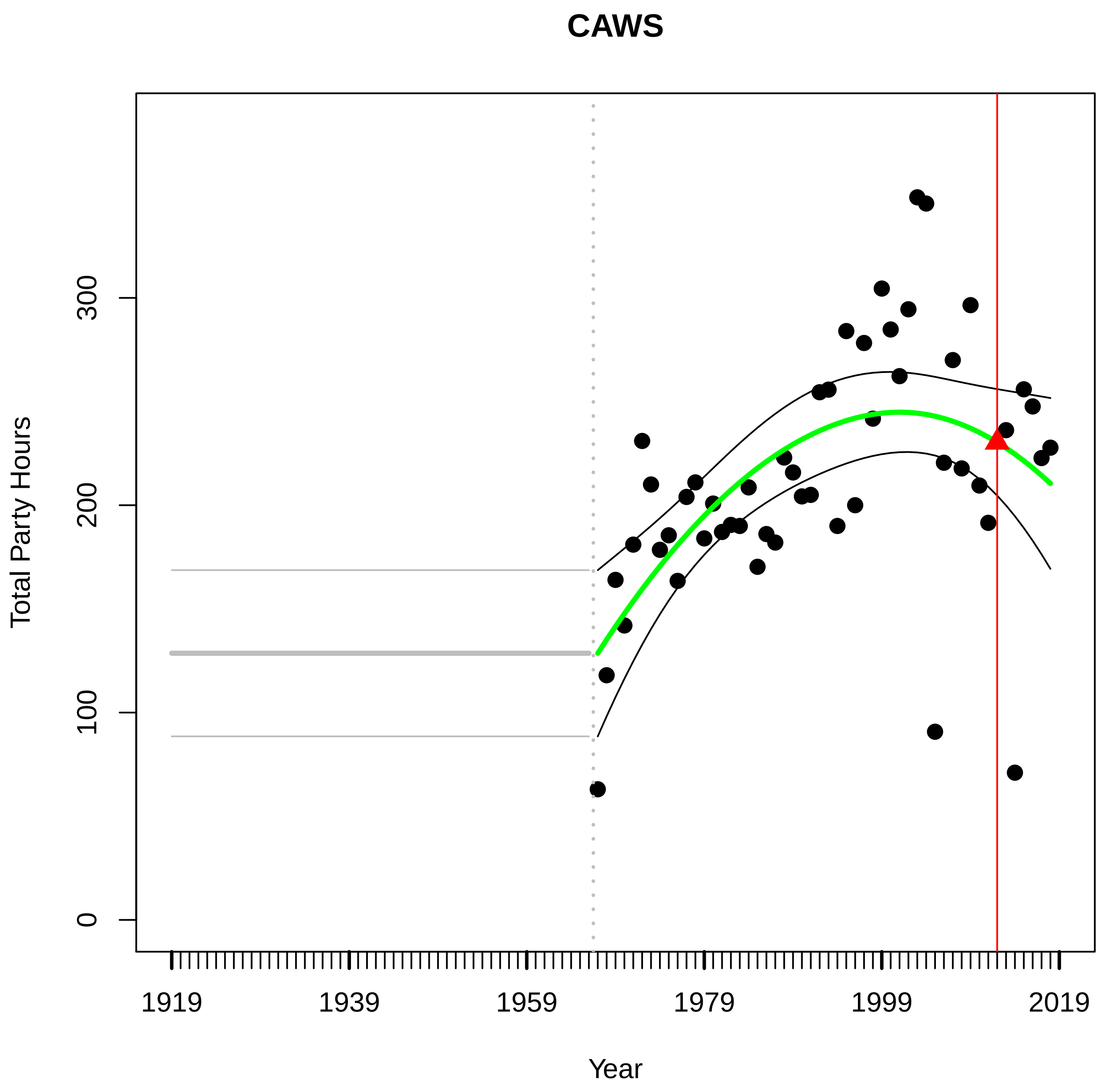

CAWS
Total Party Hours
0
100
200
300
1919
1939
1959
1979
1999
2019
Year

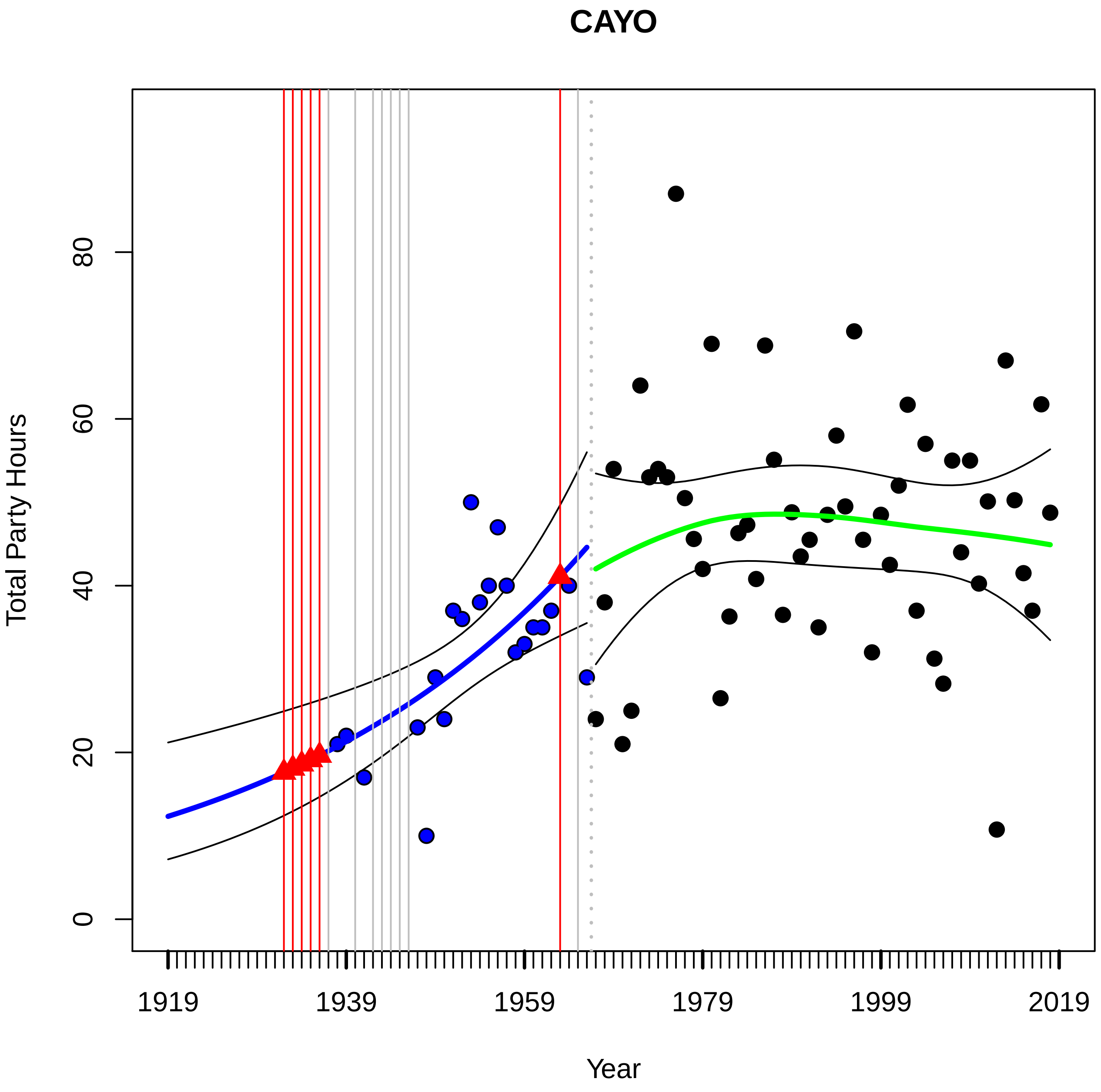
CAYO
Total Party Hours
0
20
40
60
80
1919
1939
1959
1979
1999
2019
Year

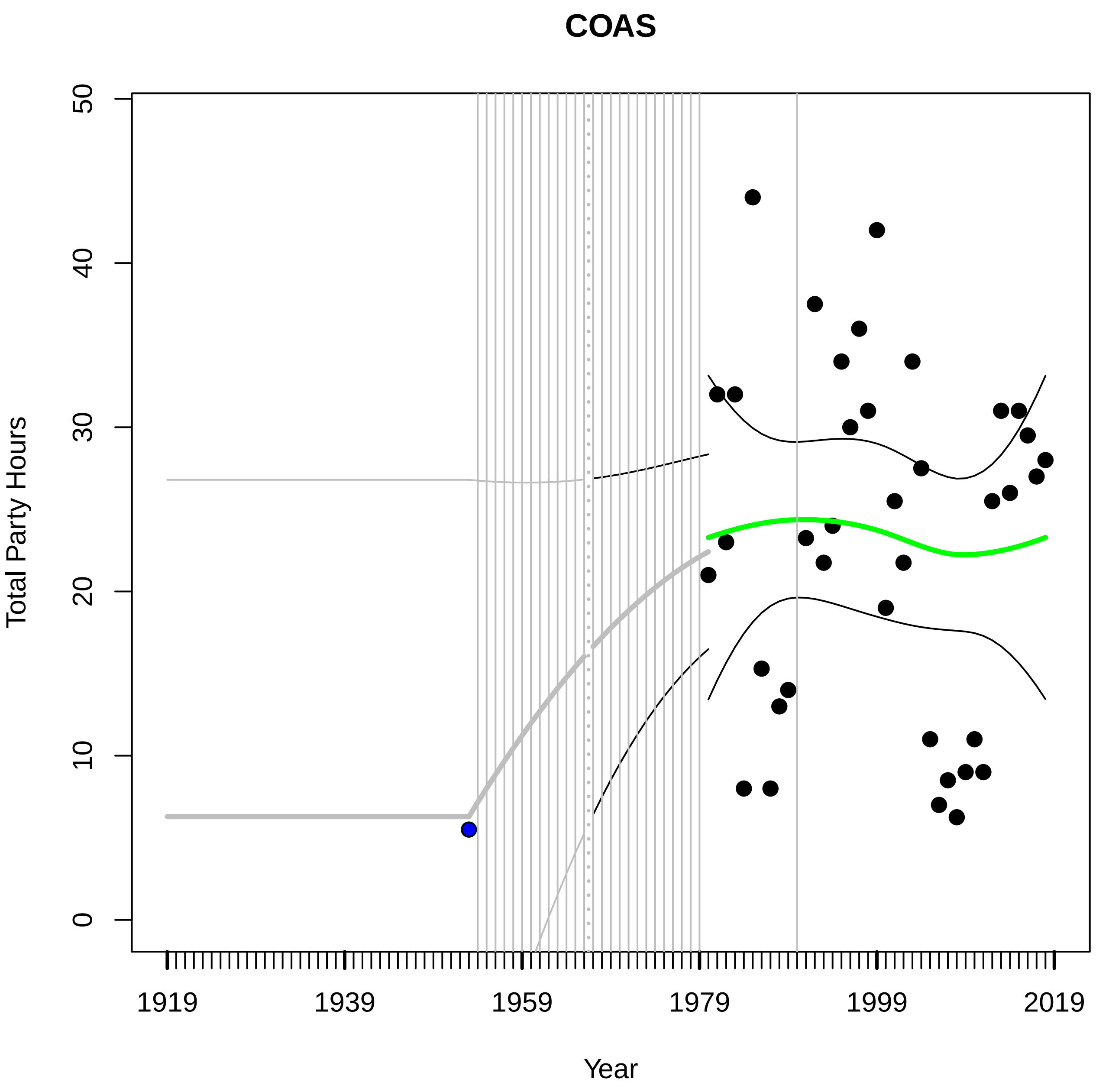
COAS
Total Party Hours
Year
0
10
20
30
40
50
1919
1939
1959
1979
1999
2019

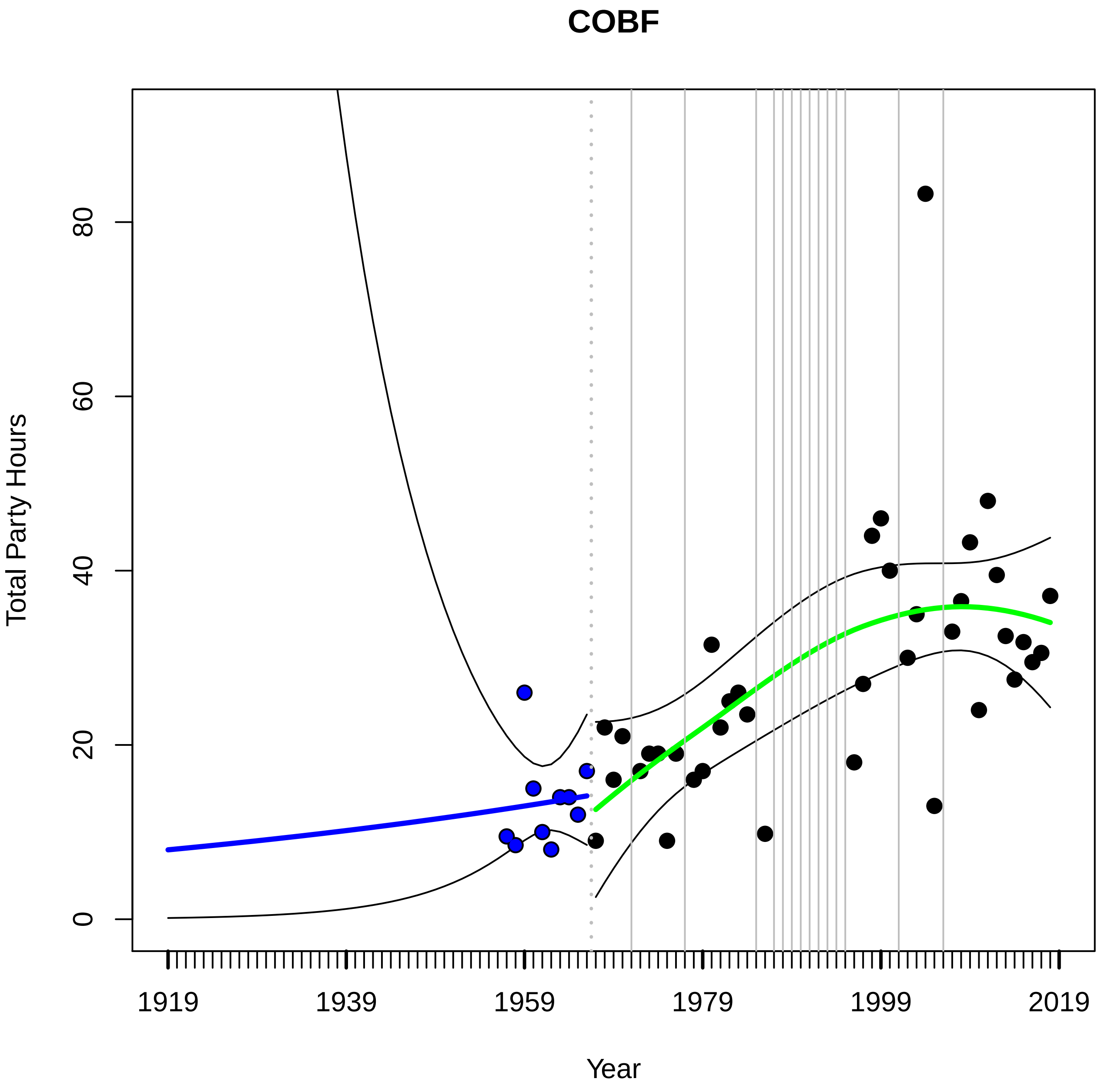
COBF
Total Party Hours
0
20
40
60
80
1919
1939
1959
1979
1999
2019
Year

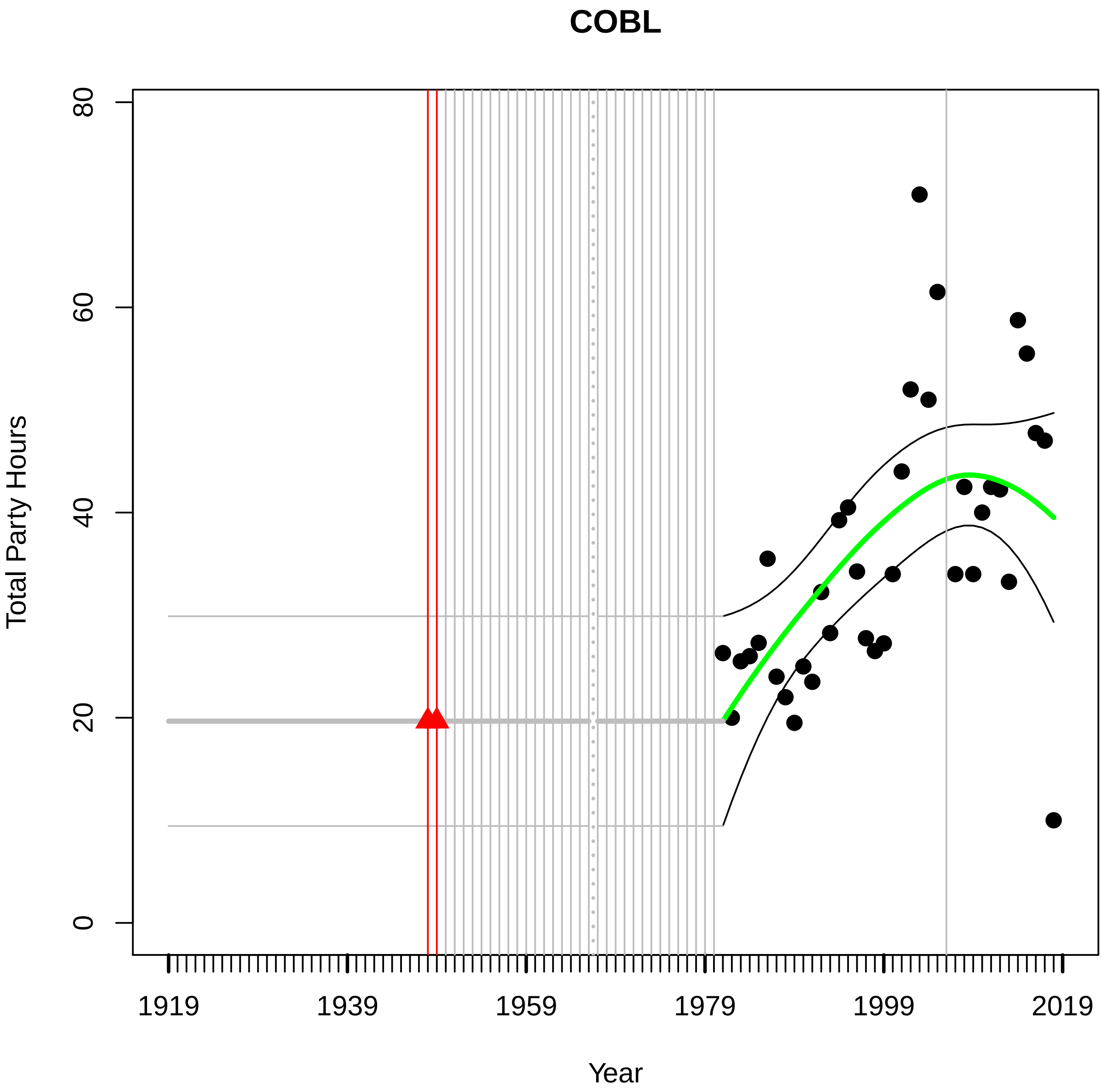

COBL
Total Party Hours
0
20
40
60
80
1919
1939
1959
1979
1999
2019
Year

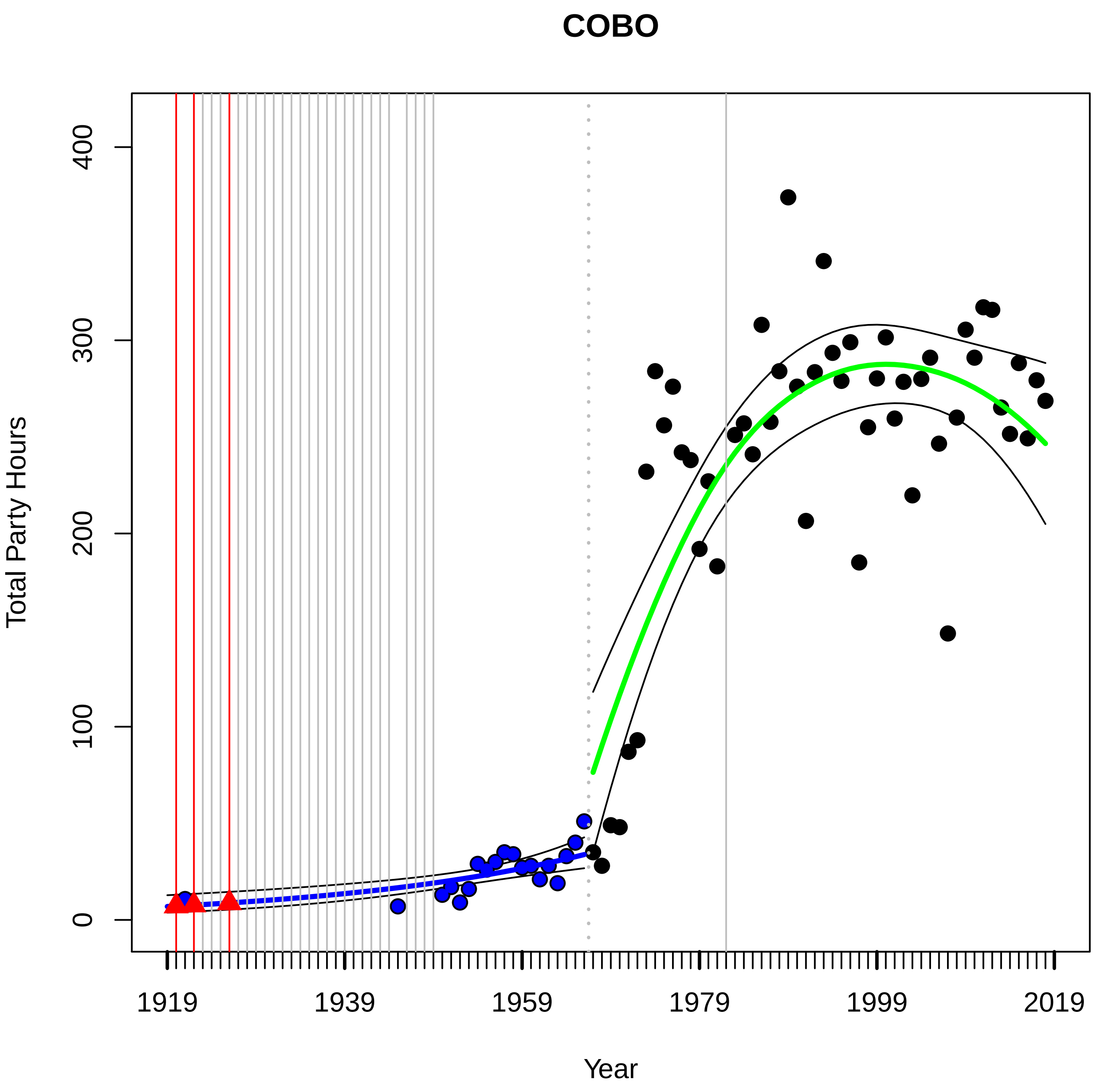

COBO
Total Party Hours
0
100
200
300
400
1919
1939
1959
1979
1999
2019
Year

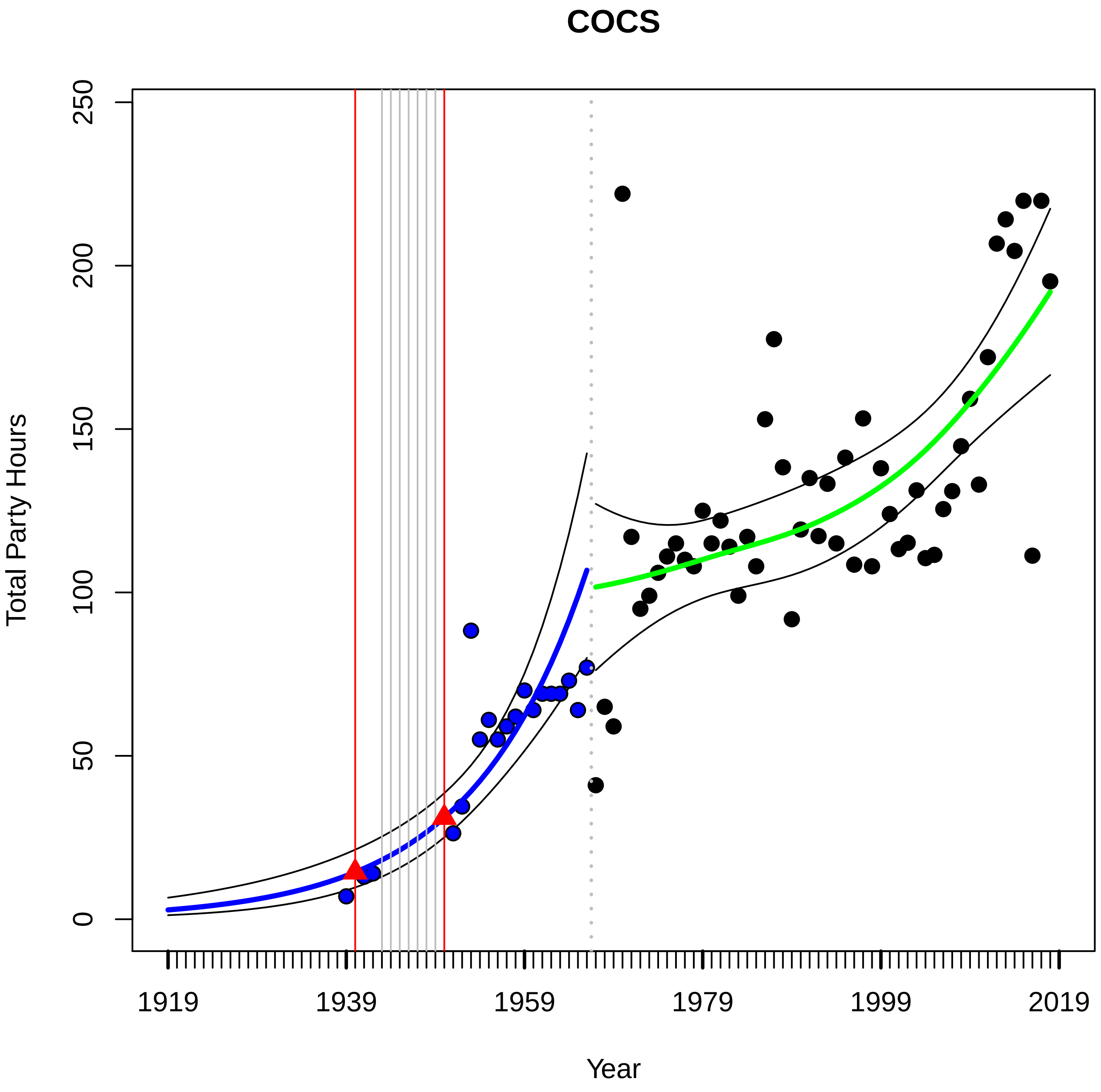
COCS
Total Party Hours
Year
0
50
100
150
200
250
1919
1939
1959
1979
1999
2019

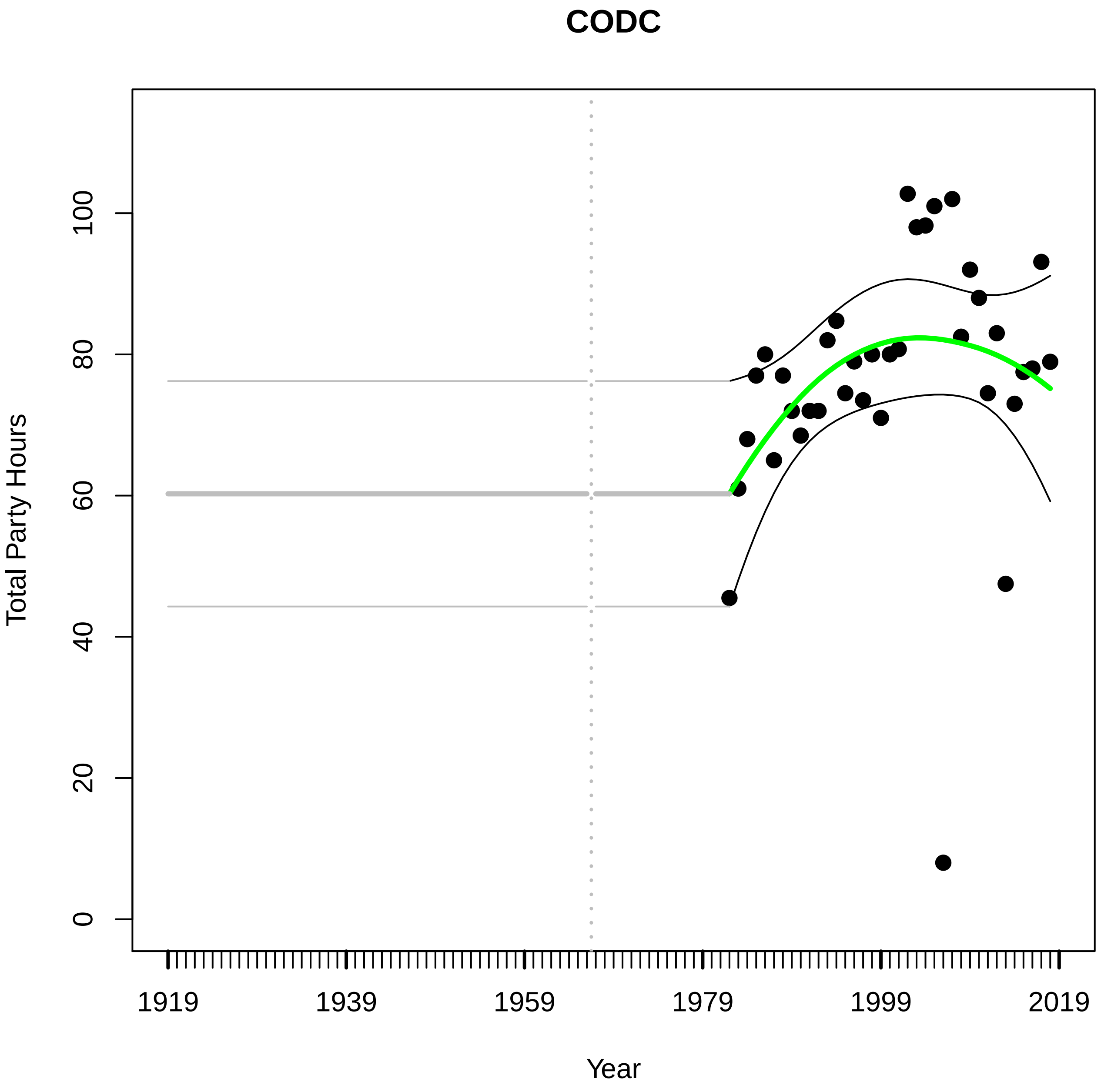

CODC
Total Party Hours
0
20
40
60
80
100
1919
1939
1959
1979
1999
2019
Year

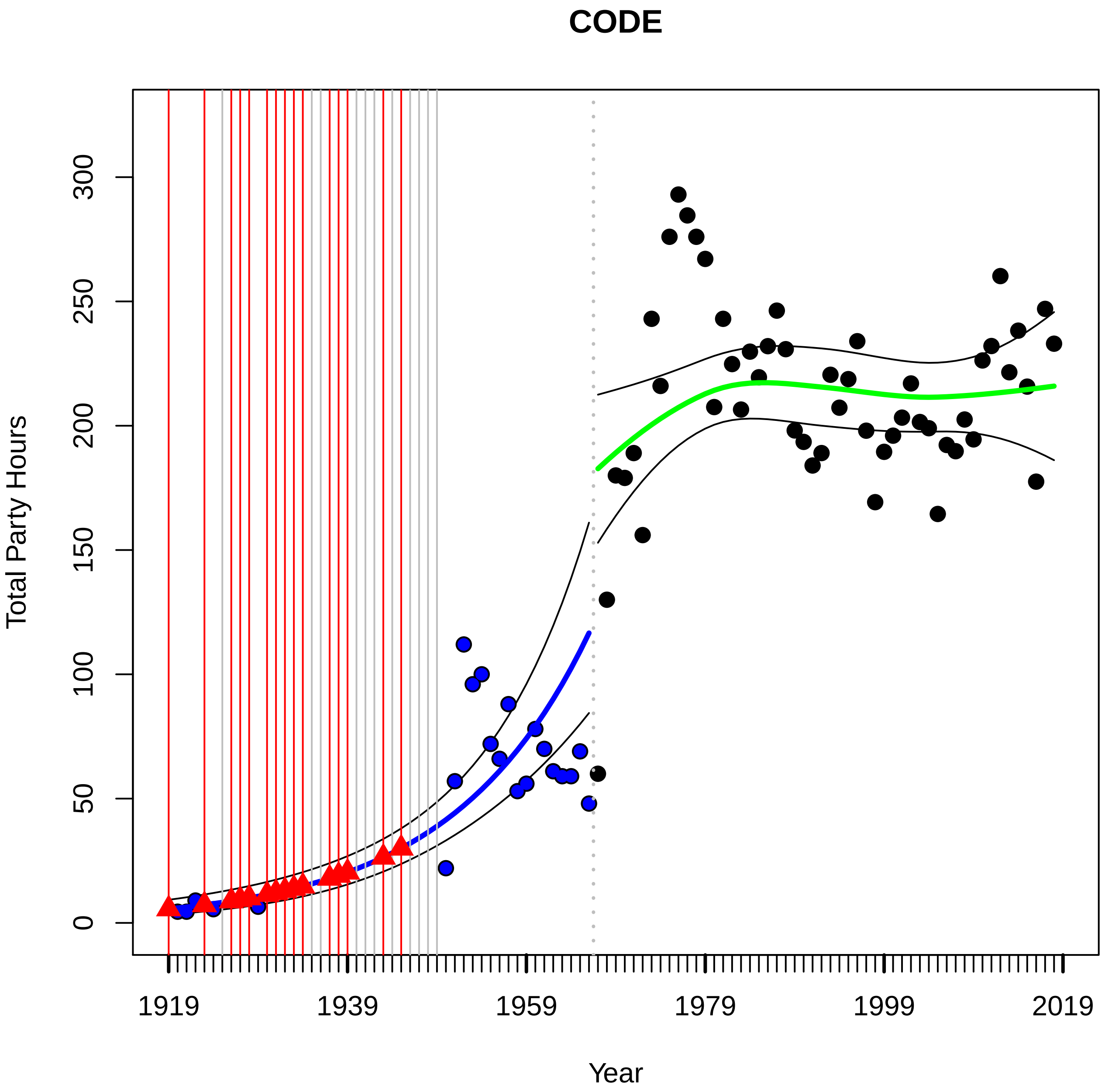
CODE
Total Party Hours
0
50
100
150
200
250
300
1919
1939
1959
1979
1999
2019
Year

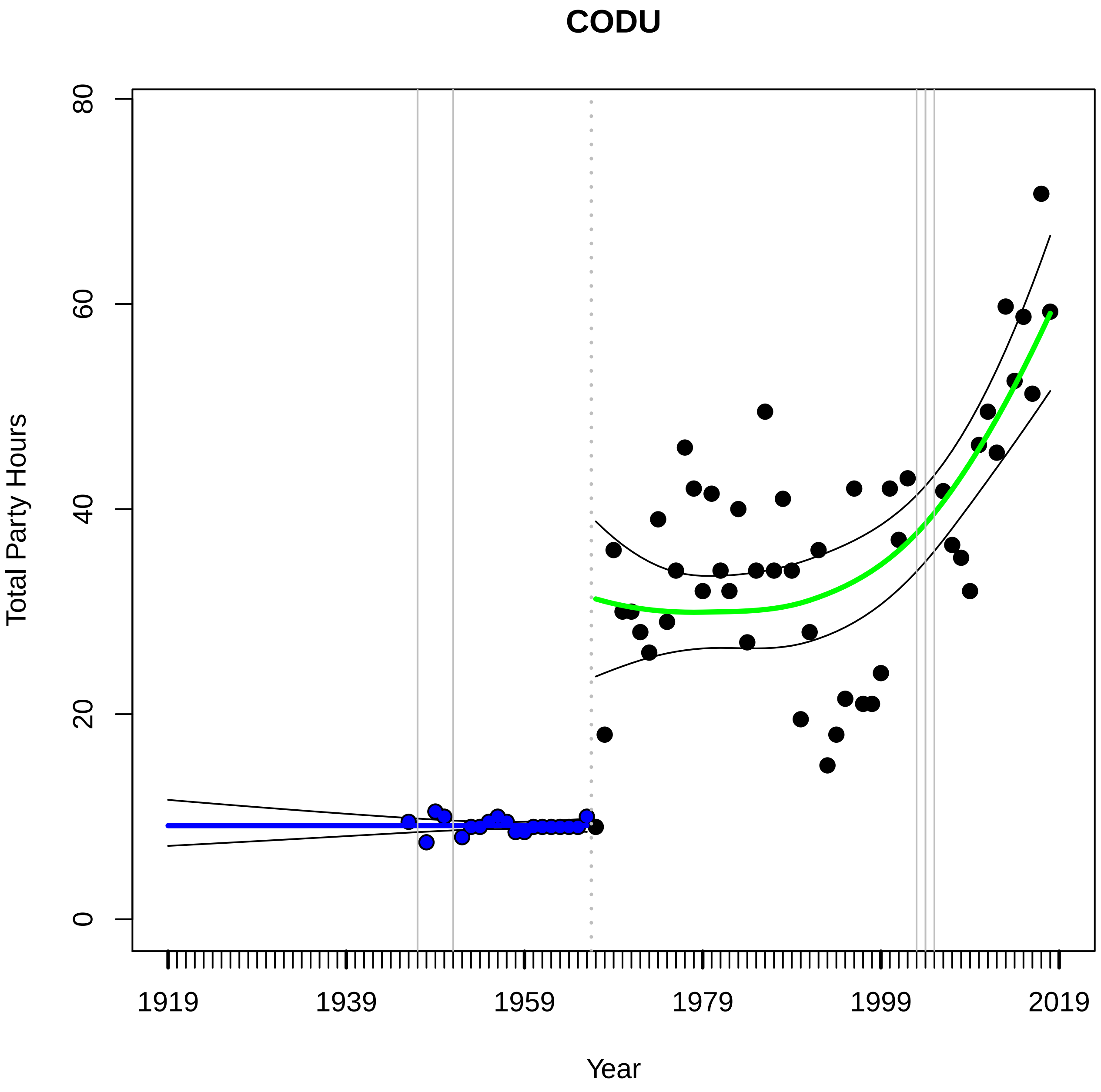

CODU
Total Party Hours
0
20
40
60
80
1919
1939
1959
1979
1999
2019
Year

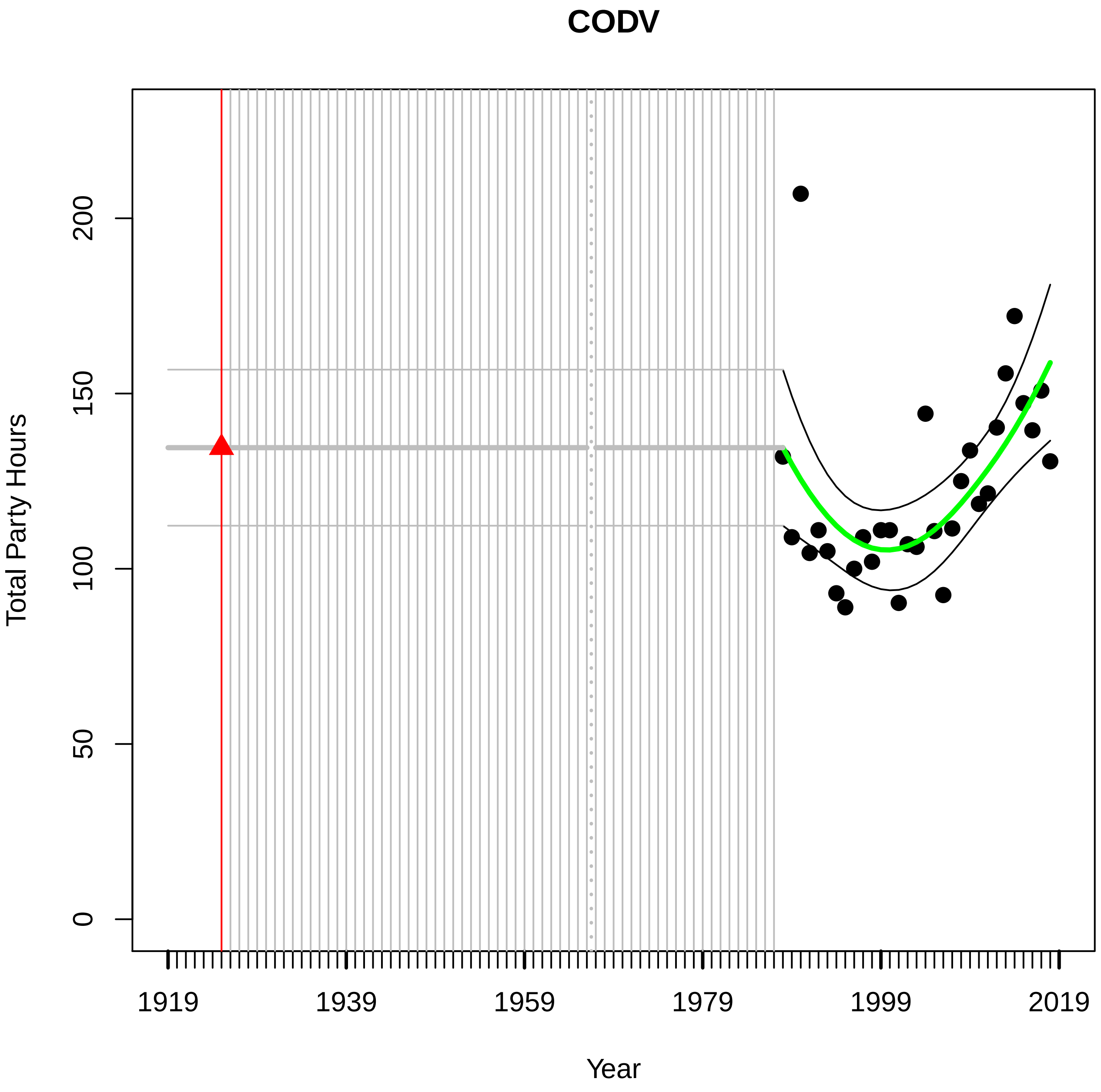

CODV
Total Party Hours
0
50
100
150
200
1919
1939
1959
1979
1999
2019
Year

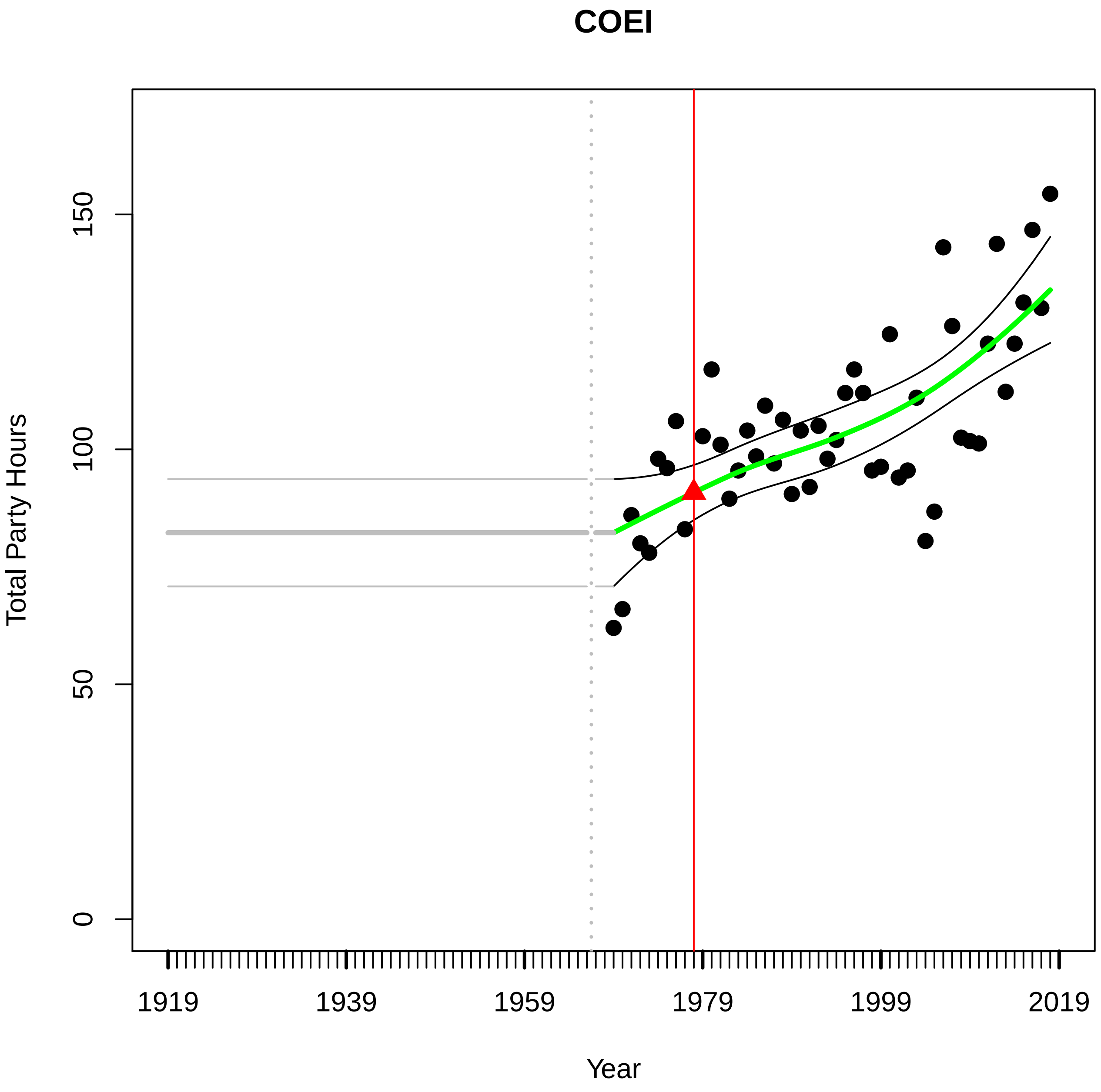

COEI
Total Party Hours
Year
0
50
100
150
1919
1939
1959
1979
1999
2019

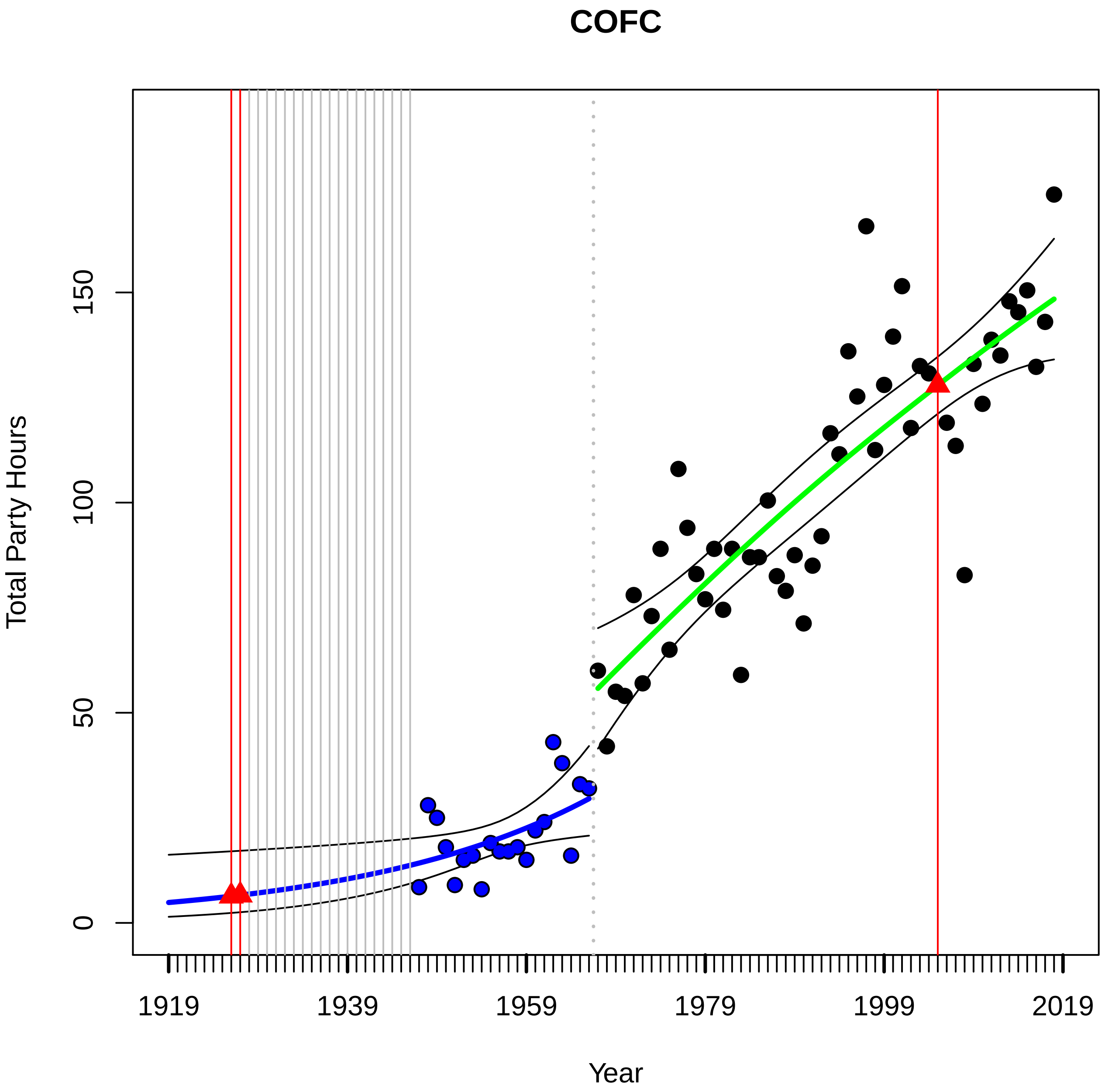

COFC
Total Party Hours
0
50
100
150
1919
1939
1959
1979
1999
2019
Year

COGJ

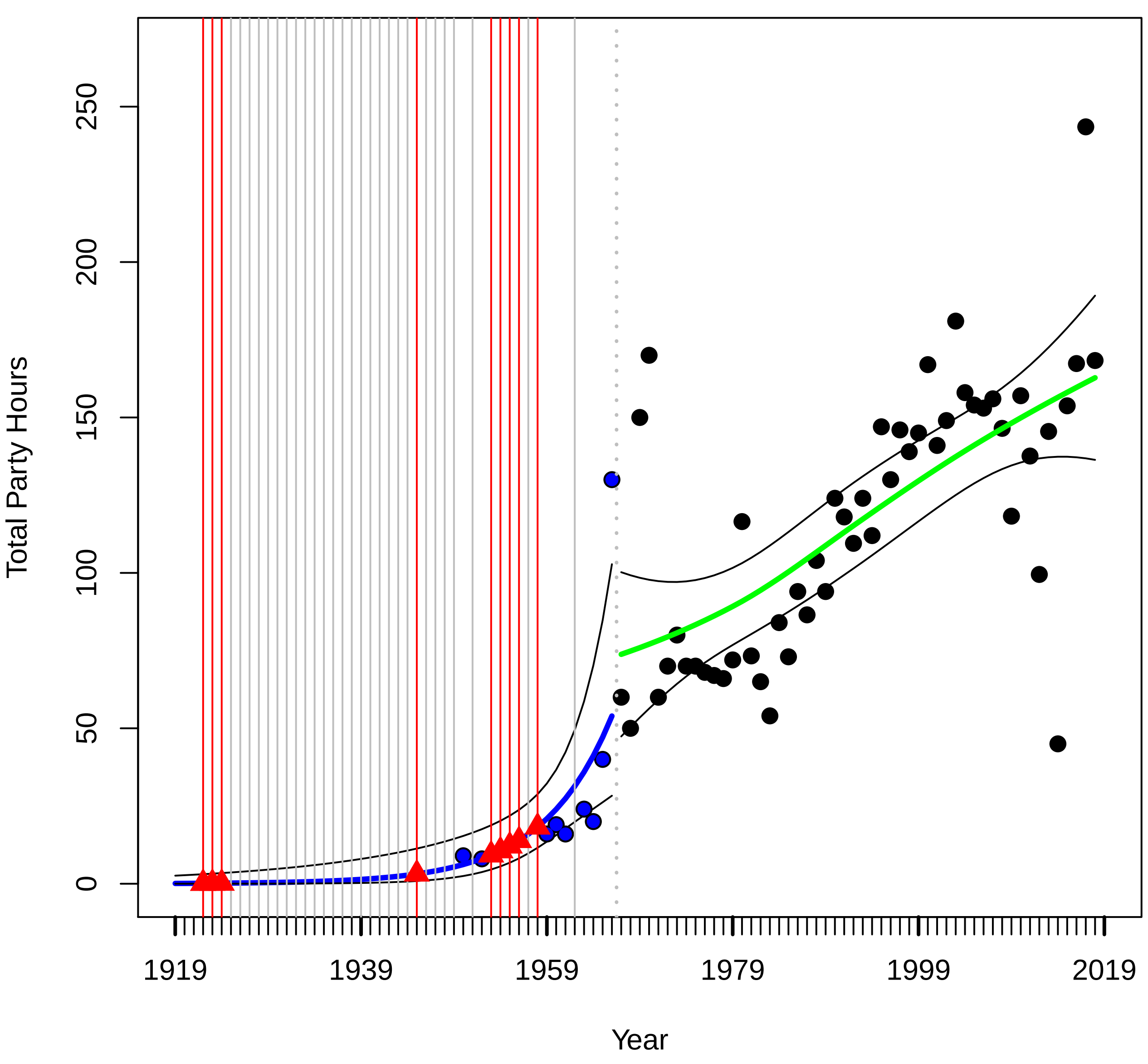

Total Party Hours
0
50
100
150
200
250
1919
1939
1959
1979
1999
2019
Year

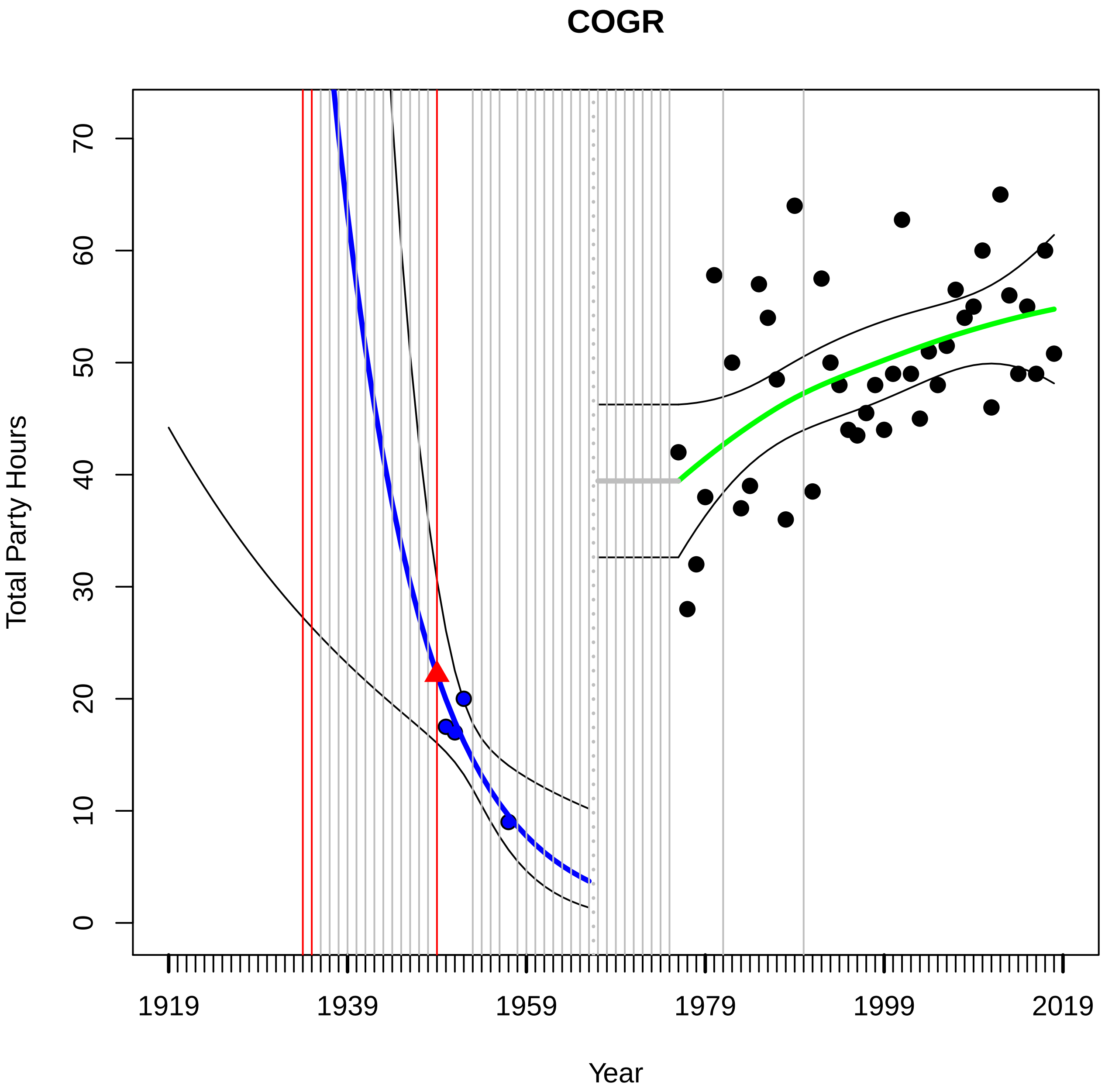

COGR
Total Party Hours
0
10
20
30
40
50
60
70
1919
1939
1959
1979
1999
2019
Year

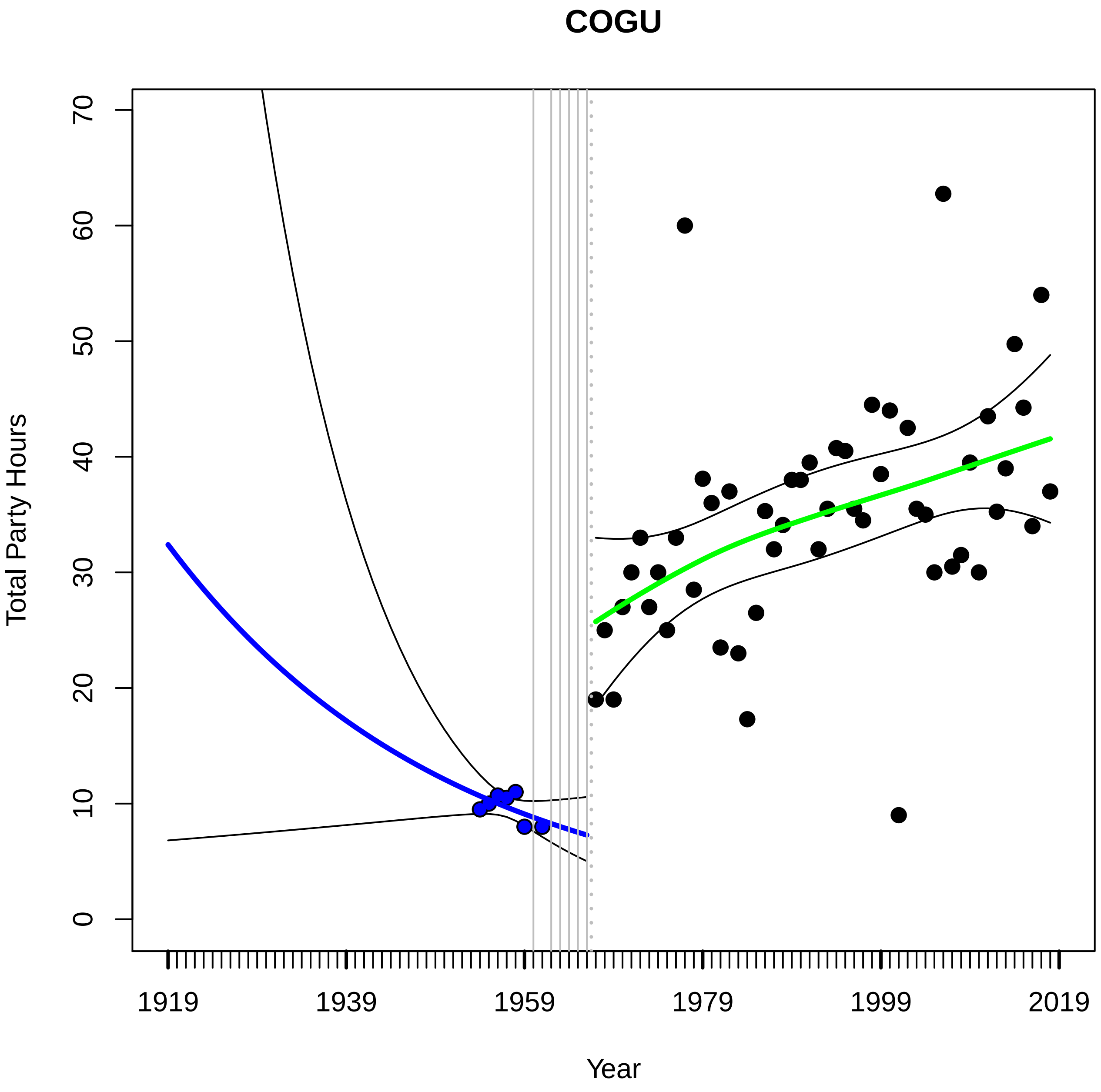
COGU
Total Party Hours
0
10
20
30
40
50
60
70
1919
1939
1959
1979
1999
2019
Year

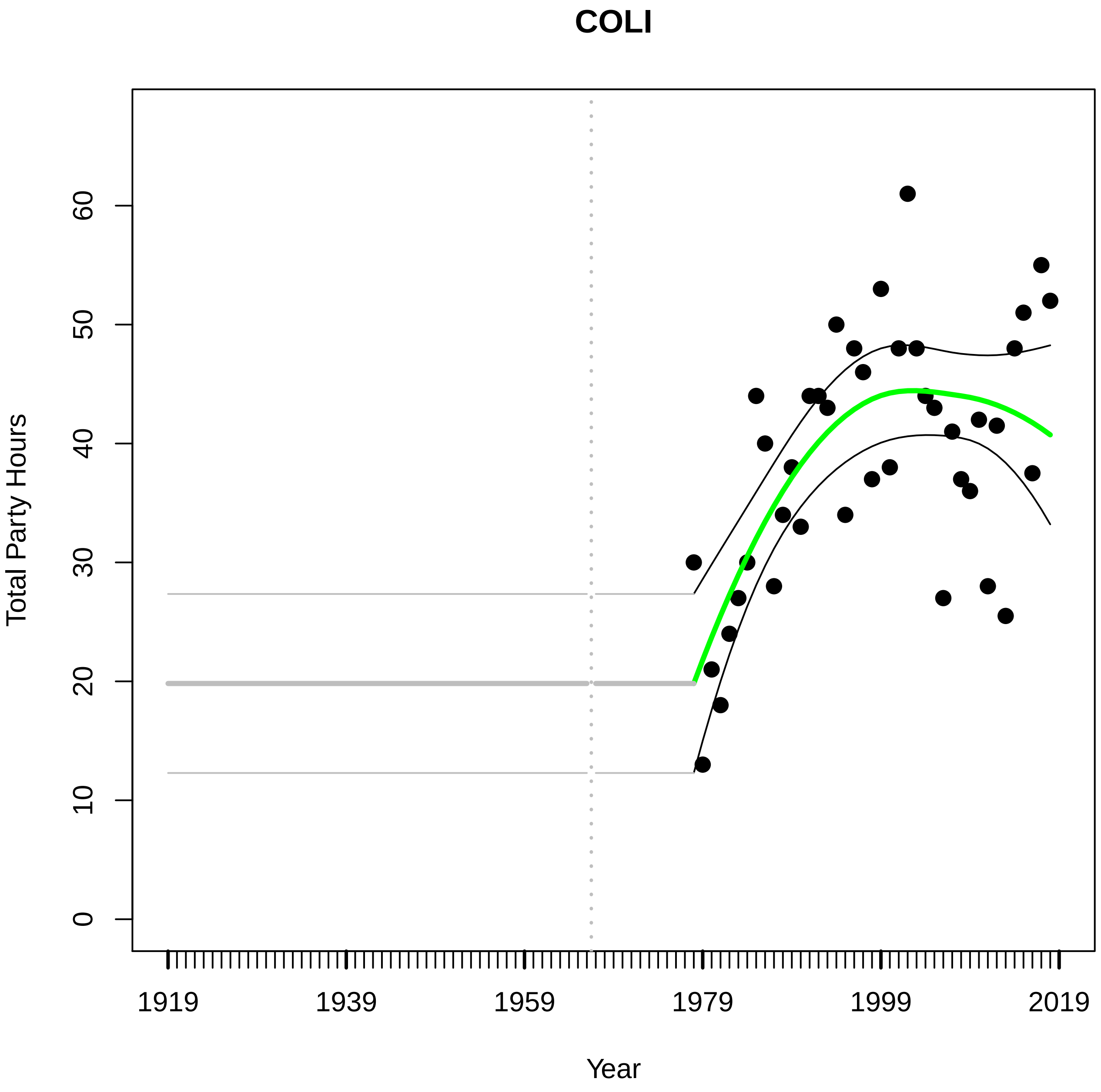

COLI
Total Party Hours
Year
0
10
20
30
40
50
60
1919
1939
1959
1979
1999
2019

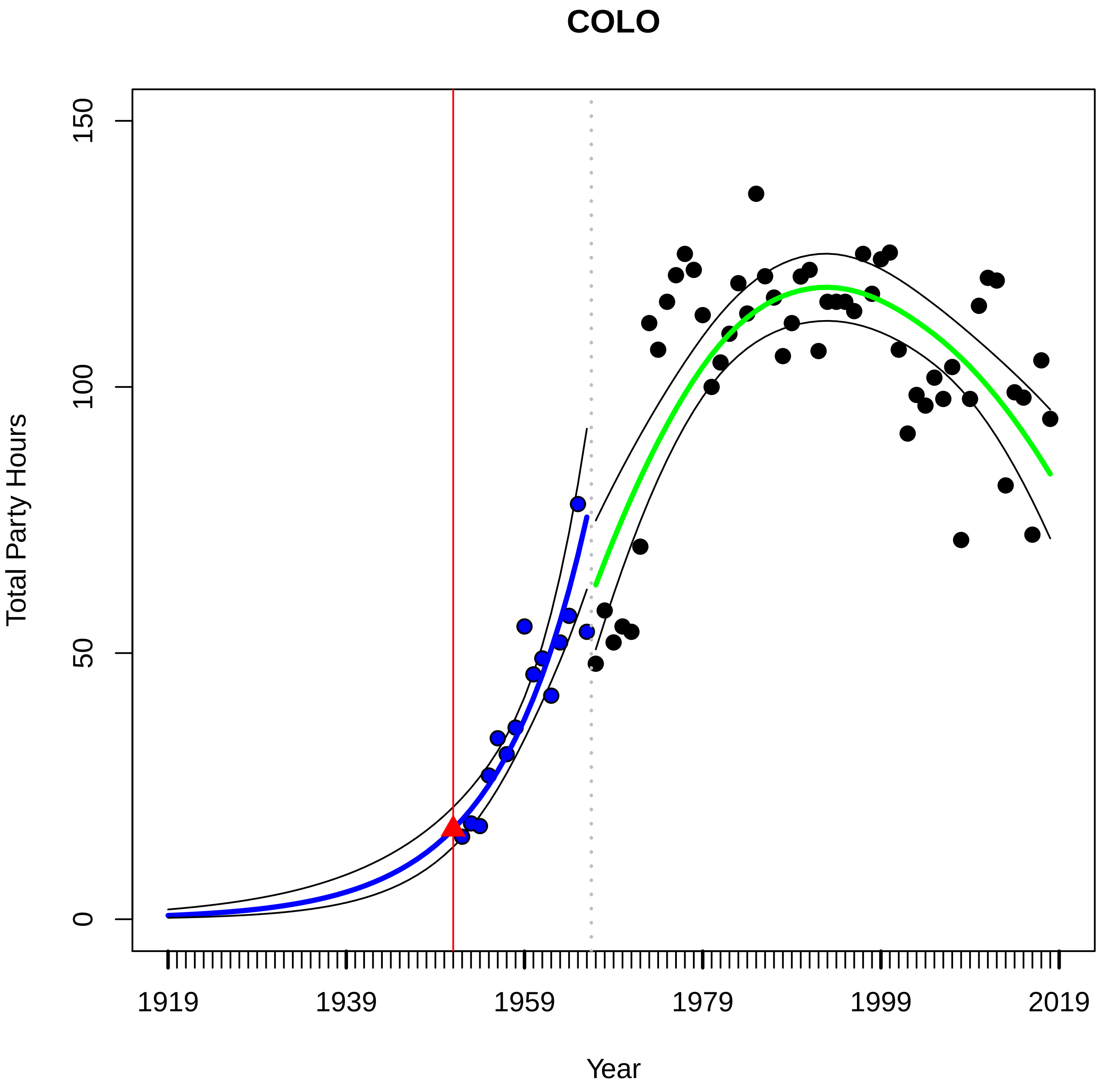

COLO
Total Party Hours
150
100
50
0
1919
1939
1959
1979
1999
2019
Year

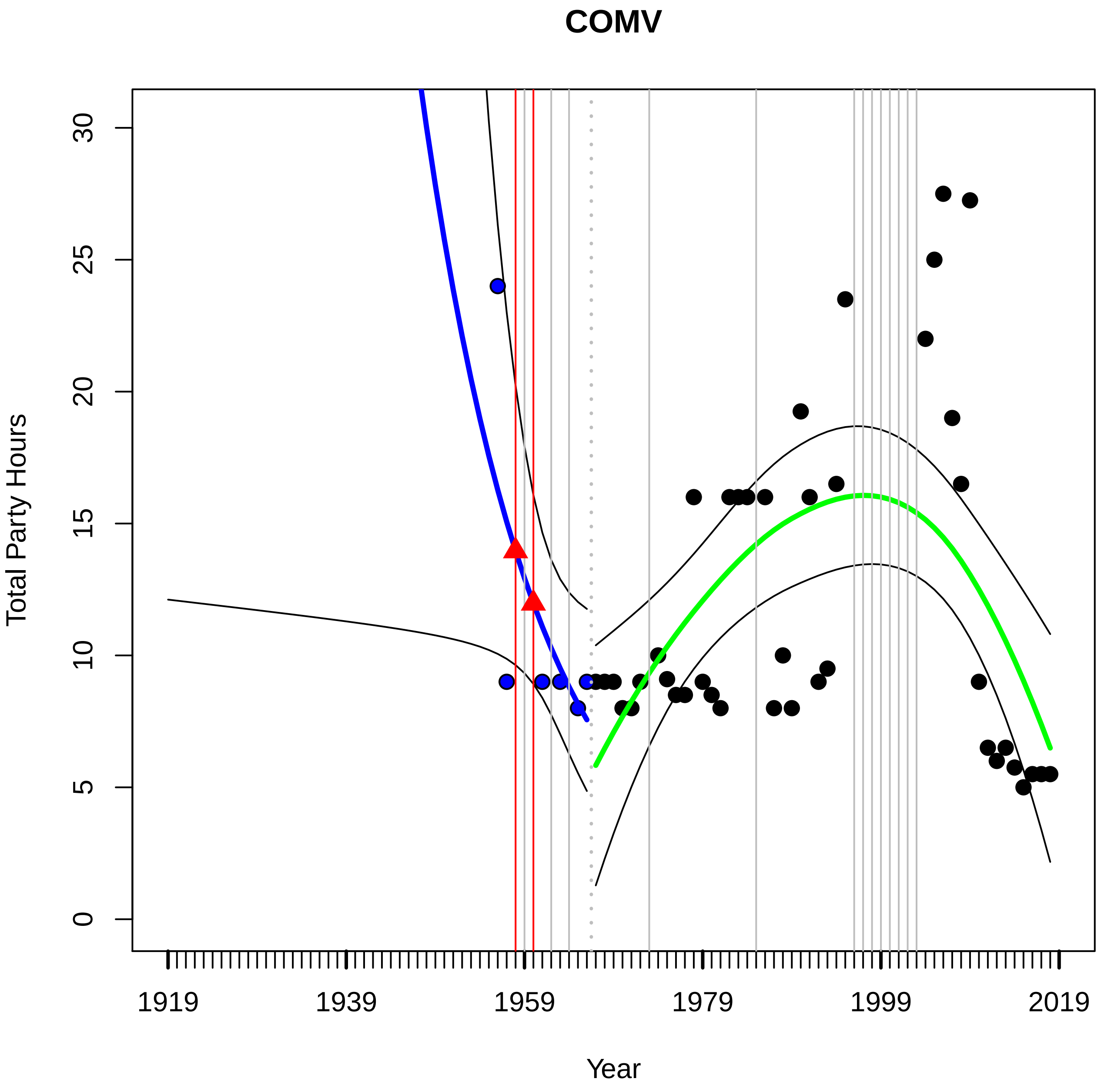
COMV
Total Party Hours
Year
0
5
10
15
20
25
30
1919
1939
1959
1979
1999
2019

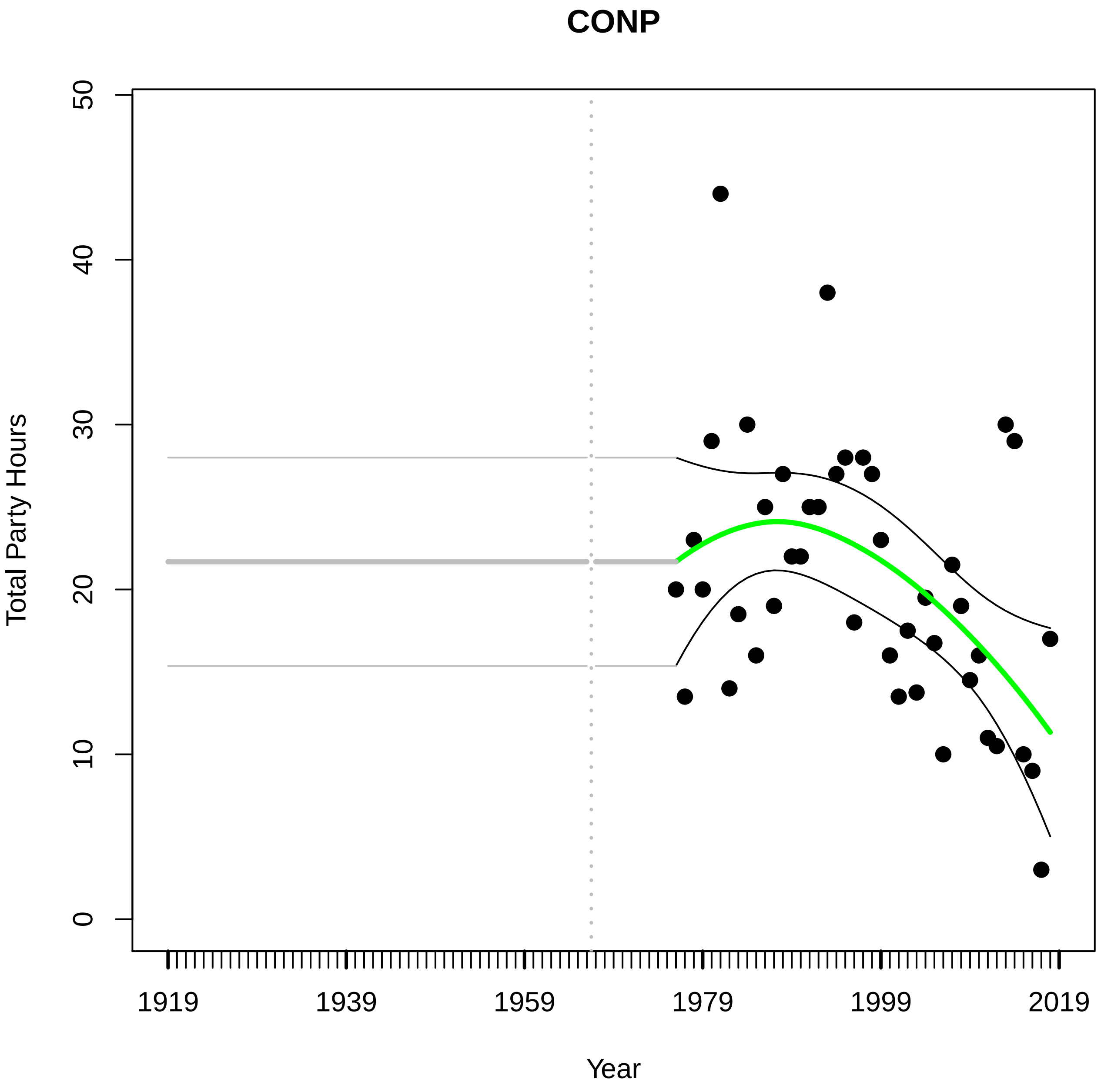
CONP
Total Party Hours
Year
0
10
20
30
40
50
1919
1939
1959
1979
1999
2019

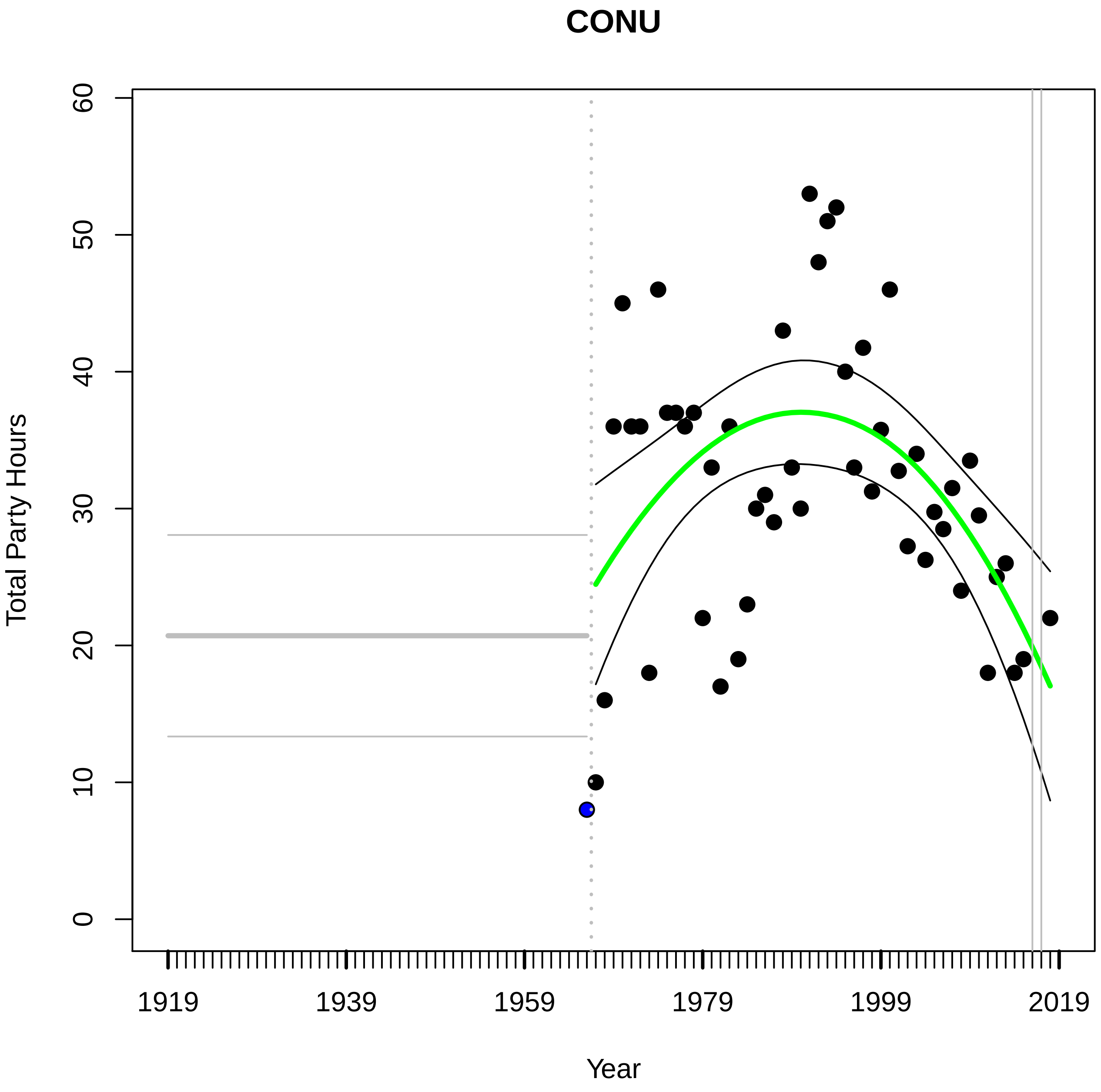

CONU
Total Party Hours
0
10
20
30
40
50
60
1919
1939
1959
1979
1999
2019
Year

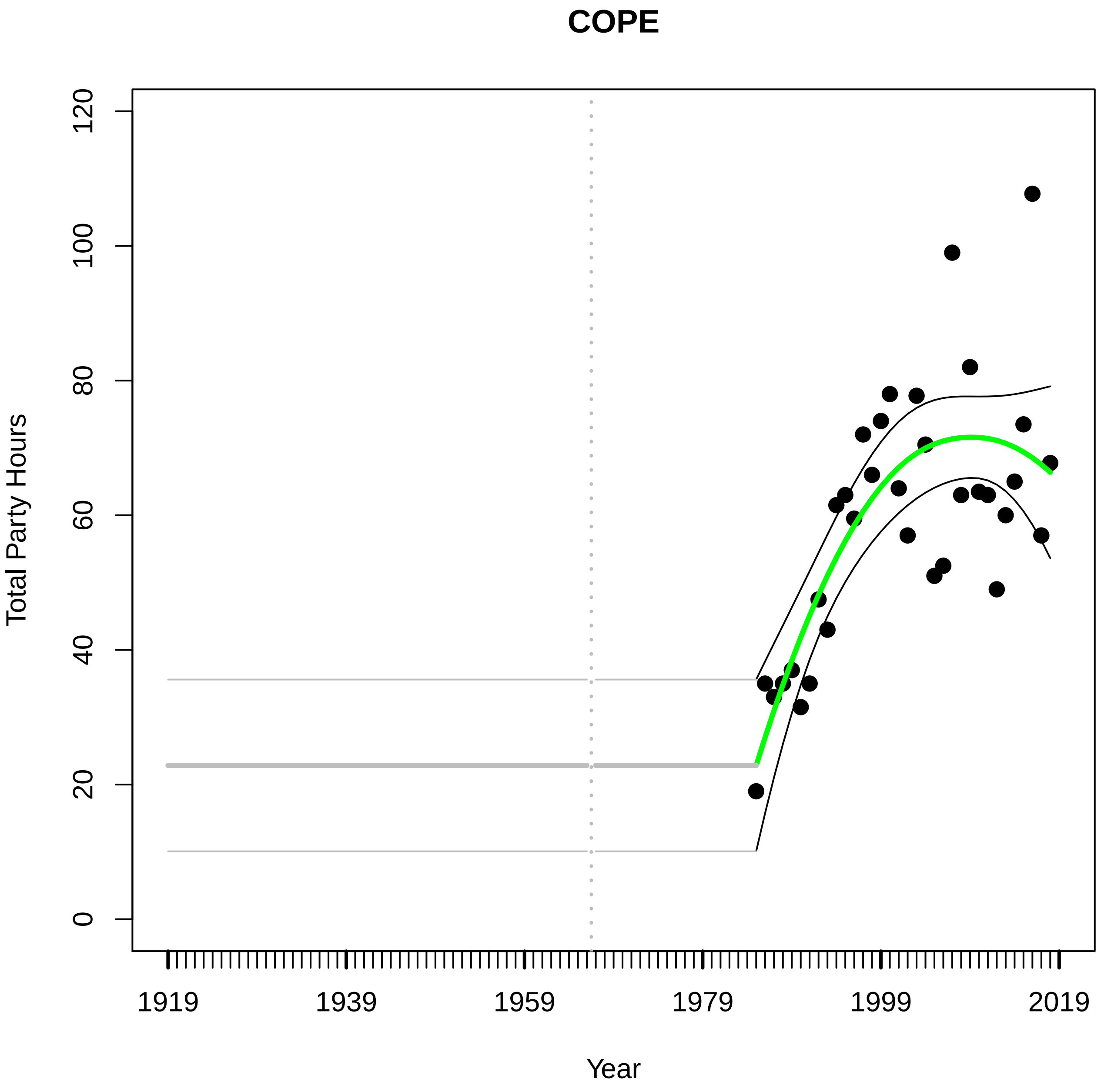
COPE
Total Party Hours
Year
0
20
40
60
80
100
120
1919
1939
1959
1979
1999
2019

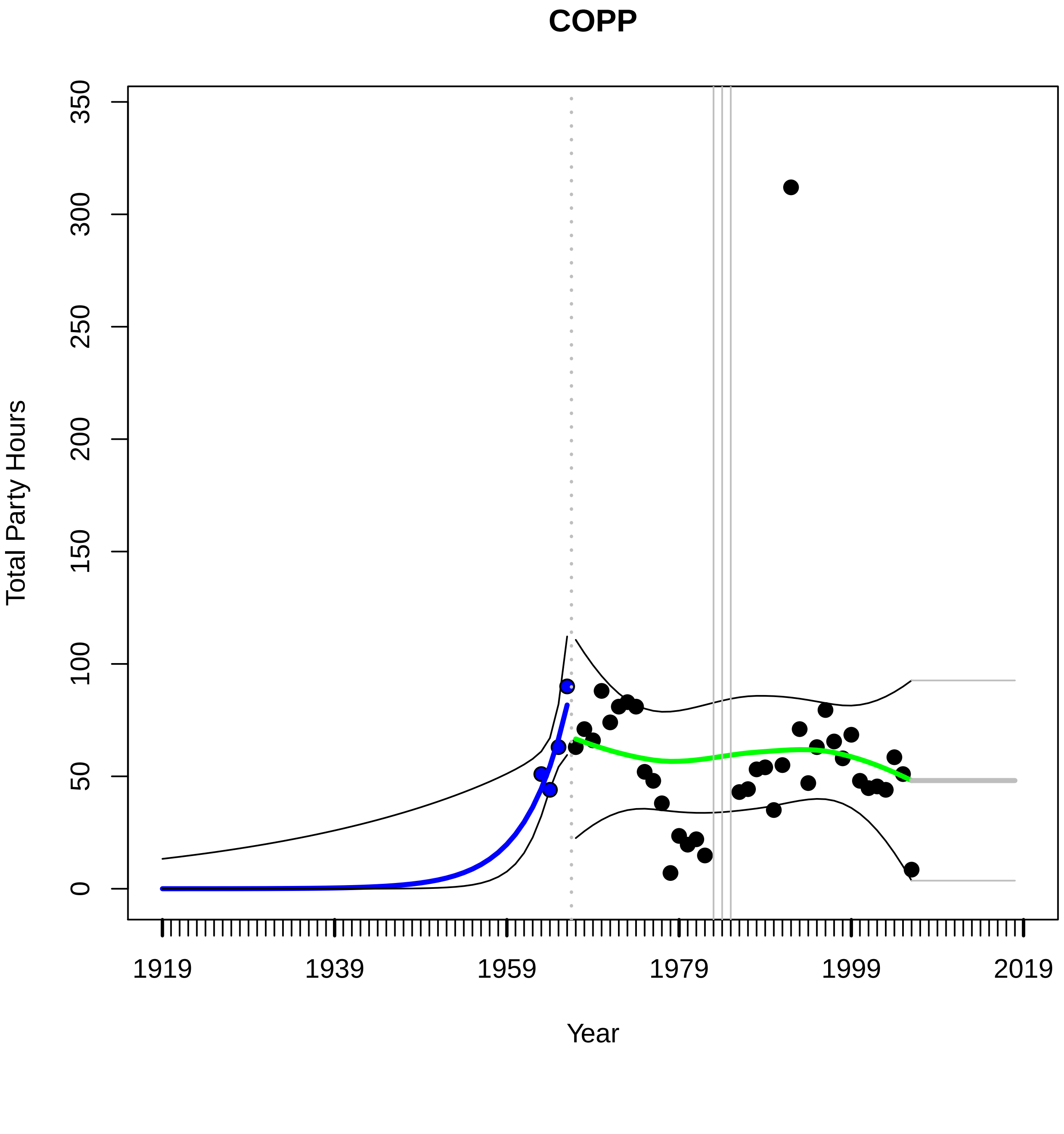
COPP
Total Party Hours
0
50
100
150
200
250
300
350
1919
1939
1959
1979
1999
2019
Year

COPR

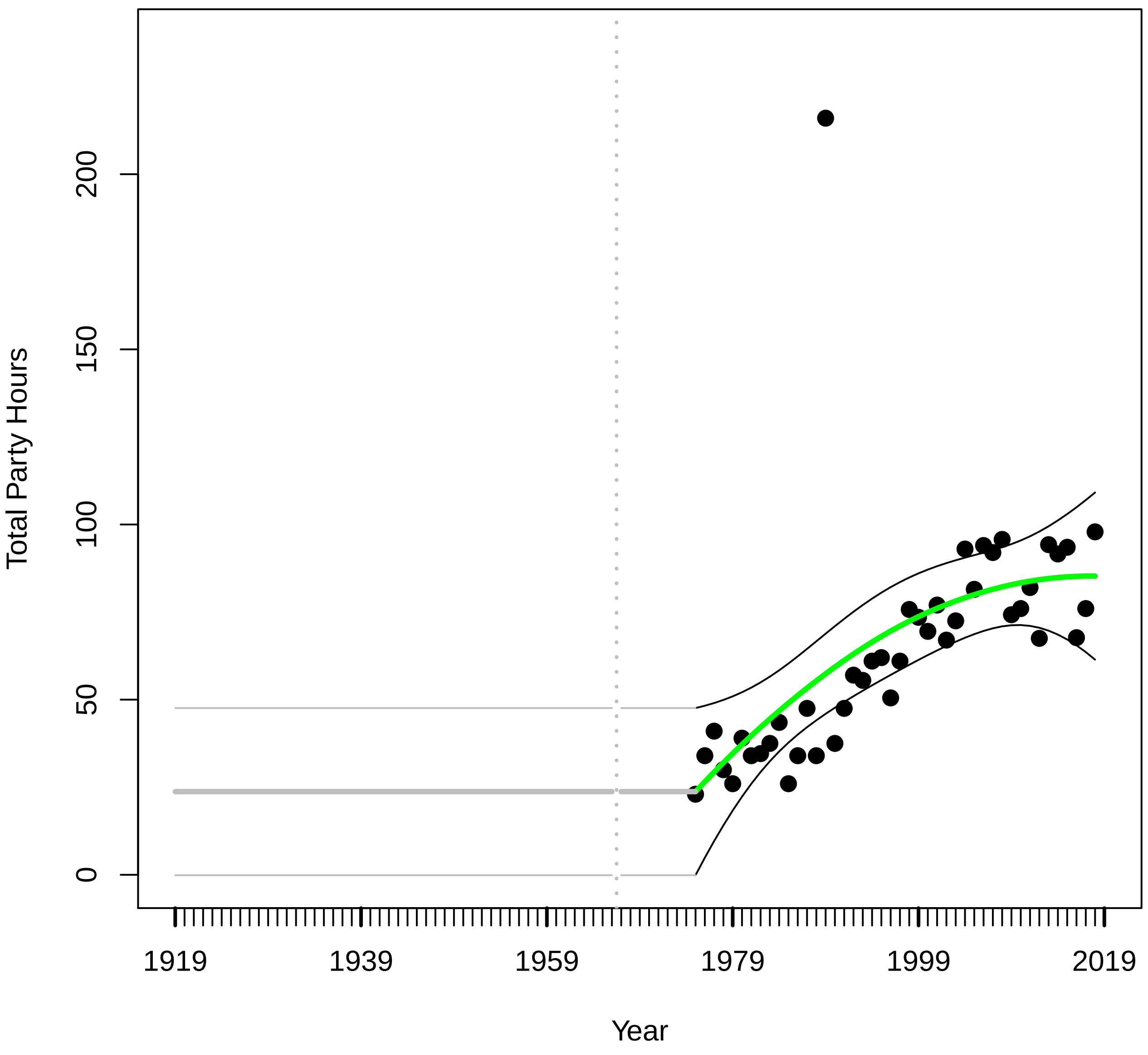

Total Party Hours
0
50
100
150
200
1919
1939
1959
1979
1999
2019
Year

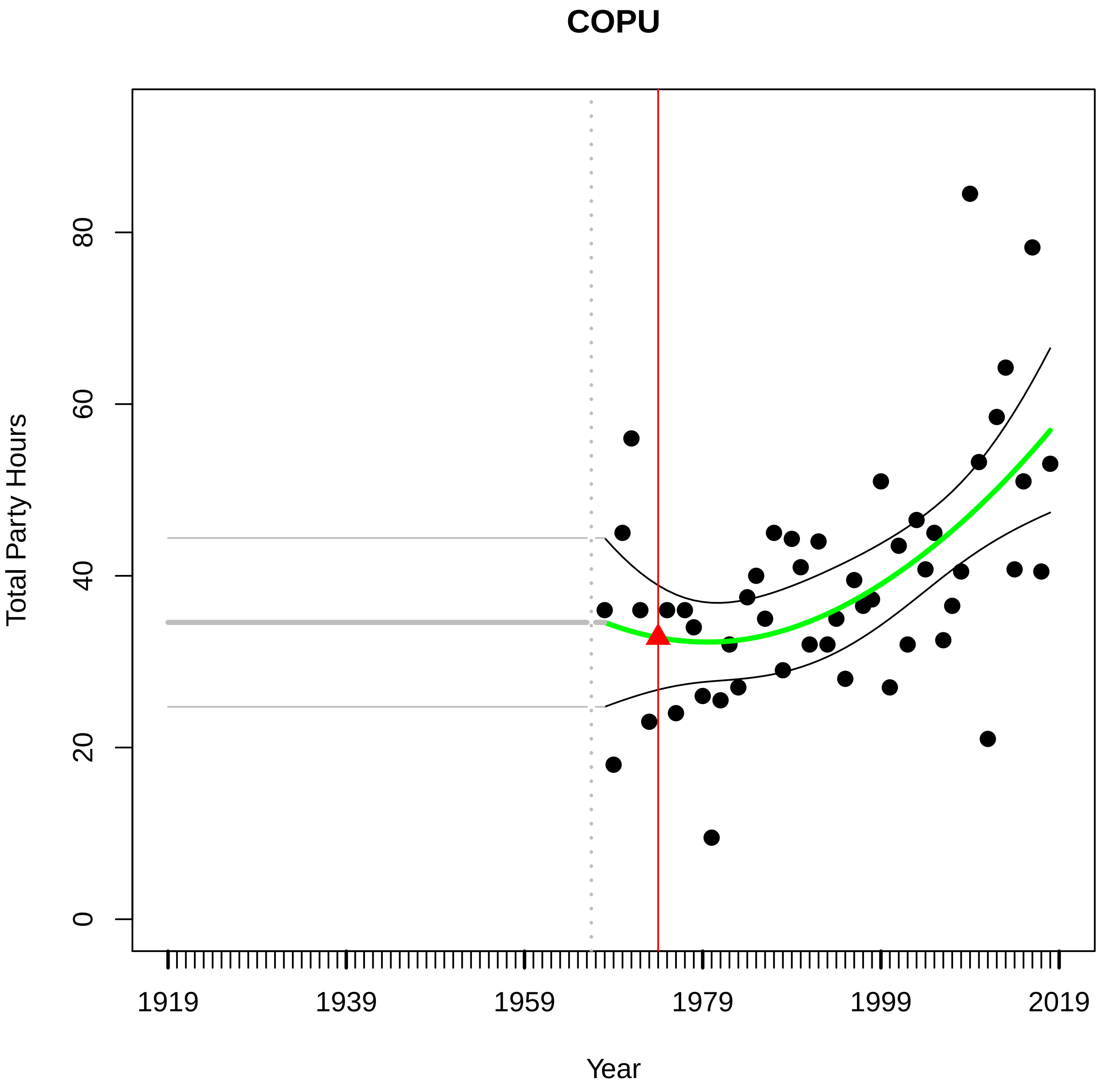
COPU
Total Party Hours
0
20
40
60
80
1919
1939
1959
1979
1999
2019
Year

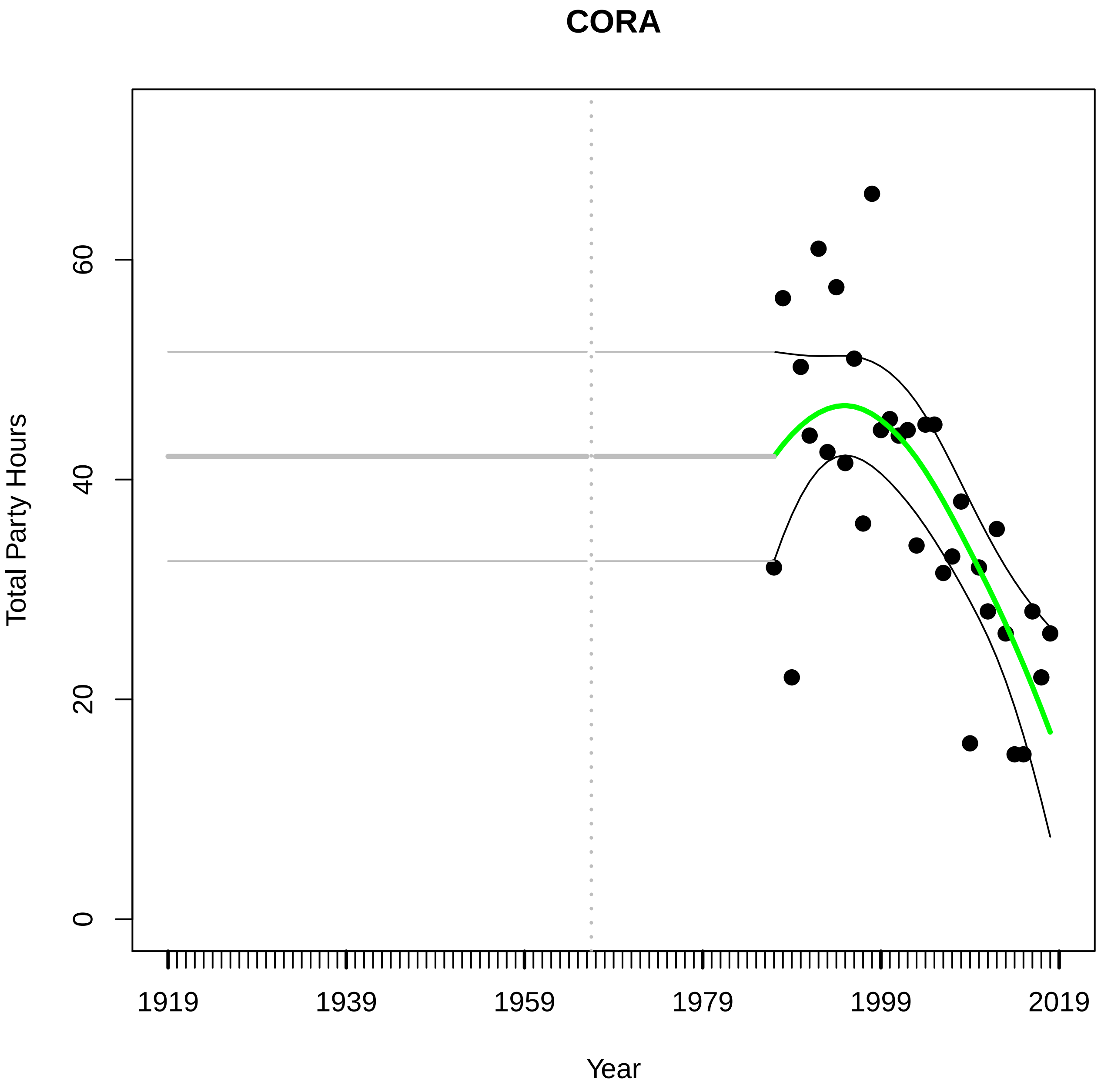
CORA
Total Party Hours
0
20
40
60
1919
1939
1959
1979
1999
2019
Year

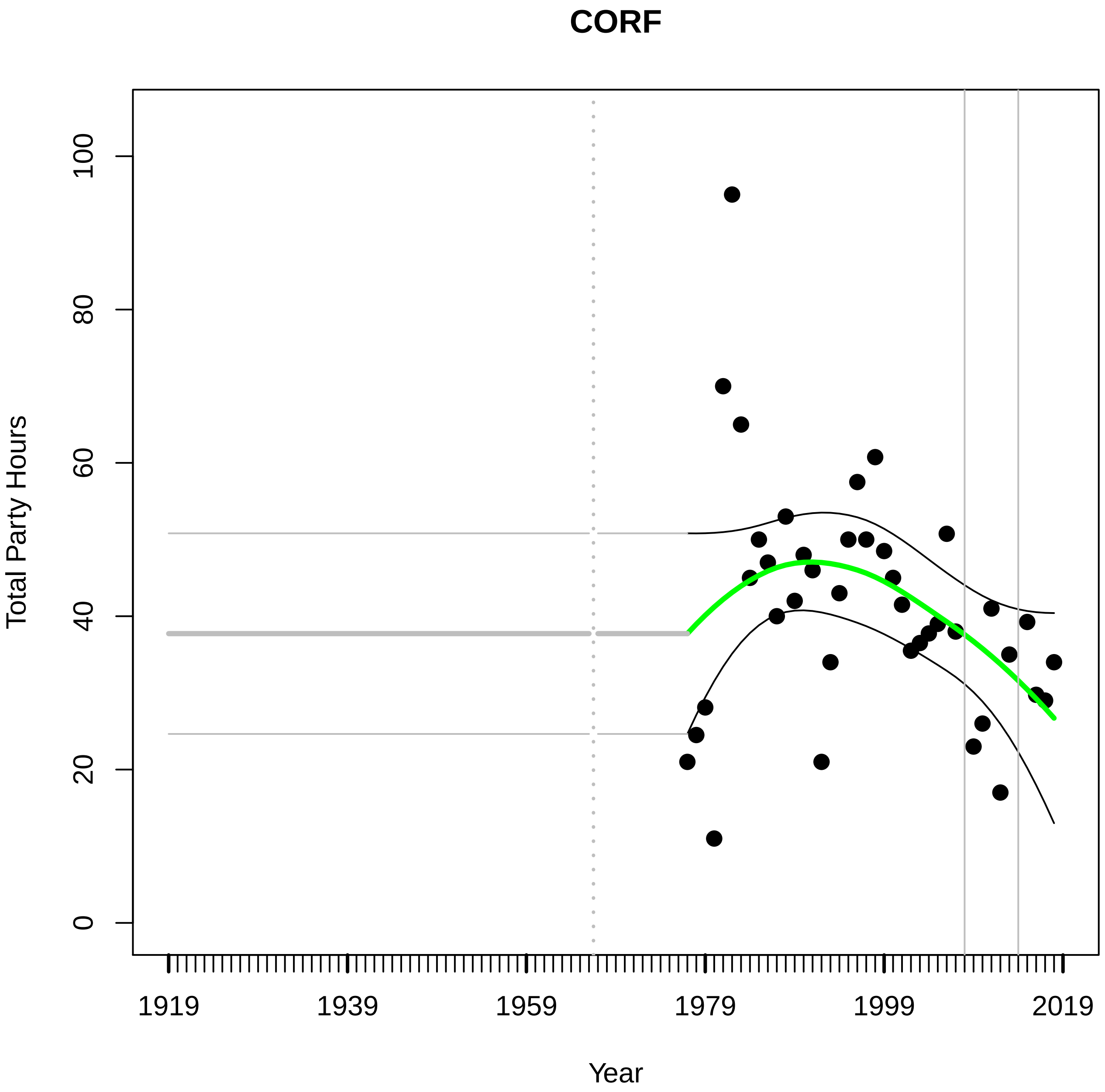

CORF
Total Party Hours
0
20
40
60
80
100
1919
1939
1959
1979
1999
2019
Year

CORM

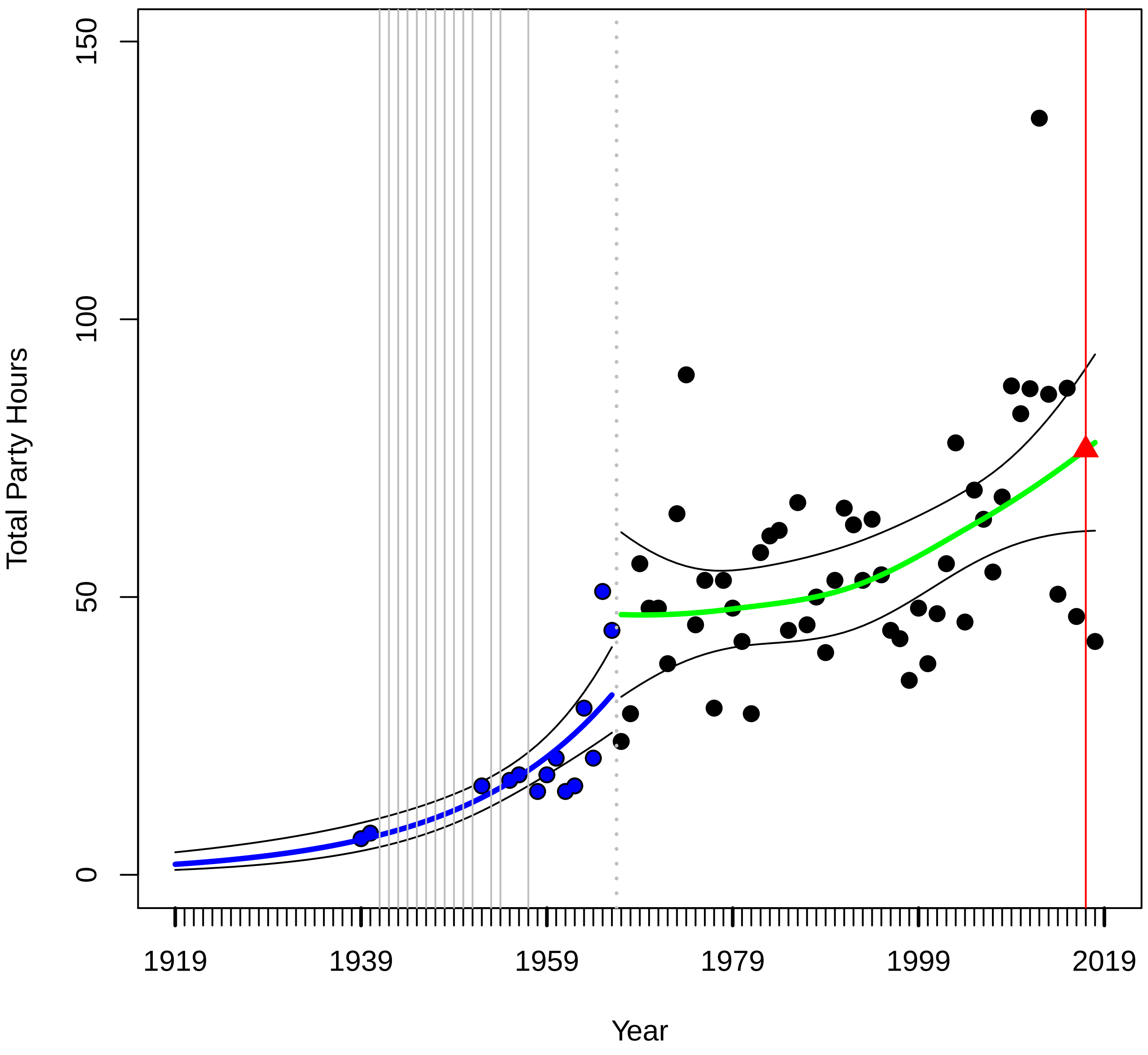

Total Party Hours
0
50
100
150
1919
1939
1959
1979
1999
2019
Year

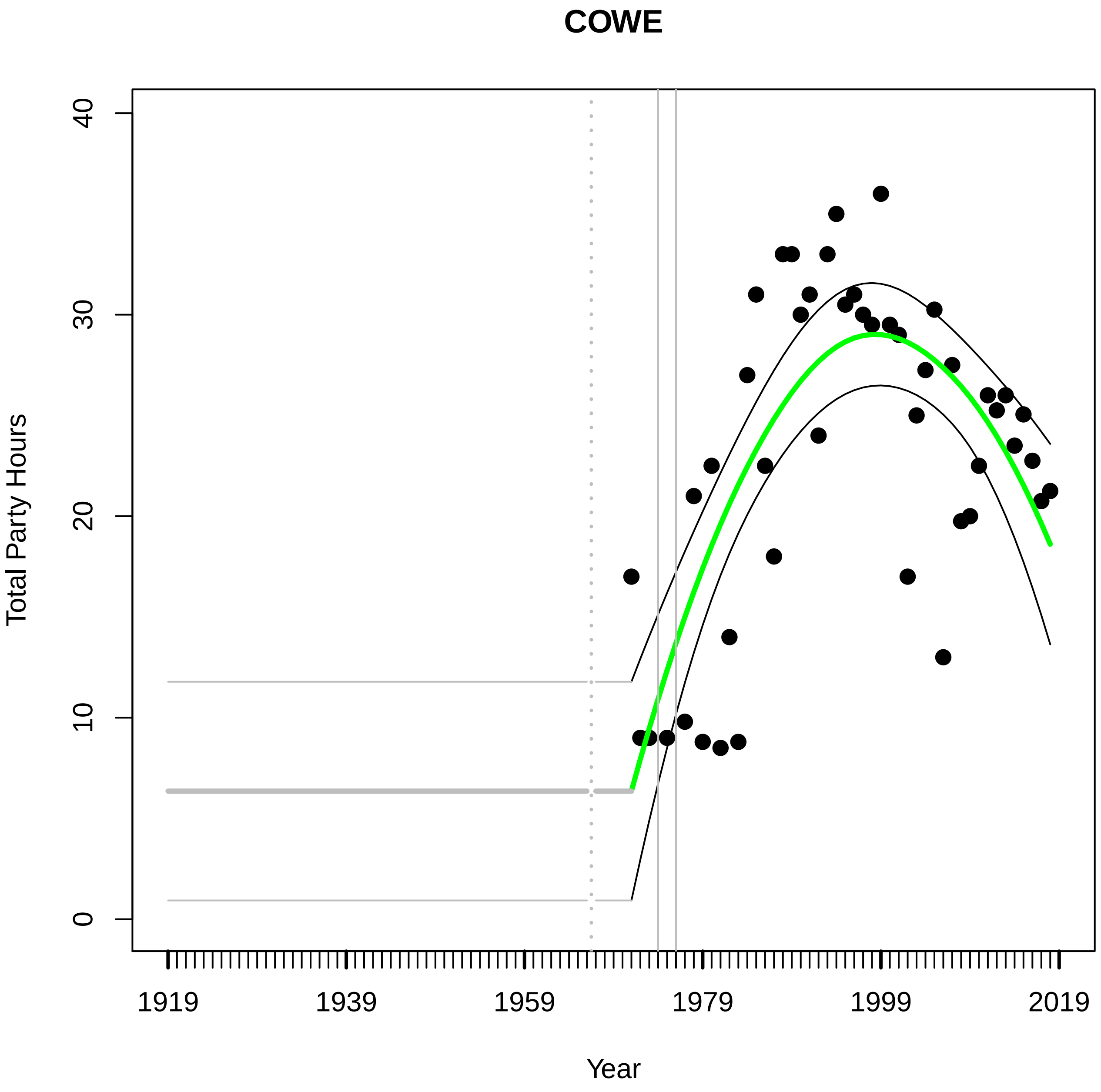

COWE
Total Party Hours
0
10
20
30
40
1919
1939
1959
1979
1999
2019
Year

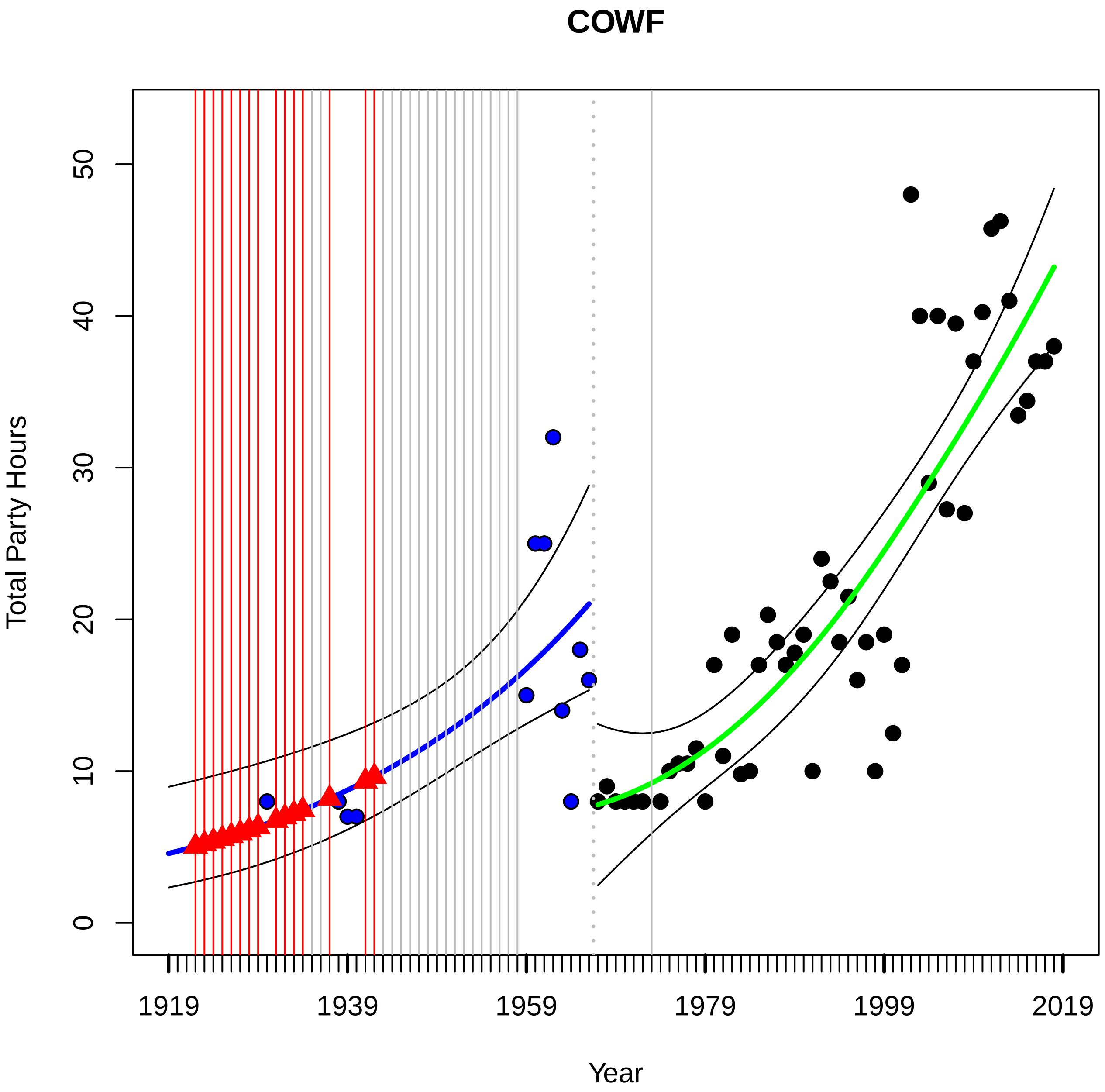

COWF
Total Party Hours
0
10
20
30
40
50
1919
1939
1959
1979
1999
2019
Year

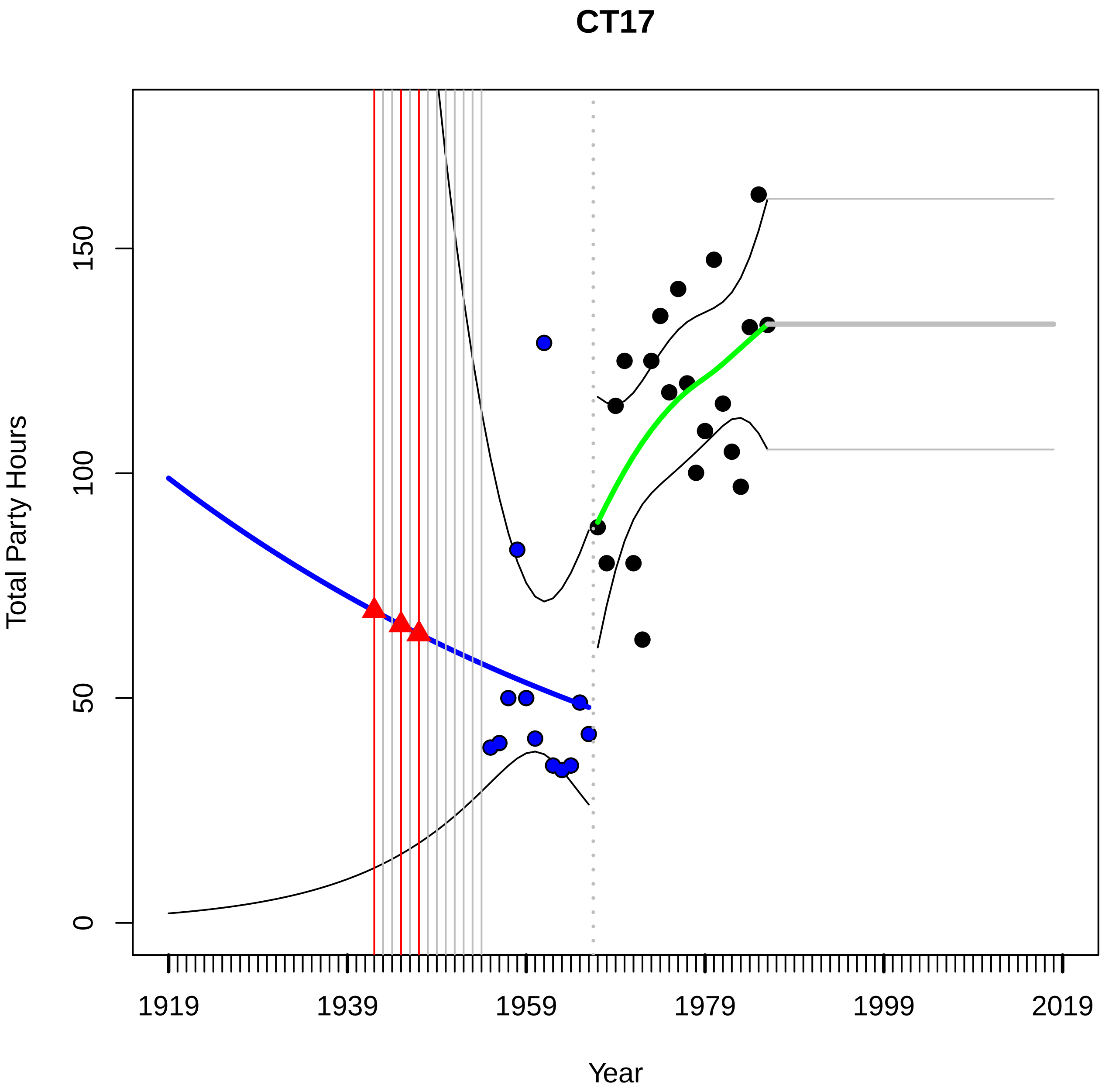

CT17
Total Party Hours
0
50
100
150
1919
1939
1959
1979
1999
2019
Year

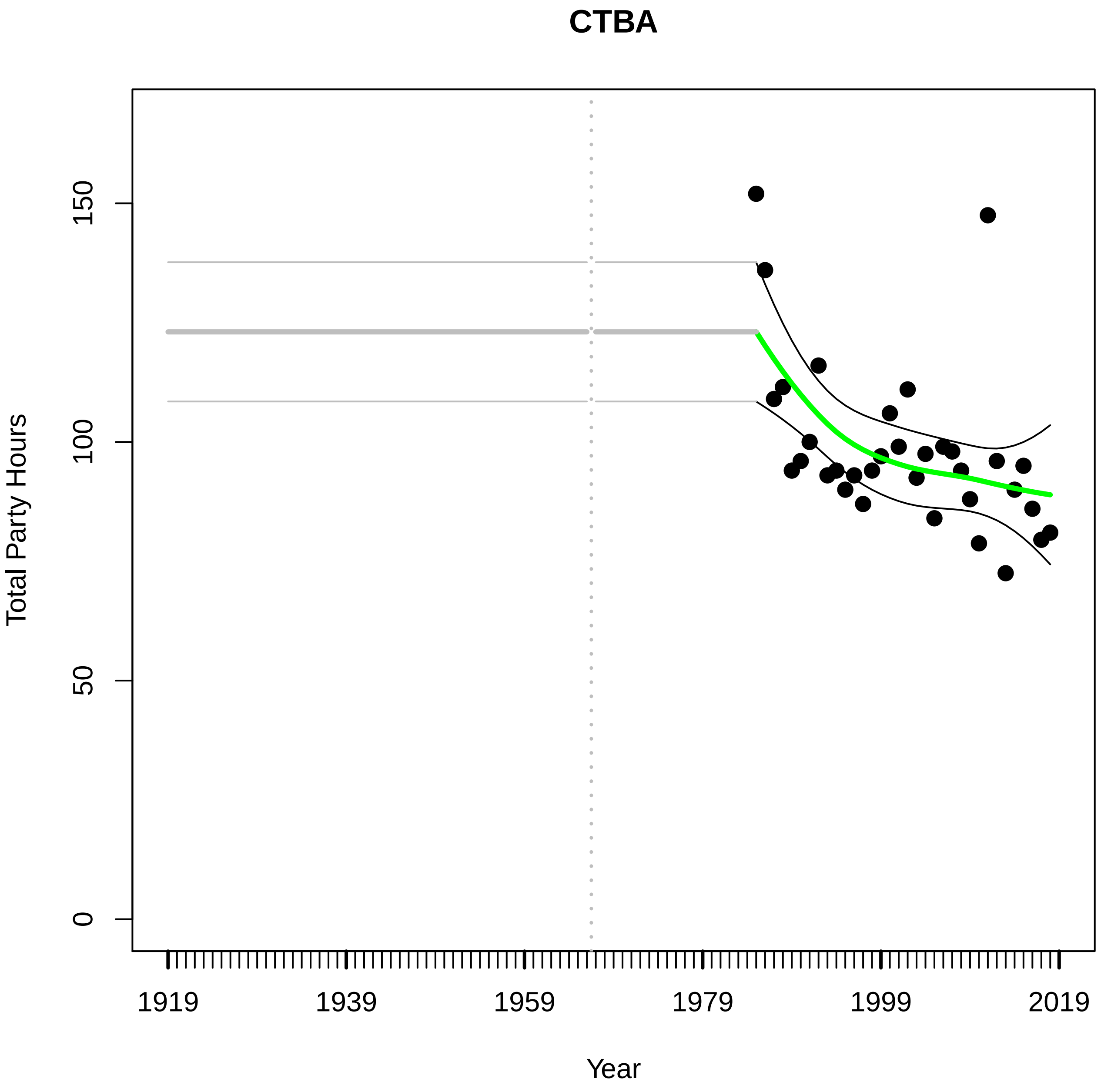

CTBA
Total Party Hours
0
50
100
150
1919
1939
1959
1979
1999
2019
Year

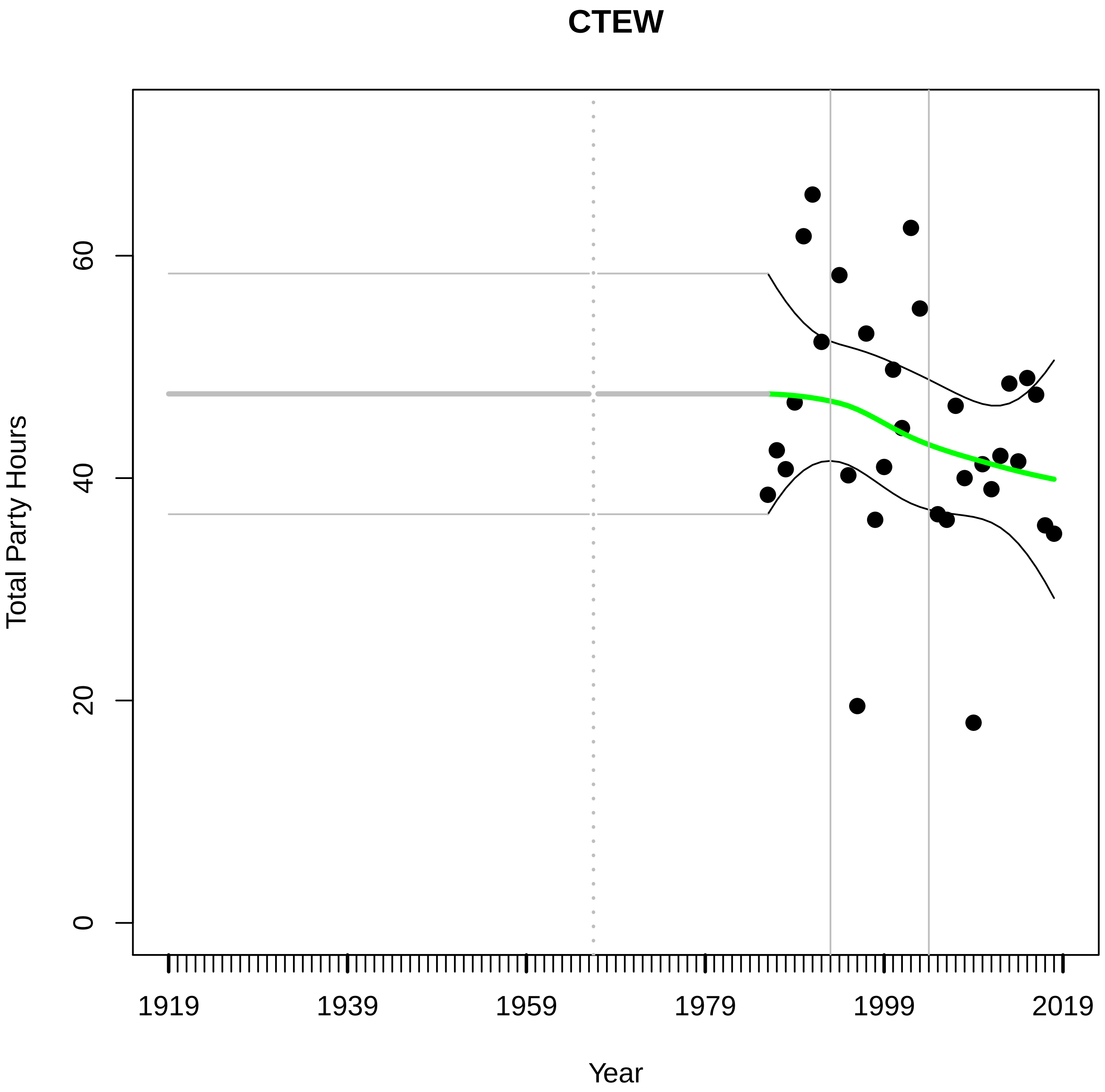

CTEW
Total Party Hours
Year
0
20
40
60
1919
1939
1959
1979
1999
2019

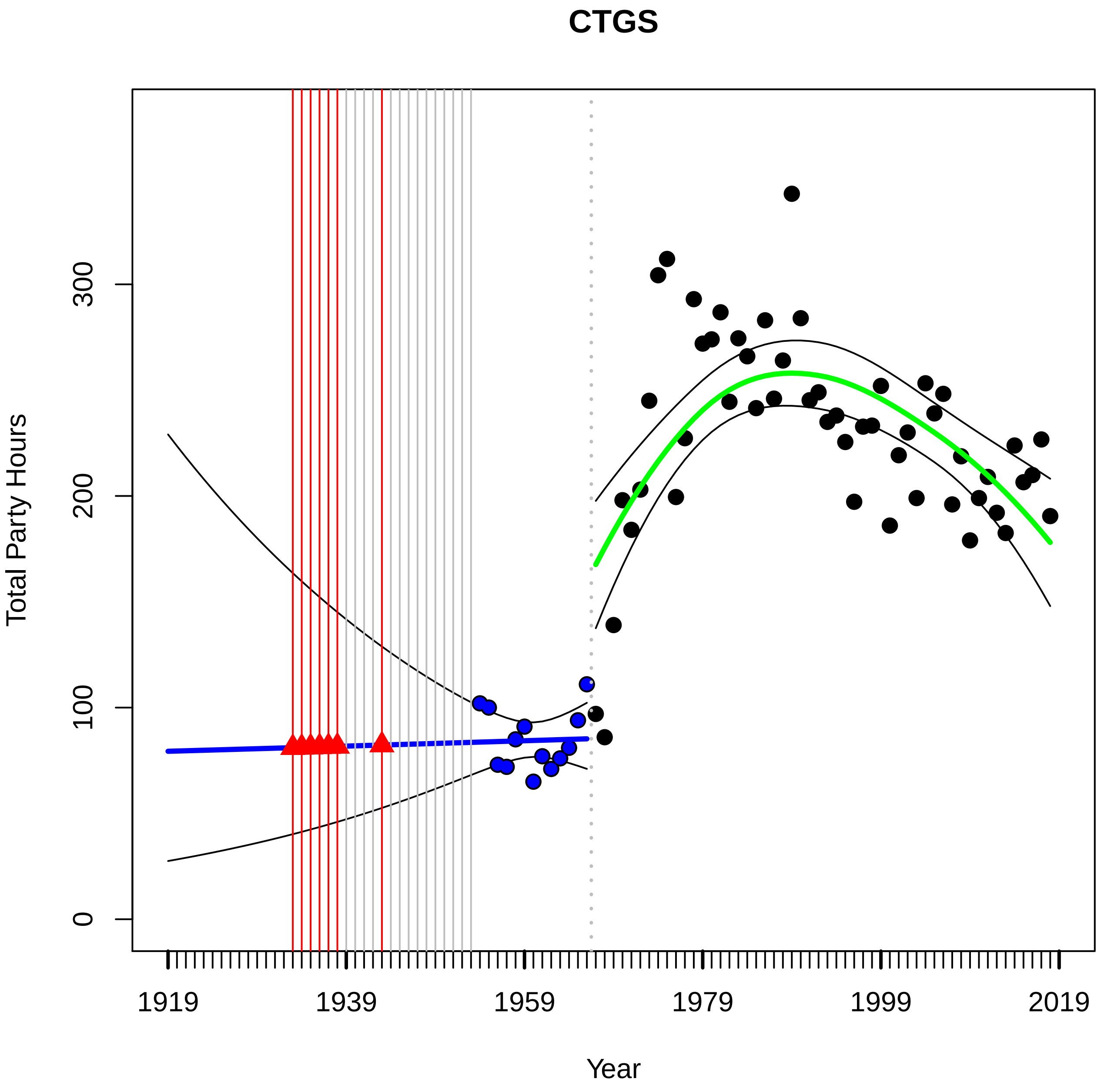

CTGS
Total Party Hours
300
200
100
0
1919
1939
1959
1979
1999
2019
Year

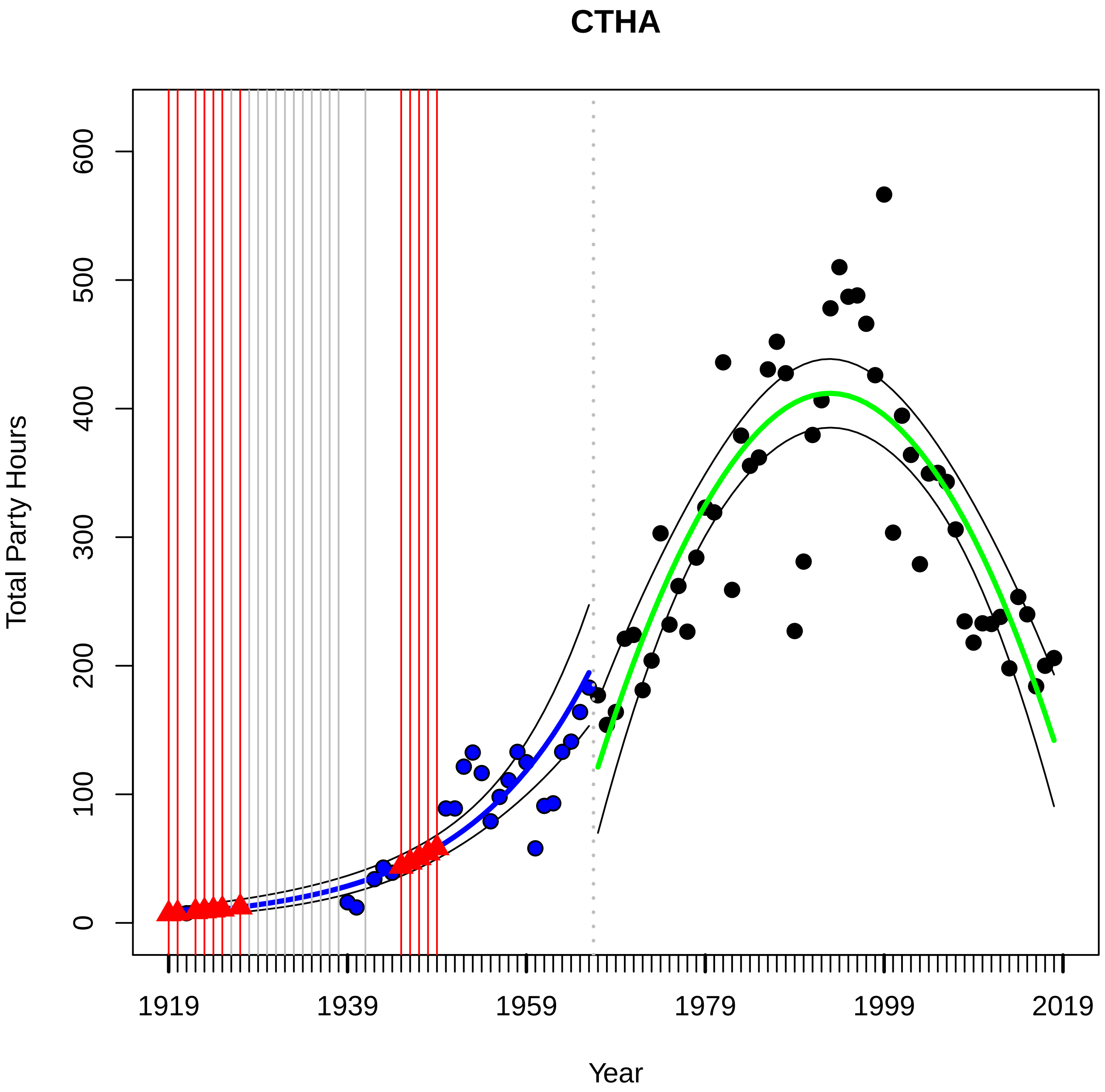

CTHA
Total Party Hours
0
100
200
300
400
500
600
1919
1939
1959
1979
1999
2019
Year

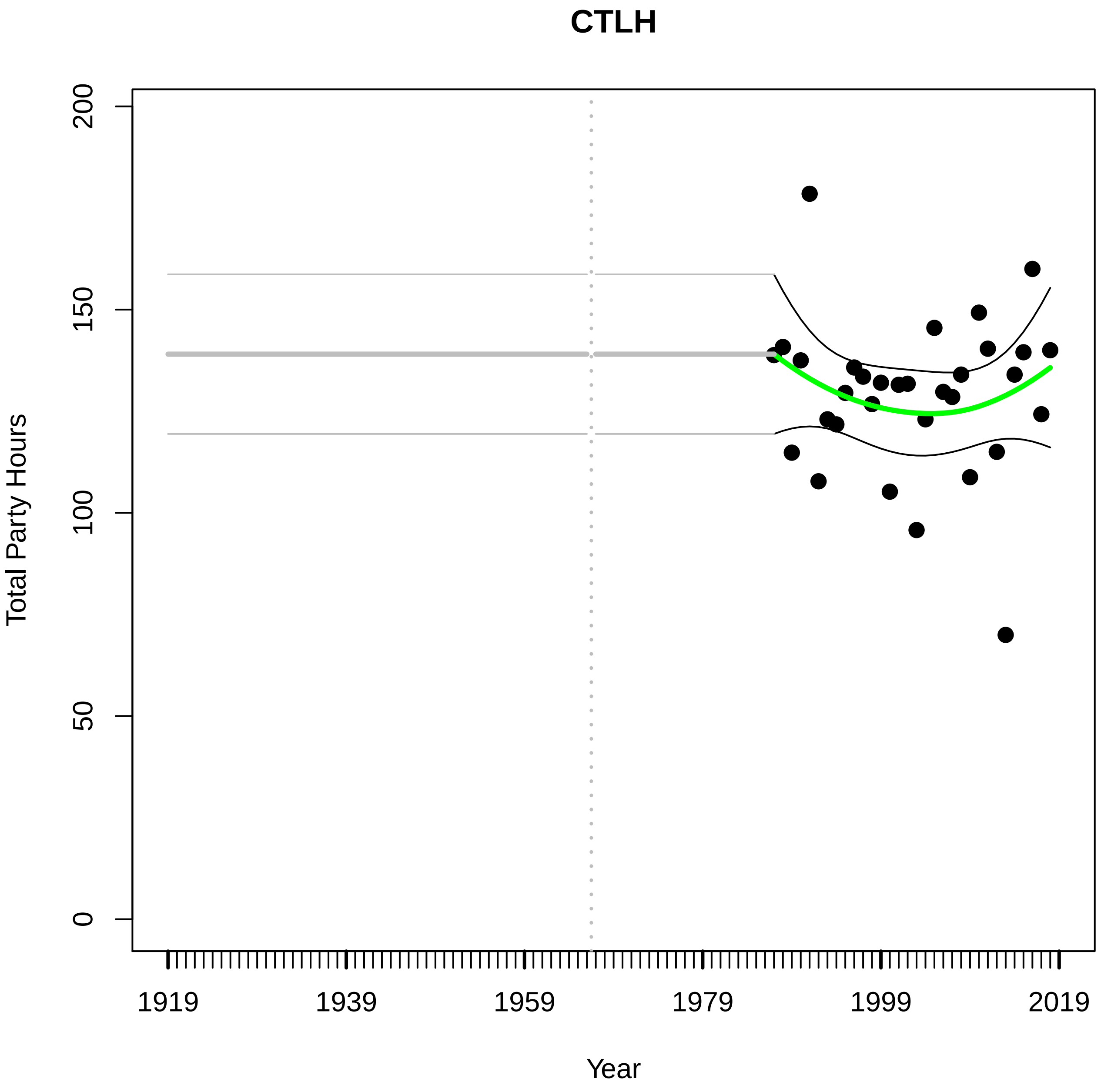

CTLH
Total Party Hours
0
50
100
150
200
Year
1919
1939
1959
1979
1999
2019

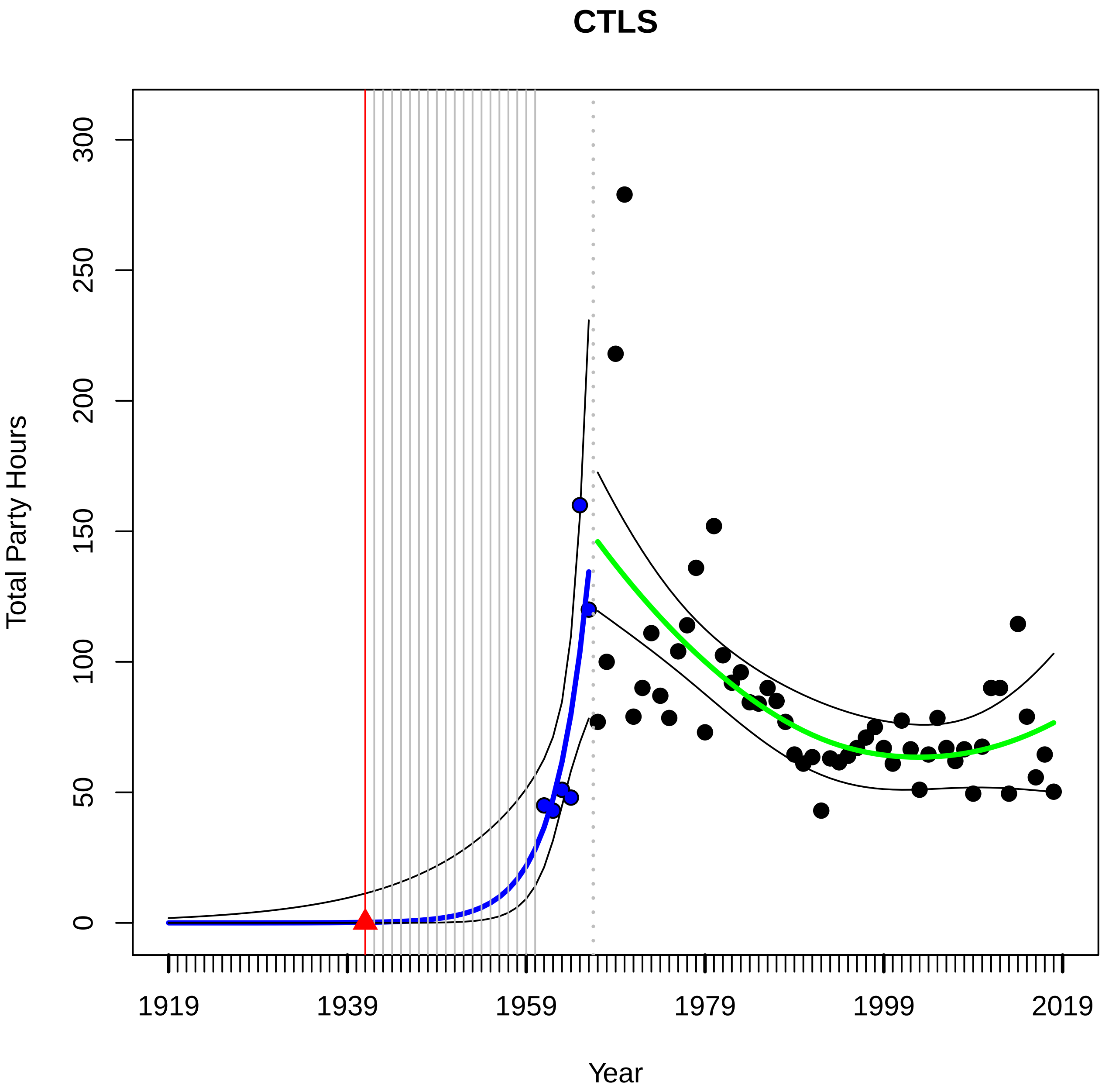

CTLS
Total Party Hours
0
50
100
150
200
250
300
1919
1939
1959
1979
1999
2019
Year

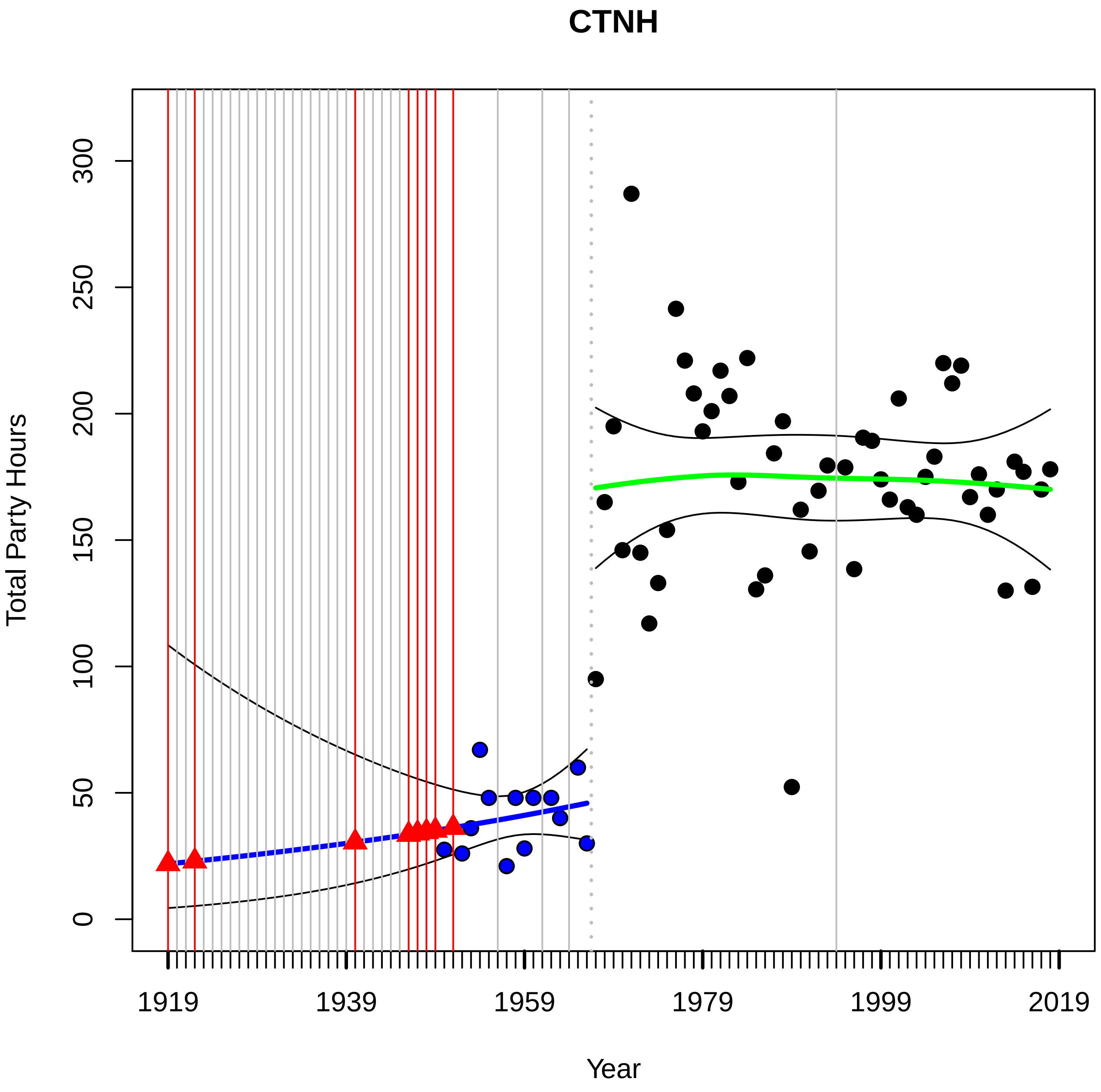

CTNH
Total Party Hours
0
50
100
150
200
250
300
1919
1939
1959
1979
1999
2019
Year

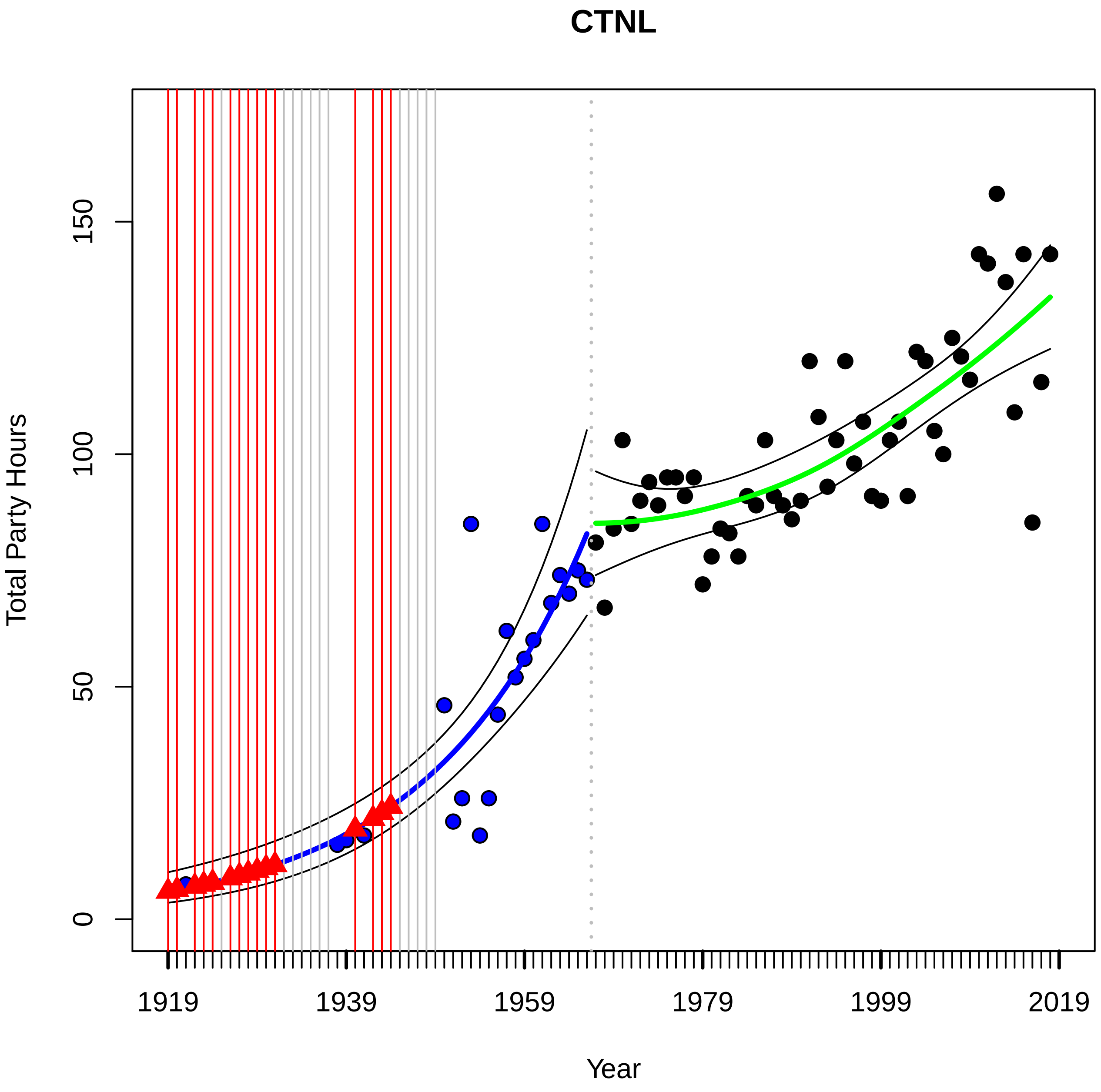

CTNL
Total Party Hours
0
50
100
150
1919
1939
1959
1979
1999
2019
Year

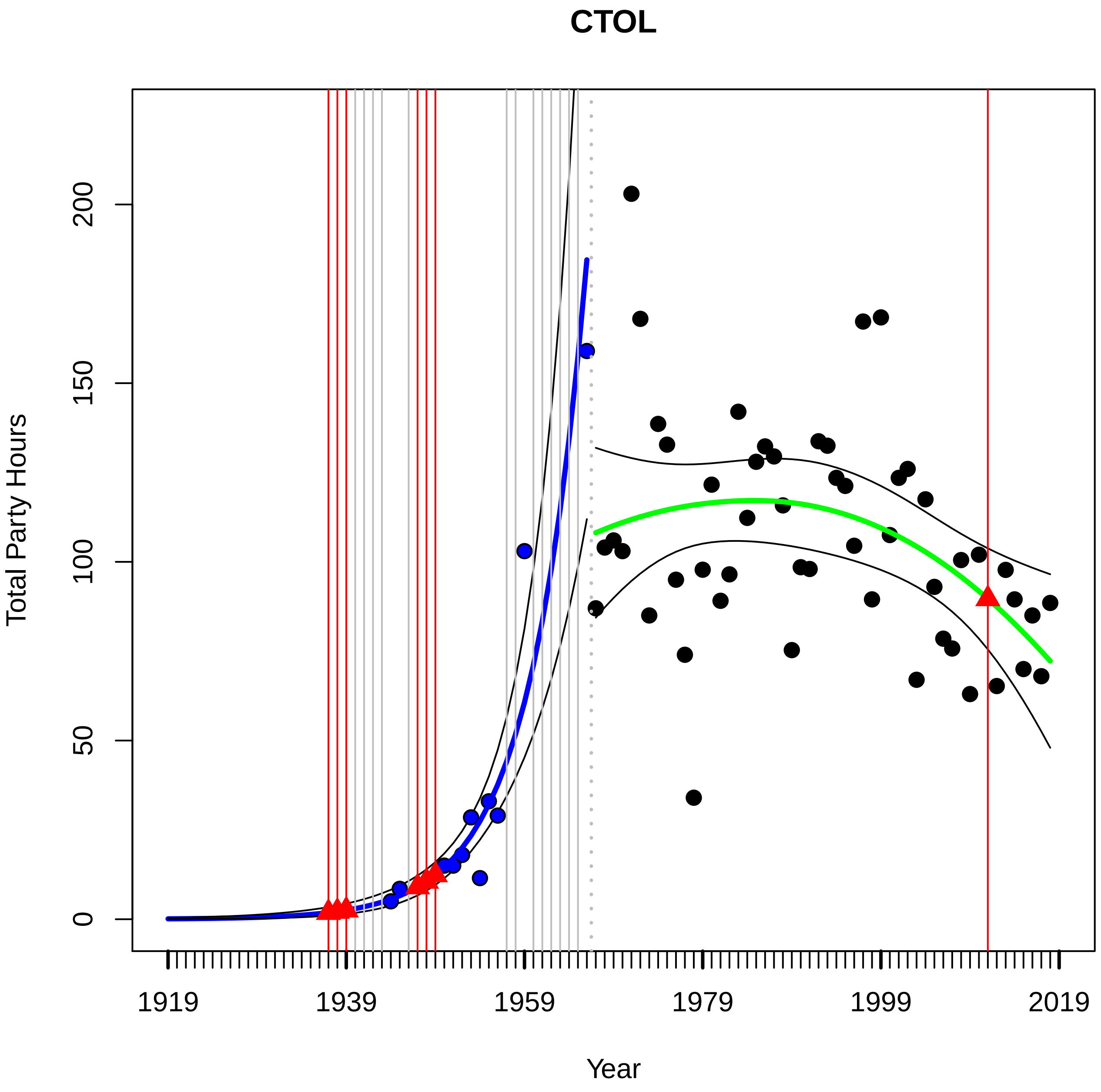

CTOL
Total Party Hours
0
50
100
150
200
1919
1939
1959
1979
1999
2019
Year

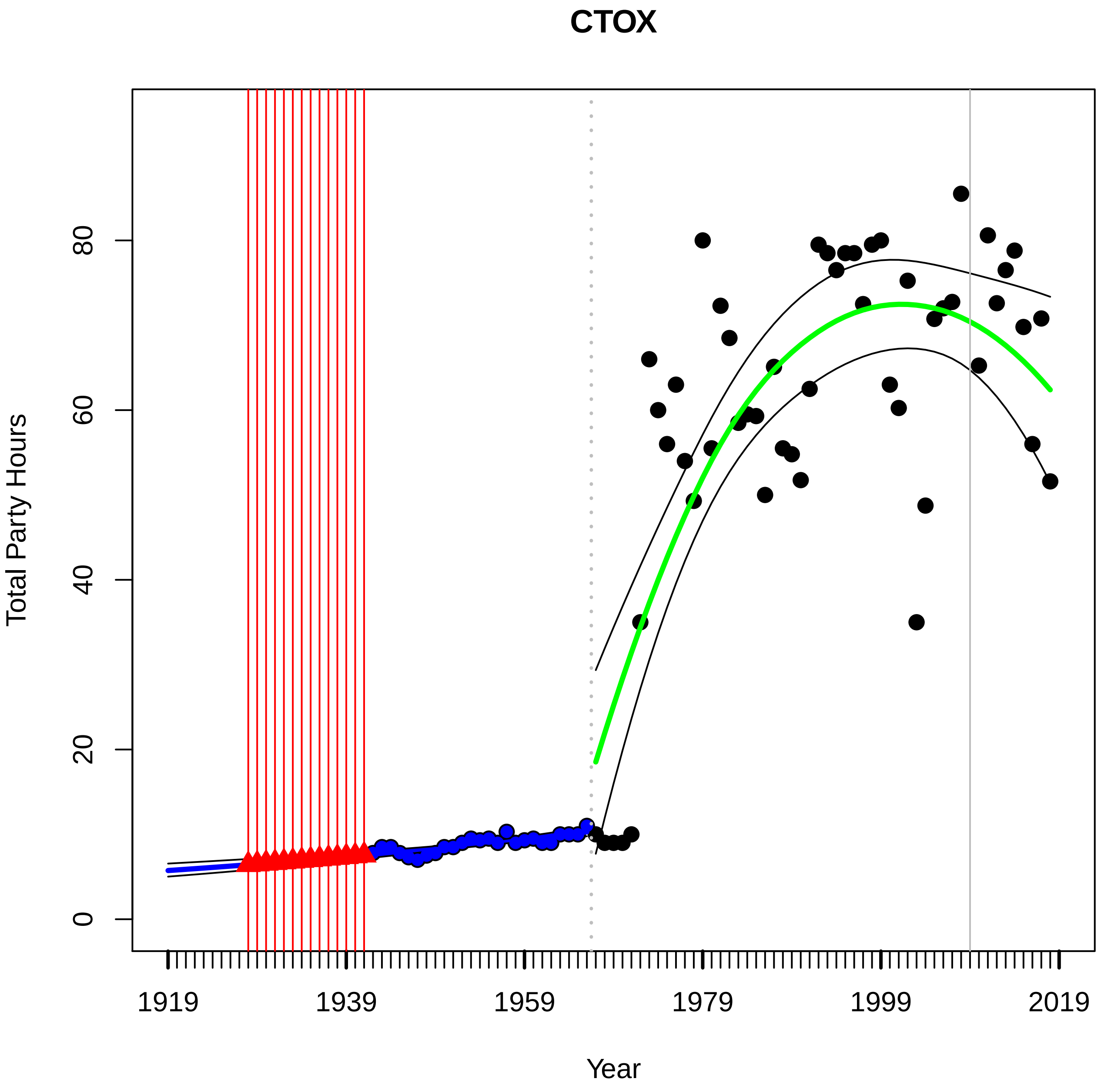
CTOX
Total Party Hours
Year
0
20
40
60
80
1919
1939
1959
1979
1999
2019

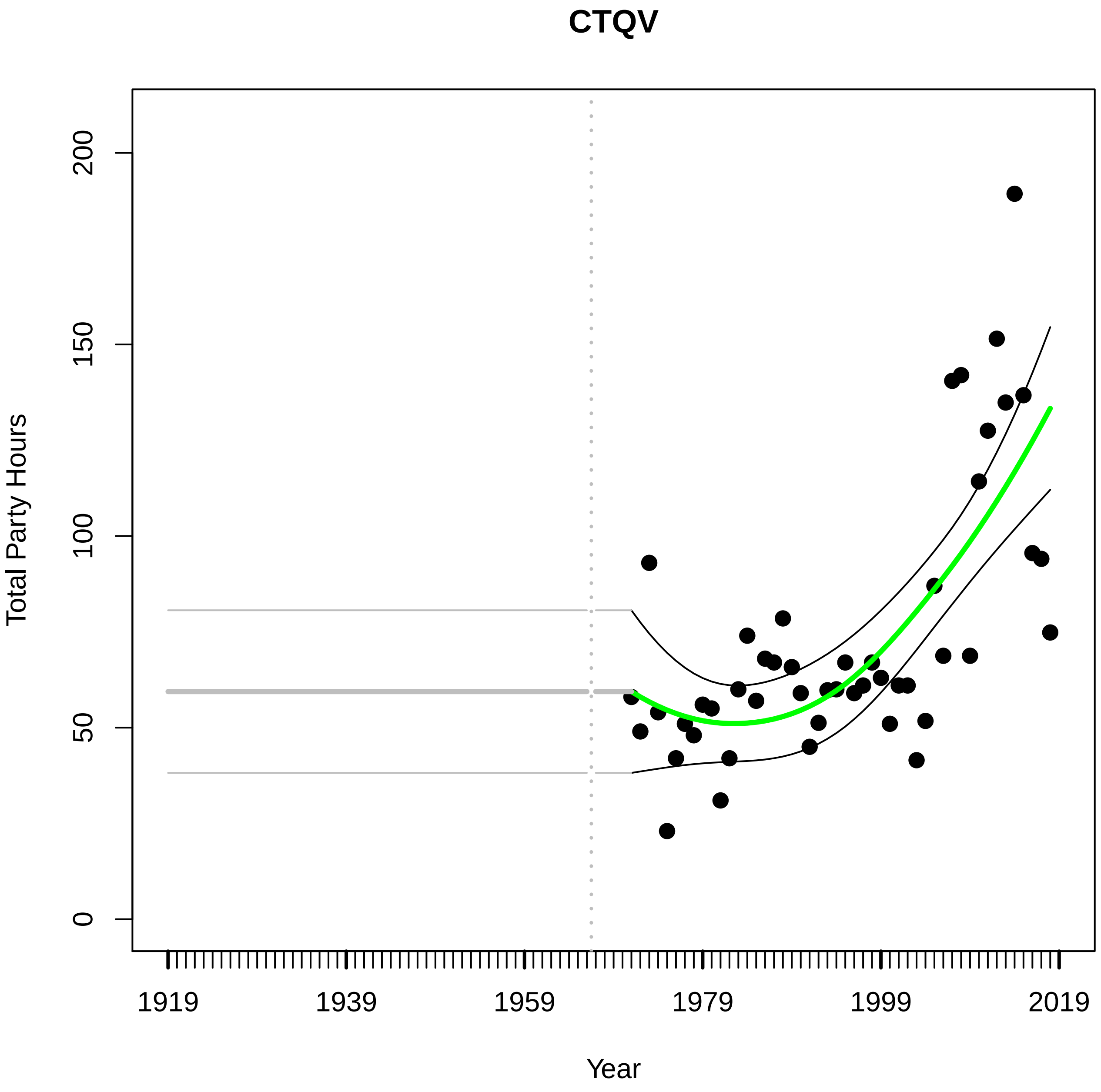

CTQV
Total Party Hours
0
50
100
150
200
1919
1939
1959
1979
1999
2019
Year

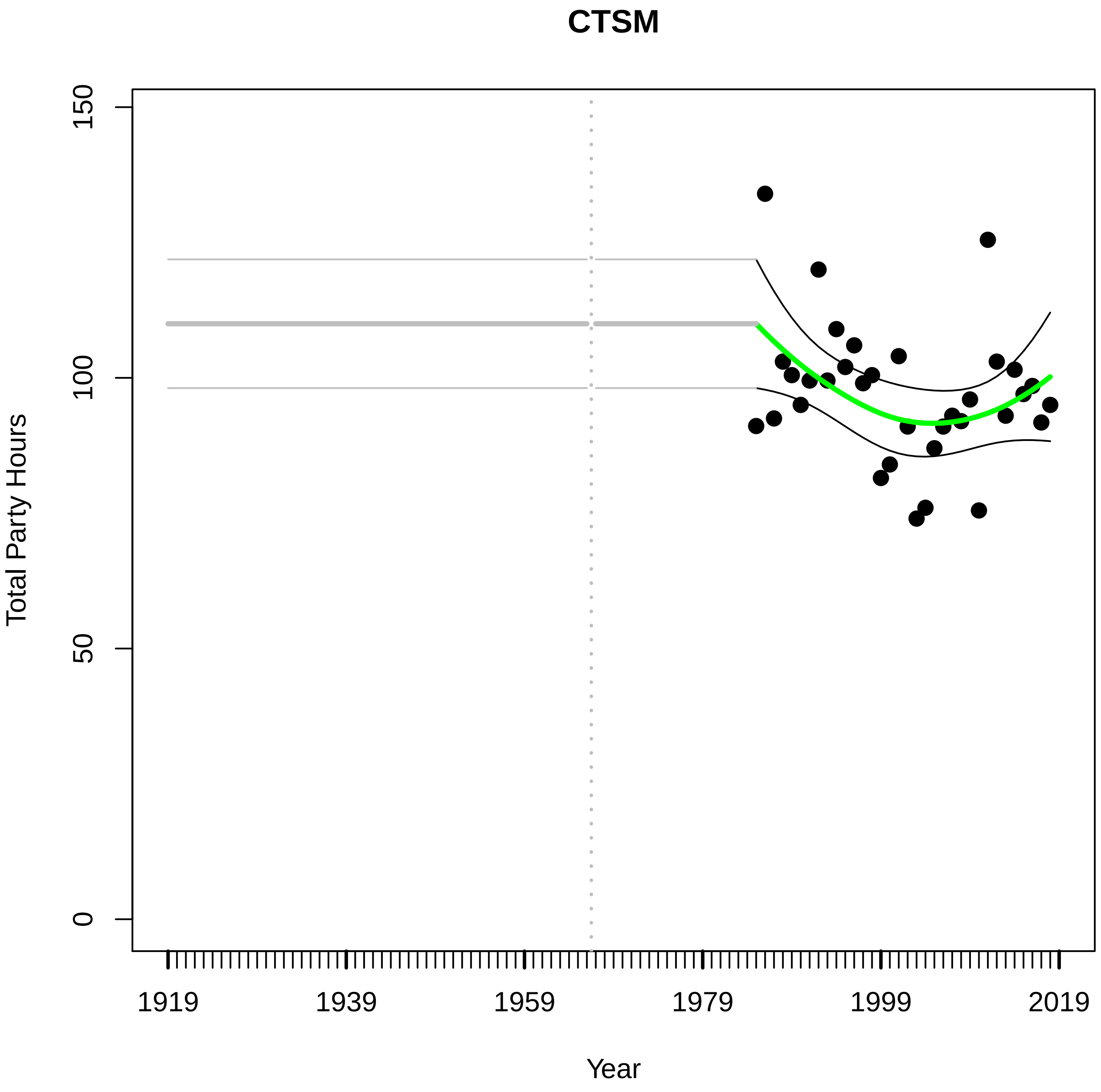
CTSM
Total Party Hours
150
100
50
0
1919
1939
1959
1979
1999
2019
Year

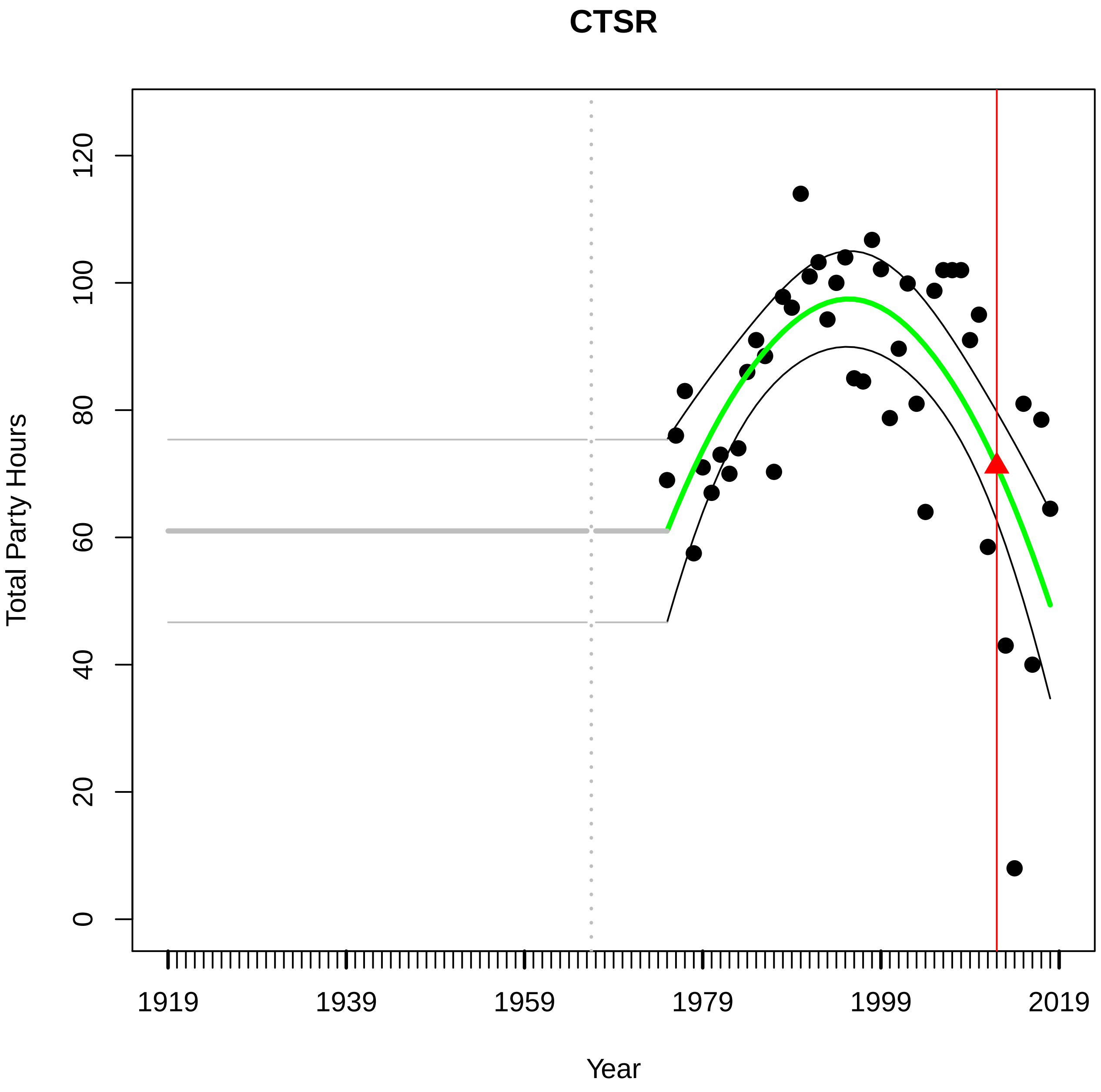
CTSR
Total Party Hours
0
20
40
60
80
100
120
1919
1939
1959
1979
1999
2019
Year

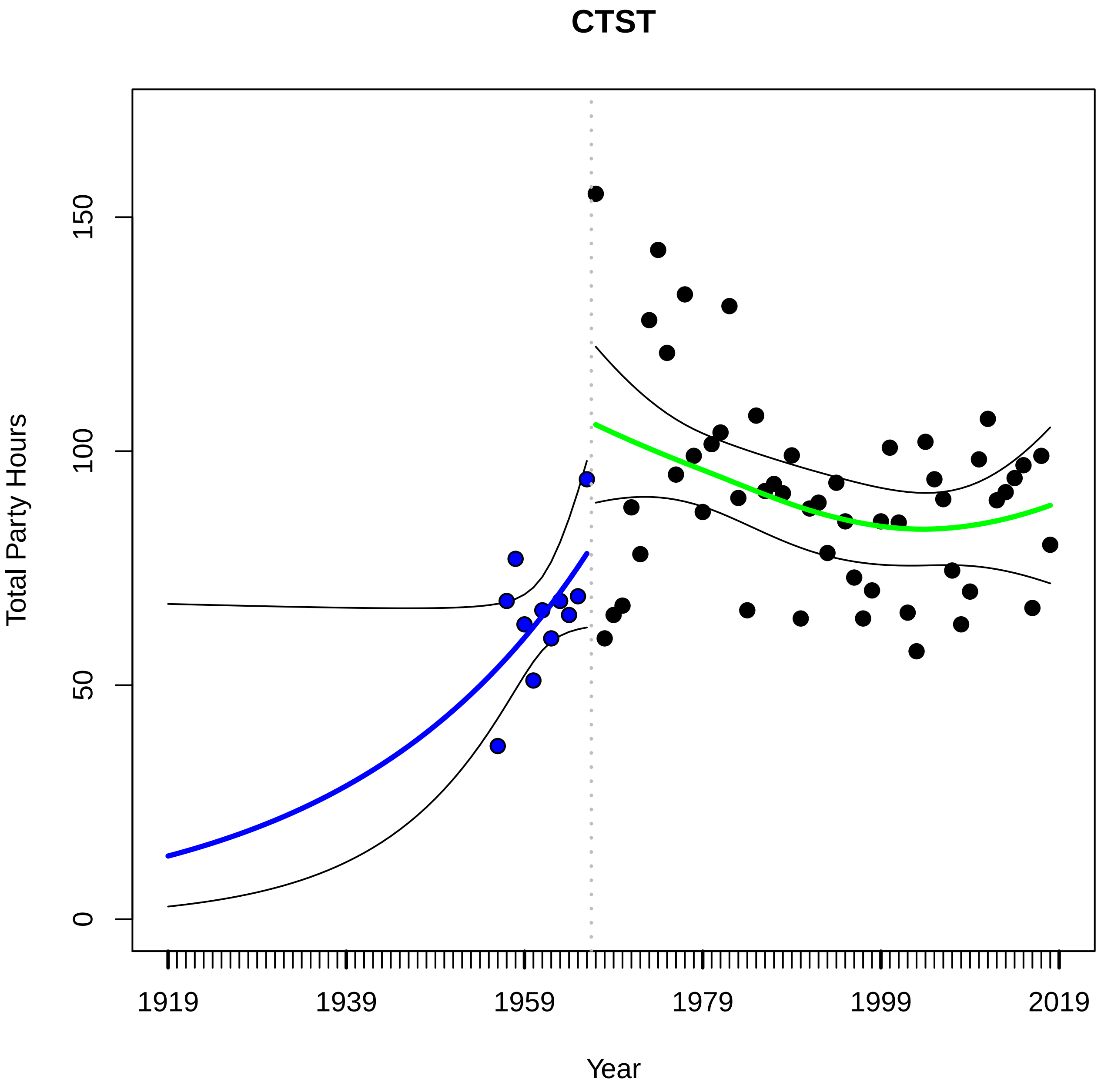

CTST
Total Party Hours
0
50
100
150
1919
1939
1959
1979
1999
2019
Year

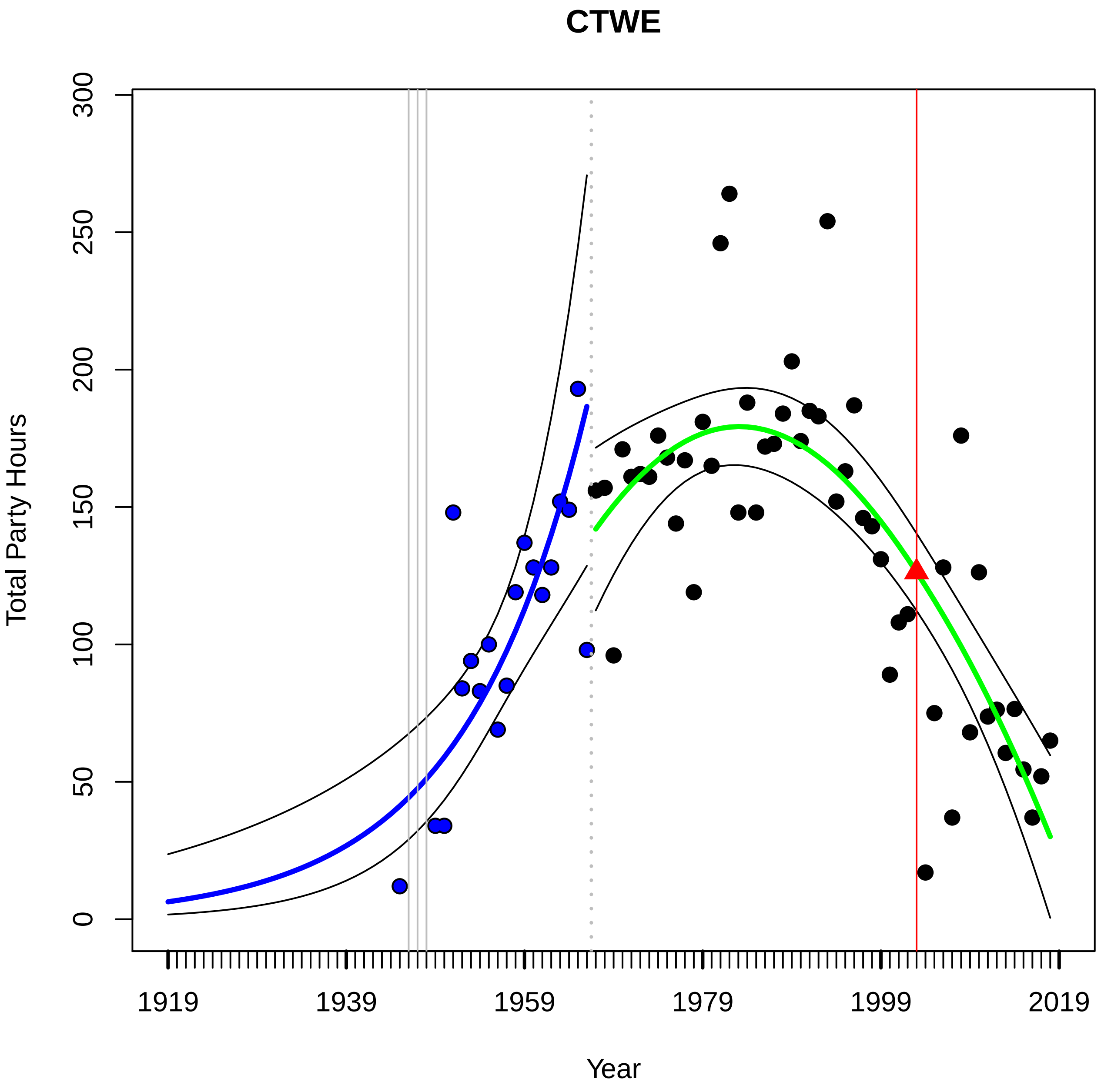

CTWE
Total Party Hours
0
50
100
150
200
250
300
1919
1939
1959
1979
1999
2019
Year

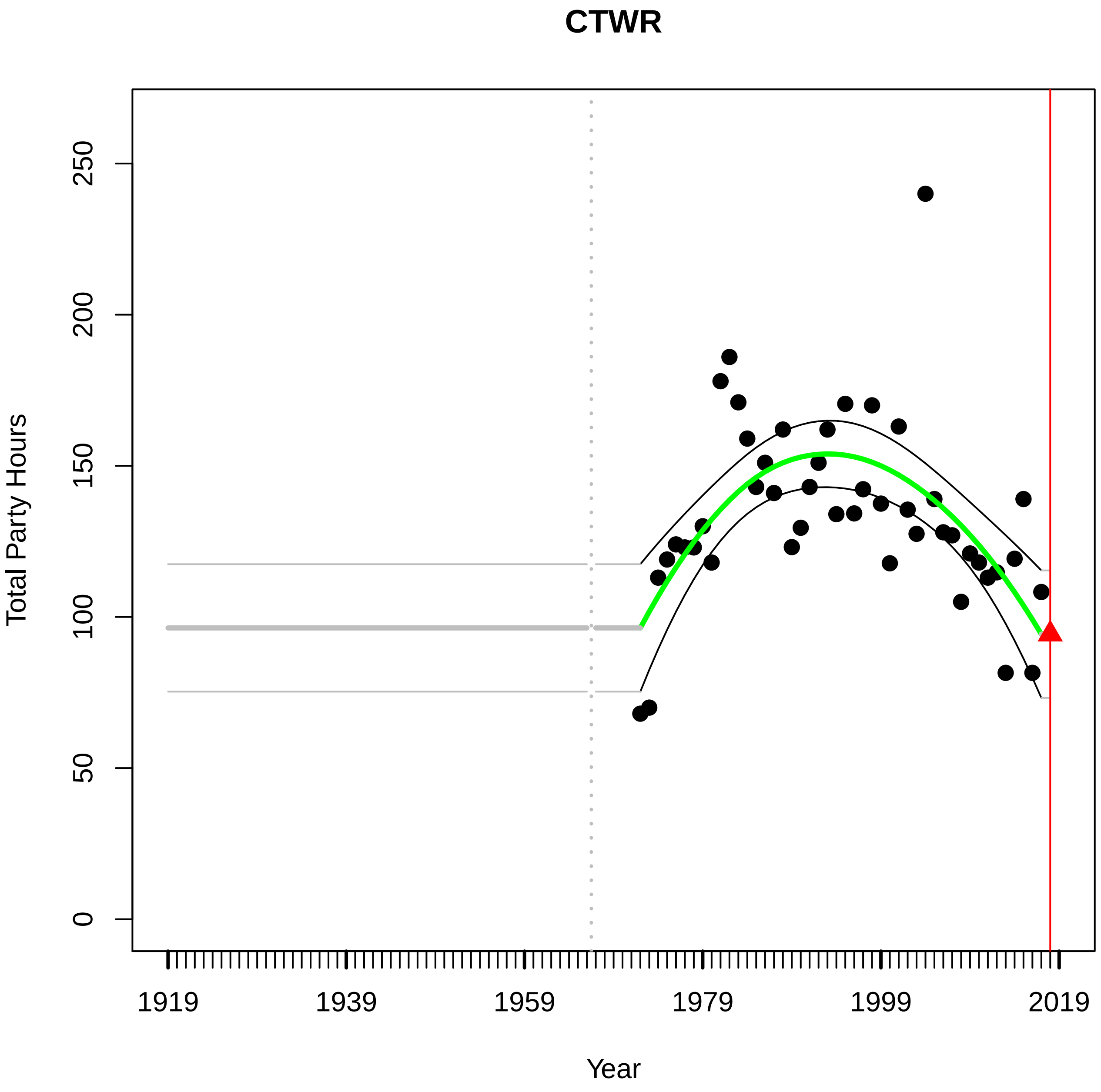

CTWR
Total Party Hours
250
200
150
100
50
0
1919
1939
1959
1979
1999
2019
Year

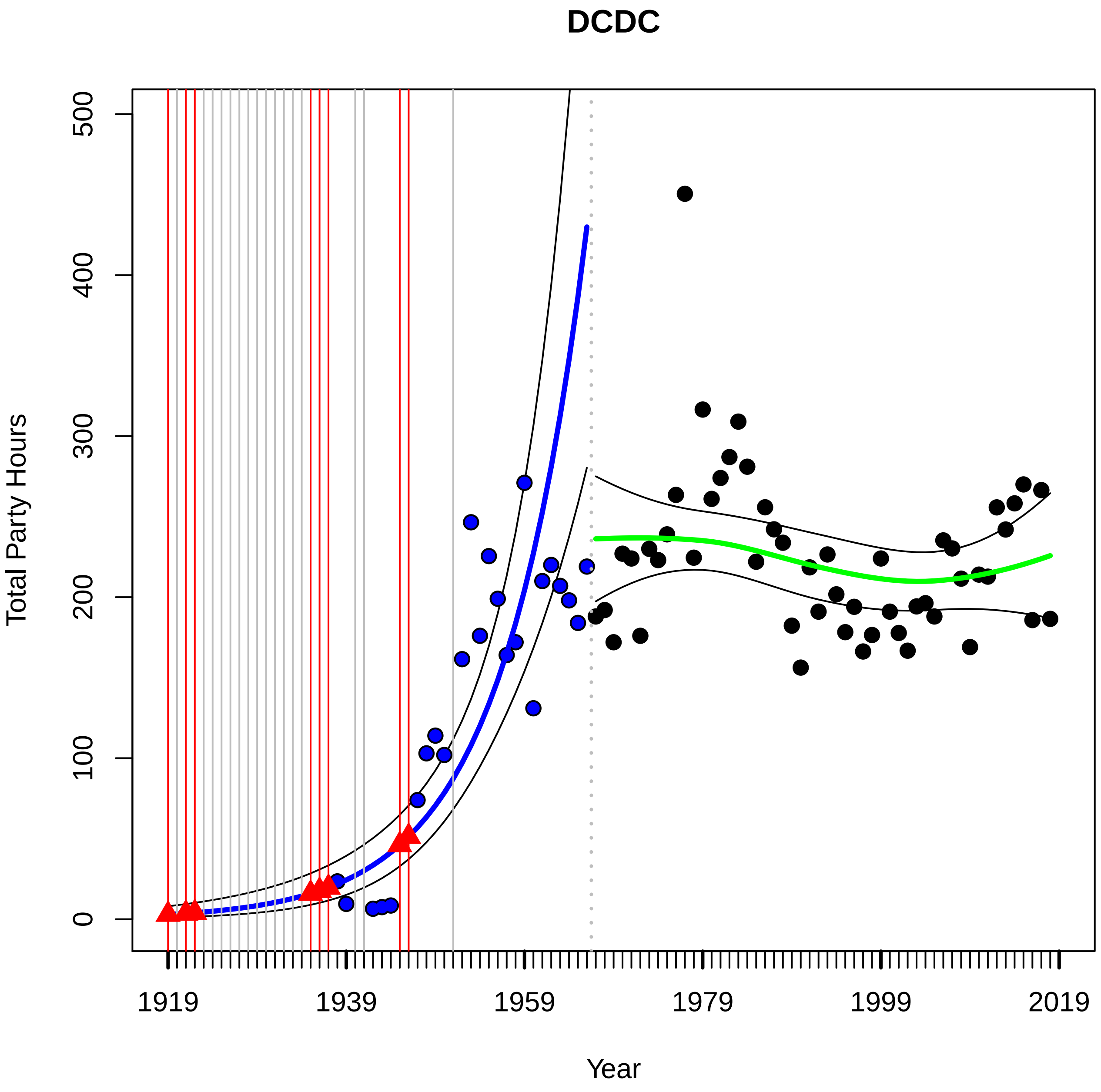

DCDC
Total Party Hours
500
400
300
200
100
0
1919
1939
1959
1979
1999
2019
Year

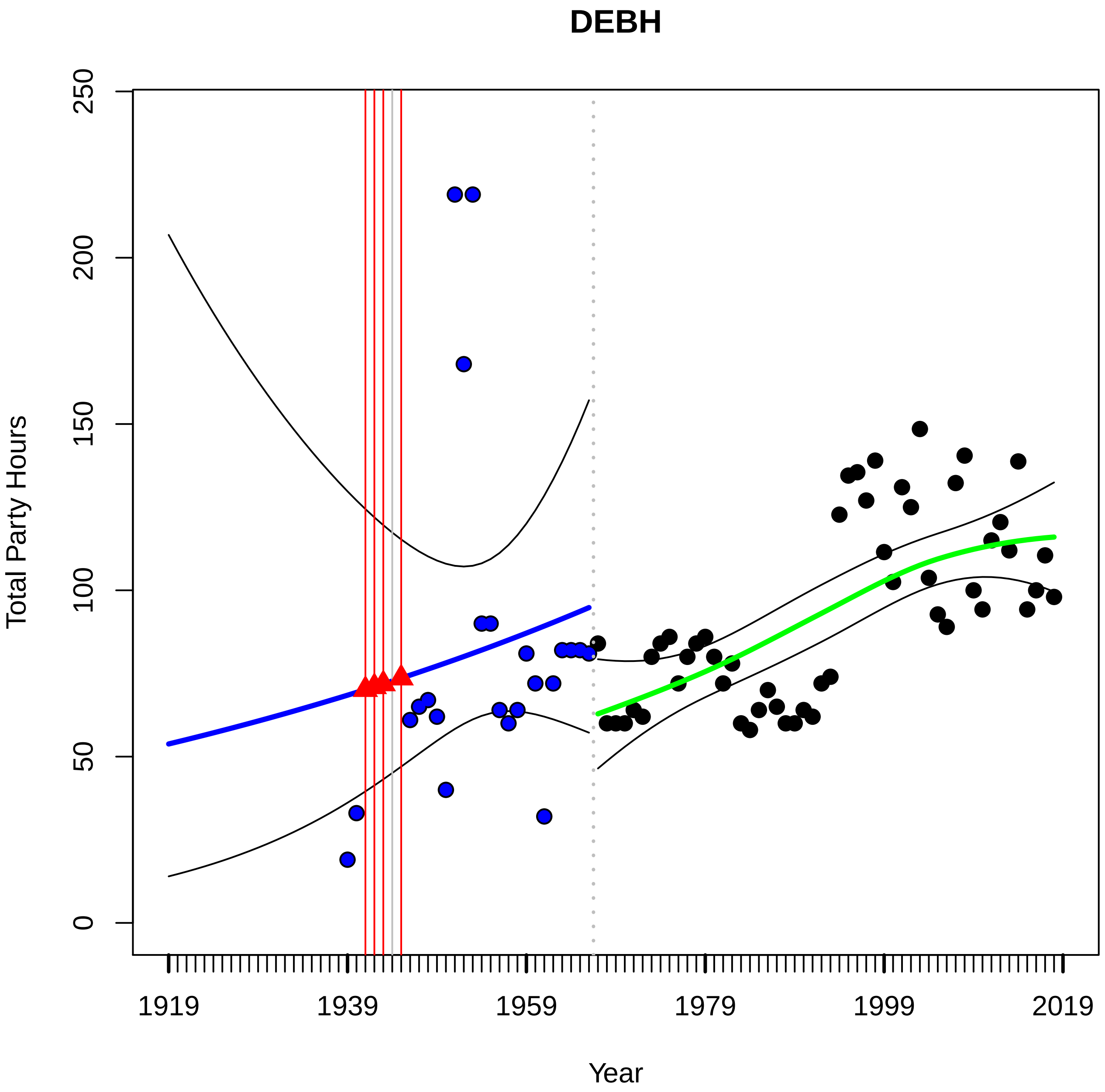

DEBH
Total Party Hours
0
50
100
150
200
250
1919
1939
1959
1979
1999
2019
Year

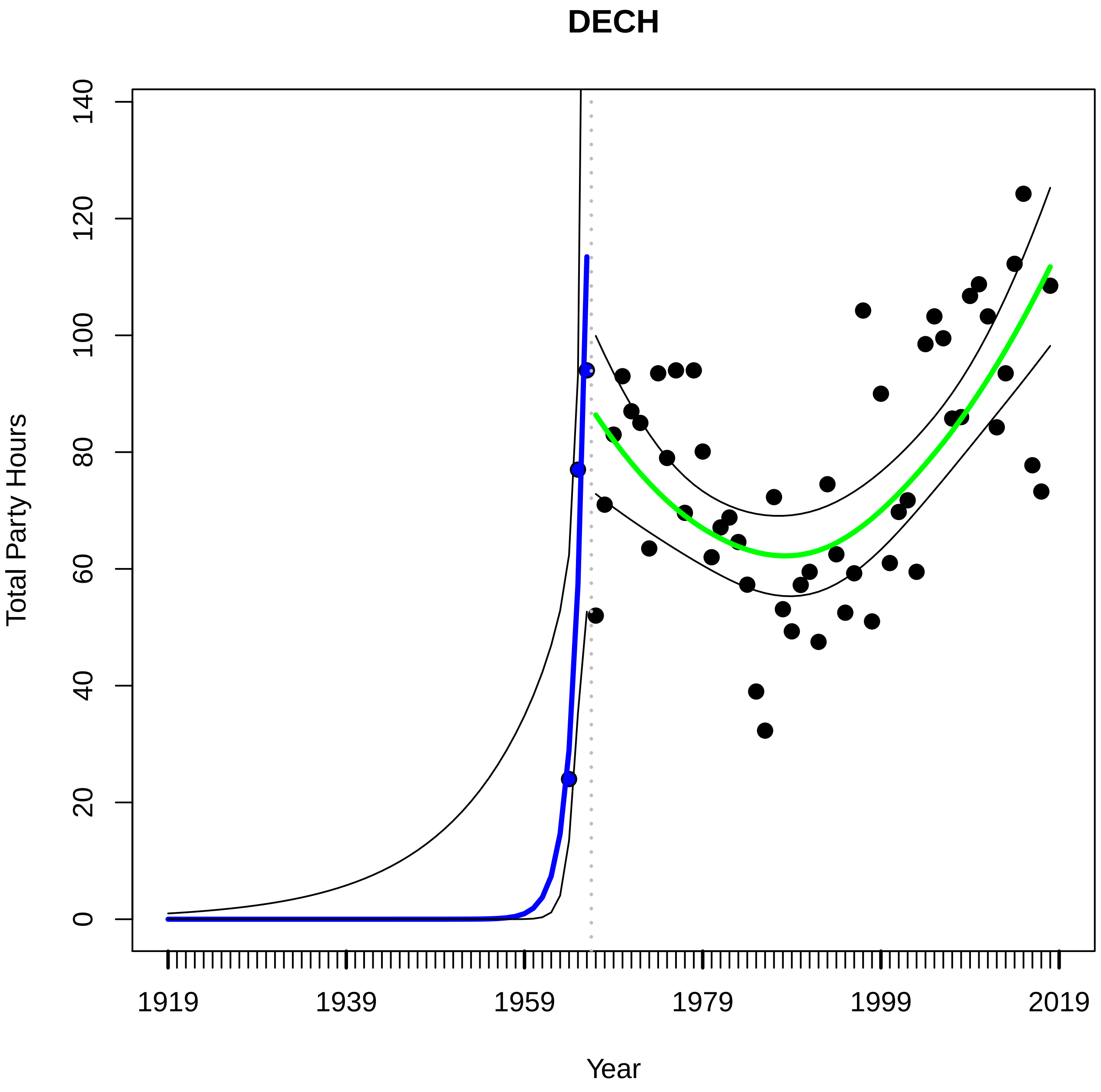

DECH
Total Party Hours
0
20
40
60
80
100
120
140
1919
1939
1959
1979
1999
2019
Year

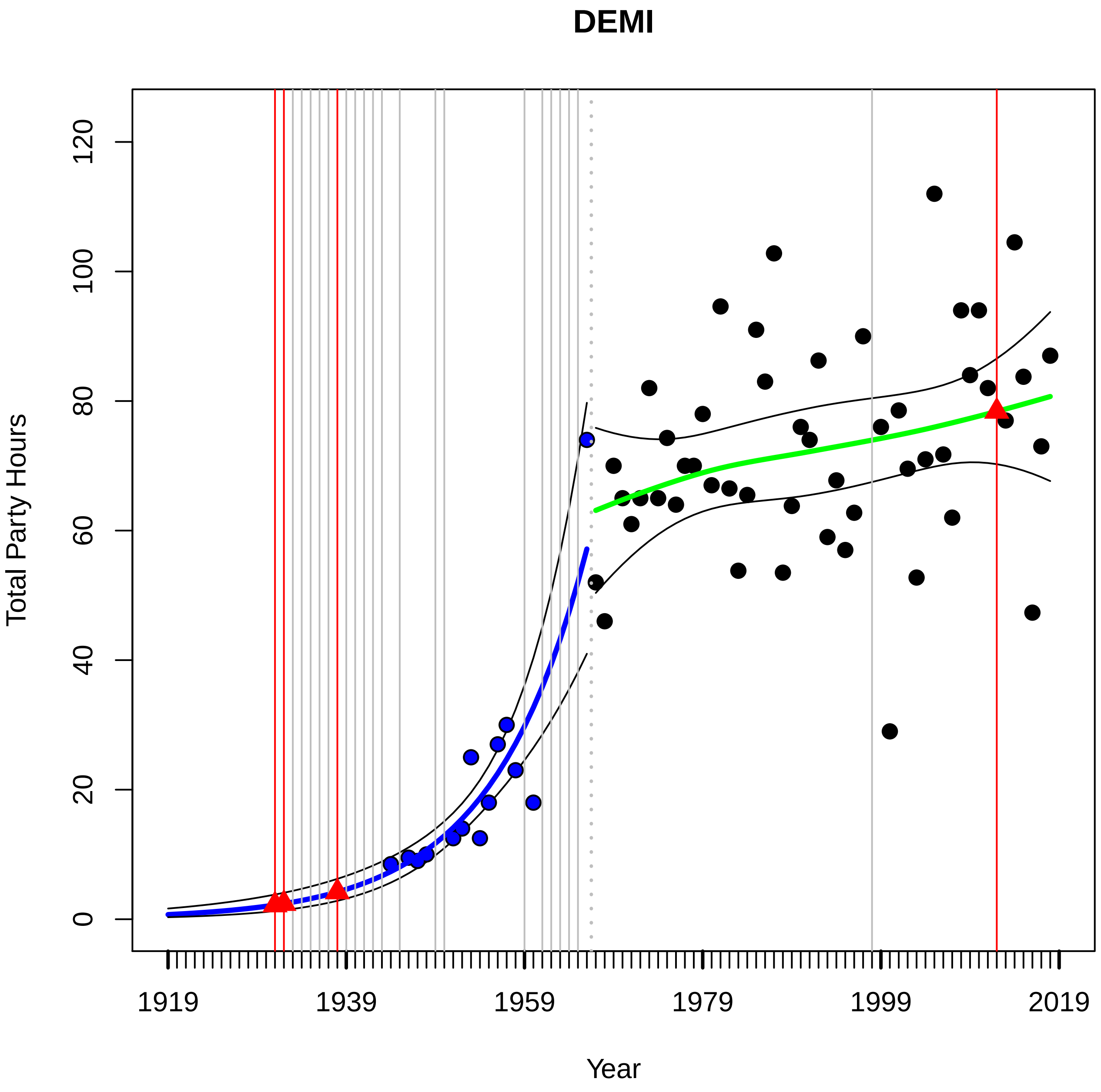

DEMI
Total Party Hours
0
20
40
60
80
100
120
1919
1939
1959
1979
1999
2019
Year

DERE

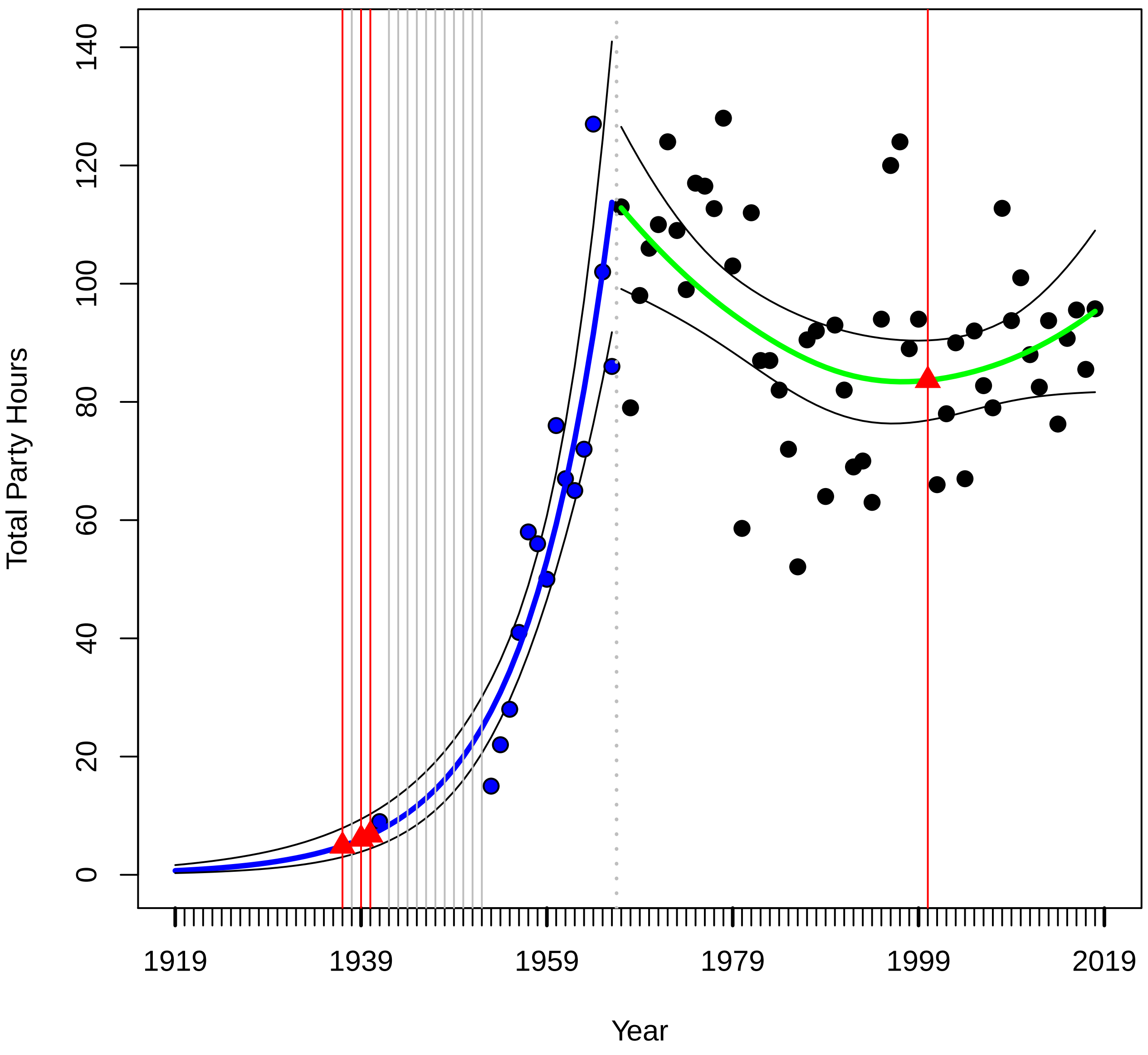

Total Party Hours
0
20
40
60
80
100
120
140
1919
1939
1959
1979
1999
2019
Year

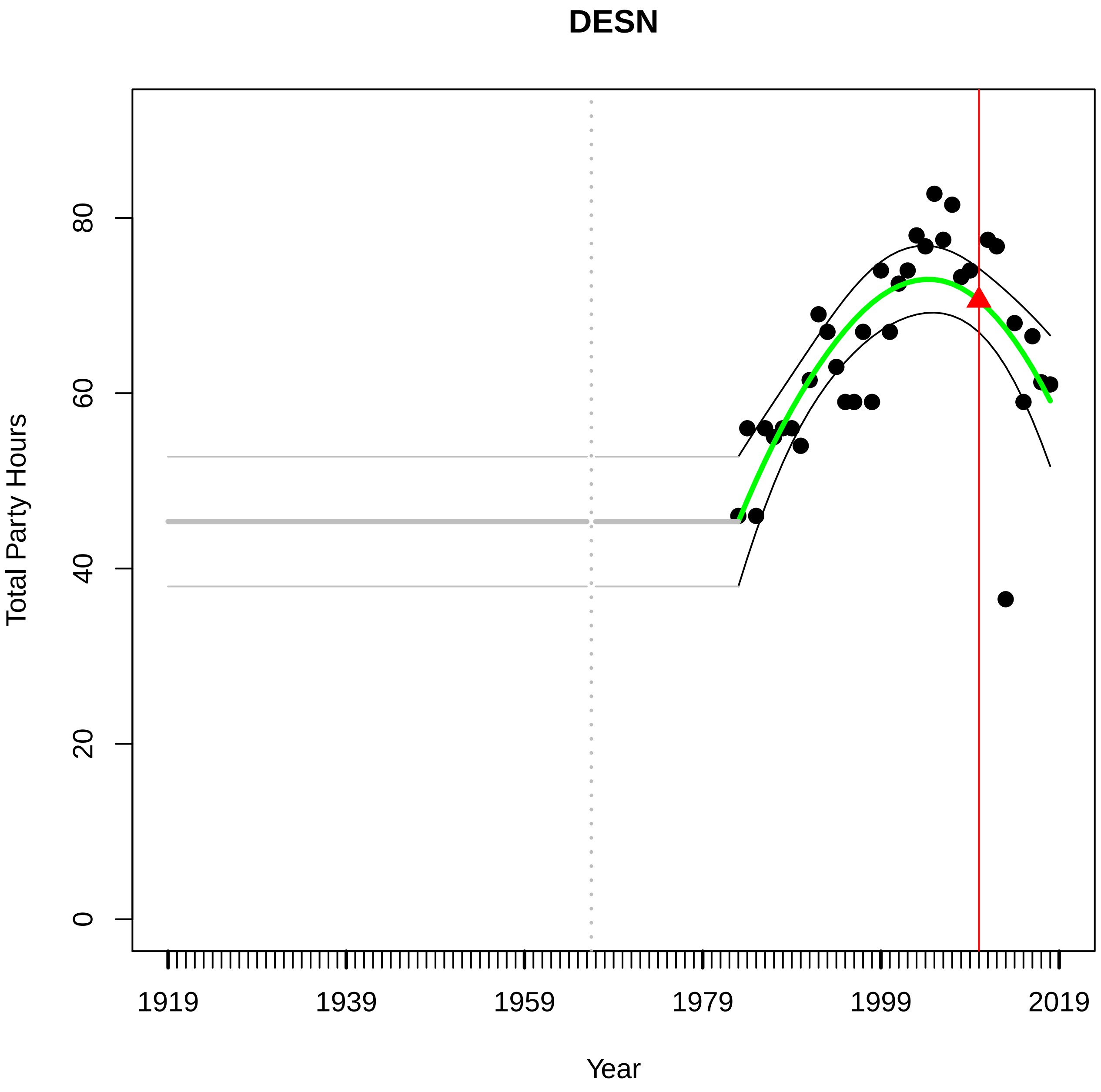

DESN
Total Party Hours
0
20
40
60
80
1919
1939
1959
1979
1999
2019
Year

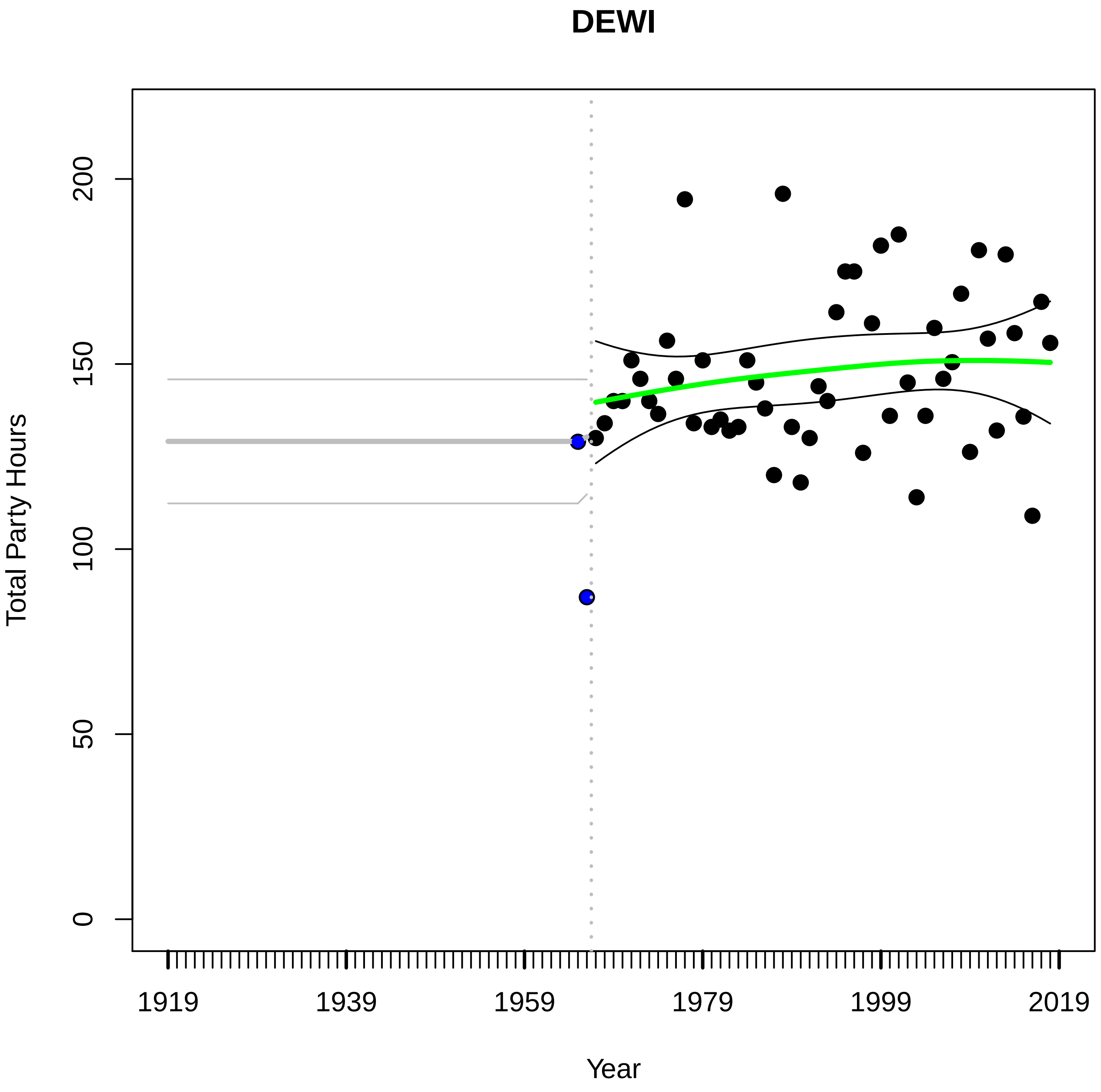
DEWI
Total Party Hours
0
50
100
150
200
1919
1939
1959
1979
1999
2019
Year

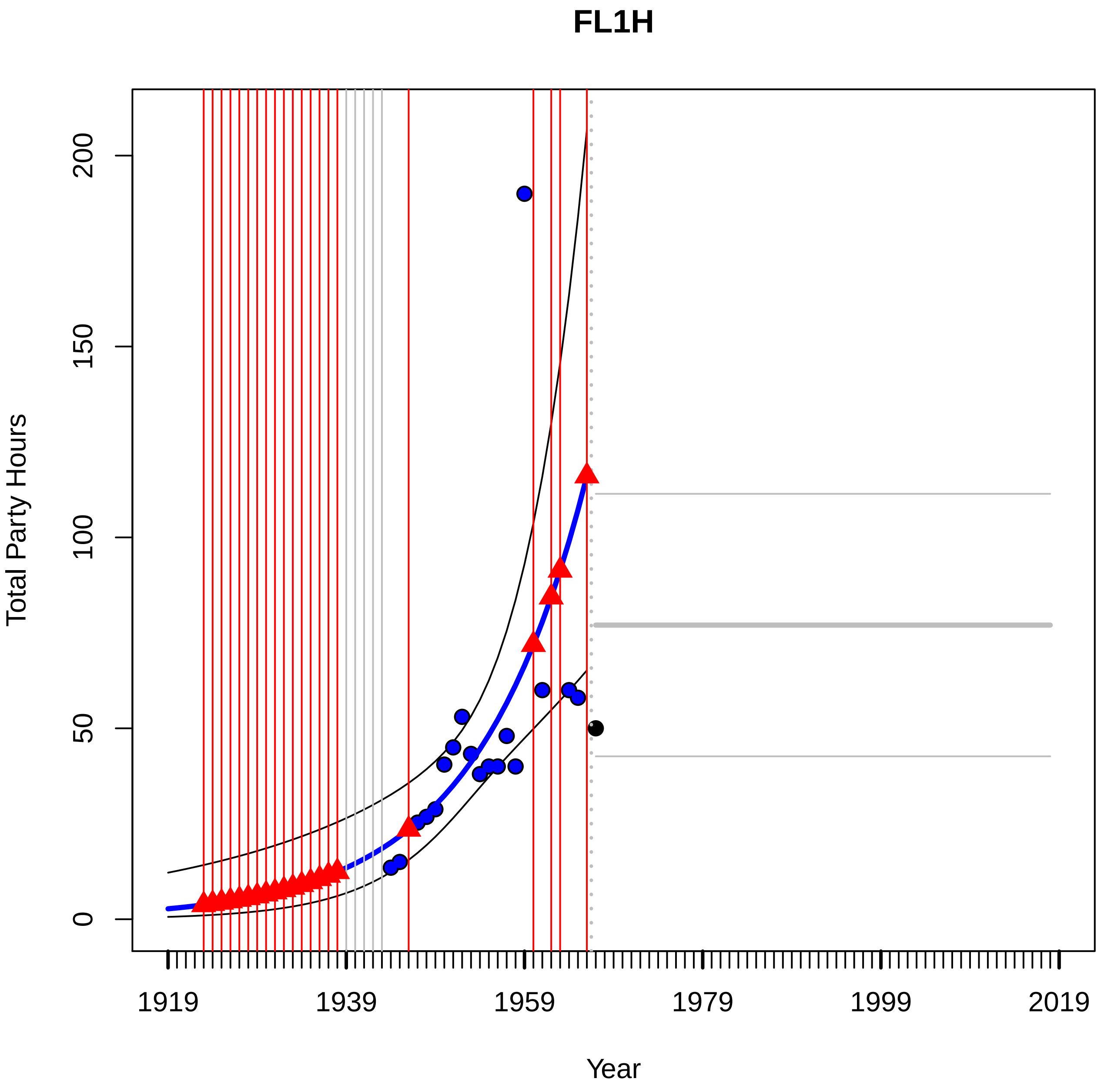

FL1H
Total Party Hours
Year
0
50
100
150
200
1919
1939
1959
1979
1999
2019

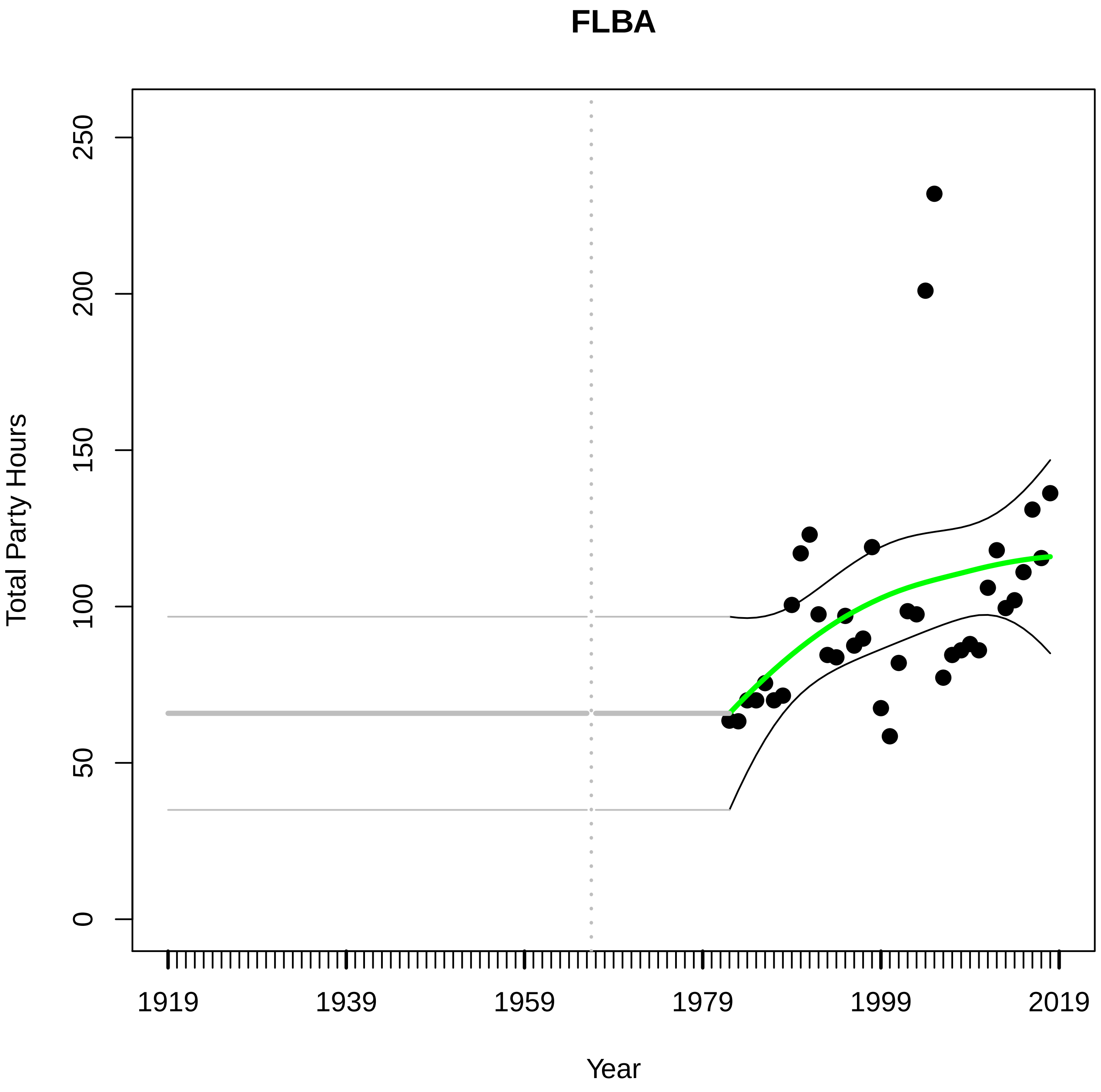

FLBA
Total Party Hours
0
50
100
150
200
250
1919
1939
1959
1979
1999
2019
Year

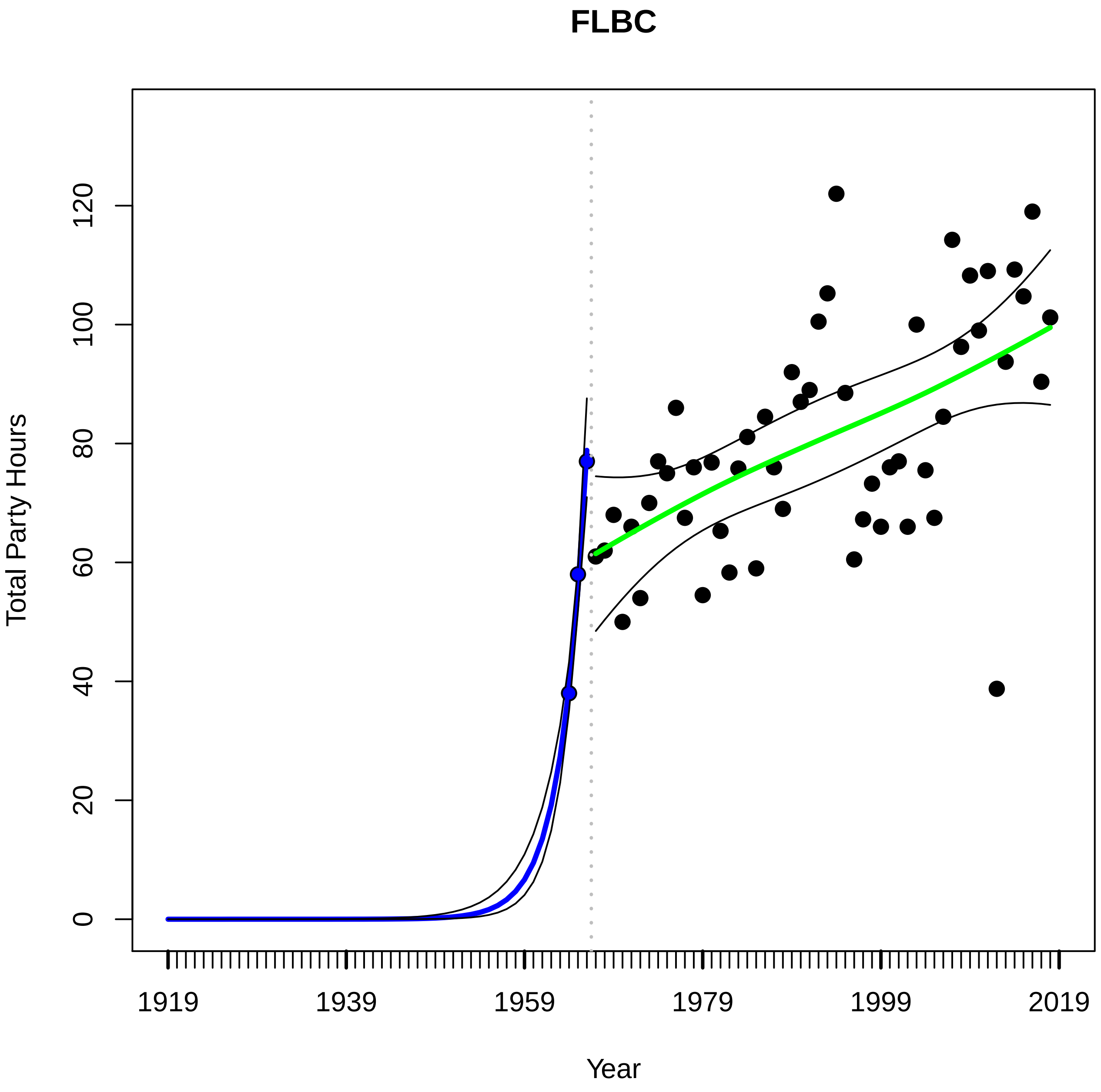

FLBC
Total Party Hours
0
20
40
60
80
100
120
1919
1939
1959
1979
1999
2019
Year

FLBI

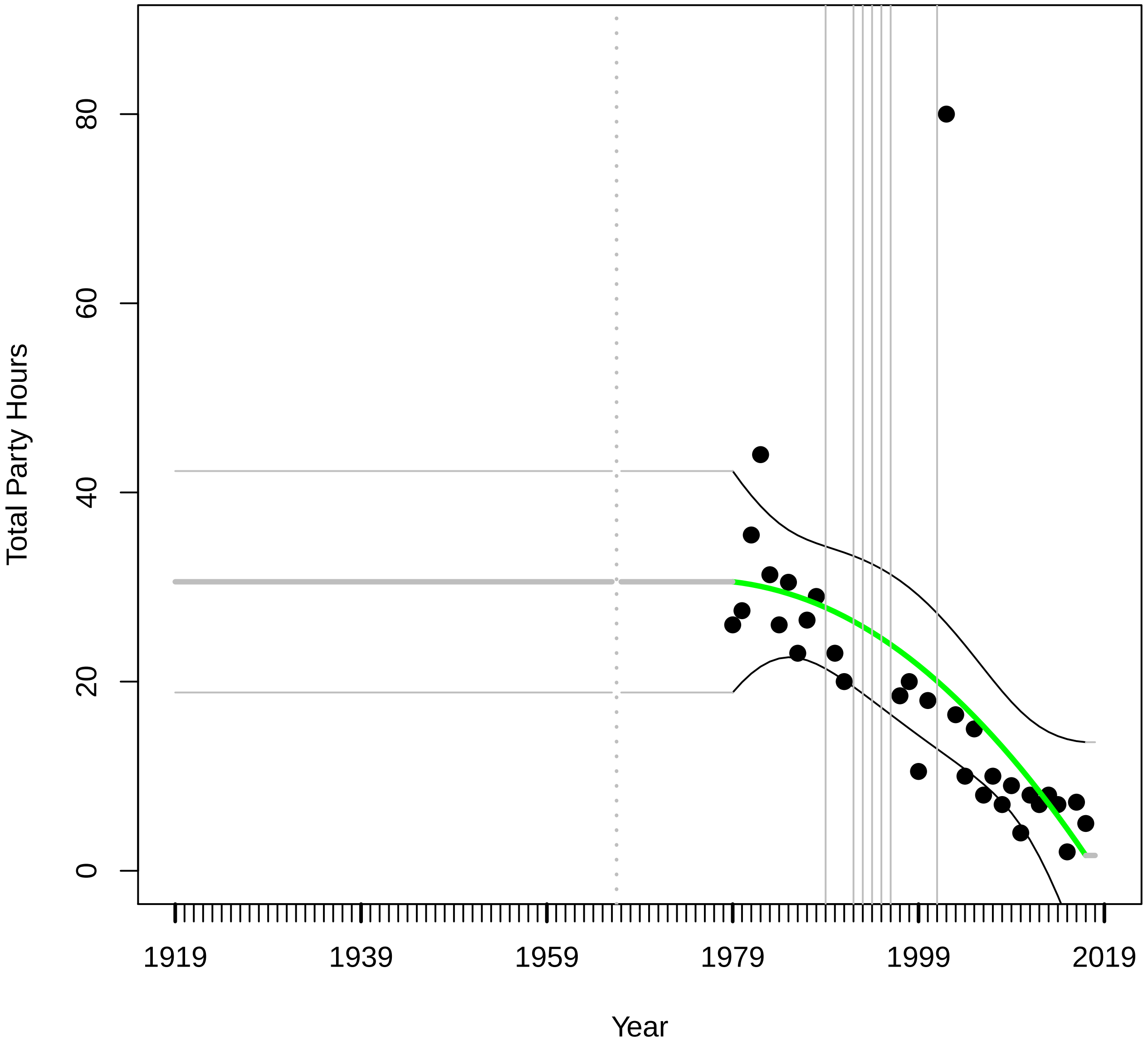

Total Party Hours
0
20
40
60
80
1919
1939
1959
1979
1999
2019
Year

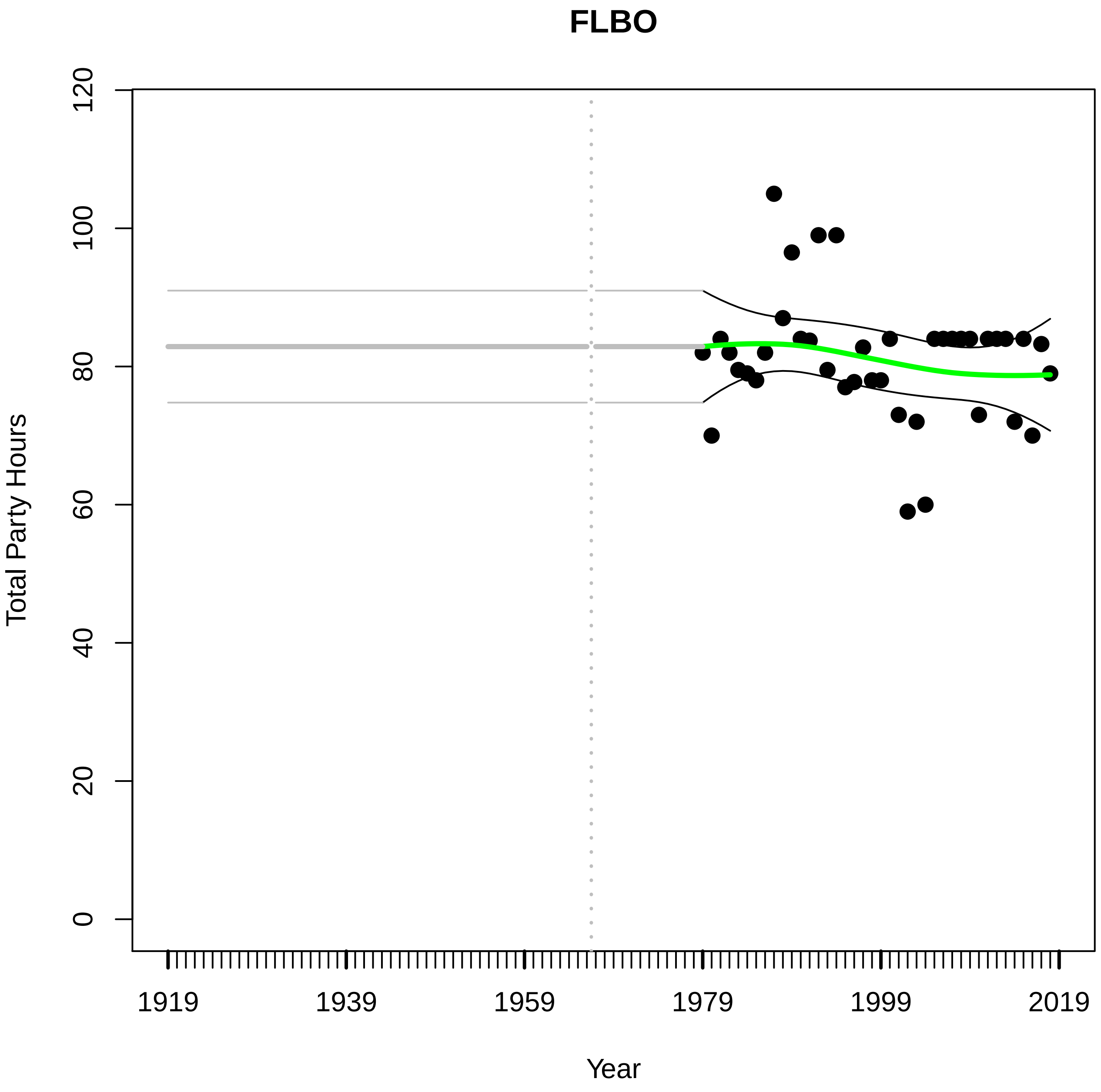
FLBO
Total Party Hours
0
20
40
60
80
100
120
1919
1939
1959
1979
1999
2019
Year

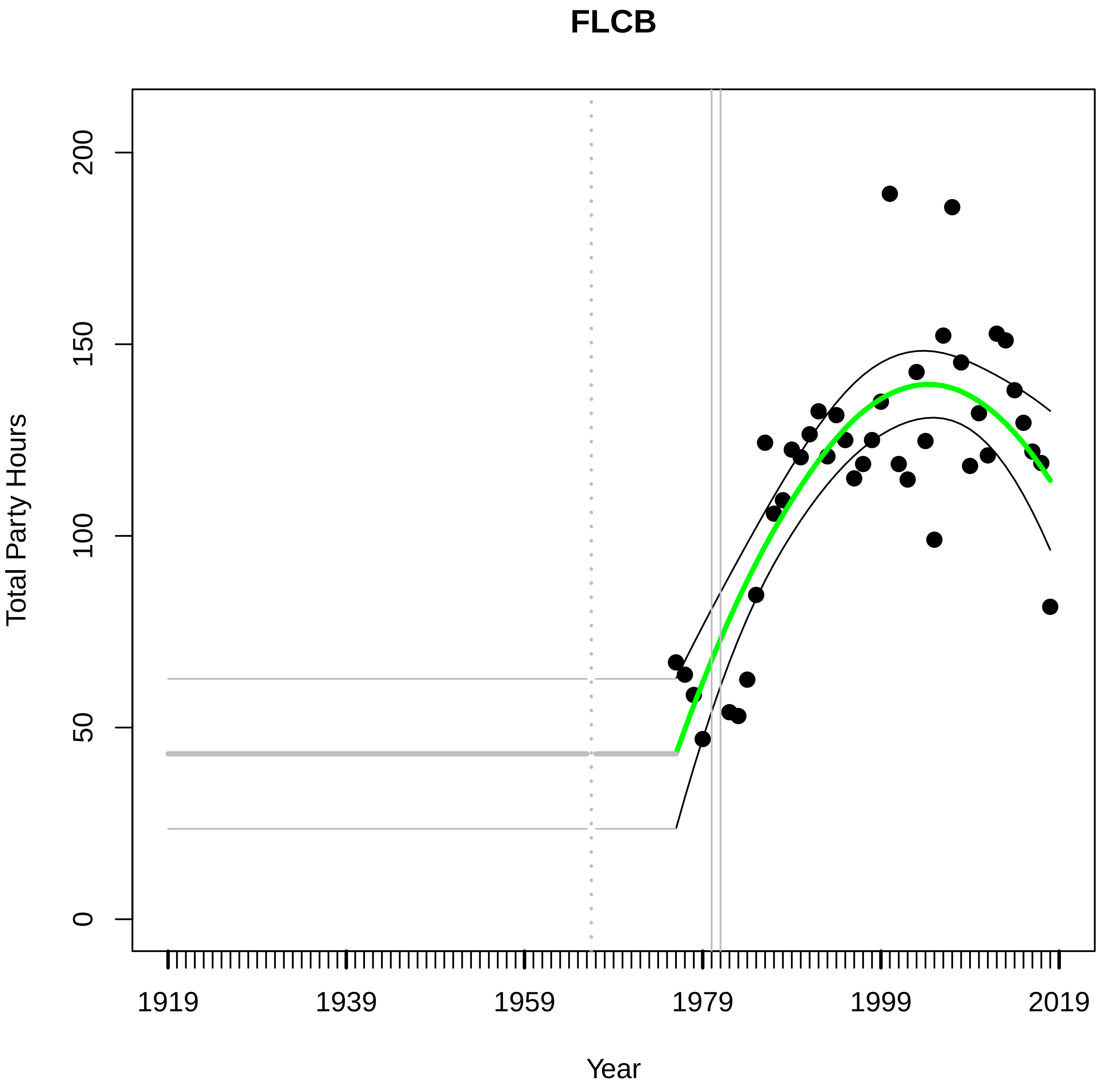

FLCB
Total Party Hours
0
50
100
150
200
1919
1939
1959
1979
1999
2019
Year

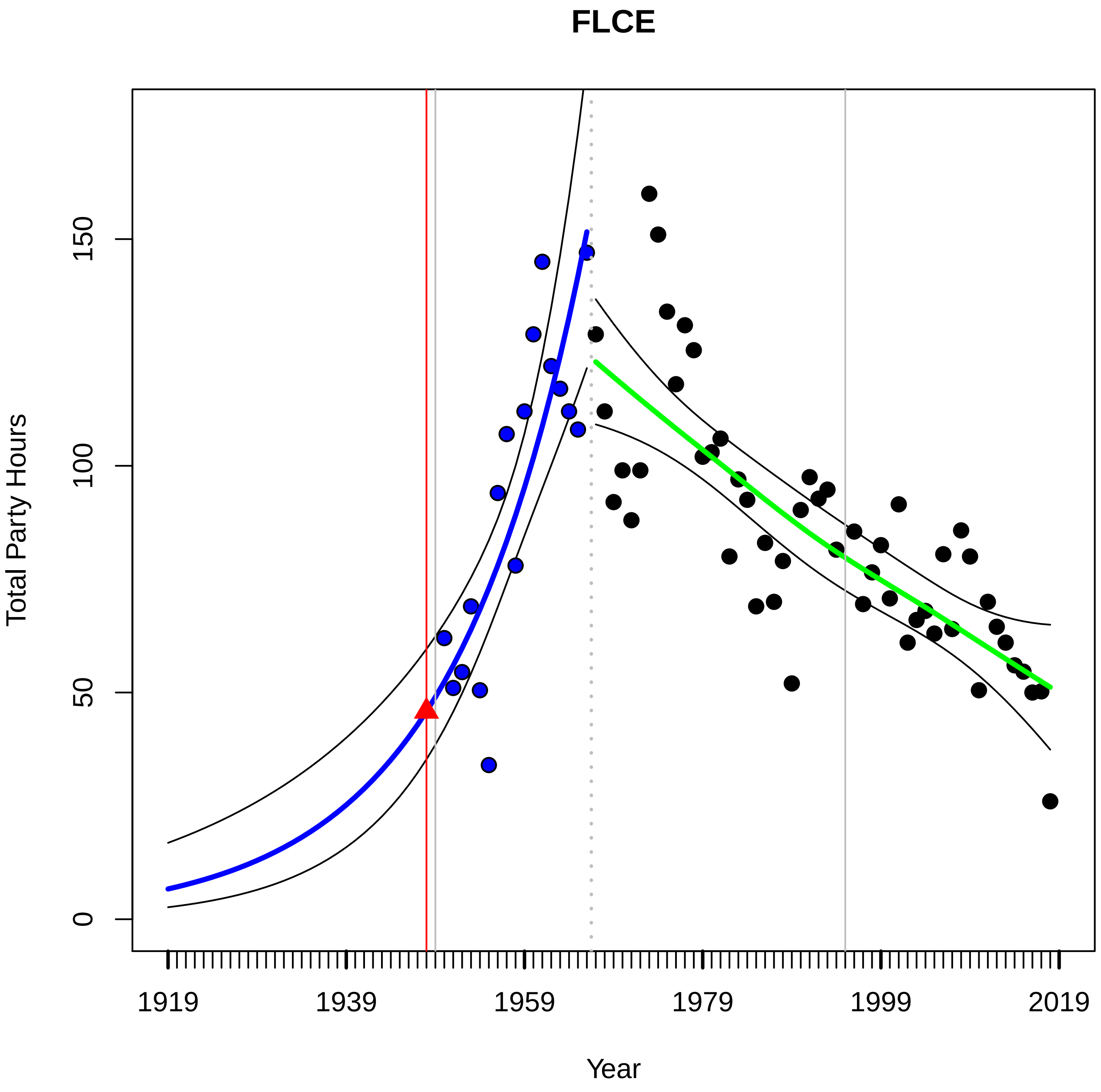
FLCE
Total Party Hours
0
50
100
150
1919
1939
1959
1979
1999
2019
Year

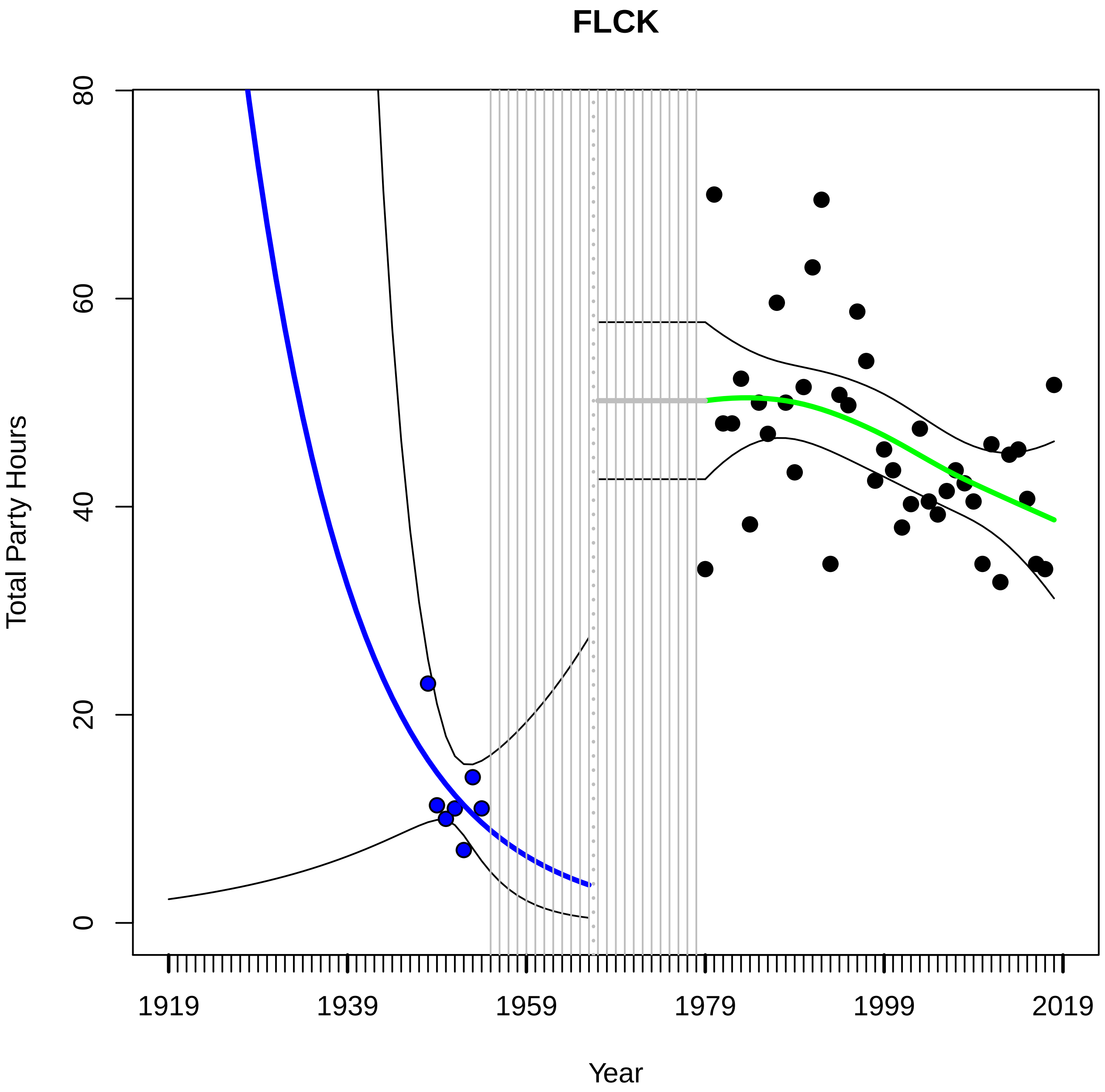
FLCK
Total Party Hours
Year
0
20
40
60
80
1919
1939
1959
1979
1999
2019

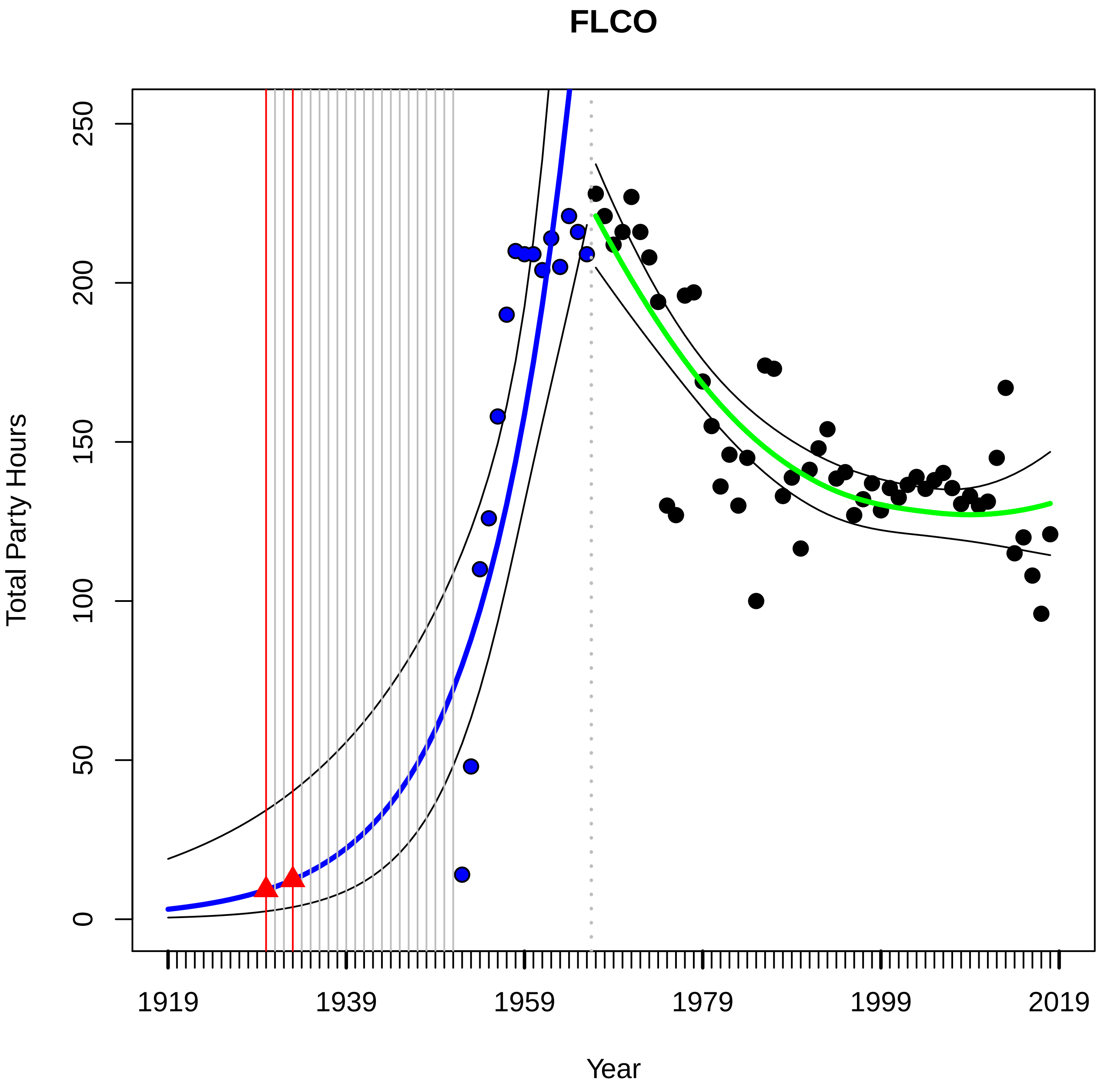

FLCO
Total Party Hours
0
50
100
150
200
250
1919
1939
1959
1979
1999
2019
Year

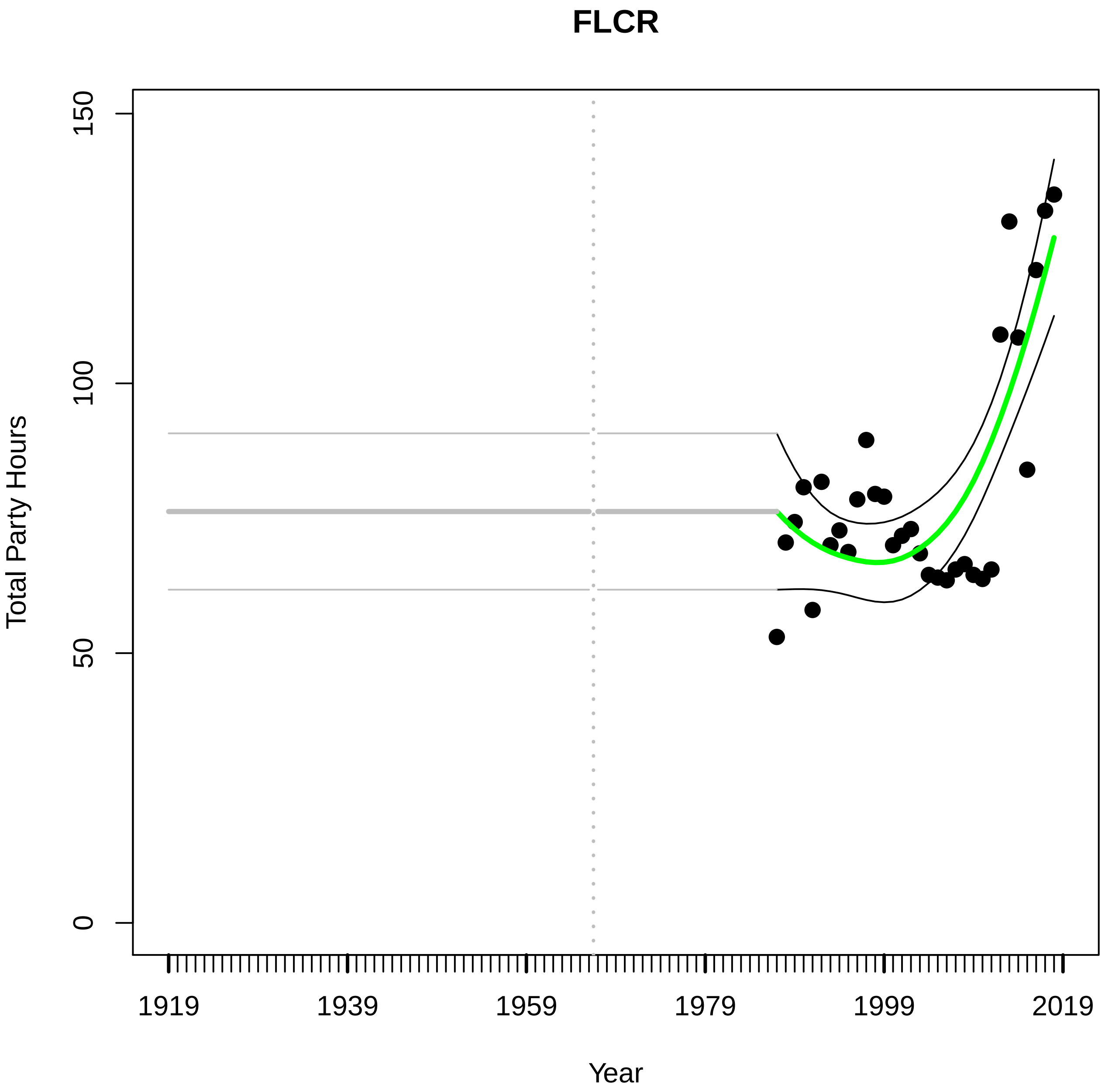

FLCR
Total Party Hours
Year
0
50
100
150
1919
1939
1959
1979
1999
2019

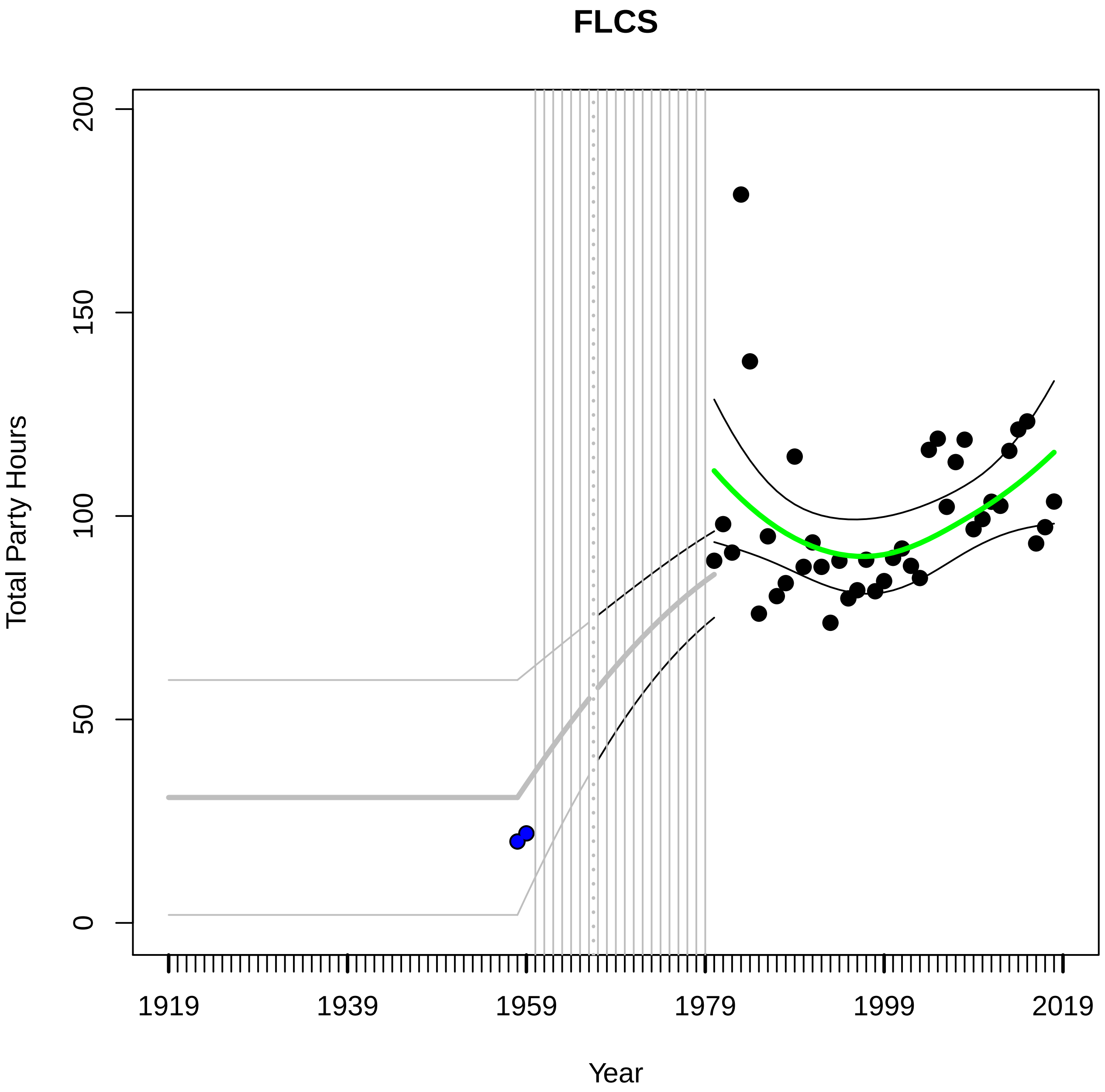

FLCS
Total Party Hours
0
50
100
150
200
1919
1939
1959
1979
1999
2019
Year

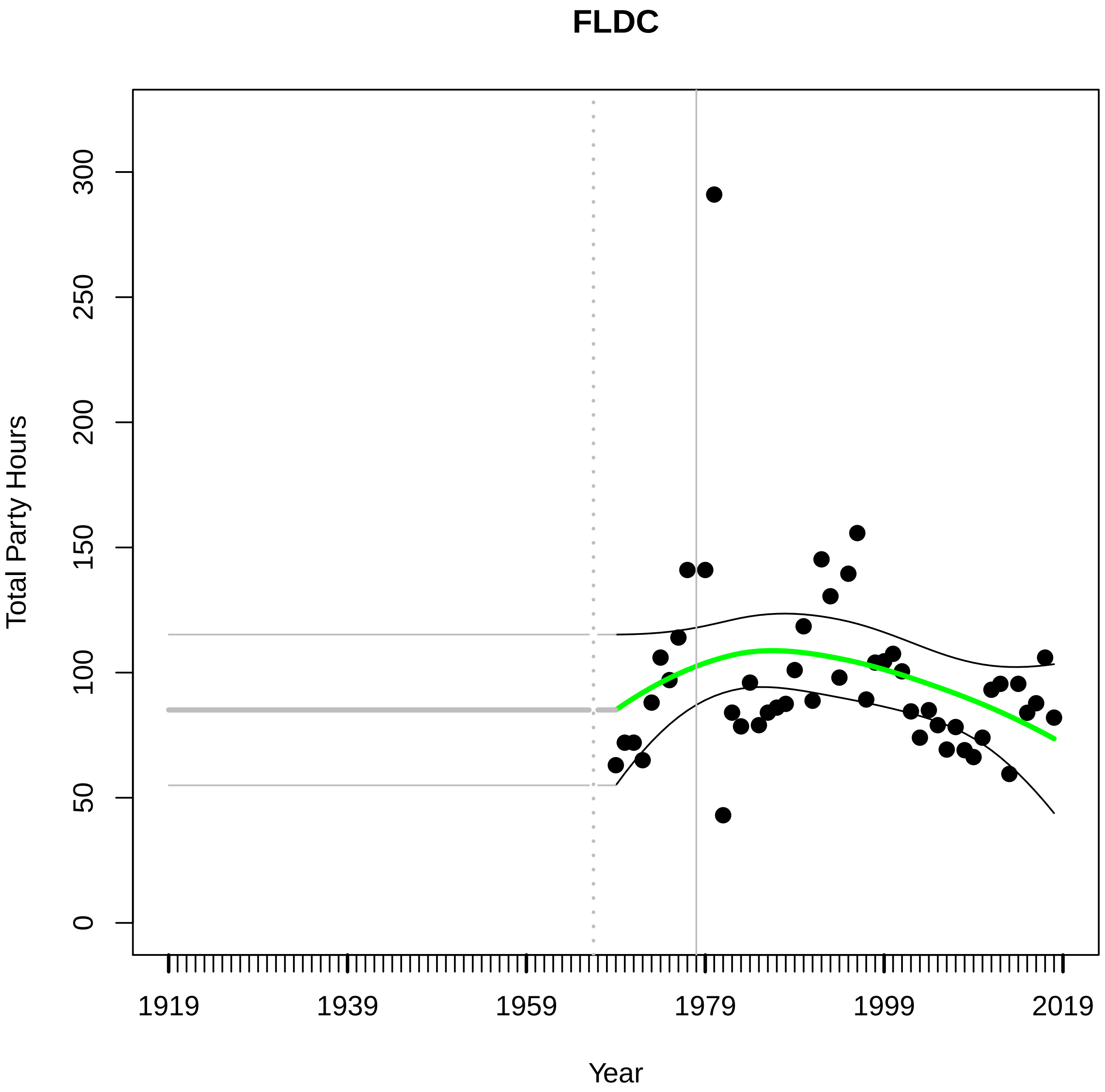

FLDC
Total Party Hours
0
50
100
150
200
250
300
1919
1939
1959
1979
1999
2019
Year

FLEC

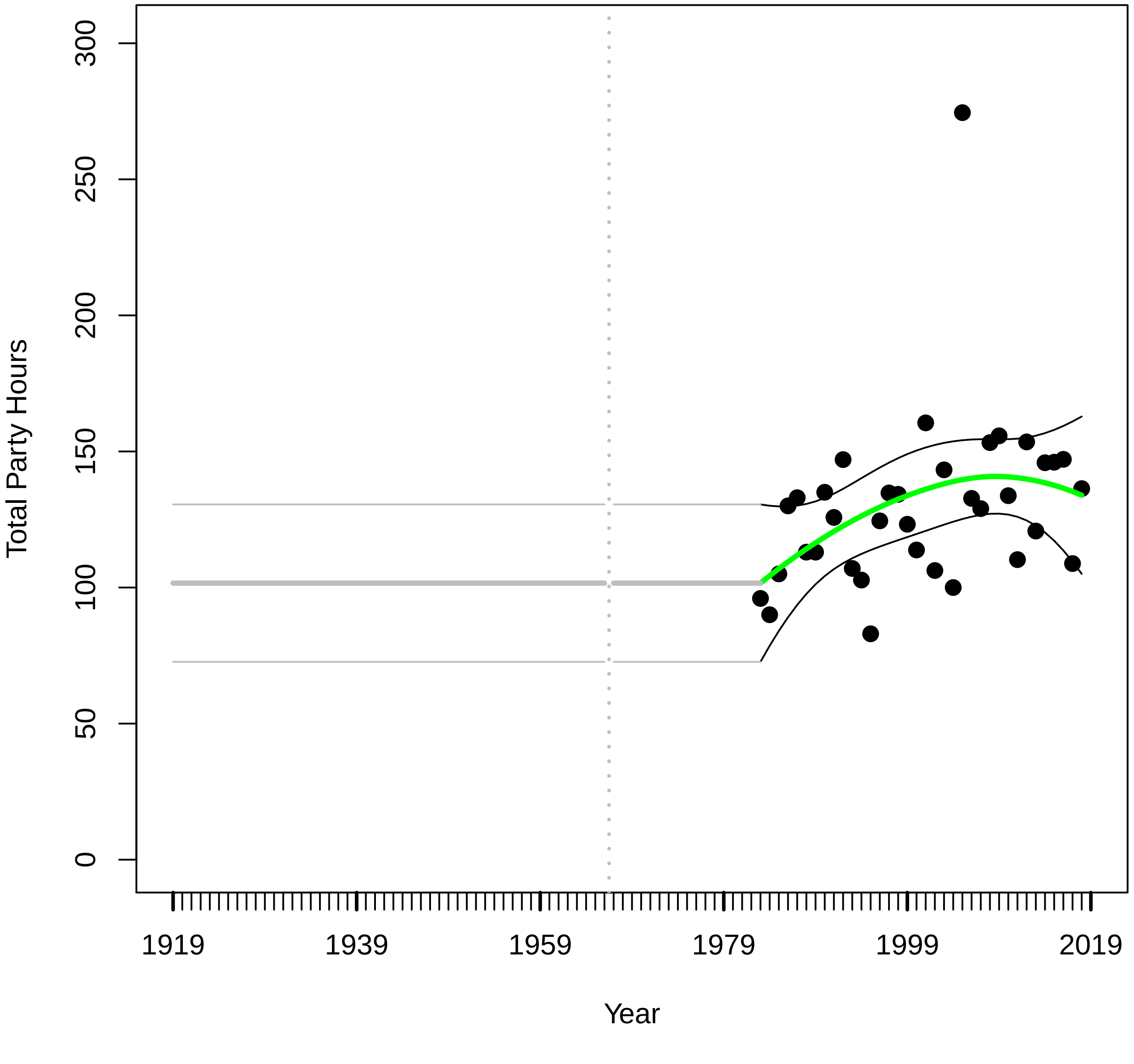

0
50
100
150
200
250
300
Total Party Hours
1919
1939
1959
1979
1999
2019
Year

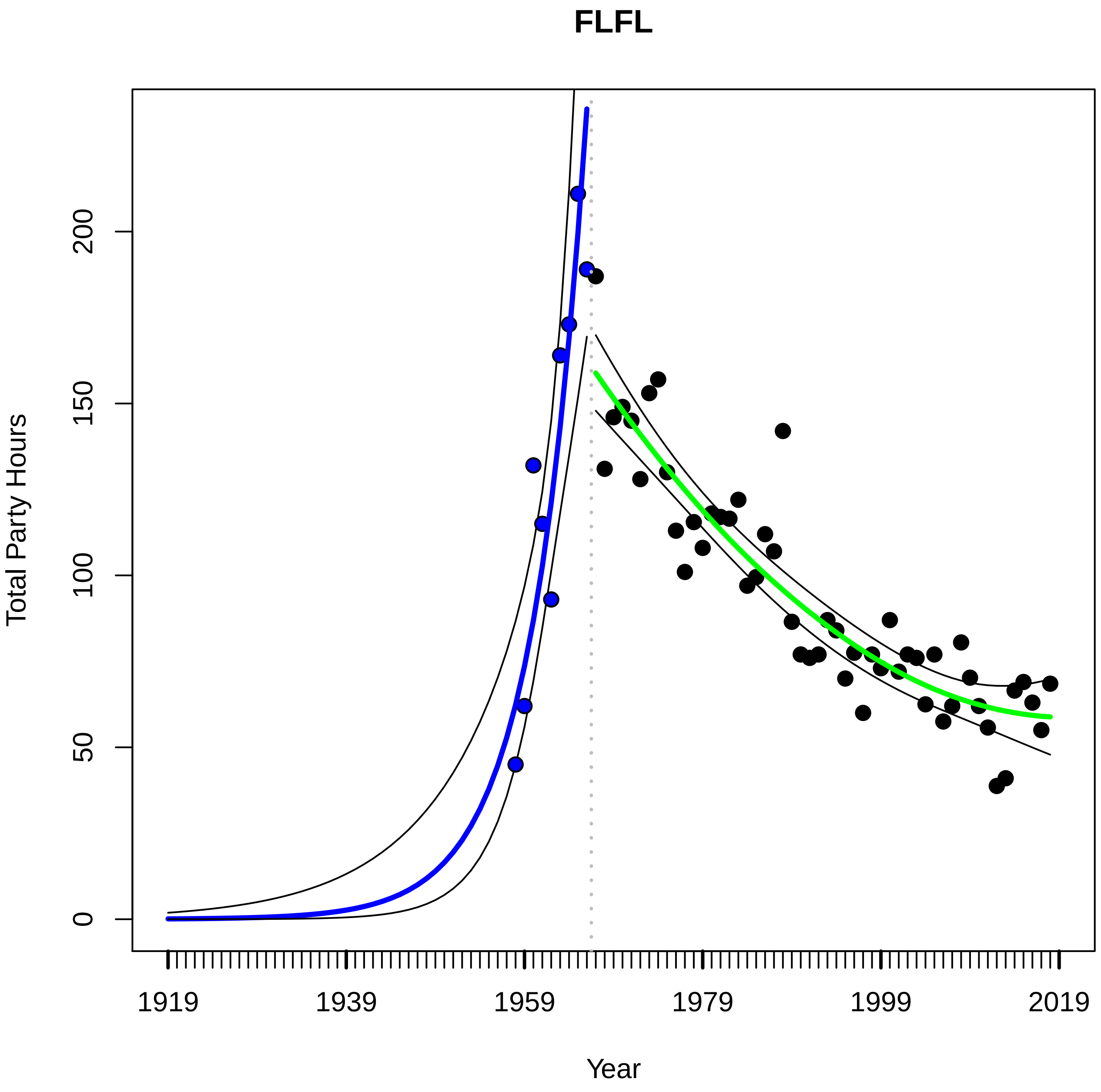

FLFL
Total Party Hours
0
50
100
150
200
Year
1919
1939
1959
1979
1999
2019

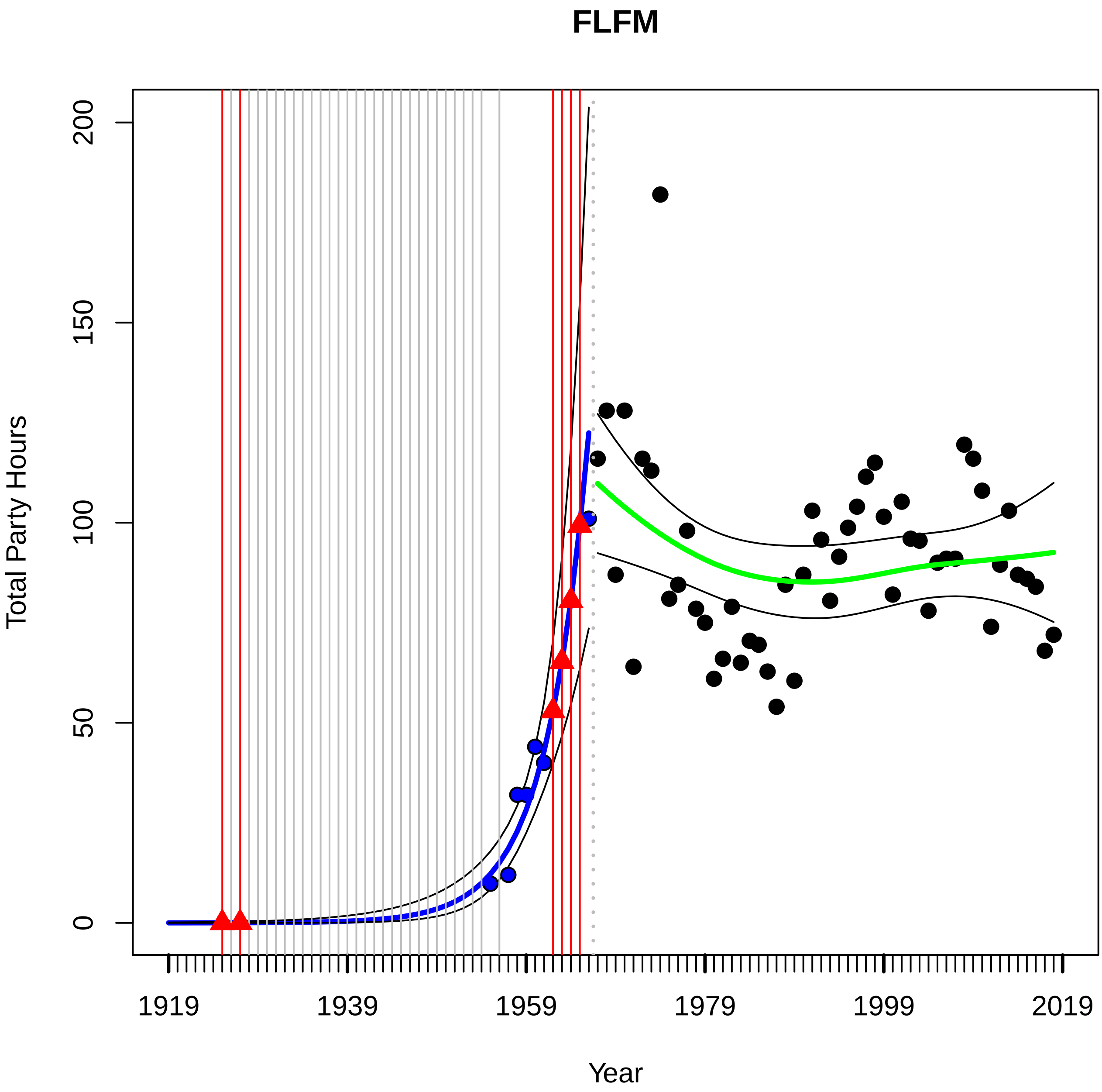

FLFM
Total Party Hours
Year
0
50
100
150
200
1919
1939
1959
1979
1999
2019

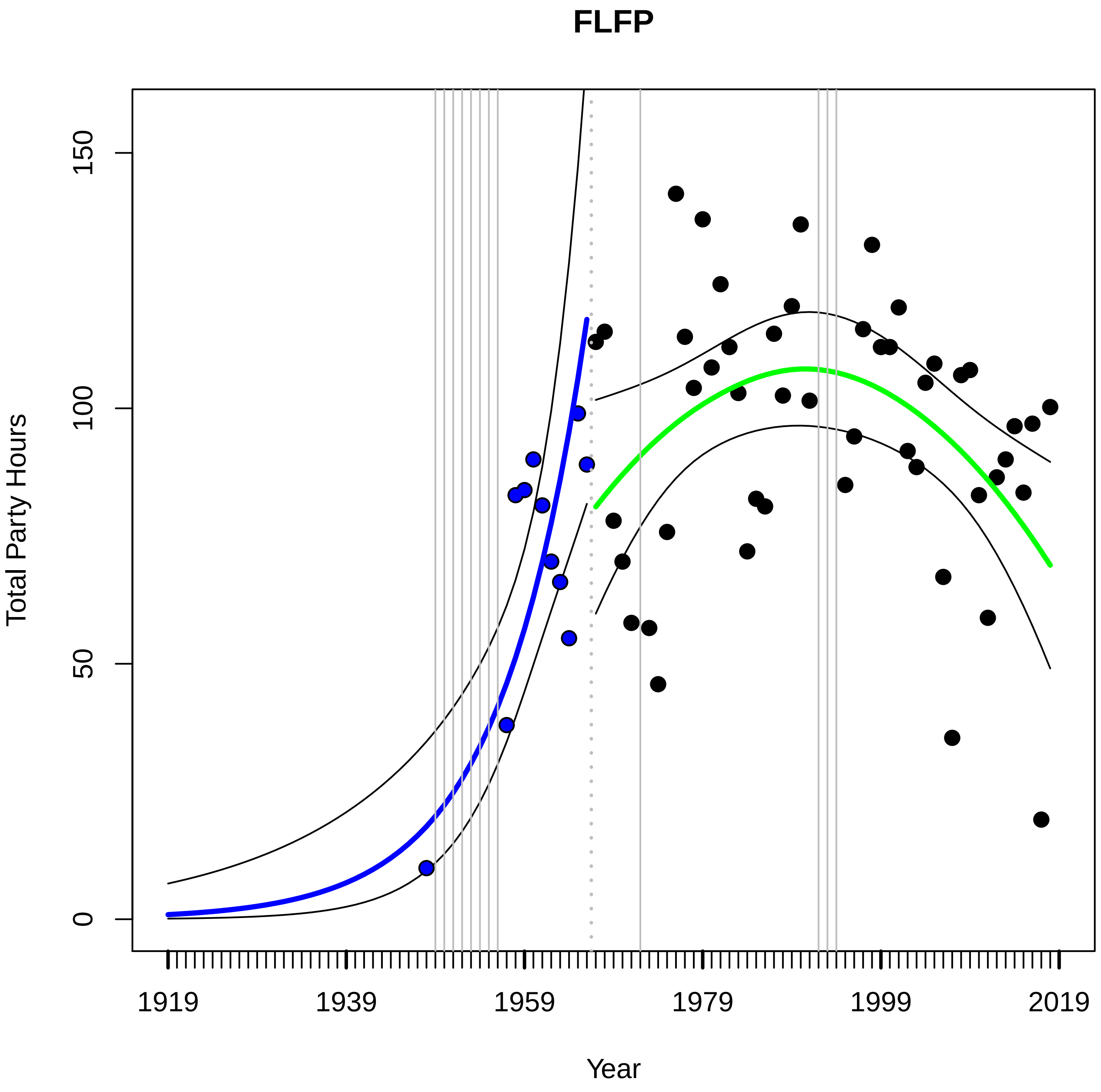

FLFP
Total Party Hours
0
50
100
150
1919
1939
1959
1979
1999
2019
Year

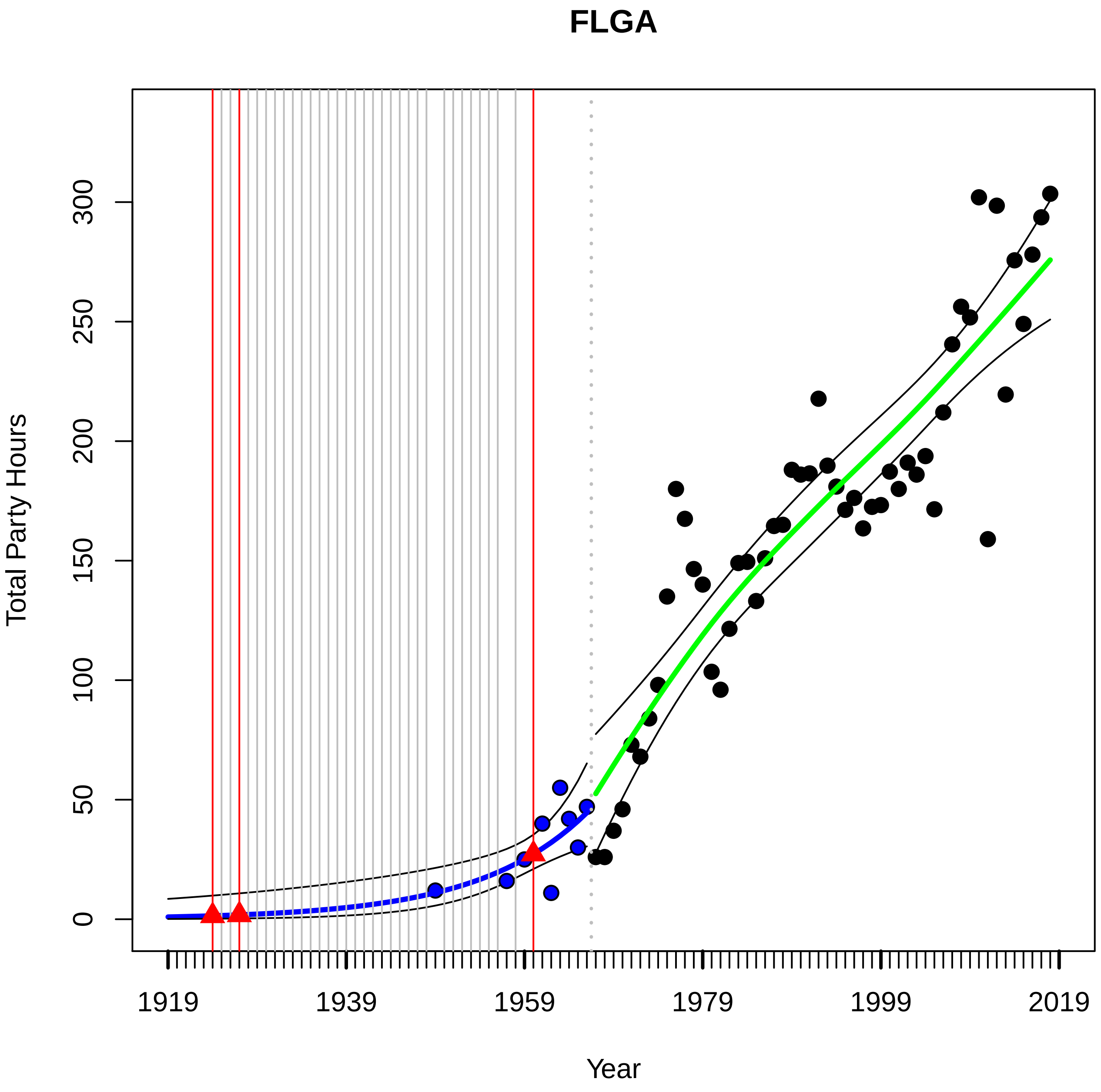

FLGA
Total Party Hours
0
50
100
150
200
250
300
Year
1919
1939
1959
1979
1999
2019

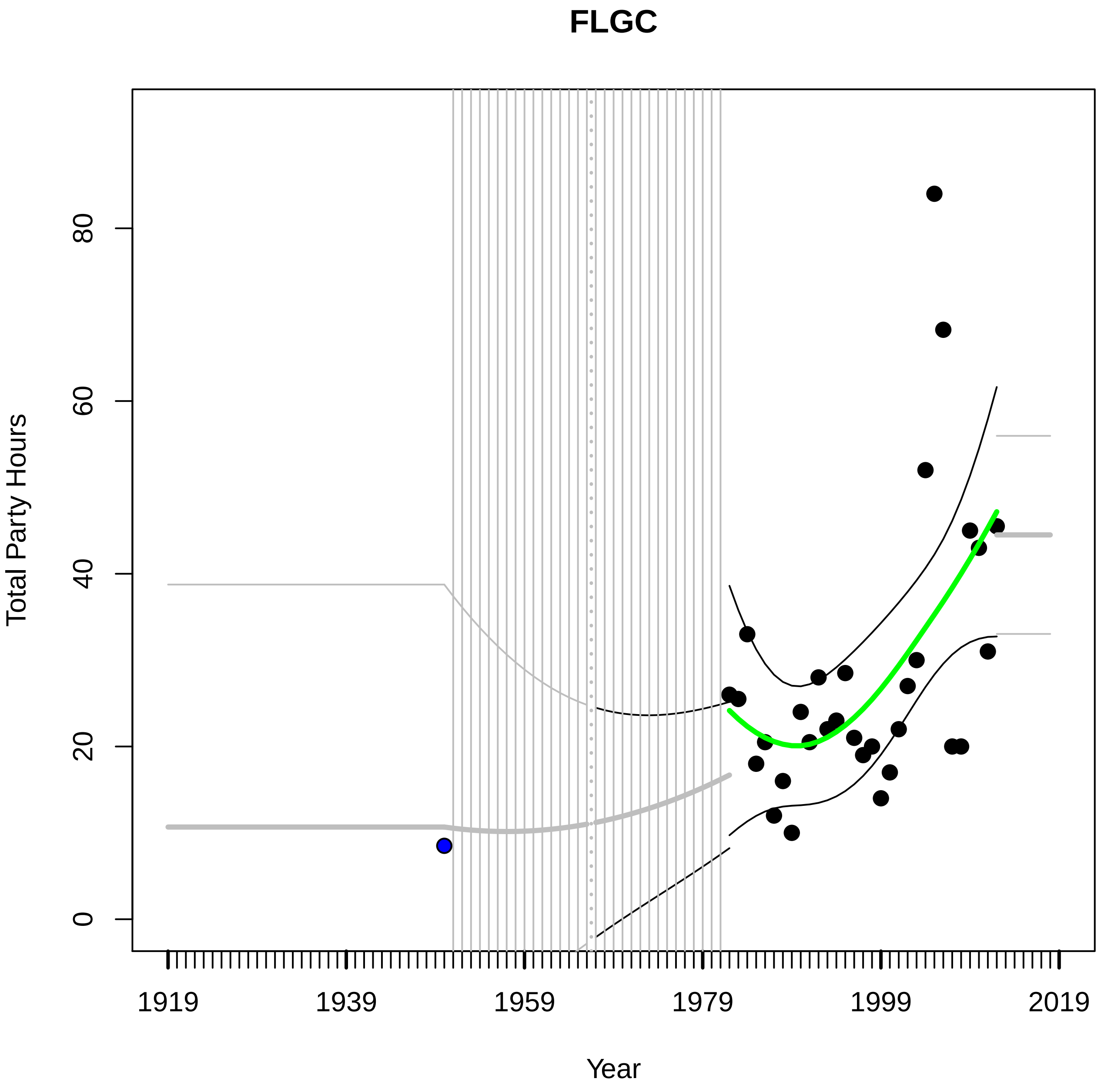

FLGC
Total Party Hours
0
20
40
60
80
1919
1939
1959
1979
1999
2019
Year

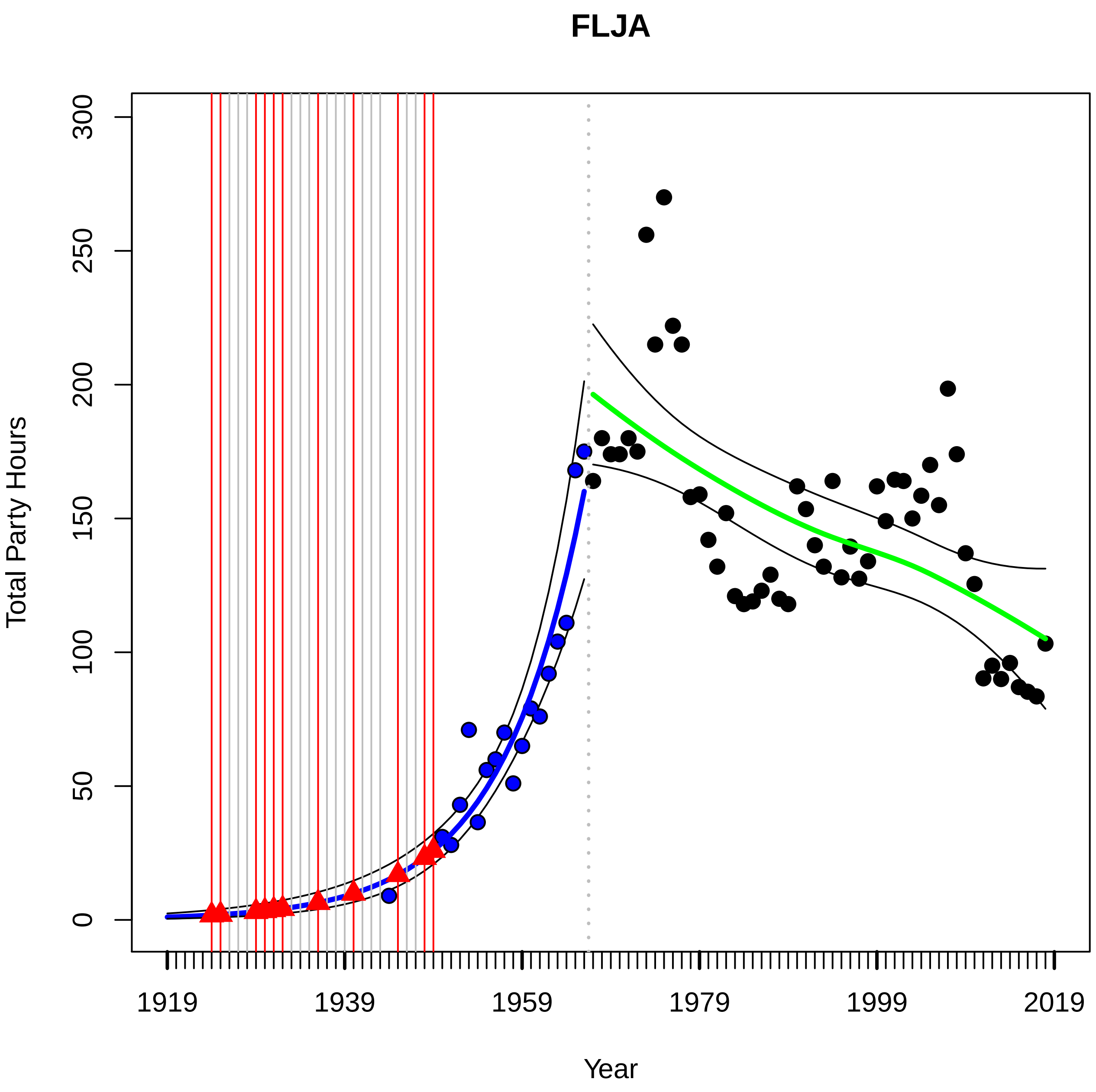

FLJA
Total Party Hours
Year
0
50
100
150
200
250
300
1919
1939
1959
1979
1999
2019

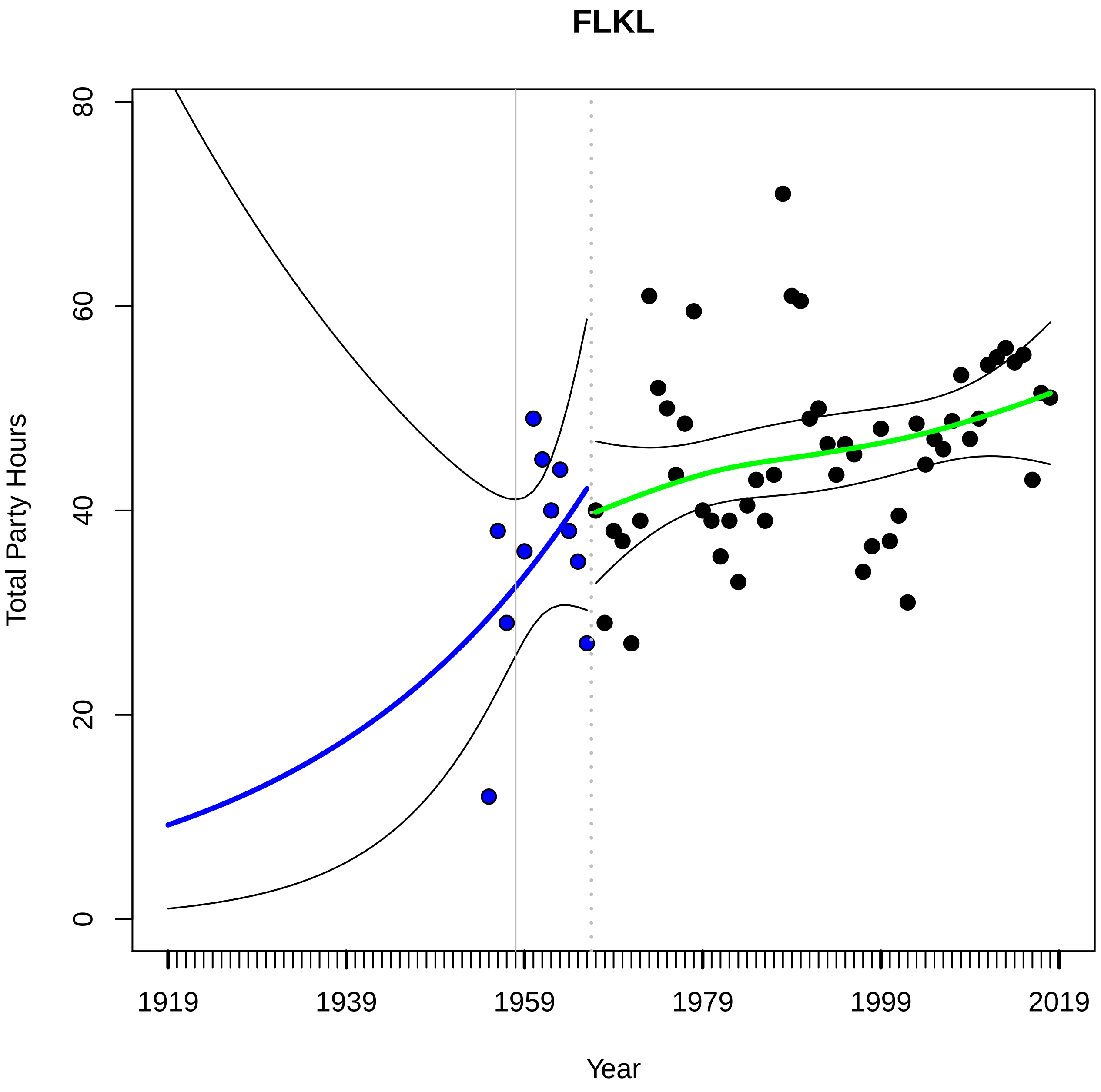

FLKL
Total Party Hours
0
20
40
60
80
1919
1939
1959
1979
1999
2019
Year

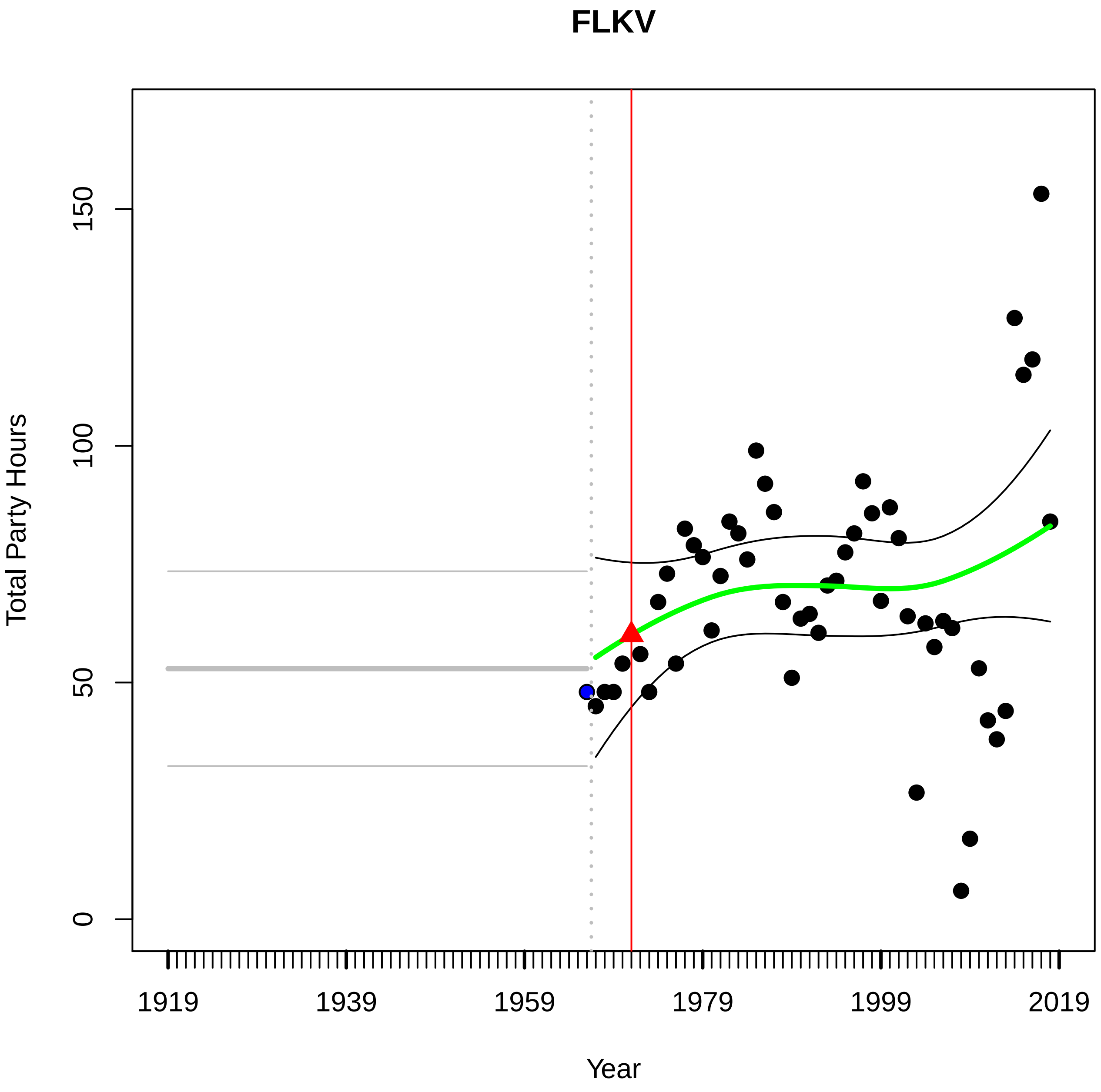

FLKV
Total Party Hours
0
50
100
150
1919
1939
1959
1979
1999
2019
Year

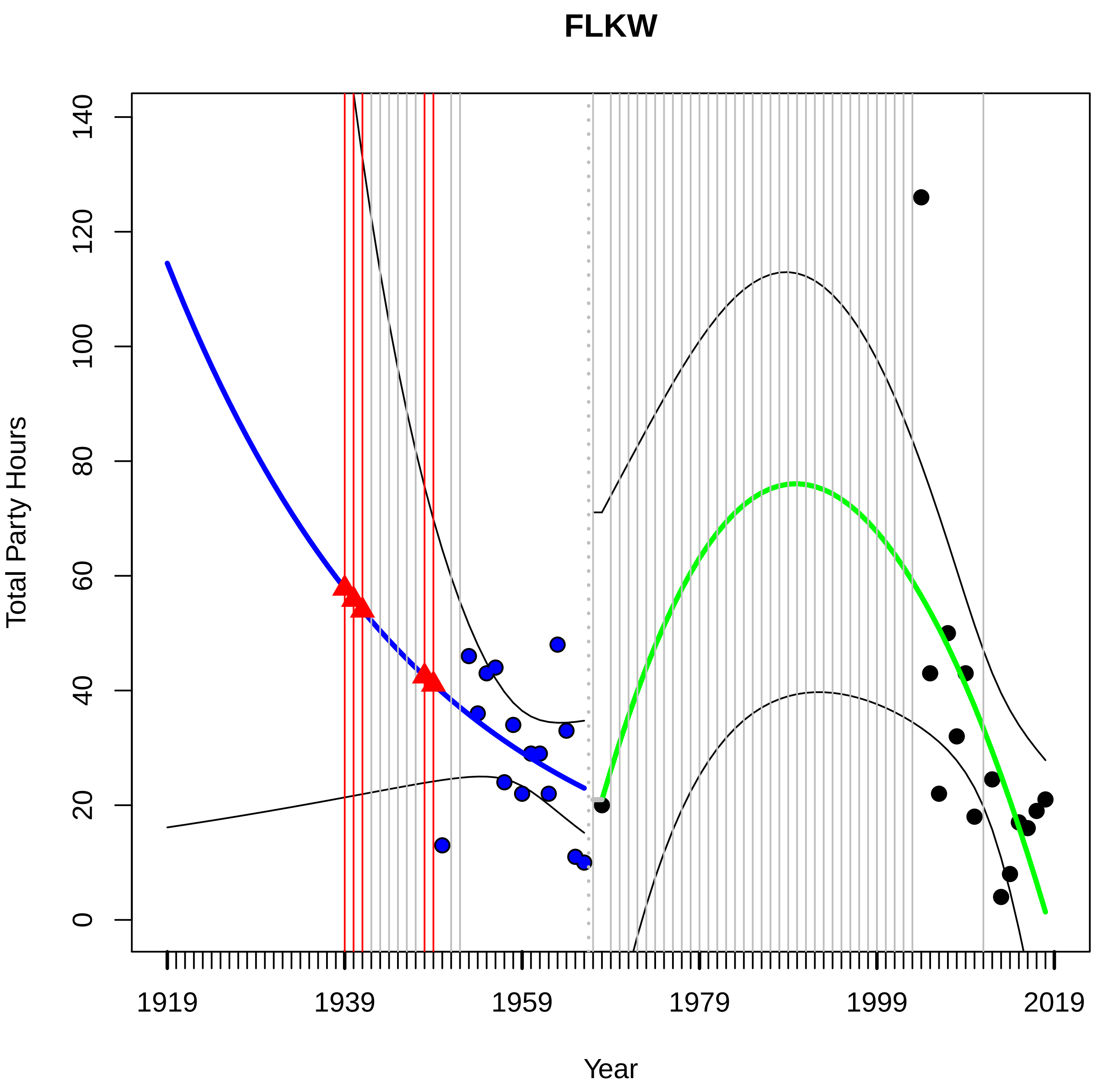

FLKW
Total Party Hours
Year
0
20
40
60
80
100
120
140
1919
1939
1959
1979
1999
2019

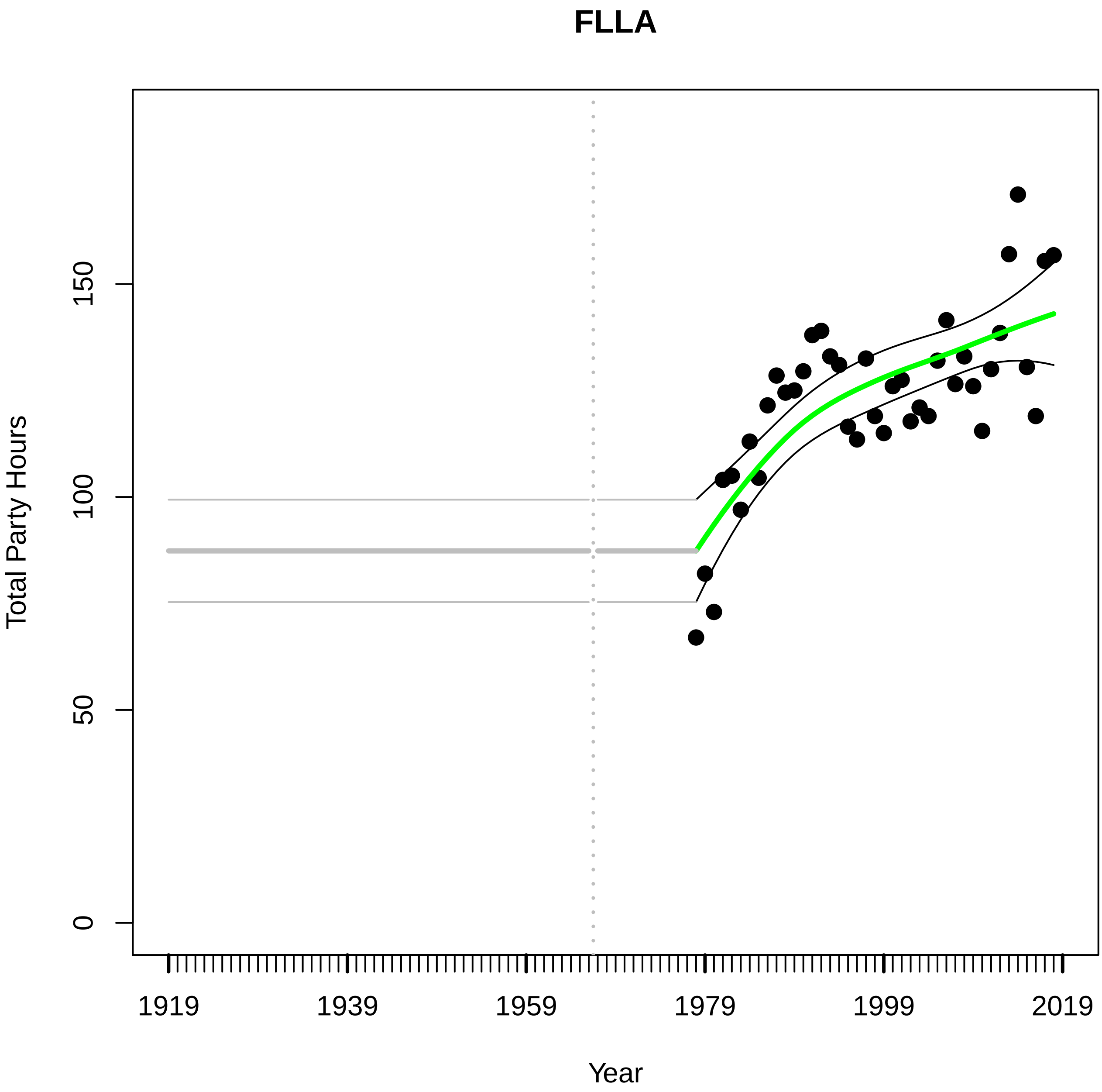

FLLA
Total Party Hours
0
50
100
150
1919
1939
1959
1979
1999
2019
Year

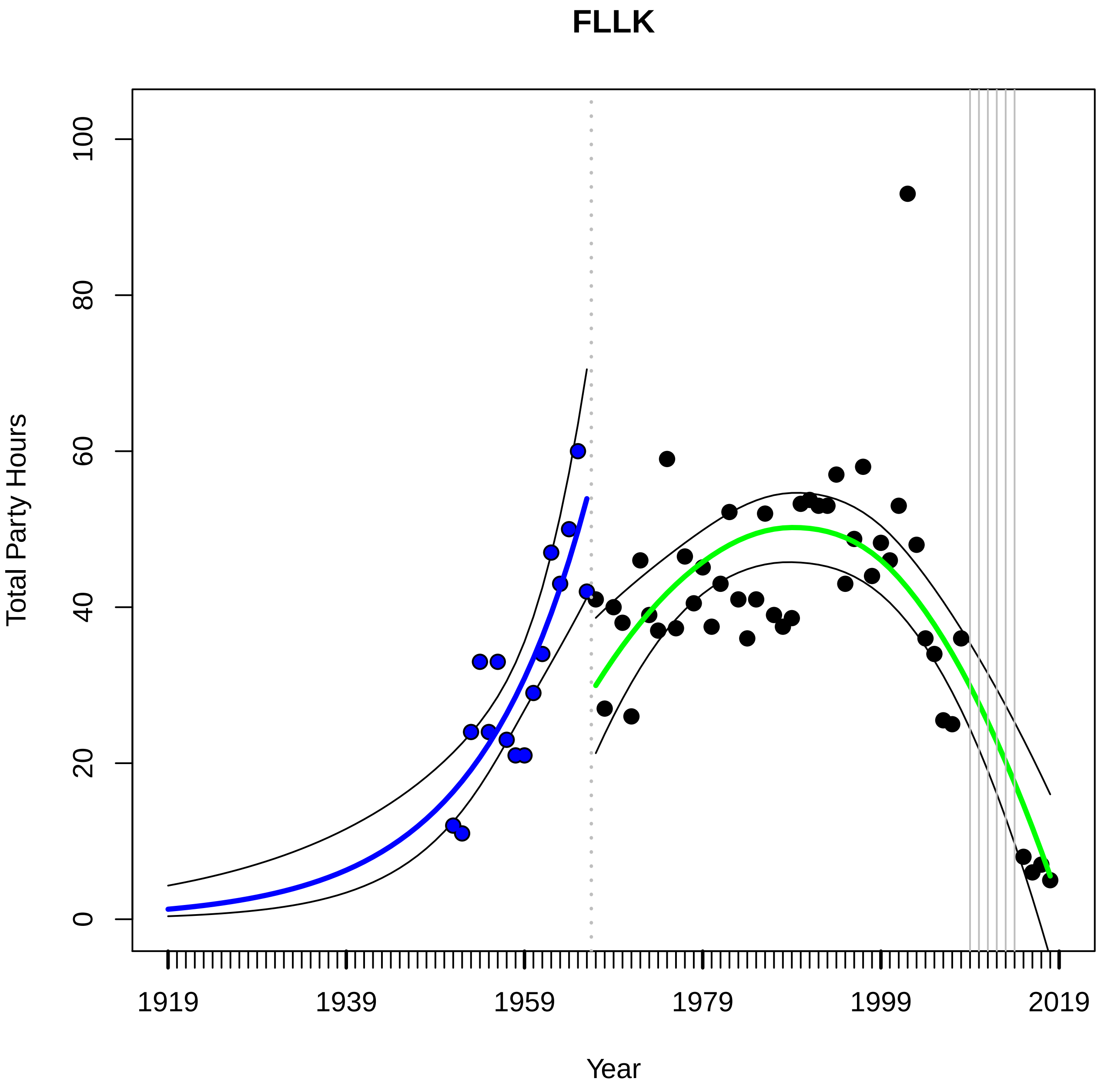

FLLK
Total Party Hours
0
20
40
60
80
100
1919
1939
1959
1979
1999
2019
Year

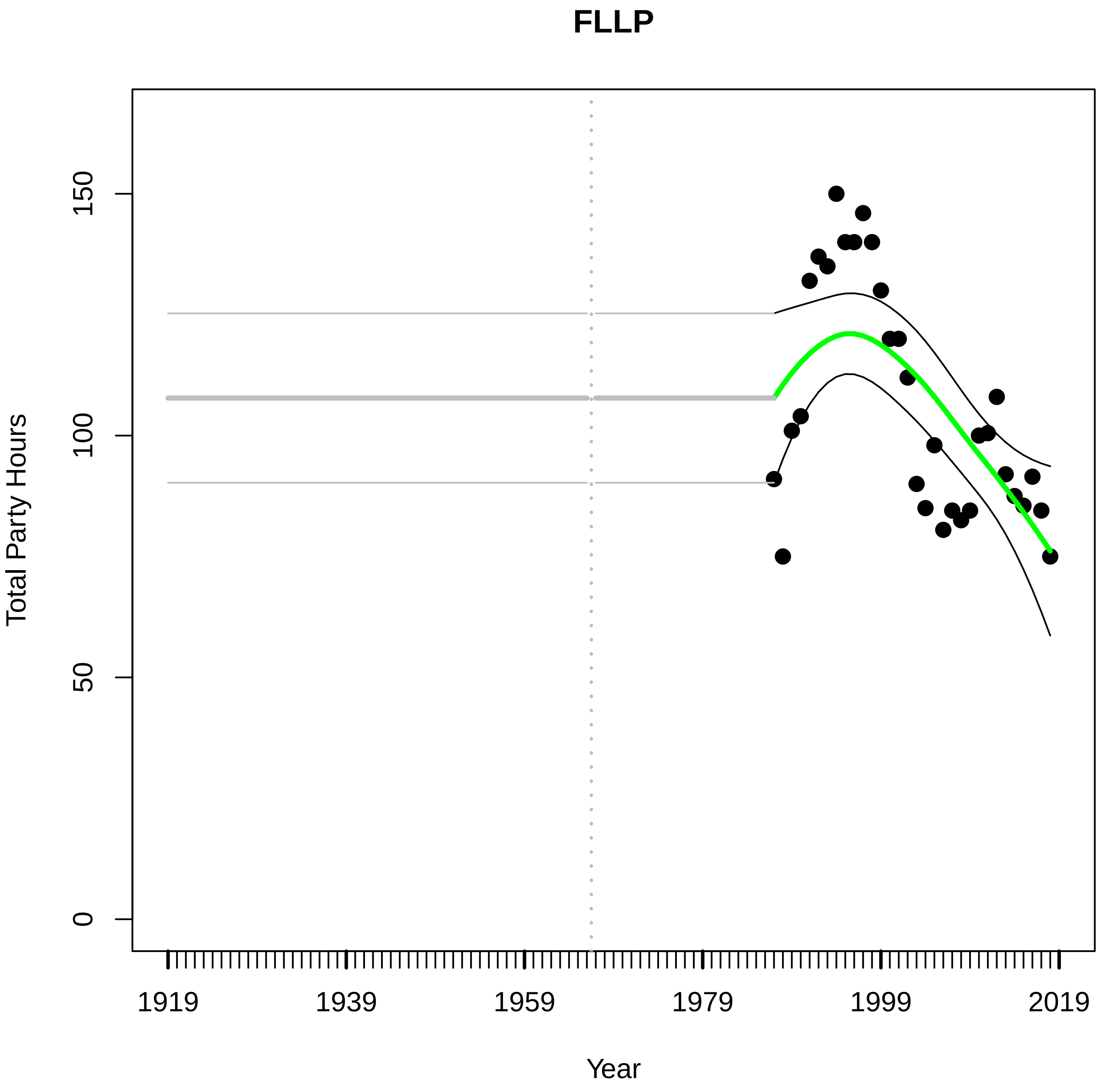

FLLP
Total Party Hours
0
50
100
150
1919
1939
1959
1979
1999
2019
Year

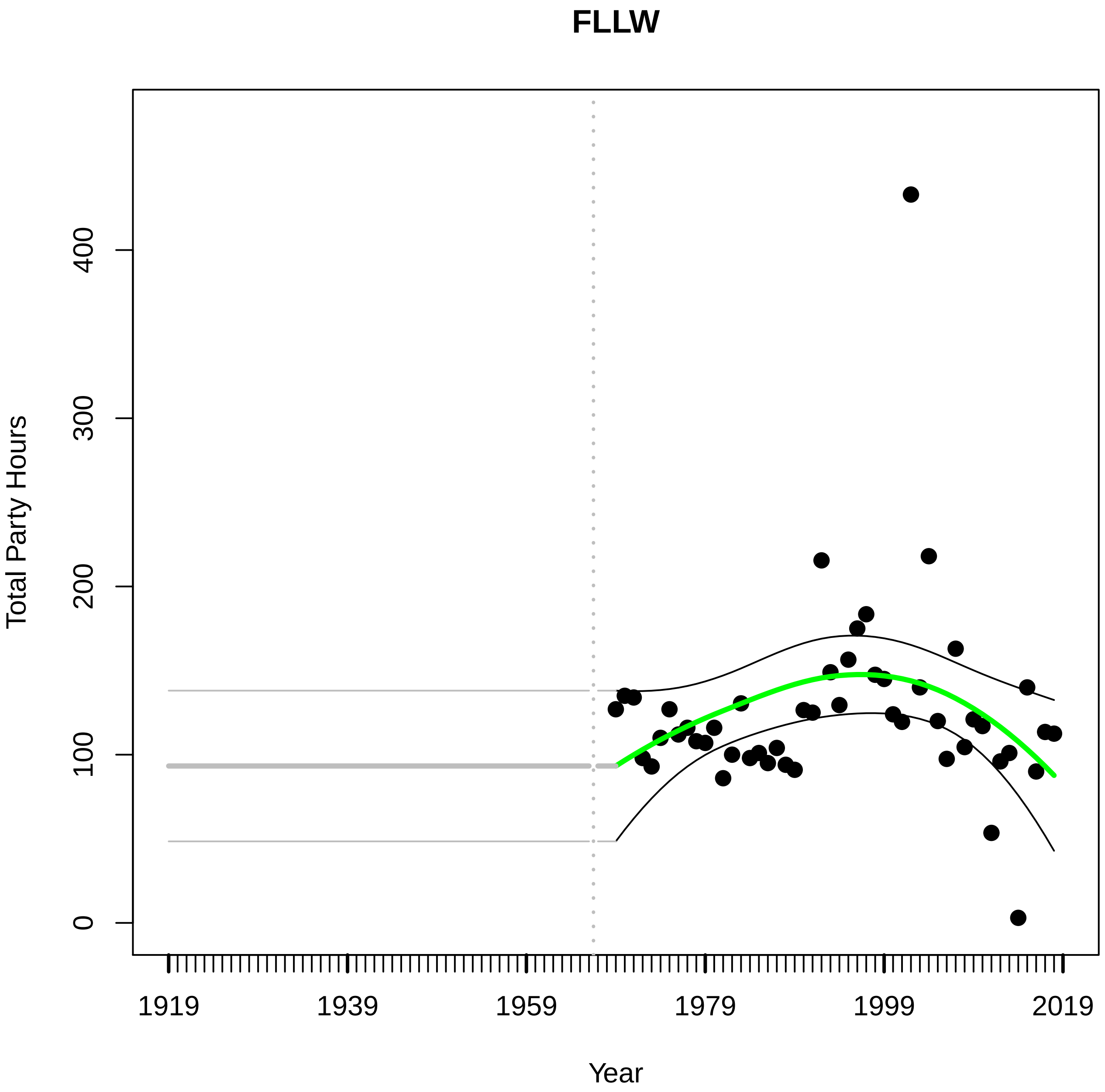

FLLW
Total Party Hours
0
100
200
300
400
1919
1939
1959
1979
1999
2019
Year

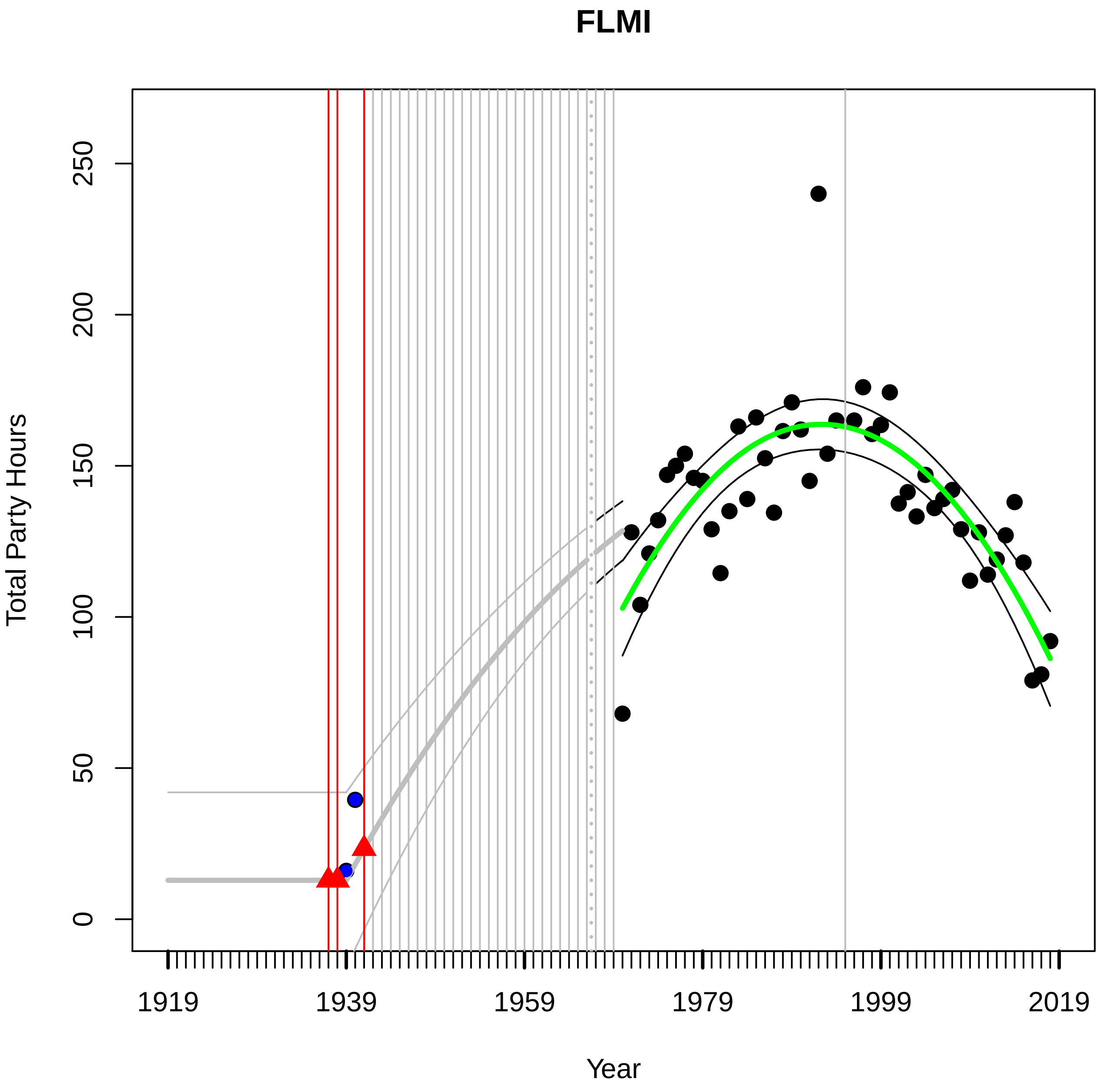

FLMI
Total Party Hours
0
50
100
150
200
250
1919
1939
1959
1979
1999
2019
Year

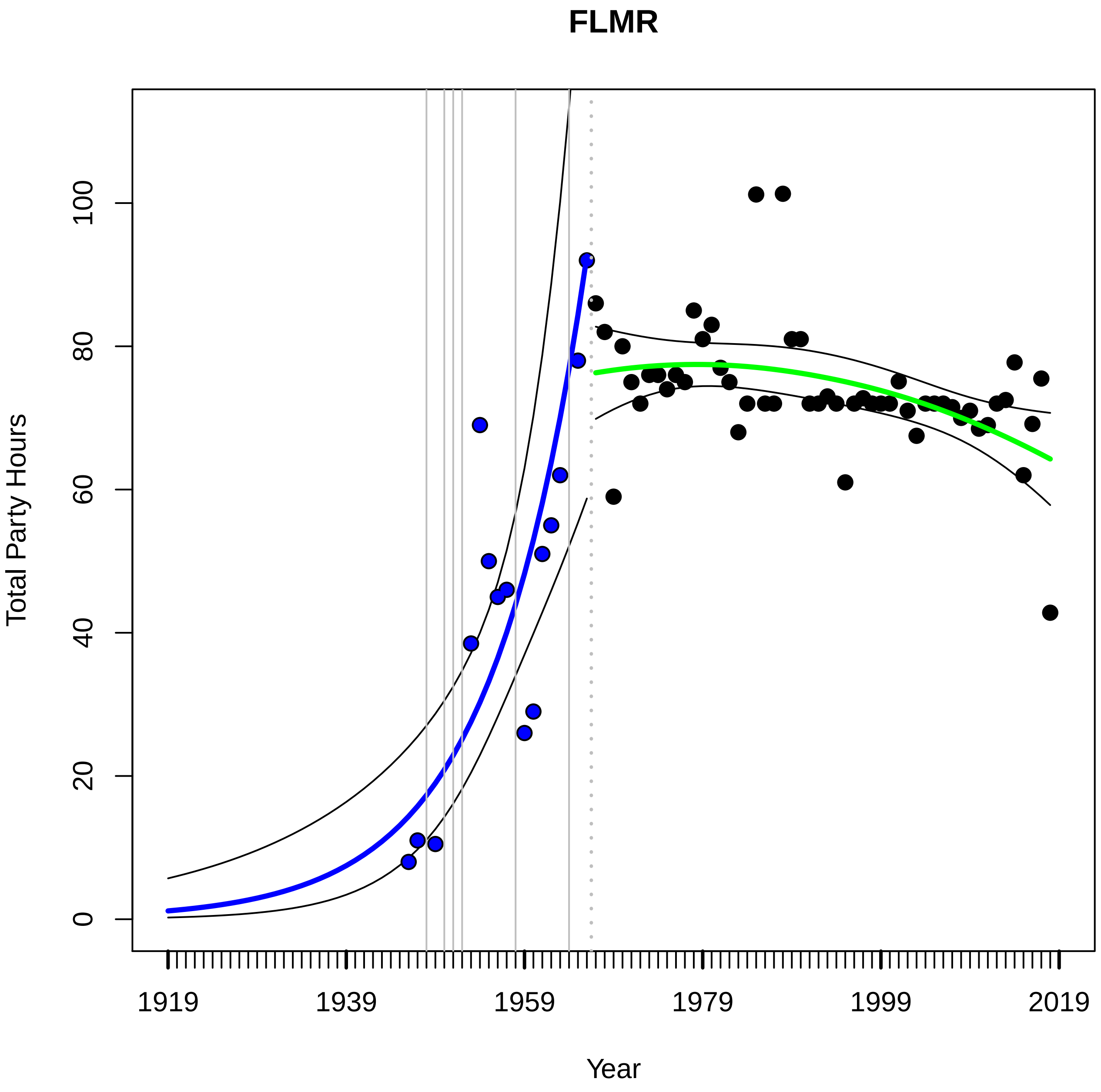

FLMR
Total Party Hours
0
20
40
60
80
100
1919
1939
1959
1979
1999
2019
Year

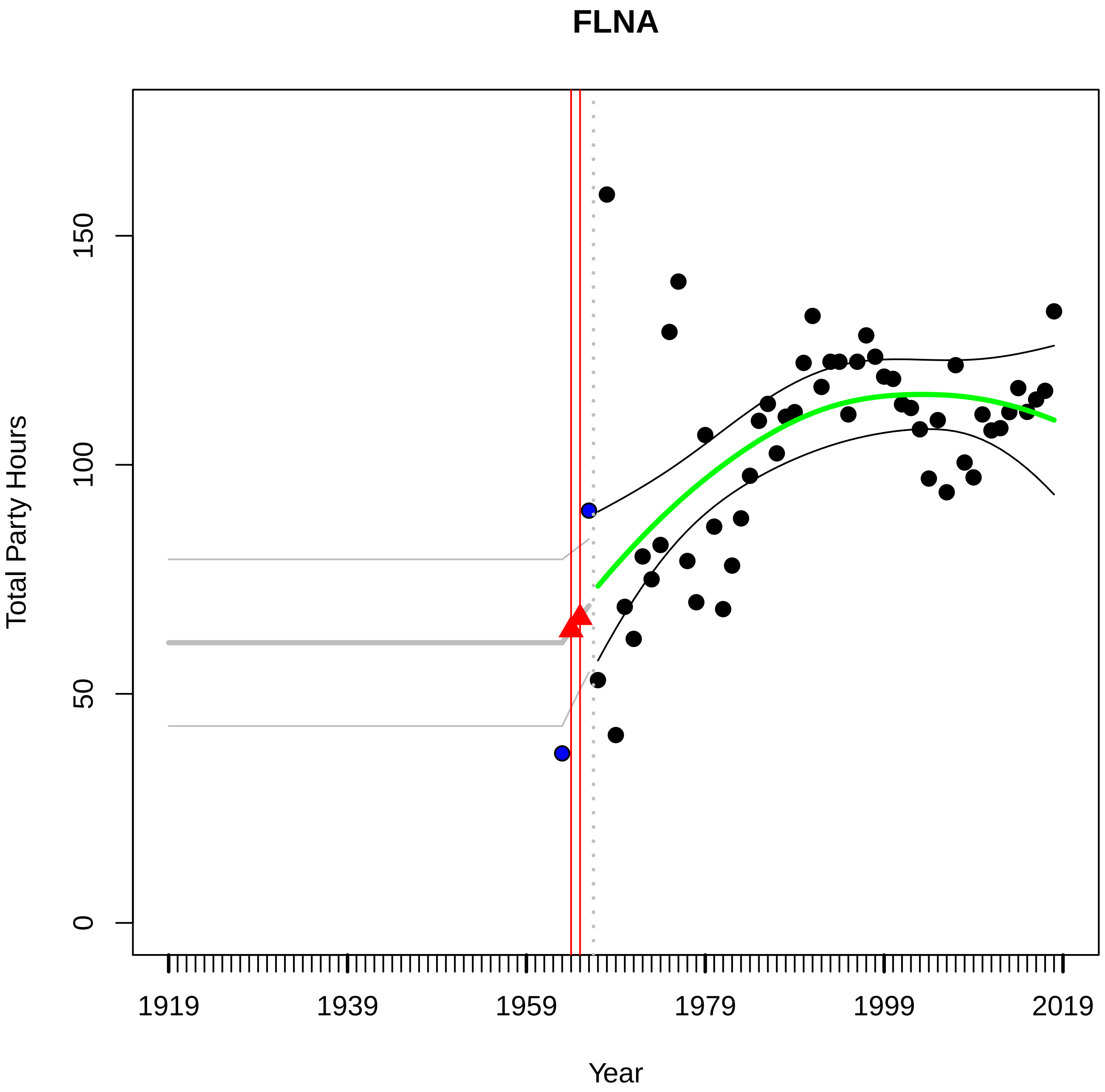

FLNA
Total Party Hours
0
50
100
150
1919
1939
1959
1979
1999
2019
Year

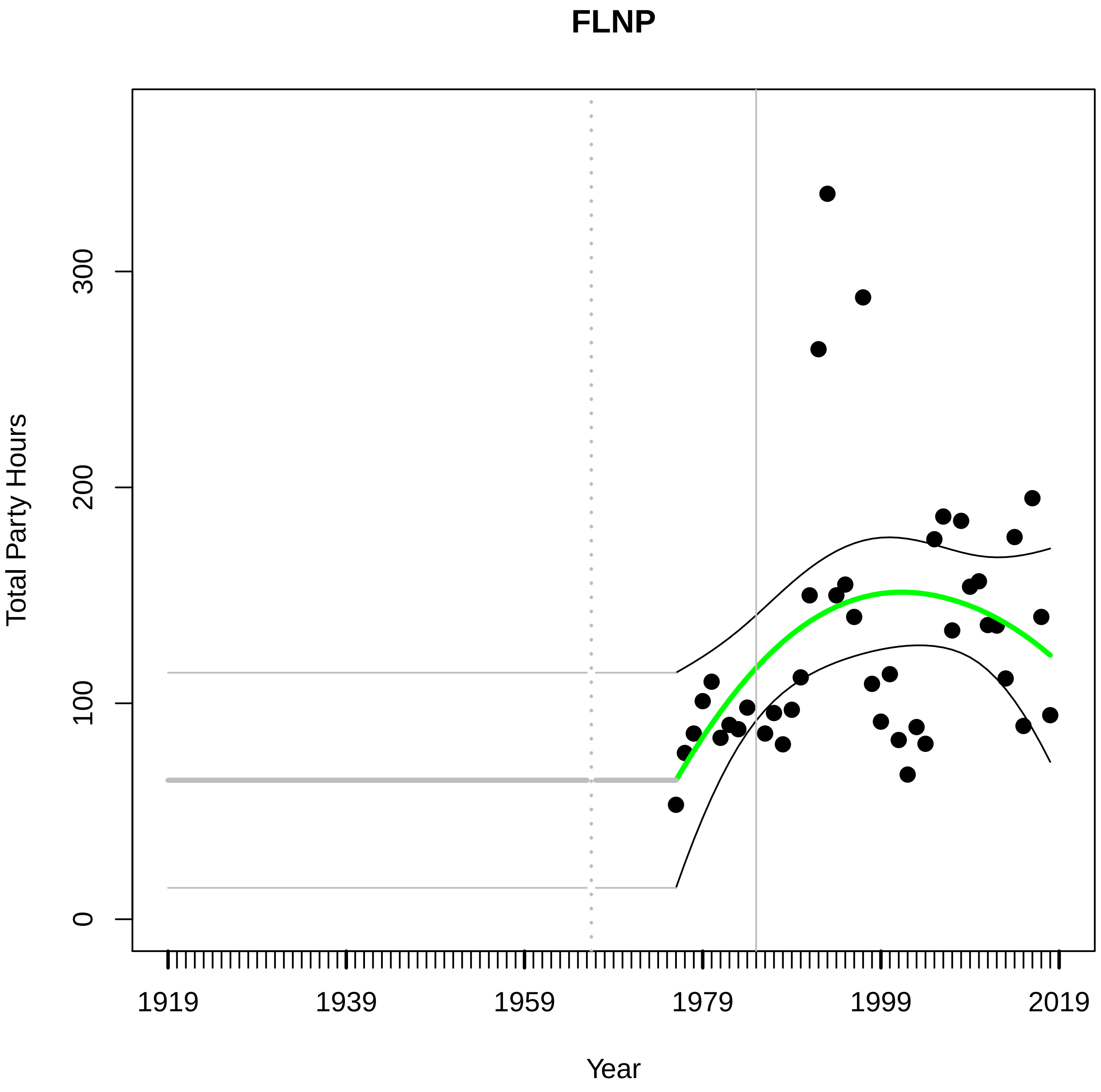

FLNP
Total Party Hours
0
100
200
300
1919
1939
1959
1979
1999
2019
Year

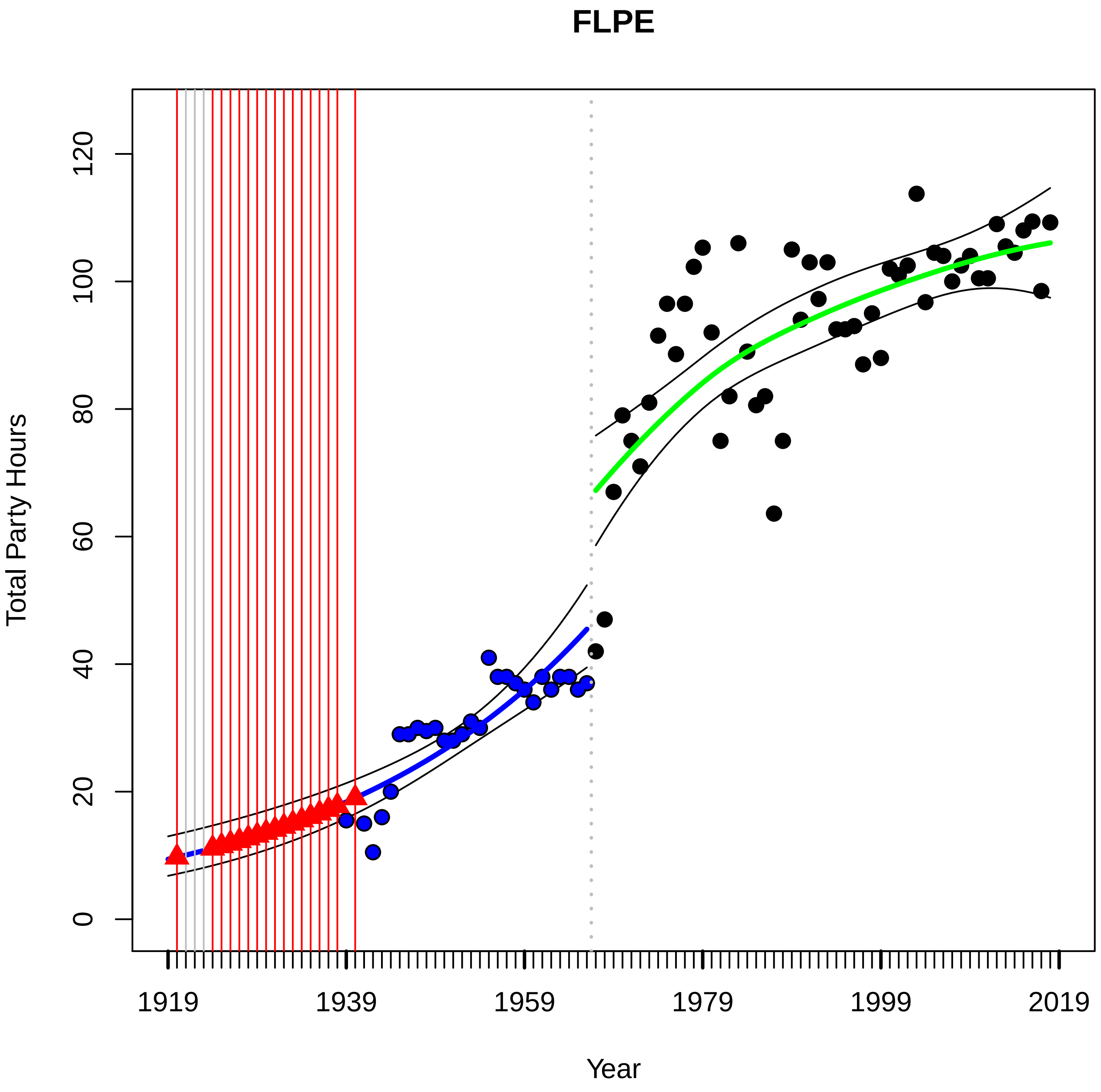
FLPE
Total Party Hours
Year
0
20
40
60
80
100
120
1919
1939
1959
1979
1999
2019

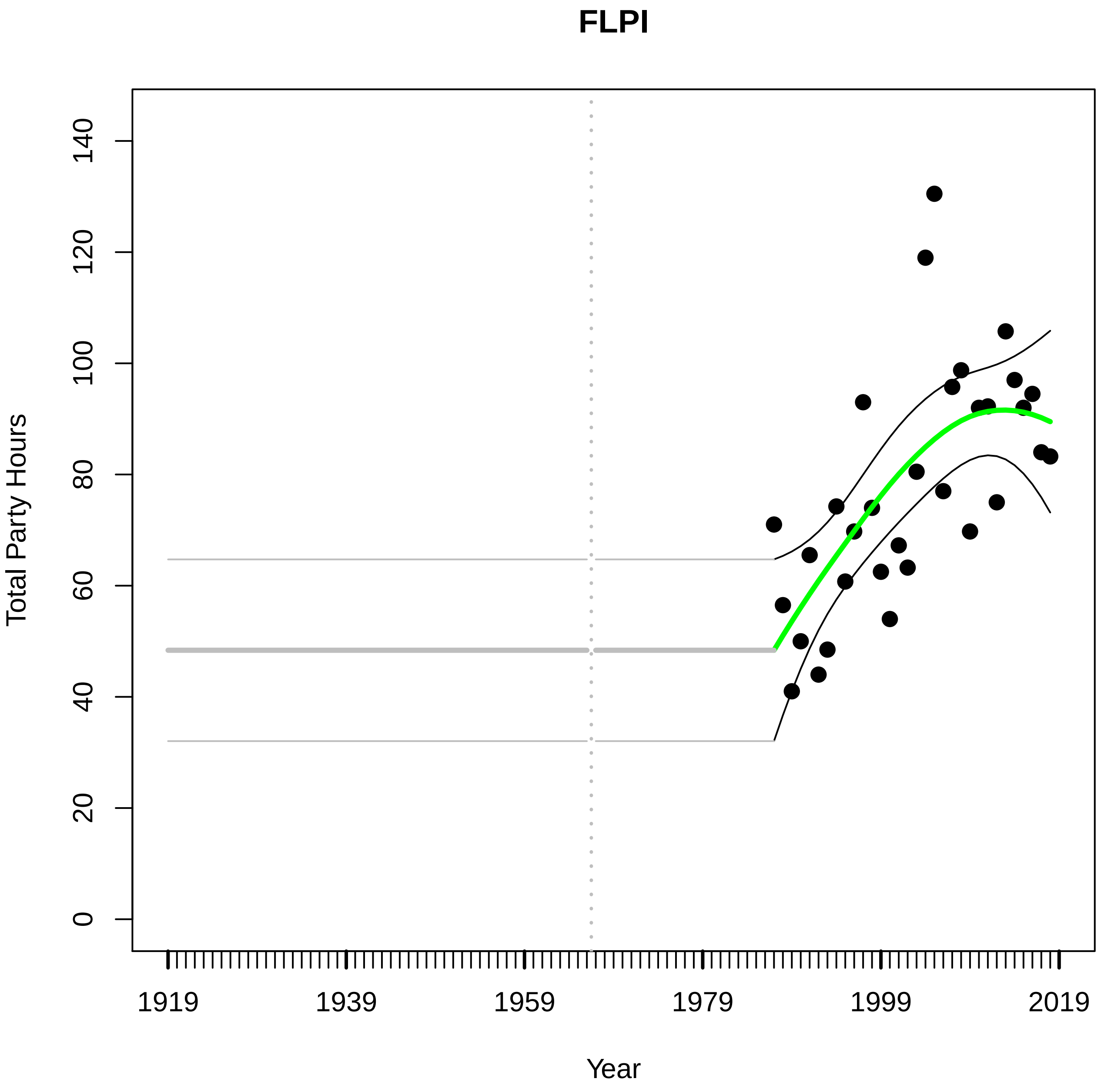

FLPI
Total Party Hours
0
20
40
60
80
100
120
140
1919
1939
1959
1979
1999
2019
Year

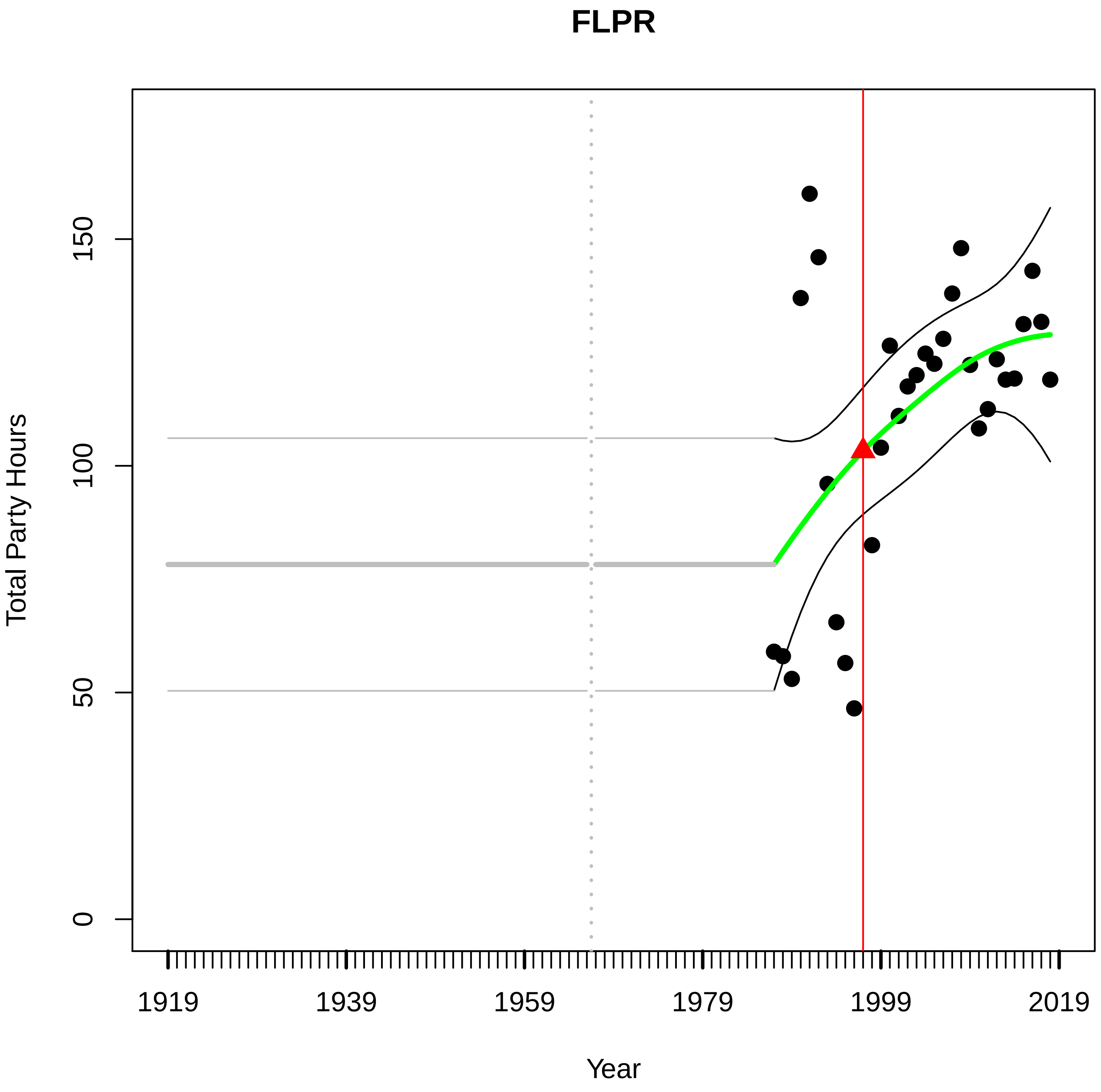

FLPR
Total Party Hours
0
50
100
150
1919
1939
1959
1979
1999
2019
Year

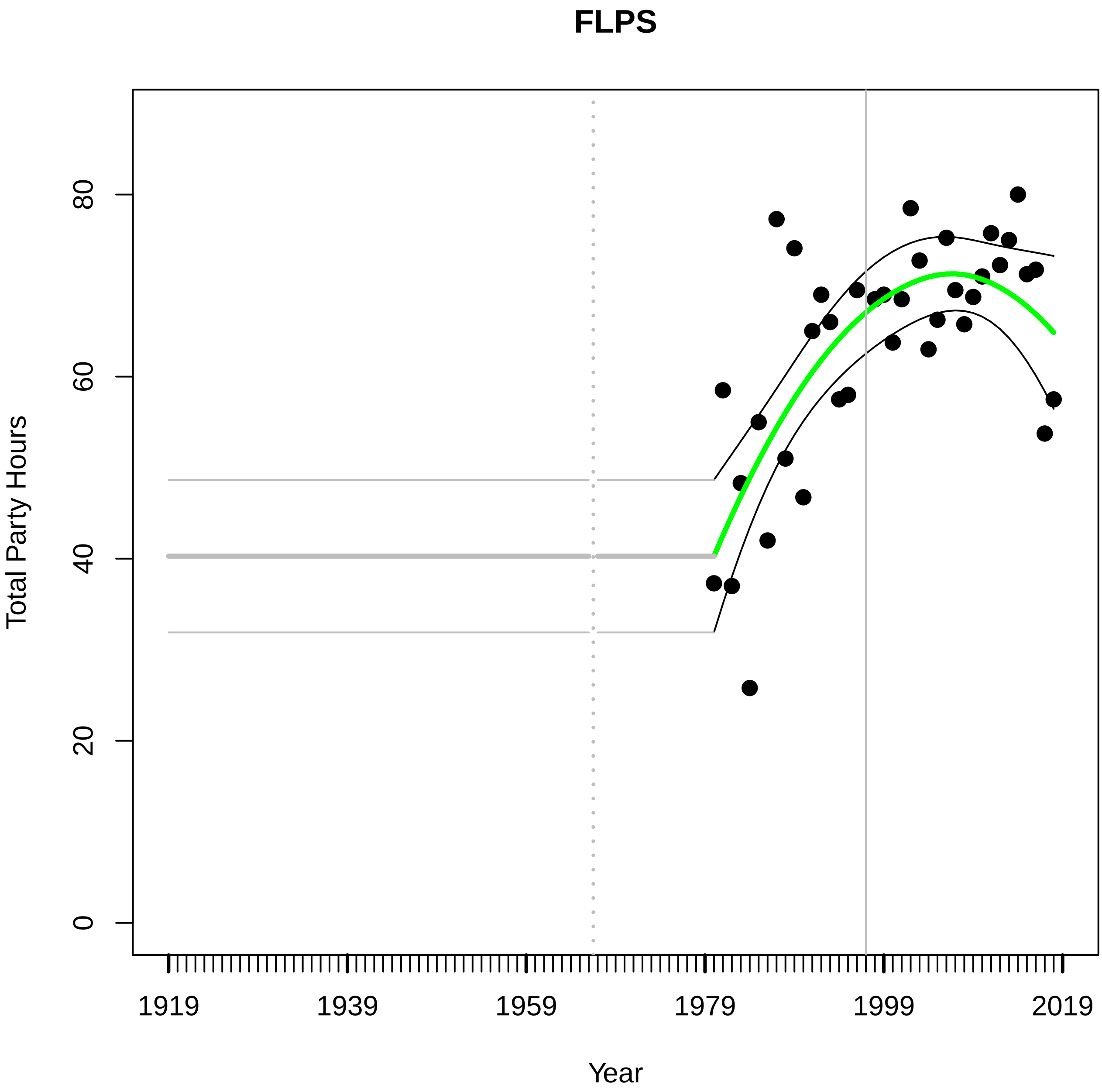

FLPS
Total Party Hours
0
20
40
60
80
1919
1939
1959
1979
1999
2019
Year

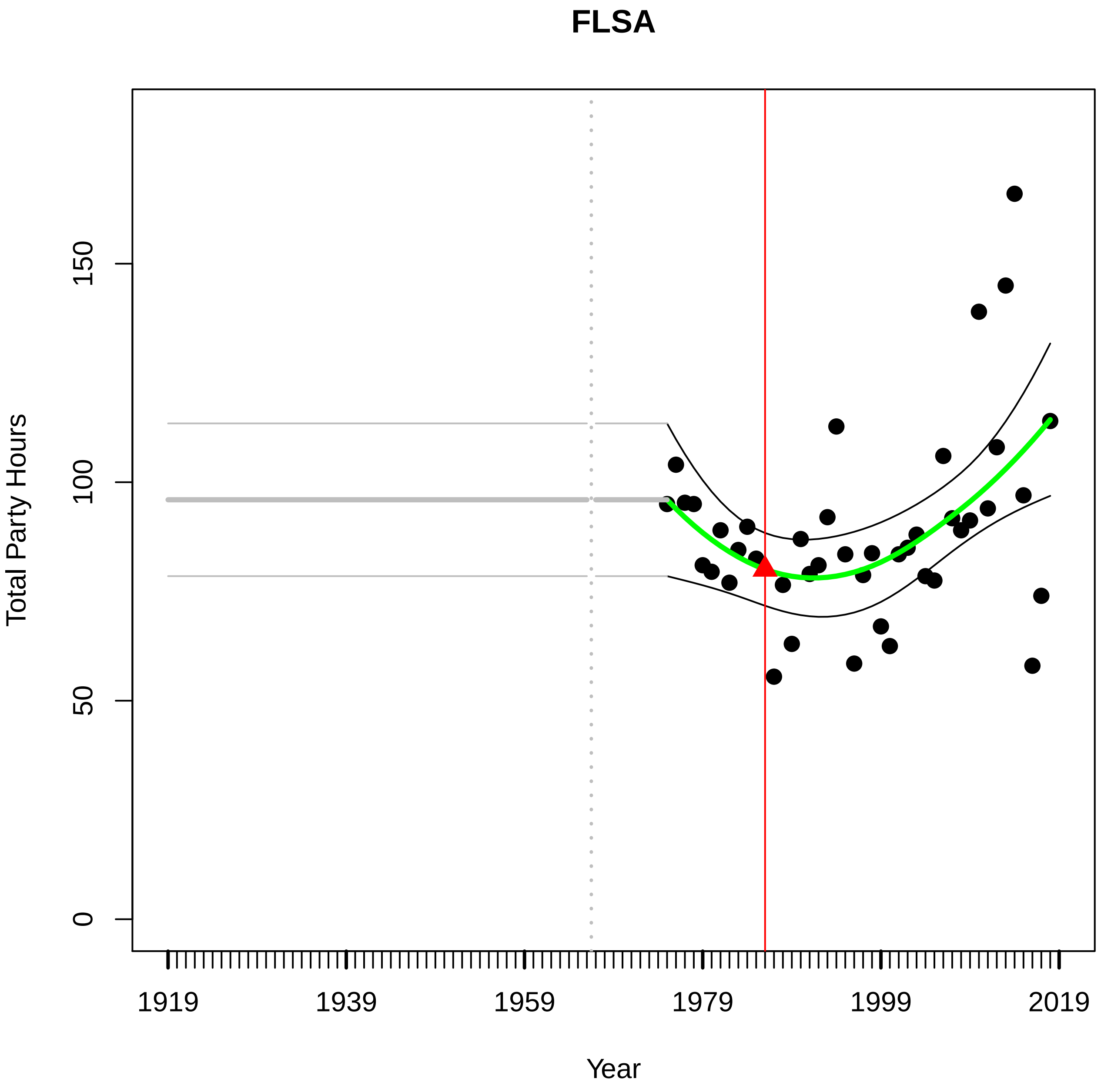

FLSA
Total Party Hours
0
50
100
150
1919
1939
1959
1979
1999
2019
Year

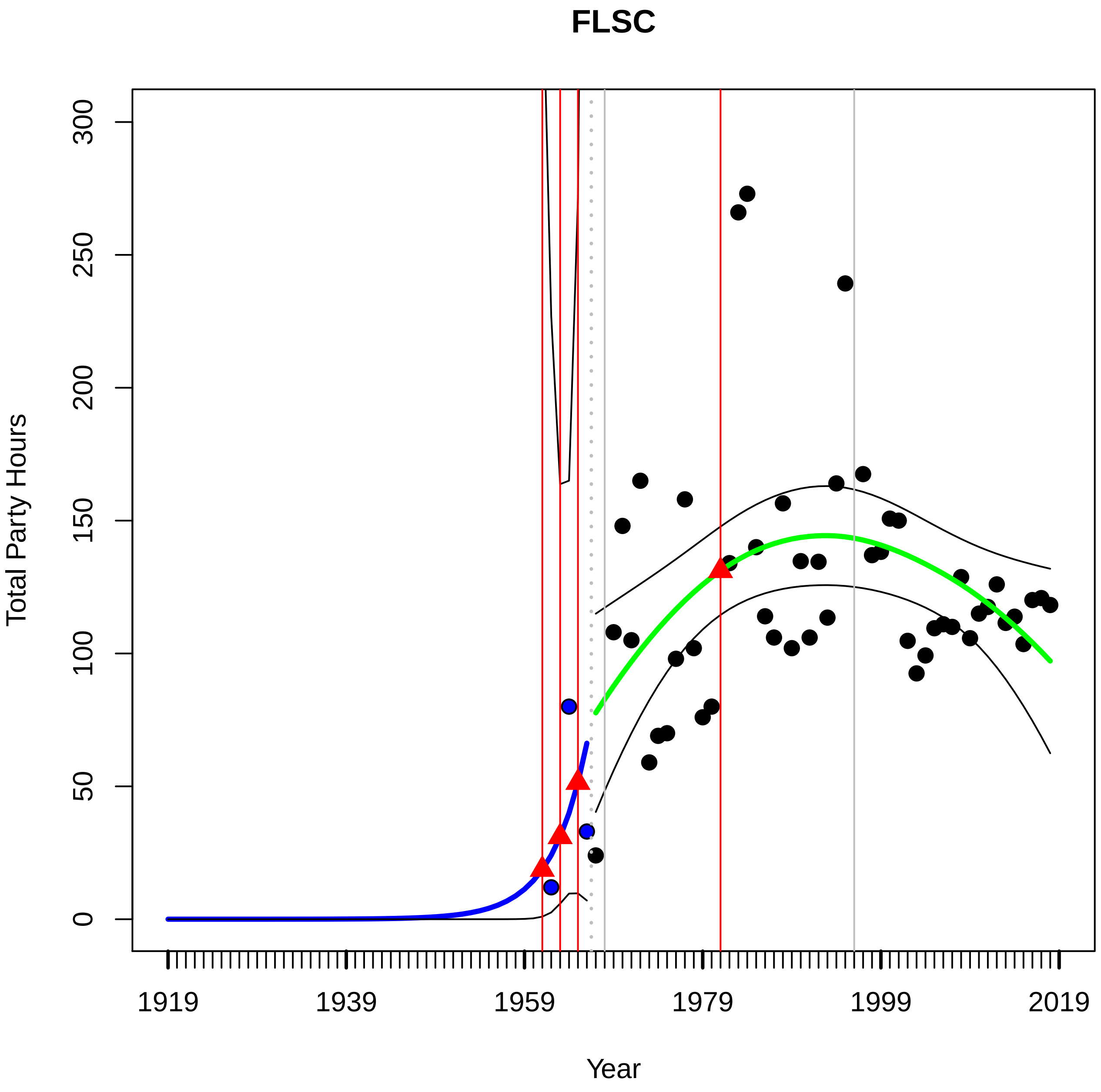
FLSC
Total Party Hours
Year
0
50
100
150
200
250
300
1919
1939
1959
1979
1999
2019

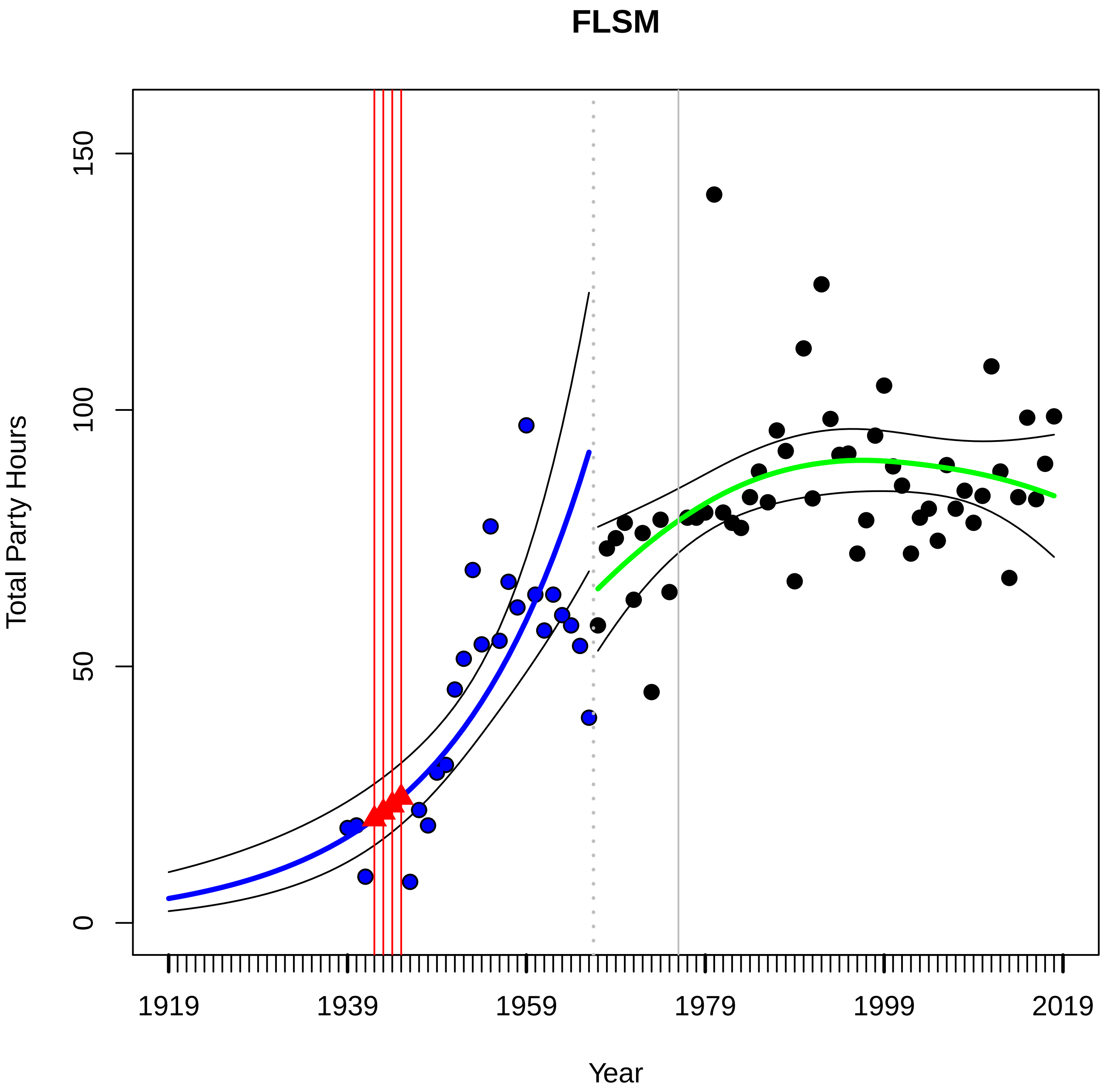

FLSM
Total Party Hours
0
50
100
150
1919
1939
1959
1979
1999
2019
Year

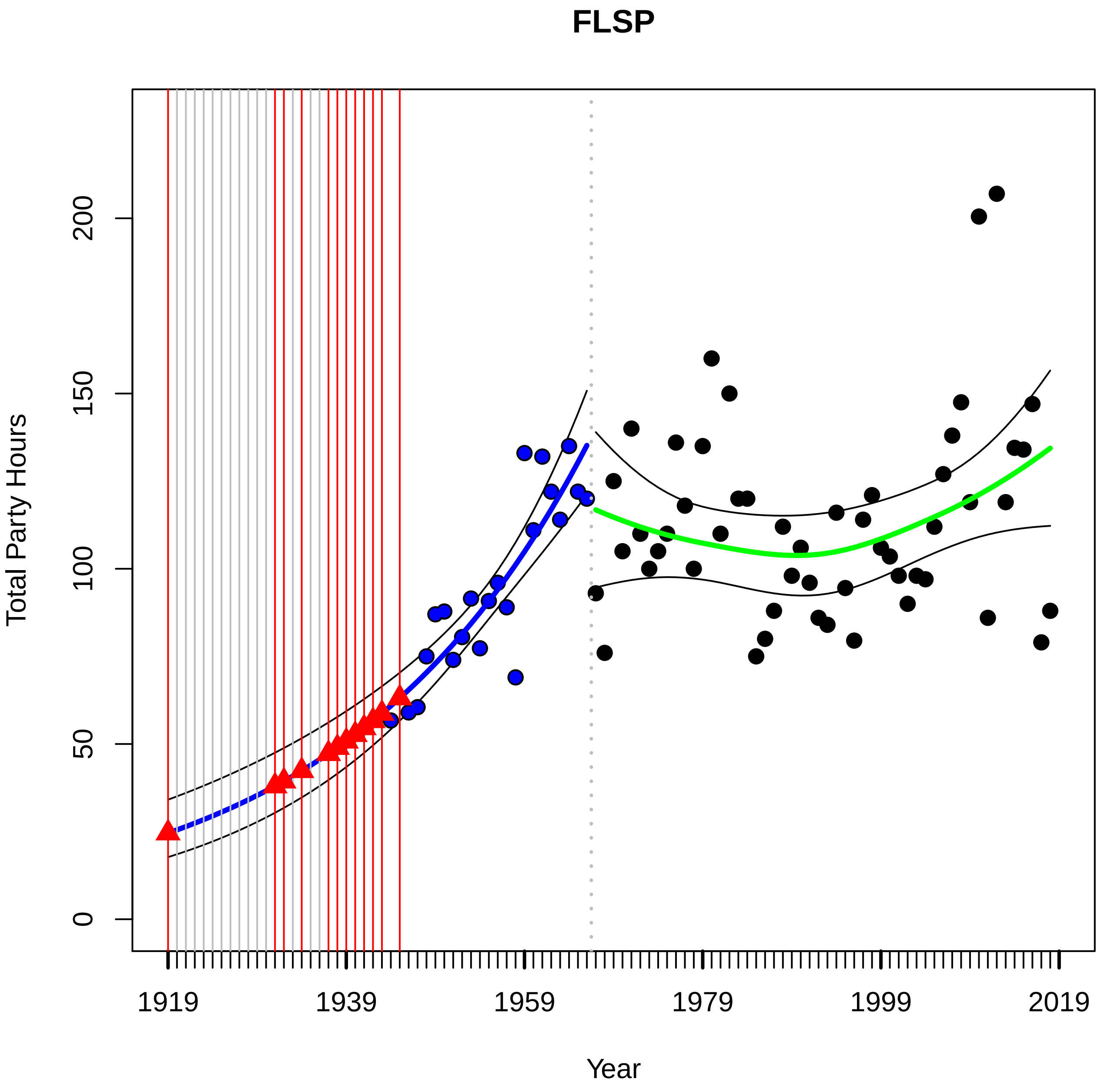
FLSP
Total Party Hours
0
50
100
150
200
1919
1939
1959
1979
1999
2019
Year

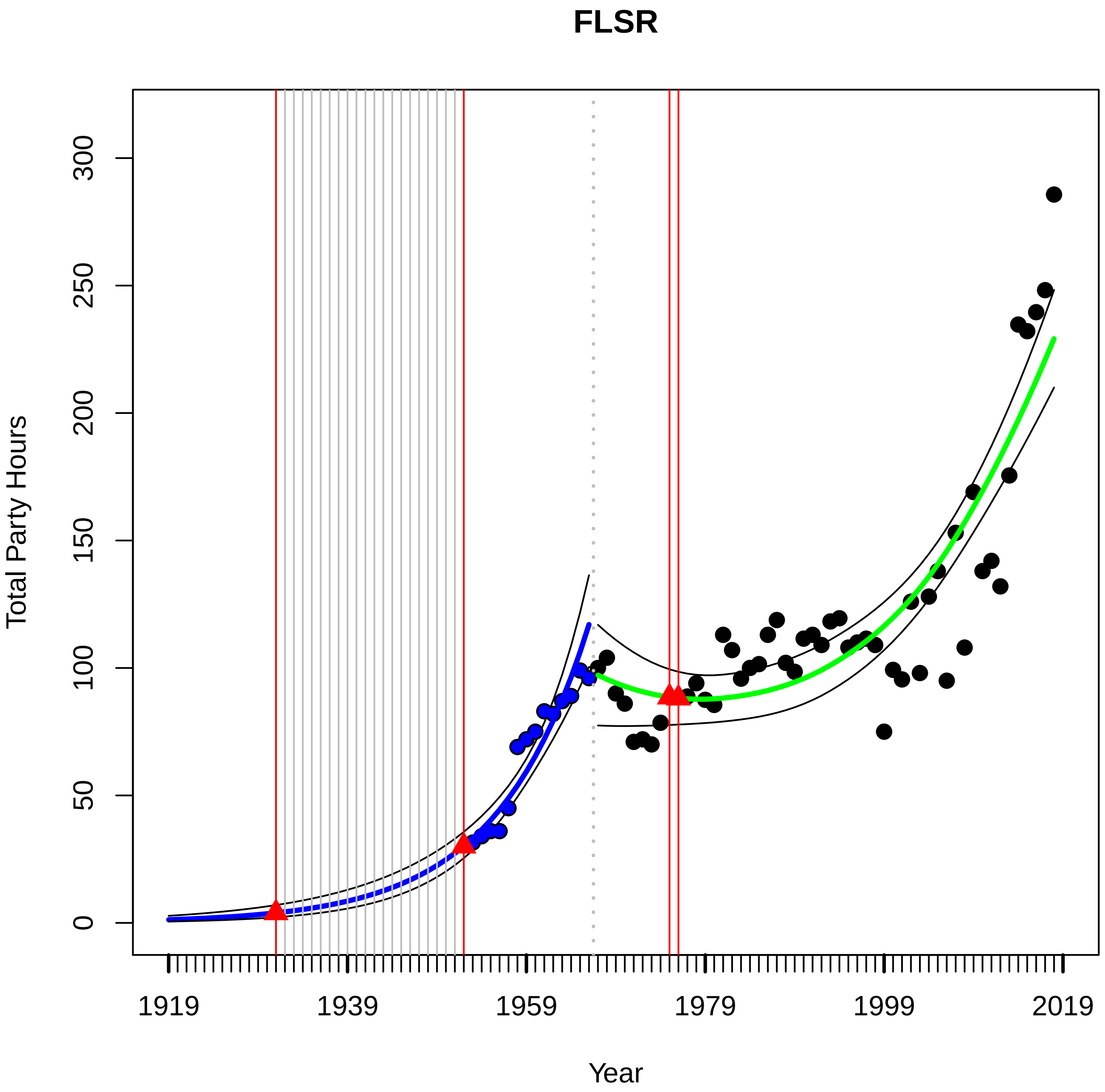

FLSR
Total Party Hours
0
50
100
150
200
250
300
1919
1939
1959
1979
1999
2019
Year

FLST

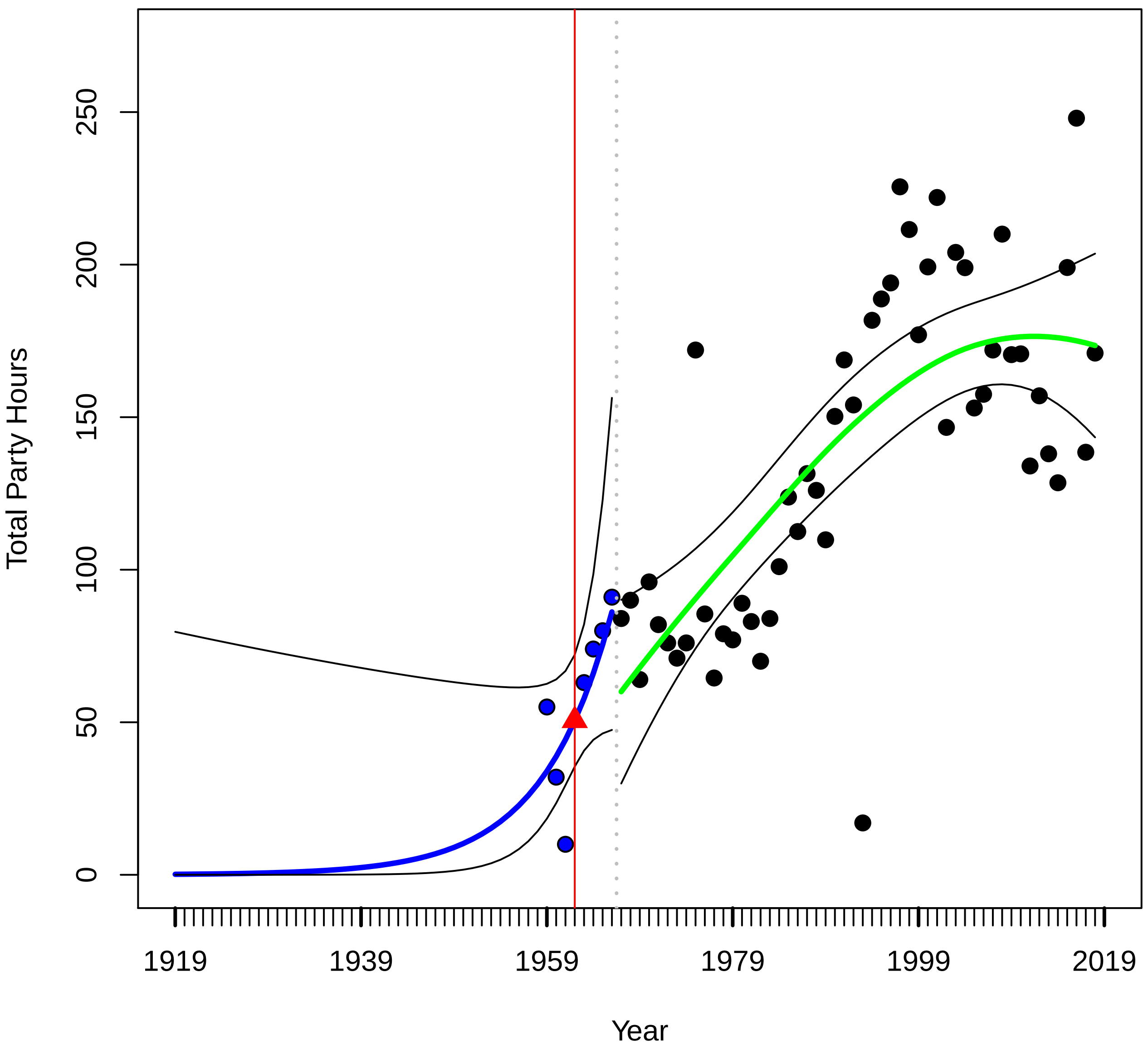

Total Party Hours
0
50
100
150
200
250
1919
1939
1959
1979
1999
2019
Year

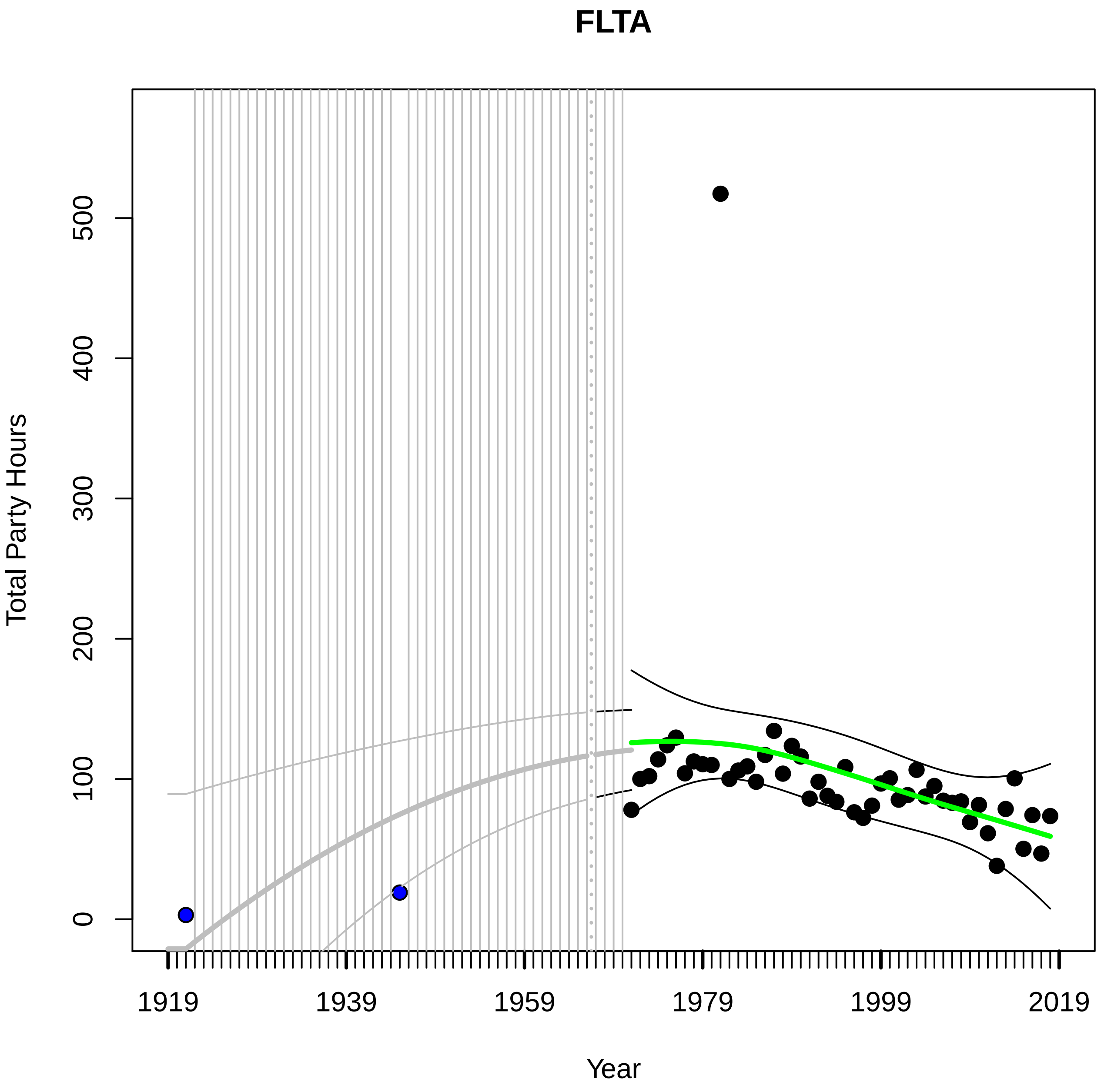
FLTA
Total Party Hours
0
100
200
300
400
500
1919
1939
1959
1979
1999
2019
Year

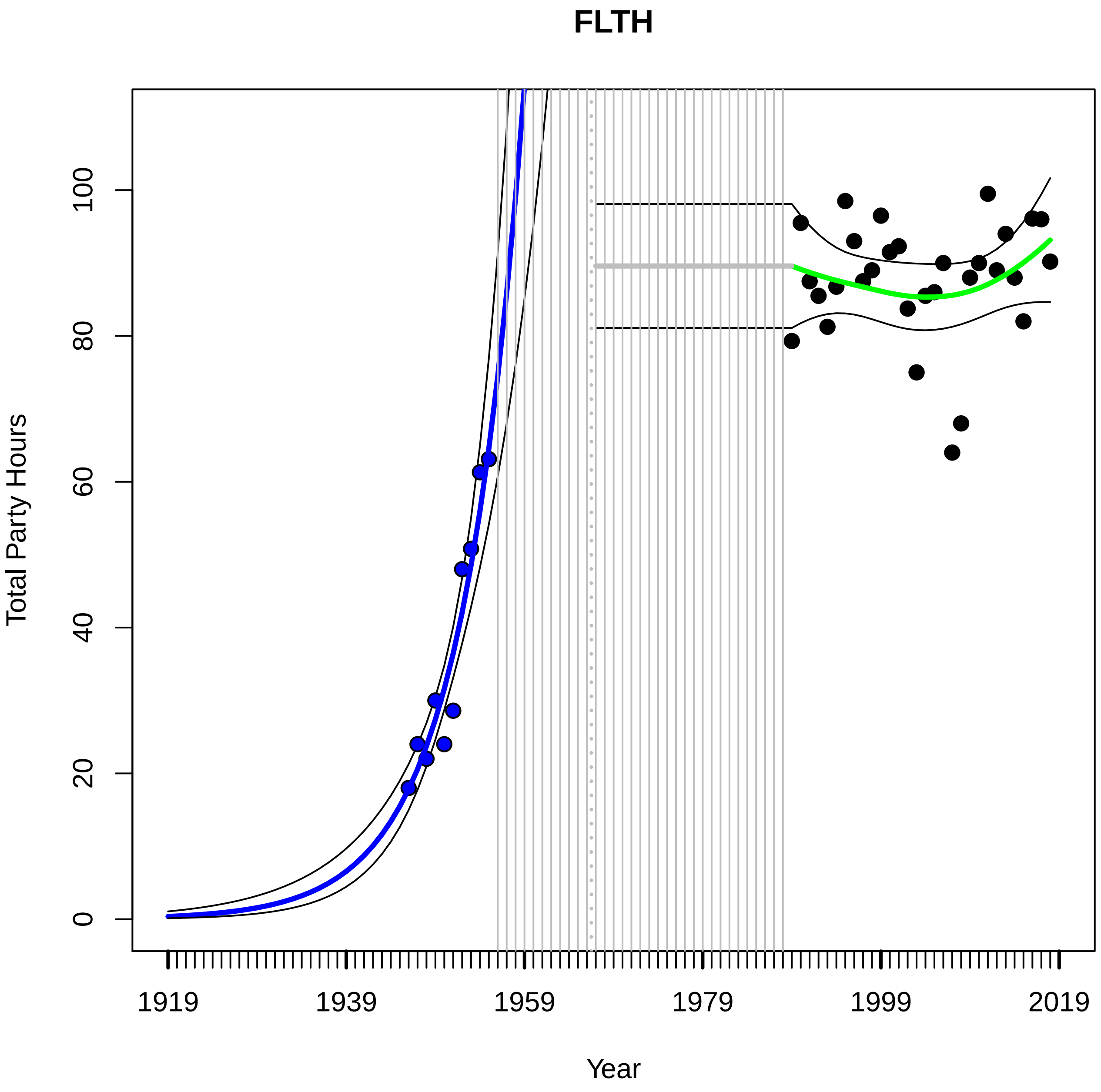
FLTH
Total Party Hours
0
20
40
60
80
100
1919
1939
1959
1979
1999
2019
Year

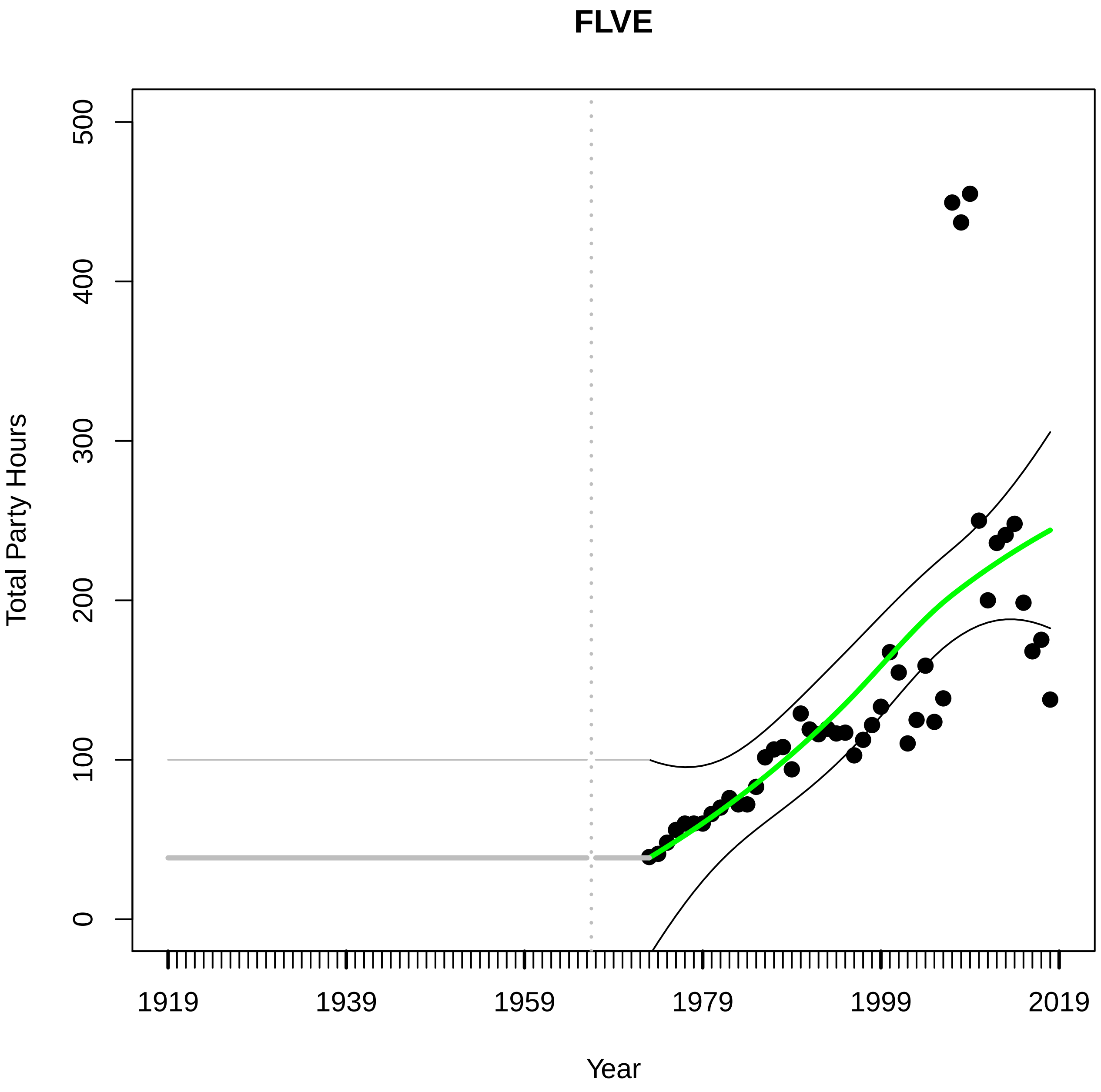

FLVE
Total Party Hours
0
100
200
300
400
500
1919
1939
1959
1979
1999
2019
Year

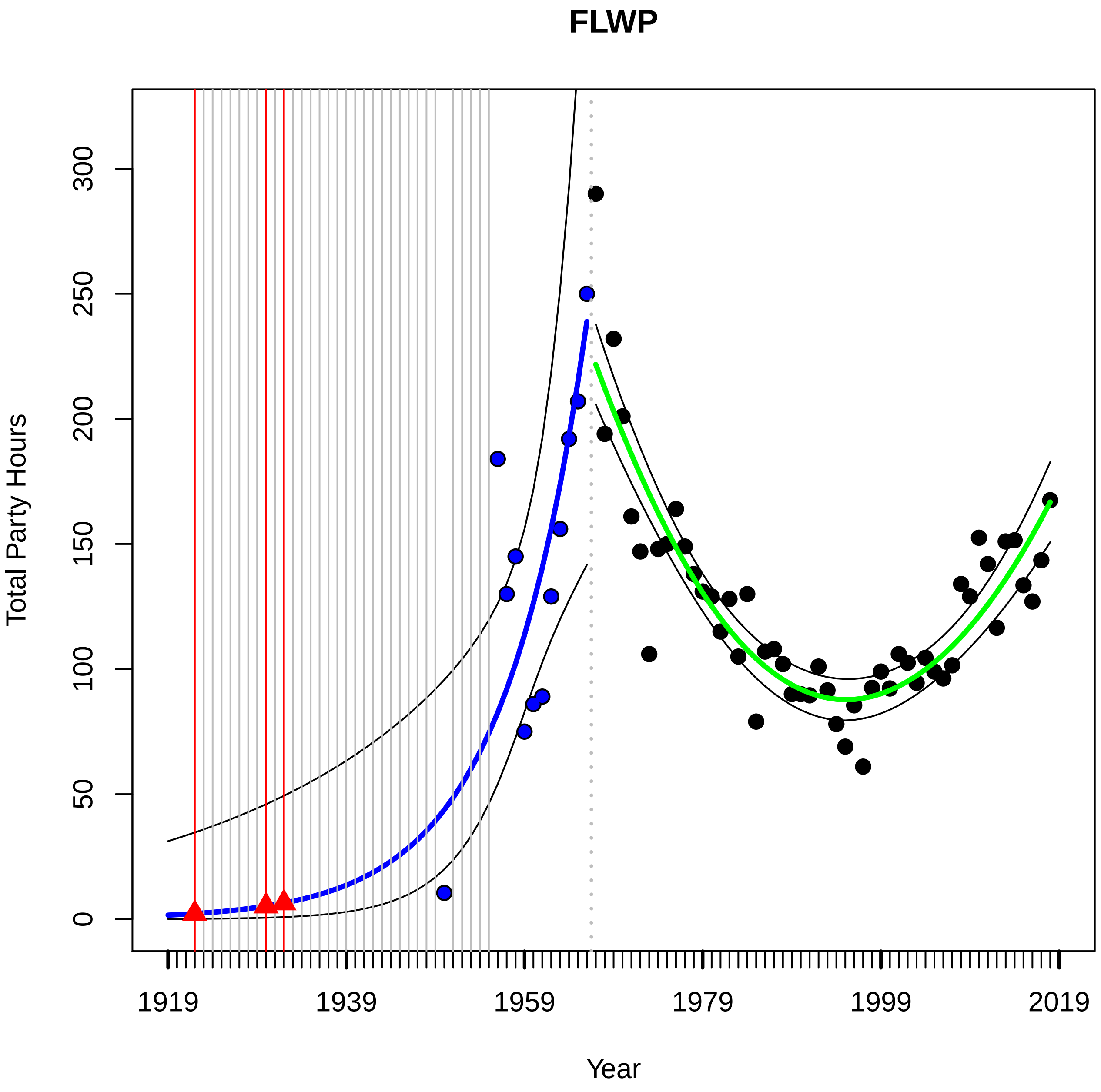

FLWP
Total Party Hours
0
50
100
150
200
250
300
1919
1939
1959
1979
1999
2019
Year

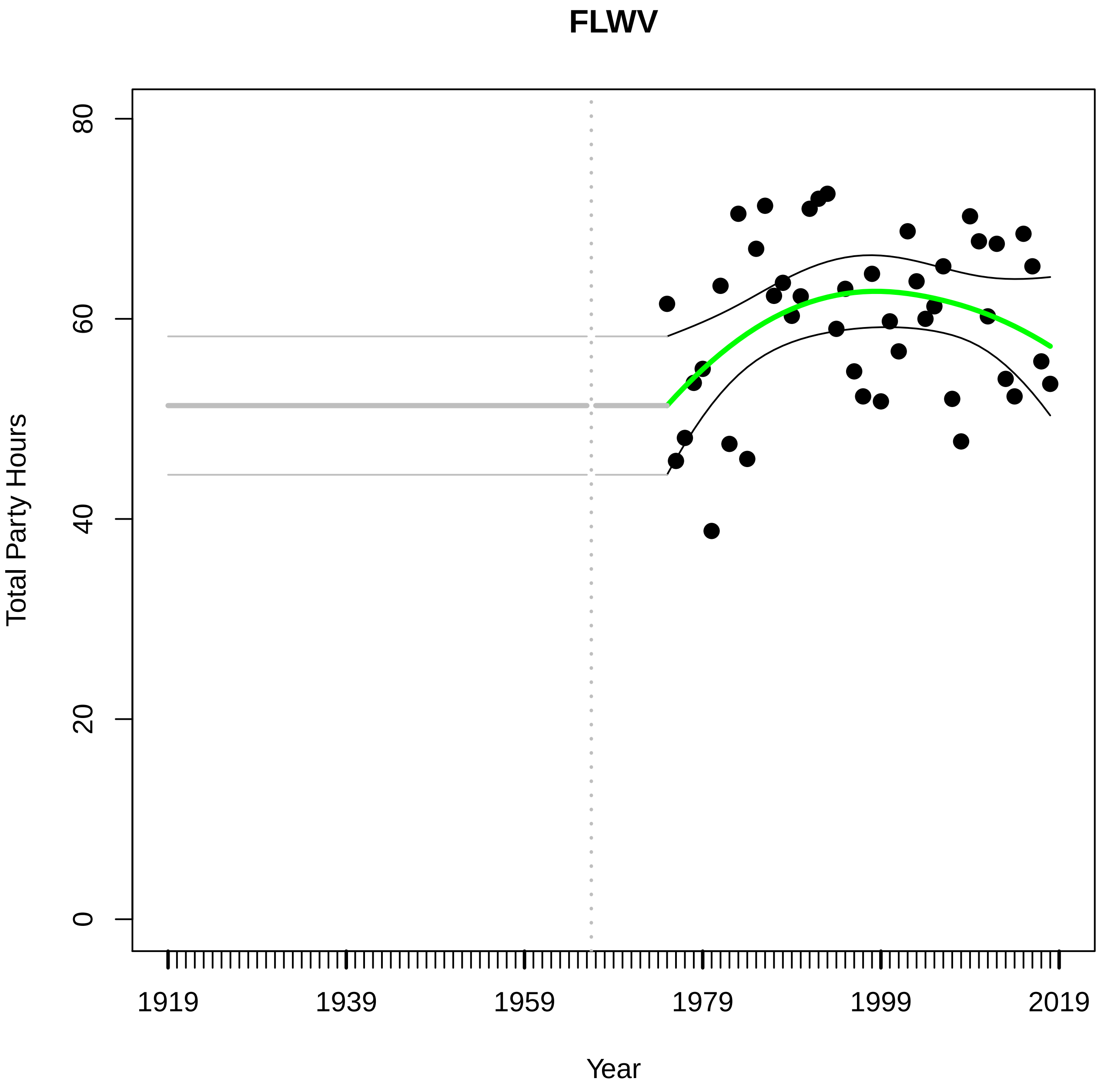
FLWV
Total Party Hours
Year
0
20
40
60
80
1919
1939
1959
1979
1999
2019

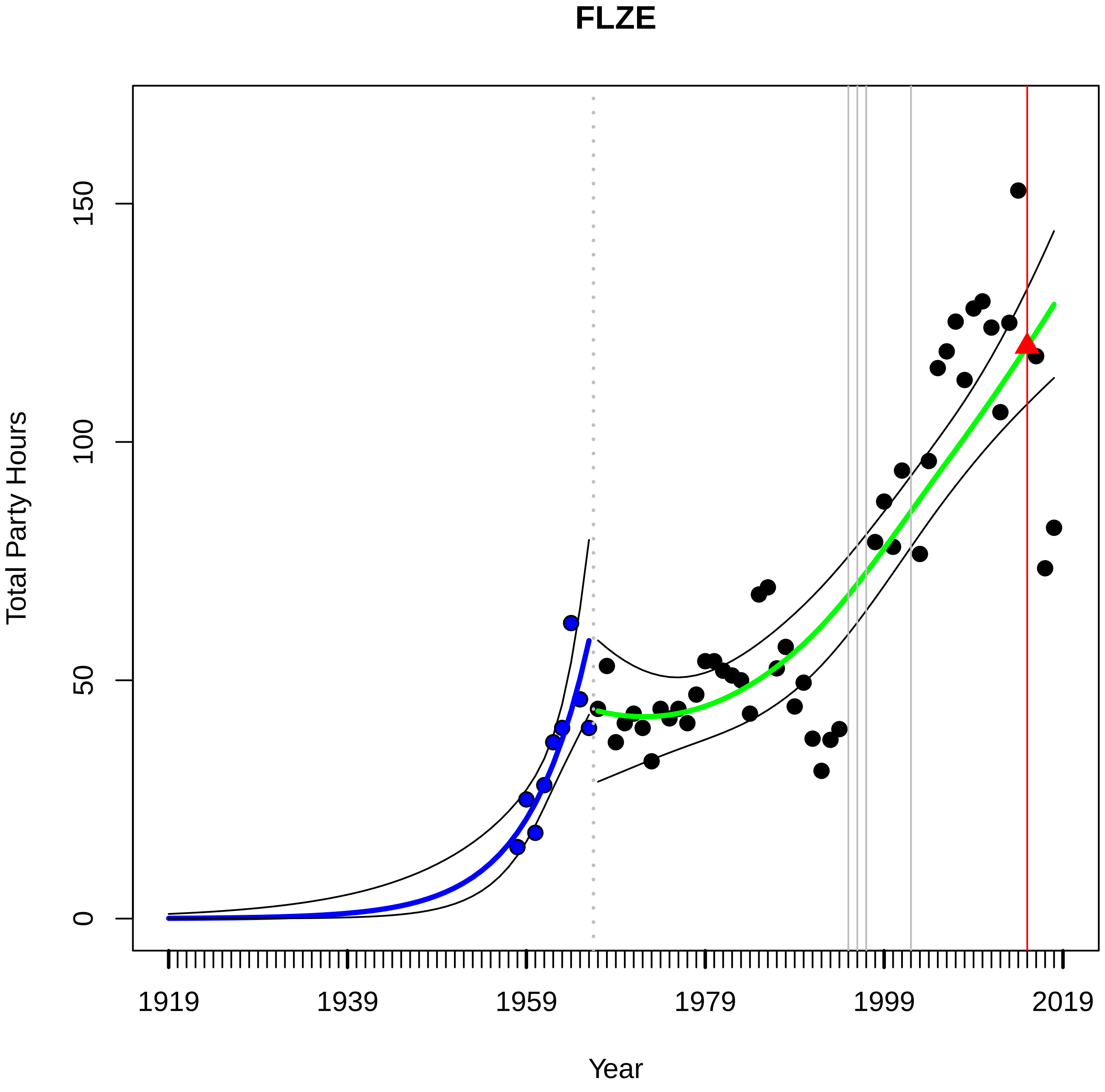

FLZE
Total Party Hours
0
50
100
150
1919
1939
1959
1979
1999
2019
Year

GA0D

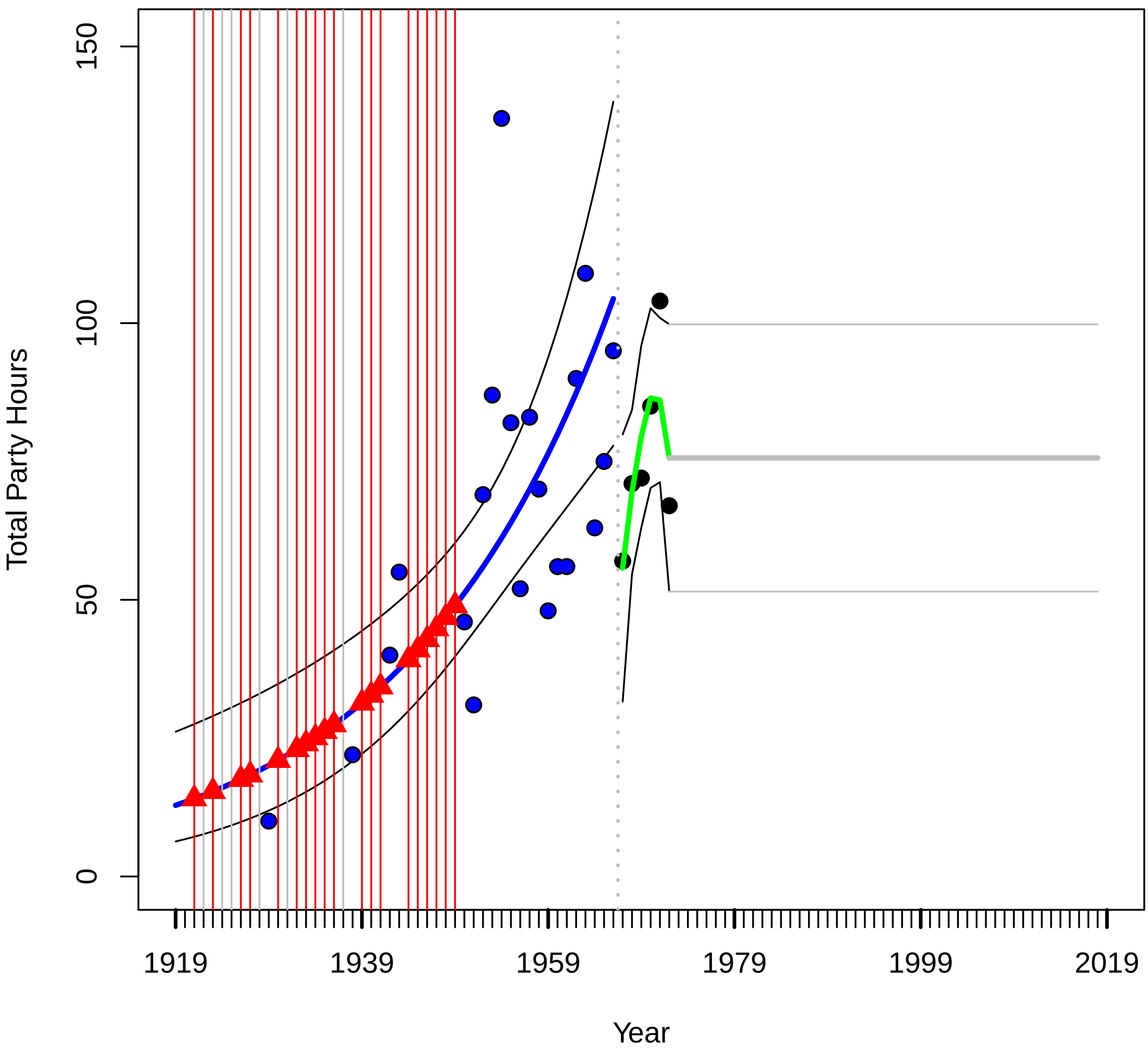

Total Party Hours
150
100
50
0
1919
1939
1959
1979
1999
2019
Year

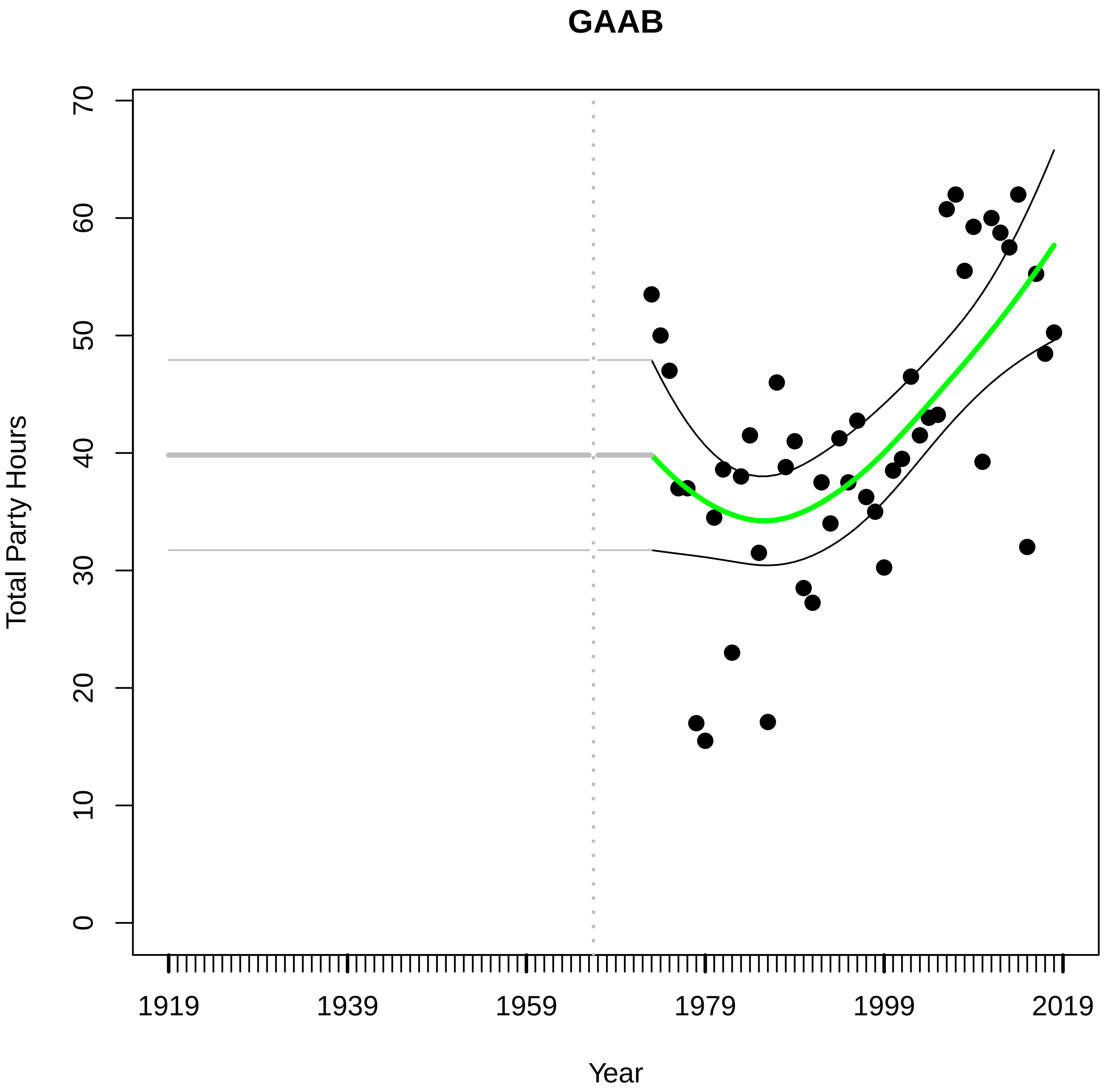
GAAB
Total Party Hours
0
10
20
30
40
50
60
70
1919
1939
1959
1979
1999
2019
Year

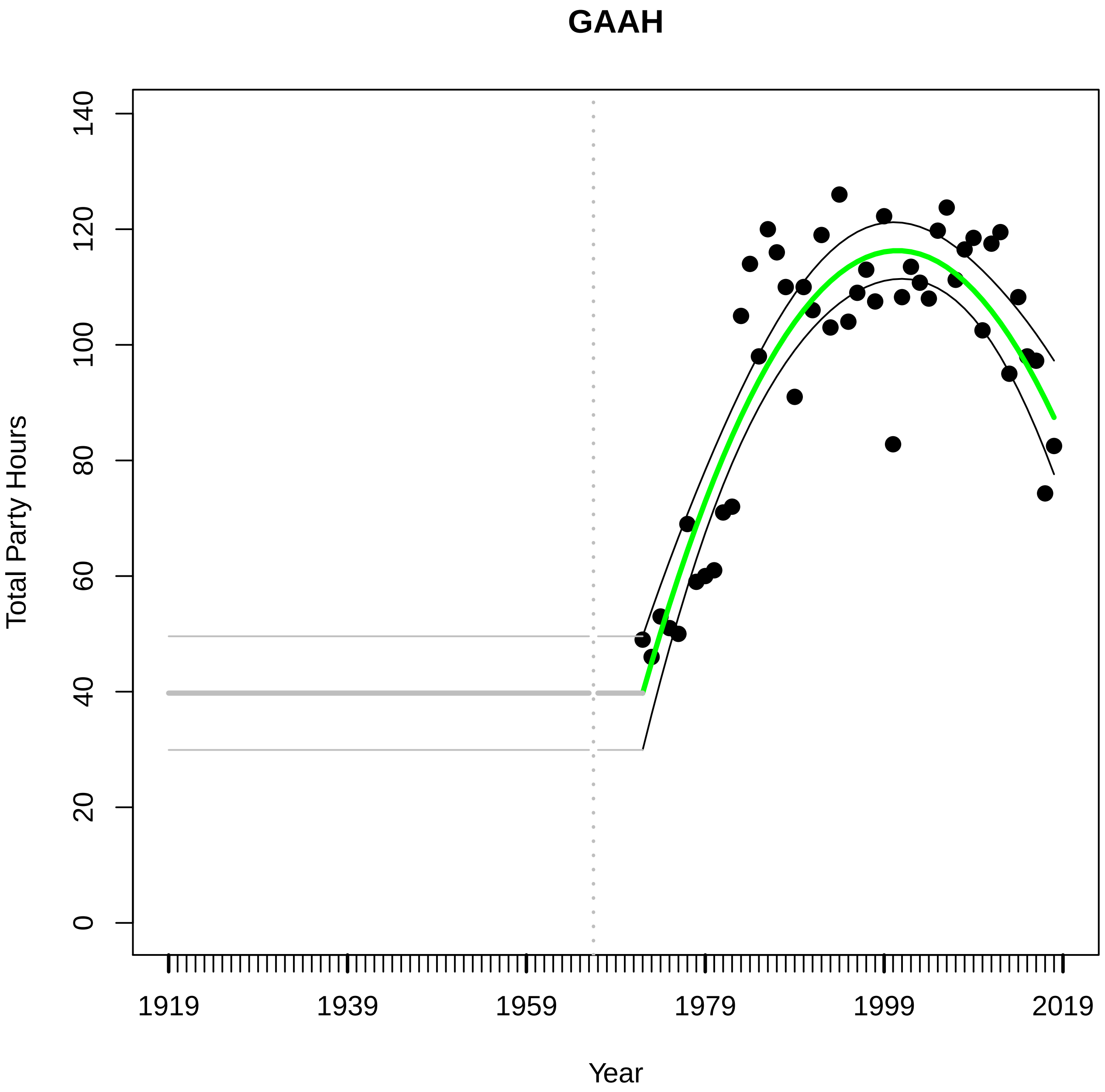

GAAH
Total Party Hours
0
20
40
60
80
100
120
140
1919
1939
1959
1979
1999
2019
Year

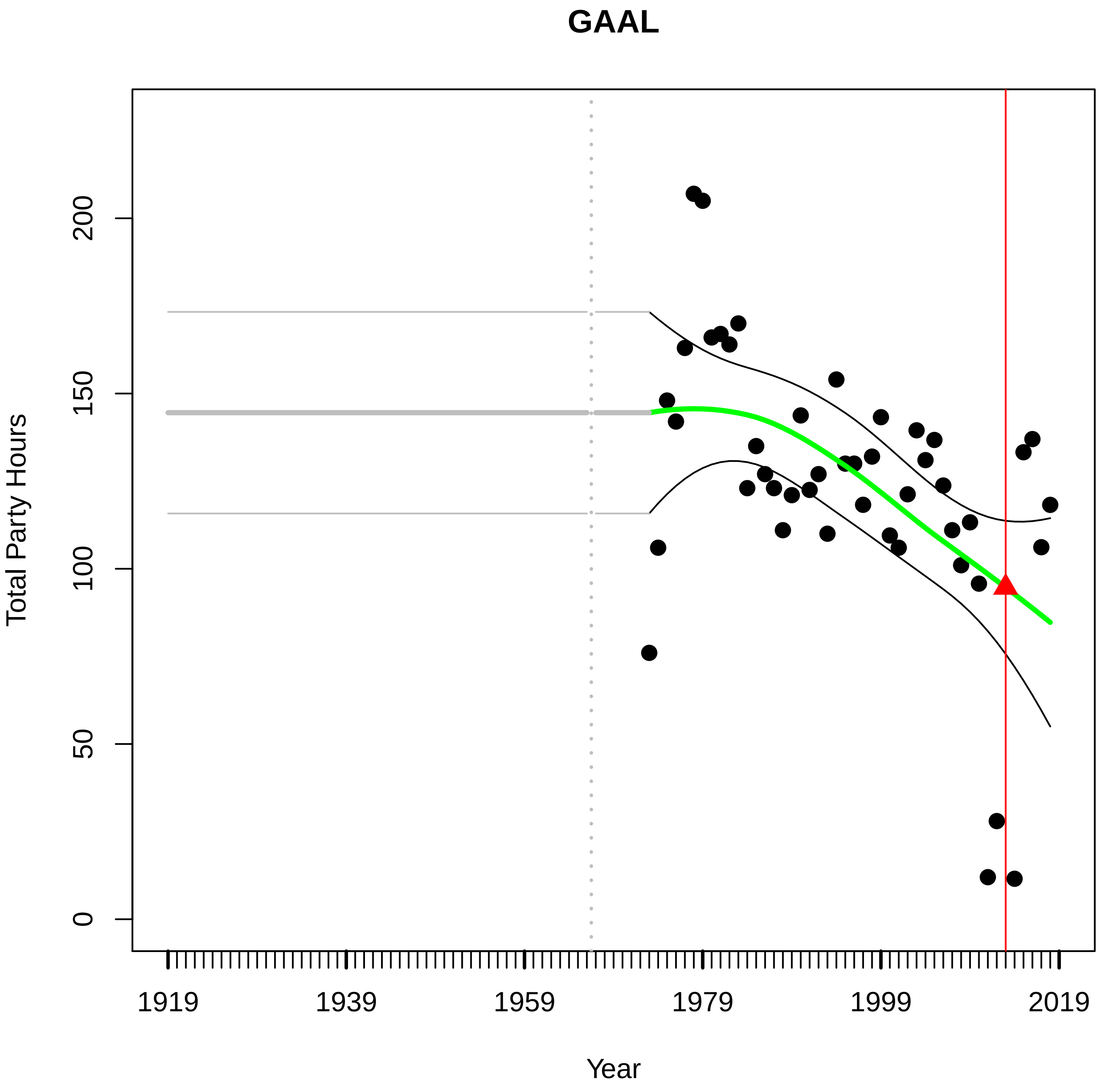
GAAL
Total Party Hours
0
50
100
150
200
1919
1939
1959
1979
1999
2019
Year

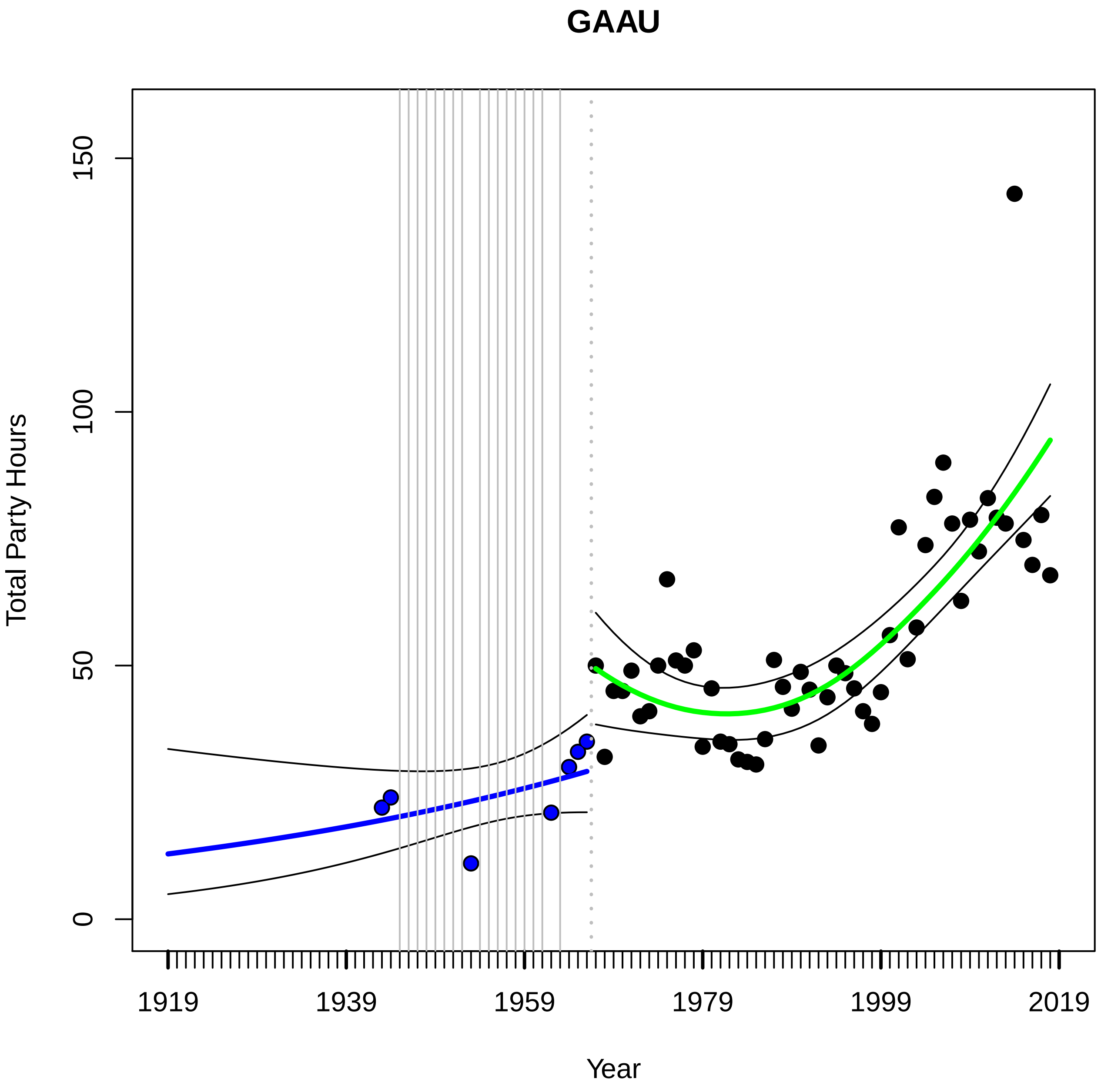

GAAU
Total Party Hours
0
50
100
150
1919
1939
1959
1979
1999
2019
Year

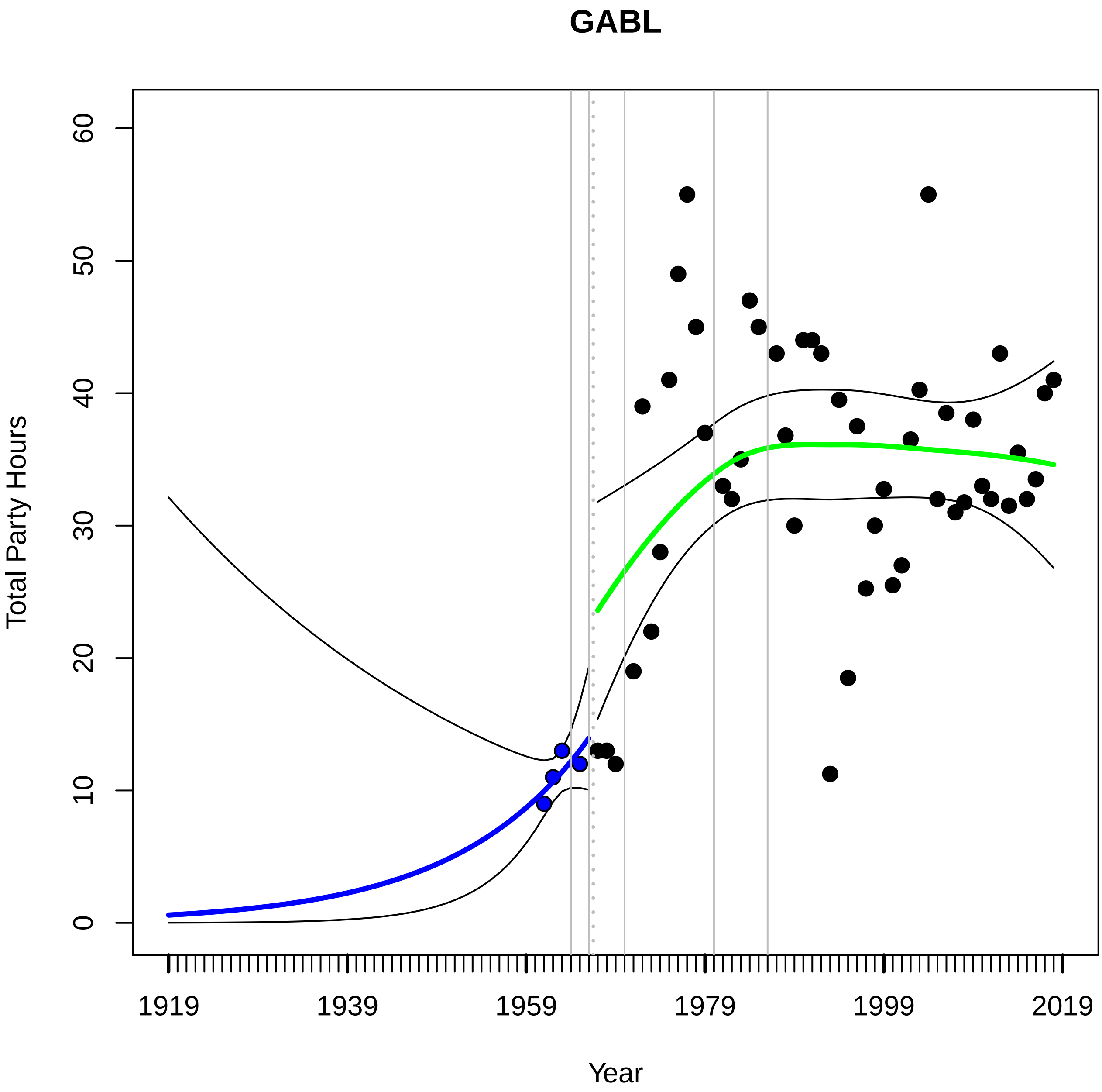
GABL
Total Party Hours
Year
0
10
20
30
40
50
60
1919
1939
1959
1979
1999
2019

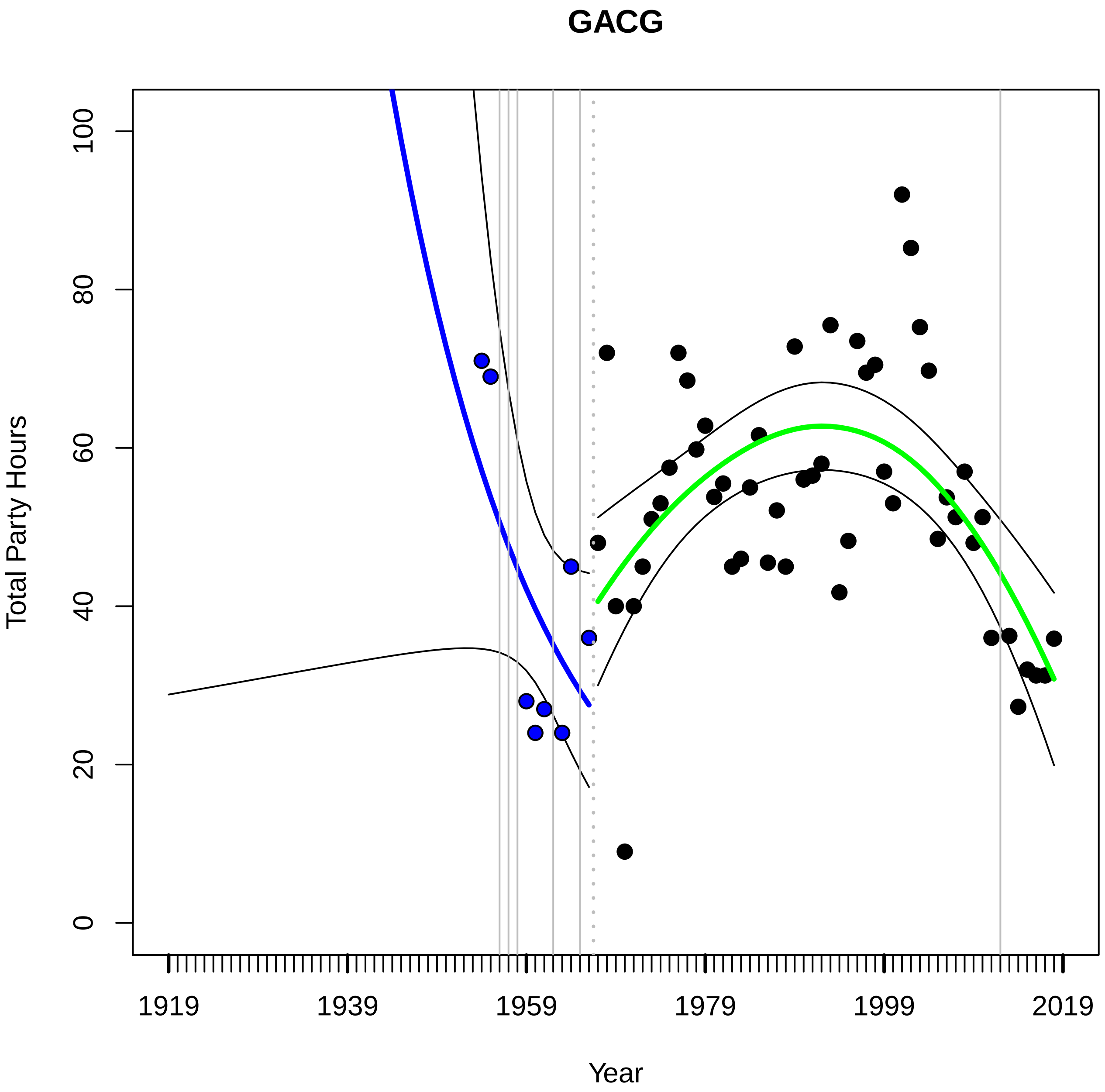
GACG
Total Party Hours
100
80
60
40
20
0
1919
1939
1959
1979
1999
2019
Year

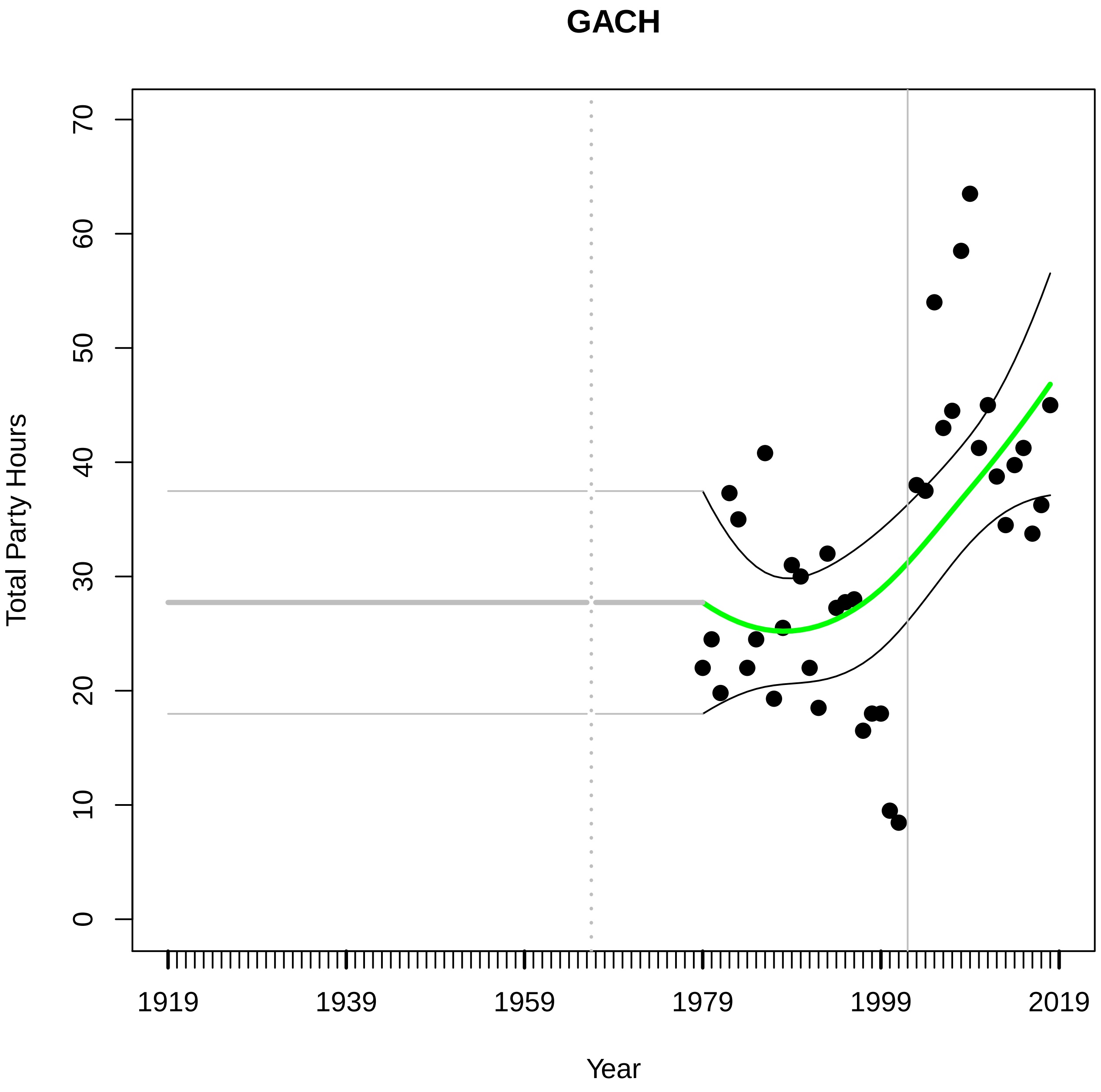

GACH
Total Party Hours
Year
0
10
20
30
40
50
60
70
1919
1939
1959
1979
1999
2019

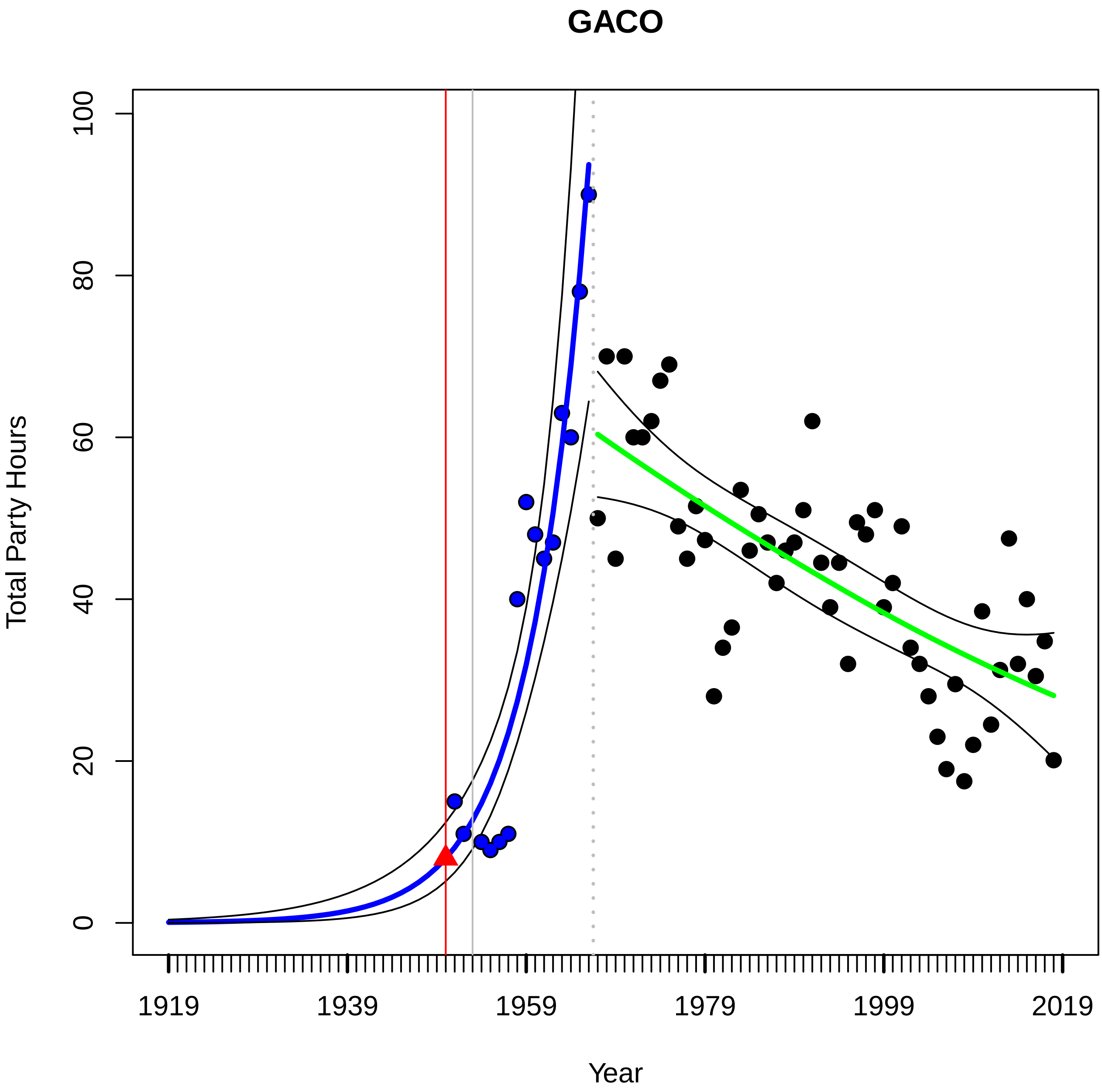

GACO
Total Party Hours
0
20
40
60
80
100
1919
1939
1959
1979
1999
2019
Year

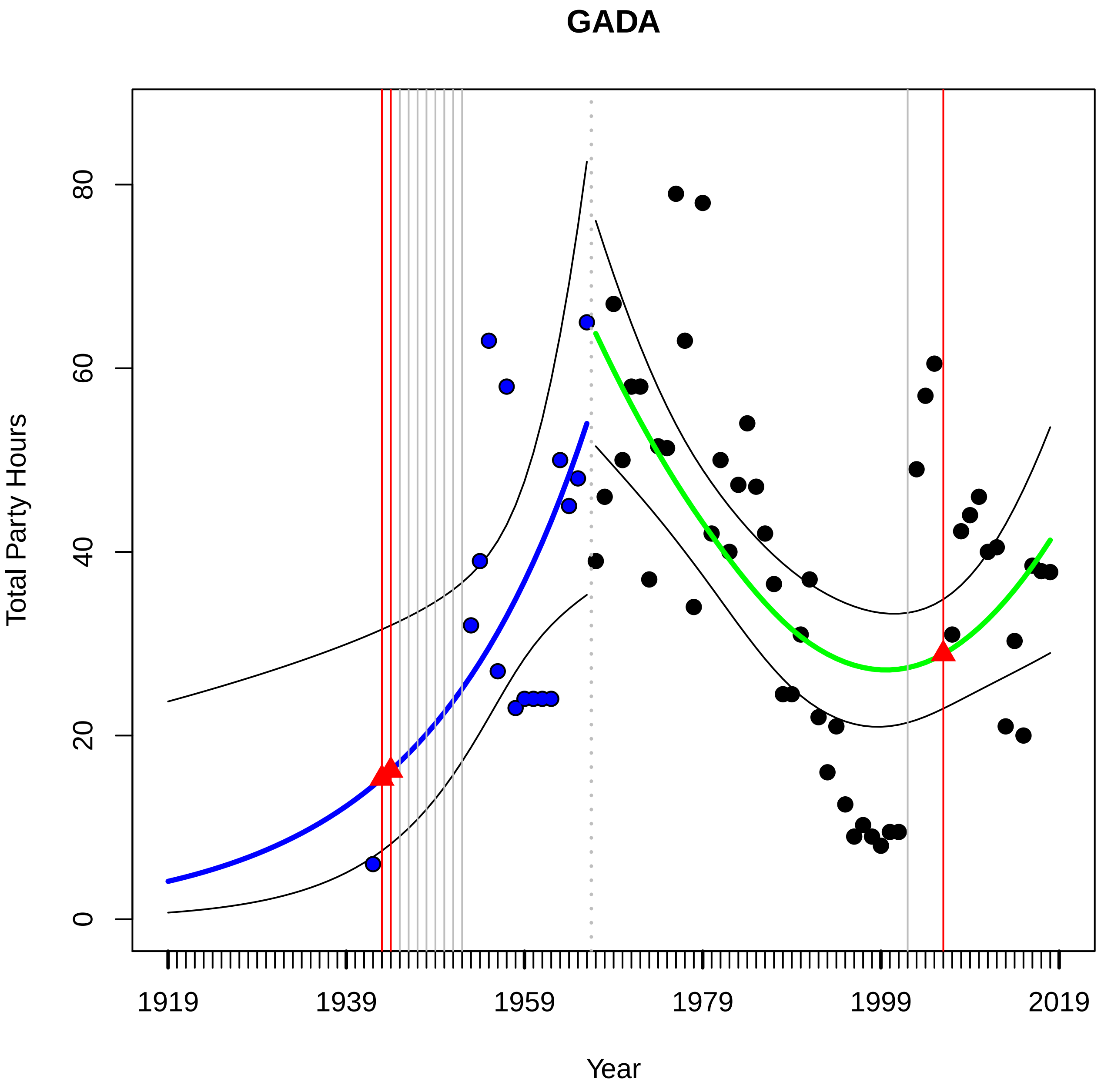
GADA
Total Party Hours
0
20
40
60
80
1919
1939
1959
1979
1999
2019
Year

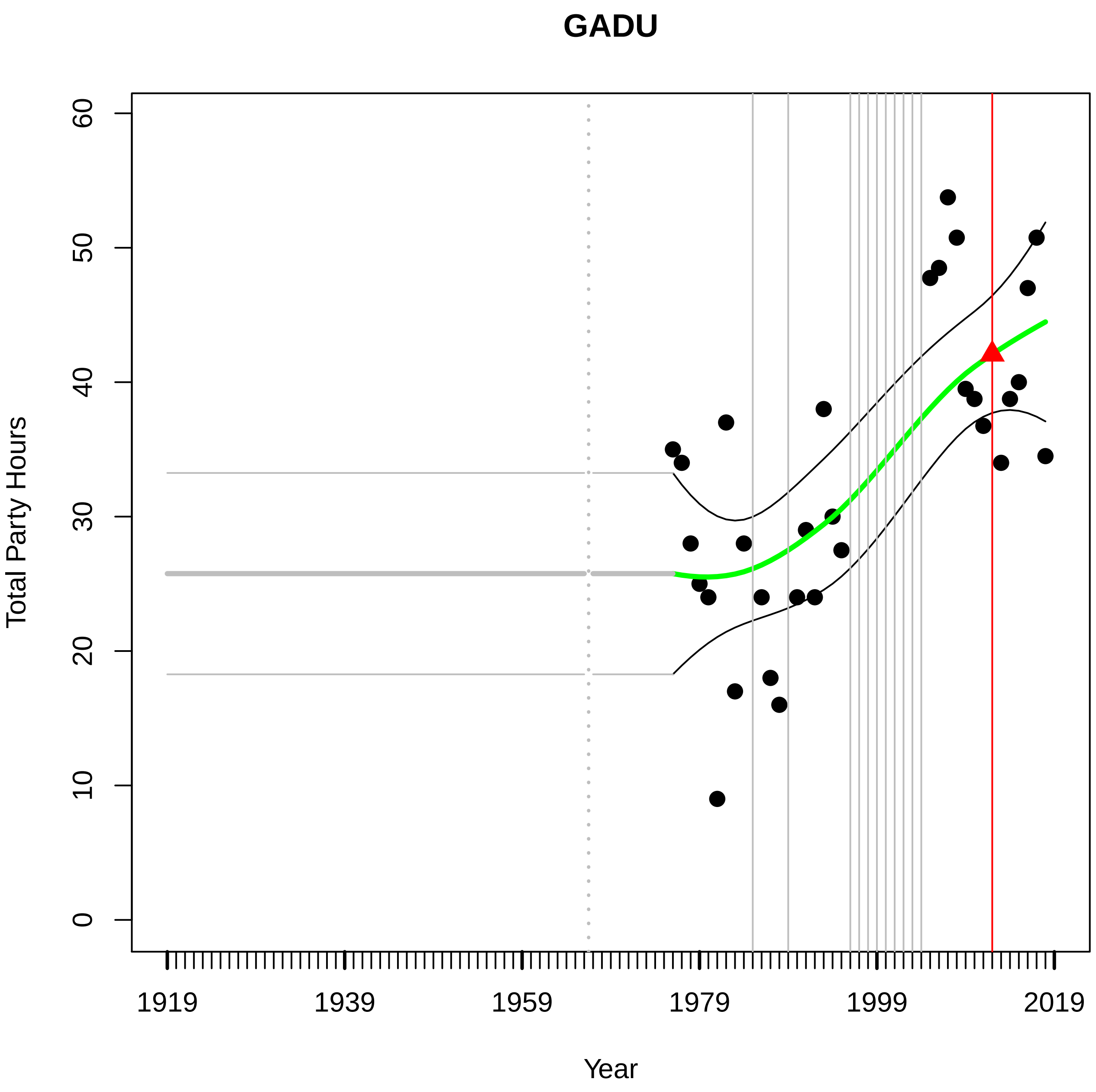

GADU
Total Party Hours
0
10
20
30
40
50
60
1919
1939
1959
1979
1999
2019
Year

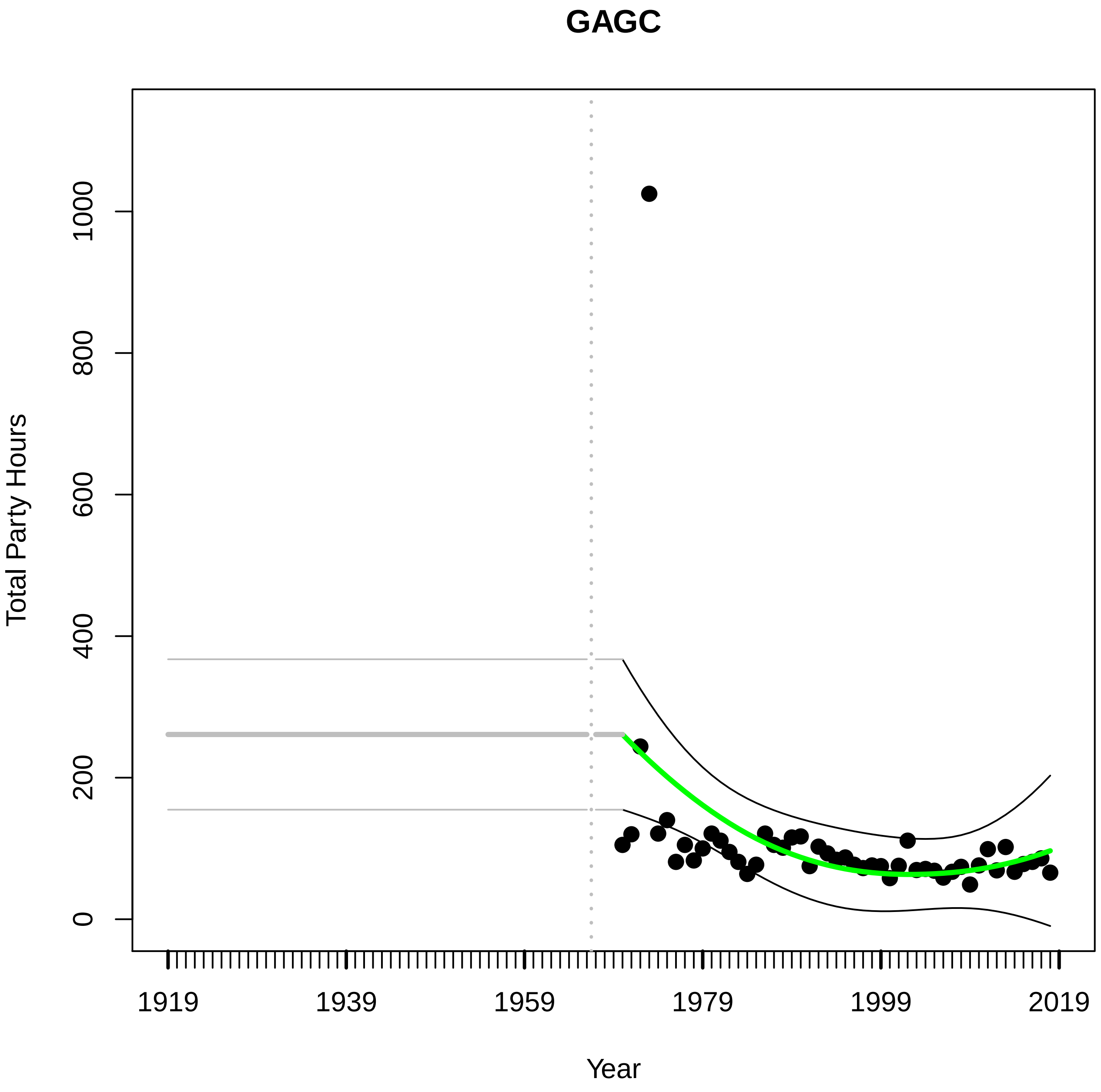
GAGC
Total Party Hours
0
200
400
600
800
1000
1919
1939
1959
1979
1999
2019
Year

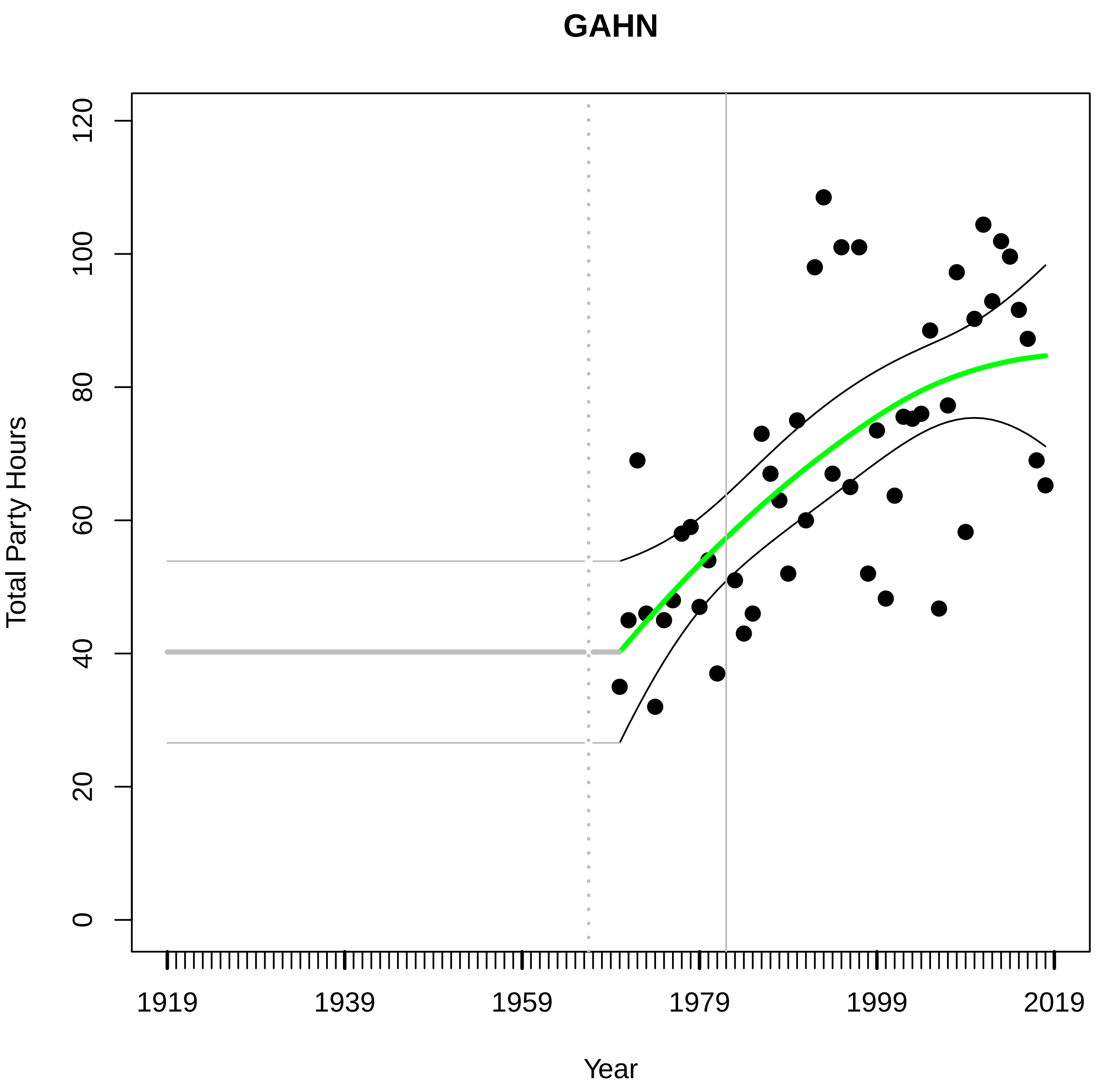

GAHN
Total Party Hours
Year
0
20
40
60
80
100
120
1919
1939
1959
1979
1999
2019

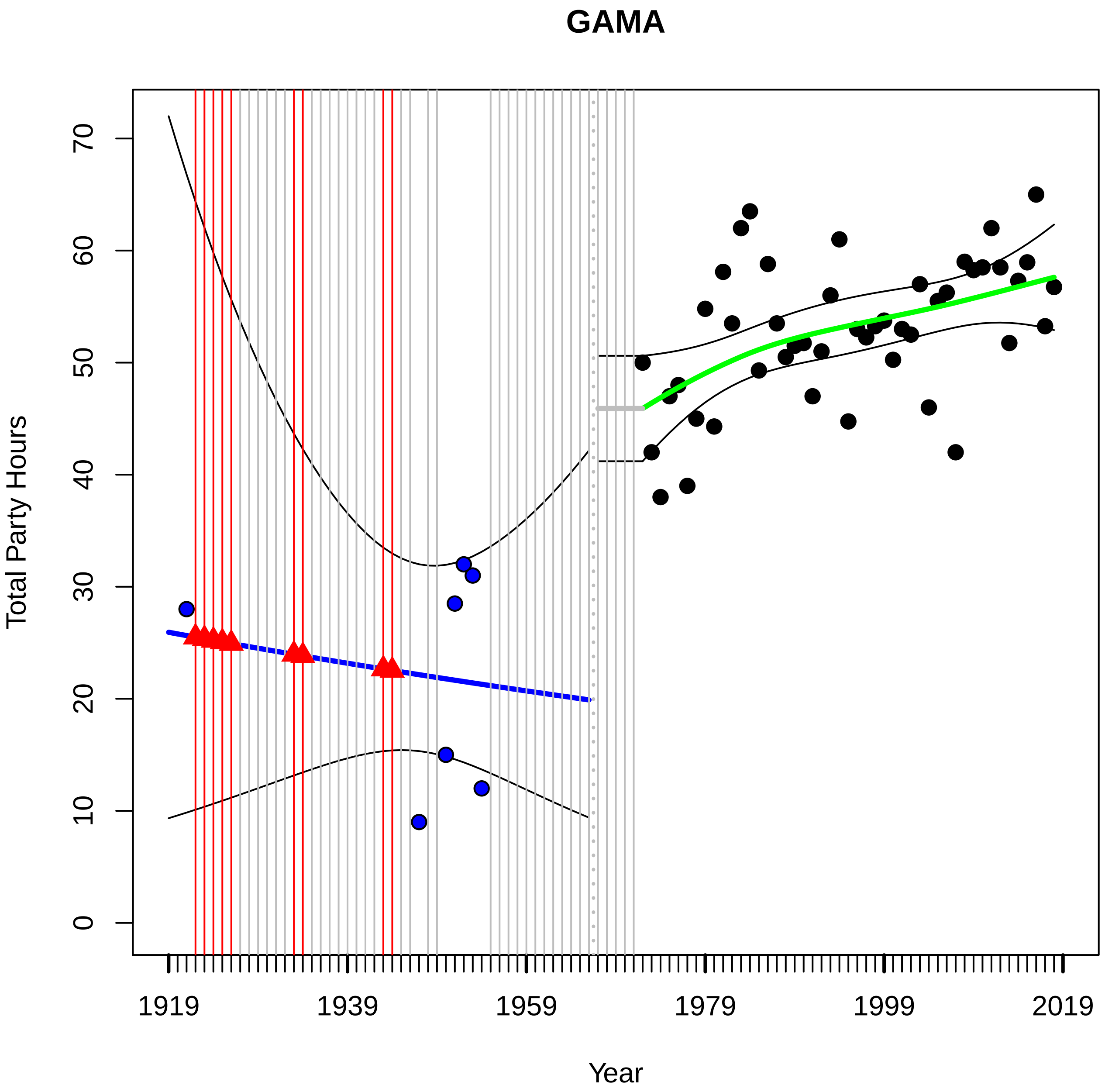
GAMA
Total Party Hours
Year
0
10
20
30
40
50
60
70
1919
1939
1959
1979
1999
2019

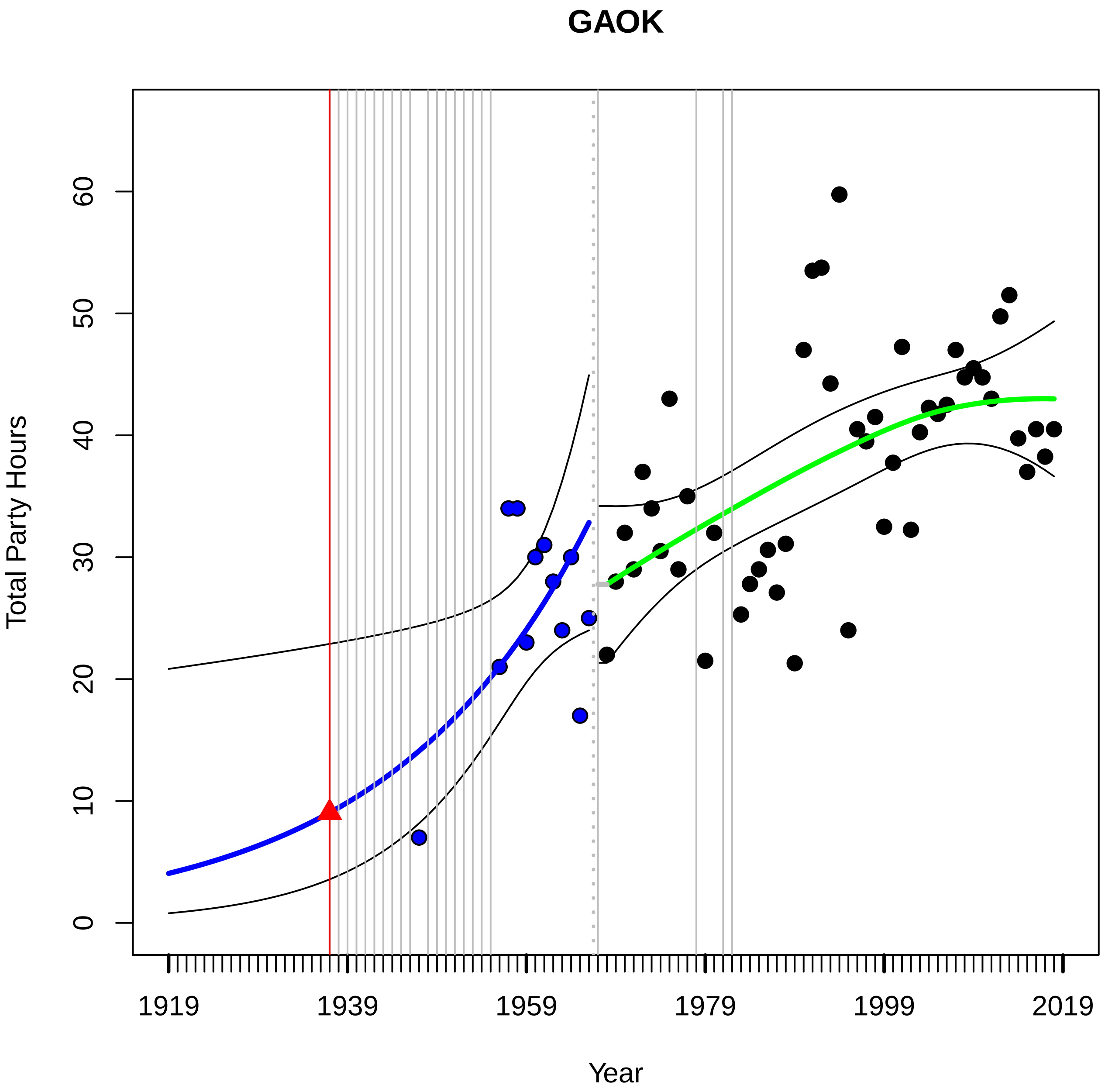

GAOK
Total Party Hours
Year
0
10
20
30
40
50
60
1919
1939
1959
1979
1999
2019

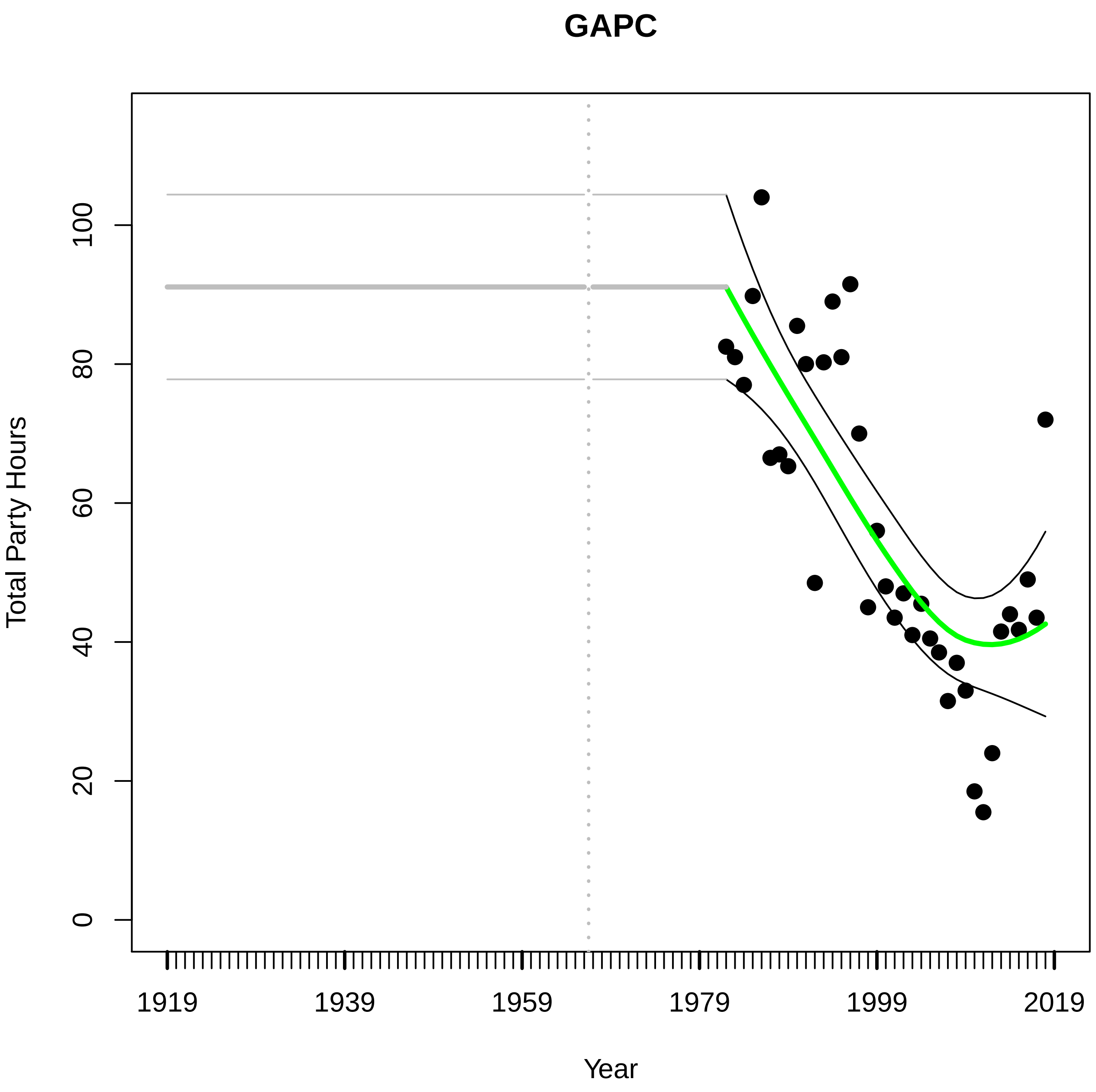
GAPC
Total Party Hours
0
20
40
60
80
100
1919
1939
1959
1979
1999
2019
Year

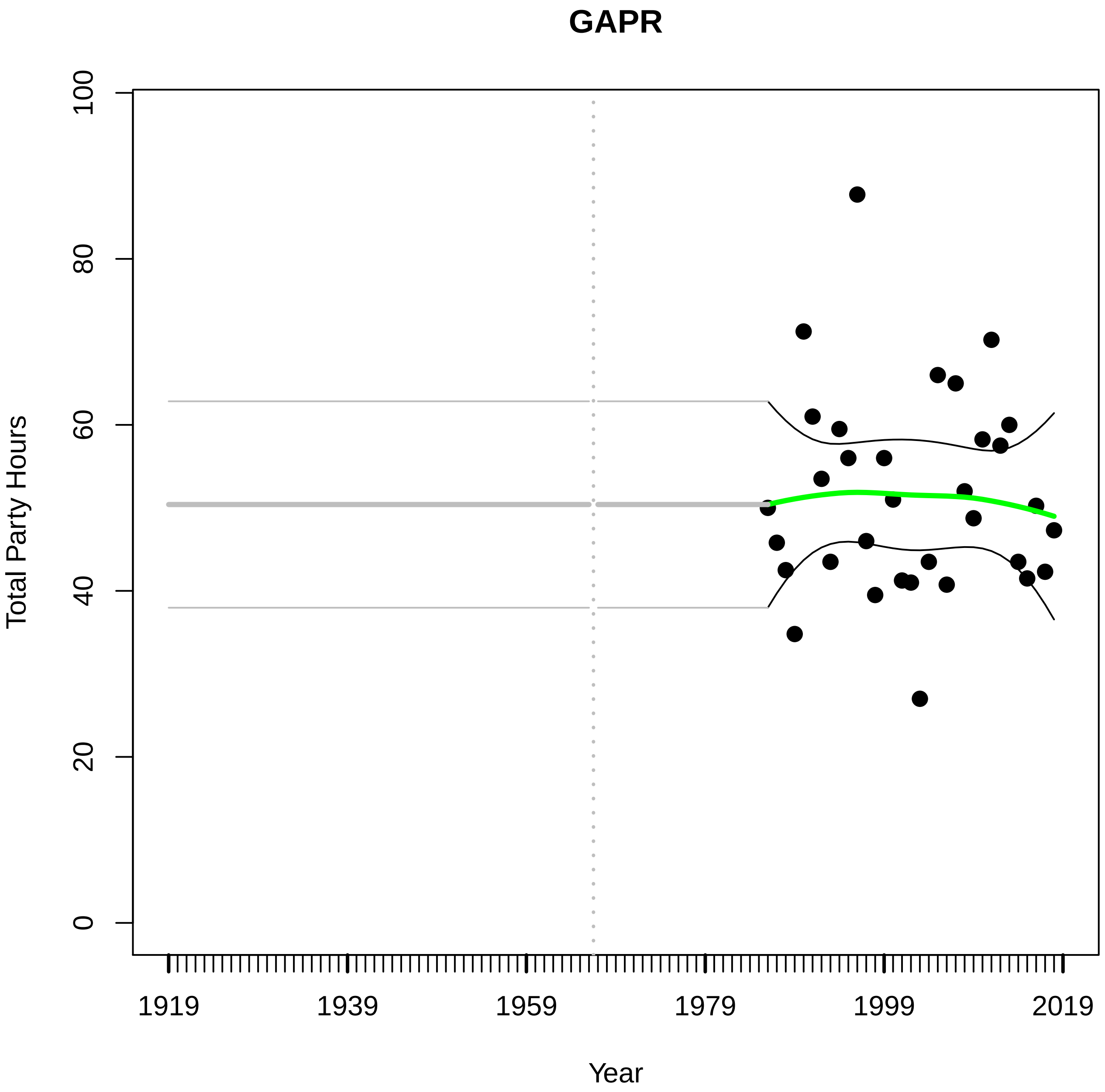

GAPR
Total Party Hours
100
80
60
40
20
0
1919
1939
1959
1979
1999
2019
Year

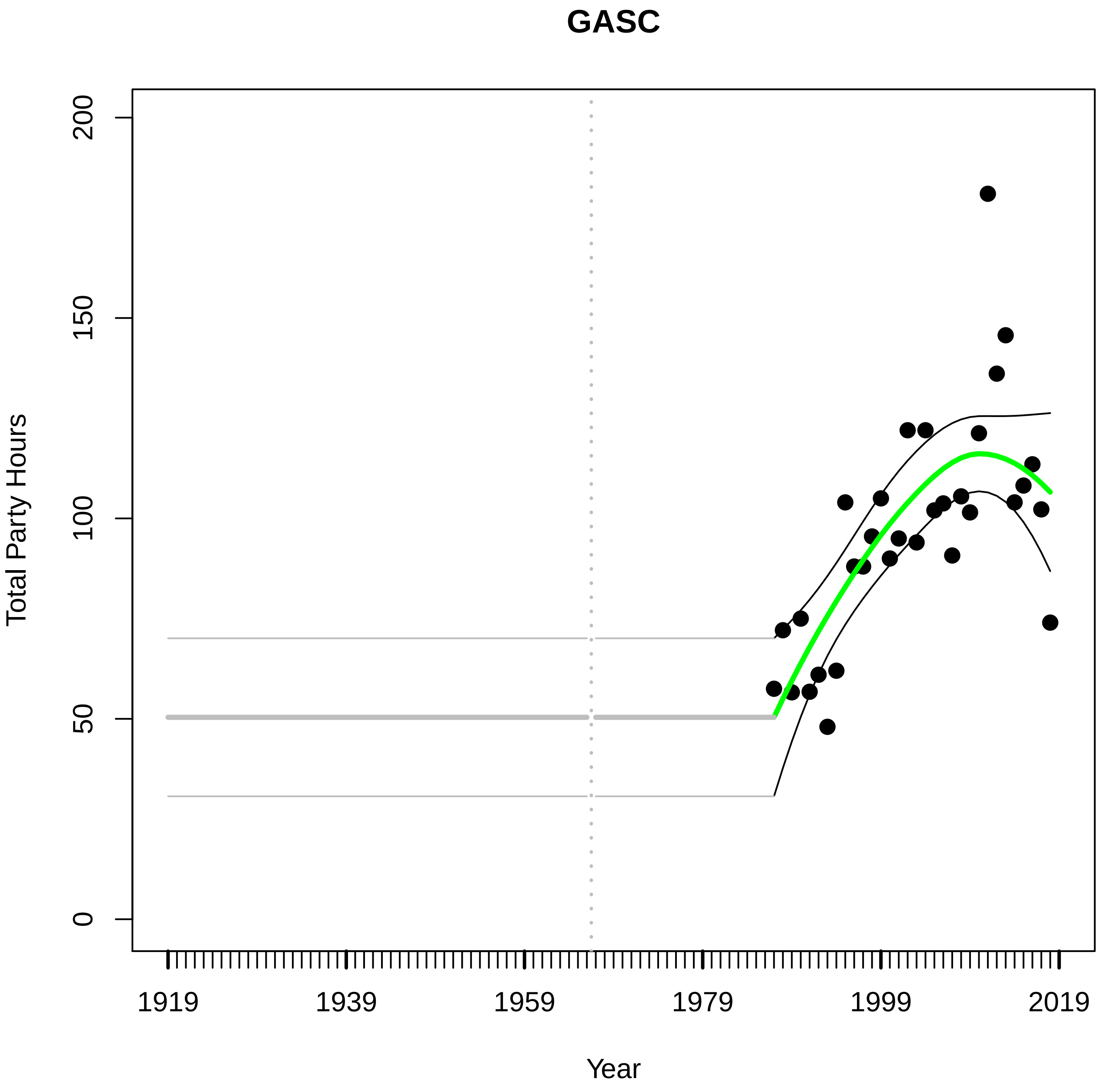
GASC
Total Party Hours
0
50
100
150
200
1919
1939
1959
1979
1999
2019
Year

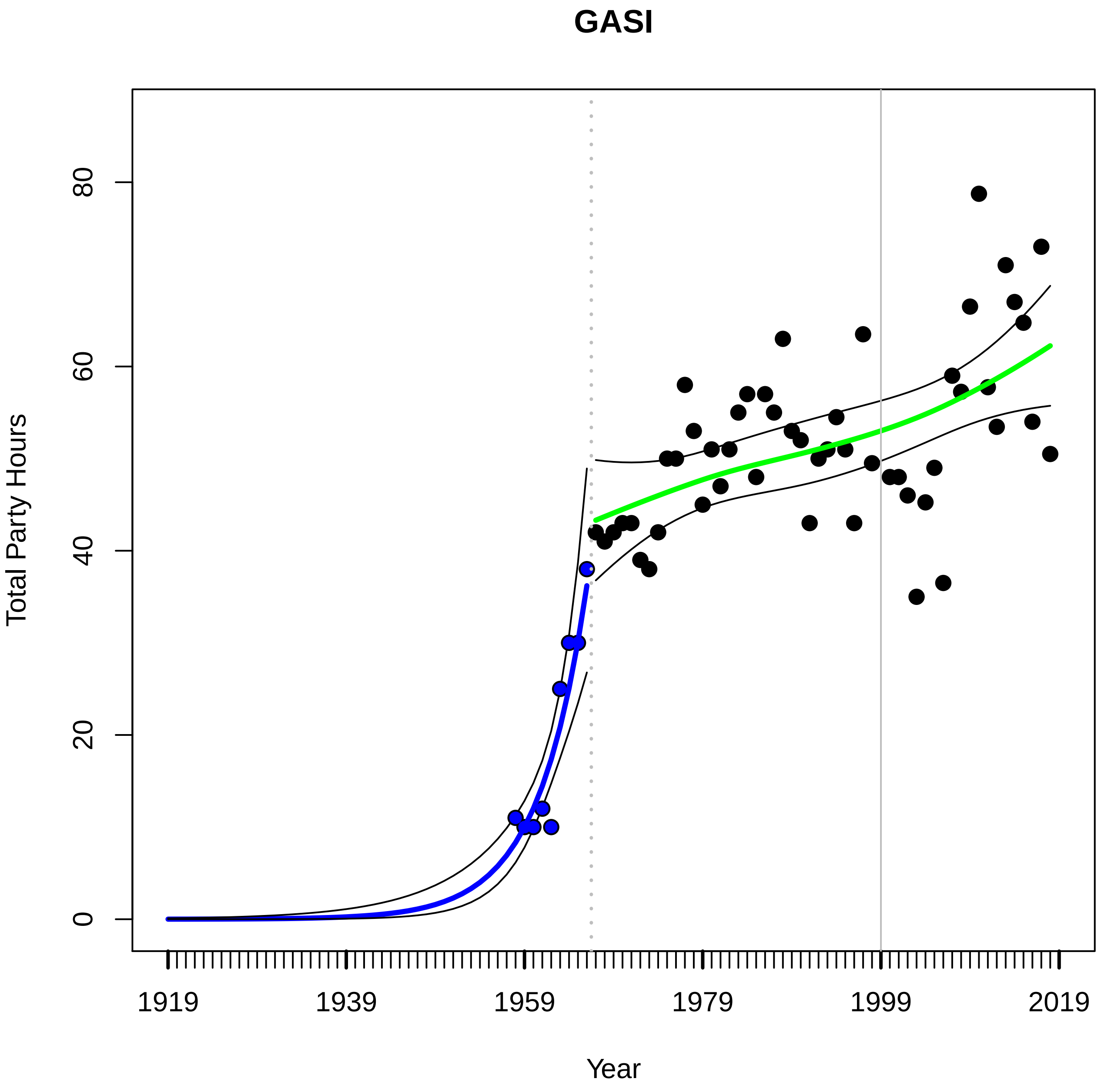
GASI
Total Party Hours
0
20
40
60
80
1919
1939
1959
1979
1999
2019
Year

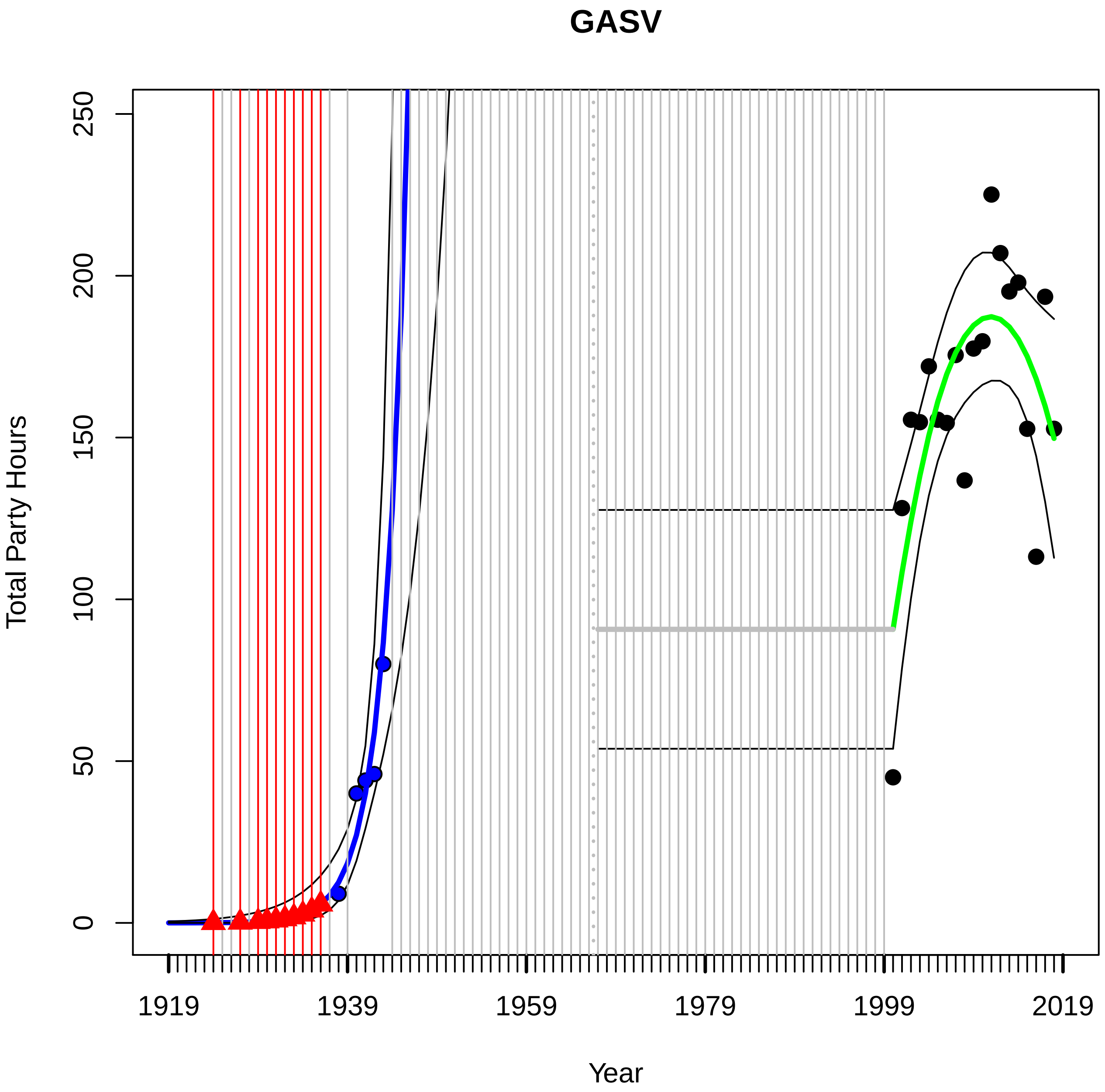

GASV
Total Party Hours
Year
0
50
100
150
200
250
1919
1939
1959
1979
1999
2019

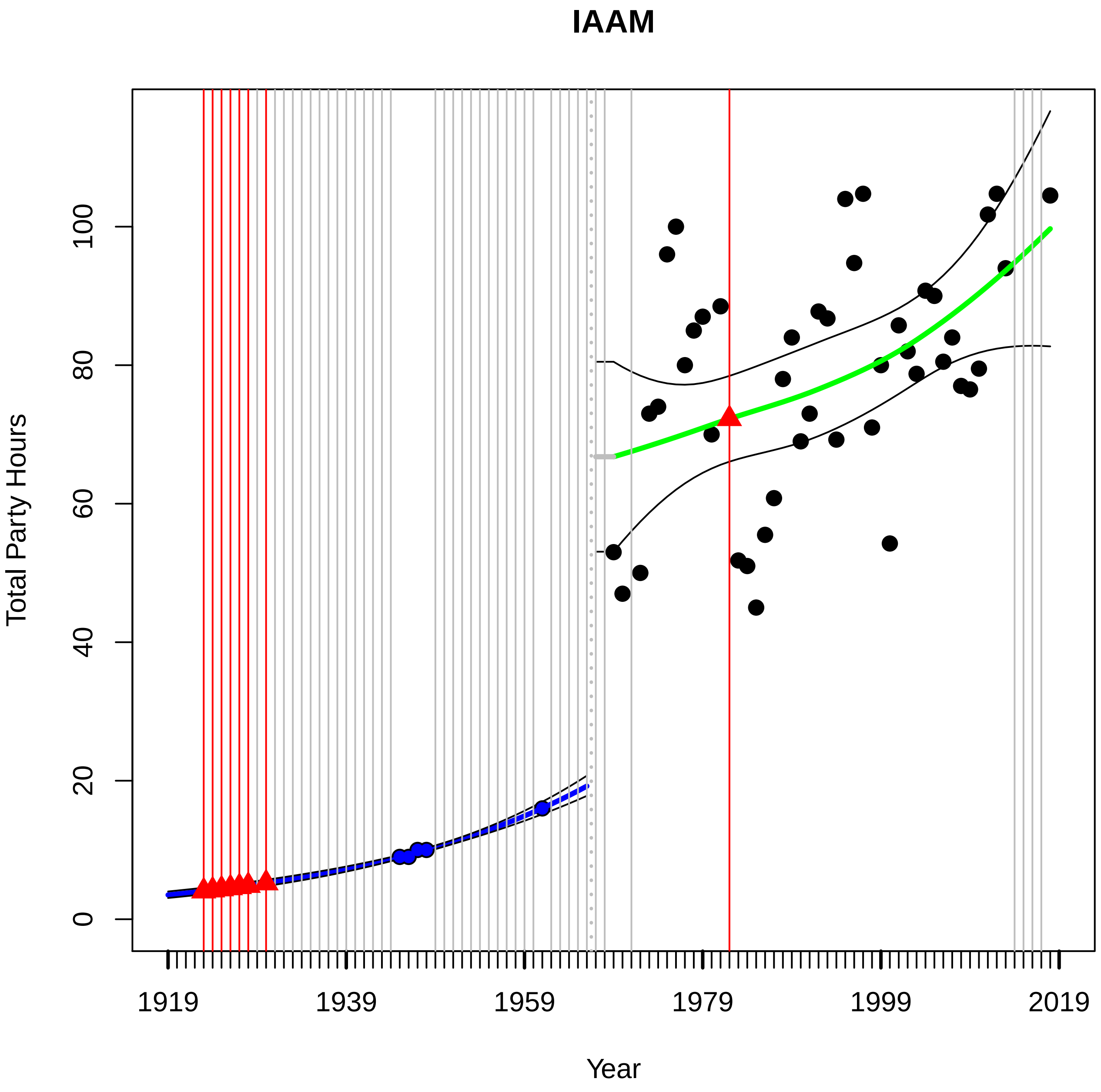
IAAM
Total Party Hours
0
20
40
60
80
100
1919
1939
1959
1979
1999
2019
Year

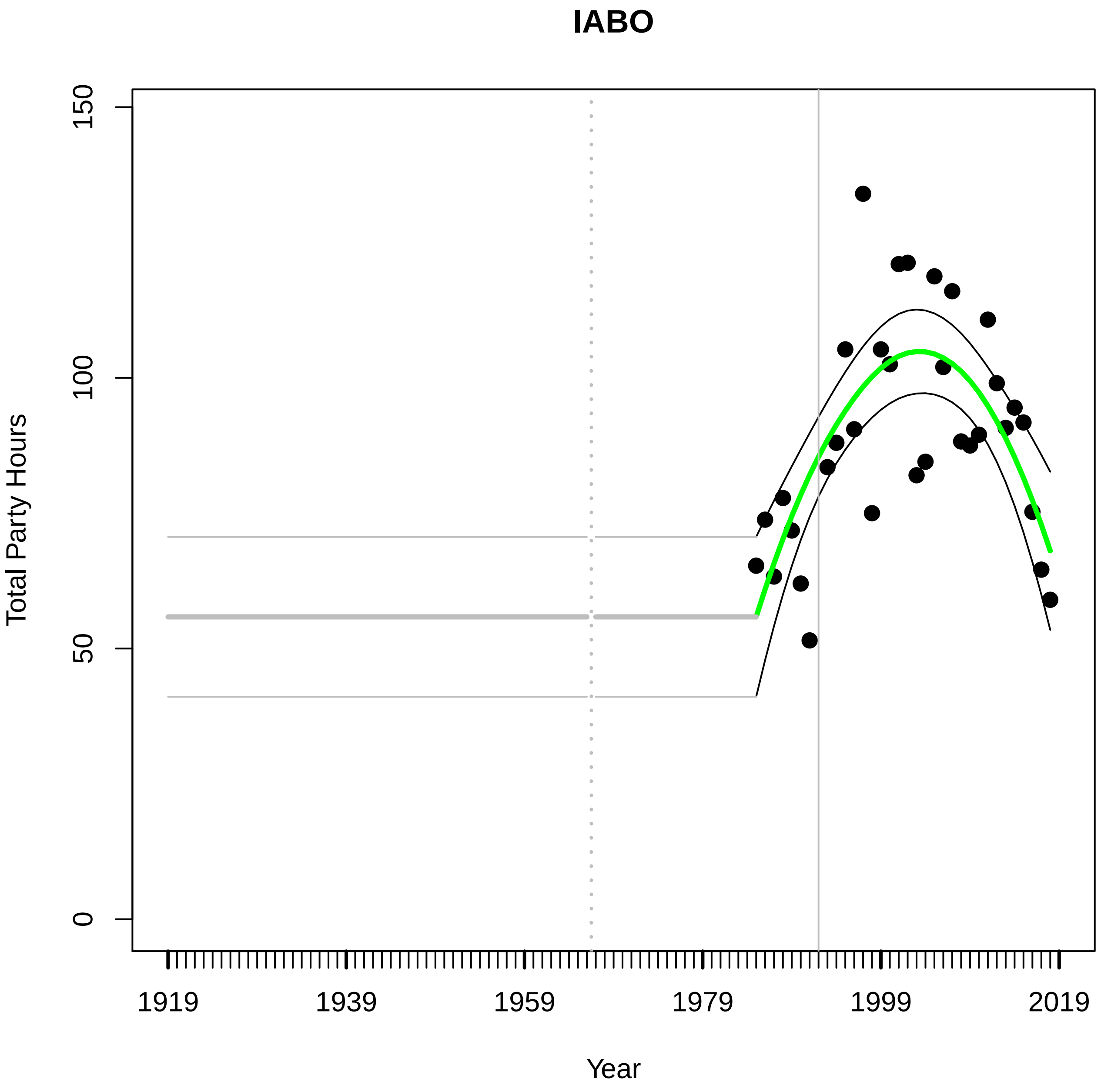

IABO
Total Party Hours
150
100
50
0
1919
1939
1959
1979
1999
2019
Year

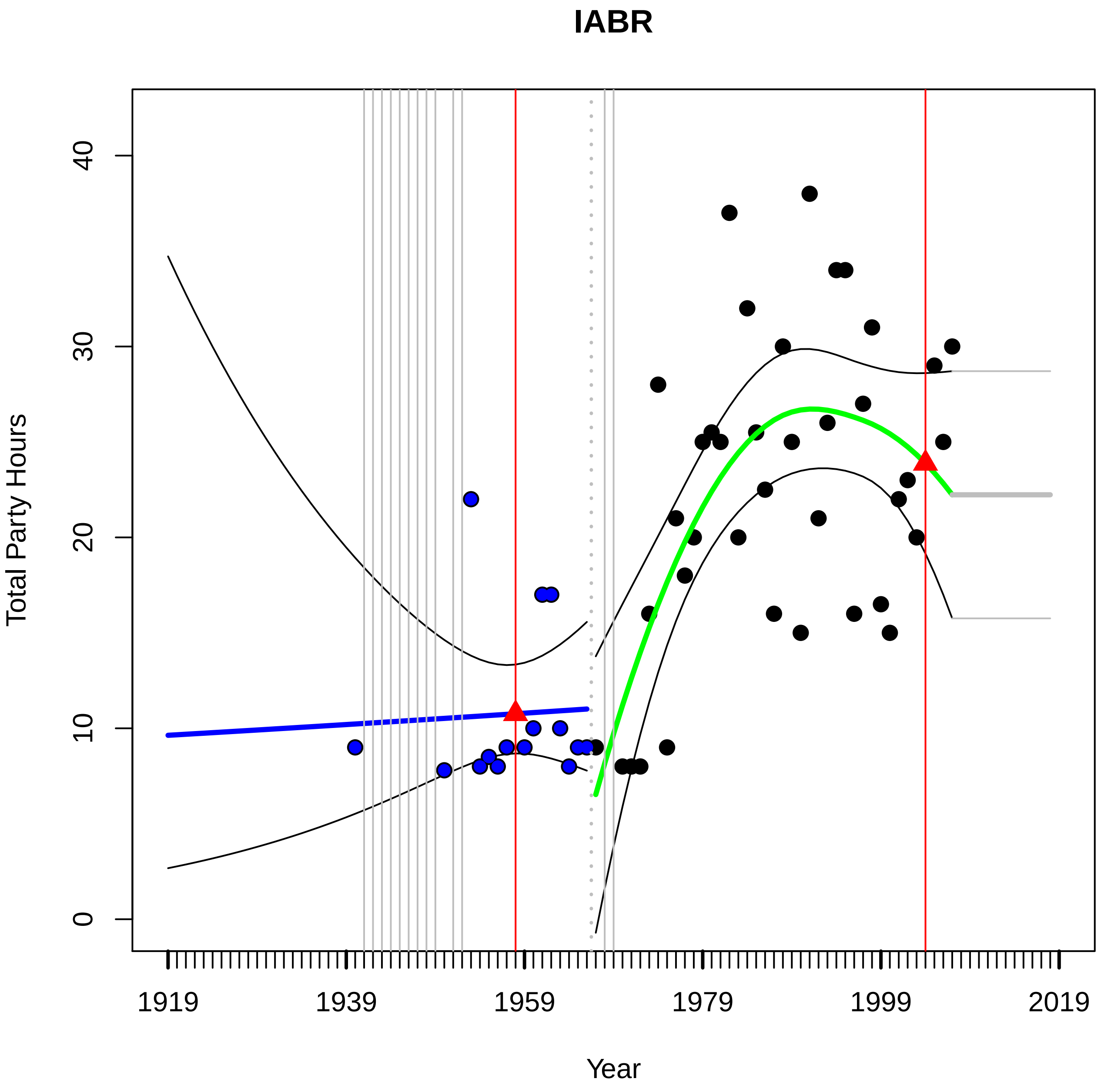

IABR
Total Party Hours
0
10
20
30
40
1919
1939
1959
1979
1999
2019
Year

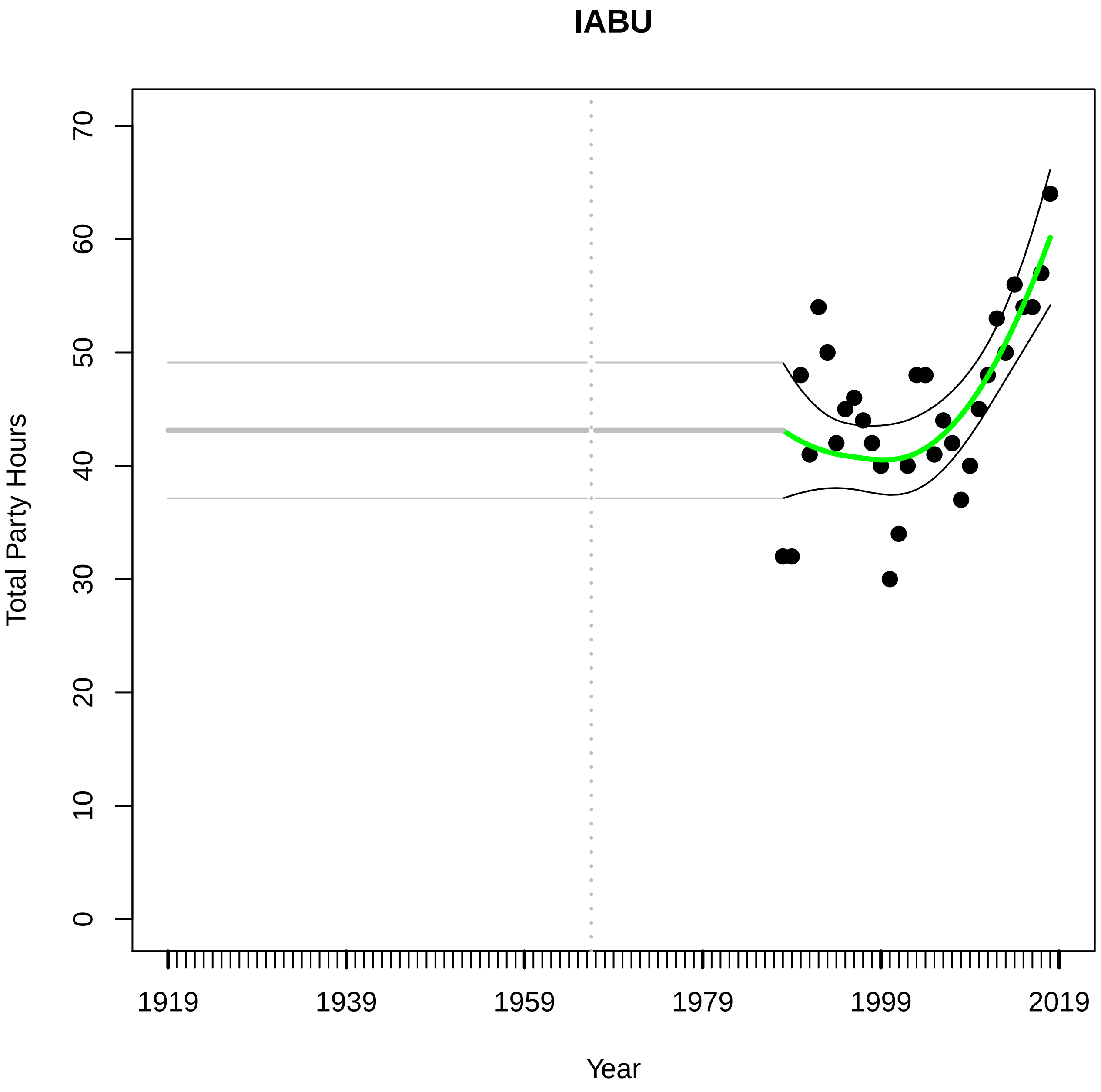

IABU
Total Party Hours
Year
0
10
20
30
40
50
60
70
1919
1939
1959
1979
1999
2019

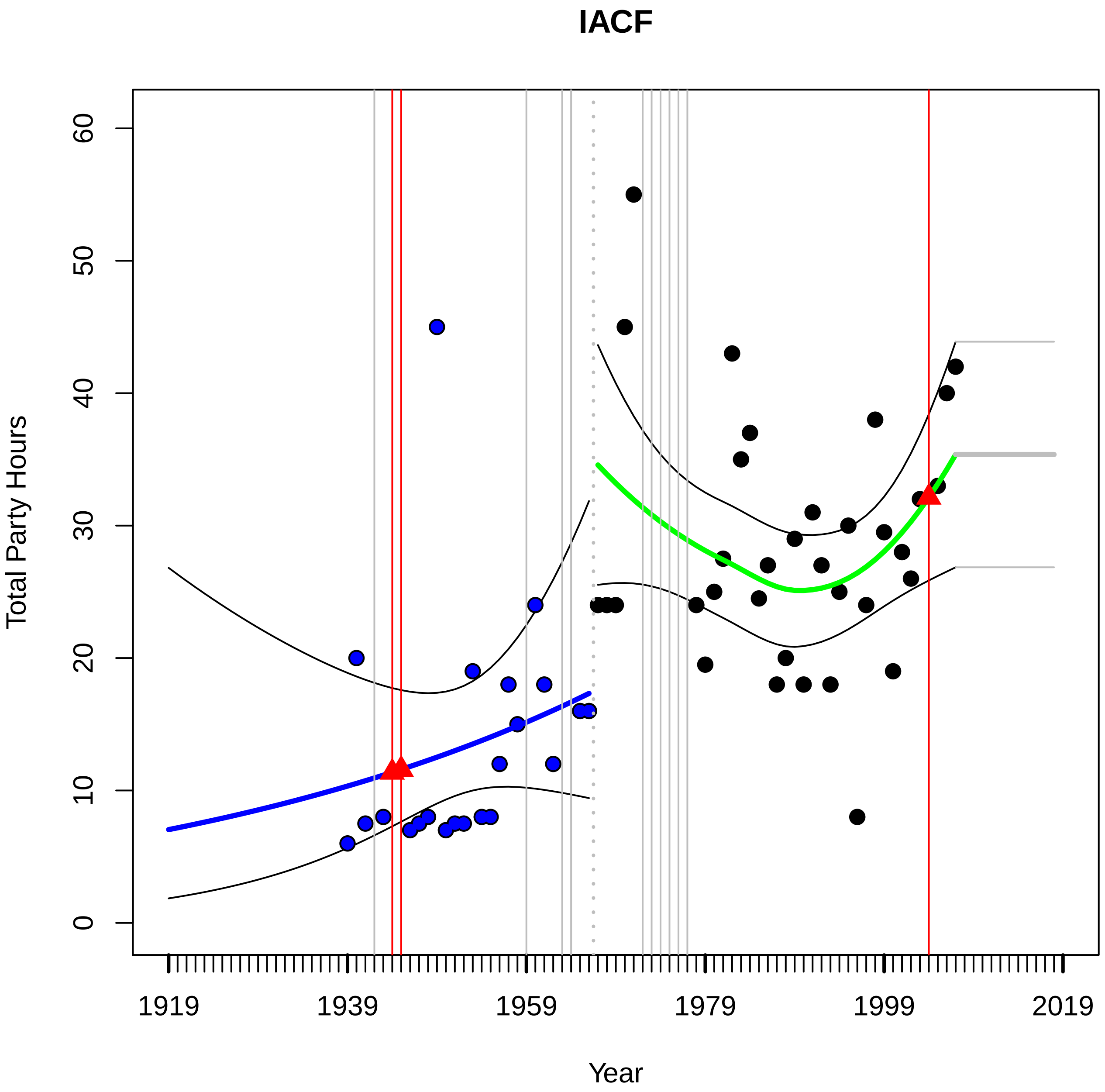
IACF
Total Party Hours
0
10
20
30
40
50
60
1919
1939
1959
1979
1999
2019
Year

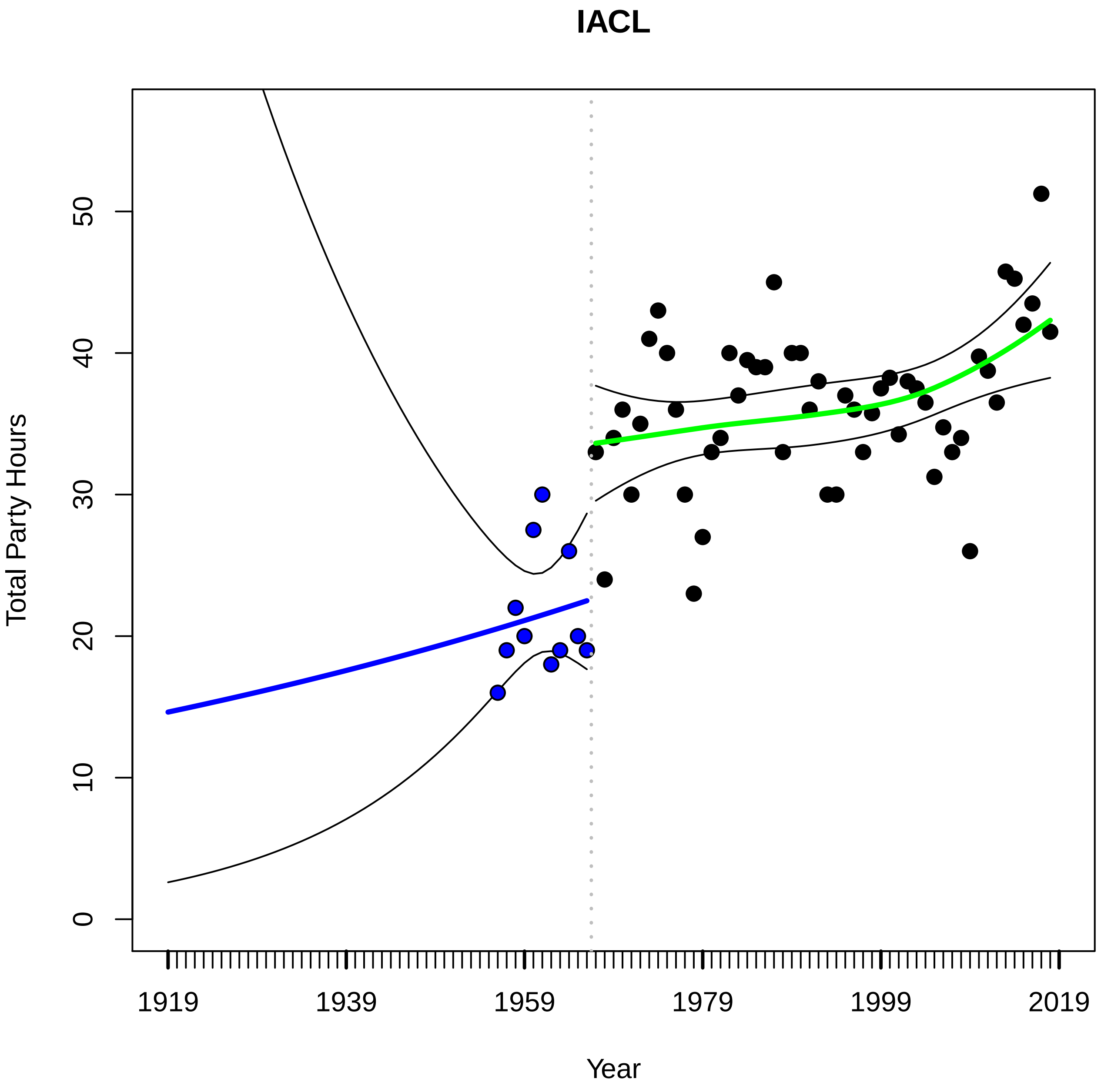
IACL
Total Party Hours
0
10
20
30
40
50
1919
1939
1959
1979
1999
2019
Year

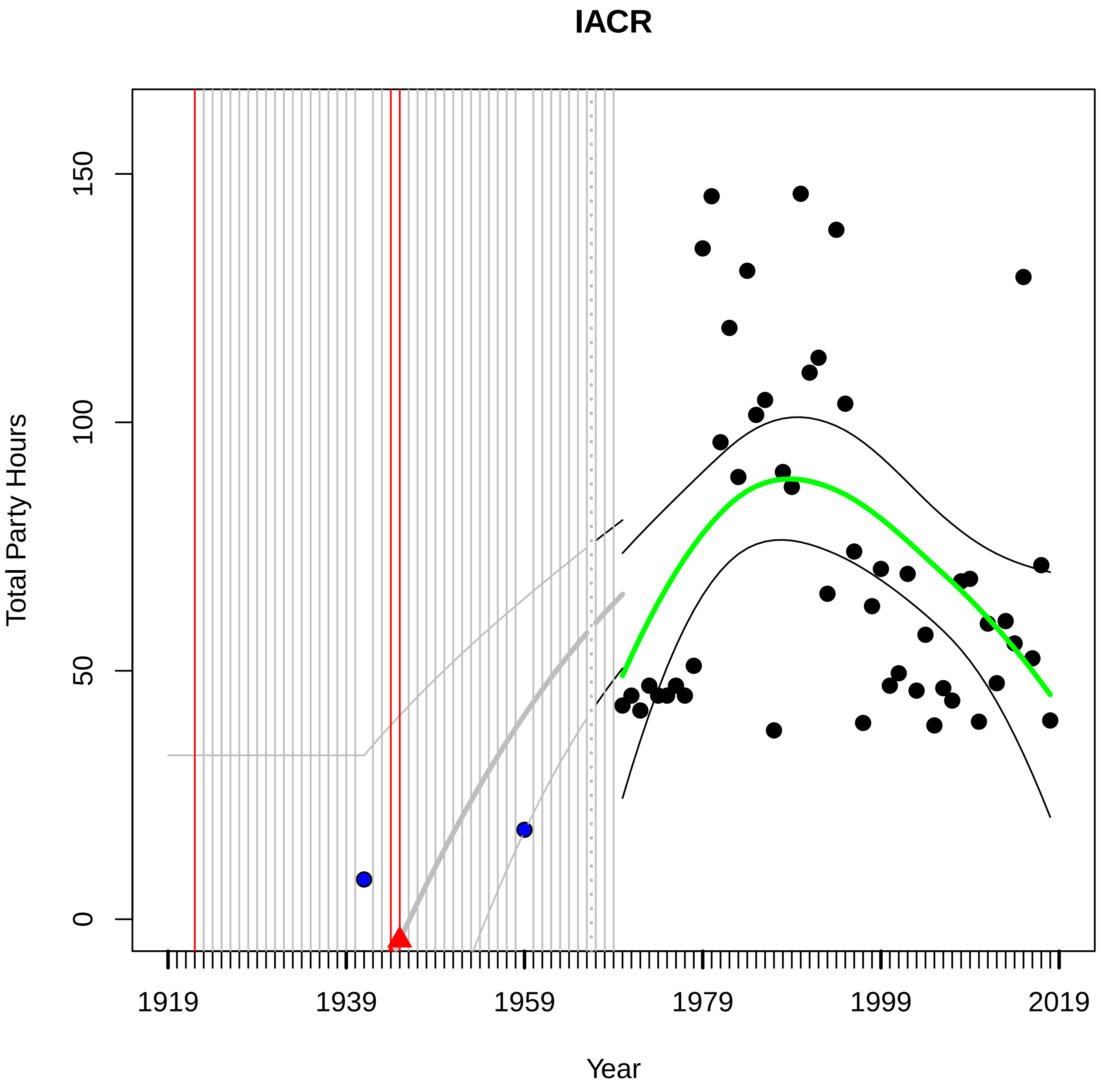
IACR
Total Party Hours
0
50
100
150
1919
1939
1959
1979
1999
2019
Year

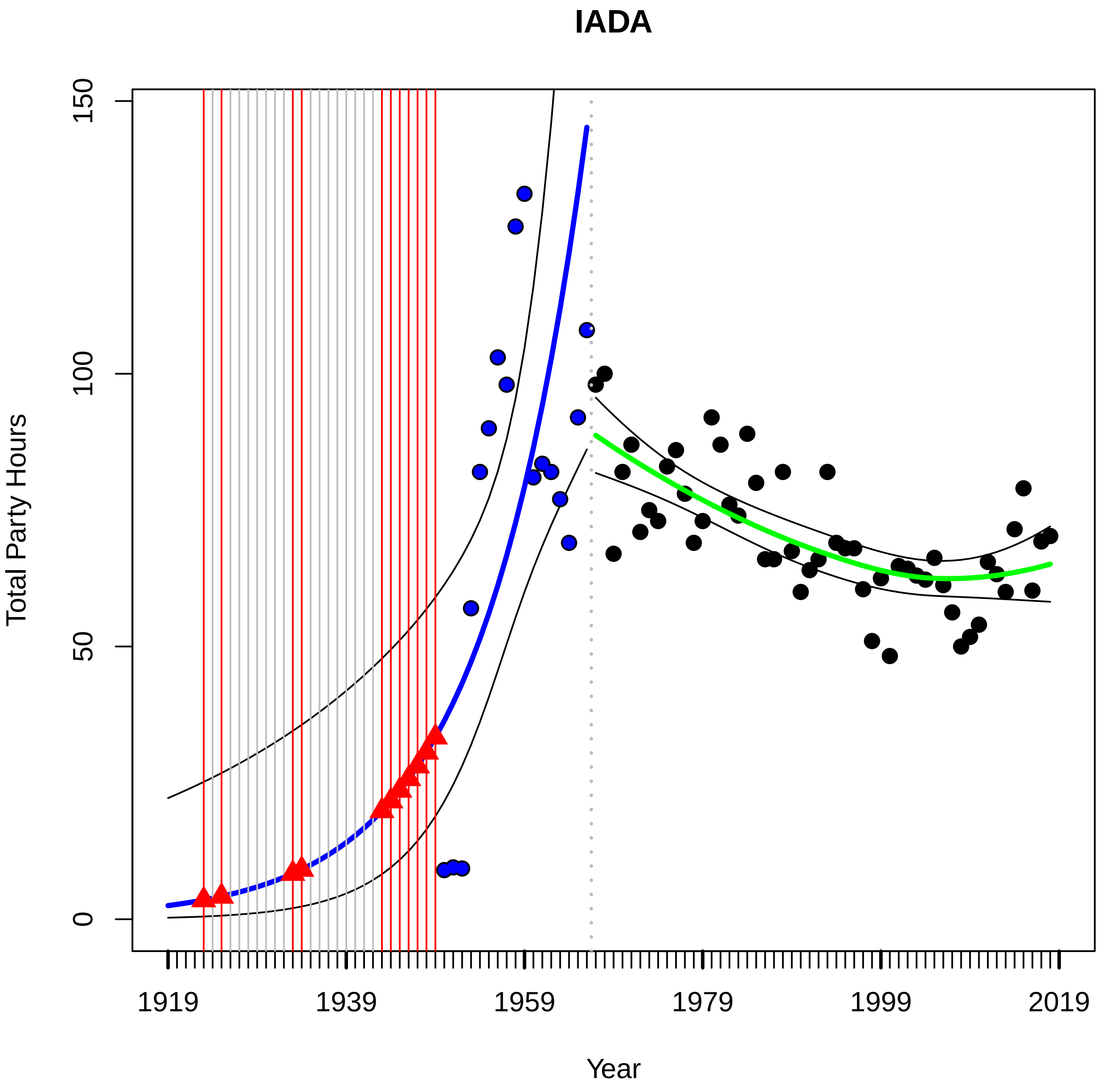

IADA
Total Party Hours
150
100
50
0
1919
1939
1959
1979
1999
2019
Year

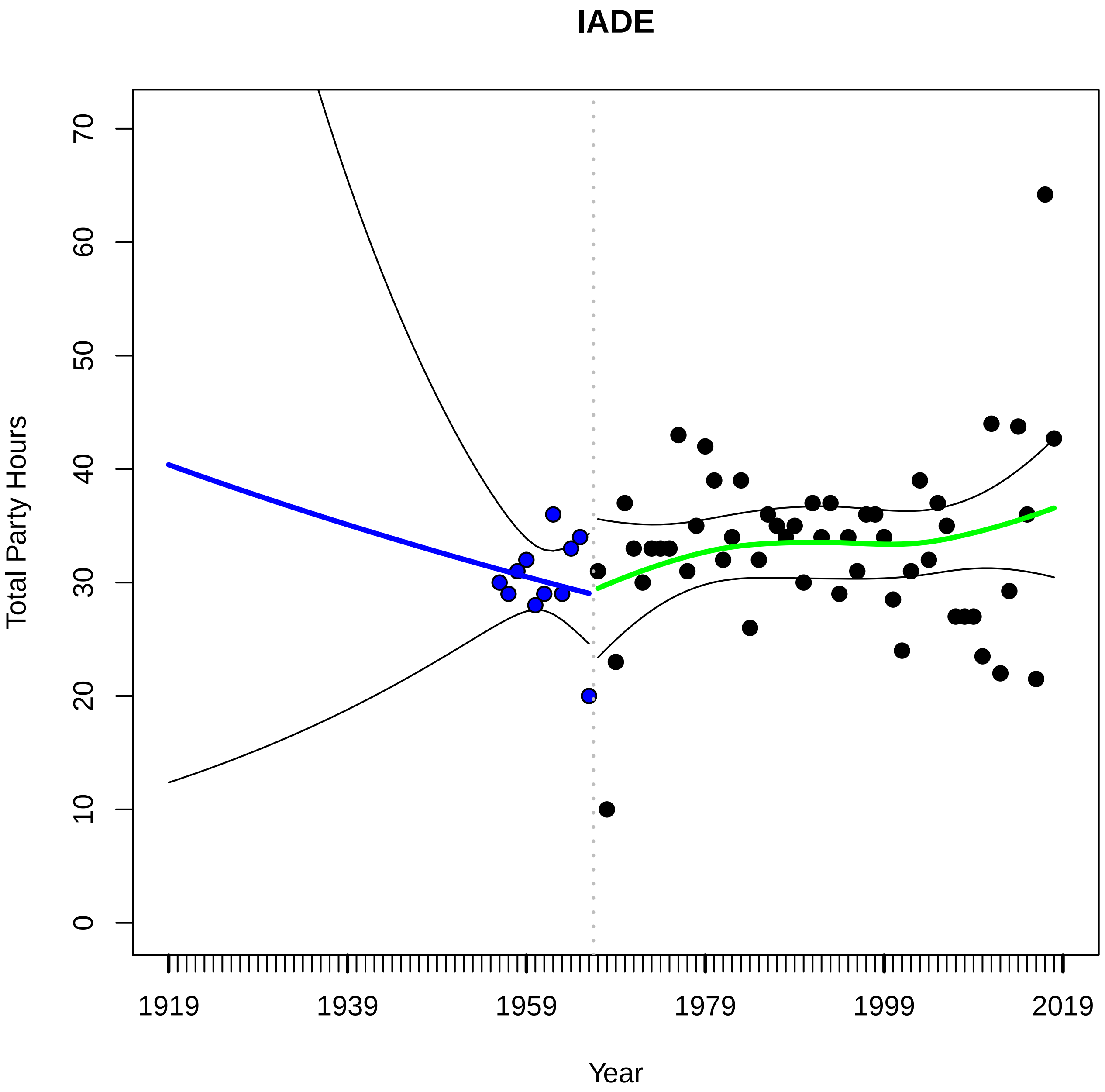

IADE
Total Party Hours
Year
0
10
20
30
40
50
60
70
1919
1939
1959
1979
1999
2019

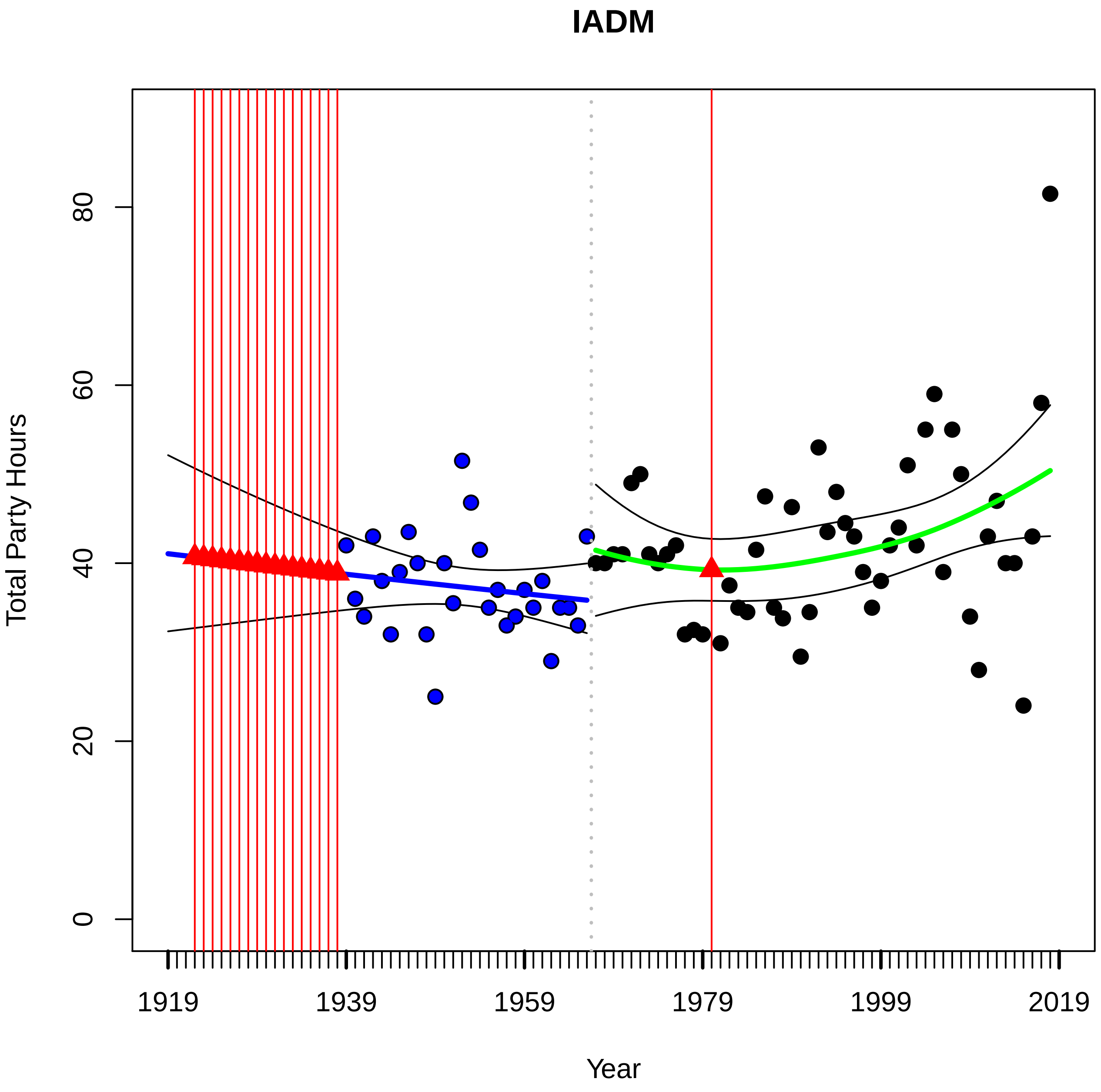

IADM
Total Party Hours
Year
0
20
40
60
80
1919
1939
1959
1979
1999
2019

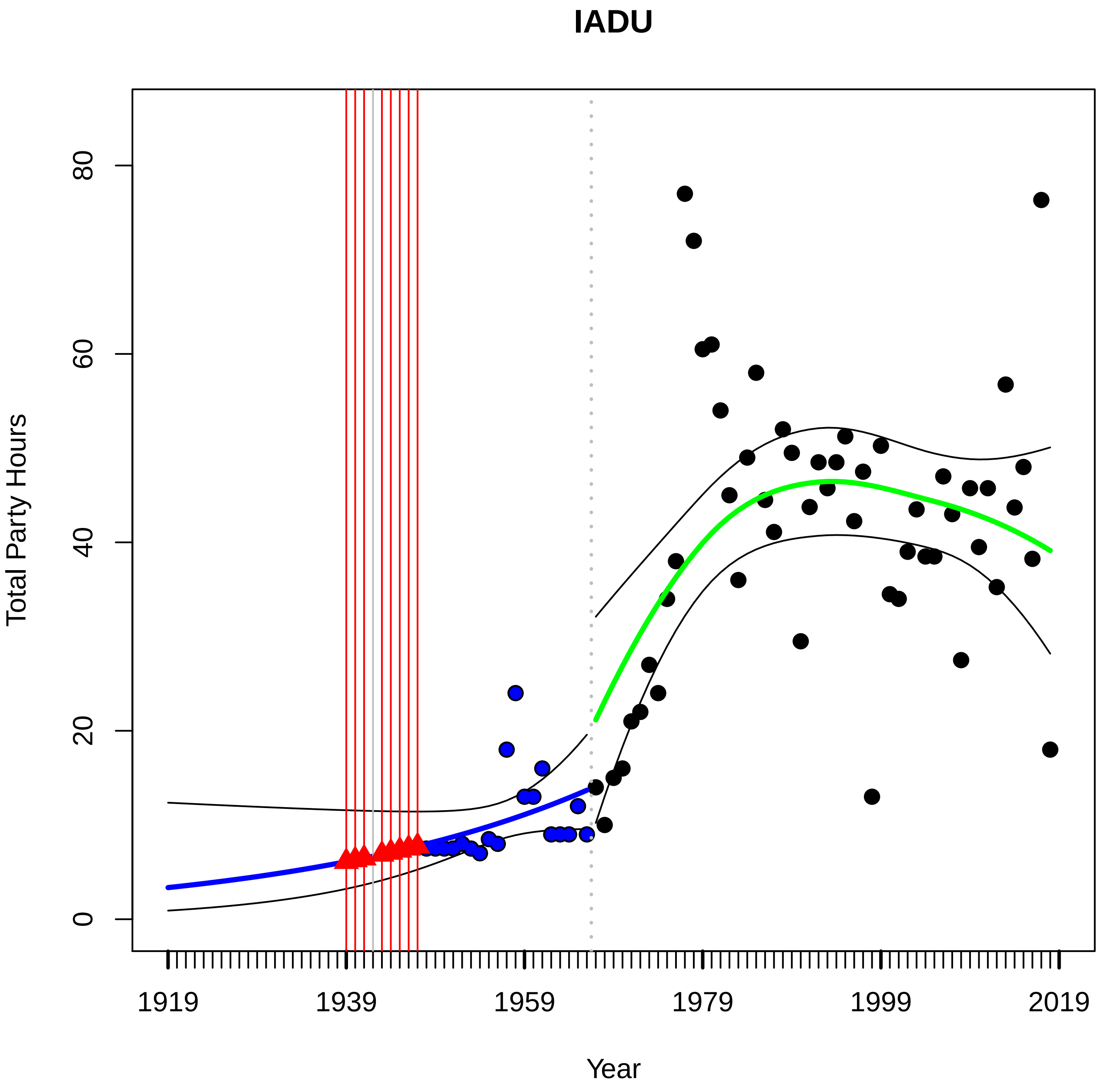
IADU
Total Party Hours
0
20
40
60
80
Year
1919
1939
1959
1979
1999
2019

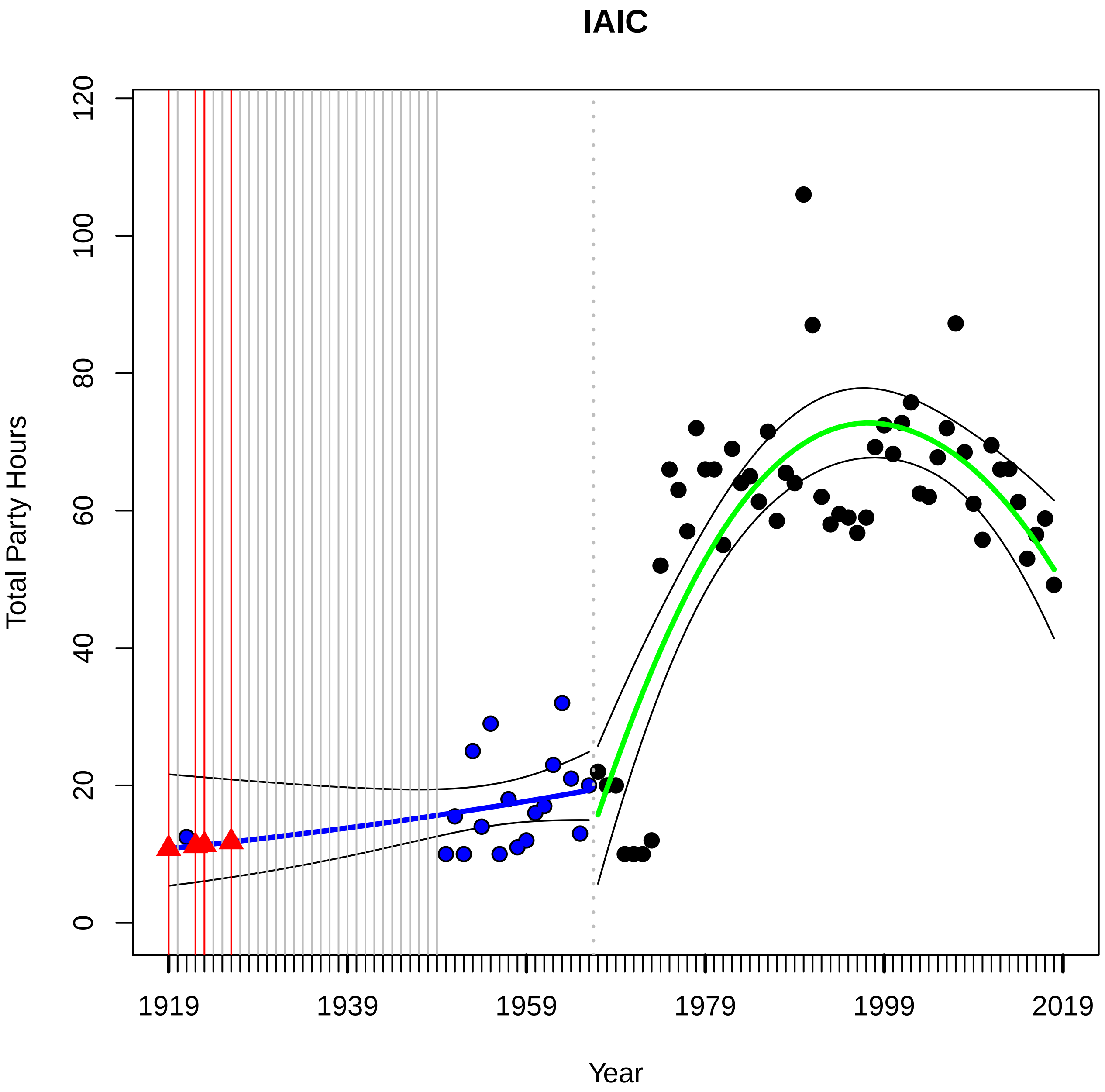

IAIC
Total Party Hours
0
20
40
60
80
100
120
1919
1939
1959
1979
1999
2019
Year

IAKE

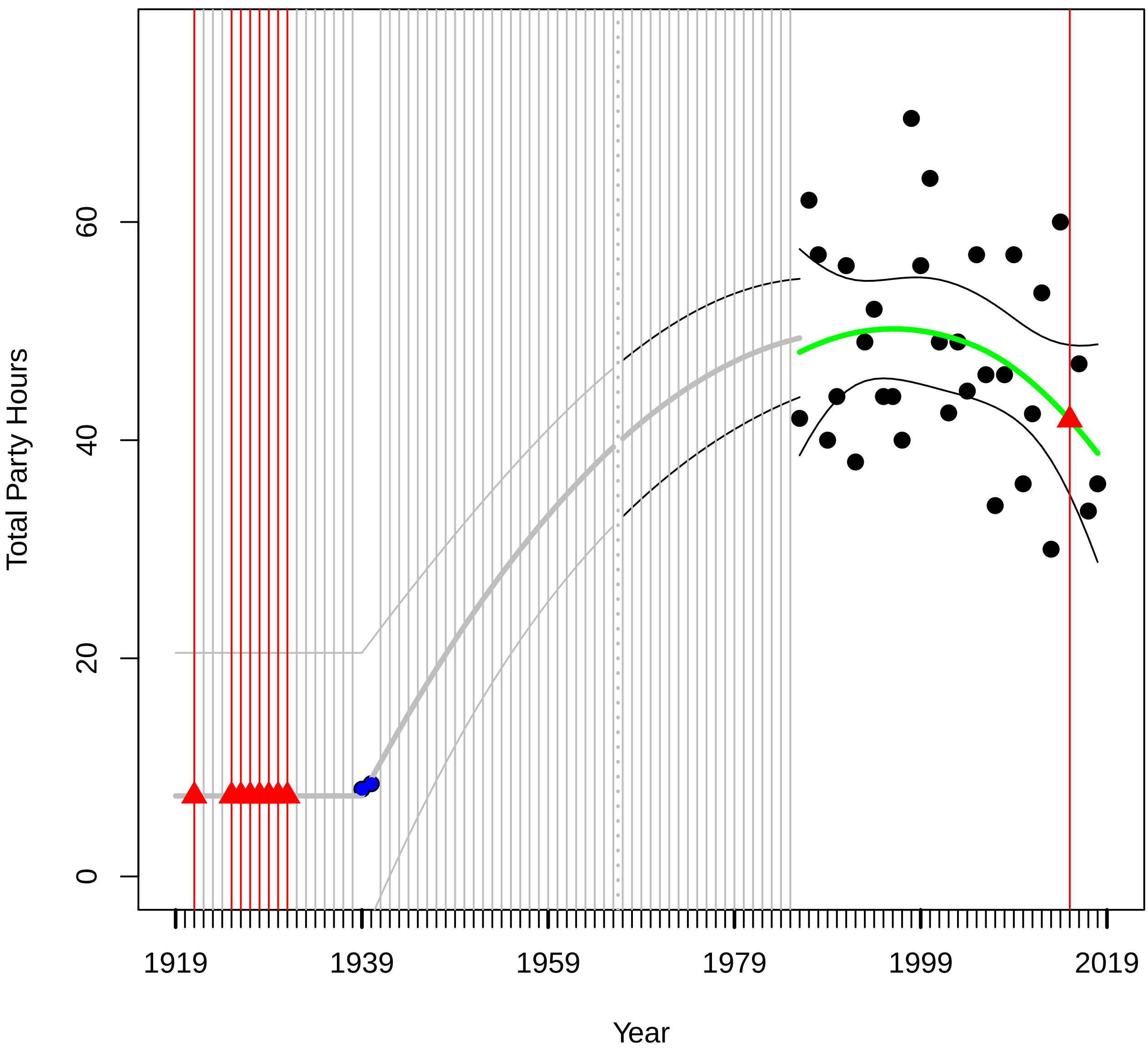

Total Party Hours
0
20
40
60
1919
1939
1959
1979
1999
2019
Year

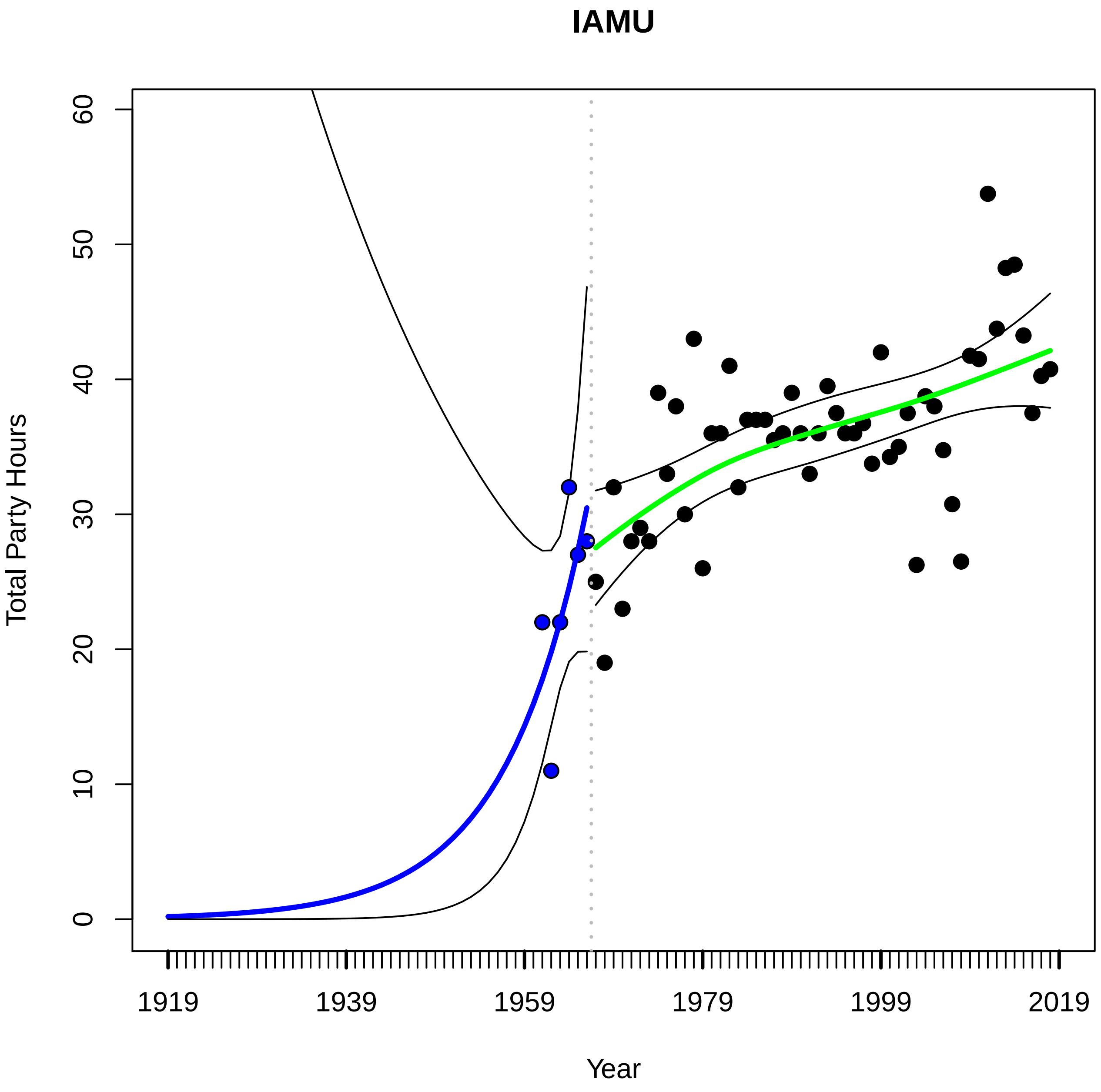

IAMU
Total Party Hours
0
10
20
30
40
50
60
Year
1919
1939
1959
1979
1999
2019

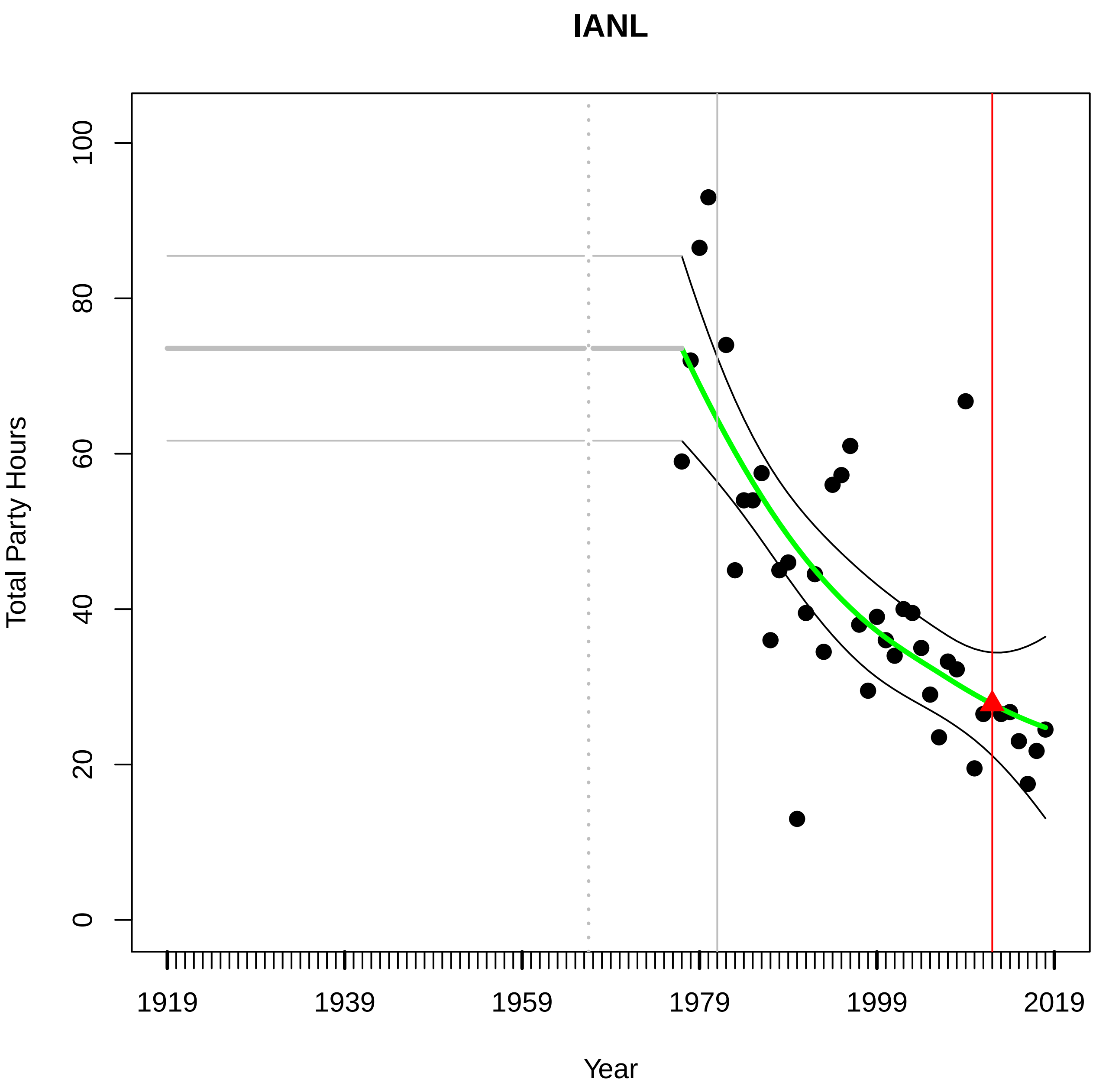
IANL
Total Party Hours
0
20
40
60
80
100
1919
1939
1959
1979
1999
2019
Year

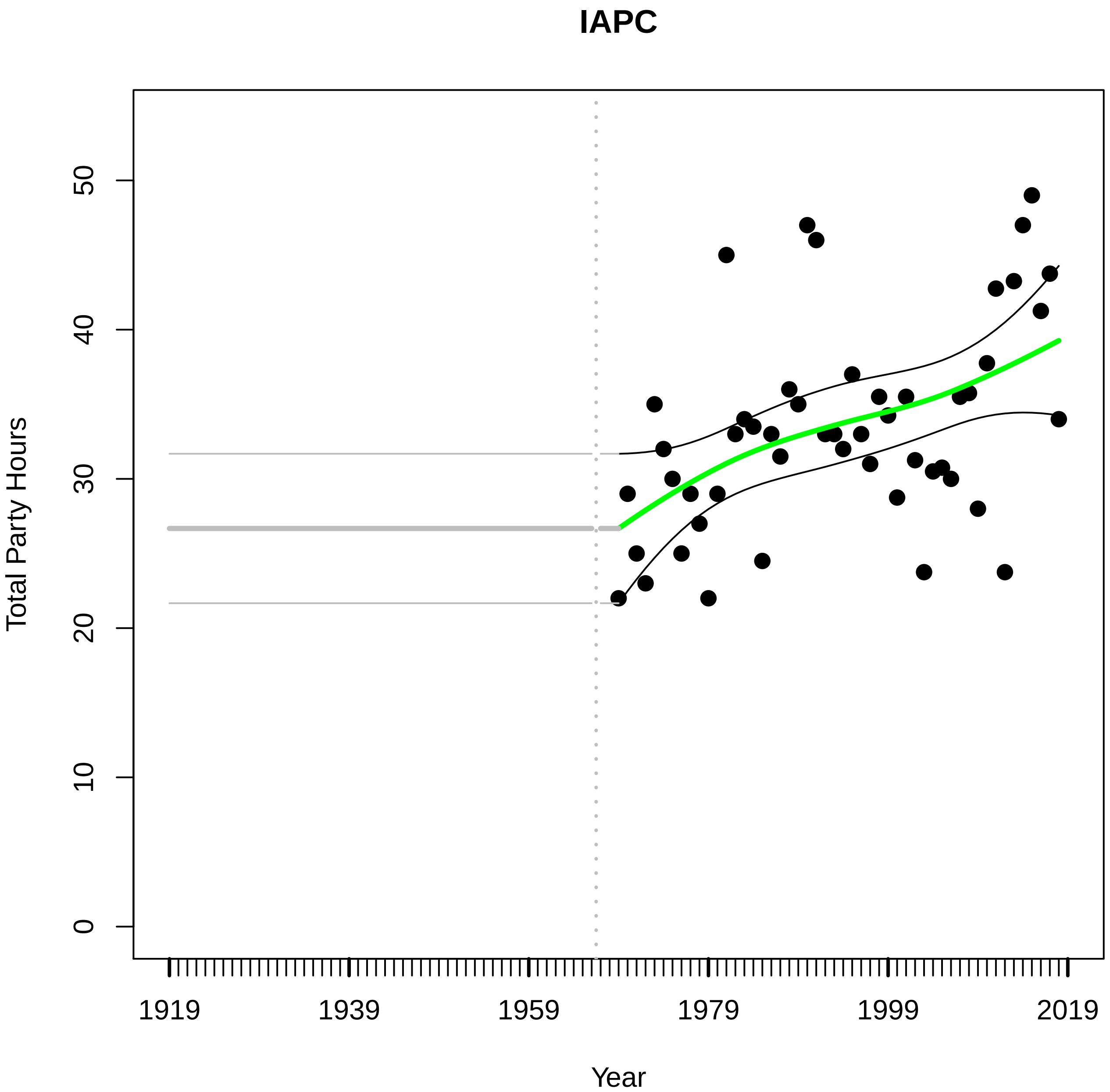

IAPC
Total Party Hours
0
10
20
30
40
50
1919
1939
1959
1979
1999
2019
Year

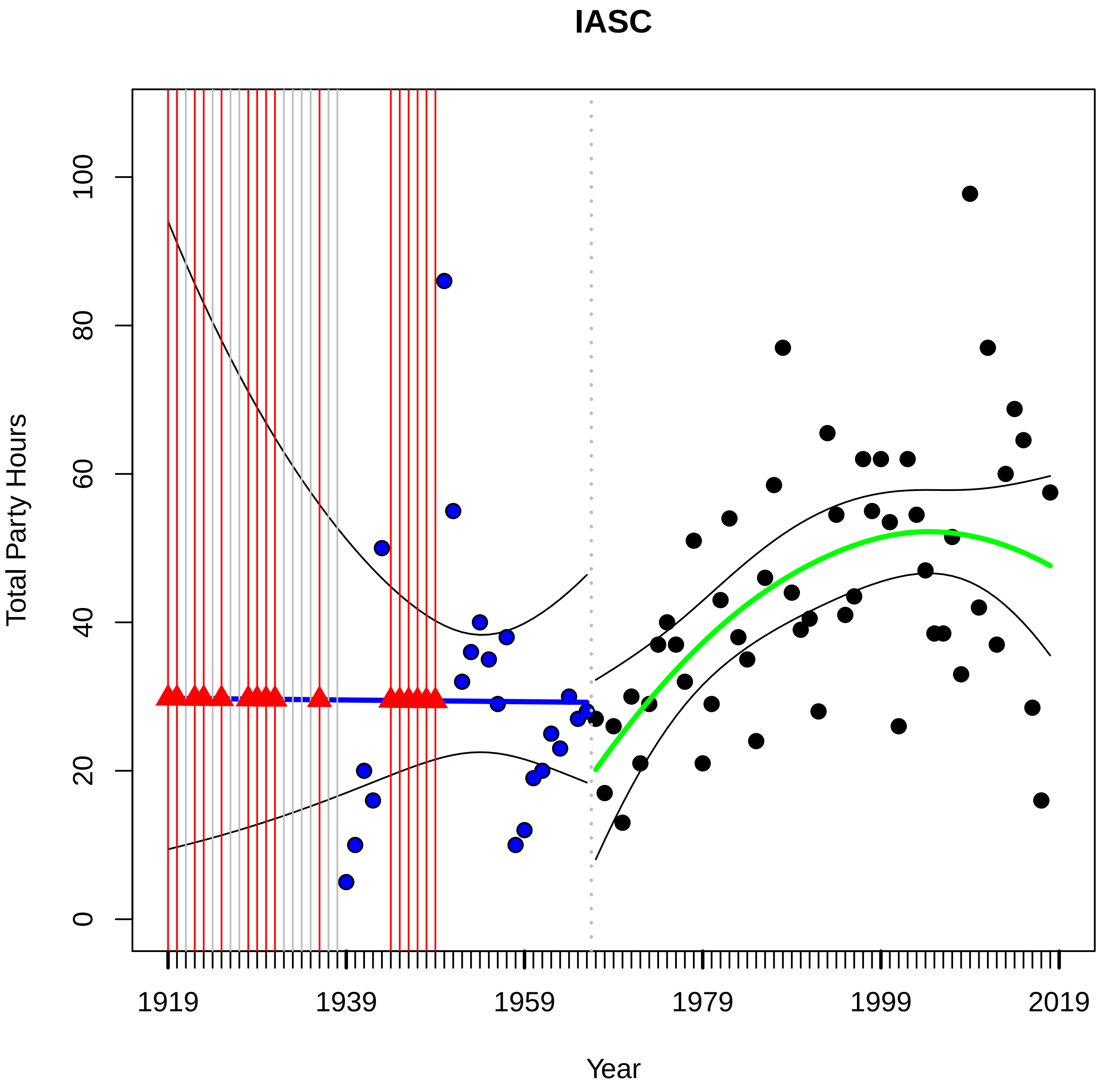

IASC
Total Party Hours
0
20
40
60
80
100
1919
1939
1959
1979
1999
2019
Year

IASH

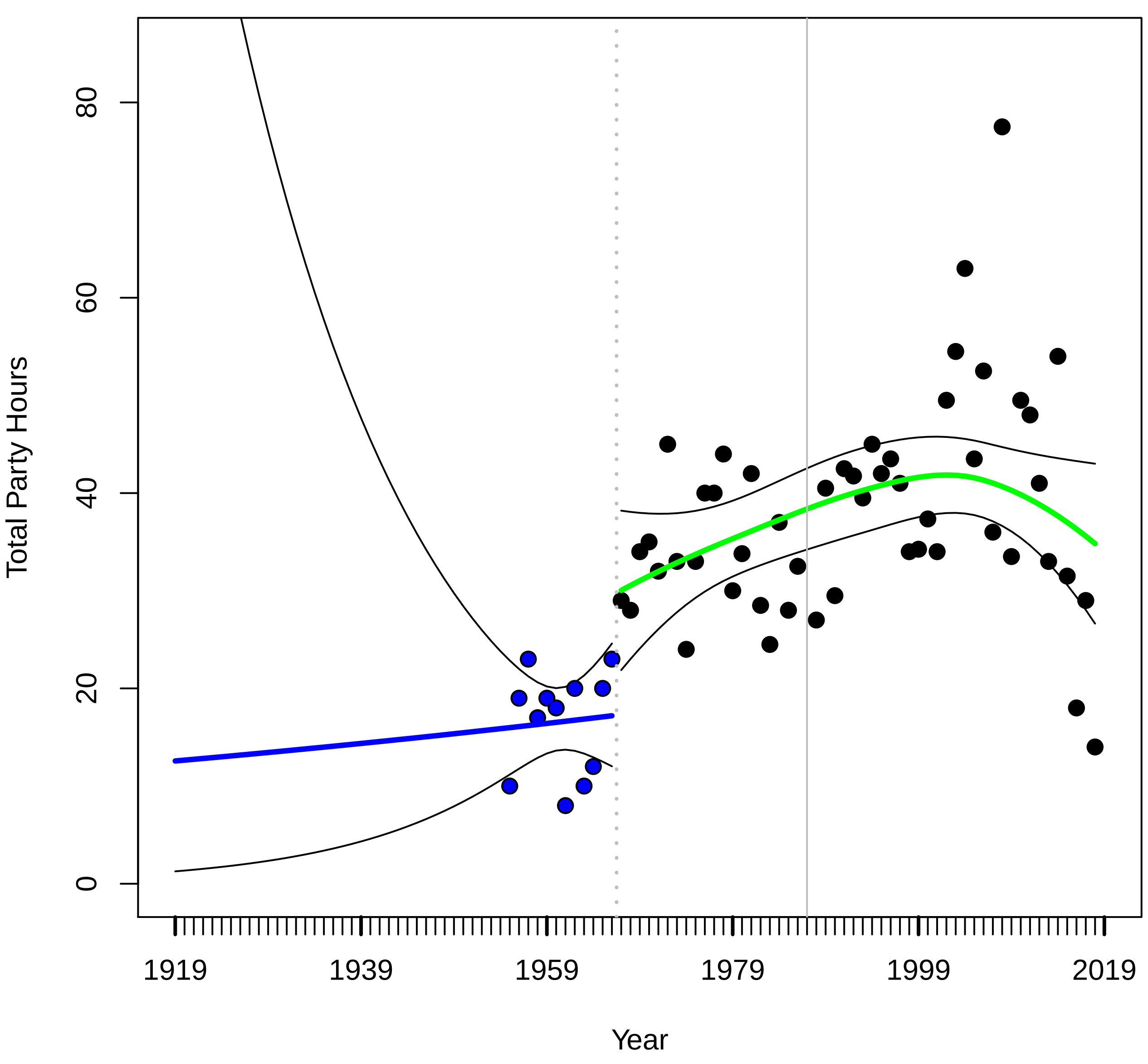

Total Party Hours
0
20
40
60
80
1919
1939
1959
1979
1999
2019
Year

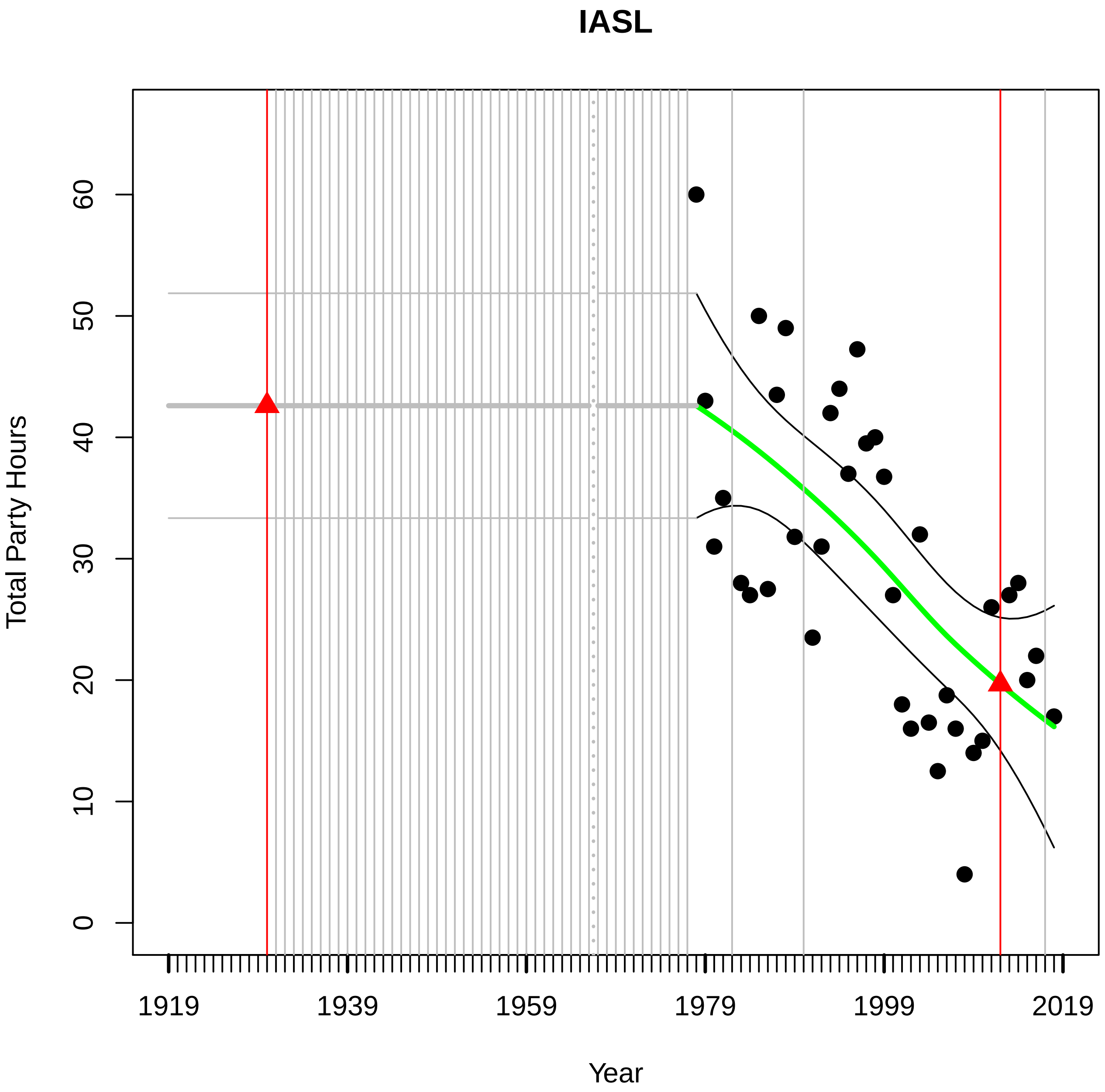

IASL
Total Party Hours
Year
0
10
20
30
40
50
60
1919
1939
1959
1979
1999
2019

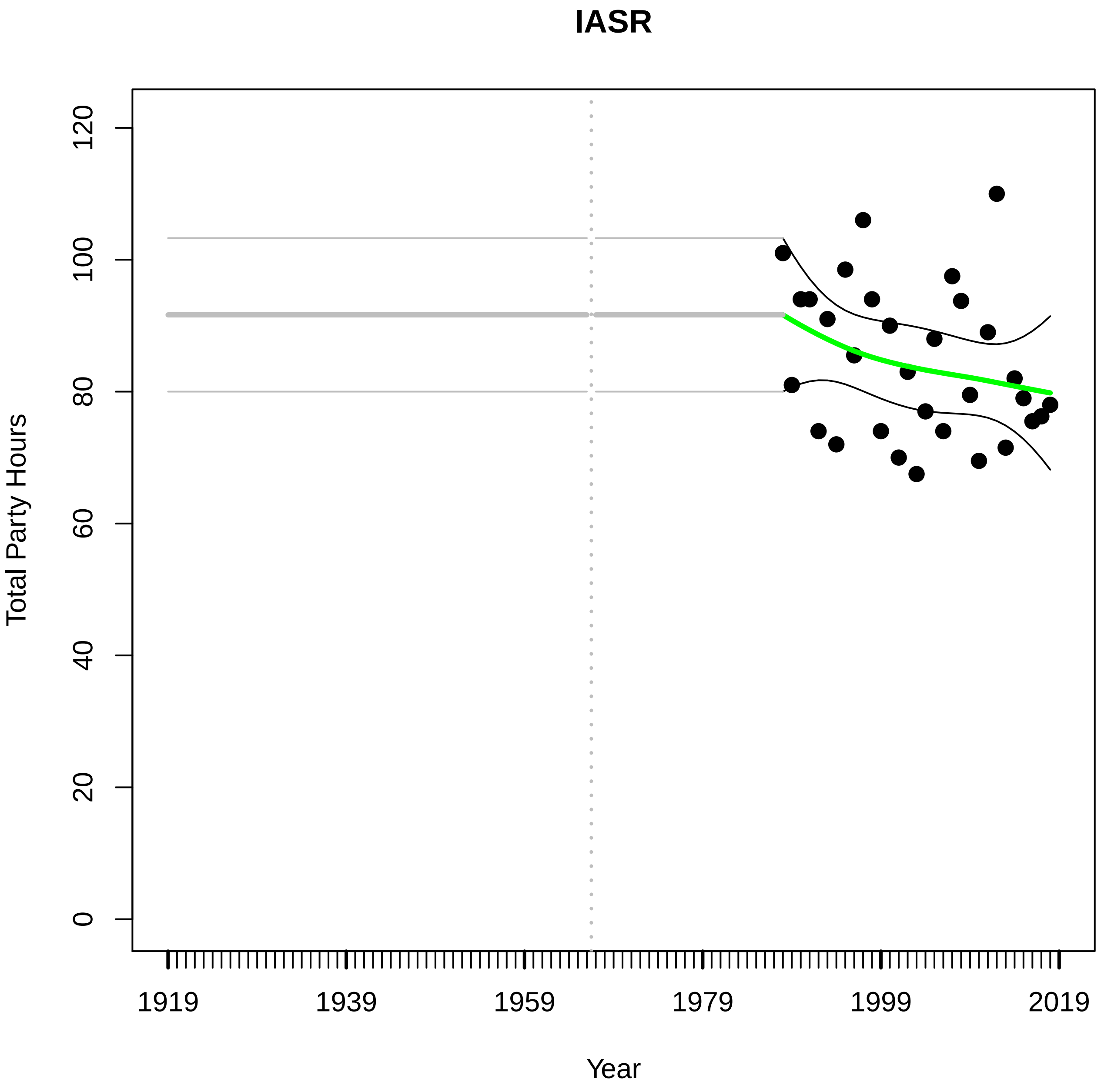

IASR
Total Party Hours
120
100
80
60
40
20
0
1919
1939
1959
1979
1999
2019
Year

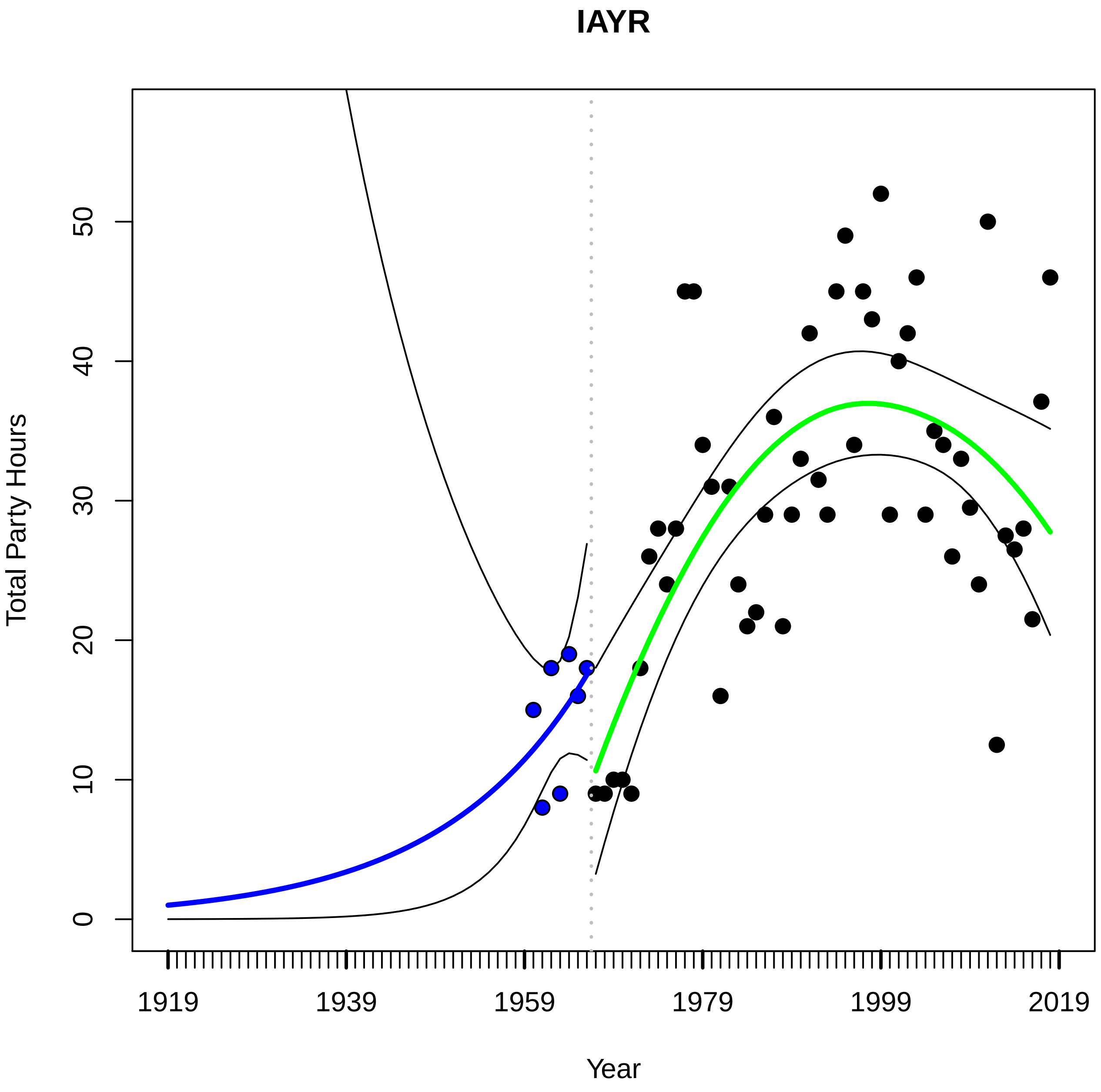
IAYR
Total Party Hours
0
10
20
30
40
50
1919
1939
1959
1979
1999
2019
Year

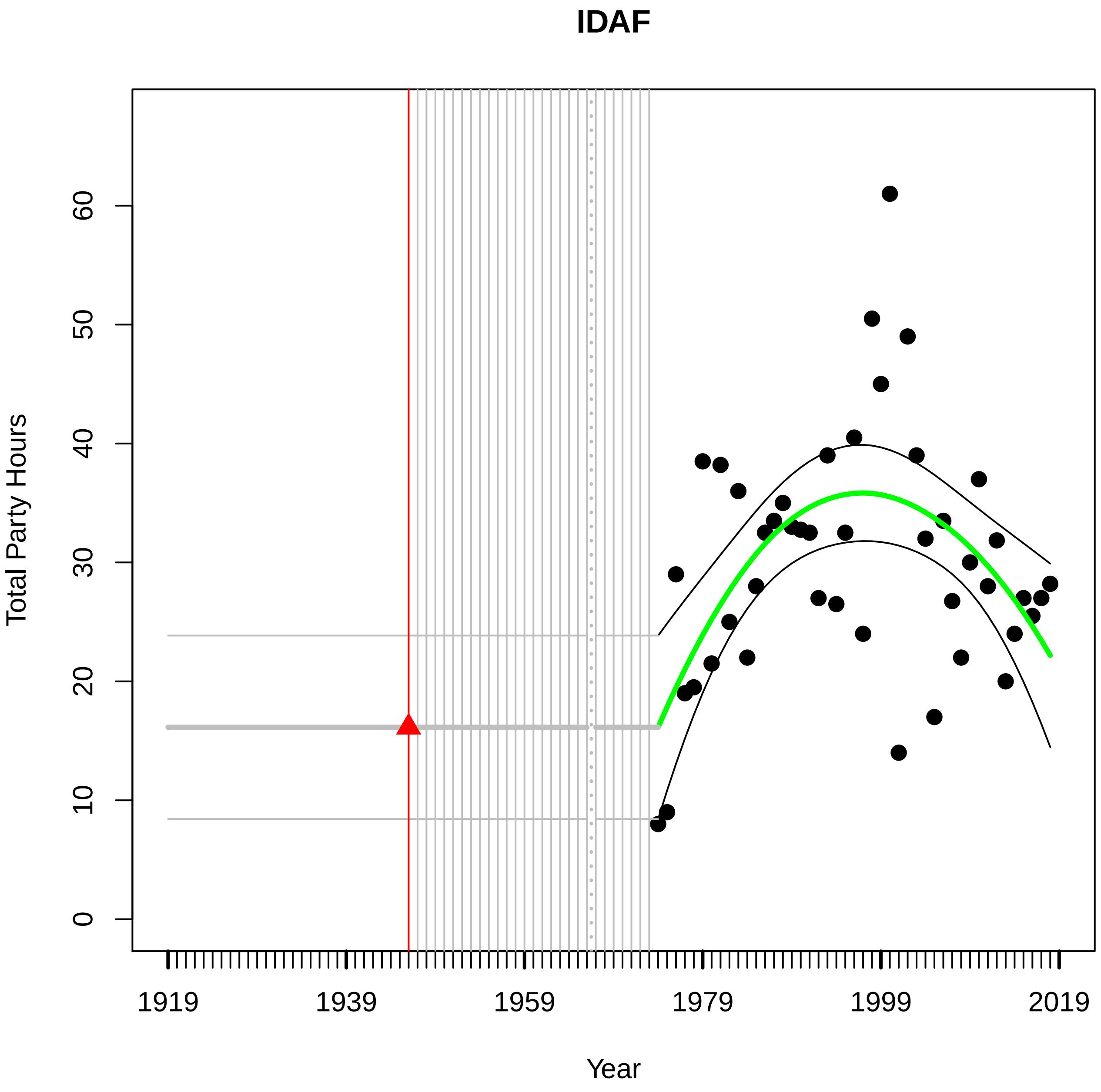

IDAF
Total Party Hours
0
10
20
30
40
50
60
1919
1939
1959
1979
1999
2019
Year

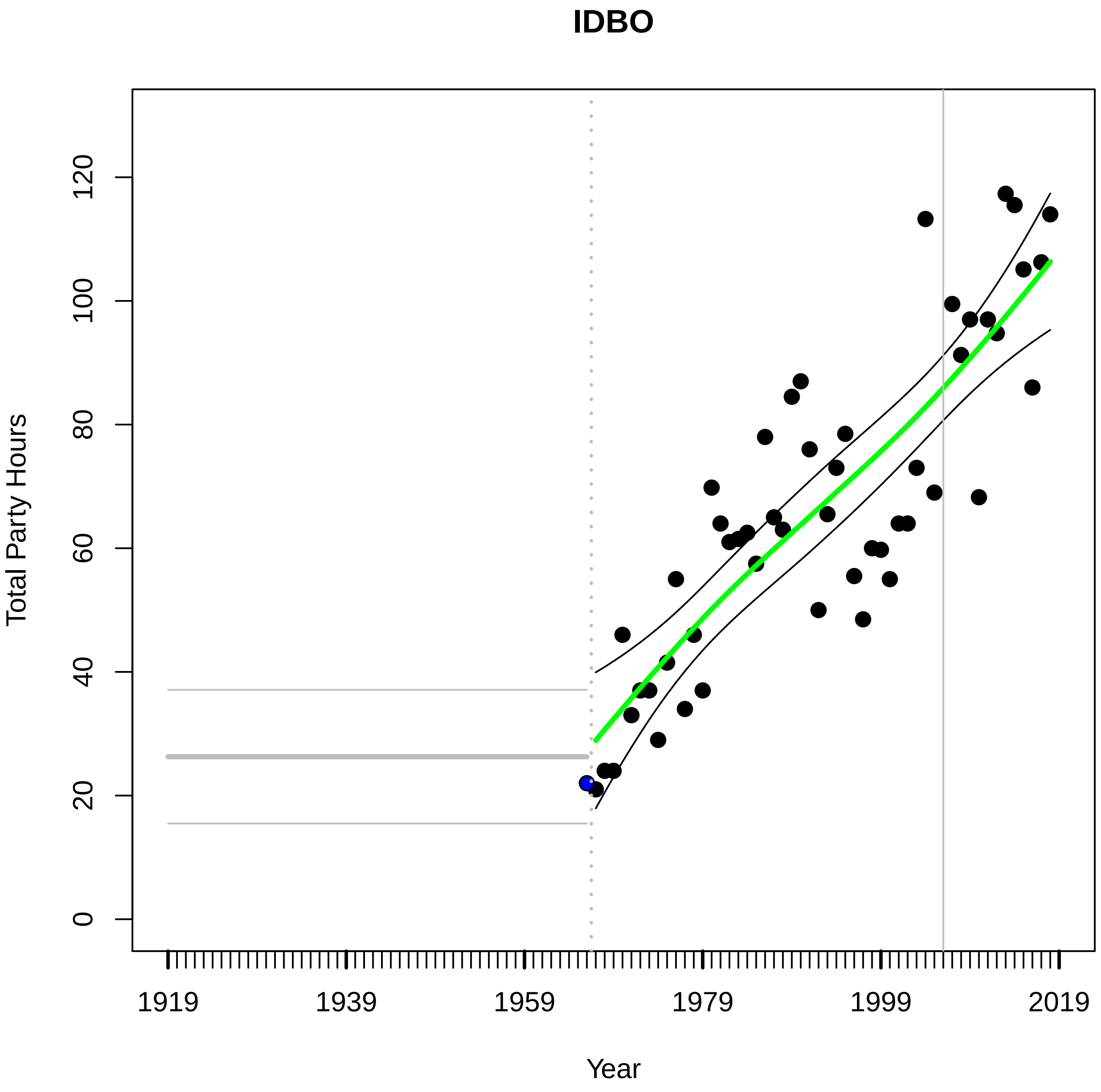

IDBO
Total Party Hours
0
20
40
60
80
100
120
1919
1939
1959
1979
1999
2019
Year

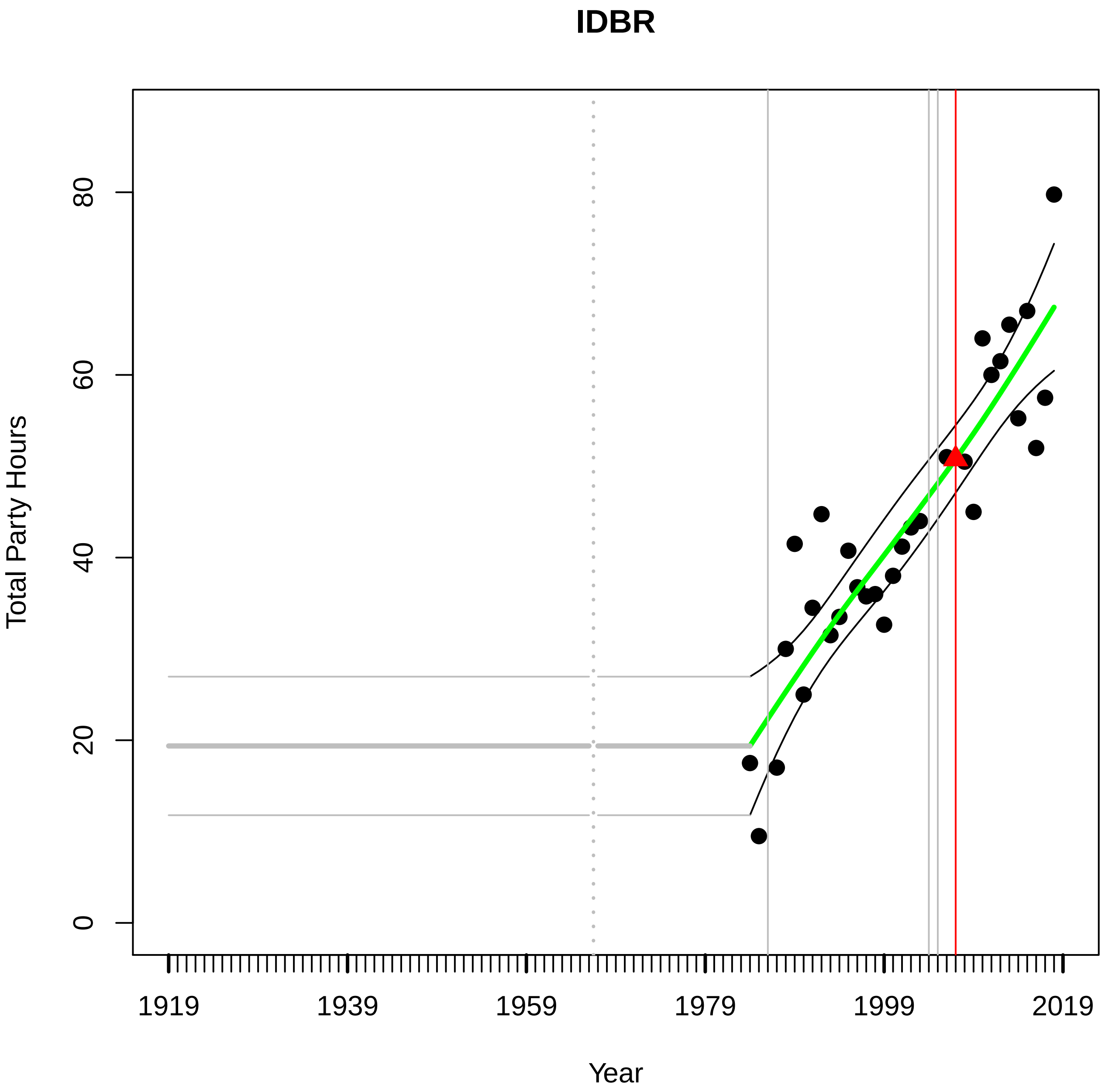

IDBR
Total Party Hours
Year
0
20
40
60
80
1919
1939
1959
1979
1999
2019

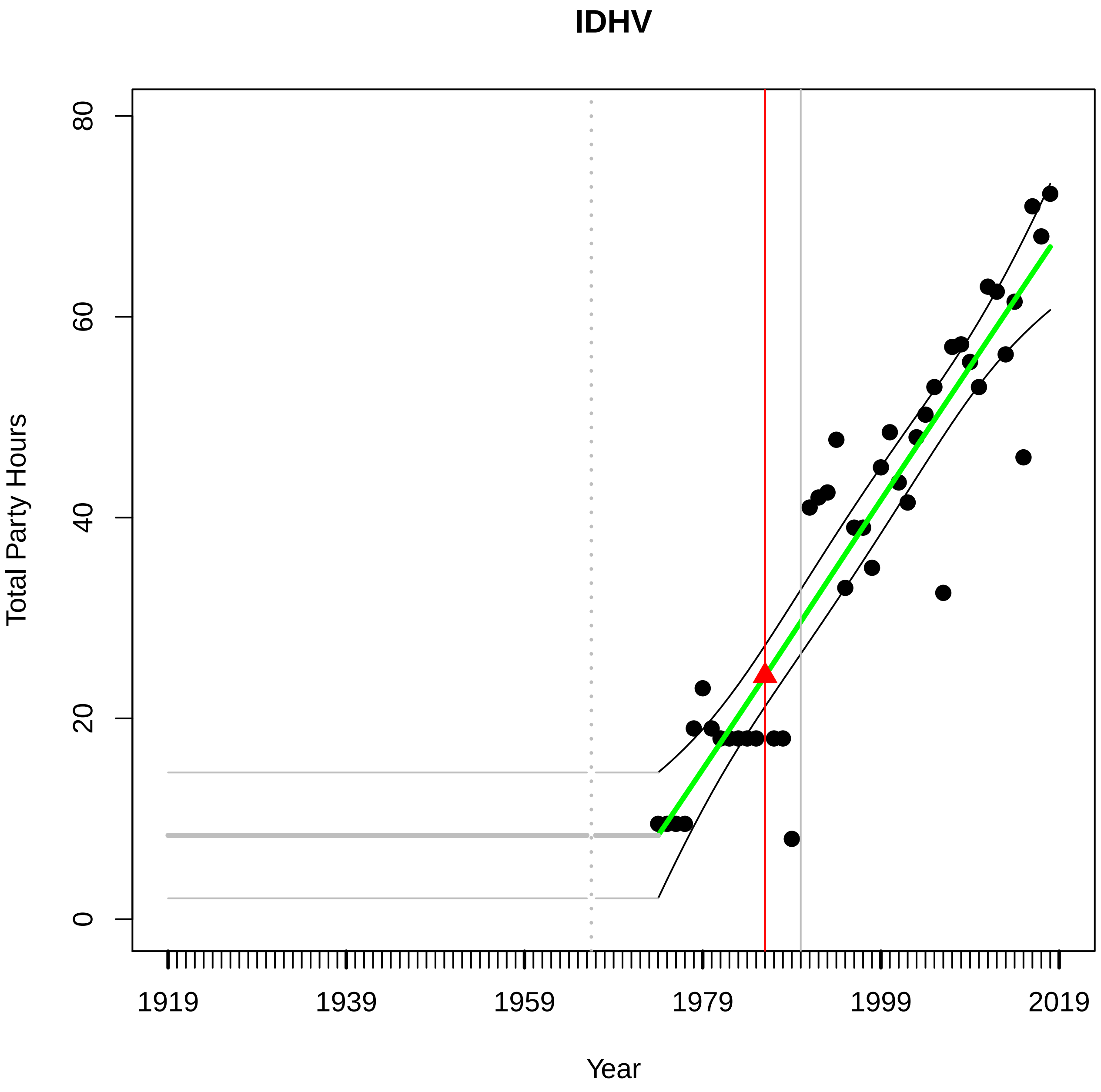

IDHV
Total Party Hours
0
20
40
60
80
1919
1939
1959
1979
1999
2019
Year

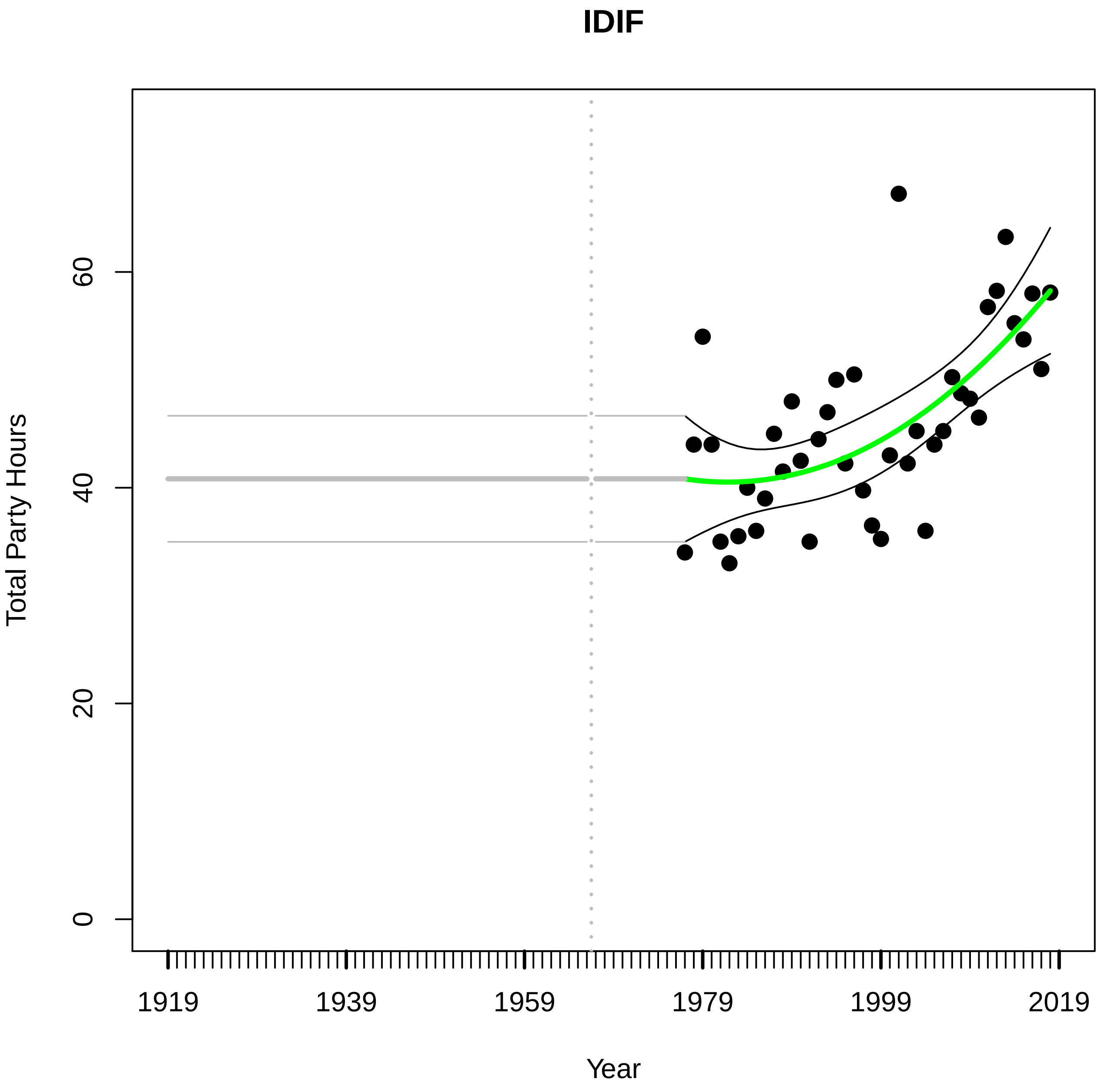

IDIF
Total Party Hours
0
20
40
60
1919
1939
1959
1979
1999
2019
Year

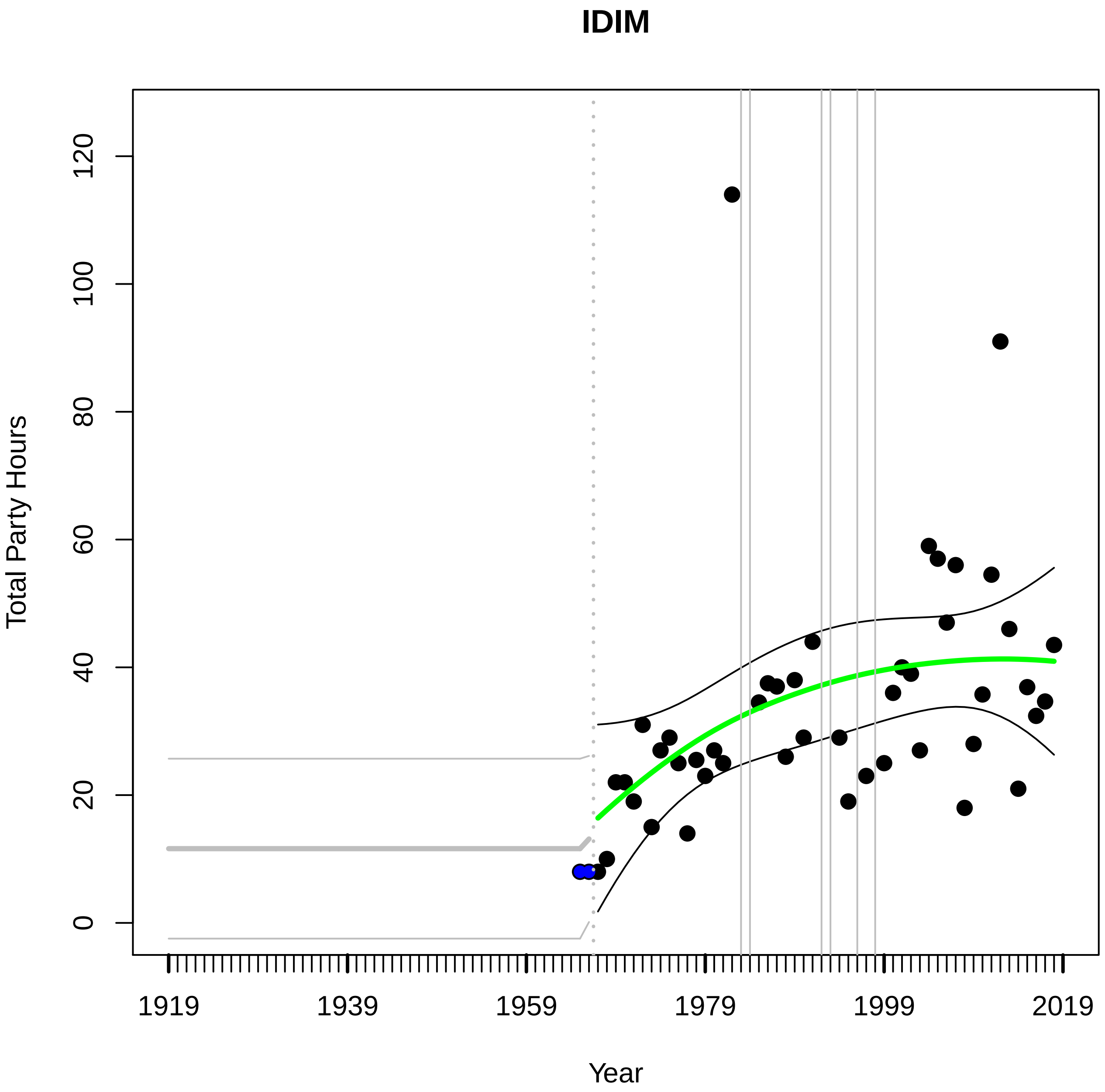

IDIM
Total Party Hours
0
20
40
60
80
100
120
Year
1919
1939
1959
1979
1999
2019

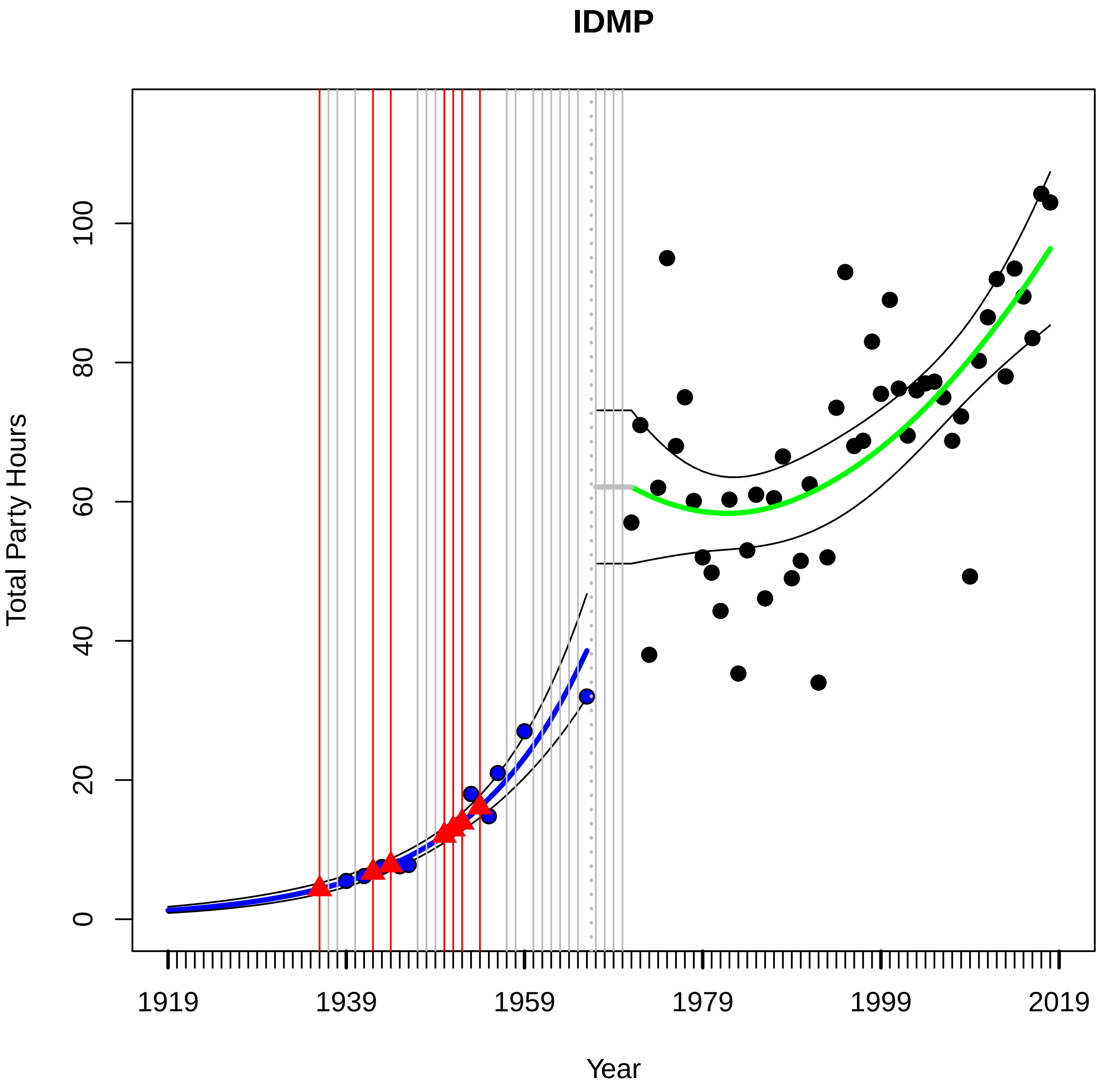
IDMP
Total Party Hours
0
20
40
60
80
100
1919
1939
1959
1979
1999
2019
Year

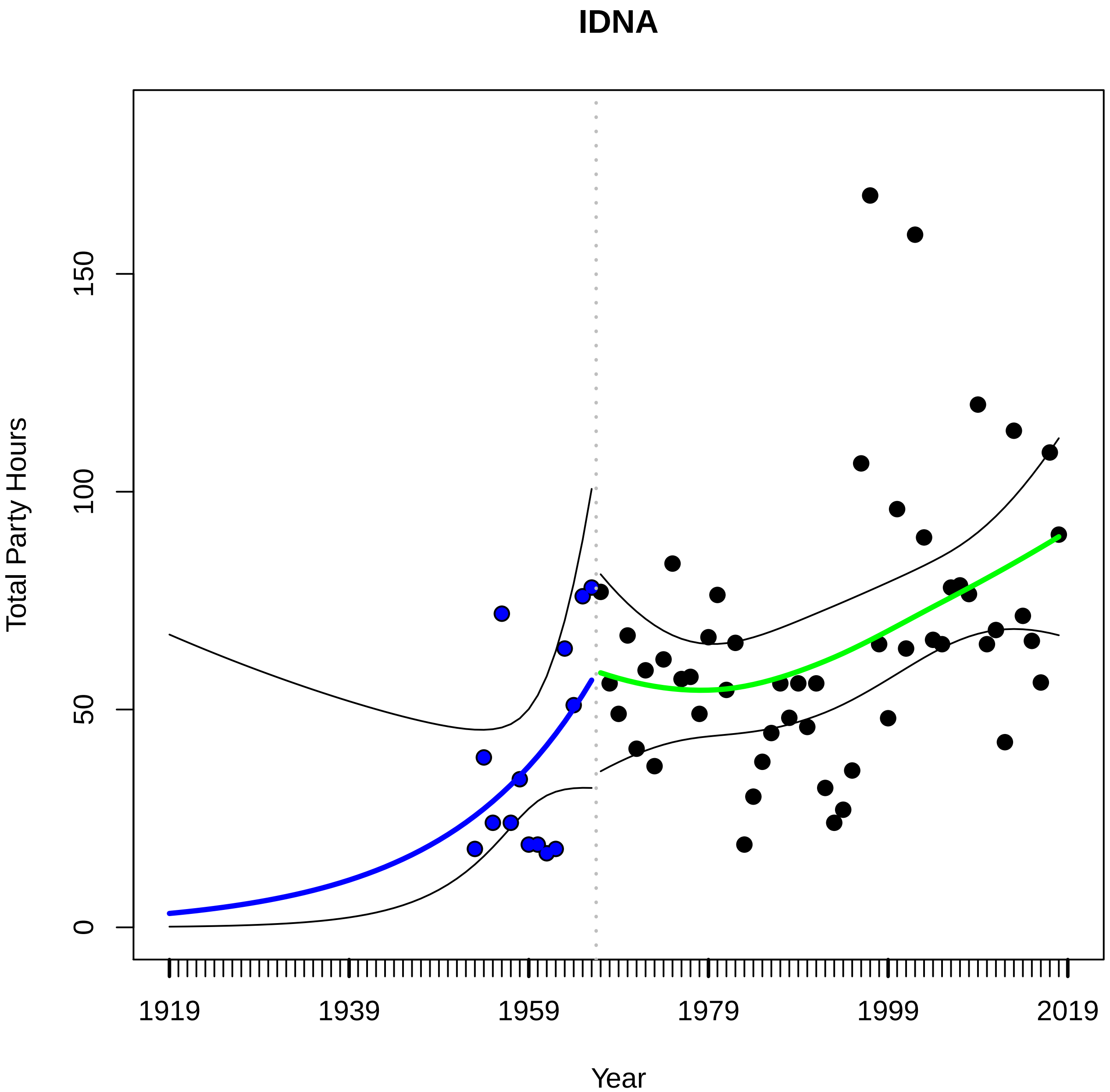
IDNA
Total Party Hours
0
50
100
150
1919
1939
1959
1979
1999
2019
Year

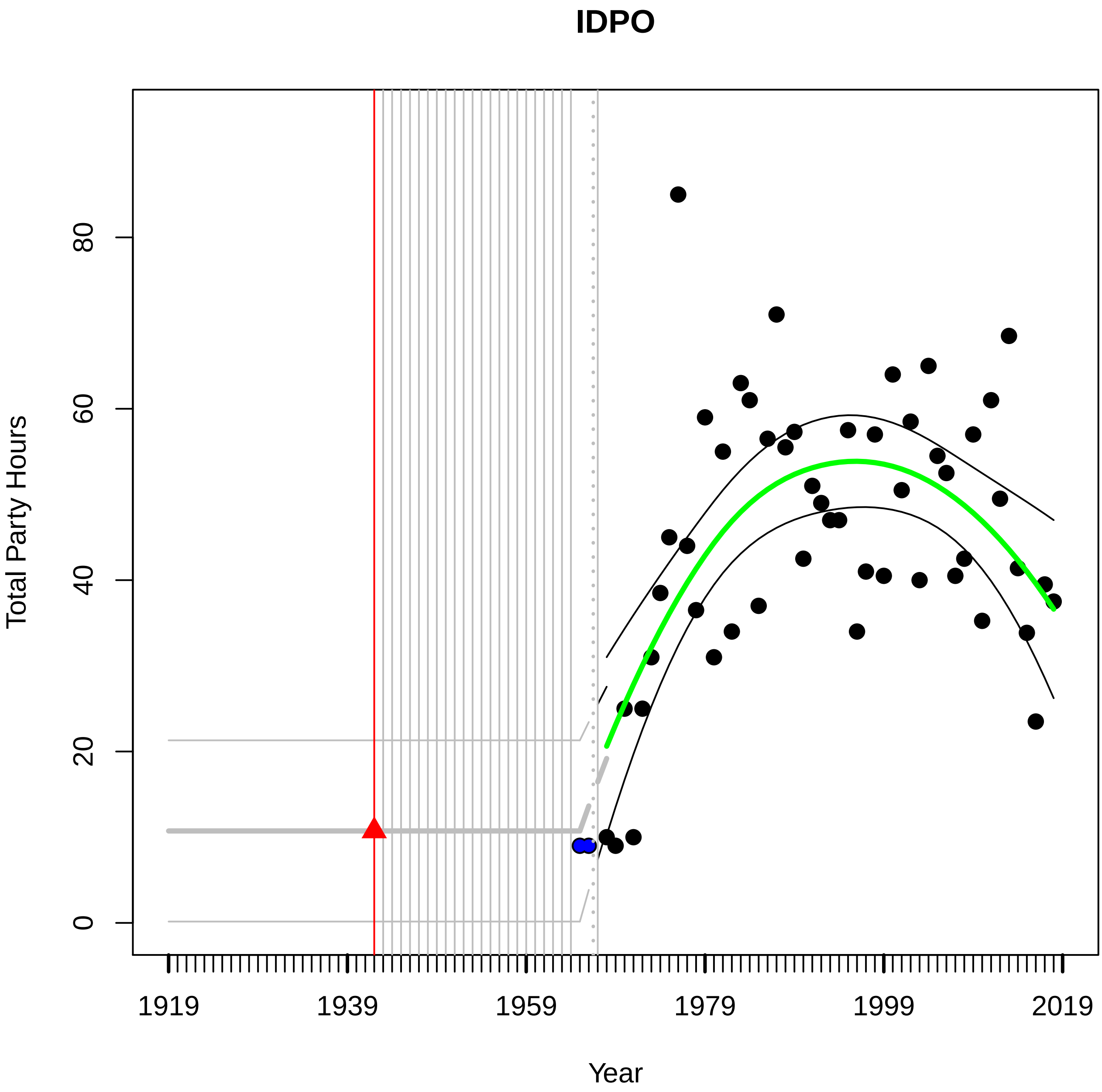
IDPO
Total Party Hours
0
20
40
60
80
1919
1939
1959
1979
1999
2019
Year

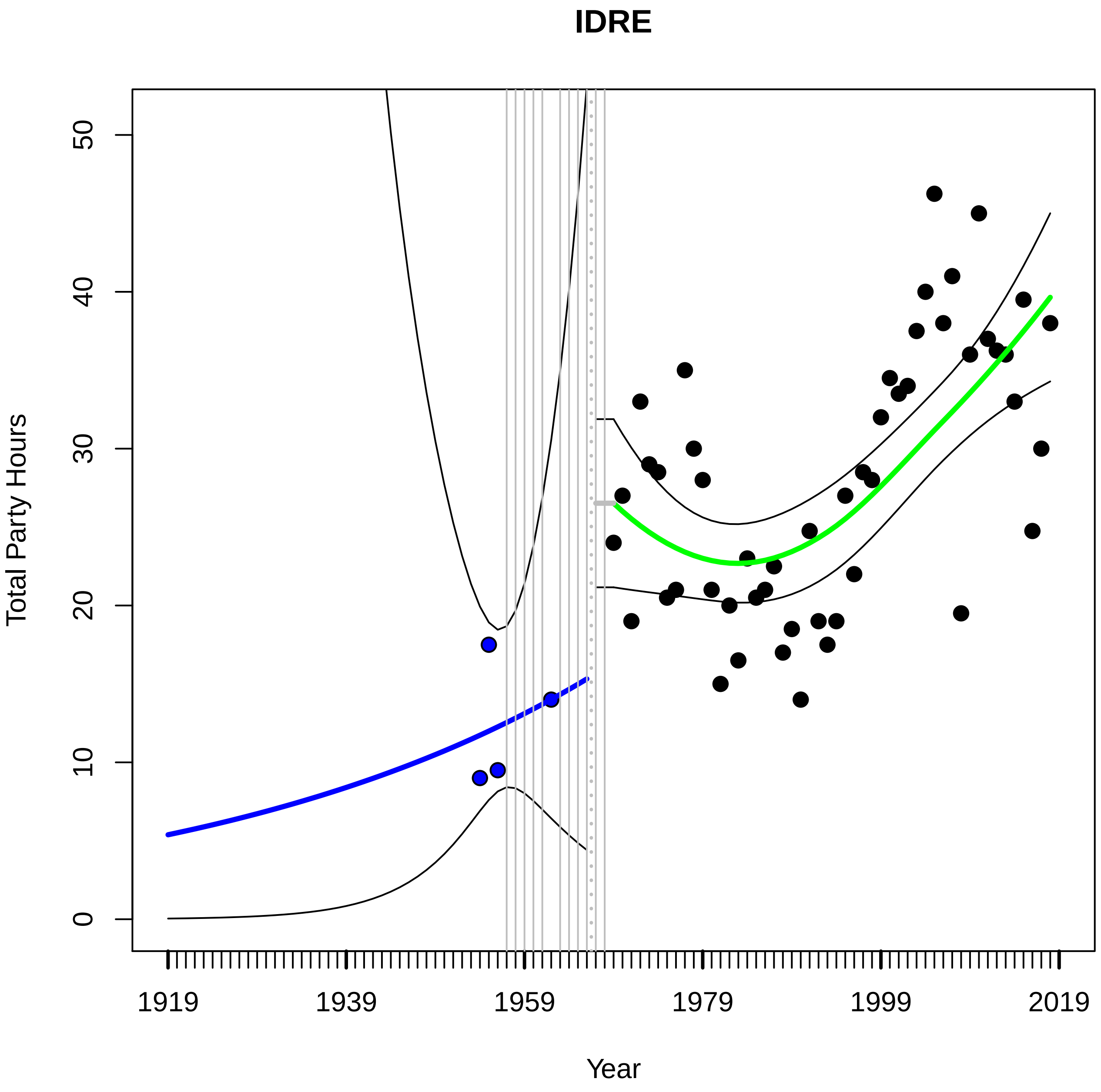

IDRE
Total Party Hours
Year
0
10
20
30
40
50
1919
1939
1959
1979
1999
2019

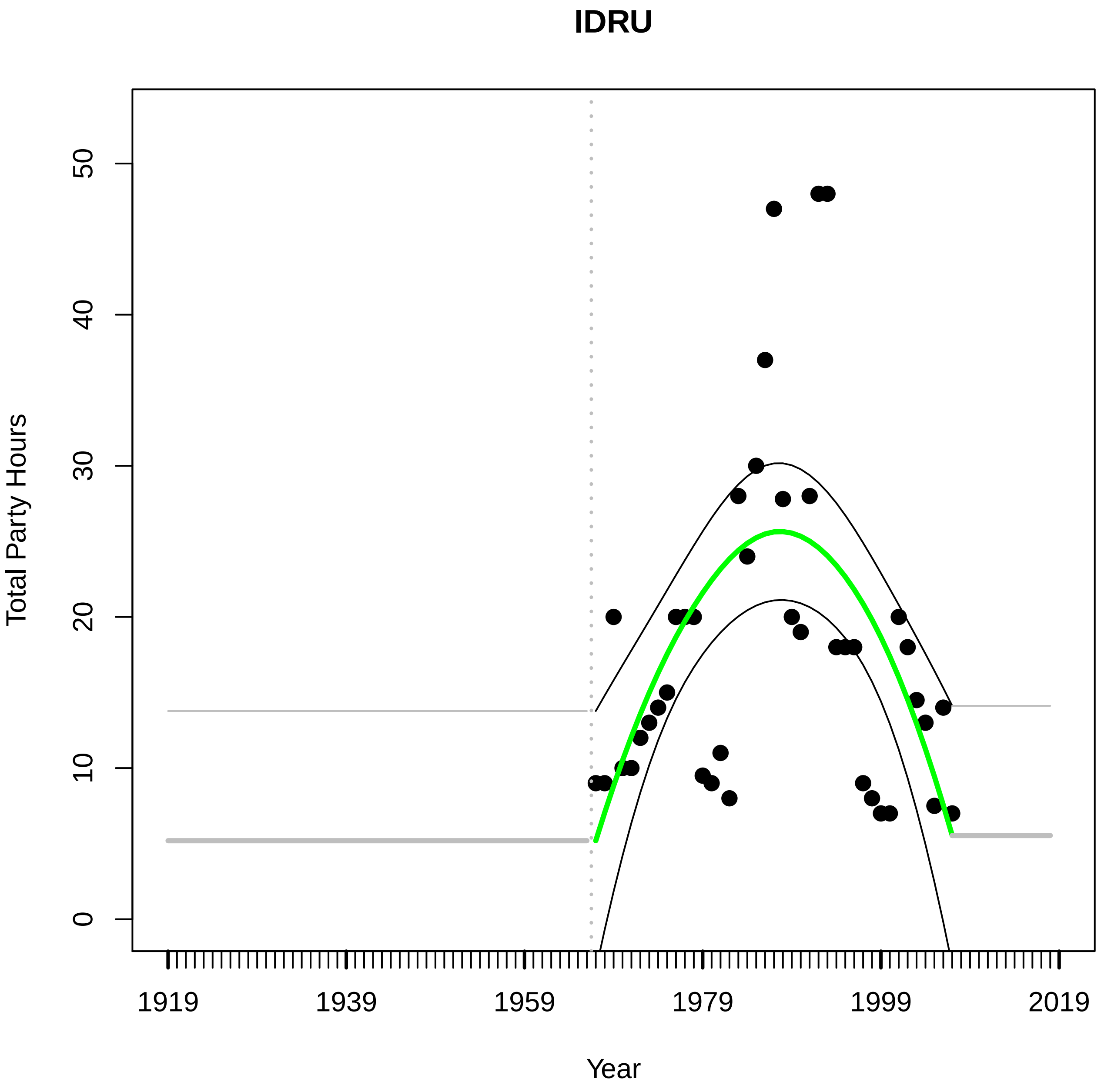

IDRU
Total Party Hours
50
40
30
20
10
0
1919
1939
1959
1979
1999
2019
Year

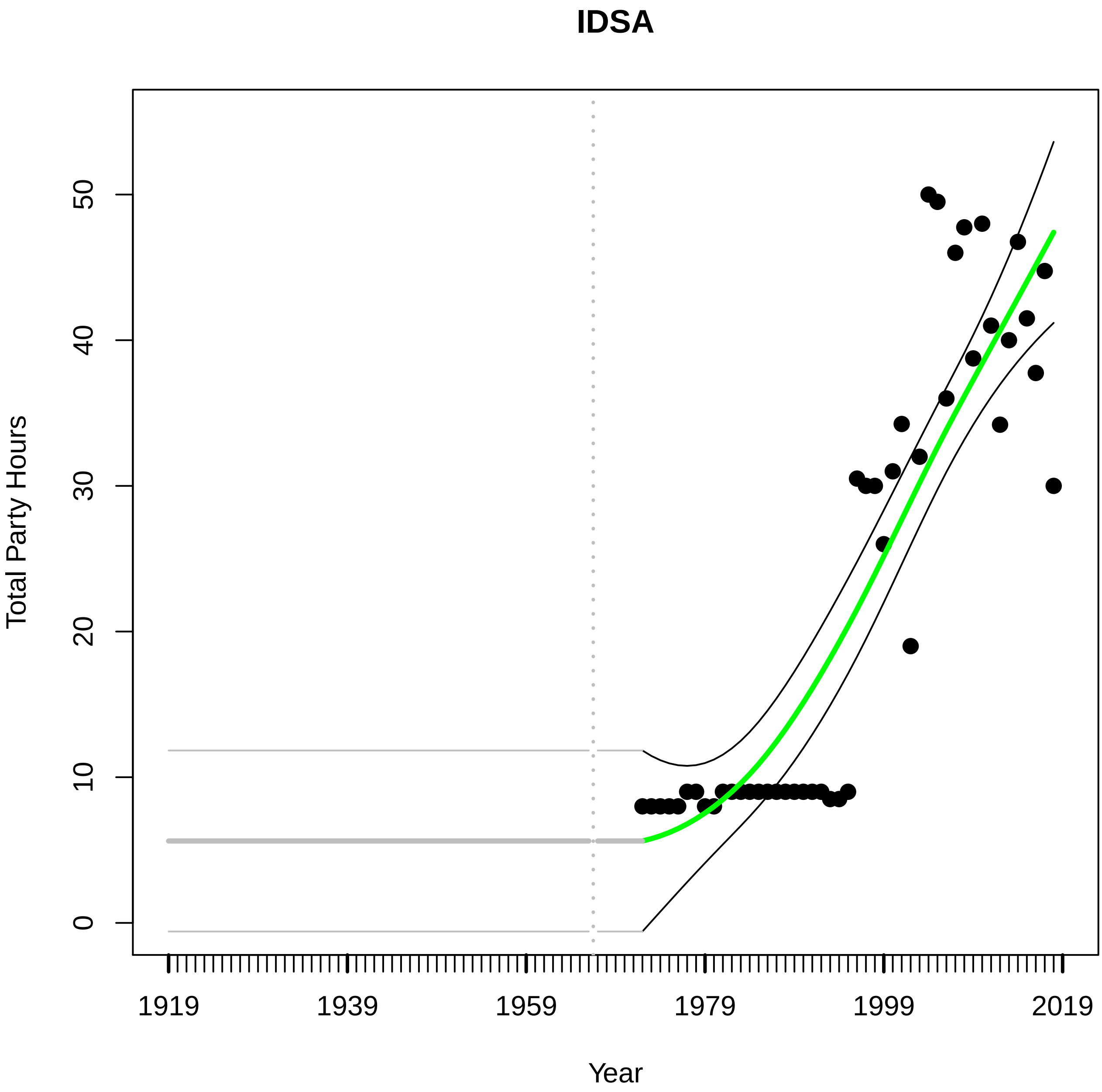

IDSA
Total Party Hours
0
10
20
30
40
50
1919
1939
1959
1979
1999
2019
Year

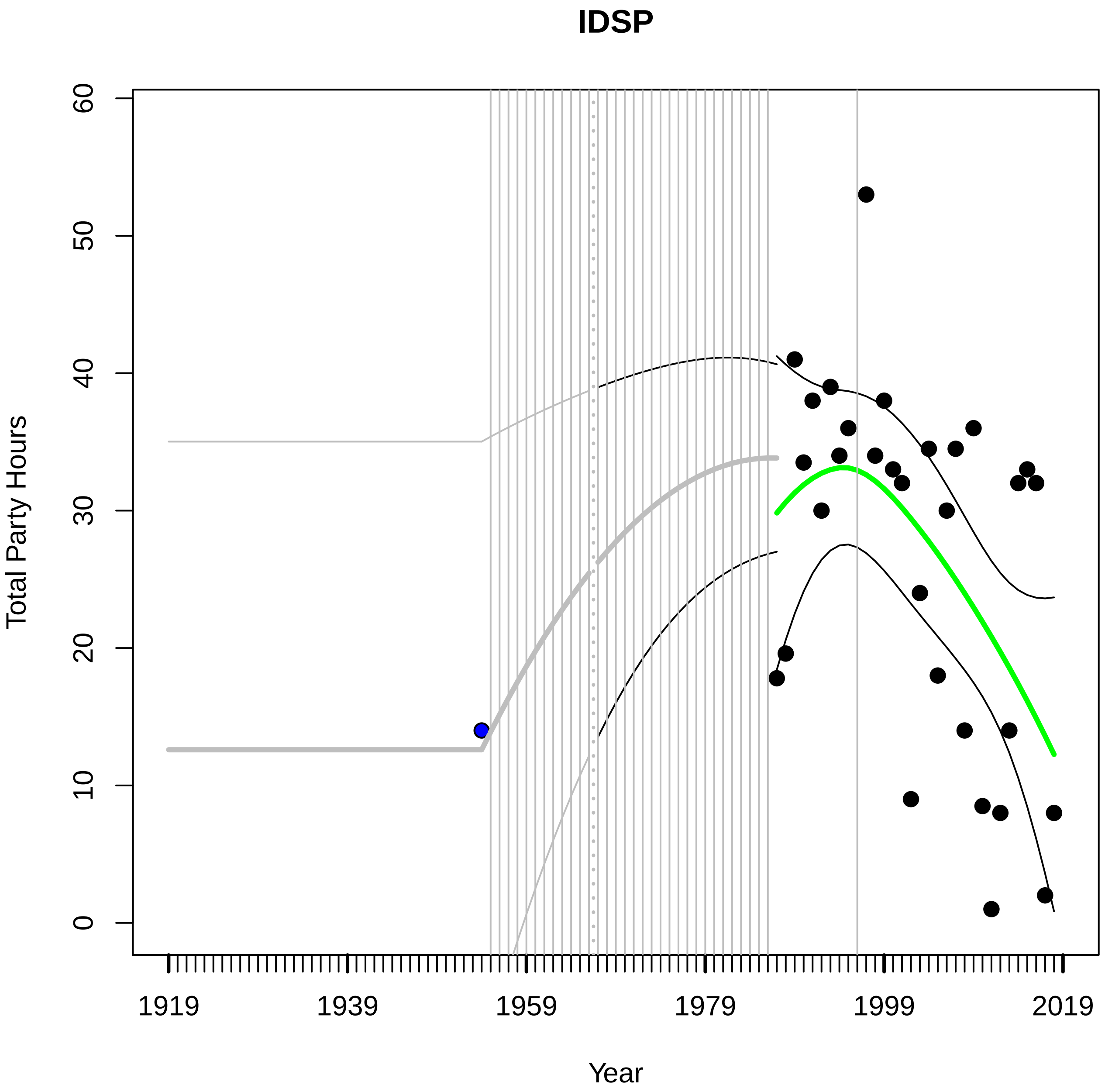

IDSP
Total Party Hours
Year
0
10
20
30
40
50
60
1919
1939
1959
1979
1999
2019

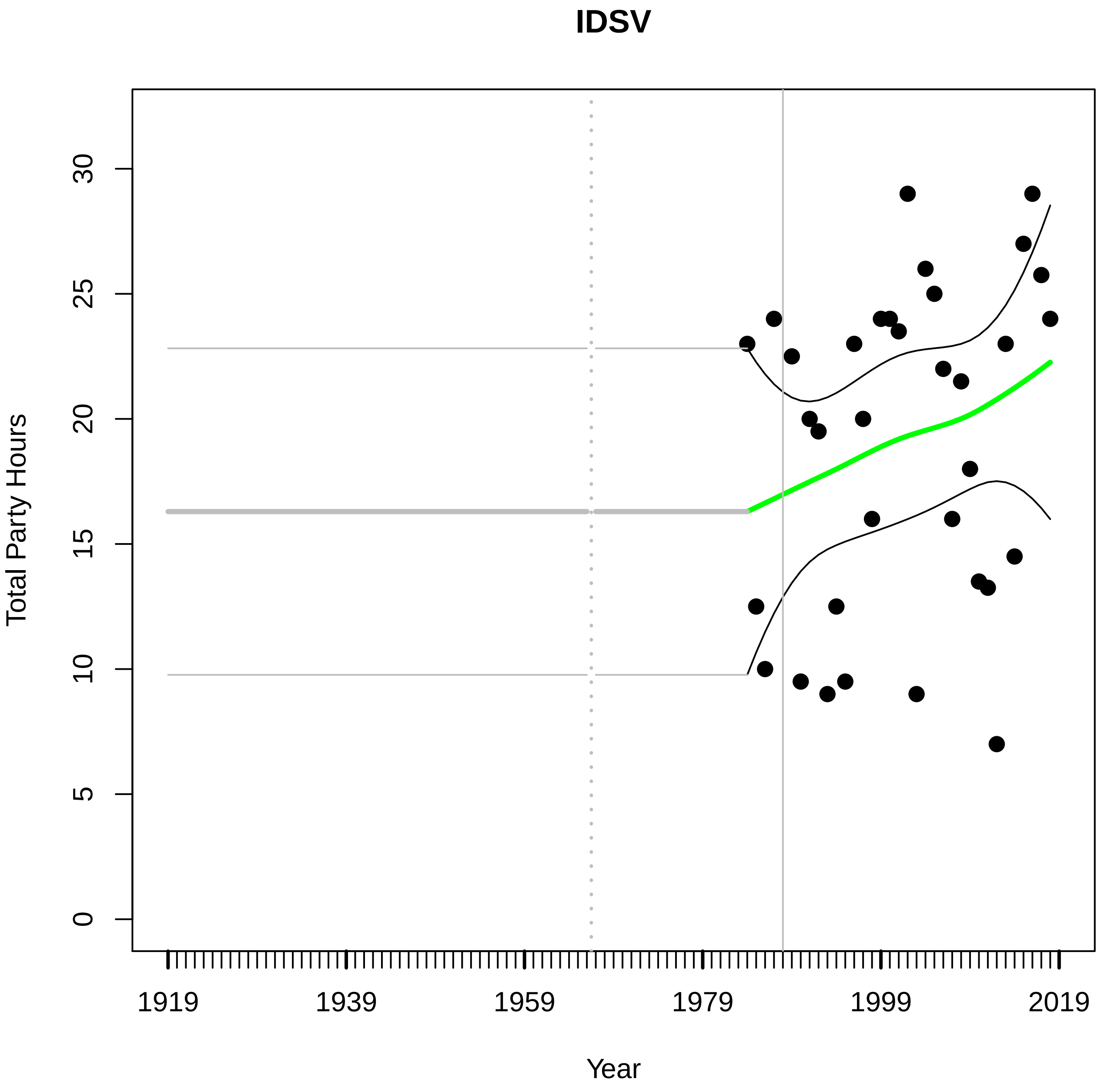

IDSV
Total Party Hours
Year
0
5
10
15
20
25
30
1919
1939
1959
1979
1999
2019

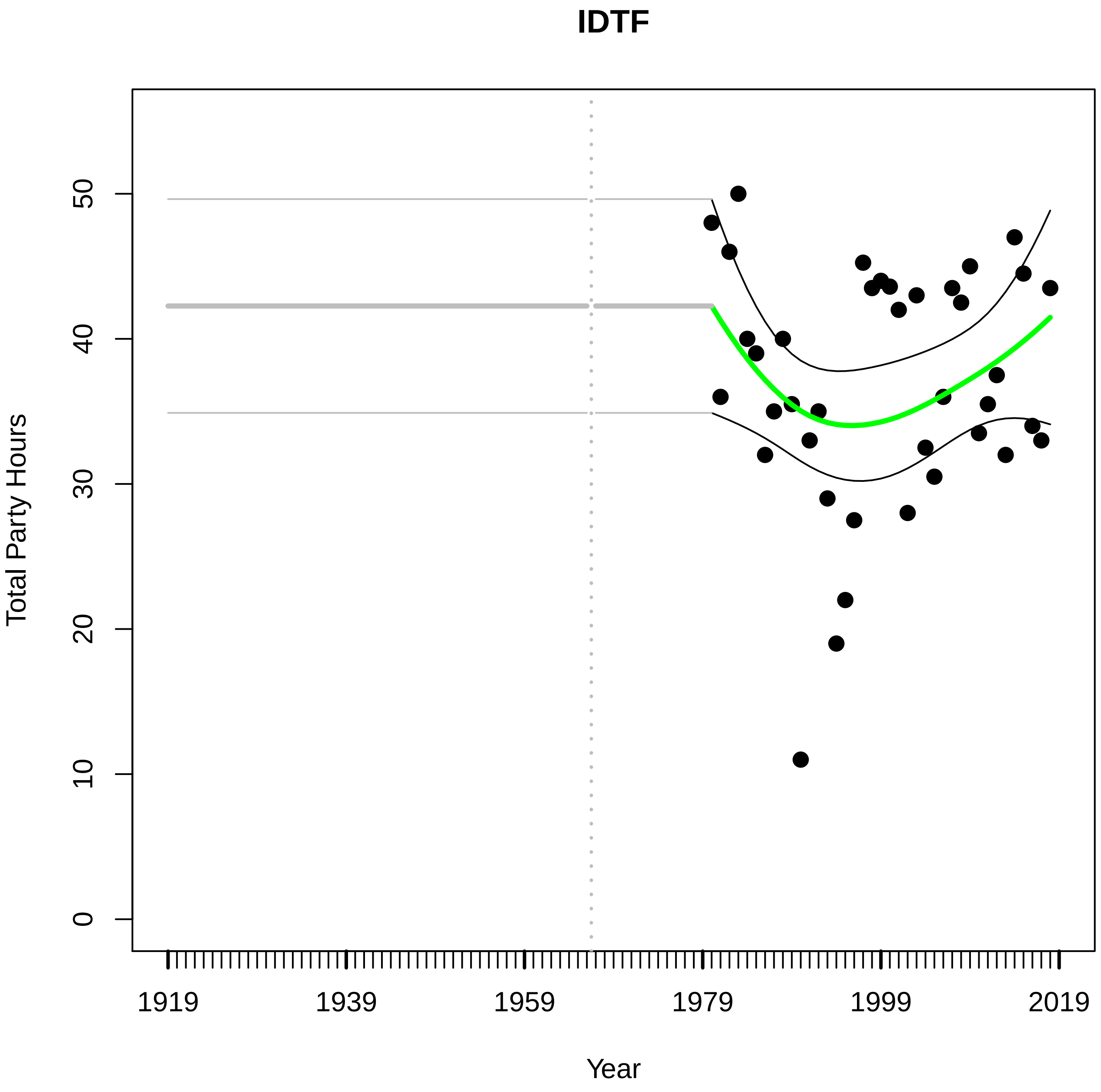
IDTF
Total Party Hours
0
10
20
30
40
50
1919
1939
1959
1979
1999
2019
Year

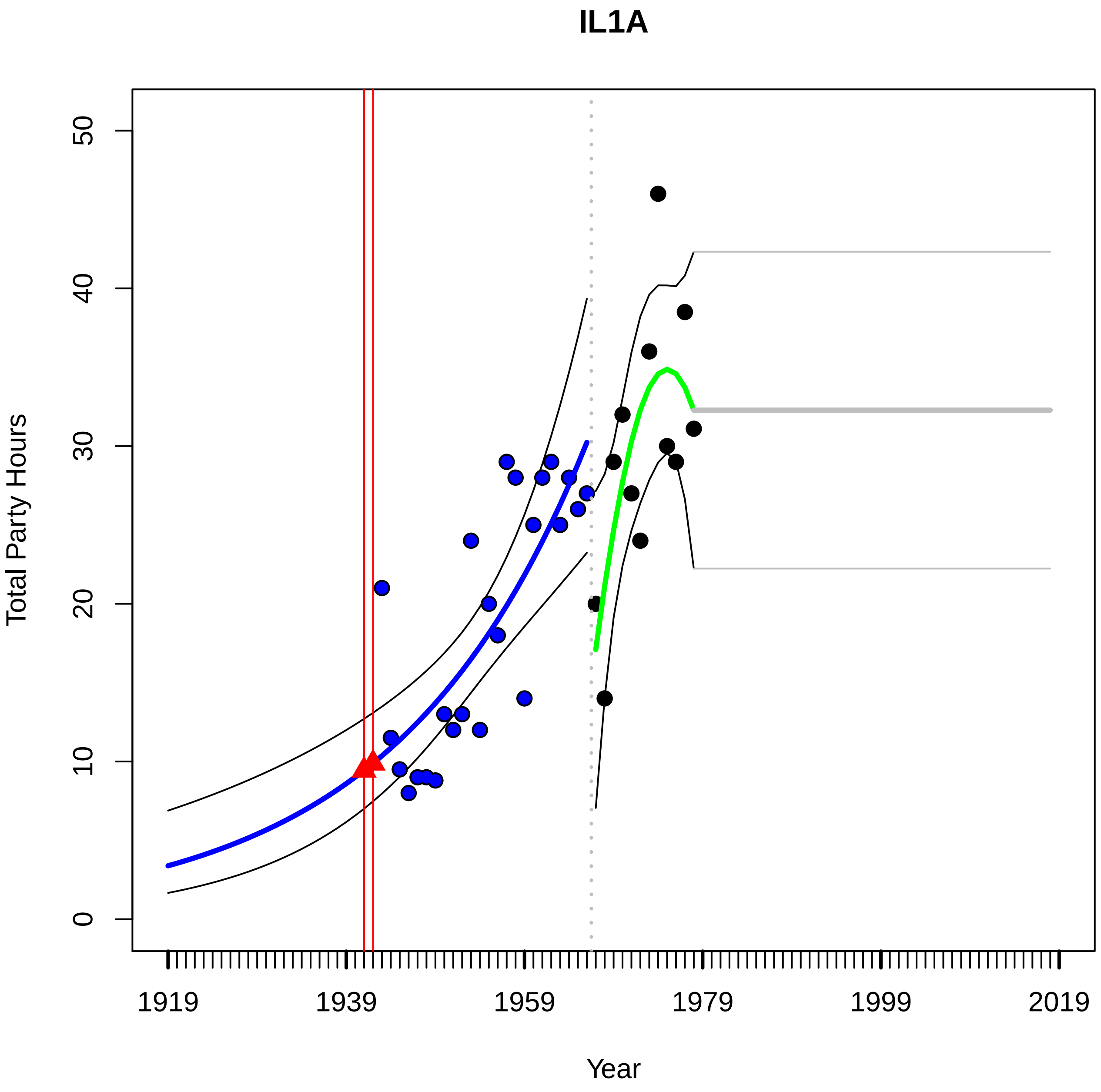

IL1A
Total Party Hours
Year
0
10
20
30
40
50
1919
1939
1959
1979
1999
2019

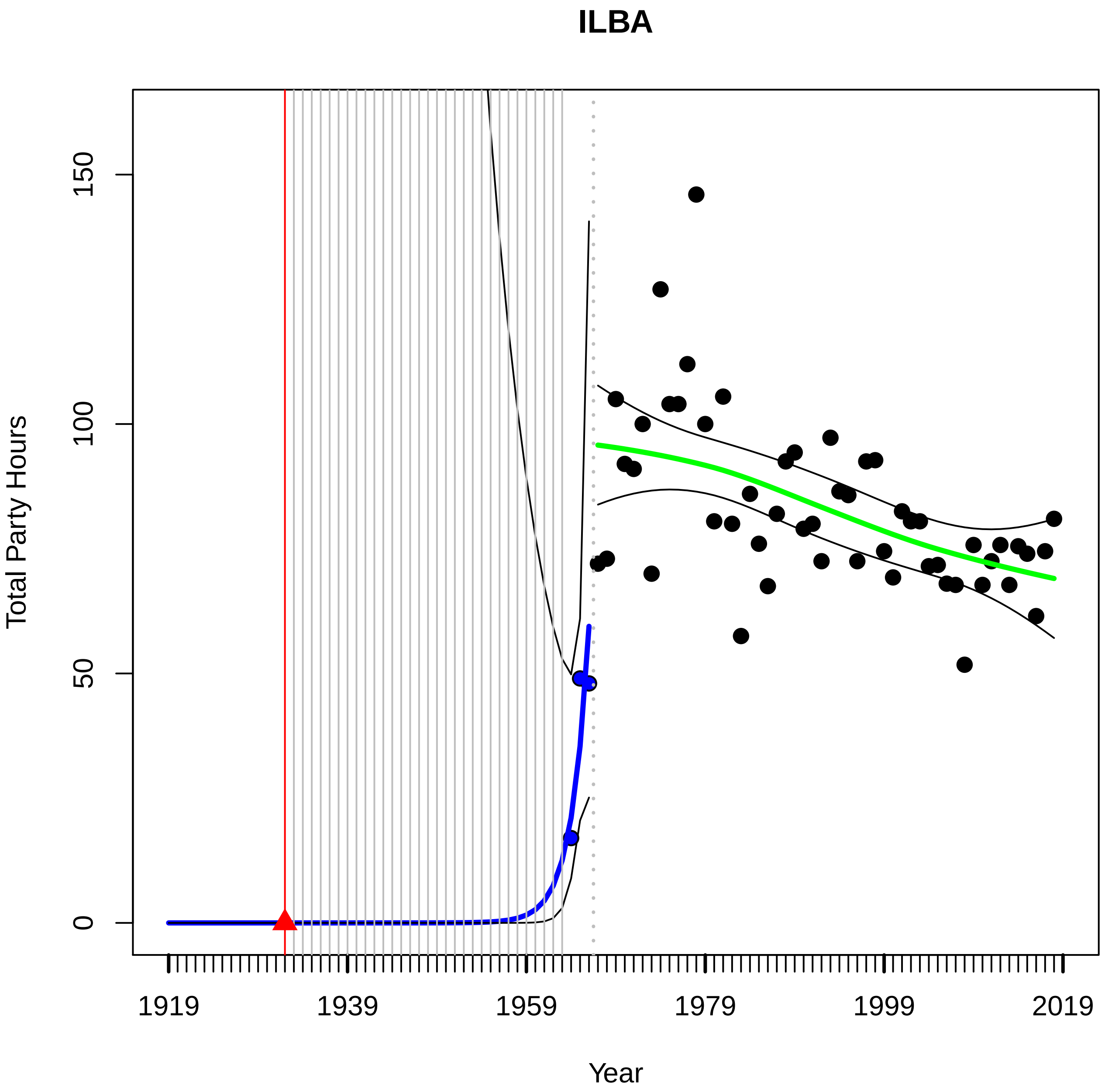

ILBA
Total Party Hours
0
50
100
150
1919
1939
1959
1979
1999
2019
Year

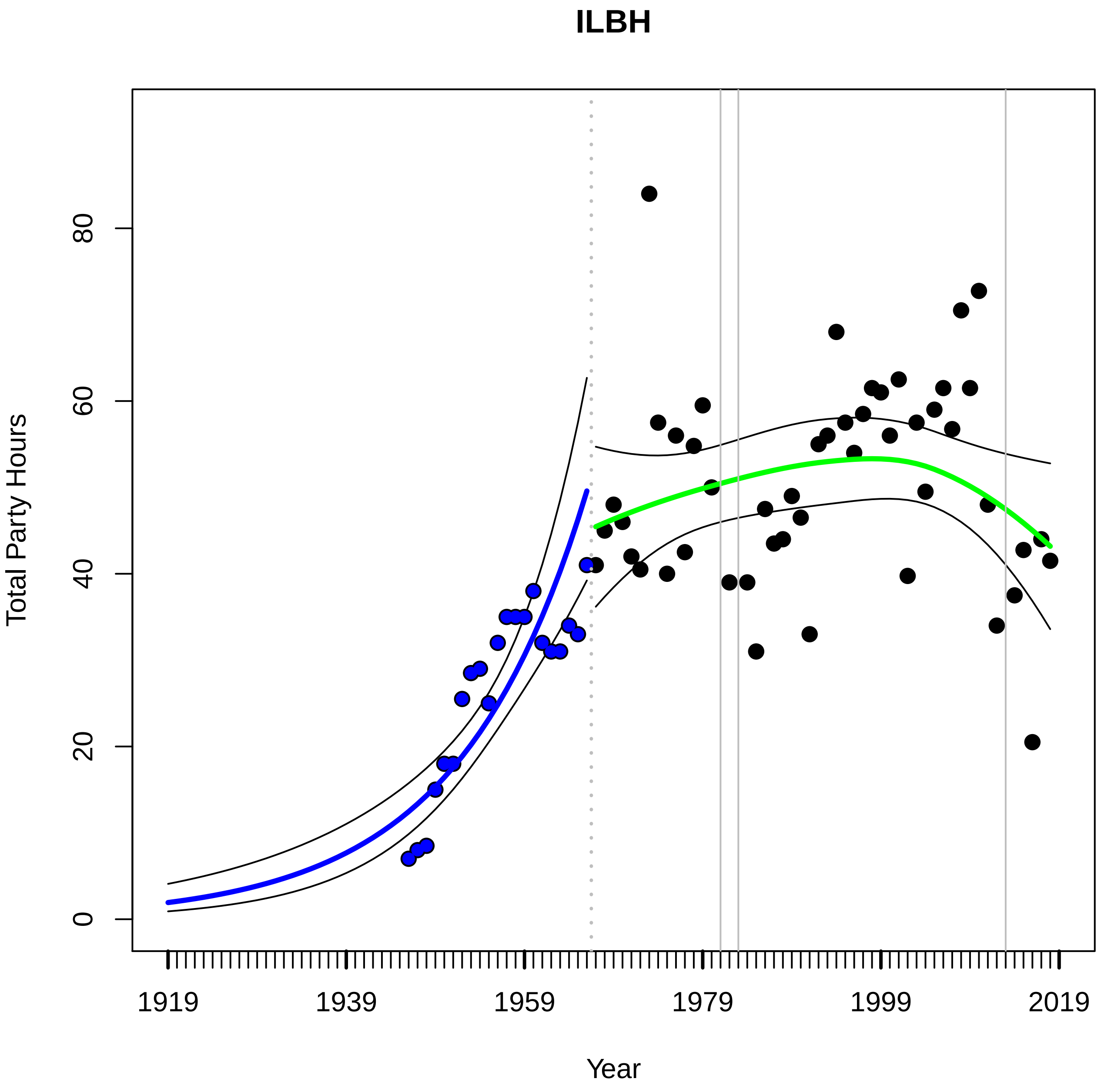
ILBH
Total Party Hours
0
20
40
60
80
1919
1939
1959
1979
1999
2019
Year

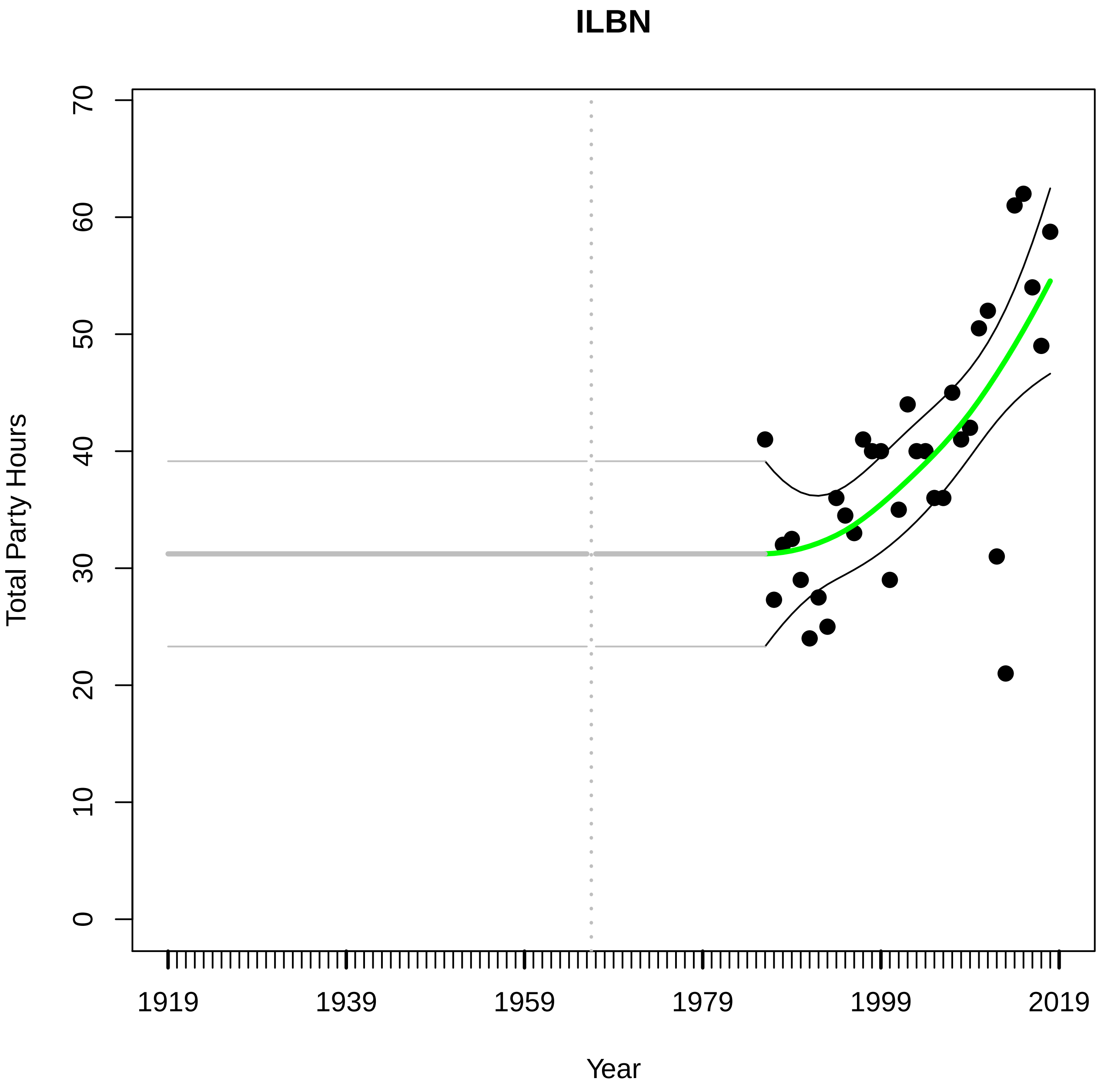
ILBN
Total Party Hours
0
10
20
30
40
50
60
70
1919
1939
1959
1979
1999
2019
Year

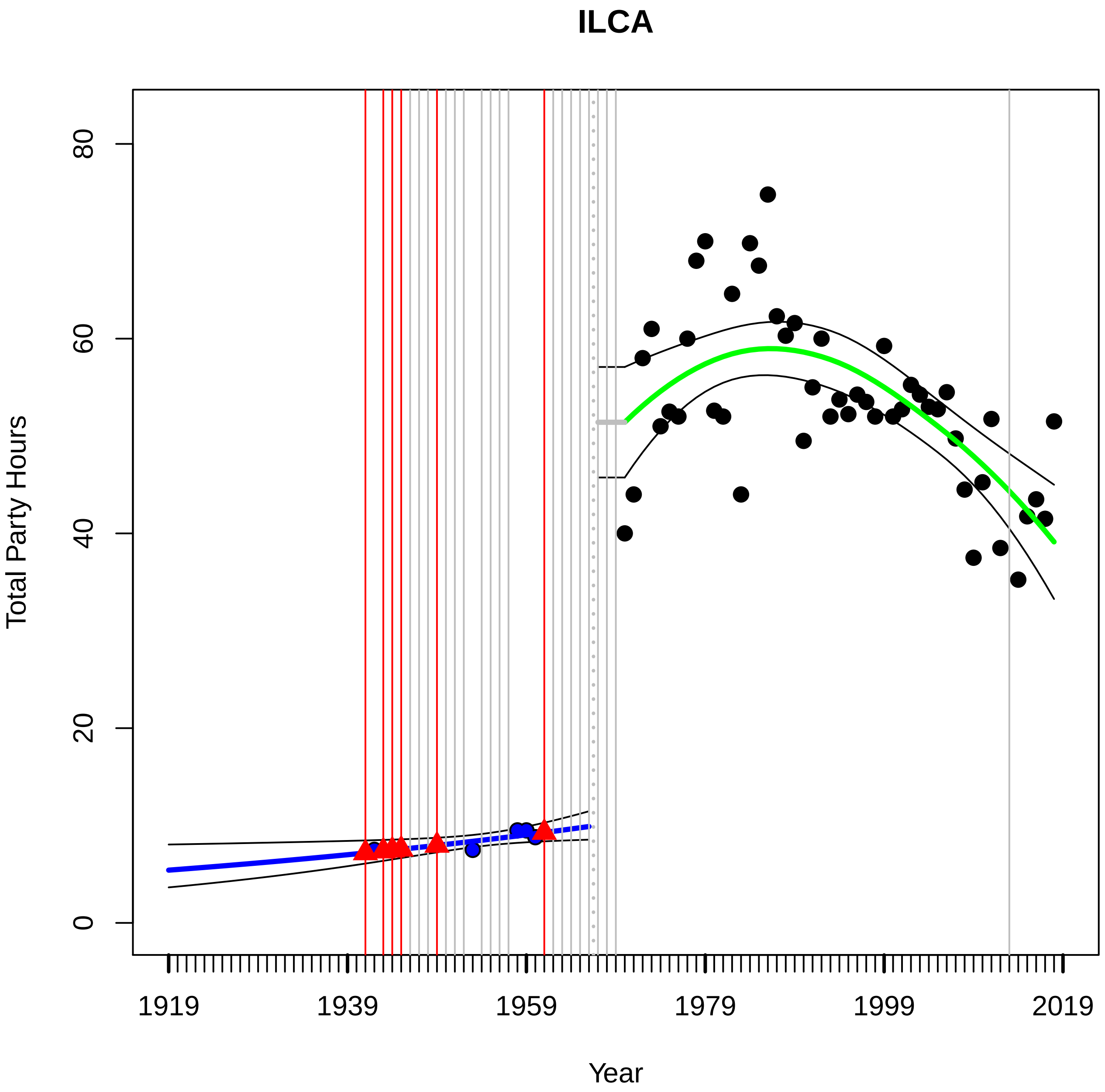

ILCA
Total Party Hours
0
20
40
60
80
1919
1939
1959
1979
1999
2019
Year

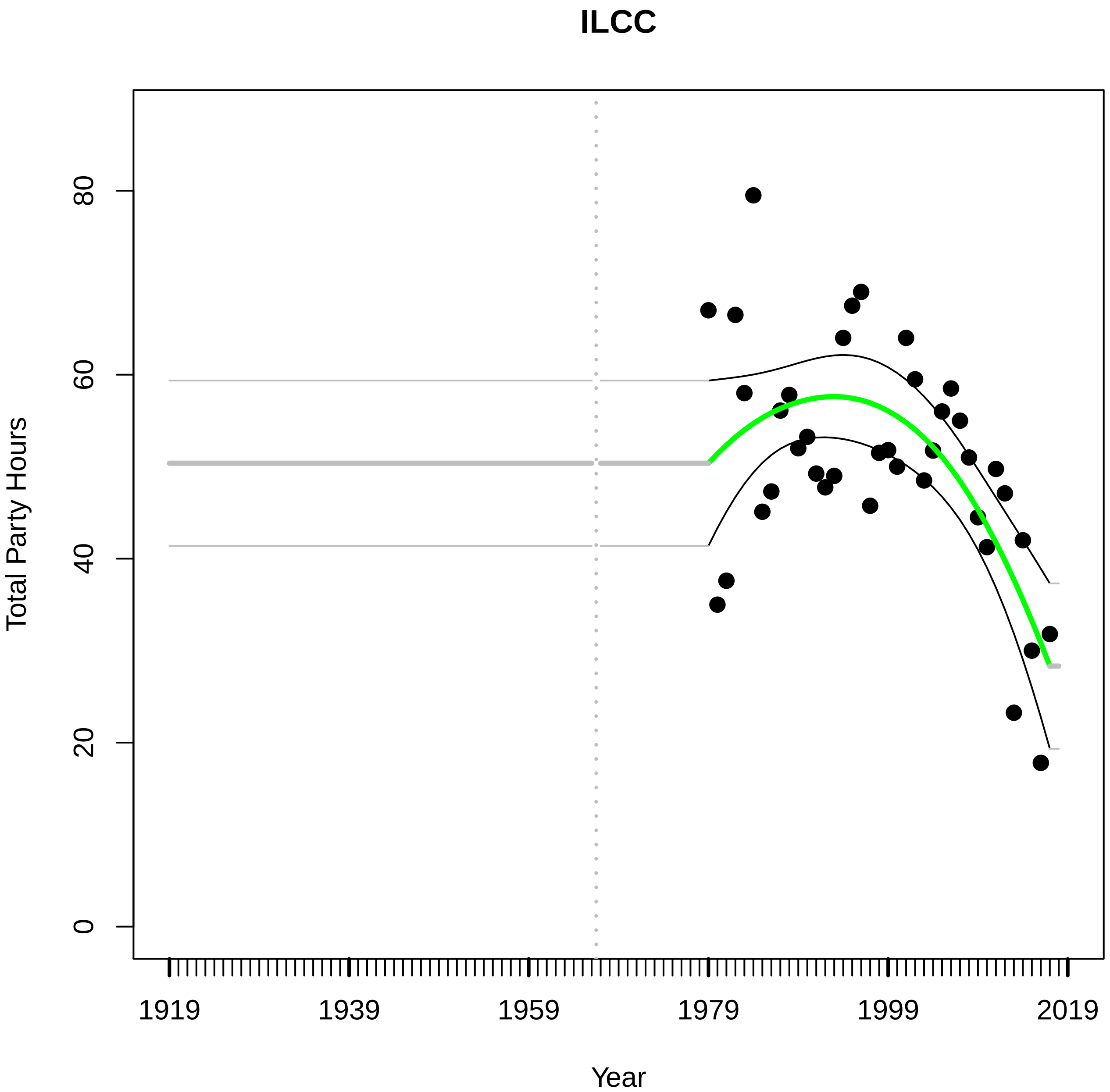

ILCC
Total Party Hours
0
20
40
60
80
1919
1939
1959
1979
1999
2019
Year

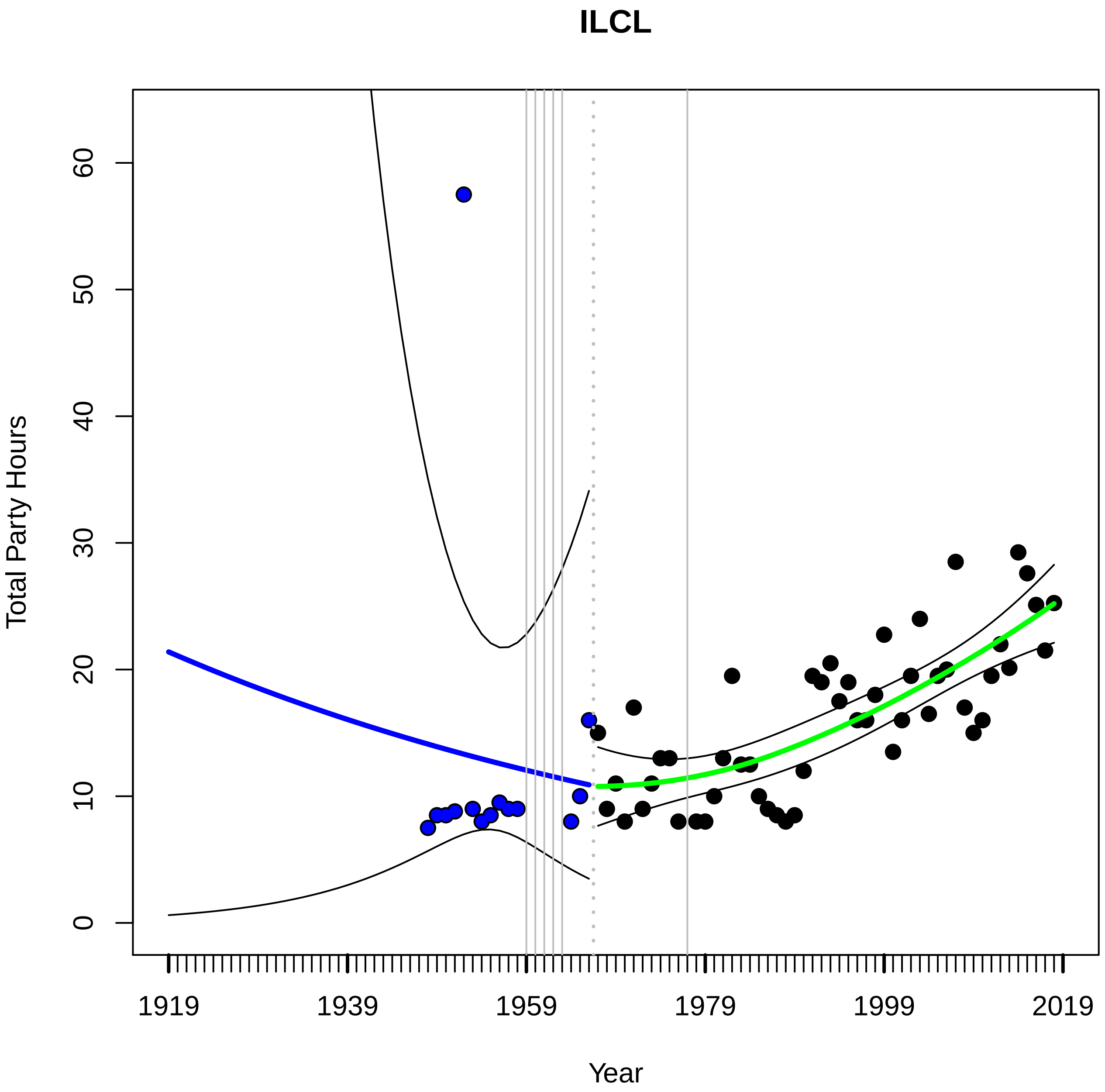

ILCL
Total Party Hours
Year
0
10
20
30
40
50
60
1919
1939
1959
1979
1999
2019

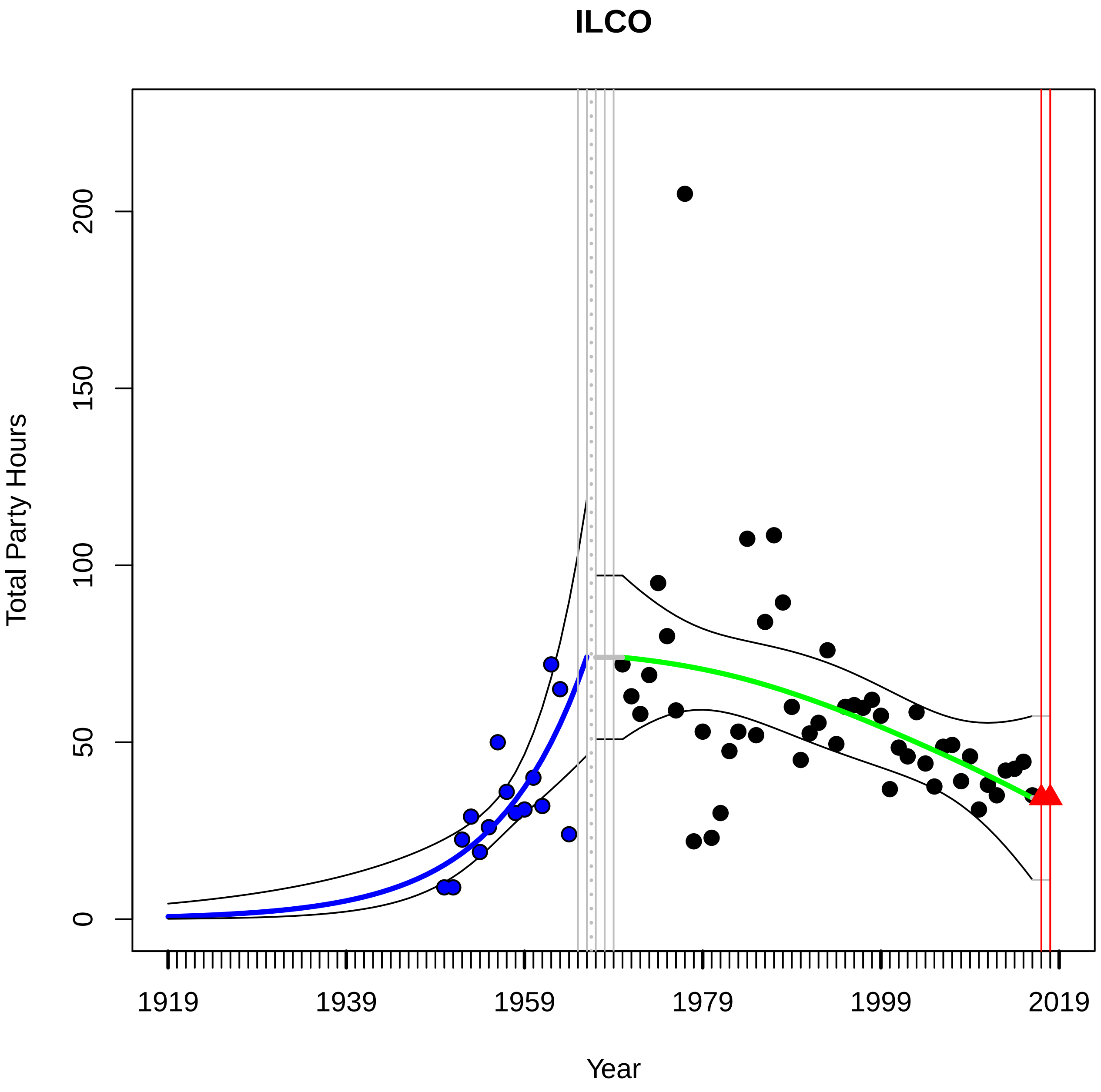
ILCO
Total Party Hours
0
50
100
150
200
1919
1939
1959
1979
1999
2019
Year

ILCR

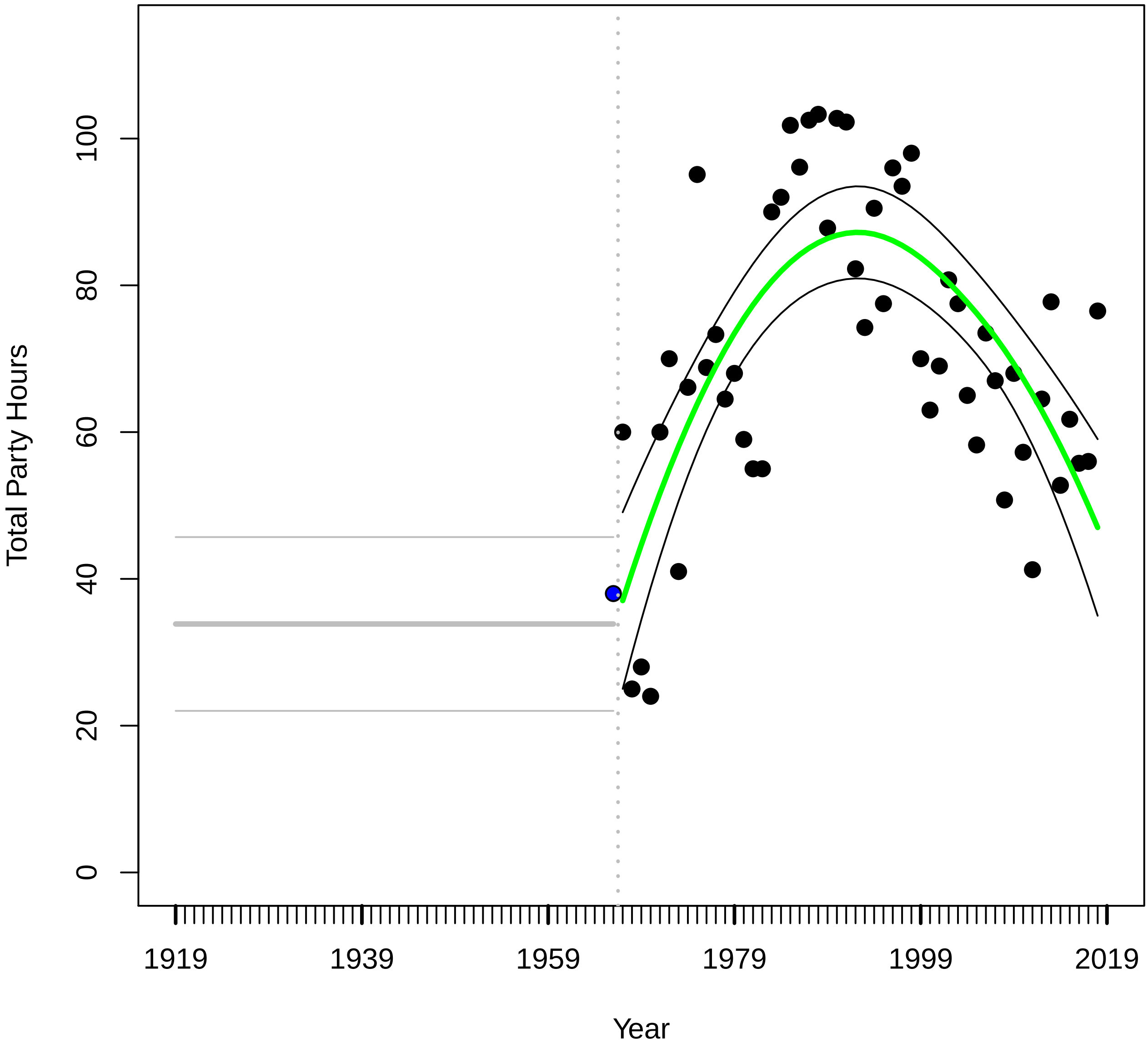

Total Party Hours
0
20
40
60
80
100
1919
1939
1959
1979
1999
2019
Year

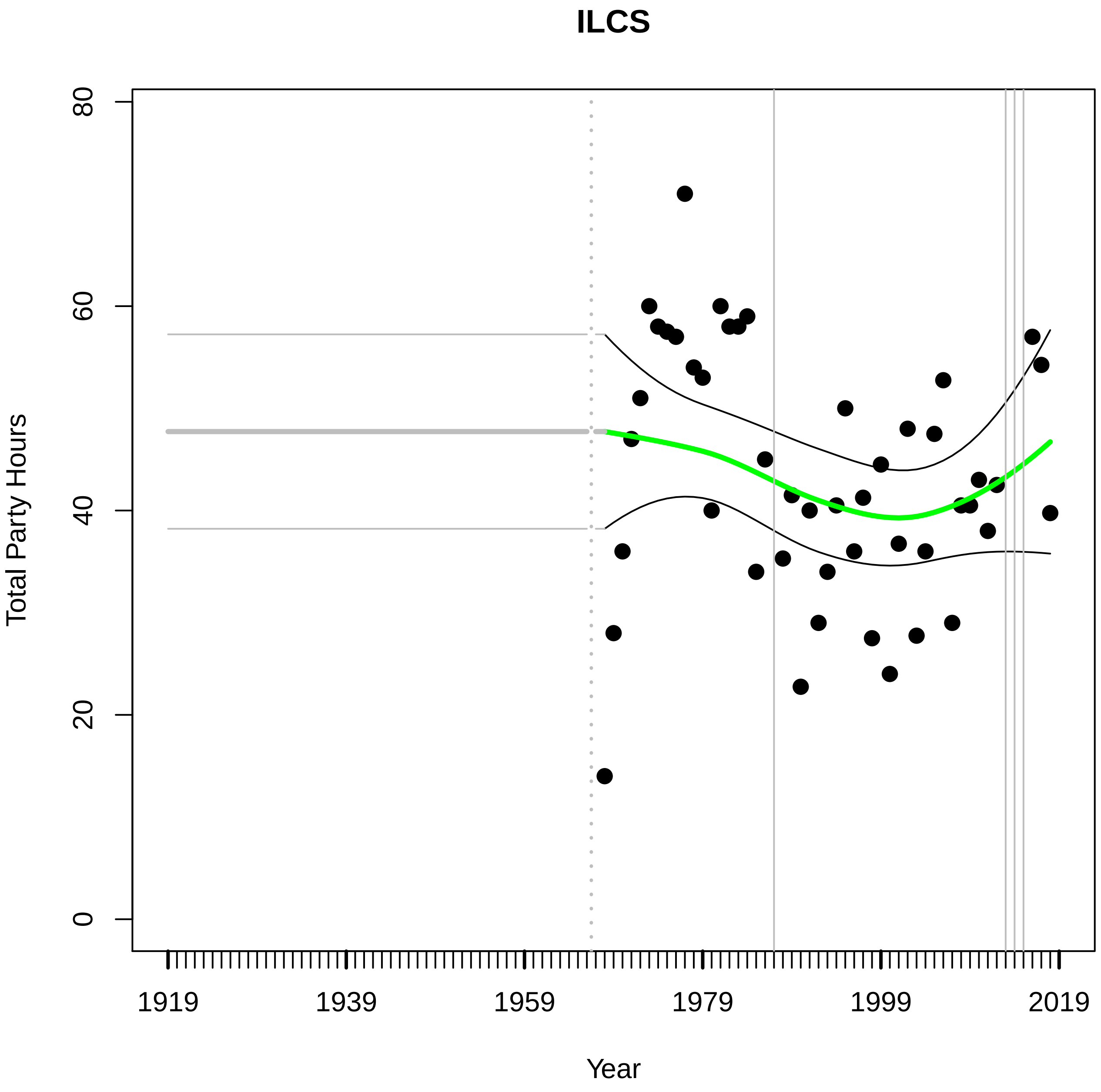
ILCS
Total Party Hours
Year
0
20
40
60
80
1919
1939
1959
1979
1999
2019

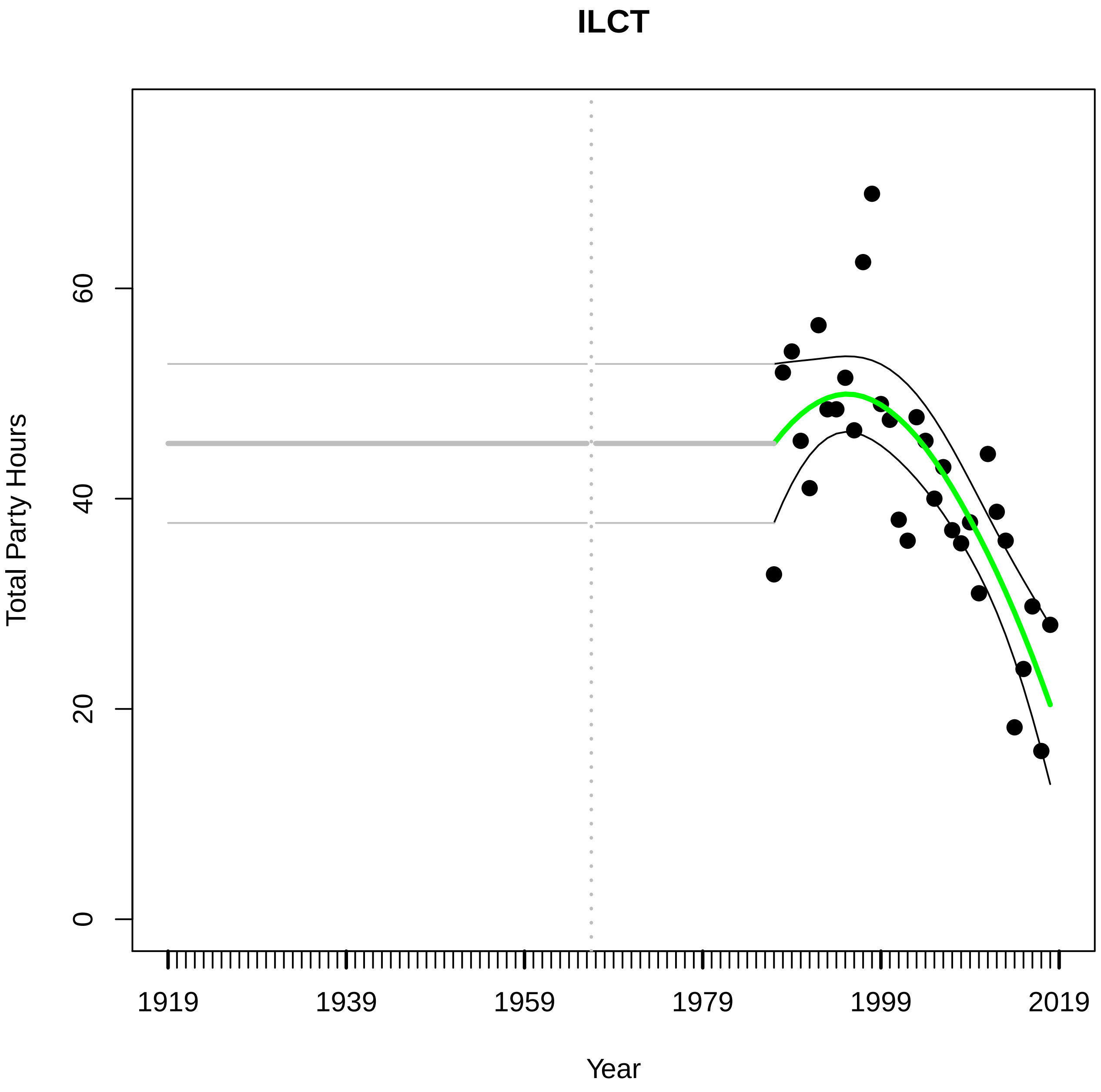

ILCT
Total Party Hours
0
20
40
60
1919
1939
1959
1979
1999
2019
Year

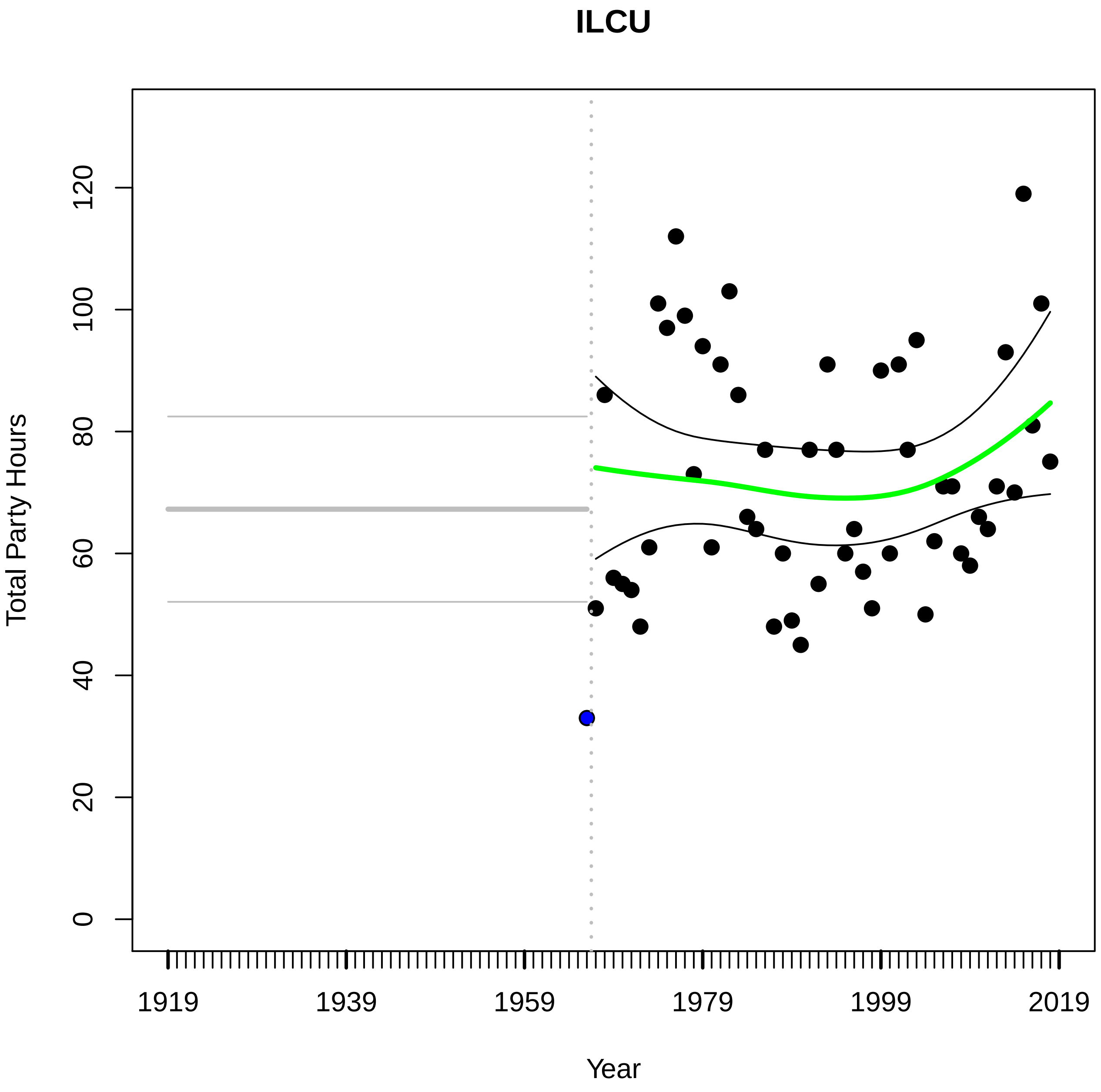

ILCU
Total Party Hours
0
20
40
60
80
100
120
1919
1939
1959
1979
1999
2019
Year

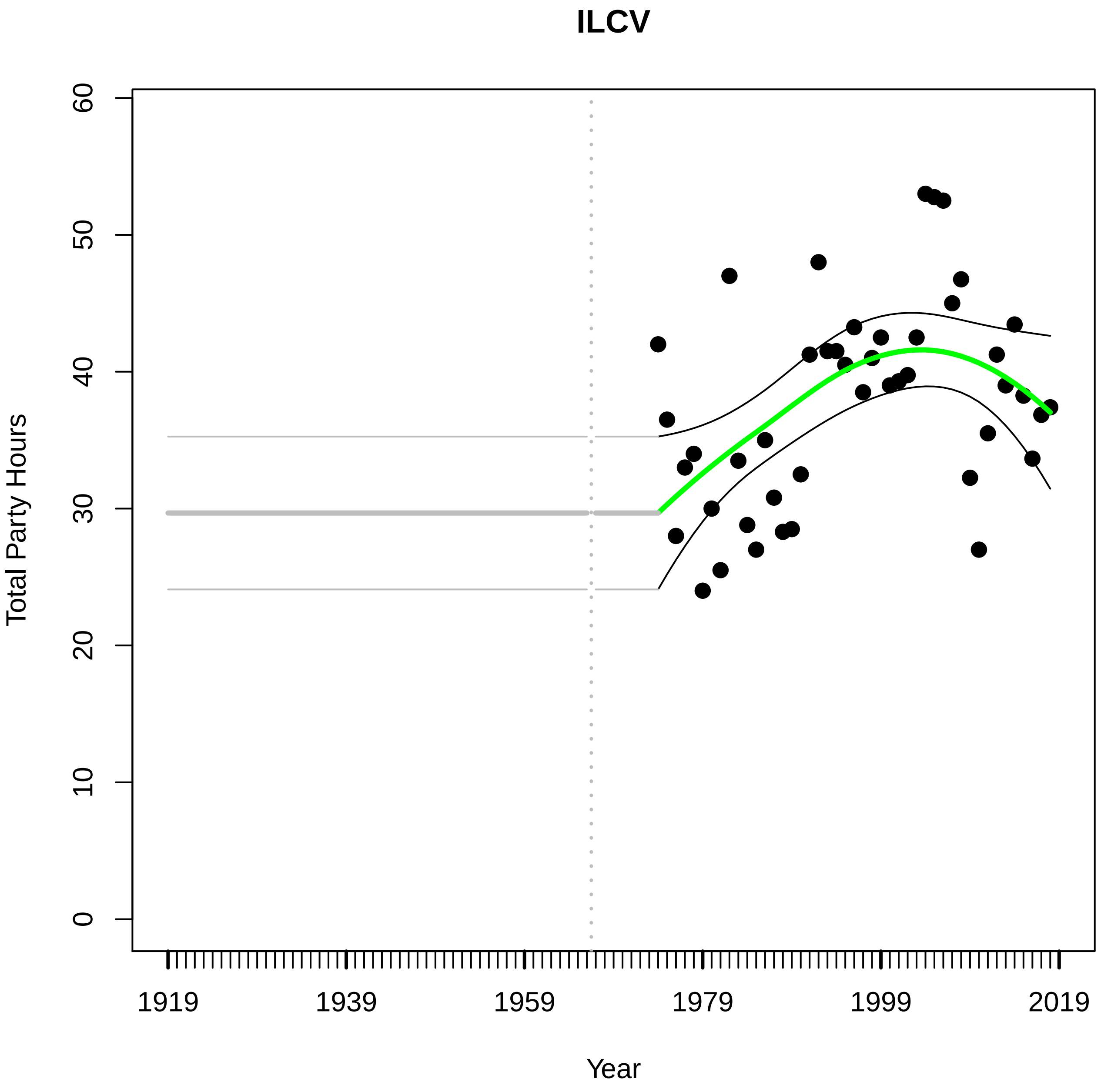

ILCV
Total Party Hours
0
10
20
30
40
50
60
1919
1939
1959
1979
1999
2019
Year

ILDE

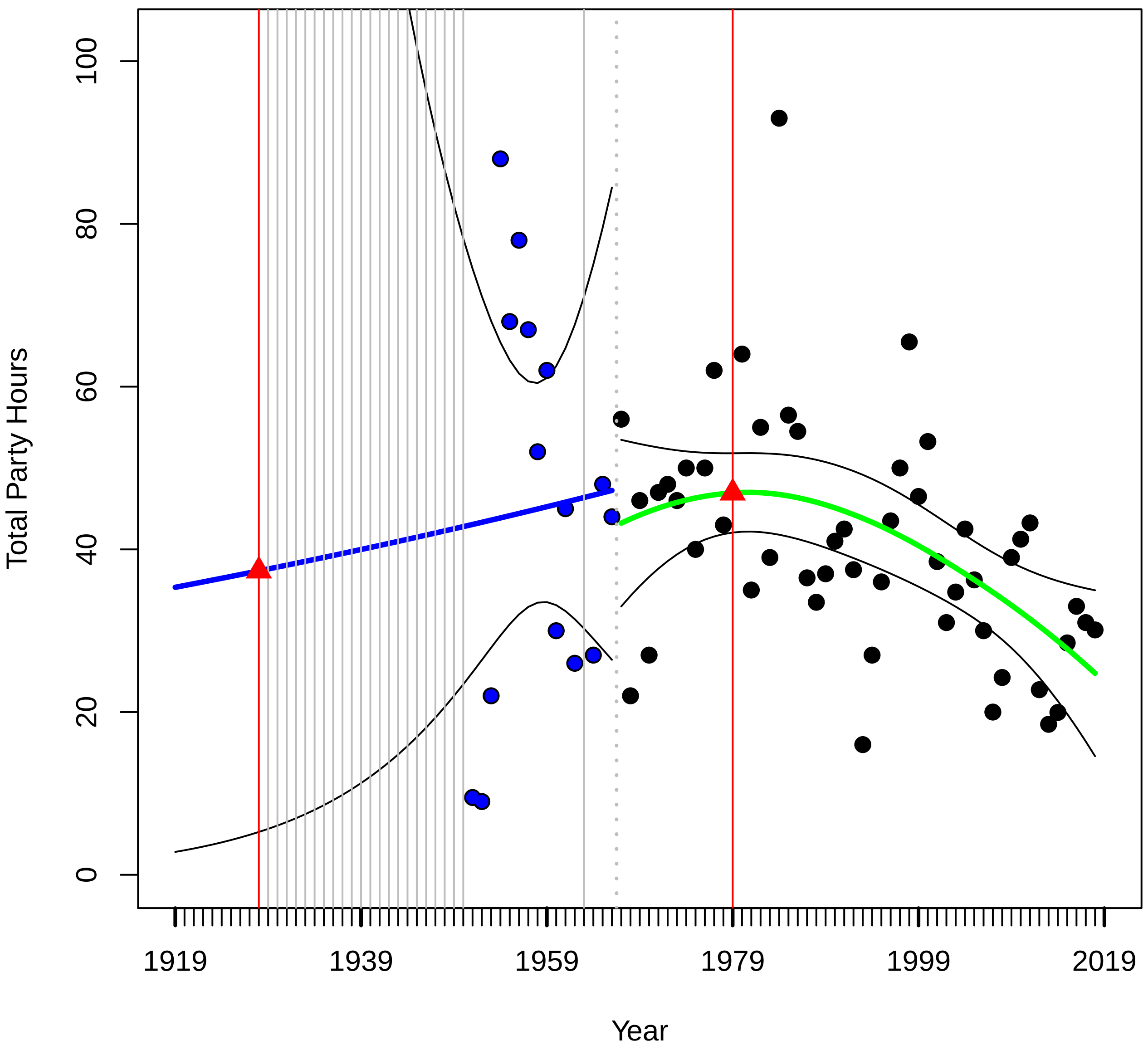

Total Party Hours
0
20
40
60
80
100
1919
1939
1959
1979
1999
2019
Year

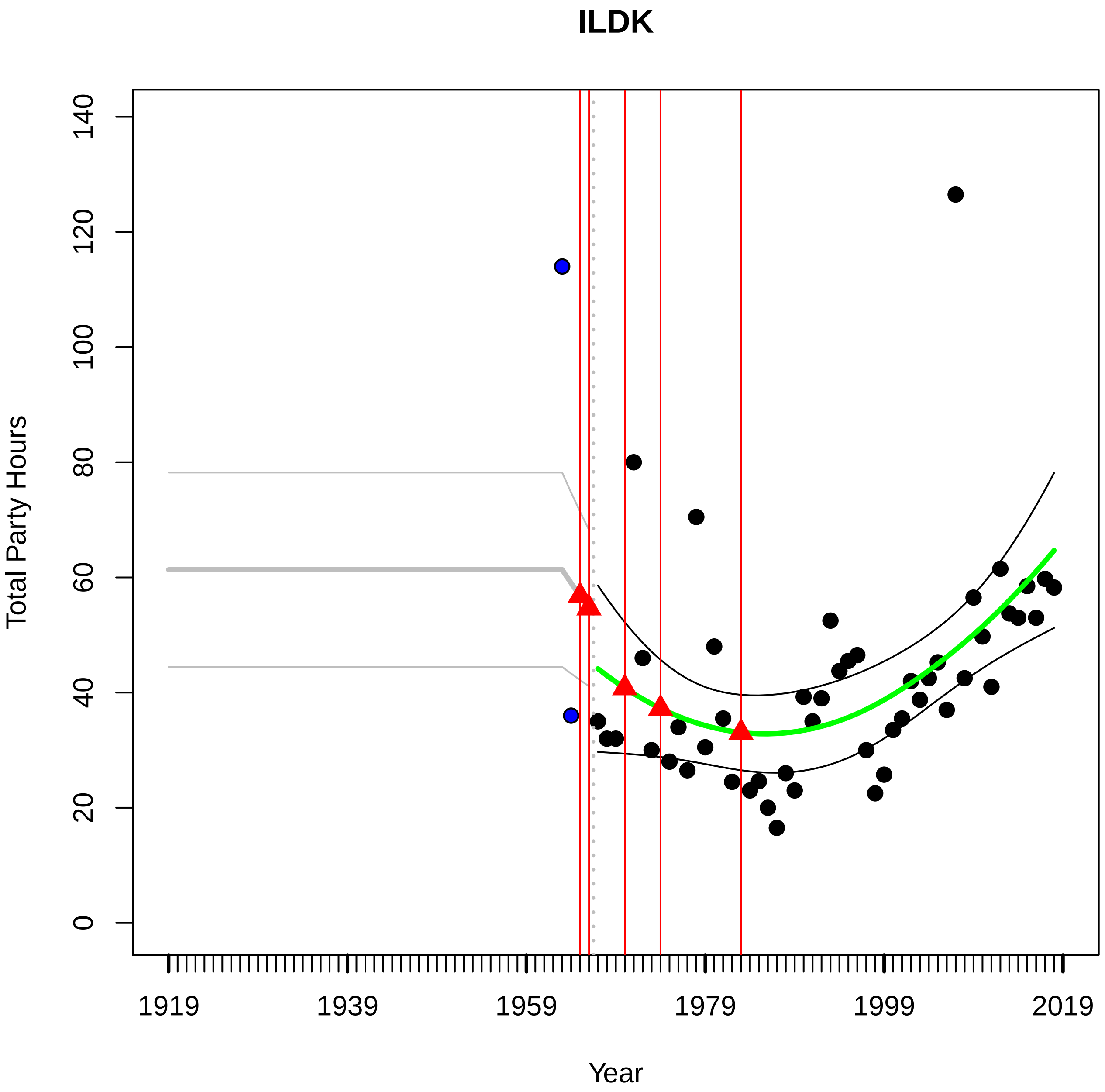

ILDK
Total Party Hours
Year
0
20
40
60
80
100
120
140
1919
1939
1959
1979
1999
2019

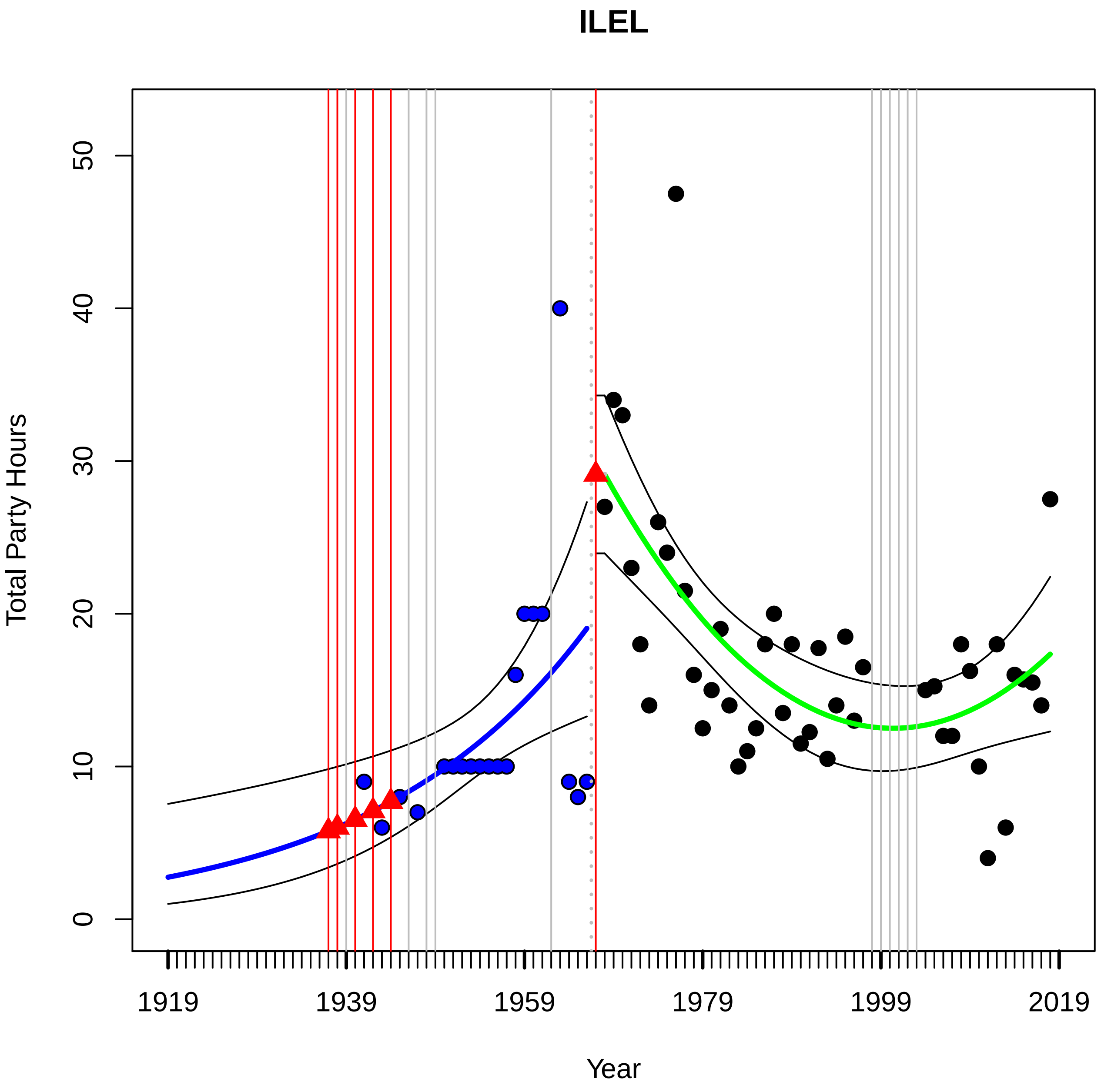

ILEL
Total Party Hours
0
10
20
30
40
50
1919
1939
1959
1979
1999
2019
Year

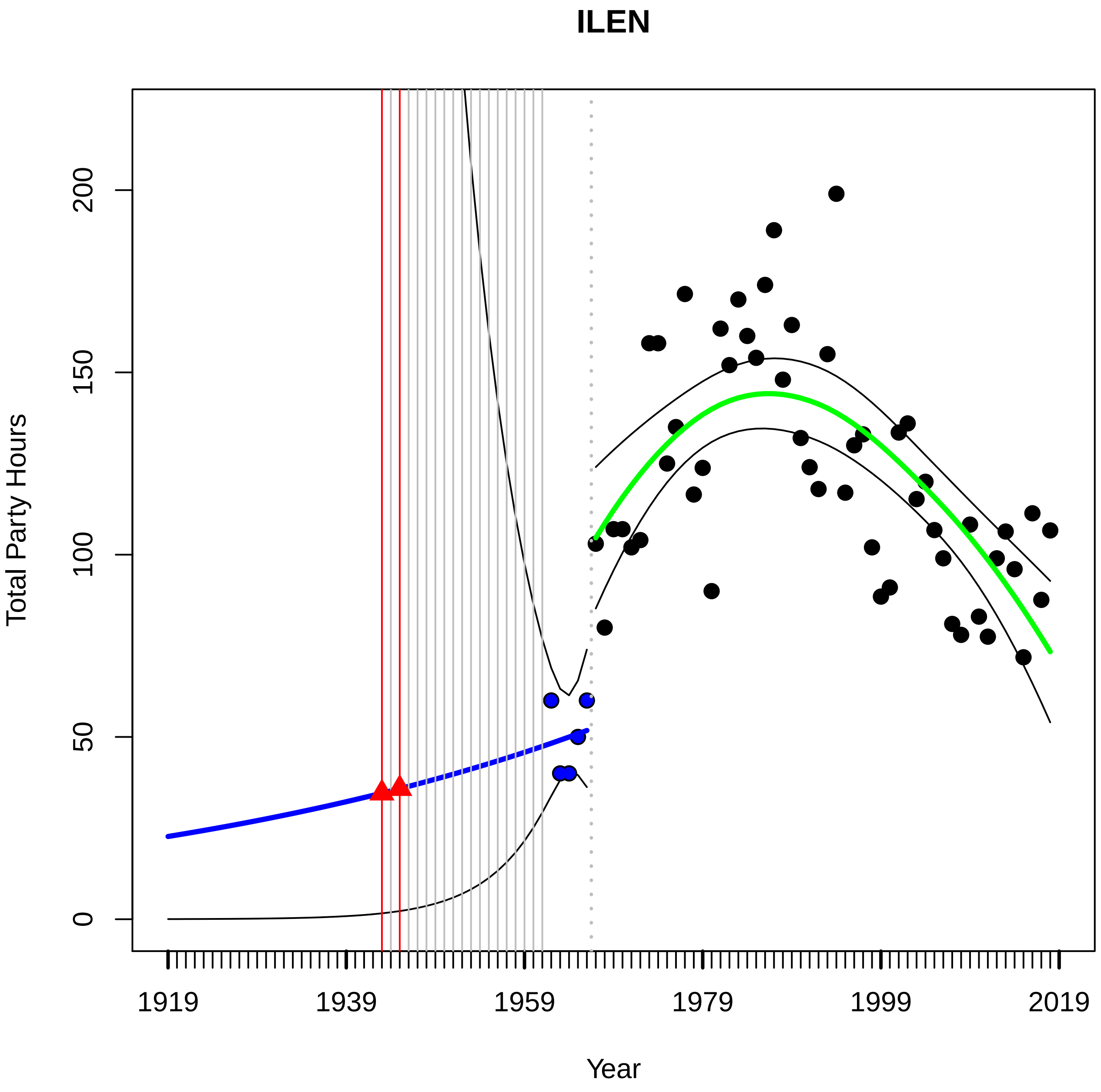

ILEN
Total Party Hours
0
50
100
150
200
1919
1939
1959
1979
1999
2019
Year

ILFB

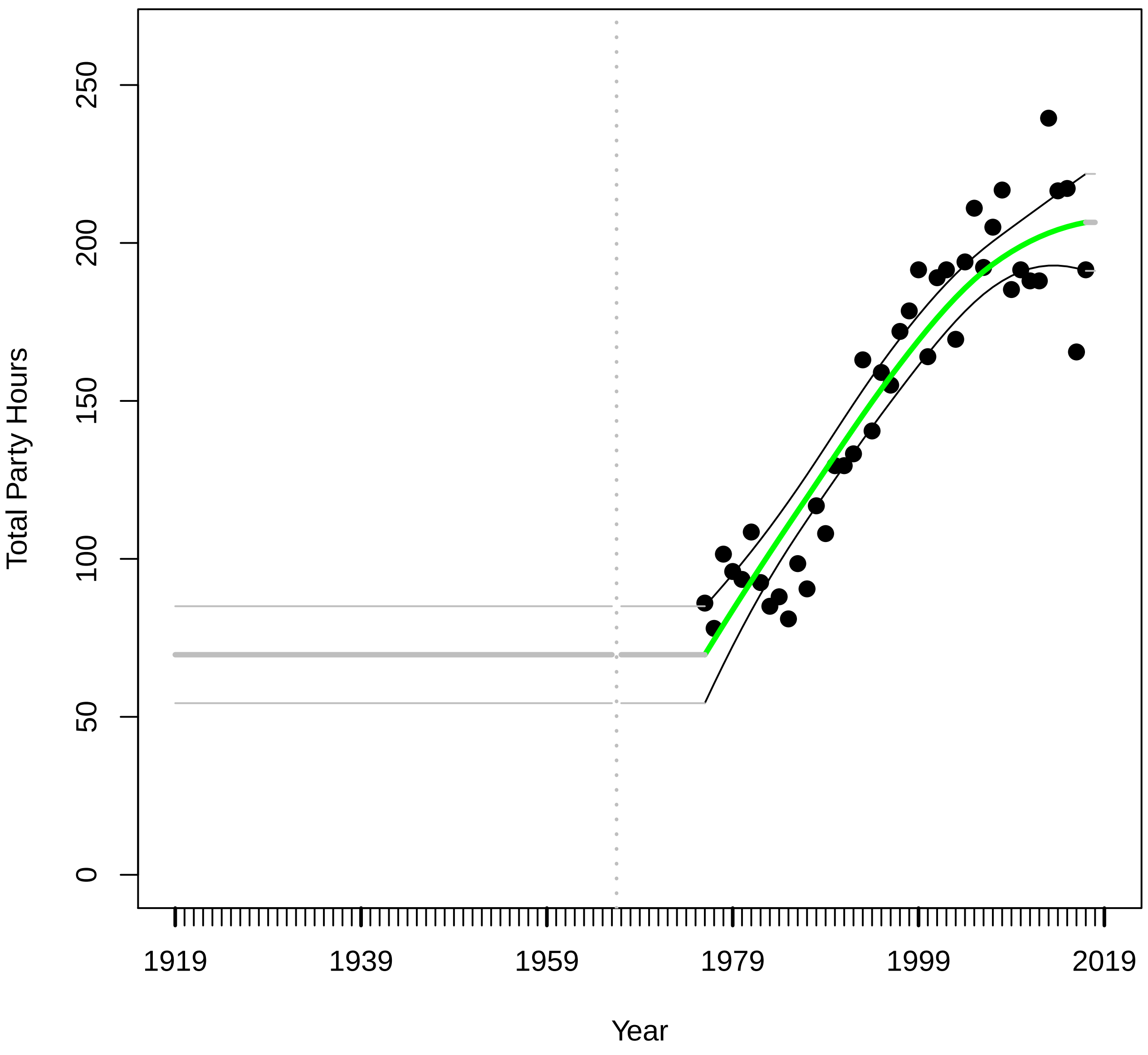

Total Party Hours
0
50
100
150
200
250
1919
1939
1959
1979
1999
2019
Year

ILFG

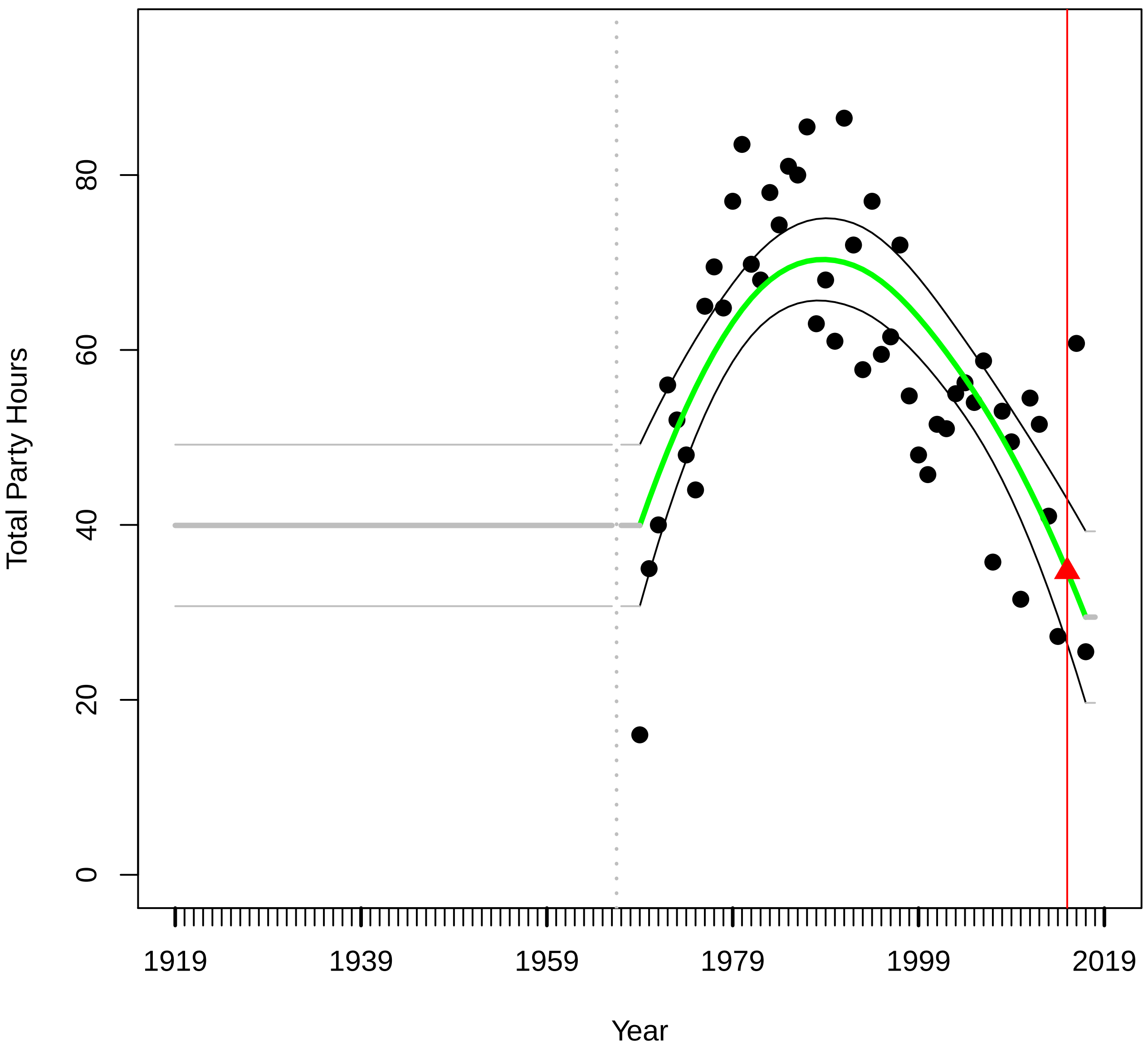
Total Party Hours
0
20
40
60
80
1919
1939
1959
1979
1999
2019
Year

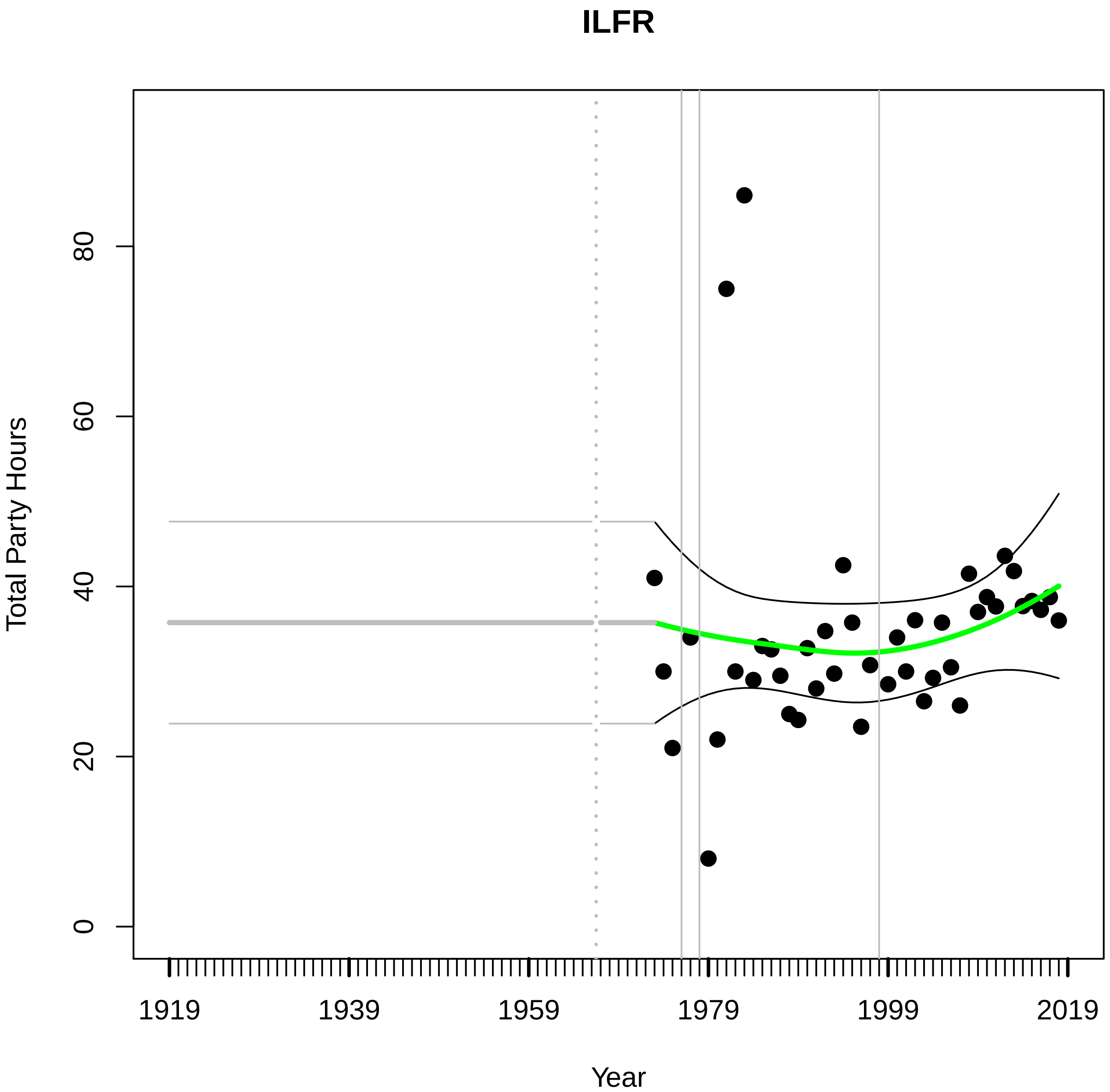

ILFR
Total Party Hours
0
20
40
60
80
Year
1919
1939
1959
1979
1999
2019

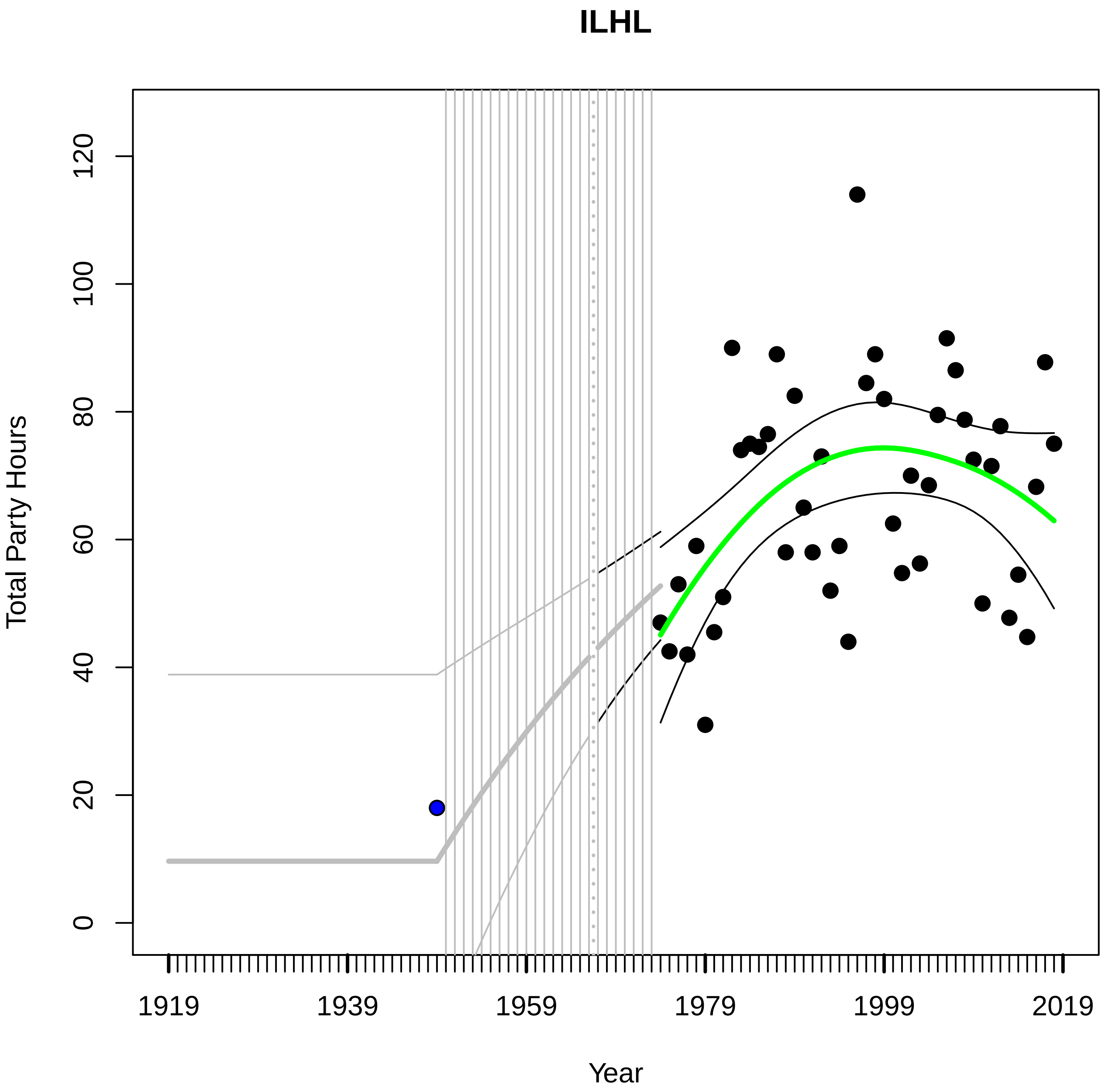

ILHL
Total Party Hours
0
20
40
60
80
100
120
Year
1919
1939
1959
1979
1999
2019

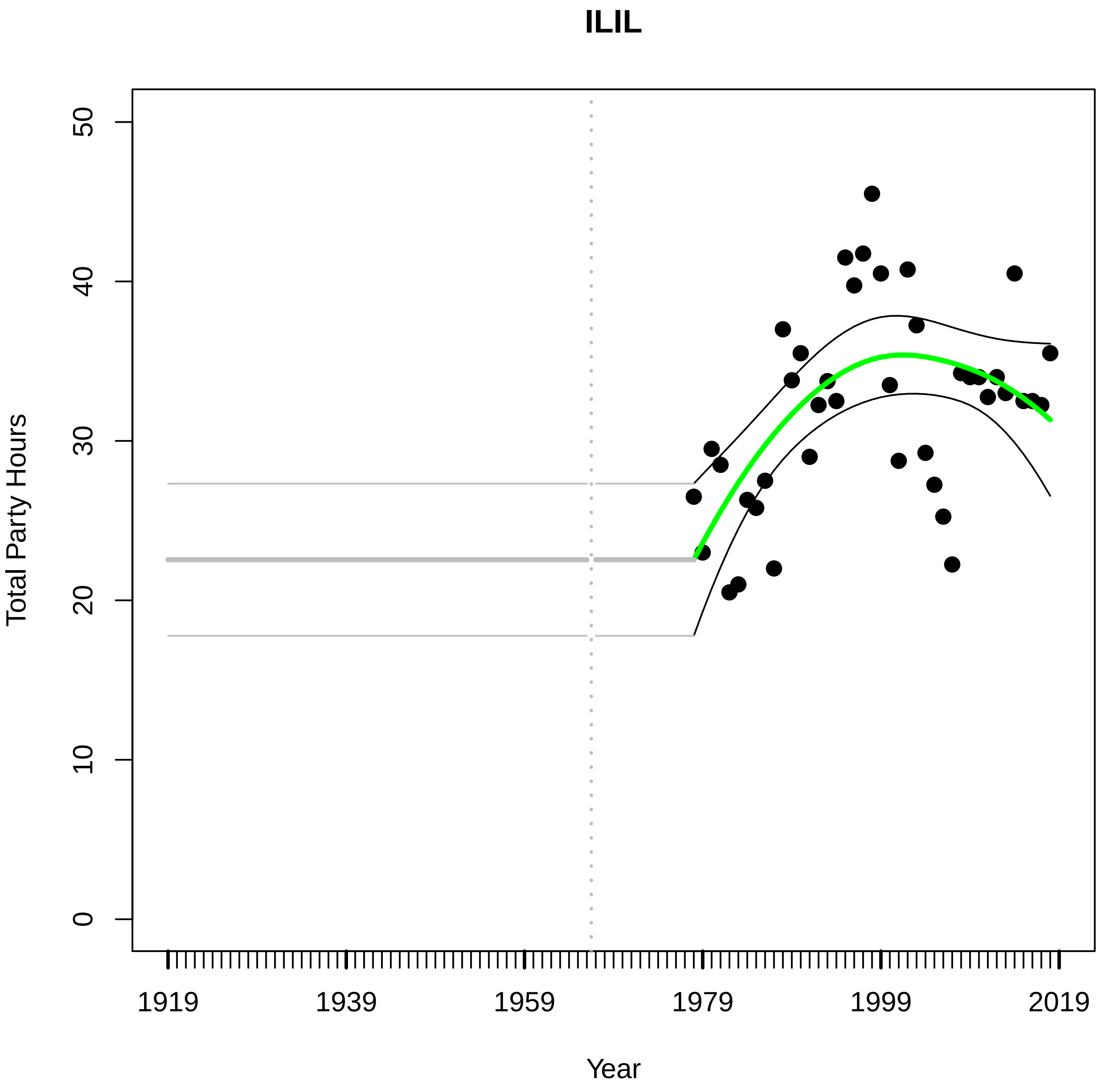
ILIL
Total Party Hours
0
10
20
30
40
50
1919
1939
1959
1979
1999
2019
Year

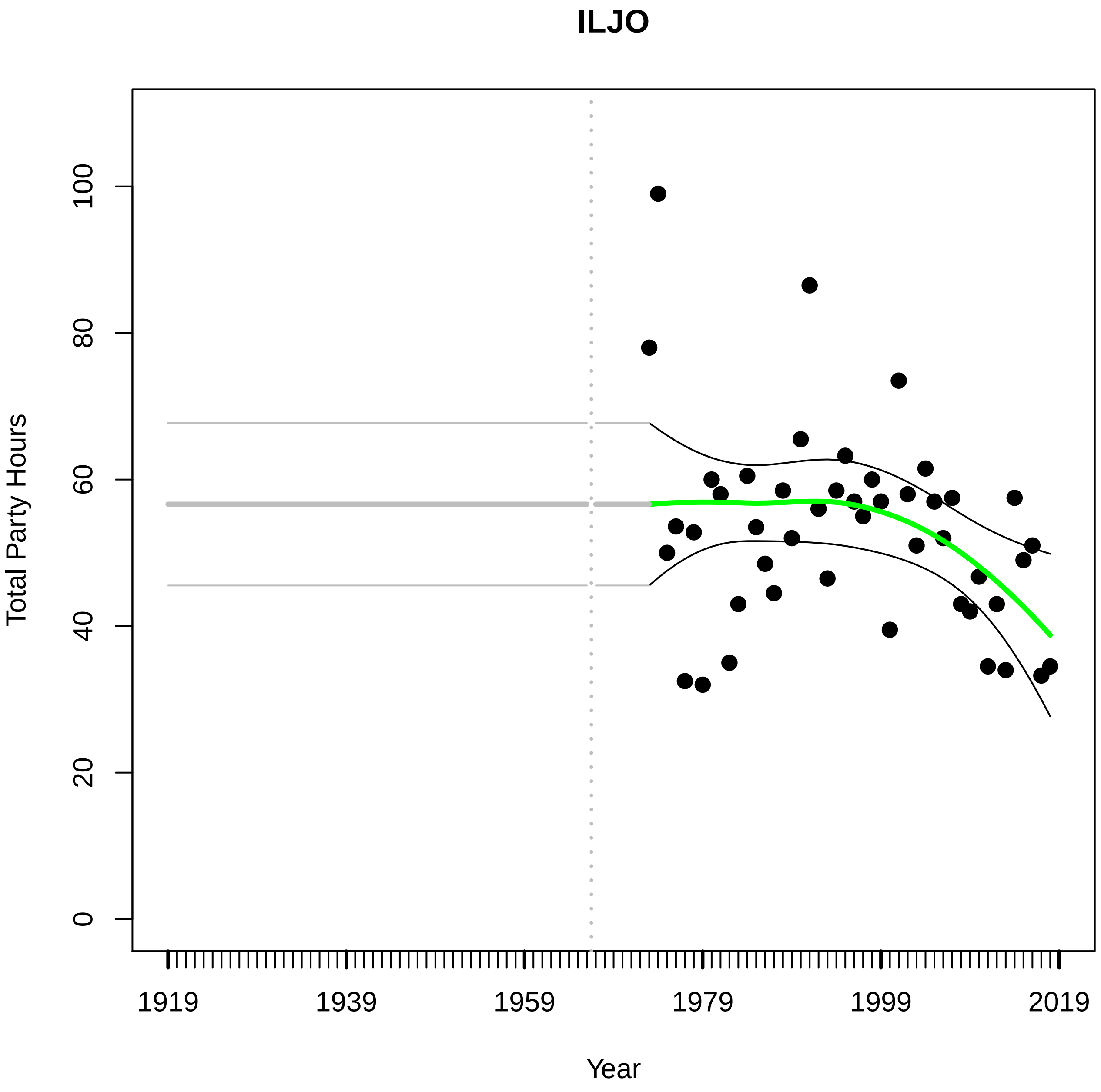
ILJO
Total Party Hours
0
20
40
60
80
100
1919
1939
1959
1979
1999
2019
Year

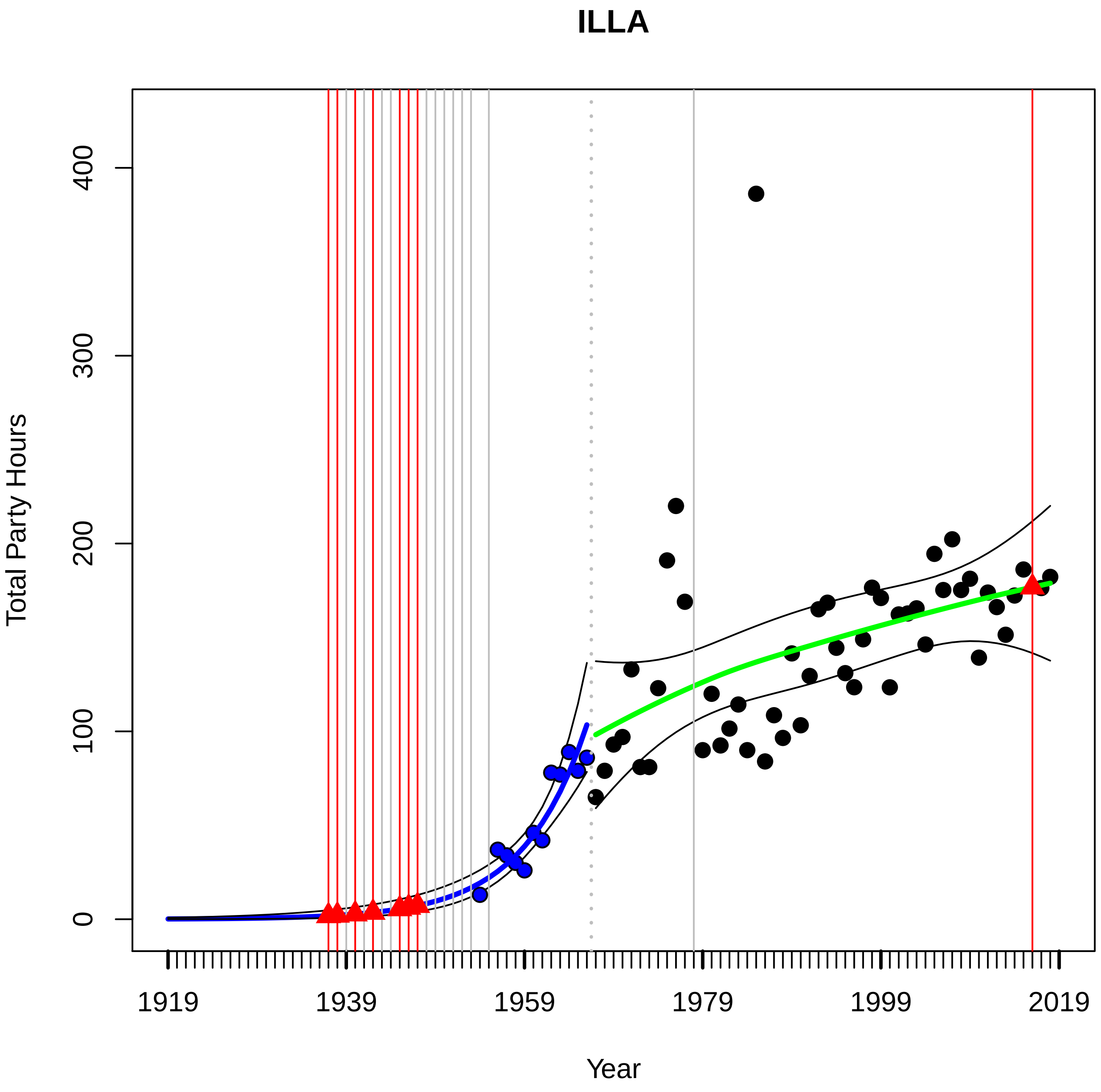

ILLA
Total Party Hours
0
100
200
300
400
1919
1939
1959
1979
1999
2019
Year

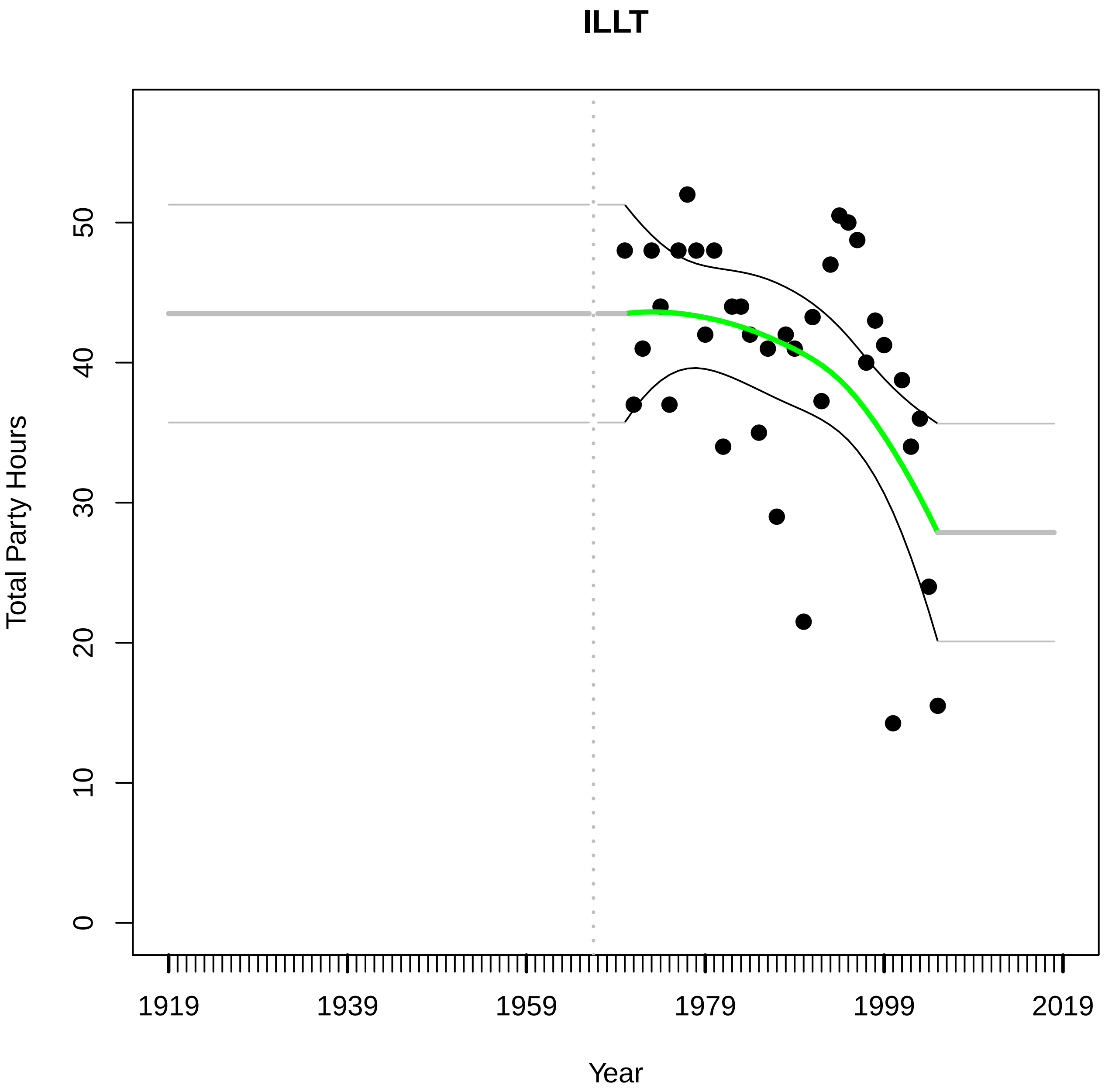
ILLT
Total Party Hours
0
10
20
30
40
50
1919
1939
1959
1979
1999
2019
Year

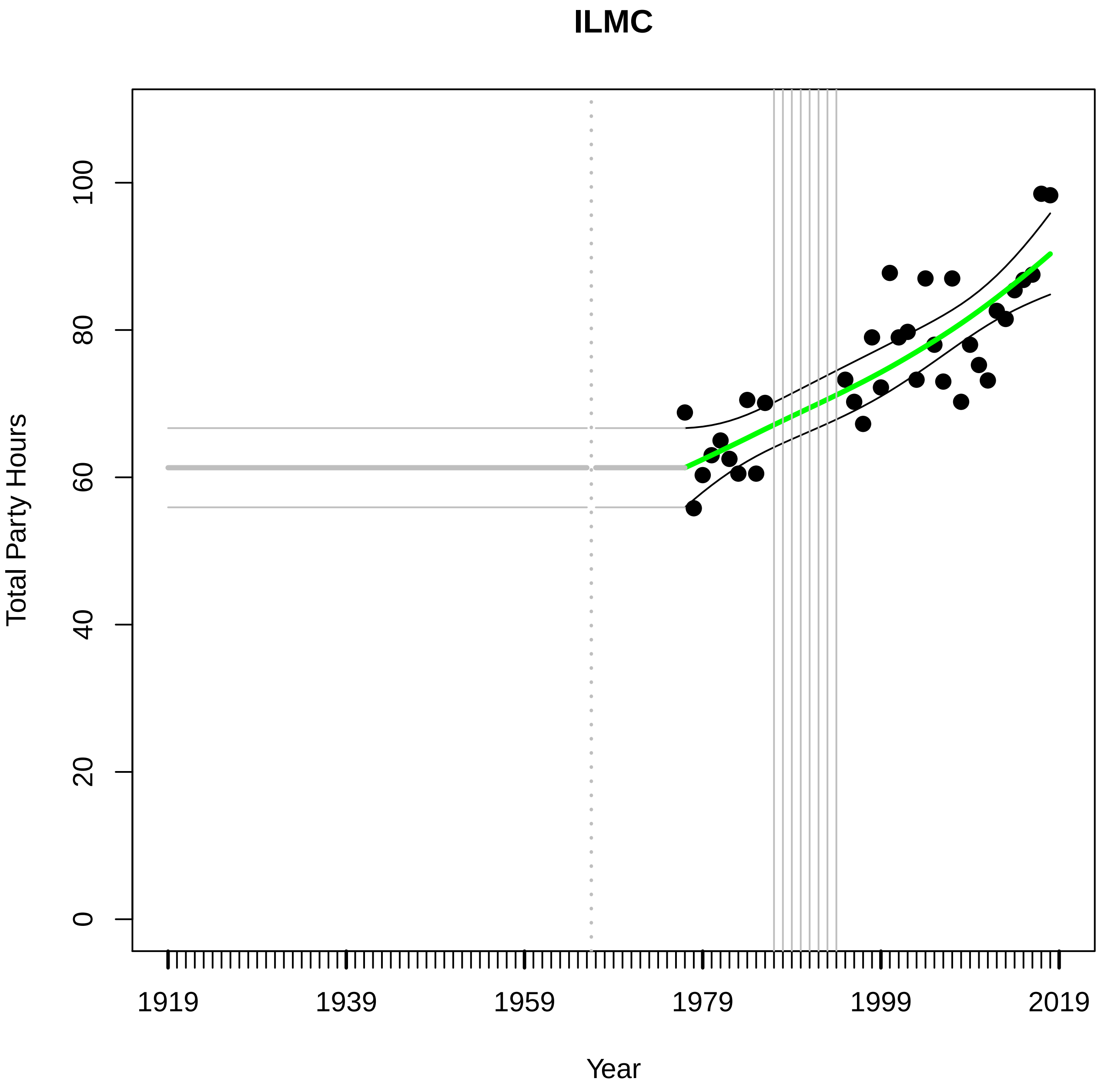
ILMC
Total Party Hours
0
20
40
60
80
100
1919
1939
1959
1979
1999
2019
Year

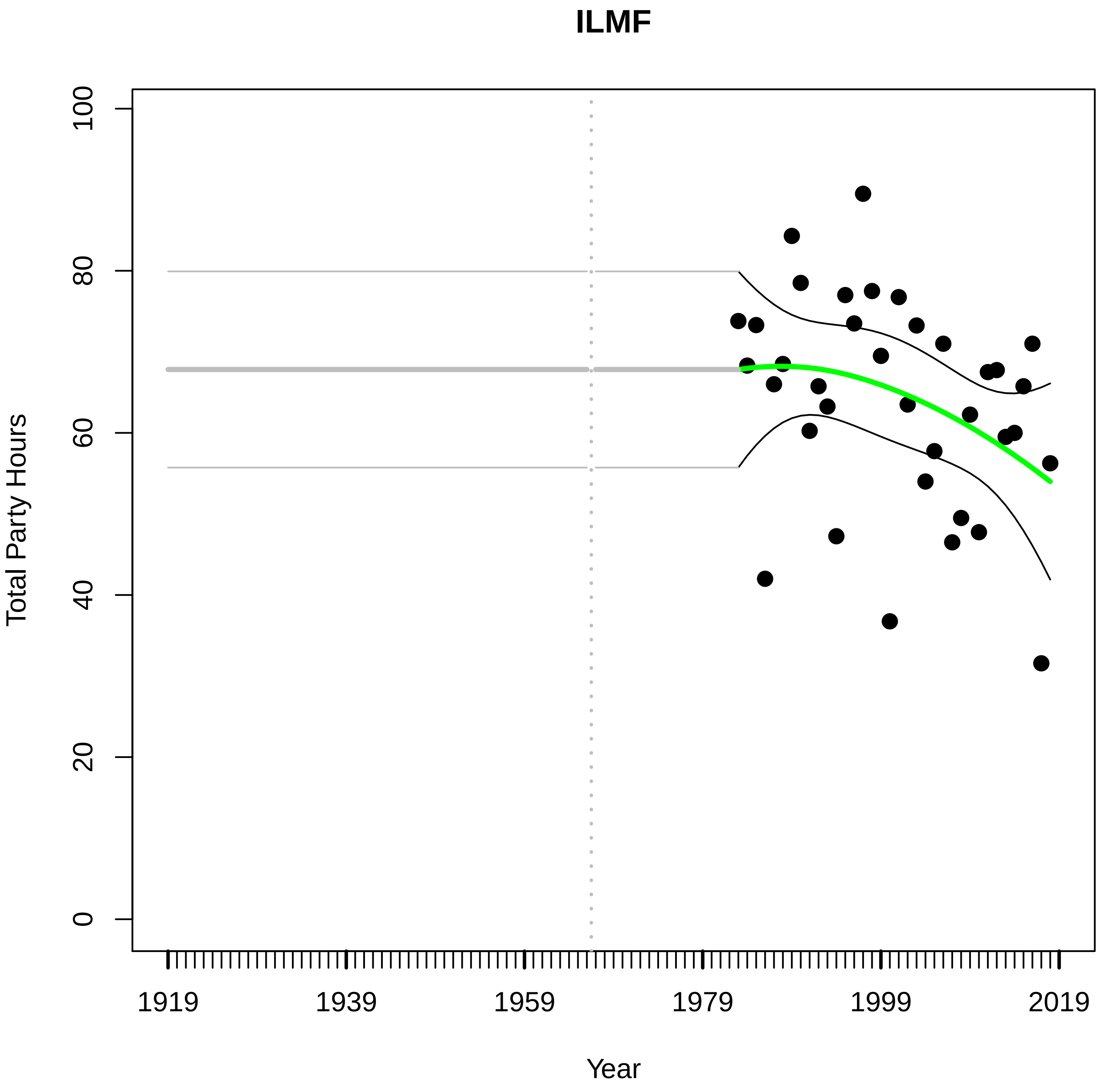

ILMF
Total Party Hours
0
20
40
60
80
100
1919
1939
1959
1979
1999
2019
Year

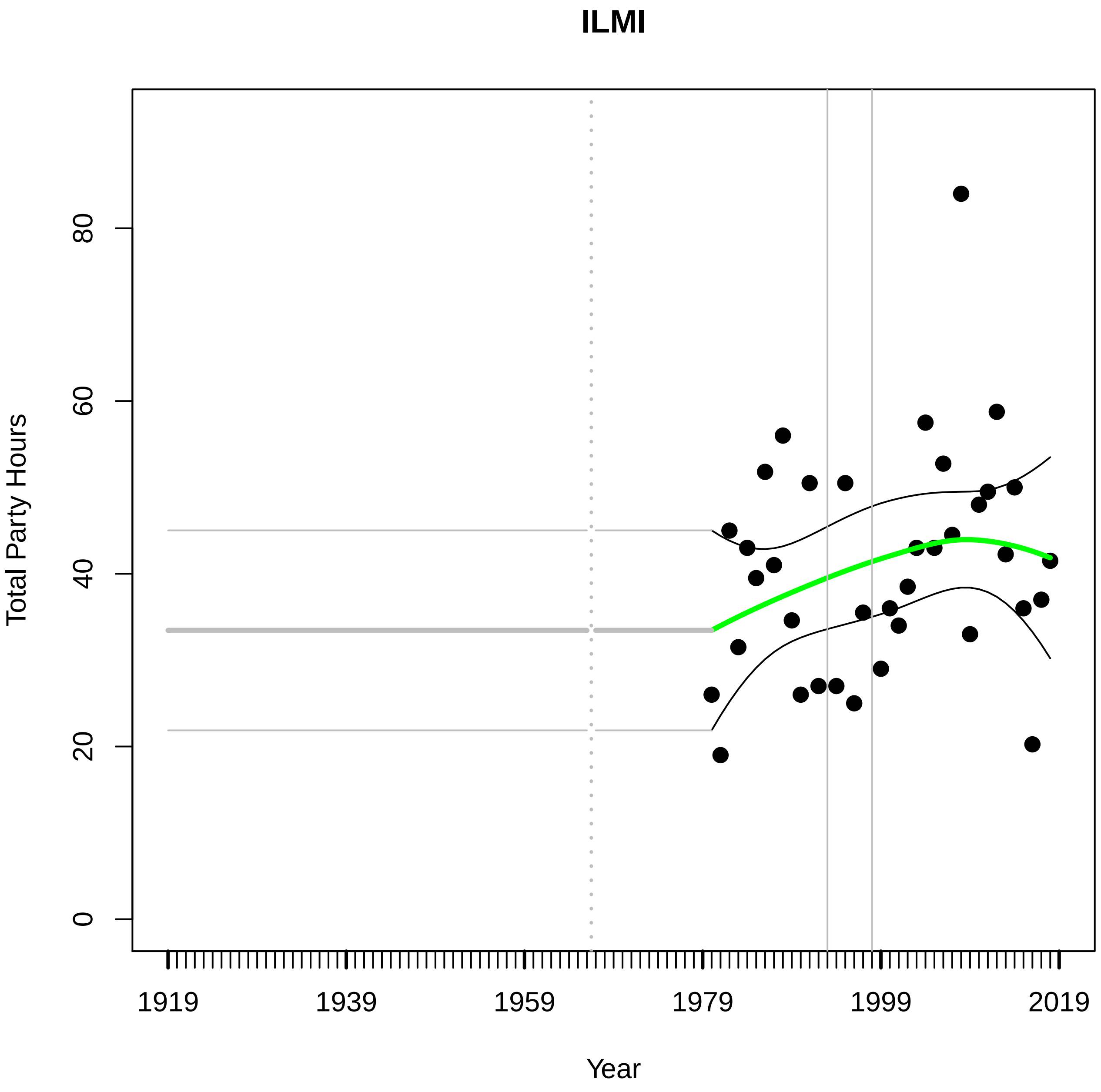
ILMI
Total Party Hours
0
20
40
60
80
1919
1939
1959
1979
1999
2019
Year

ILMW

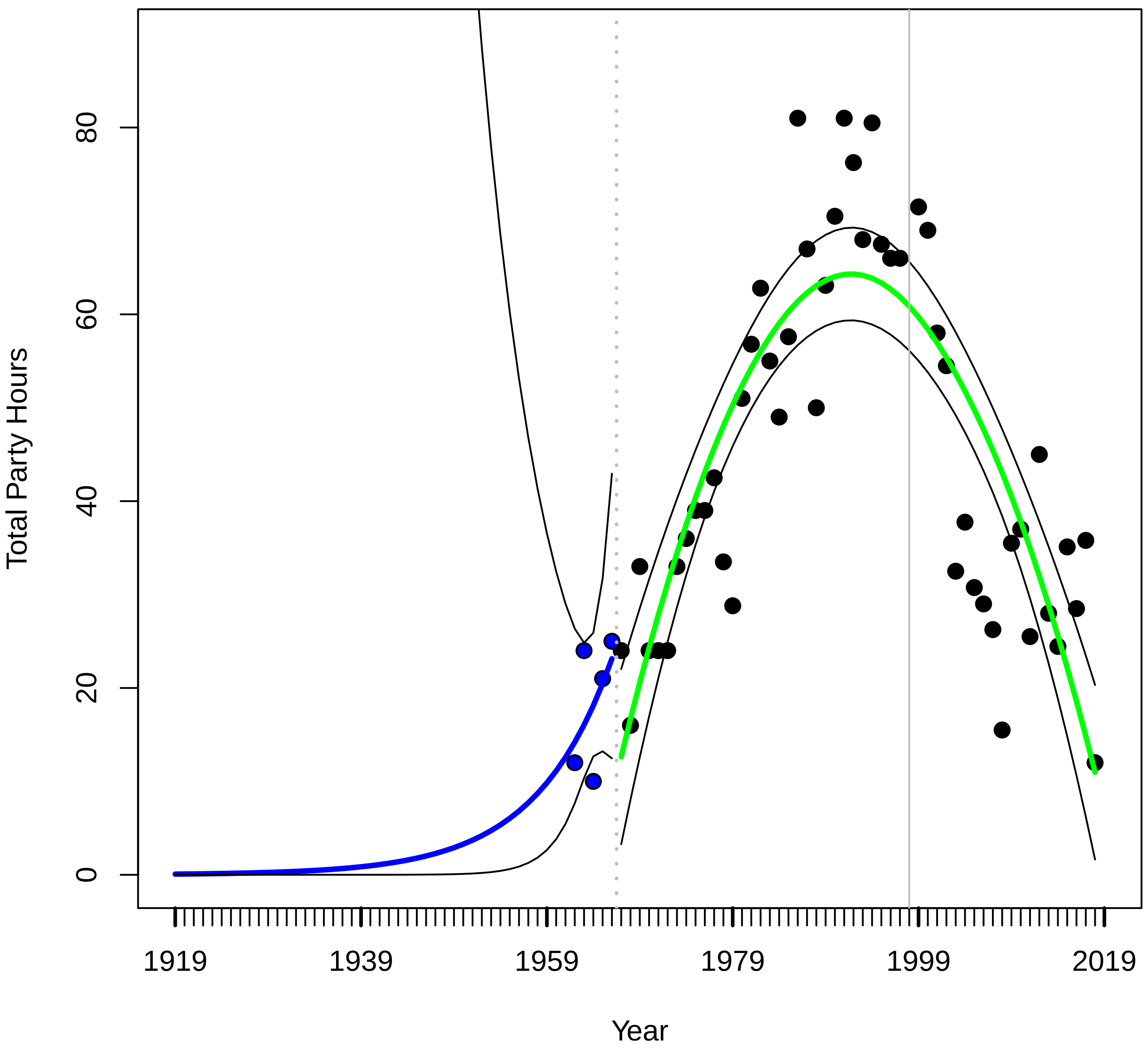

Total Party Hours
0
20
40
60
80
1919
1939
1959
1979
1999
2019
Year

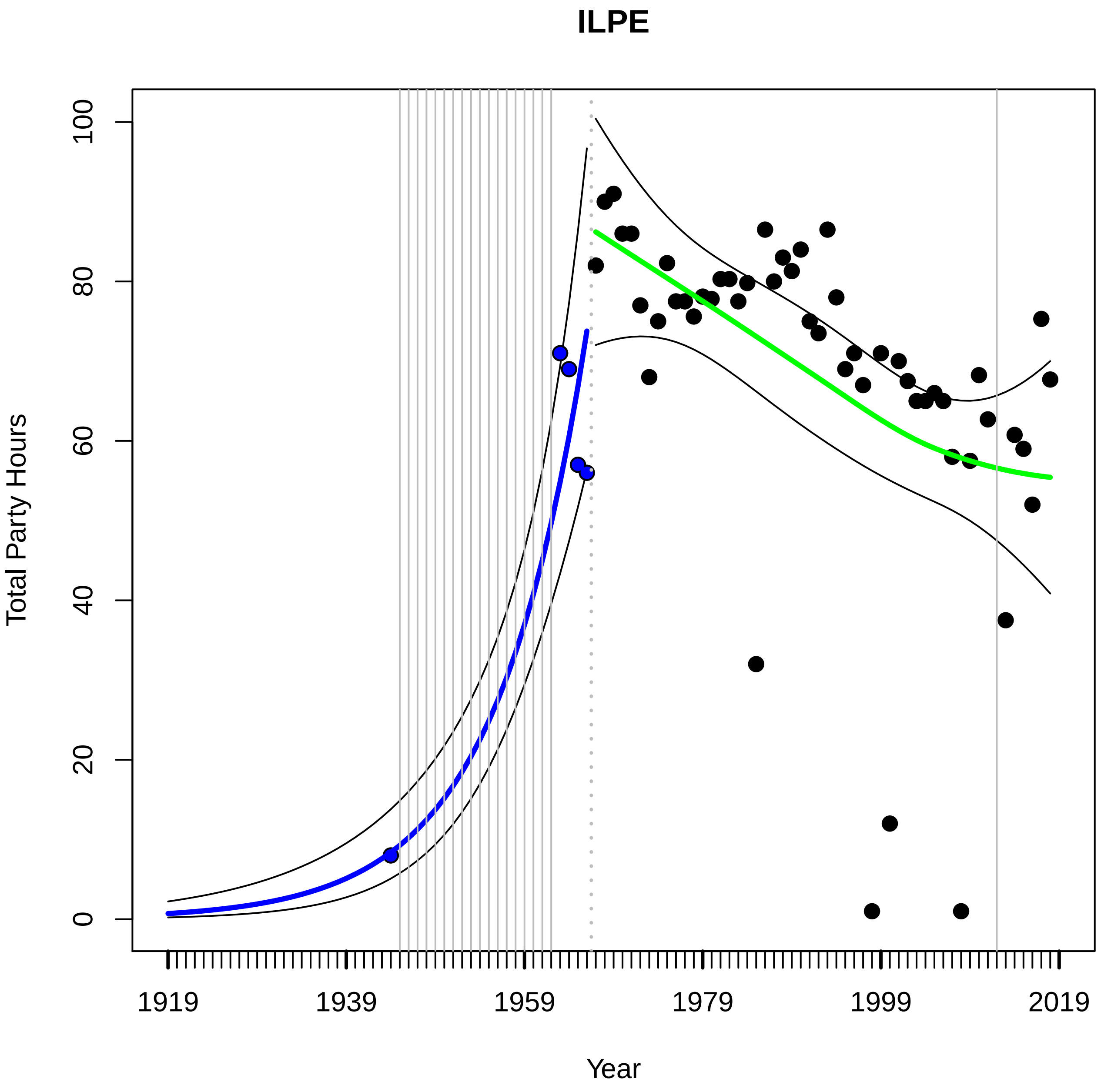

ILPE
Total Party Hours
0
20
40
60
80
100
1919
1939
1959
1979
1999
2019
Year

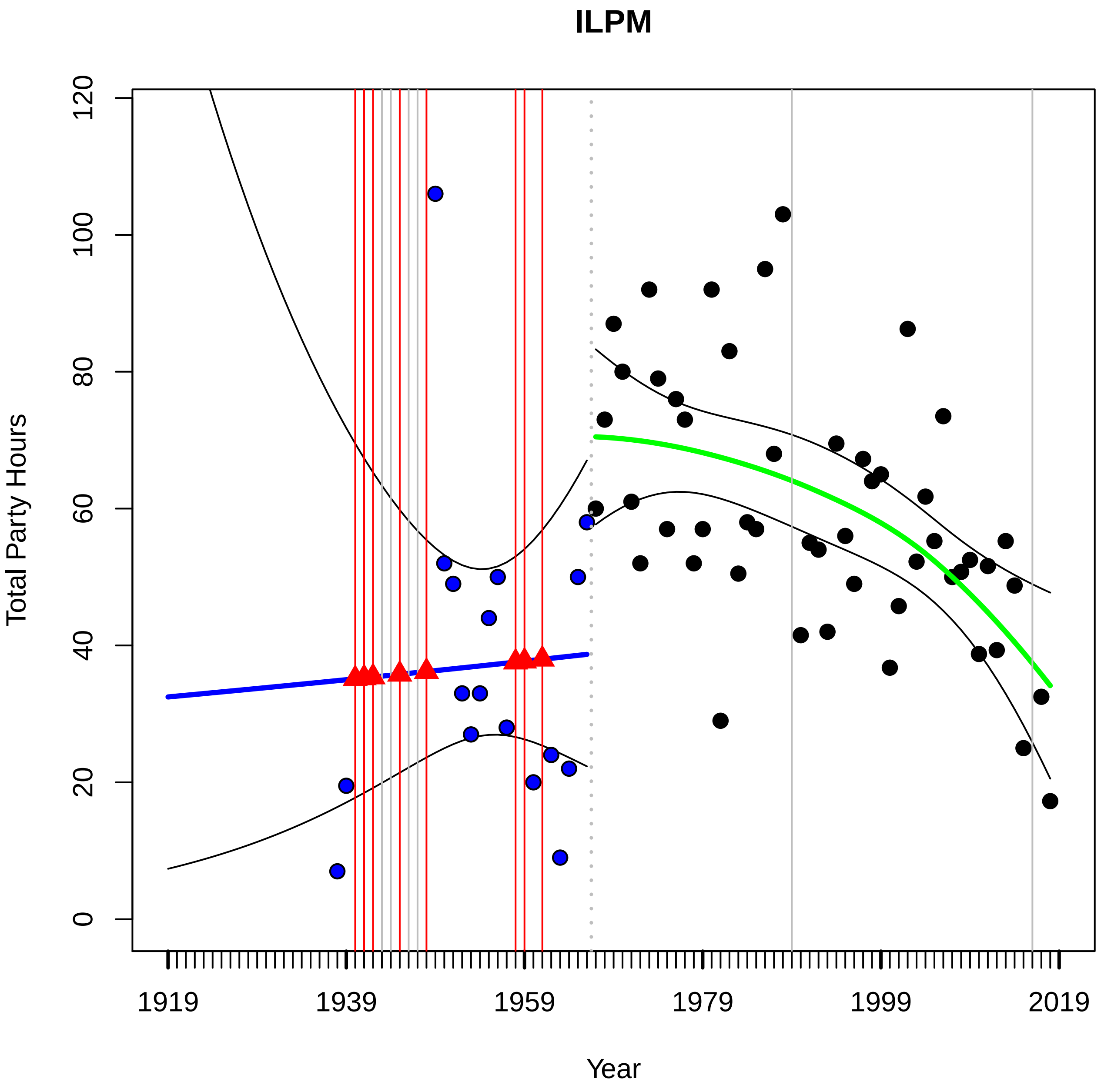

ILPM
Total Party Hours
Year
0
20
40
60
80
100
120
1919
1939
1959
1979
1999
2019

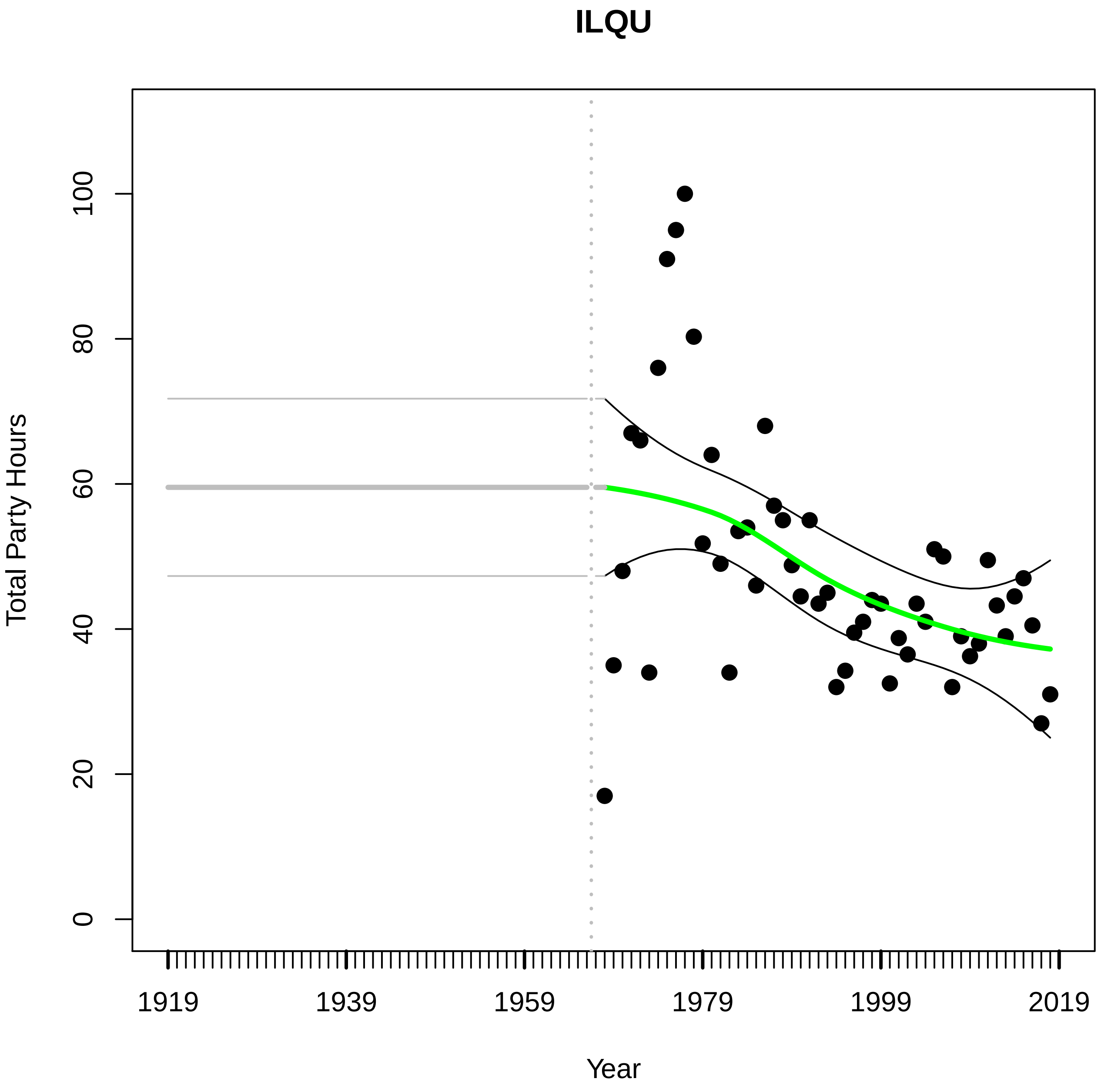

ILQU
Total Party Hours
0
20
40
60
80
100
1919
1939
1959
1979
1999
2019
Year

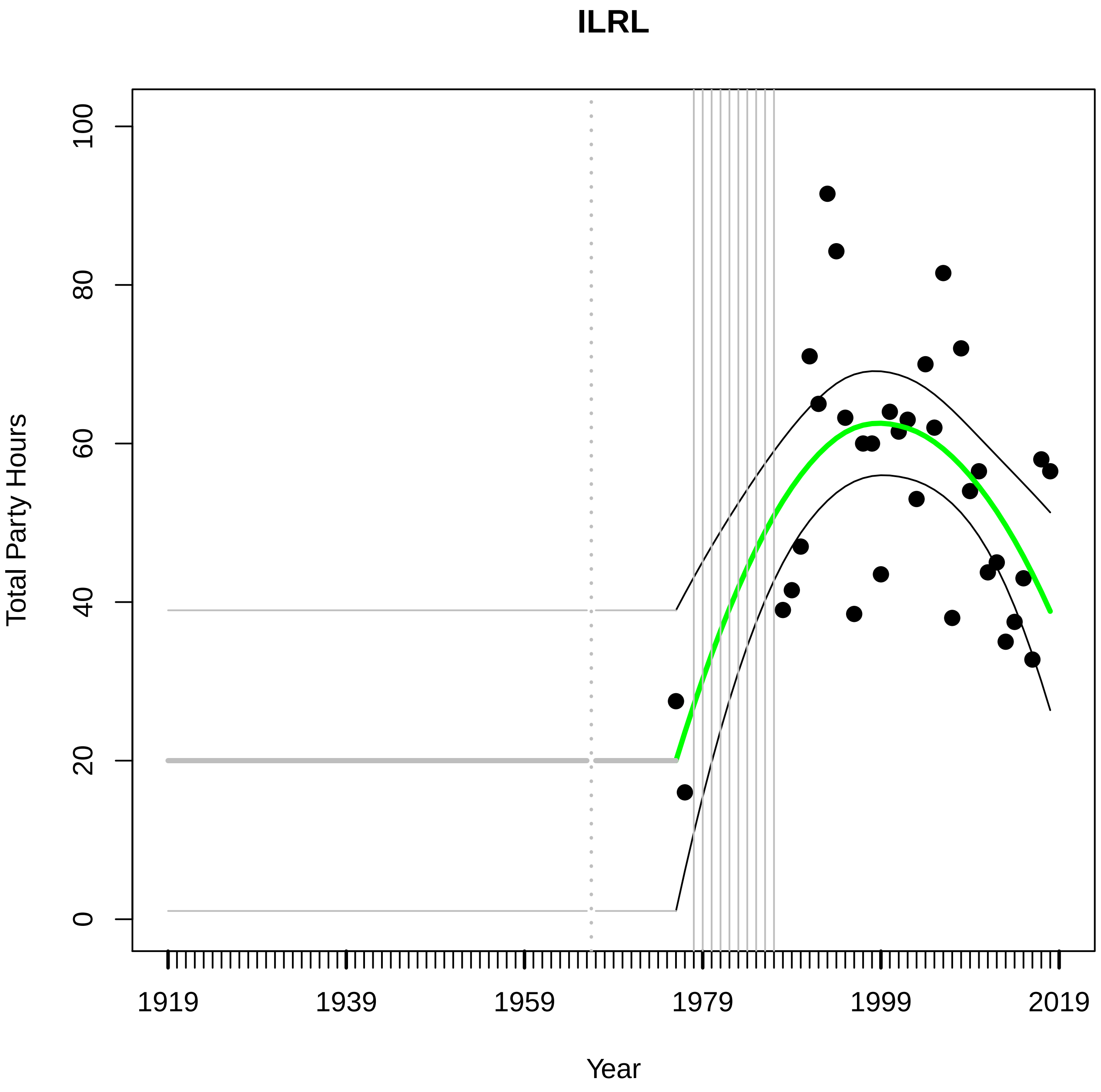

ILRL
Total Party Hours
0
20
40
60
80
100
1919
1939
1959
1979
1999
2019
Year

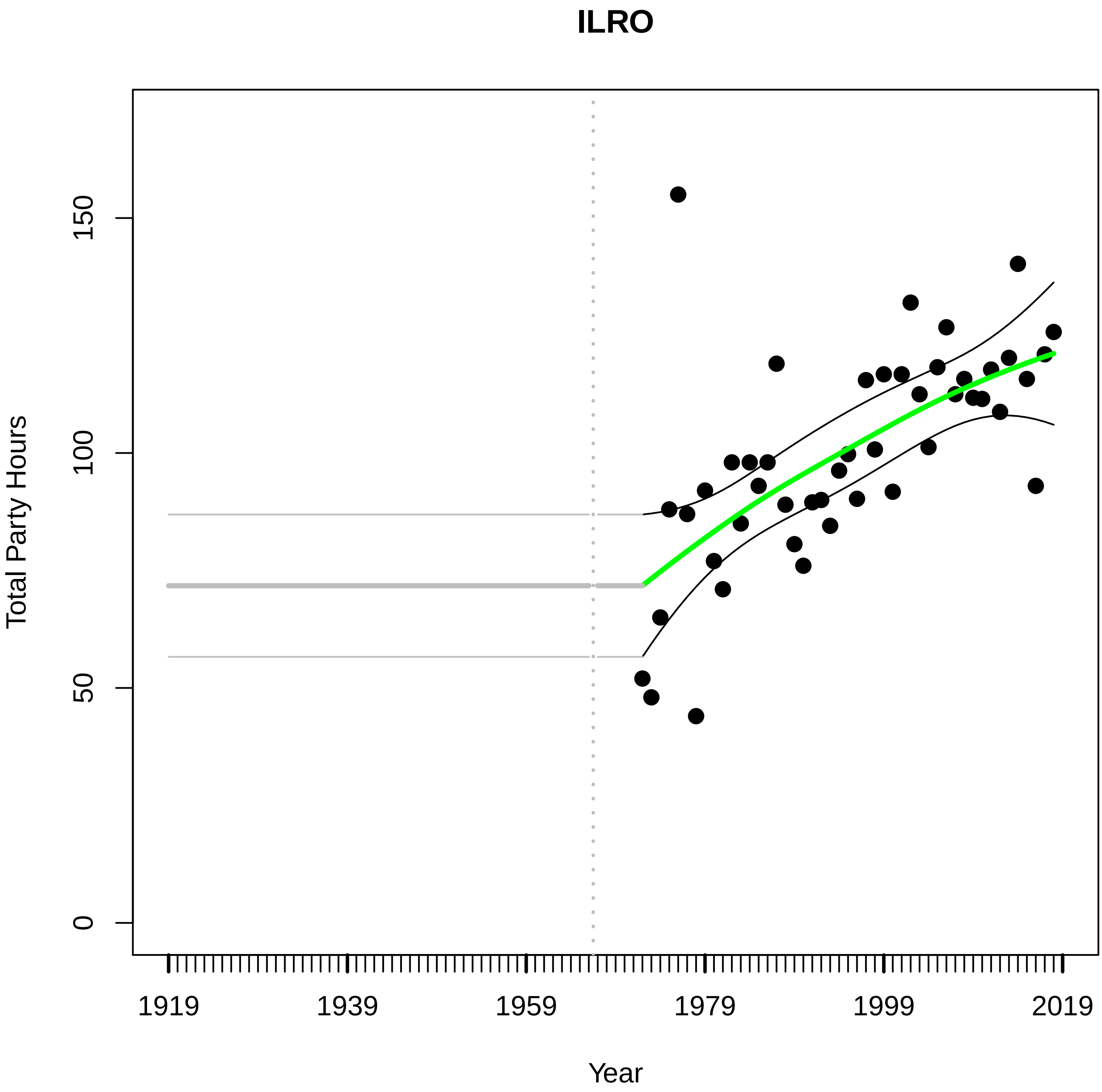
ILRO
Total Party Hours
Year
0
50
100
150
1919
1939
1959
1979
1999
2019

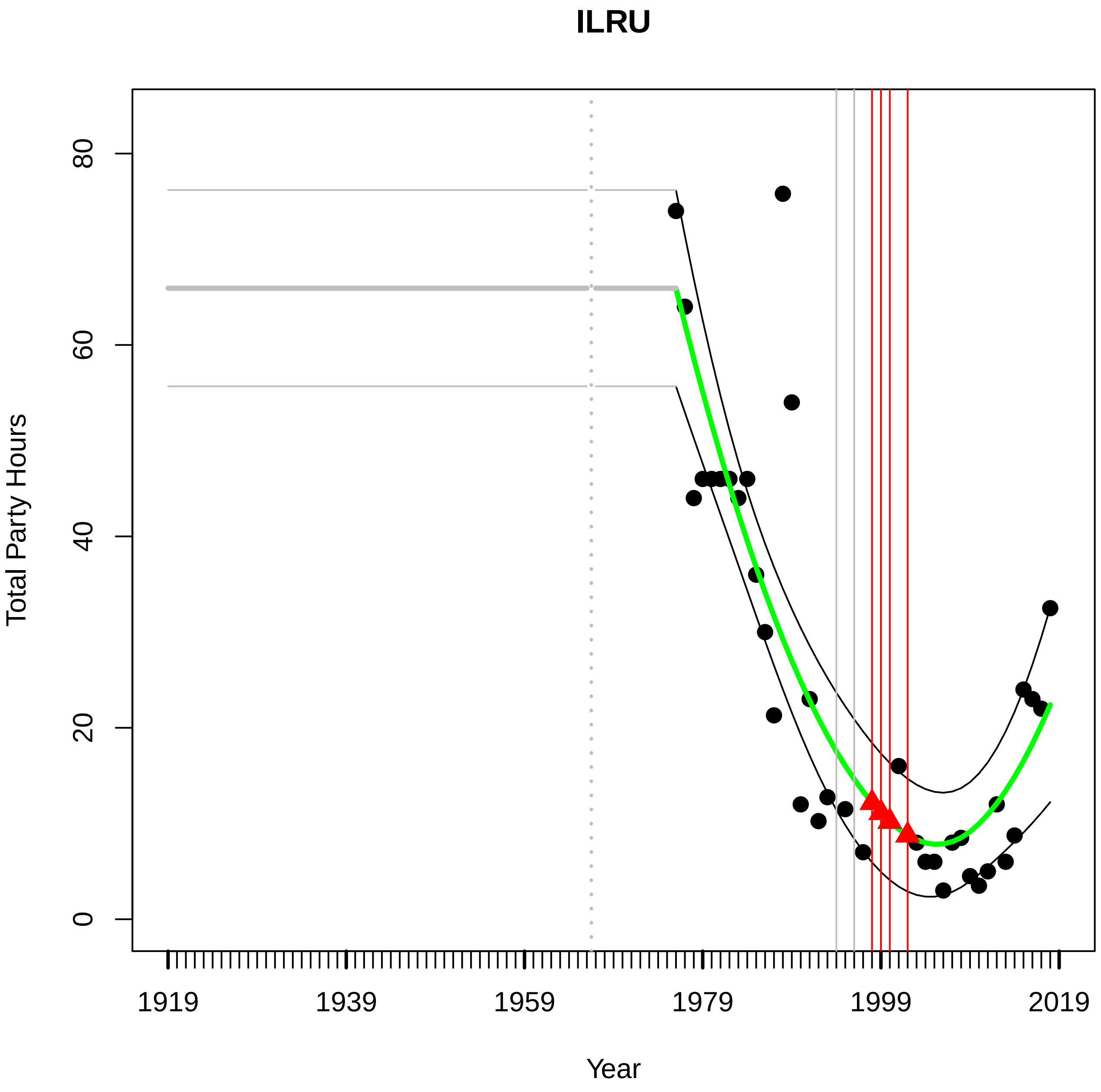

ILRU
Total Party Hours
0
20
40
60
80
1919
1939
1959
1979
1999
2019
Year

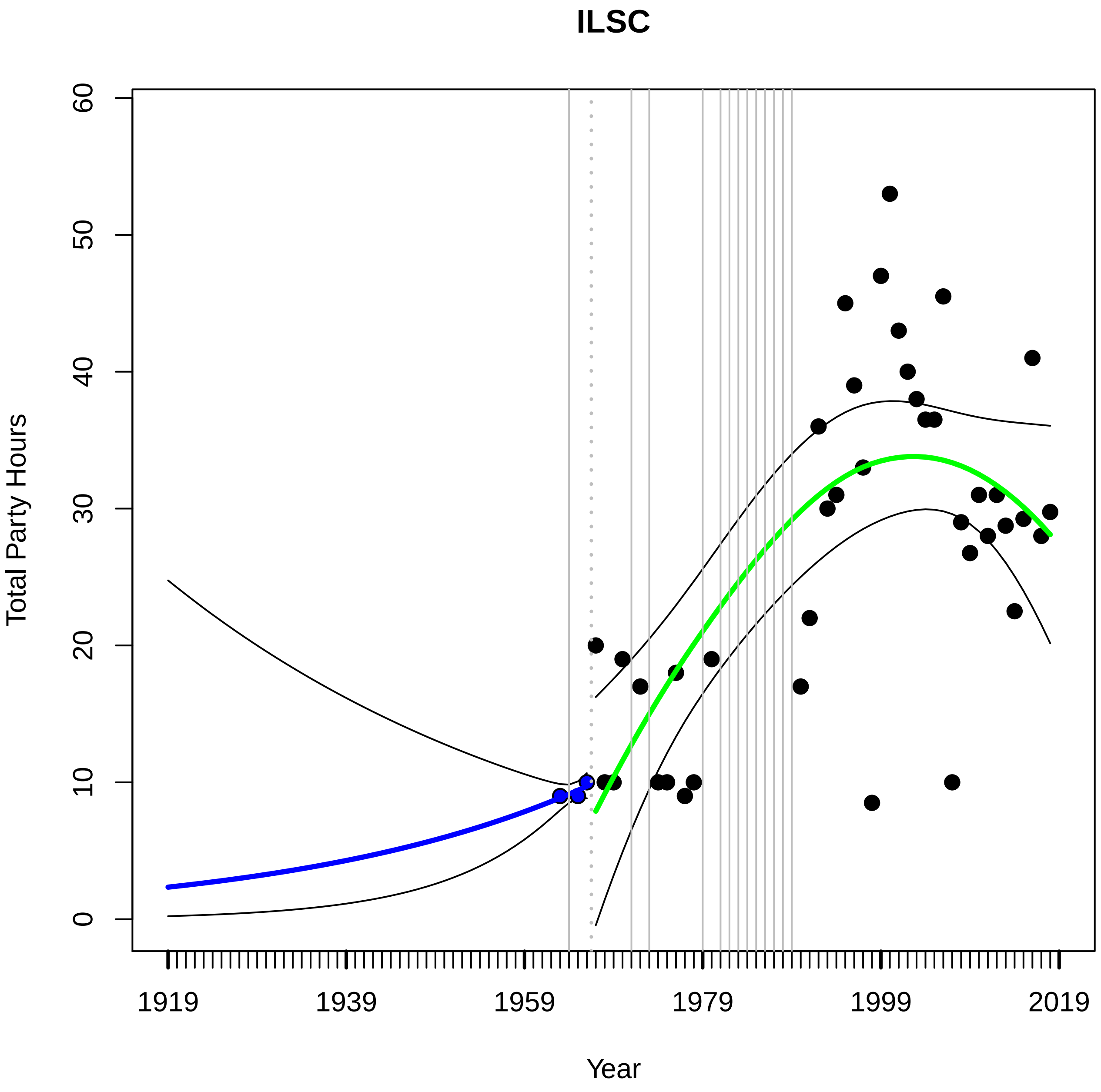

ILSC
Total Party Hours
Year
0
10
20
30
40
50
60
1919
1939
1959
1979
1999
2019

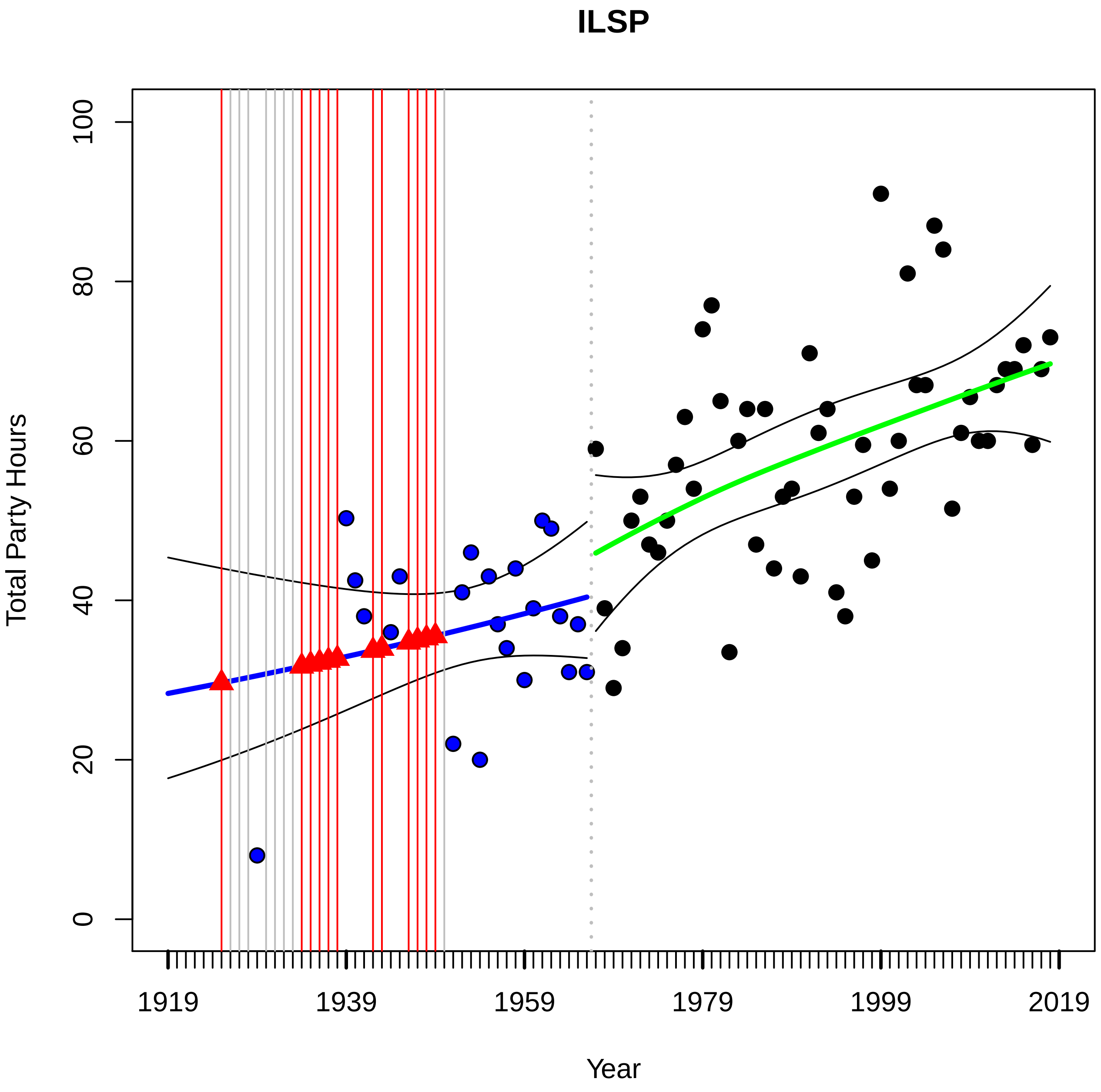

ILSP
Total Party Hours
0
20
40
60
80
100
1919
1939
1959
1979
1999
2019
Year

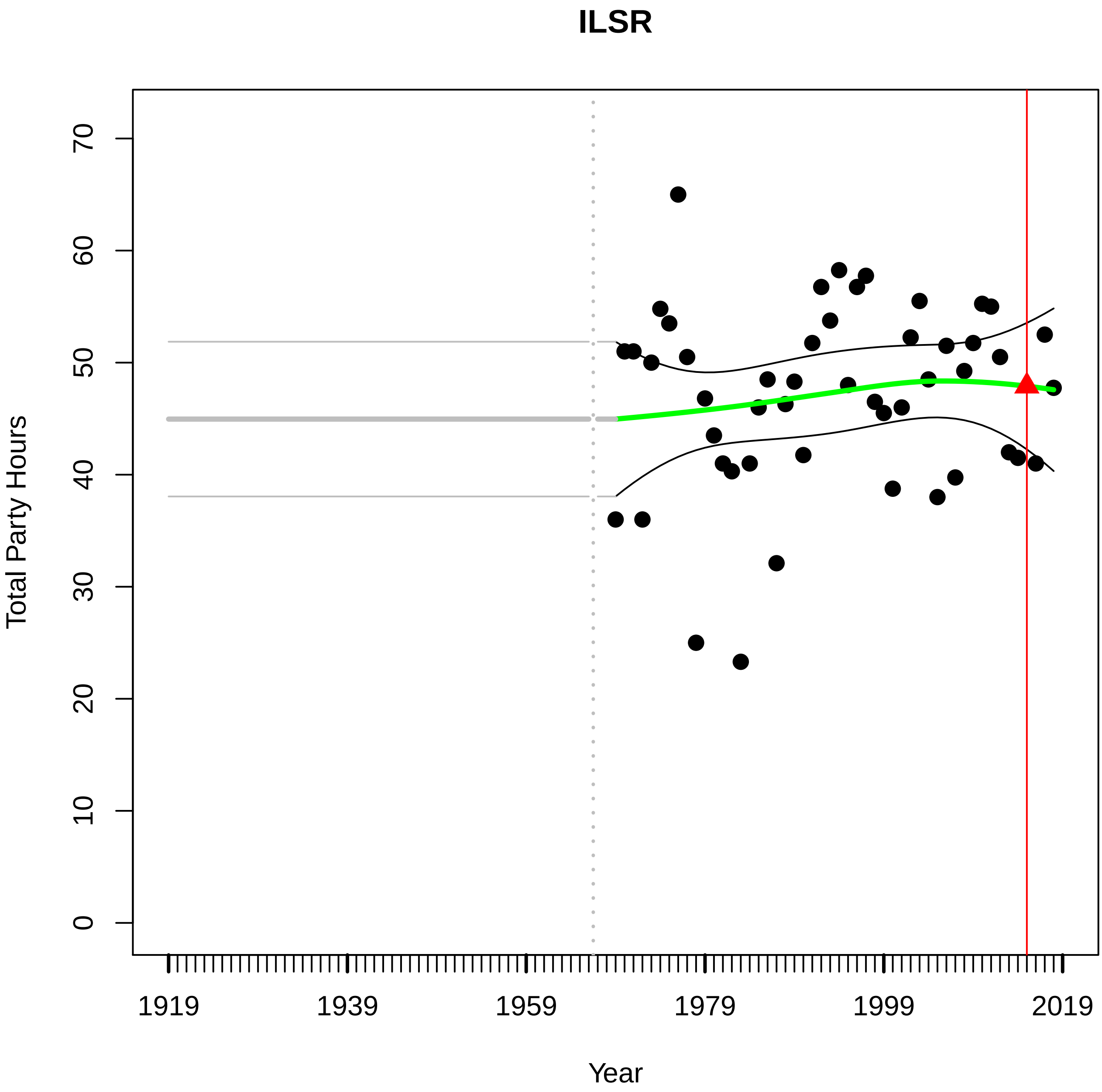
ILSR
Total Party Hours
Year
0
10
20
30
40
50
60
70
1919
1939
1959
1979
1999
2019

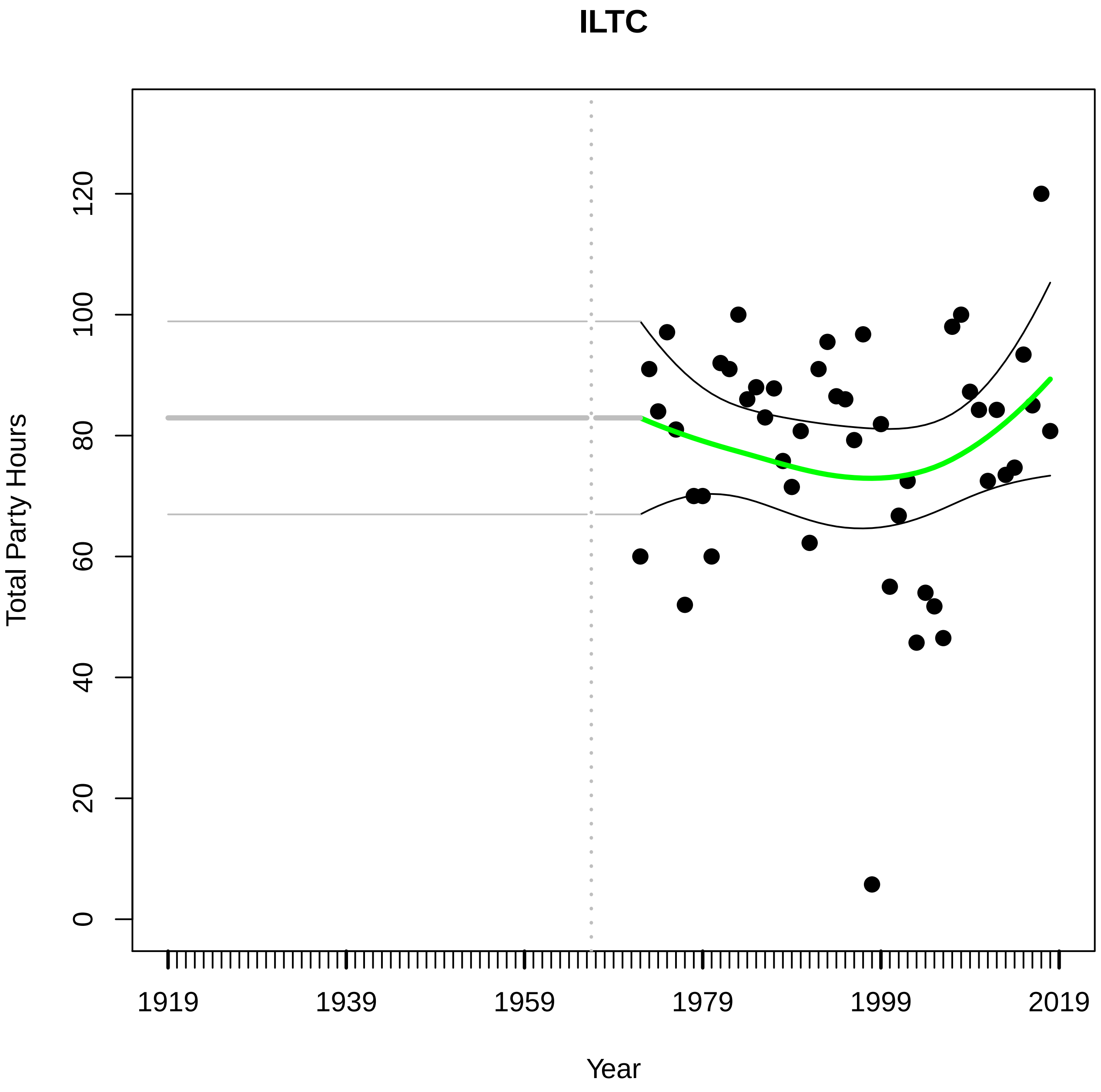
ILTC
Total Party Hours
0
20
40
60
80
100
120
1919
1939
1959
1979
1999
2019
Year

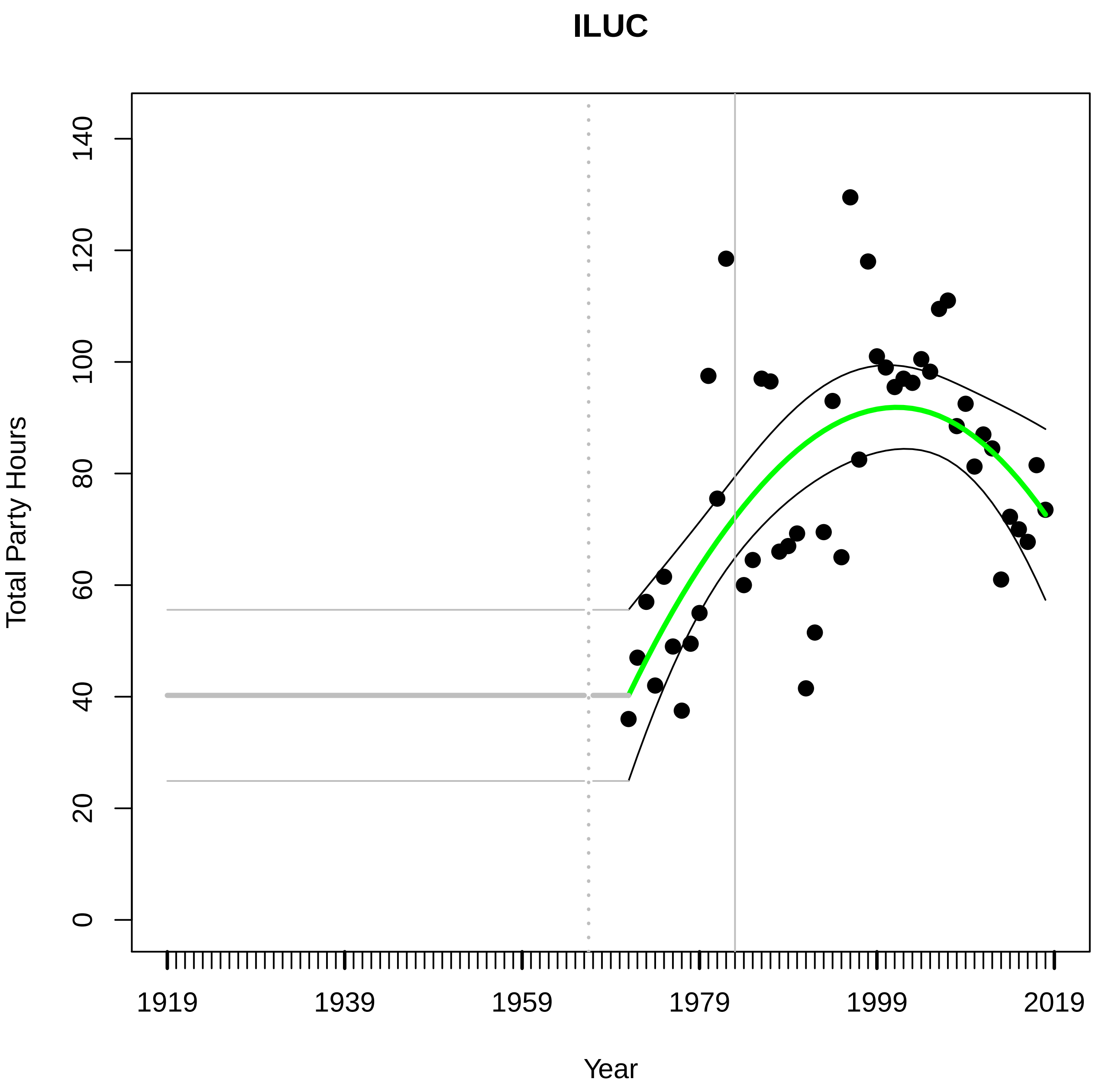
ILUC
Total Party Hours
0
20
40
60
80
100
120
140
1919
1939
1959
1979
1999
2019
Year

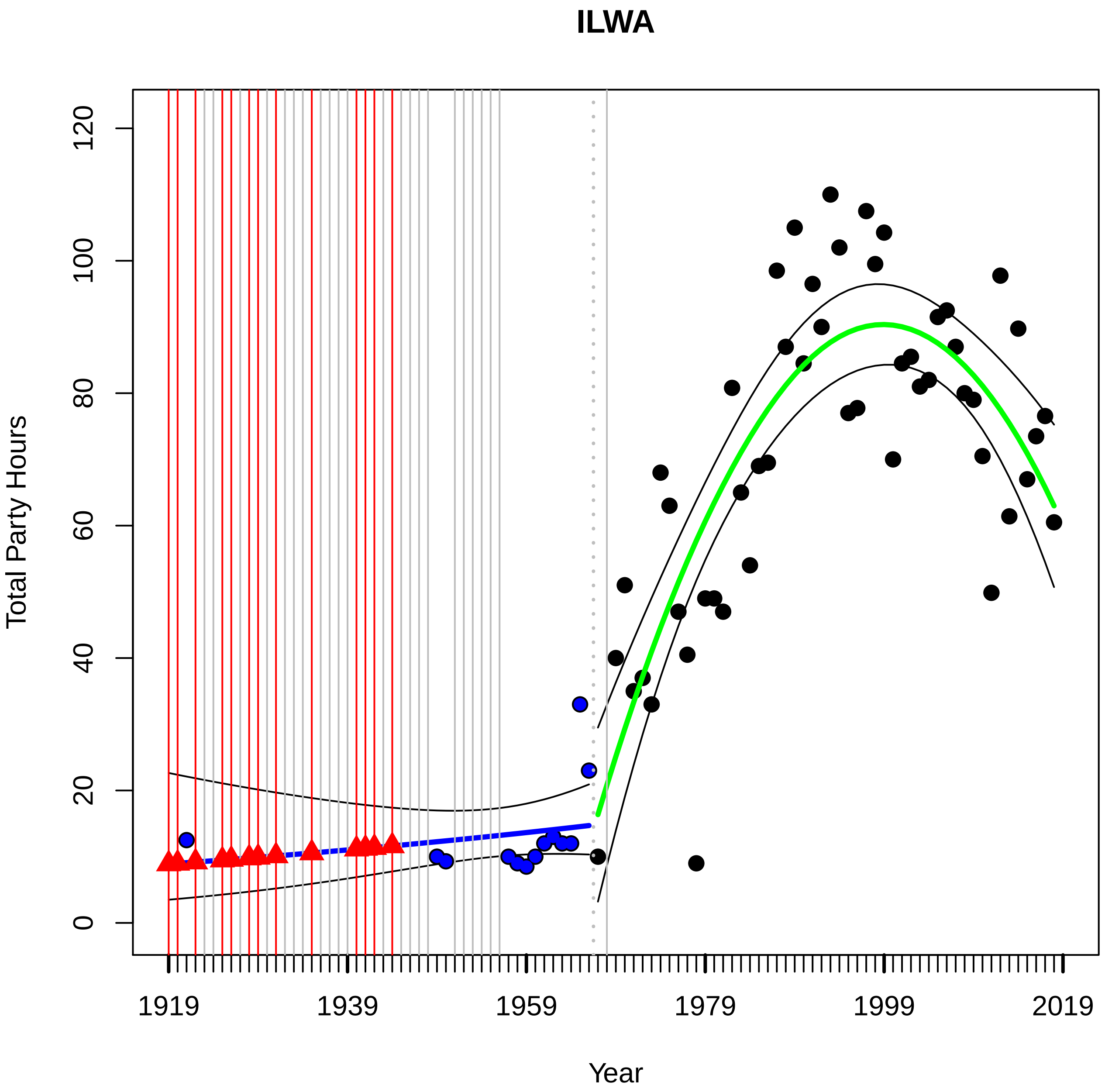

ILWA
Total Party Hours
0
20
40
60
80
100
120
1919
1939
1959
1979
1999
2019
Year

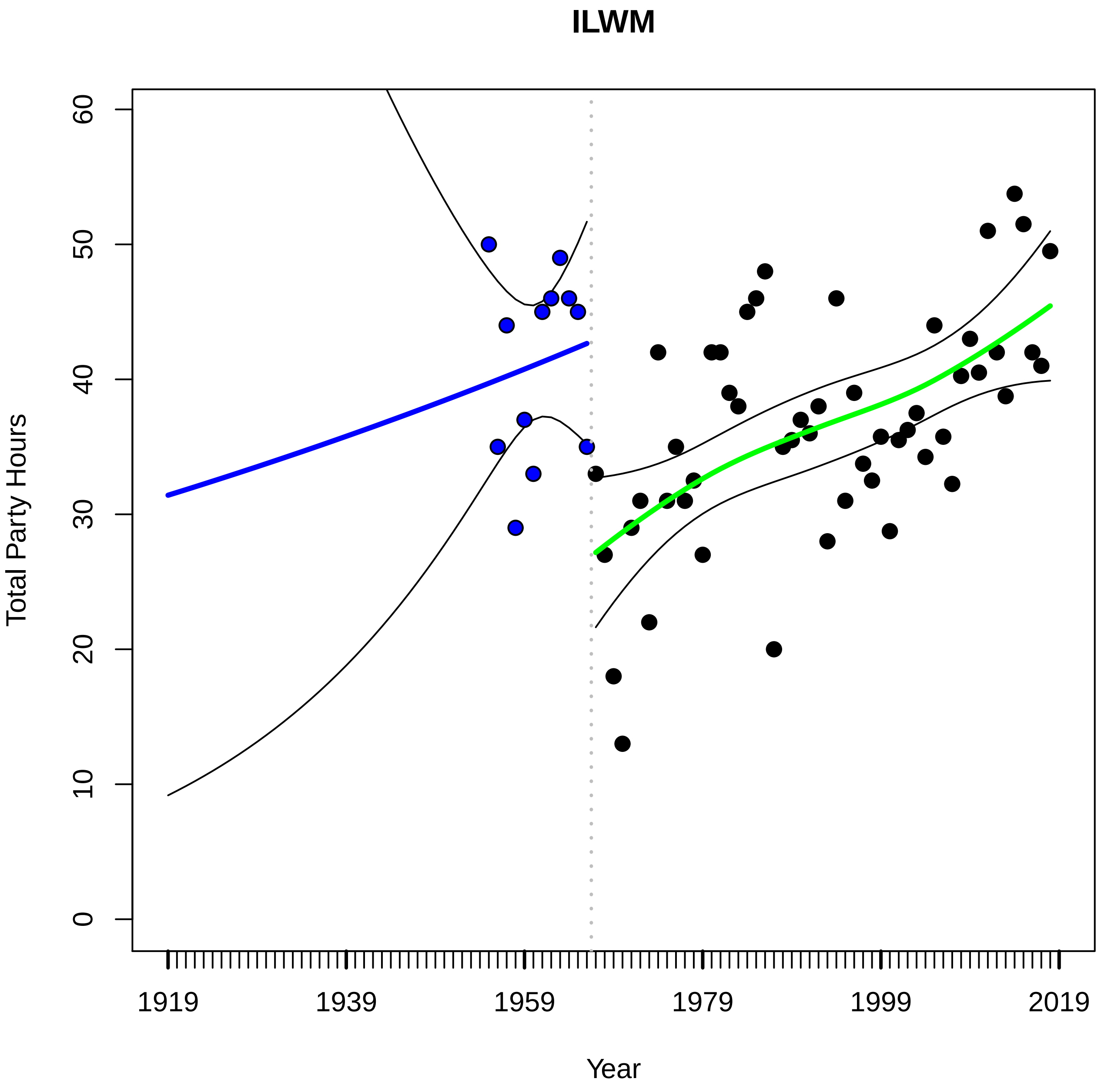
ILWM
Total Party Hours
Year
0
10
20
30
40
50
60
1919
1939
1959
1979
1999
2019

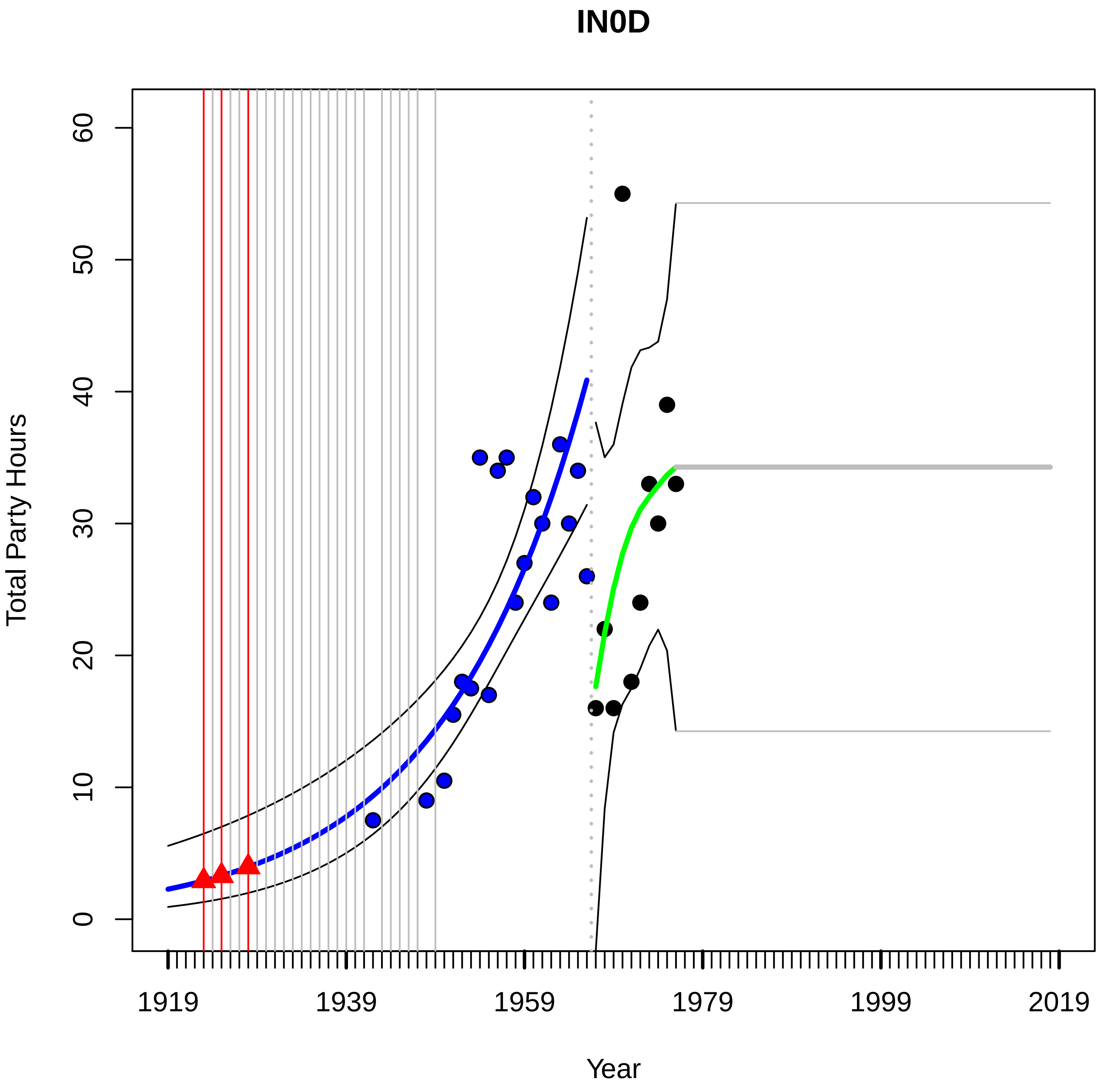
IN0D
Total Party Hours
Year
0
10
20
30
40
50
60
1919
1939
1959
1979
1999
2019

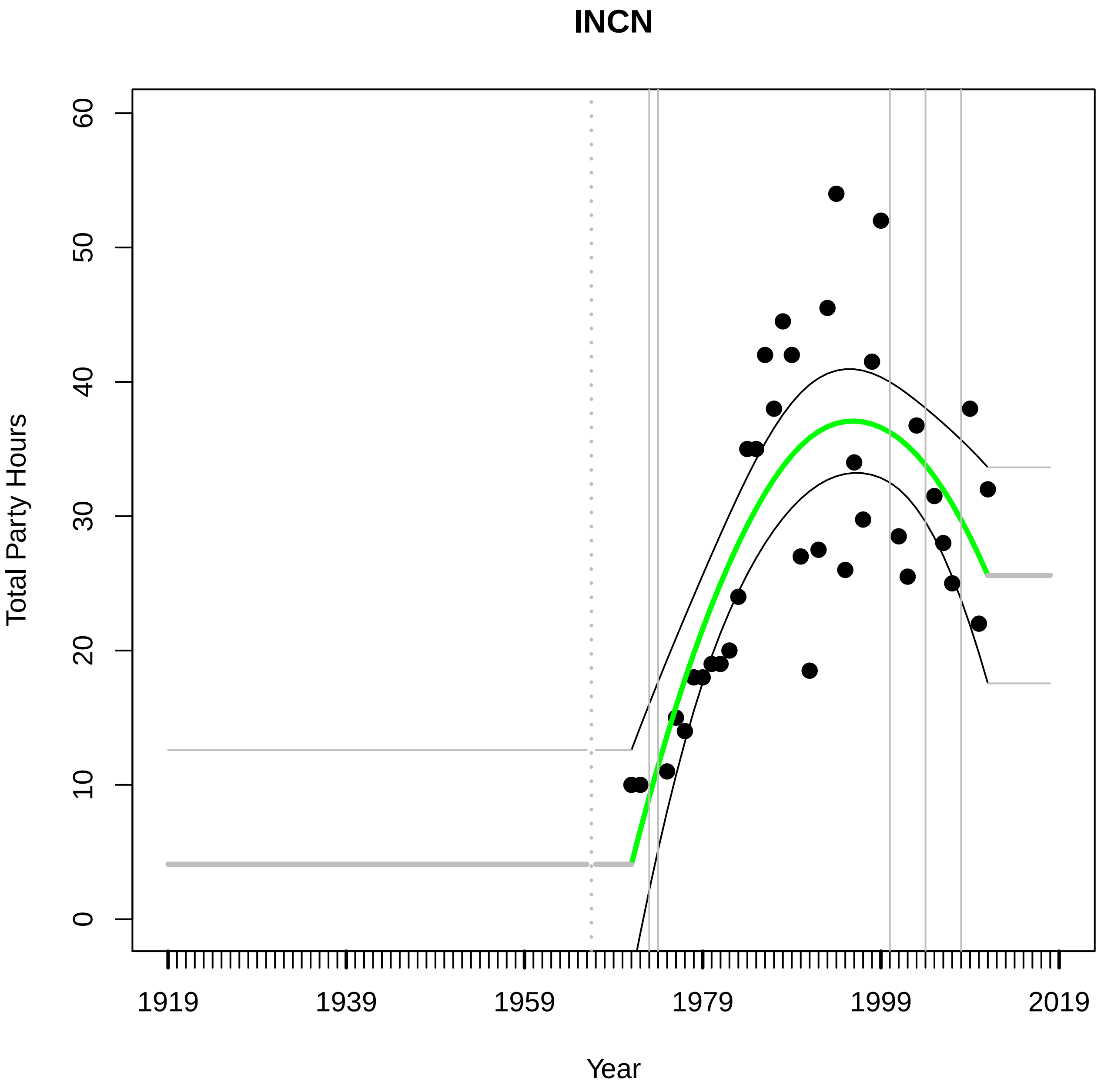

INCN
Total Party Hours
Year
0
10
20
30
40
50
60
1919
1939
1959
1979
1999
2019

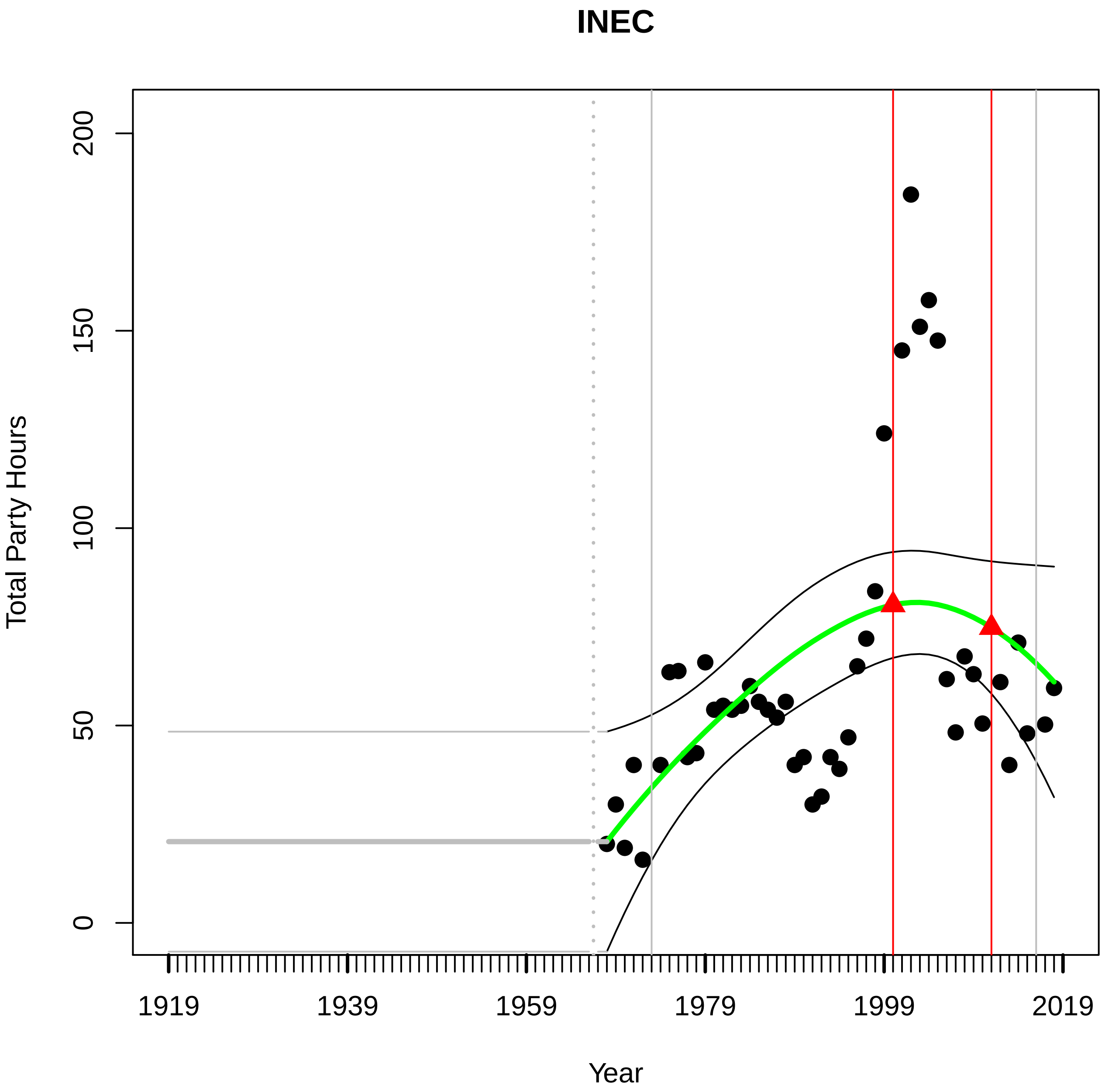

INEC
Total Party Hours
0
50
100
150
200
1919
1939
1959
1979
1999
2019
Year

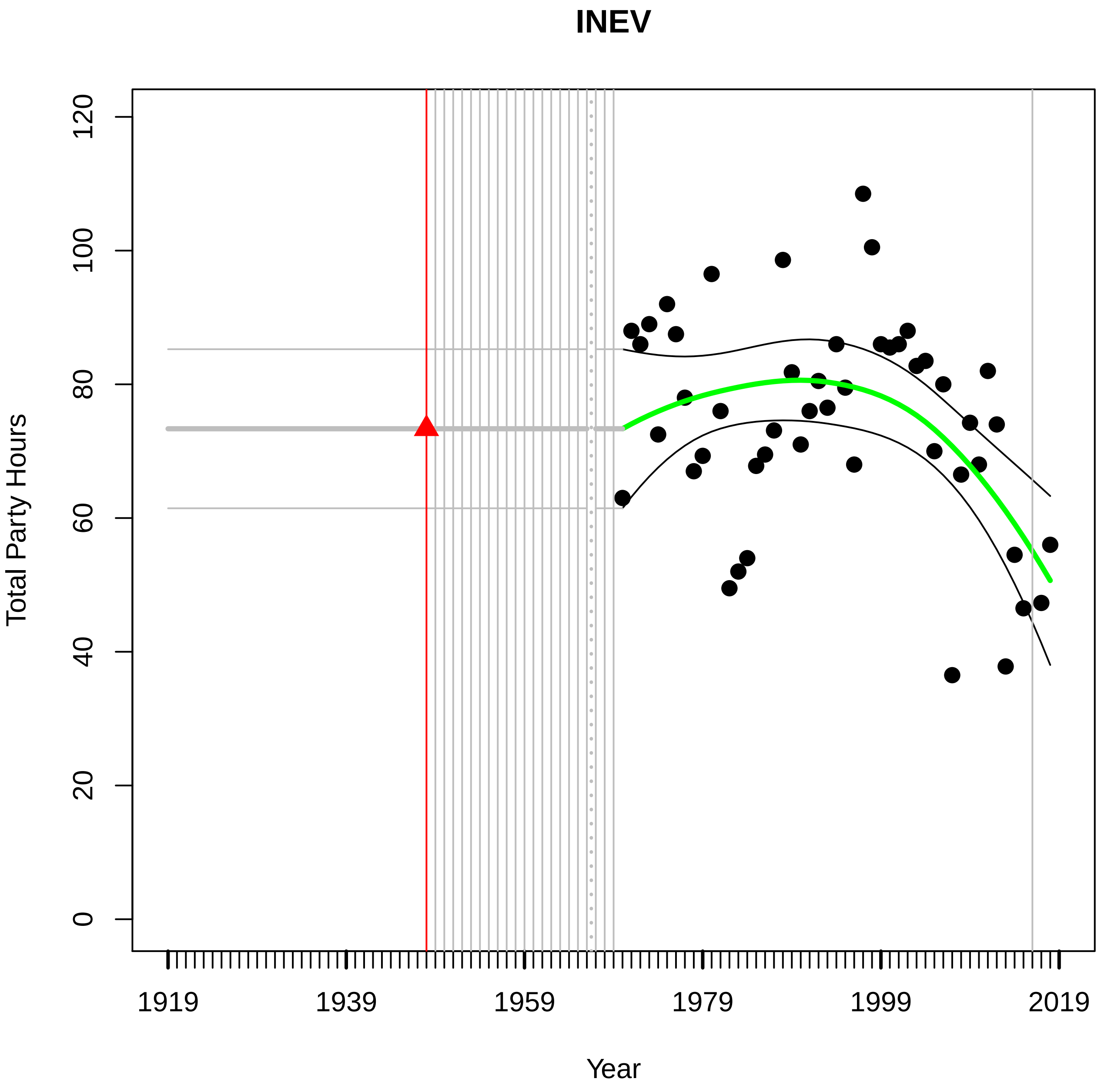
INEV
Total Party Hours
0
20
40
60
80
100
120
1919
1939
1959
1979
1999
2019
Year

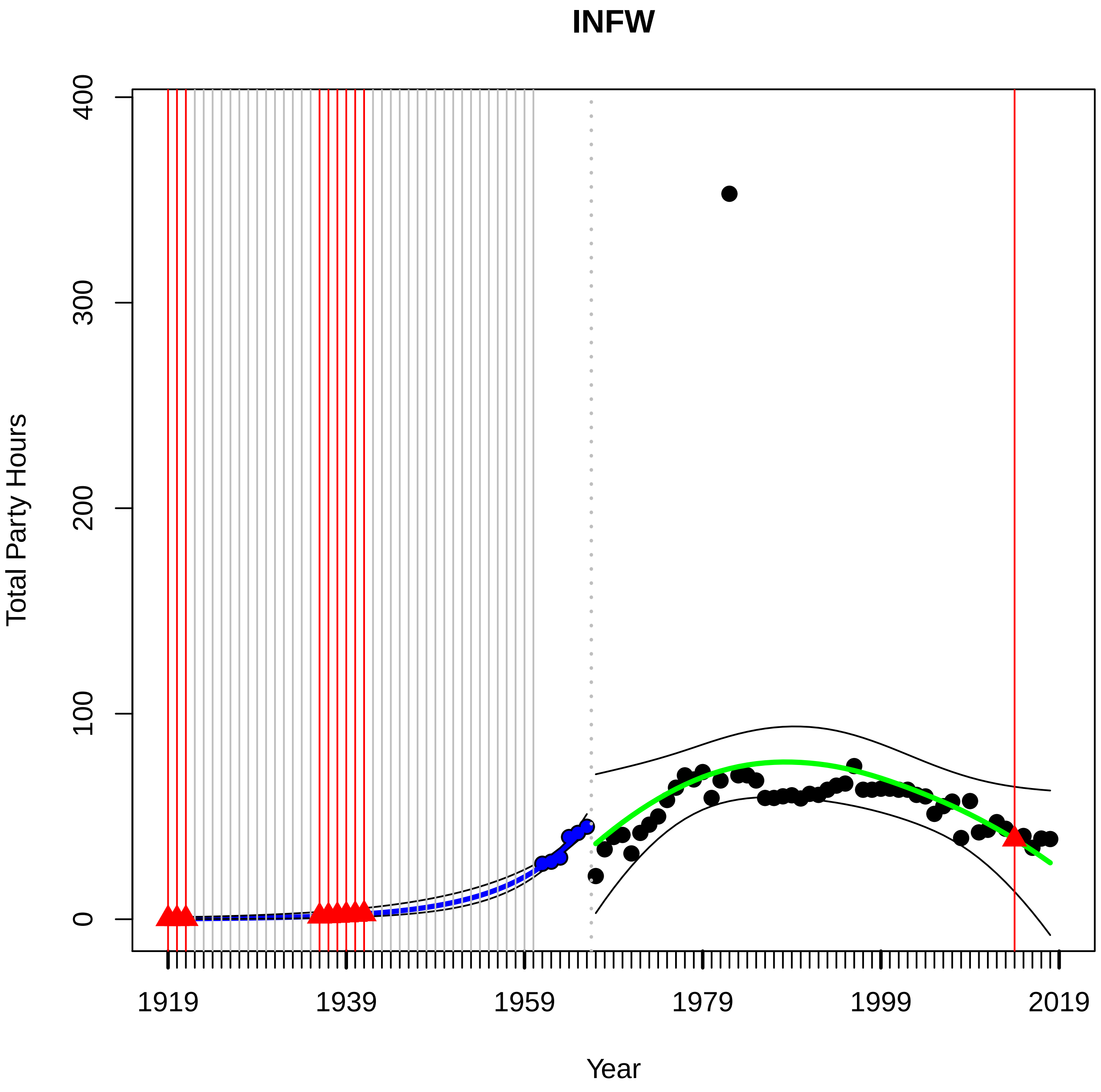

INFW
Total Party Hours
0
100
200
300
400
1919
1939
1959
1979
1999
2019
Year

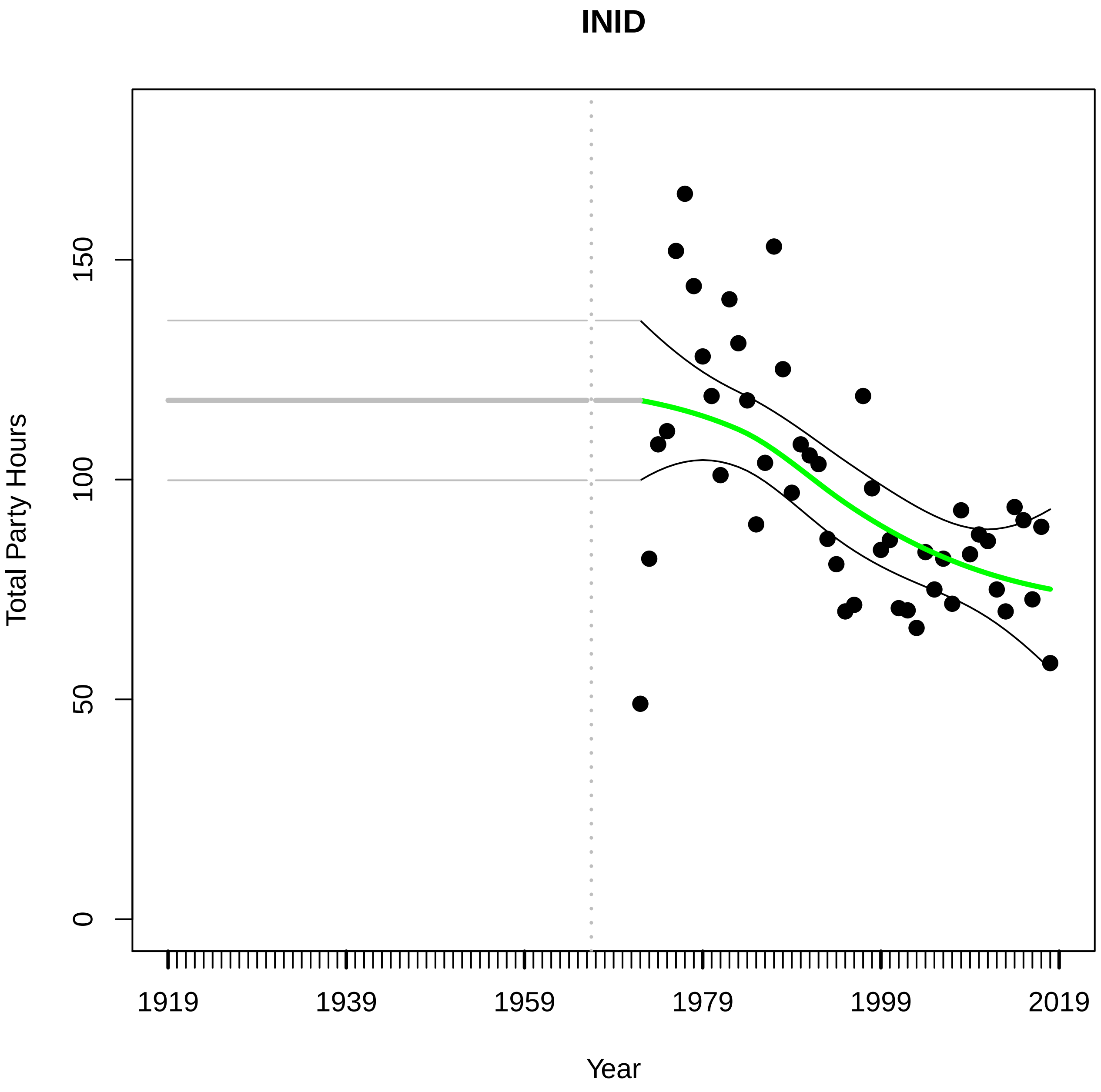

INID
Total Party Hours
0
50
100
150
1919
1939
1959
1979
1999
2019
Year

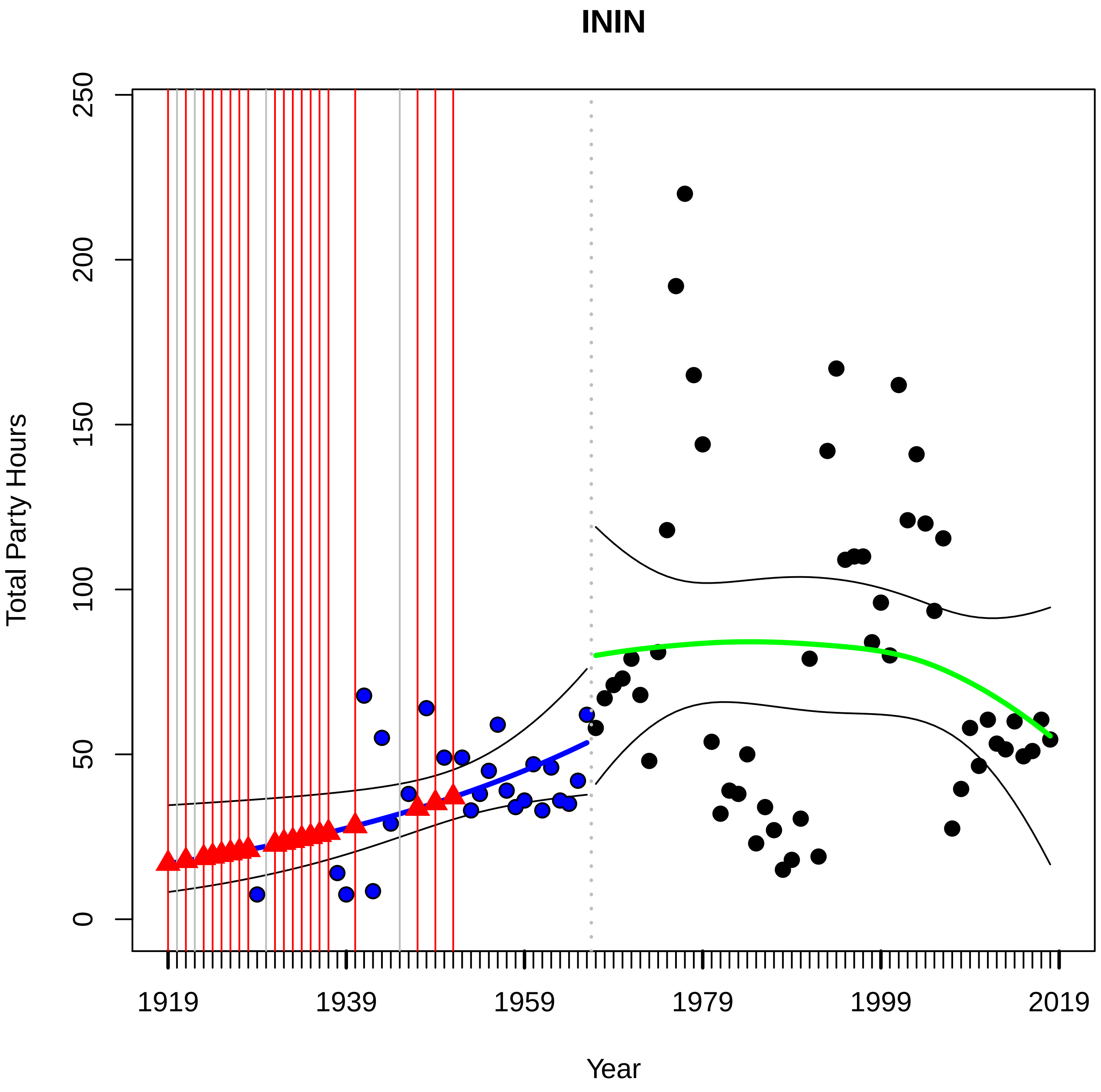
ININ
Total Party Hours
0
50
100
150
200
250
1919
1939
1959
1979
1999
2019
Year

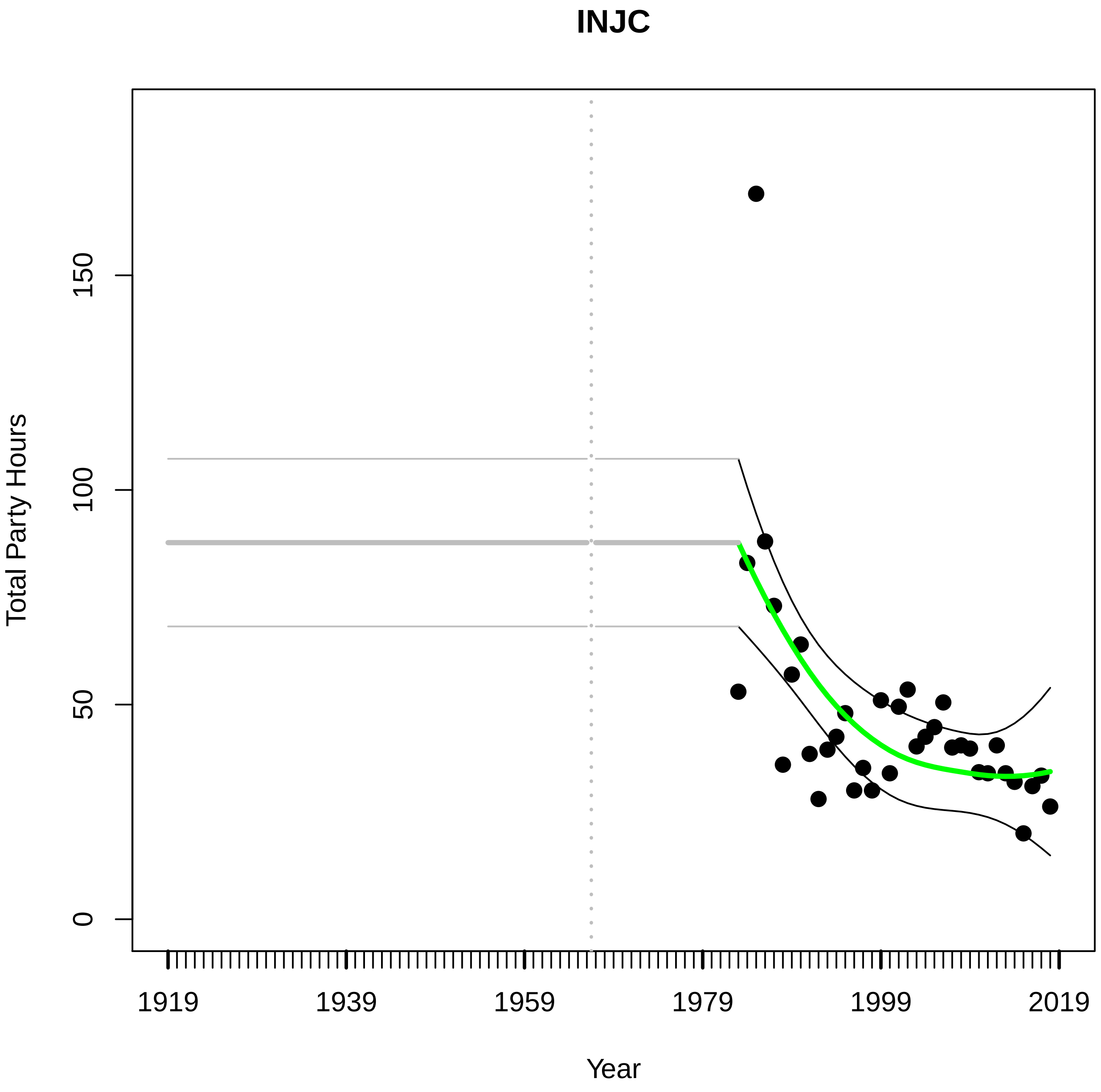

INJC
Total Party Hours
0
50
100
150
1919
1939
1959
1979
1999
2019
Year

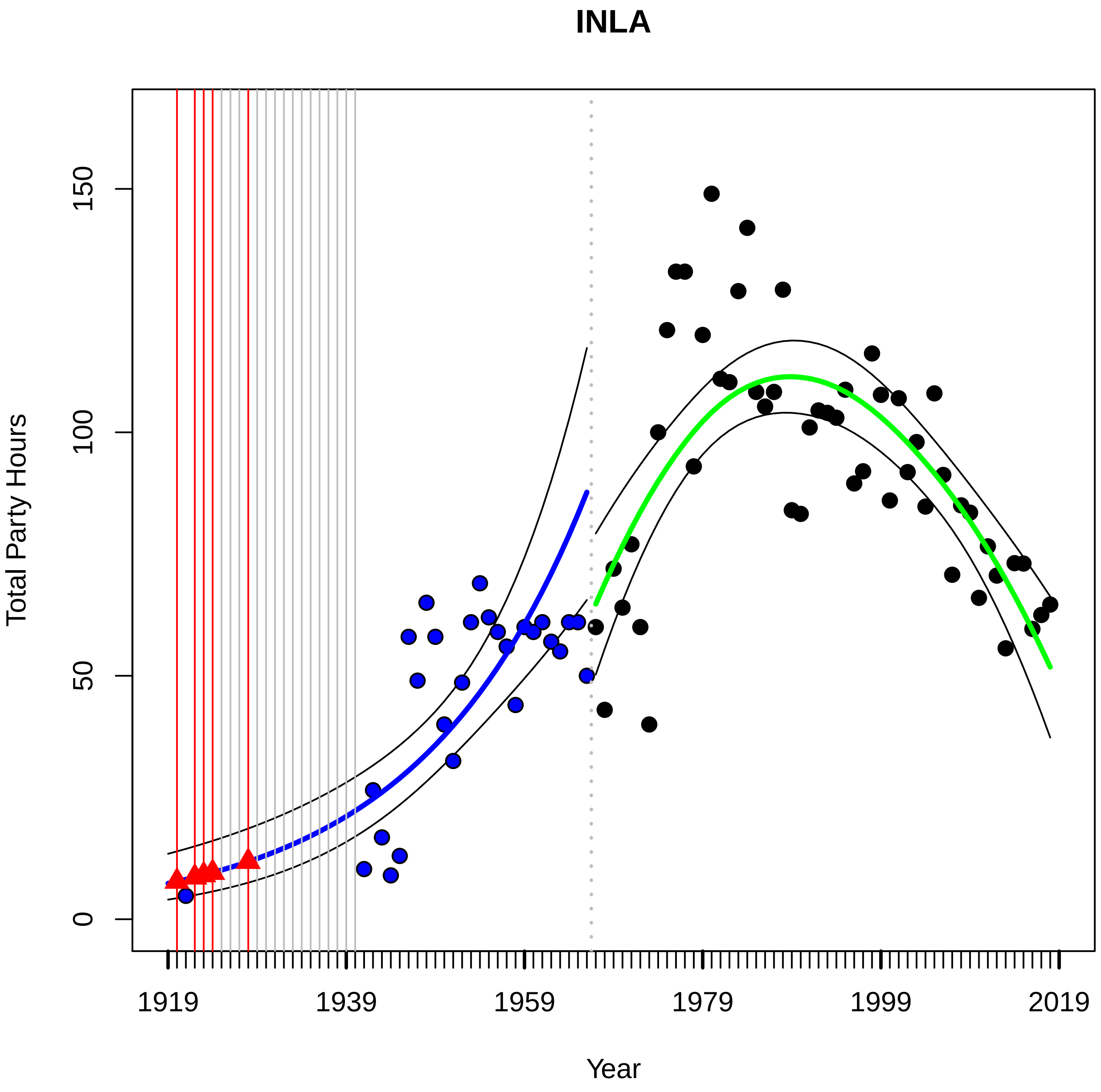

INLA
Total Party Hours
0
50
100
150
1919
1939
1959
1979
1999
2019
Year

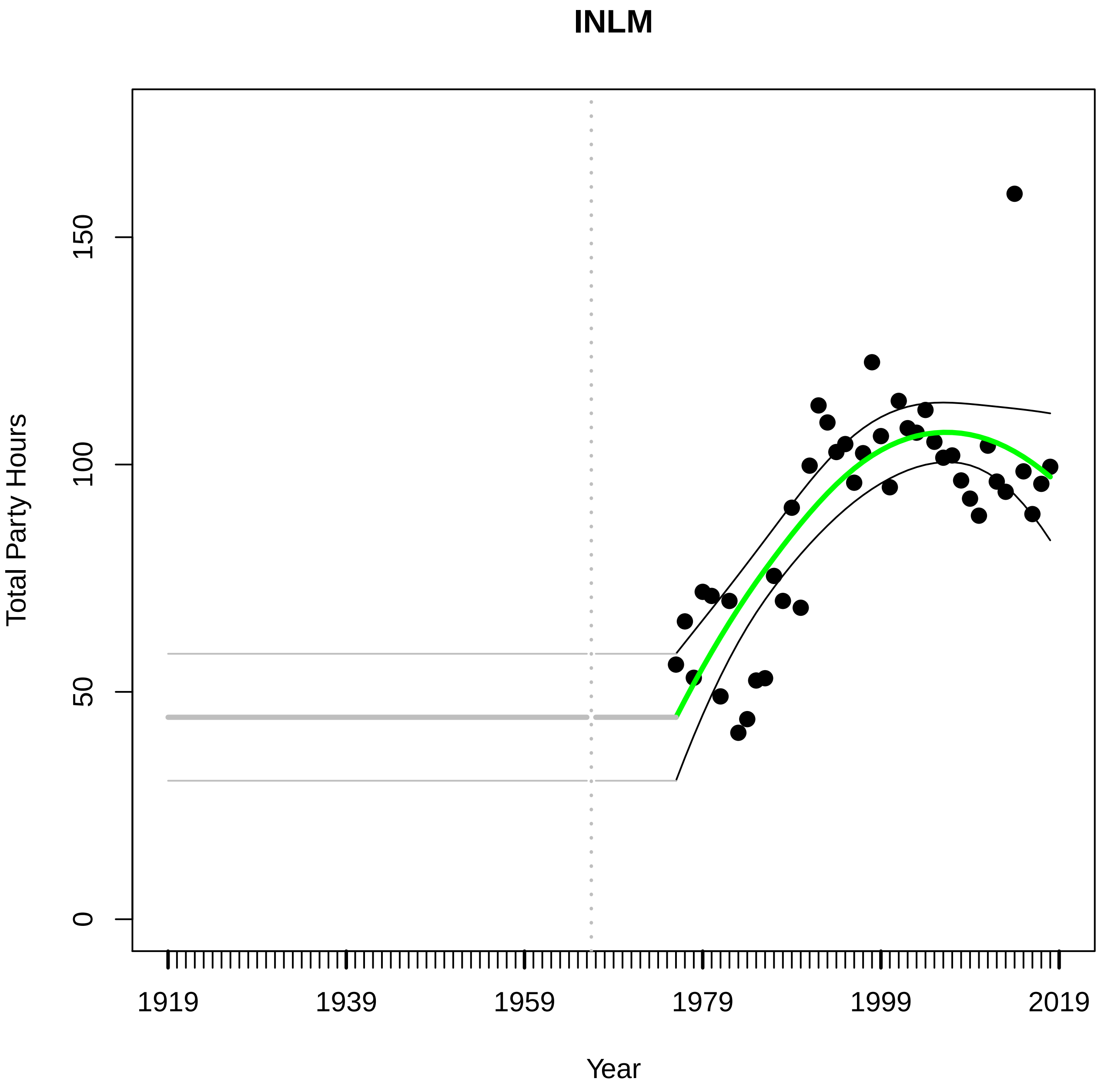

INLM
Total Party Hours
0
50
100
150
1919
1939
1959
1979
1999
2019
Year

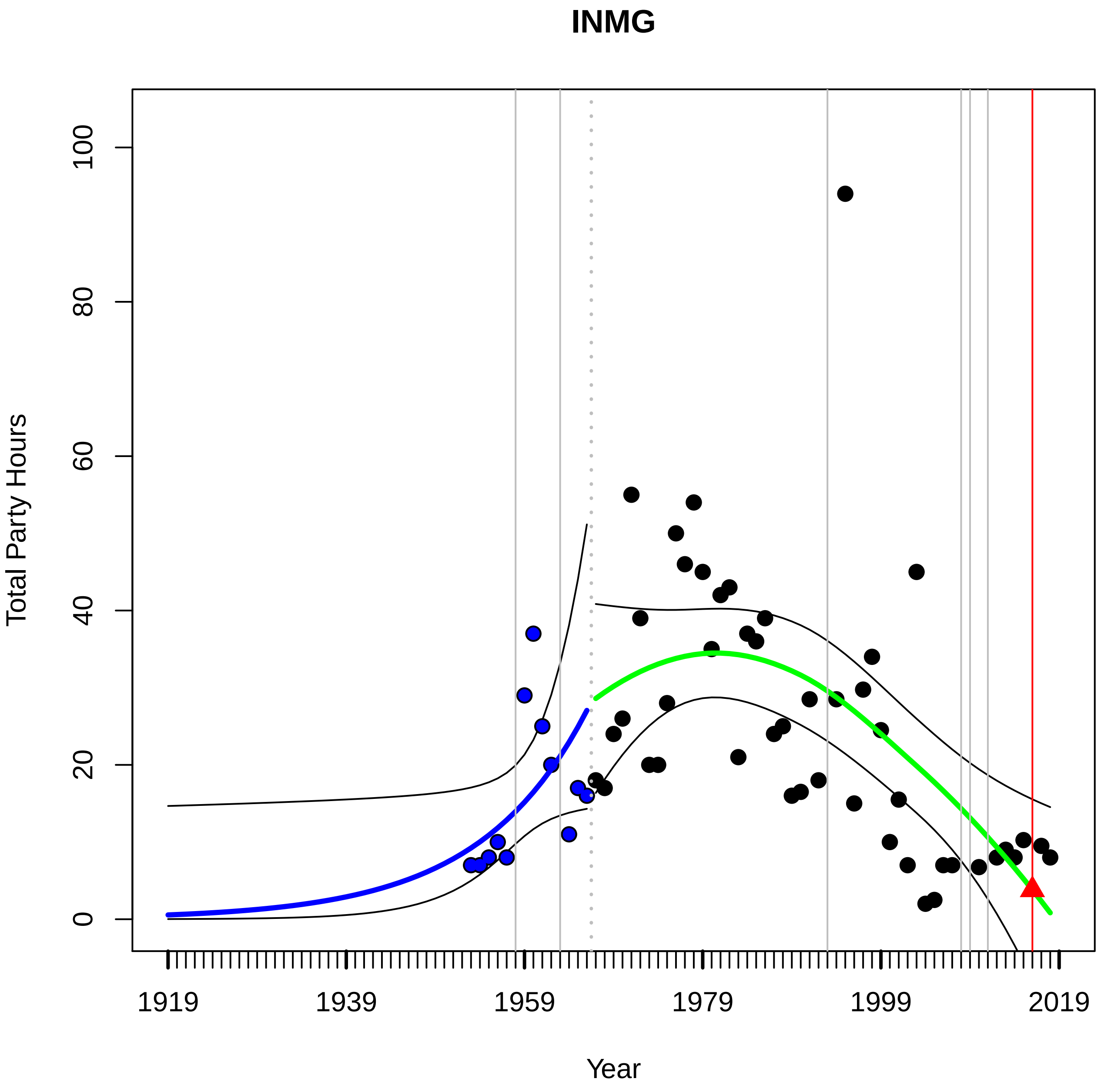
INMG
Total Party Hours
0
20
40
60
80
100
1919
1939
1959
1979
1999
2019
Year

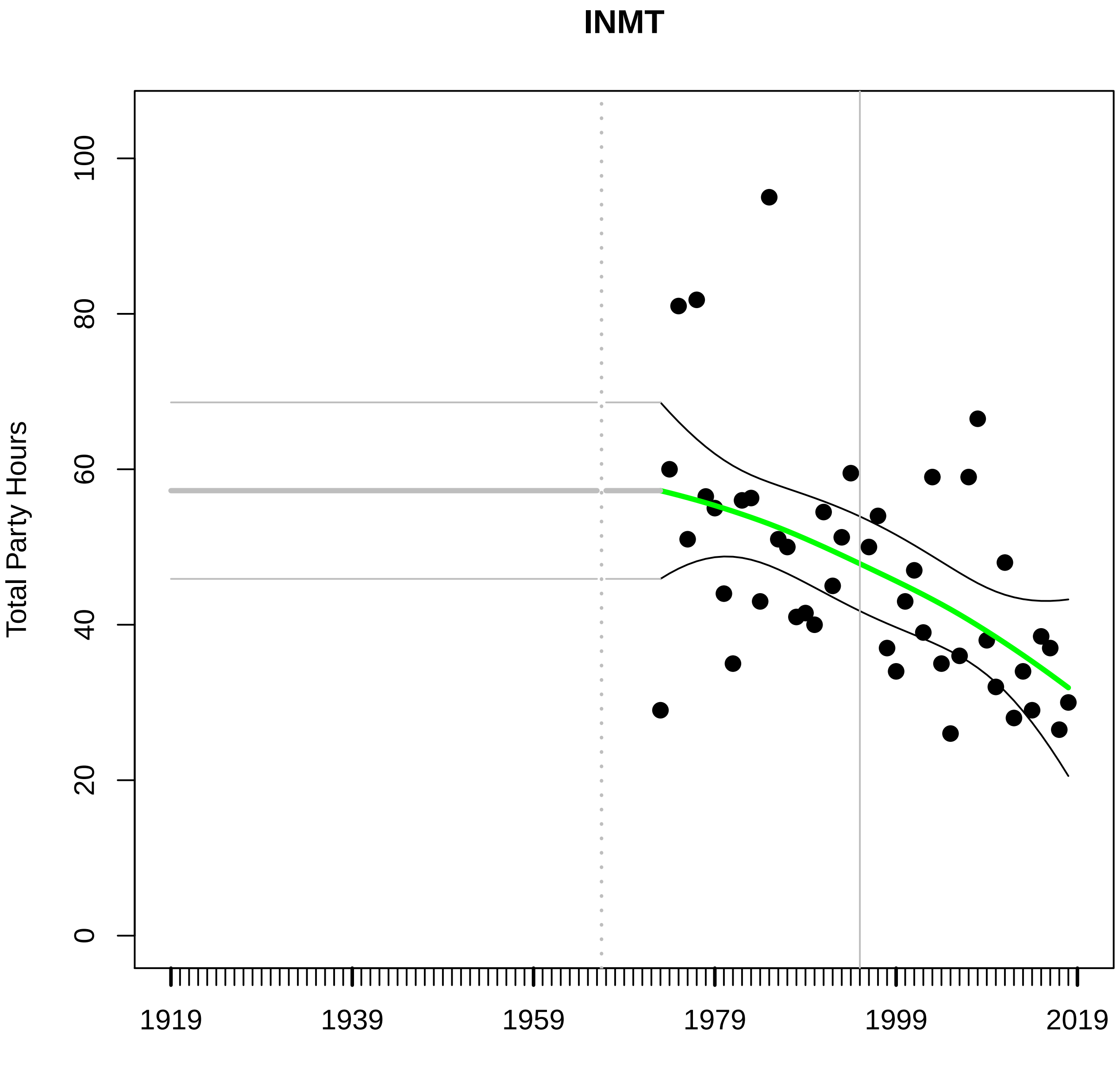

INMT
Total Party Hours
0
20
40
60
80
100
1919
1939
1959
1979
1999
2019
Year

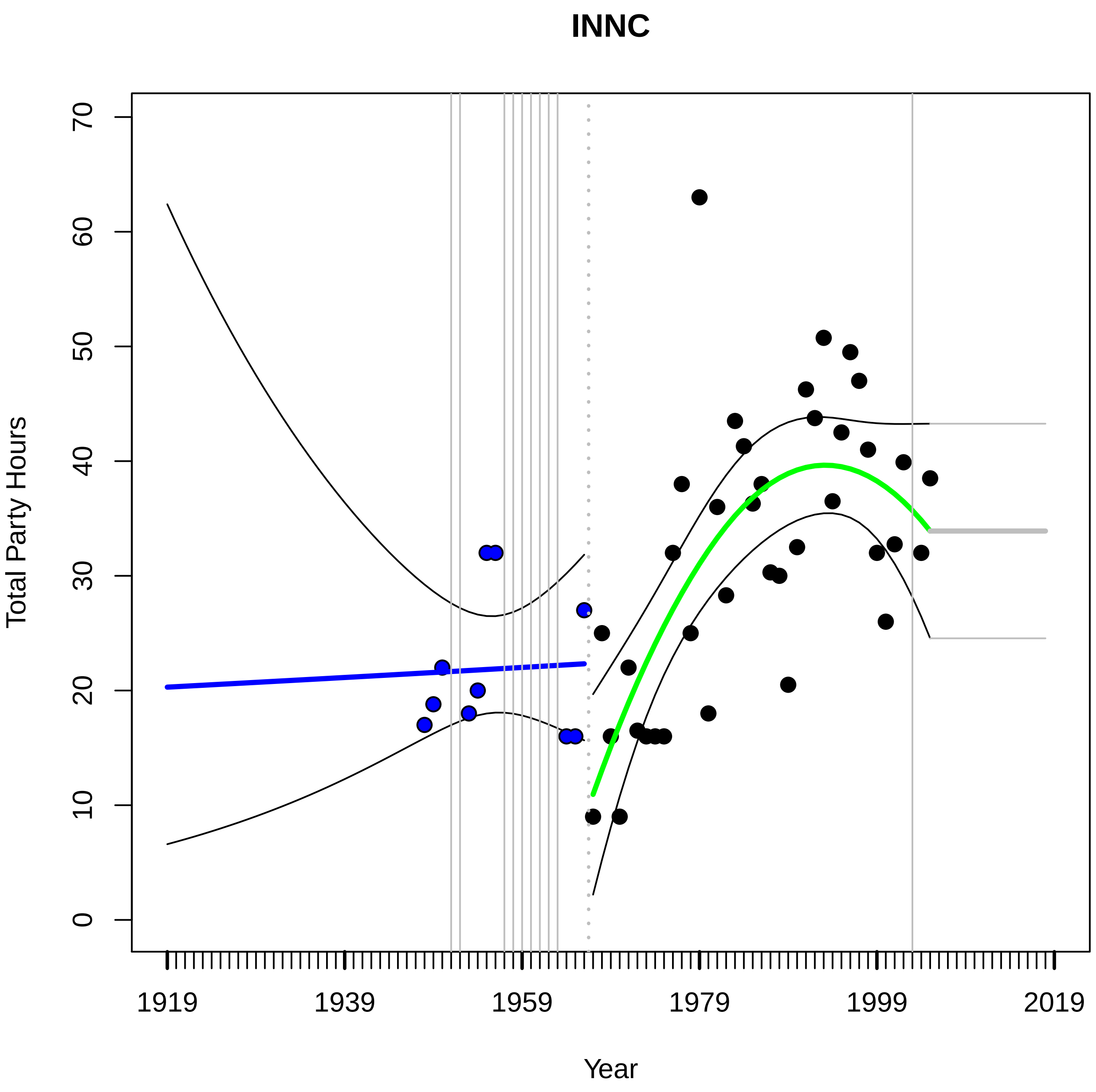

INNC
Total Party Hours
0
10
20
30
40
50
60
70
1919
1939
1959
1979
1999
2019
Year

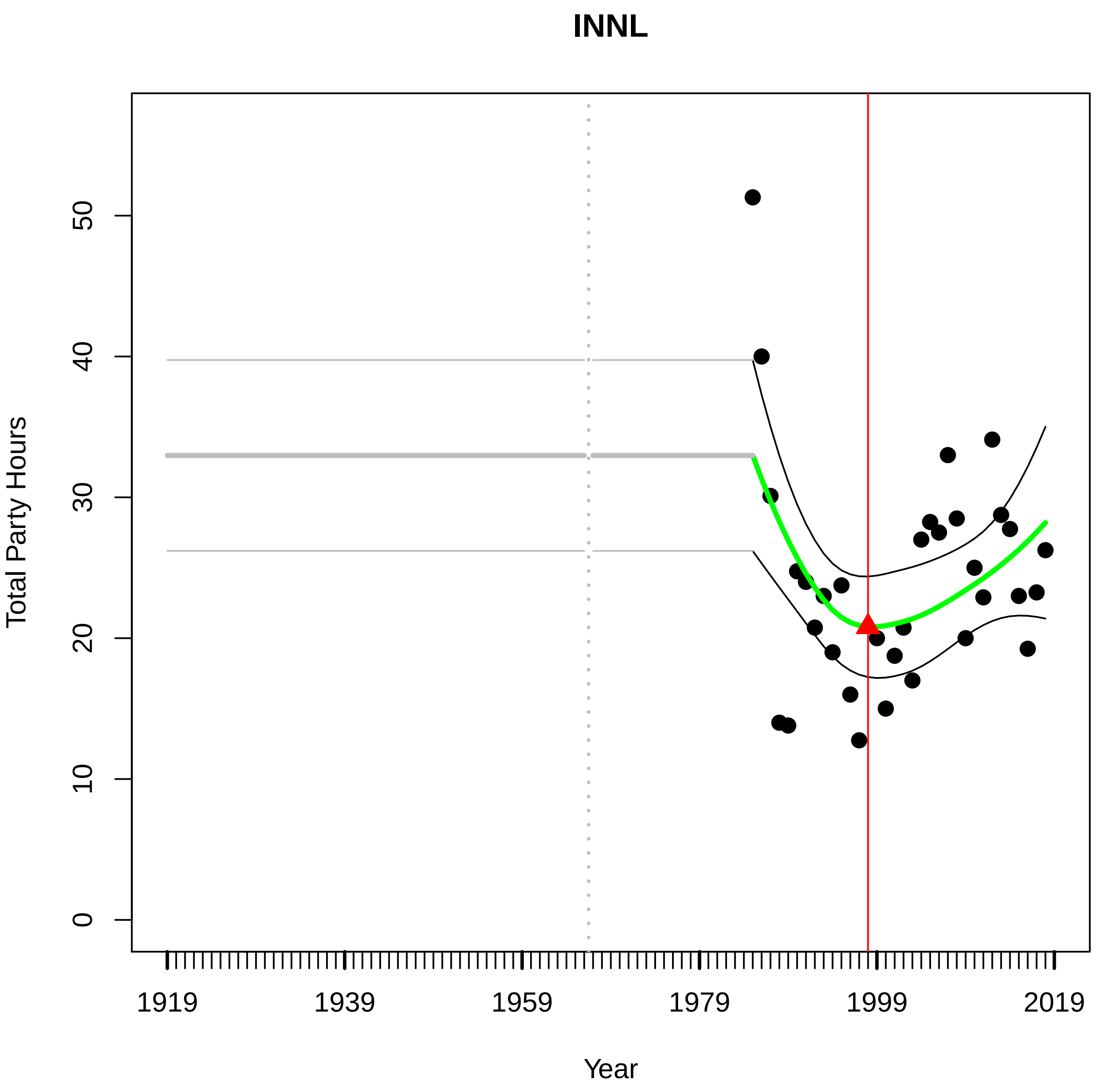

INNL
Total Party Hours
0
10
20
30
40
50
Year
1919
1939
1959
1979
1999
2019

INOC

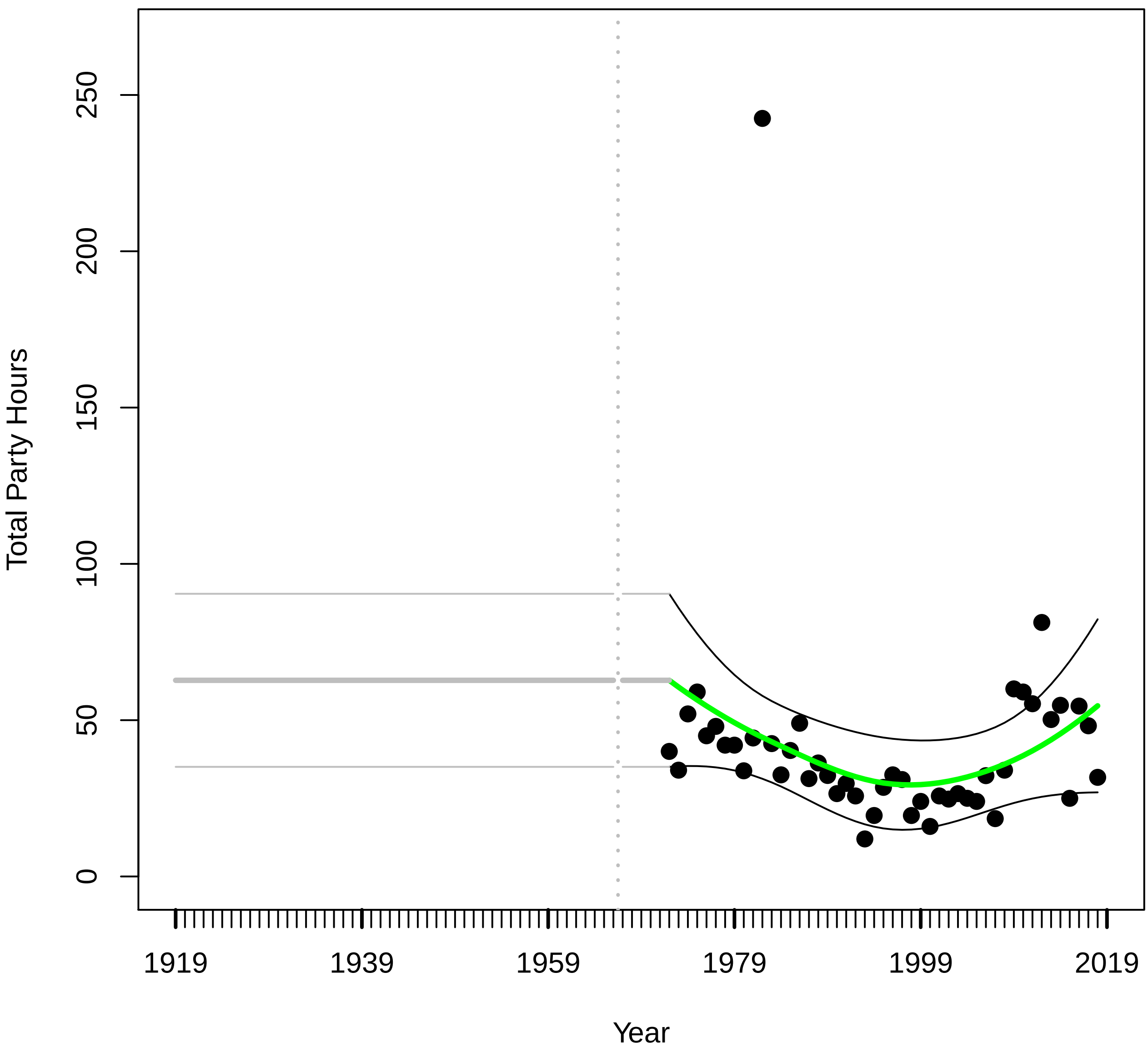

Total Party Hours
0
50
100
150
200
250
1919
1939
1959
1979
1999
2019
Year

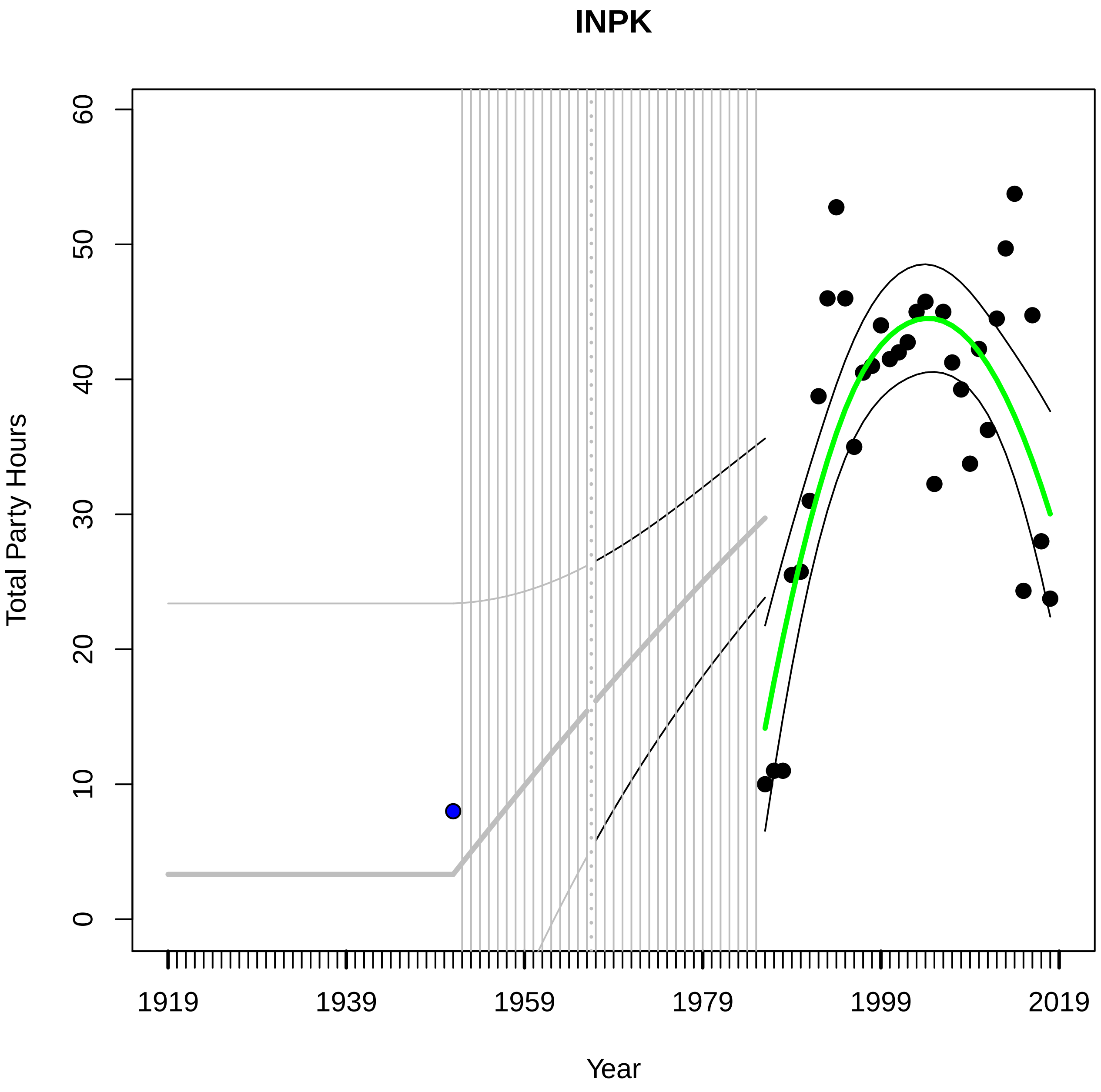
INPK
Total Party Hours
0
10
20
30
40
50
60
1919
1939
1959
1979
1999
2019
Year

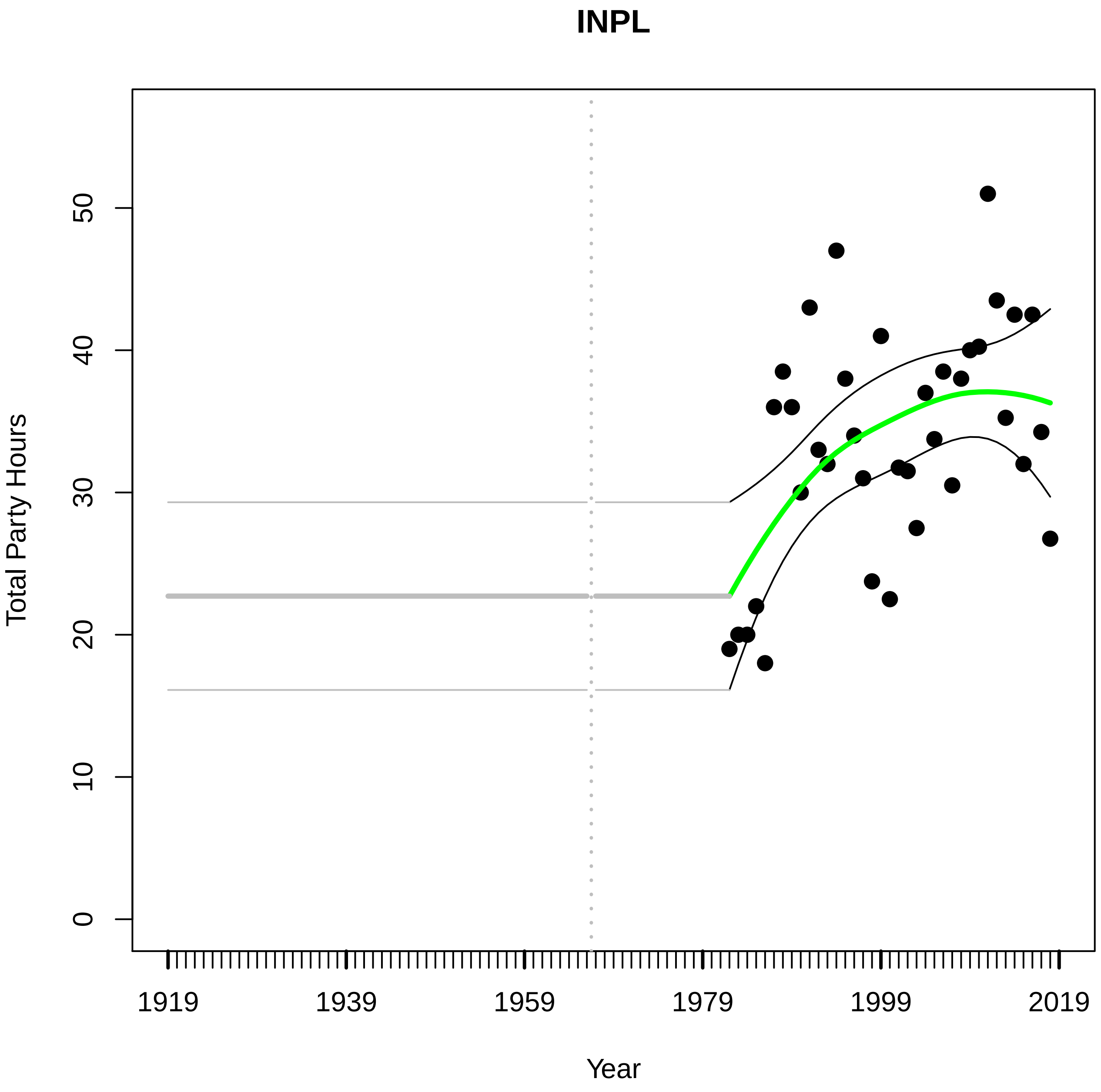
INPL
Total Party Hours
0
10
20
30
40
50
1919
1939
1959
1979
1999
2019
Year

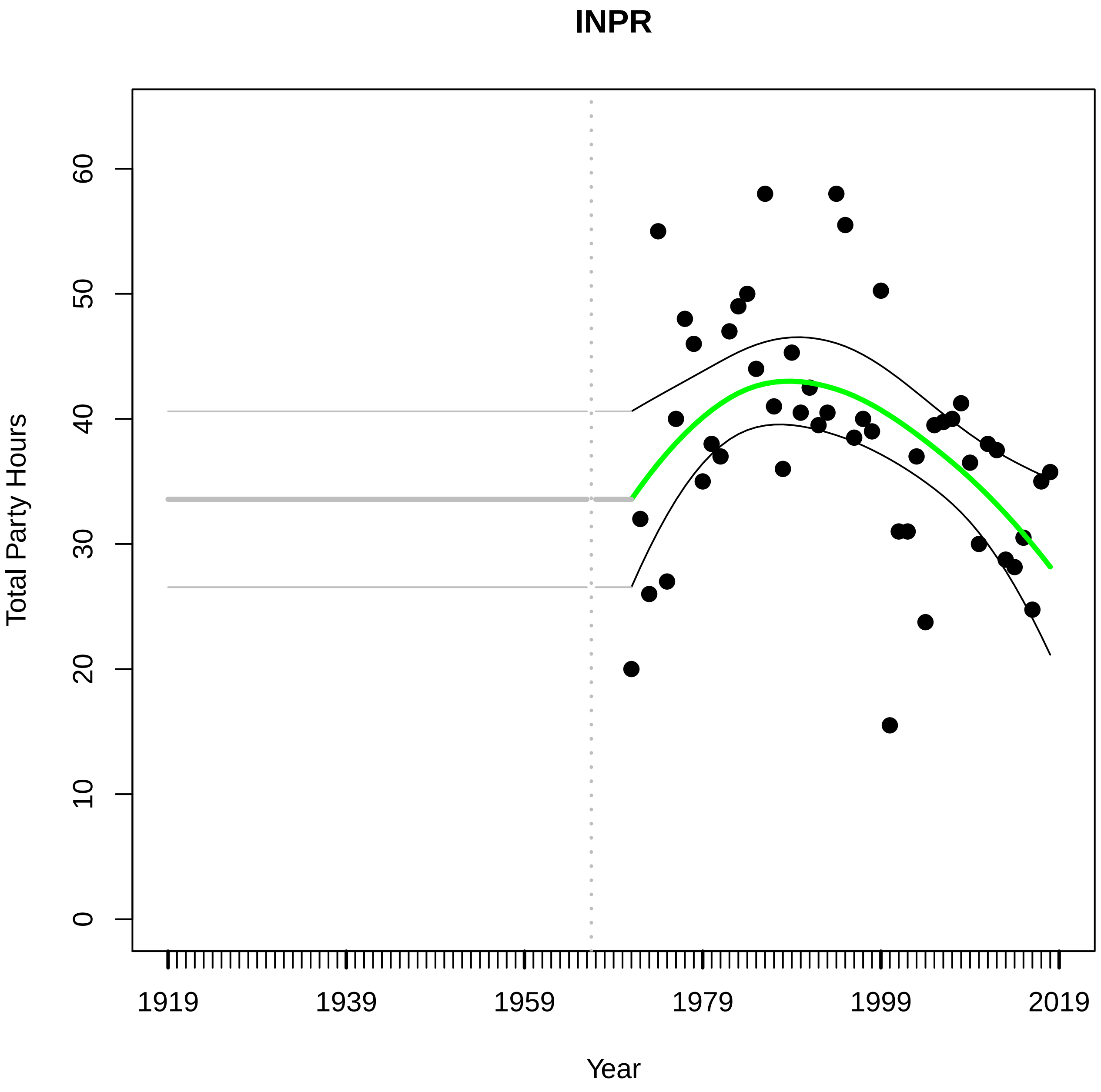

INPR
Total Party Hours
0
10
20
30
40
50
60
1919
1939
1959
1979
1999
2019
Year

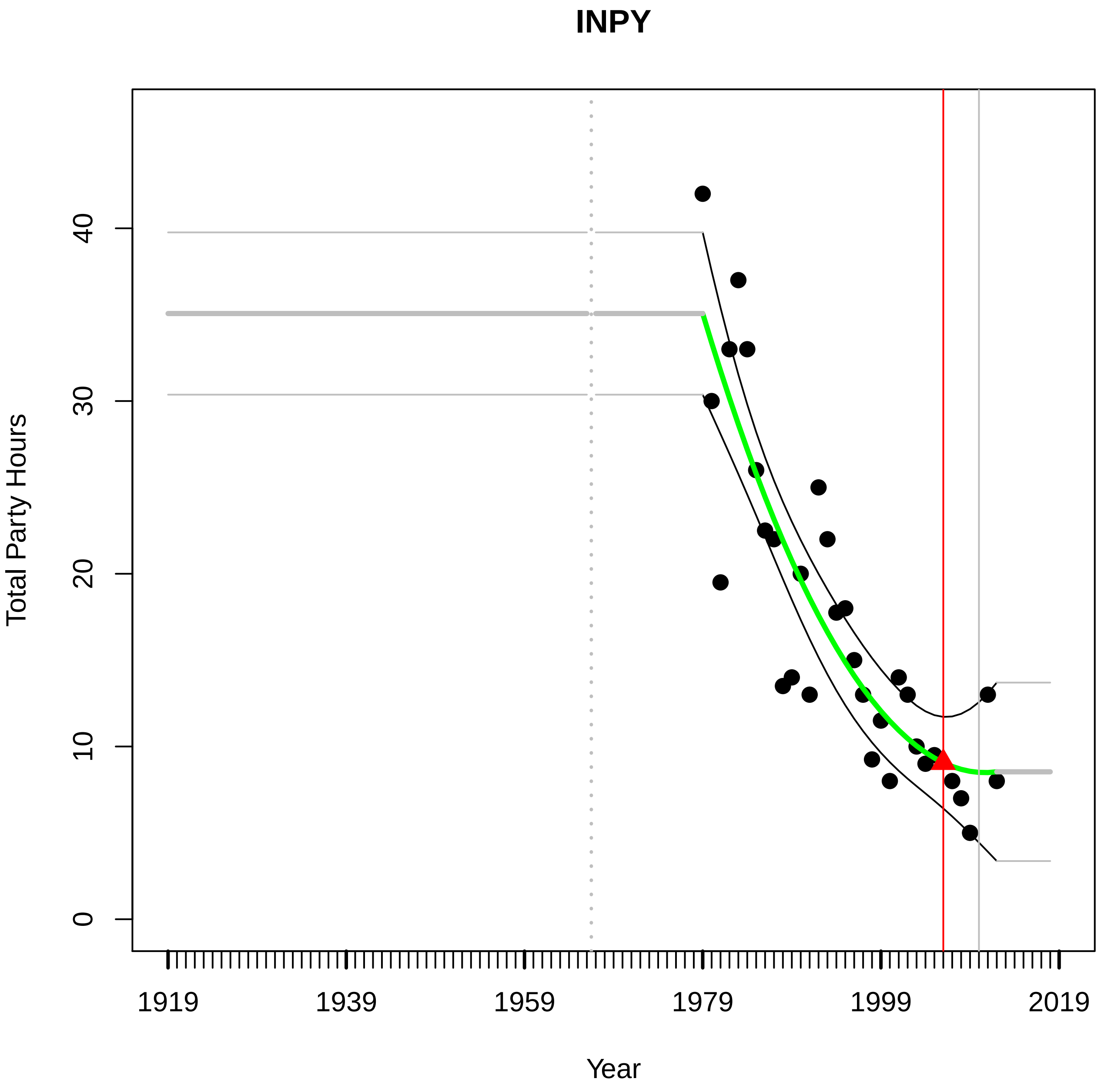
INPY
Total Party Hours
0
10
20
30
40
1919
1939
1959
1979
1999
2019
Year

INRI

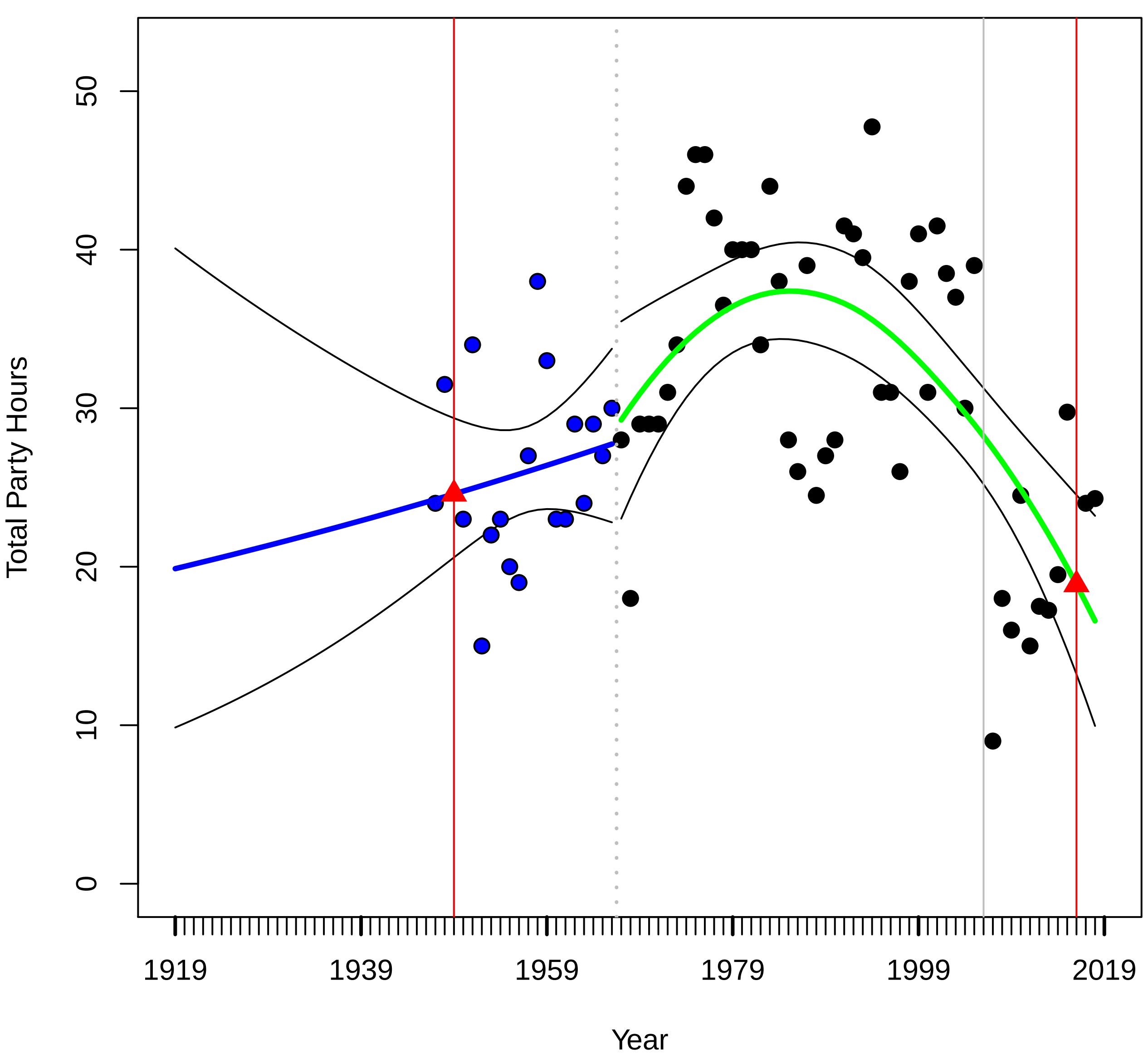

Total Party Hours
0
10
20
30
40
50
1919
1939
1959
1979
1999
2019
Year

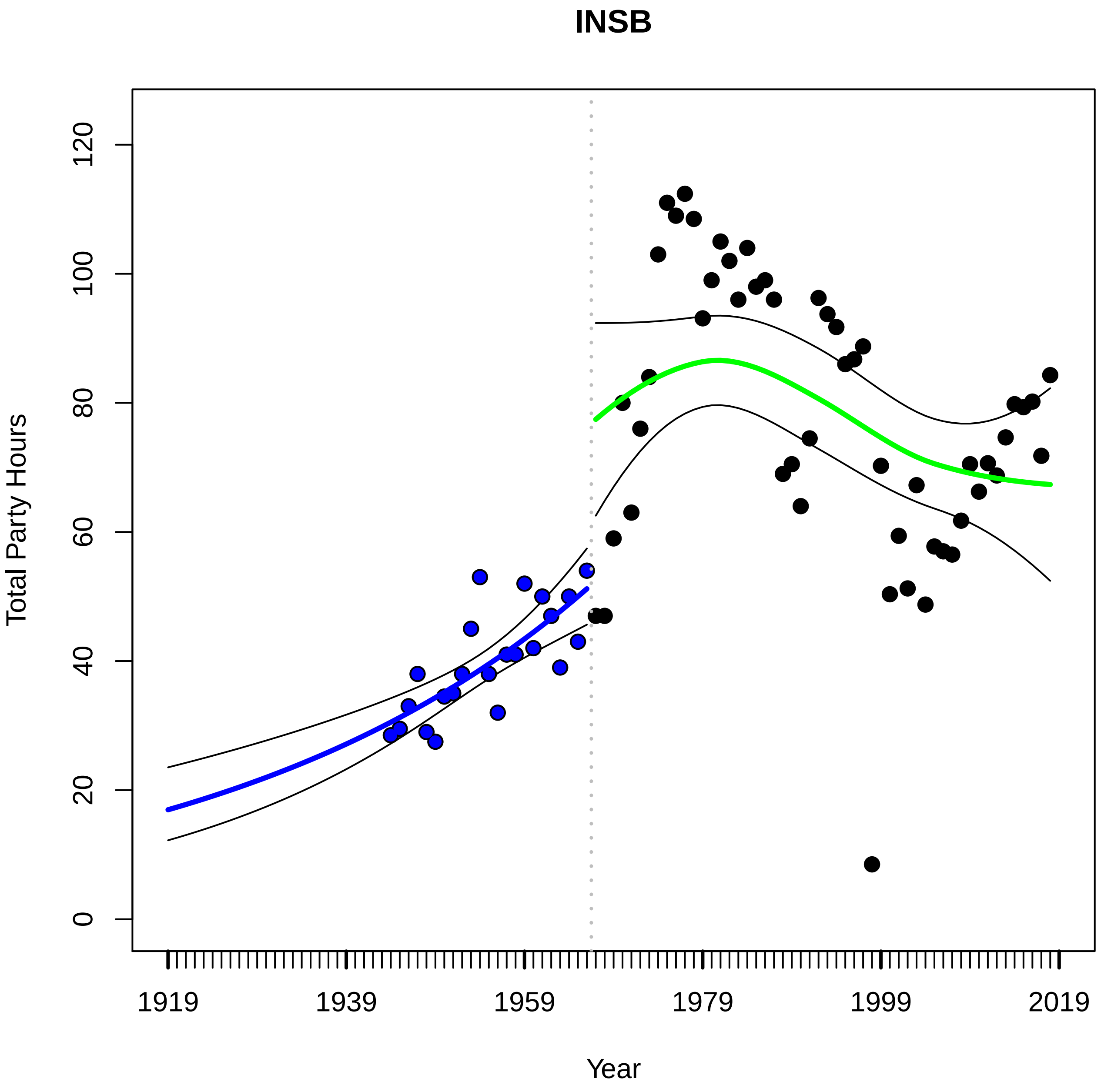

INSB
Total Party Hours
0
20
40
60
80
100
120
1919
1939
1959
1979
1999
2019
Year

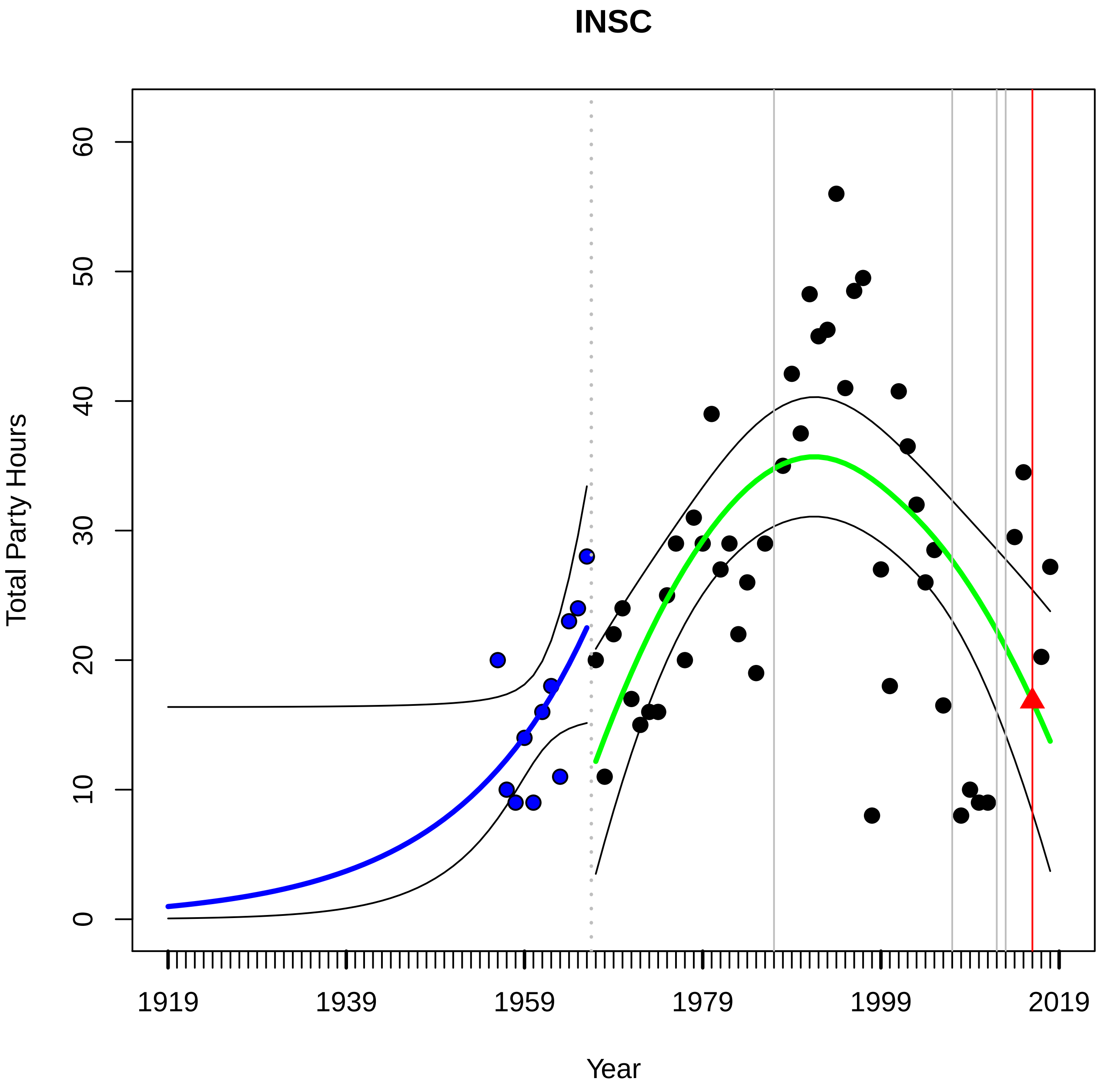

INSC
Total Party Hours
0
10
20
30
40
50
60
1919
1939
1959
1979
1999
2019
Year

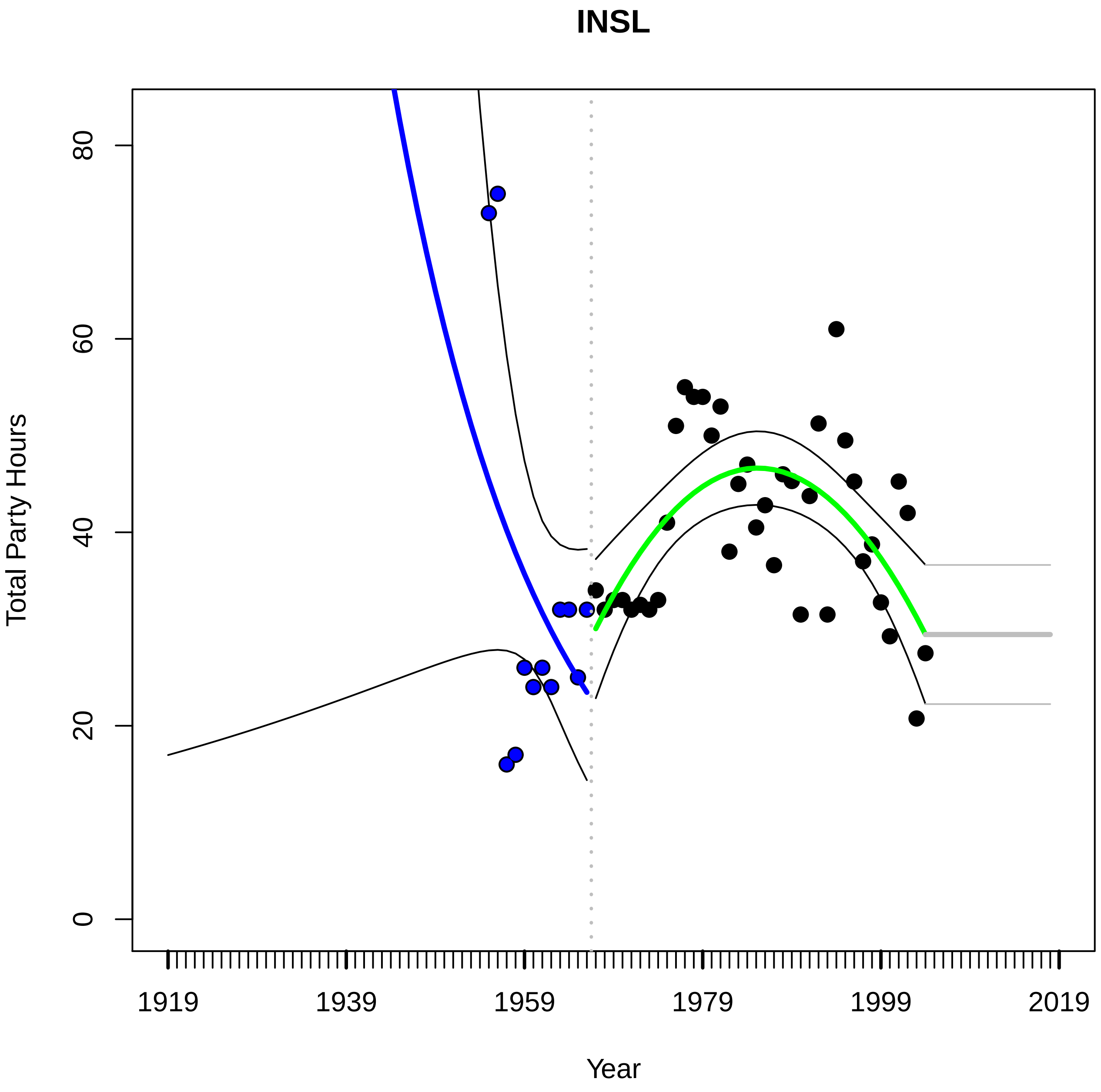

INSL
Total Party Hours
0
20
40
60
80
Year
1919
1939
1959
1979
1999
2019

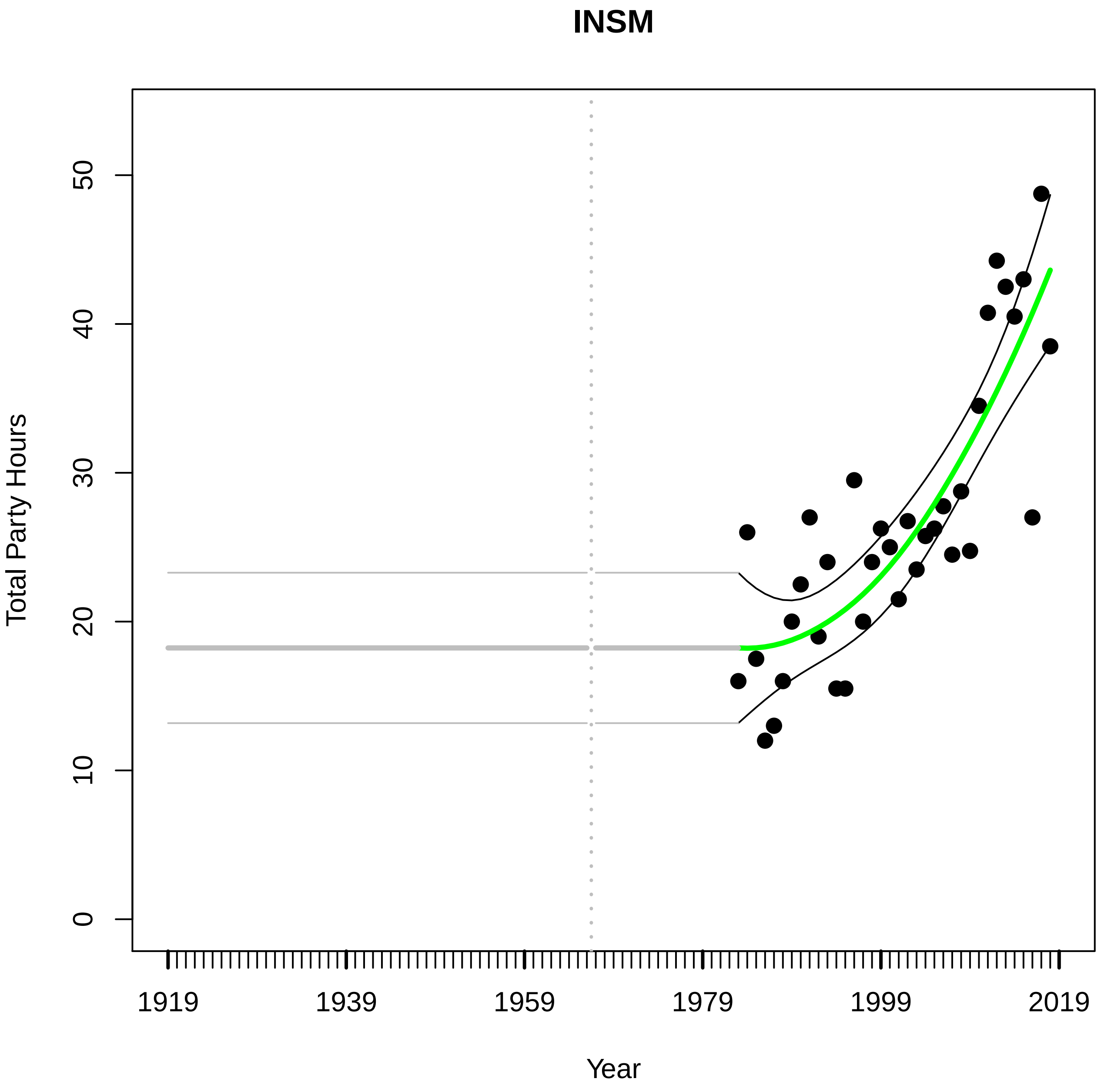

INSM
Total Party Hours
0
10
20
30
40
50
1919
1939
1959
1979
1999
2019
Year

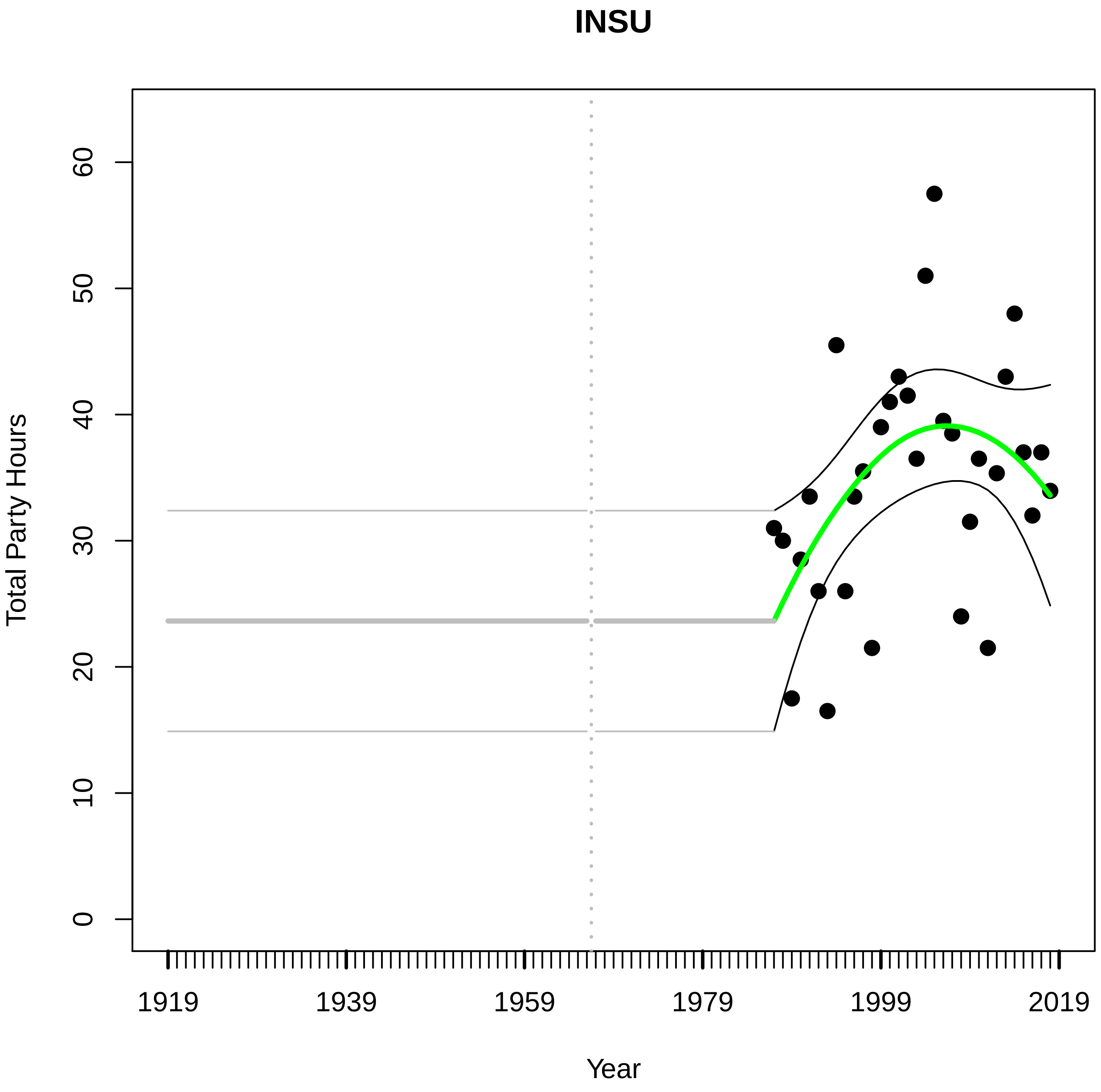
INSU
Total Party Hours
Year
0
10
20
30
40
50
60
1919
1939
1959
1979
1999
2019

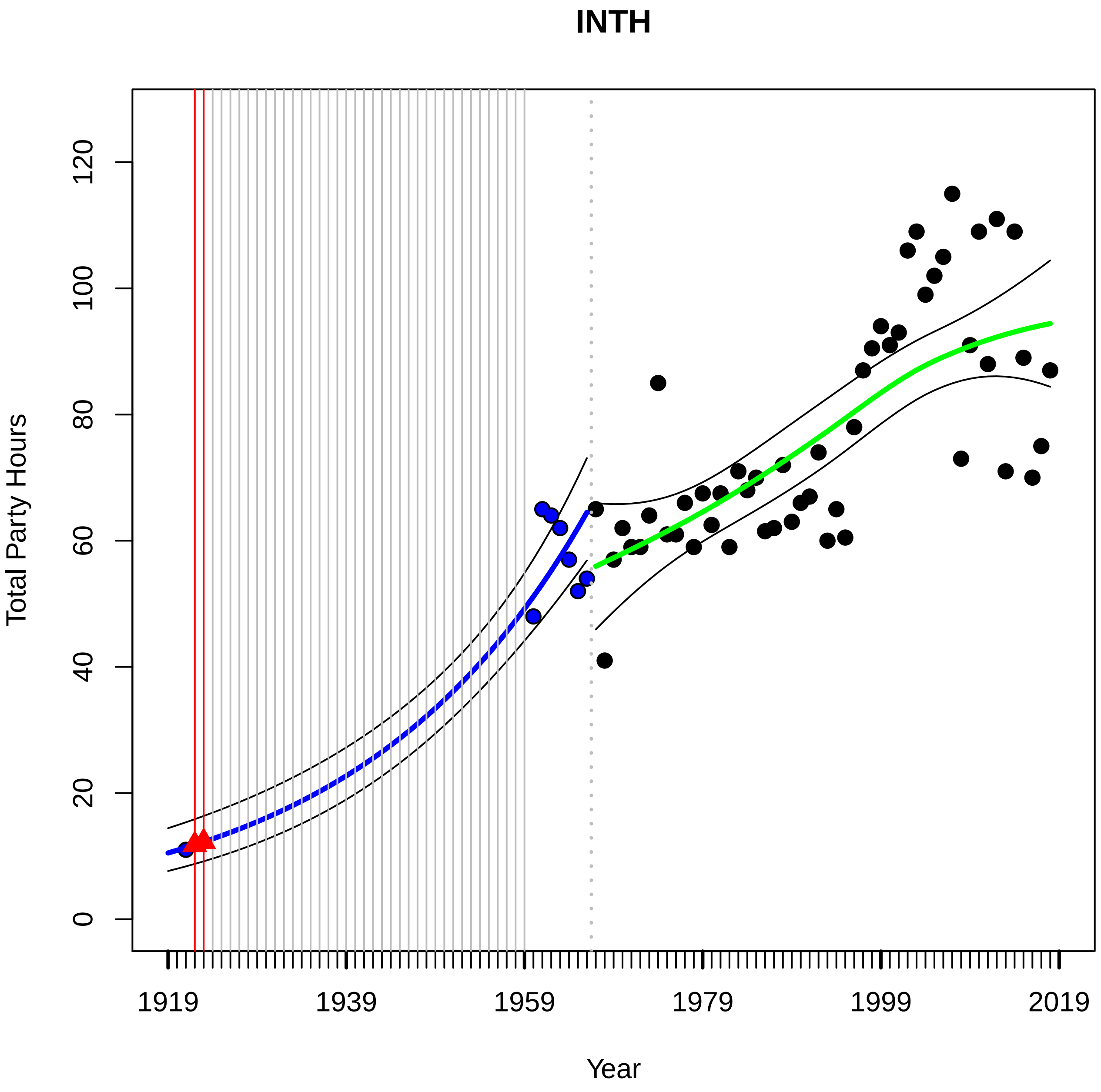

INTH
Total Party Hours
0
20
40
60
80
100
120
1919
1939
1959
1979
1999
2019
Year

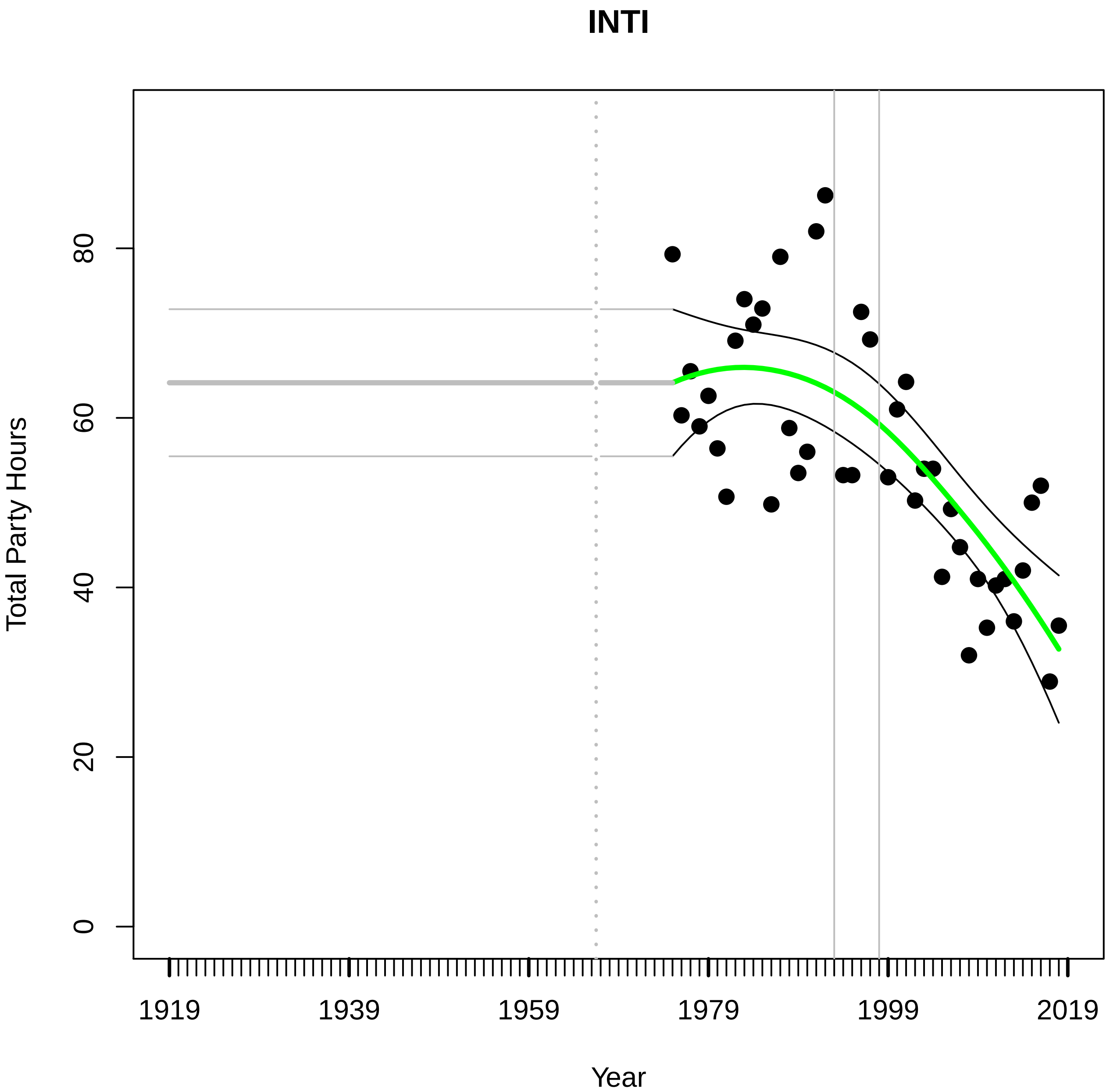
INTI
Total Party Hours
Year
0
20
40
60
80
1919
1939
1959
1979
1999
2019

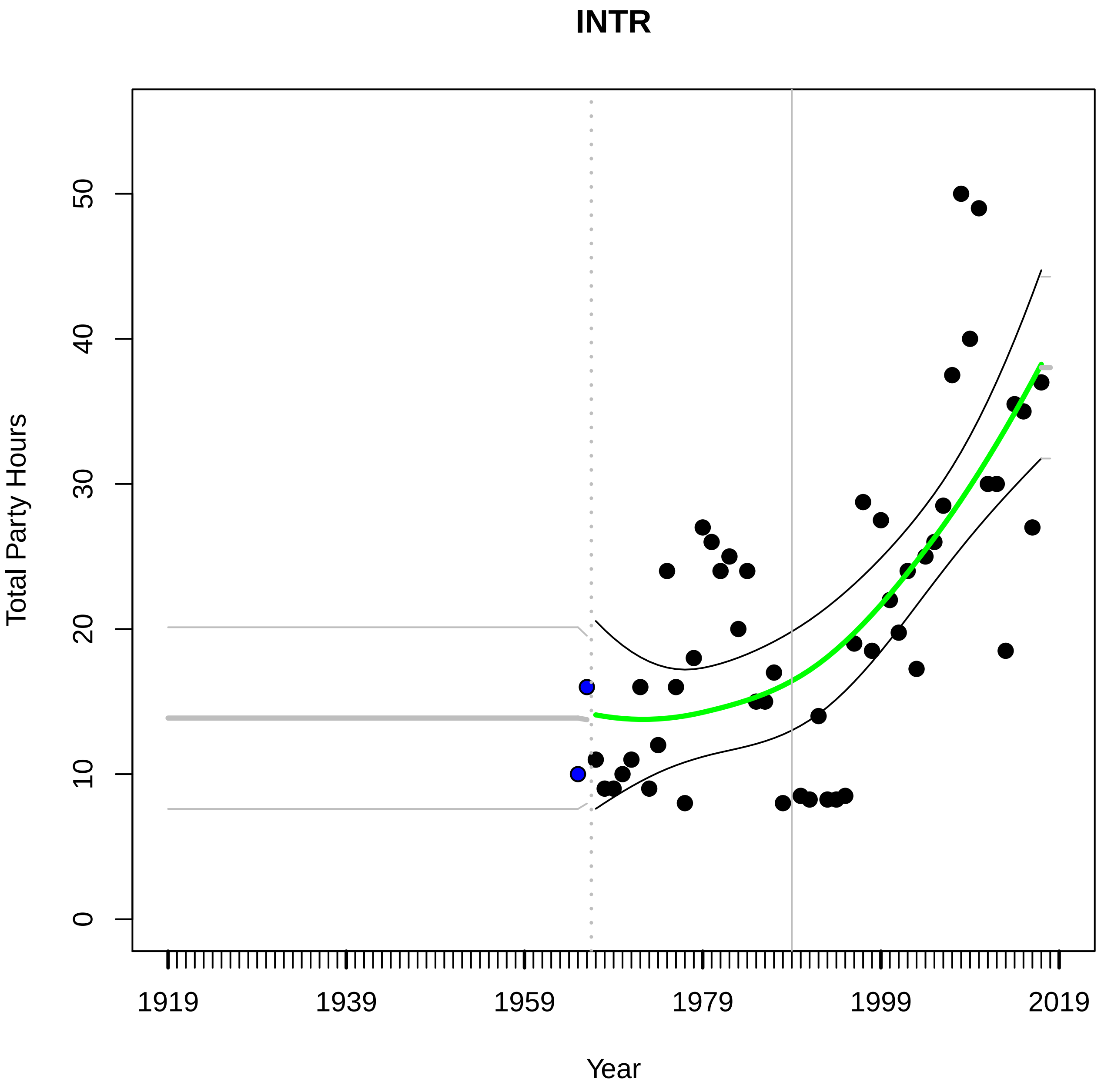
INTR
Total Party Hours
0
10
20
30
40
50
1919
1939
1959
1979
1999
2019
Year

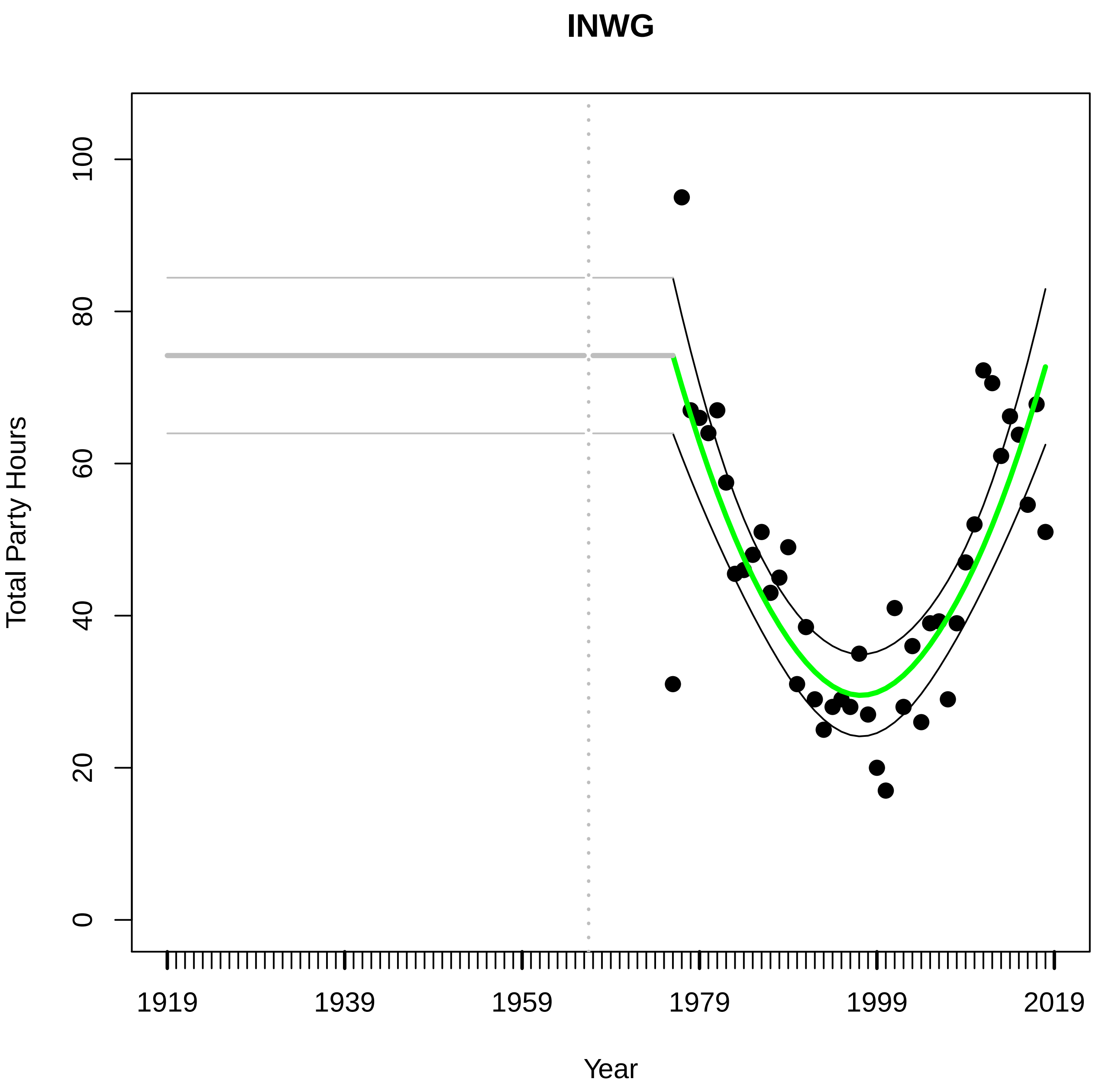

INWG
Total Party Hours
0
20
40
60
80
100
1919
1939
1959
1979
1999
2019
Year

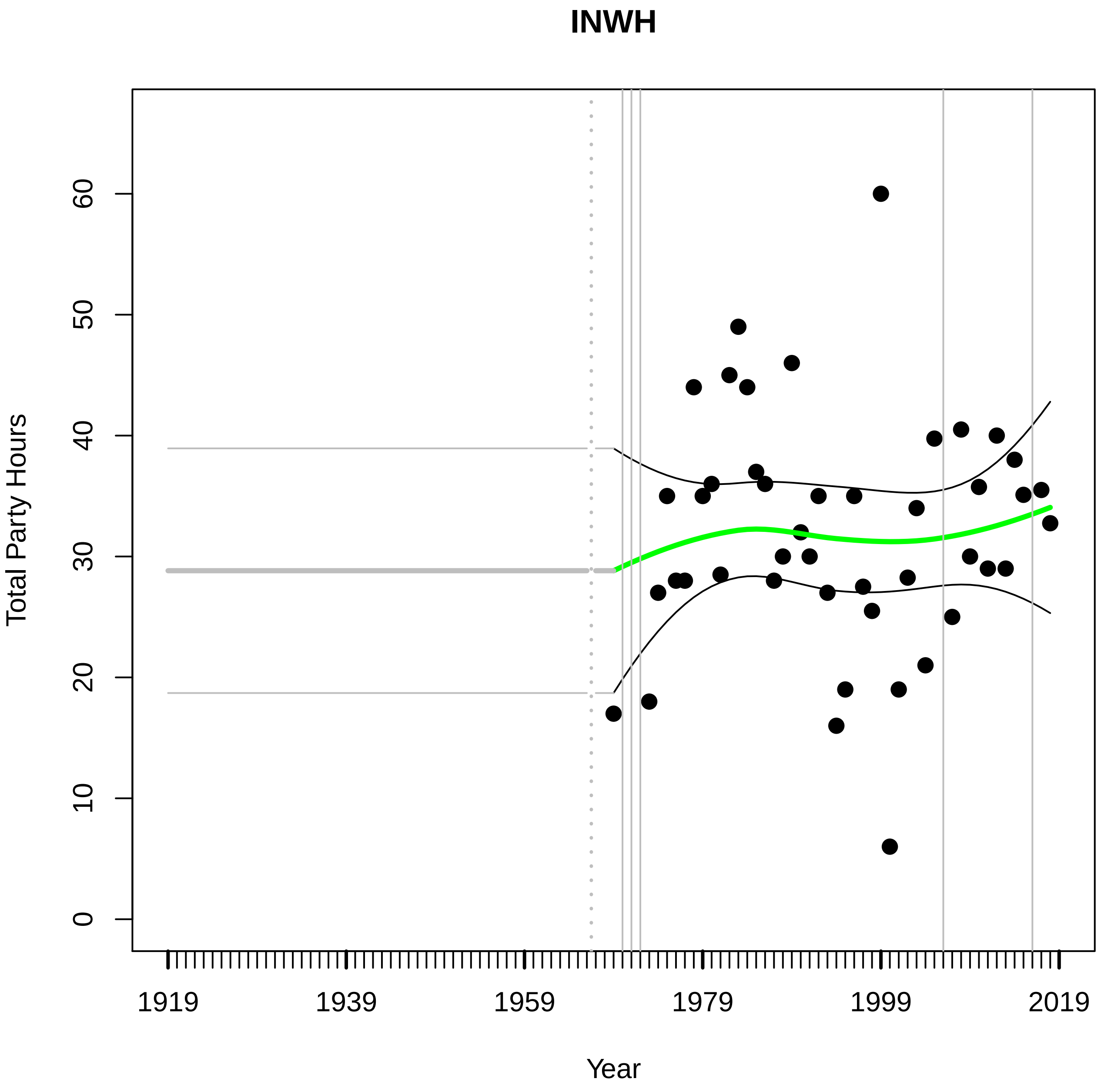

INWH
Total Party Hours
0
10
20
30
40
50
60
1919
1939
1959
1979
1999
2019
Year

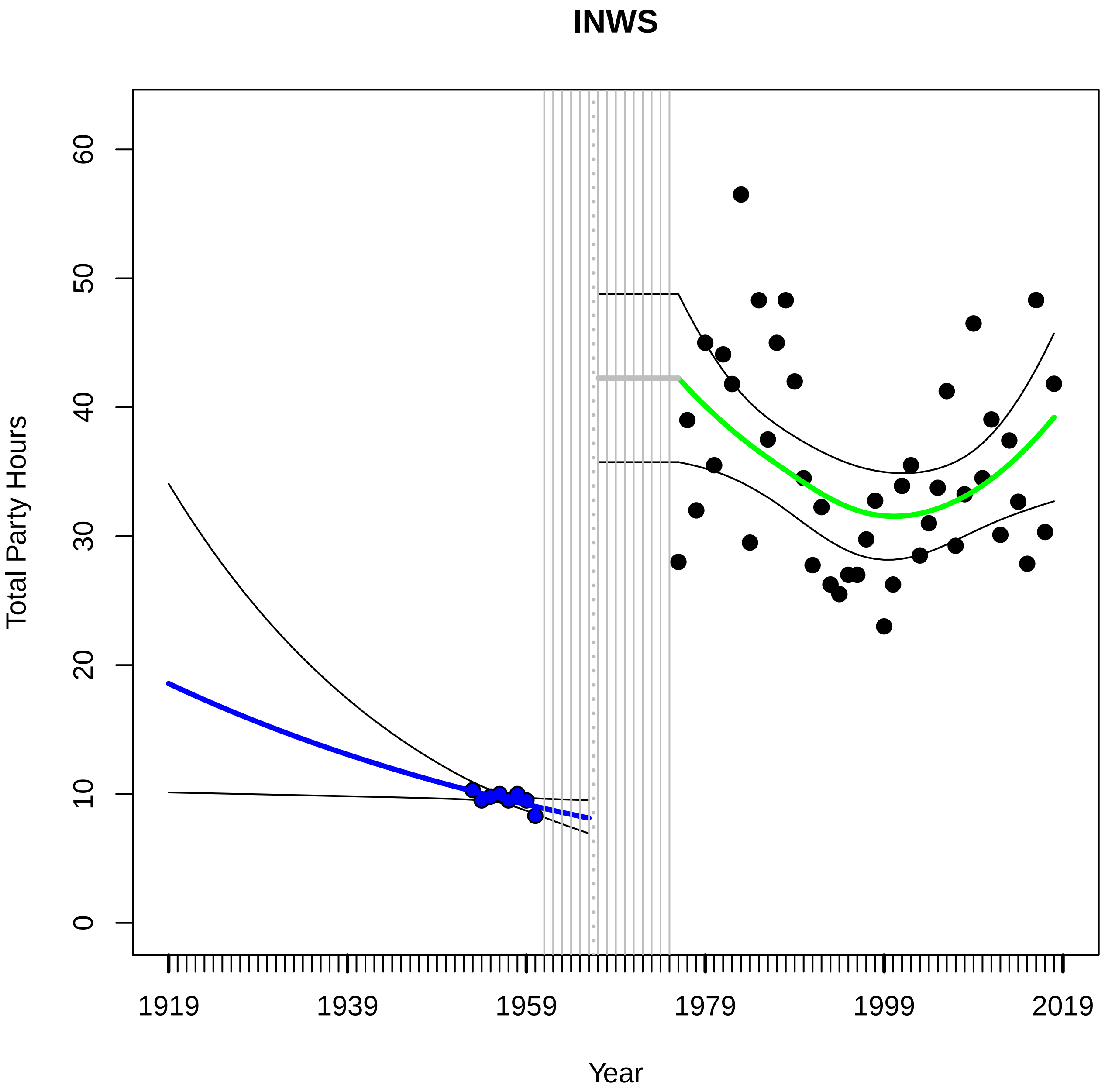

INWS
Total Party Hours
0
10
20
30
40
50
60
Year
1919
1939
1959
1979
1999
2019

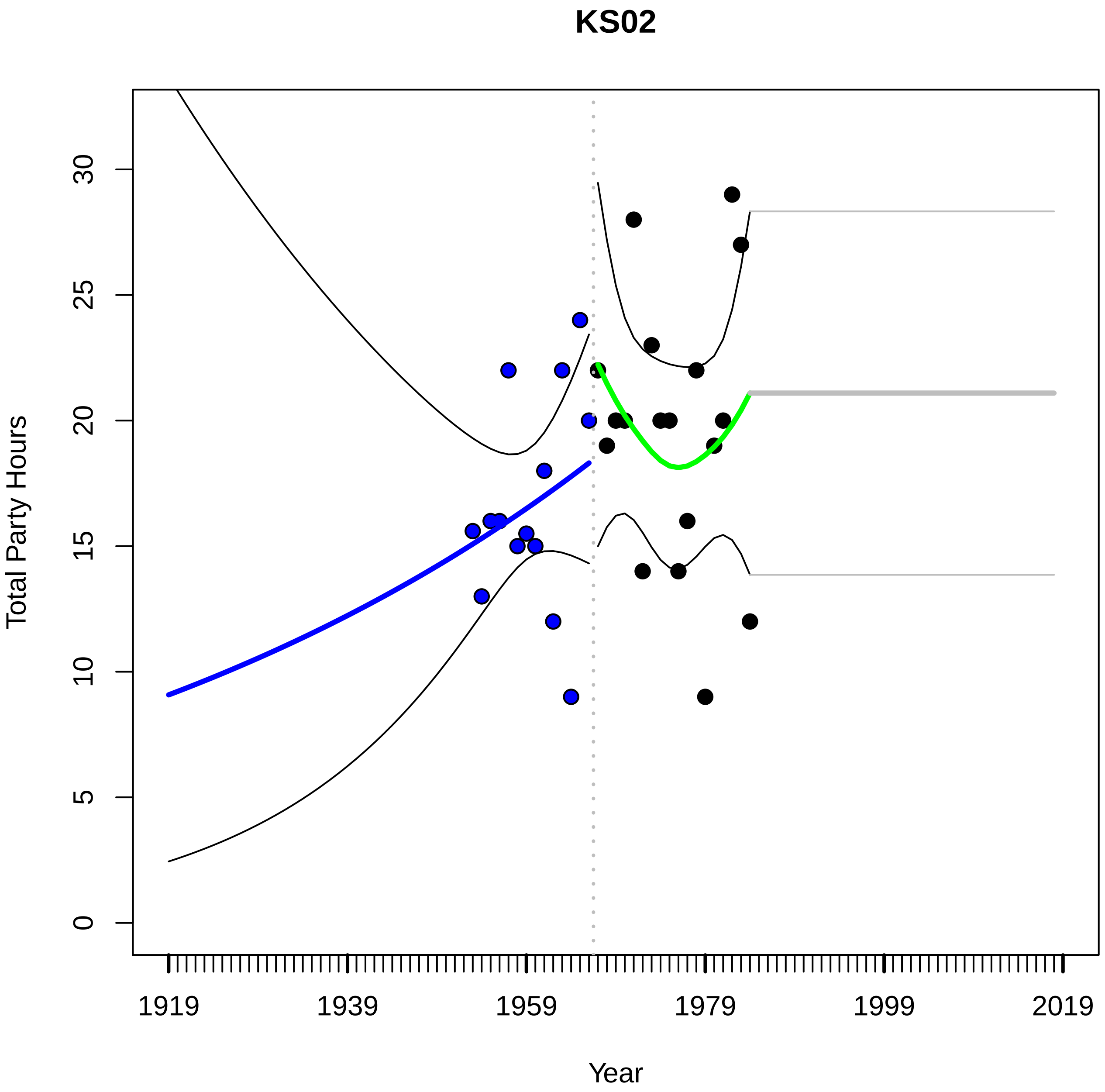

KS02
Total Party Hours
0
5
10
15
20
25
30
Year
1919
1939
1959
1979
1999
2019

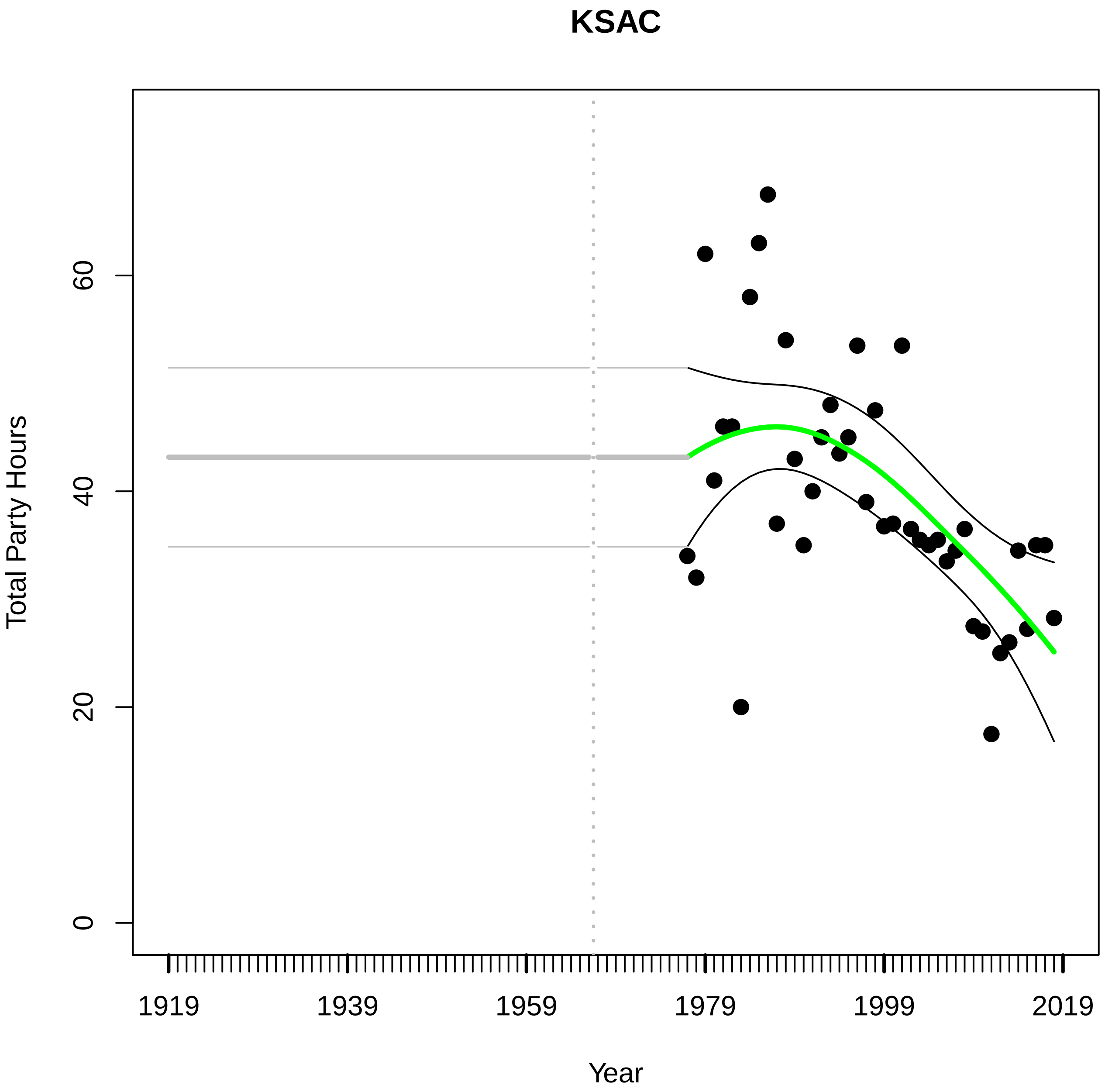
KSAC
Total Party Hours
0
20
40
60
1919
1939
1959
1979
1999
2019
Year

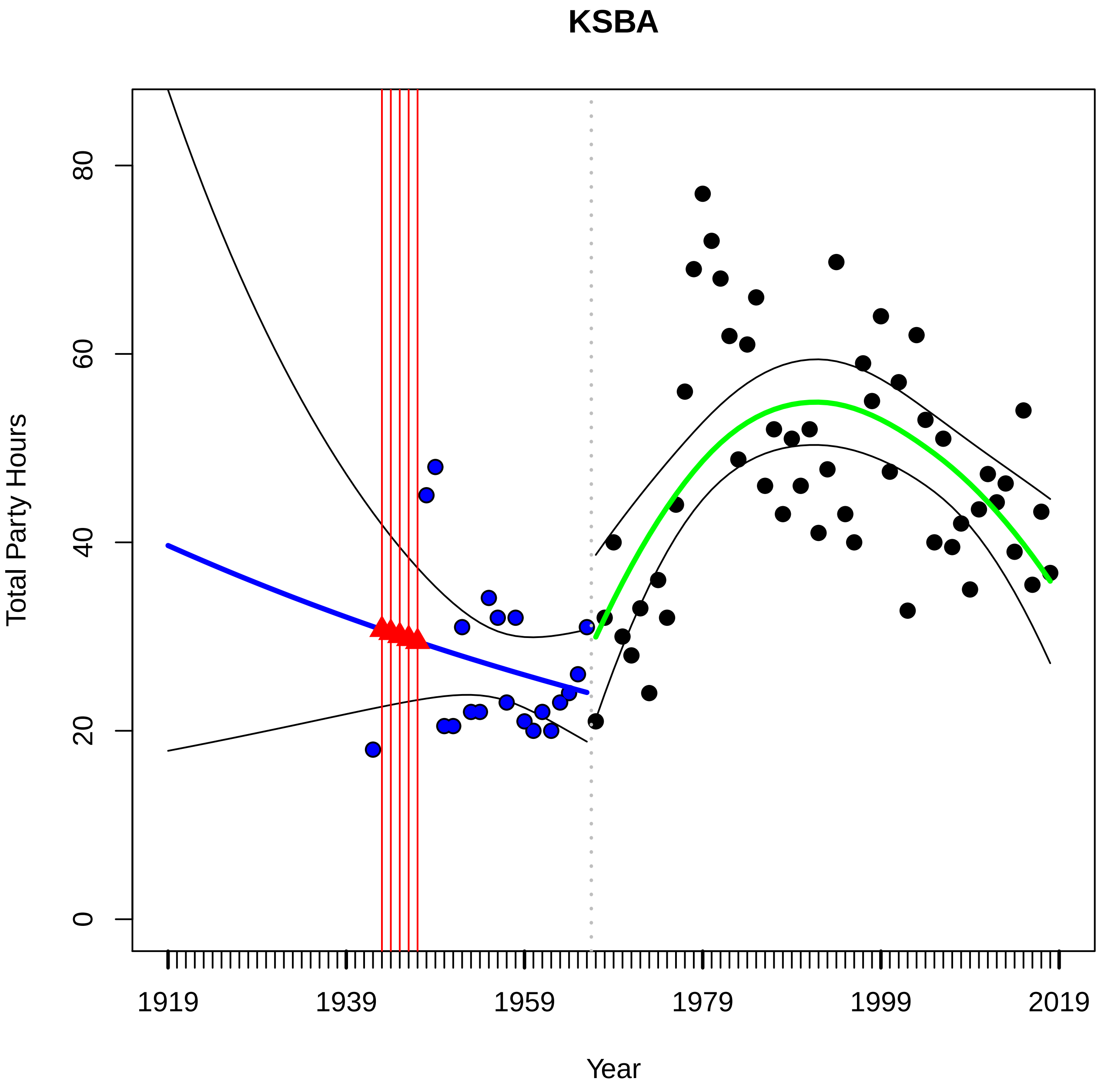

KSBA
Total Party Hours
0
20
40
60
80
1919
1939
1959
1979
1999
2019
Year

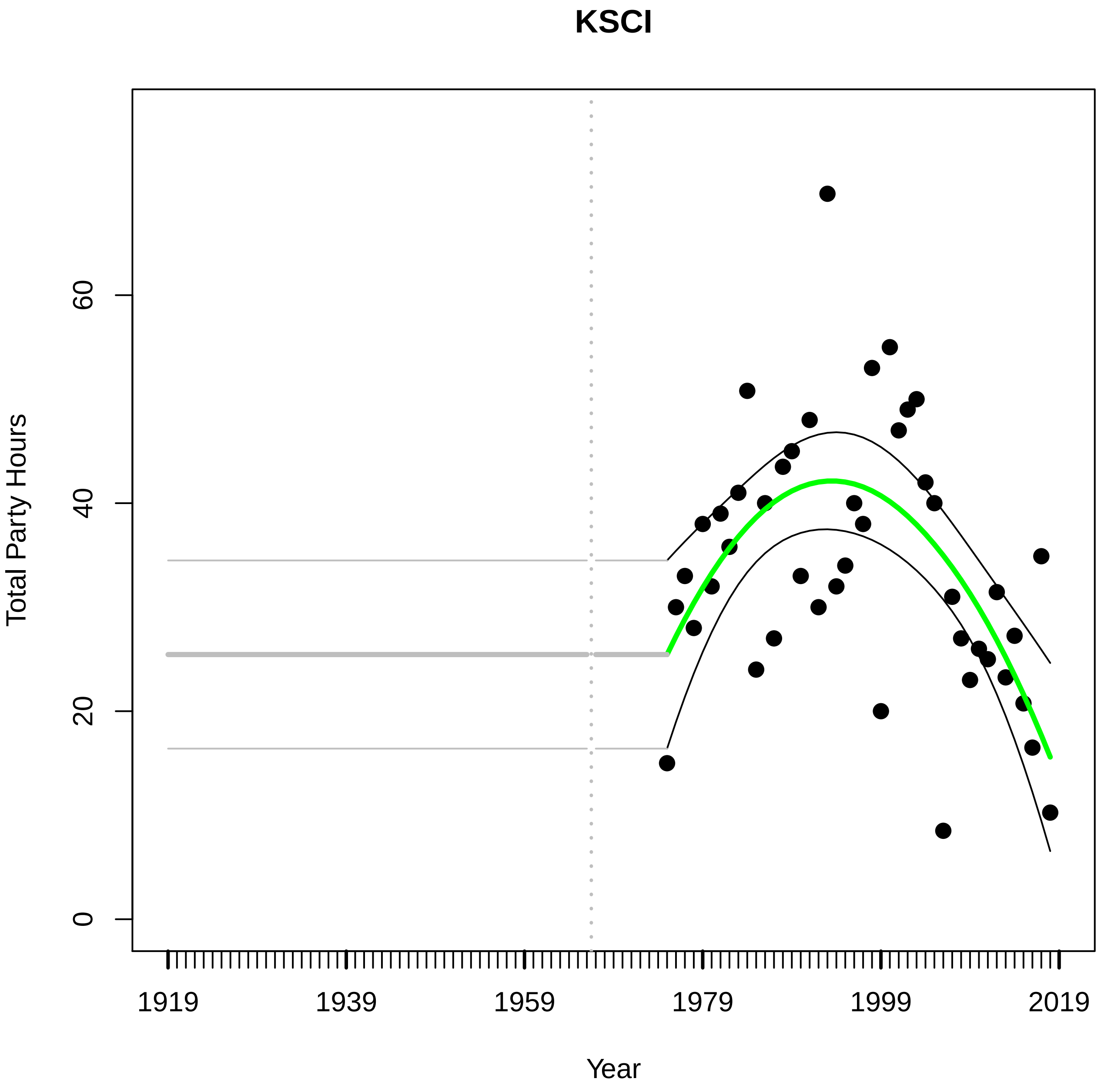

KSCI
Total Party Hours
0
20
40
60
1919
1939
1959
1979
1999
2019
Year

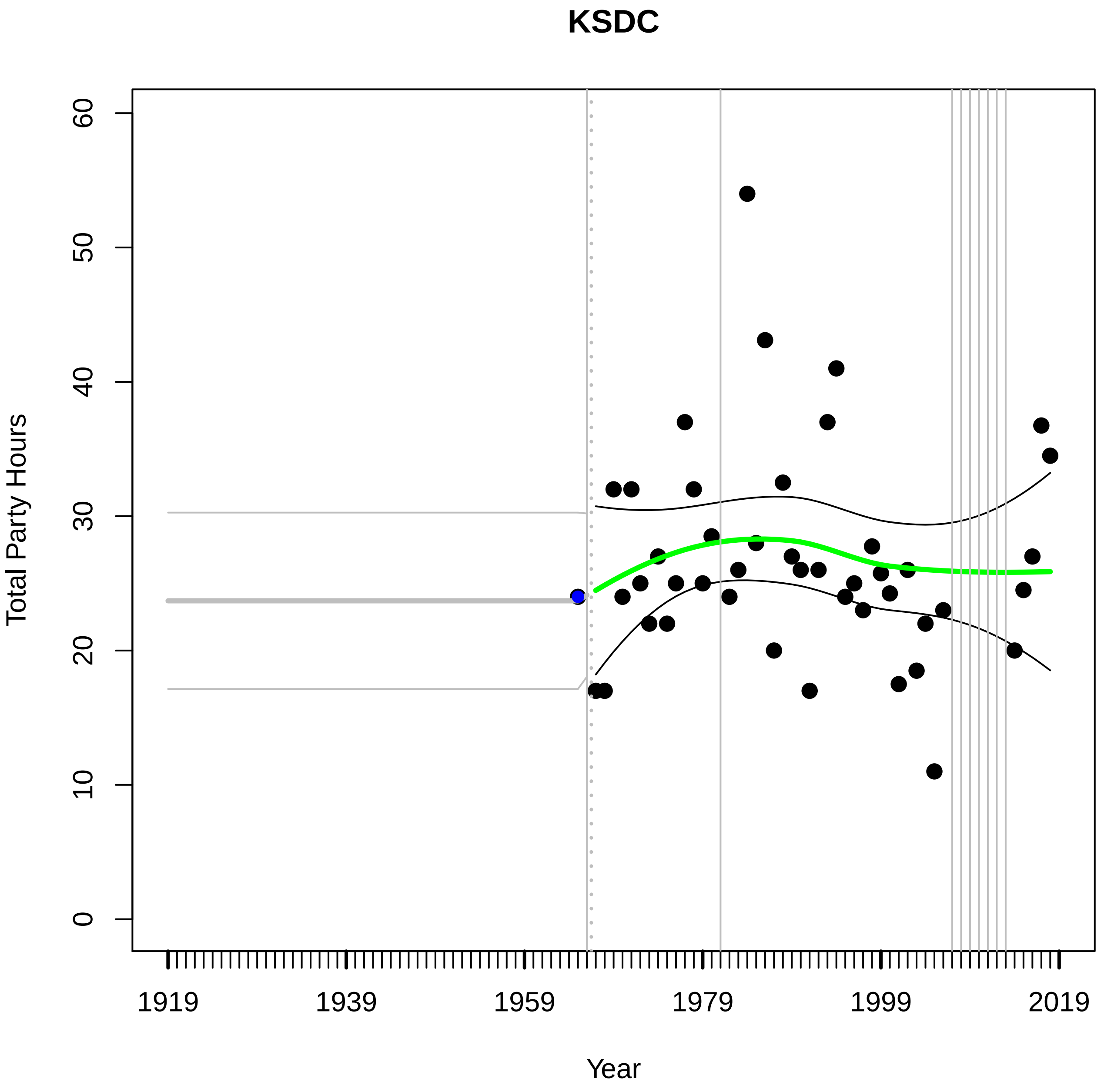
KSDC
Total Party Hours
0
10
20
30
40
50
60
1919
1939
1959
1979
1999
2019
Year

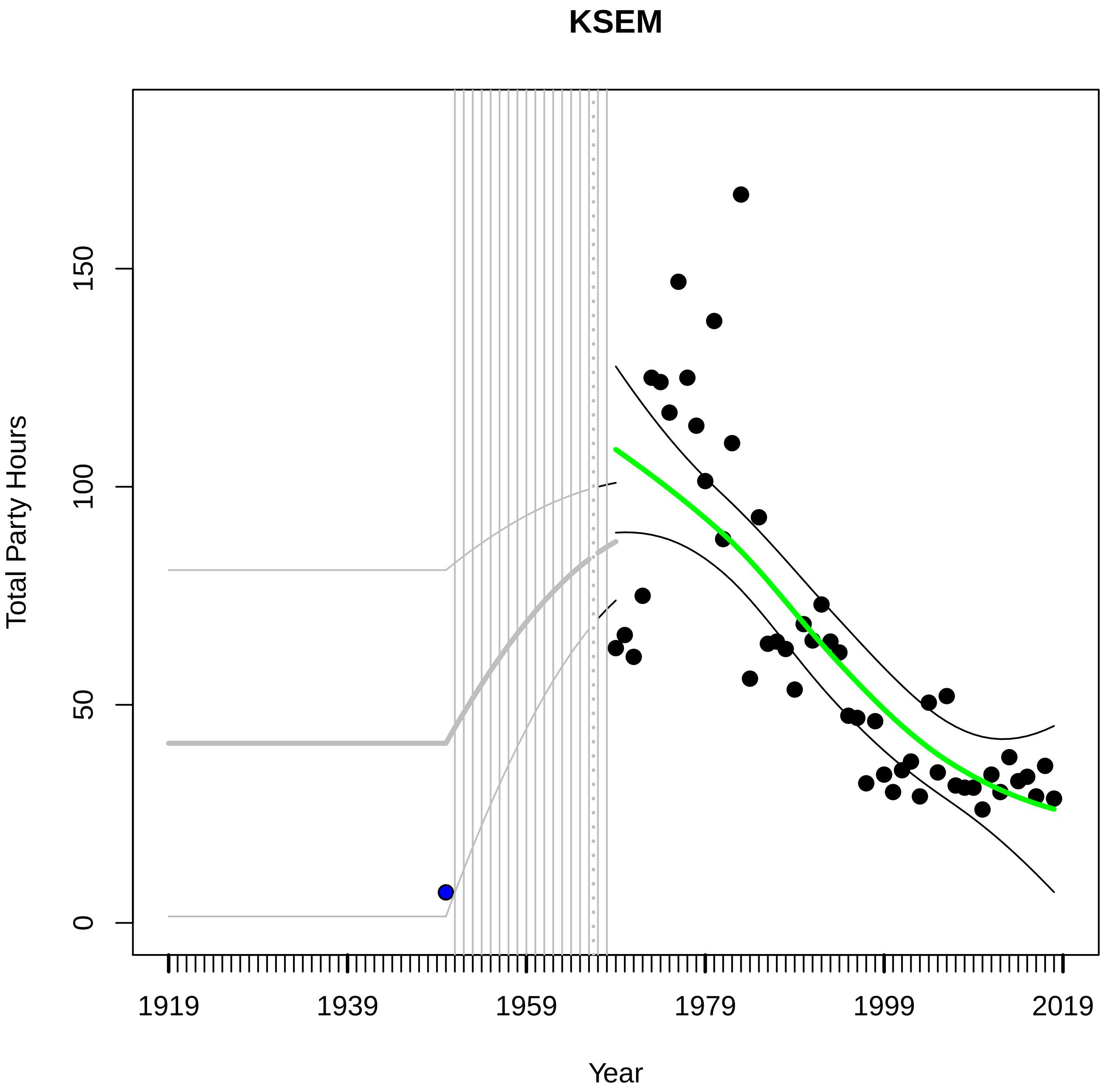
KSEM
Total Party Hours
0
50
100
150
1919
1939
1959
1979
1999
2019
Year

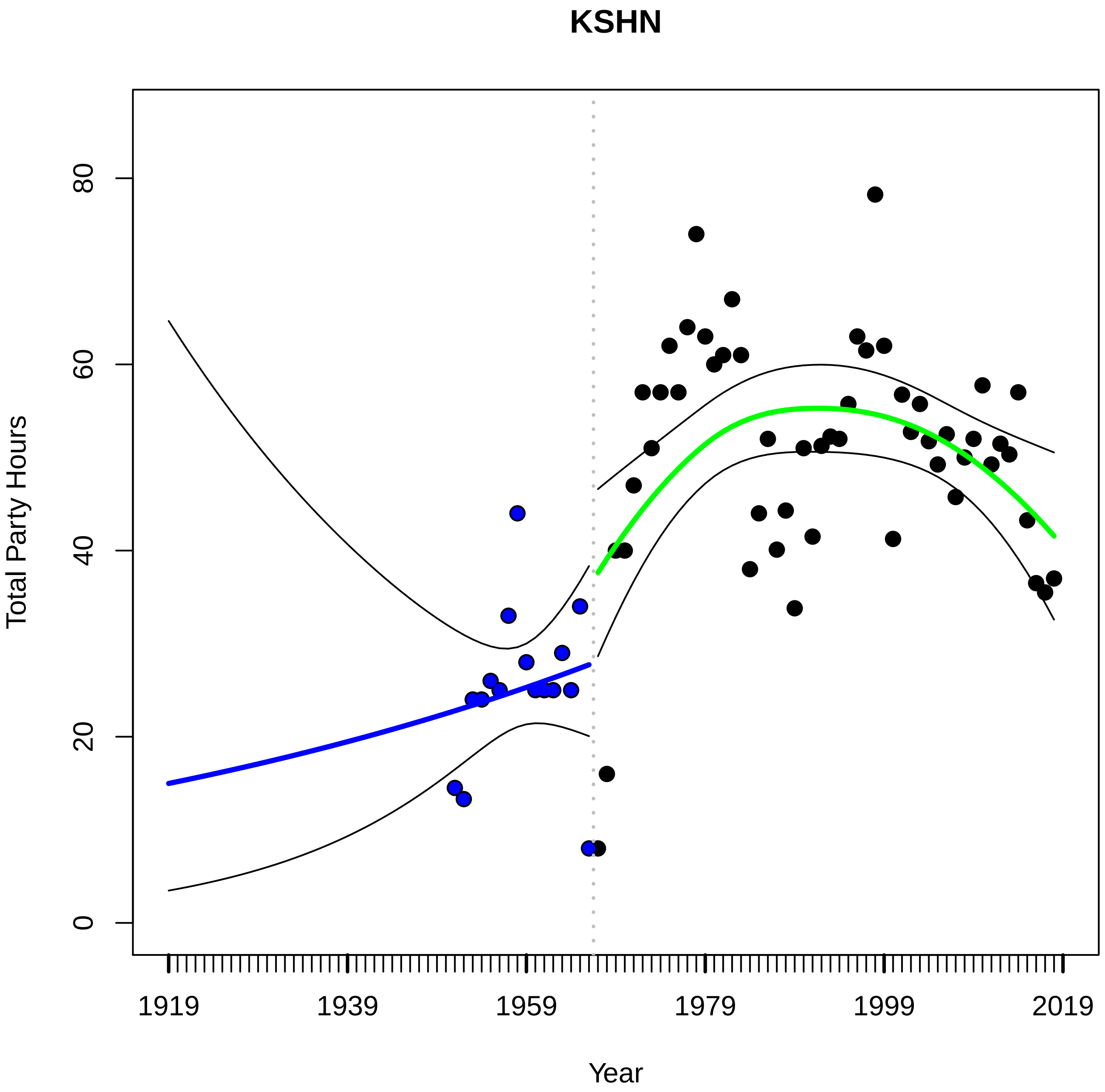

KSHN
Total Party Hours
0
20
40
60
80
1919
1939
1959
1979
1999
2019
Year

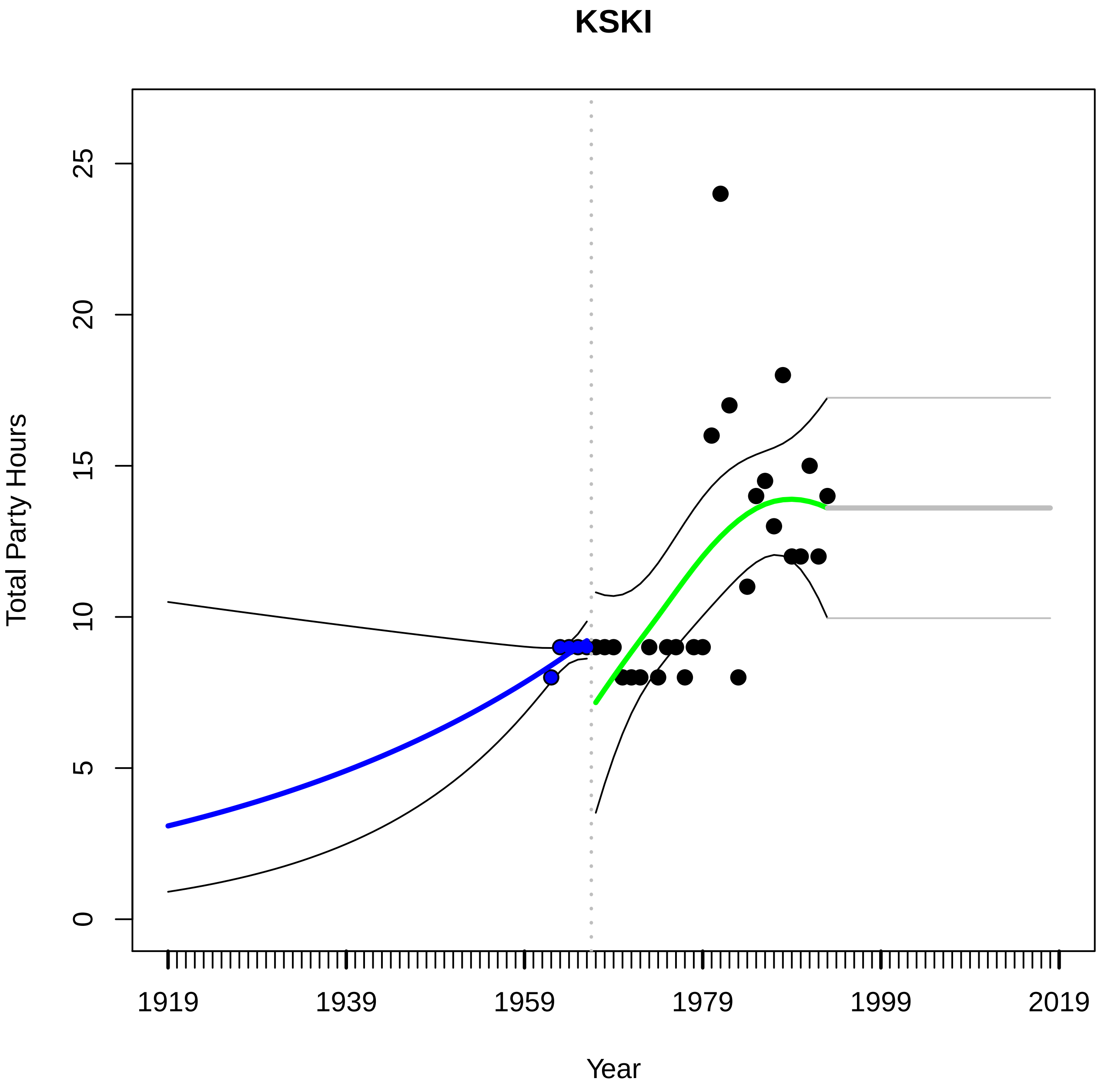

KSKI
Total Party Hours
Year
0
5
10
15
20
25
1919
1939
1959
1979
1999
2019

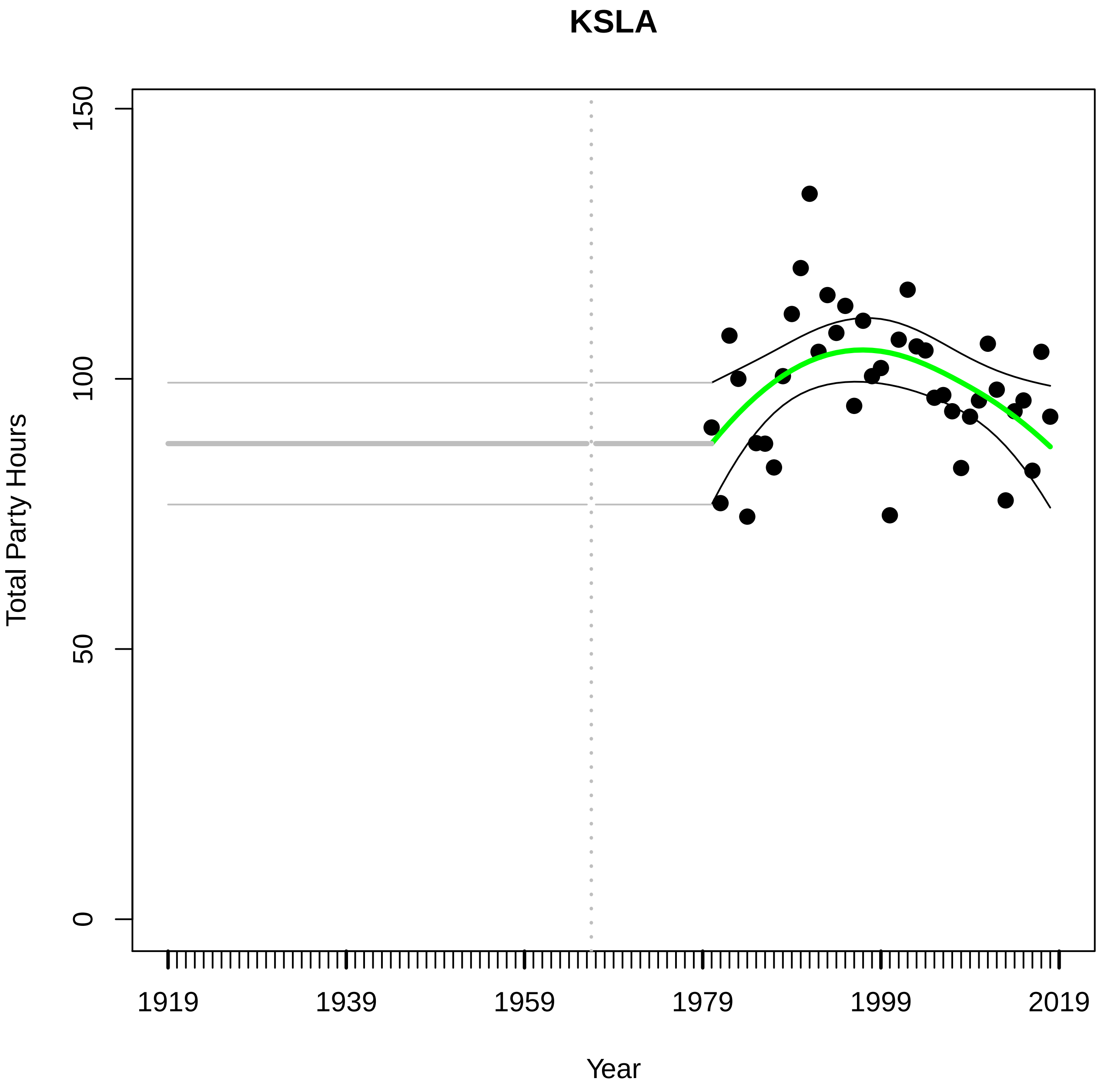

KSLA
Total Party Hours
0
50
100
150
1919
1939
1959
1979
1999
2019
Year

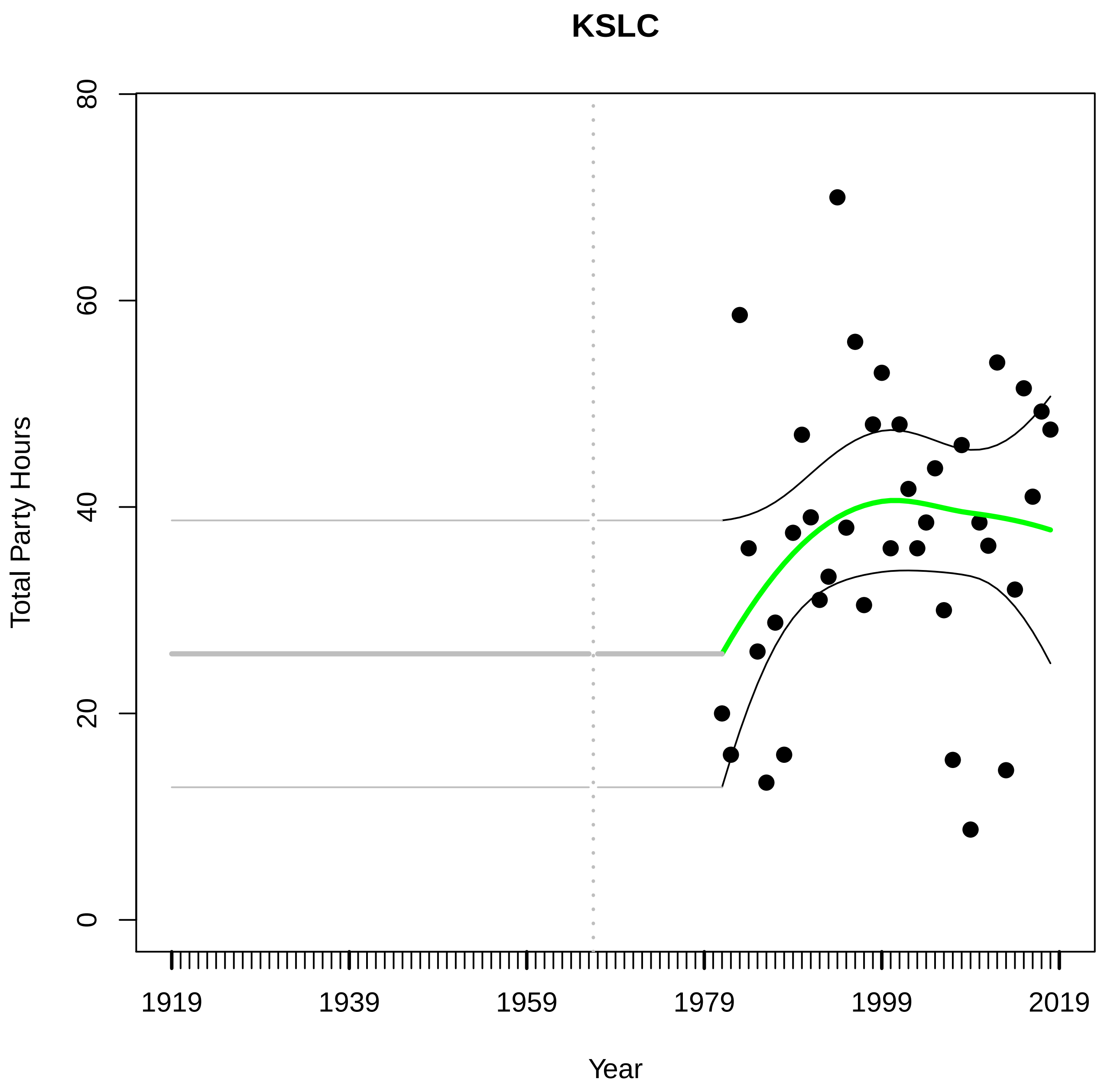
KSLC
Total Party Hours
0
20
40
60
80
1919
1939
1959
1979
1999
2019
Year

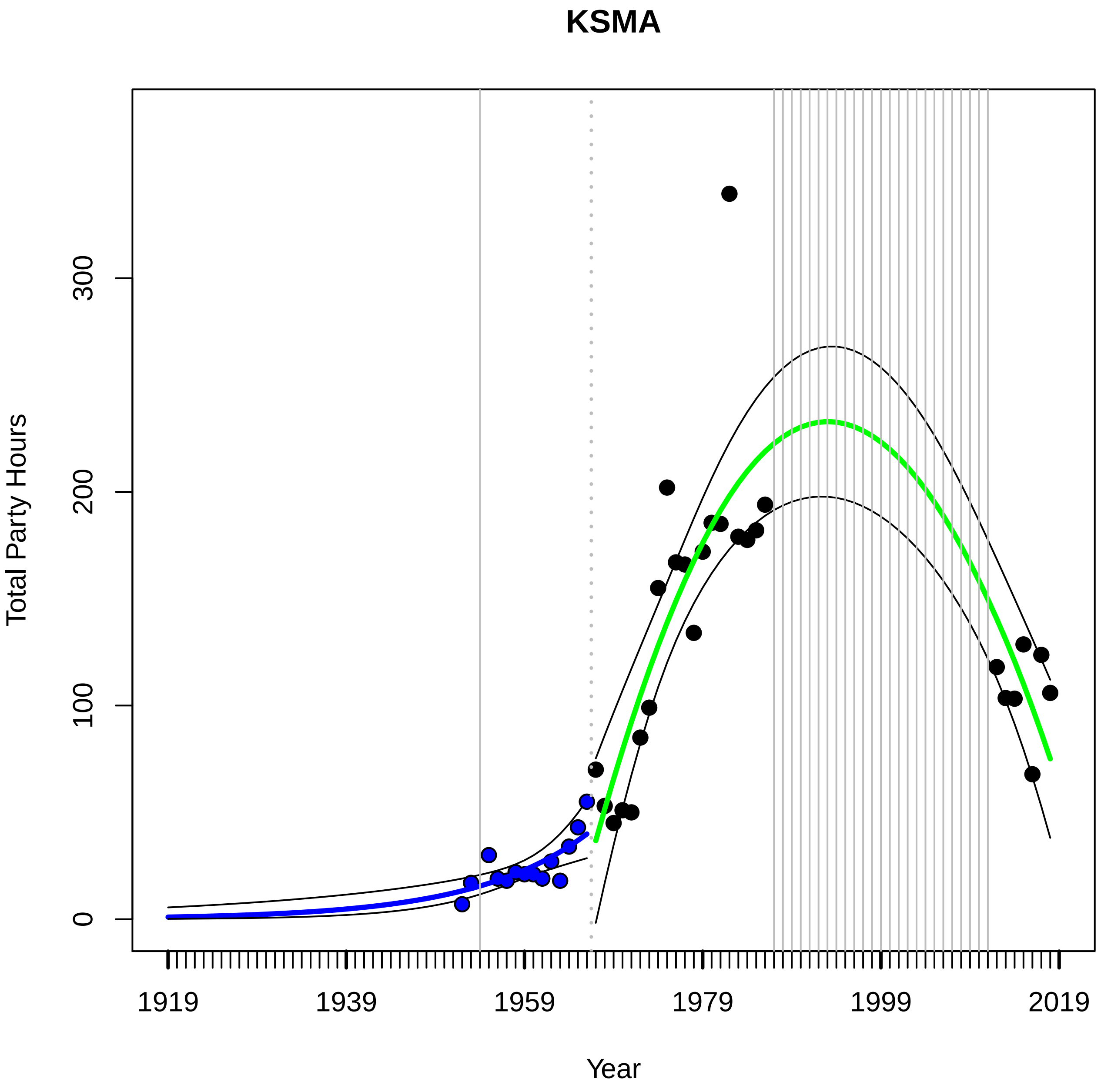

KSMA
Total Party Hours
0
100
200
300
1919
1939
1959
1979
1999
2019
Year

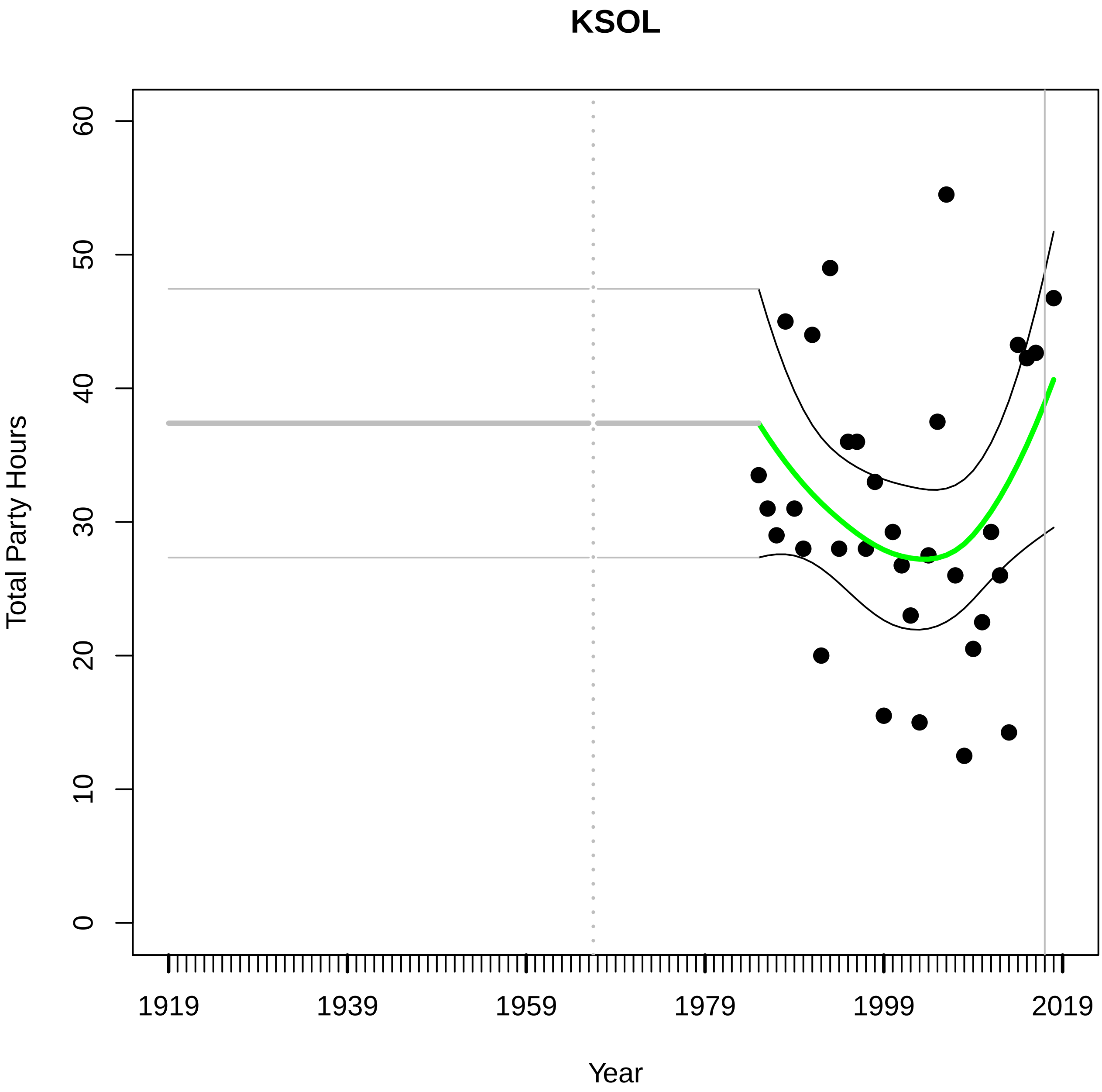

KSOL
Total Party Hours
0
10
20
30
40
50
60
1919
1939
1959
1979
1999
2019
Year

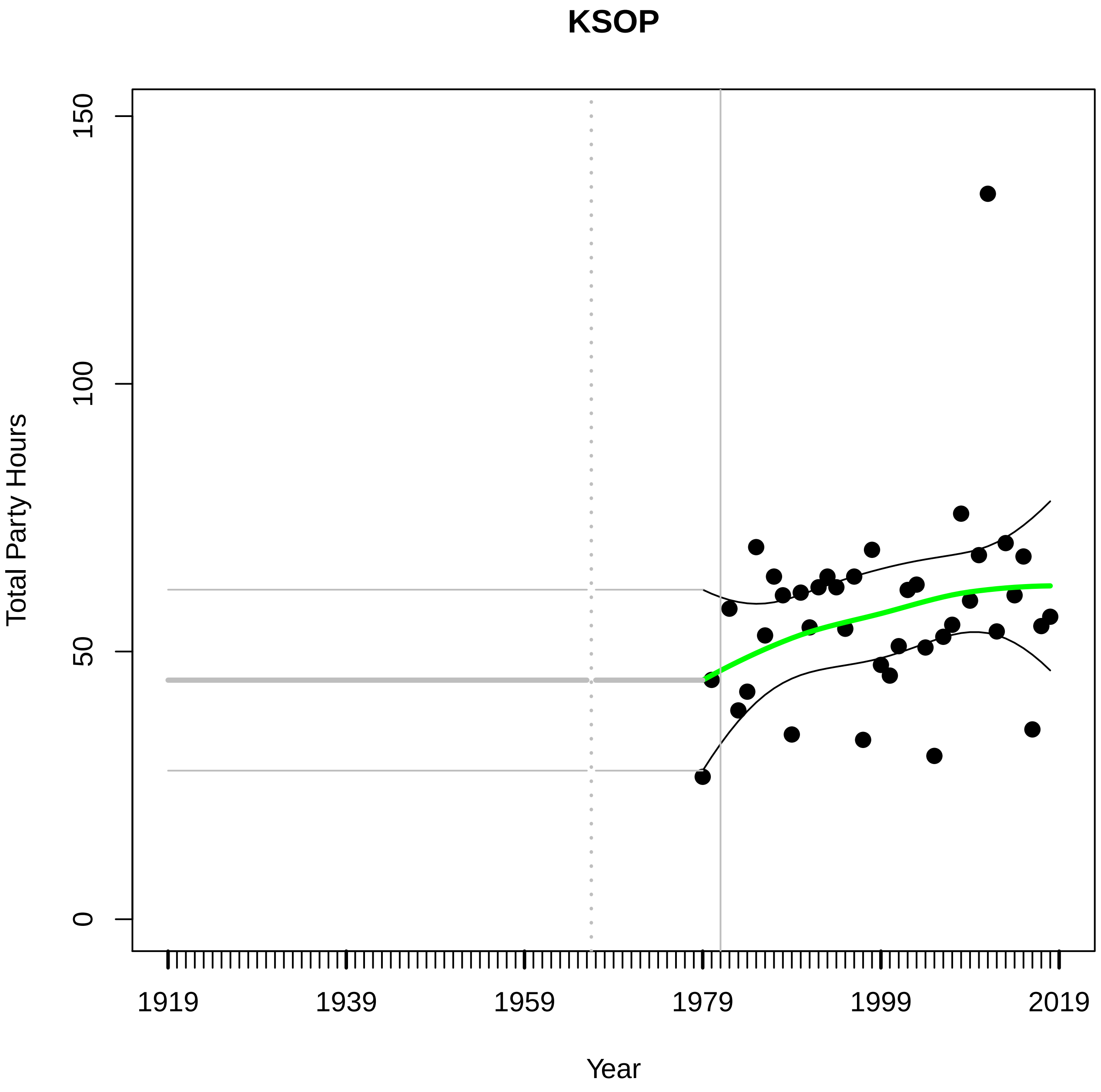
KSOP
Total Party Hours
0
50
100
150
1919
1939
1959
1979
1999
2019
Year

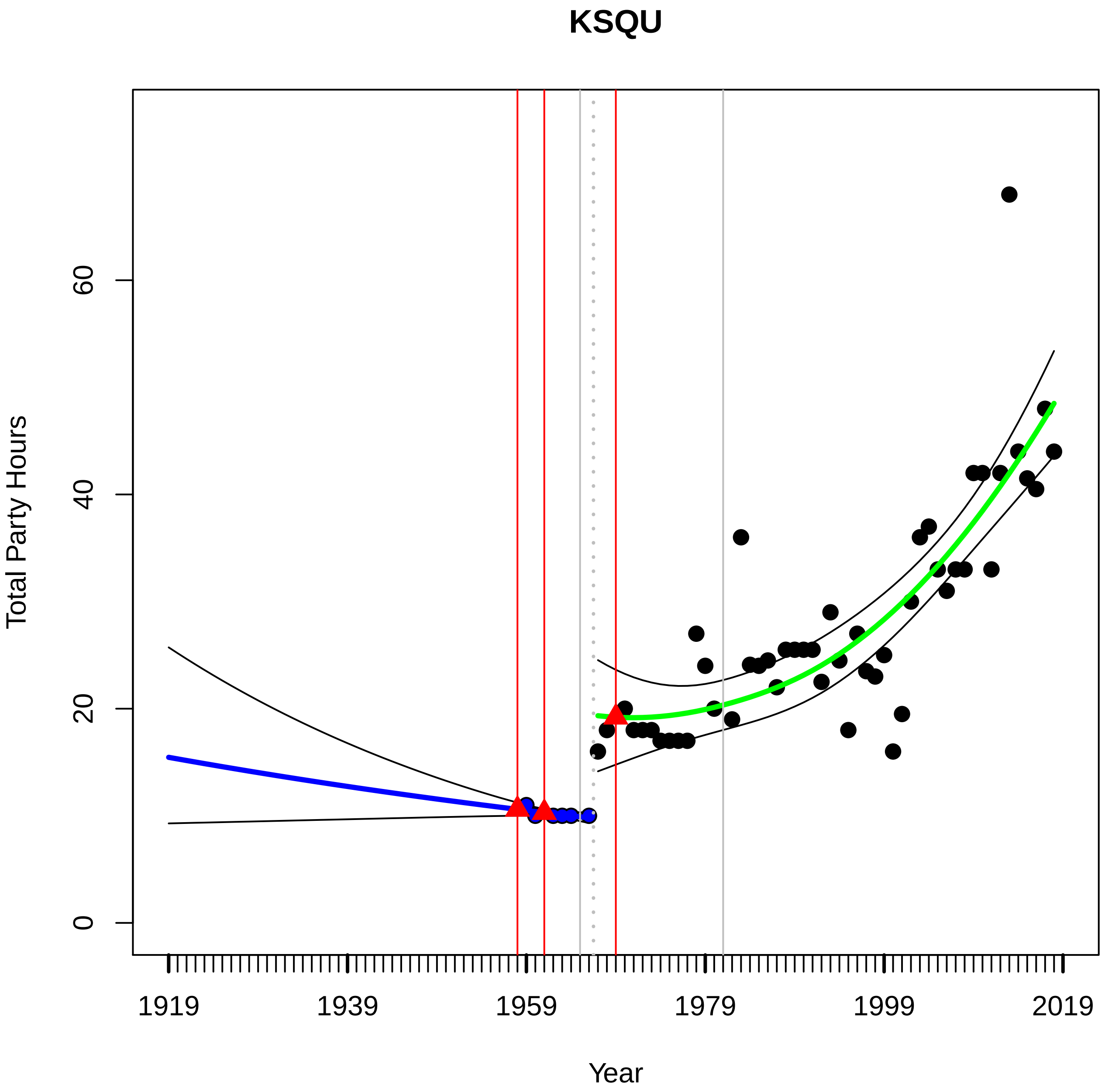

KSQU
Total Party Hours
0
20
40
60
1919
1939
1959
1979
1999
2019
Year

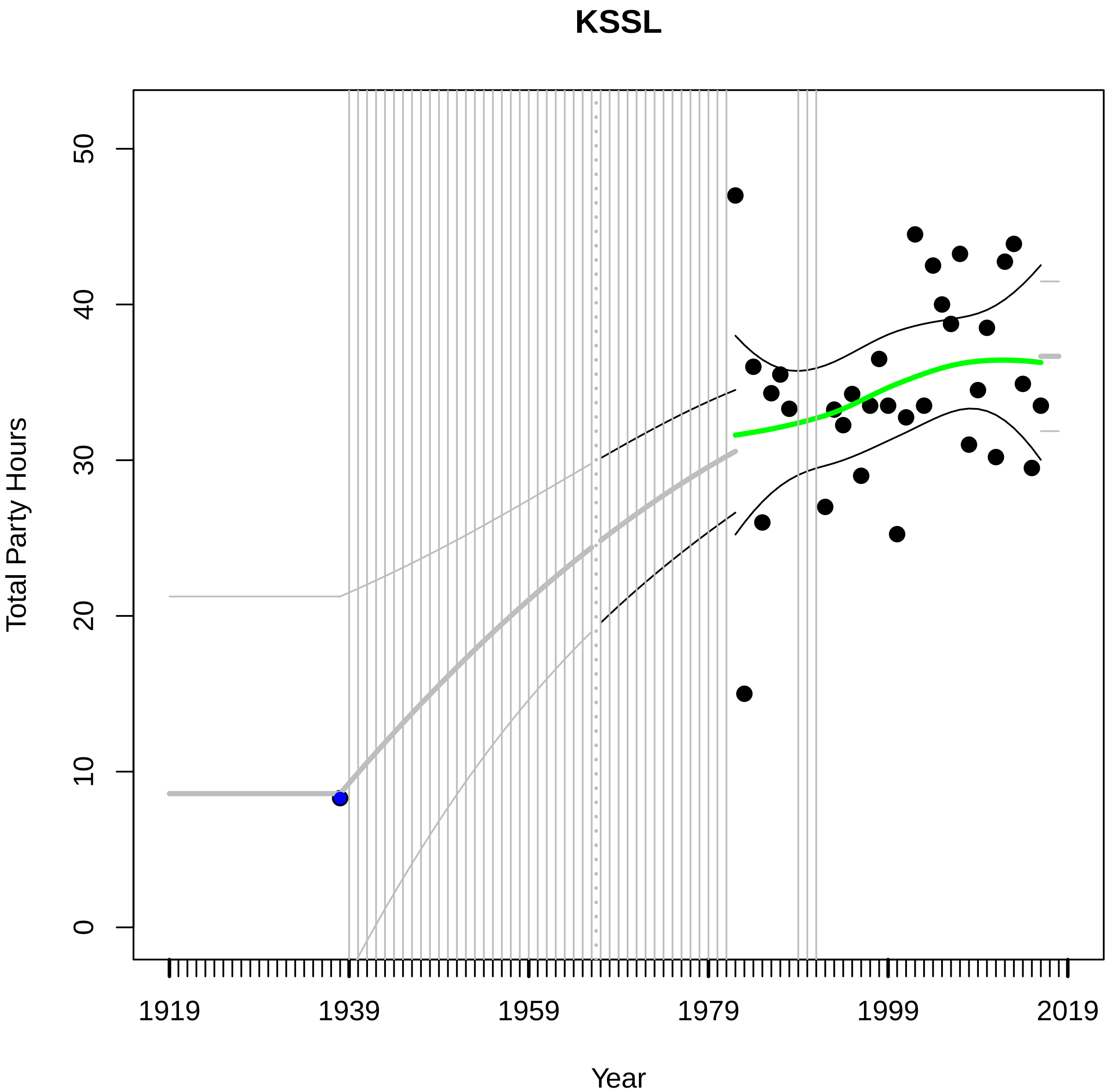

KSSL
Total Party Hours
Year
0
10
20
30
40
50
1919
1939
1959
1979
1999
2019

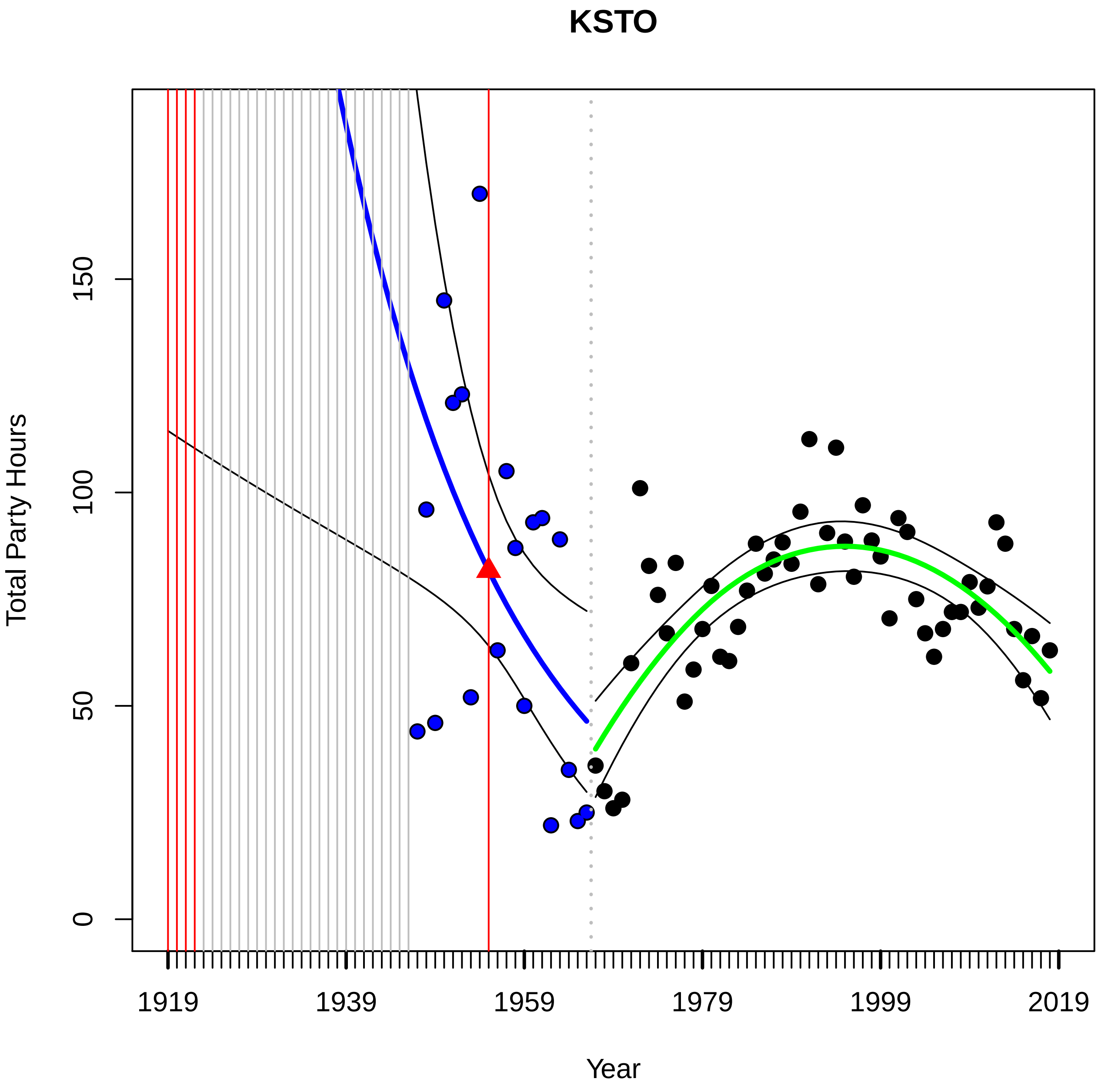

KSTO
Total Party Hours
0
50
100
150
Year
1919
1939
1959
1979
1999
2019

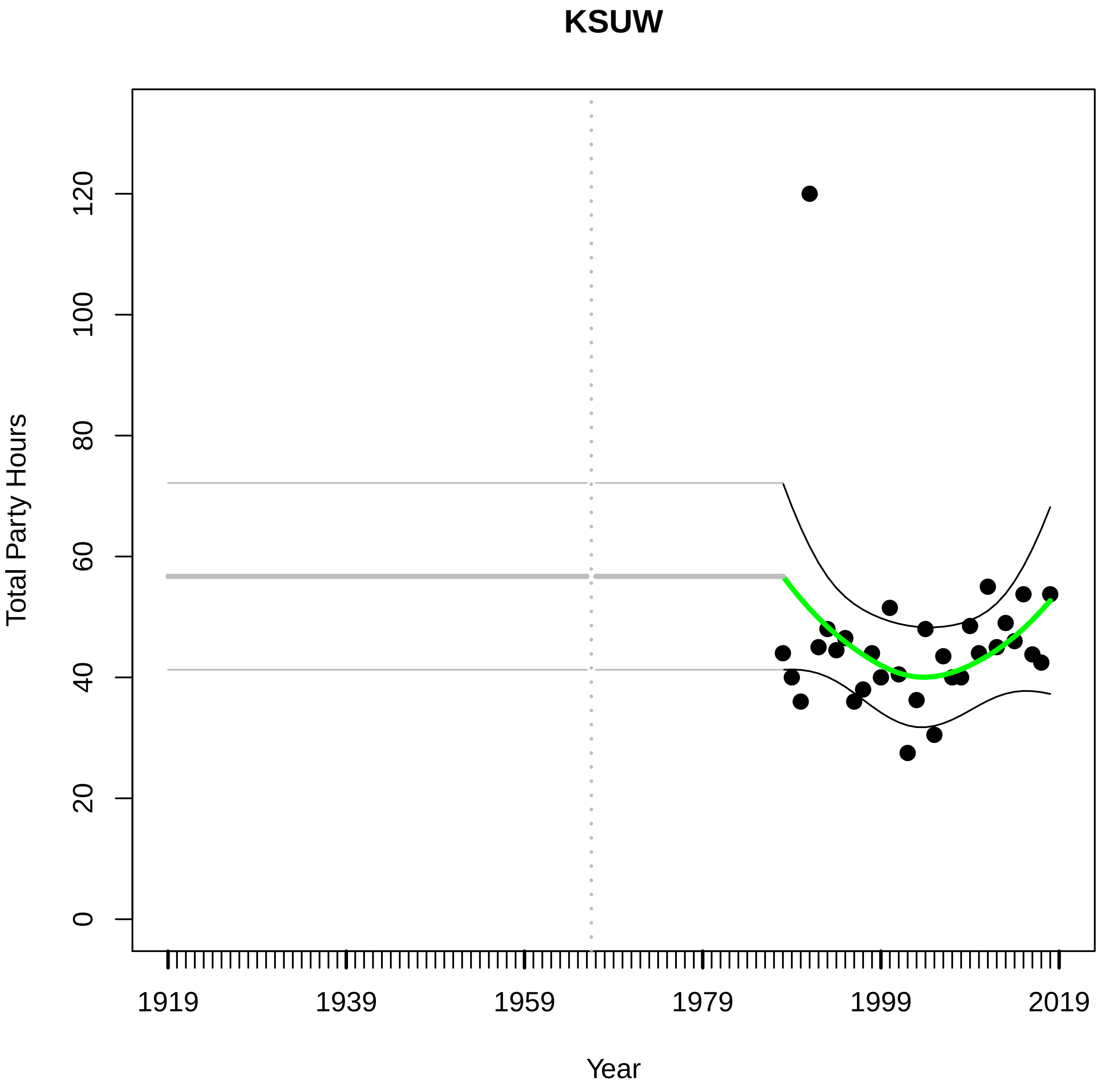

KSUW
Total Party Hours
0
20
40
60
80
100
120
1919
1939
1959
1979
1999
2019
Year

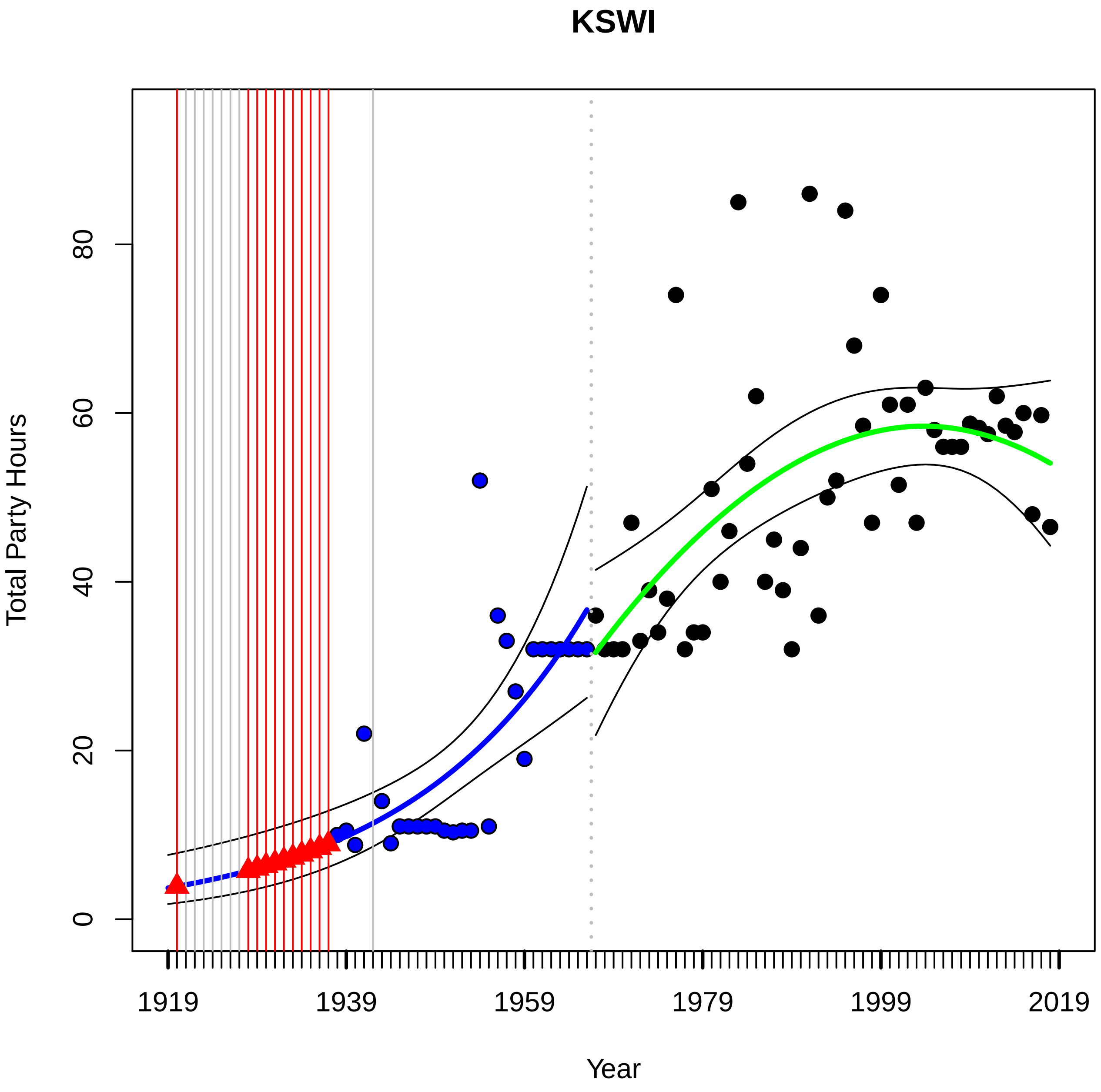
KSWI
Total Party Hours
0
20
40
60
80
1919
1939
1959
1979
1999
2019
Year

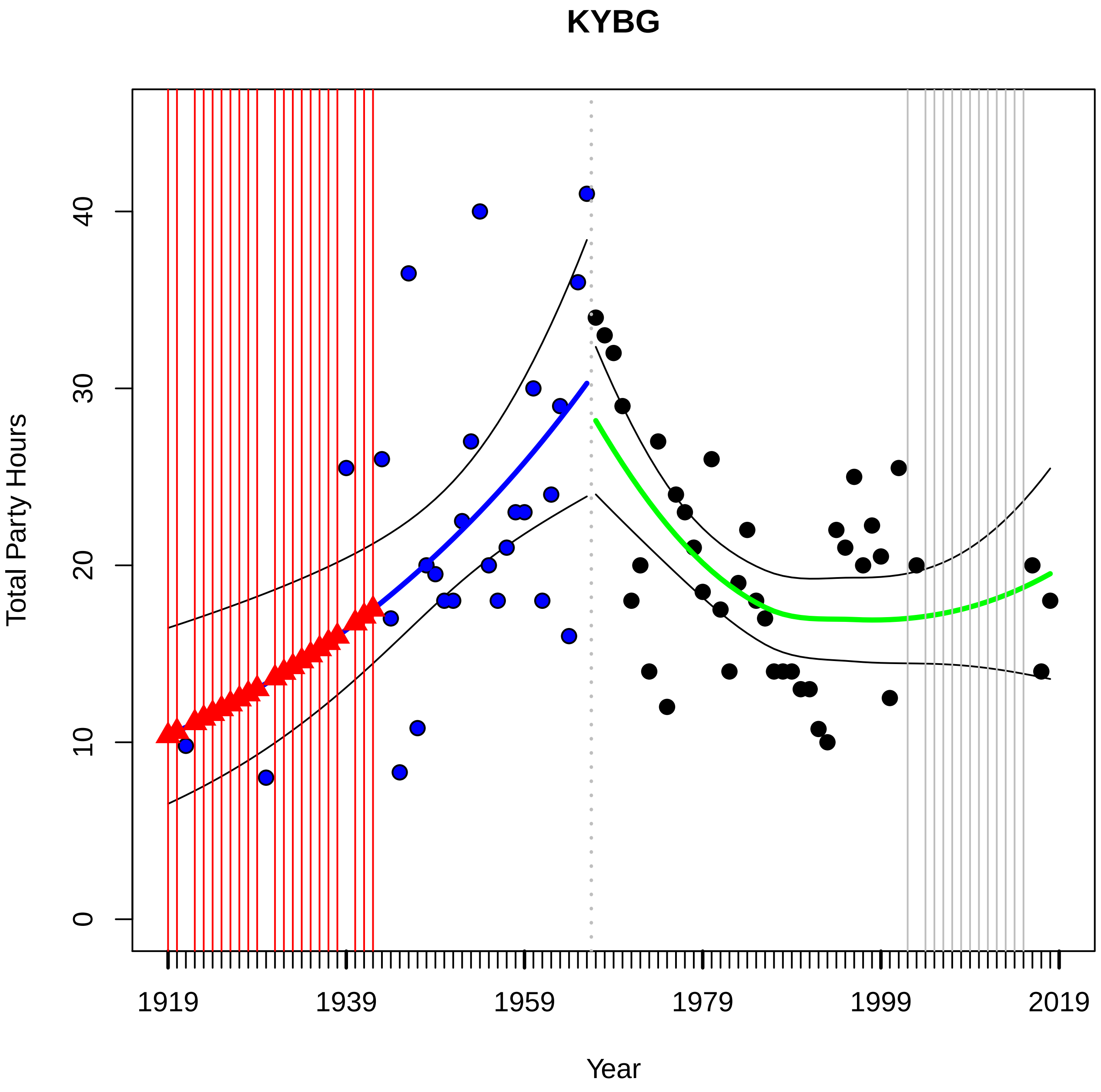
KYBG
Total Party Hours
40
30
20
10
0
1919
1939
1959
1979
1999
2019
Year

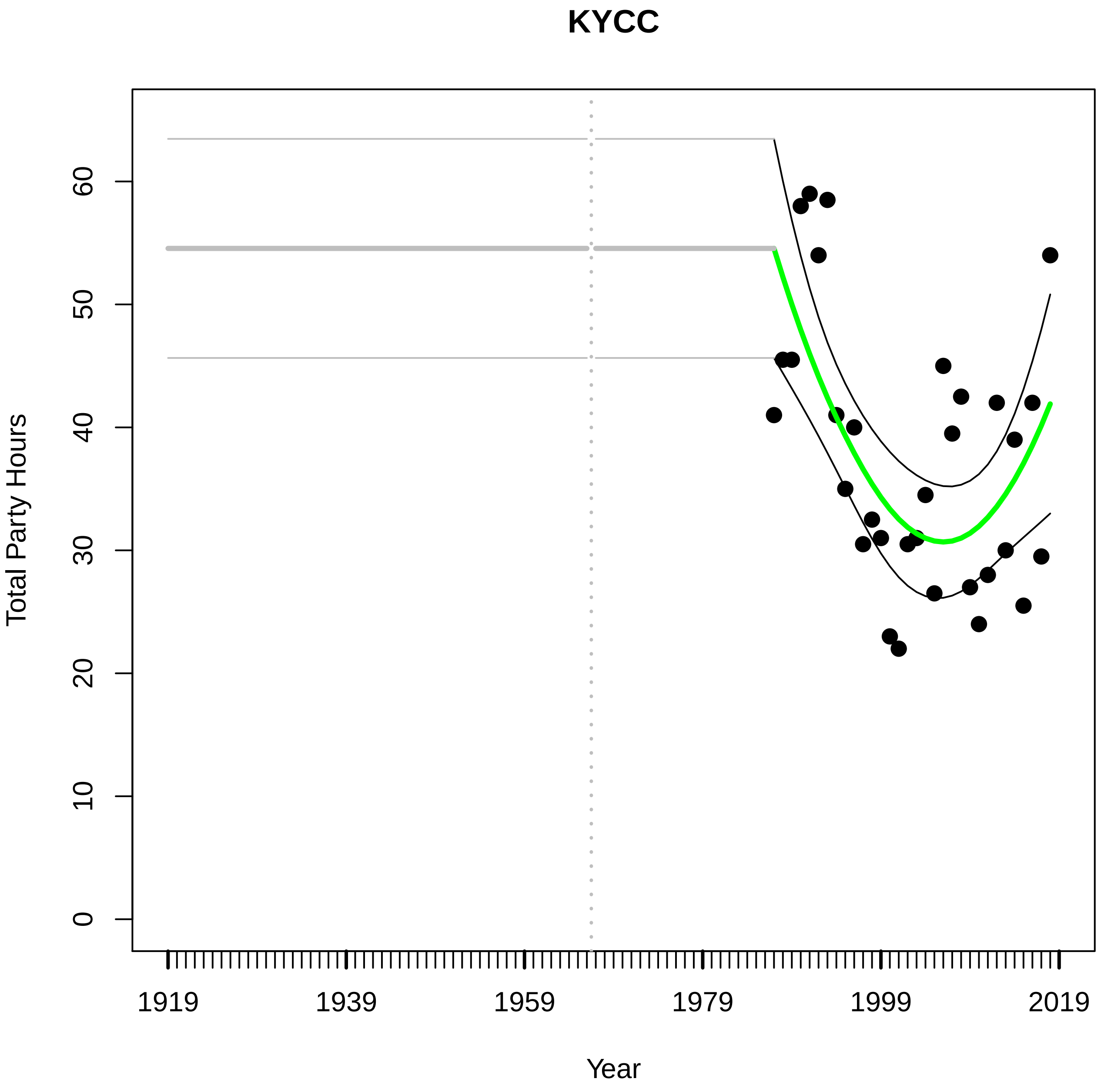

KYCC
Total Party Hours
0
10
20
30
40
50
60
1919
1939
1959
1979
1999
2019
Year

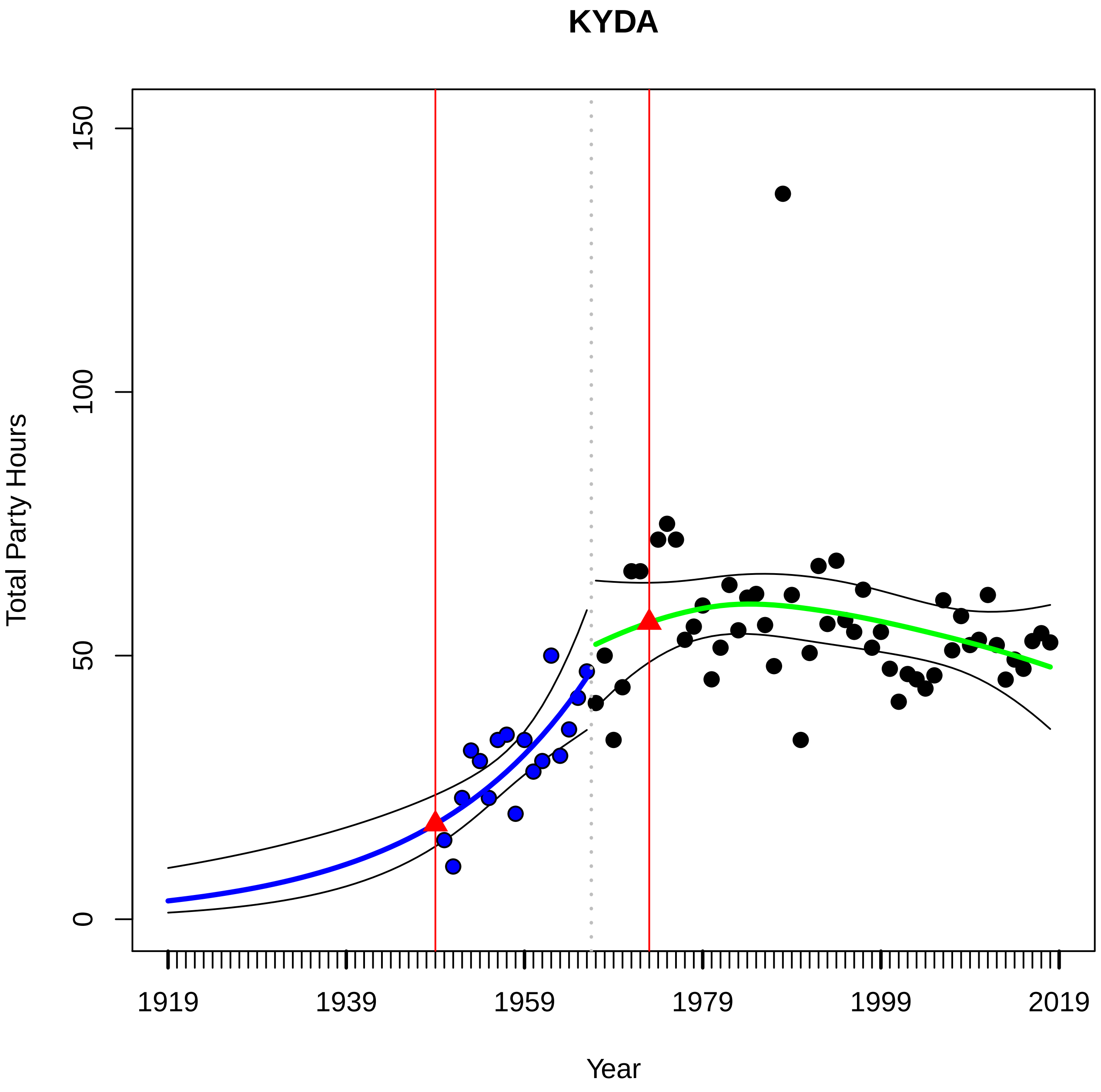

KYDA
Total Party Hours
150
100
50
0
1919
1939
1959
1979
1999
2019
Year

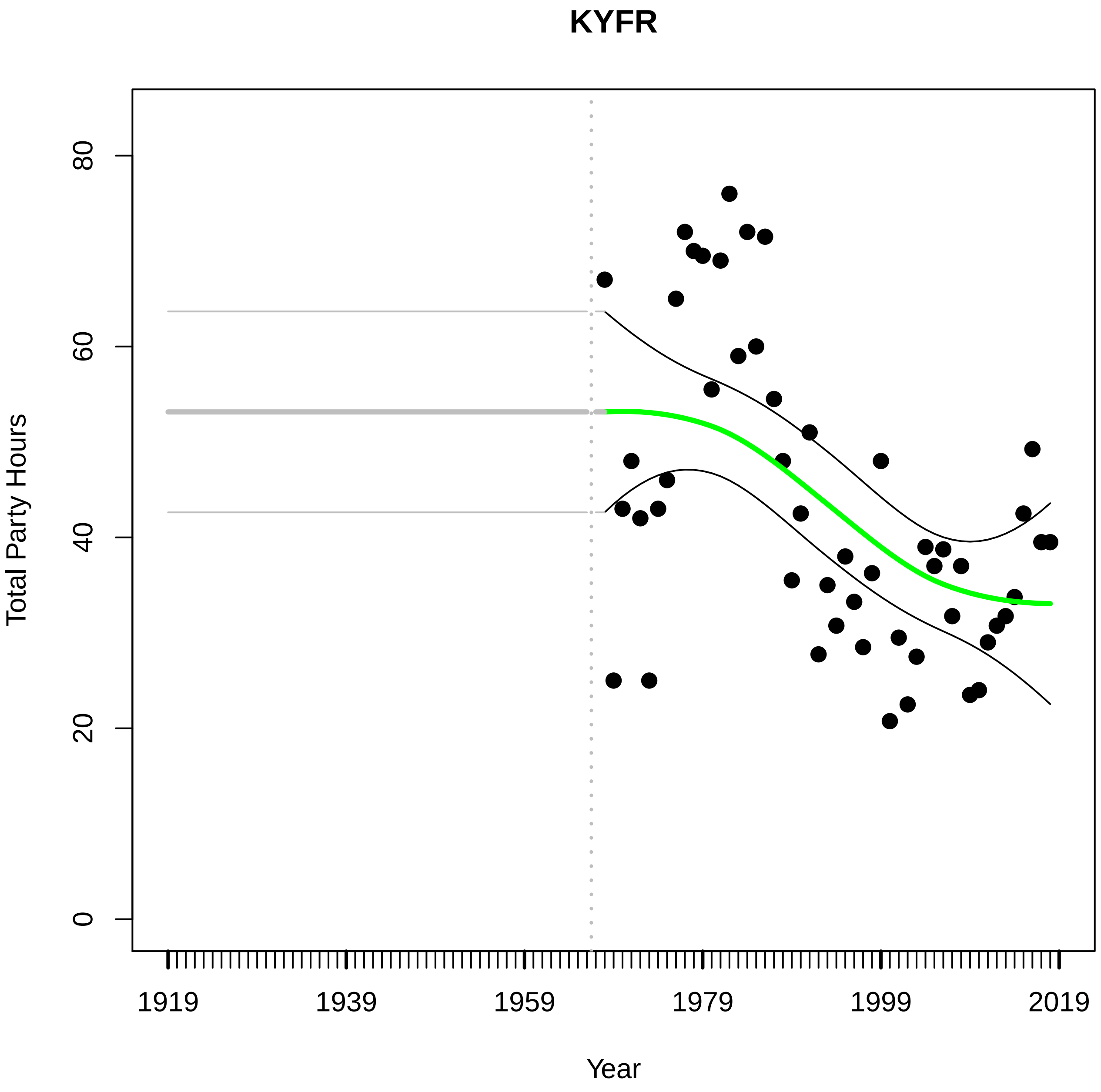

KYFR
Total Party Hours
0
20
40
60
80
1919
1939
1959
1979
1999
2019
Year

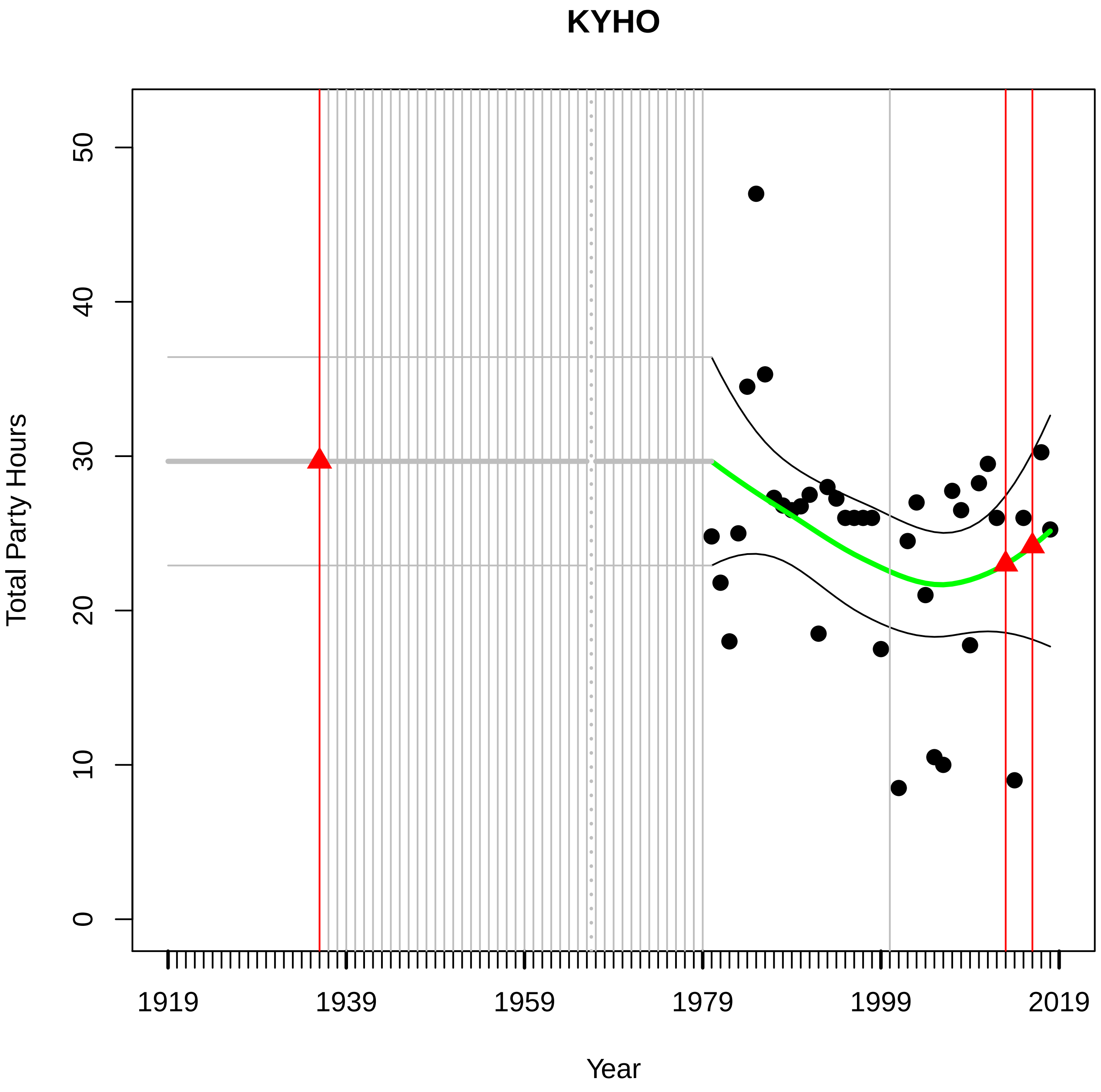

KYHO
Total Party Hours
0
10
20
30
40
50
1919
1939
1959
1979
1999
2019
Year

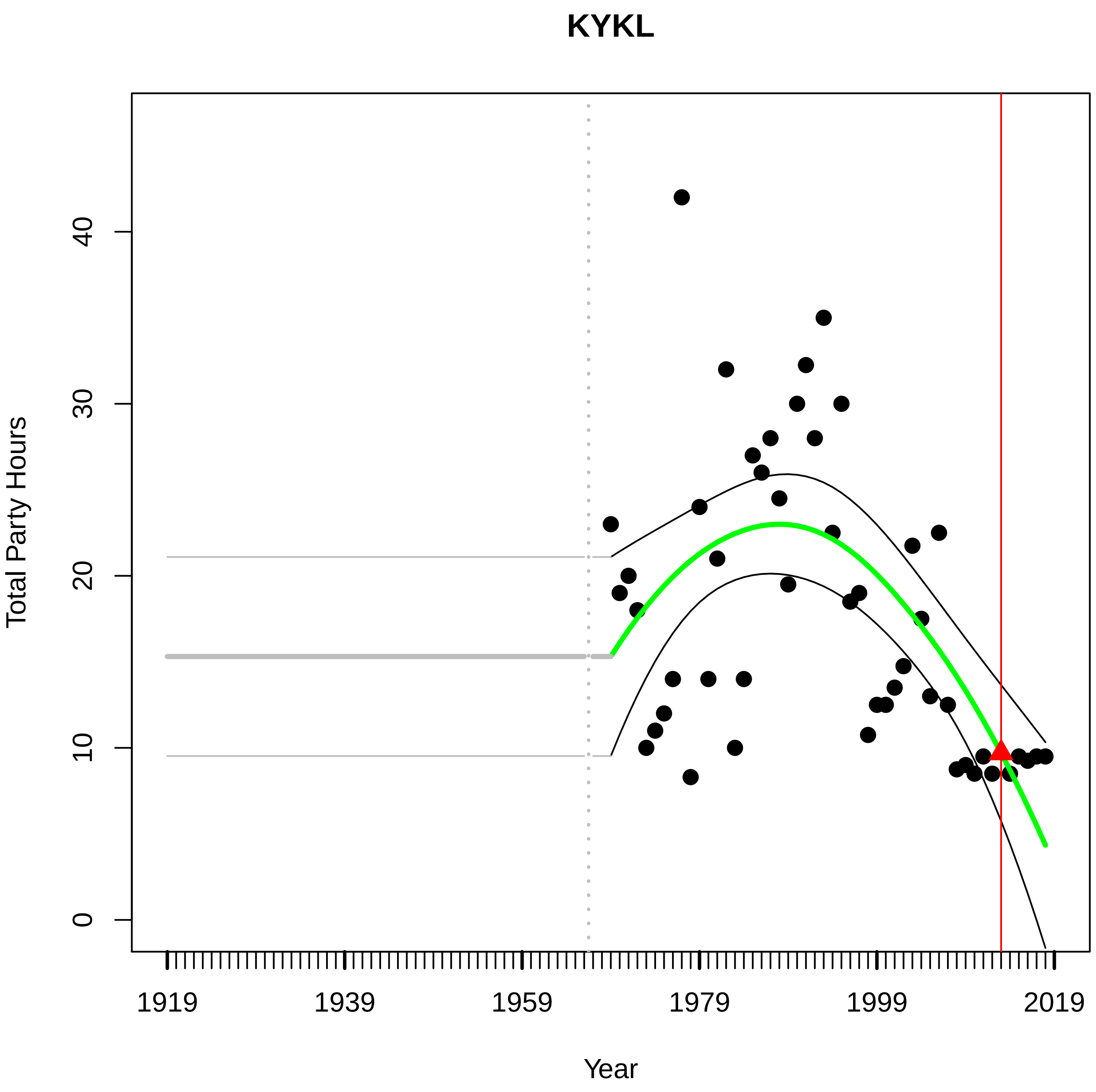
KYKL
Total Party Hours
0
10
20
30
40
1919
1939
1959
1979
1999
2019
Year

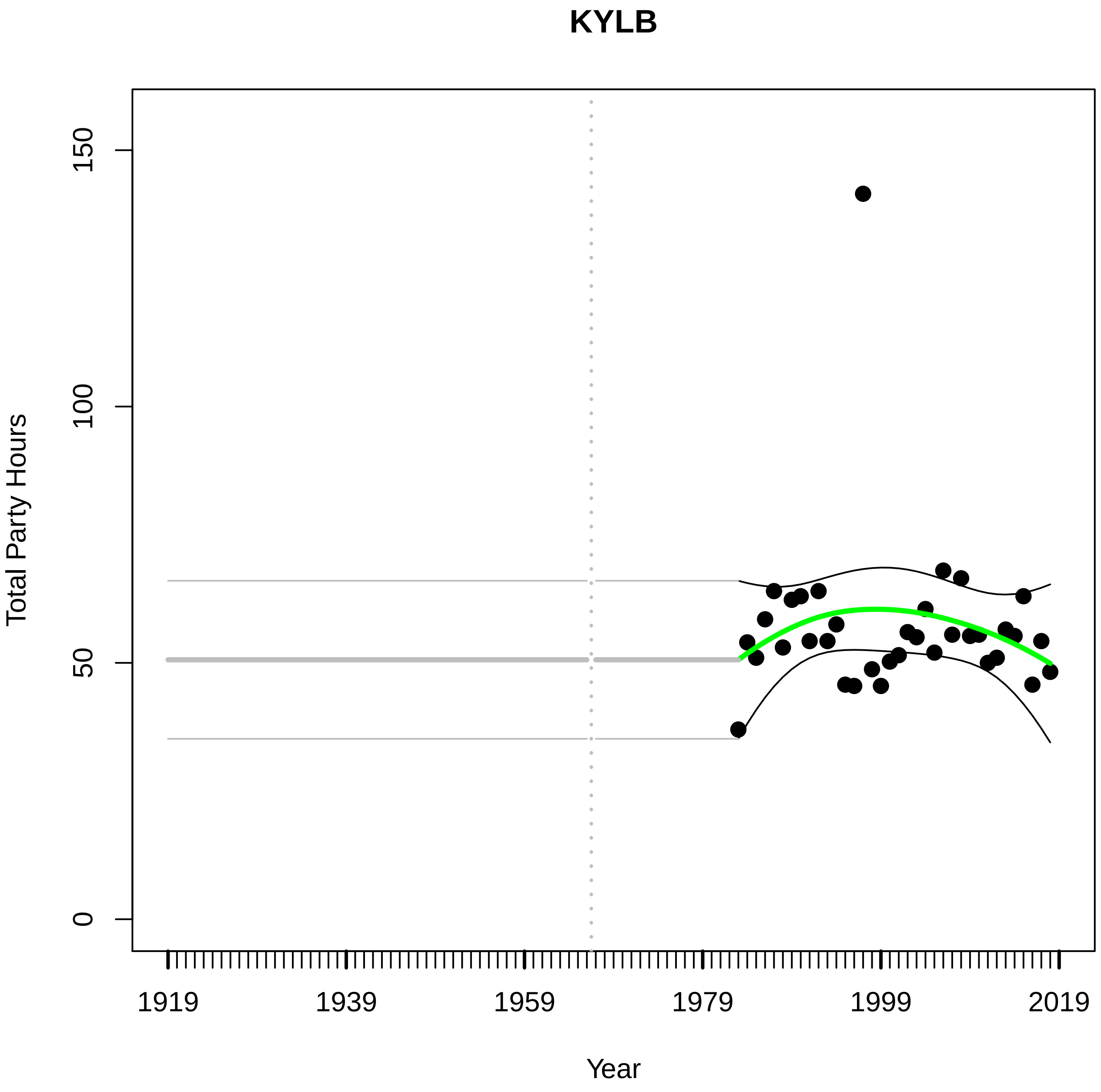
KYLB
Total Party Hours
0
50
100
150
1919
1939
1959
1979
1999
2019
Year

KYLE

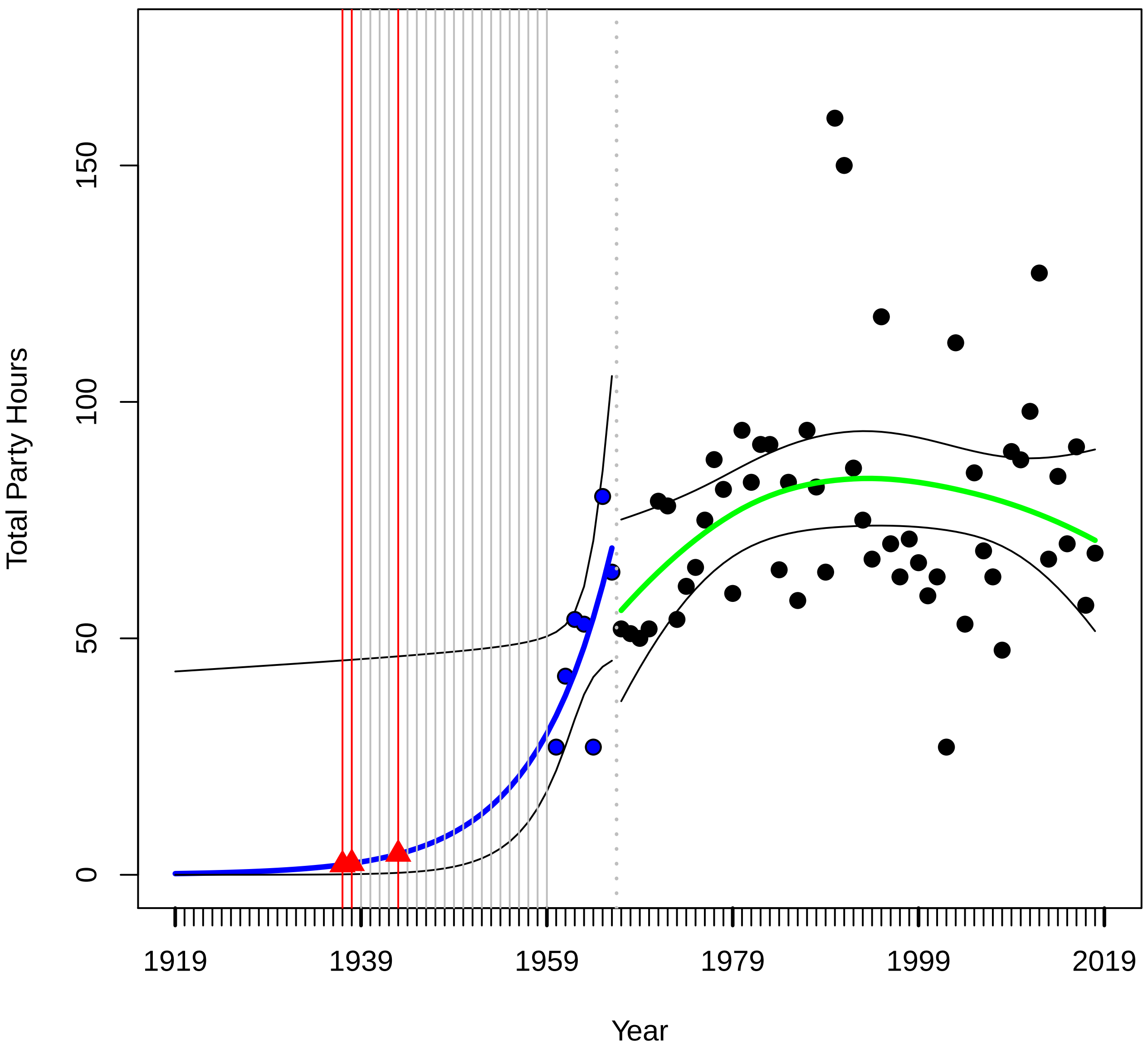

Total Party Hours
0
50
100
150
1919
1939
1959
1979
1999
2019
Year

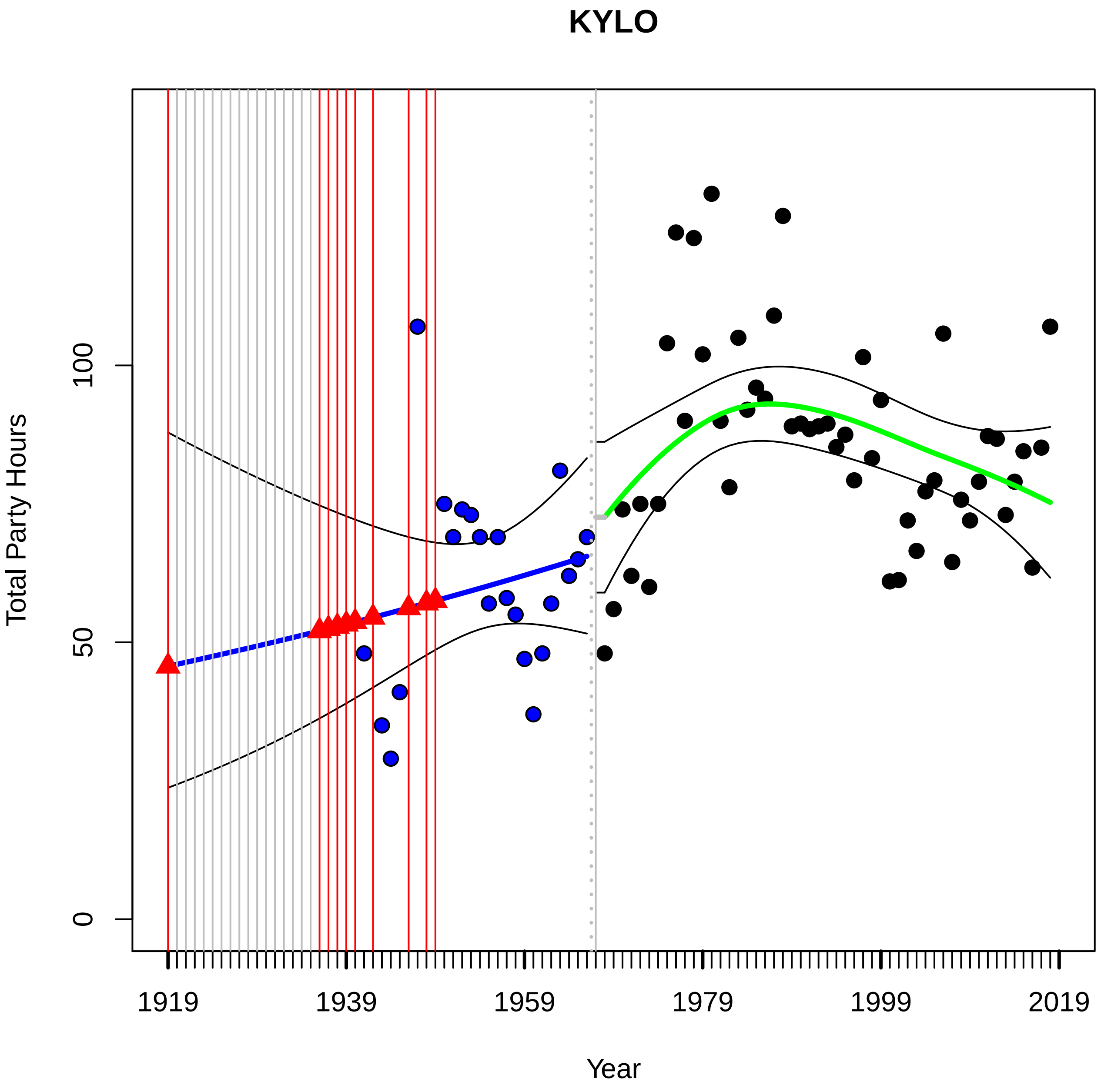

KYLO
Total Party Hours
0
50
100
Year
1919
1939
1959
1979
1999
2019

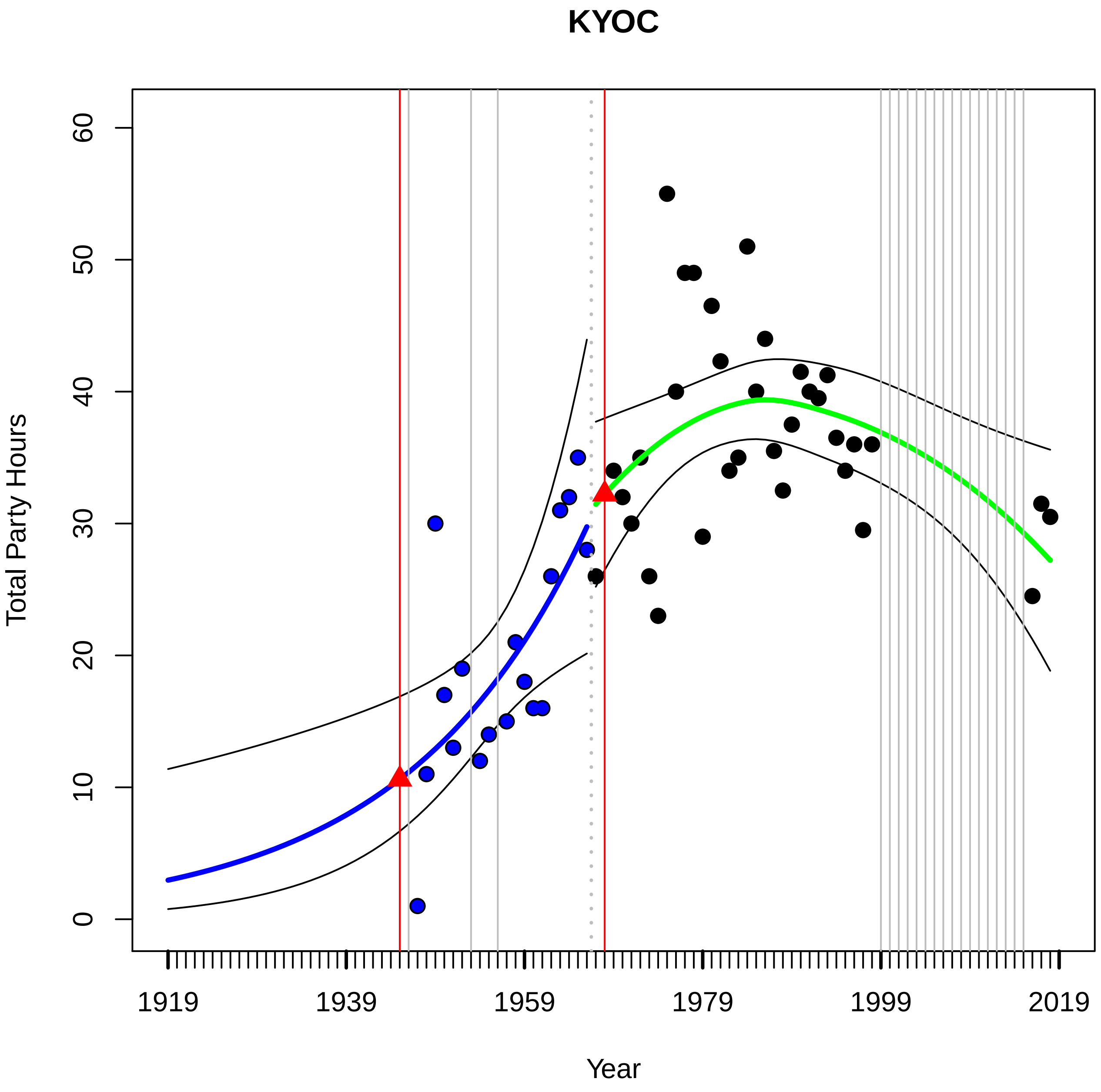

KYOC
Total Party Hours
Year
0
10
20
30
40
50
60
1919
1939
1959
1979
1999
2019

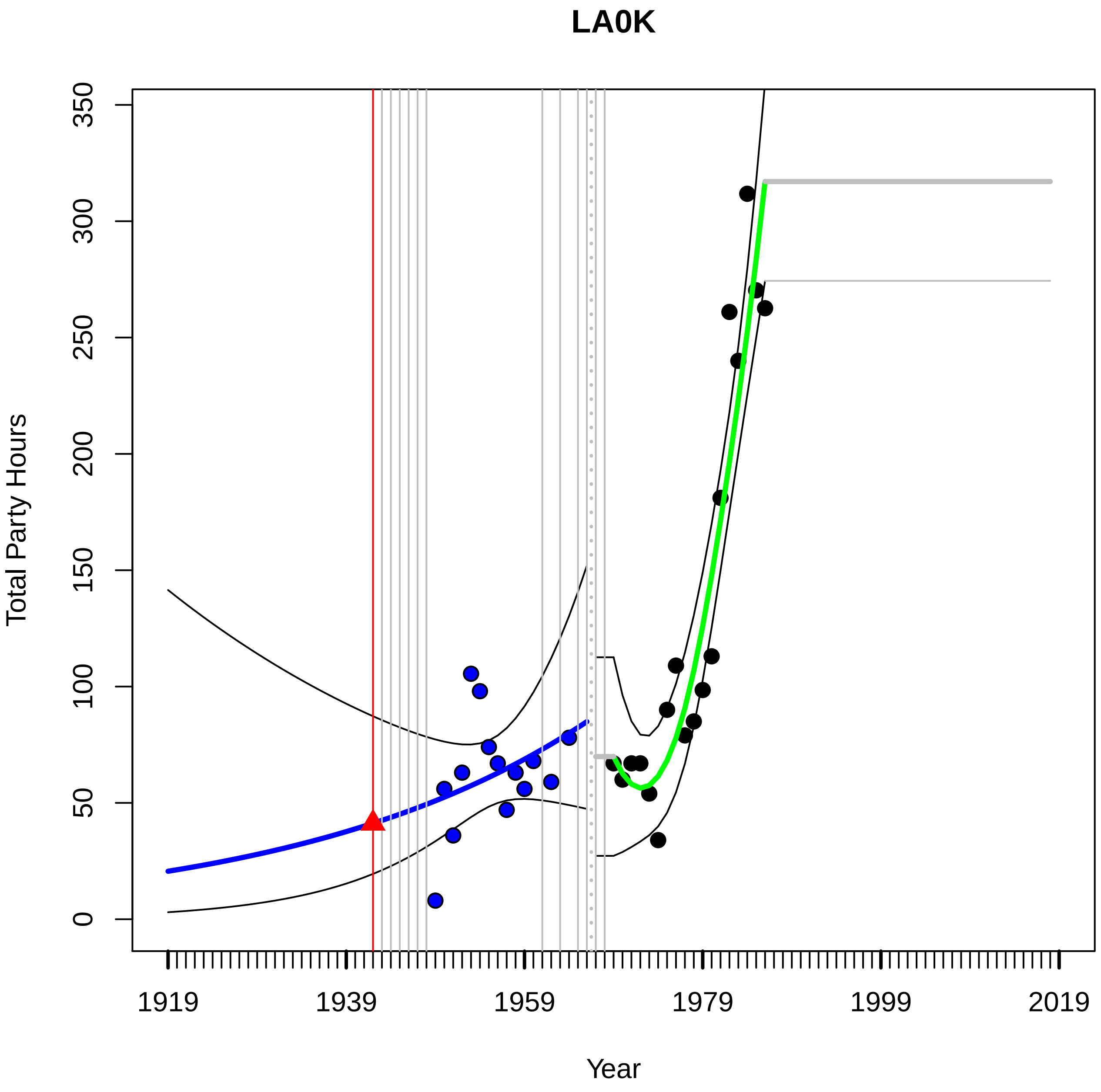
LA0K
Total Party Hours
0
50
100
150
200
250
300
350
1919
1939
1959
1979
1999
2019
Year

LA0P

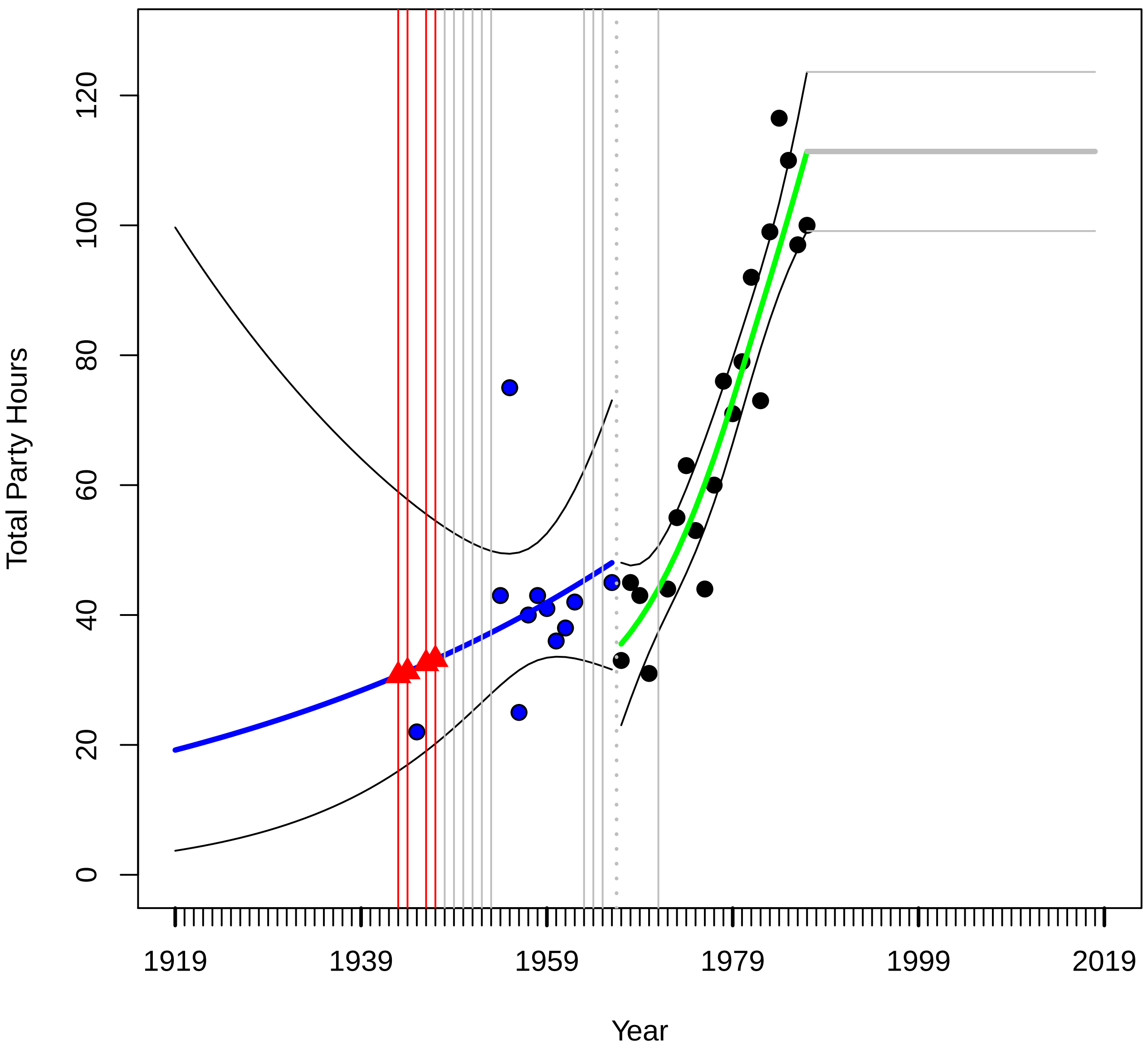

Total Party Hours
0
20
40
60
80
100
120
1919
1939
1959
1979
1999
2019
Year

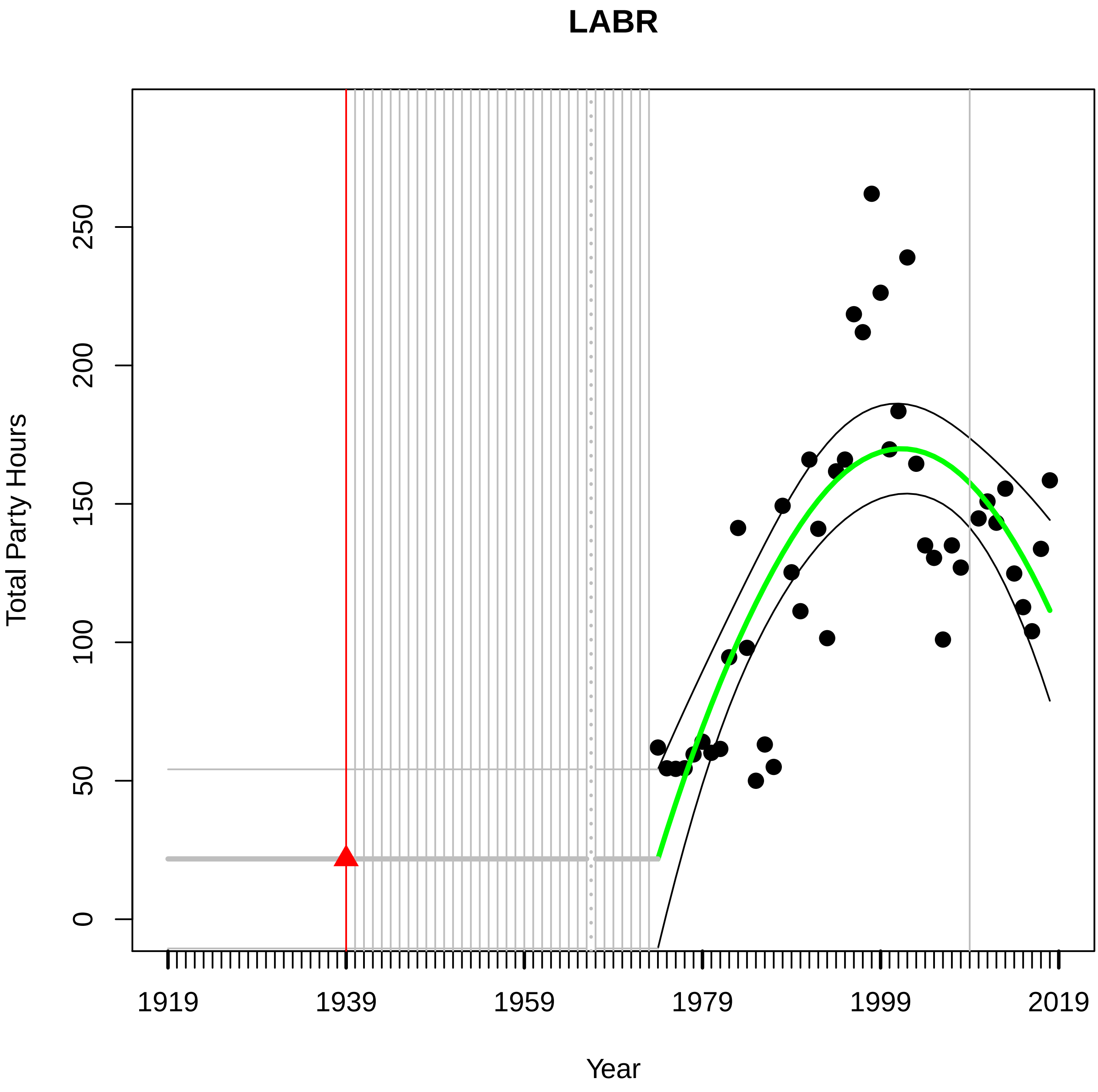

LABR
Total Party Hours
0
50
100
150
200
250
1919
1939
1959
1979
1999
2019
Year

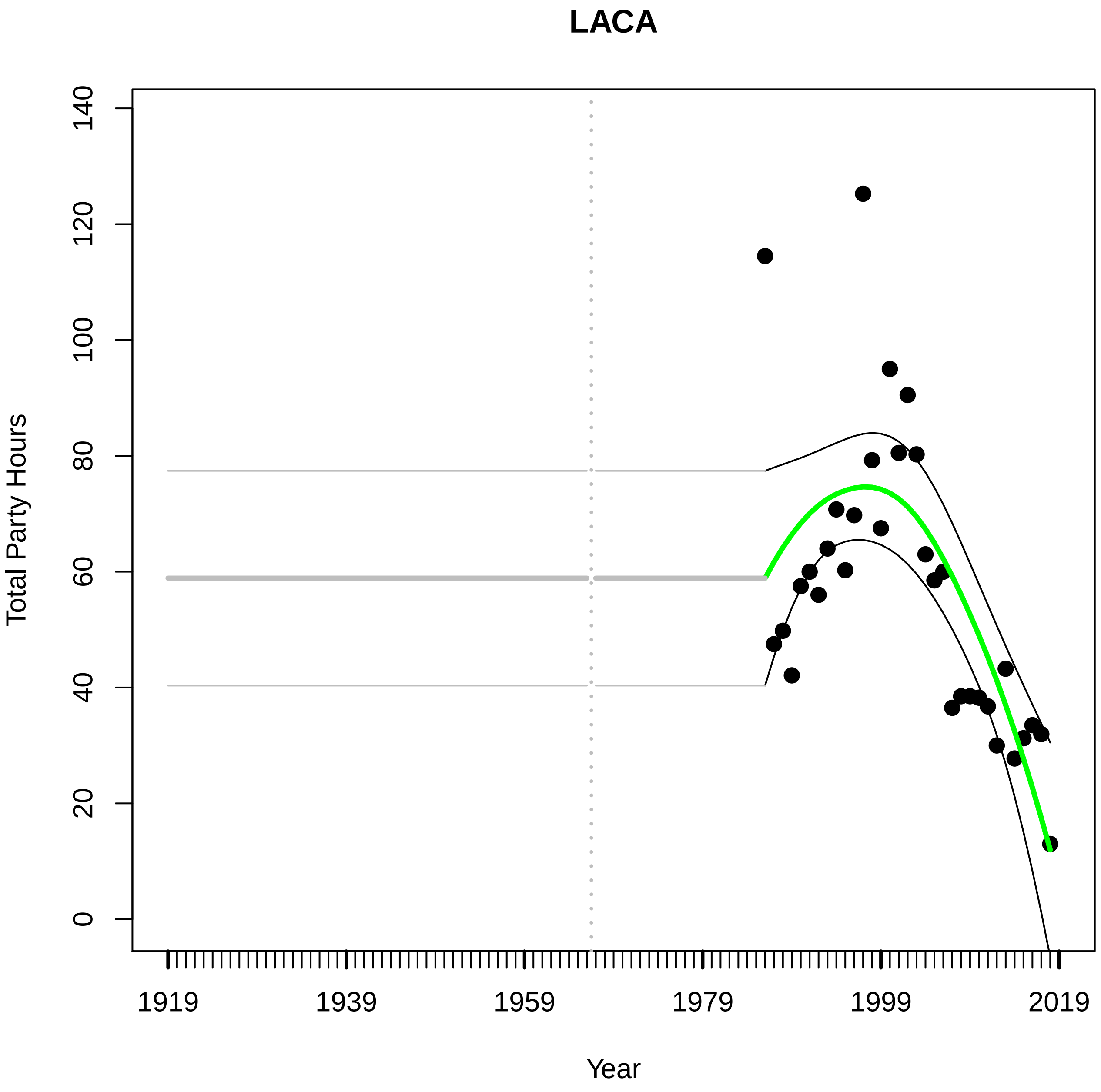

LACA
Total Party Hours
0
20
40
60
80
100
120
140
Year
1919
1939
1959
1979
1999
2019

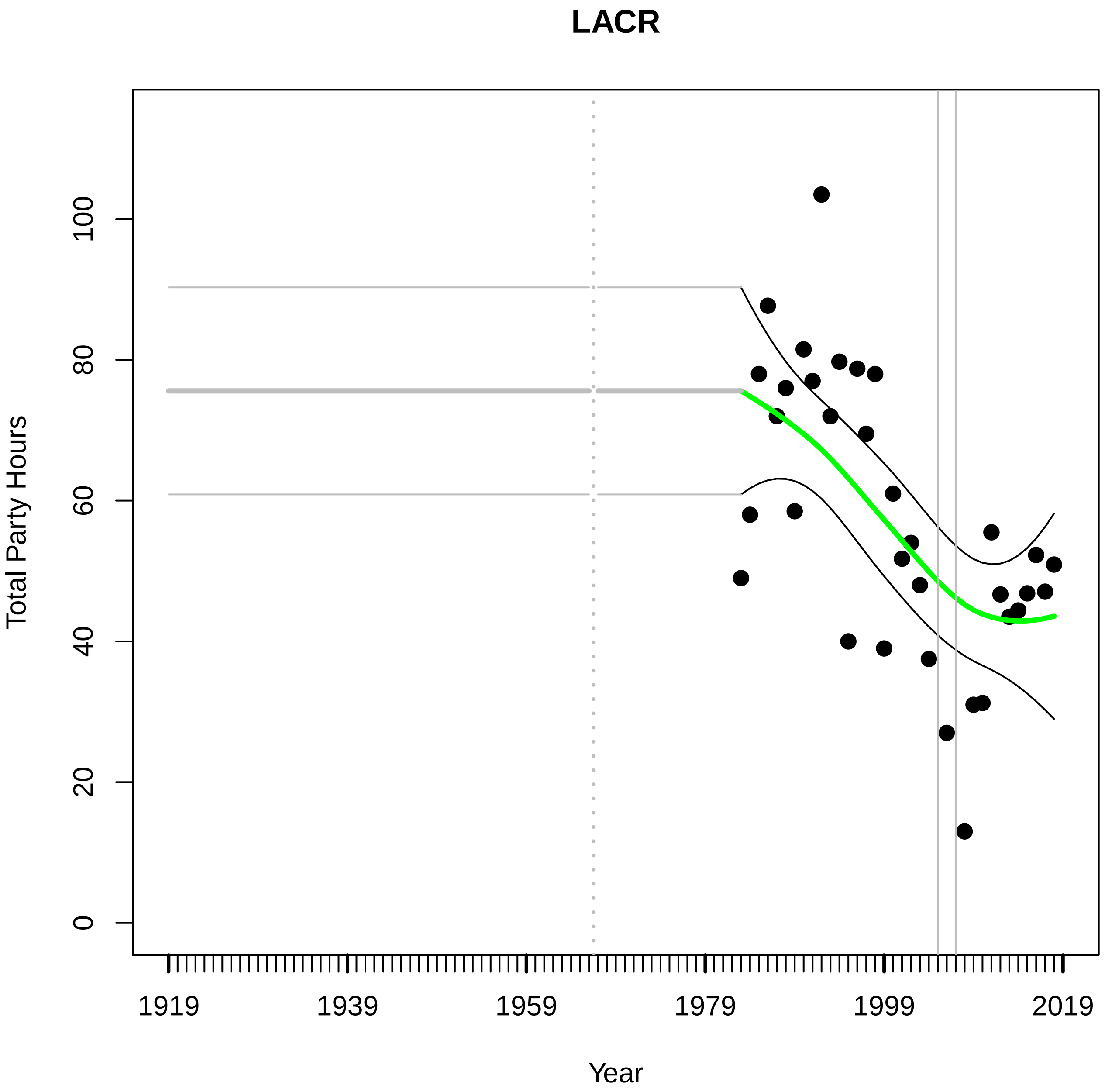

LACR
Total Party Hours
0
20
40
60
80
100
1919
1939
1959
1979
1999
2019
Year

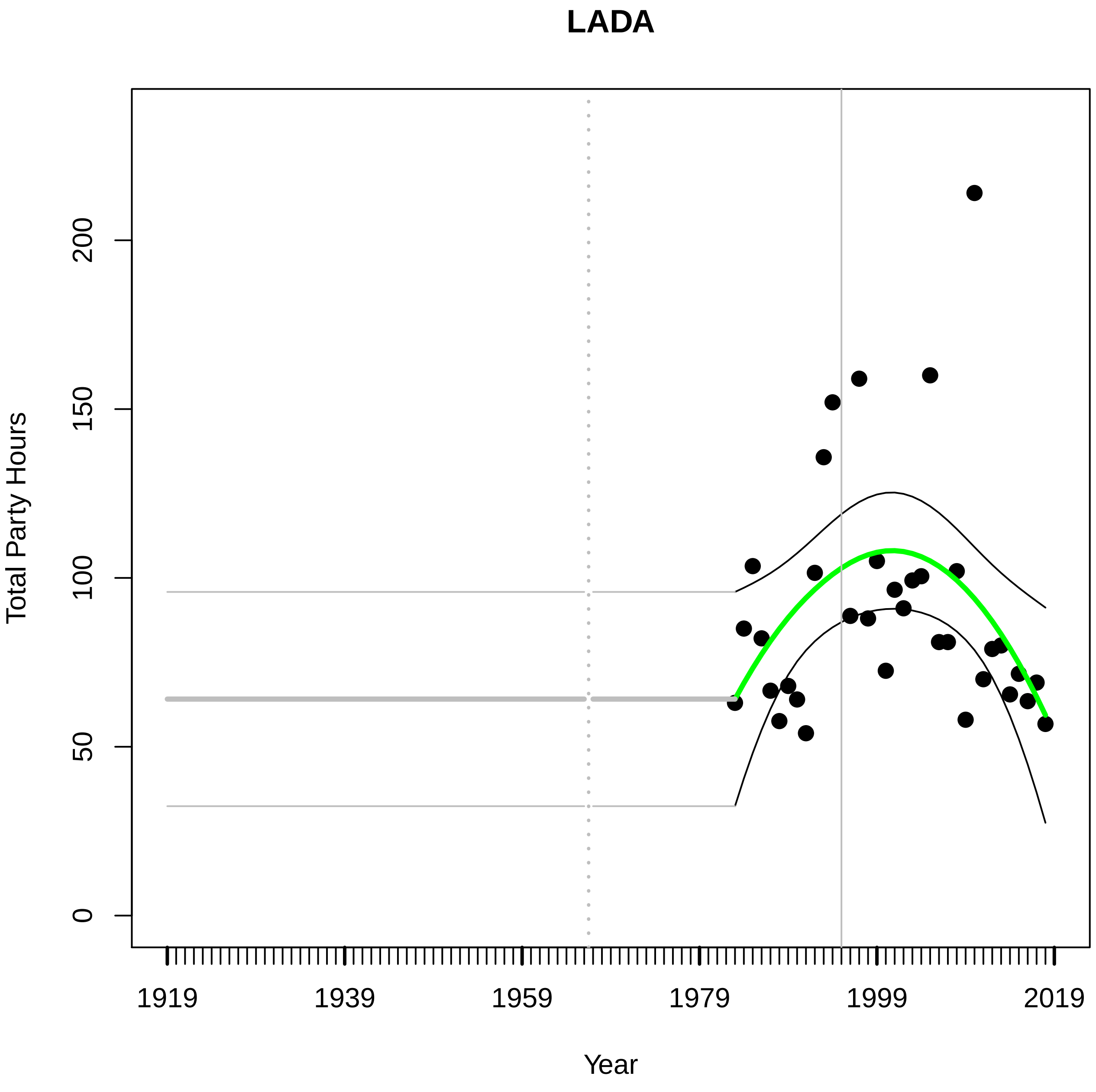
LADA
Total Party Hours
0
50
100
150
200
1919
1939
1959
1979
1999
2019
Year

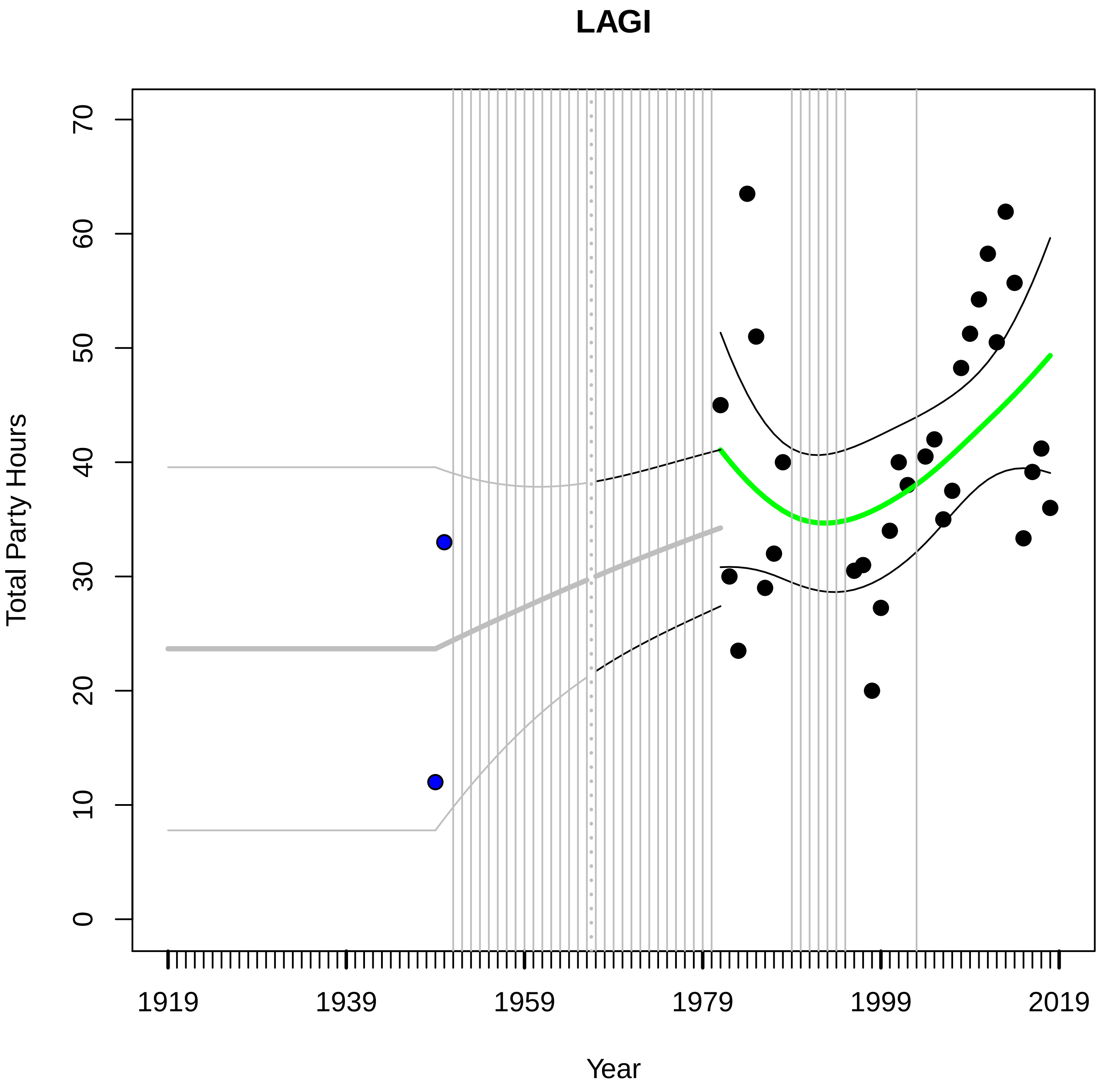

LAGI
Total Party Hours
0
10
20
30
40
50
60
70
1919
1939
1959
1979
1999
2019
Year

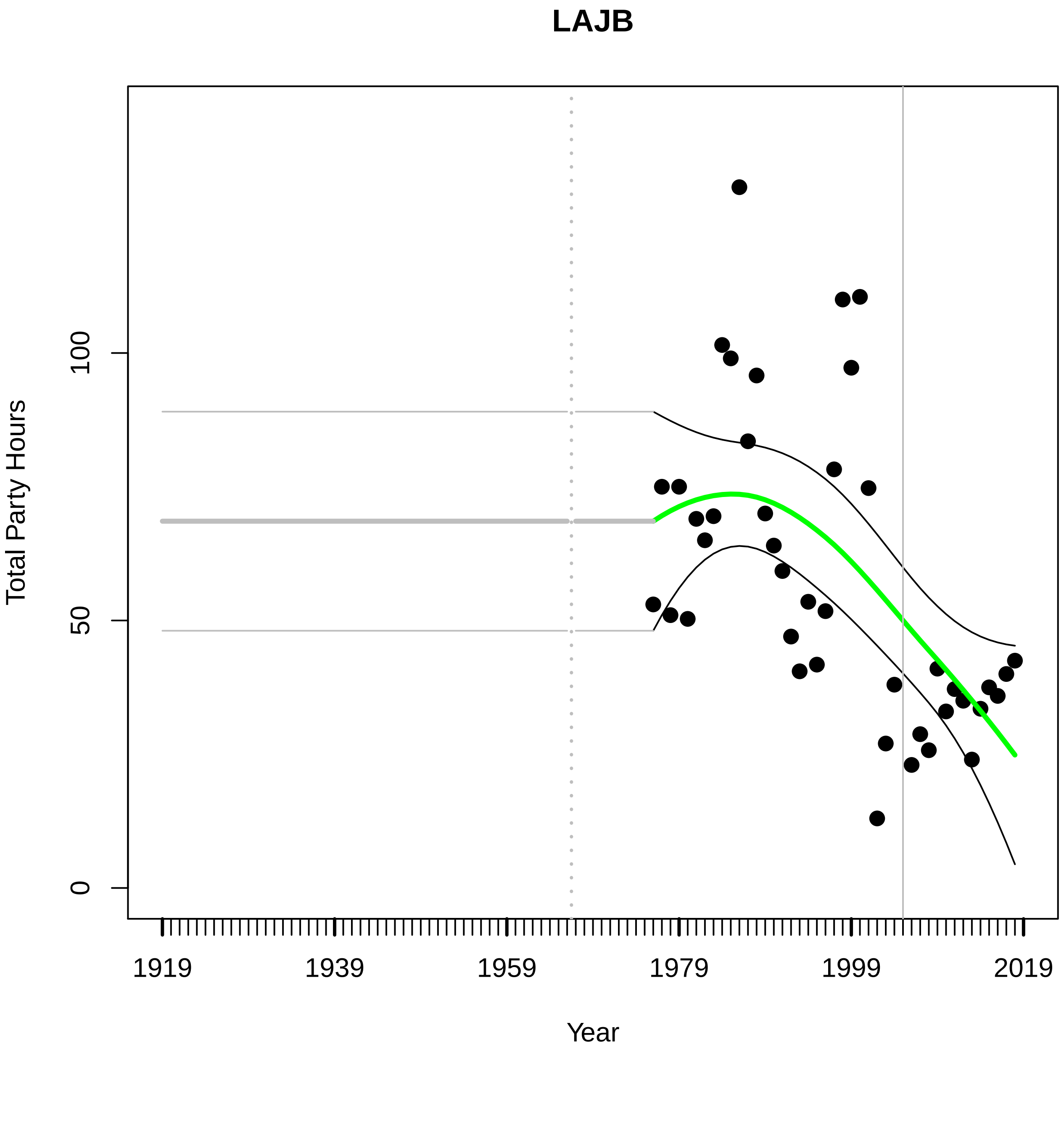
LAJB
Total Party Hours
0
50
100
1919
1939
1959
1979
1999
2019
Year

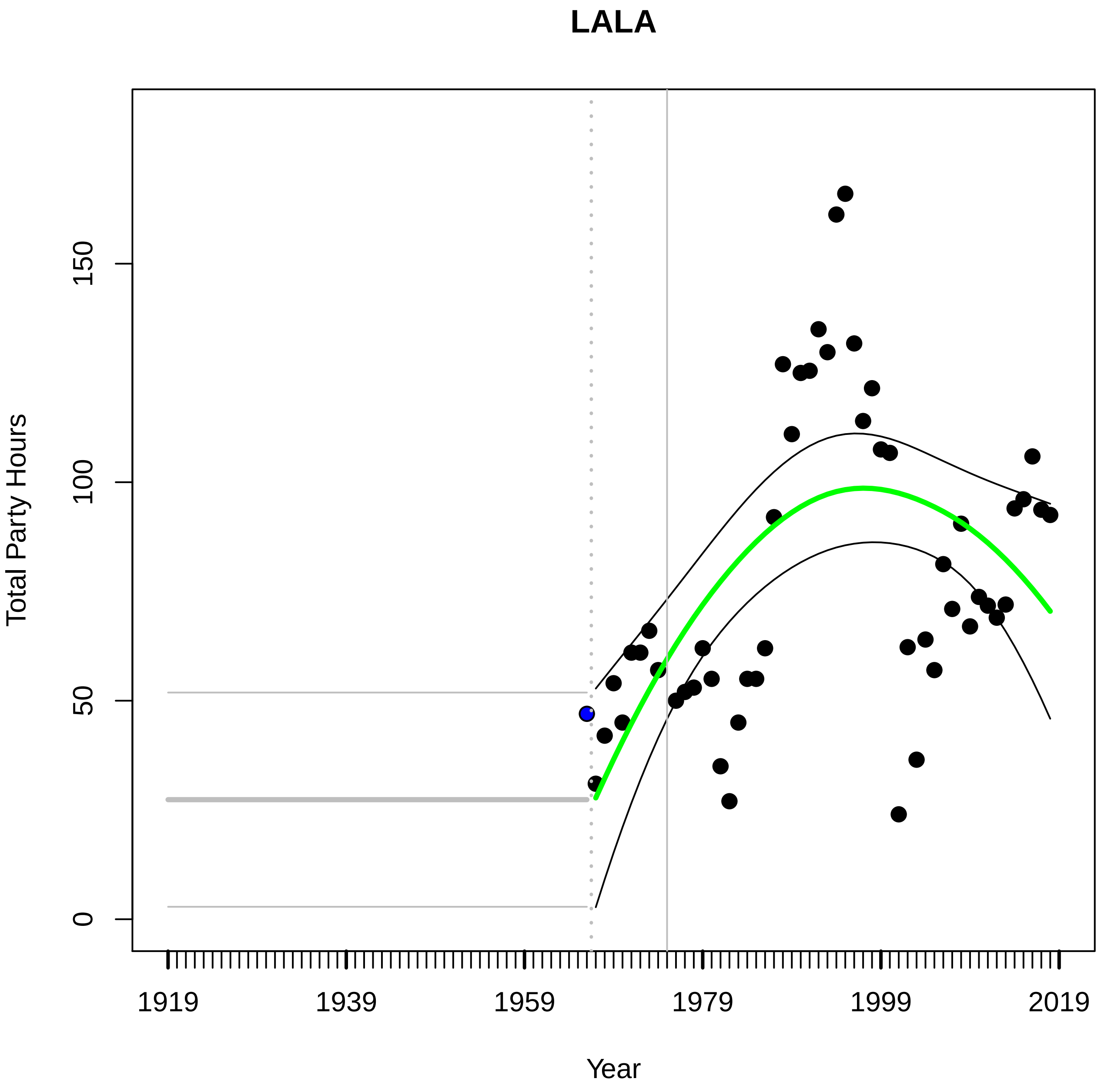

LALA
Total Party Hours
0
50
100
150
Year
1919
1939
1959
1979
1999
2019

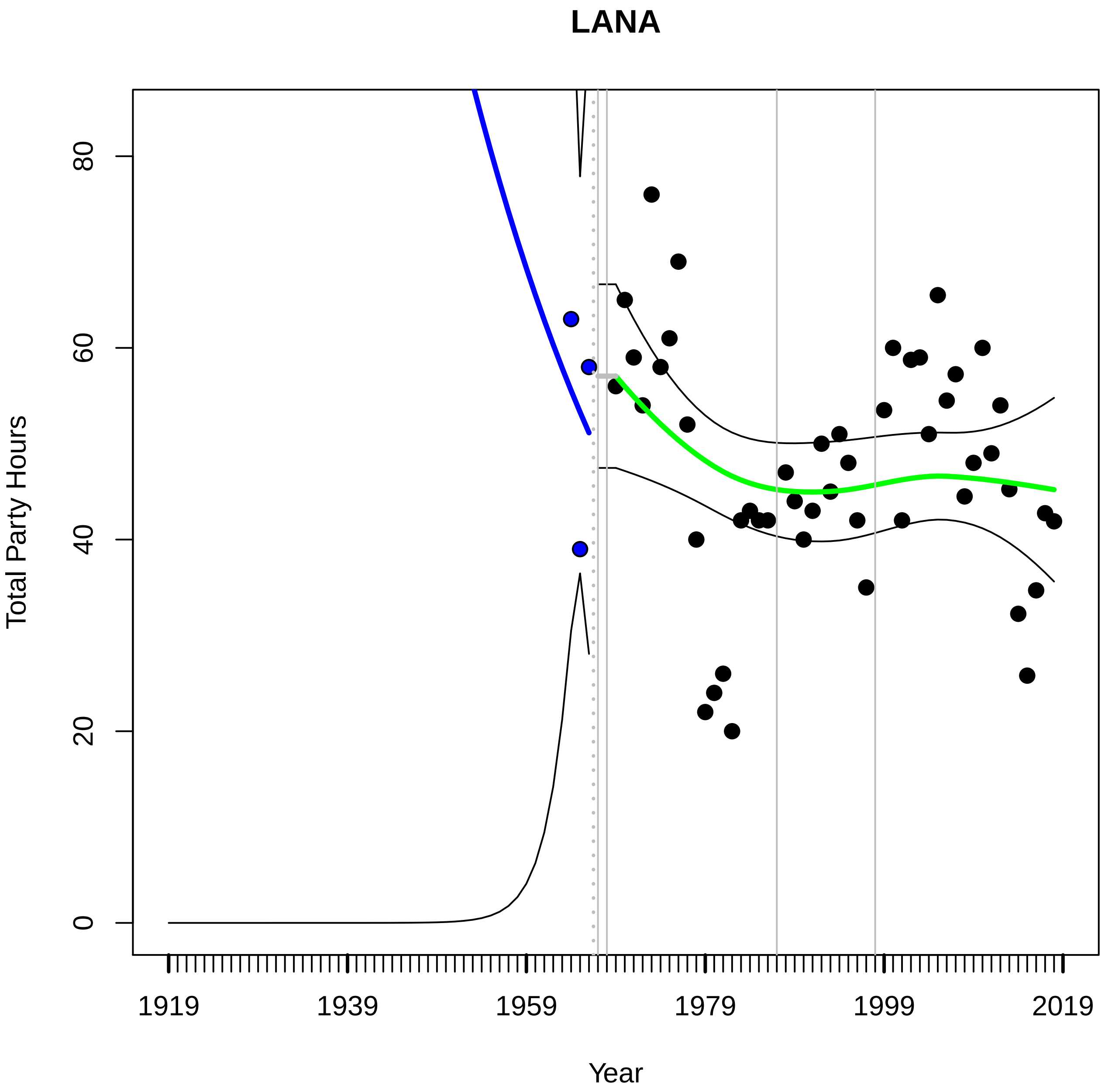

LANA
Total Party Hours
0
20
40
60
80
1919
1939
1959
1979
1999
2019
Year

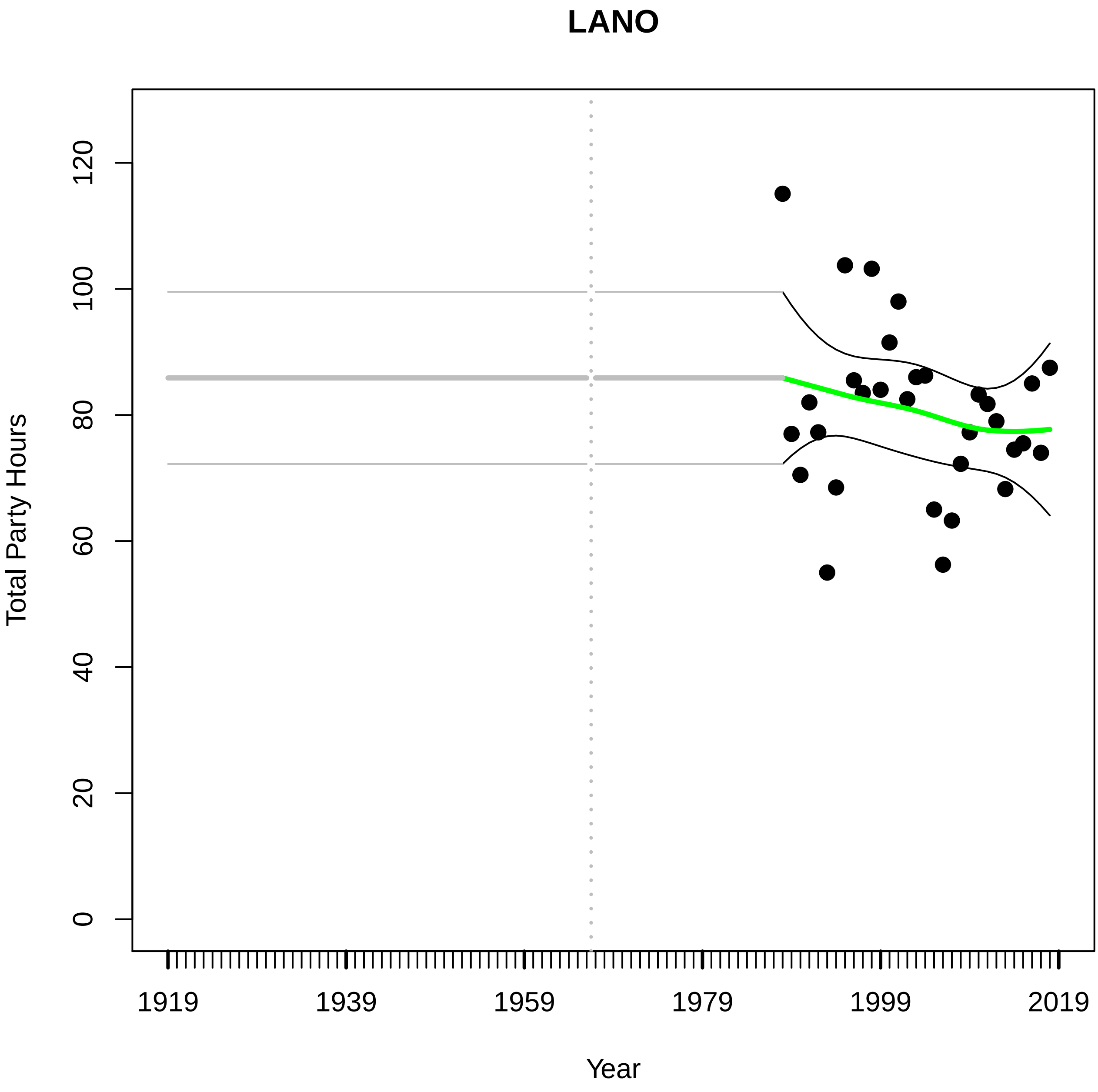

LANO
Total Party Hours
0
20
40
60
80
100
120
1919
1939
1959
1979
1999
2019
Year

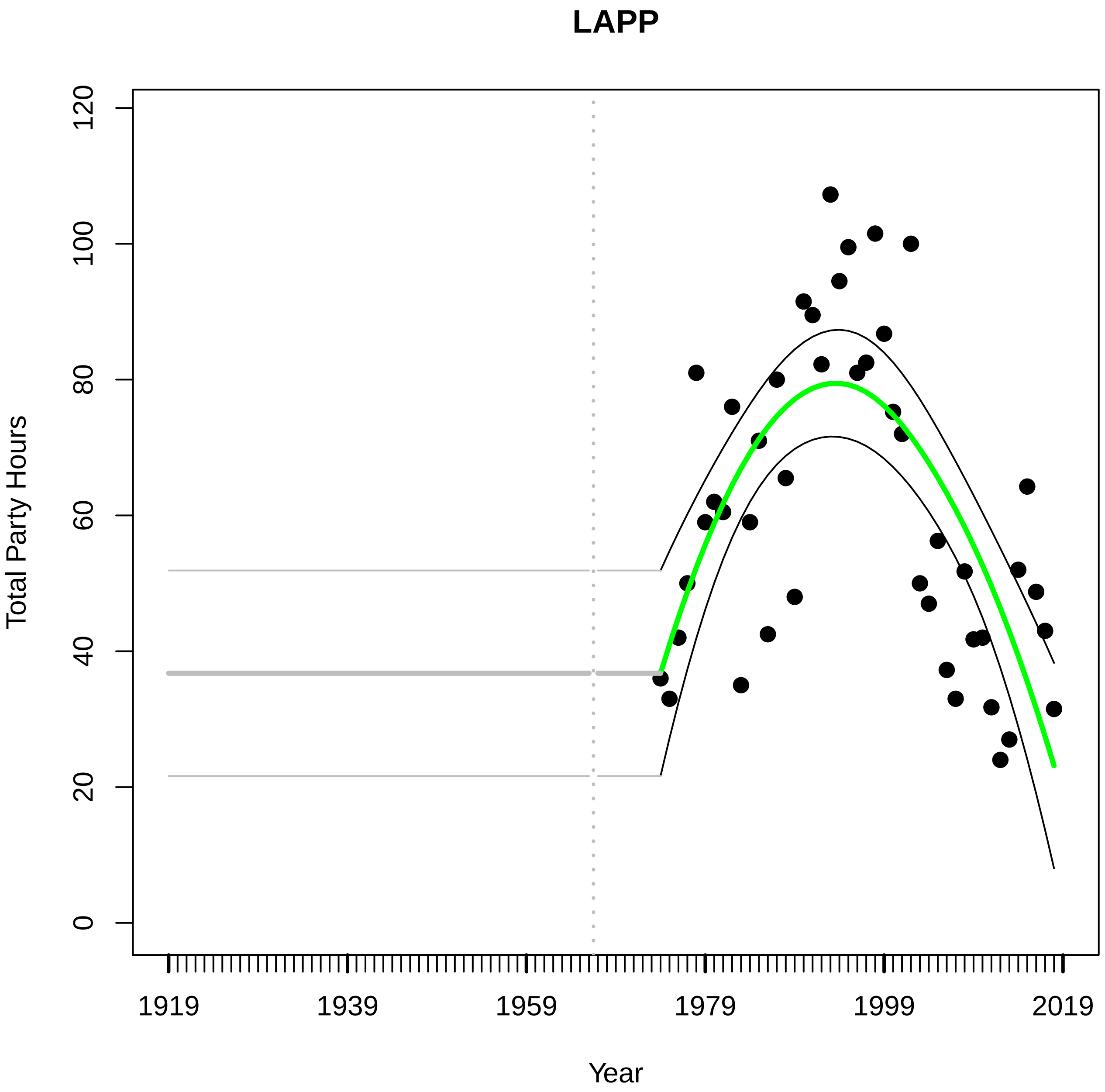

LAPP
Total Party Hours
0
20
40
60
80
100
120
1919
1939
1959
1979
1999
2019
Year

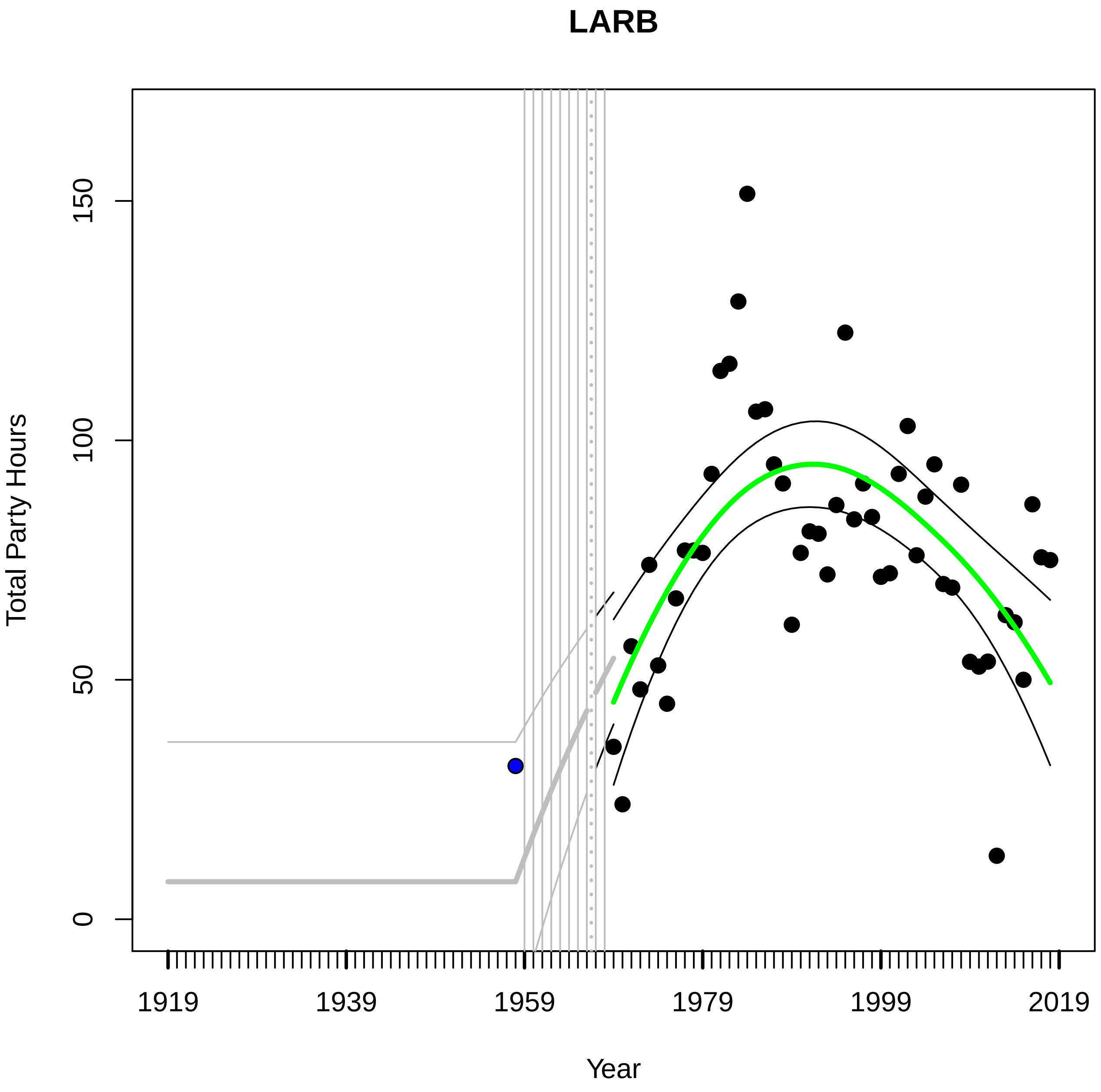

LARB
Total Party Hours
0
50
100
150
1919
1939
1959
1979
1999
2019
Year

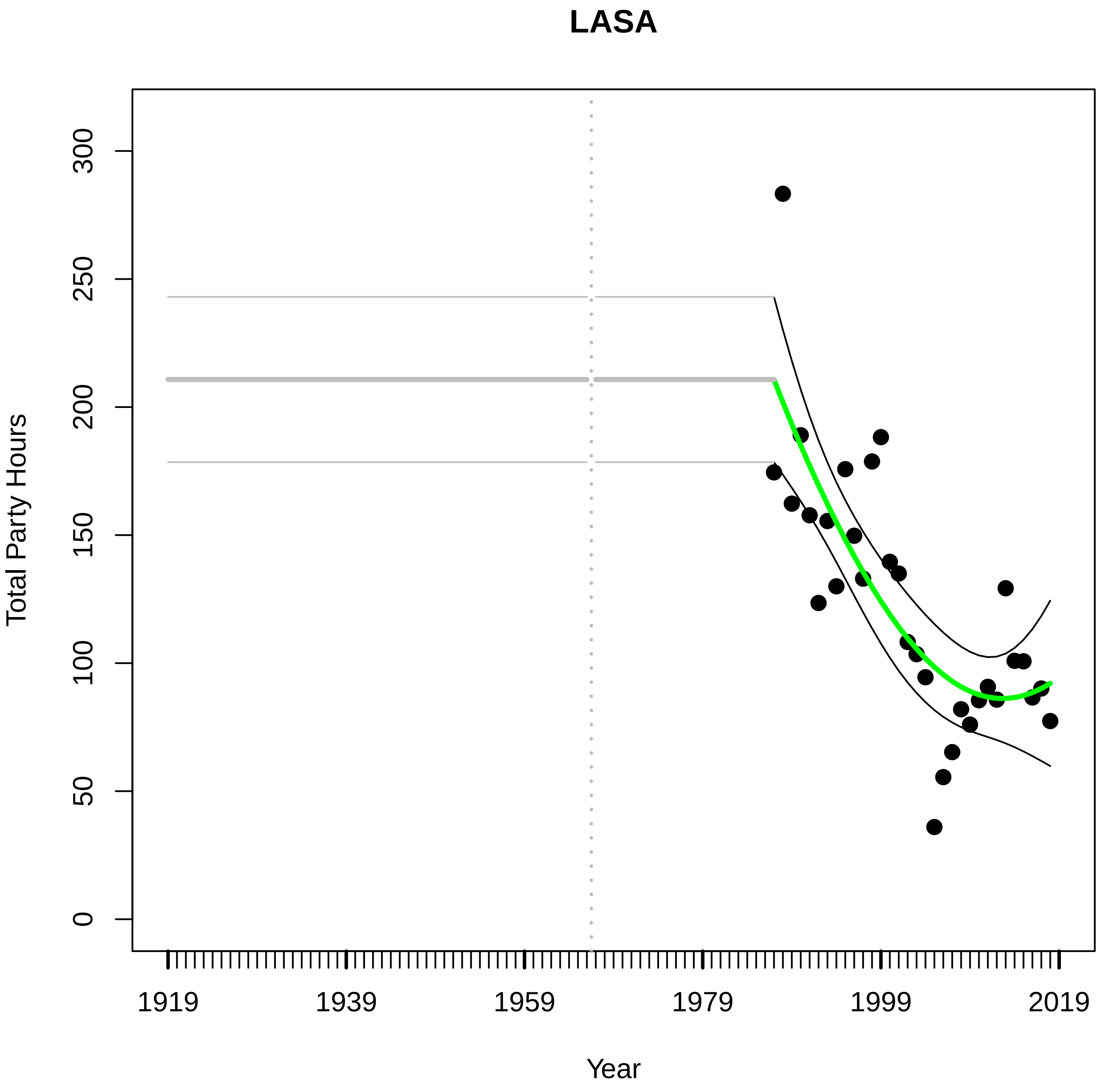

LASA
Total Party Hours
0
50
100
150
200
250
300
1919
1939
1959
1979
1999
2019
Year

LASH

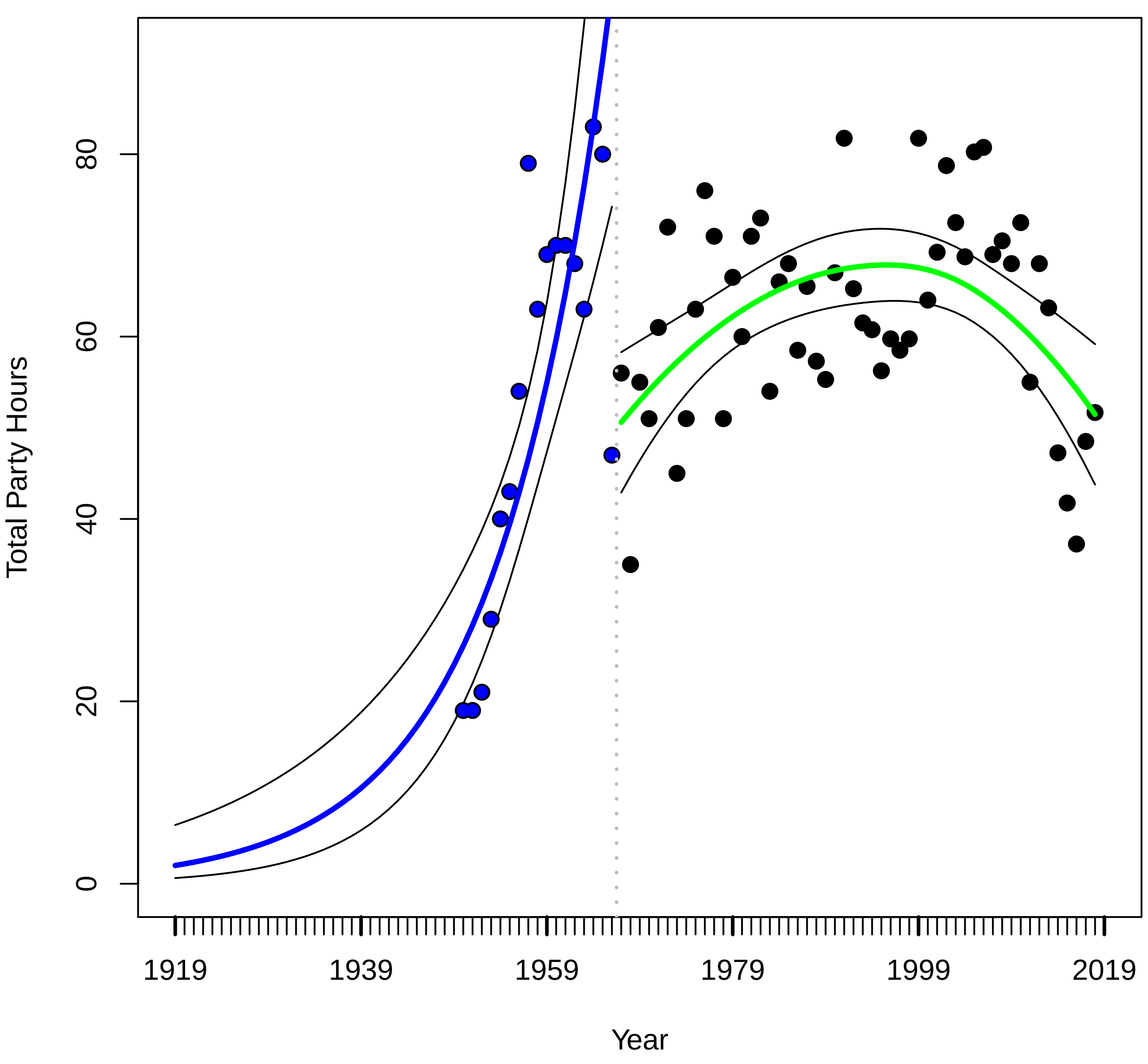

Total Party Hours
0
20
40
60
80
1919
1939
1959
1979
1999
2019
Year

LAST

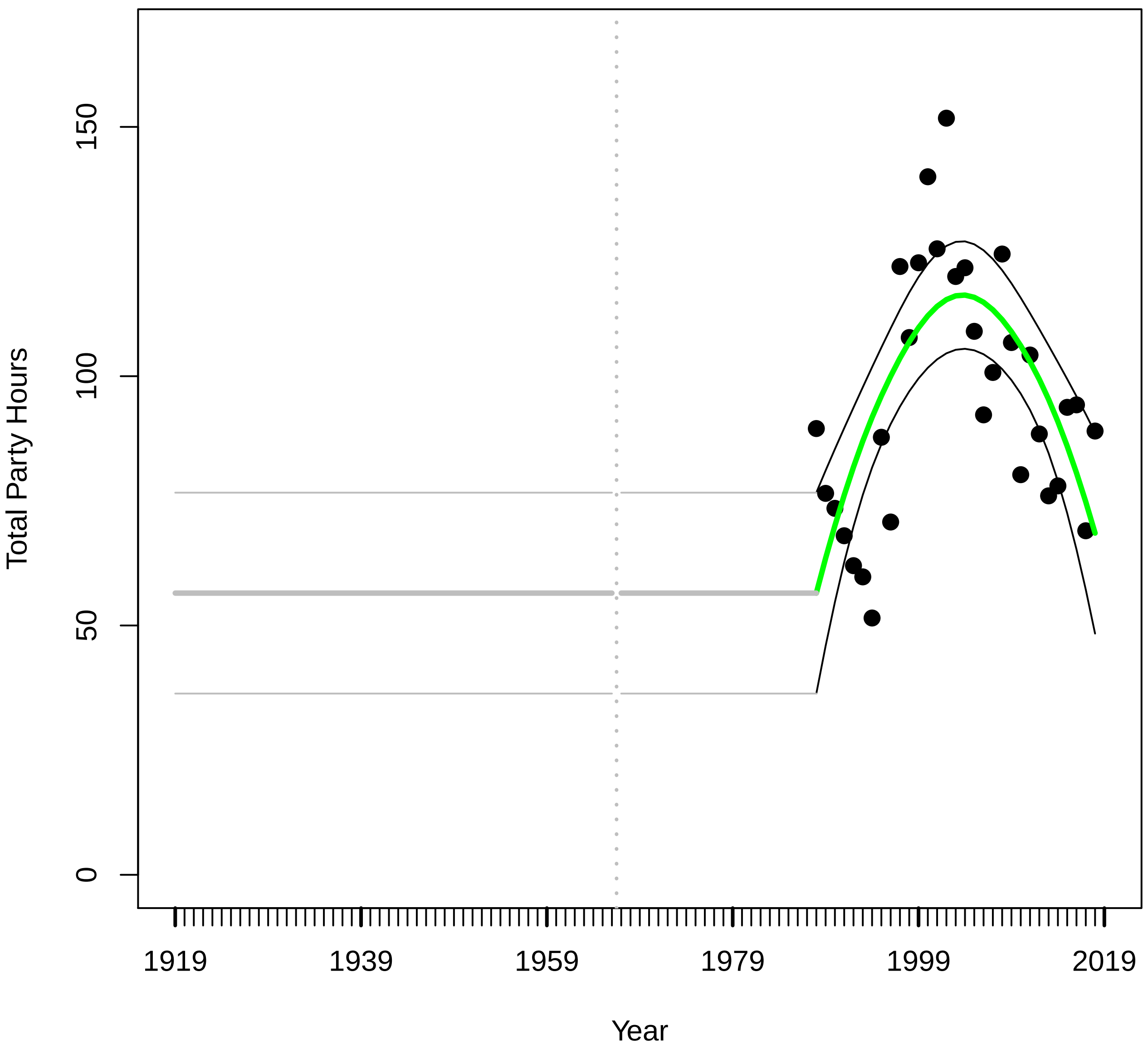

Total Party Hours
0
50
100
150
1919
1939
1959
1979
1999
2019
Year

MA0J

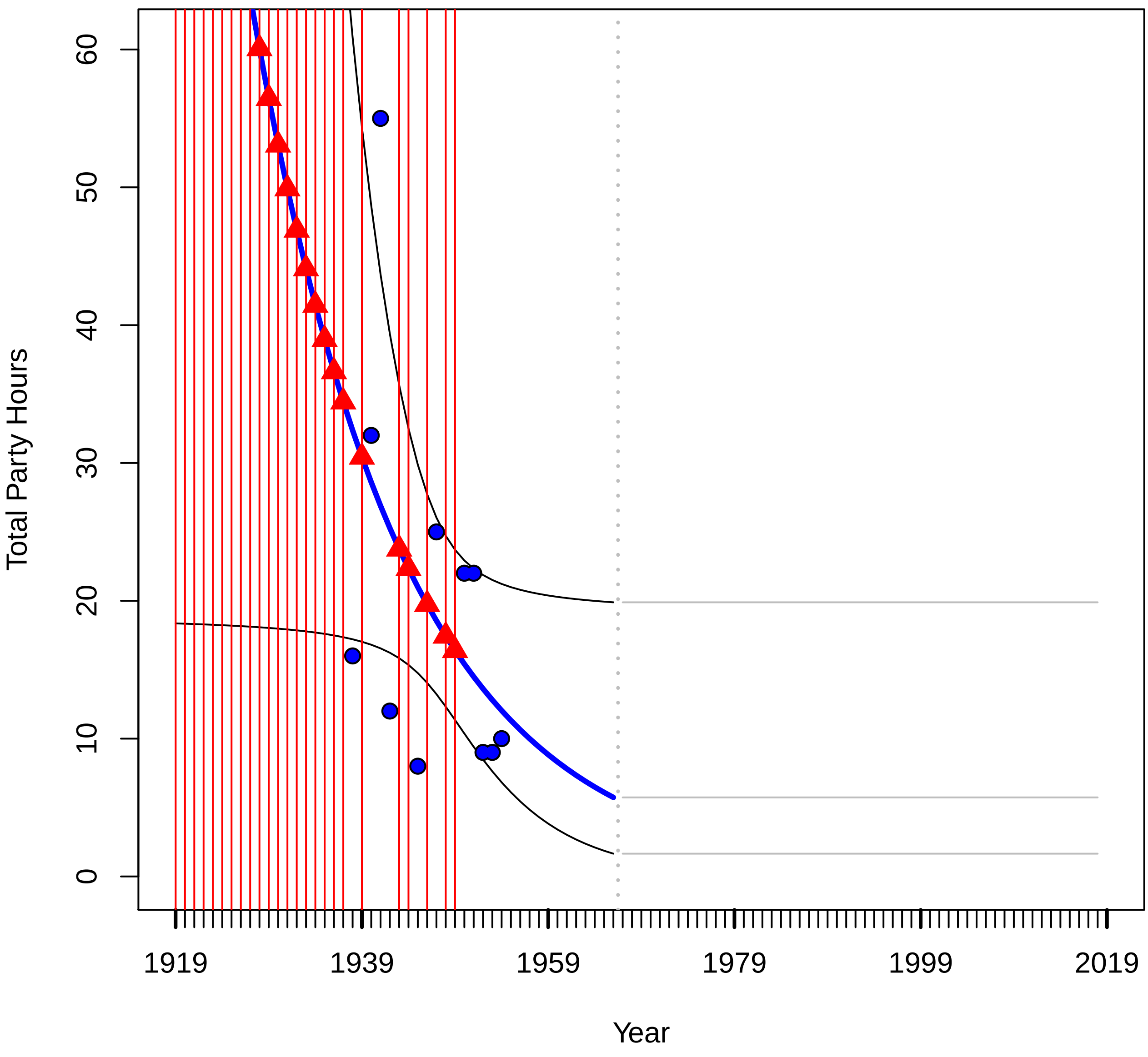

Total Party Hours
0
10
20
30
40
50
60
1919
1939
1959
1979
1999
2019
Year

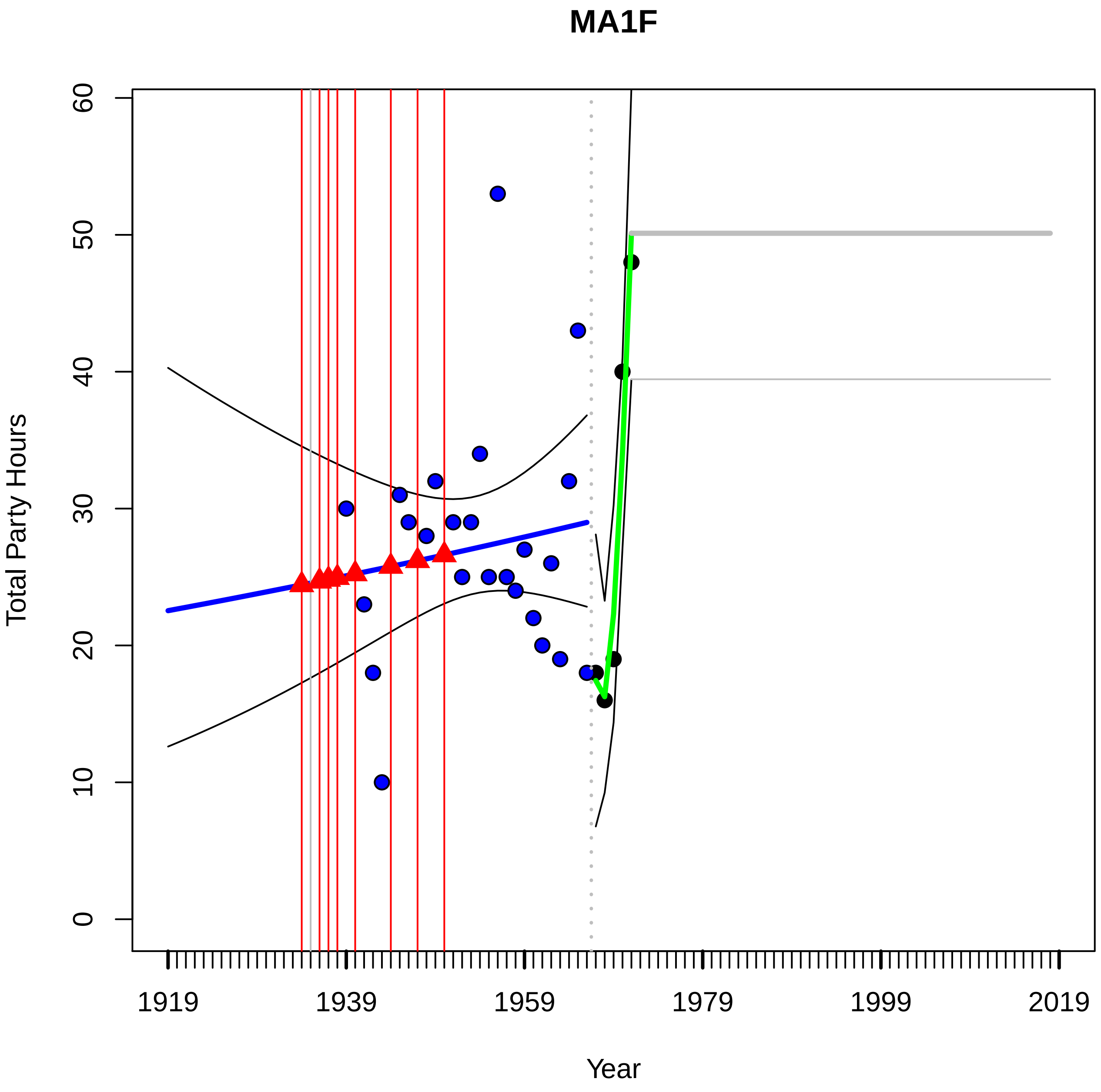
MA1F
Total Party Hours
Year
0
10
20
30
40
50
60
1919
1939
1959
1979
1999
2019

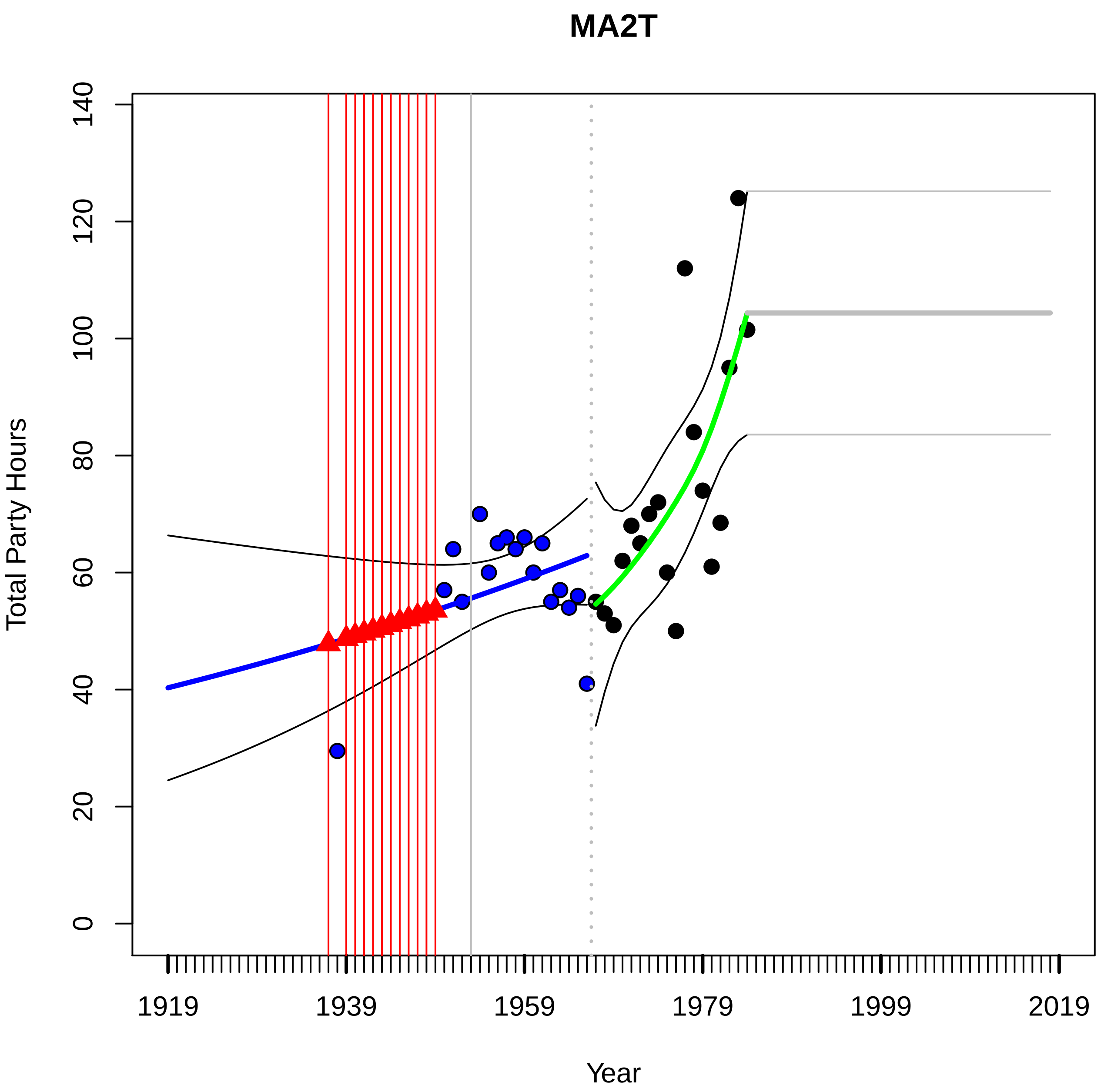

MA2T
Total Party Hours
Year
0
20
40
60
80
100
120
140
1919
1939
1959
1979
1999
2019

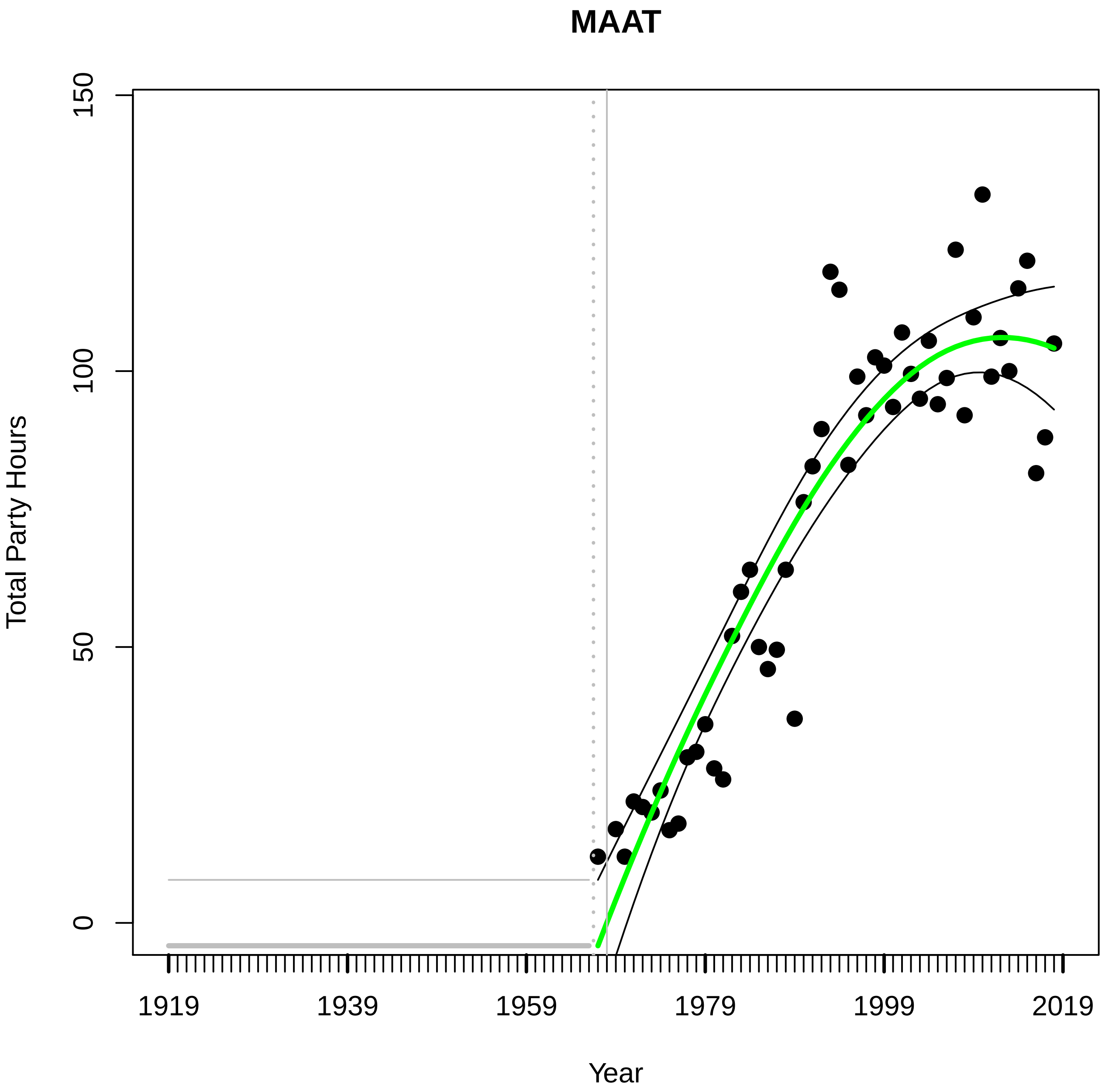
MAAT
Total Party Hours
0
50
100
150
1919
1939
1959
1979
1999
2019
Year

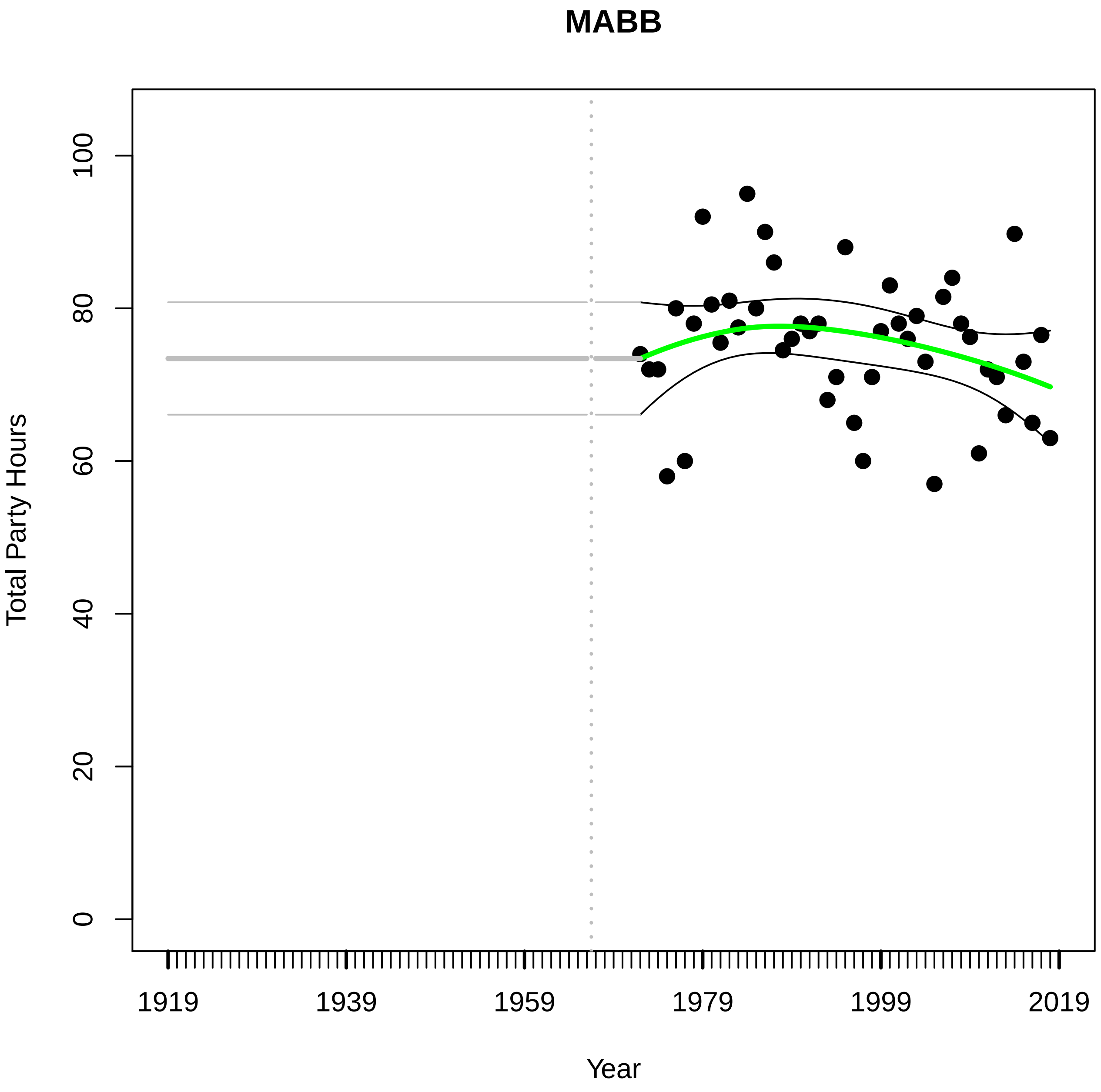

MABB
Total Party Hours
0
20
40
60
80
100
1919
1939
1959
1979
1999
2019
Year

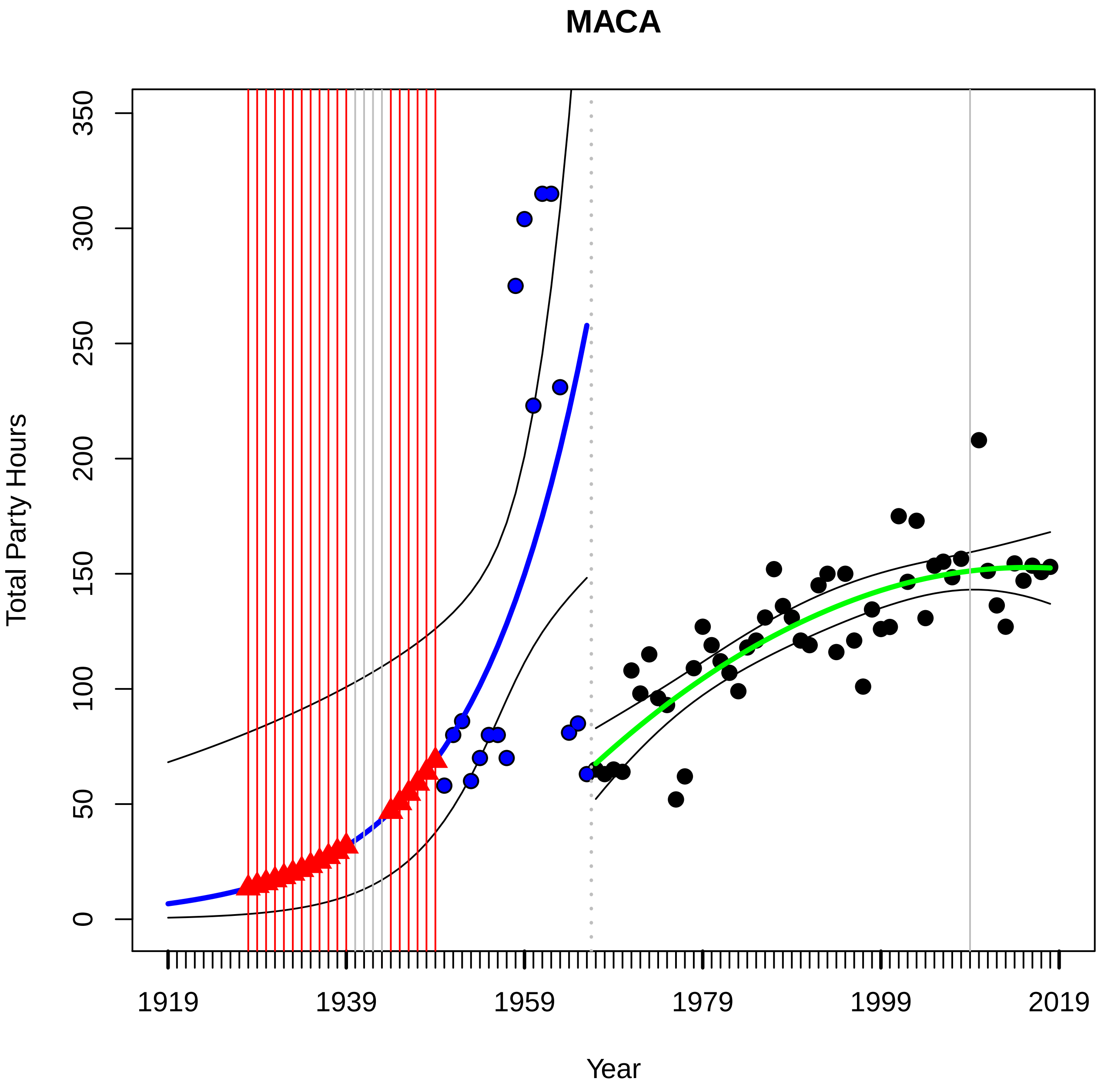

MACA
Total Party Hours
Year
0
50
100
150
200
250
300
350
1919
1939
1959
1979
1999
2019

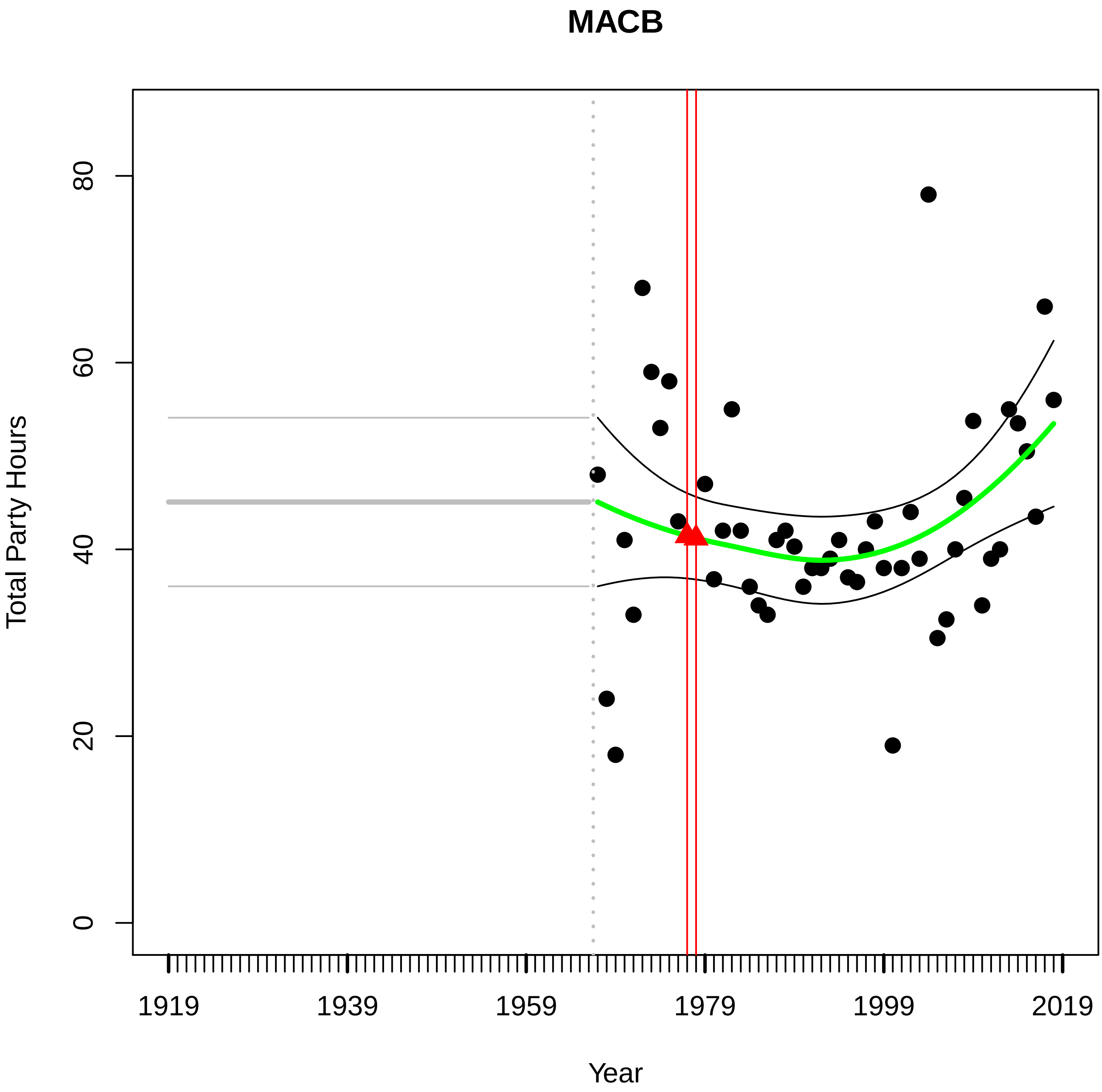

MACB
Total Party Hours
0
20
40
60
80
1919
1939
1959
1979
1999
2019
Year

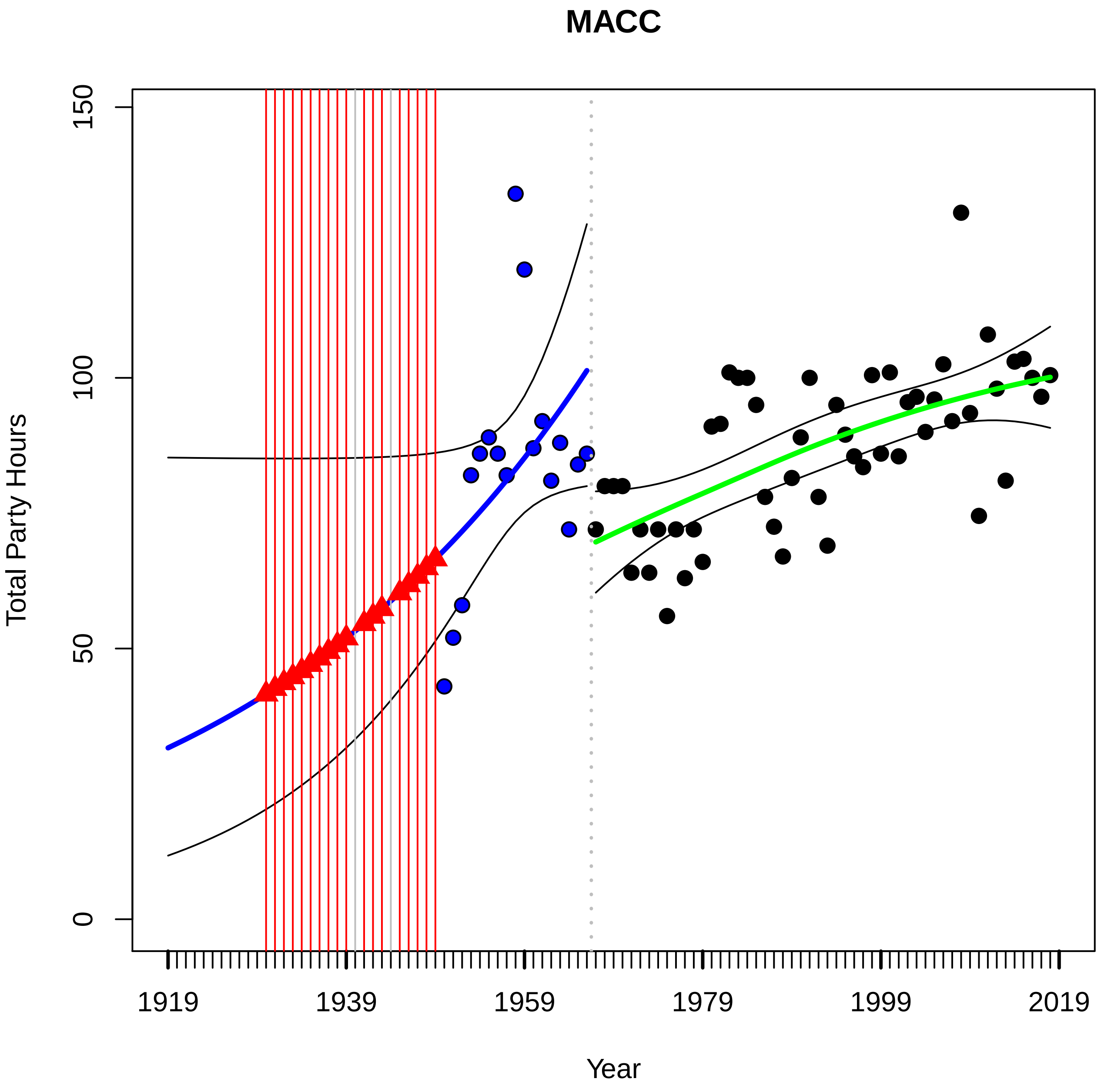

MACC
Total Party Hours
0
50
100
150
1919
1939
1959
1979
1999
2019
Year

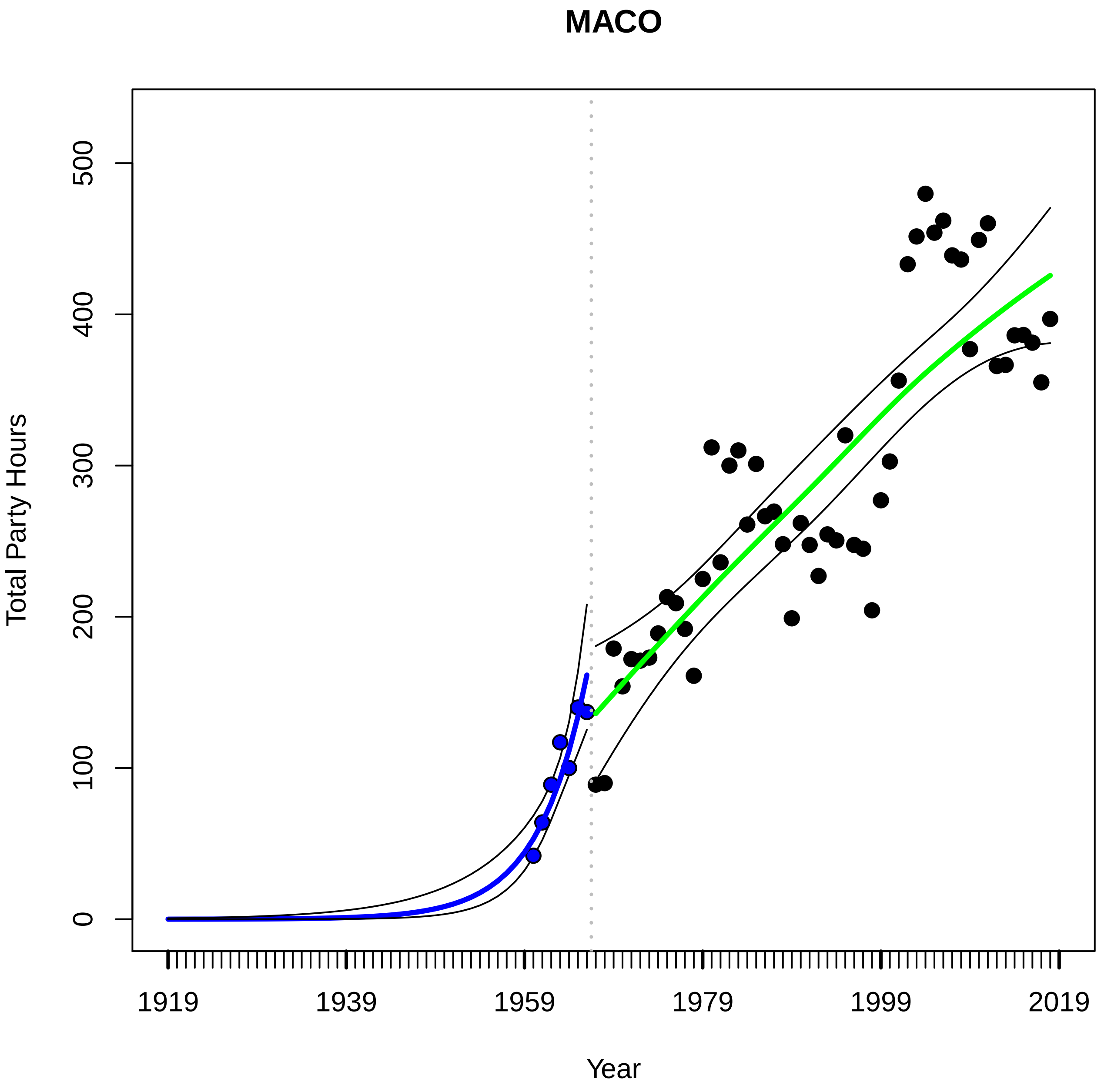
MACO
Total Party Hours
0
100
200
300
400
500
1919
1939
1959
1979
1999
2019
Year

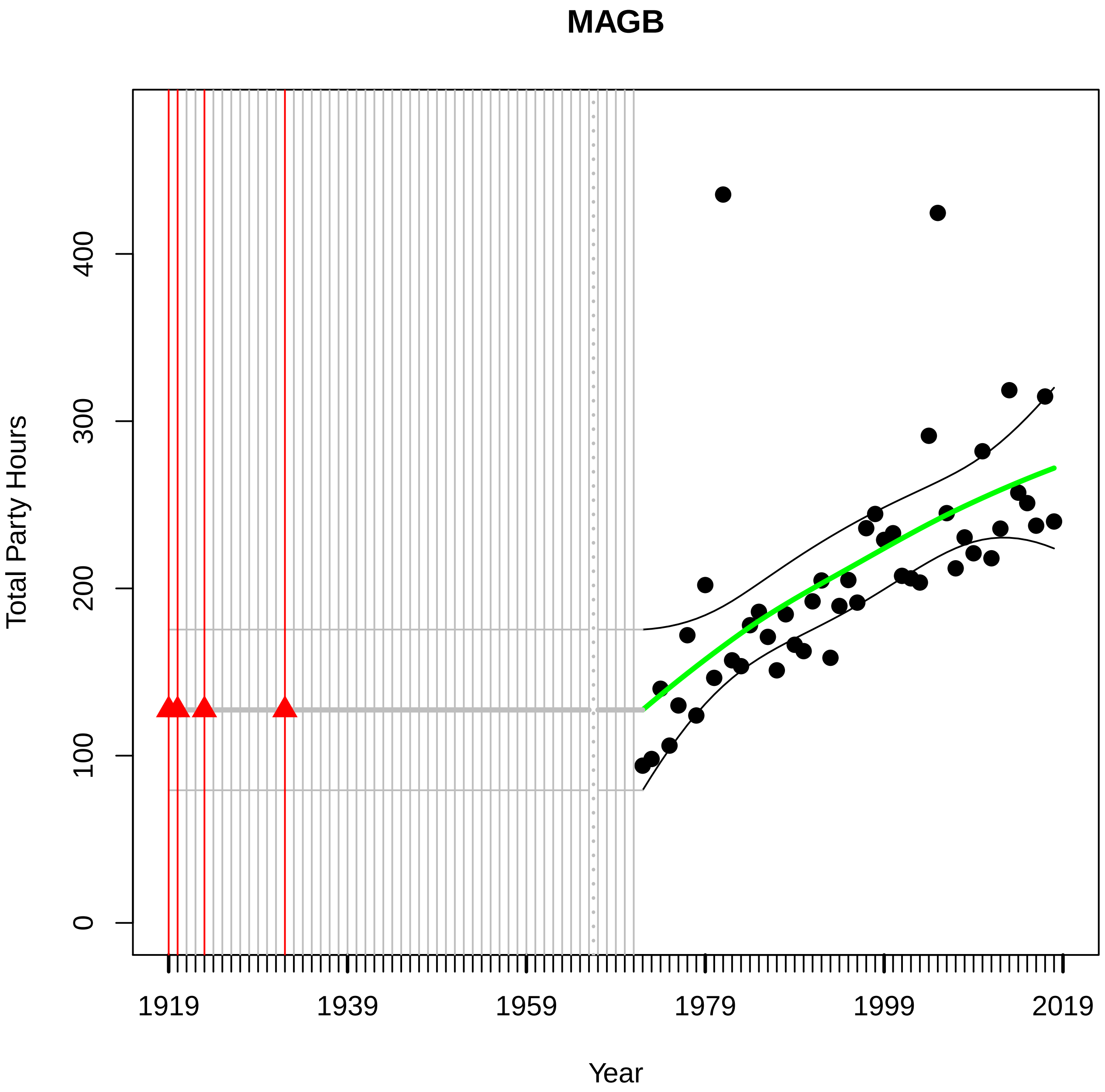
MAGB
Total Party Hours
0
100
200
300
400
Year
1919
1939
1959
1979
1999
2019

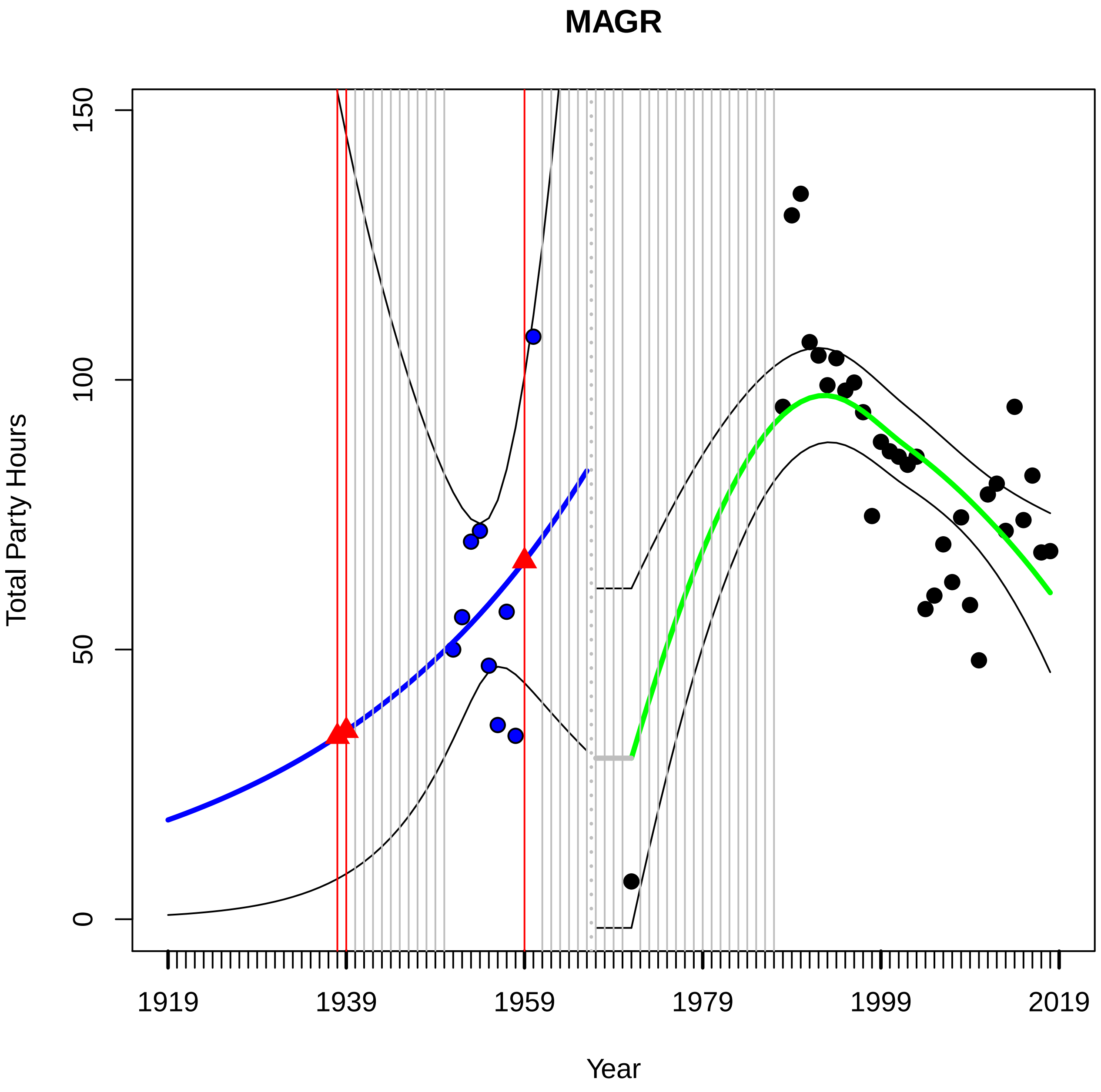

MAGR
Total Party Hours
Year
0
50
100
150
1919
1939
1959
1979
1999
2019

MAMA

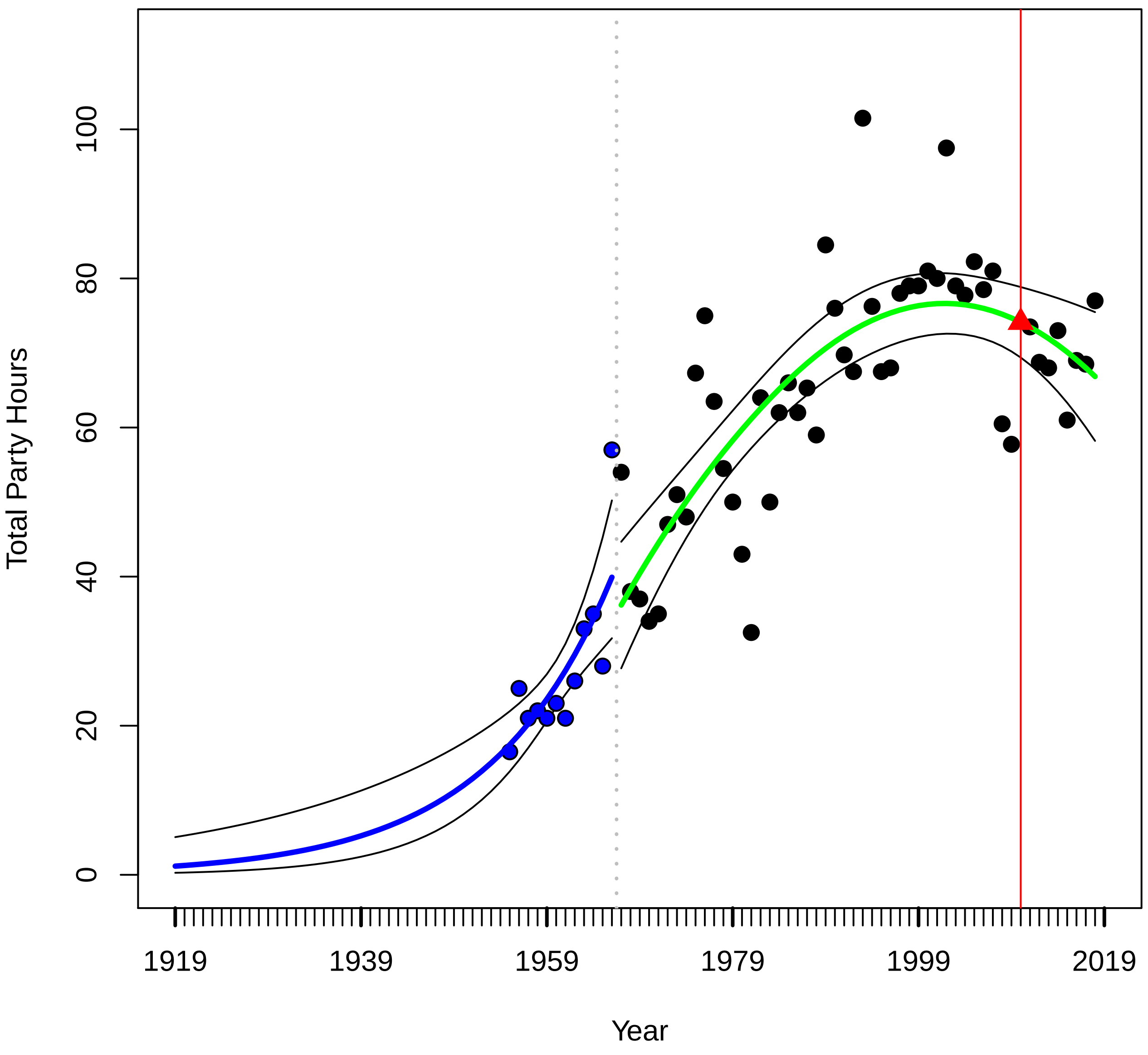

Total Party Hours
0
20
40
60
80
100
1919
1939
1959
1979
1999
2019
Year

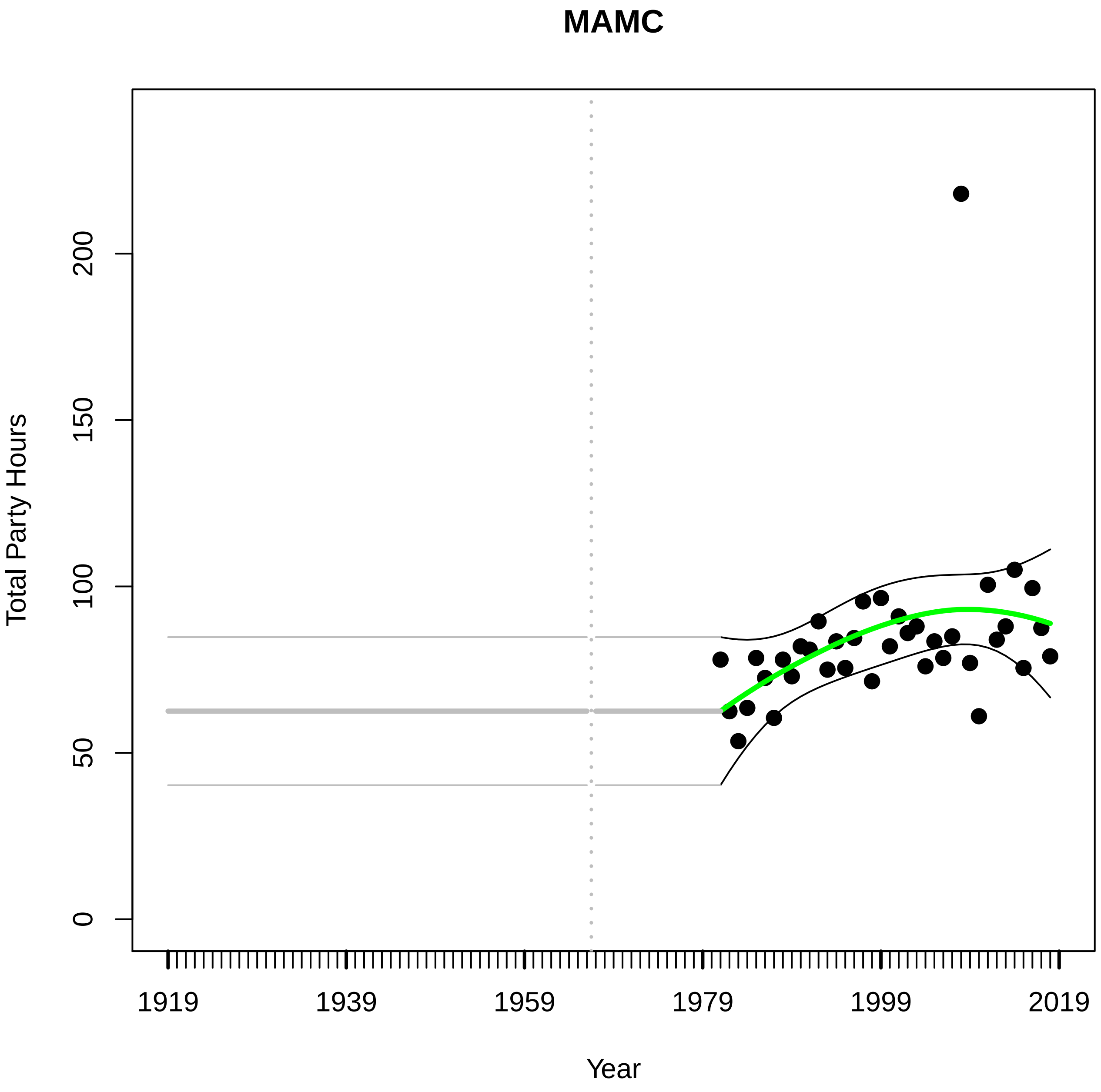
MAMC
Total Party Hours
0
50
100
150
200
1919
1939
1959
1979
1999
2019
Year

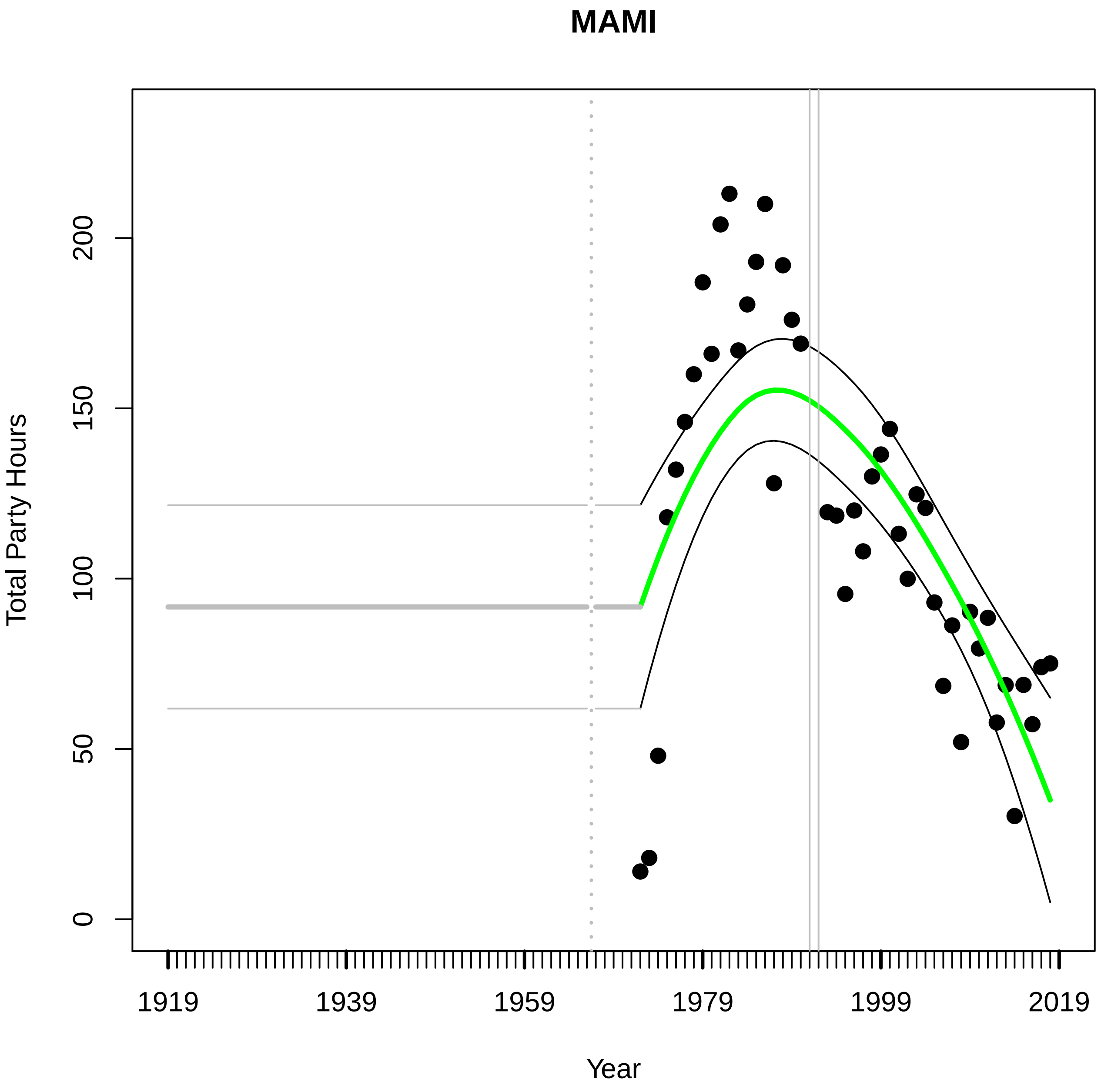

MAMI
Total Party Hours
Year
0
50
100
150
200
1919
1939
1959
1979
1999
2019

MAMV

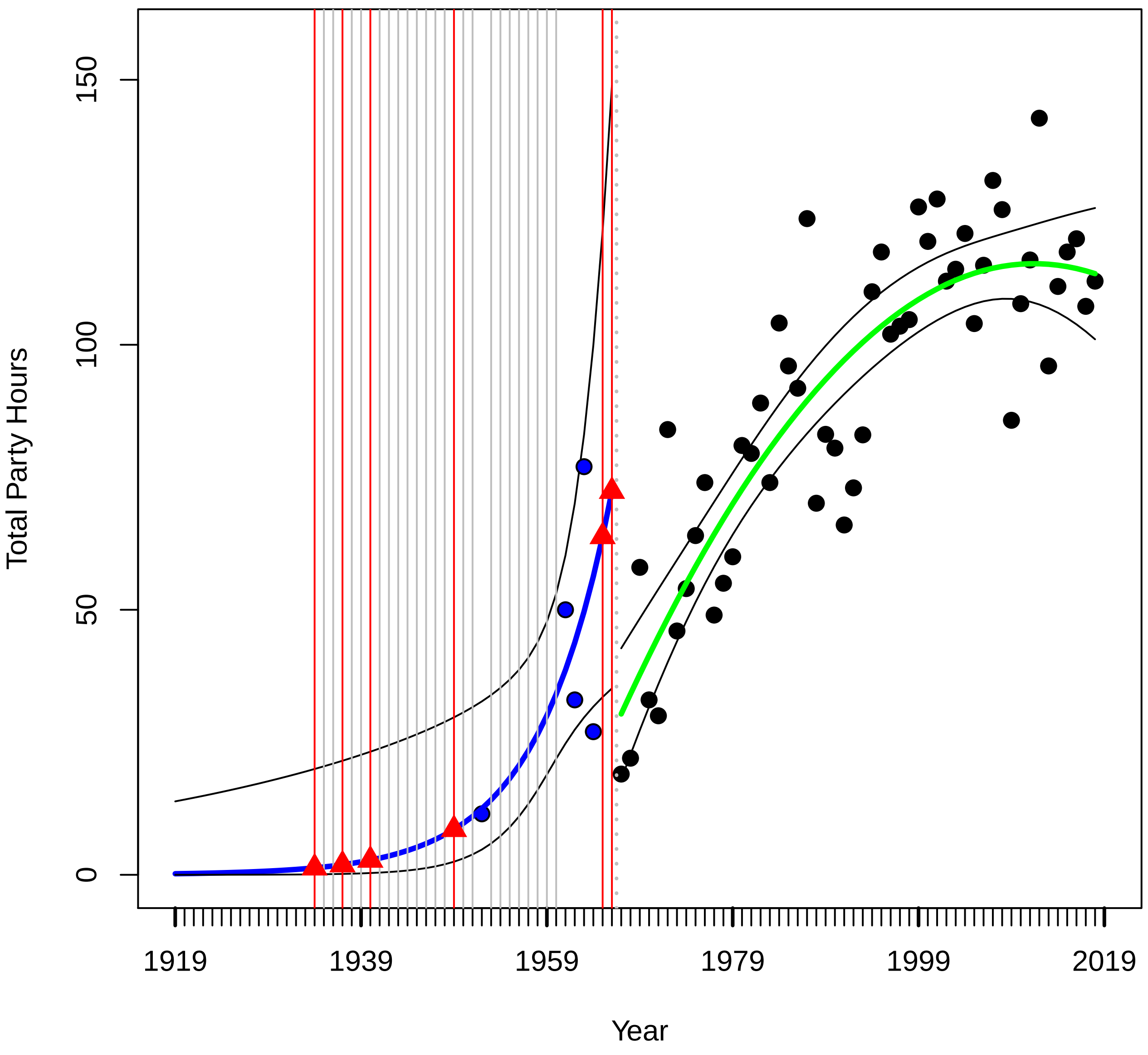

Total Party Hours
150
100
50
0
1919
1939
1959
1979
1999
2019
Year

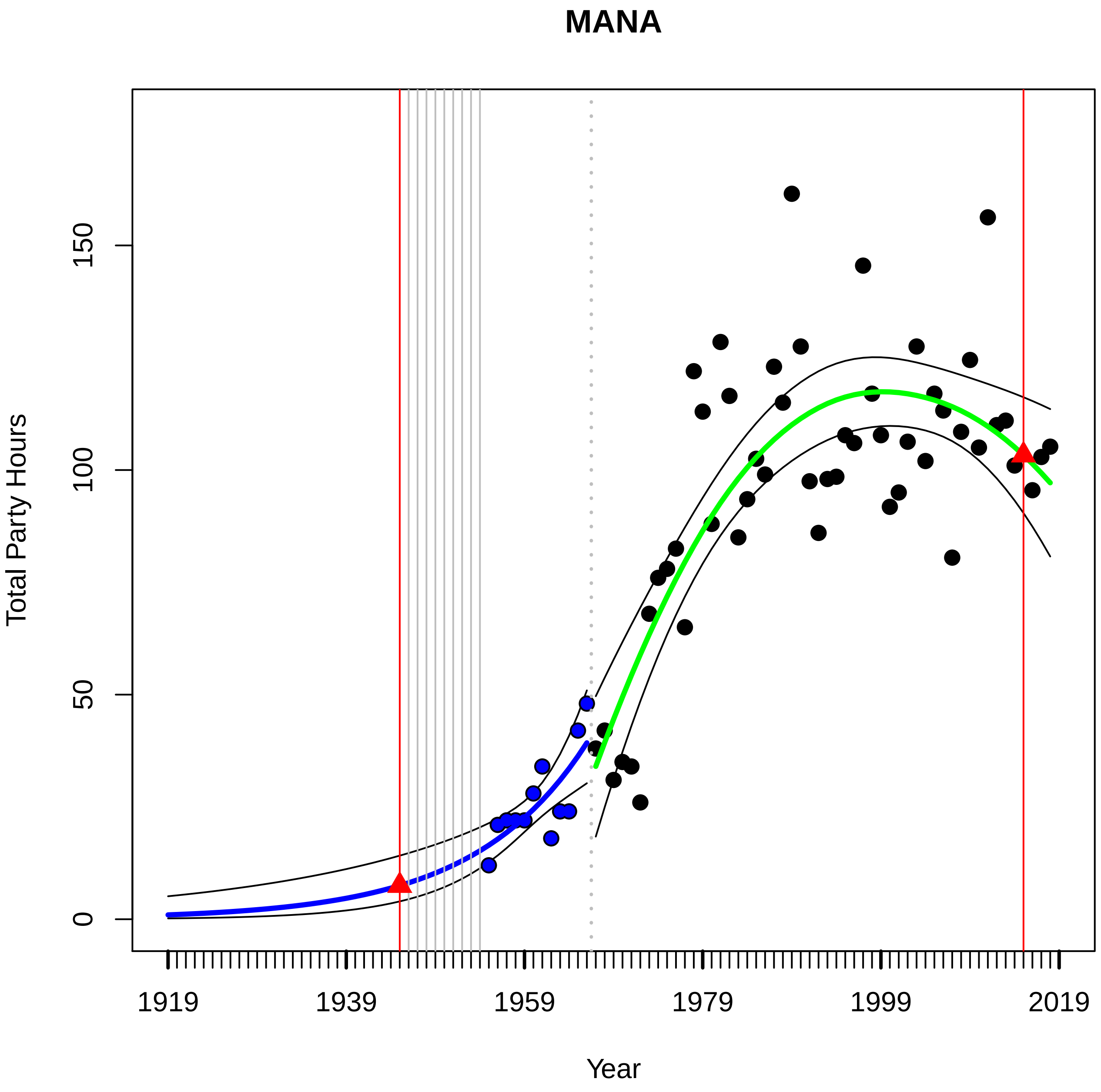

MANA
Total Party Hours
150
100
50
0
1919
1939
1959
1979
1999
2019
Year

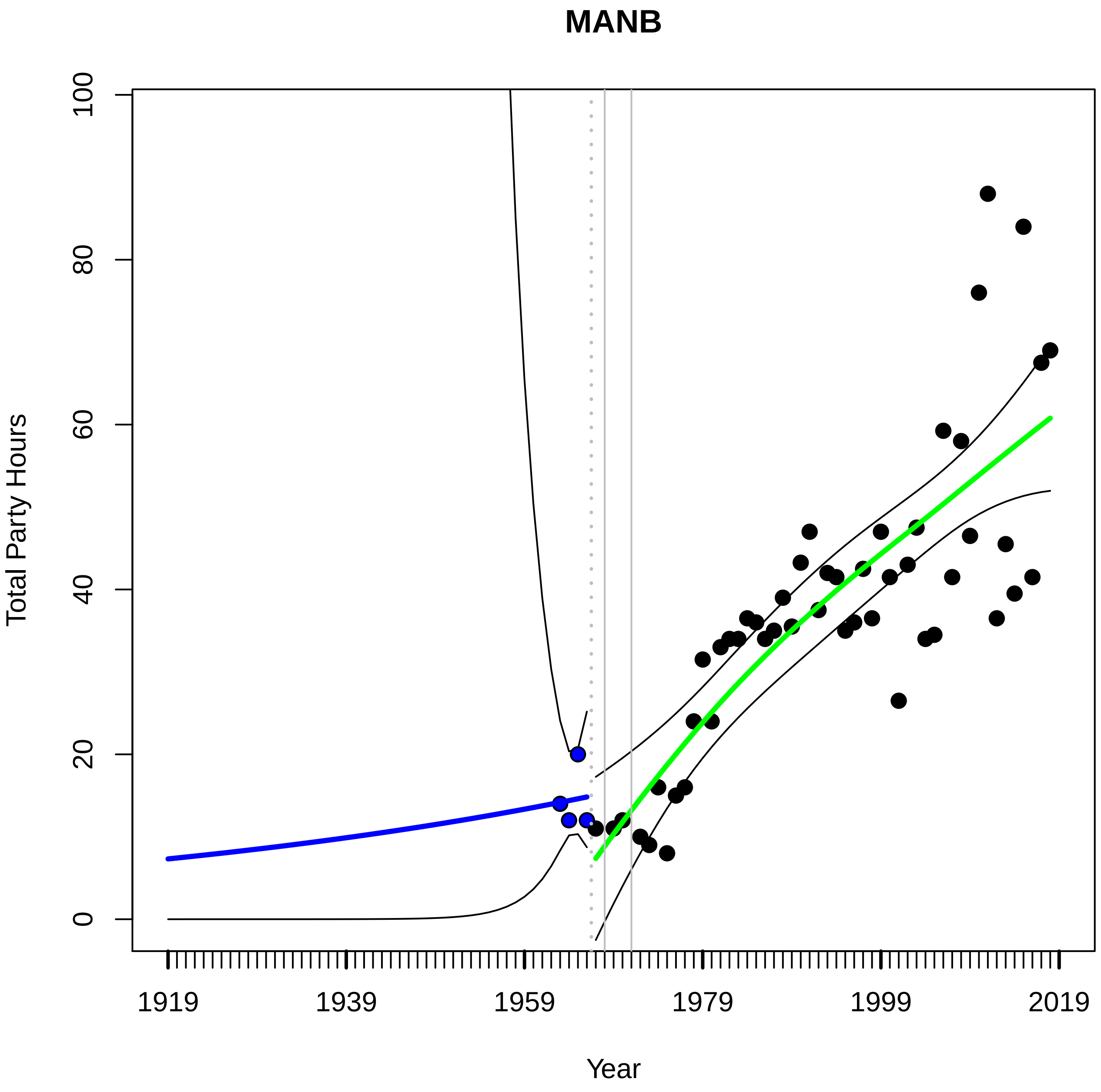
MANB
Total Party Hours
Year
0
20
40
60
80
100
1919
1939
1959
1979
1999
2019

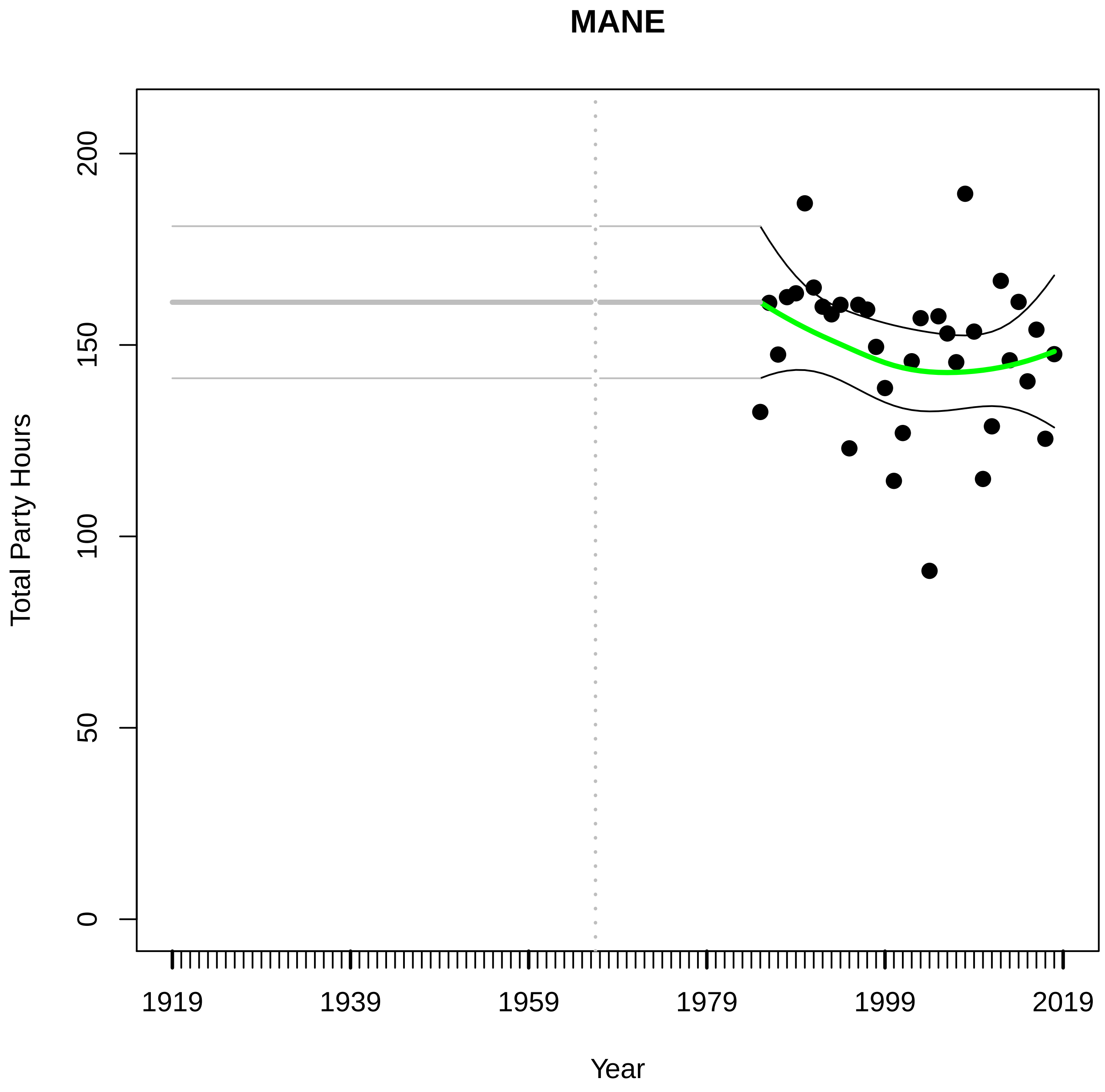

MANE
Total Party Hours
0
50
100
150
200
1919
1939
1959
1979
1999
2019
Year

MANF

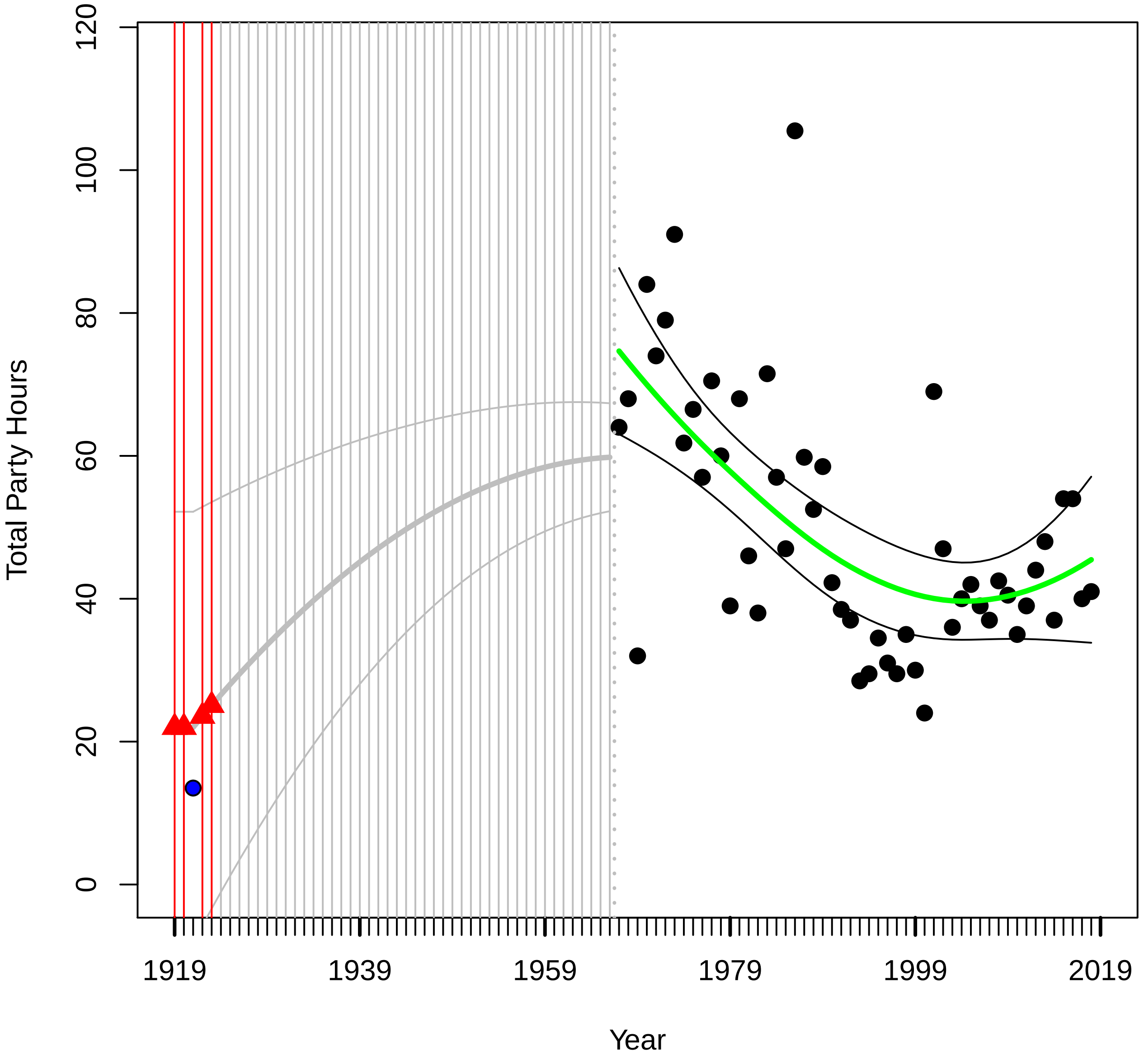

Total Party Hours
120
100
80
60
40
20
0
1919
1939
1959
1979
1999
2019
Year

MANO

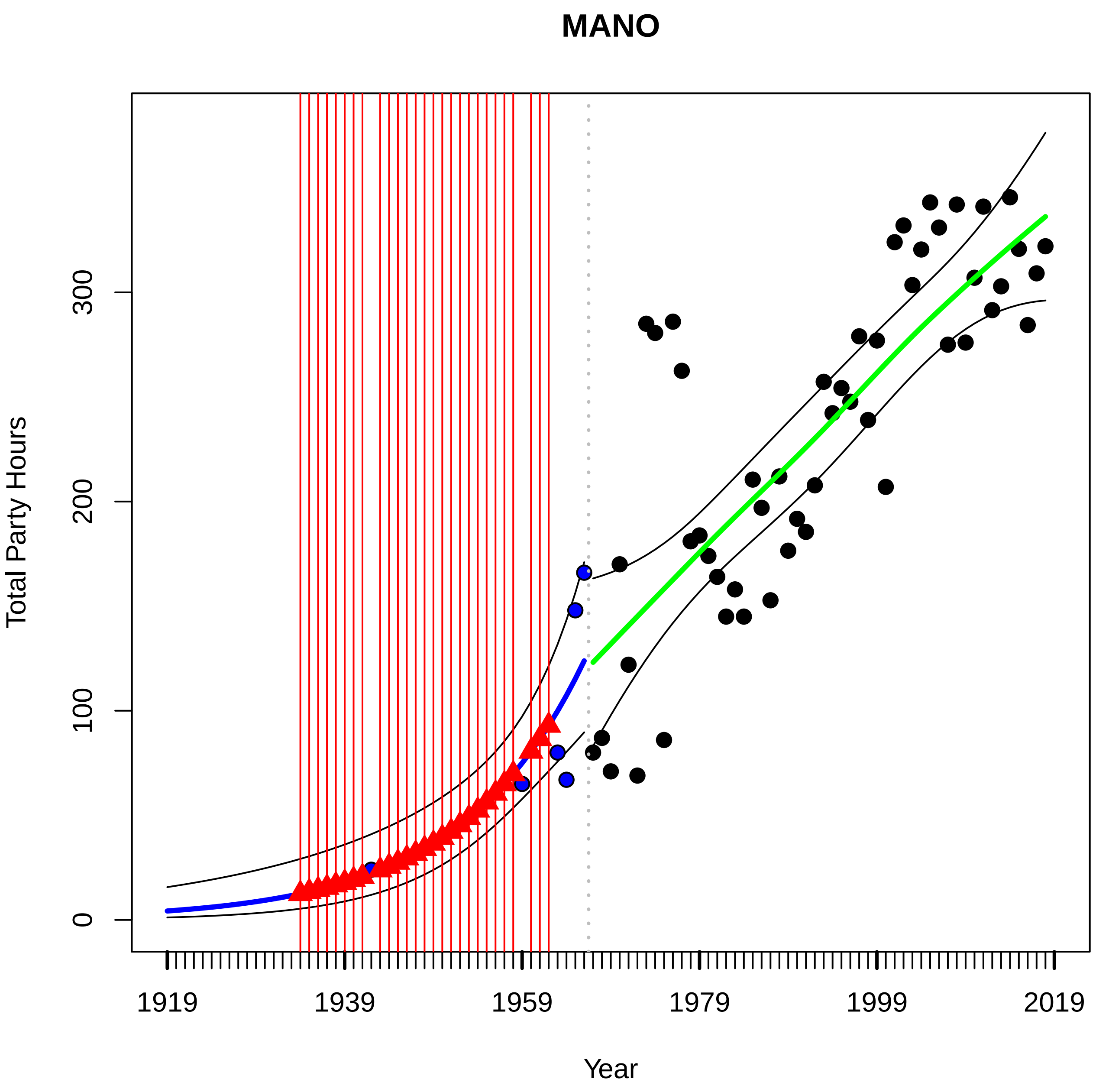

MANO
Total Party Hours
0
100
200
300
Year
1919
1939
1959
1979
1999
2019

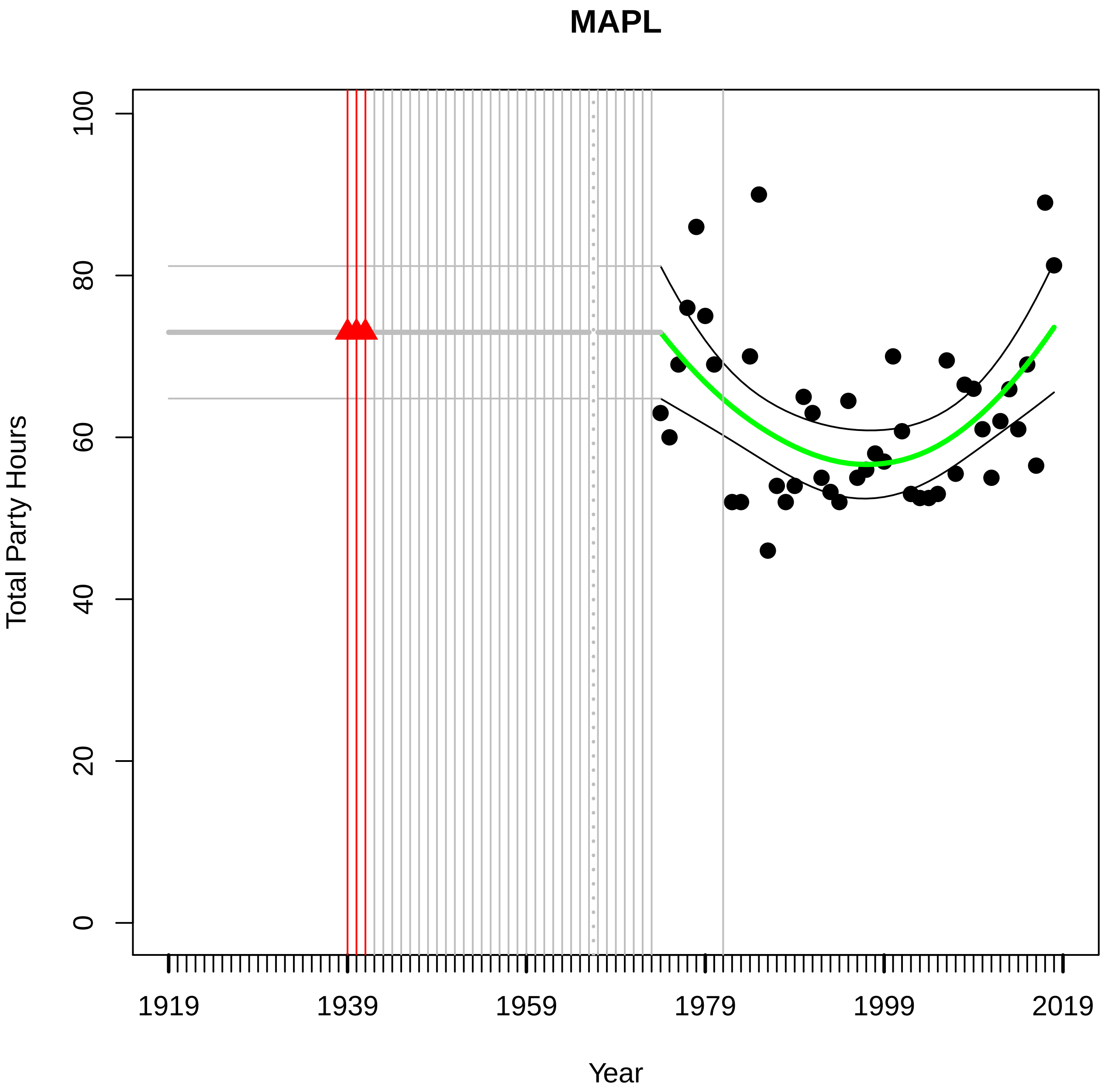
MAPL
Total Party Hours
0
20
40
60
80
100
1919
1939
1959
1979
1999
2019
Year

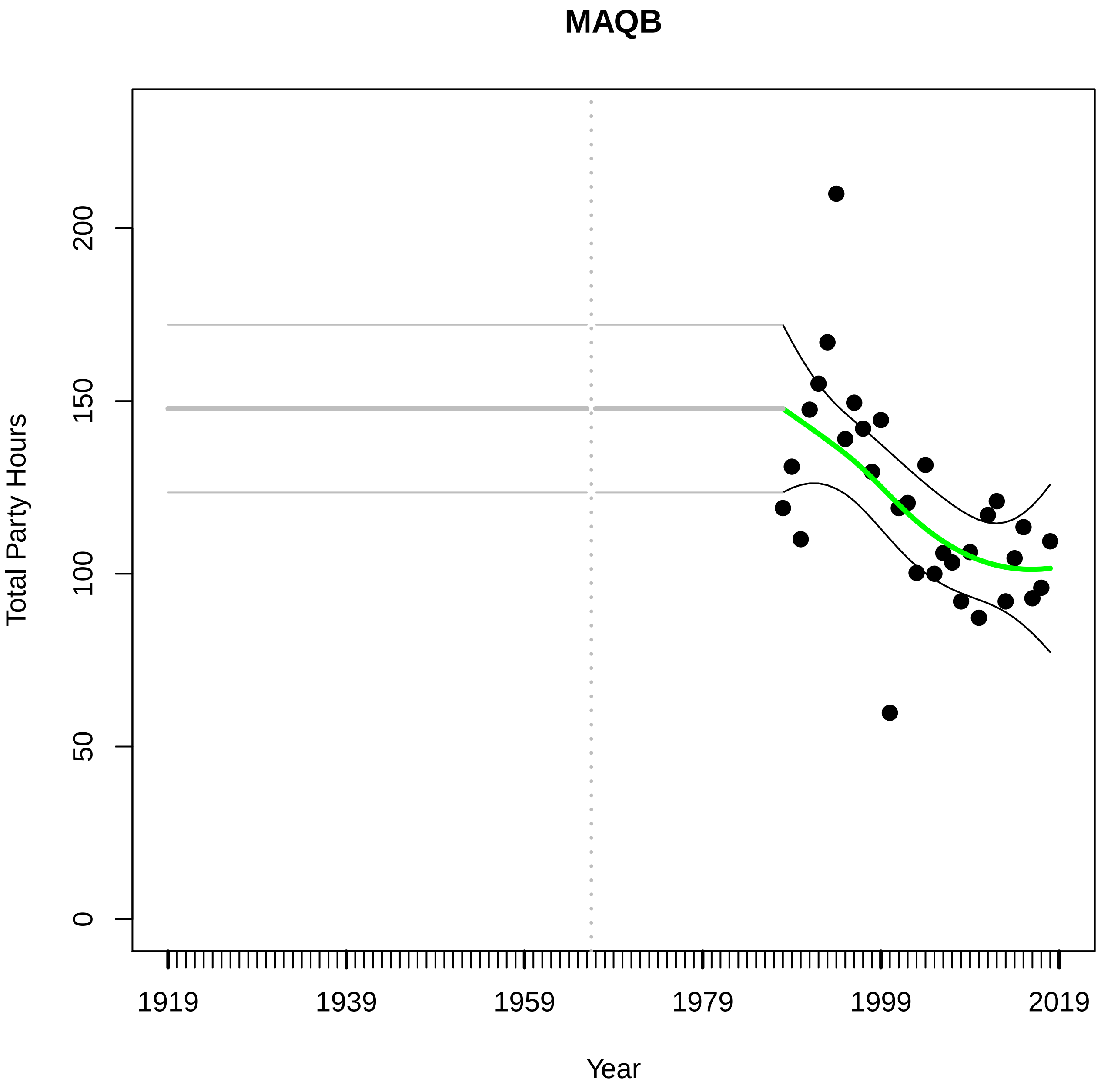

MAQB
Total Party Hours
0
50
100
150
200
Year
1919
1939
1959
1979
1999
2019

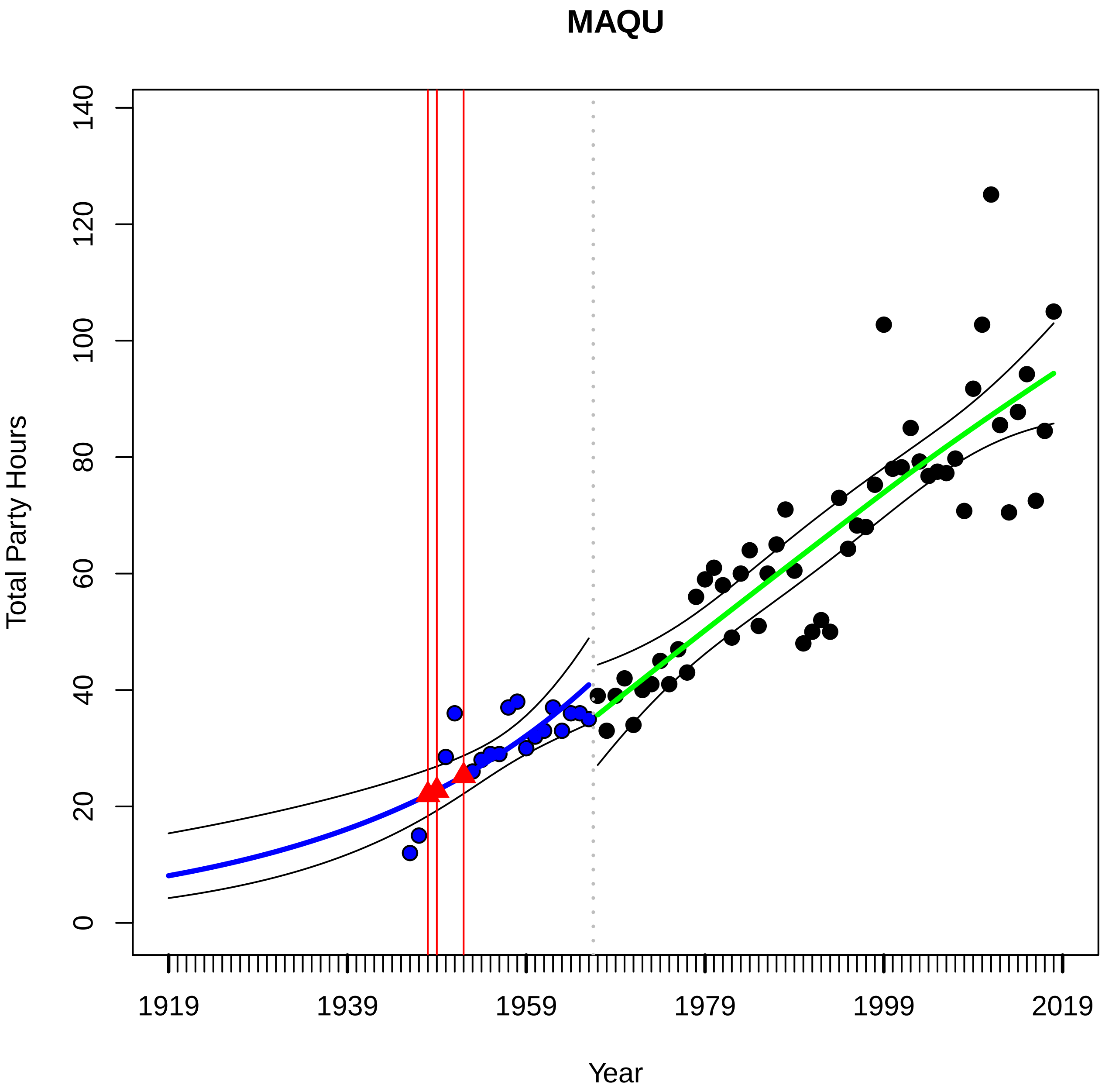
MAQU
Total Party Hours
Year
0
20
40
60
80
100
120
140
1919
1939
1959
1979
1999
2019

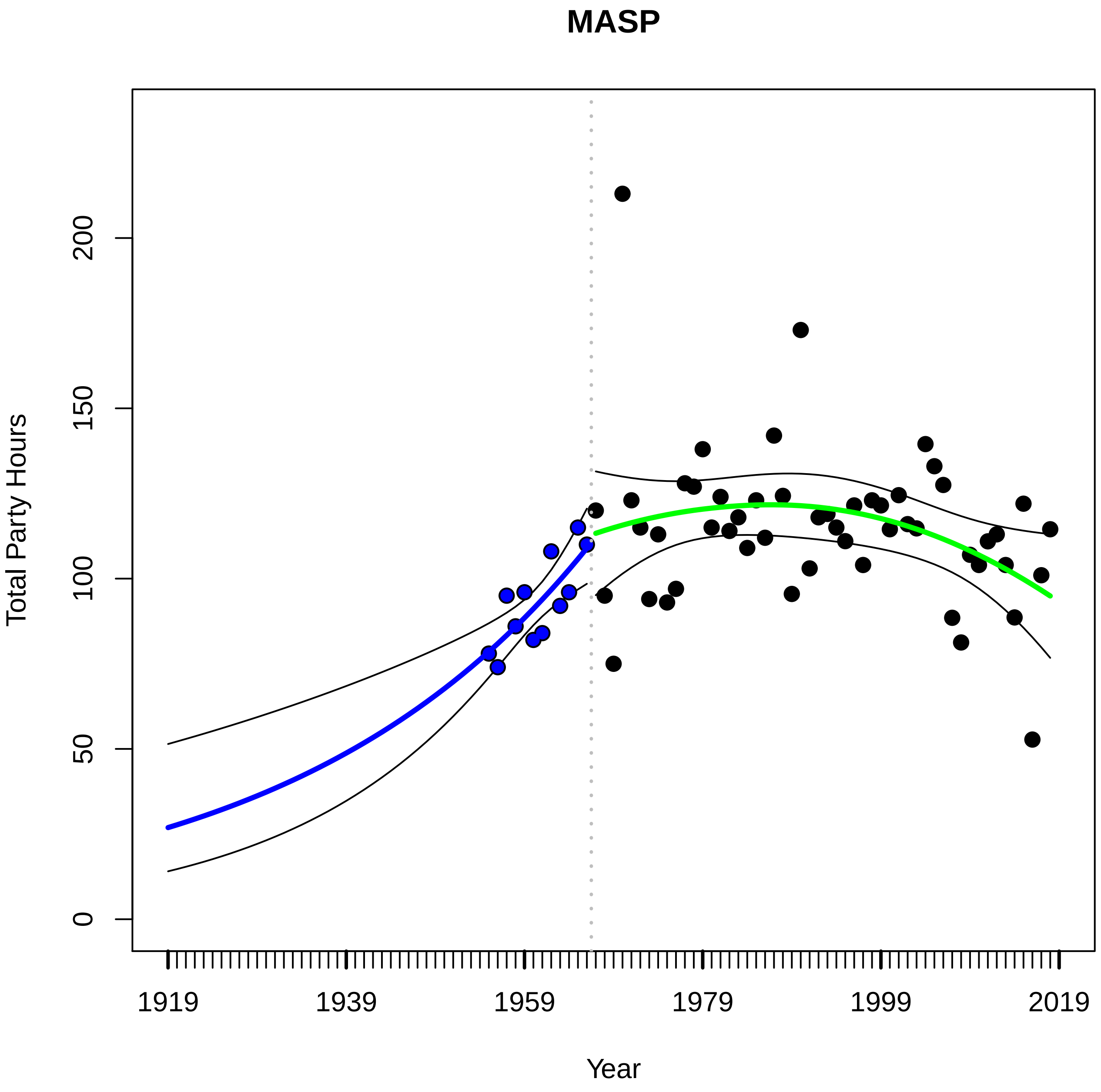
MASP
Total Party Hours
0
50
100
150
200
1919
1939
1959
1979
1999
2019
Year

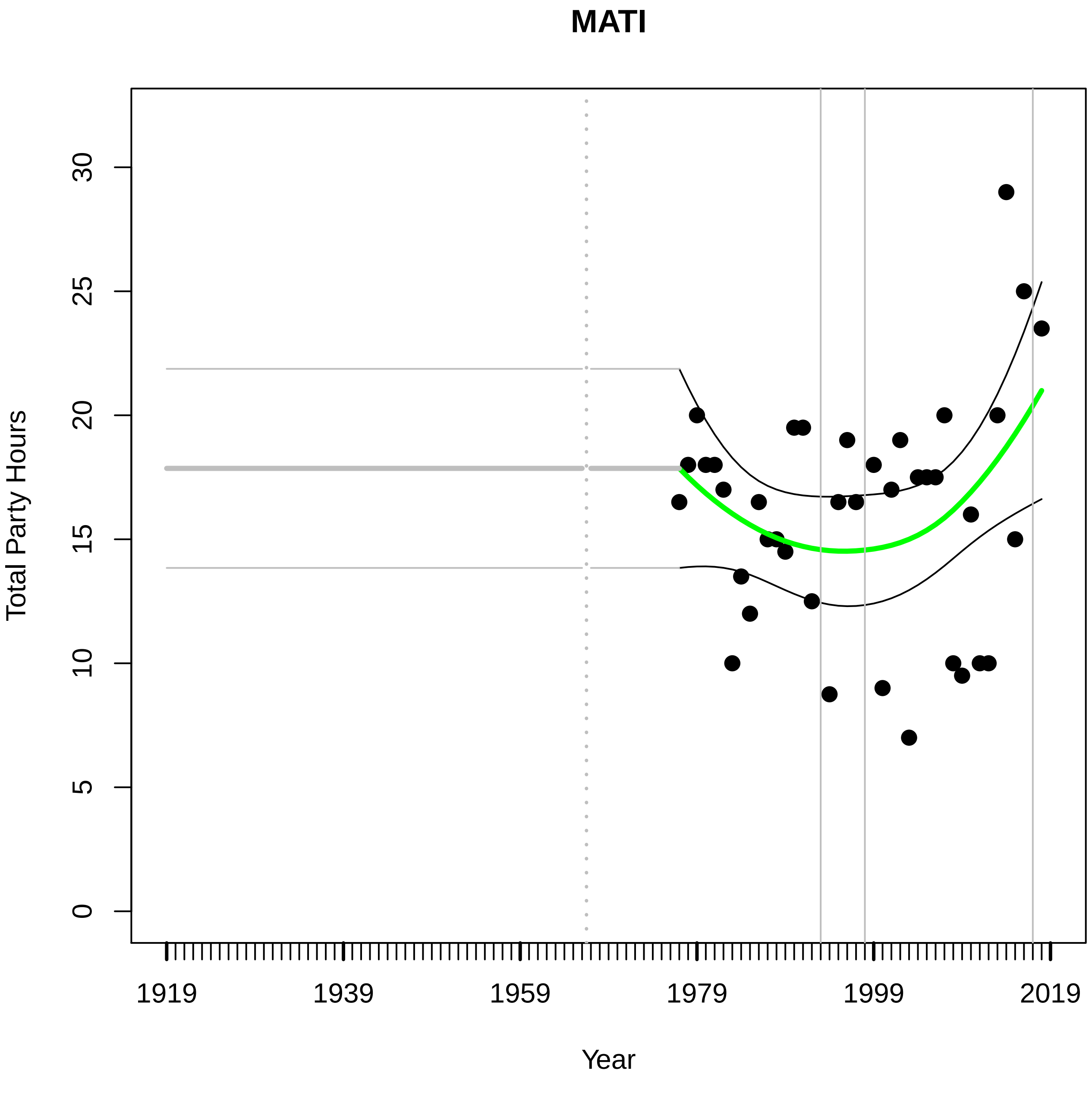

MATI
Total Party Hours
0
5
10
15
20
25
30
1919
1939
1959
1979
1999
2019
Year

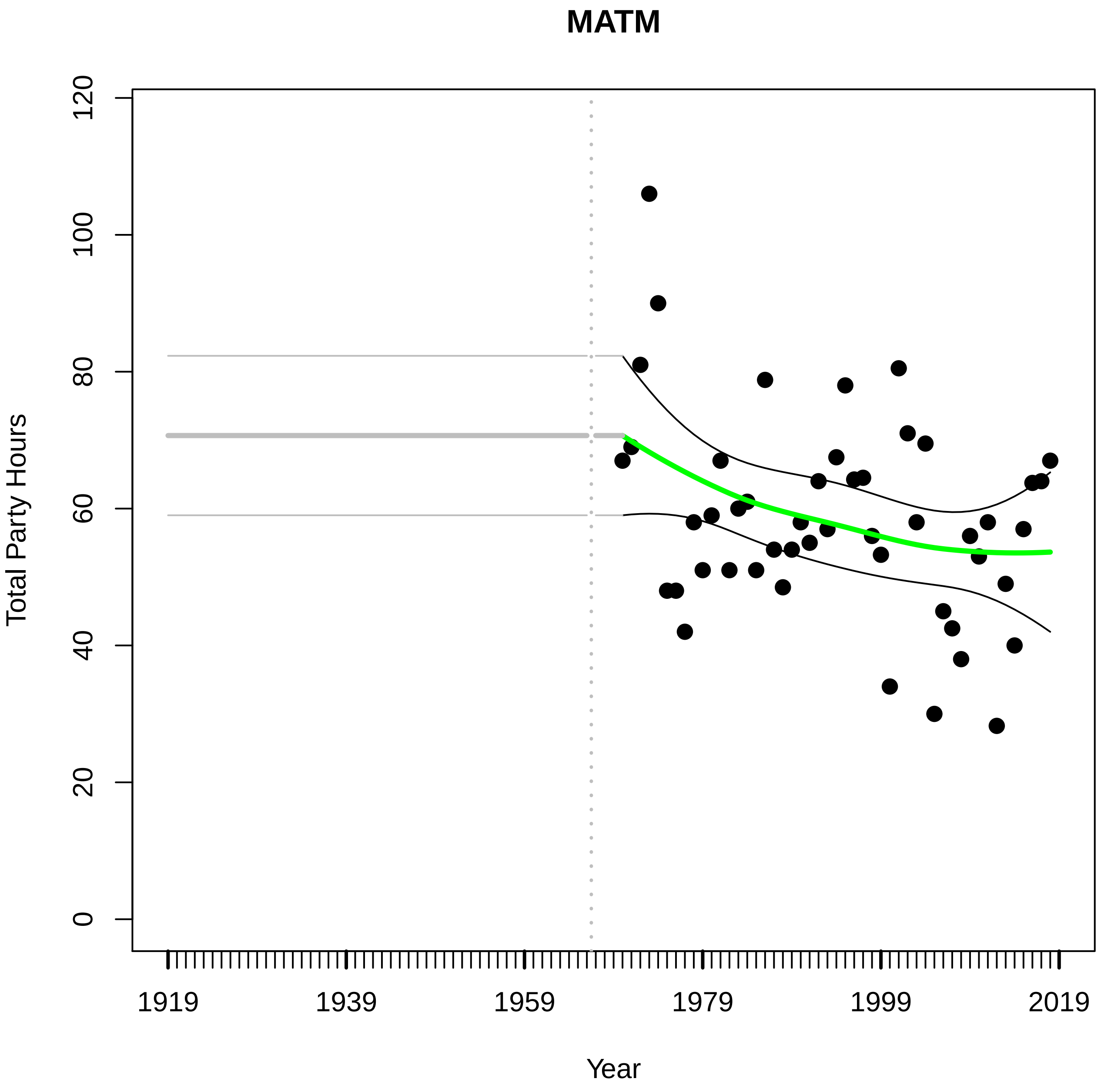

MATM
Total Party Hours
120
100
80
60
40
20
0
1919
1939
1959
1979
1999
2019
Year

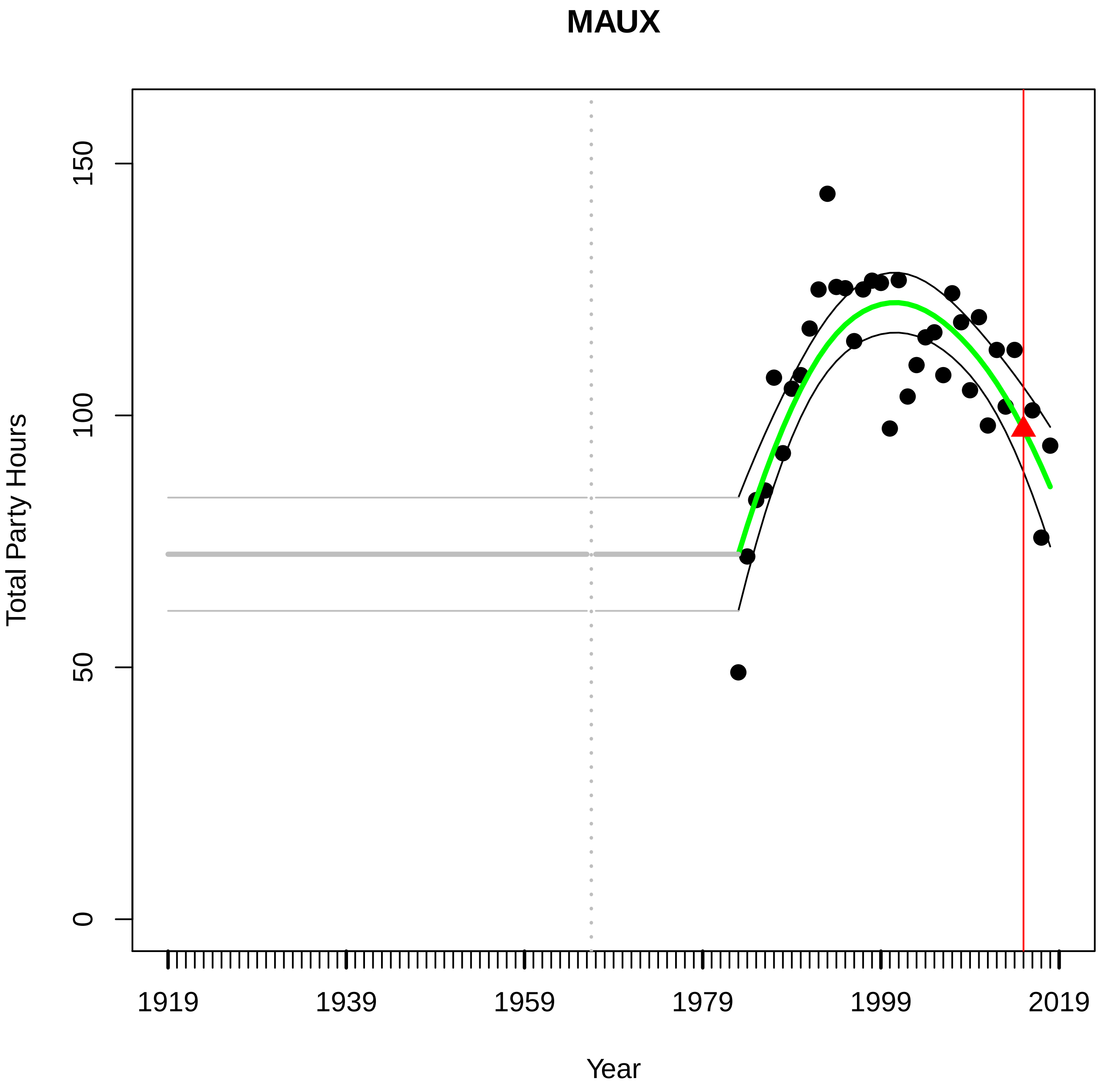

MAUX
Total Party Hours
0
50
100
150
1919
1939
1959
1979
1999
2019
Year

MAWE

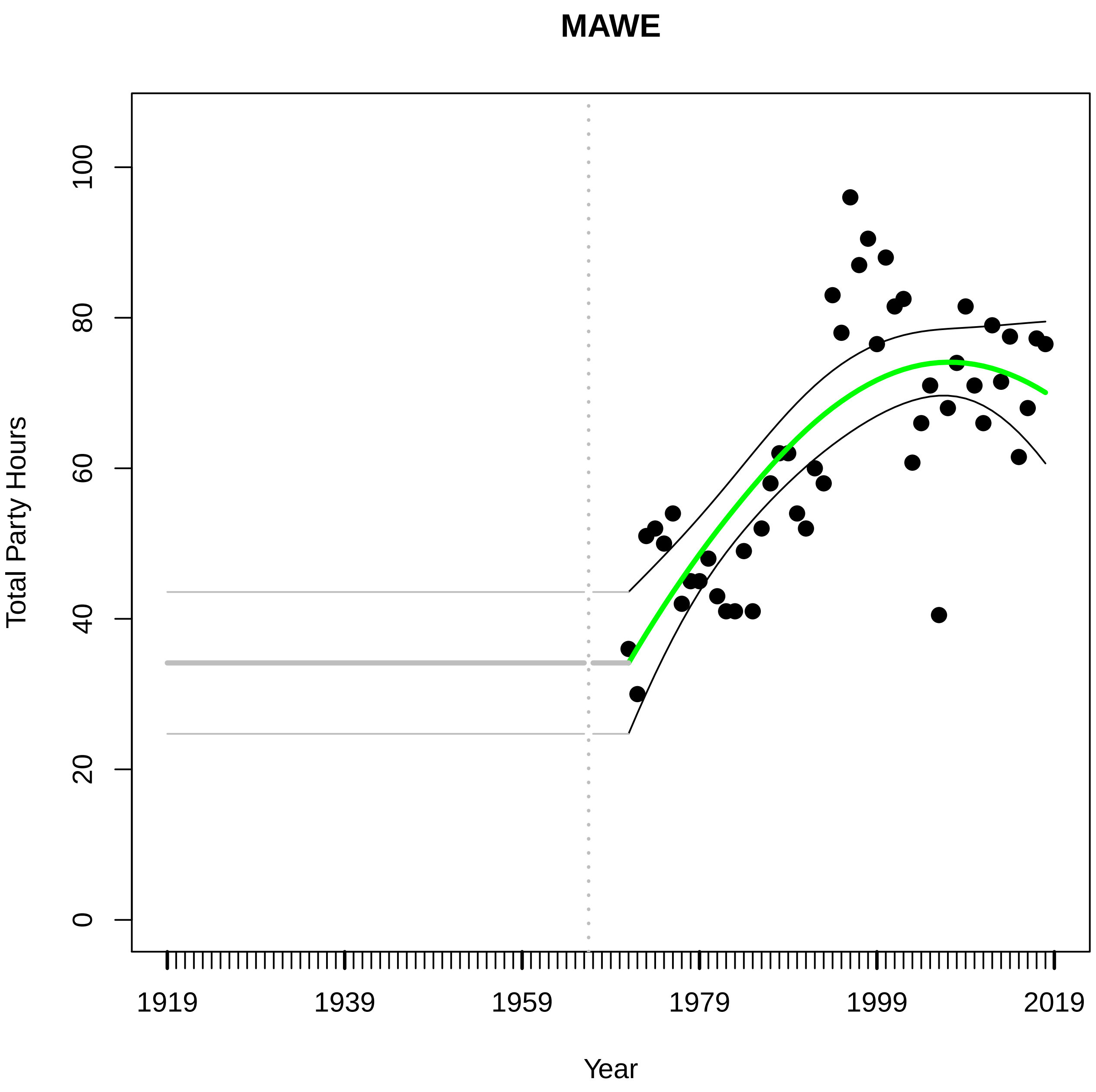

MAWE
Total Party Hours
0
20
40
60
80
100
1919
1939
1959
1979
1999
2019
Year

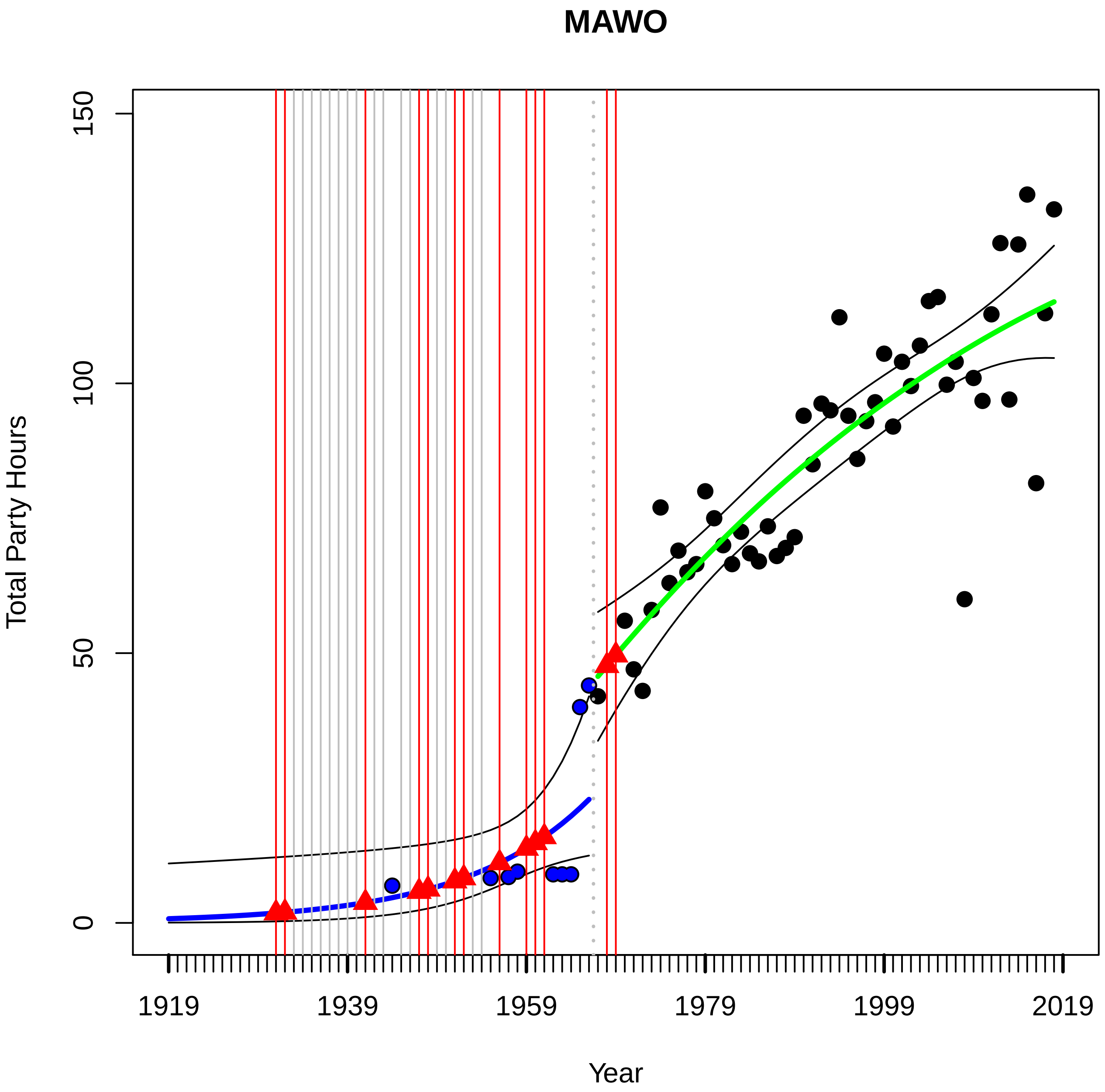
MAWO
Total Party Hours
0
50
100
150
1919
1939
1959
1979
1999
2019
Year

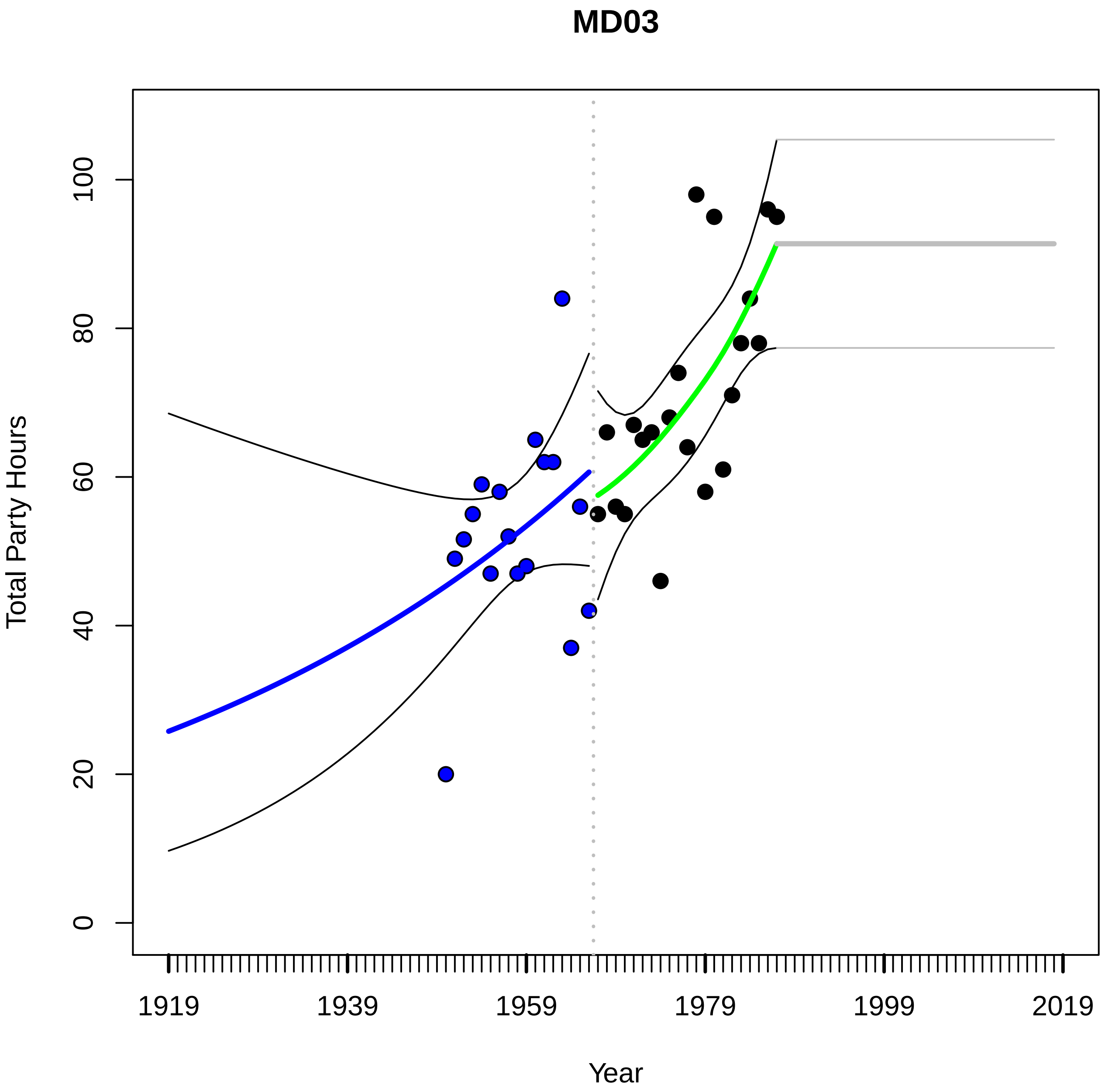
MD03
Total Party Hours
0
20
40
60
80
100
1919
1939
1959
1979
1999
2019
Year

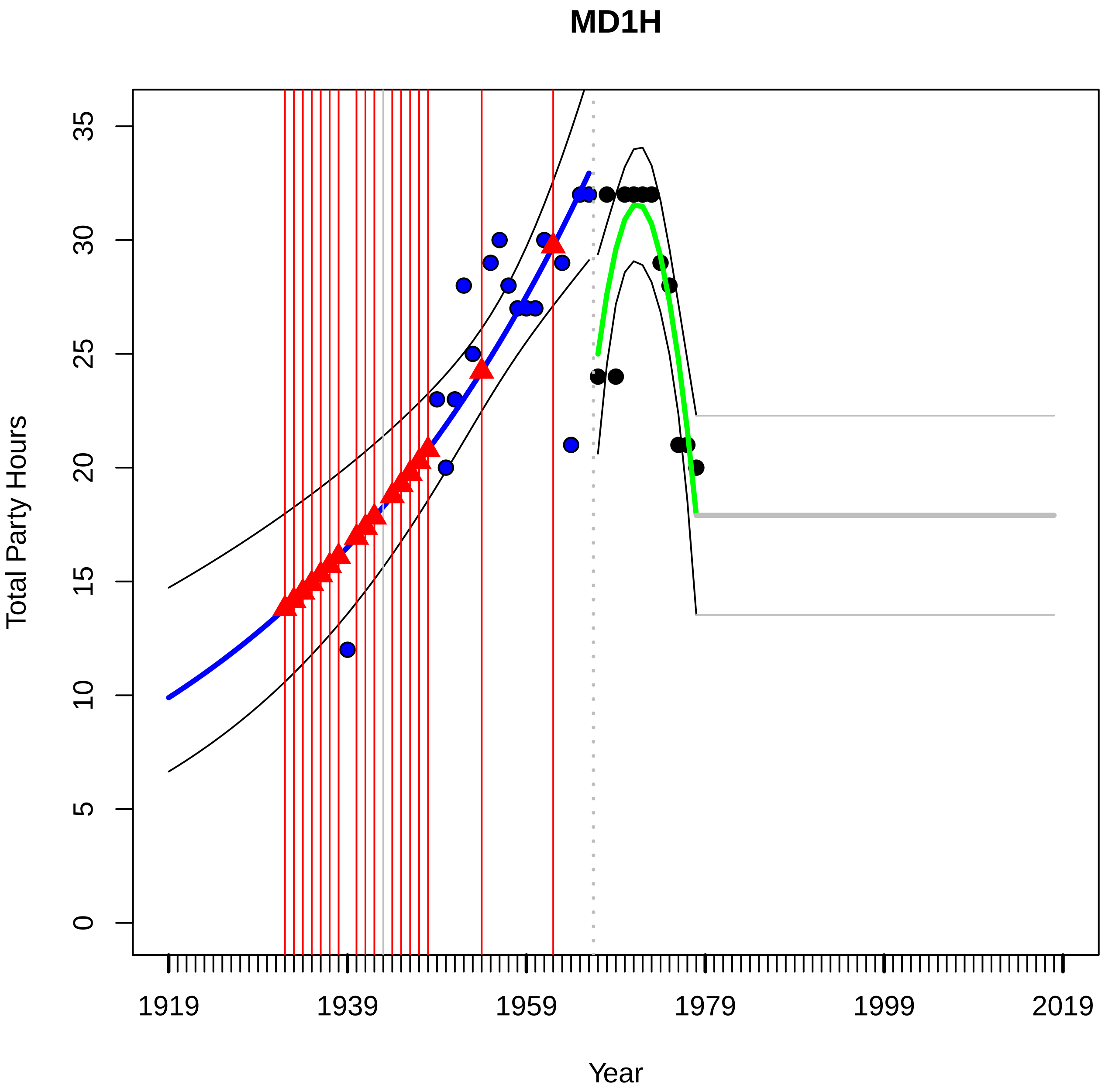
MD1H
Total Party Hours
Year
0
5
10
15
20
25
30
35
1919
1939
1959
1979
1999
2019

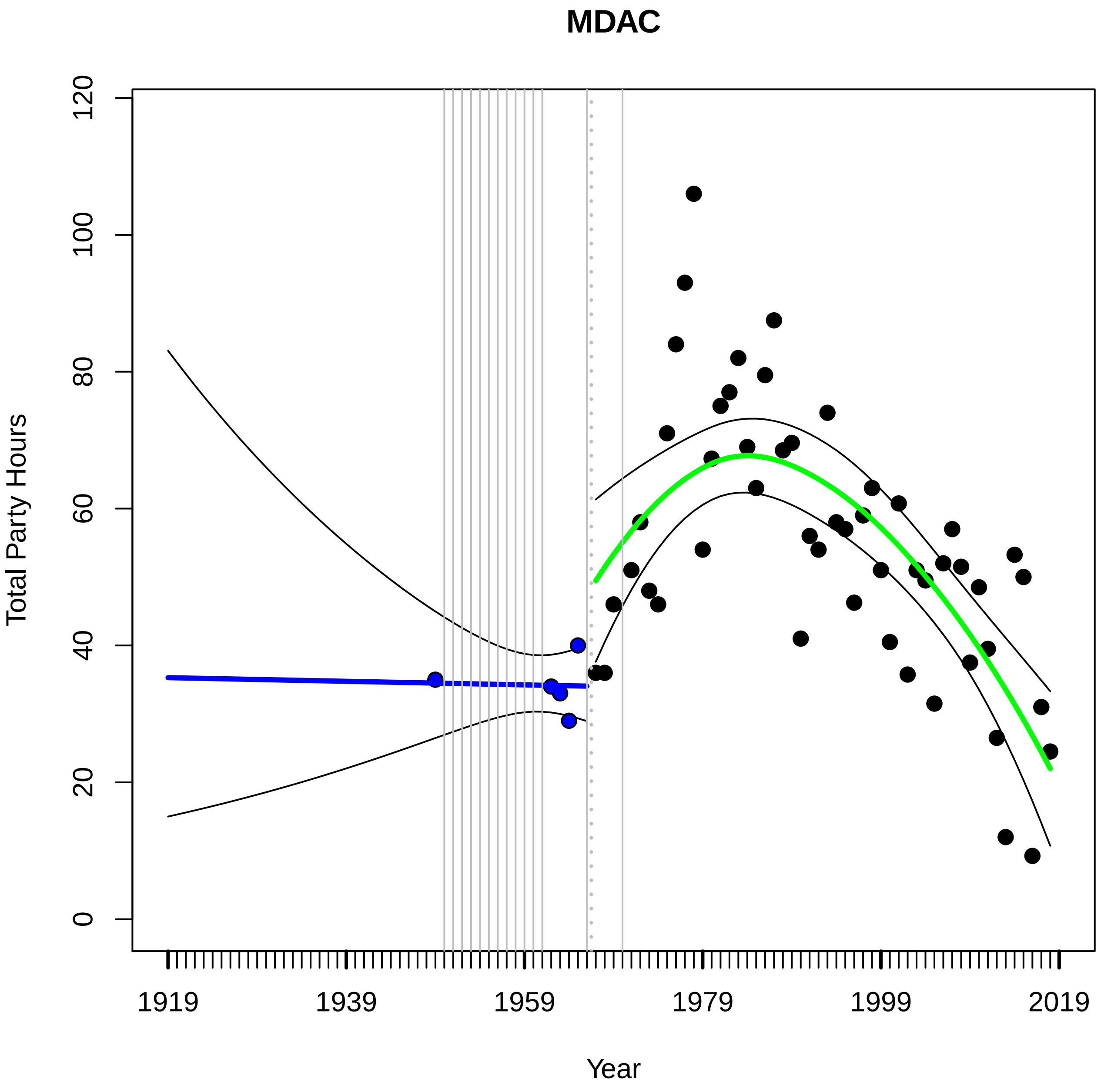

MDAC
Total Party Hours
Year
0
20
40
60
80
100
120
1919
1939
1959
1979
1999
2019

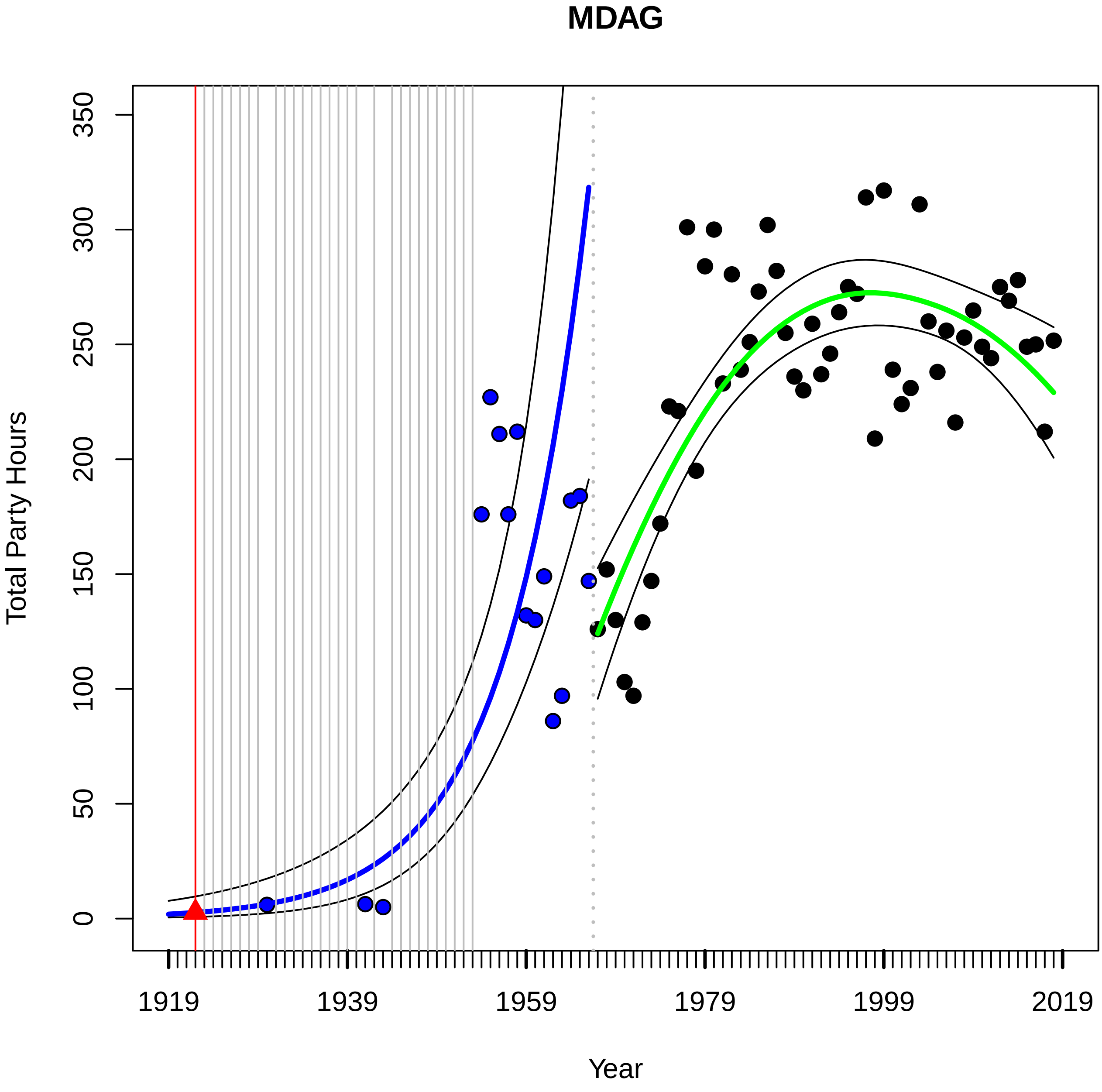
MDAG
Total Party Hours
0
50
100
150
200
250
300
350
1919
1939
1959
1979
1999
2019
Year

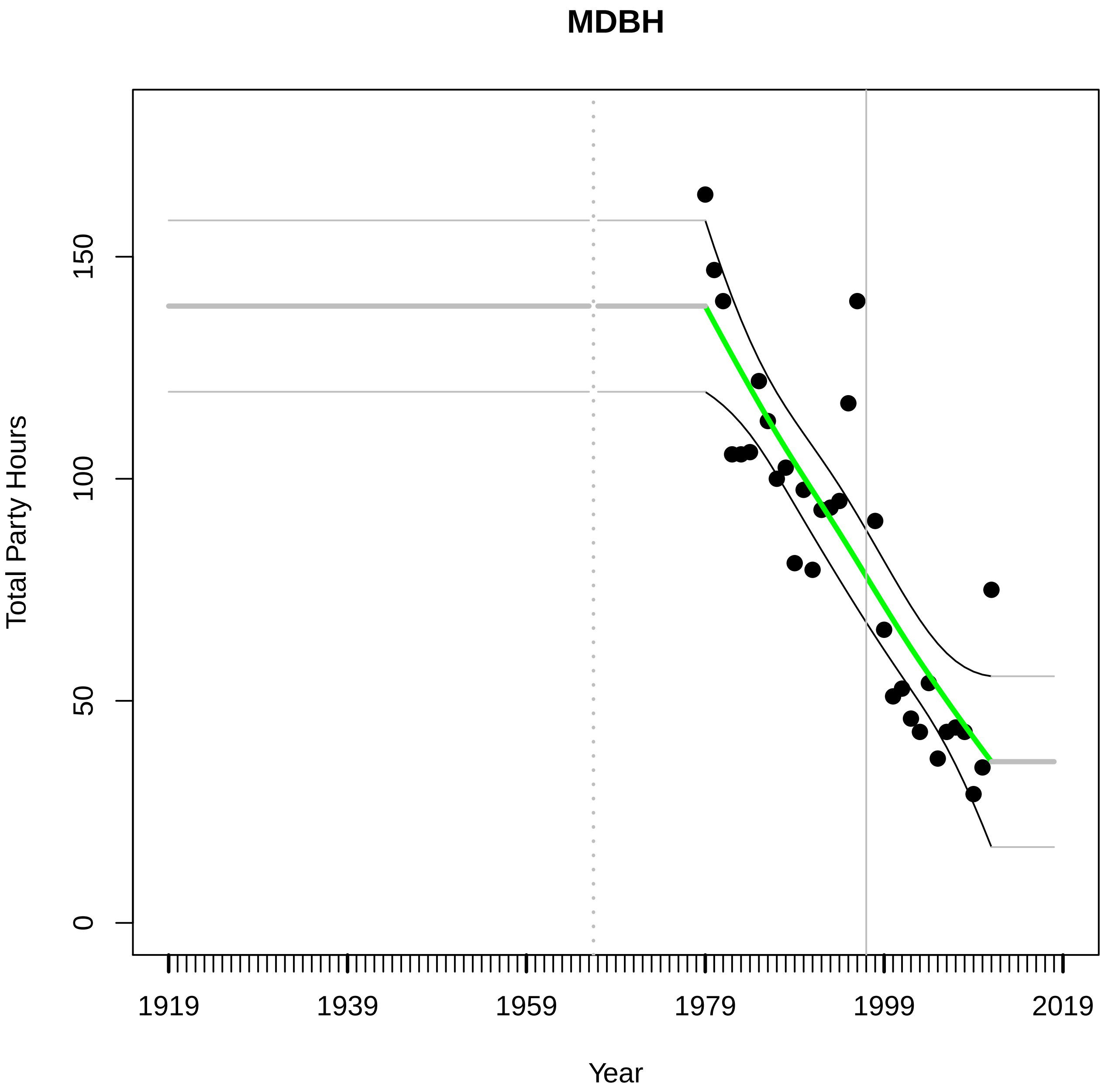
MDBH
Total Party Hours
0
50
100
150
1919
1939
1959
1979
1999
2019
Year

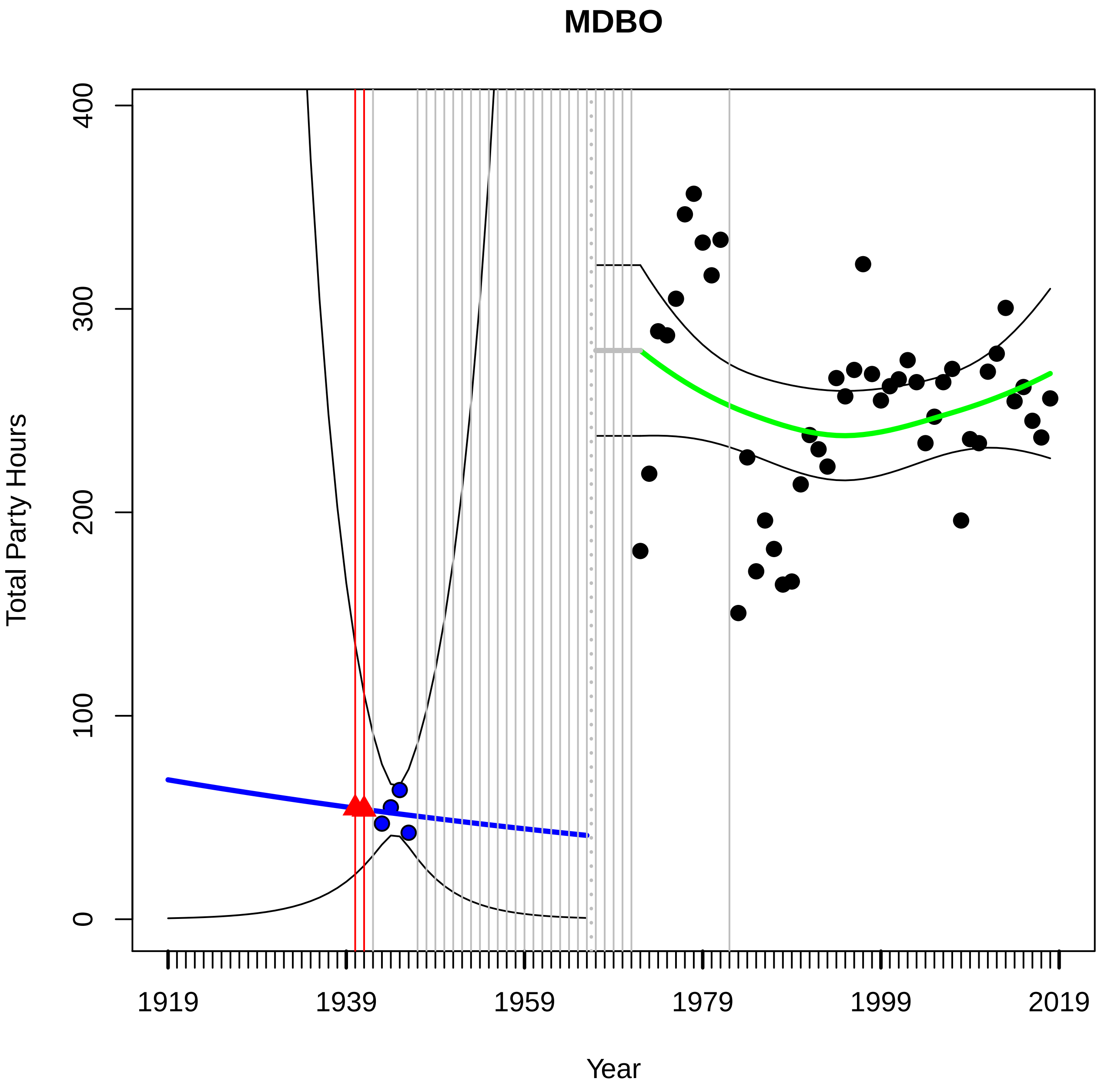

MDBO
Total Party Hours
Year
0
100
200
300
400
1919
1939
1959
1979
1999
2019

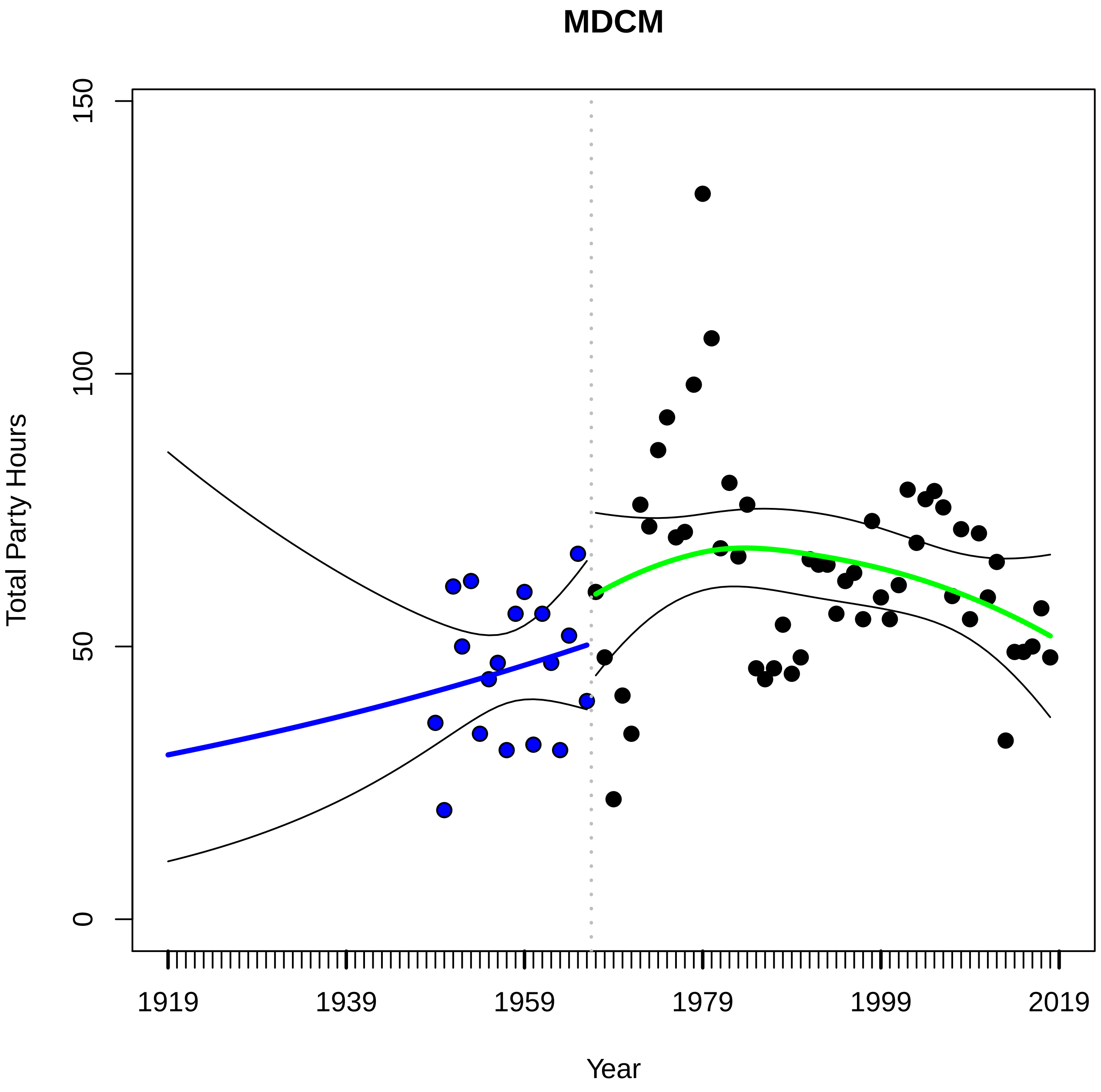
MDCM
Total Party Hours
0
50
100
150
1919
1939
1959
1979
1999
2019
Year

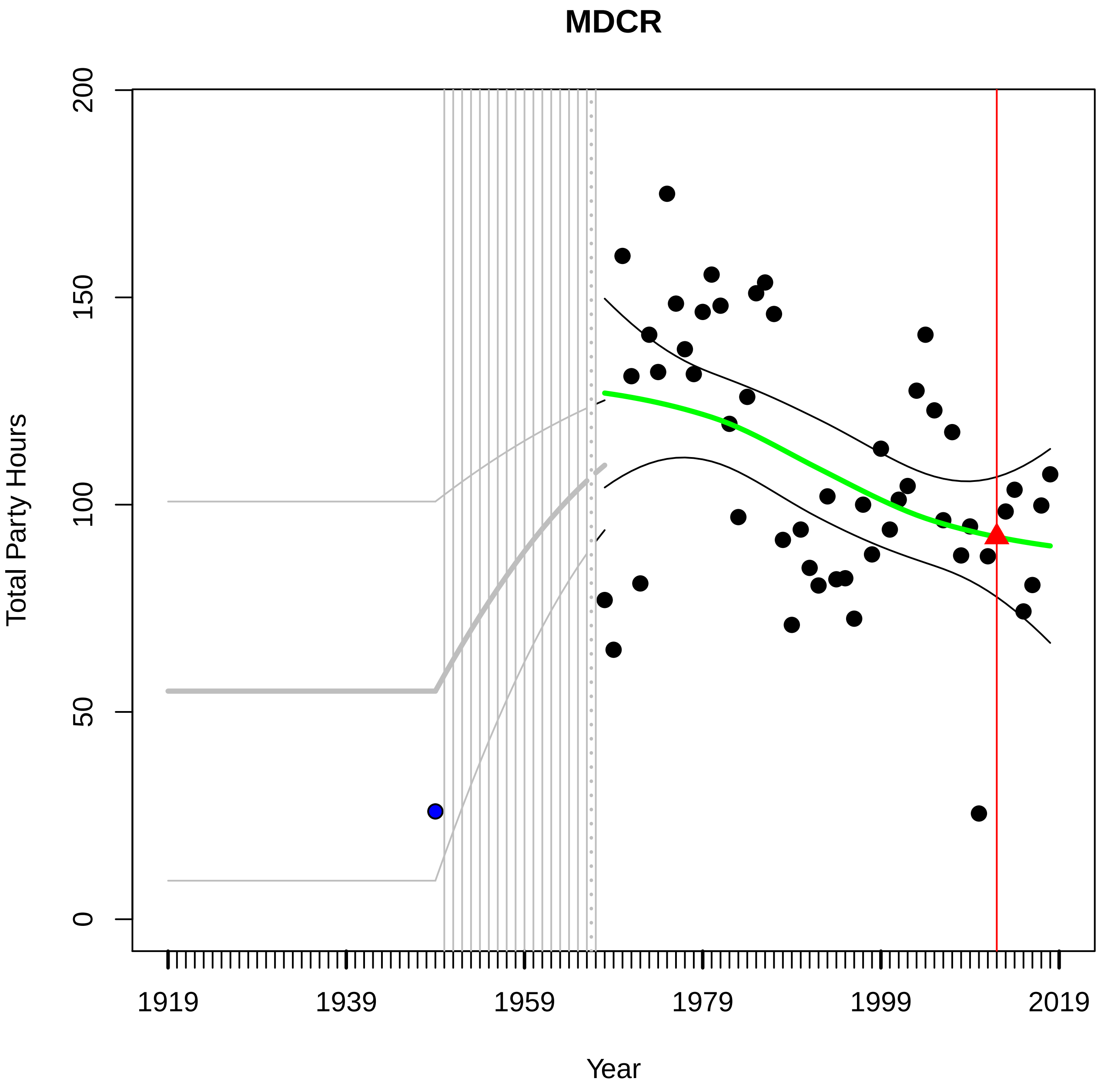

MDCR
Total Party Hours
0
50
100
150
200
Year
1919
1939
1959
1979
1999
2019

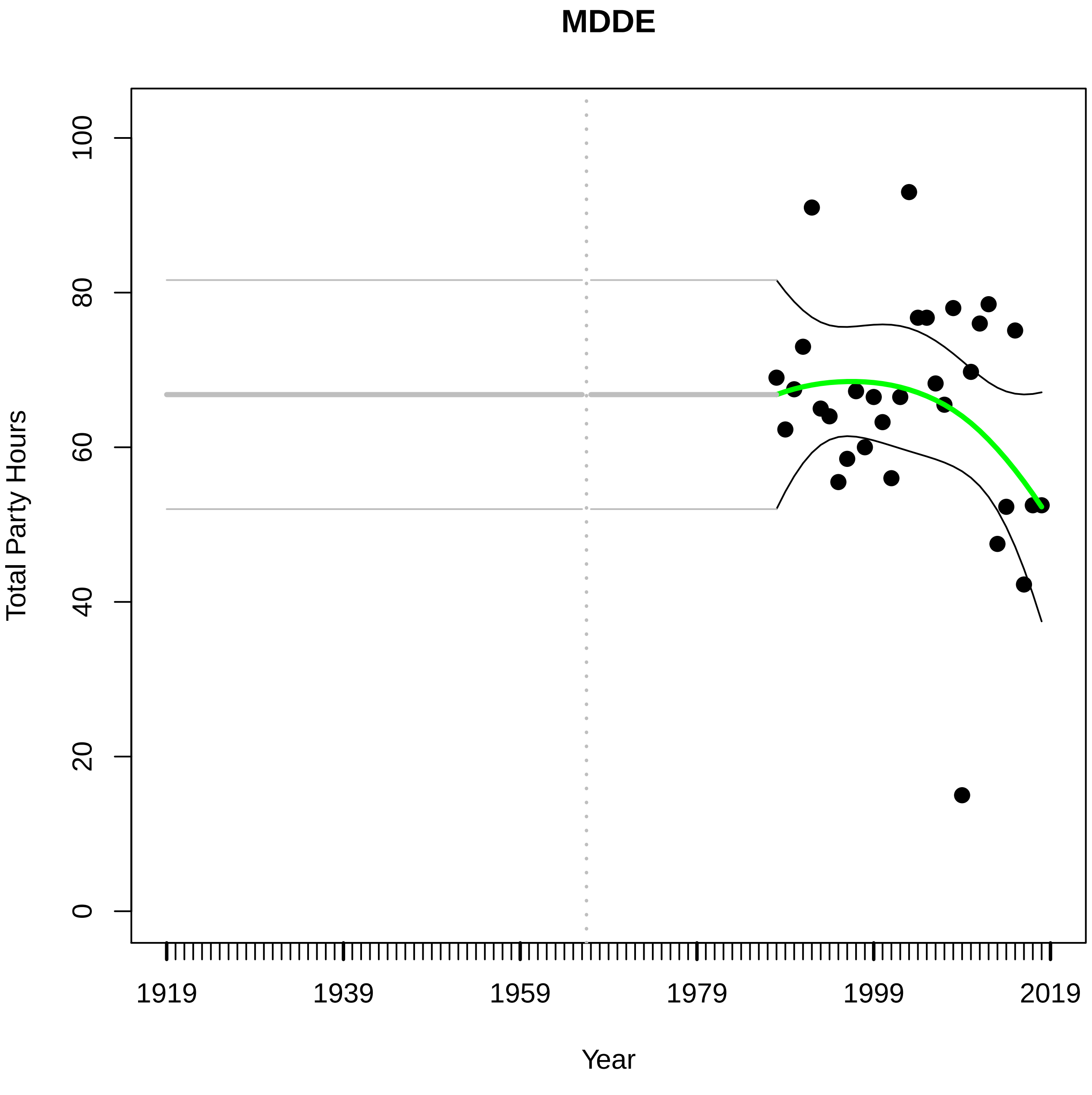
MDDE
Total Party Hours
0
20
40
60
80
100
1919
1939
1959
1979
1999
2019
Year

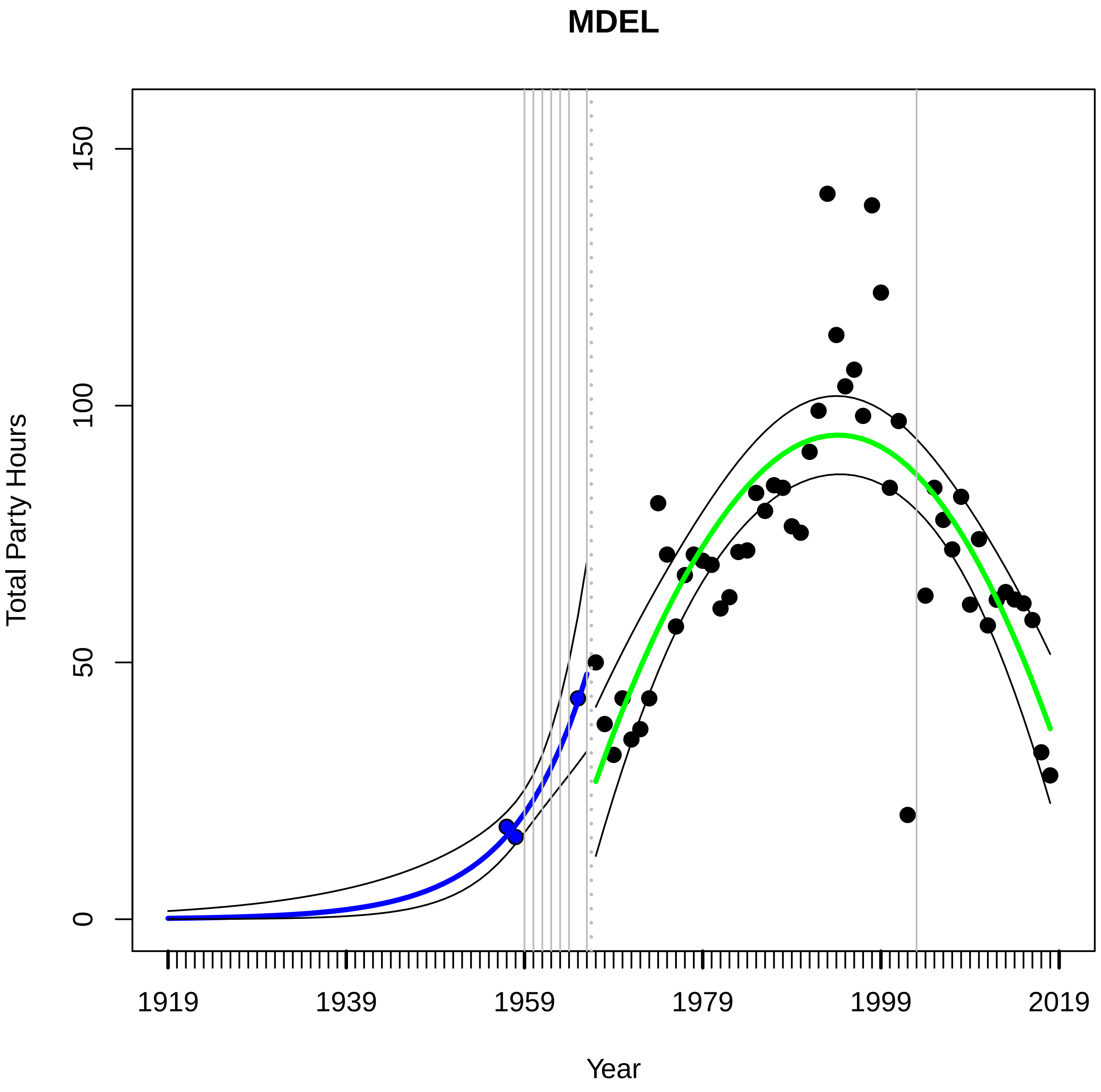

MDEL
Total Party Hours
0
50
100
150
1919
1939
1959
1979
1999
2019
Year

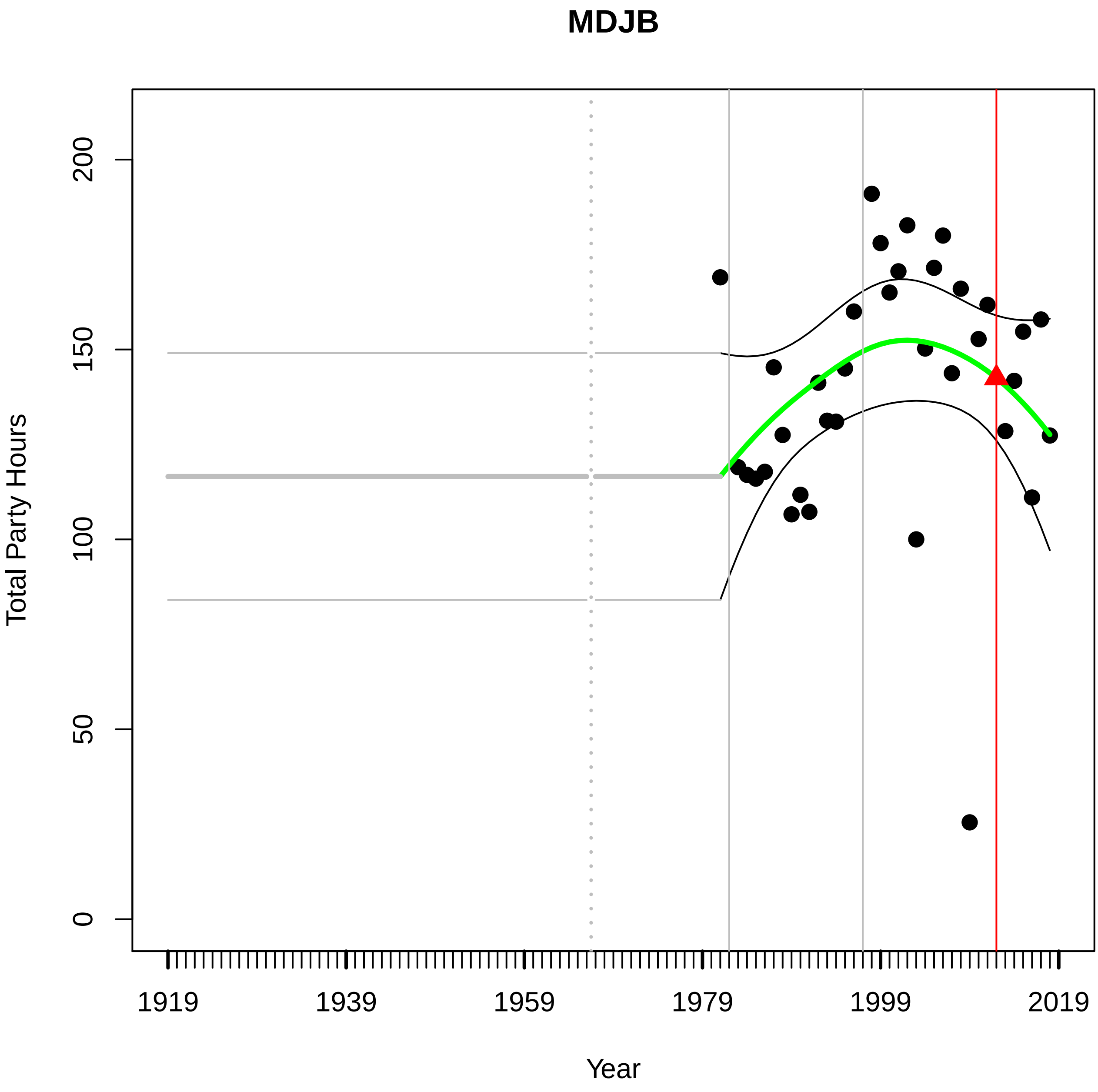

MDJB
Total Party Hours
0
50
100
150
200
1919
1939
1959
1979
1999
2019
Year

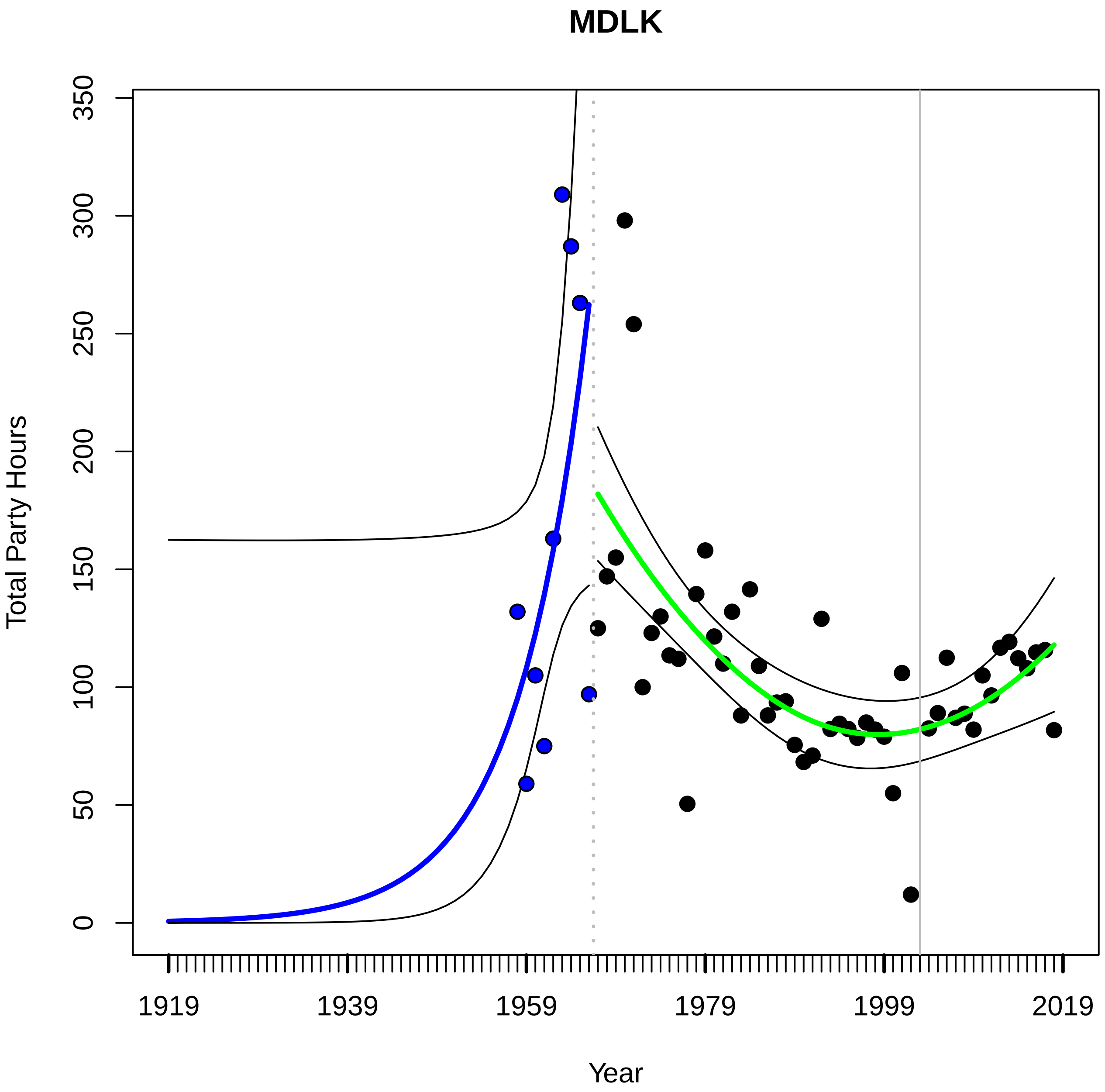

MDLK
Total Party Hours
Year
0
50
100
150
200
250
300
350
1919
1939
1959
1979
1999
2019

MDOA

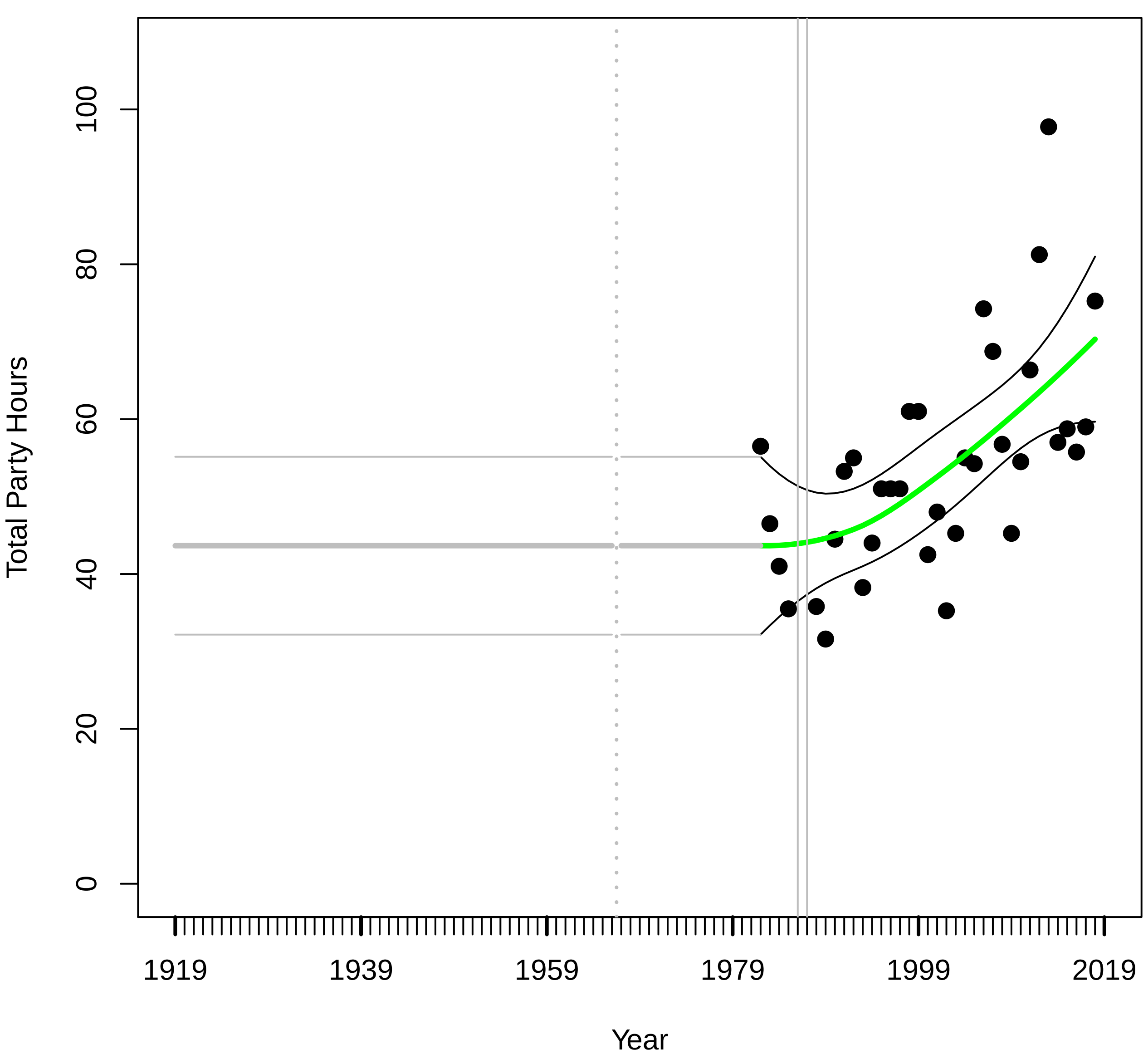

100
80
60
40
20
0
Total Party Hours
1919
1939
1959
1979
1999
2019
Year

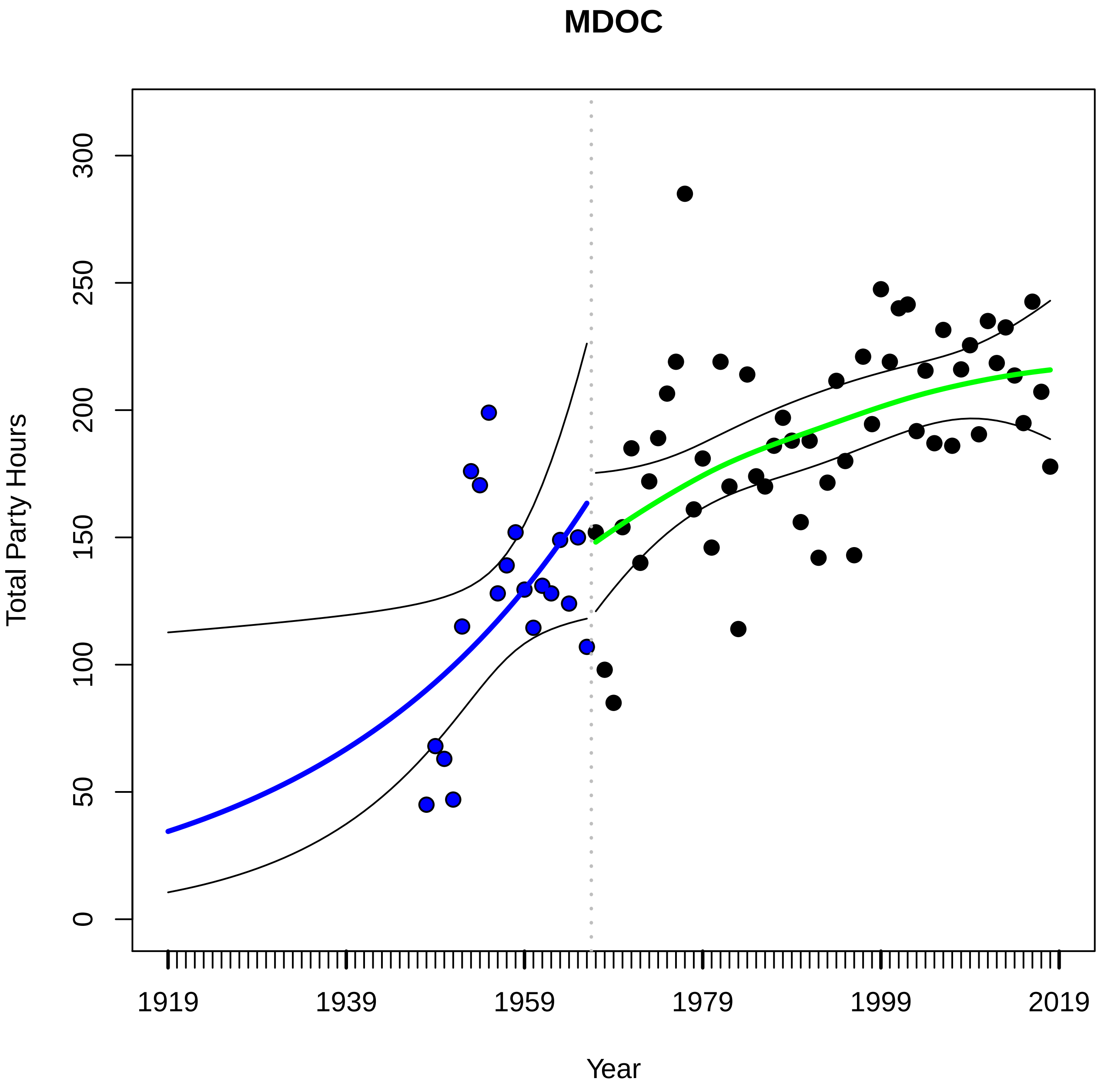

MDOC
Total Party Hours
0
50
100
150
200
250
300
1919
1939
1959
1979
1999
2019
Year

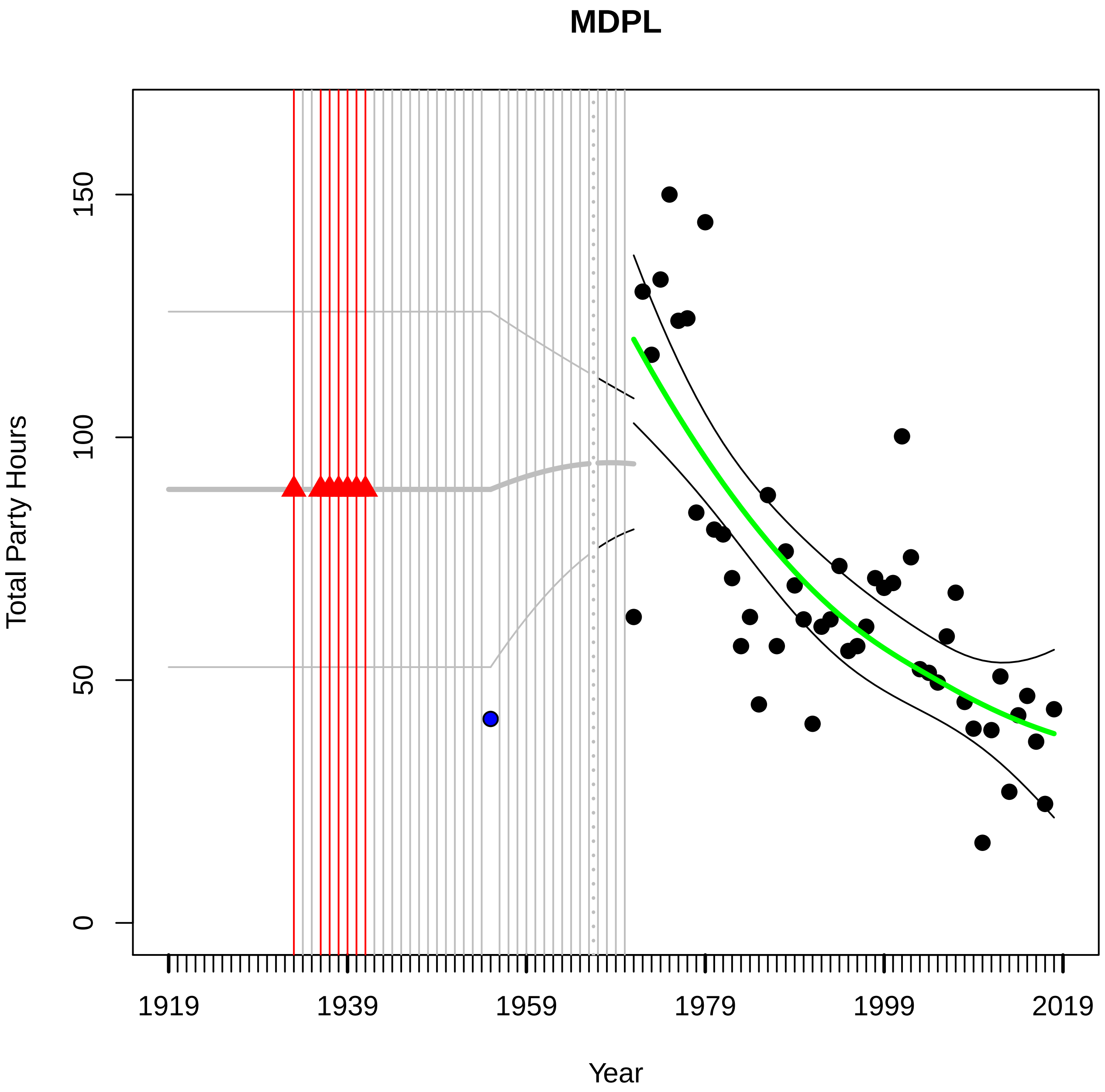

MDPL
Total Party Hours
150
100
50
0
1919
1939
1959
1979
1999
2019
Year

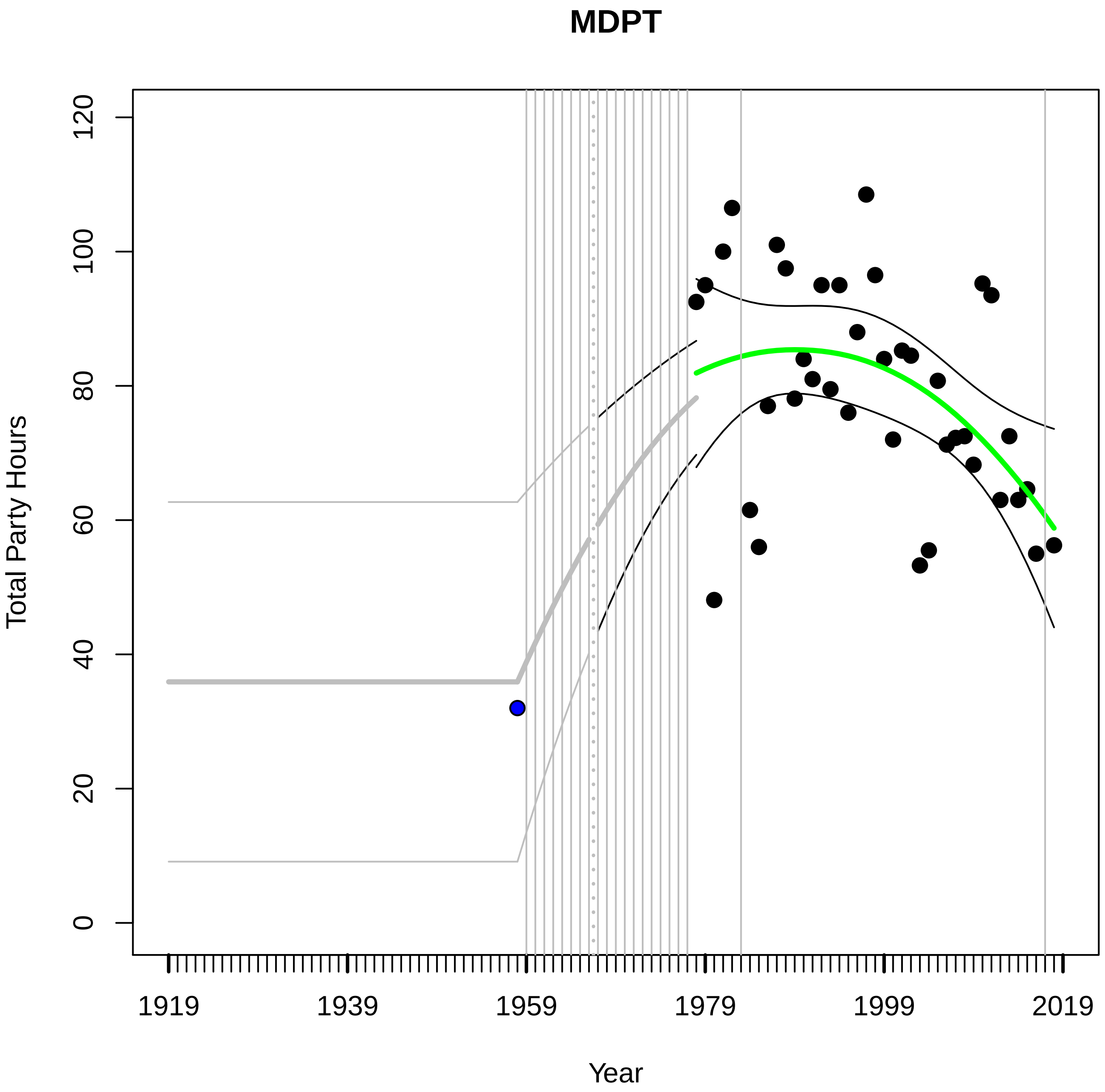

MDPT
Total Party Hours
Year
0
20
40
60
80
100
120
1919
1939
1959
1979
1999
2019

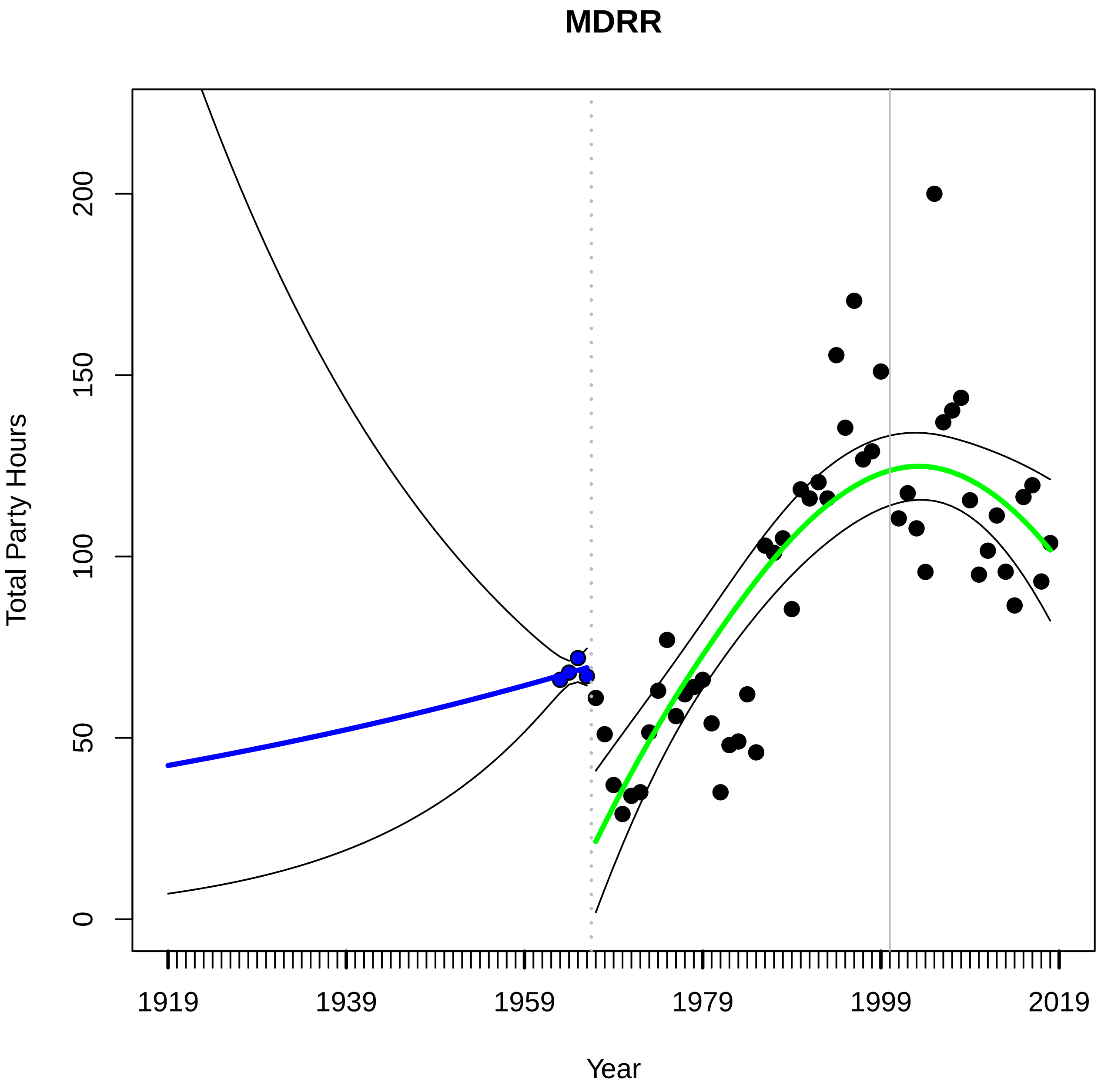

MDRR
Total Party Hours
0
50
100
150
200
1919
1939
1959
1979
1999
2019
Year

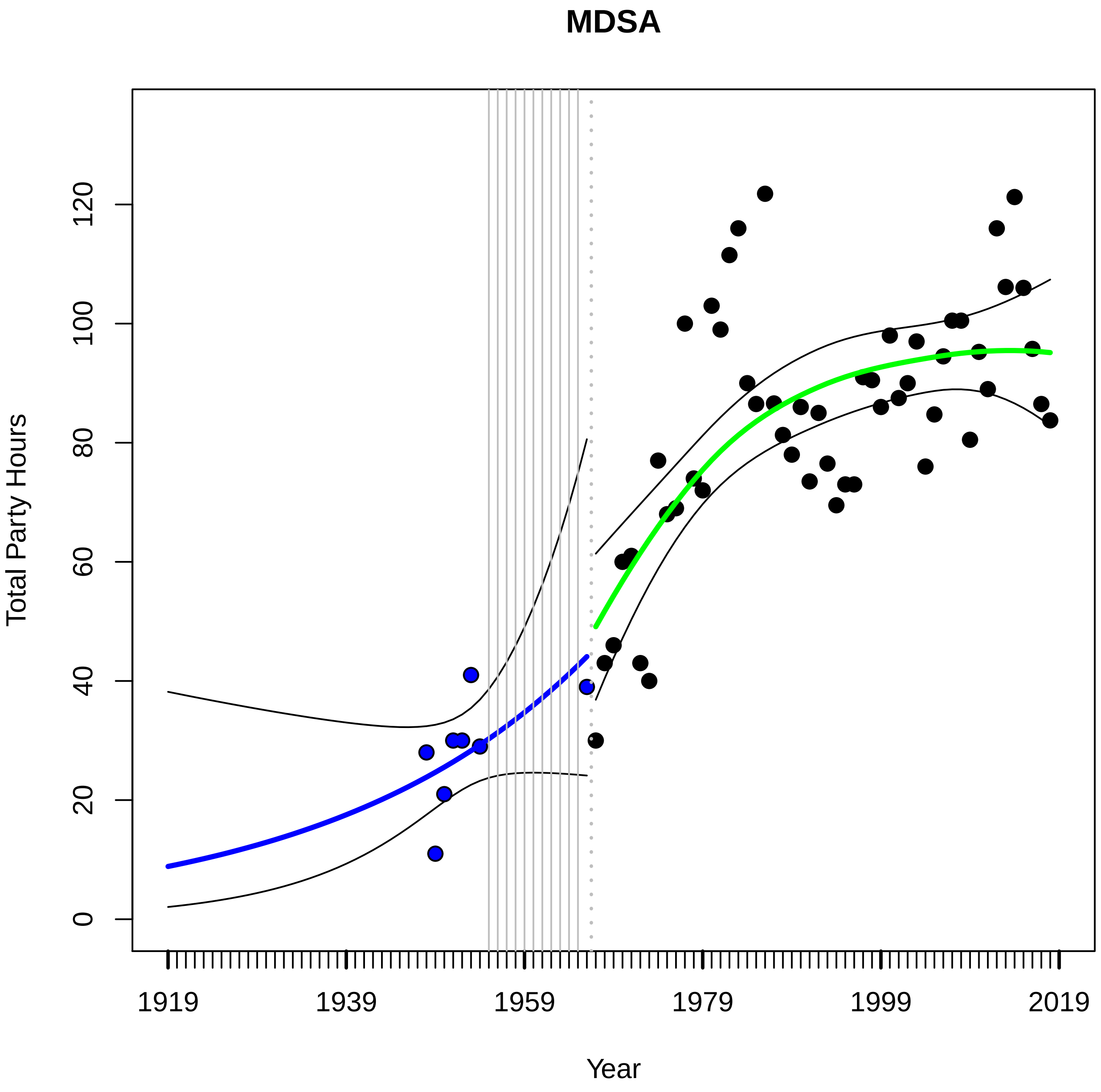

MDSA
Total Party Hours
0
20
40
60
80
100
120
1919
1939
1959
1979
1999
2019
Year

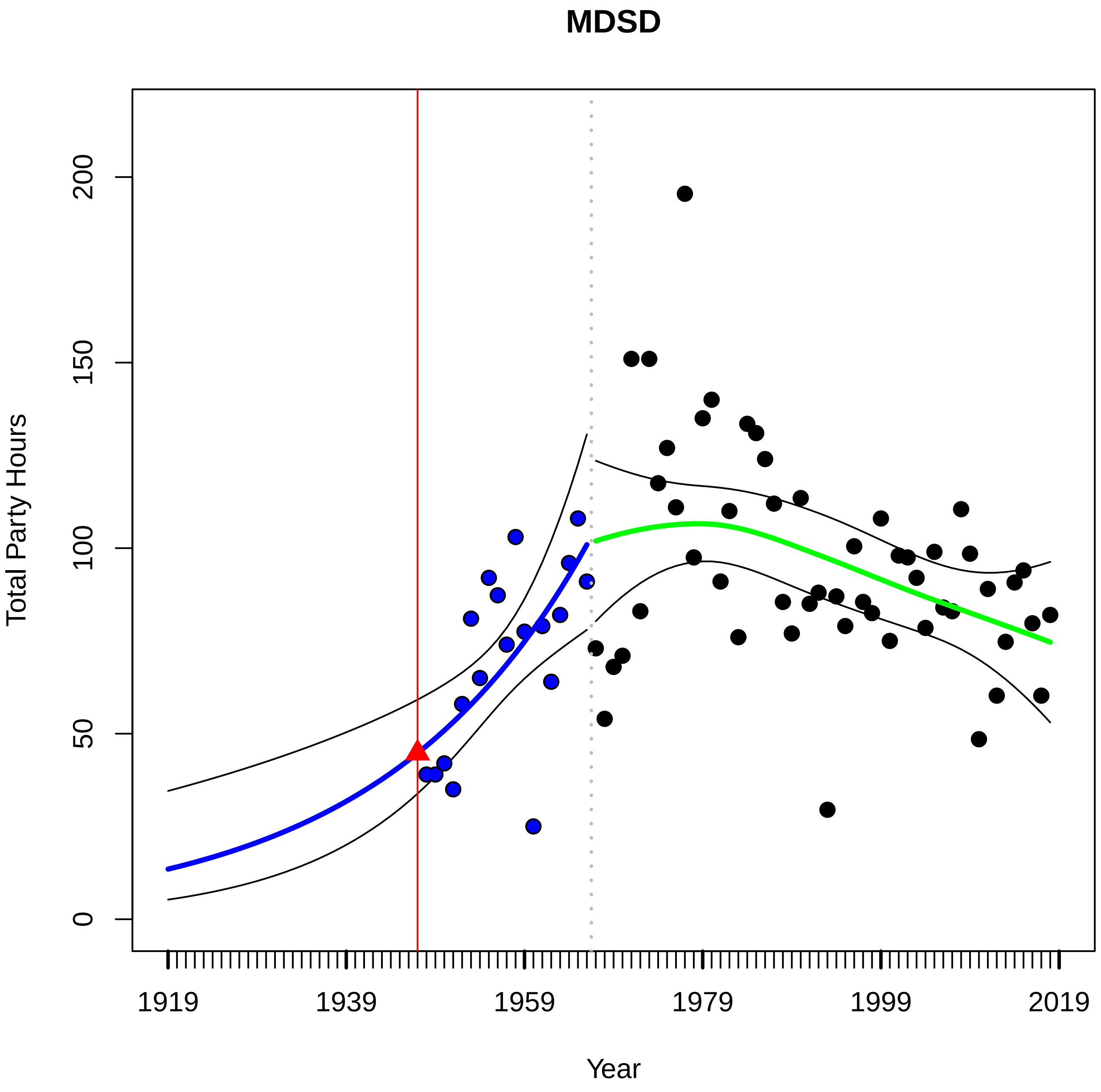

MDSD
Total Party Hours
0
50
100
150
200
1919
1939
1959
1979
1999
2019
Year

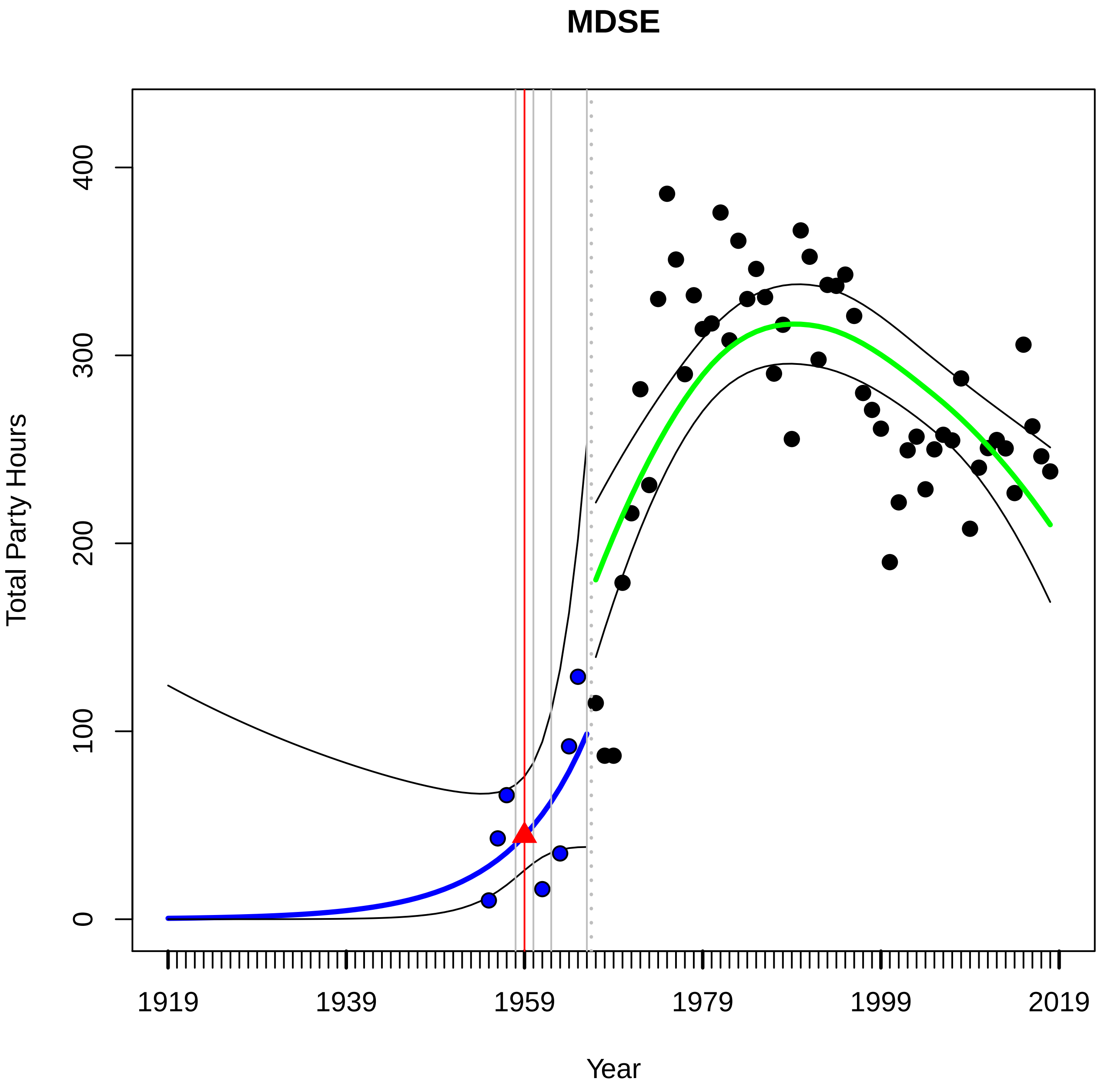

MDSE
Total Party Hours
0
100
200
300
400
1919
1939
1959
1979
1999
2019
Year

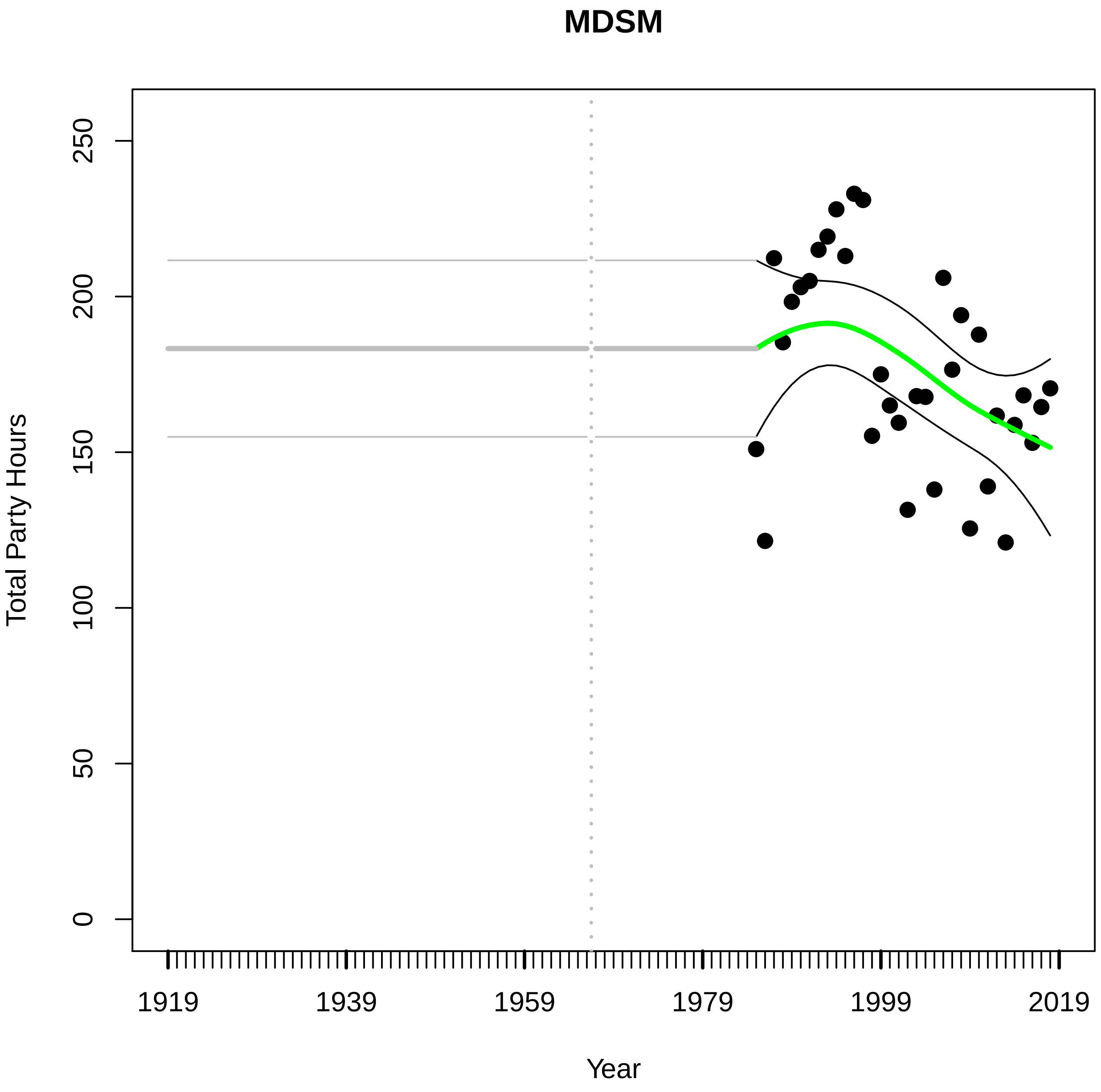
MDSM
Total Party Hours
0
50
100
150
200
250
1919
1939
1959
1979
1999
2019
Year

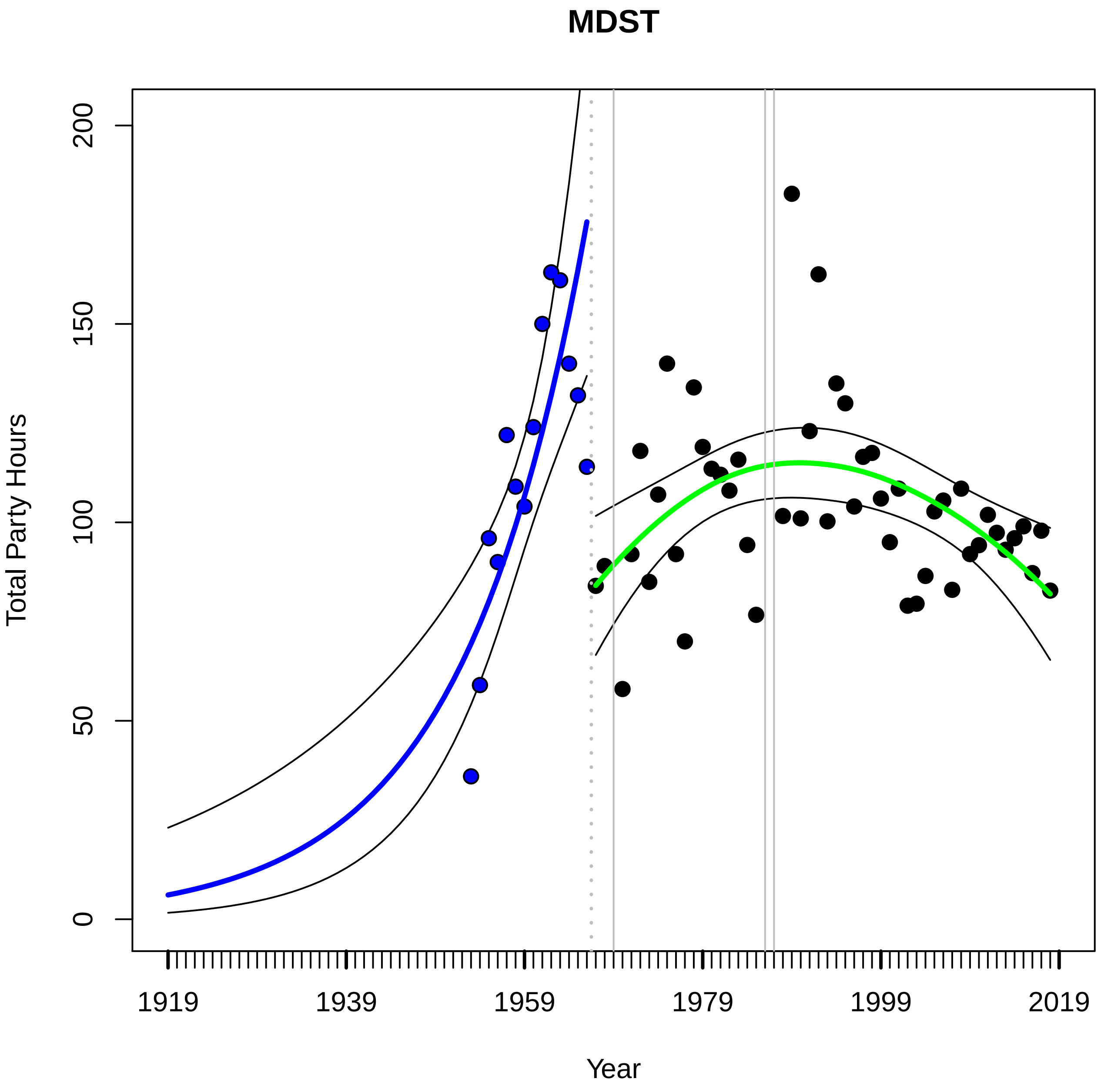

MDST
Total Party Hours
200
150
100
50
0
1919
1939
1959
1979
1999
2019
Year

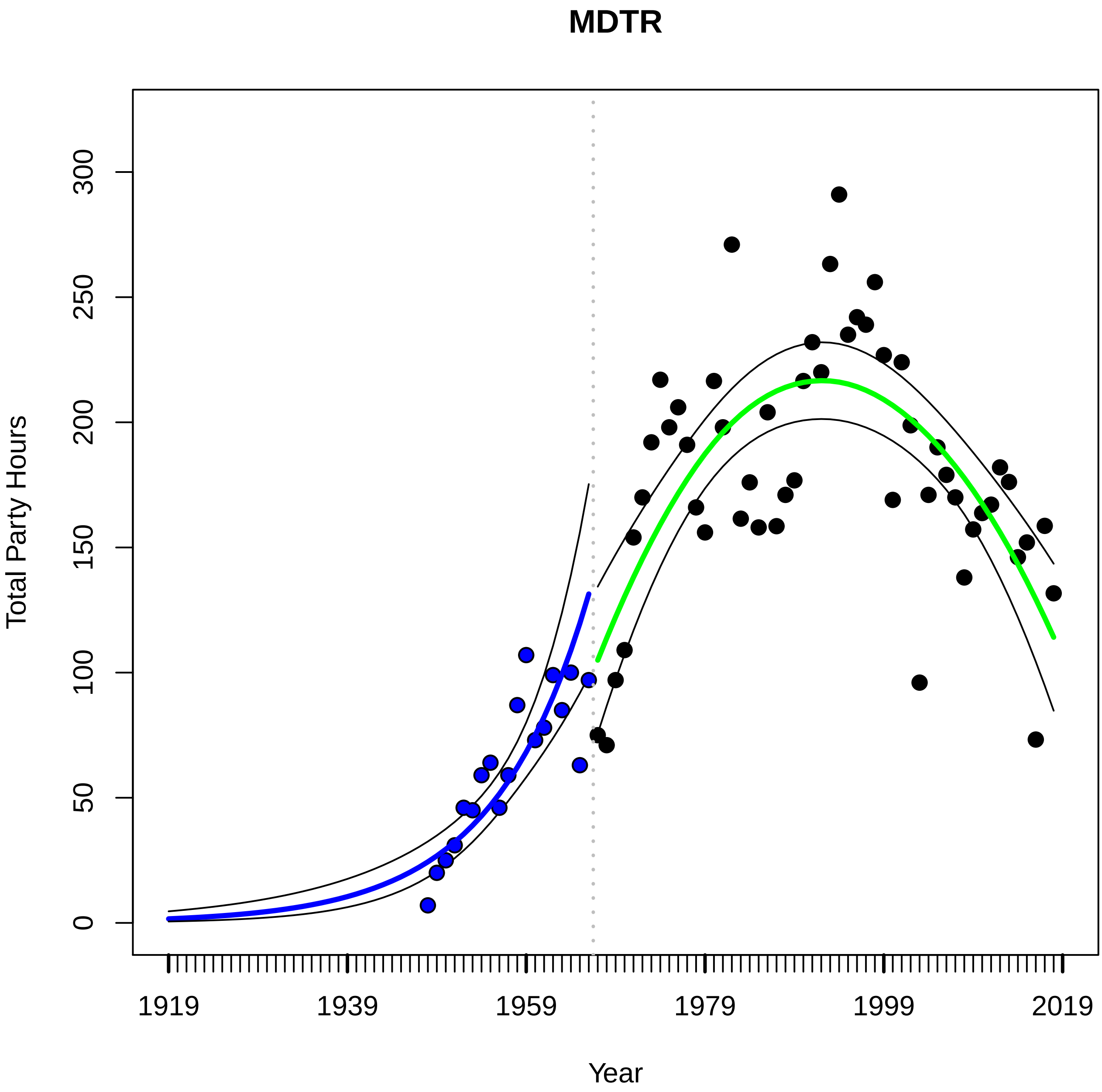
MDTR
Total Party Hours
Year
0
50
100
150
200
250
300
1919
1939
1959
1979
1999
2019

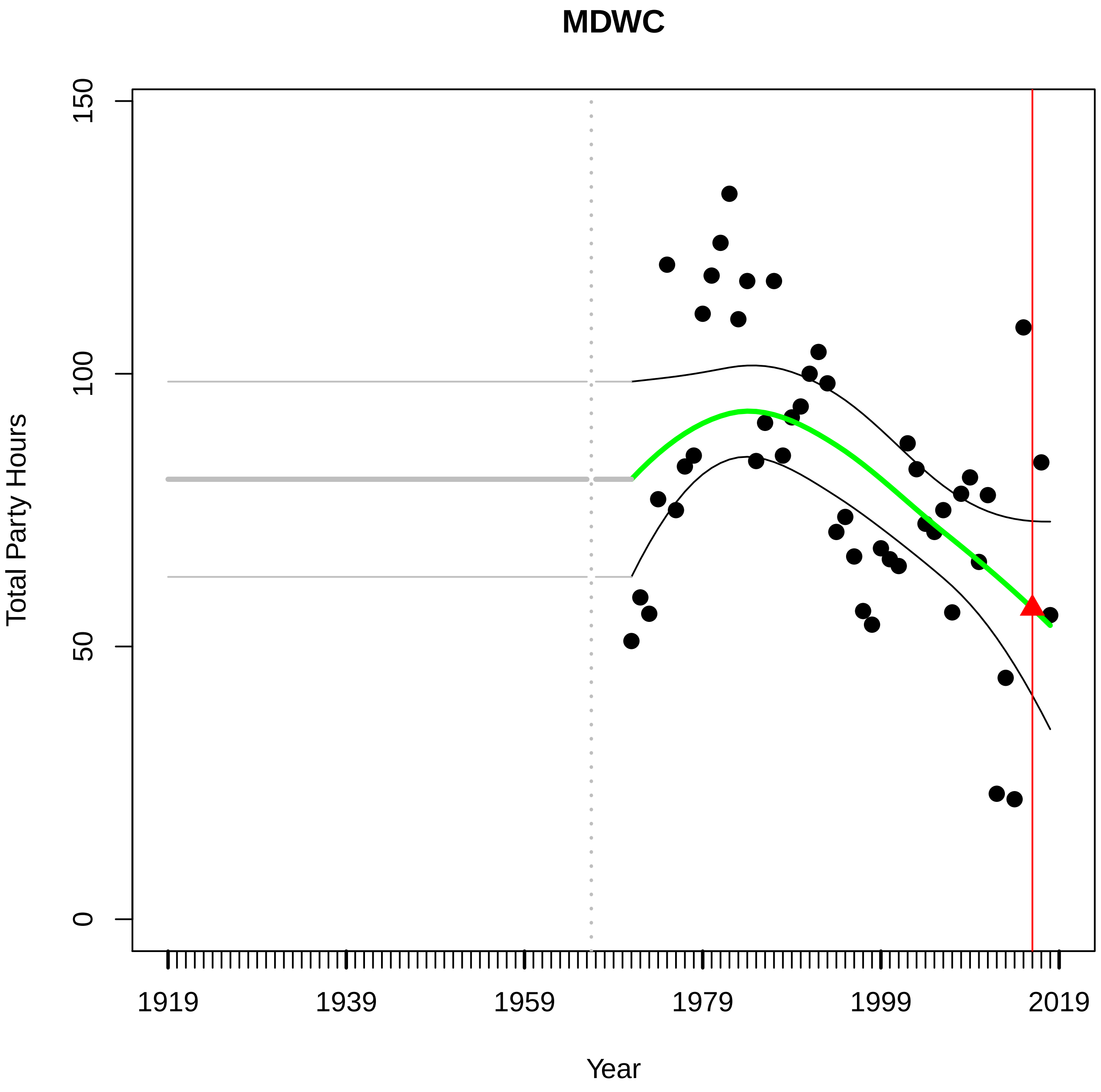
MDWC
Total Party Hours
0
50
100
150
1919
1939
1959
1979
1999
2019
Year

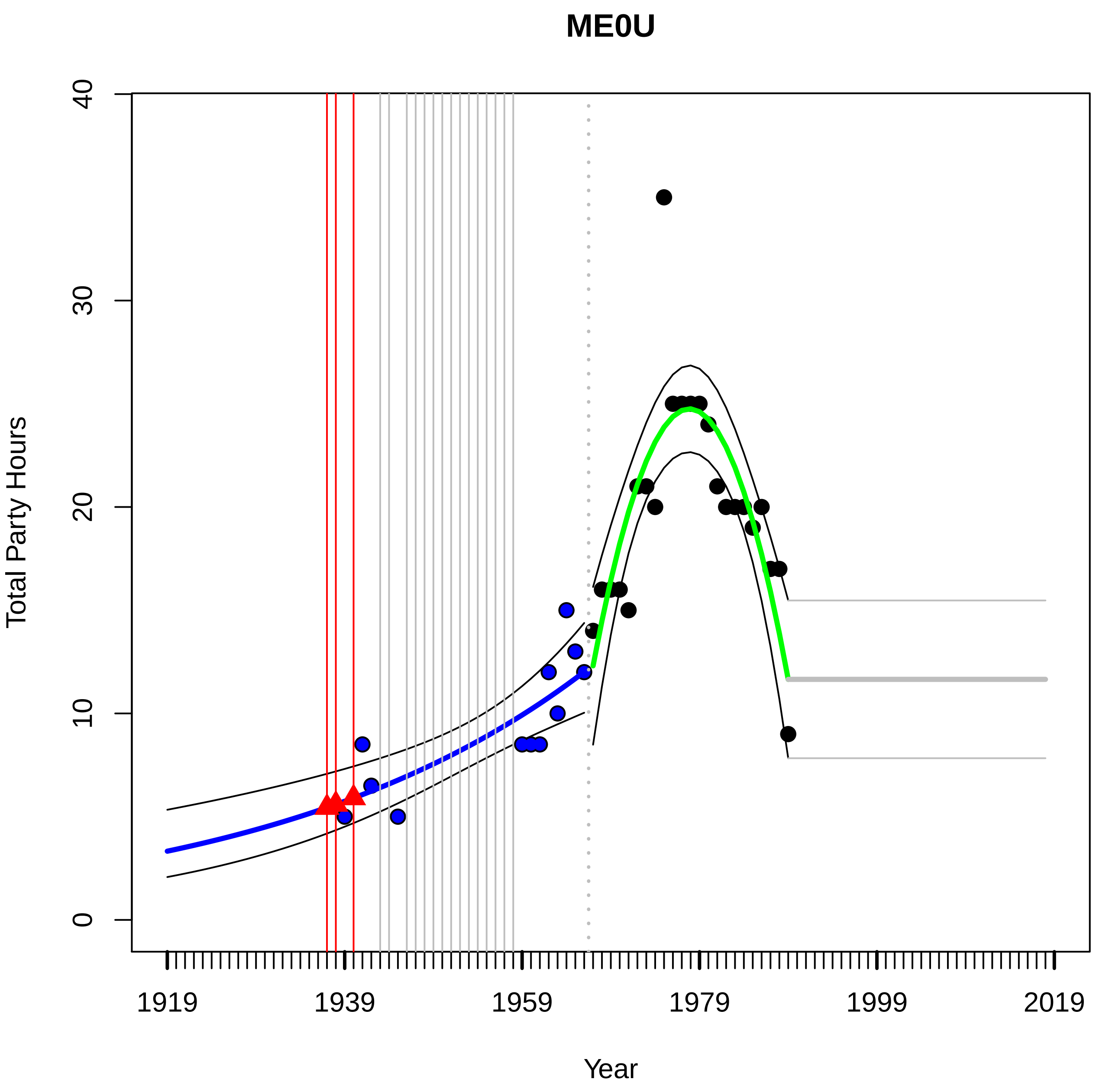

ME0U
Total Party Hours
0
10
20
30
40
Year
1919
1939
1959
1979
1999
2019

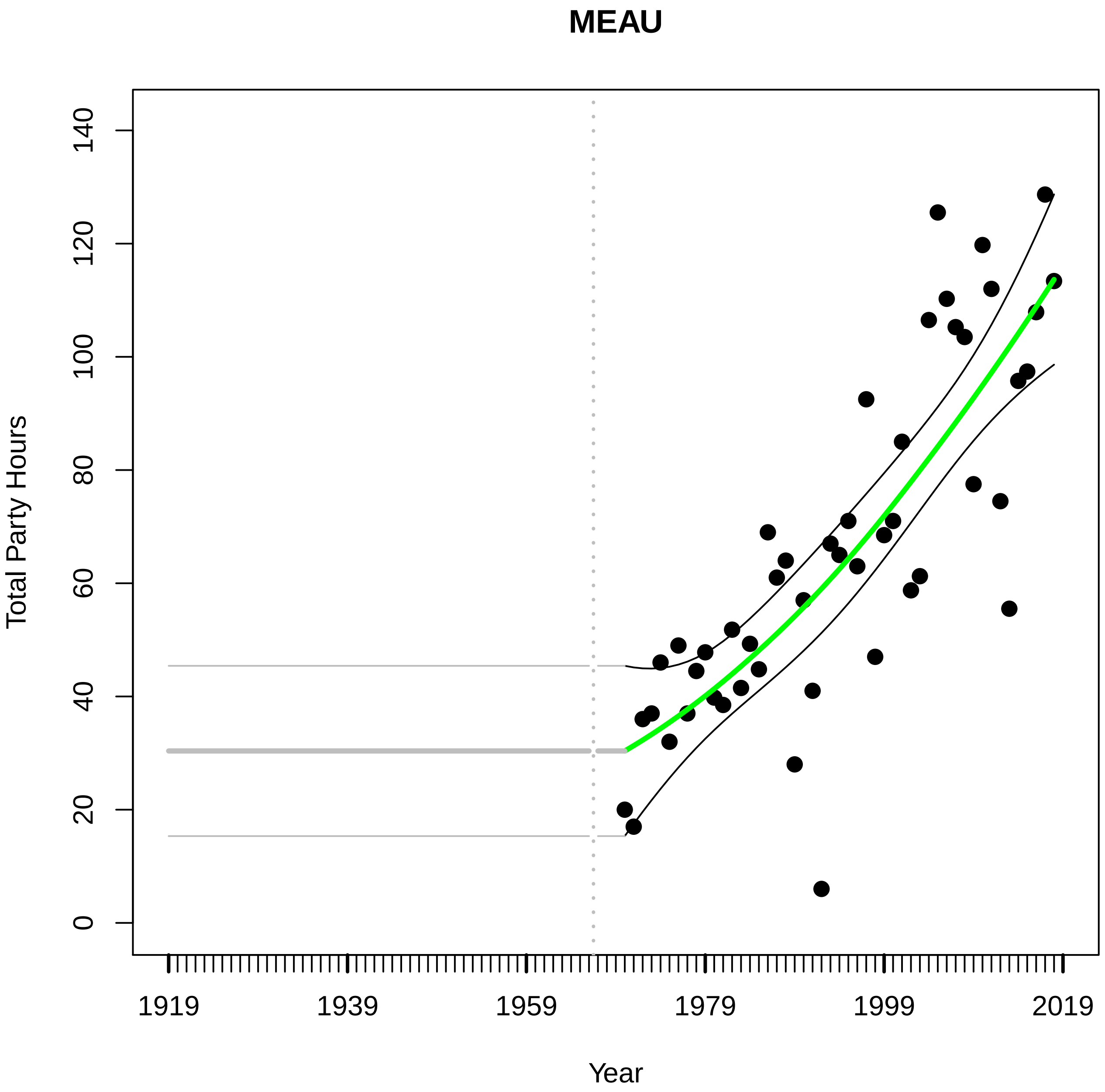

MEAU
Total Party Hours
0
20
40
60
80
100
120
140
1919
1939
1959
1979
1999
2019
Year

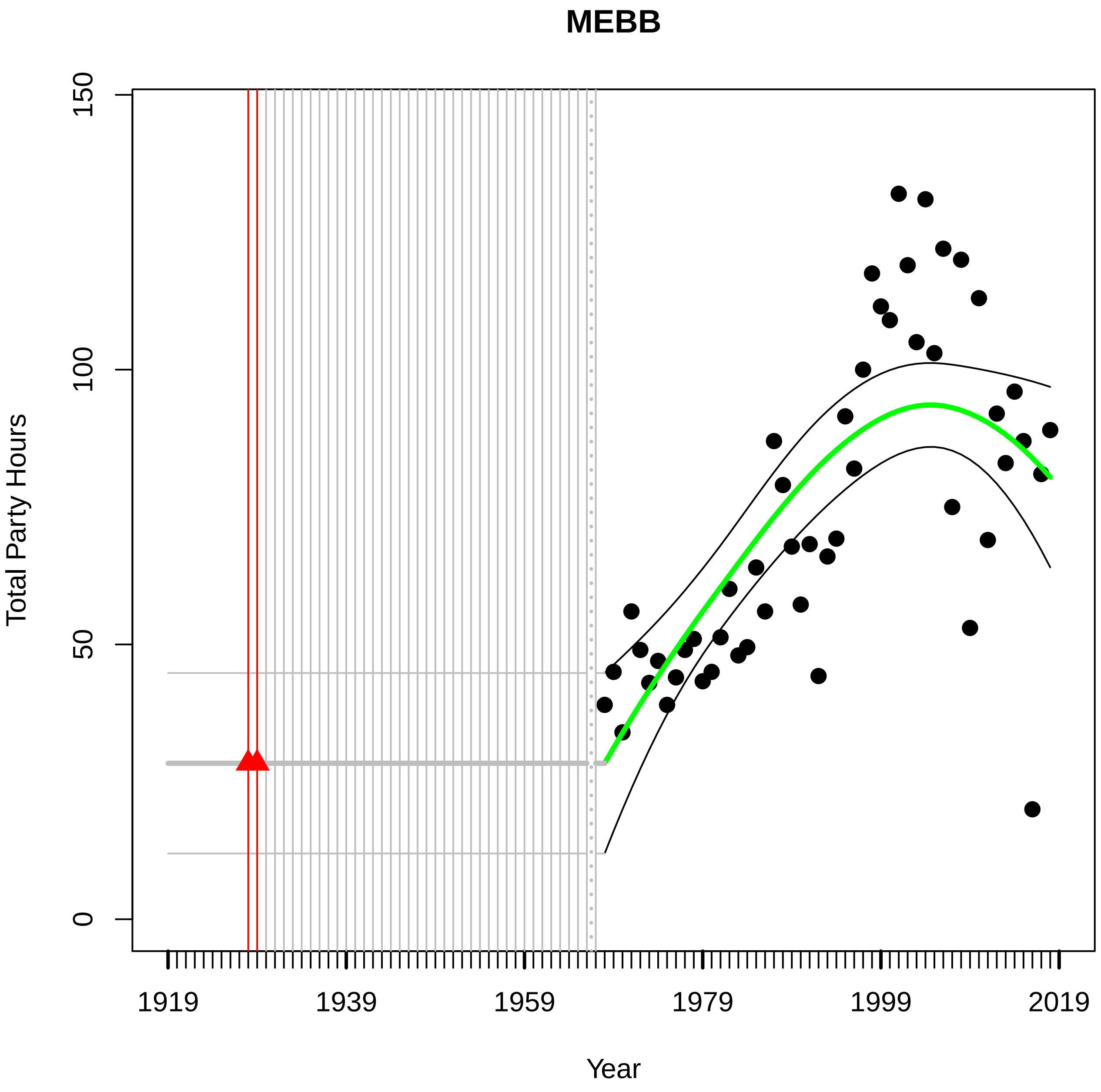

MEBB
Total Party Hours
0
50
100
150
1919
1939
1959
1979
1999
2019
Year

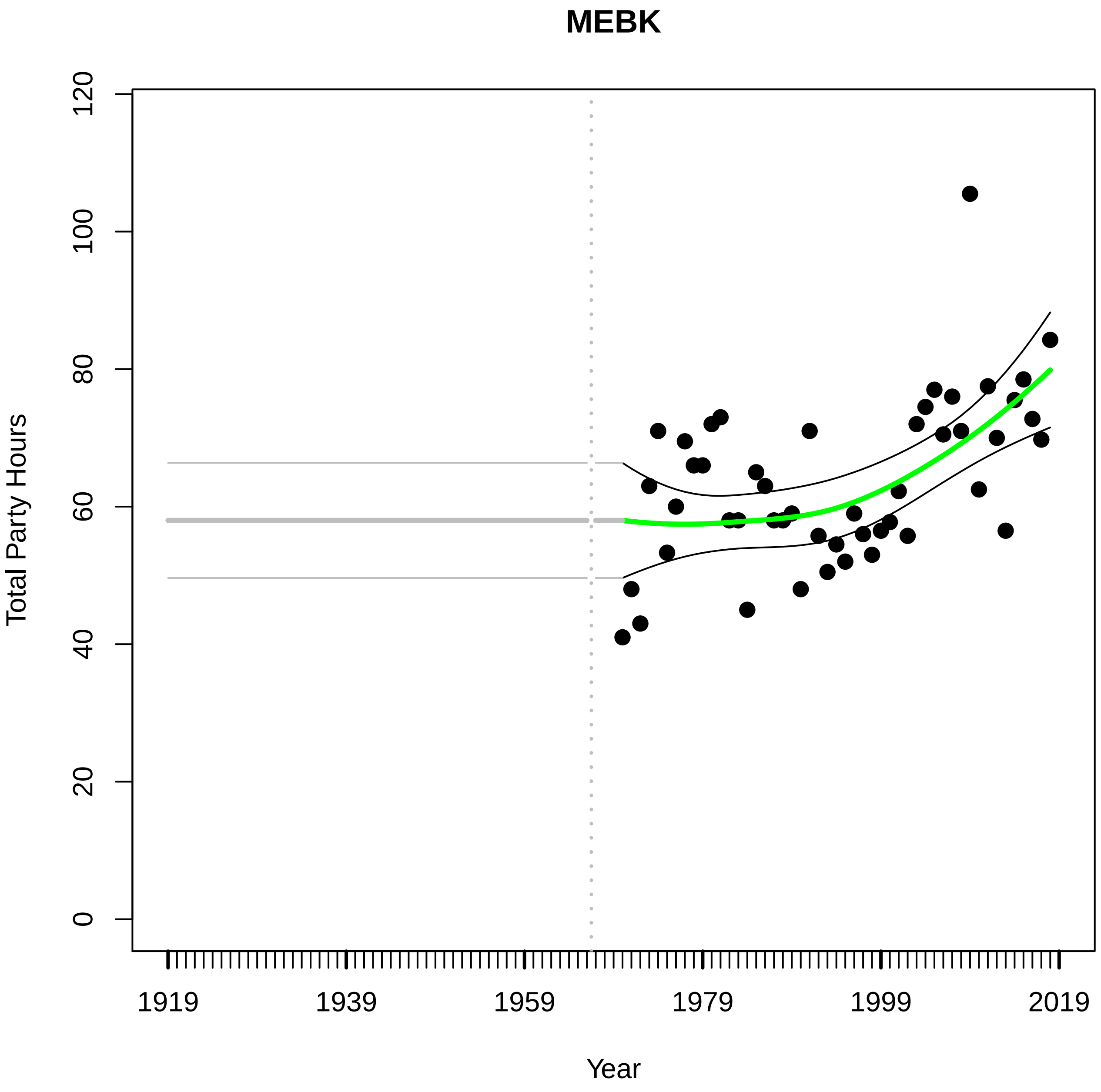

MEBK
Total Party Hours
Year
0
20
40
60
80
100
120
1919
1939
1959
1979
1999
2019

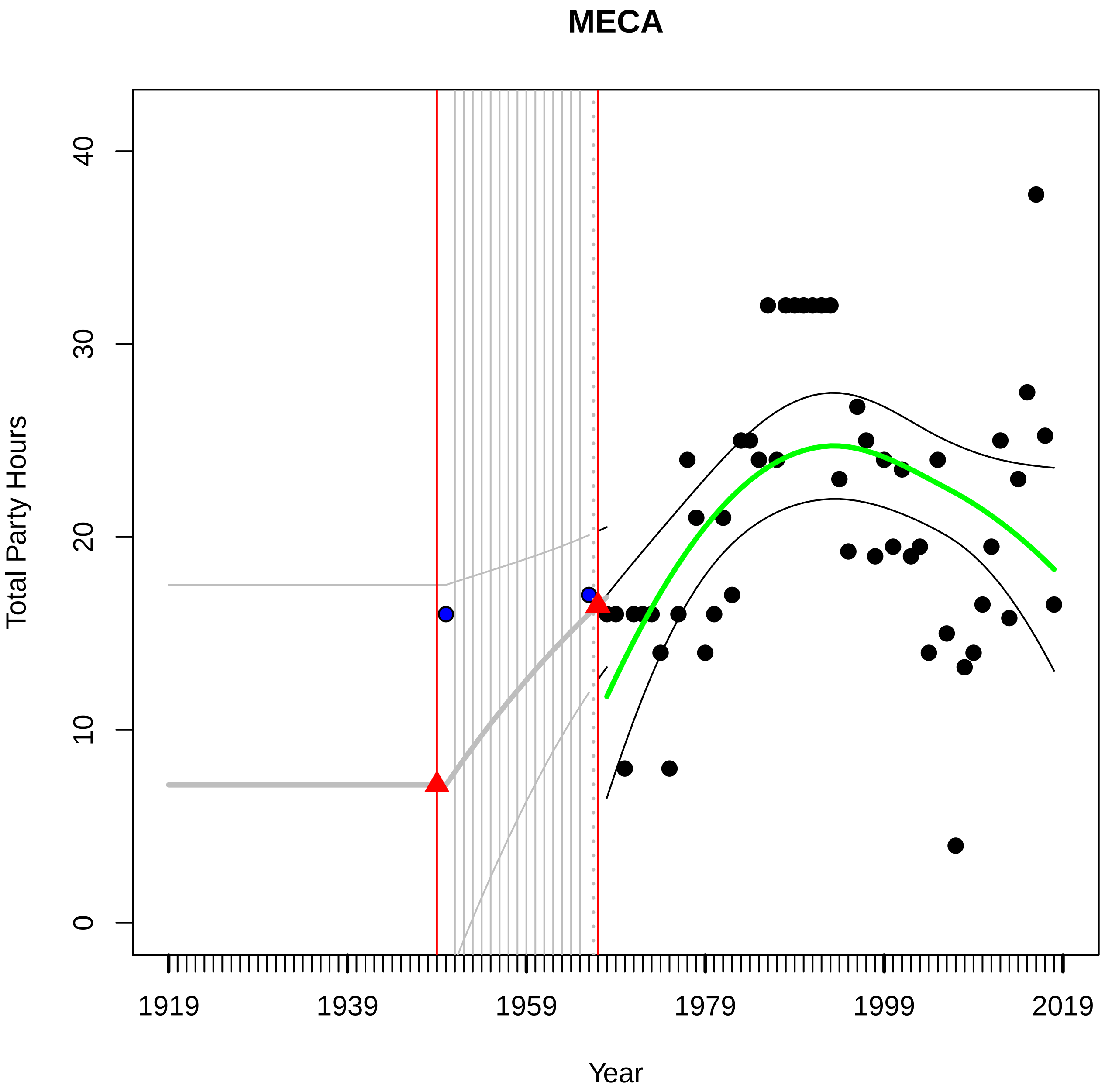

MECA
Total Party Hours
Year
0
10
20
30
40
1919
1939
1959
1979
1999
2019

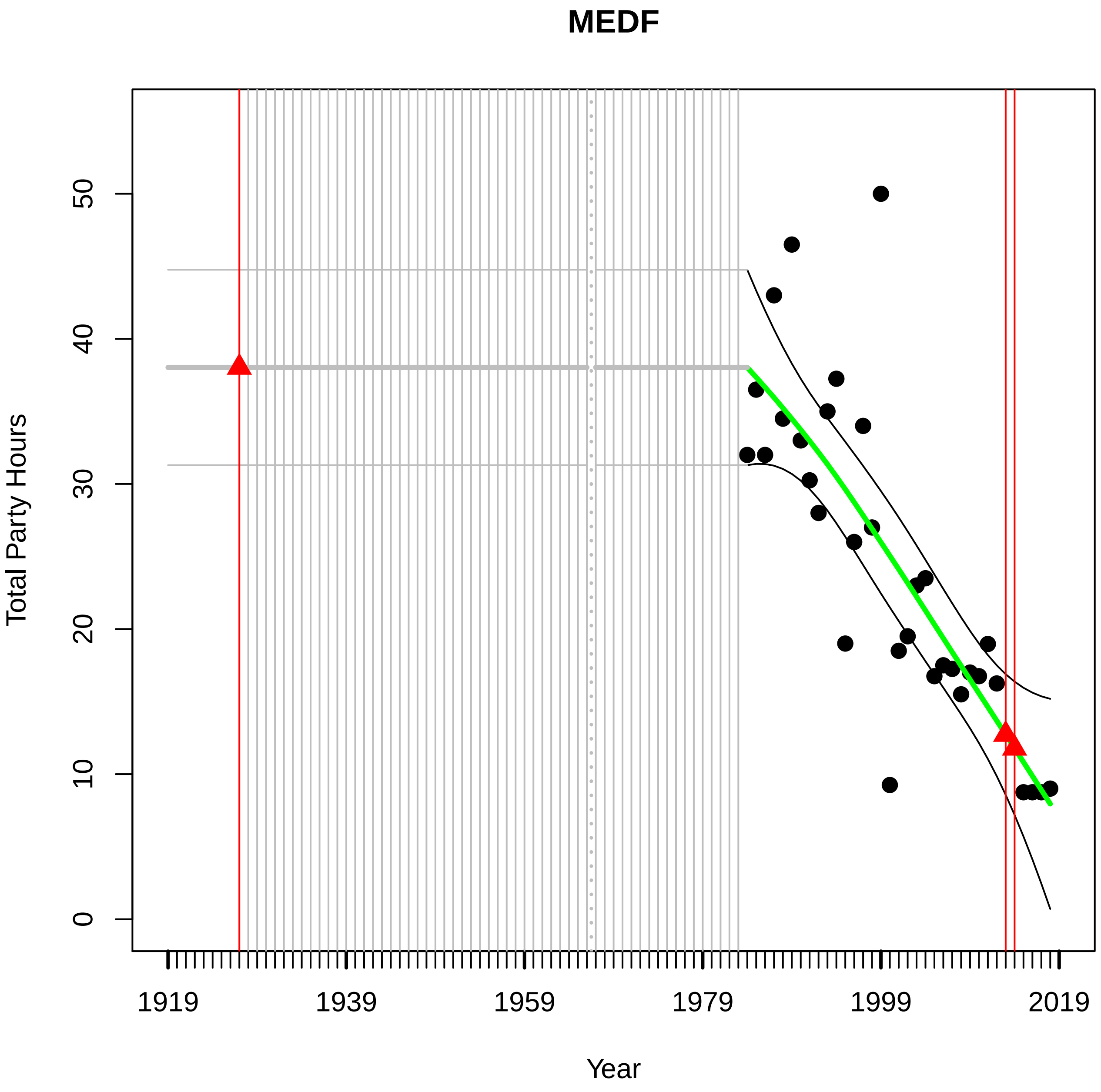

MEDF
Total Party Hours
0
10
20
30
40
50
1919
1939
1959
1979
1999
2019
Year

MEDI

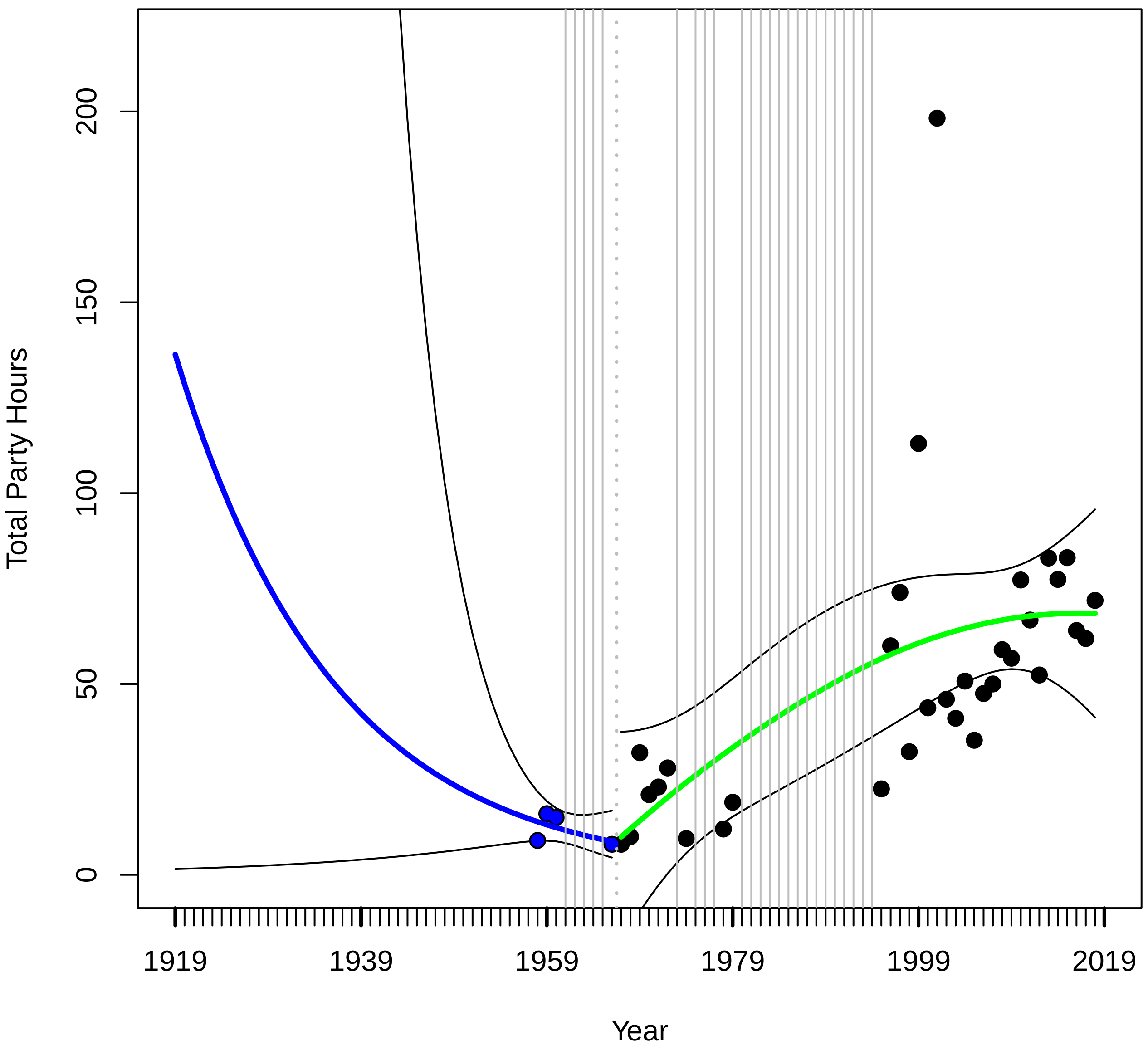

Total Party Hours
0
50
100
150
200
1919
1939
1959
1979
1999
2019
Year

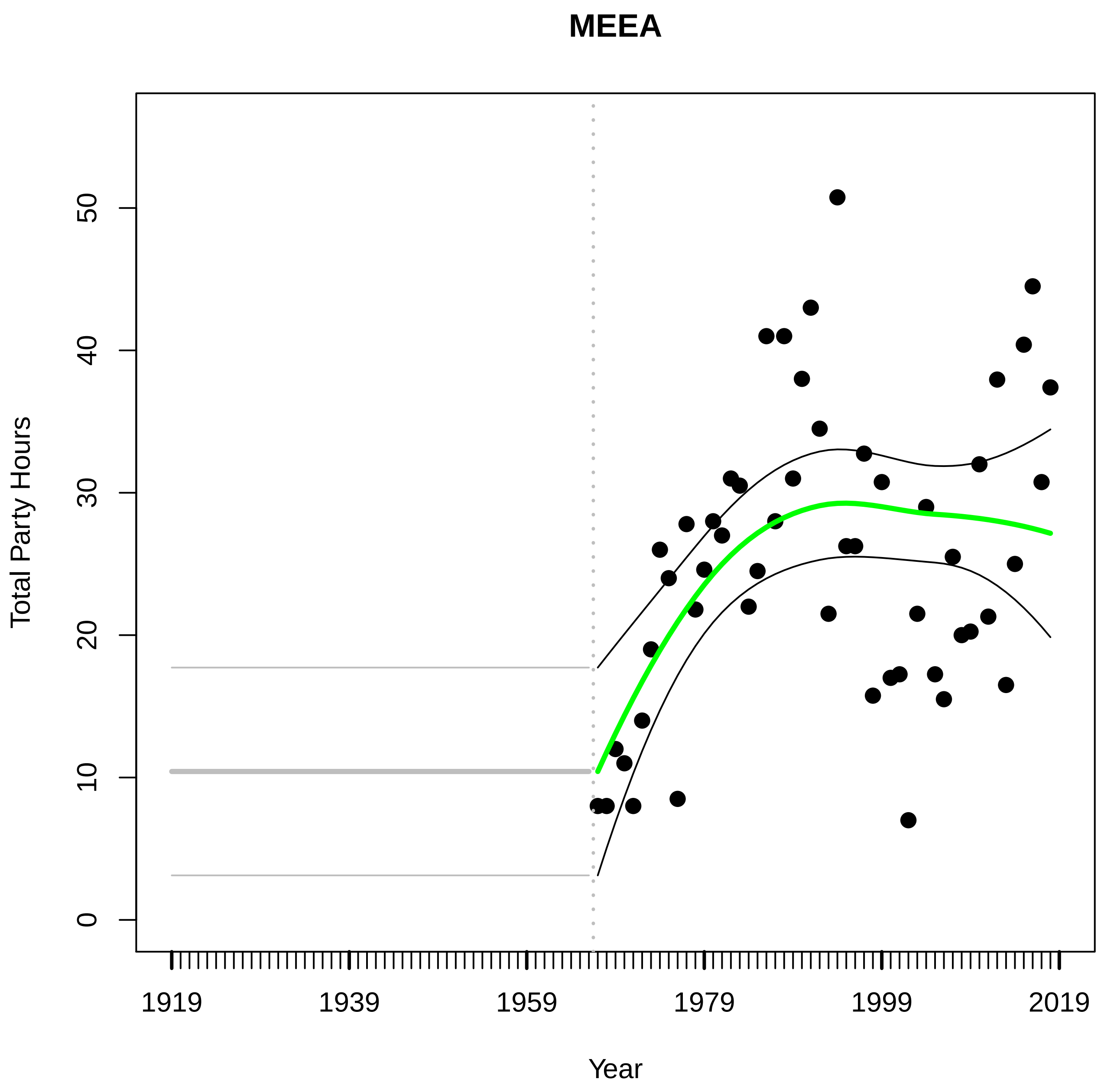

MEEA
Total Party Hours
0
10
20
30
40
50
1919
1939
1959
1979
1999
2019
Year

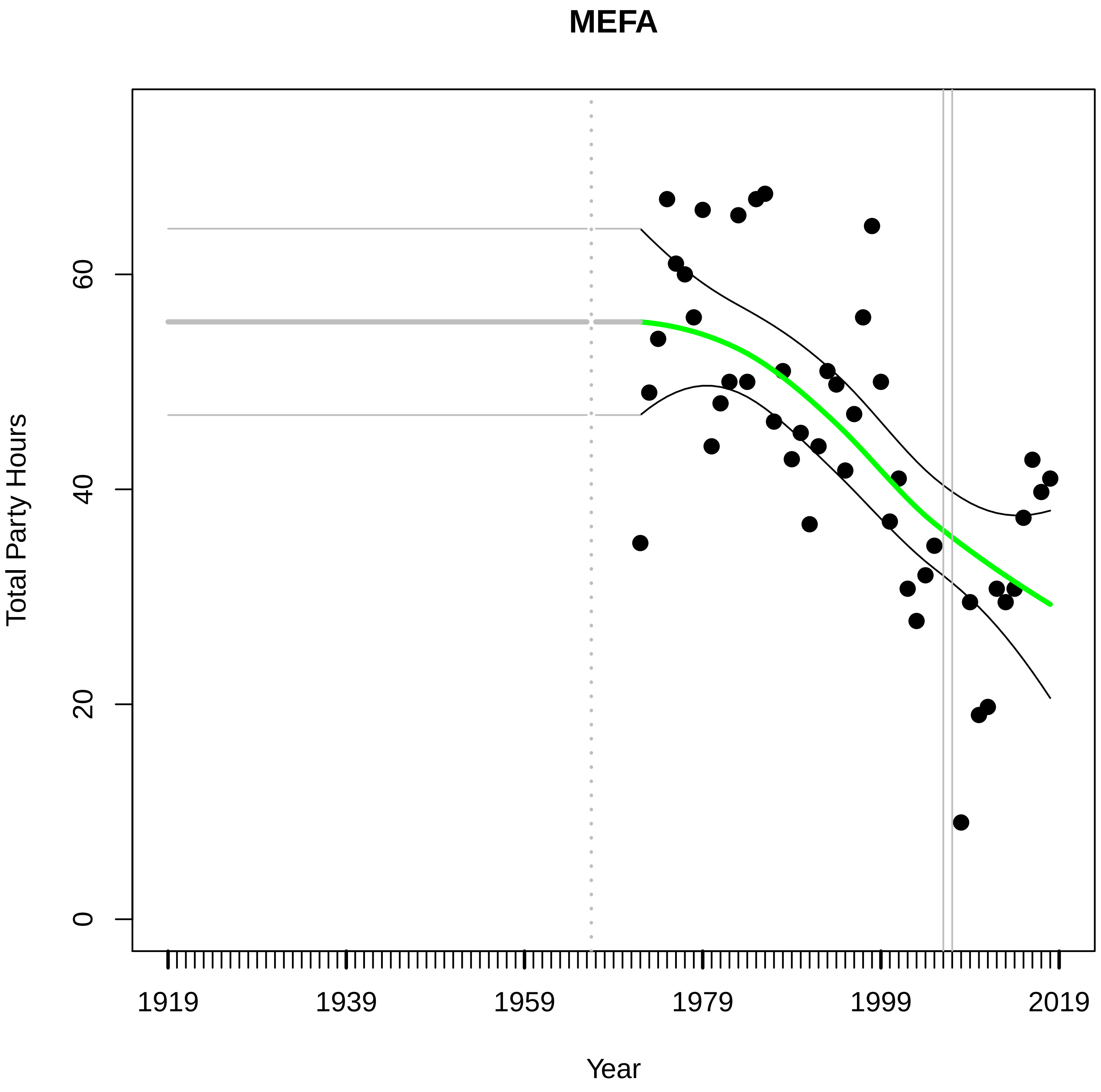
MEFA
Total Party Hours
0
20
40
60
Year
1919
1939
1959
1979
1999
2019

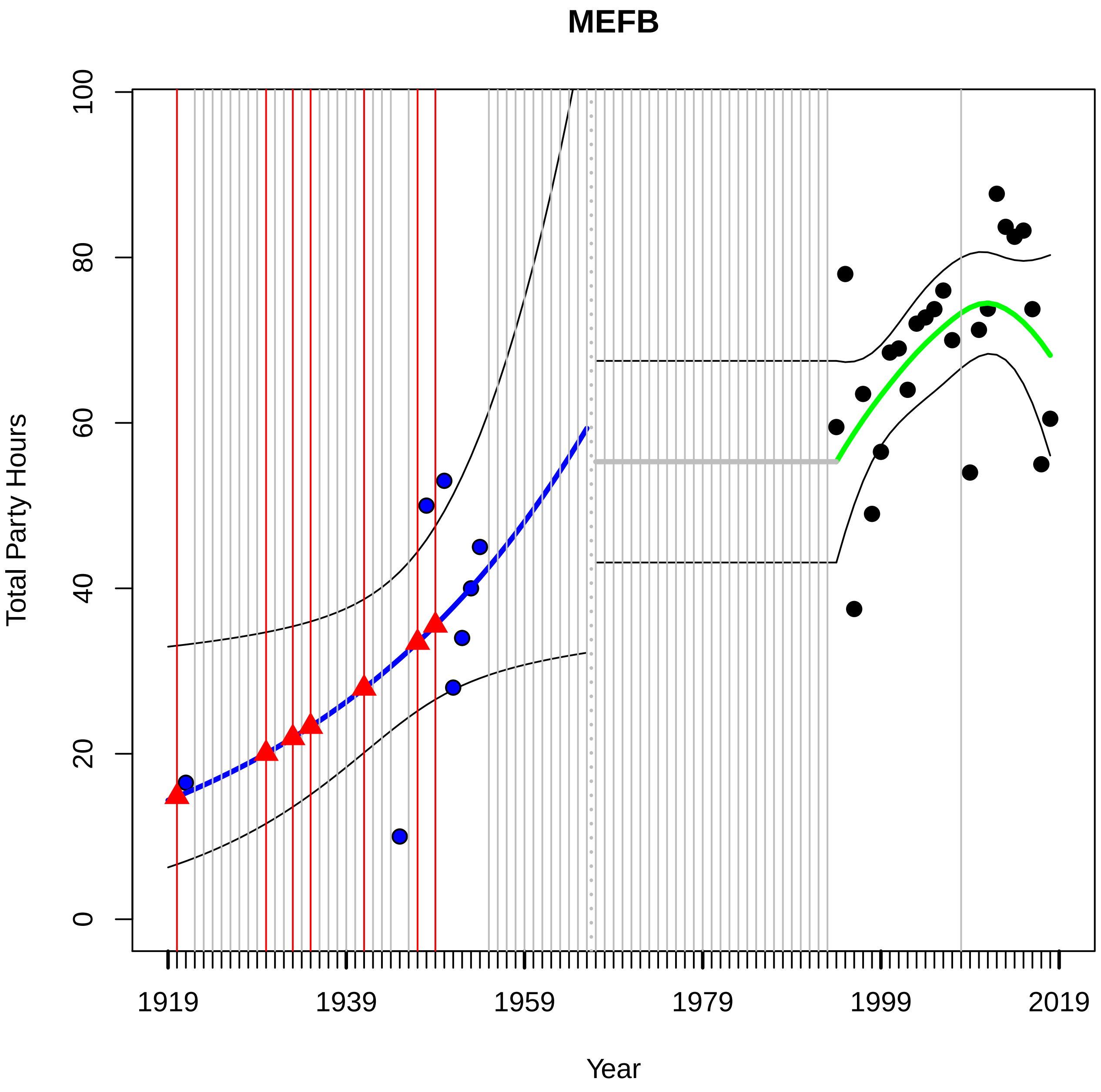

MEFB
Total Party Hours
0
20
40
60
80
100
1919
1939
1959
1979
1999
2019
Year

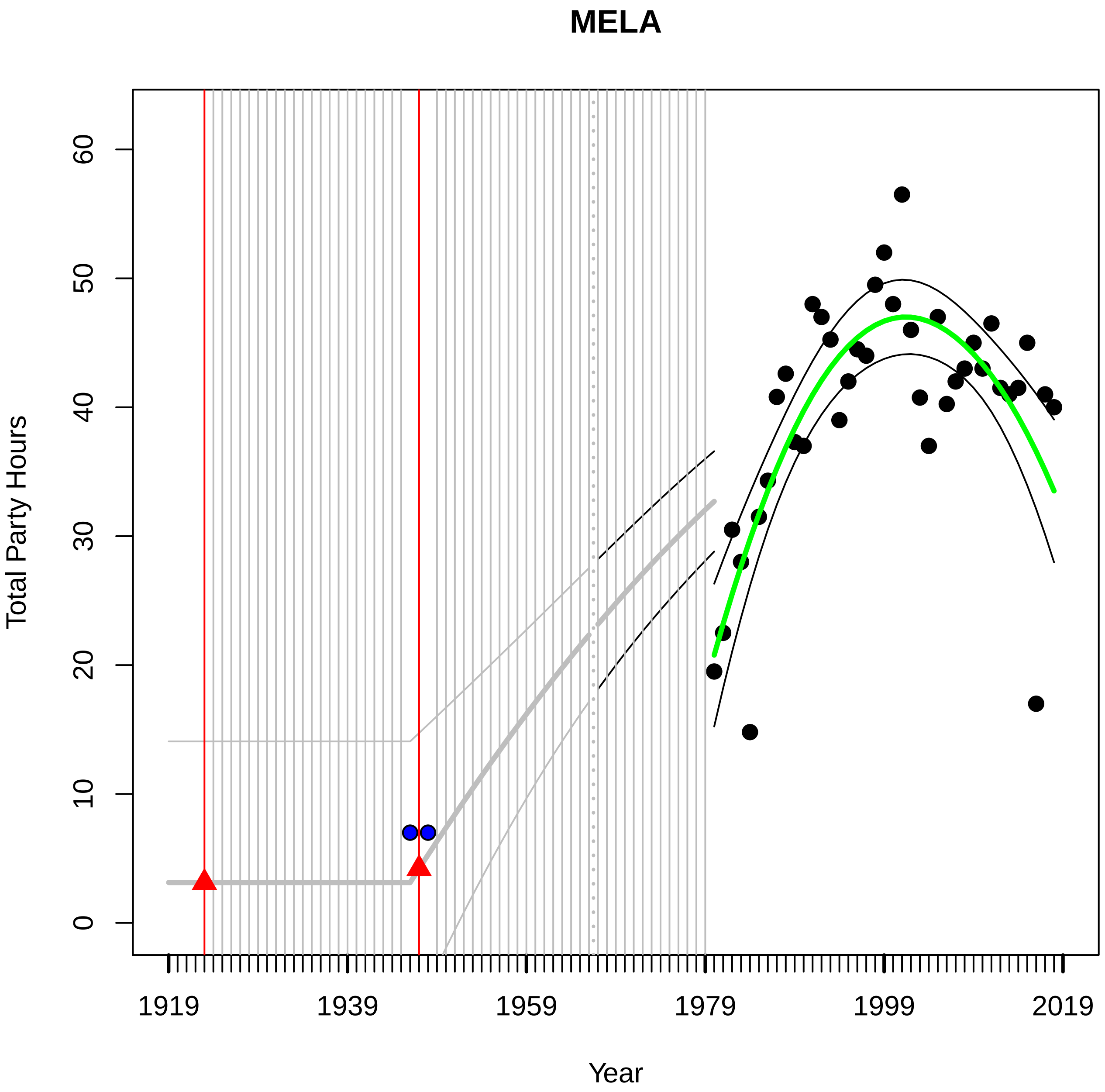

MELA
Total Party Hours
0
10
20
30
40
50
60
1919
1939
1959
1979
1999
2019
Year

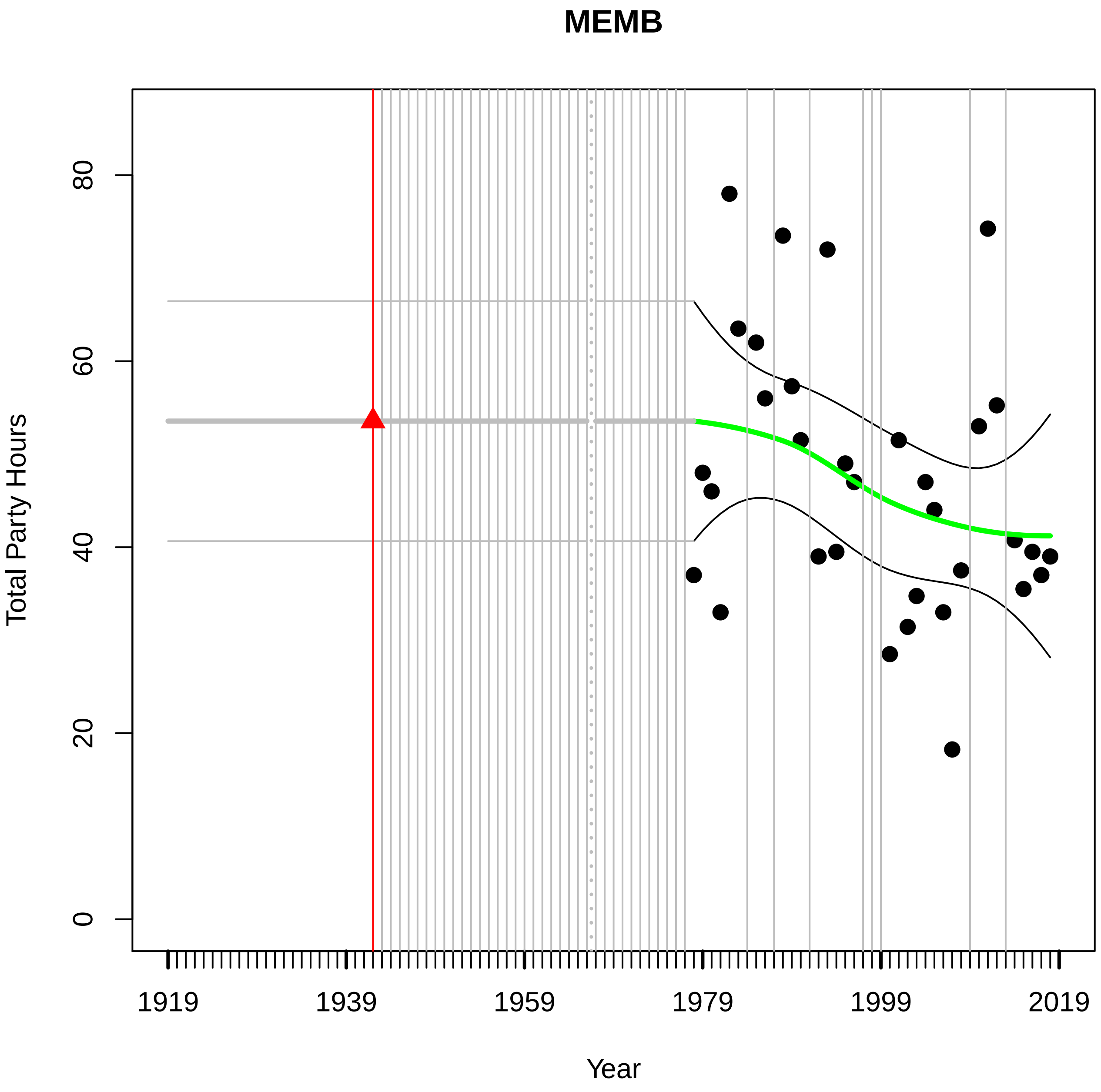

MEMB
Total Party Hours
0
20
40
60
80
1919
1939
1959
1979
1999
2019
Year

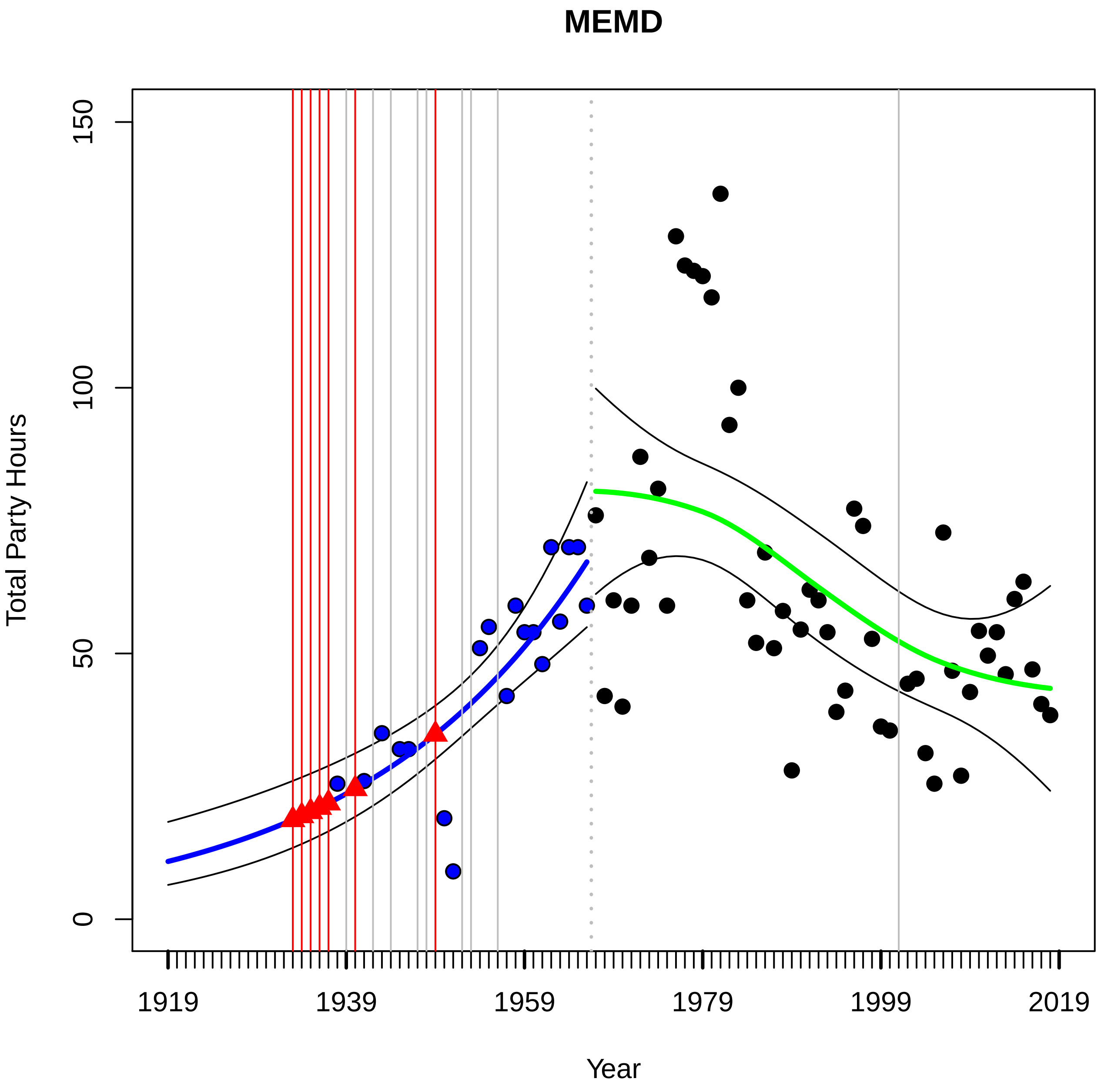
MEMD
Total Party Hours
0
50
100
150
1919
1939
1959
1979
1999
2019
Year

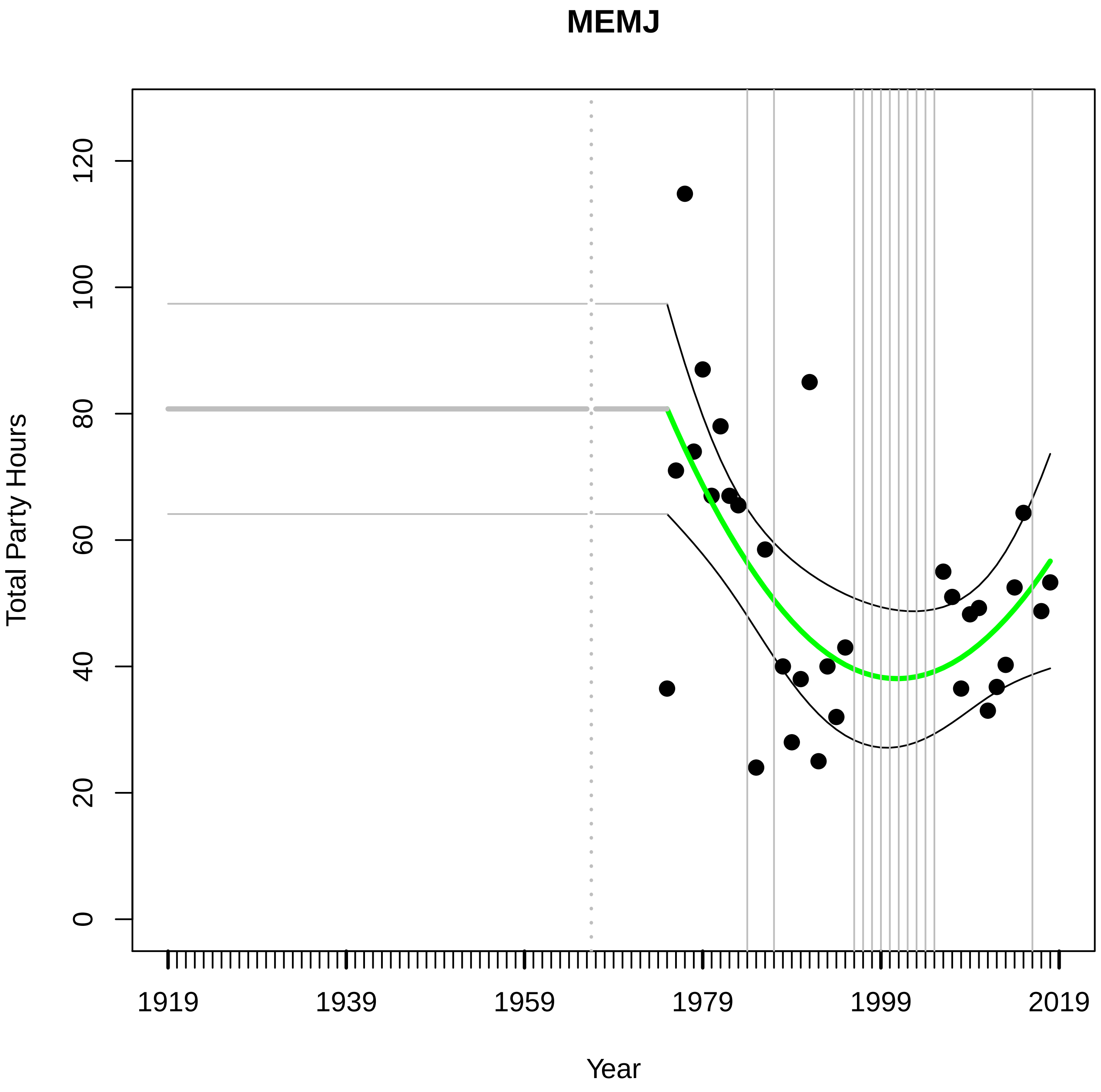
MEMJ
Total Party Hours
0
20
40
60
80
100
120
1919
1939
1959
1979
1999
2019
Year

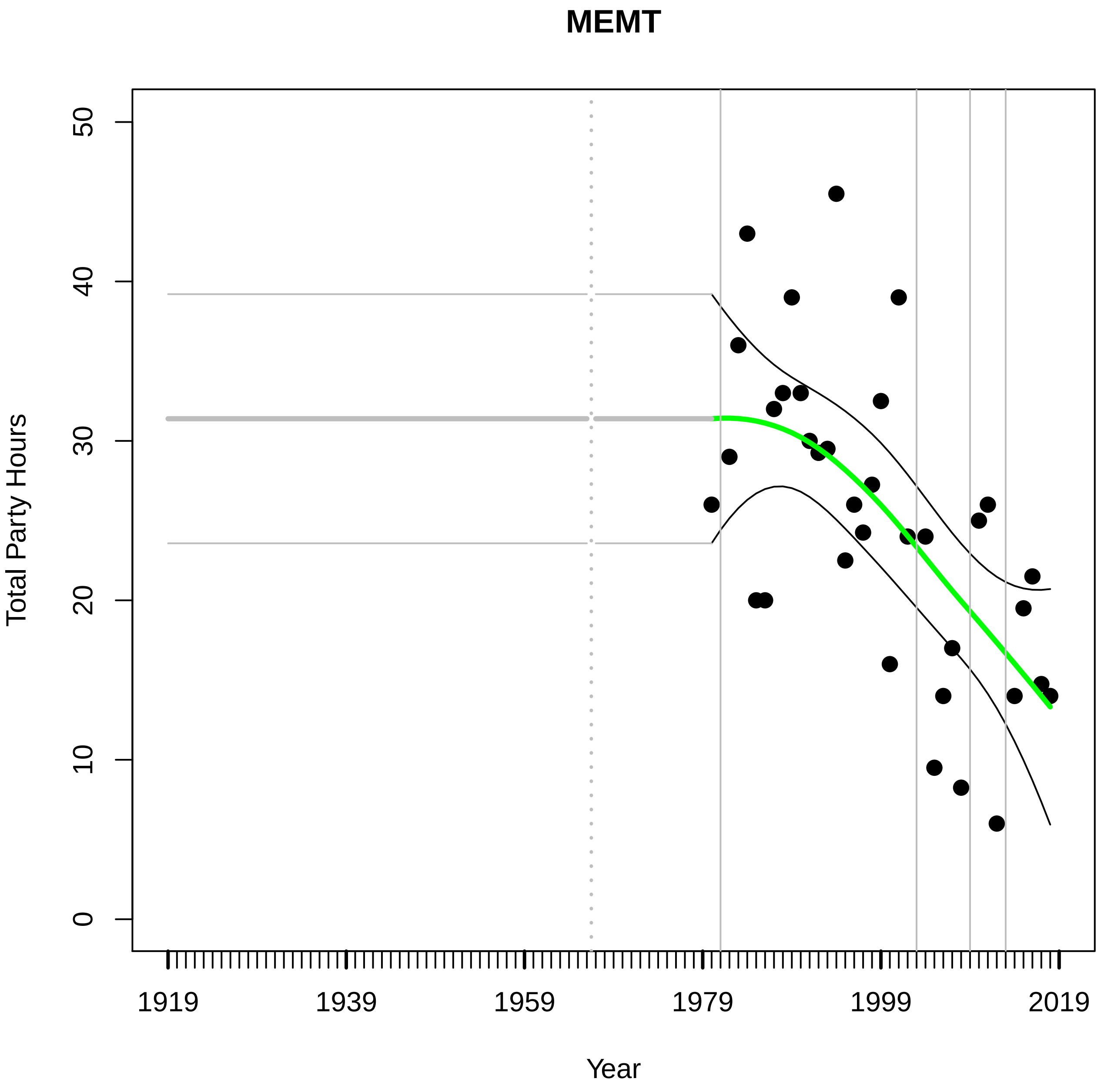

MEMT
Total Party Hours
0
10
20
30
40
50
1919
1939
1959
1979
1999
2019
Year

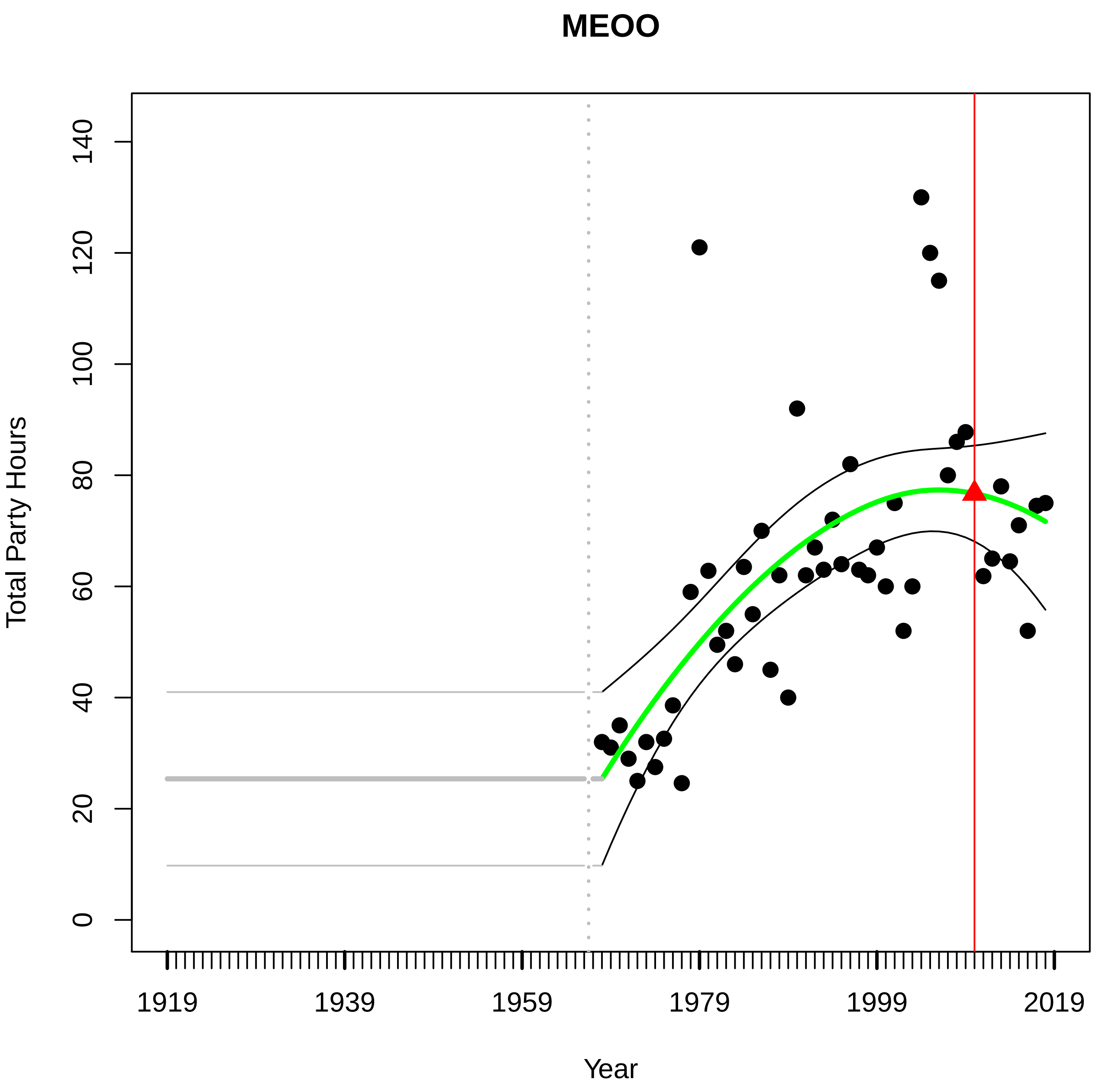
MEOO
Total Party Hours
0
20
40
60
80
100
120
140
1919
1939
1959
1979
1999
2019
Year

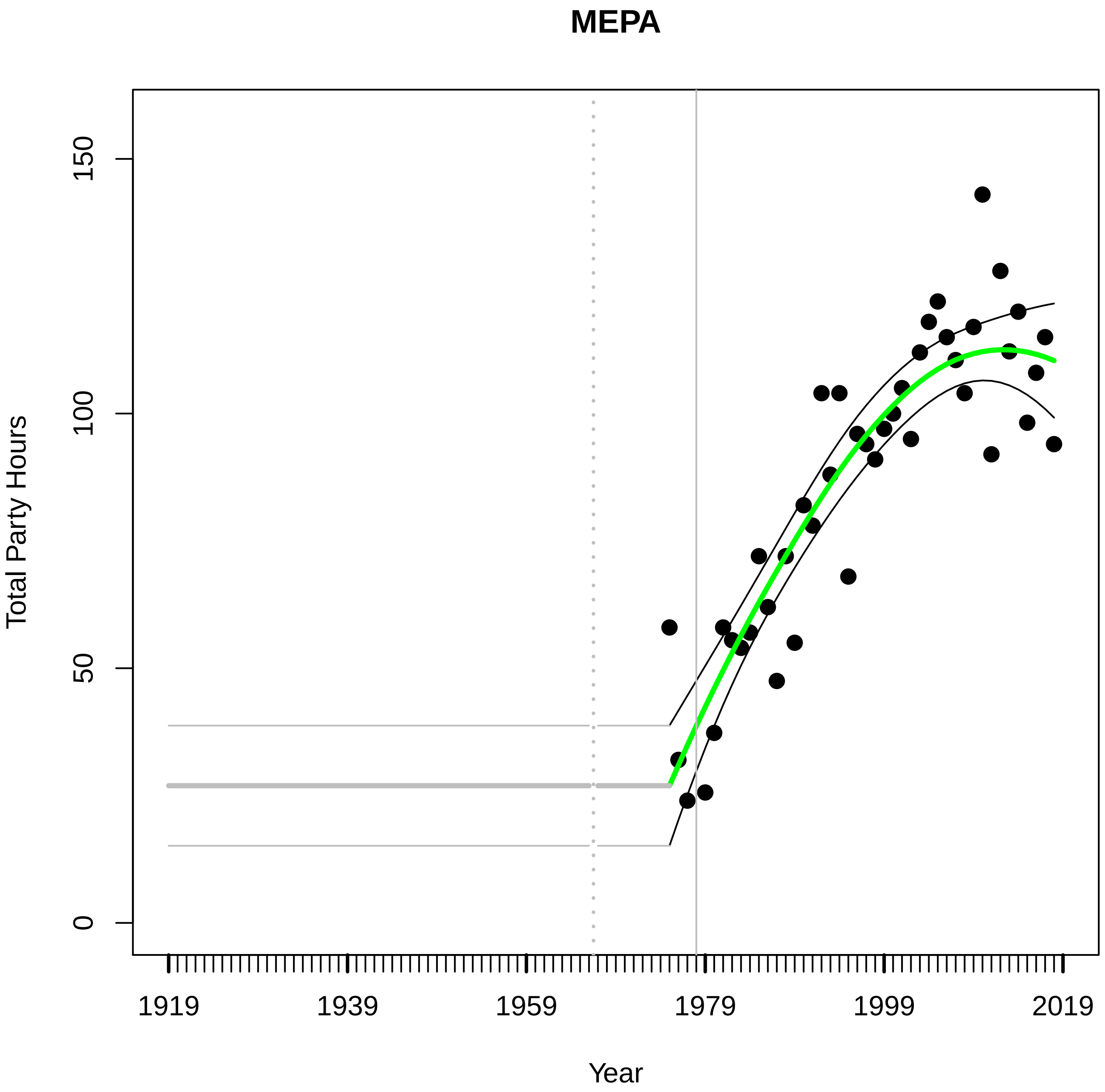
MEPA
Total Party Hours
0
50
100
150
1919
1939
1959
1979
1999
2019
Year

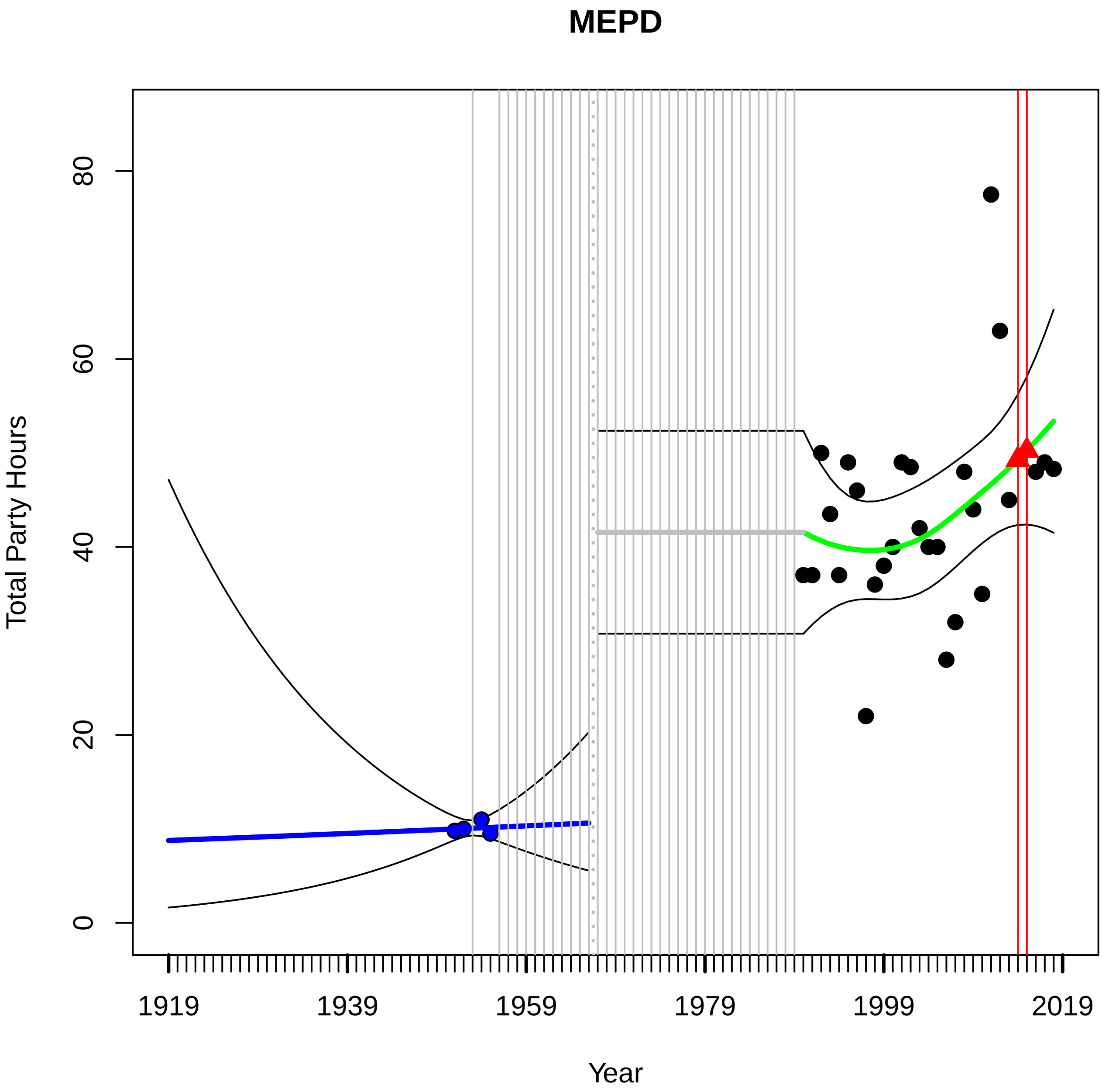

MEPD
Total Party Hours
0
20
40
60
80
1919
1939
1959
1979
1999
2019
Year

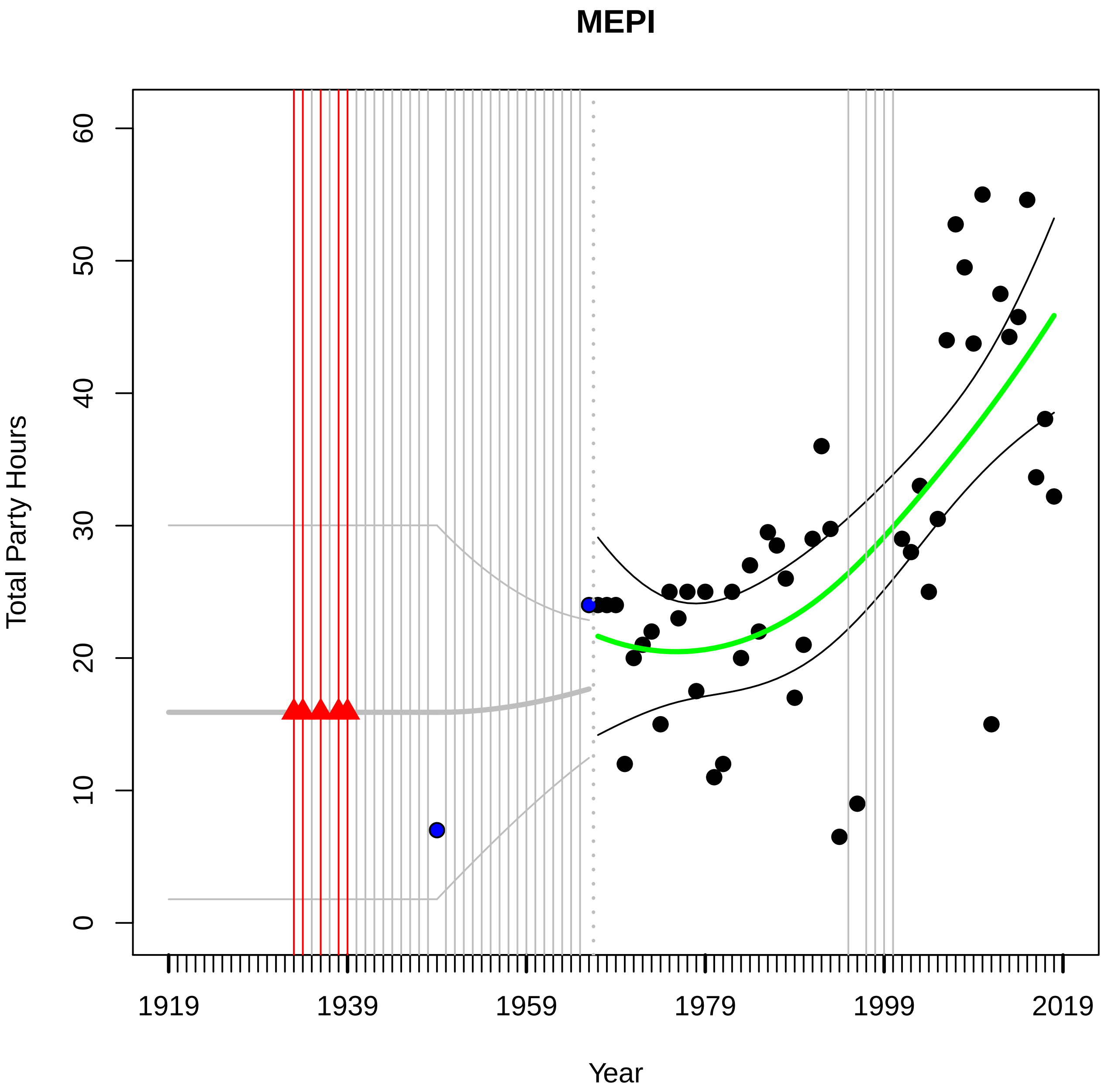
MEPI
Total Party Hours
0
10
20
30
40
50
60
1919
1939
1959
1979
1999
2019
Year

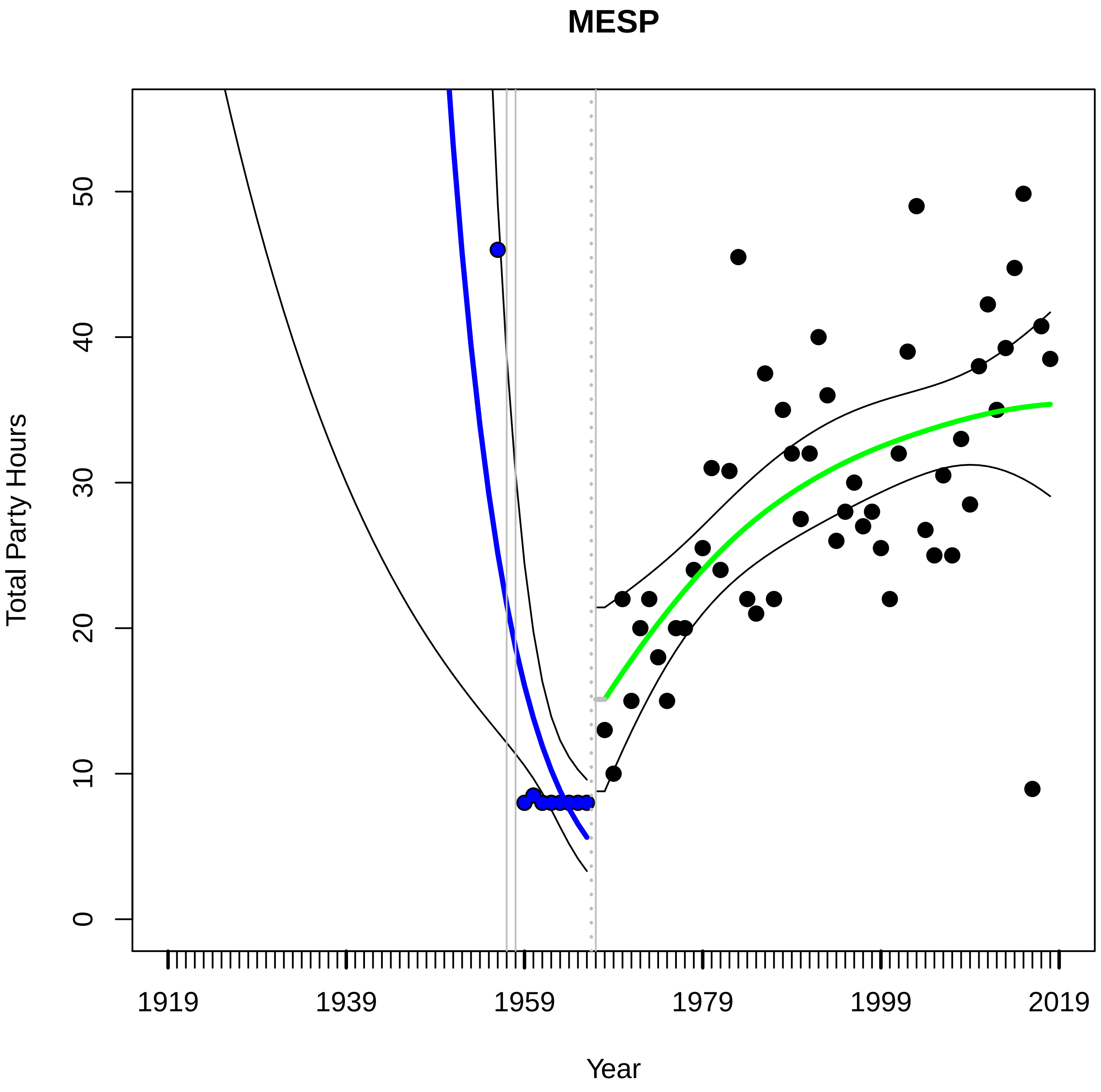

MESP
Total Party Hours
Year
0
10
20
30
40
50
1919
1939
1959
1979
1999
2019

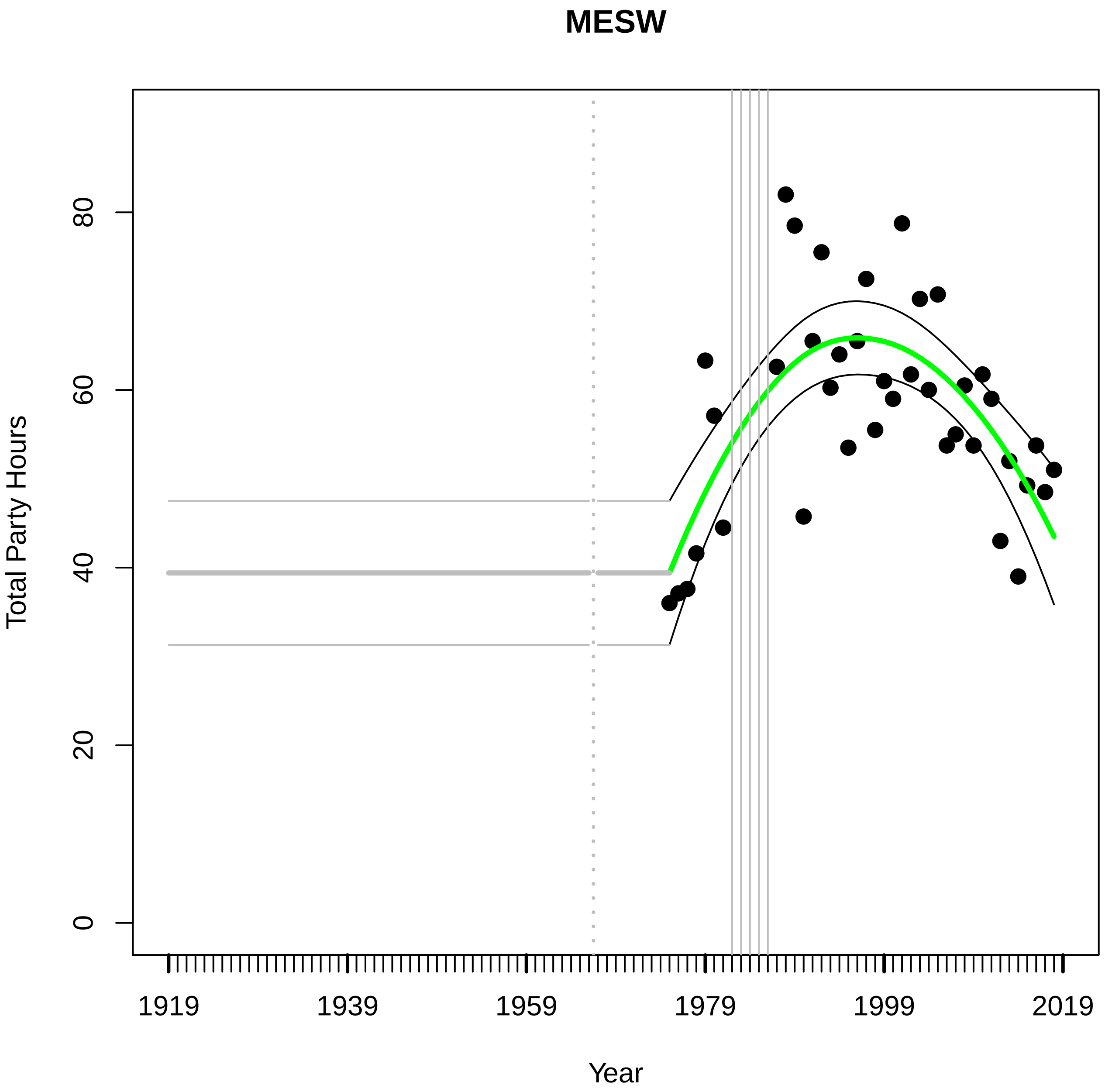

MESW
Total Party Hours
0
20
40
60
80
1919
1939
1959
1979
1999
2019
Year

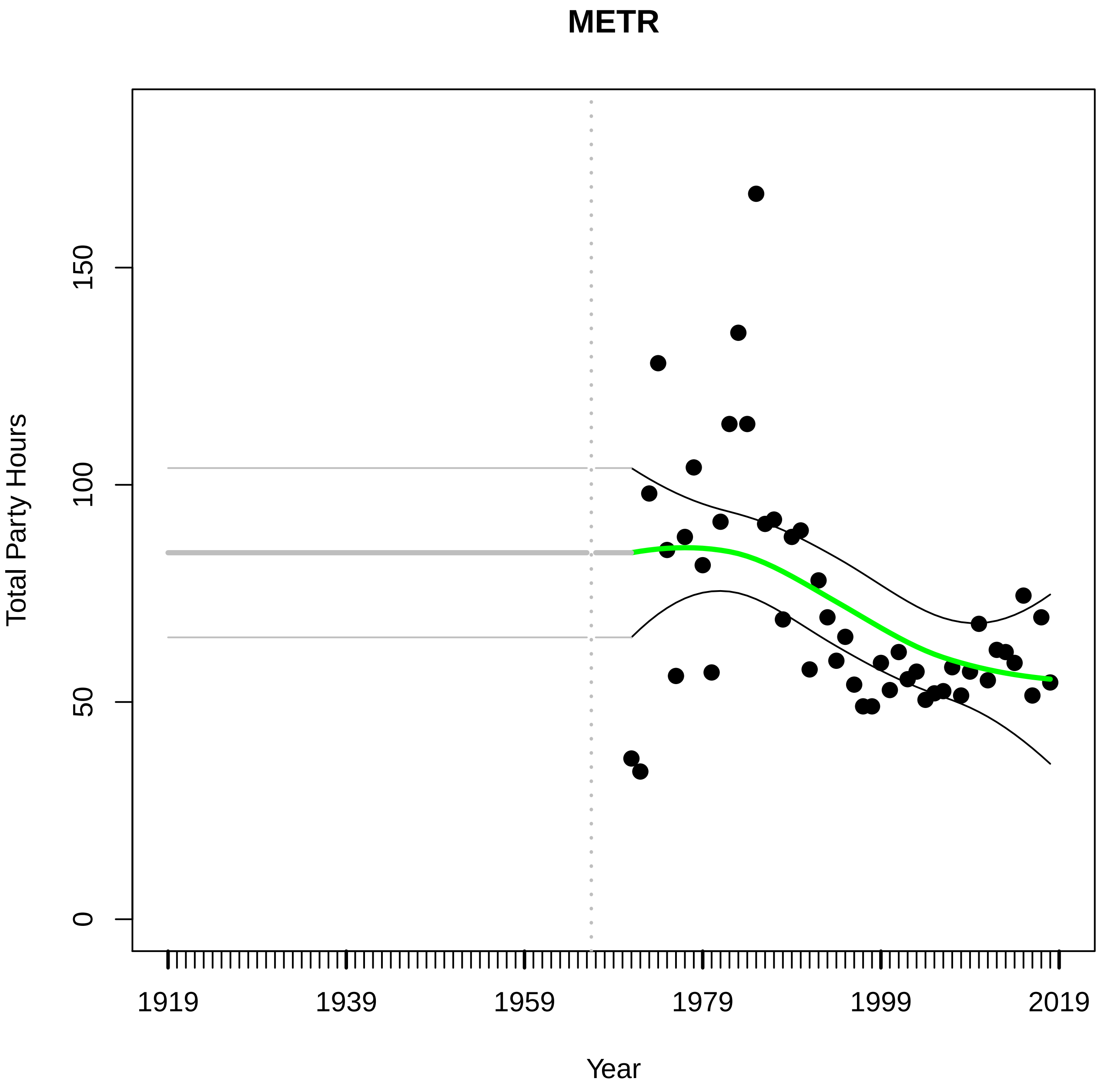
METR
Total Party Hours
0
50
100
150
Year
1919
1939
1959
1979
1999
2019

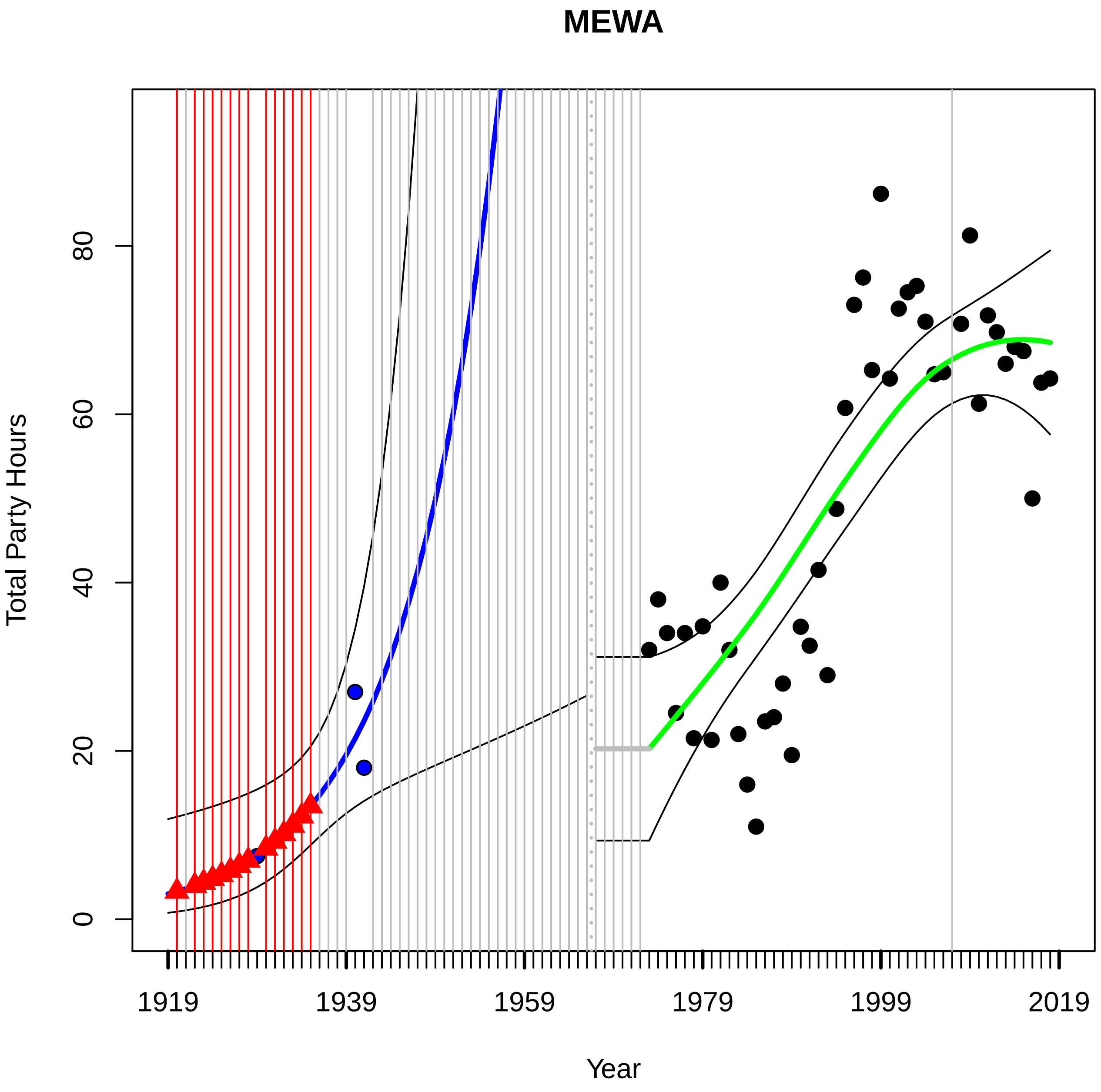
MEWA
Total Party Hours
0
20
40
60
80
Year
1919
1939
1959
1979
1999
2019

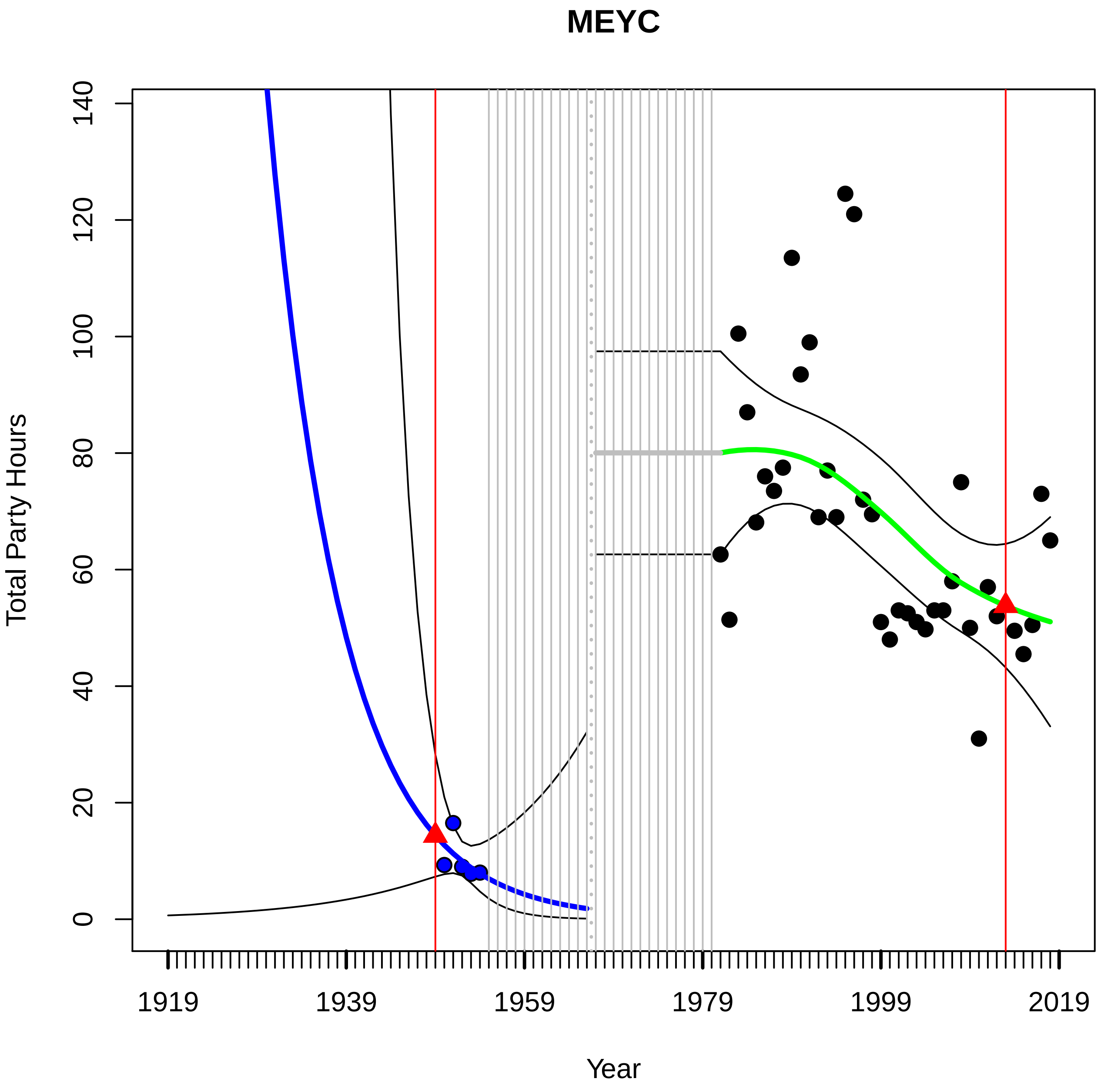
MEYC
Total Party Hours
0
20
40
60
80
100
120
140
1919
1939
1959
1979
1999
2019
Year

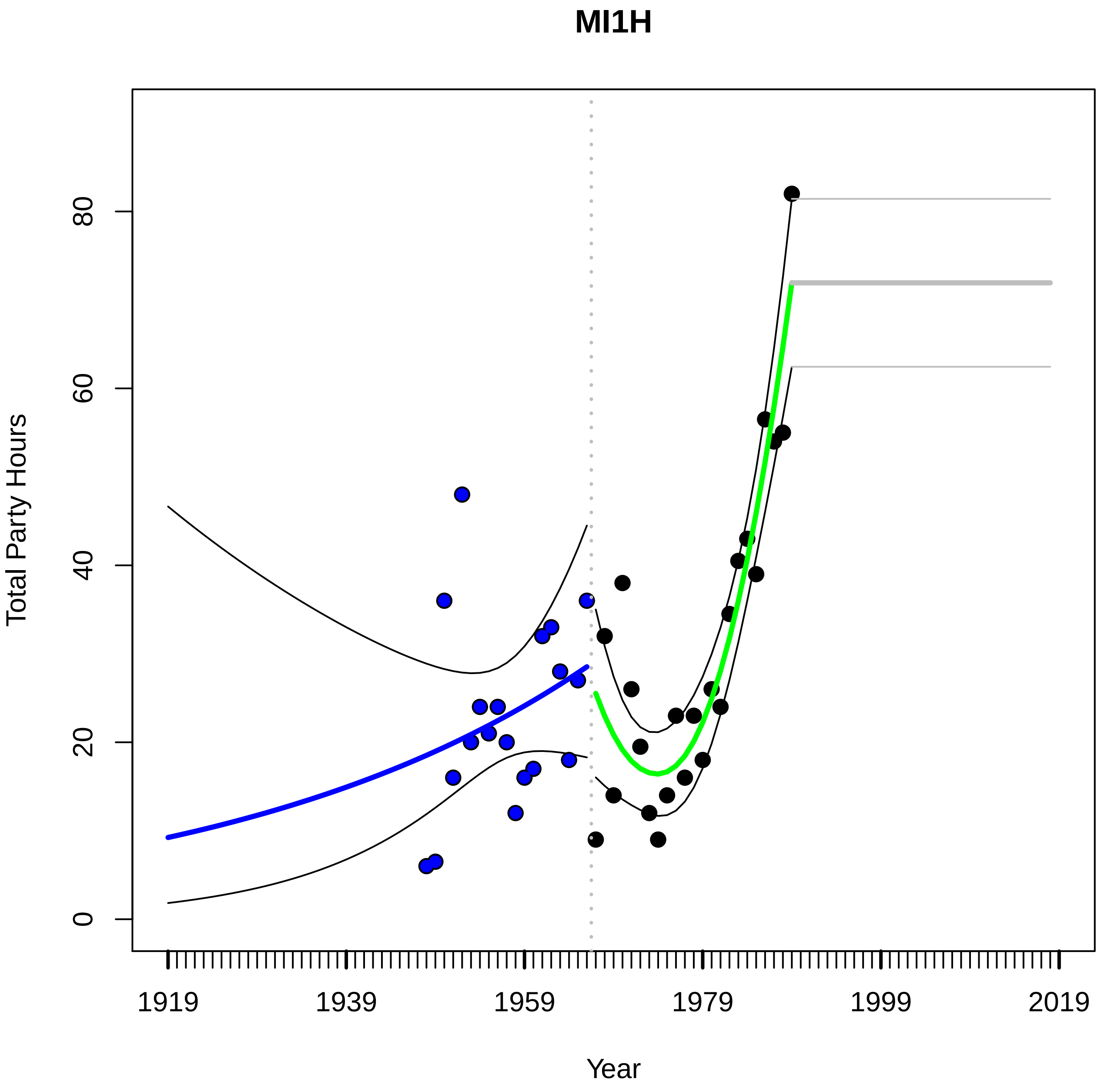

MI1H
Total Party Hours
Year
0
20
40
60
80
1919
1939
1959
1979
1999
2019

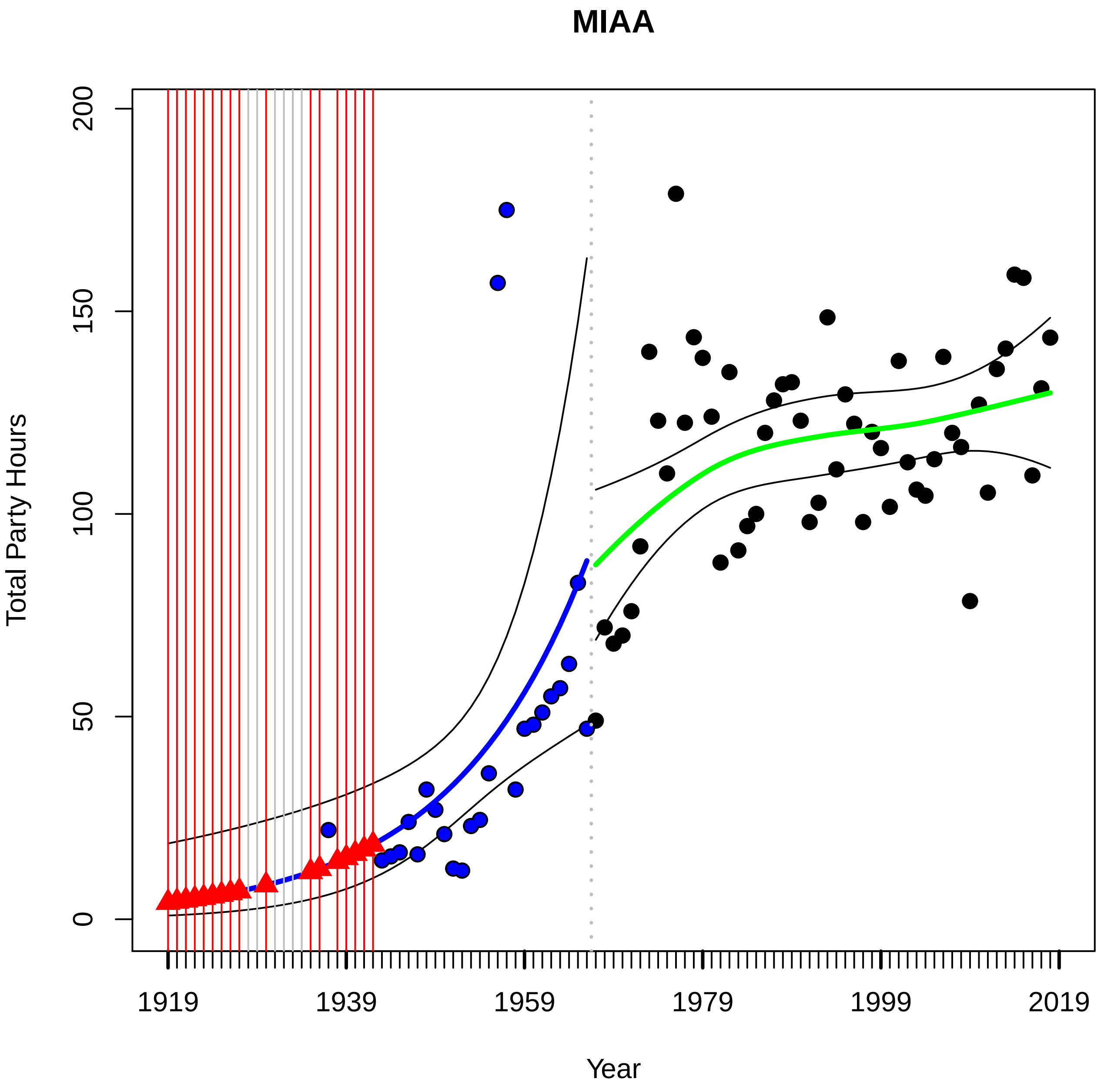
MIAA
Total Party Hours
0
50
100
150
200
1919
1939
1959
1979
1999
2019
Year

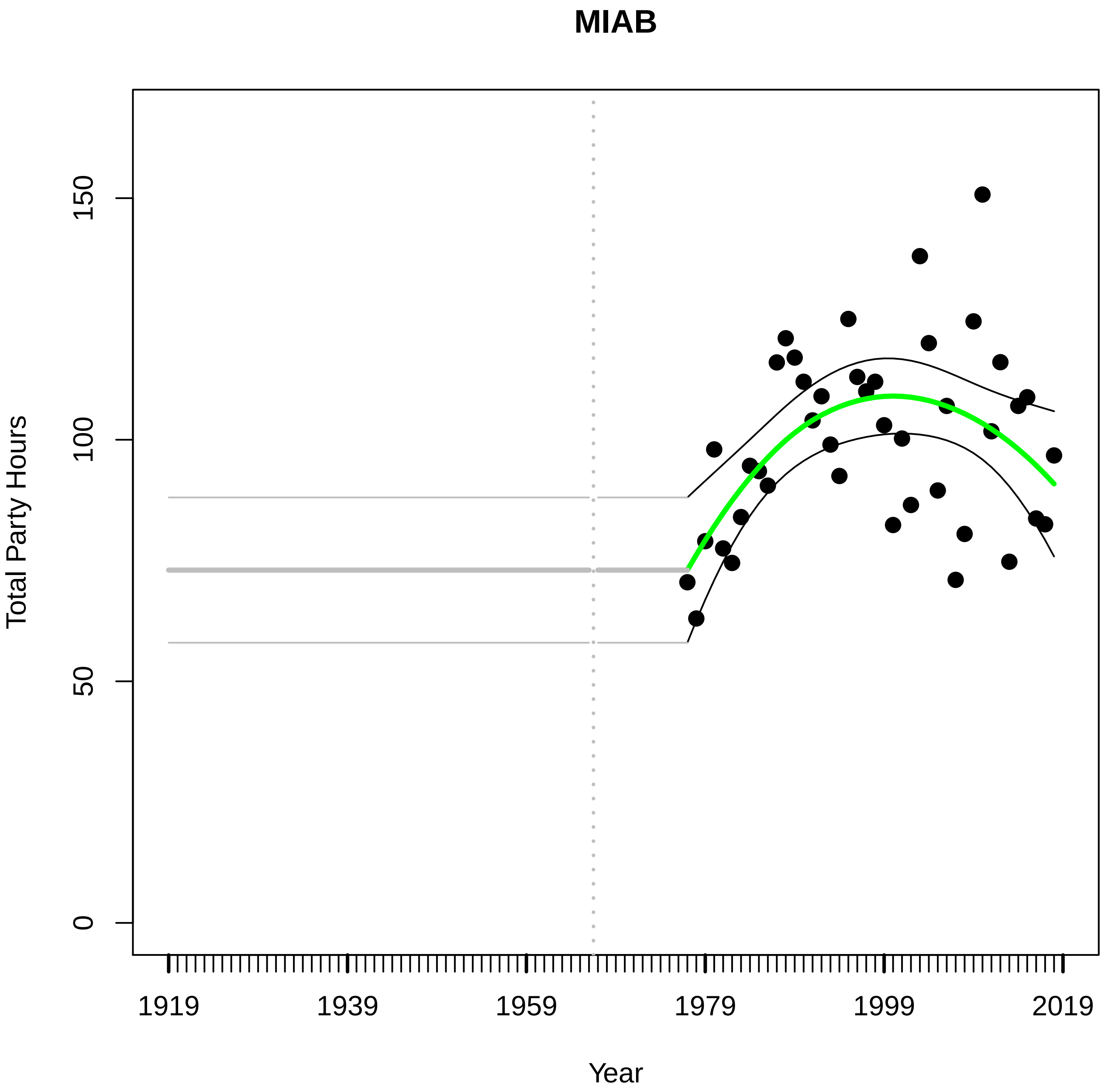

MIAB
Total Party Hours
0
50
100
150
1919
1939
1959
1979
1999
2019
Year

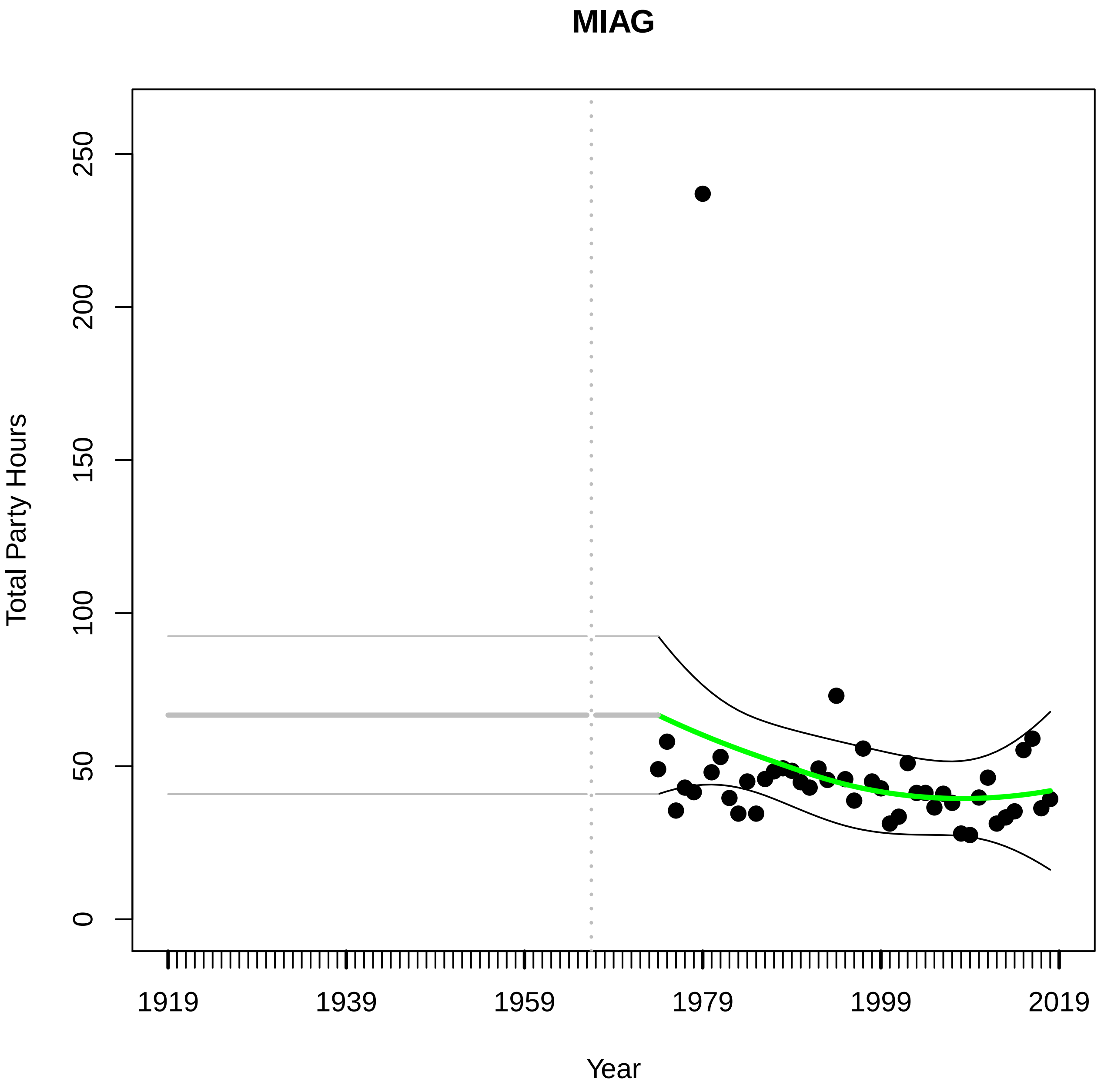
MIAG
Total Party Hours
0
50
100
150
200
250
1919
1939
1959
1979
1999
2019
Year

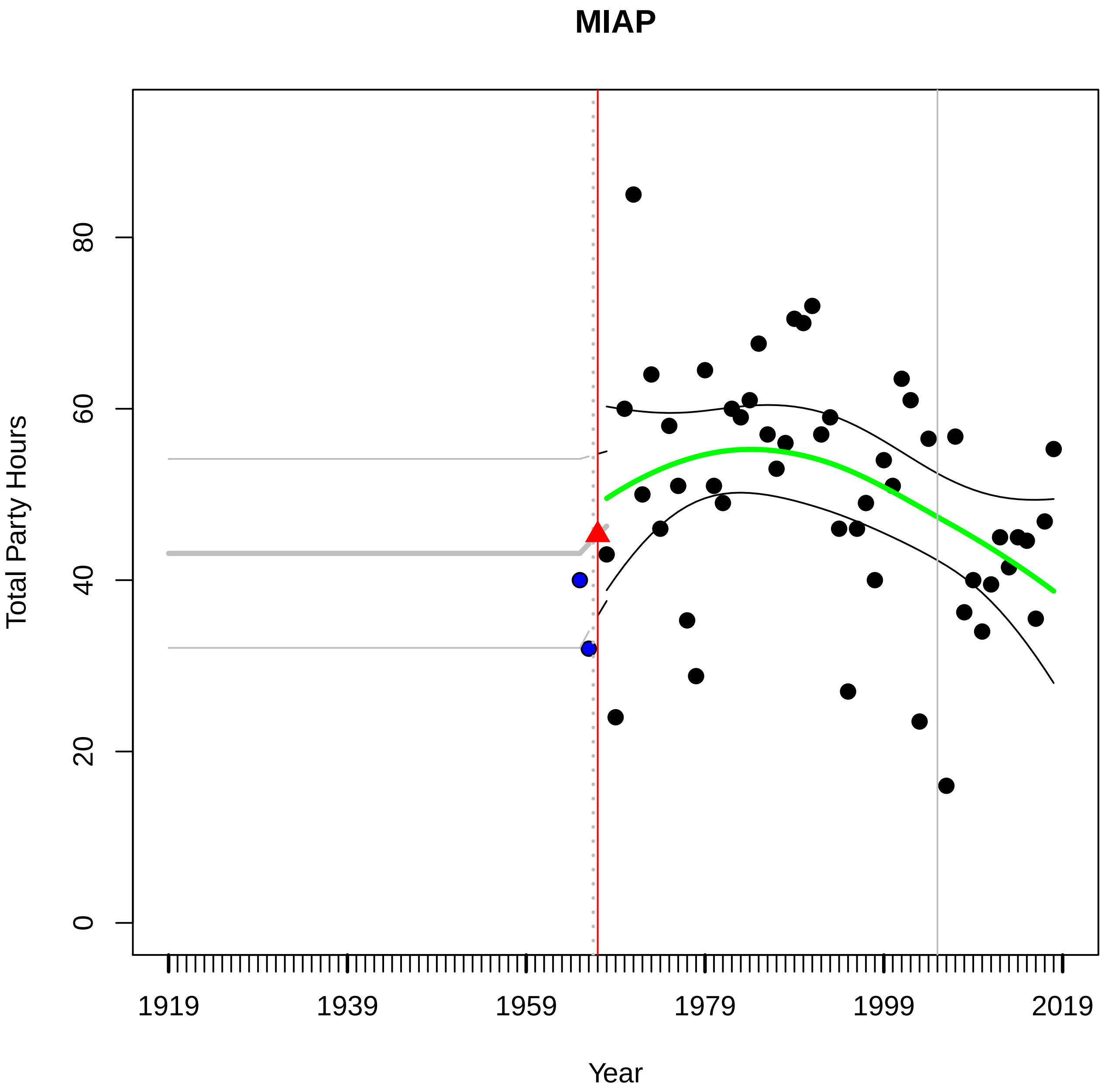

MIAP
Total Party Hours
0
20
40
60
80
1919
1939
1959
1979
1999
2019
Year

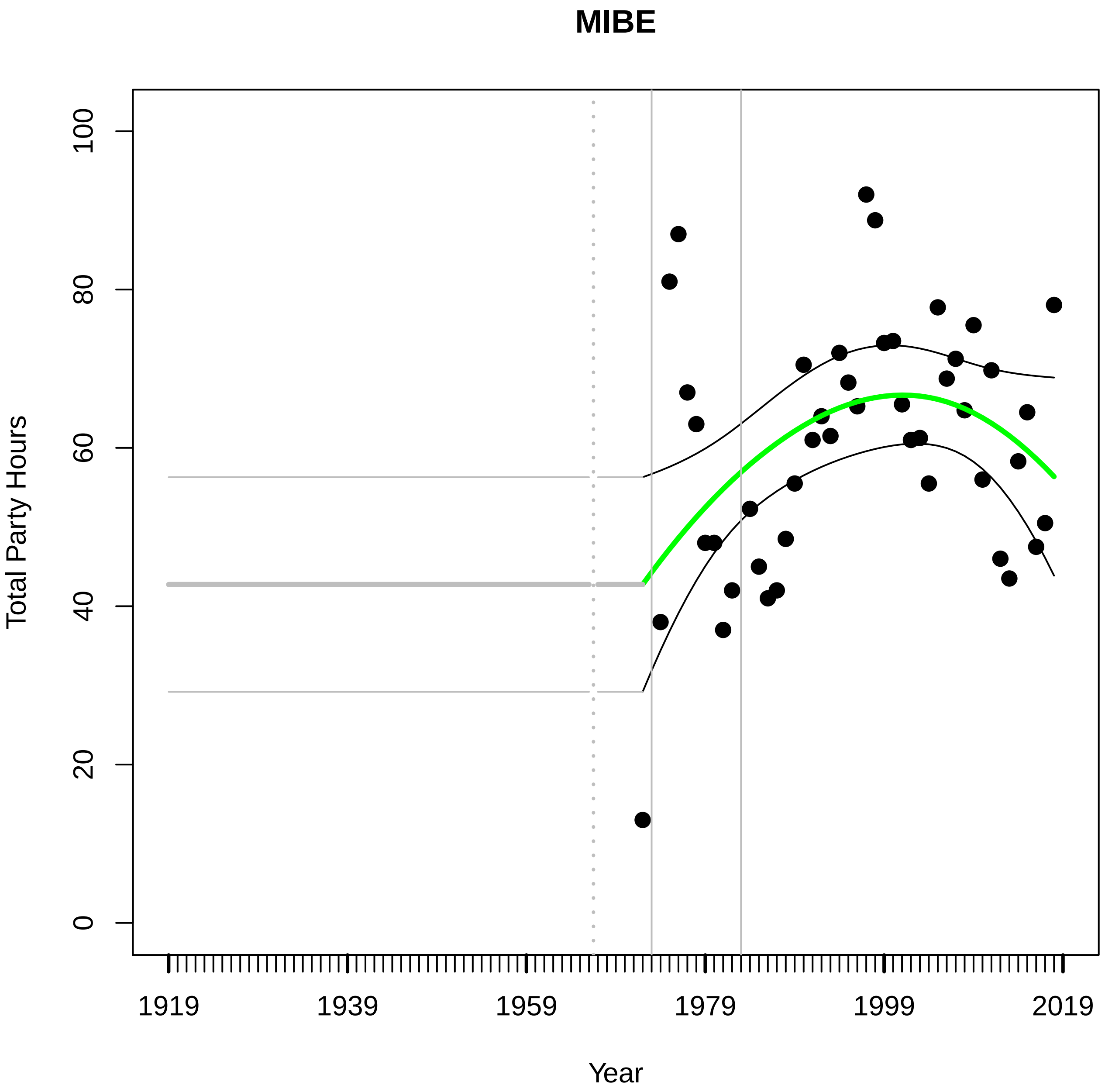

MIBE
Total Party Hours
0
20
40
60
80
100
1919
1939
1959
1979
1999
2019
Year

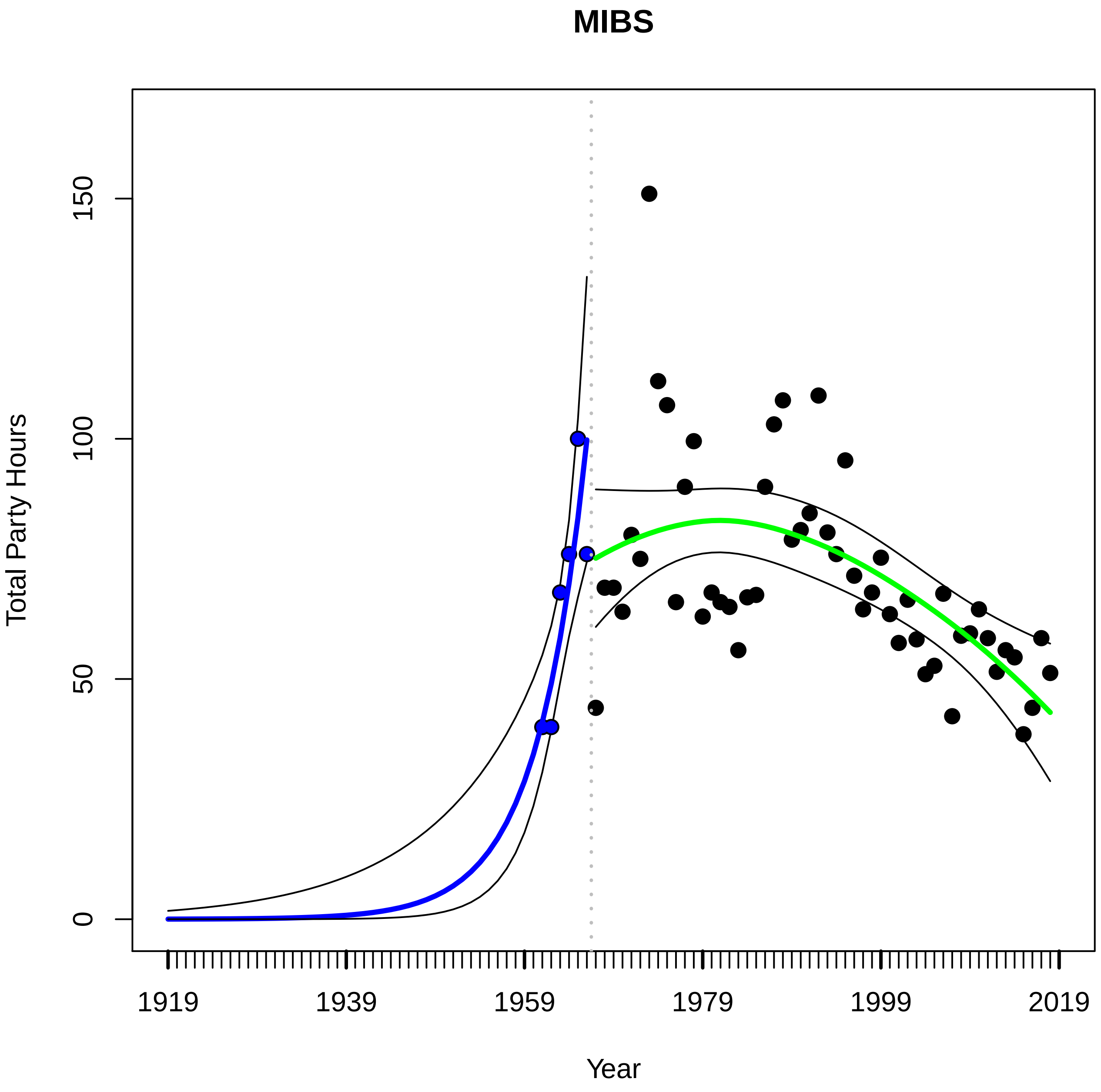

MIBS
Total Party Hours
Year
0
50
100
150
1919
1939
1959
1979
1999
2019

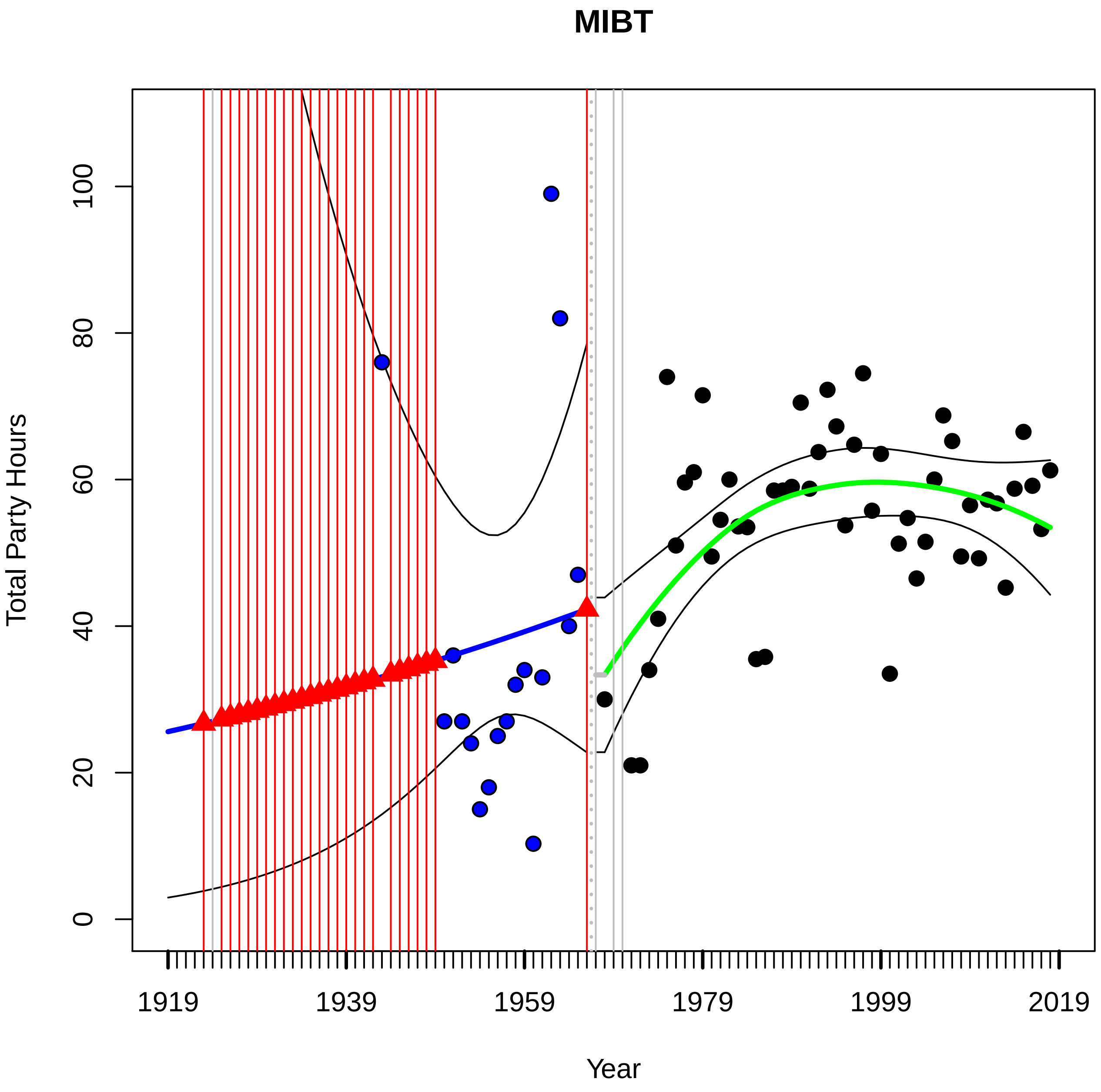

MIBT
Total Party Hours
Year
0
20
40
60
80
100
1919
1939
1959
1979
1999
2019

MICO

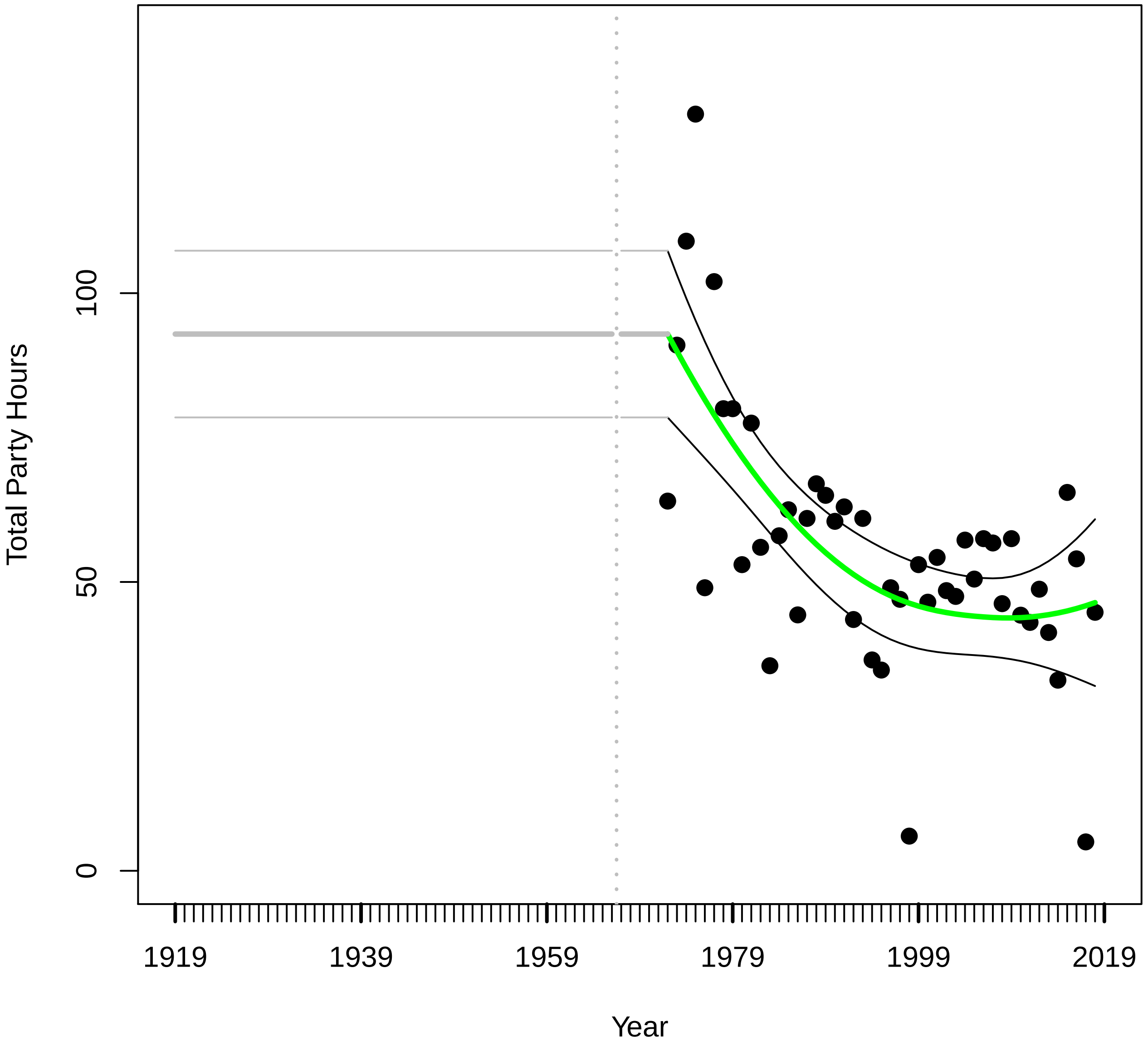

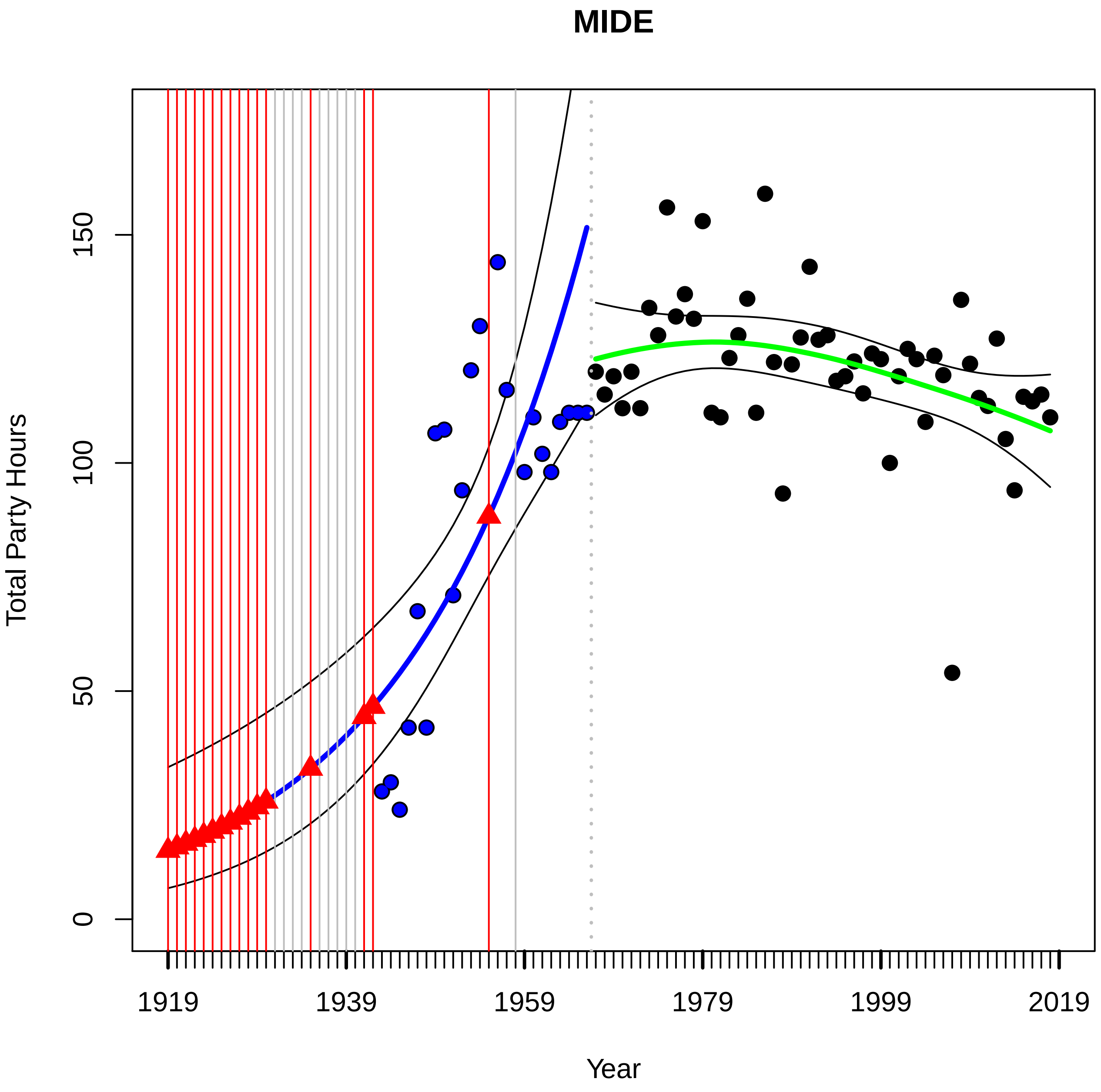

MIDE
Total Party Hours
0
50
100
150
1919
1939
1959
1979
1999
2019
Year

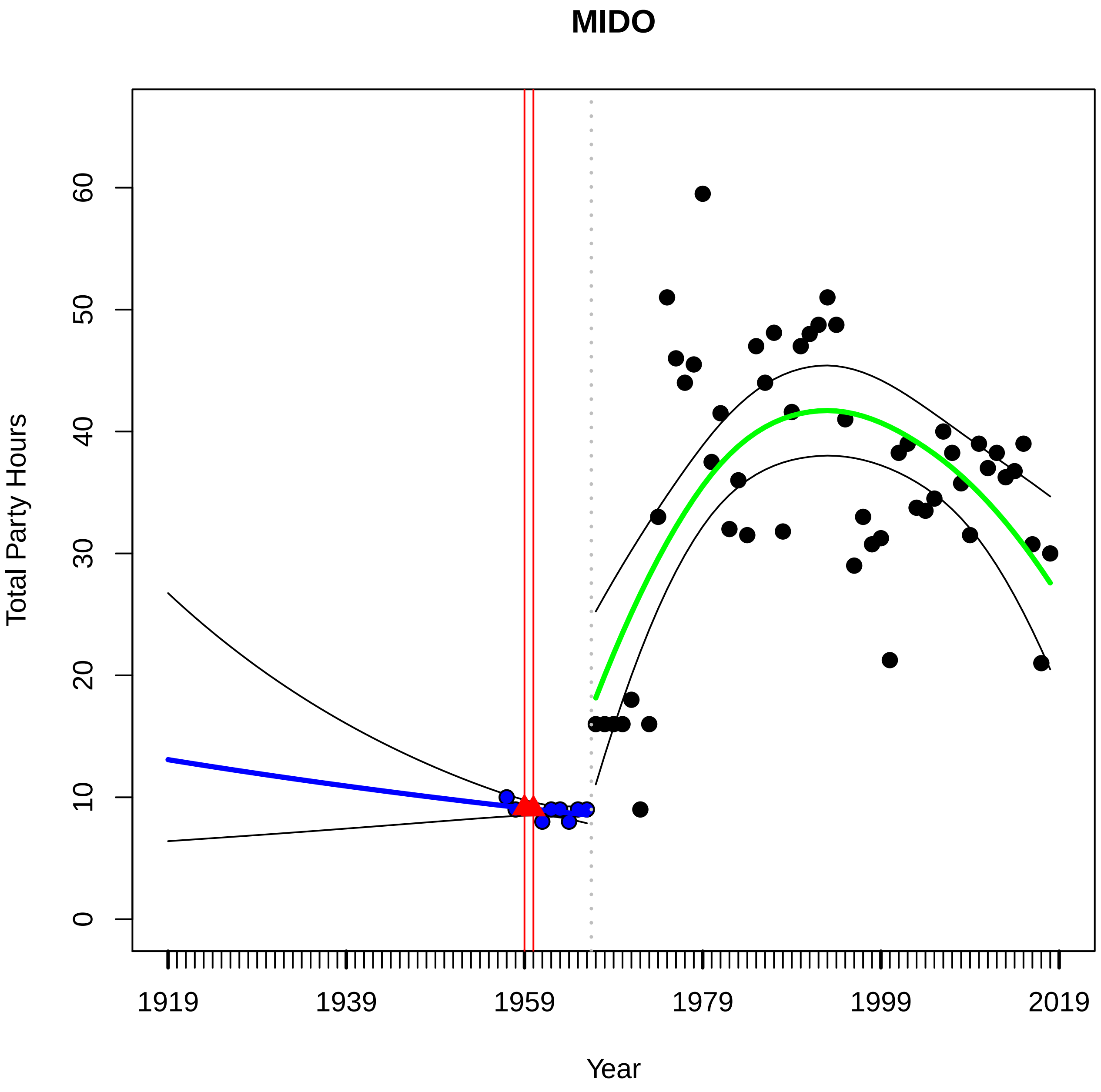
MIDO
Total Party Hours
Year
0
10
20
30
40
50
60
1919
1939
1959
1979
1999
2019

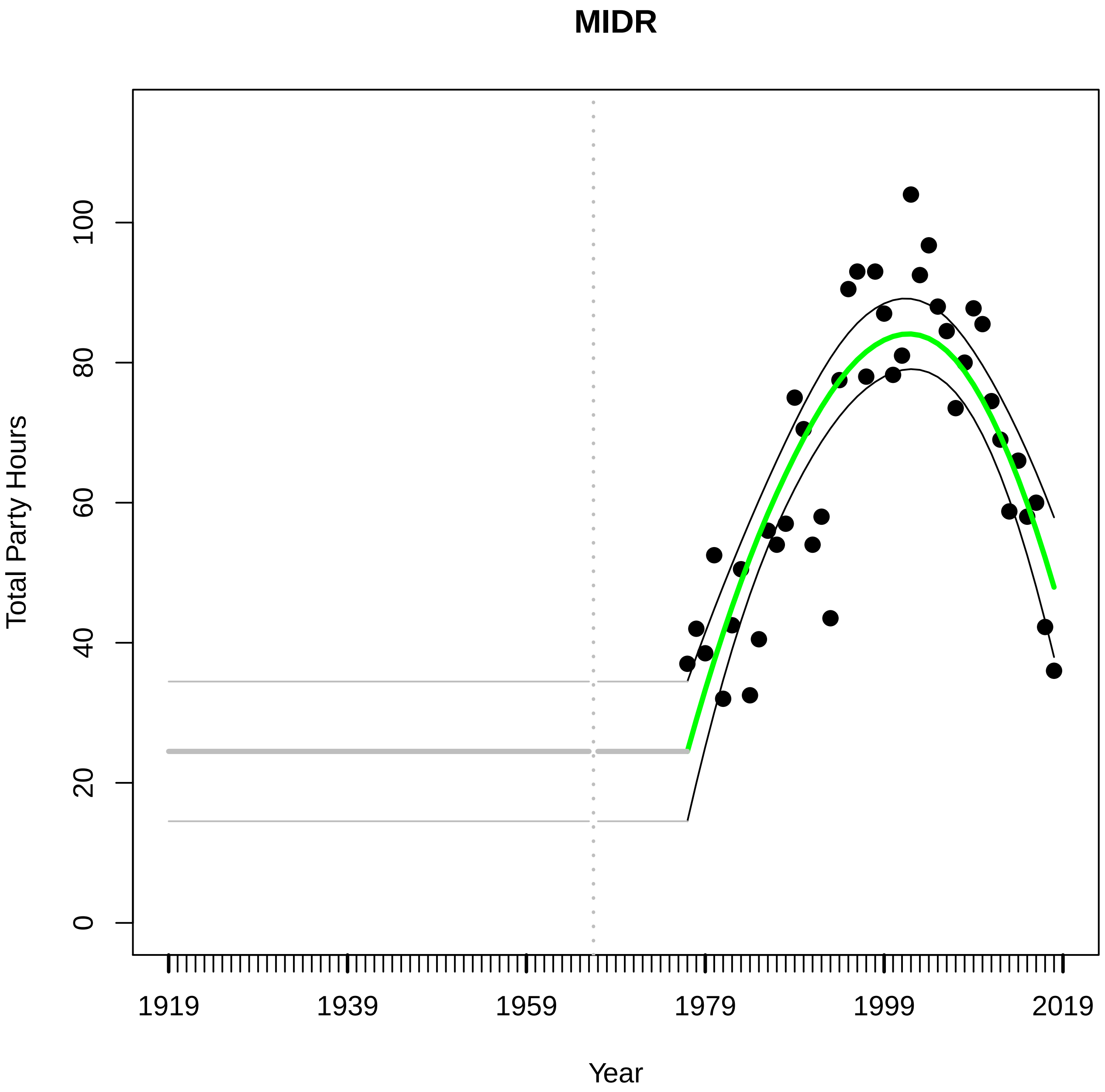
MIDR
Total Party Hours
0
20
40
60
80
100
1919
1939
1959
1979
1999
2019
Year

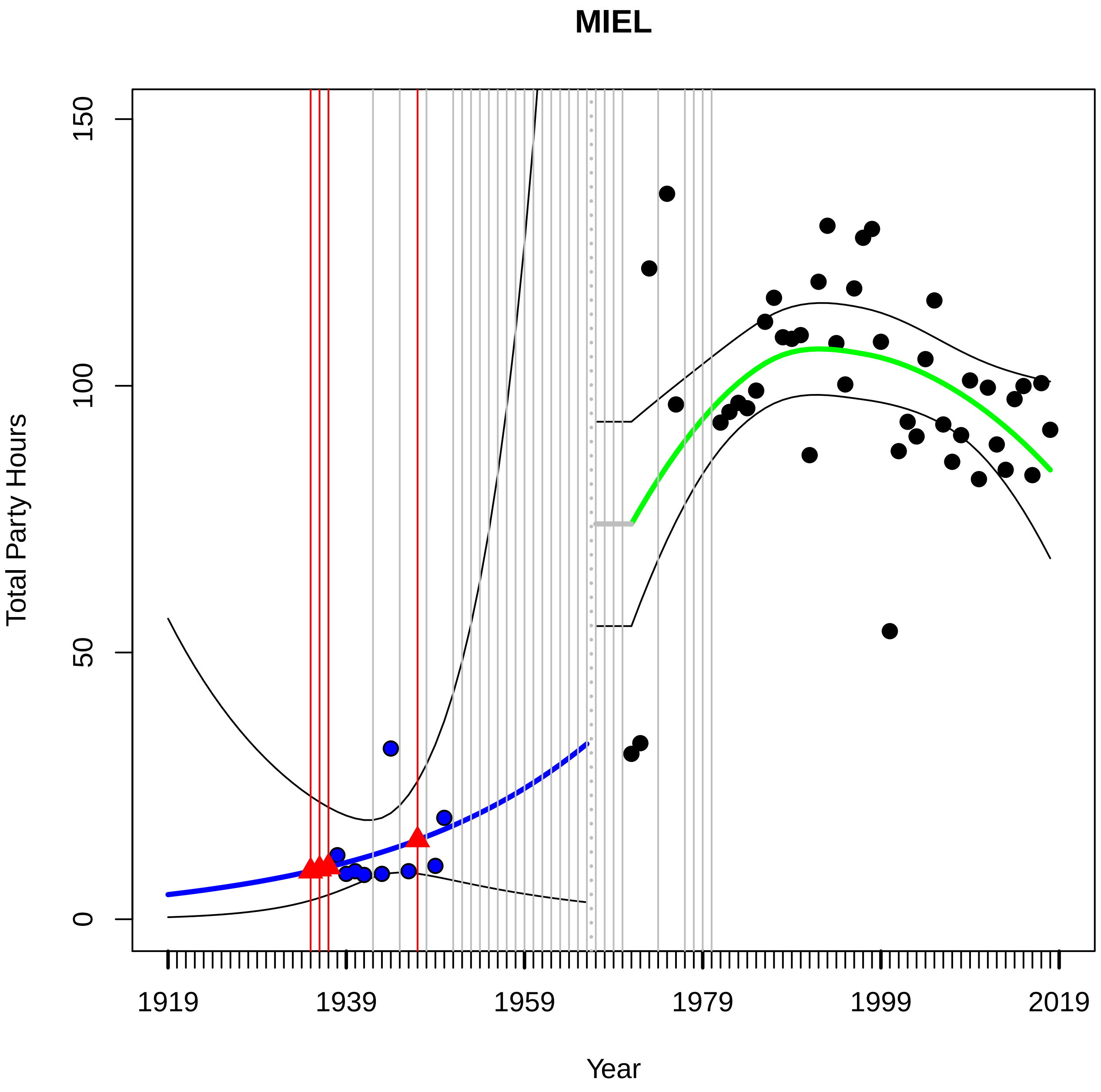

MIEL
Total Party Hours
Year
0
50
100
150
1919
1939
1959
1979
1999
2019

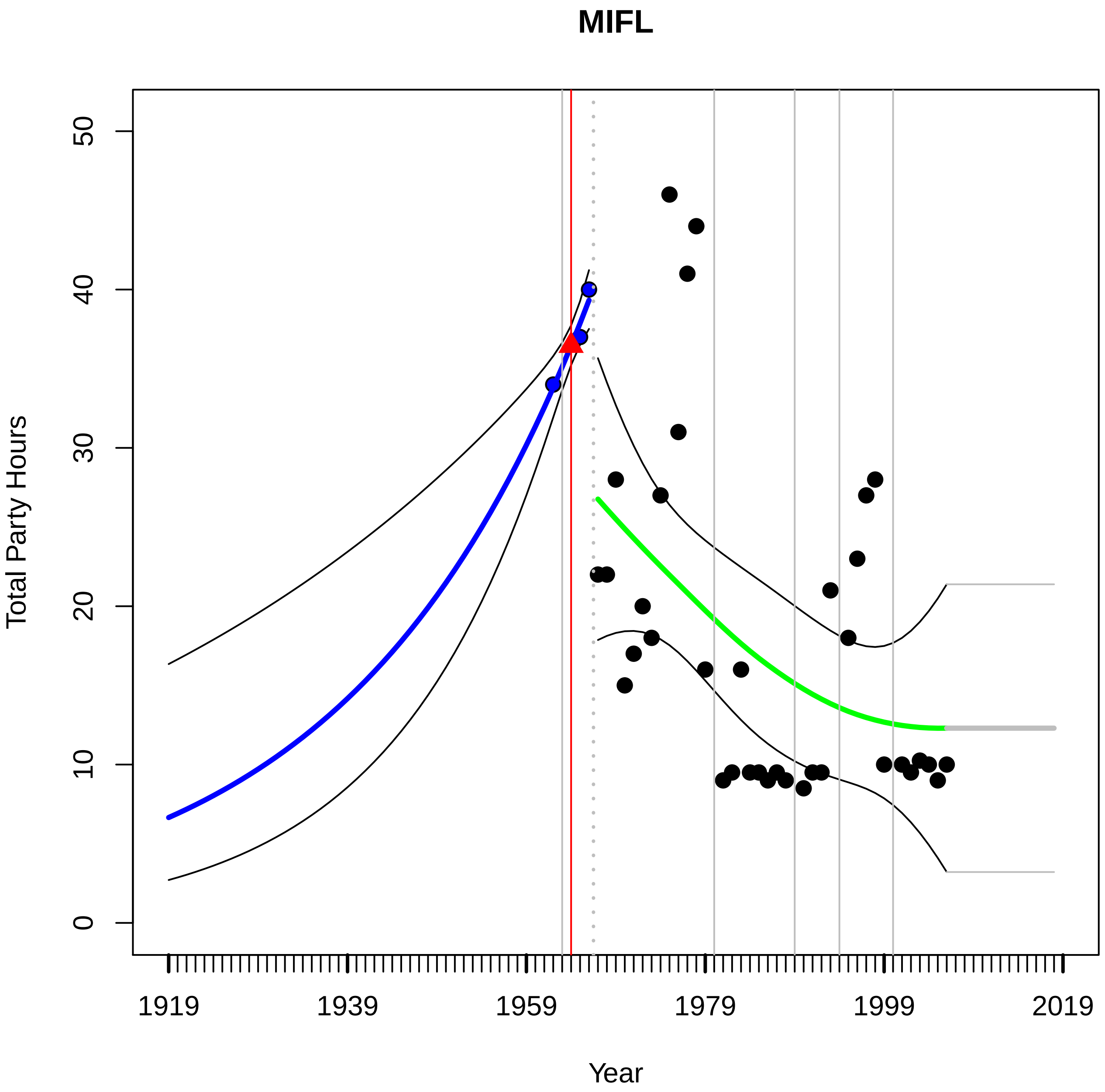

MIFL
Total Party Hours
0
10
20
30
40
50
1919
1939
1959
1979
1999
2019
Year

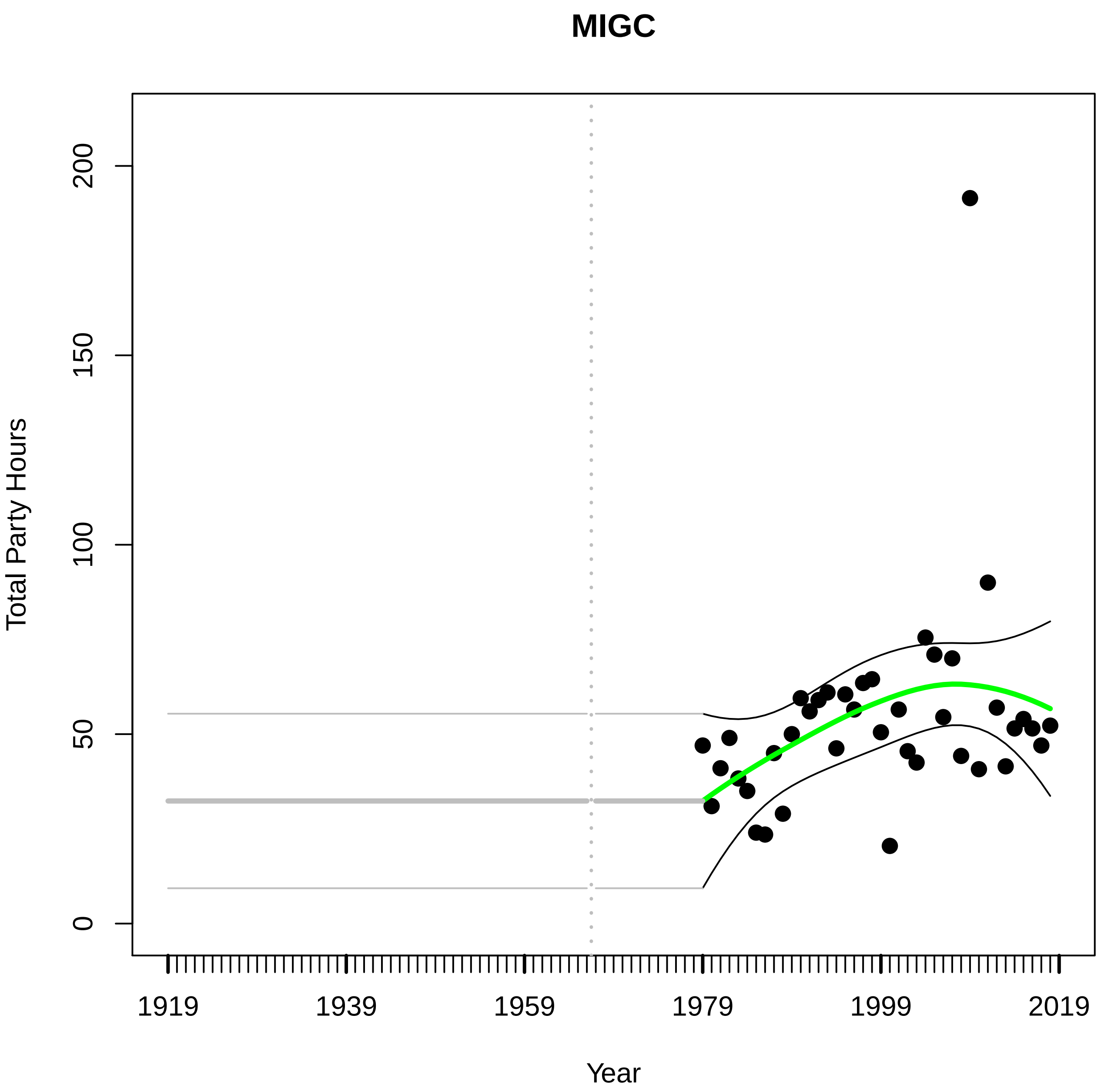

MIGC
Total Party Hours
0
50
100
150
200
1919
1939
1959
1979
1999
2019
Year

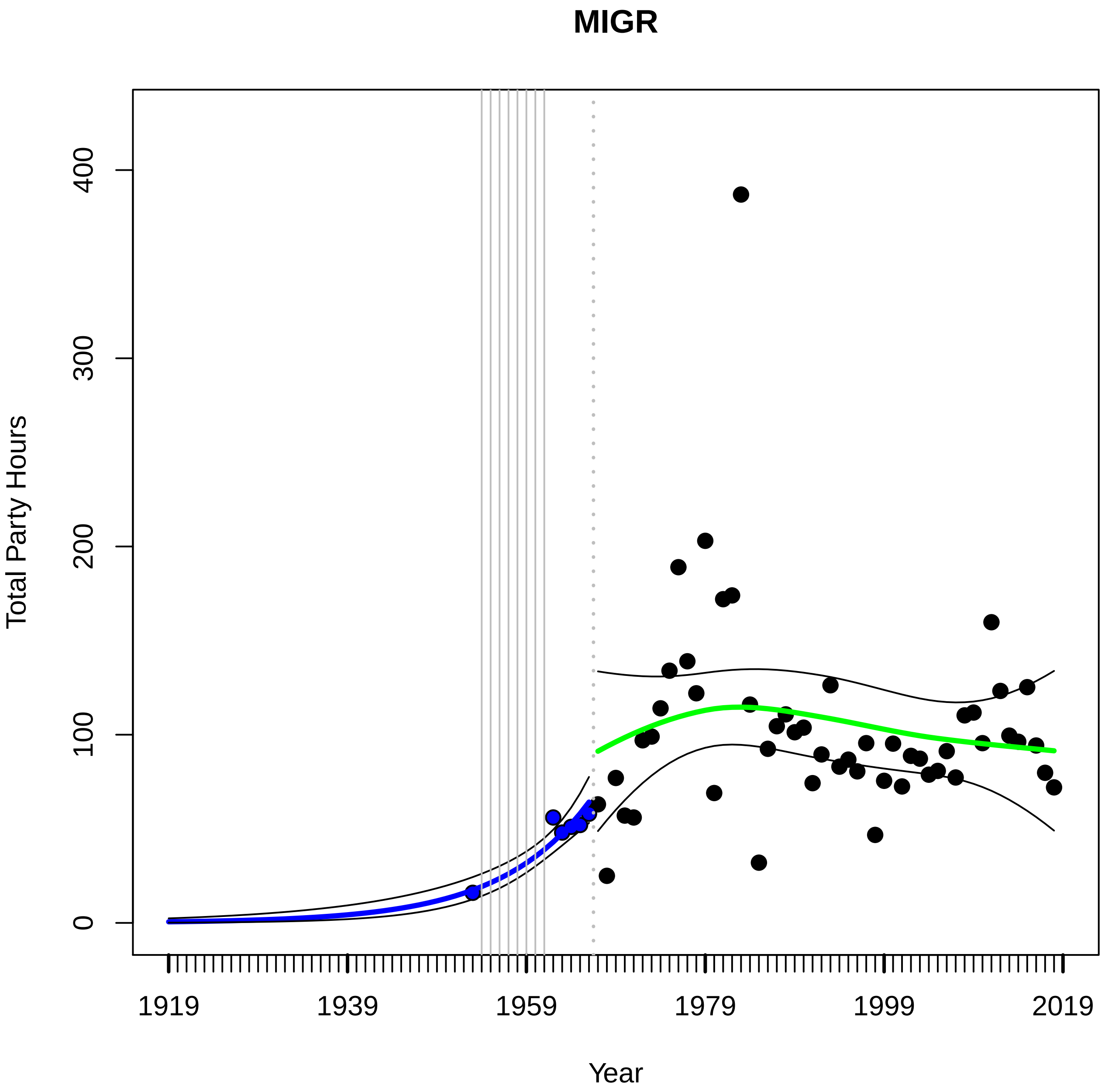

MIGR
Total Party Hours
0
100
200
300
400
1919
1939
1959
1979
1999
2019
Year

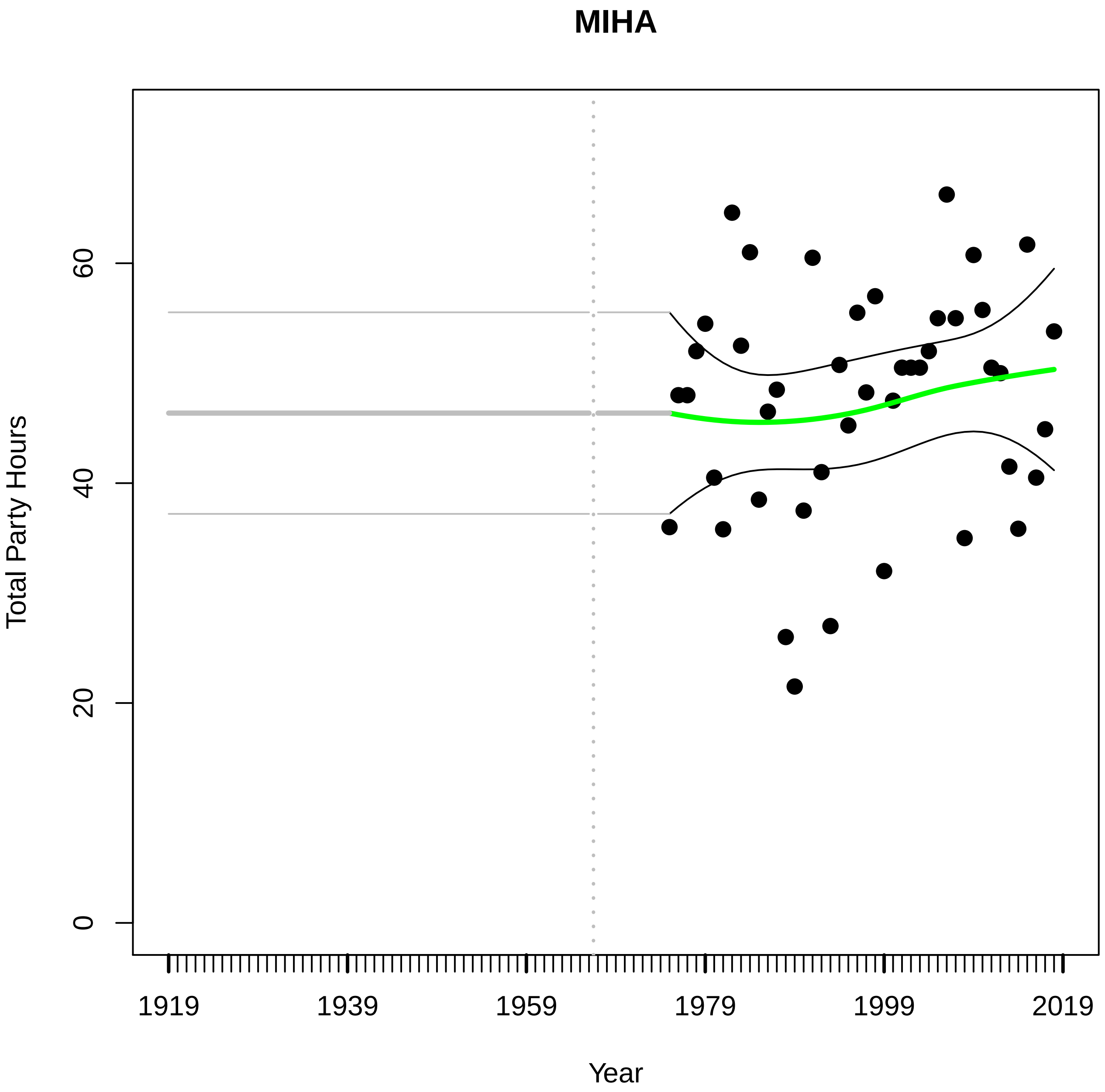

MIHA
Total Party Hours
Year
0
20
40
60
1919
1939
1959
1979
1999
2019

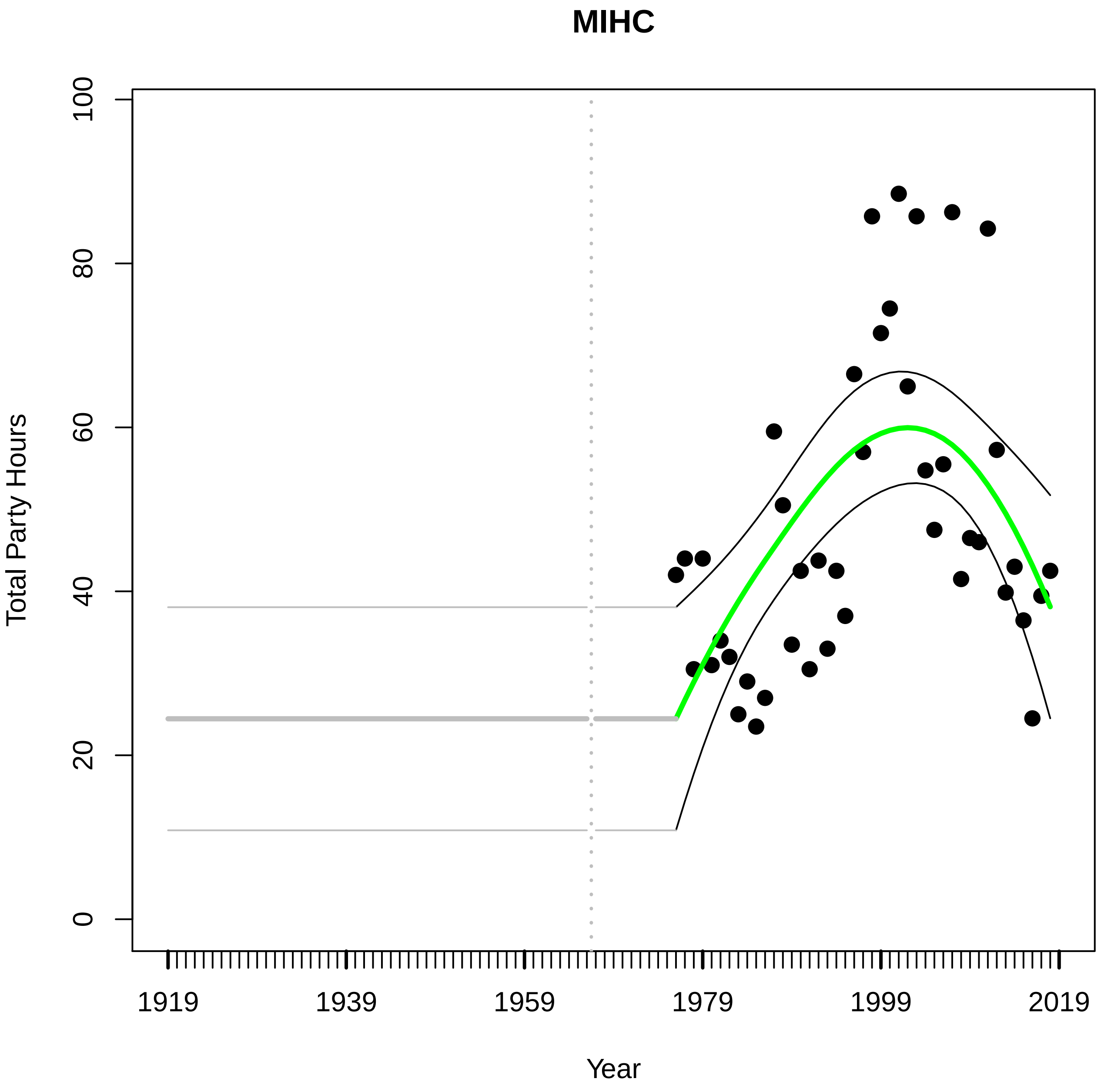

MIHC
Total Party Hours
0
20
40
60
80
100
1919
1939
1959
1979
1999
2019
Year

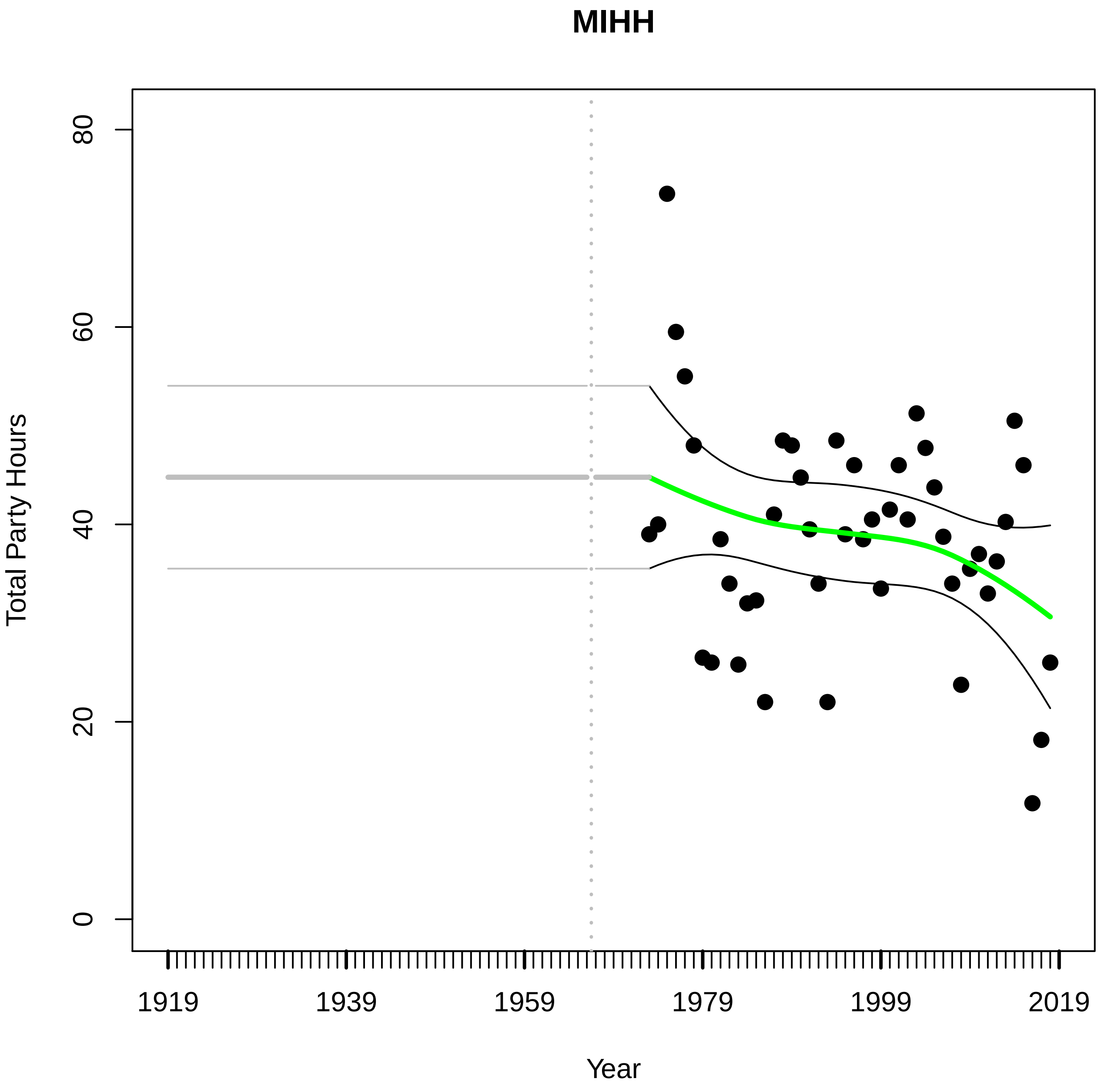
MIHH
Total Party Hours
0
20
40
60
80
1919
1939
1959
1979
1999
2019
Year

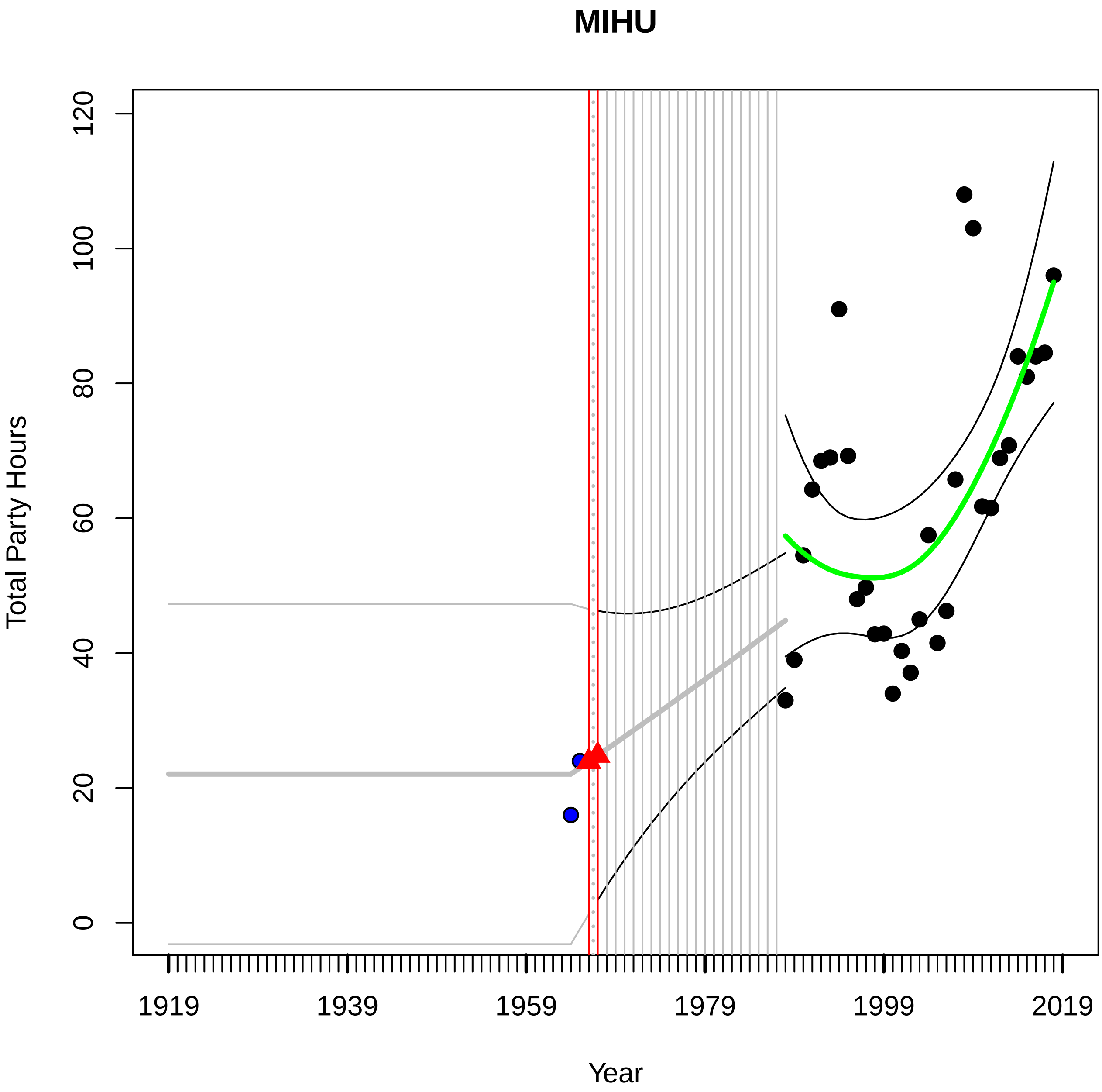

MIHU
Total Party Hours
0
20
40
60
80
100
120
1919
1939
1959
1979
1999
2019
Year

MIIR

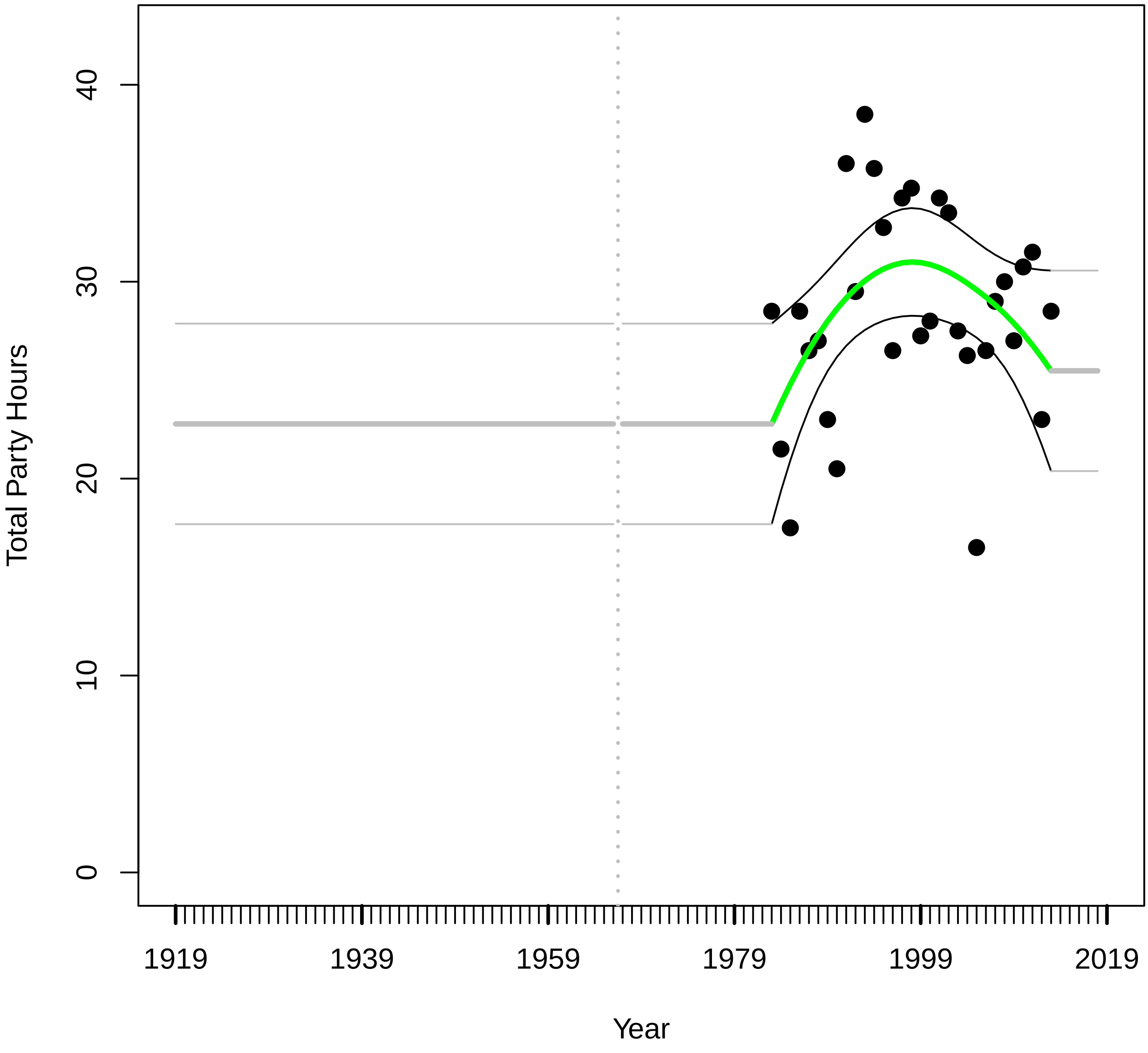

40
30
20
10
0
Total Party Hours
1919
1939
1959
1979
1999
2019
Year

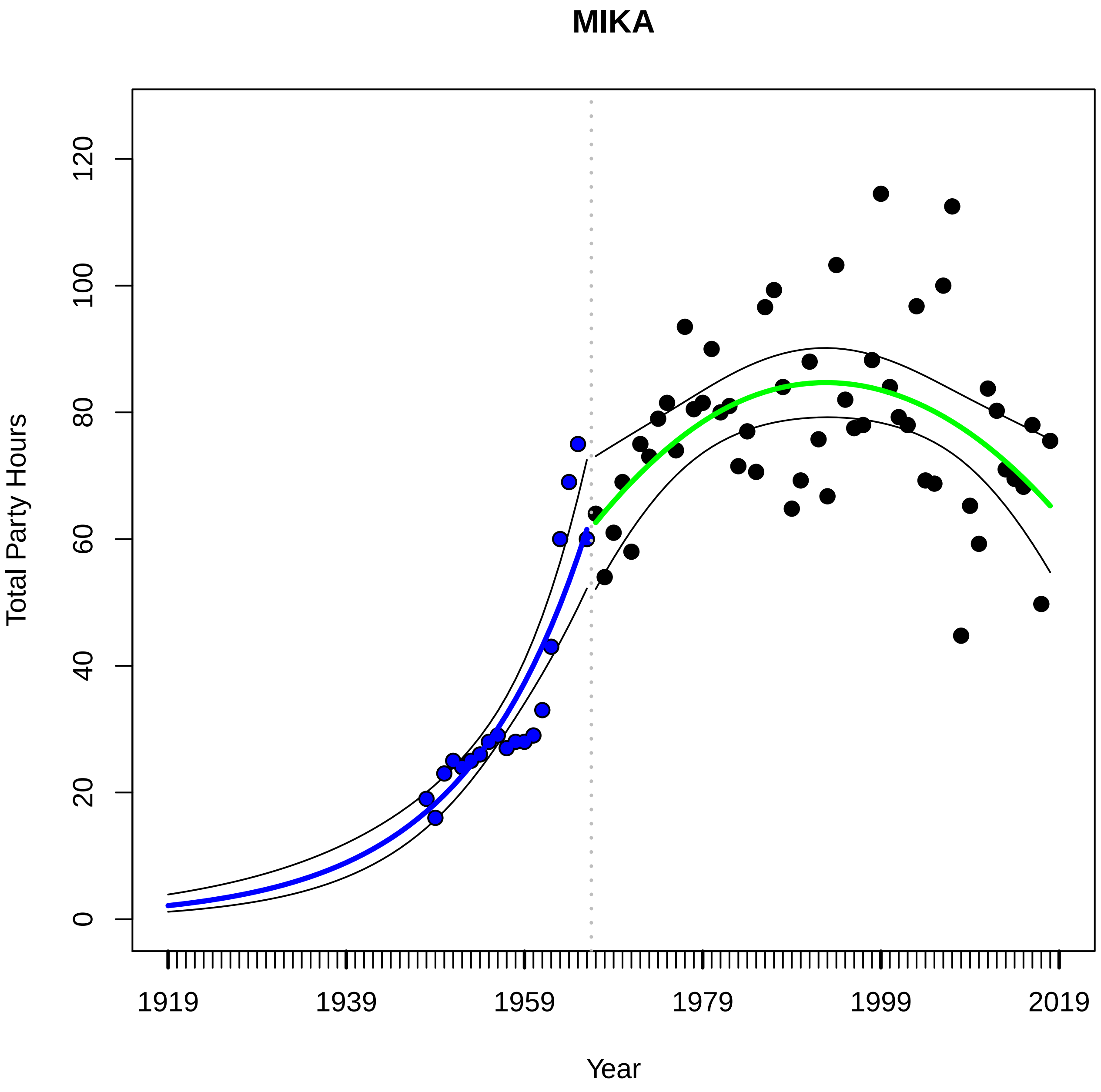

MIKA
Total Party Hours
0
20
40
60
80
100
120
1919
1939
1959
1979
1999
2019
Year

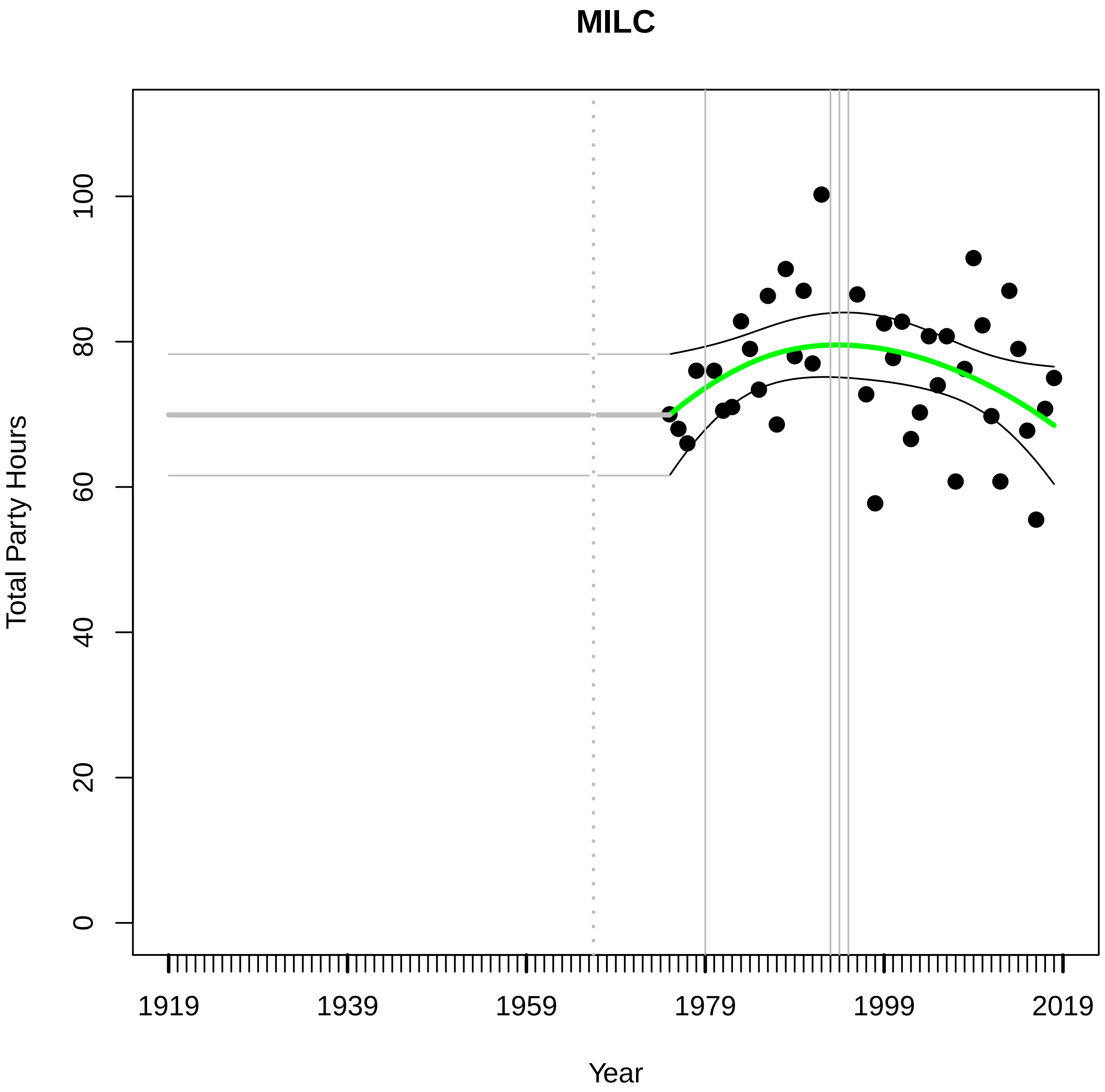

MILC
Total Party Hours
Year
0
20
40
60
80
100
1919
1939
1959
1979
1999
2019

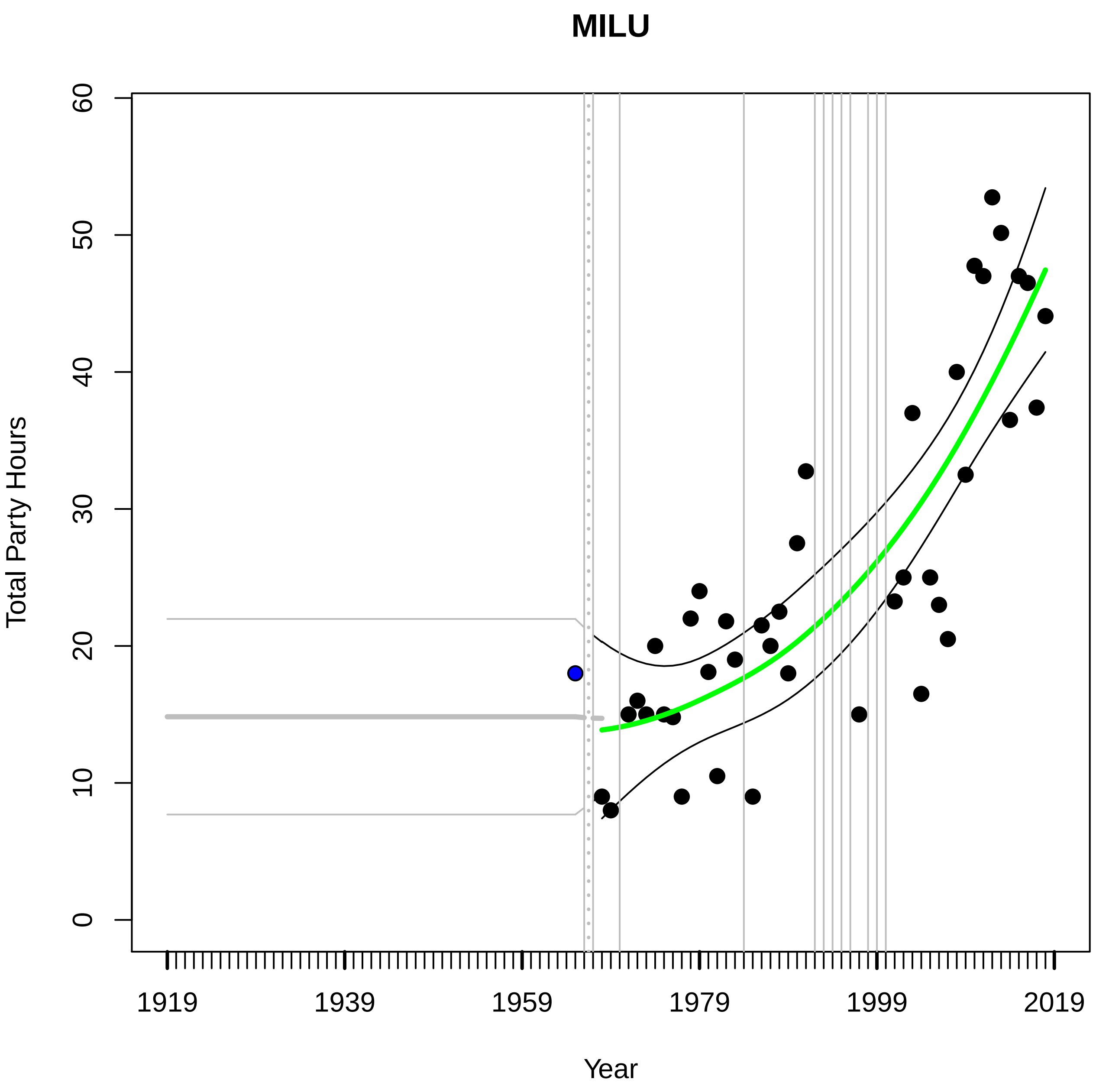

MILU
Total Party Hours
0
10
20
30
40
50
60
1919
1939
1959
1979
1999
2019
Year

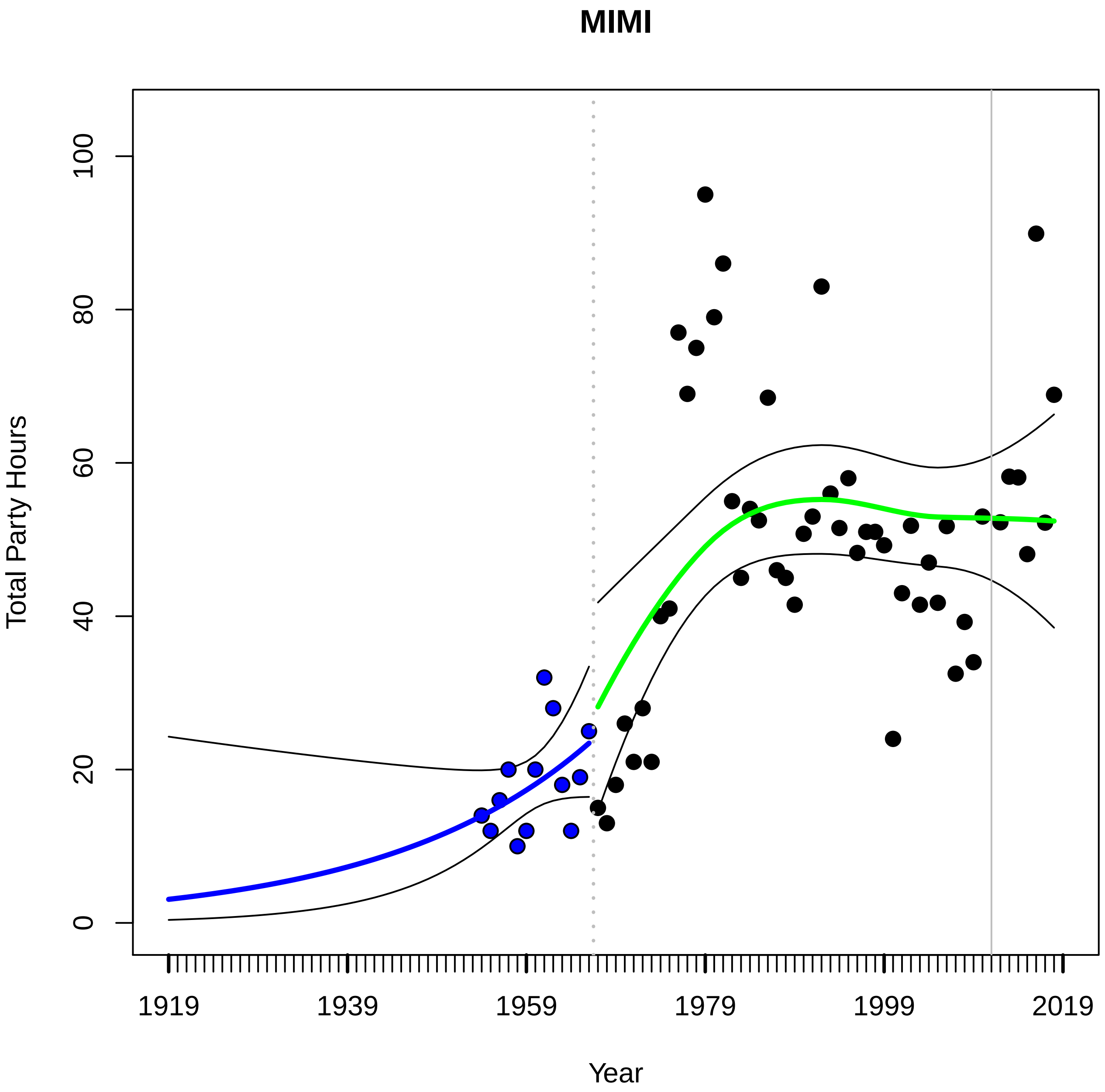

MIMI
Total Party Hours
0
20
40
60
80
100
1919
1939
1959
1979
1999
2019
Year

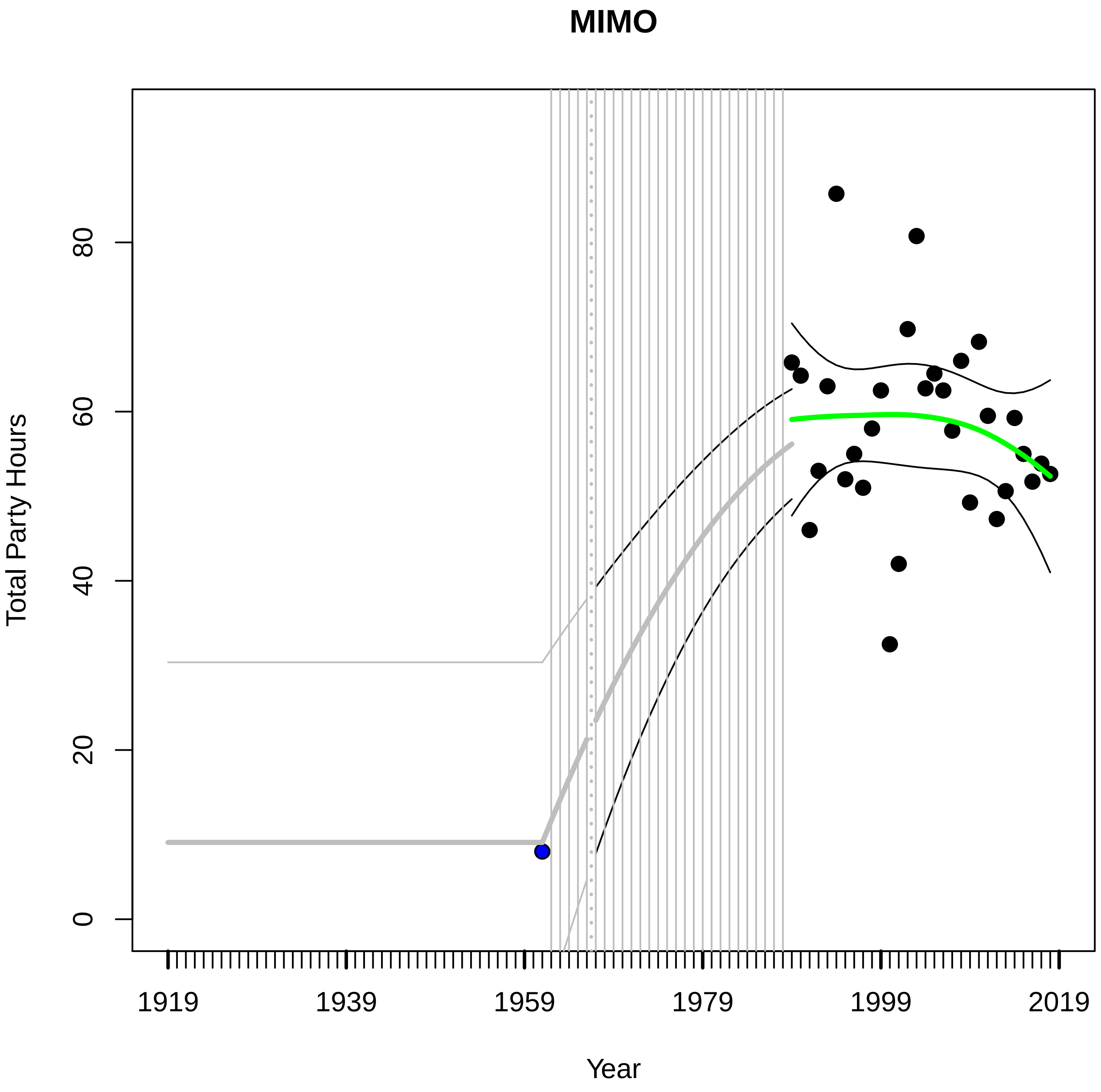
MIMO
Total Party Hours
0
20
40
60
80
1919
1939
1959
1979
1999
2019
Year

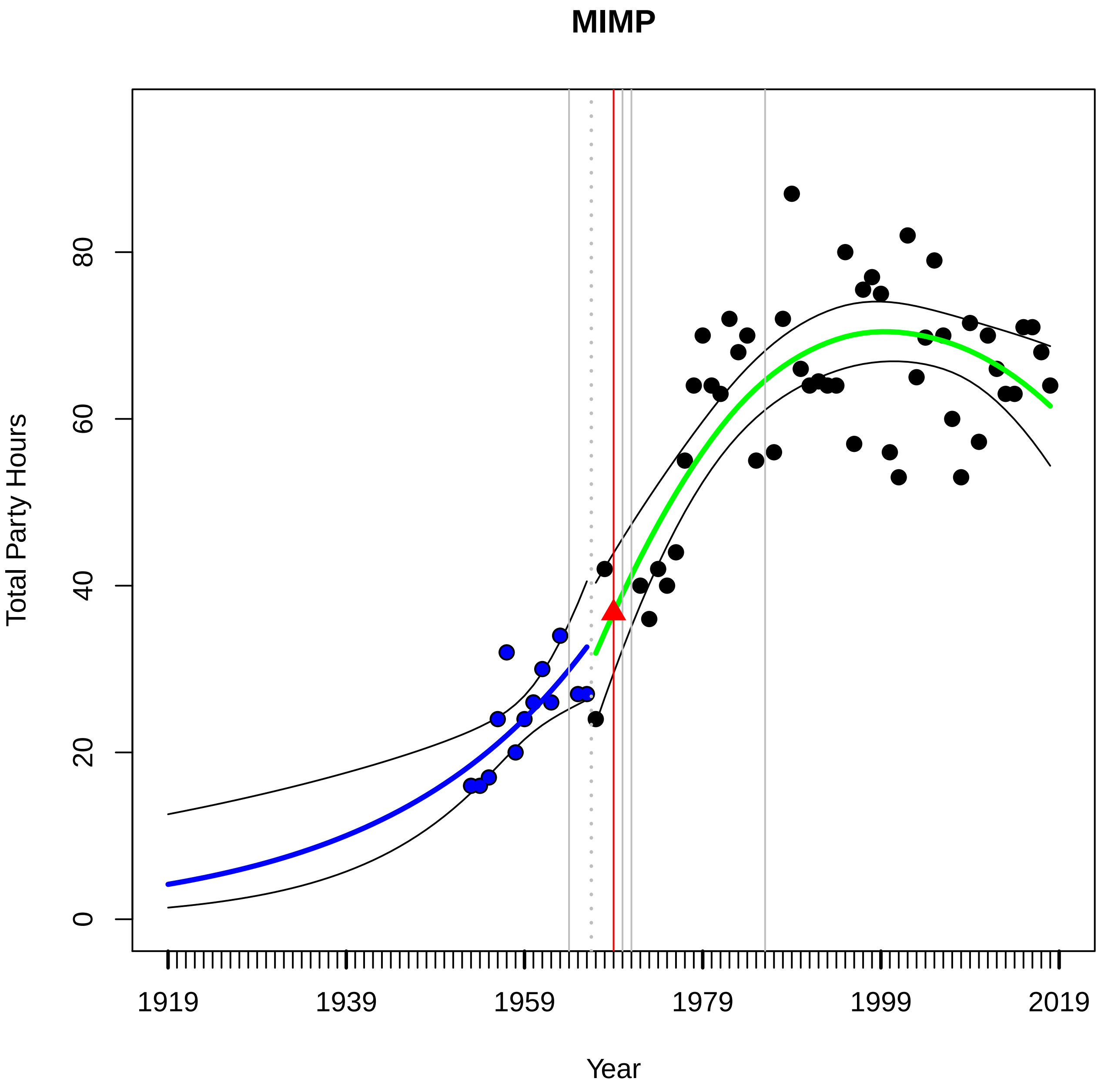

MIMP
Total Party Hours
0
20
40
60
80
1919
1939
1959
1979
1999
2019
Year

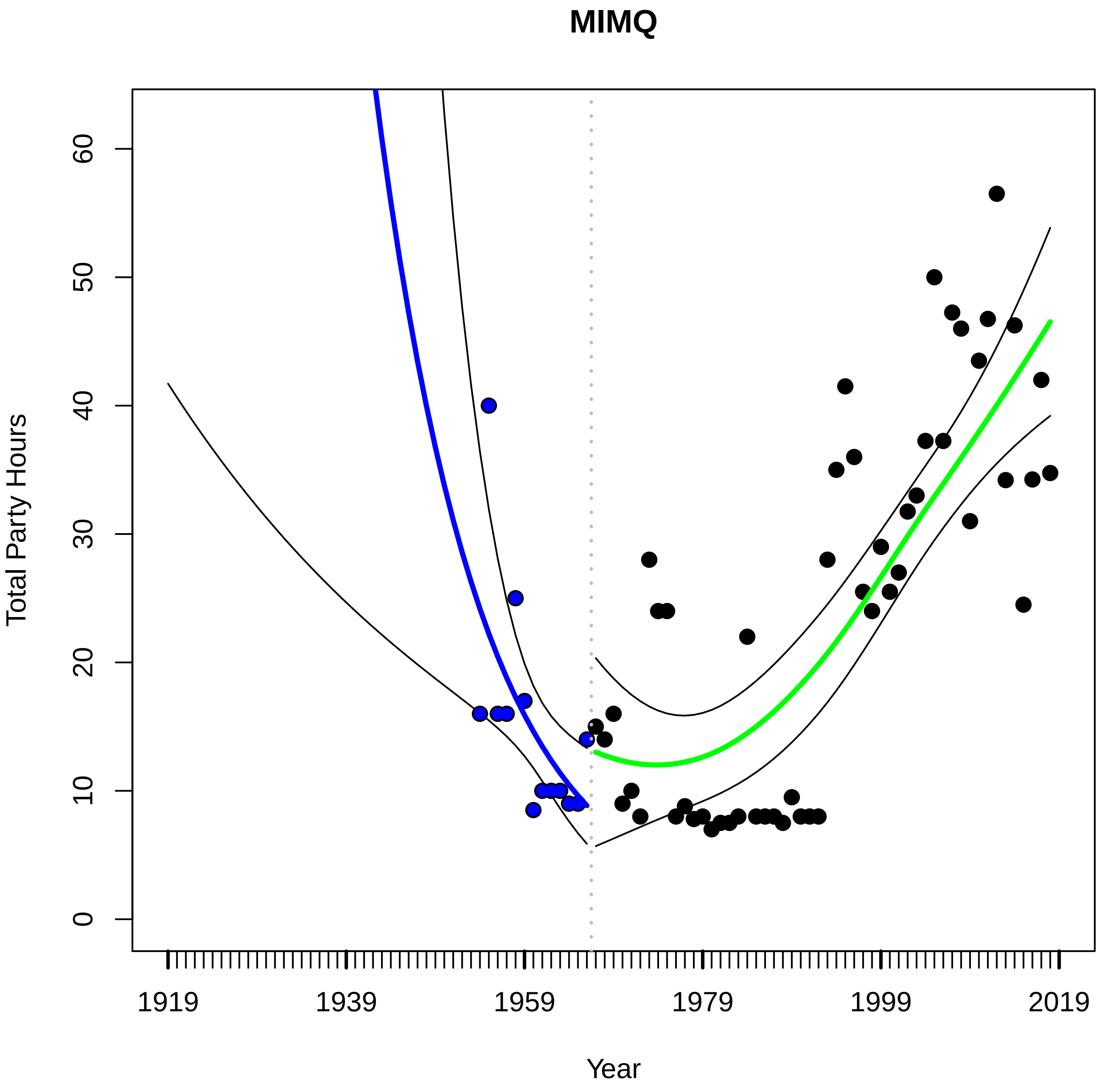

MIMQ
Total Party Hours
Year
0
10
20
30
40
50
60
1919
1939
1959
1979
1999
2019

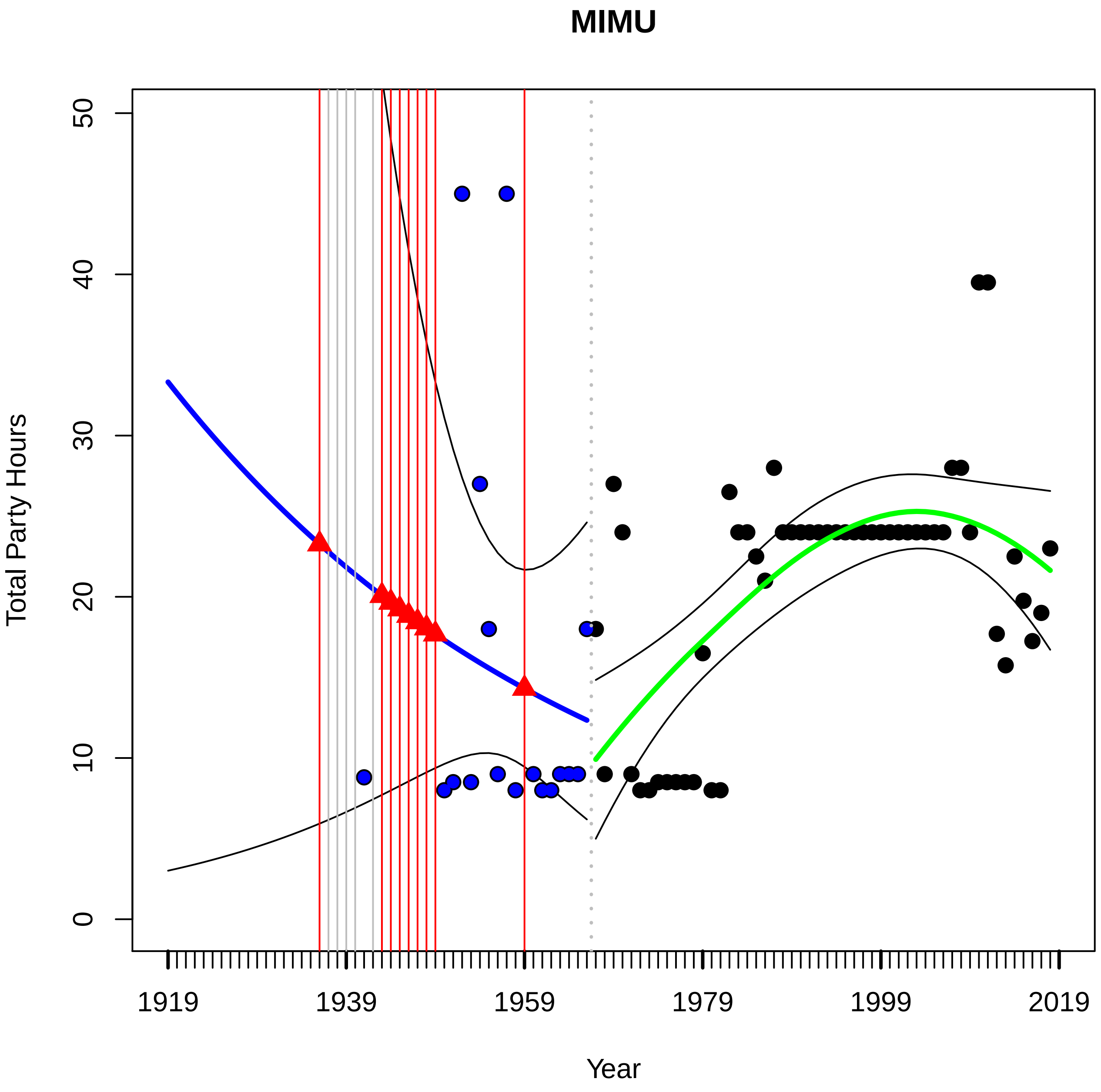
MIMU
Total Party Hours
0
10
20
30
40
50
1919
1939
1959
1979
1999
2019
Year

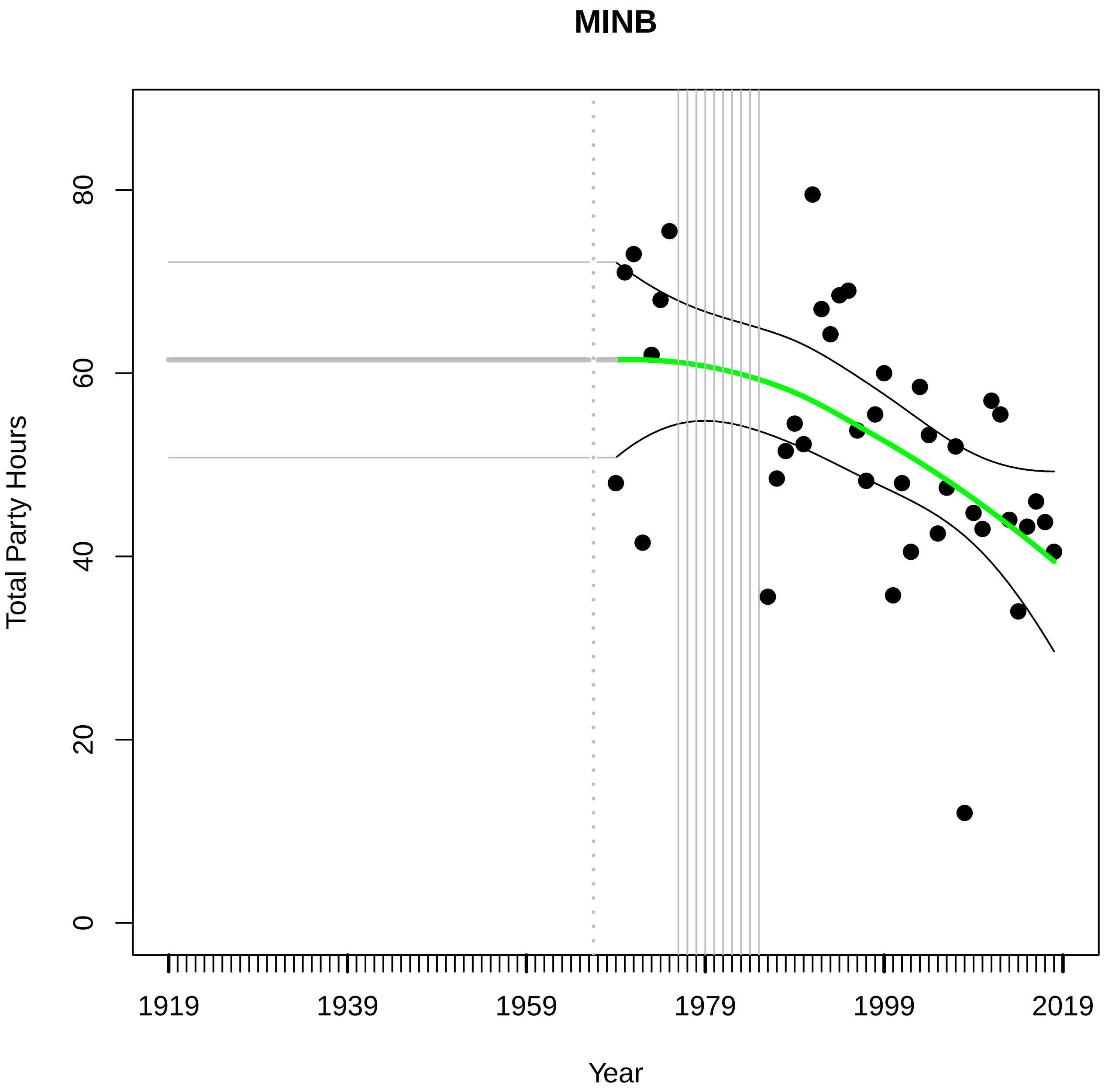
MINB
Total Party Hours
0
20
40
60
80
Year
1919
1939
1959
1979
1999
2019

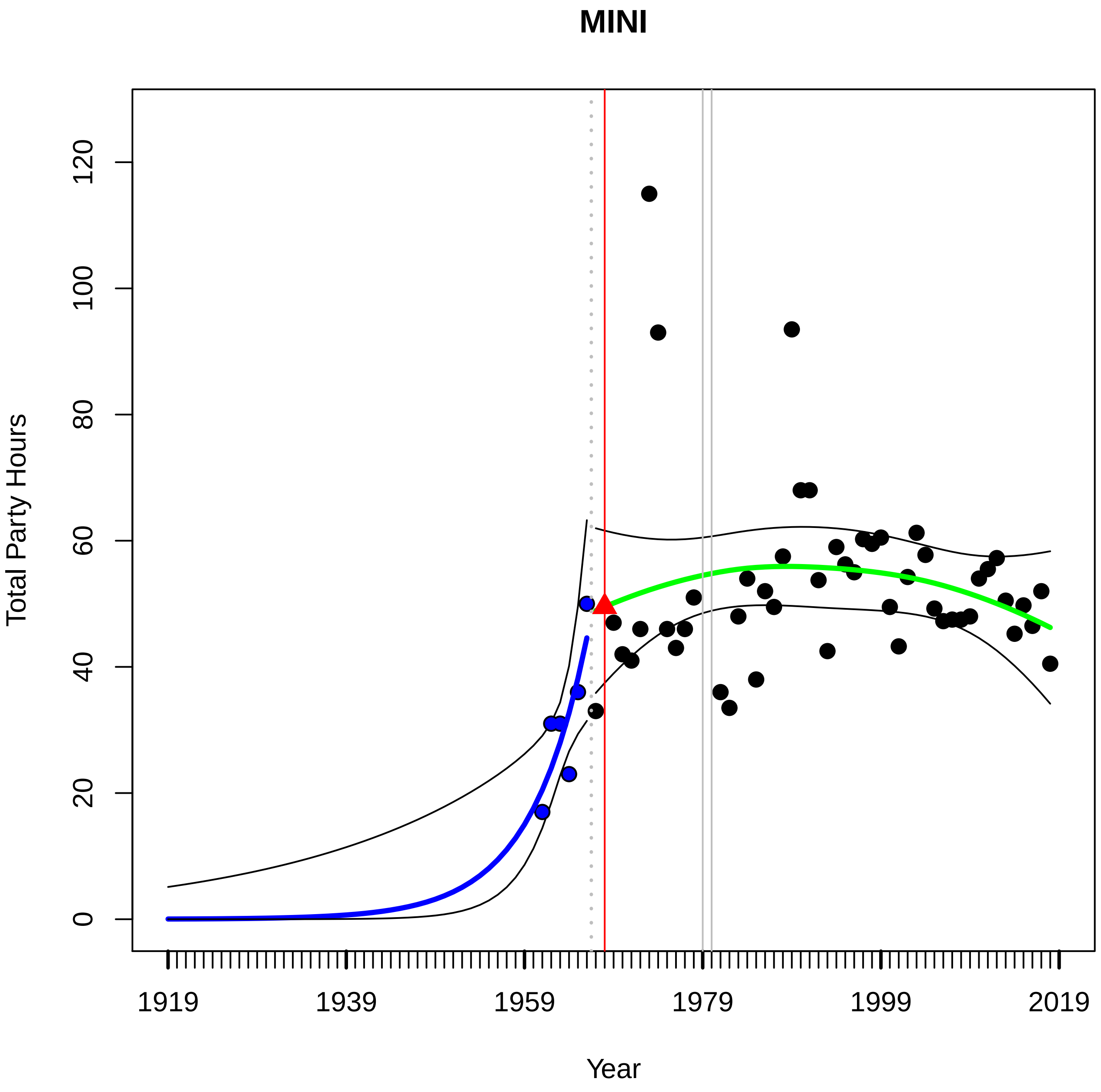
MINI
Total Party Hours
0
20
40
60
80
100
120
1919
1939
1959
1979
1999
2019
Year

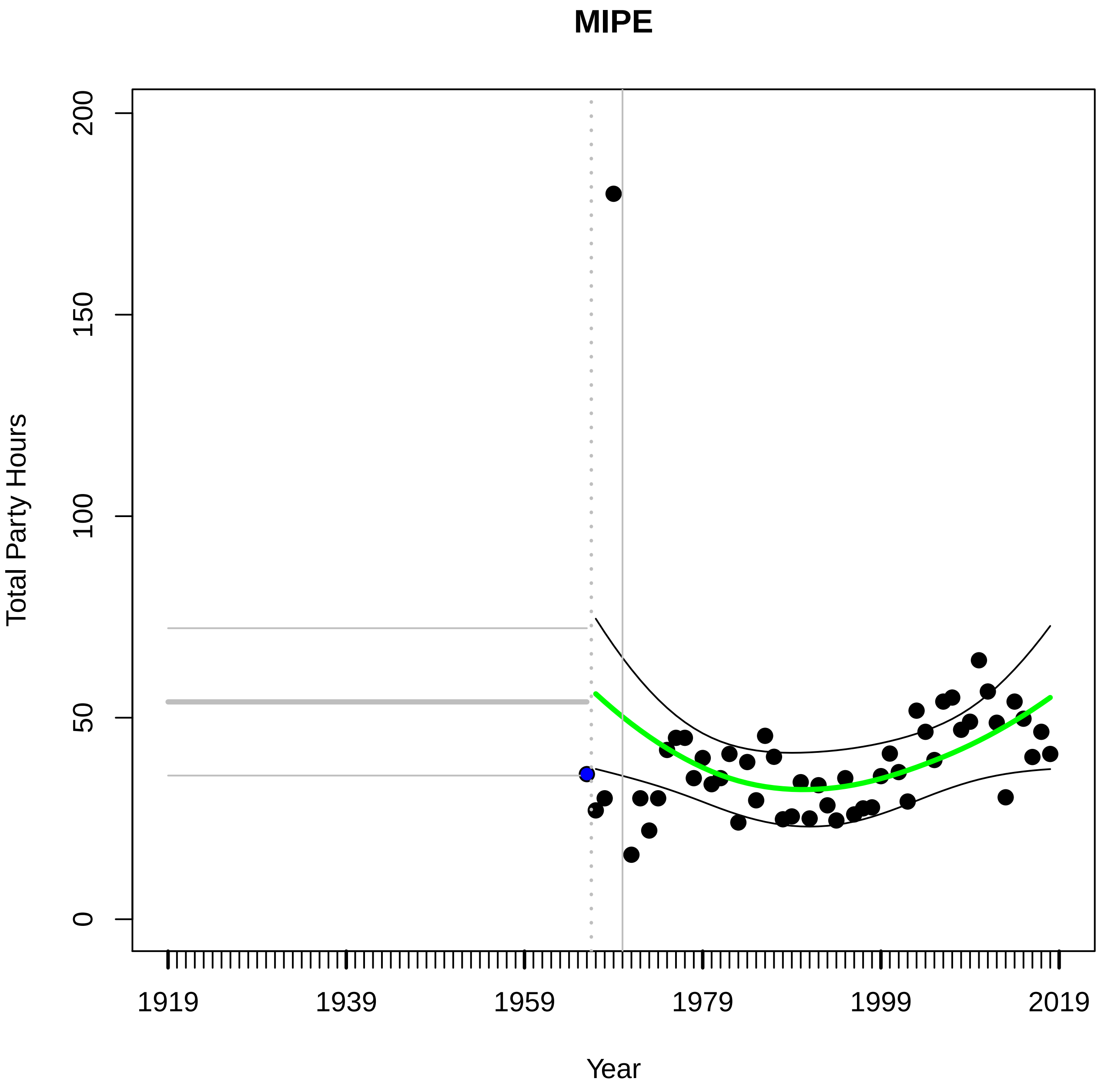

MIPE
Total Party Hours
200
150
100
50
0
1919
1939
1959
1979
1999
2019
Year

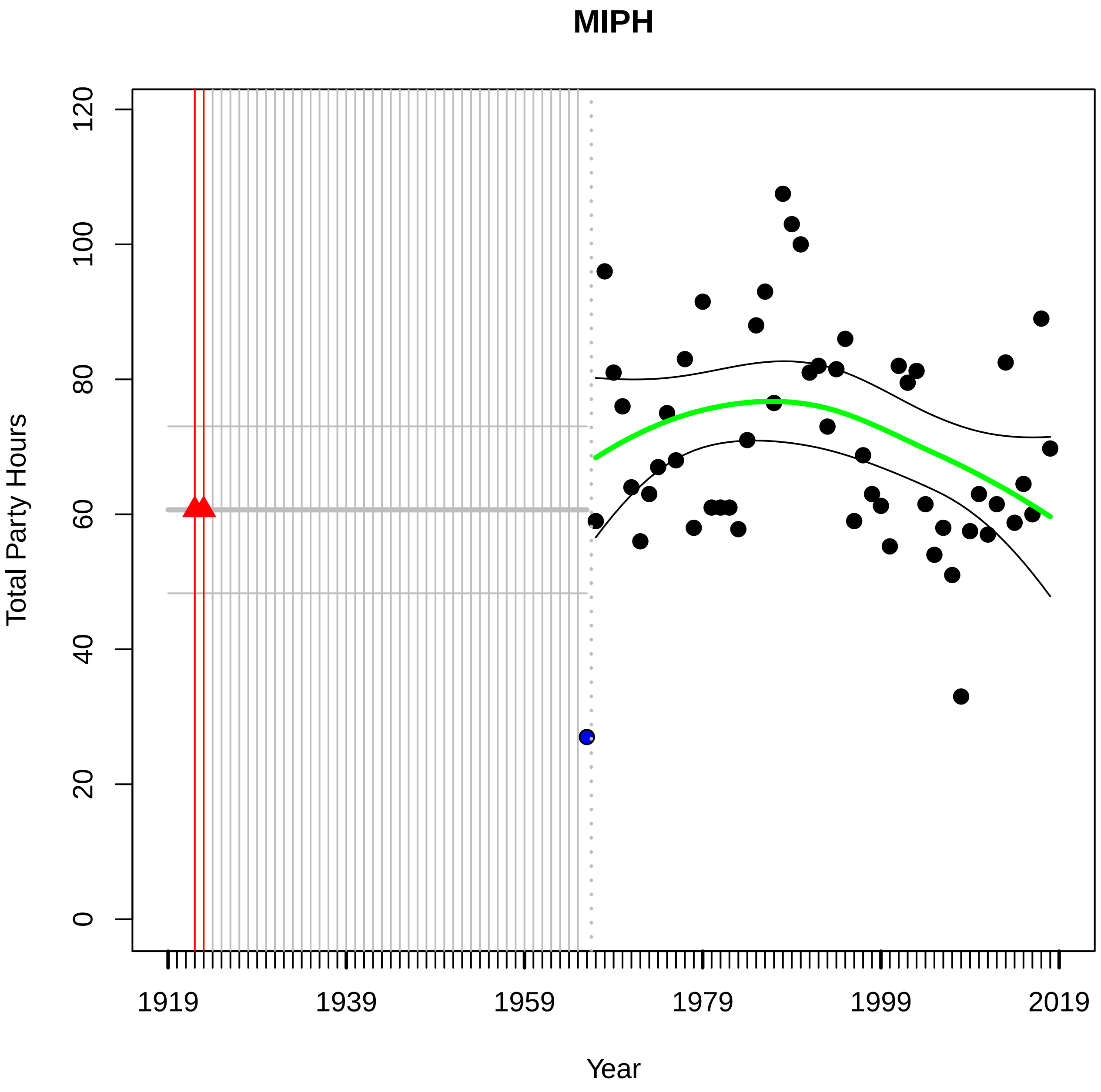

MIPH
Total Party Hours
0
20
40
60
80
100
120
Year
1919
1939
1959
1979
1999
2019

MIPO

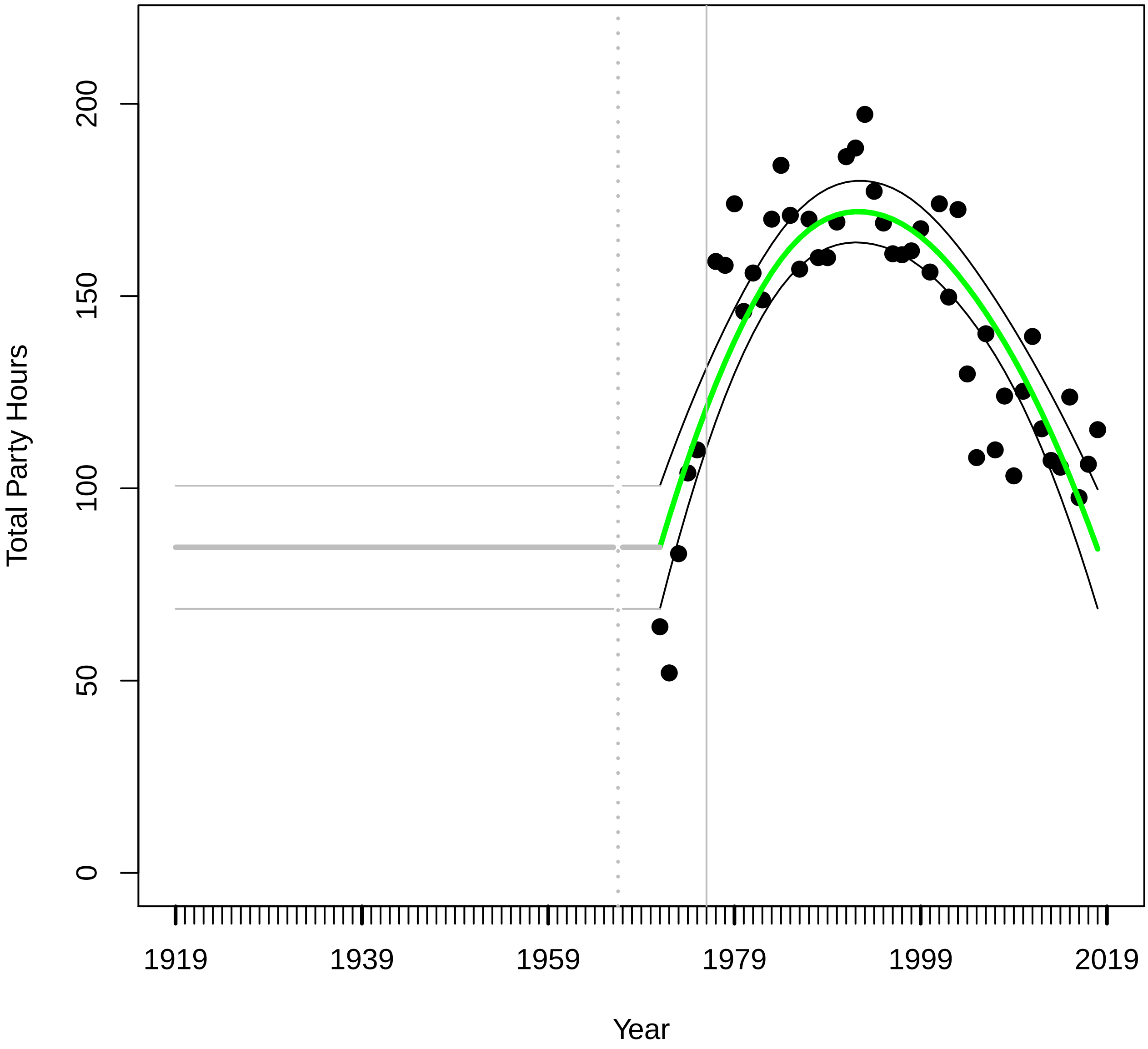

Total Party Hours
200
150
100
50
0
1919
1939
1959
1979
1999
2019
Year

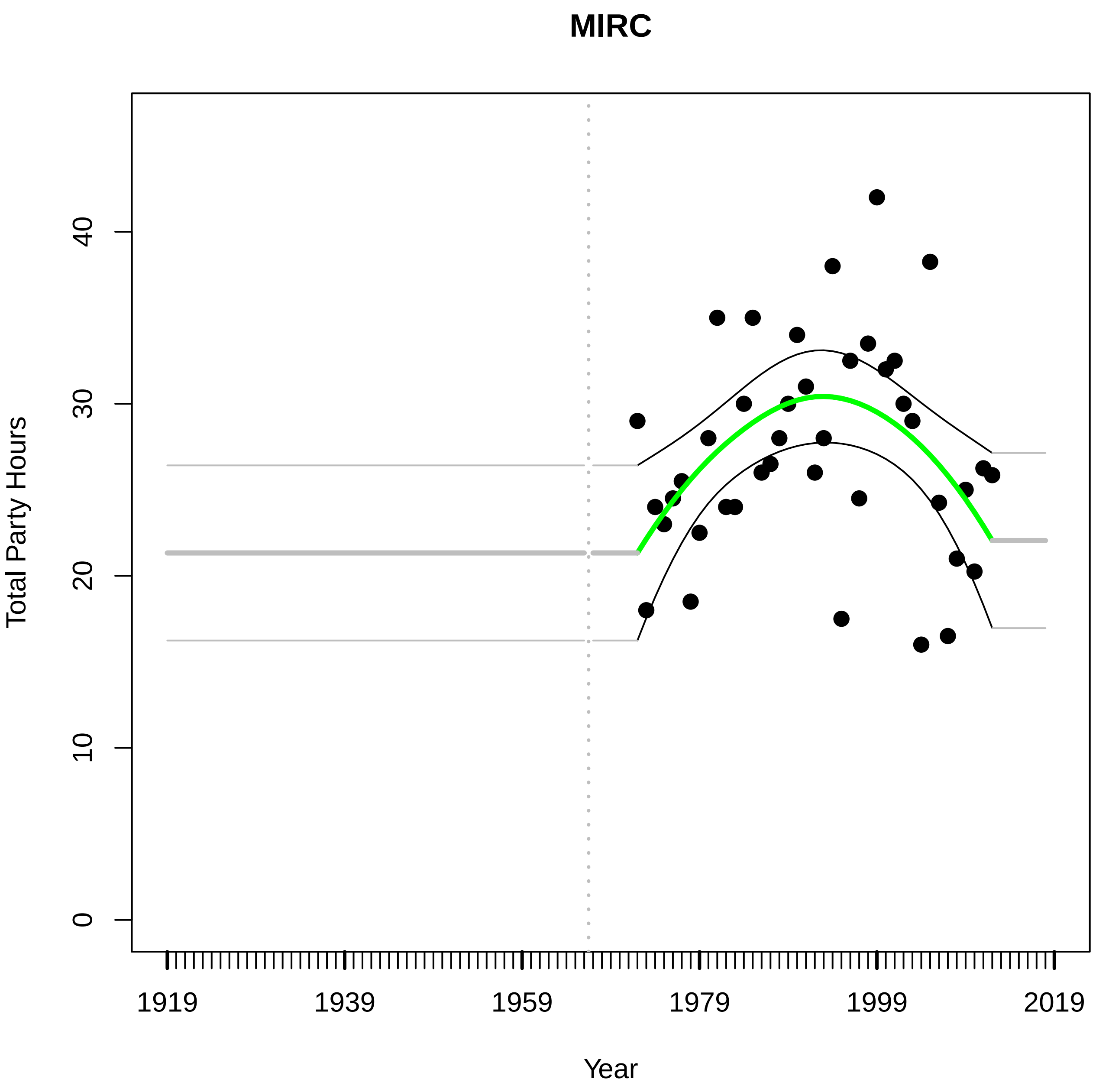
MIRC
Total Party Hours
0
10
20
30
40
1919
1939
1959
1979
1999
2019
Year

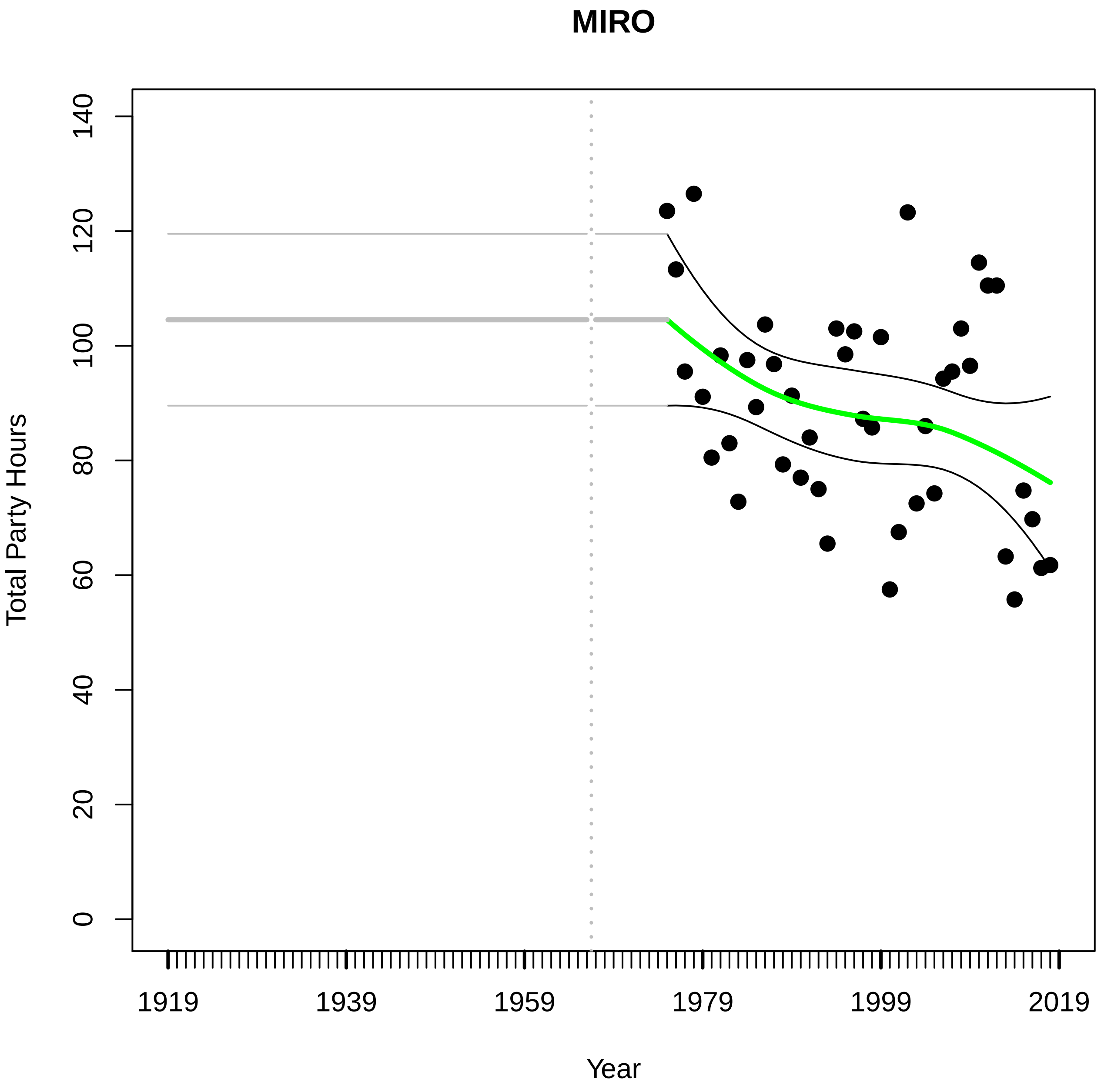

MIRO
Total Party Hours
0
20
40
60
80
100
120
140
1919
1939
1959
1979
1999
2019
Year

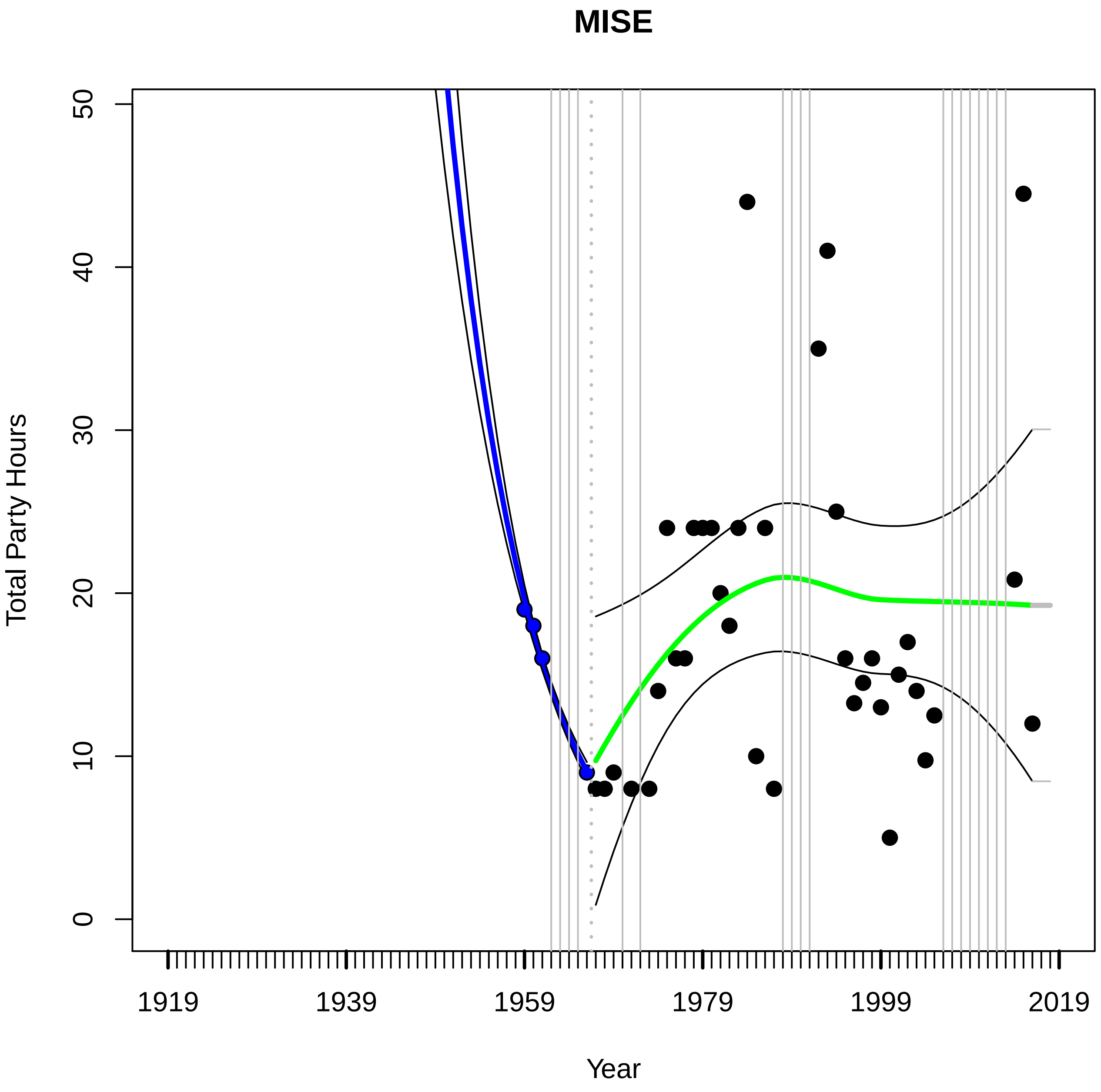

MISE
Total Party Hours
0
10
20
30
40
50
1919
1939
1959
1979
1999
2019
Year

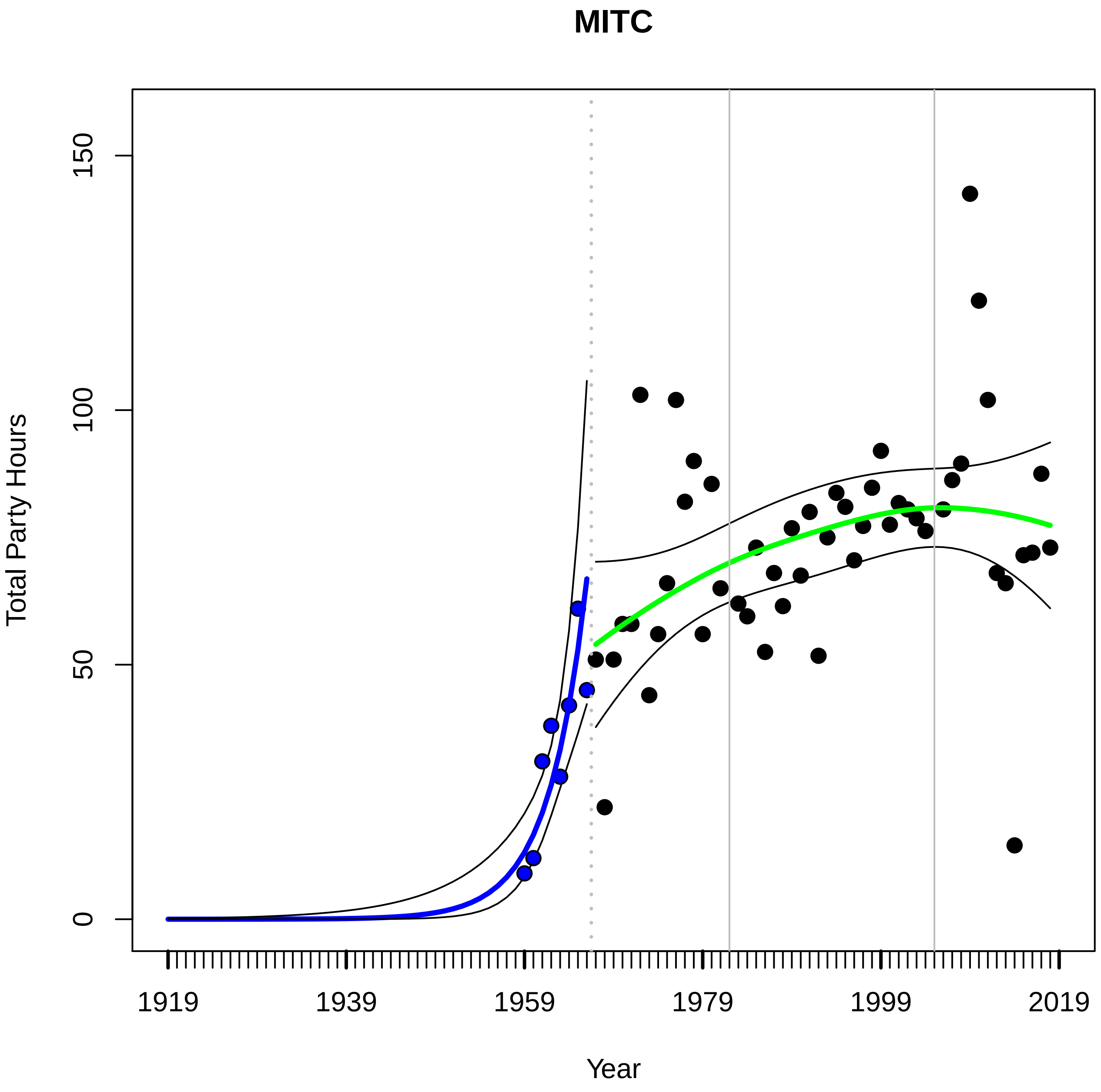

MITC
Total Party Hours
0
50
100
150
1919
1939
1959
1979
1999
2019
Year

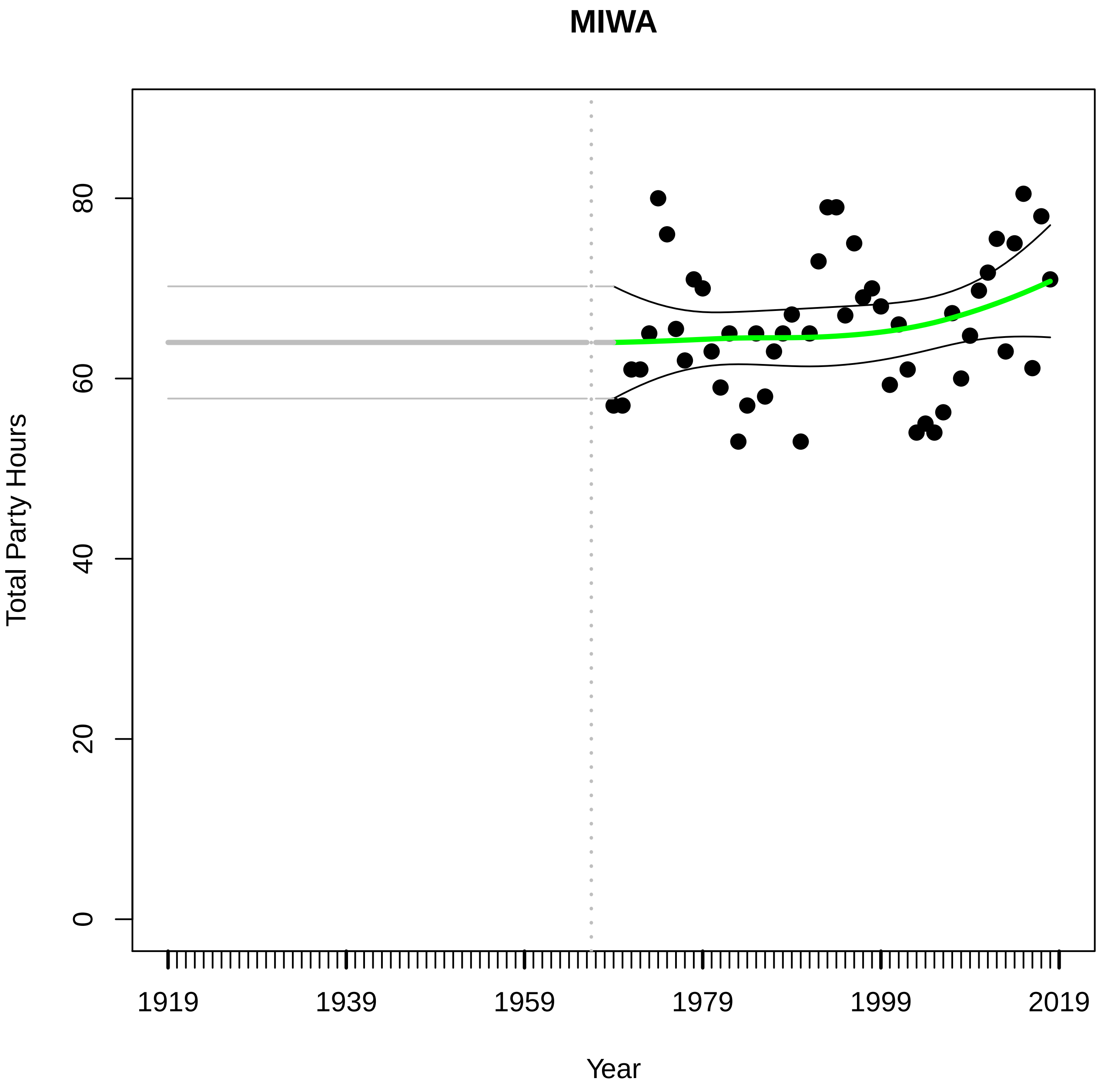

MIWA
Total Party Hours
0
20
40
60
80
1919
1939
1959
1979
1999
2019
Year

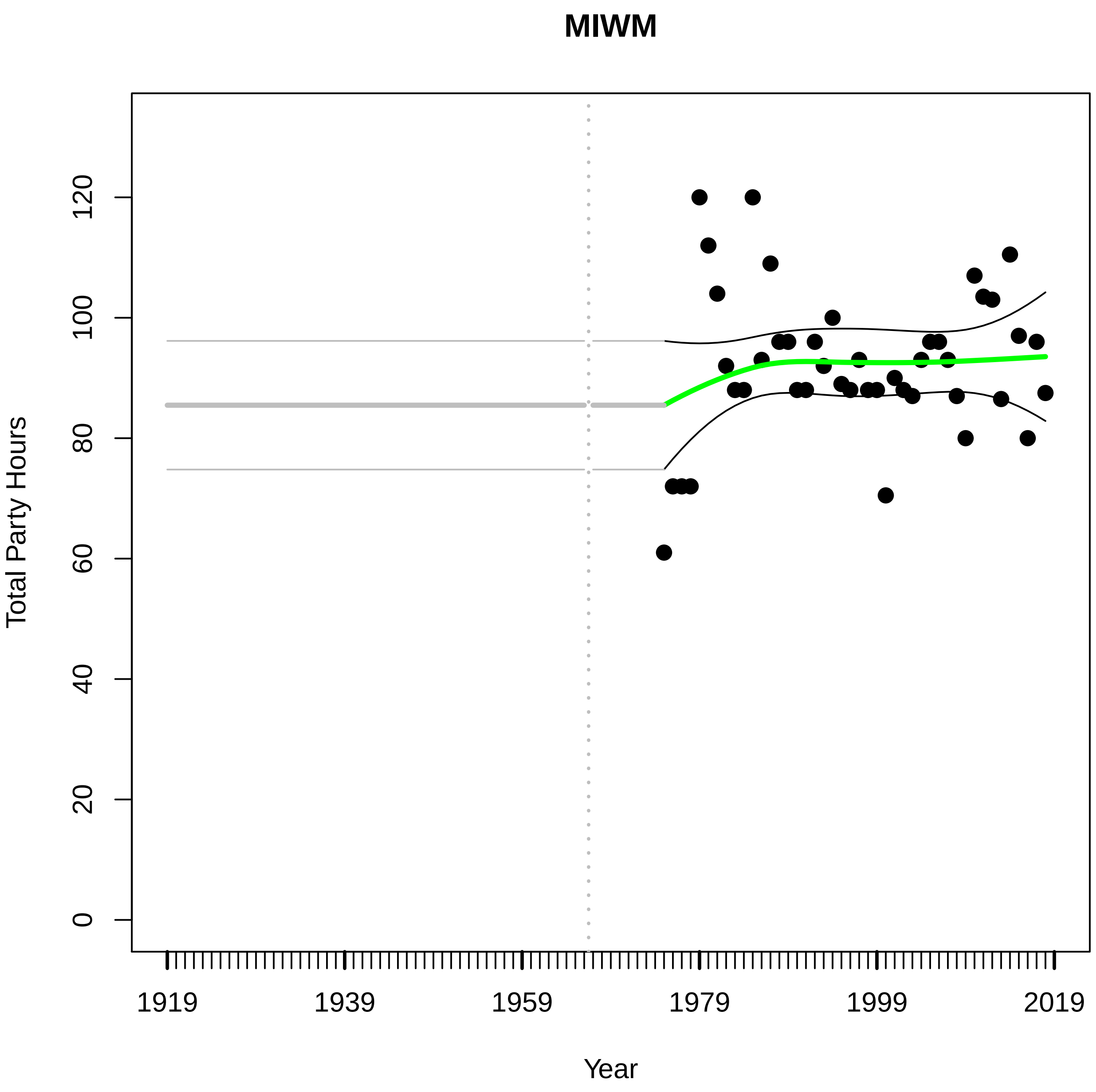

MIWM
Total Party Hours
Year
0
20
40
60
80
100
120
1919
1939
1959
1979
1999
2019

MN01

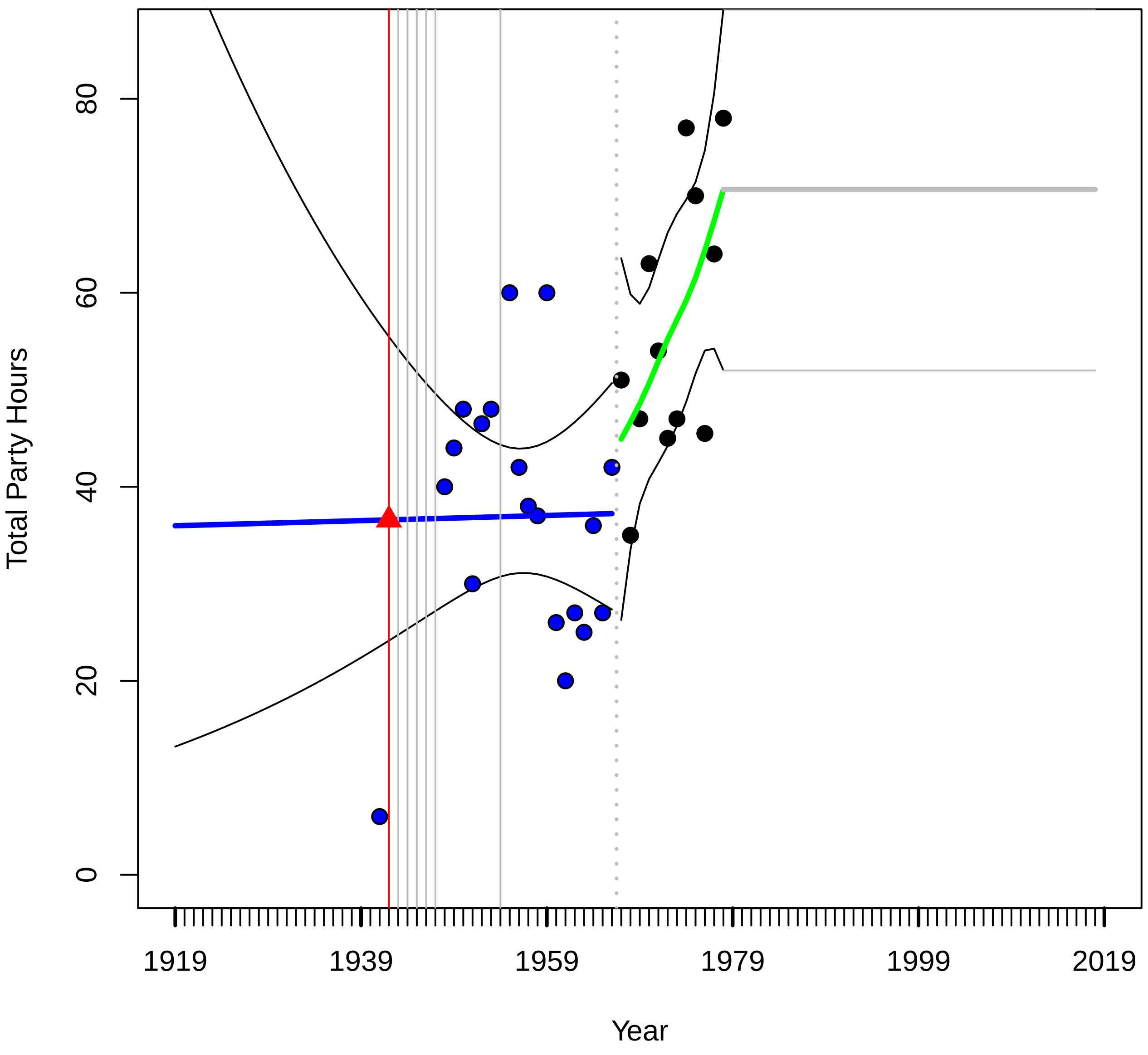

Total Party Hours
0
20
40
60
80
1919
1939
1959
1979
1999
2019
Year

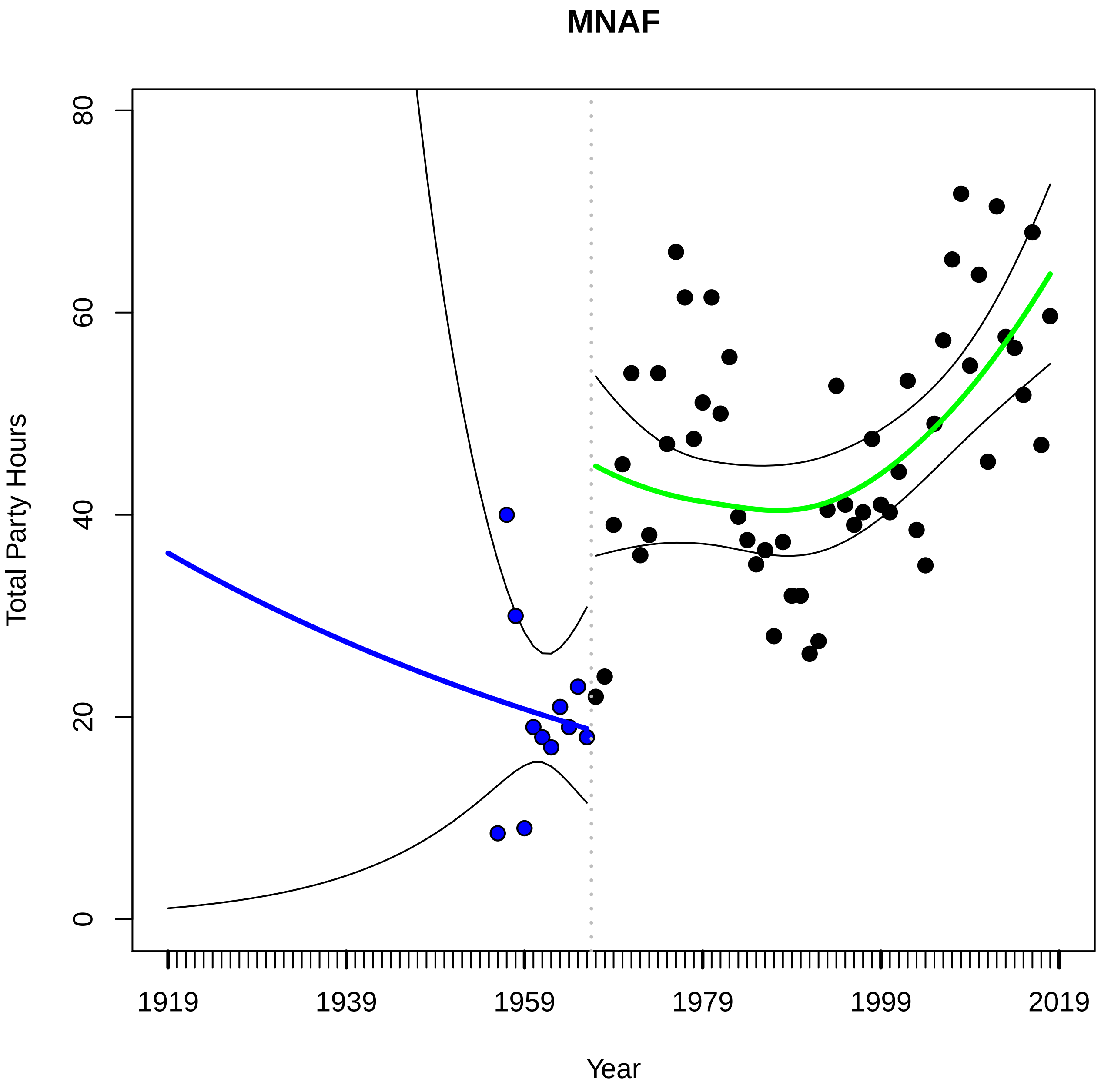

MNAF
Total Party Hours
0
20
40
60
80
1919
1939
1959
1979
1999
2019
Year

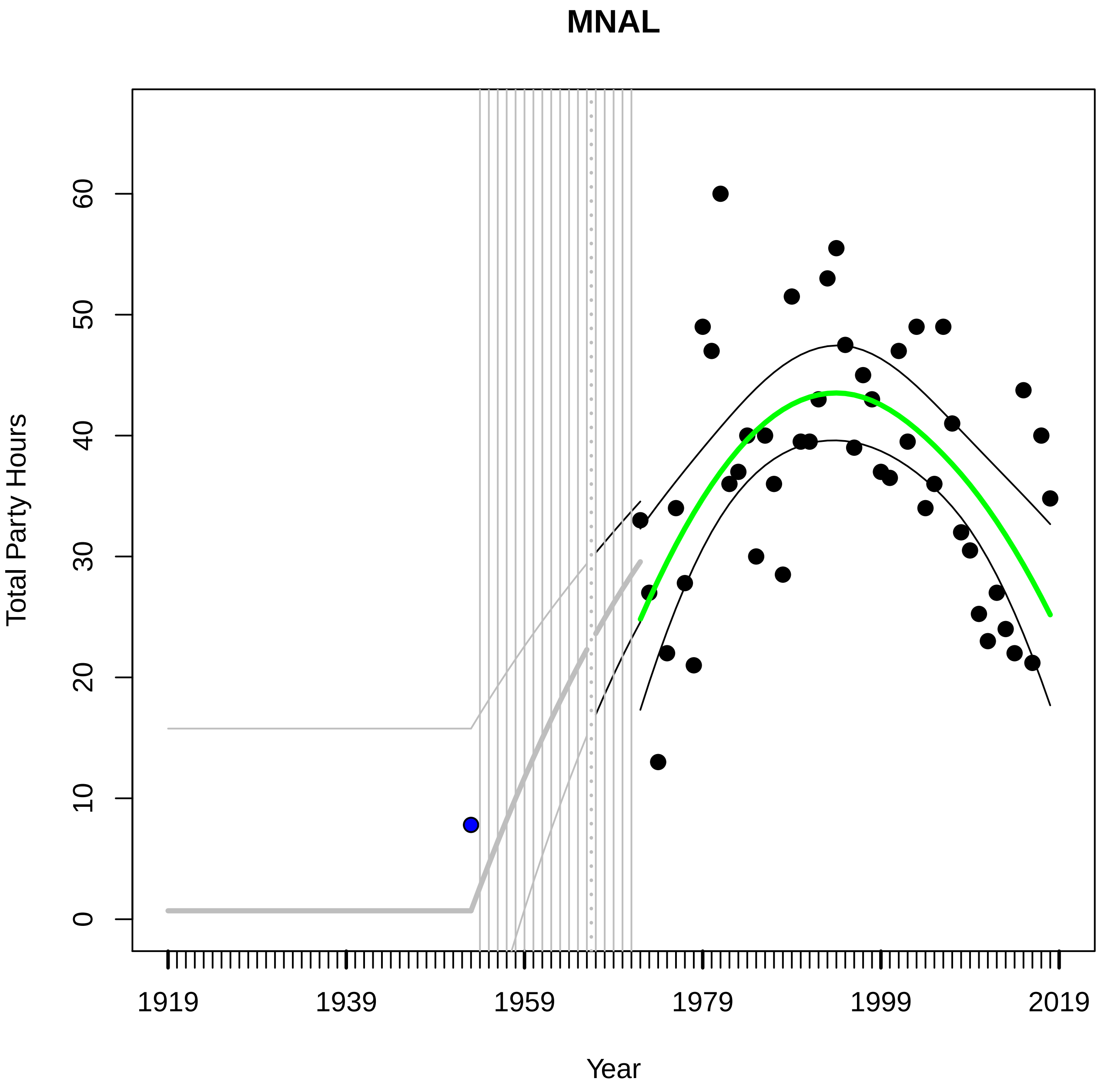
MNAL
Total Party Hours
0
10
20
30
40
50
60
1919
1939
1959
1979
1999
2019
Year

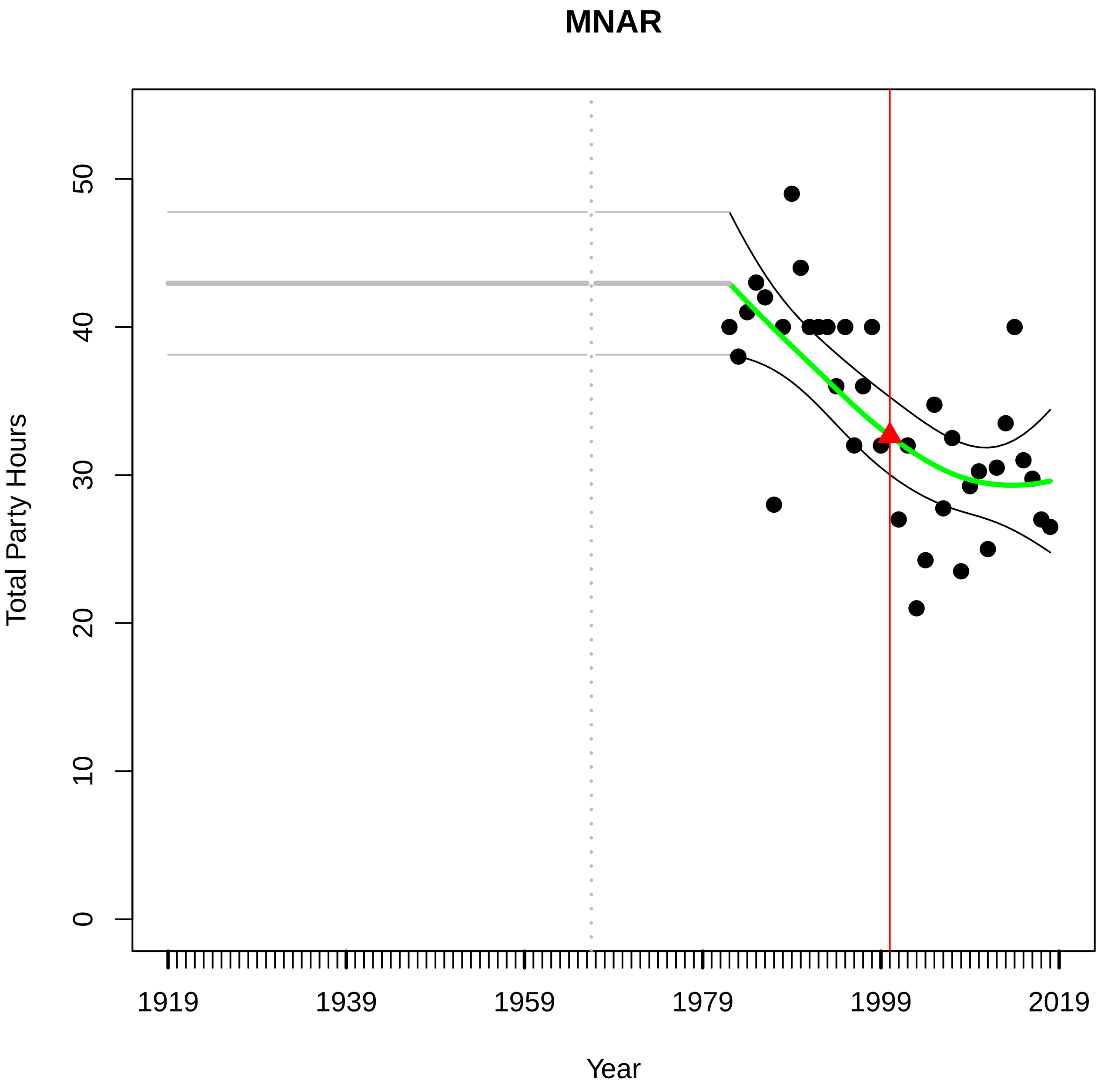

MNAR
Total Party Hours
0
10
20
30
40
50
1919
1939
1959
1979
1999
2019
Year

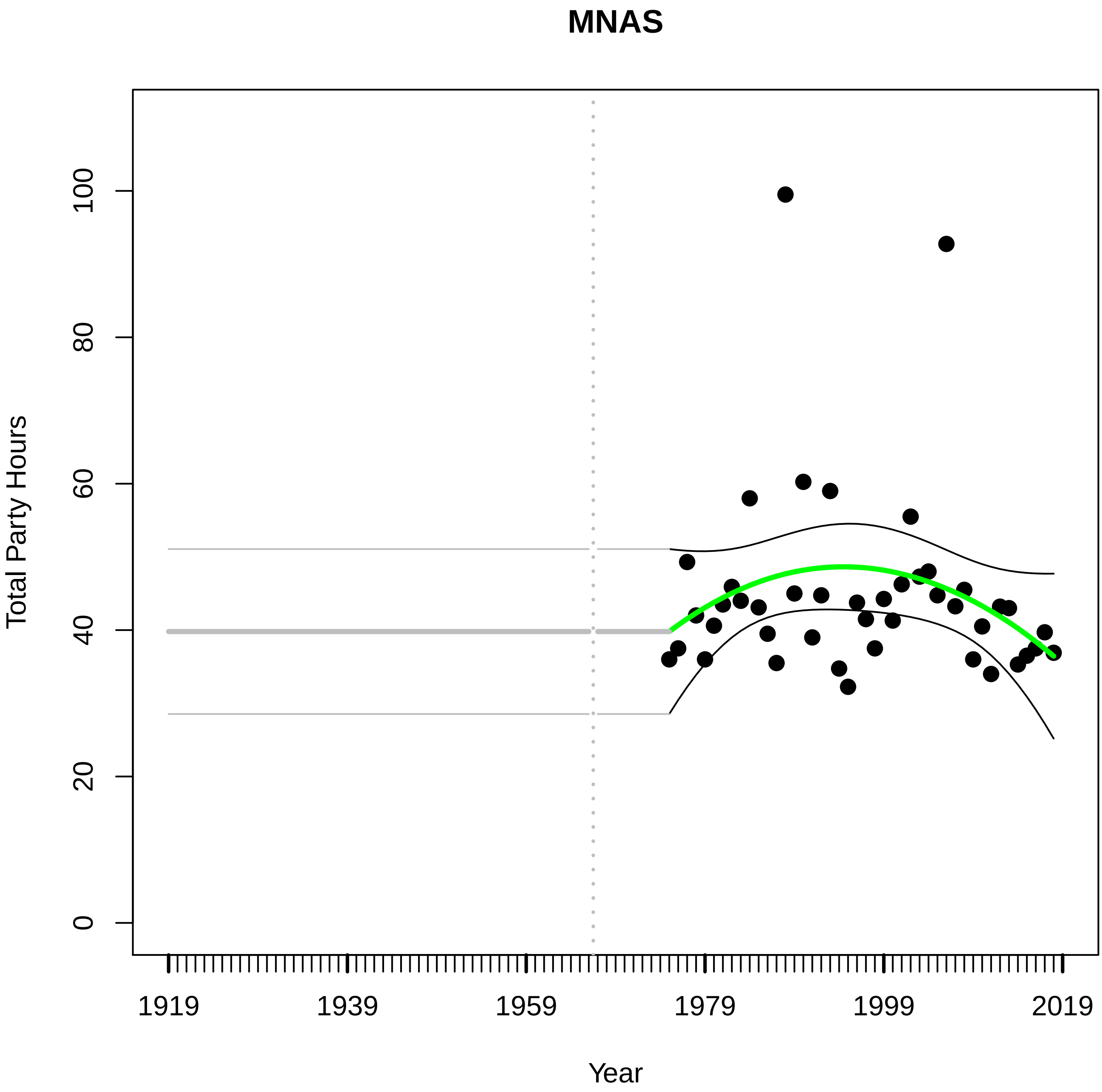

MNAS
Total Party Hours
0
20
40
60
80
100
1919
1939
1959
1979
1999
2019
Year

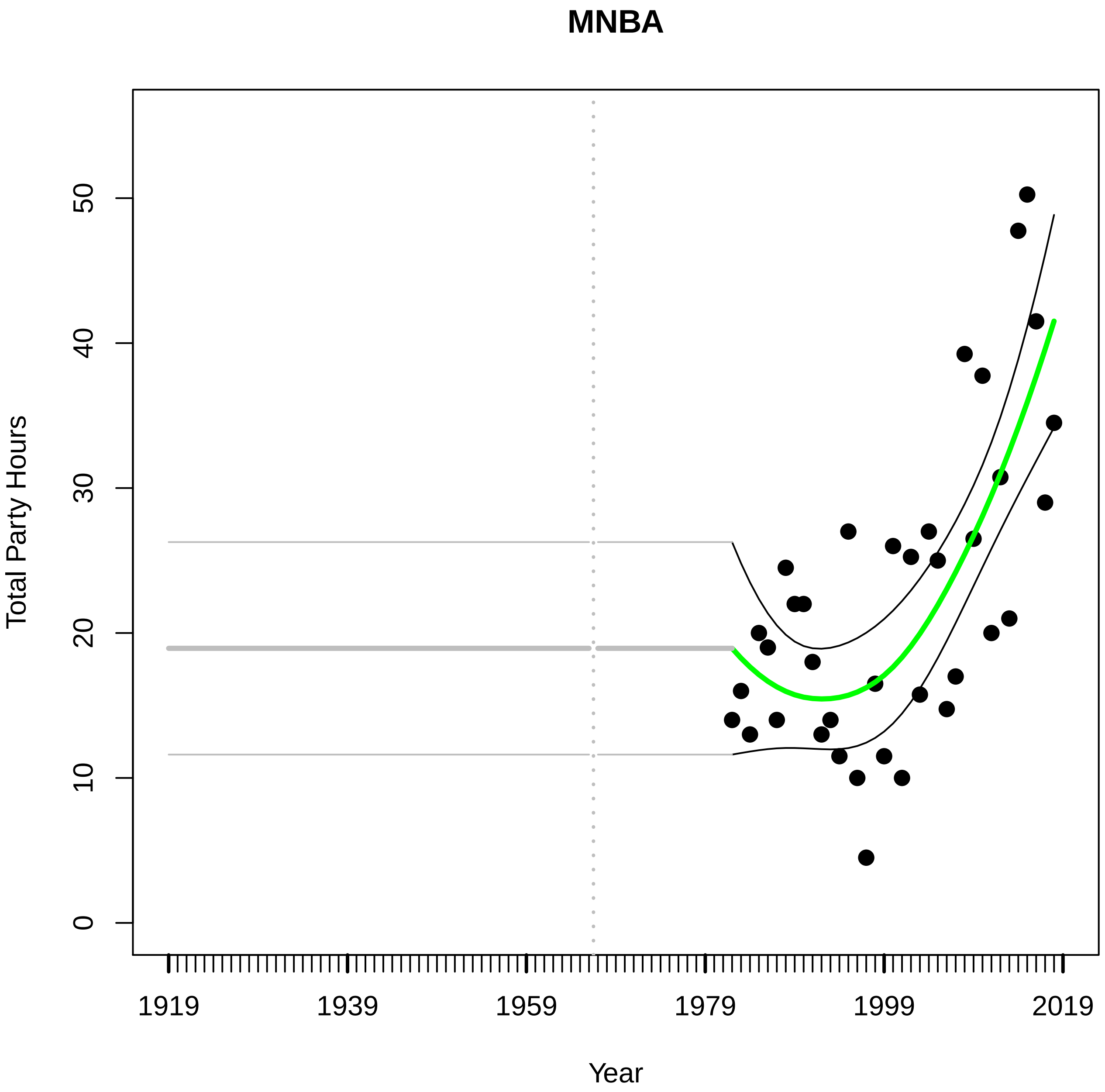

MNBA
Total Party Hours
0
10
20
30
40
50
1919
1939
1959
1979
1999
2019
Year

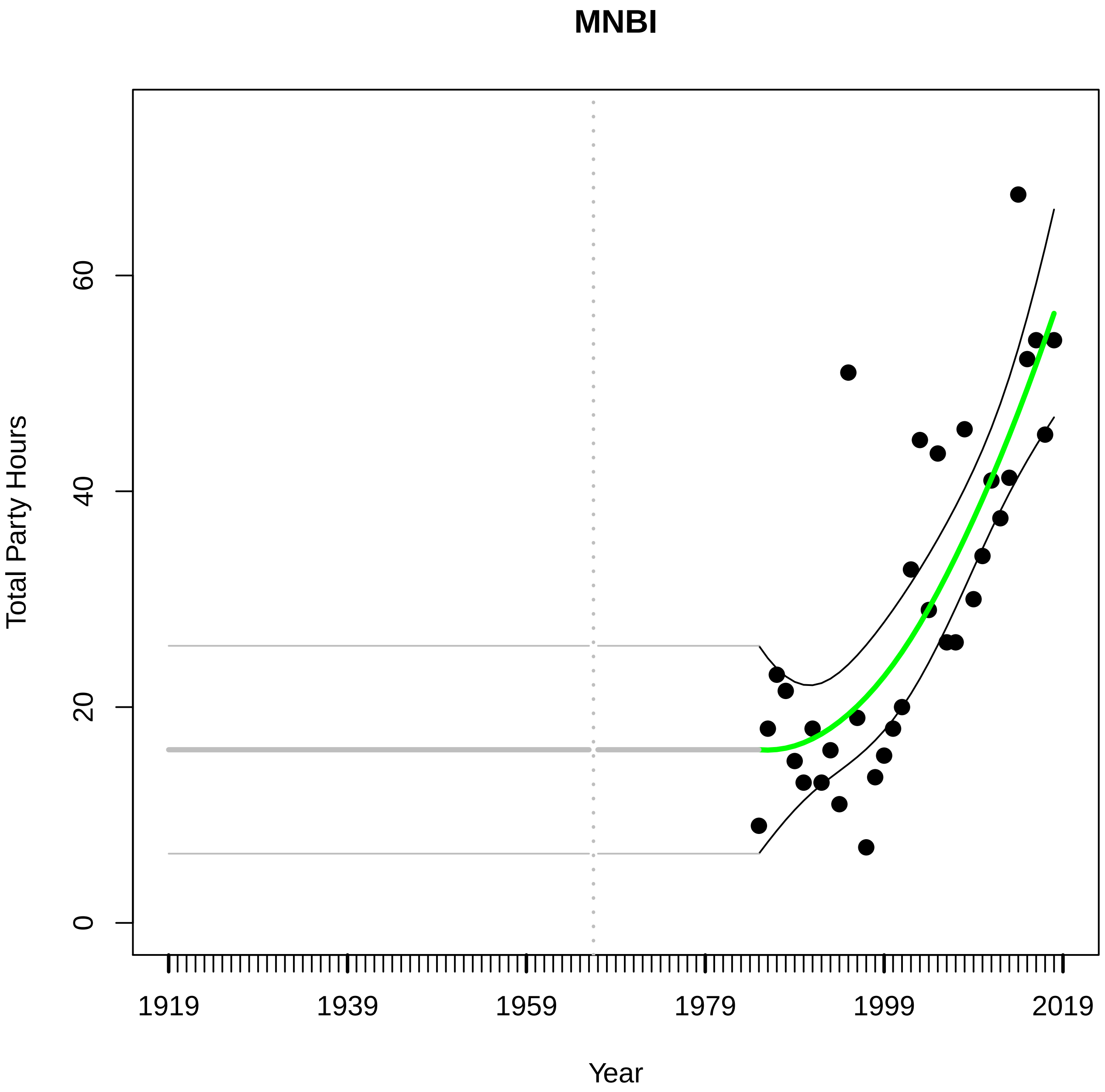

MNBI
Total Party Hours
0
20
40
60
1919
1939
1959
1979
1999
2019
Year

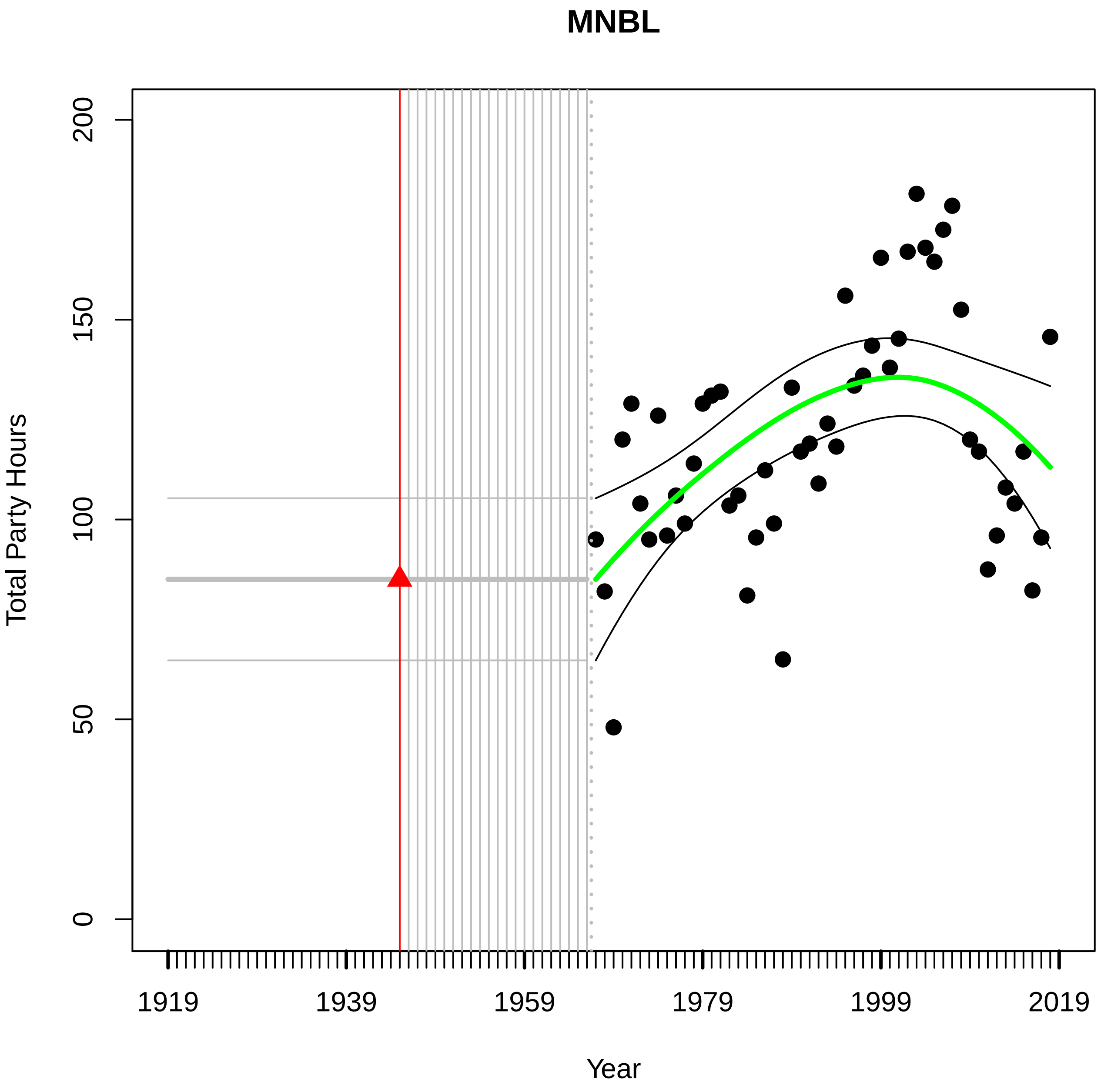

MNBL
Total Party Hours
200
150
100
50
0
1919
1939
1959
1979
1999
2019
Year

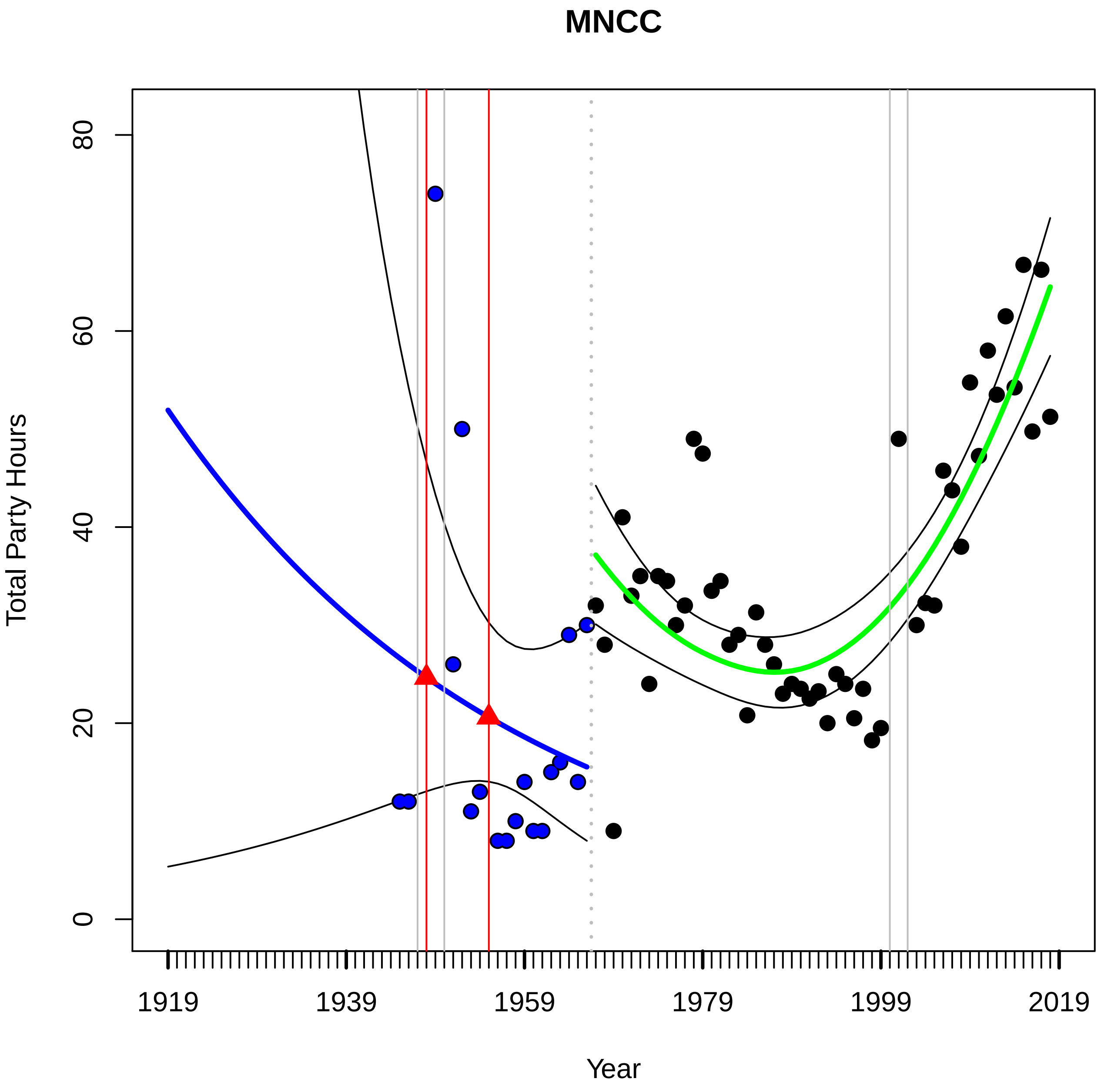
MNCC
Total Party Hours
Year
0
20
40
60
80
1919
1939
1959
1979
1999
2019

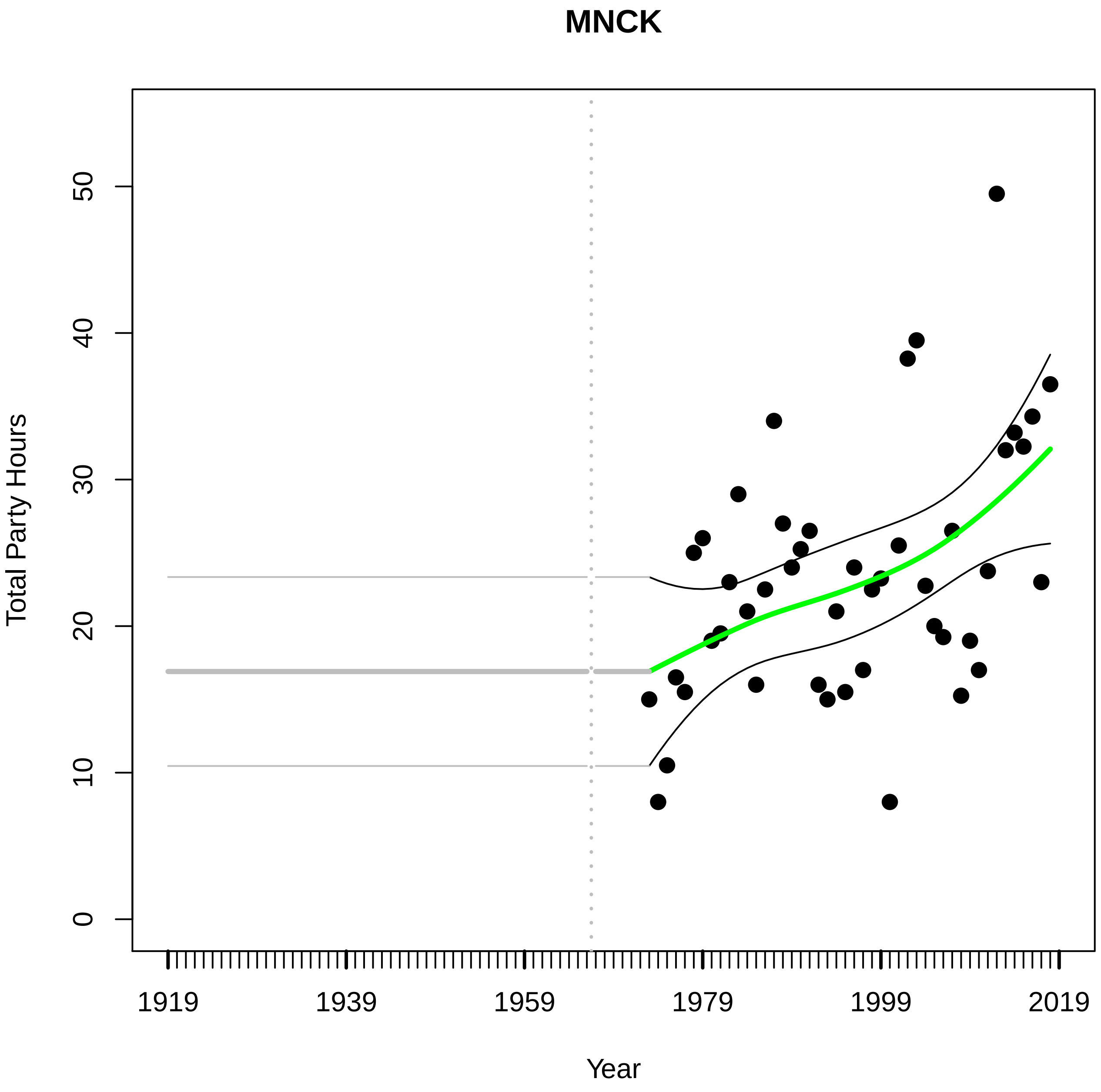

MNCK
Total Party Hours
0
10
20
30
40
50
1919
1939
1959
1979
1999
2019
Year

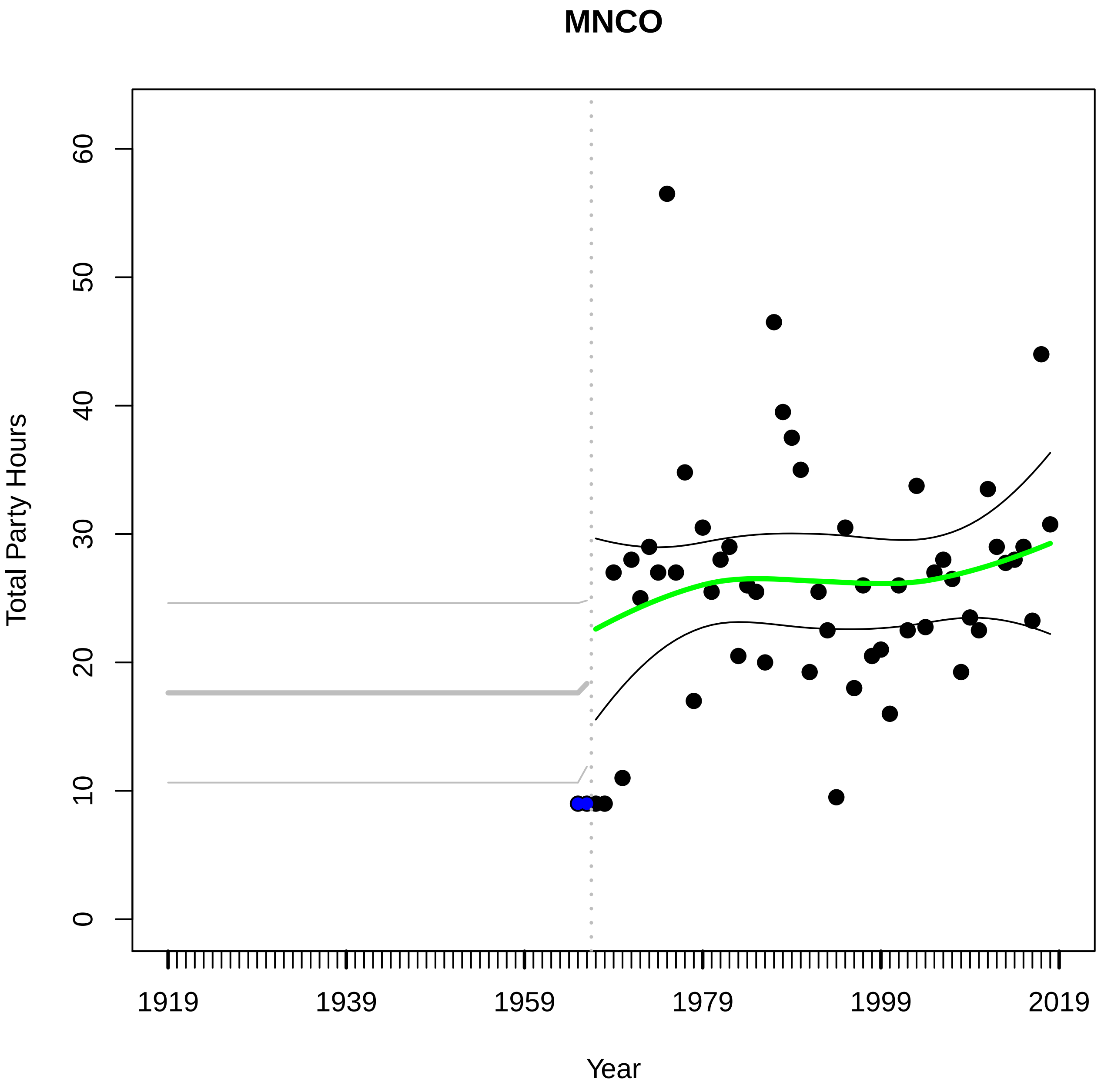

MNCO
Total Party Hours
0
10
20
30
40
50
60
1919
1939
1959
1979
1999
2019
Year

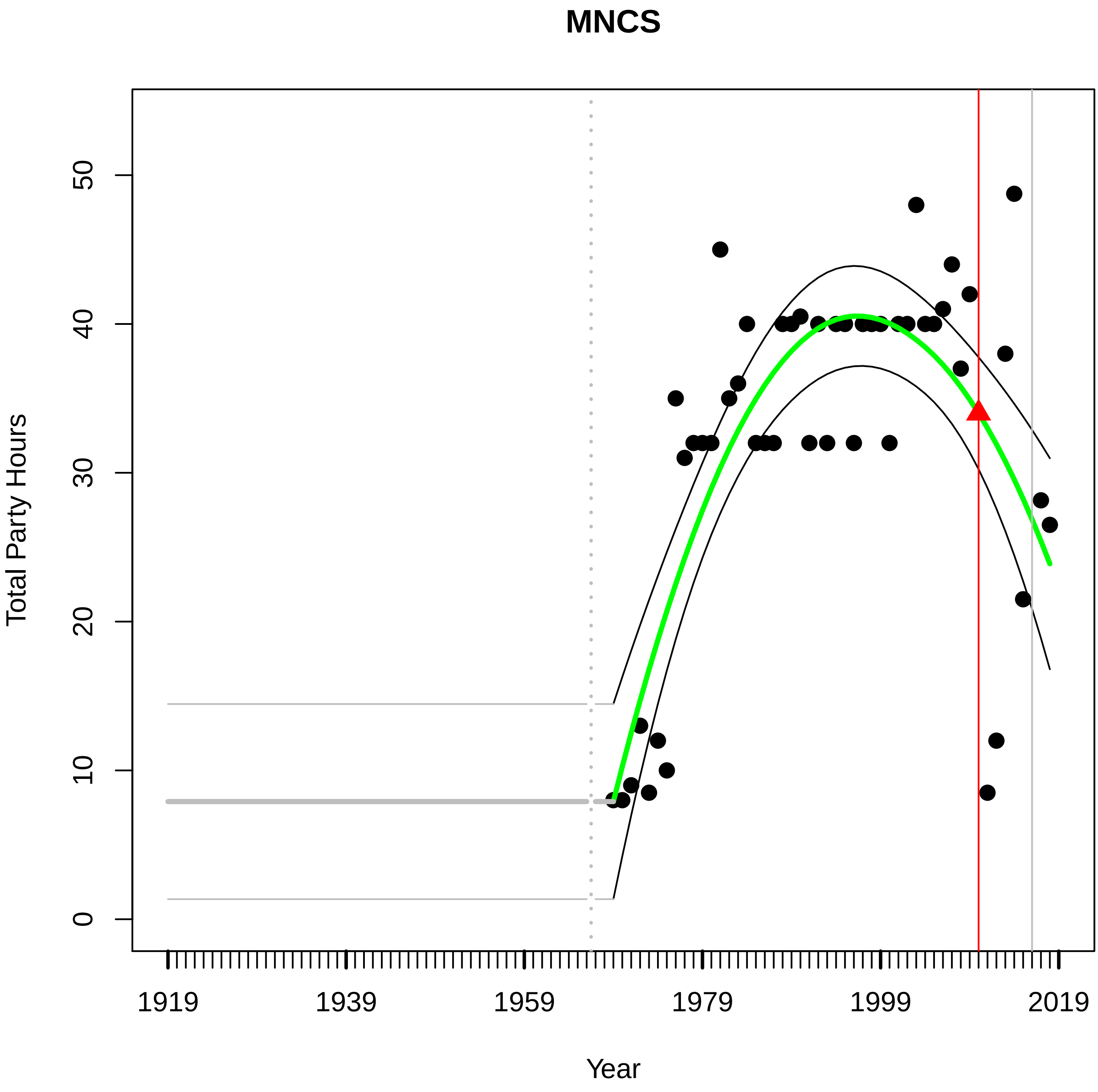
MNCS
Total Party Hours
0
10
20
30
40
50
1919
1939
1959
1979
1999
2019
Year

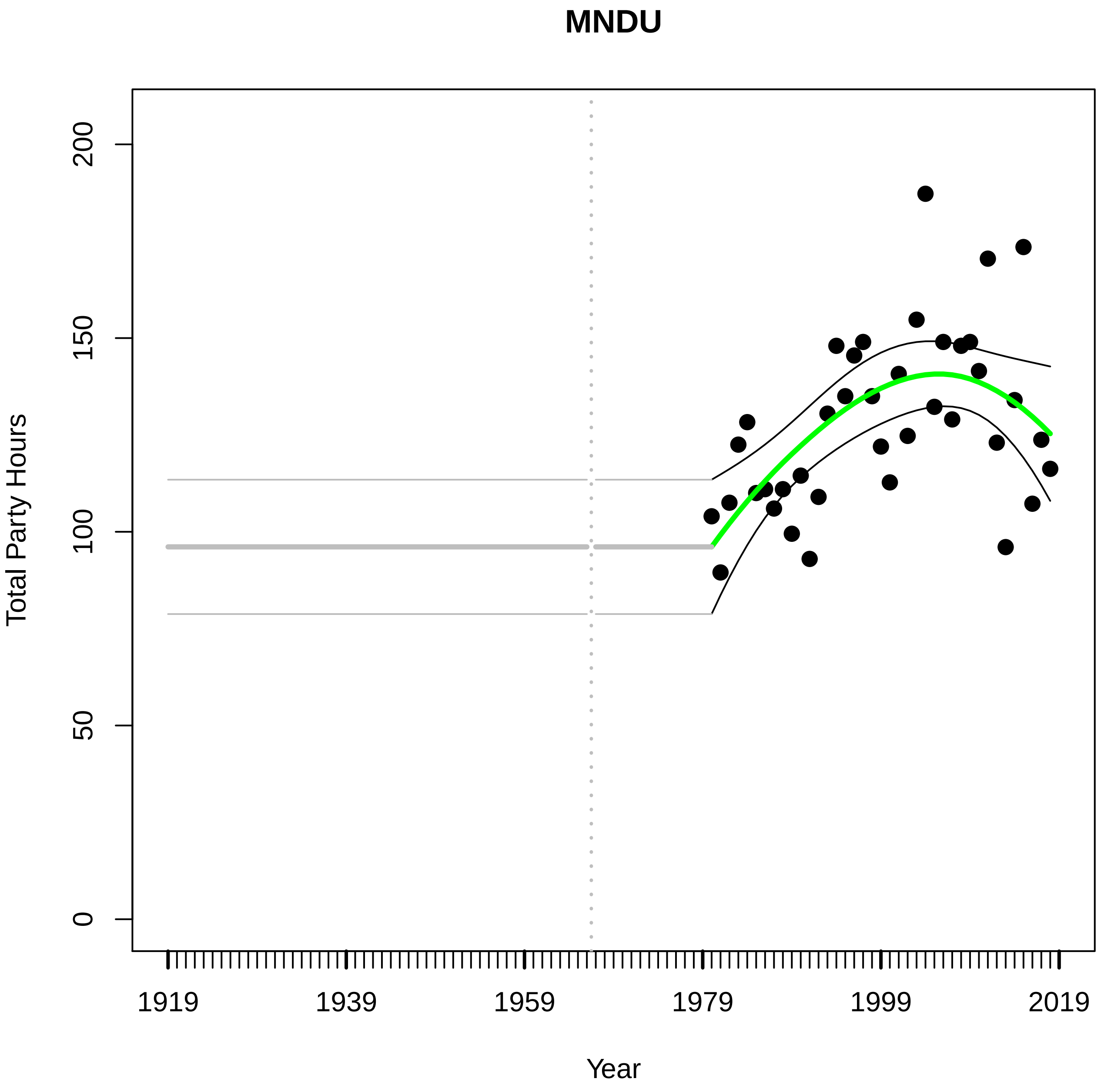

MNDU
Total Party Hours
0
50
100
150
200
Year
1919
1939
1959
1979
1999
2019

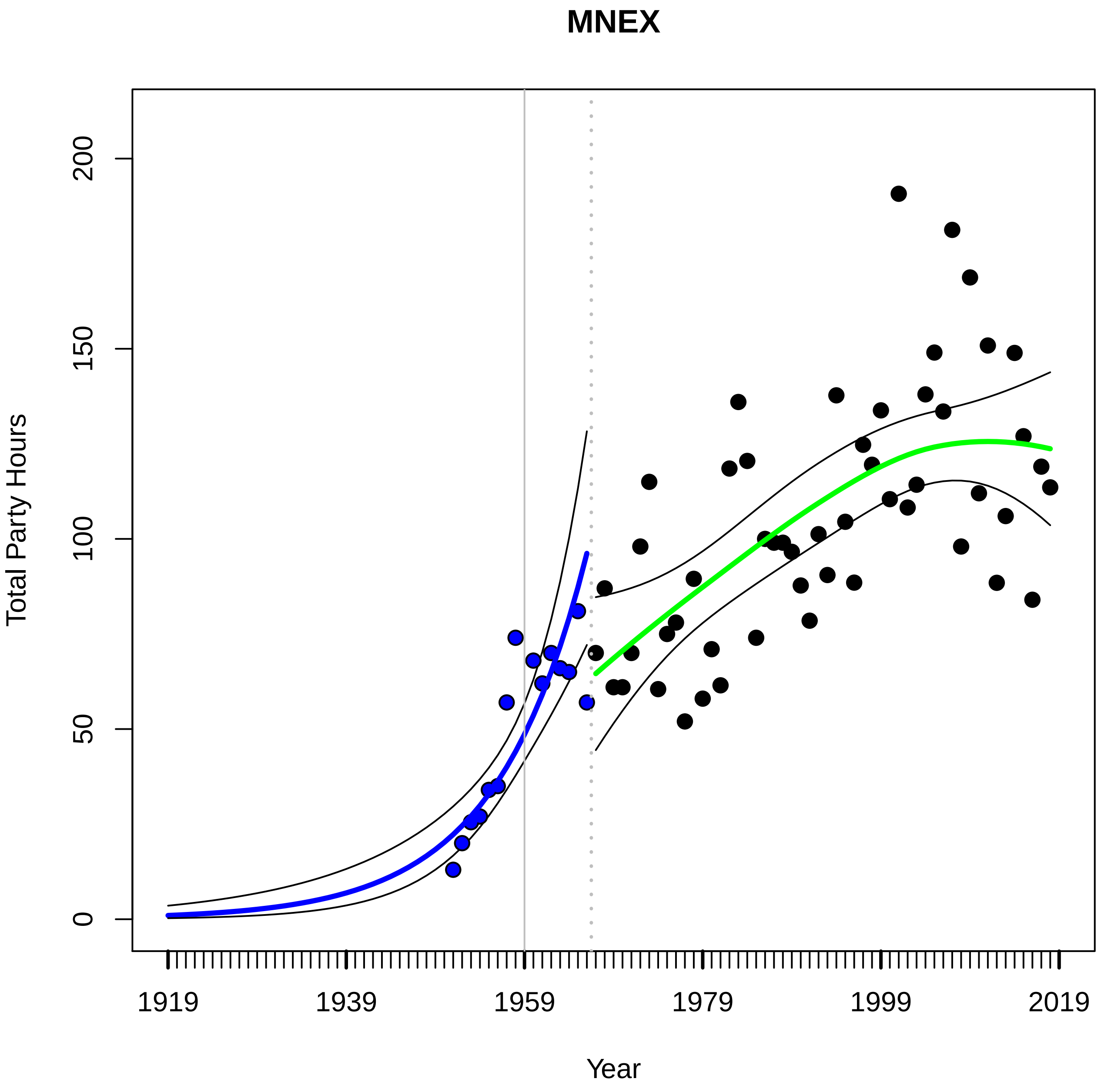

MNEX
Total Party Hours
0
50
100
150
200
1919
1939
1959
1979
1999
2019
Year

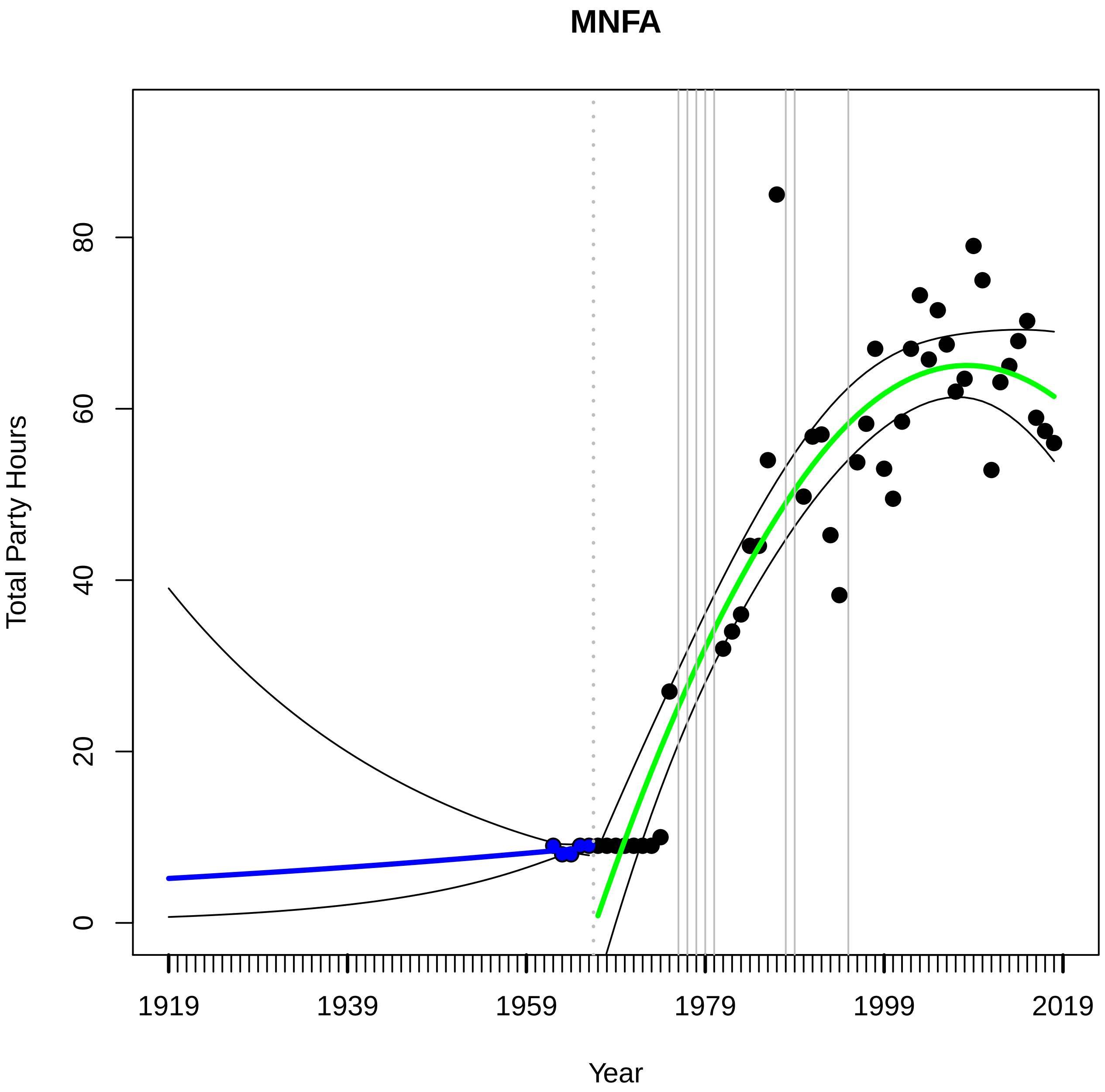

MNFA
Total Party Hours
0
20
40
60
80
1919
1939
1959
1979
1999
2019
Year

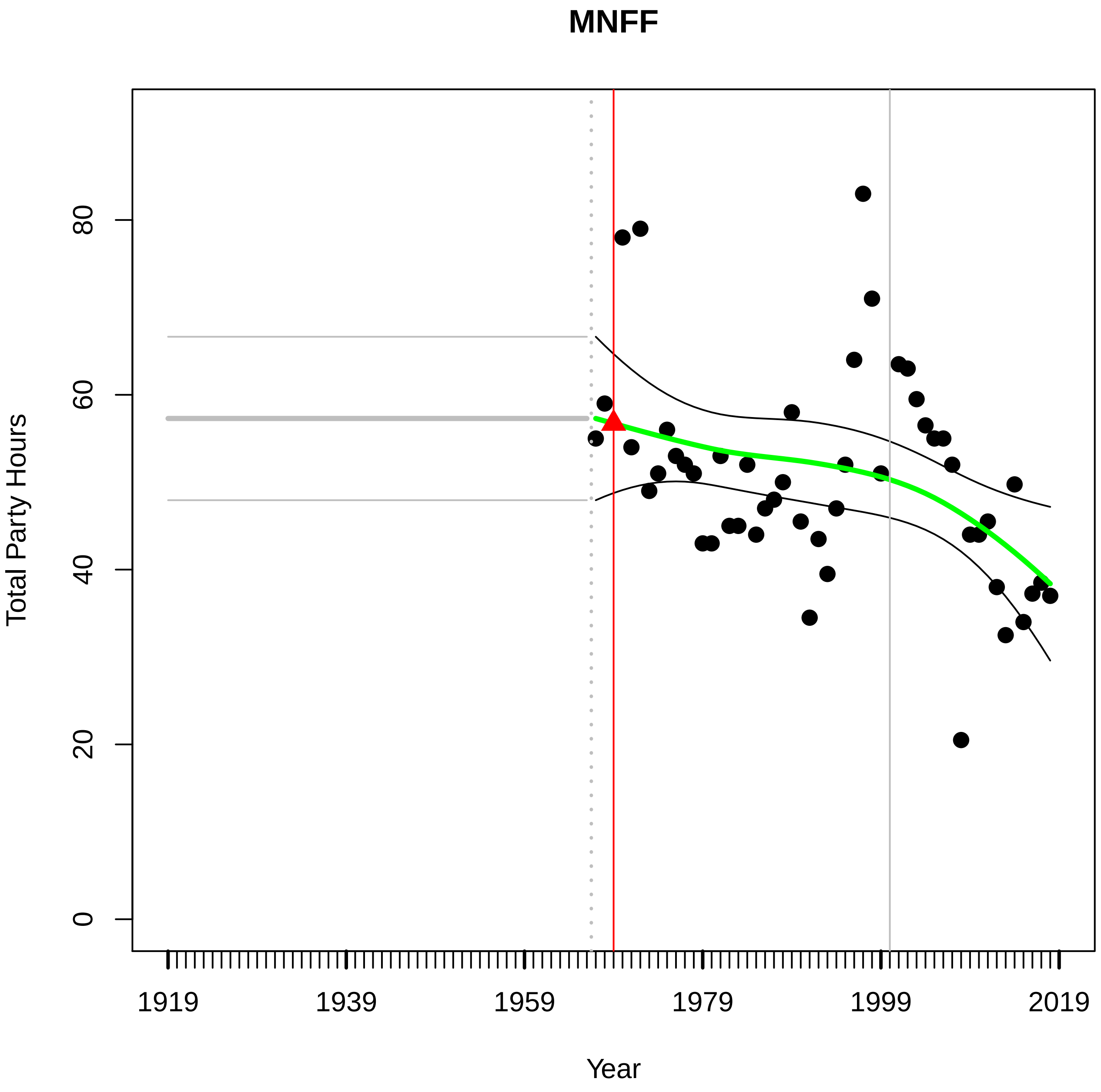

MNFF
Total Party Hours
0
20
40
60
80
1919
1939
1959
1979
1999
2019
Year

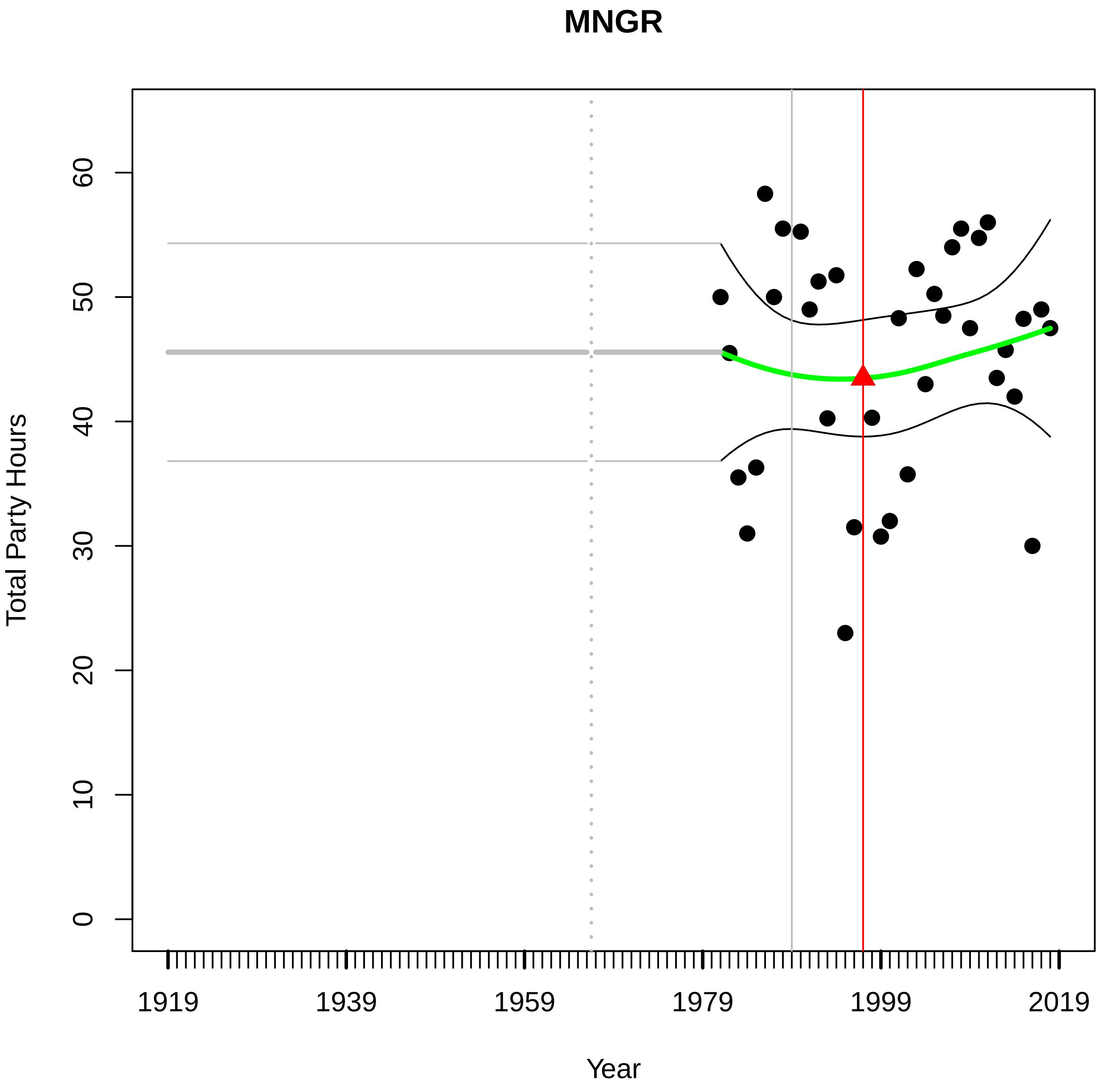

MNGR
Total Party Hours
0
10
20
30
40
50
60
1919
1939
1959
1979
1999
2019
Year

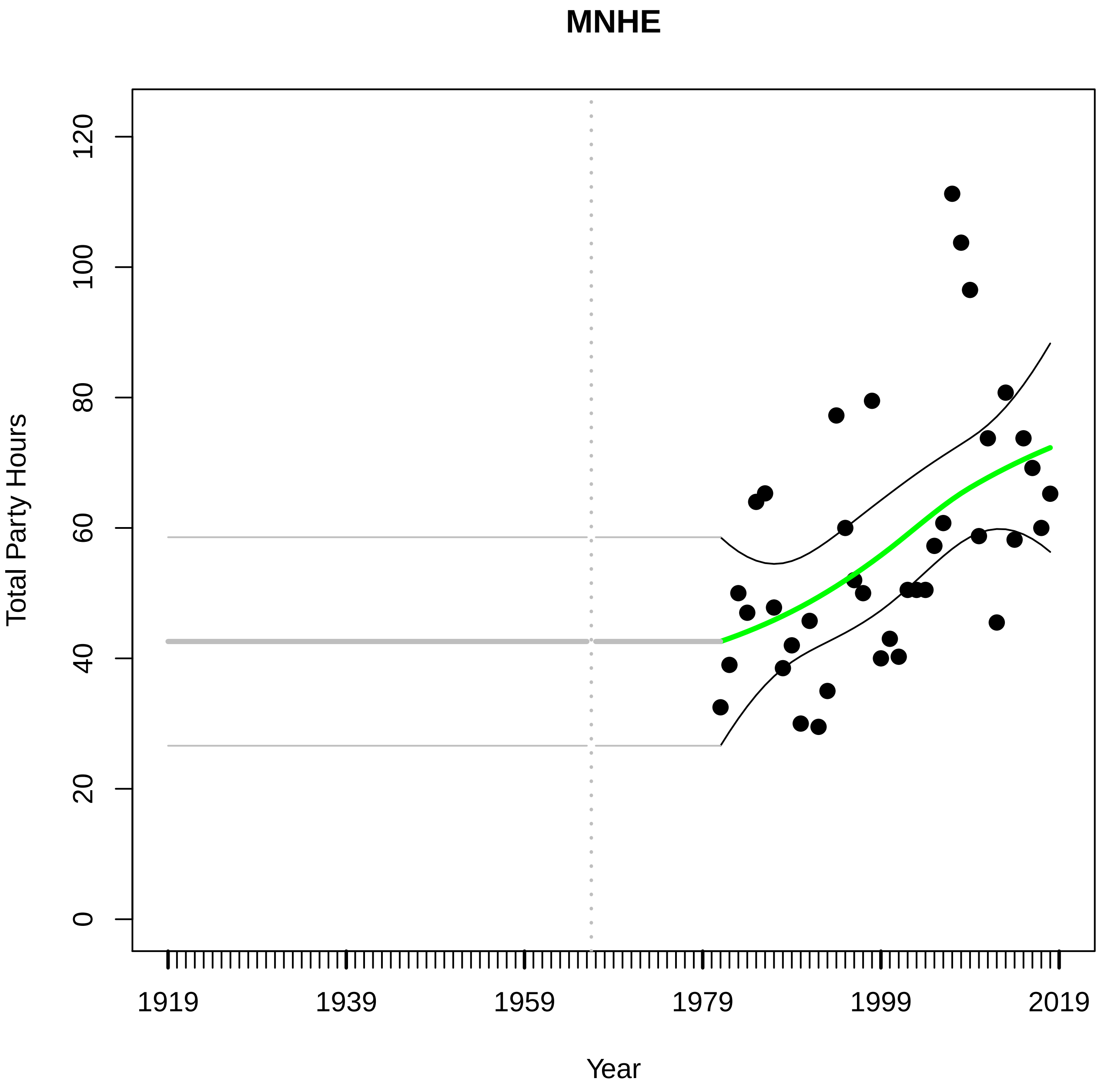

MNHE
Total Party Hours
Year
0
20
40
60
80
100
120
1919
1939
1959
1979
1999
2019

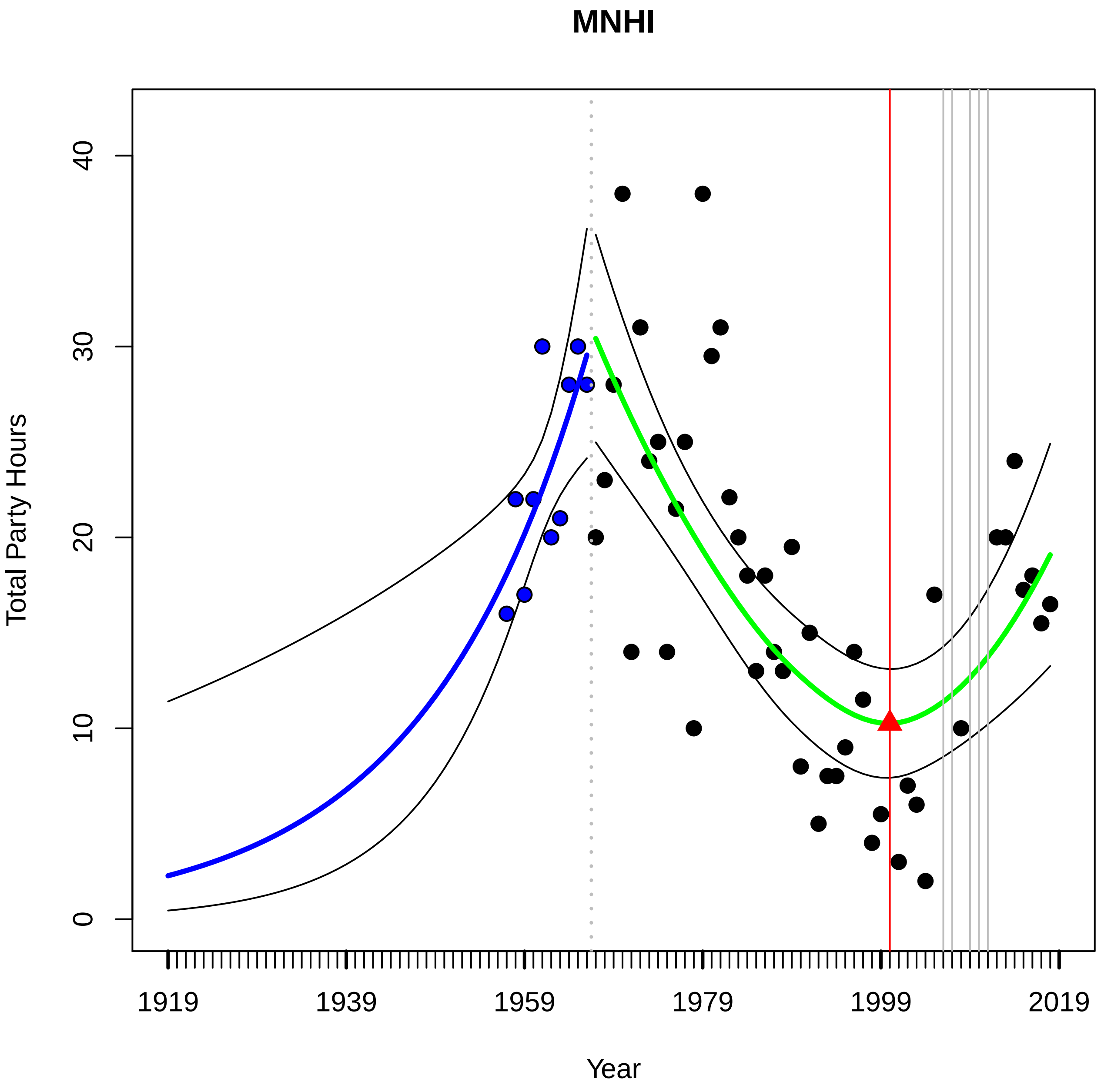
MNHI
Total Party Hours
0
10
20
30
40
1919
1939
1959
1979
1999
2019
Year

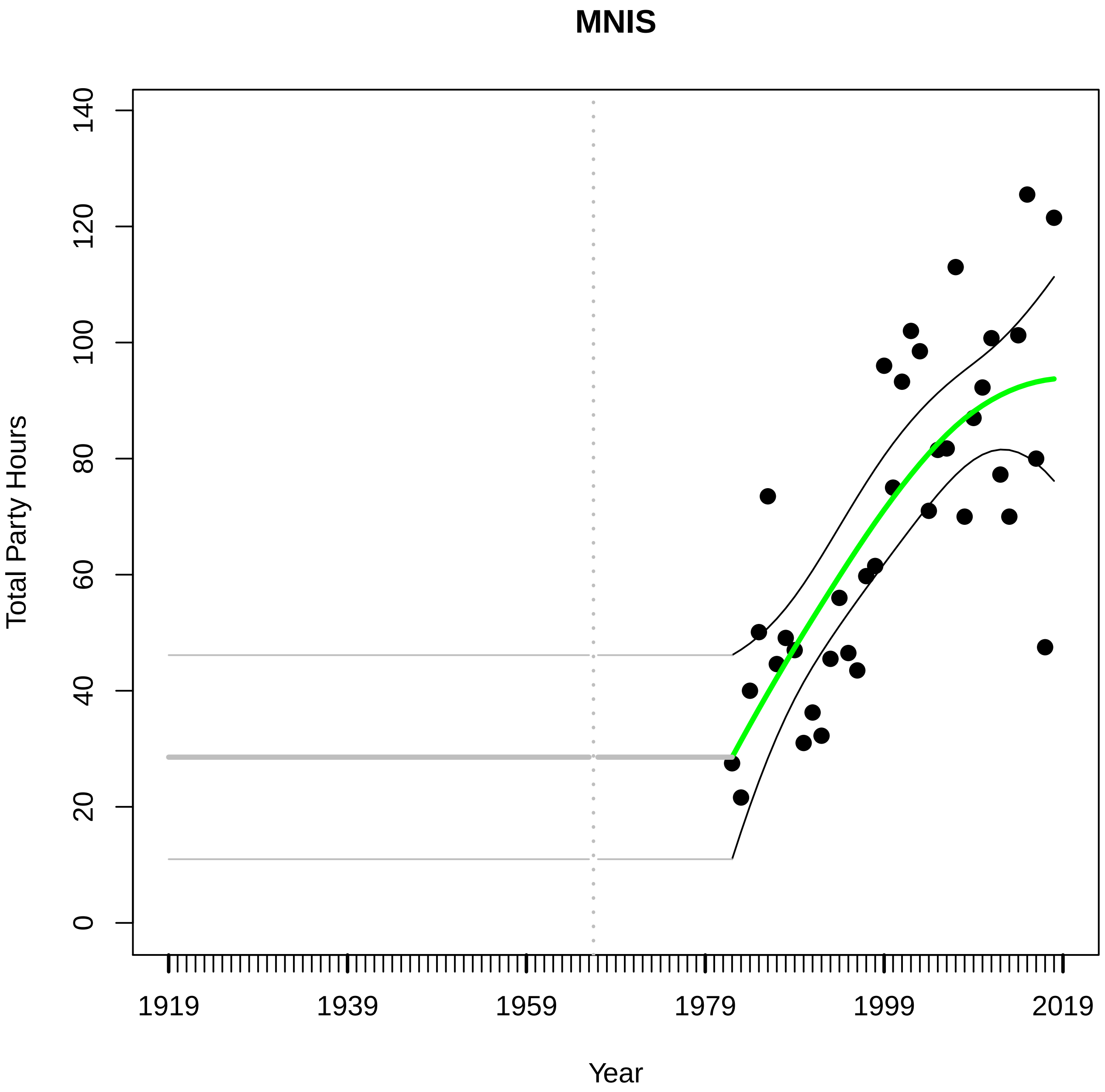
MNIS
Total Party Hours
Year
0
20
40
60
80
100
120
140
1919
1939
1959
1979
1999
2019

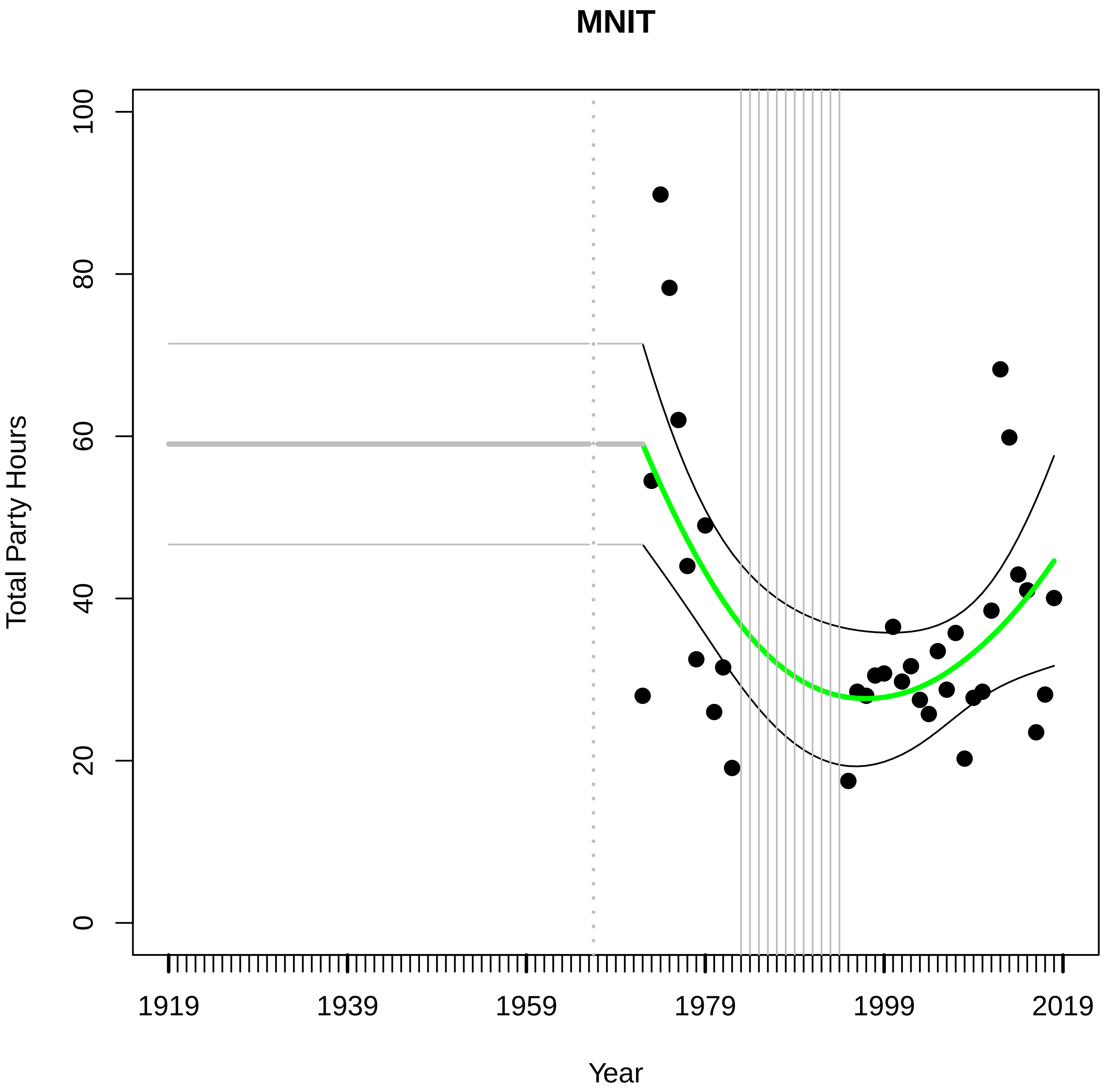

MNIT
Total Party Hours
Year
0
20
40
60
80
100
1919
1939
1959
1979
1999
2019

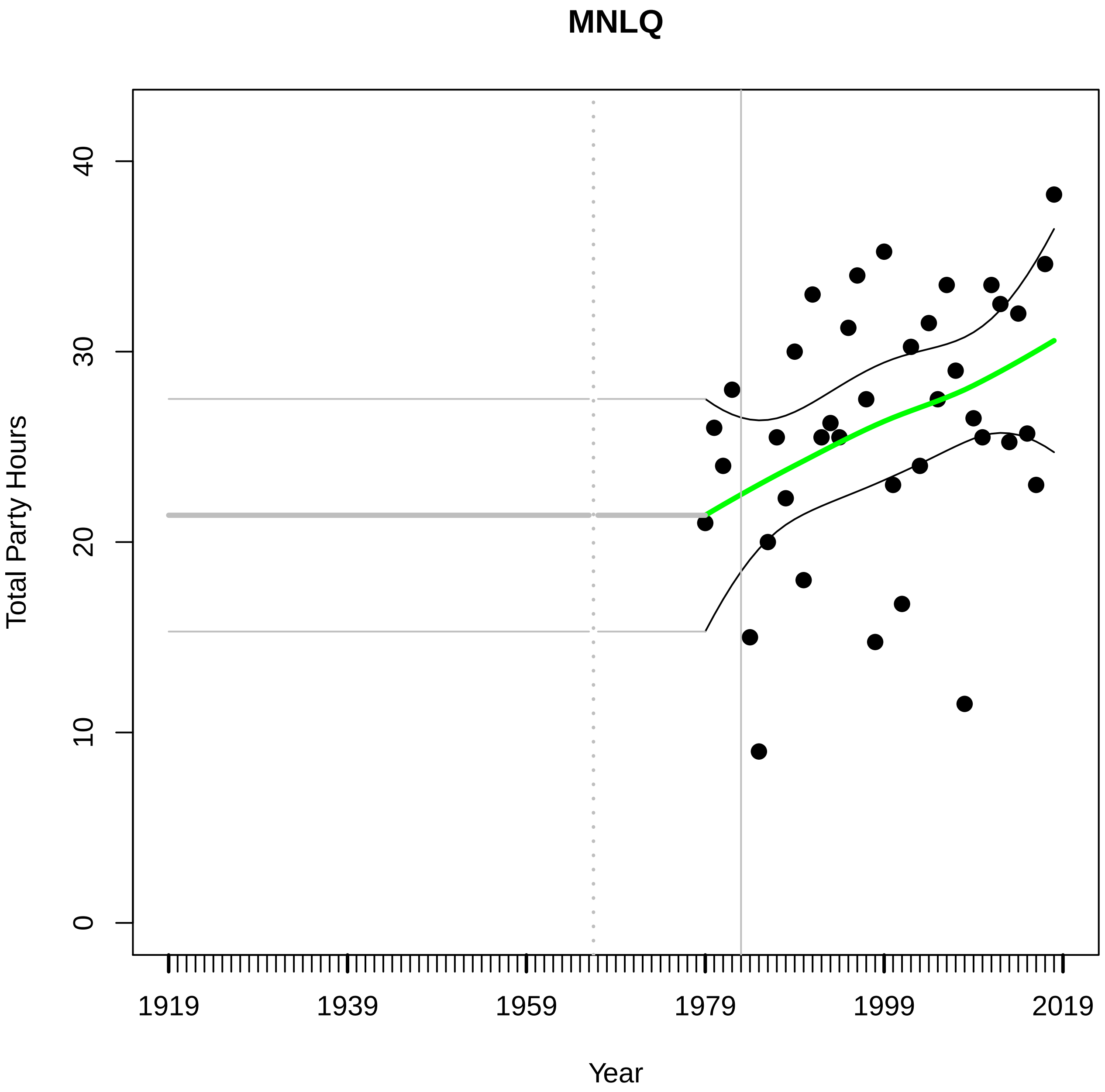

MNLQ
Total Party Hours
0
10
20
30
40
1919
1939
1959
1979
1999
2019
Year

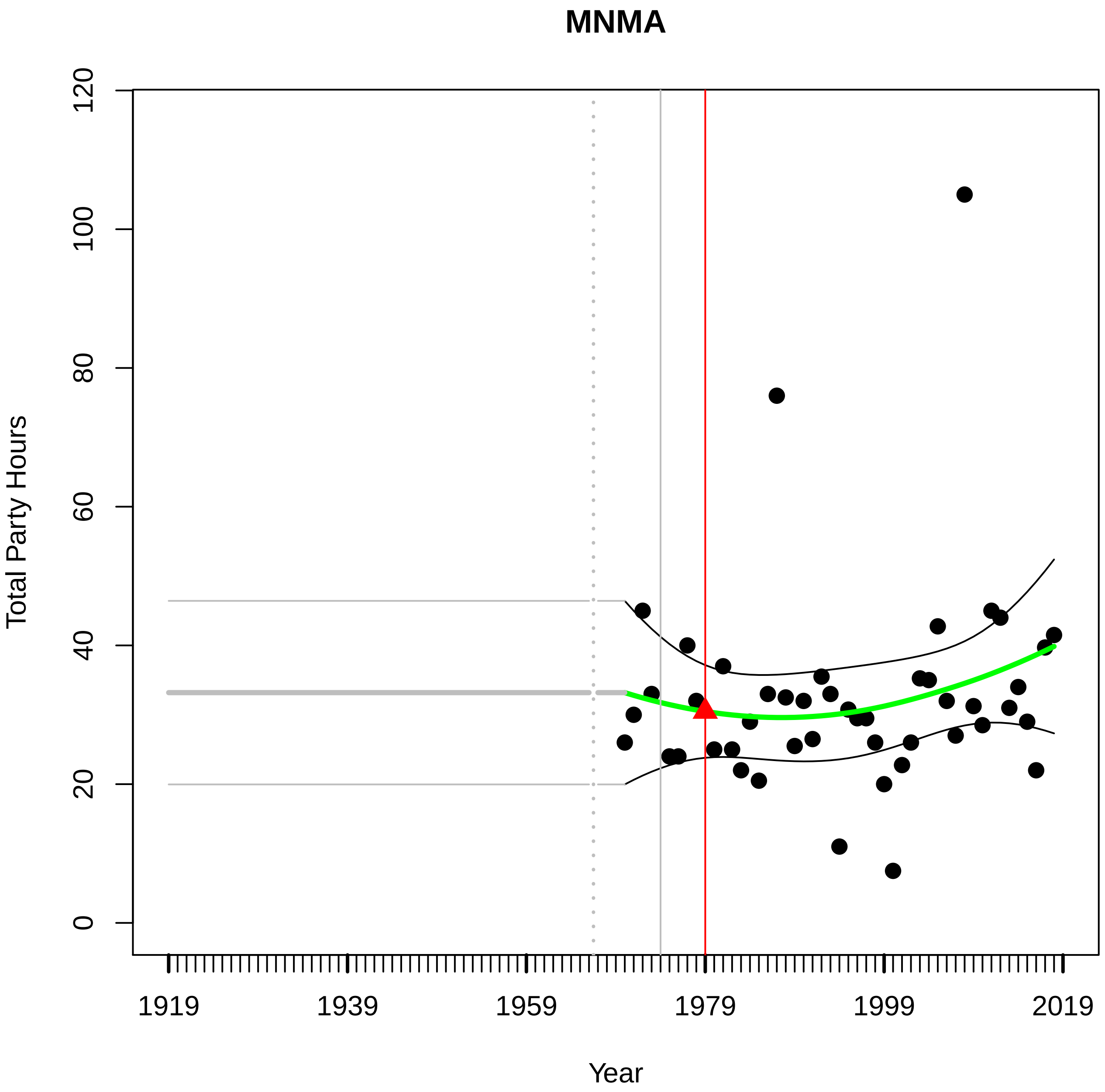

MNMA
Total Party Hours
0
20
40
60
80
100
120
1919
1939
1959
1979
1999
2019
Year

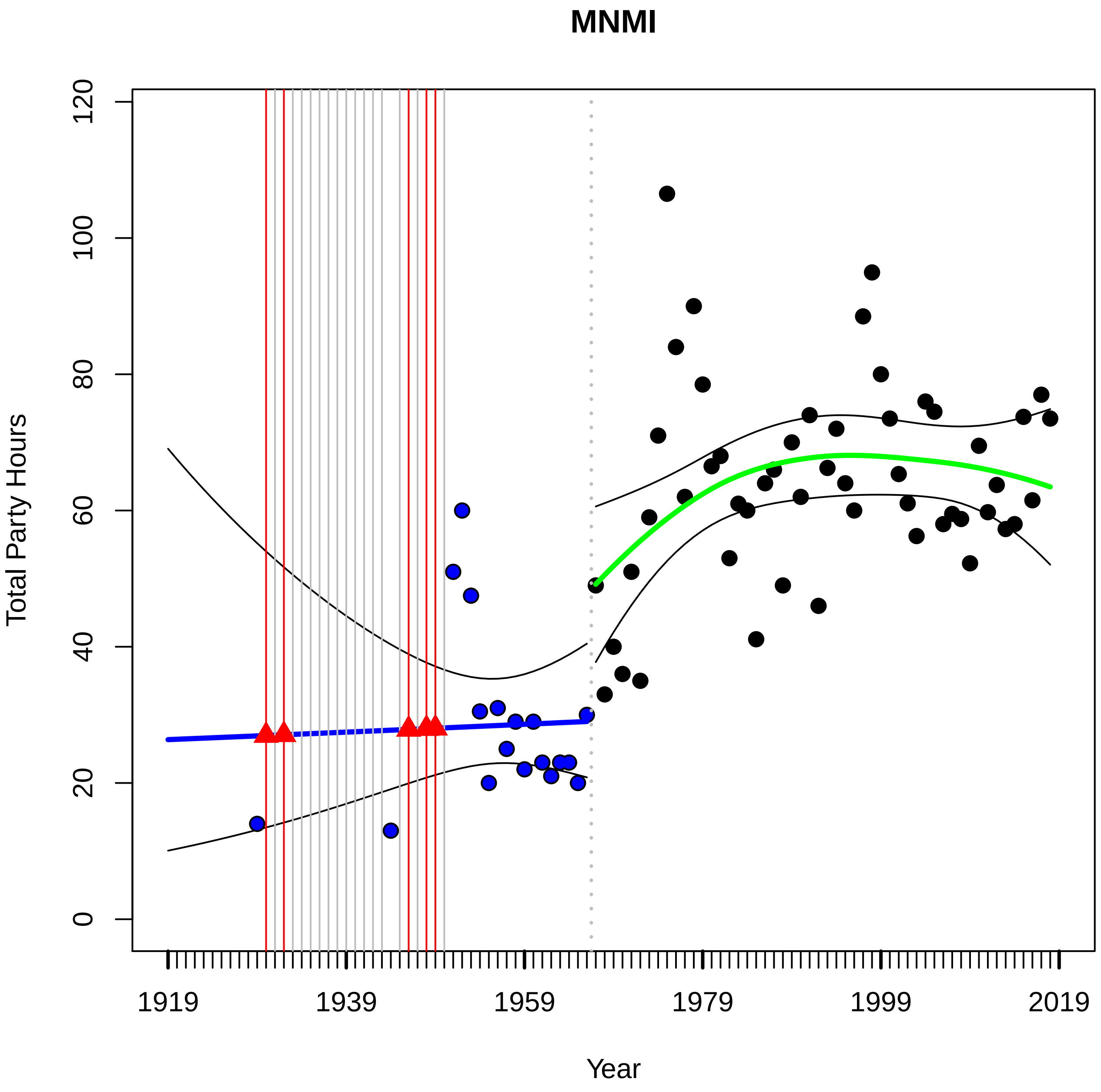
MNMI
Total Party Hours
0
20
40
60
80
100
120
1919
1939
1959
1979
1999
2019
Year

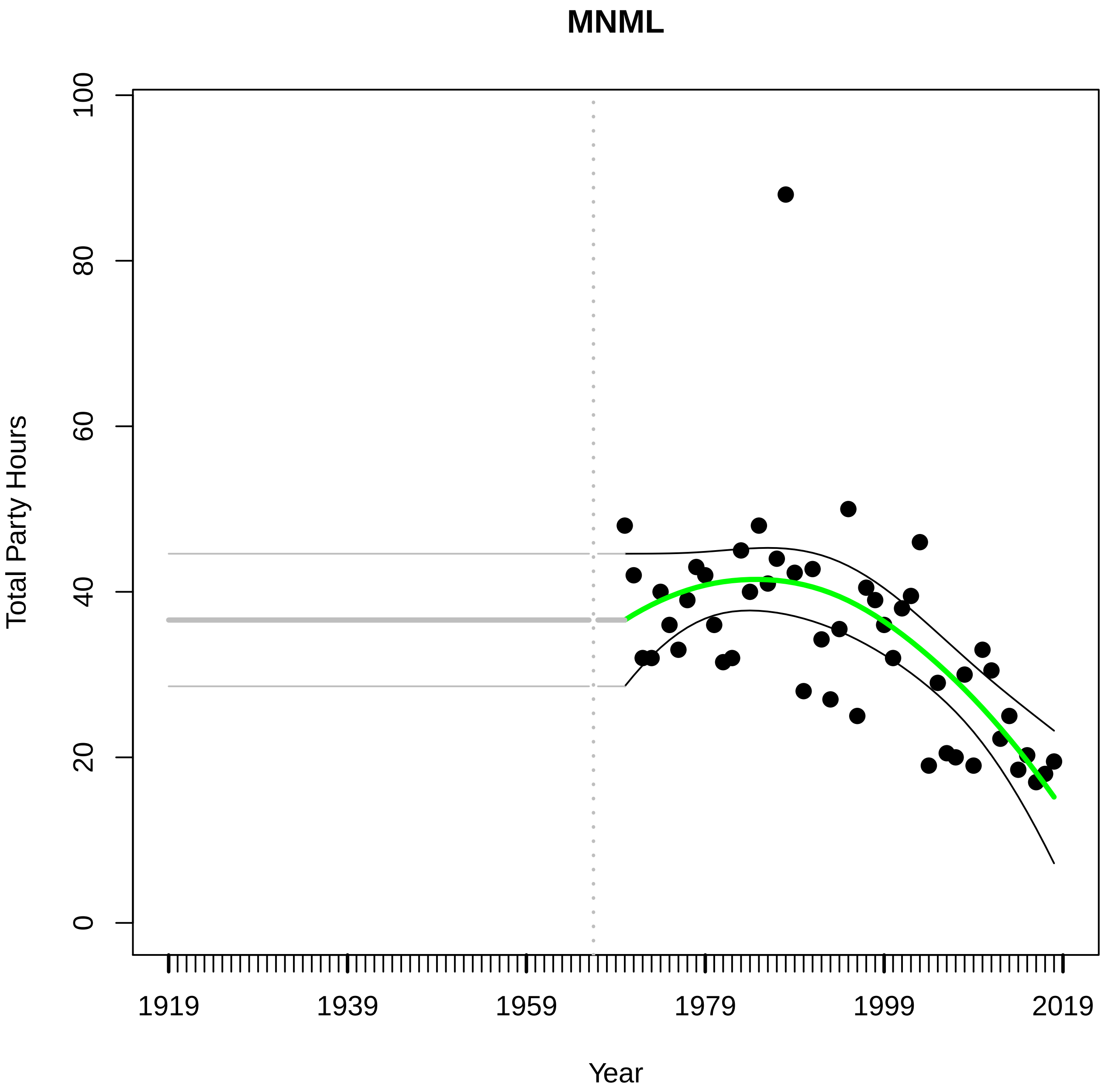

MNML
Total Party Hours
100
80
60
40
20
0
1919
1939
1959
1979
1999
2019
Year

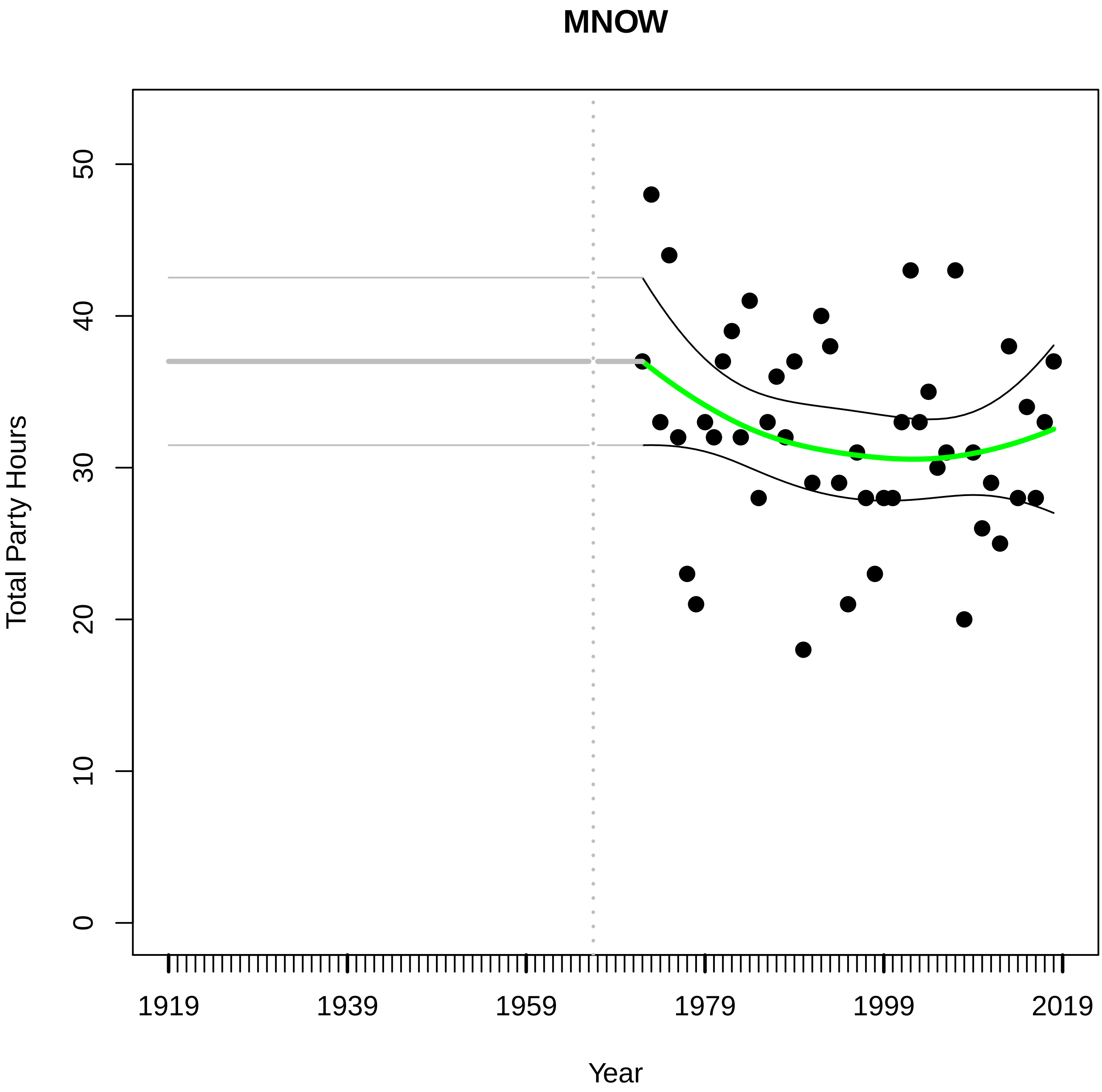

MNOW
Total Party Hours
0
10
20
30
40
50
1919
1939
1959
1979
1999
2019
Year

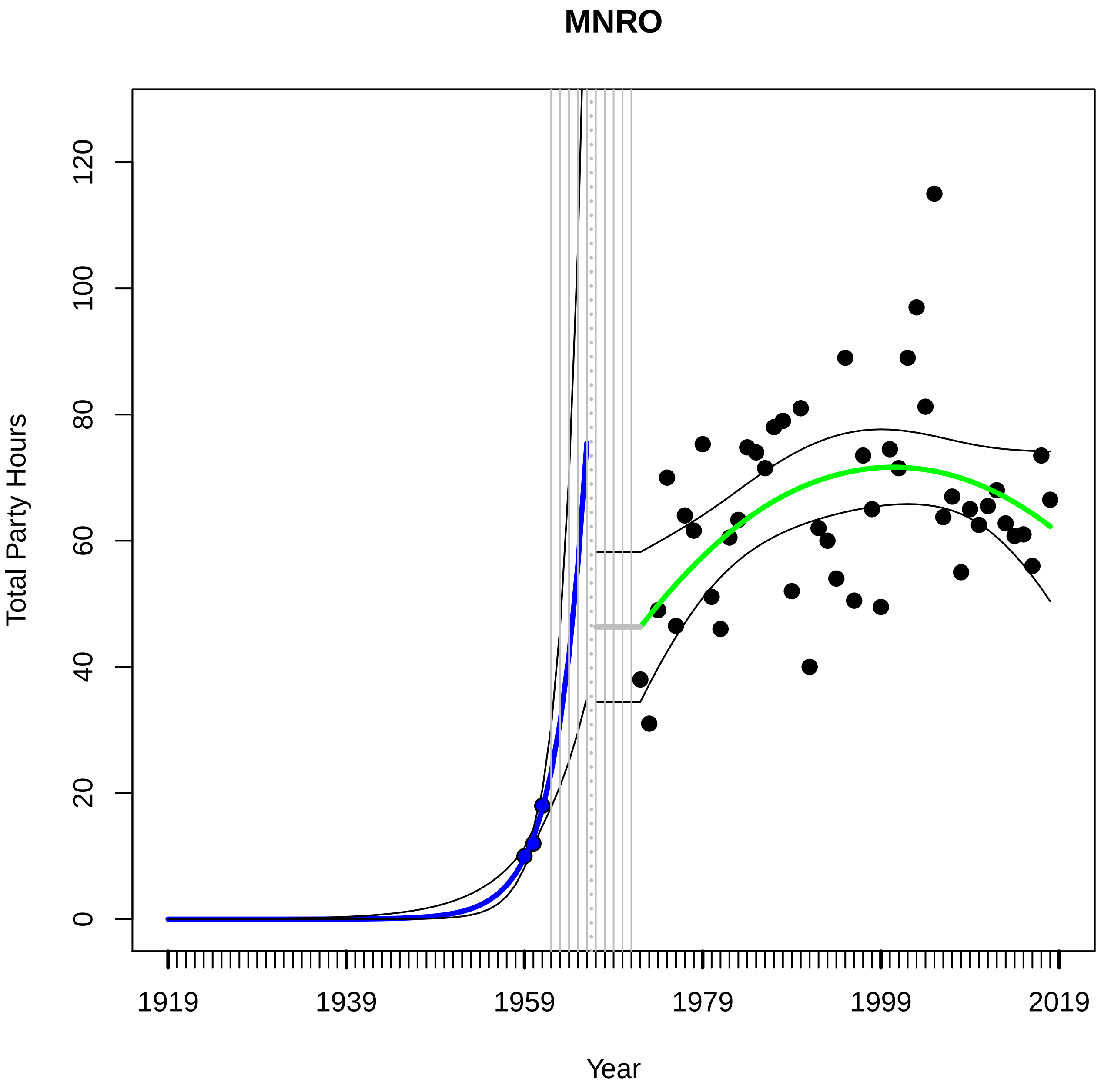
MNRO
Total Party Hours
0
20
40
60
80
100
120
1919
1939
1959
1979
1999
2019
Year

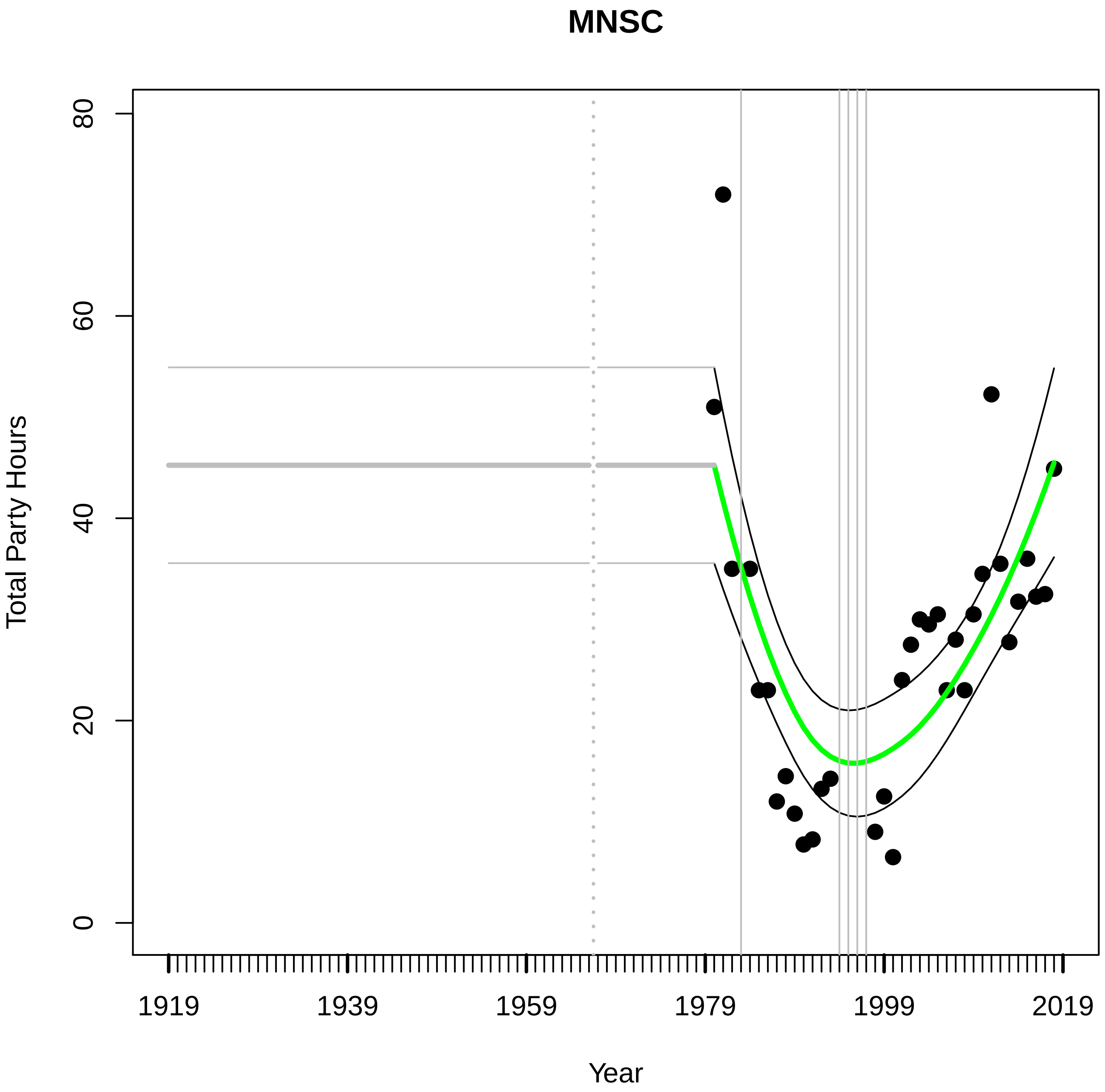
MNSC
Total Party Hours
0
20
40
60
80
1919
1939
1959
1979
1999
2019
Year

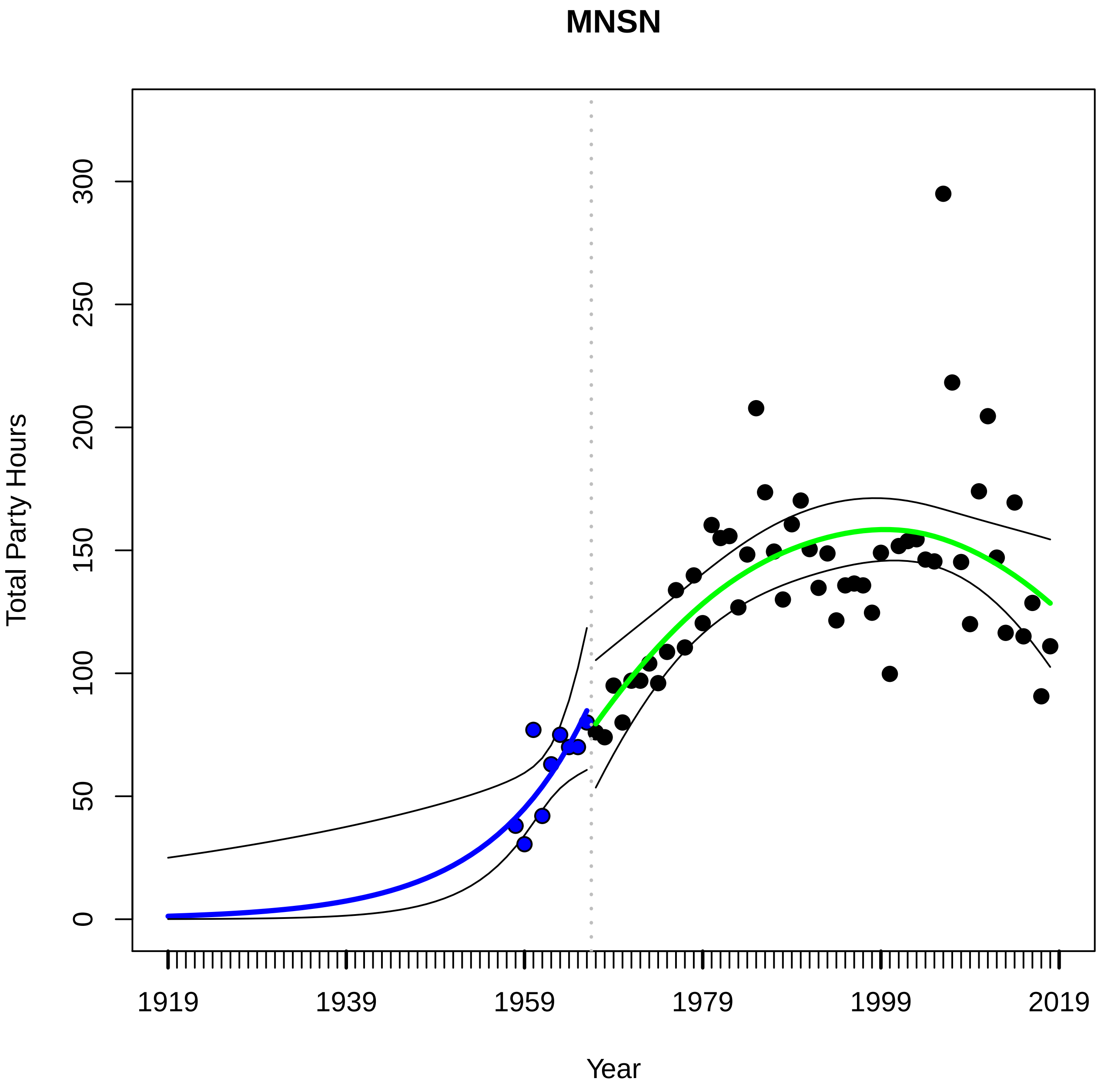
MNSN
Total Party Hours
0
50
100
150
200
250
300
1919
1939
1959
1979
1999
2019
Year

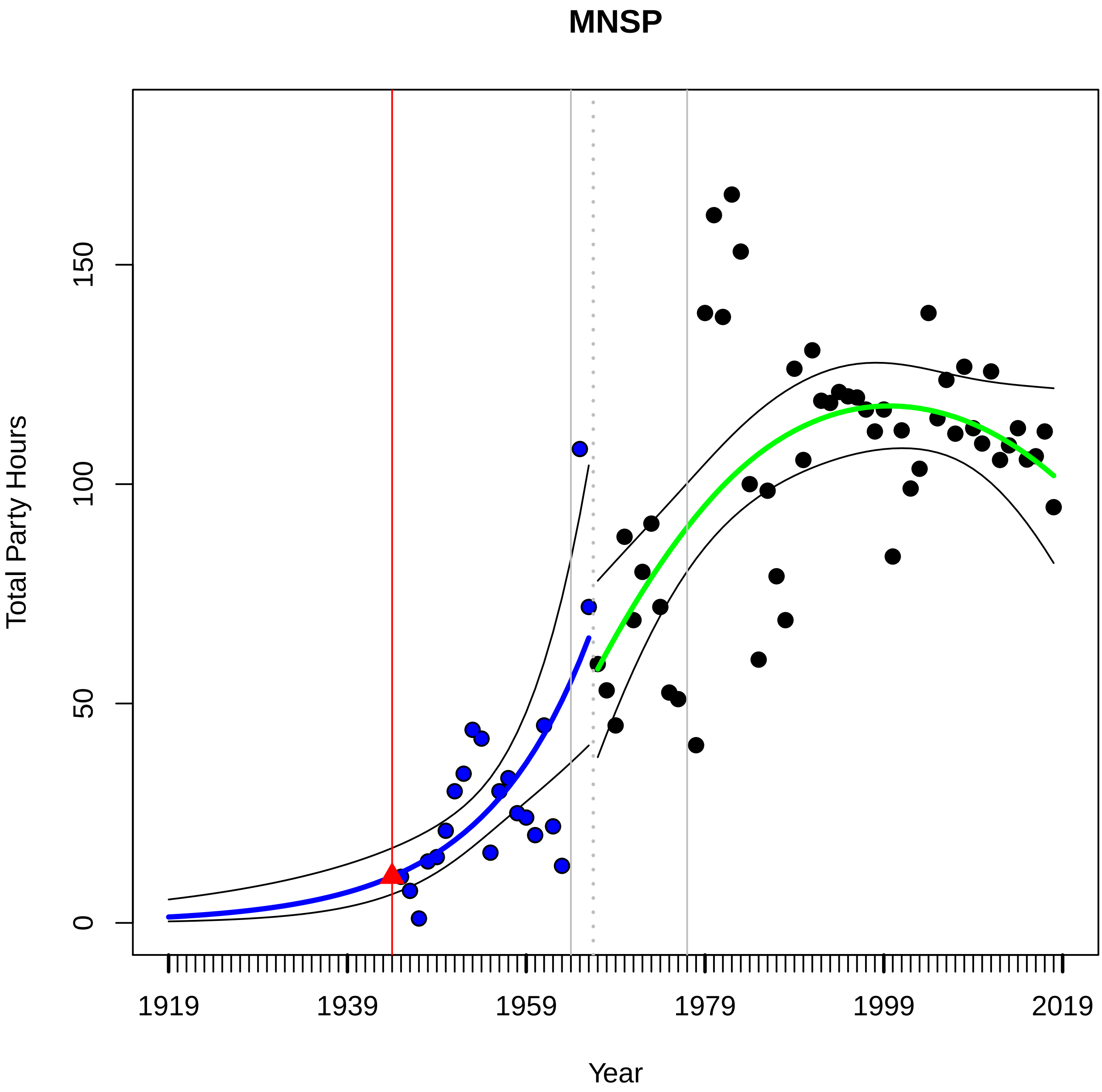

MNSP
Total Party Hours
0
50
100
150
1919
1939
1959
1979
1999
2019
Year

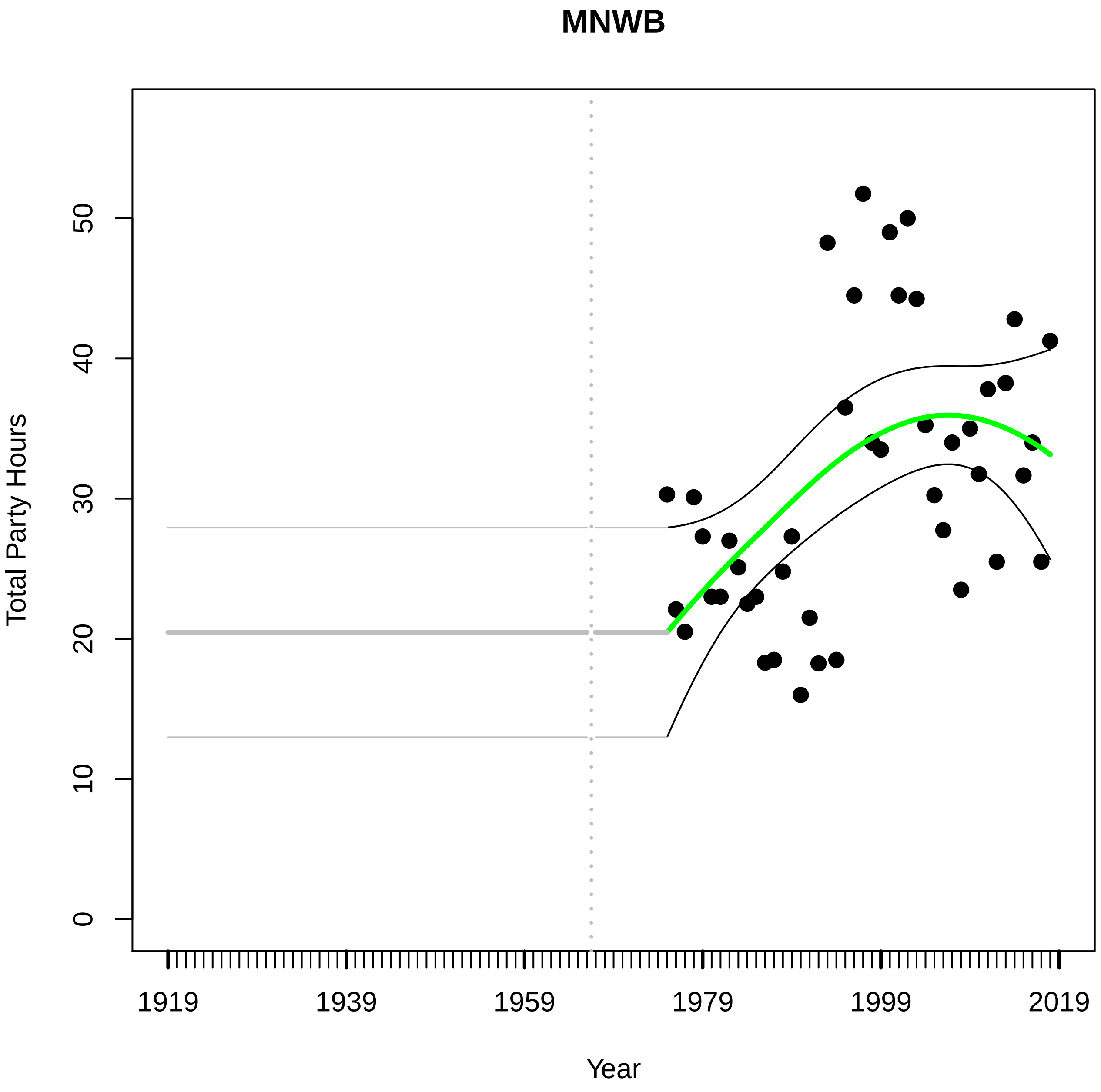
MNWB
Total Party Hours
0
10
20
30
40
50
1919
1939
1959
1979
1999
2019
Year

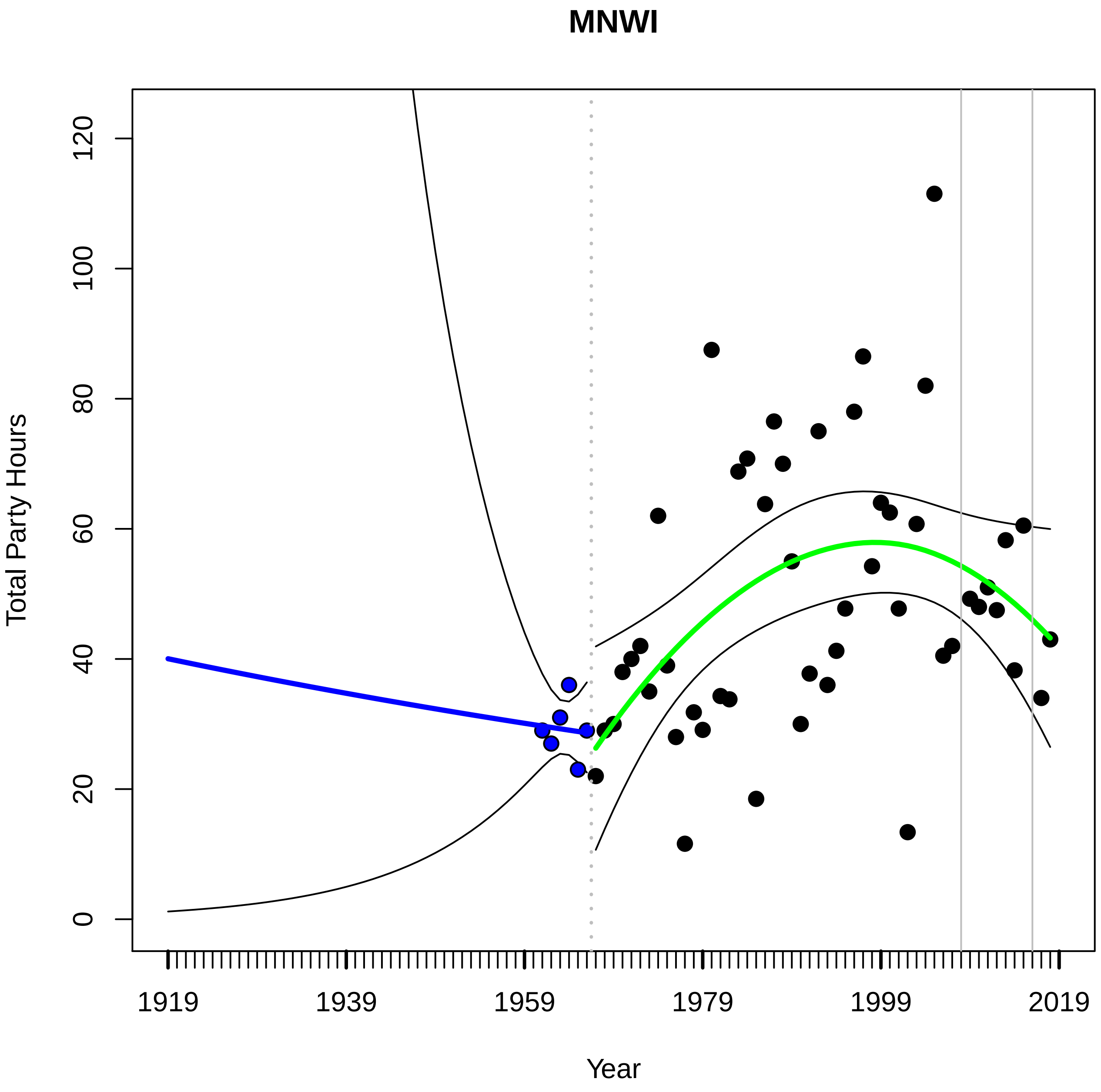

MNWI
Total Party Hours
0
20
40
60
80
100
120
1919
1939
1959
1979
1999
2019
Year

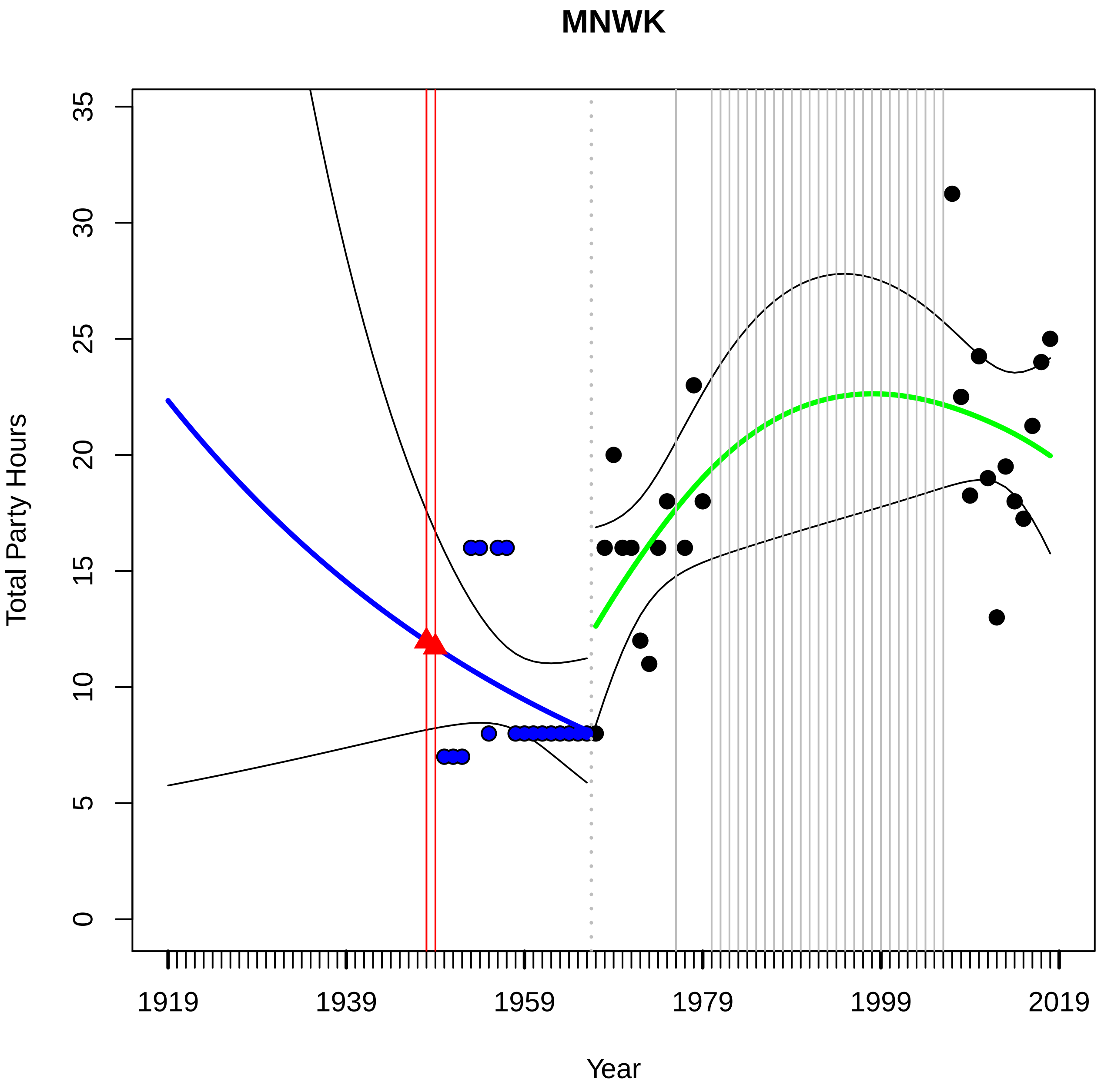
MNWK
Total Party Hours
Year
0
5
10
15
20
25
30
35
1919
1939
1959
1979
1999
2019

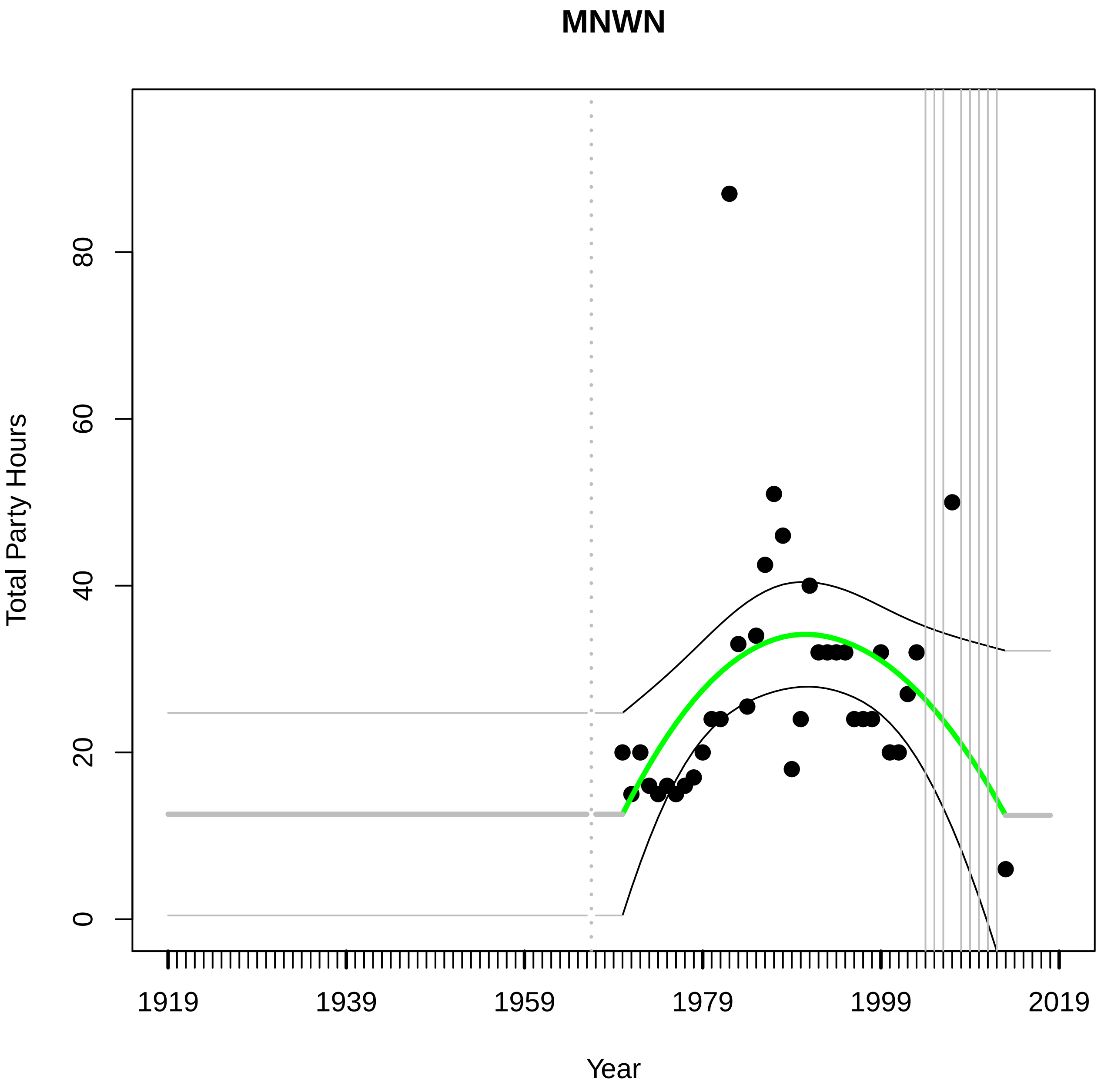

MNWN
Total Party Hours
0
20
40
60
80
1919
1939
1959
1979
1999
2019
Year

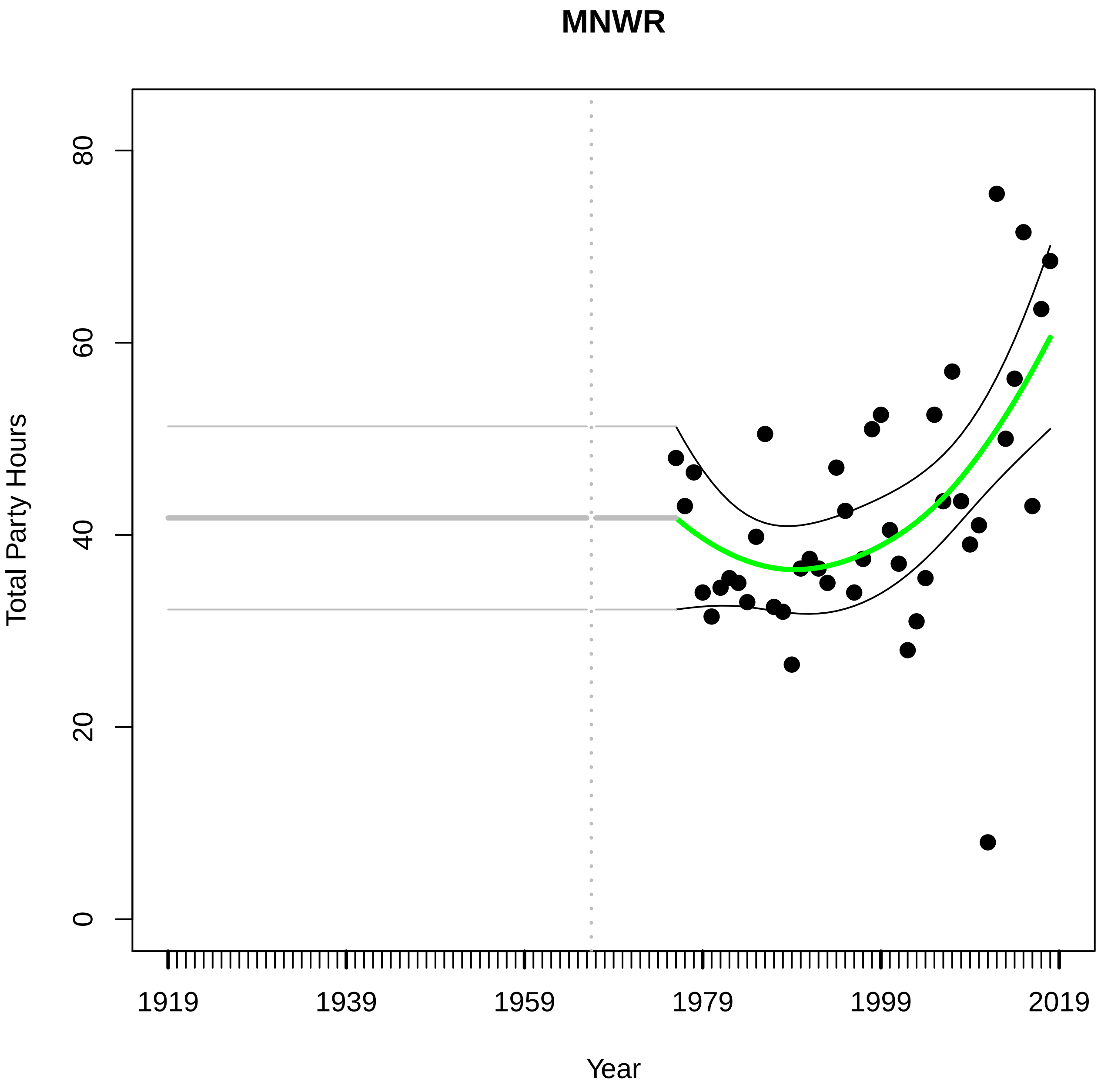

MNWR
Total Party Hours
0
20
40
60
80
1919
1939
1959
1979
1999
2019
Year

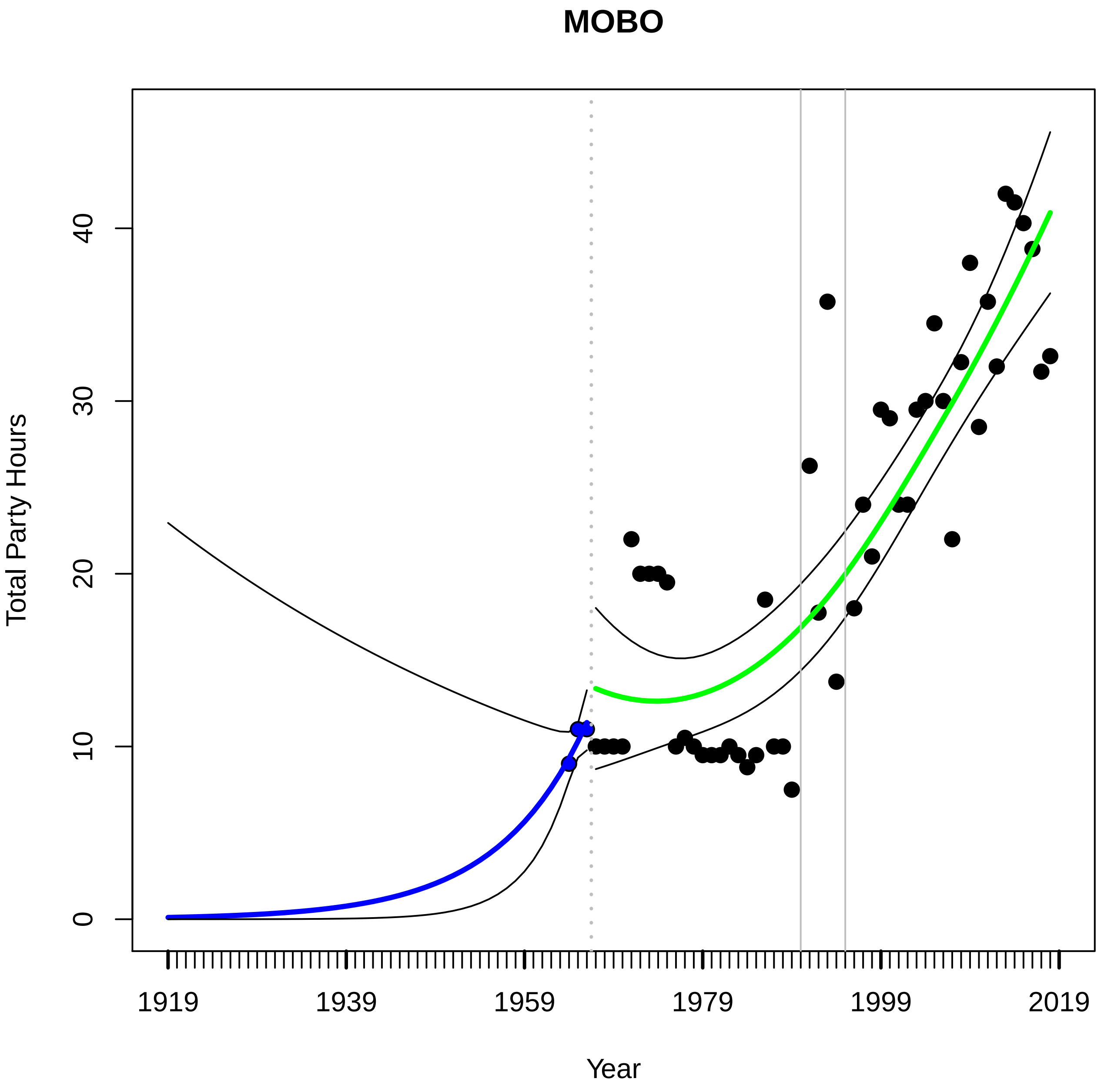

MOBO
Total Party Hours
Year
0
10
20
30
40
1919
1939
1959
1979
1999
2019

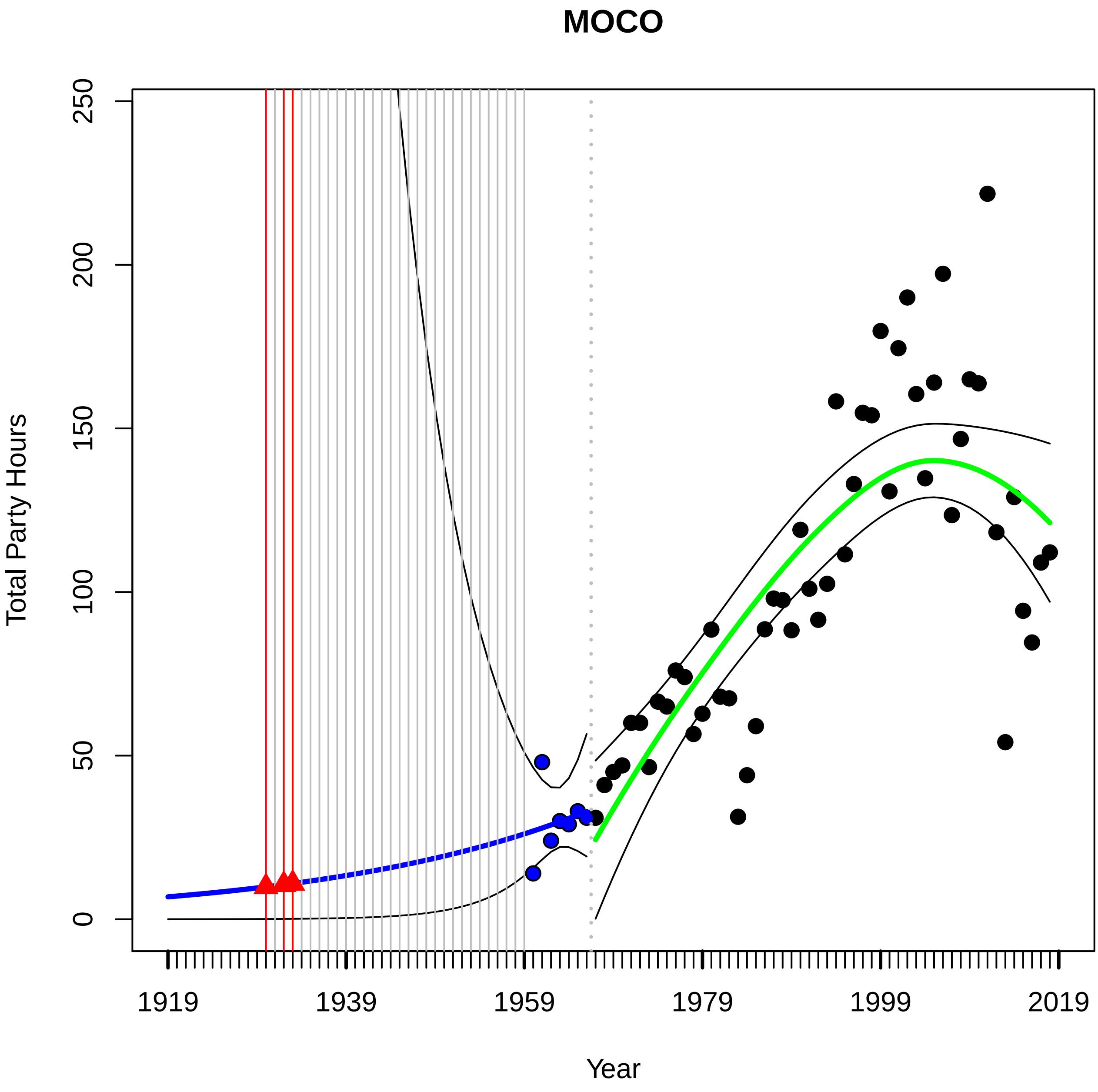

MOCO
Total Party Hours
0
50
100
150
200
250
1919
1939
1959
1979
1999
2019
Year

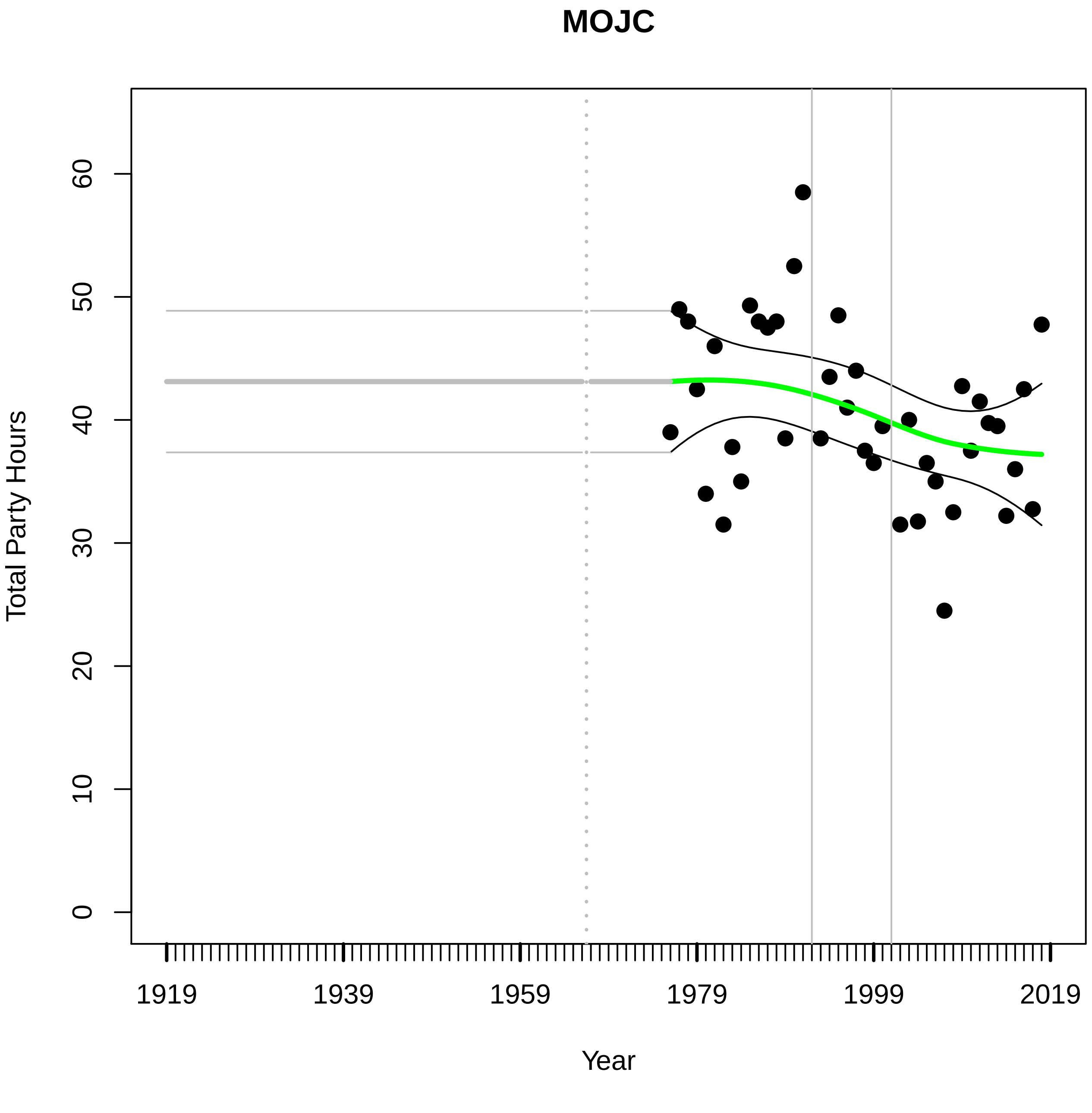

MOJC
Total Party Hours
Year
0
10
20
30
40
50
60
1919
1939
1959
1979
1999
2019

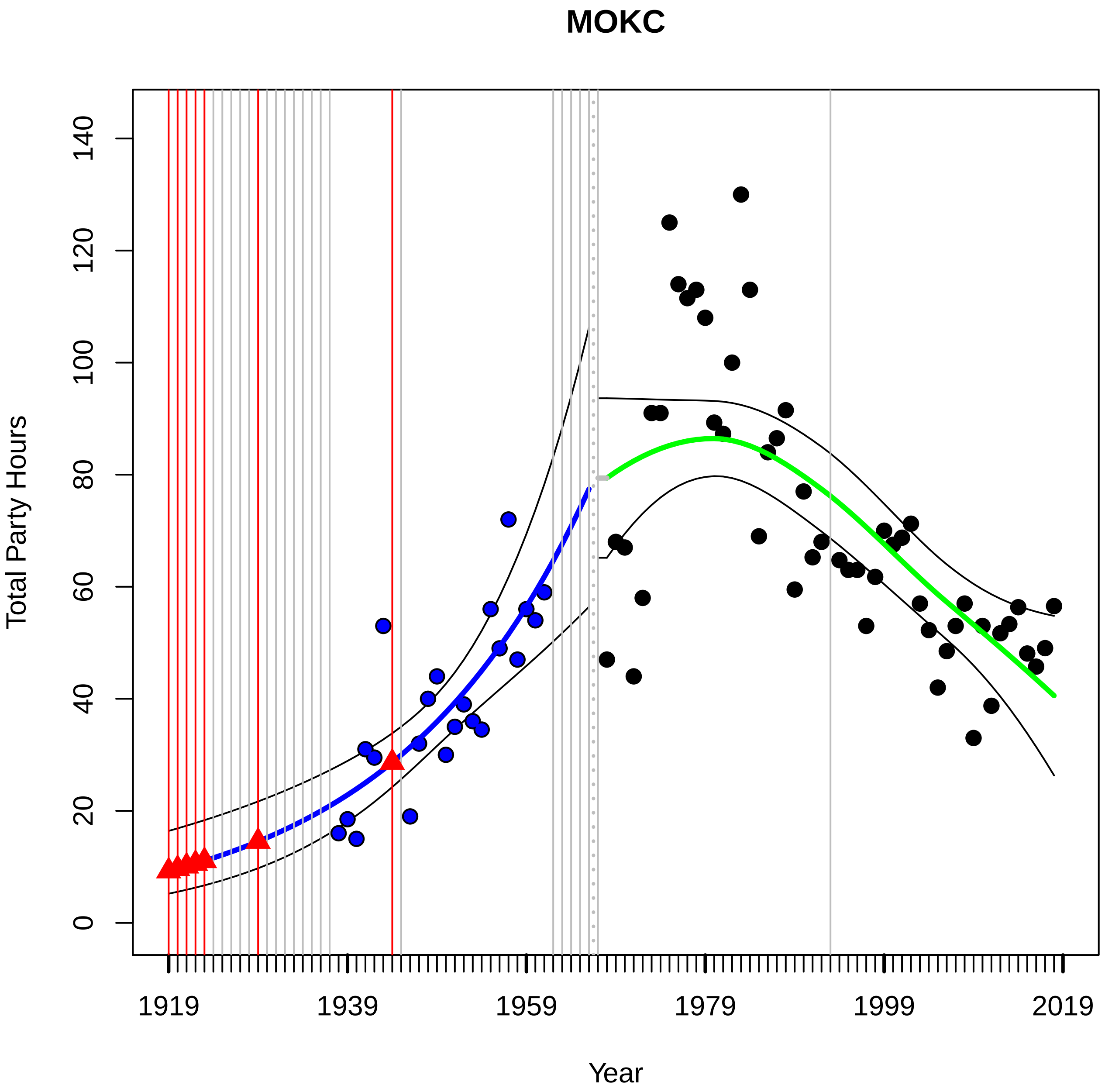

MOKC
Total Party Hours
Year
0
20
40
60
80
100
120
140
1919
1939
1959
1979
1999
2019

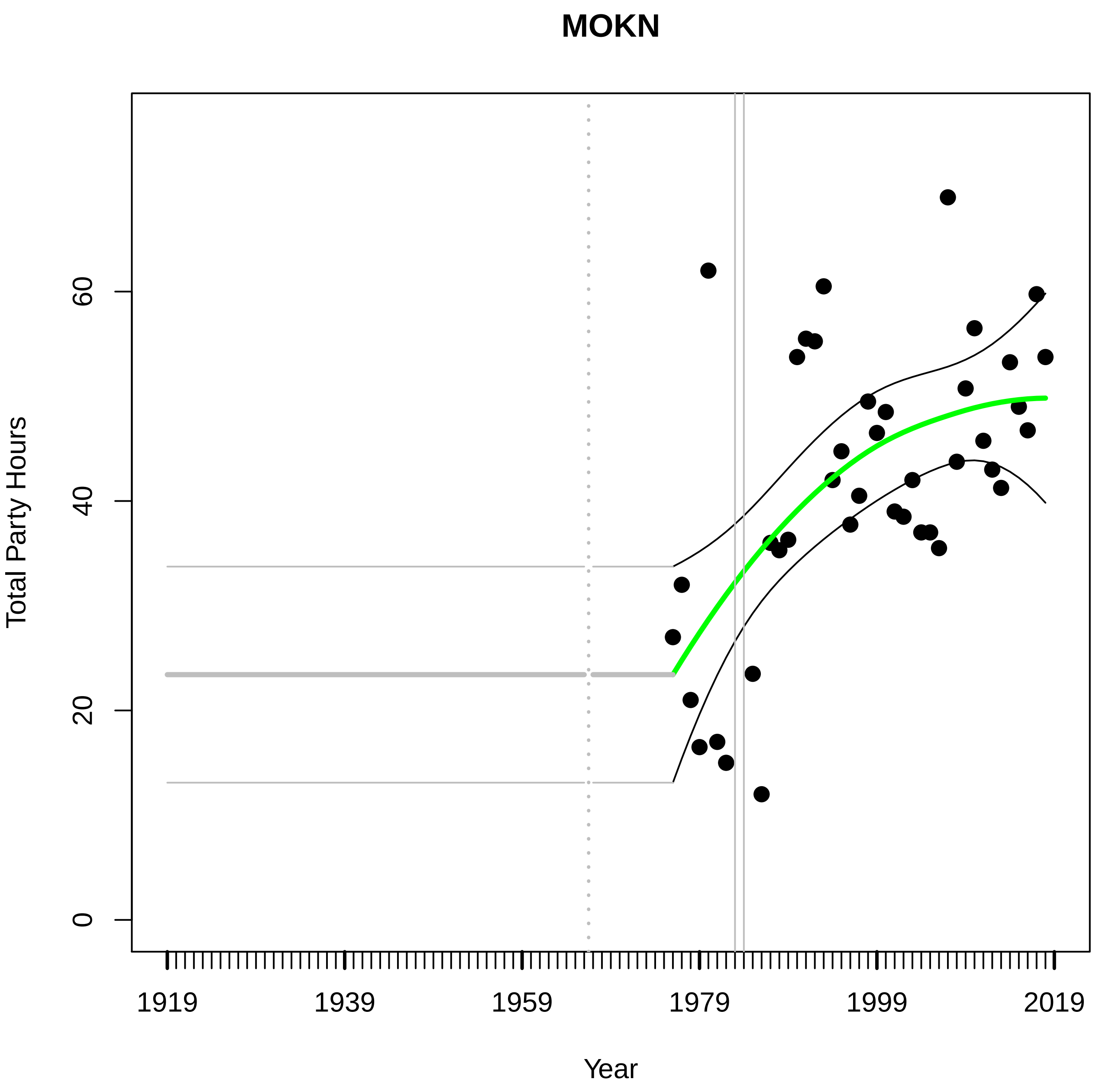

MOKN
Total Party Hours
0
20
40
60
1919
1939
1959
1979
1999
2019
Year

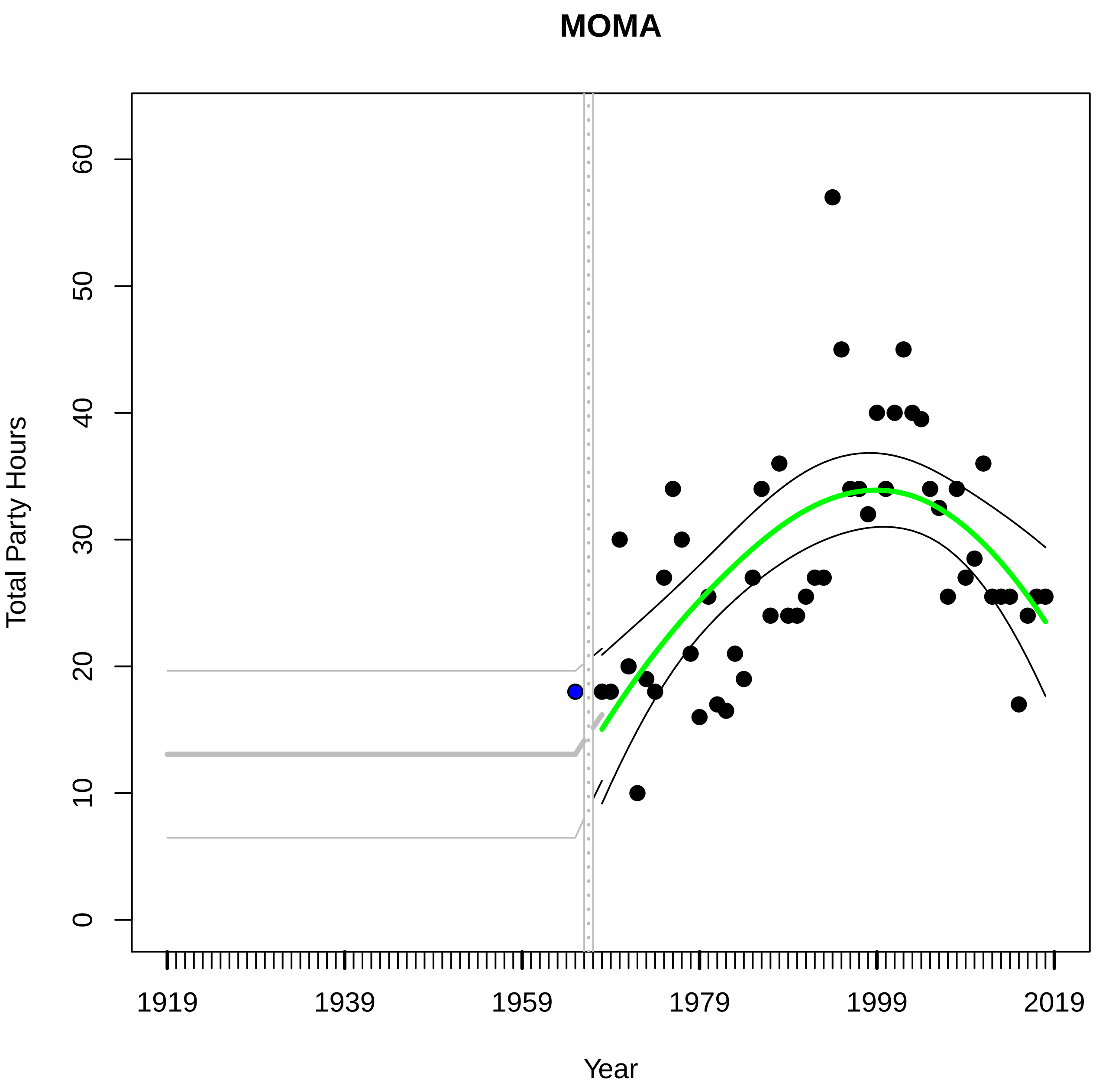
MOMA
Total Party Hours
0
10
20
30
40
50
60
1919
1939
1959
1979
1999
2019
Year

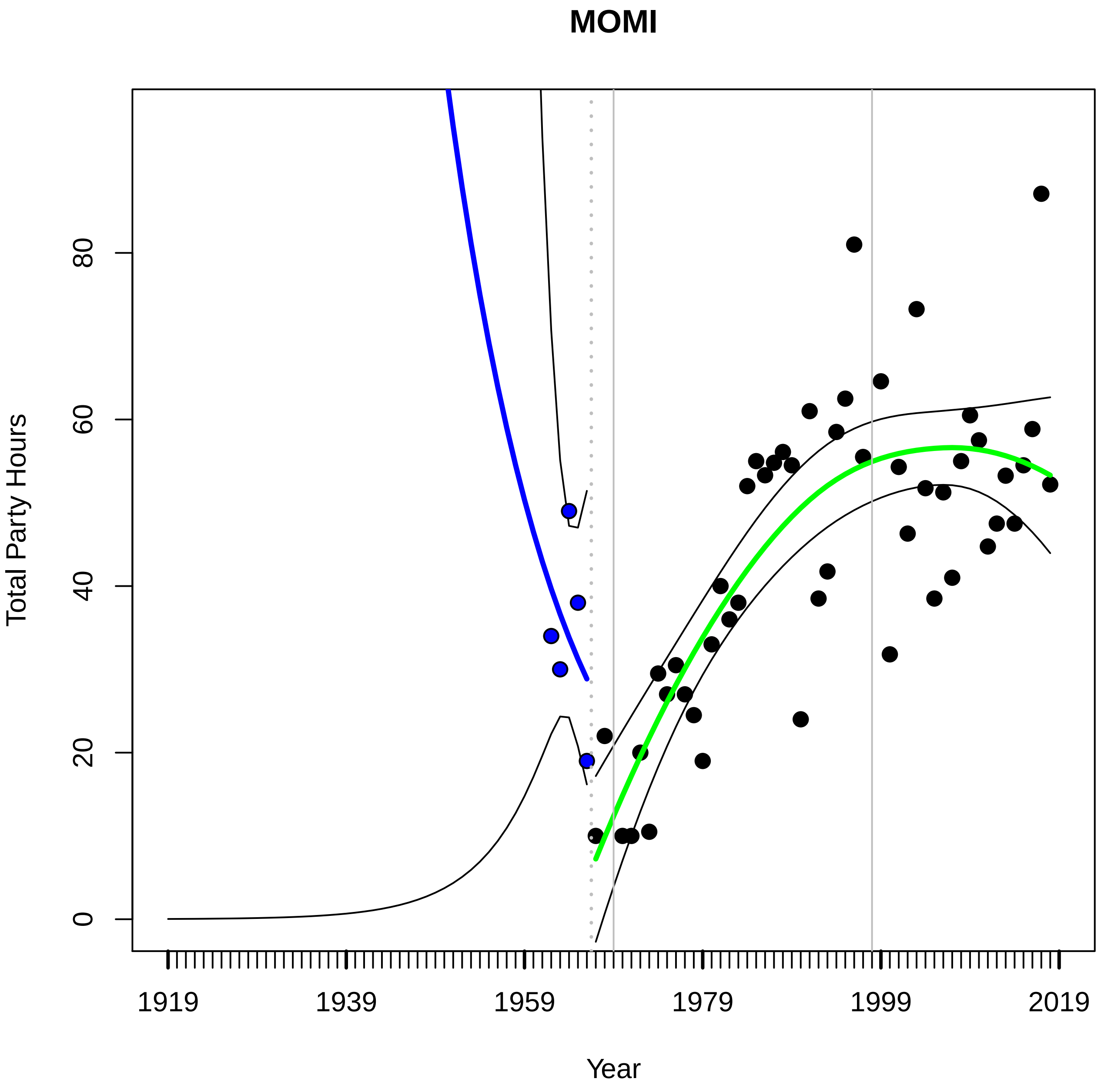

MOMI
Total Party Hours
0
20
40
60
80
Year
1919
1939
1959
1979
1999
2019

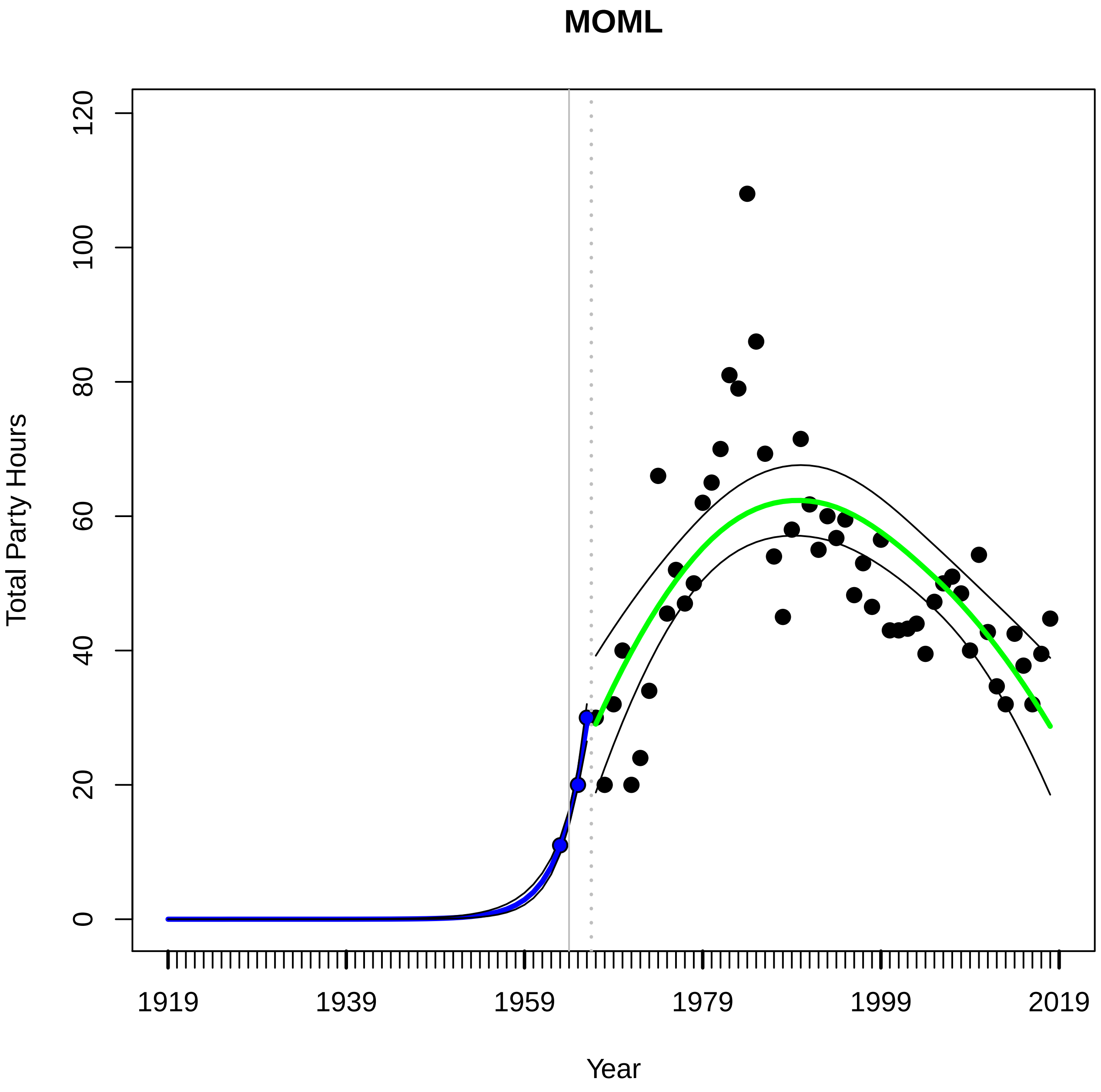
MOML
Total Party Hours
0
20
40
60
80
100
120
1919
1939
1959
1979
1999
2019
Year

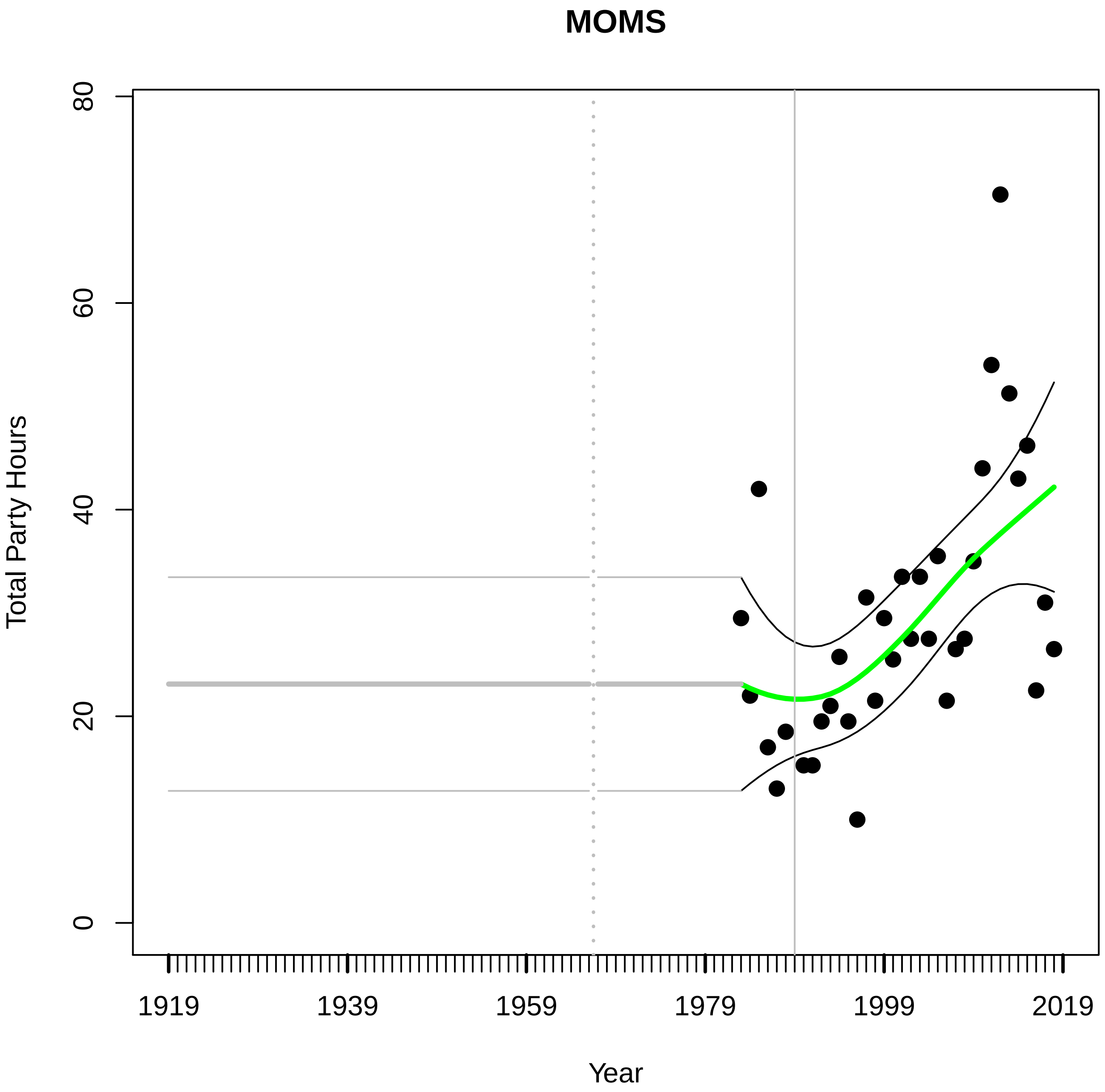
MOMS
Total Party Hours
0
20
40
60
80
1919
1939
1959
1979
1999
2019
Year

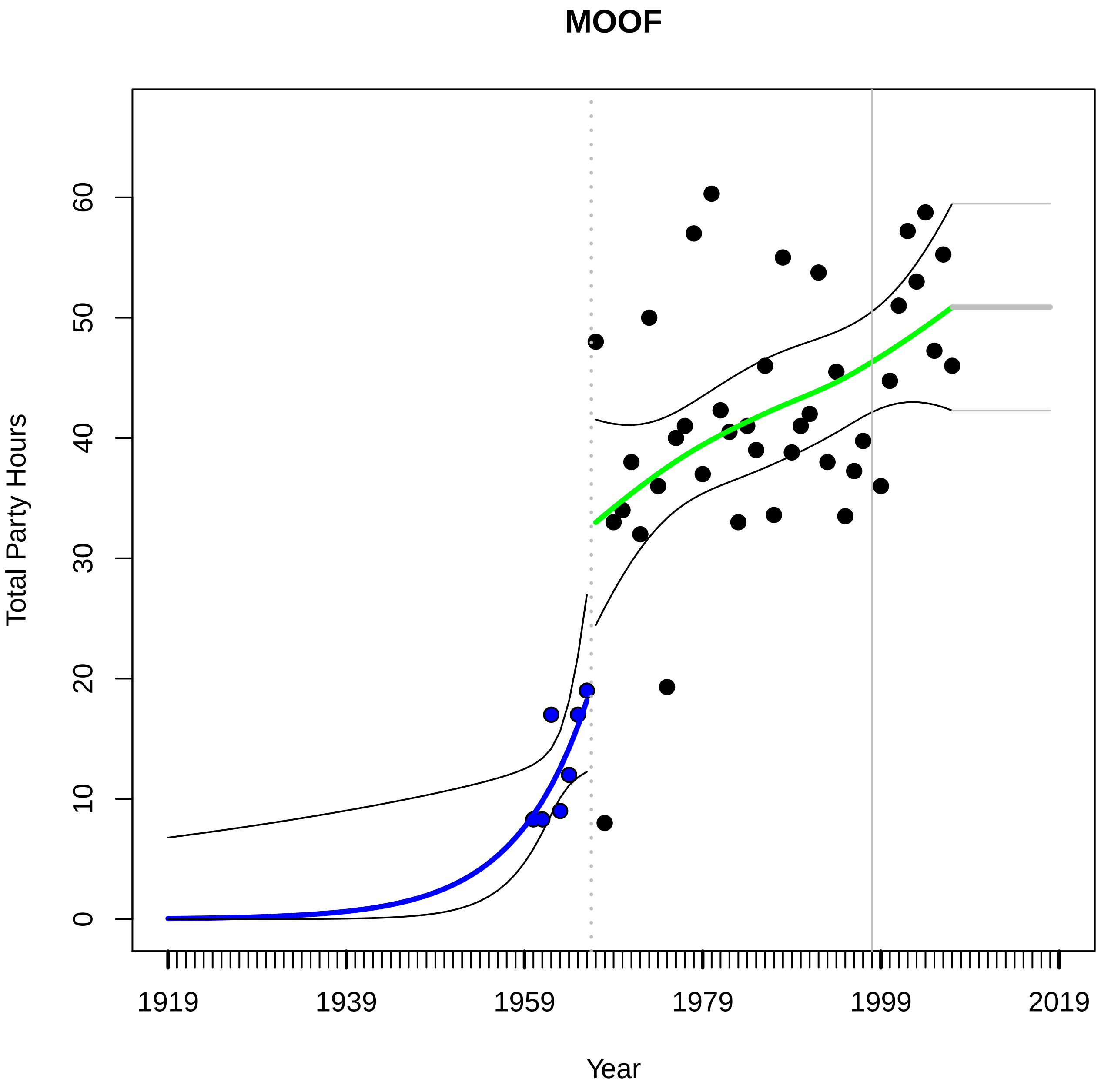
MOOF
Total Party Hours
0
10
20
30
40
50
60
1919
1939
1959
1979
1999
2019
Year

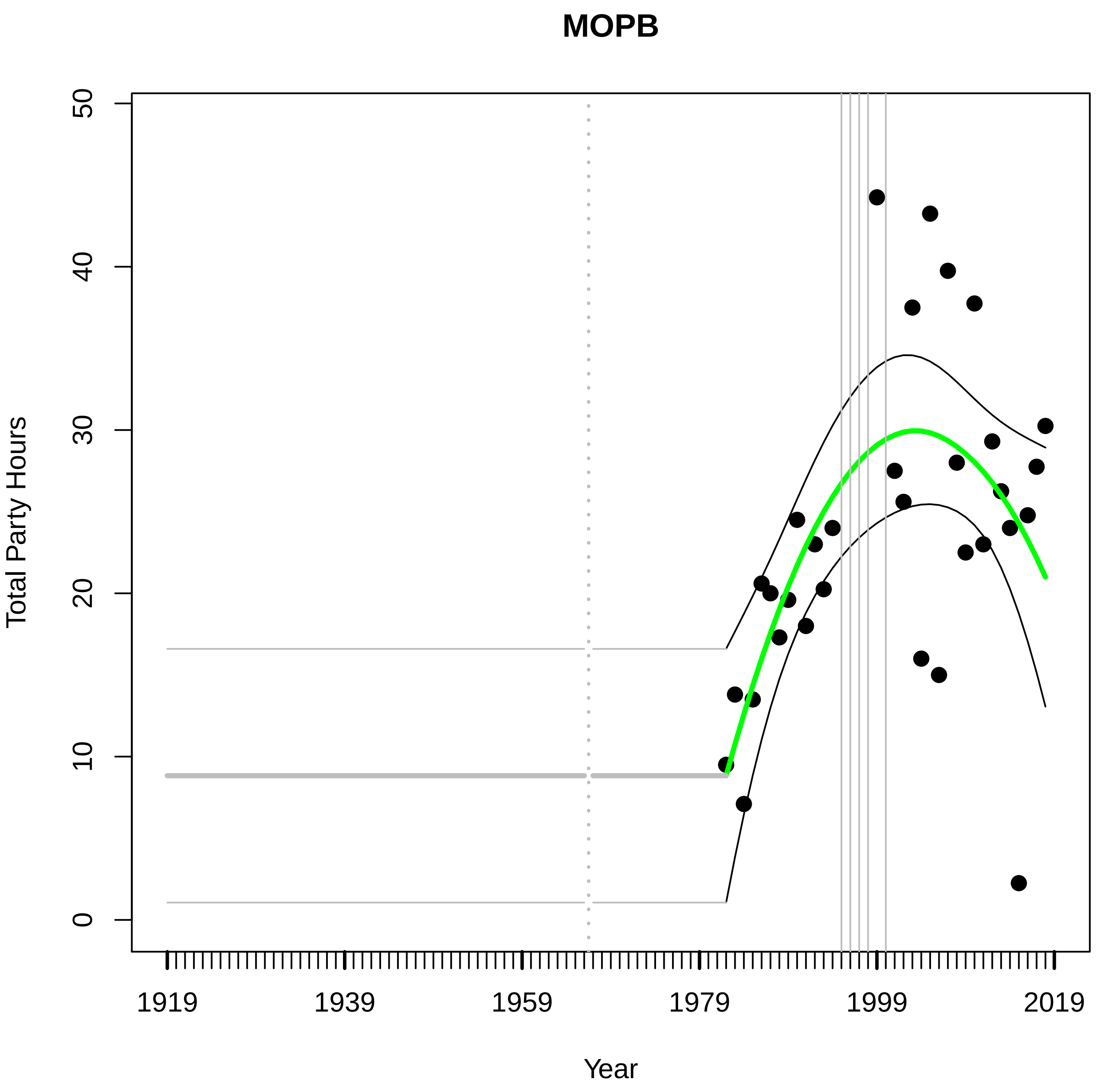
MOPB
Total Party Hours
Year
0
10
20
30
40
50
1919
1939
1959
1979
1999
2019

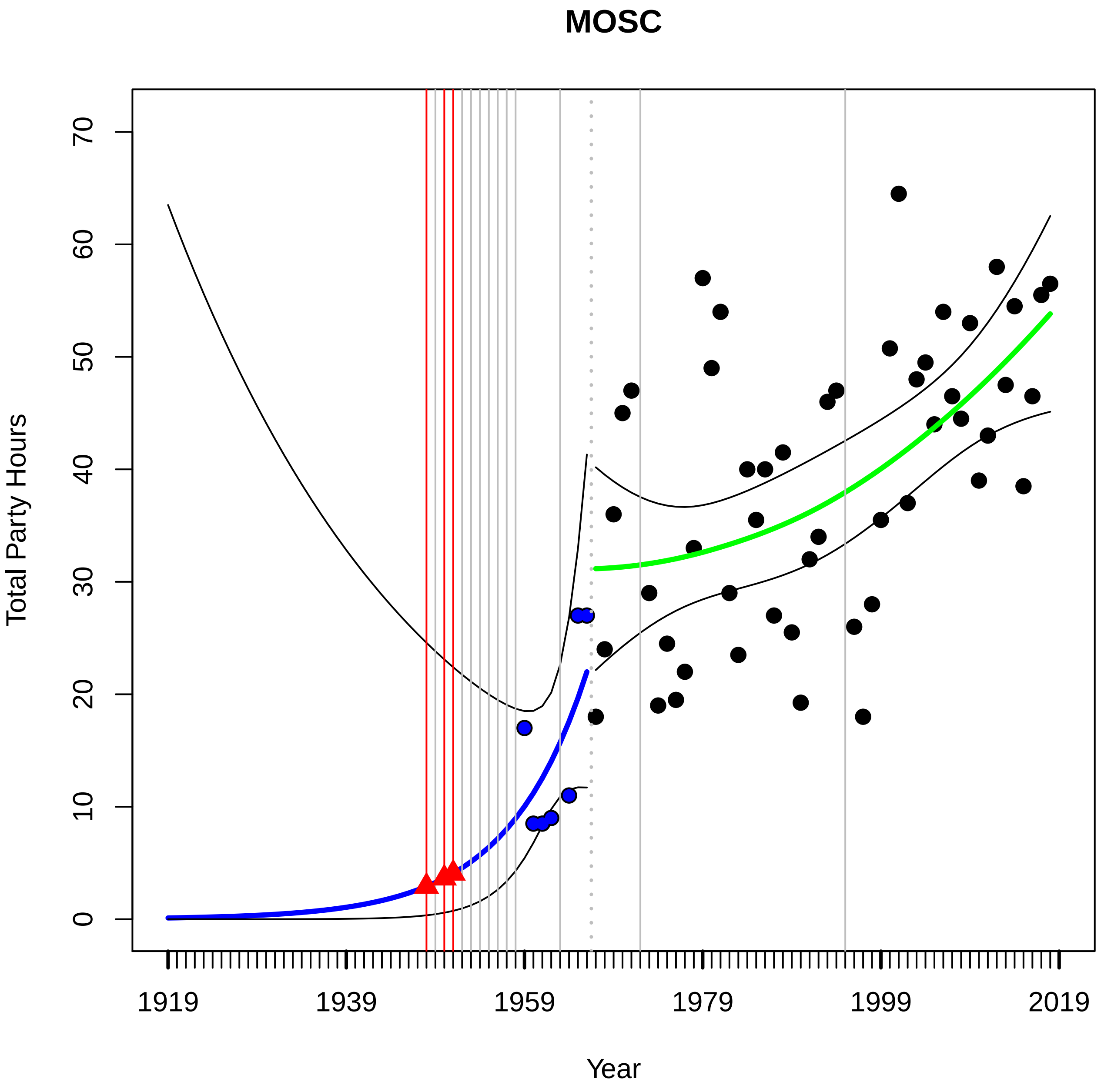

MOSC
Total Party Hours
Year
0
10
20
30
40
50
60
70
1919
1939
1959
1979
1999
2019

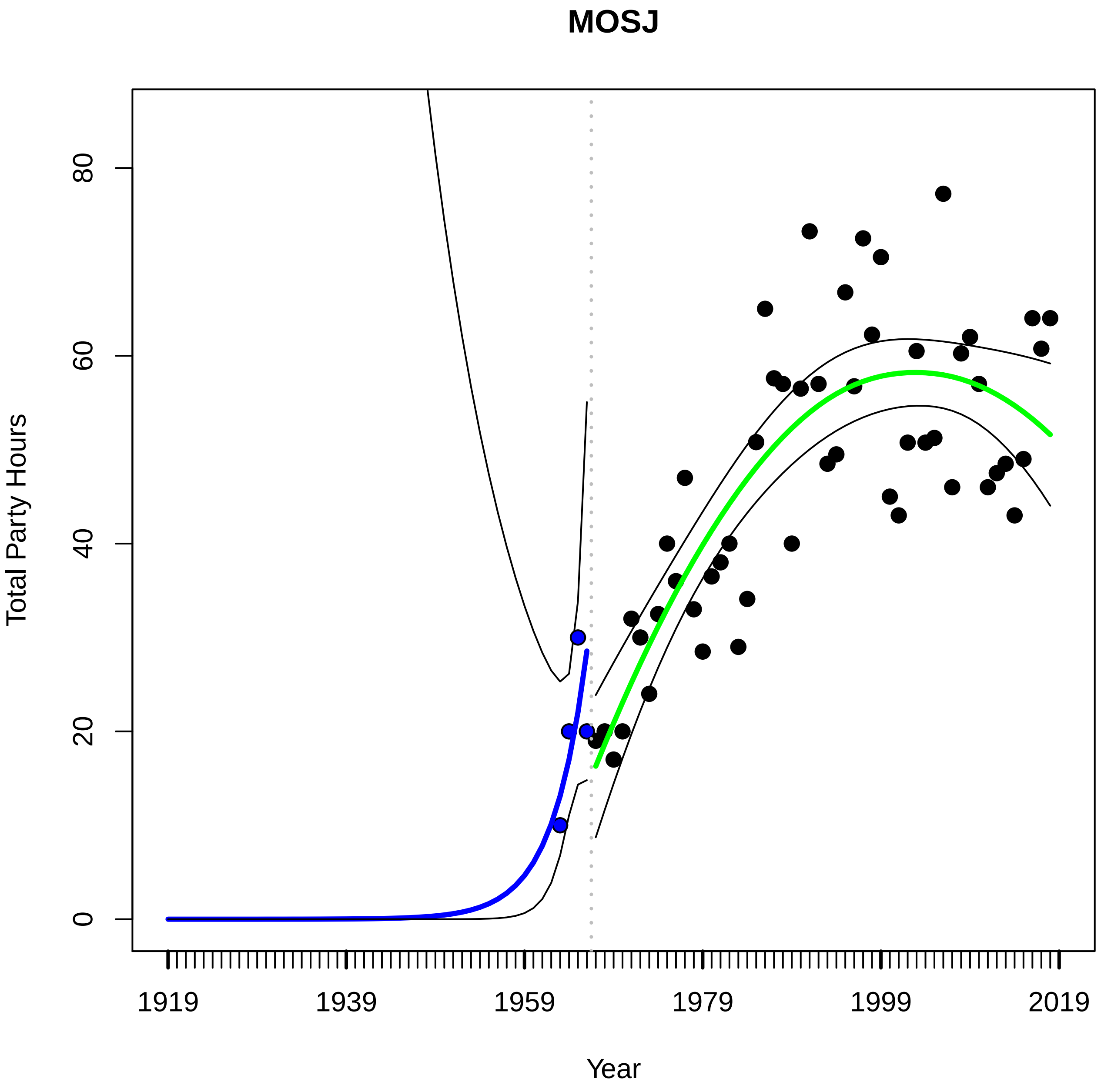

MOSJ
Total Party Hours
0
20
40
60
80
1919
1939
1959
1979
1999
2019
Year

MOSL

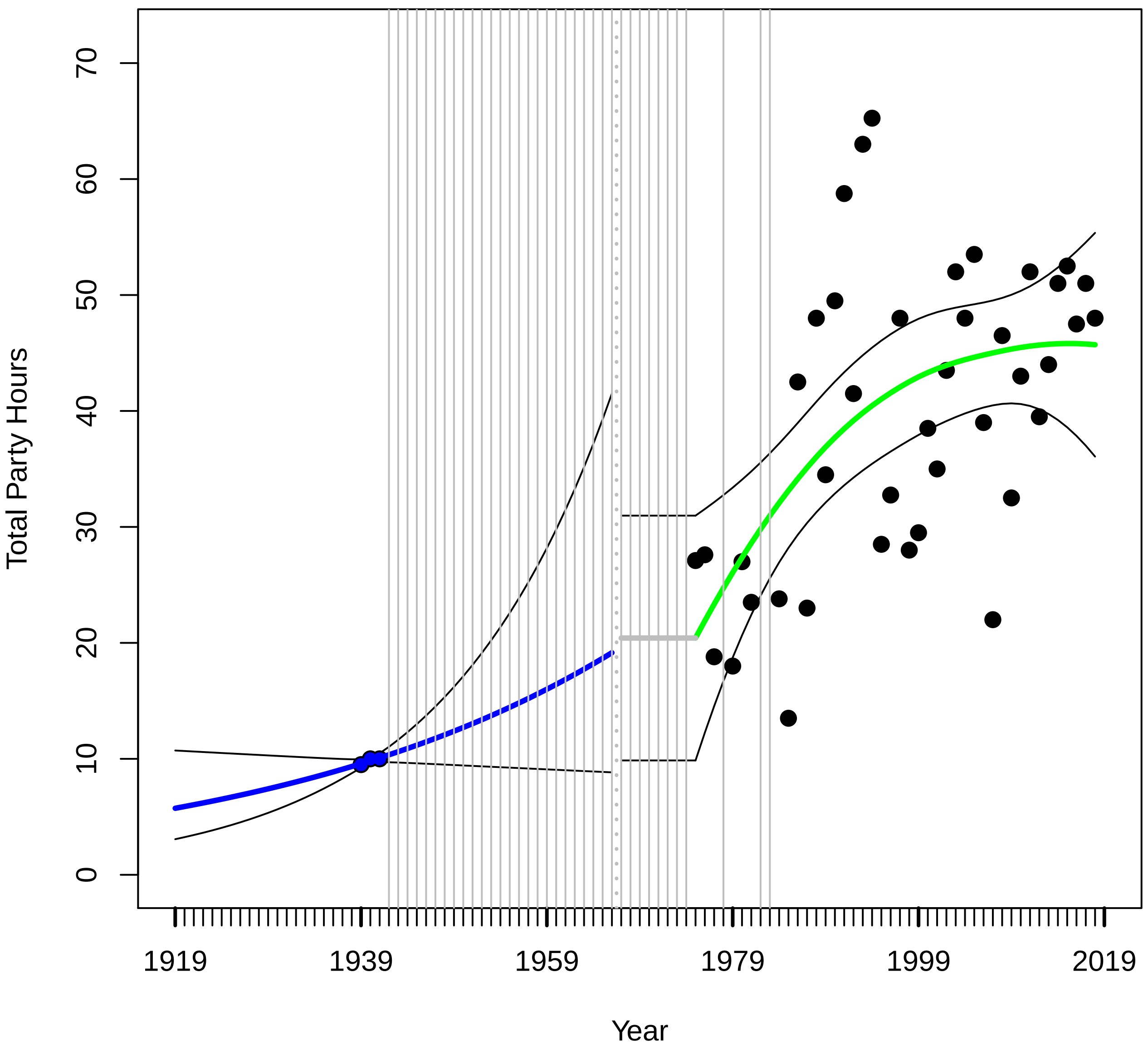

Total Party Hours
0
10
20
30
40
50
60
70
1919
1939
1959
1979
1999
2019
Year

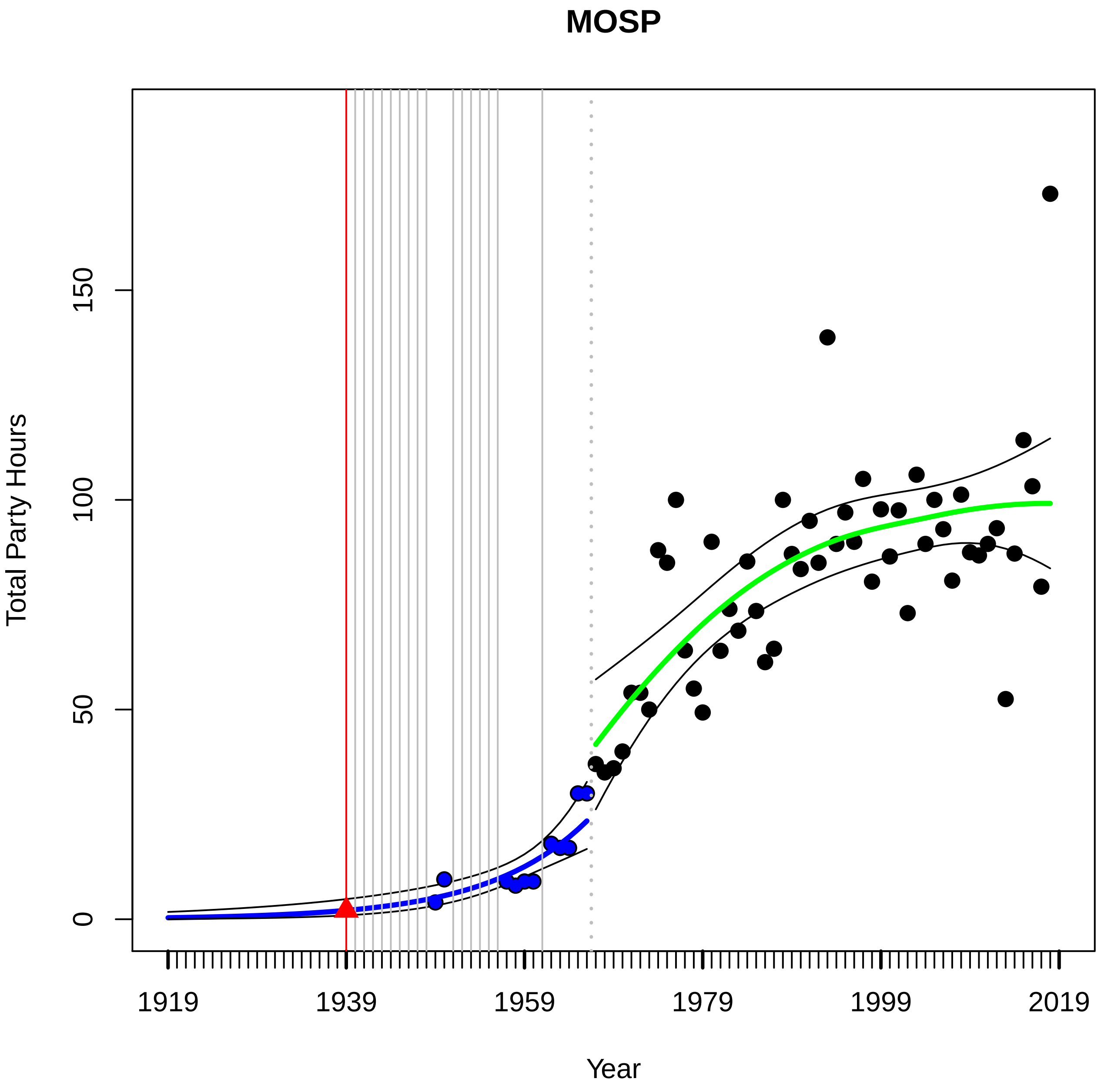
MOSP
Total Party Hours
0
50
100
150
1919
1939
1959
1979
1999
2019
Year

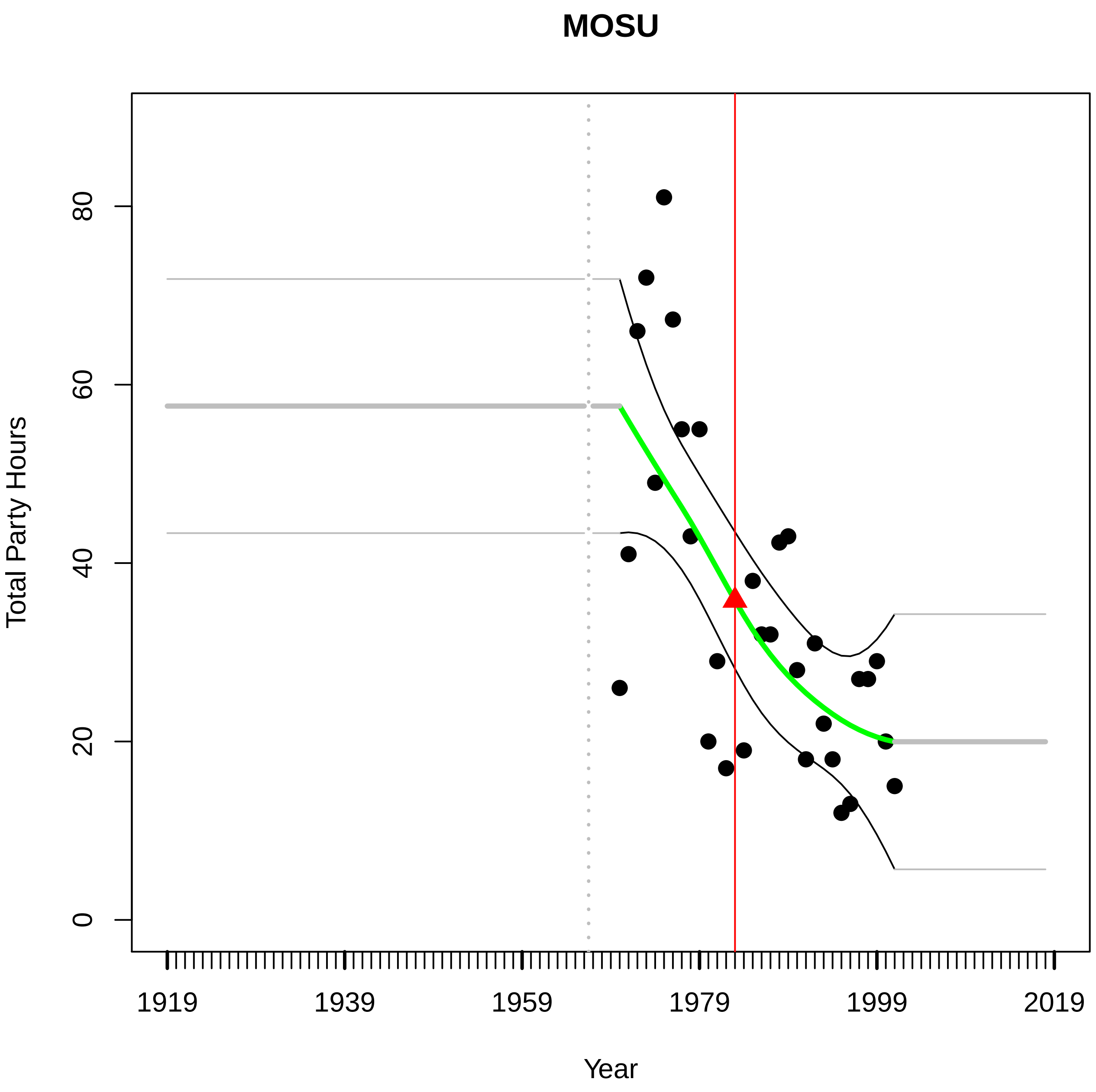
MOSU
Total Party Hours
0
20
40
60
80
1919
1939
1959
1979
1999
2019
Year

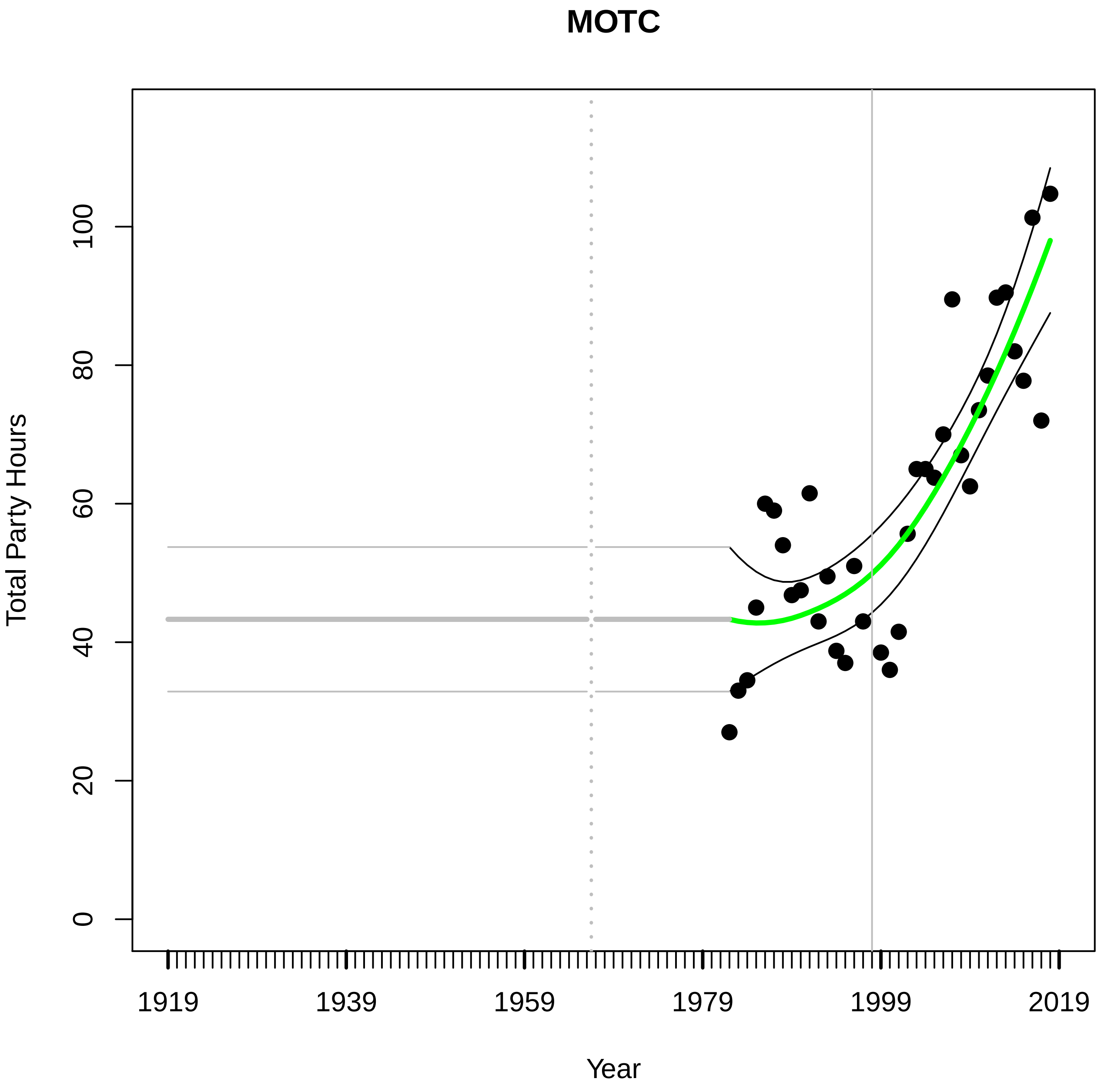

MOTC
Total Party Hours
0
20
40
60
80
100
1919
1939
1959
1979
1999
2019
Year

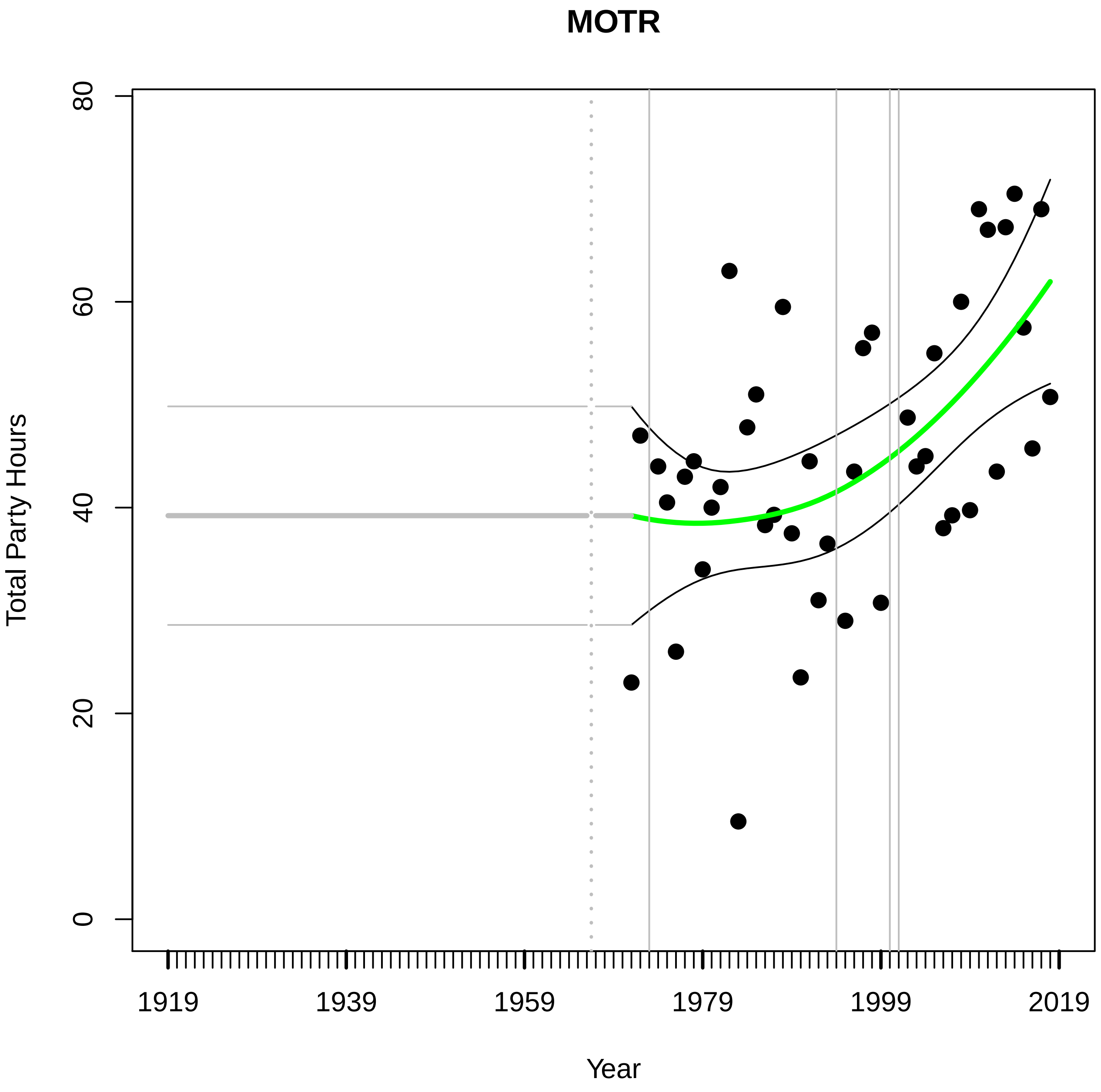
MOTR
Total Party Hours
0
20
40
60
80
1919
1939
1959
1979
1999
2019
Year

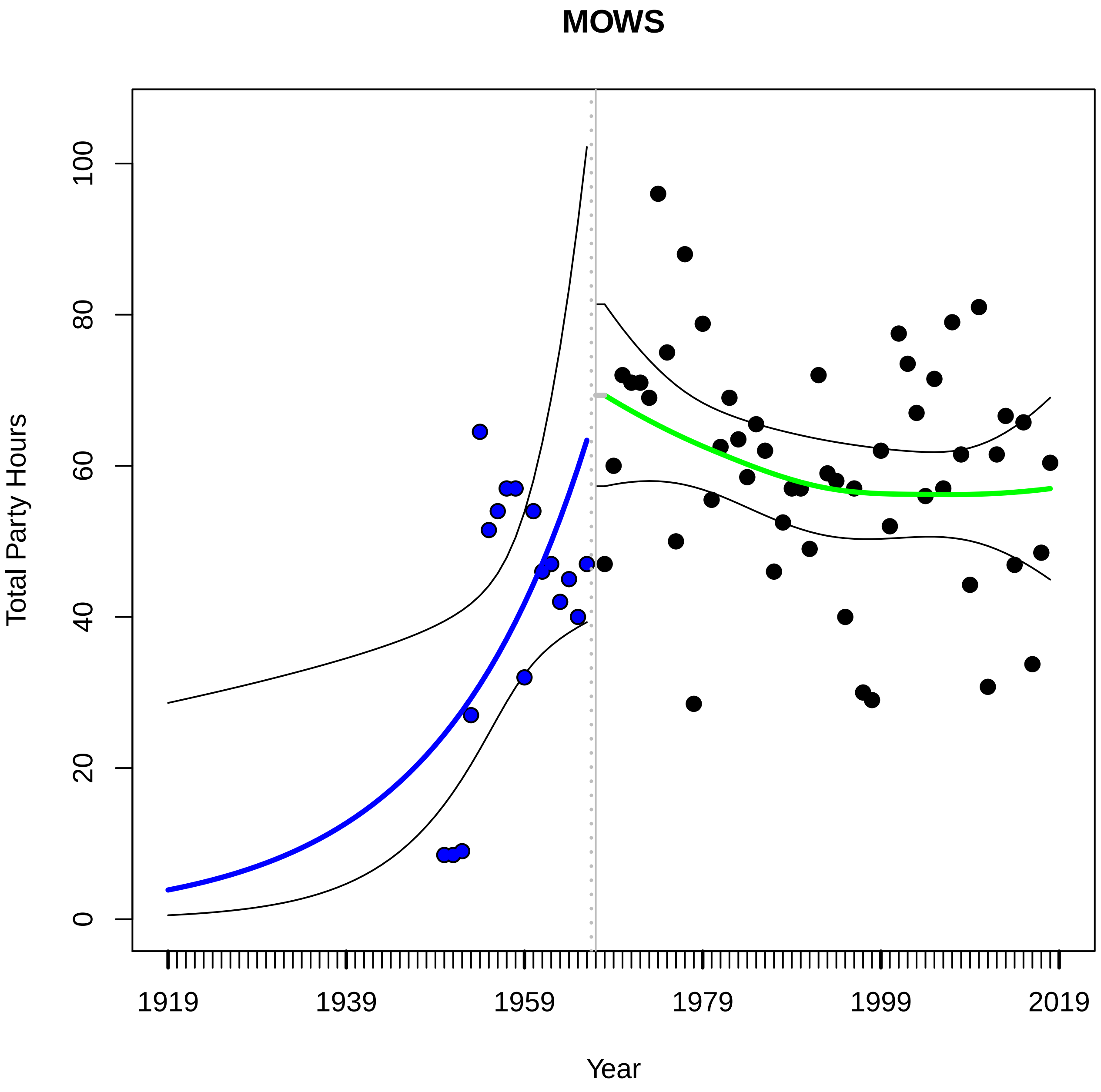

MOWS
Total Party Hours
0
20
40
60
80
100
1919
1939
1959
1979
1999
2019
Year

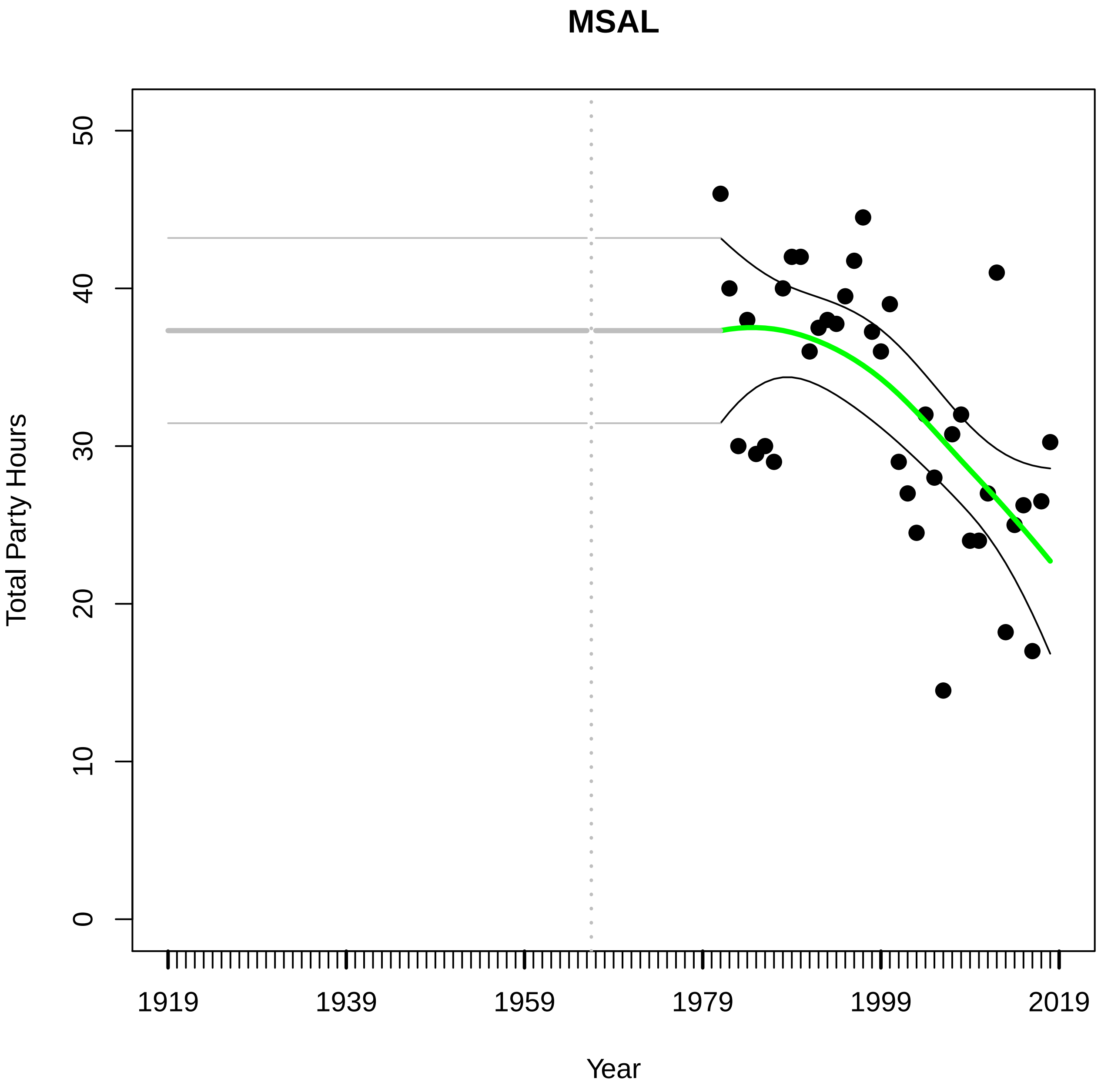
MSAL
Total Party Hours
0
10
20
30
40
50
1919
1939
1959
1979
1999
2019
Year

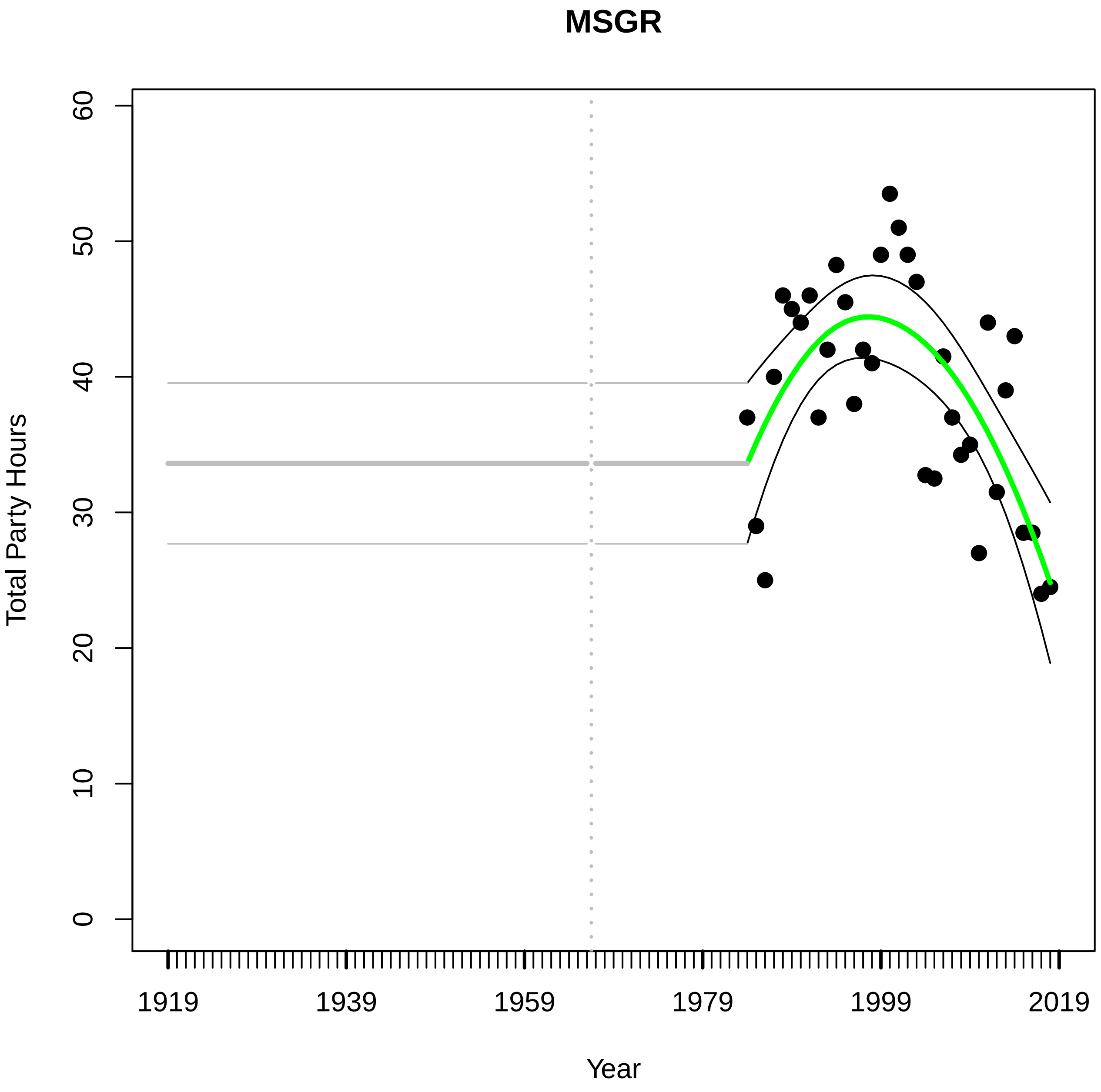

MSGR
Total Party Hours
0
10
20
30
40
50
60
1919
1939
1959
1979
1999
2019
Year

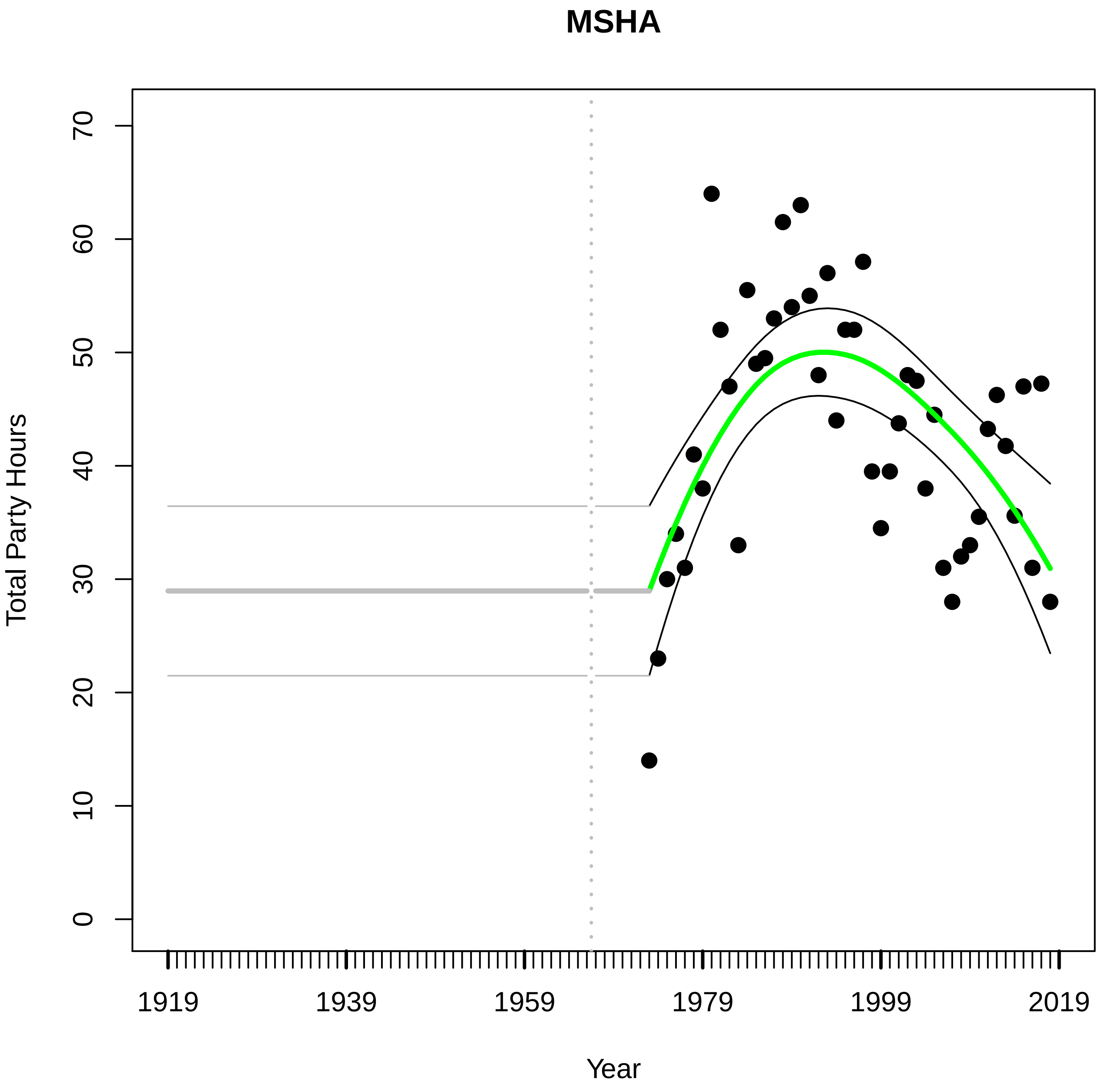

MSHA
Total Party Hours
0
10
20
30
40
50
60
70
1919
1939
1959
1979
1999
2019
Year

MSJA

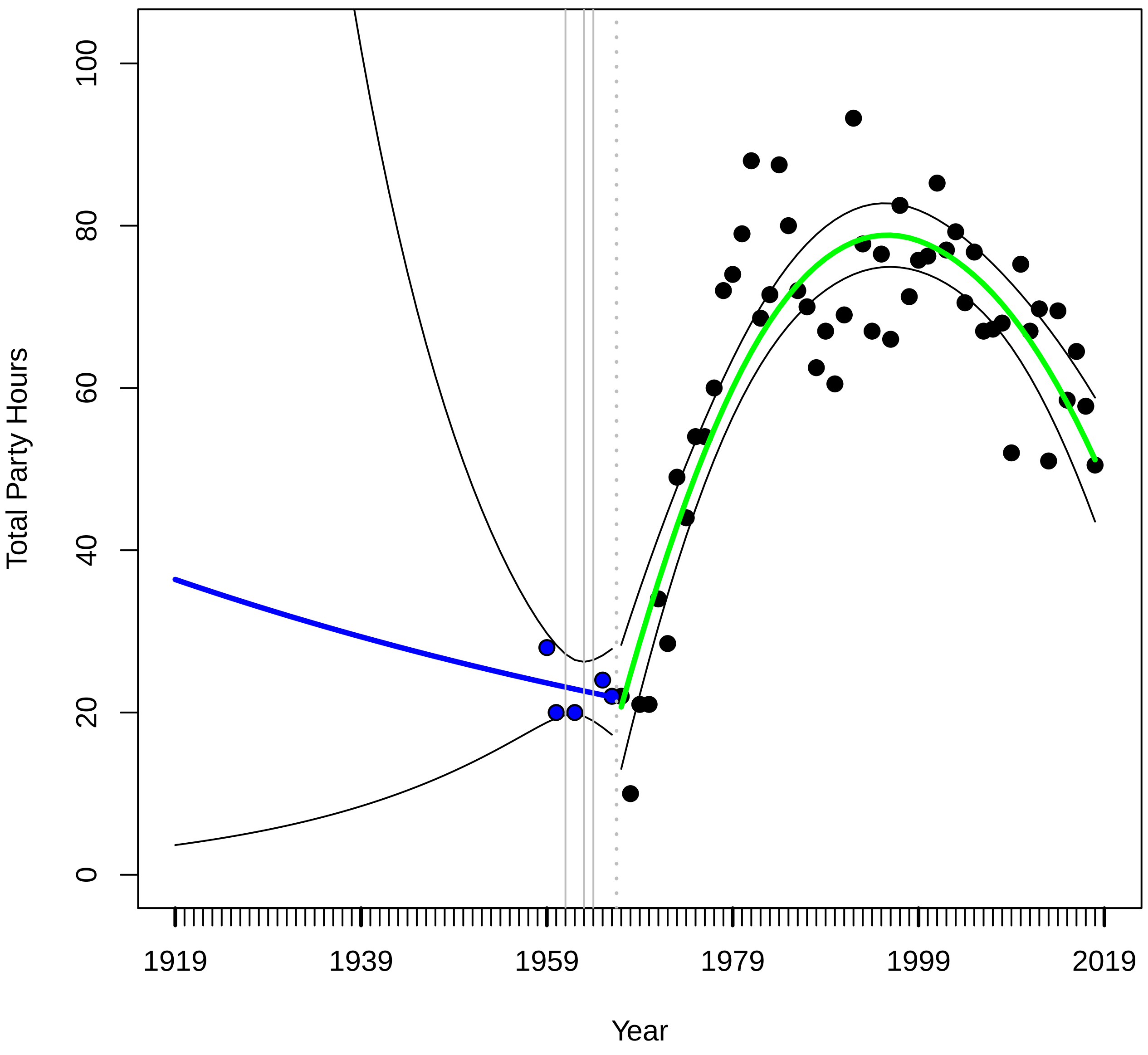

Total Party Hours
0
20
40
60
80
100
1919
1939
1959
1979
1999
2019
Year

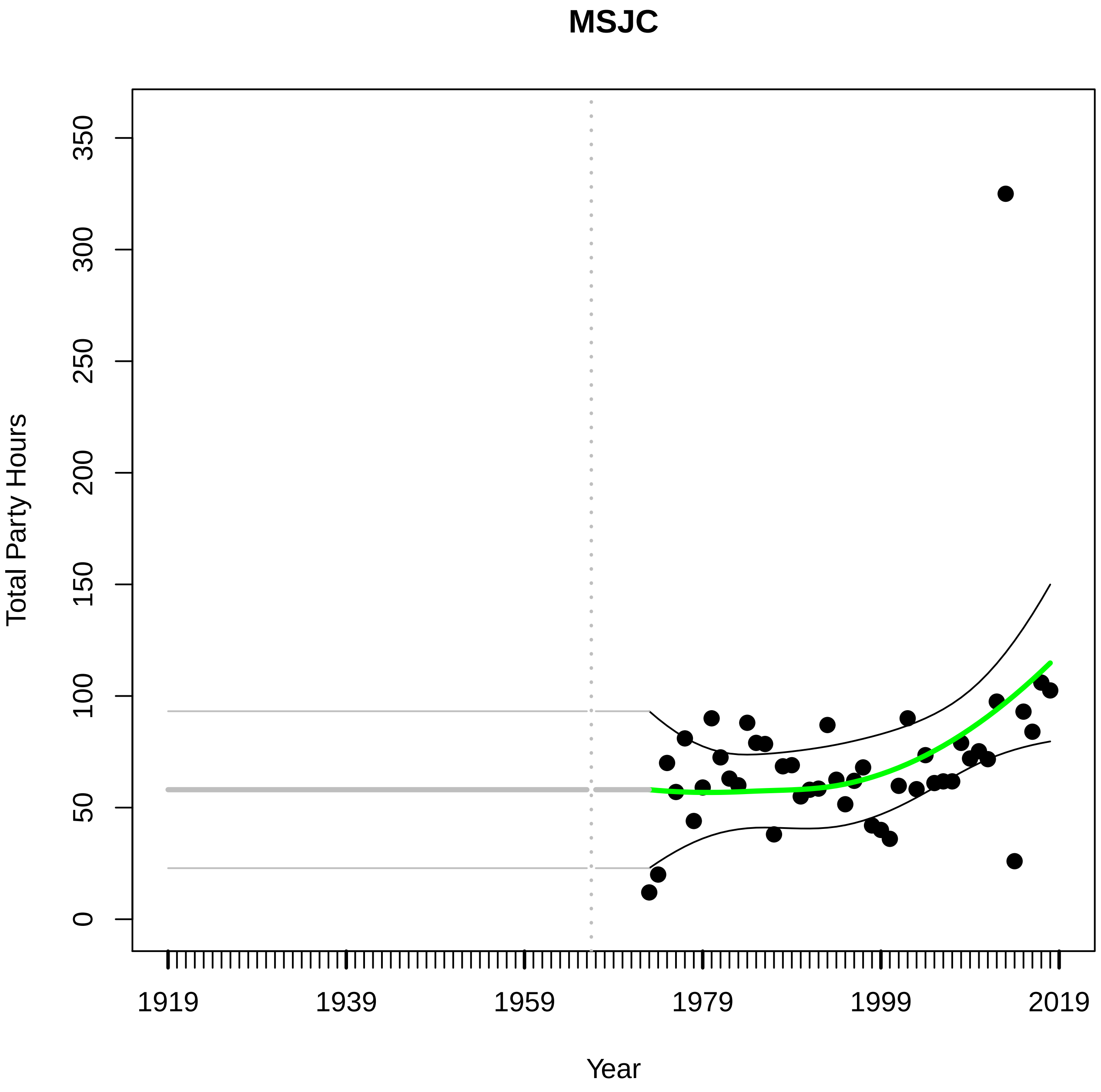
MSJC
Total Party Hours
0
50
100
150
200
250
300
350
1919
1939
1959
1979
1999
2019
Year

MSML

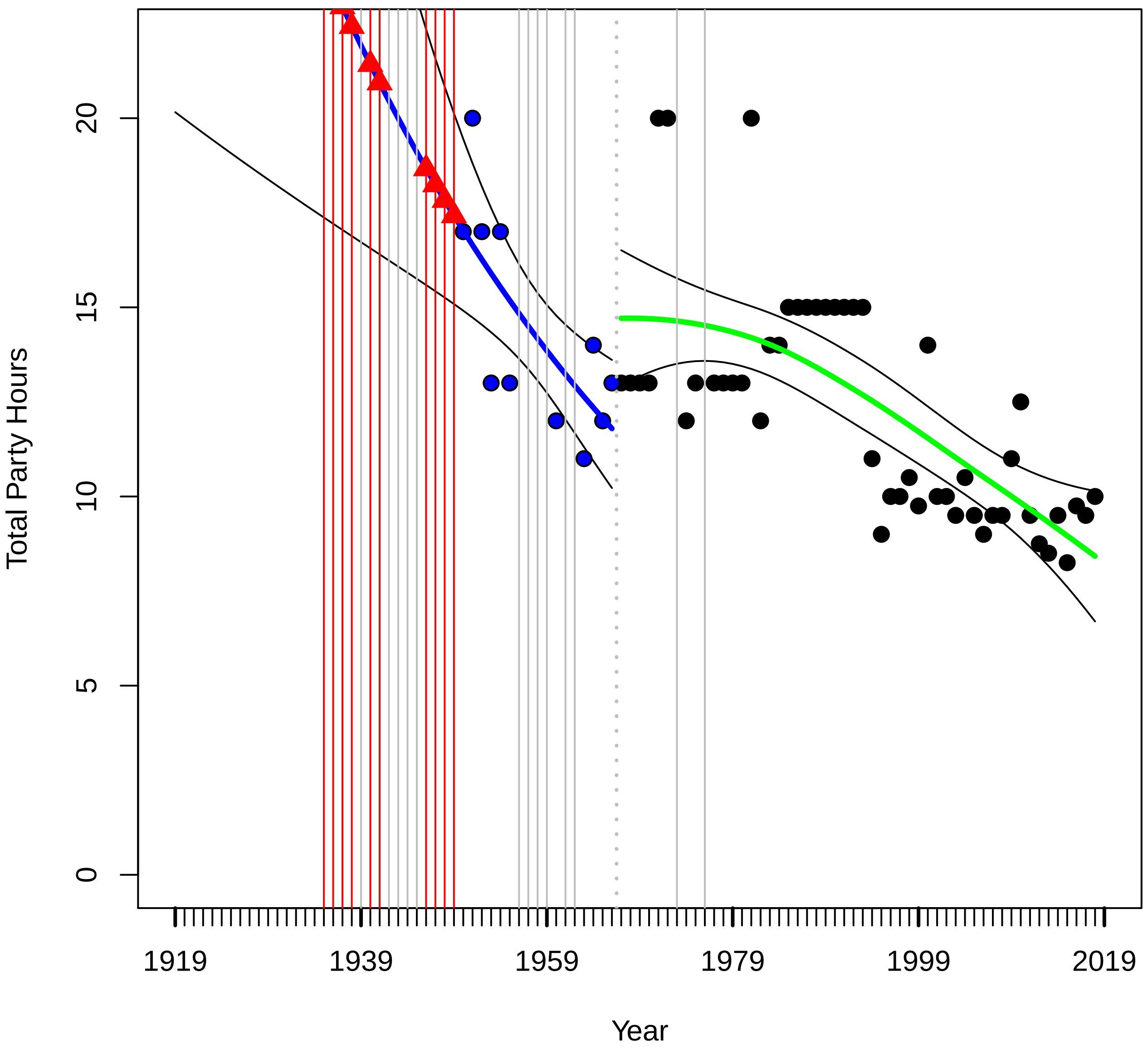

Total Party Hours
Year
0
5
10
15
20
1919
1939
1959
1979
1999
2019

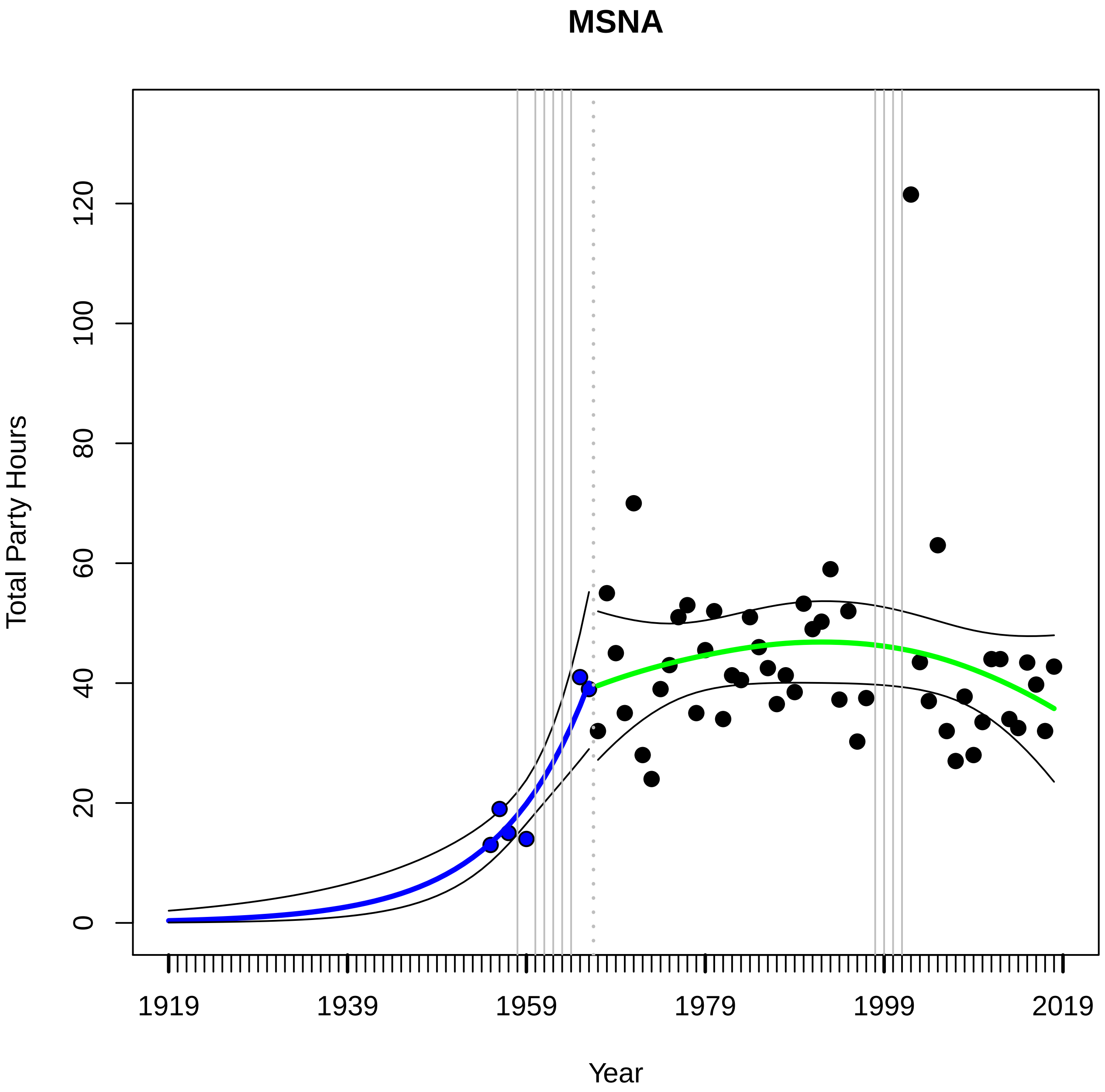

MSNA
Total Party Hours
0
20
40
60
80
100
120
1919
1939
1959
1979
1999
2019
Year

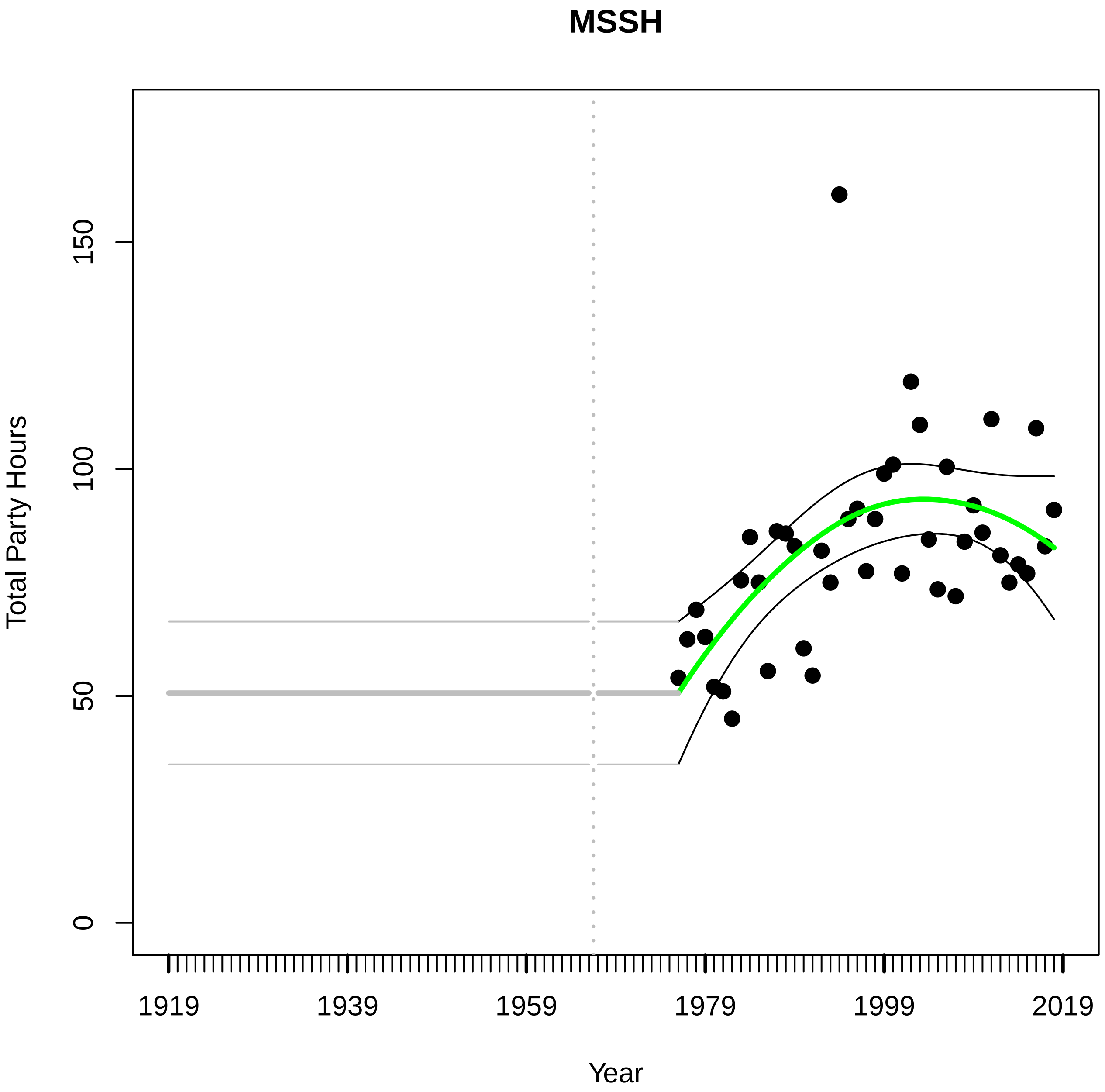
MSSH
Total Party Hours
0
50
100
150
1919
1939
1959
1979
1999
2019
Year

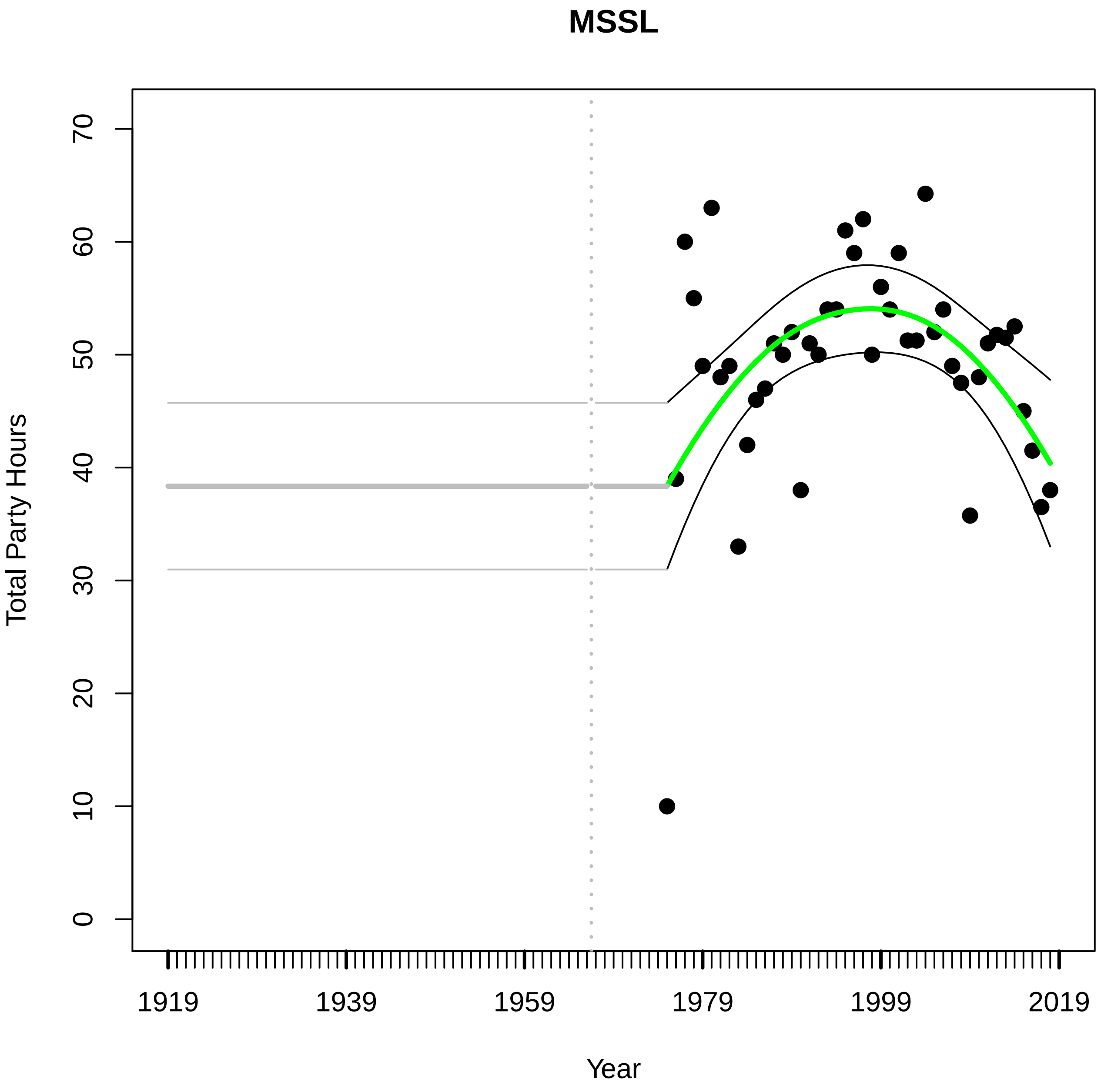

MSSL
Total Party Hours
Year
0
10
20
30
40
50
60
70
1919
1939
1959
1979
1999
2019

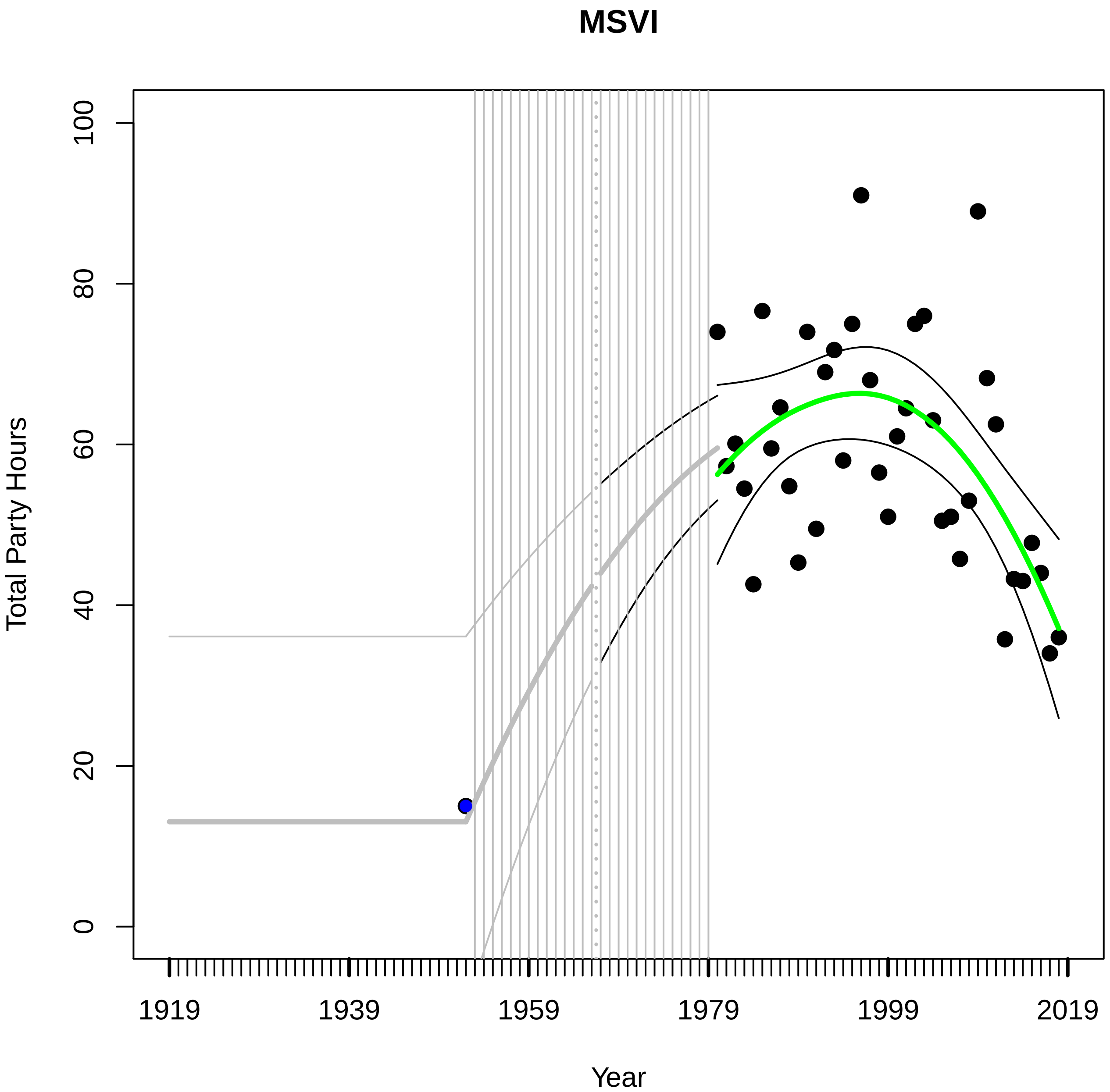
MSVI
Total Party Hours
100
80
60
40
20
0
1919
1939
1959
1979
1999
2019
Year

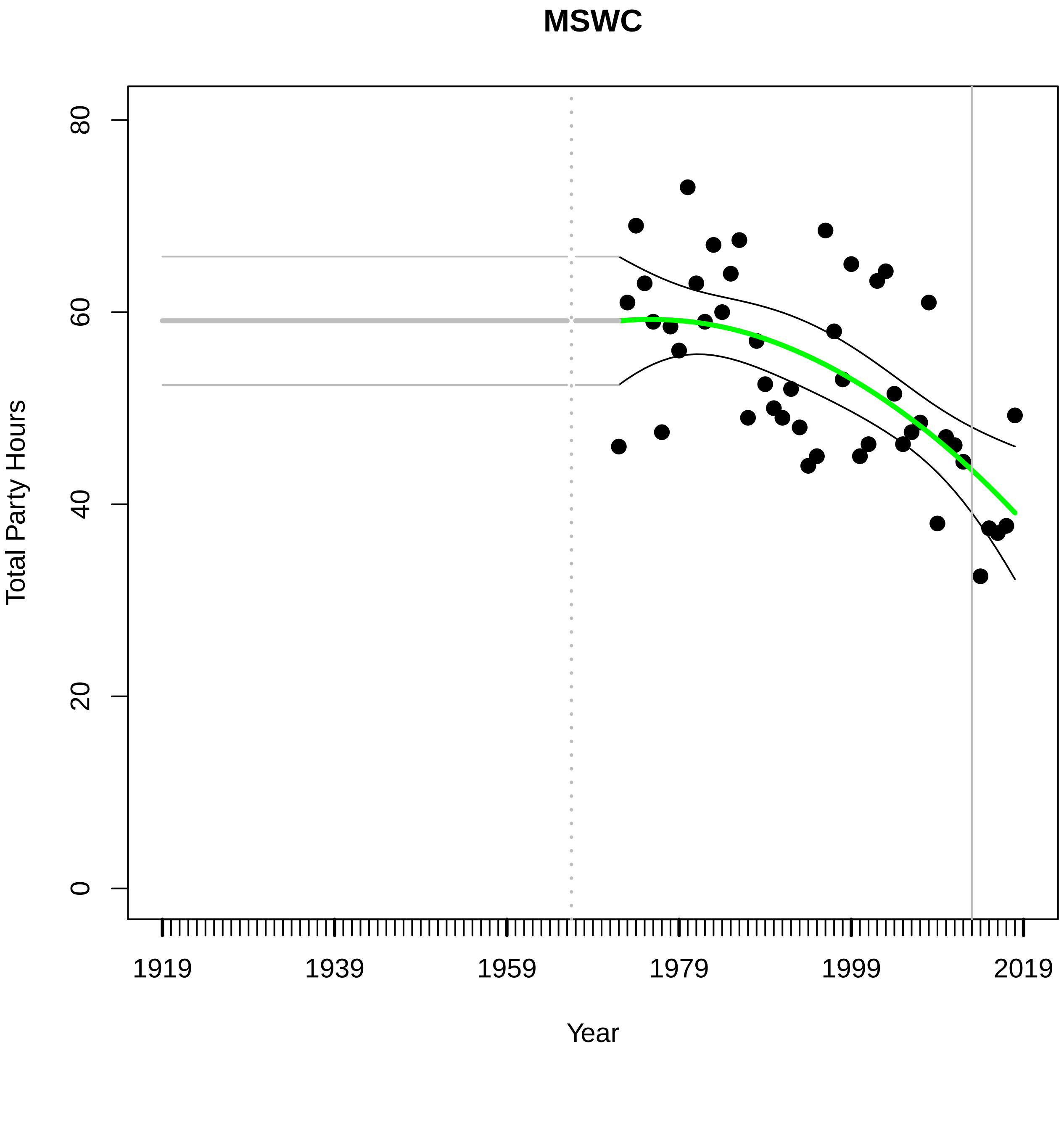

MSWC
Total Party Hours
0
20
40
60
80
1919
1939
1959
1979
1999
2019
Year

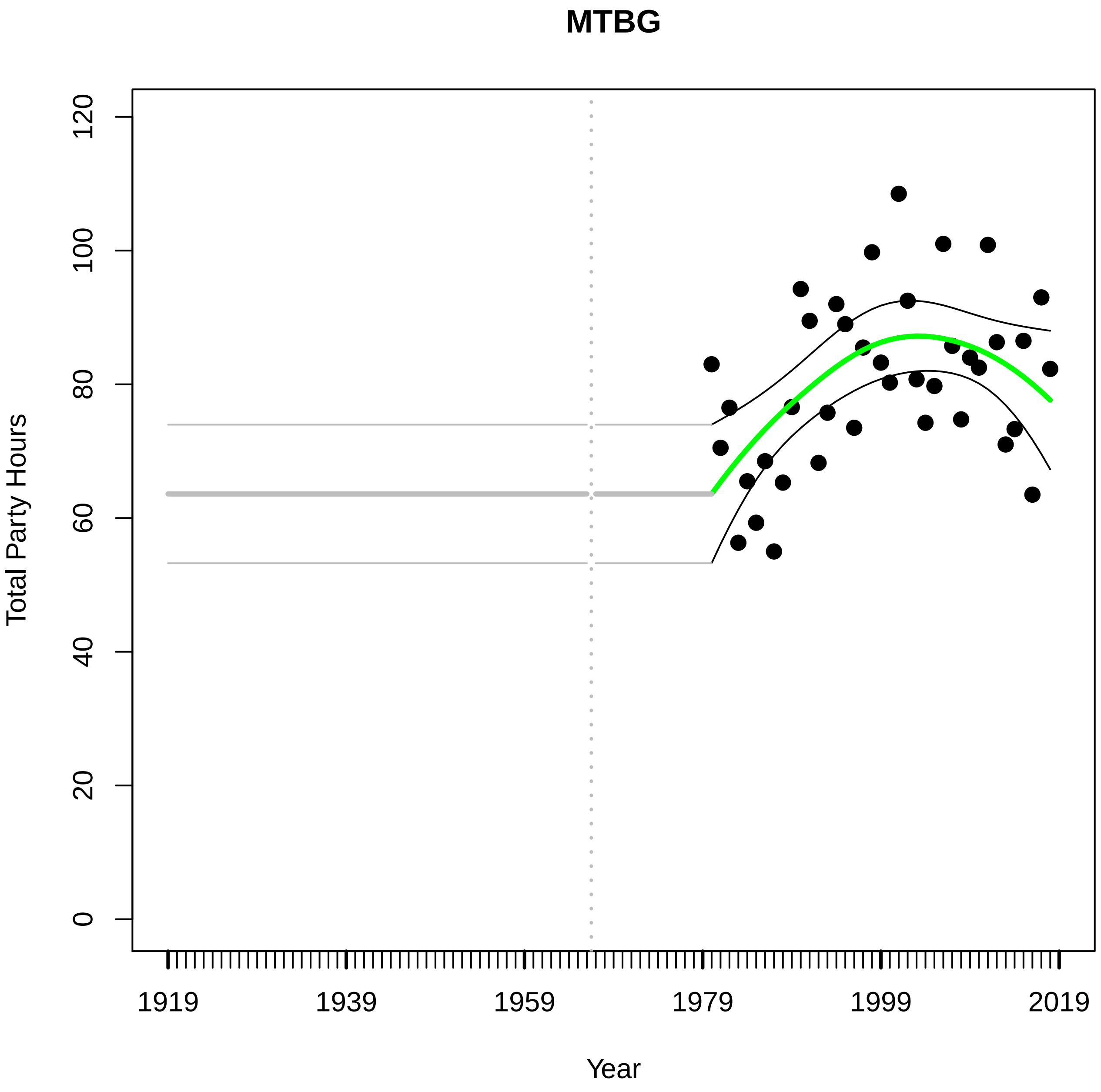
MTBG
Total Party Hours
0
20
40
60
80
100
120
1919
1939
1959
1979
1999
2019
Year

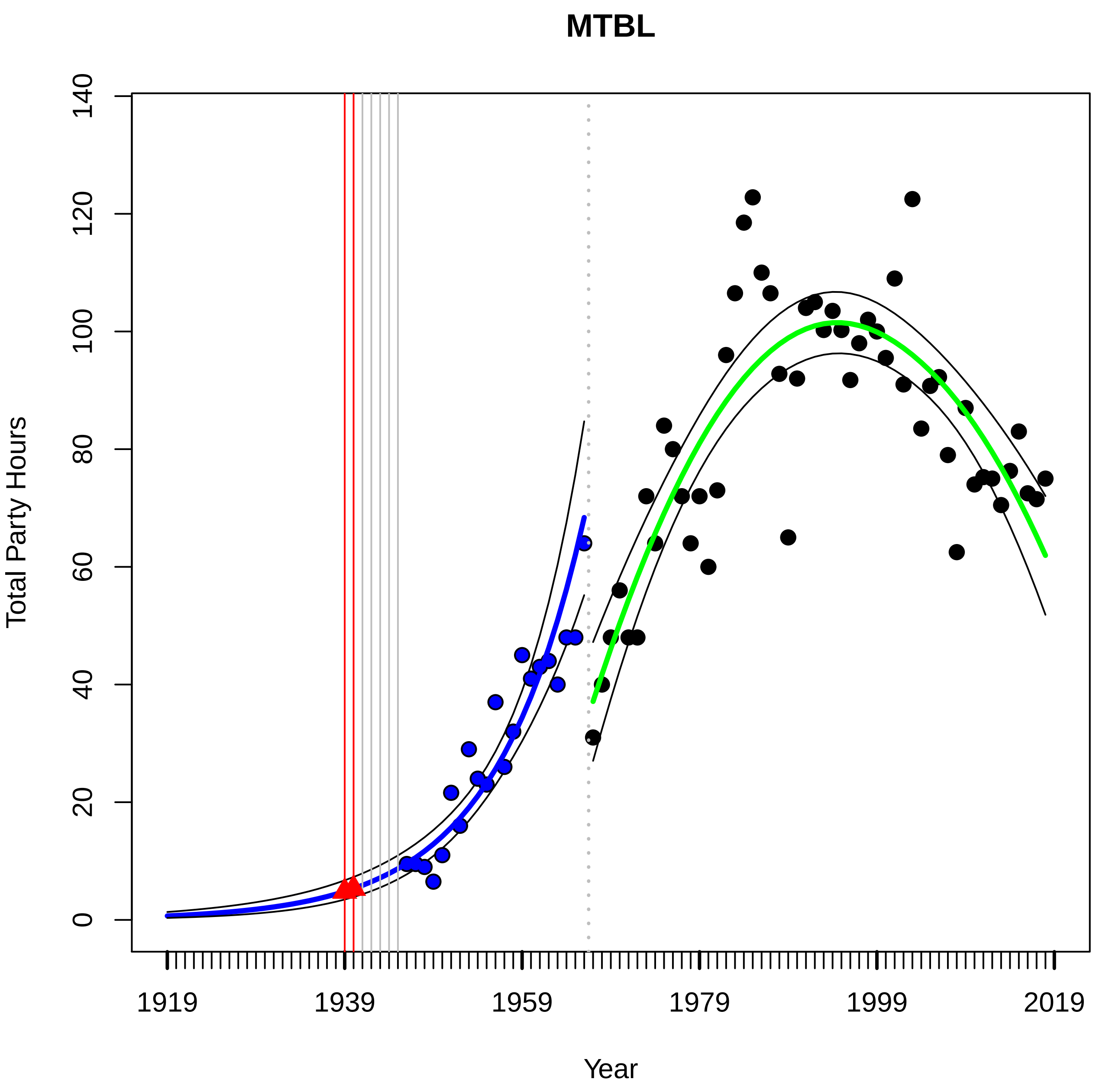

MTBL
Total Party Hours
0
20
40
60
80
100
120
140
1919
1939
1959
1979
1999
2019
Year

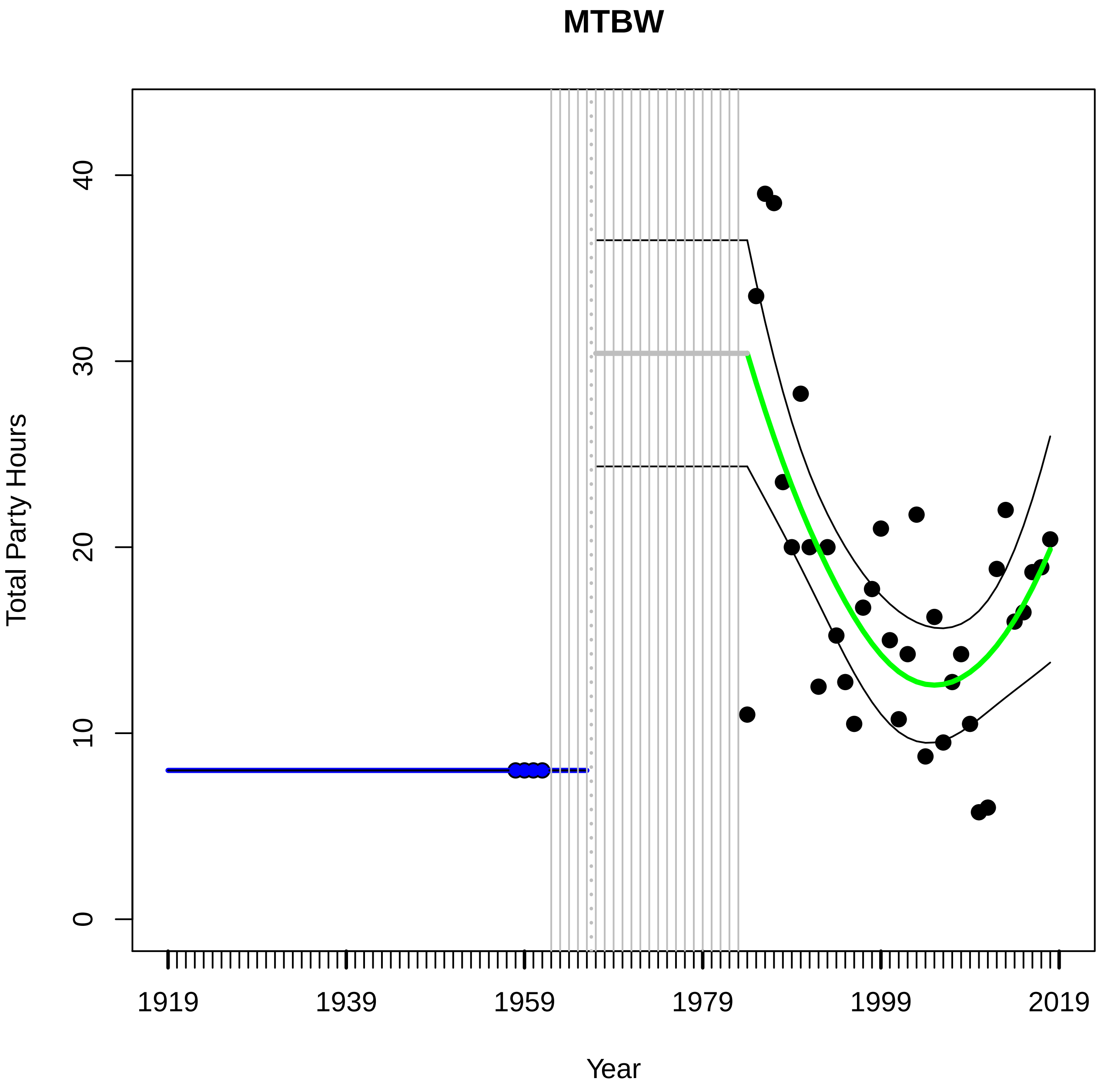

MTBW
Total Party Hours
Year
0
10
20
30
40
1919
1939
1959
1979
1999
2019

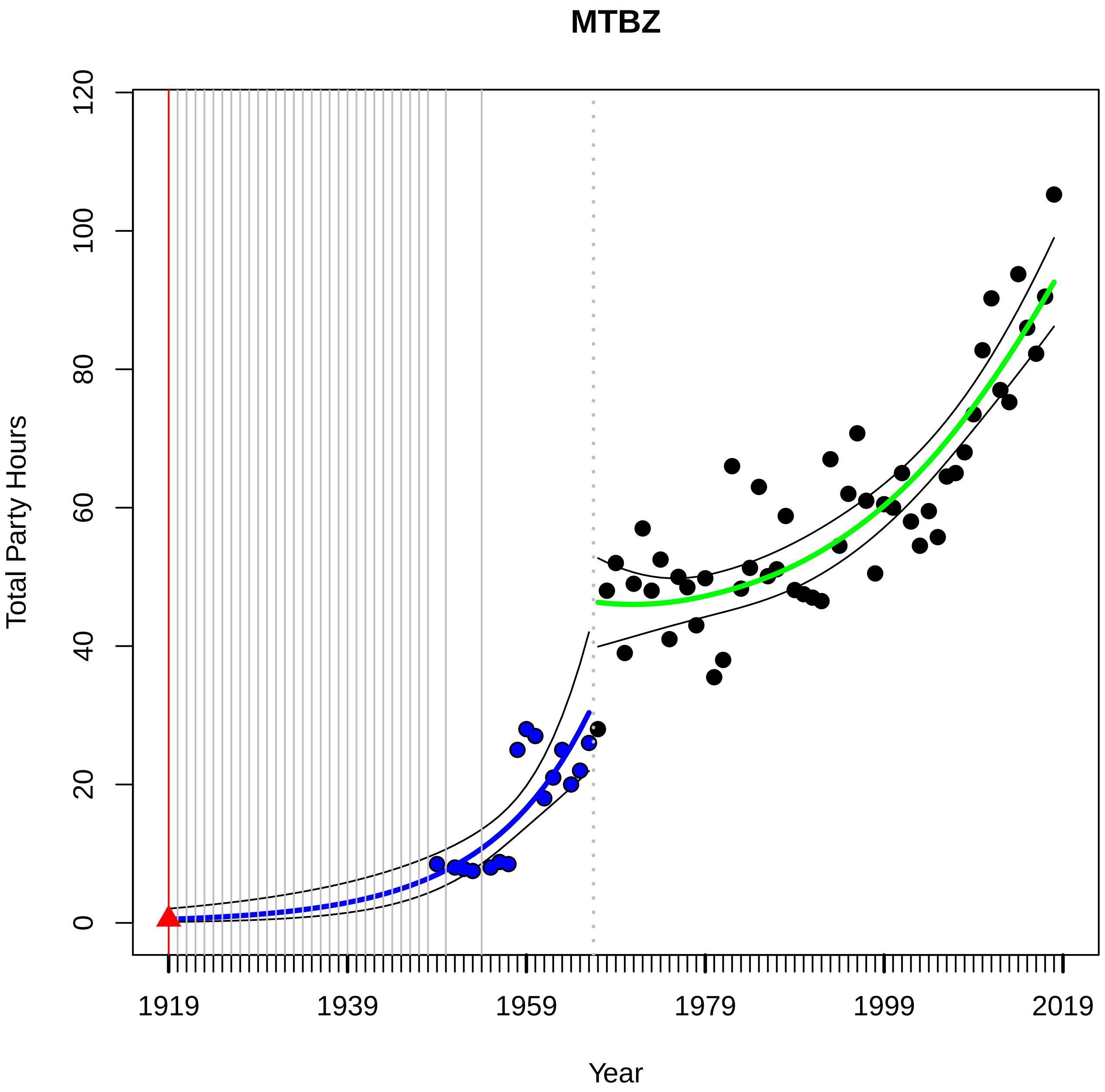

MTBZ
Total Party Hours
Year
0
20
40
60
80
100
120
1919
1939
1959
1979
1999
2019

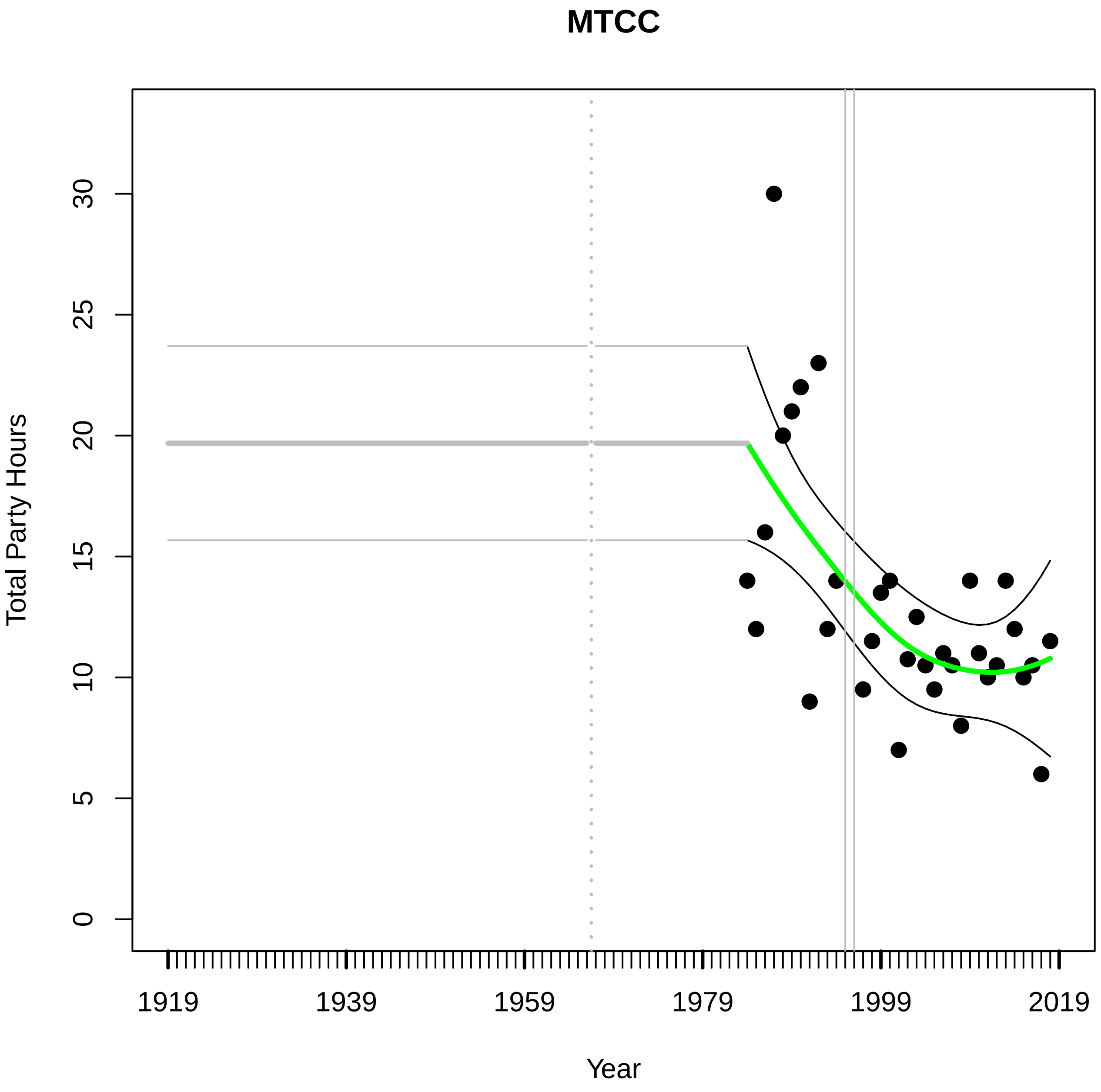

MTCC
Total Party Hours
0
5
10
15
20
25
30
1919
1939
1959
1979
1999
2019
Year

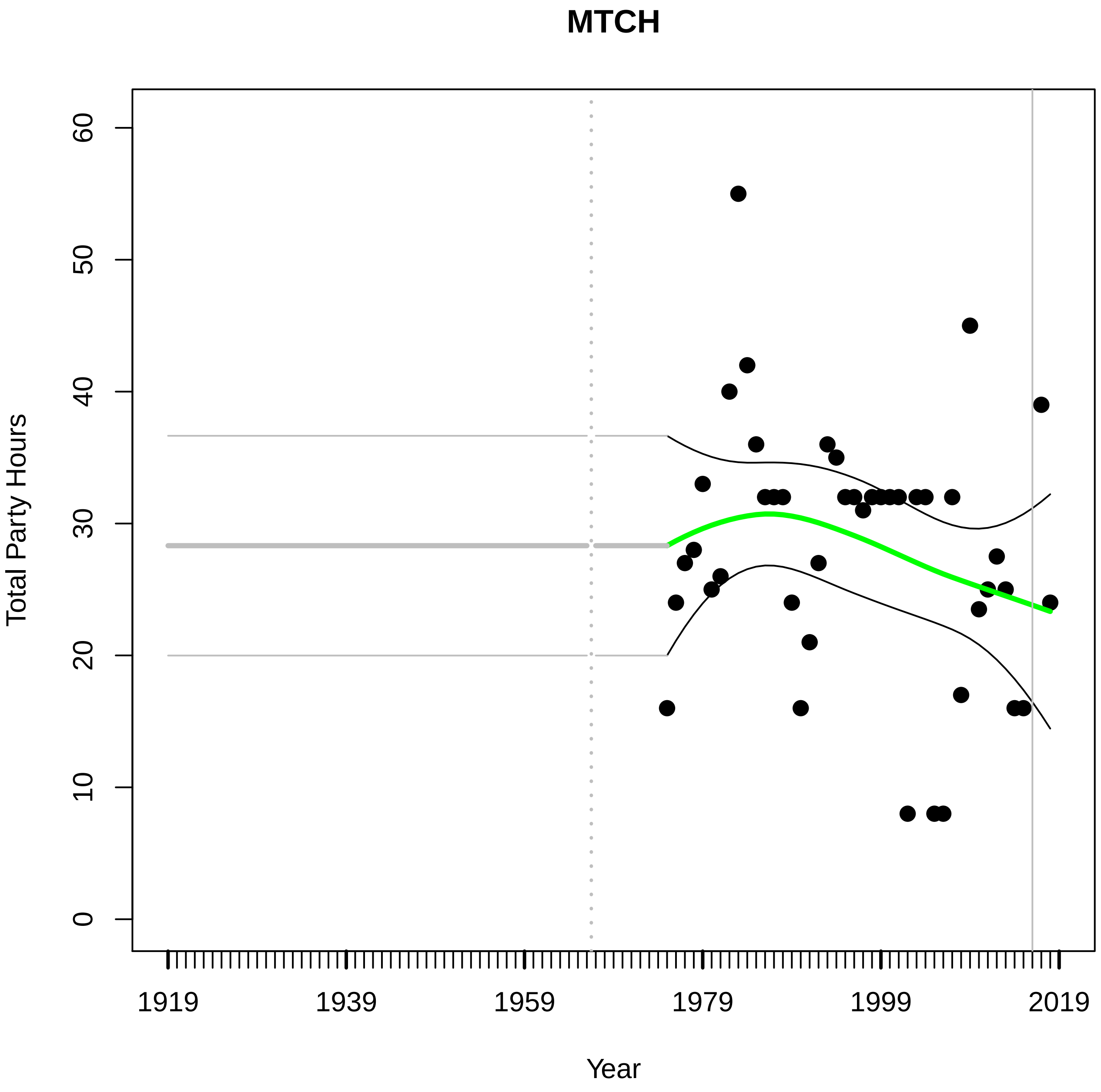

MTCH
Total Party Hours
Year
0
10
20
30
40
50
60
1919
1939
1959
1979
1999
2019

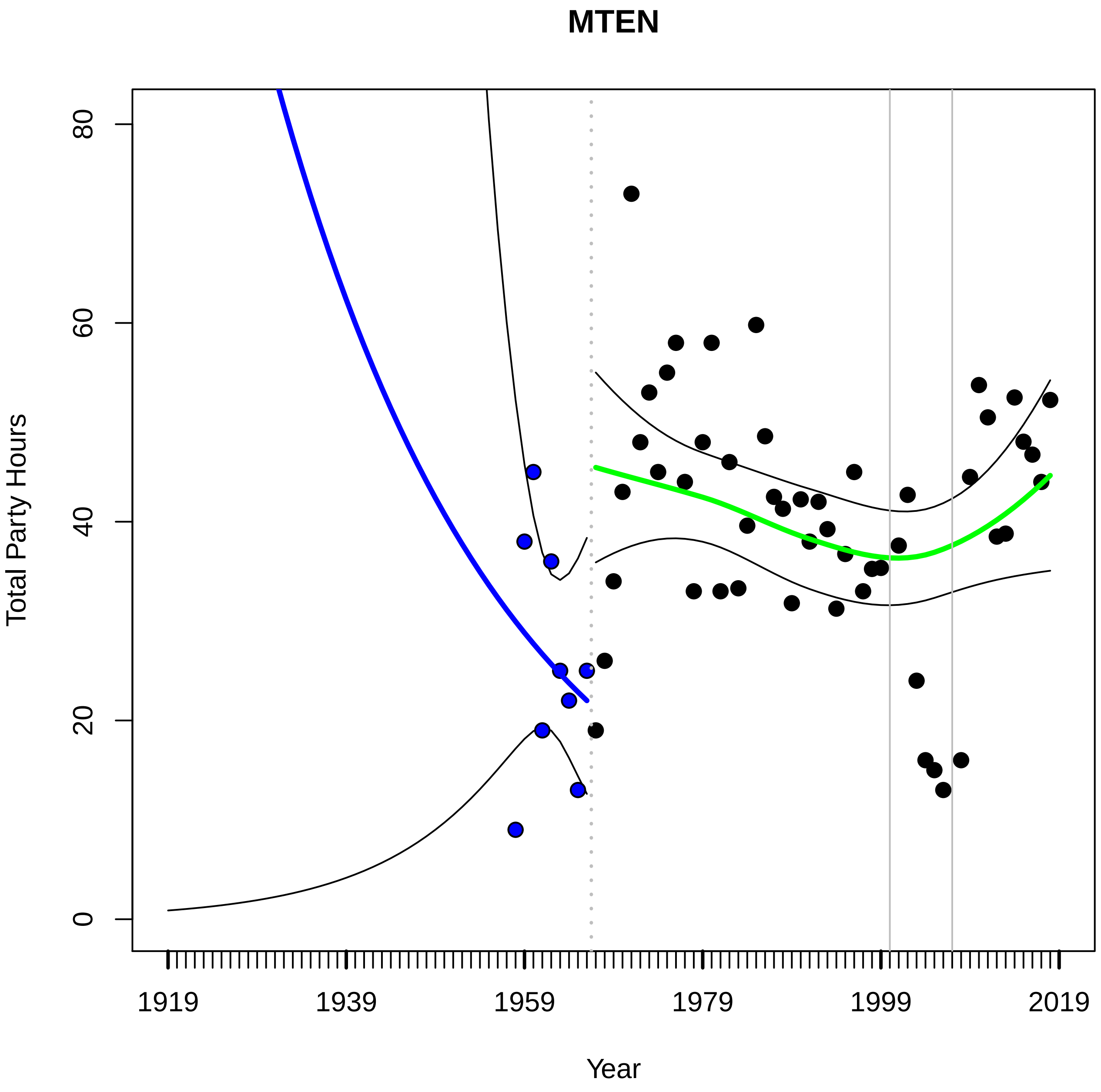
MTEN
Total Party Hours
0
20
40
60
80
1919
1939
1959
1979
1999
2019
Year

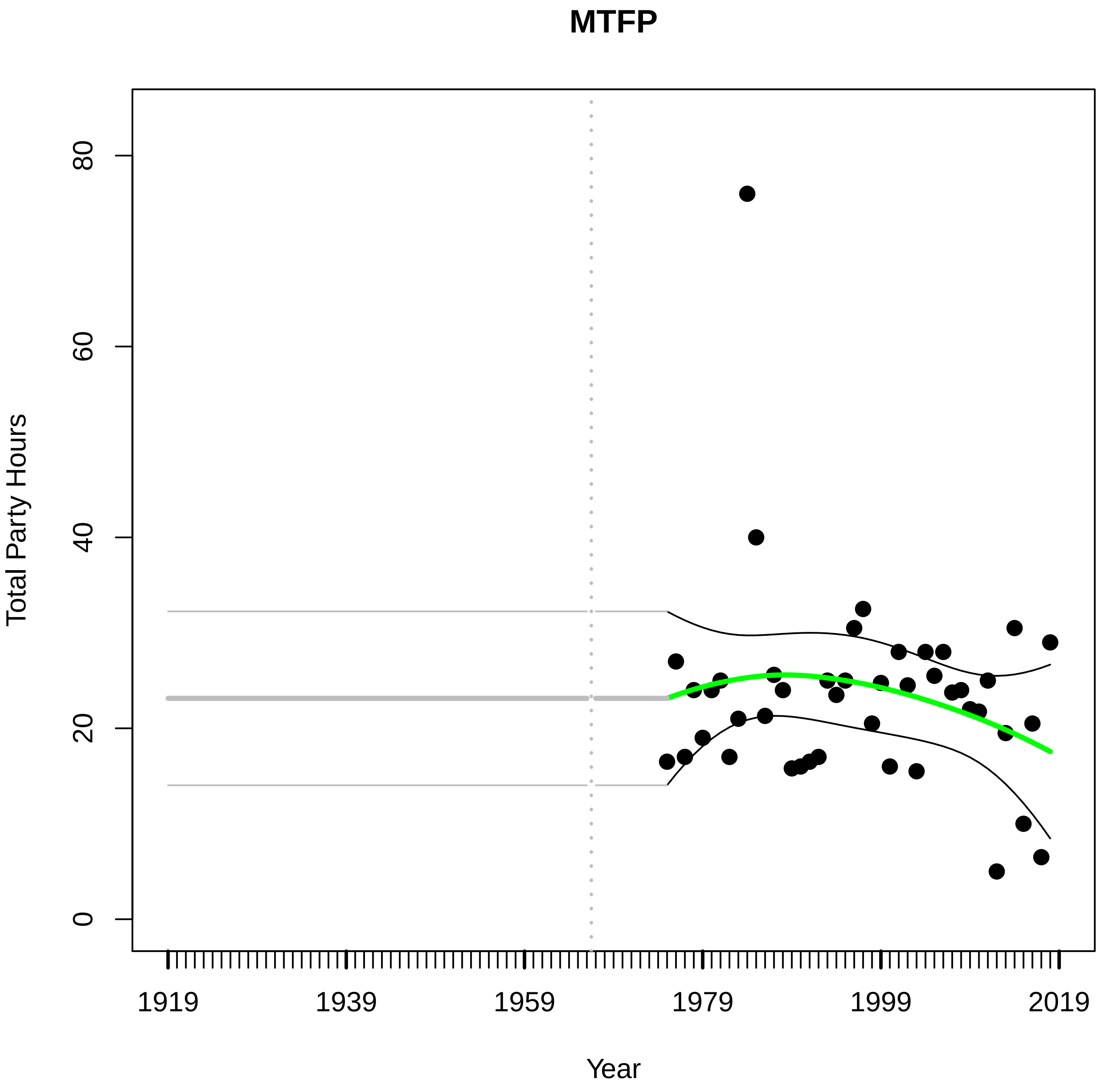
MTFP
Total Party Hours
0
20
40
60
80
1919
1939
1959
1979
1999
2019
Year

MTGF

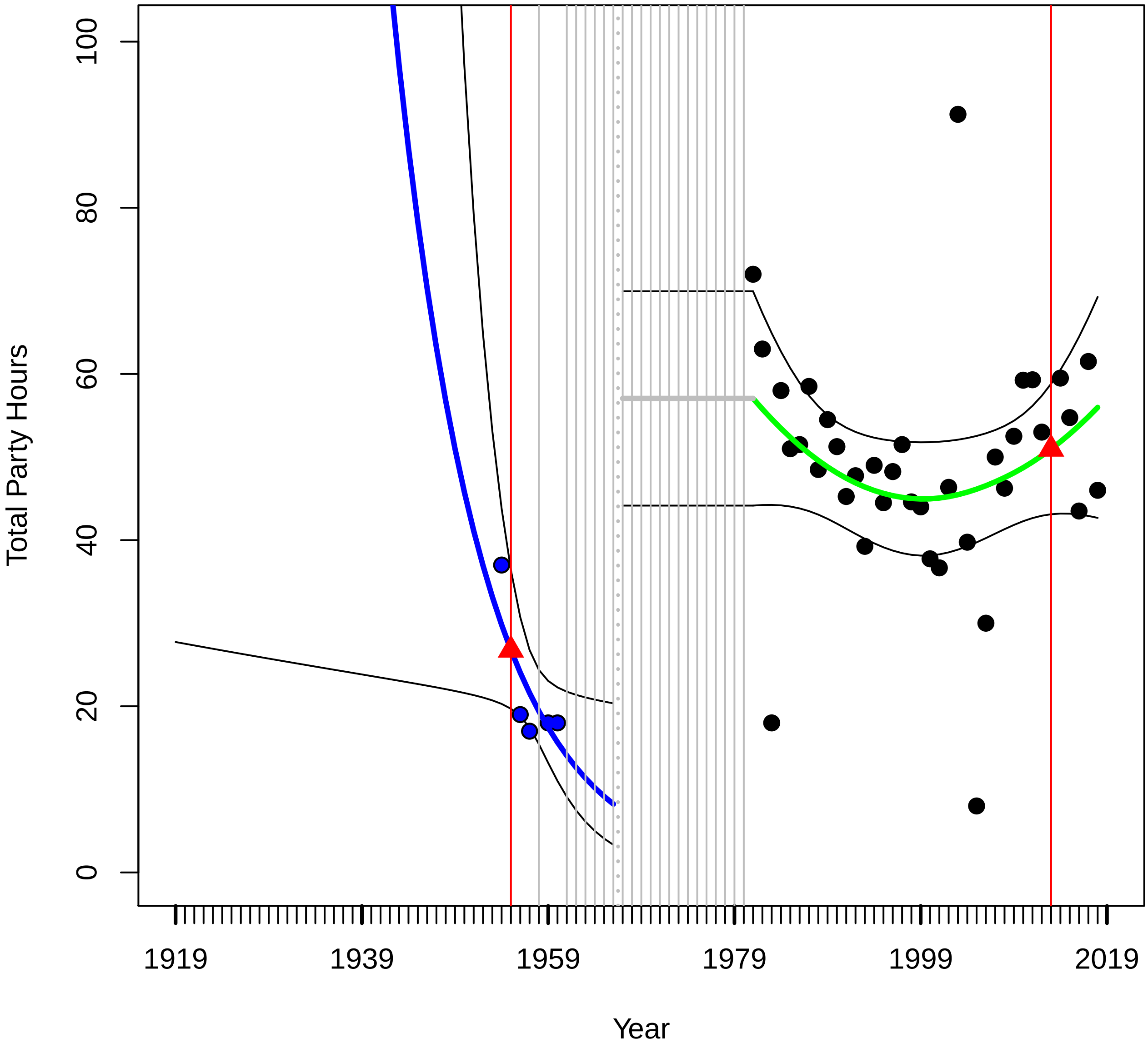

Total Party Hours
0
20
40
60
80
100
1919
1939
1959
1979
1999
2019
Year

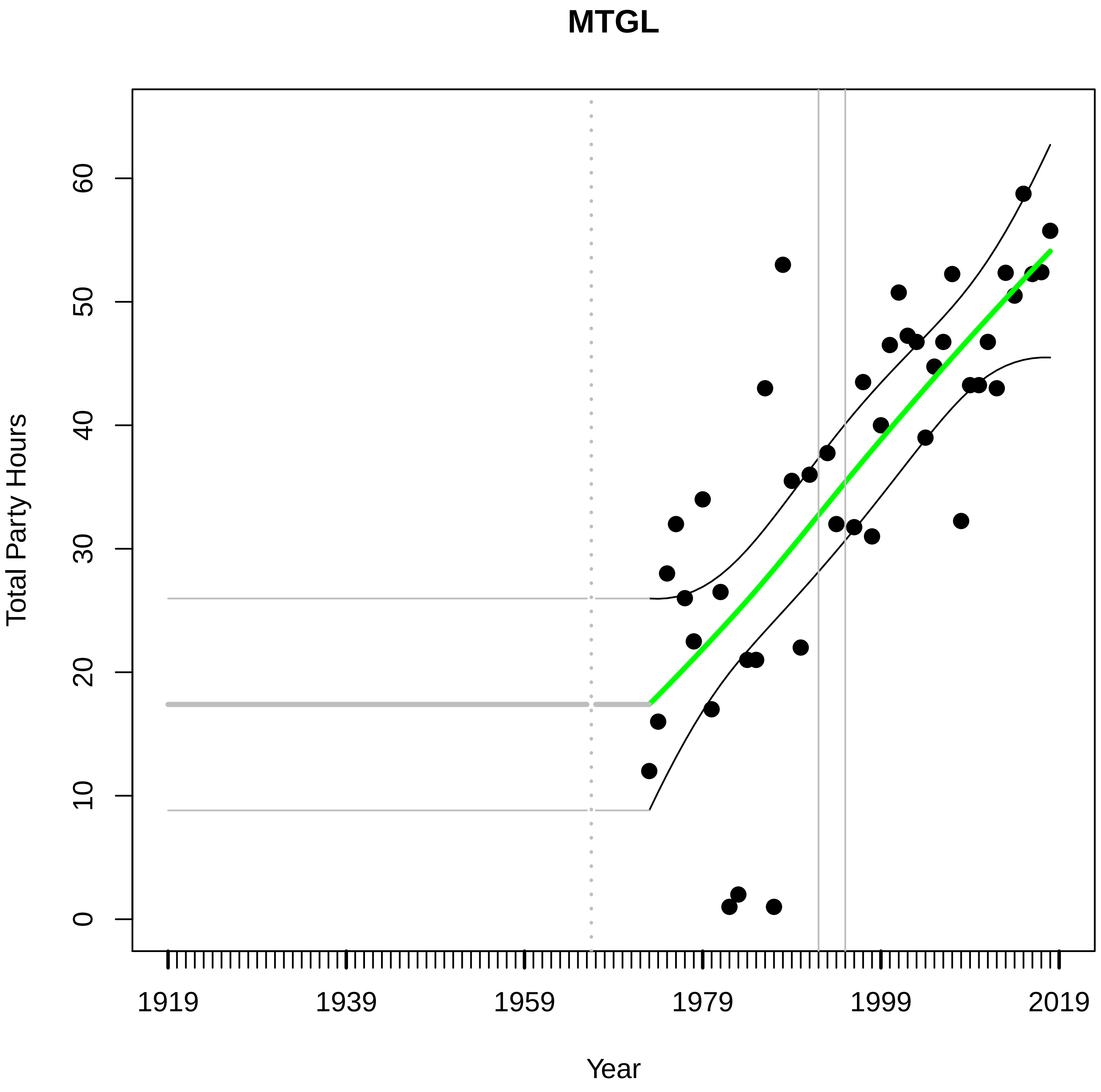

MTGL
Total Party Hours
0
10
20
30
40
50
60
1919
1939
1959
1979
1999
2019
Year

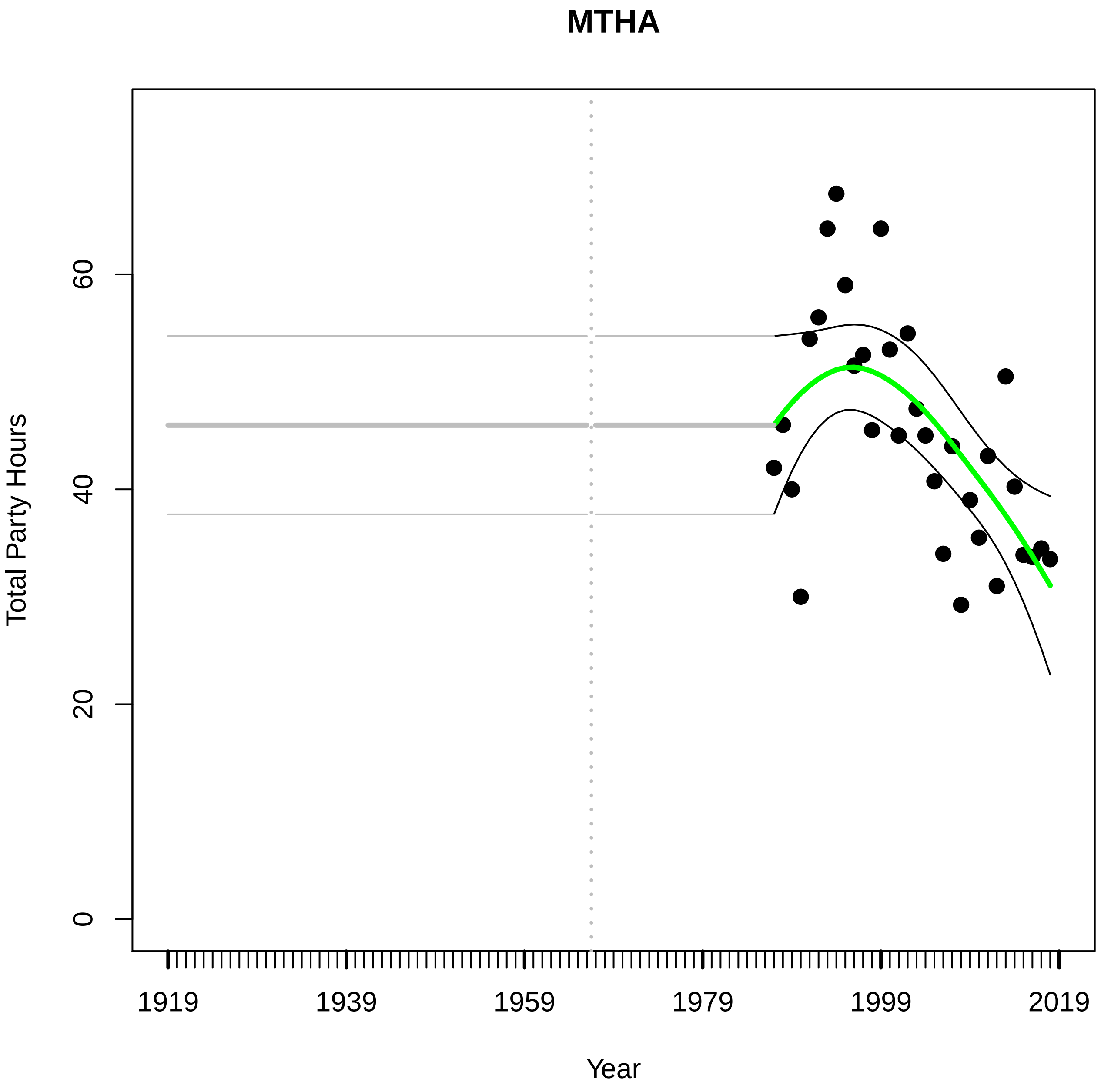
MTHA
Total Party Hours
0
20
40
60
1919
1939
1959
1979
1999
2019
Year

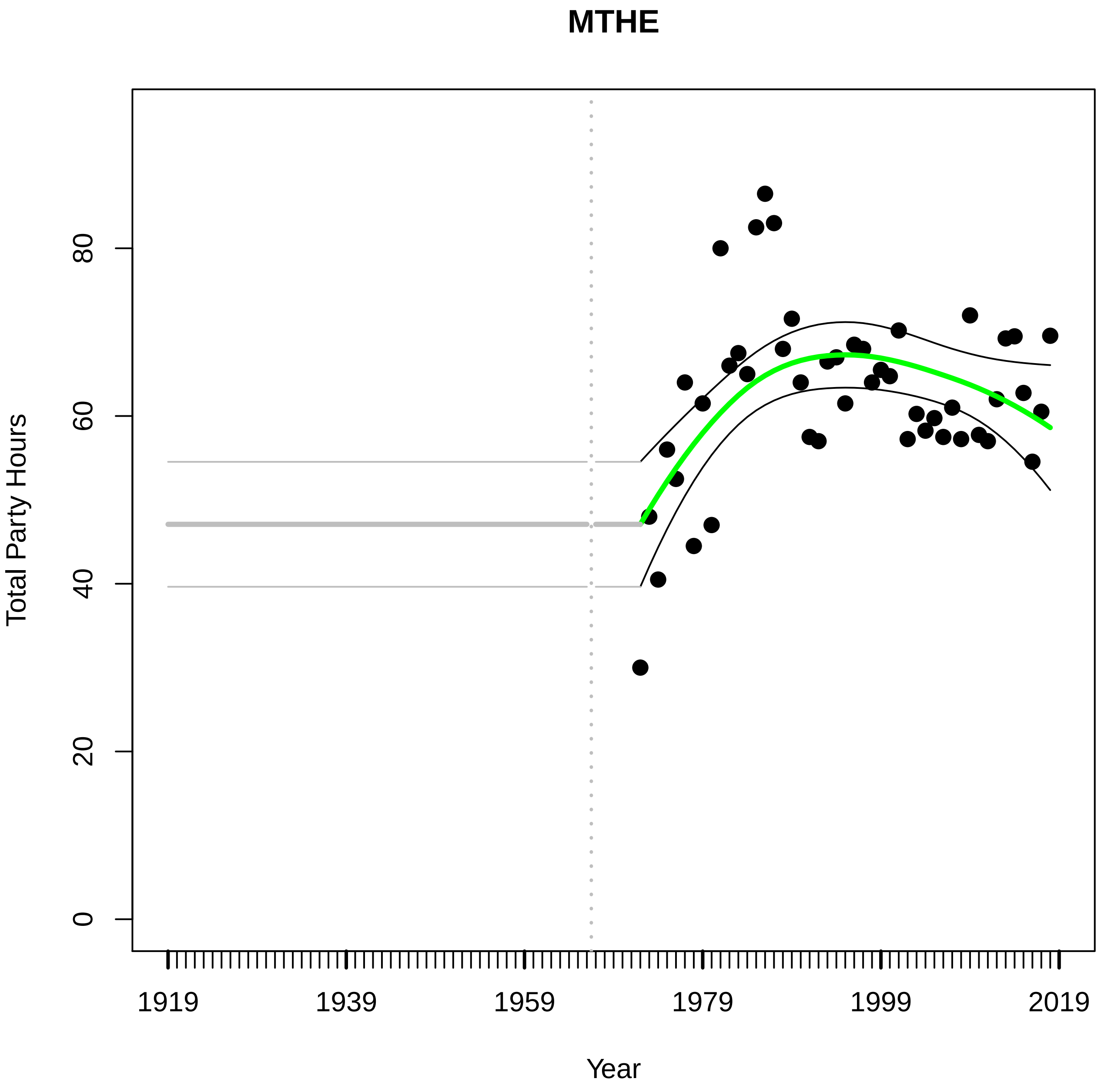
MTHE
Total Party Hours
Year
0
20
40
60
80
1919
1939
1959
1979
1999
2019

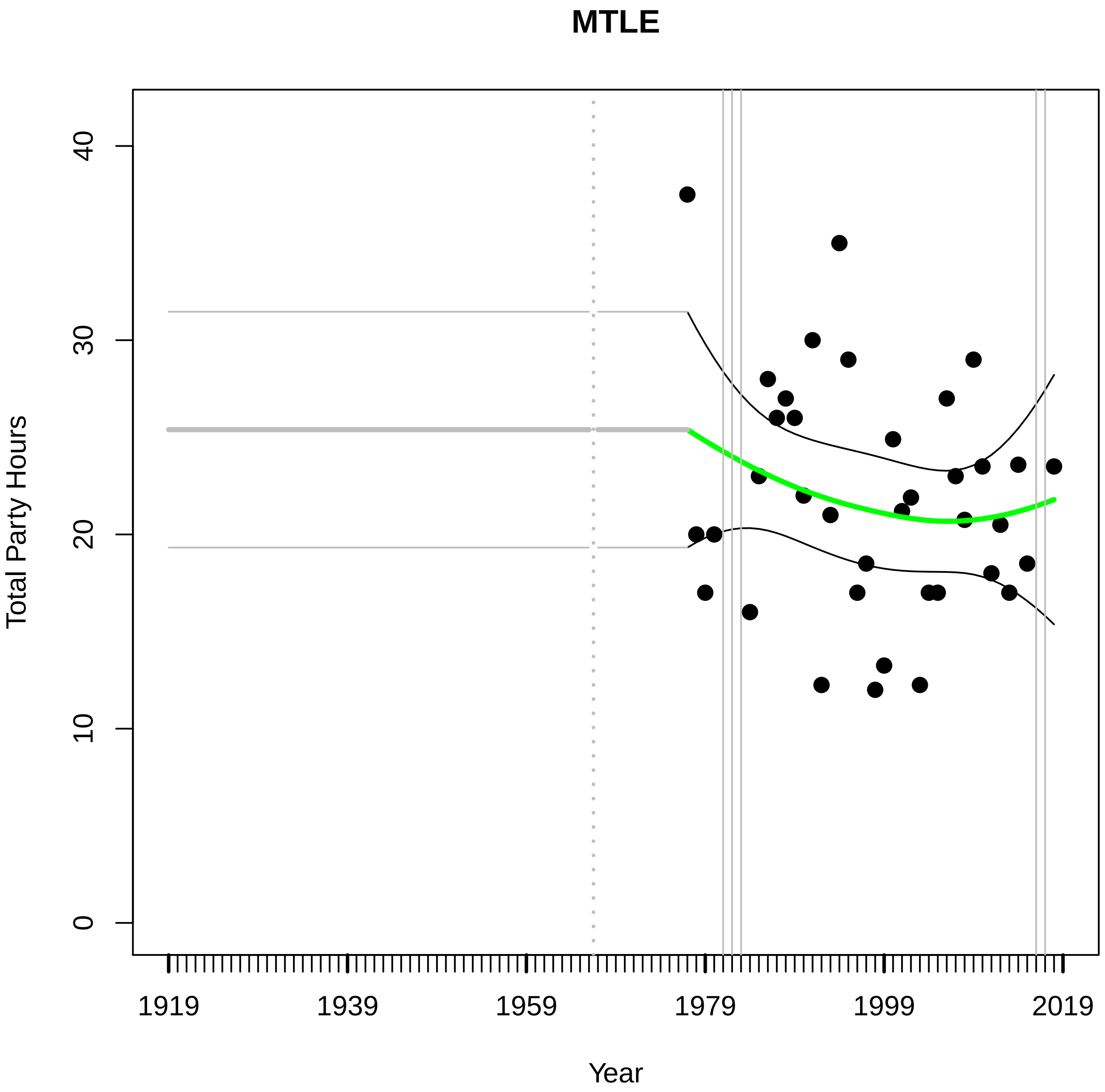

MTLE
Total Party Hours
0
10
20
30
40
1919
1939
1959
1979
1999
2019
Year

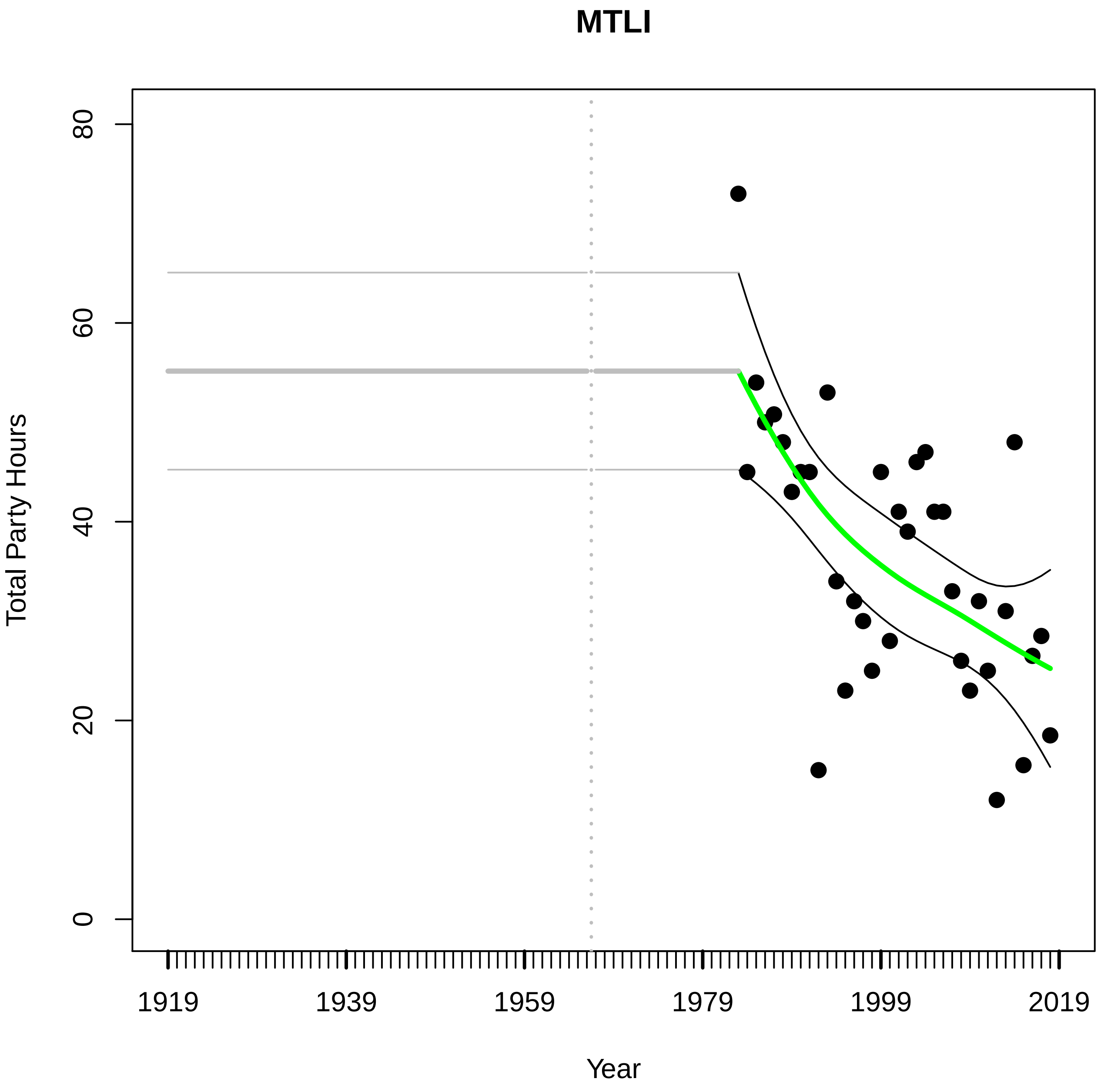
MTLI
Total Party Hours
0
20
40
60
80
1919
1939
1959
1979
1999
2019
Year

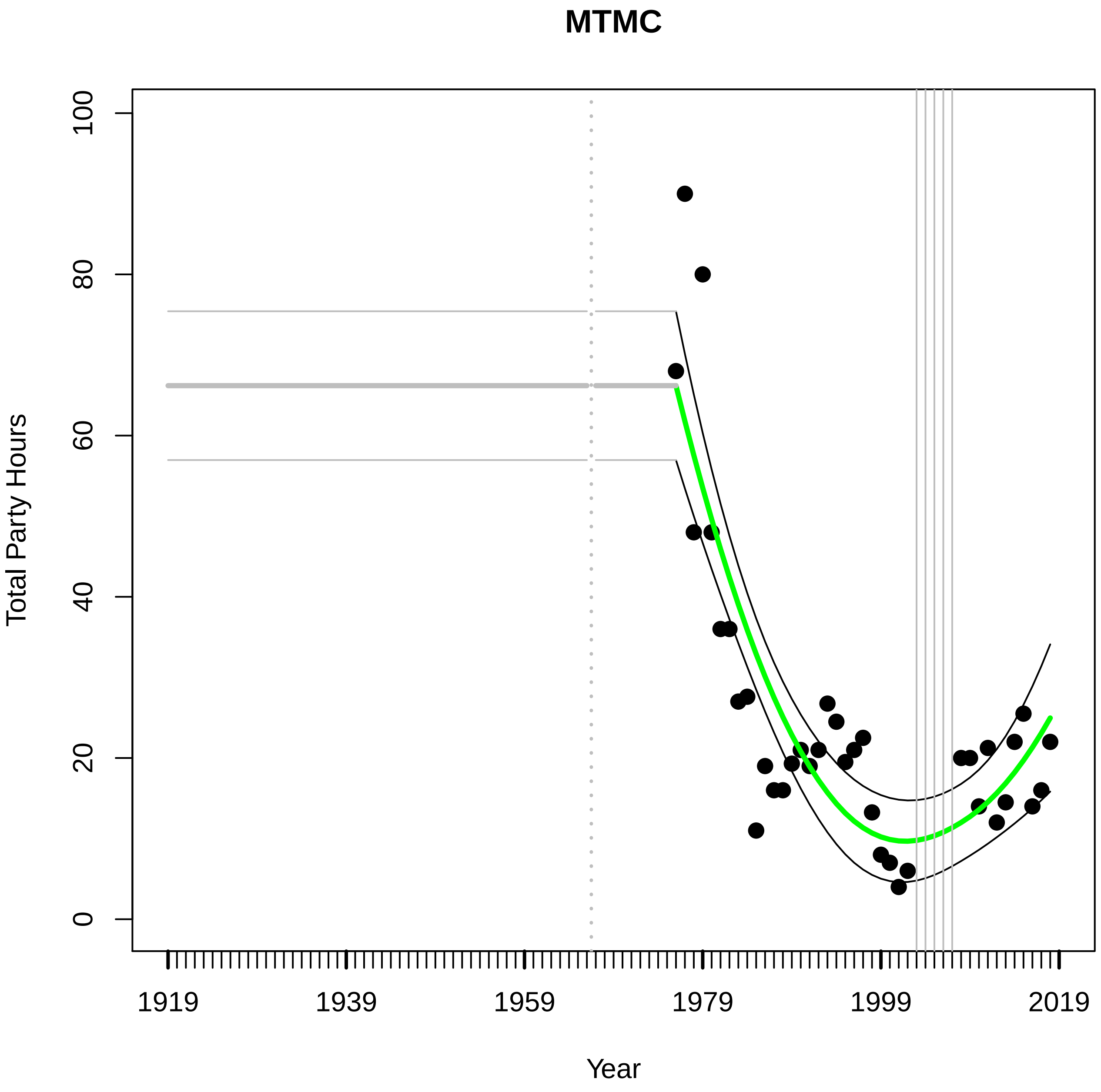
MTMC
Total Party Hours
0
20
40
60
80
100
Year
1919
1939
1959
1979
1999
2019

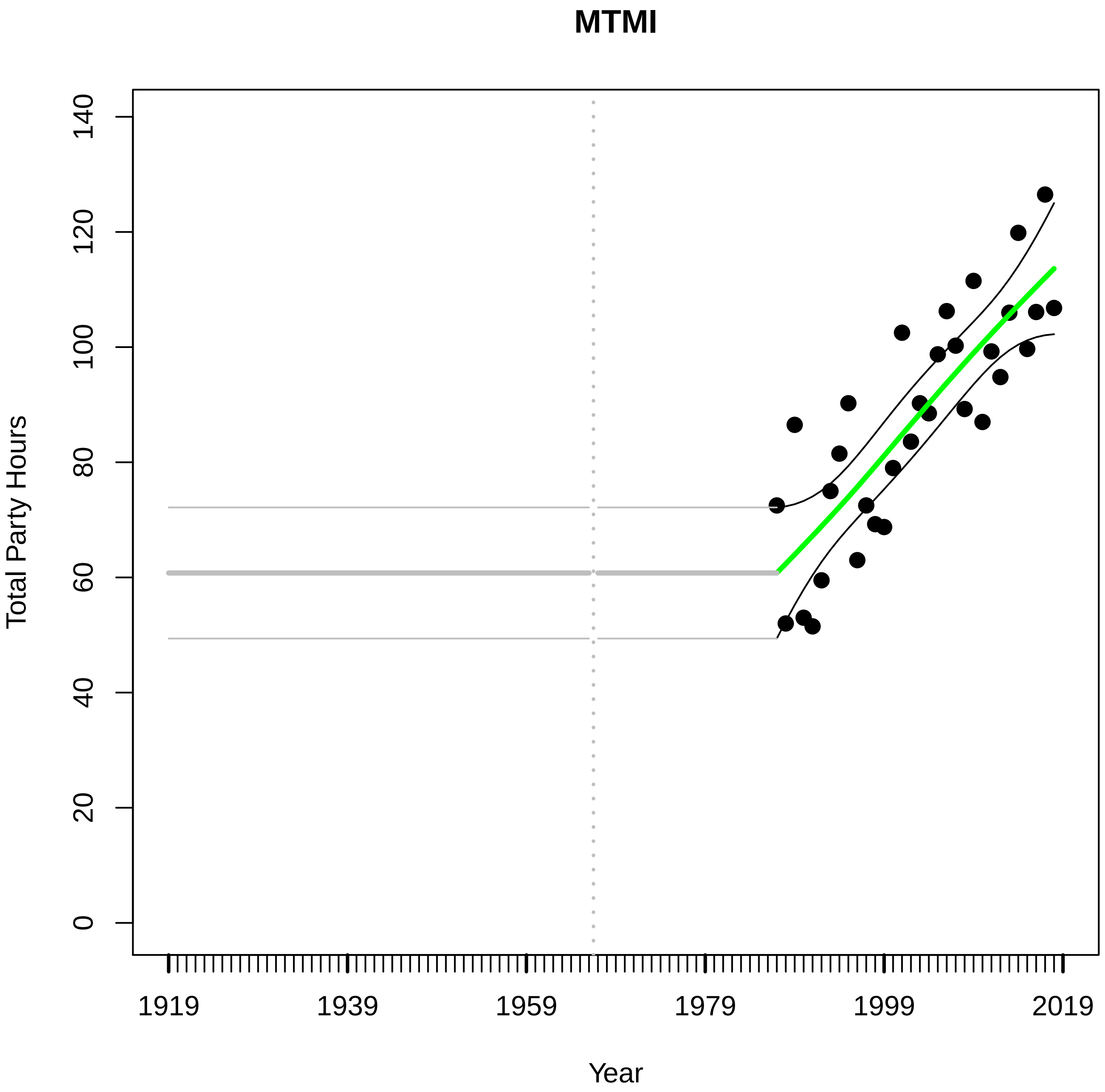

MTMI
Total Party Hours
0
20
40
60
80
100
120
140
1919
1939
1959
1979
1999
2019
Year

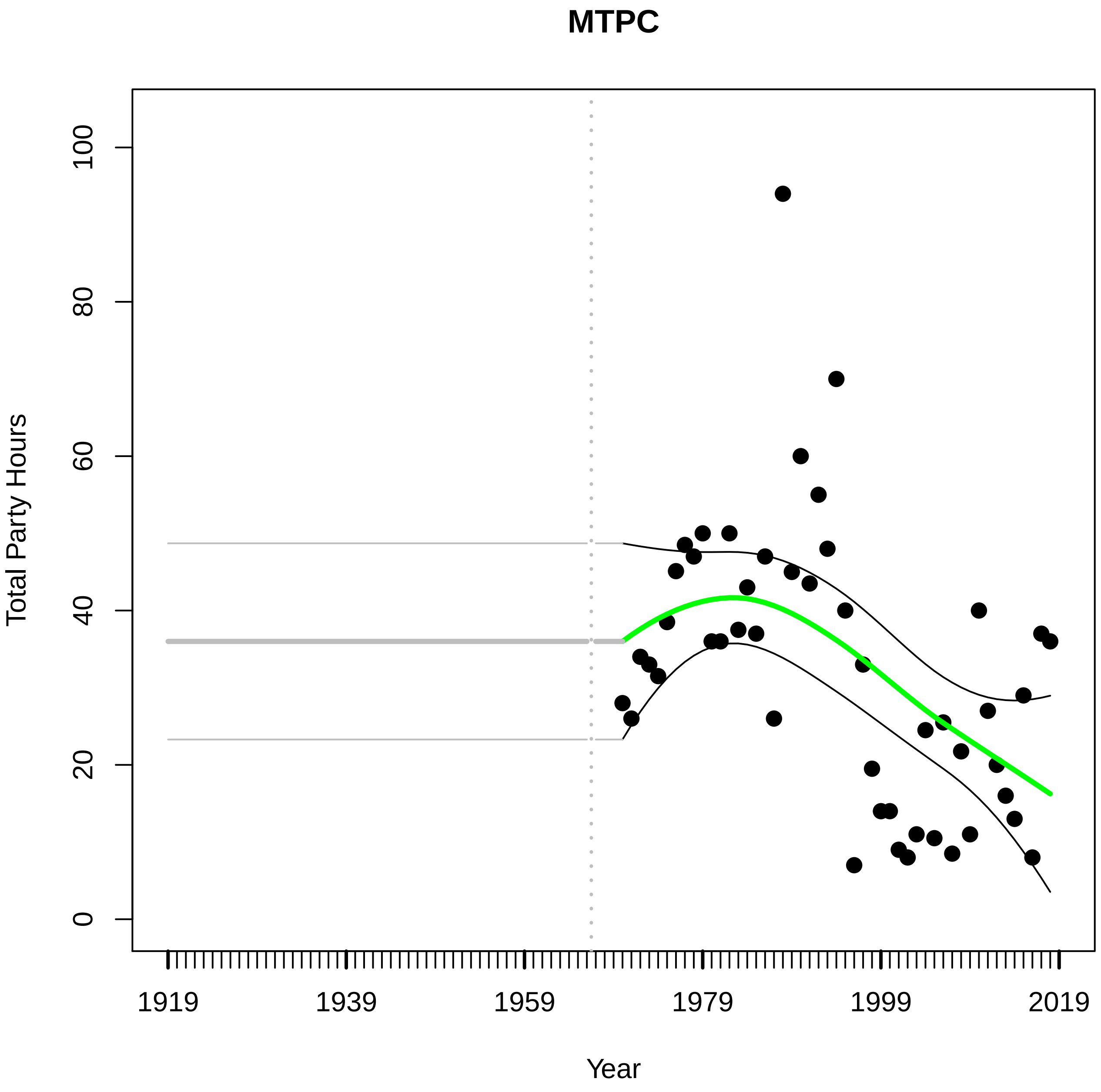

MTPC
Total Party Hours
0
20
40
60
80
100
1919
1939
1959
1979
1999
2019
Year

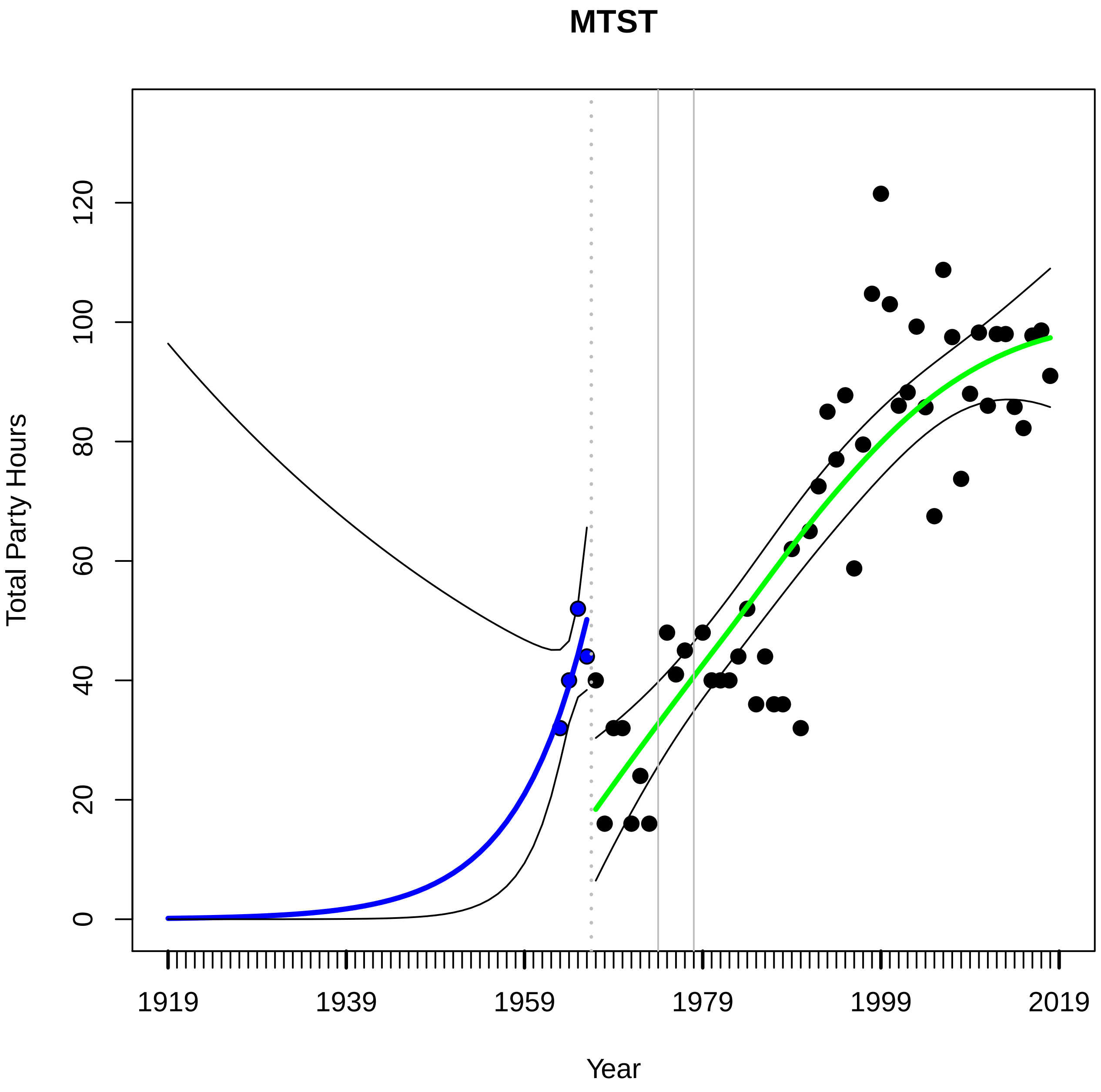
MTST
Total Party Hours
Year
0
20
40
60
80
100
120
1919
1939
1959
1979
1999
2019

MTTF

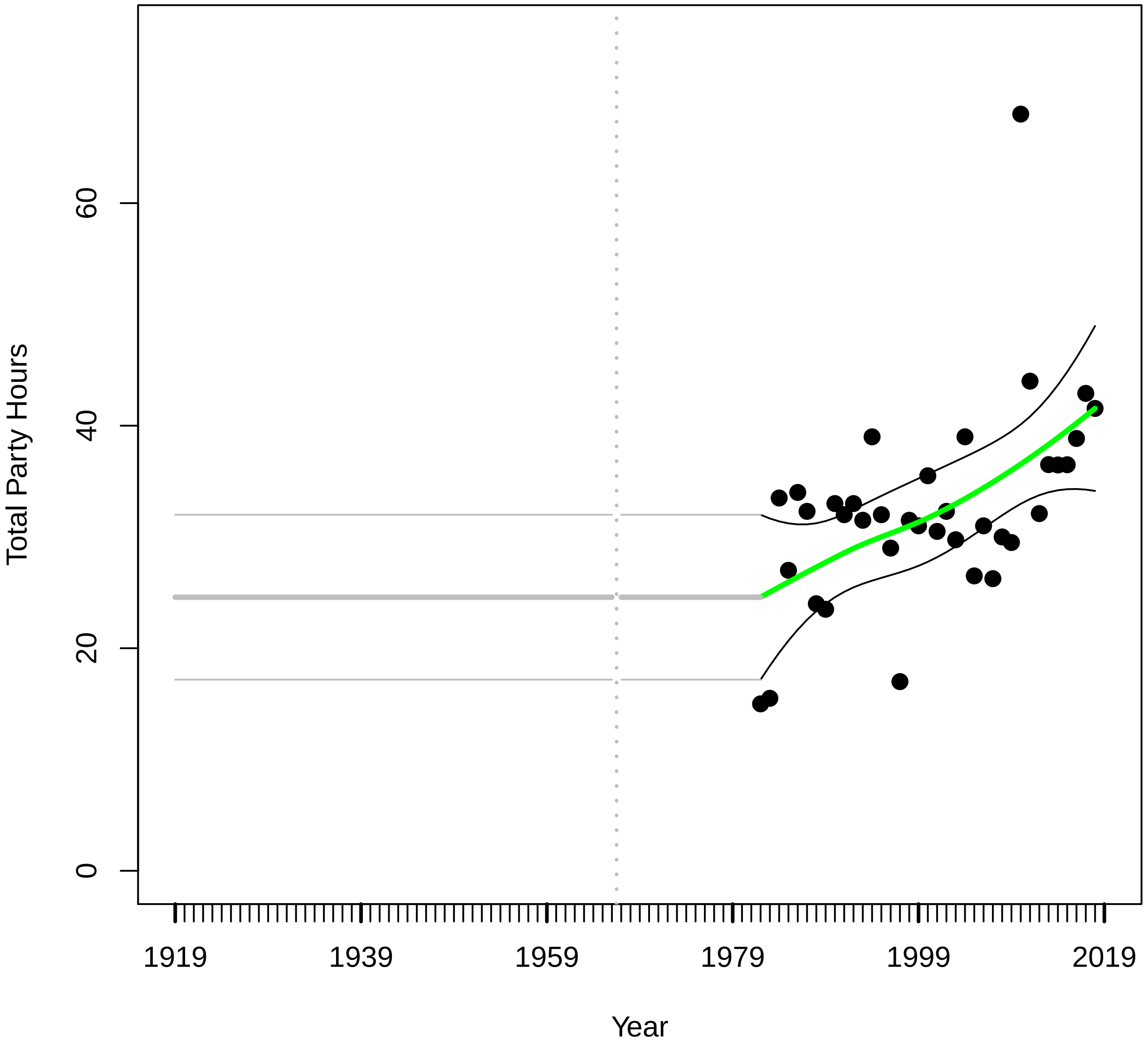

Total Party Hours
0
20
40
60
1919
1939
1959
1979
1999
2019
Year

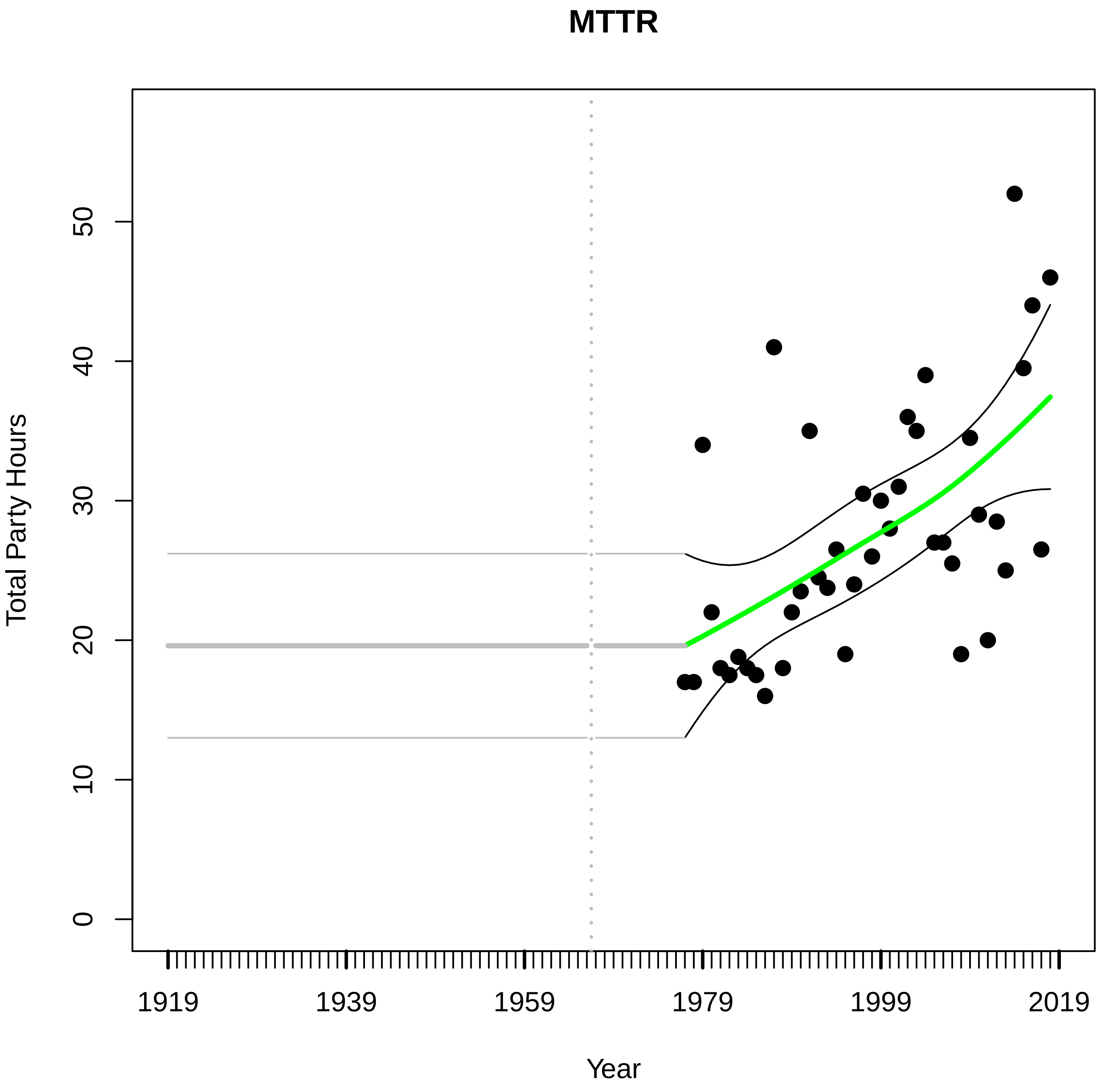

MTTR
Total Party Hours
Year
0
10
20
30
40
50
1919
1939
1959
1979
1999
2019

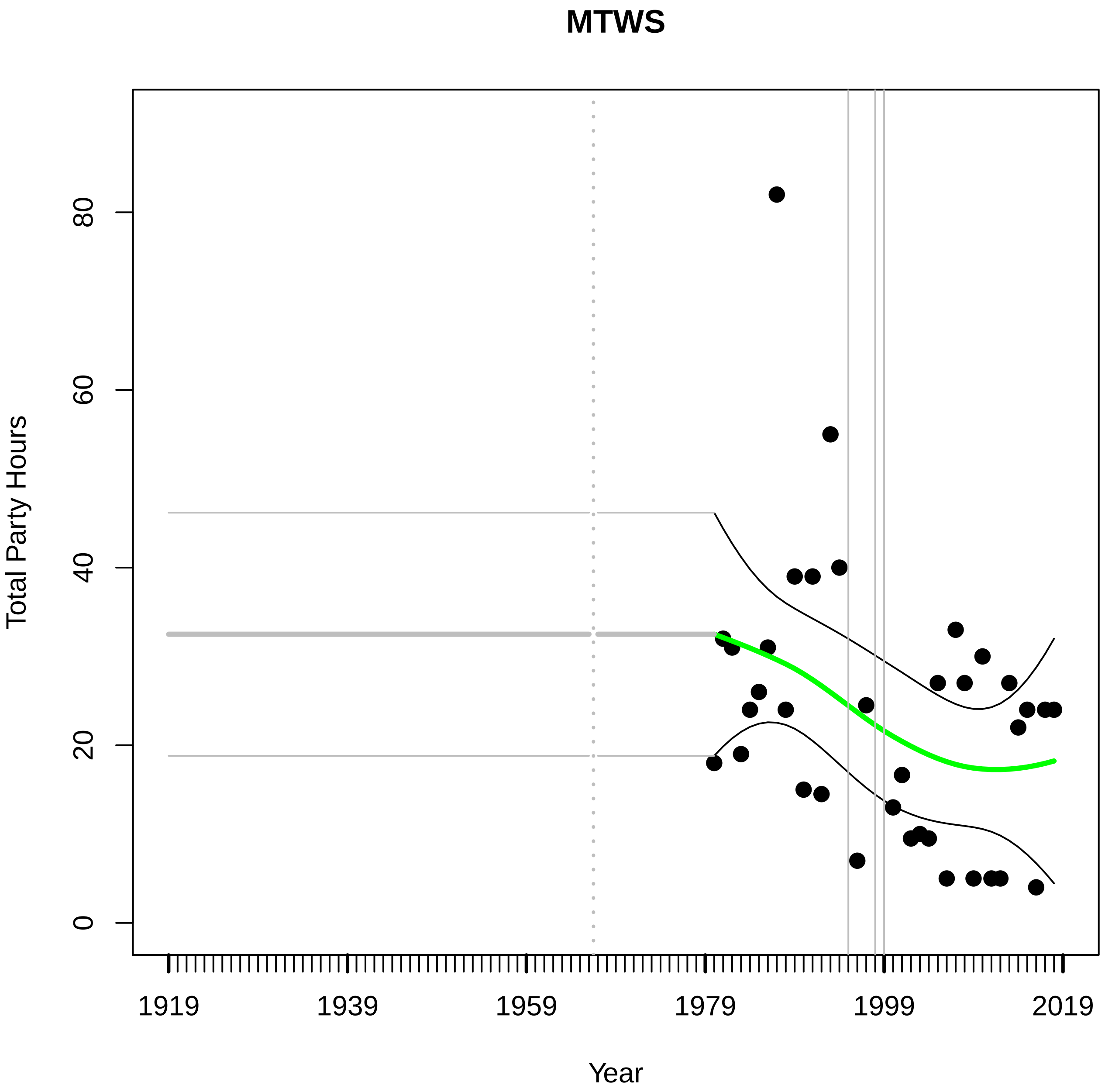

MTWS
Total Party Hours
0
20
40
60
80
1919
1939
1959
1979
1999
2019
Year

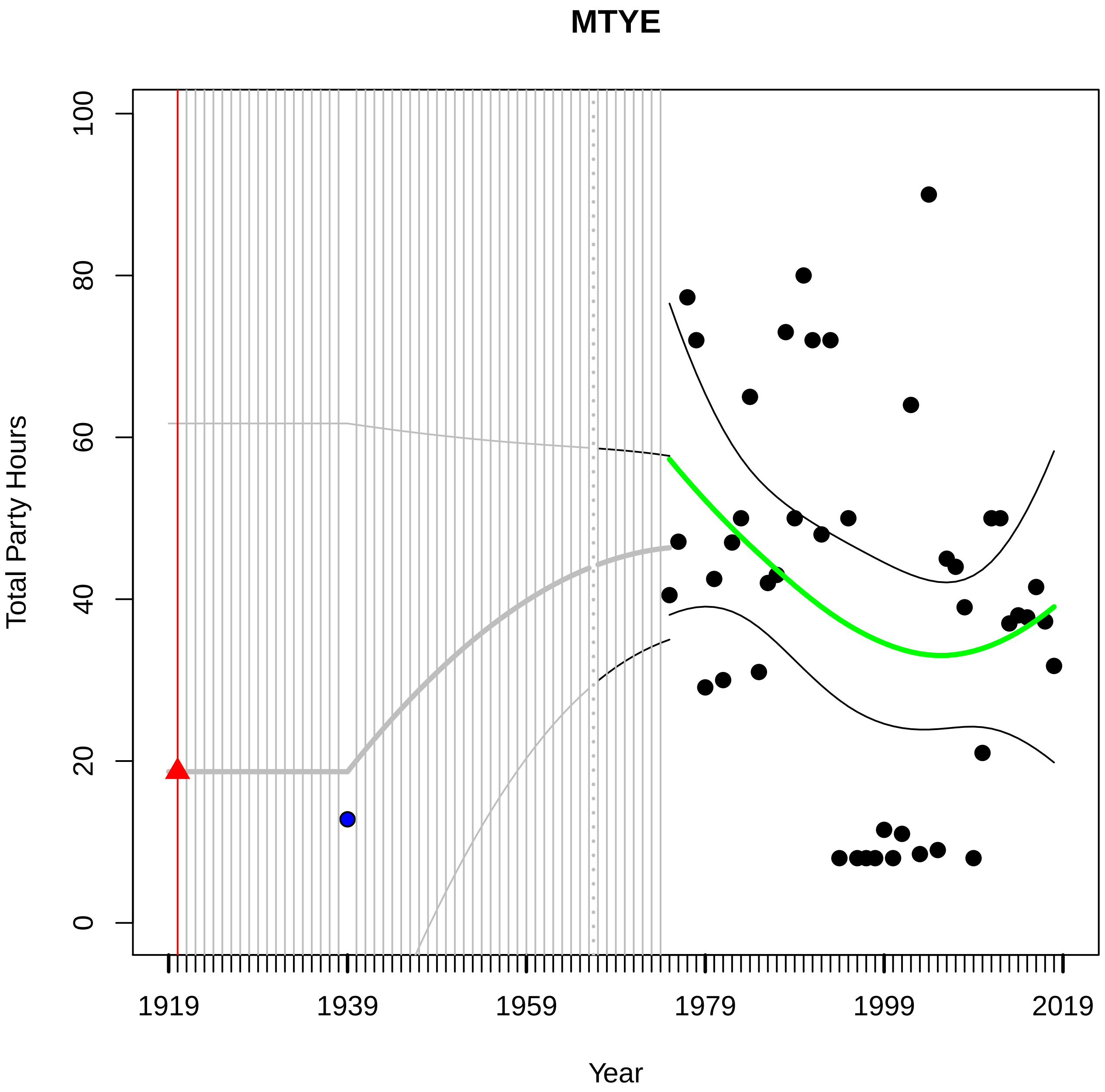

MTYE
Total Party Hours
Year
0
20
40
60
80
100
1919
1939
1959
1979
1999
2019

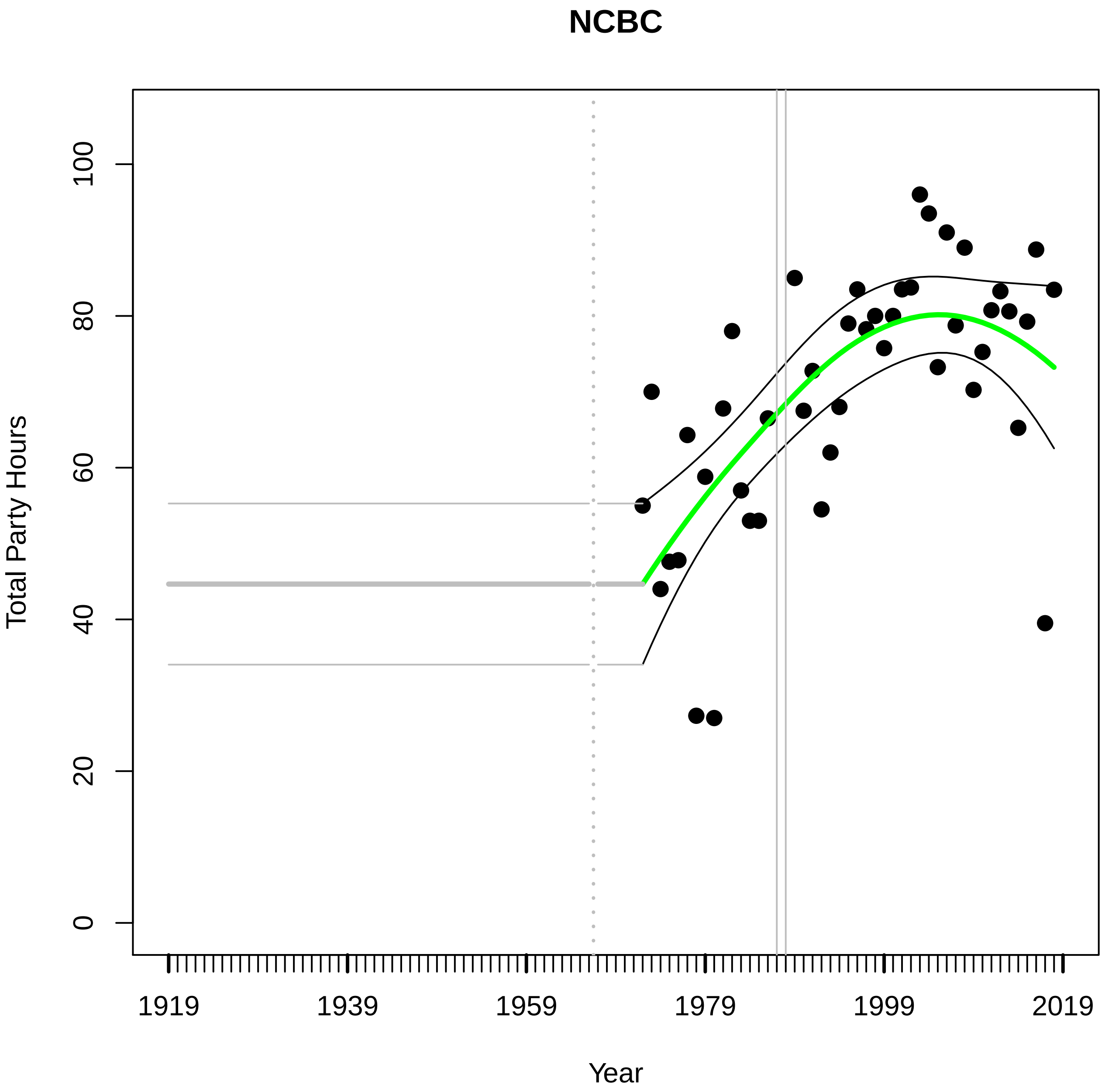

NCBC
Total Party Hours
0
20
40
60
80
100
1919
1939
1959
1979
1999
2019
Year

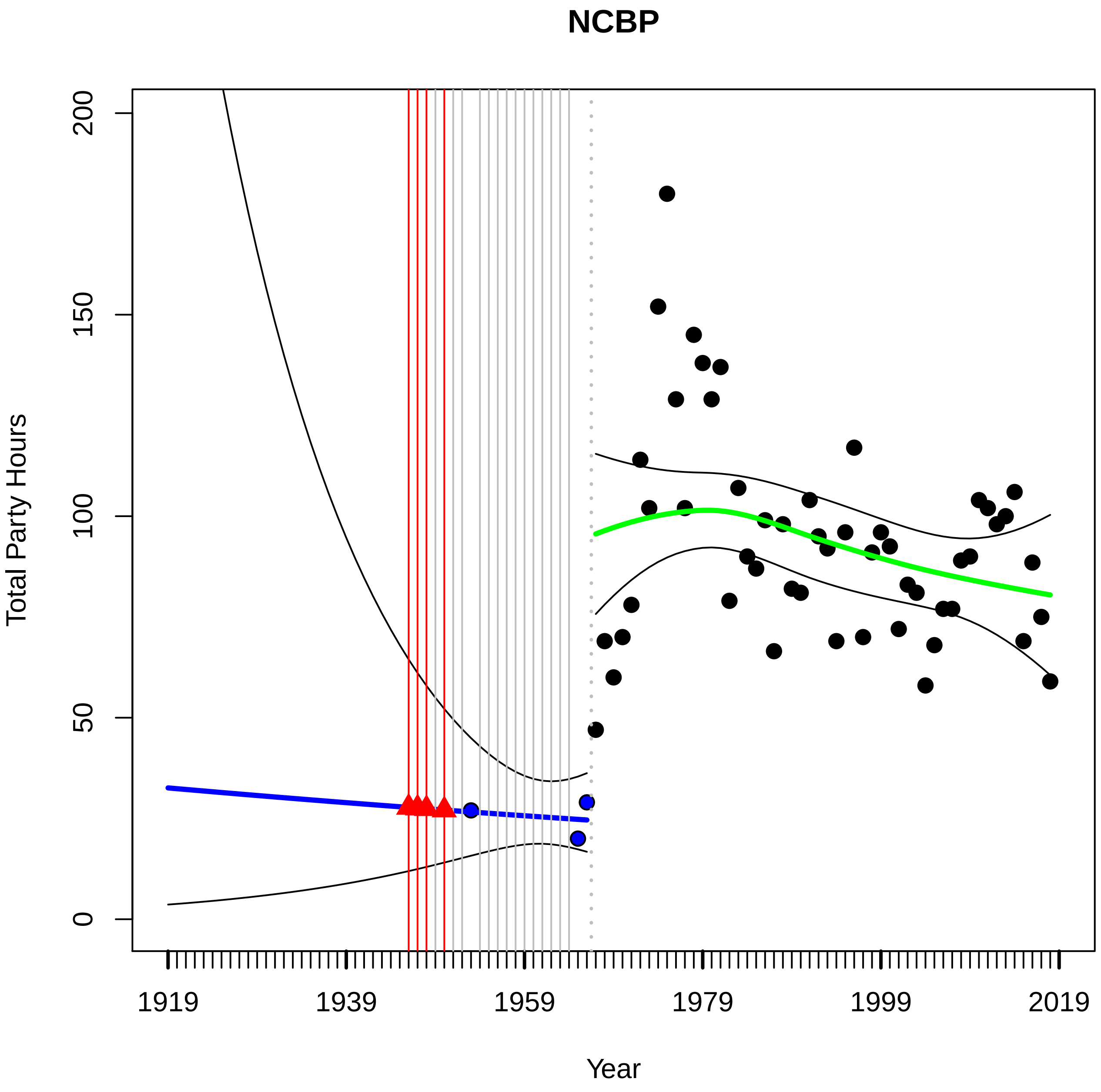
NCBP
Total Party Hours
Year
0
50
100
150
200
1919
1939
1959
1979
1999
2019

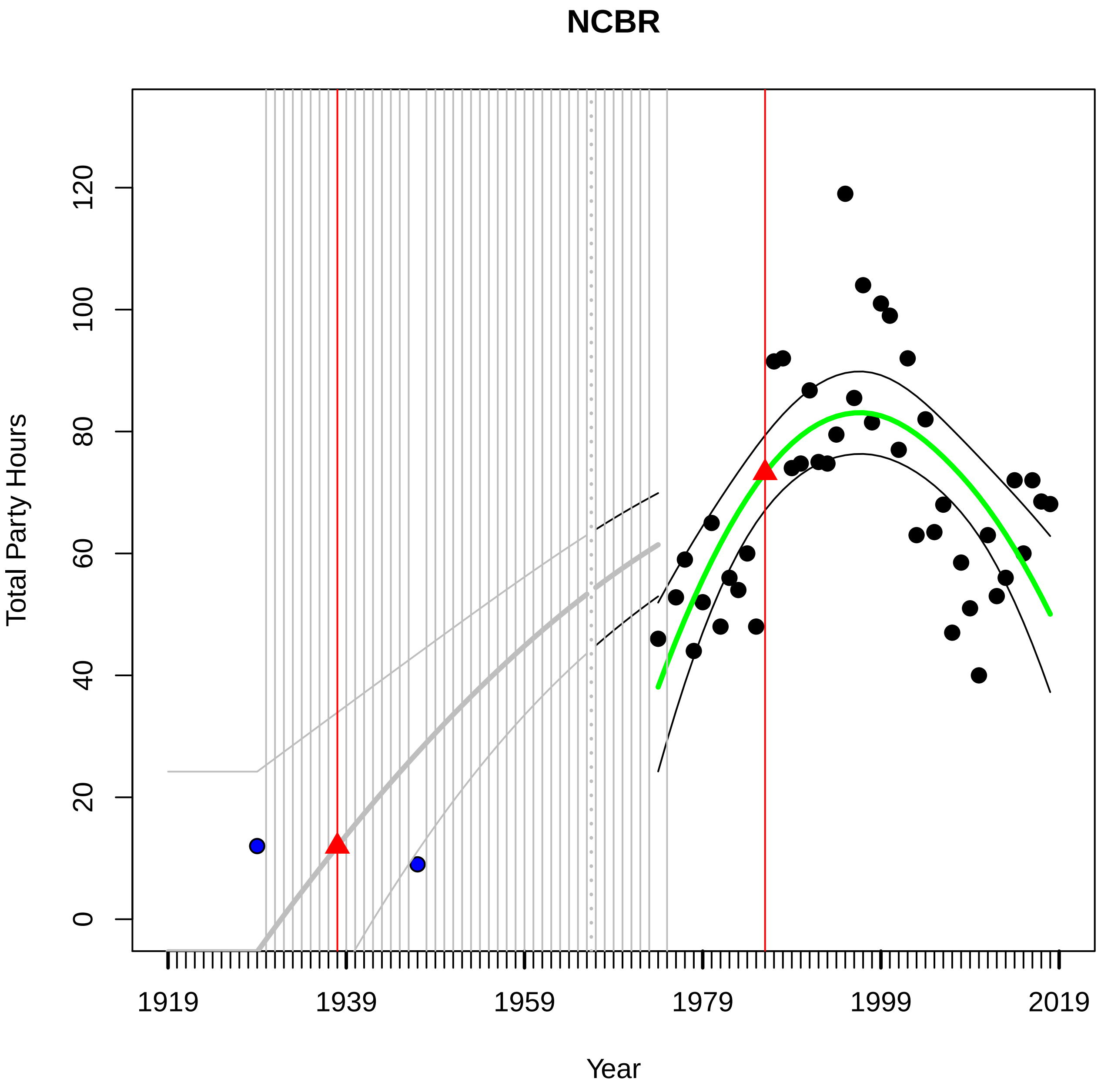

NCBR
Total Party Hours
Year
0
20
40
60
80
100
120
1919
1939
1959
1979
1999
2019

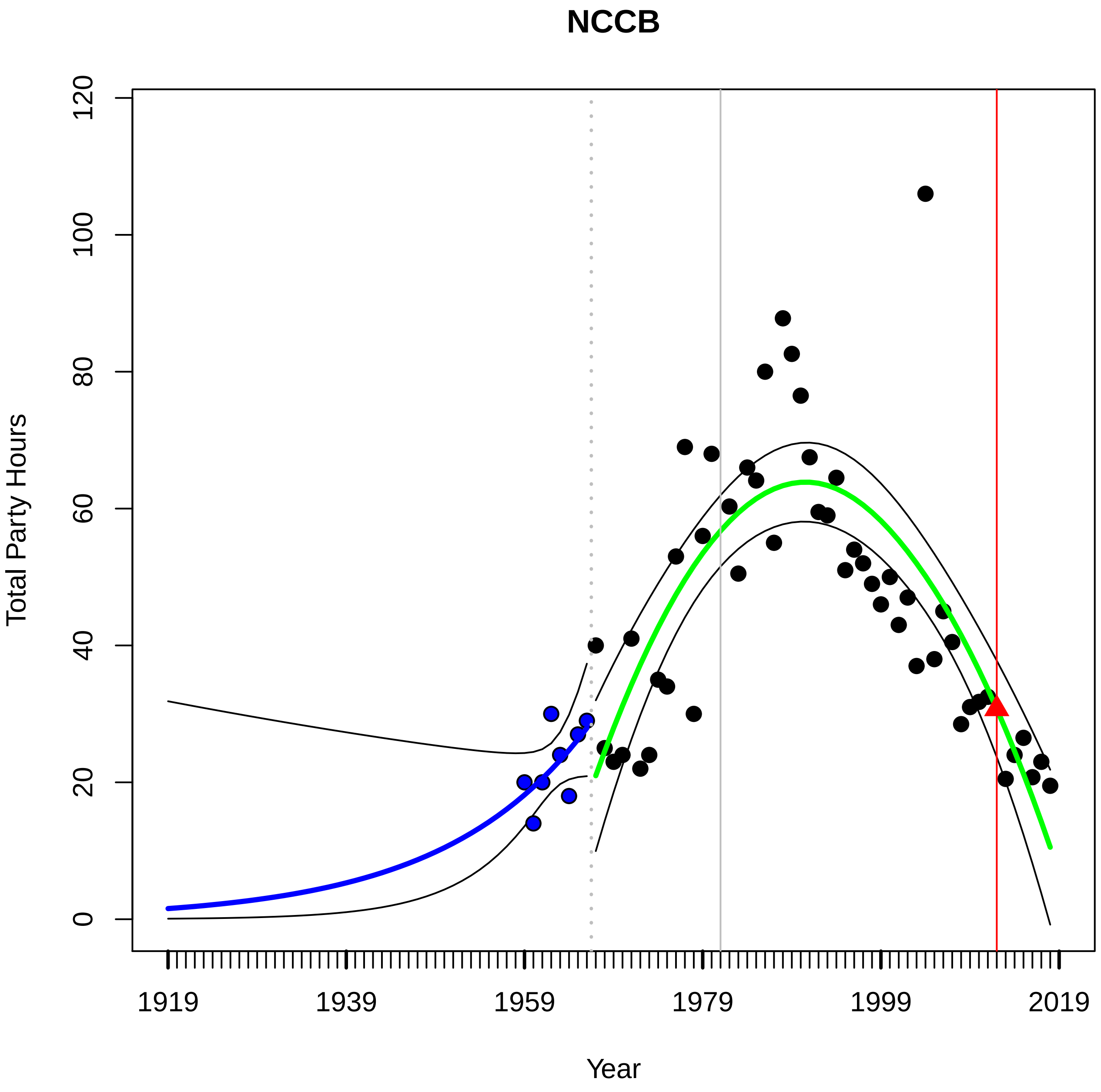
NCCB
Total Party Hours
0
20
40
60
80
100
120
Year
1919
1939
1959
1979
1999
2019

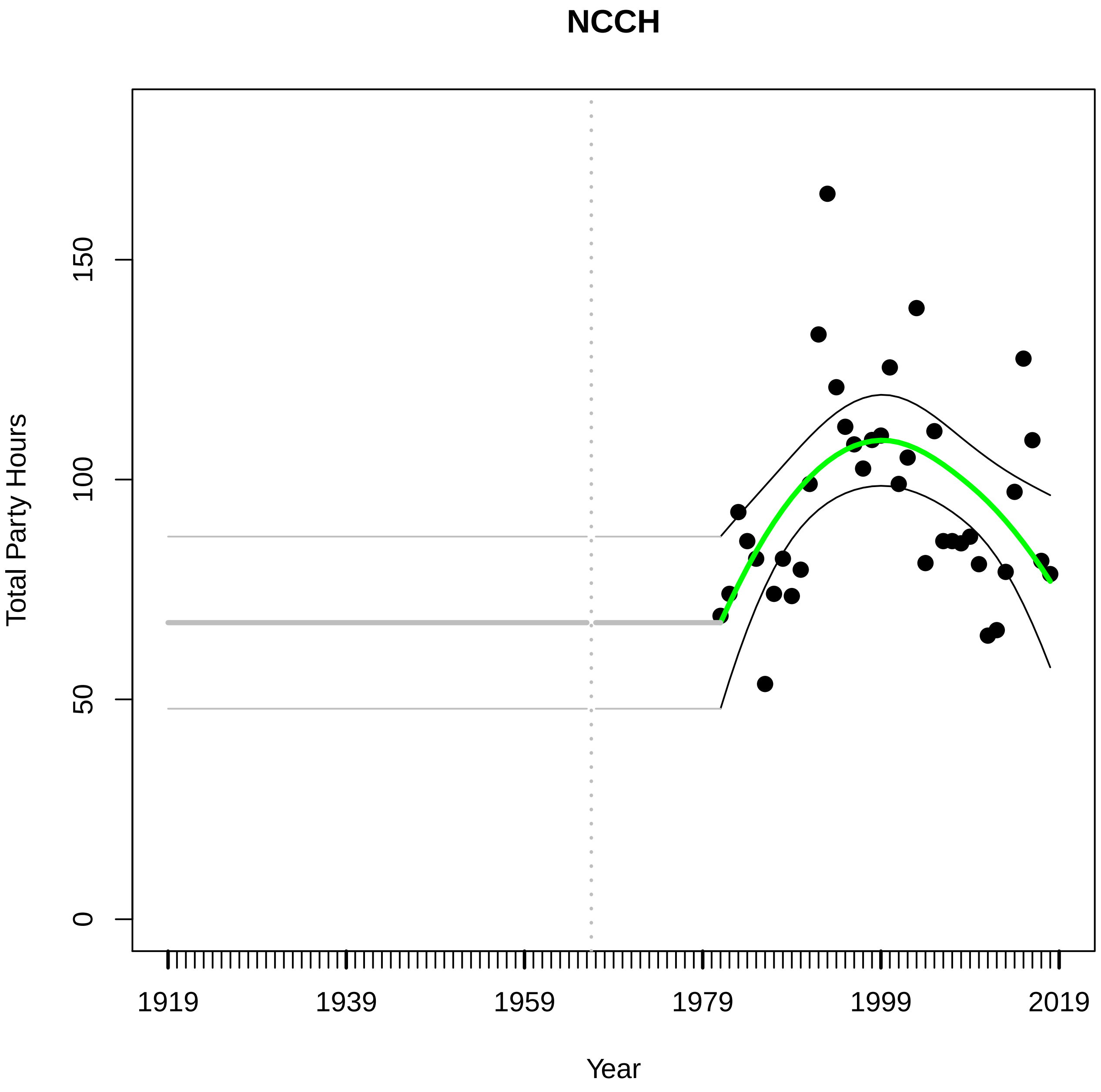

NCCH
Total Party Hours
0
50
100
150
Year
1919
1939
1959
1979
1999
2019

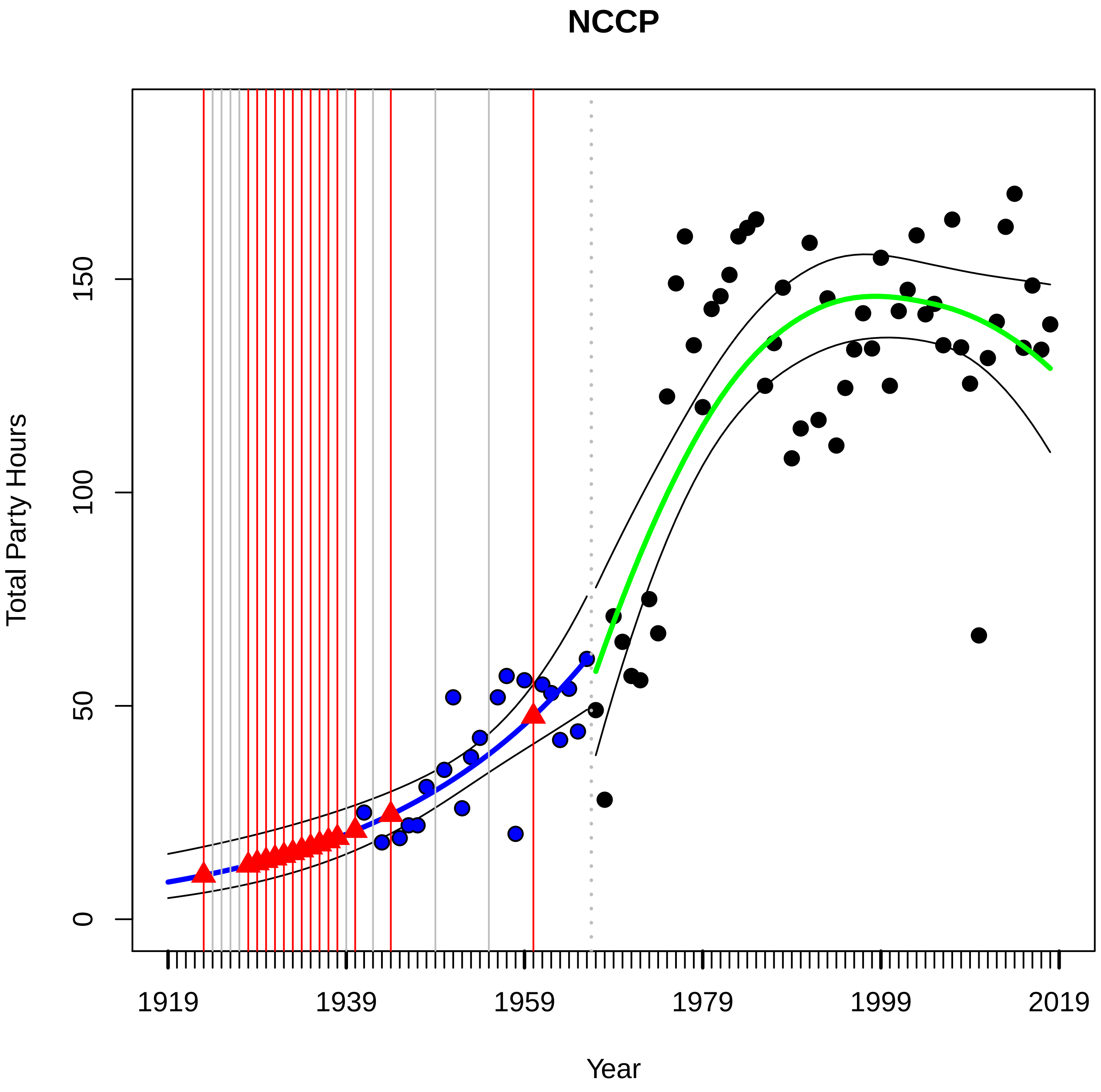

NCCP
Total Party Hours
0
50
100
150
1919
1939
1959
1979
1999
2019
Year

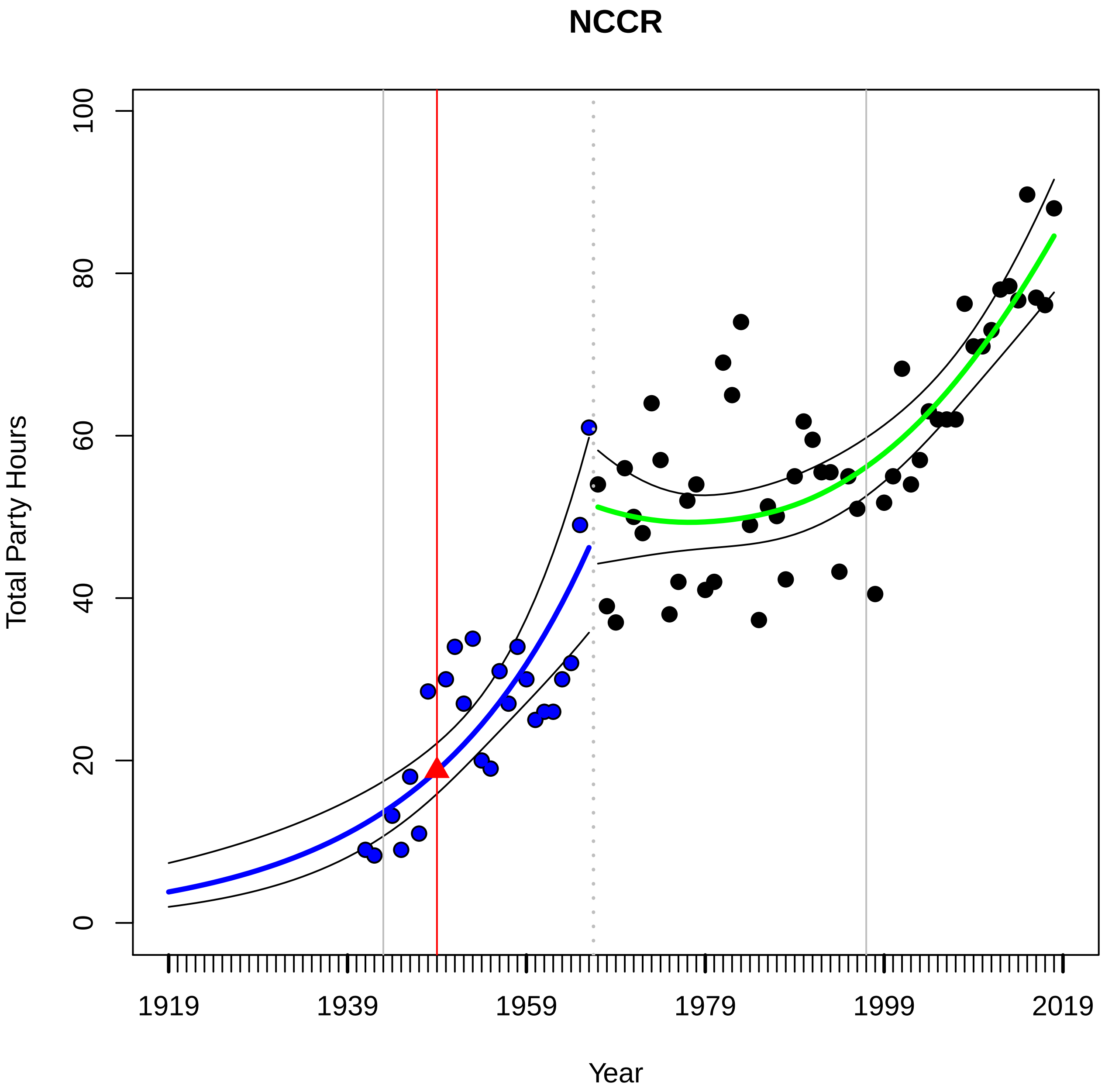

NCCR
Total Party Hours
Year
0
20
40
60
80
100
1919
1939
1959
1979
1999
2019

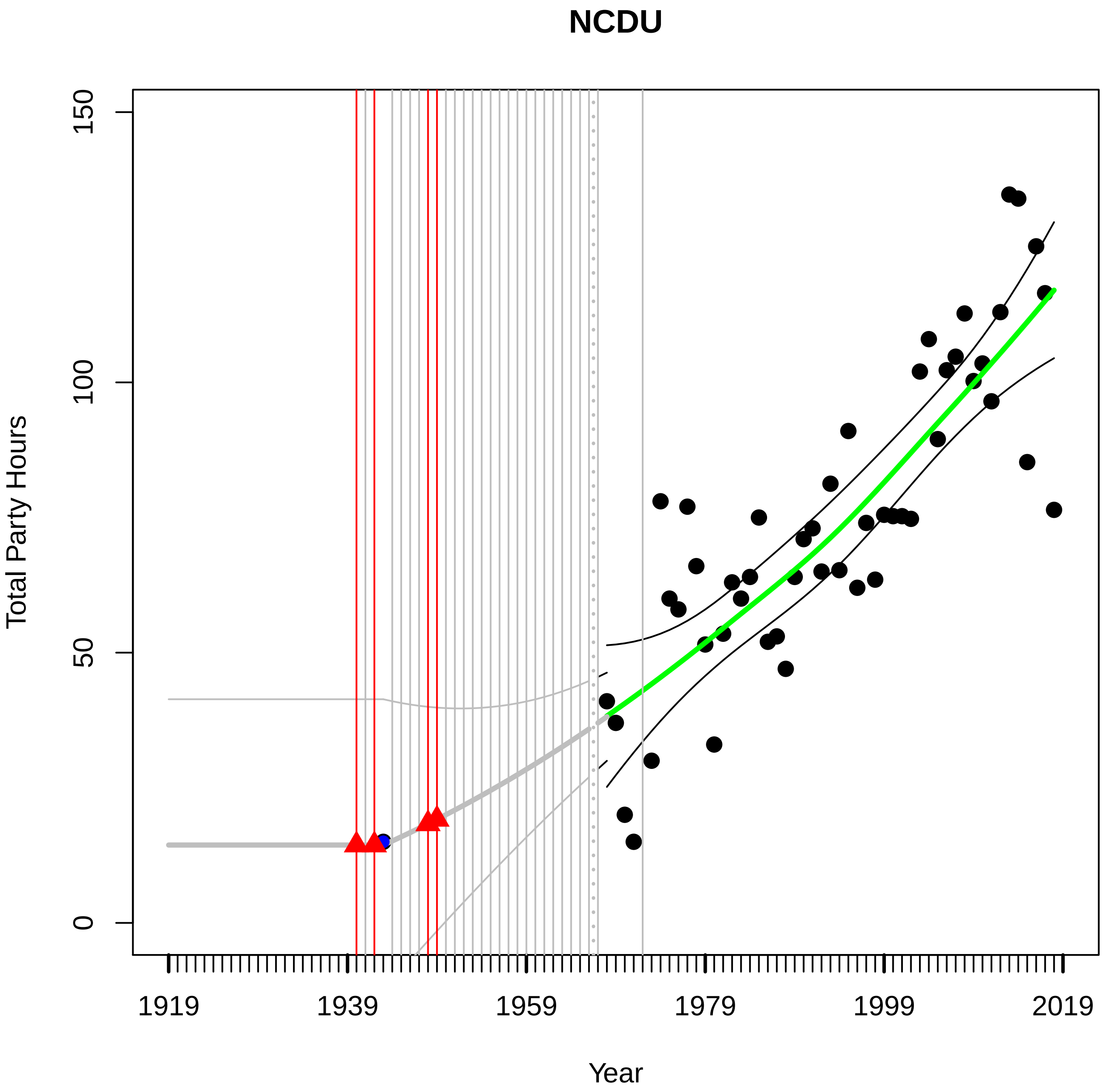

NCDU
Total Party Hours
150
100
50
0
1919
1939
1959
1979
1999
2019
Year

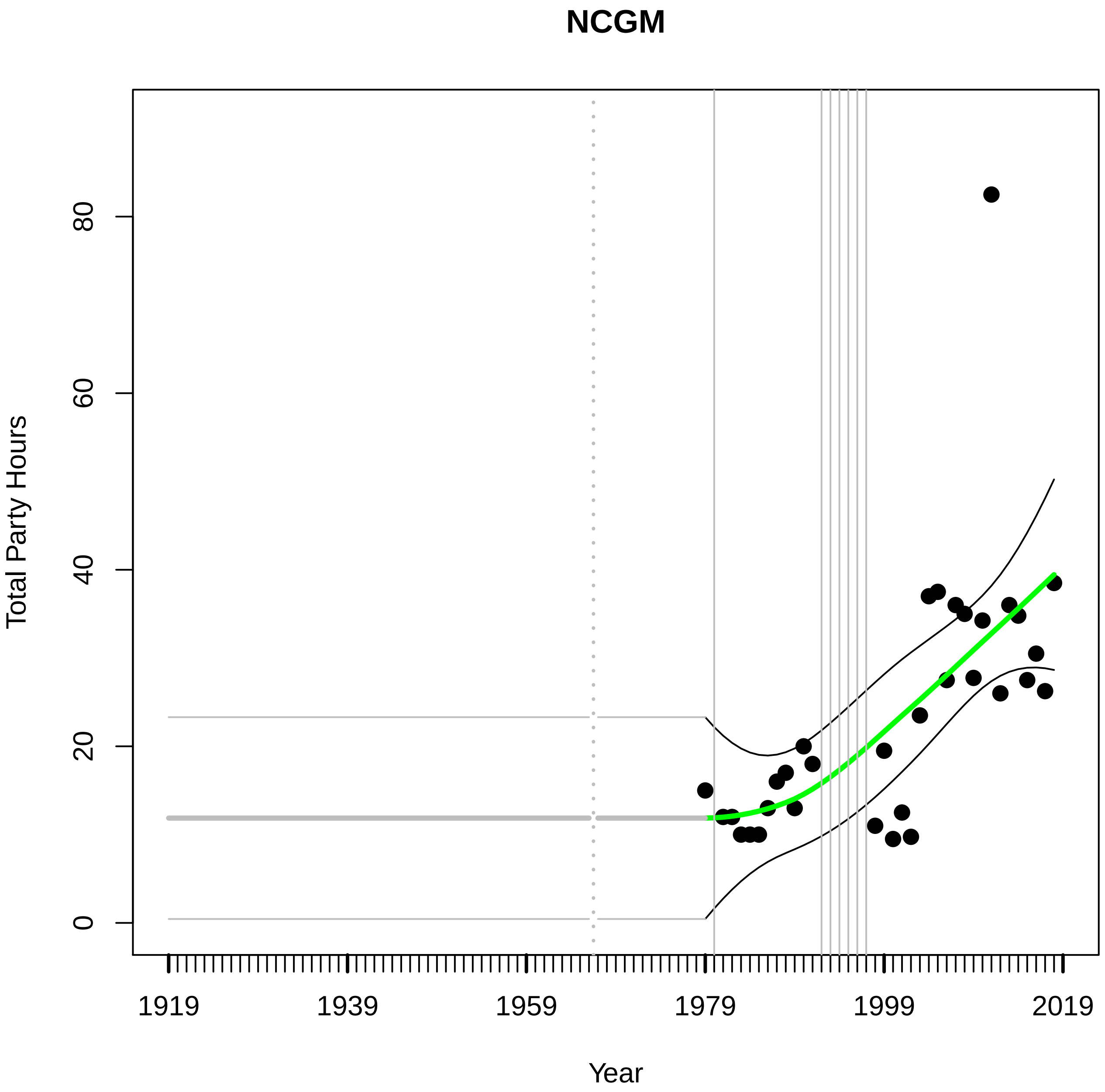
NCGM
Total Party Hours
0
20
40
60
80
1919
1939
1959
1979
1999
2019
Year

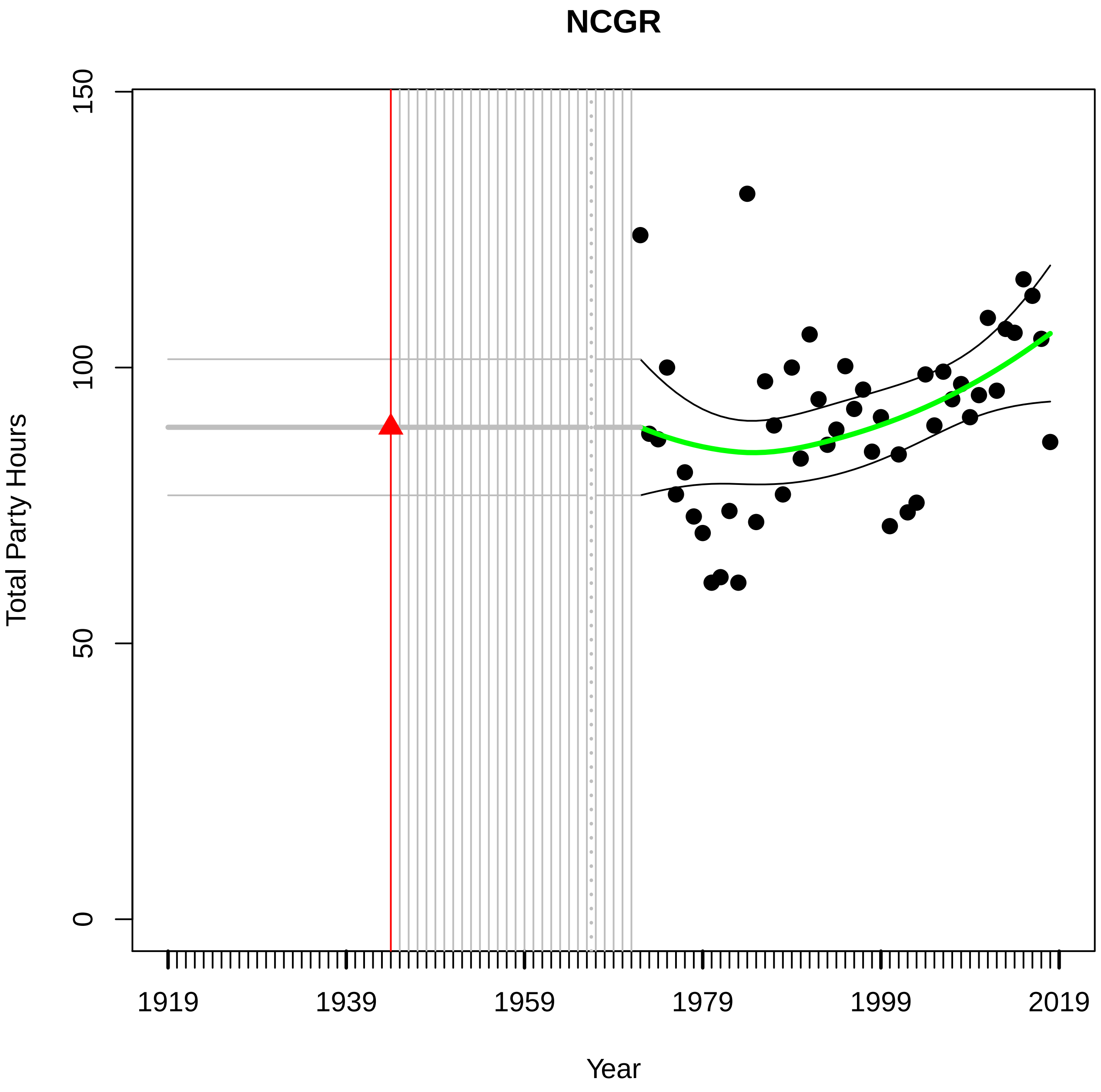
NCGR
Total Party Hours
150
100
50
0
1919
1939
1959
1979
1999
2019
Year

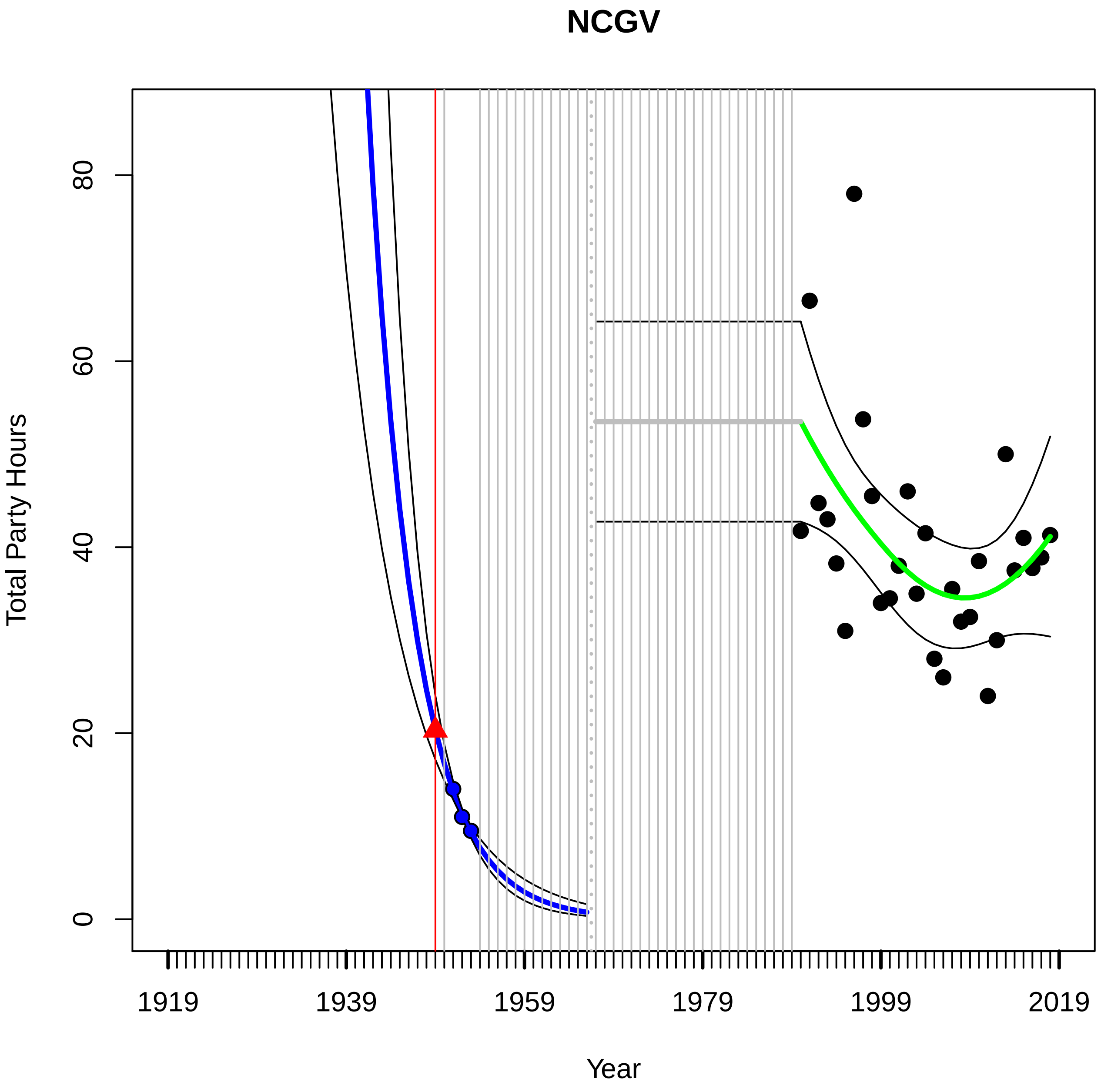
NCGV
Total Party Hours
0
20
40
60
80
1919
1939
1959
1979
1999
2019
Year

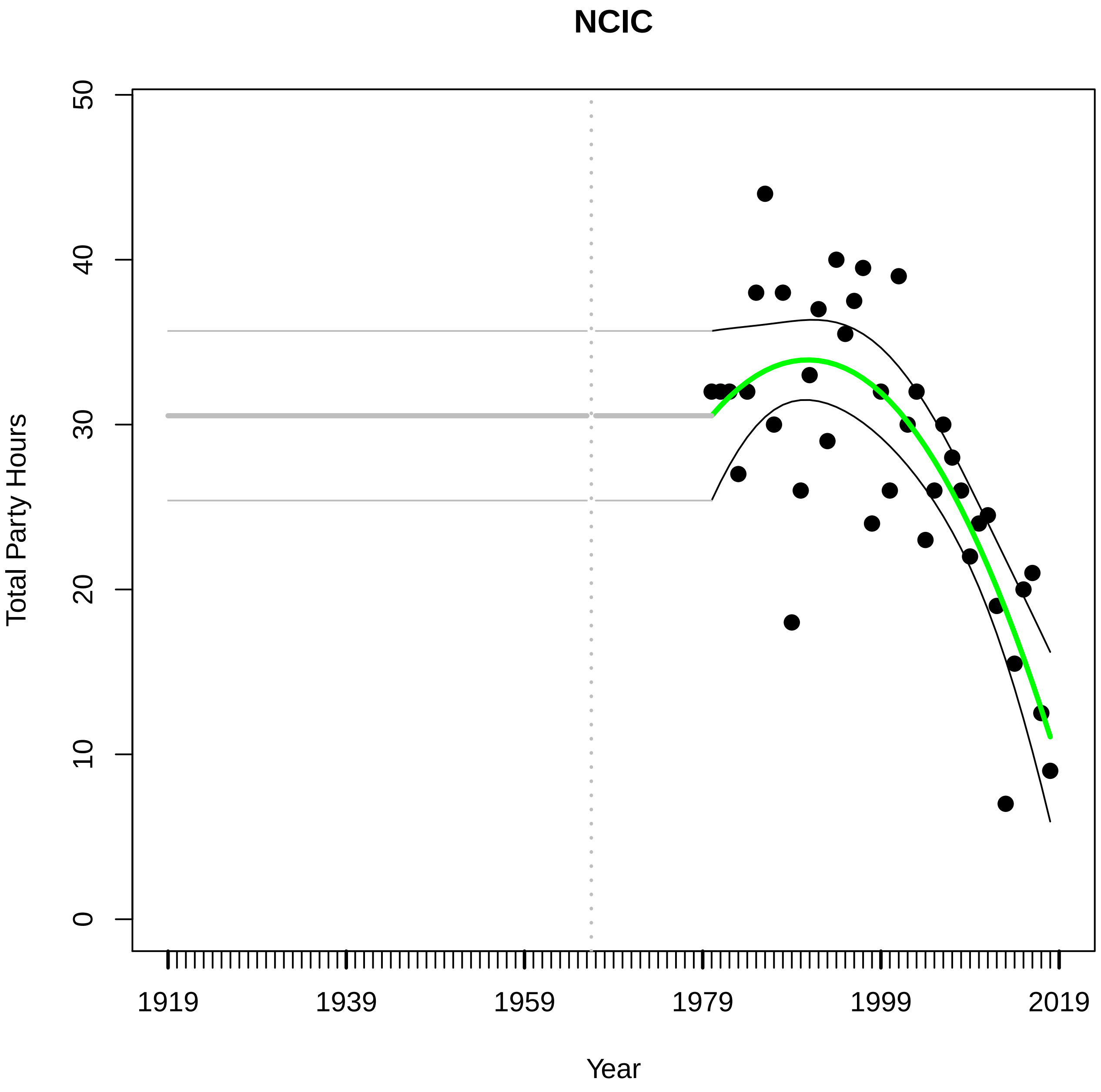

NCIC
Total Party Hours
0
10
20
30
40
50
1919
1939
1959
1979
1999
2019
Year

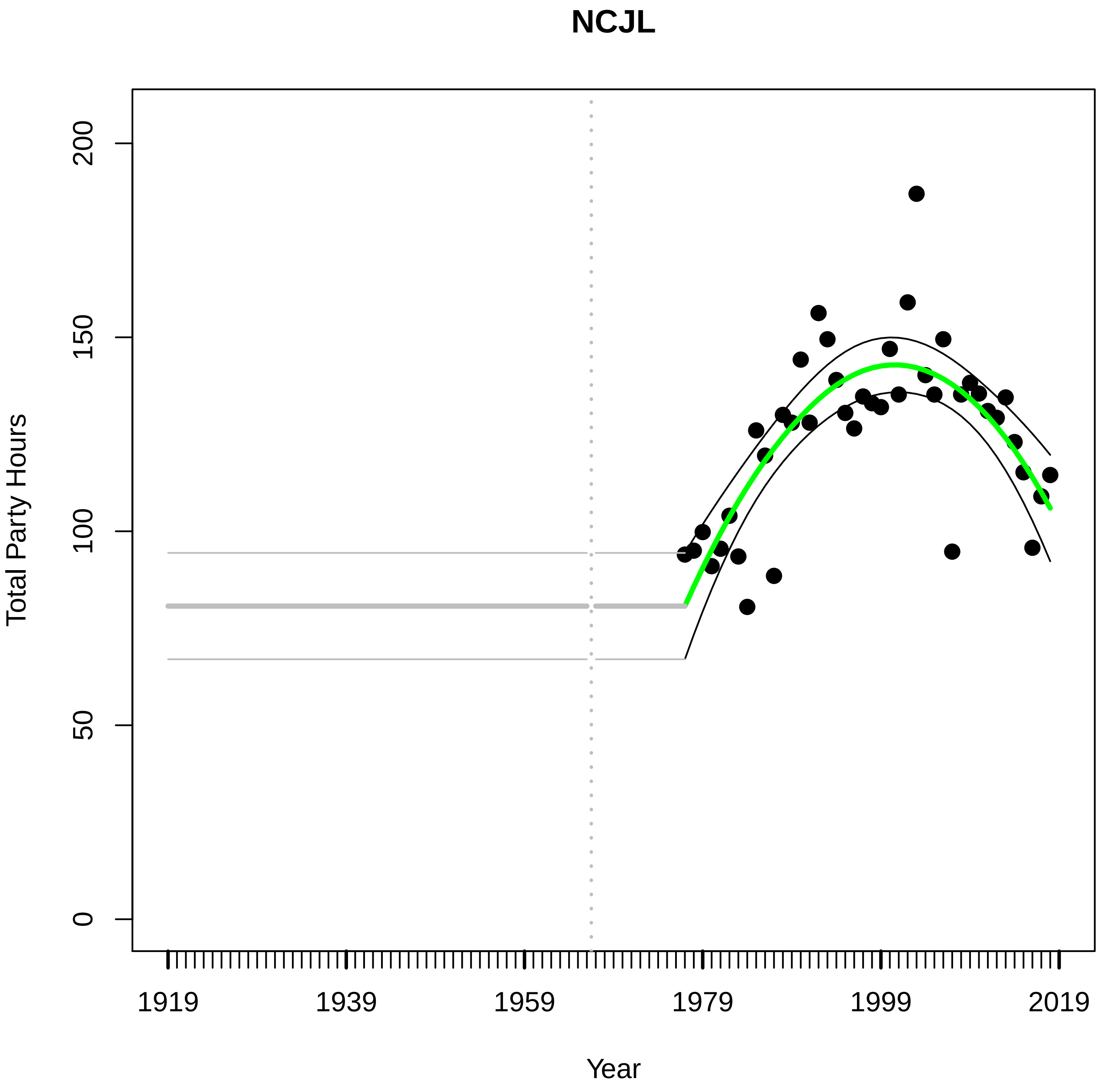

NCJL
Total Party Hours
200
150
100
50
0
1919
1939
1959
1979
1999
2019
Year

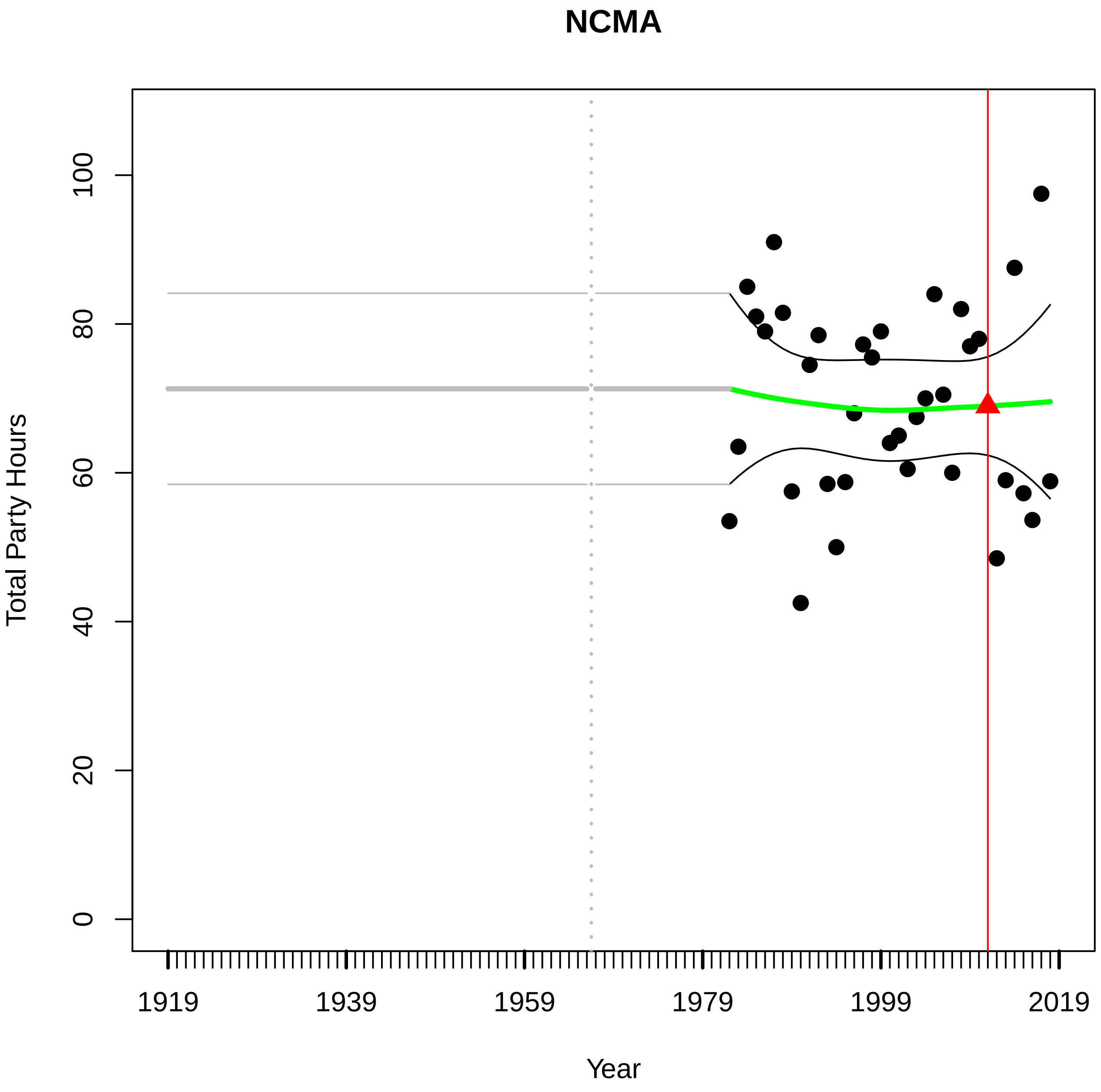

NCMA
Total Party Hours
0
20
40
60
80
100
1919
1939
1959
1979
1999
2019
Year

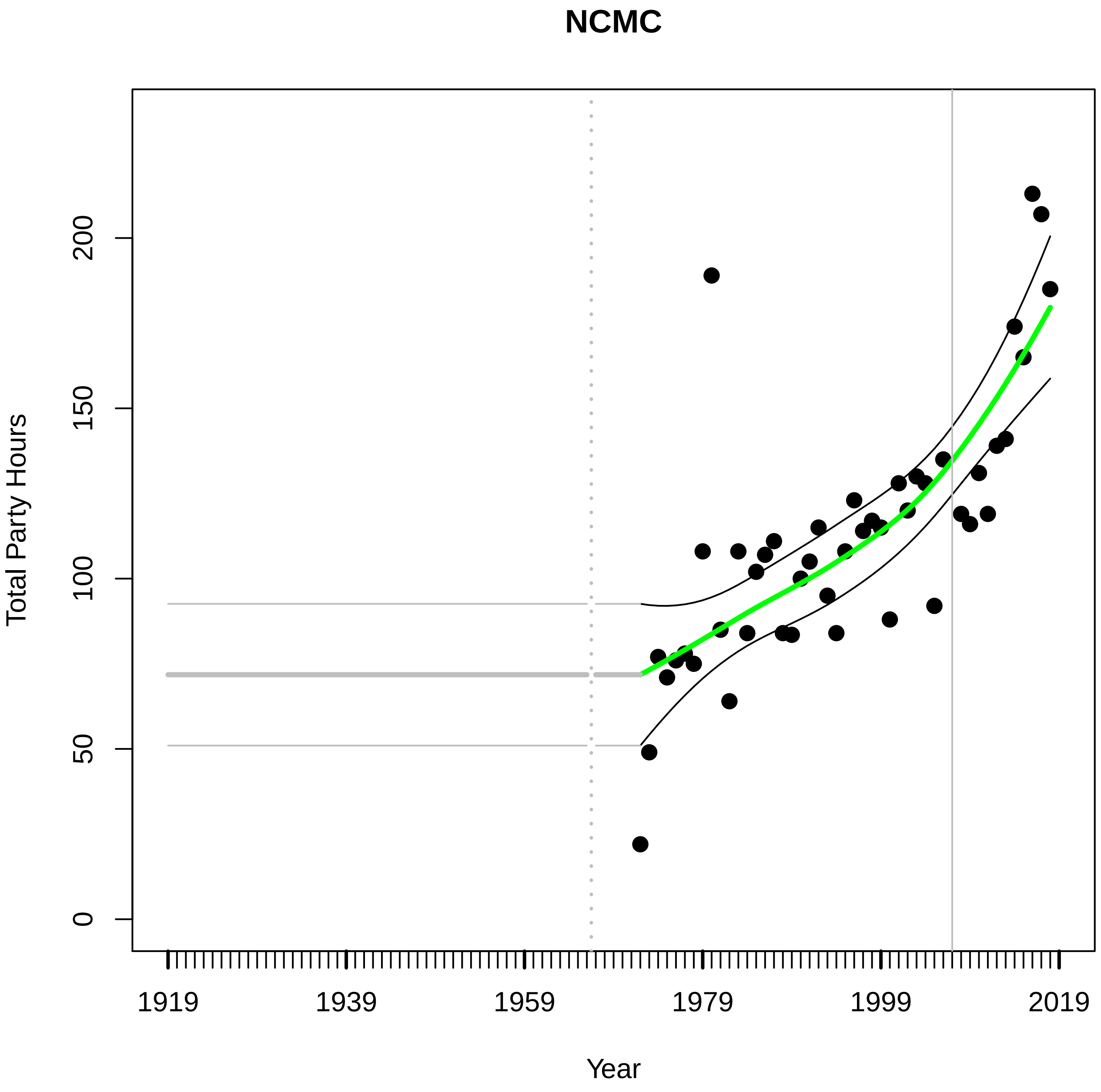

NCMC
Total Party Hours
0
50
100
150
200
Year
1919
1939
1959
1979
1999
2019

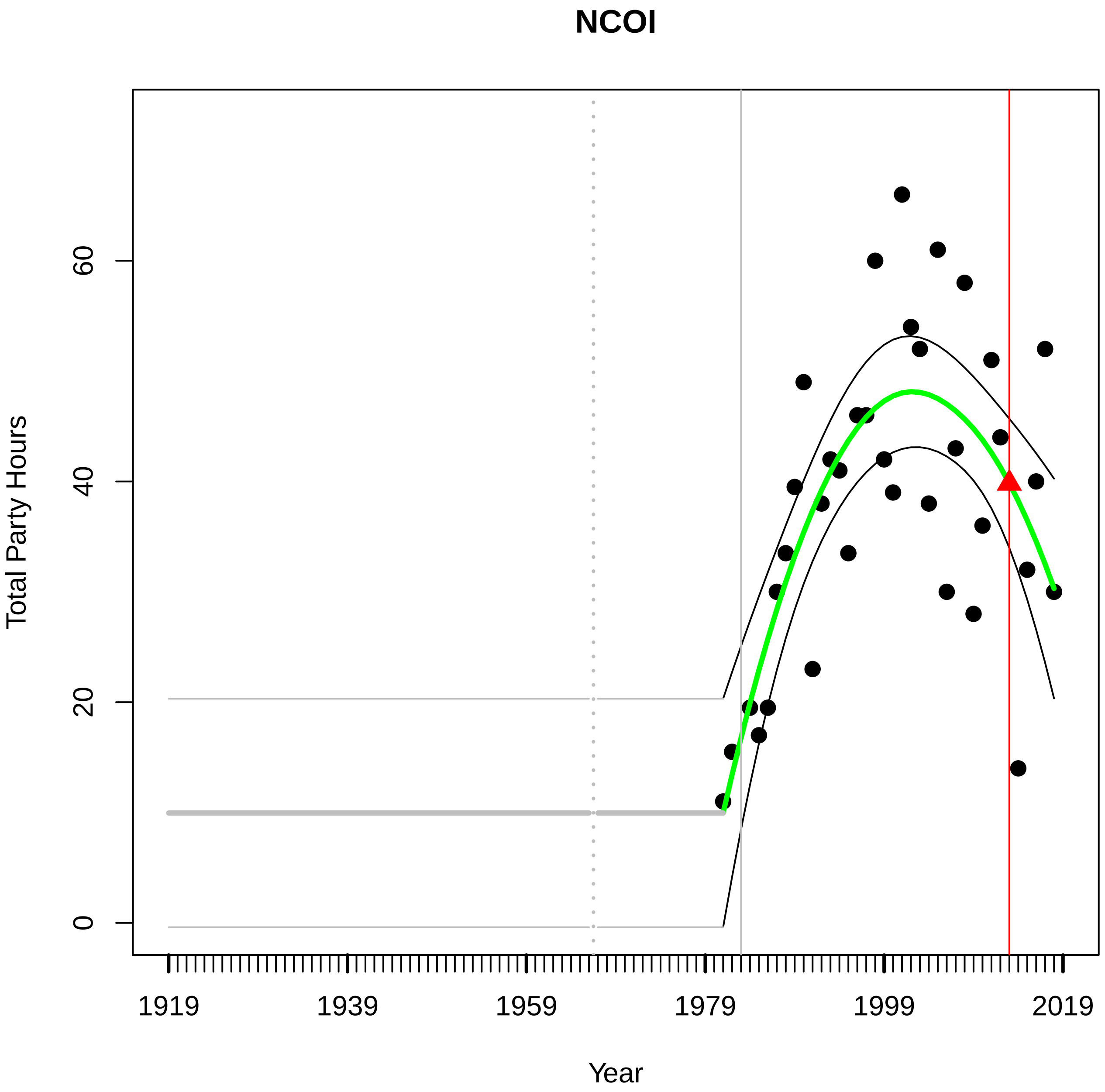
NCOI
Total Party Hours
0
20
40
60
1919
1939
1959
1979
1999
2019
Year

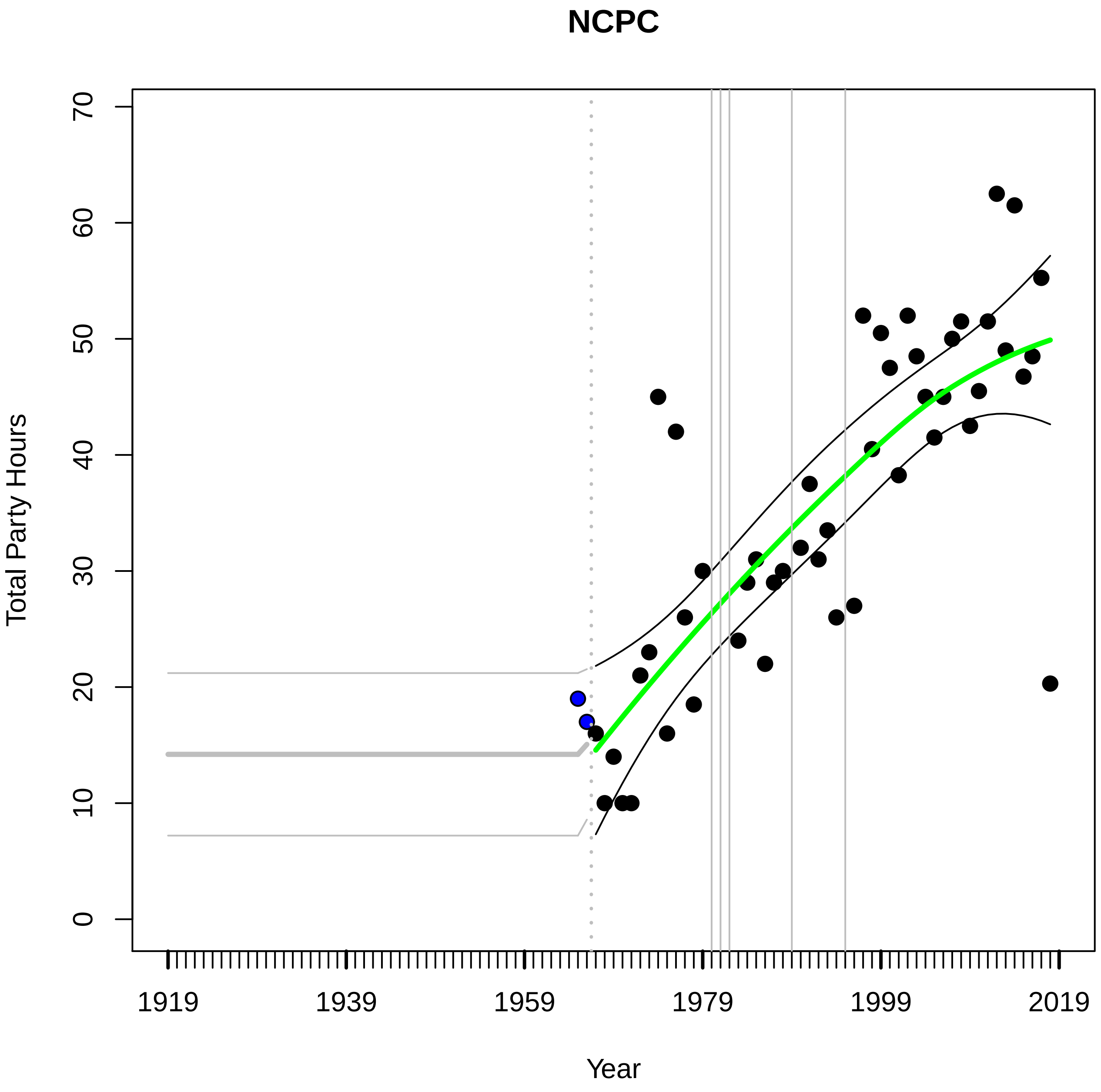
NCPC
Total Party Hours
Year
0
10
20
30
40
50
60
70
1919
1939
1959
1979
1999
2019

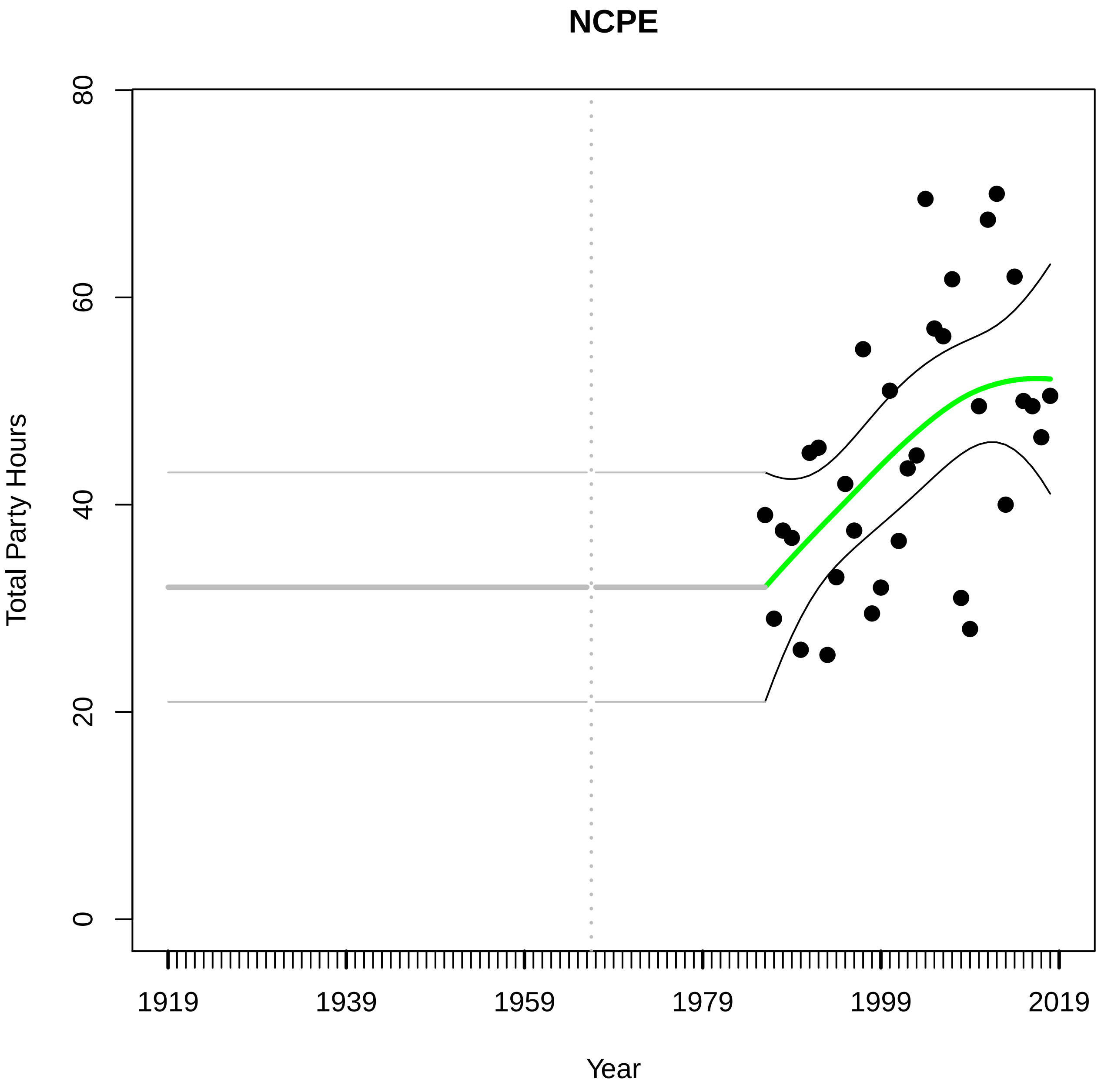
NCPE
Total Party Hours
0
20
40
60
80
1919
1939
1959
1979
1999
2019
Year

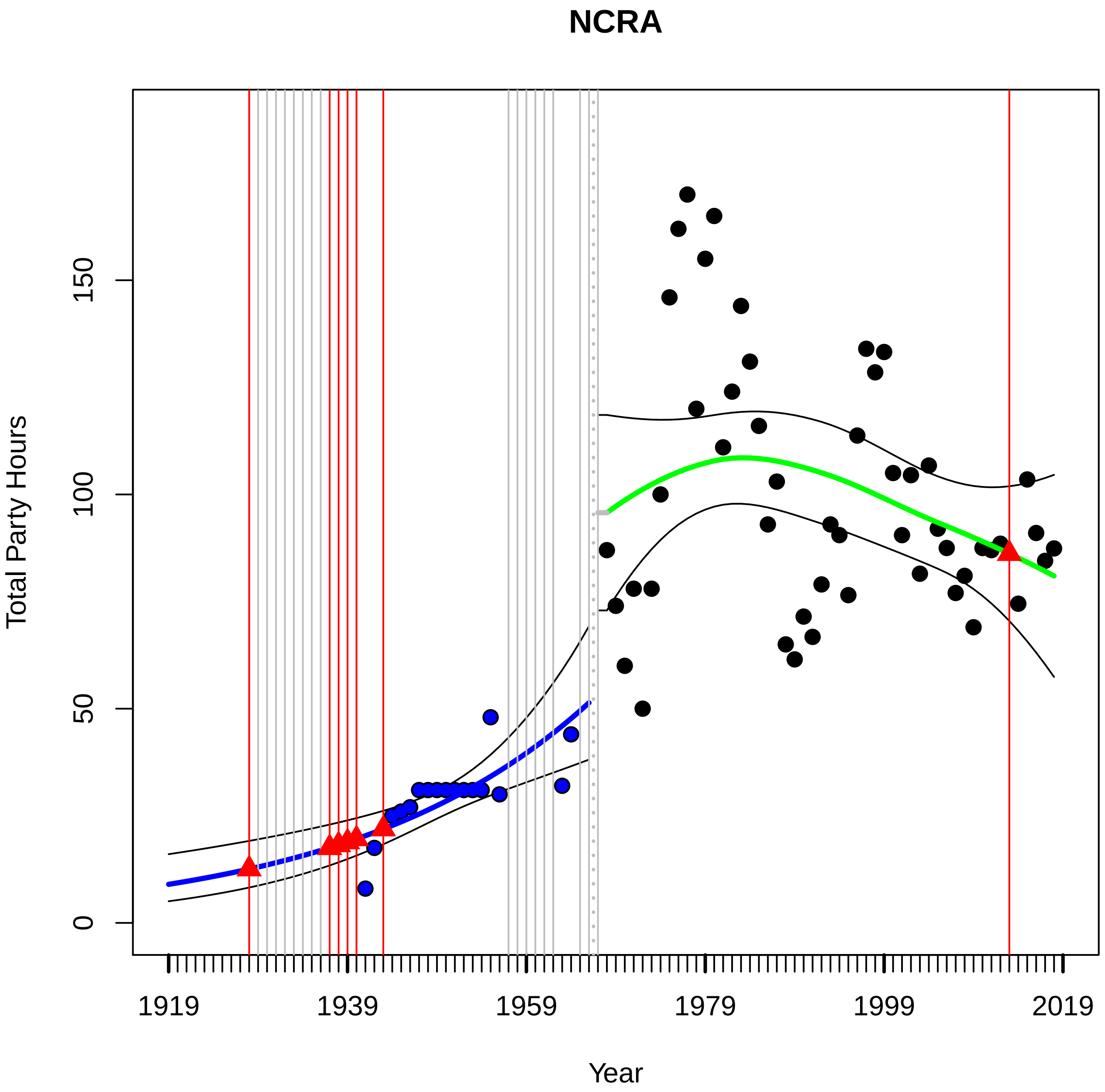

NCRA
Total Party Hours
0
50
100
150
1919
1939
1959
1979
1999
2019
Year

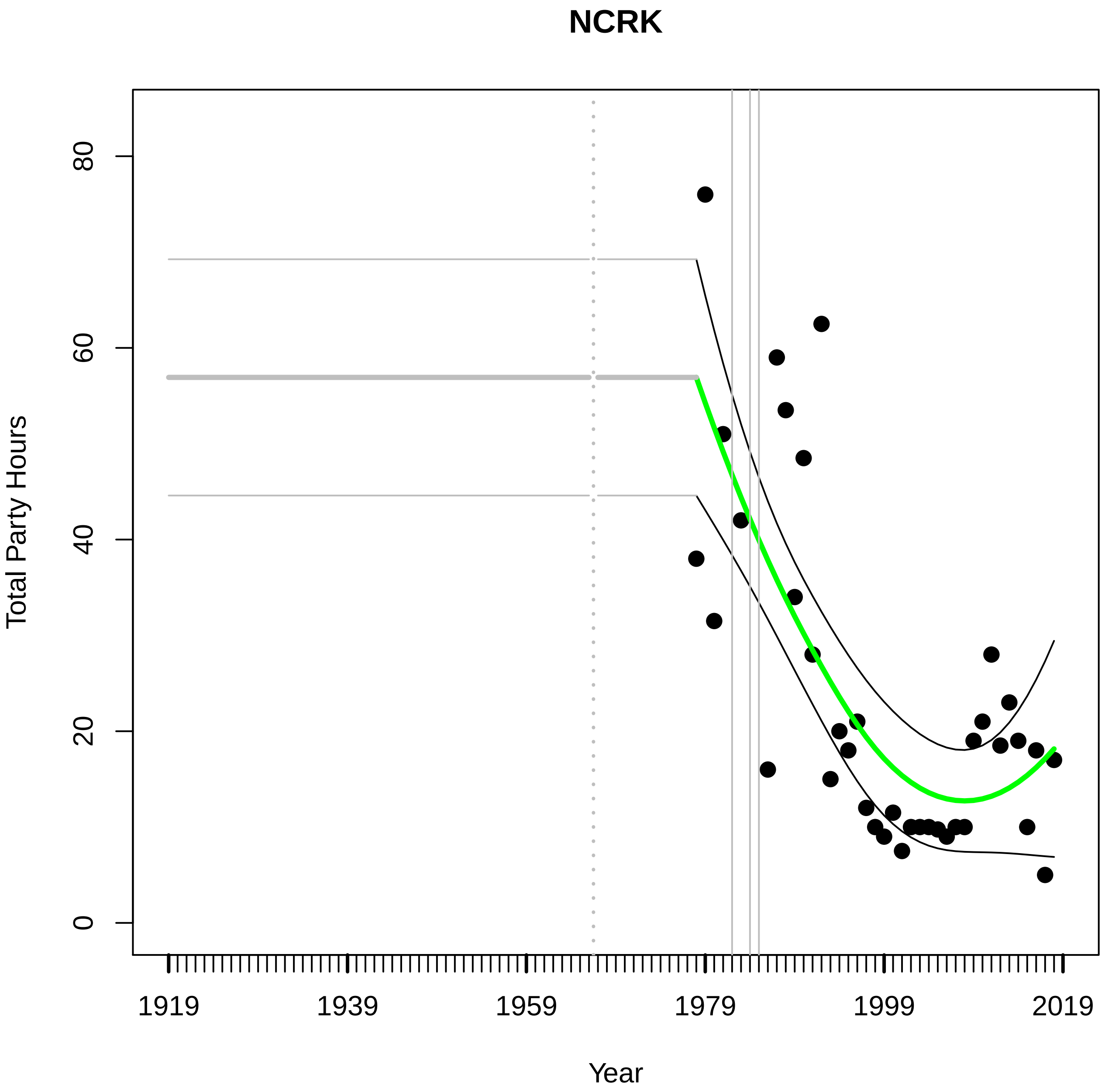
NCRK
Total Party Hours
80
60
40
20
0
1919
1939
1959
1979
1999
2019
Year

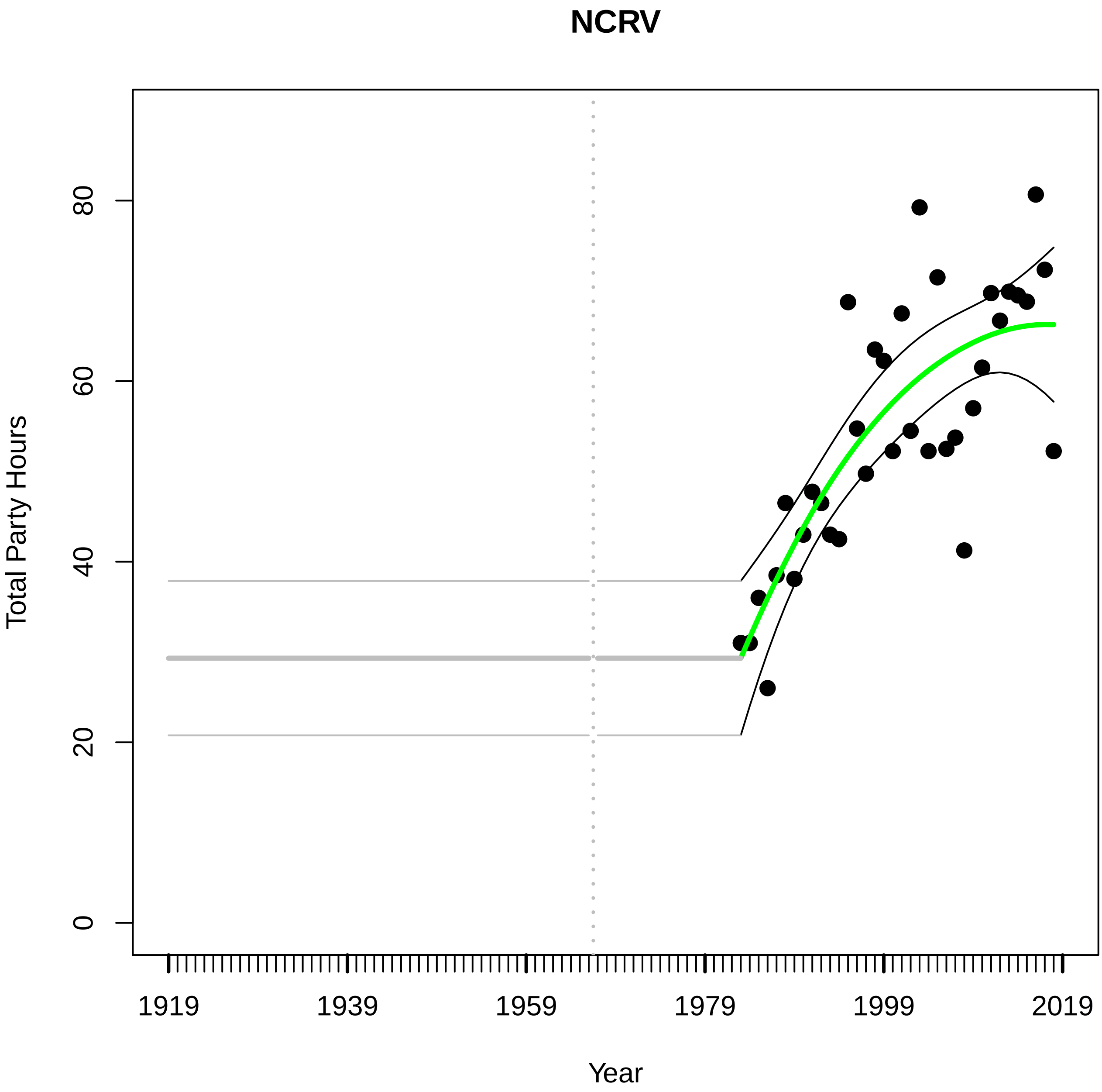

NCRV
Total Party Hours
0
20
40
60
80
1919
1939
1959
1979
1999
2019
Year

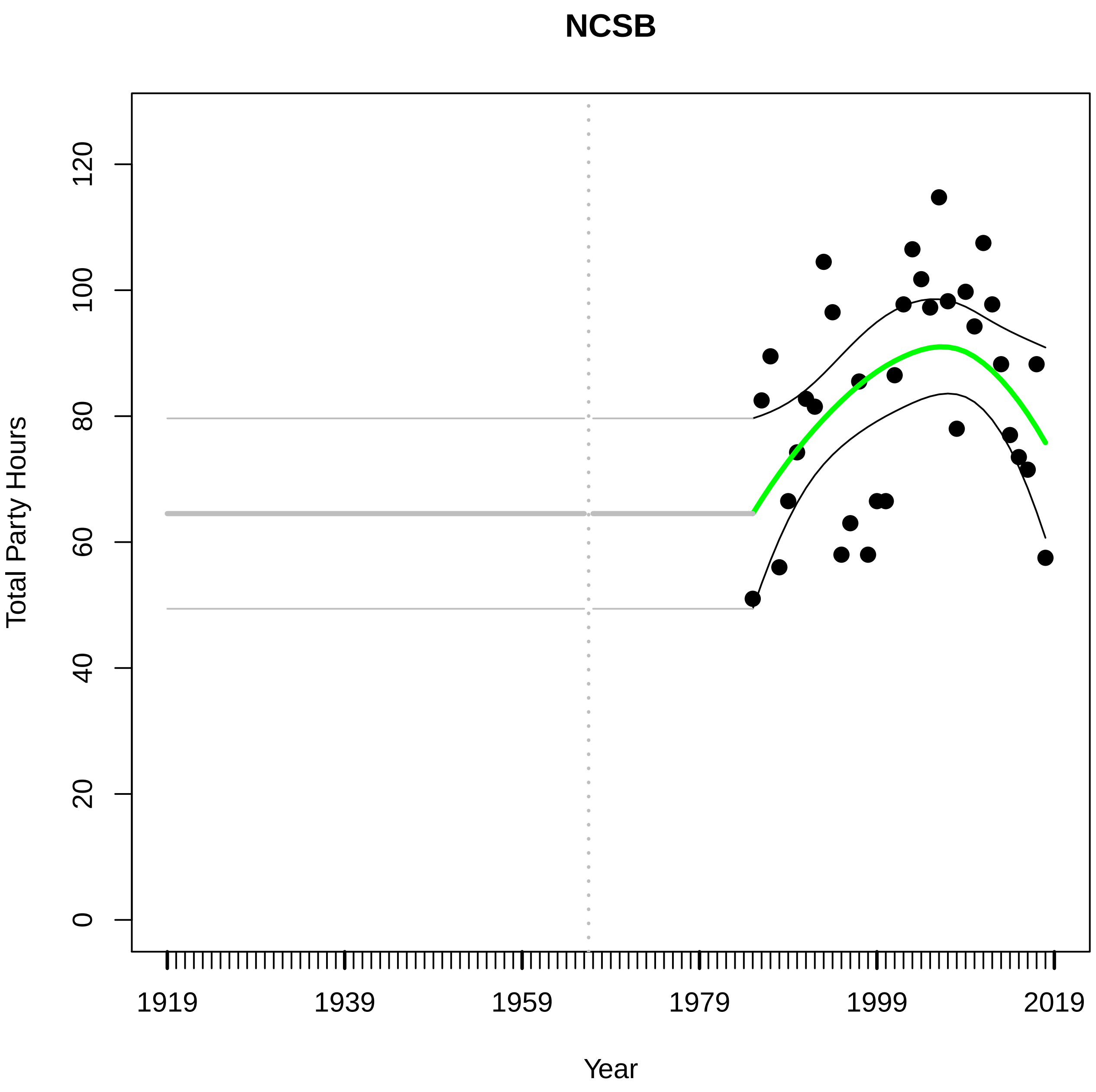

NCSB
Total Party Hours
0
20
40
60
80
100
120
1919
1939
1959
1979
1999
2019
Year

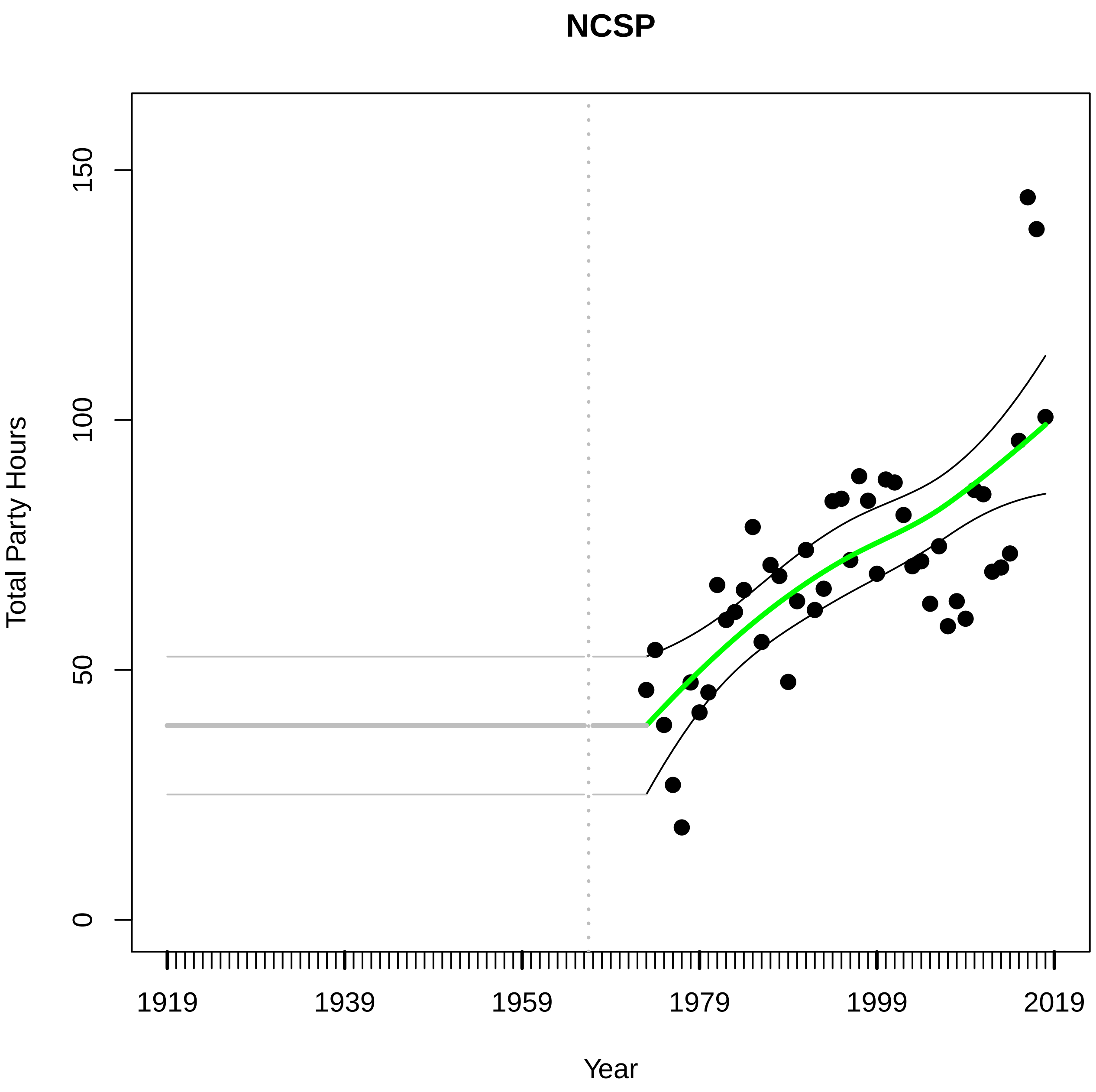
NCSP
Total Party Hours
0
50
100
150
1919
1939
1959
1979
1999
2019
Year

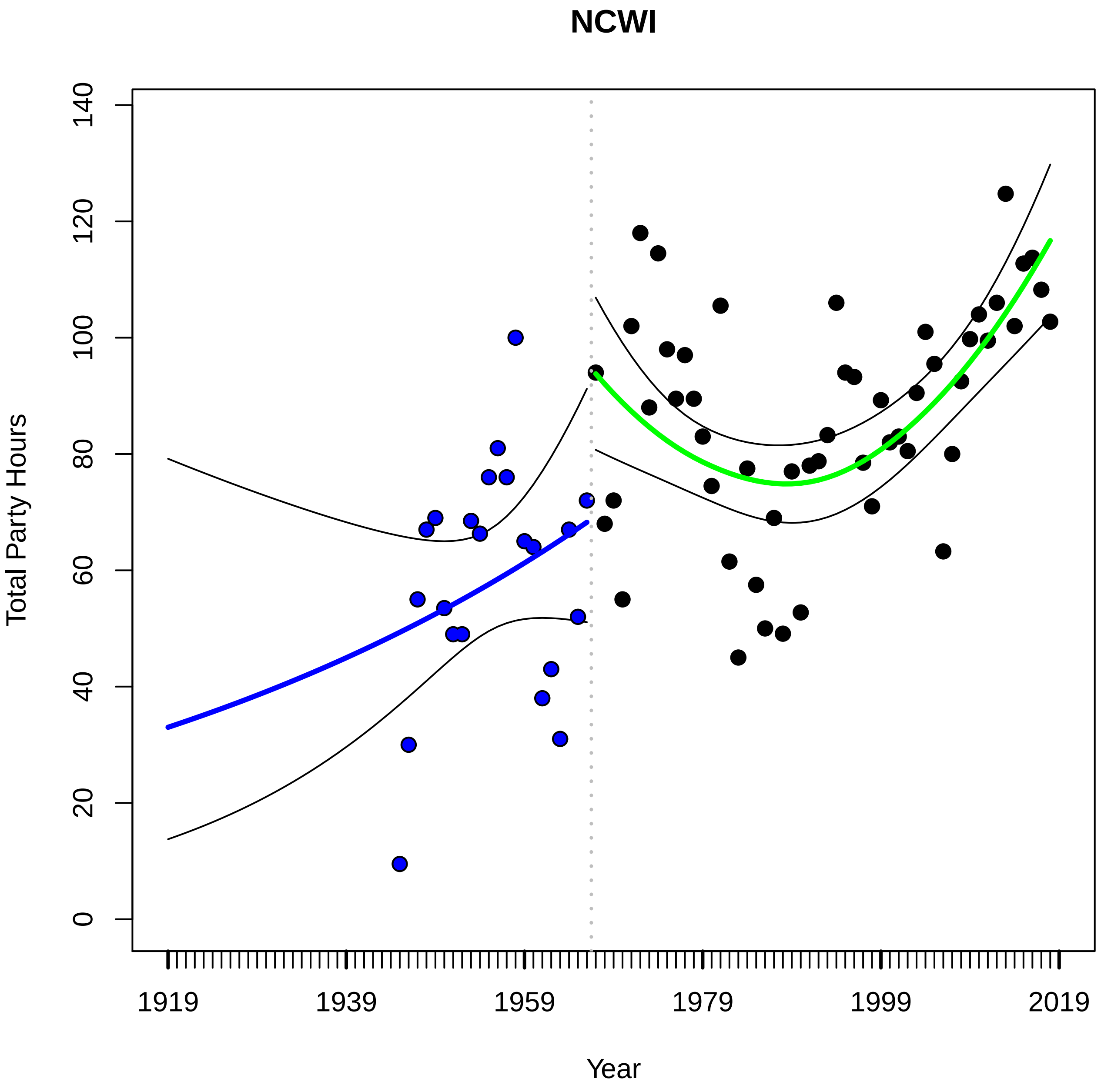
NCWI
Total Party Hours
0
20
40
60
80
100
120
140
1919
1939
1959
1979
1999
2019
Year

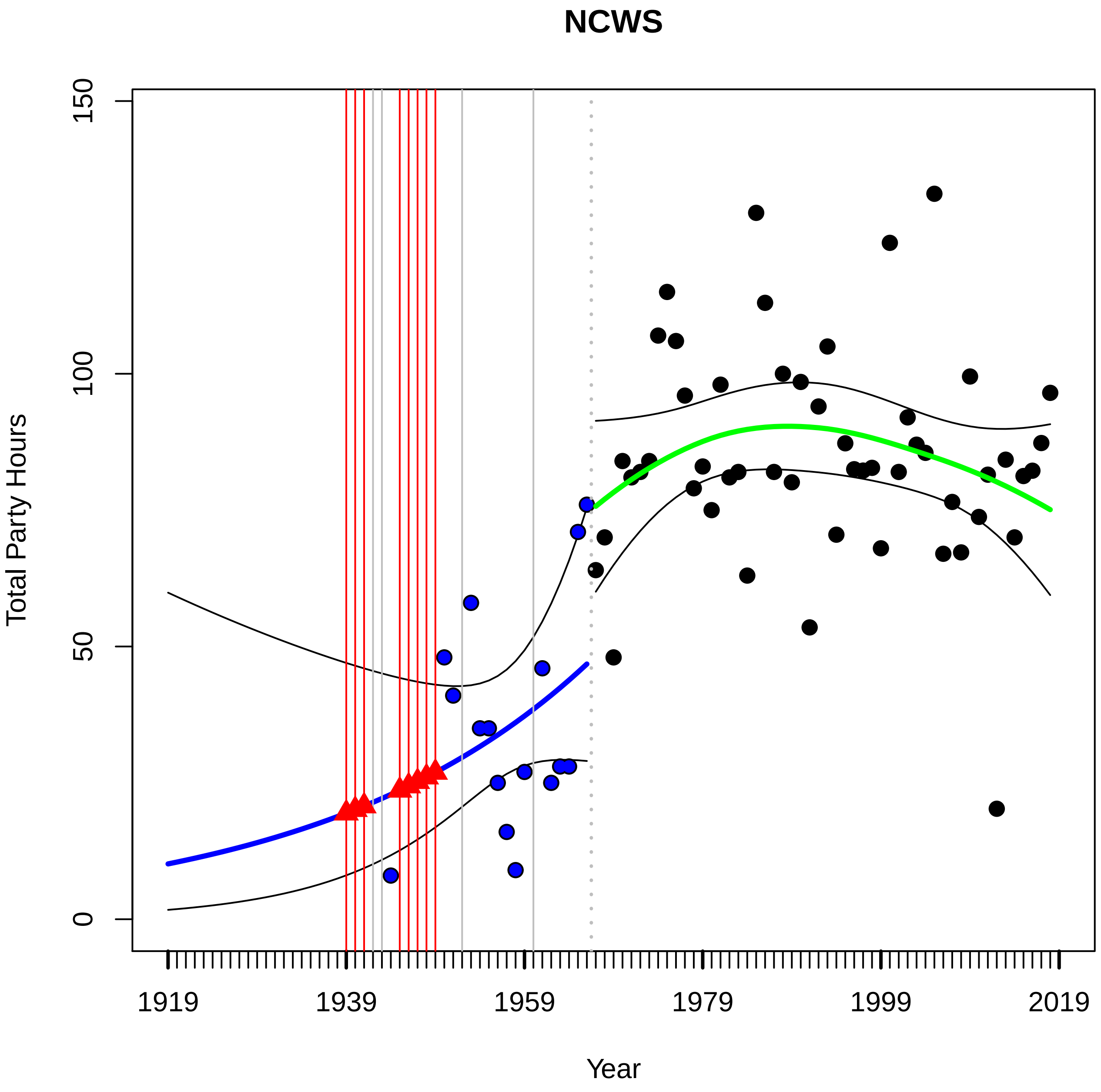

NCWS
Total Party Hours
Year
0
50
100
150
1919
1939
1959
1979
1999
2019

NDBM

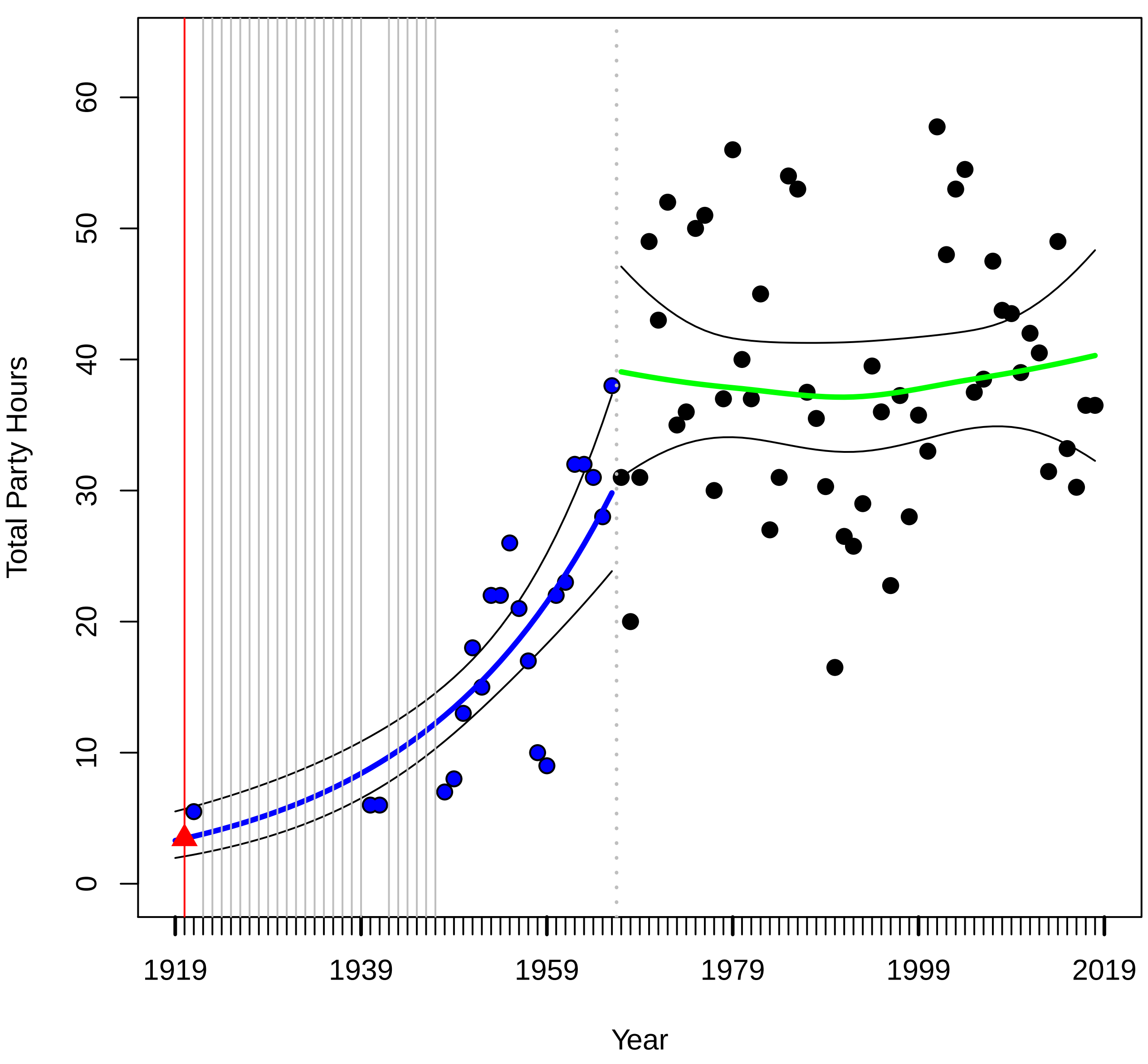

Total Party Hours
0
10
20
30
40
50
60
1919
1939
1959
1979
1999
2019
Year

NDDL

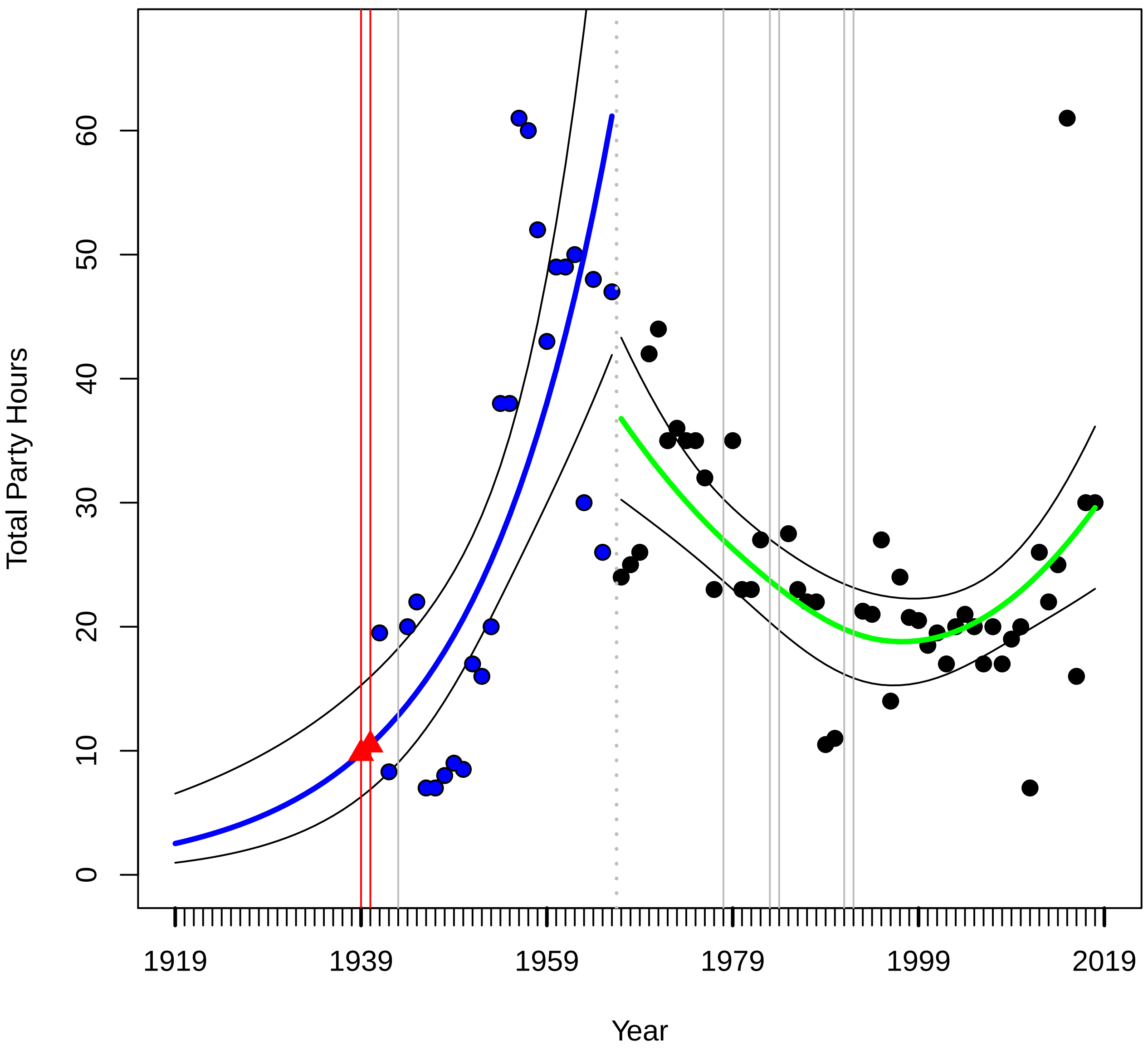

Total Party Hours
0
10
20
30
40
50
60
Year
1919
1939
1959
1979
1999
2019

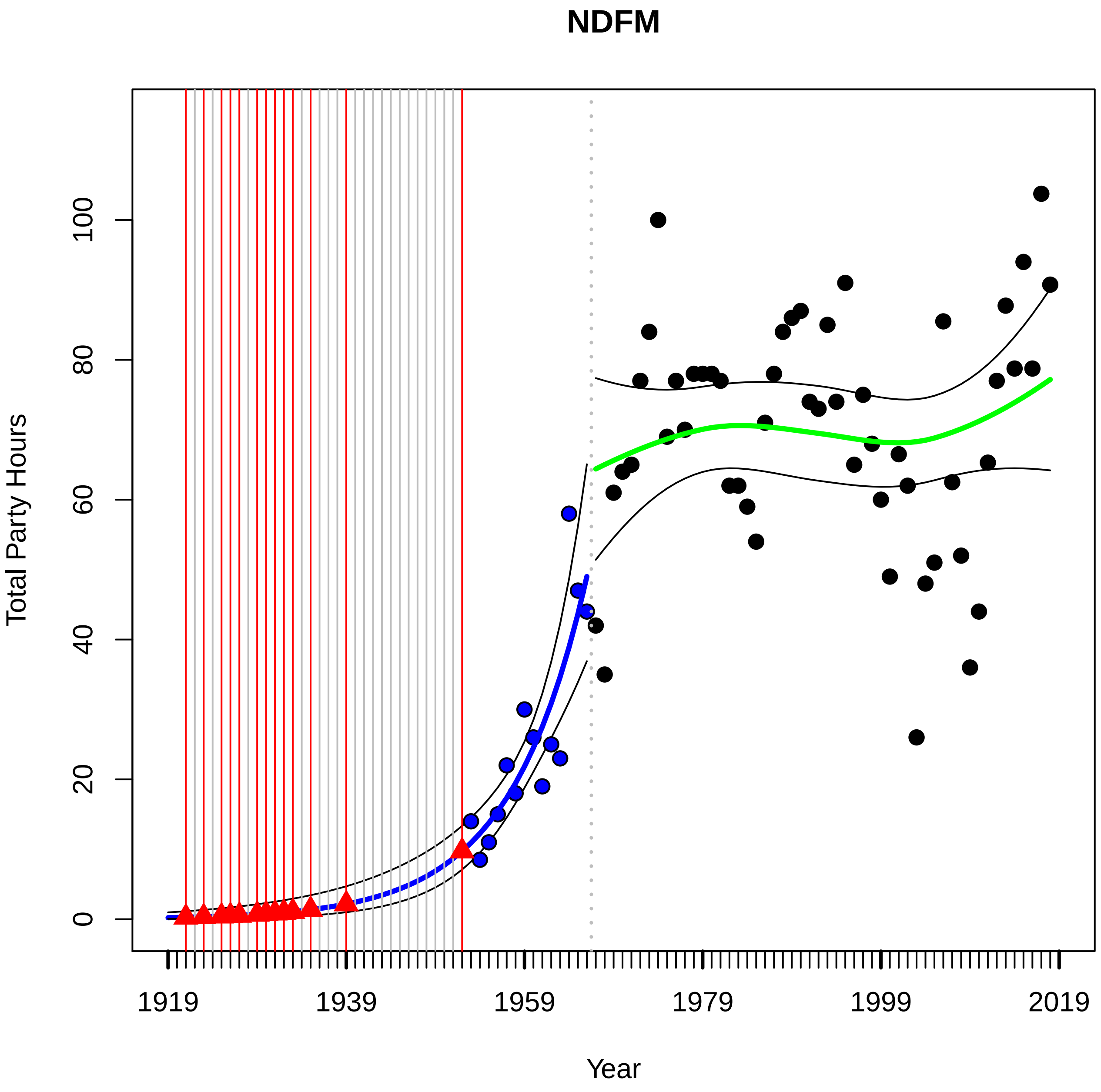
NDFM
Total Party Hours
0
20
40
60
80
100
1919
1939
1959
1979
1999
2019
Year

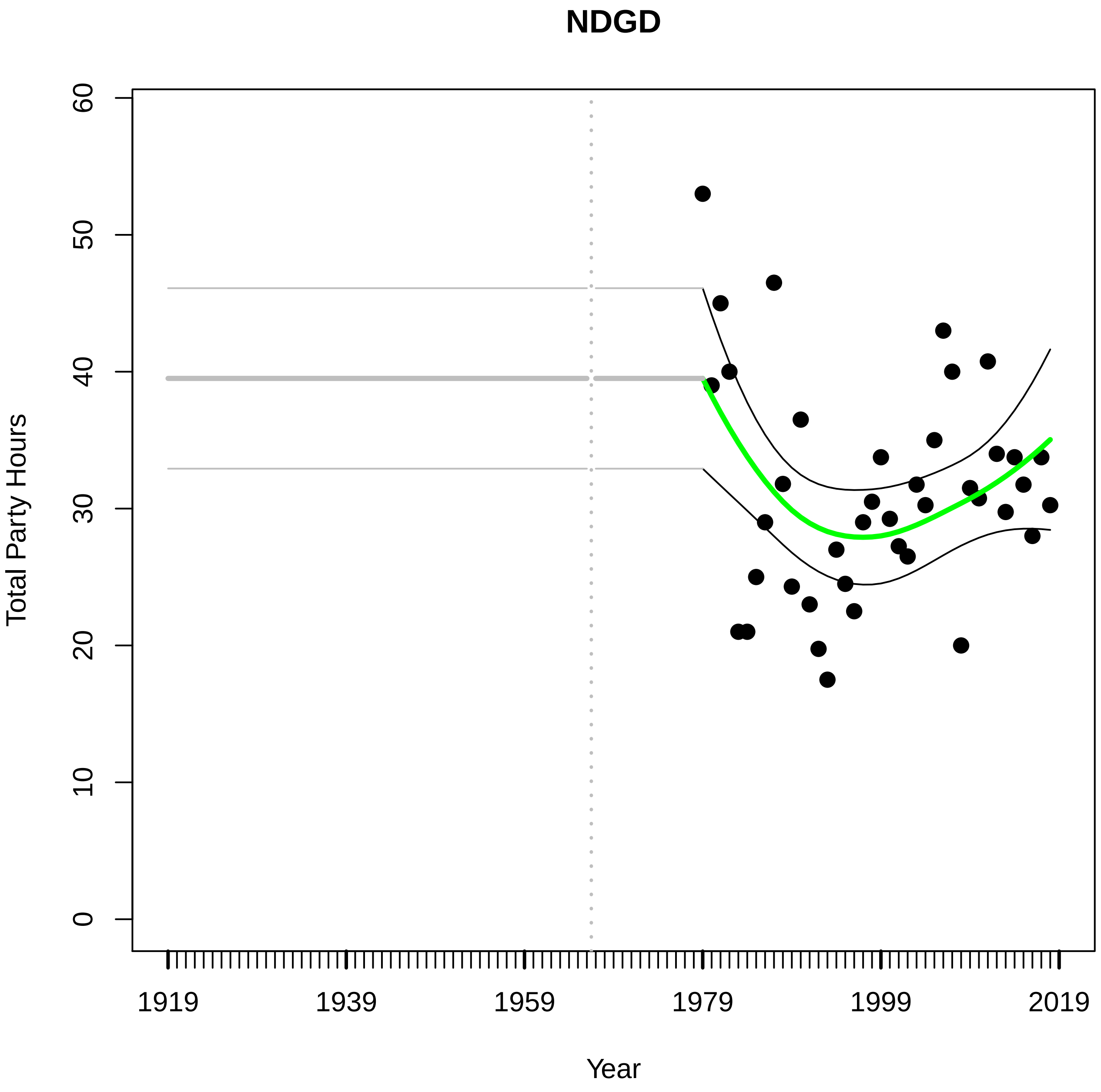
NDGD
Total Party Hours
0
10
20
30
40
50
60
1919
1939
1959
1979
1999
2019
Year

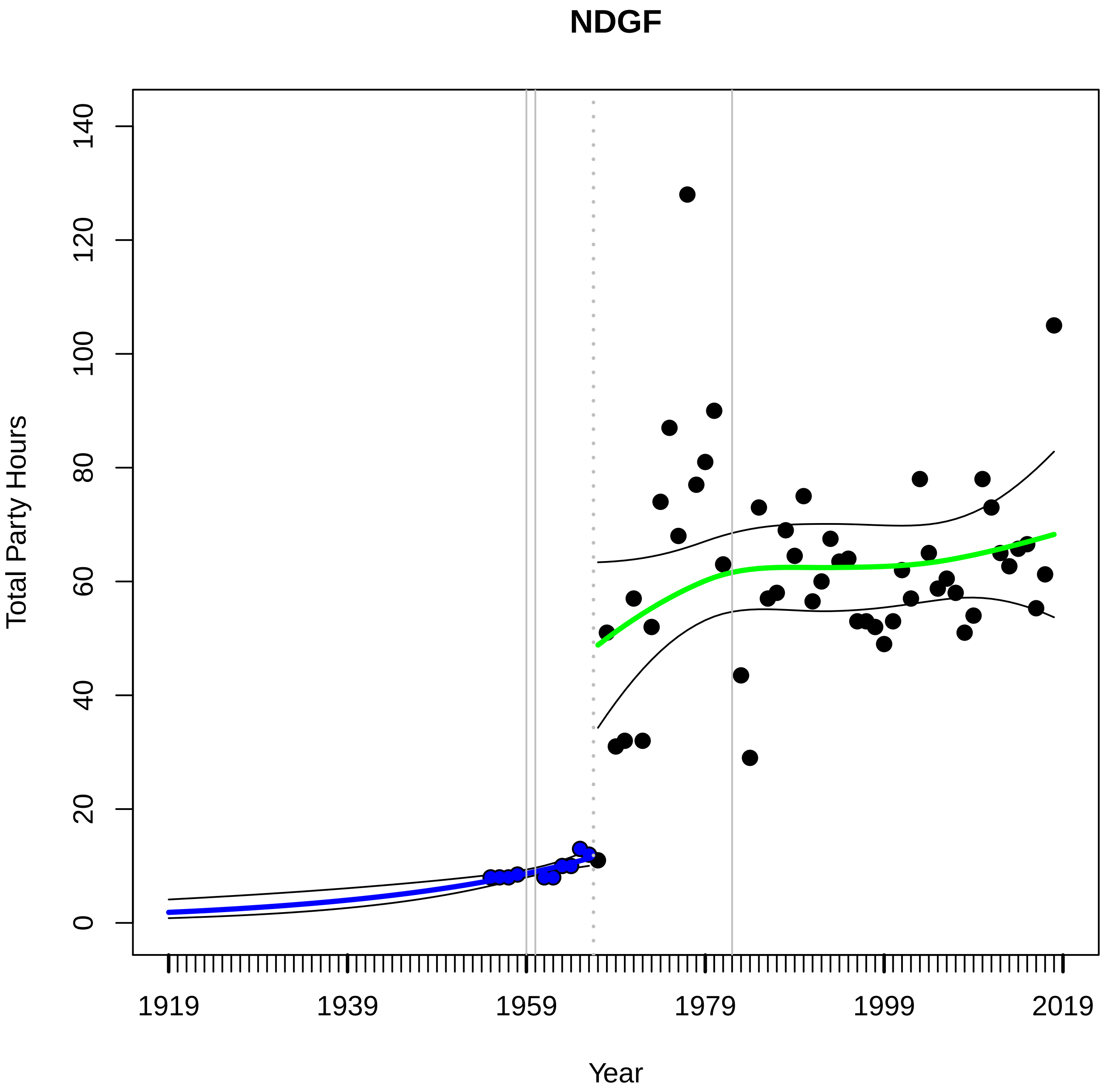

NDGF
Total Party Hours
Year
0
20
40
60
80
100
120
140
1919
1939
1959
1979
1999
2019

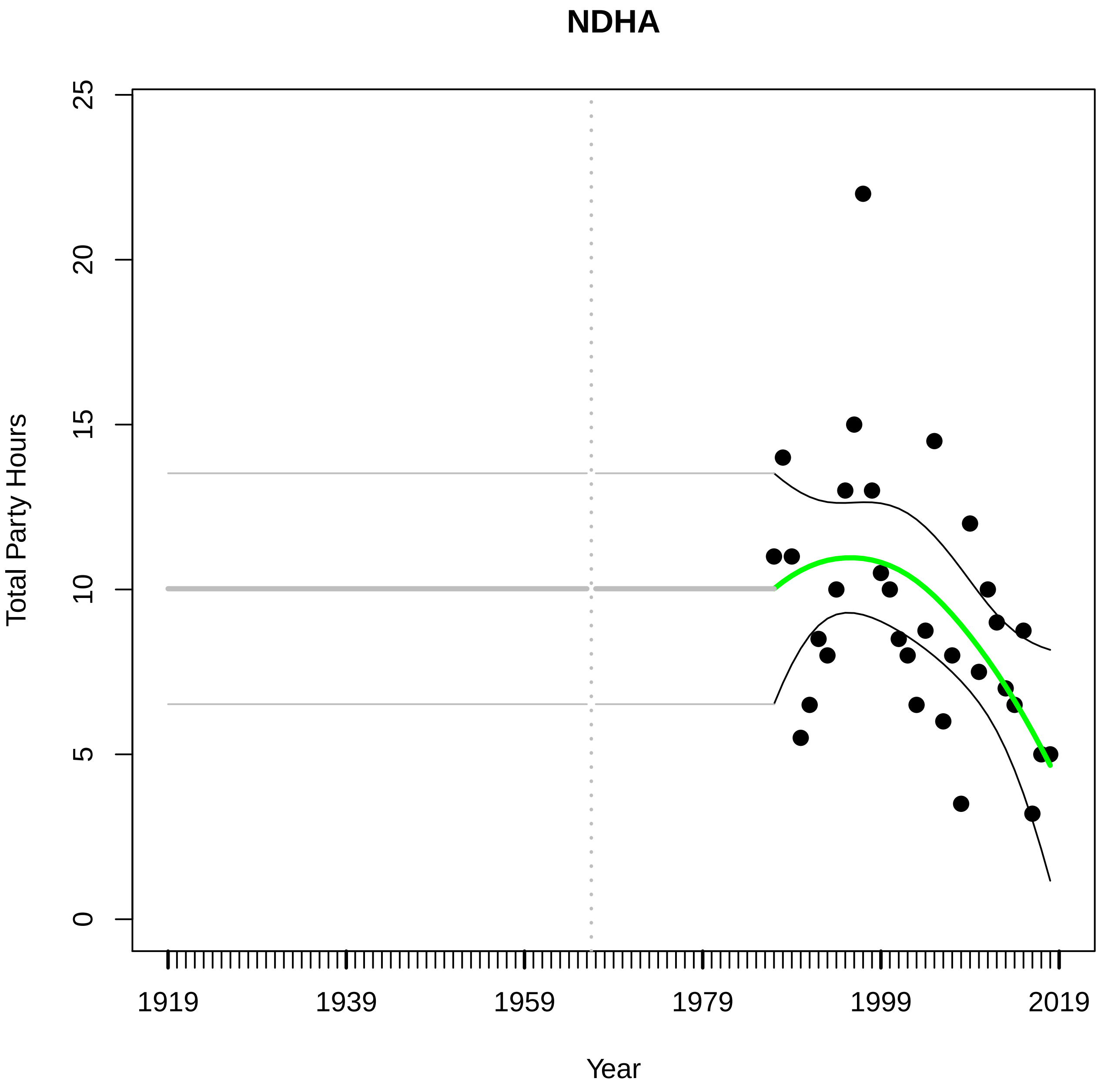

NDHA
Total Party Hours
Year
0
5
10
15
20
25
1919
1939
1959
1979
1999
2019

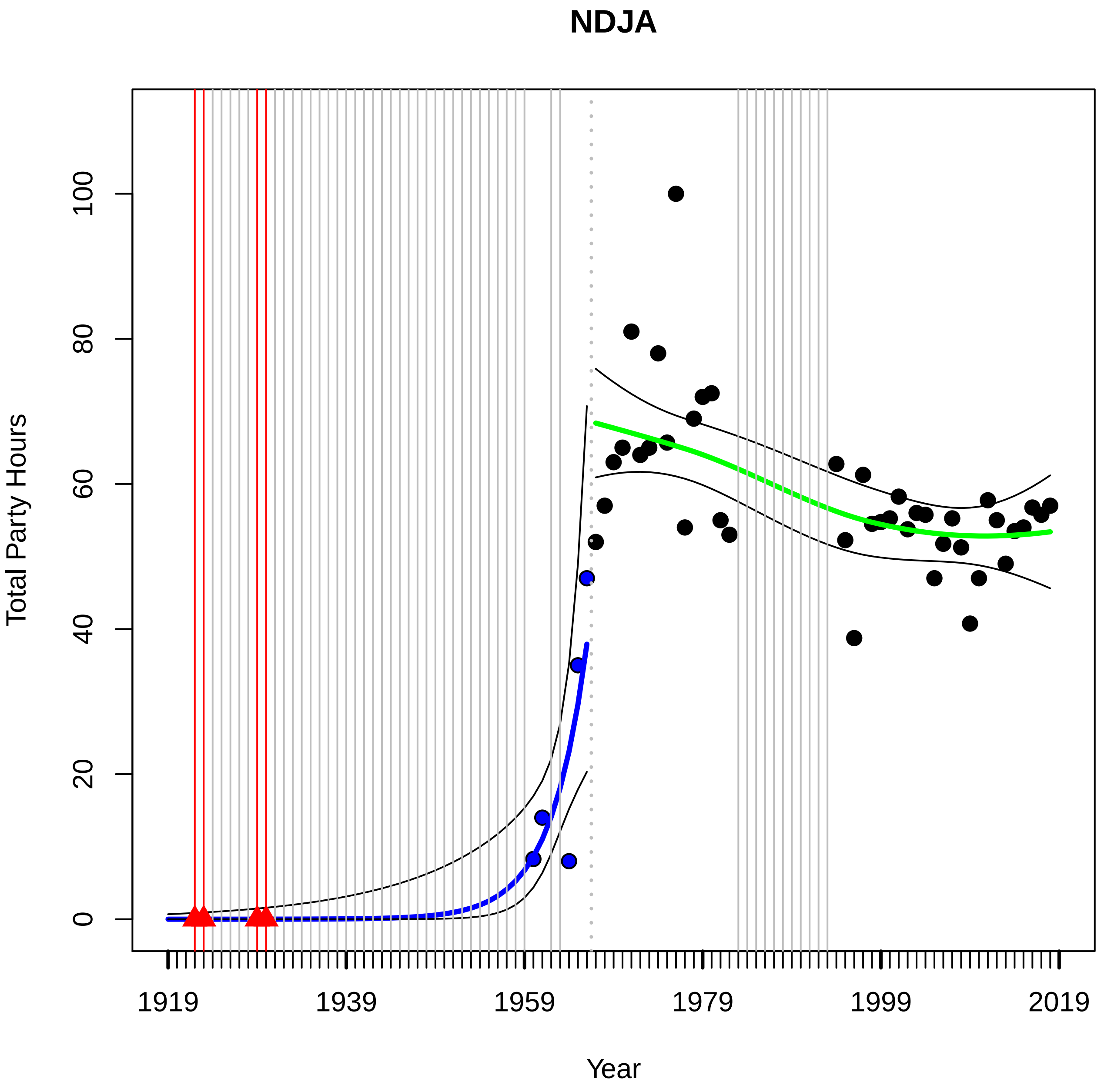

NDJA
Total Party Hours
0
20
40
60
80
100
1919
1939
1959
1979
1999
2019
Year

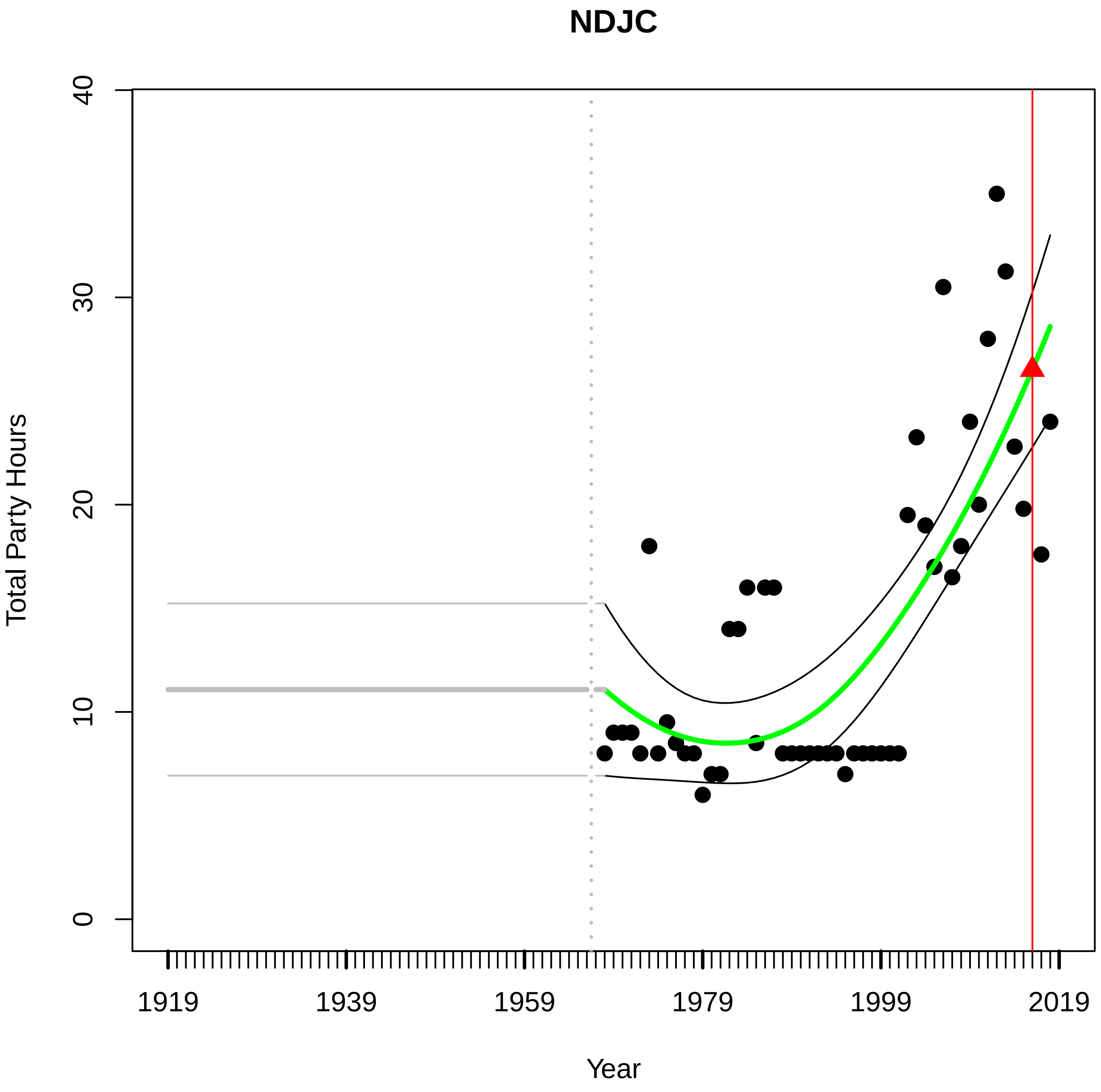
NDJC
Total Party Hours
0
10
20
30
40
1919
1939
1959
1979
1999
2019
Year

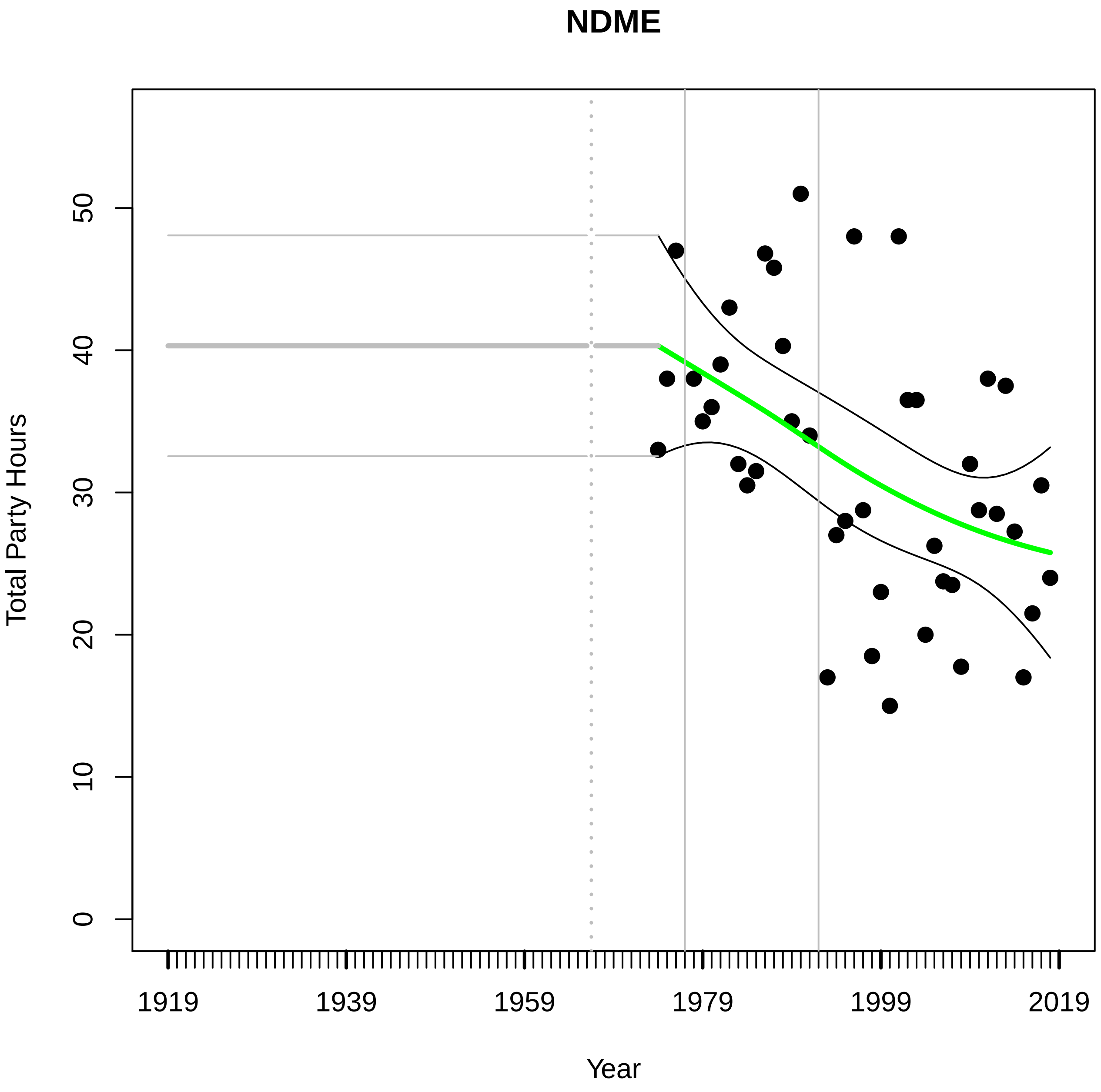
NDME
Total Party Hours
Year
0
10
20
30
40
50
1919
1939
1959
1979
1999
2019

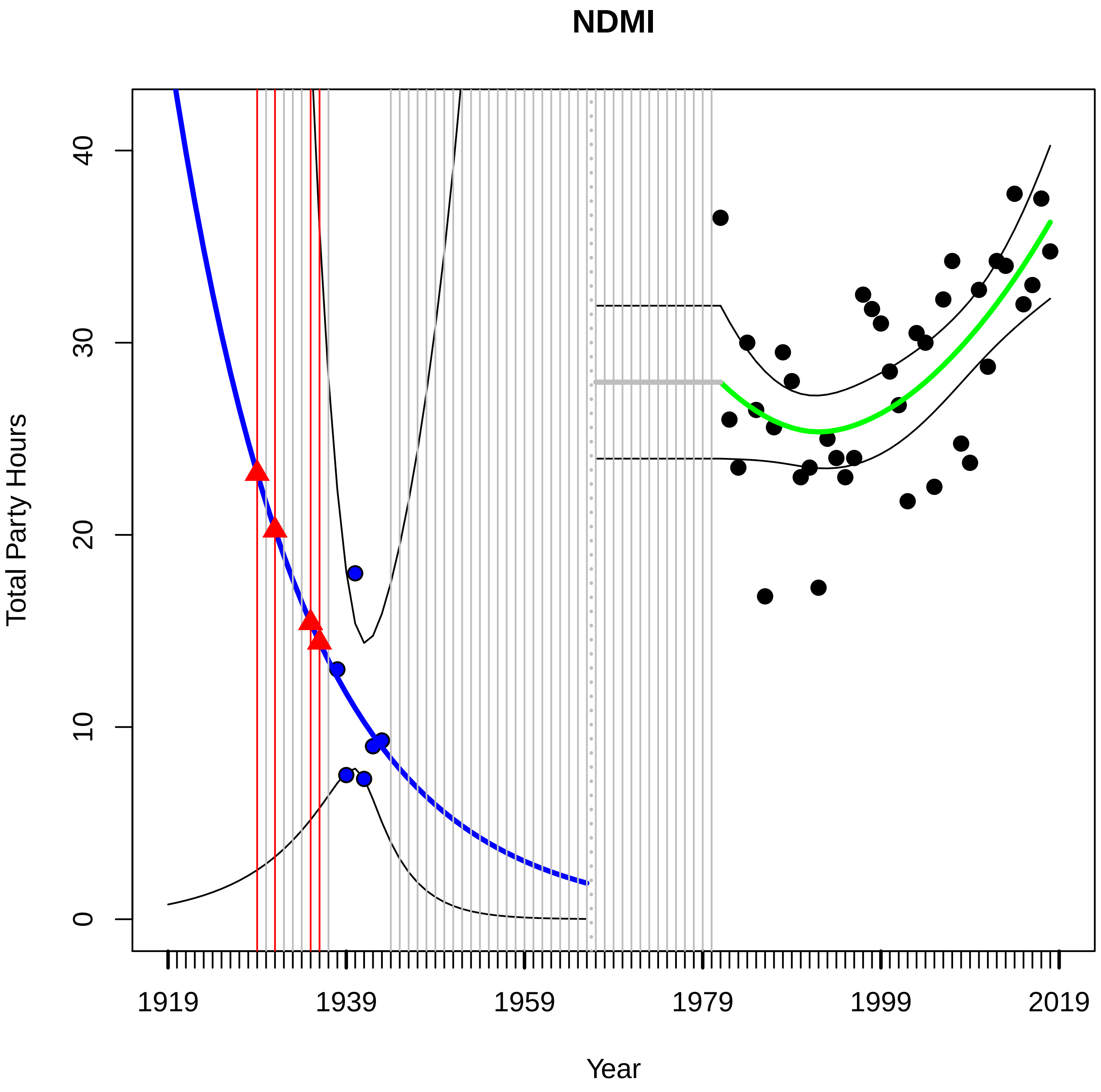

NDMI
Total Party Hours
0
10
20
30
40
1919
1939
1959
1979
1999
2019
Year

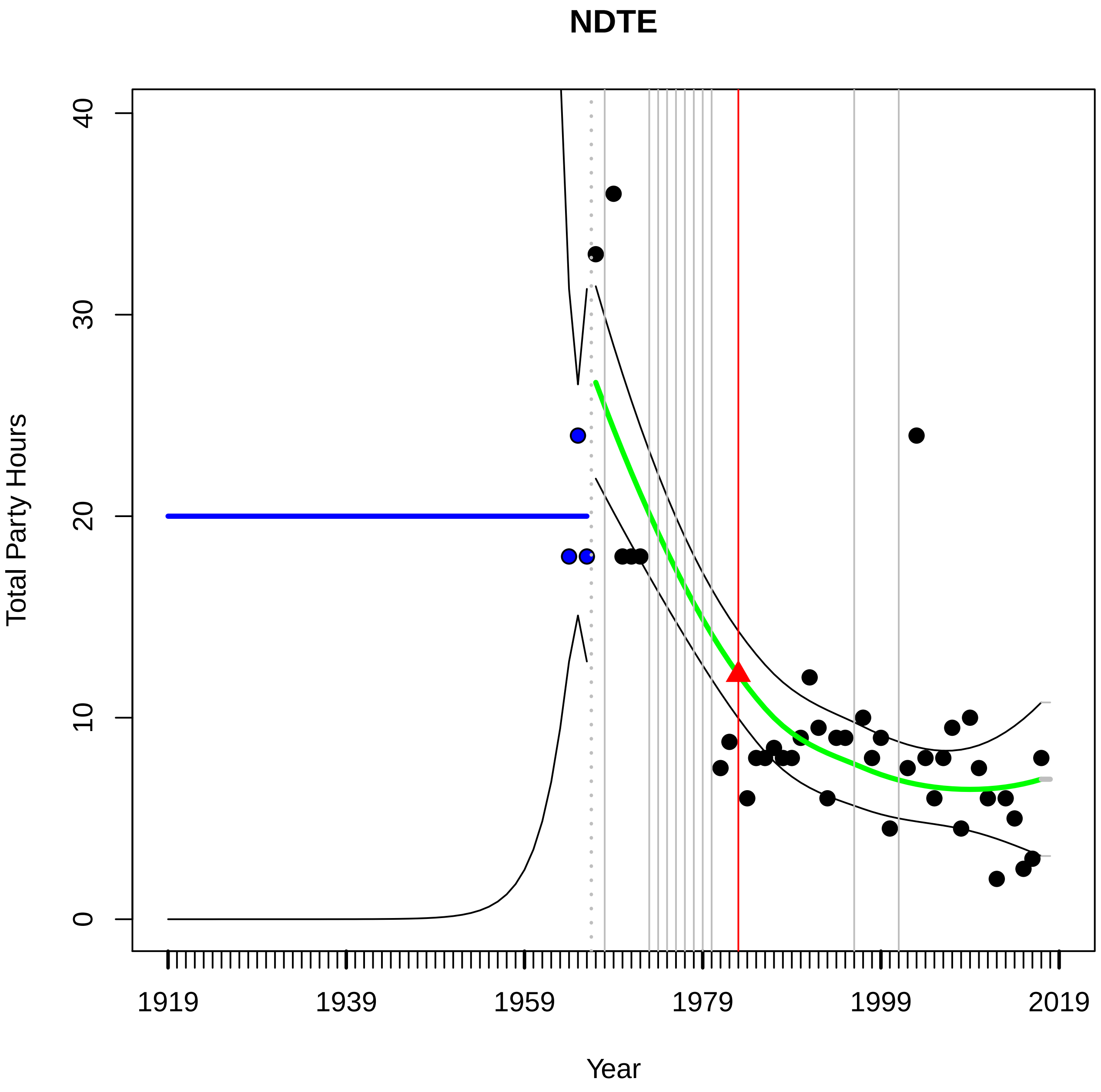

NDTE
Total Party Hours
Year
0
10
20
30
40
1919
1939
1959
1979
1999
2019

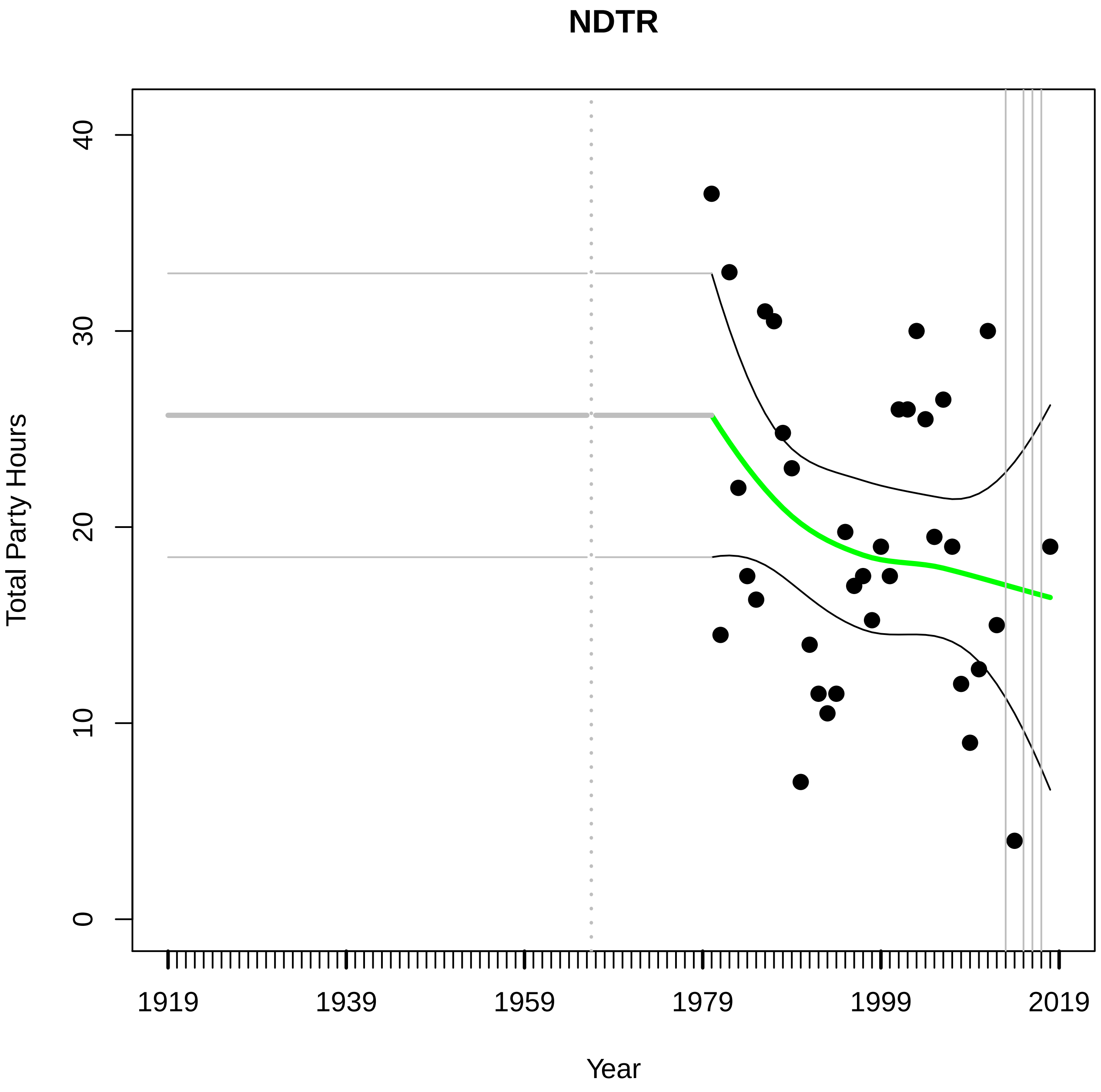
NDTR
Total Party Hours
0
10
20
30
40
Year
1919
1939
1959
1979
1999
2019

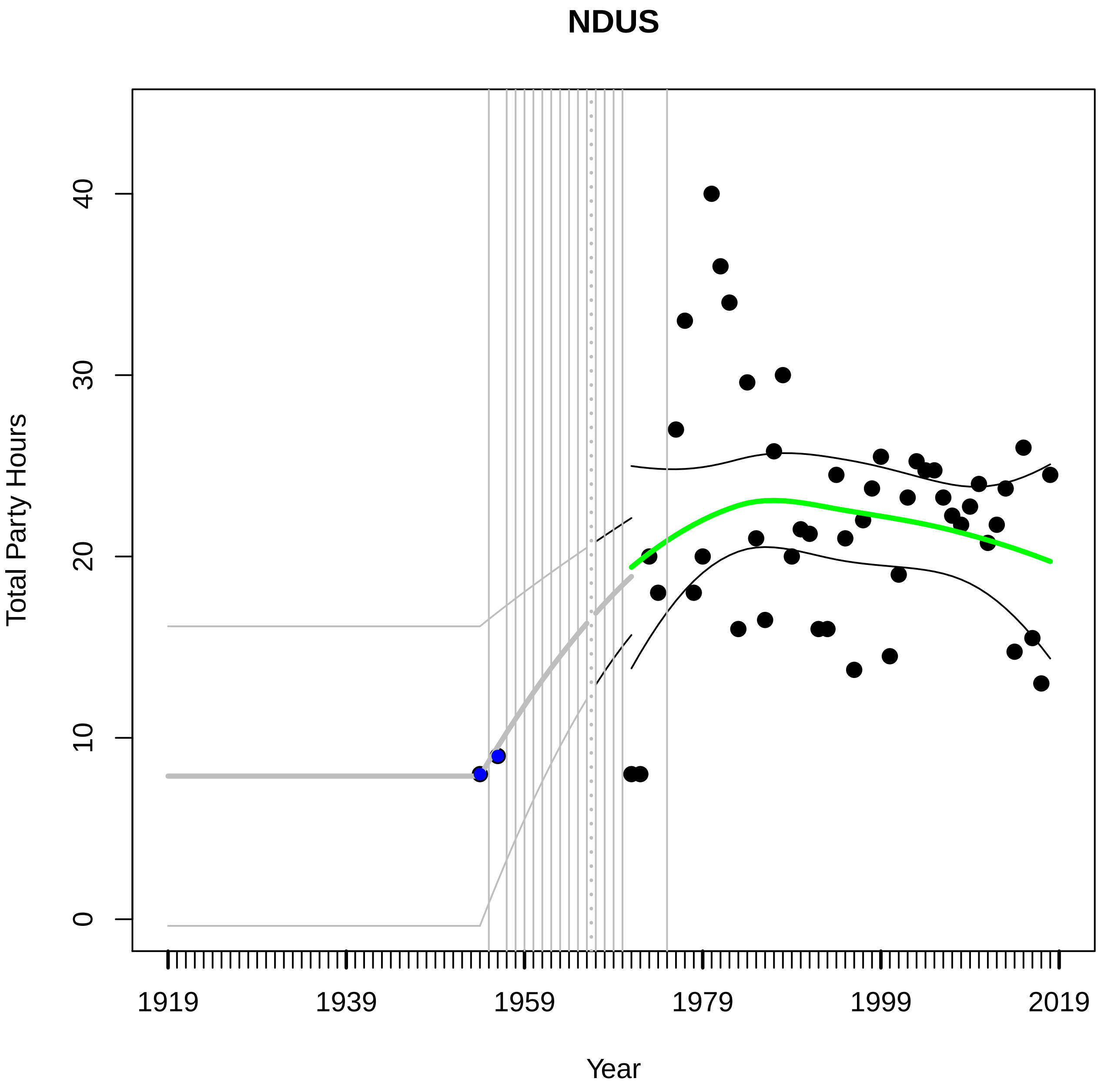
NDUS
Total Party Hours
0
10
20
30
40
1919
1939
1959
1979
1999
2019
Year

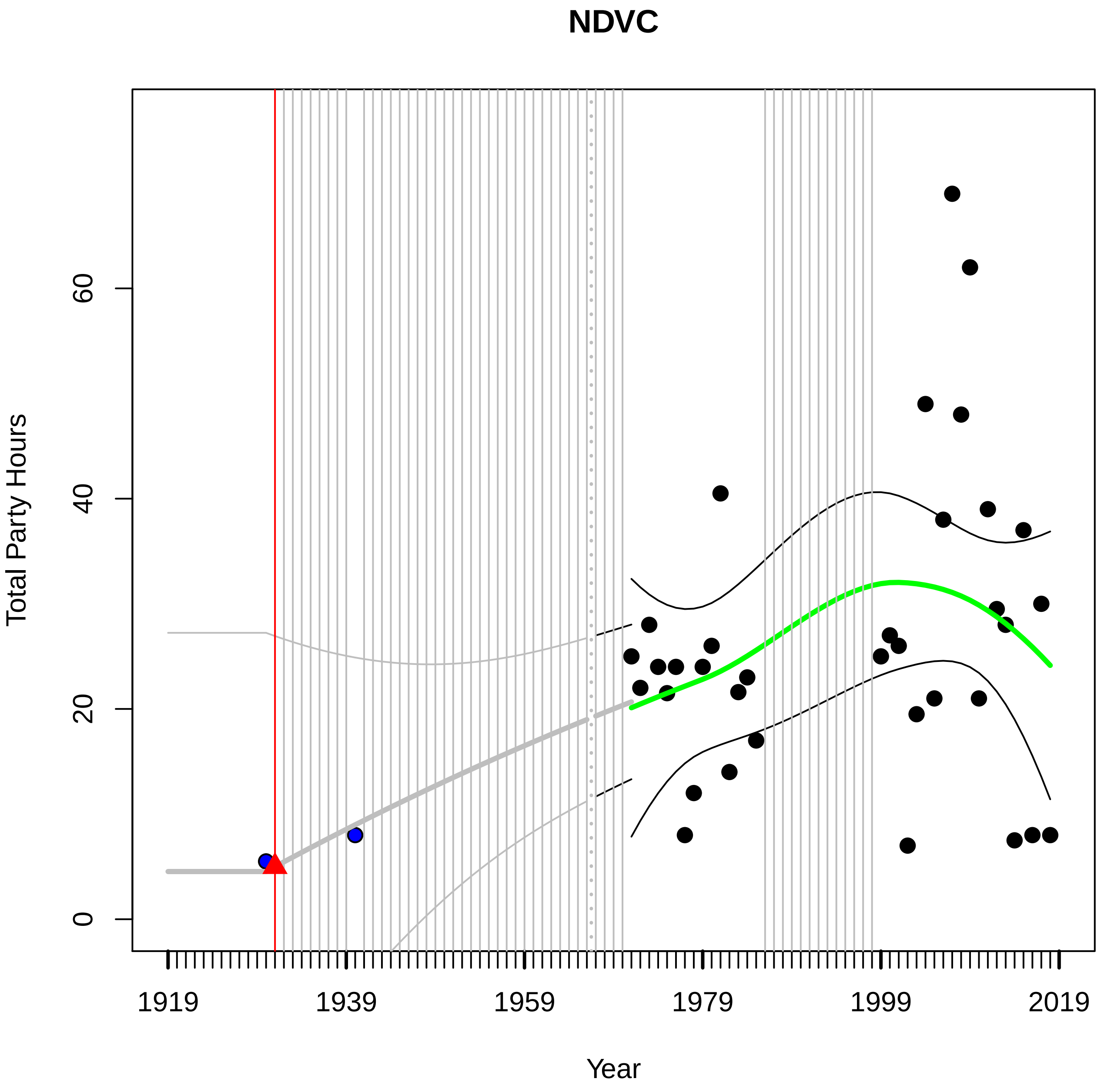

NDVC
Total Party Hours
Year
0
20
40
60
1919
1939
1959
1979
1999
2019

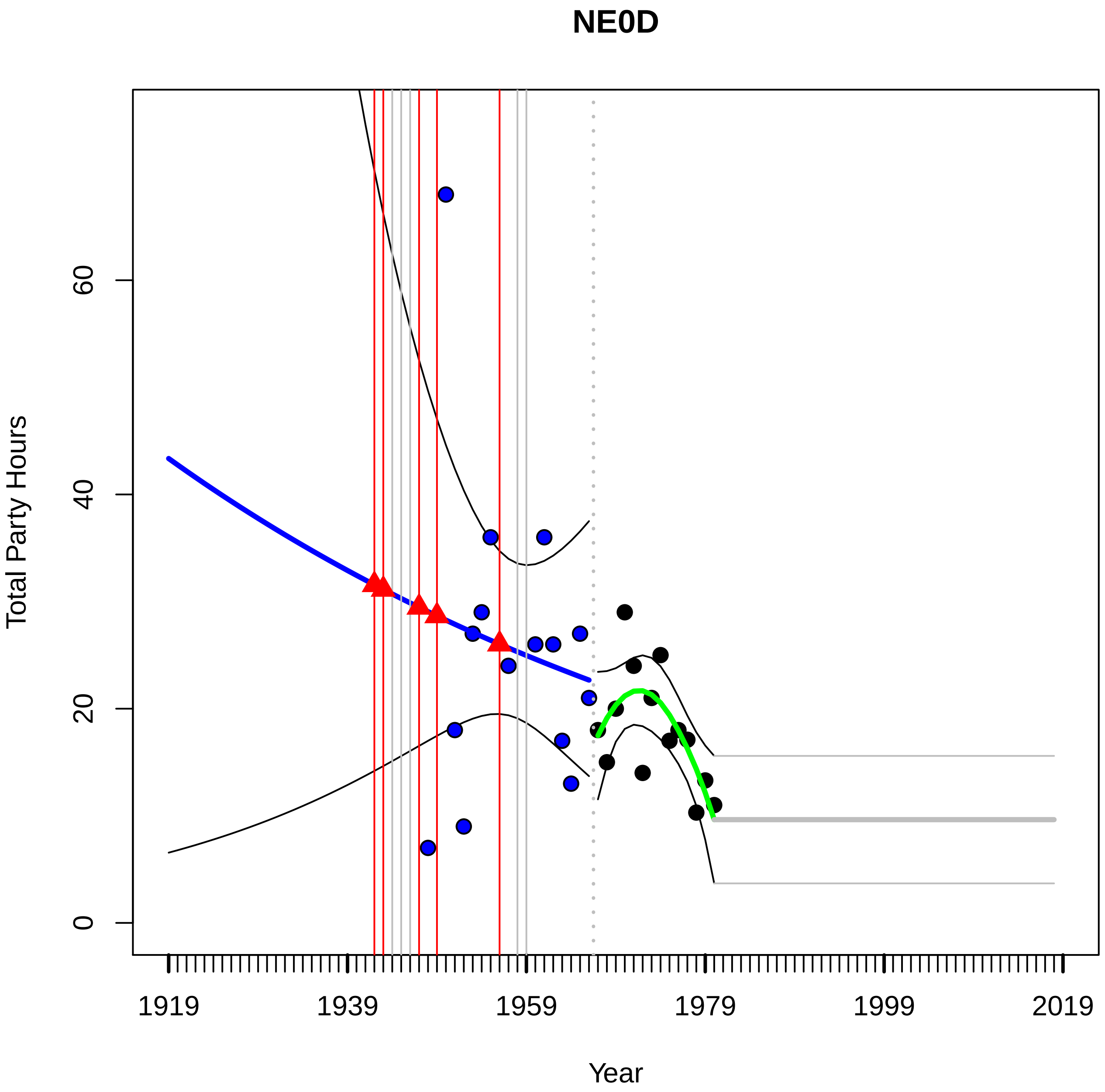

NE0D
Total Party Hours
Year
0
20
40
60
1919
1939
1959
1979
1999
2019

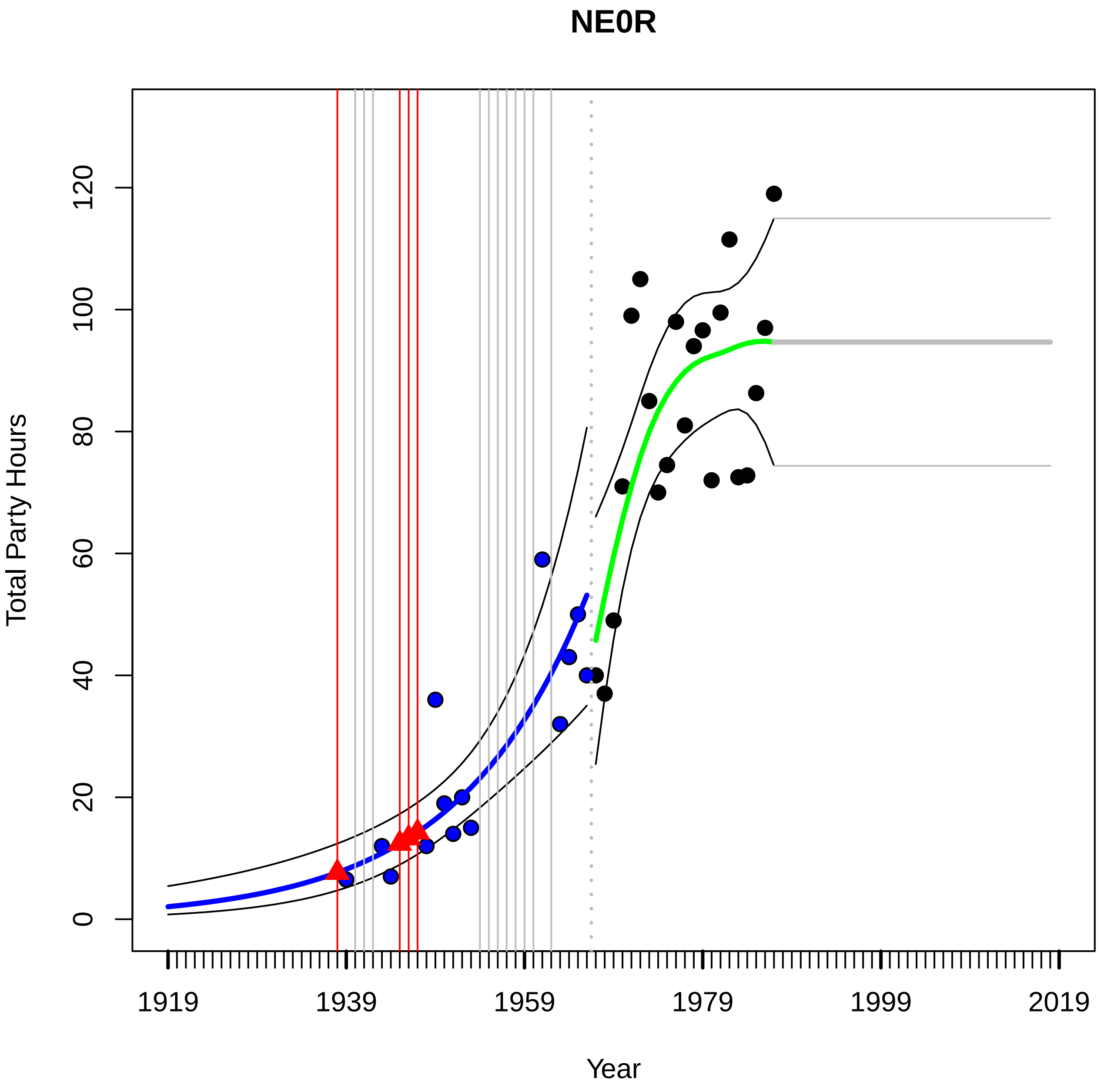

NE0R
Total Party Hours
0
20
40
60
80
100
120
1919
1939
1959
1979
1999
2019
Year

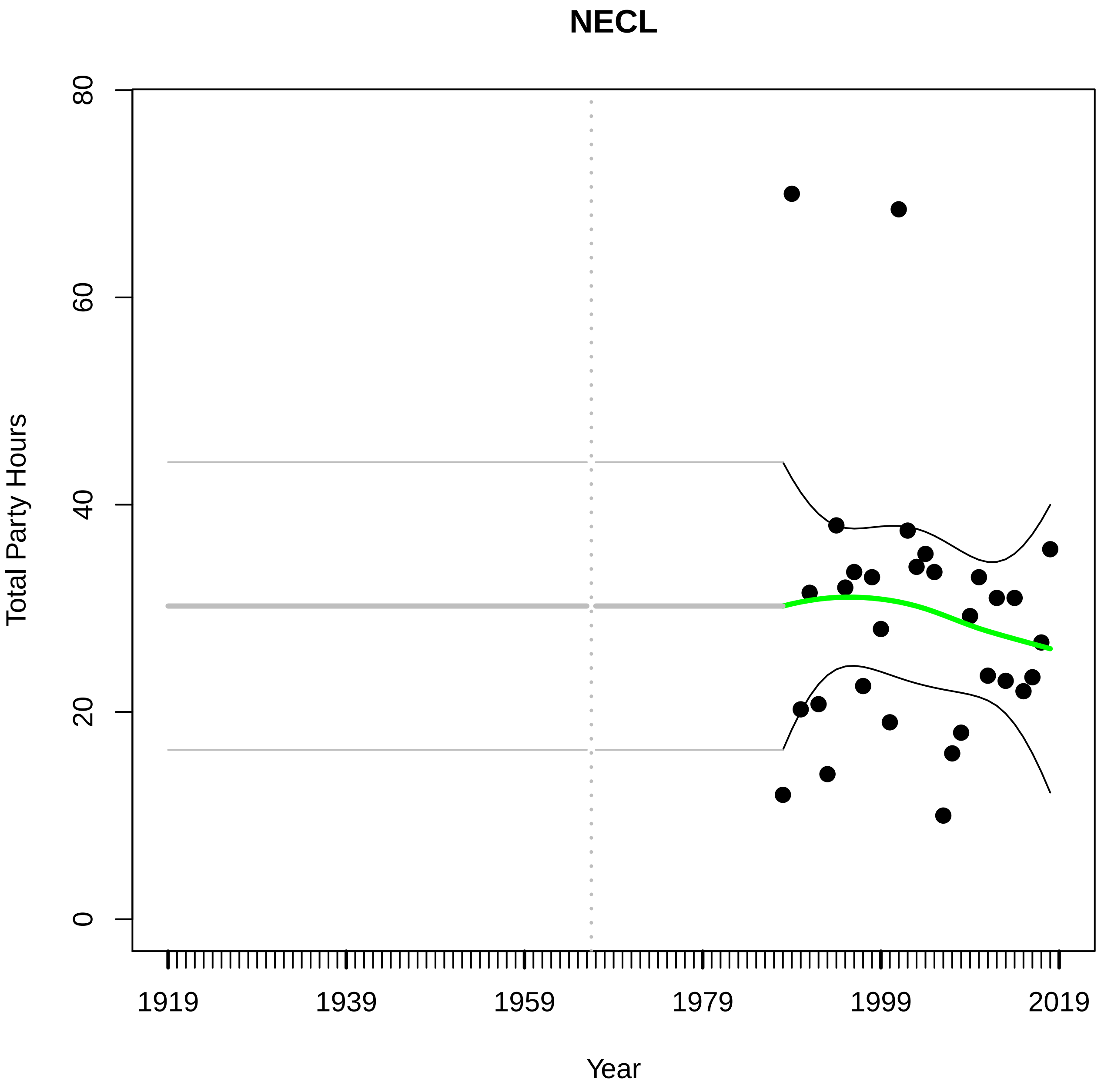
NECL
Total Party Hours
0
20
40
60
80
Year
1919
1939
1959
1979
1999
2019

NELI

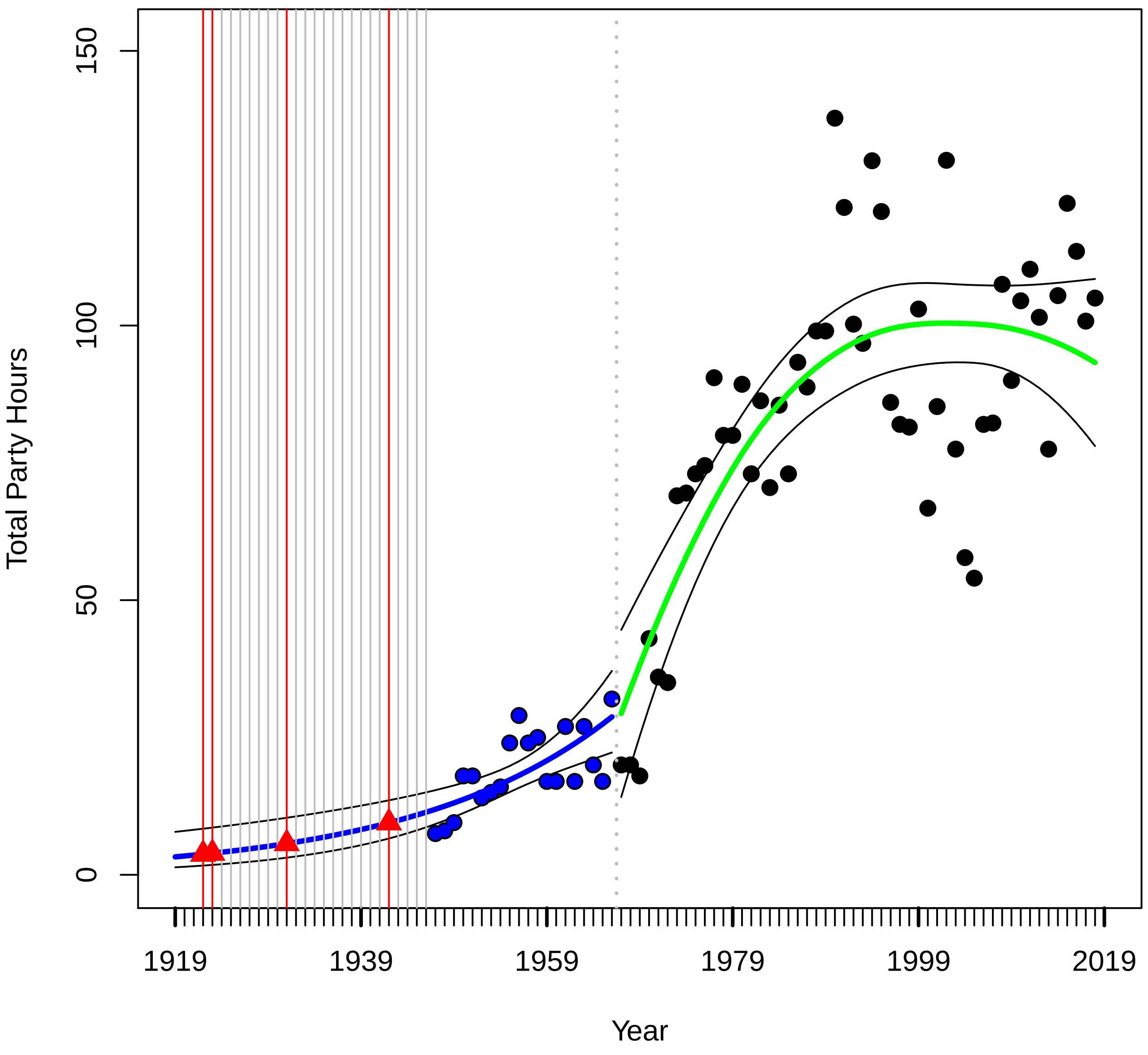

150
100
50
0
Total Party Hours
1919
1939
1959
1979
1999
2019
Year

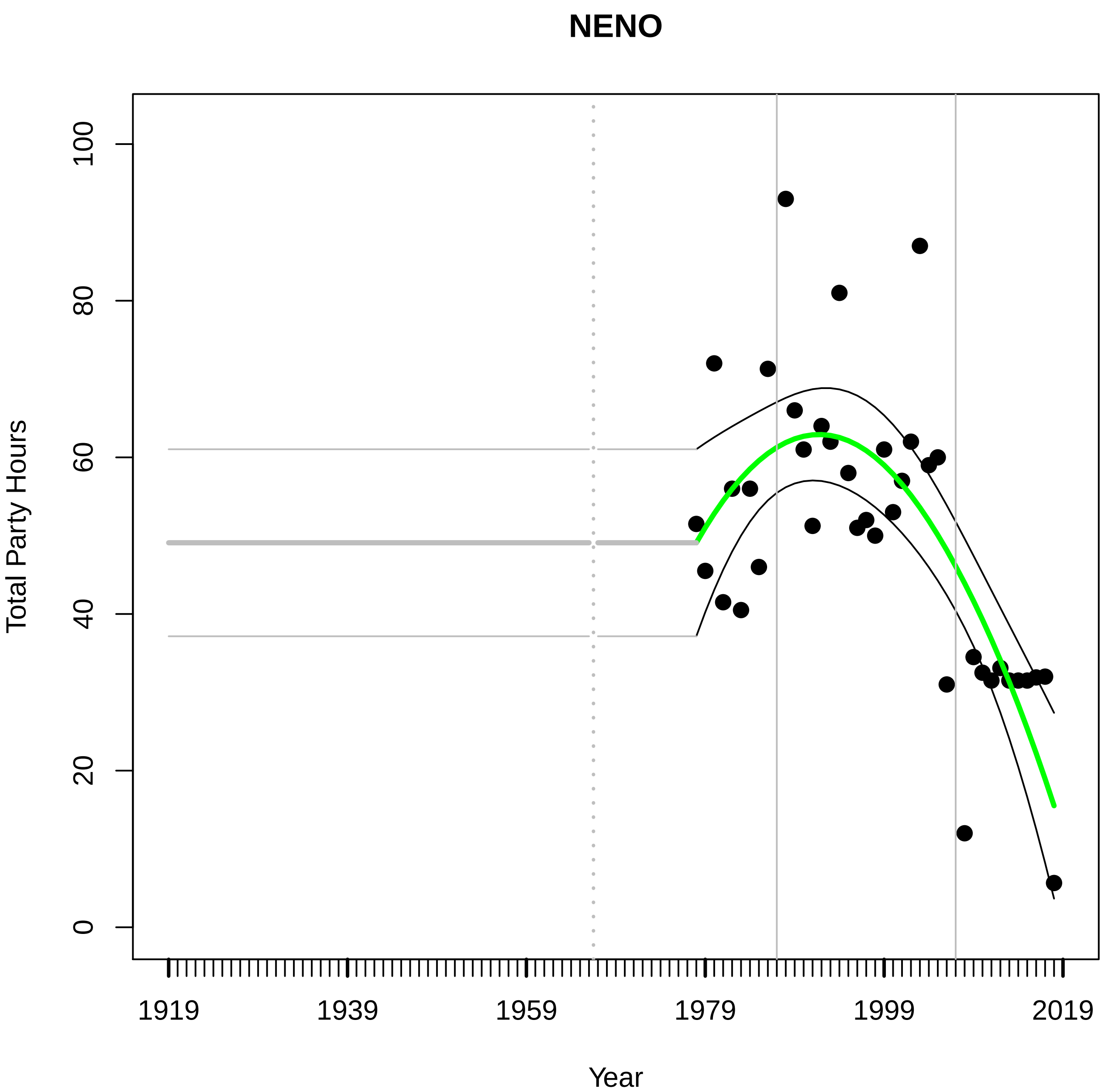
NENO
Total Party Hours
0
20
40
60
80
100
1919
1939
1959
1979
1999
2019
Year

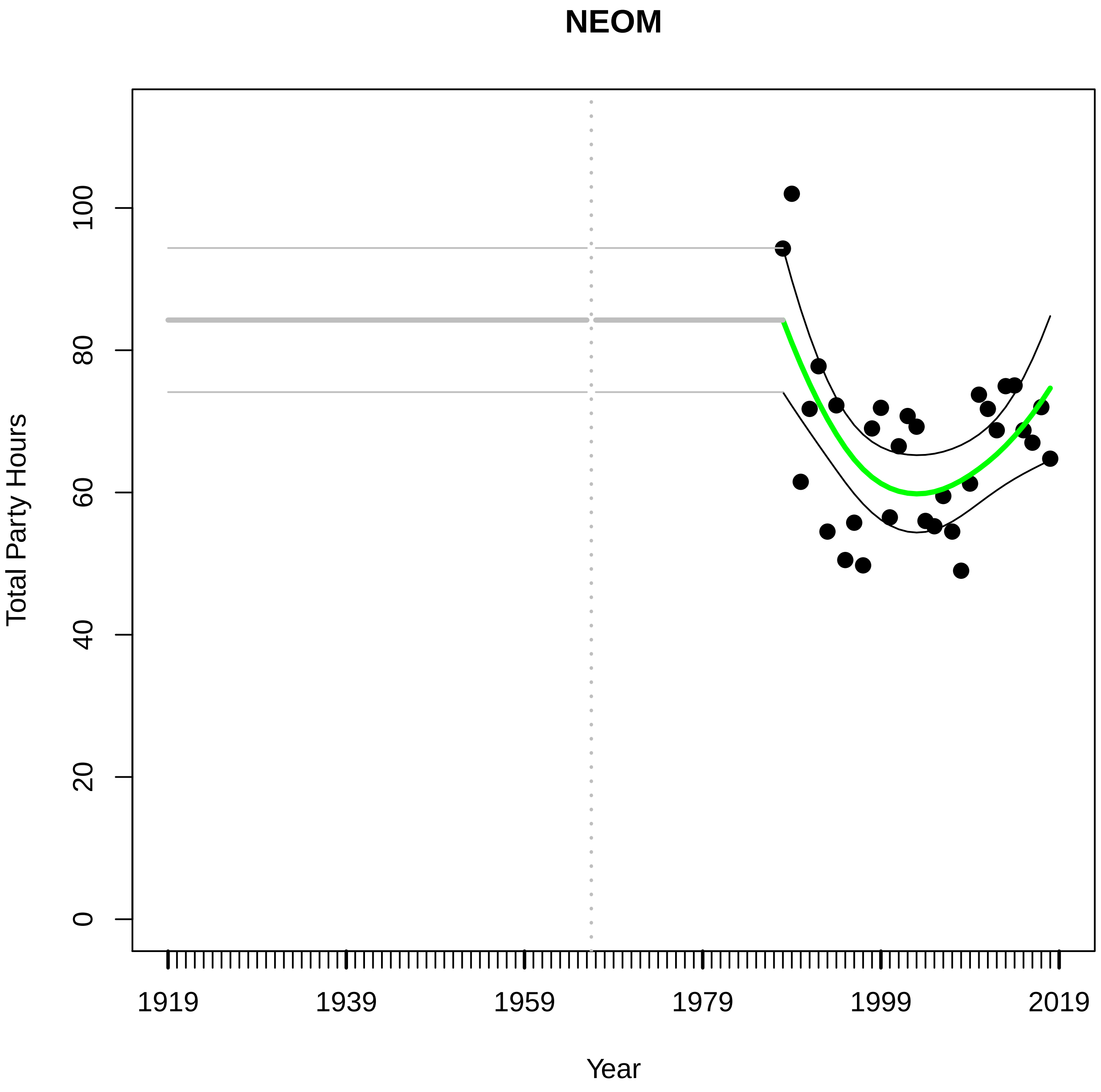
NEOM
Total Party Hours
0
20
40
60
80
100
1919
1939
1959
1979
1999
2019
Year

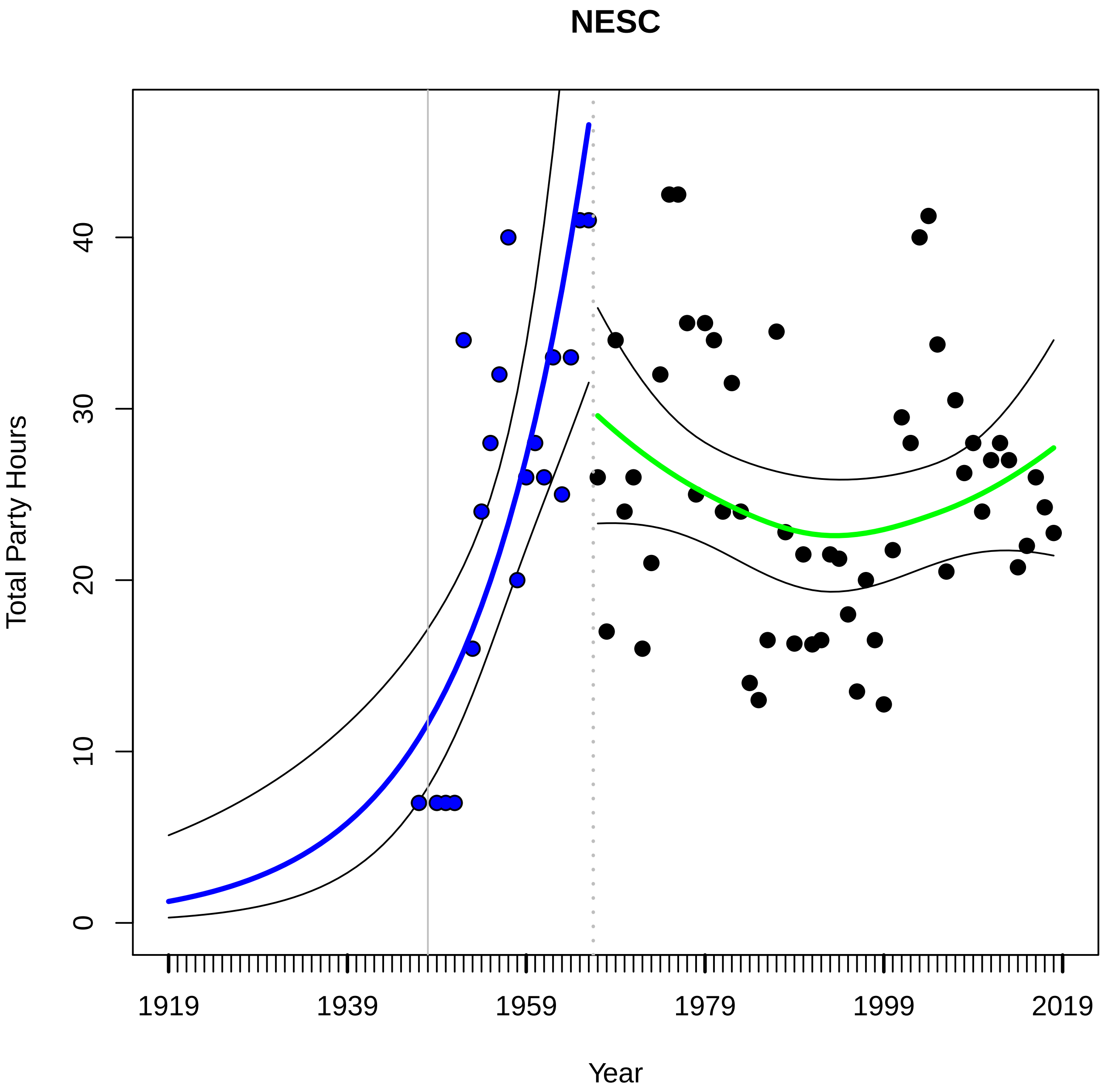
NESC
Total Party Hours
0
10
20
30
40
1919
1939
1959
1979
1999
2019
Year

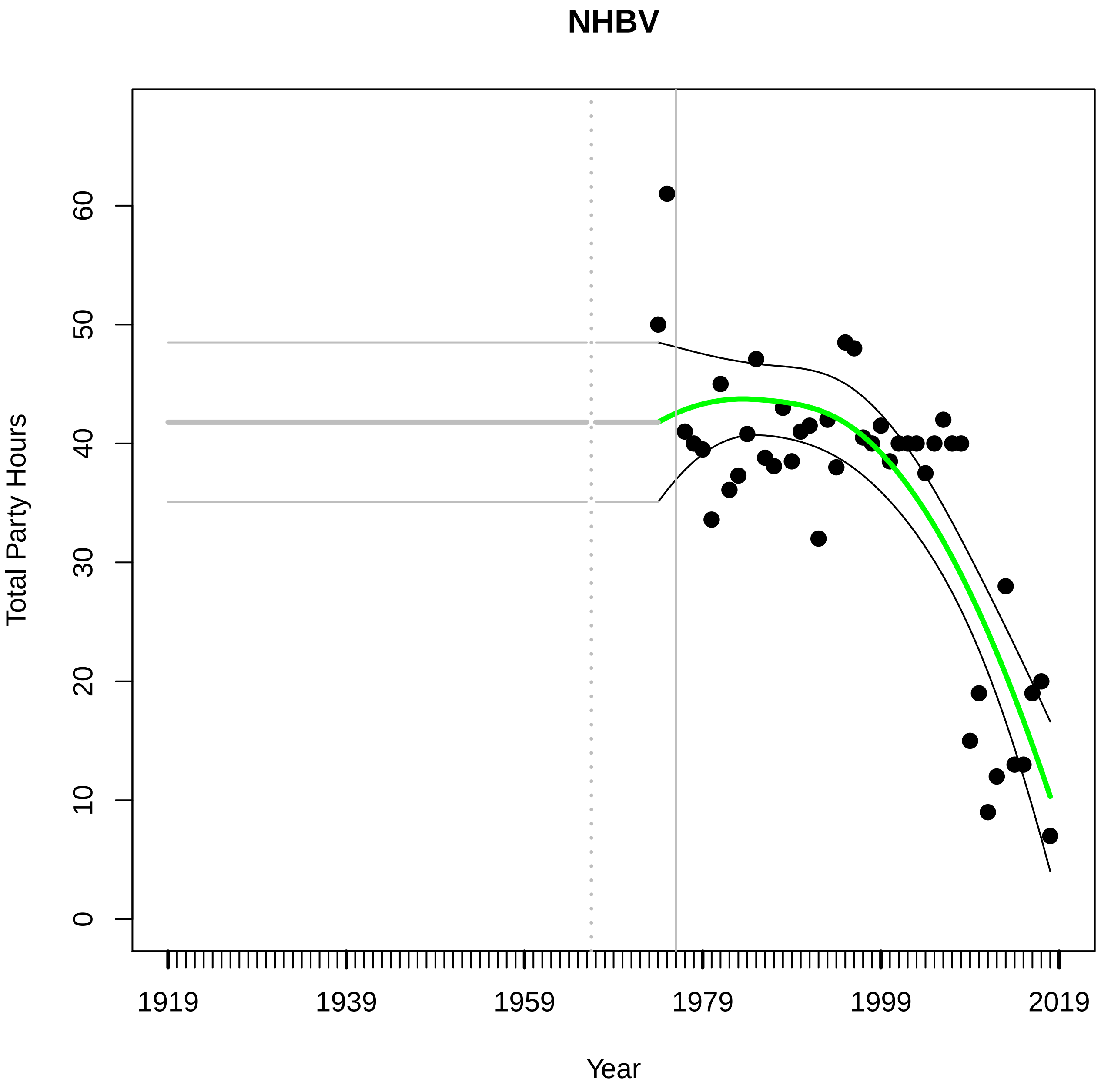
NHBV
Total Party Hours
0
10
20
30
40
50
60
1919
1939
1959
1979
1999
2019
Year

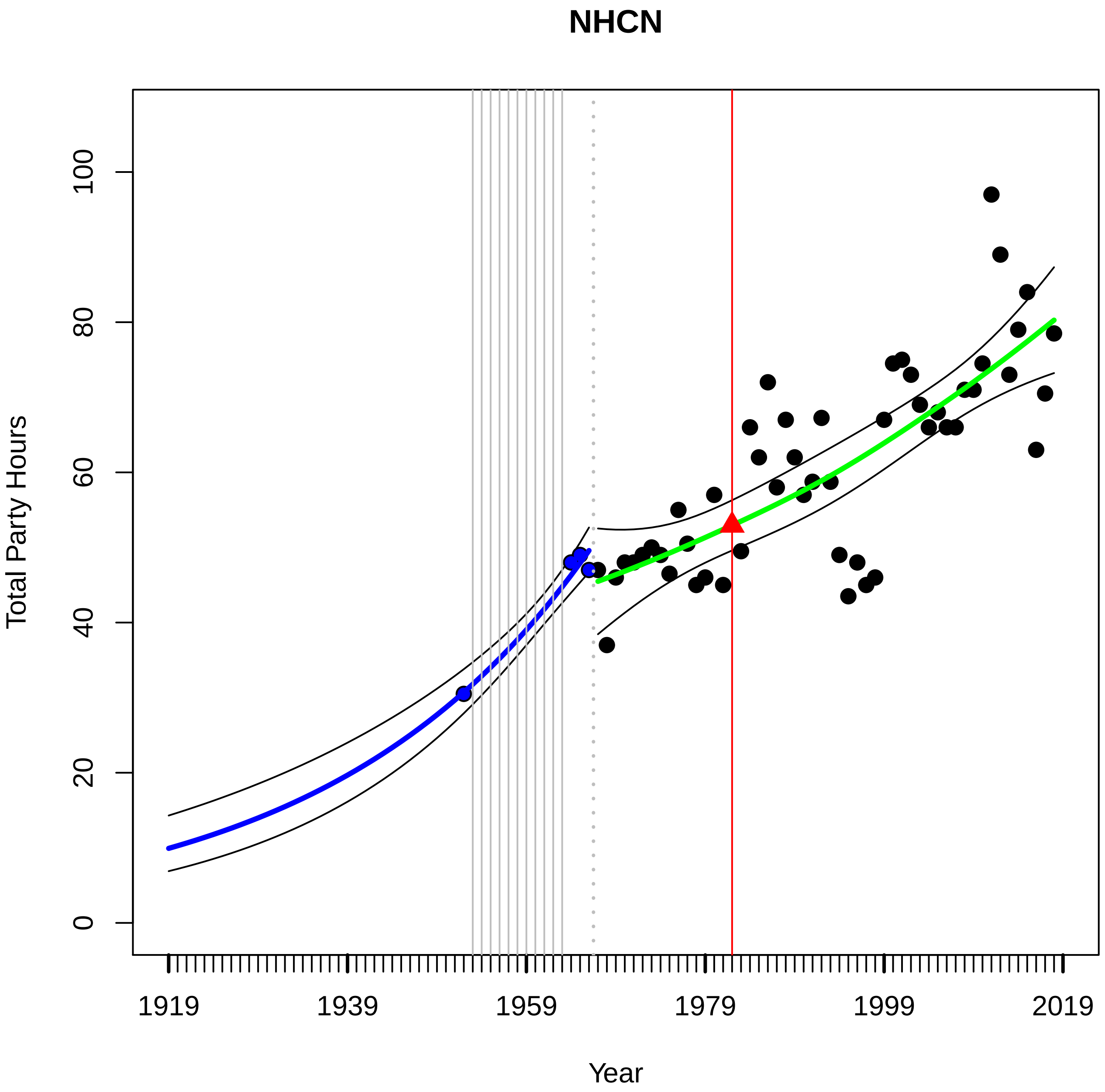
NHCN
Total Party Hours
Year
0
20
40
60
80
100
1919
1939
1959
1979
1999
2019

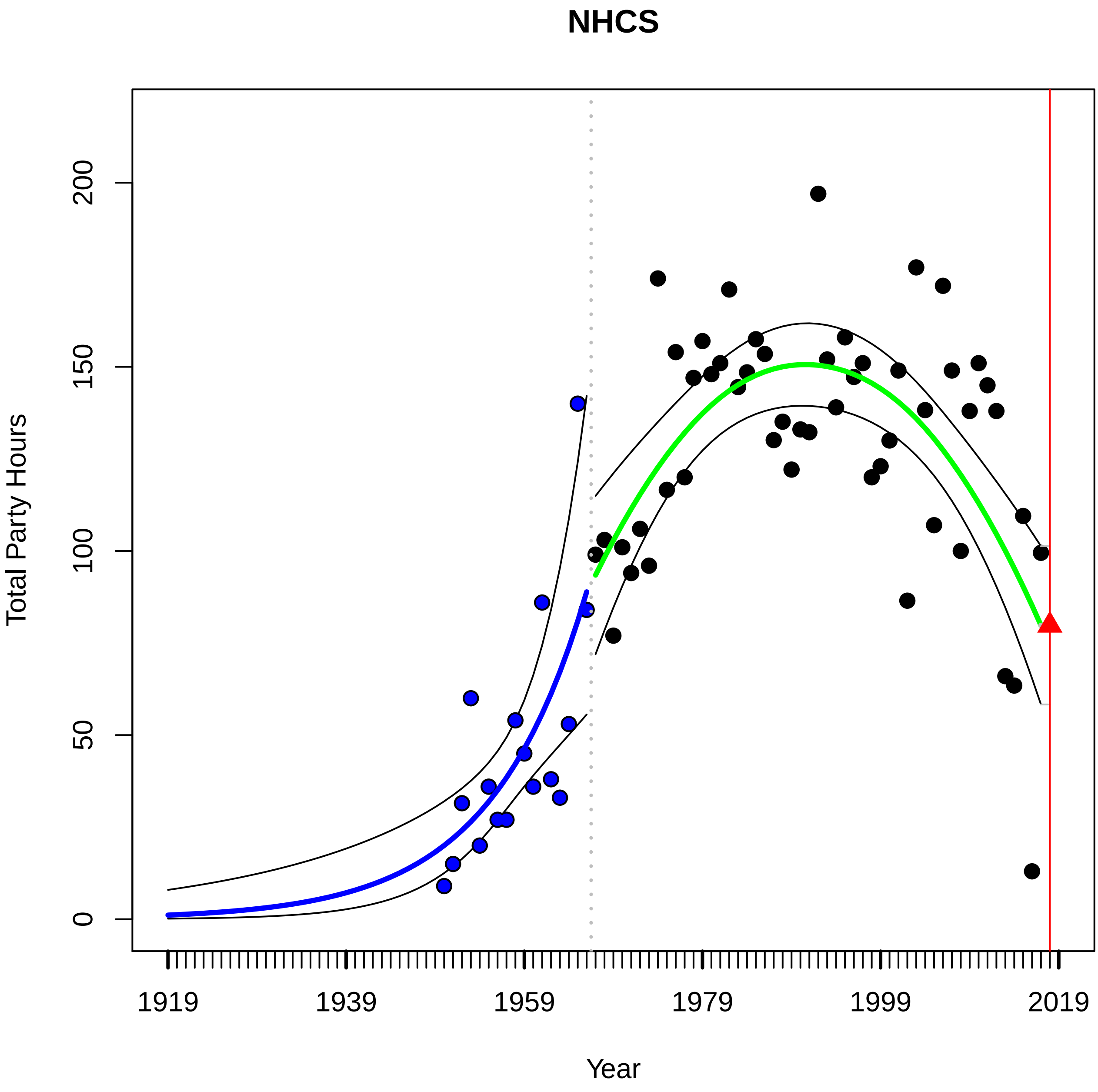

NHCS
Total Party Hours
0
50
100
150
200
1919
1939
1959
1979
1999
2019
Year

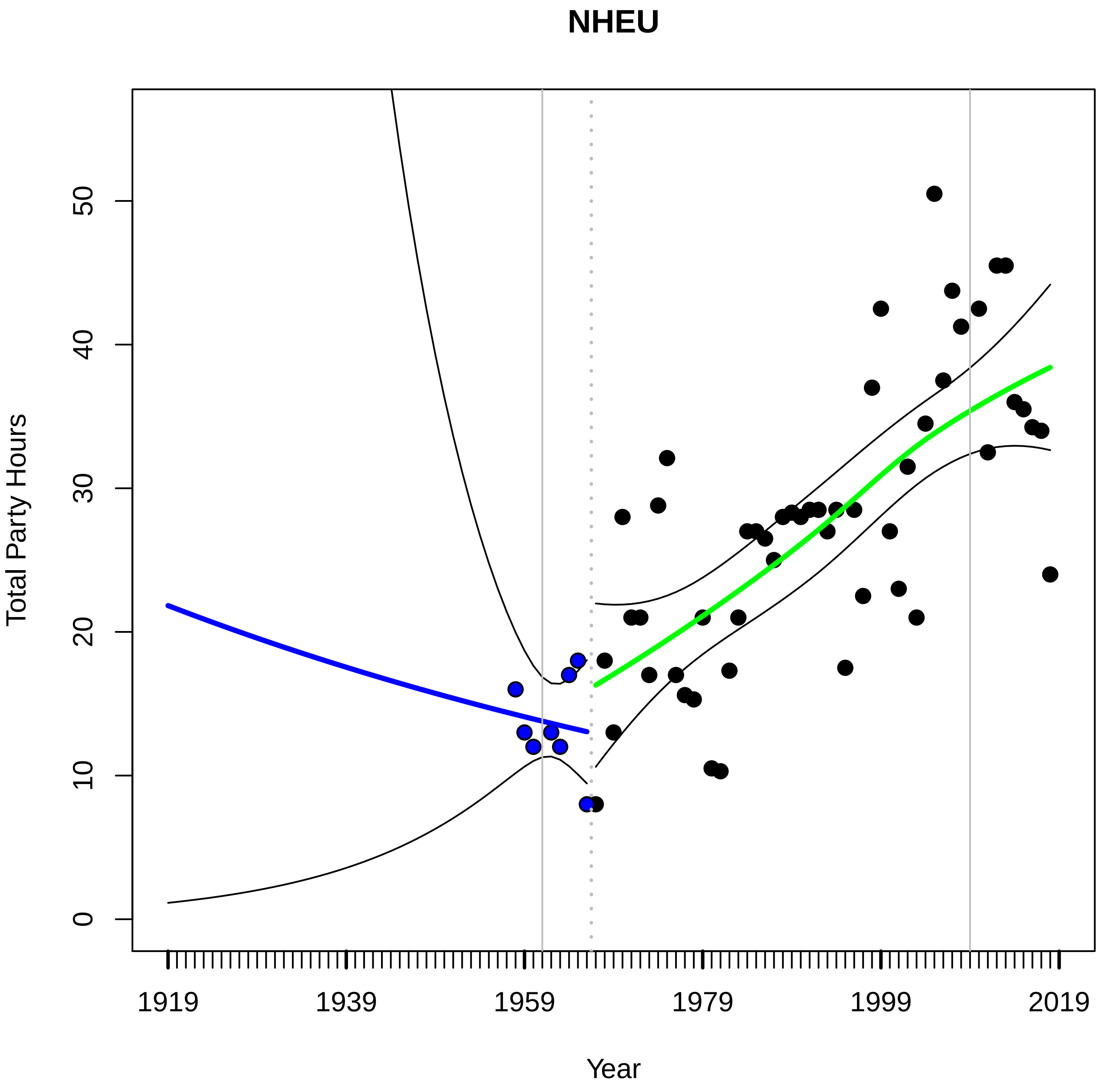

NHEU
Total Party Hours
0
10
20
30
40
50
1919
1939
1959
1979
1999
2019
Year

NHHN

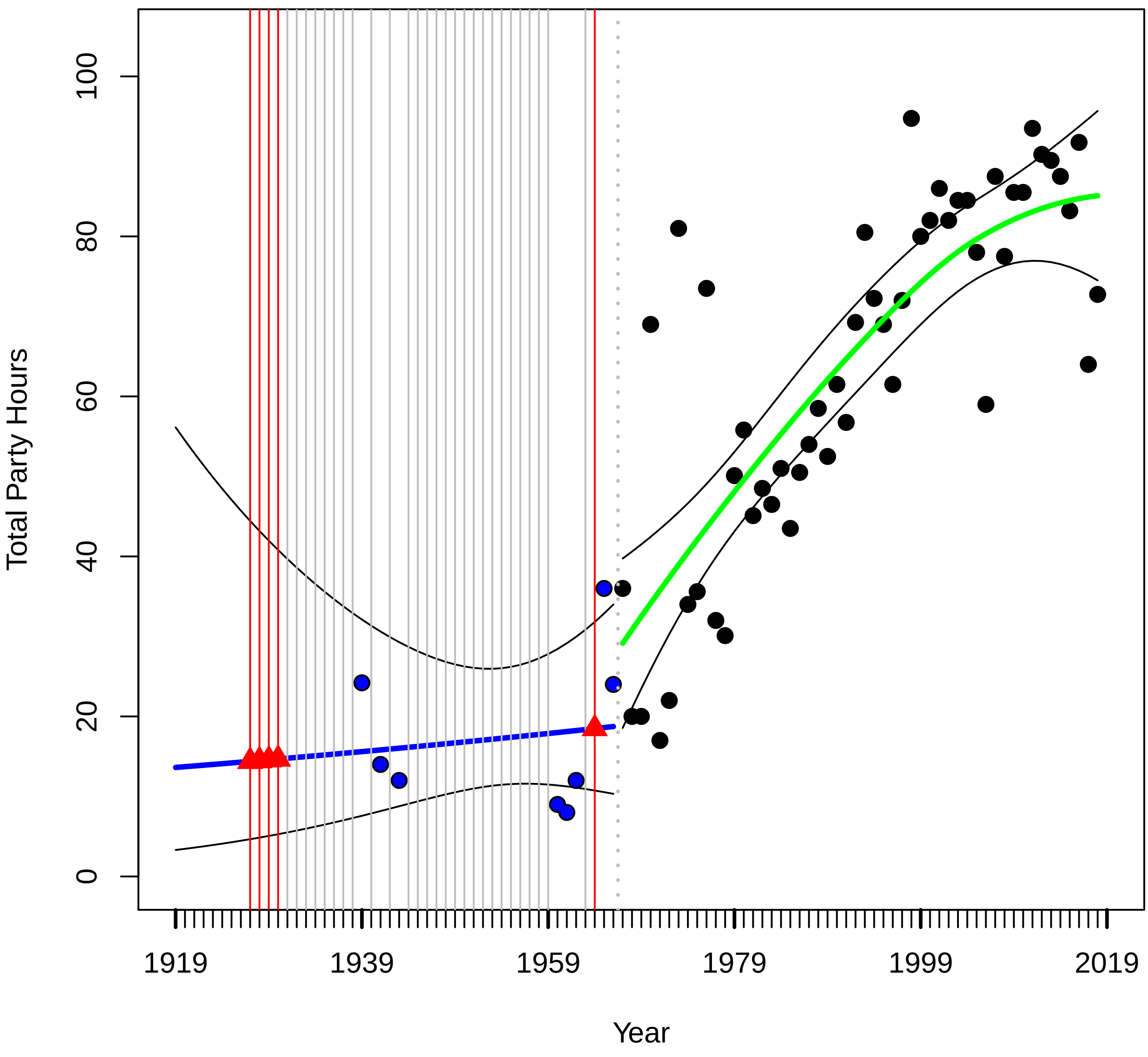

Total Party Hours
0
20
40
60
80
100
1919
1939
1959
1979
1999
2019
Year

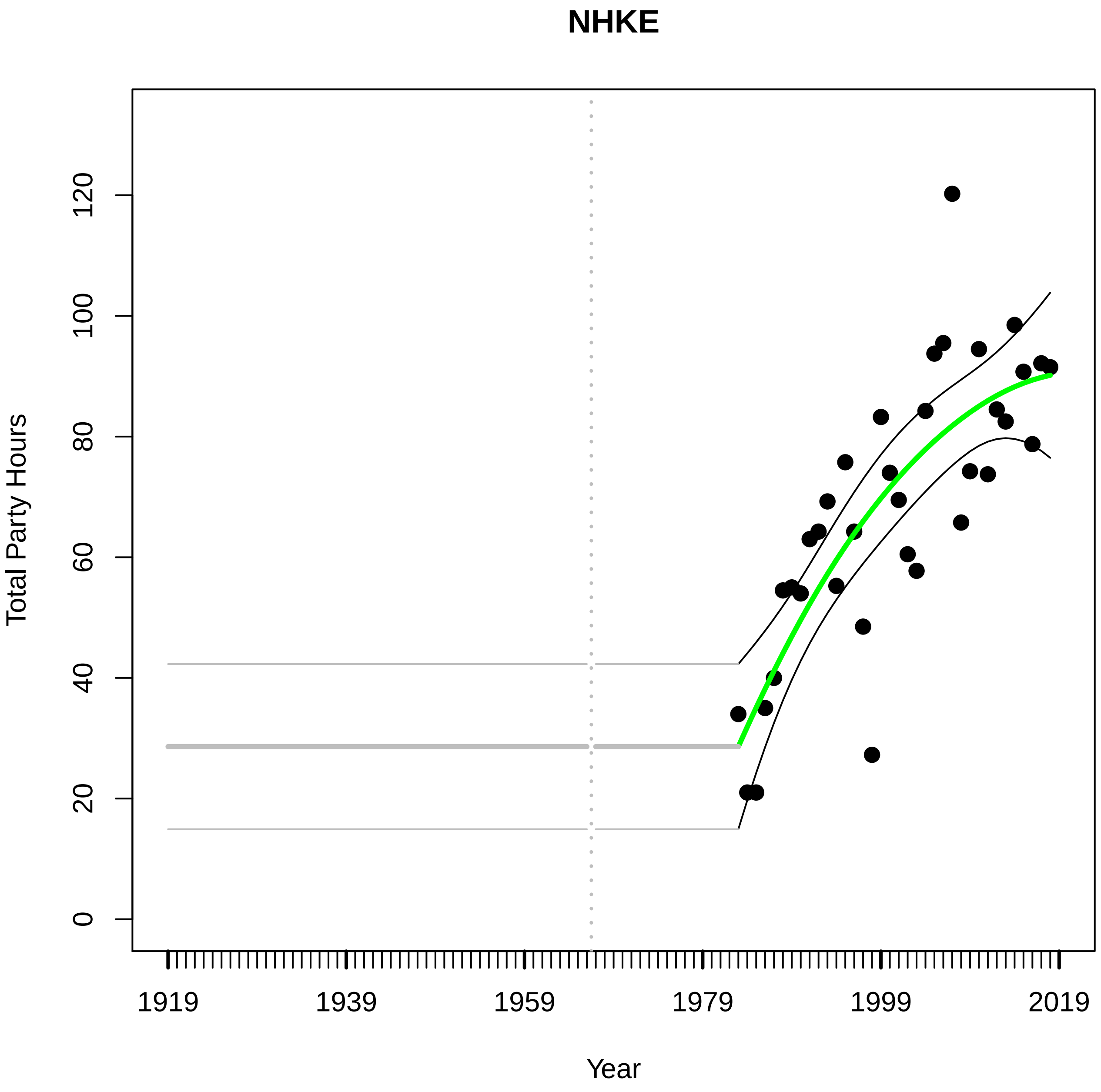

NHKE
Total Party Hours
0
20
40
60
80
100
120
1919
1939
1959
1979
1999
2019
Year

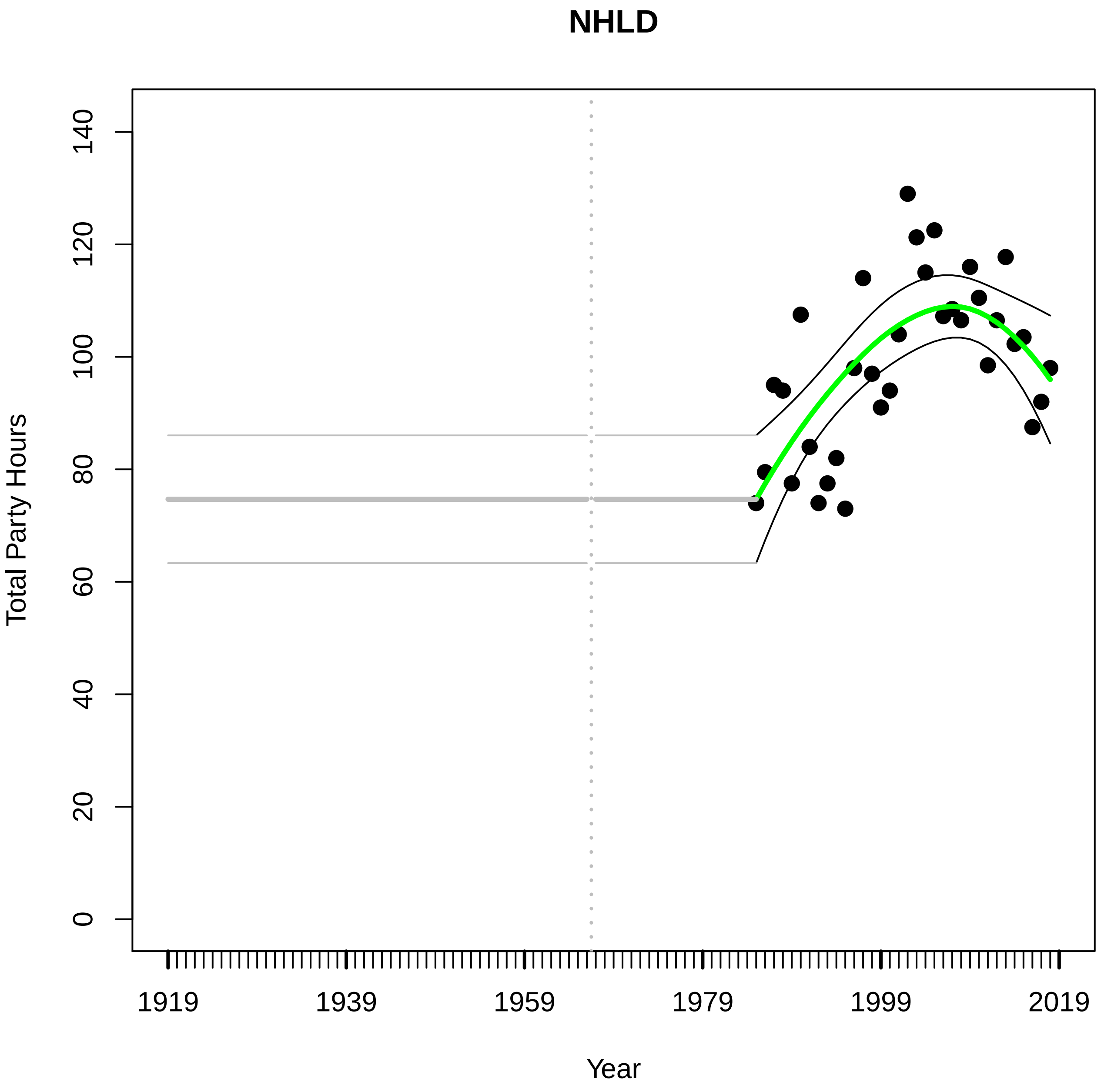

NHLD
Total Party Hours
0
20
40
60
80
100
120
140
1919
1939
1959
1979
1999
2019
Year

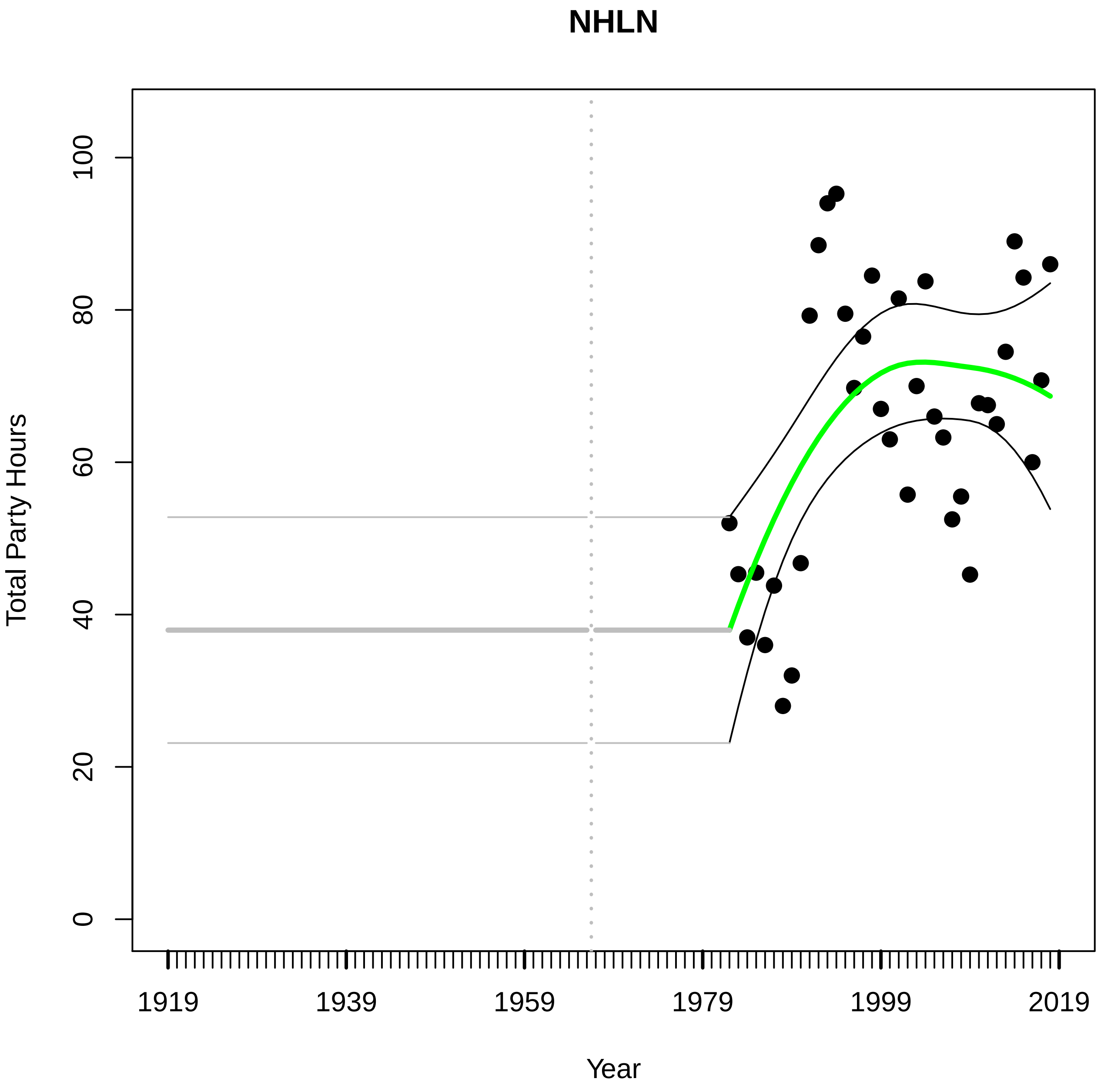
NHLN
Total Party Hours
0
20
40
60
80
100
1919
1939
1959
1979
1999
2019
Year

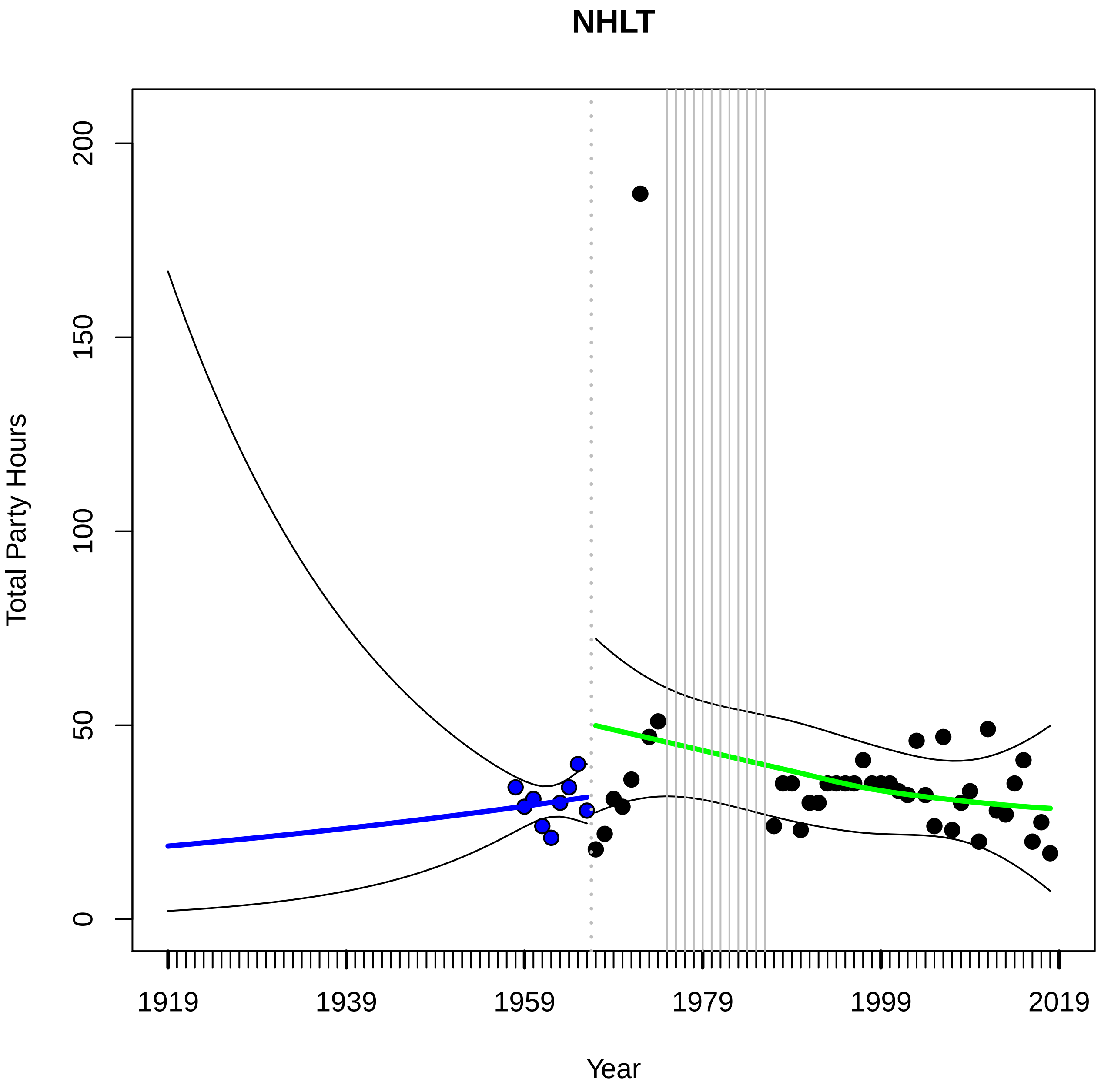

NHLT
Total Party Hours
0
50
100
150
200
1919
1939
1959
1979
1999
2019
Year

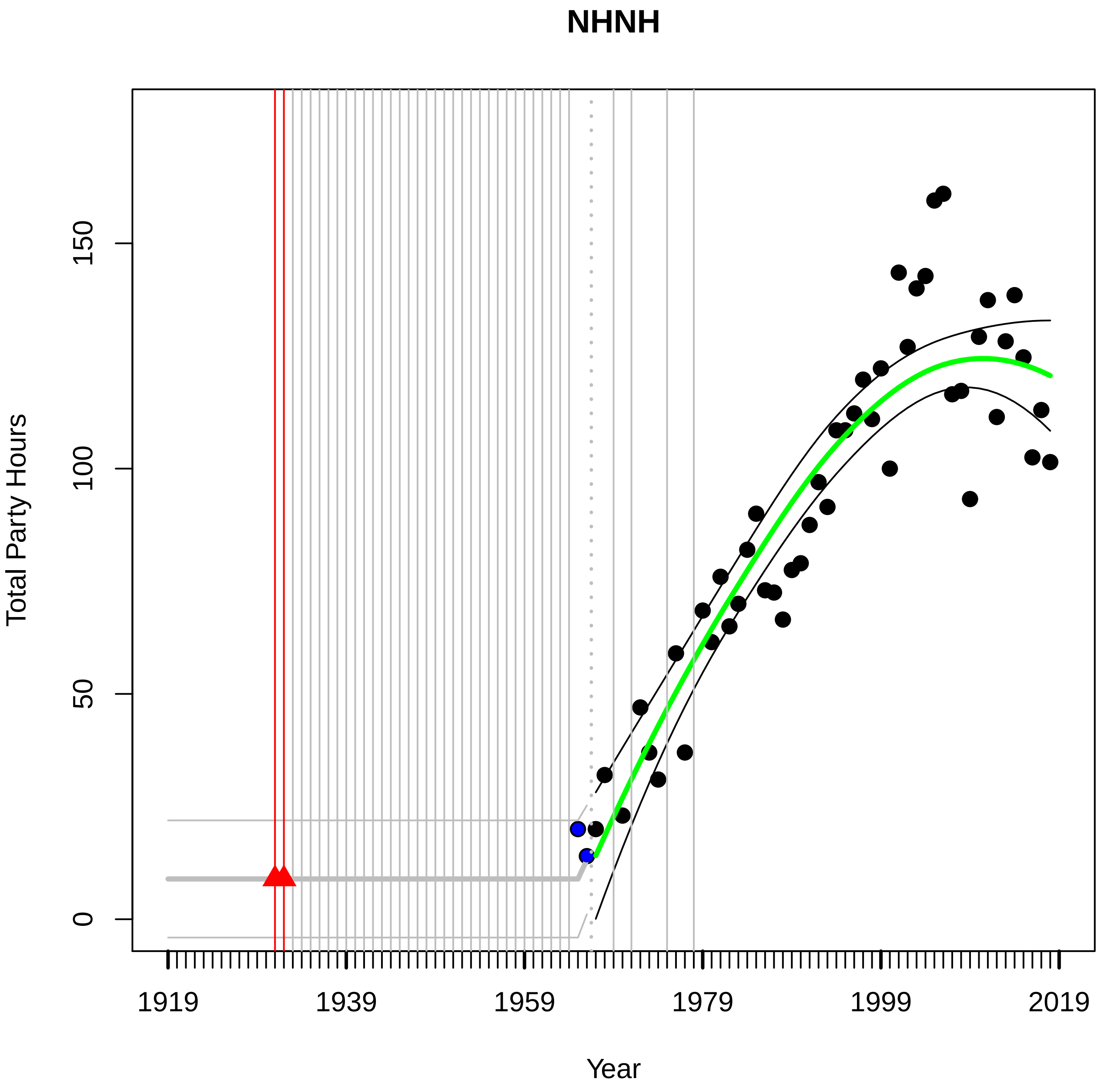

NHNH
Total Party Hours
0
50
100
150
1919
1939
1959
1979
1999
2019
Year

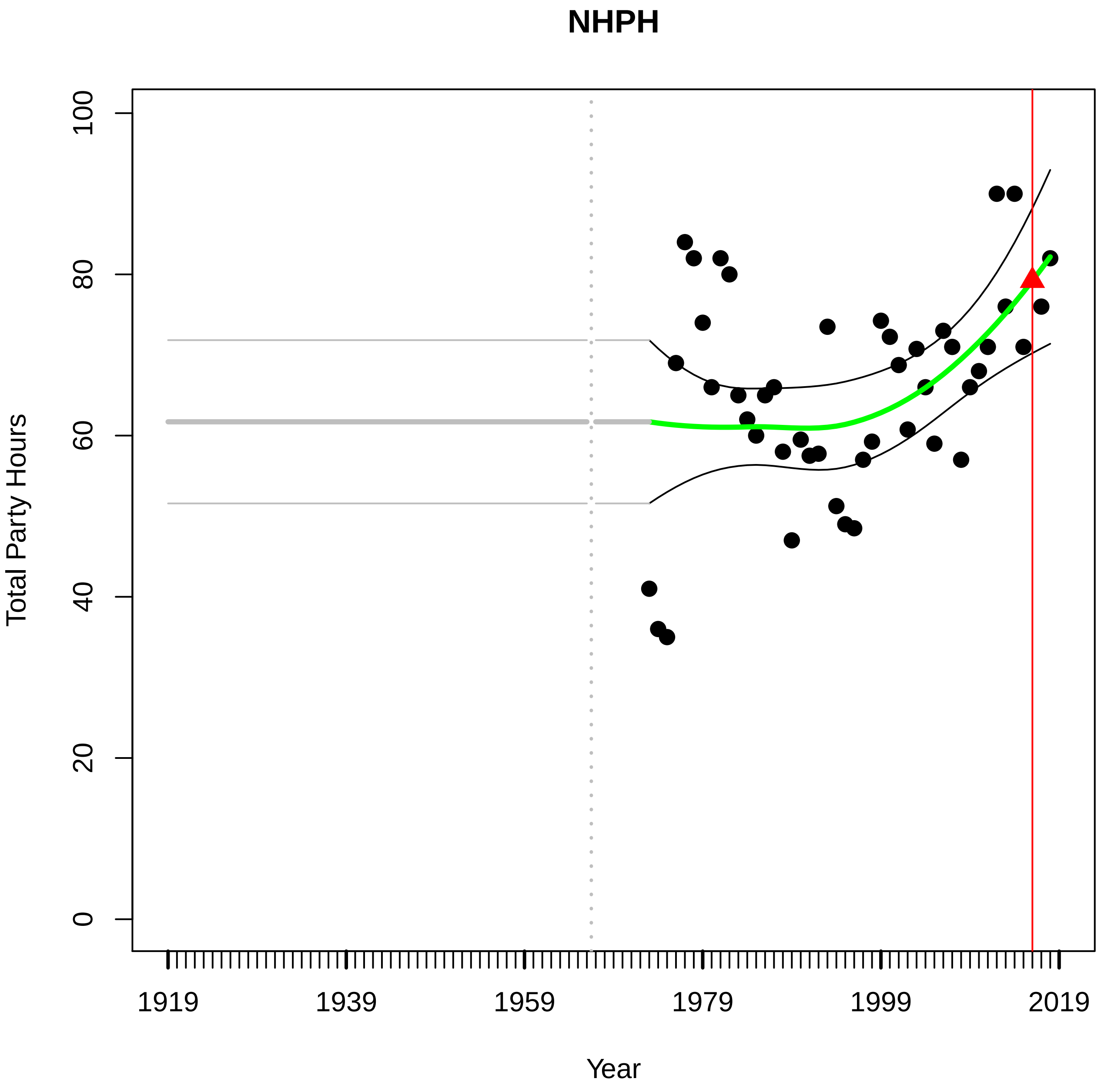

NHPH
Total Party Hours
0
20
40
60
80
100
1919
1939
1959
1979
1999
2019
Year

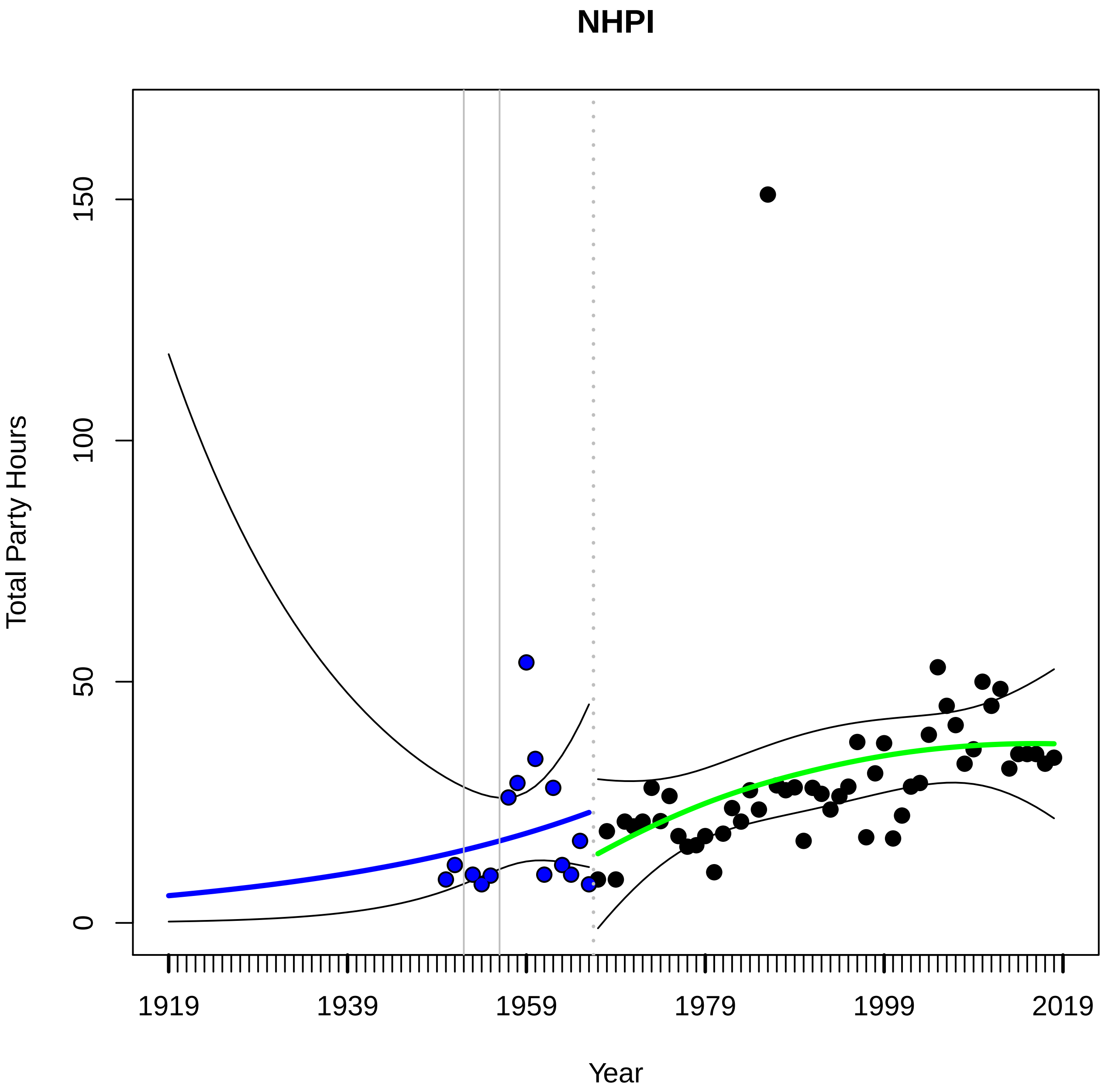

NHPI
Total Party Hours
0
50
100
150
1919
1939
1959
1979
1999
2019
Year

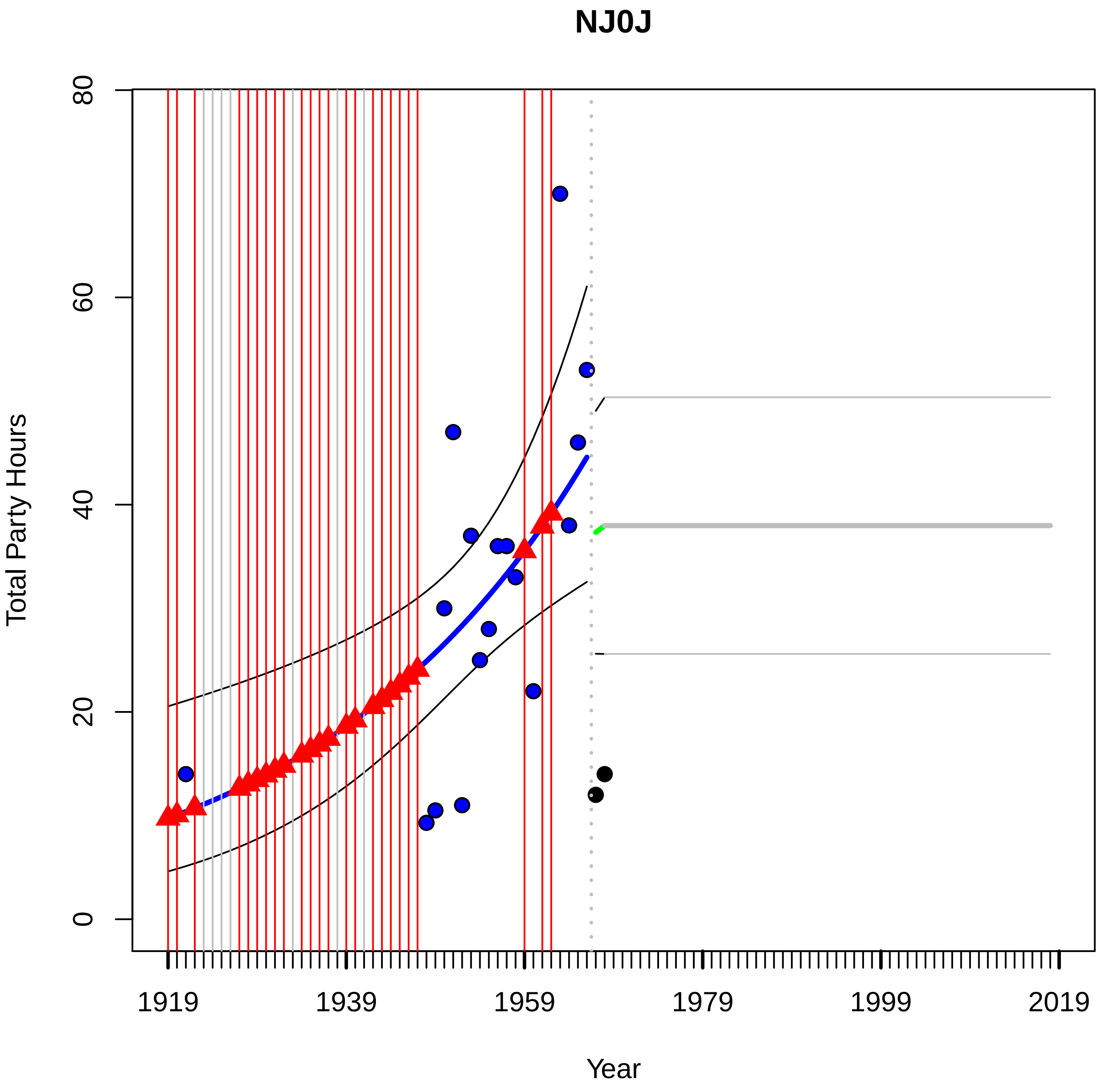

NJ0J
Total Party Hours
0
20
40
60
80
1919
1939
1959
1979
1999
2019
Year

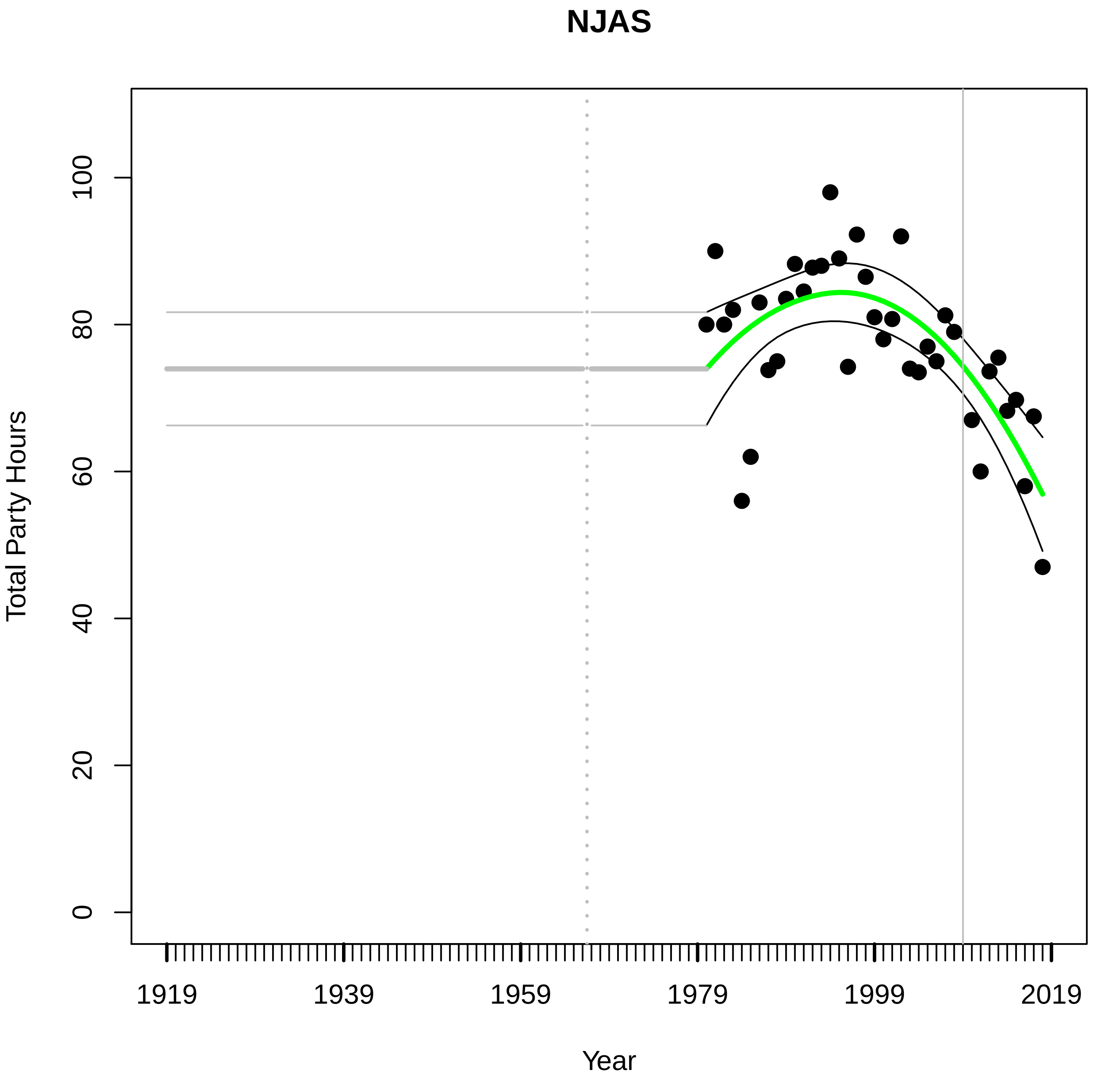
NJAS
Total Party Hours
0
20
40
60
80
100
1919
1939
1959
1979
1999
2019
Year

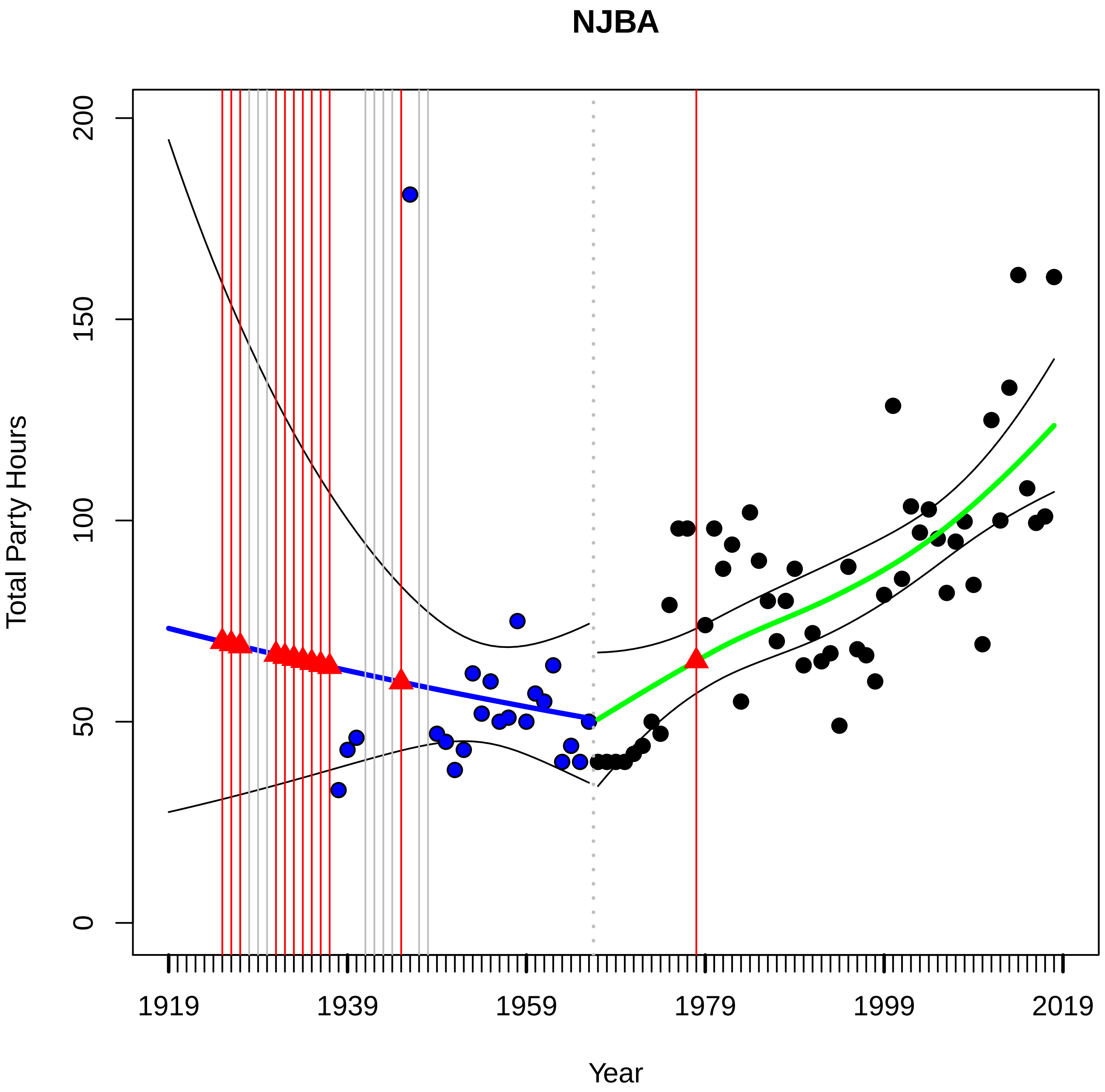

NJBA
Total Party Hours
Year
0
50
100
150
200
1919
1939
1959
1979
1999
2019

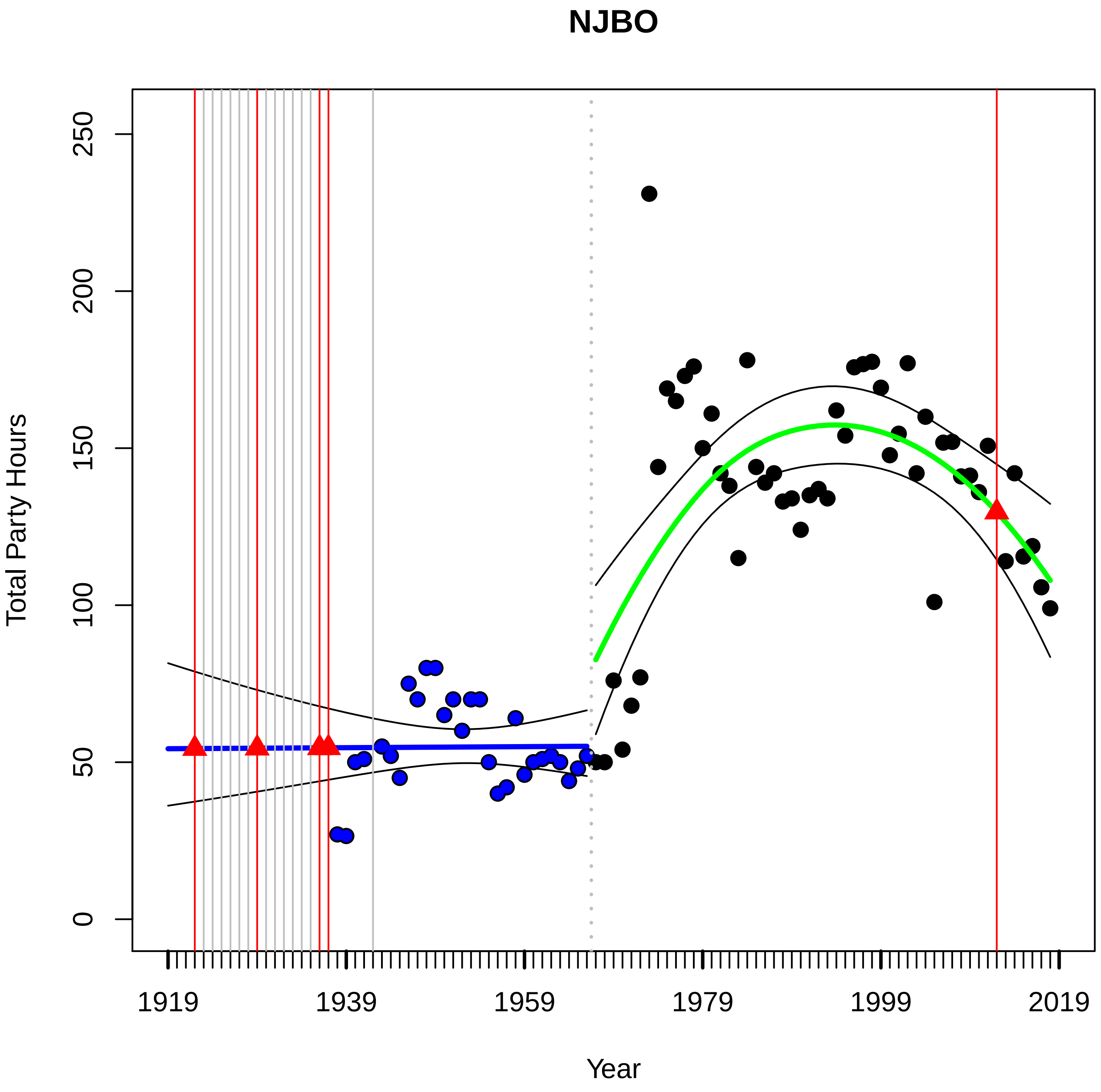

NJBO
Total Party Hours
0
50
100
150
200
250
1919
1939
1959
1979
1999
2019
Year

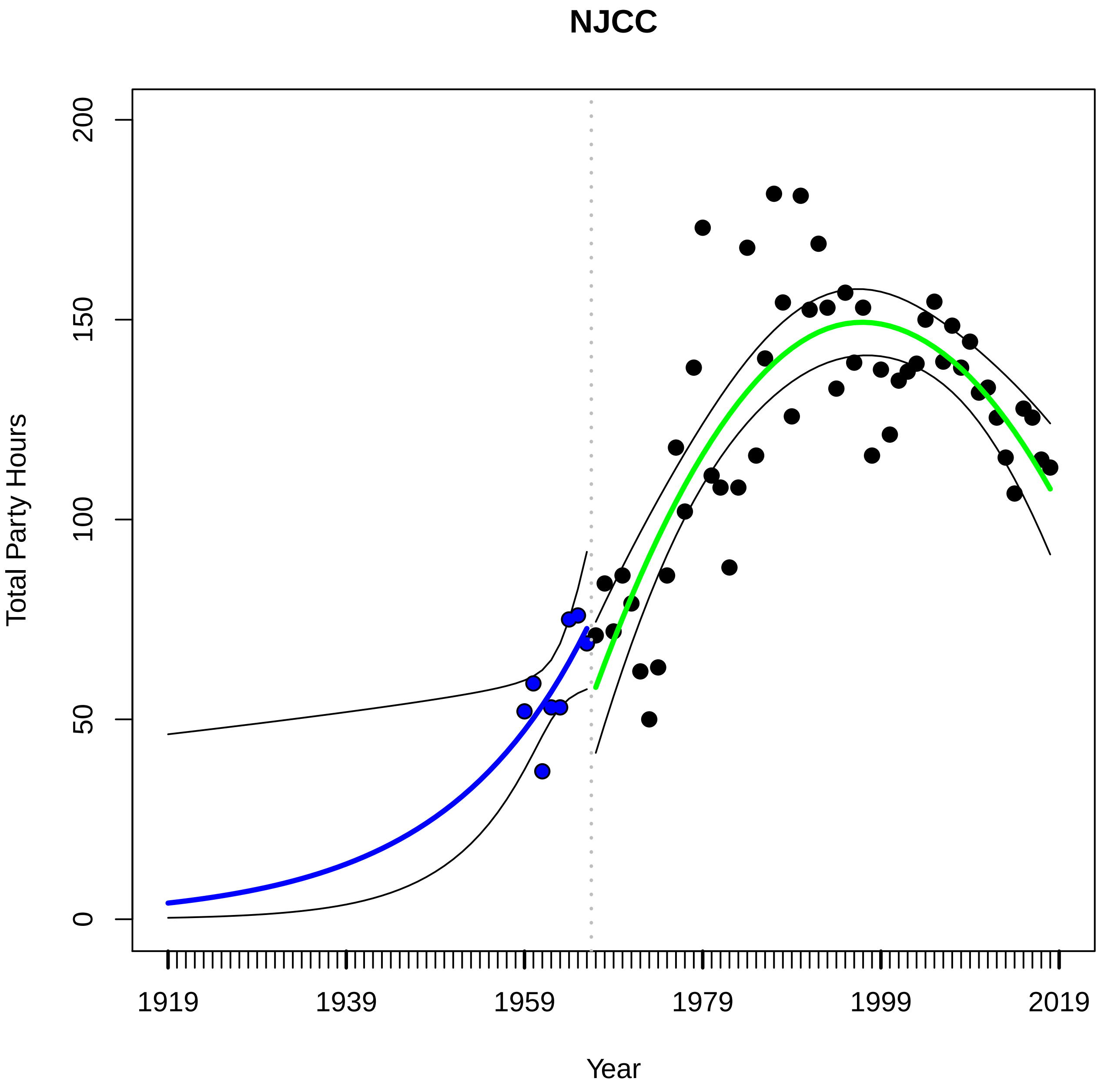

NJCC
Total Party Hours
0
50
100
150
200
1919
1939
1959
1979
1999
2019
Year

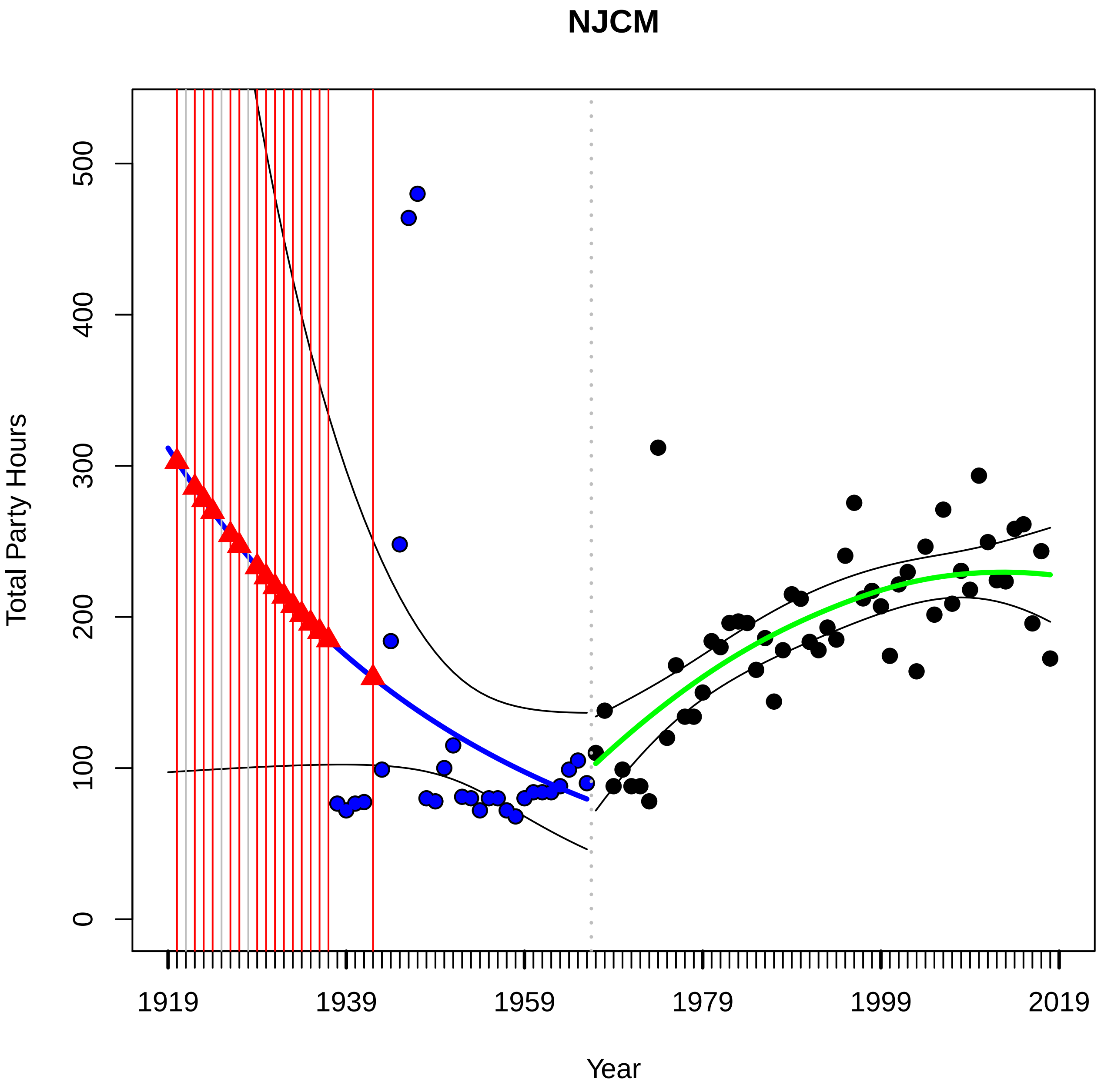

NJCM
Total Party Hours
0
100
200
300
400
500
Year
1919
1939
1959
1979
1999
2019

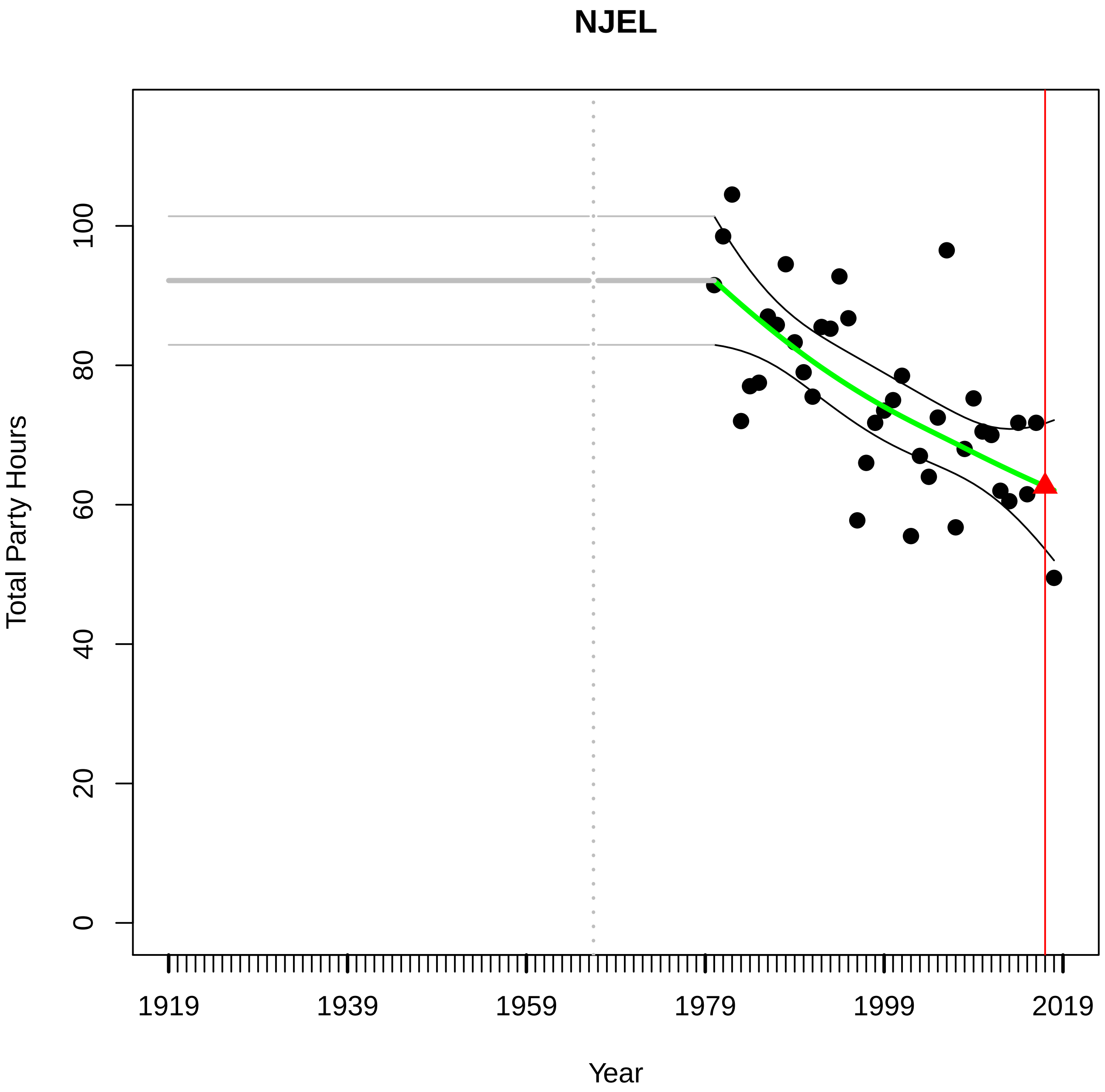

NJEL
Total Party Hours
0
20
40
60
80
100
1919
1939
1959
1979
1999
2019
Year

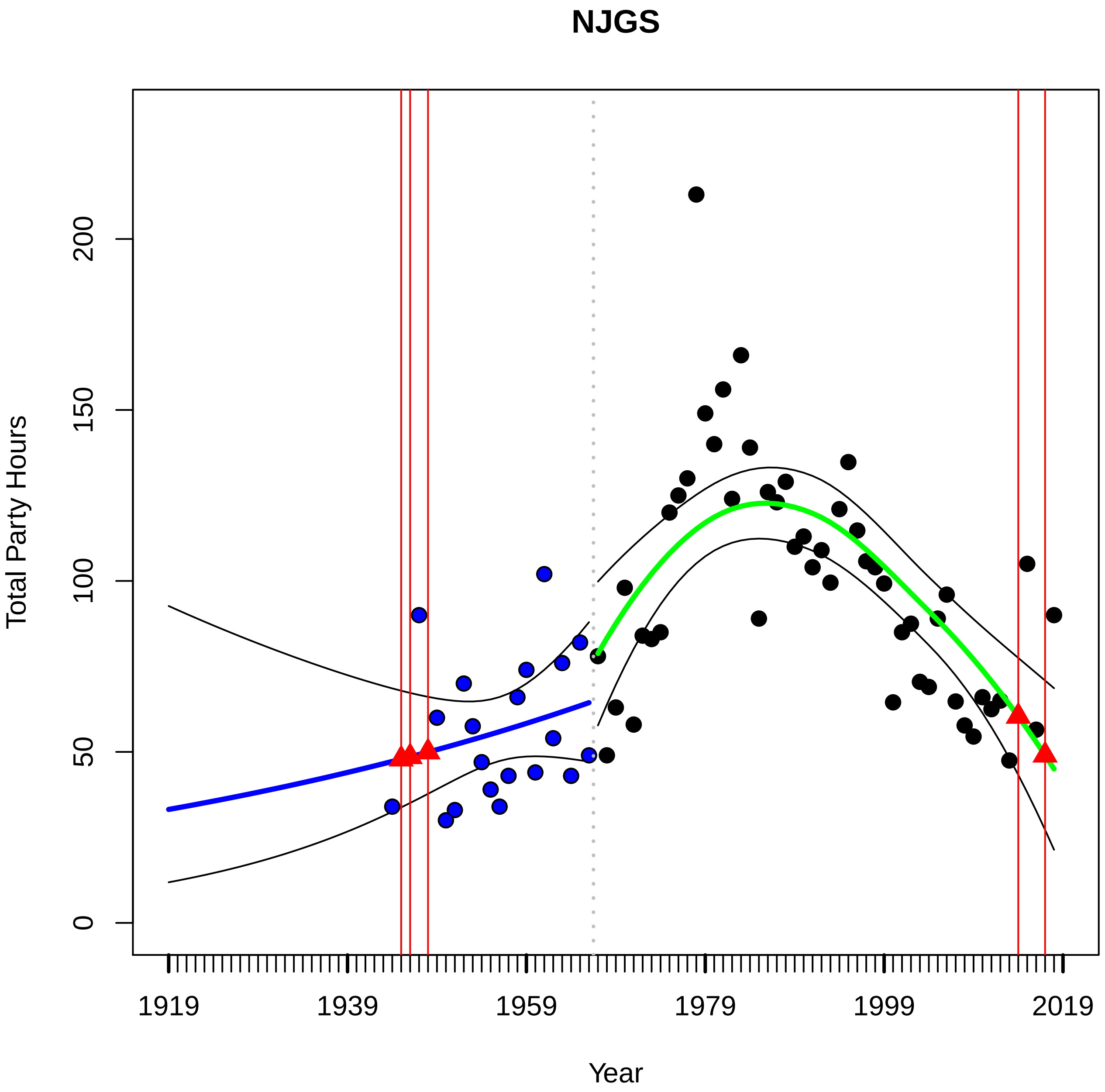

NJGS
Total Party Hours
0
50
100
150
200
1919
1939
1959
1979
1999
2019
Year

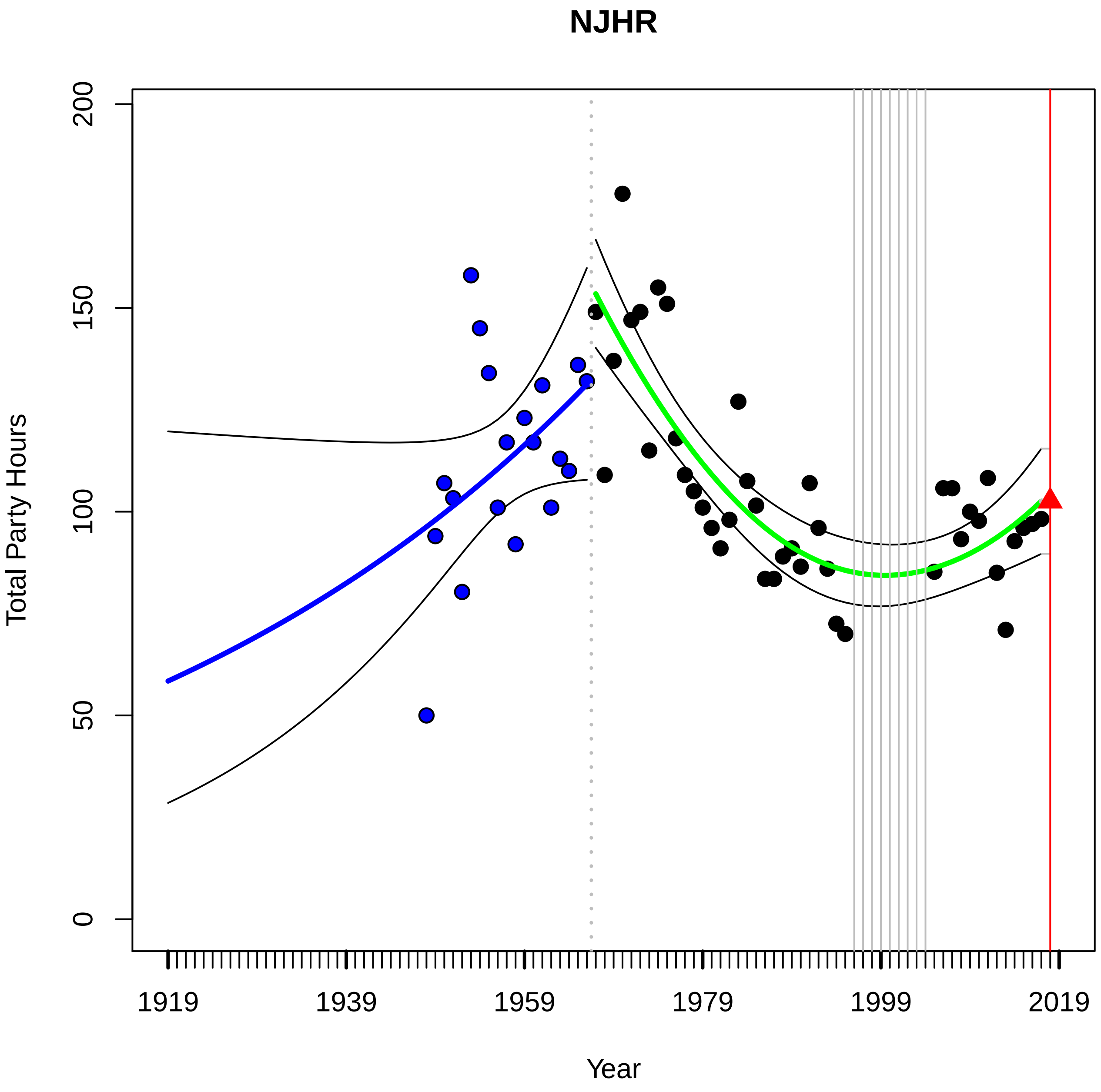

NJHR
Total Party Hours
Year
0
50
100
150
200
1919
1939
1959
1979
1999
2019

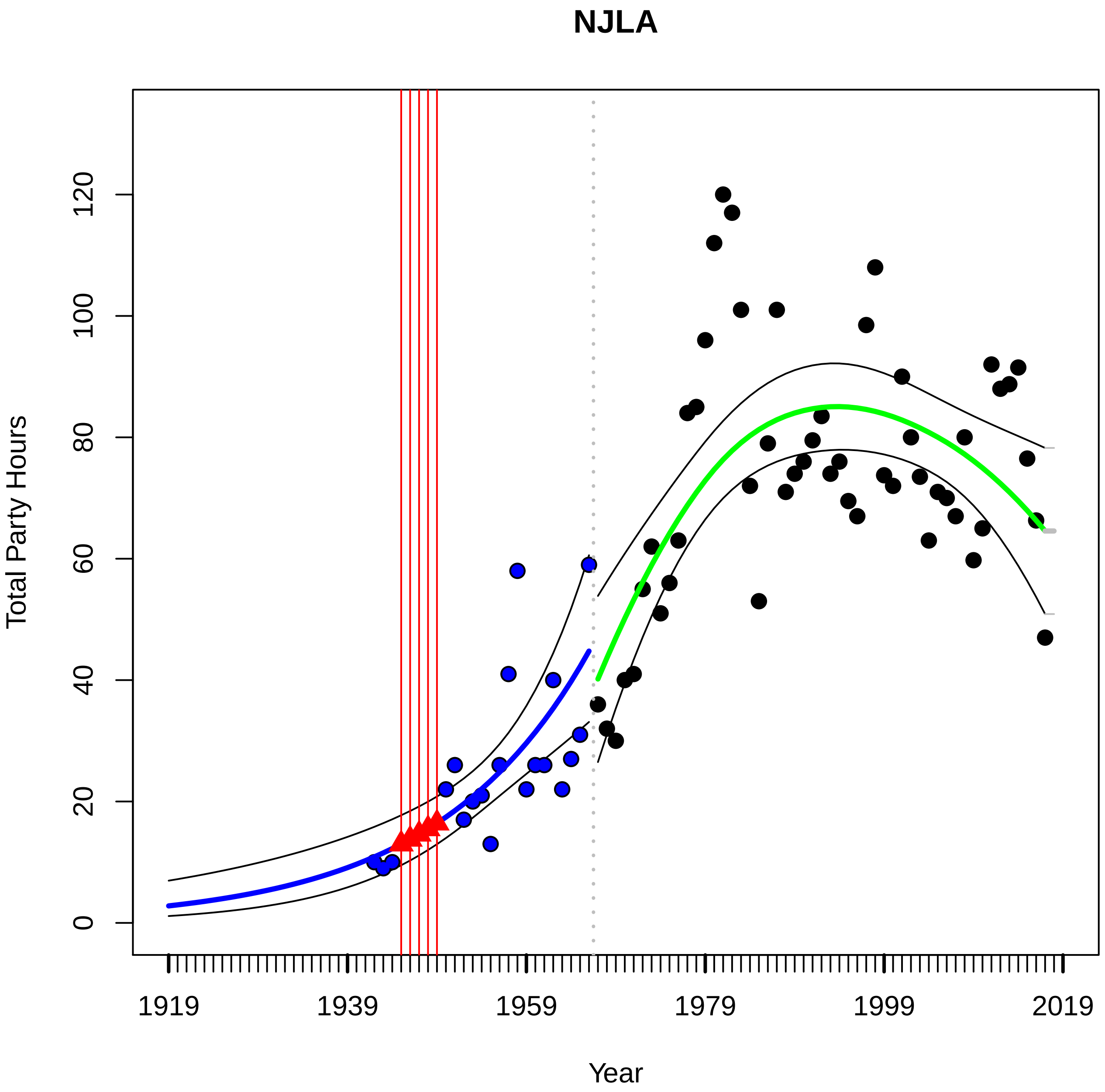

NJLA
Total Party Hours
Year
0
20
40
60
80
100
120
1919
1939
1959
1979
1999
2019

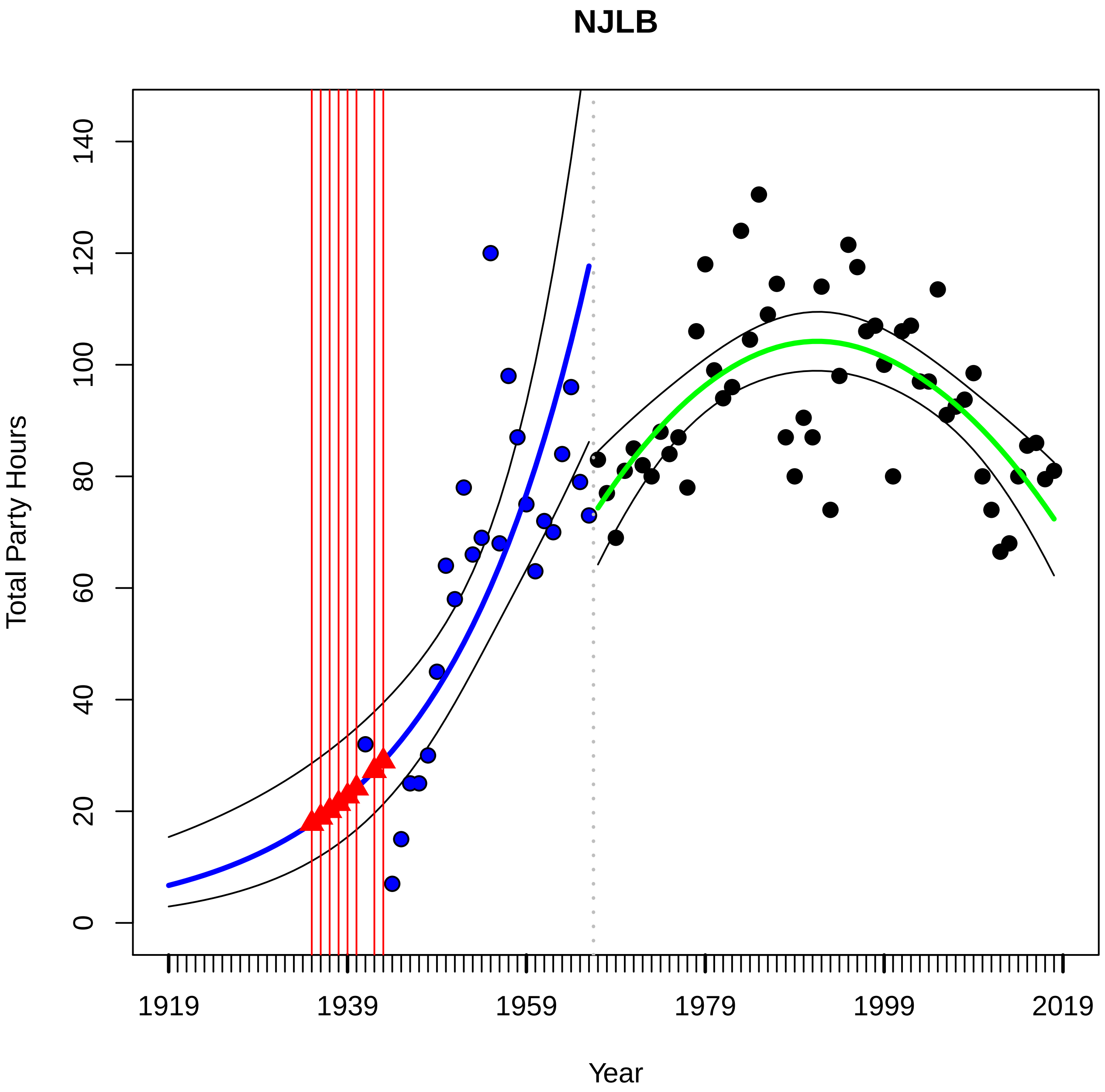
NJLB
Total Party Hours
0
20
40
60
80
100
120
140
1919
1939
1959
1979
1999
2019
Year

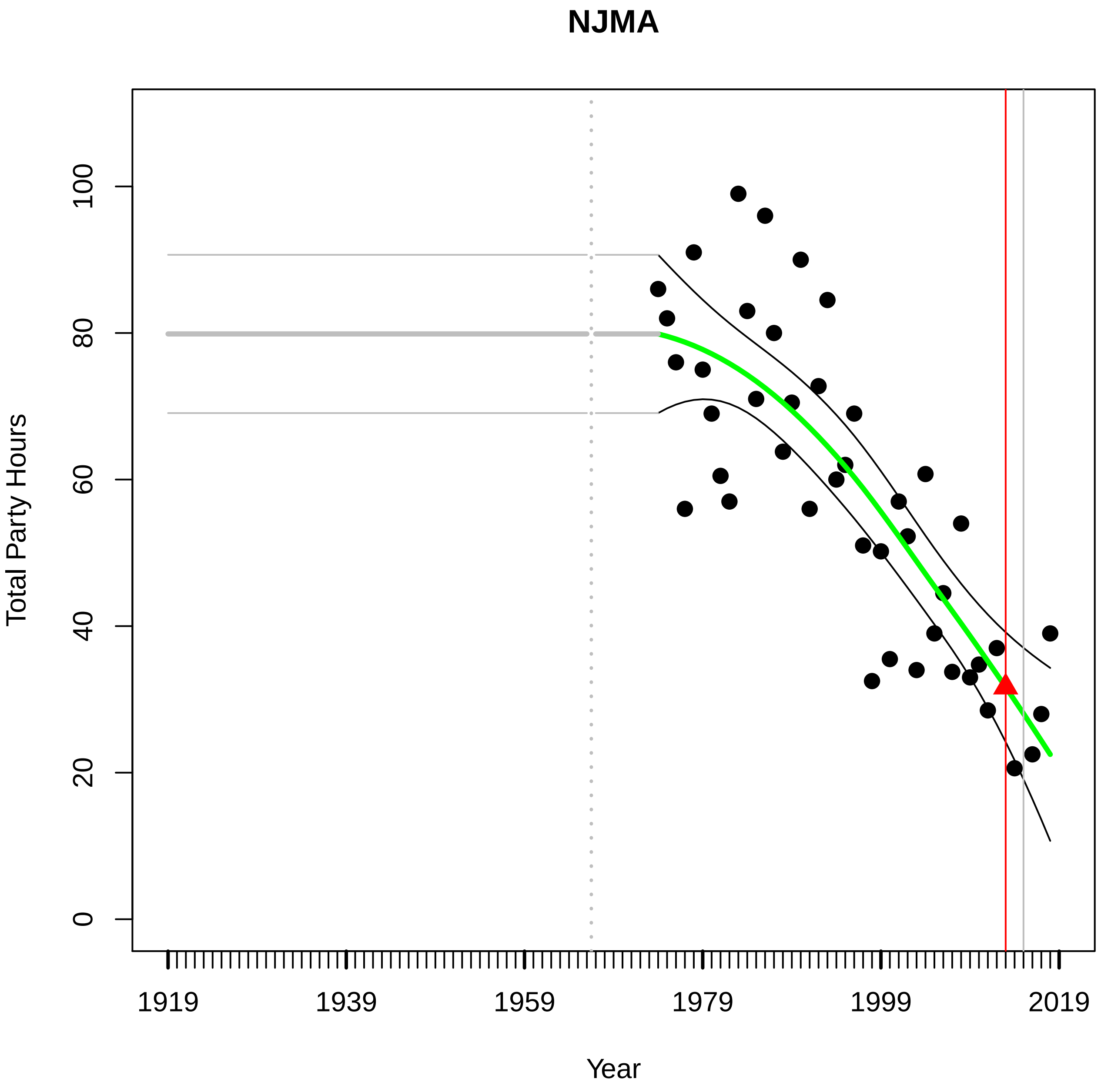

NJMA
Total Party Hours
0
20
40
60
80
100
1919
1939
1959
1979
1999
2019
Year

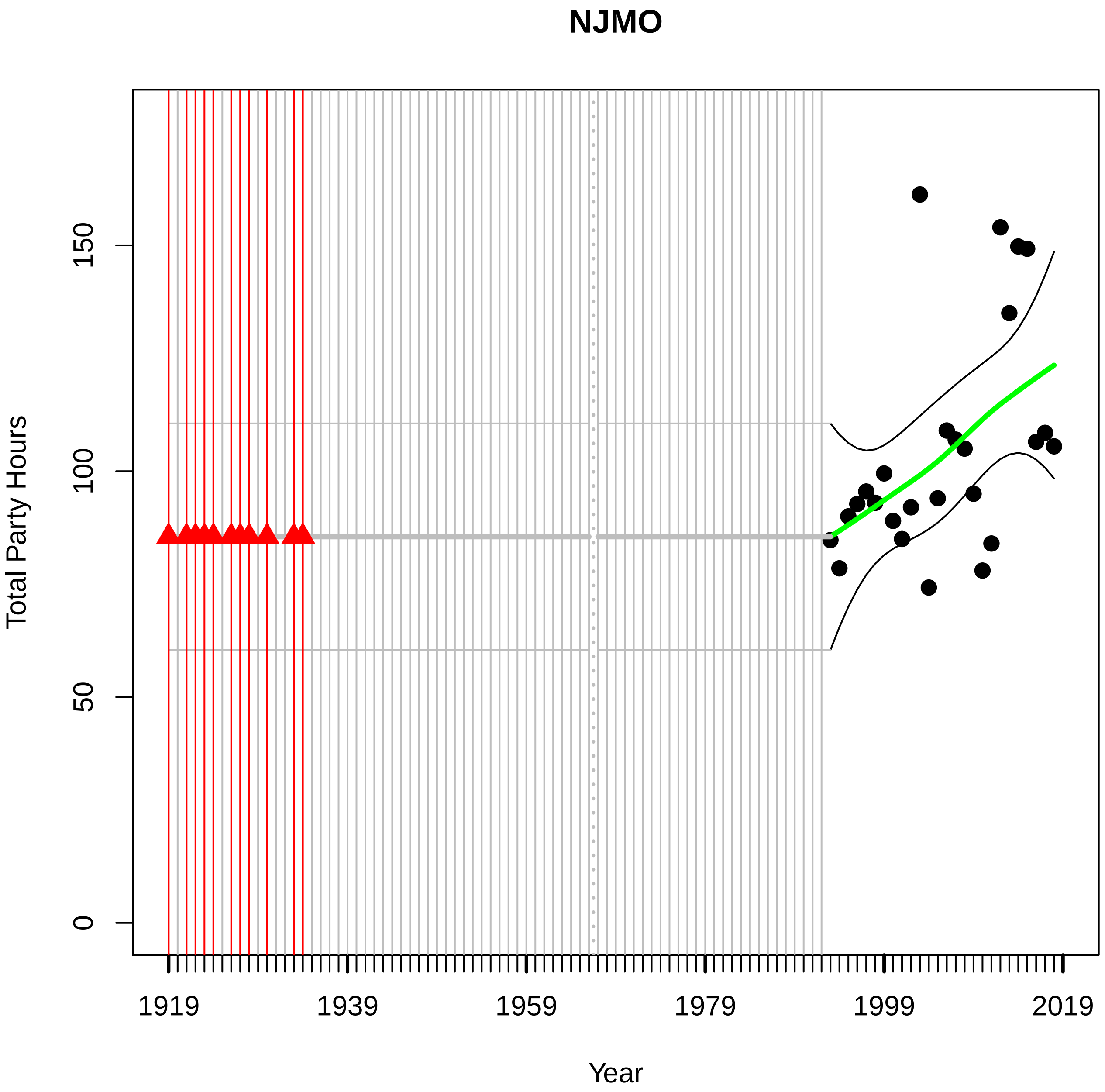
NJMO
Total Party Hours
0
50
100
150
1919
1939
1959
1979
1999
2019
Year

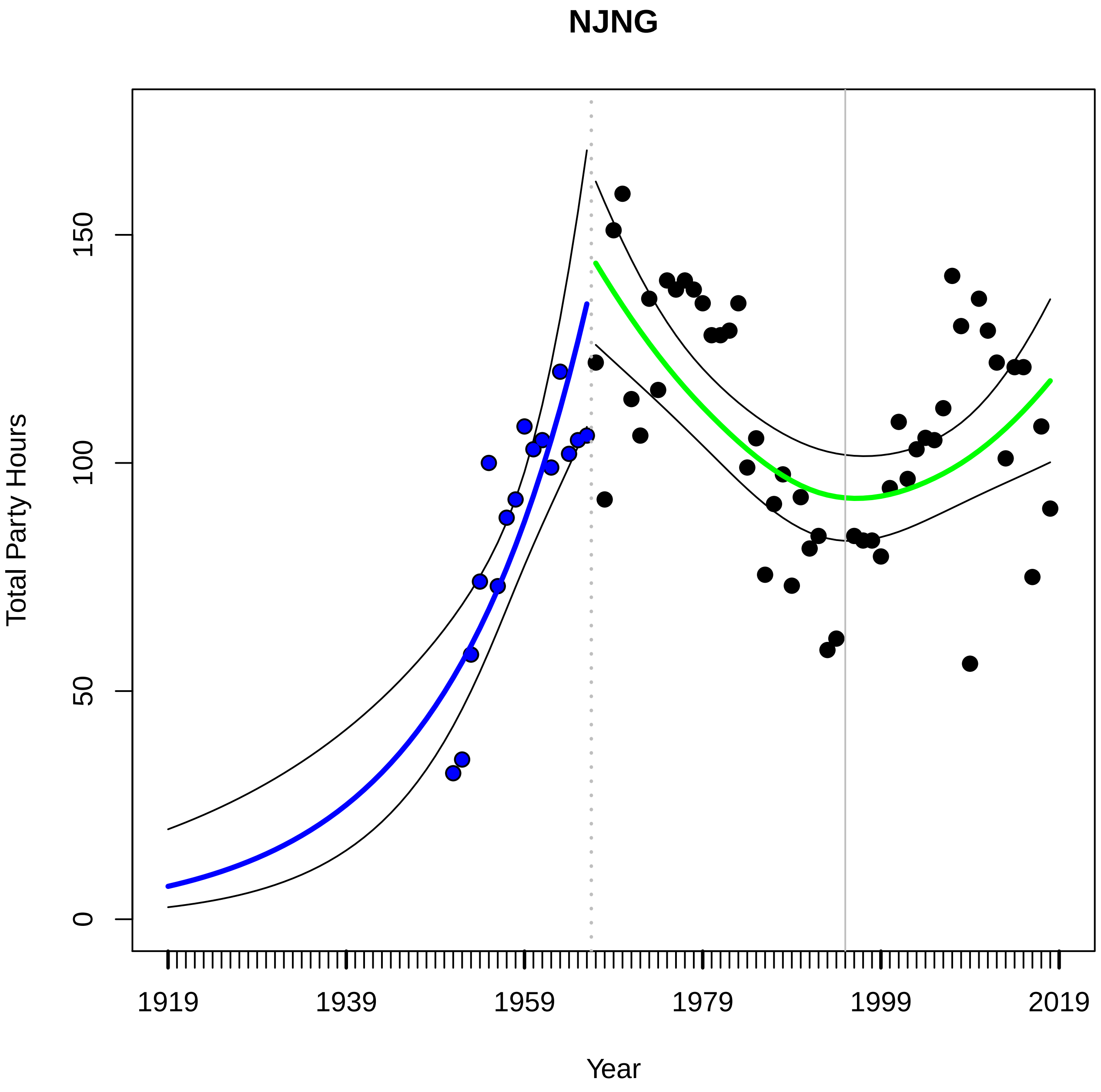
NJNG
Total Party Hours
0
50
100
150
1919
1939
1959
1979
1999
2019
Year

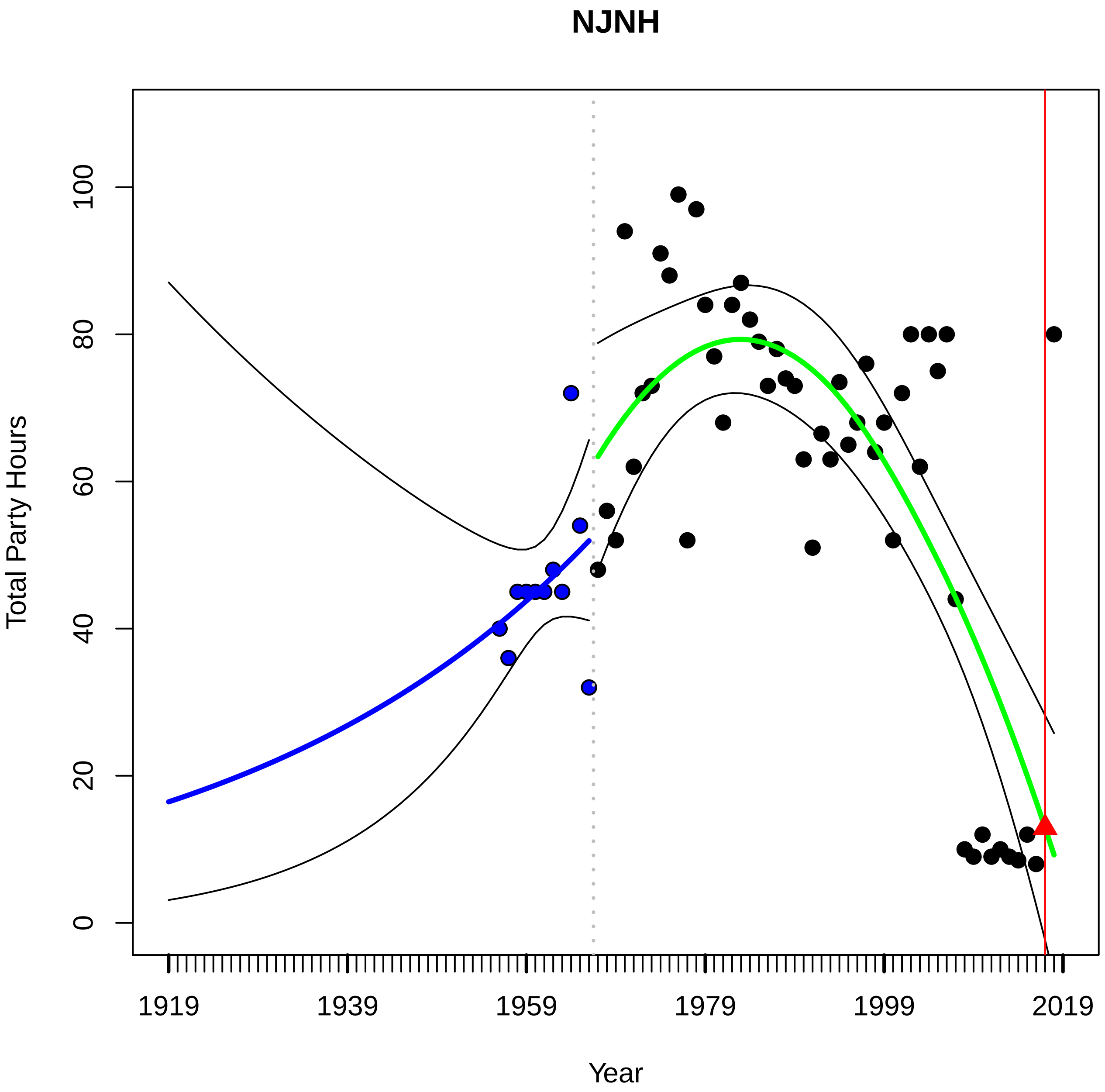

NJNH
Total Party Hours
Year
0
20
40
60
80
100
1919
1939
1959
1979
1999
2019

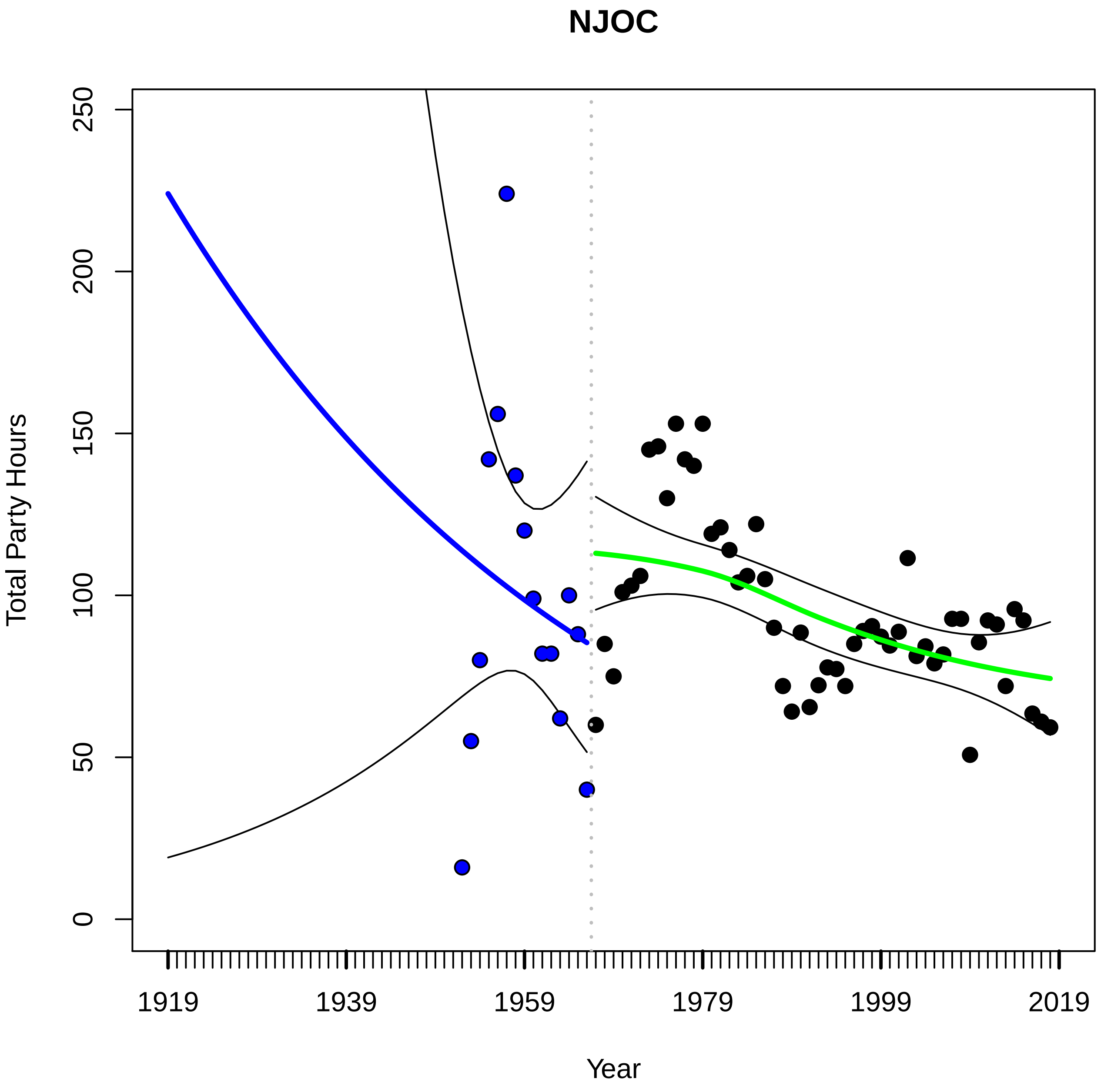
NJOC
Total Party Hours
Year
0
50
100
150
200
250
1919
1939
1959
1979
1999
2019

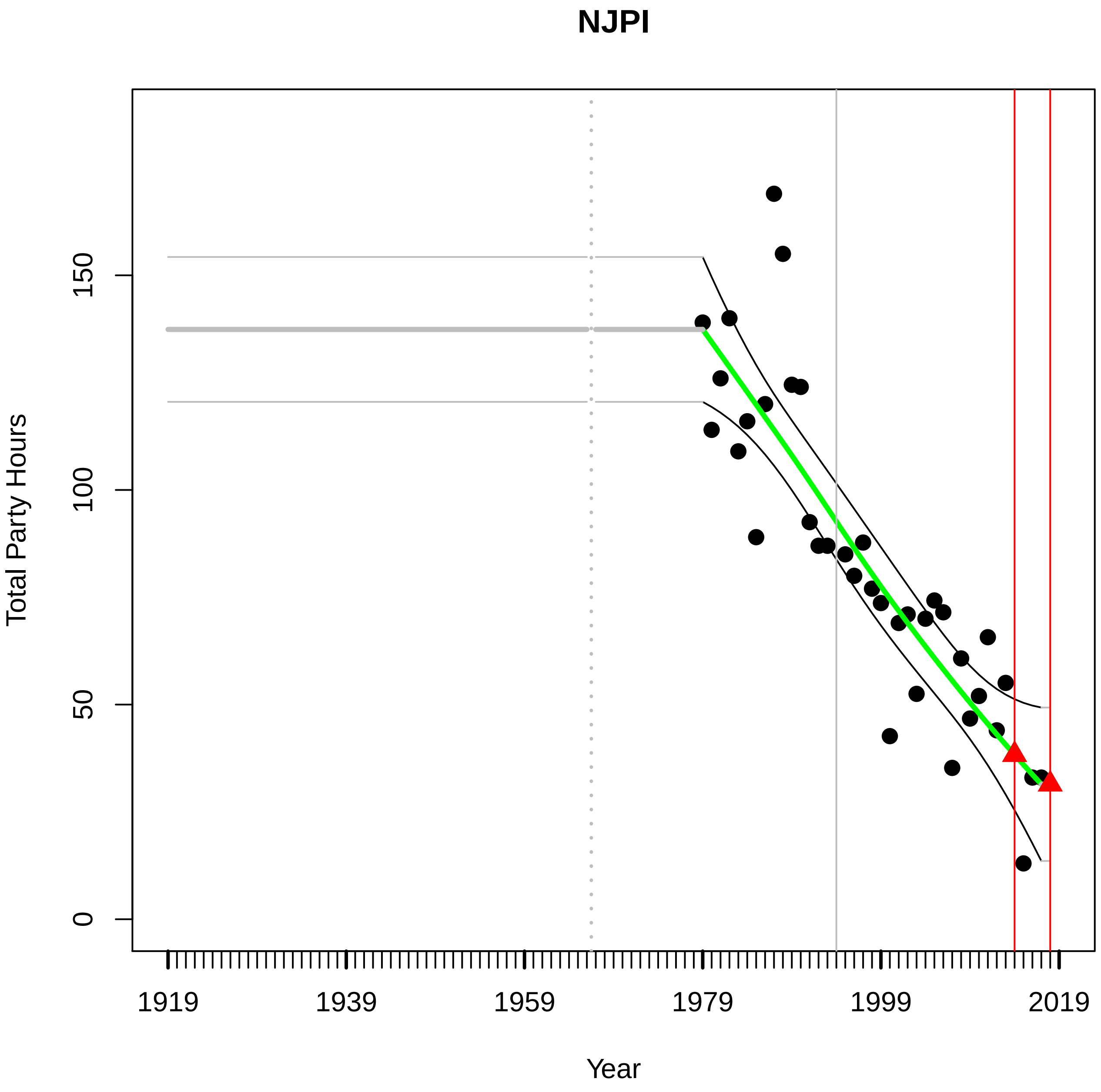

NJPI
Total Party Hours
0
50
100
150
1919
1939
1959
1979
1999
2019
Year

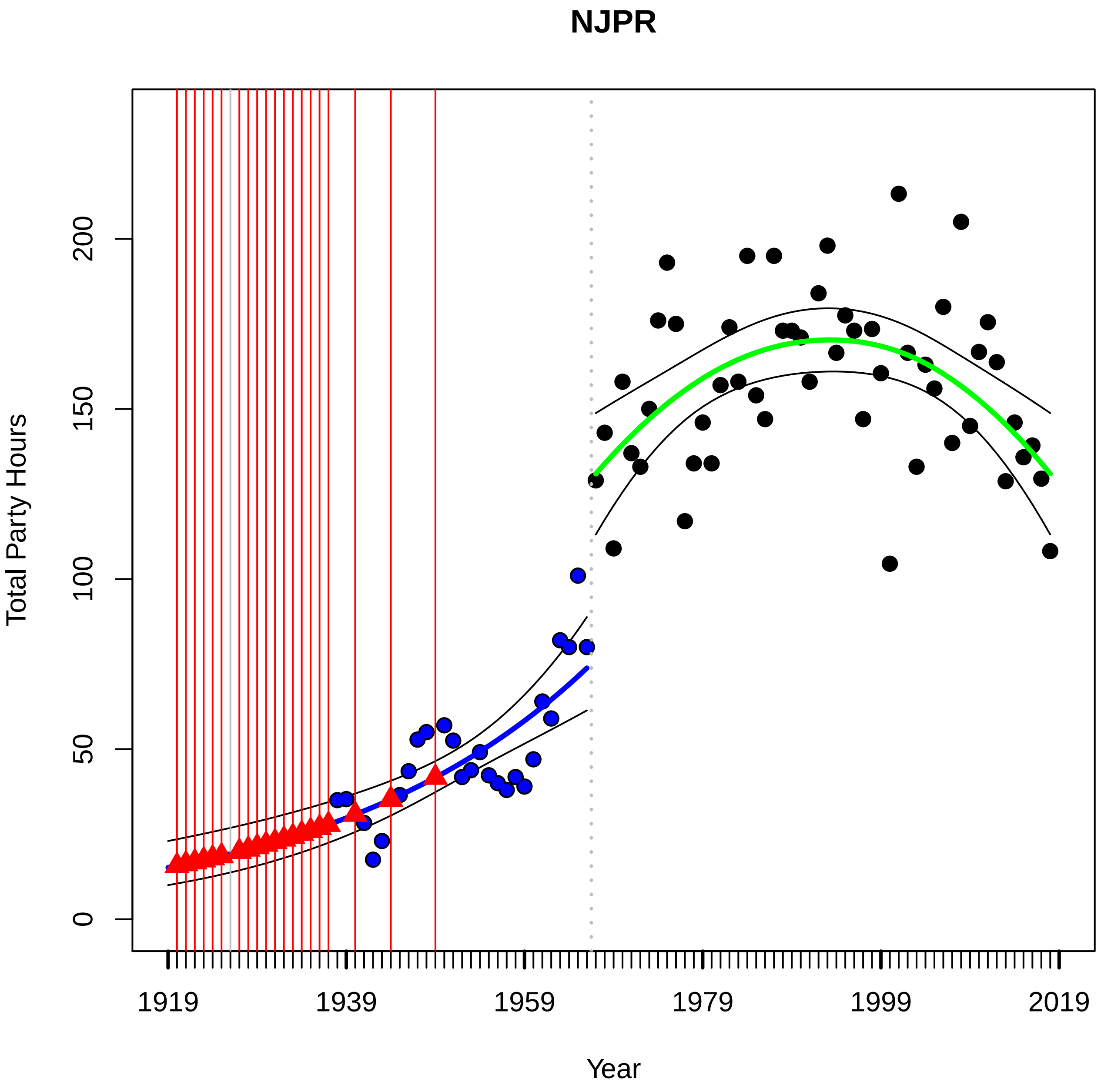

NJPR
Total Party Hours
0
50
100
150
200
Year
1919
1939
1959
1979
1999
2019

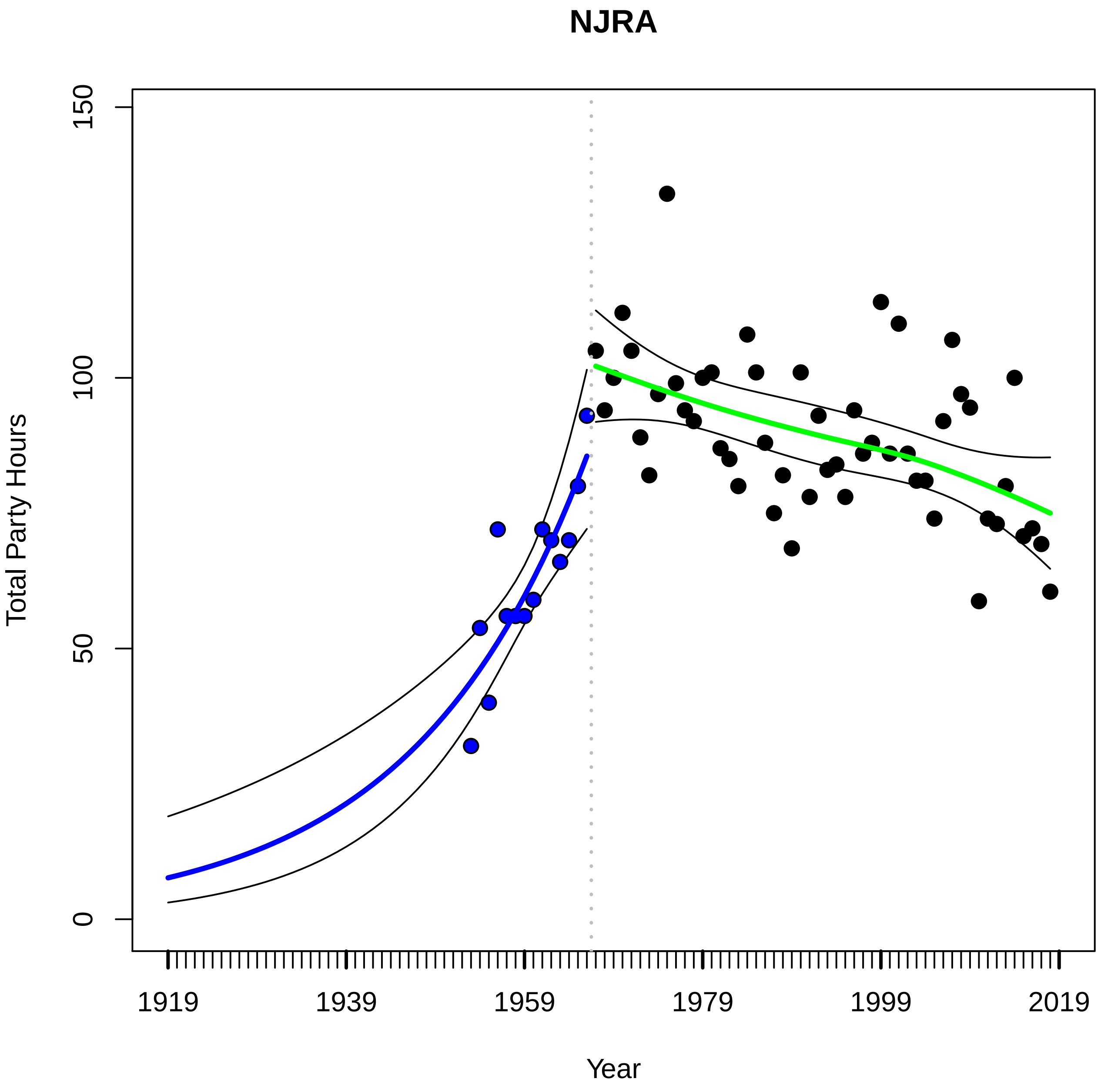

NJRA
Total Party Hours
Year
0
50
100
150
1919
1939
1959
1979
1999
2019

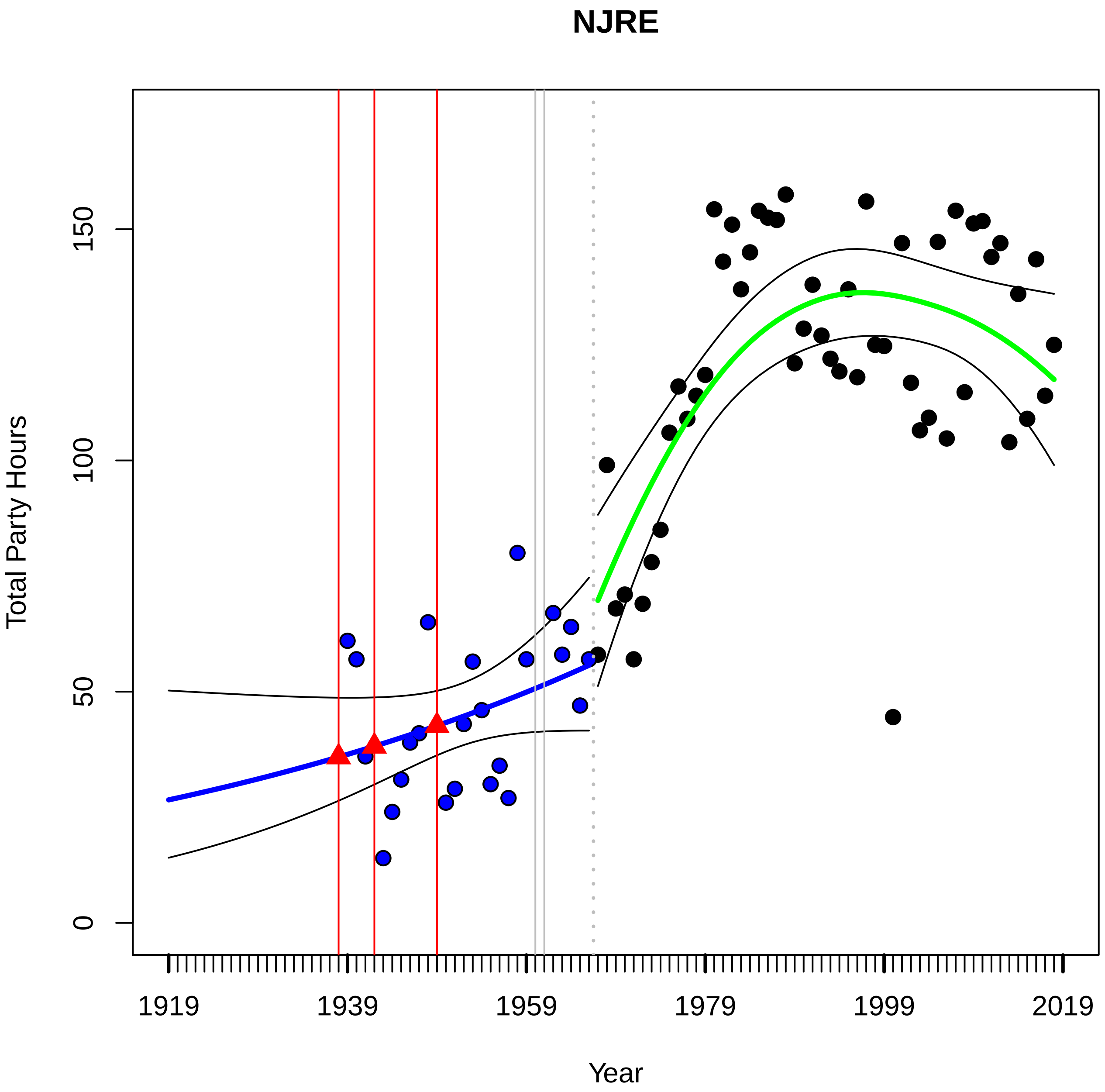
NJRE
Total Party Hours
0
50
100
150
1919
1939
1959
1979
1999
2019
Year

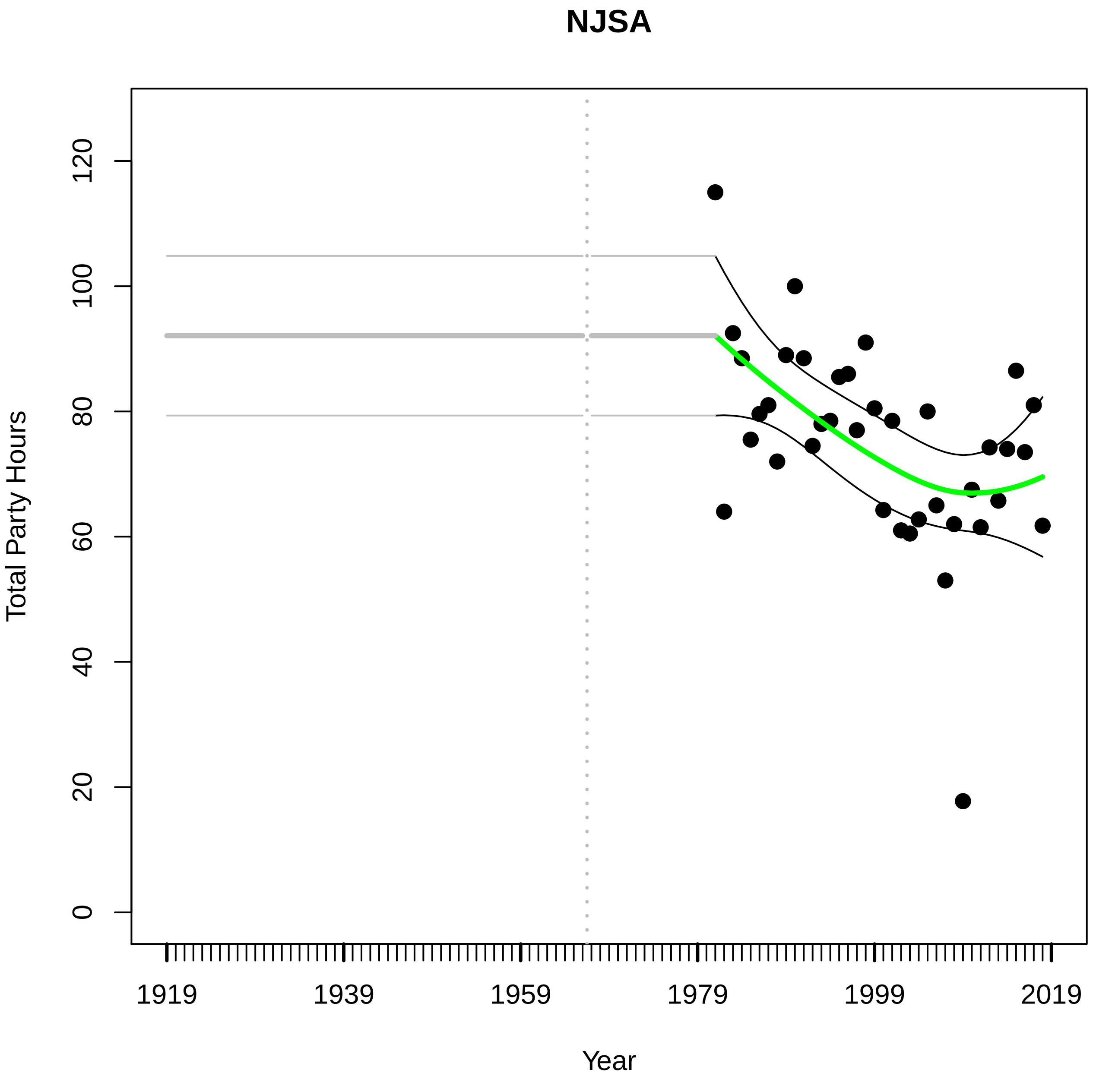

NJSA
Total Party Hours
0
20
40
60
80
100
120
1919
1939
1959
1979
1999
2019
Year

NJSH

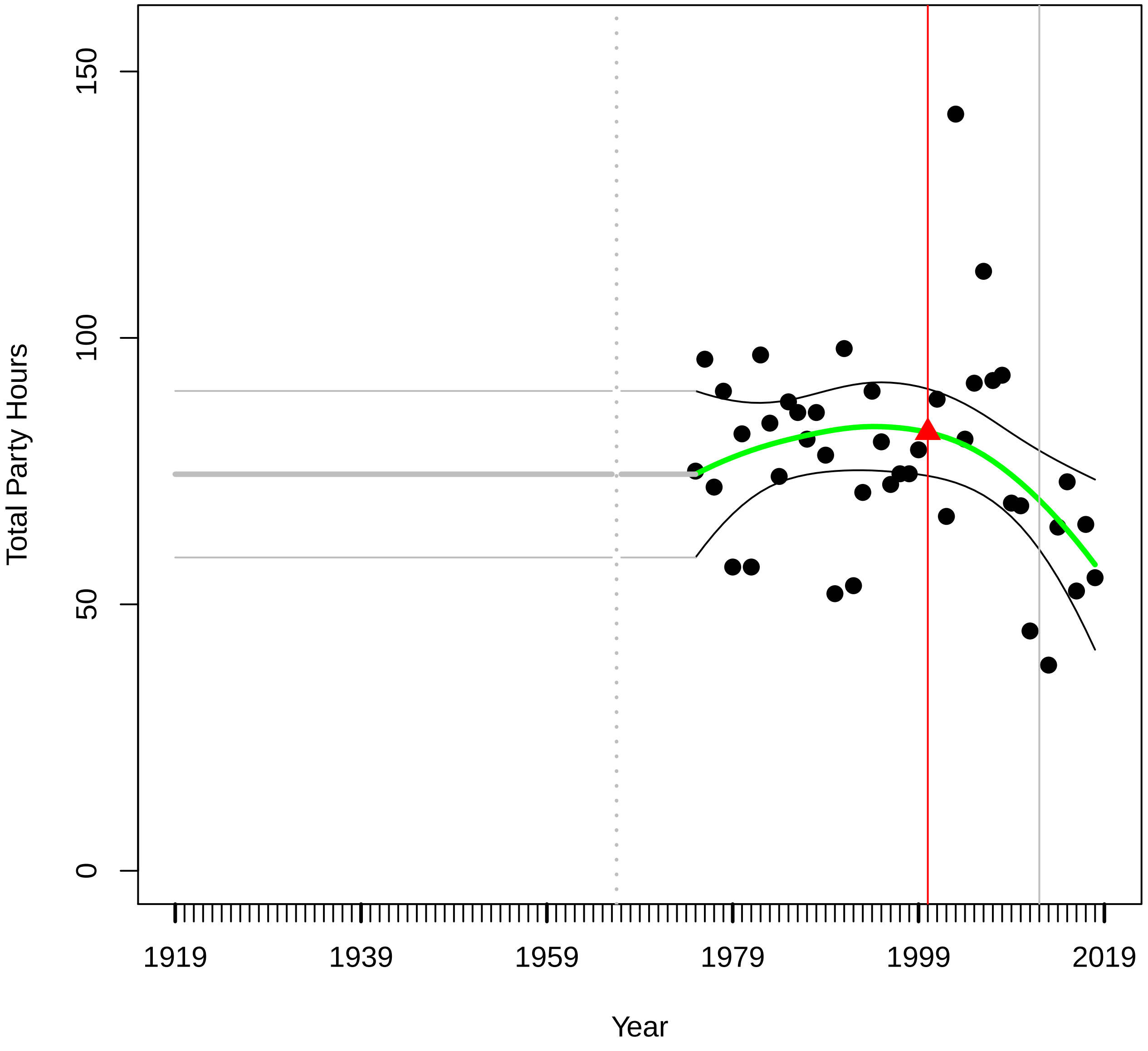

Total Party Hours
0
50
100
150
1919
1939
1959
1979
1999
2019
Year

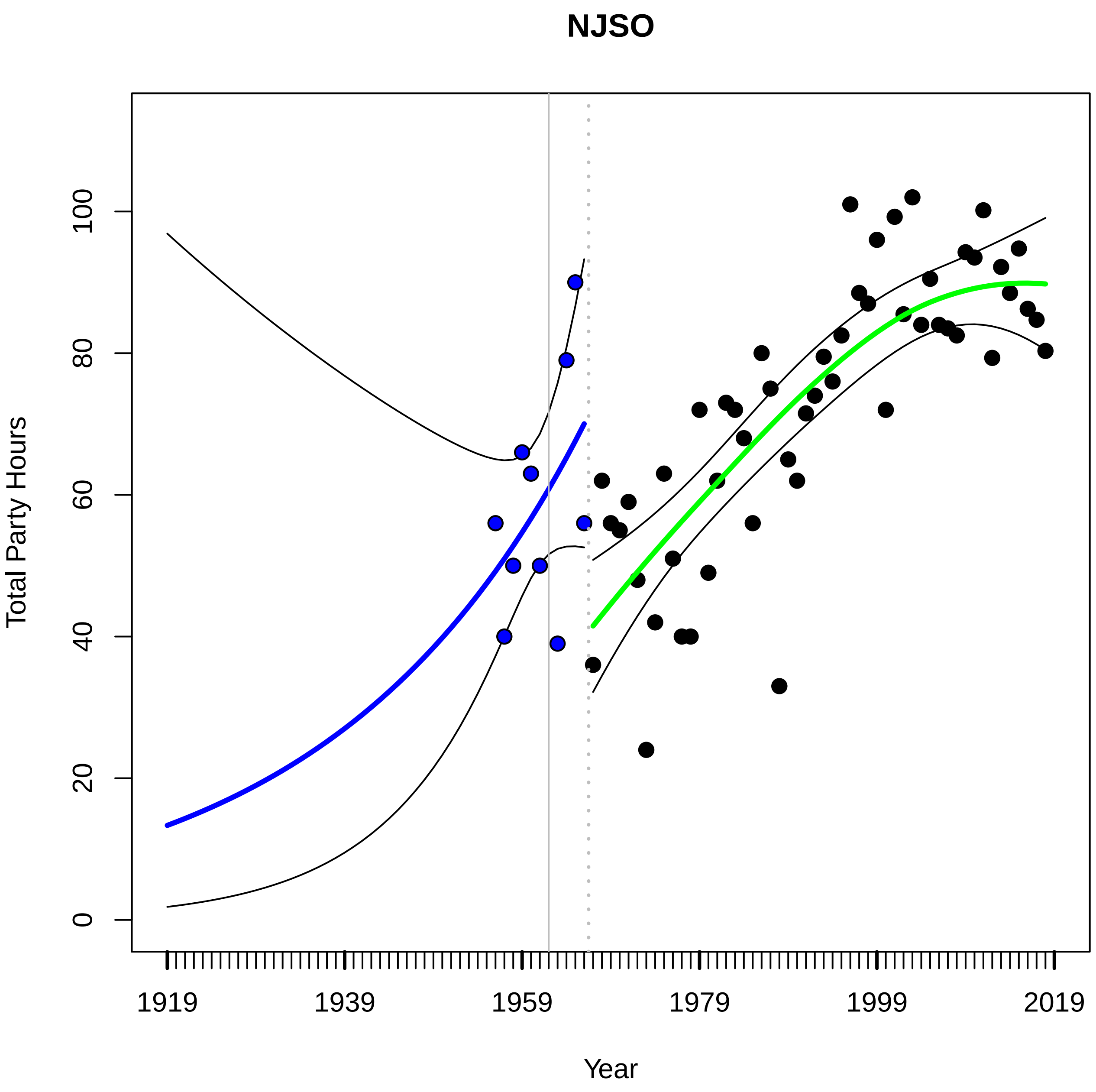

NJSO
Total Party Hours
0
20
40
60
80
100
1919
1939
1959
1979
1999
2019
Year

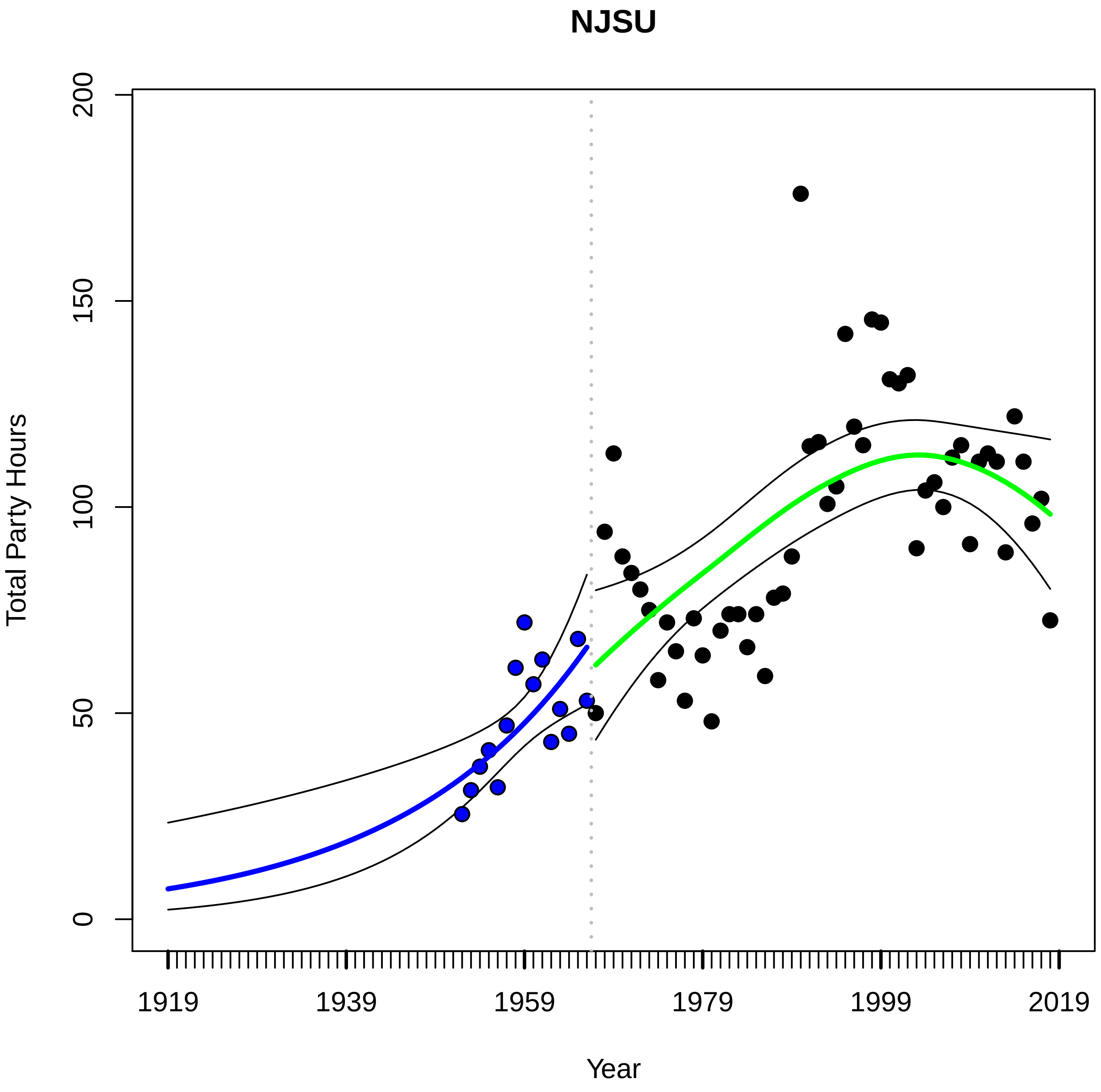

NJSU
Total Party Hours
0
50
100
150
200
1919
1939
1959
1979
1999
2019
Year

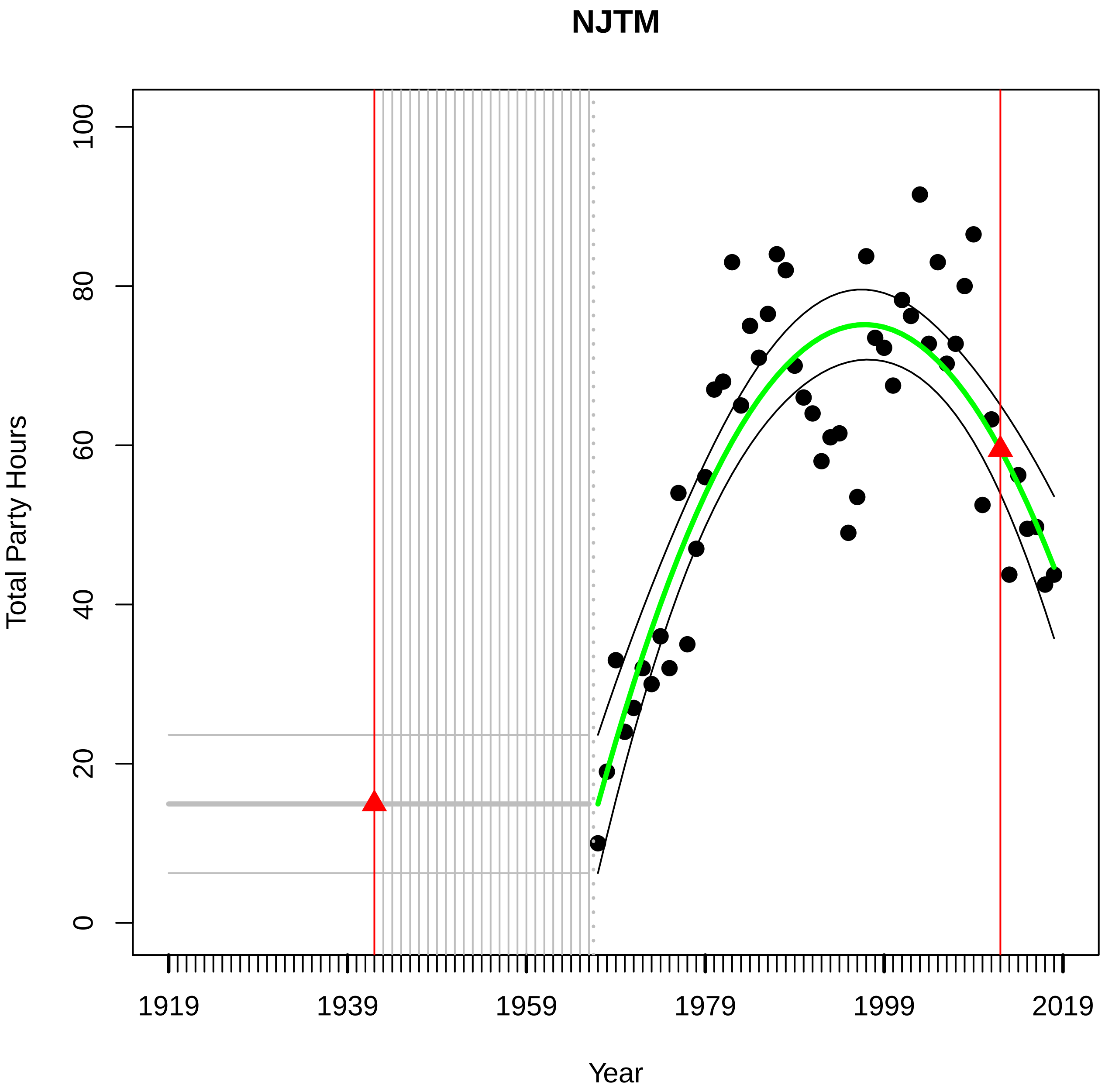

NJTM
Total Party Hours
0
20
40
60
80
100
1919
1939
1959
1979
1999
2019
Year

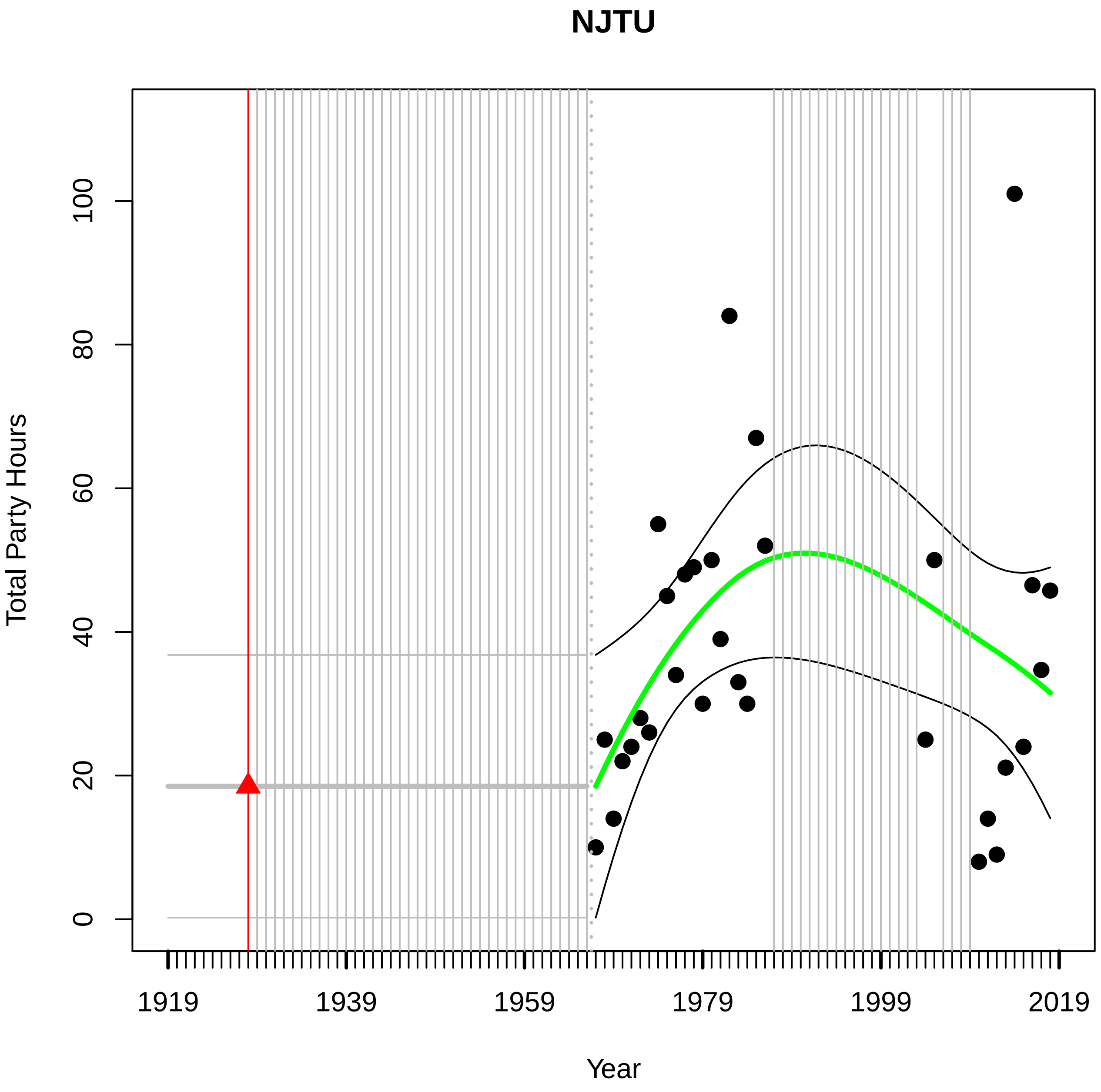
NJTU
Total Party Hours
0
20
40
60
80
100
1919
1939
1959
1979
1999
2019
Year

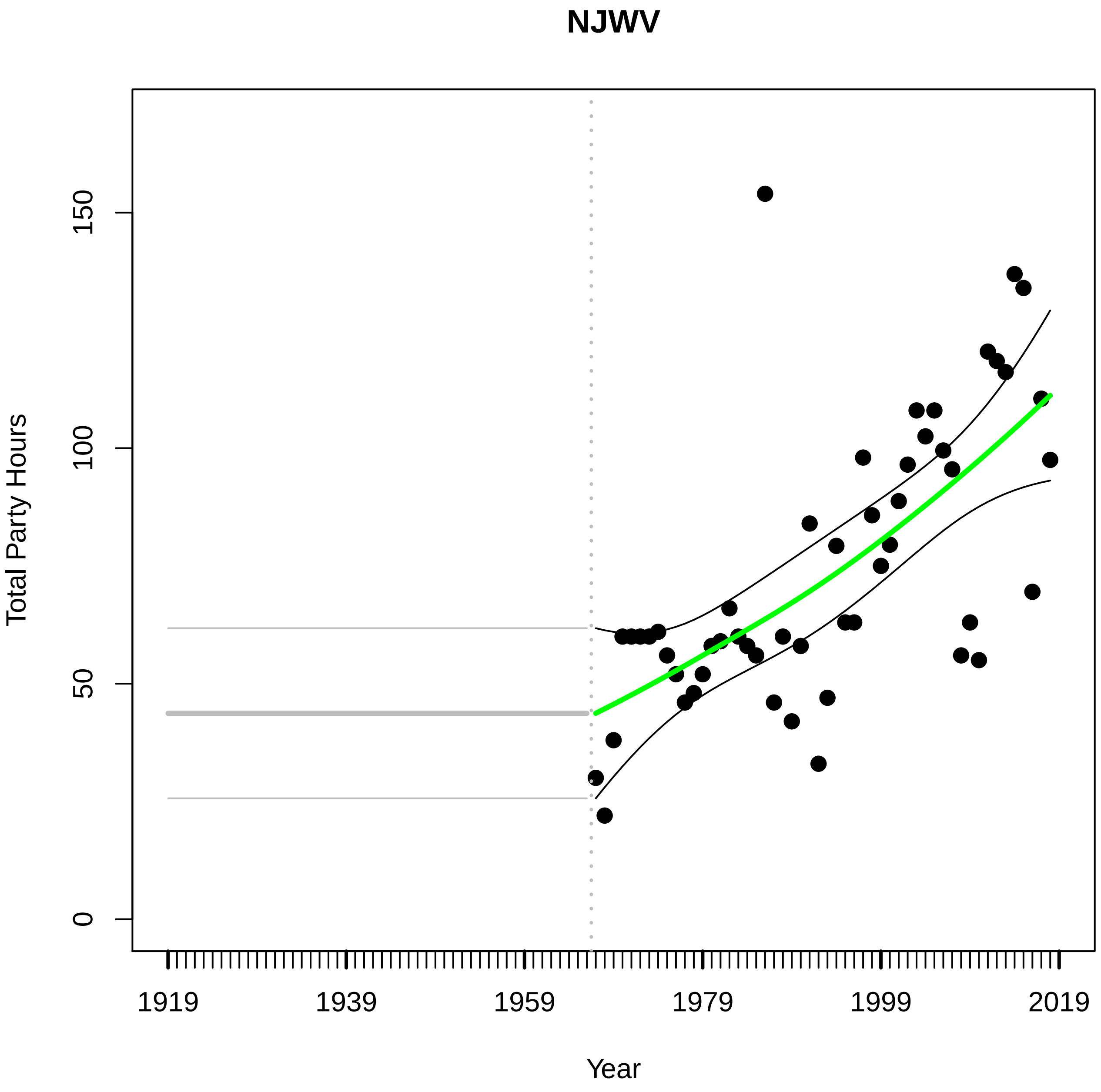

NJWV
Total Party Hours
0
50
100
150
1919
1939
1959
1979
1999
2019
Year

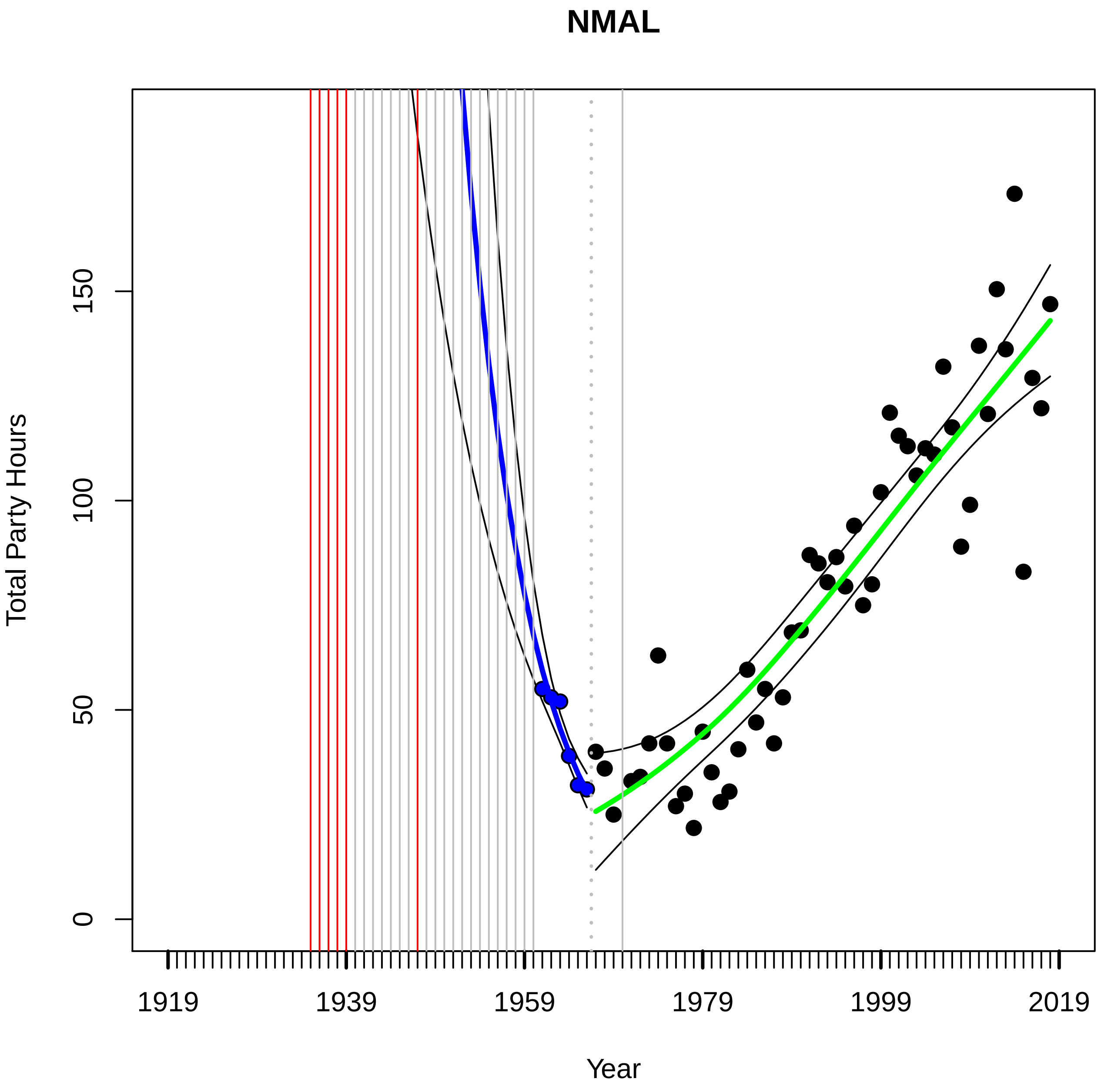

NMAL
Total Party Hours
0
50
100
150
1919
1939
1959
1979
1999
2019
Year

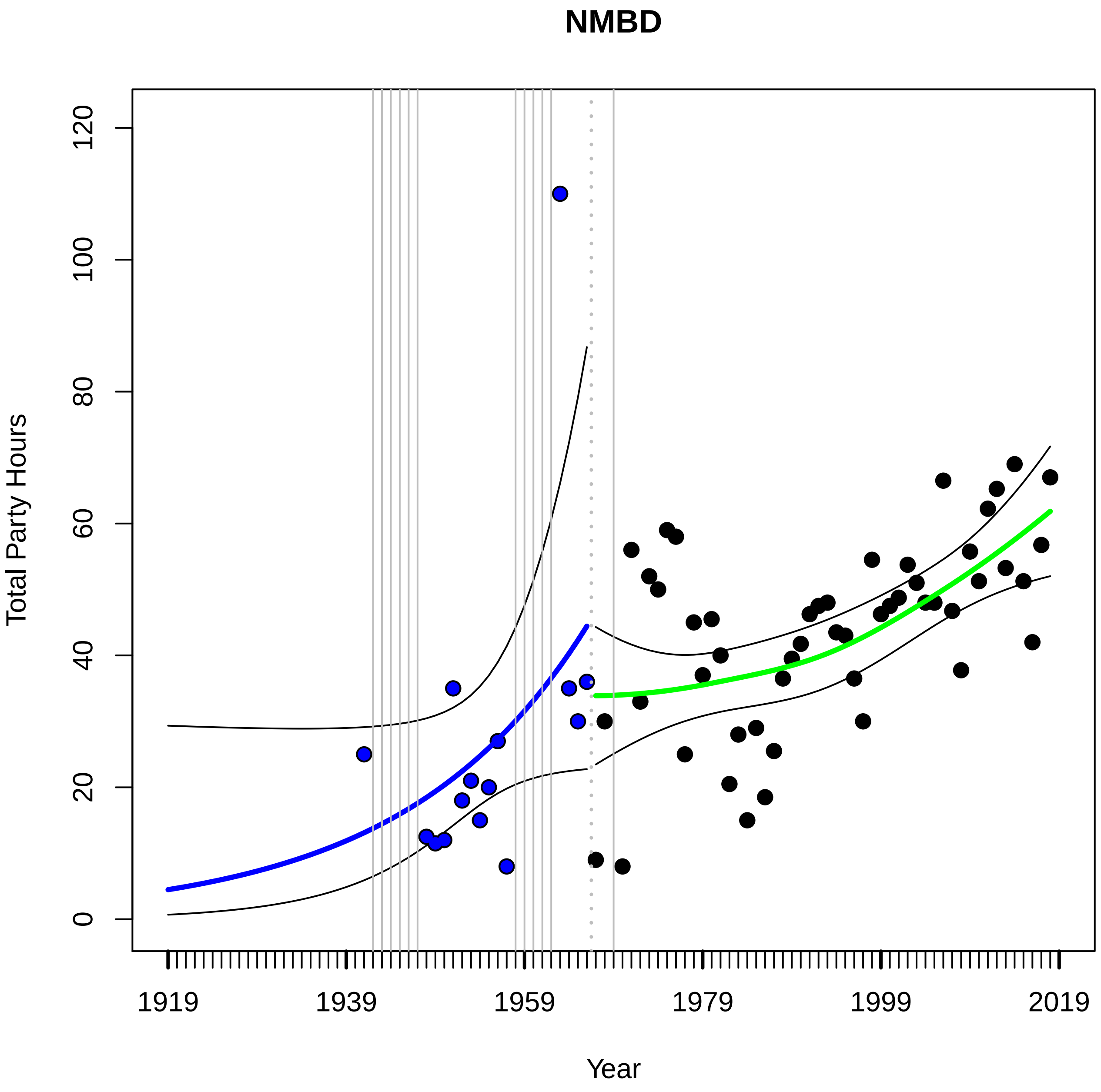

NMBD
Total Party Hours
Year
0
20
40
60
80
100
120
1919
1939
1959
1979
1999
2019

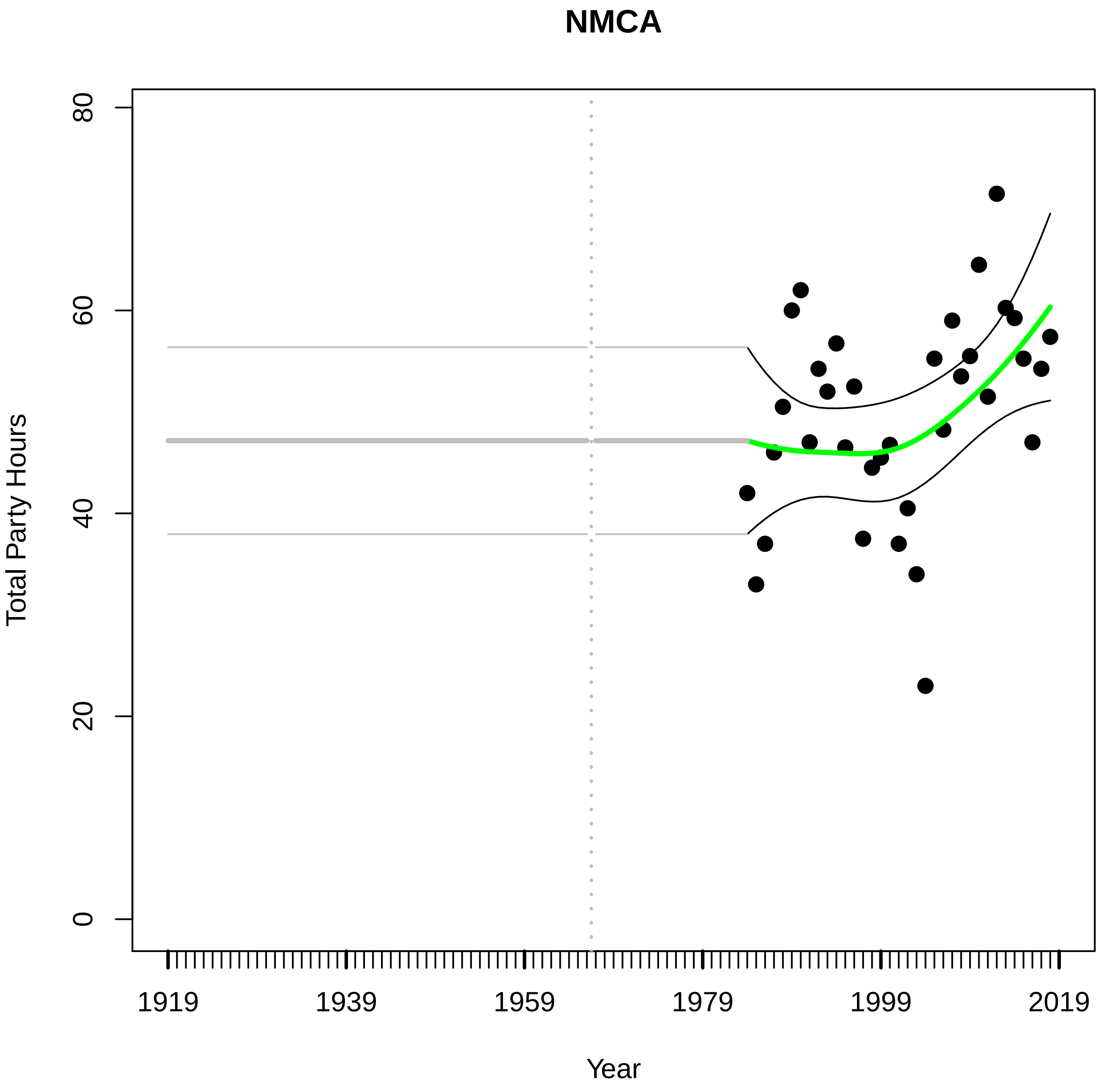

NMCA
Total Party Hours
0
20
40
60
80
1919
1939
1959
1979
1999
2019
Year

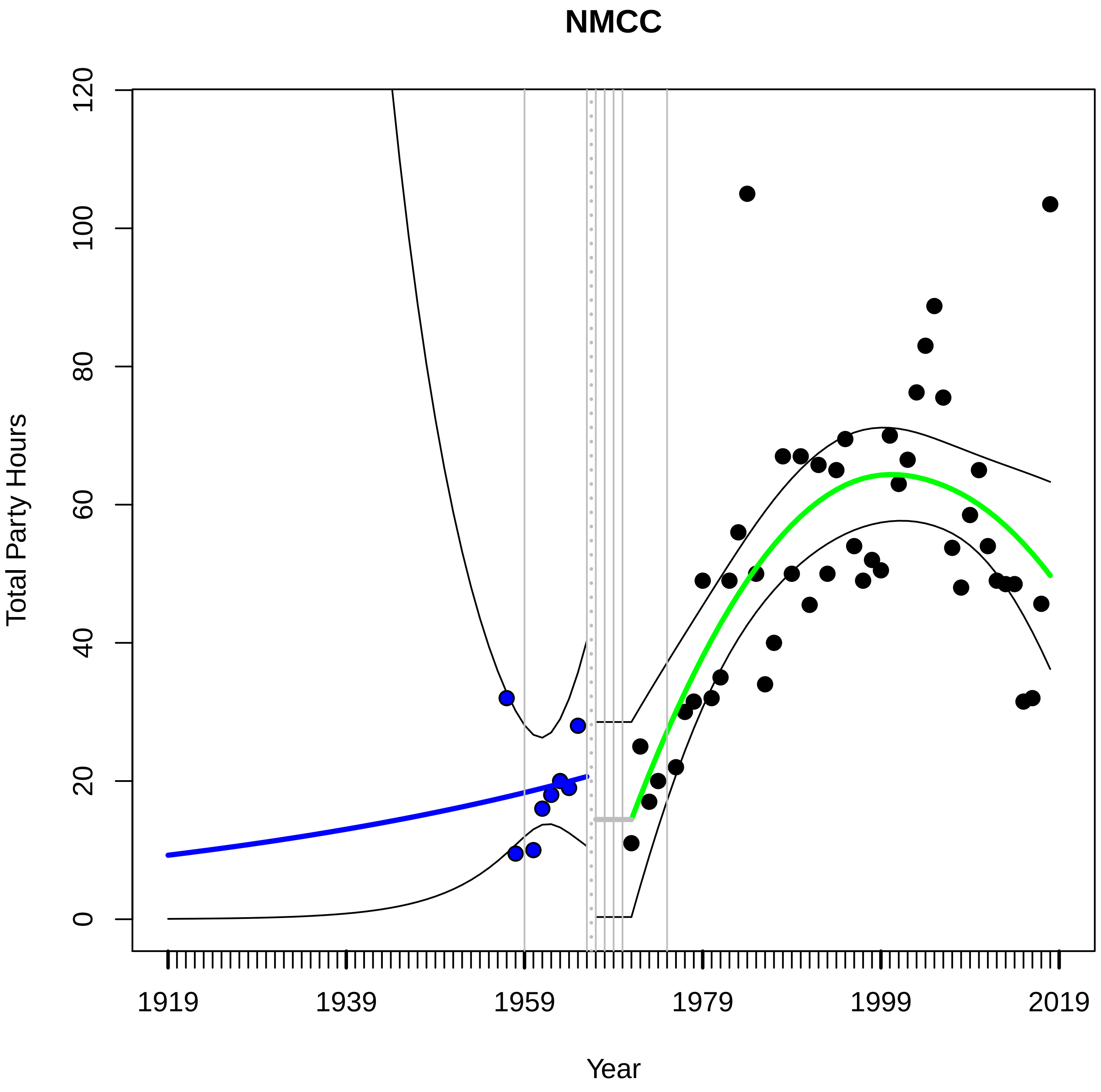

NMCC
Total Party Hours
Year
0
20
40
60
80
100
120
1919
1939
1959
1979
1999
2019

NMCL

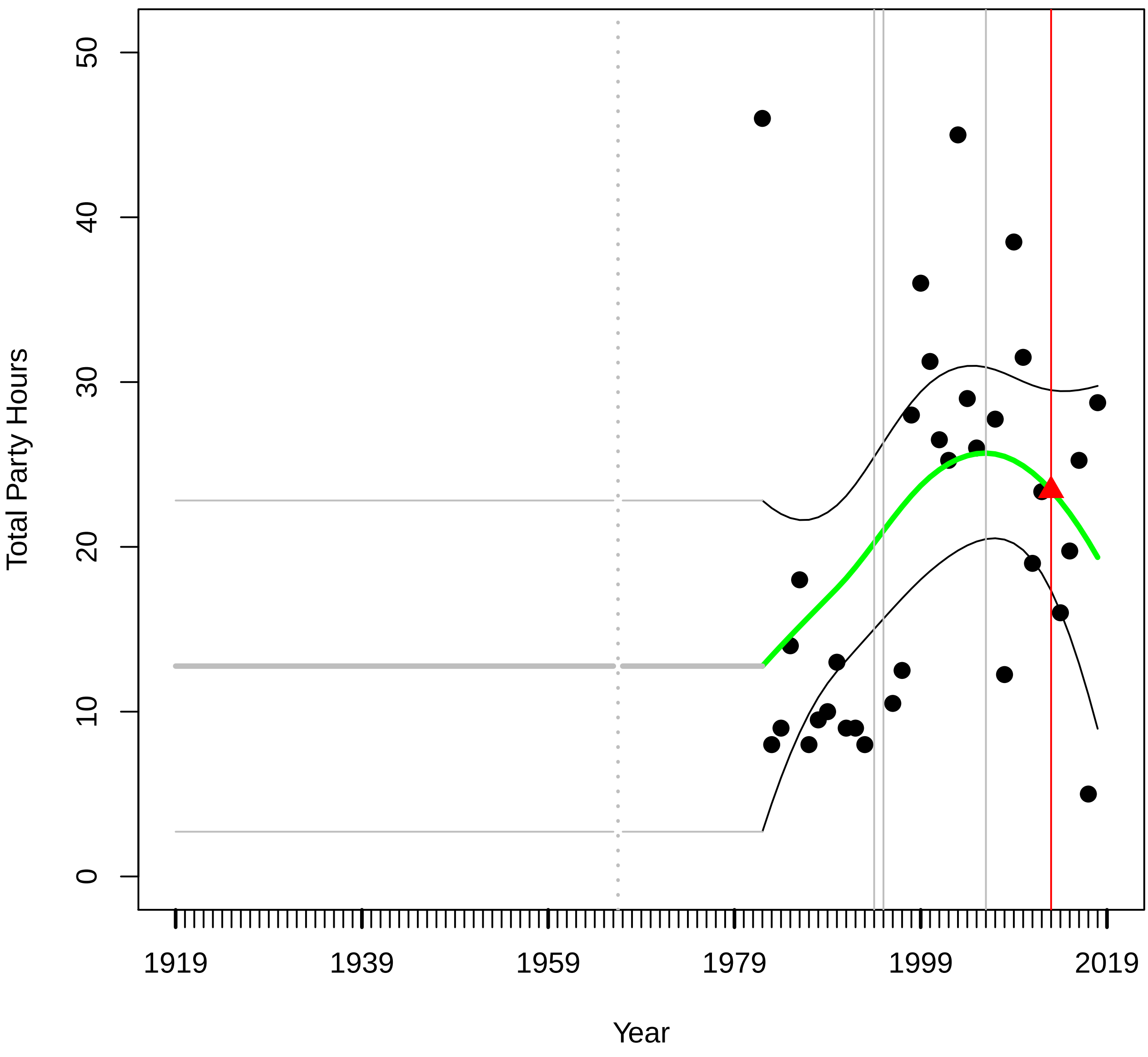

Total Party Hours
0
10
20
30
40
50
1919
1939
1959
1979
1999
2019
Year

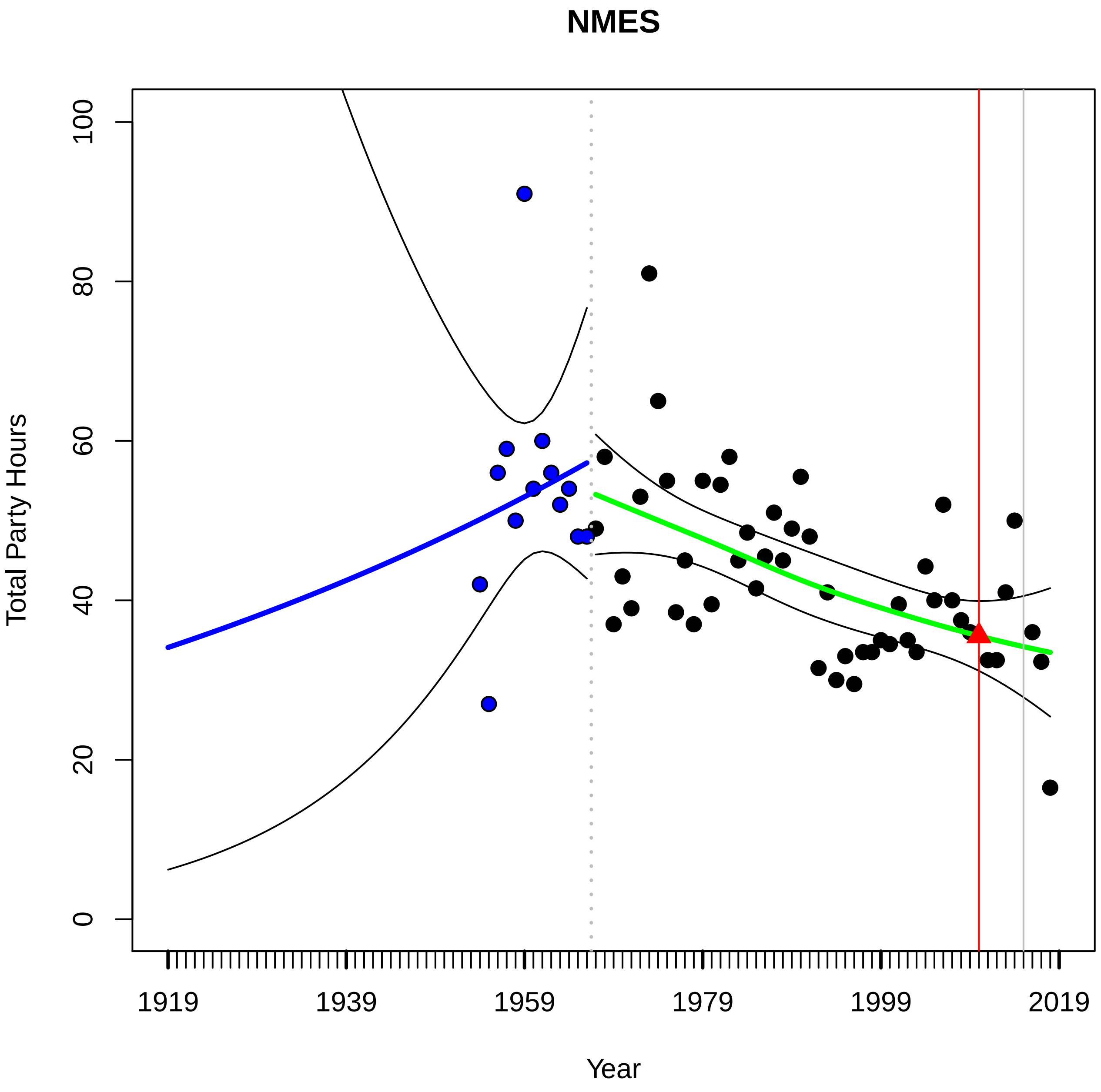
NMES
Total Party Hours
0
20
40
60
80
100
1919
1939
1959
1979
1999
2019
Year

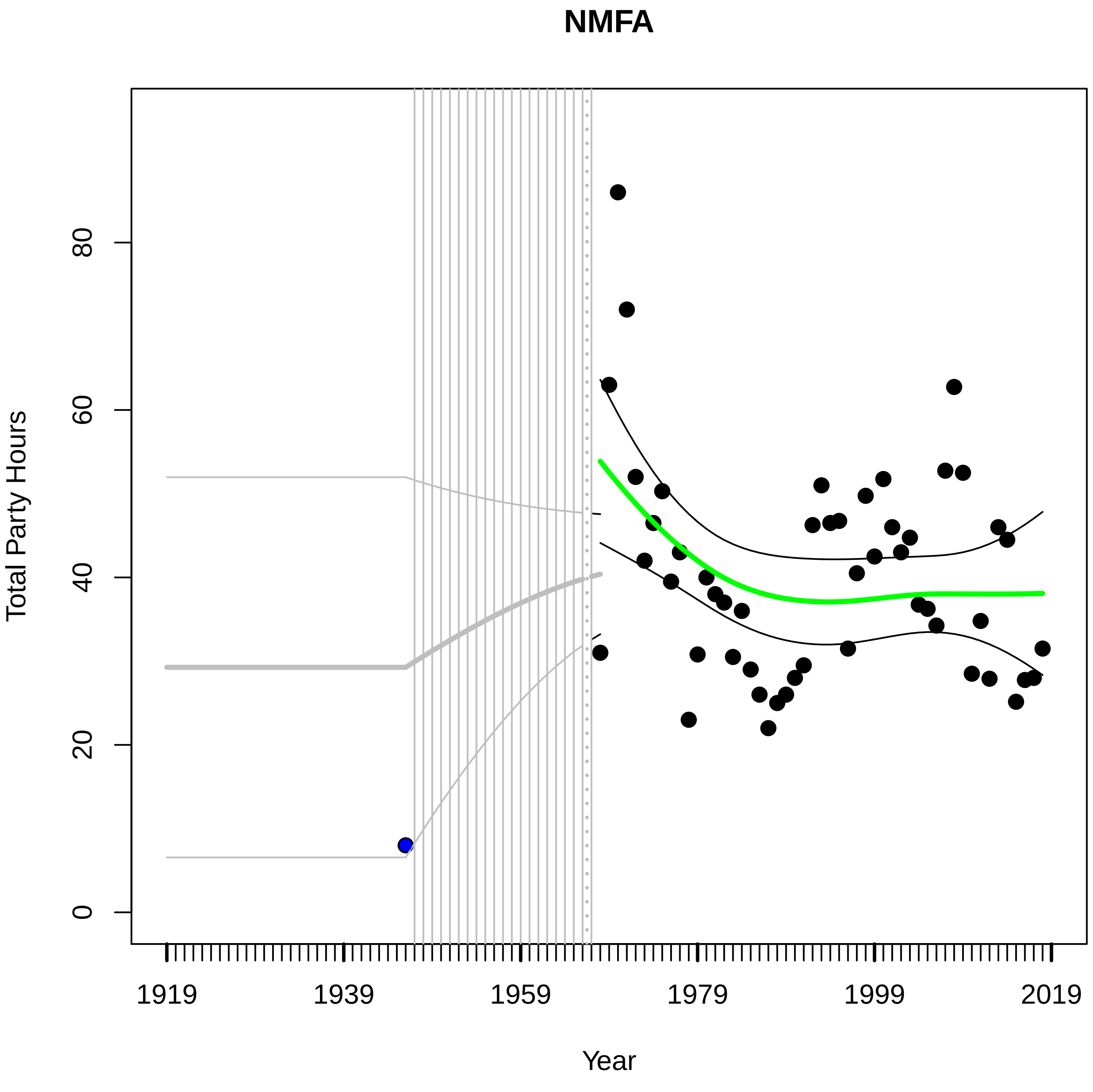

NMFA
Total Party Hours
0
20
40
60
80
1919
1939
1959
1979
1999
2019
Year

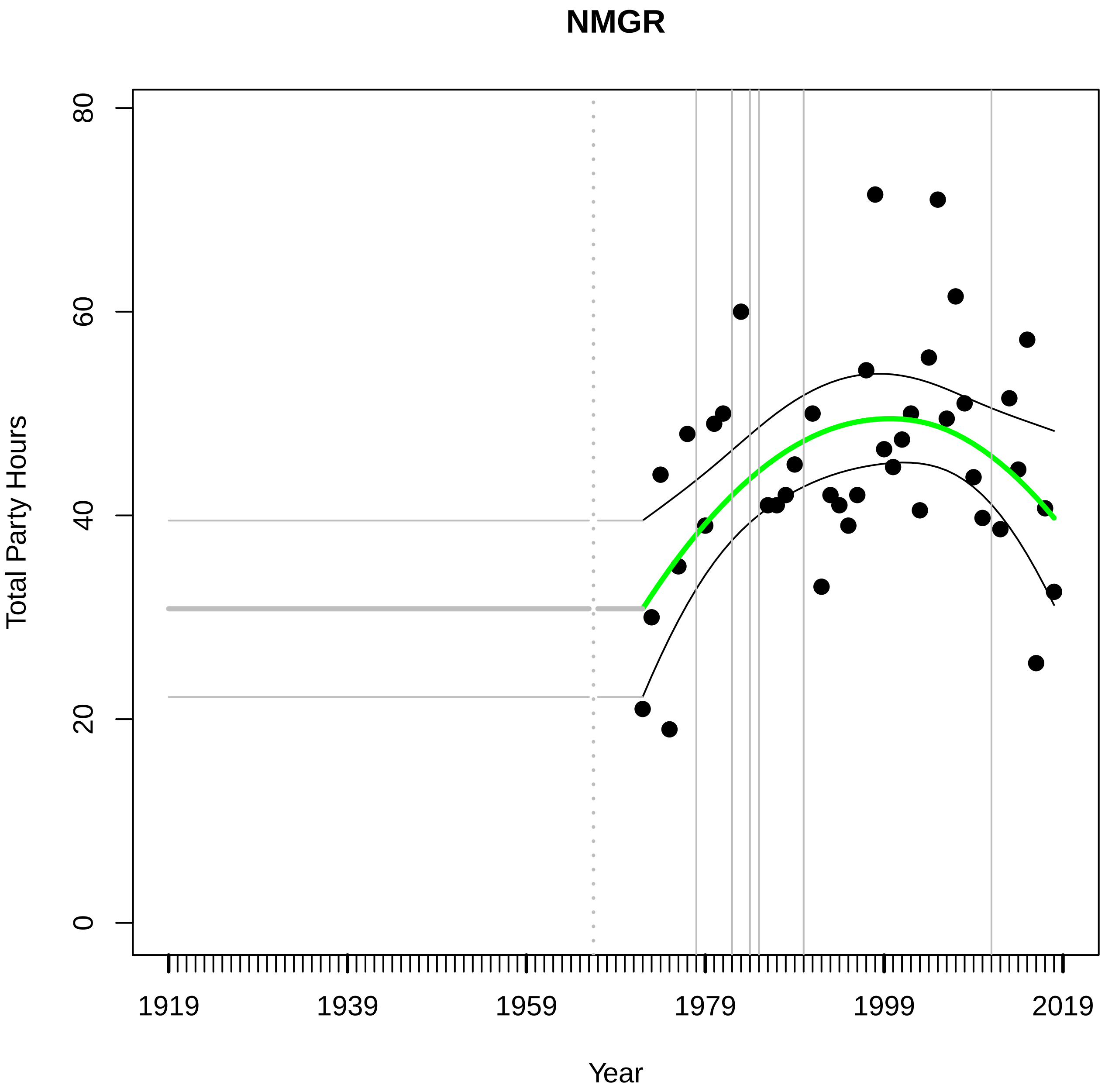

NMGR
Total Party Hours
Year
0
20
40
60
80
1919
1939
1959
1979
1999
2019

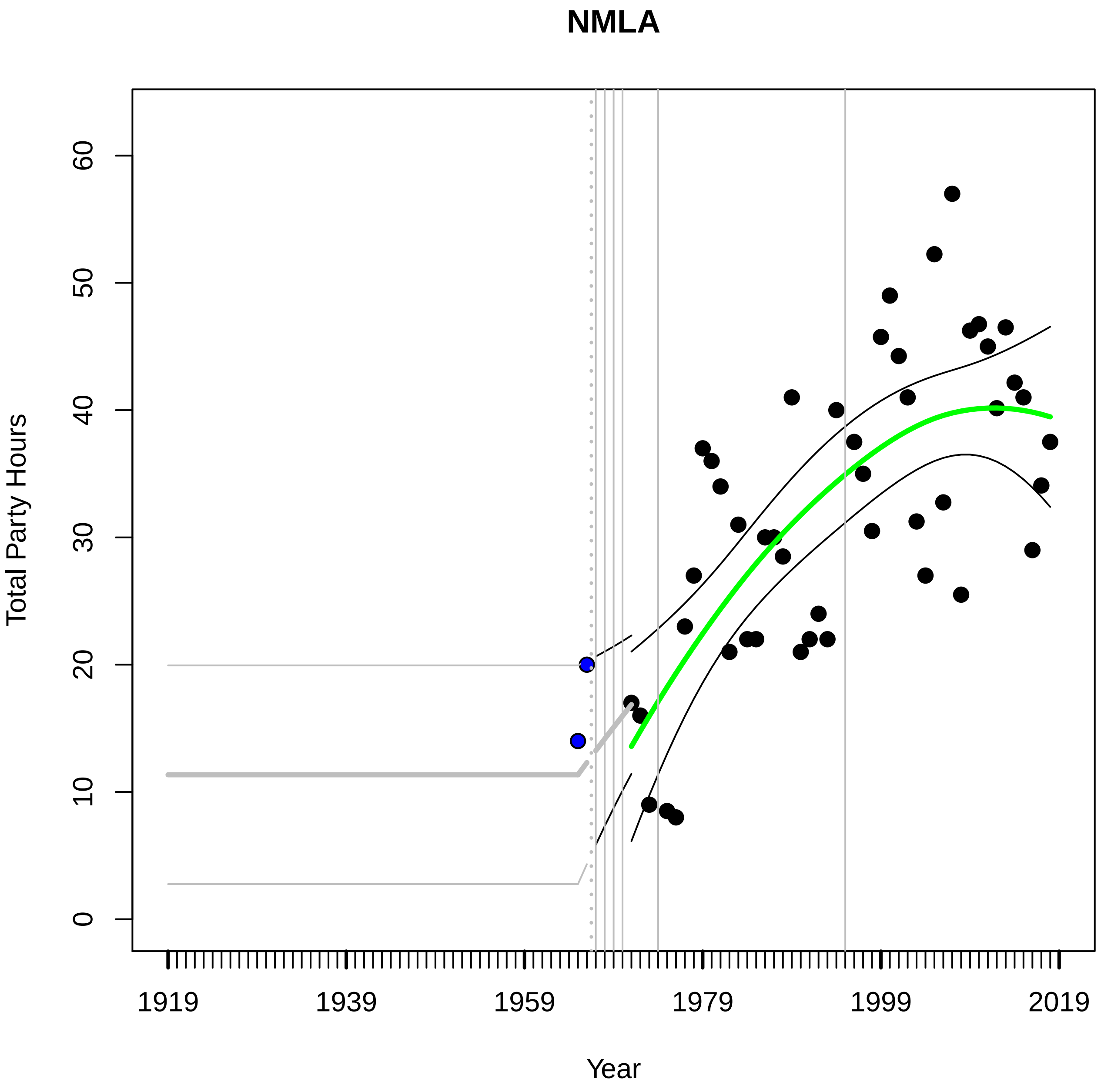

NMLA
Total Party Hours
0
10
20
30
40
50
60
1919
1939
1959
1979
1999
2019
Year

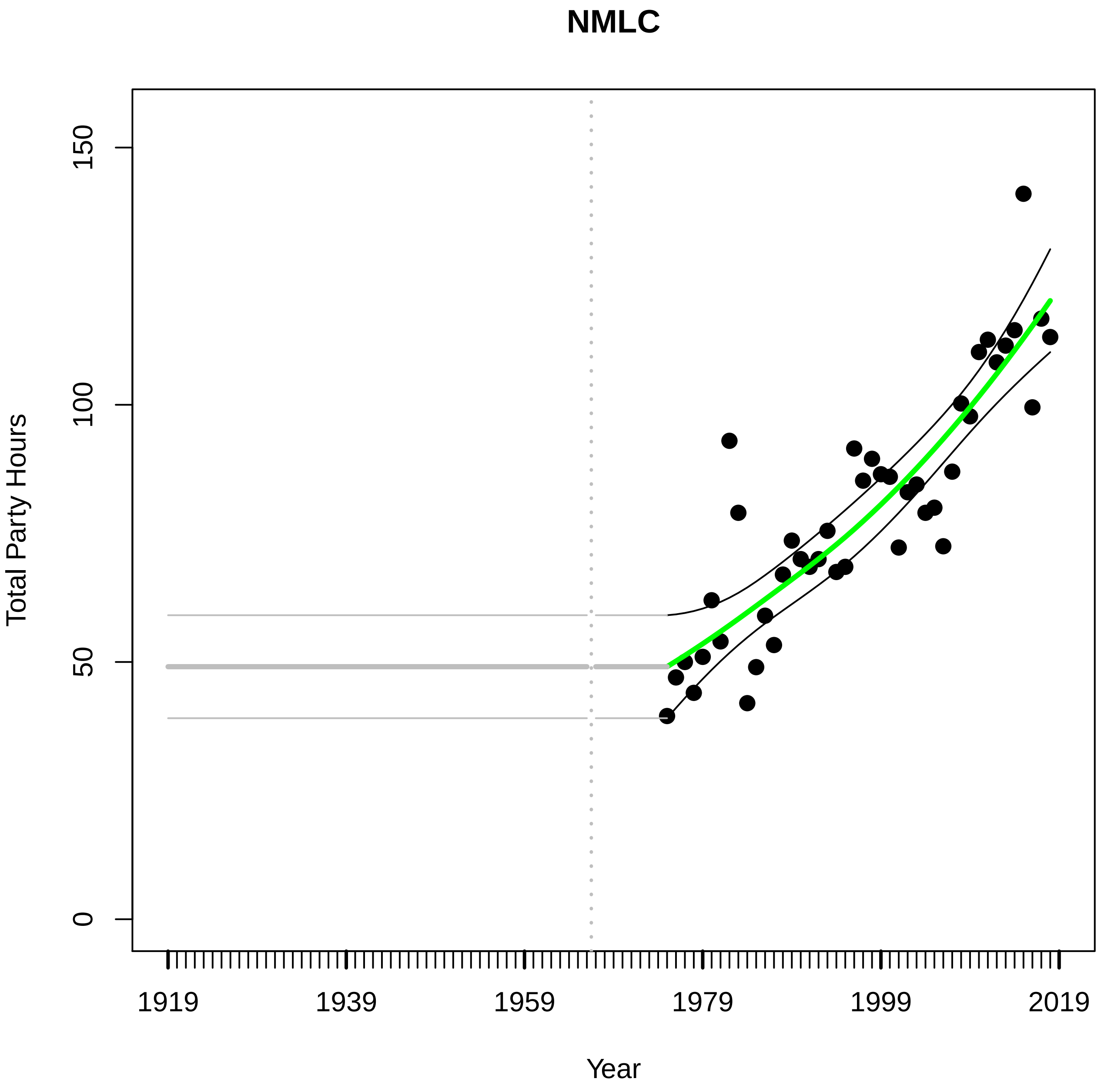

NMLC
Total Party Hours
0
50
100
150
1919
1939
1959
1979
1999
2019
Year

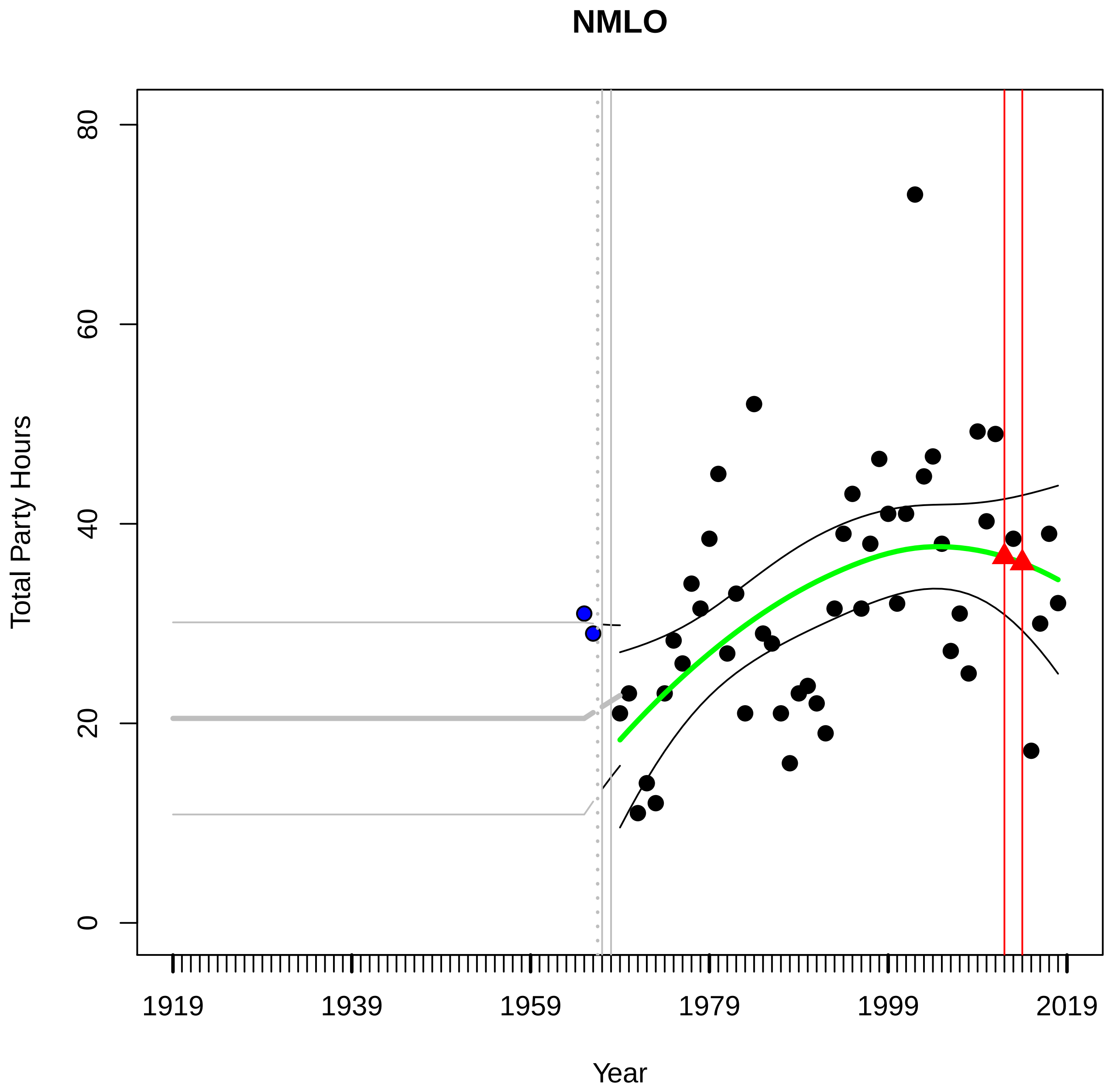

NMLO
Total Party Hours
0
20
40
60
80
1919
1939
1959
1979
1999
2019
Year

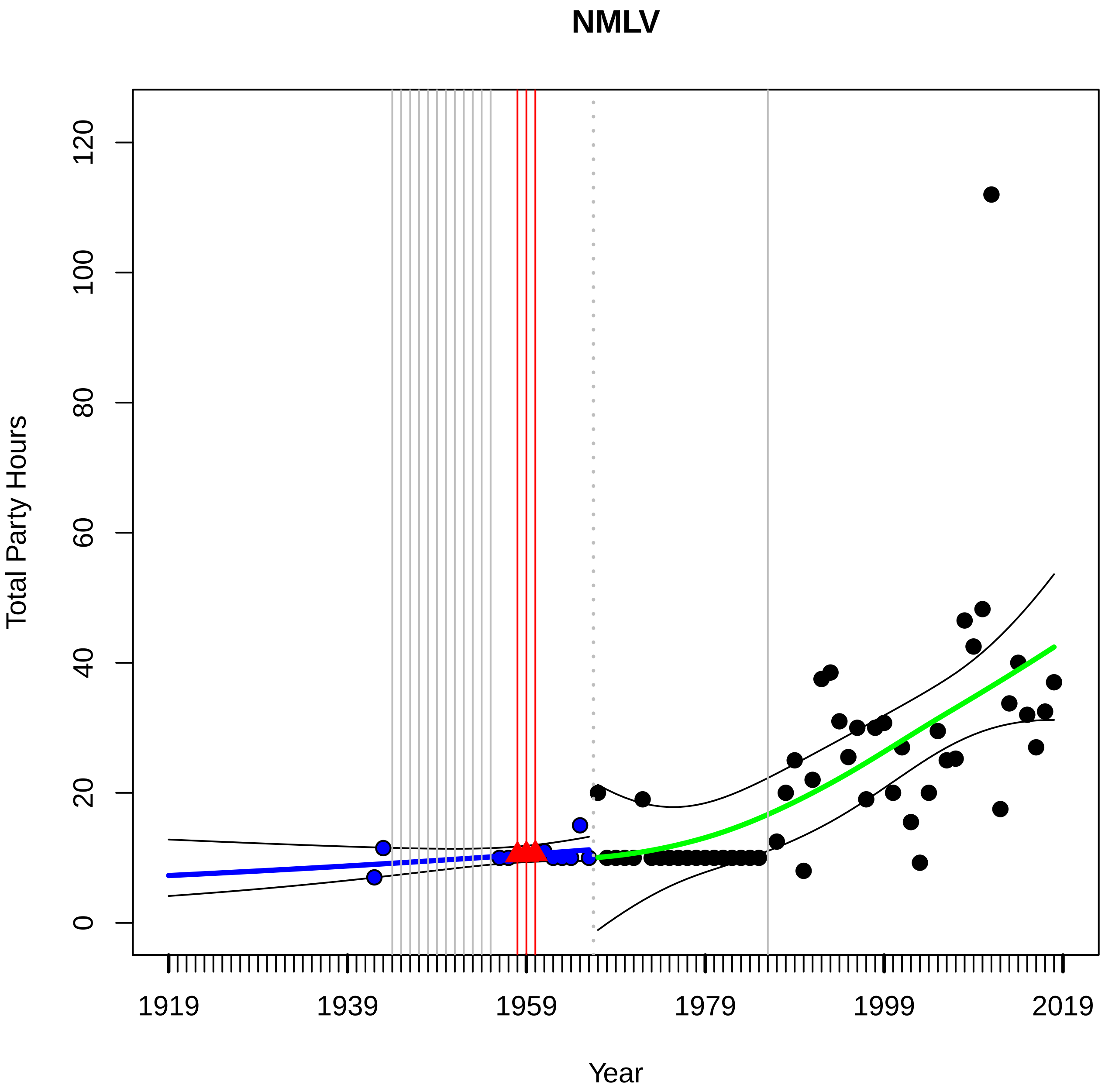
NMLV
Total Party Hours
0
20
40
60
80
100
120
1919
1939
1959
1979
1999
2019
Year

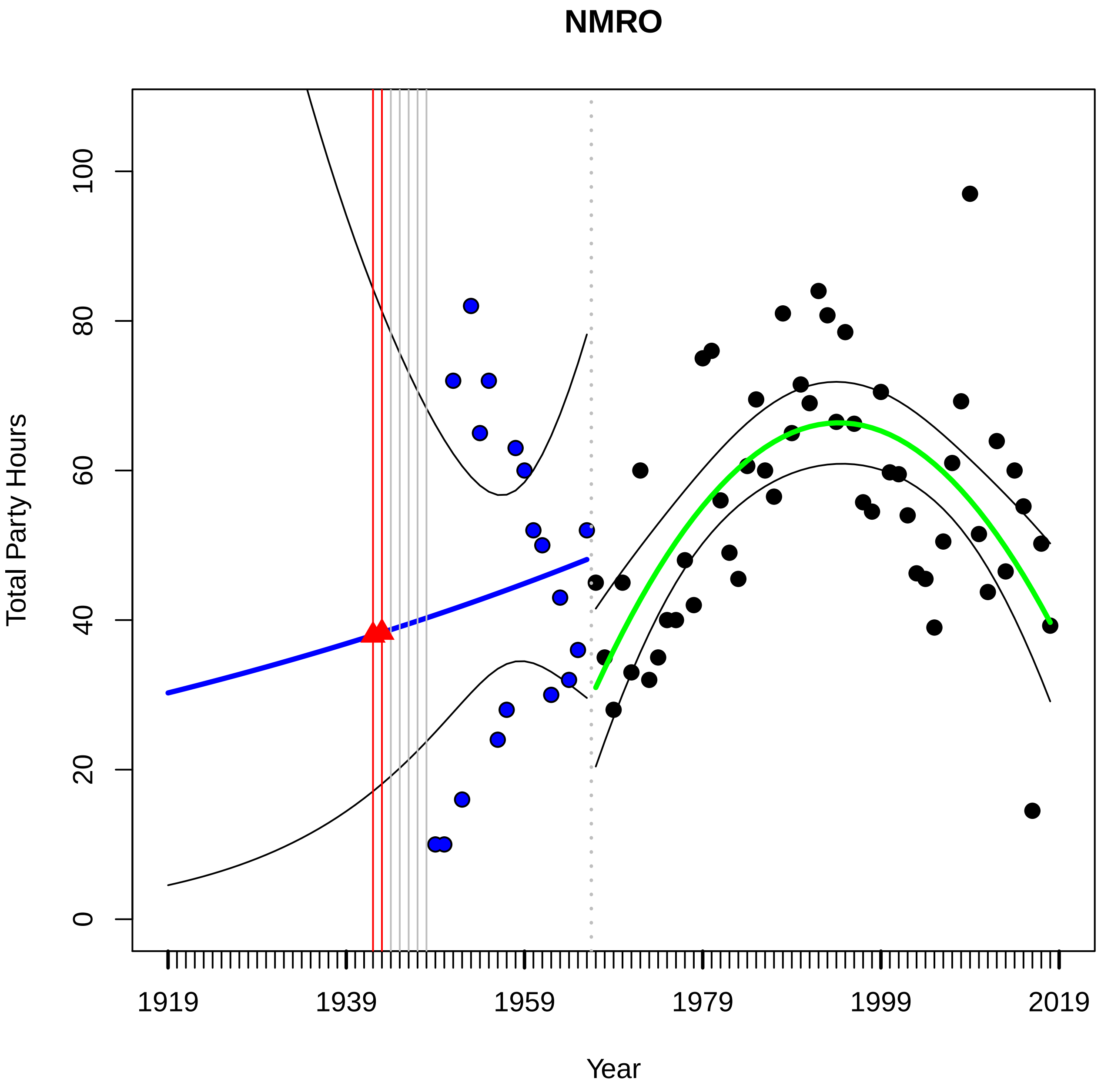

NMRO
Total Party Hours
Year
0
20
40
60
80
100
1919
1939
1959
1979
1999
2019

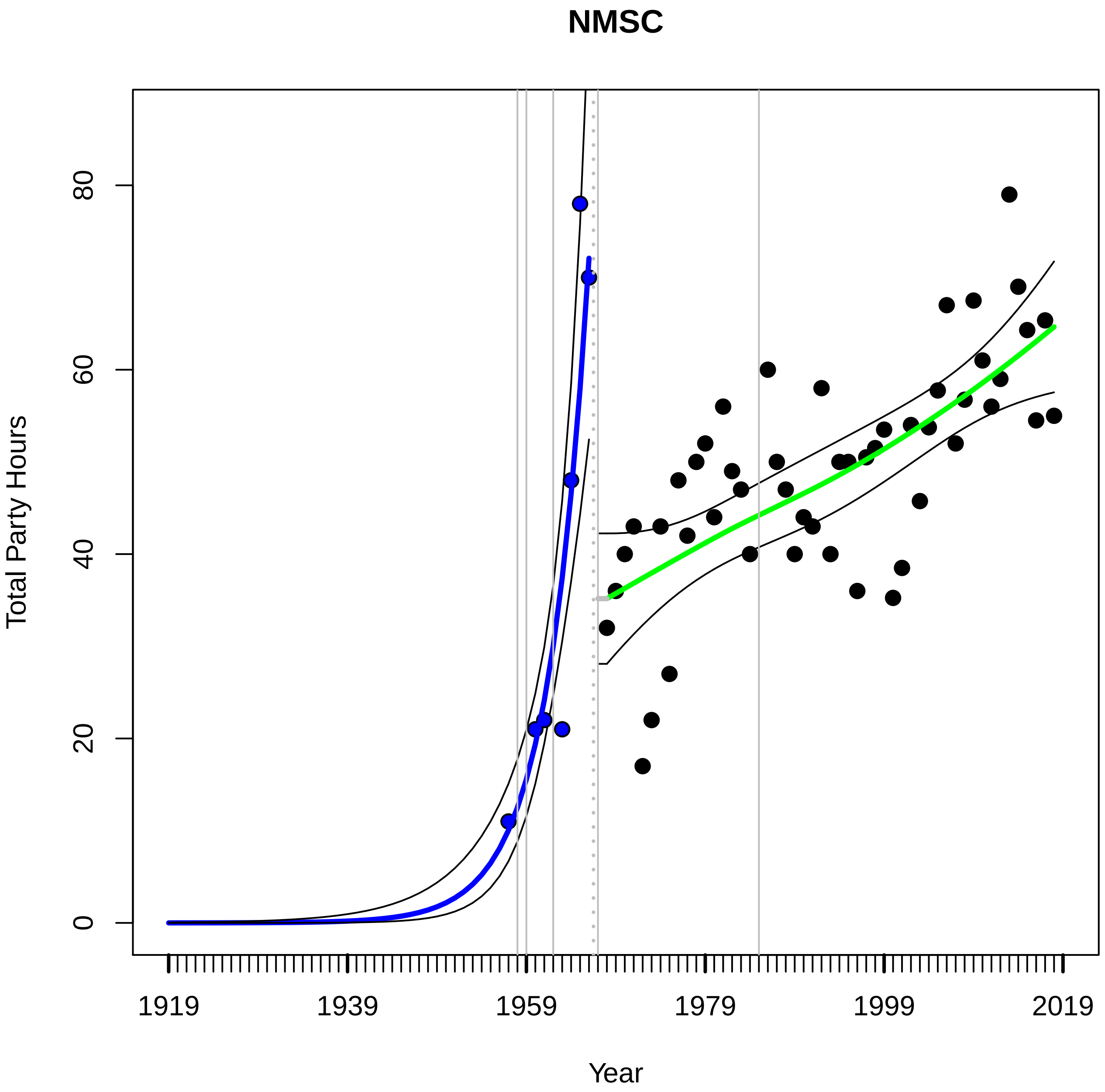
NMSC
Total Party Hours
0
20
40
60
80
1919
1939
1959
1979
1999
2019
Year

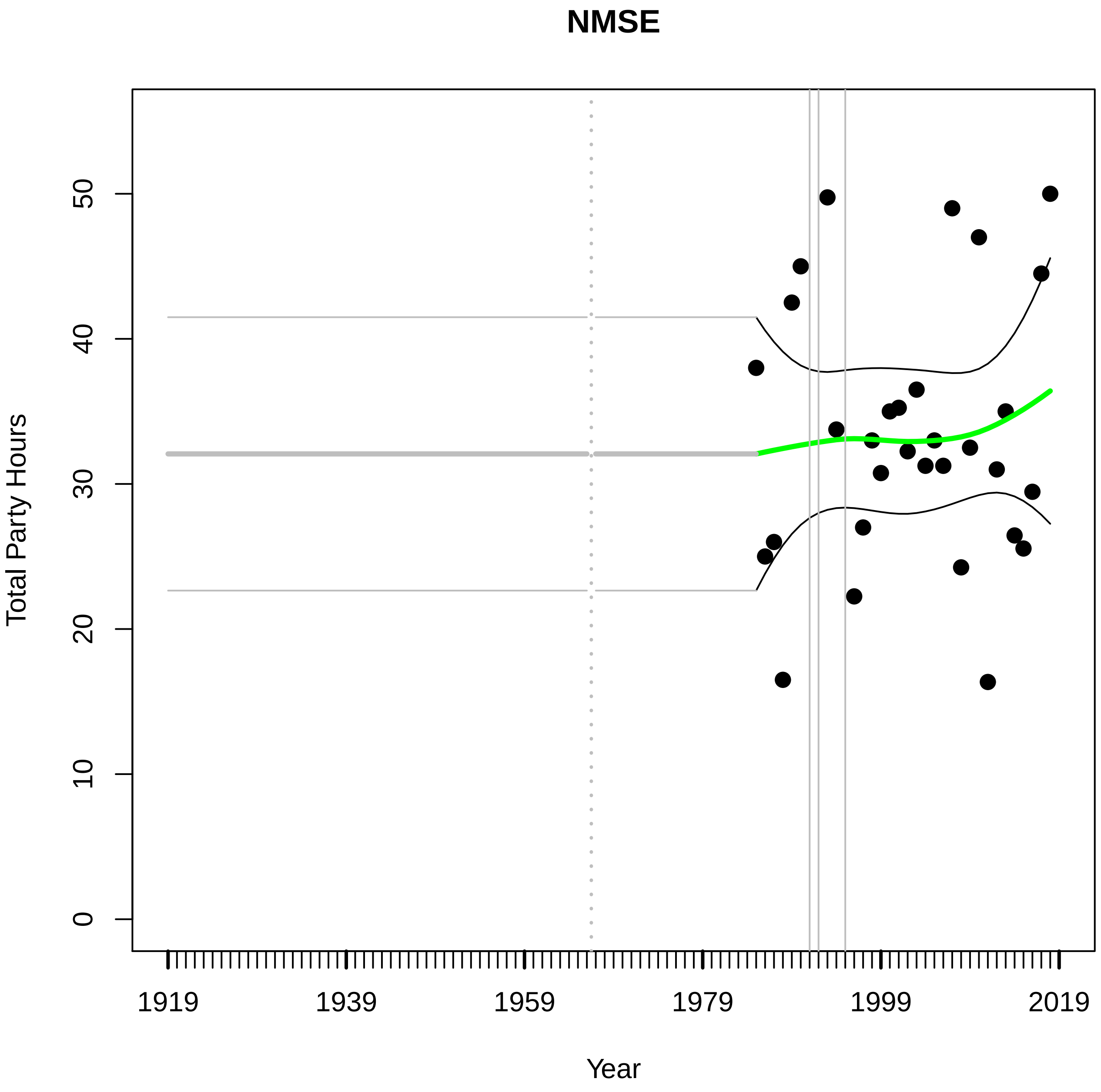

NMSE
Total Party Hours
Year
0
10
20
30
40
50
1919
1939
1959
1979
1999
2019

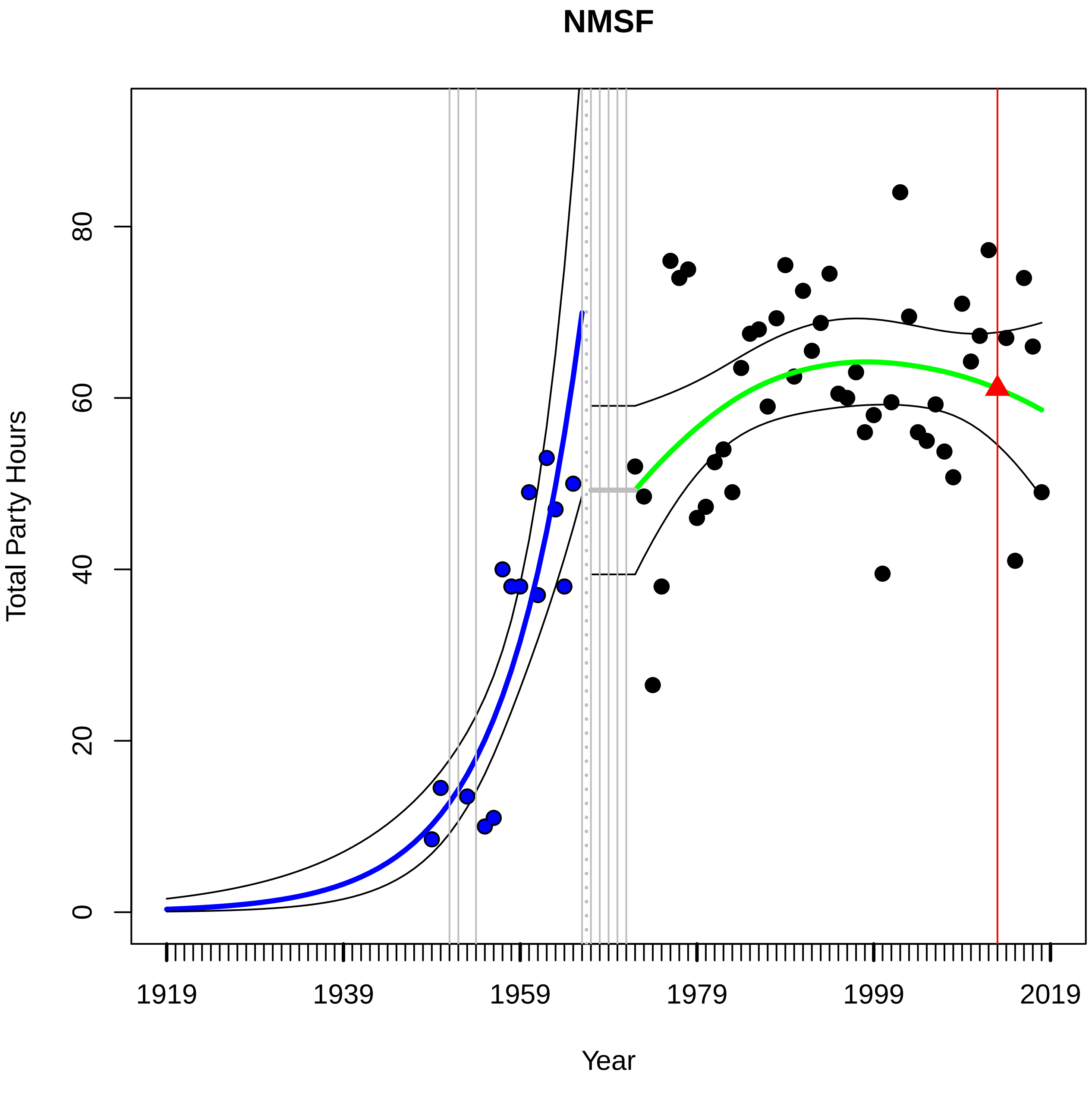
NMSF
Total Party Hours
Year
0
20
40
60
80
1919
1939
1959
1979
1999
2019

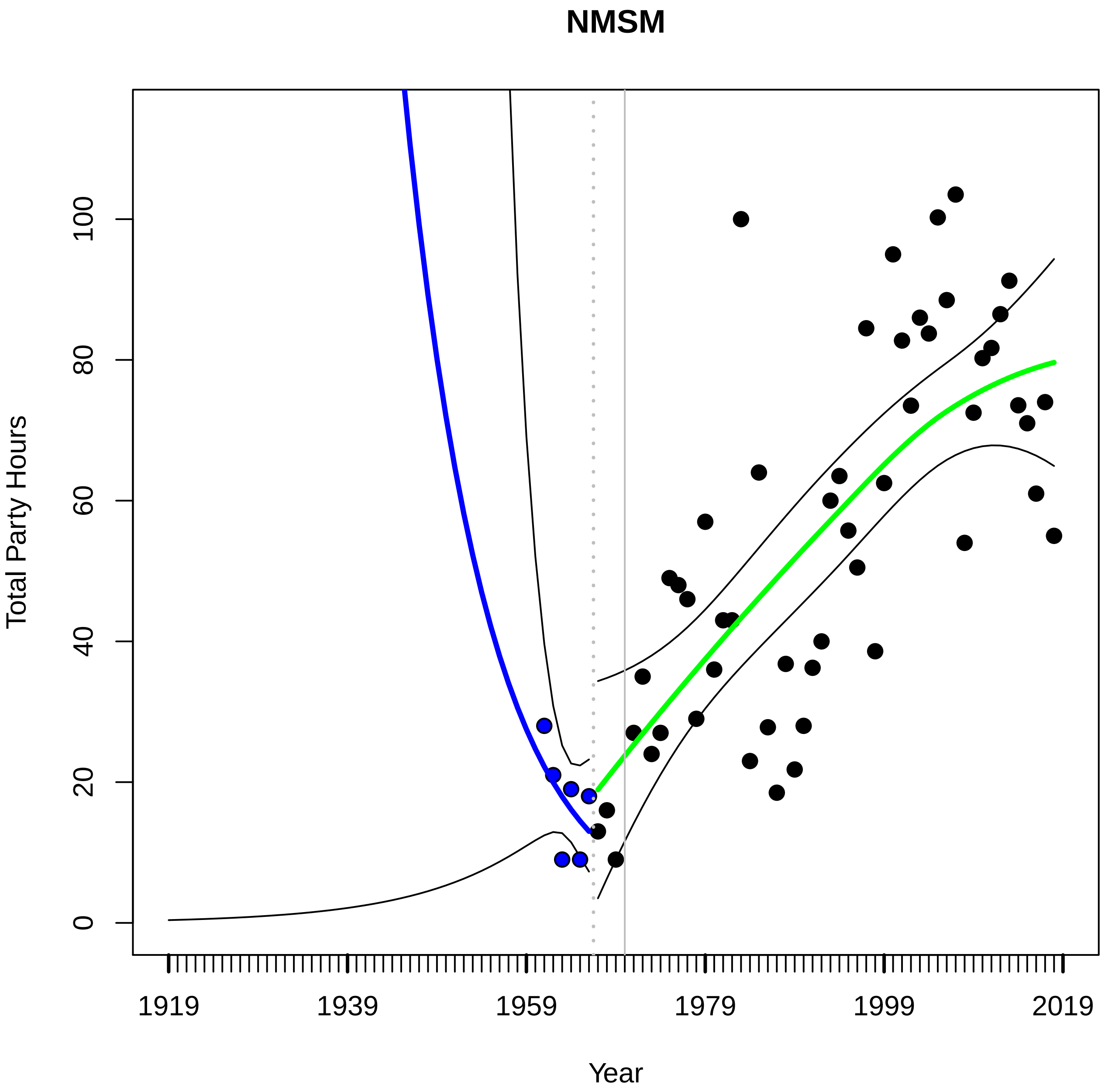
NMSM
Total Party Hours
0
20
40
60
80
100
Year
1919
1939
1959
1979
1999
2019

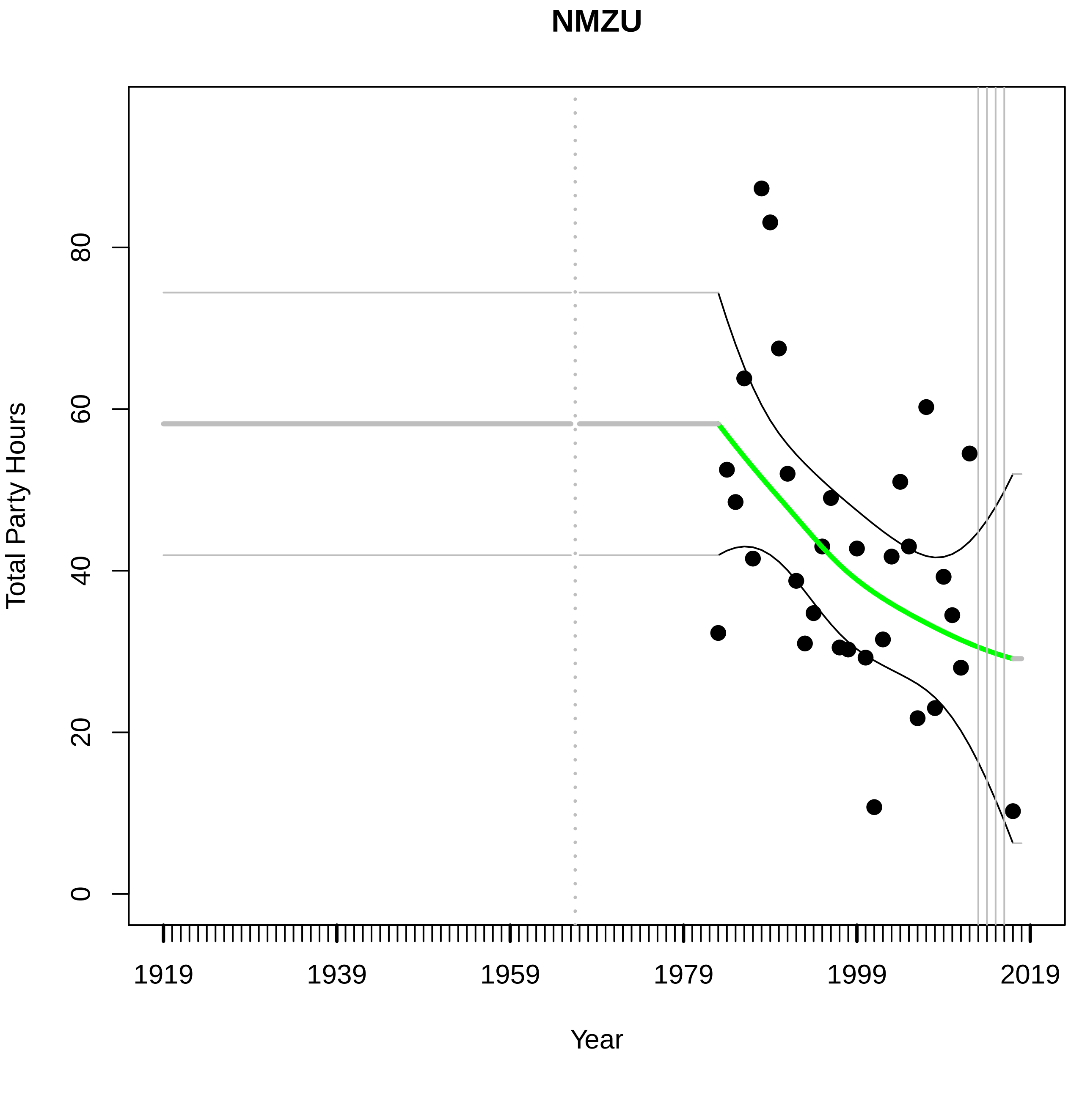
NMZU
Total Party Hours
0
20
40
60
80
1919
1939
1959
1979
1999
2019
Year

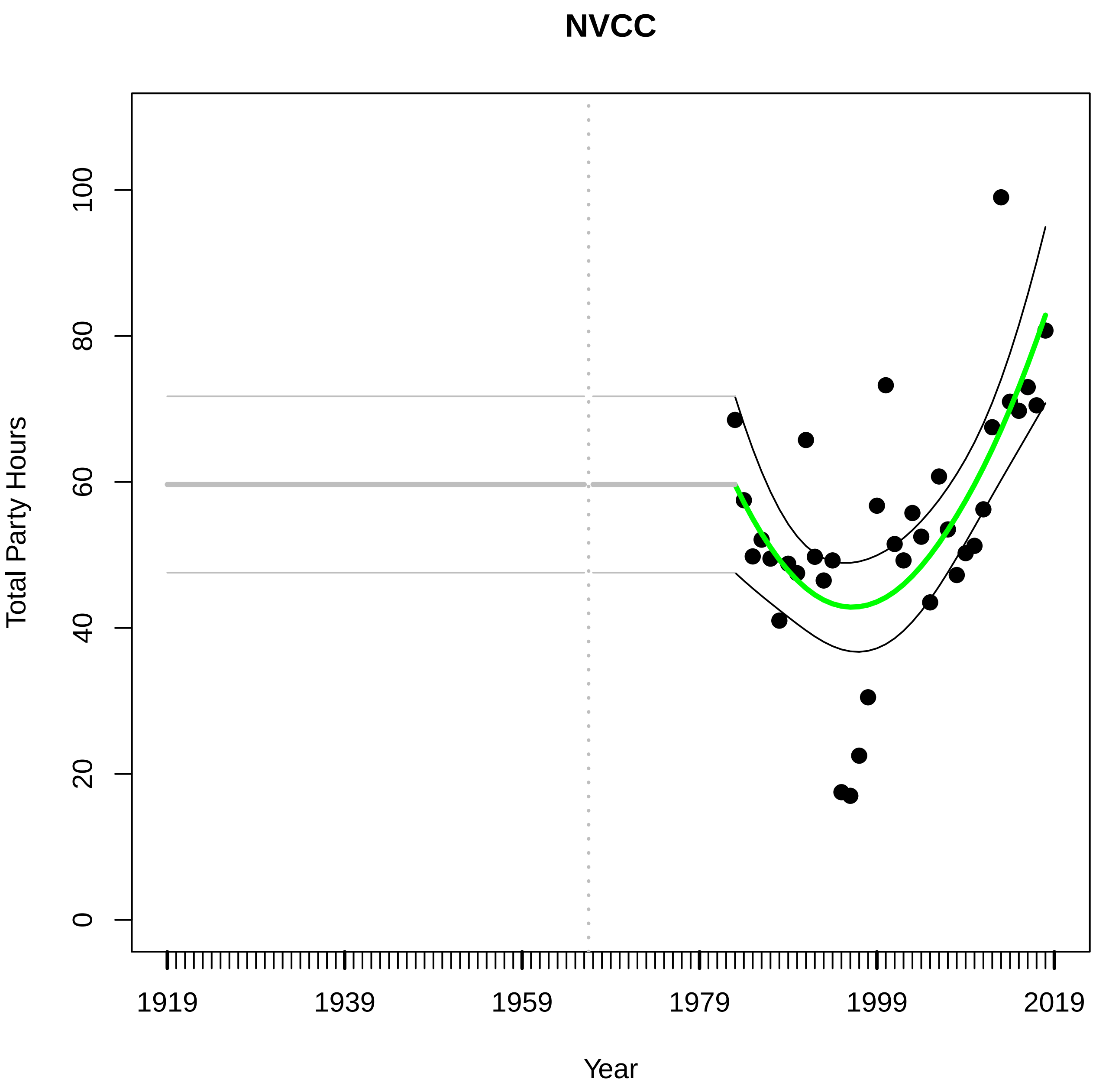

NVCC
Total Party Hours
0
20
40
60
80
100
1919
1939
1959
1979
1999
2019
Year

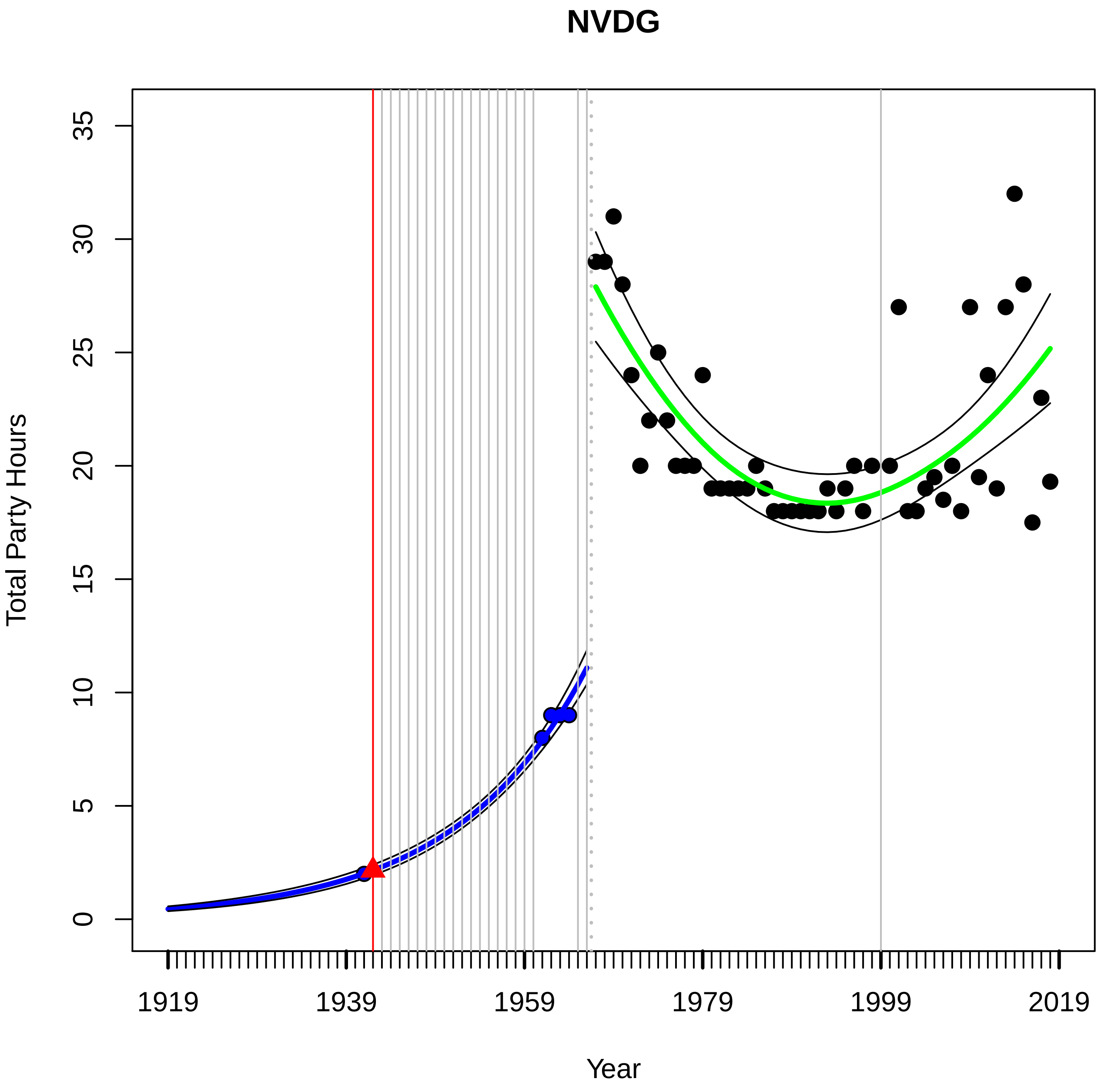

NVDG
Total Party Hours
Year
0
5
10
15
20
25
30
35
1919
1939
1959
1979
1999
2019

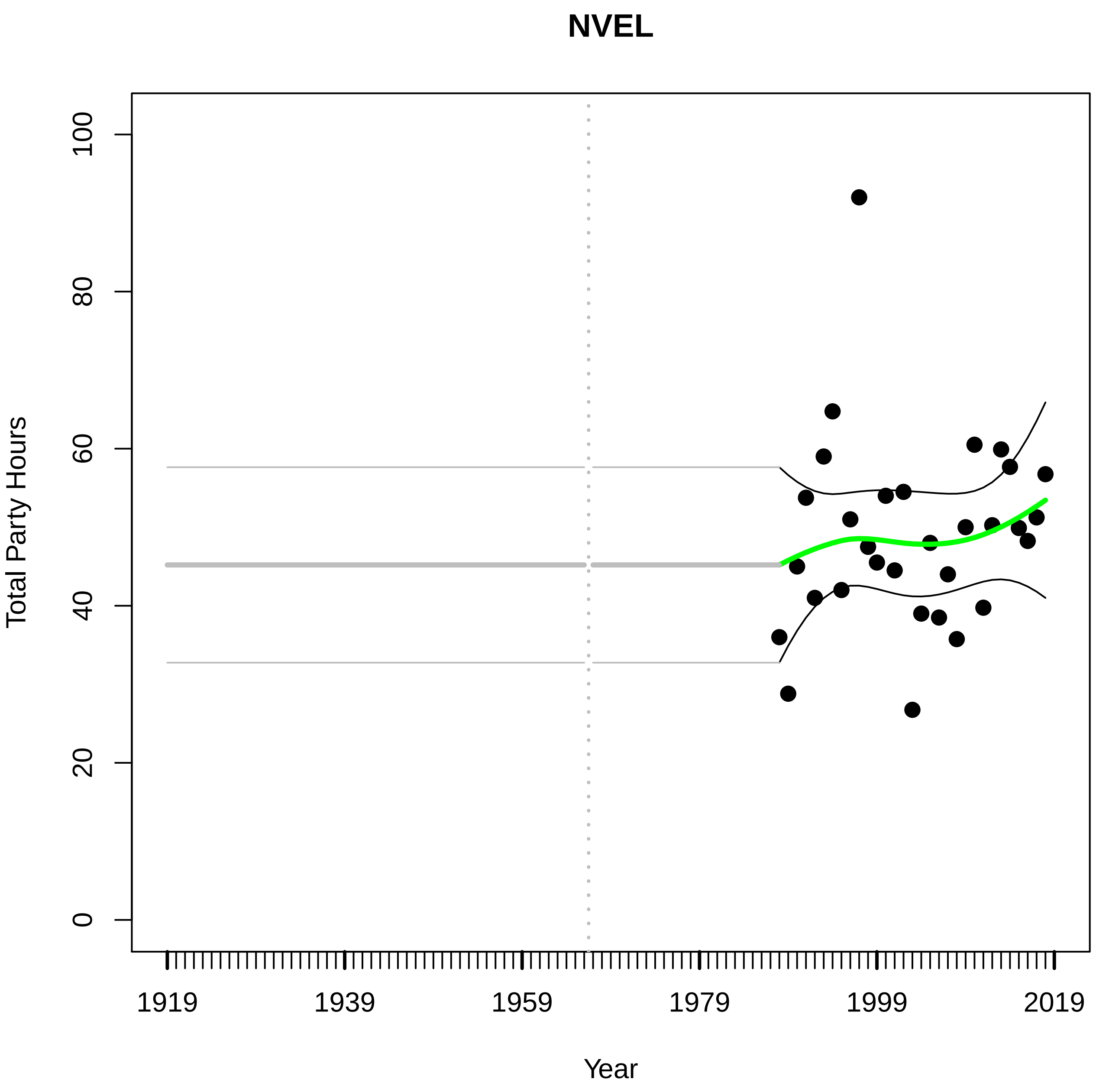

NVEL
Total Party Hours
0
20
40
60
80
100
1919
1939
1959
1979
1999
2019
Year

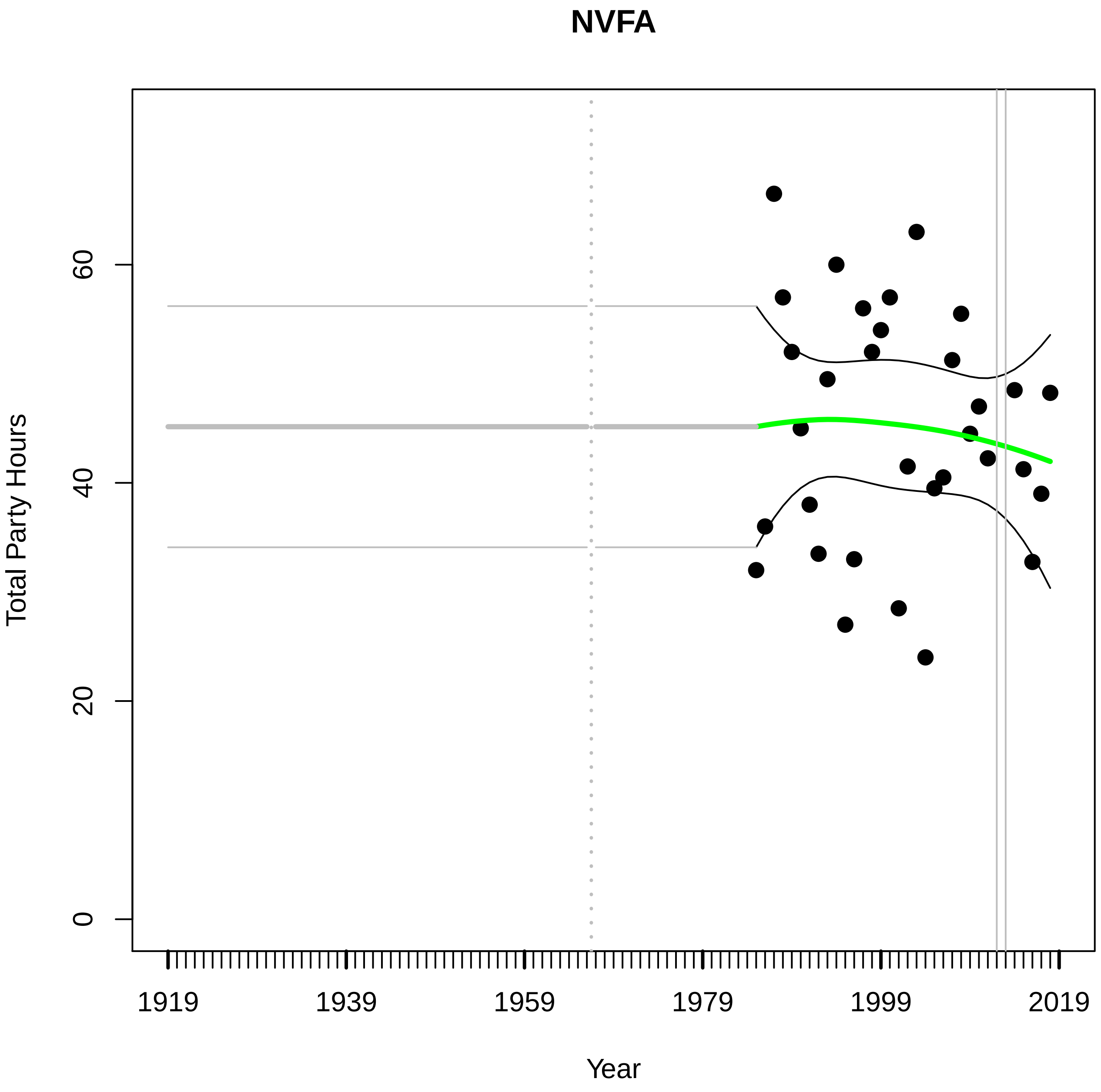
NVFA
Total Party Hours
0
20
40
60
1919
1939
1959
1979
1999
2019
Year

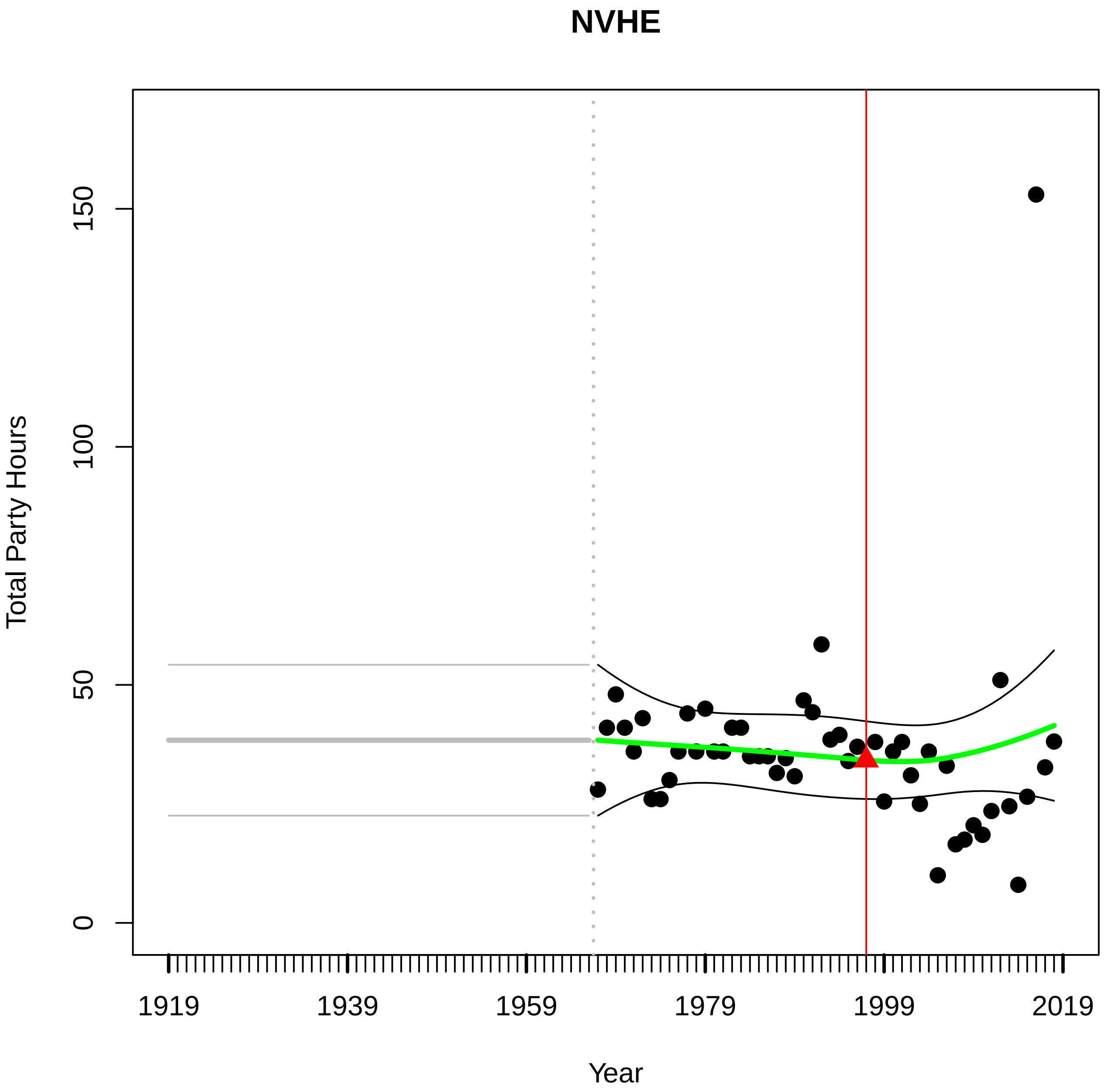
NVHE
Total Party Hours
150
100
50
0
1919
1939
1959
1979
1999
2019
Year

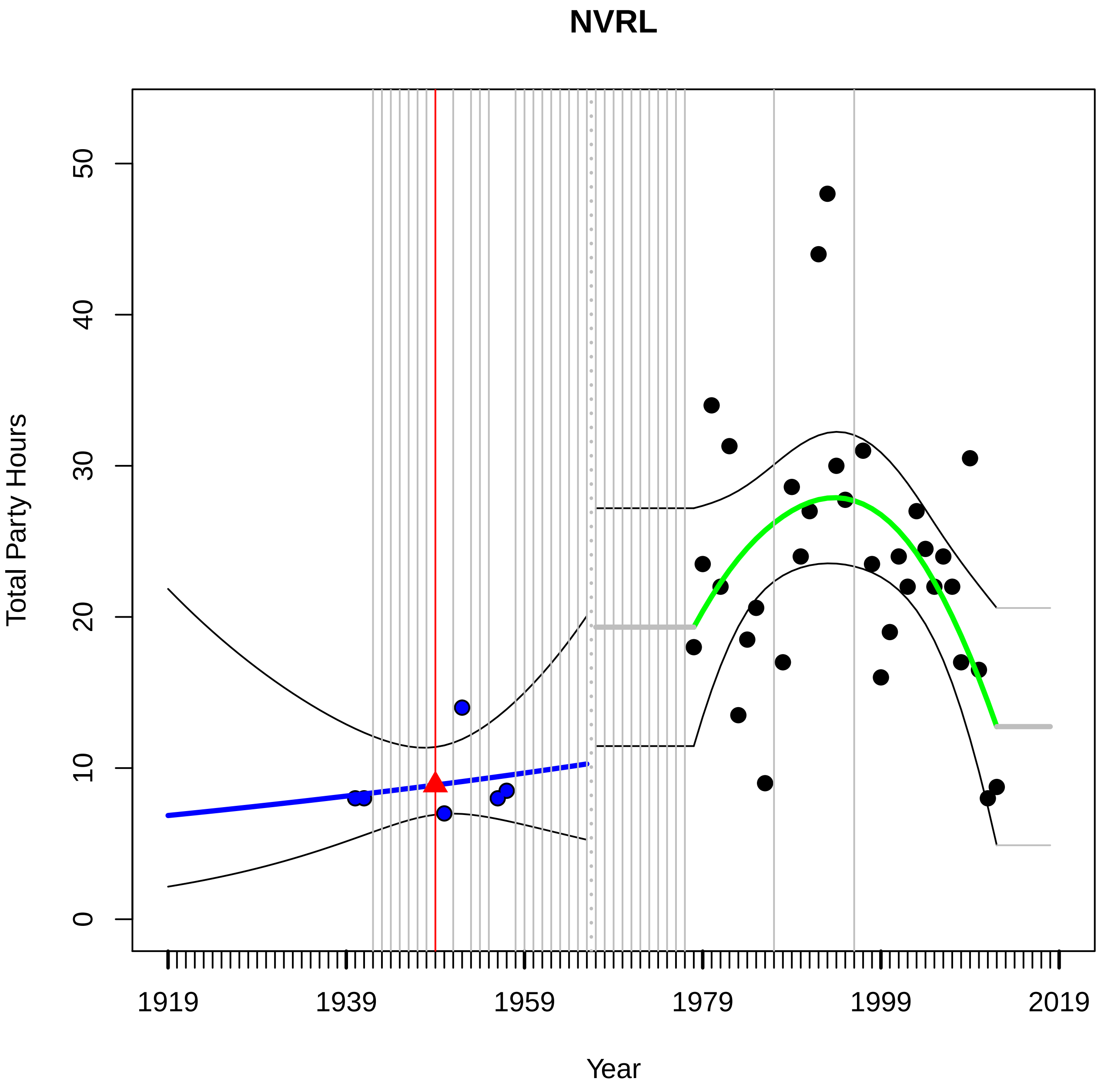
NVRL
Total Party Hours
0
10
20
30
40
50
Year
1919
1939
1959
1979
1999
2019

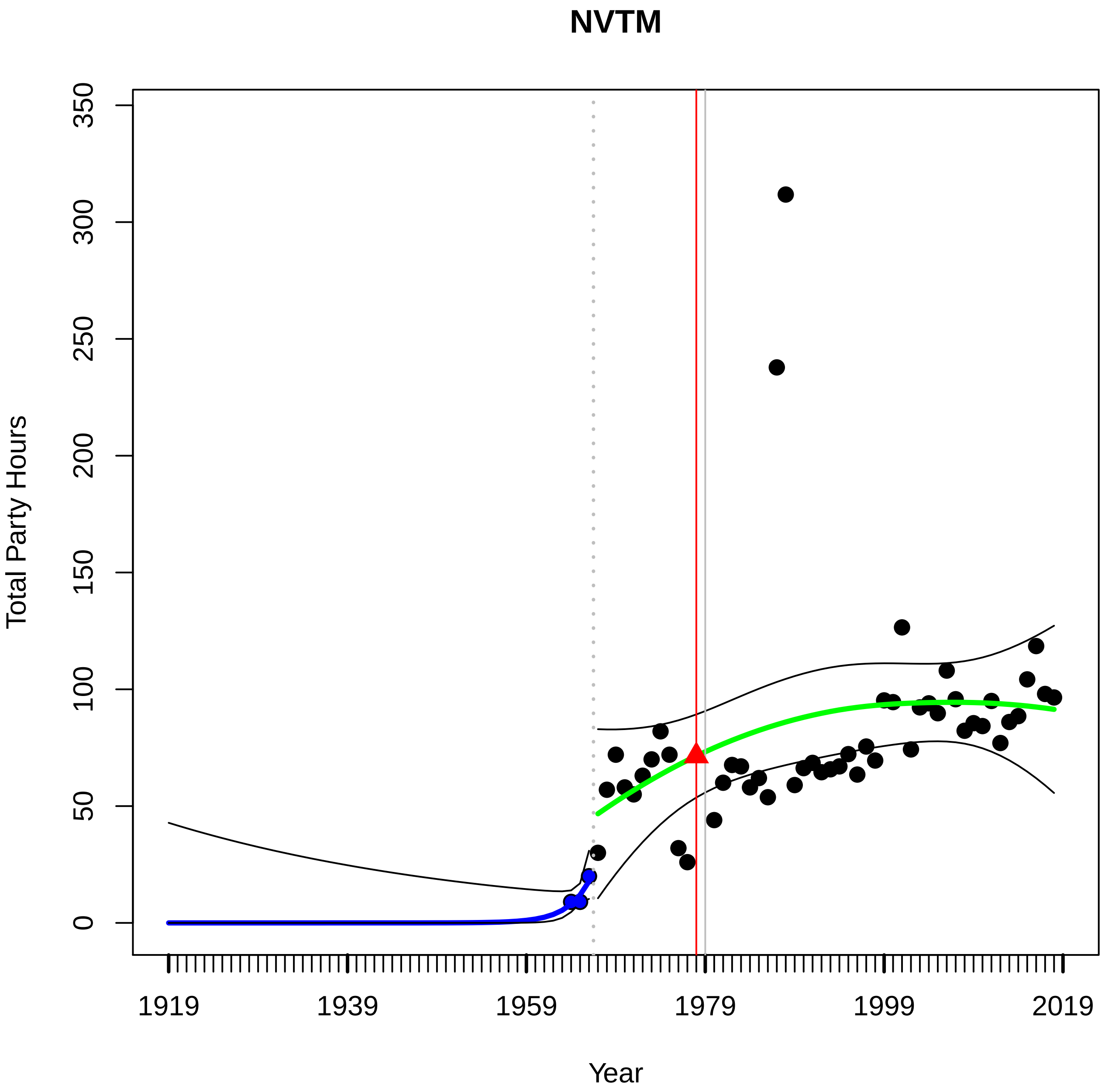

NVTM
Total Party Hours
Year
0
50
100
150
200
250
300
350
1919
1939
1959
1979
1999
2019

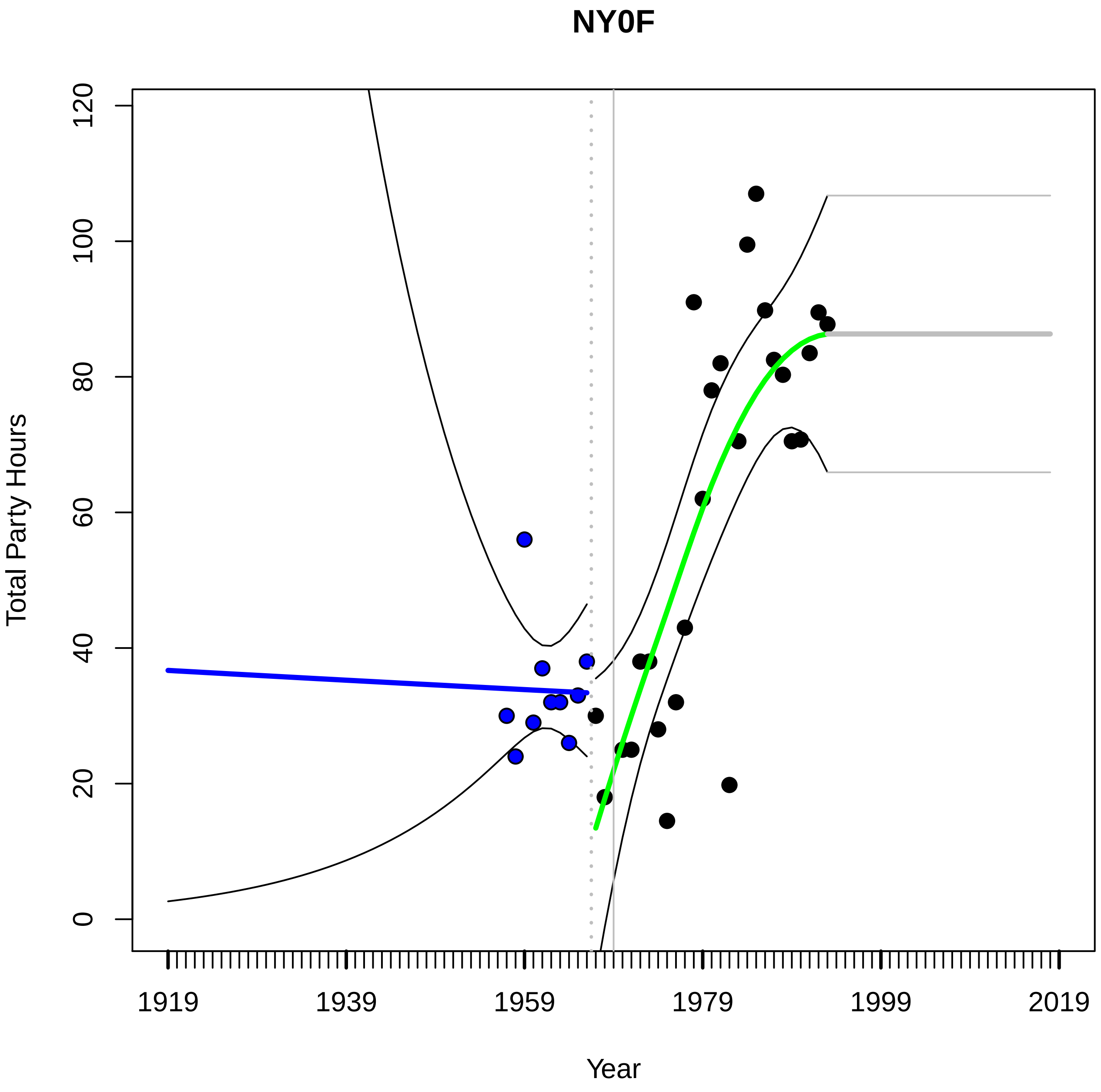

NY0F
Total Party Hours
0
20
40
60
80
100
120
1919
1939
1959
1979
1999
2019
Year

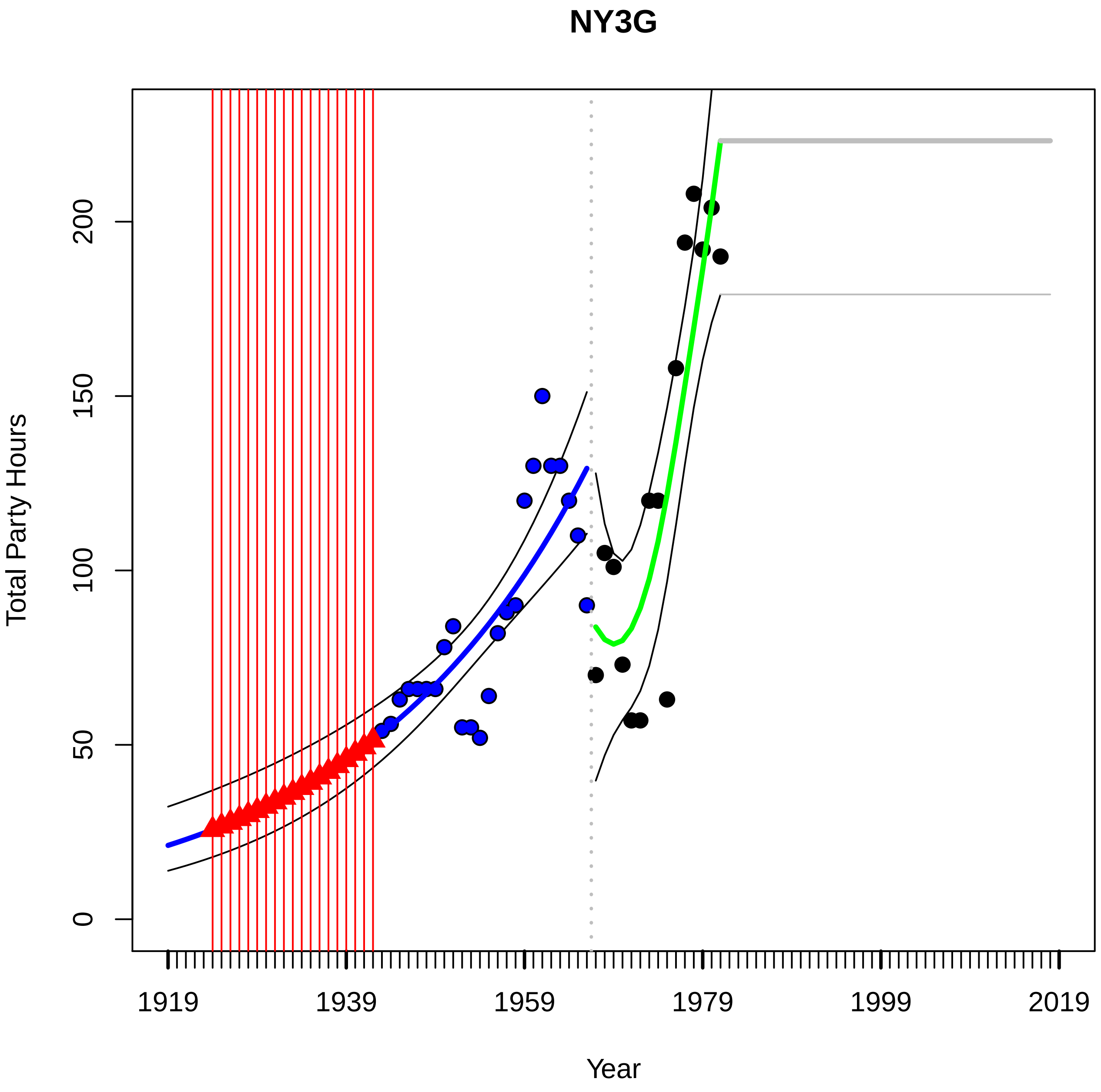
NY3G
Total Party Hours
0
50
100
150
200
1919
1939
1959
1979
1999
2019
Year

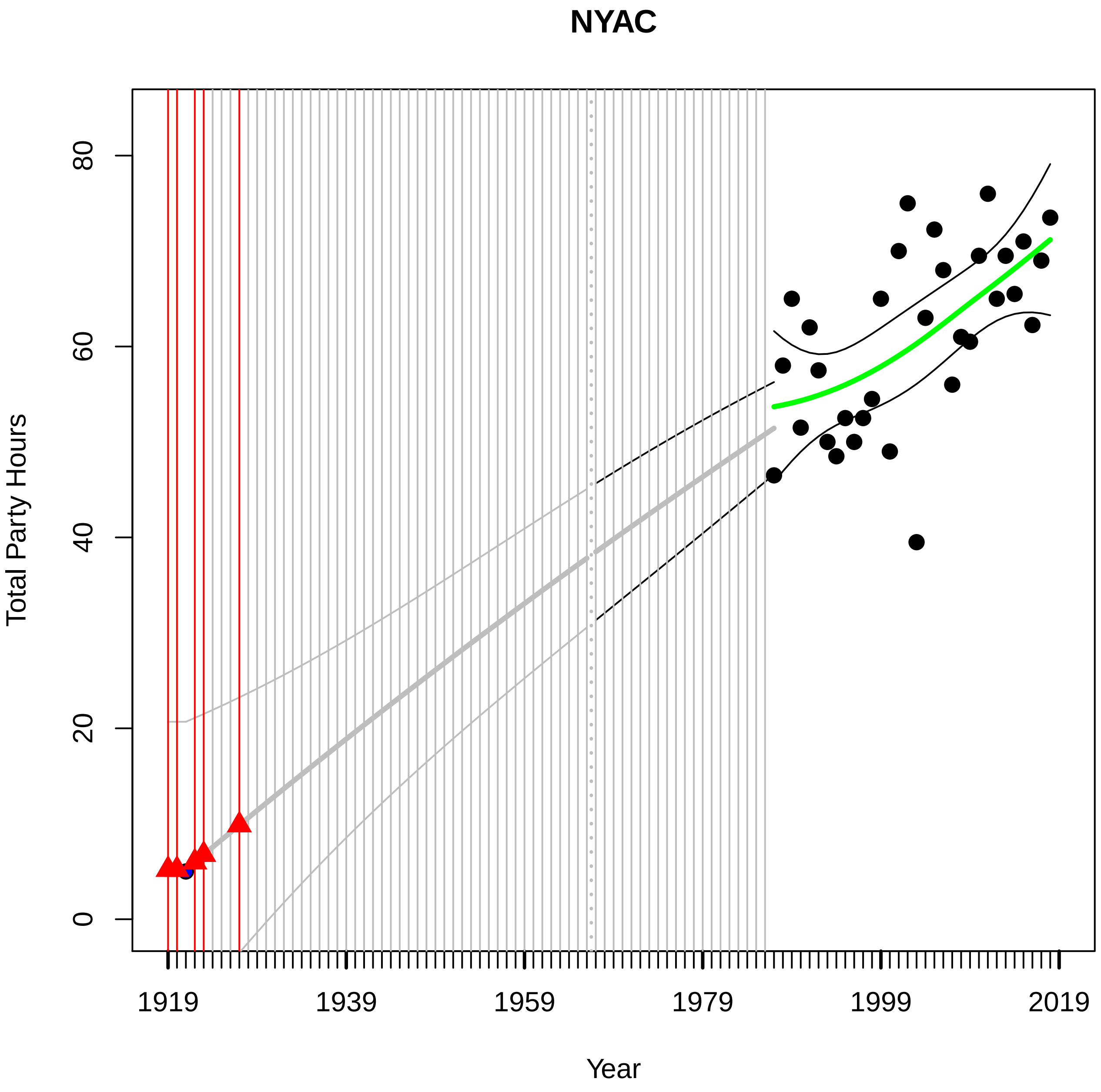
NYAC
Total Party Hours
0
20
40
60
80
1919
1939
1959
1979
1999
2019
Year

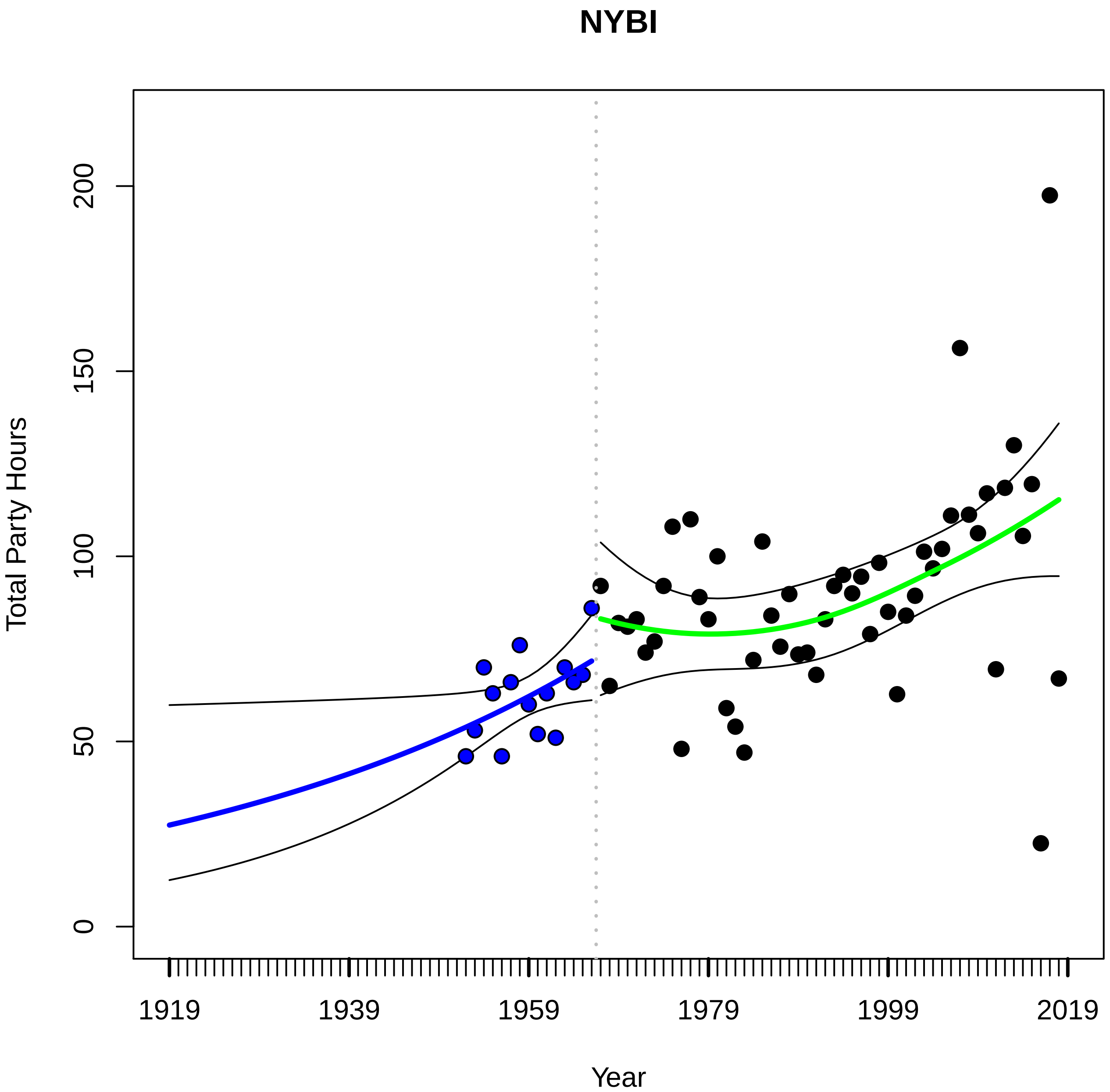

NYBI
Total Party Hours
0
50
100
150
200
1919
1939
1959
1979
1999
2019
Year

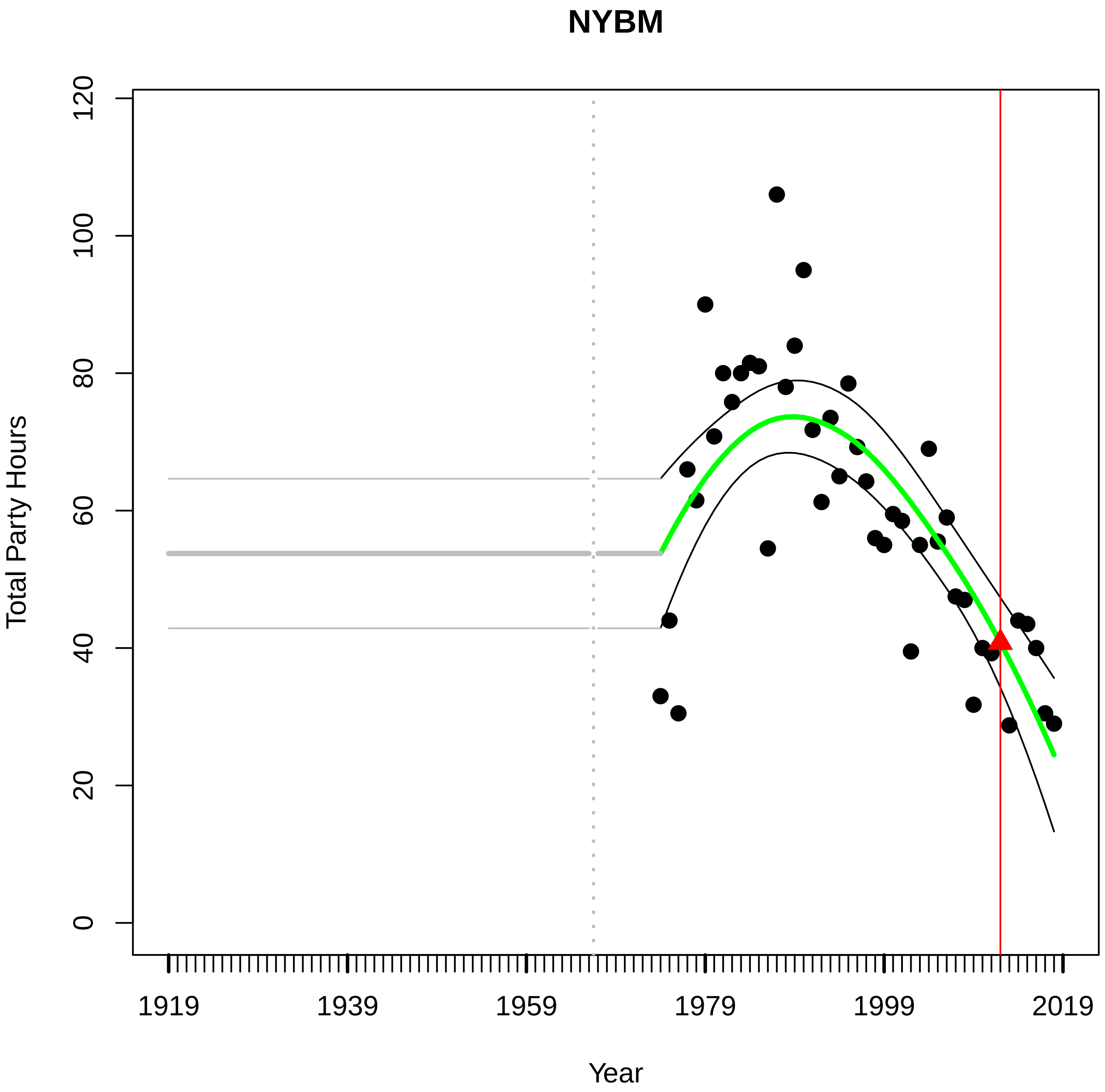

NYBM
Total Party Hours
0
20
40
60
80
100
120
1919
1939
1959
1979
1999
2019
Year

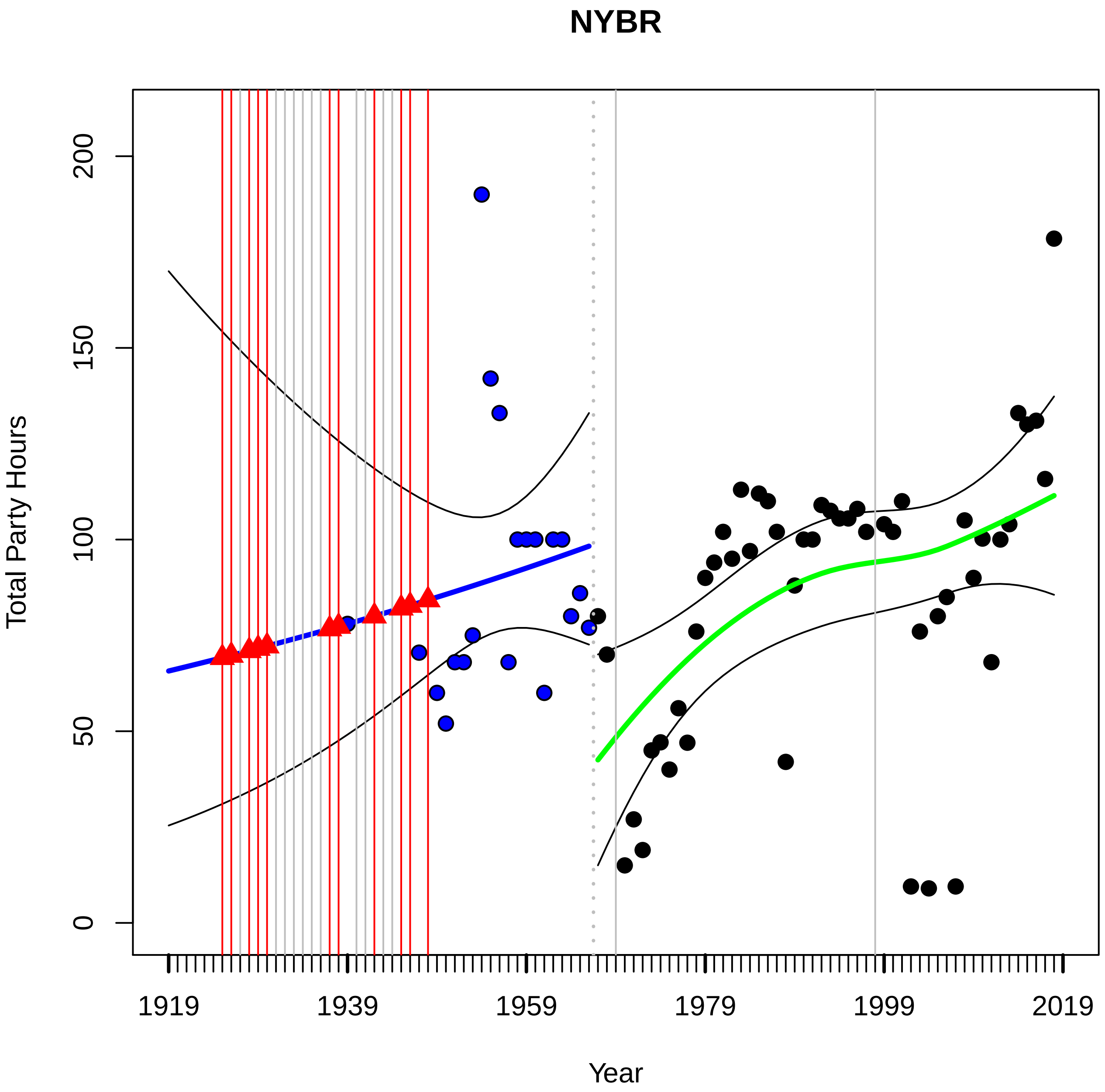

NYBR
Total Party Hours
0
50
100
150
200
1919
1939
1959
1979
1999
2019
Year

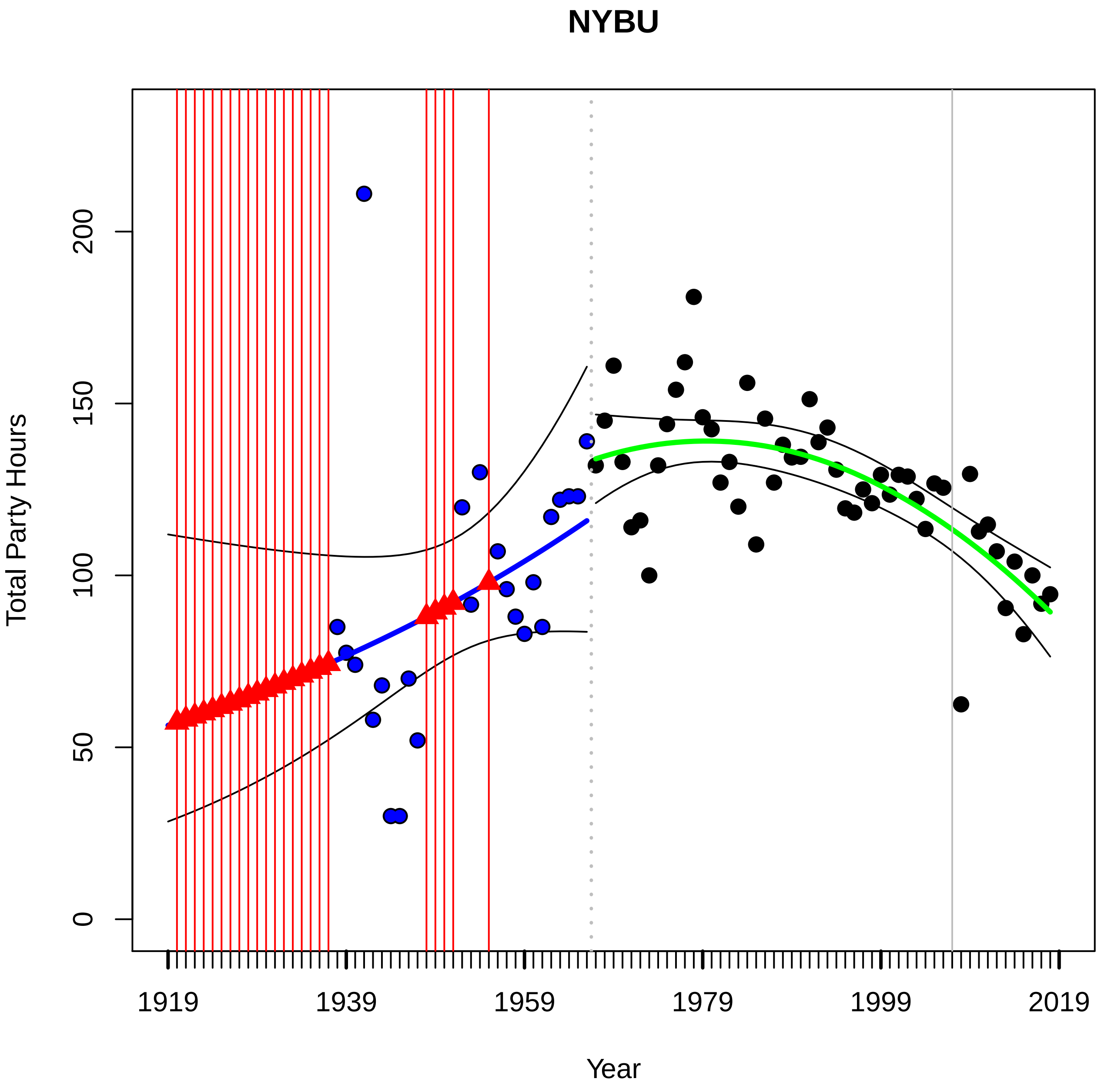
NYBU
Total Party Hours
Year
0
50
100
150
200
1919
1939
1959
1979
1999
2019

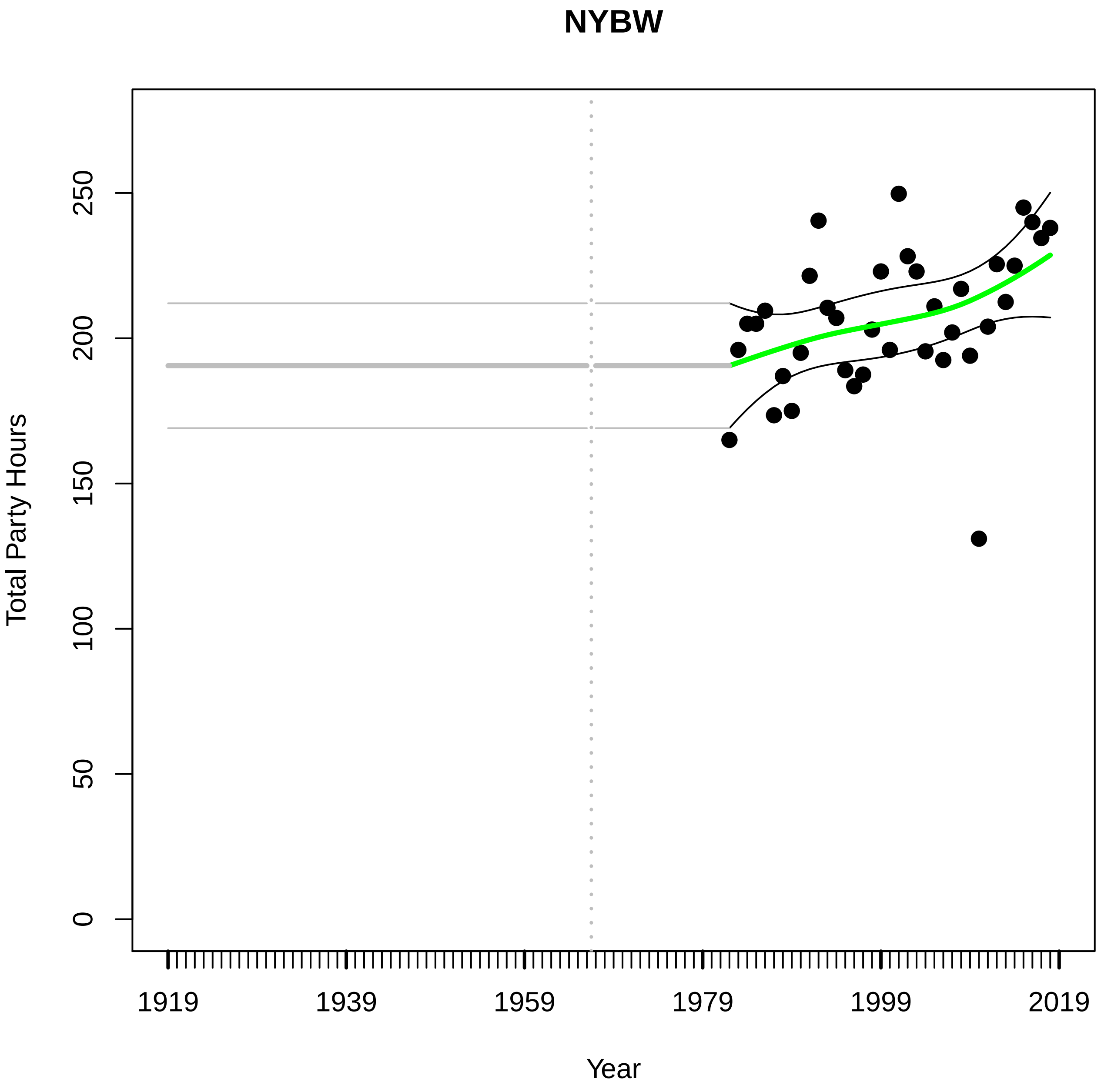

NYBW
Total Party Hours
0
50
100
150
200
250
1919
1939
1959
1979
1999
2019
Year

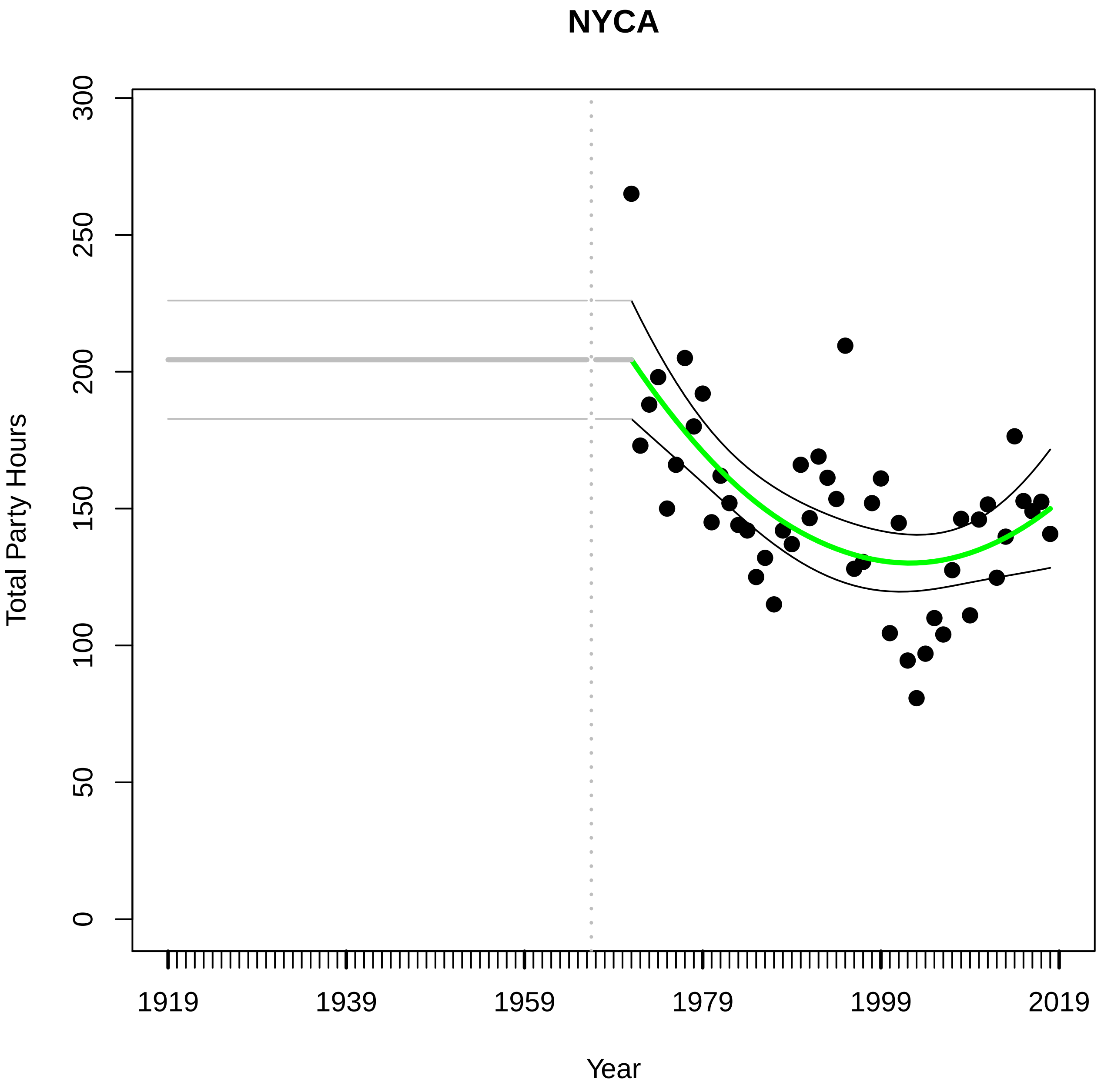

NYCA
Total Party Hours
Year
0
50
100
150
200
250
300
1919
1939
1959
1979
1999
2019

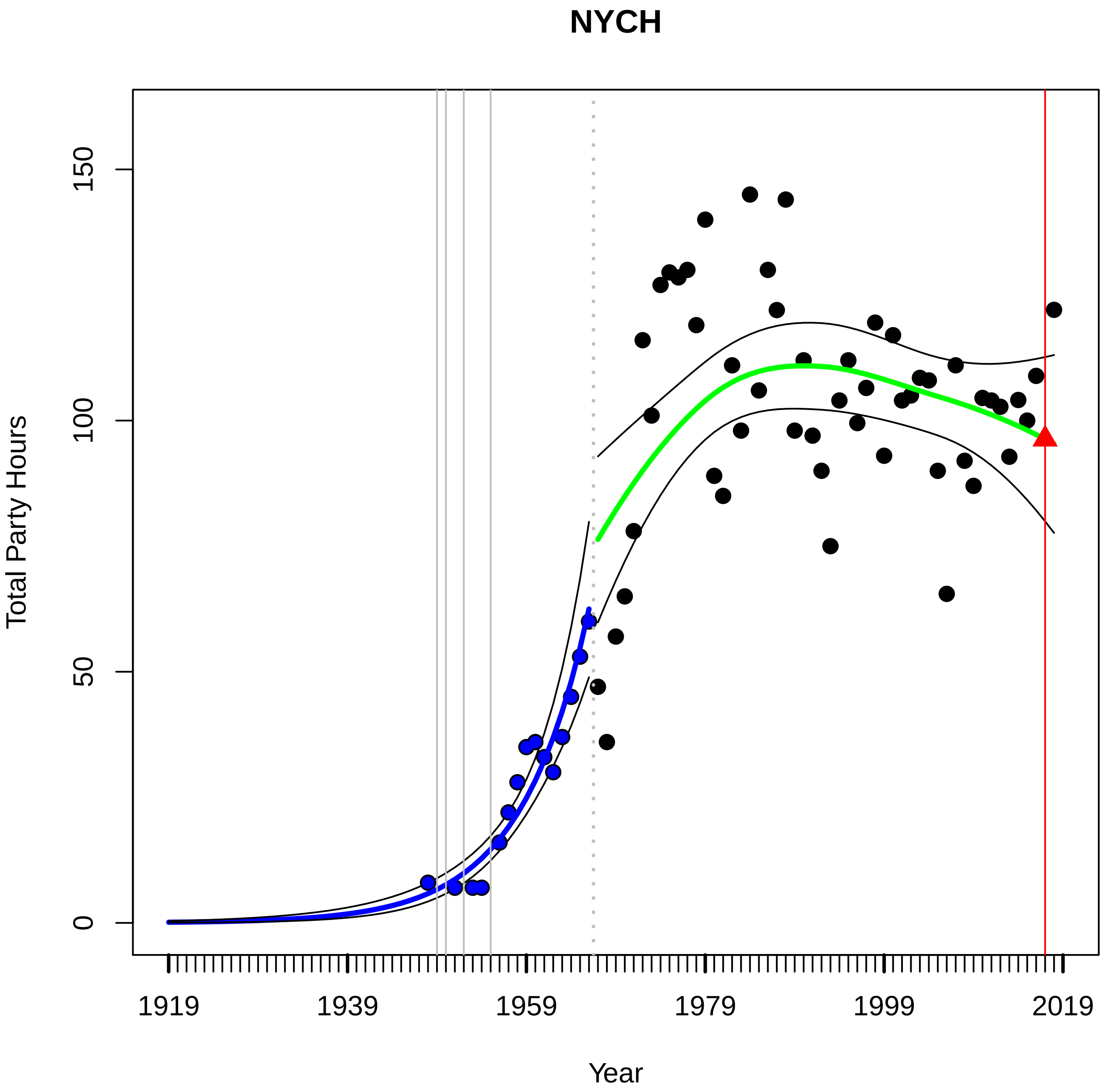

NYCH
Total Party Hours
0
50
100
150
1919
1939
1959
1979
1999
2019
Year

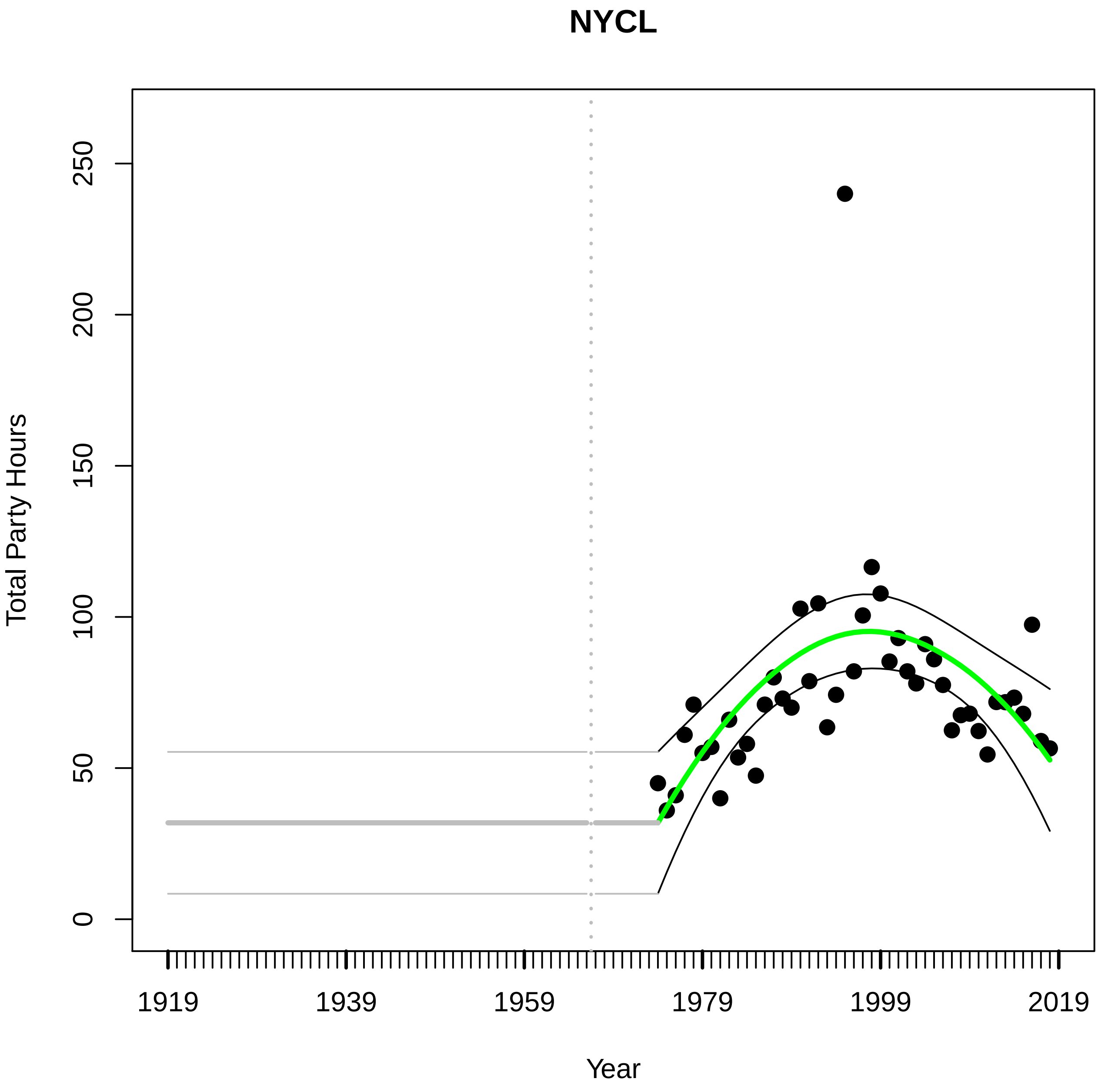

NYCL
Total Party Hours
Year
0
50
100
150
200
250
1919
1939
1959
1979
1999
2019

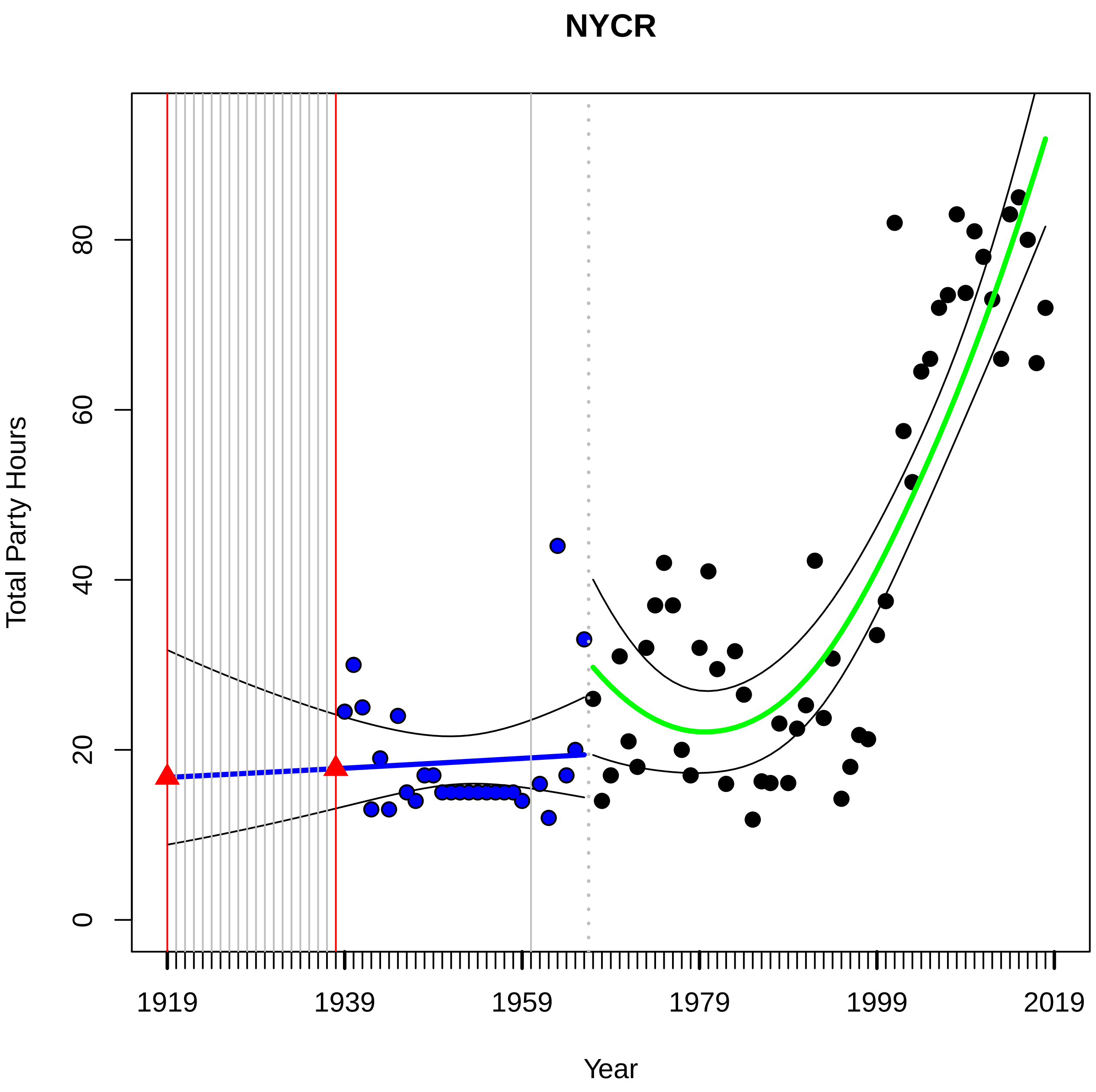

NYCR
Total Party Hours
0
20
40
60
80
1919
1939
1959
1979
1999
2019
Year

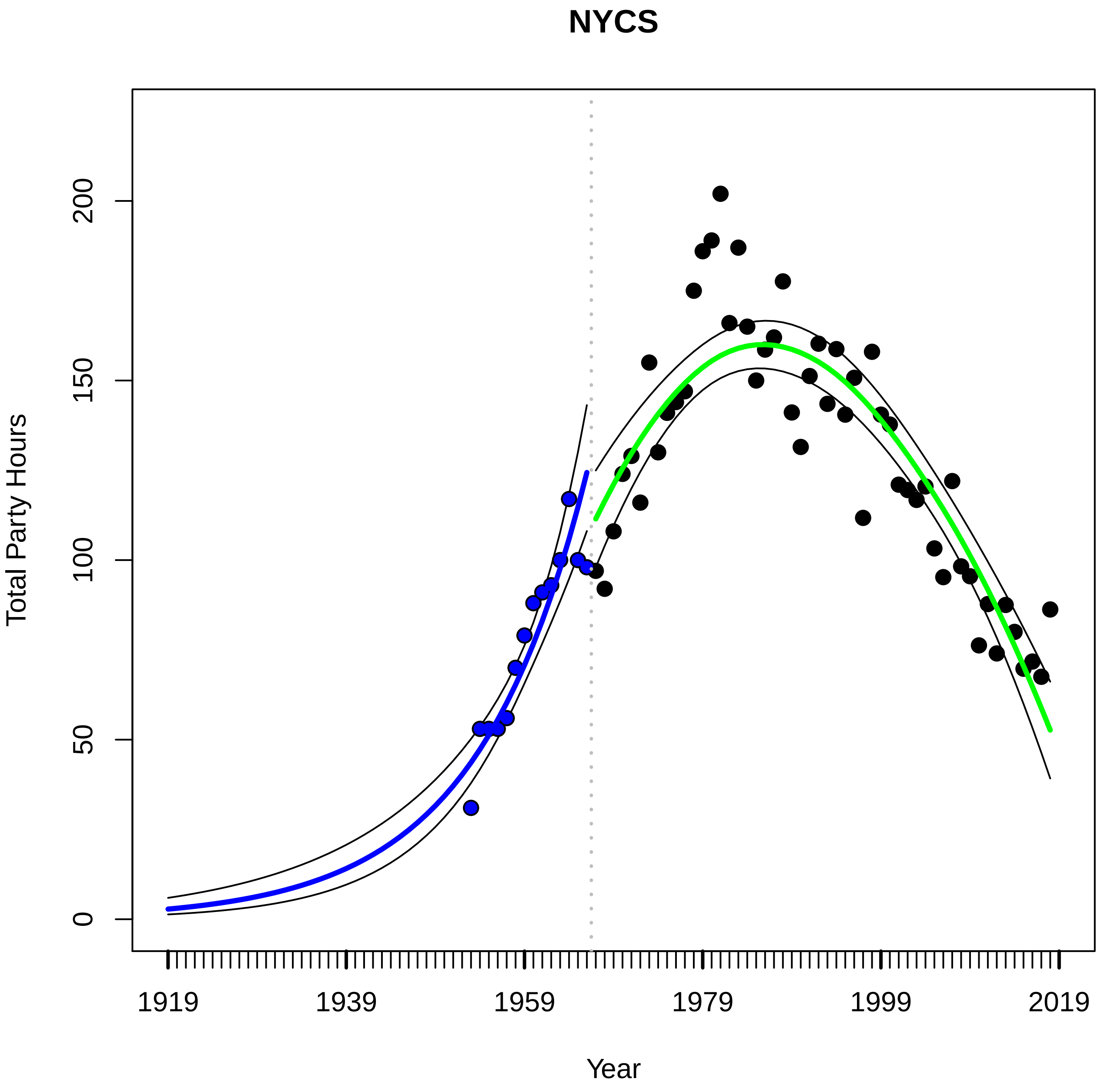

NYCS
Total Party Hours
Year
0
50
100
150
200
1919
1939
1959
1979
1999
2019

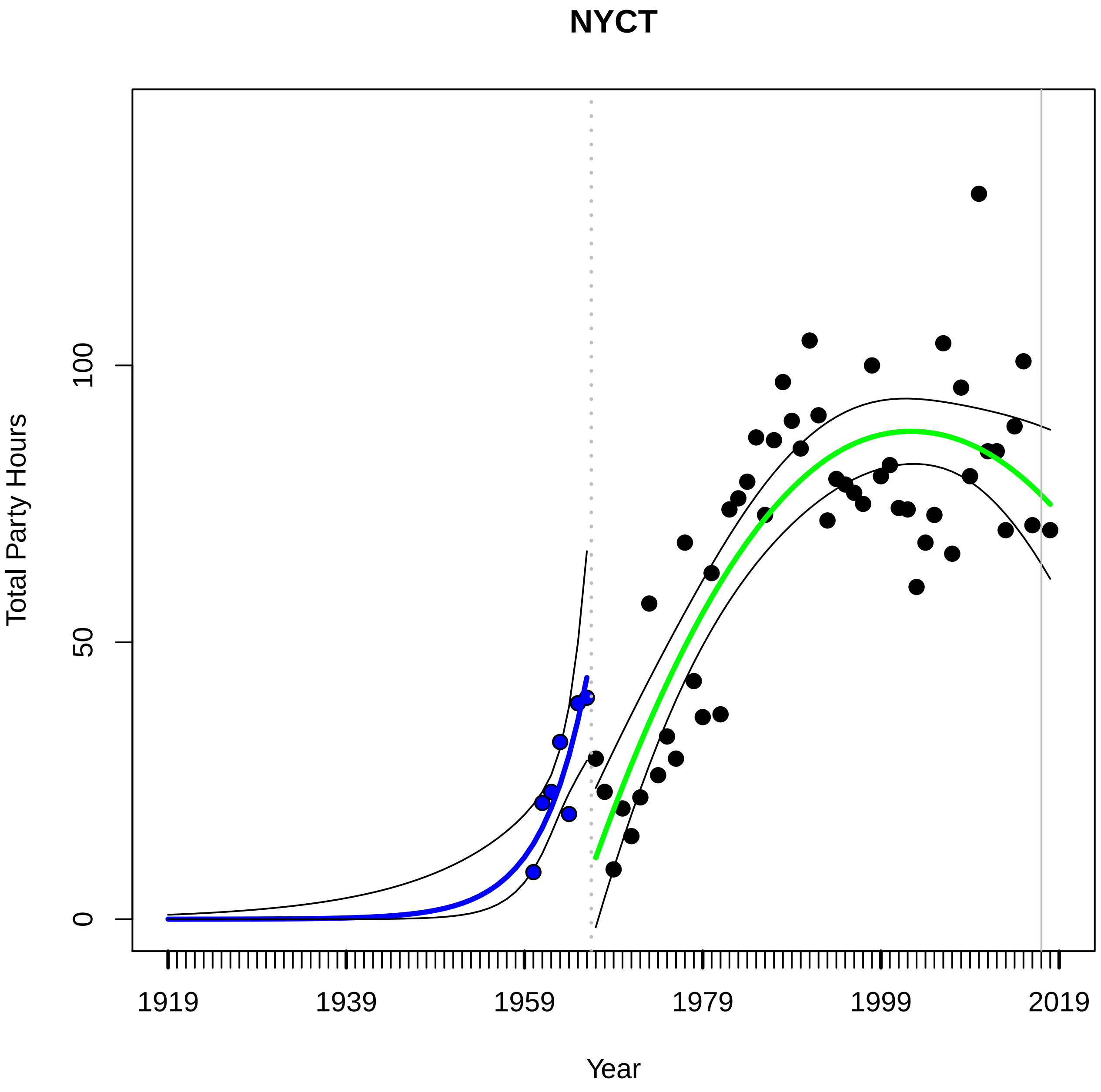
NYCT
Total Party Hours
0
50
100
1919
1939
1959
1979
1999
2019
Year

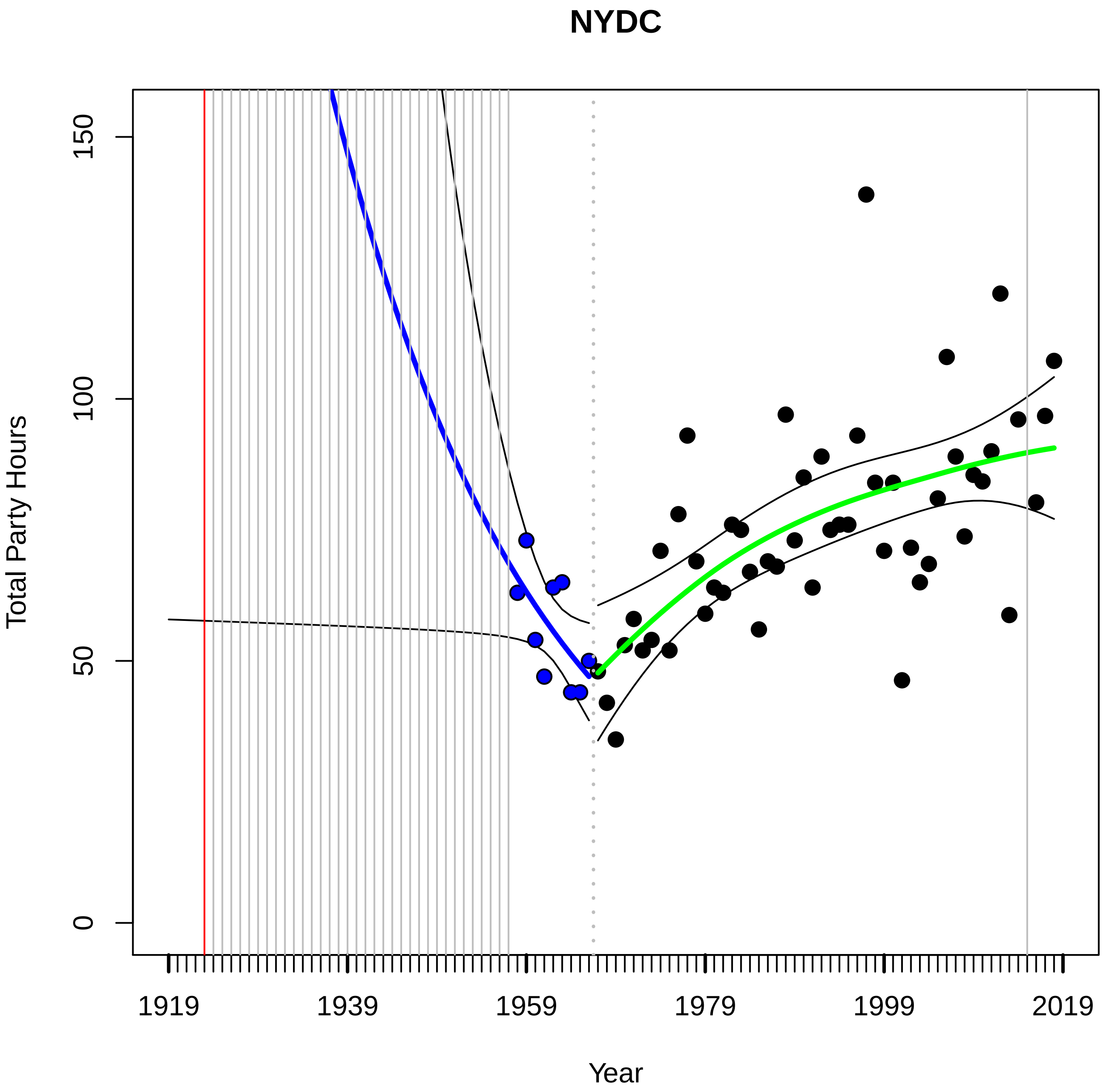
NYDC
Total Party Hours
0
50
100
150
1919
1939
1959
1979
1999
2019
Year

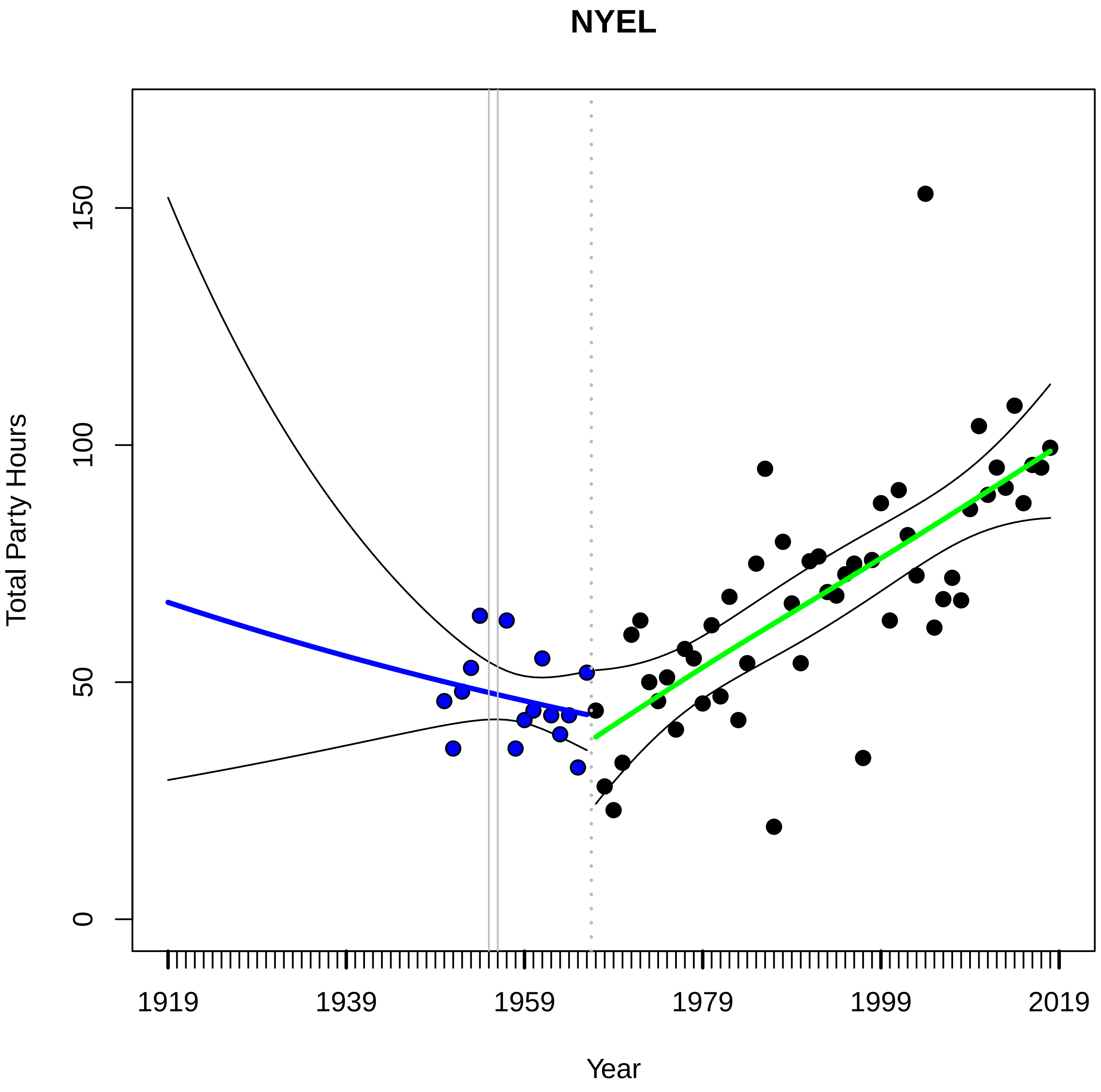
NYEL
Total Party Hours
0
50
100
150
1919
1939
1959
1979
1999
2019
Year

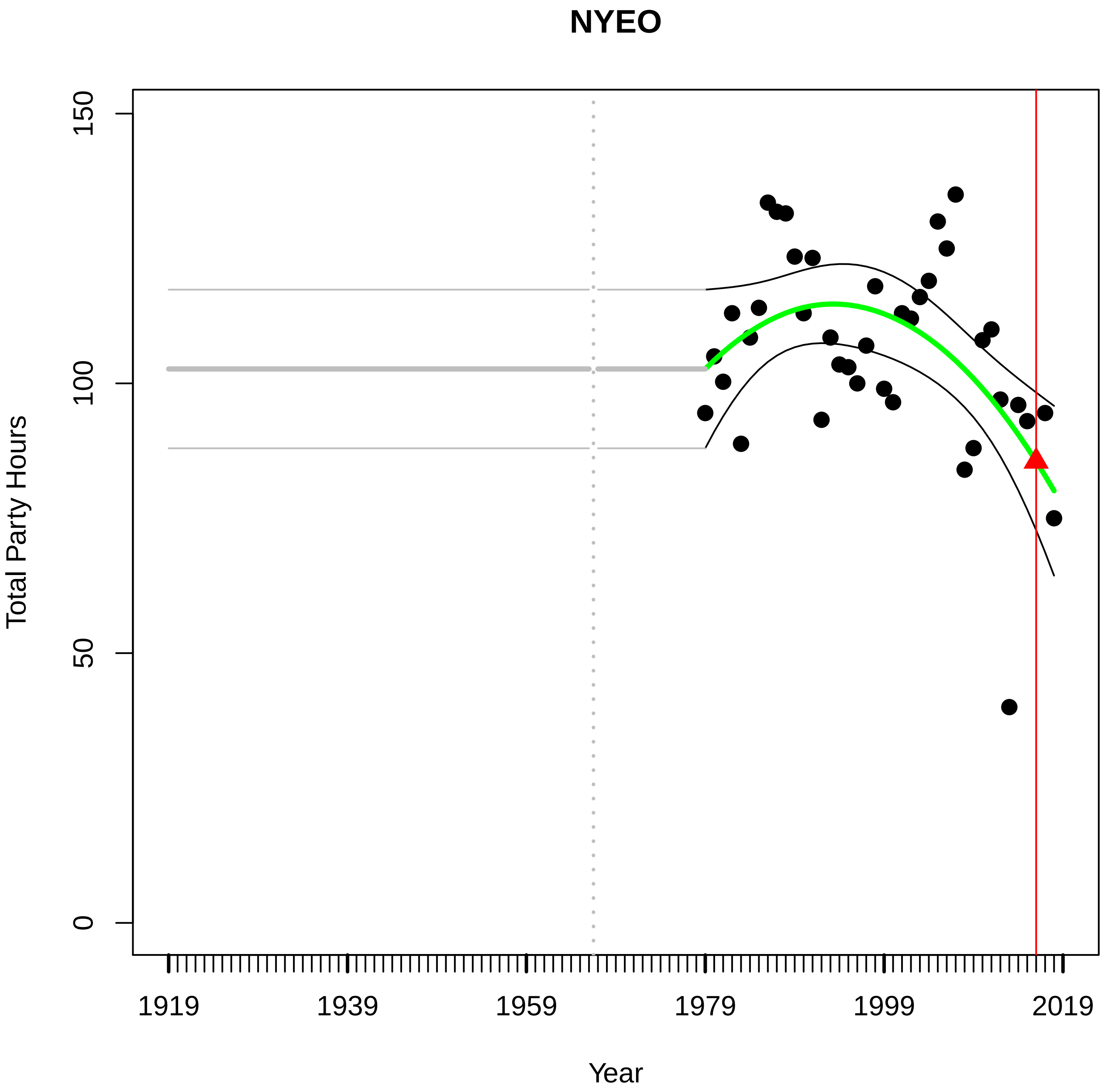

NYEO
Total Party Hours
150
100
50
0
1919
1939
1959
1979
1999
2019
Year

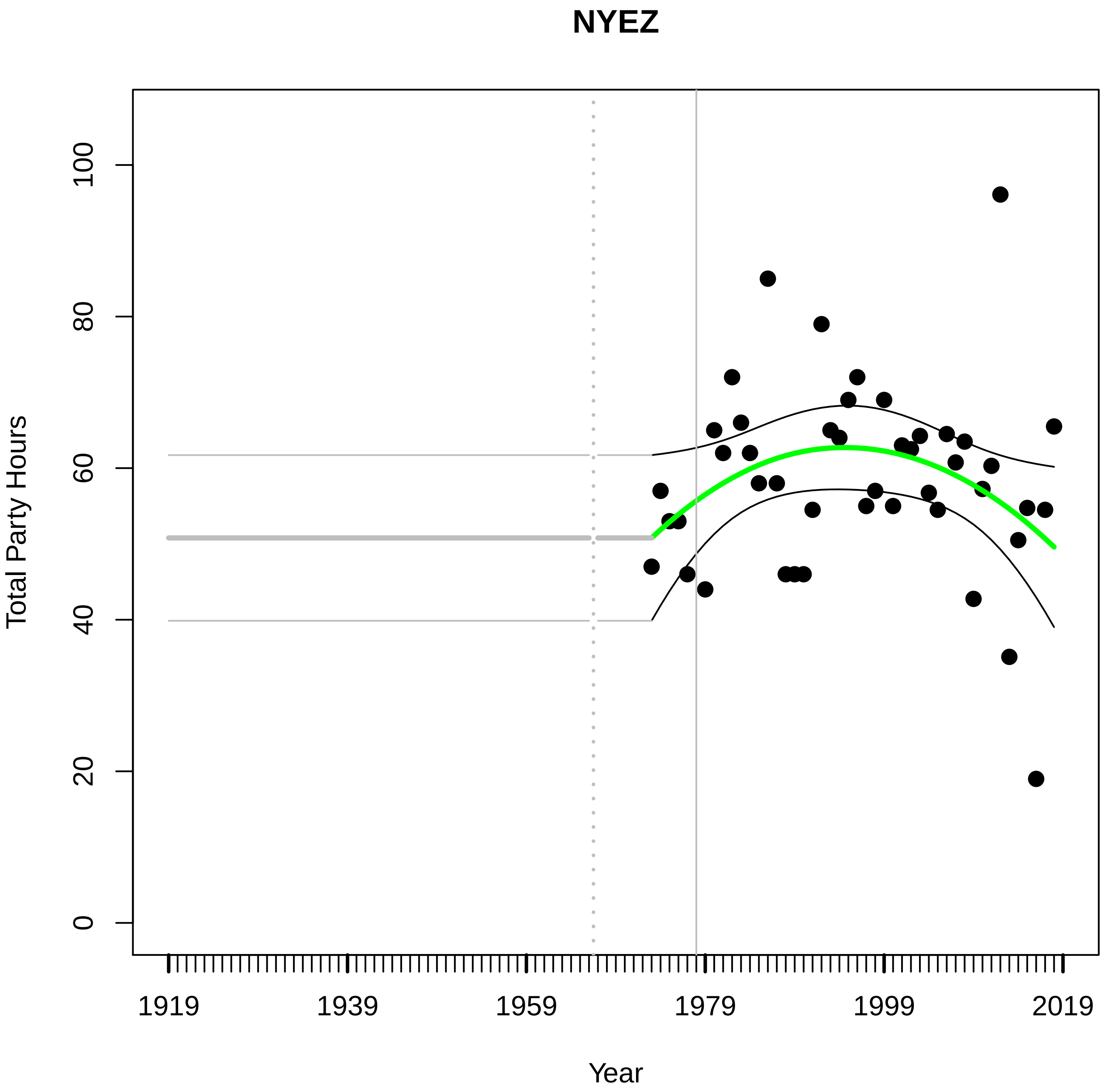

NYEZ
Total Party Hours
0
20
40
60
80
100
Year
1919
1939
1959
1979
1999
2019

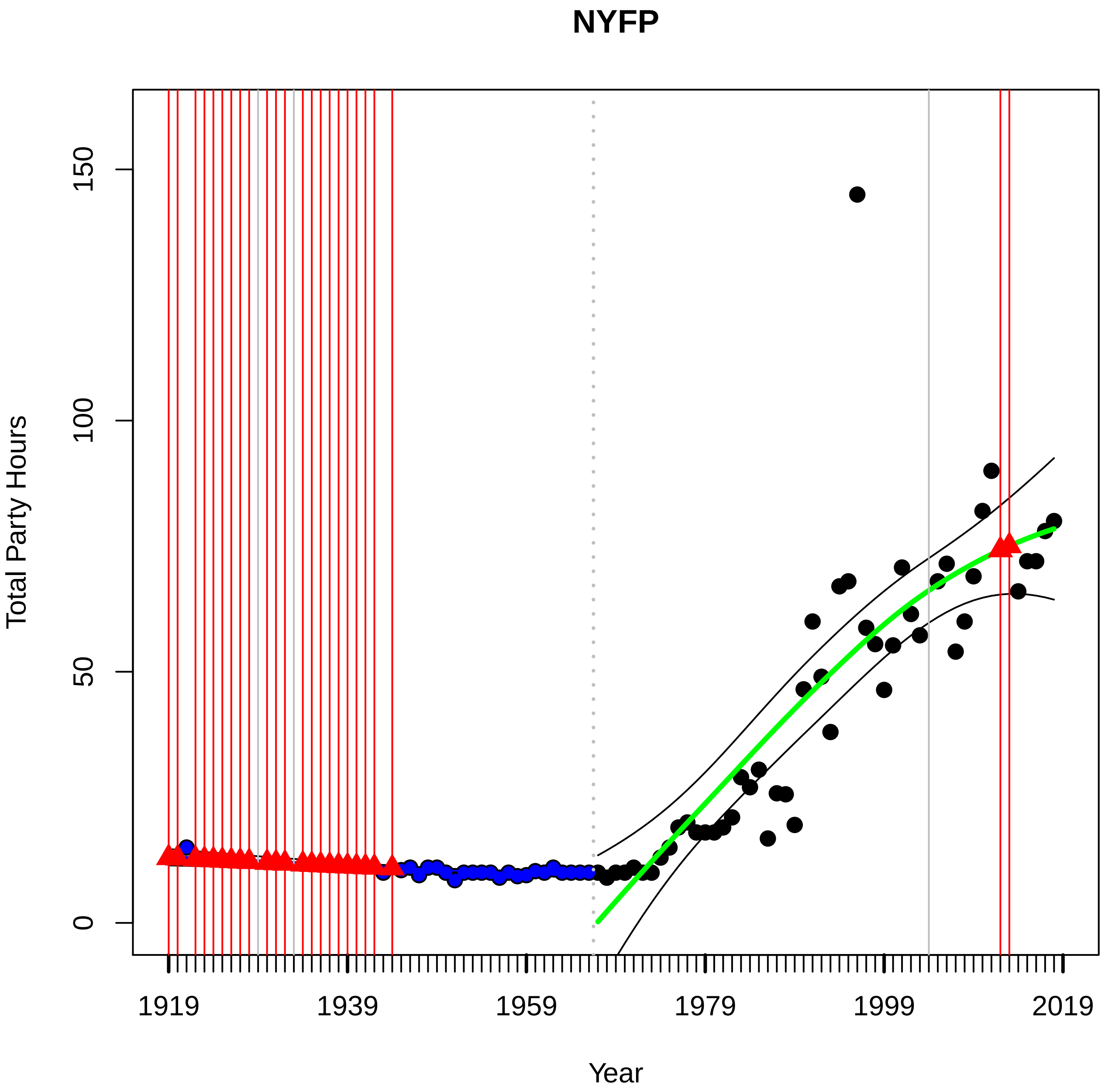

NYFP
Total Party Hours
0
50
100
150
1919
1939
1959
1979
1999
2019
Year

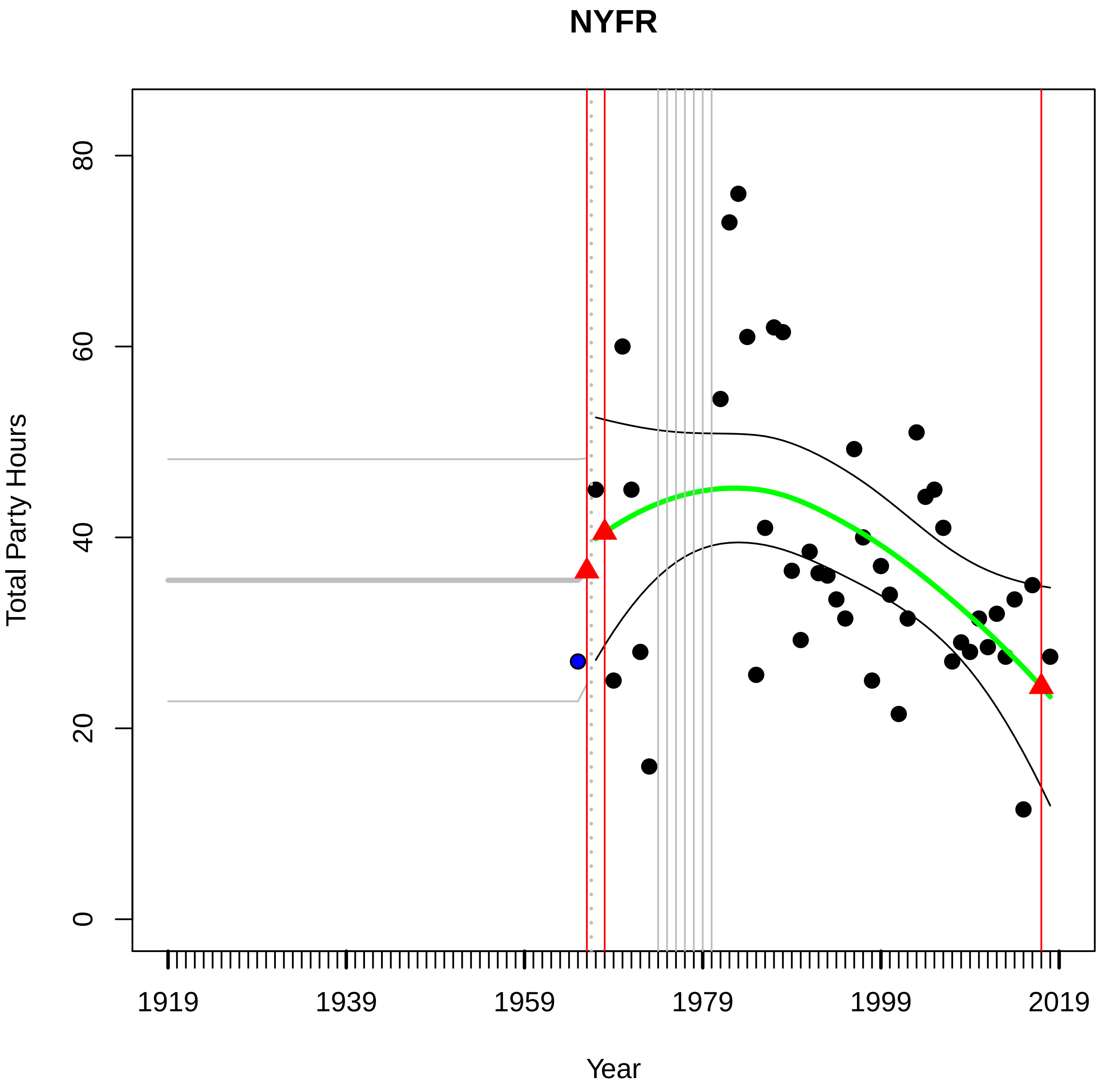
NYFR
Total Party Hours
0
20
40
60
80
1919
1939
1959
1979
1999
2019
Year

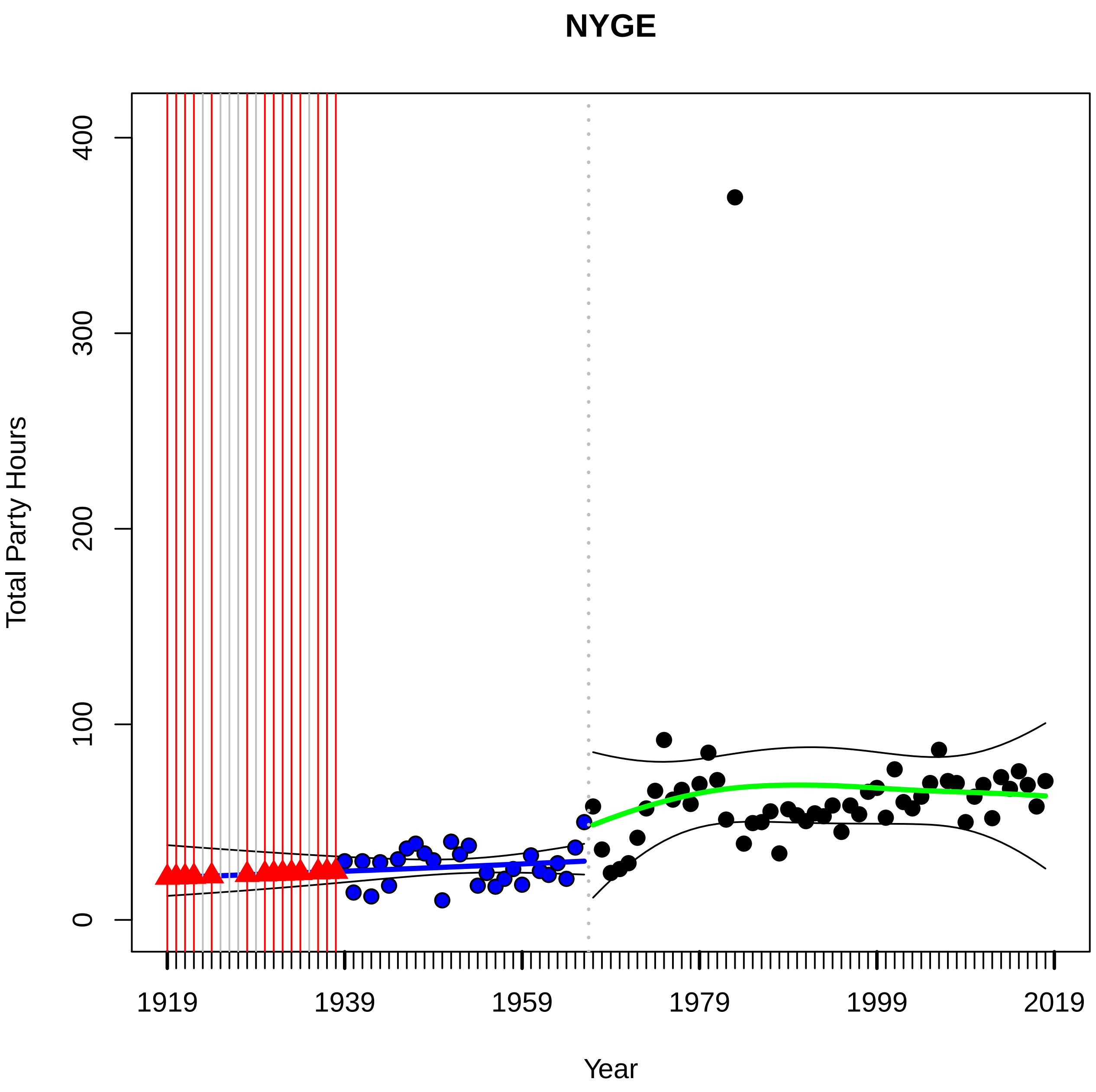

NYGE
Total Party Hours
0
100
200
300
400
Year
1919
1939
1959
1979
1999
2019

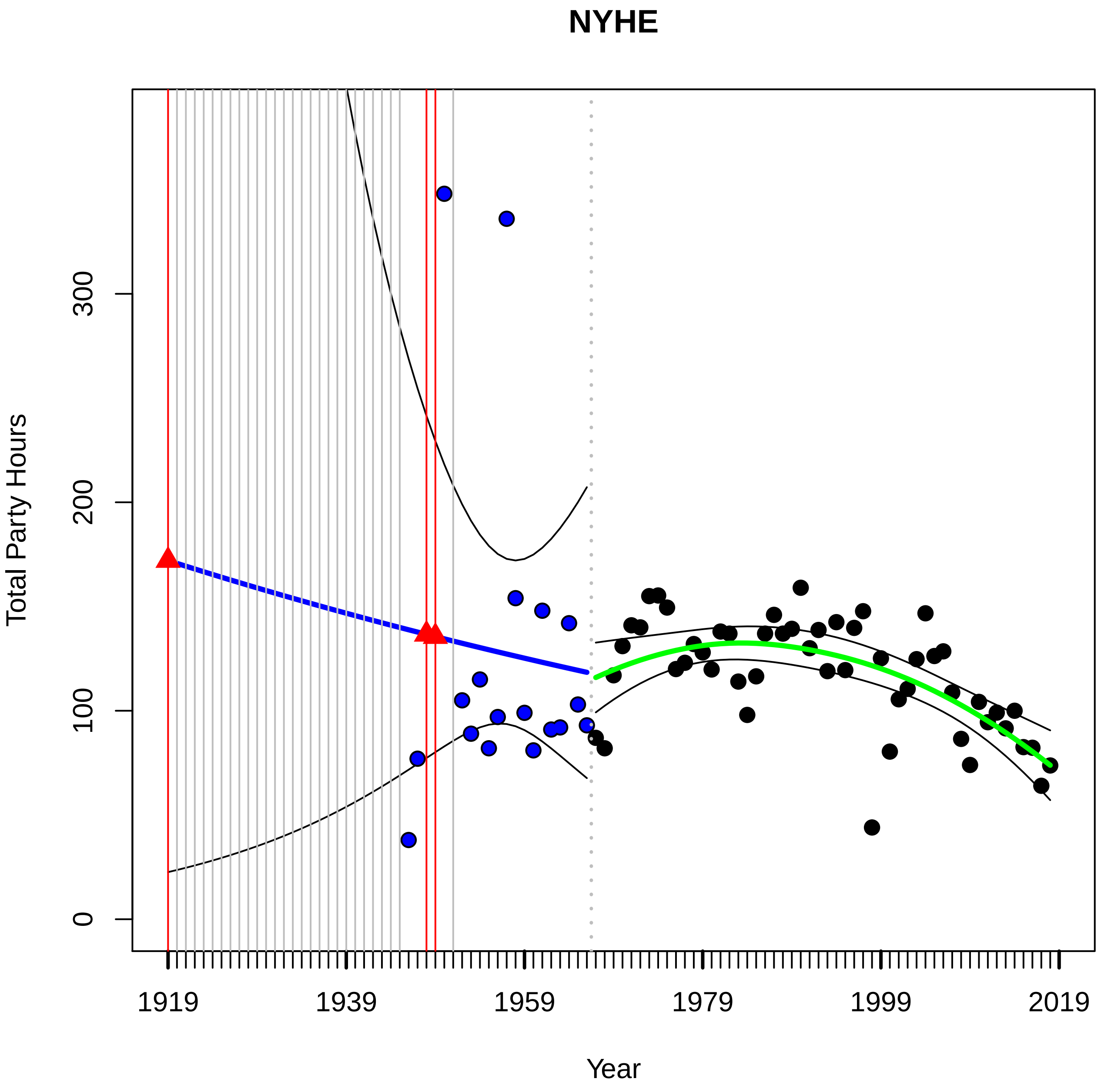
NYHE
Total Party Hours
0
100
200
300
1919
1939
1959
1979
1999
2019
Year

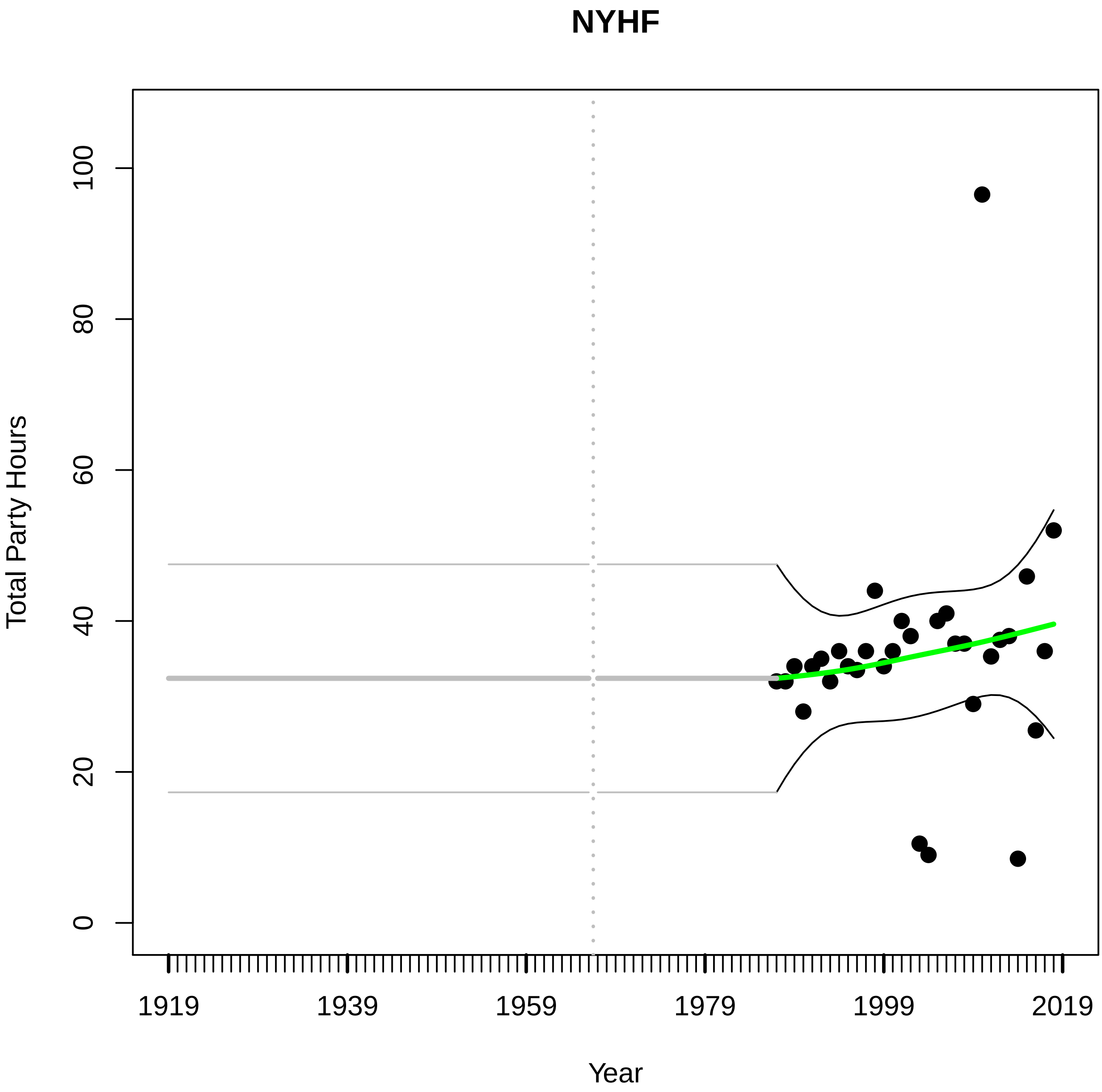
NYHF
Total Party Hours
0
20
40
60
80
100
1919
1939
1959
1979
1999
2019
Year

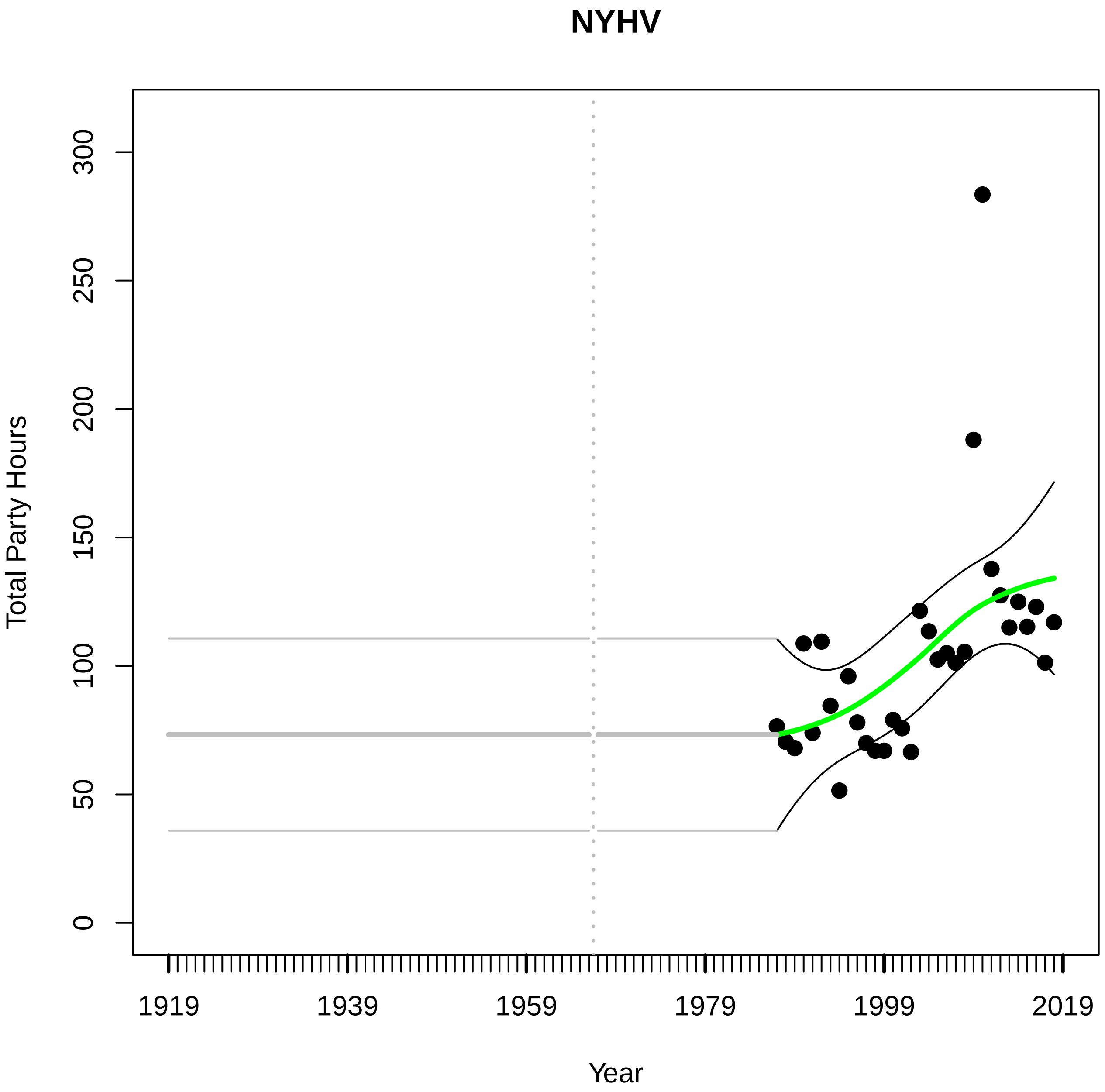
NYHV
Total Party Hours
Year
0
50
100
150
200
250
300
1919
1939
1959
1979
1999
2019

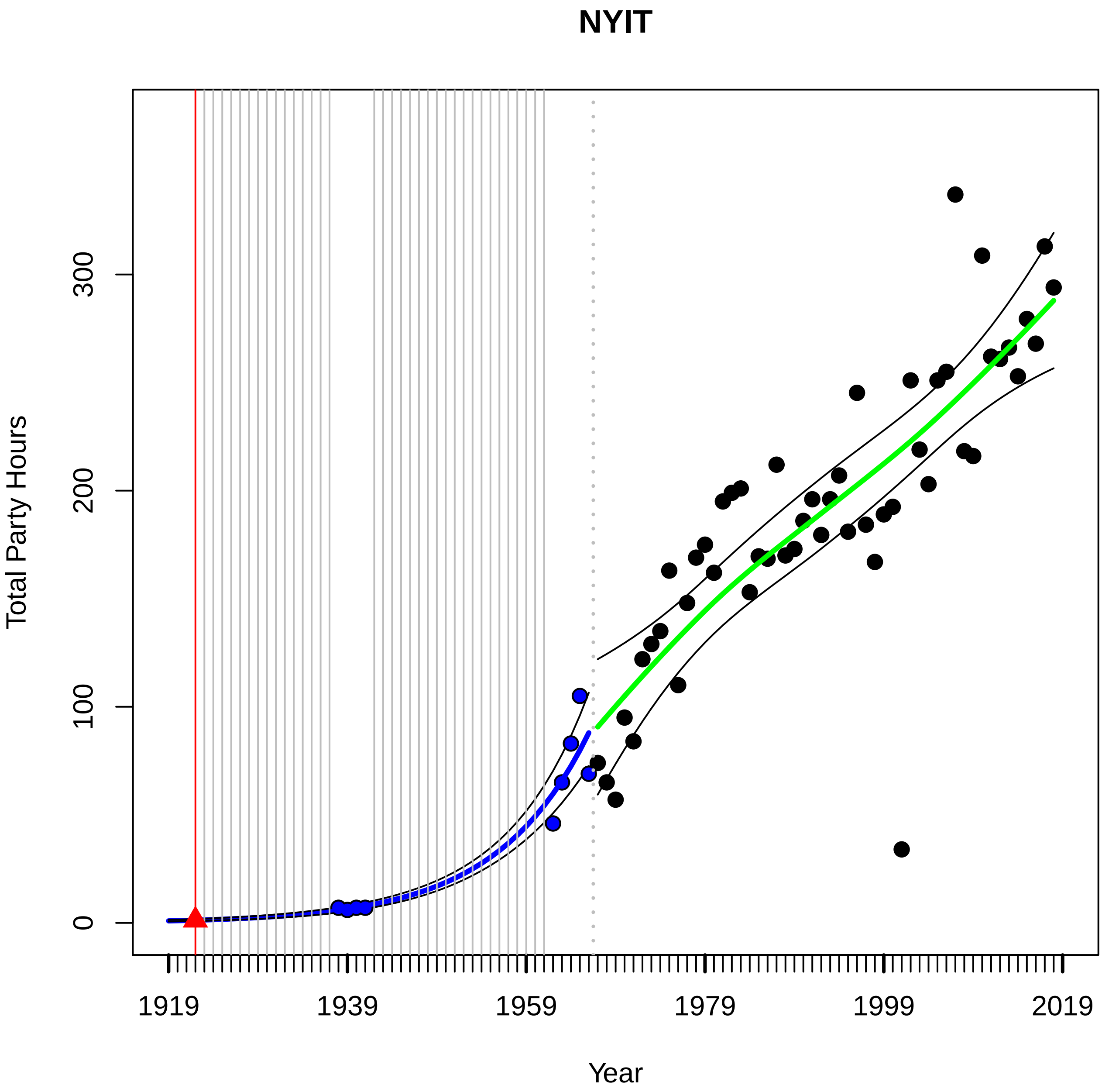
NYIT
Total Party Hours
0
100
200
300
1919
1939
1959
1979
1999
2019
Year

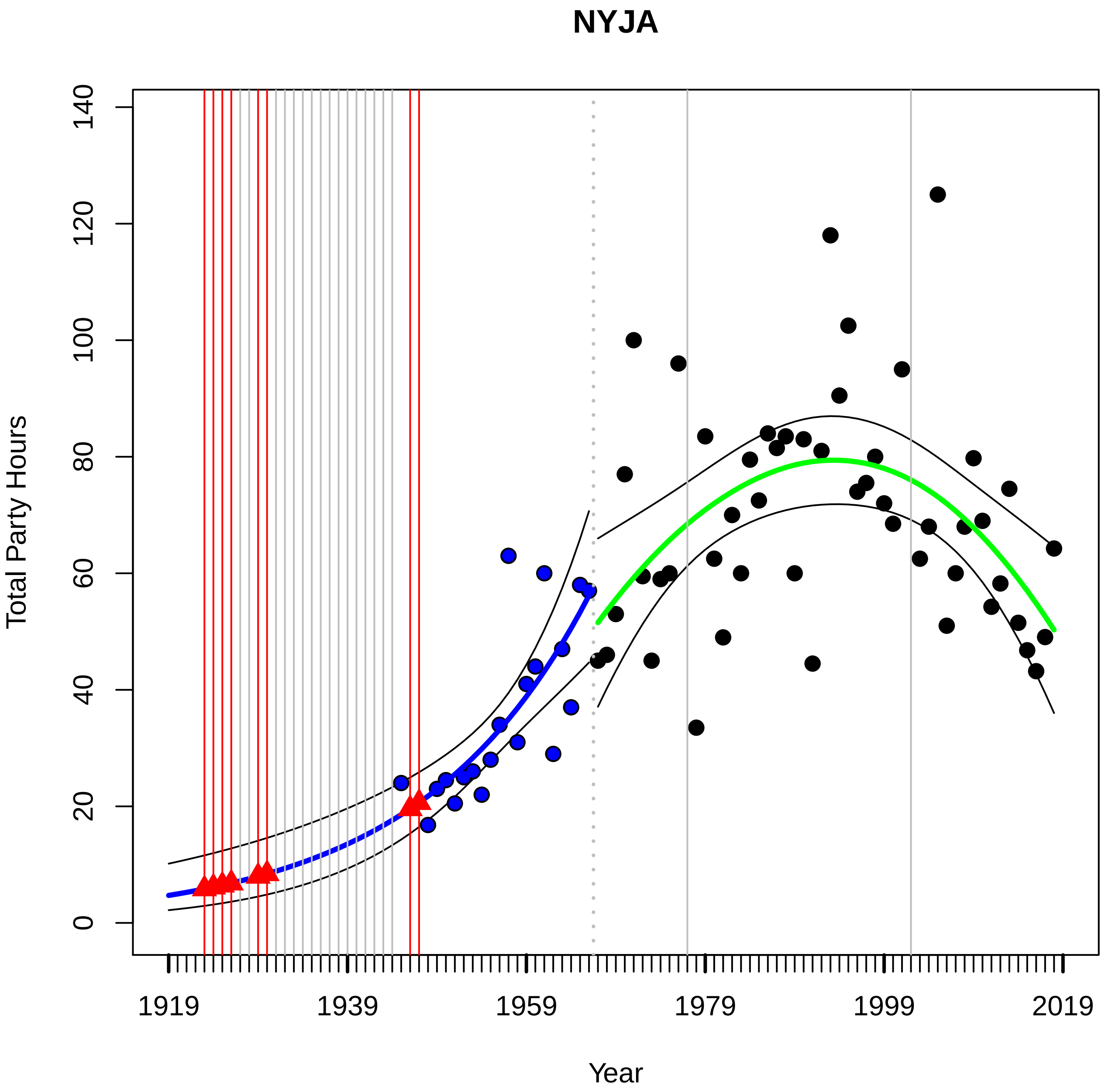

NYJA
Total Party Hours
0
20
40
60
80
100
120
140
1919
1939
1959
1979
1999
2019
Year

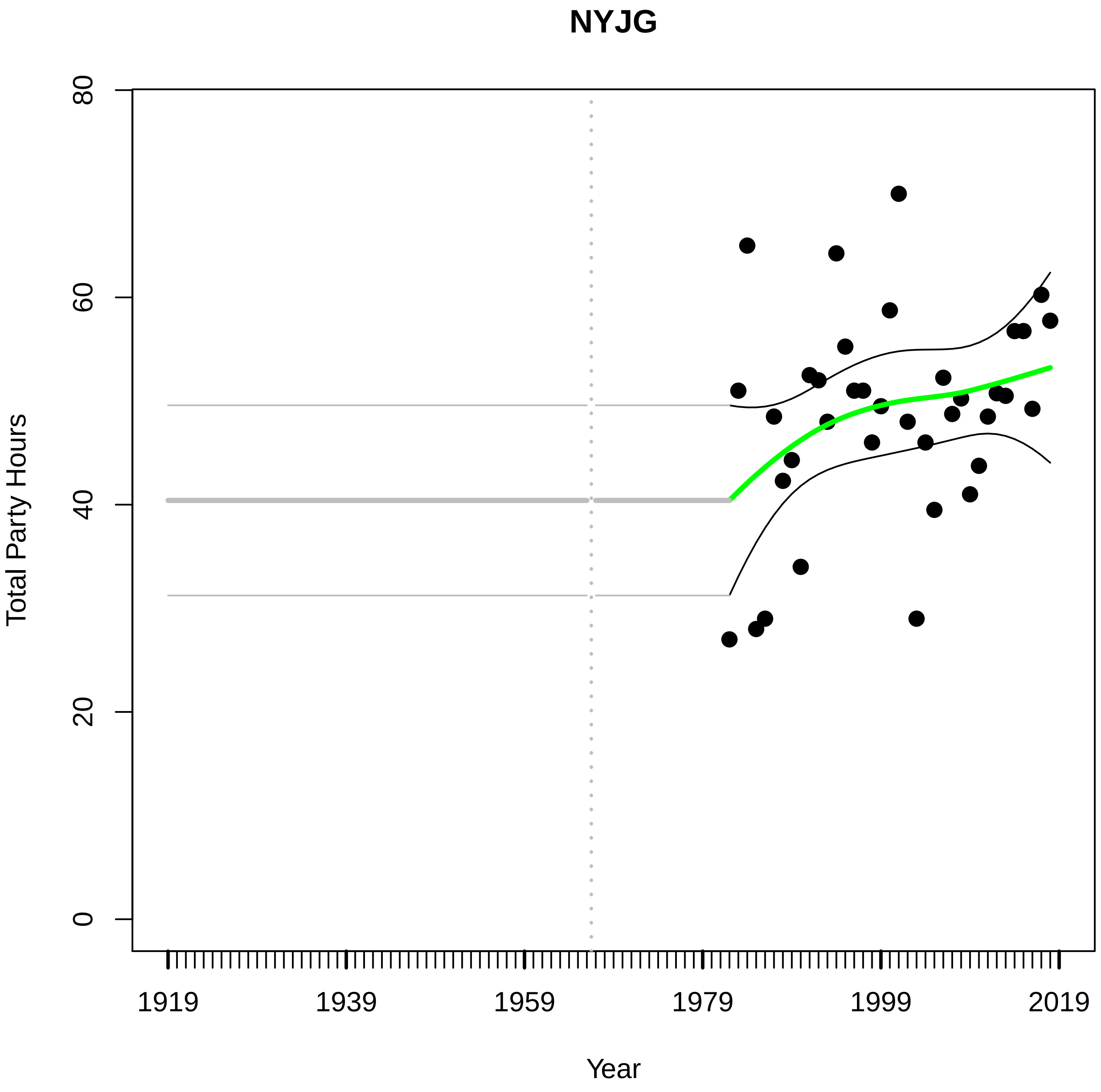

NYJG
Total Party Hours
80
60
40
20
0
1919
1939
1959
1979
1999
2019
Year

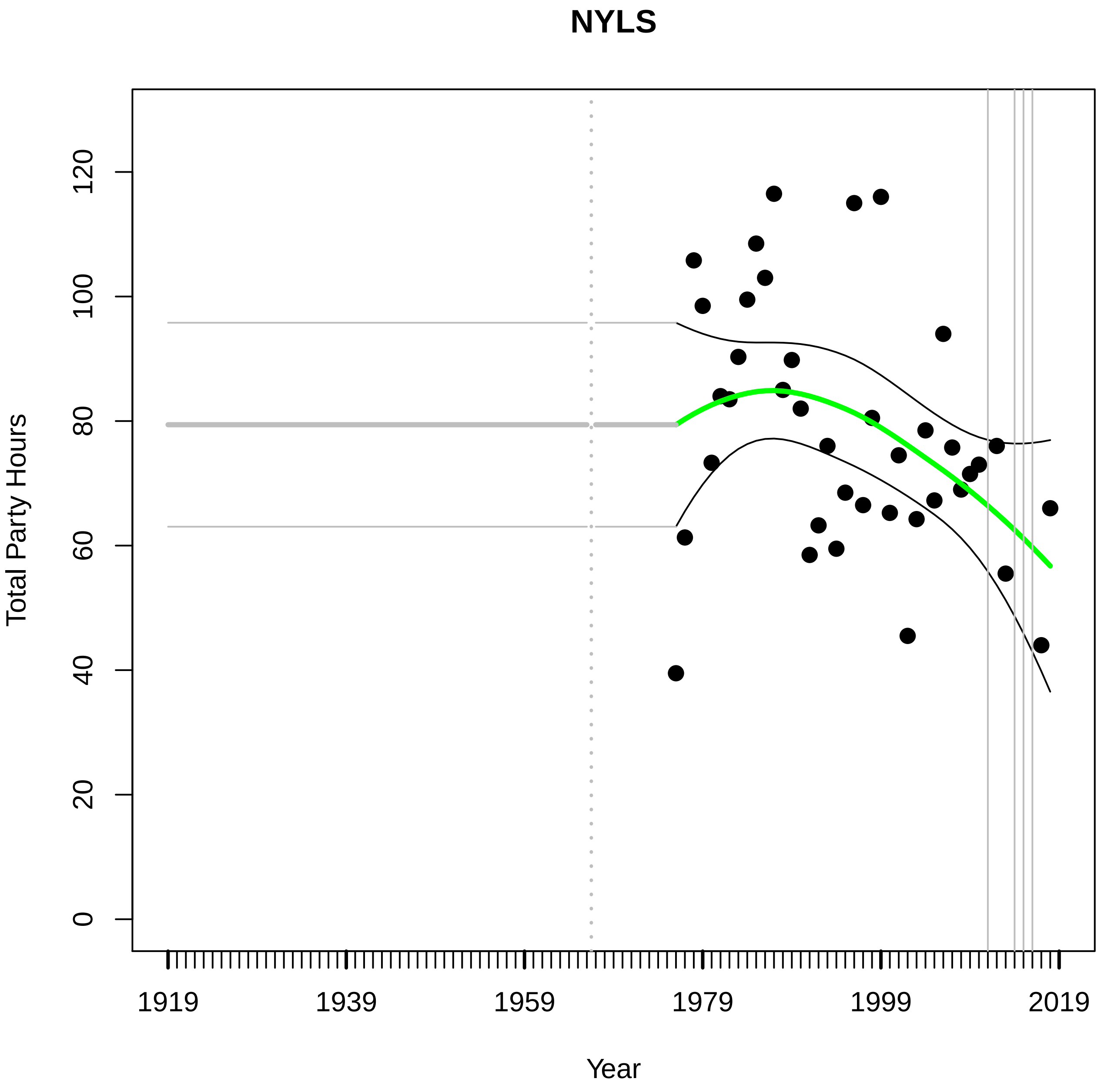

NYLS
Total Party Hours
0
20
40
60
80
100
120
1919
1939
1959
1979
1999
2019
Year

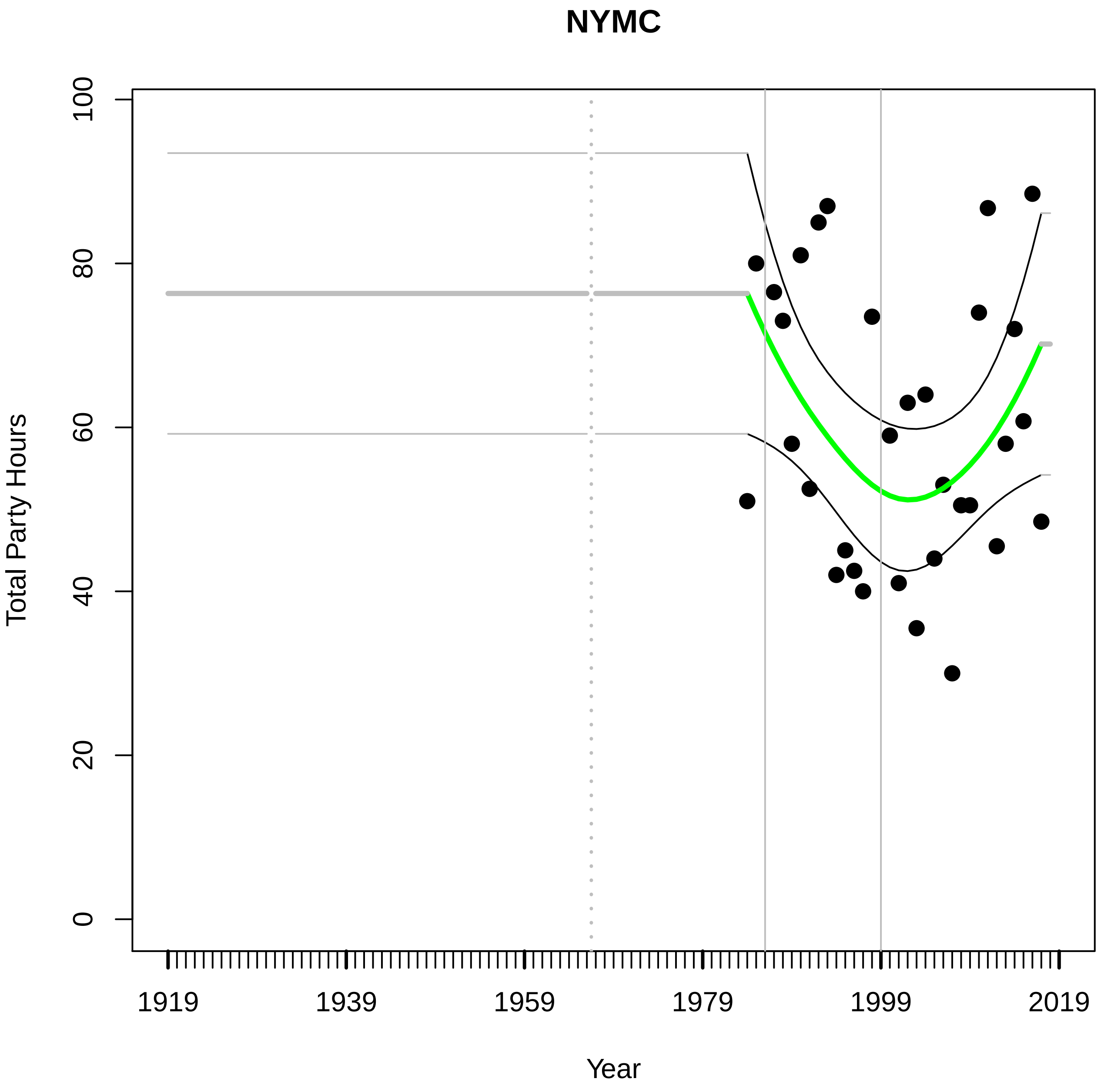

NYMC
Total Party Hours
0
20
40
60
80
100
1919
1939
1959
1979
1999
2019
Year

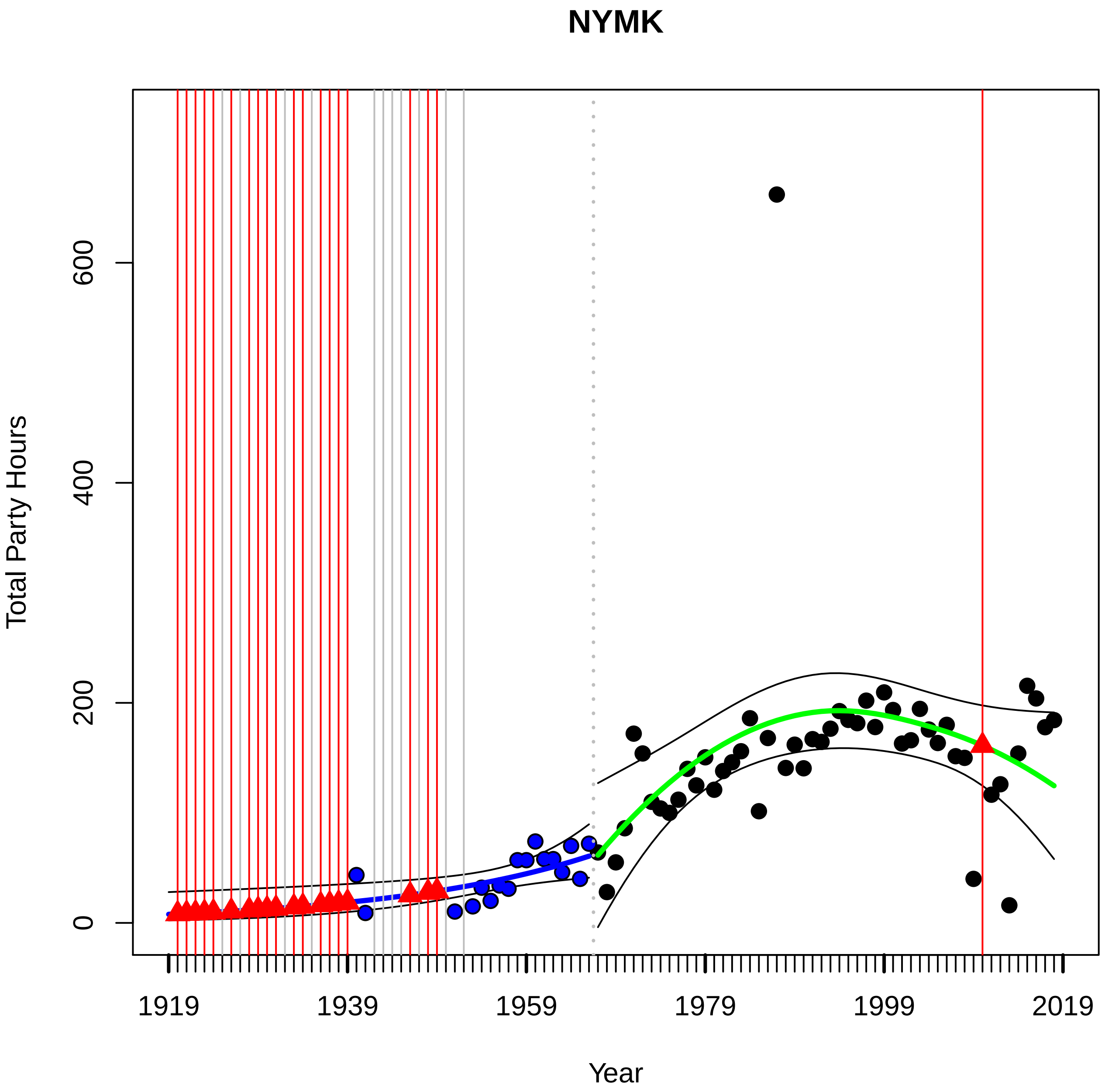

NYMK
Total Party Hours
0
200
400
600
1919
1939
1959
1979
1999
2019
Year

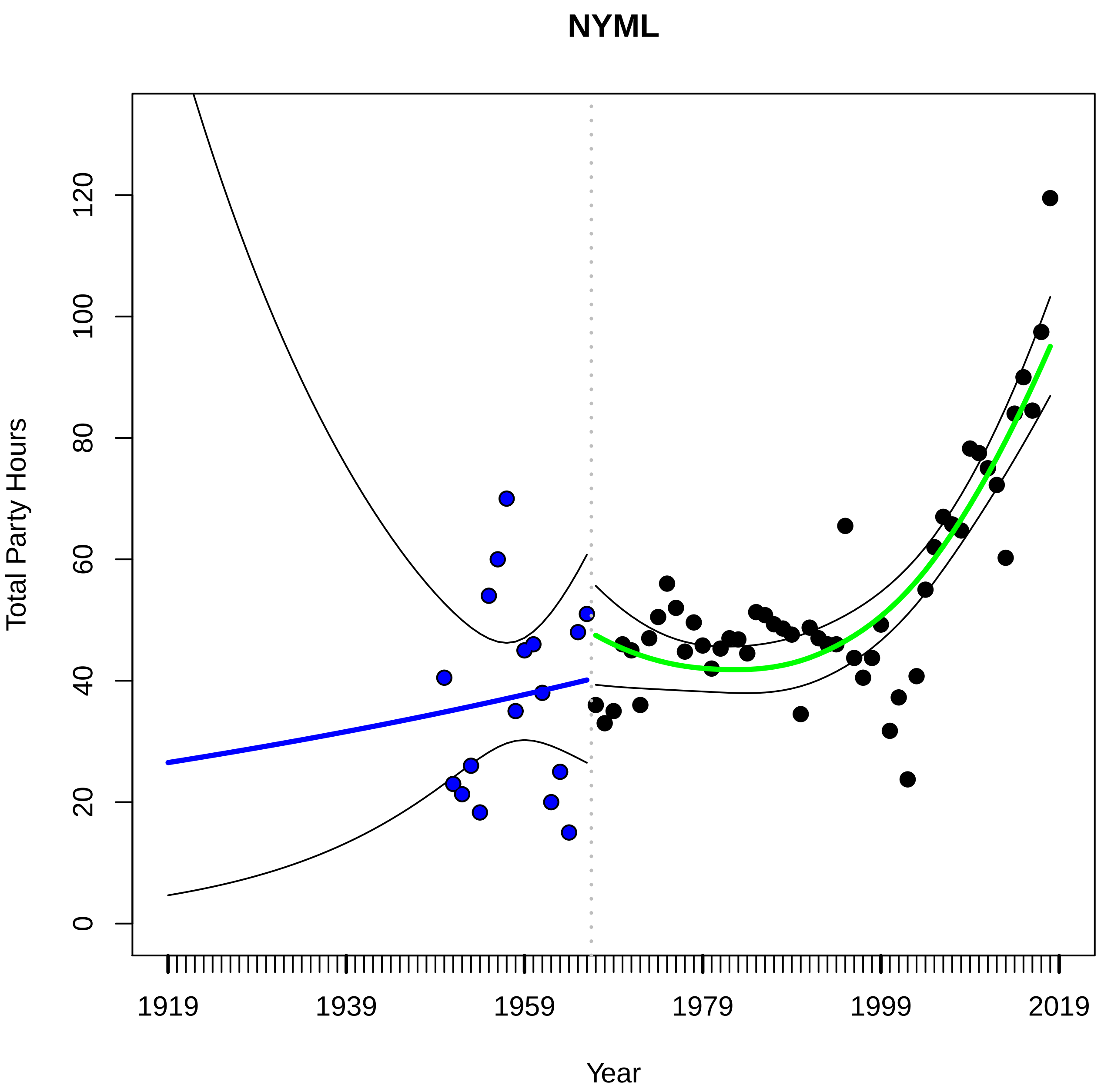
NYML
Total Party Hours
Year
0
20
40
60
80
100
120
1919
1939
1959
1979
1999
2019

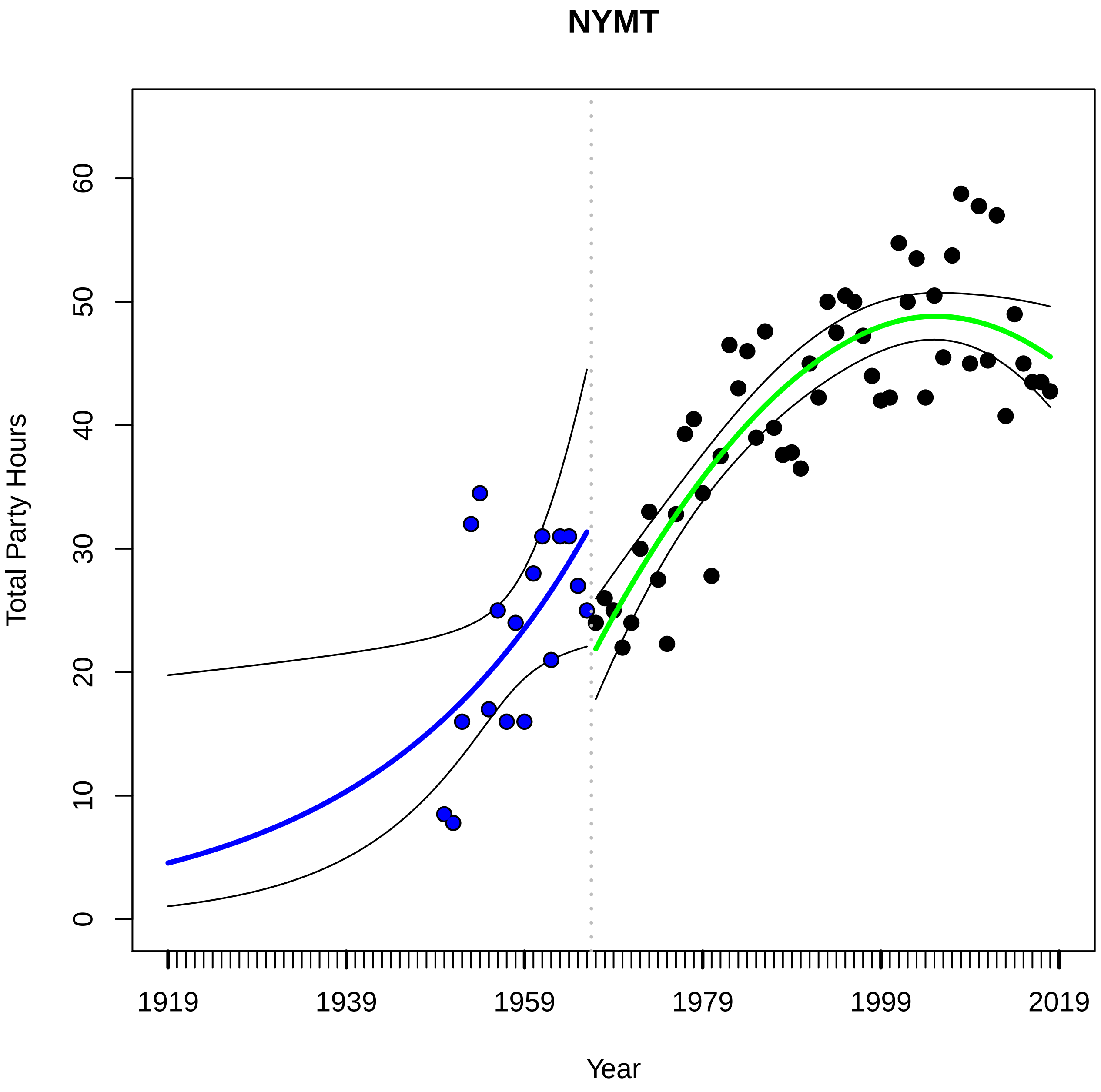
NYMT
Total Party Hours
0
10
20
30
40
50
60
1919
1939
1959
1979
1999
2019
Year

NYMZ

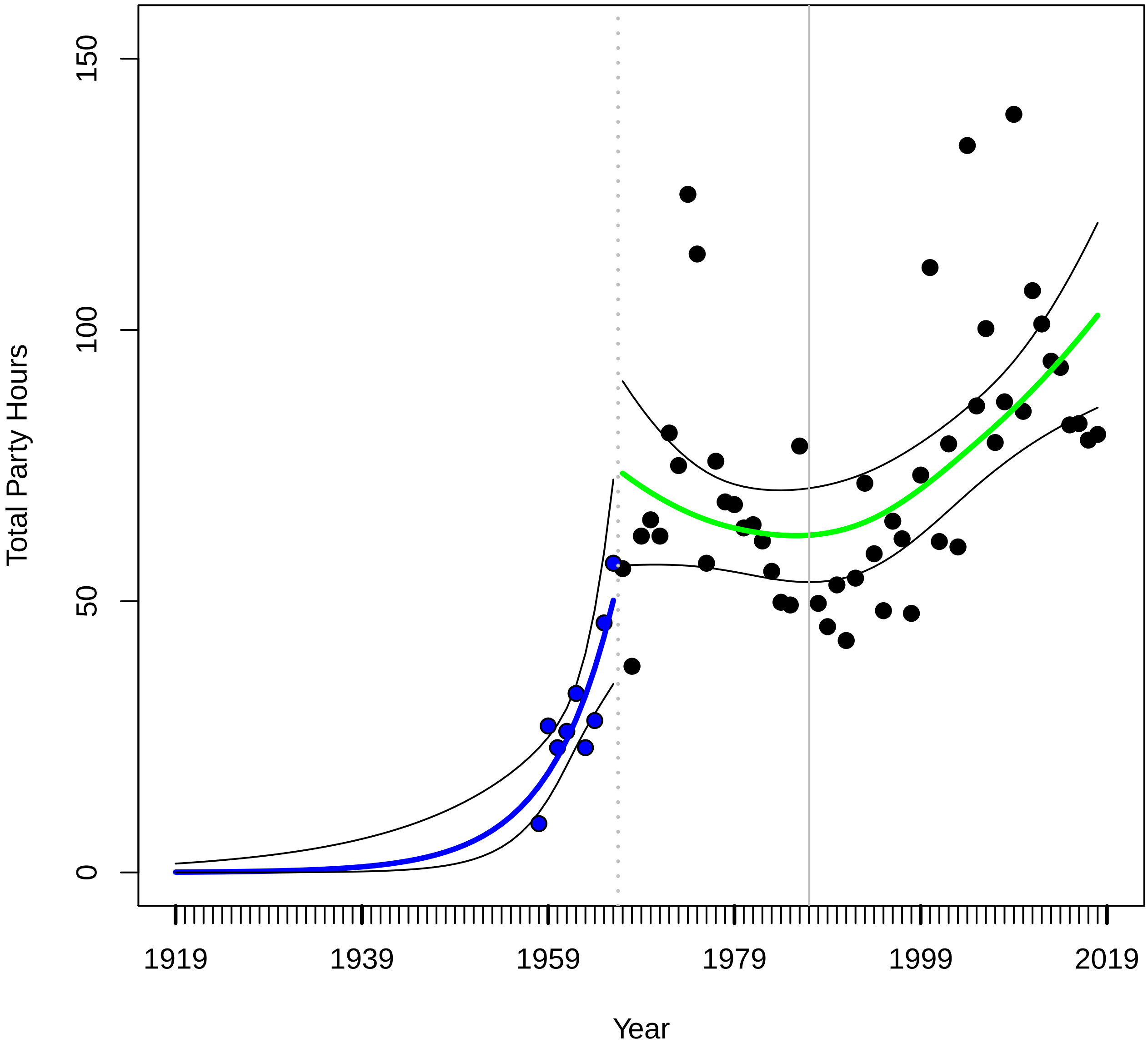

Total Party Hours
0
50
100
150
1919
1939
1959
1979
1999
2019
Year

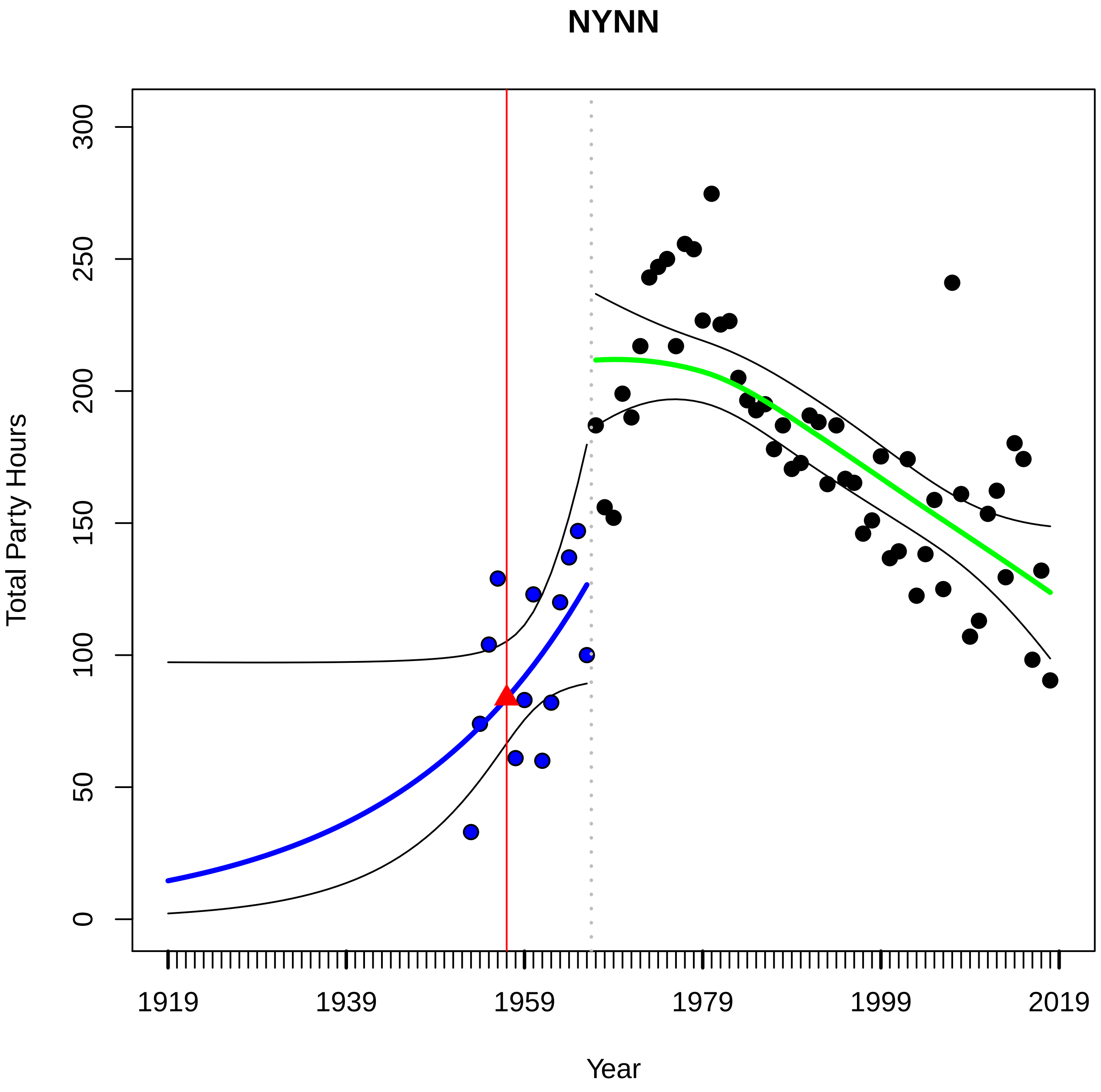
NYNN
Total Party Hours
0
50
100
150
200
250
300
1919
1939
1959
1979
1999
2019
Year

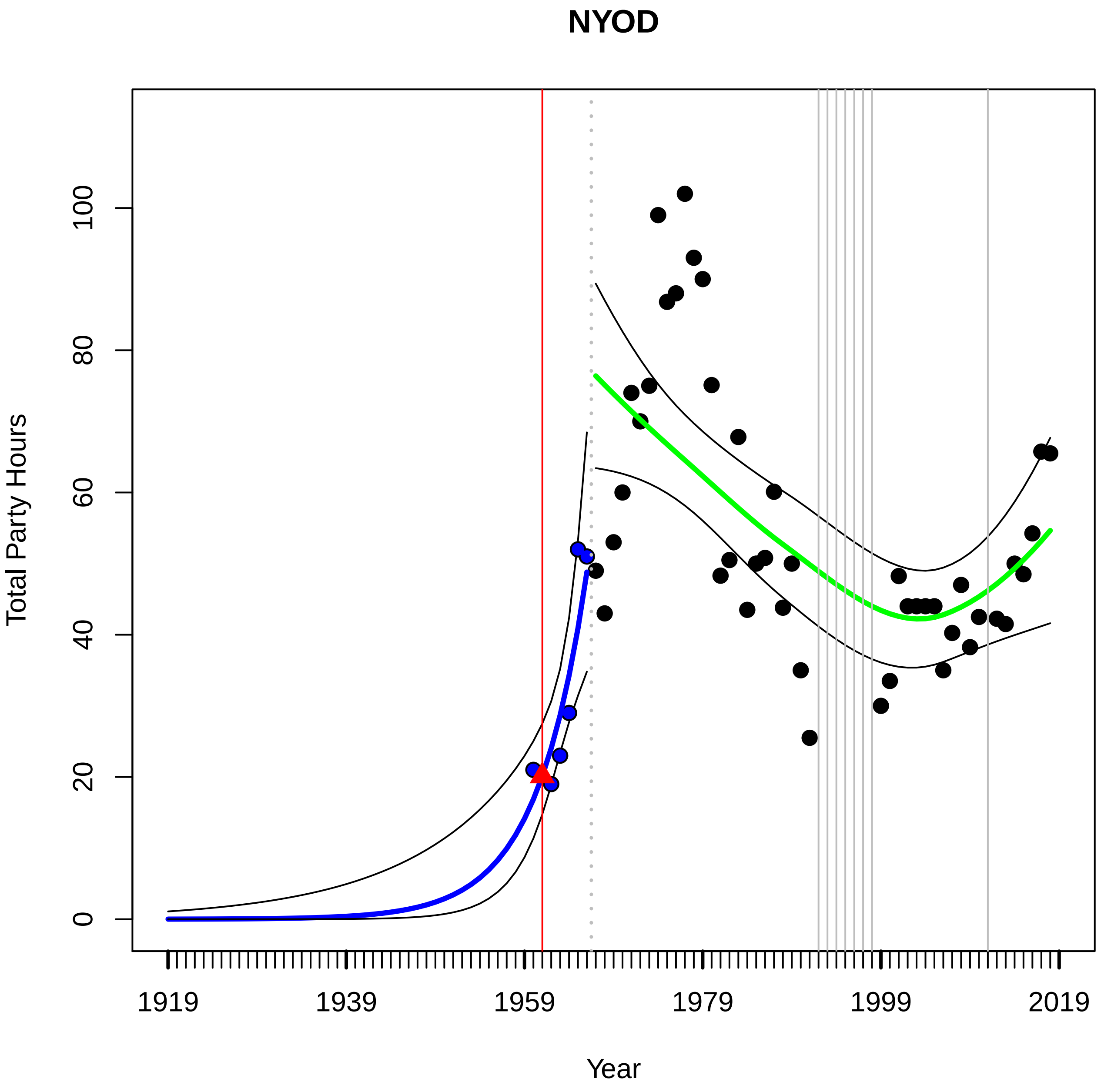
NYOD
Total Party Hours
Year
0
20
40
60
80
100
1919
1939
1959
1979
1999
2019

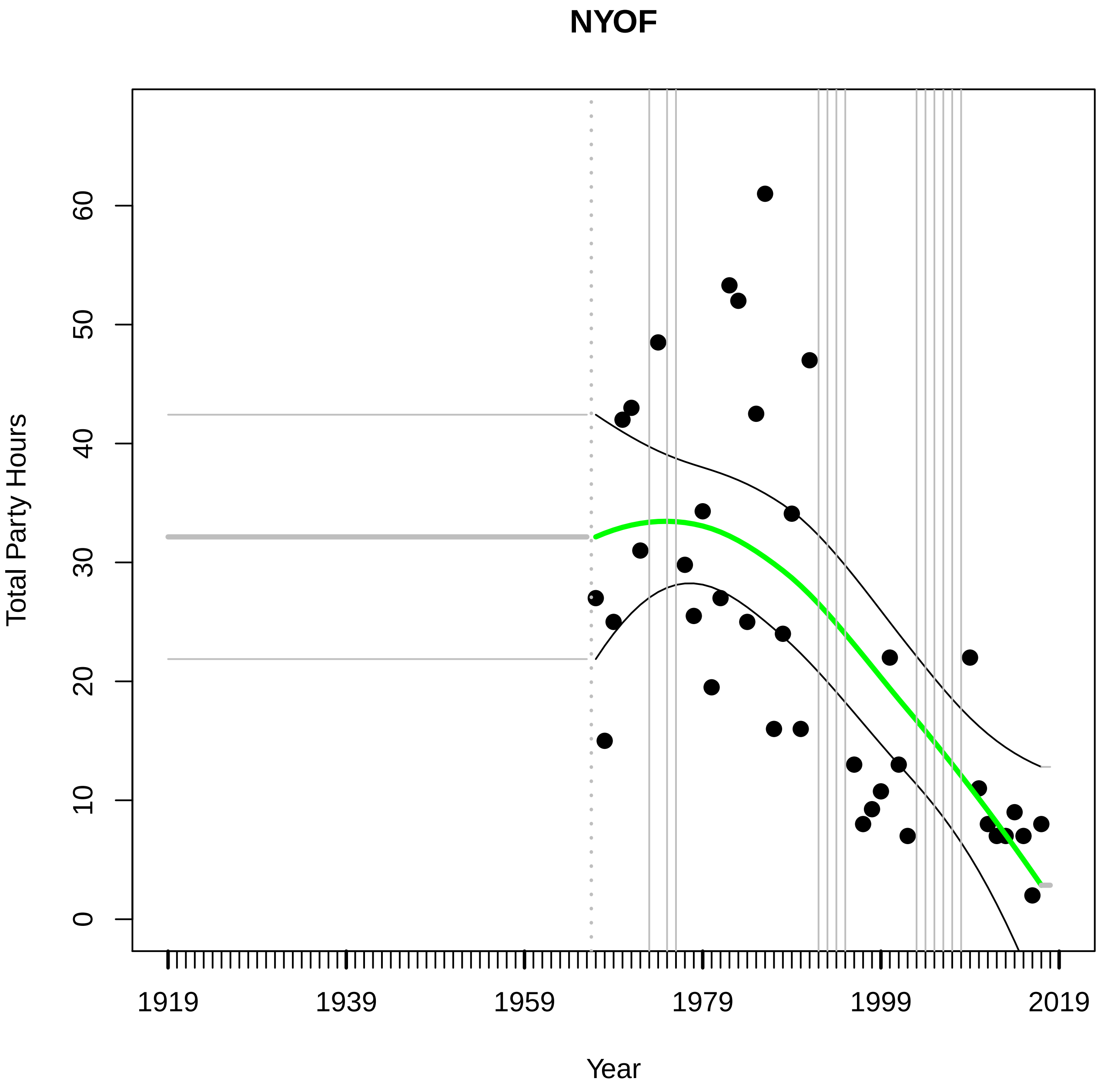
NYOF
Total Party Hours
0
10
20
30
40
50
60
1919
1939
1959
1979
1999
2019
Year

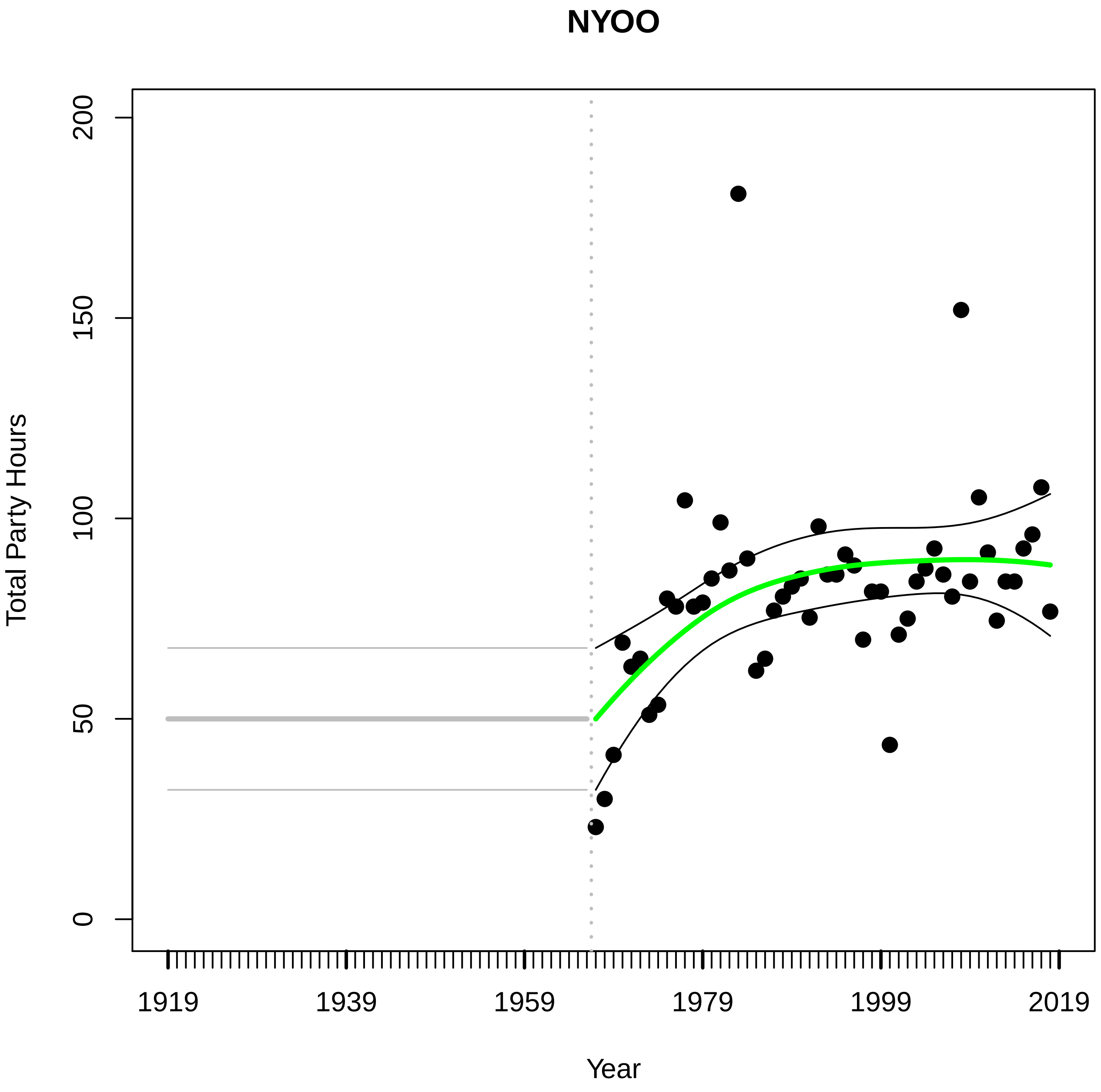

NYOO
Total Party Hours
200
150
100
50
0
1919
1939
1959
1979
1999
2019
Year

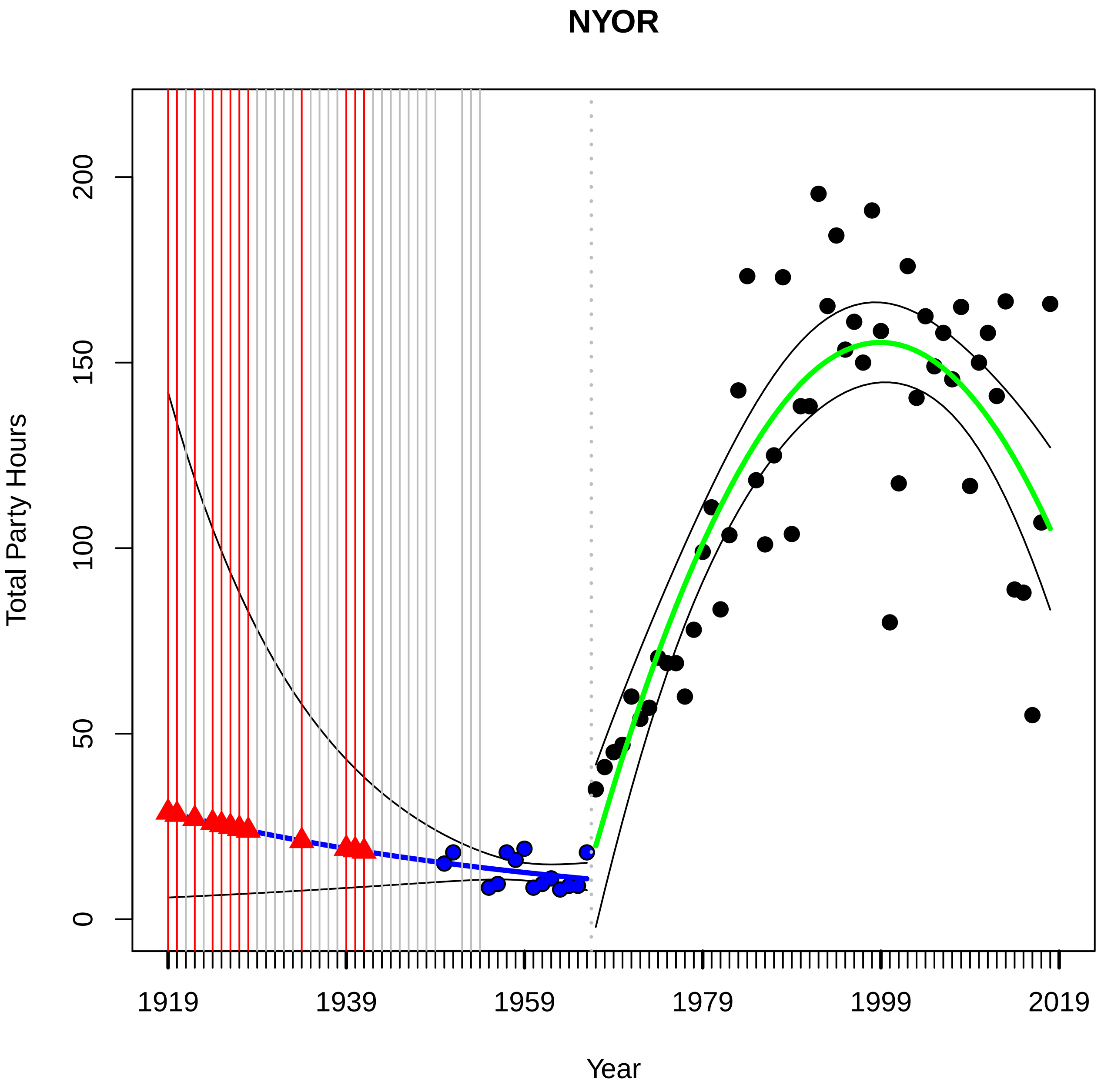
NYOR
Total Party Hours
0
50
100
150
200
1919
1939
1959
1979
1999
2019
Year

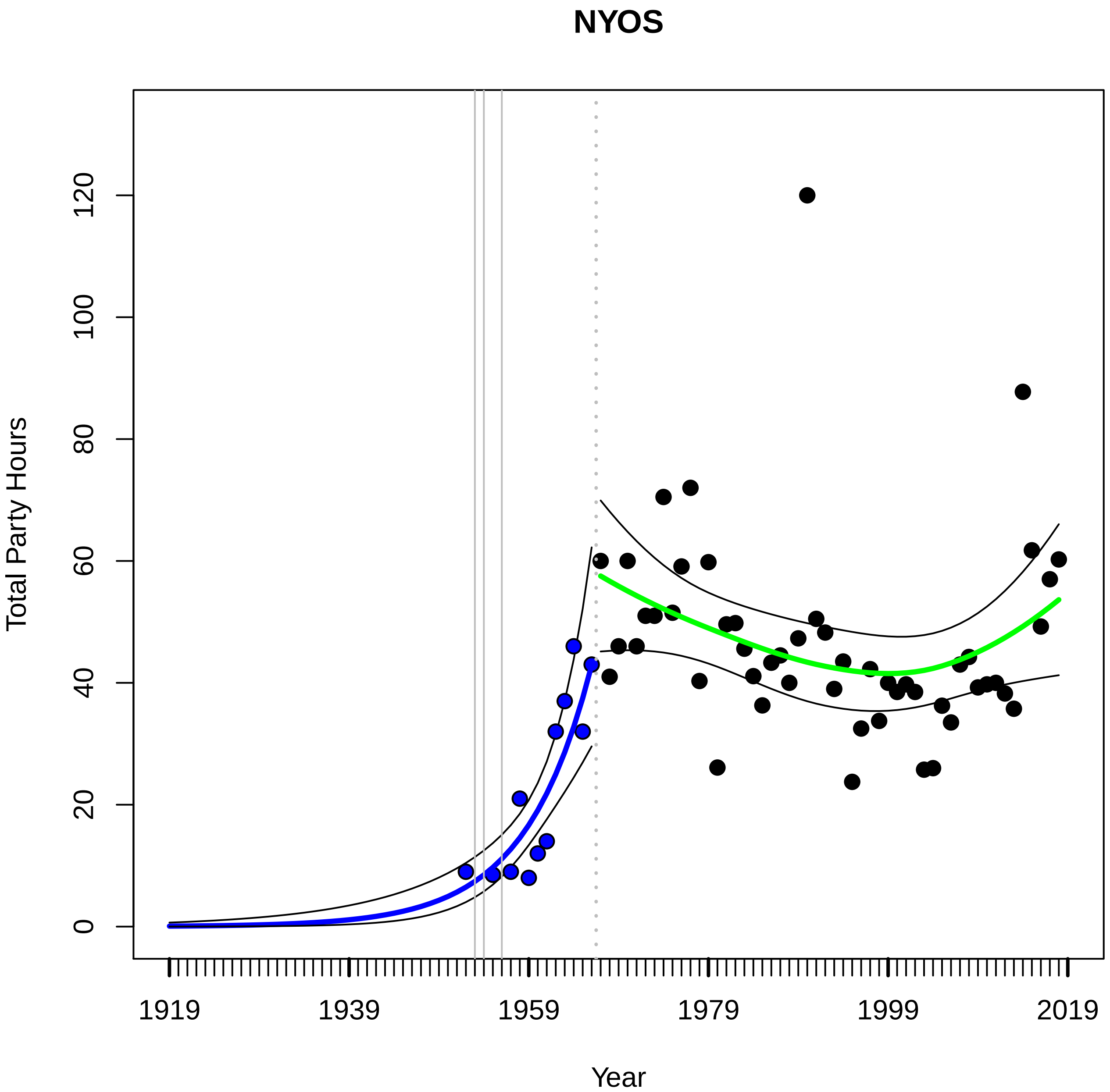

NYOS
Total Party Hours
0
20
40
60
80
100
120
1919
1939
1959
1979
1999
2019
Year

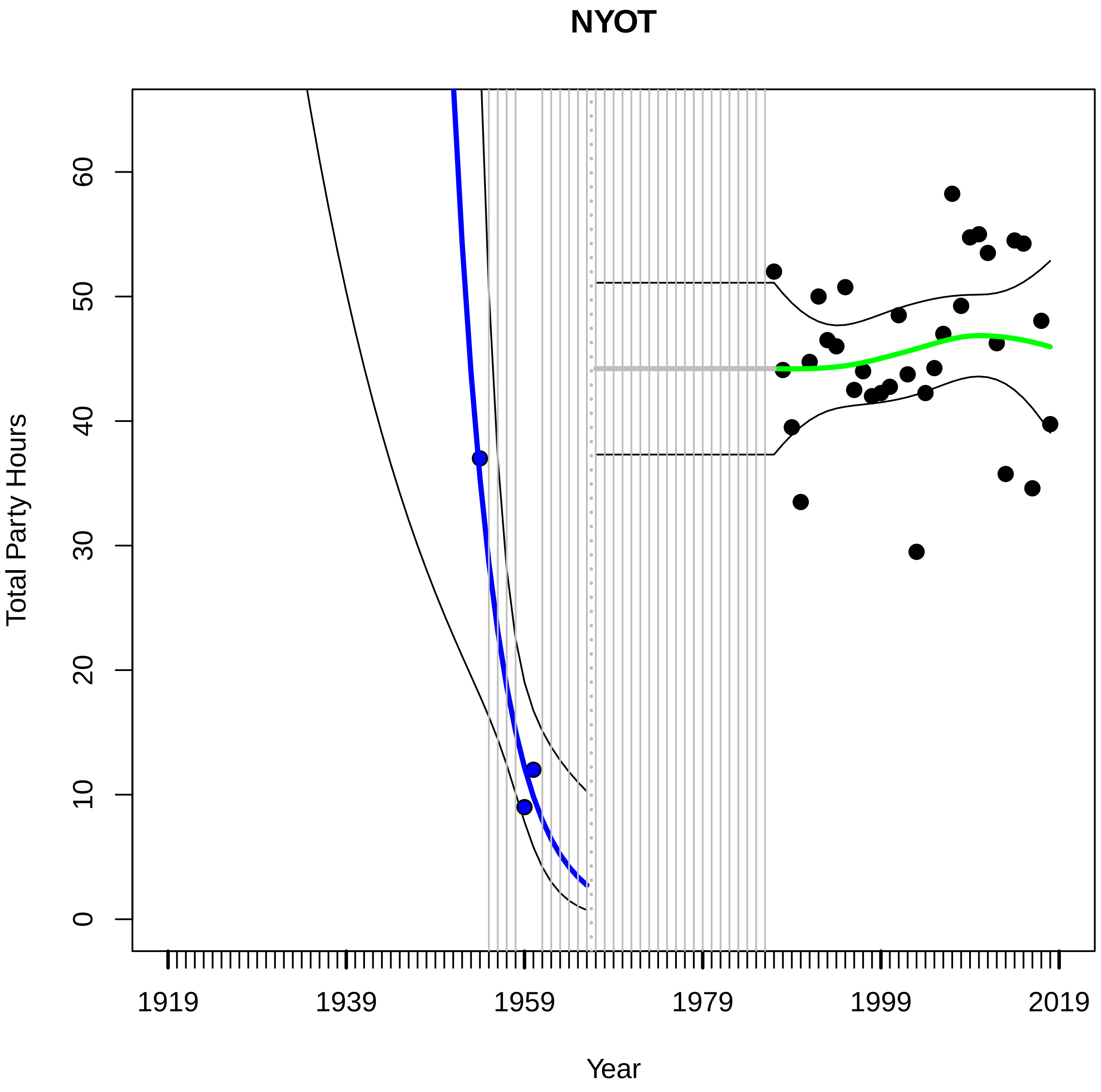

NYOT
Total Party Hours
0
10
20
30
40
50
60
1919
1939
1959
1979
1999
2019
Year

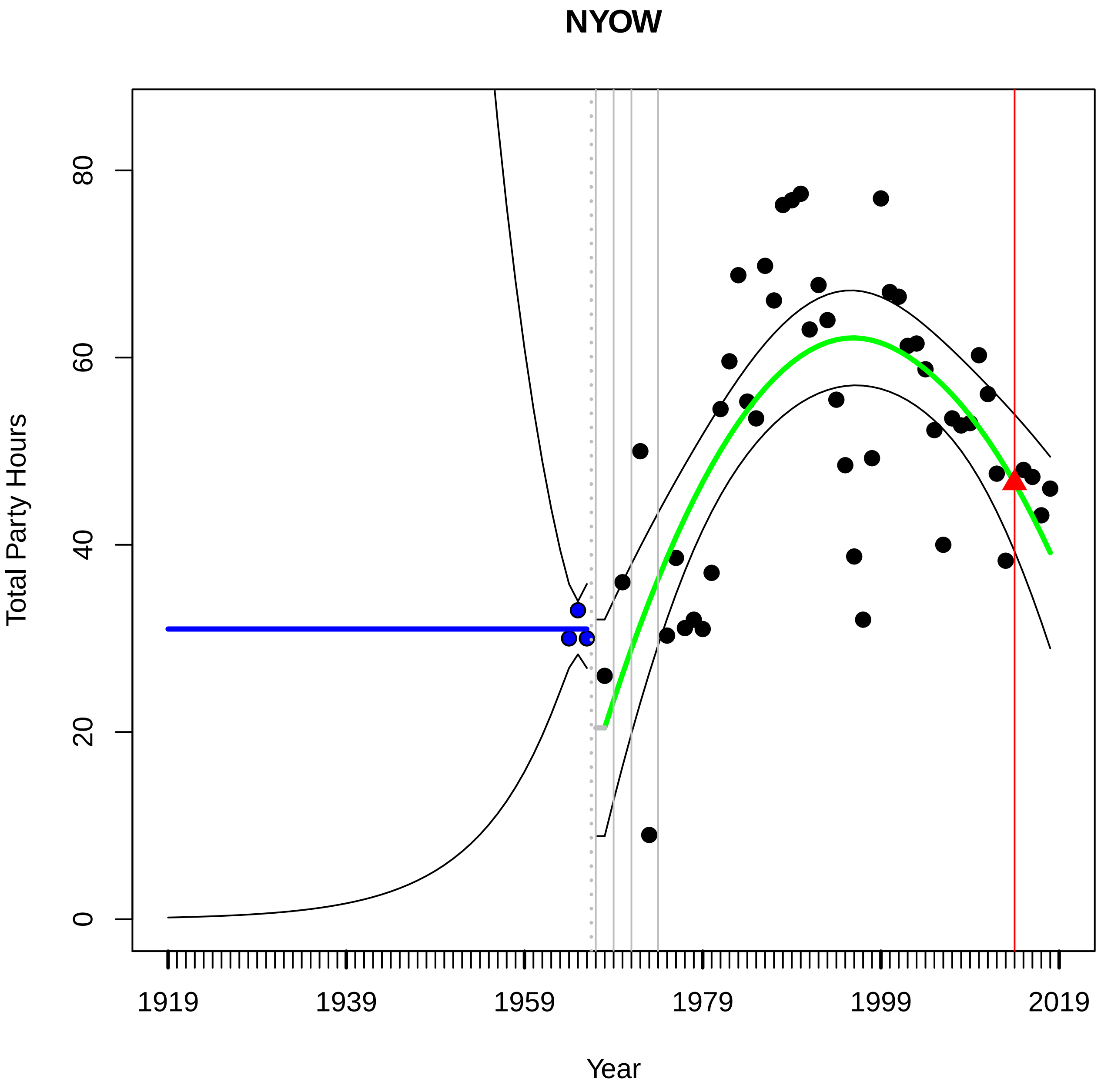

NYOW
Total Party Hours
0
20
40
60
80
1919
1939
1959
1979
1999
2019
Year

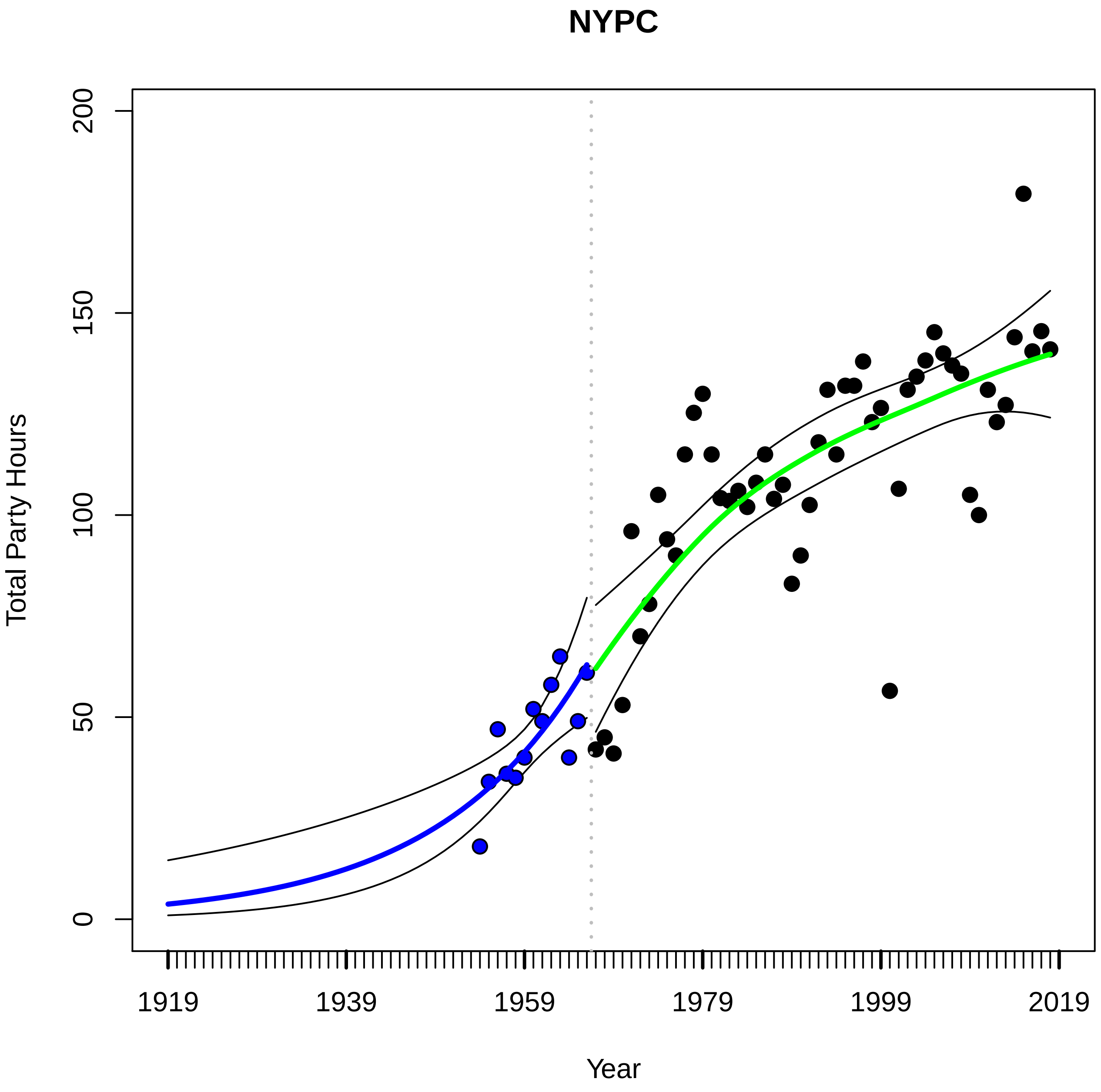

NYPC
Total Party Hours
0
50
100
150
200
1919
1939
1959
1979
1999
2019
Year

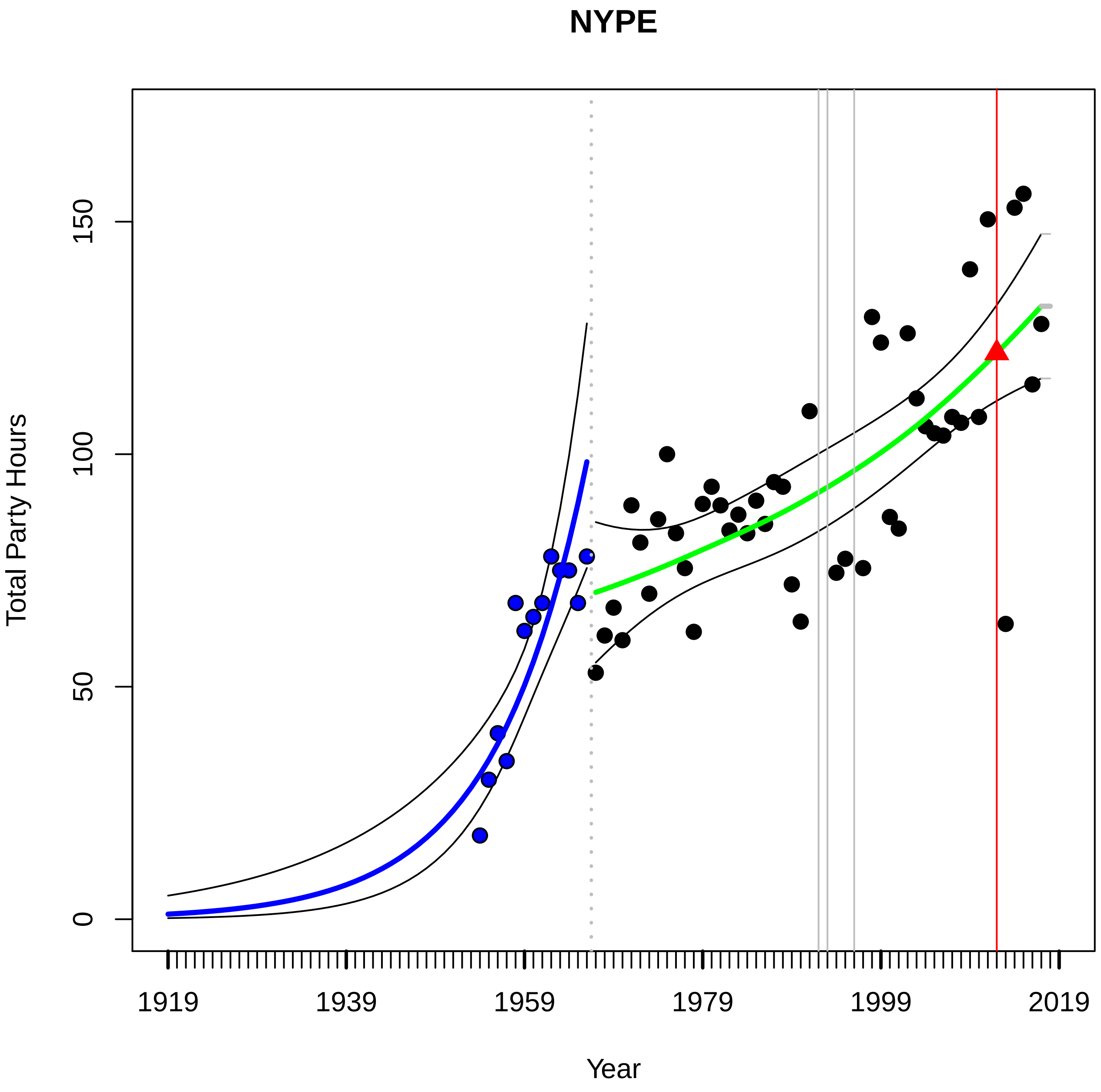
NYPE
Total Party Hours
0
50
100
150
1919
1939
1959
1979
1999
2019
Year

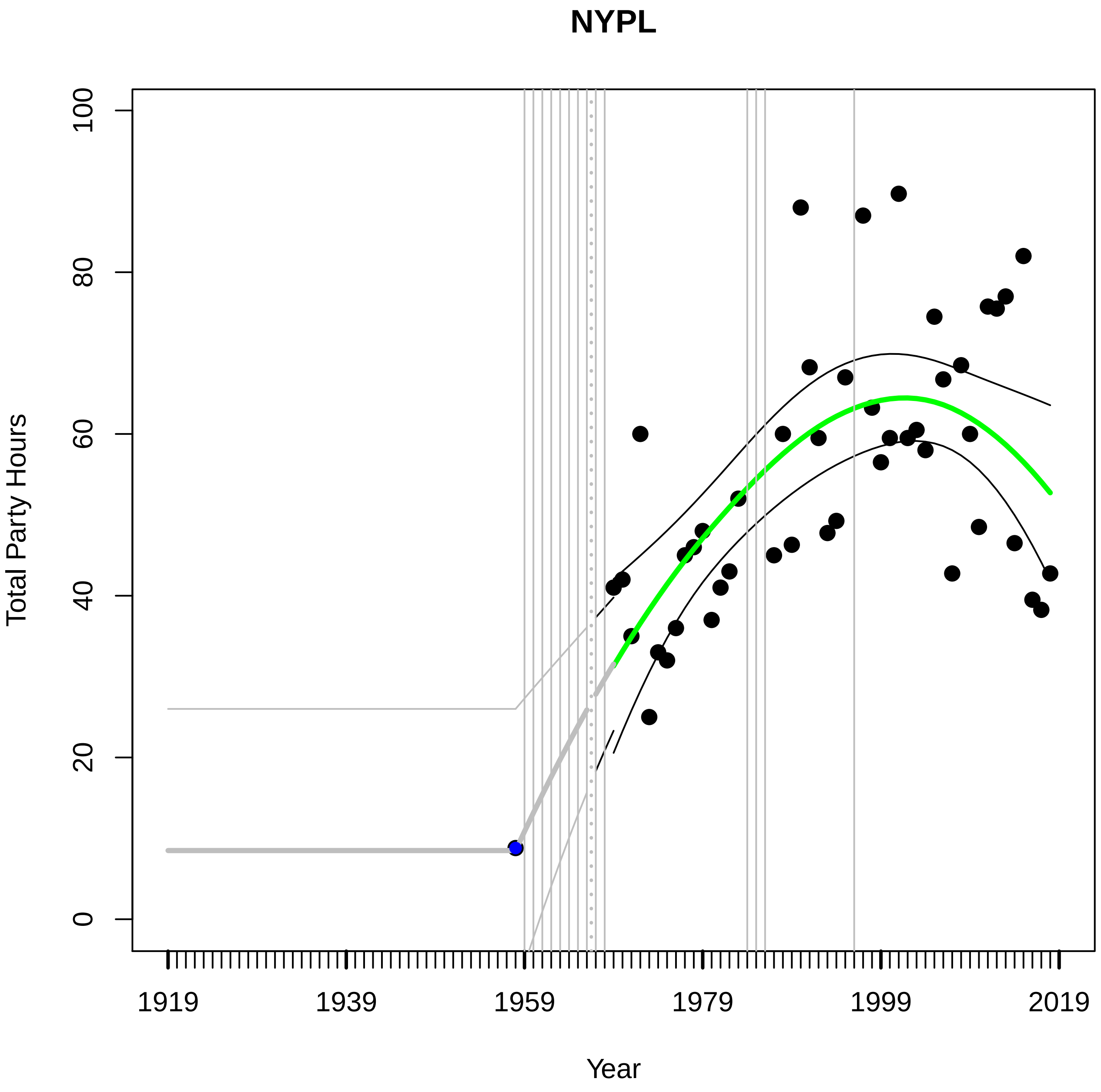

NYPL
Total Party Hours
Year
0
20
40
60
80
100
1919
1939
1959
1979
1999
2019

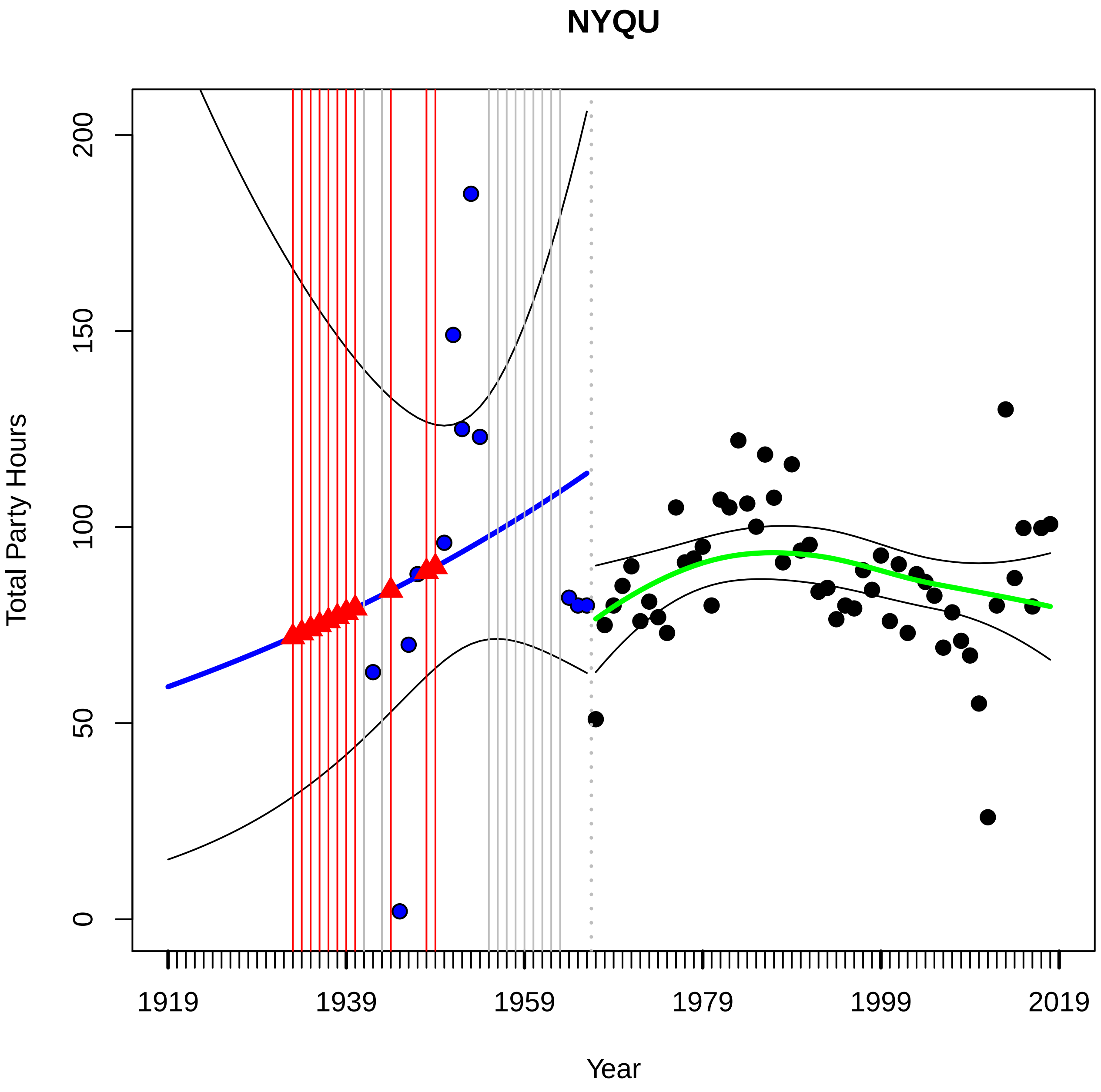
NYQU
Total Party Hours
0
50
100
150
200
Year
1919
1939
1959
1979
1999
2019

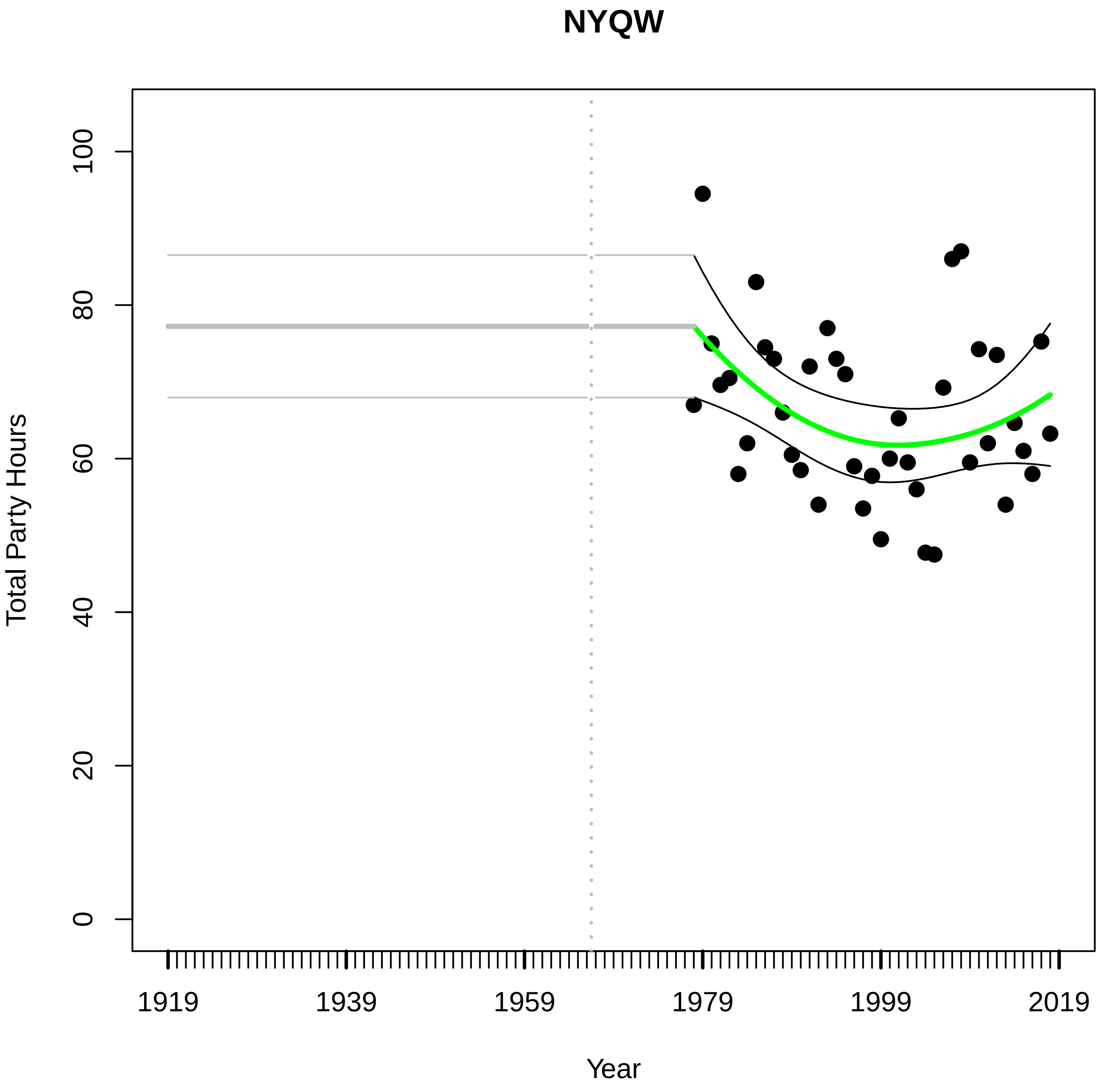
NYQW
Total Party Hours
0
20
40
60
80
100
1919
1939
1959
1979
1999
2019
Year

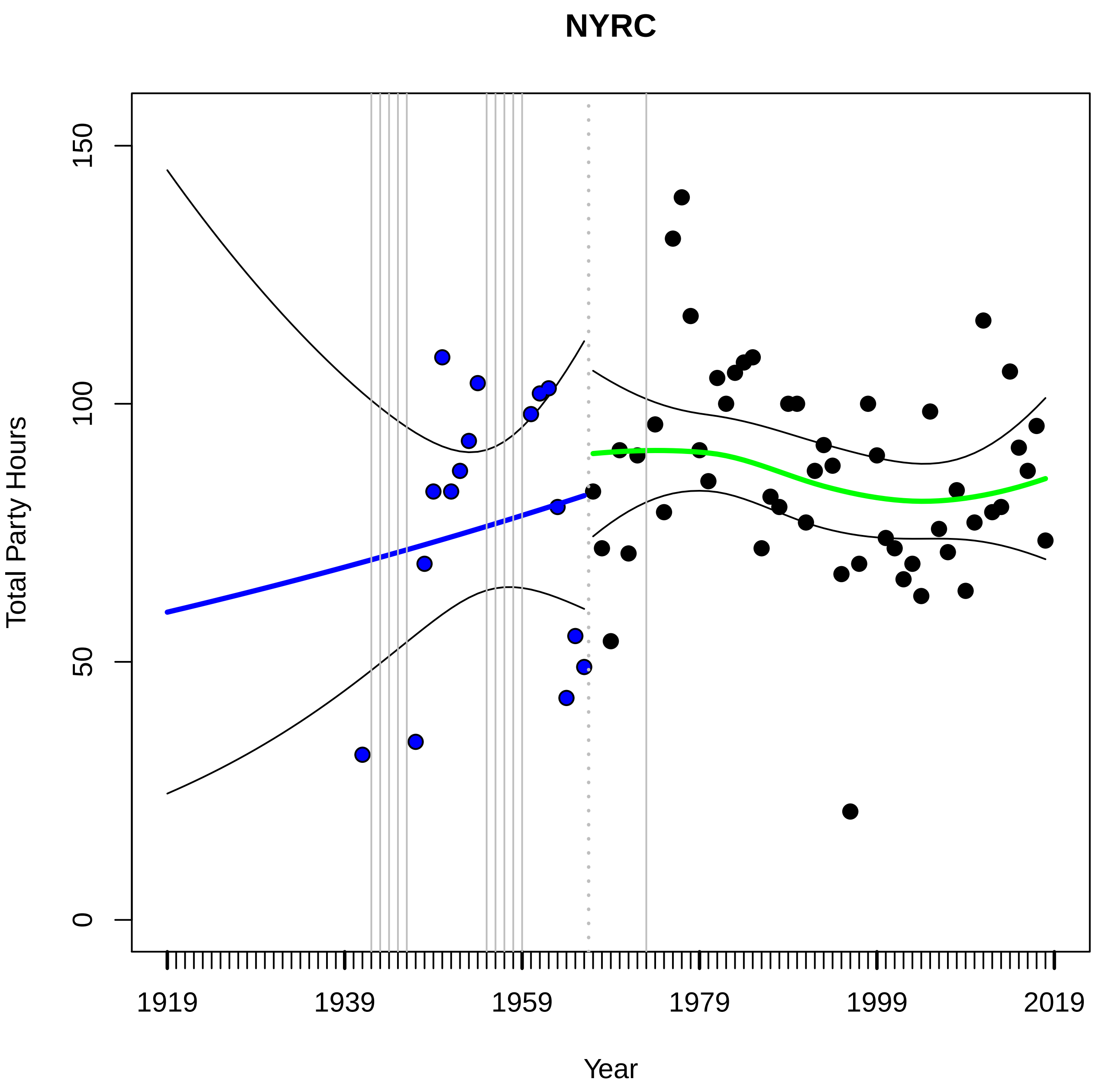
NYRC
Total Party Hours
0
50
100
150
1919
1939
1959
1979
1999
2019
Year

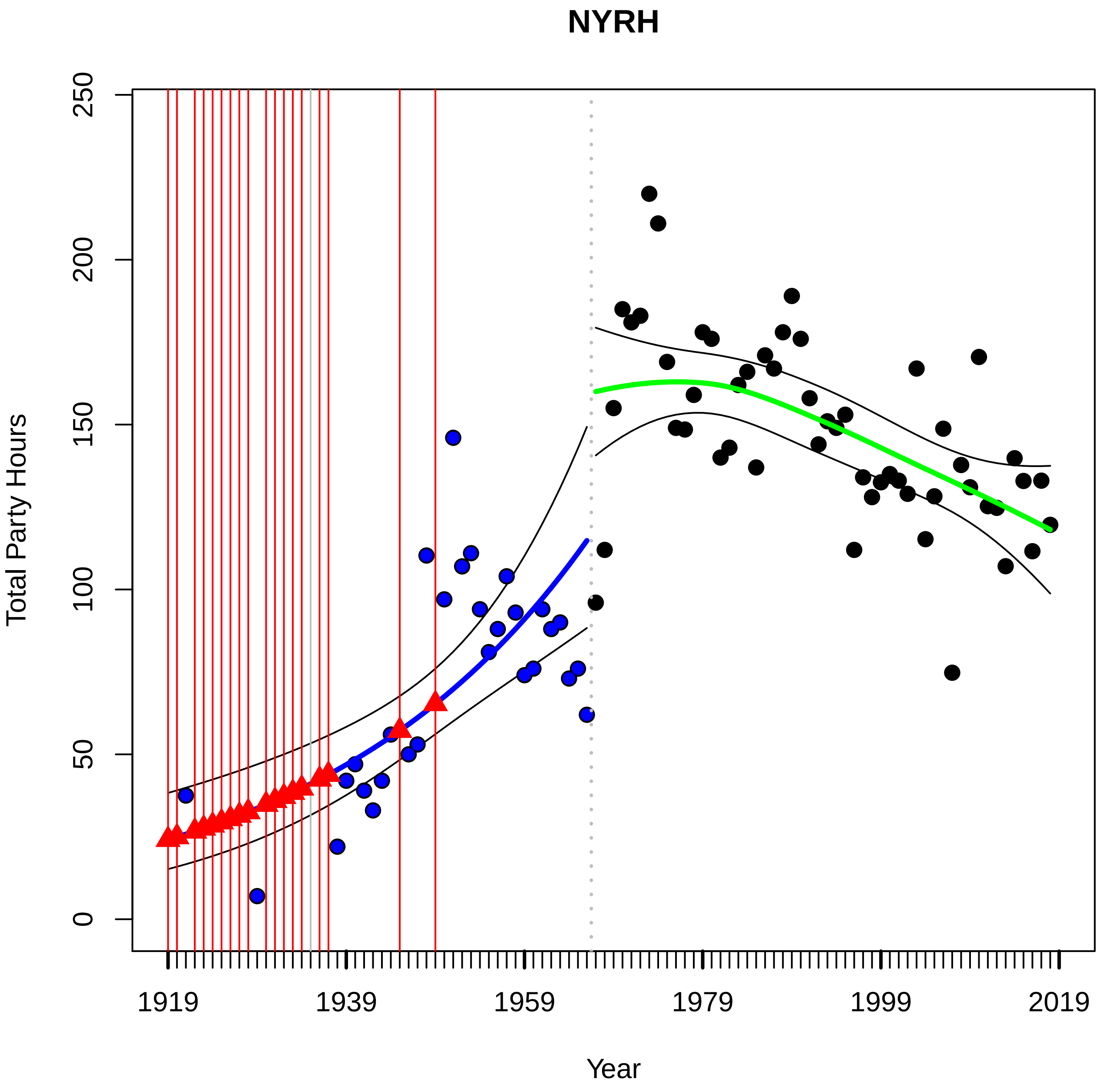

NYRH
Total Party Hours
0
50
100
150
200
250
1919
1939
1959
1979
1999
2019
Year

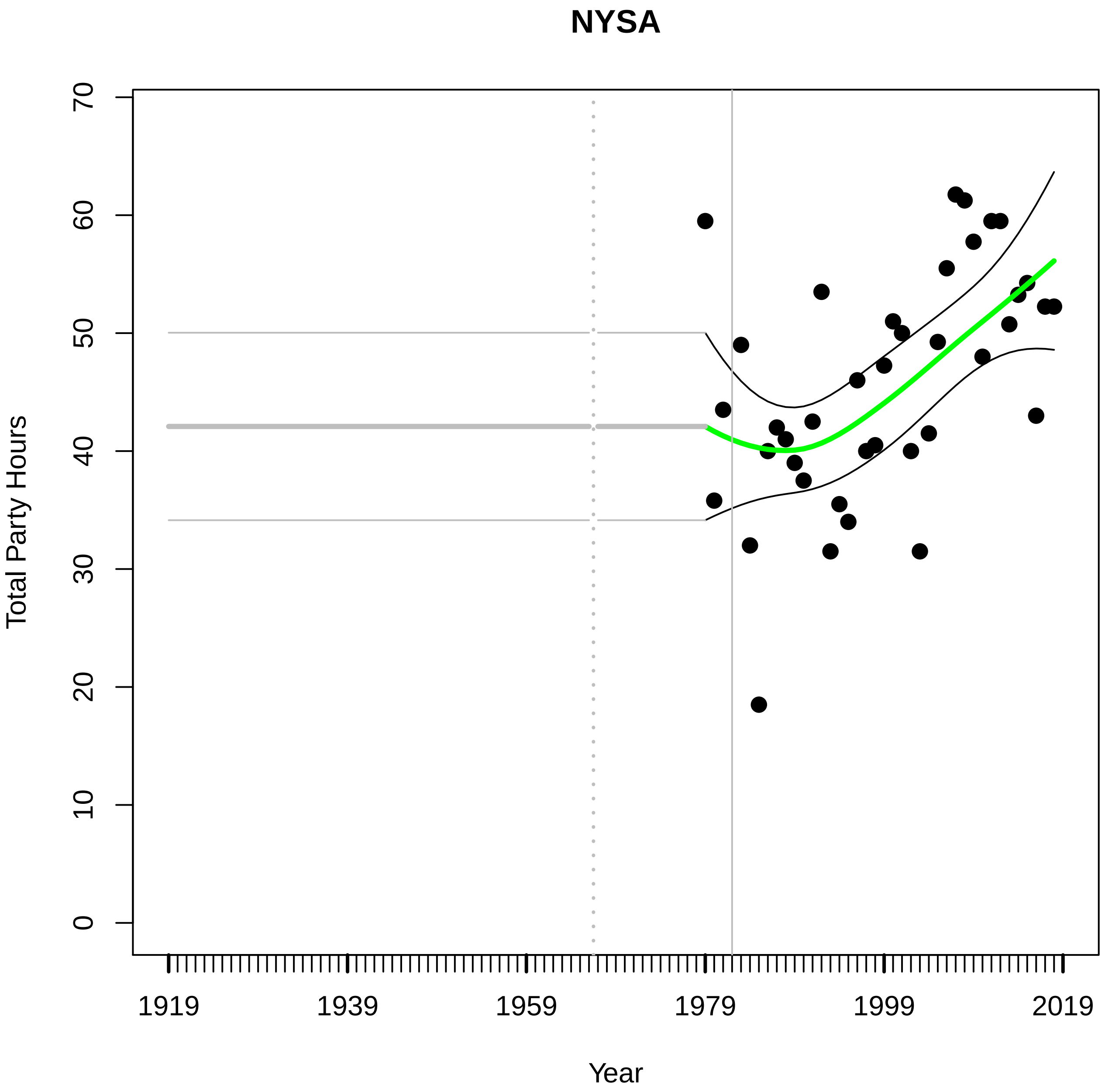

NYSA
Total Party Hours
0
10
20
30
40
50
60
70
1919
1939
1959
1979
1999
2019
Year

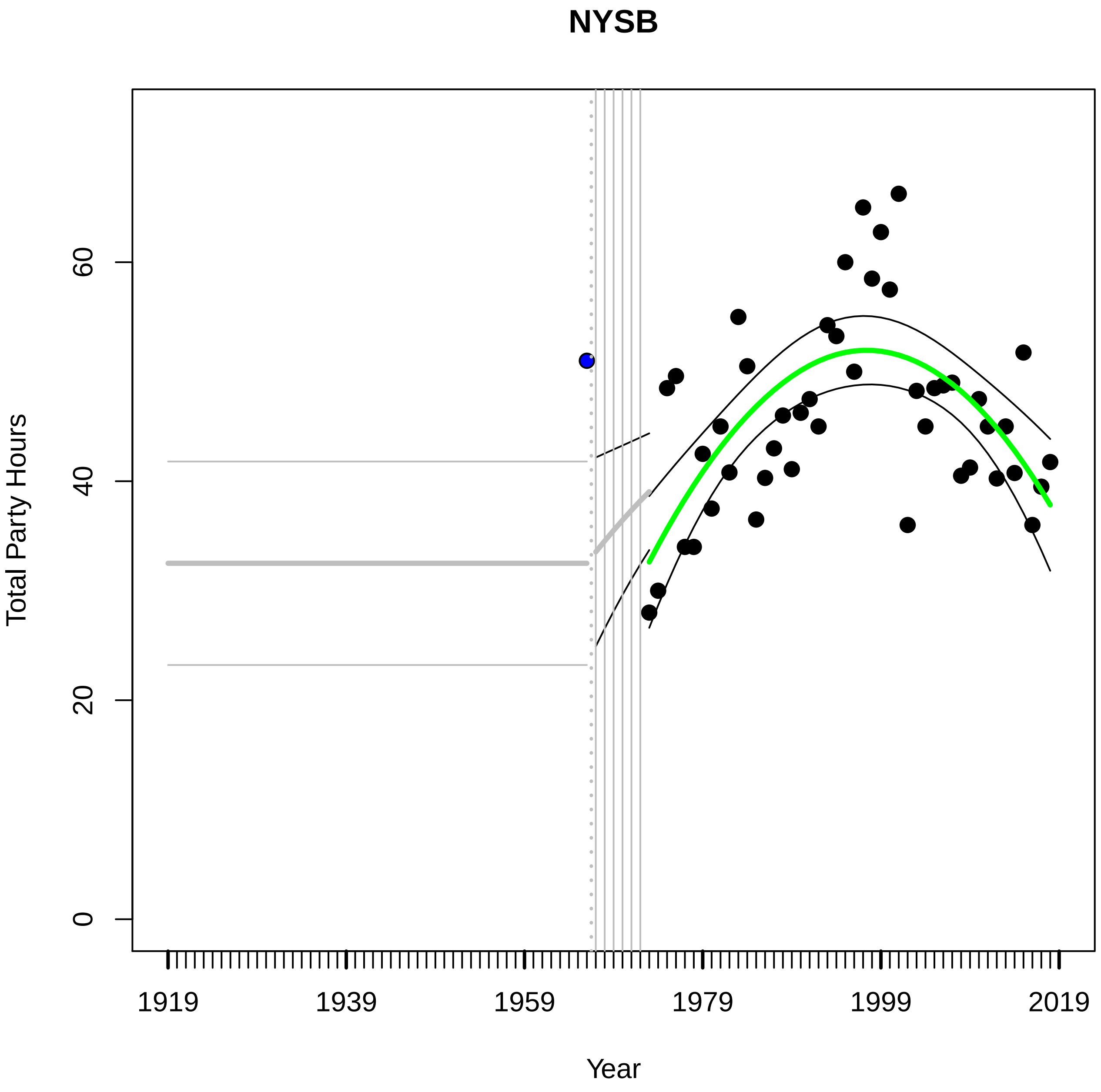
NYSB
Total Party Hours
0
20
40
60
Year
1919
1939
1959
1979
1999
2019

NYSH

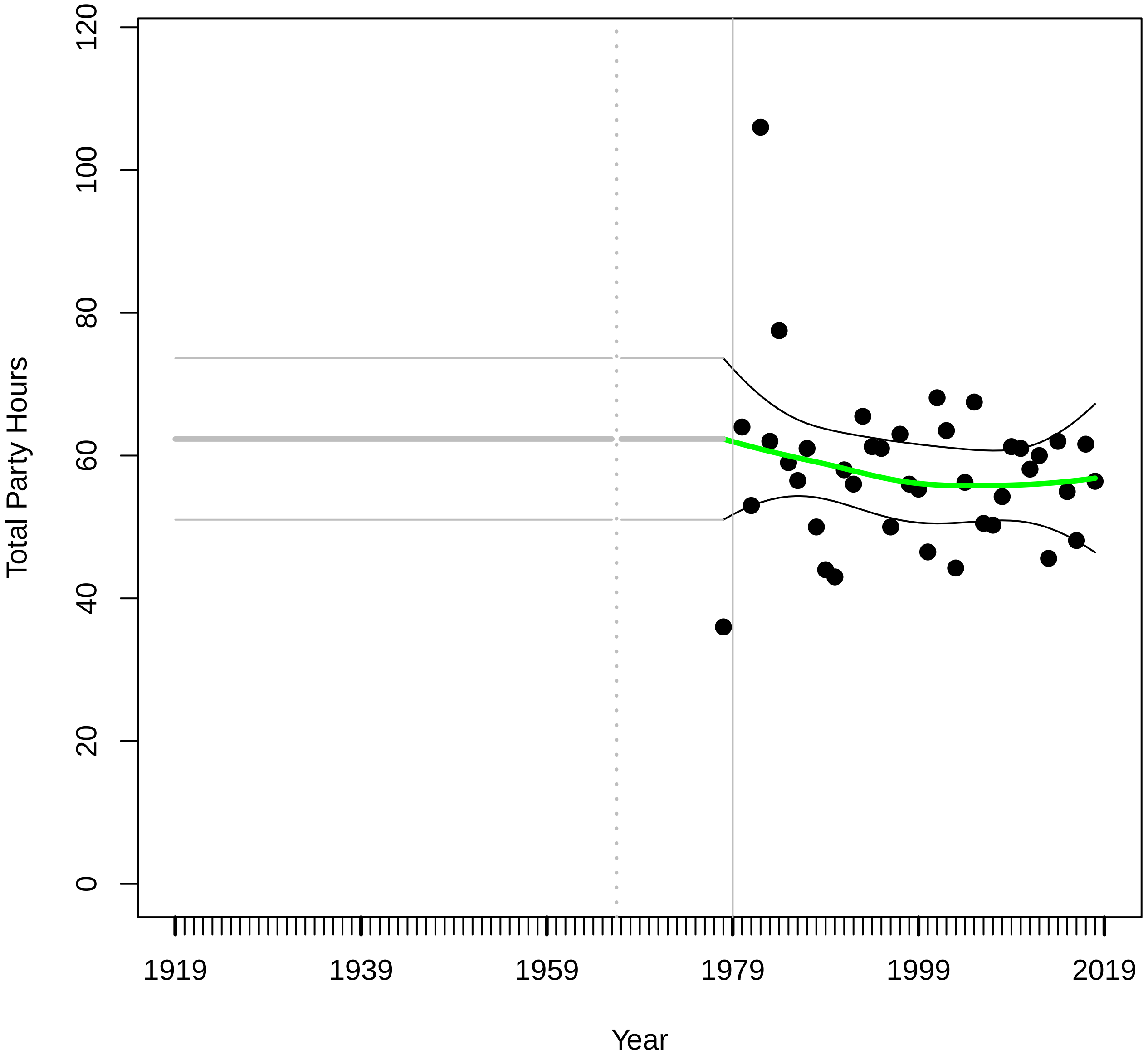

Total Party Hours
0
20
40
60
80
100
120
1919
1939
1959
1979
1999
2019
Year

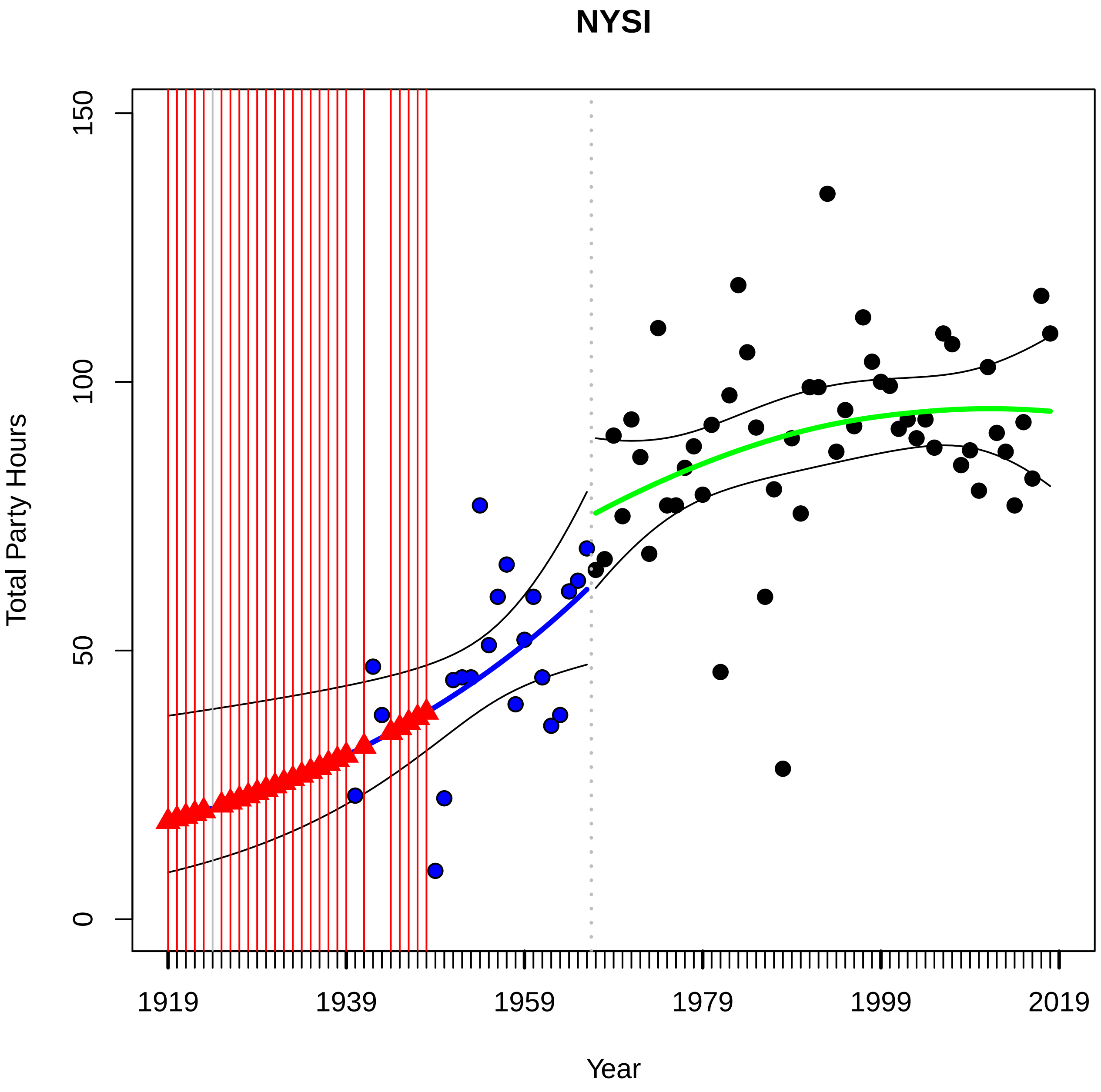
NYSI
Total Party Hours
0
50
100
150
1919
1939
1959
1979
1999
2019
Year

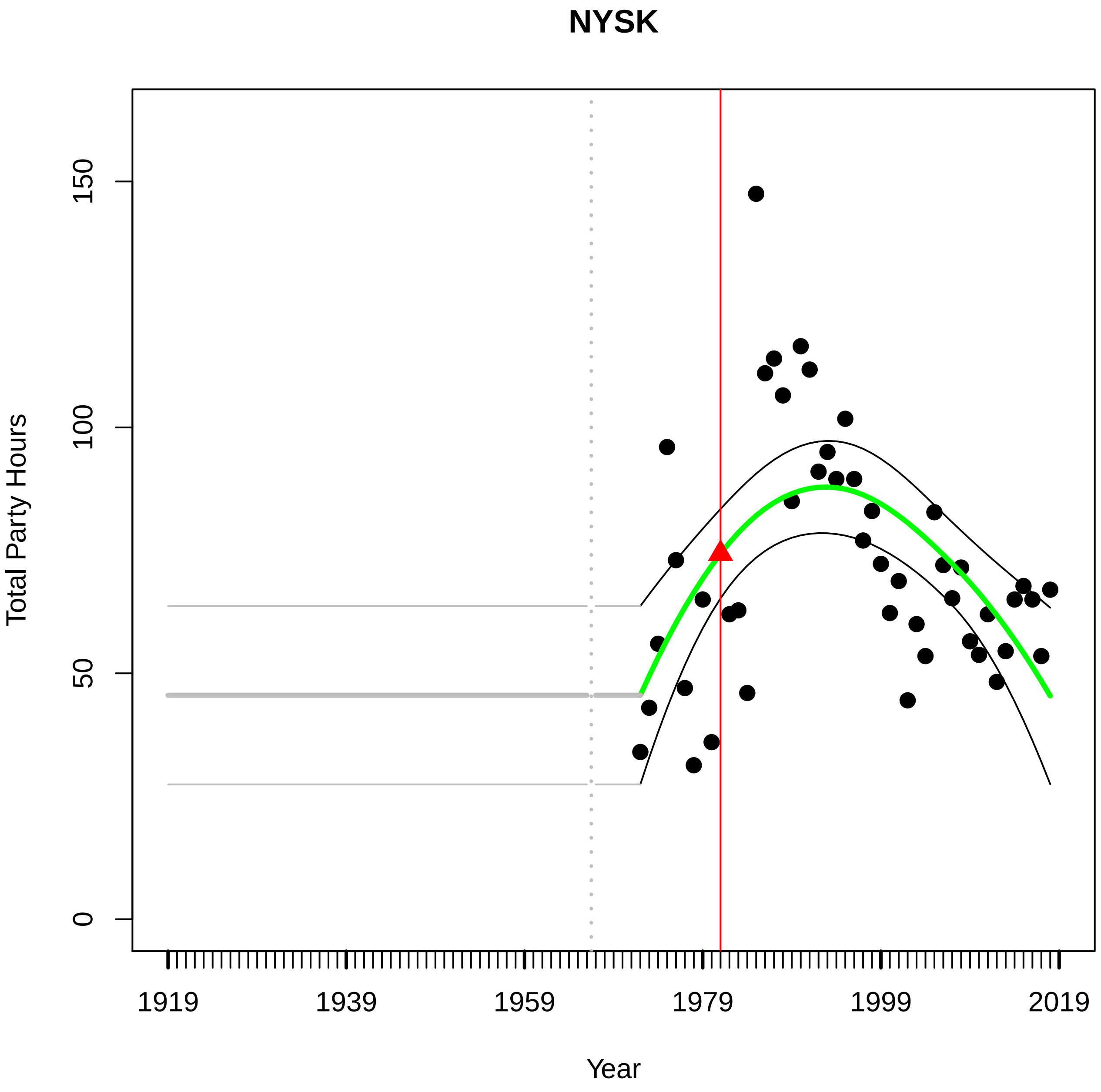
NYSK
Total Party Hours
0
50
100
150
1919
1939
1959
1979
1999
2019
Year

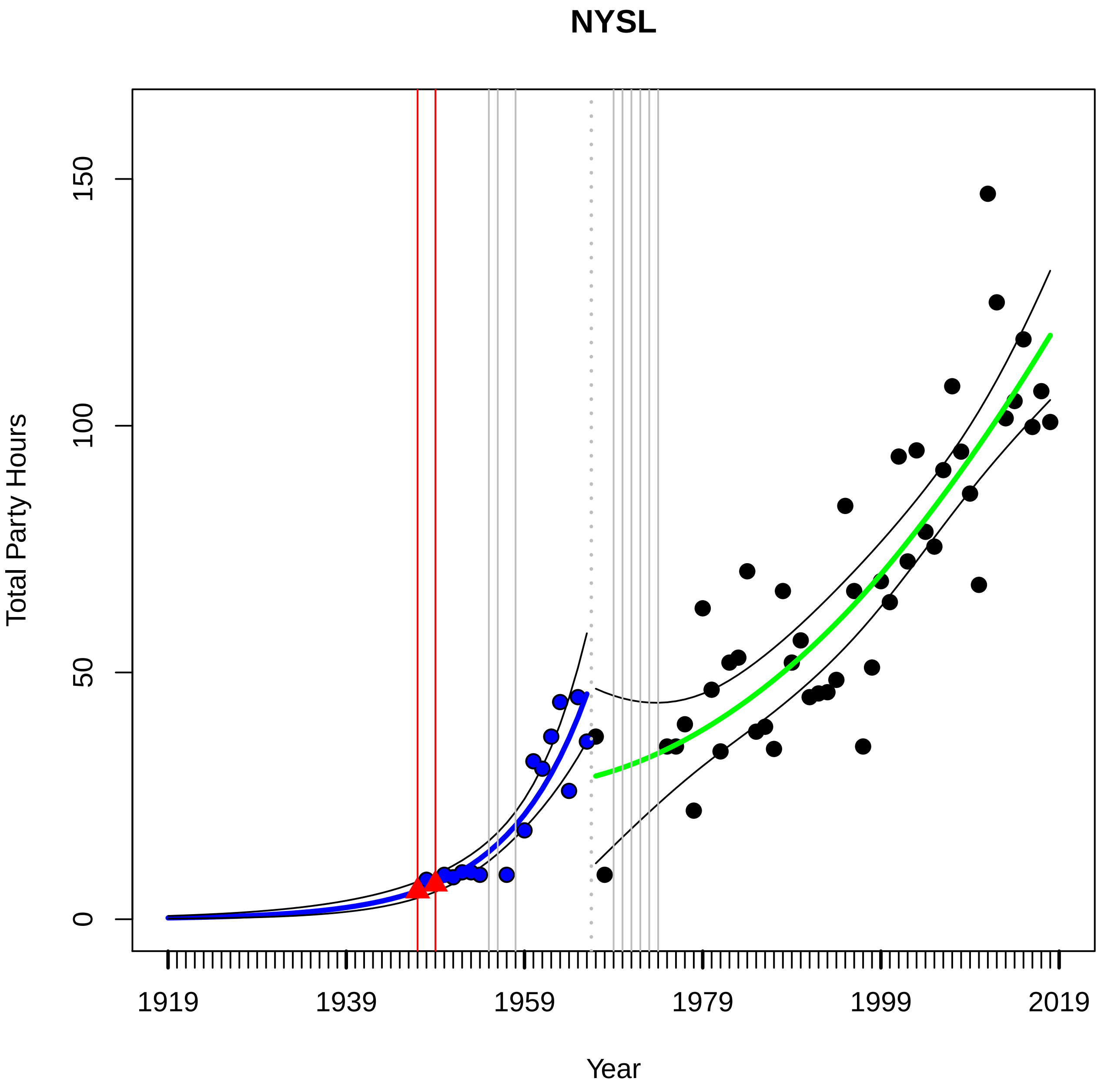

NYSL
Total Party Hours
0
50
100
150
1919
1939
1959
1979
1999
2019
Year

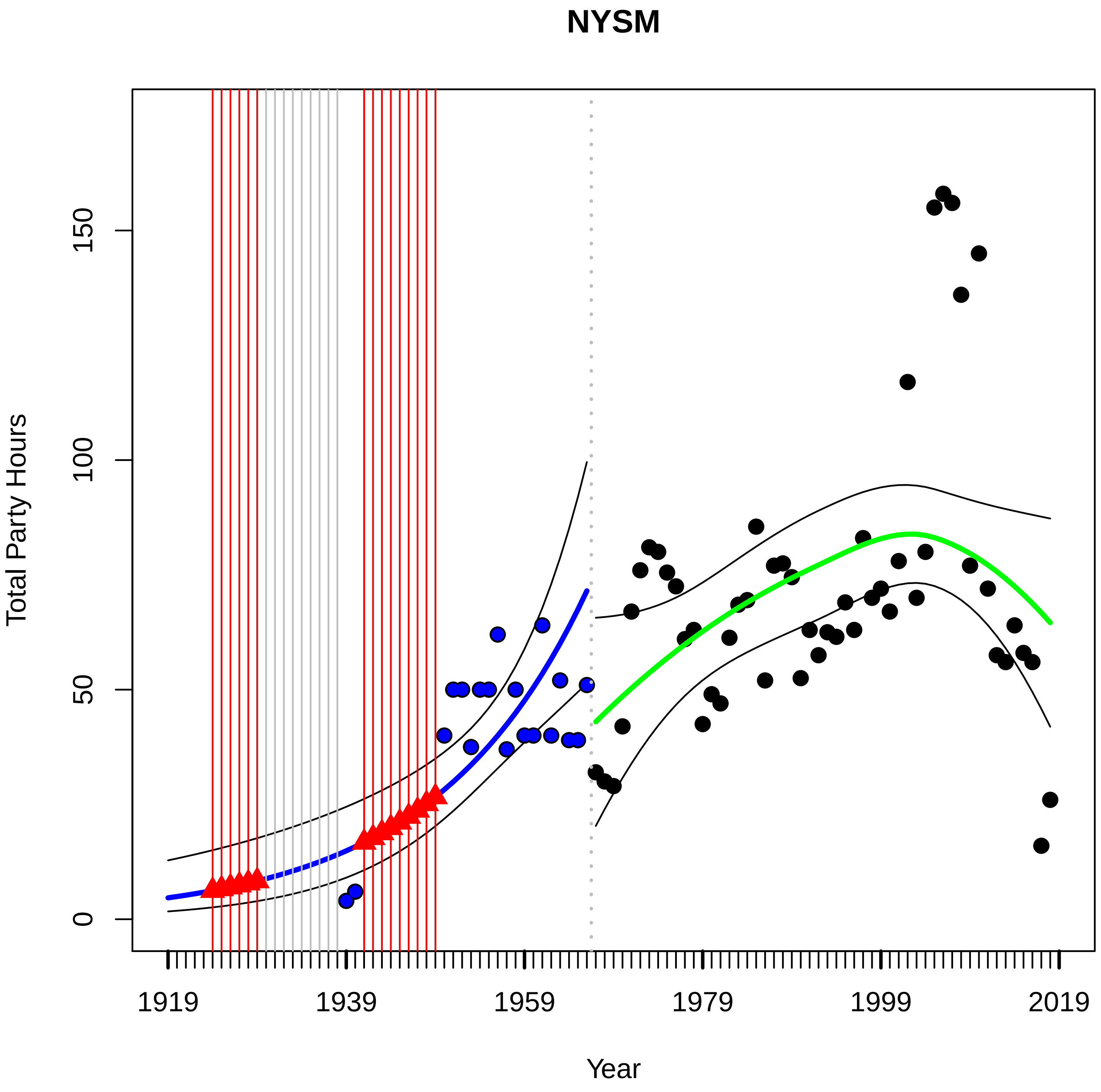

NYSM
Total Party Hours
0
50
100
150
1919
1939
1959
1979
1999
2019
Year

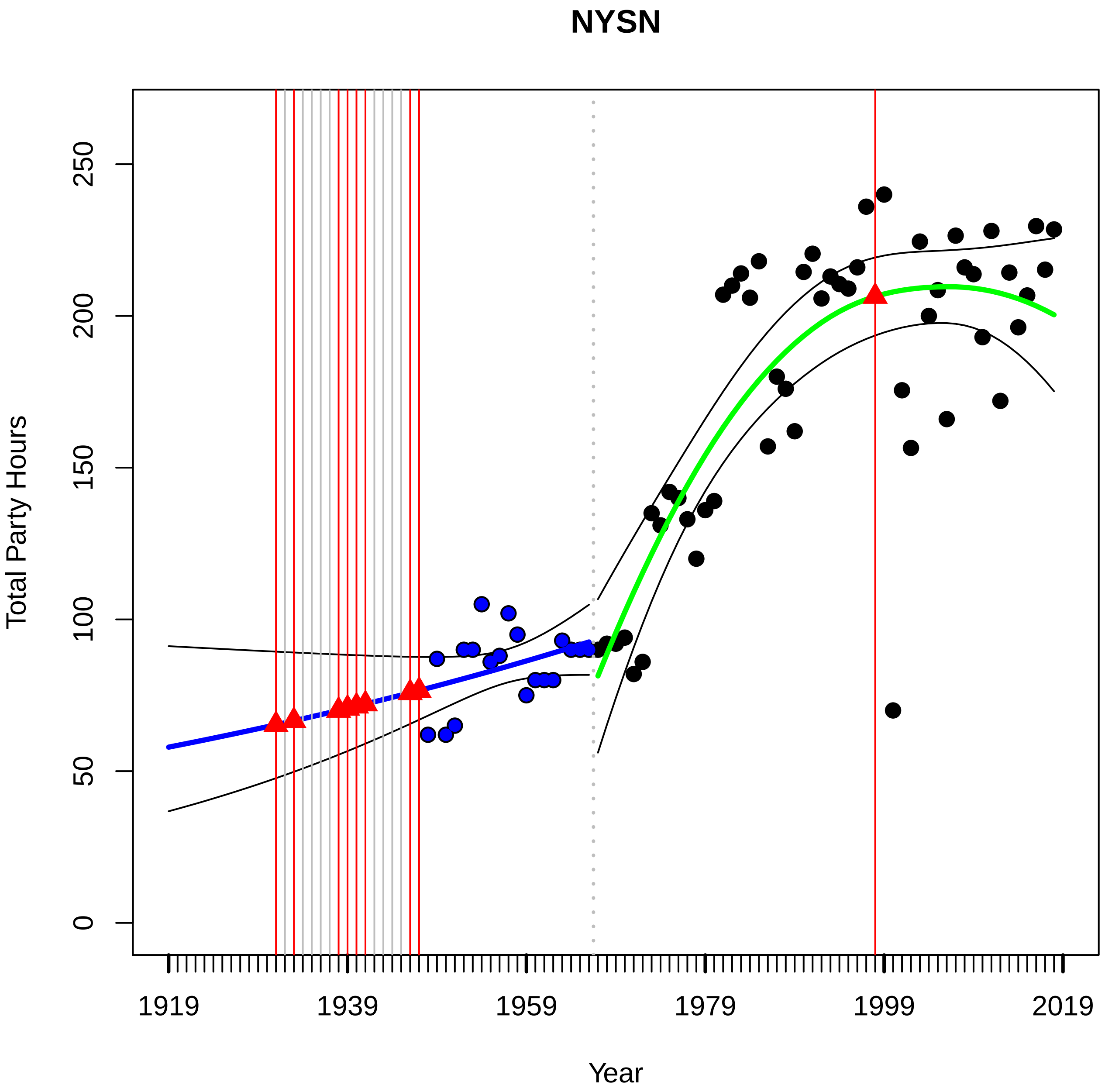

NYSN
Total Party Hours
Year
0
50
100
150
200
250
1919
1939
1959
1979
1999
2019

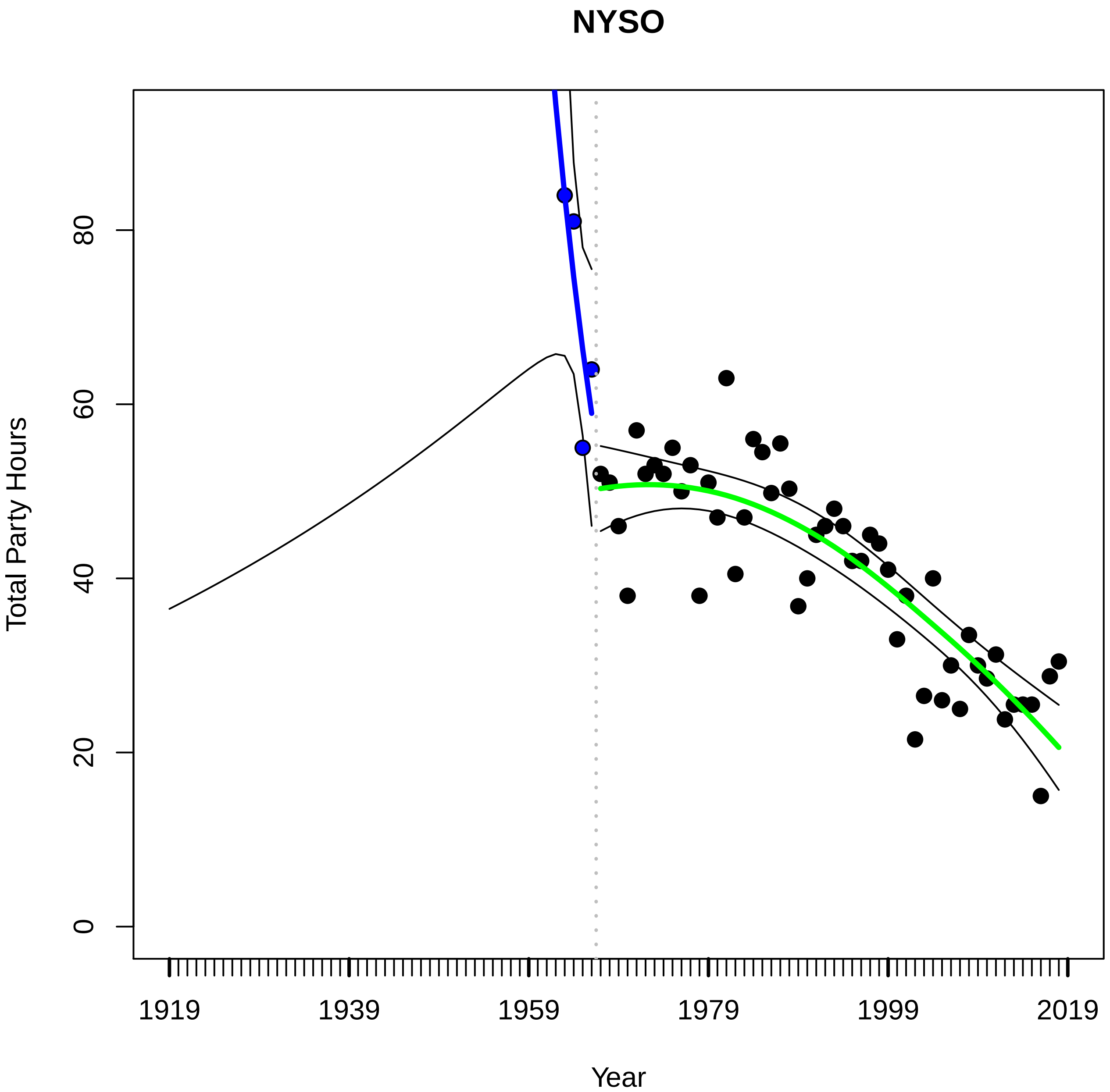
NYSO
Total Party Hours
0
20
40
60
80
1919
1939
1959
1979
1999
2019
Year

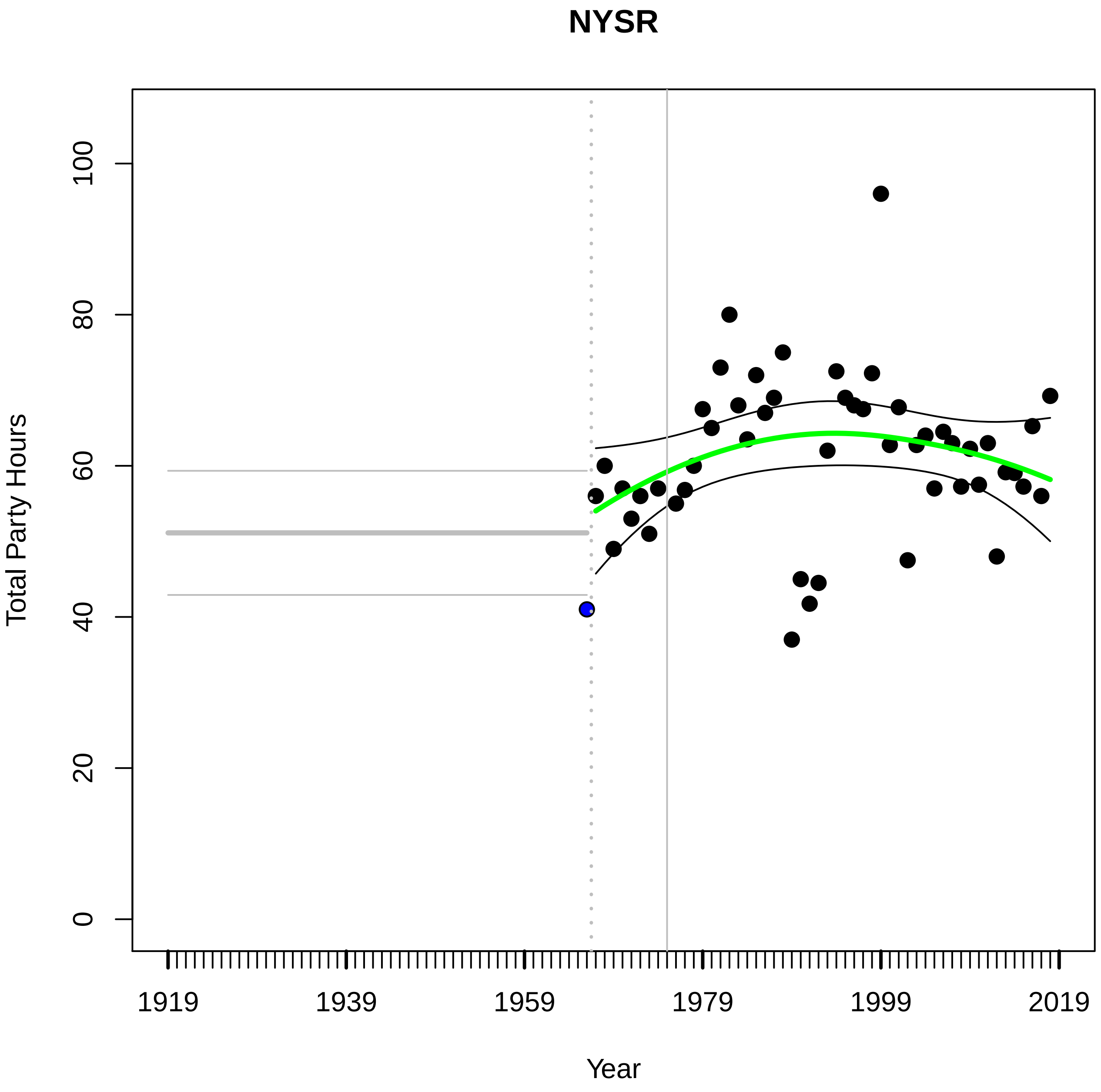

NYSR
Total Party Hours
Year
0
20
40
60
80
100
1919
1939
1959
1979
1999
2019

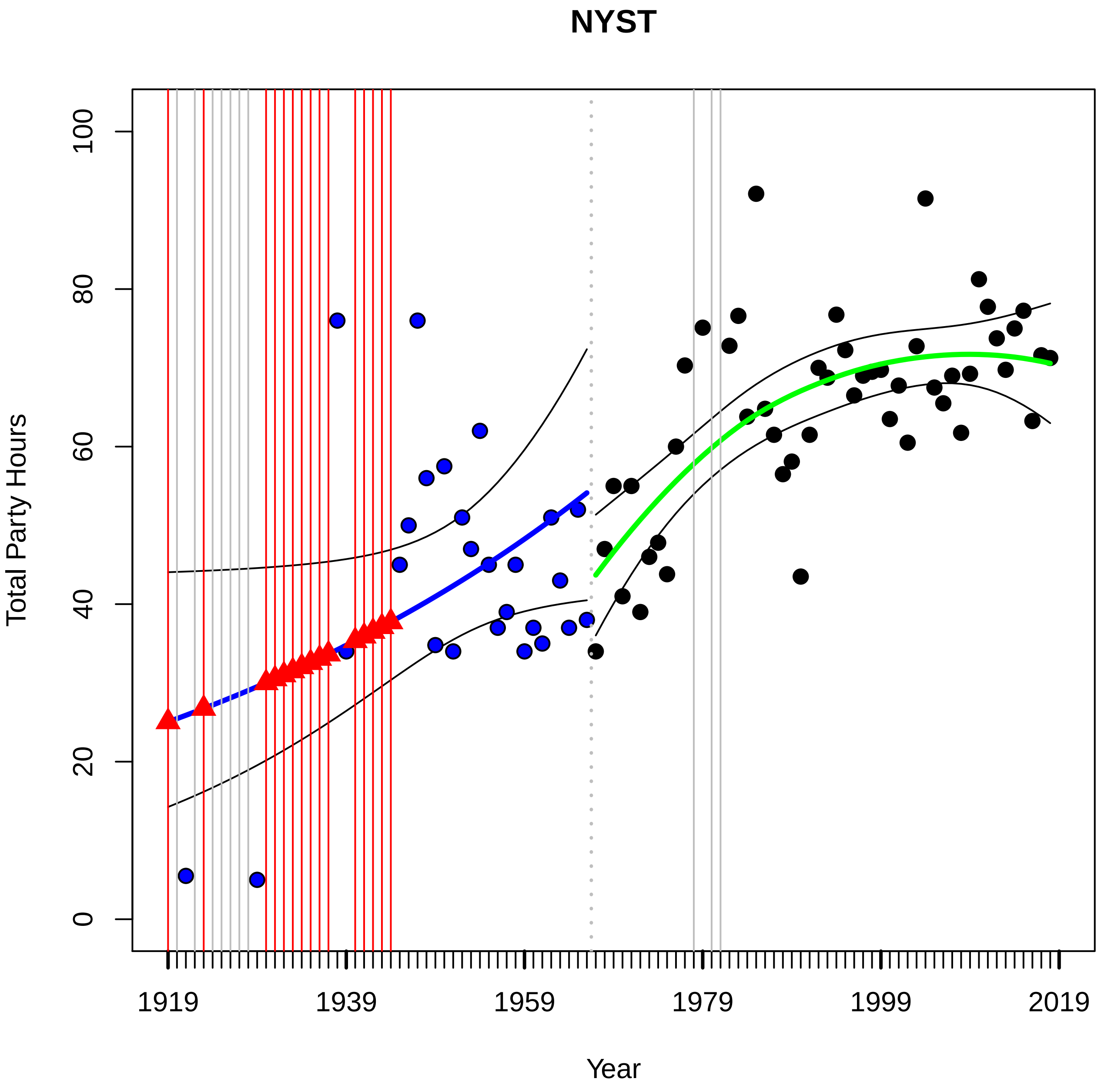
NYST
Total Party Hours
Year
0
20
40
60
80
100
1919
1939
1959
1979
1999
2019

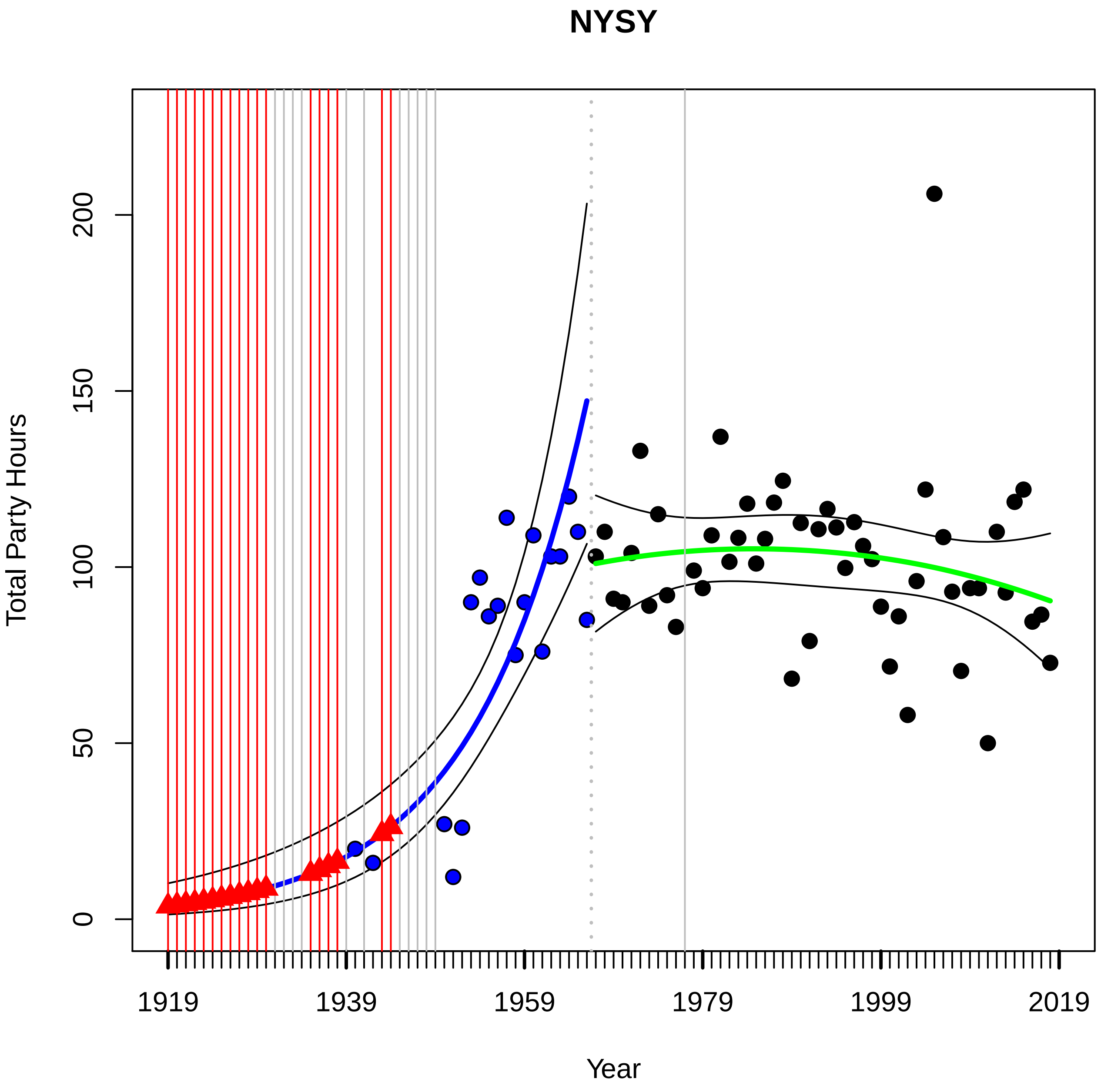

NYSY
Total Party Hours
0
50
100
150
200
1919
1939
1959
1979
1999
2019
Year

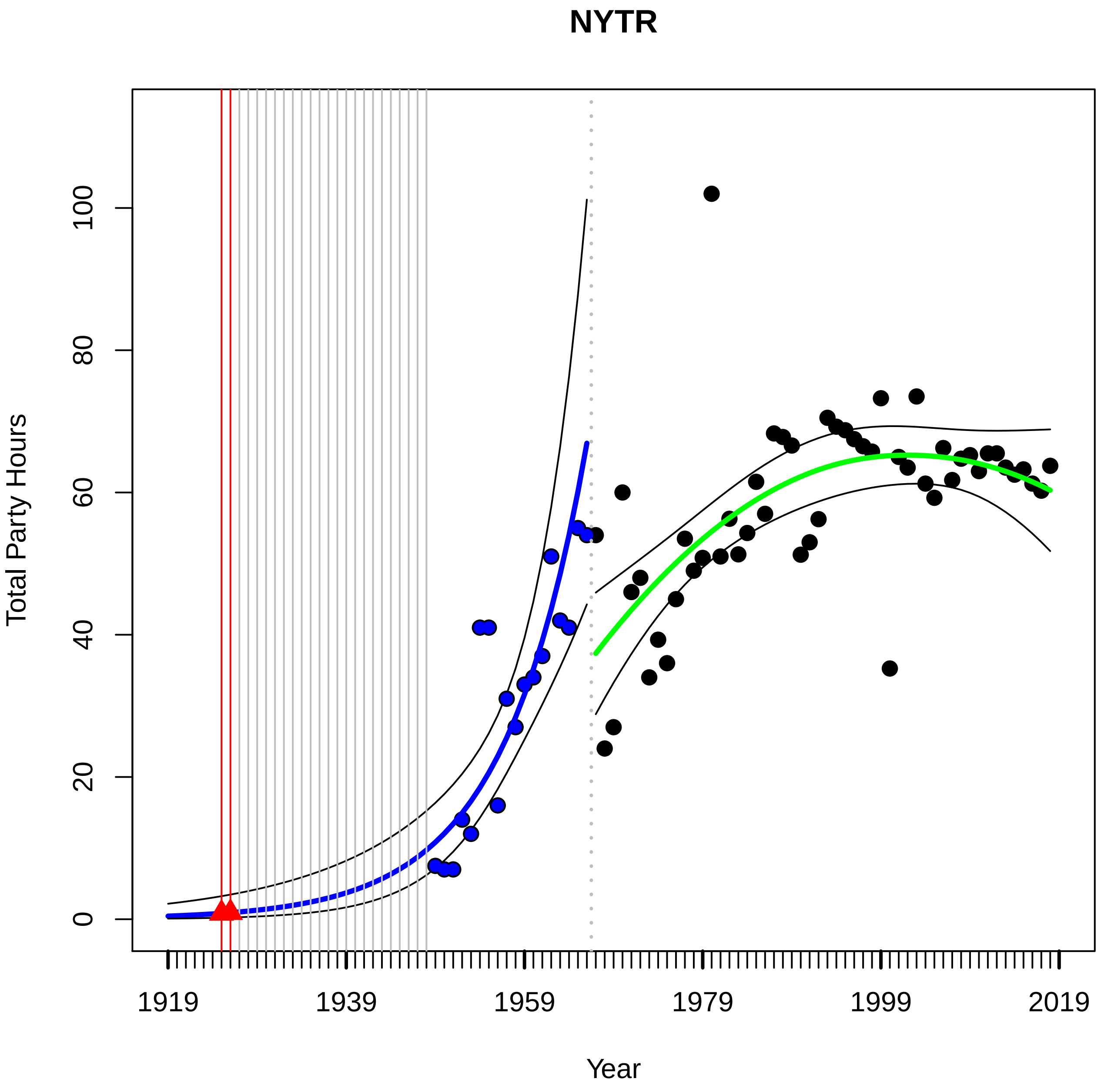

NYTR
Total Party Hours
Year
0
20
40
60
80
100
1919
1939
1959
1979
1999
2019

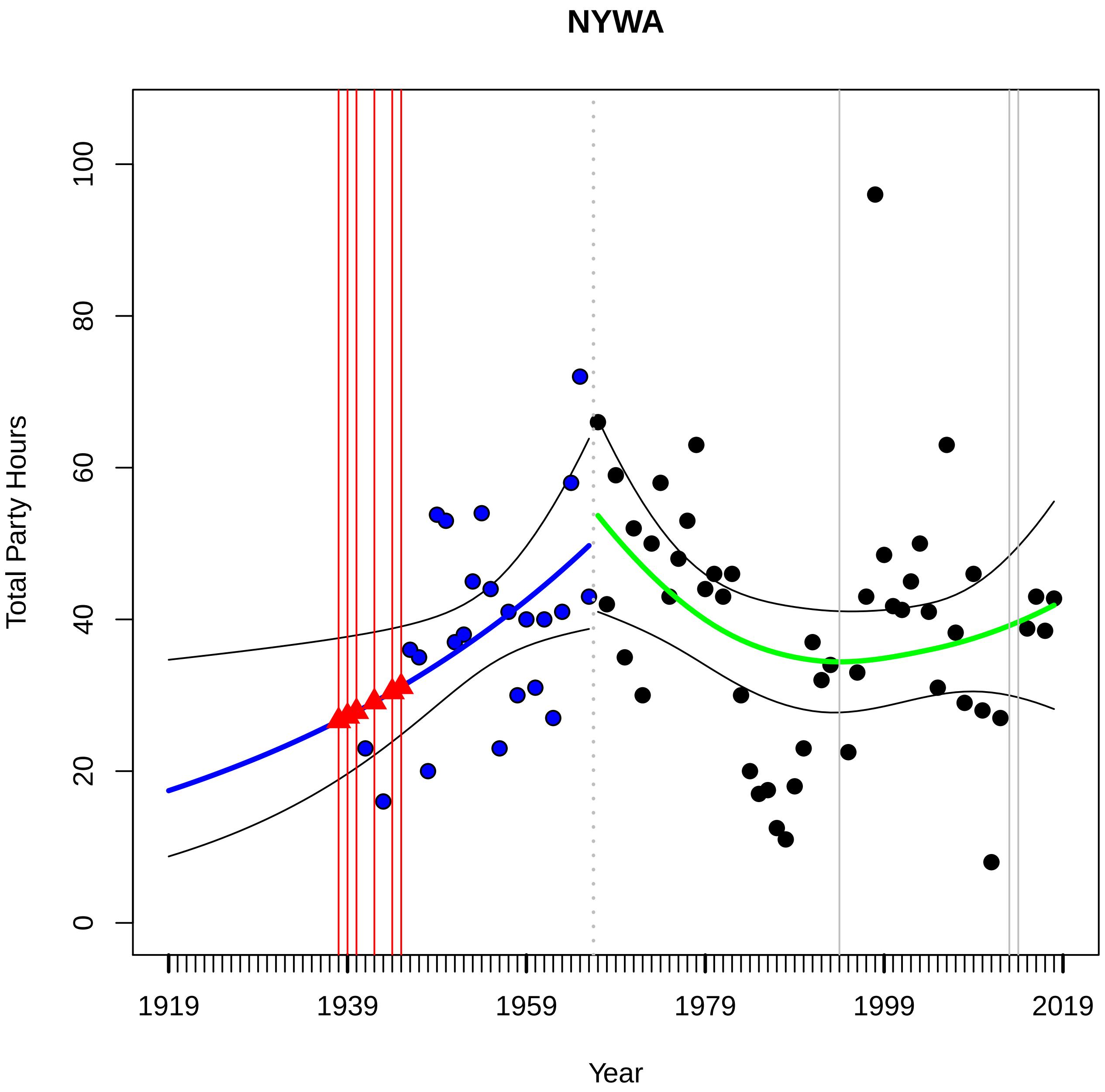

NYWA
Total Party Hours
Year
0
20
40
60
80
100
1919
1939
1959
1979
1999
2019

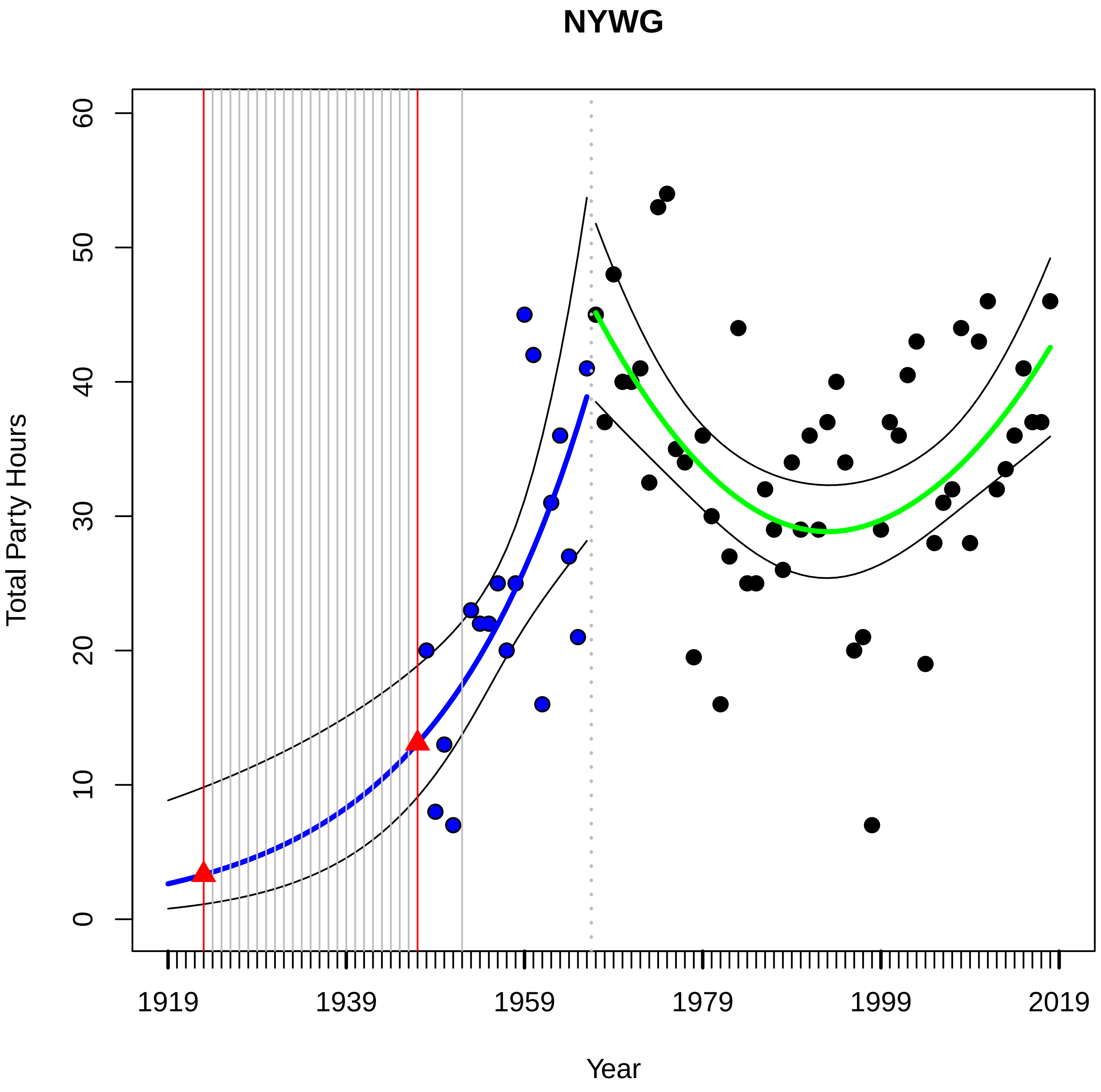

NYWG
Total Party Hours
0
10
20
30
40
50
60
1919
1939
1959
1979
1999
2019
Year

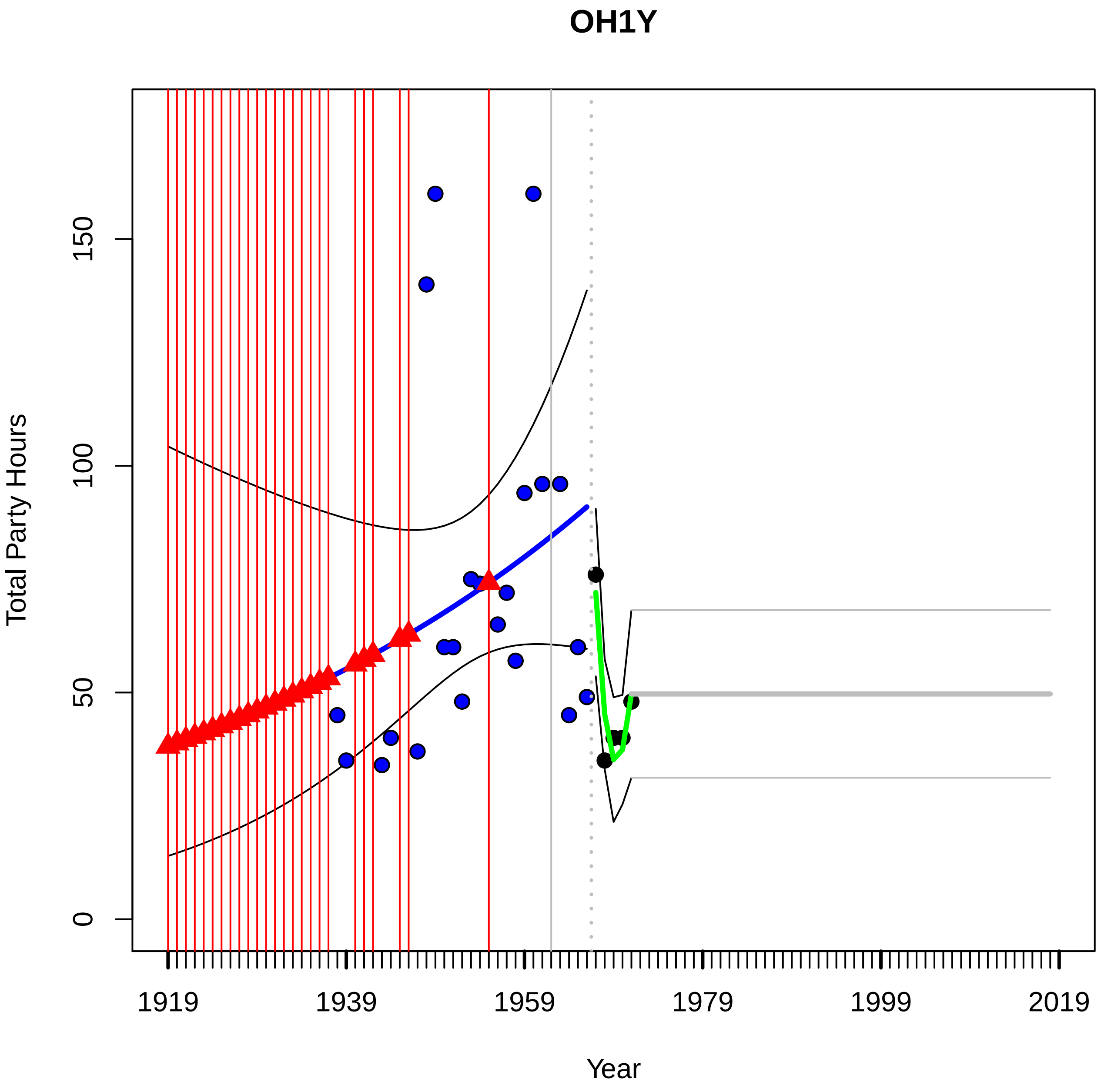
OH1Y
Total Party Hours
0
50
100
150
1919
1939
1959
1979
1999
2019
Year

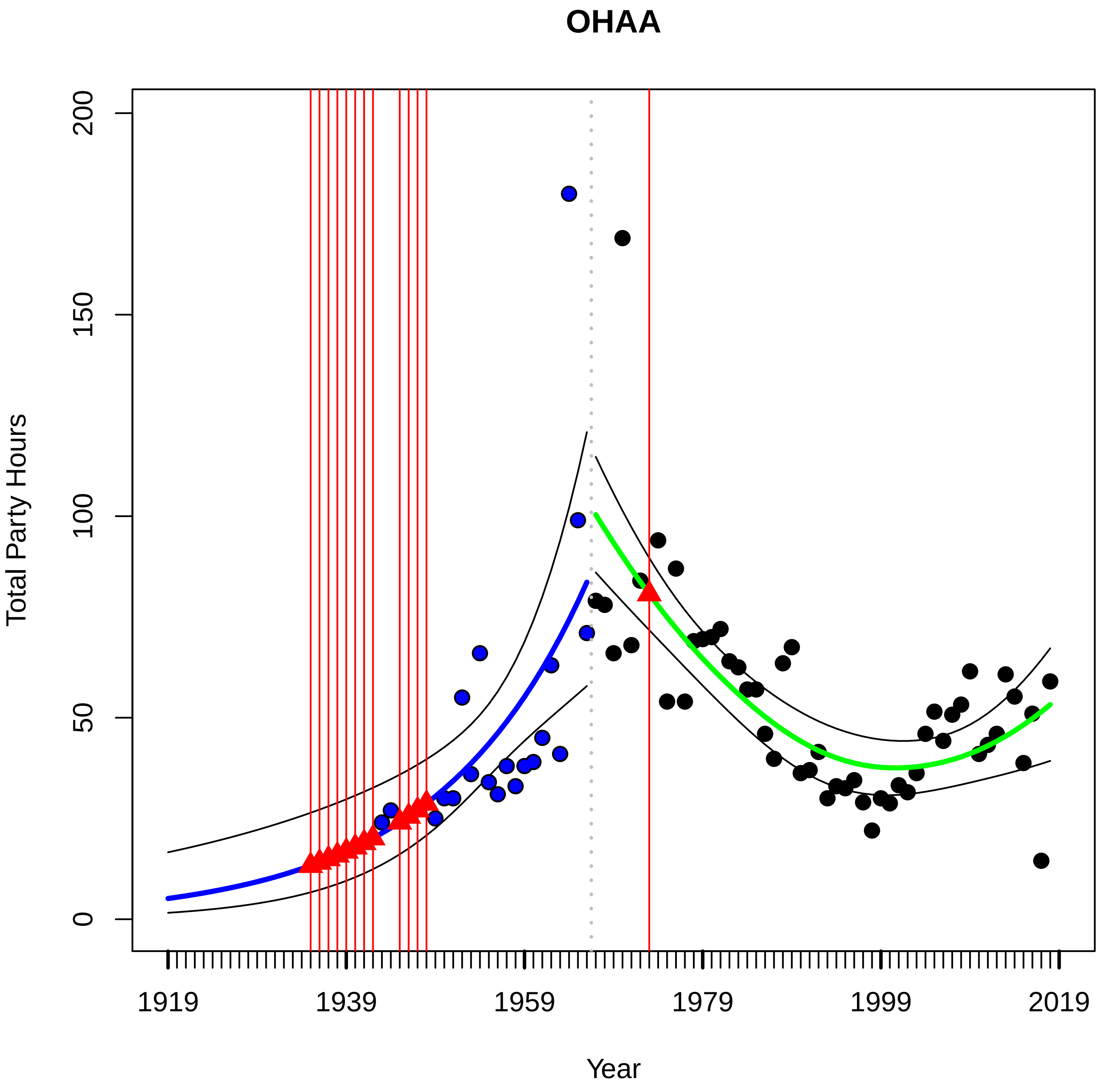
OHAA
Total Party Hours
0
50
100
150
200
1919
1939
1959
1979
1999
2019
Year

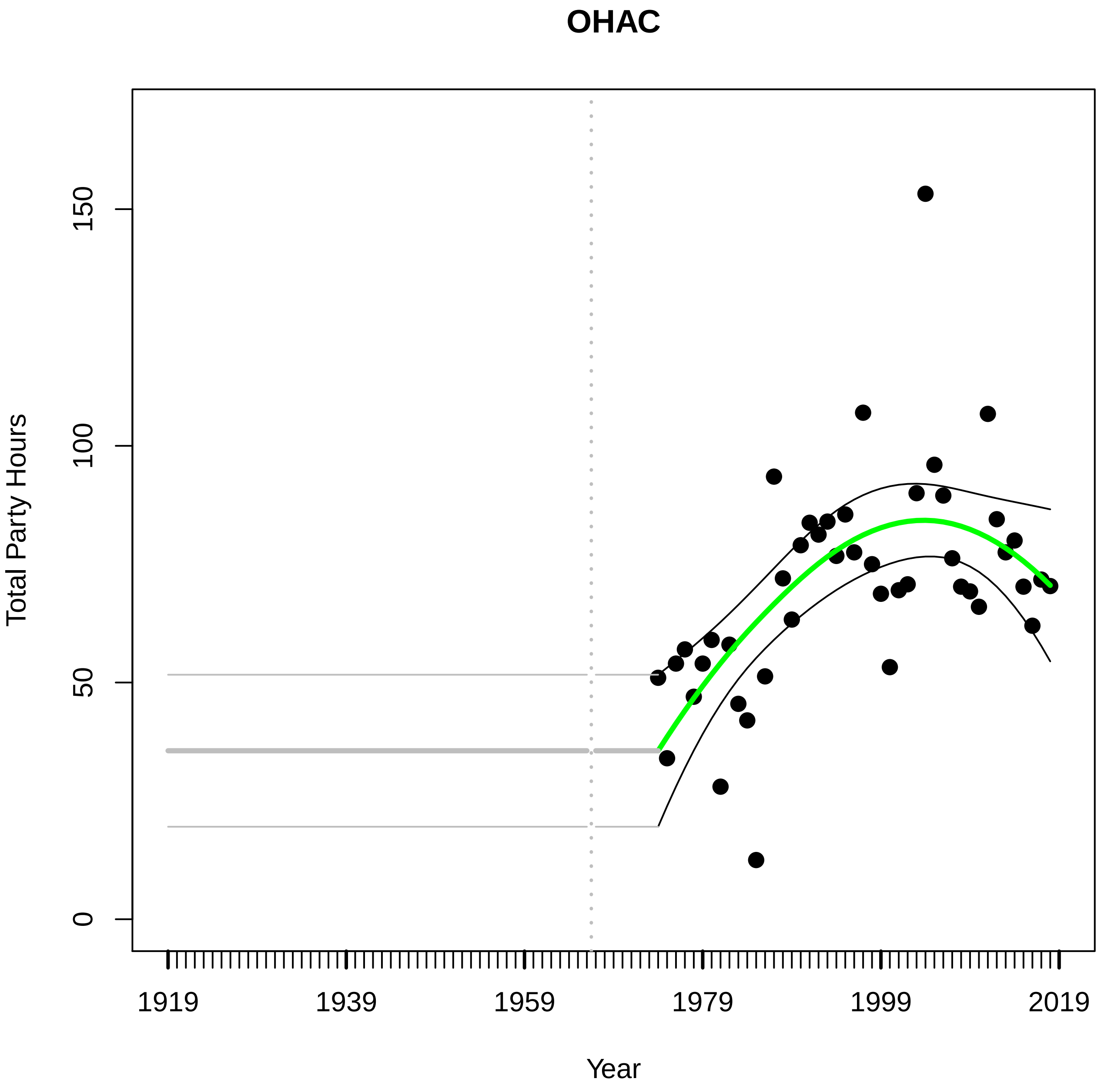
OHAC
Total Party Hours
0
50
100
150
1919
1939
1959
1979
1999
2019
Year

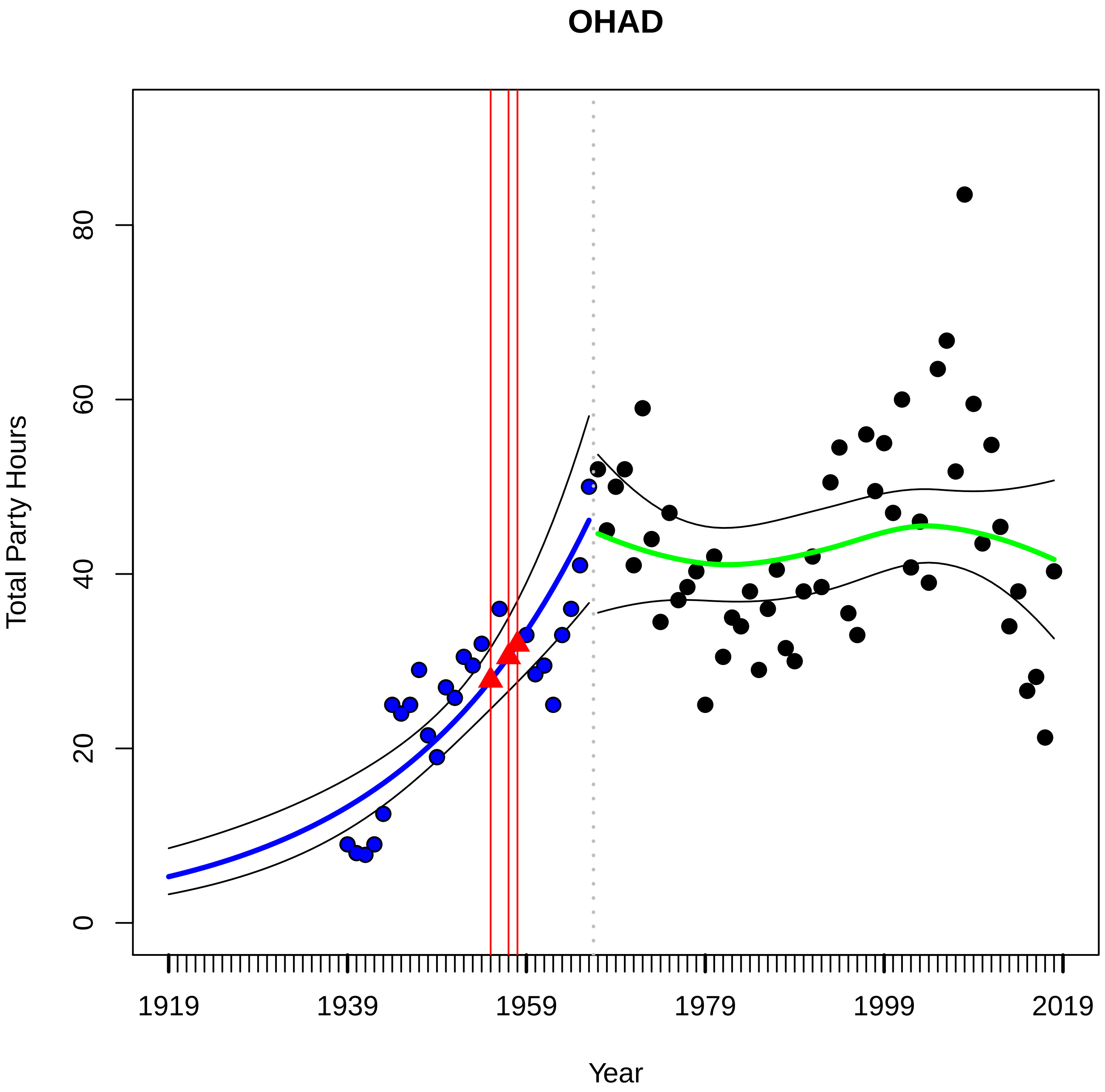

OHAD
Total Party Hours
Year
0
20
40
60
80
1919
1939
1959
1979
1999
2019

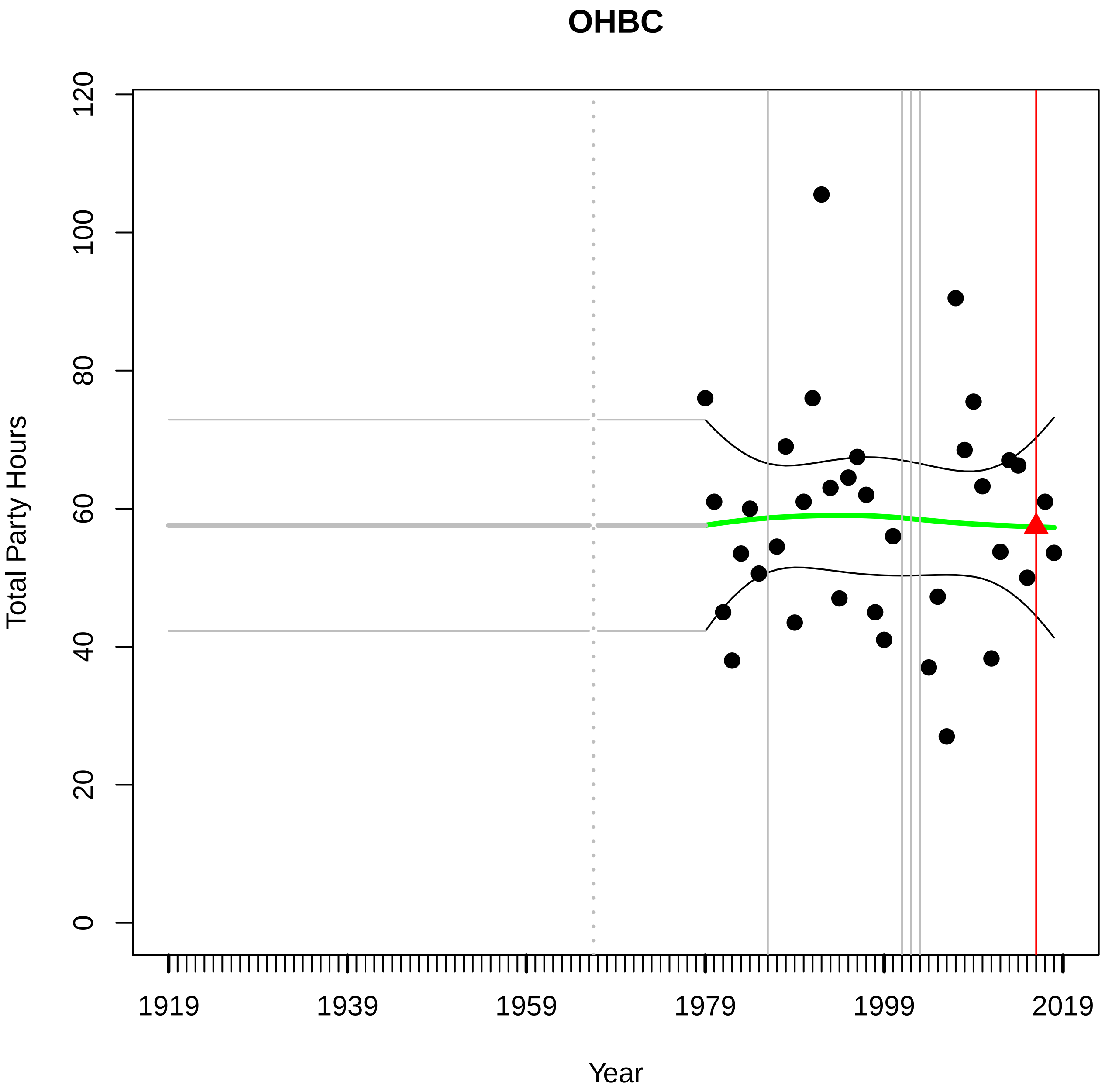

OHBC
Total Party Hours
Year
0
20
40
60
80
100
120
1919
1939
1959
1979
1999
2019

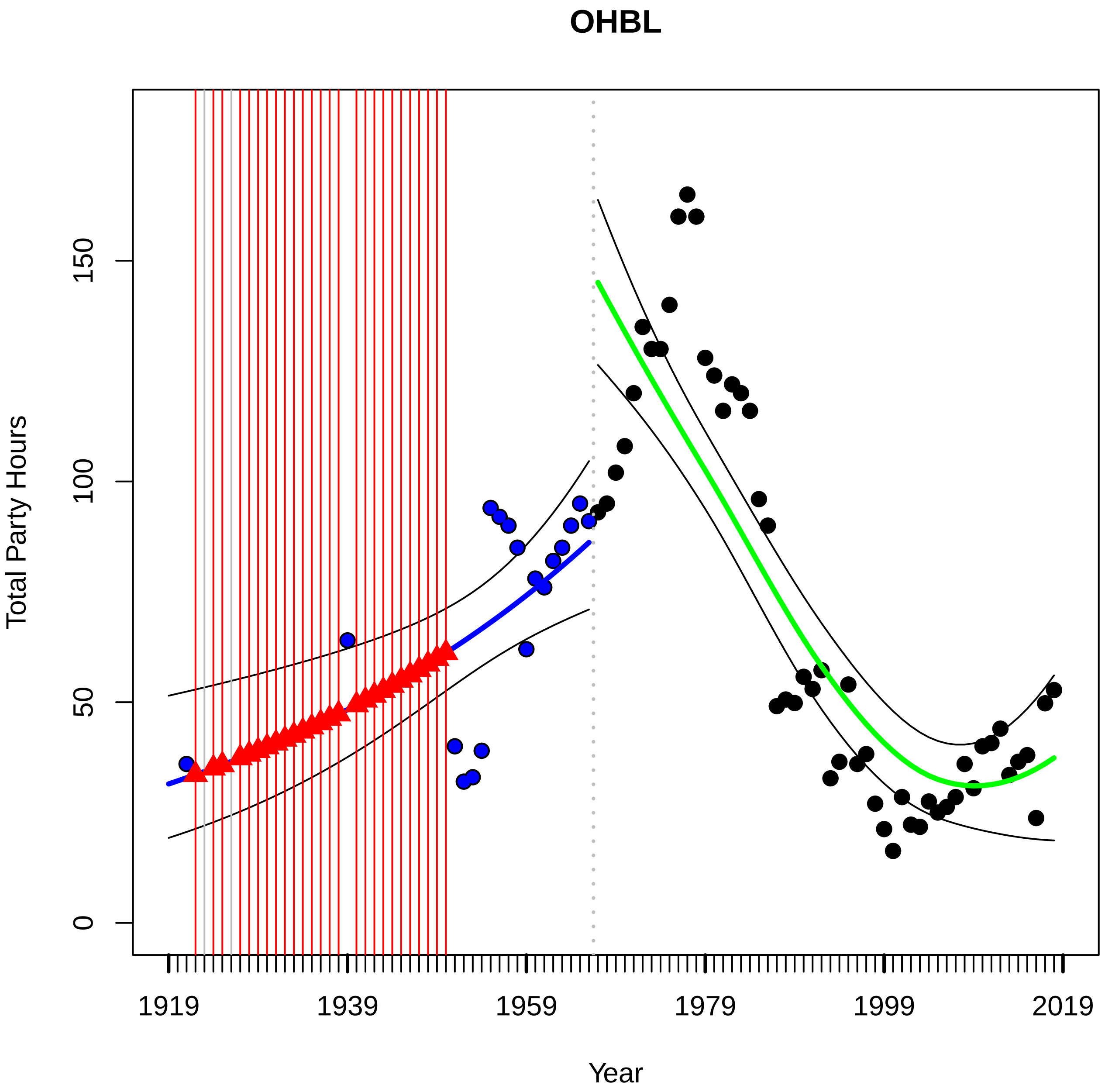
OHBL
Total Party Hours
0
50
100
150
1919
1939
1959
1979
1999
2019
Year

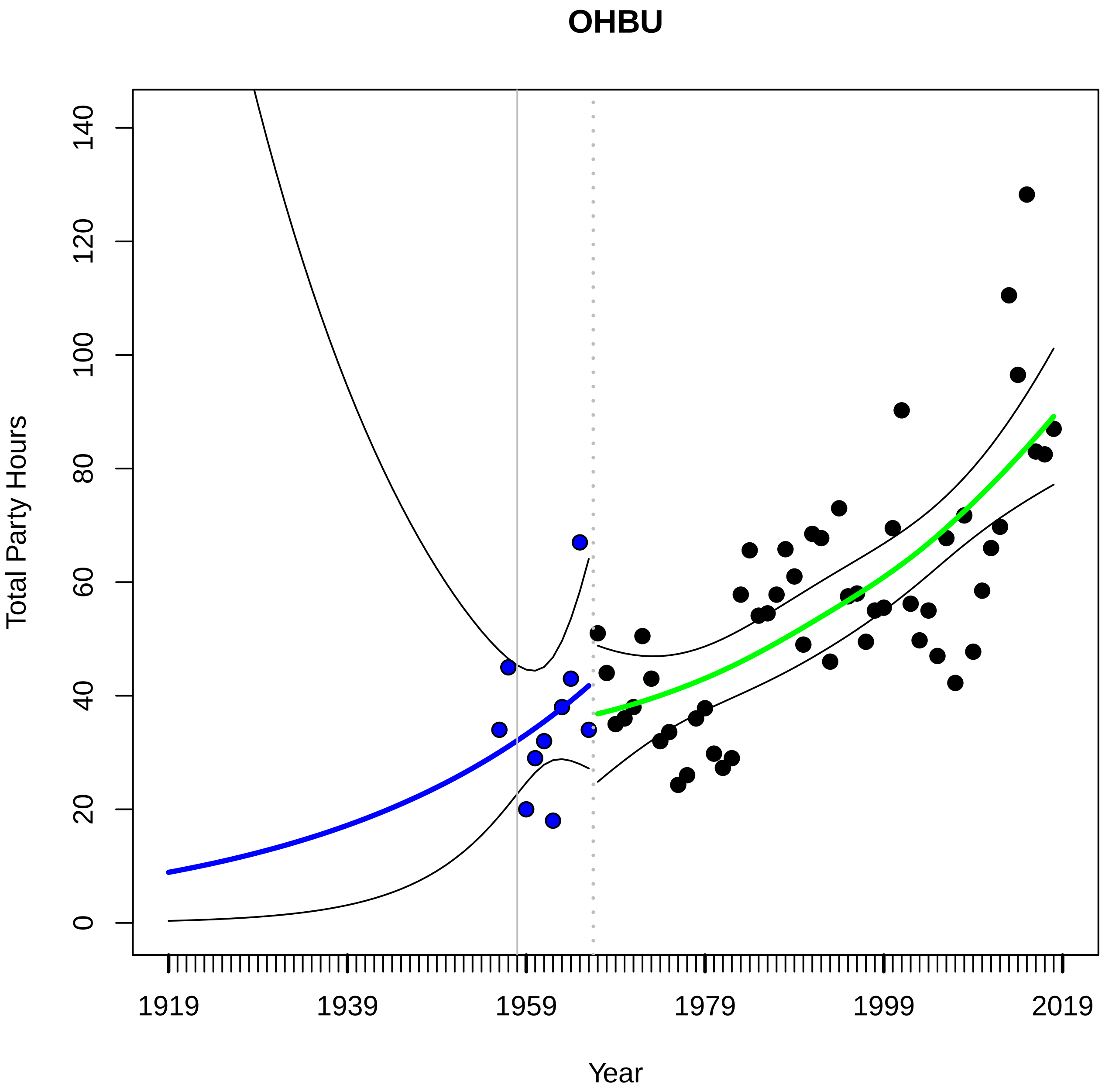

OHBU
Total Party Hours
0
20
40
60
80
100
120
140
1919
1939
1959
1979
1999
2019
Year

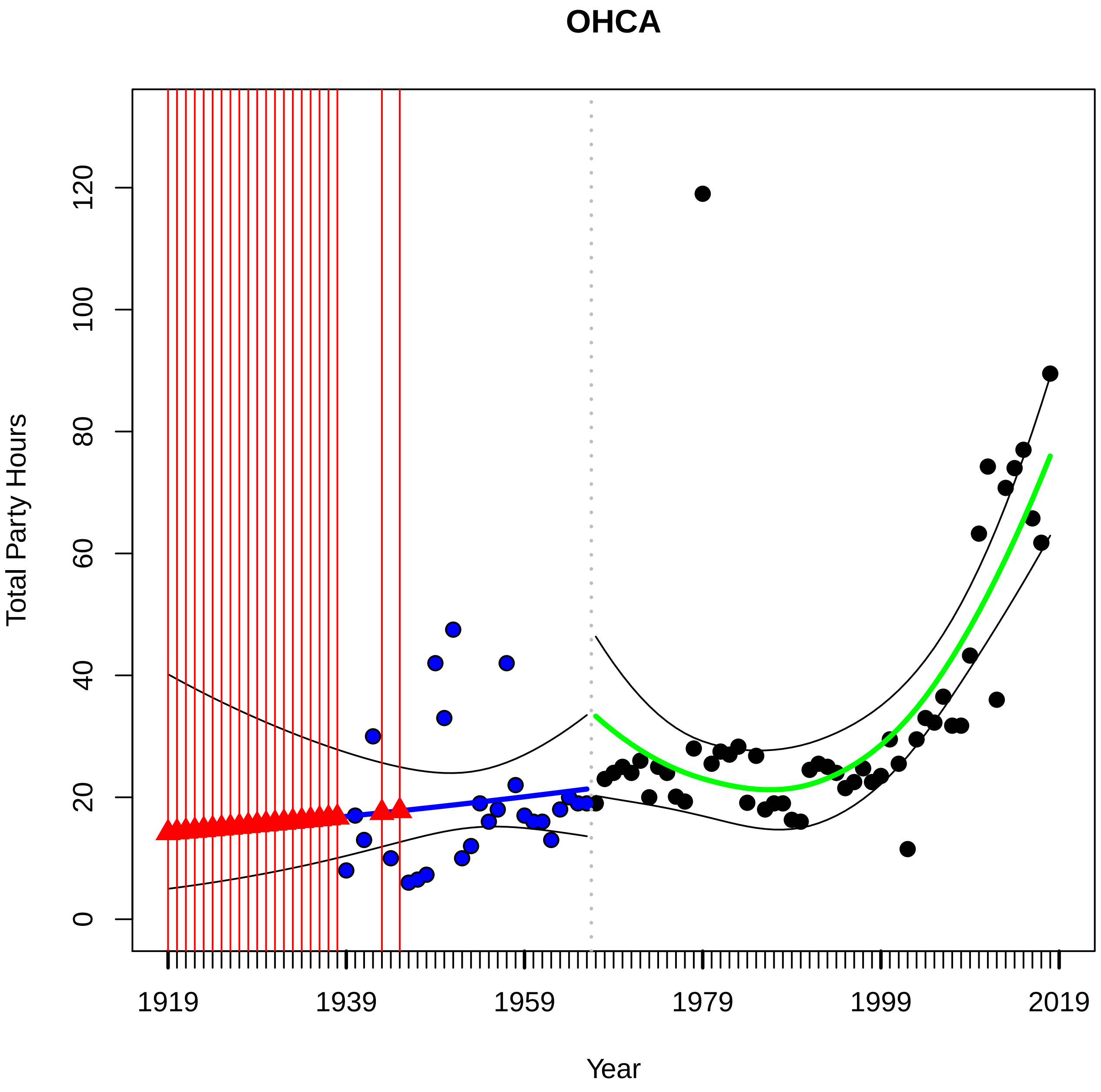

OHCA
Total Party Hours
0
20
40
60
80
100
120
1919
1939
1959
1979
1999
2019
Year

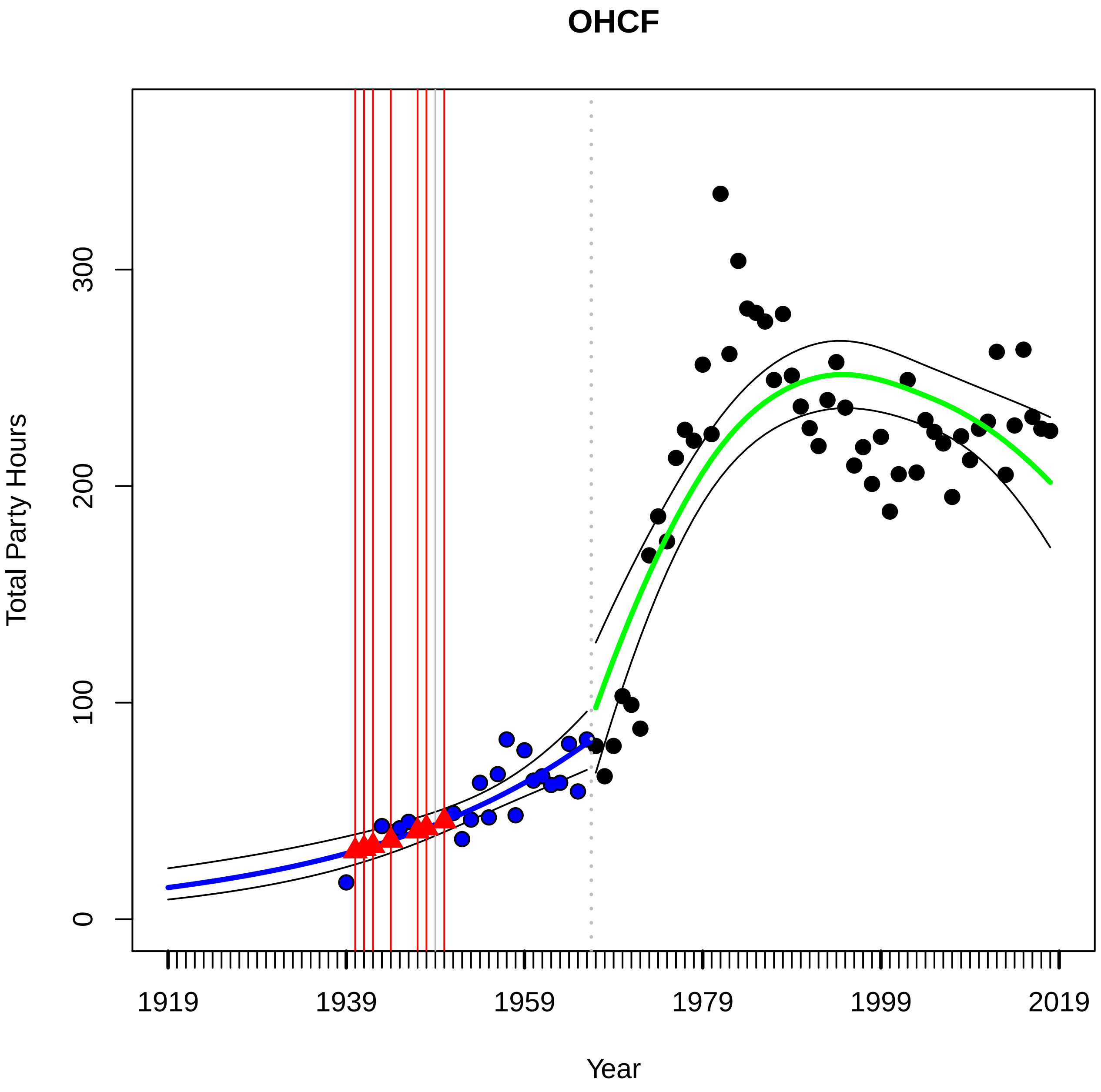

OHCF
Total Party Hours
0
100
200
300
Year
1919
1939
1959
1979
1999
2019

OHCI

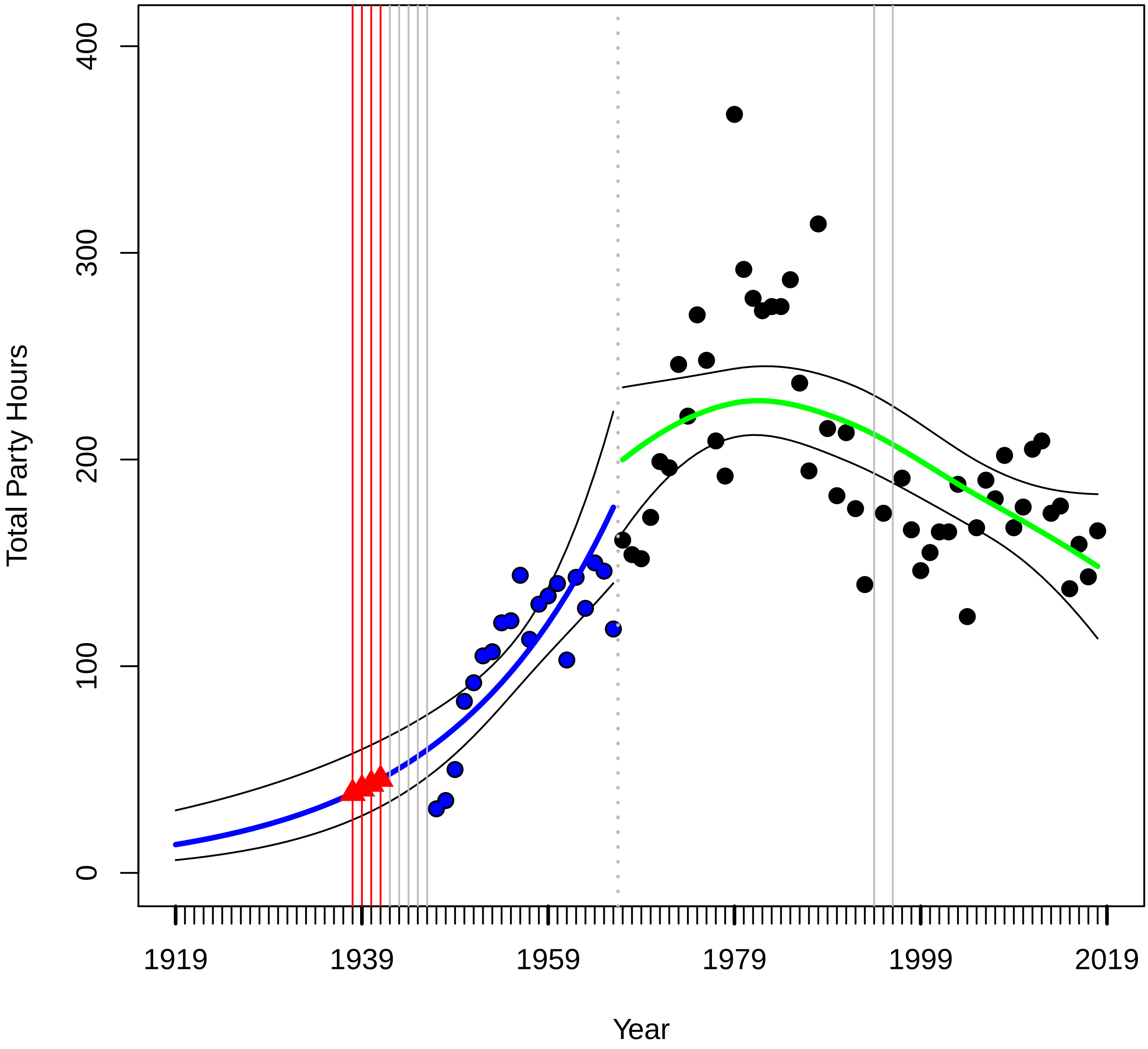

Total Party Hours
0
100
200
300
400
1919
1939
1959
1979
1999
2019
Year

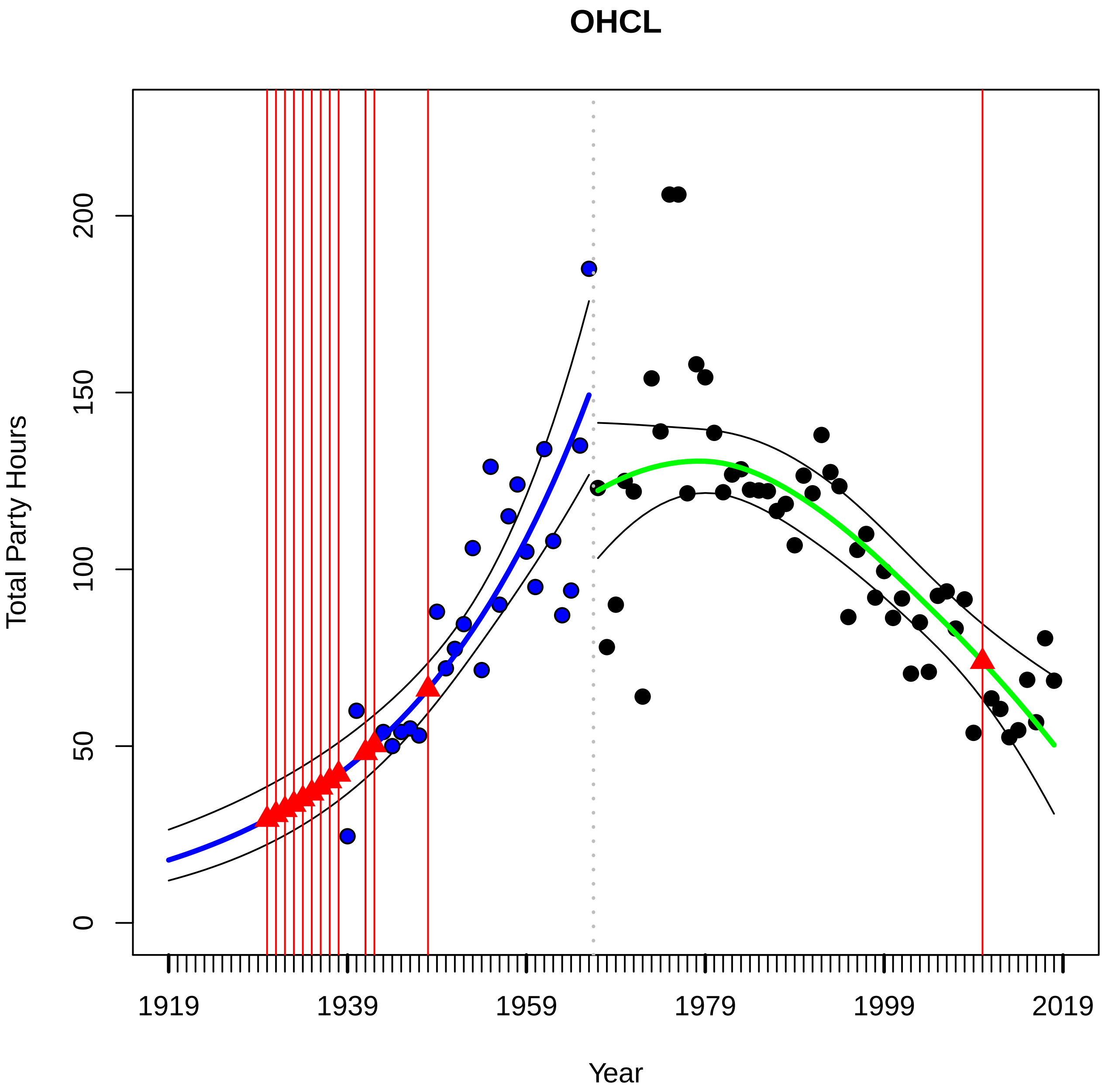

OHCL
Total Party Hours
0
50
100
150
200
1919
1939
1959
1979
1999
2019
Year

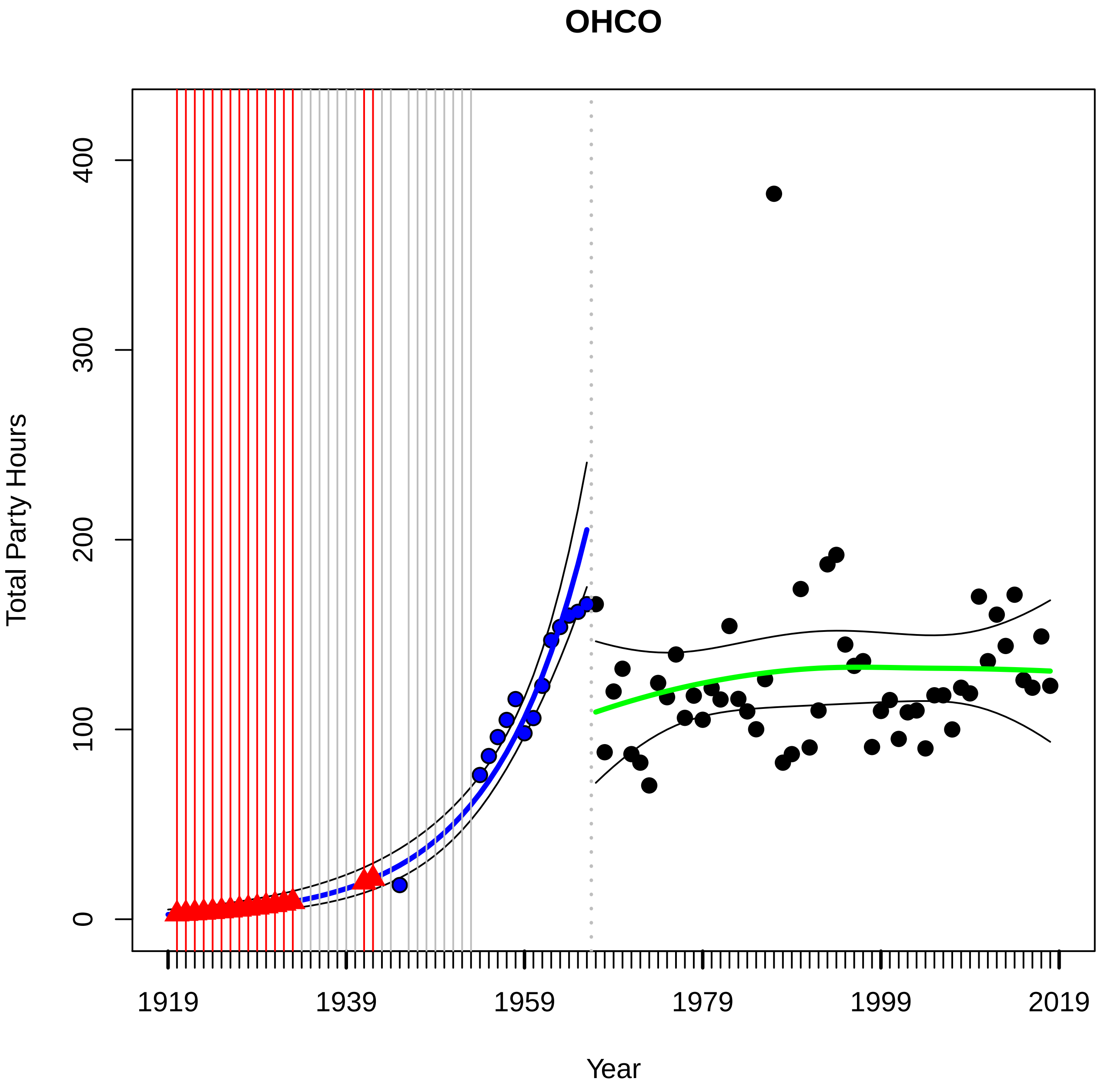

OHCO
Total Party Hours
0
100
200
300
400
1919
1939
1959
1979
1999
2019
Year

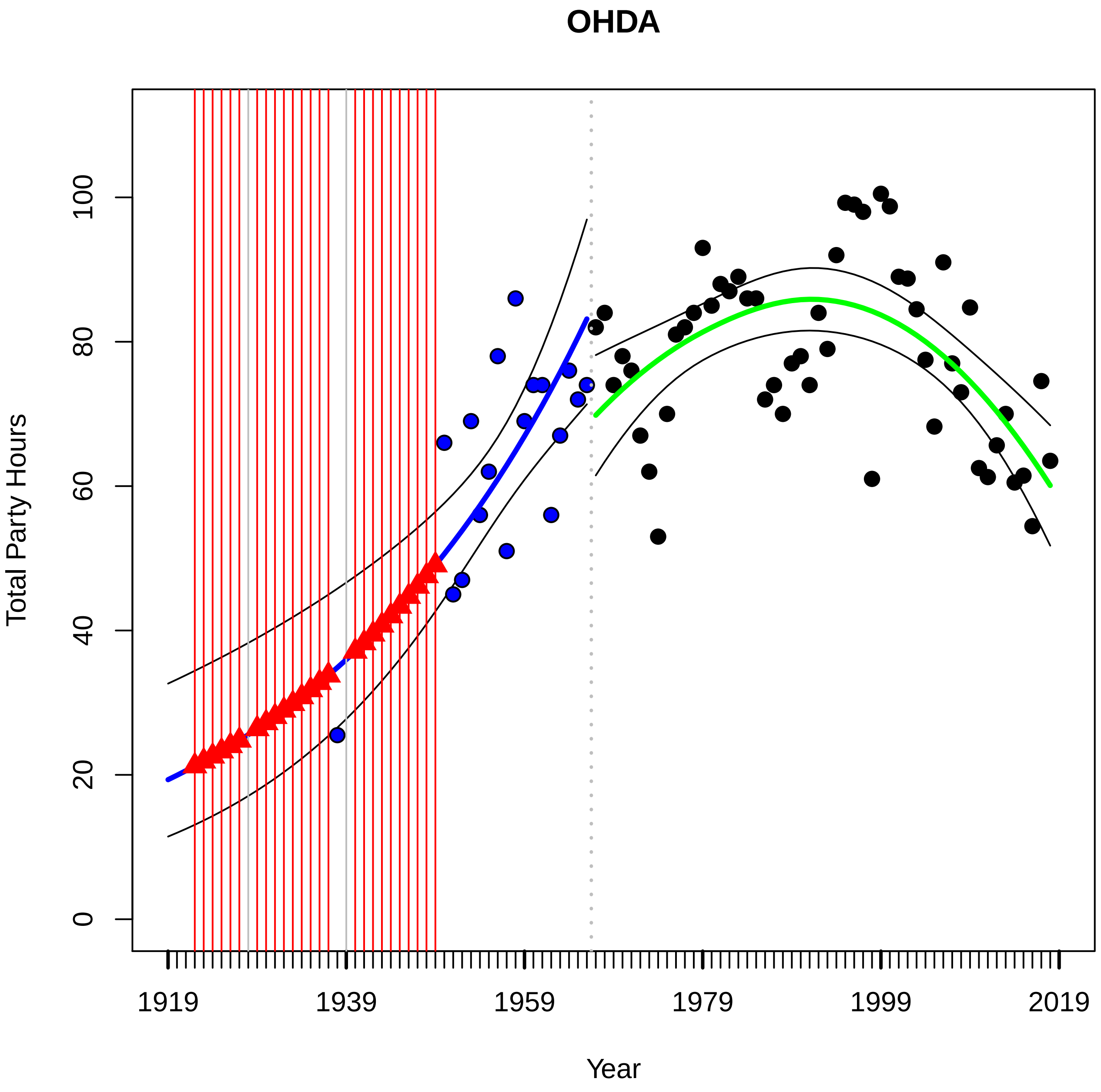
OHDA
Total Party Hours
0
20
40
60
80
100
1919
1939
1959
1979
1999
2019
Year

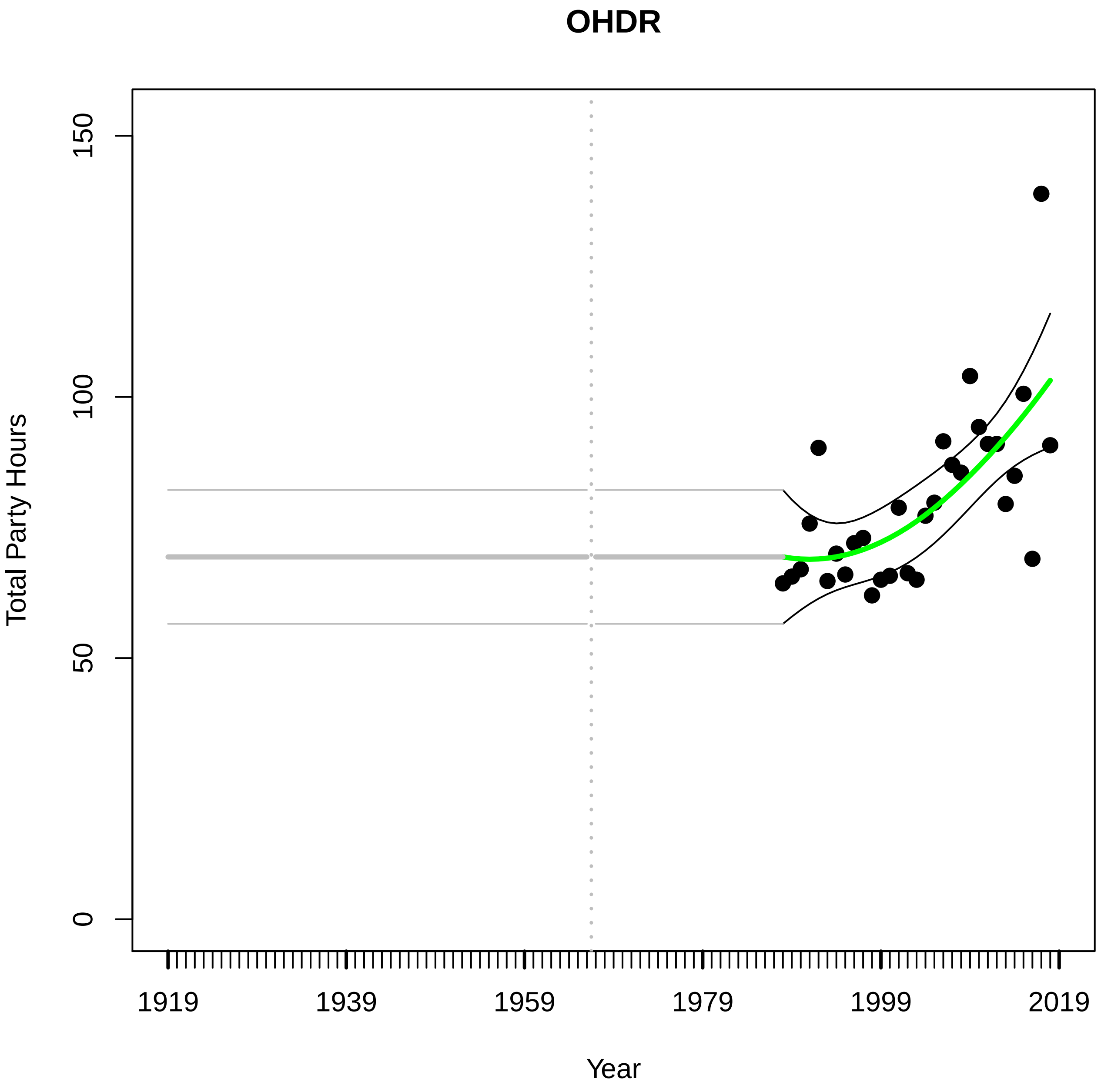
OHDR
Total Party Hours
Year
0
50
100
150
1919
1939
1959
1979
1999
2019

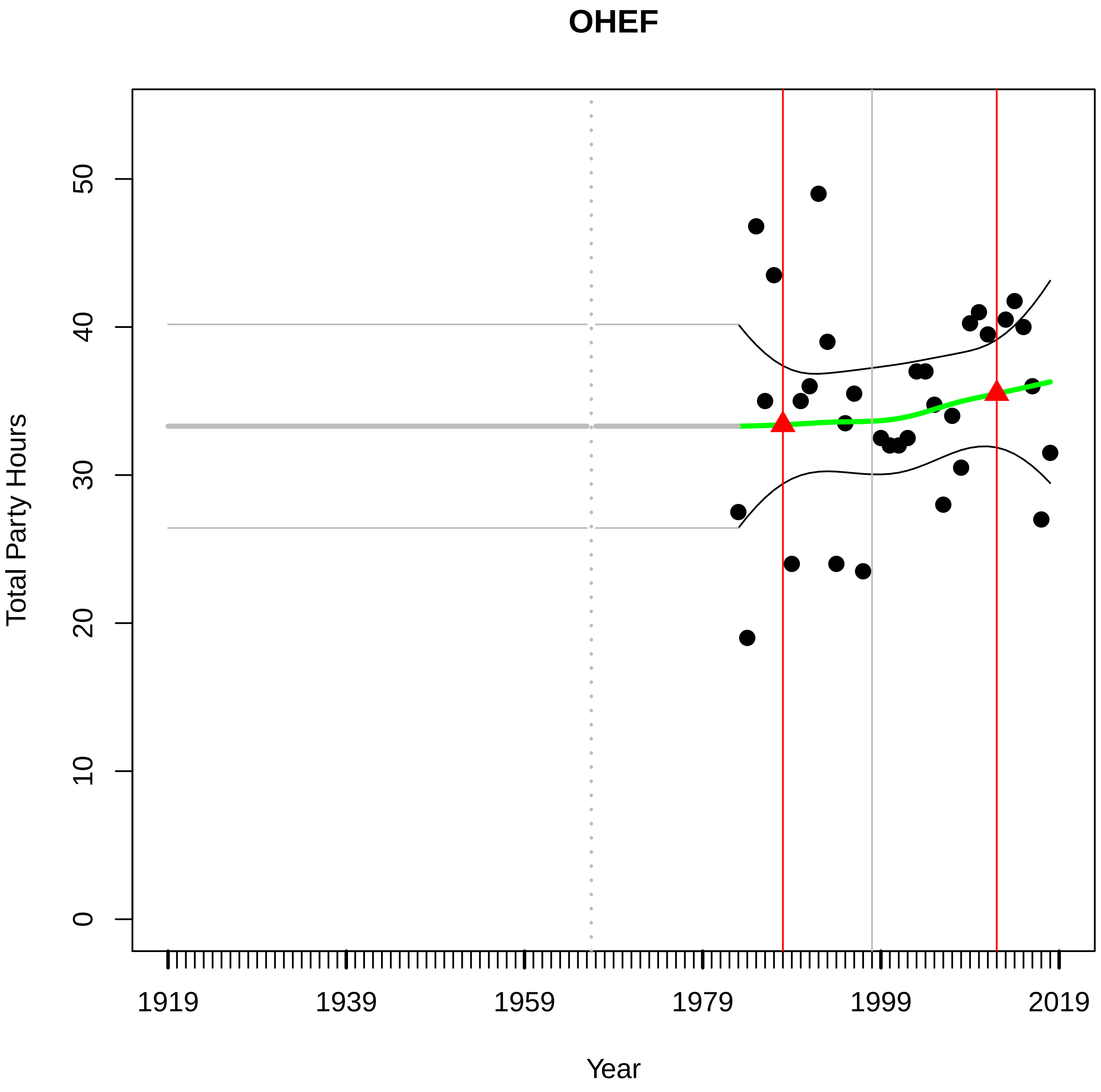

OHEF
Total Party Hours
Year
0
10
20
30
40
50
1919
1939
1959
1979
1999
2019

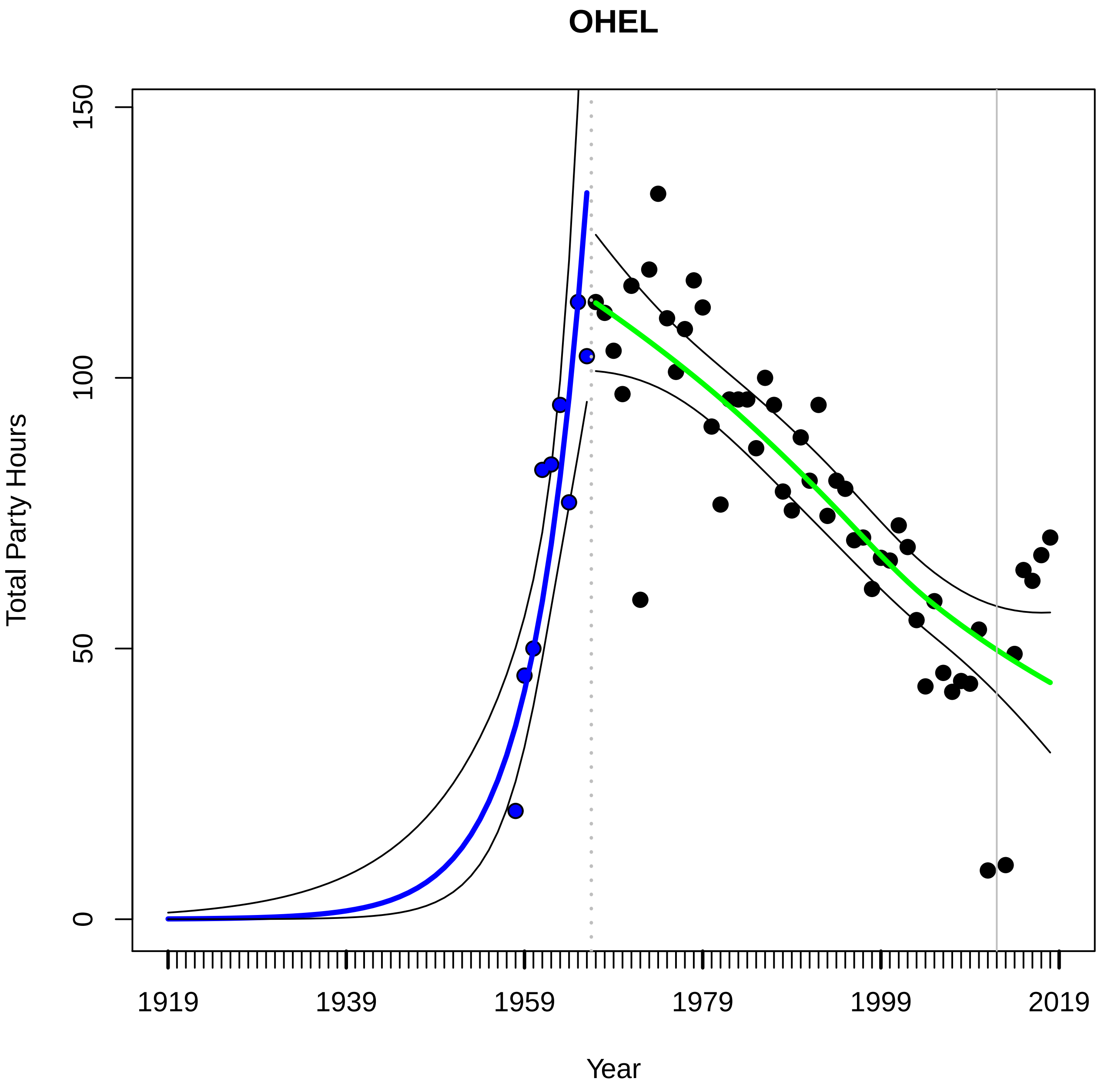

OHEL
Total Party Hours
0
50
100
150
1919
1939
1959
1979
1999
2019
Year

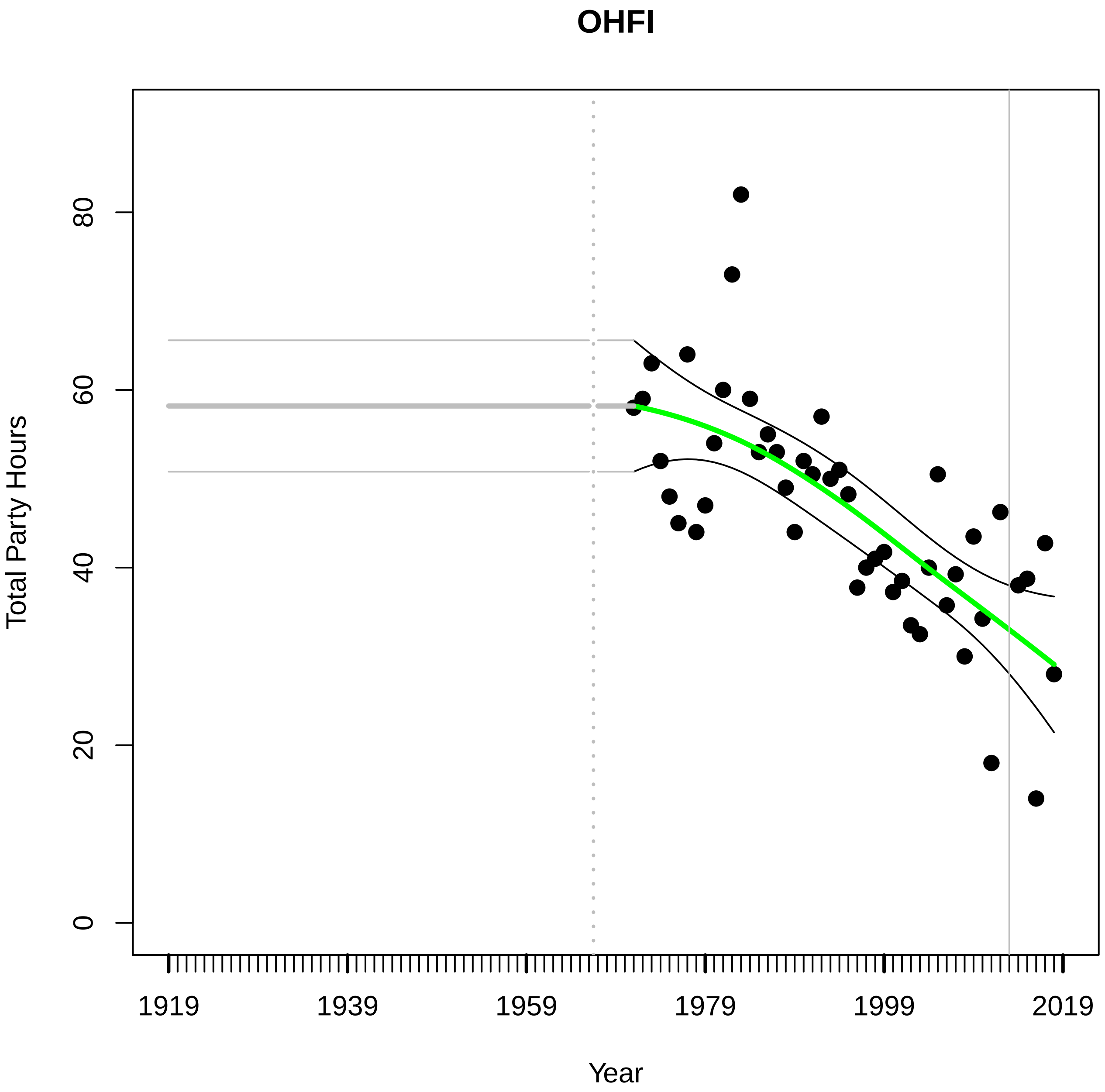

OHFI
Total Party Hours
0
20
40
60
80
1919
1939
1959
1979
1999
2019
Year

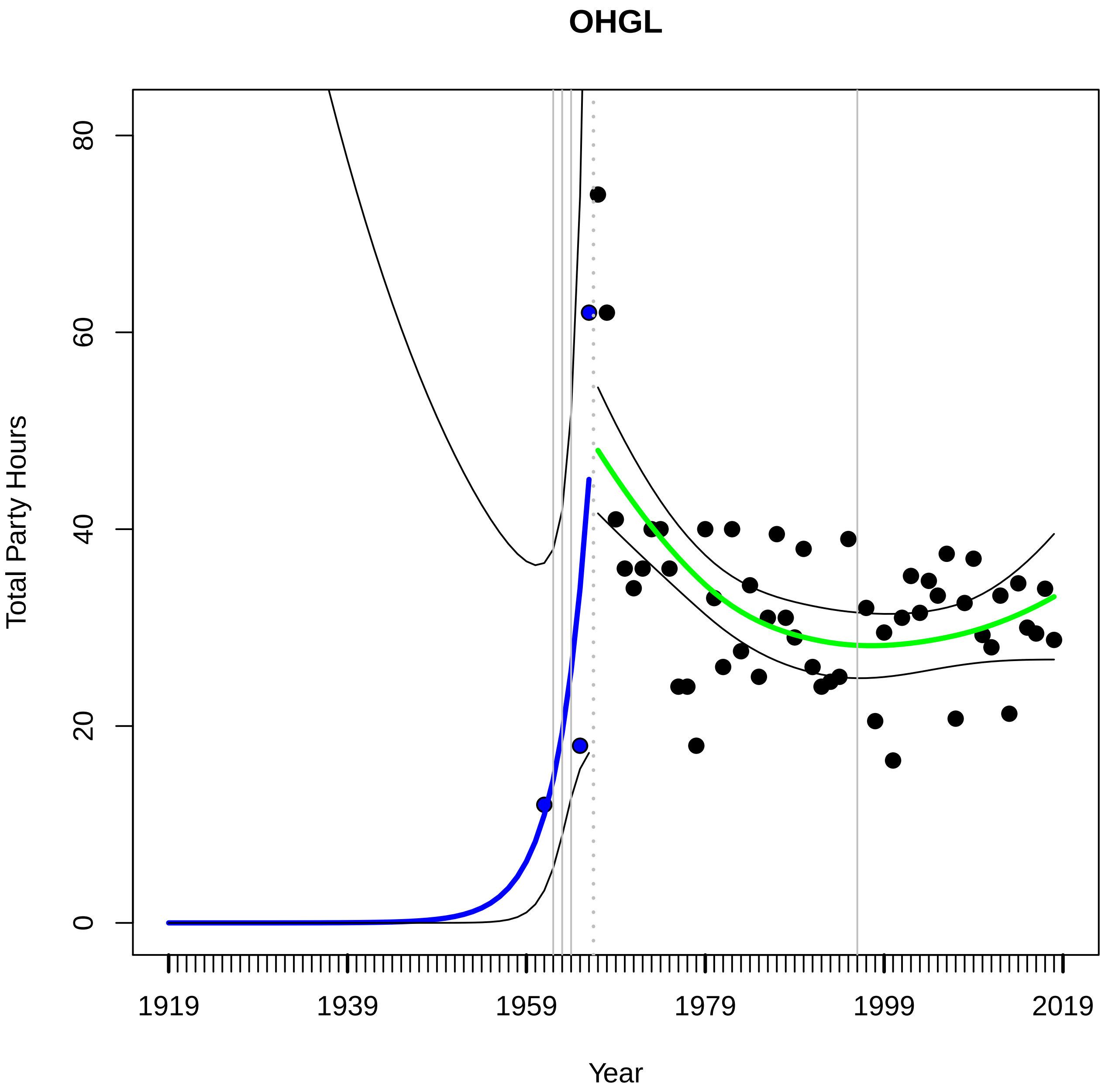
OHGL
Total Party Hours
0
20
40
60
80
1919
1939
1959
1979
1999
2019
Year

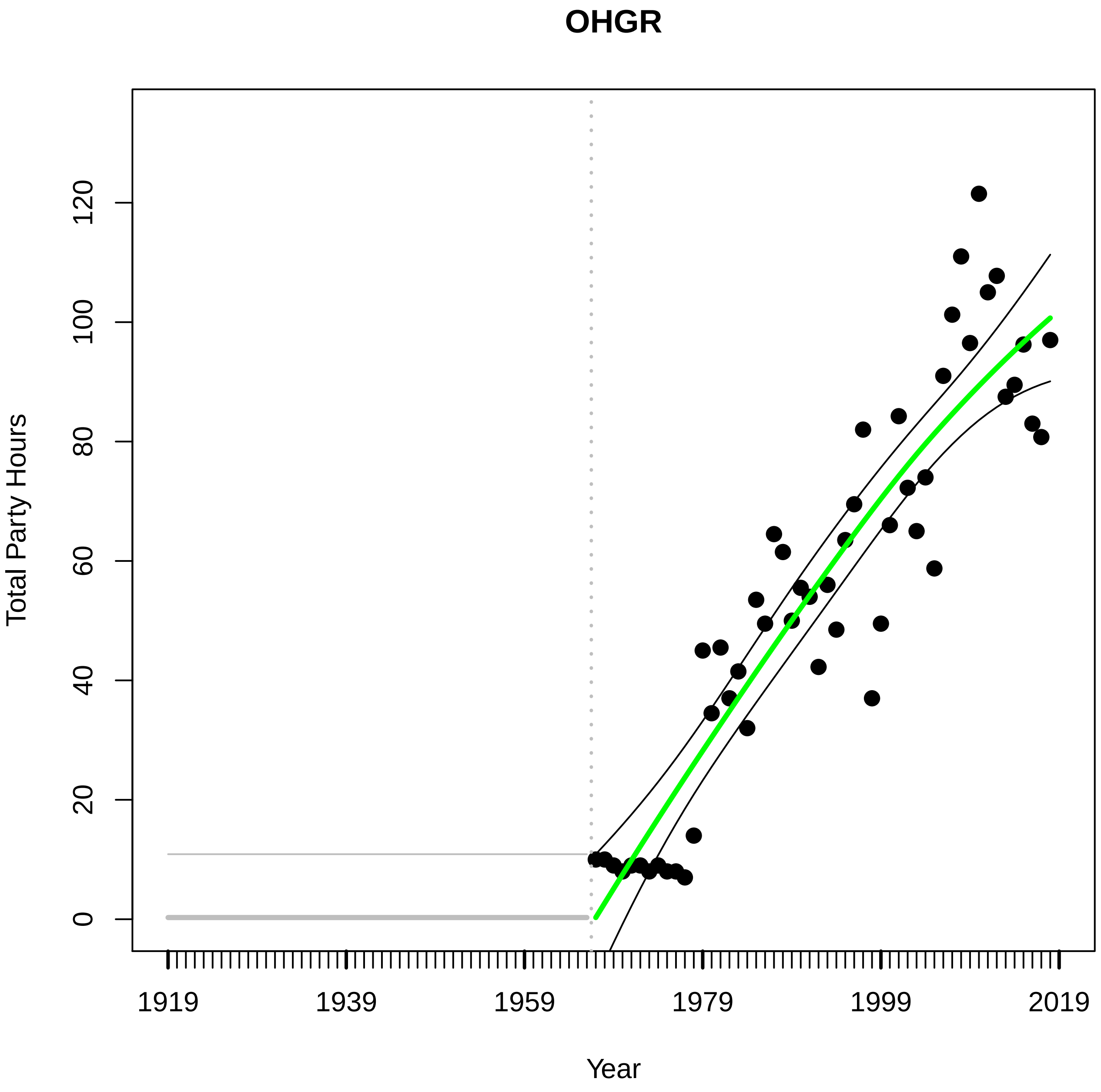
OHGR
Total Party Hours
0
20
40
60
80
100
120
1919
1939
1959
1979
1999
2019
Year

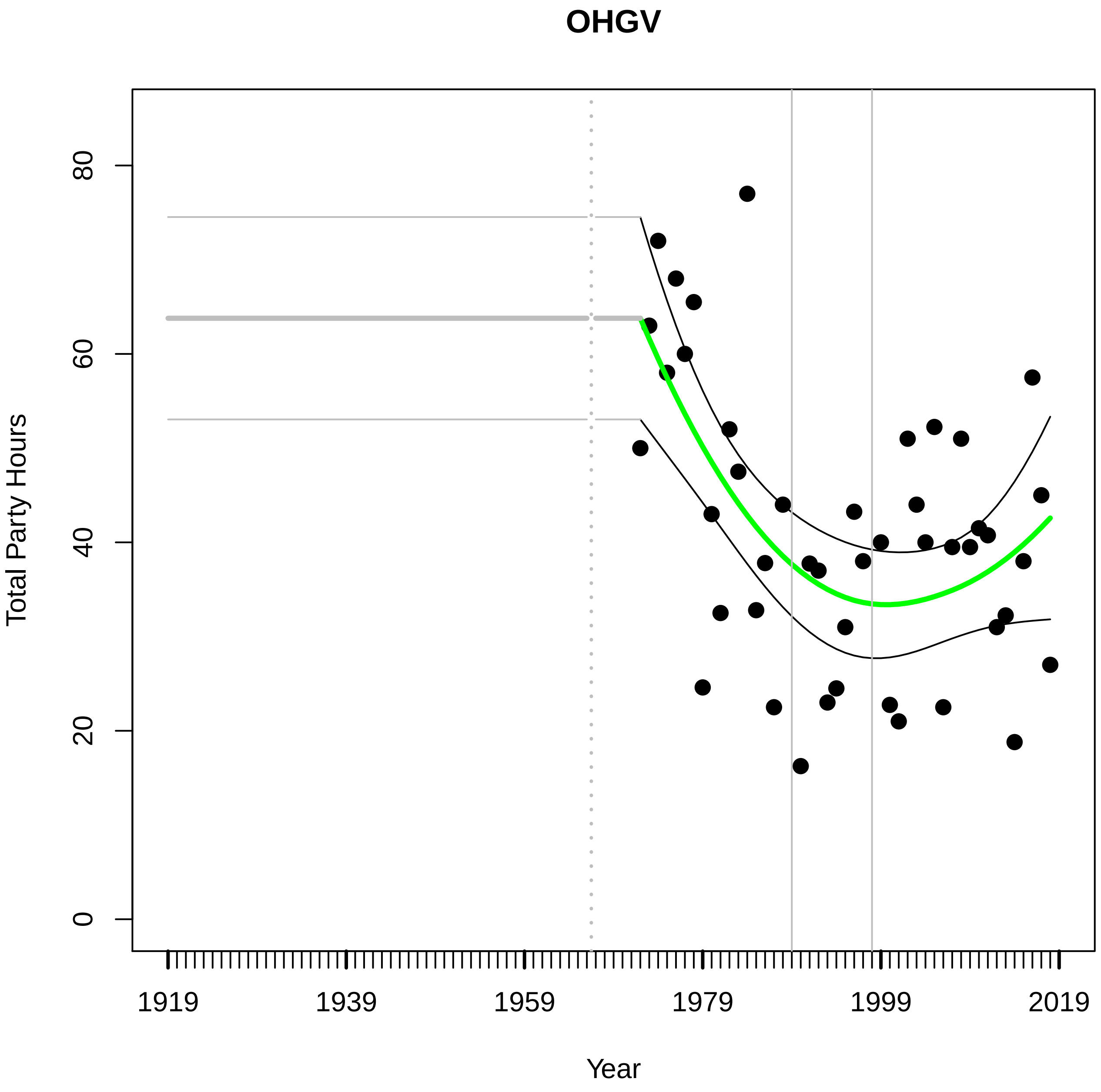
OHGV
Total Party Hours
80
60
40
20
0
1919
1939
1959
1979
1999
2019
Year

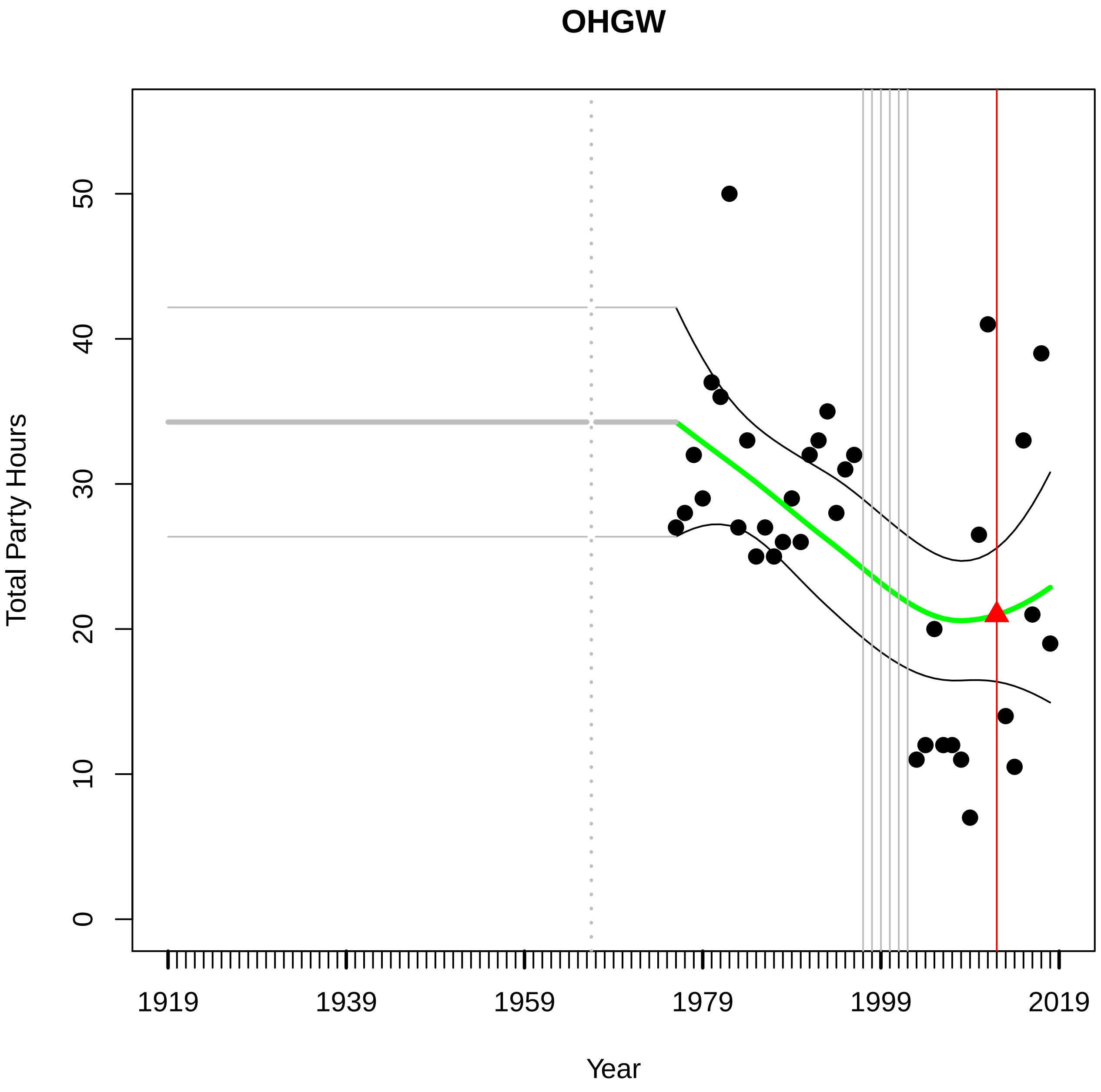
OHGW
Total Party Hours
0
10
20
30
40
50
1919
1939
1959
1979
1999
2019
Year

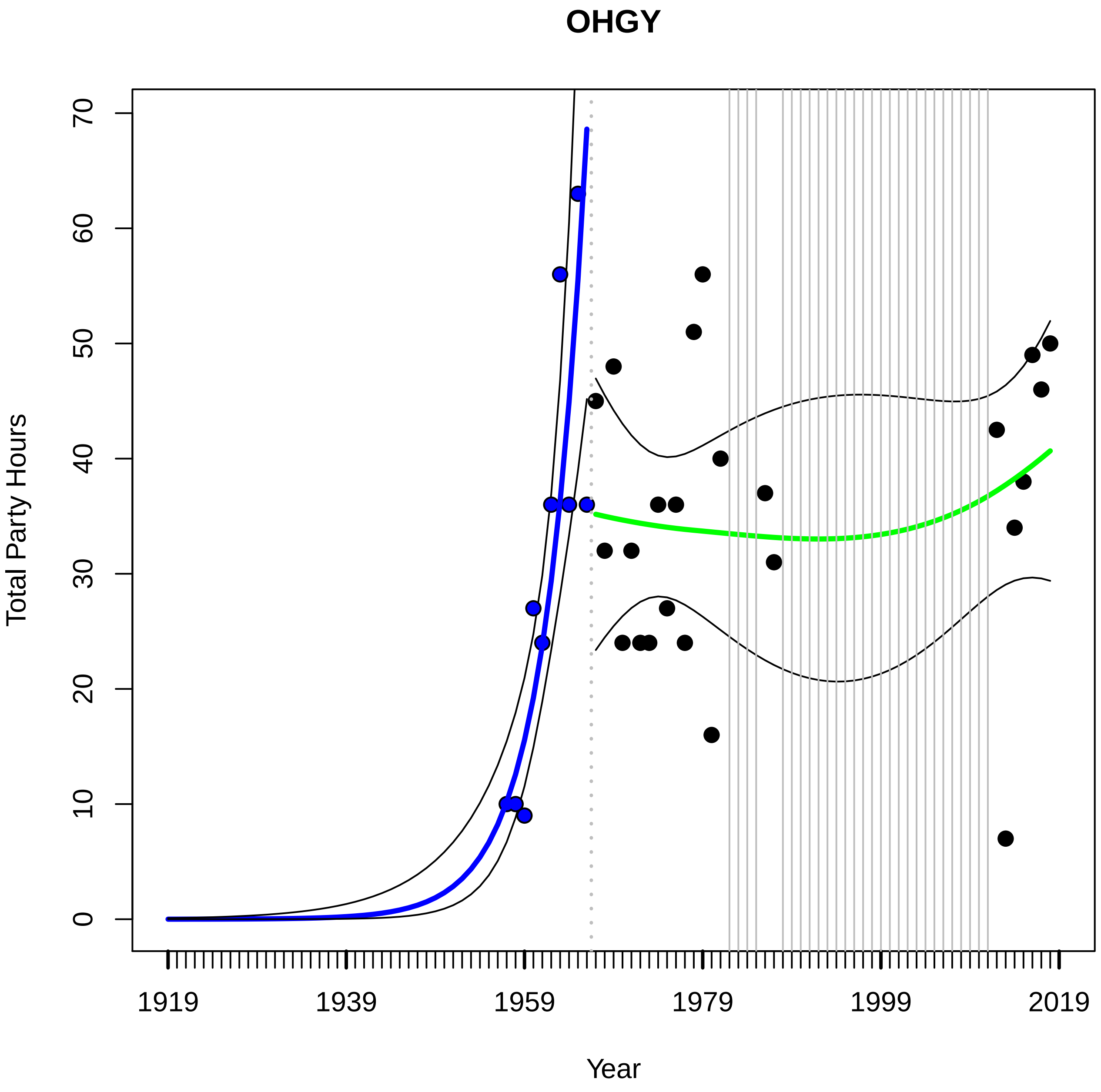

OHGY
Total Party Hours
Year
0
10
20
30
40
50
60
70
1919
1939
1959
1979
1999
2019

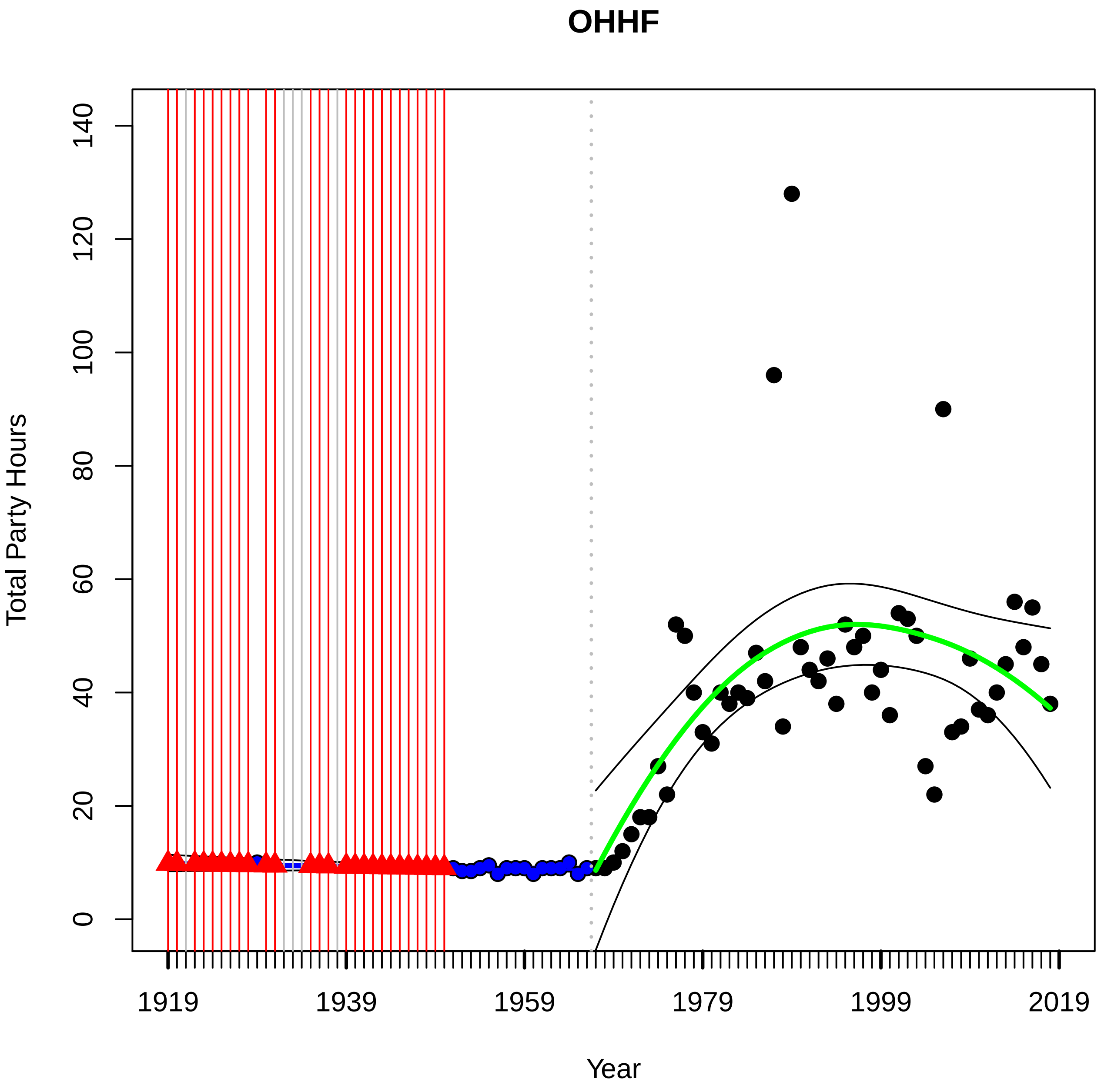
OHHF
Total Party Hours
0
20
40
60
80
100
120
140
1919
1939
1959
1979
1999
2019
Year

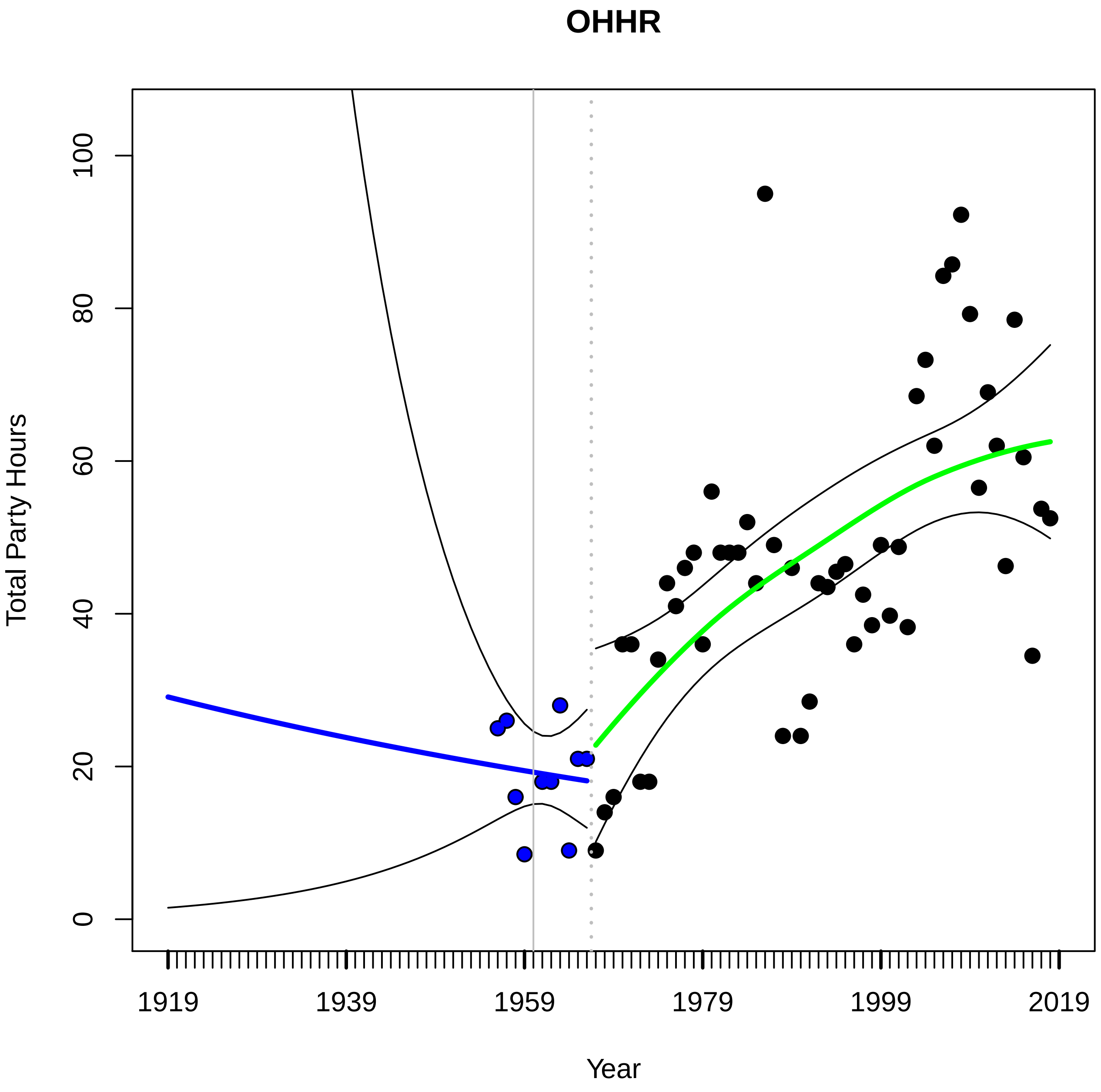
OHHR
Total Party Hours
0
20
40
60
80
100
1919
1939
1959
1979
1999
2019
Year

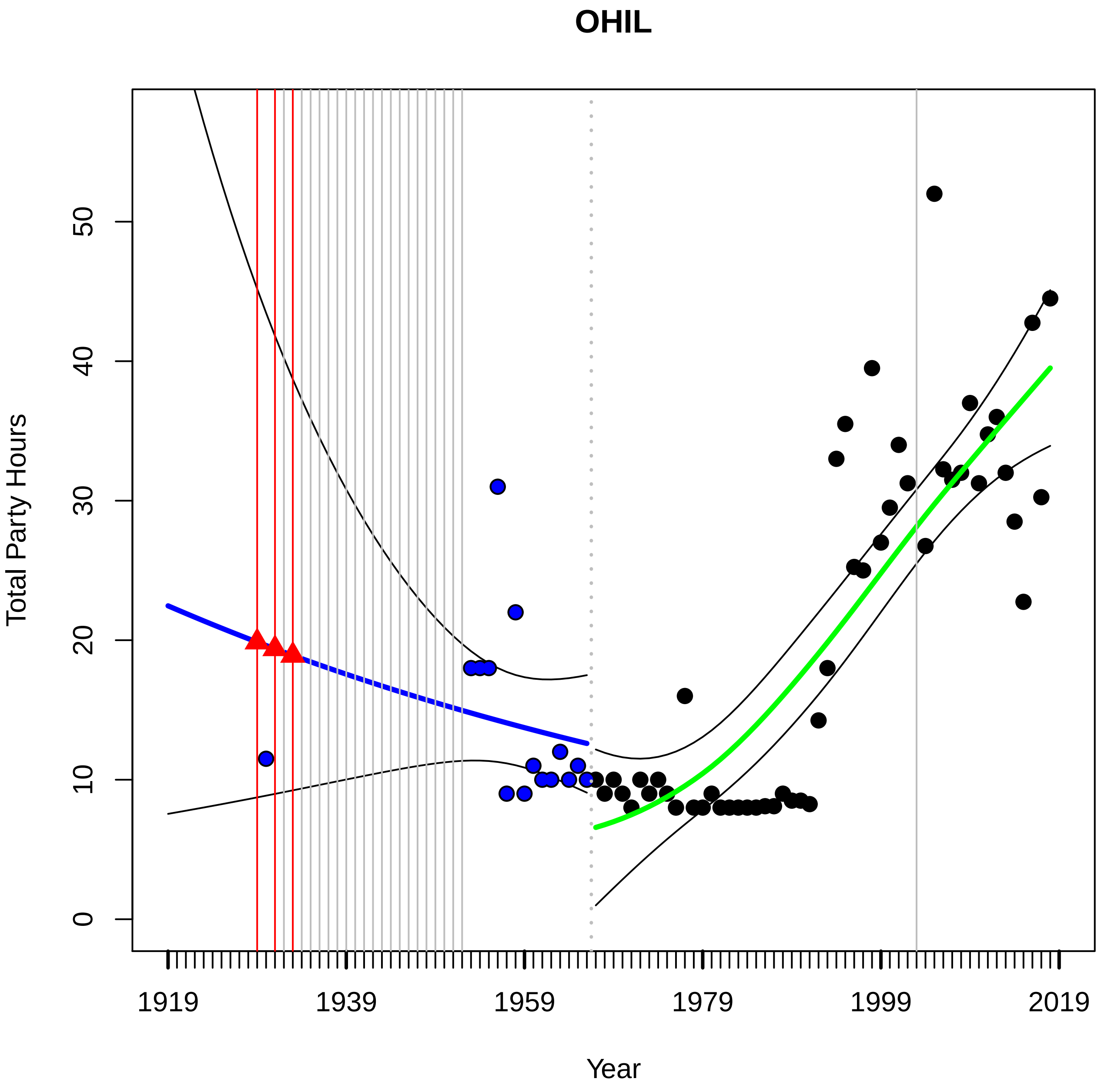

OHIL
Total Party Hours
0
10
20
30
40
50
1919
1939
1959
1979
1999
2019
Year

OHKI

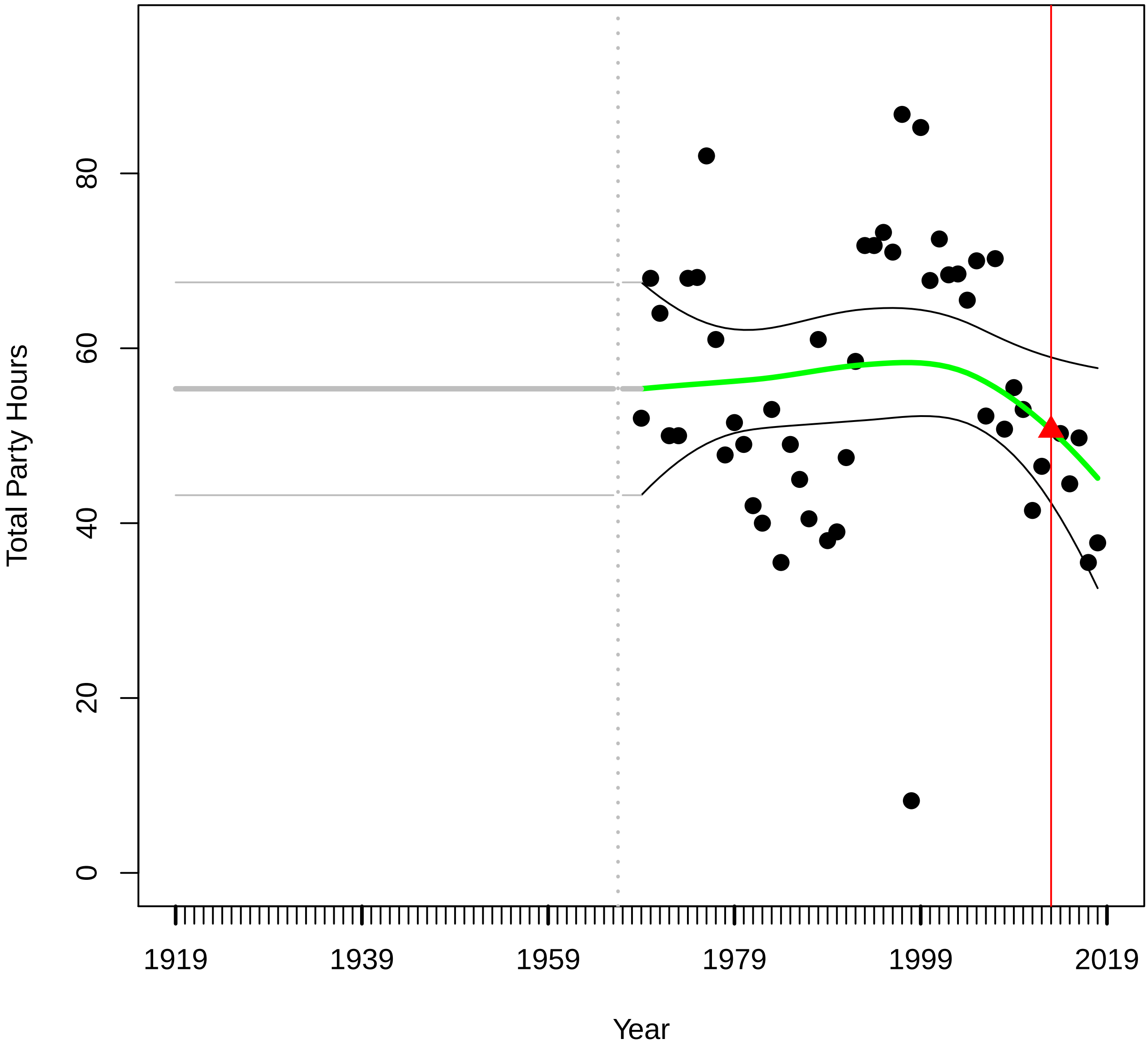

Total Party Hours
0
20
40
60
80
1919
1939
1959
1979
1999
2019
Year

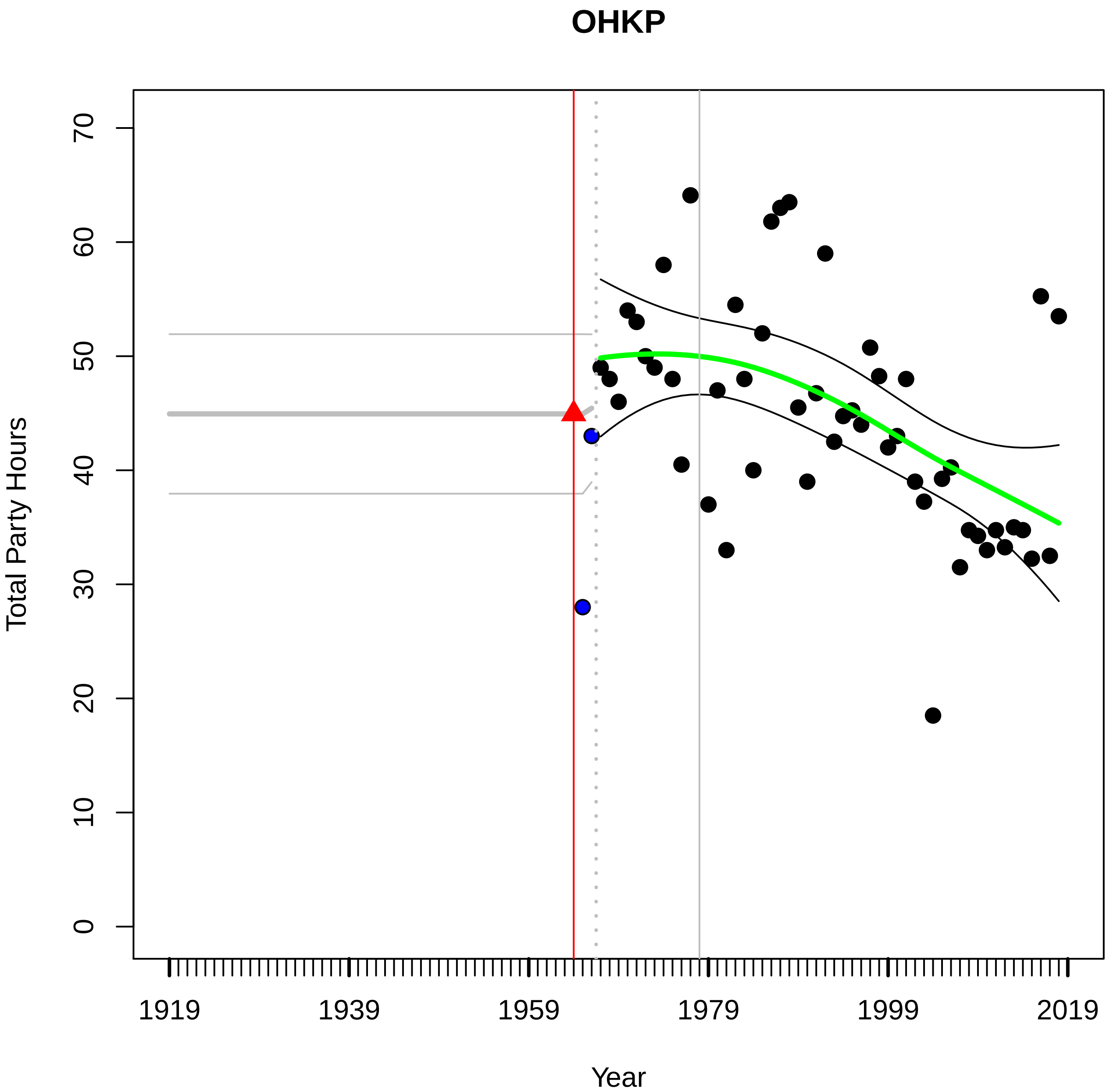

OHKP
Total Party Hours
Year
0
10
20
30
40
50
60
70
1919
1939
1959
1979
1999
2019

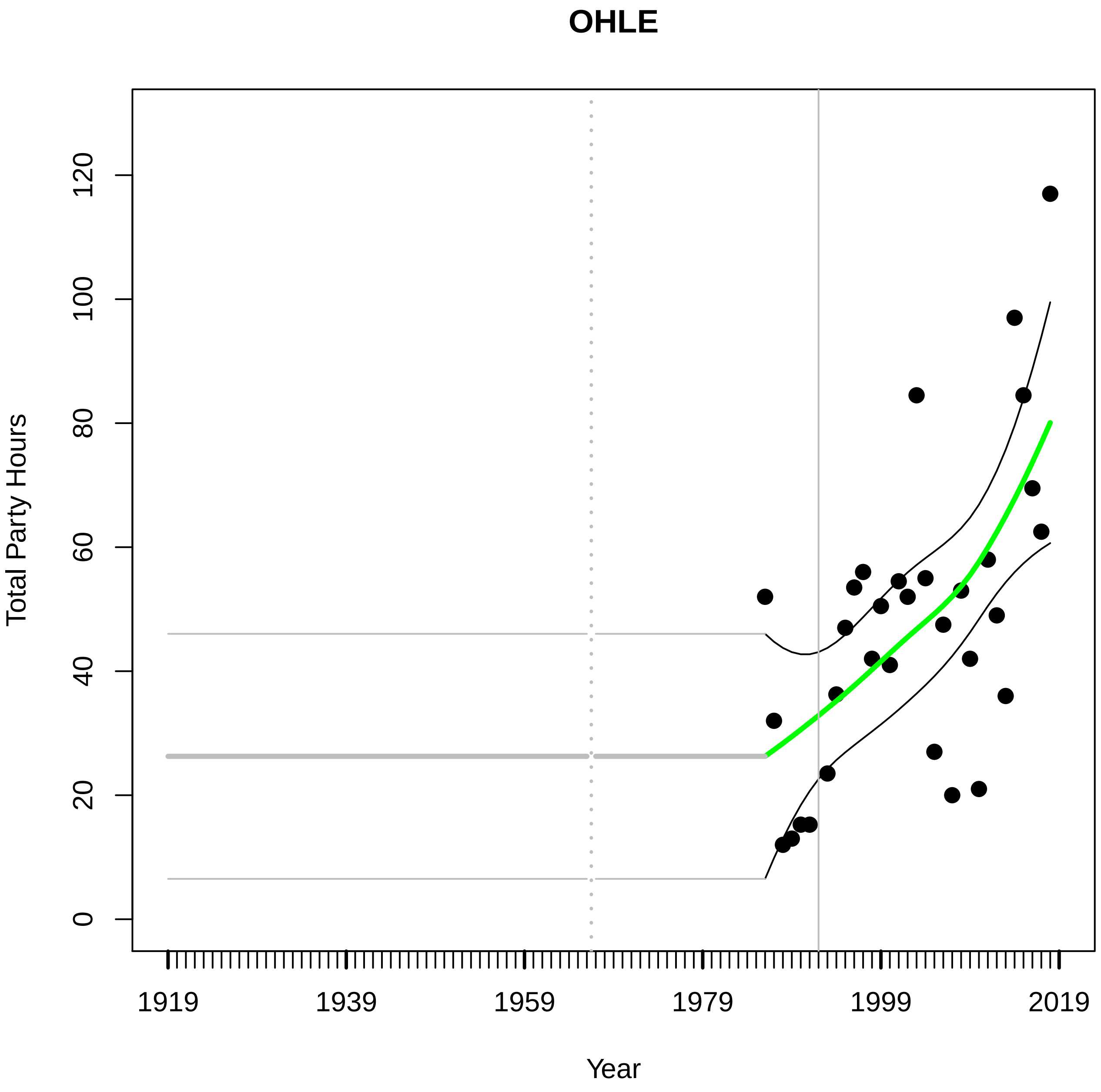

OHLE
Total Party Hours
120
100
80
60
40
20
0
1919
1939
1959
1979
1999
2019
Year

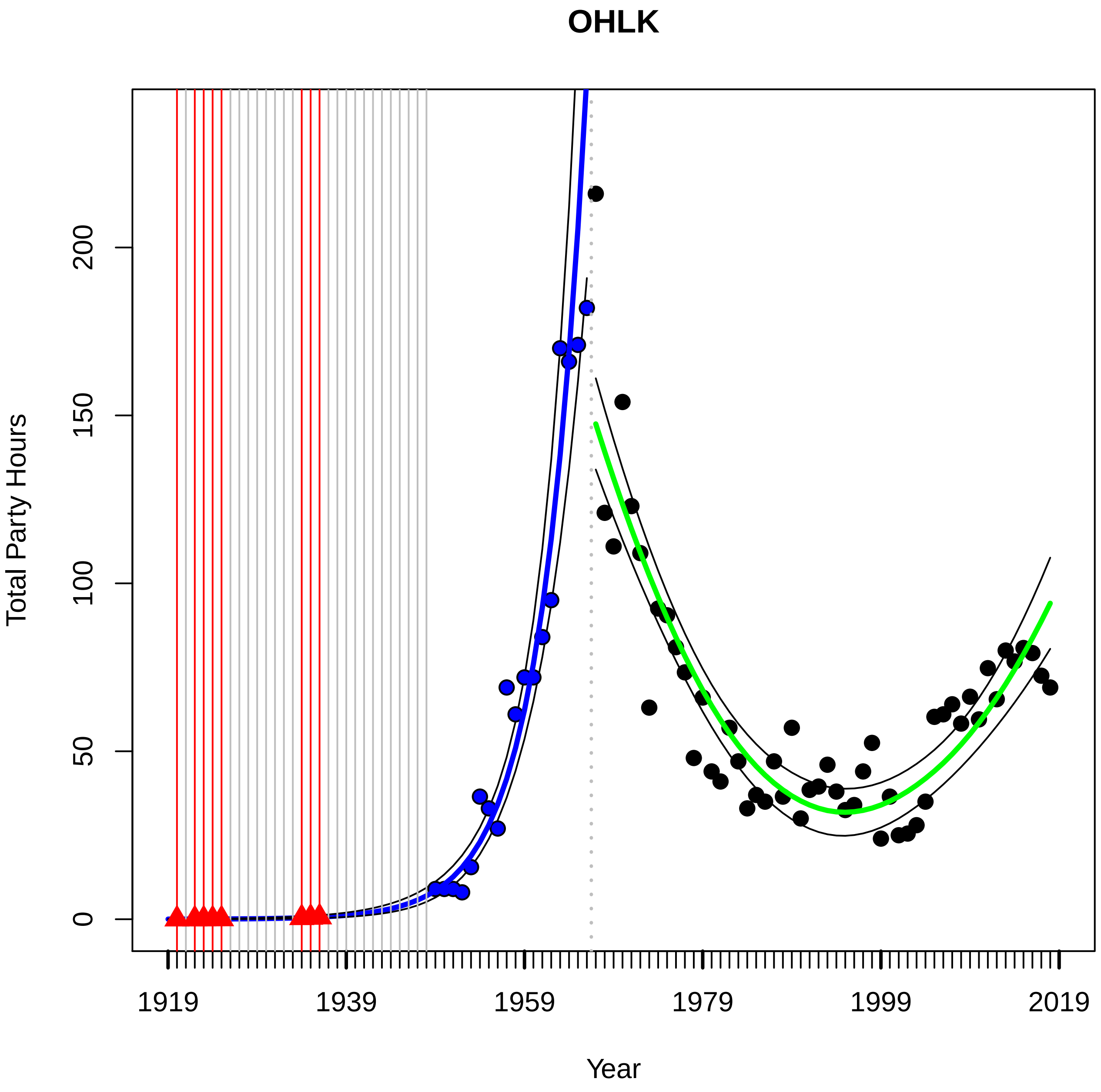

OHLK
Total Party Hours
0
50
100
150
200
1919
1939
1959
1979
1999
2019
Year

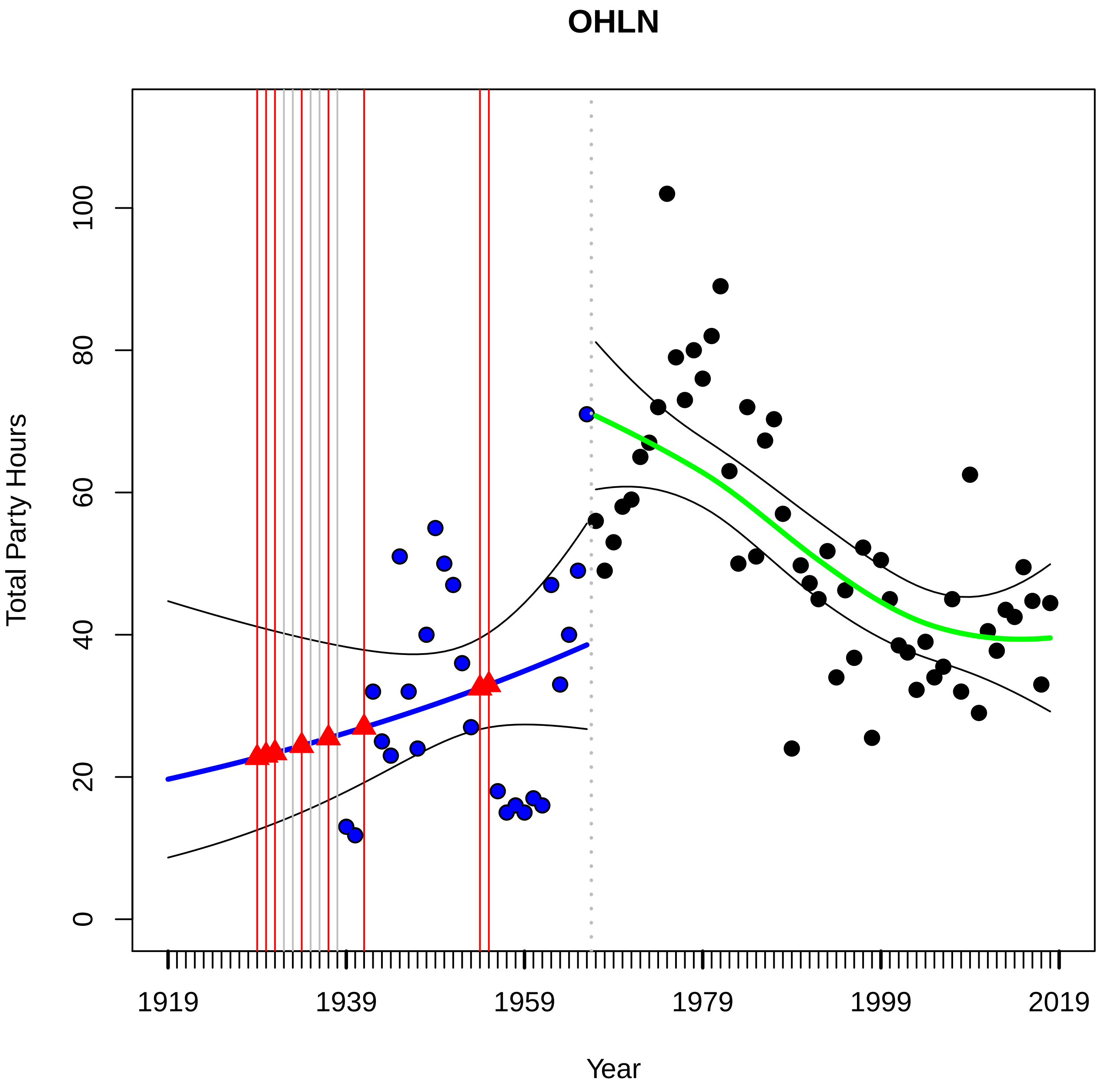
OHLN
Total Party Hours
0
20
40
60
80
100
Year
1919
1939
1959
1979
1999
2019

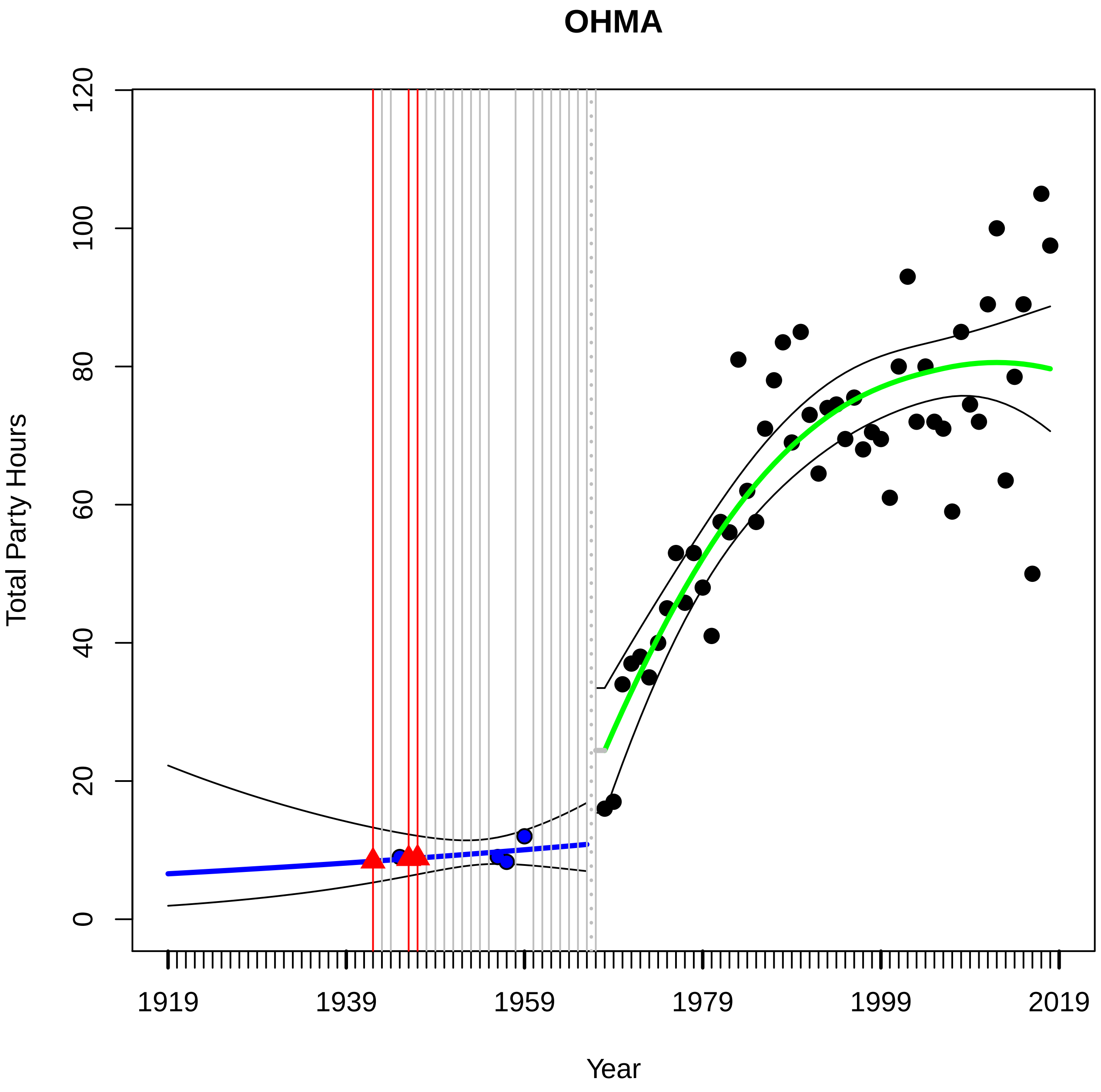
OHMA
Total Party Hours
Year
0
20
40
60
80
100
120
1919
1939
1959
1979
1999
2019

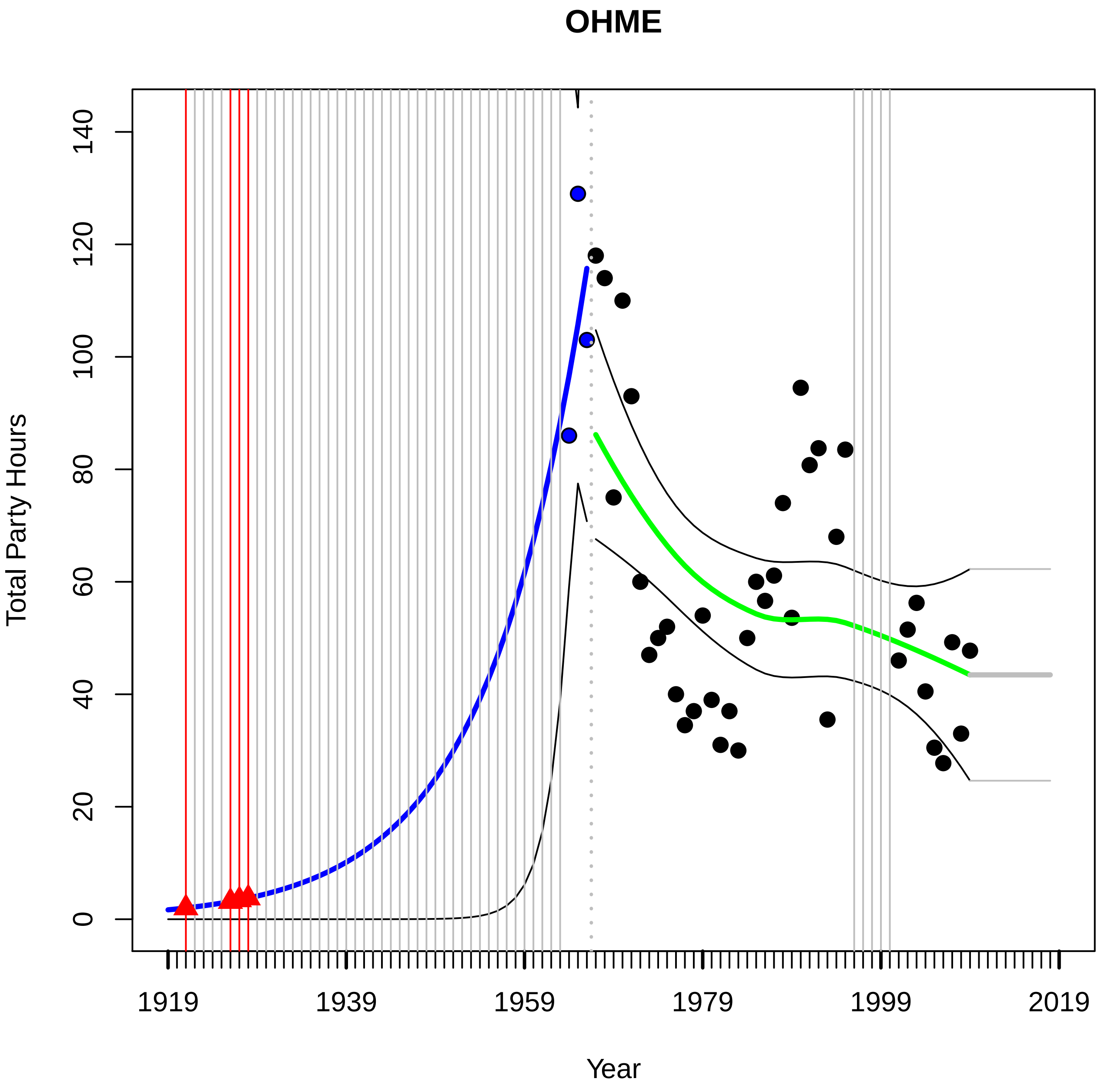

OHME
Total Party Hours
Year
0
20
40
60
80
100
120
140
1919
1939
1959
1979
1999
2019

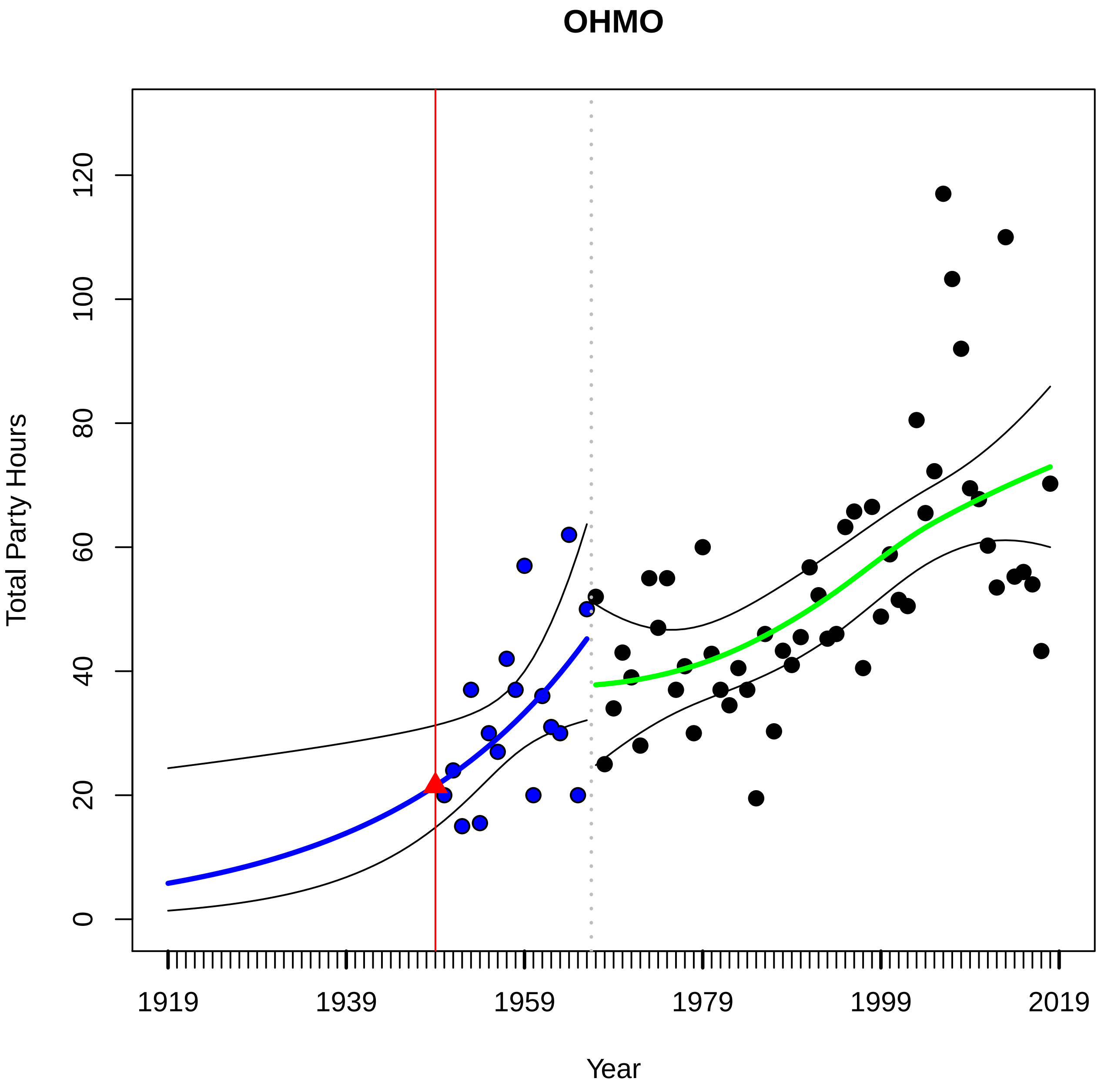

OHMO
Total Party Hours
0
20
40
60
80
100
120
1919
1939
1959
1979
1999
2019
Year

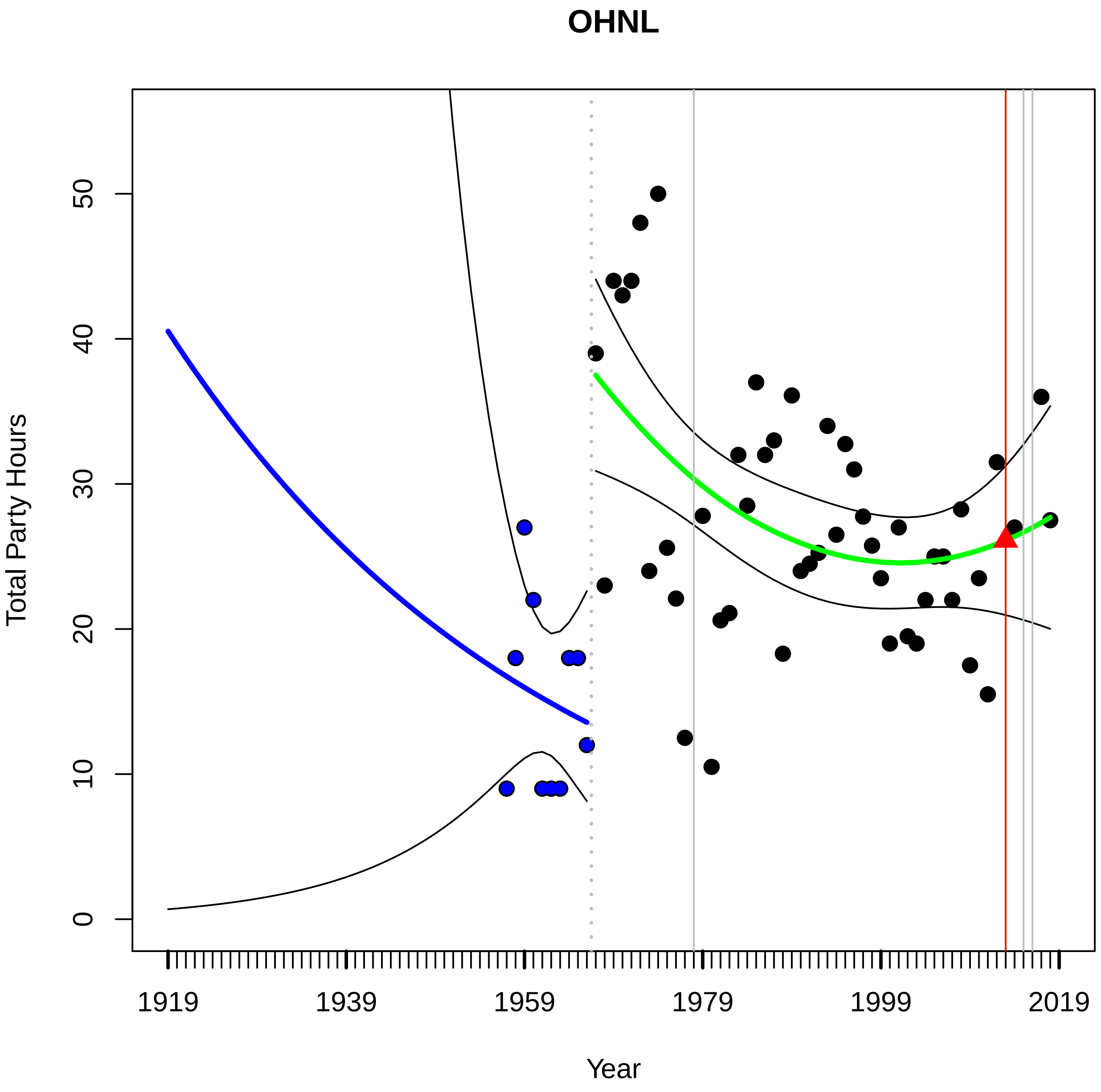
OHNL
Total Party Hours
0
10
20
30
40
50
1919
1939
1959
1979
1999
2019
Year

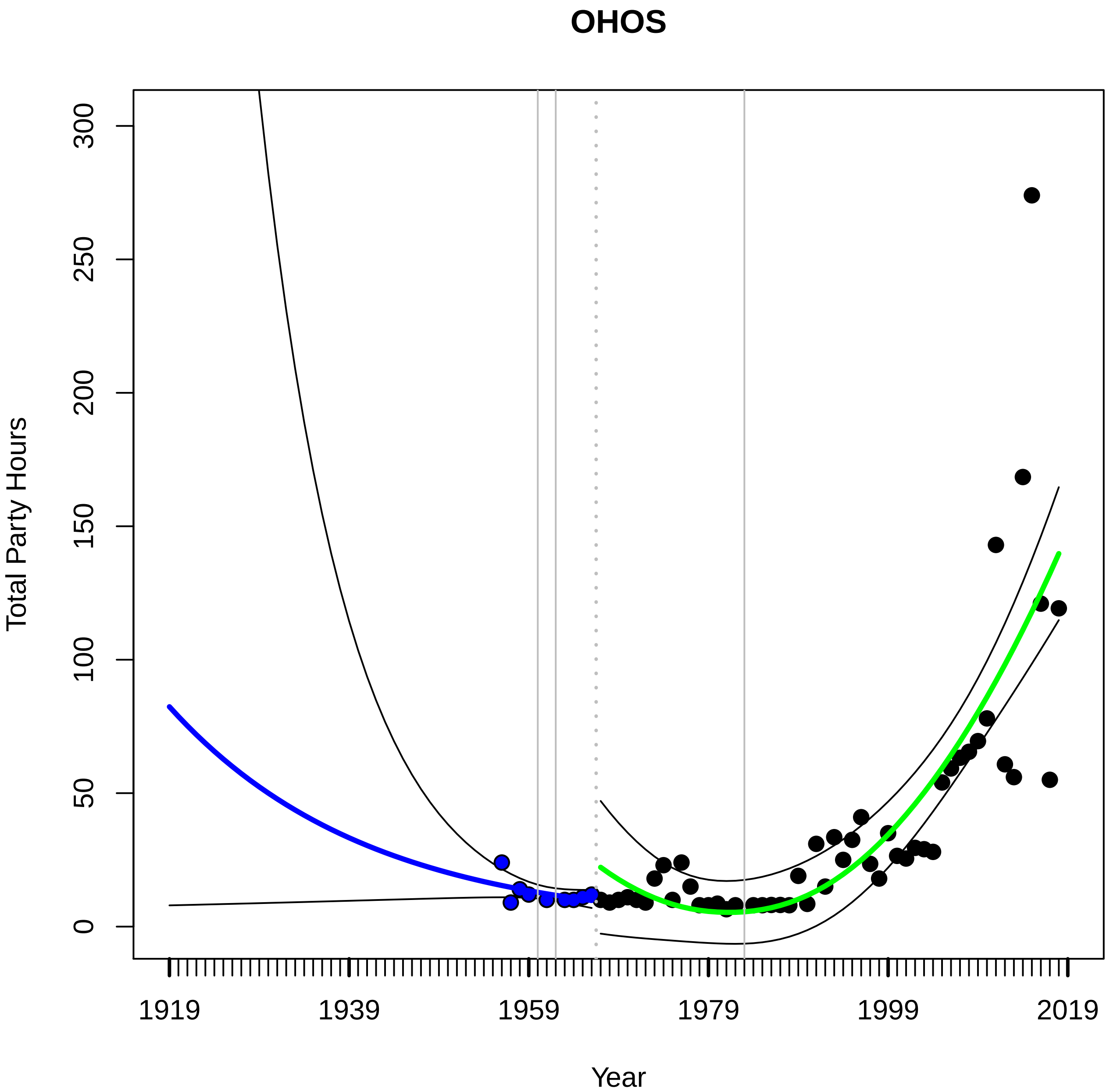

OHOS
Total Party Hours
0
50
100
150
200
250
300
1919
1939
1959
1979
1999
2019
Year

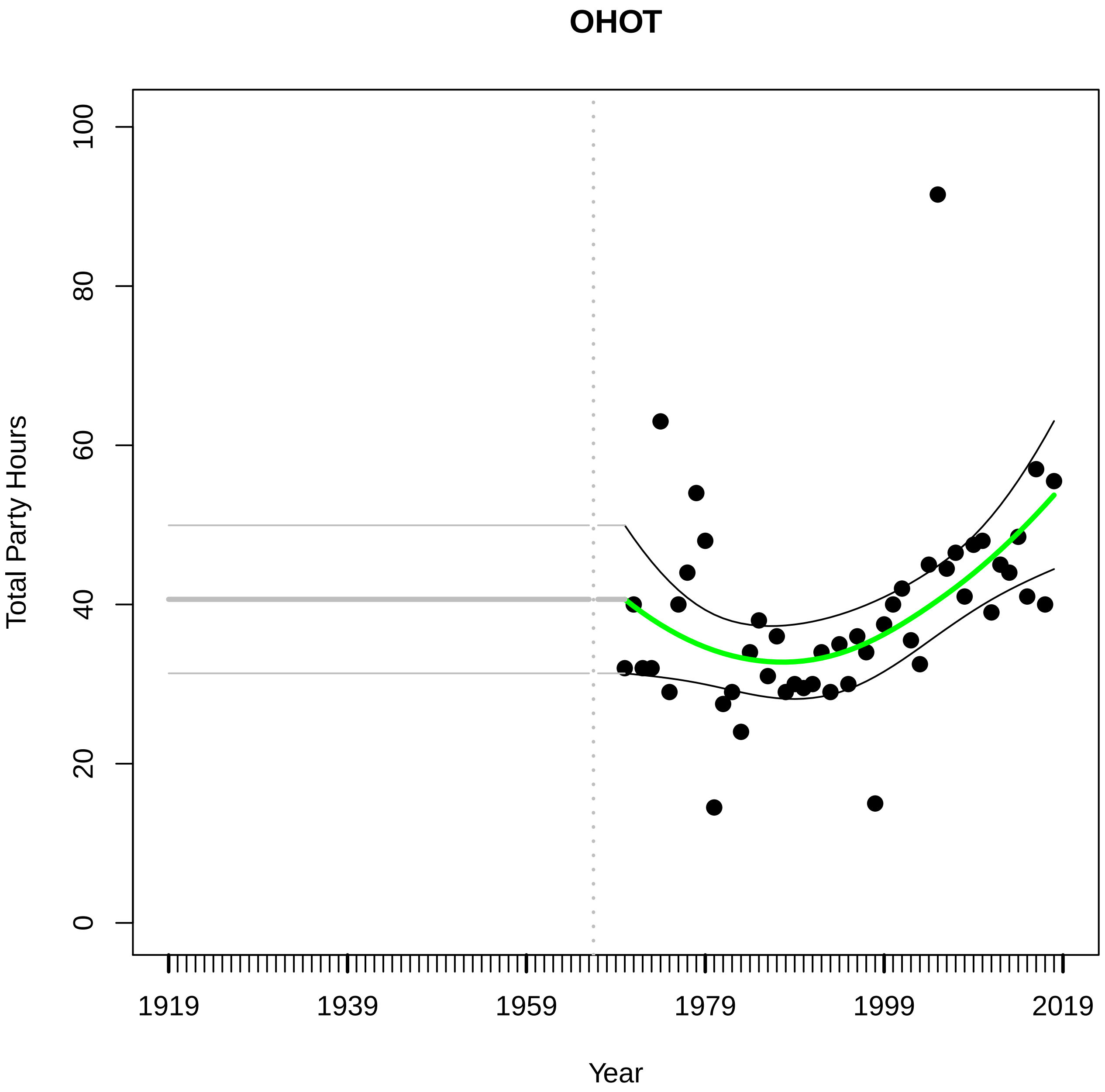

OHOT
Total Party Hours
0
20
40
60
80
100
1919
1939
1959
1979
1999
2019
Year

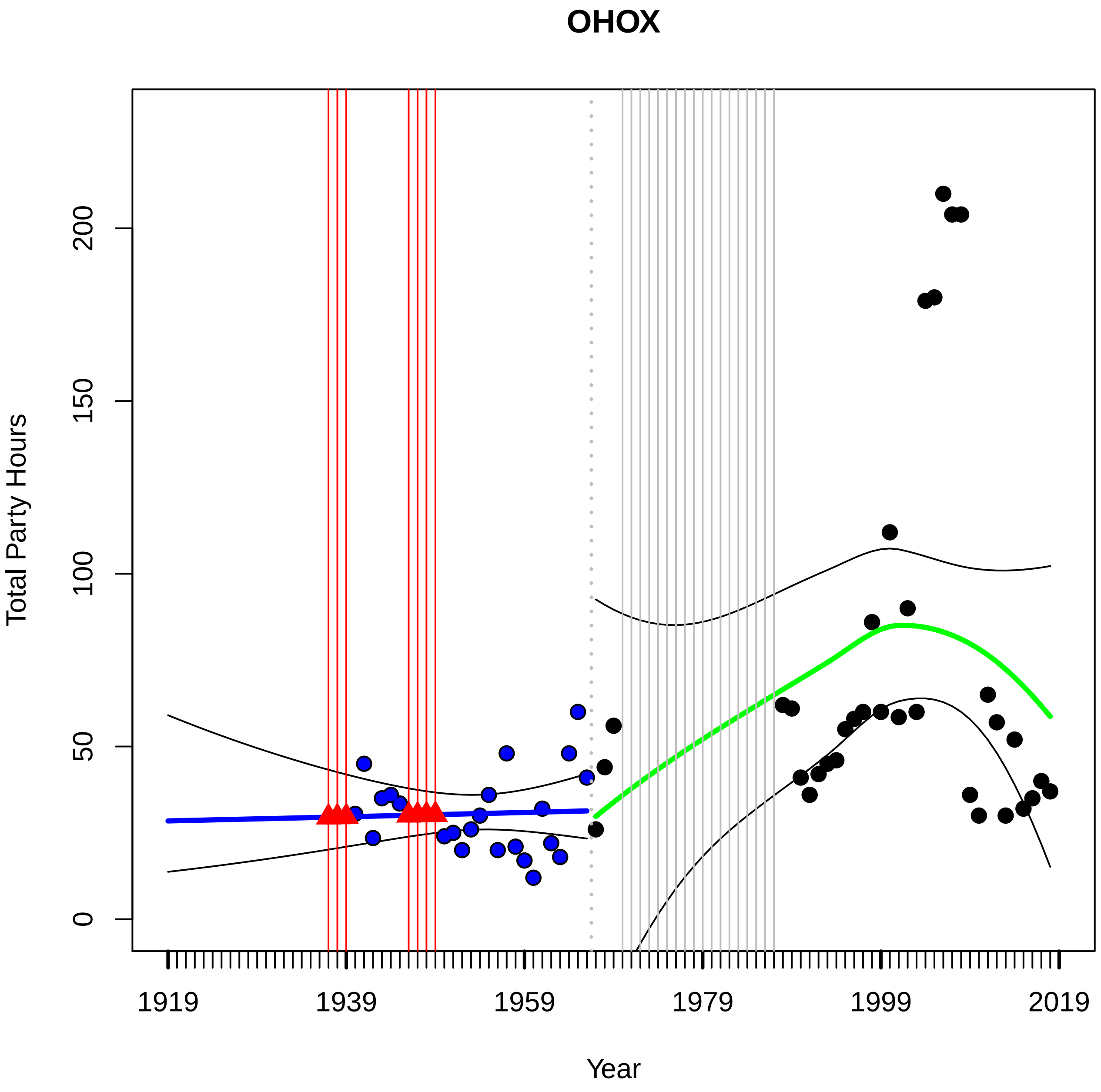

OHOX
Total Party Hours
0
50
100
150
200
1919
1939
1959
1979
1999
2019
Year

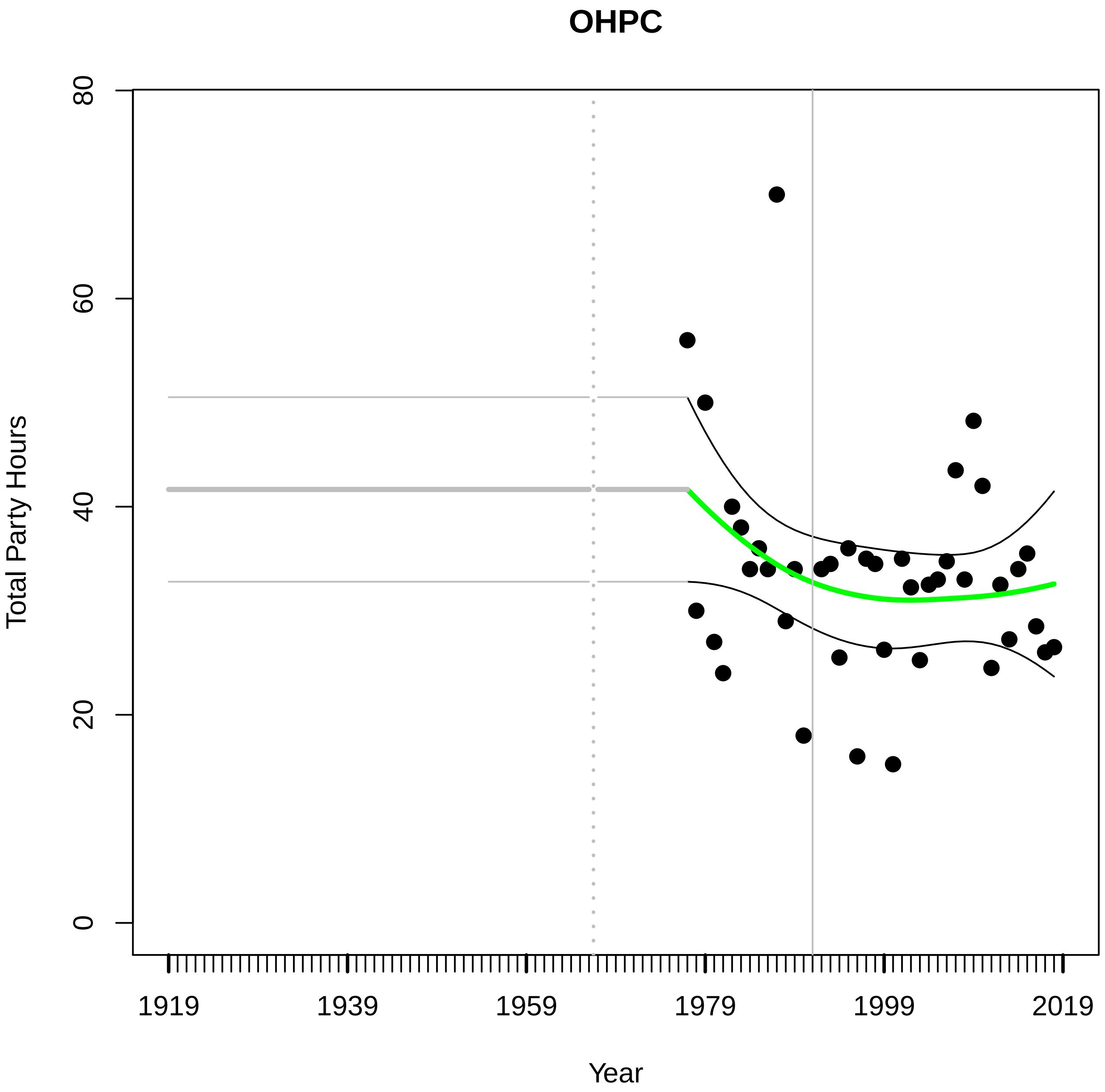

OHPC
Total Party Hours
Year
0
20
40
60
80
1919
1939
1959
1979
1999
2019

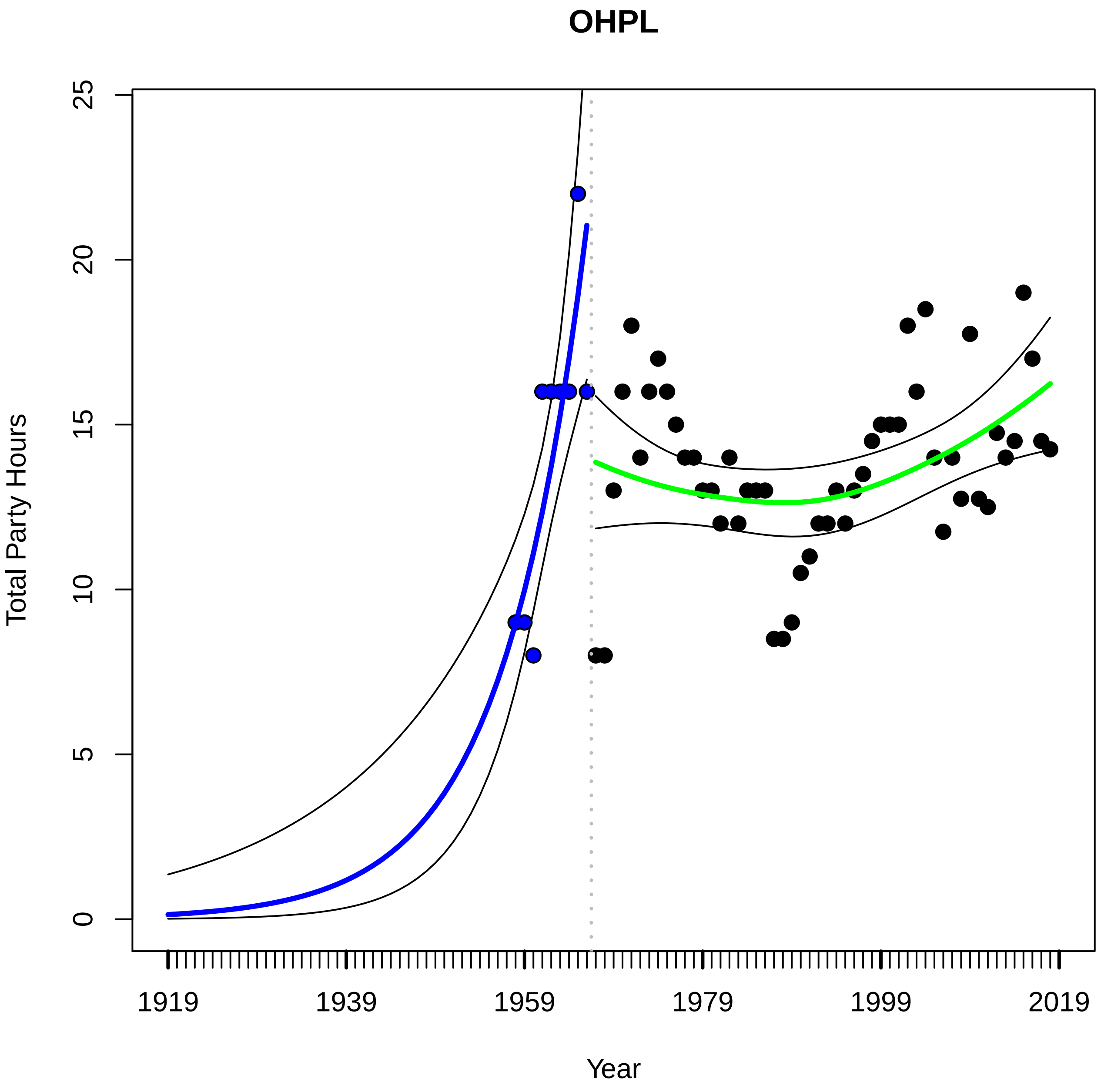

OHPL
Total Party Hours
Year
0
5
10
15
20
25
1919
1939
1959
1979
1999
2019

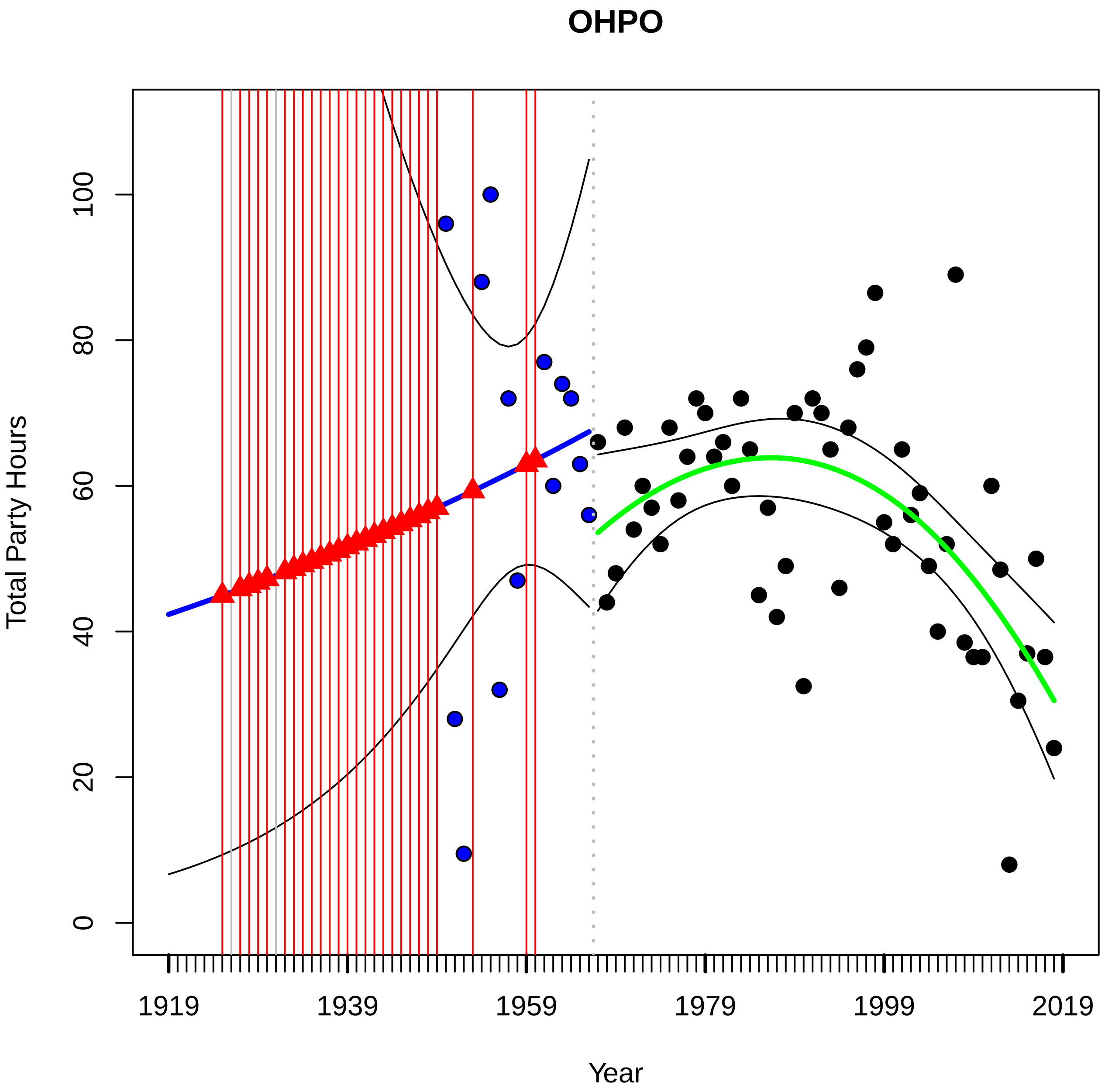

OHPO
Total Party Hours
Year
0
20
40
60
80
100
1919
1939
1959
1979
1999
2019

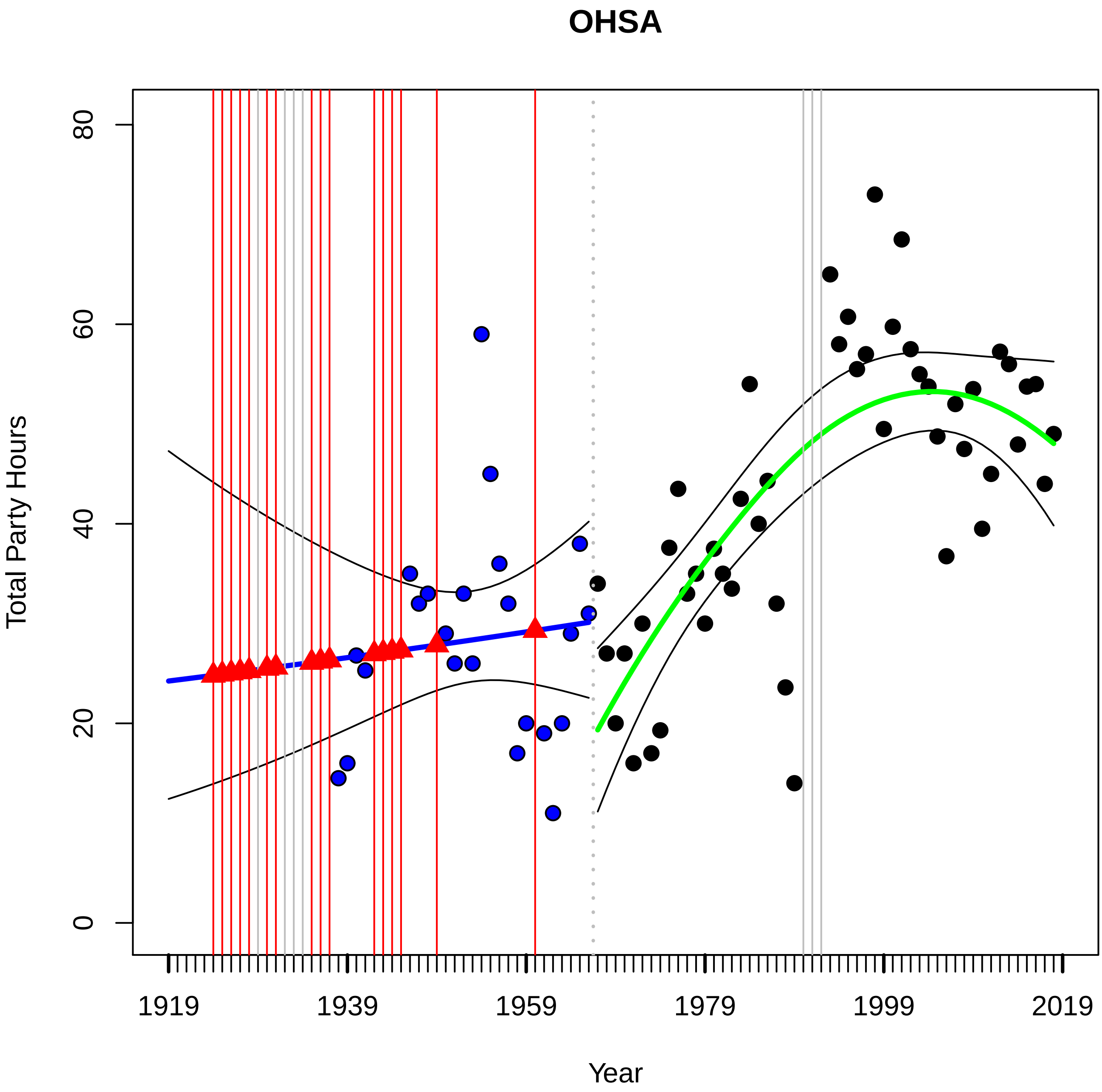

OHSA
Total Party Hours
0
20
40
60
80
1919
1939
1959
1979
1999
2019
Year

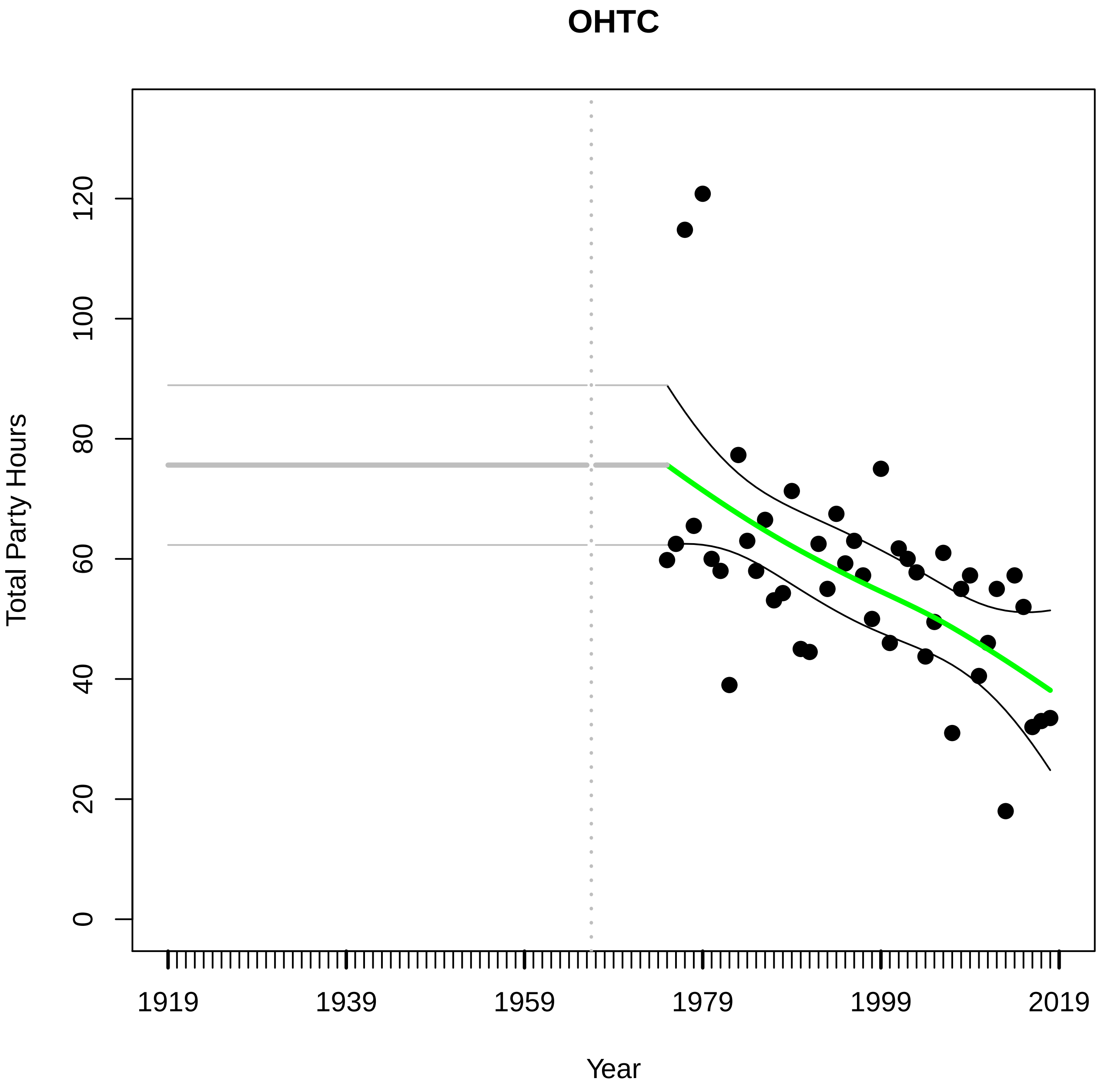

OHTC
Total Party Hours
0
20
40
60
80
100
120
1919
1939
1959
1979
1999
2019
Year

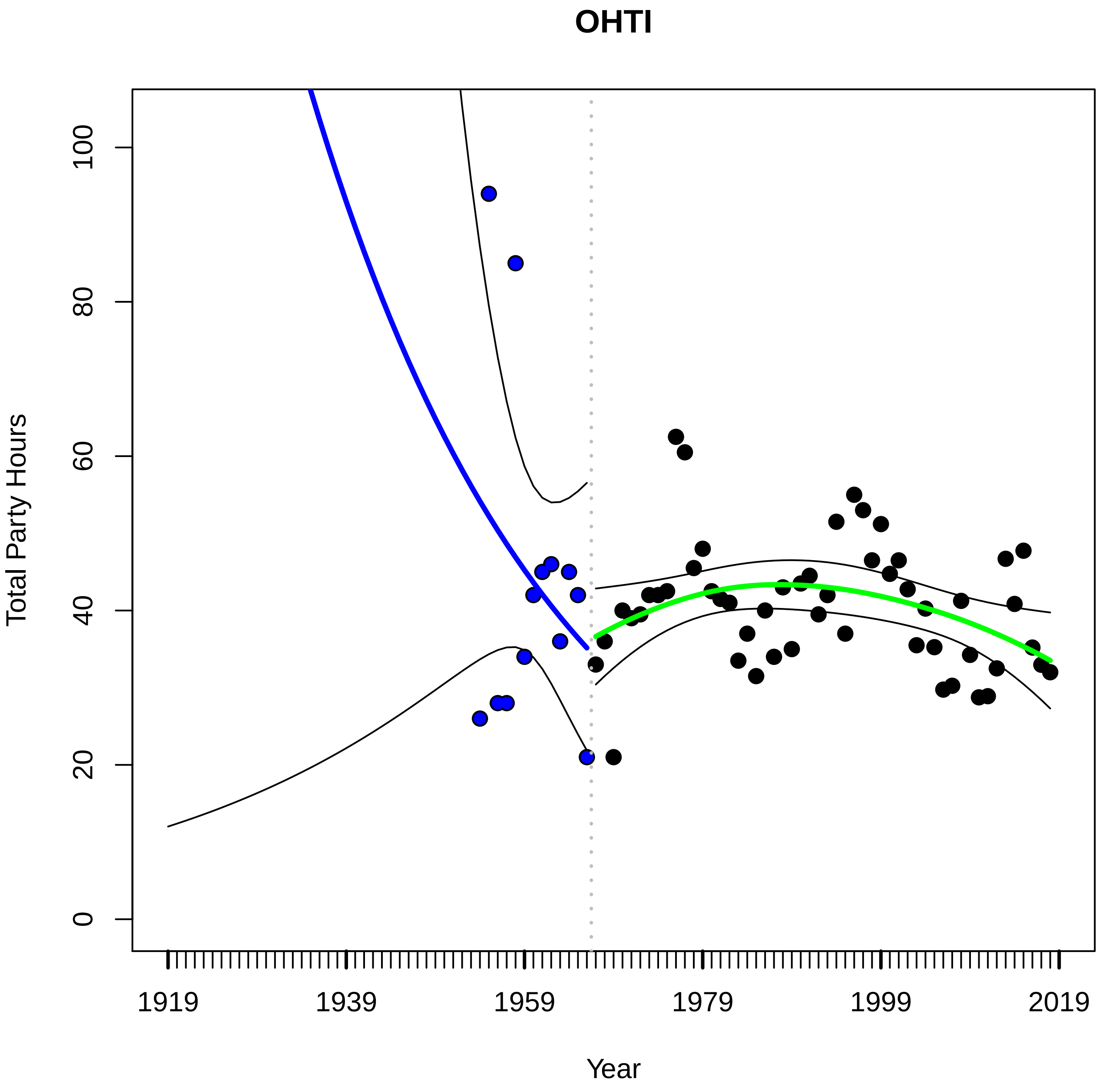

OHTI
Total Party Hours
0
20
40
60
80
100
1919
1939
1959
1979
1999
2019
Year

OHTO

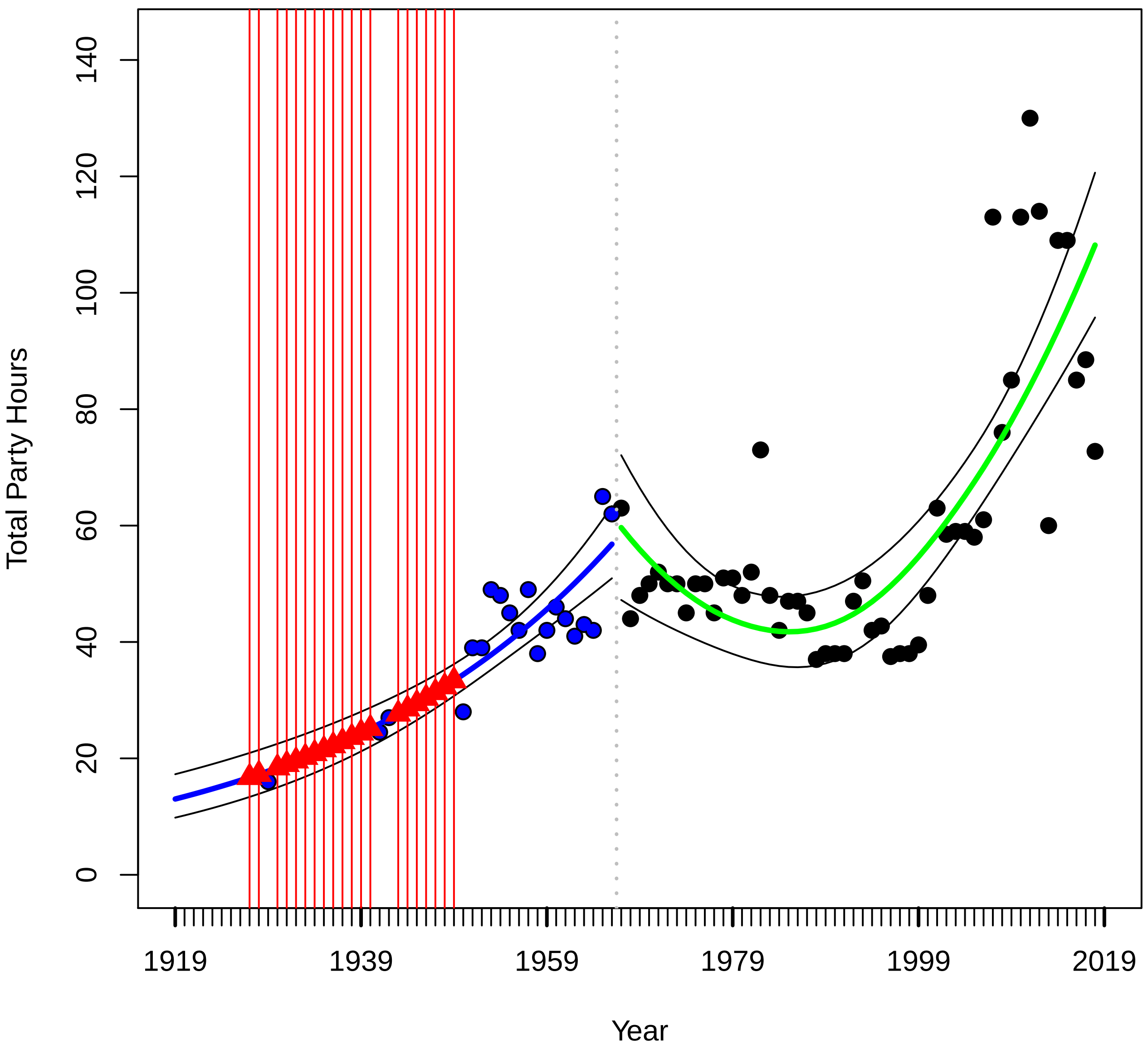

Total Party Hours
0
20
40
60
80
100
120
140
1919
1939
1959
1979
1999
2019
Year

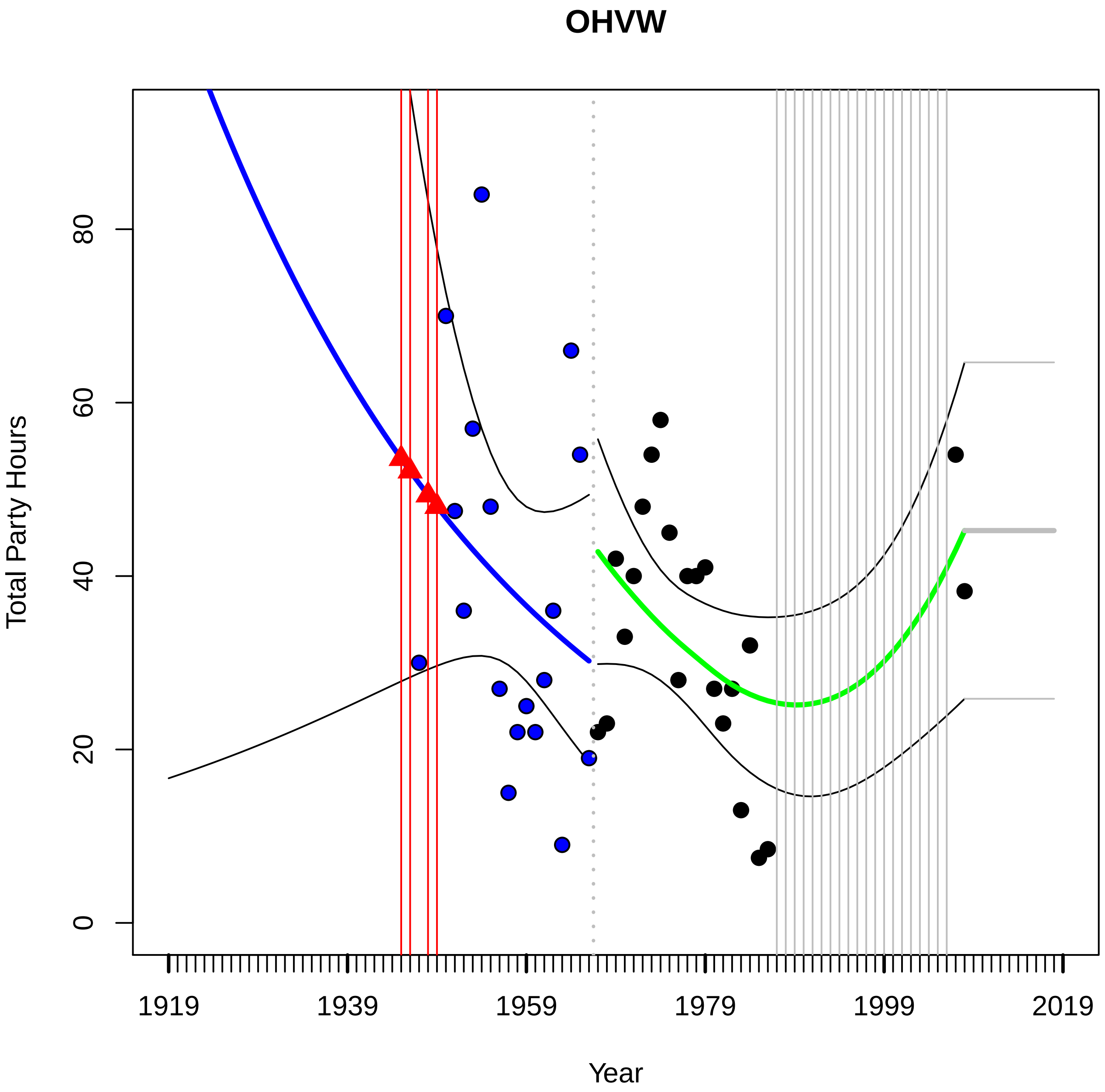

OHVW
Total Party Hours
Year
0
20
40
60
80
1919
1939
1959
1979
1999
2019

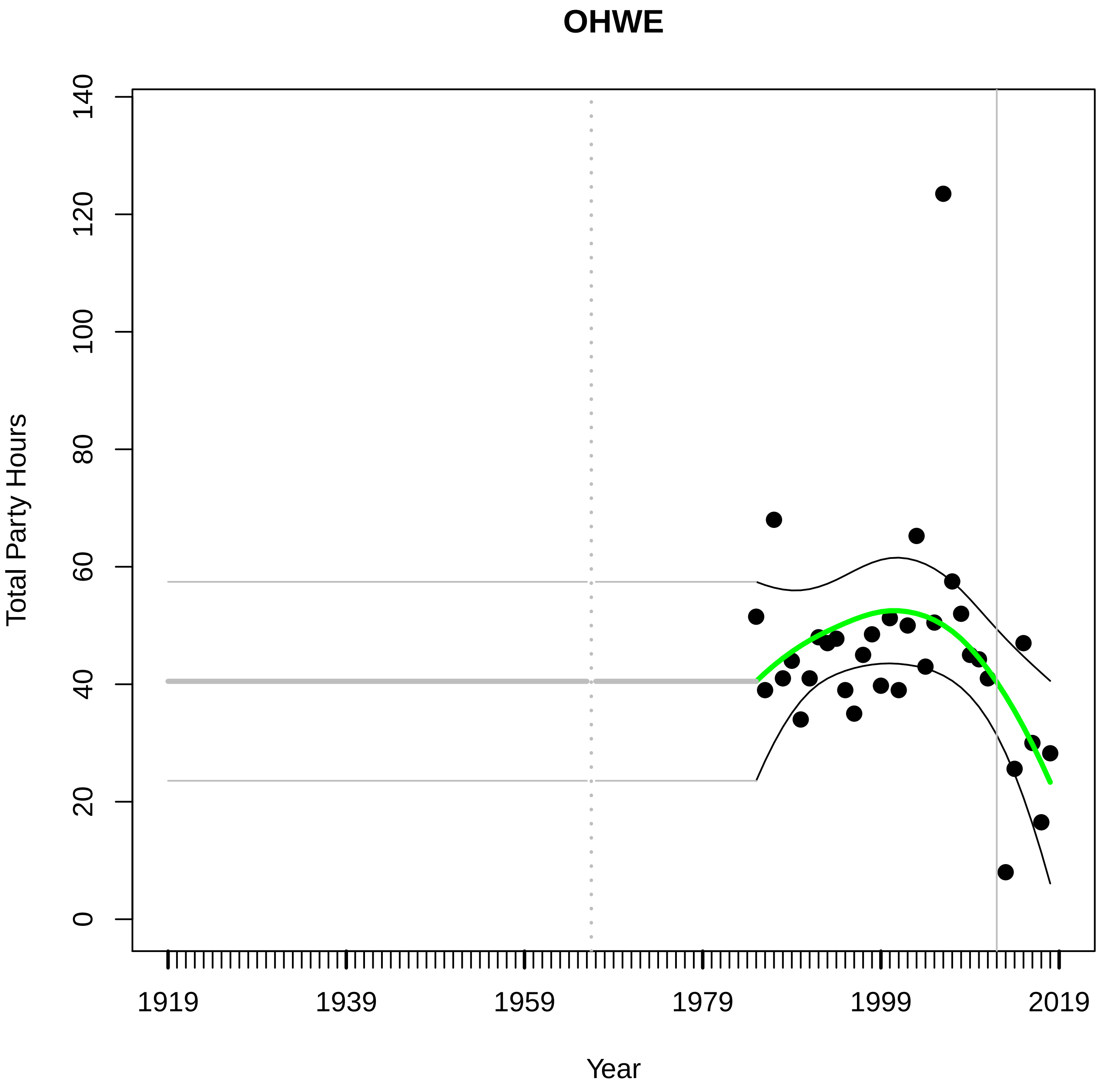
OHWE
Total Party Hours
0
20
40
60
80
100
120
140
1919
1939
1959
1979
1999
2019
Year

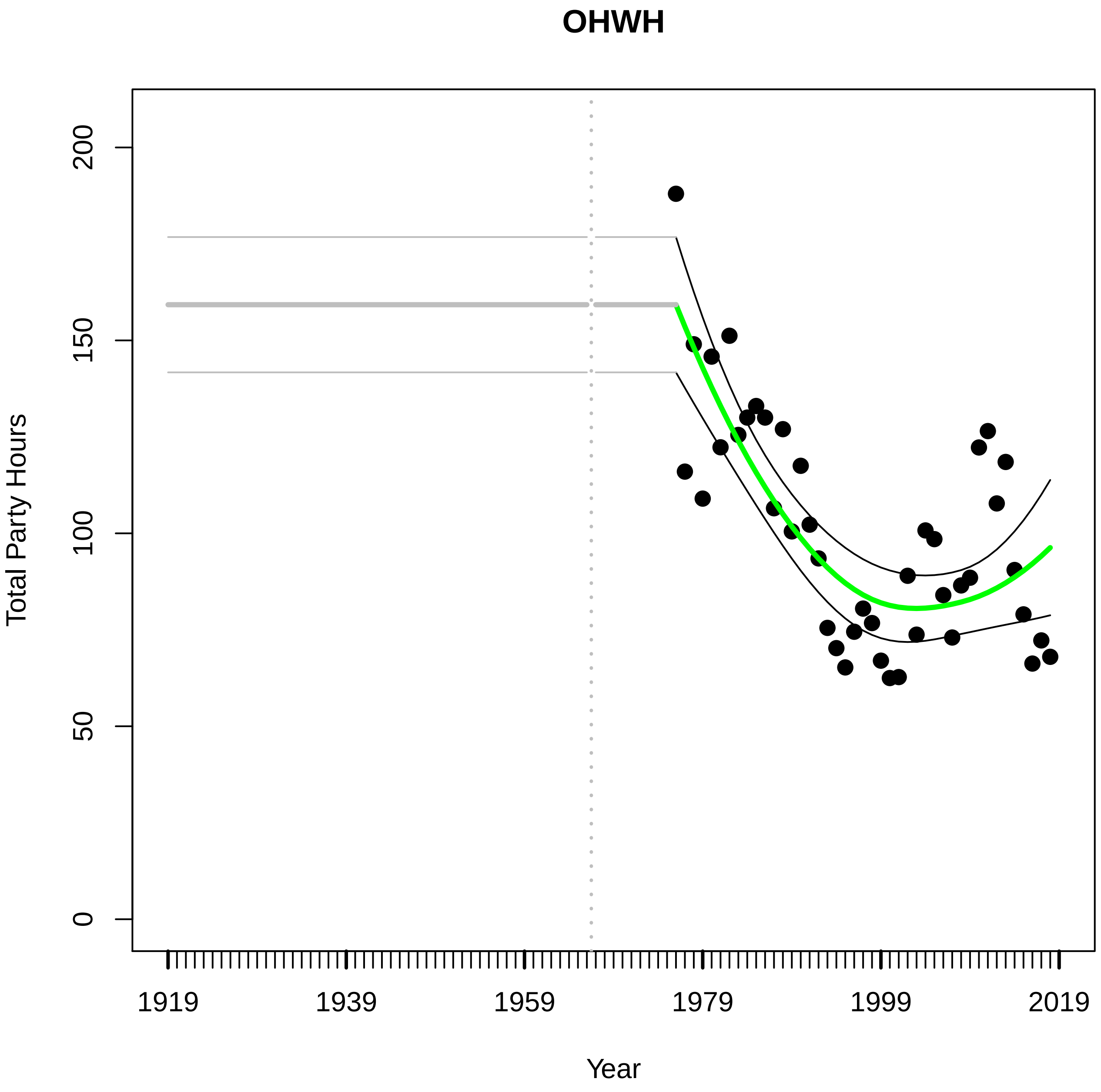

OHWH
200
150
100
50
0
Total Party Hours
1919
1939
1959
1979
1999
2019
Year

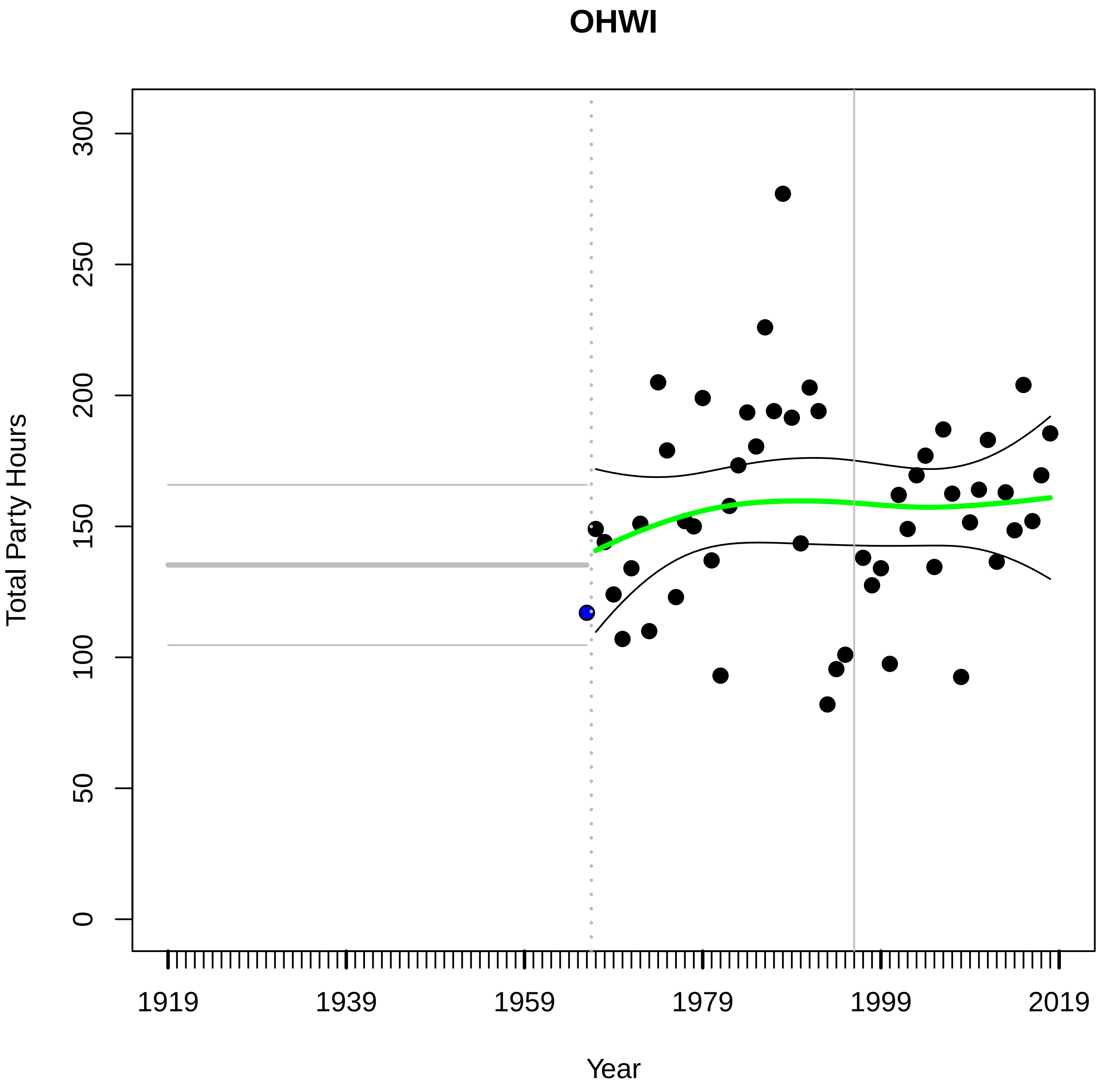

OHWI
Total Party Hours
0
50
100
150
200
250
300
1919
1939
1959
1979
1999
2019
Year

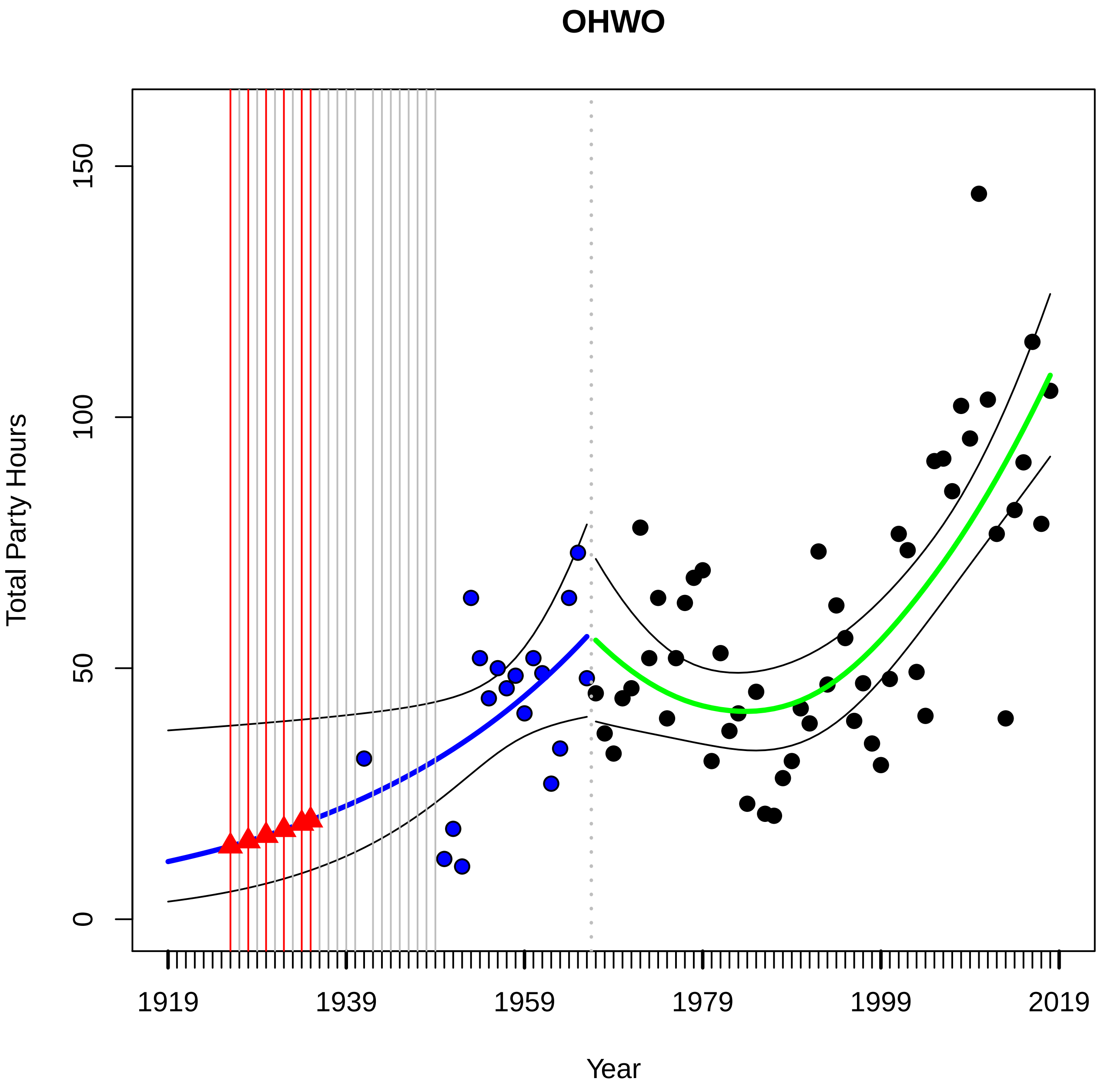

OHWO
Total Party Hours
0
50
100
150
Year
1919
1939
1959
1979
1999
2019

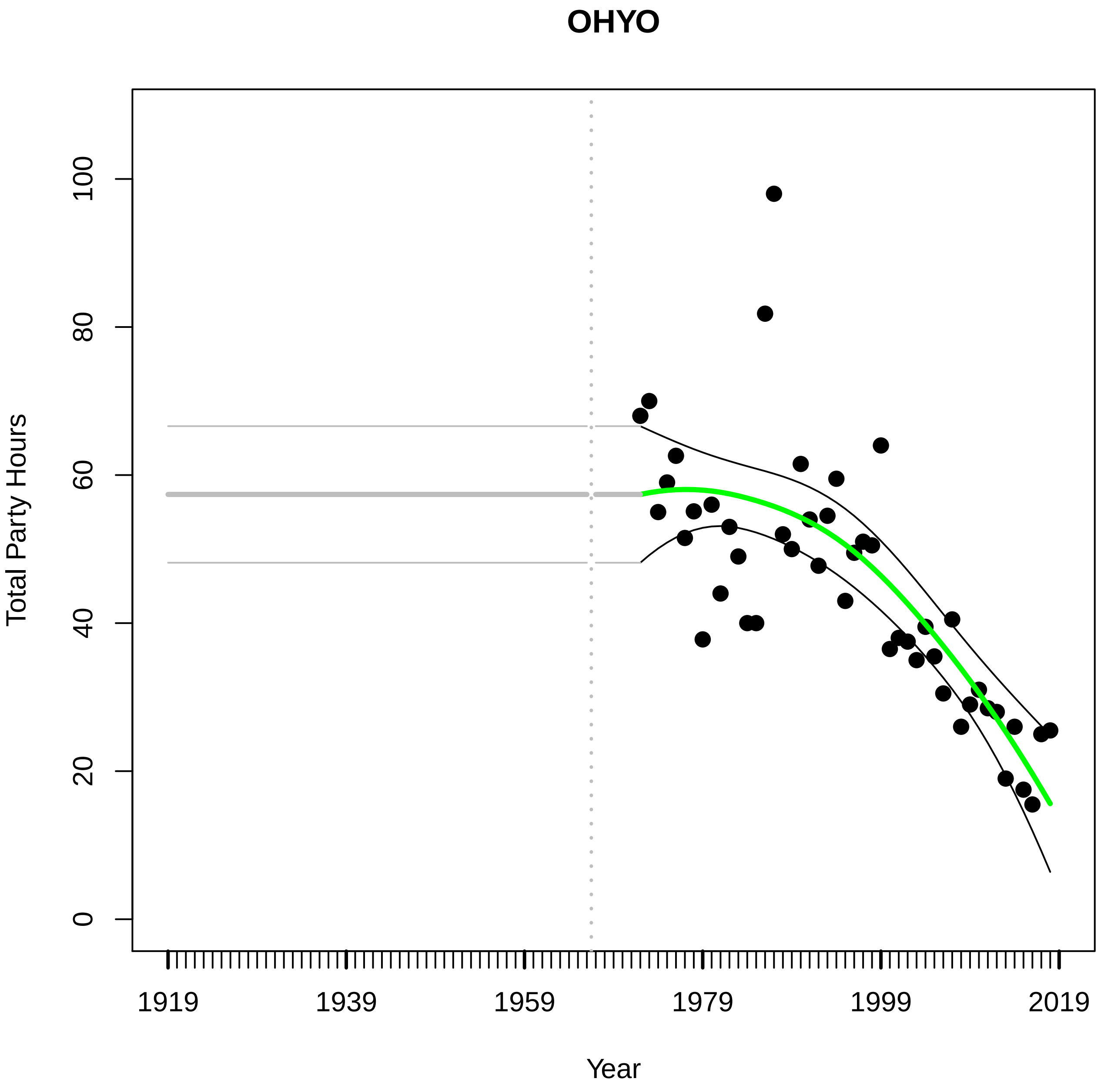
OHYO
Total Party Hours
0
20
40
60
80
100
1919
1939
1959
1979
1999
2019
Year

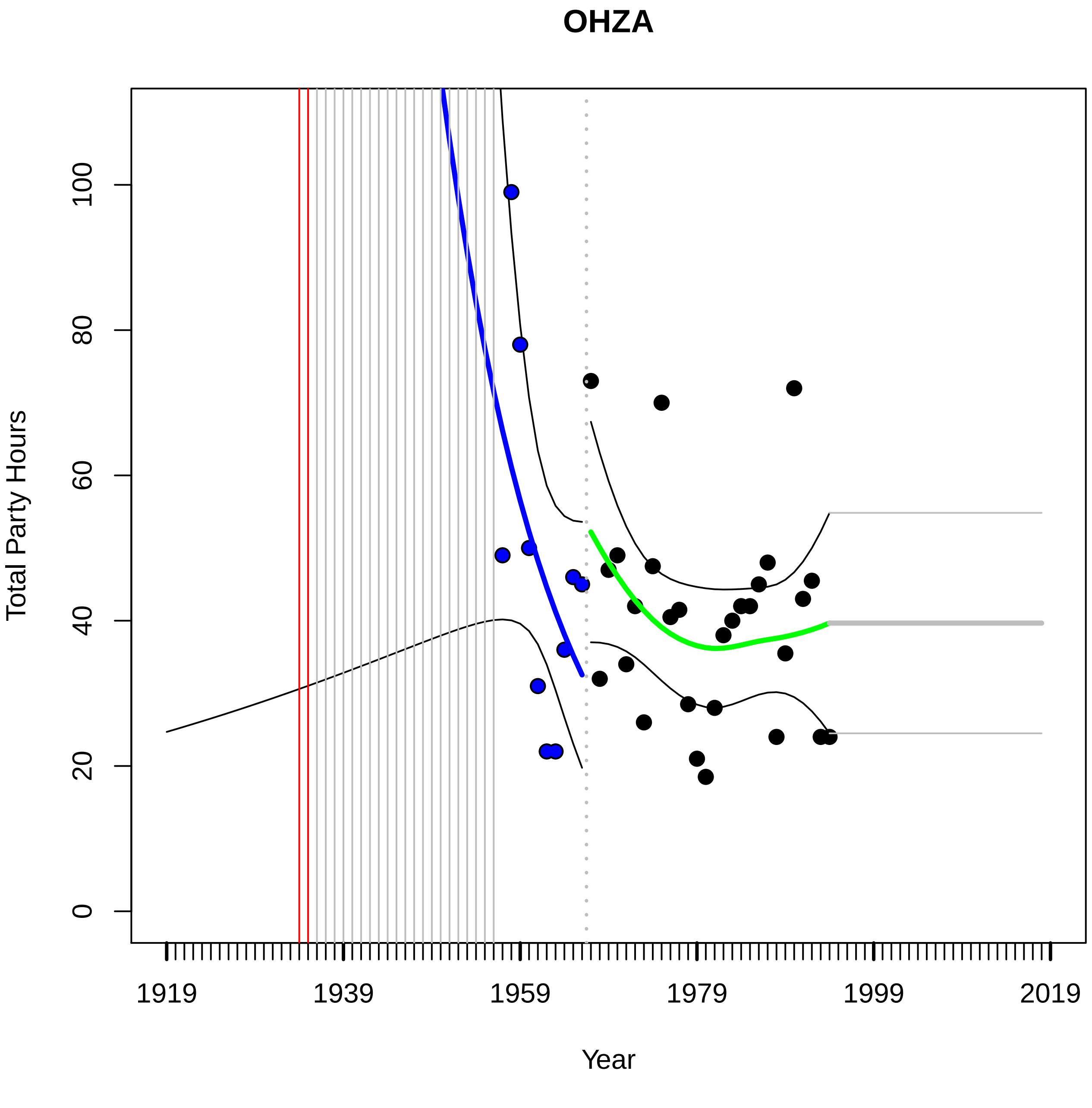

OHZA
Total Party Hours
Year
0
20
40
60
80
100
1919
1939
1959
1979
1999
2019

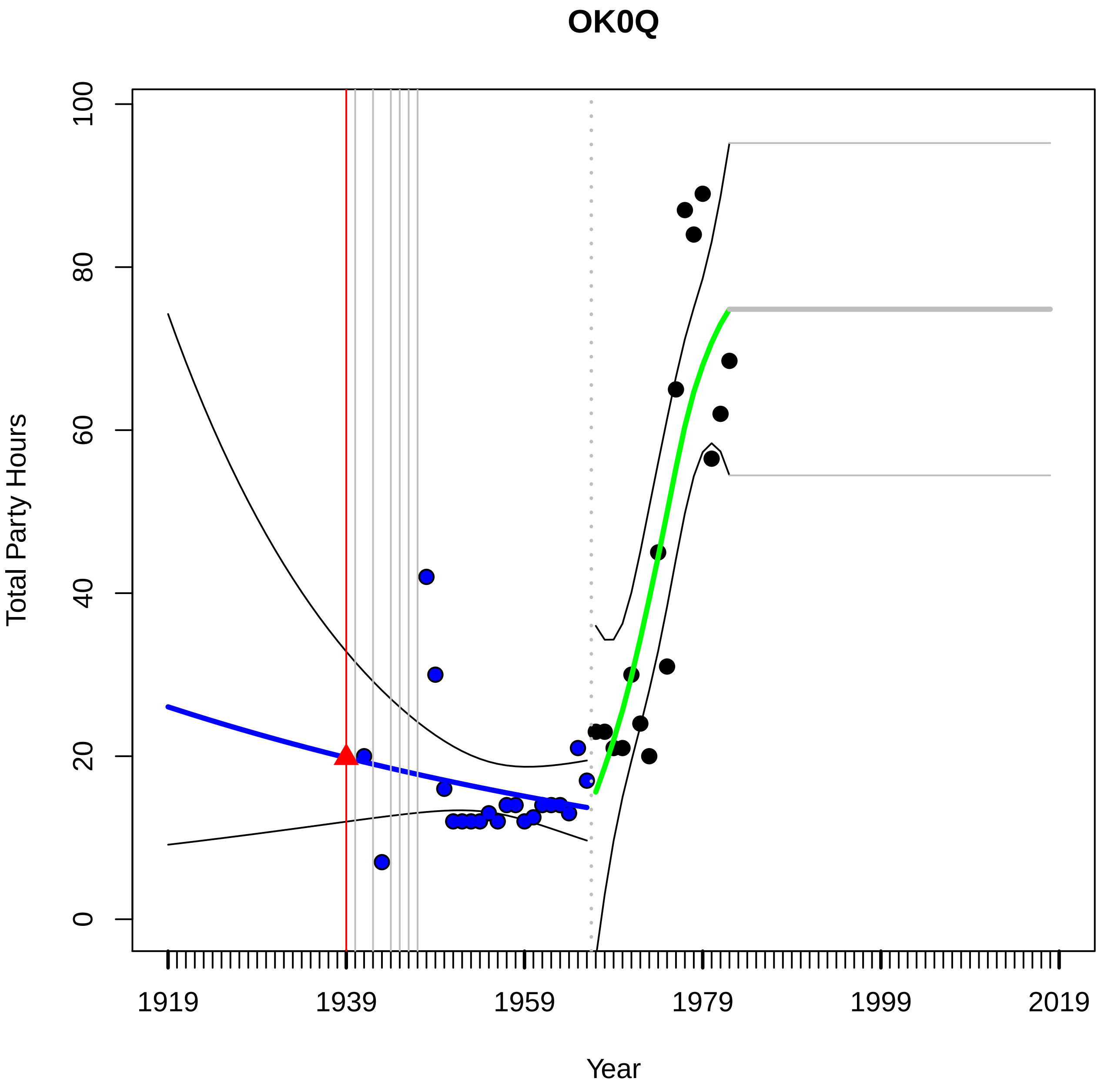
OK0Q
Total Party Hours
0
20
40
60
80
100
1919
1939
1959
1979
1999
2019
Year

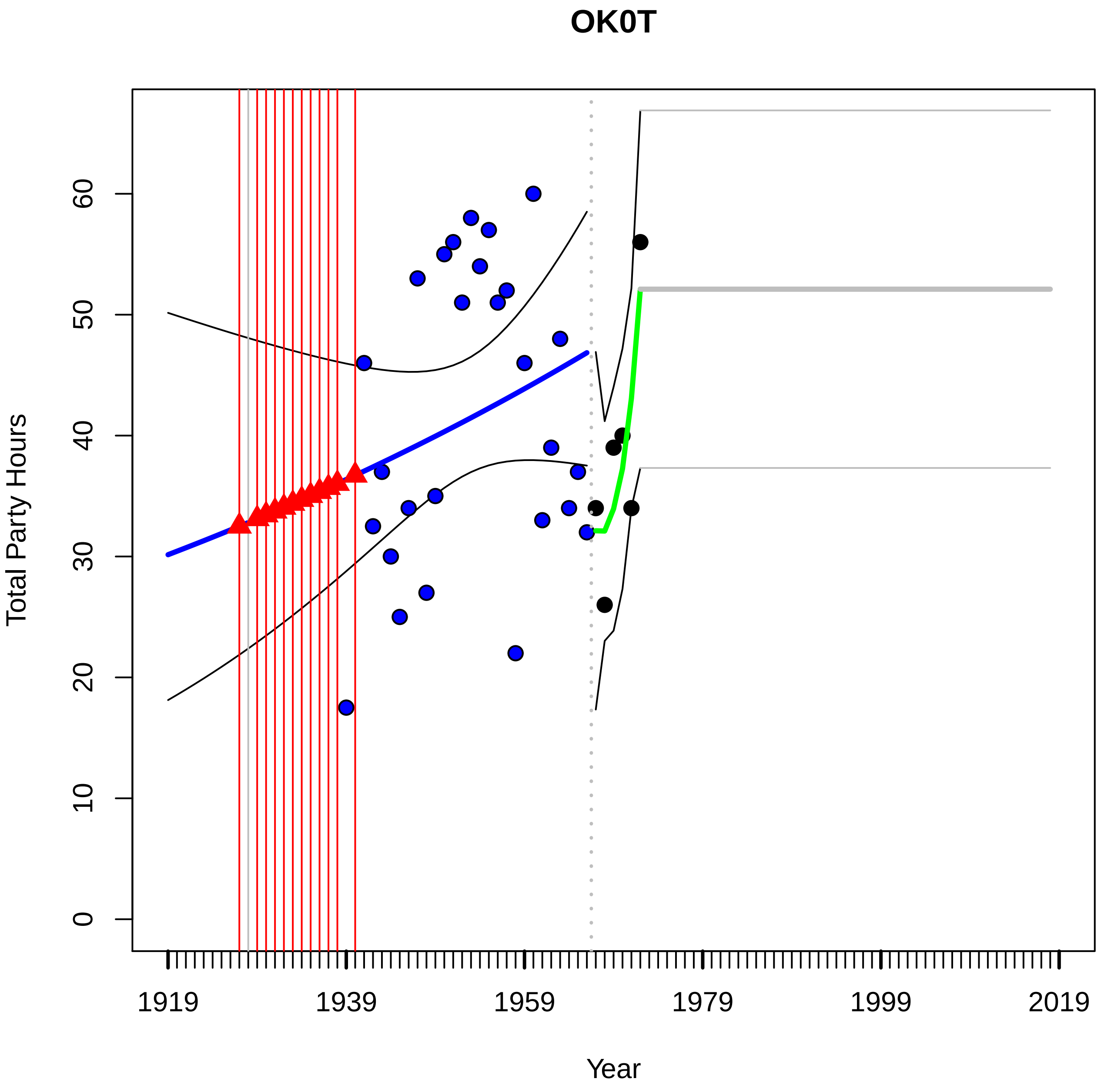

OK0T
Total Party Hours
Year
0
10
20
30
40
50
60
1919
1939
1959
1979
1999
2019

OKAN

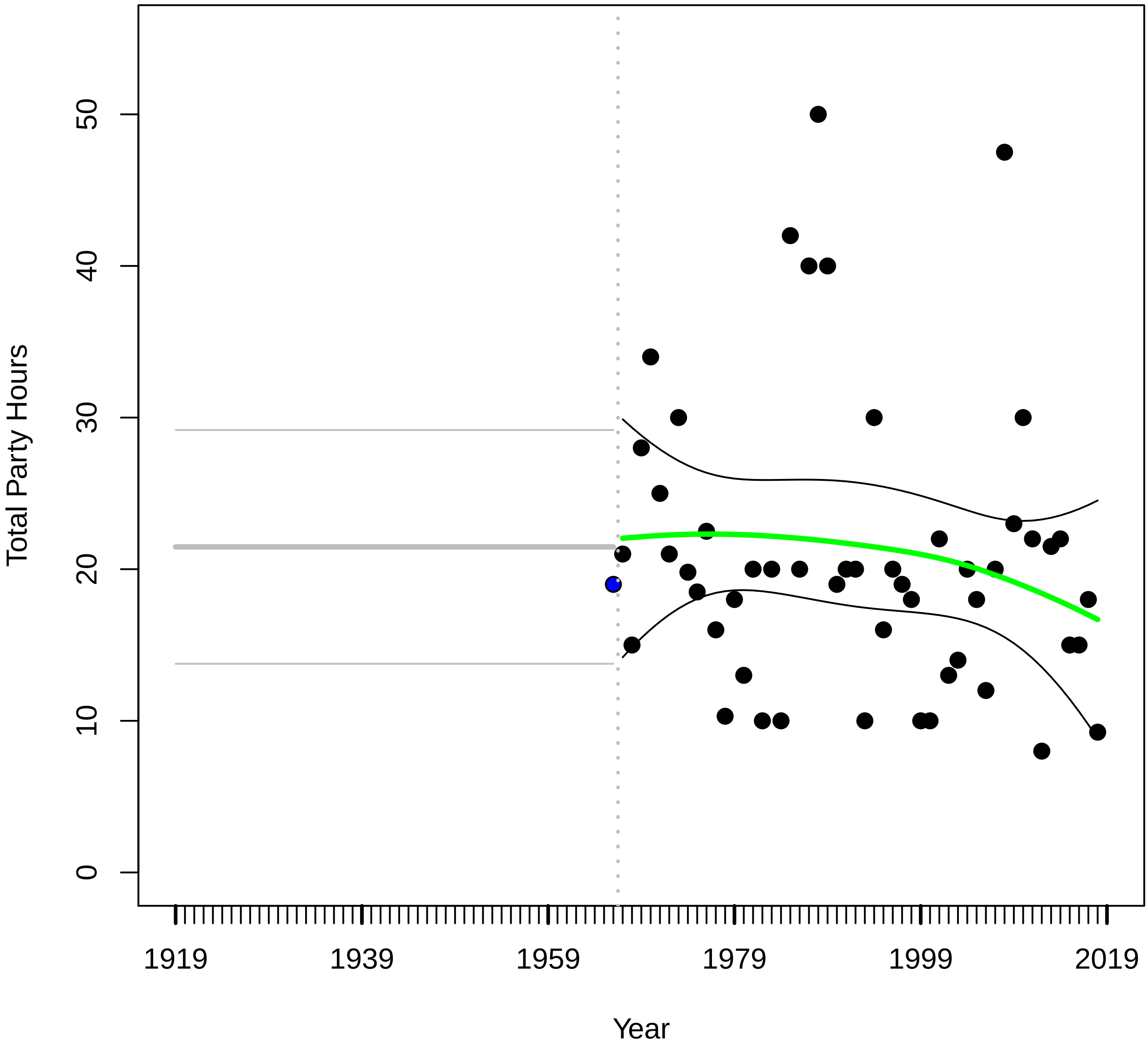

Total Party Hours
0
10
20
30
40
50
1919
1939
1959
1979
1999
2019
Year

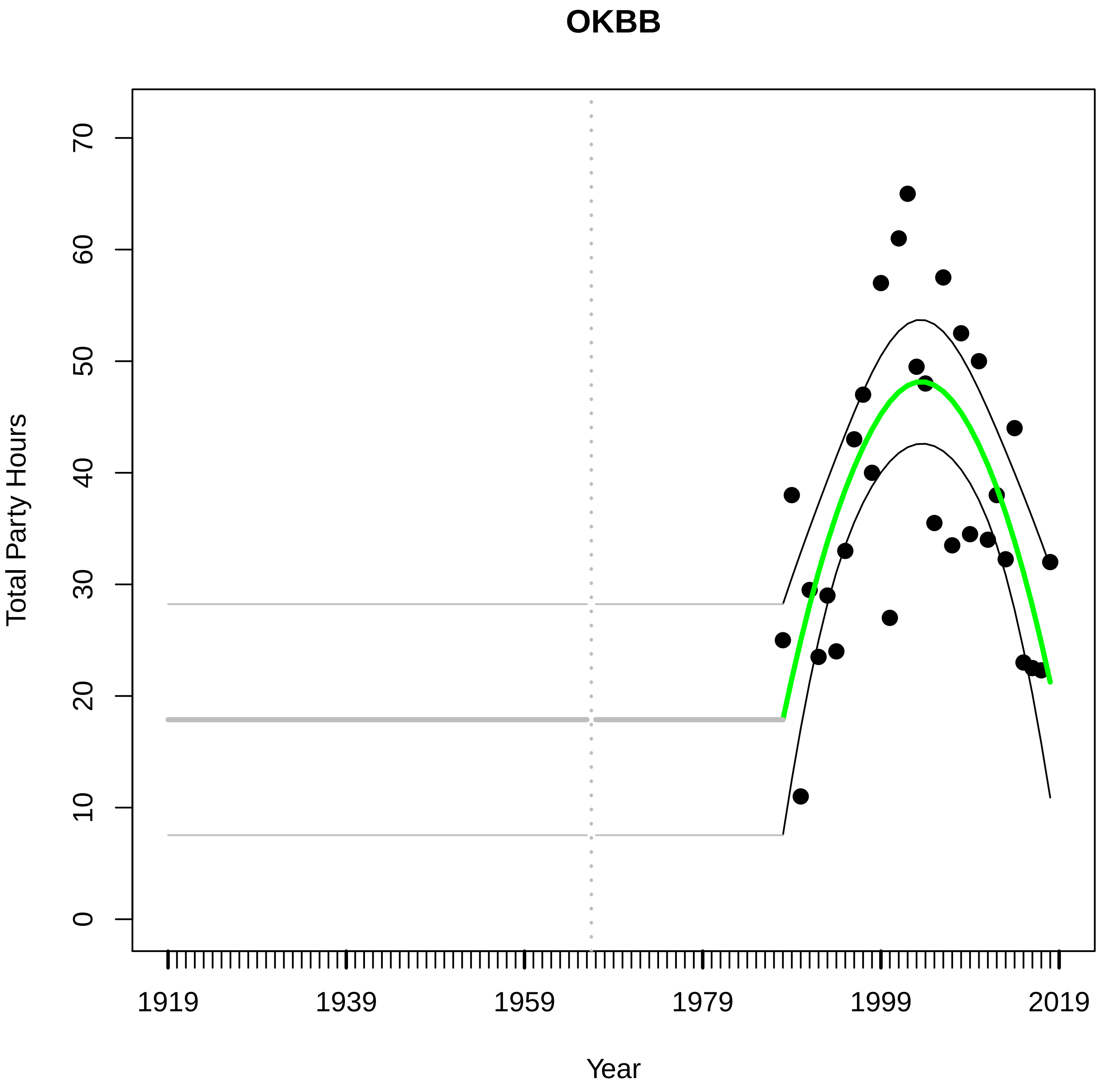

OKBB
Total Party Hours
0
10
20
30
40
50
60
70
Year
1919
1939
1959
1979
1999
2019

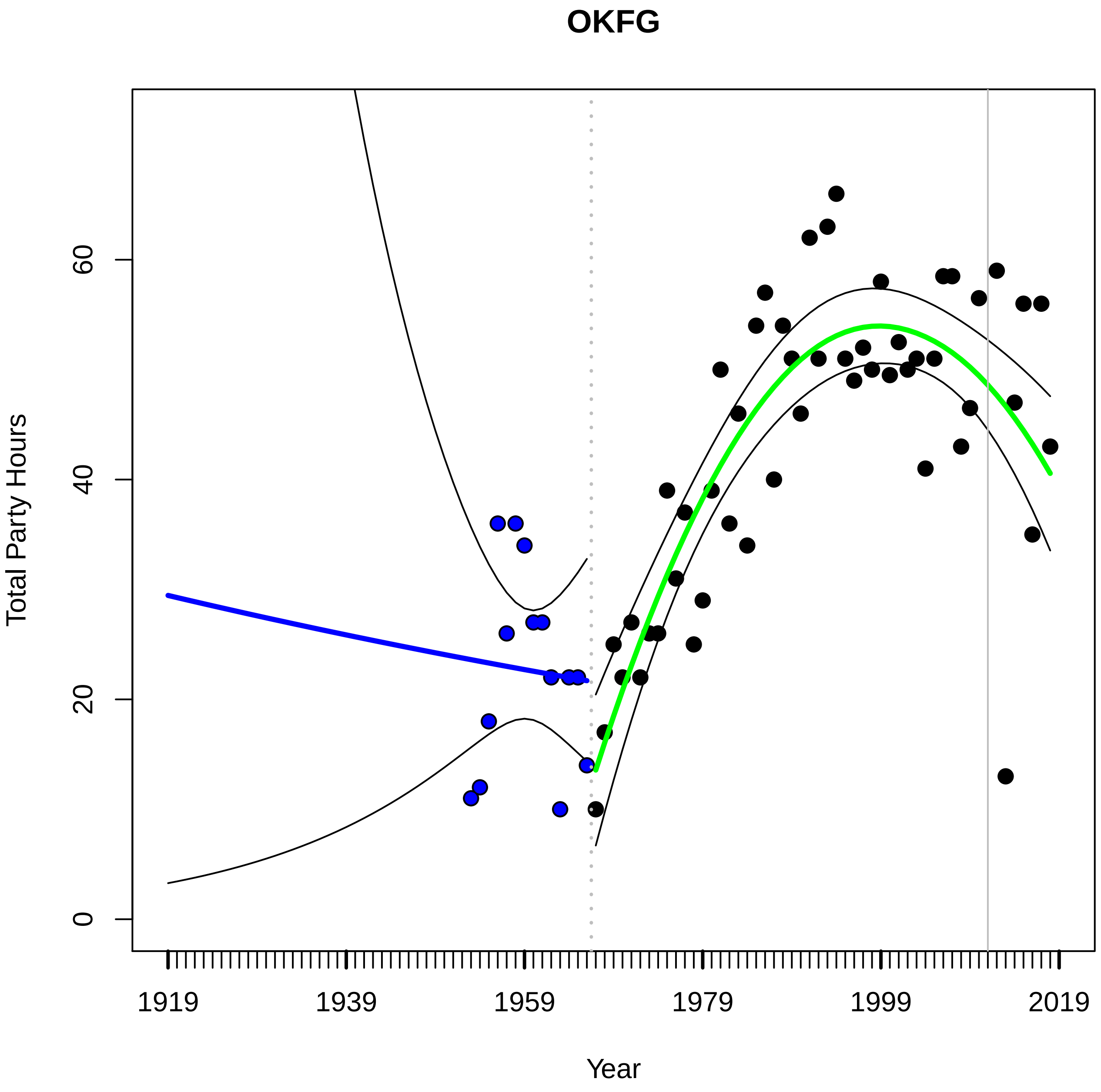
OKFG
Total Party Hours
Year
0
20
40
60
1919
1939
1959
1979
1999
2019

OKHR

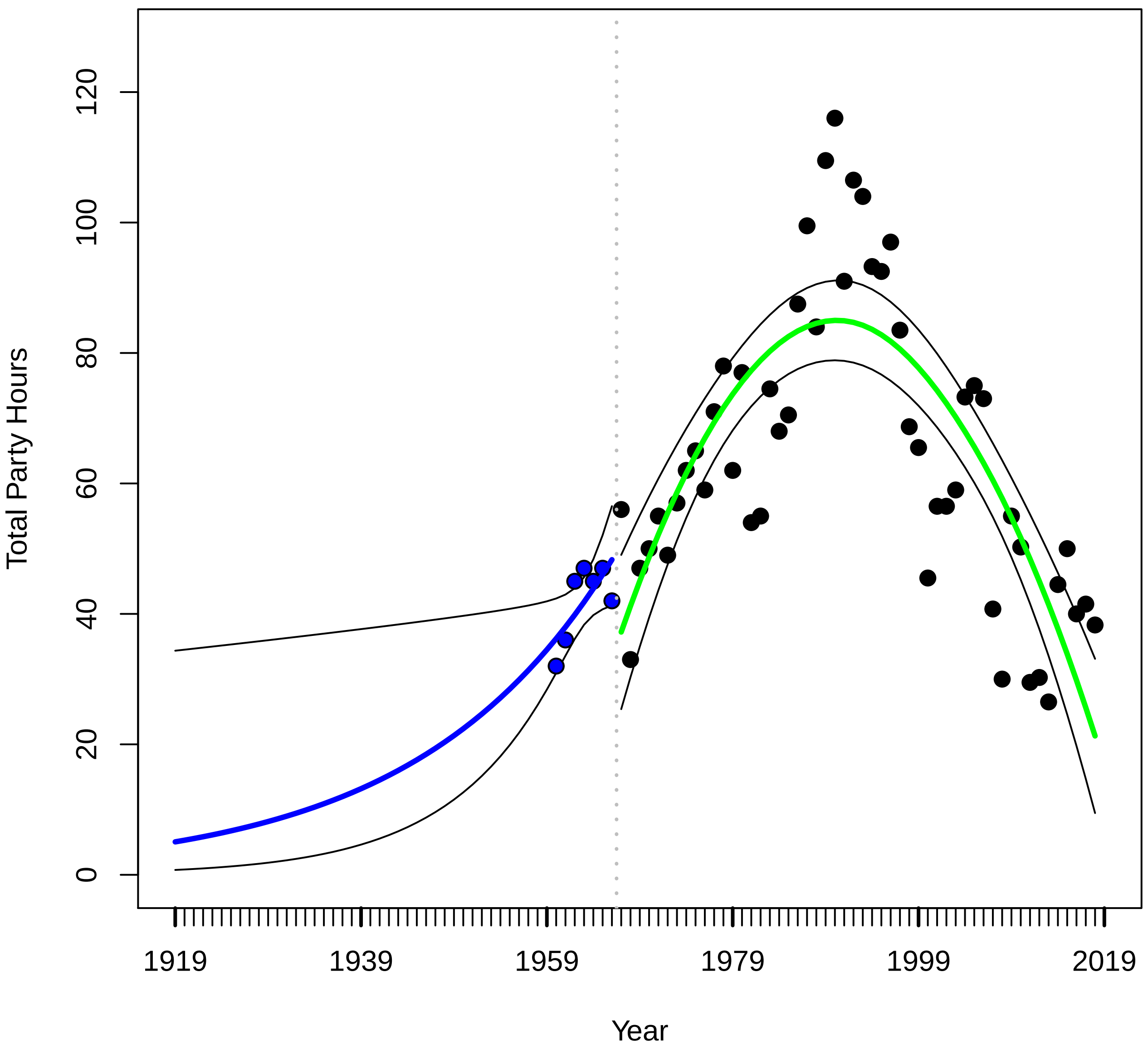

Total Party Hours
0
20
40
60
80
100
120
1919
1939
1959
1979
1999
2019
Year

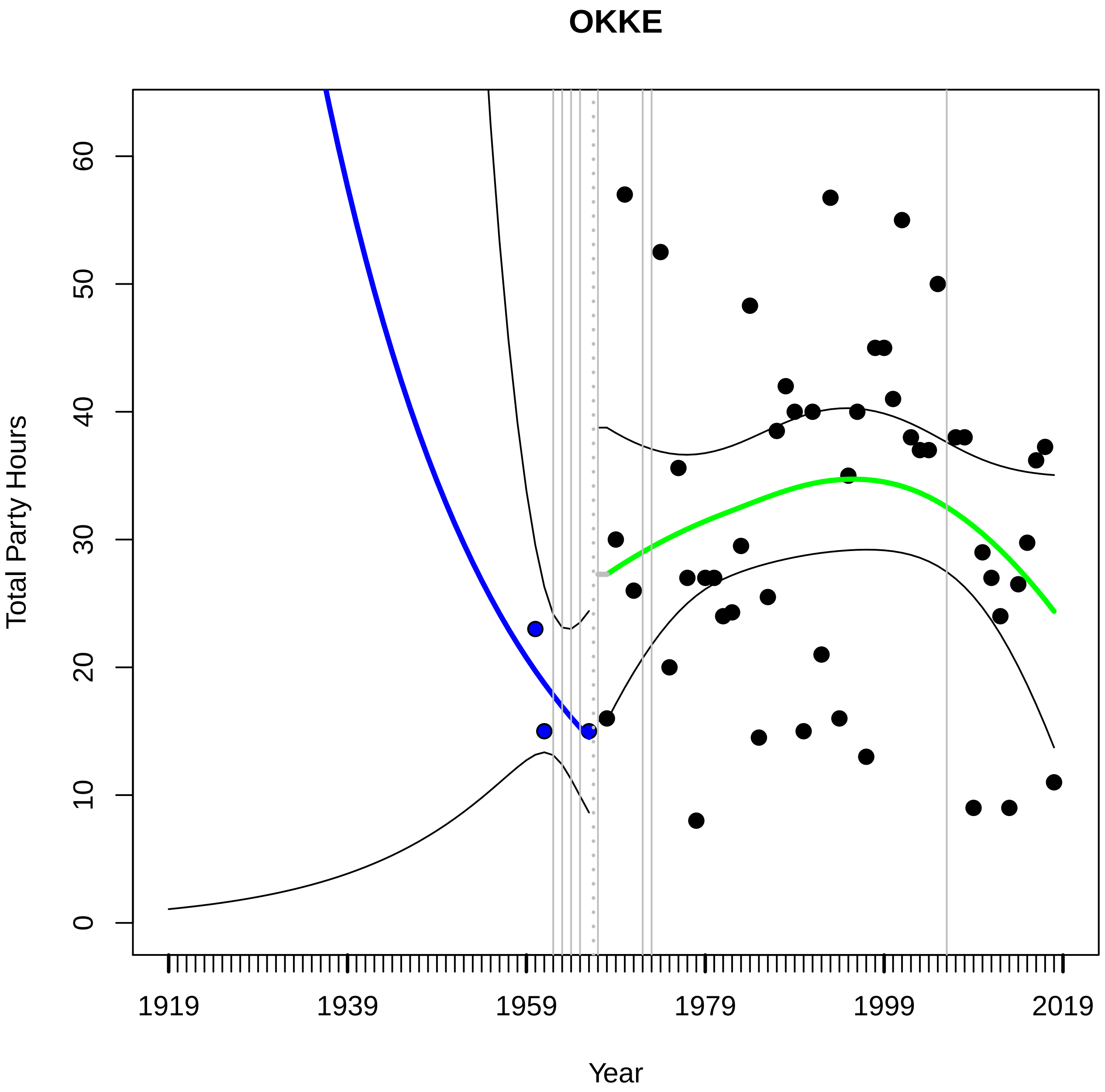

OKKE
Total Party Hours
0
10
20
30
40
50
60
Year
1919
1939
1959
1979
1999
2019

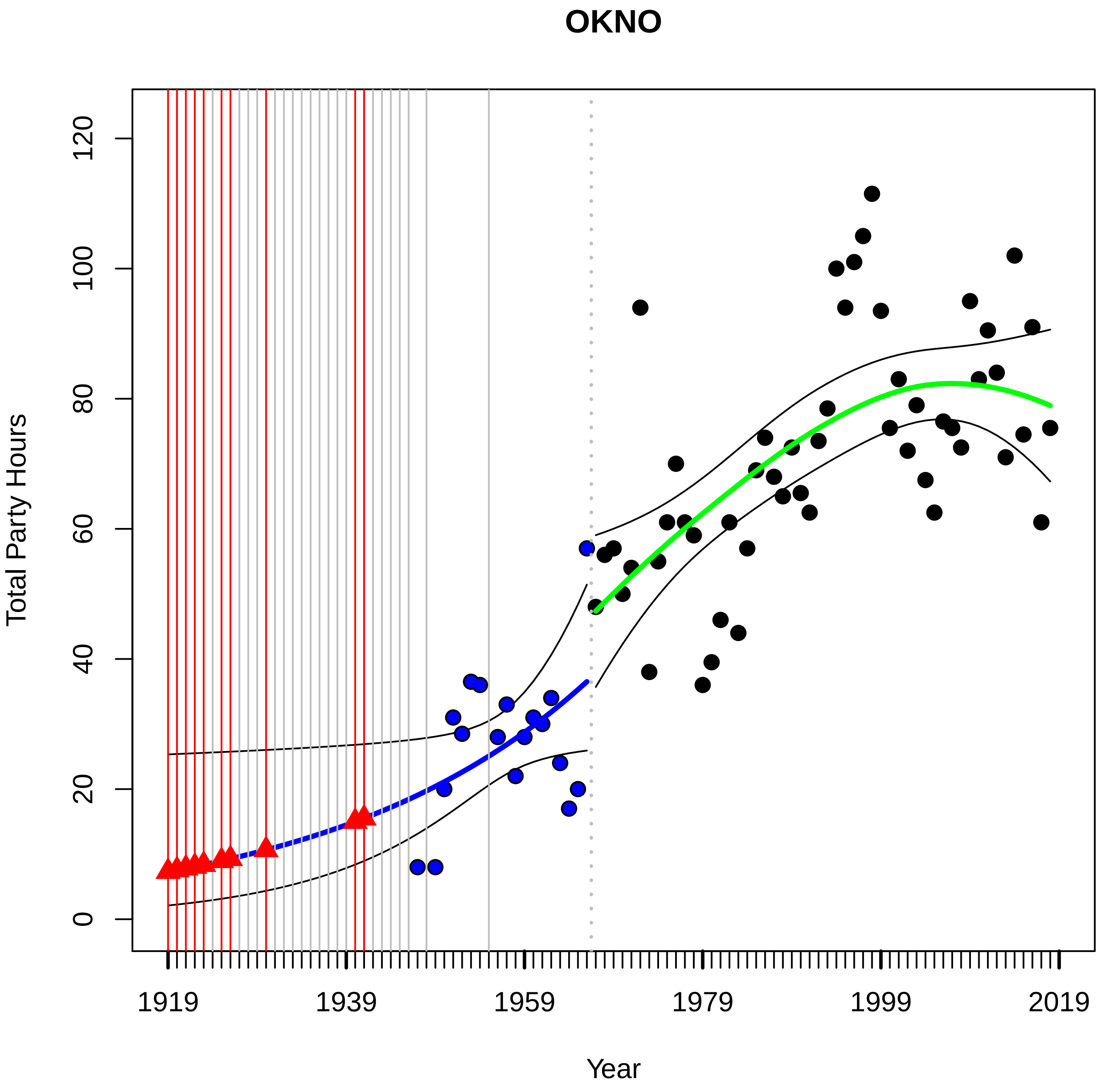
OKNO
Total Party Hours
0
20
40
60
80
100
120
1919
1939
1959
1979
1999
2019
Year

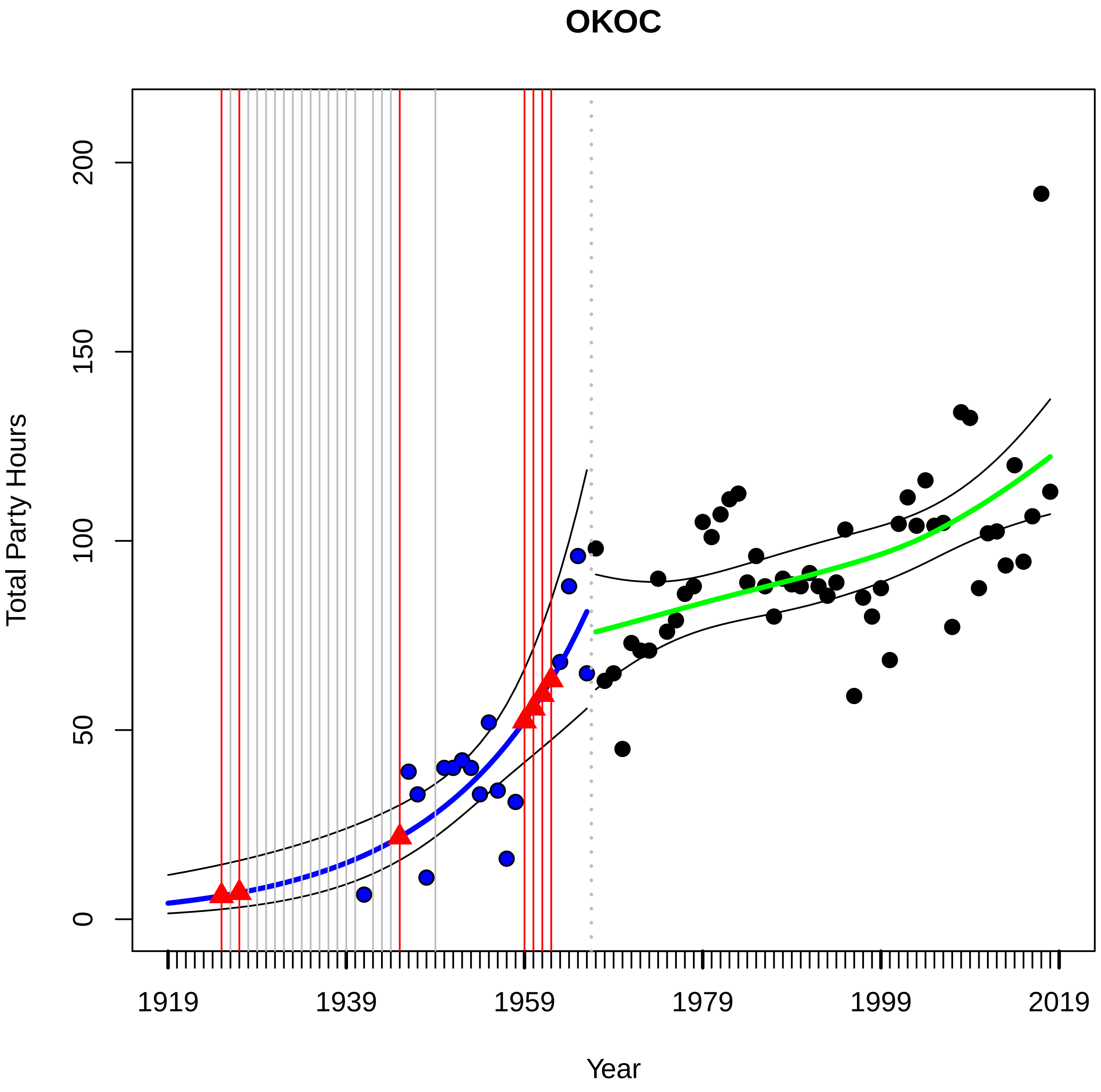

OKOC
Total Party Hours
0
50
100
150
200
1919
1939
1959
1979
1999
2019
Year

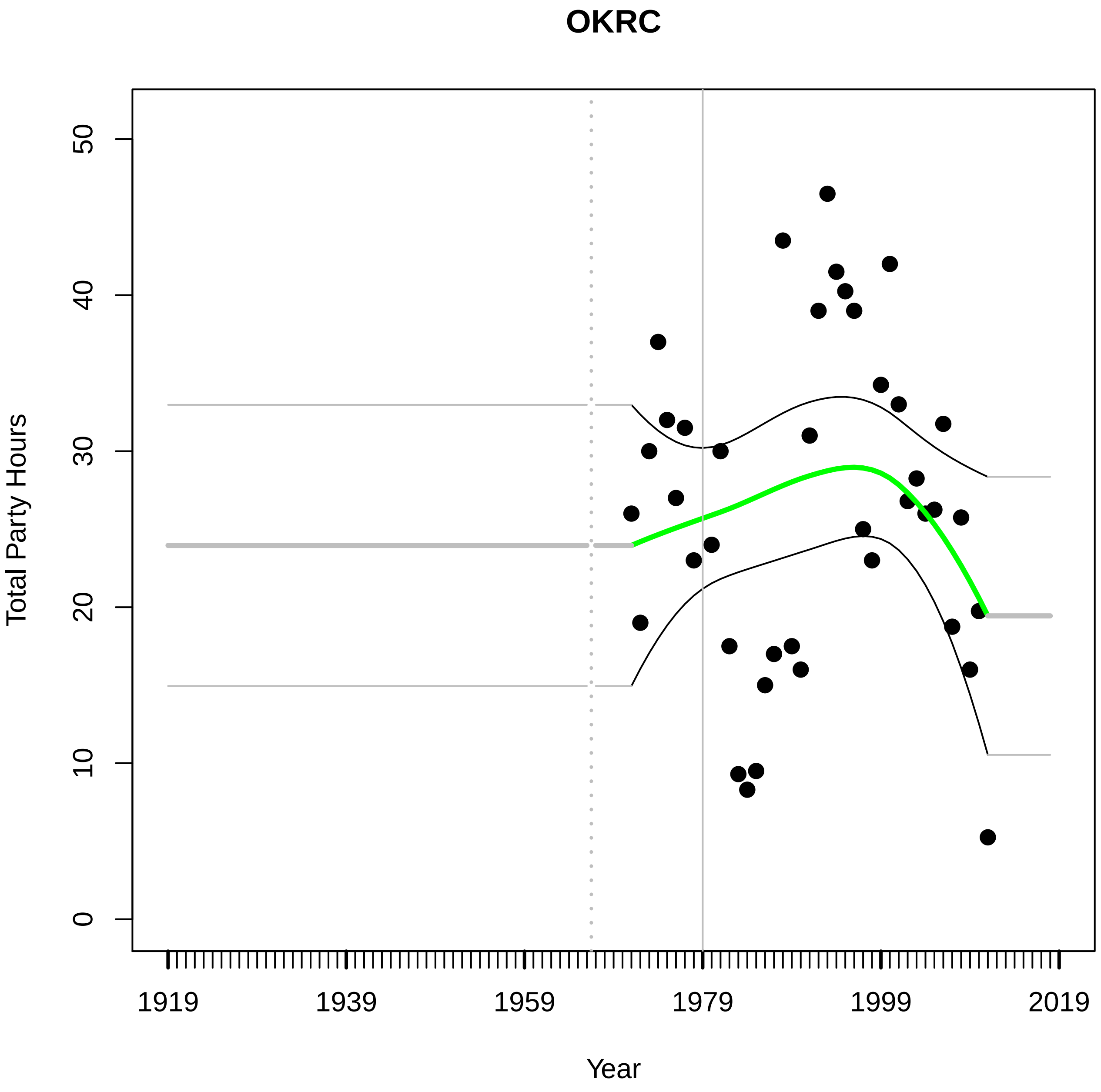
OKRC
Total Party Hours
0
10
20
30
40
50
1919
1939
1959
1979
1999
2019
Year

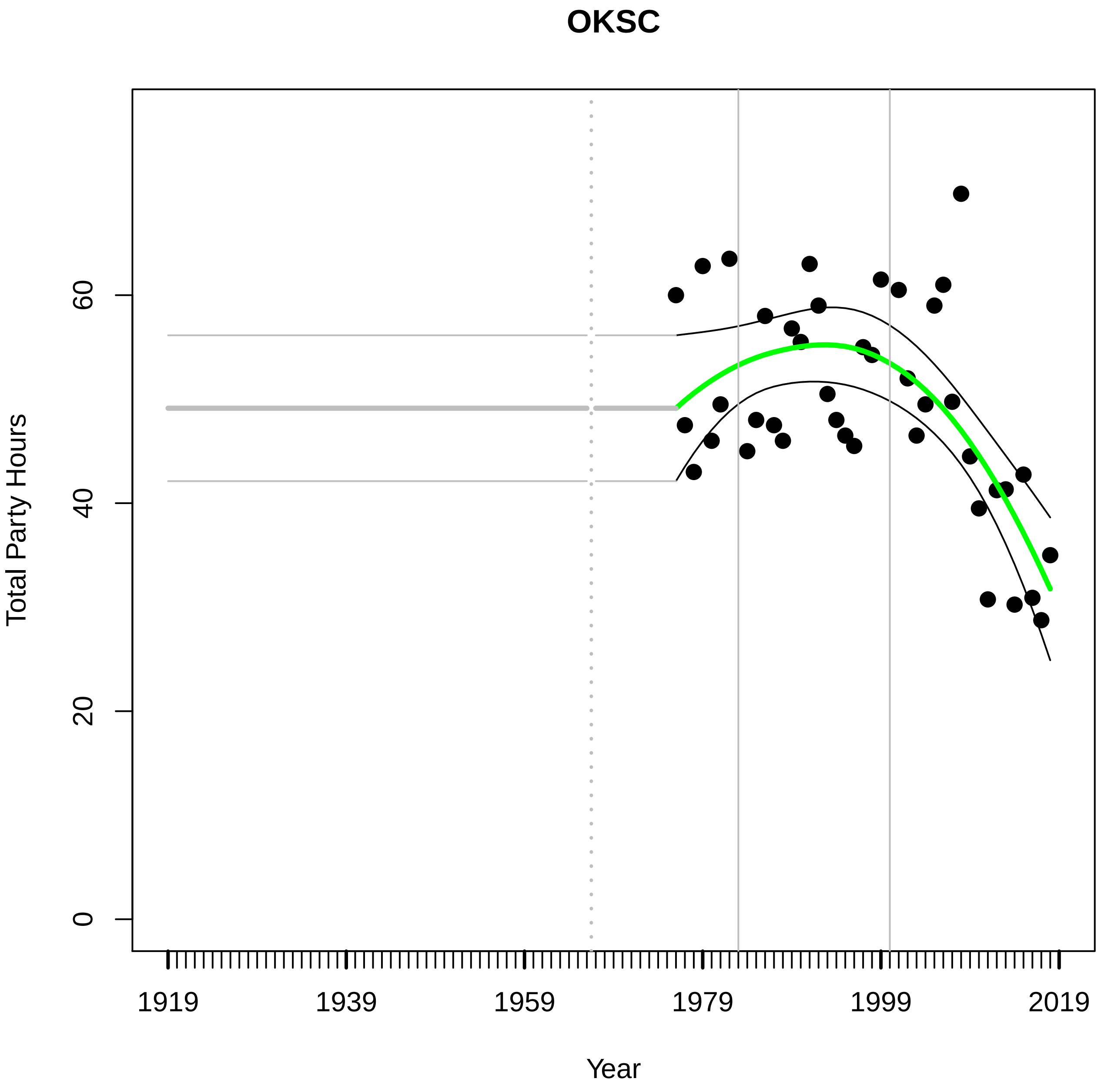
OKSC
Total Party Hours
0
20
40
60
1919
1939
1959
1979
1999
2019
Year

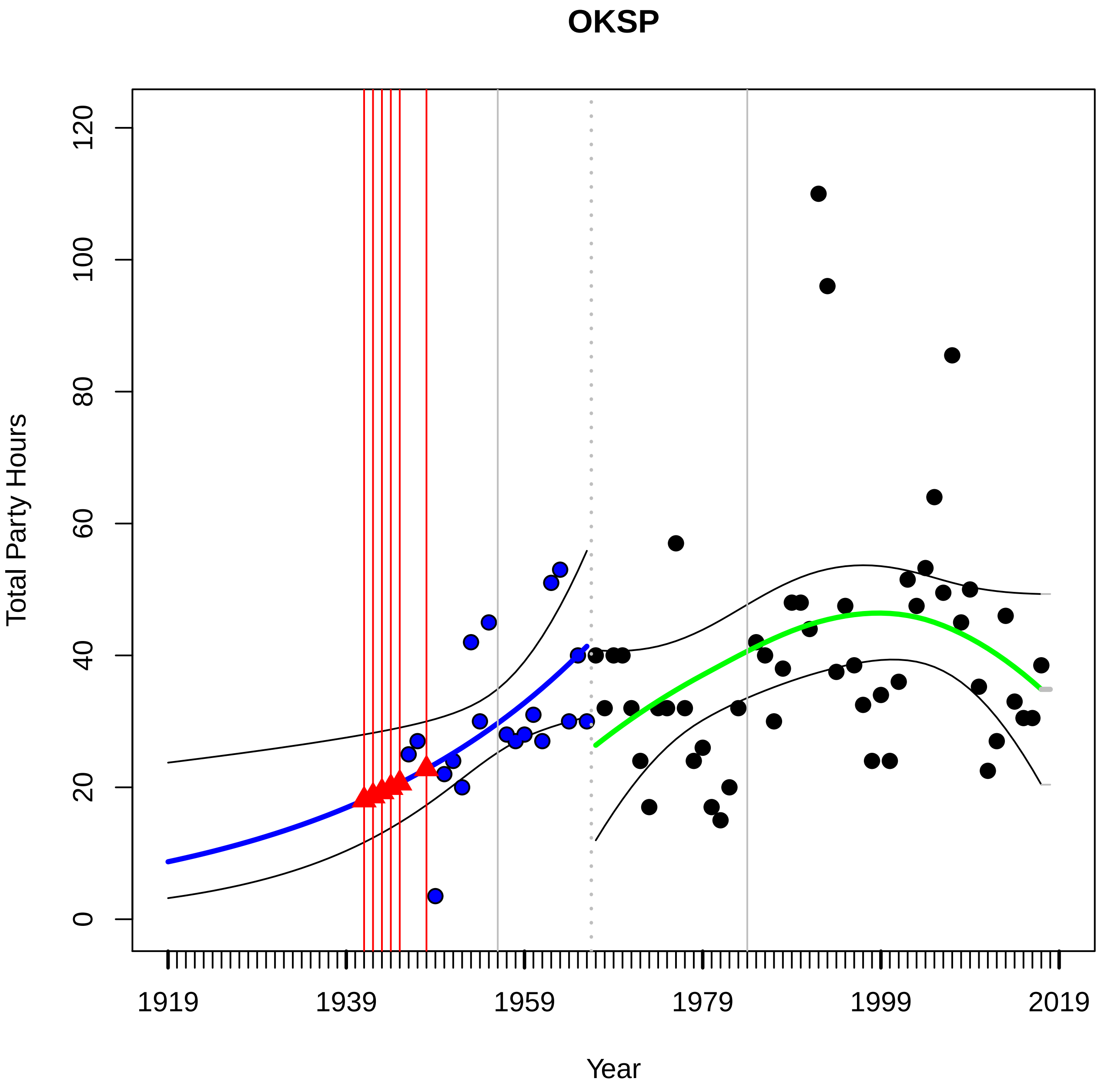

OKSP
Total Party Hours
0
20
40
60
80
100
120
1919
1939
1959
1979
1999
2019
Year

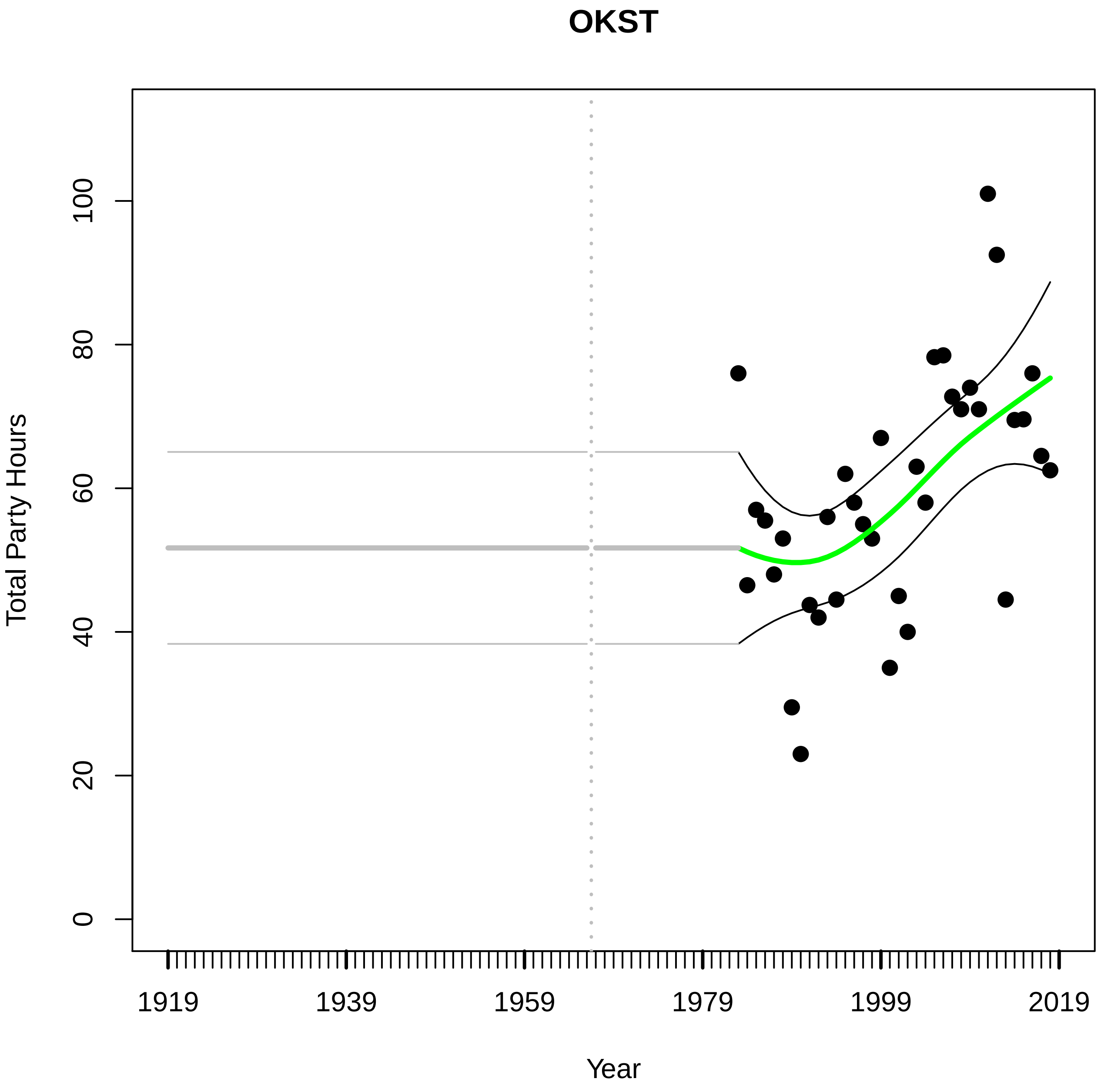

OKST
Total Party Hours
0
20
40
60
80
100
1919
1939
1959
1979
1999
2019
Year

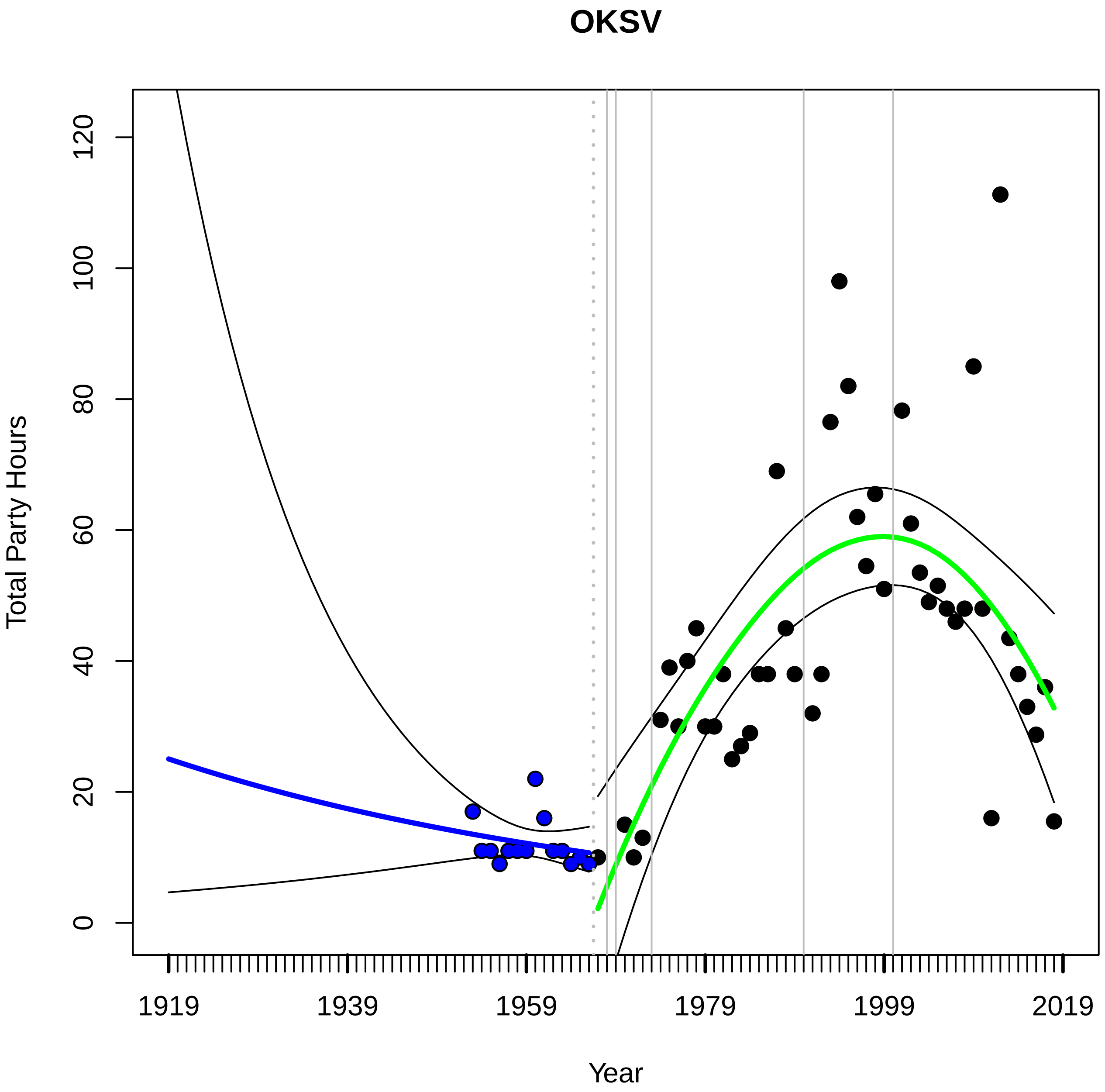

OKSV
Total Party Hours
0
20
40
60
80
100
120
1919
1939
1959
1979
1999
2019
Year

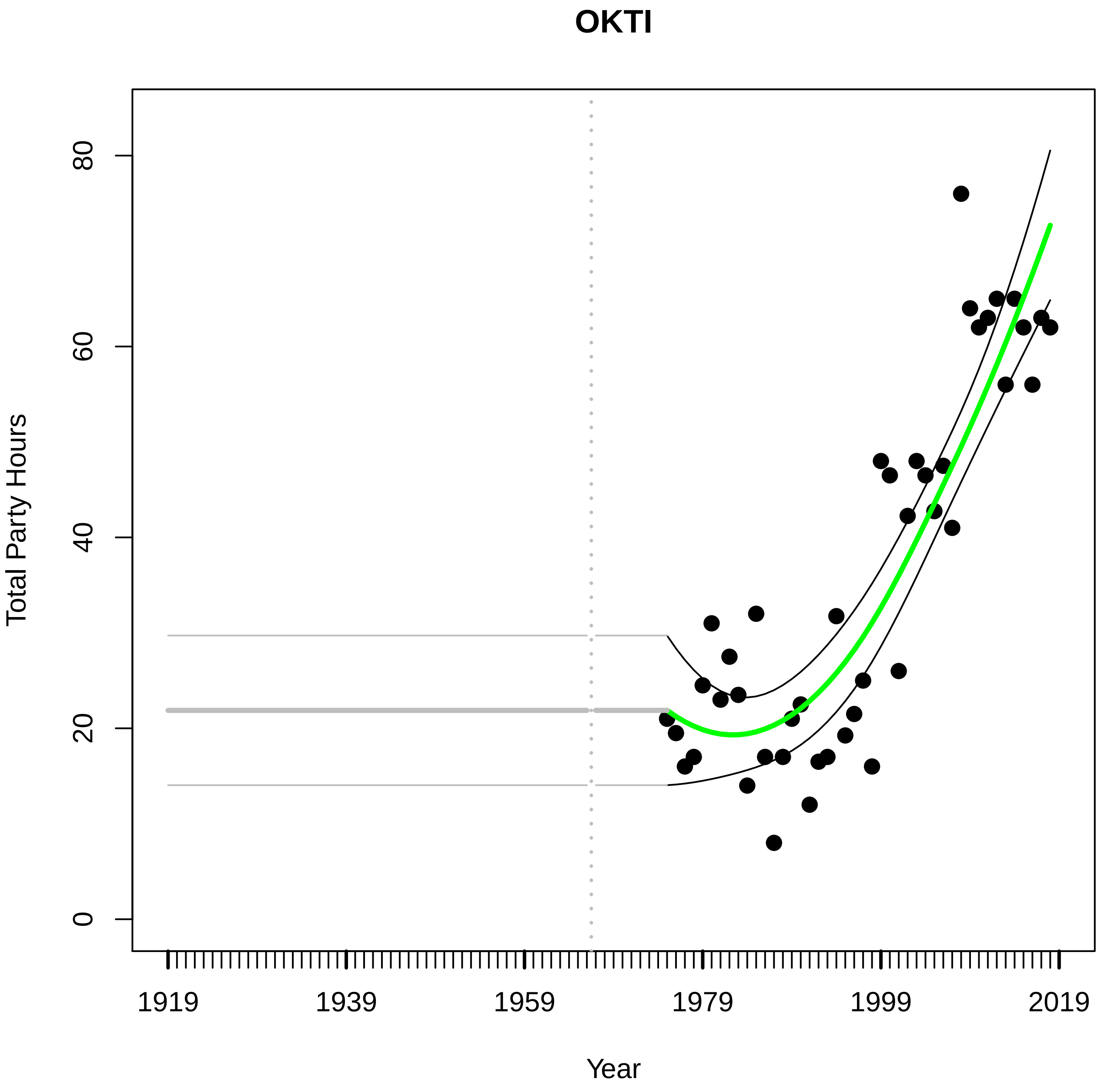

OKTI
Total Party Hours
0
20
40
60
80
1919
1939
1959
1979
1999
2019
Year

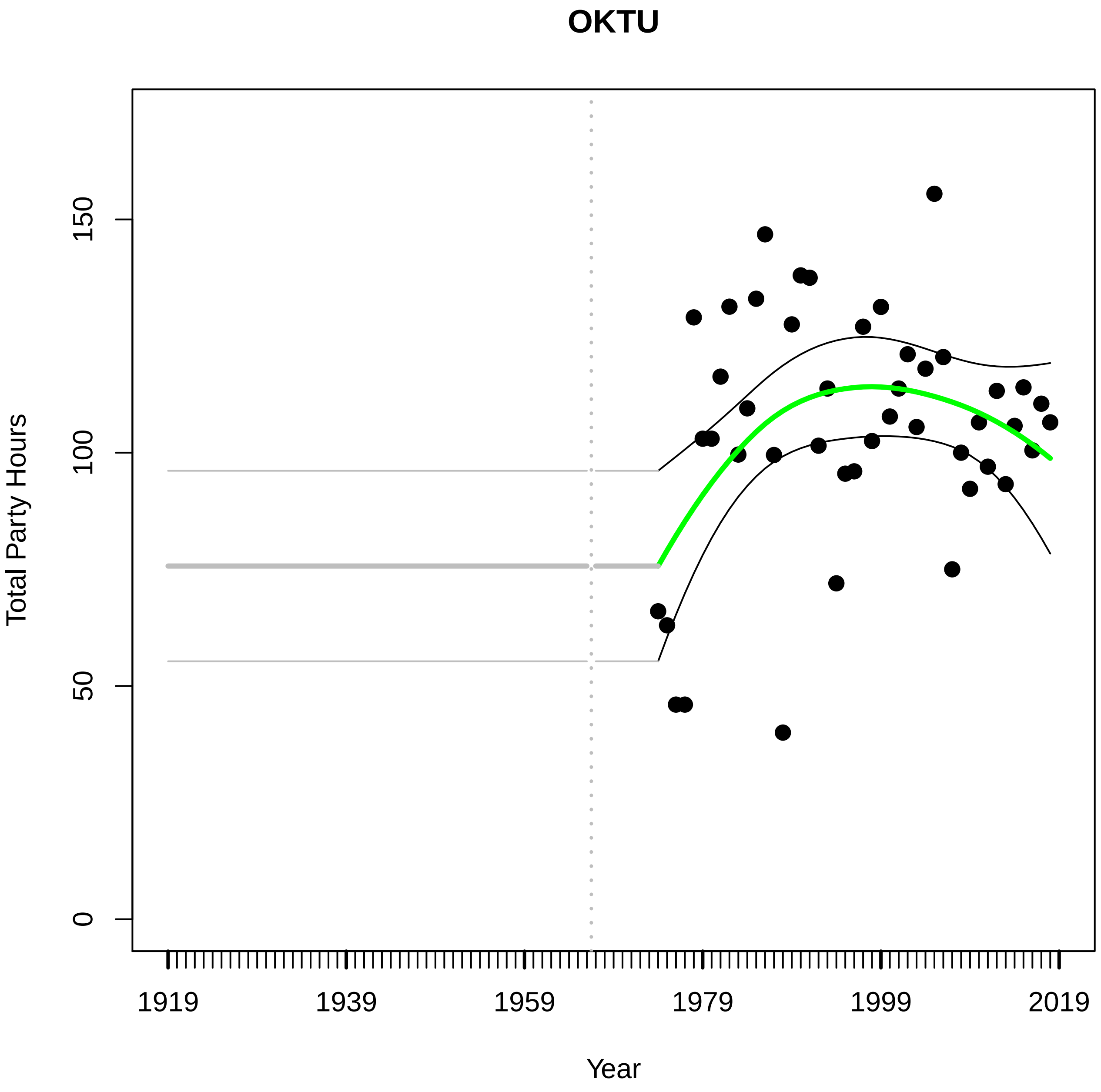

OKTU
Total Party Hours
0
50
100
150
1919
1939
1959
1979
1999
2019
Year

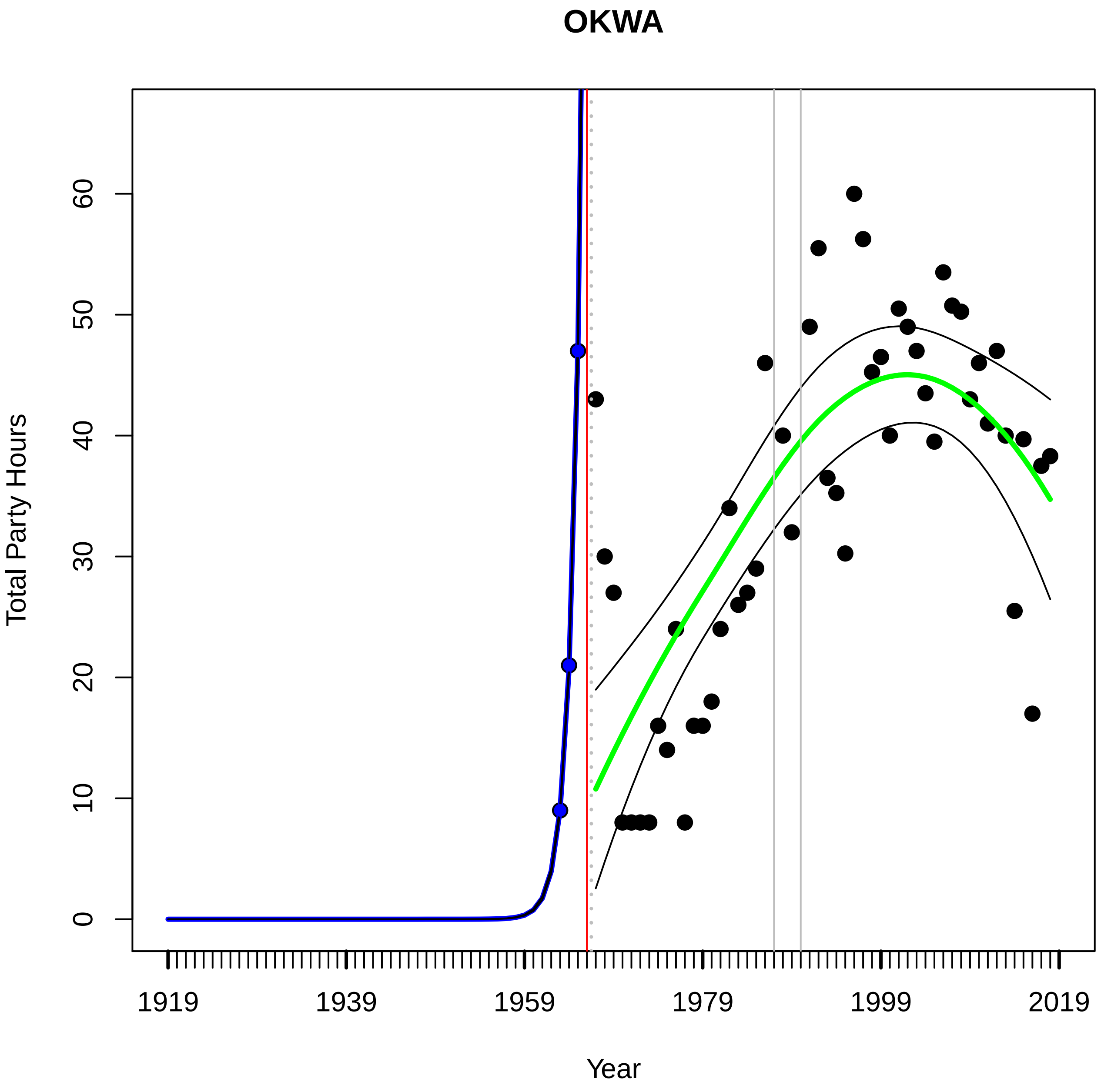

OKWA
Total Party Hours
0
10
20
30
40
50
60
1919
1939
1959
1979
1999
2019
Year

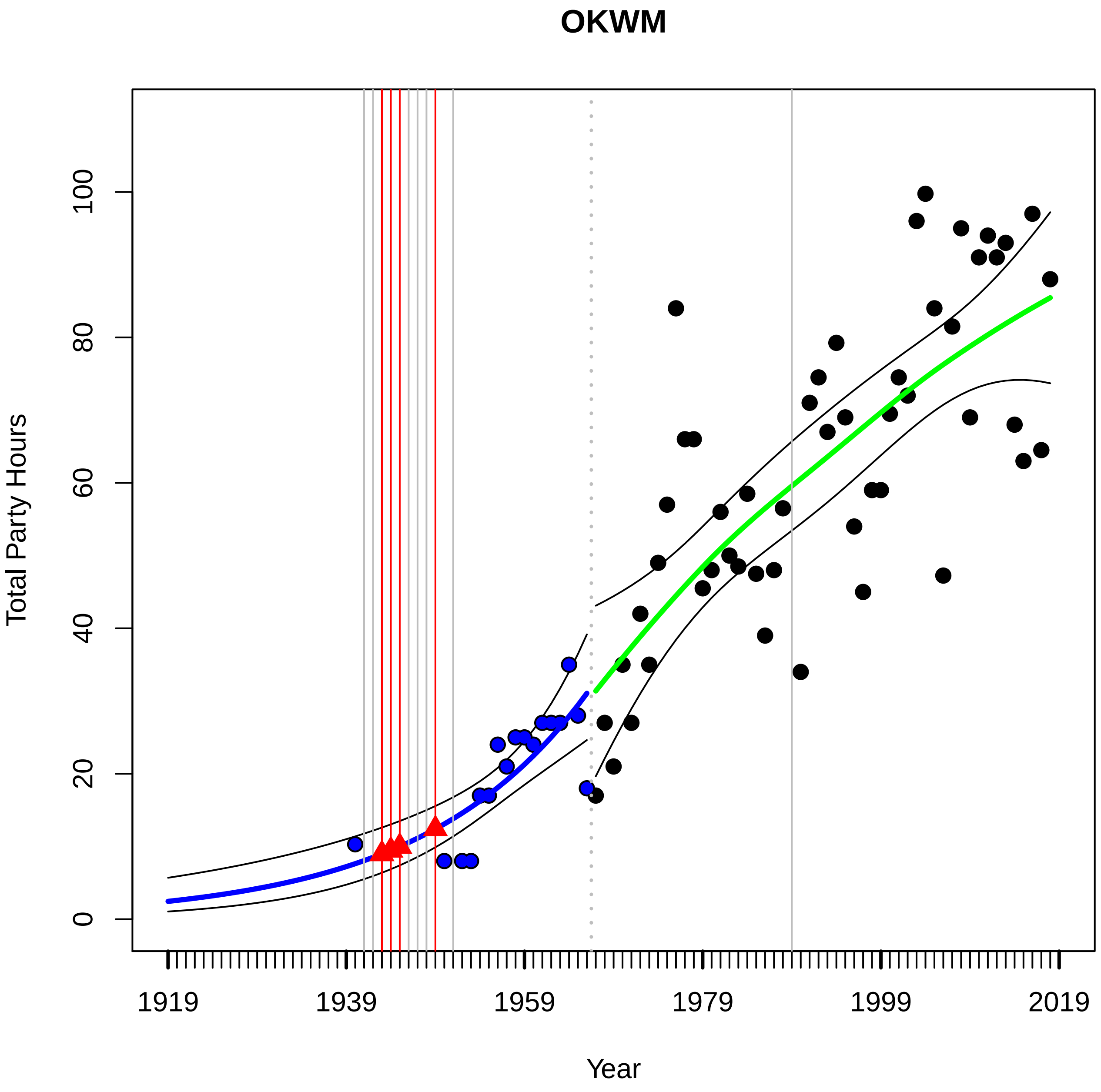

OKWM
Total Party Hours
0
20
40
60
80
100
1919
1939
1959
1979
1999
2019
Year

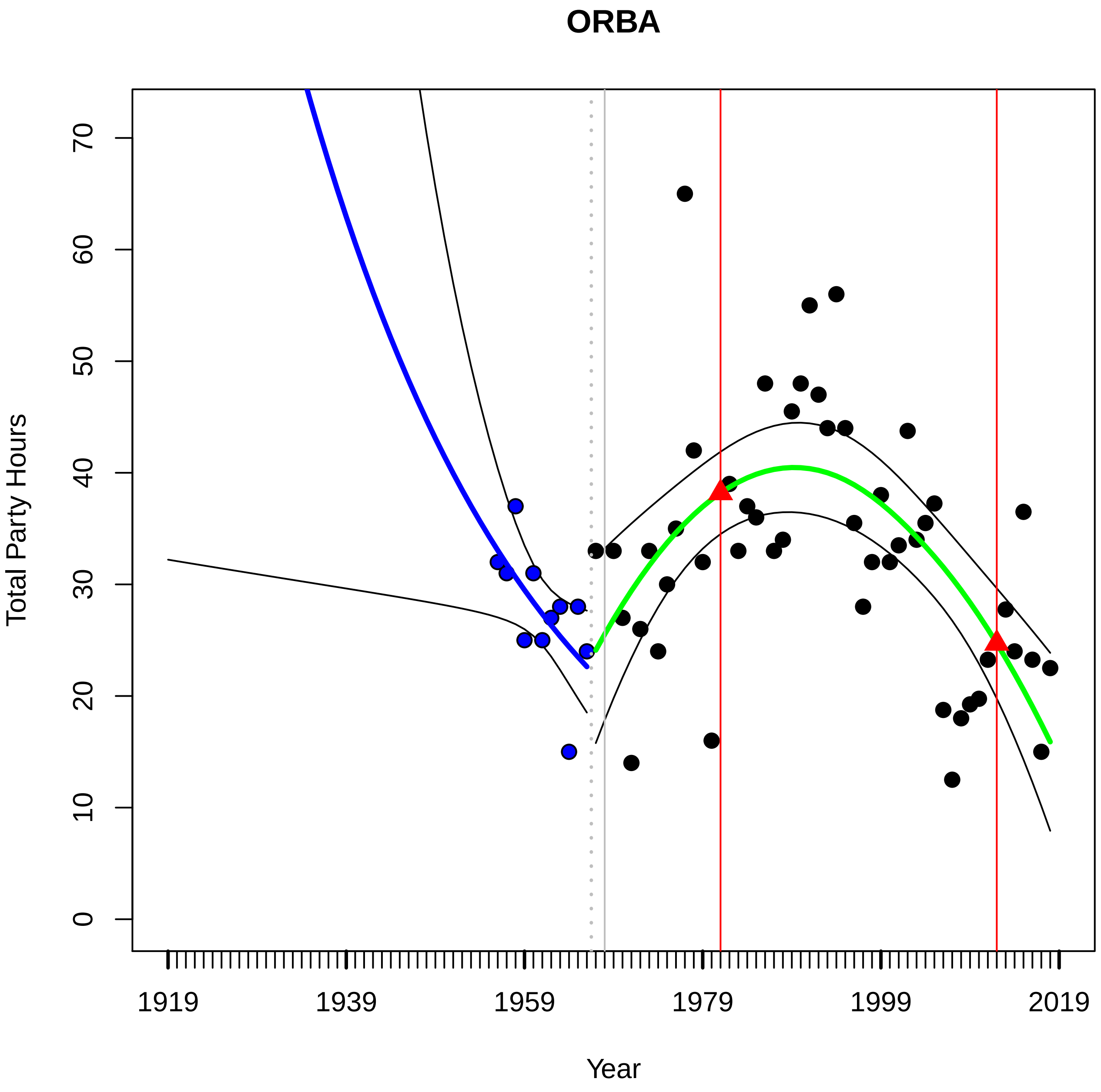

ORBA
Total Party Hours
0
10
20
30
40
50
60
70
1919
1939
1959
1979
1999
2019
Year

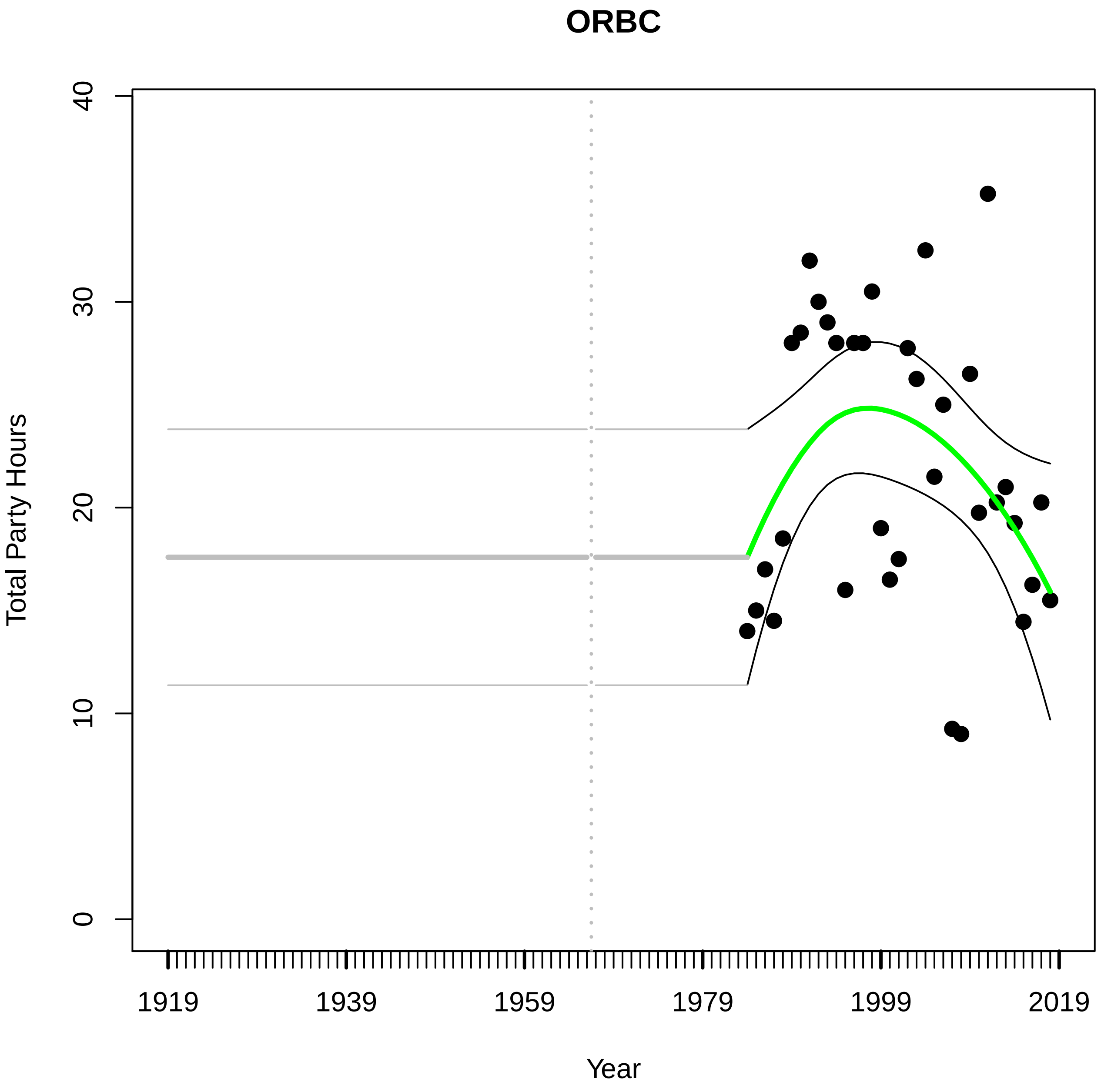

ORBC
Total Party Hours
0
10
20
30
40
1919
1939
1959
1979
1999
2019
Year

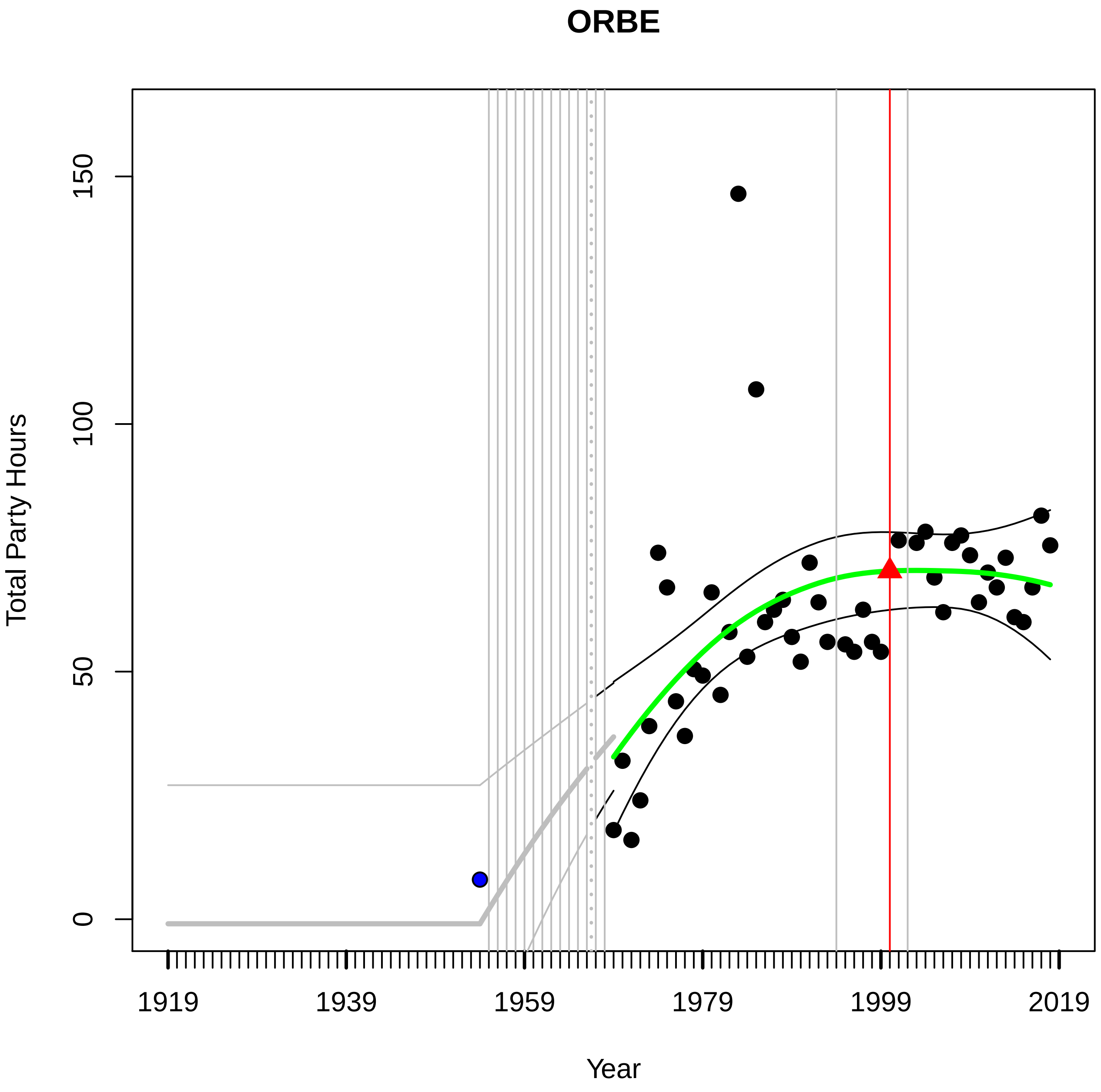

ORBE
Total Party Hours
0
50
100
150
1919
1939
1959
1979
1999
2019
Year

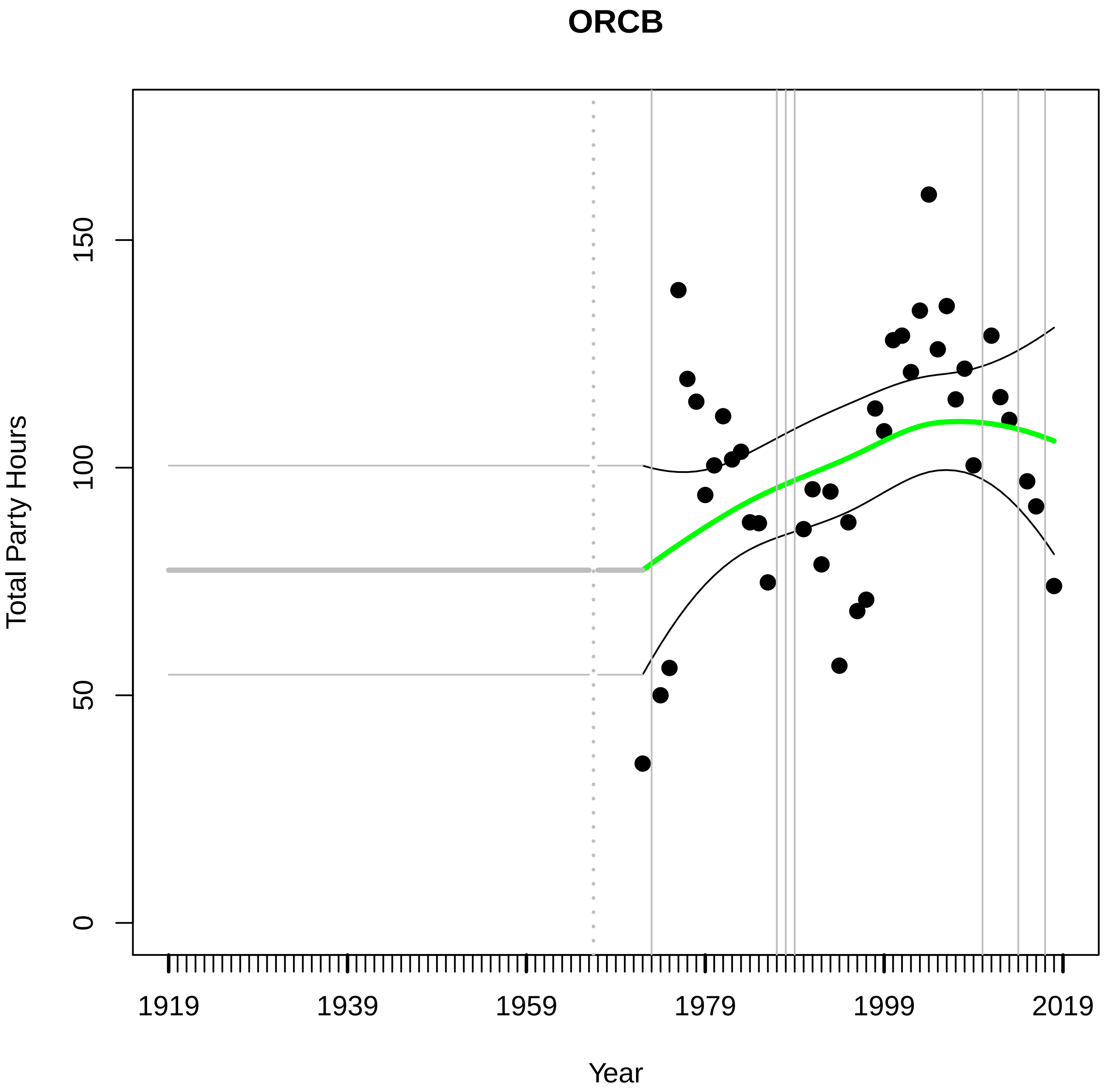

ORCB
Total Party Hours
Year
0
50
100
150
1919
1939
1959
1979
1999
2019

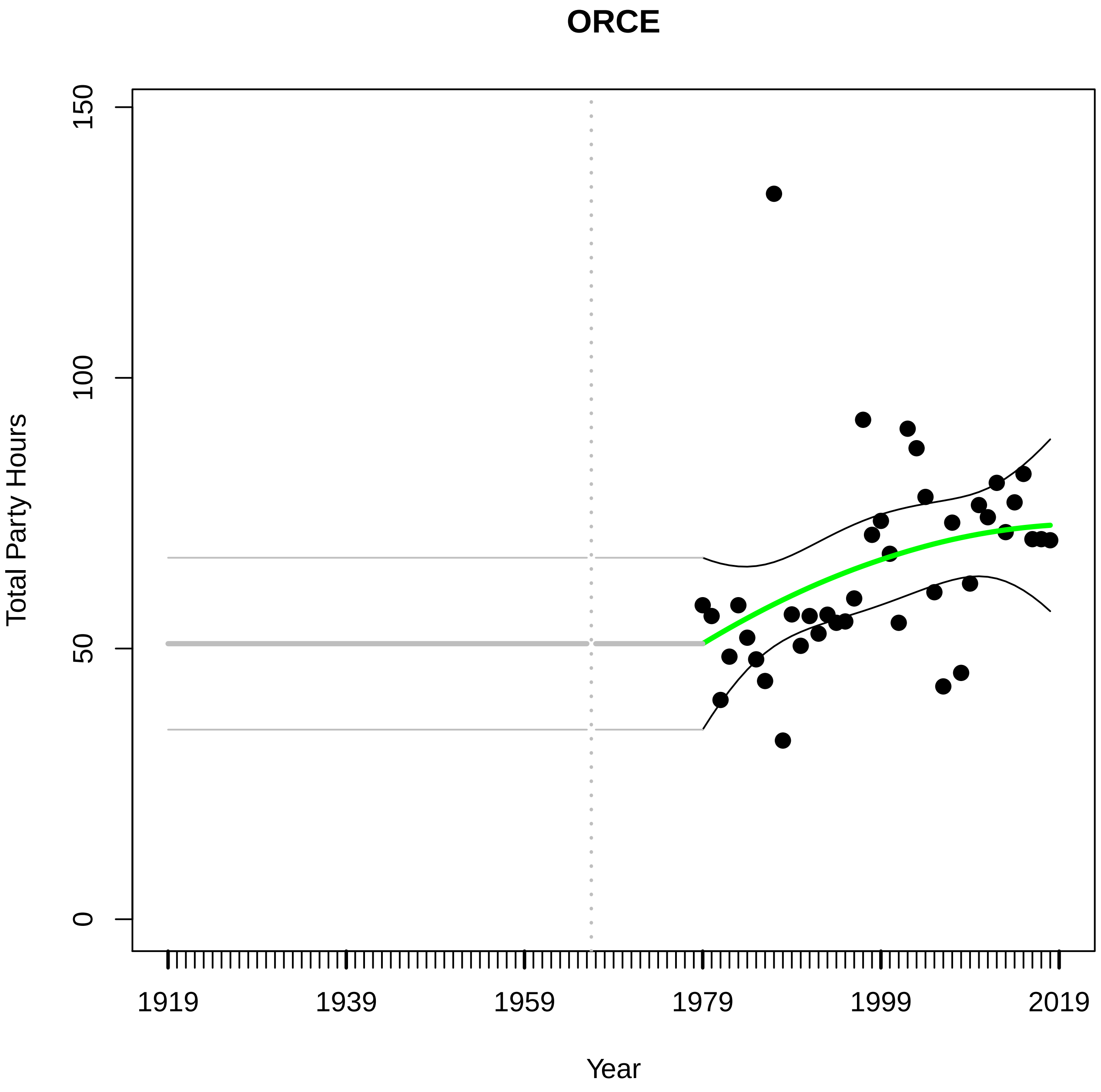

ORCE
Total Party Hours
0
50
100
150
1919
1939
1959
1979
1999
2019
Year

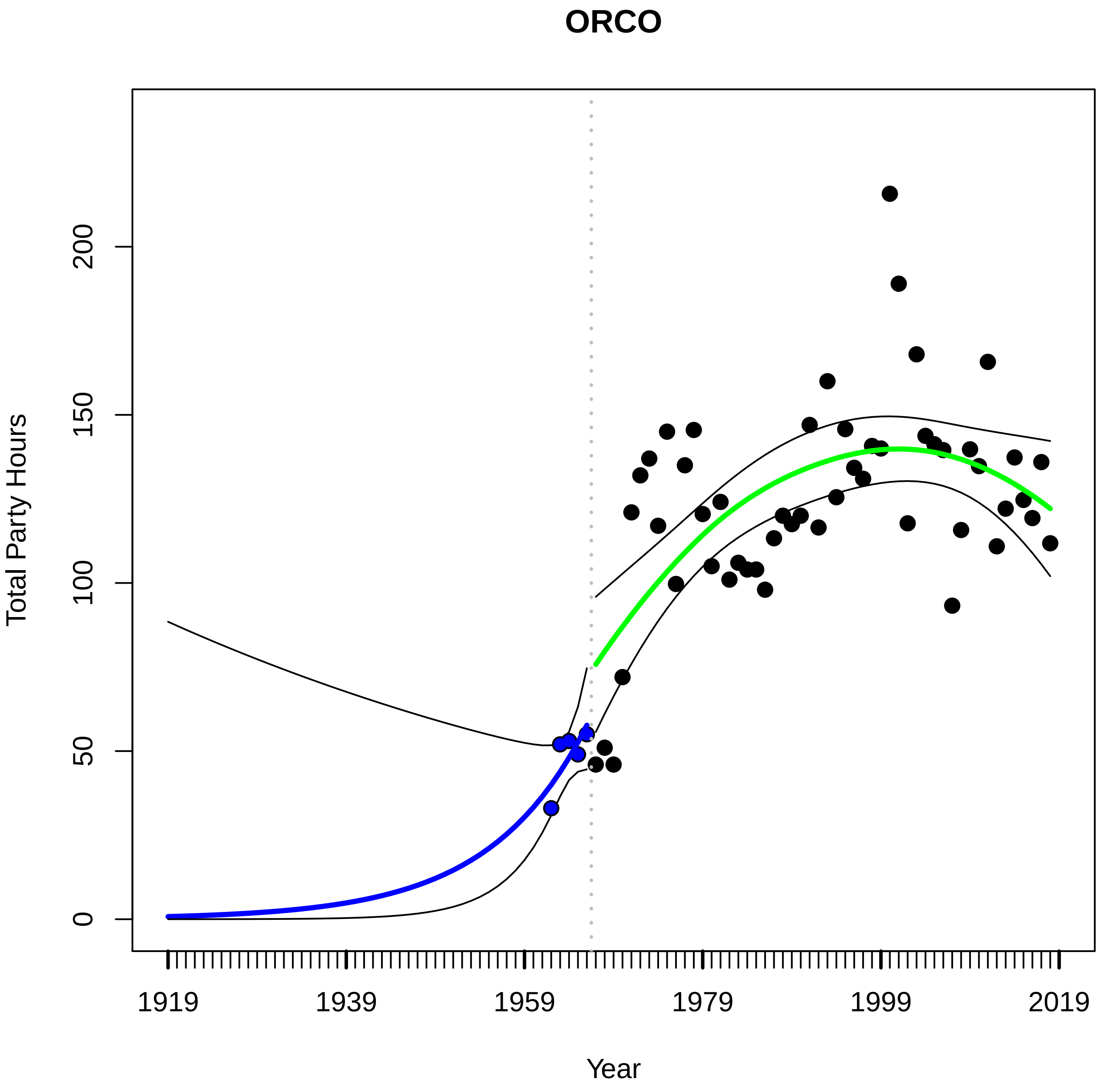

ORCO
Total Party Hours
0
50
100
150
200
1919
1939
1959
1979
1999
2019
Year

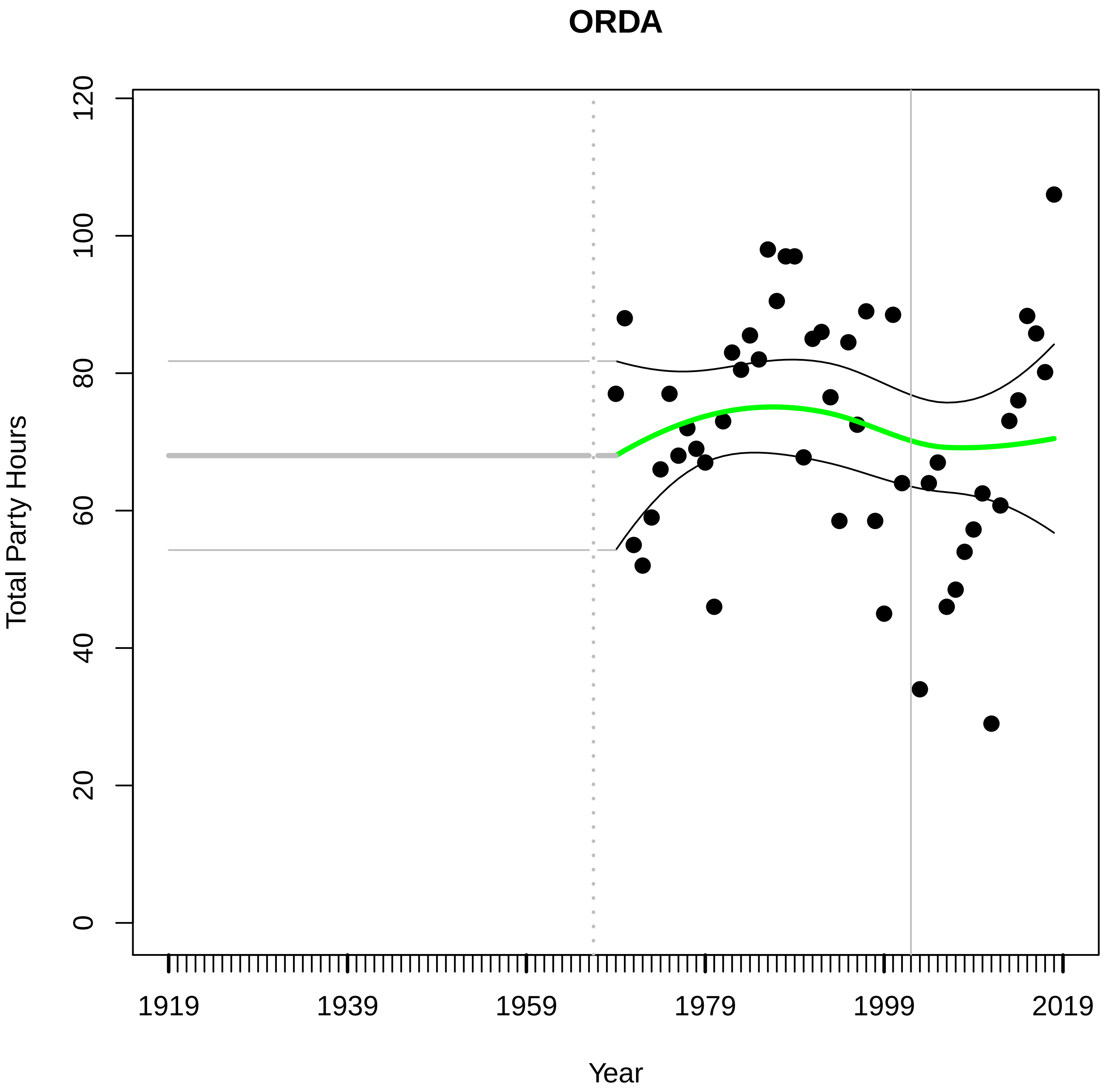

ORDA
Total Party Hours
0
20
40
60
80
100
120
1919
1939
1959
1979
1999
2019
Year

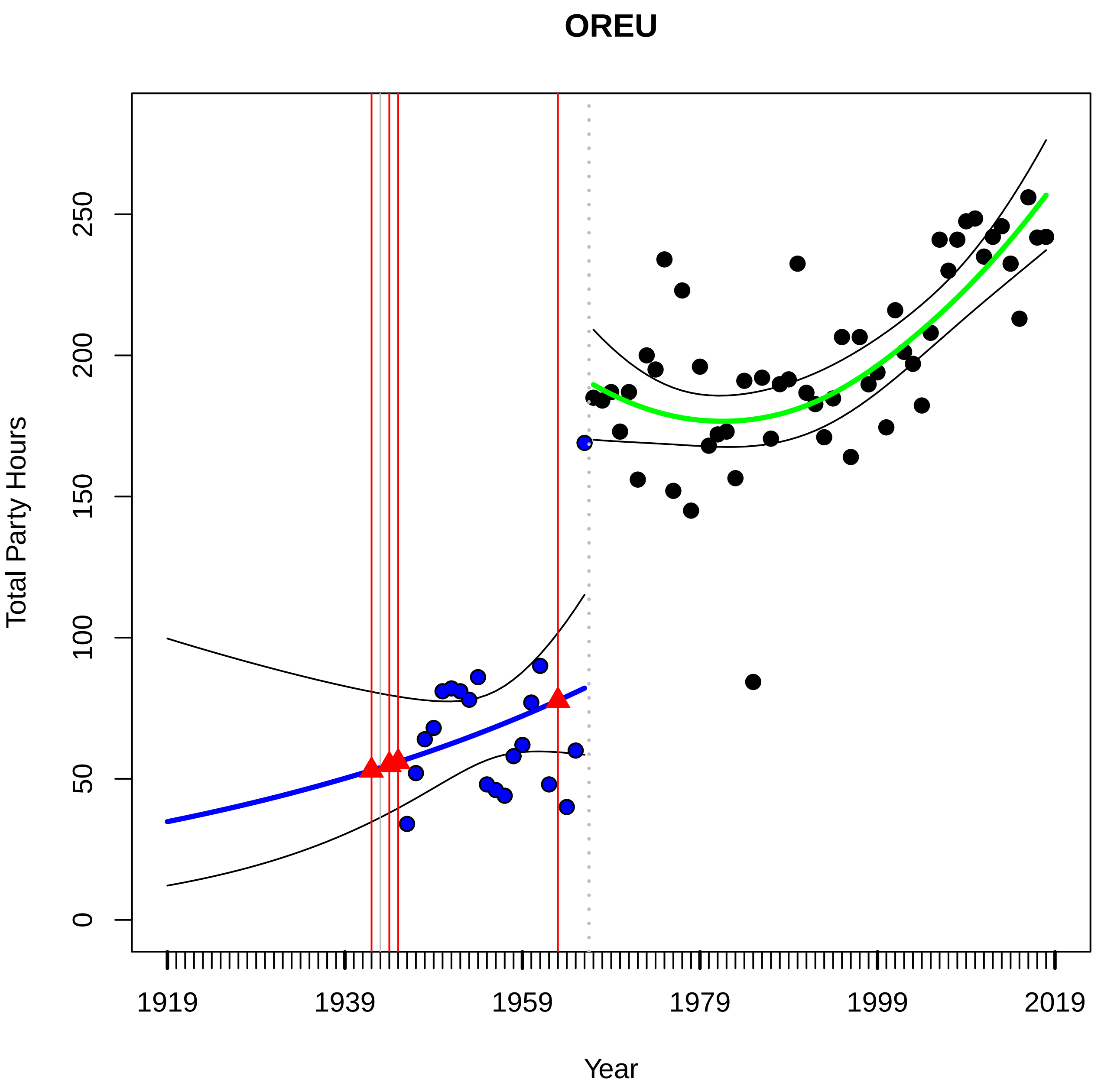

OREU
Total Party Hours
0
50
100
150
200
250
1919
1939
1959
1979
1999
2019
Year

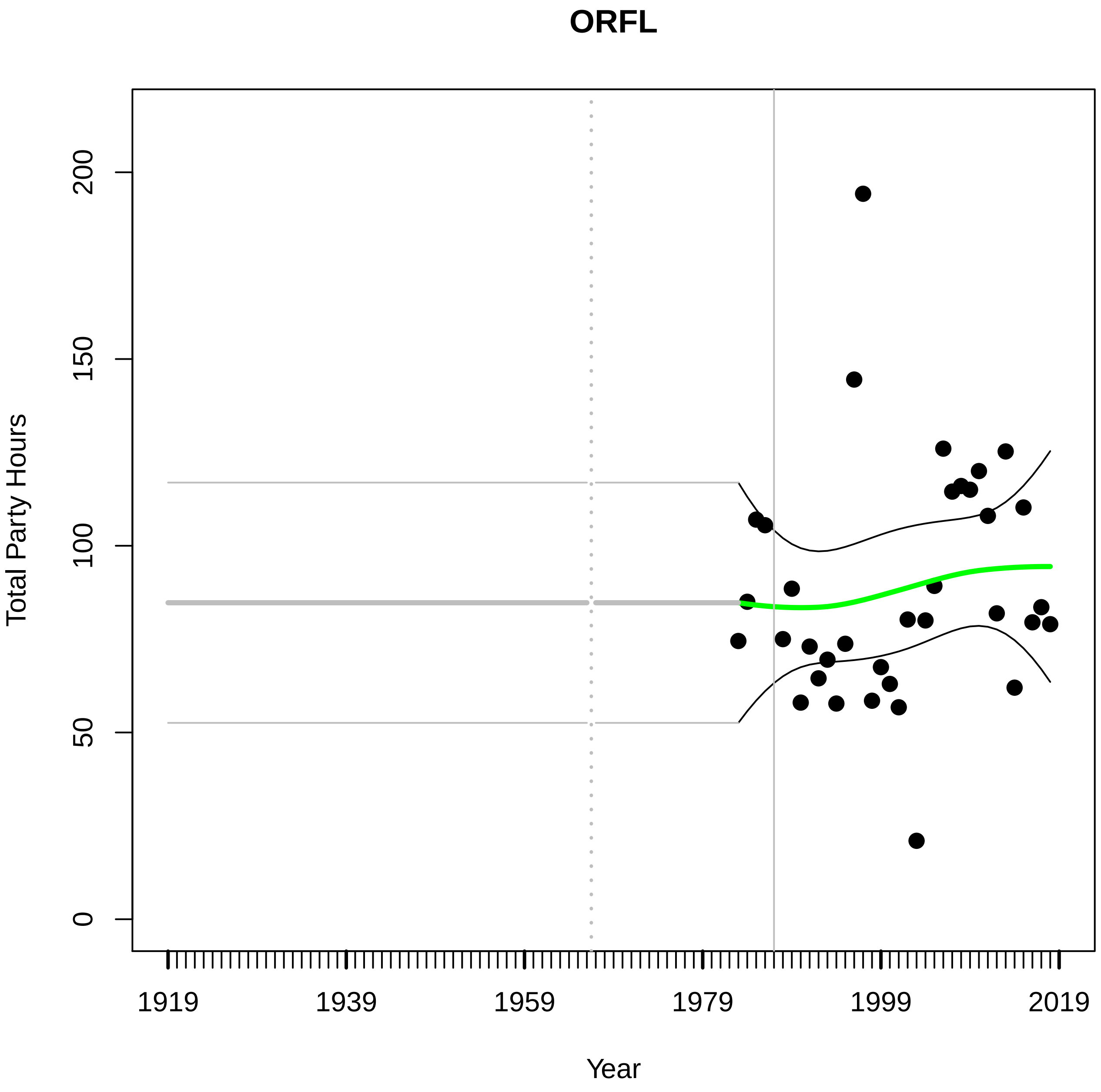

ORFL
Total Party Hours
0
50
100
150
200
1919
1939
1959
1979
1999
2019
Year

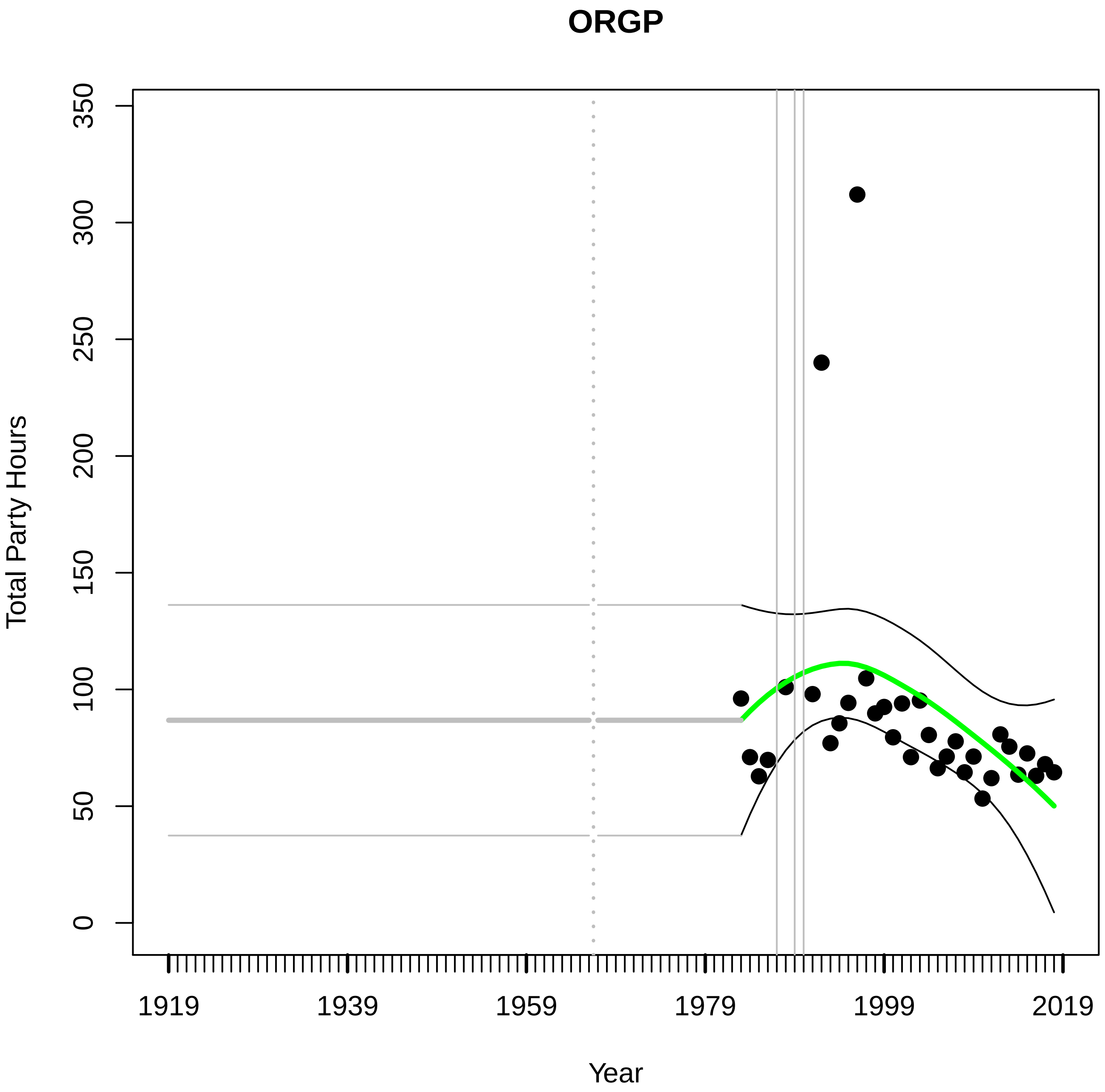

ORGP
Total Party Hours
Year
0
50
100
150
200
250
300
350
1919
1939
1959
1979
1999
2019

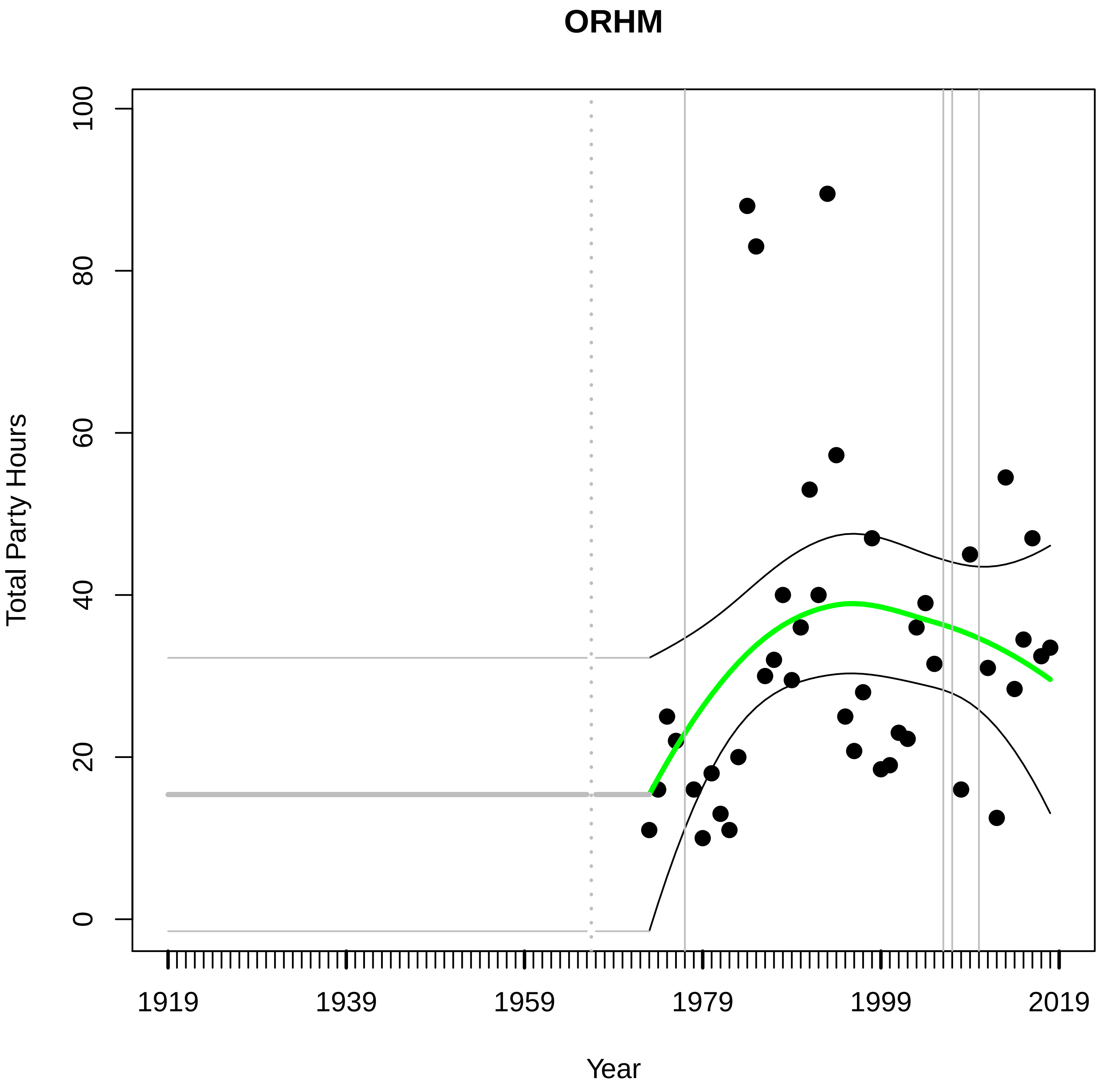

ORHM
Total Party Hours
0
20
40
60
80
100
1919
1939
1959
1979
1999
2019
Year

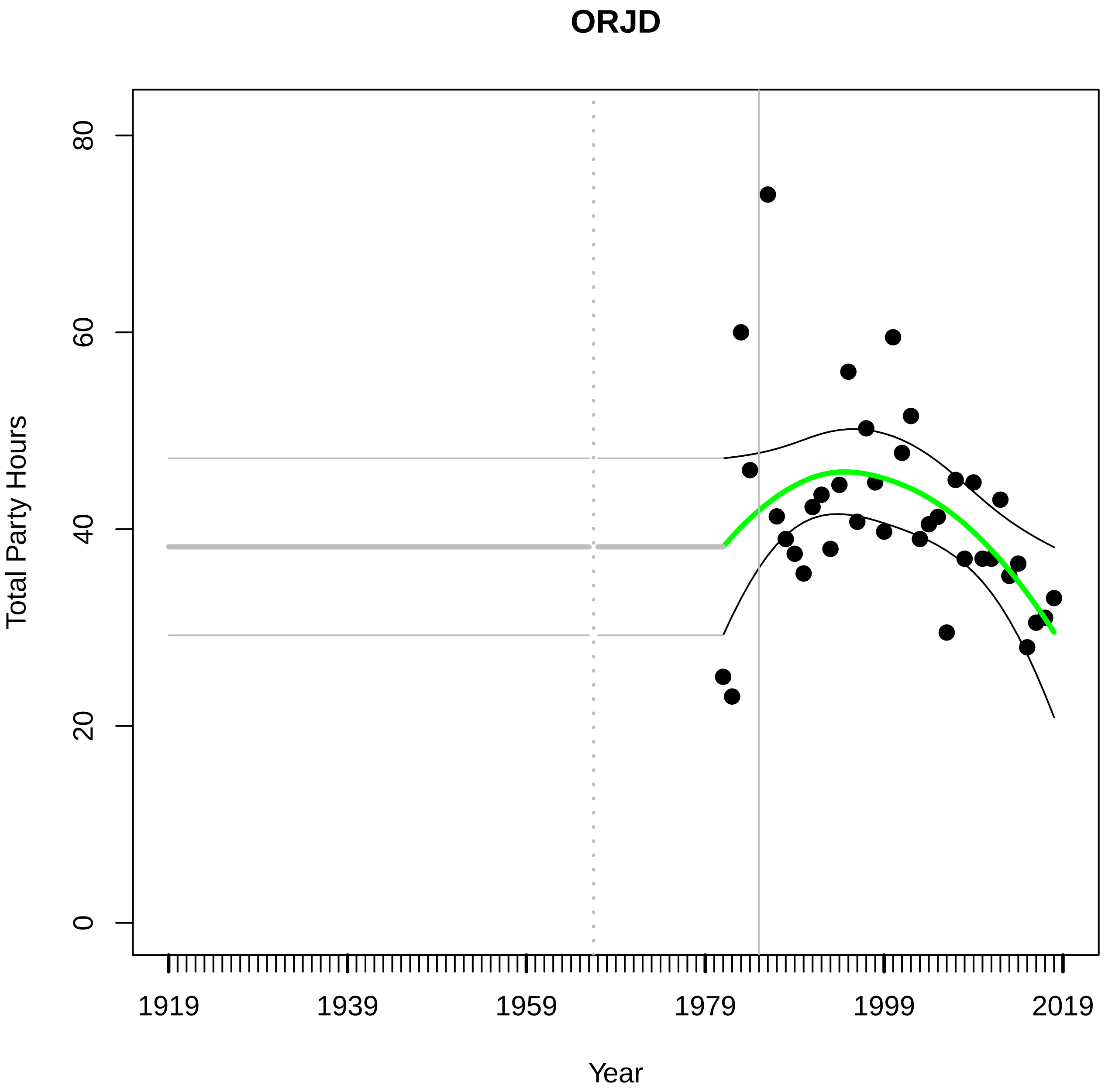

ORJD
Total Party Hours
0
20
40
60
80
1919
1939
1959
1979
1999
2019
Year

ORKF

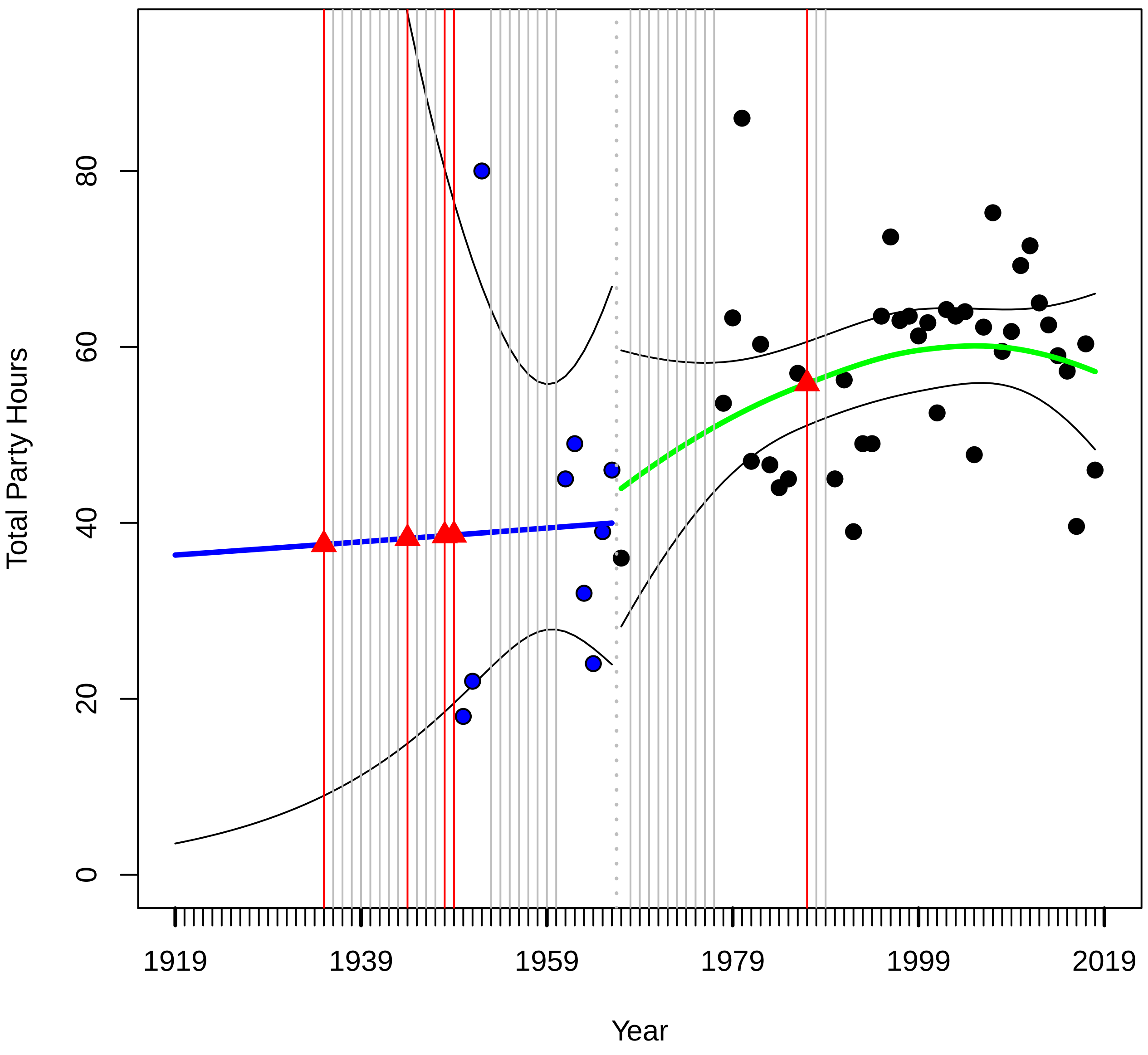

Total Party Hours
0
20
40
60
80
1919
1939
1959
1979
1999
2019
Year

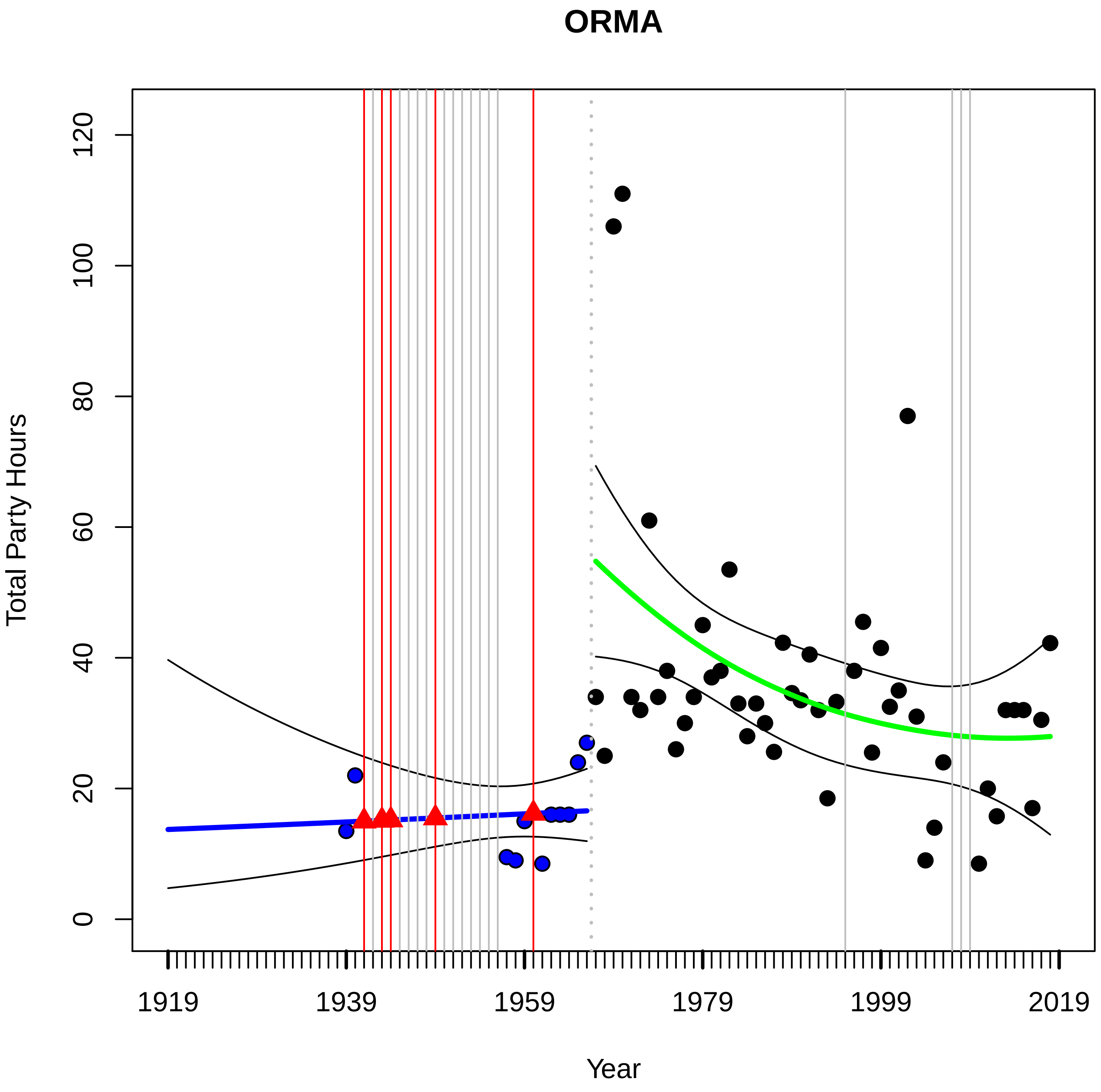
ORMA
Total Party Hours
0
20
40
60
80
100
120
1919
1939
1959
1979
1999
2019
Year

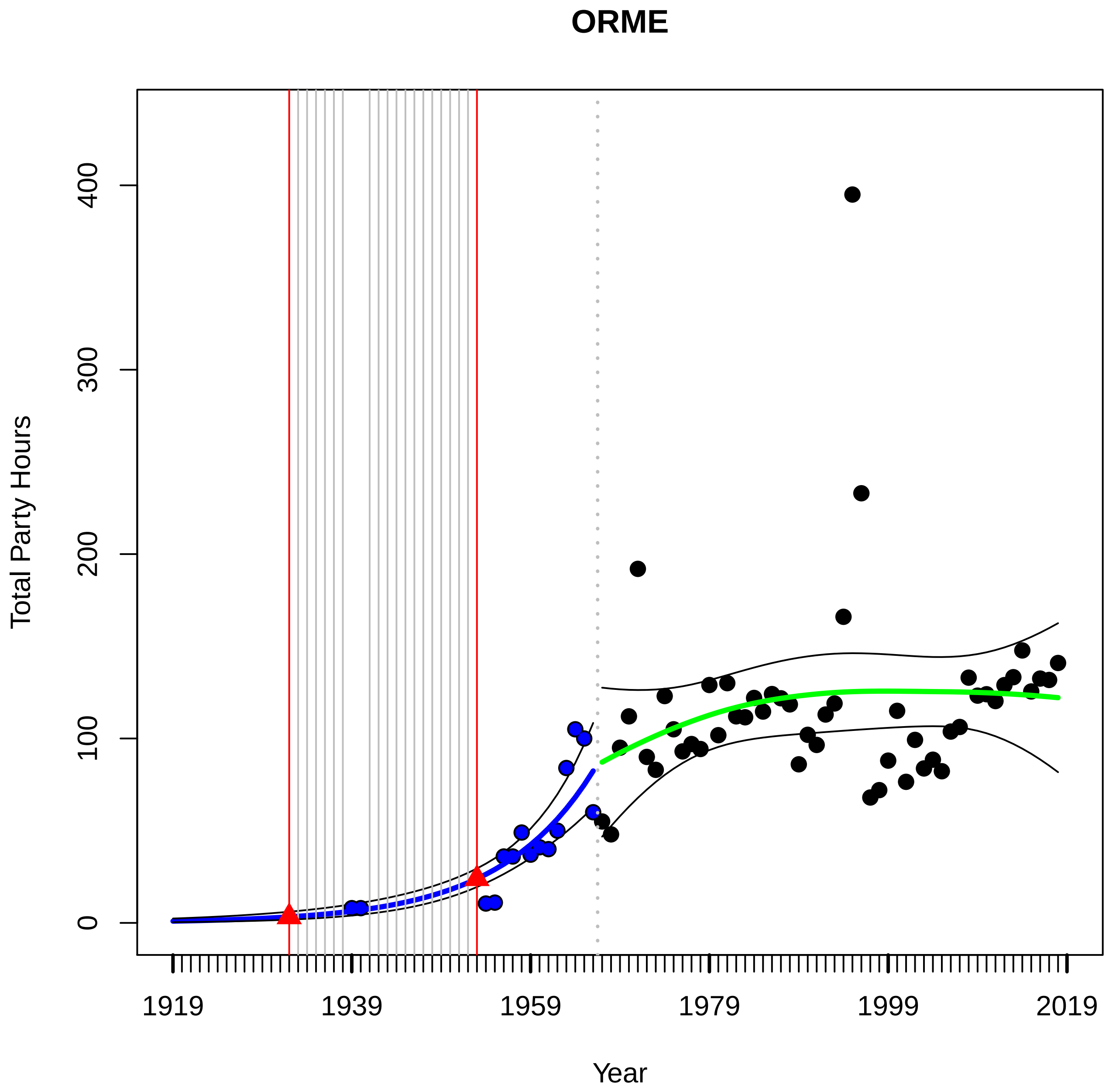

ORME
Total Party Hours
0
100
200
300
400
1919
1939
1959
1979
1999
2019
Year

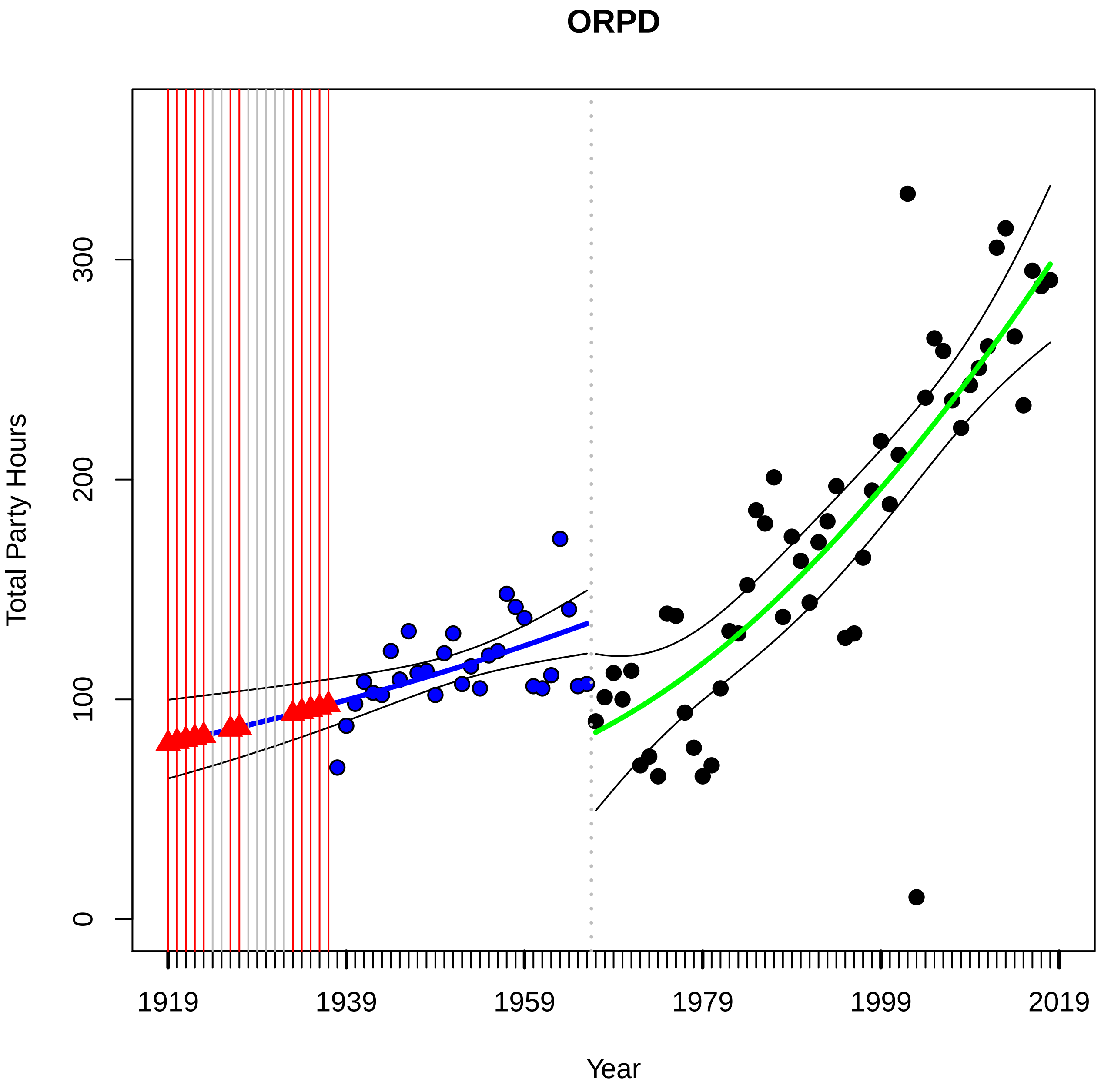

ORPD
Total Party Hours
0
100
200
300
1919
1939
1959
1979
1999
2019
Year

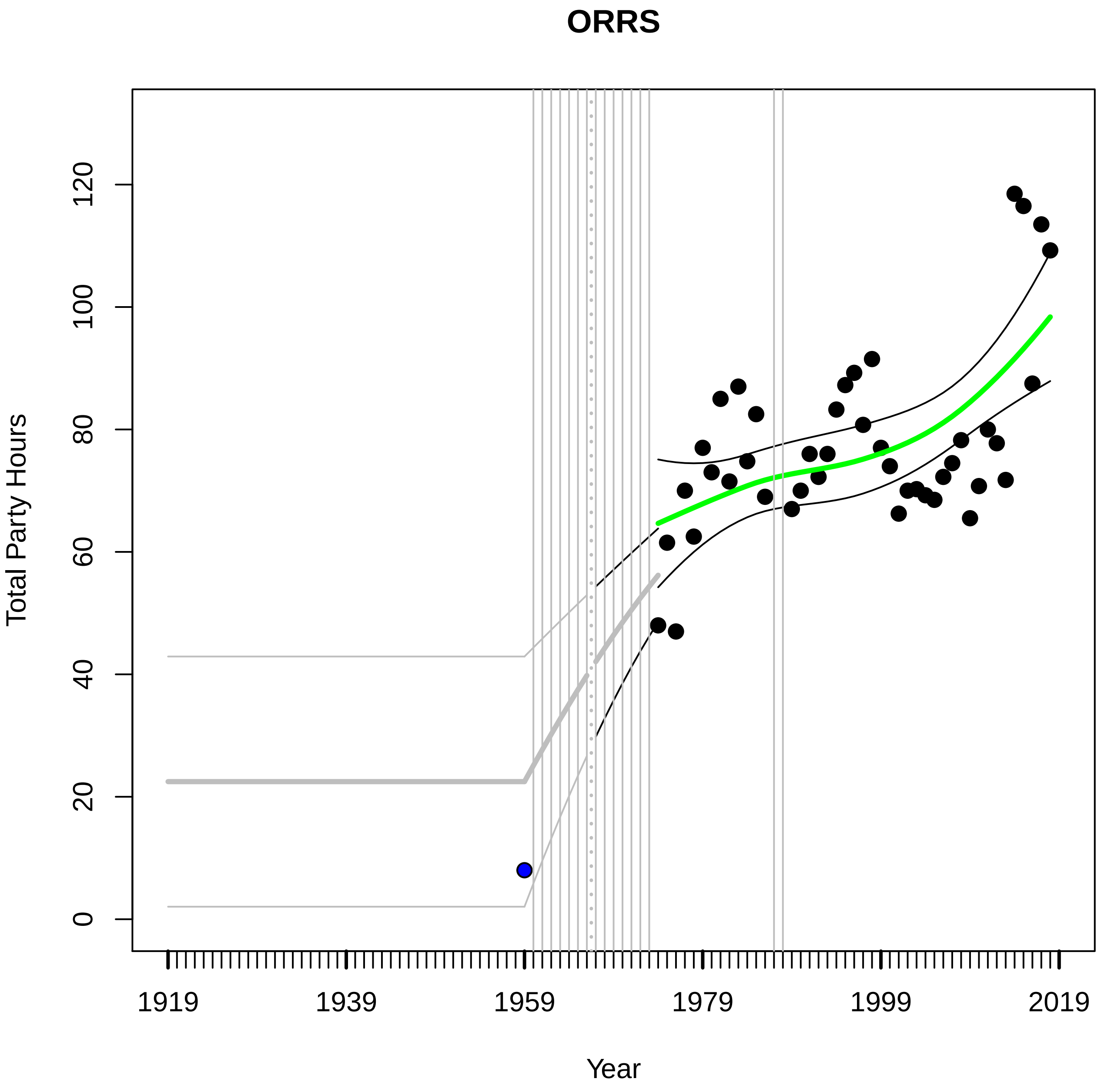
ORRS
Total Party Hours
0
20
40
60
80
100
120
1919
1939
1959
1979
1999
2019
Year

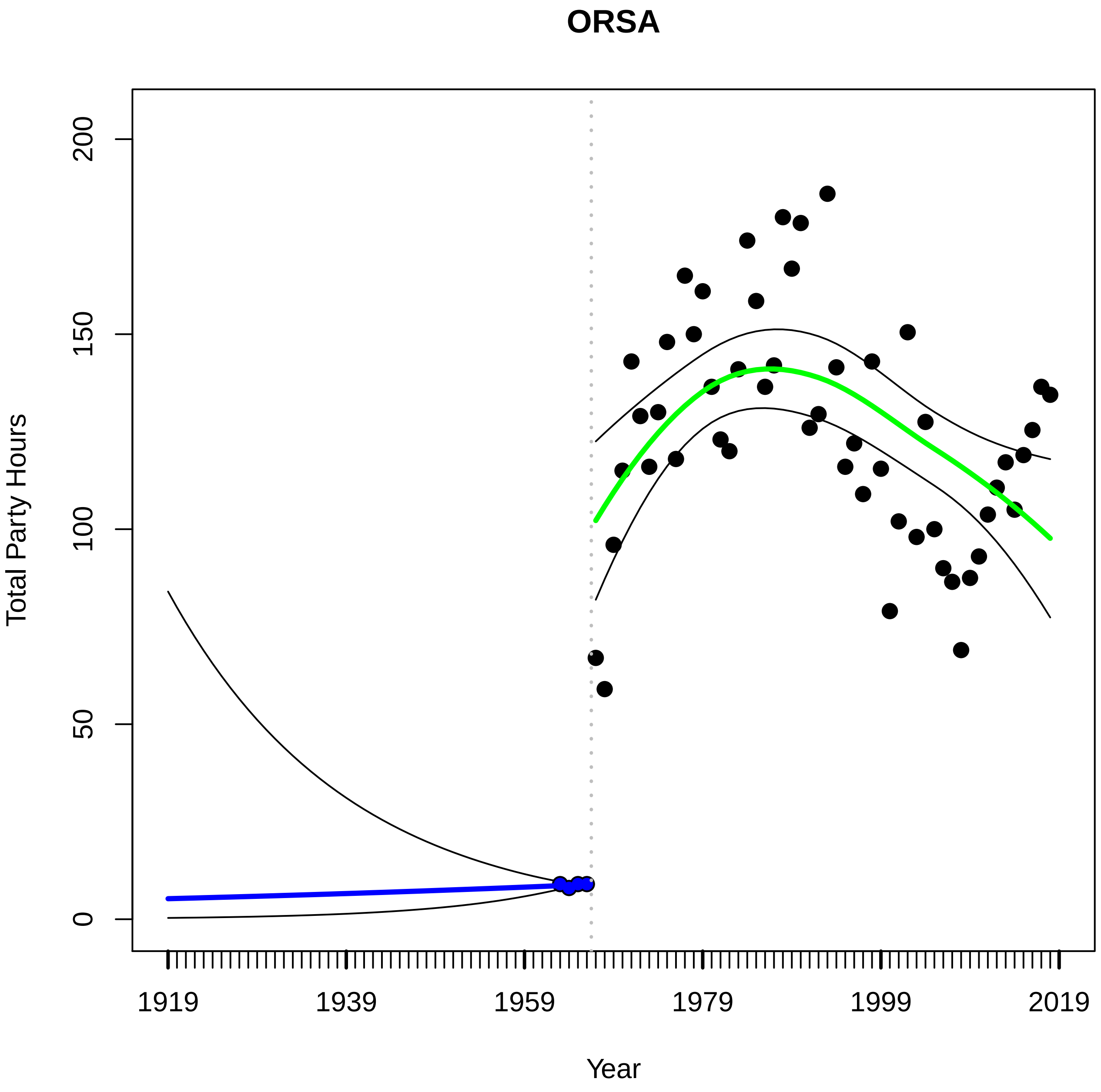

ORSA
Total Party Hours
200
150
100
50
0
1919
1939
1959
1979
1999
2019
Year

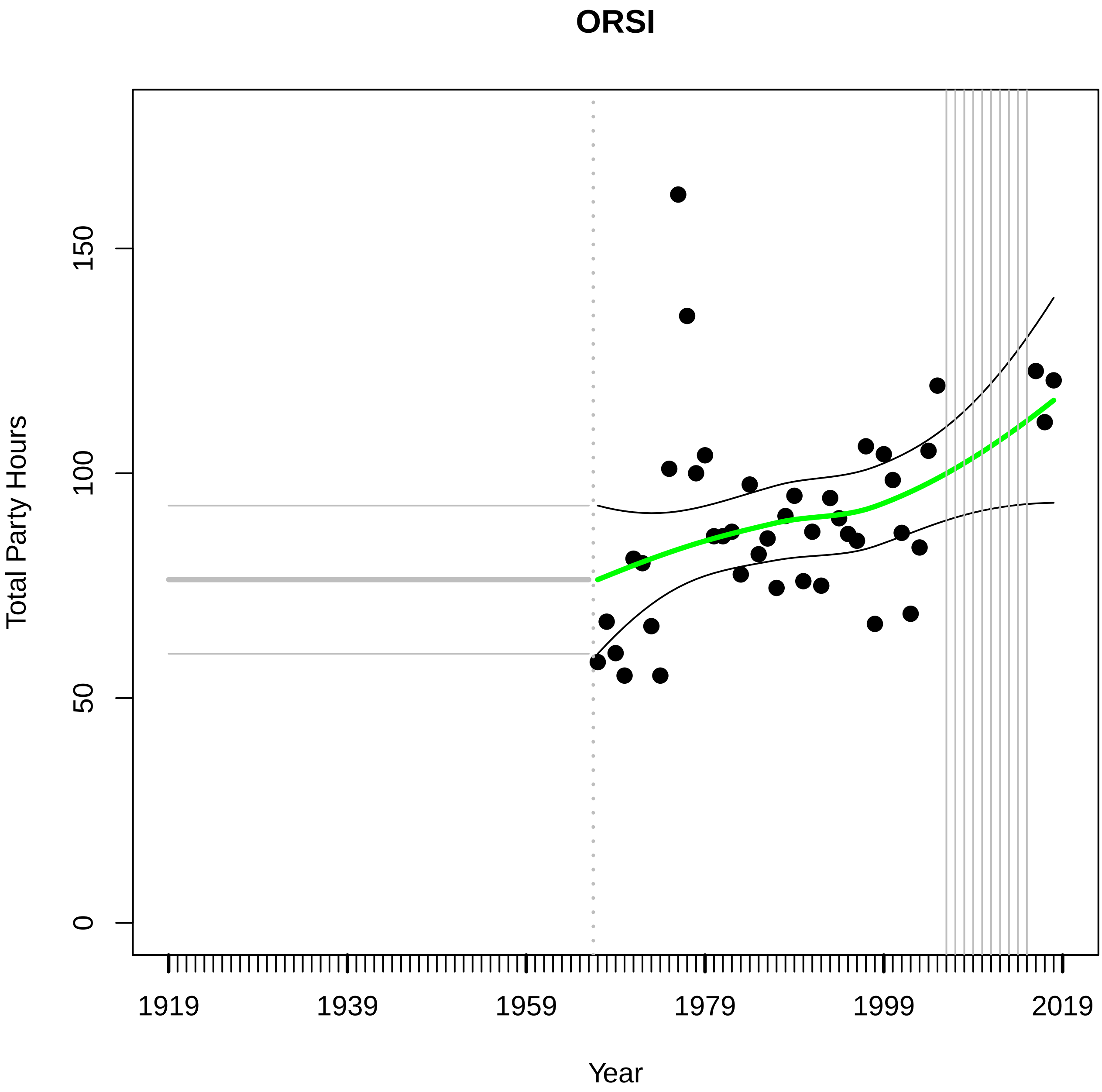

ORSI
Total Party Hours
0
50
100
150
1919
1939
1959
1979
1999
2019
Year

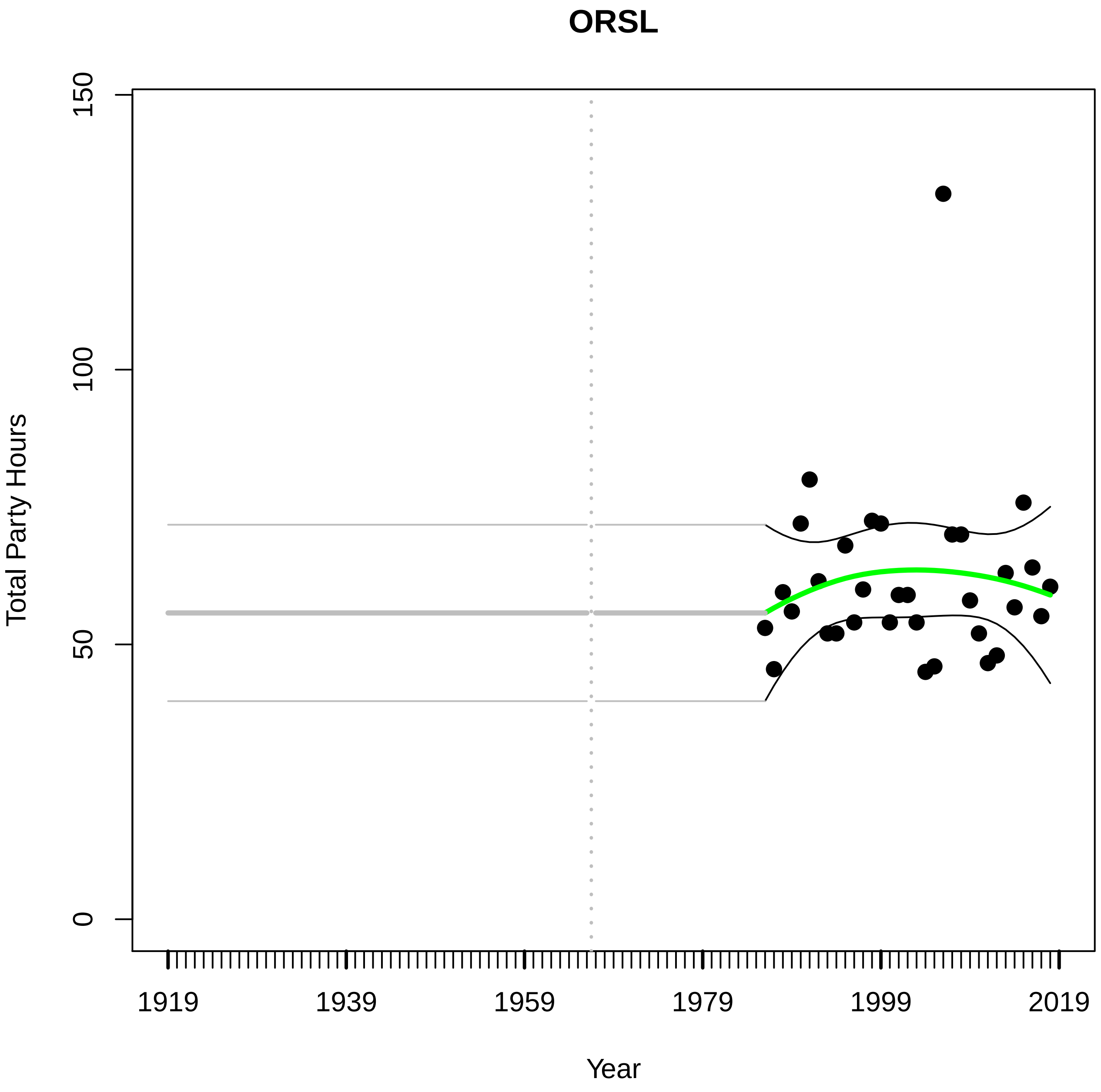

ORSL
150
100
50
0
Total Party Hours
1919
1939
1959
1979
1999
2019
Year

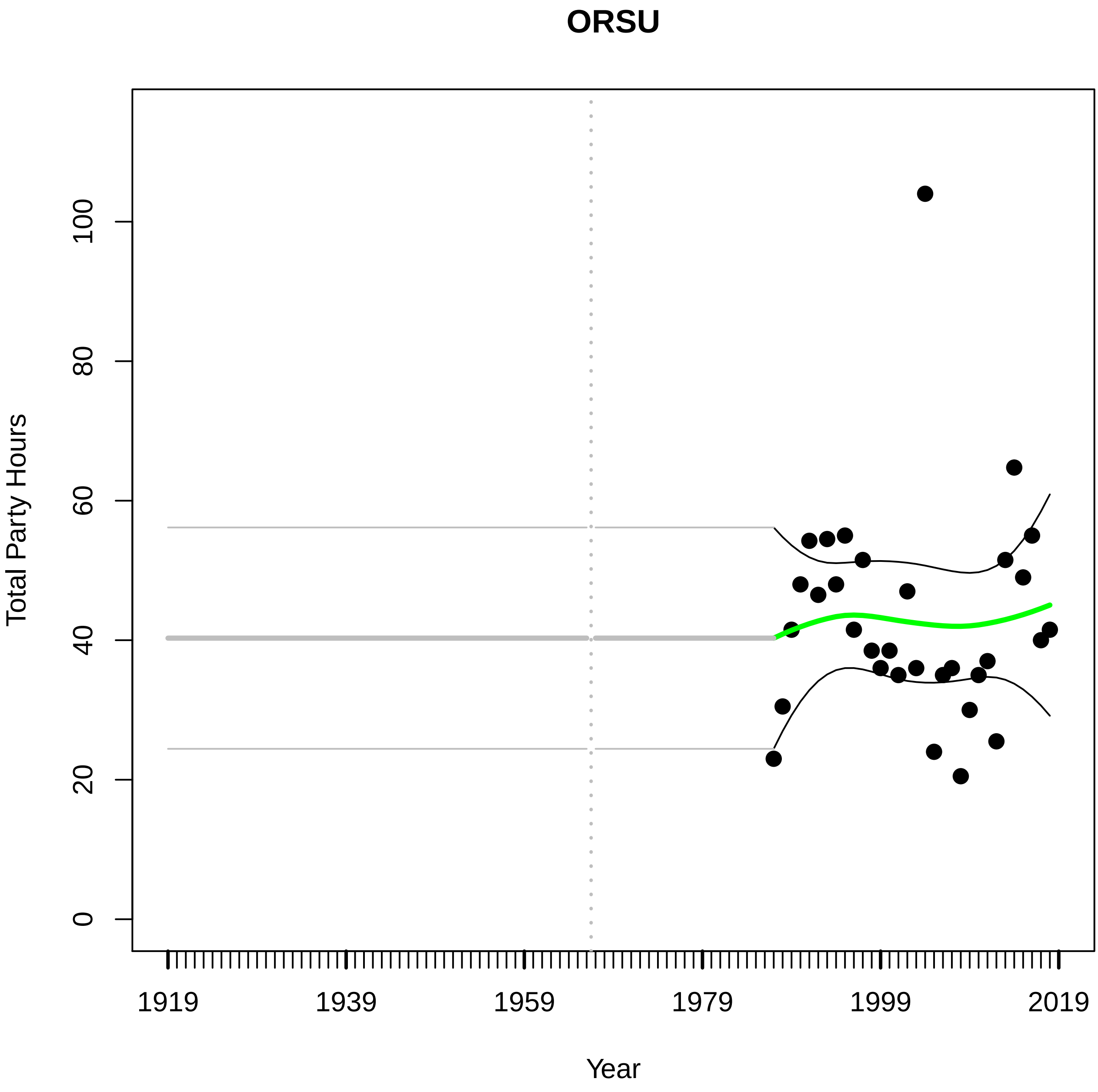

ORSU
Total Party Hours
0
20
40
60
80
100
1919
1939
1959
1979
1999
2019
Year

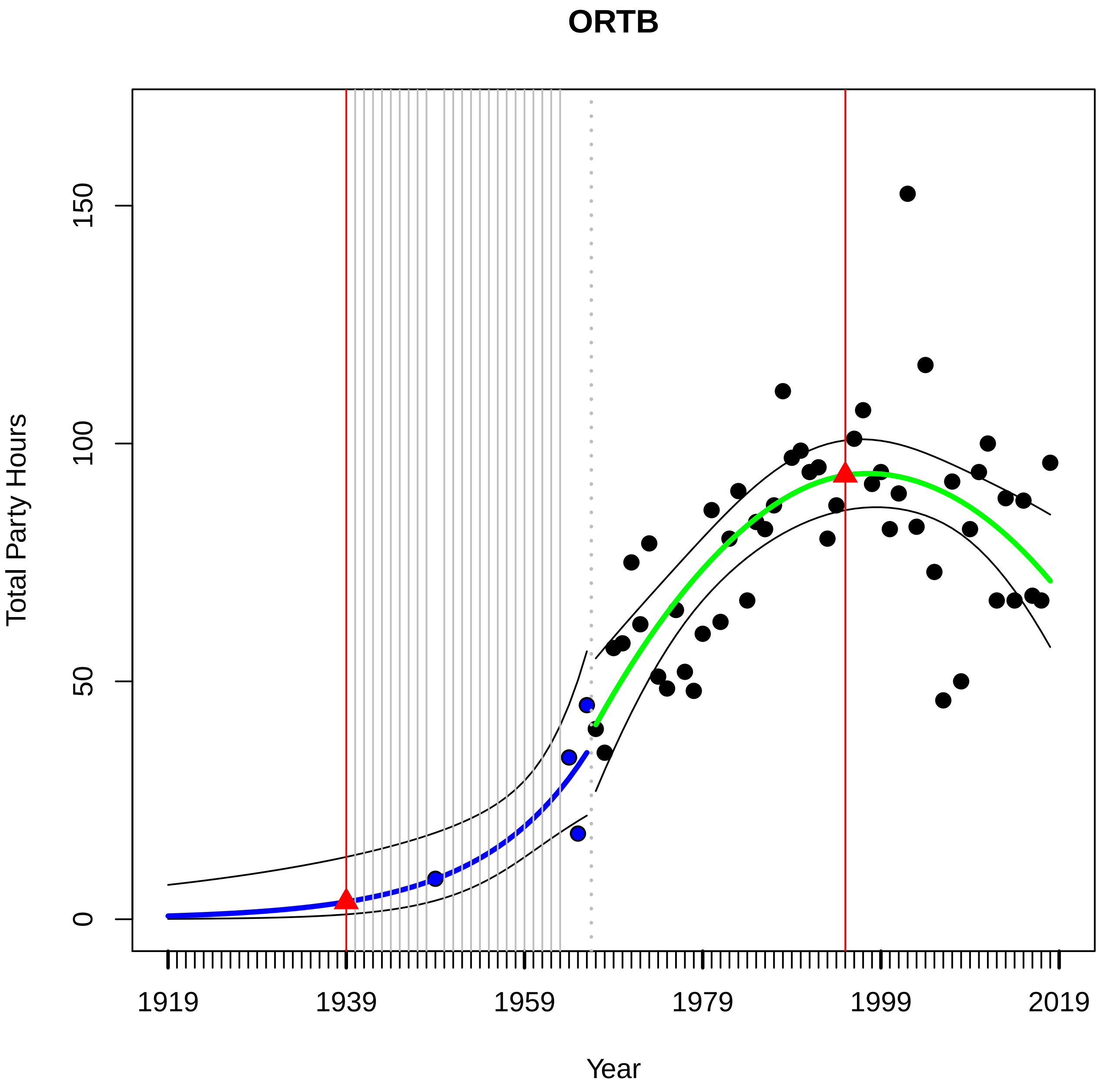

ORTB
Total Party Hours
0
50
100
150
1919
1939
1959
1979
1999
2019
Year

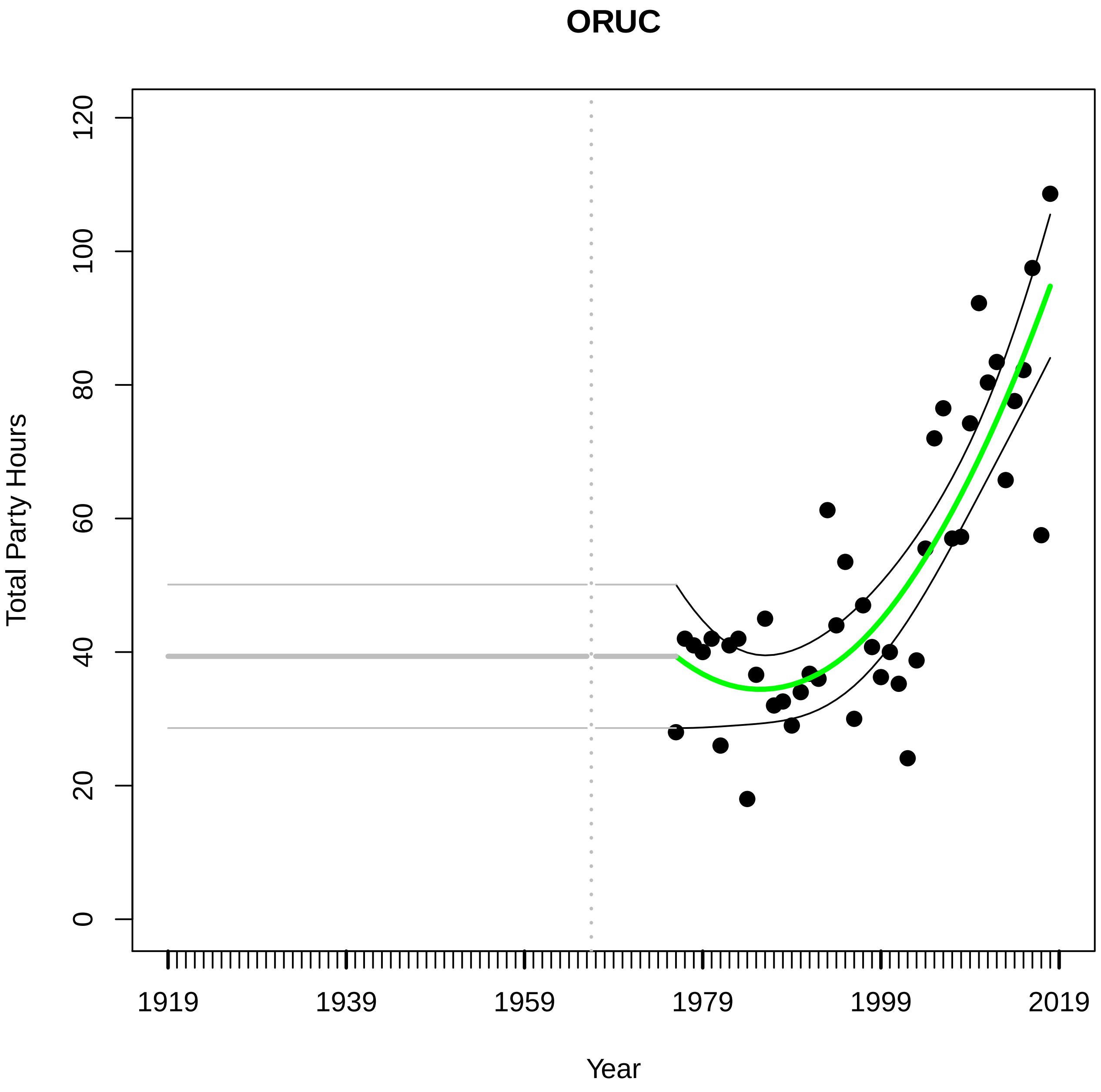

ORUC
Total Party Hours
Year
0
20
40
60
80
100
120
1919
1939
1959
1979
1999
2019

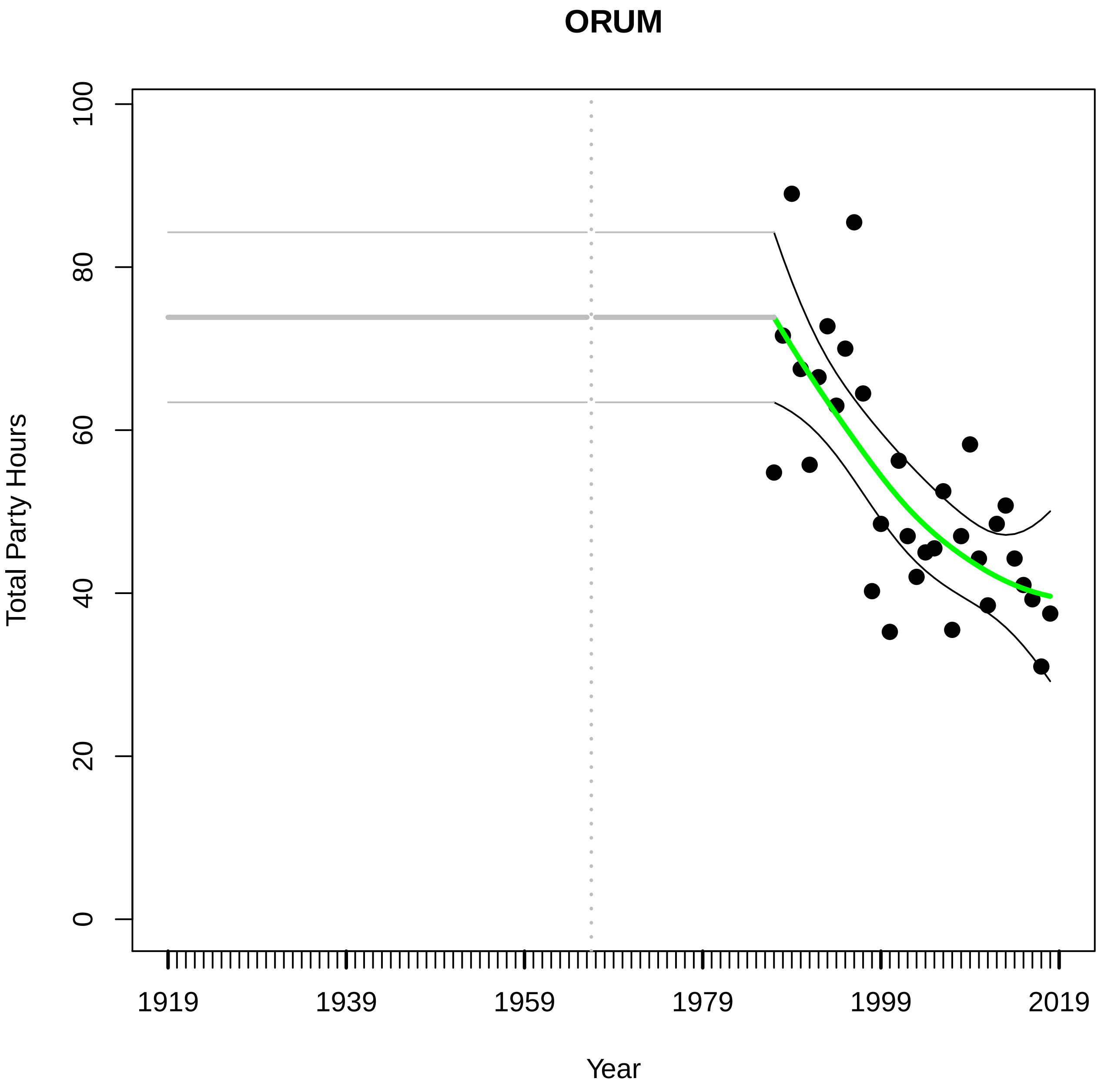

ORUM
Total Party Hours
0
20
40
60
80
100
1919
1939
1959
1979
1999
2019
Year

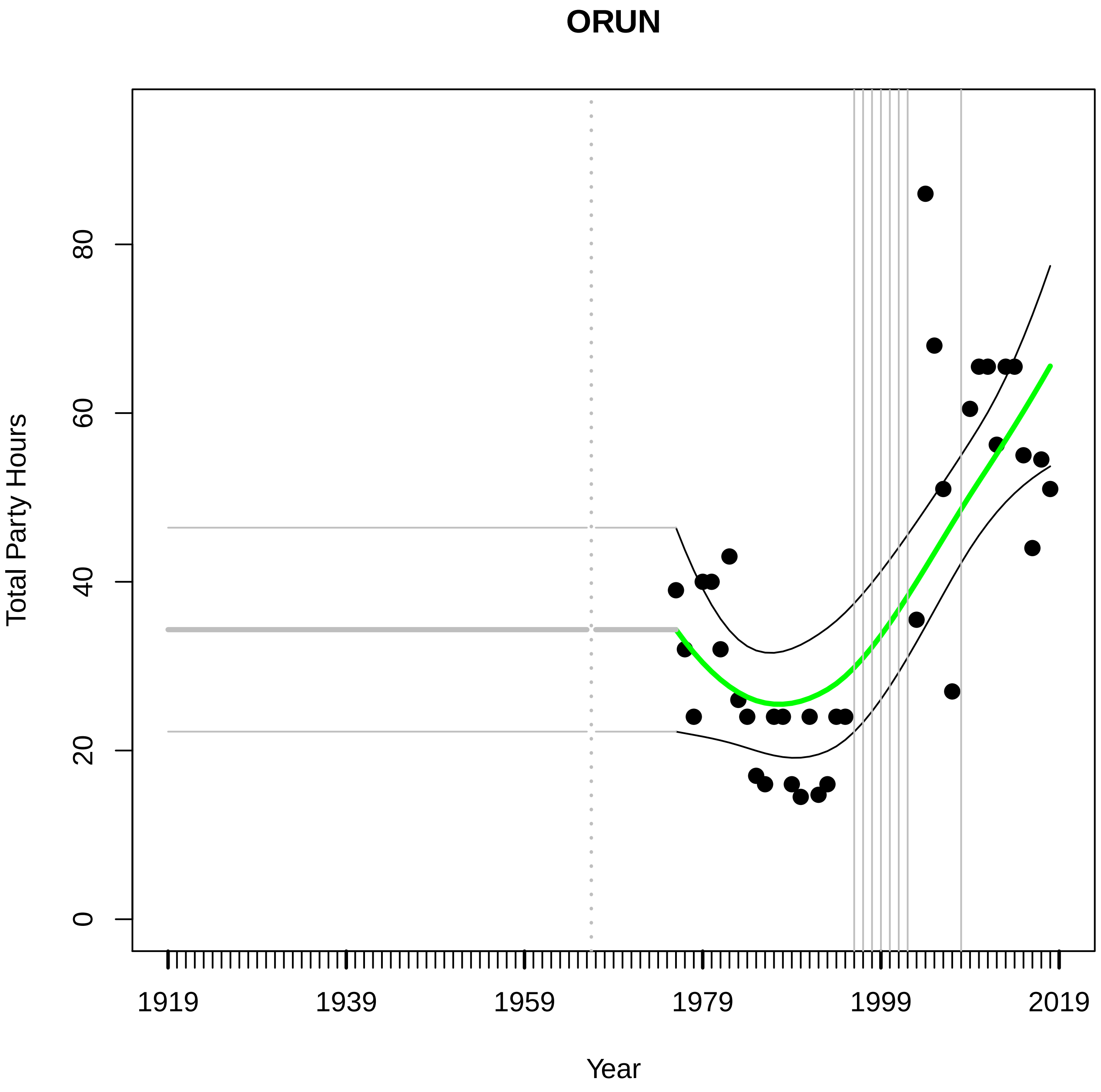

ORUN
Total Party Hours
0
20
40
60
80
1919
1939
1959
1979
1999
2019
Year

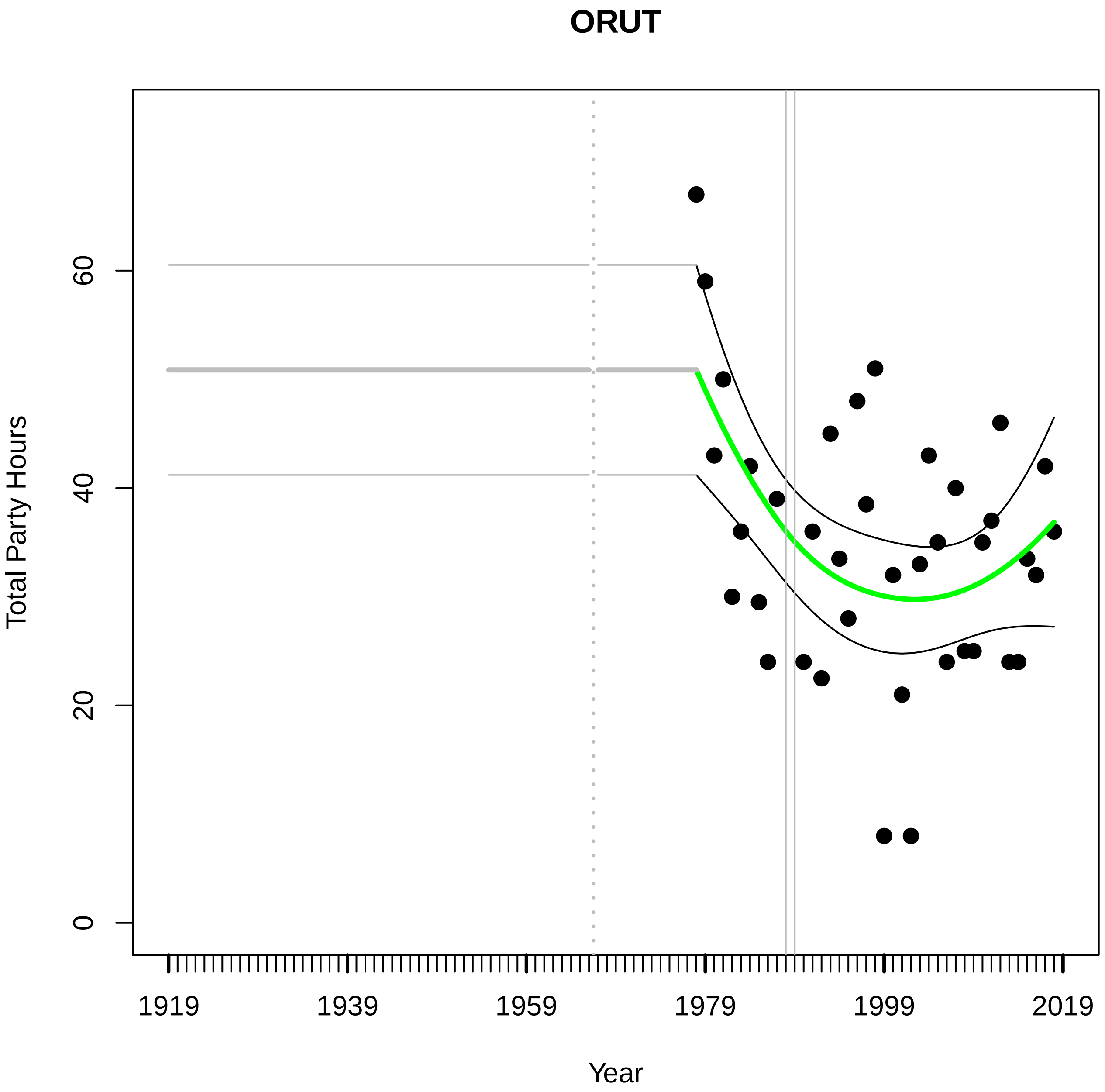

ORUT
Total Party Hours
0
20
40
60
1919
1939
1959
1979
1999
2019
Year

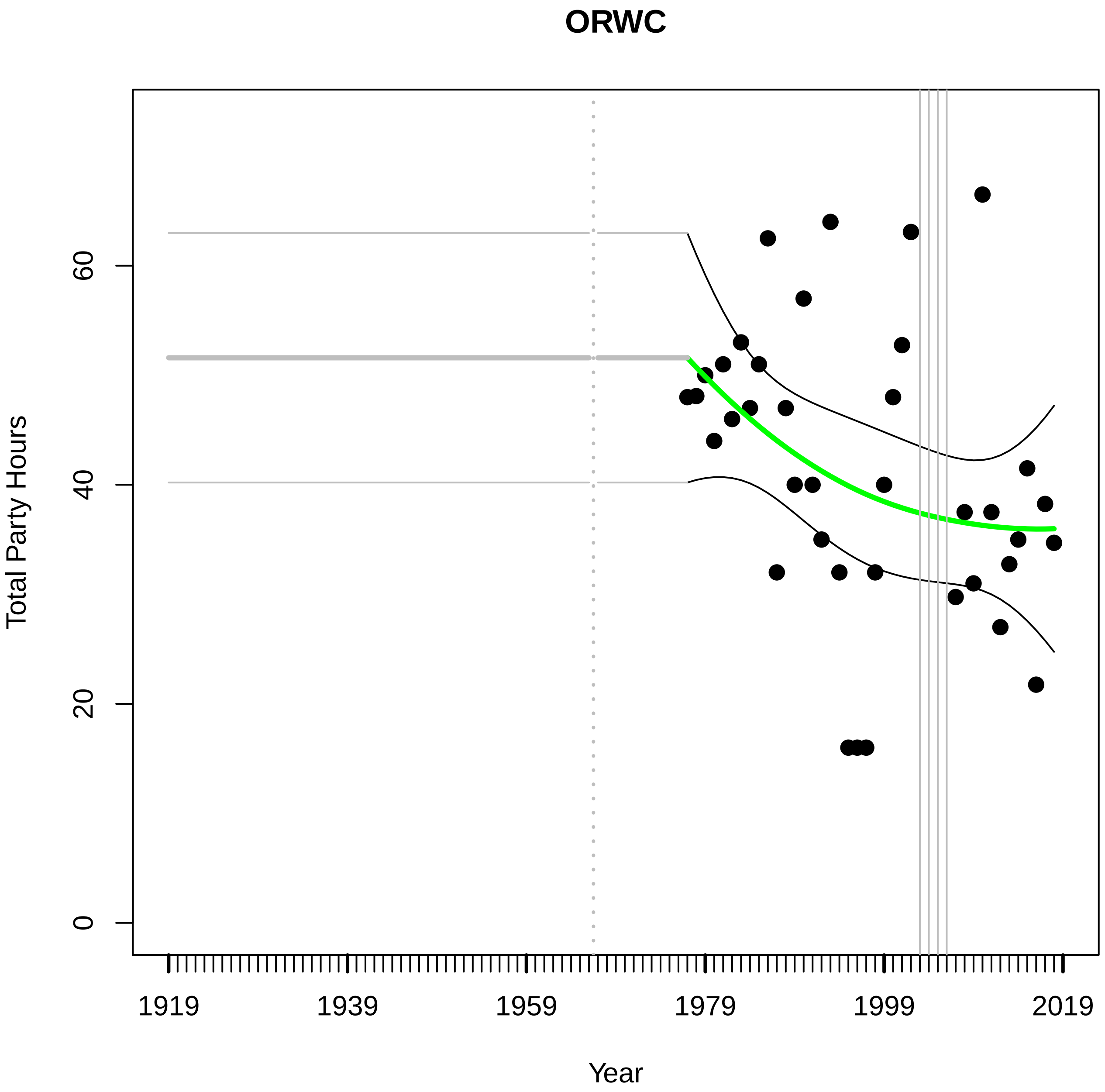

ORWC
Total Party Hours
0
20
40
60
1919
1939
1959
1979
1999
2019
Year

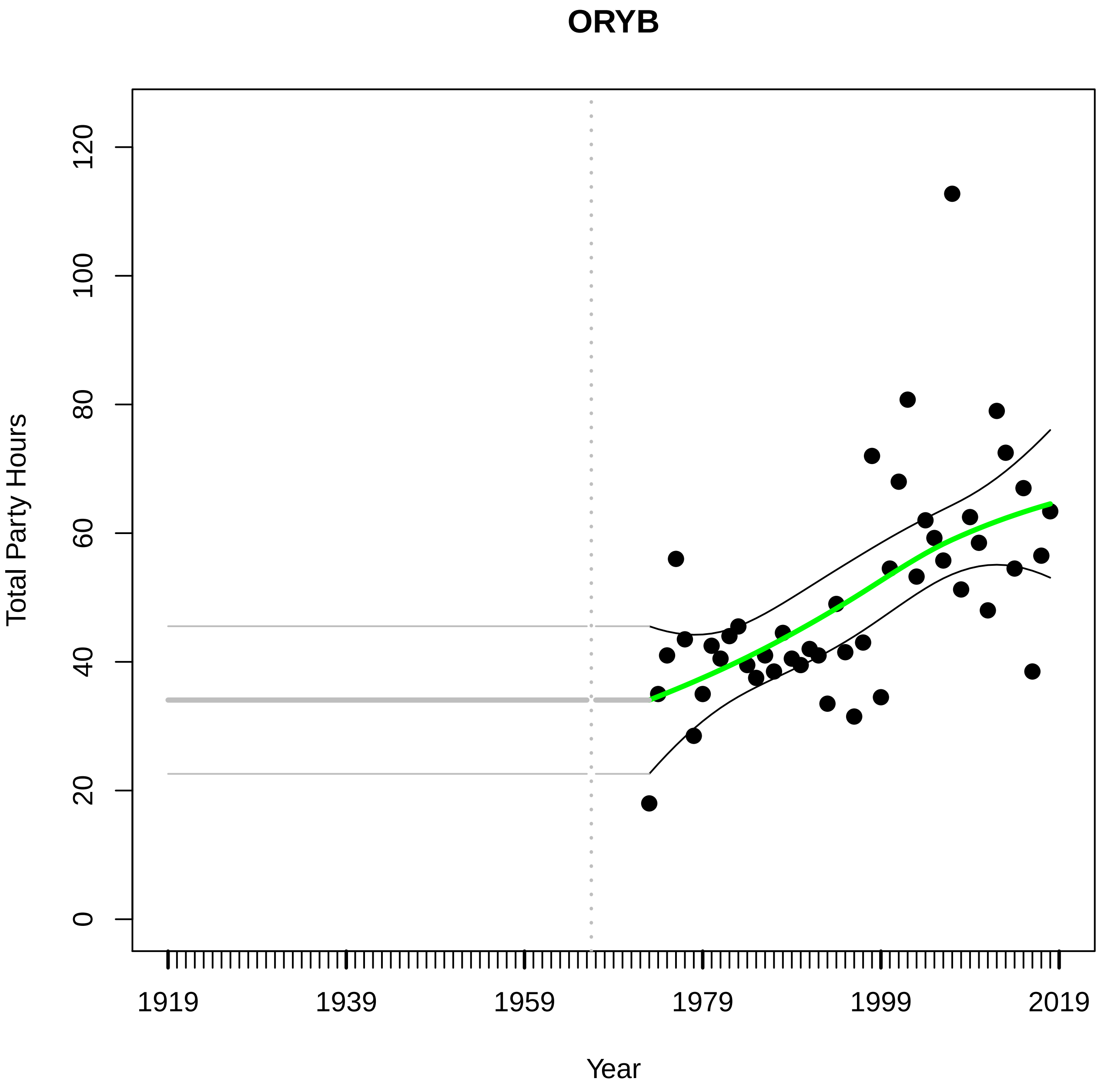

ORYB
Total Party Hours
0
20
40
60
80
100
120
1919
1939
1959
1979
1999
2019
Year

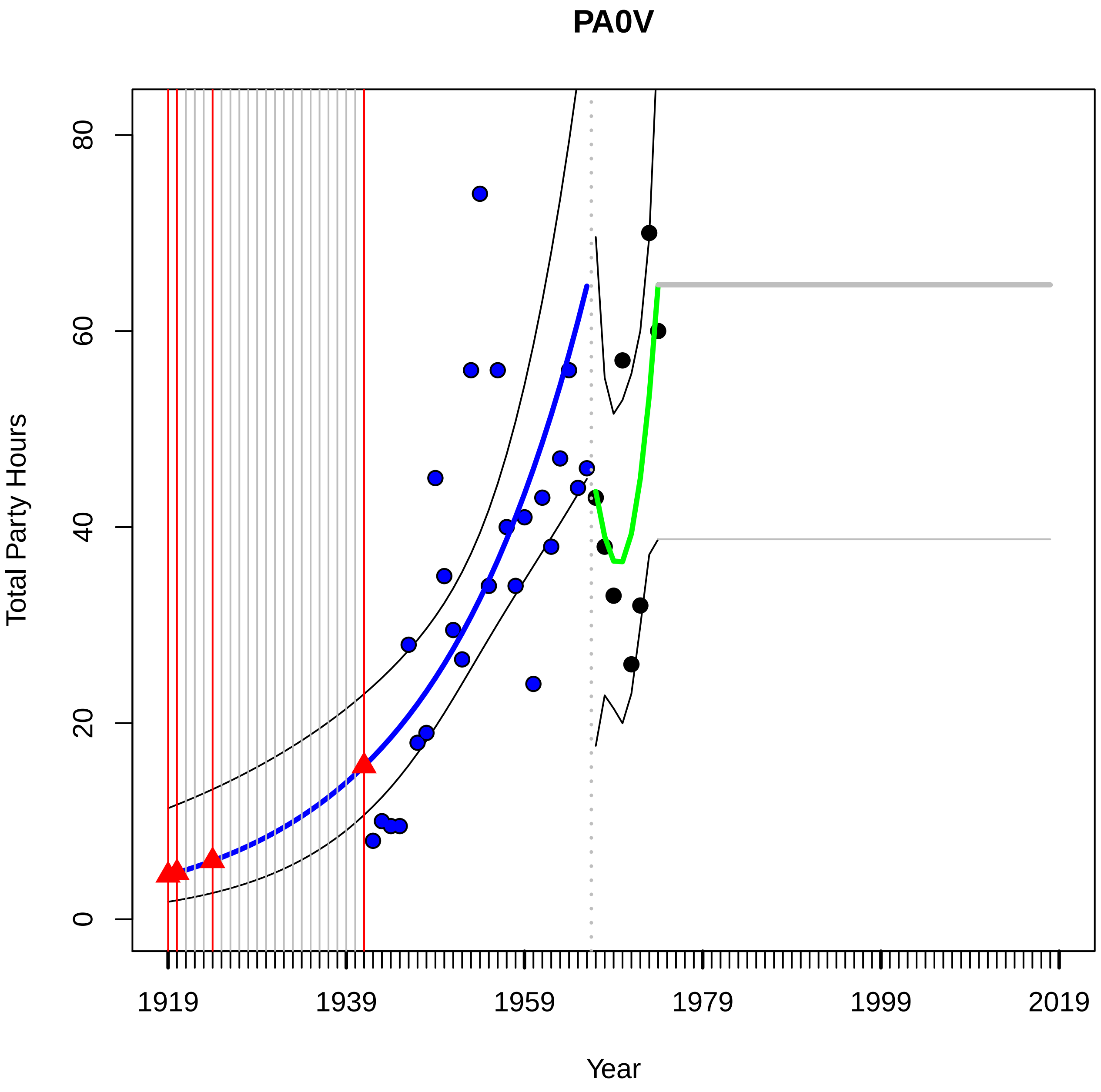

PA0V
Total Party Hours
0
20
40
60
80
1919
1939
1959
1979
1999
2019
Year

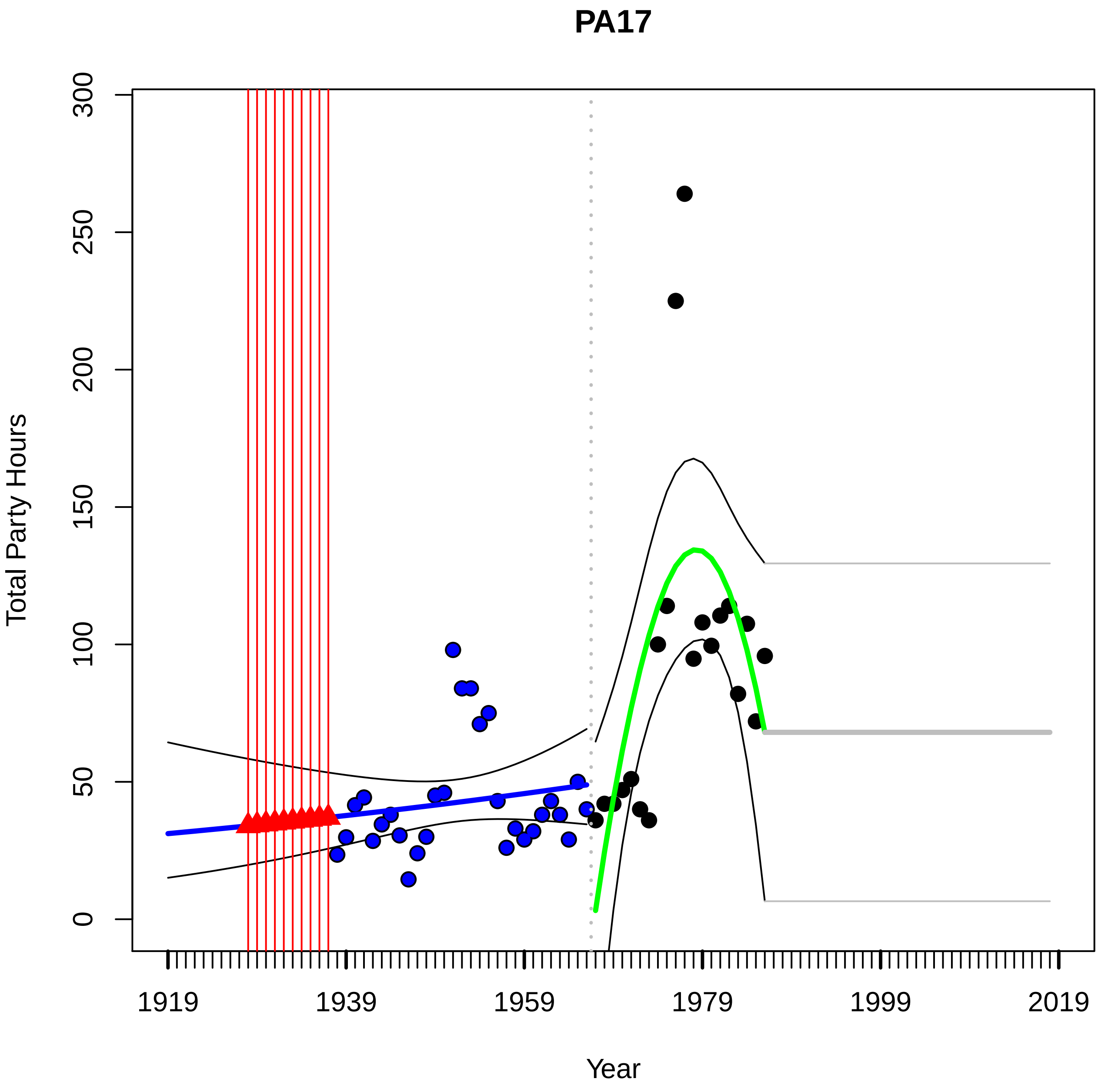

PA17
Total Party Hours
Year
300
250
200
150
100
50
0
1919
1939
1959
1979
1999
2019

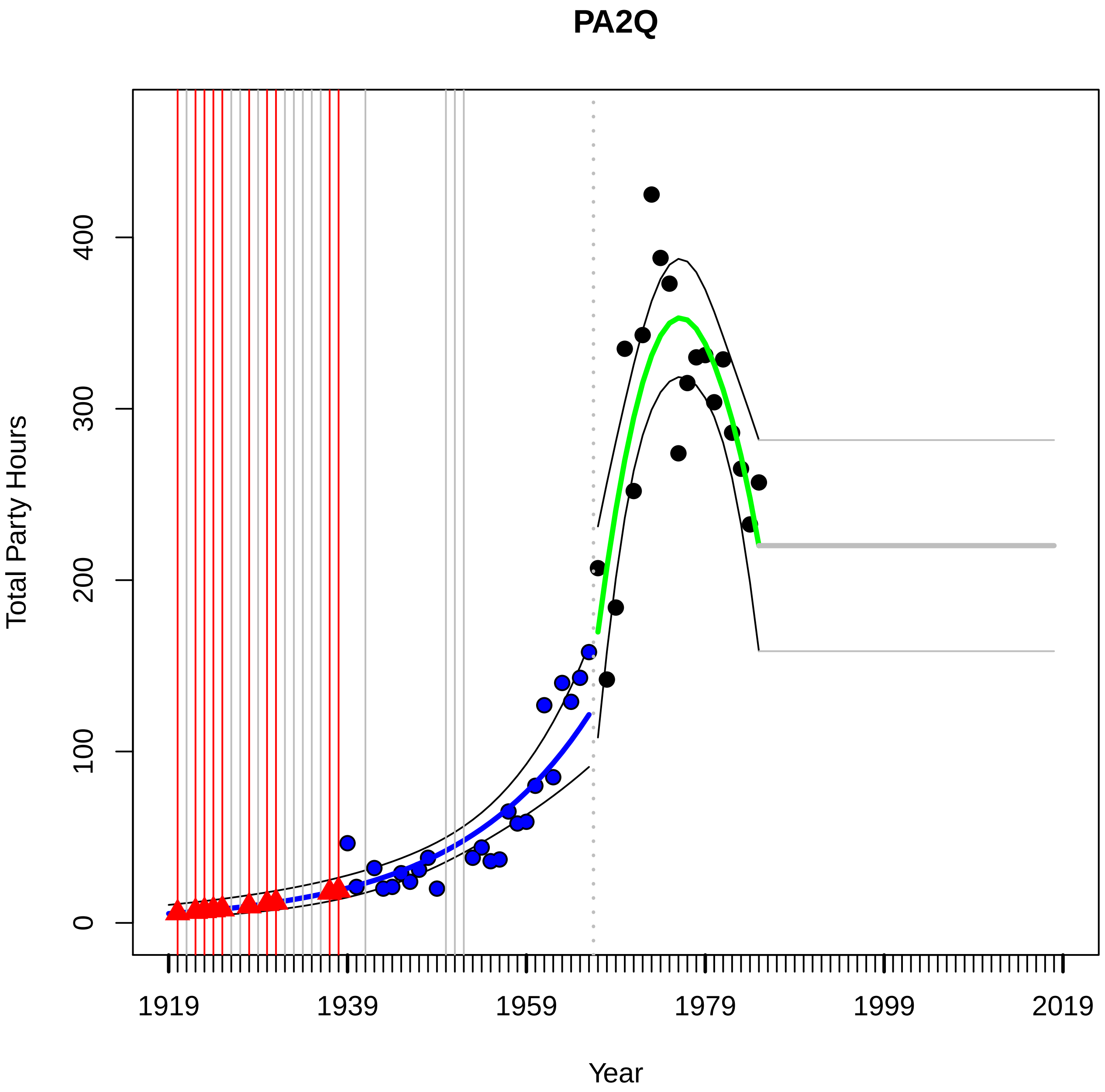
PA2Q
Total Party Hours
Year
0
100
200
300
400
1919
1939
1959
1979
1999
2019

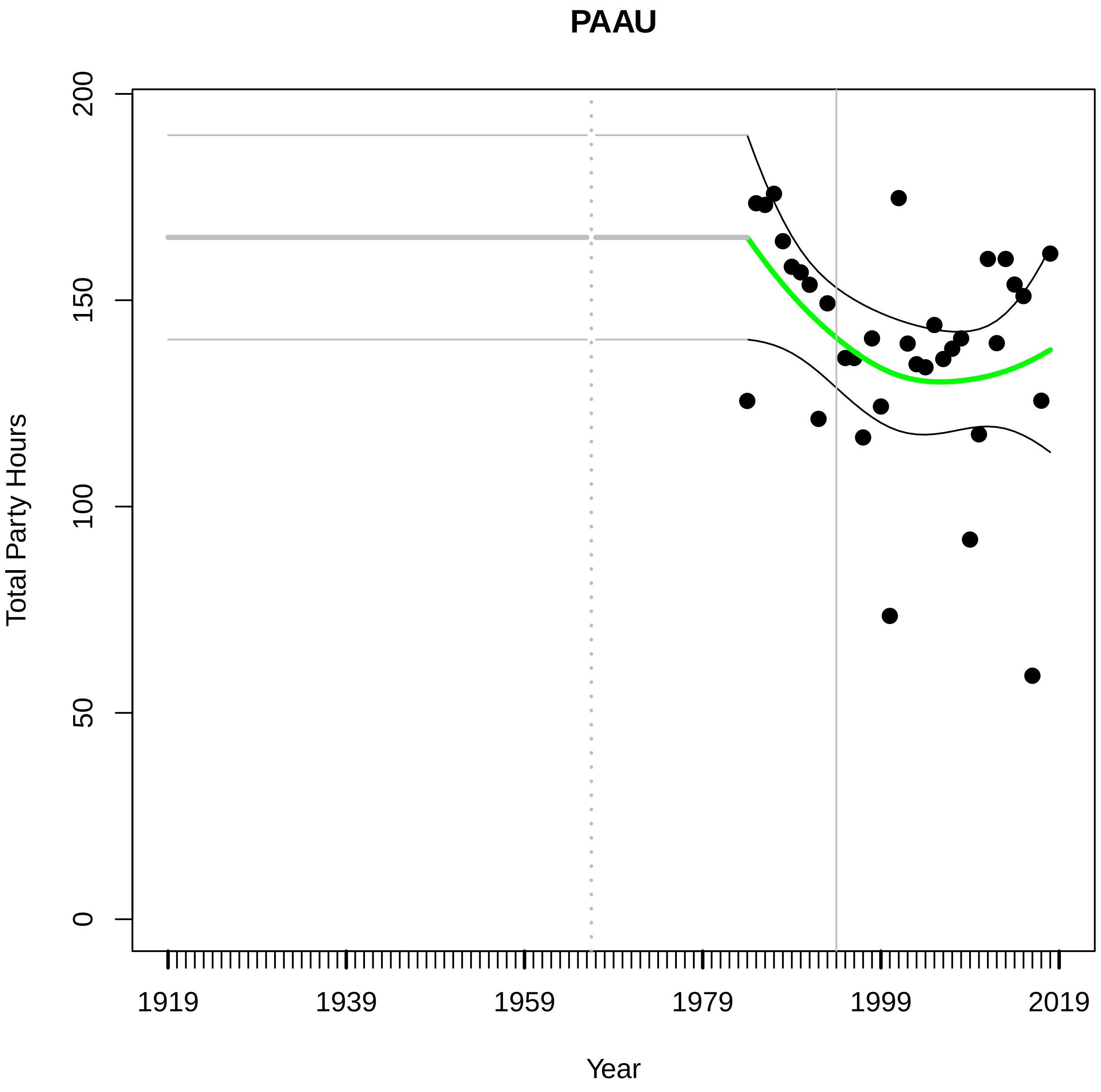

PAAU
Total Party Hours
Year
0
50
100
150
200
1919
1939
1959
1979
1999
2019

PABC

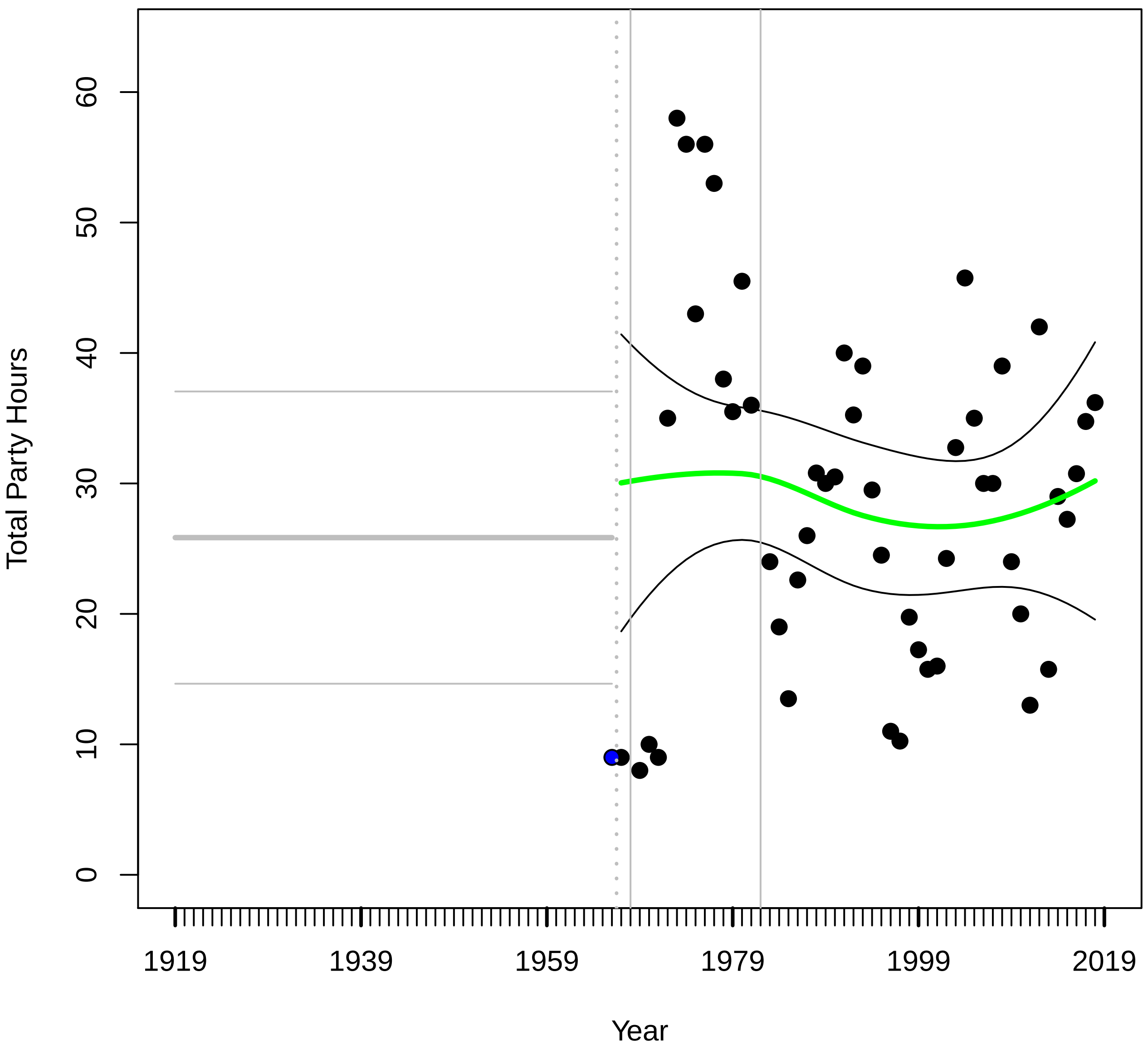

Total Party Hours
0
10
20
30
40
50
60
1919
1939
1959
1979
1999
2019
Year

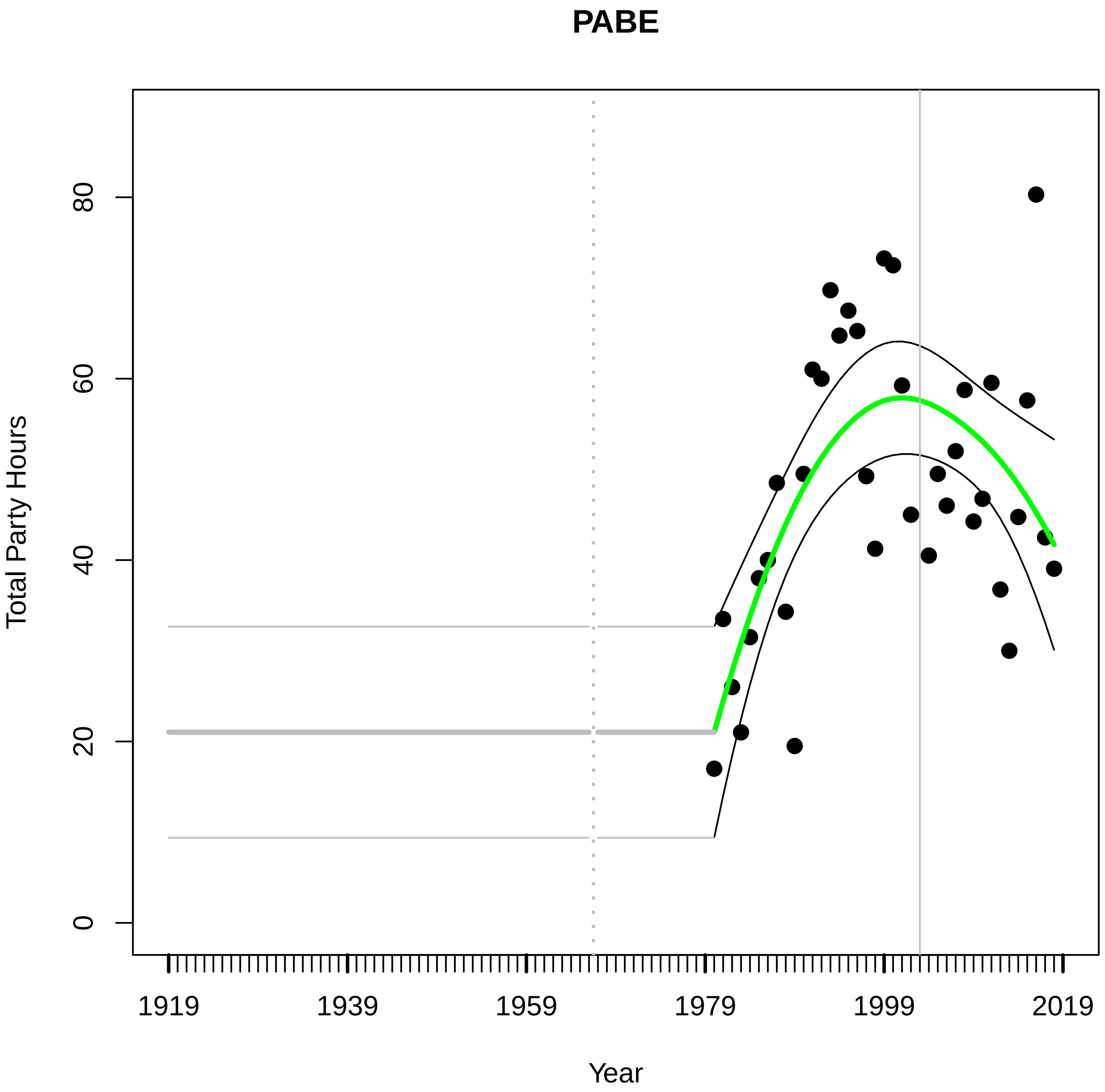
PABE
Total Party Hours
0
20
40
60
80
1919
1939
1959
1979
1999
2019
Year

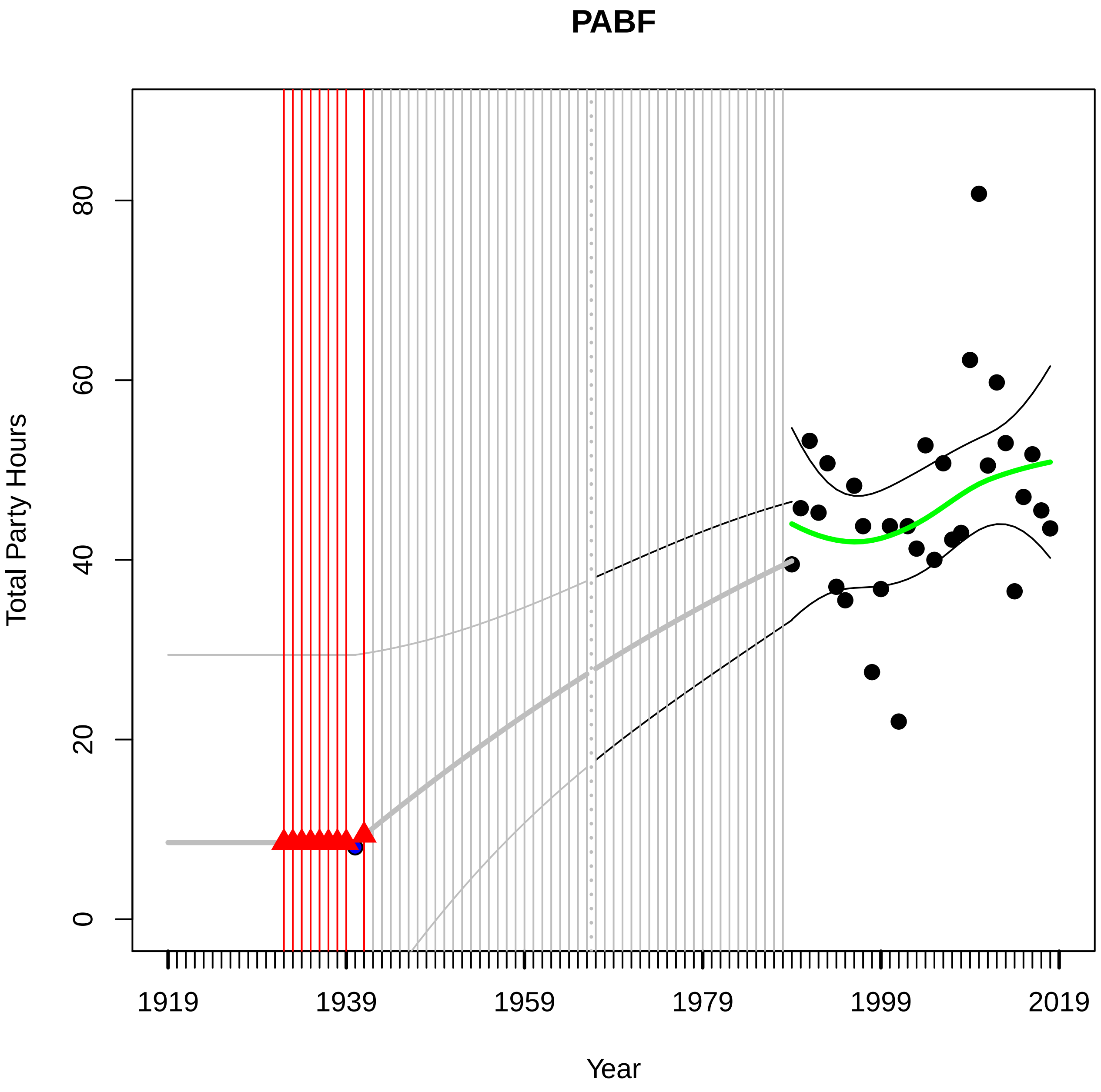
PABF
Total Party Hours
0
20
40
60
80
1919
1939
1959
1979
1999
2019
Year

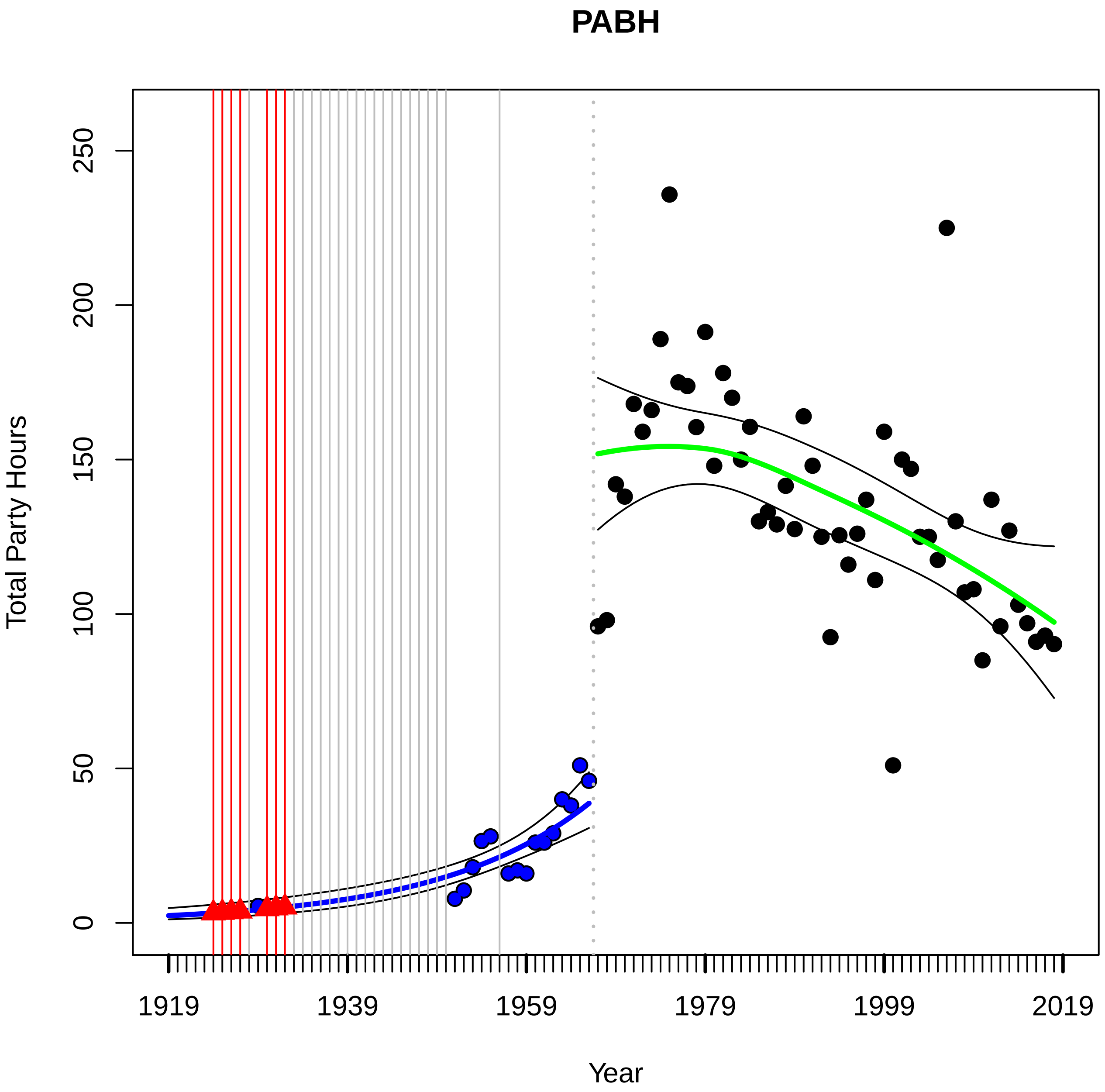

PABH
Total Party Hours
Year
0
50
100
150
200
250
1919
1939
1959
1979
1999
2019

PABL

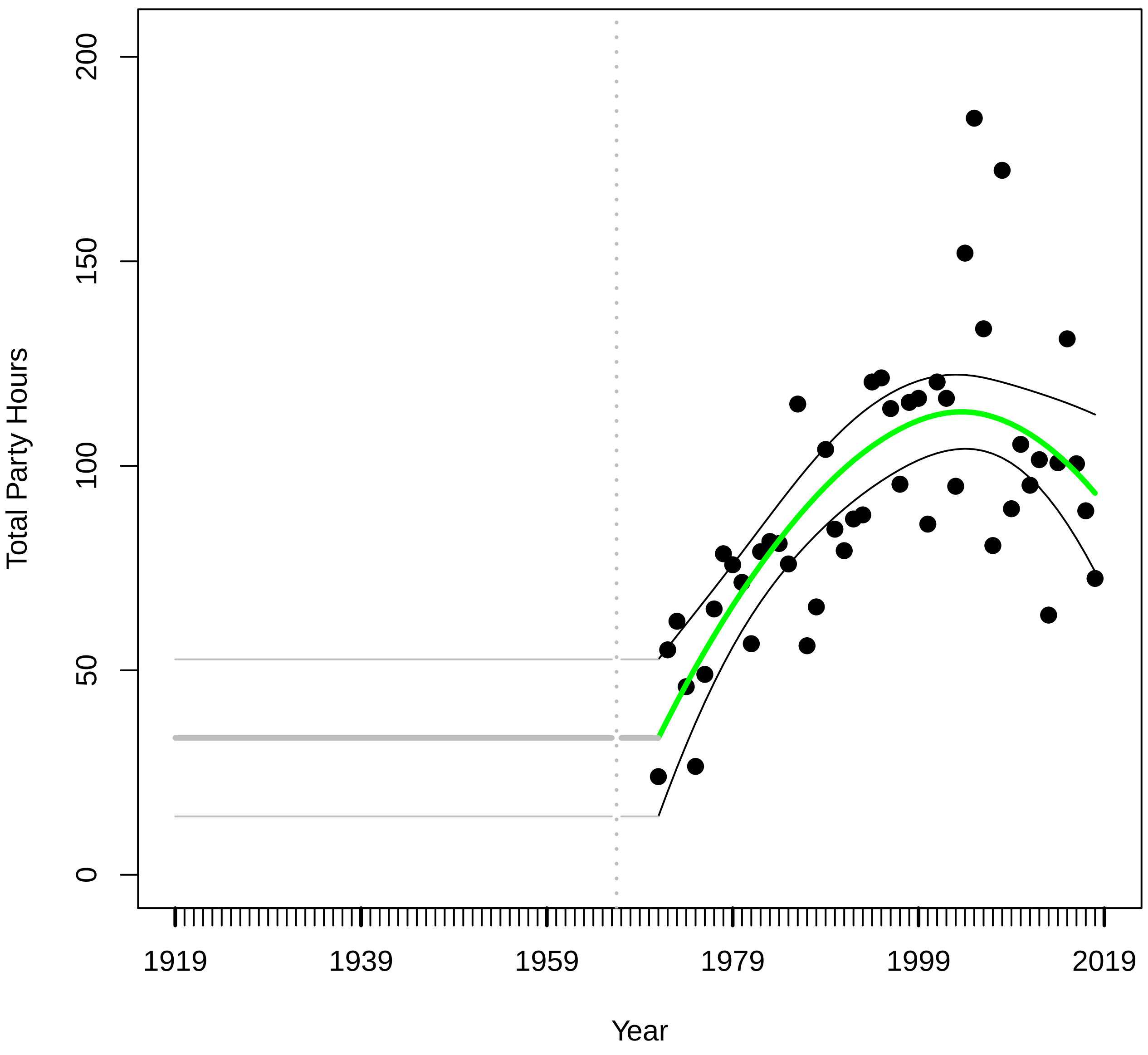

Total Party Hours
0
50
100
150
200
1919
1939
1959
1979
1999
2019
Year

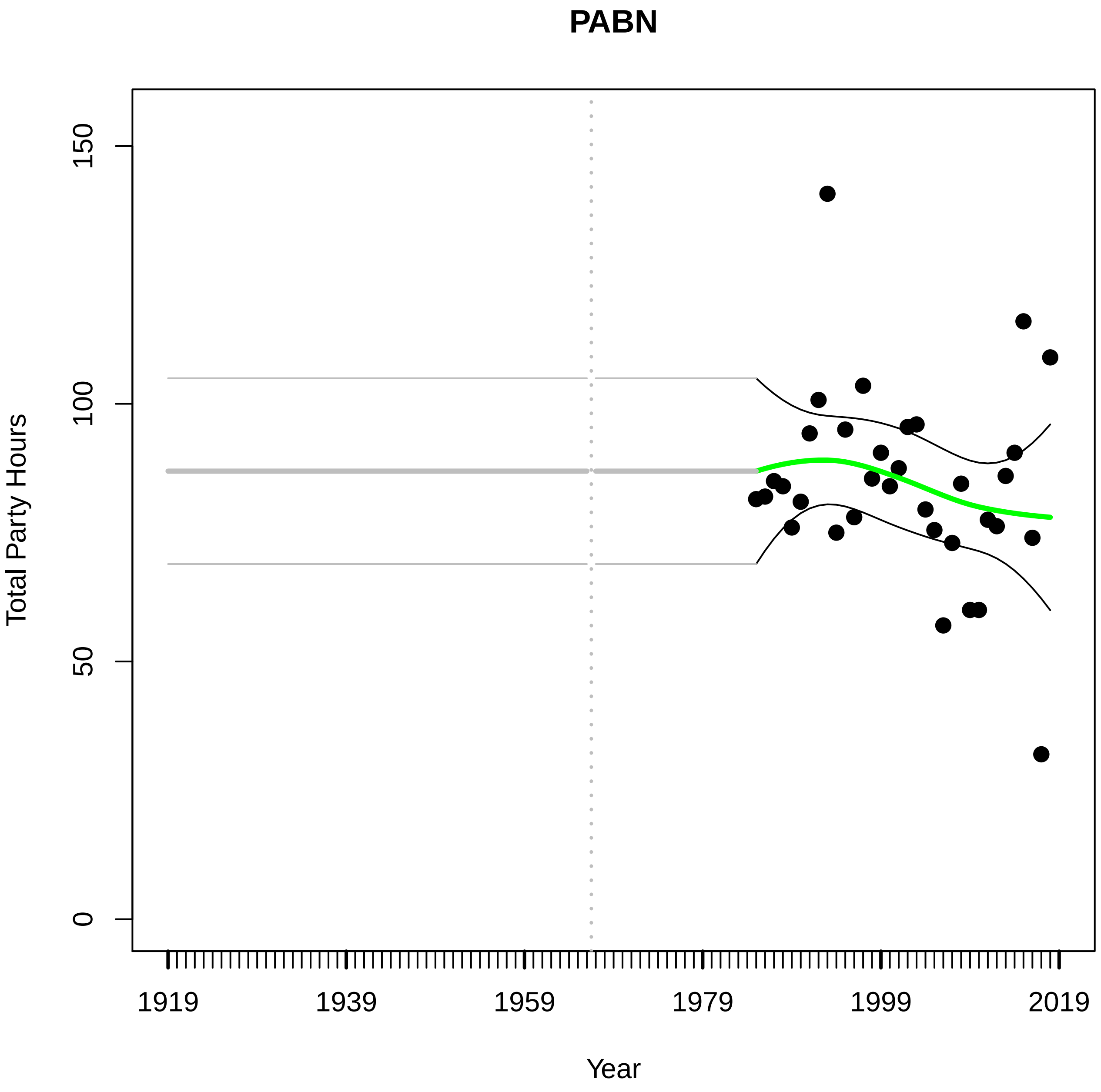

PABN
Total Party Hours
150
100
50
0
1919
1939
1959
1979
1999
2019
Year

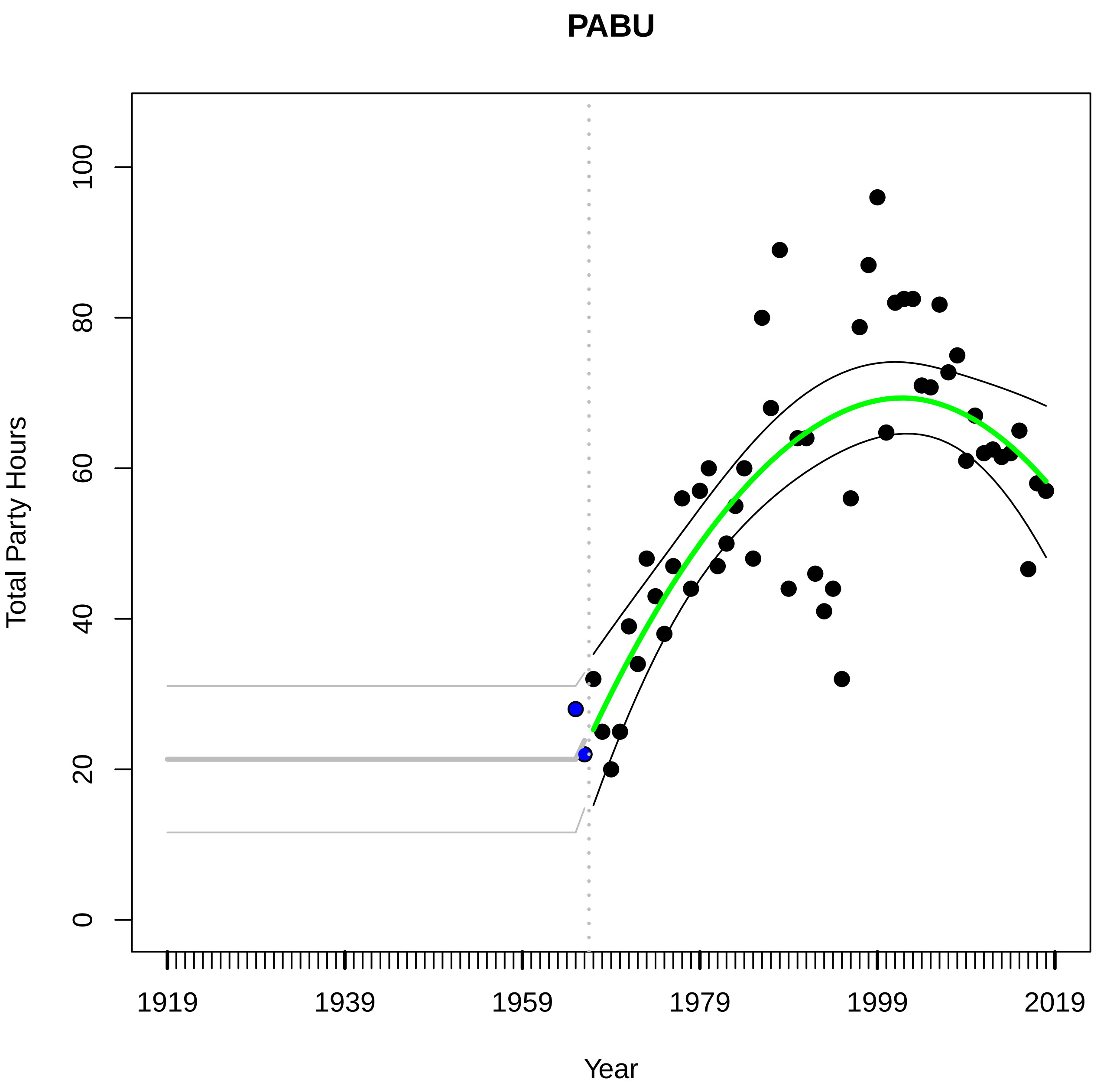

PABU
Total Party Hours
0
20
40
60
80
100
1919
1939
1959
1979
1999
2019
Year

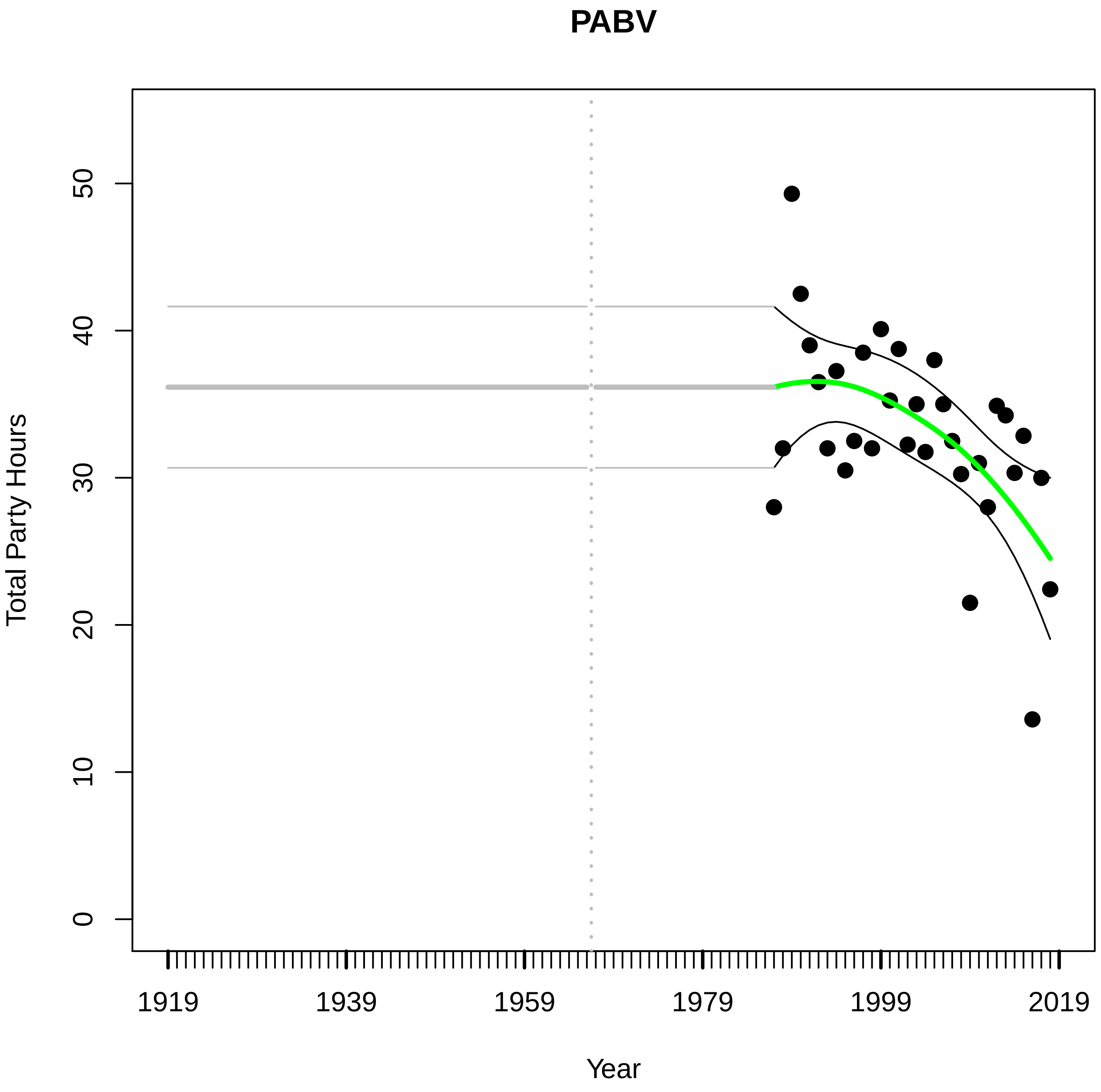

PABV
Total Party Hours
0
10
20
30
40
50
1919
1939
1959
1979
1999
2019
Year

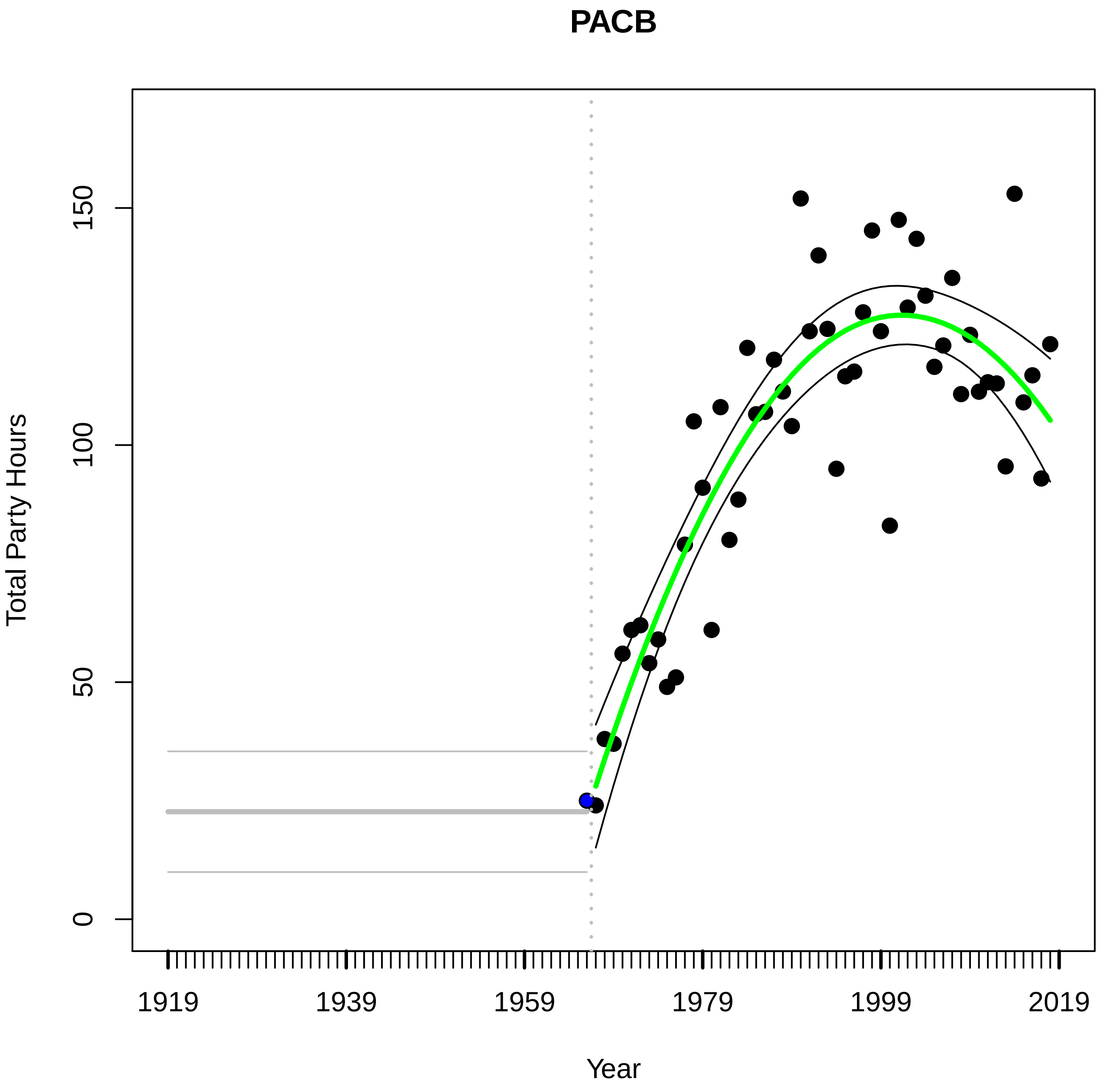

PACB
Total Party Hours
0
50
100
150
1919
1939
1959
1979
1999
2019
Year

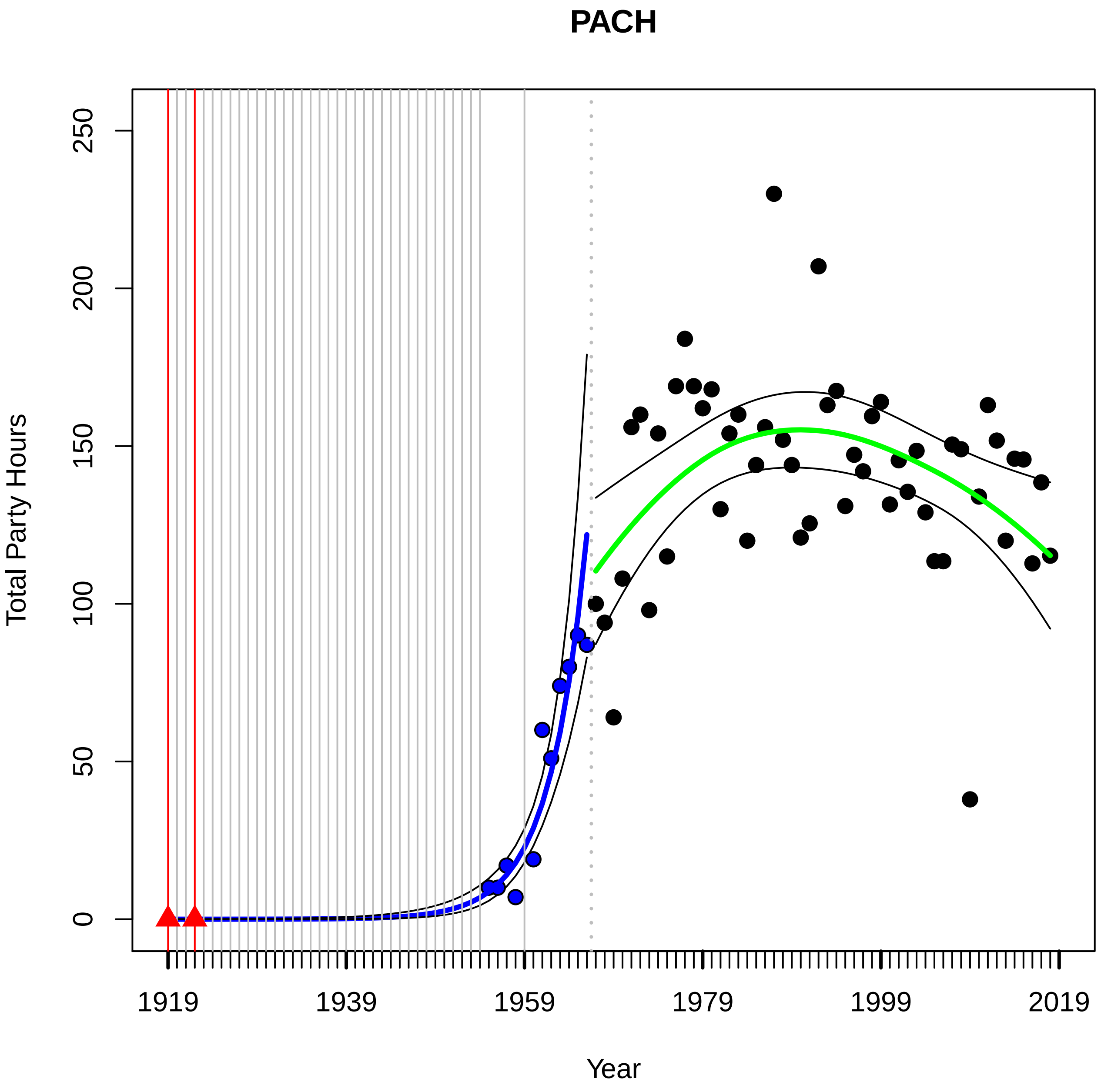
PACH
Total Party Hours
Year
0
50
100
150
200
250
1919
1939
1959
1979
1999
2019

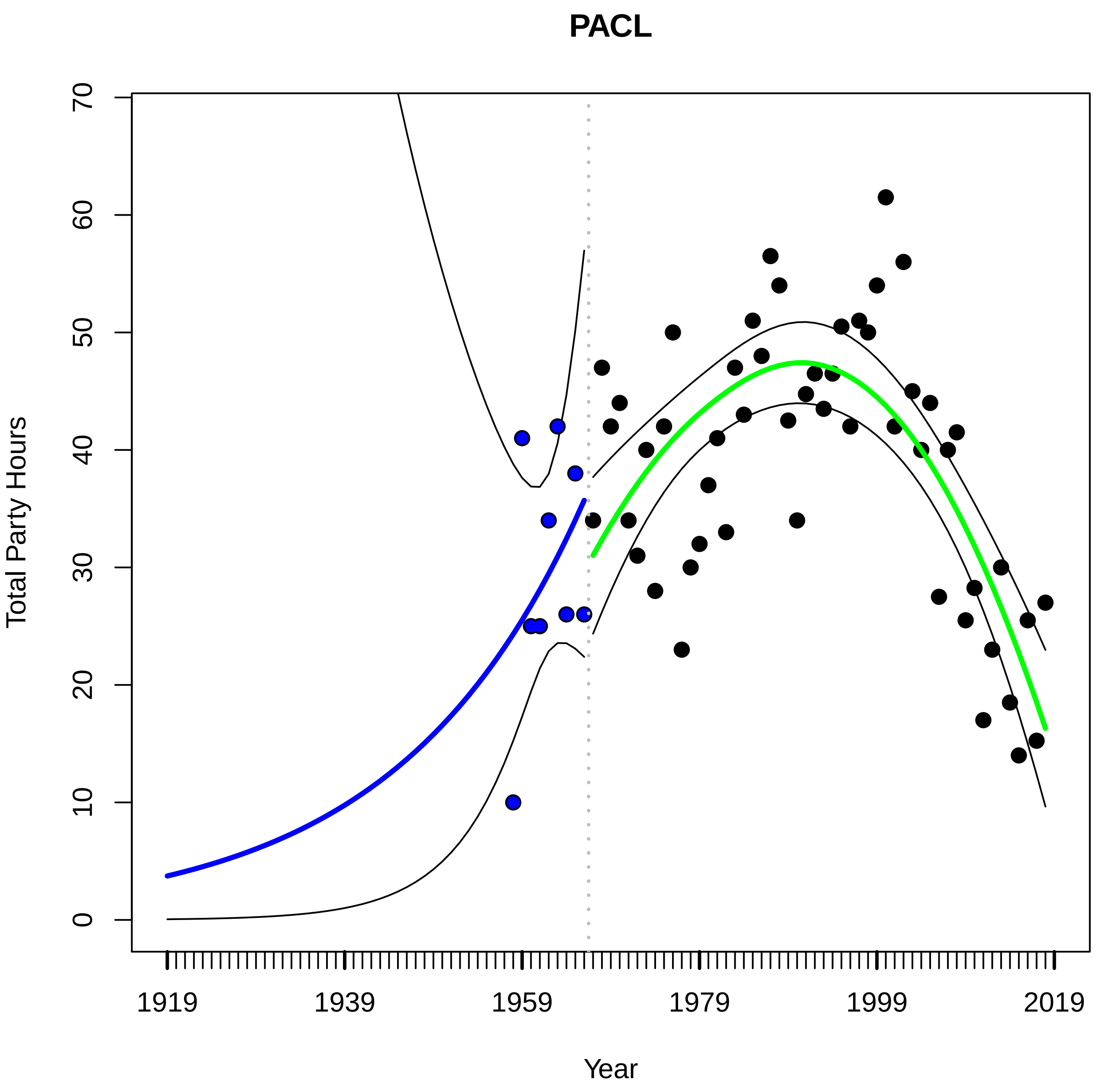
PACL
Total Party Hours
Year
0
10
20
30
40
50
60
70
1919
1939
1959
1979
1999
2019

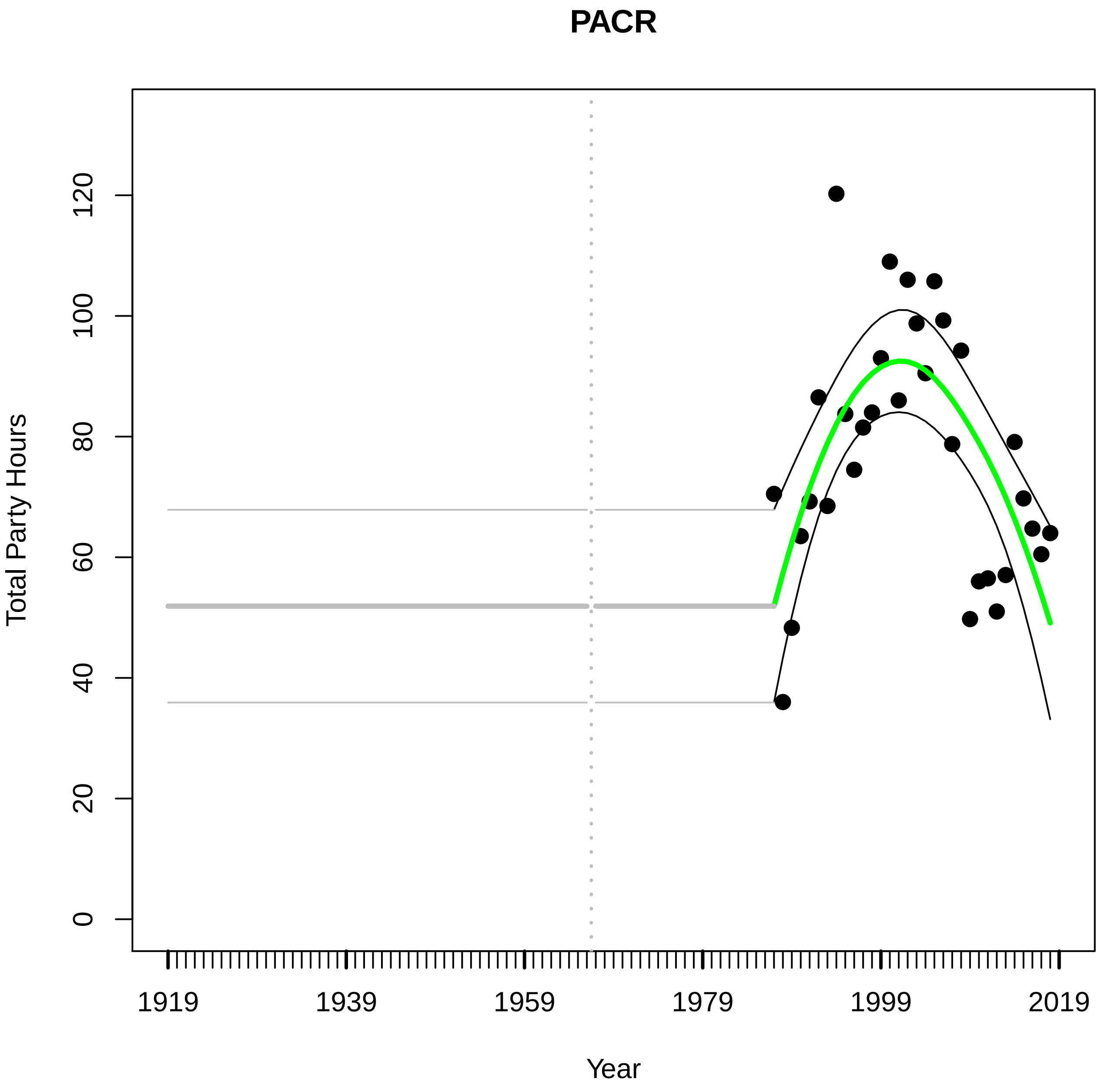

PACR
Total Party Hours
0
20
40
60
80
100
120
1919
1939
1959
1979
1999
2019
Year

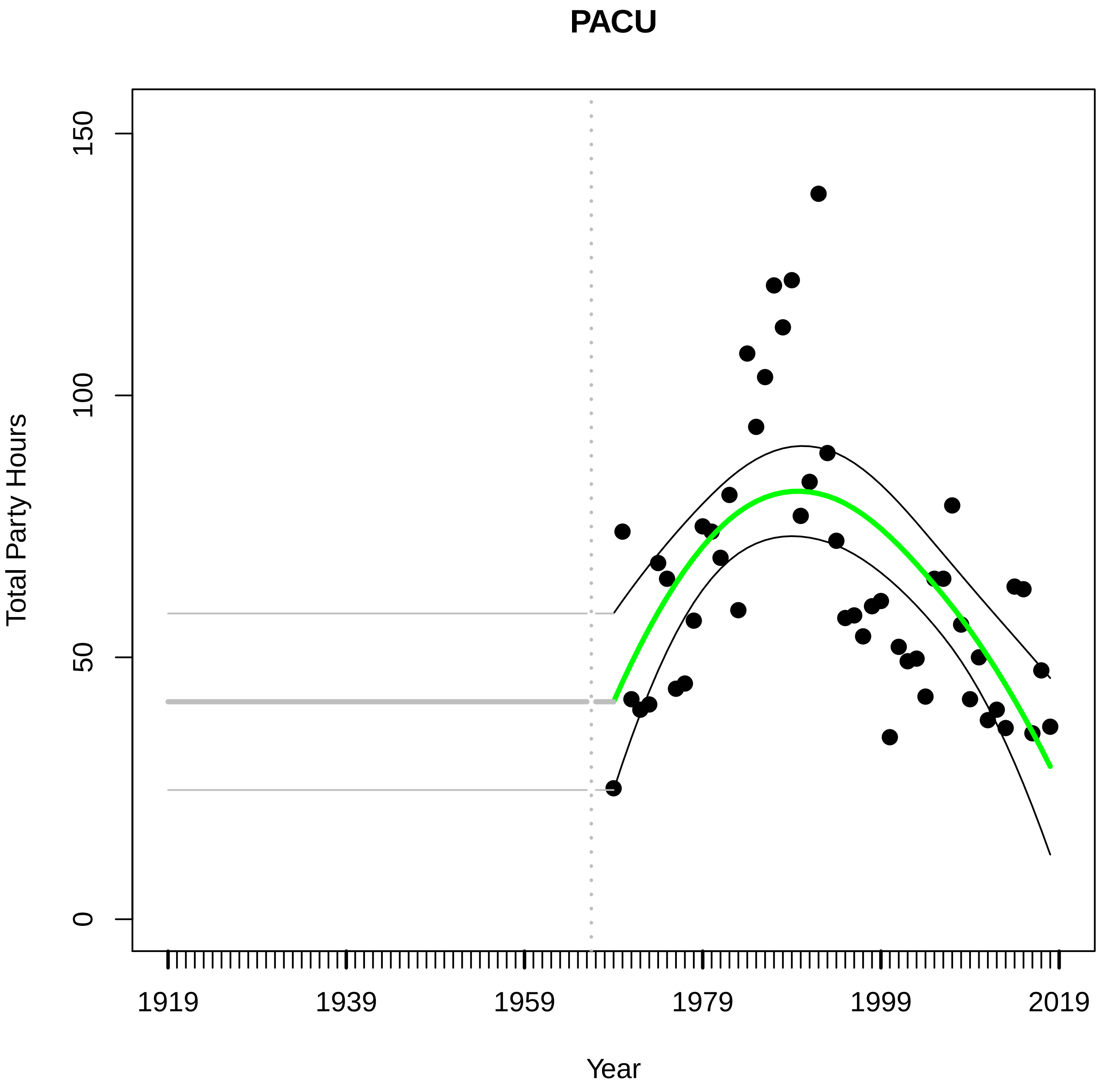
PACU
Total Party Hours
0
50
100
150
1919
1939
1959
1979
1999
2019
Year

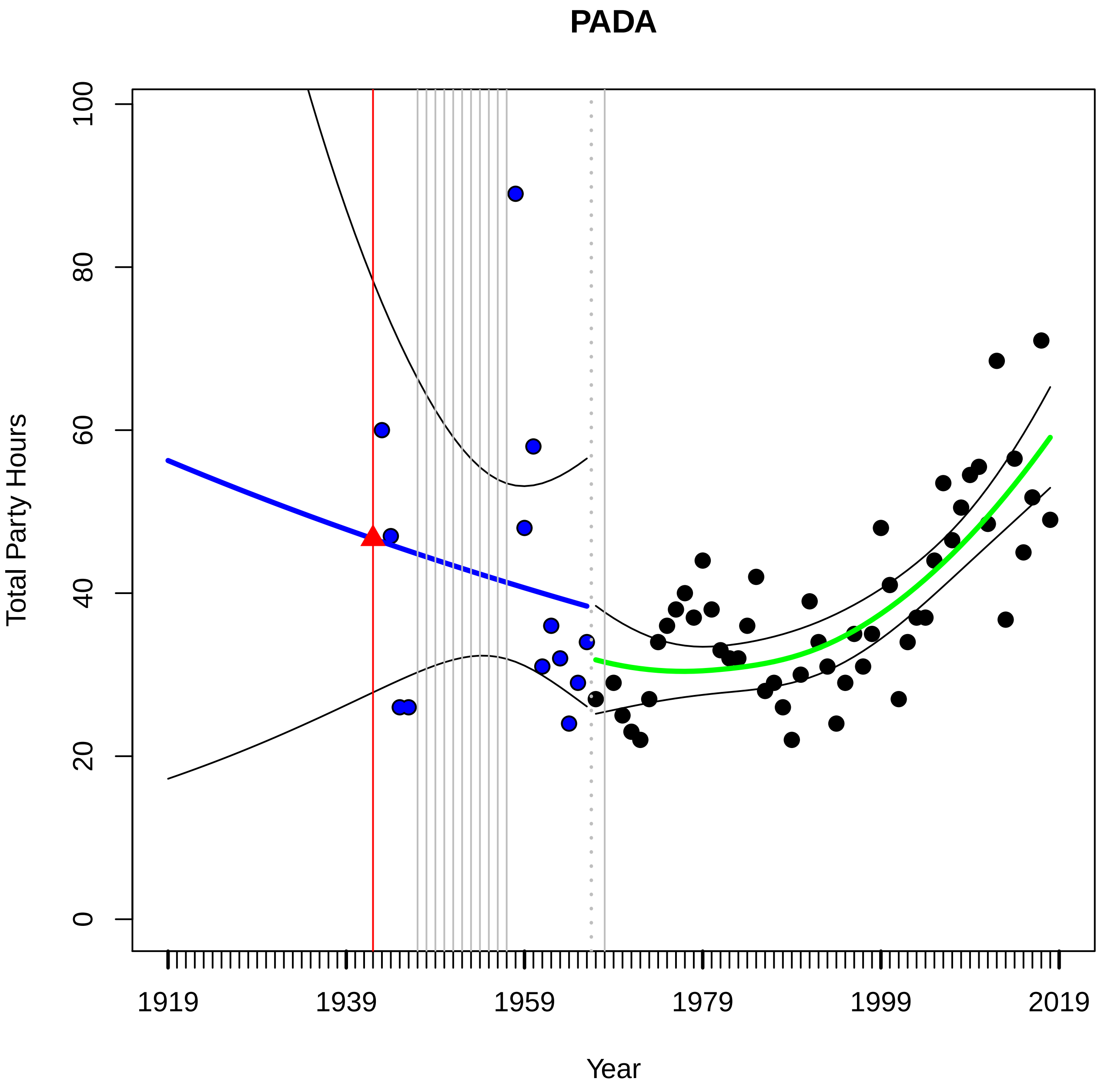

PADA
Total Party Hours
Year
0
20
40
60
80
100
1919
1939
1959
1979
1999
2019

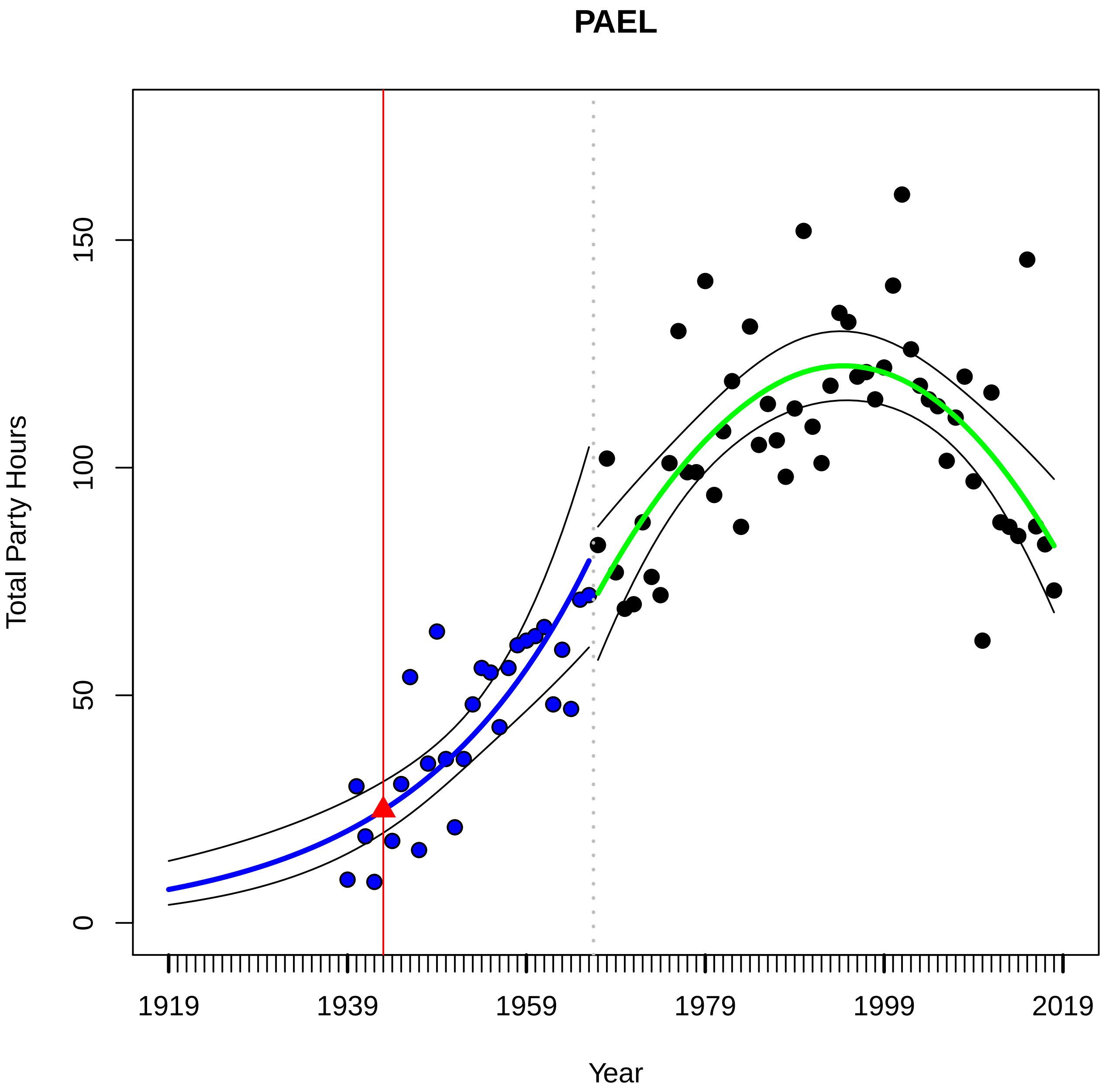

PAEL
Total Party Hours
0
50
100
150
1919
1939
1959
1979
1999
2019
Year

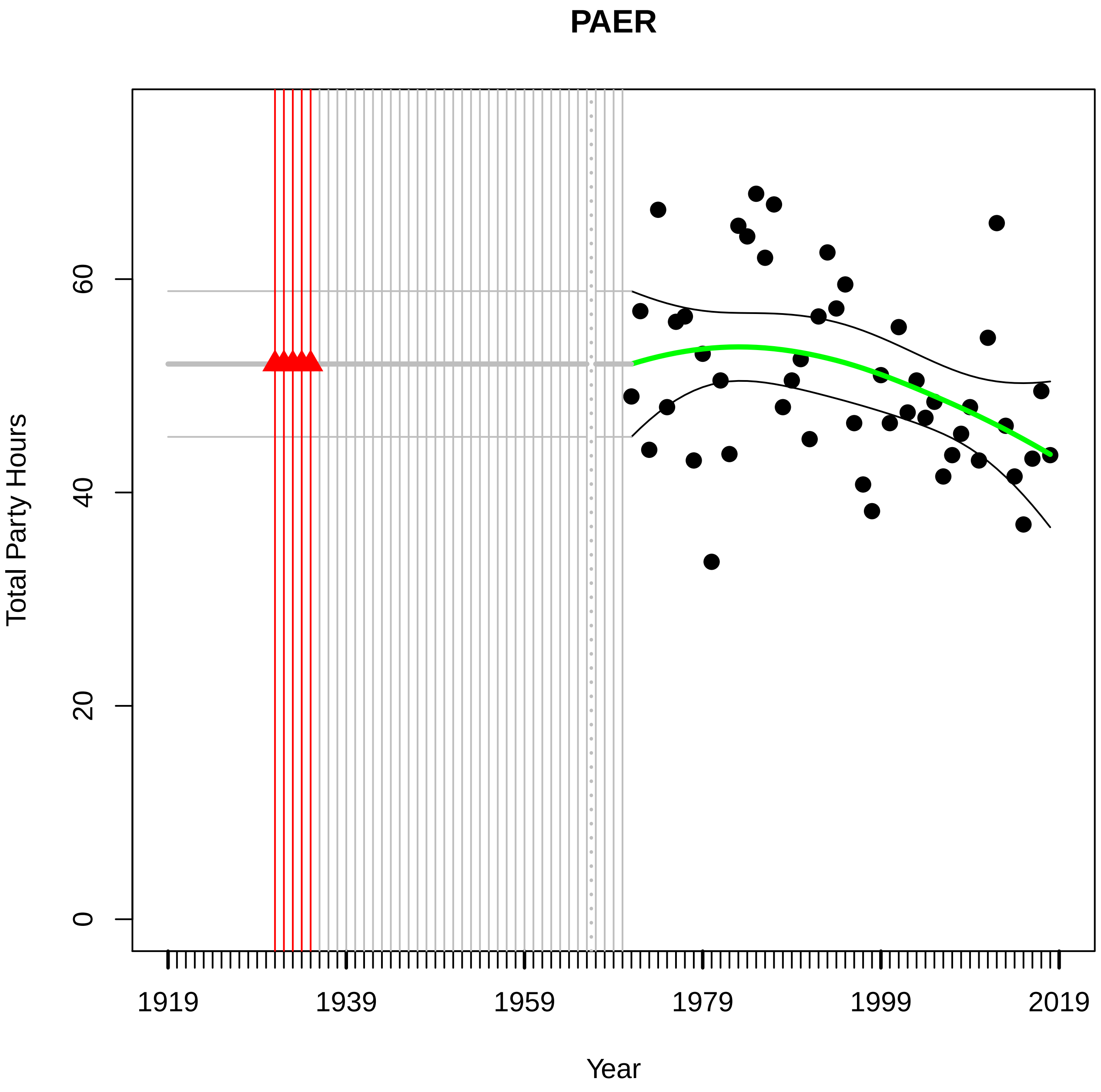
PAER
Total Party Hours
Year
0
20
40
60
1919
1939
1959
1979
1999
2019

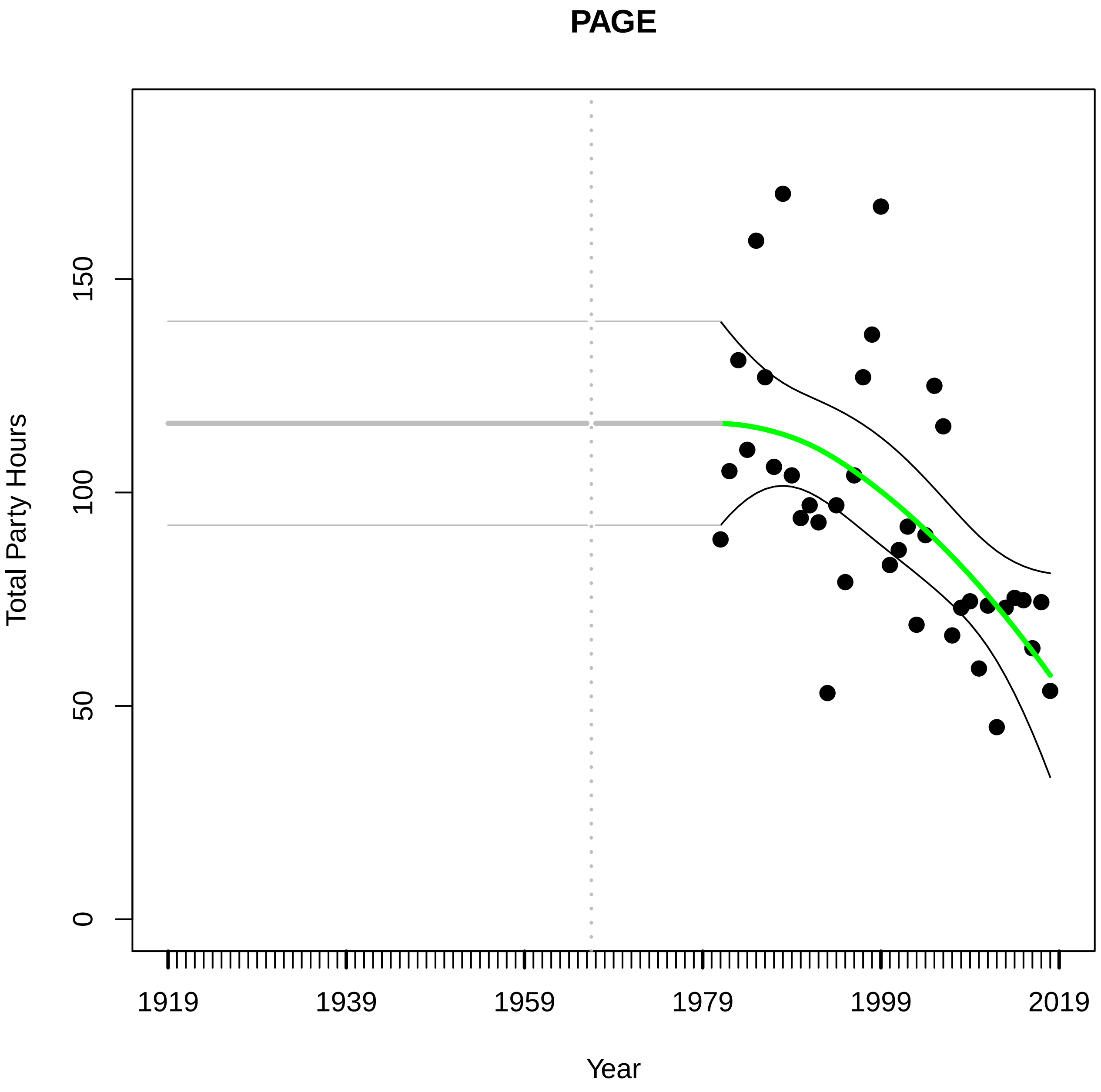

PAGE
Total Party Hours
0
50
100
150
1919
1939
1959
1979
1999
2019
Year

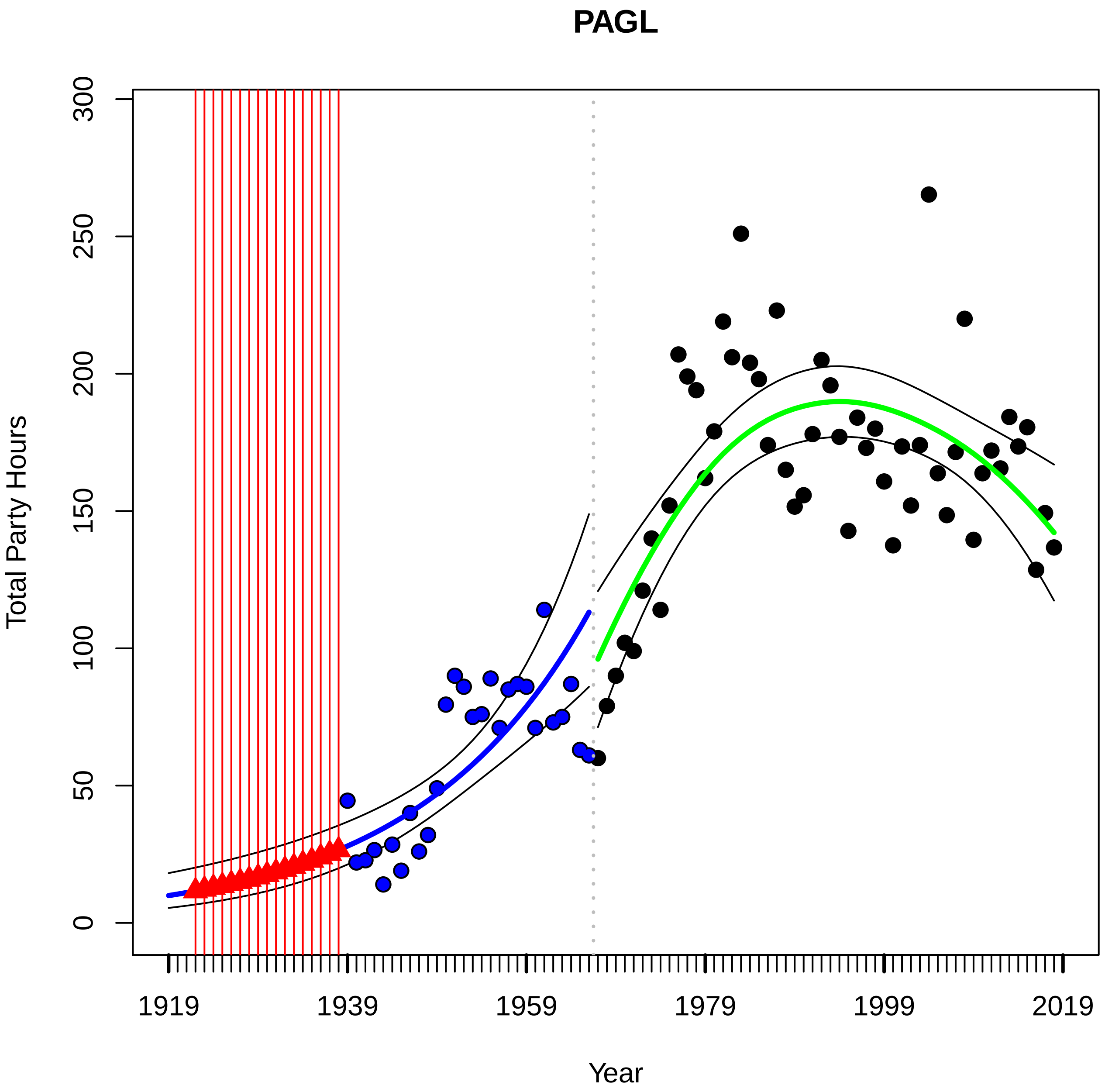

PAGL
Total Party Hours
0
50
100
150
200
250
300
1919
1939
1959
1979
1999
2019
Year

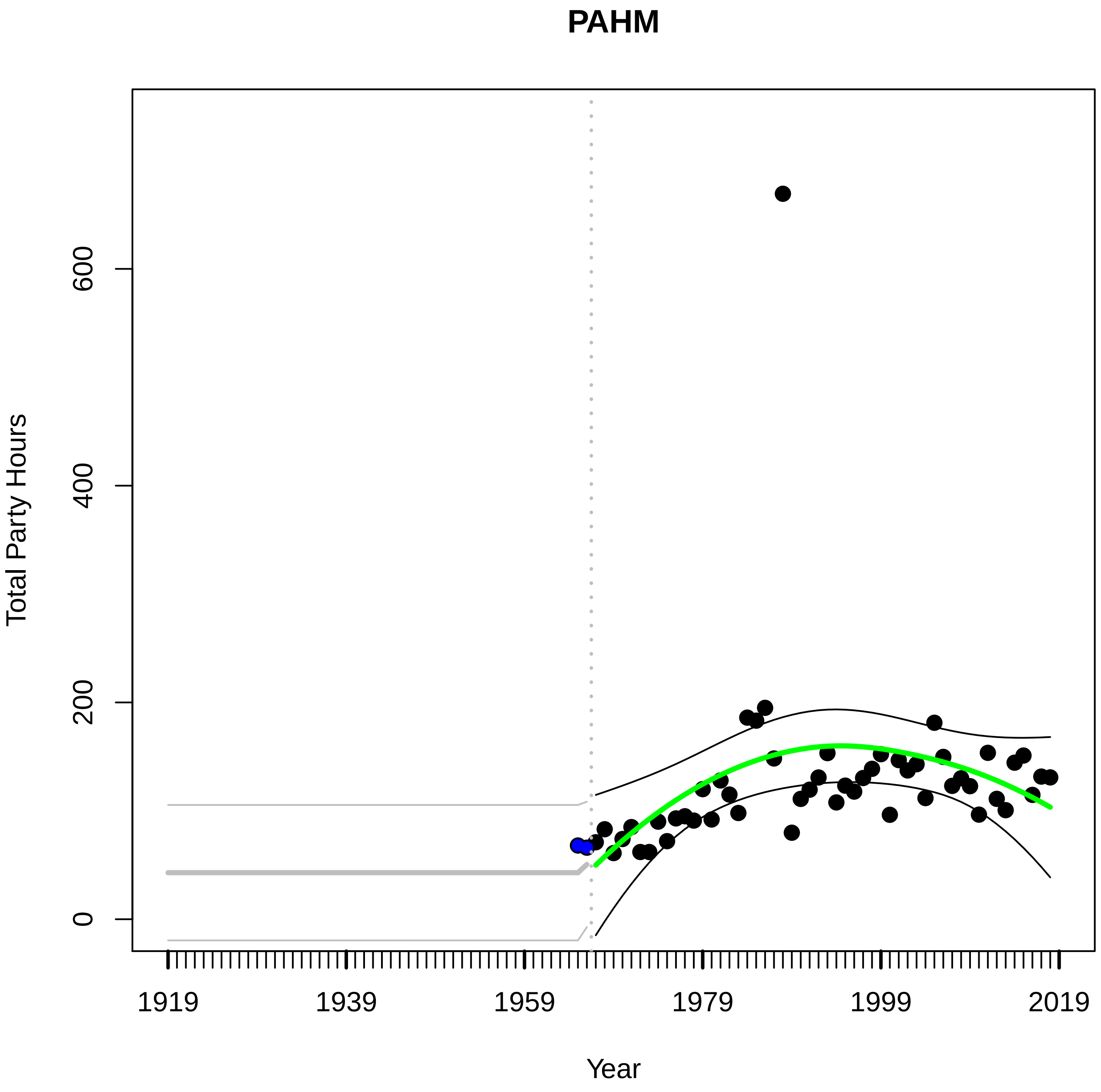
PAHM
Total Party Hours
0
200
400
600
1919
1939
1959
1979
1999
2019
Year

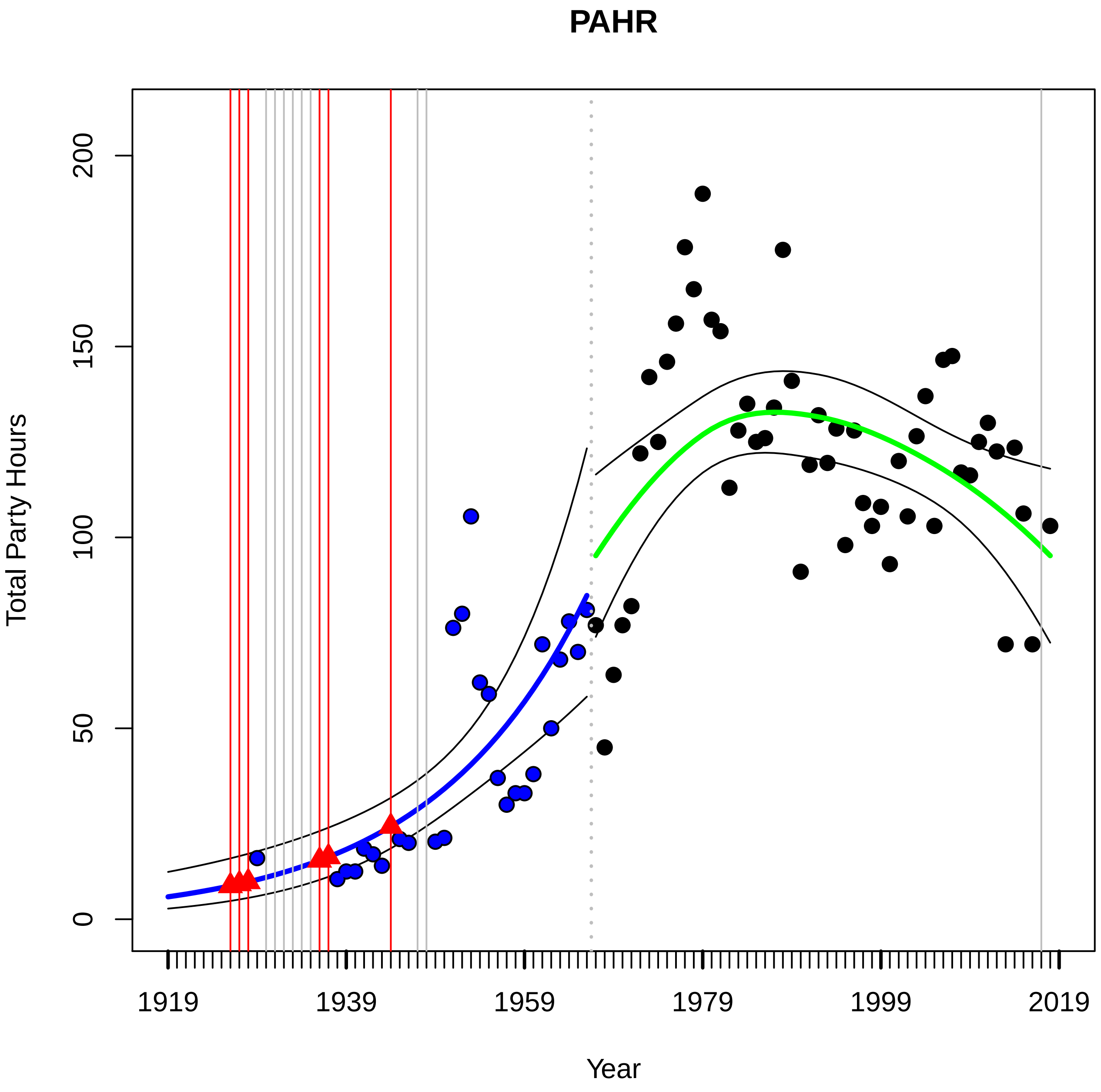
PAHR
Total Party Hours
Year
0
50
100
150
200
1919
1939
1959
1979
1999
2019

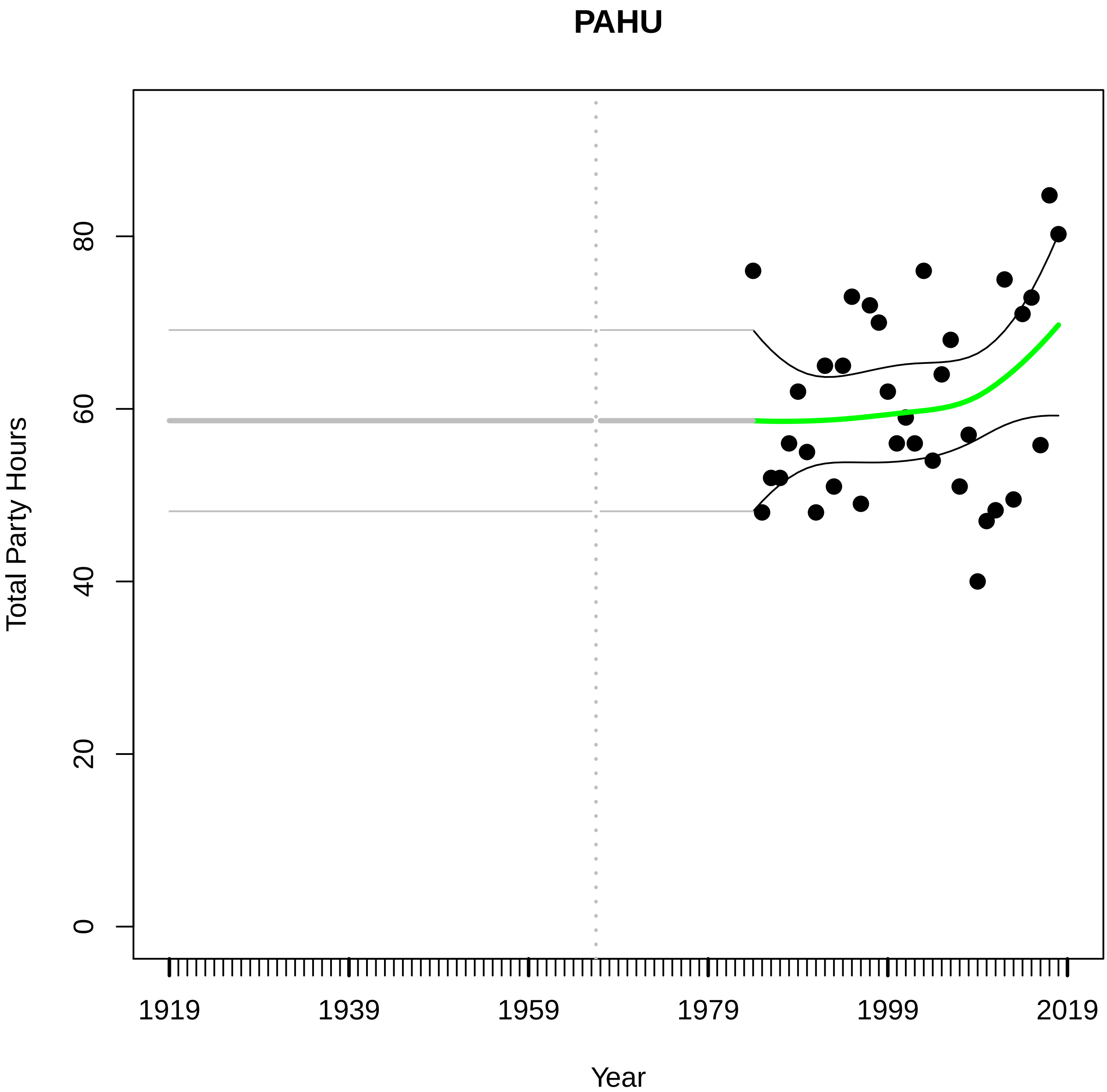
PAHU
Total Party Hours
0
20
40
60
80
1919
1939
1959
1979
1999
2019
Year

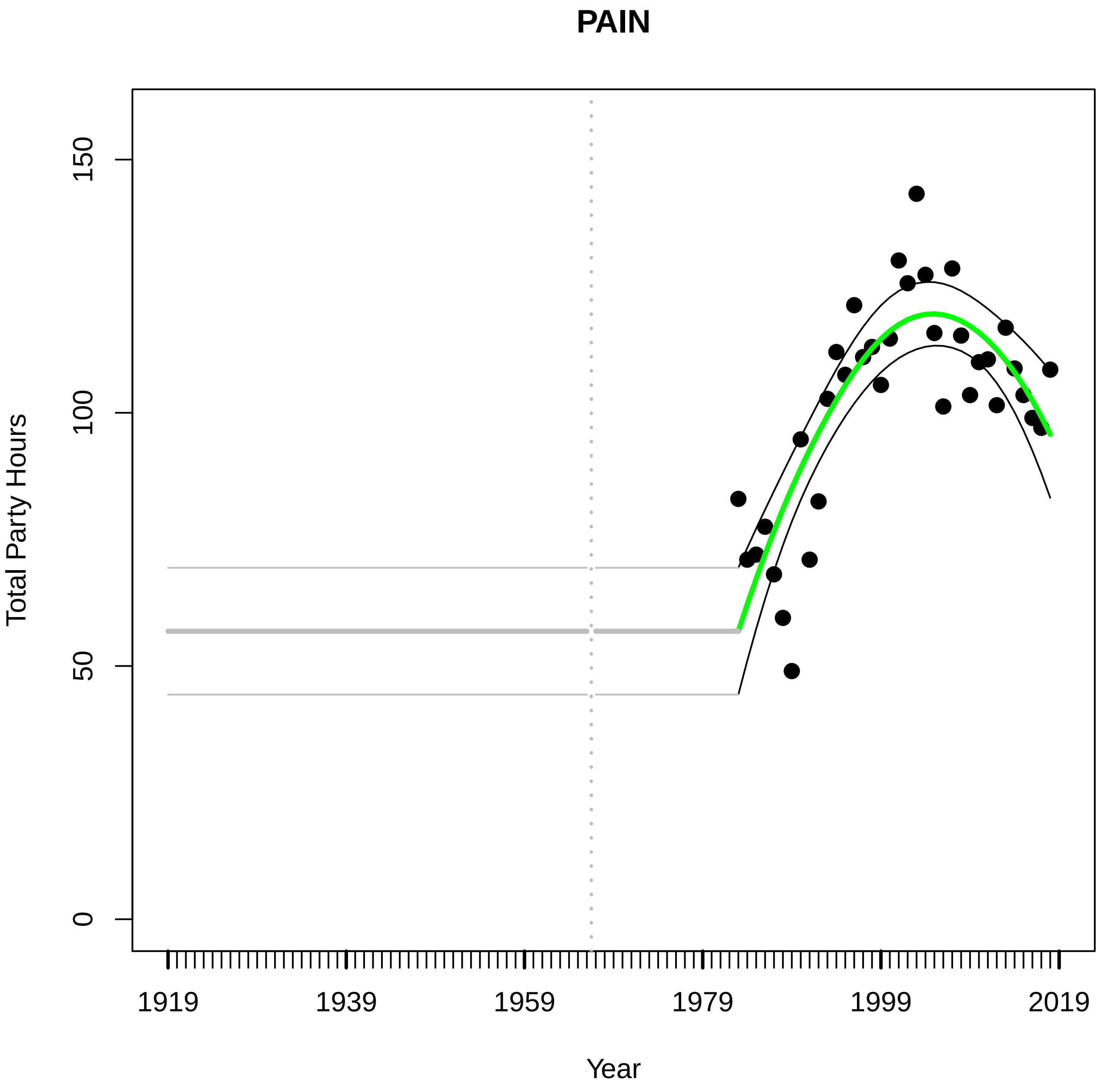
PAIN
Total Party Hours
0
50
100
150
1919
1939
1959
1979
1999
2019
Year

PAJO

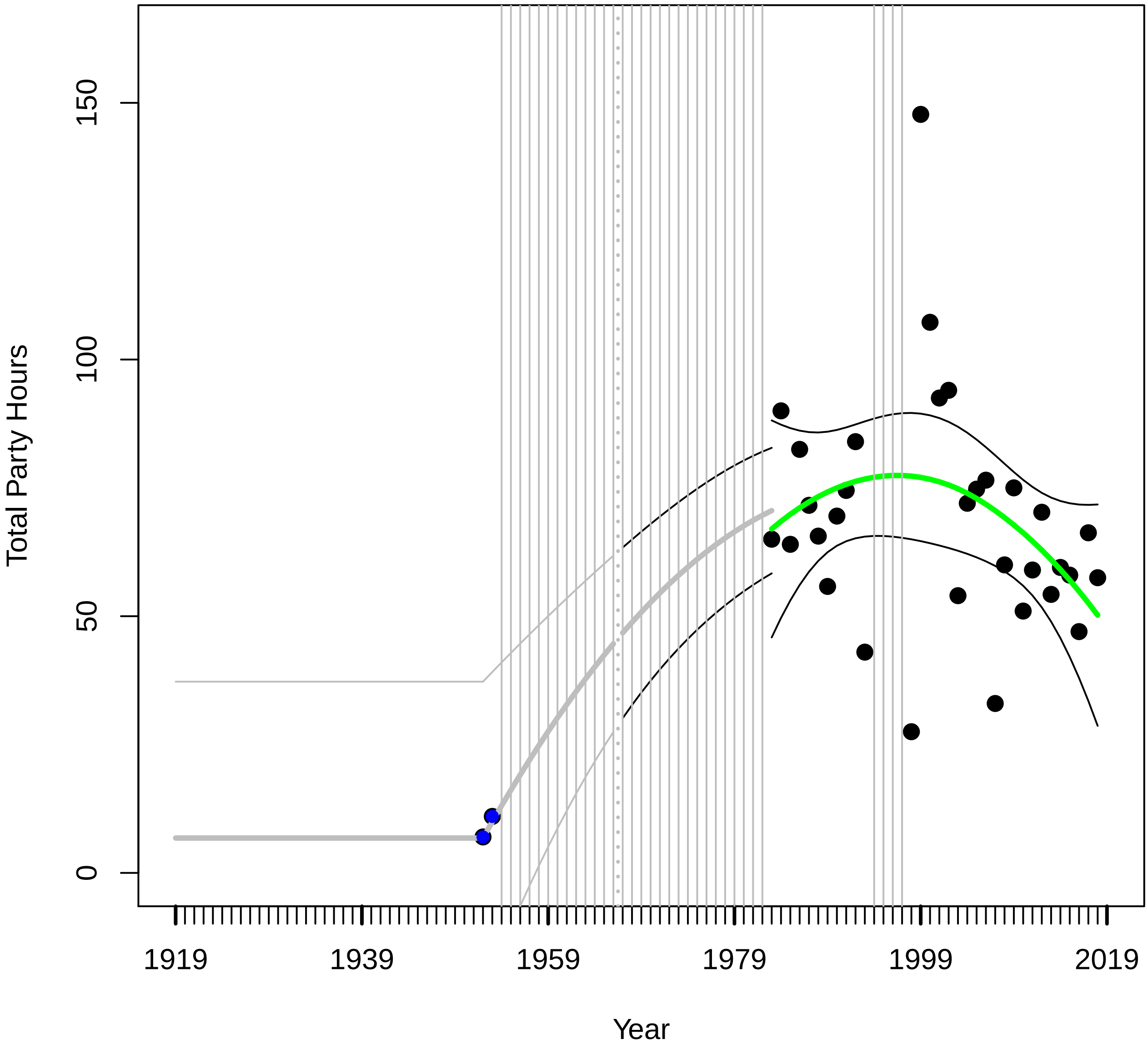

Total Party Hours
0
50
100
150
1919
1939
1959
1979
1999
2019
Year

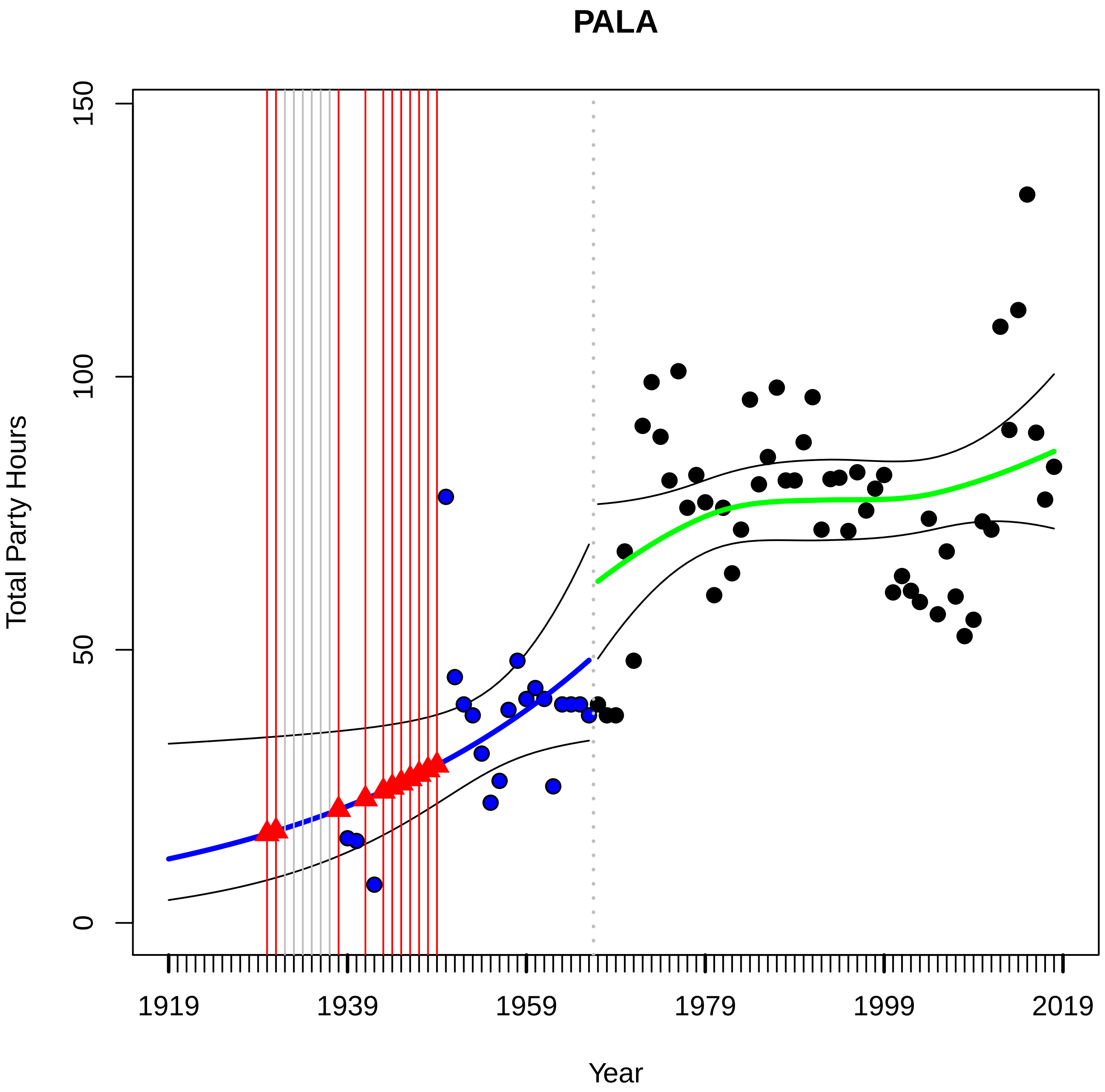
PALA
Total Party Hours
0
50
100
150
1919
1939
1959
1979
1999
2019
Year

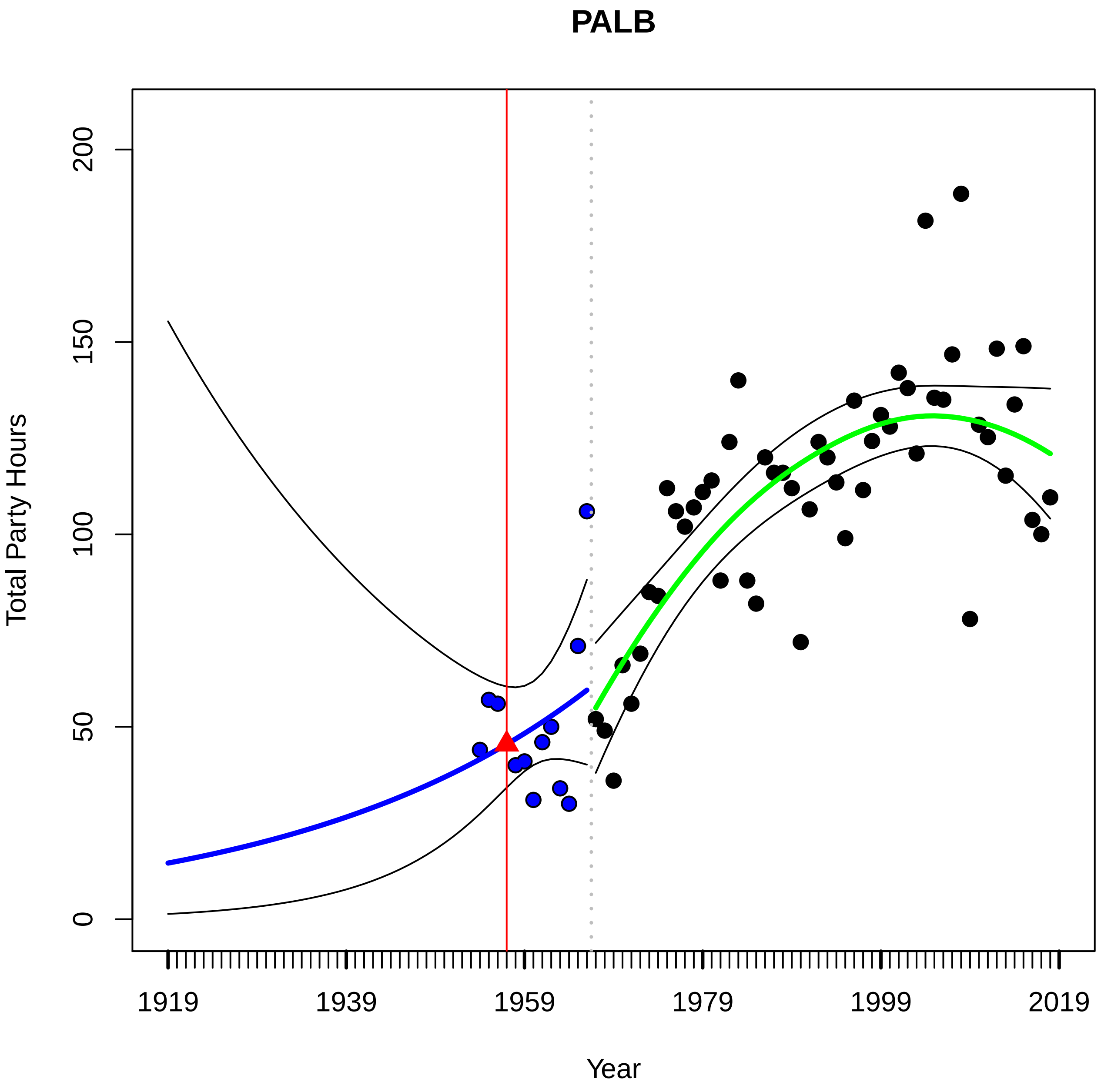
PALB
Total Party Hours
0
50
100
150
200
1919
1939
1959
1979
1999
2019
Year

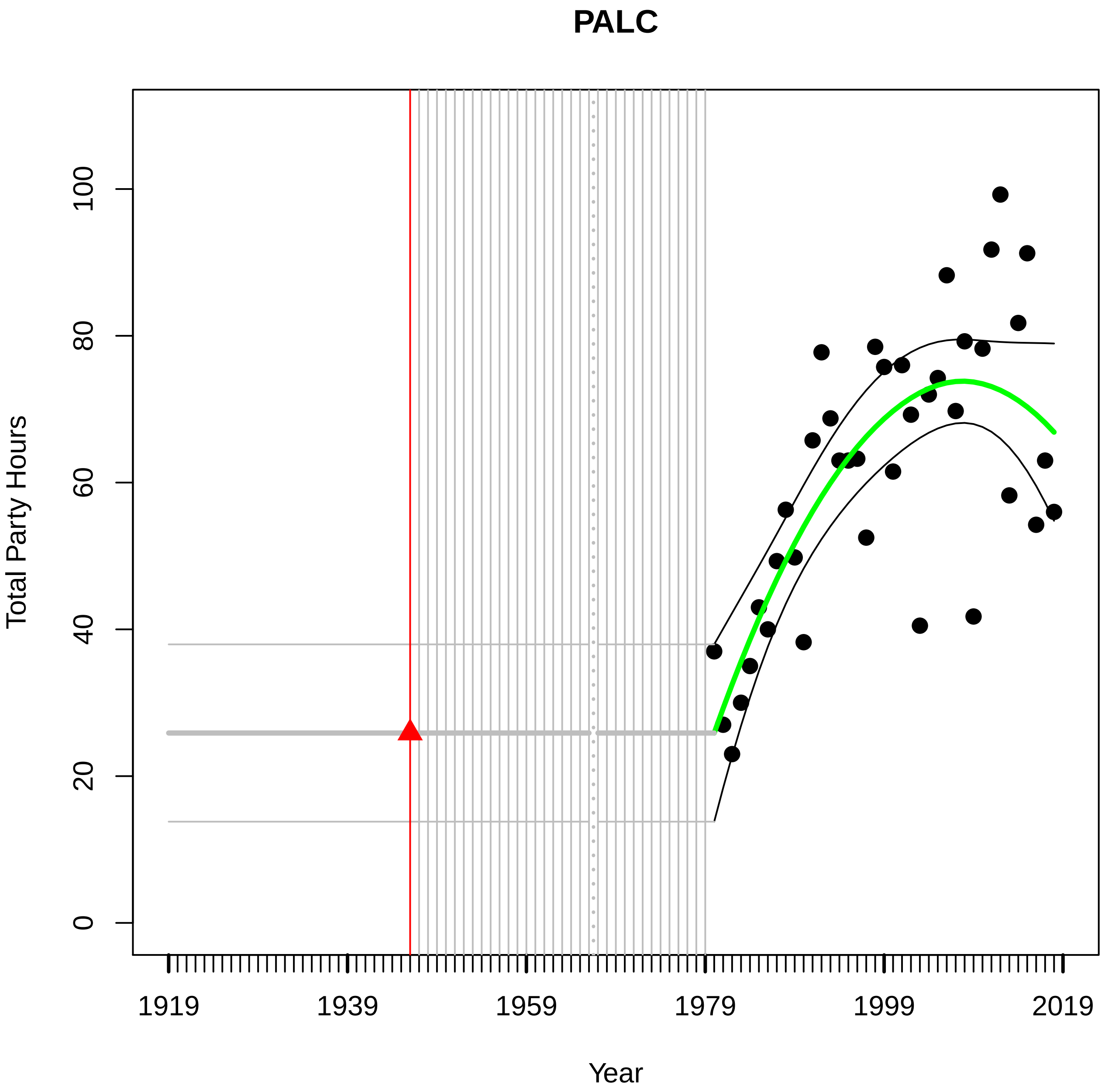
PALC
Total Party Hours
0
20
40
60
80
100
1919
1939
1959
1979
1999
2019
Year

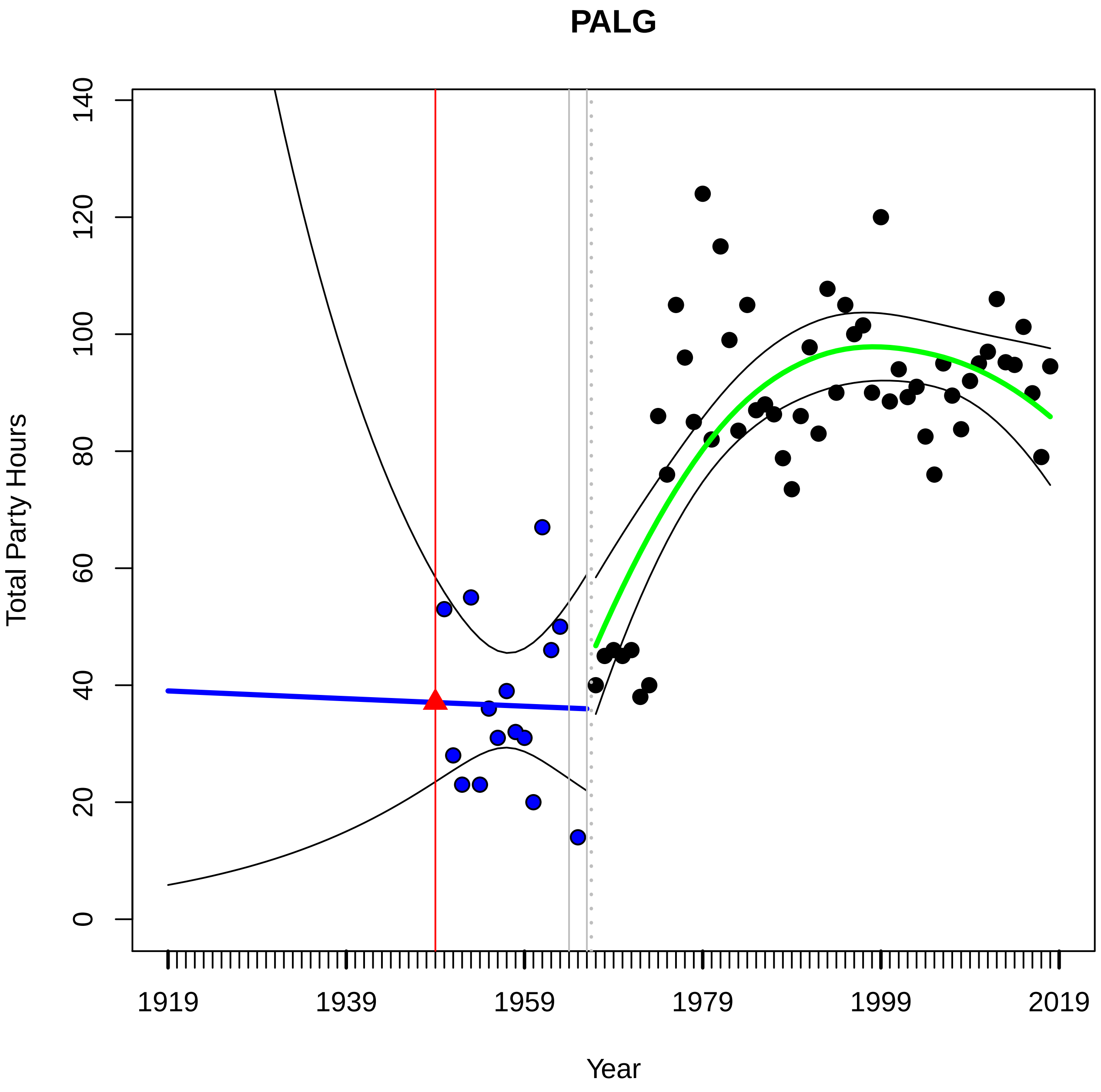
PALG
Total Party Hours
0
20
40
60
80
100
120
140
1919
1939
1959
1979
1999
2019
Year

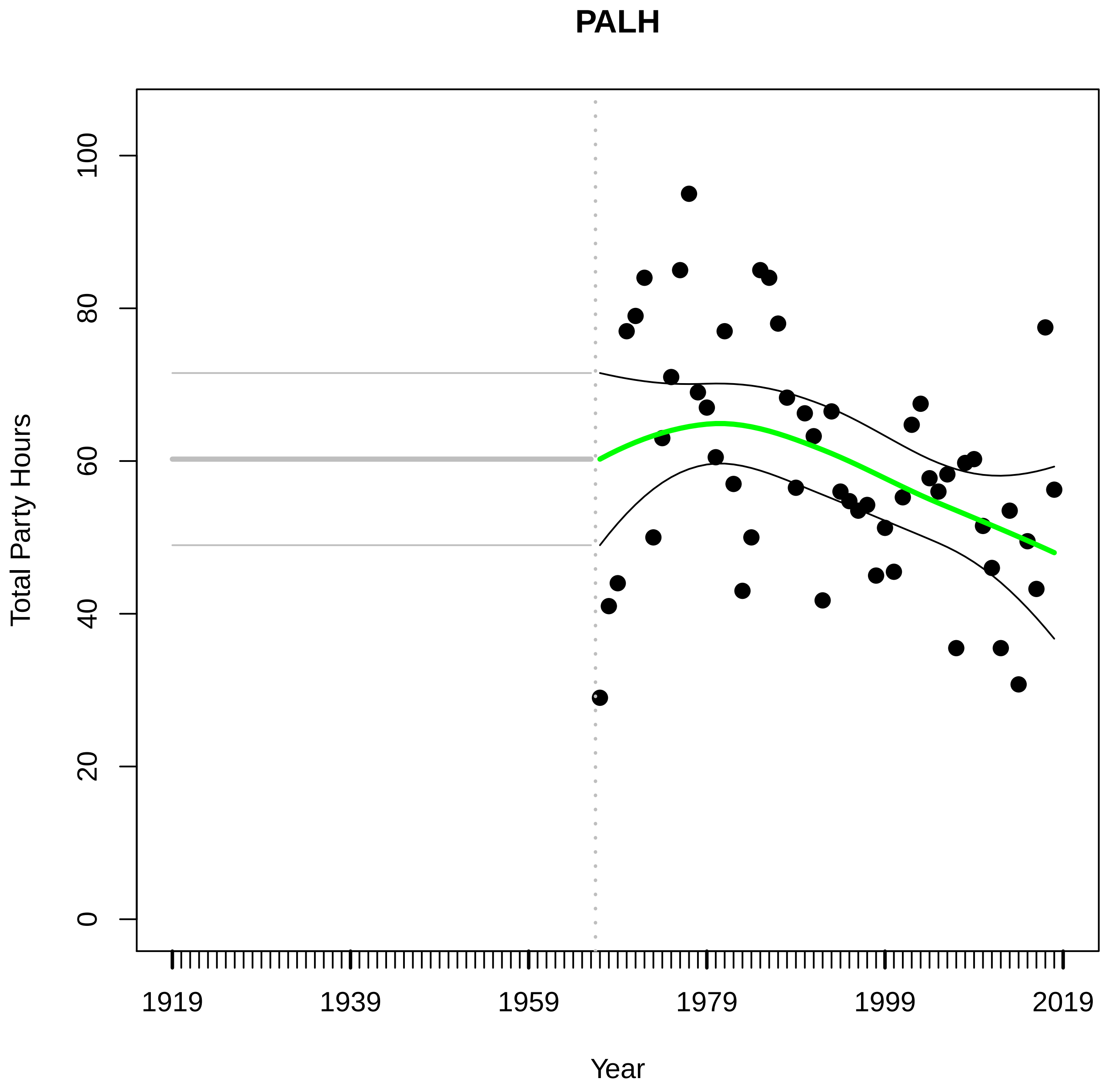

PALH
Total Party Hours
0
20
40
60
80
100
1919
1939
1959
1979
1999
2019
Year

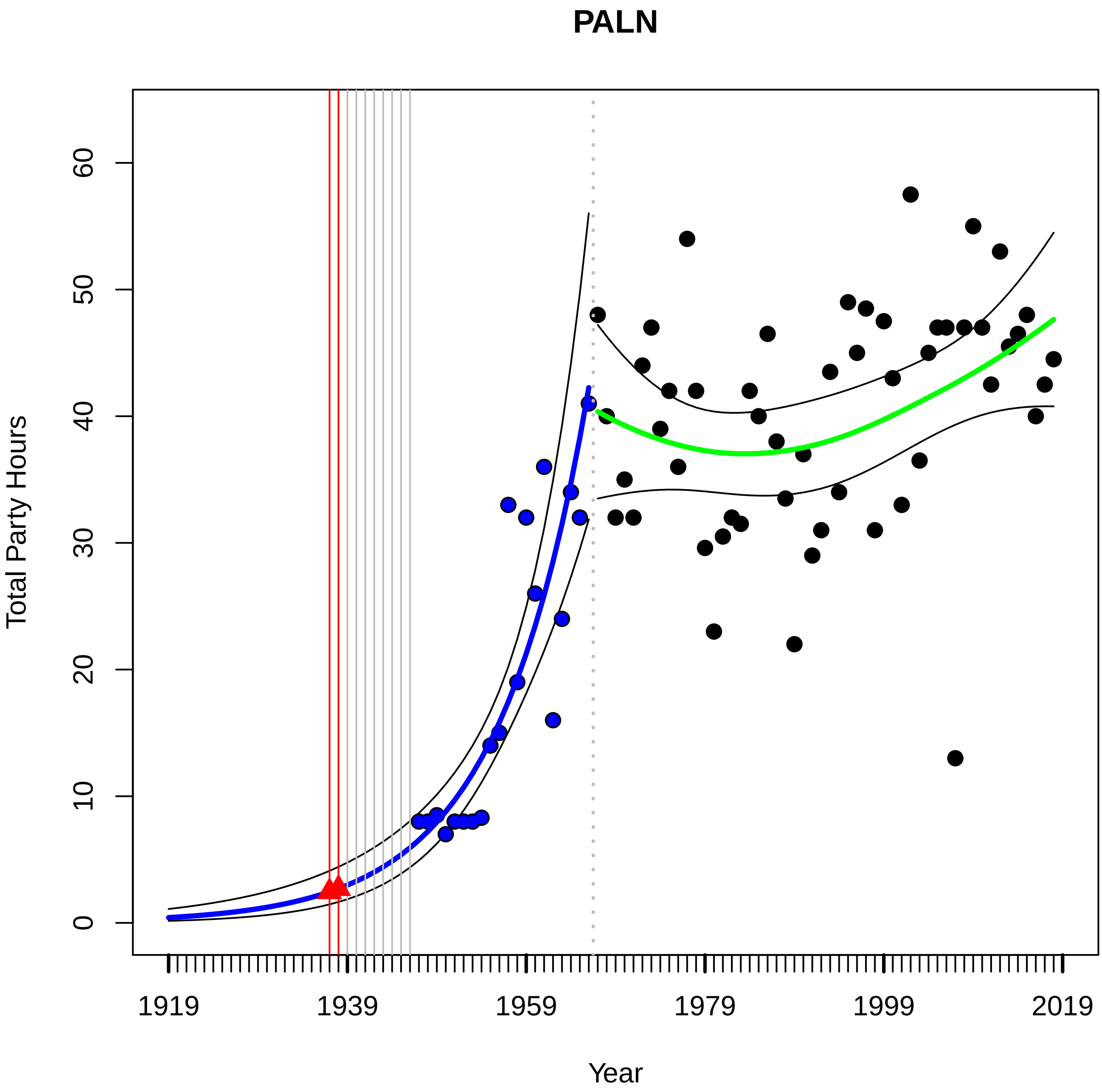

PALN
Total Party Hours
Year
0
10
20
30
40
50
60
1919
1939
1959
1979
1999
2019

PALT

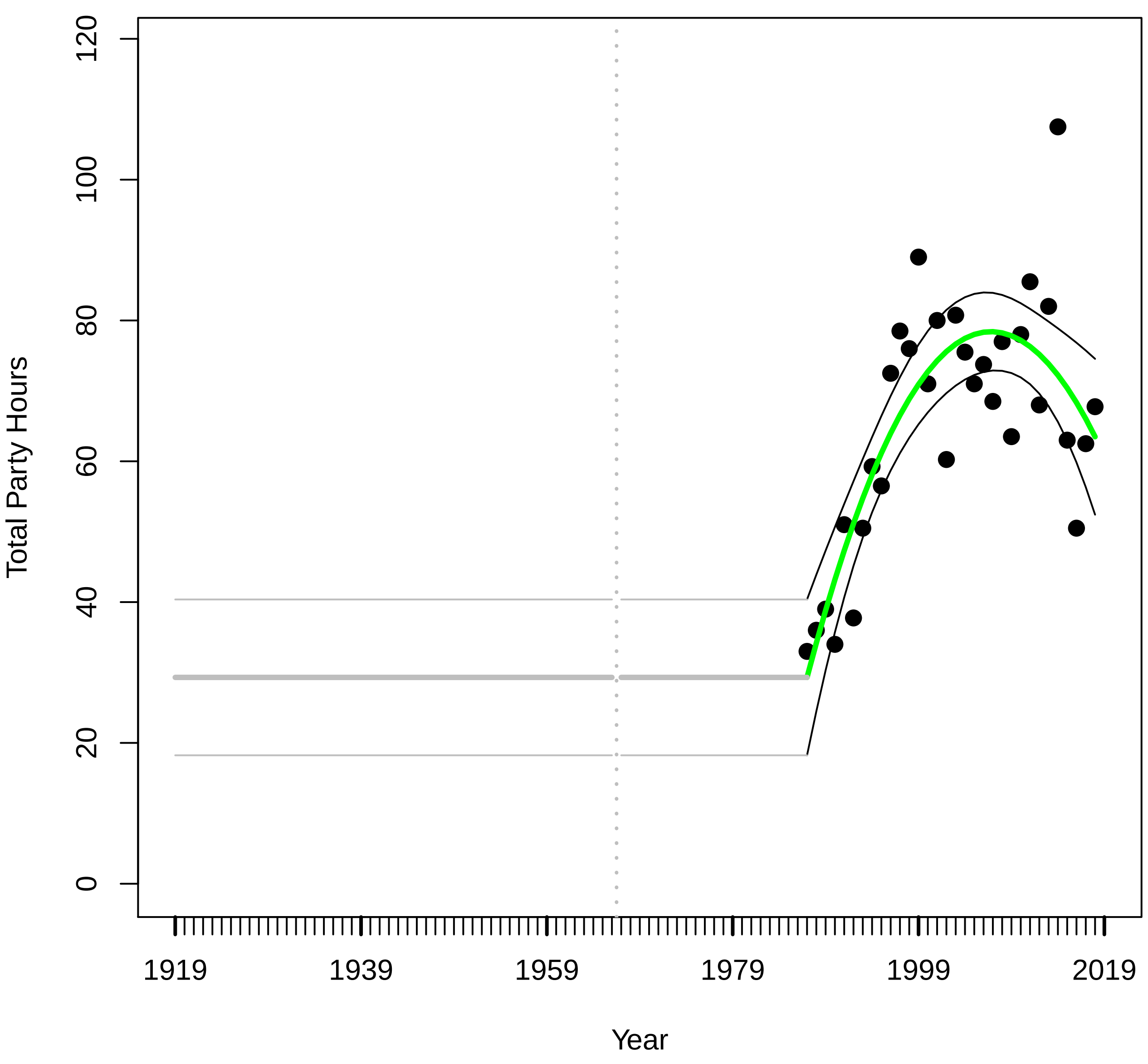

Total Party Hours
0
20
40
60
80
100
120
1919
1939
1959
1979
1999
2019
Year

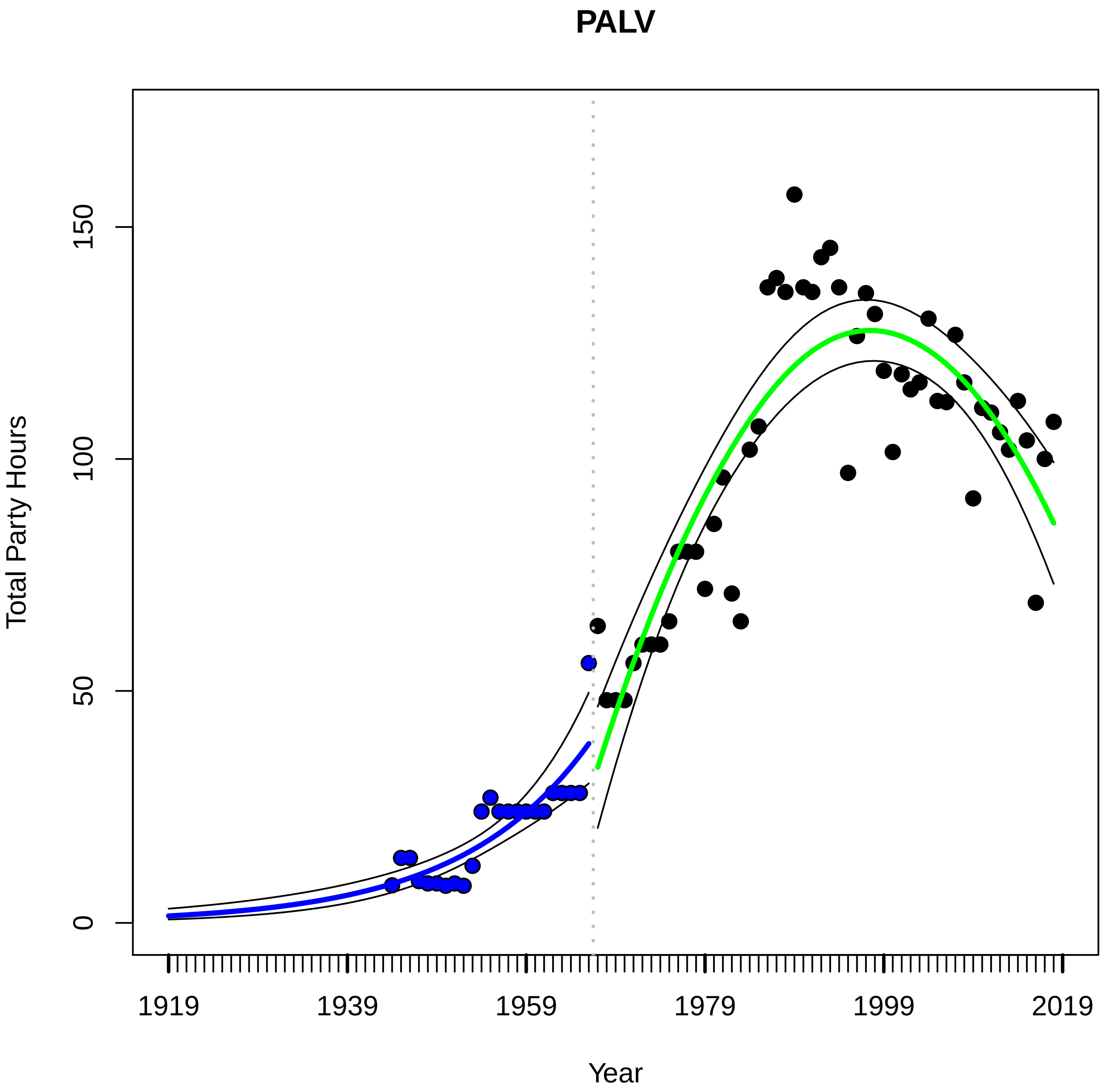
PALV
Total Party Hours
Year
0
50
100
150
1919
1939
1959
1979
1999
2019

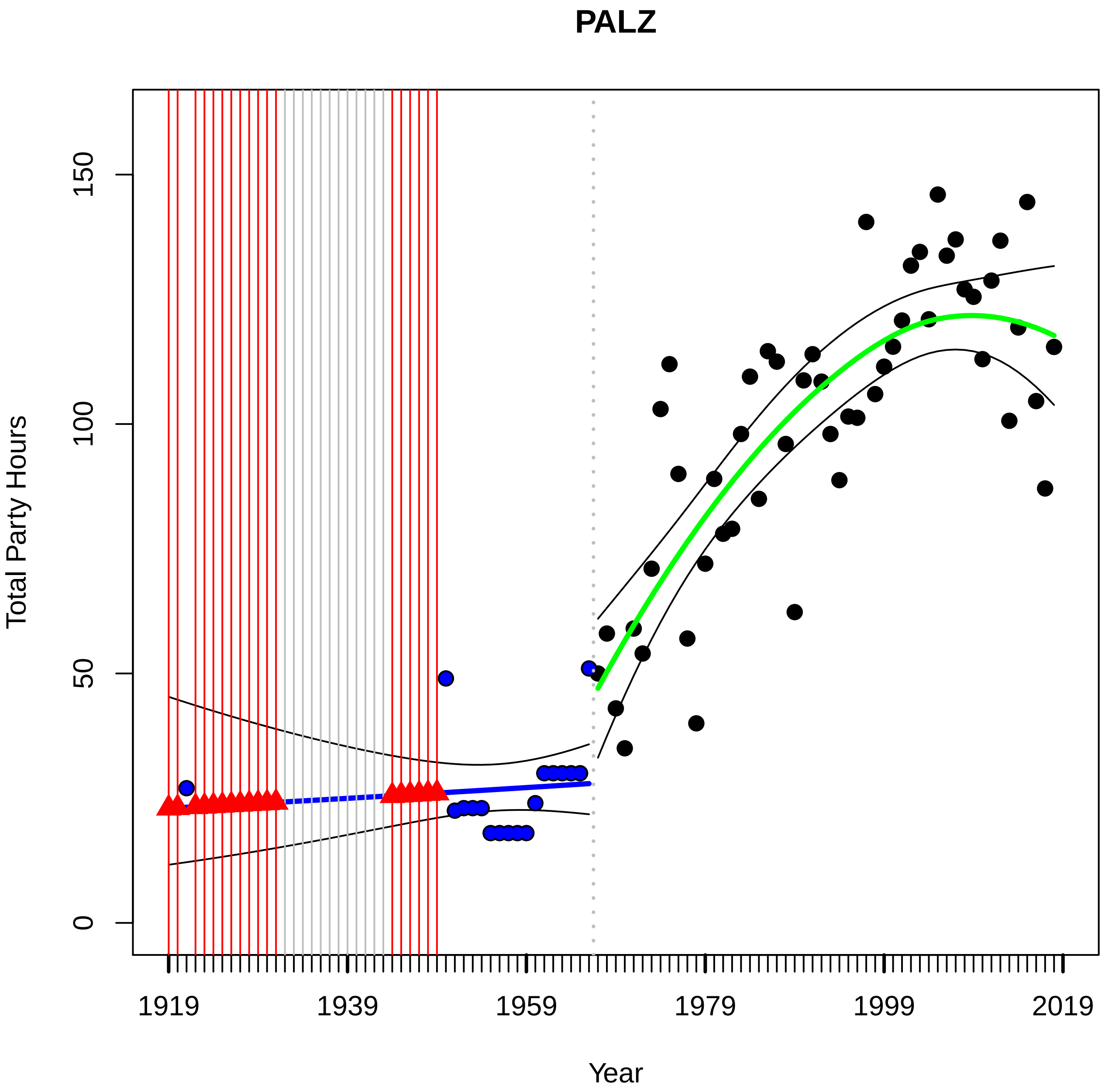

PALZ
Total Party Hours
0
50
100
150
1919
1939
1959
1979
1999
2019
Year

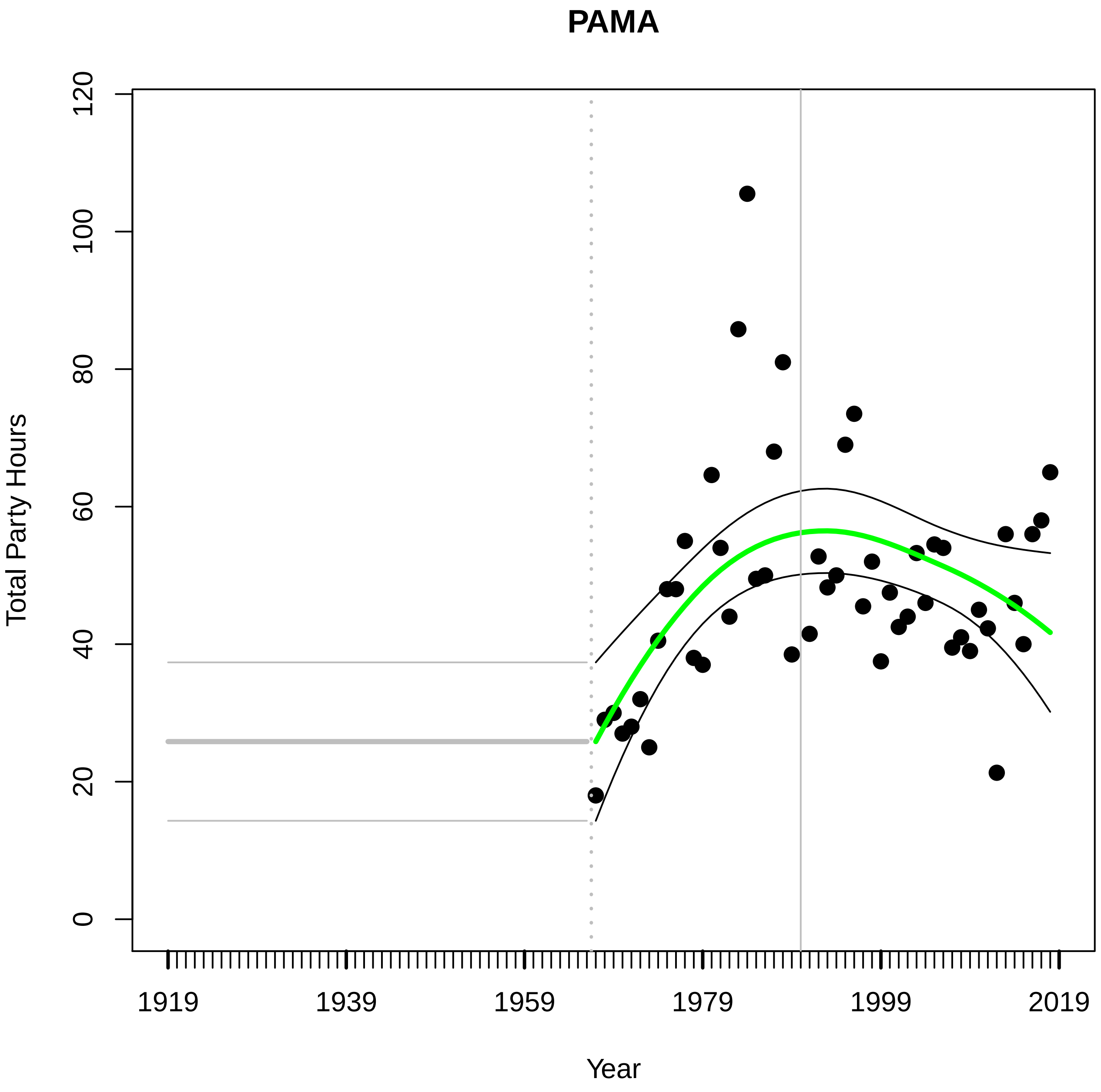

PAMA
Total Party Hours
0
20
40
60
80
100
120
1919
1939
1959
1979
1999
2019
Year

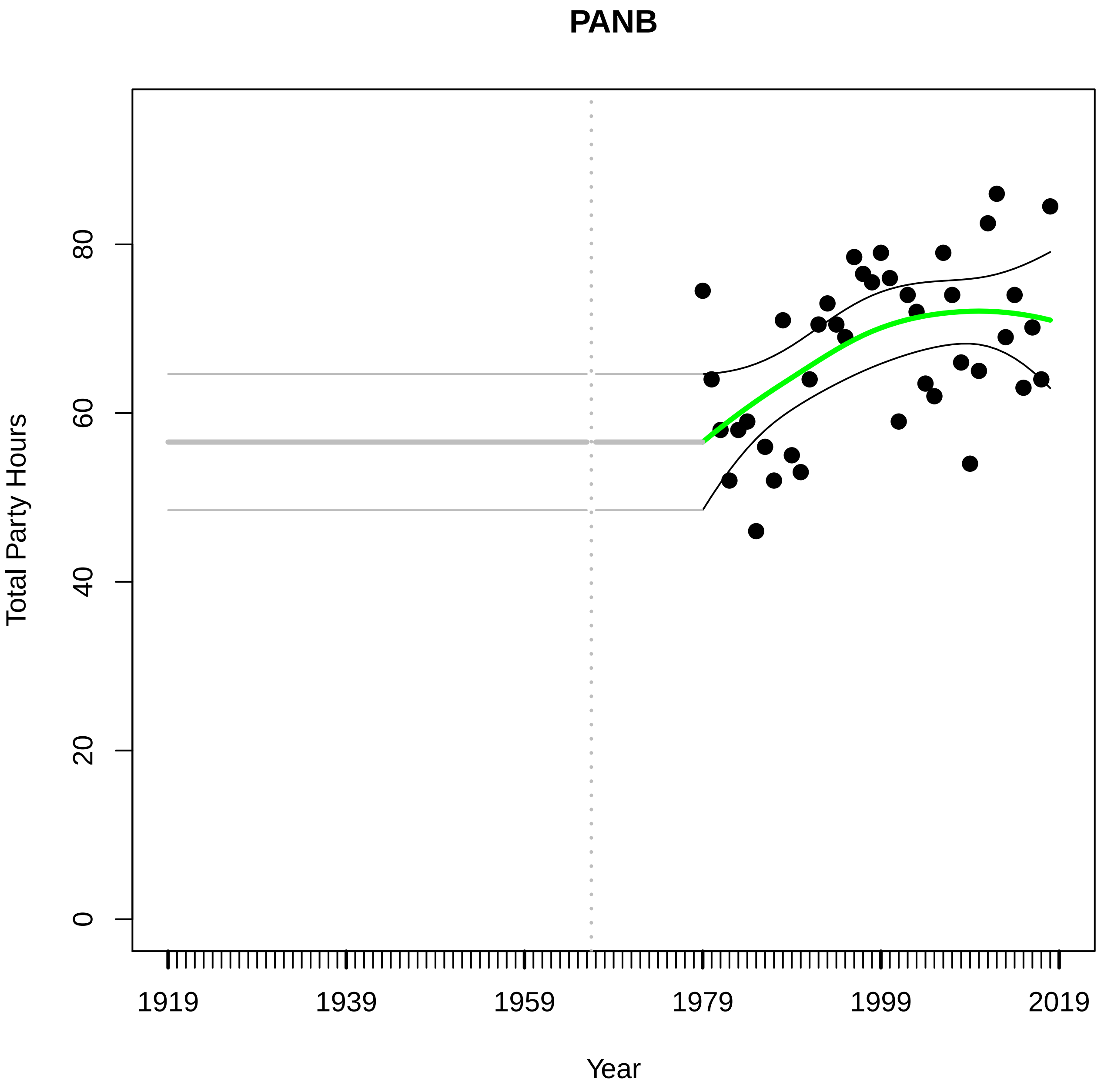

PANB
Total Party Hours
0
20
40
60
80
Year
1919
1939
1959
1979
1999
2019

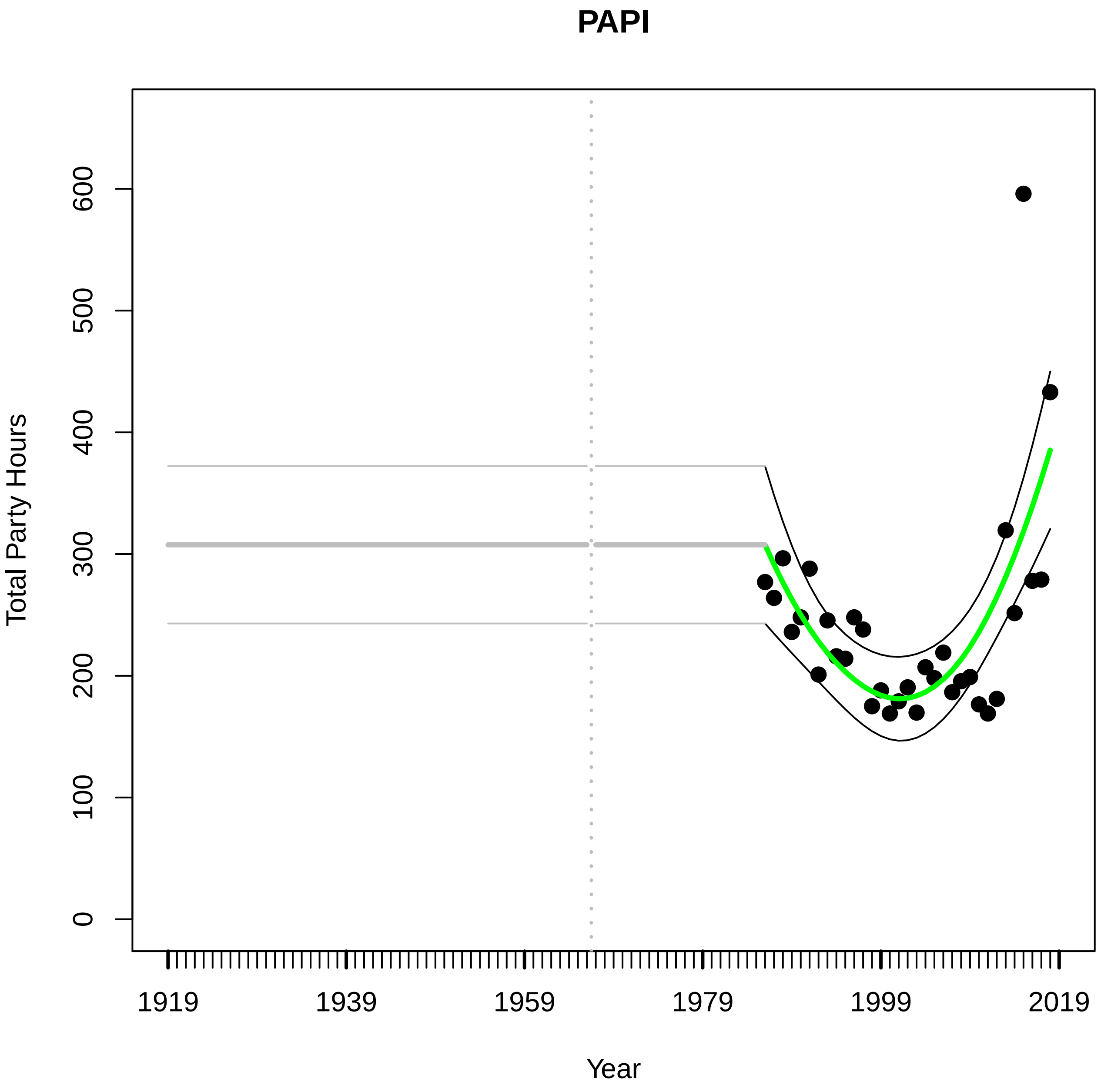

PAPI
Total Party Hours
0
100
200
300
400
500
600
1919
1939
1959
1979
1999
2019
Year

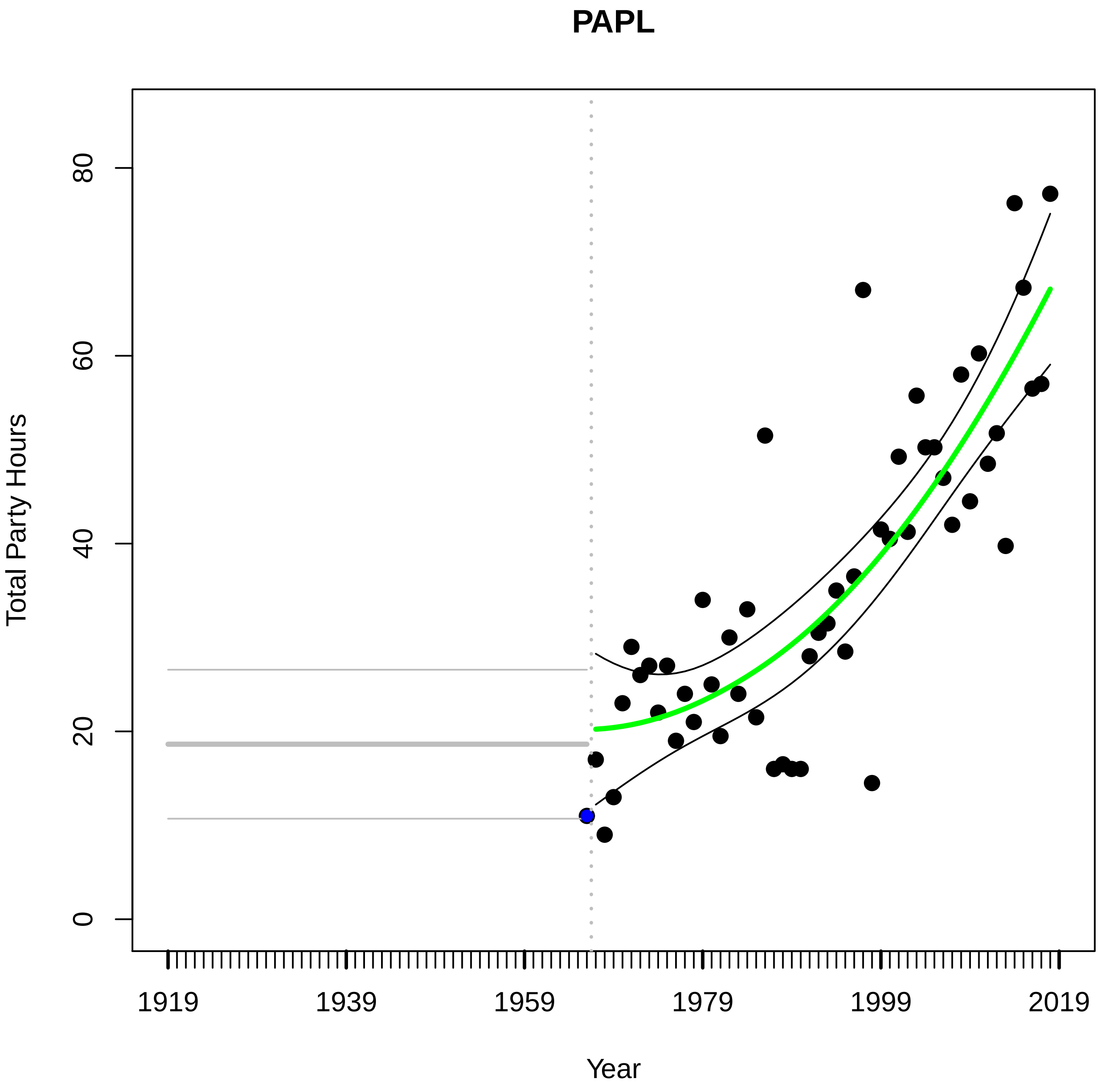

PAPL
Total Party Hours
0
20
40
60
80
1919
1939
1959
1979
1999
2019
Year

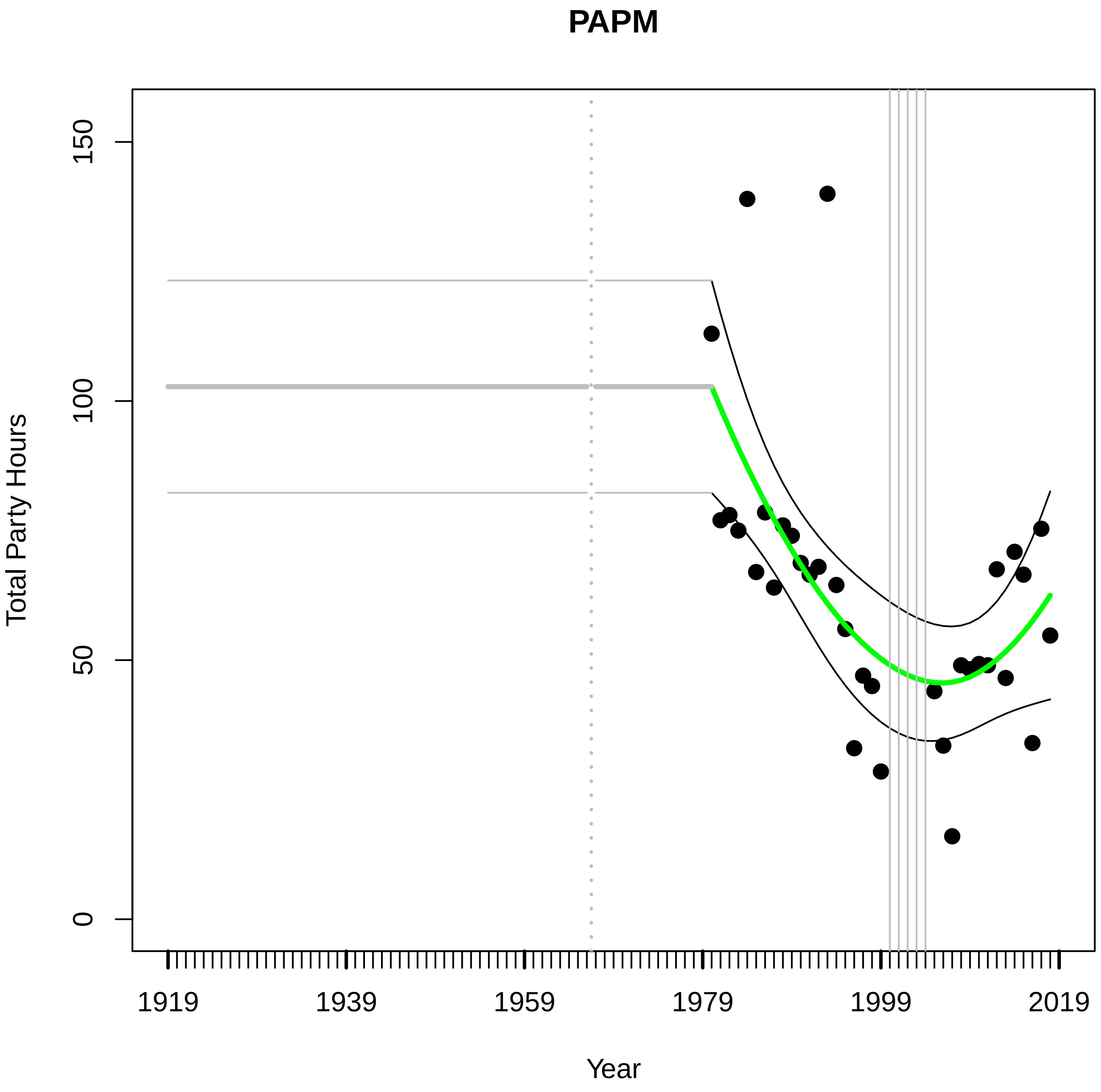
PAPM
Total Party Hours
0
50
100
150
1919
1939
1959
1979
1999
2019
Year

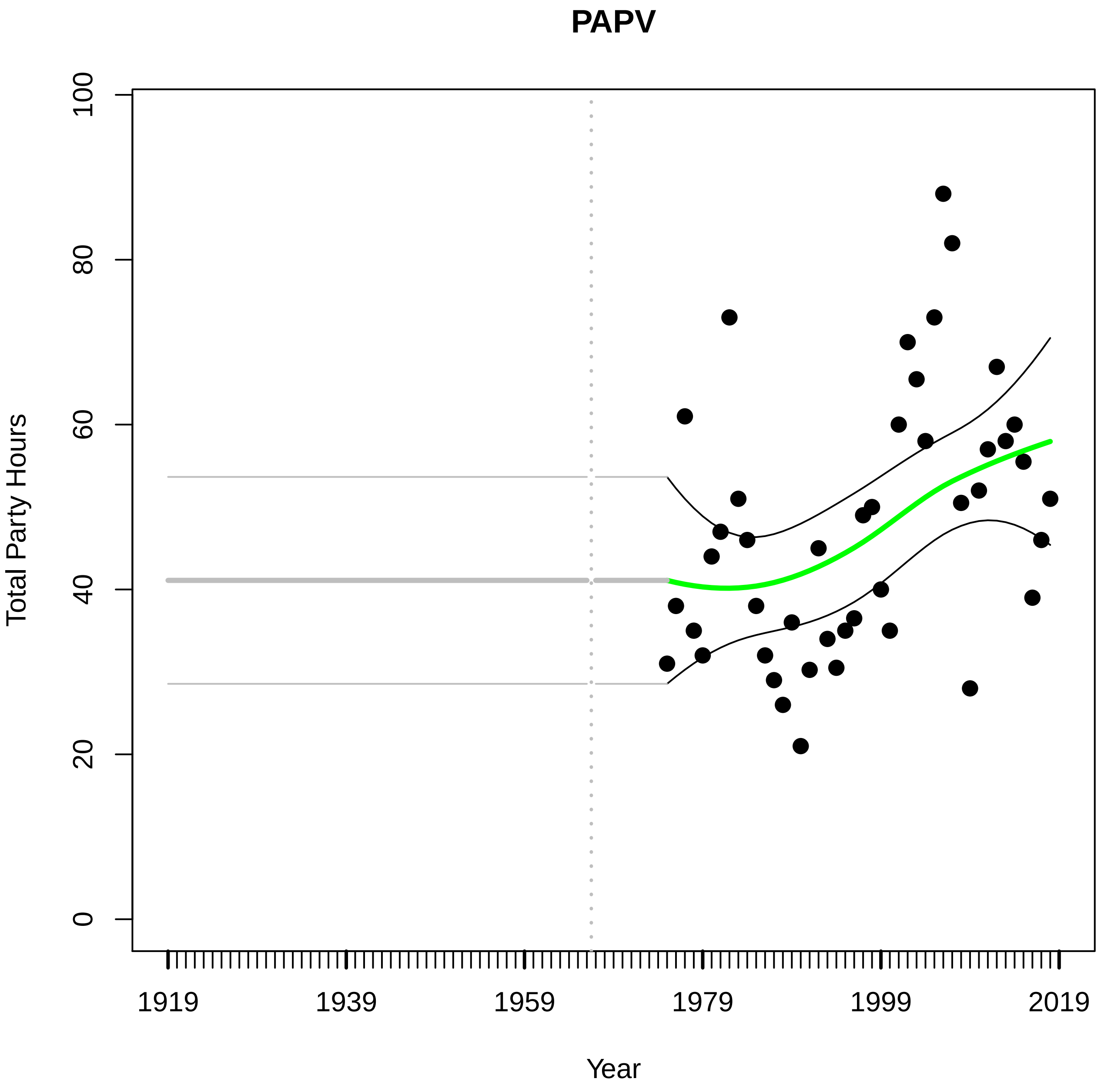

PAPV
Total Party Hours
Year
0
20
40
60
80
100
1919
1939
1959
1979
1999
2019

PARC

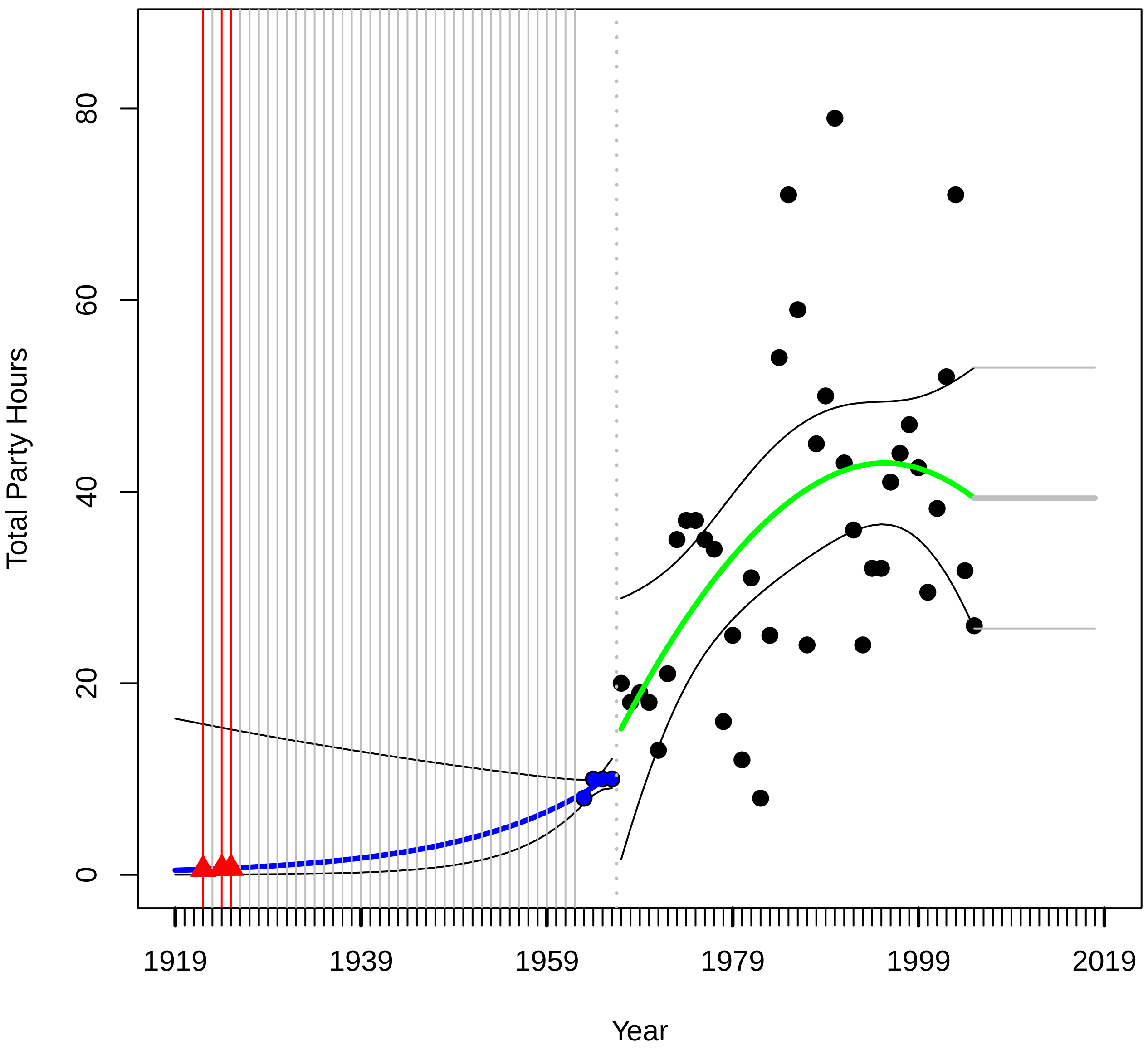

Total Party Hours
0
20
40
60
80
1919
1939
1959
1979
1999
2019
Year

PARD

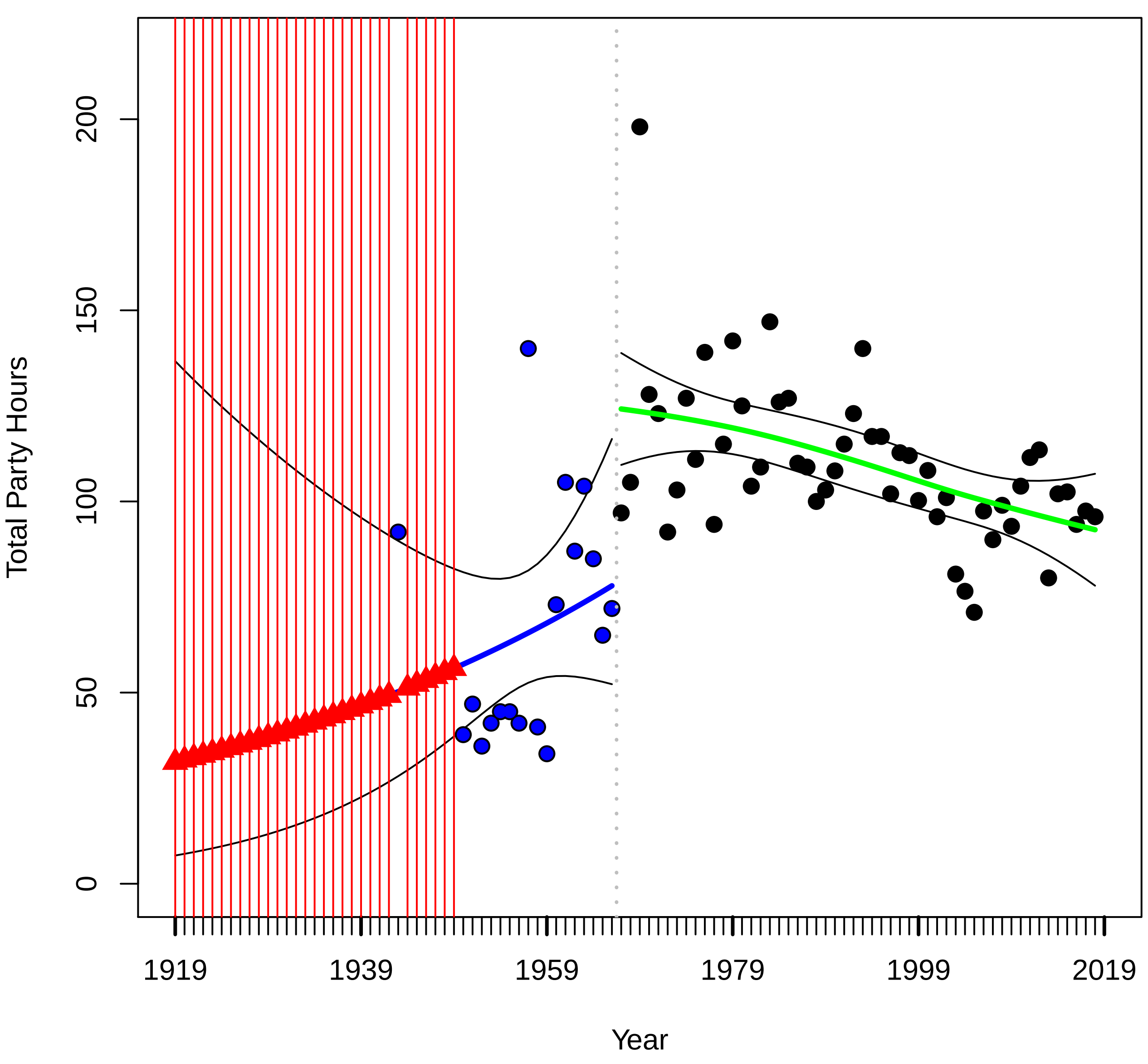

Total Party Hours
0
50
100
150
200
1919
1939
1959
1979
1999
2019
Year

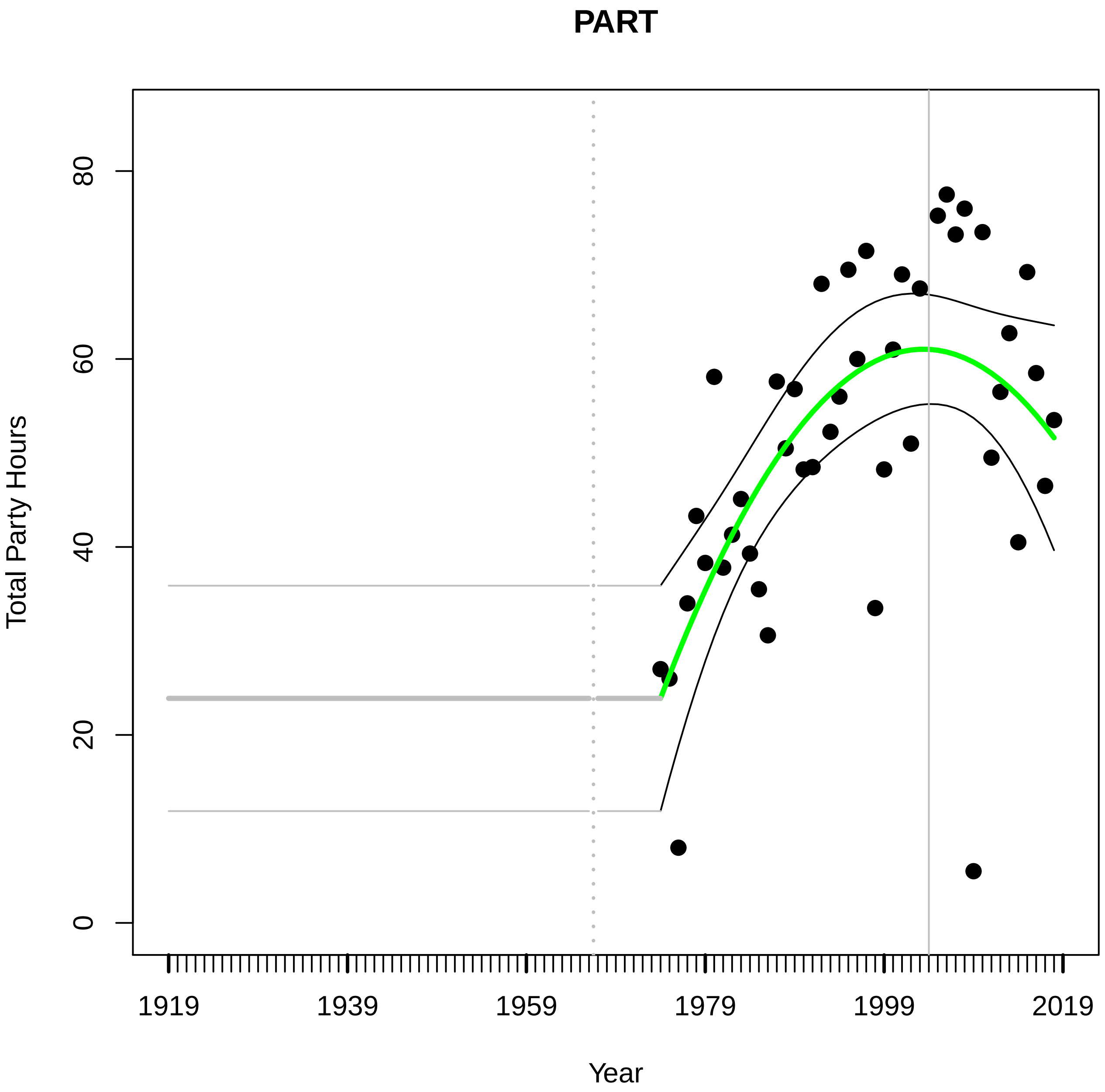

PART
Total Party Hours
0
20
40
60
80
1919
1939
1959
1979
1999
2019
Year

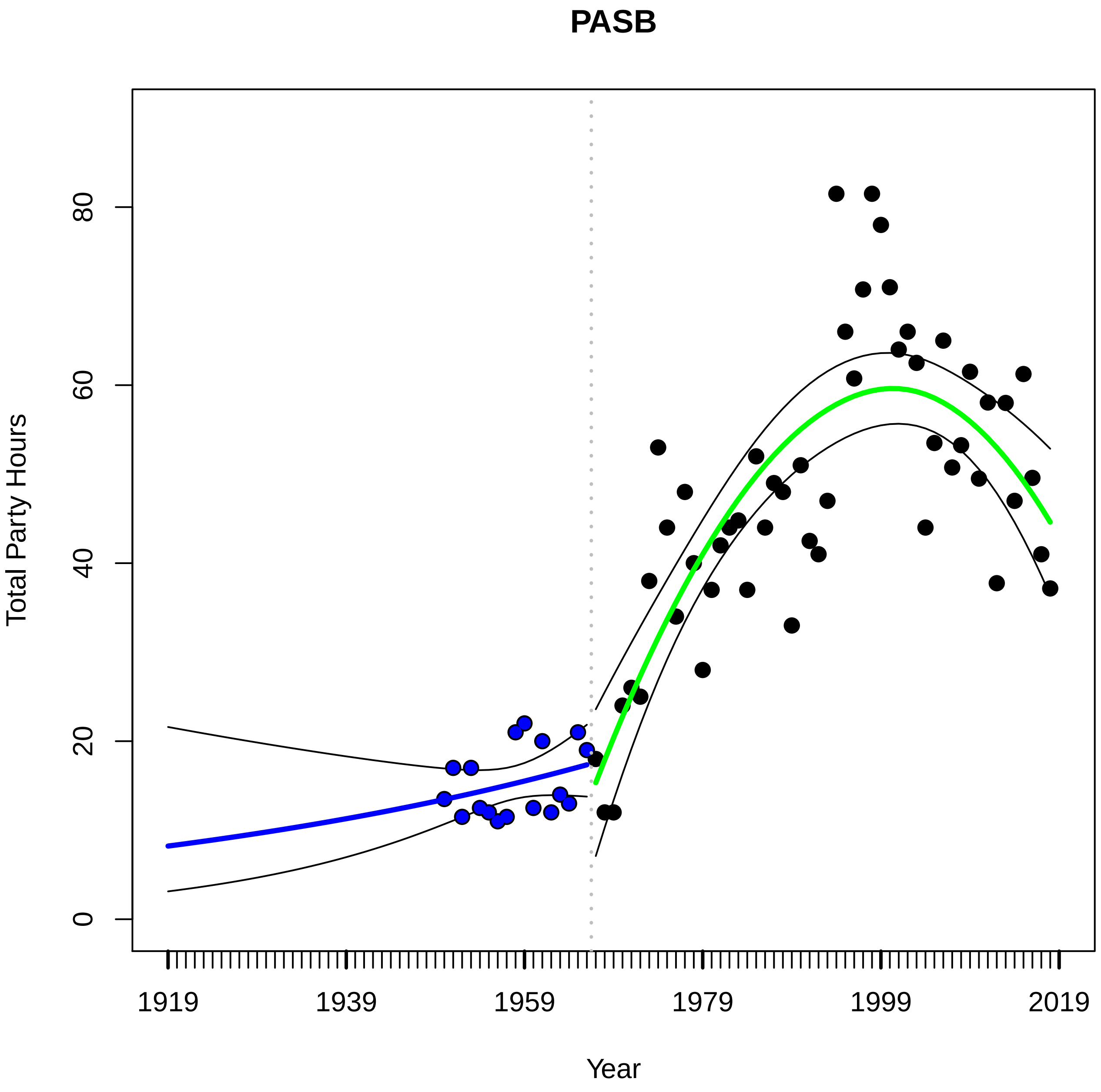

PASB
Total Party Hours
0
20
40
60
80
1919
1939
1959
1979
1999
2019
Year

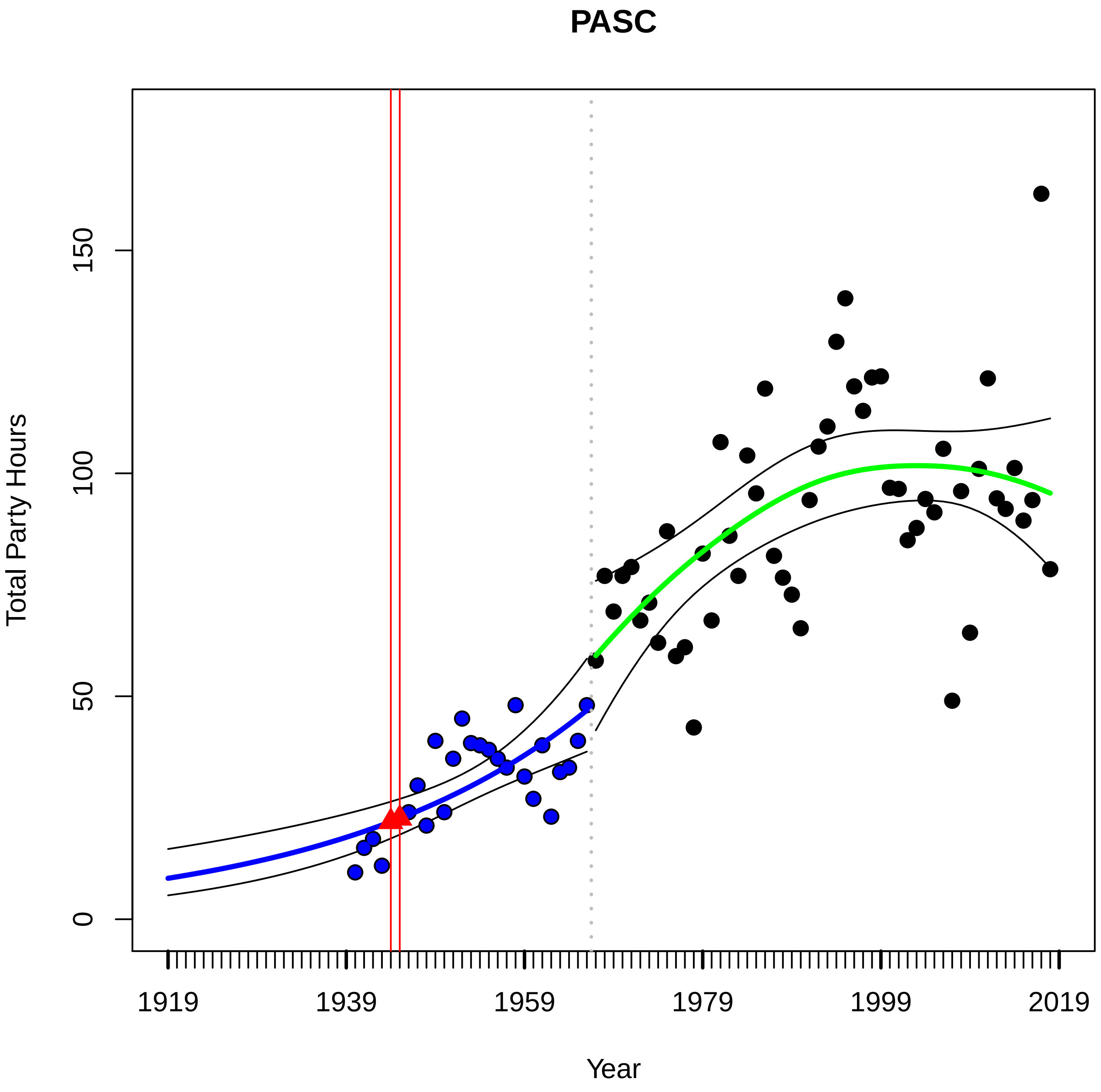

PASC
Total Party Hours
0
50
100
150
Year
1919
1939
1959
1979
1999
2019

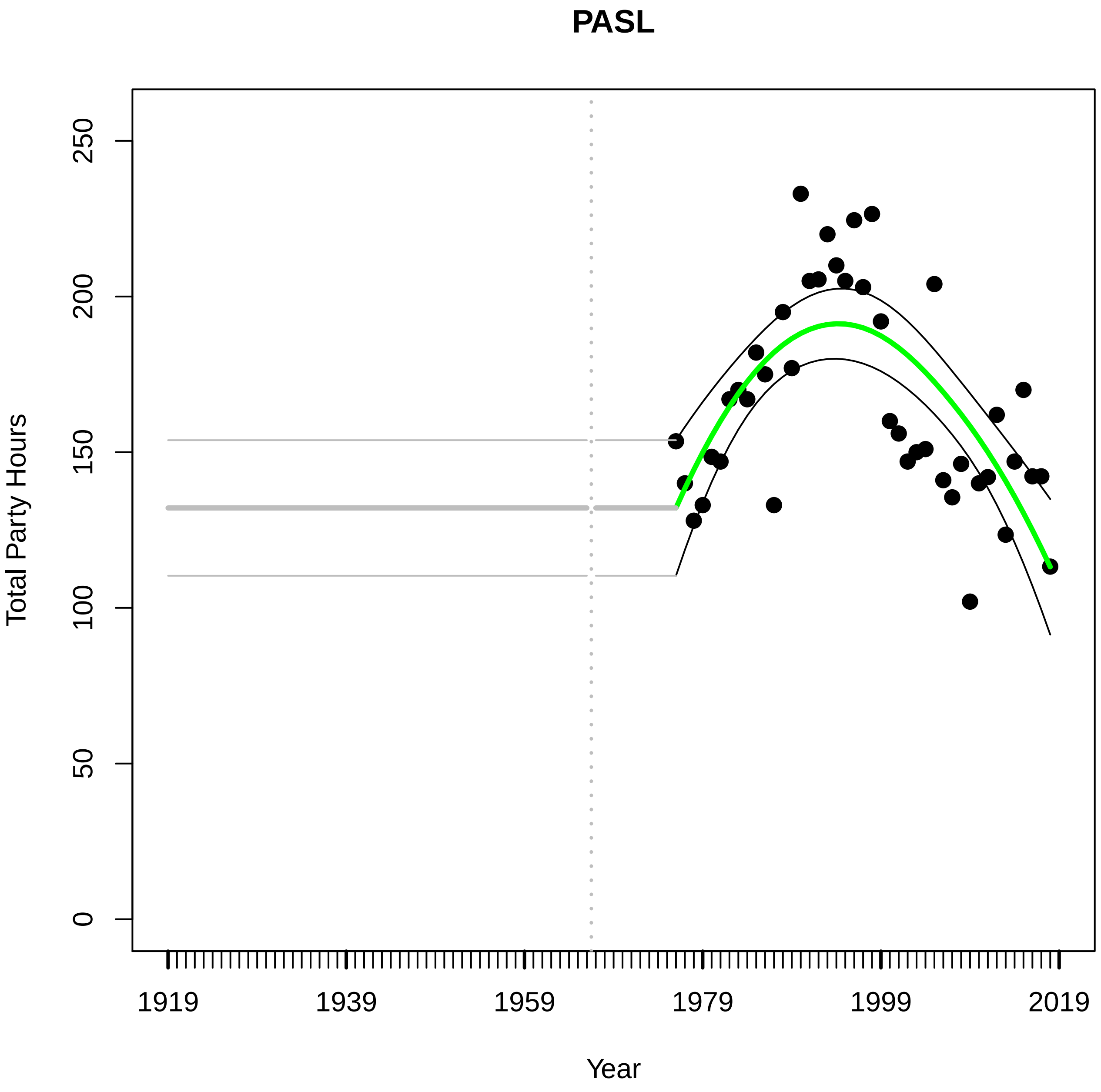
PASL
Total Party Hours
0
50
100
150
200
250
1919
1939
1959
1979
1999
2019
Year

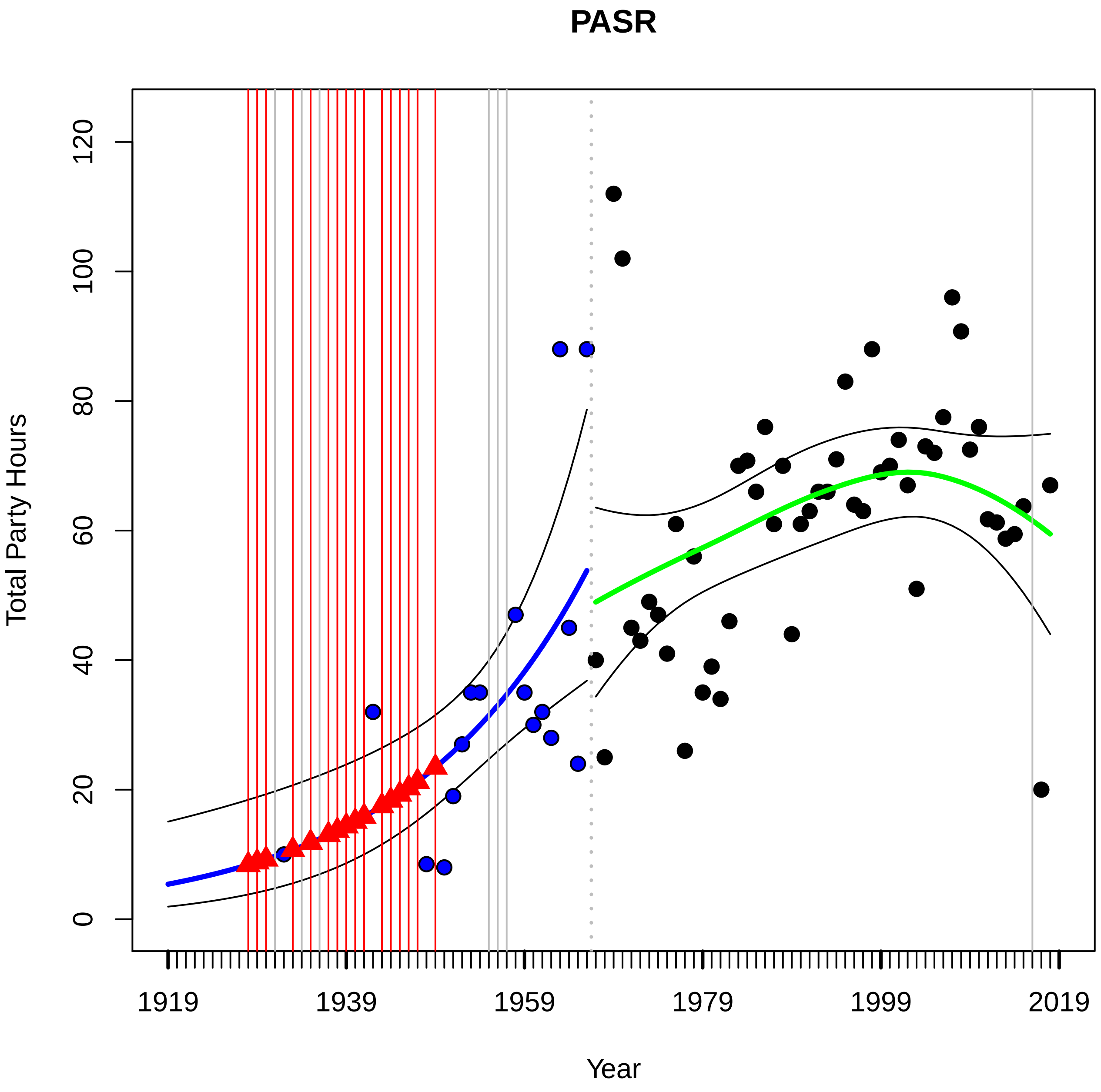

PASR
Total Party Hours
0
20
40
60
80
100
120
1919
1939
1959
1979
1999
2019
Year

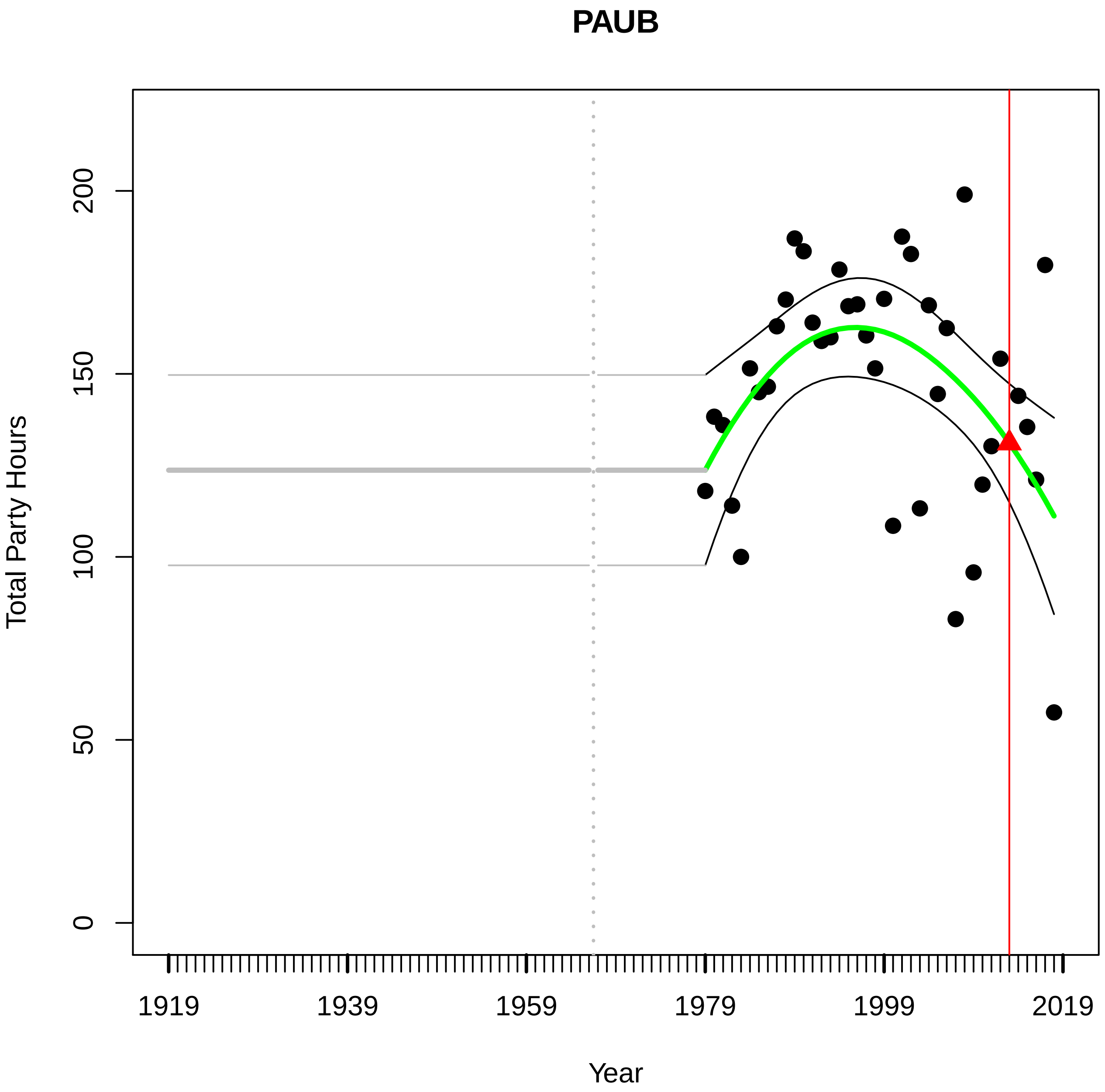

PAUB
Total Party Hours
Year
0
50
100
150
200
1919
1939
1959
1979
1999
2019

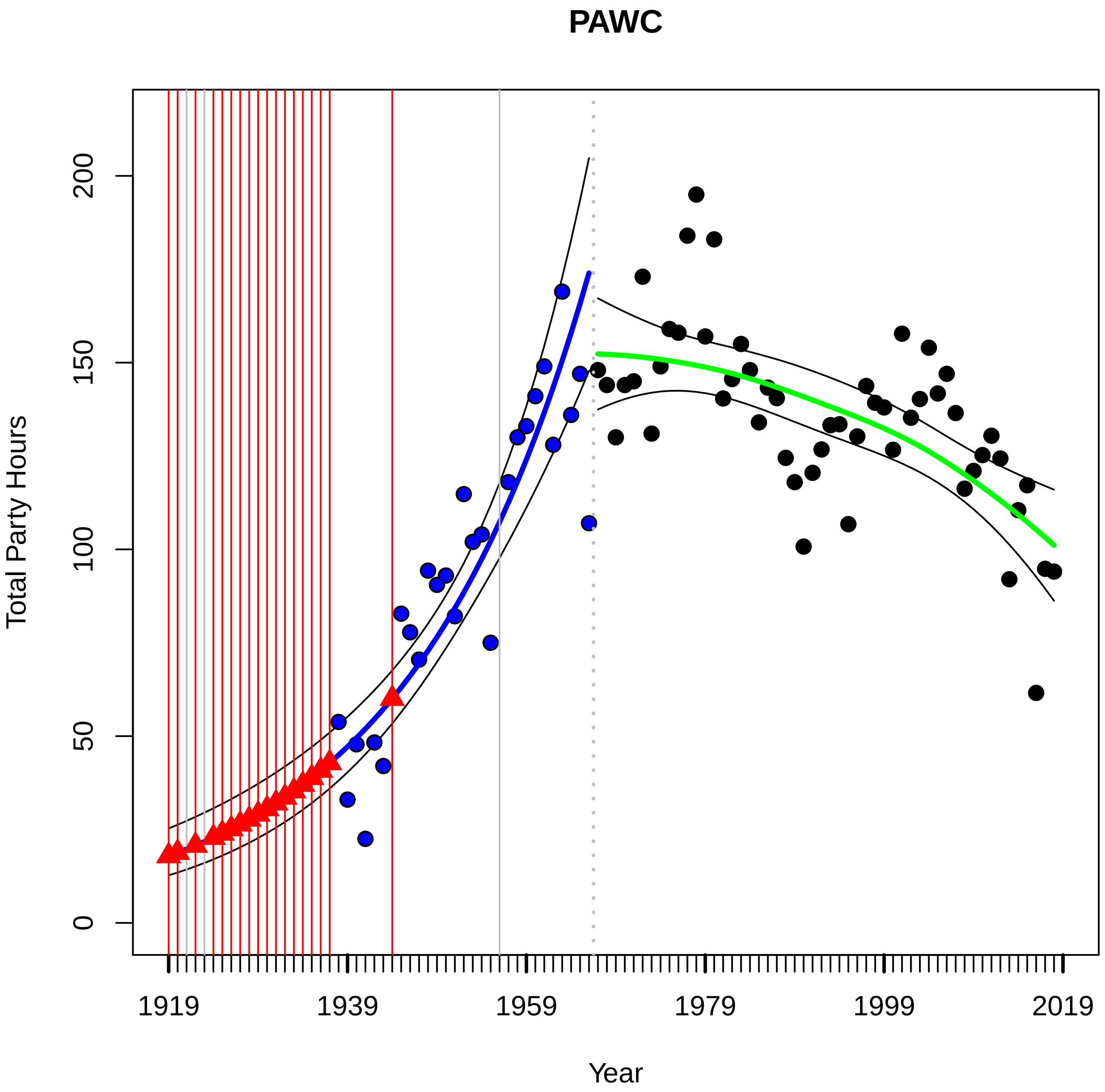
PAWC
Total Party Hours
0
50
100
150
200
1919
1939
1959
1979
1999
2019
Year

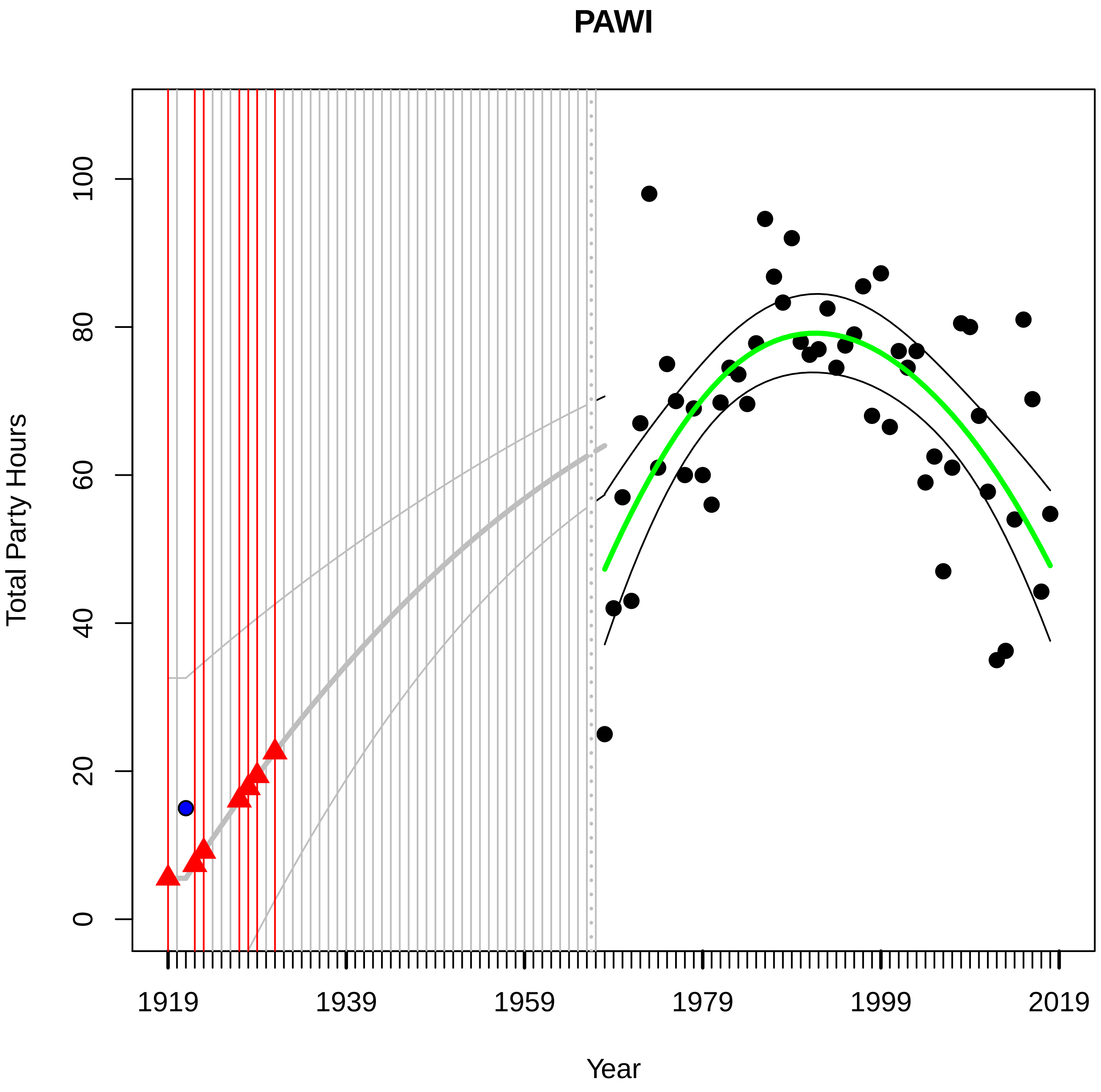

PAWI
Total Party Hours
Year
0
20
40
60
80
100
1919
1939
1959
1979
1999
2019

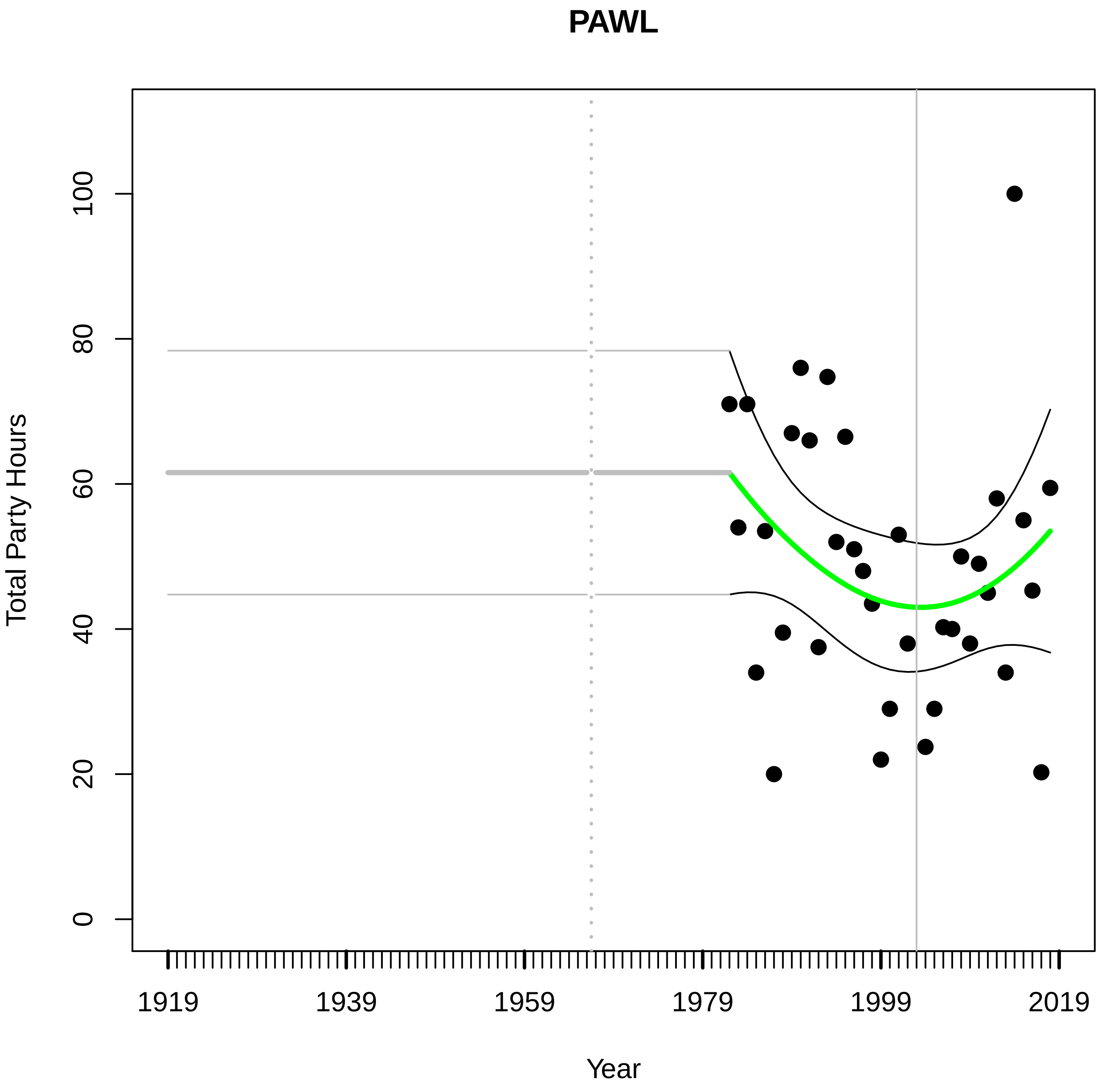
PAWL
Total Party Hours
0
20
40
60
80
100
1919
1939
1959
1979
1999
2019
Year

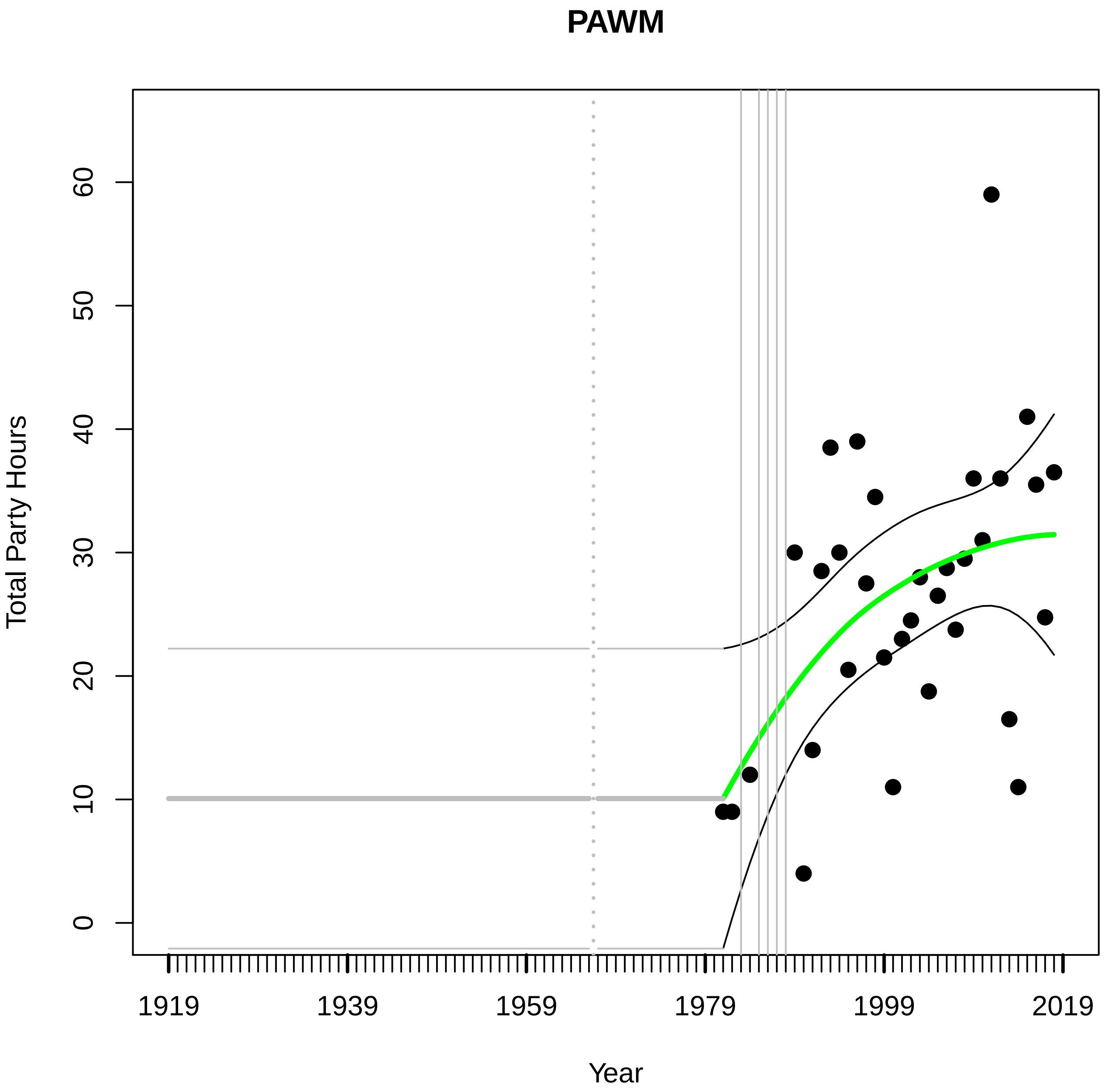

PAWM
Total Party Hours
0
10
20
30
40
50
60
1919
1939
1959
1979
1999
2019
Year

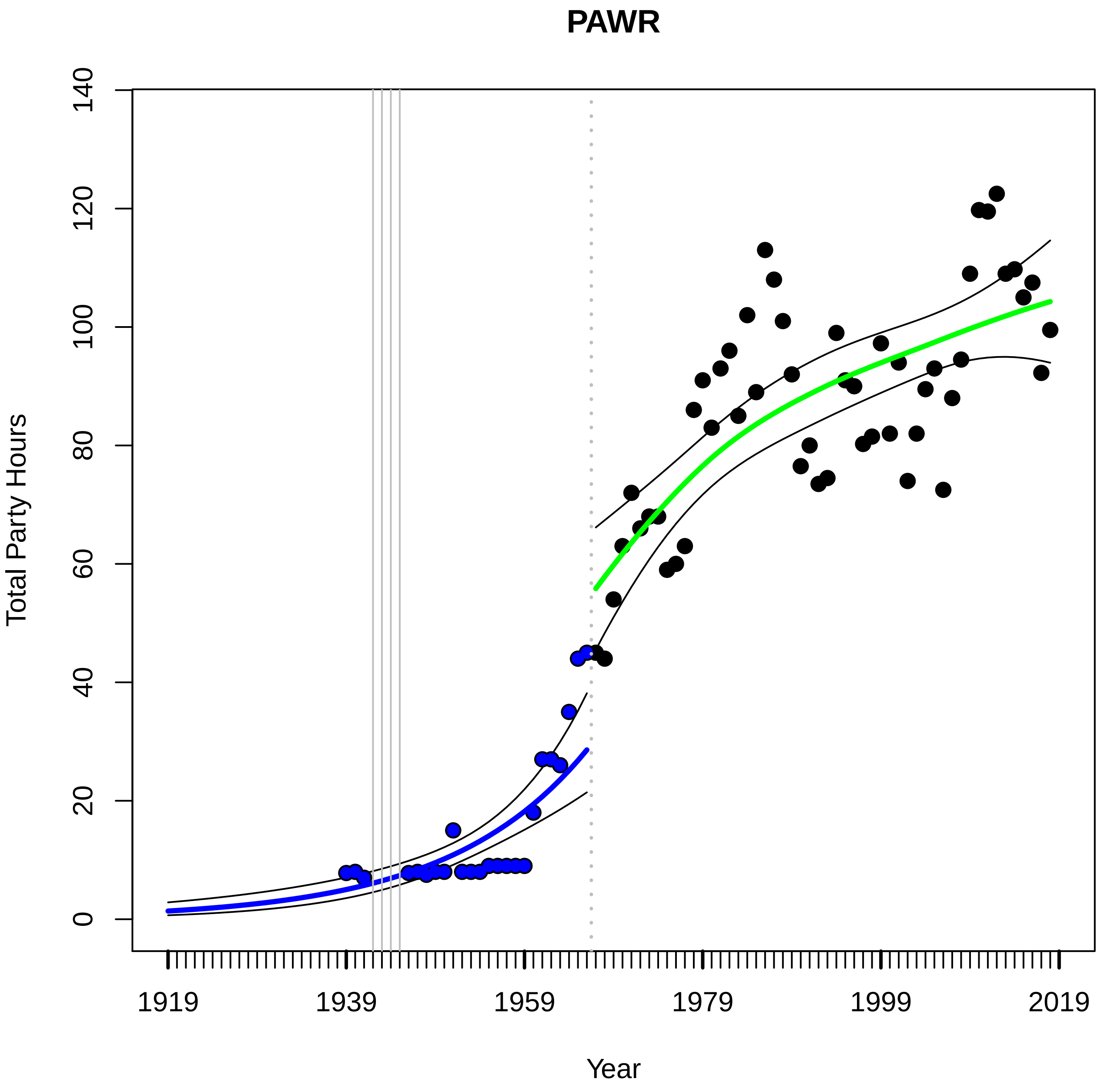

PAWR
Total Party Hours
0
20
40
60
80
100
120
140
1919
1939
1959
1979
1999
2019
Year

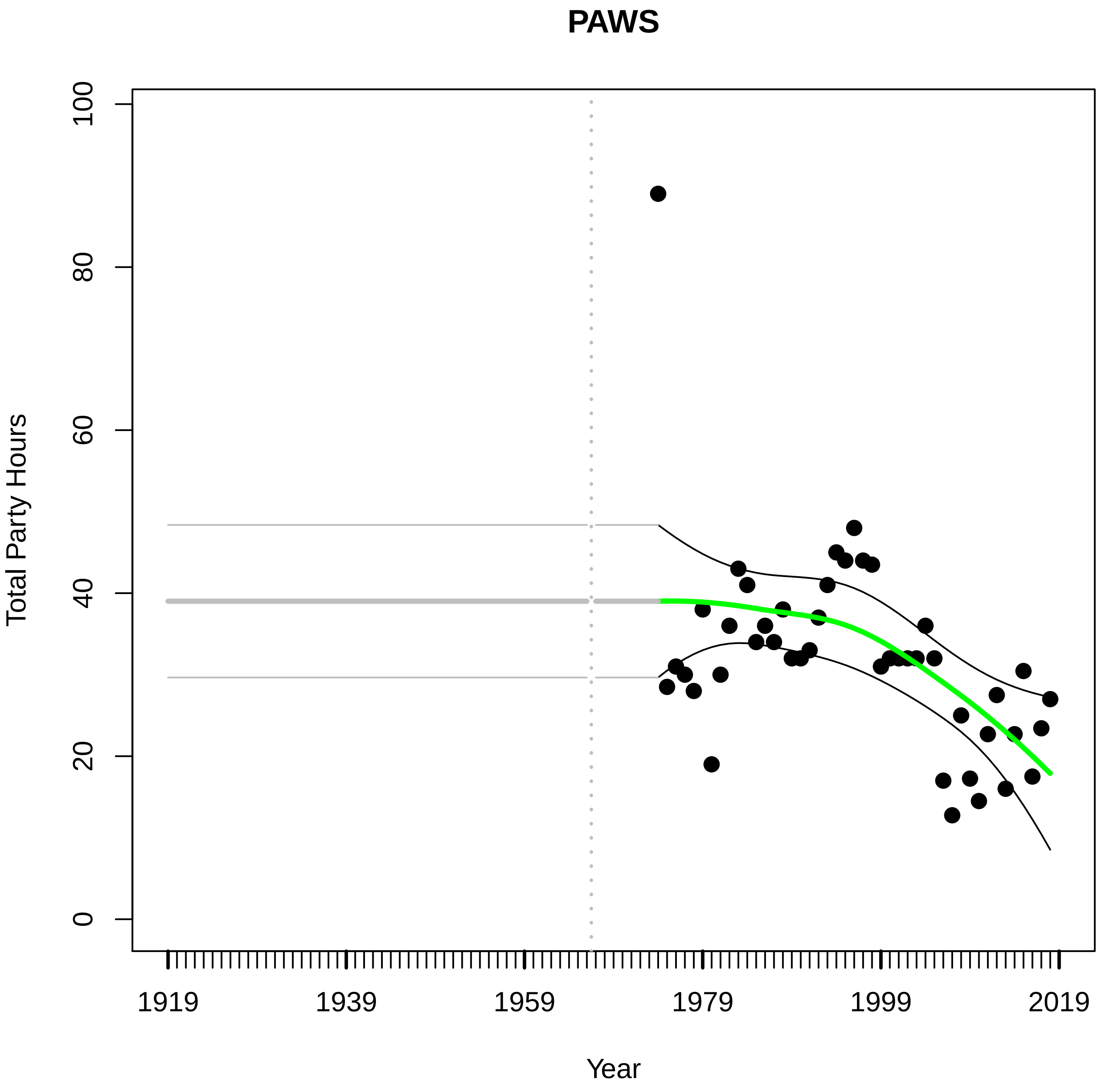

PAWS
Total Party Hours
0
20
40
60
80
100
1919
1939
1959
1979
1999
2019
Year

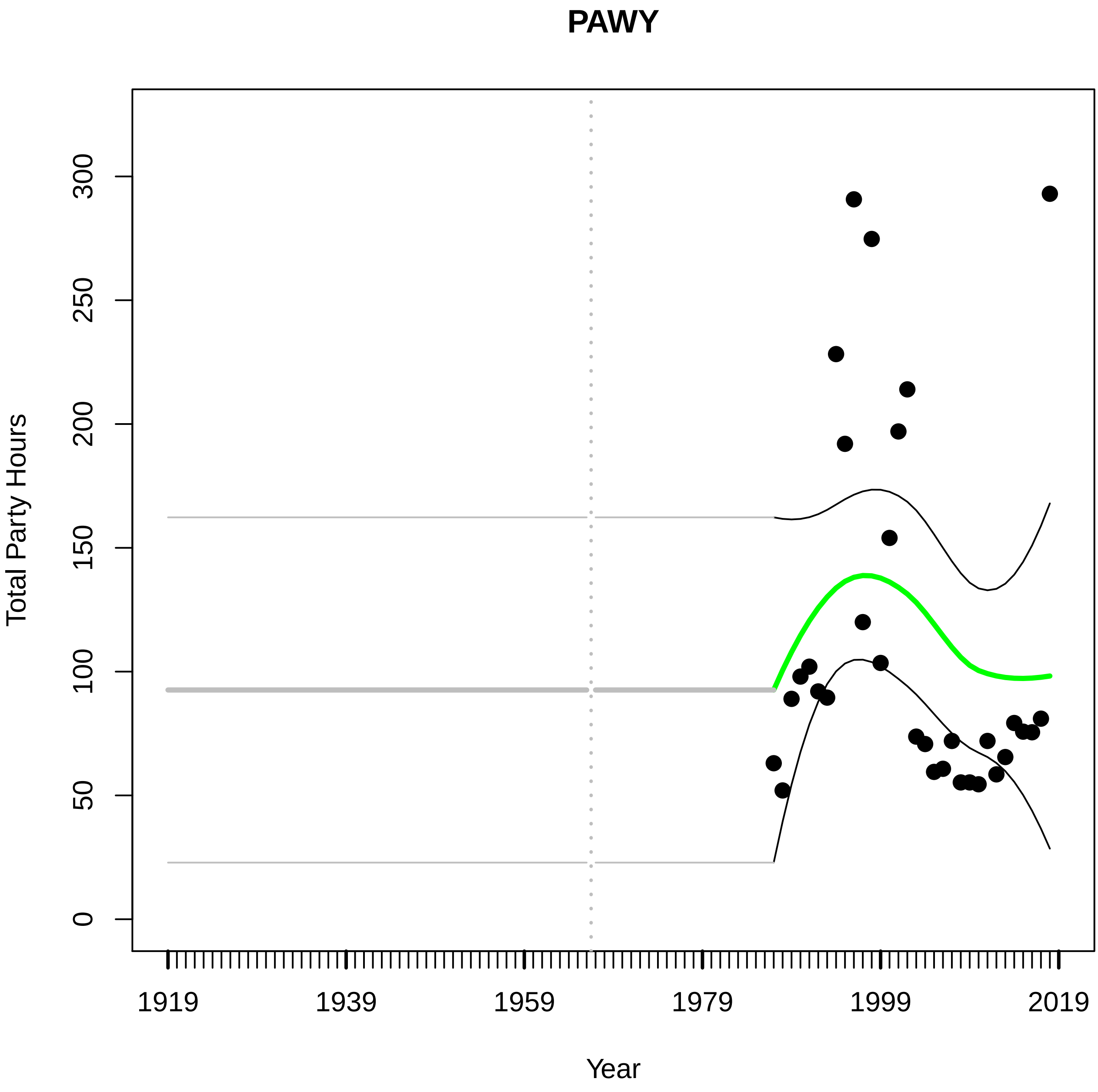
PAWY
Total Party Hours
0
50
100
150
200
250
300
1919
1939
1959
1979
1999
2019
Year

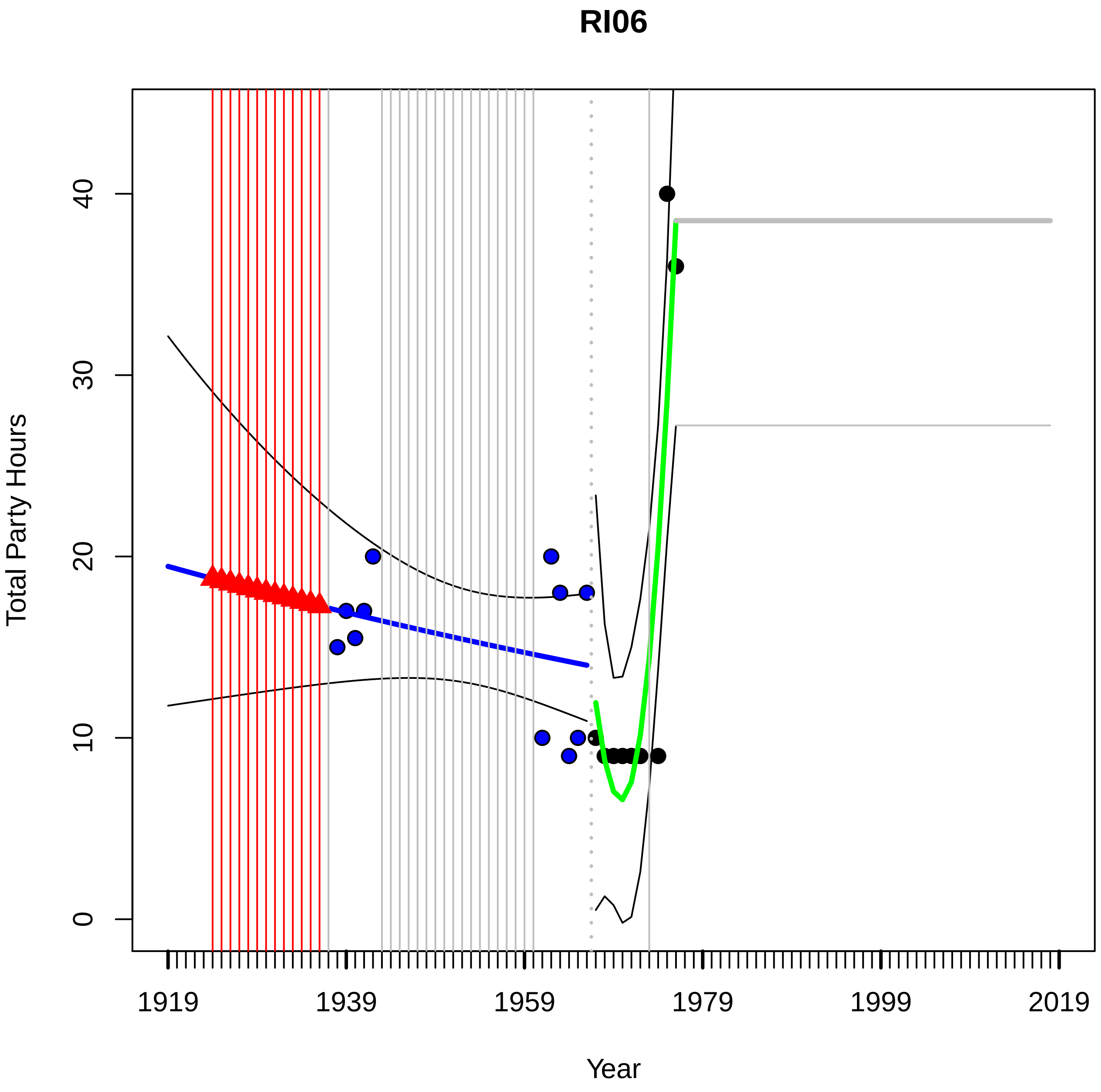
RI06
Total Party Hours
0
10
20
30
40
1919
1939
1959
1979
1999
2019
Year

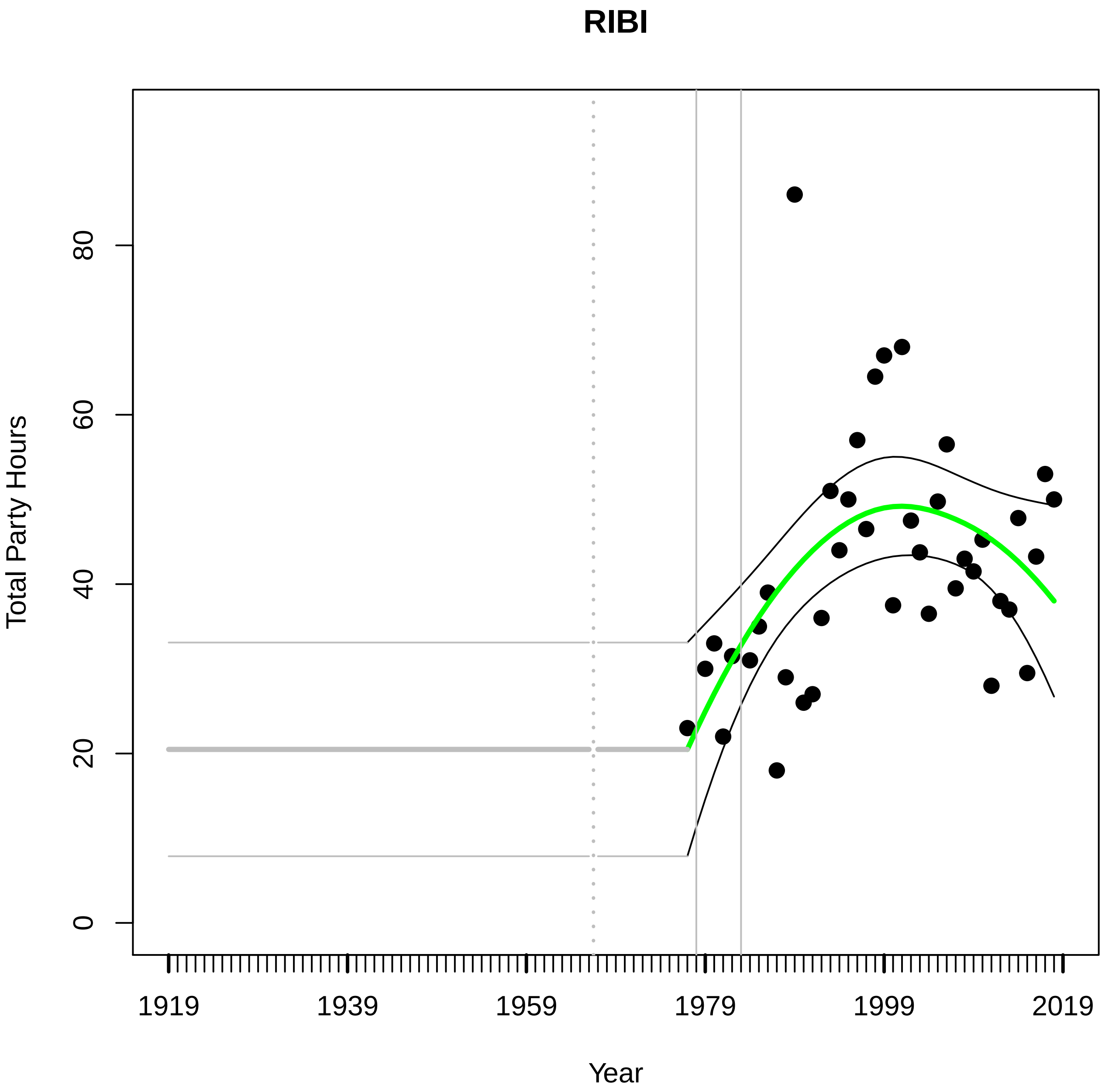
RIBI
Total Party Hours
0
20
40
60
80
1919
1939
1959
1979
1999
2019
Year

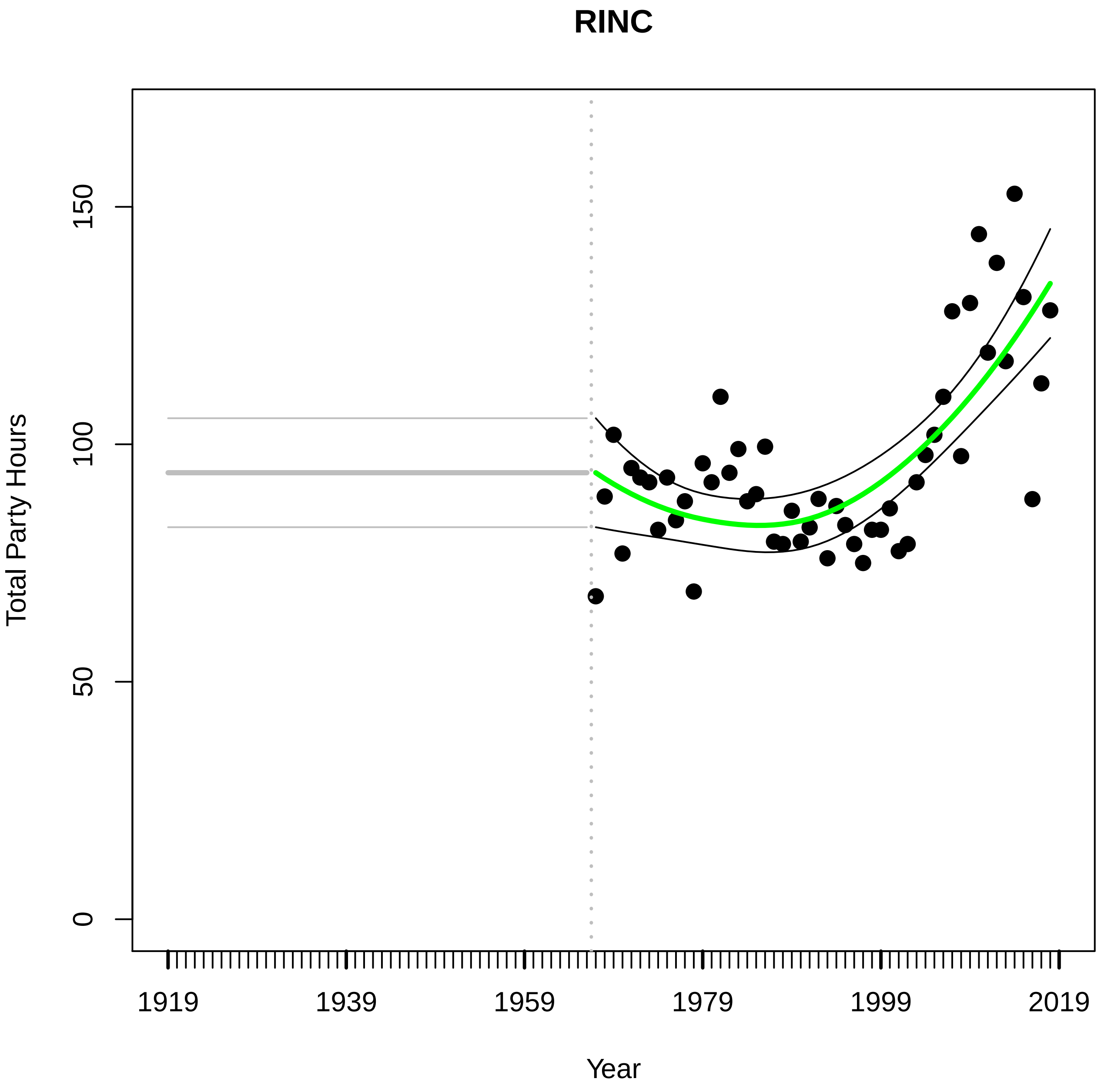

RINC
Total Party Hours
150
100
50
0
1919
1939
1959
1979
1999
2019
Year

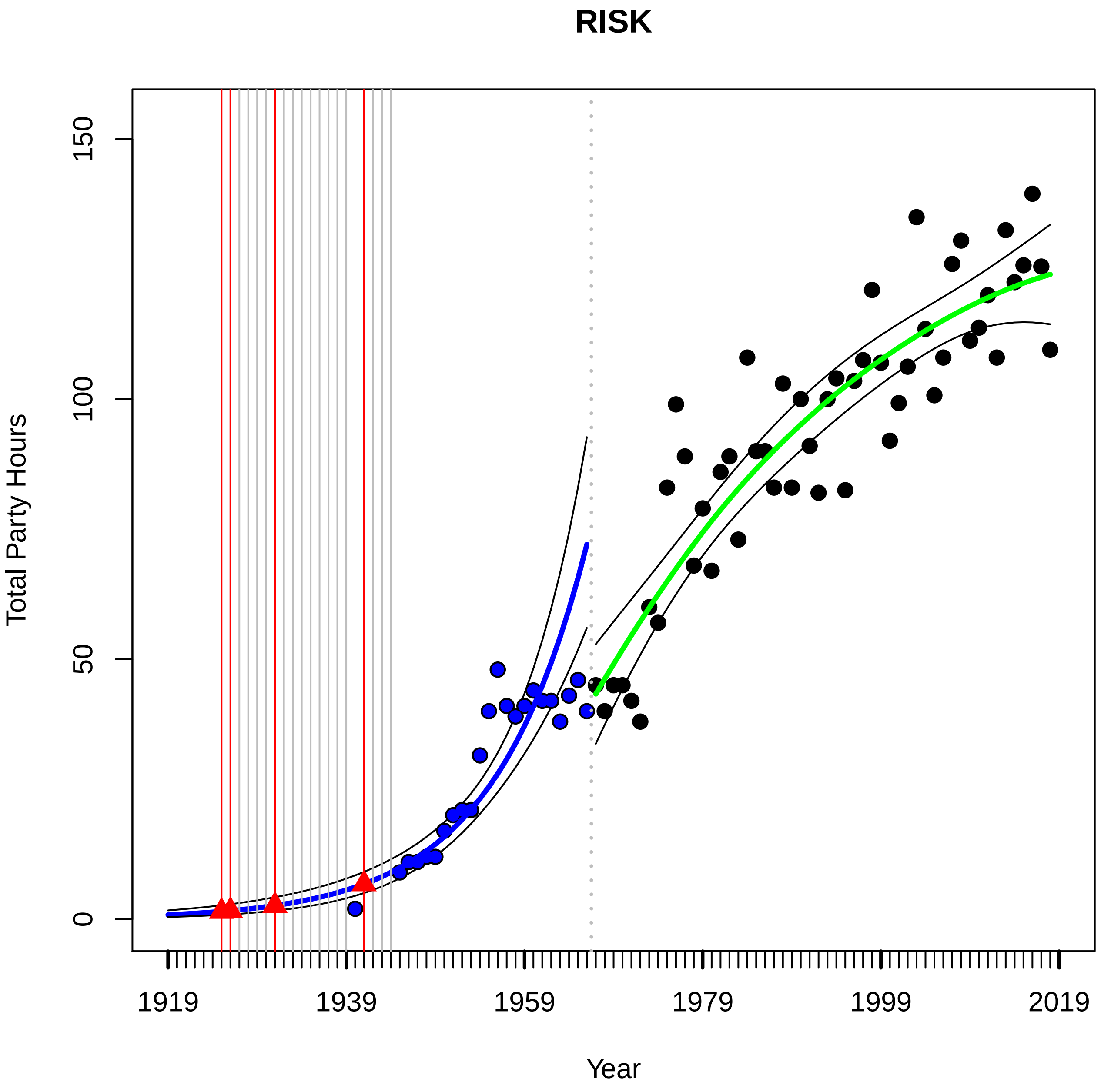

RISK
Total Party Hours
Year
0
50
100
150
1919
1939
1959
1979
1999
2019

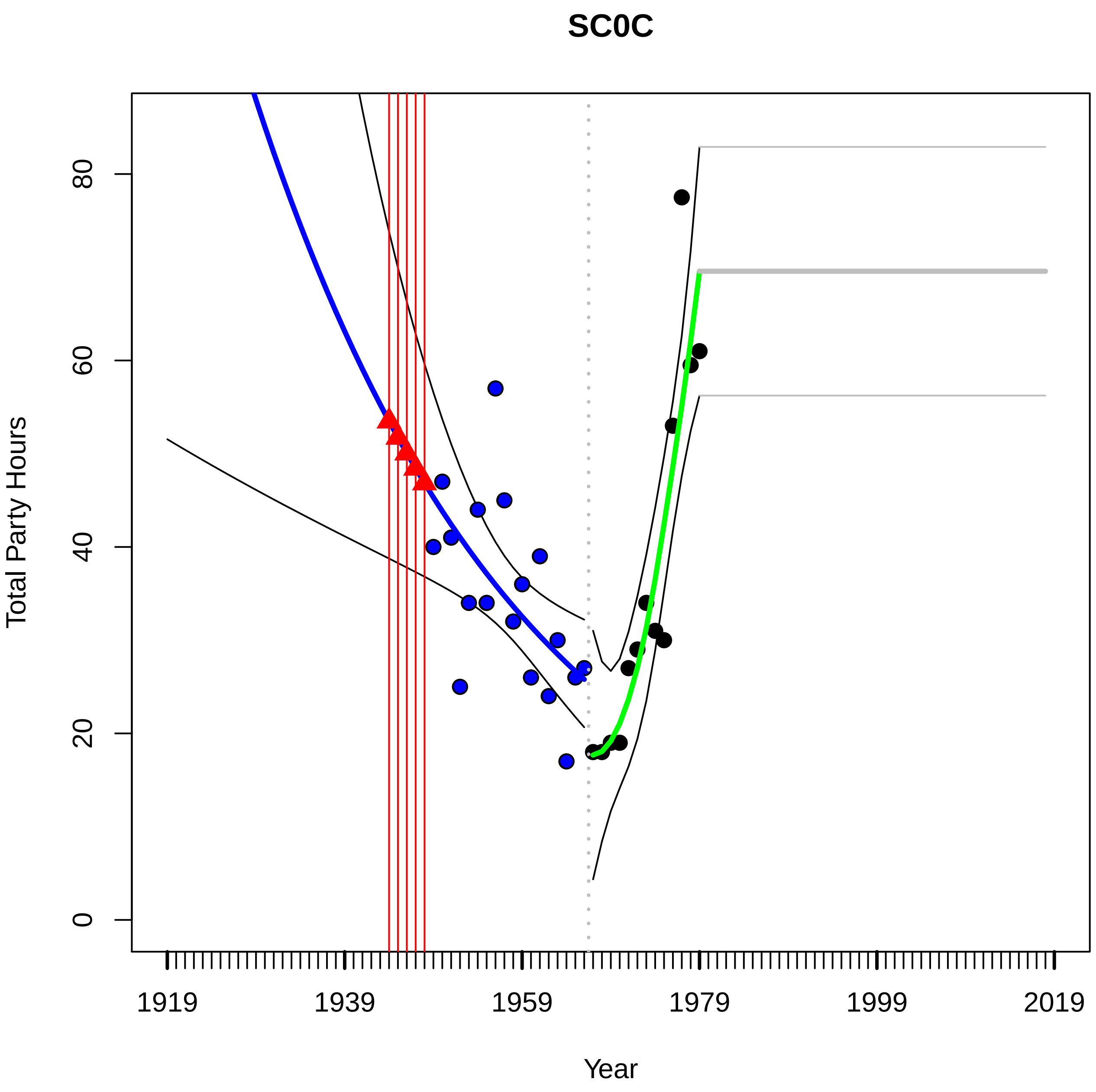

SC0C
Total Party Hours
0
20
40
60
80
1919
1939
1959
1979
1999
2019
Year

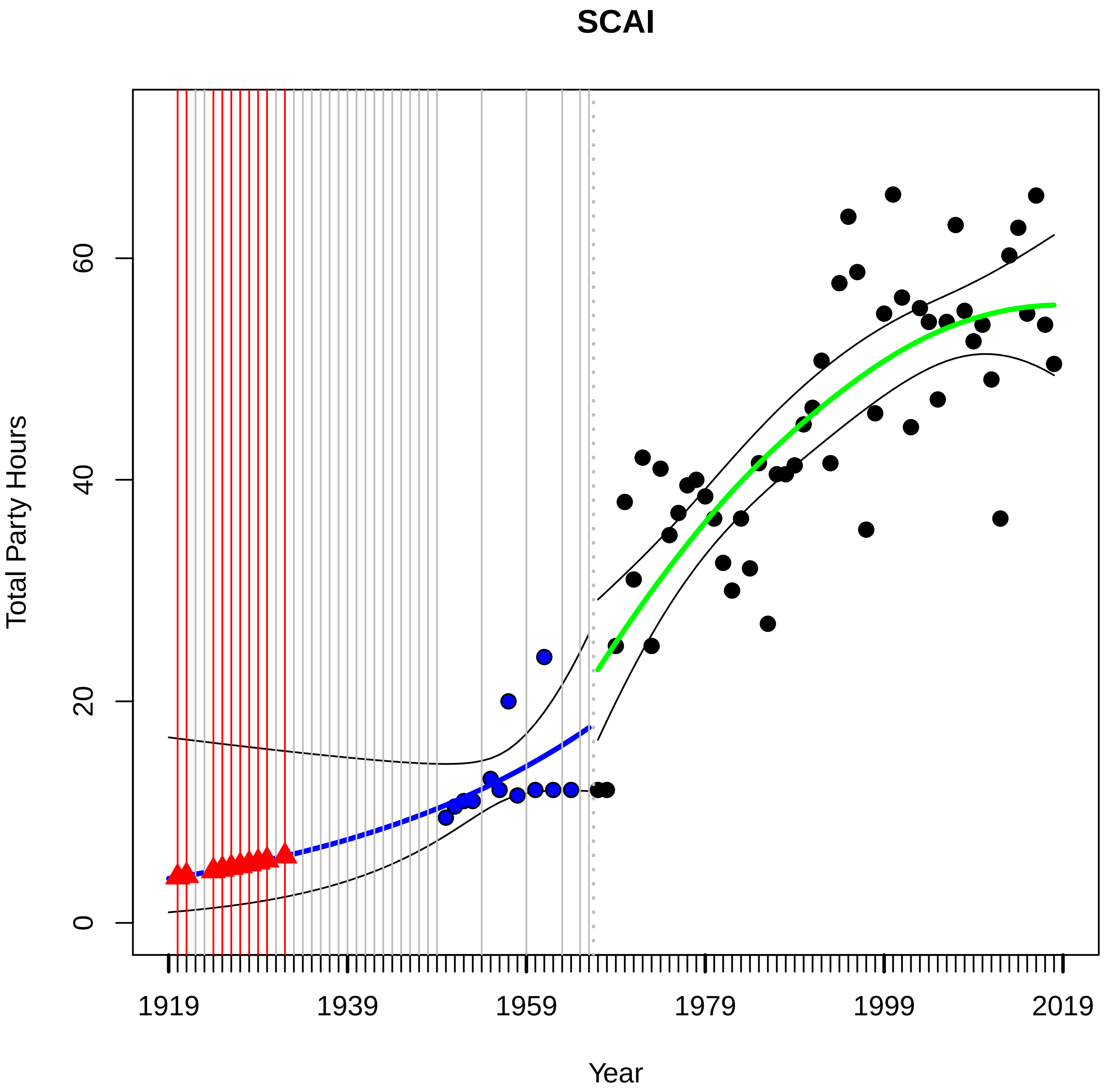
SCAI
Total Party Hours
0
20
40
60
1919
1939
1959
1979
1999
2019
Year

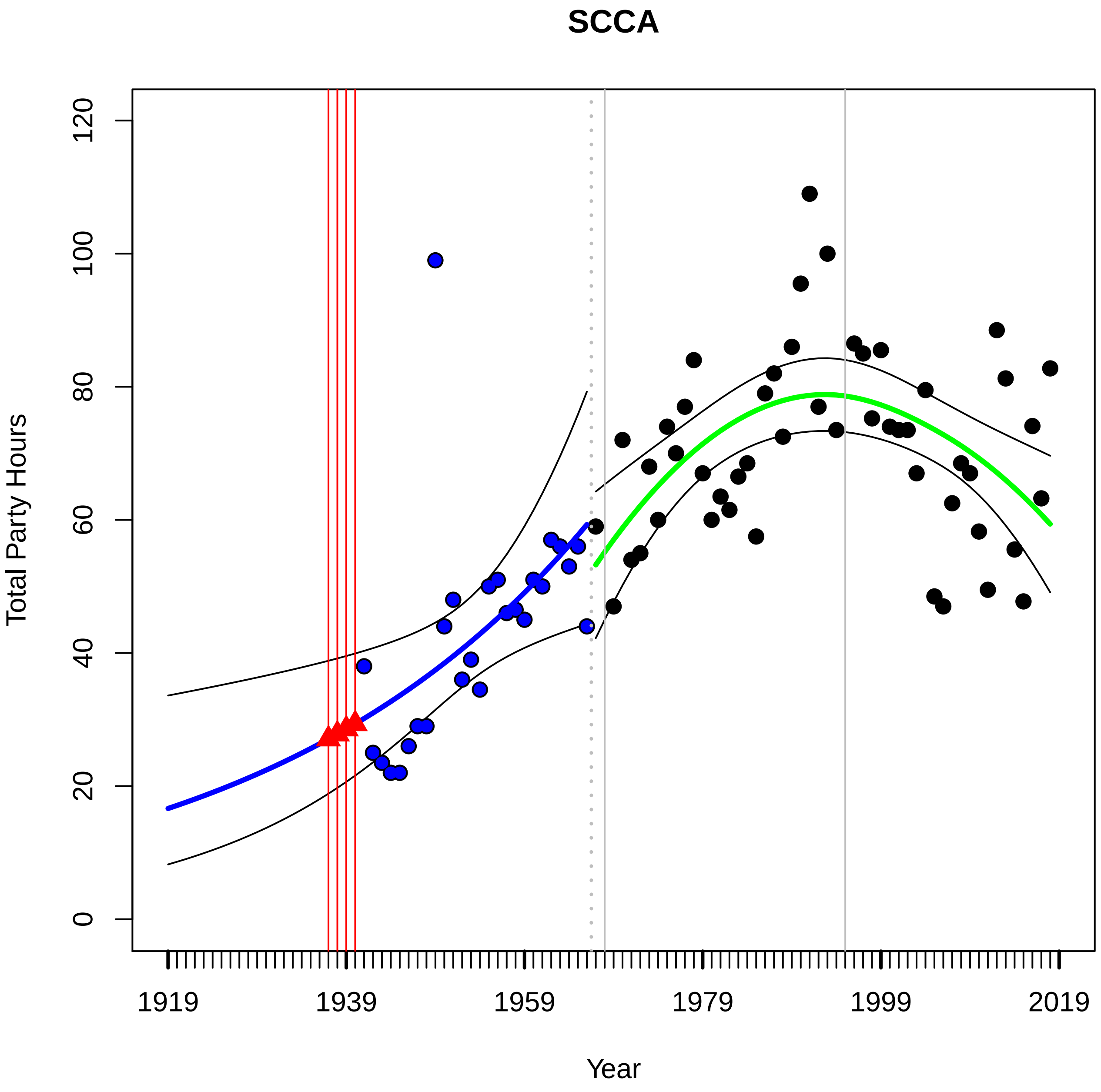

SCCA
Total Party Hours
Year
0
20
40
60
80
100
120
1919
1939
1959
1979
1999
2019

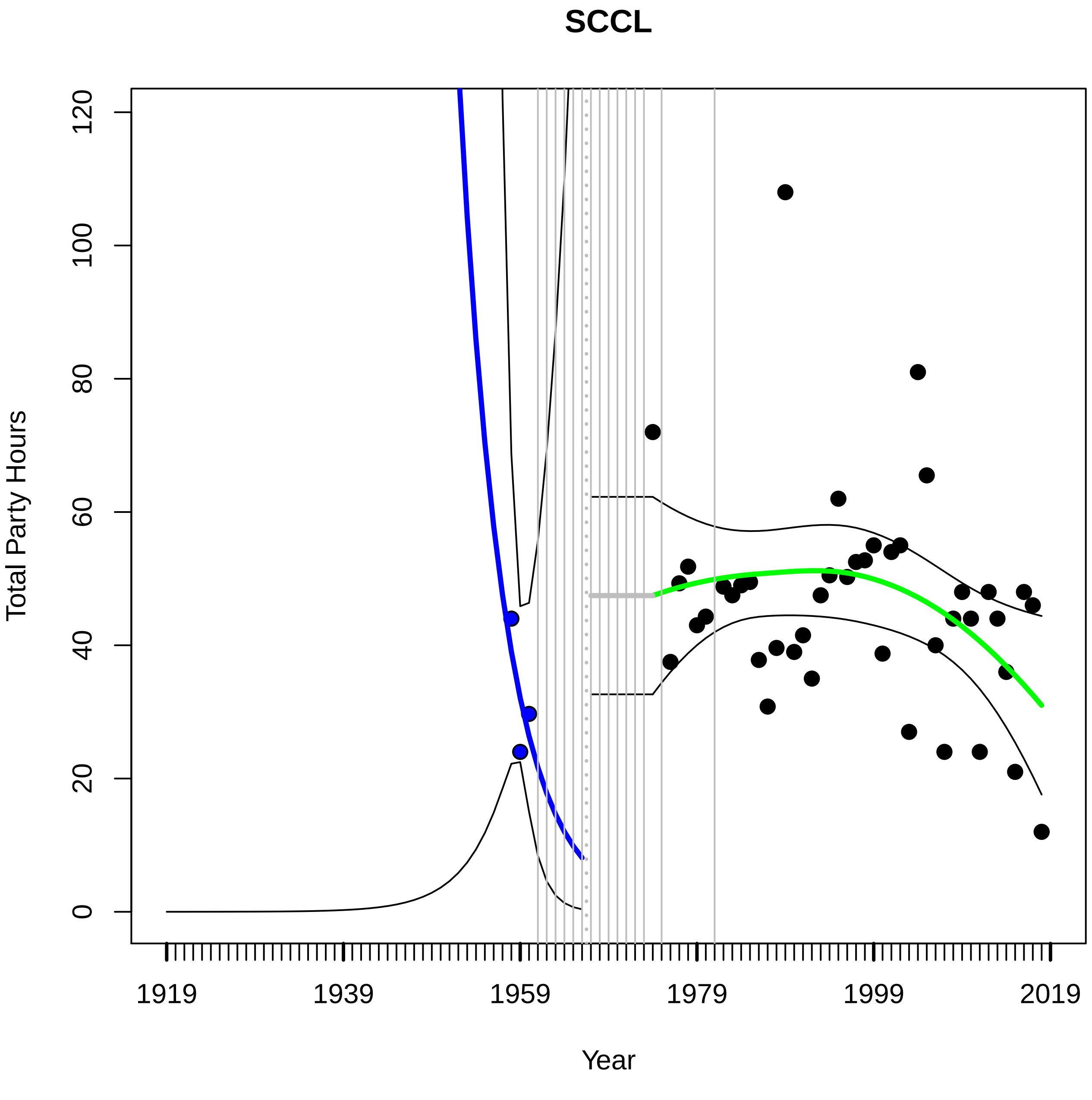
SCCL
Total Party Hours
Year
0
20
40
60
80
100
120
1919
1939
1959
1979
1999
2019

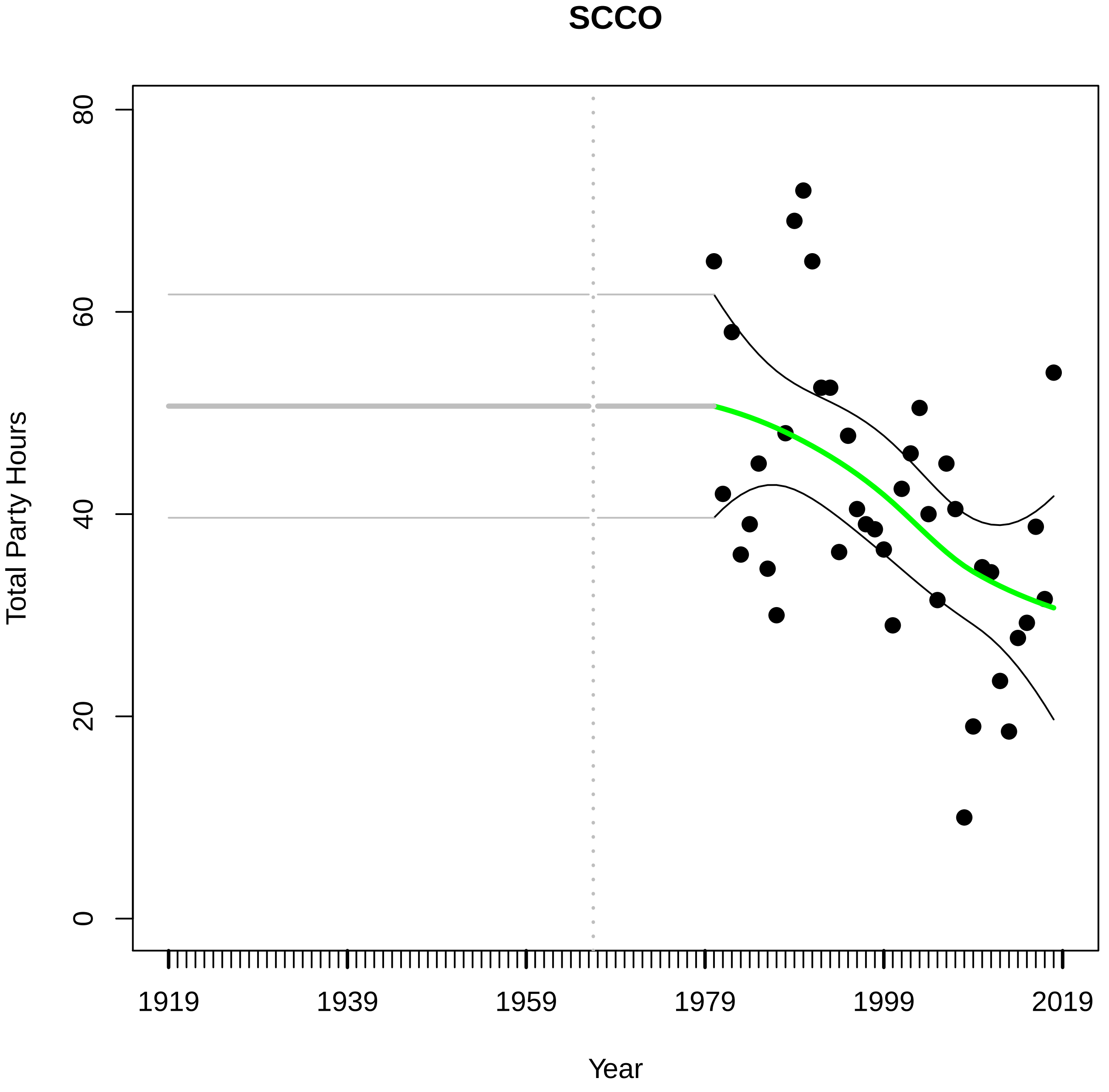
SCCO
Total Party Hours
0
20
40
60
80
1919
1939
1959
1979
1999
2019
Year

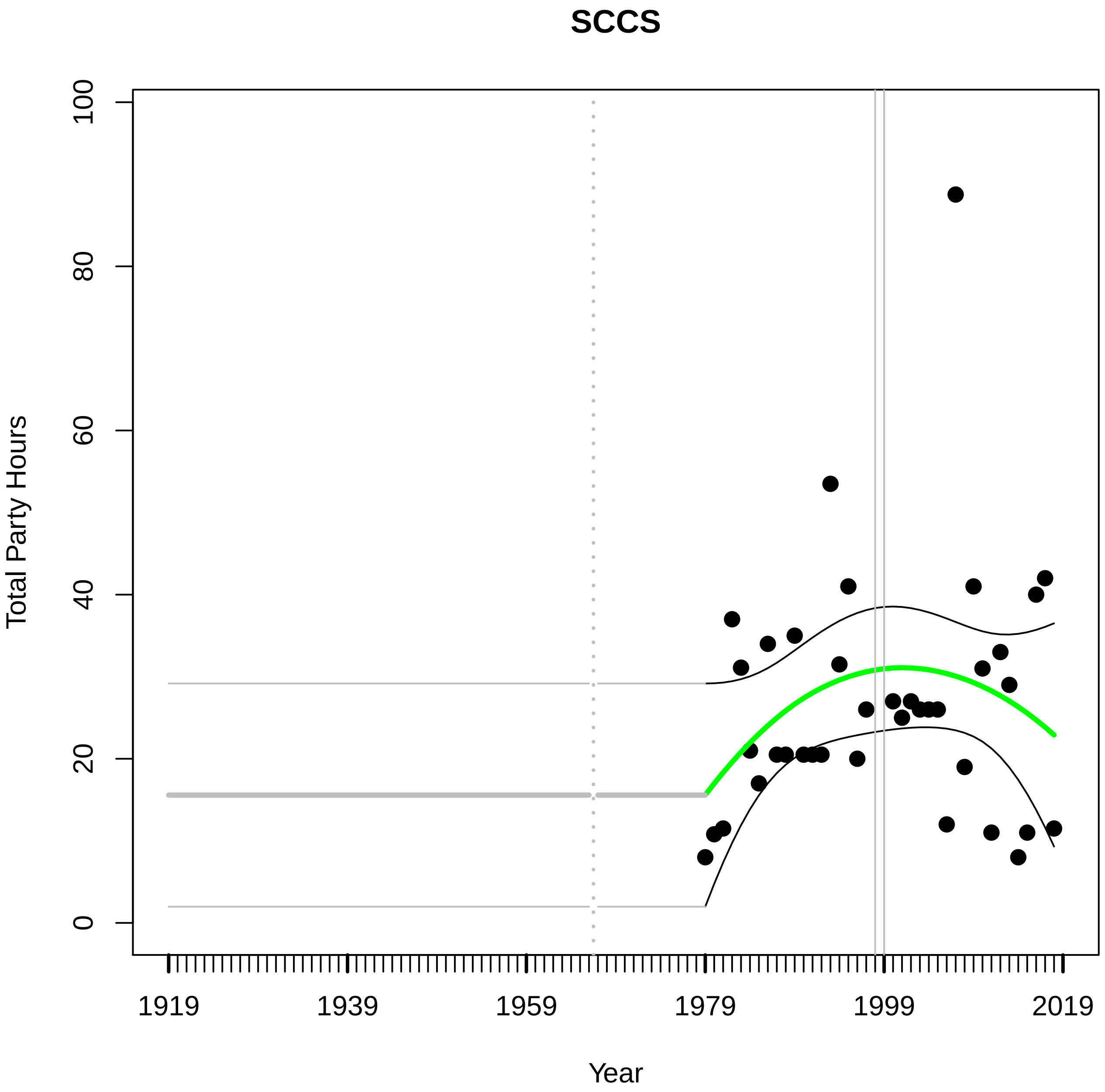

SCCS
Total Party Hours
0
20
40
60
80
100
1919
1939
1959
1979
1999
2019
Year

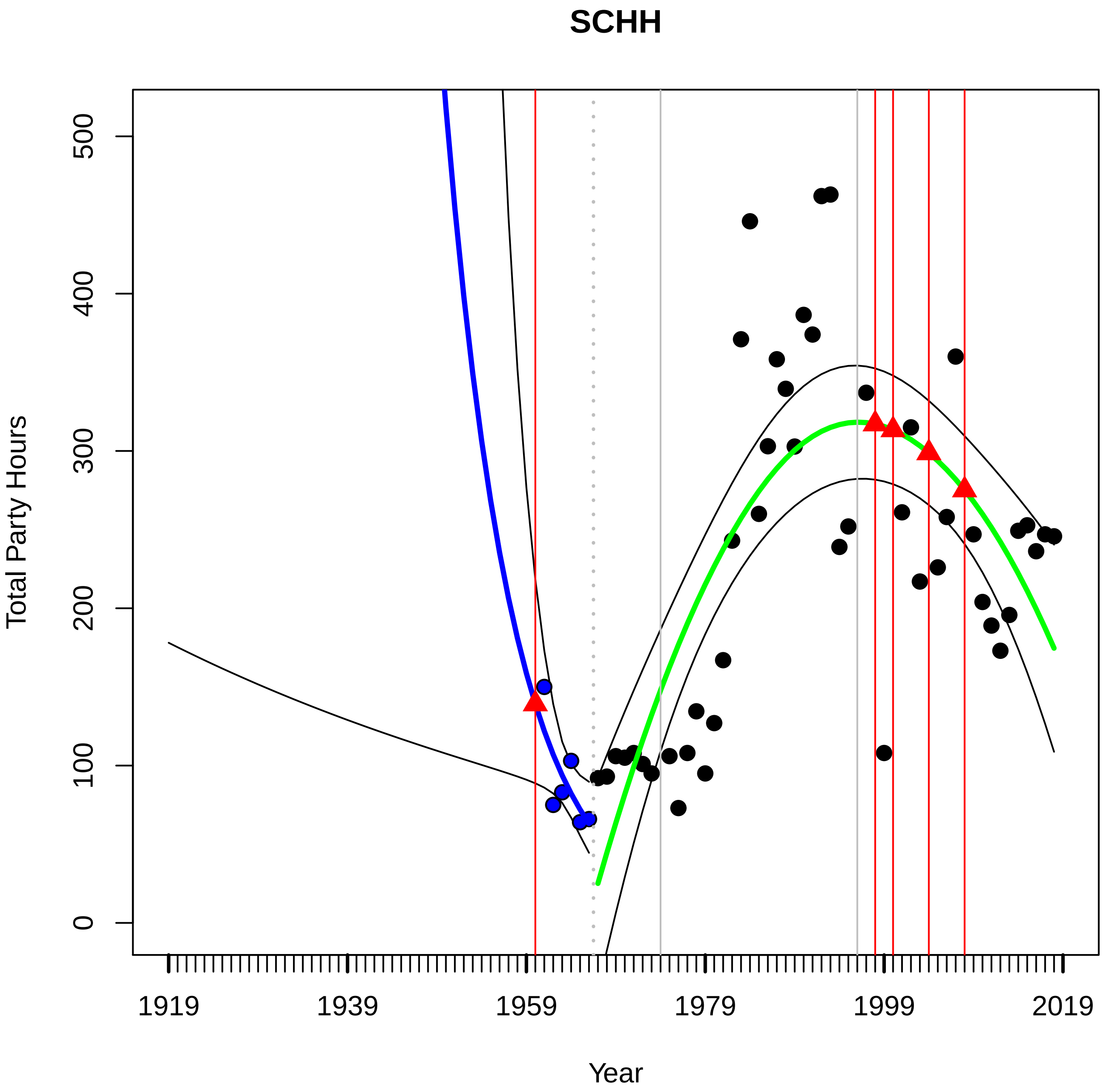

SCHH
Total Party Hours
0
100
200
300
400
500
1919
1939
1959
1979
1999
2019
Year

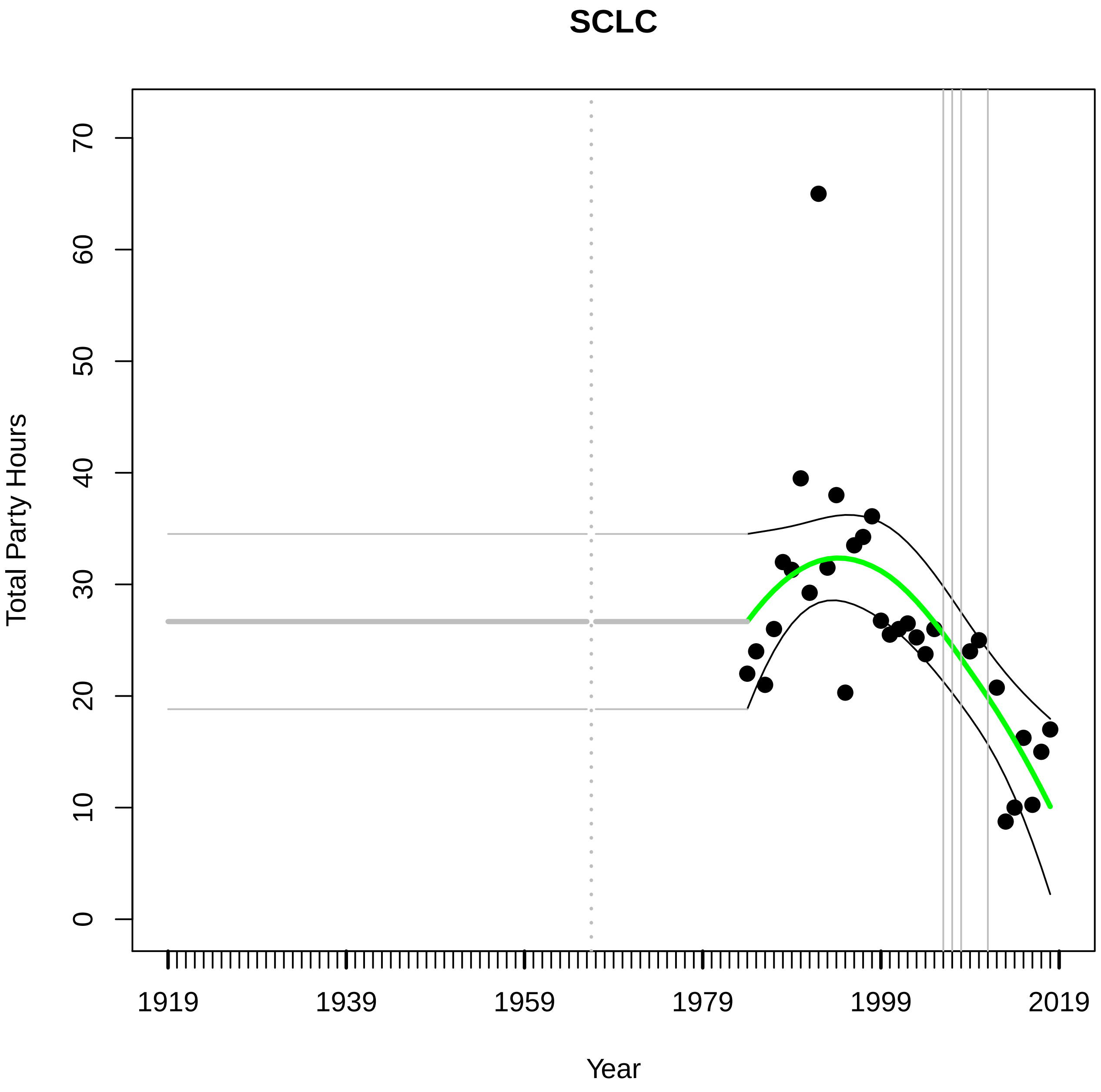
SCLC
Total Party Hours
Year
0
10
20
30
40
50
60
70
1919
1939
1959
1979
1999
2019

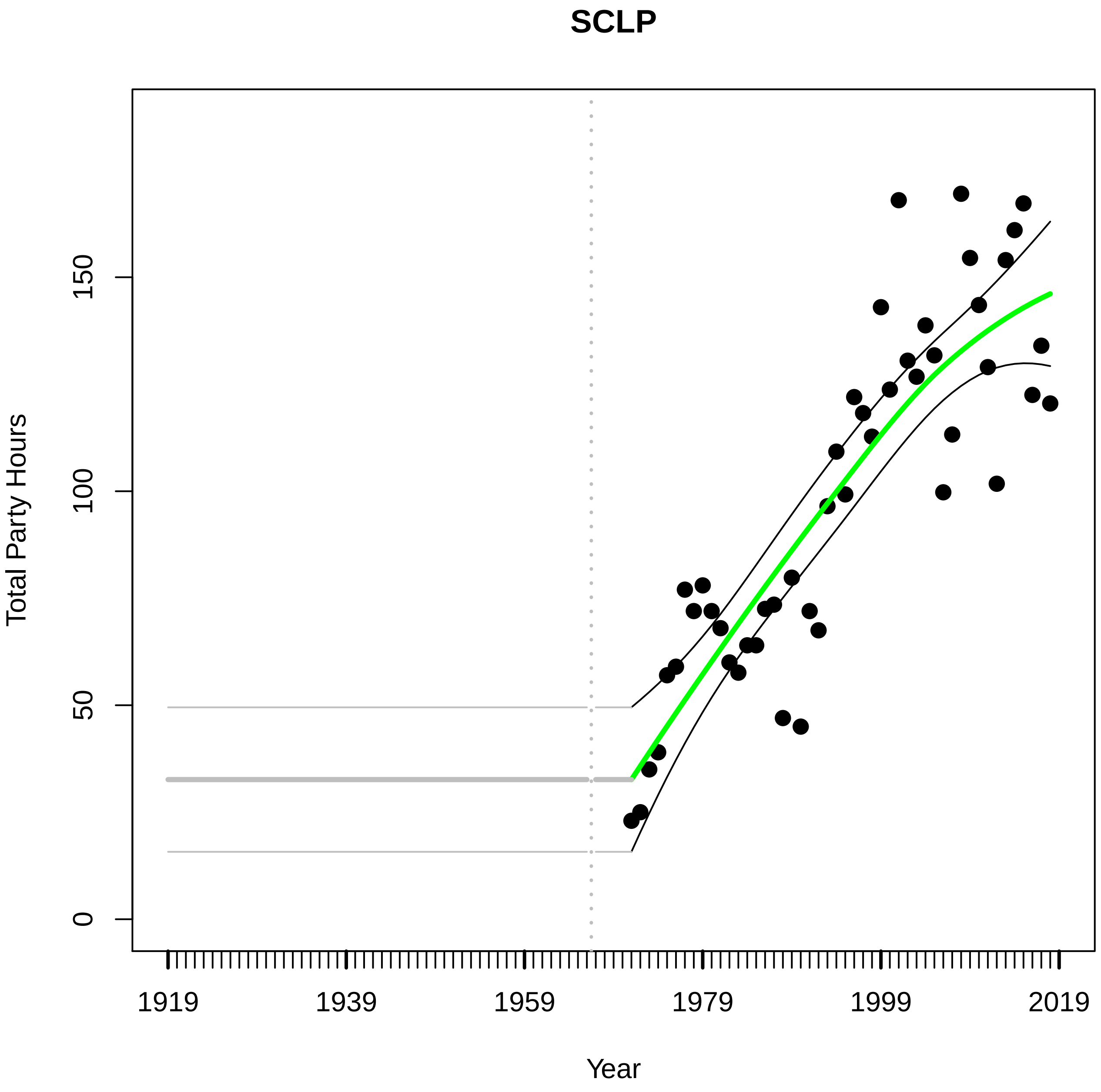

SCLP
Total Party Hours
0
50
100
150
1919
1939
1959
1979
1999
2019
Year

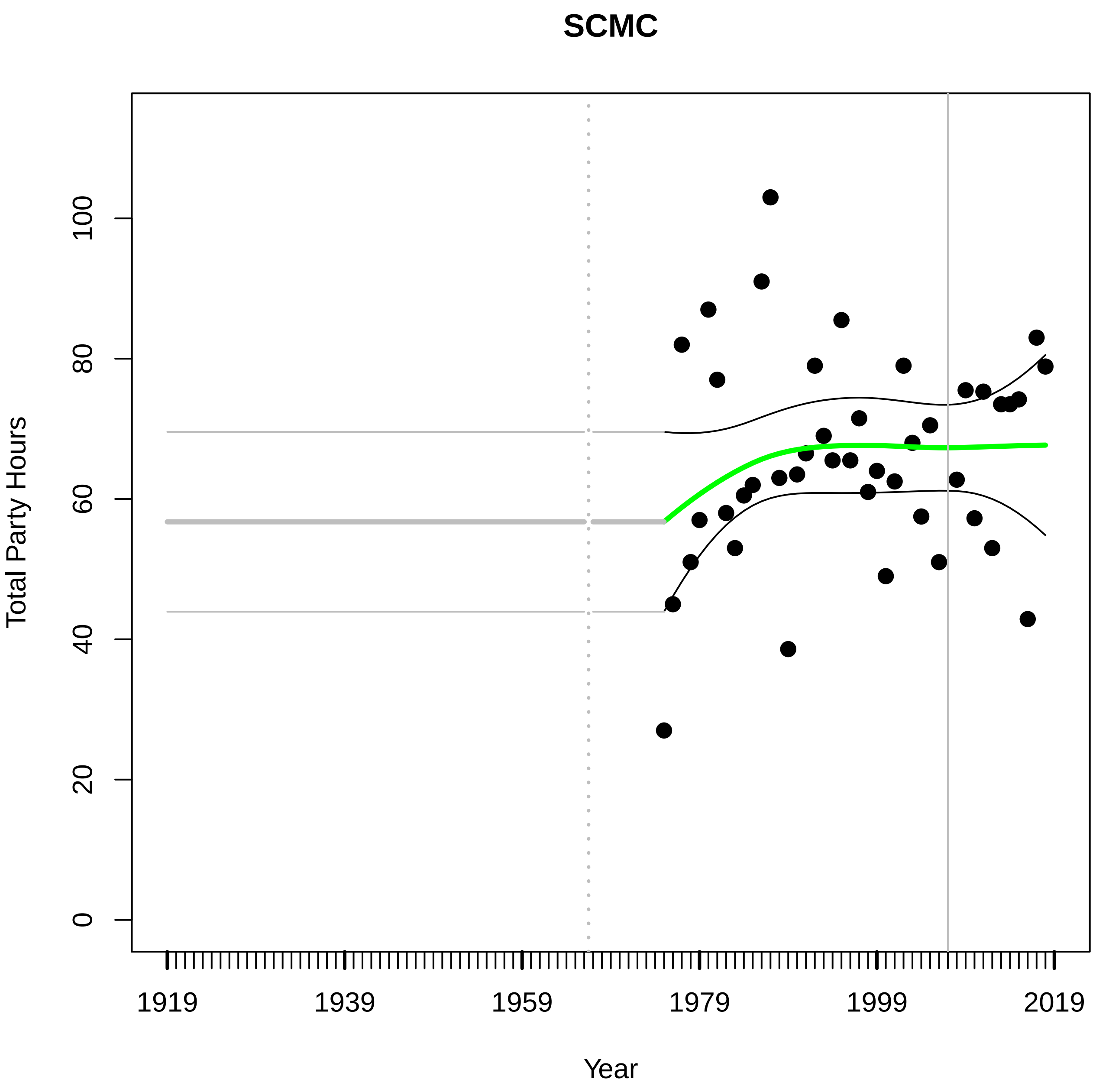

SCMC
Total Party Hours
Year
0
20
40
60
80
100
1919
1939
1959
1979
1999
2019

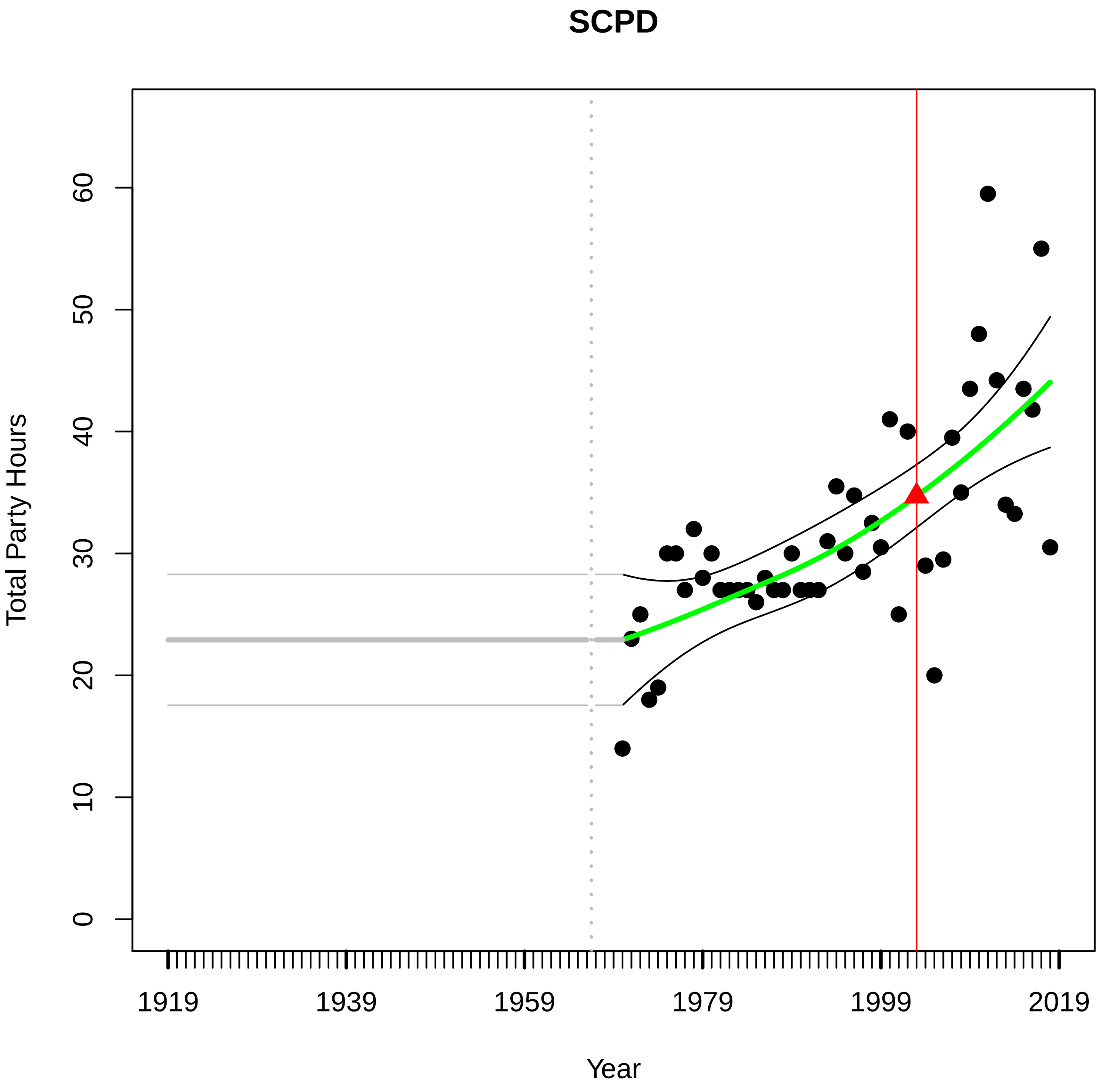

SCPD
Total Party Hours
0
10
20
30
40
50
60
1919
1939
1959
1979
1999
2019
Year

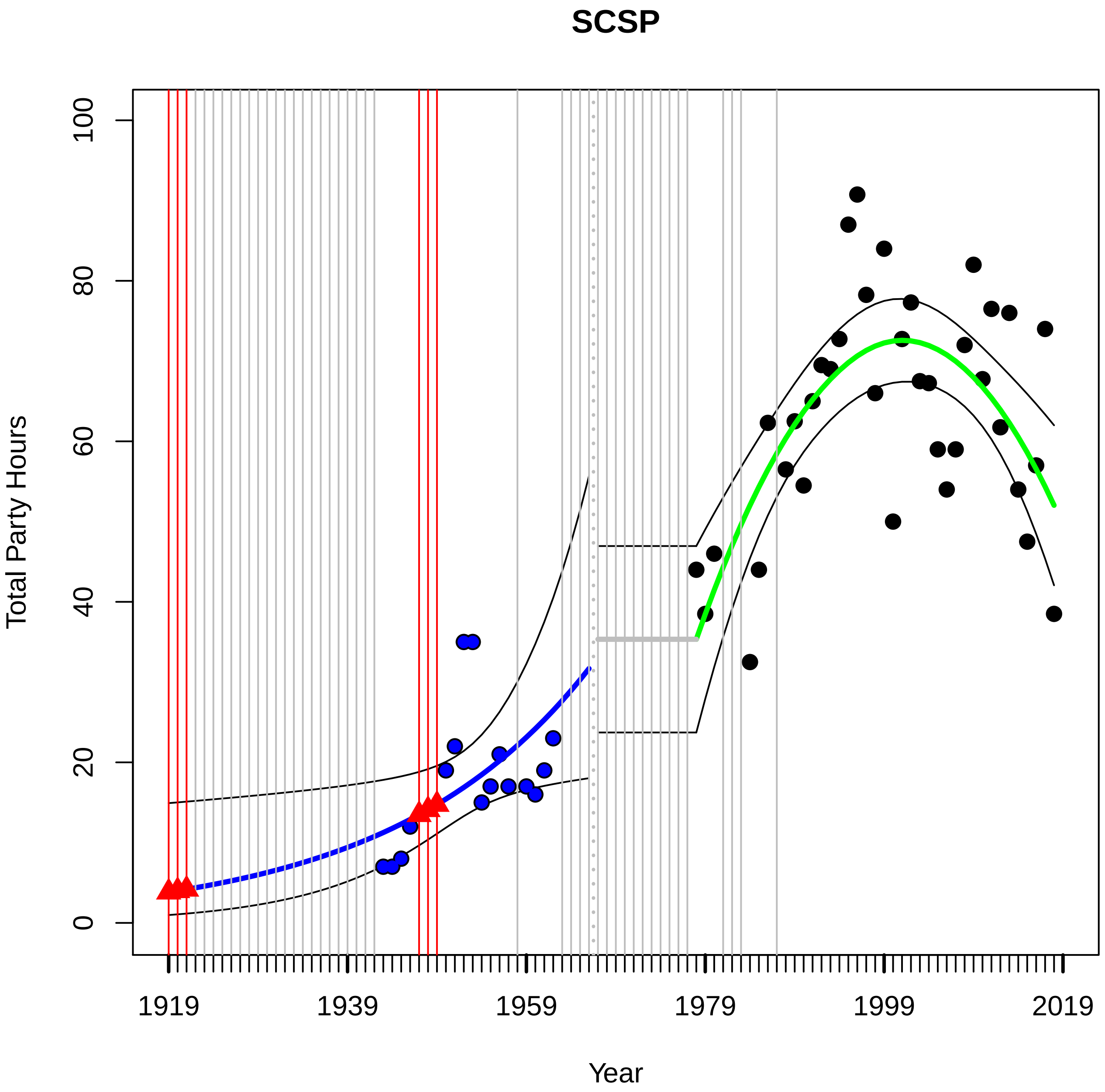

SCSP
Total Party Hours
0
20
40
60
80
100
1919
1939
1959
1979
1999
2019
Year

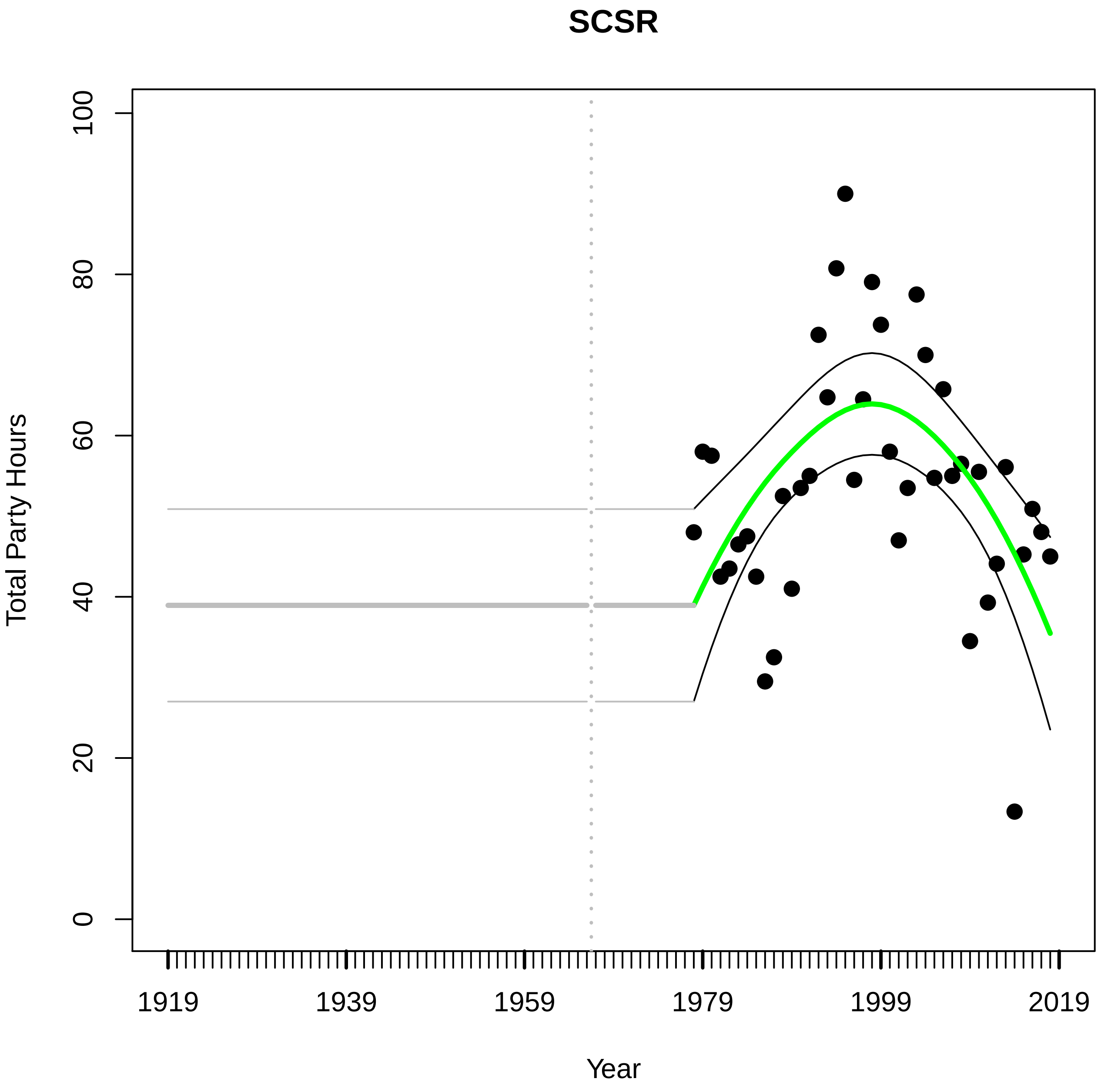
SCSR
Total Party Hours
Year
0
20
40
60
80
100
1919
1939
1959
1979
1999
2019

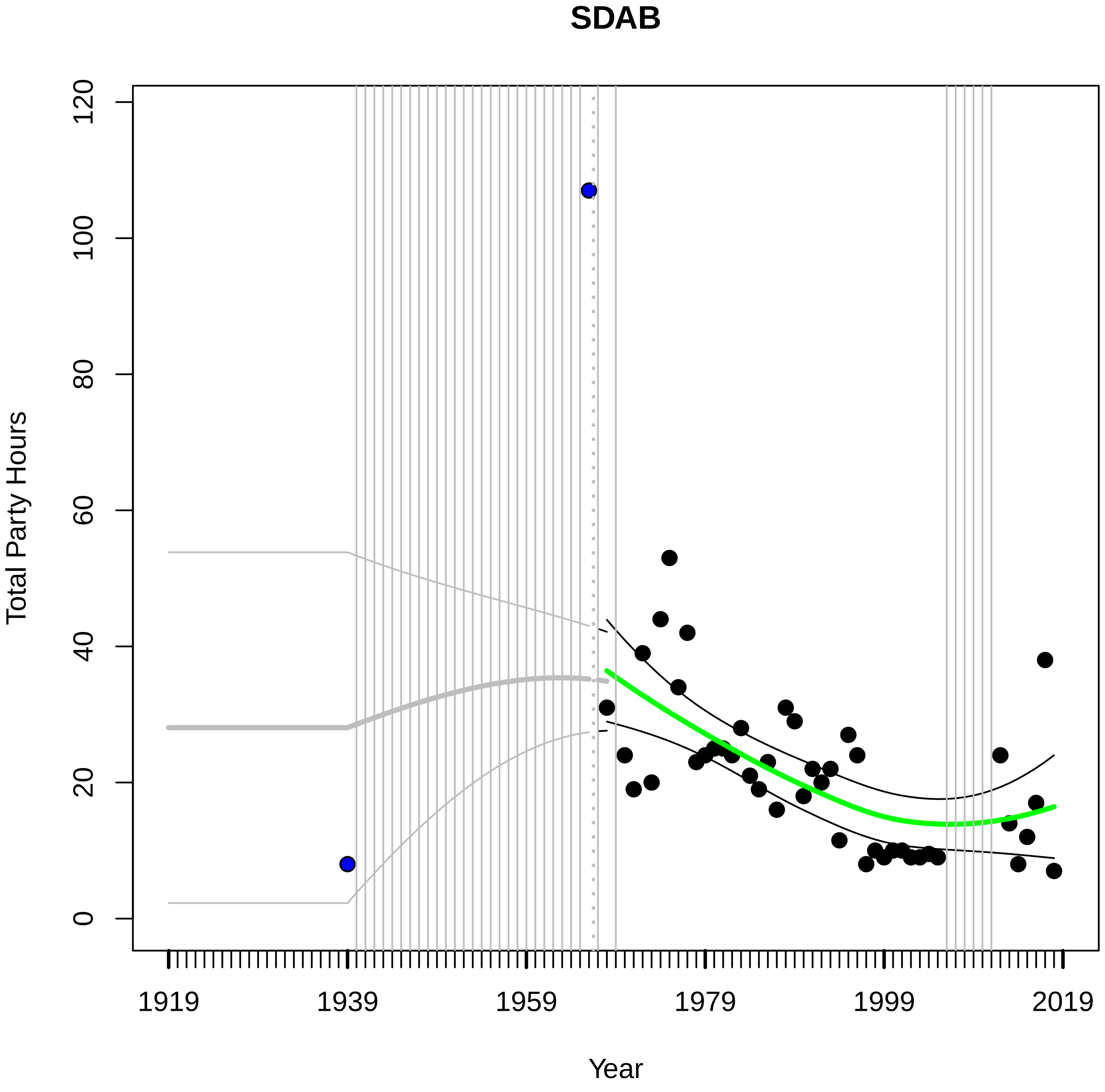

SDAB
Total Party Hours
Year
0
20
40
60
80
100
120
1919
1939
1959
1979
1999
2019

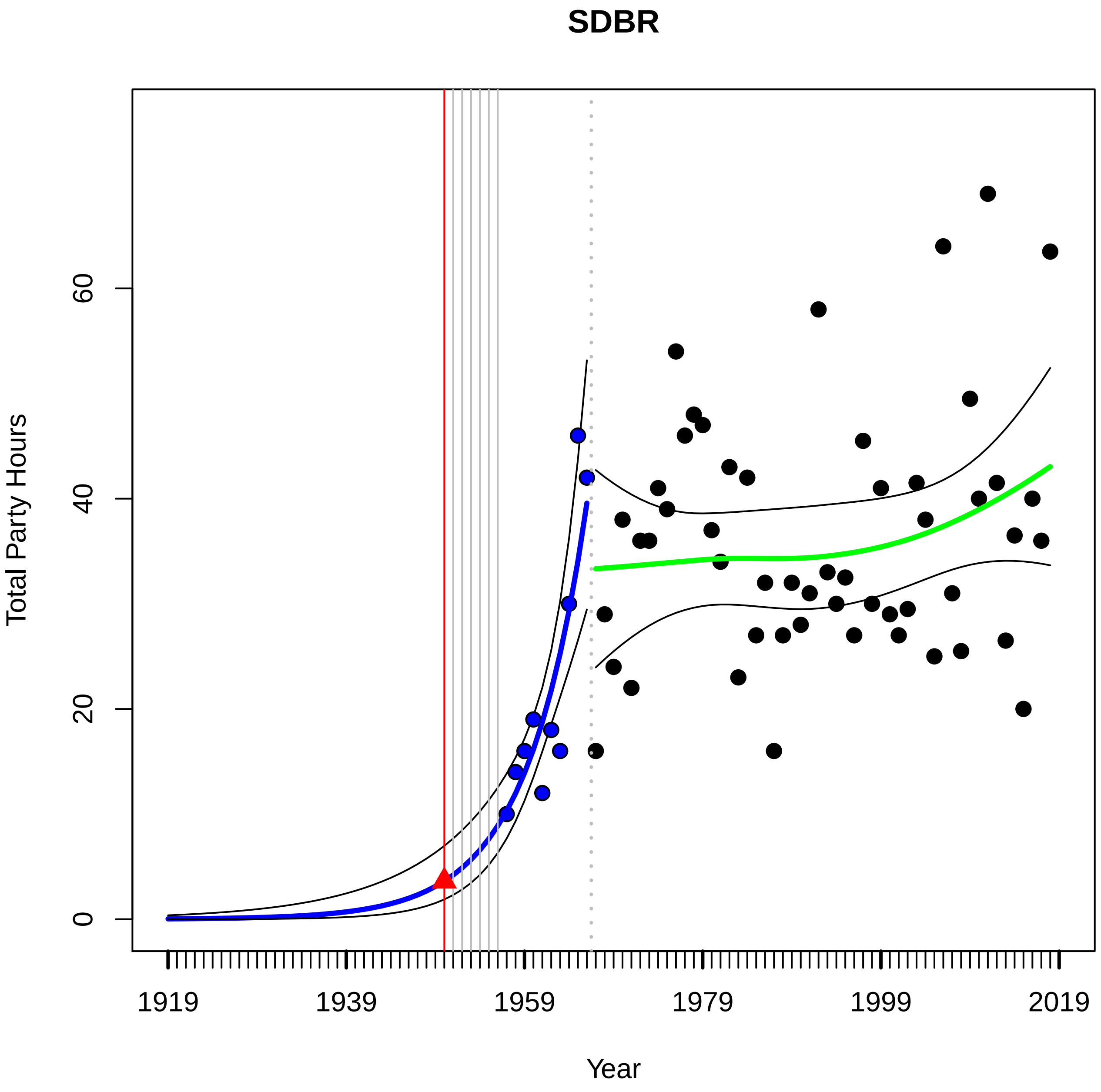

SDBR
Total Party Hours
0
20
40
60
1919
1939
1959
1979
1999
2019
Year

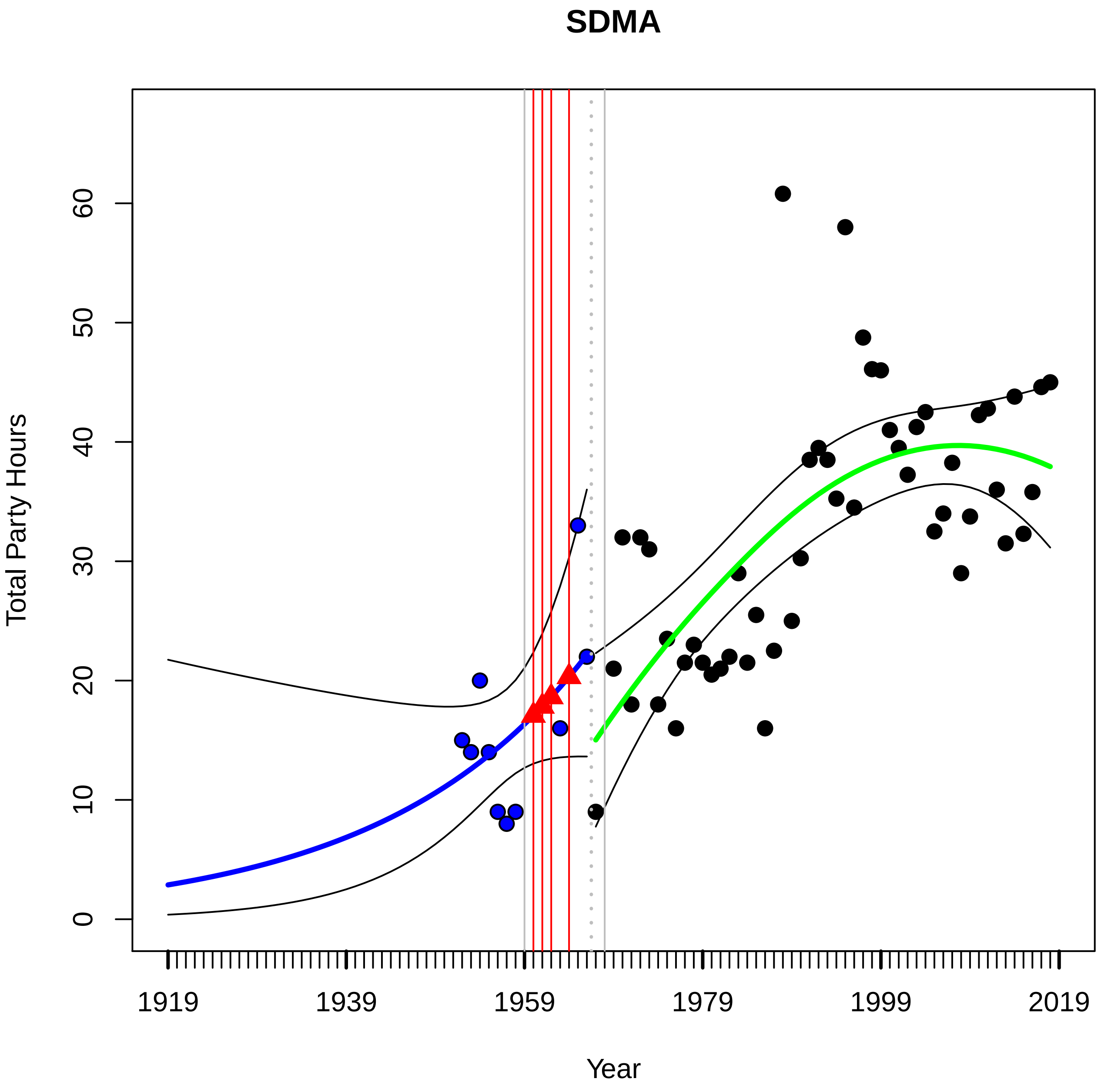
SDMA
Total Party Hours
0
10
20
30
40
50
60
1919
1939
1959
1979
1999
2019
Year

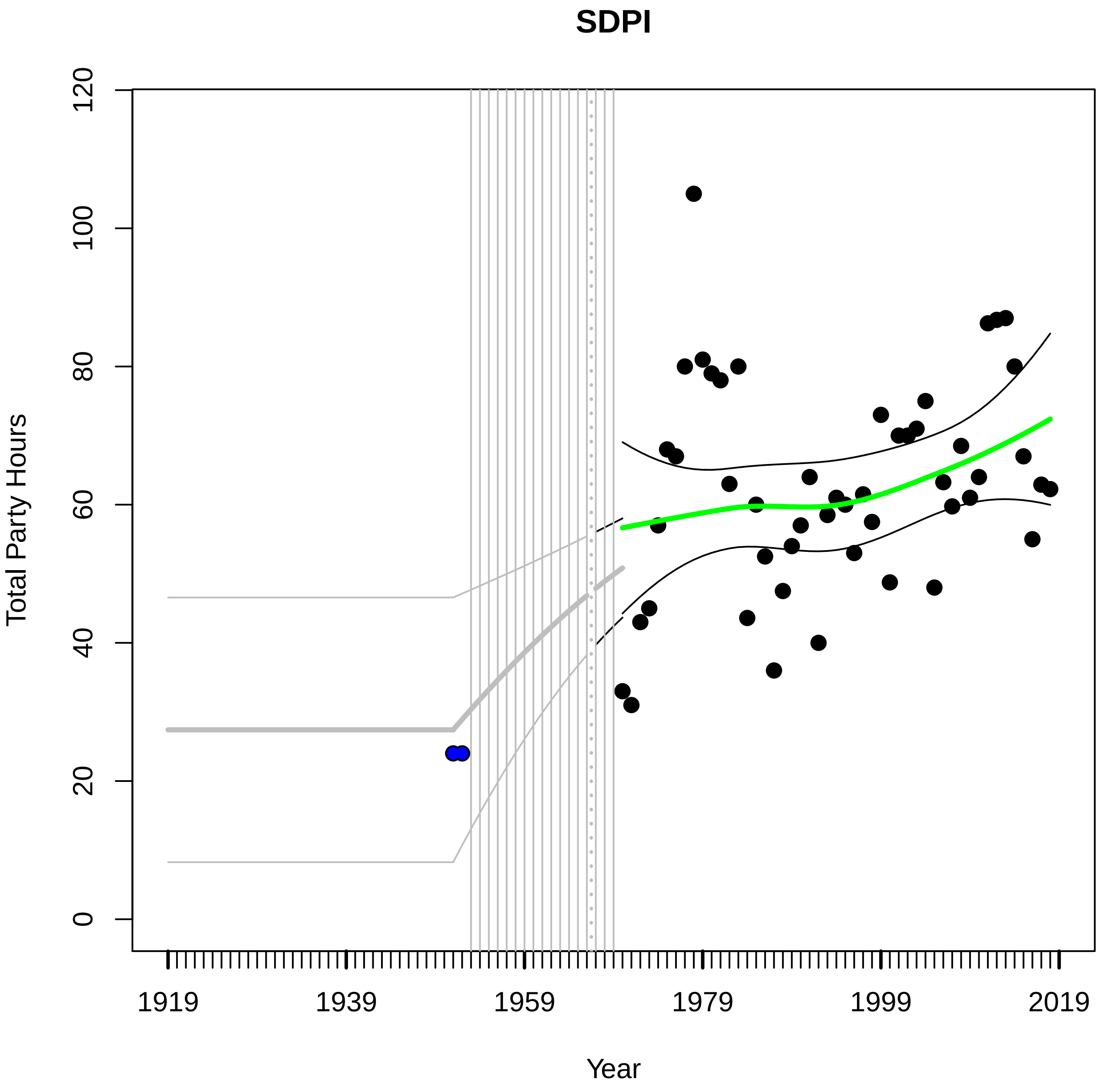

SDPI
Total Party Hours
0
20
40
60
80
100
120
1919
1939
1959
1979
1999
2019
Year

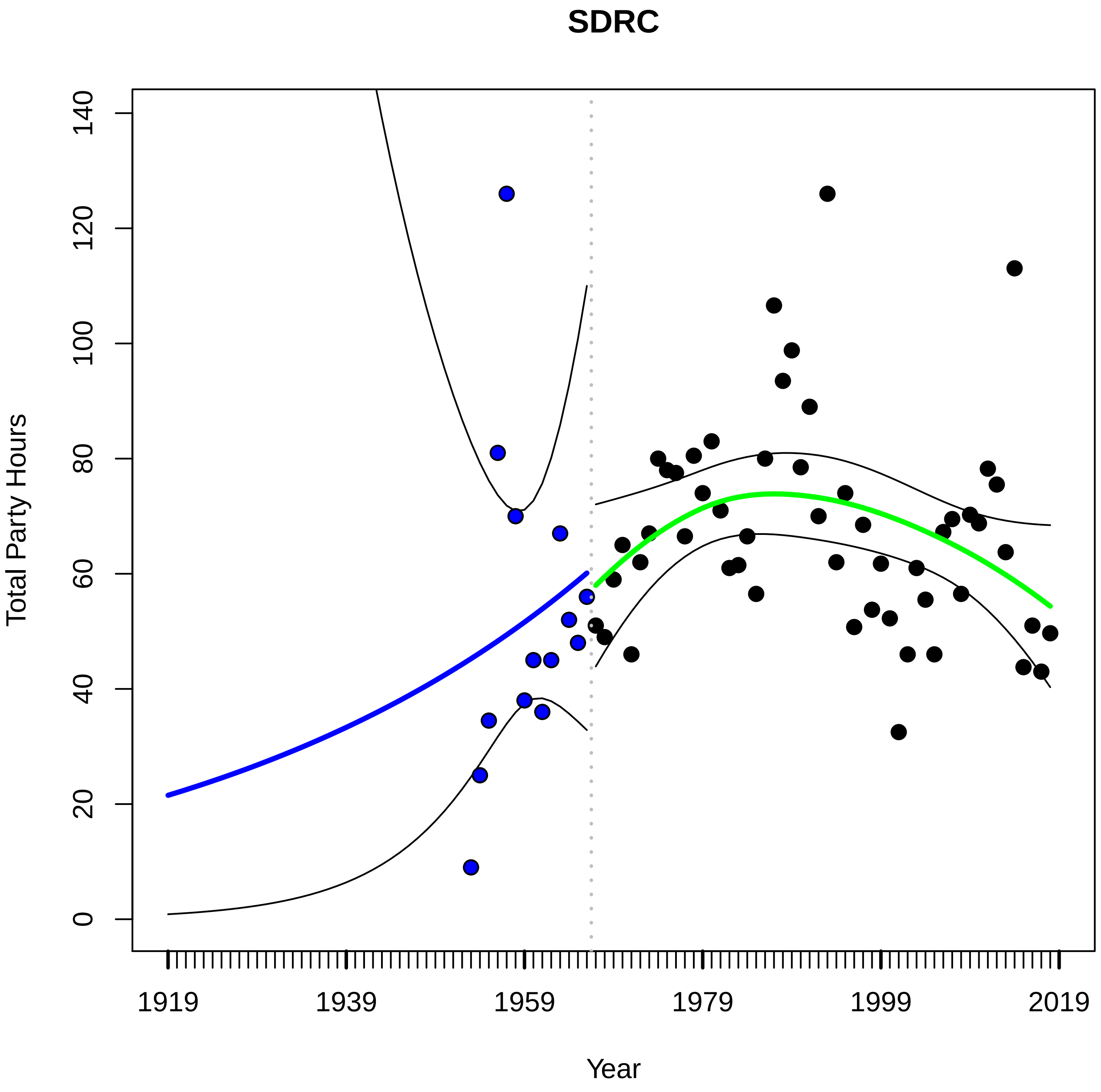

SDRC
Total Party Hours
Year
0
20
40
60
80
100
120
140
1919
1939
1959
1979
1999
2019

SDSF

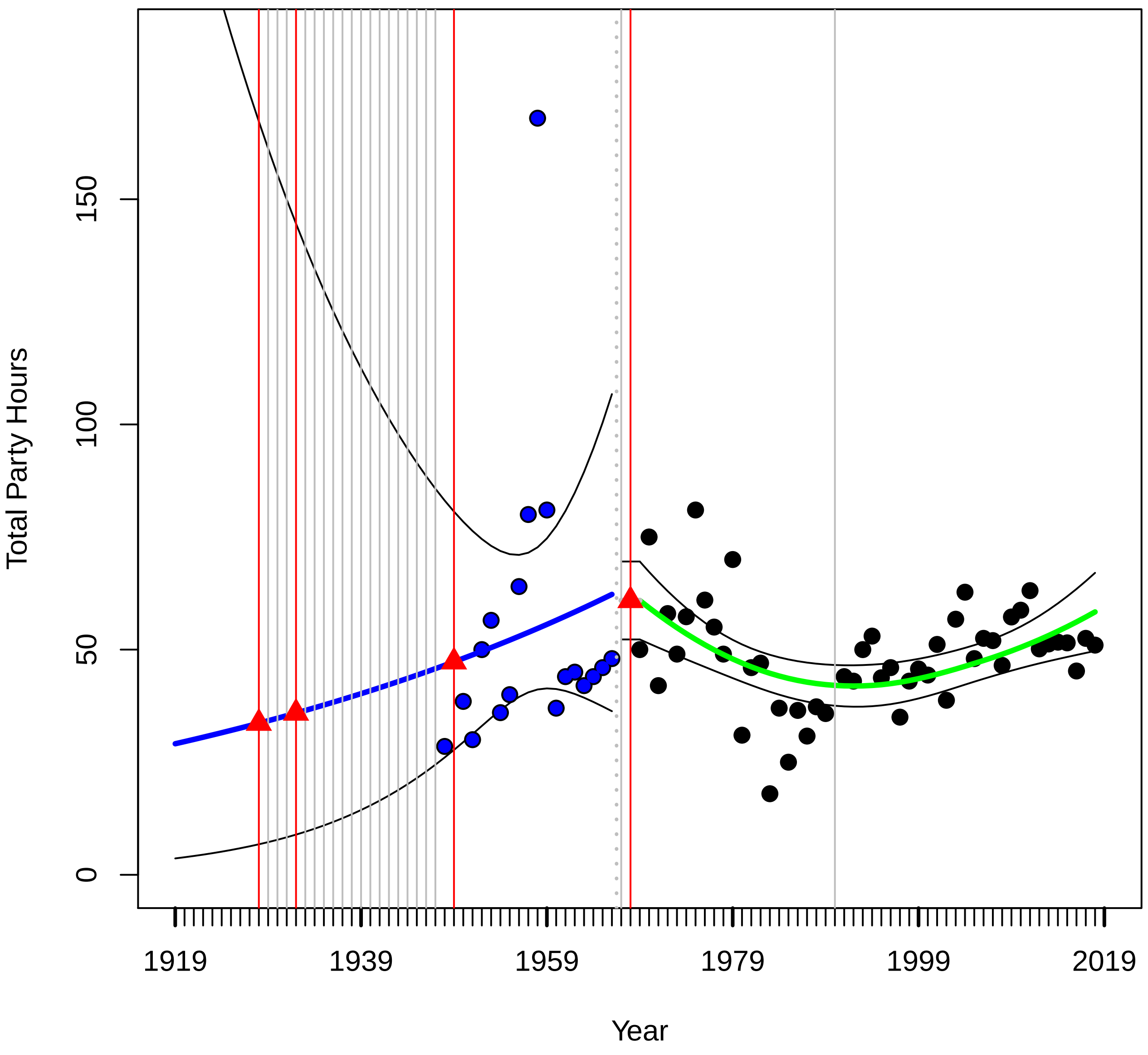

Total Party Hours
0
50
100
150
1919
1939
1959
1979
1999
2019
Year

SDSL

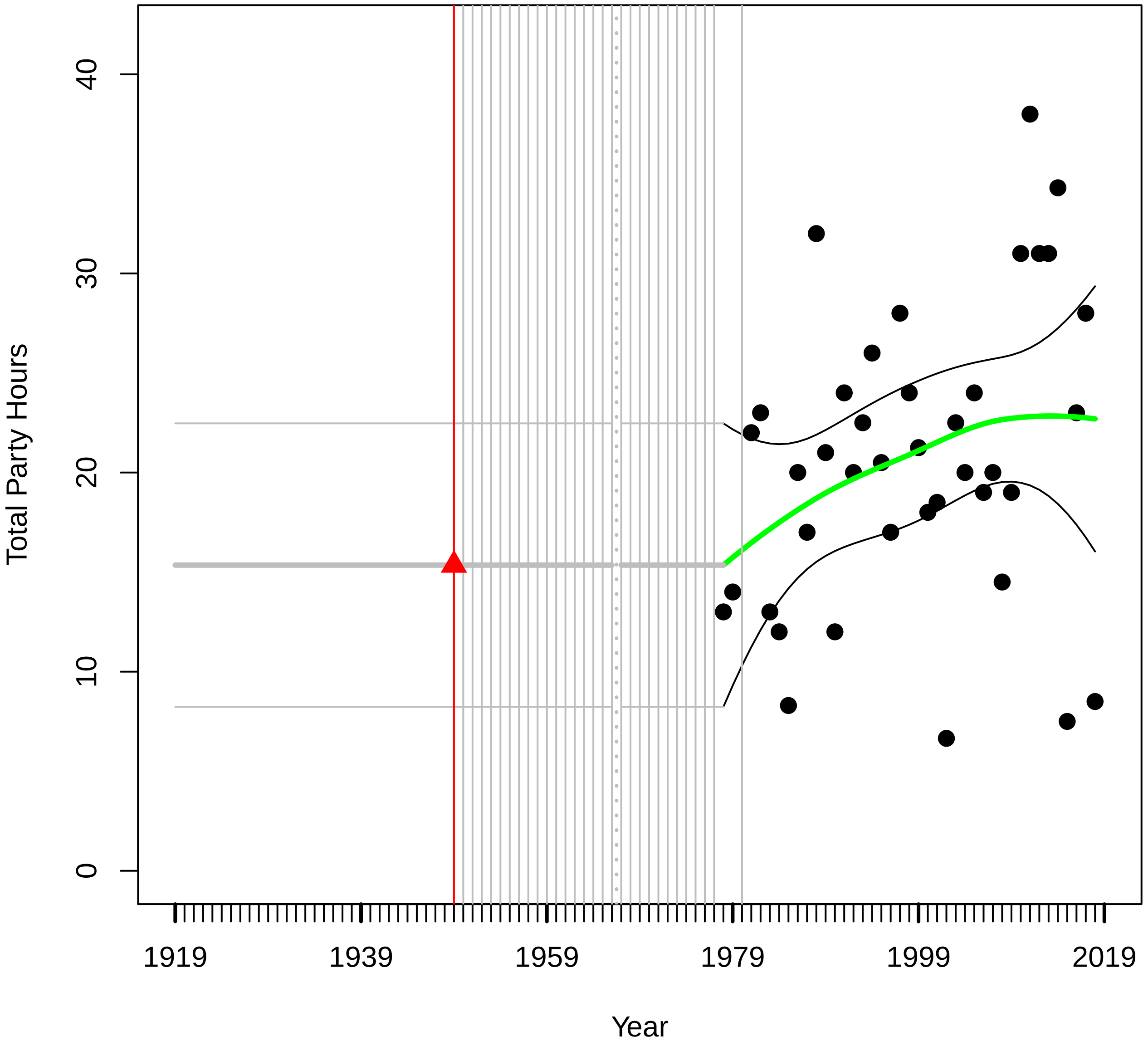

Total Party Hours
0
10
20
30
40
1919
1939
1959
1979
1999
2019
Year

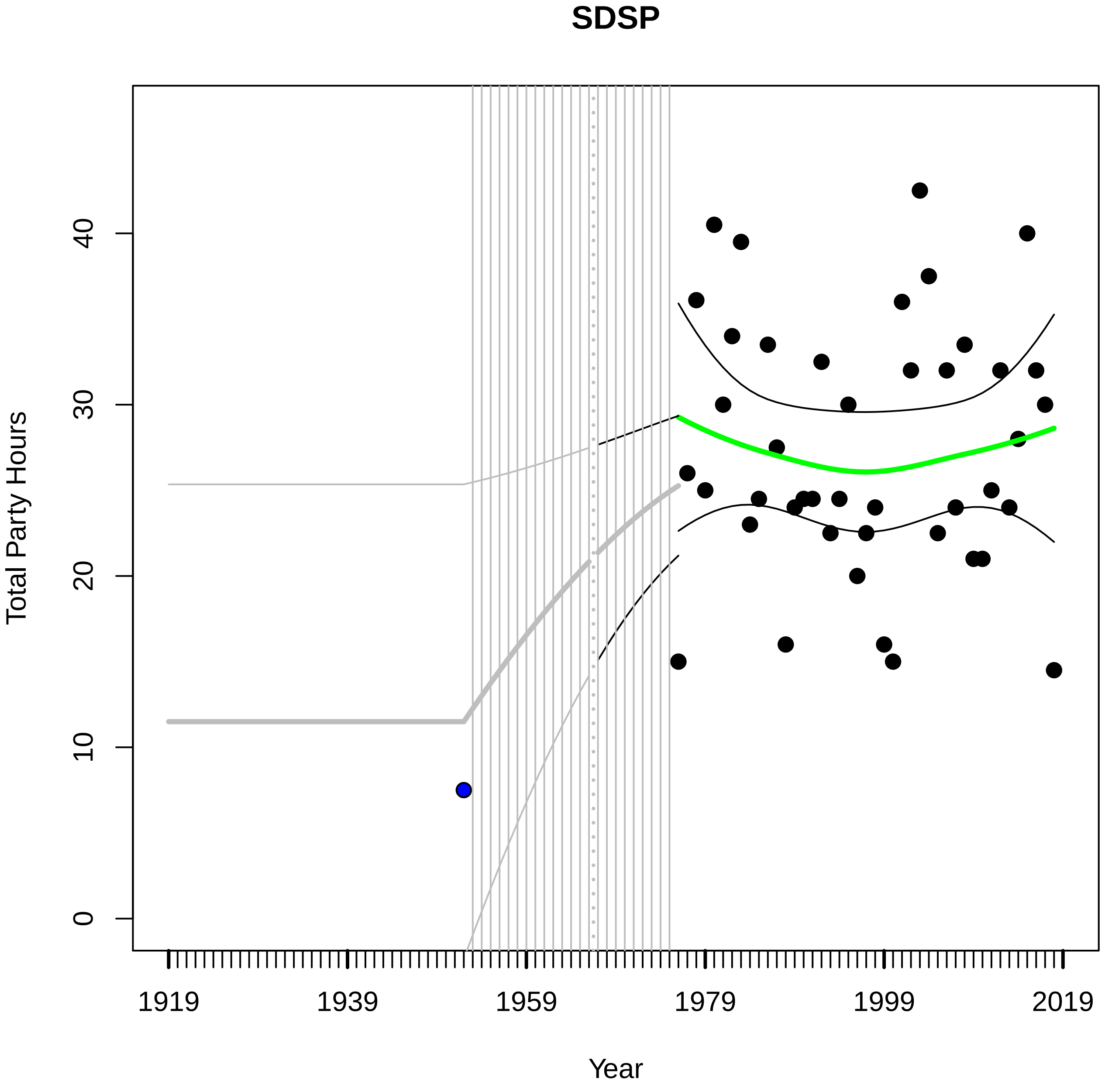

SDSP
Total Party Hours
Year
0
10
20
30
40
1919
1939
1959
1979
1999
2019

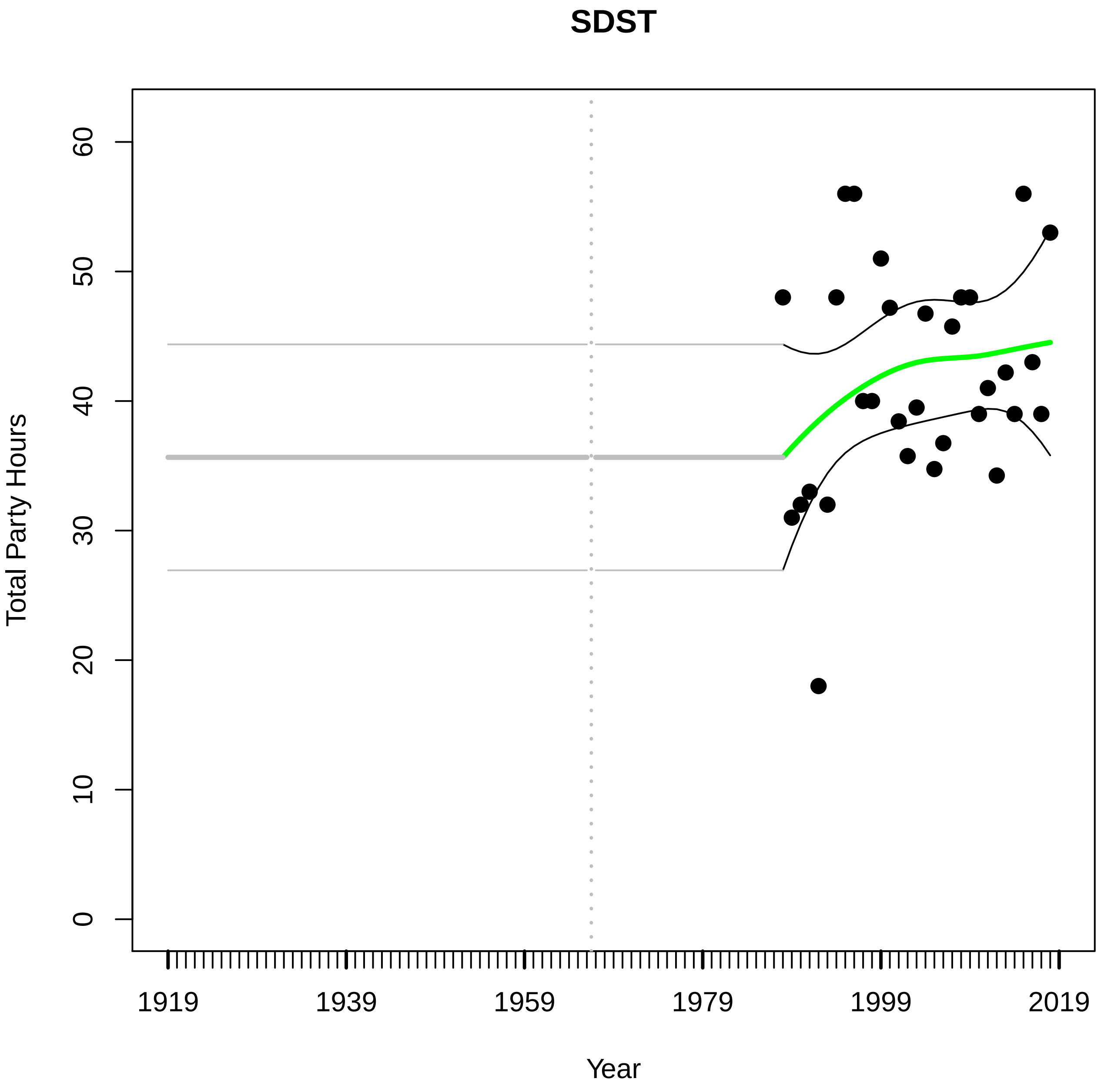

SDST
Total Party Hours
Year
0
10
20
30
40
50
60
1919
1939
1959
1979
1999
2019

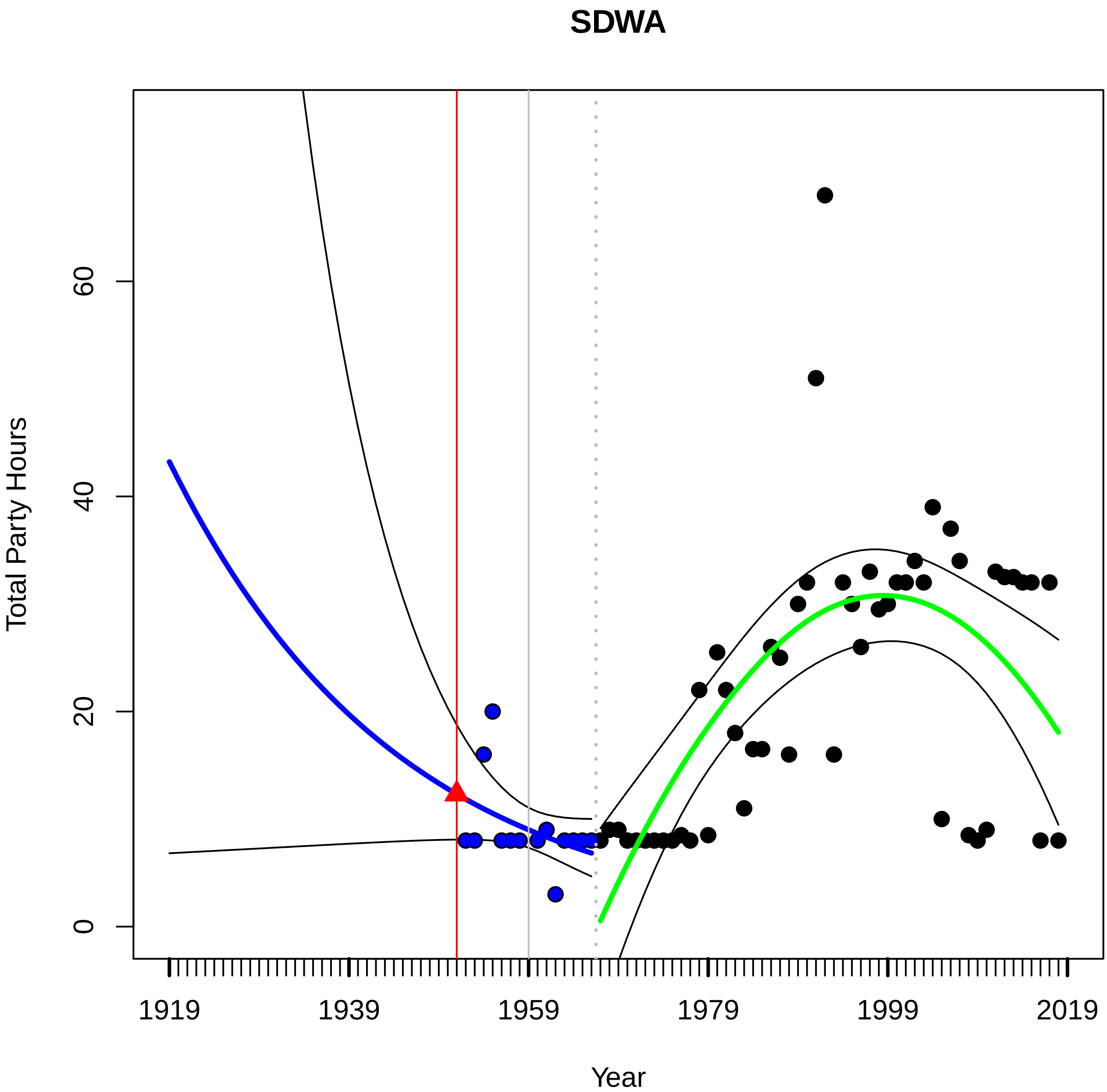

SDWA
Total Party Hours
0
20
40
60
1919
1939
1959
1979
1999
2019
Year

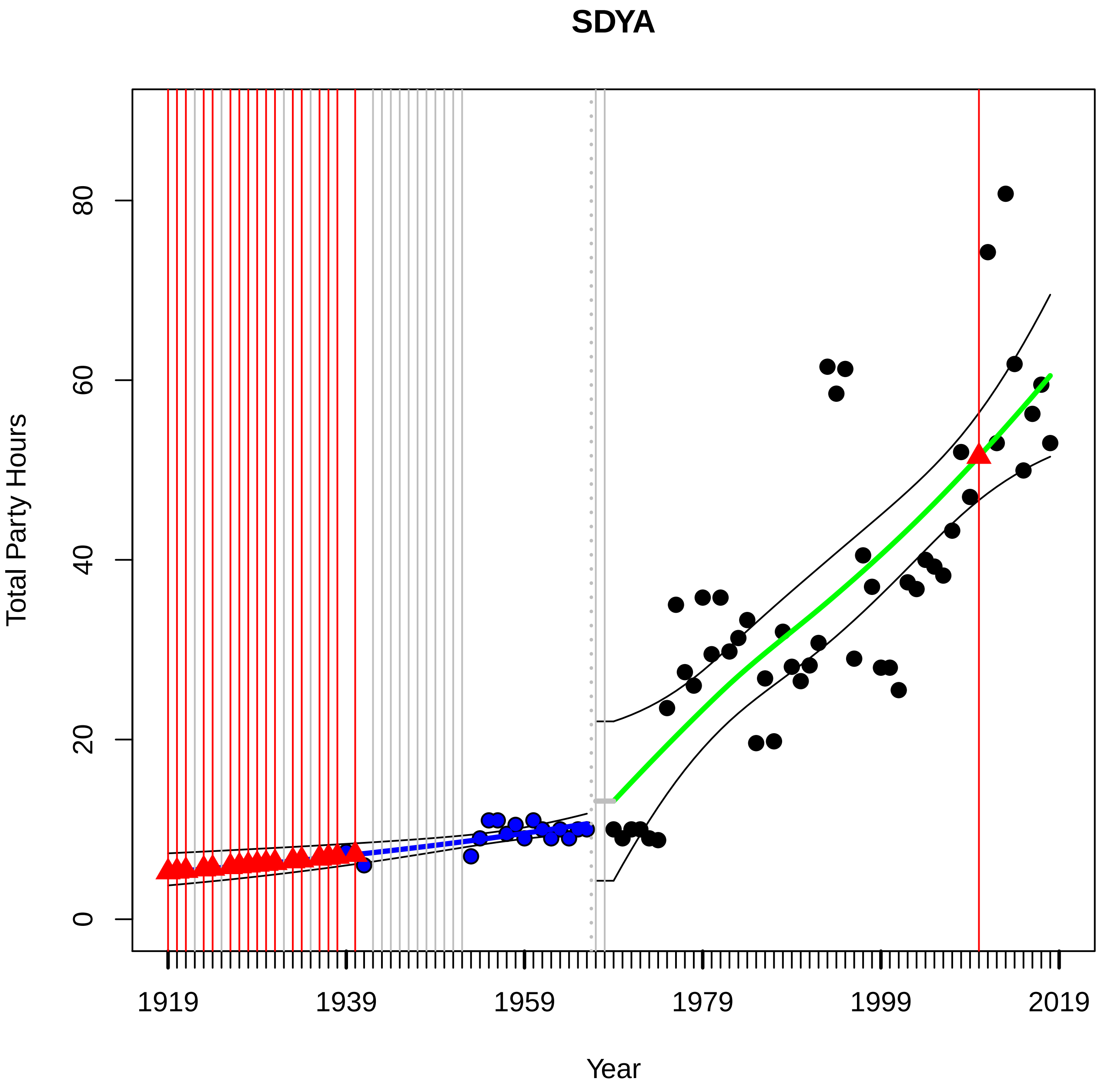
SDYA
Total Party Hours
Year
0
20
40
60
80
1919
1939
1959
1979
1999
2019

TN09

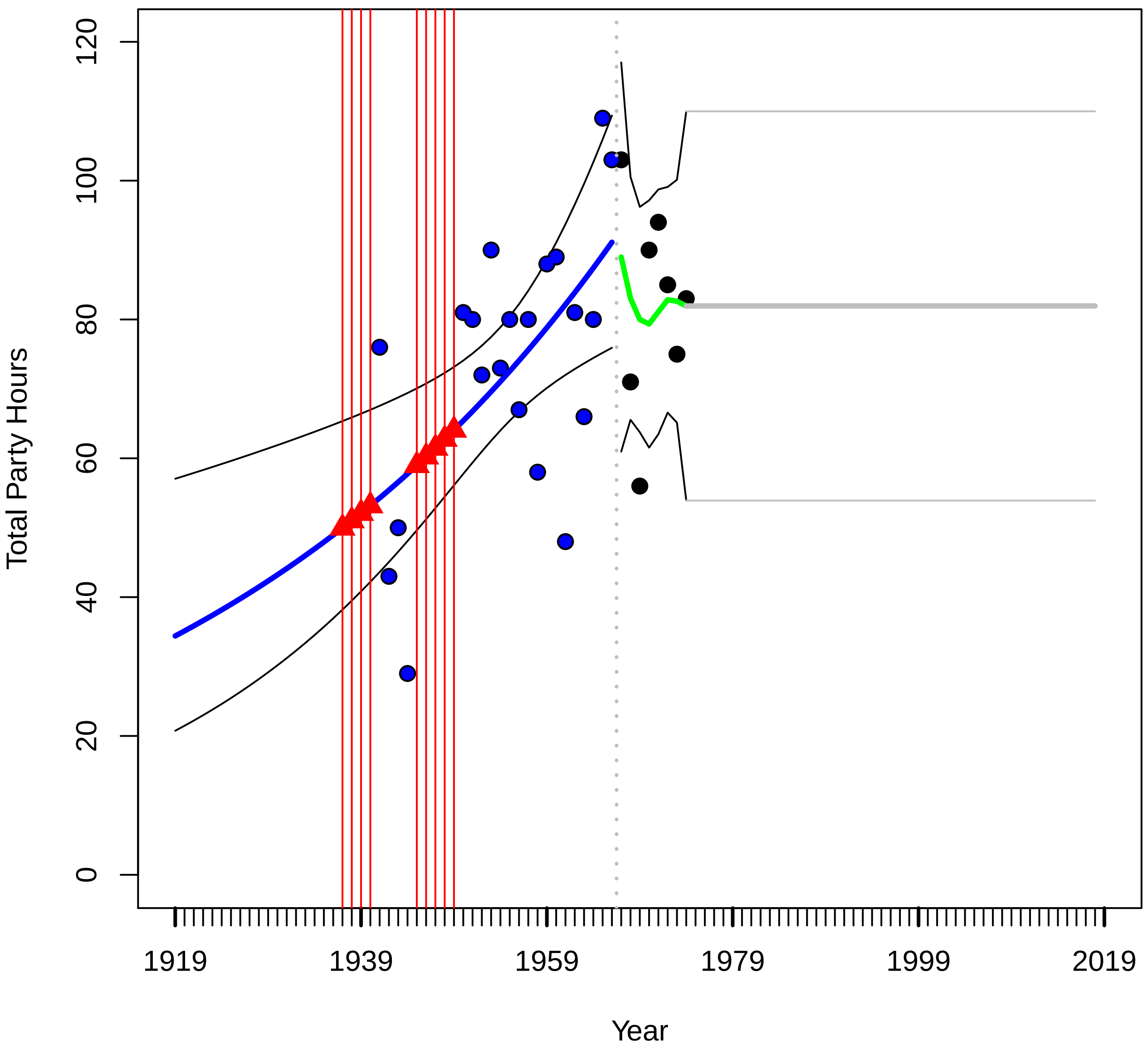

Total Party Hours
0
20
40
60
80
100
120
1919
1939
1959
1979
1999
2019
Year

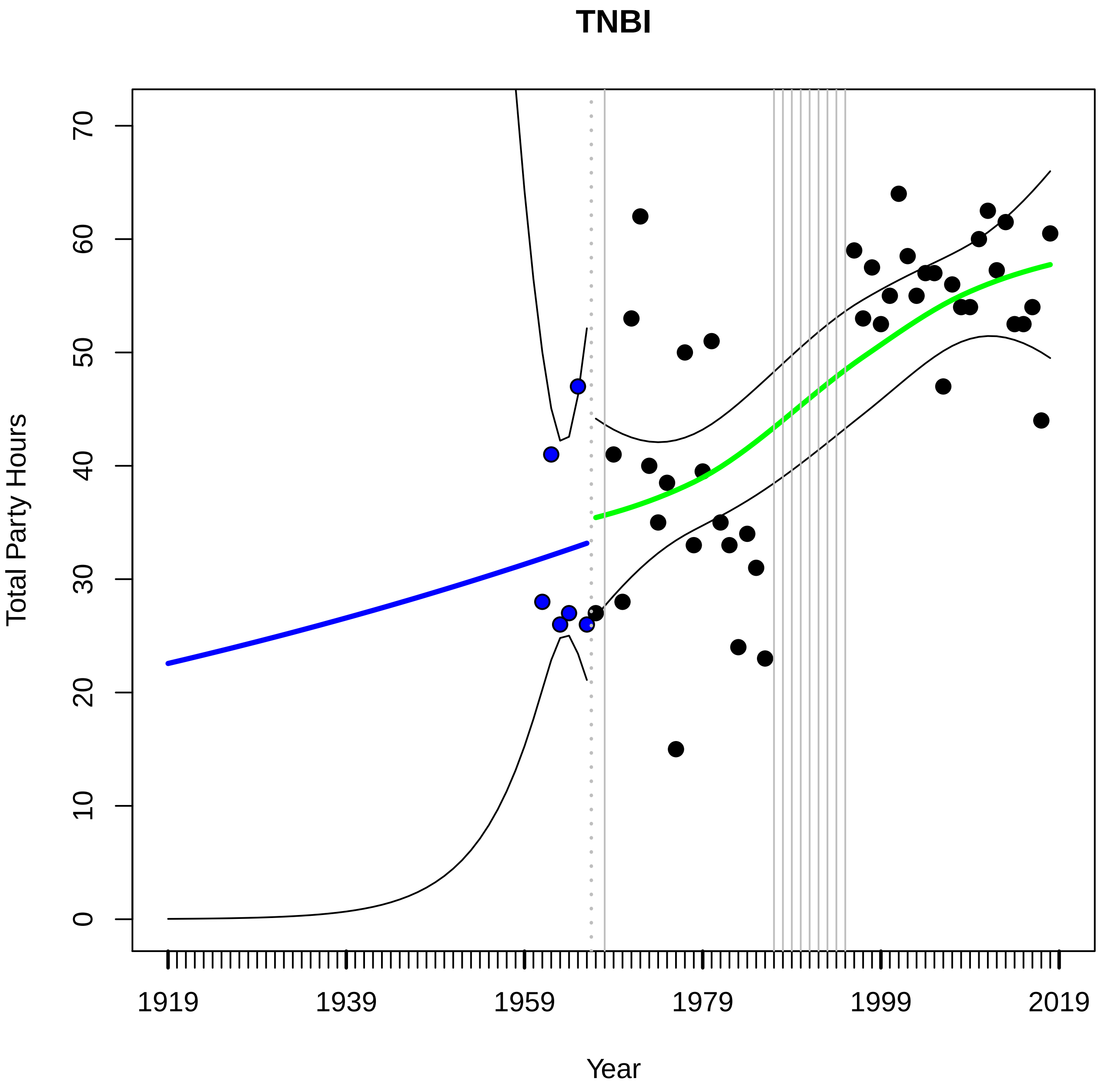

TNBI
Total Party Hours
Year
0
10
20
30
40
50
60
70
1919
1939
1959
1979
1999
2019

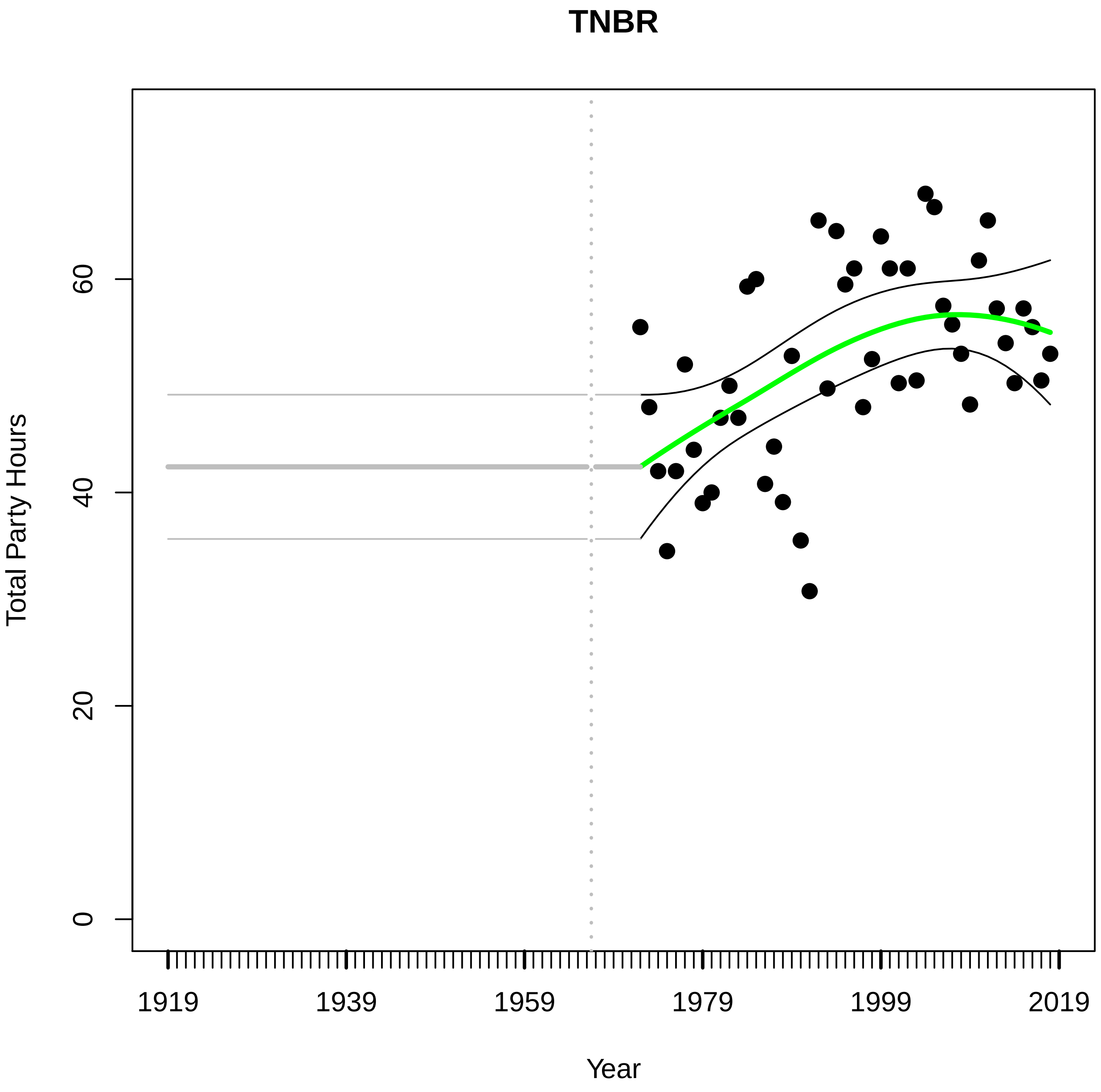
TNBR
Total Party Hours
0
20
40
60
1919
1939
1959
1979
1999
2019
Year

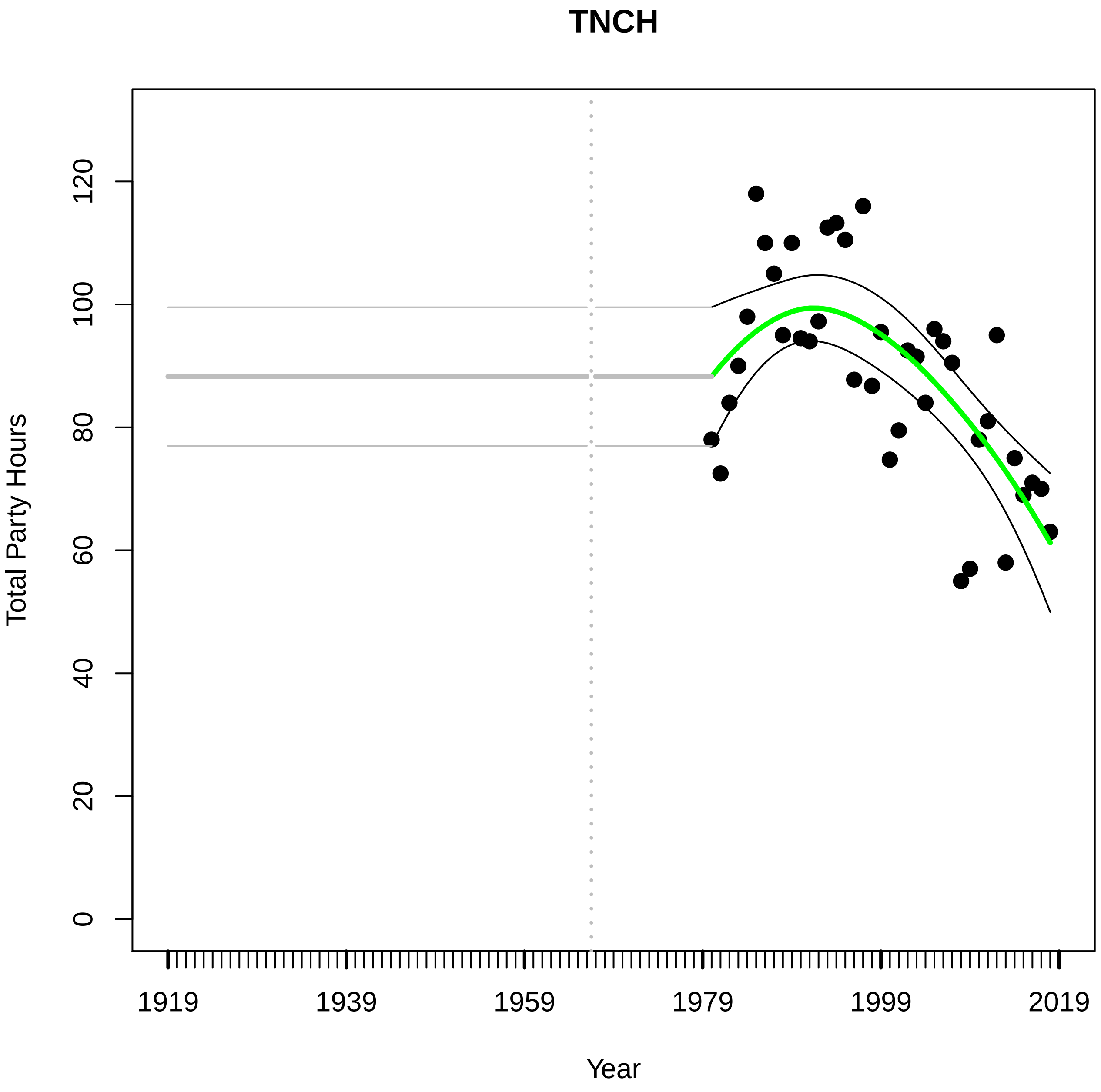

TNCH
Total Party Hours
0
20
40
60
80
100
120
1919
1939
1959
1979
1999
2019
Year

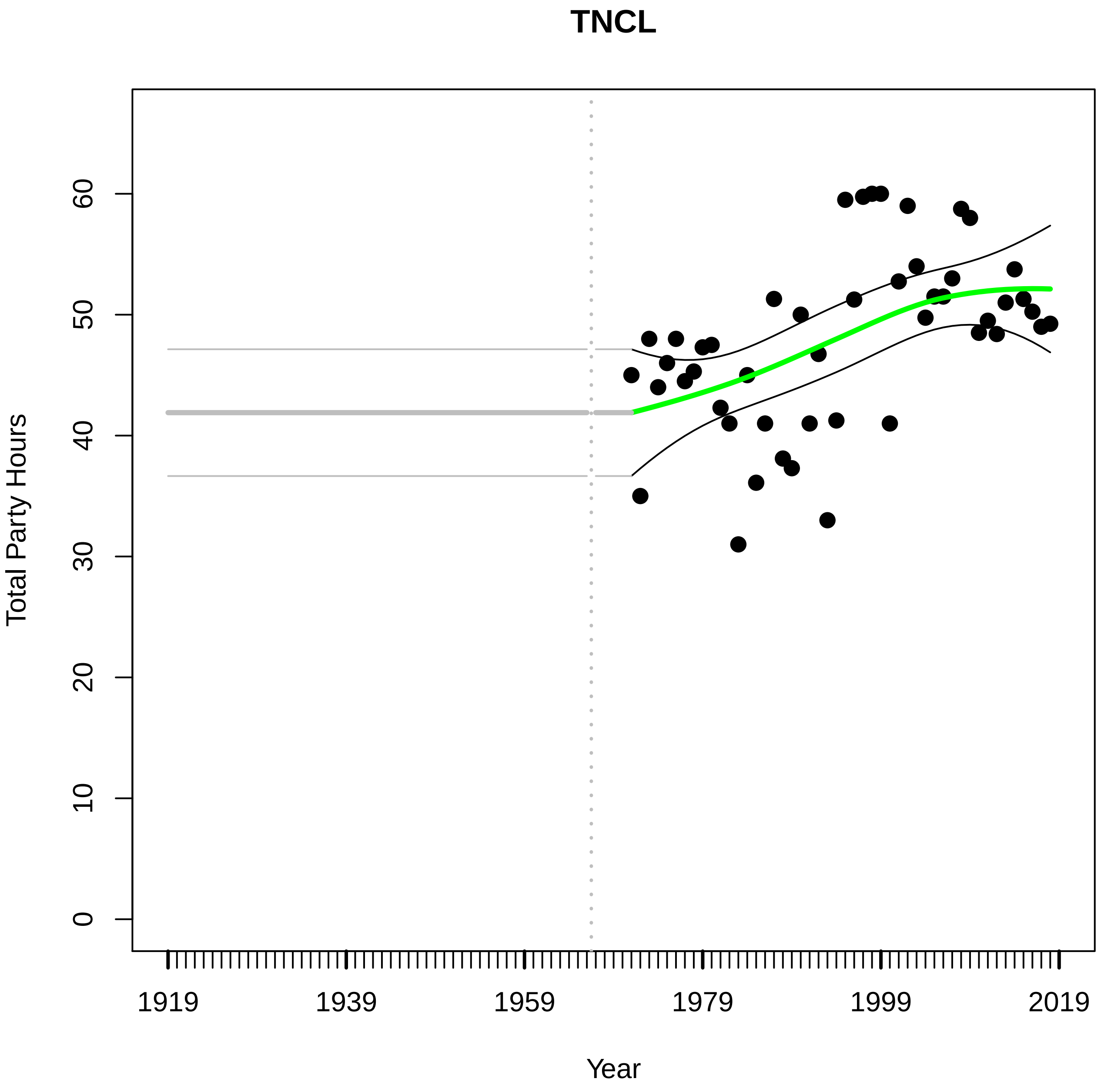

TNCL
Total Party Hours
Year
0
10
20
30
40
50
60
1919
1939
1959
1979
1999
2019

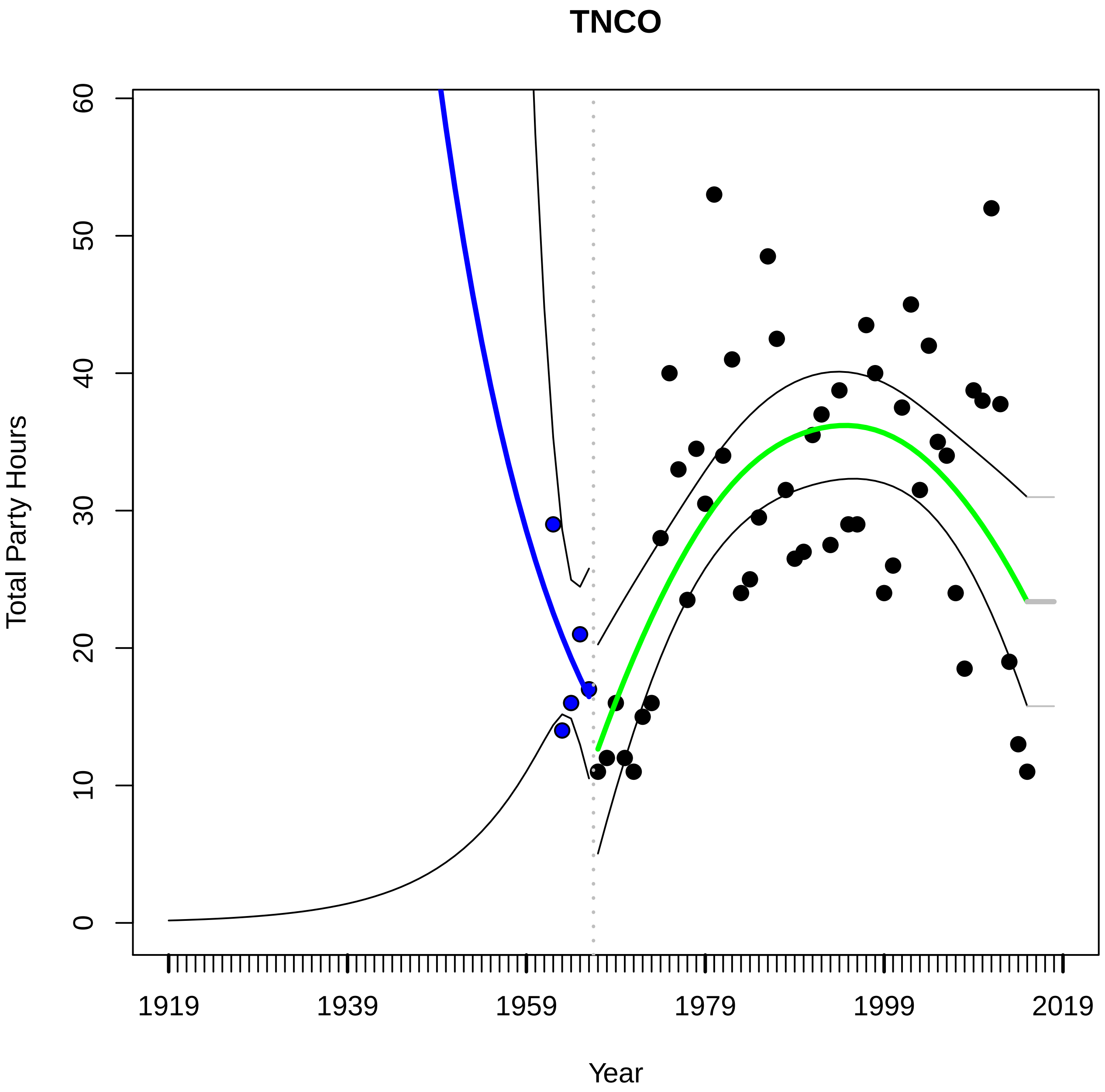
TNCO
Total Party Hours
0
10
20
30
40
50
60
1919
1939
1959
1979
1999
2019
Year

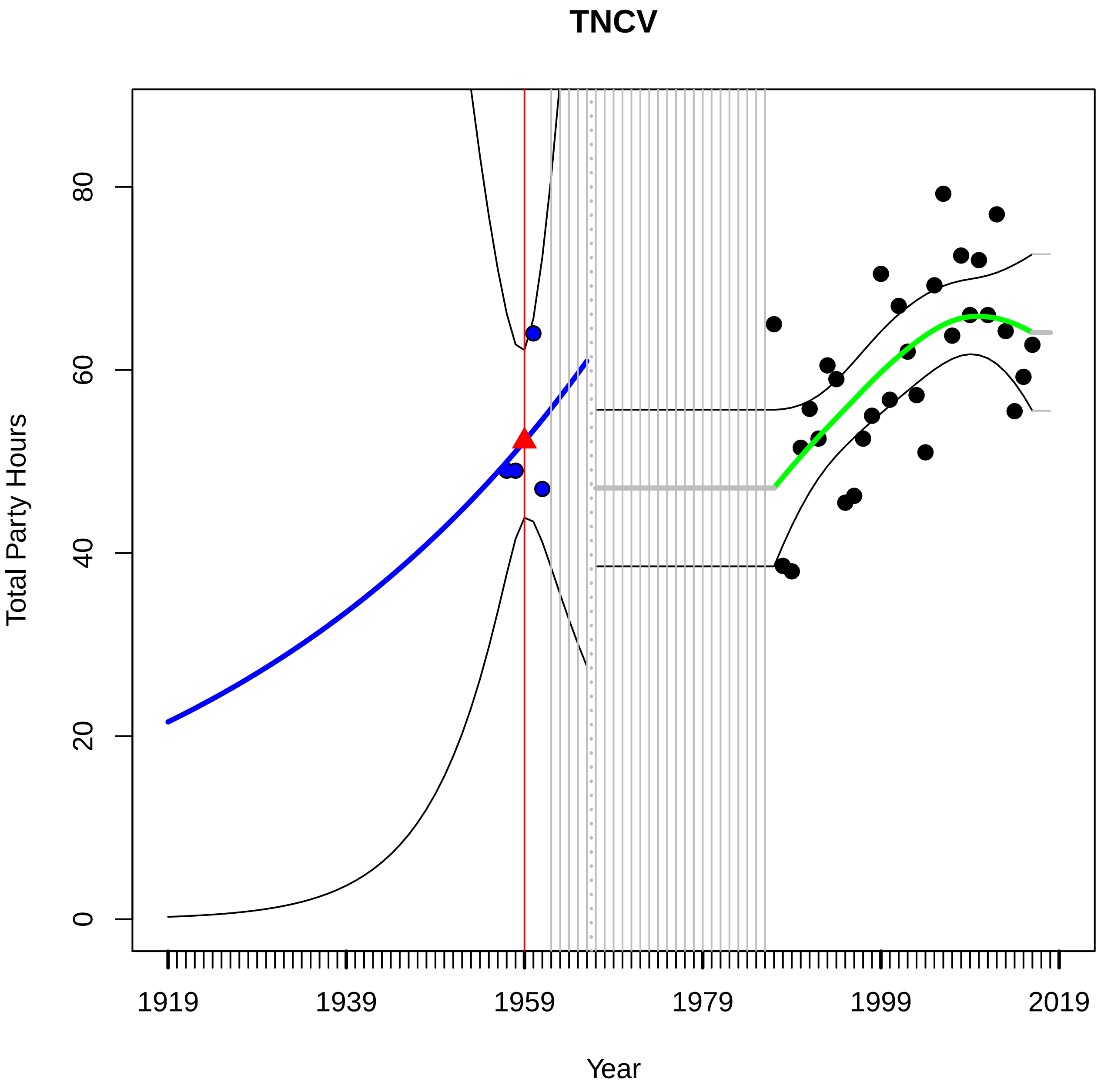
TNCV
Total Party Hours
0
20
40
60
80
1919
1939
1959
1979
1999
2019
Year

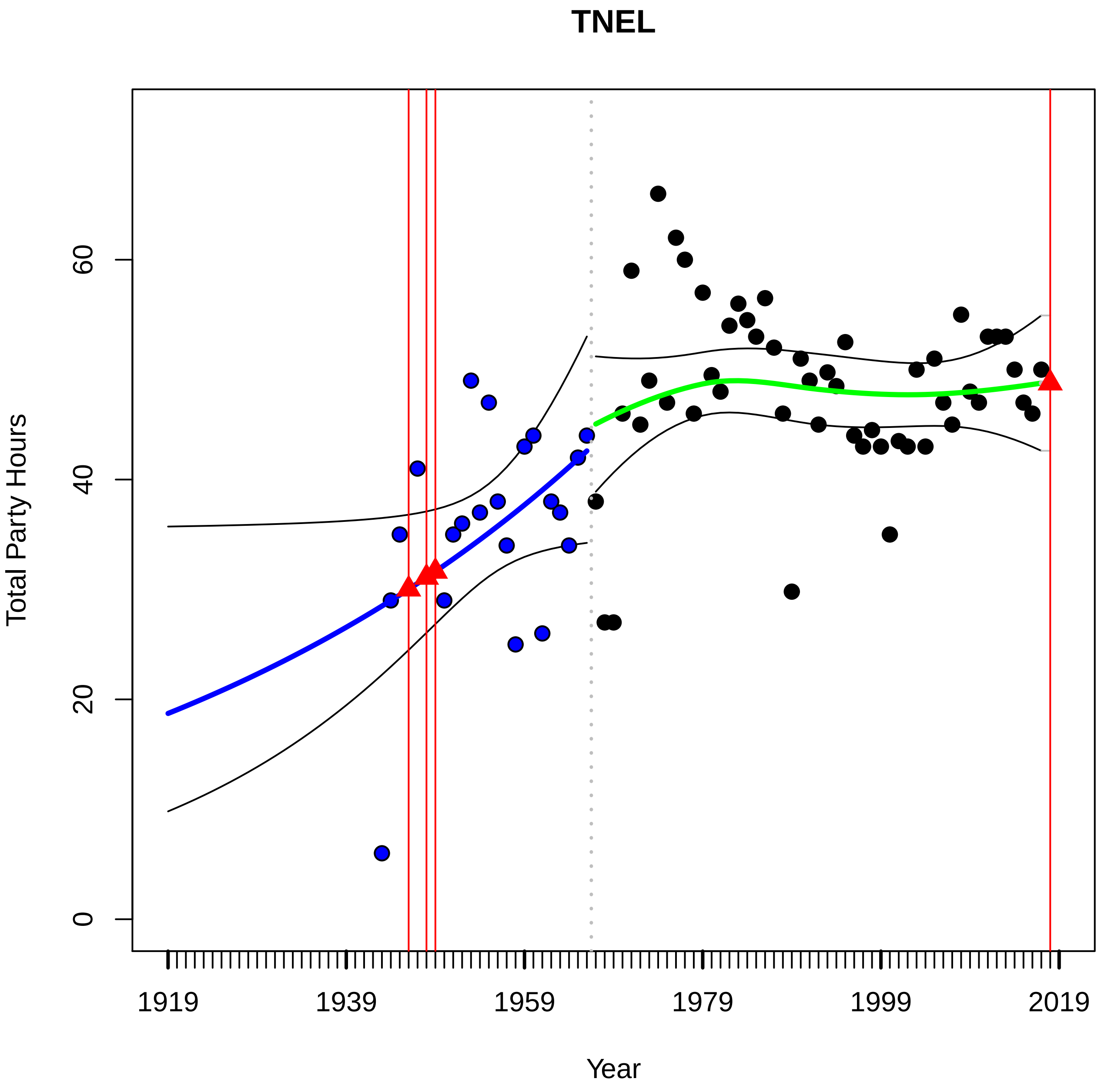

TNEL
Total Party Hours
0
20
40
60
1919
1939
1959
1979
1999
2019
Year

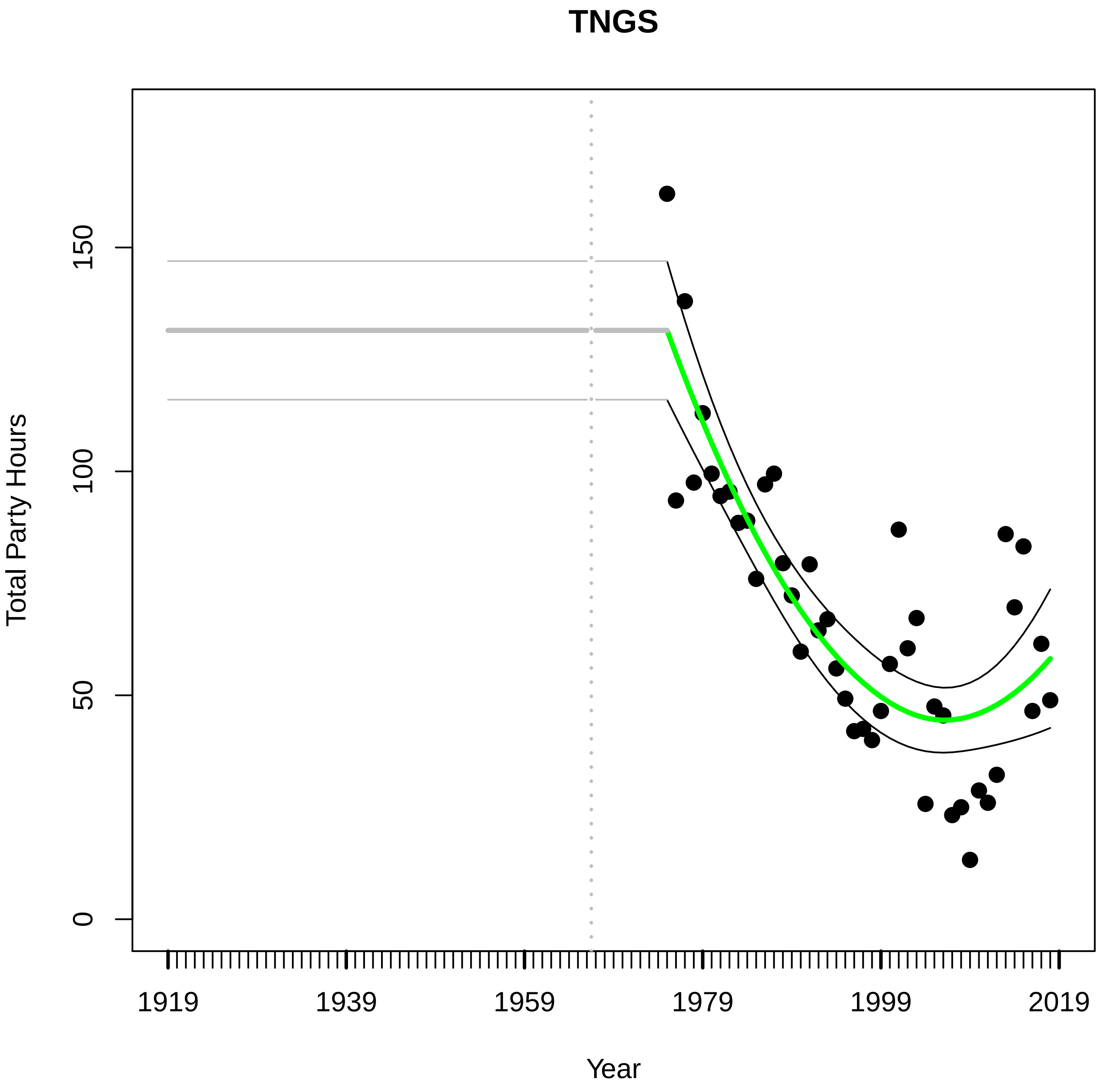
TNGS
Total Party Hours
0
50
100
150
1919
1939
1959
1979
1999
2019
Year

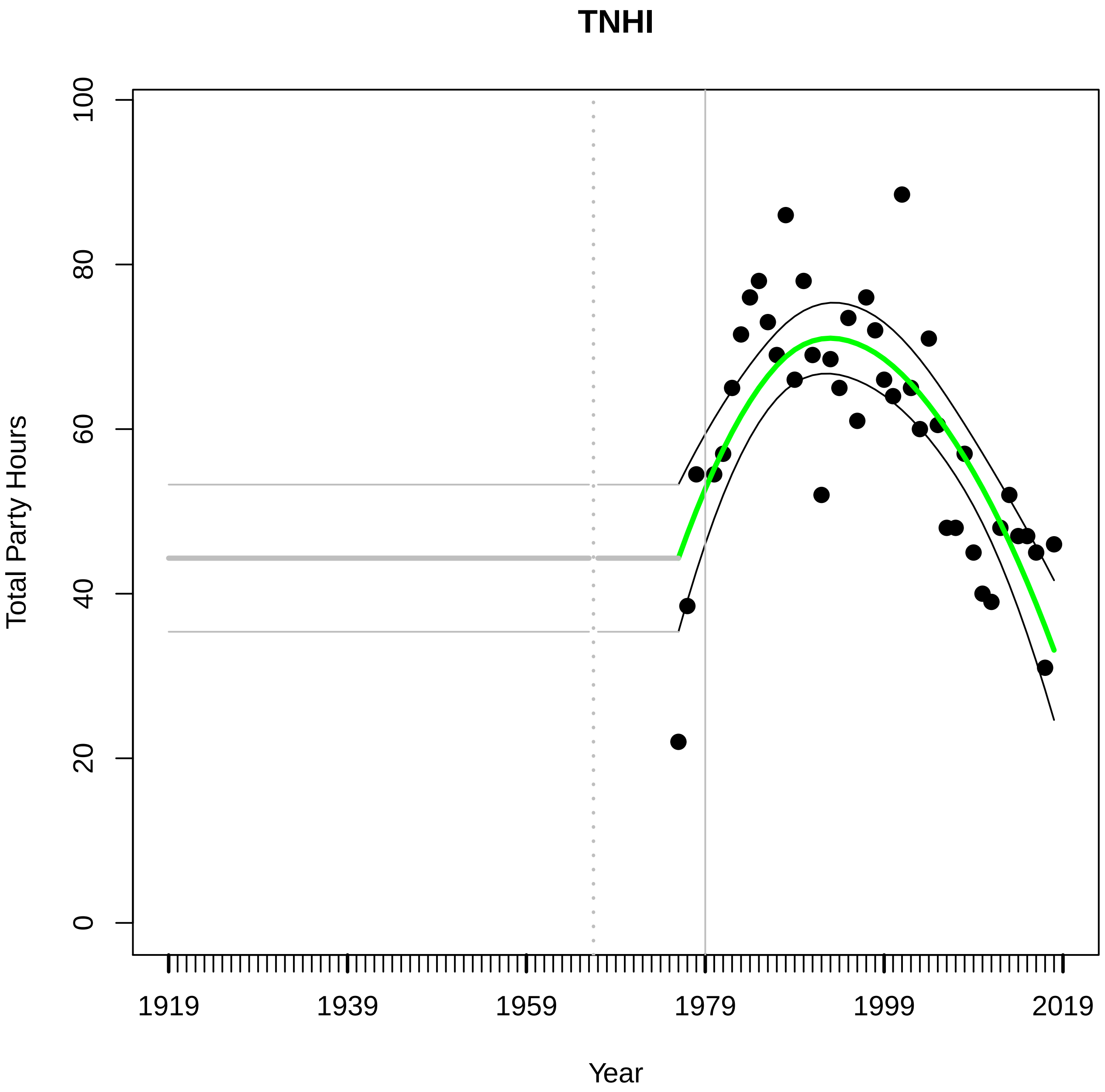
TNHI
Total Party Hours
Year
0
20
40
60
80
100
1919
1939
1959
1979
1999
2019

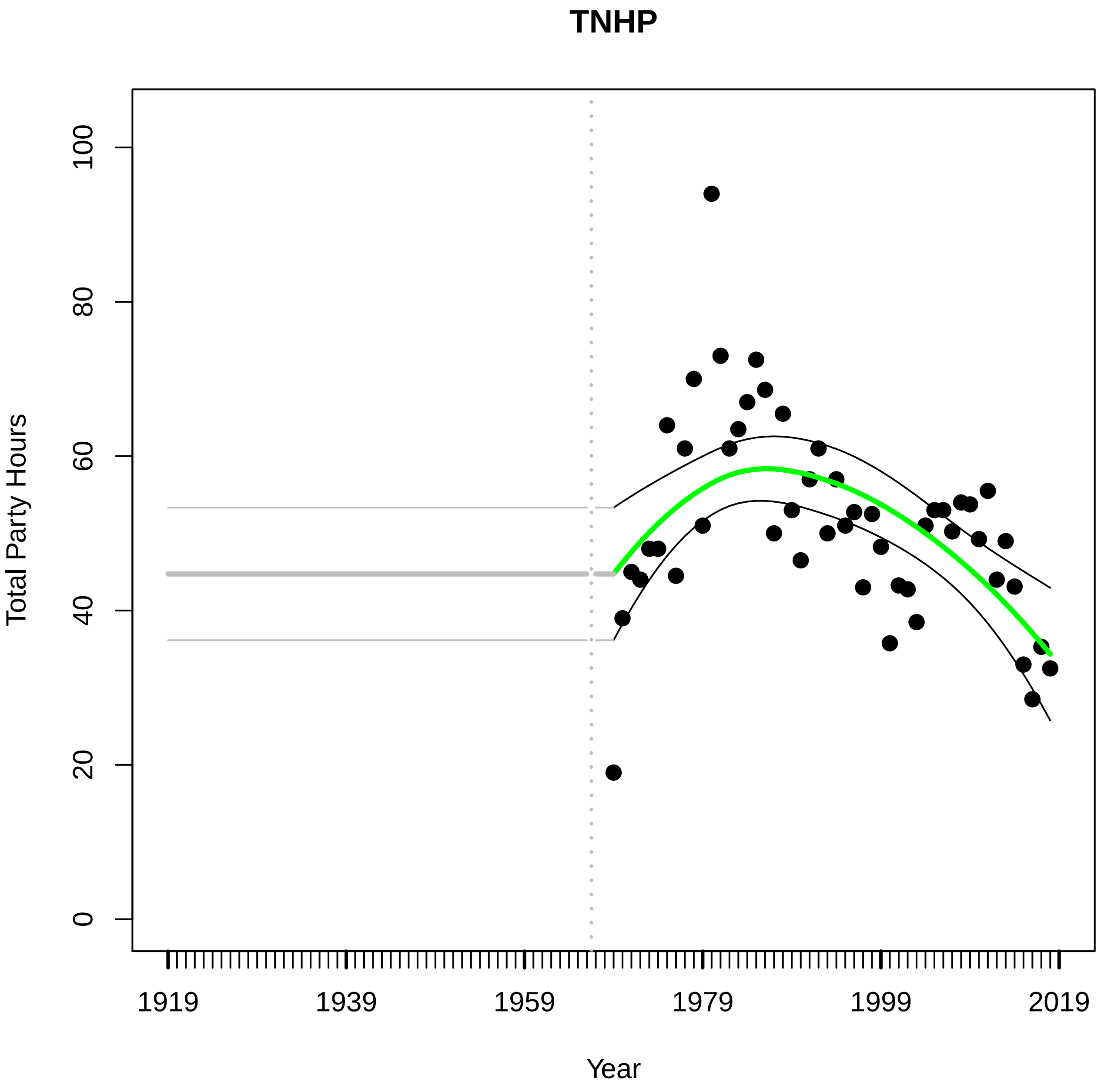

TNHP
Total Party Hours
0
20
40
60
80
100
1919
1939
1959
1979
1999
2019
Year

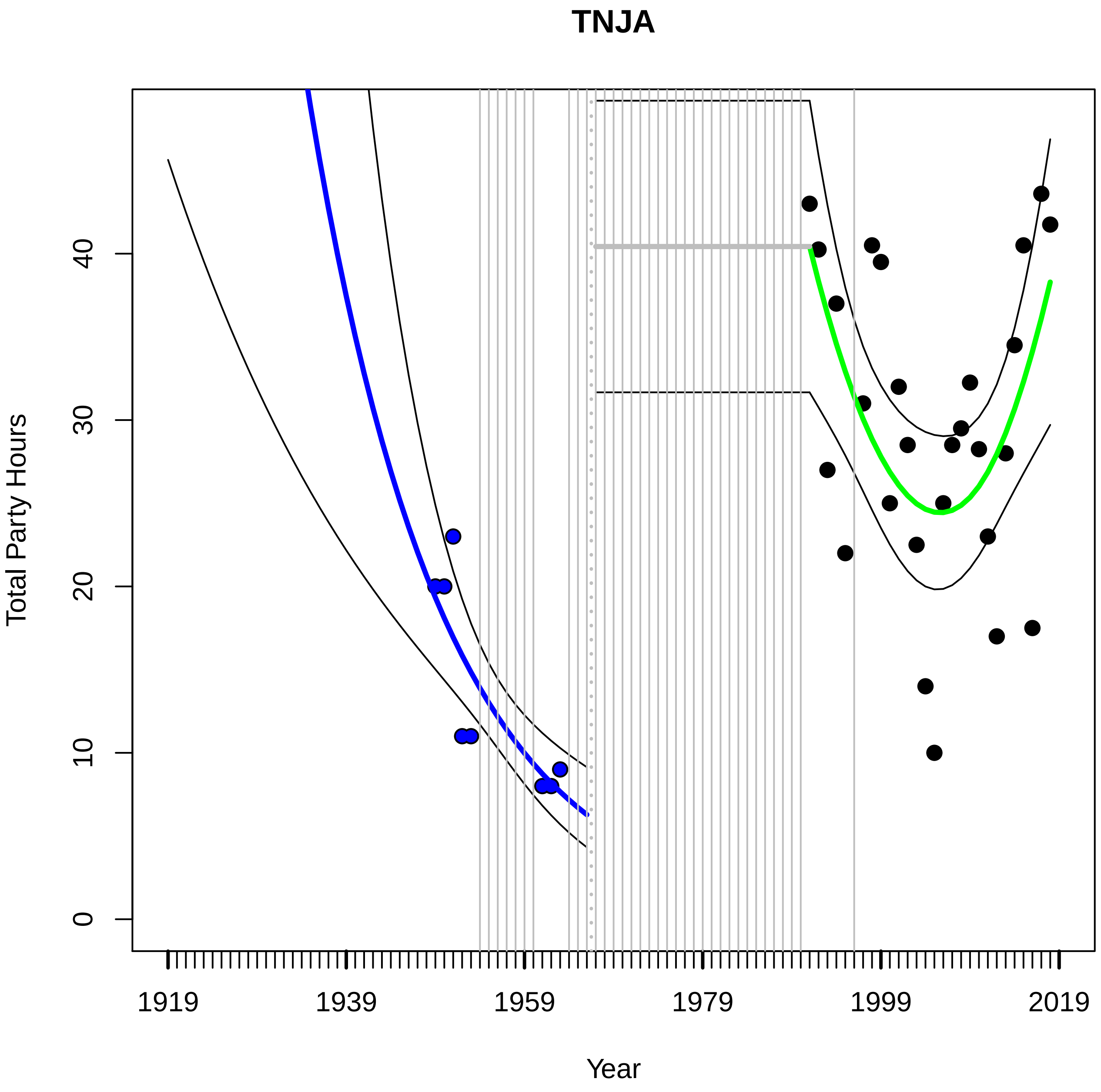
TNJA
Total Party Hours
0
10
20
30
40
Year
1919
1939
1959
1979
1999
2019

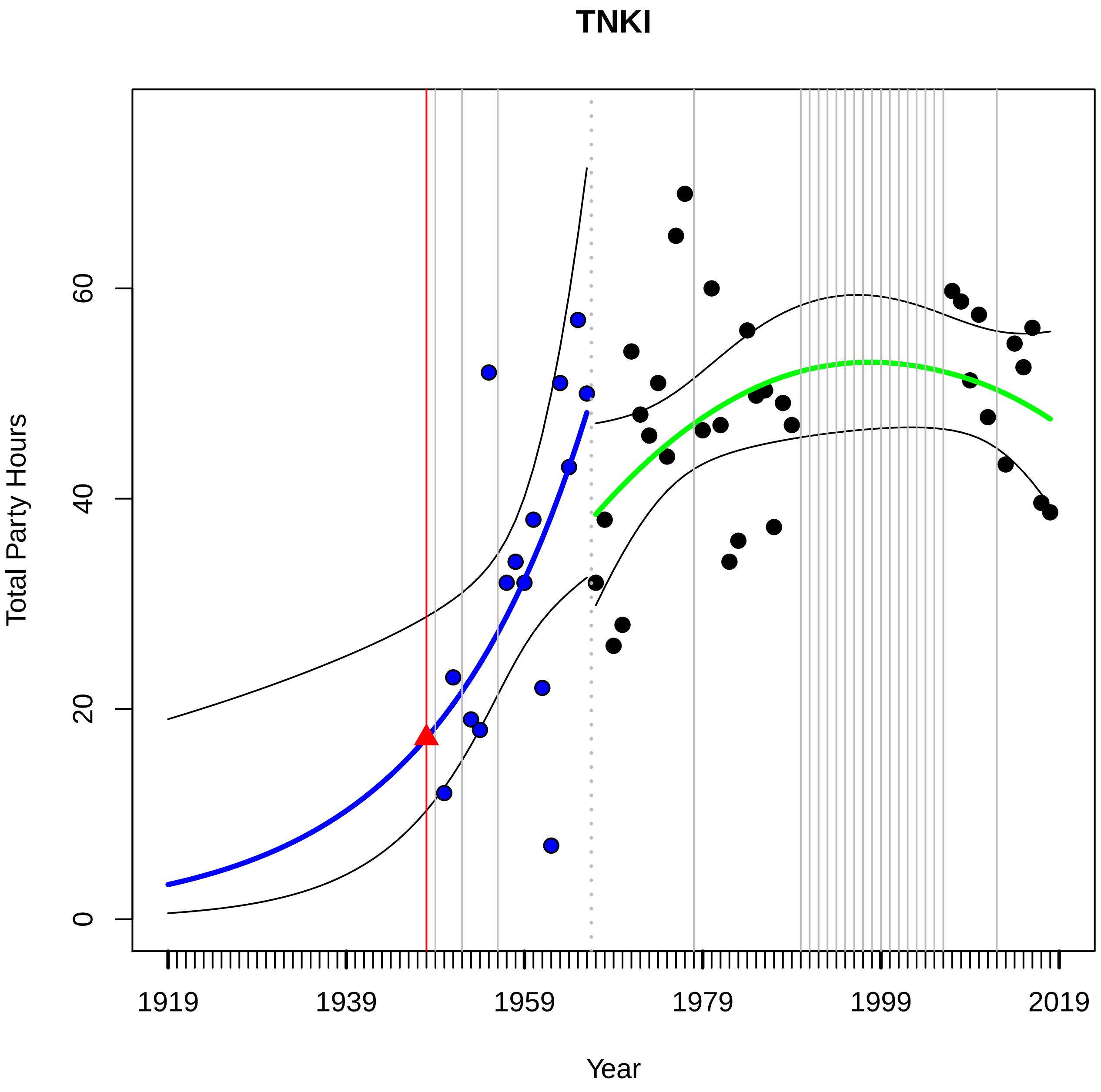

TNKI
Total Party Hours
0
20
40
60
1919
1939
1959
1979
1999
2019
Year

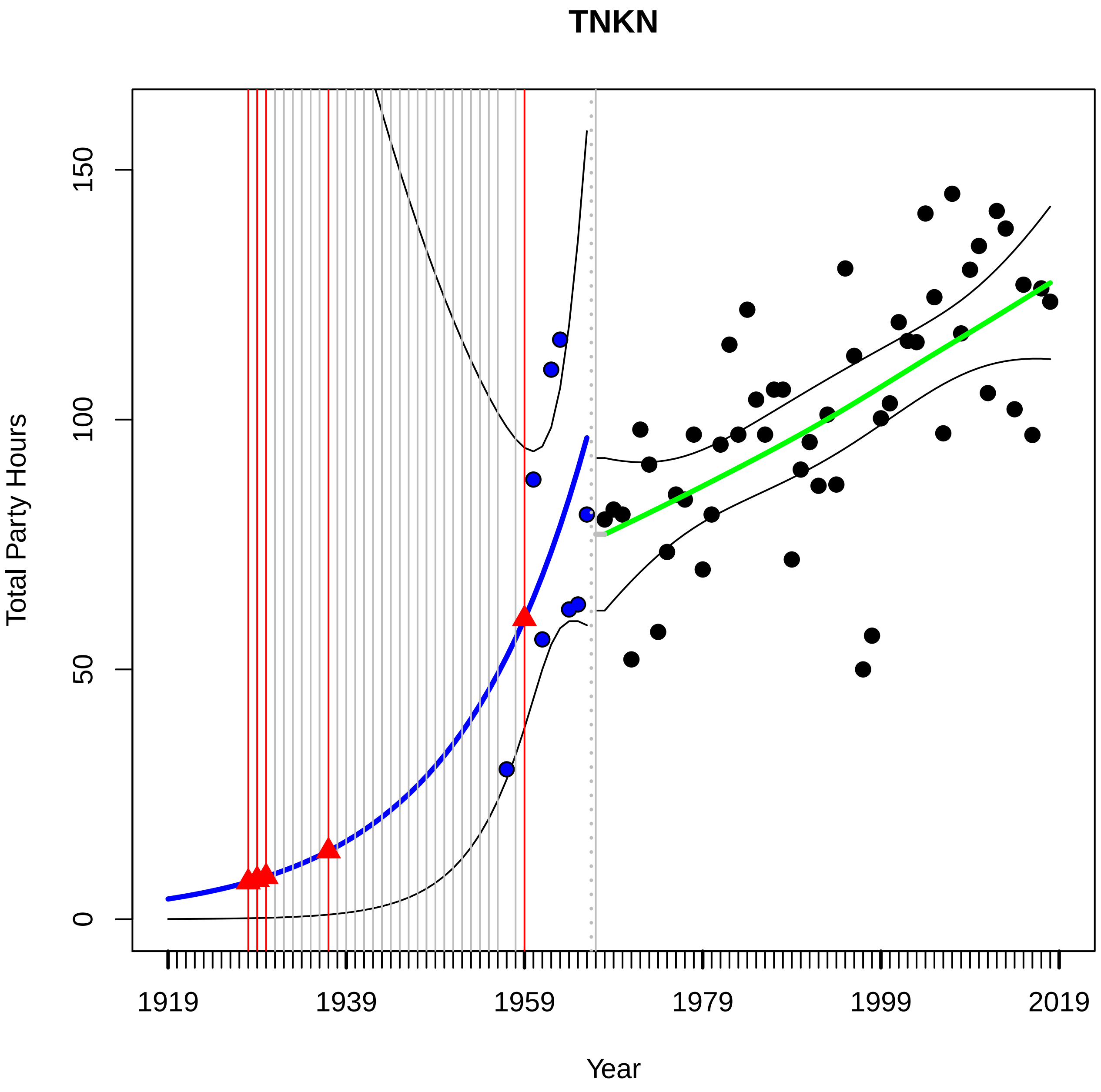
TNKN
Total Party Hours
Year
0
50
100
150
1919
1939
1959
1979
1999
2019

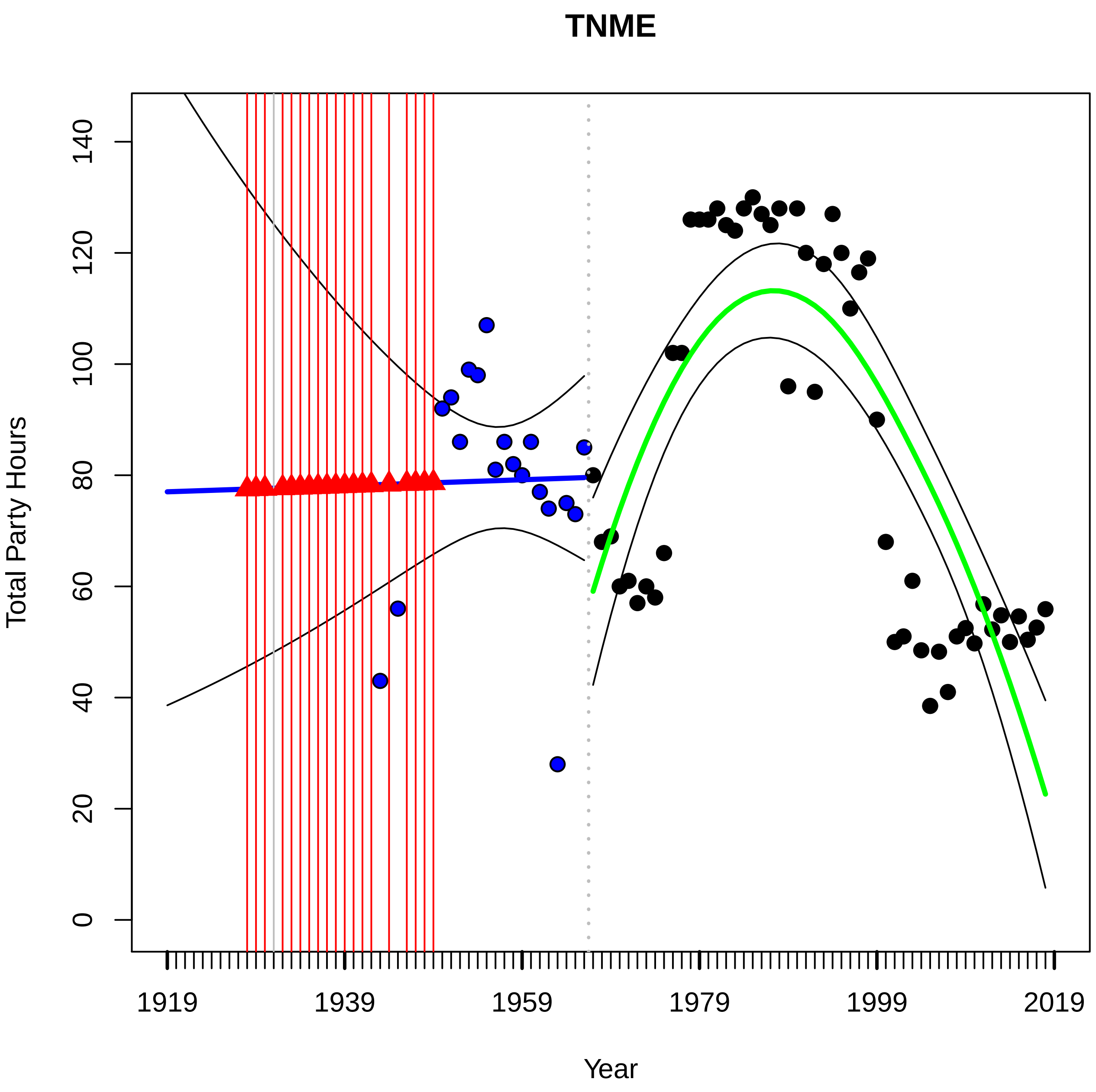
TNME
Total Party Hours
0
20
40
60
80
100
120
140
1919
1939
1959
1979
1999
2019
Year

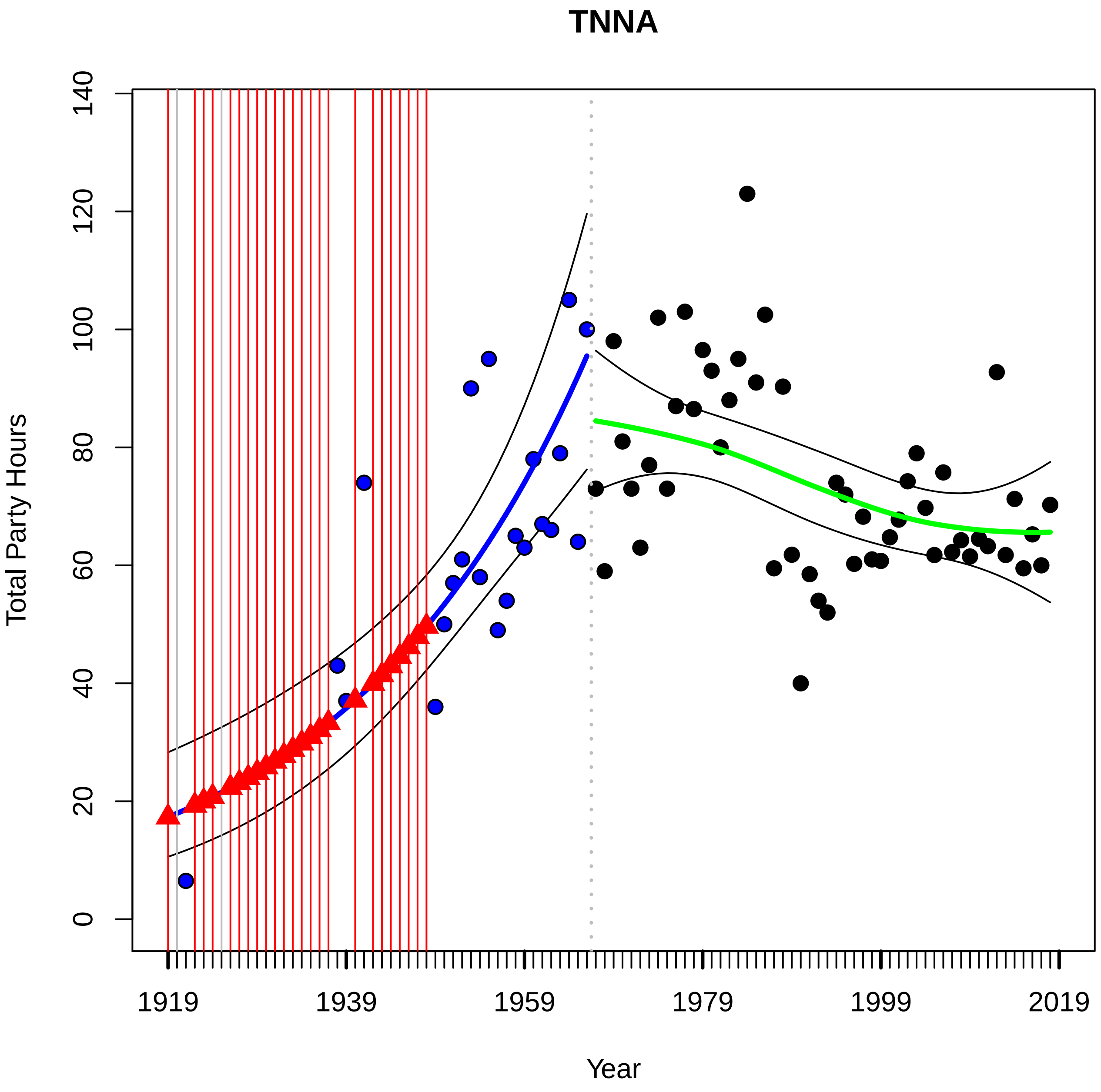
TNNA
Total Party Hours
Year
0
20
40
60
80
100
120
140
1919
1939
1959
1979
1999
2019

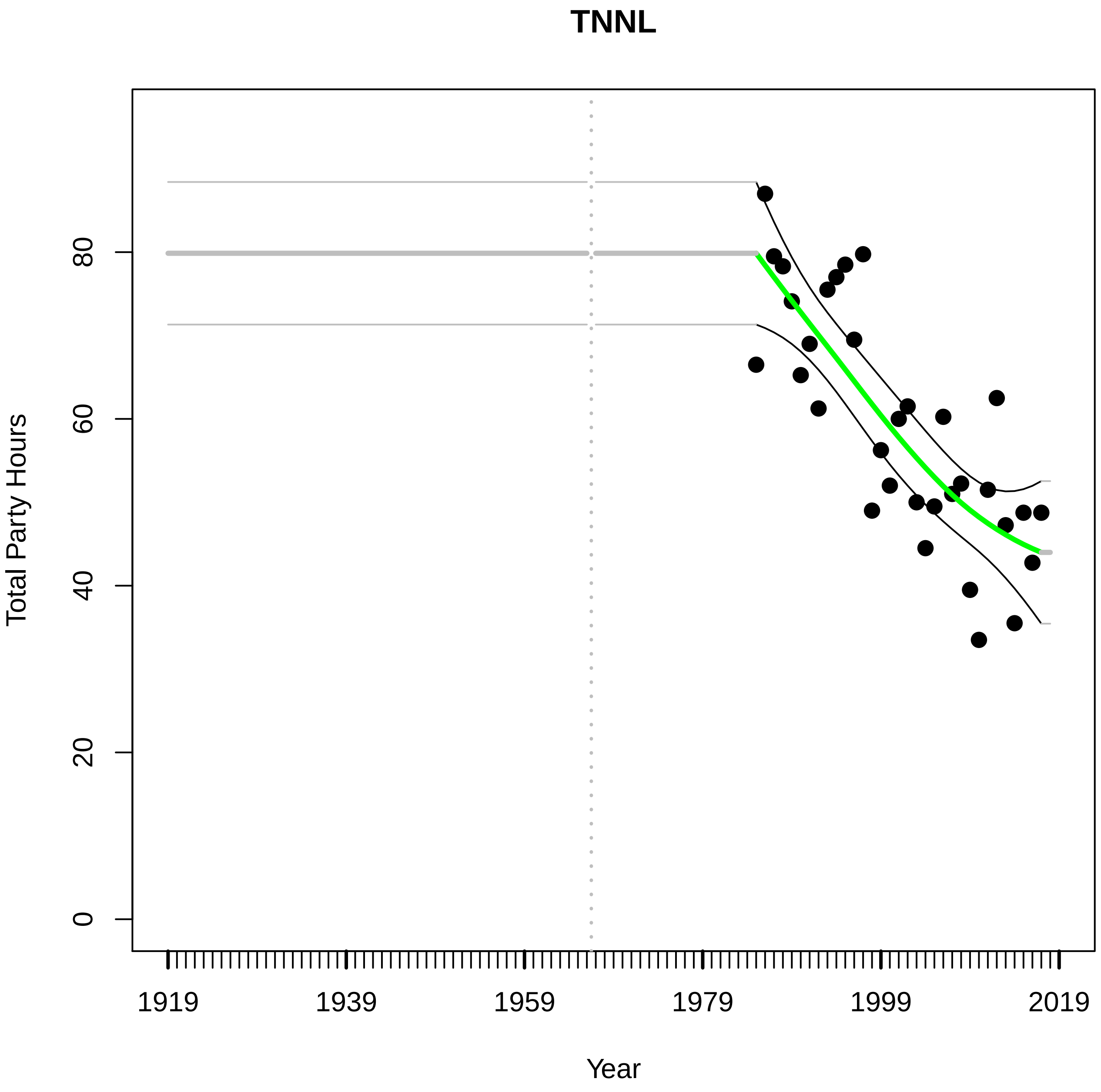

TNNL
Total Party Hours
0
20
40
60
80
1919
1939
1959
1979
1999
2019
Year

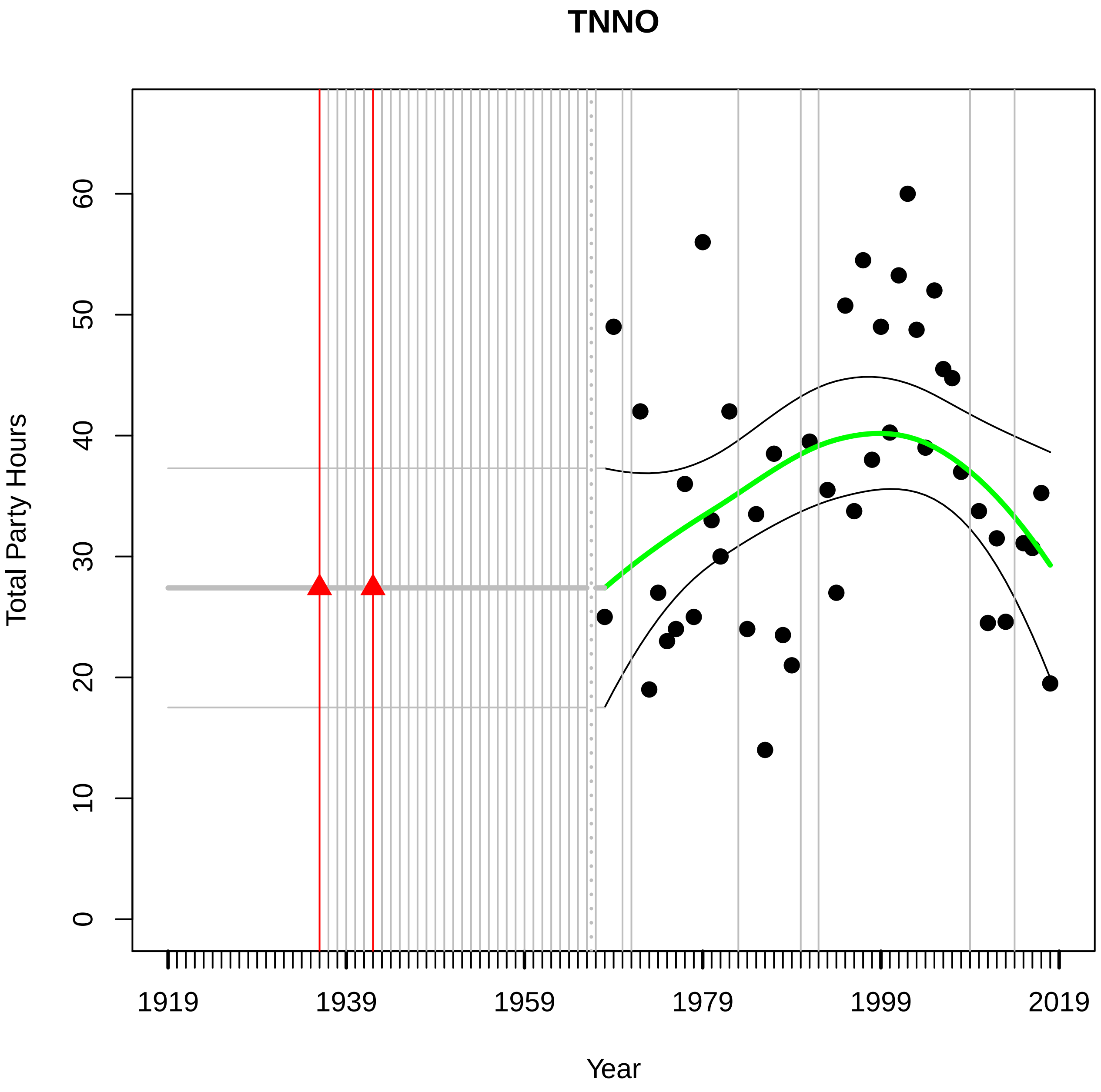
TNNO
Total Party Hours
Year
0
10
20
30
40
50
60
1919
1939
1959
1979
1999
2019

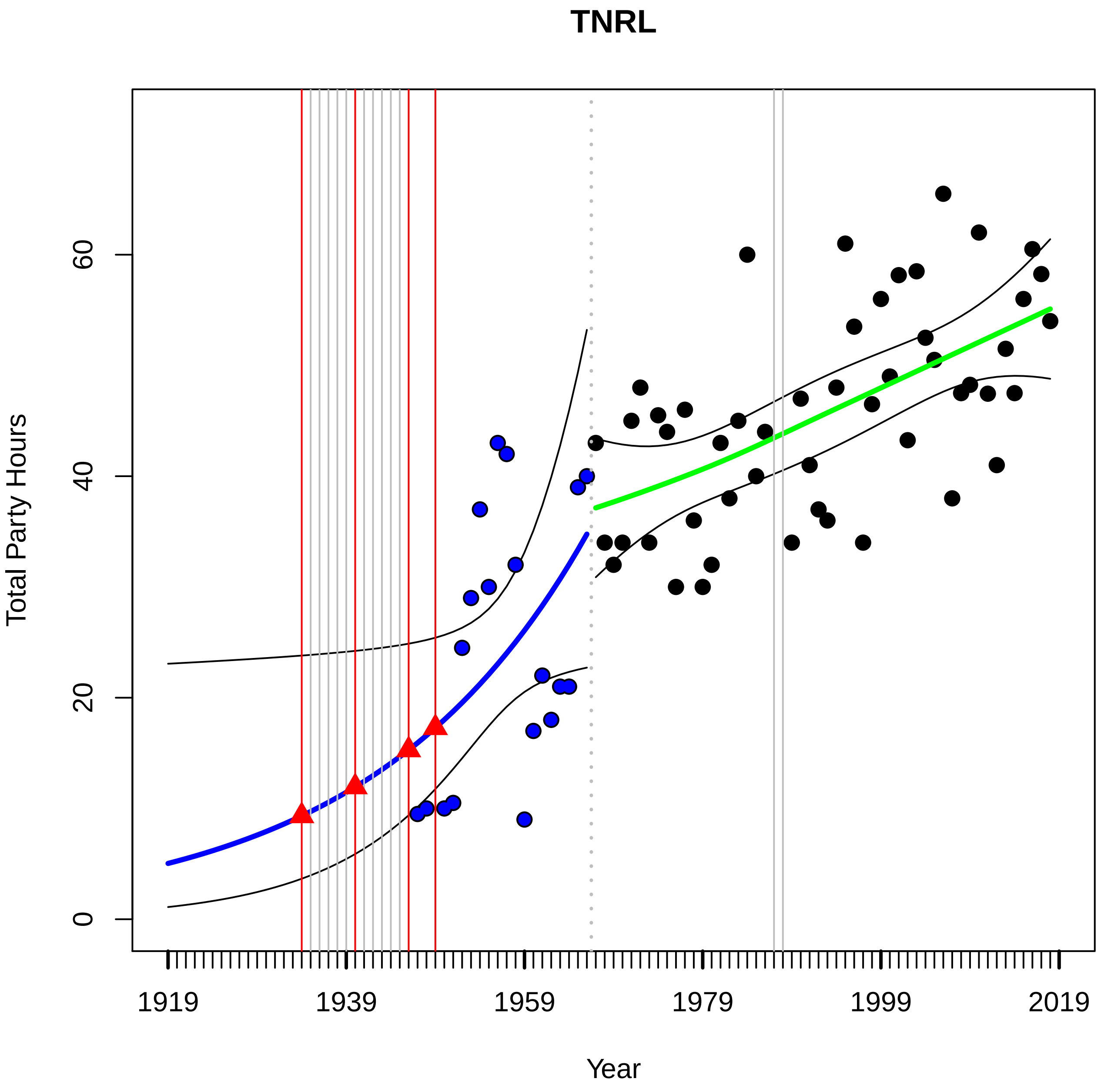
TNRL
Total Party Hours
0
20
40
60
1919
1939
1959
1979
1999
2019
Year

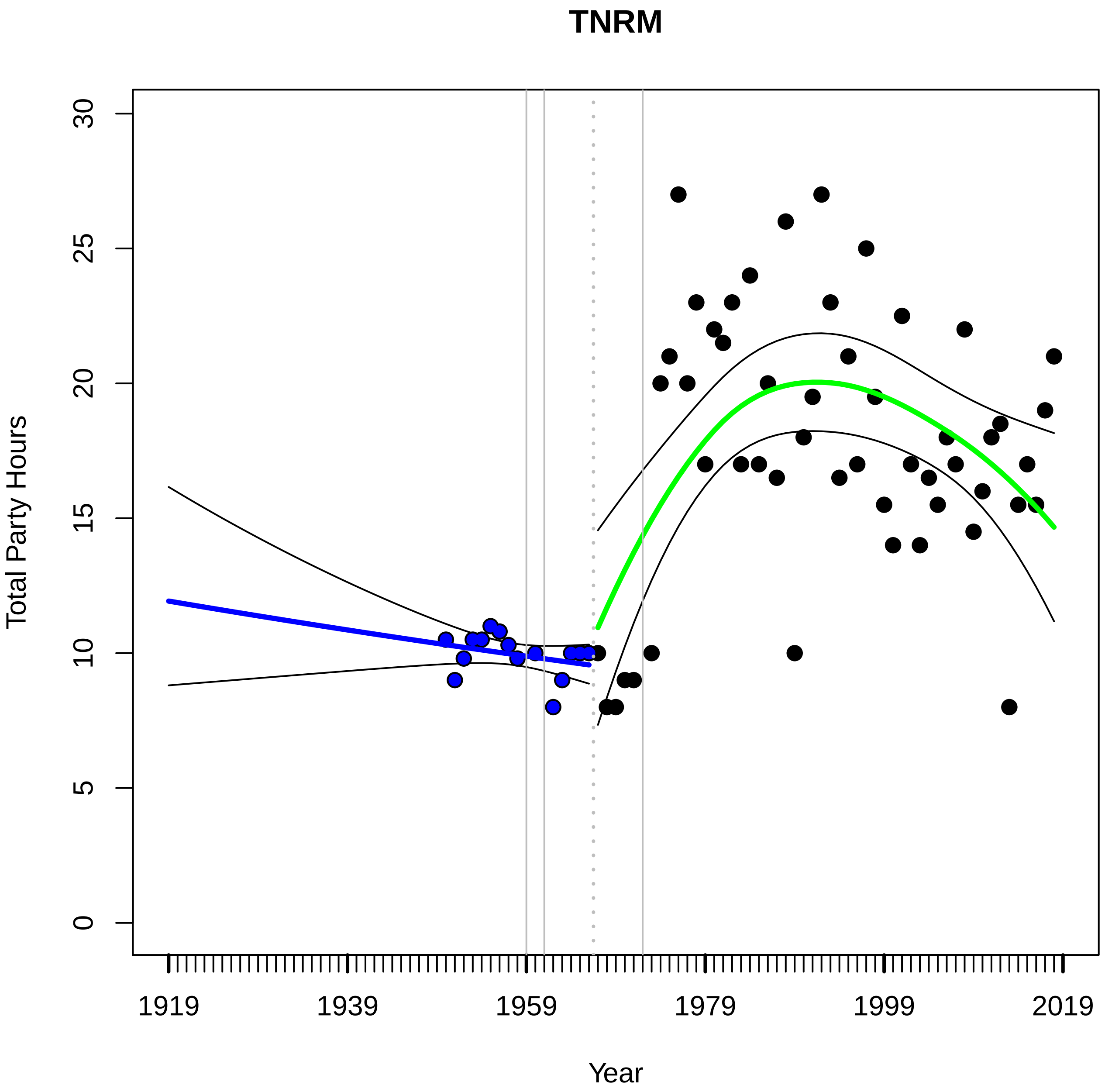

TNRM
Total Party Hours
0
5
10
15
20
25
30
1919
1939
1959
1979
1999
2019
Year

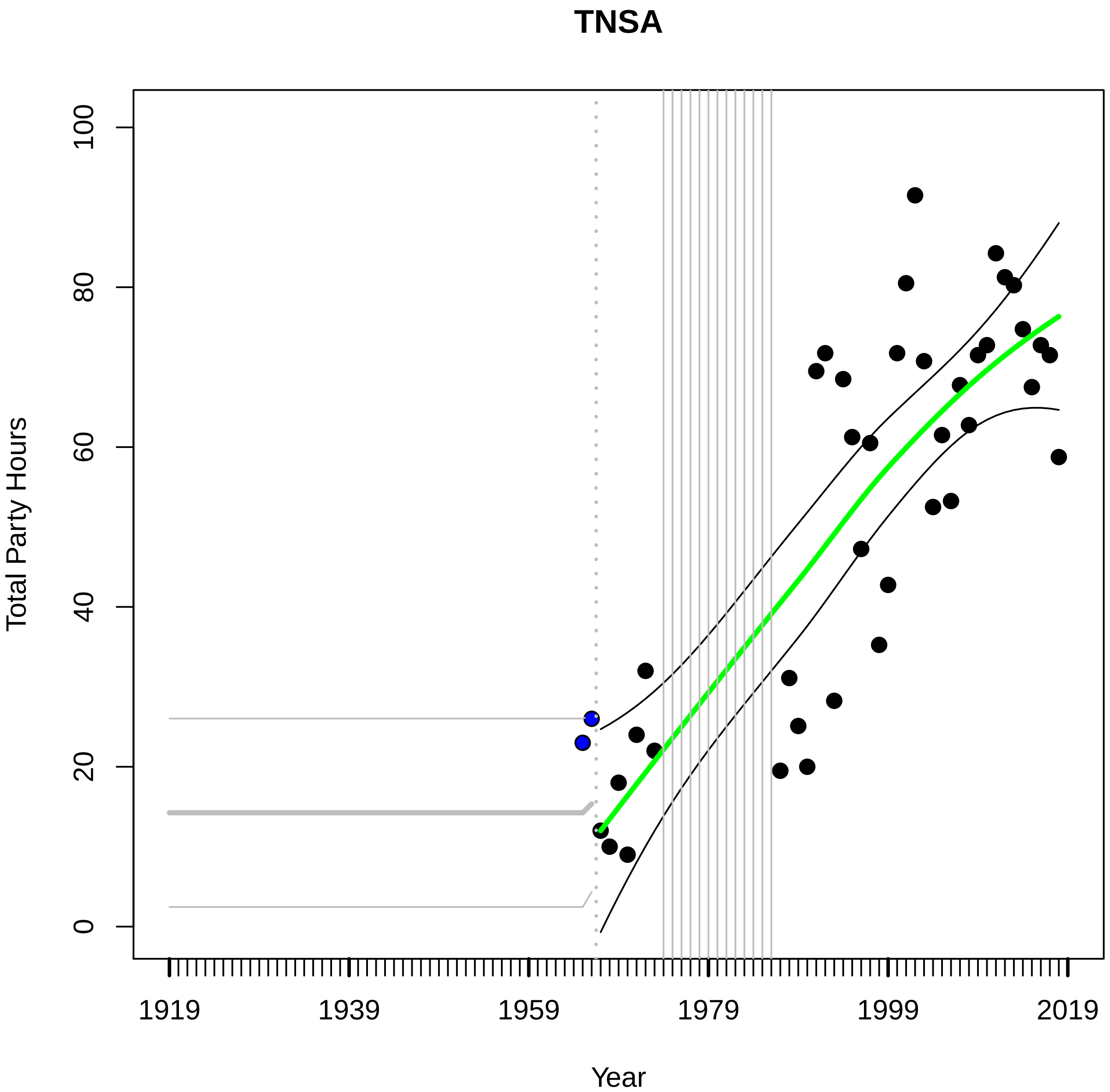

TNSA
Total Party Hours
0
20
40
60
80
100
1919
1939
1959
1979
1999
2019
Year

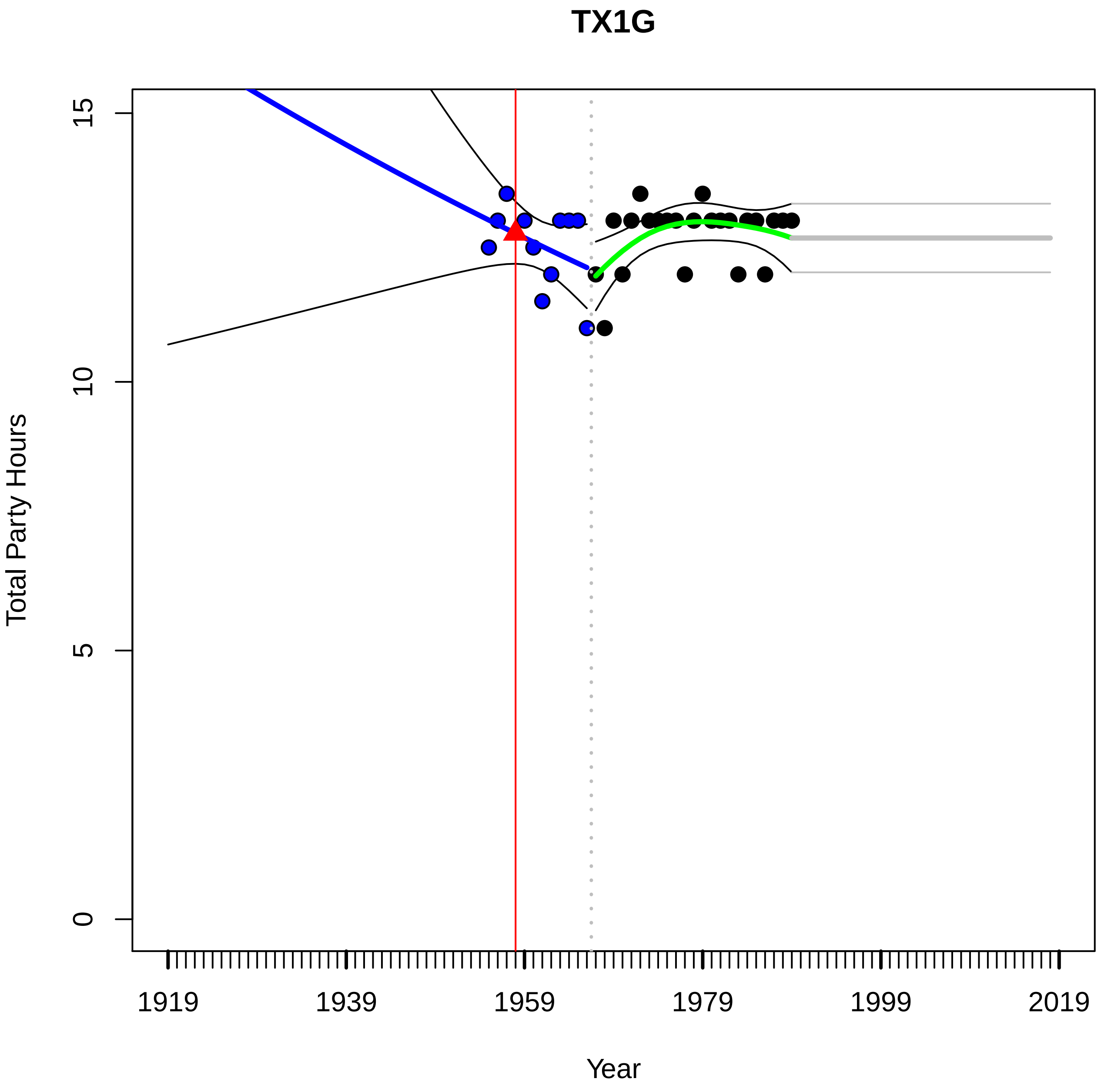
TX1G
Total Party Hours
0
5
10
15
1919
1939
1959
1979
1999
2019
Year

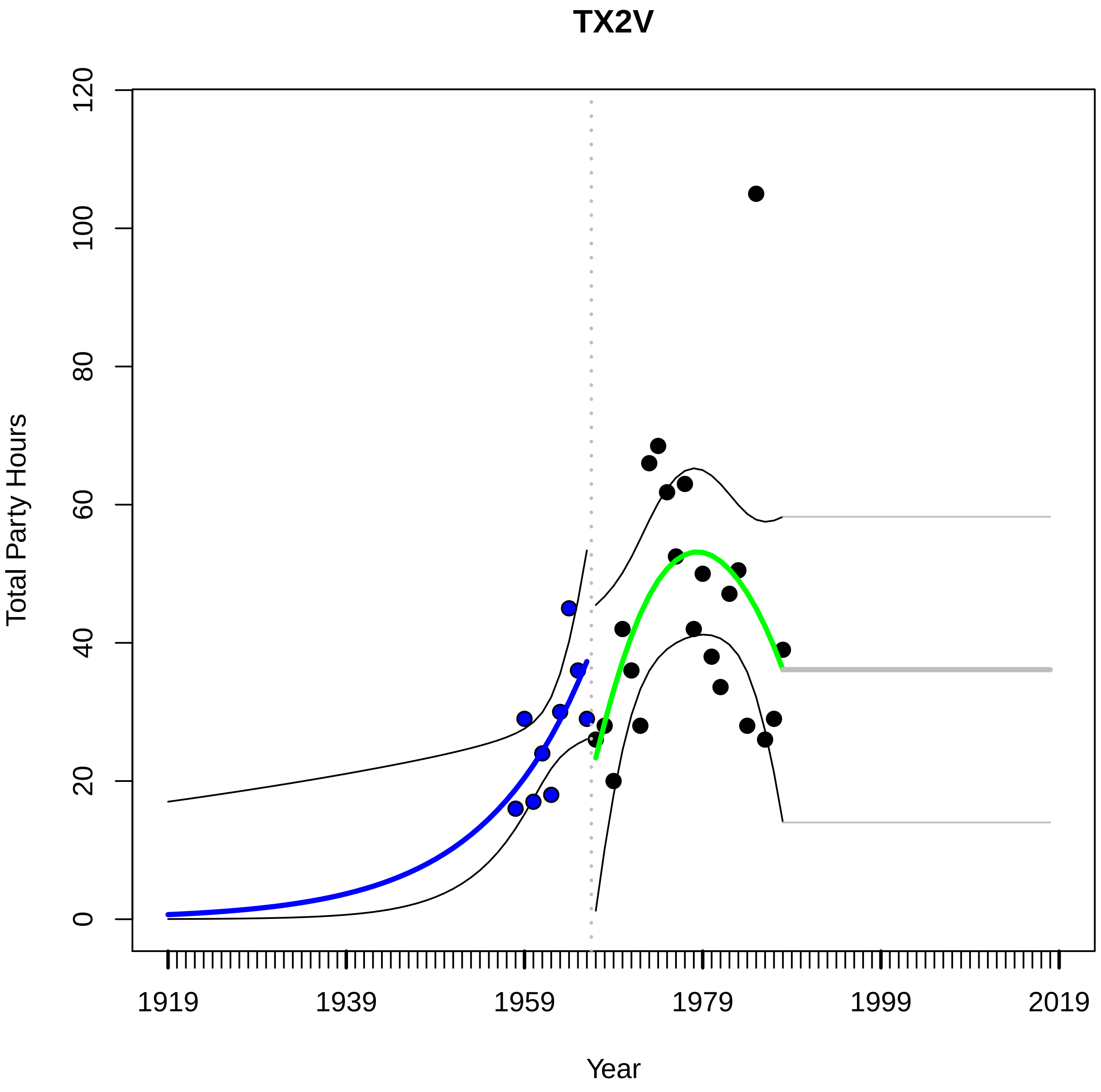
TX2V
Total Party Hours
0
20
40
60
80
100
120
1919
1939
1959
1979
1999
2019
Year

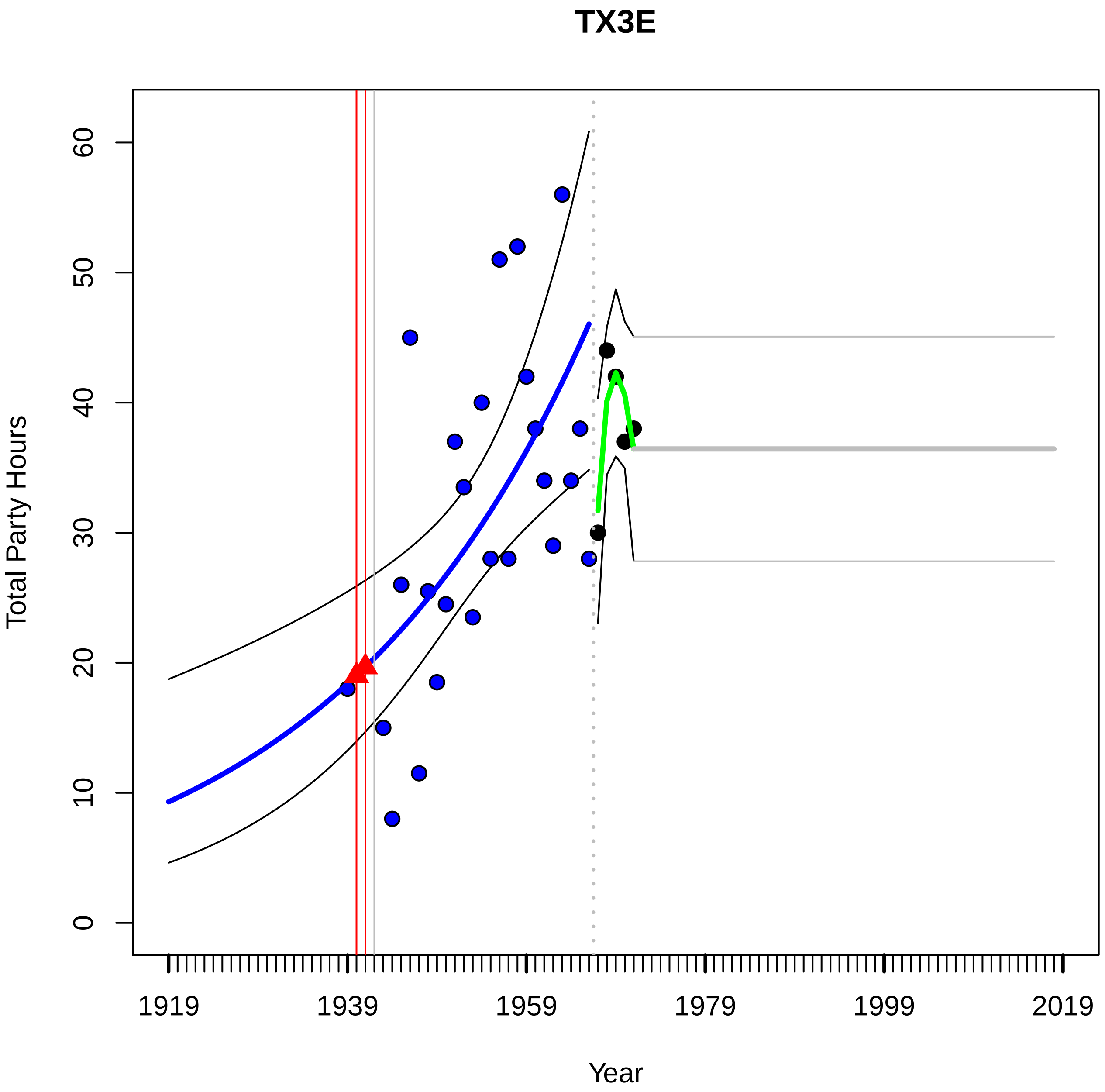

TX3E
Total Party Hours
0
10
20
30
40
50
60
1919
1939
1959
1979
1999
2019
Year

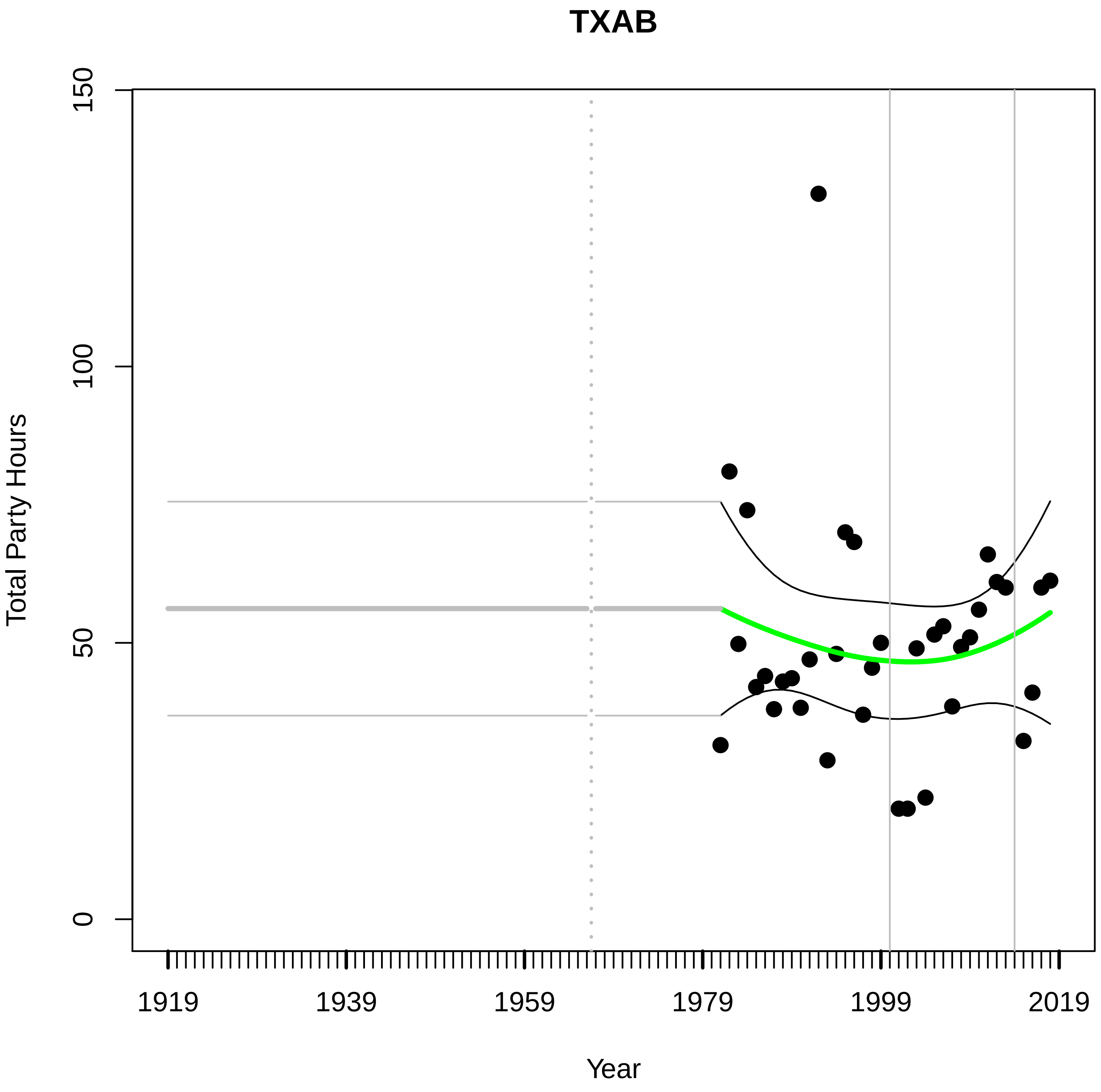

TXAB
Total Party Hours
150
100
50
0
1919
1939
1959
1979
1999
2019
Year

TXAM

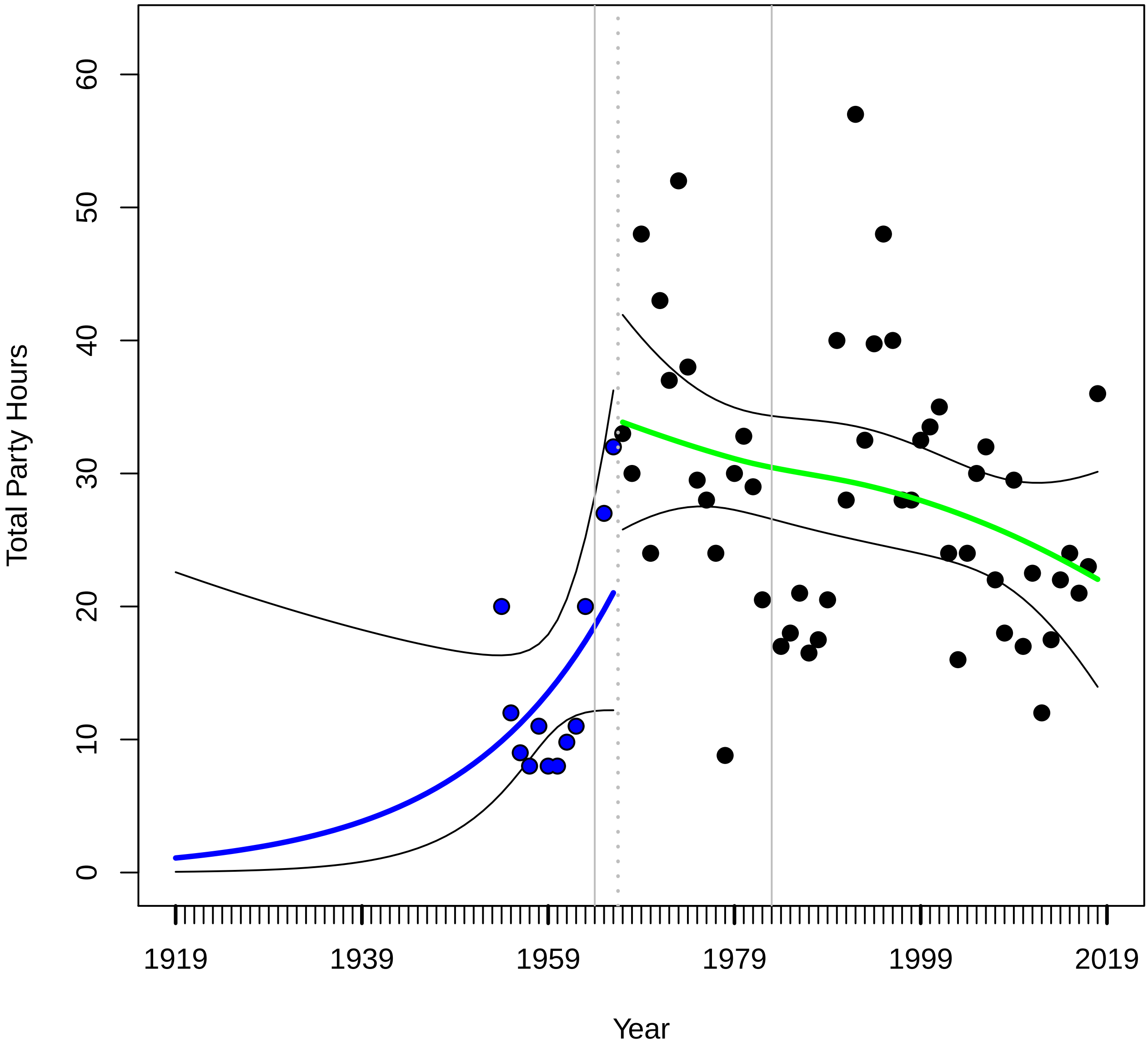

Total Party Hours
0
10
20
30
40
50
60
1919
1939
1959
1979
1999
2019
Year

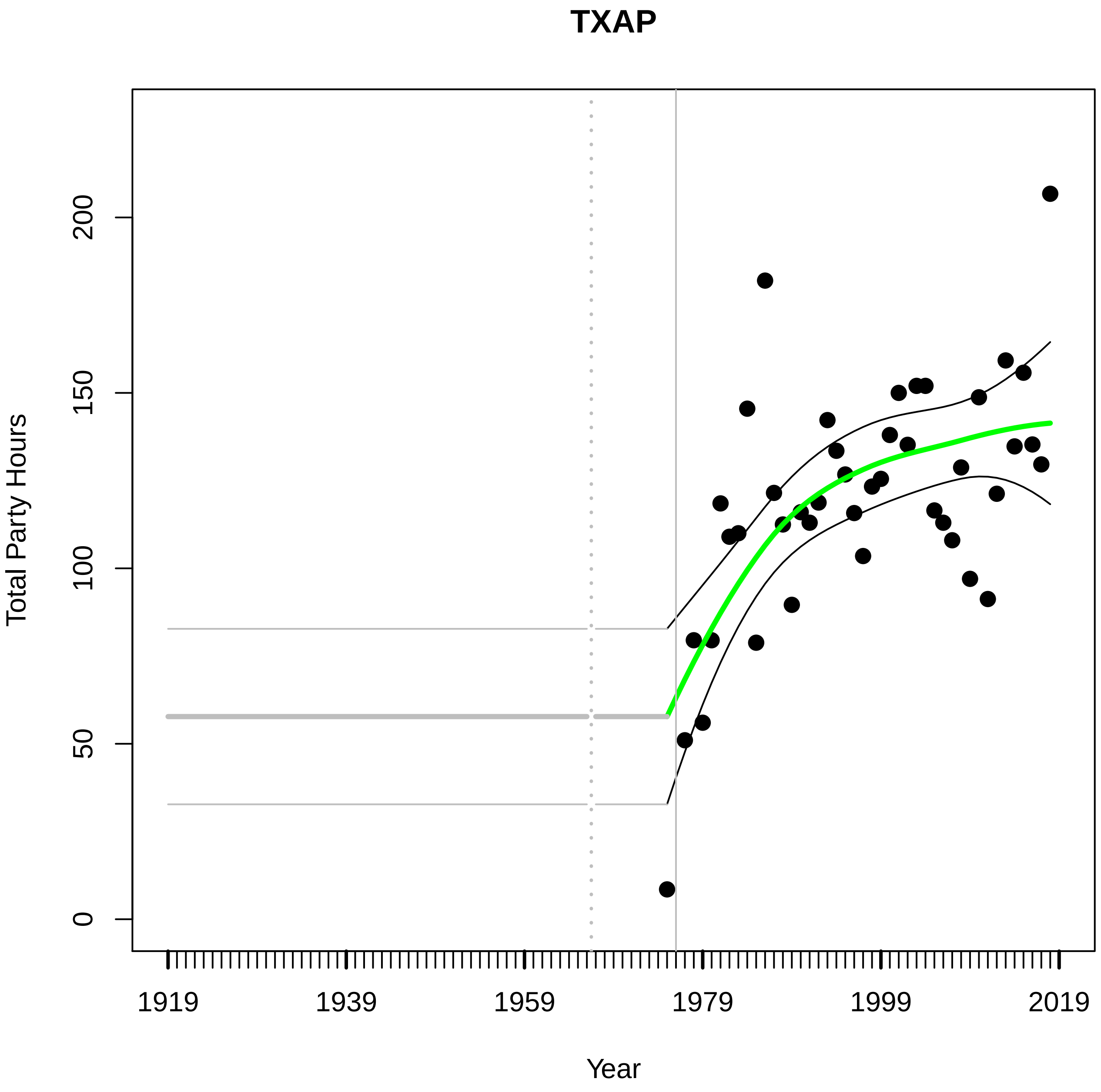

TXAP
Total Party Hours
0
50
100
150
200
1919
1939
1959
1979
1999
2019
Year

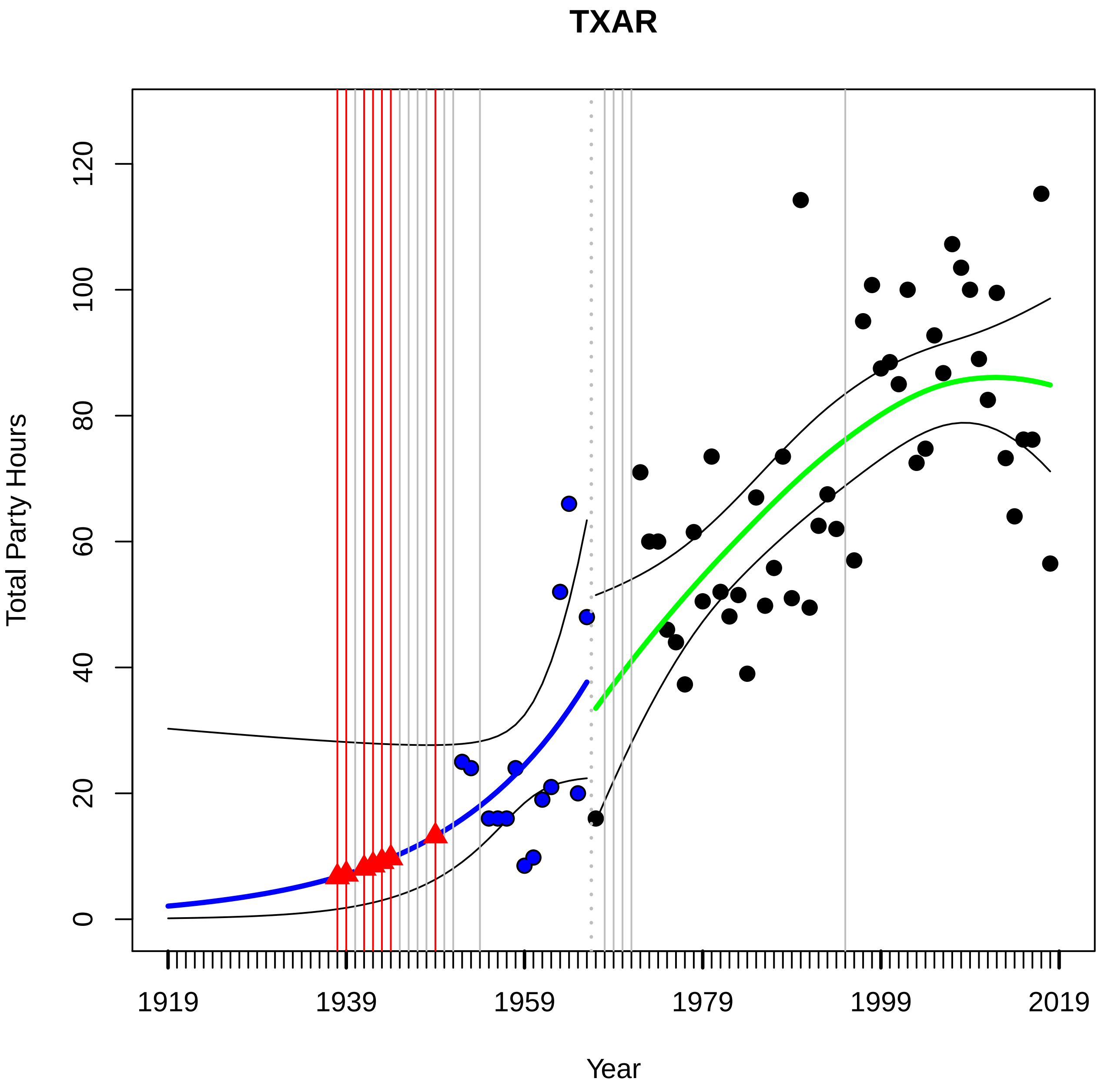
TXAR
Total Party Hours
0
20
40
60
80
100
120
1919
1939
1959
1979
1999
2019
Year

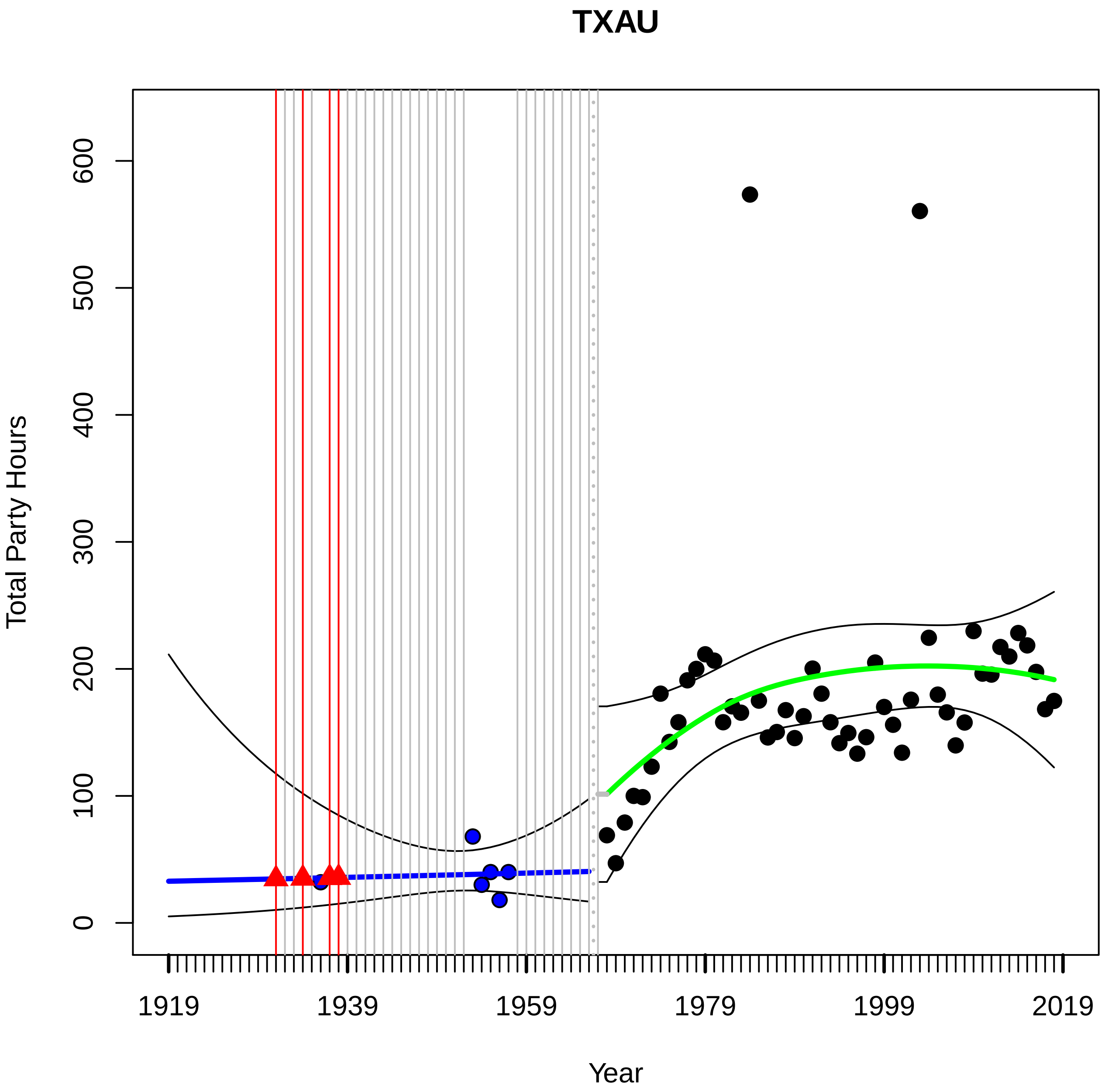

TXAU
Total Party Hours
0
100
200
300
400
500
600
1919
1939
1959
1979
1999
2019
Year

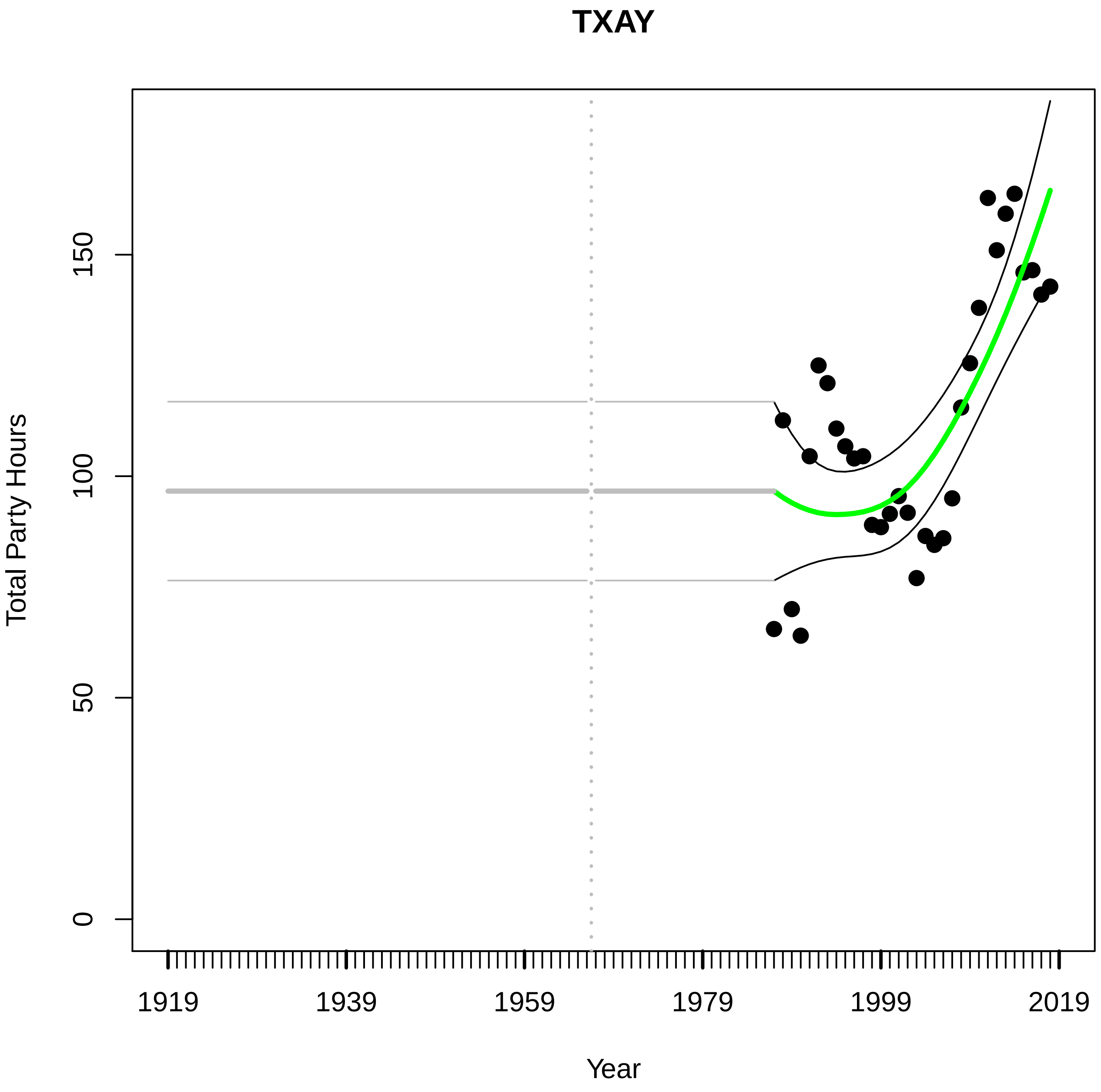

TXAY
Total Party Hours
0
50
100
150
1919
1939
1959
1979
1999
2019
Year

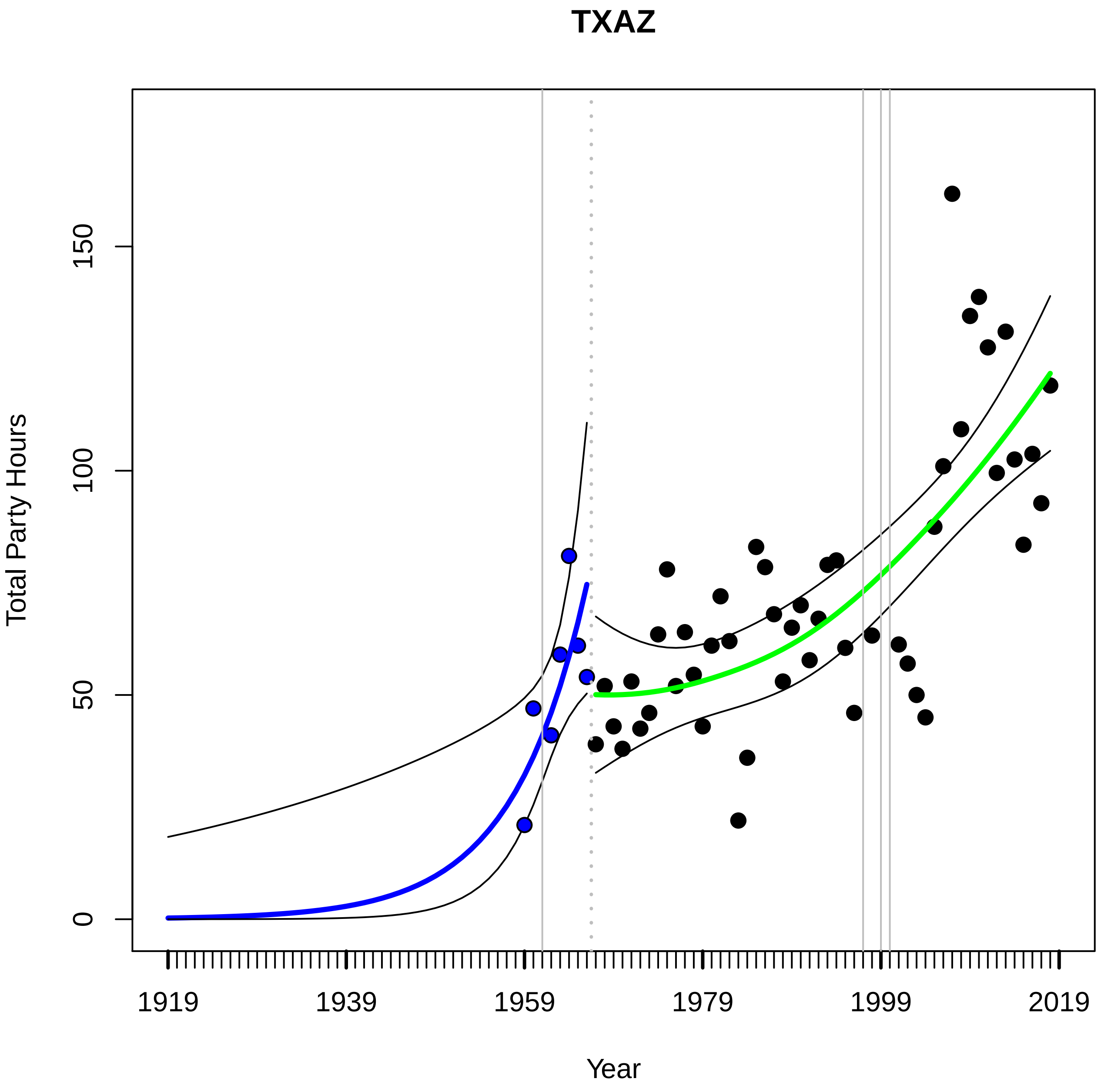
TXAZ
Total Party Hours
0
50
100
150
Year
1919
1939
1959
1979
1999
2019

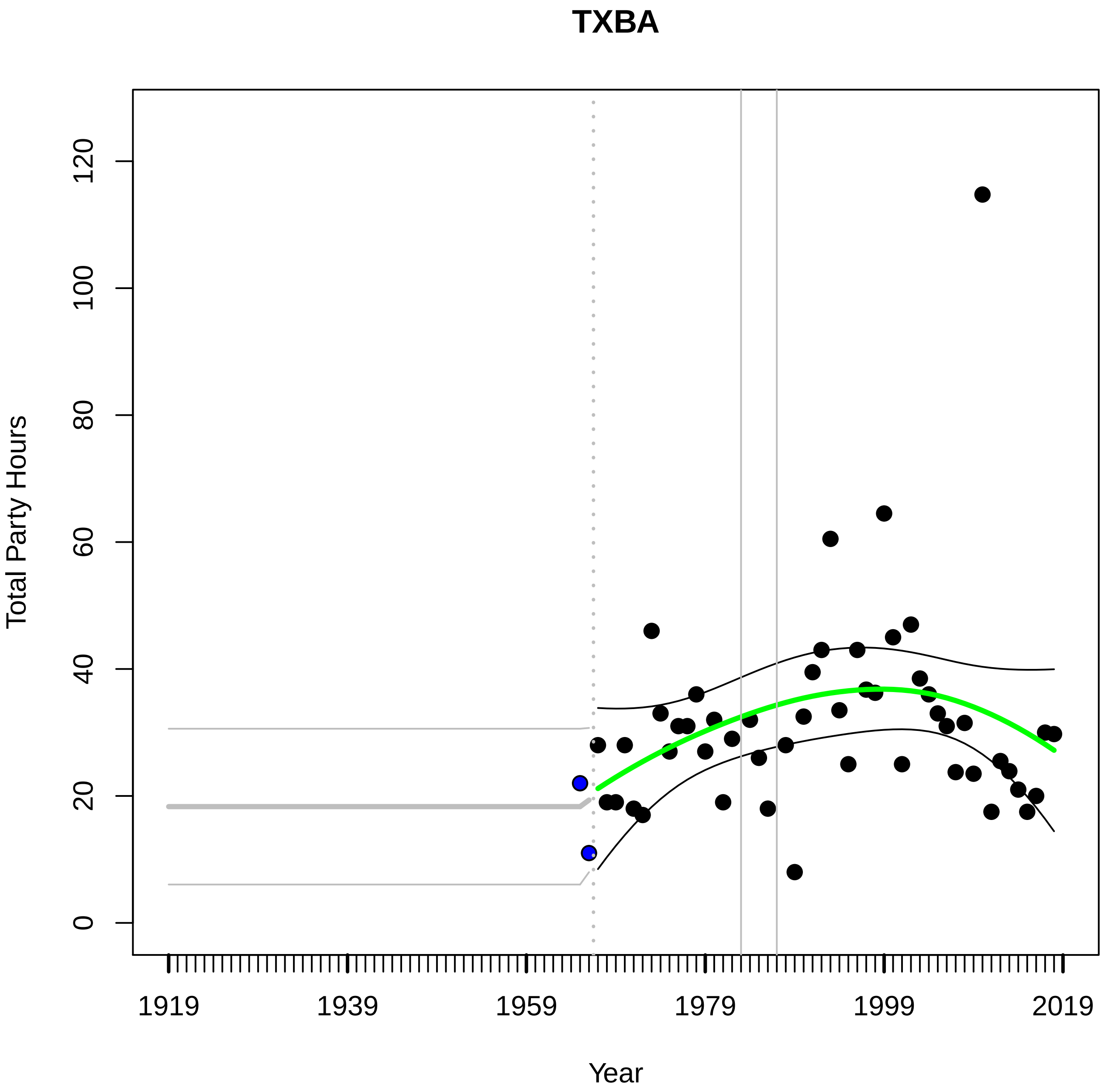

TXBA
Total Party Hours
0
20
40
60
80
100
120
1919
1939
1959
1979
1999
2019
Year

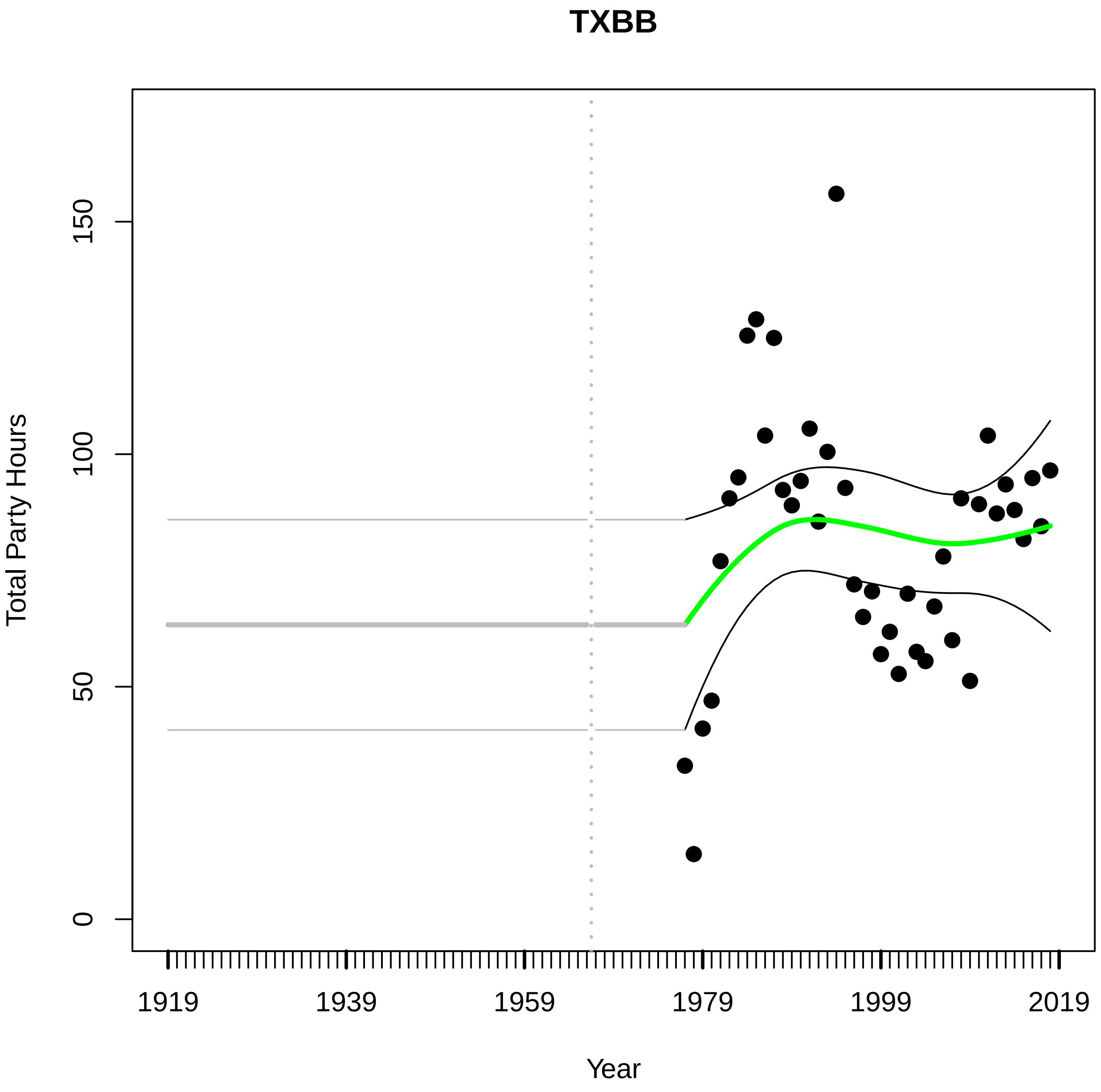

TXBB
Total Party Hours
0
50
100
150
1919
1939
1959
1979
1999
2019
Year

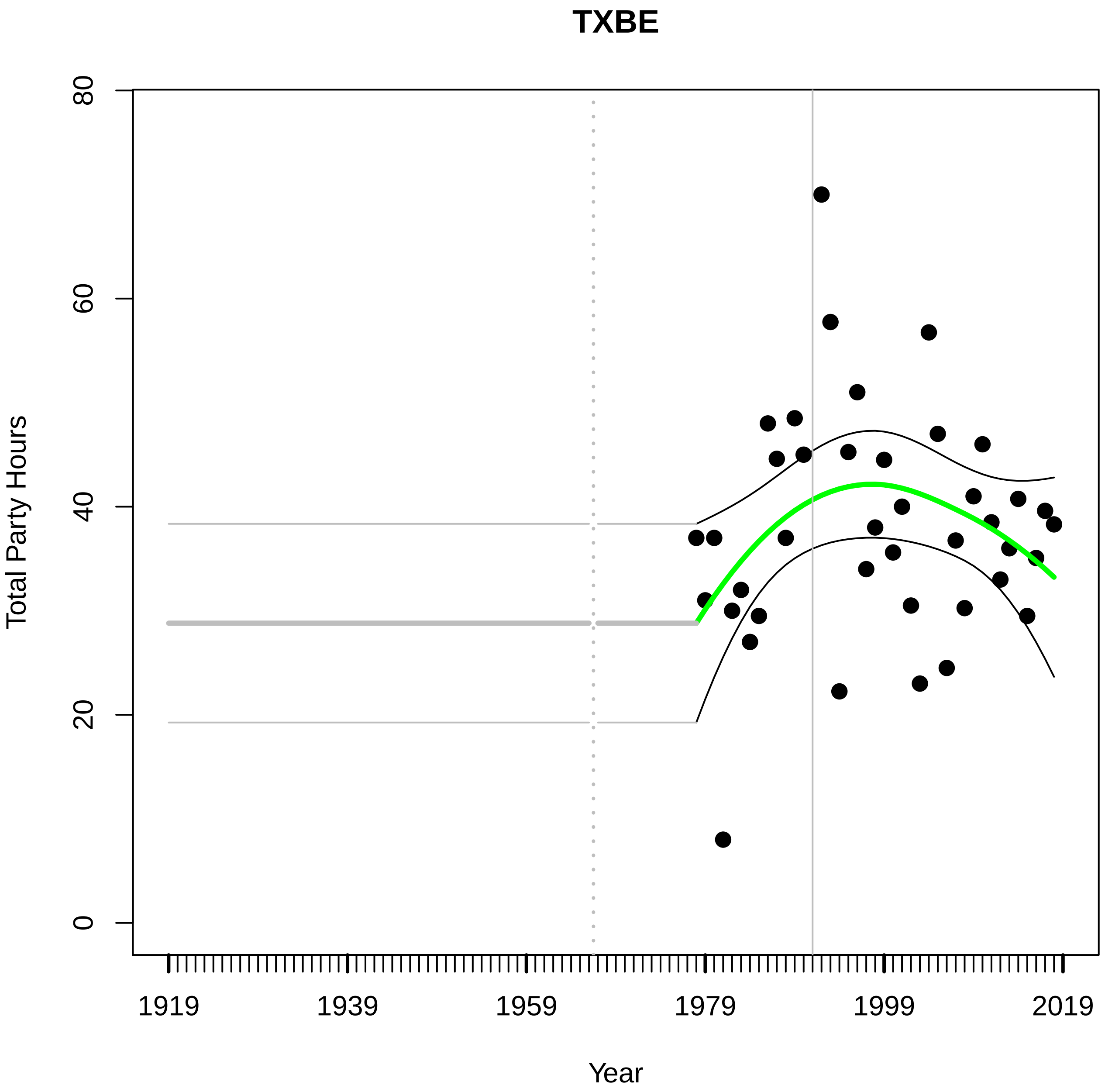
TXBE
Total Party Hours
0
20
40
60
80
Year
1919
1939
1959
1979
1999
2019

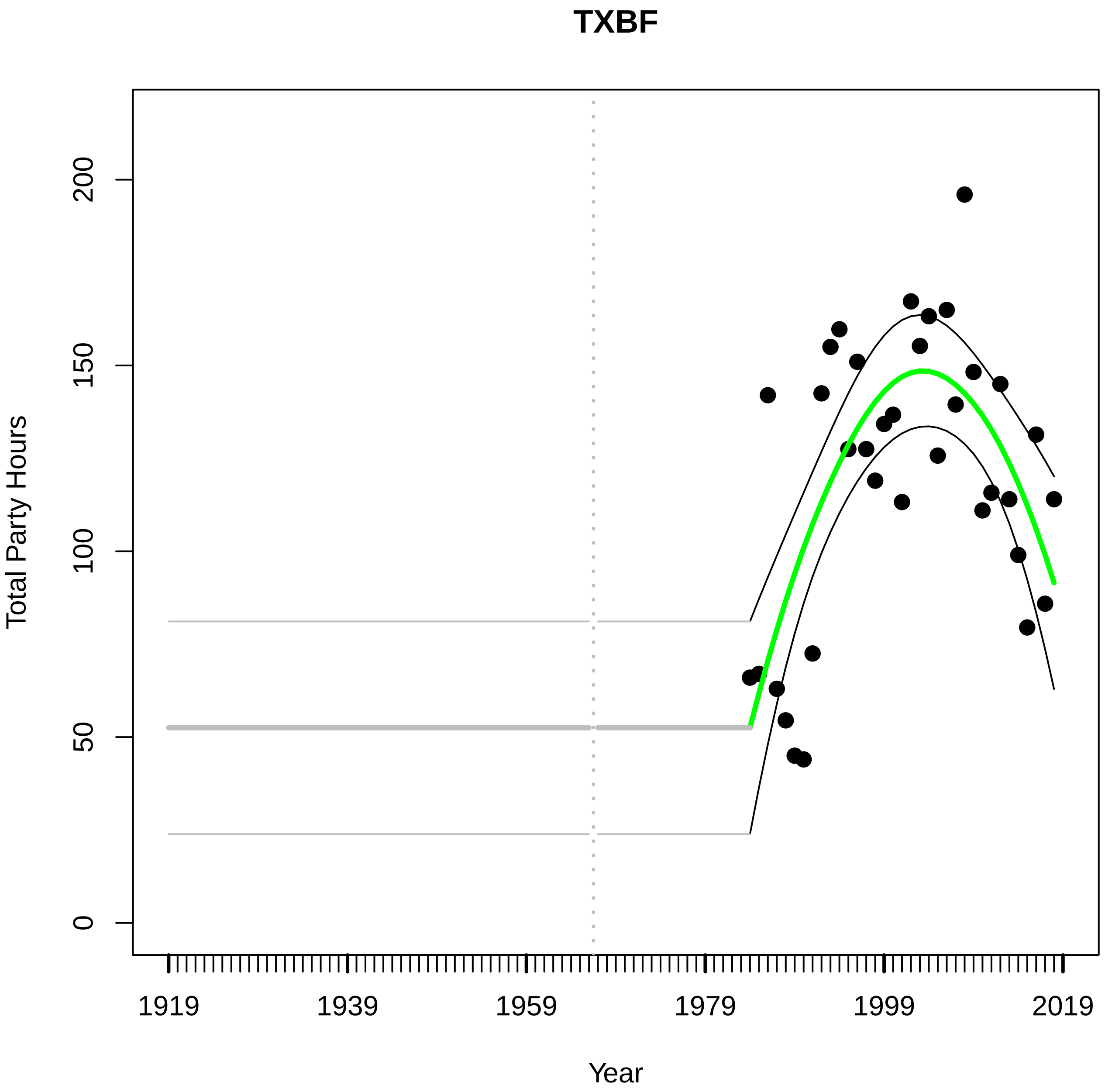

TXBF
Total Party Hours
0
50
100
150
200
1919
1939
1959
1979
1999
2019
Year

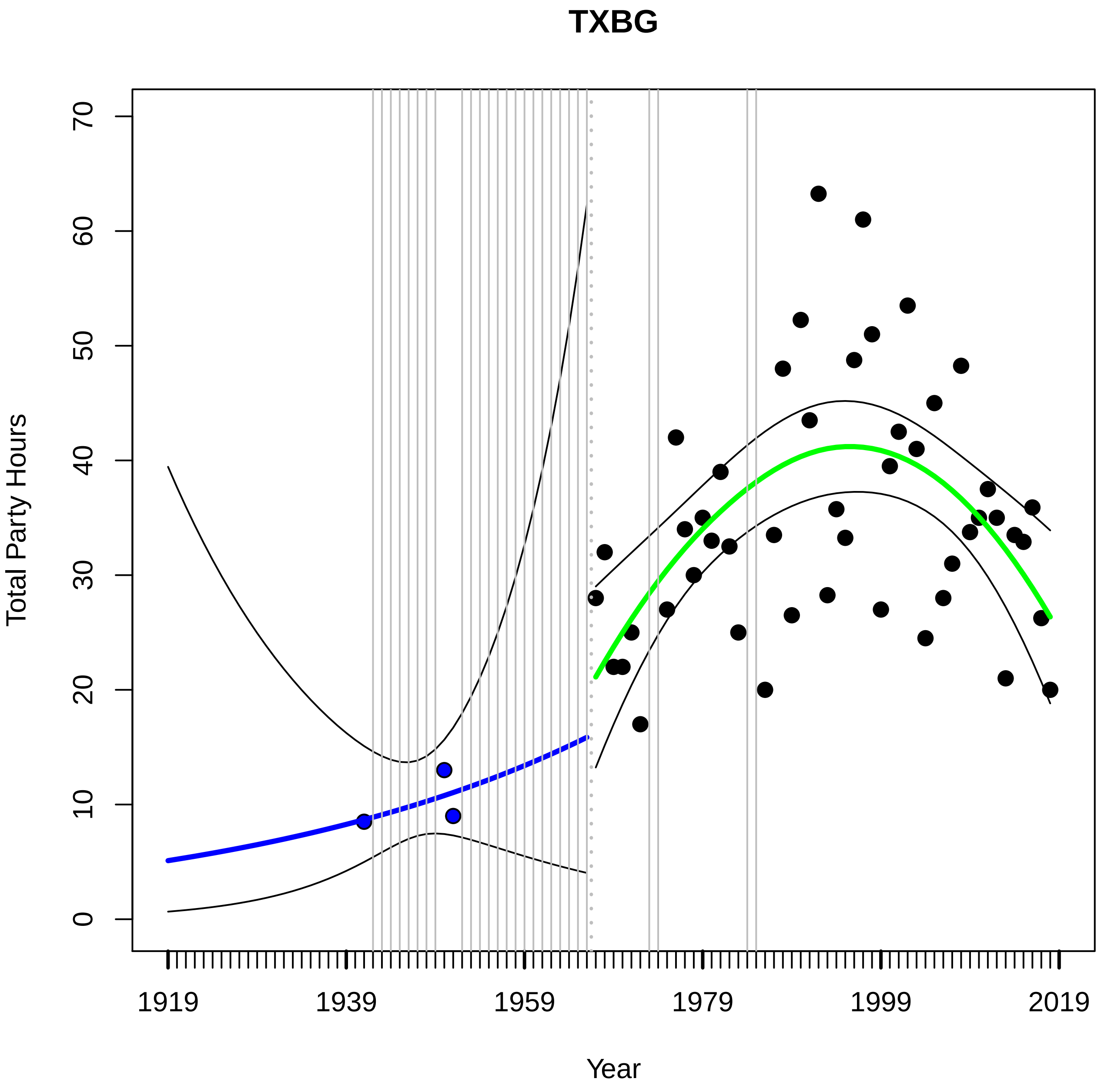

TXBG
Total Party Hours
0
10
20
30
40
50
60
70
1919
1939
1959
1979
1999
2019
Year

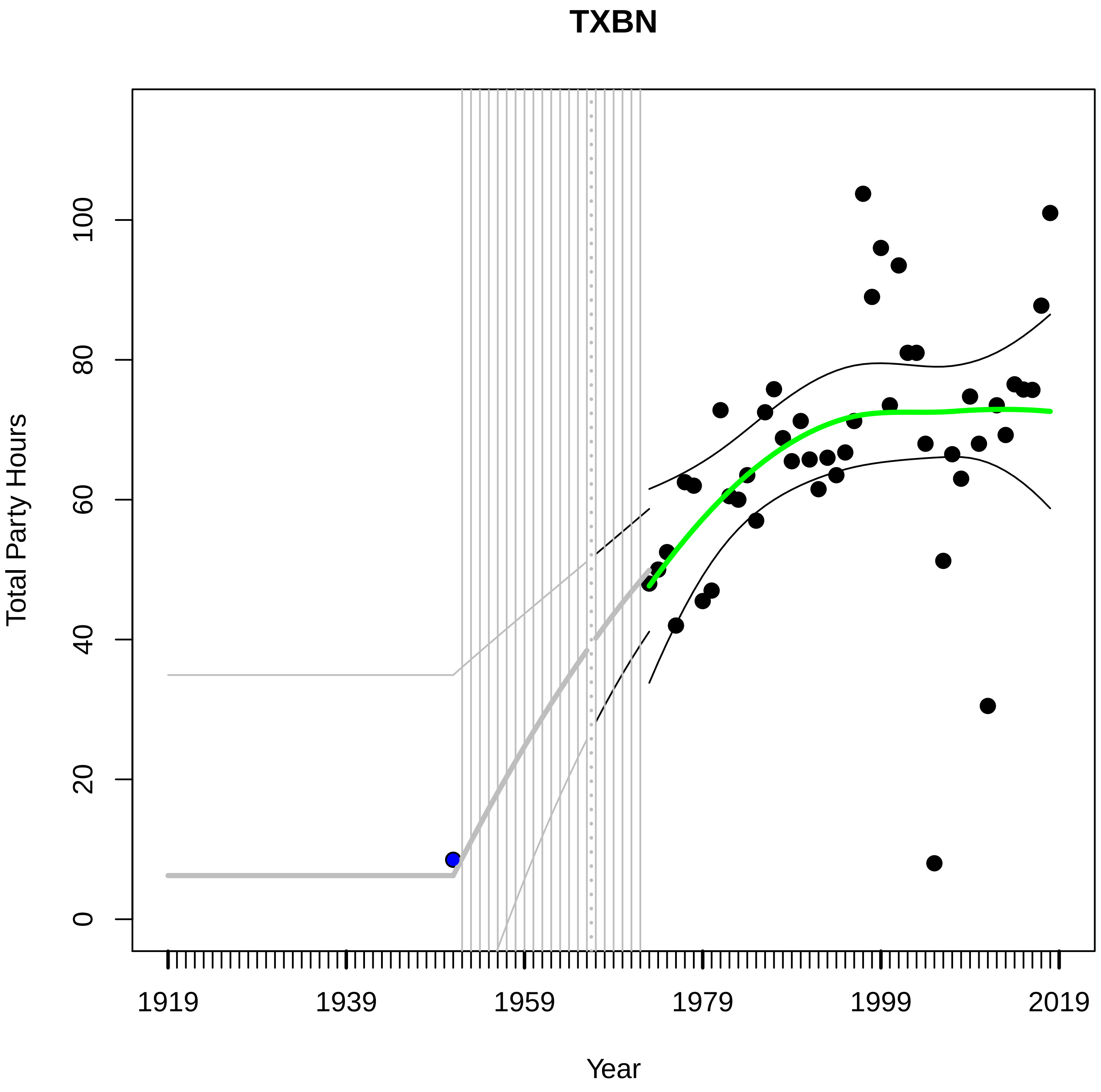

TXBN
Total Party Hours
0
20
40
60
80
100
1919
1939
1959
1979
1999
2019
Year

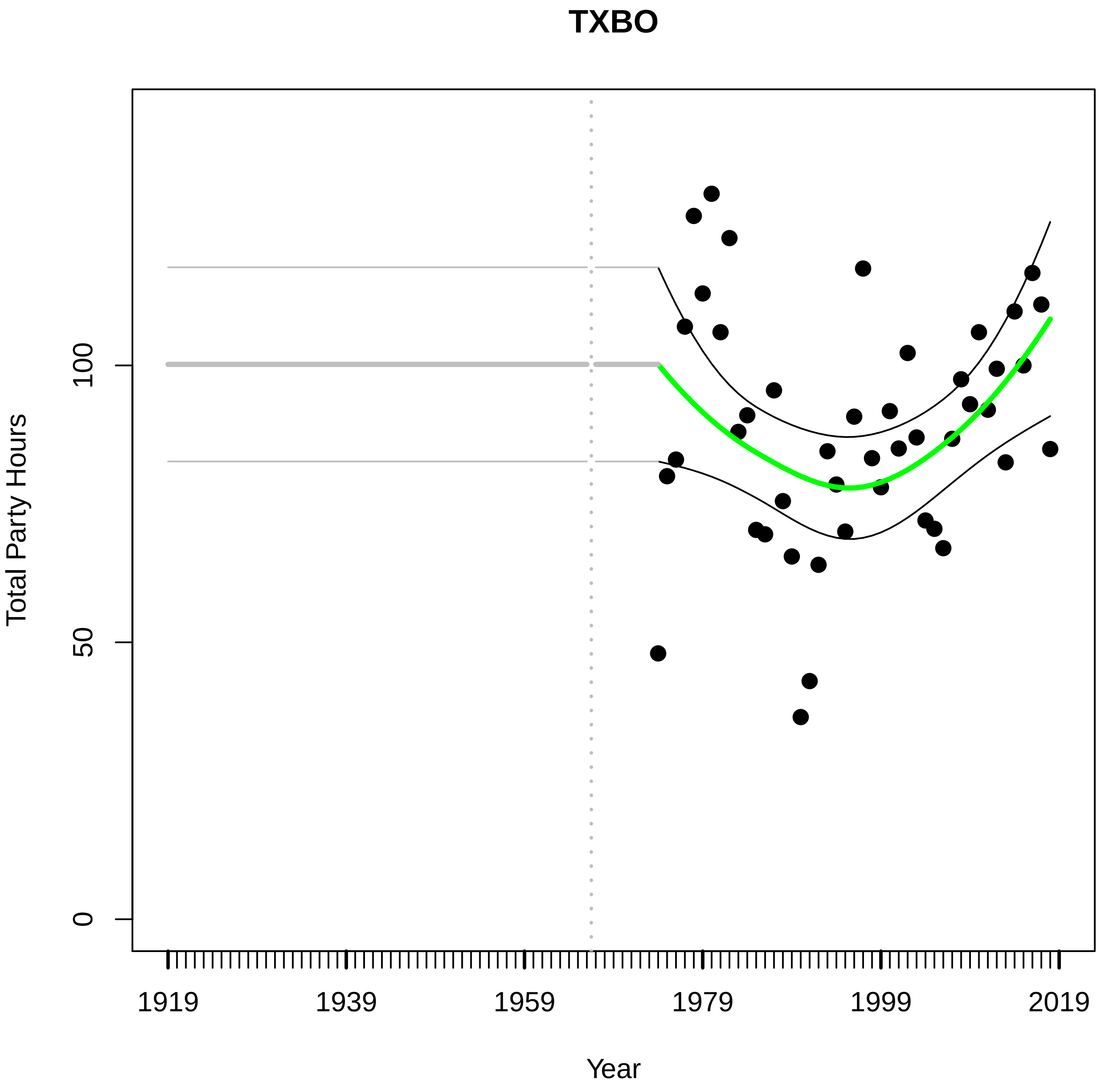
TXBO
Total Party Hours
0
50
100
1919
1939
1959
1979
1999
2019
Year

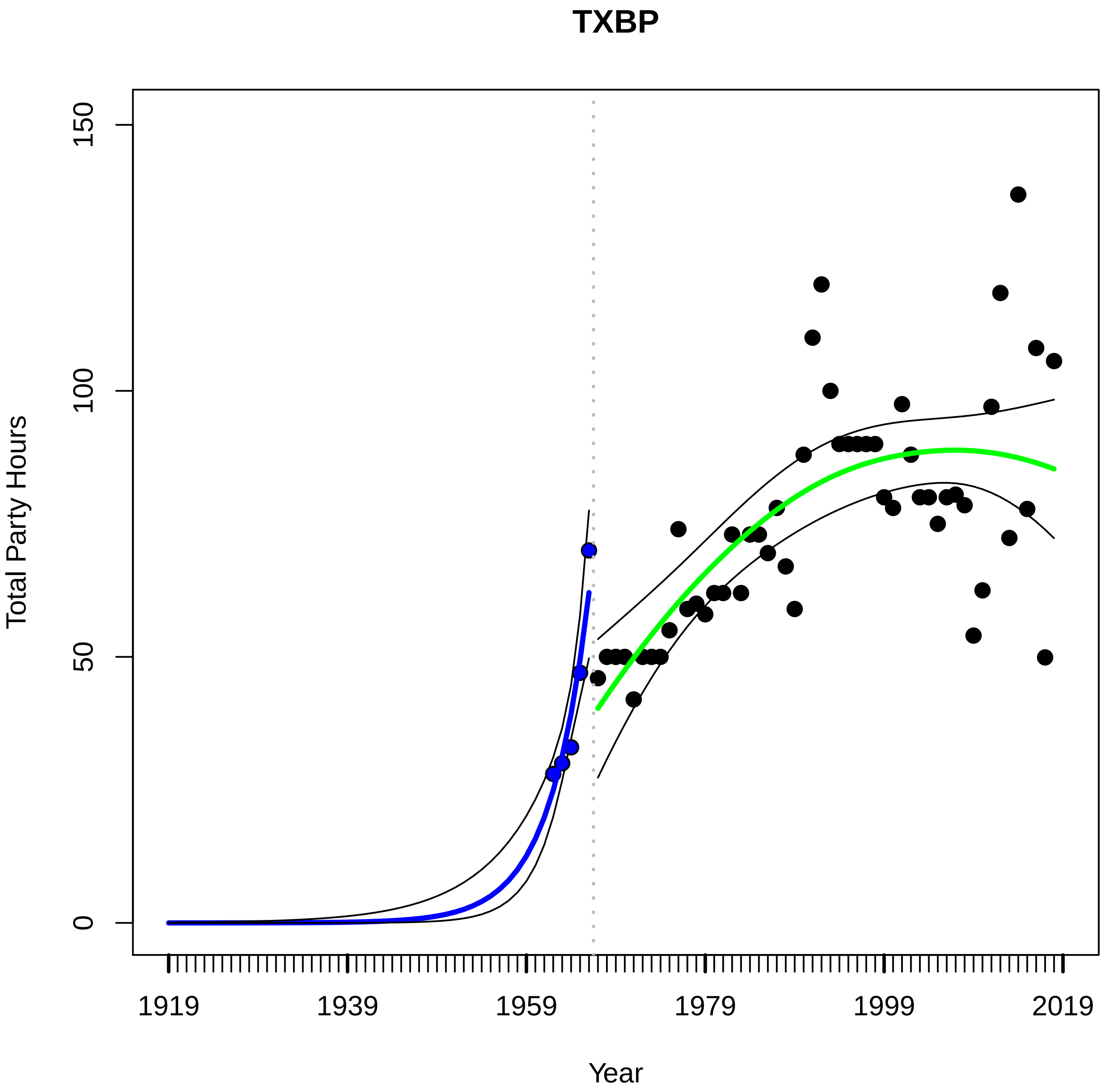
TXBP
Total Party Hours
0
50
100
150
1919
1939
1959
1979
1999
2019
Year

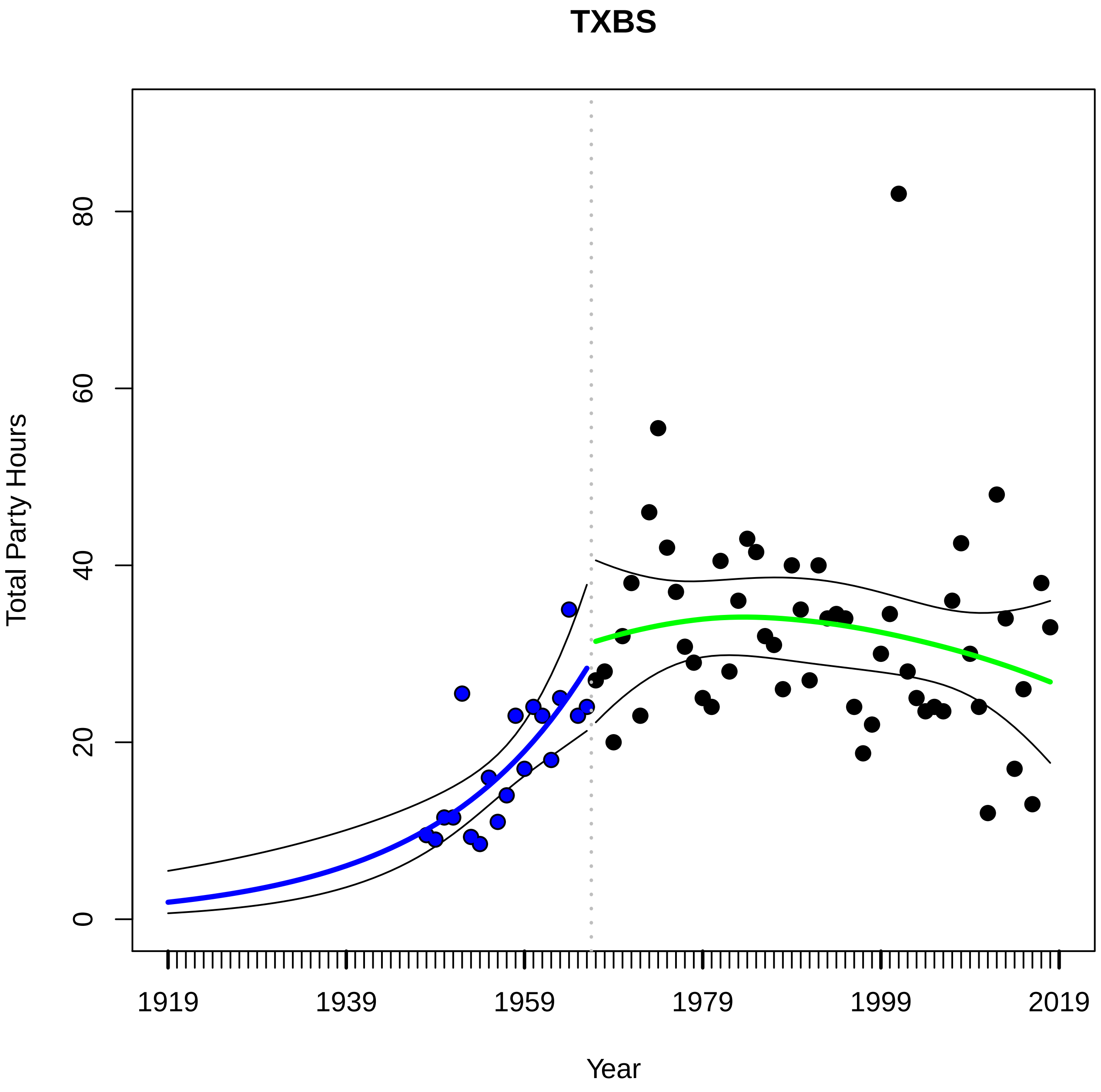
TXBS
Total Party Hours
0
20
40
60
80
Year
1919
1939
1959
1979
1999
2019

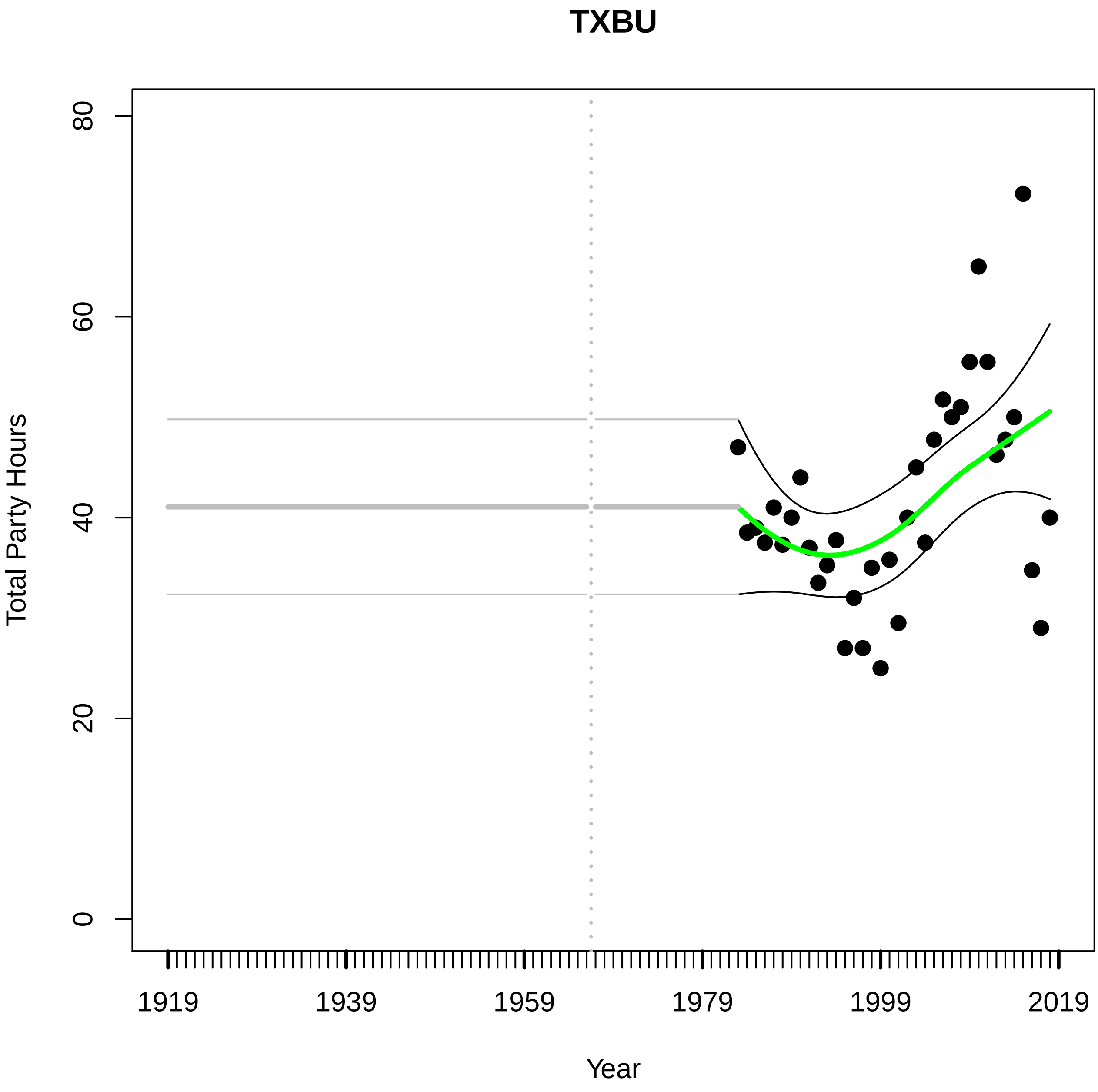
TXBU
Total Party Hours
0
20
40
60
80
1919
1939
1959
1979
1999
2019
Year

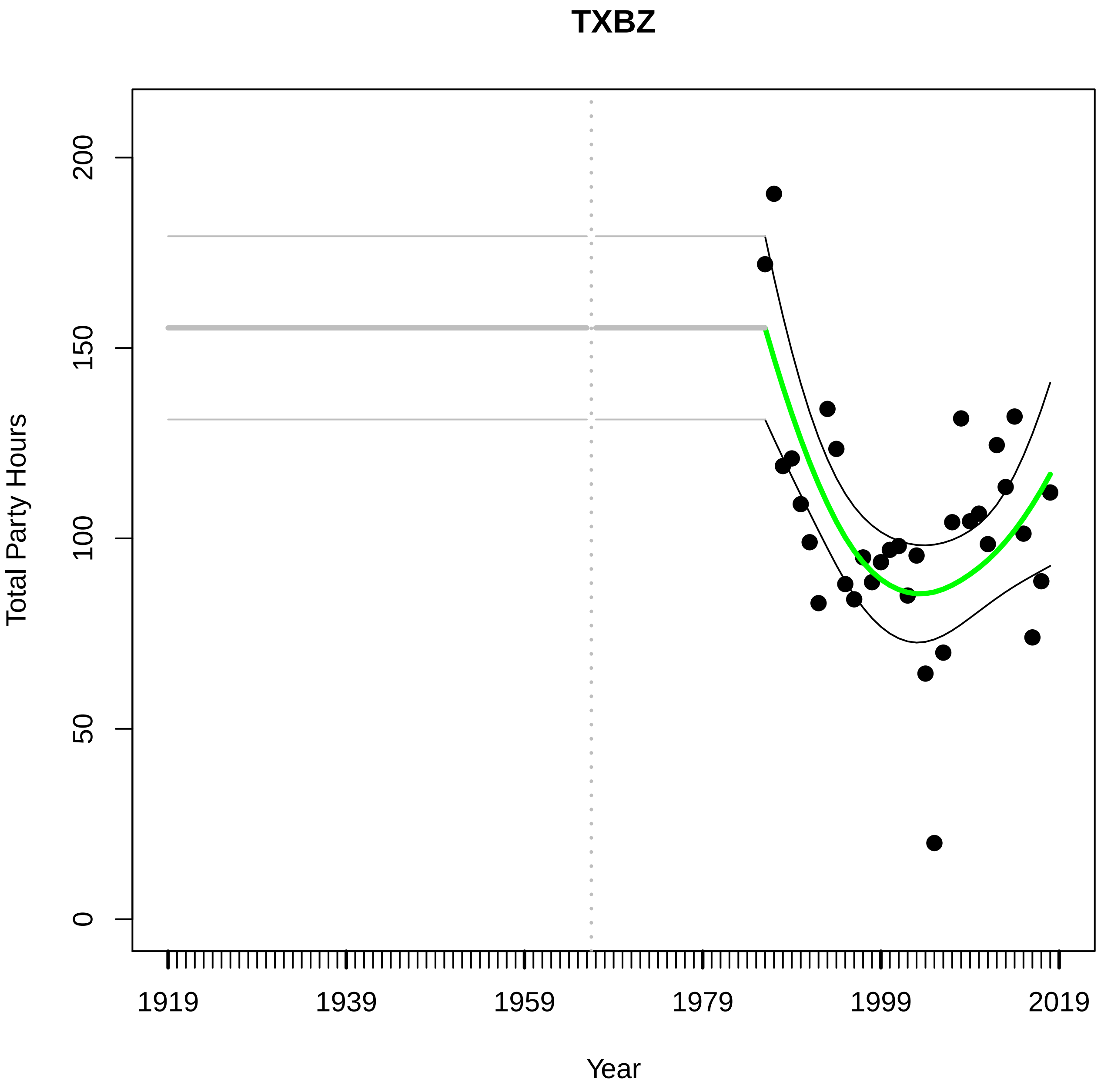
TXBZ
Total Party Hours
0
50
100
150
200
1919
1939
1959
1979
1999
2019
Year

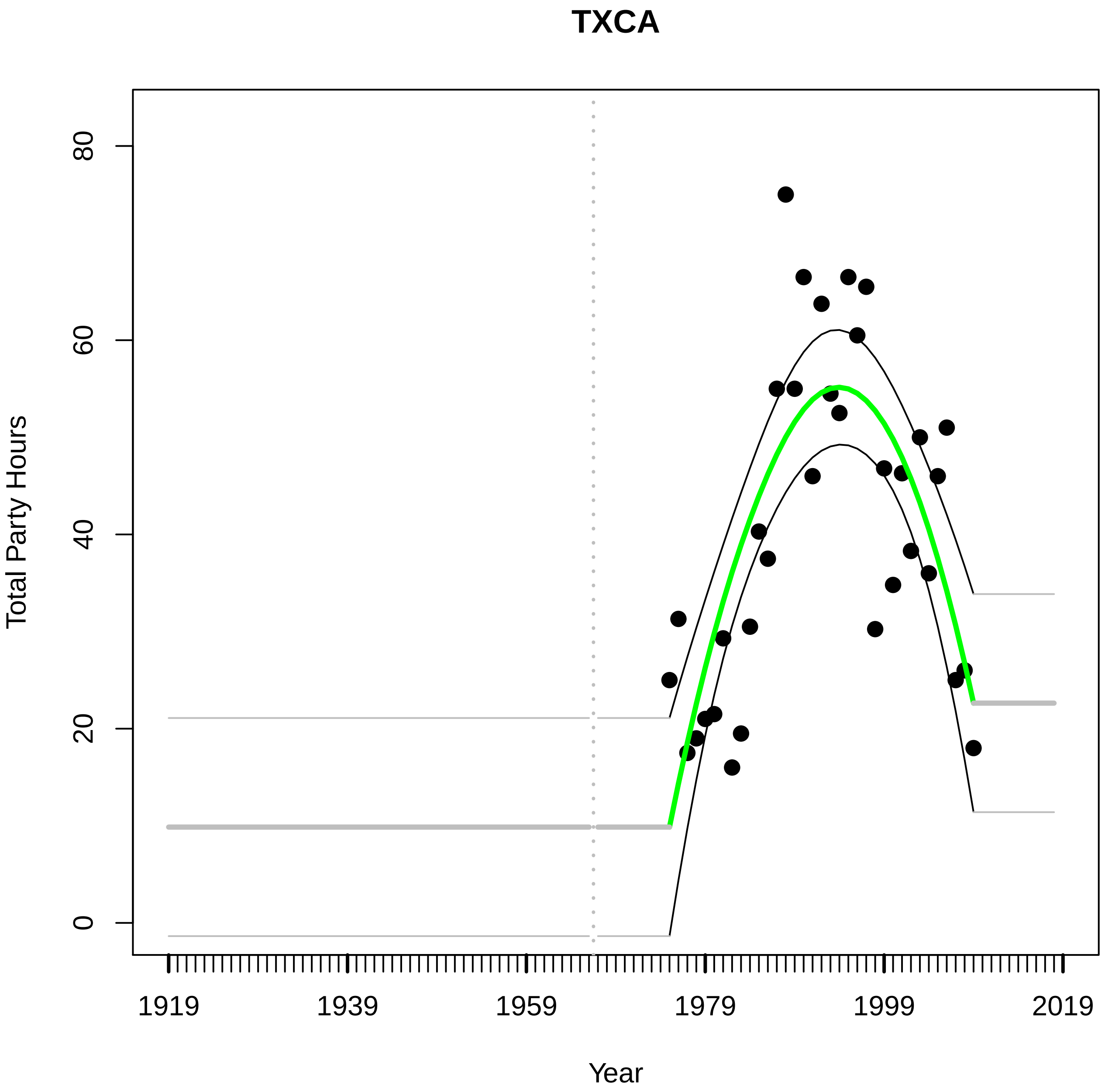

TXCA
Total Party Hours
0
20
40
60
80
1919
1939
1959
1979
1999
2019
Year

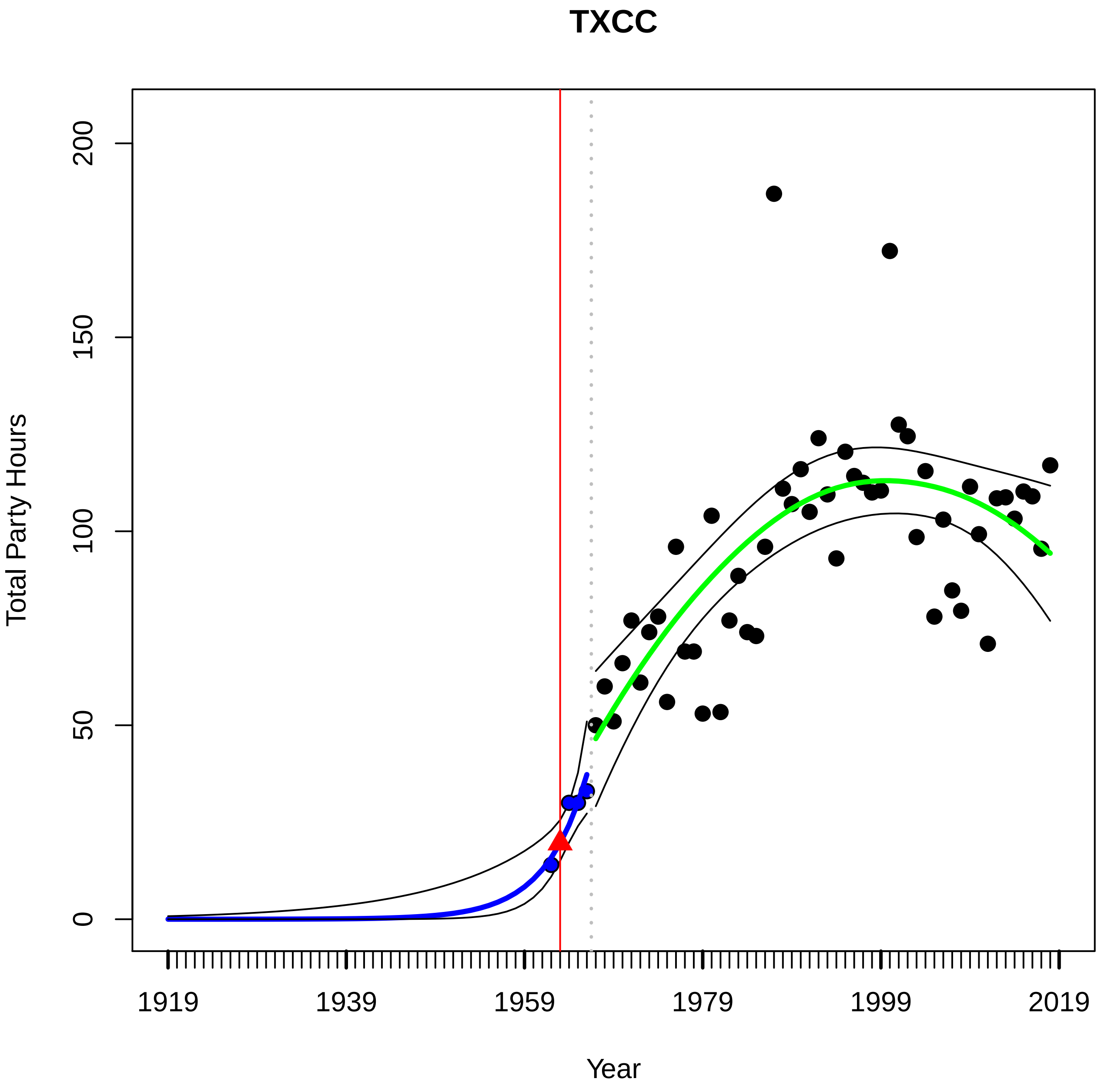
TXCC
Total Party Hours
0
50
100
150
200
1919
1939
1959
1979
1999
2019
Year

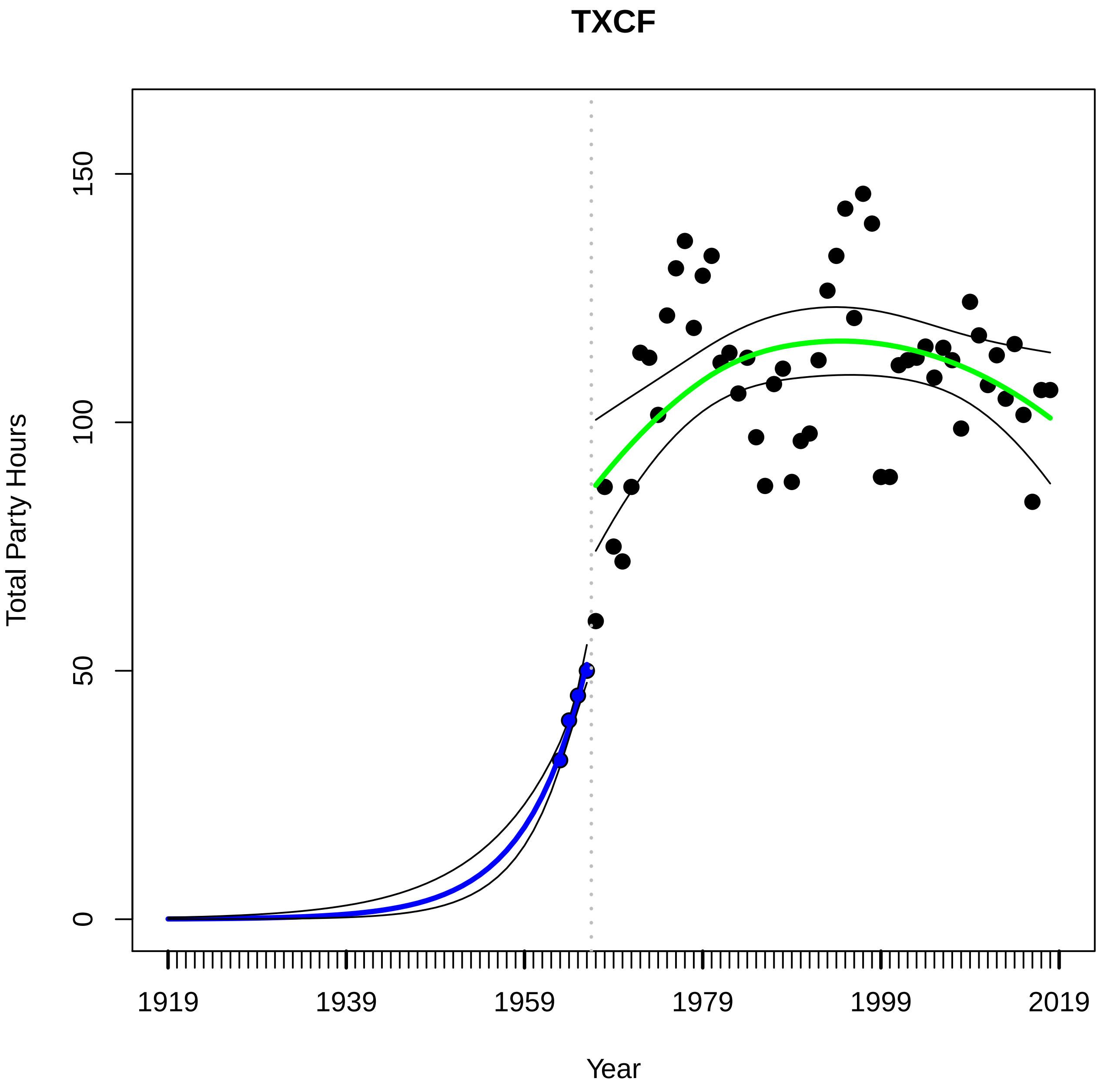
TXCF
Total Party Hours
0
50
100
150
1919
1939
1959
1979
1999
2019
Year

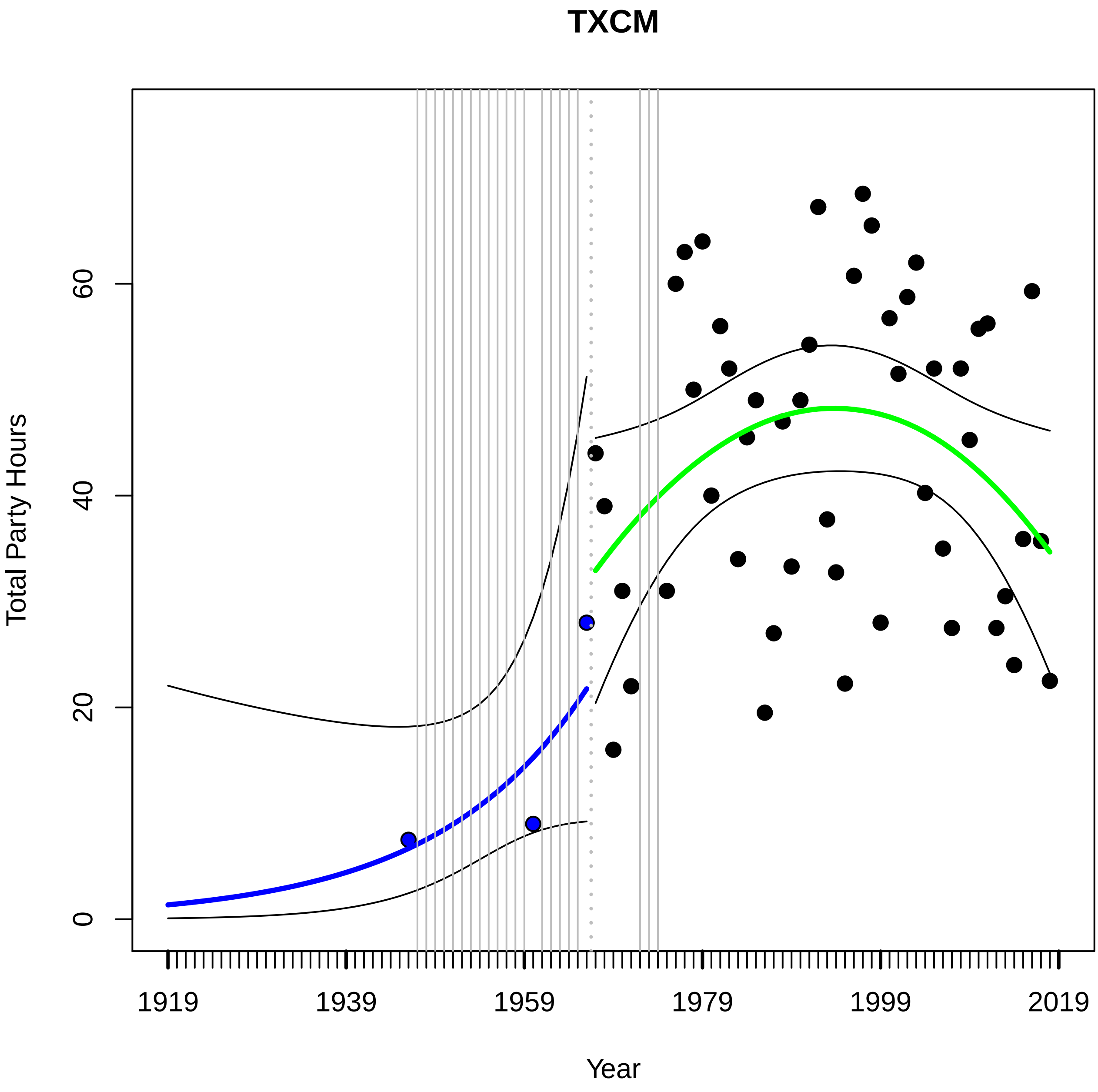

TXCM
Total Party Hours
0
20
40
60
1919
1939
1959
1979
1999
2019
Year

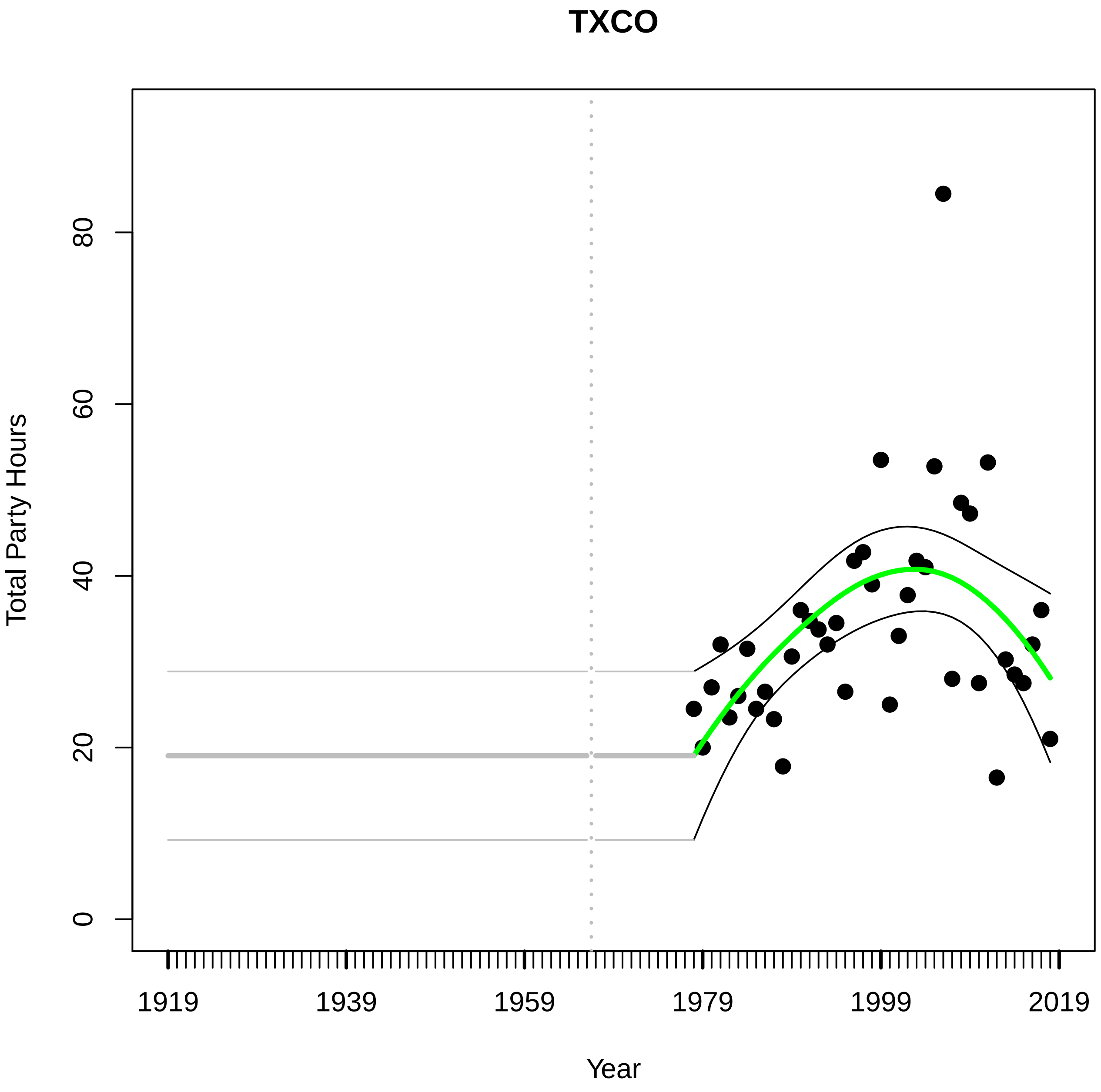

TXCO
Total Party Hours
0
20
40
60
80
1919
1939
1959
1979
1999
2019
Year

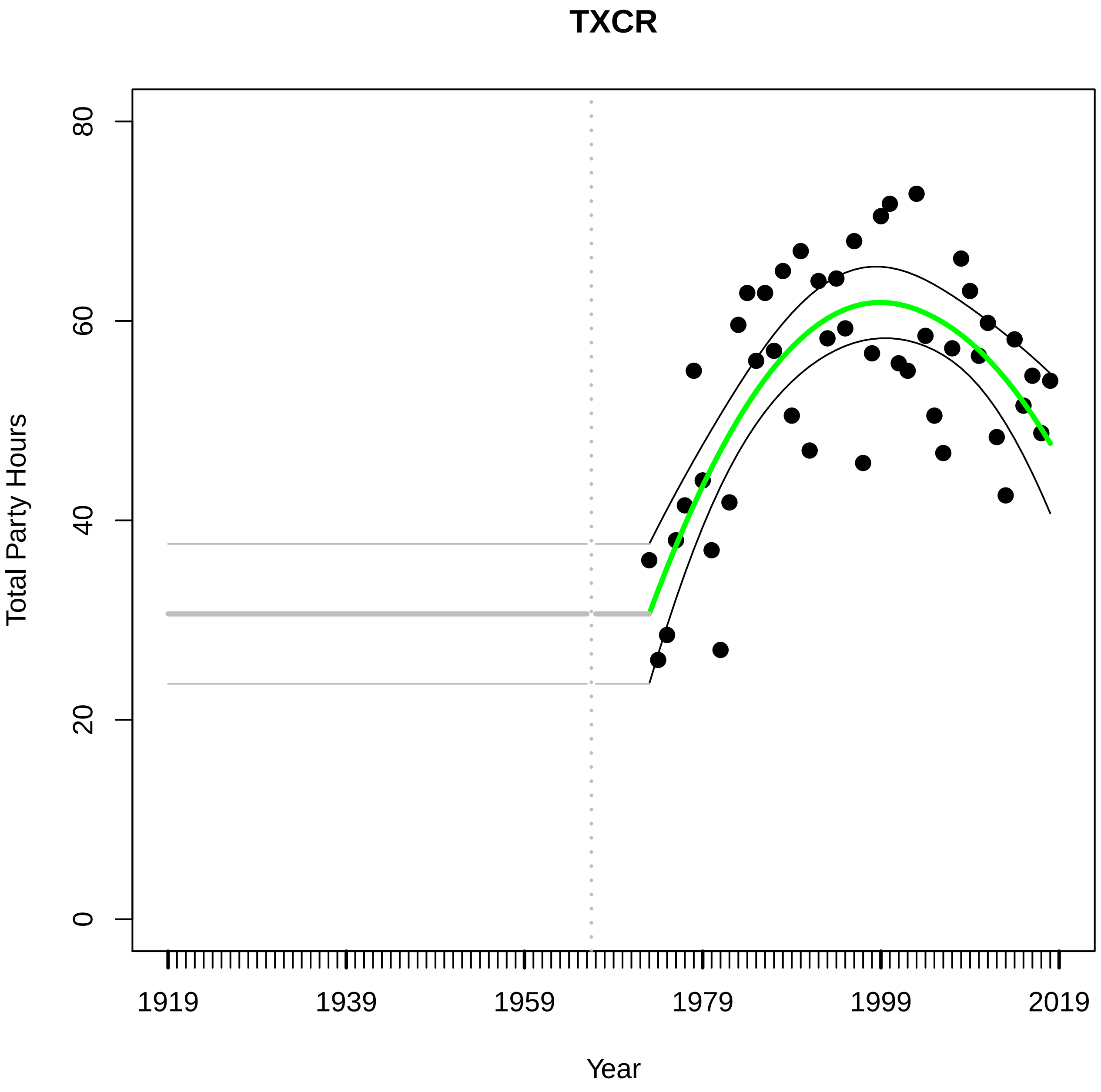
TXCR
Total Party Hours
0
20
40
60
80
Year
1919
1939
1959
1979
1999
2019

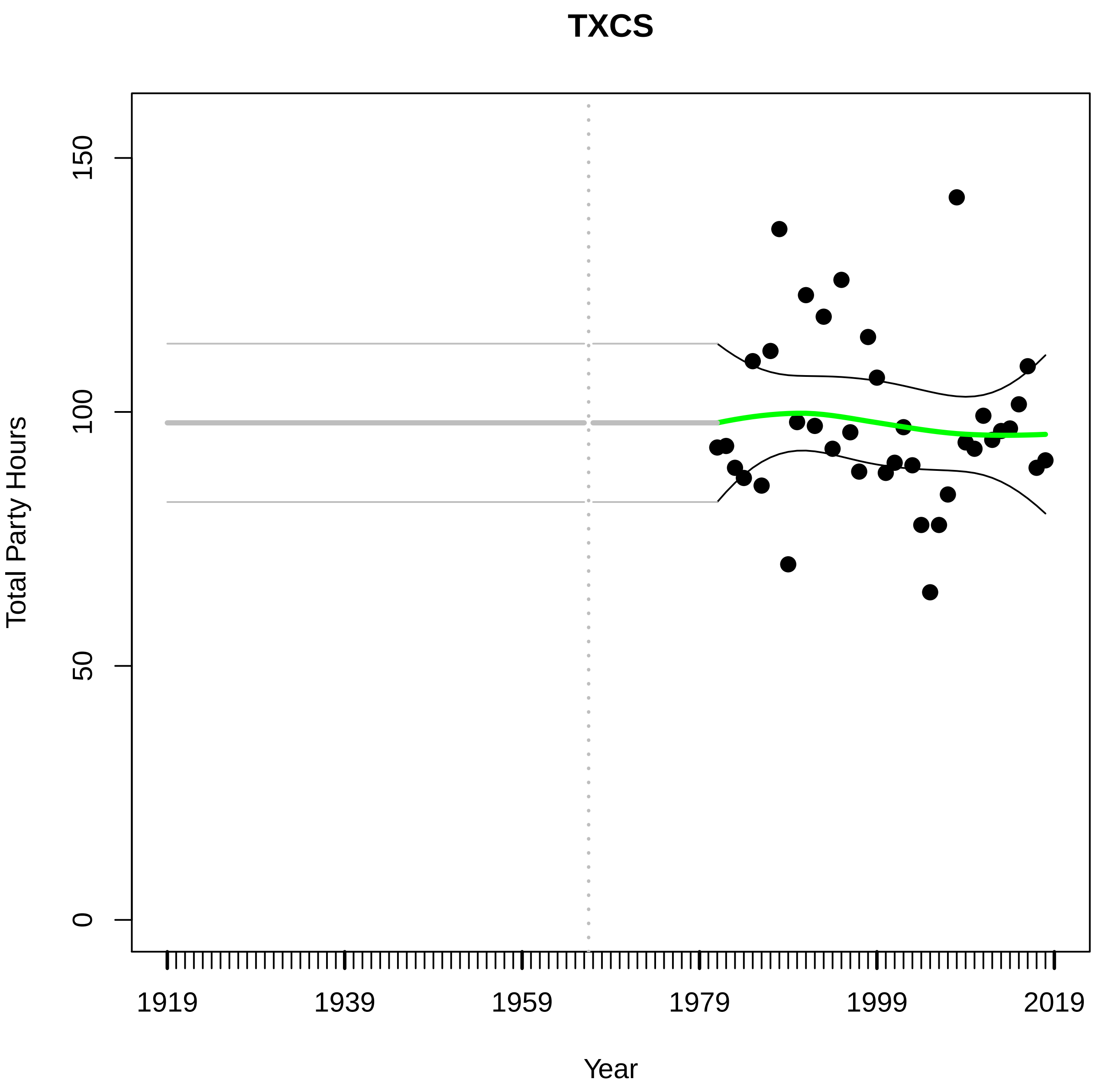

TXCS
Total Party Hours
0
50
100
150
1919
1939
1959
1979
1999
2019
Year

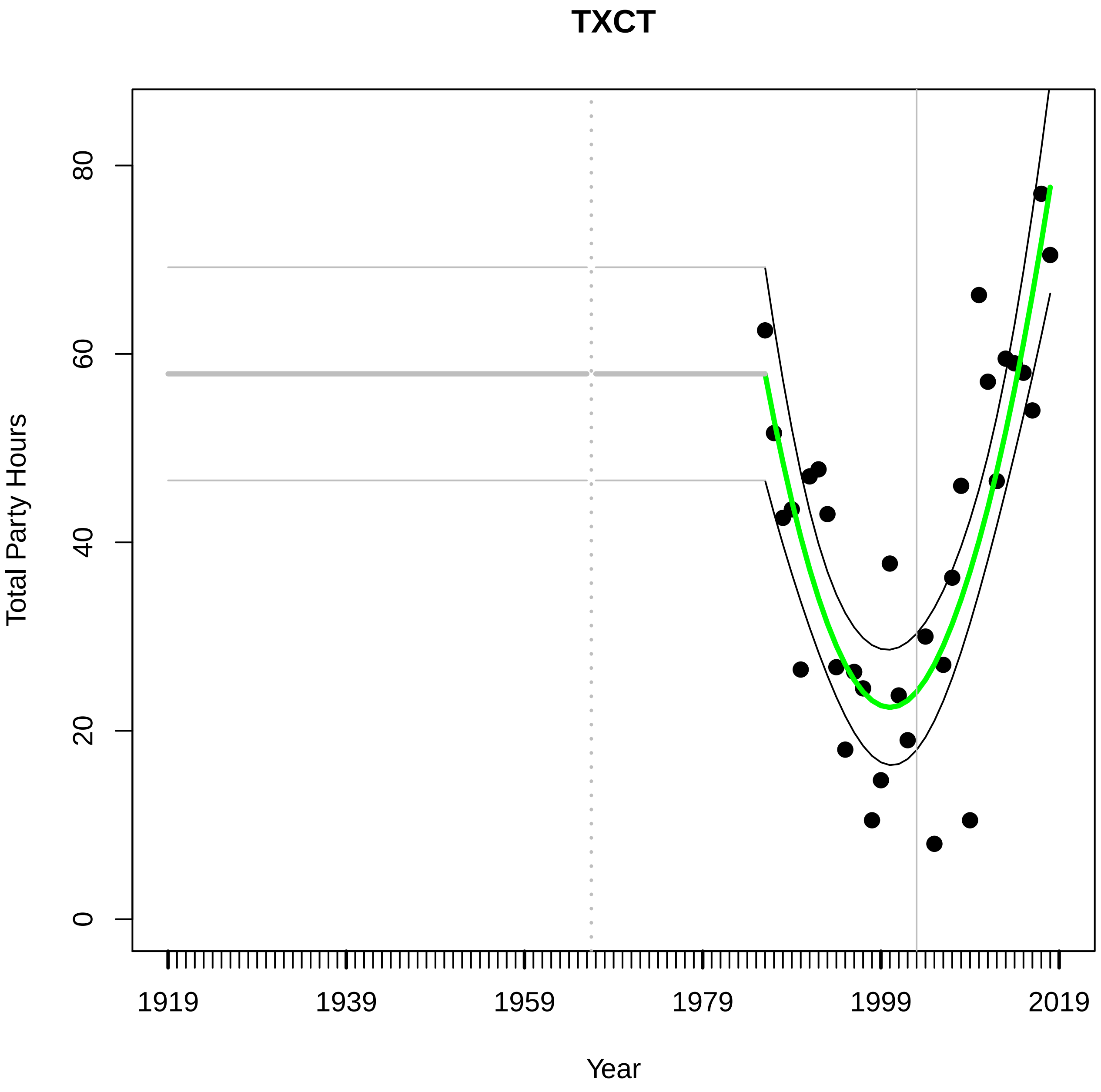
TXCT
Total Party Hours
0
20
40
60
80
1919
1939
1959
1979
1999
2019
Year

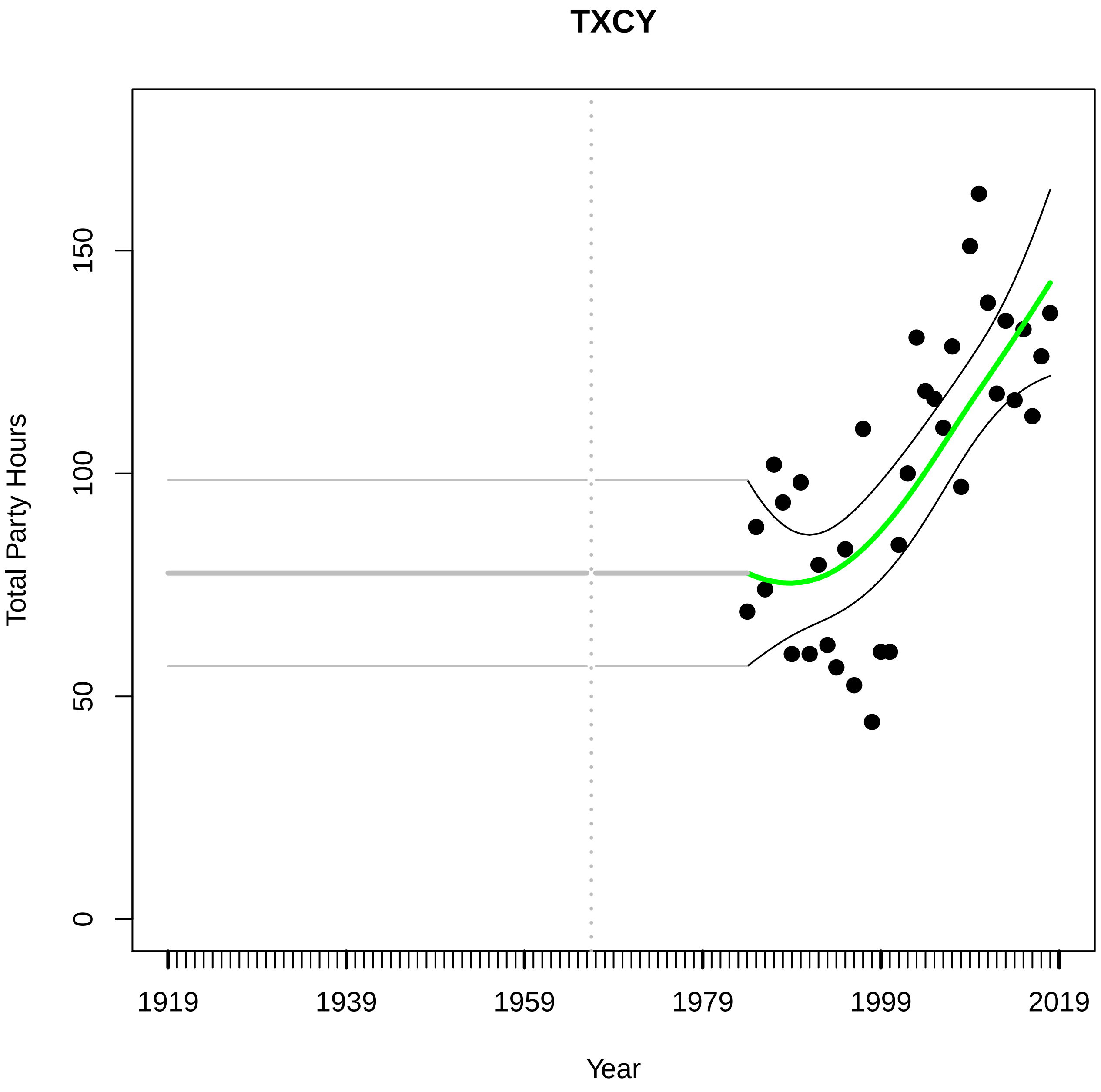

TXCY
Total Party Hours
0
50
100
150
1919
1939
1959
1979
1999
2019
Year

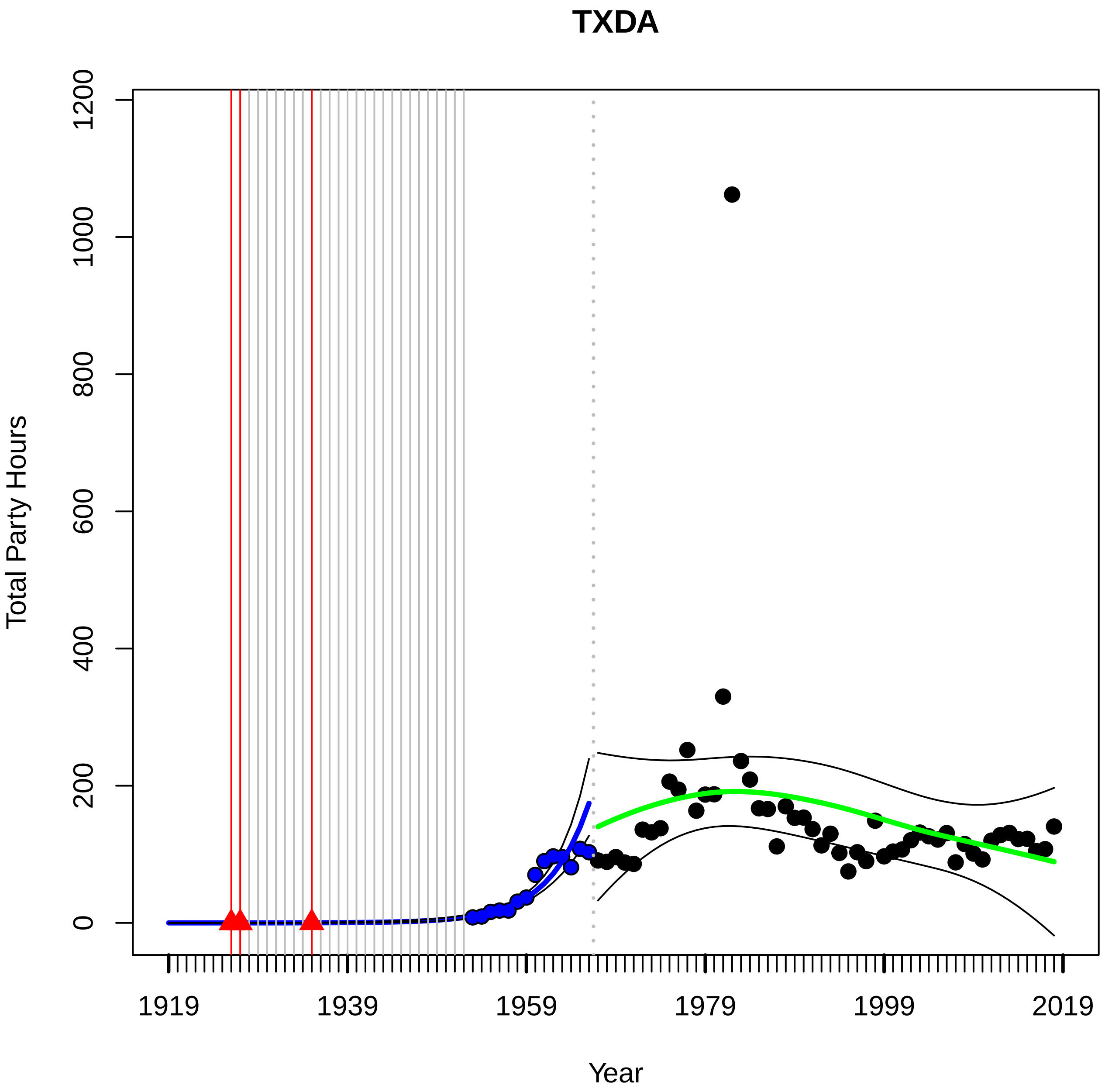

TXDA
Total Party Hours
Year
0
200
400
600
800
1000
1200
1919
1939
1959
1979
1999
2019

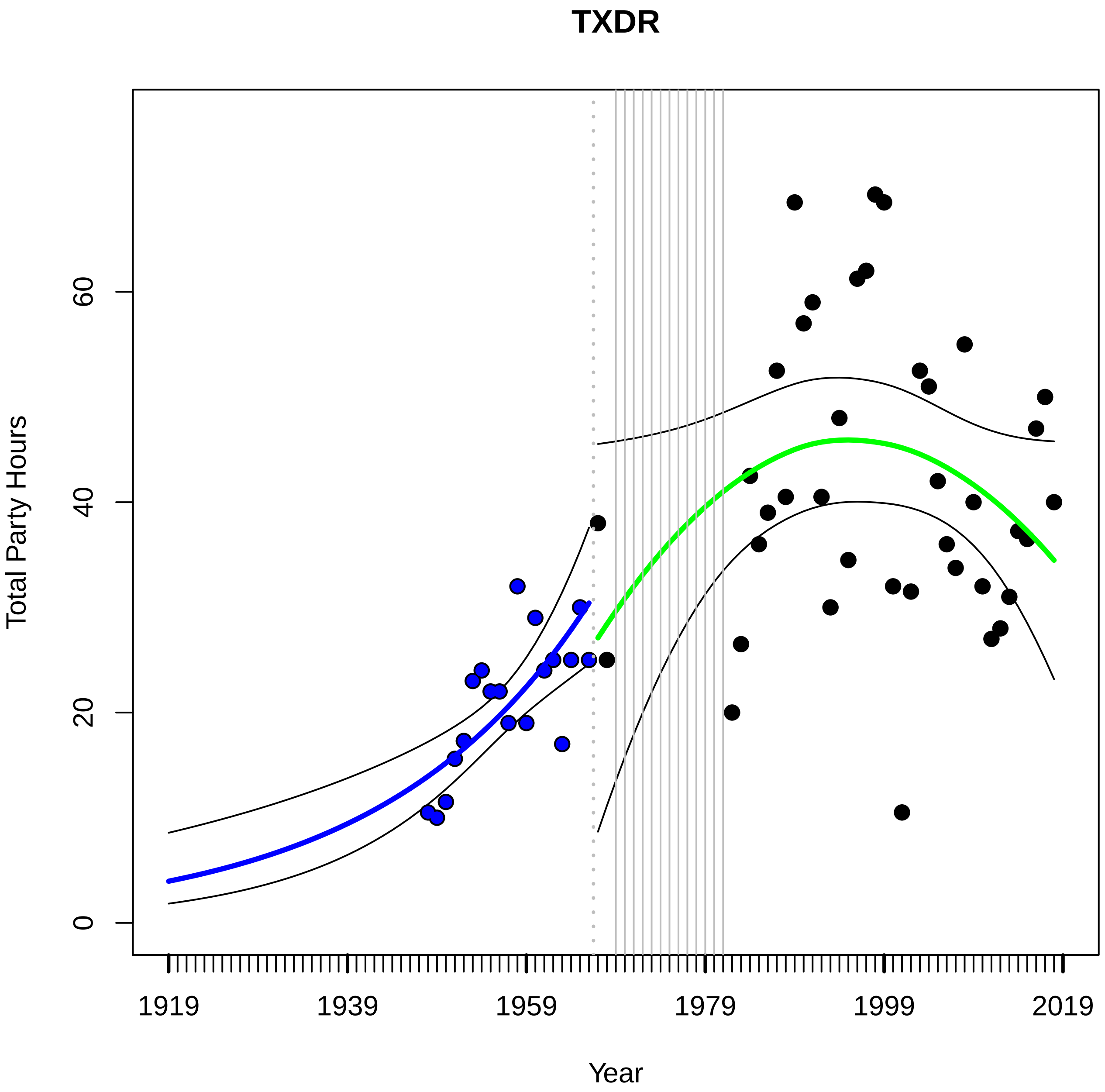
TXDR
Total Party Hours
Year
0
20
40
60
1919
1939
1959
1979
1999
2019

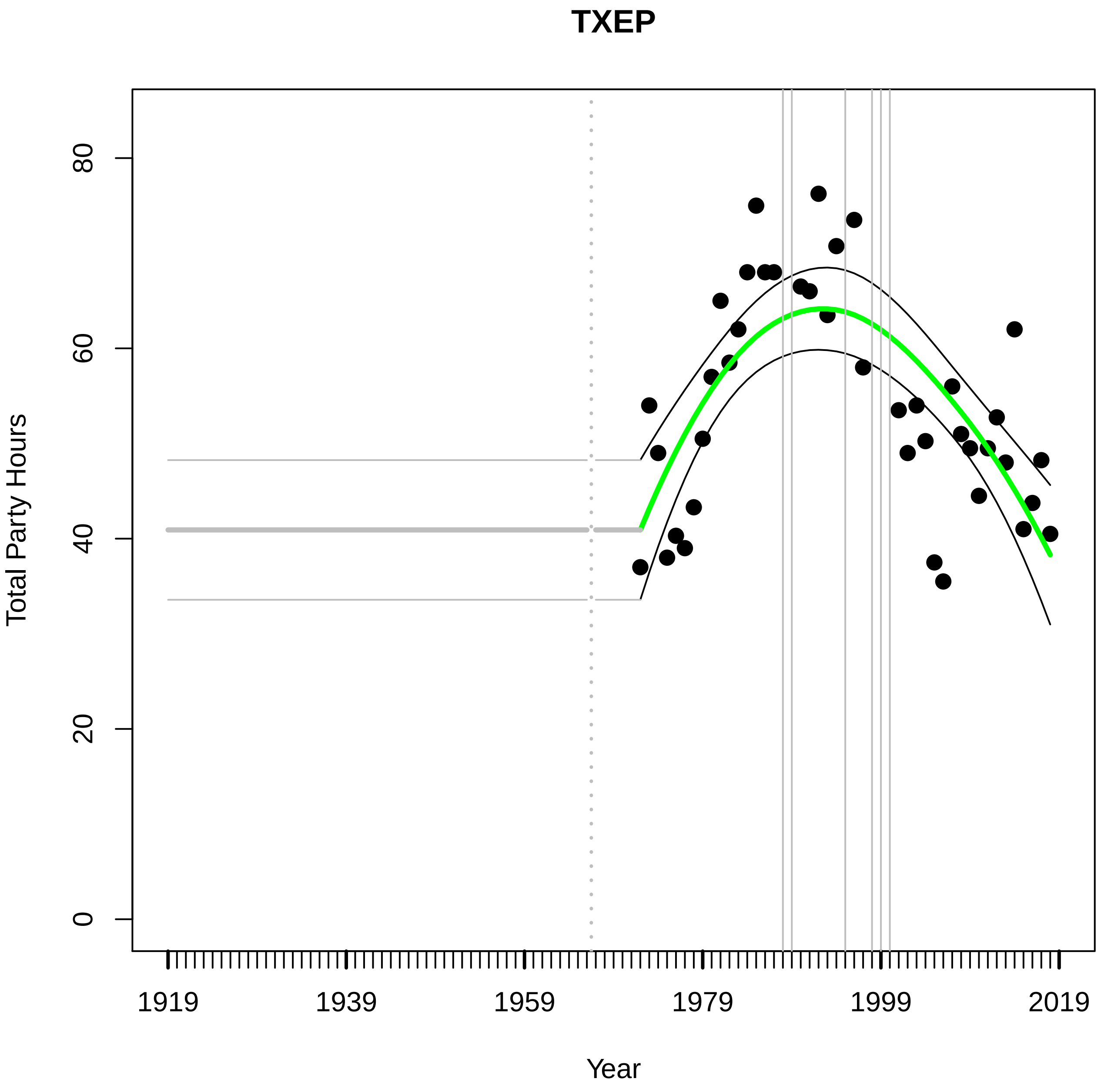

TXEP
Total Party Hours
0
20
40
60
80
1919
1939
1959
1979
1999
2019
Year

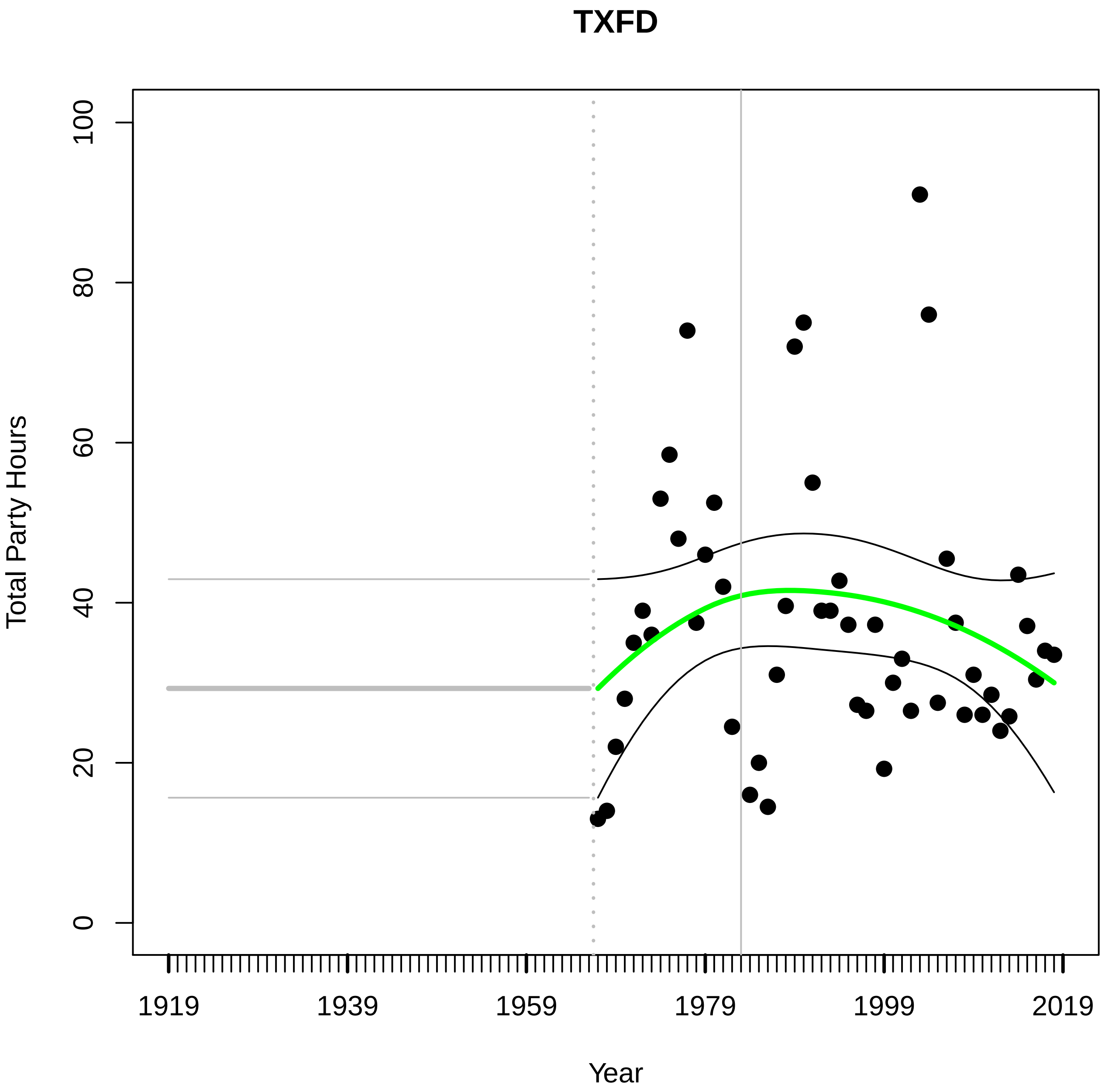

TXFD
Total Party Hours
Year
0
20
40
60
80
100
1919
1939
1959
1979
1999
2019

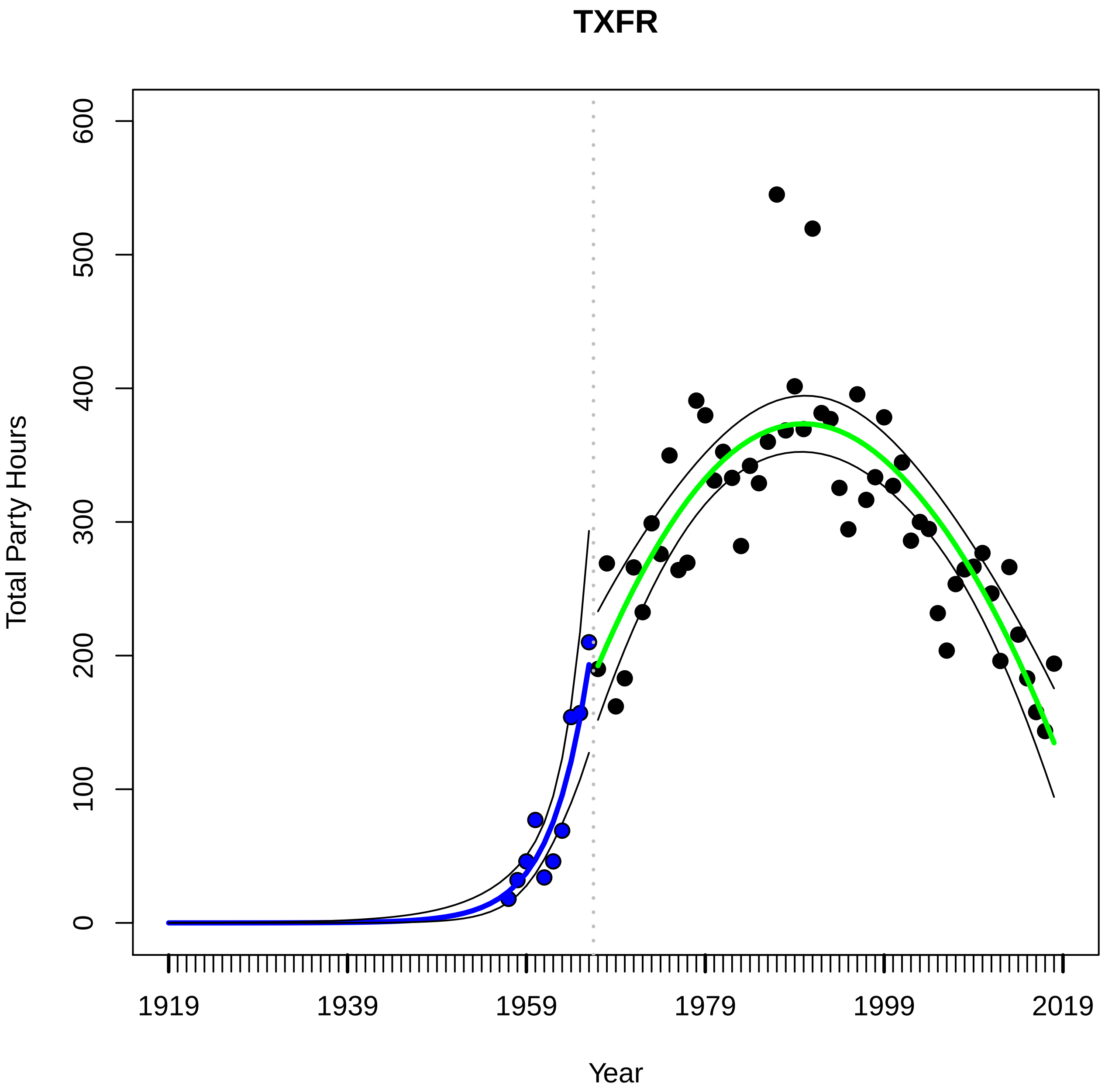

TXFR
Total Party Hours
0
100
200
300
400
500
600
Year
1919
1939
1959
1979
1999
2019

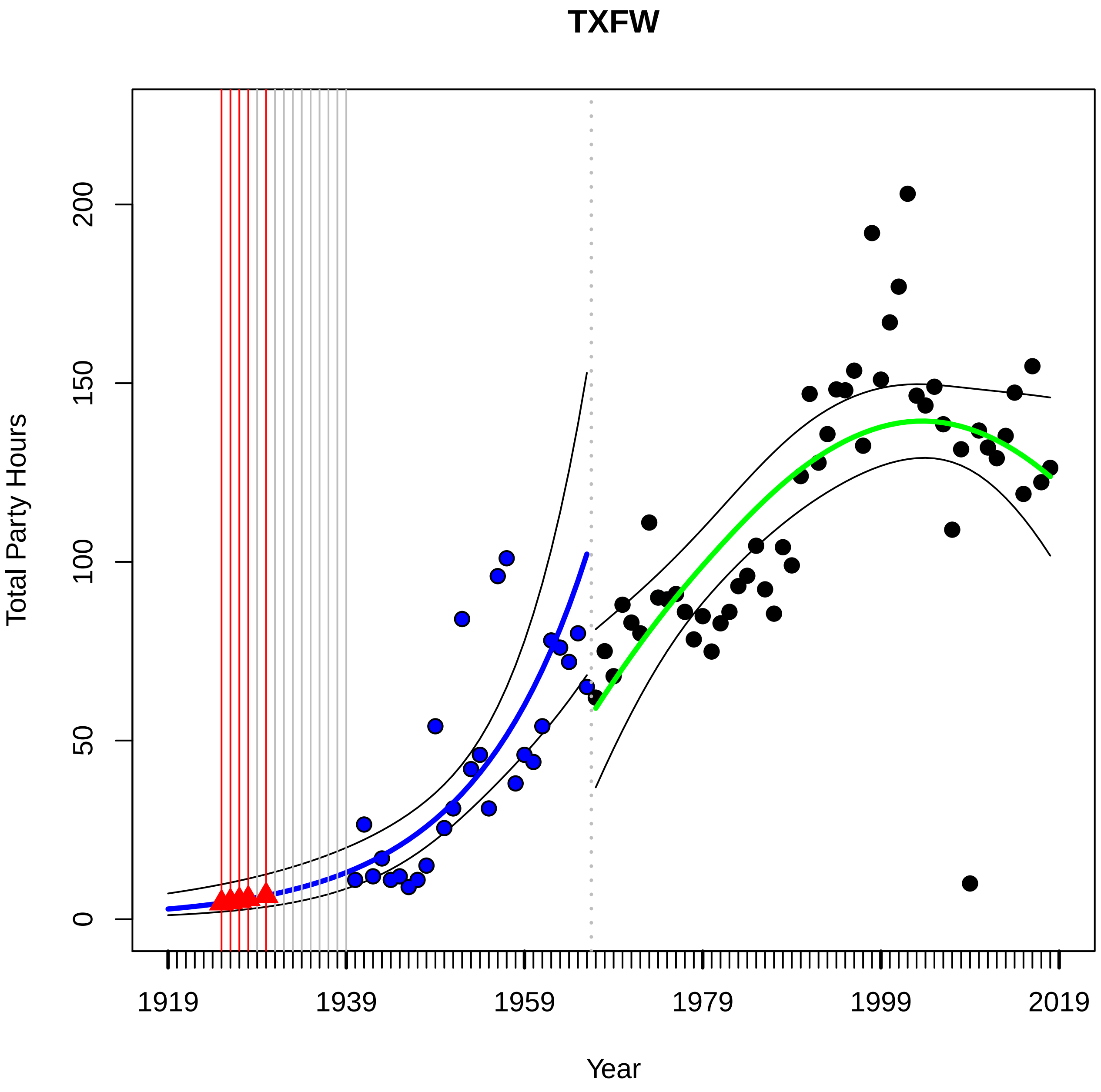
TXFW
Total Party Hours
Year
0
50
100
150
200
1919
1939
1959
1979
1999
2019

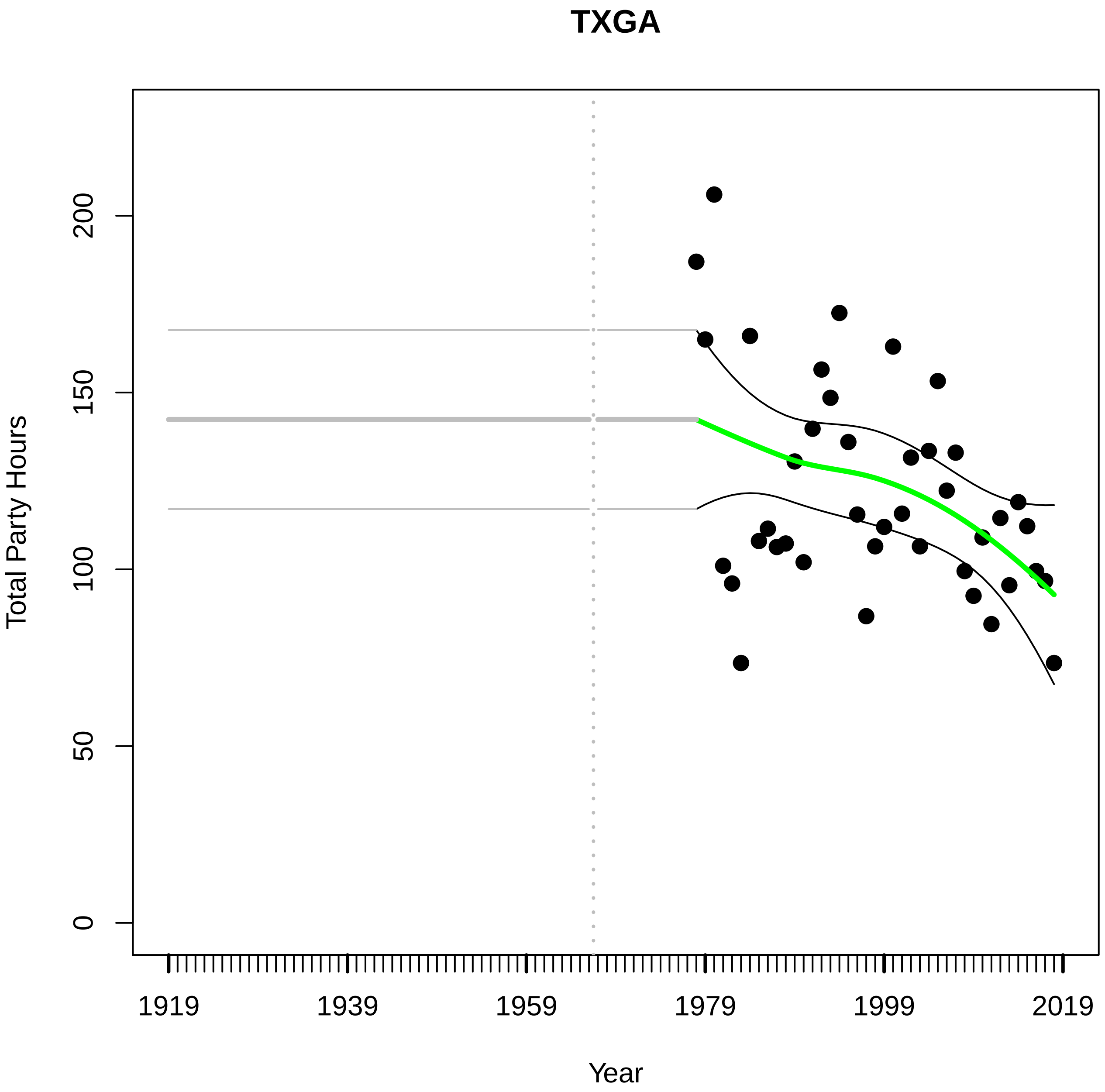

TXGA
Total Party Hours
0
50
100
150
200
Year
1919
1939
1959
1979
1999
2019

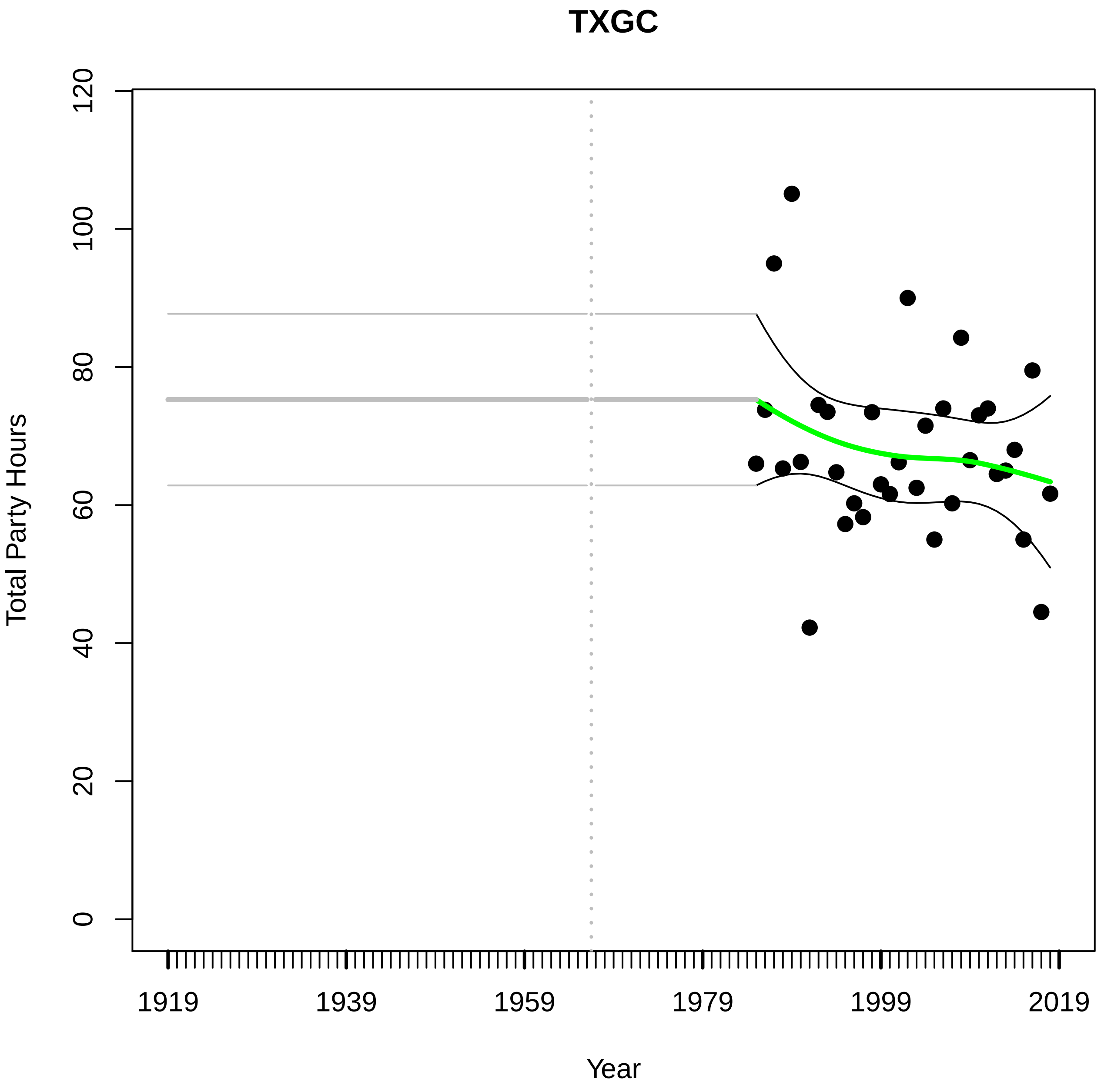
TXGC
Total Party Hours
Year
0
20
40
60
80
100
120
1919
1939
1959
1979
1999
2019

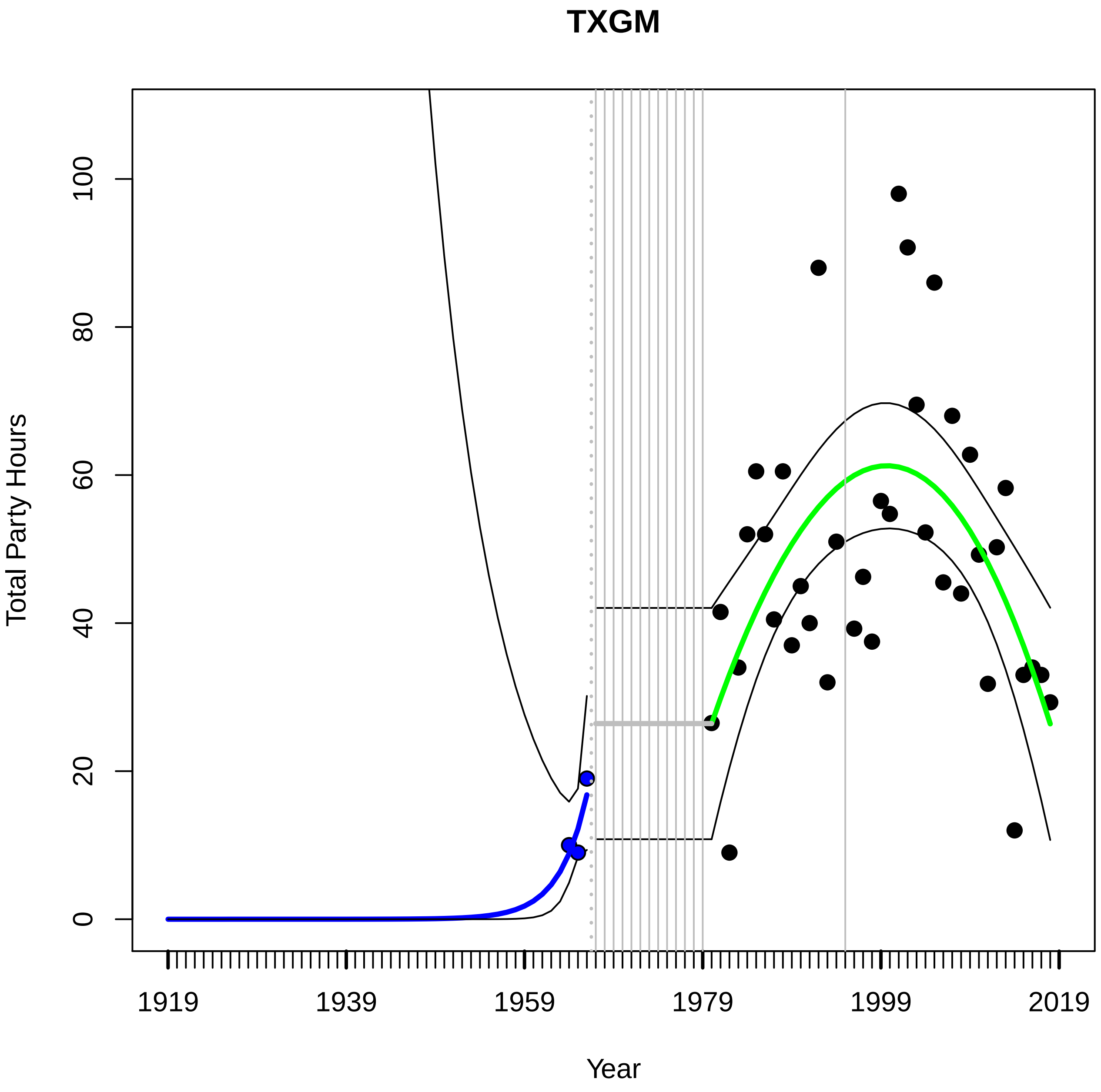

TXGM
Total Party Hours
0
20
40
60
80
100
1919
1939
1959
1979
1999
2019
Year

TXHA

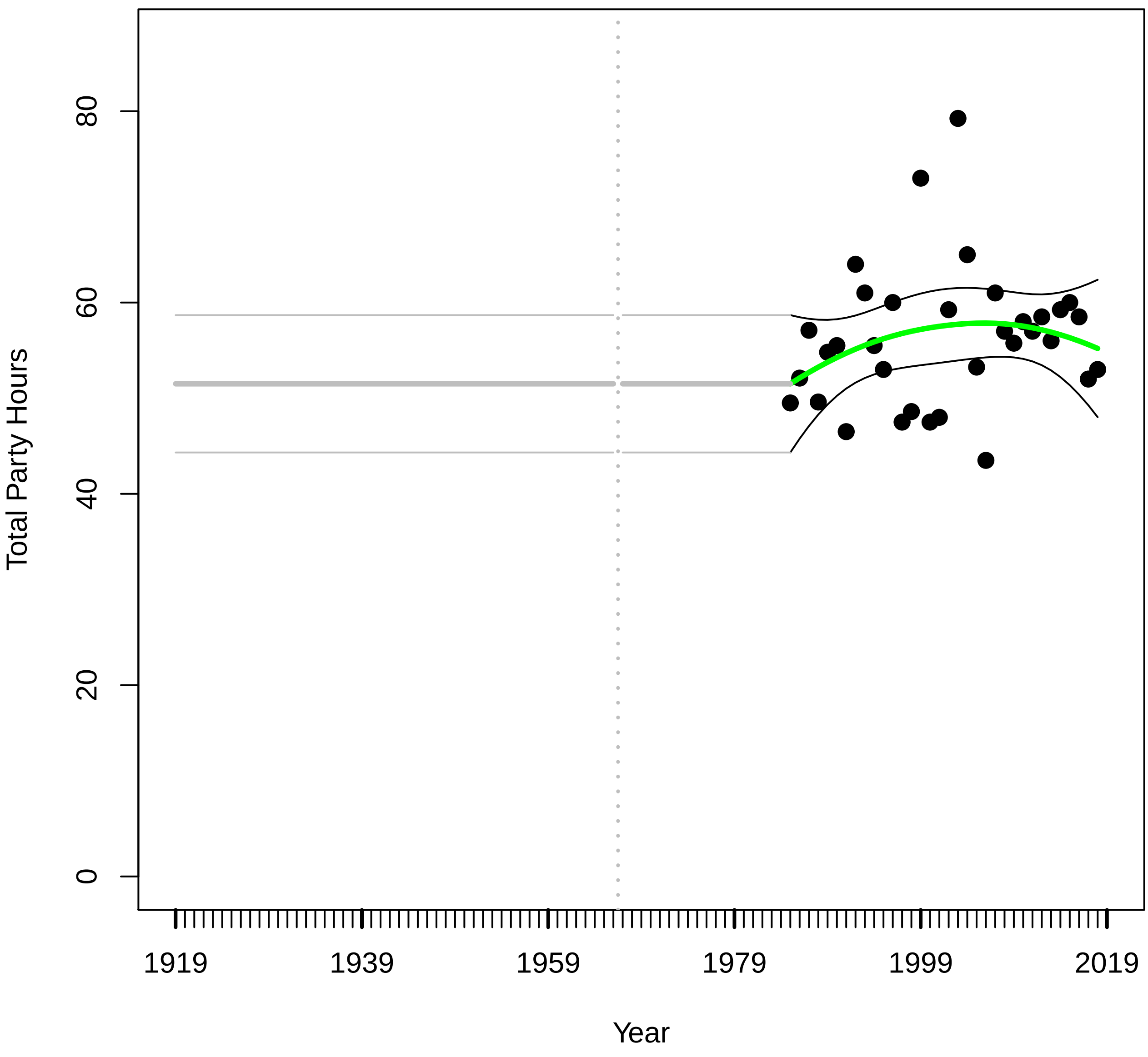

Total Party Hours
0
20
40
60
80
1919
1939
1959
1979
1999
2019
Year

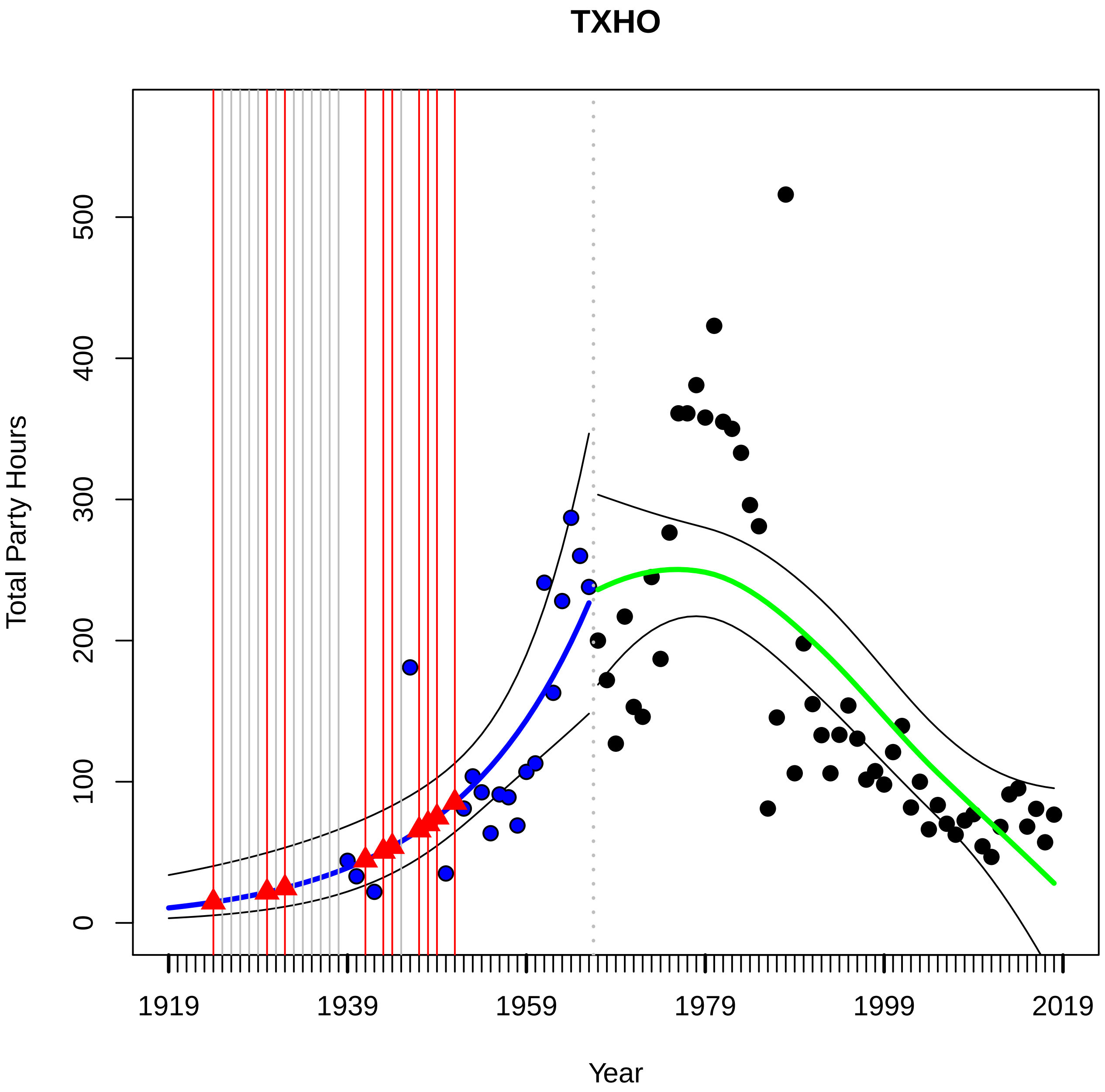

TXHO
Total Party Hours
0
100
200
300
400
500
1919
1939
1959
1979
1999
2019
Year

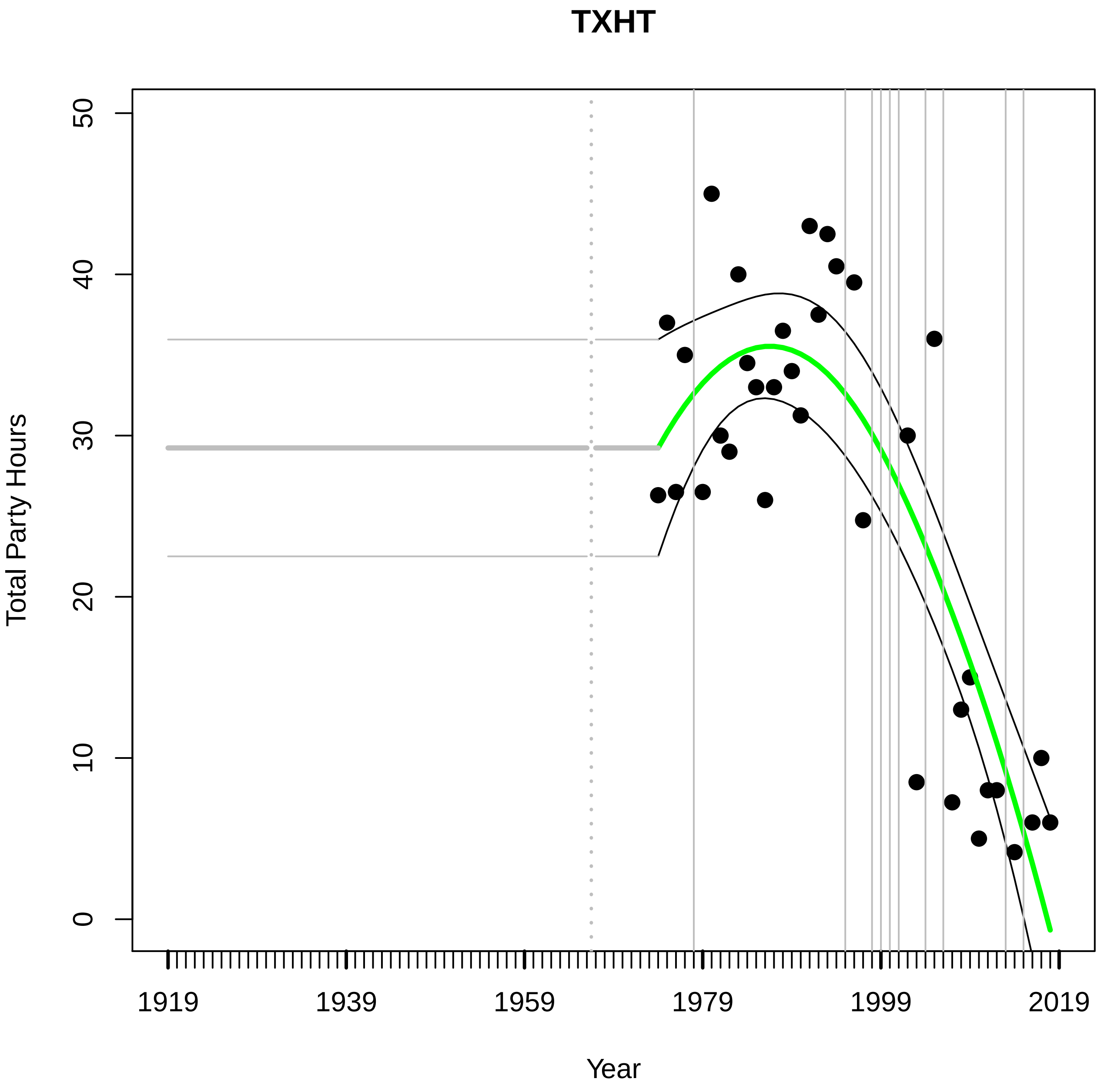

TXHT
Total Party Hours
0
10
20
30
40
50
1919
1939
1959
1979
1999
2019
Year

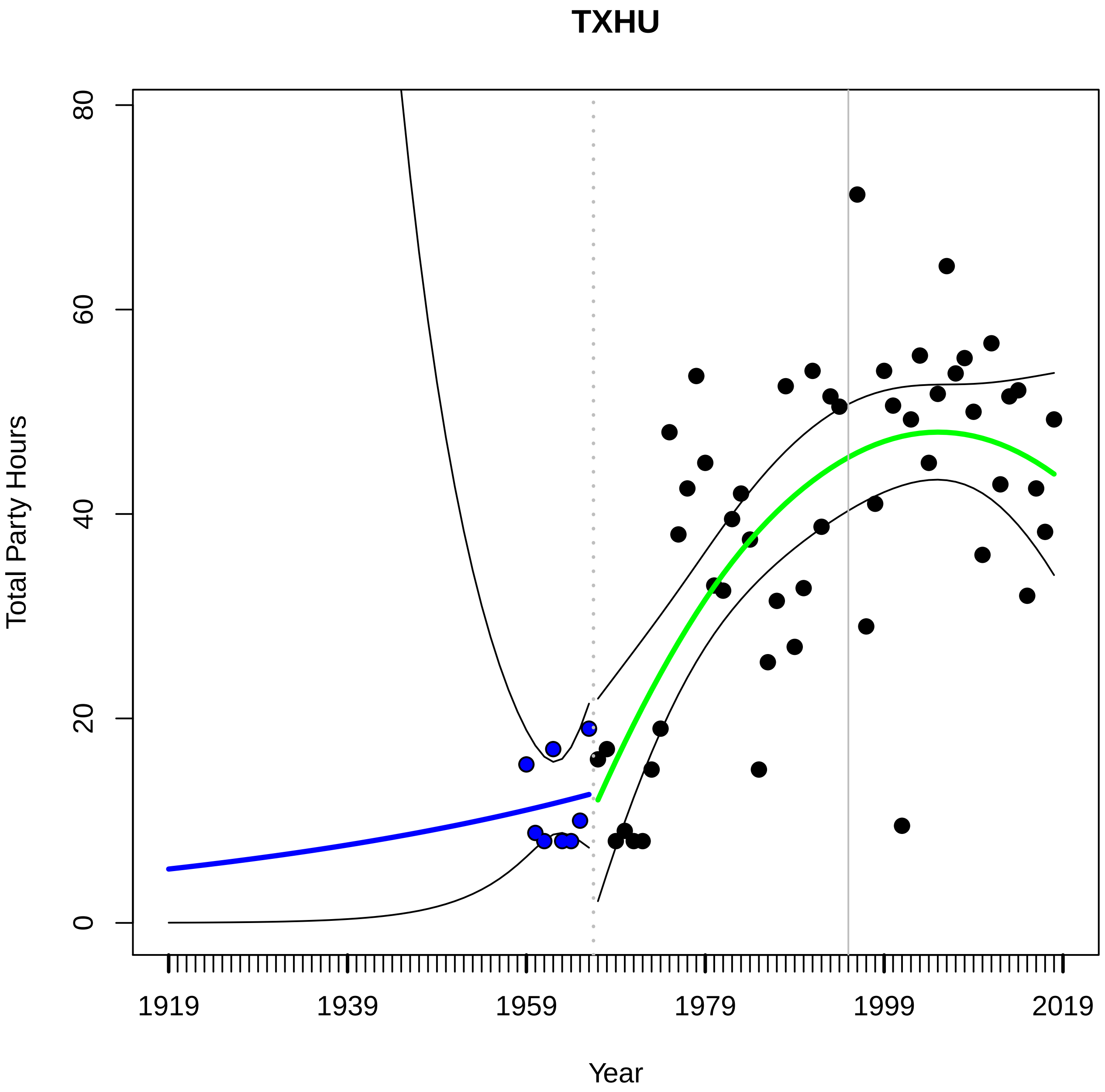

TXHU
Total Party Hours
0
20
40
60
80
Year
1919
1939
1959
1979
1999
2019

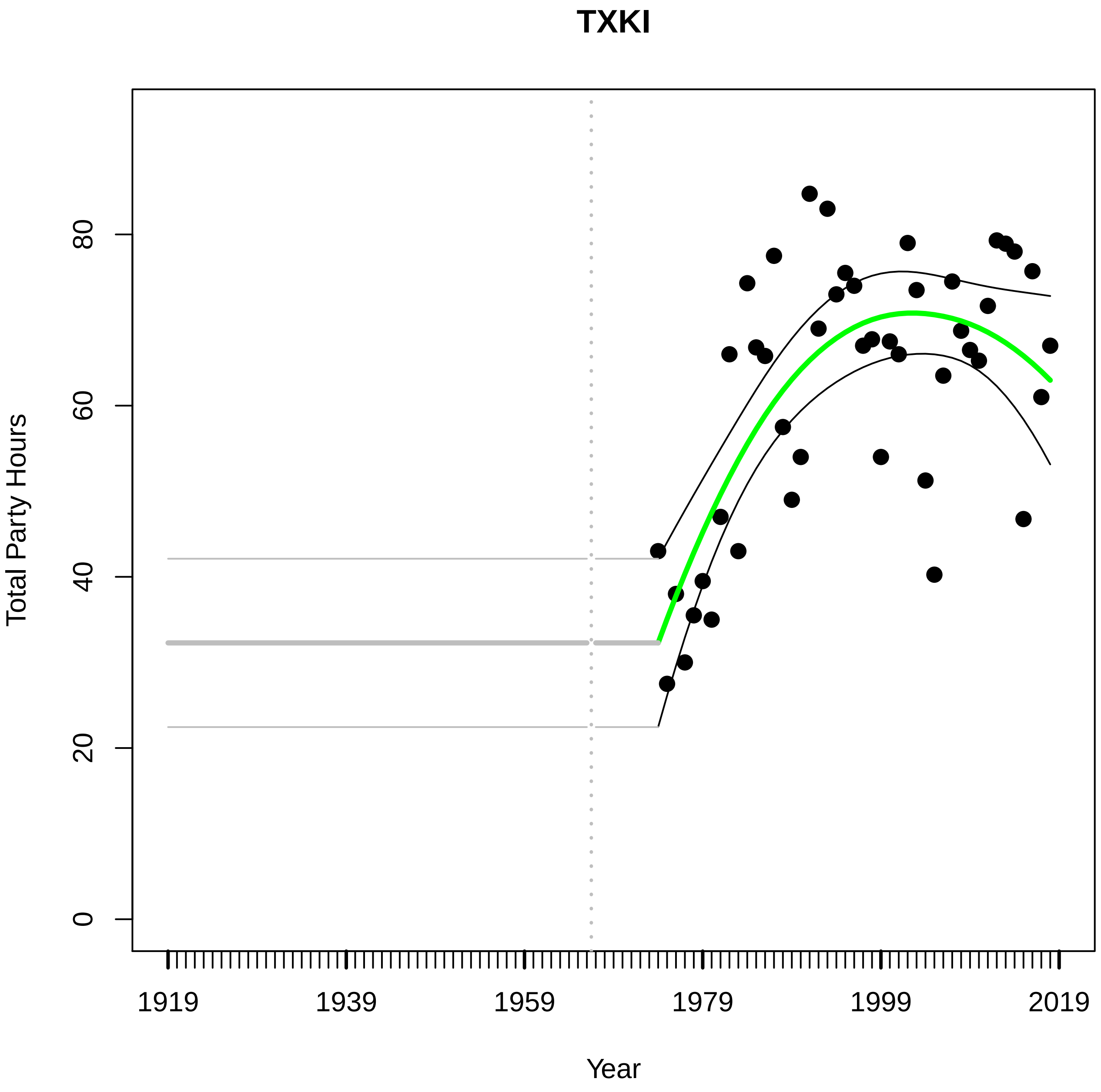

TXKI
Total Party Hours
0
20
40
60
80
Year
1919
1939
1959
1979
1999
2019

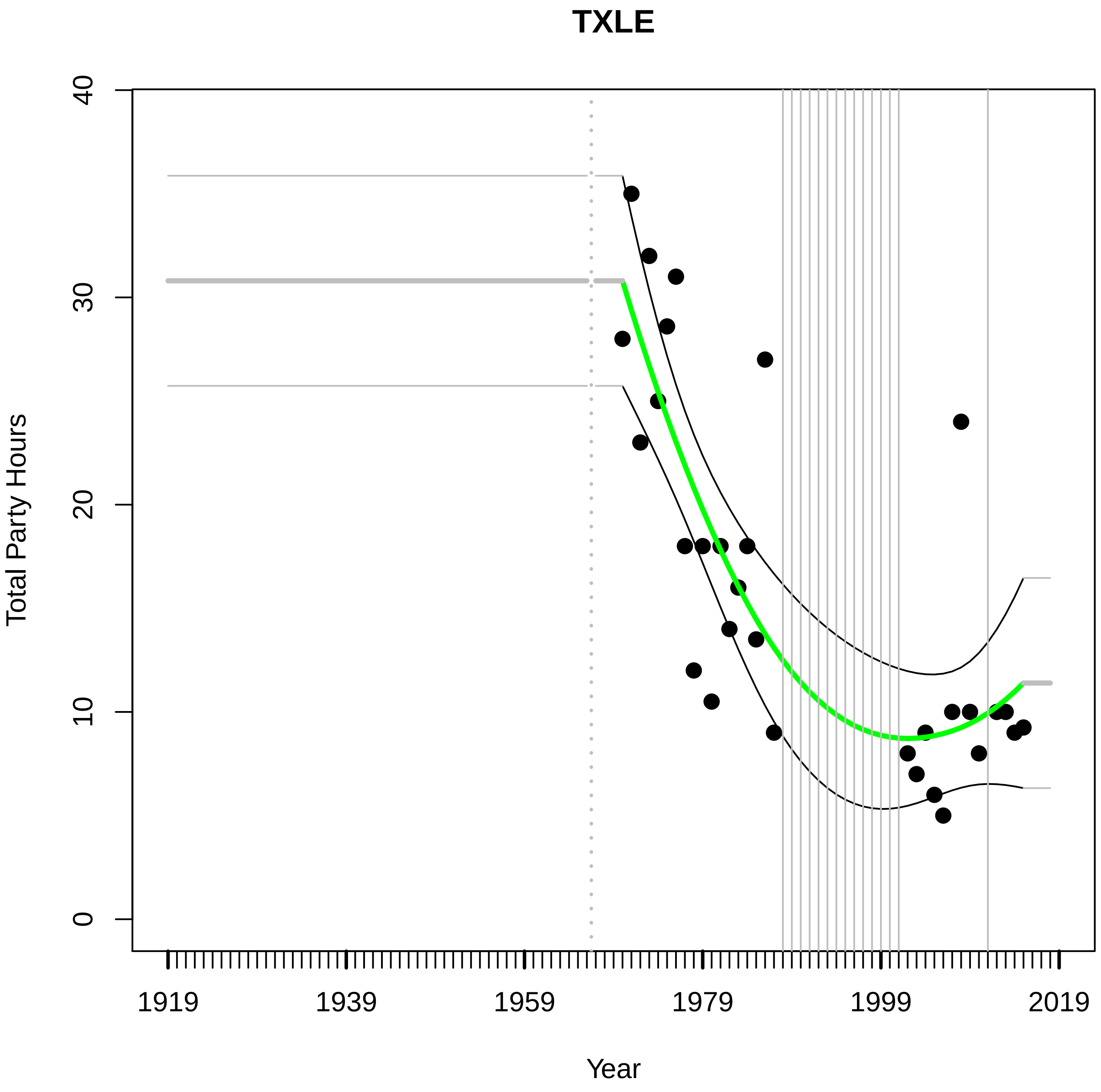
TXLE
Total Party Hours
0
10
20
30
40
Year
1919
1939
1959
1979
1999
2019

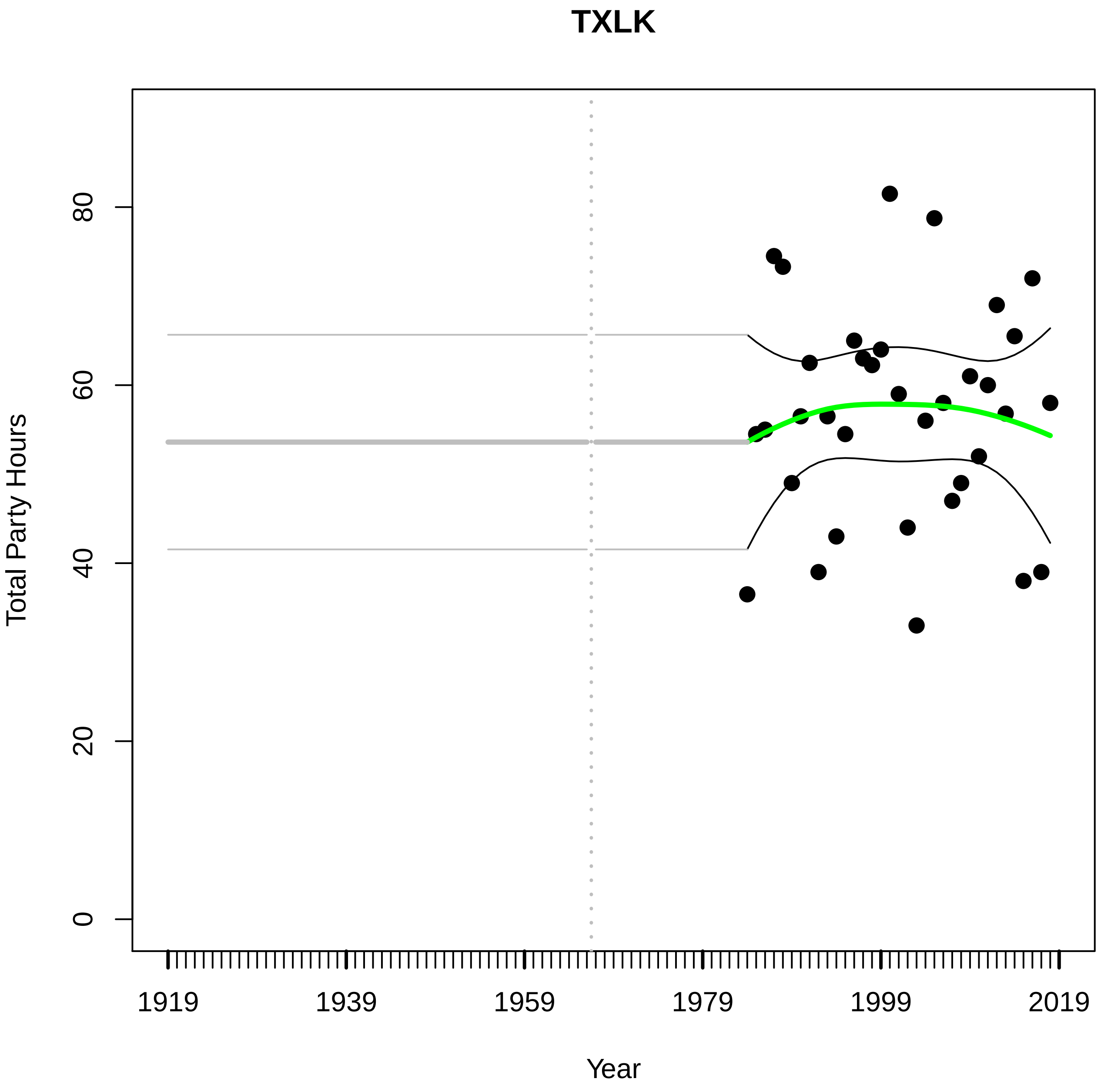

TXLK
Total Party Hours
0
20
40
60
80
1919
1939
1959
1979
1999
2019
Year

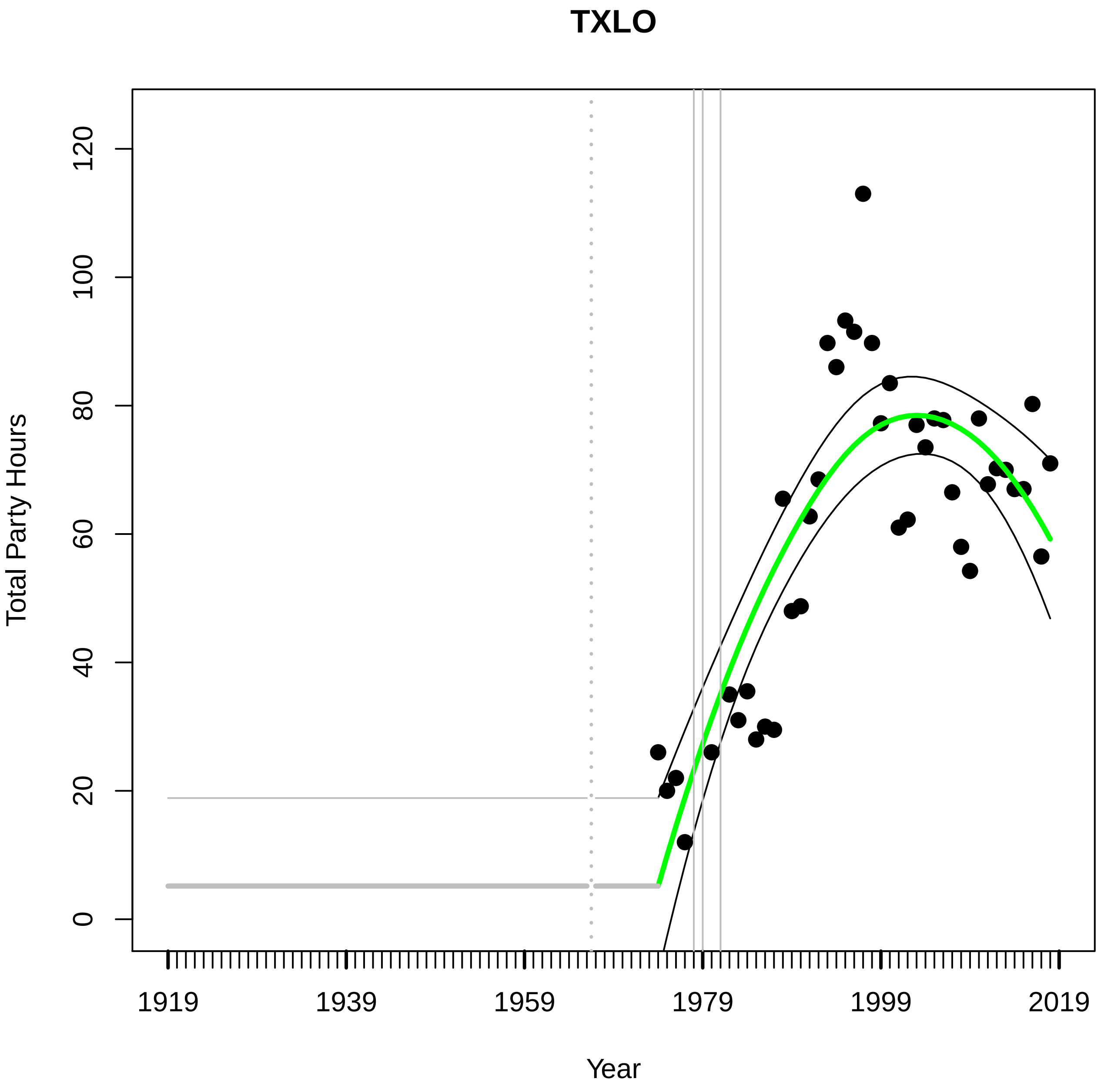
TXLO
Total Party Hours
0
20
40
60
80
100
120
1919
1939
1959
1979
1999
2019
Year

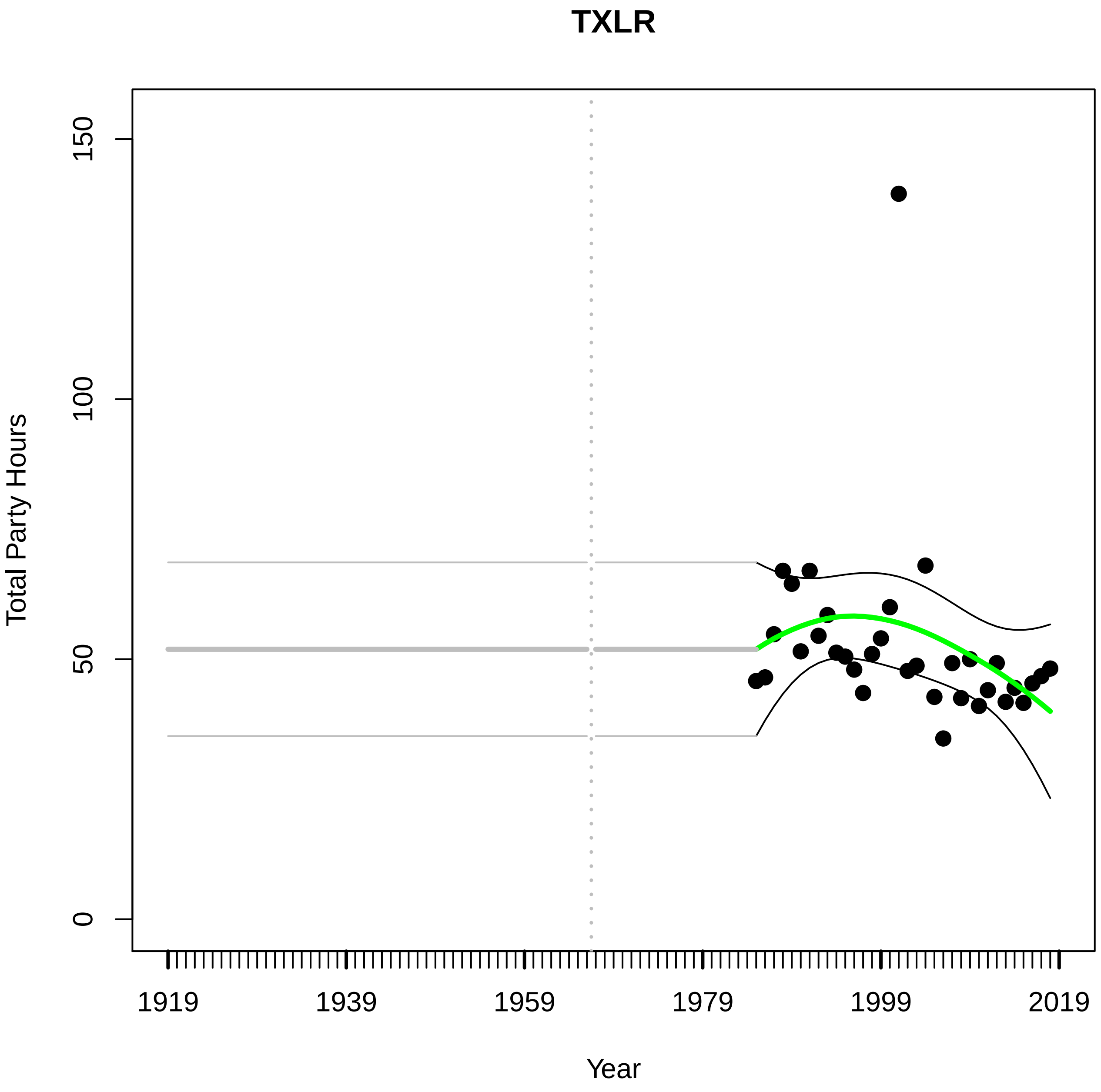
TXLR
Total Party Hours
150
100
50
0
1919
1939
1959
1979
1999
2019
Year

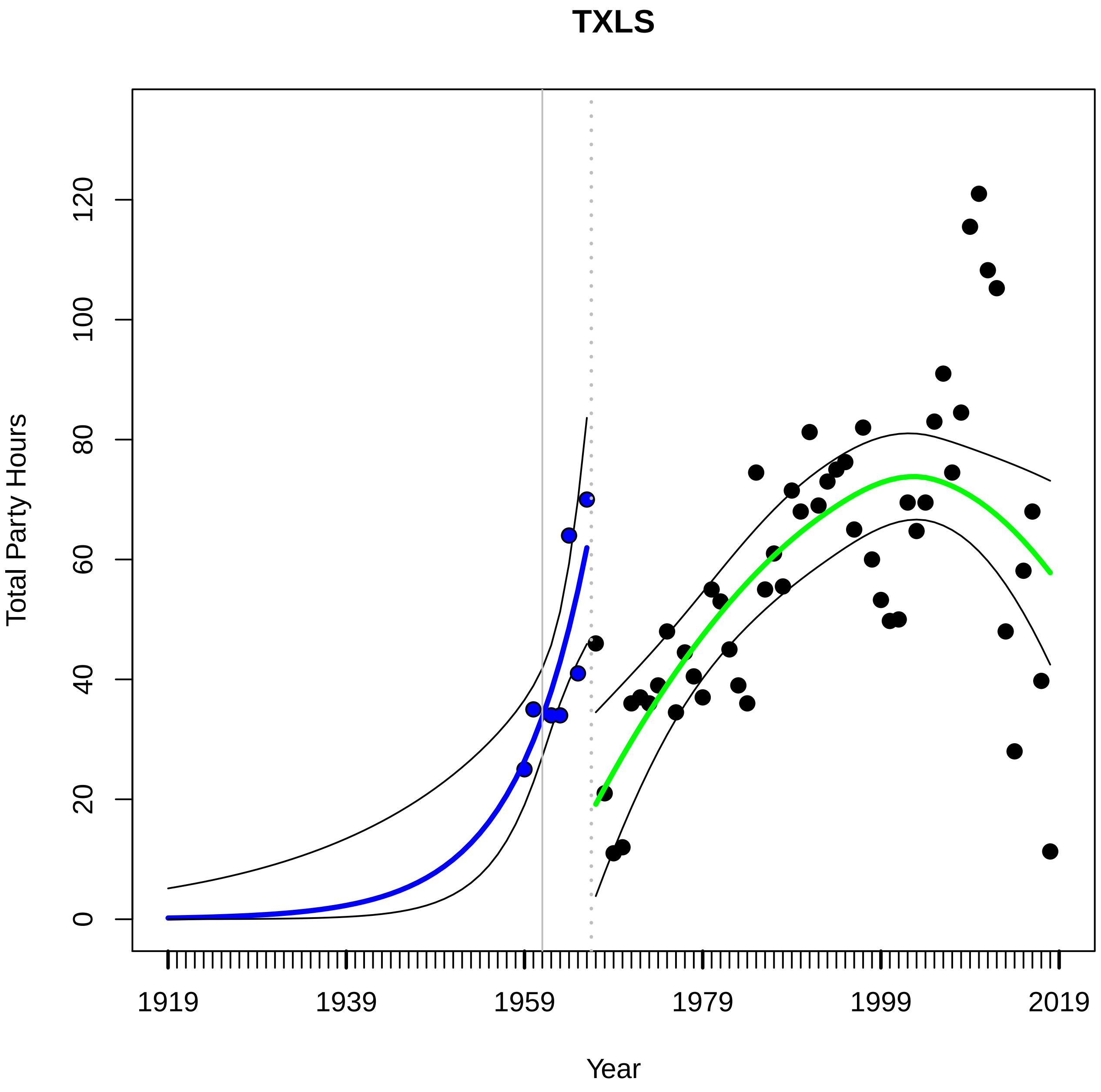

TXLS
Total Party Hours
0
20
40
60
80
100
120
1919
1939
1959
1979
1999
2019
Year

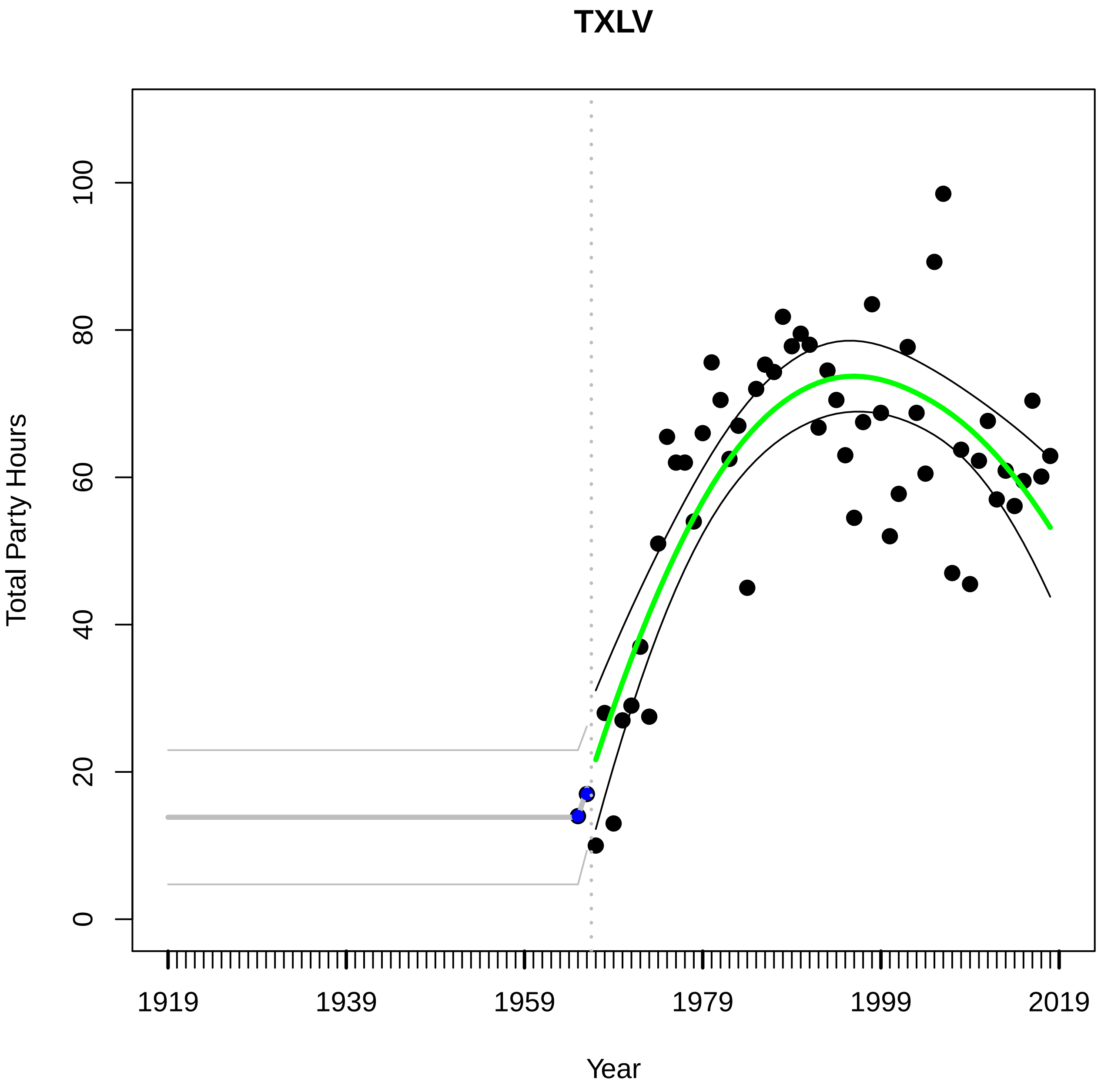

TXLV
Total Party Hours
Year
0
20
40
60
80
100
1919
1939
1959
1979
1999
2019

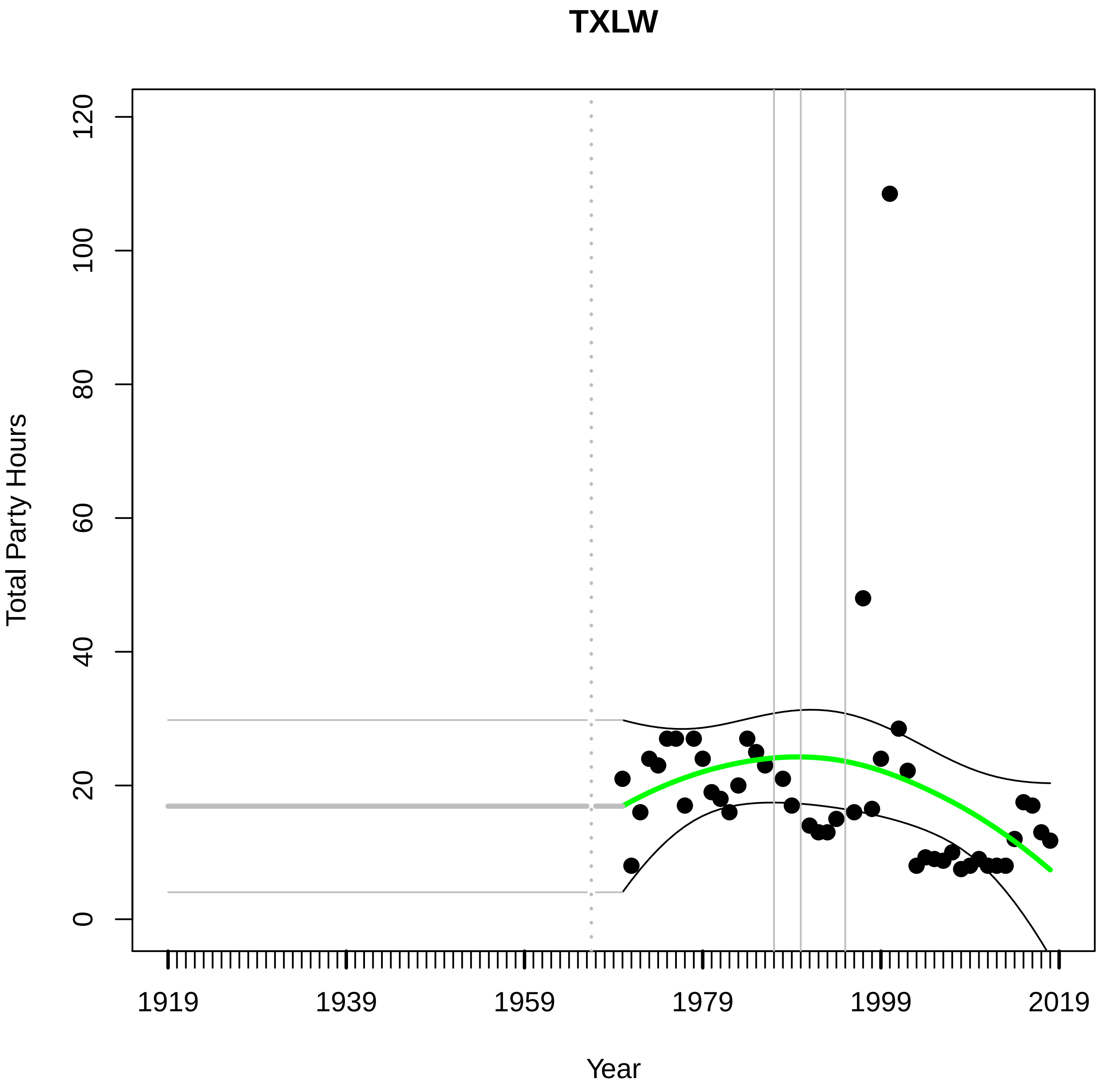

TXLW
Total Party Hours
0
20
40
60
80
100
120
1919
1939
1959
1979
1999
2019
Year

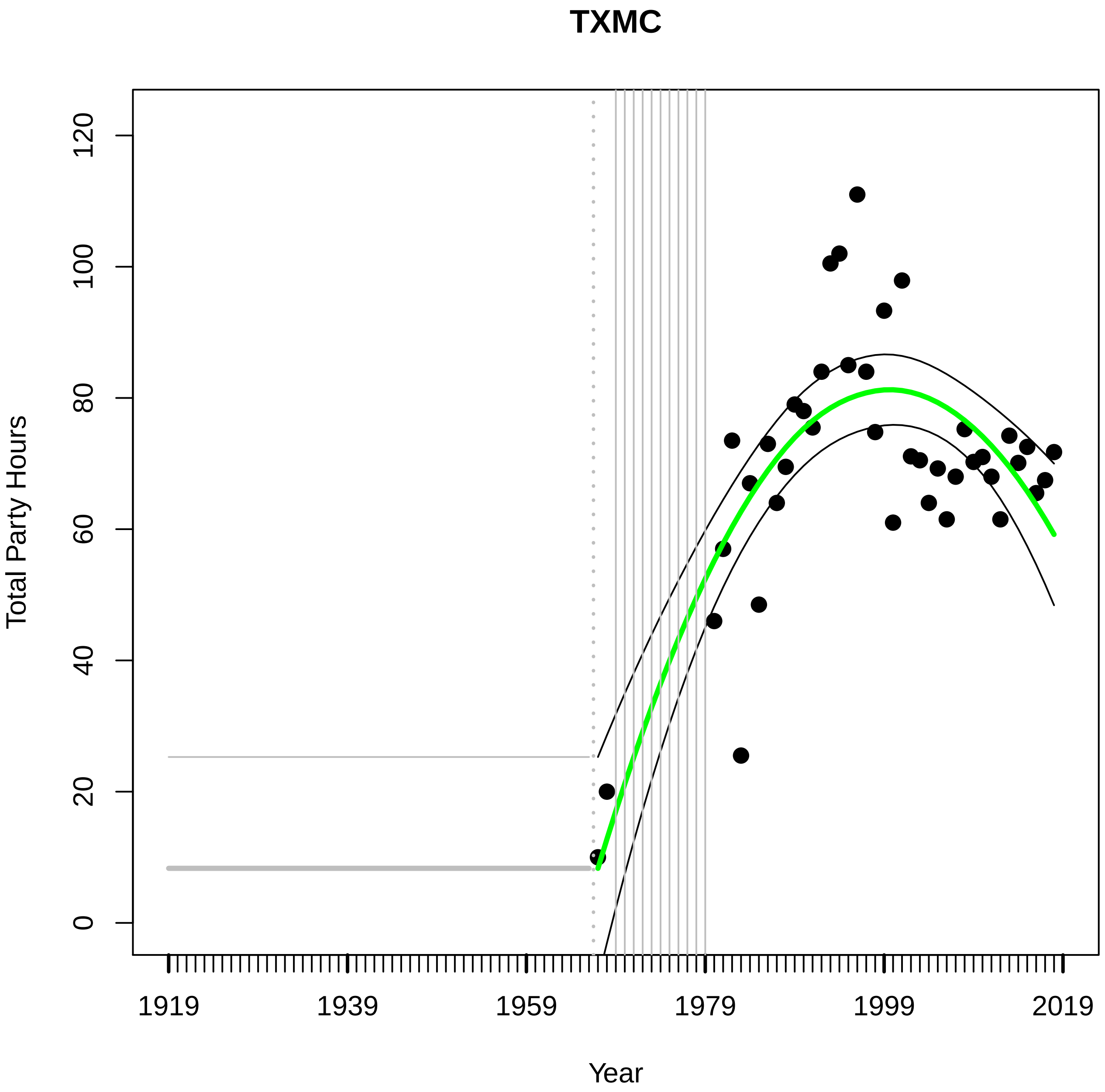

TXMC
Total Party Hours
120
100
80
60
40
20
0
1919
1939
1959
1979
1999
2019
Year

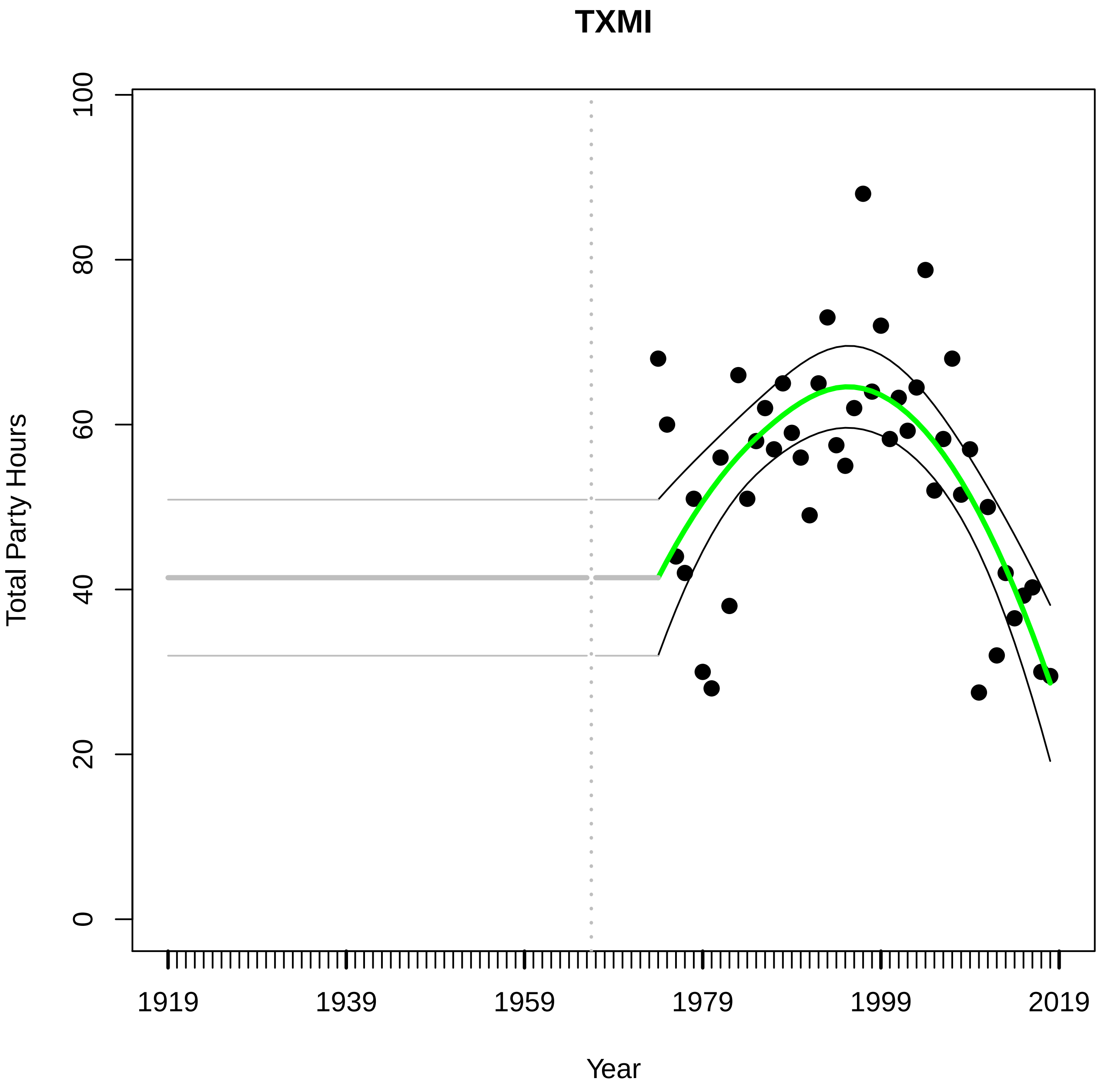
TXMI
Total Party Hours
0
20
40
60
80
100
1919
1939
1959
1979
1999
2019
Year

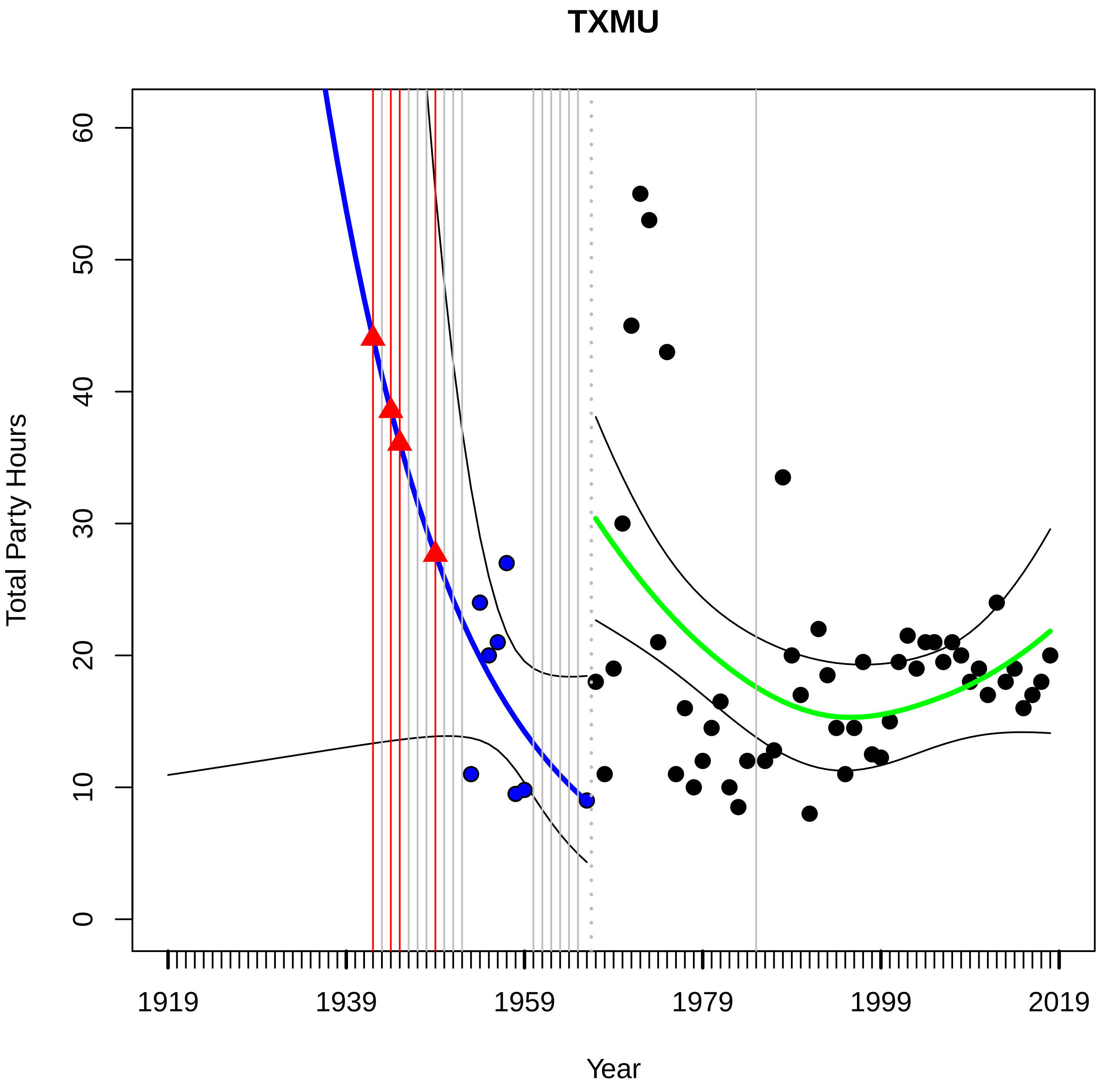

TXMU
Total Party Hours
Year
0
10
20
30
40
50
60
1919
1939
1959
1979
1999
2019

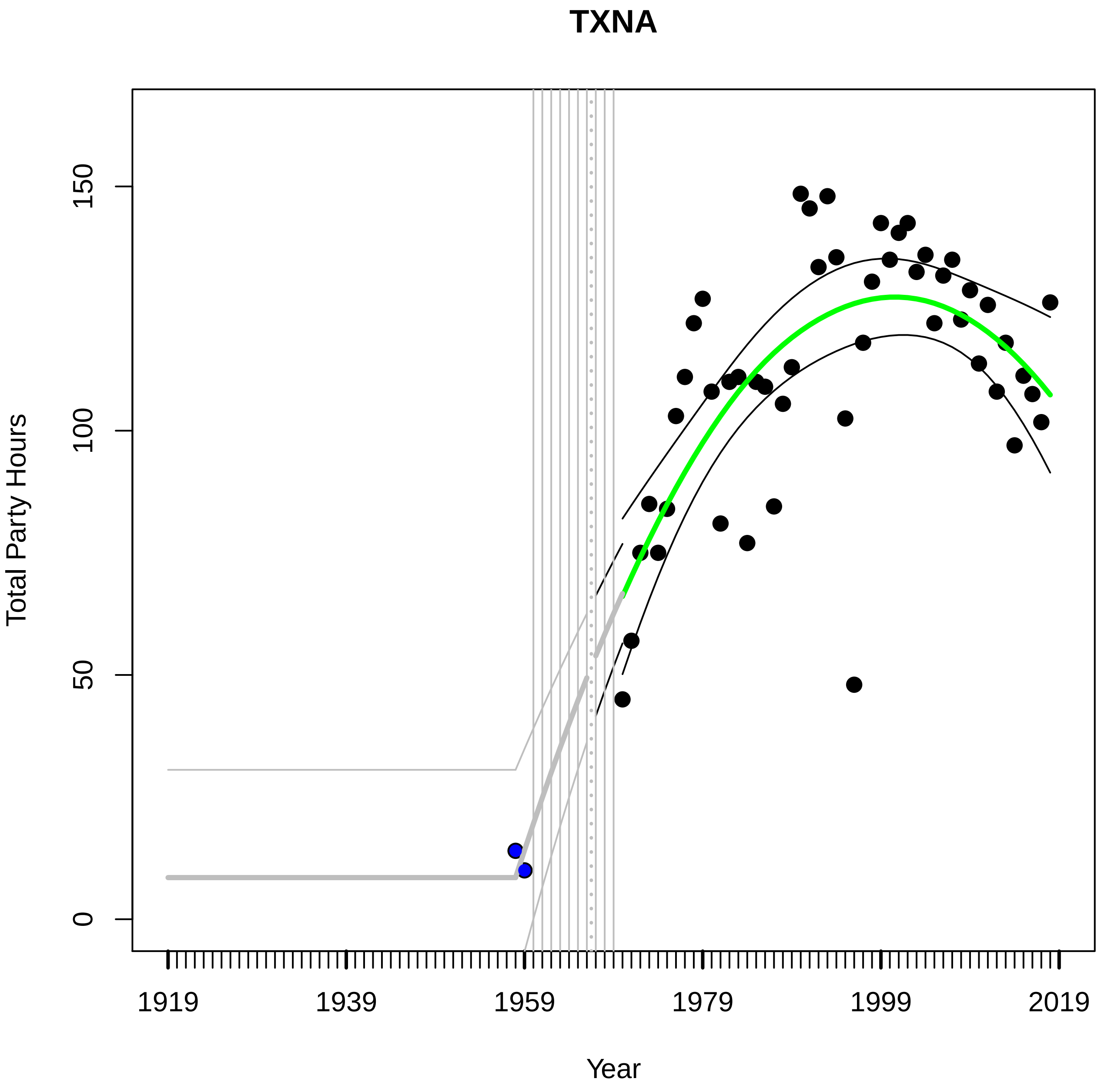

TXNA
Total Party Hours
150
100
50
0
1919
1939
1959
1979
1999
2019
Year

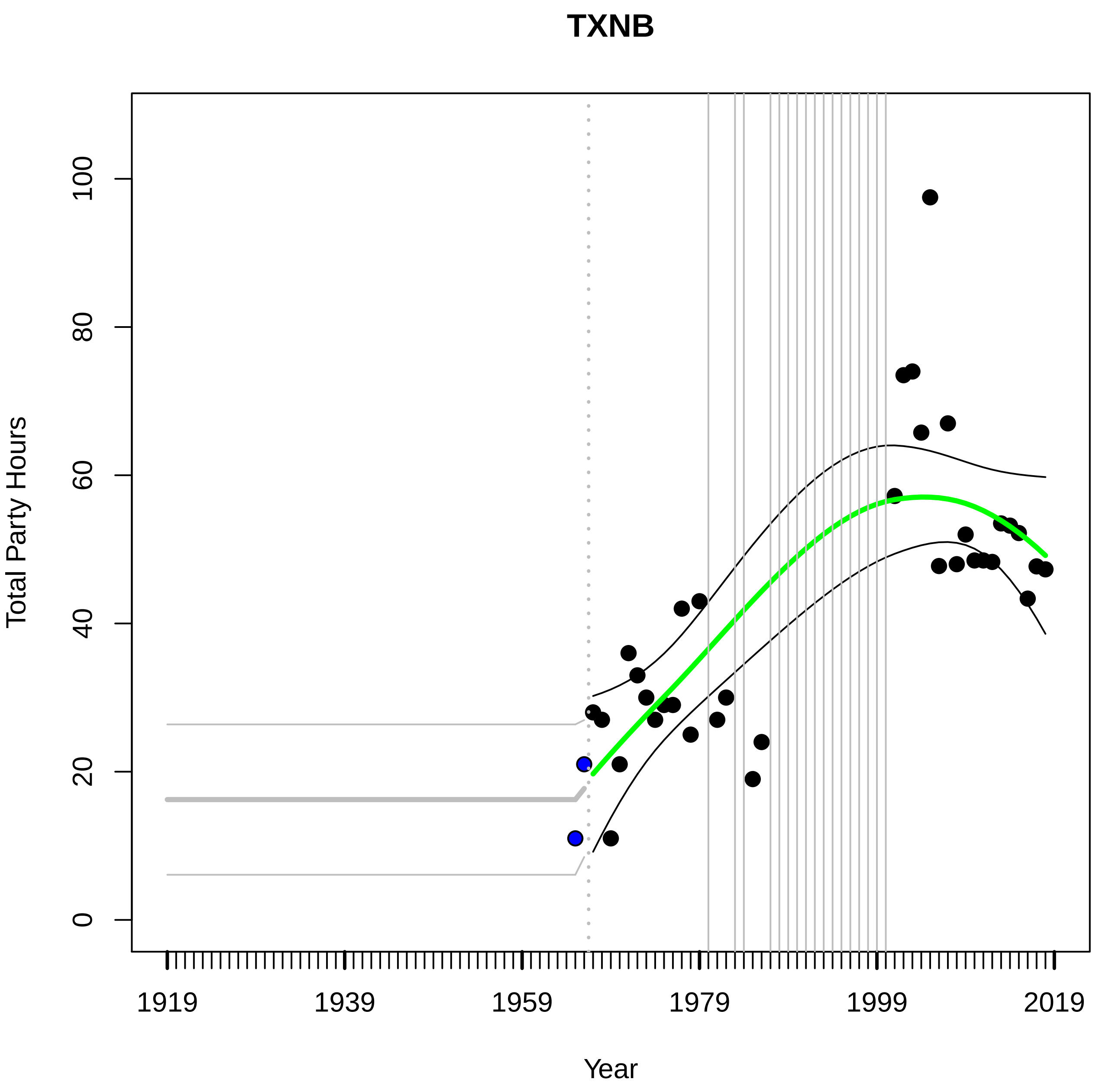

TXNB
Total Party Hours
Year
0
20
40
60
80
100
1919
1939
1959
1979
1999
2019

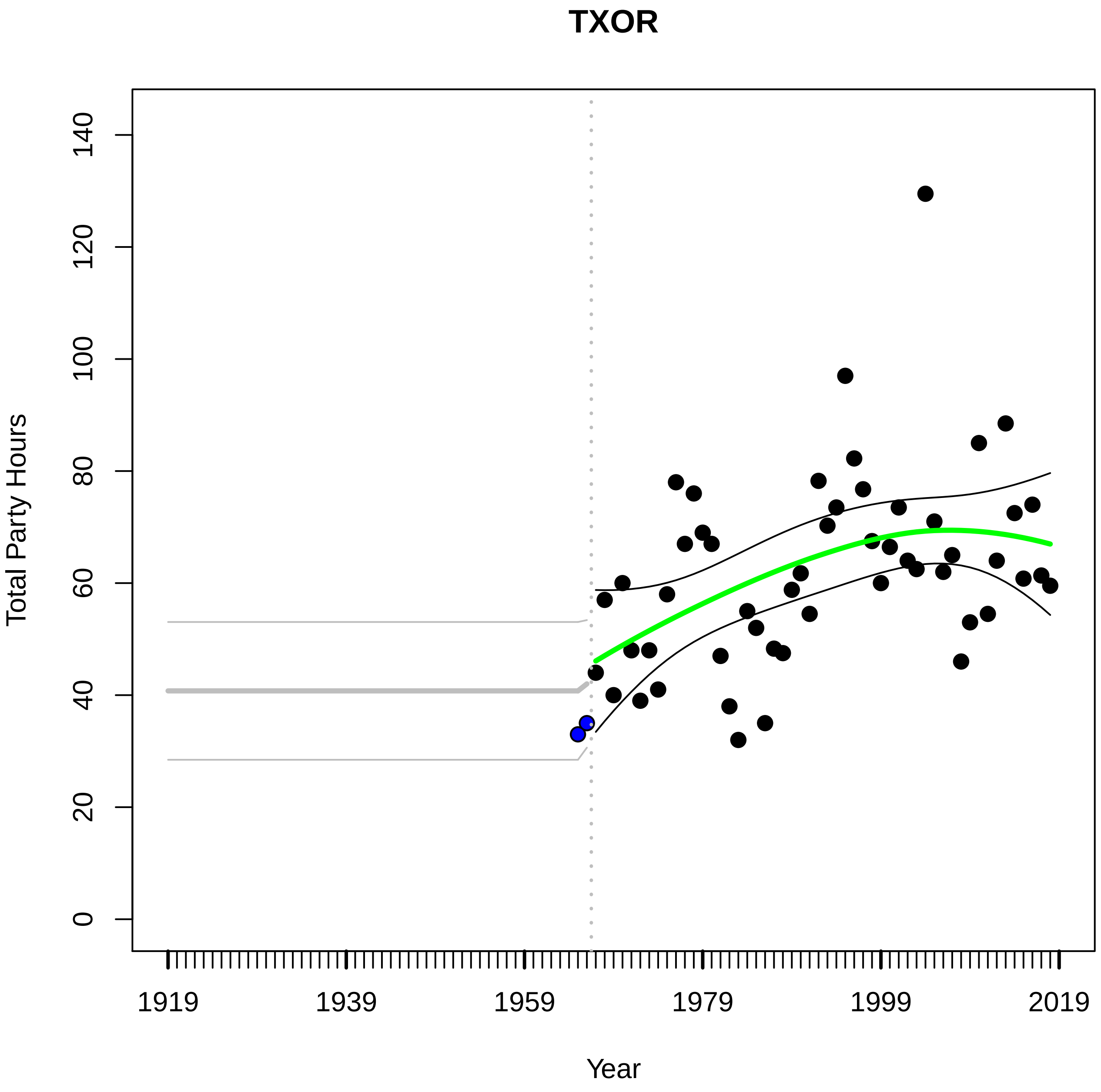
TXOR
Total Party Hours
0
20
40
60
80
100
120
140
1919
1939
1959
1979
1999
2019
Year

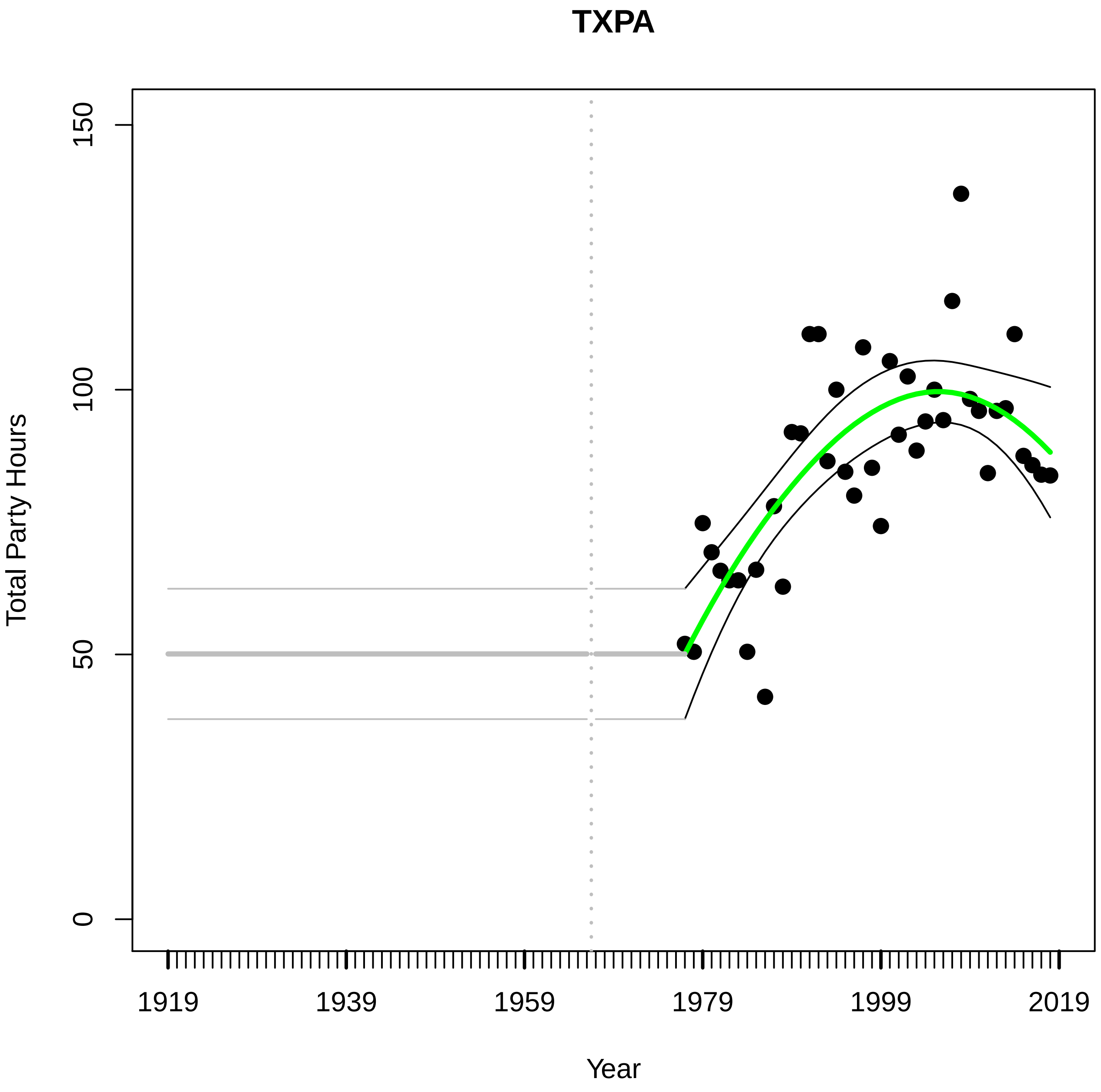
TXPA
Total Party Hours
Year
0
50
100
150
1919
1939
1959
1979
1999
2019

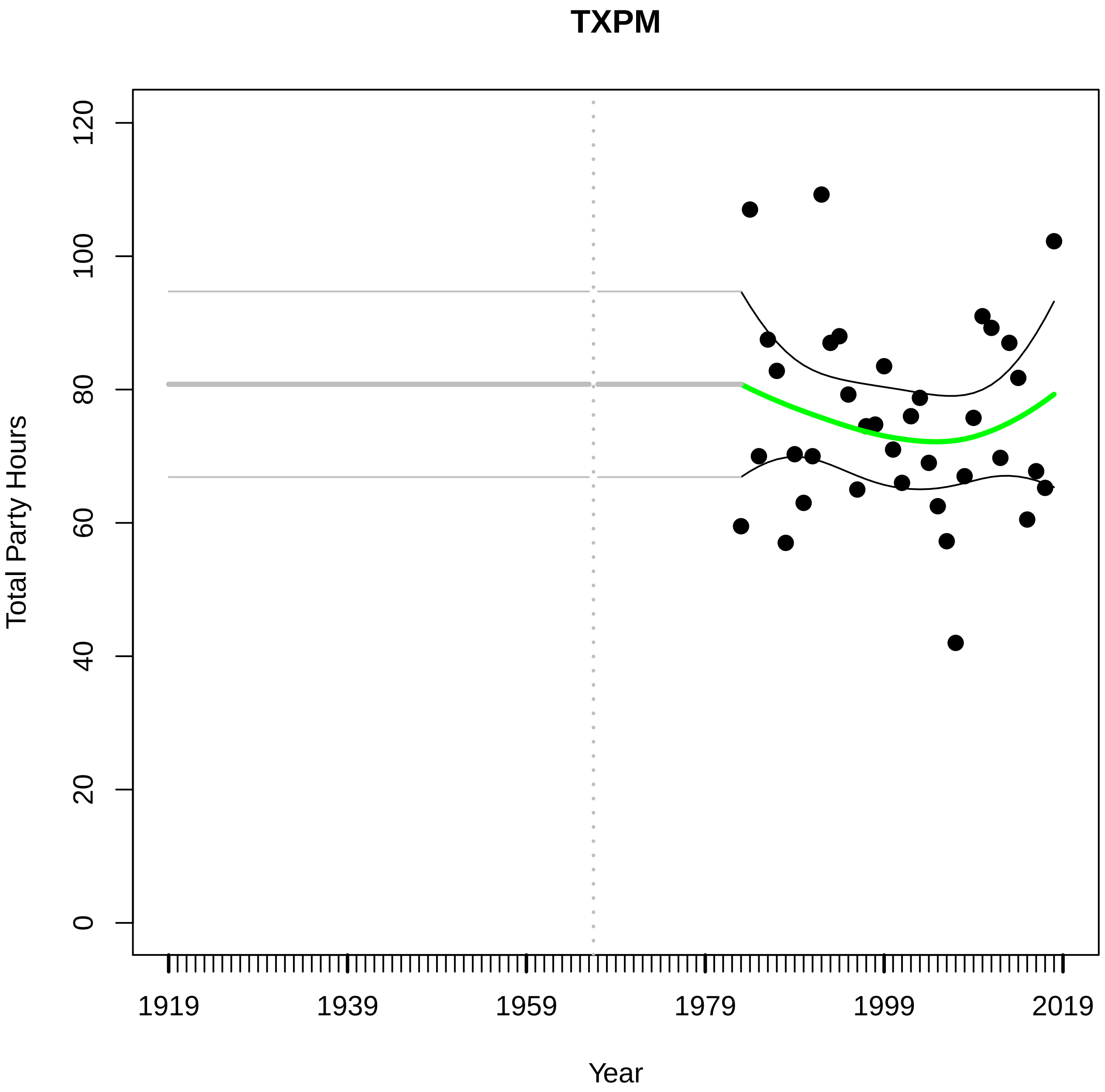
TXPM
Total Party Hours
Year
0
20
40
60
80
100
120
1919
1939
1959
1979
1999
2019

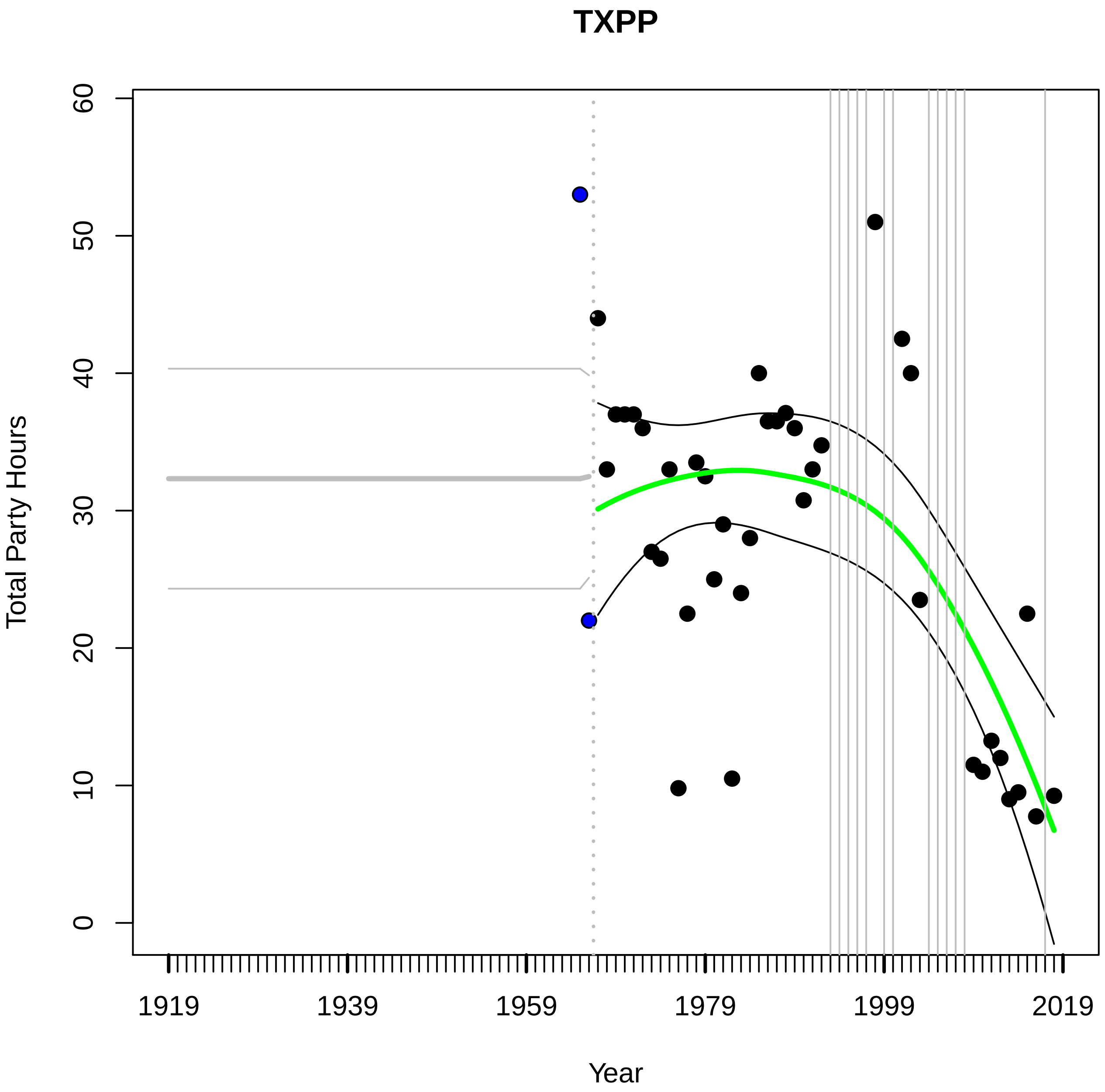

TXPP
Total Party Hours
Year
0
10
20
30
40
50
60
1919
1939
1959
1979
1999
2019

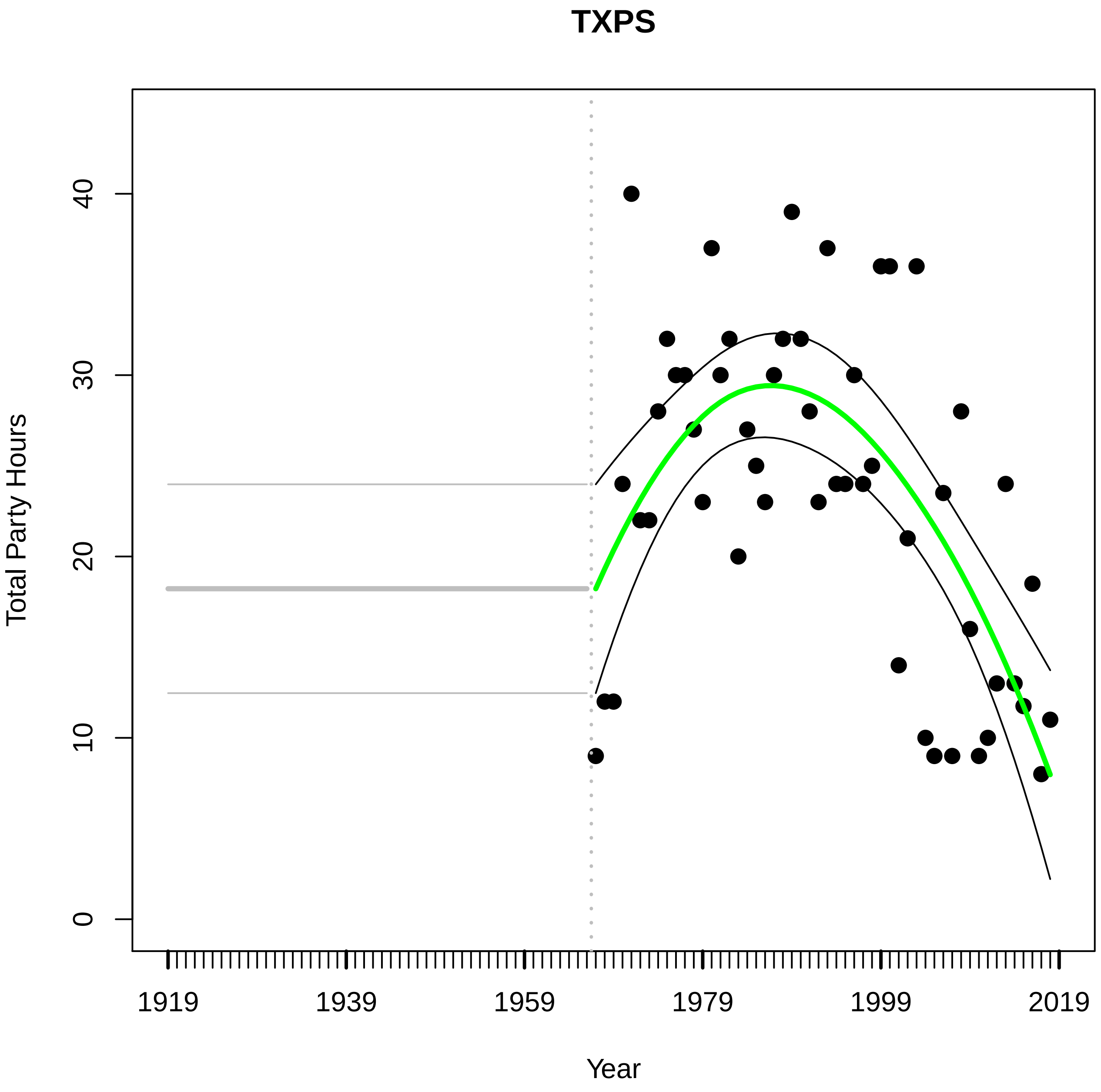

TXPS
Total Party Hours
0
10
20
30
40
1919
1939
1959
1979
1999
2019
Year

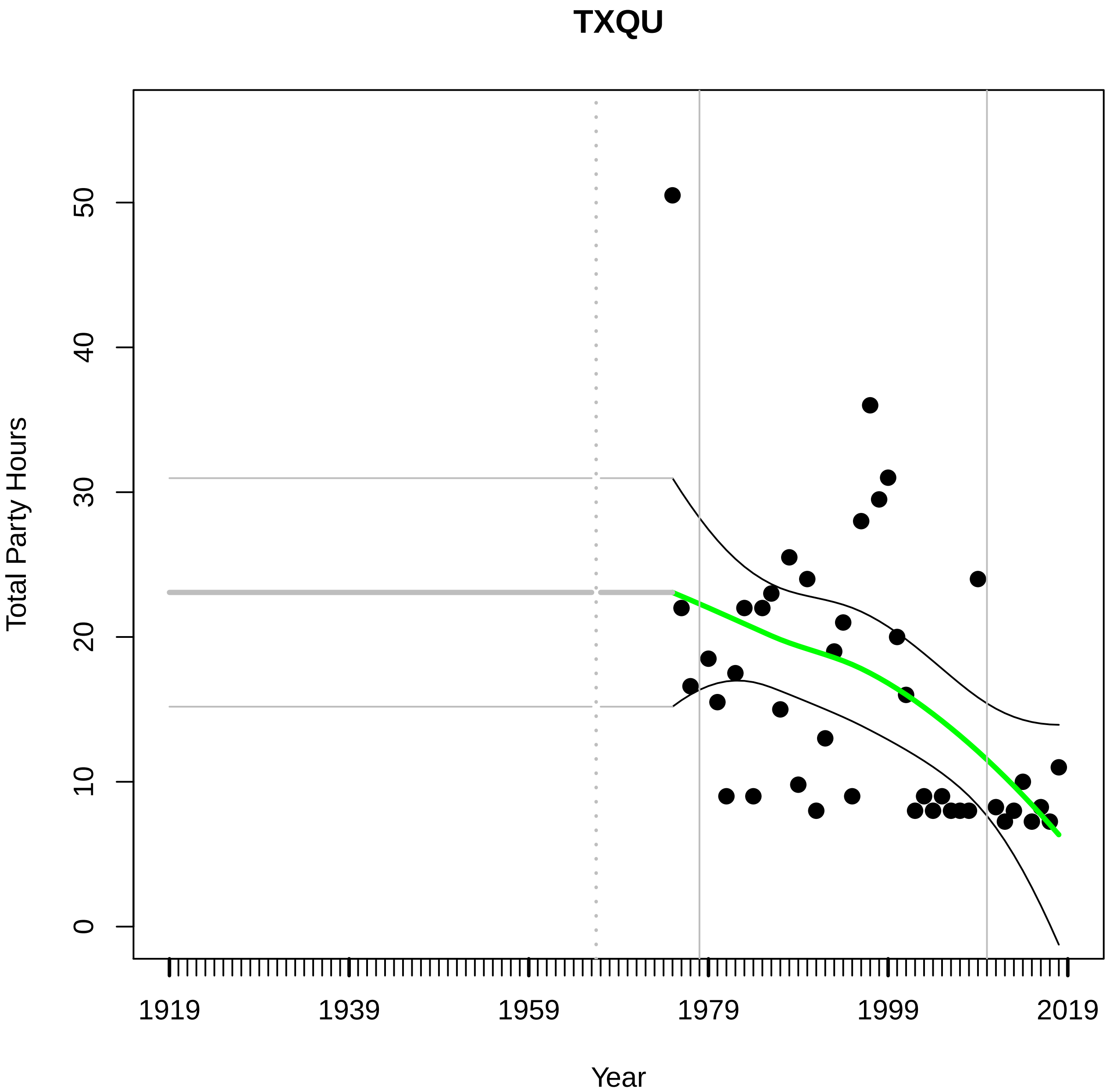

TXQU
Total Party Hours
Year
0
10
20
30
40
50
1919
1939
1959
1979
1999
2019

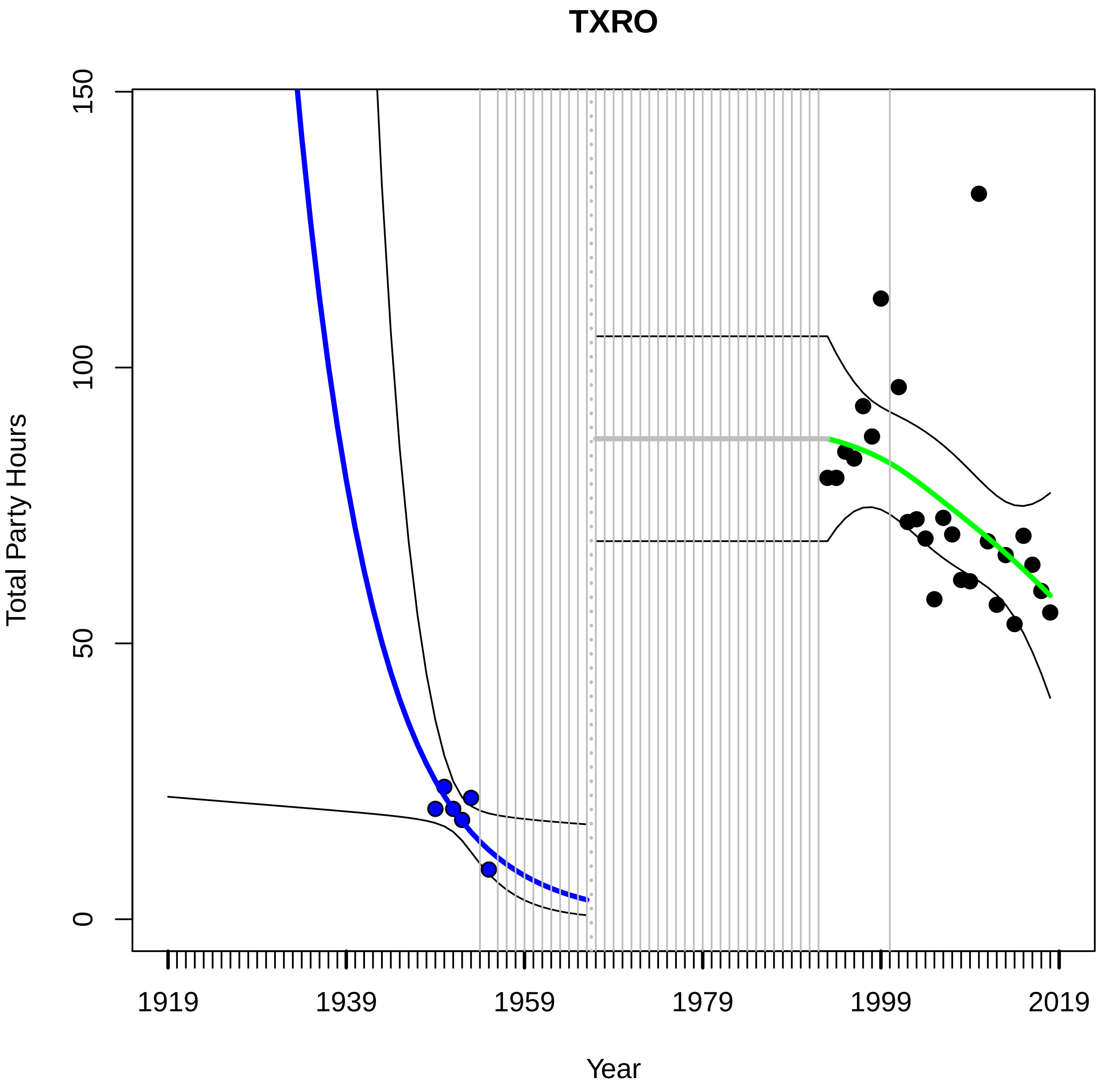

TXRO
Total Party Hours
150
100
50
0
1919
1939
1959
1979
1999
2019
Year

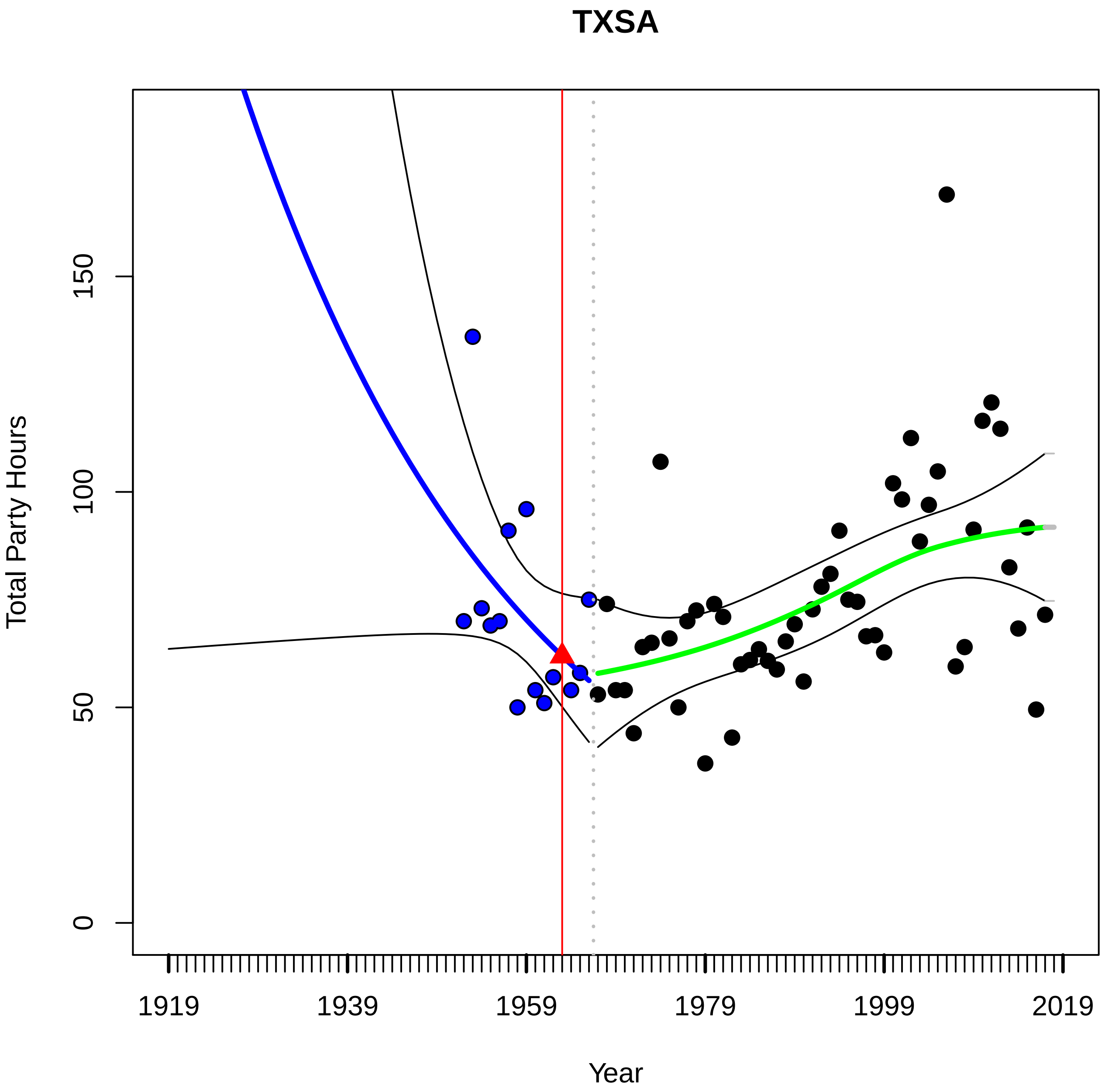
TXSA
Total Party Hours
Year
0
50
100
150
1919
1939
1959
1979
1999
2019

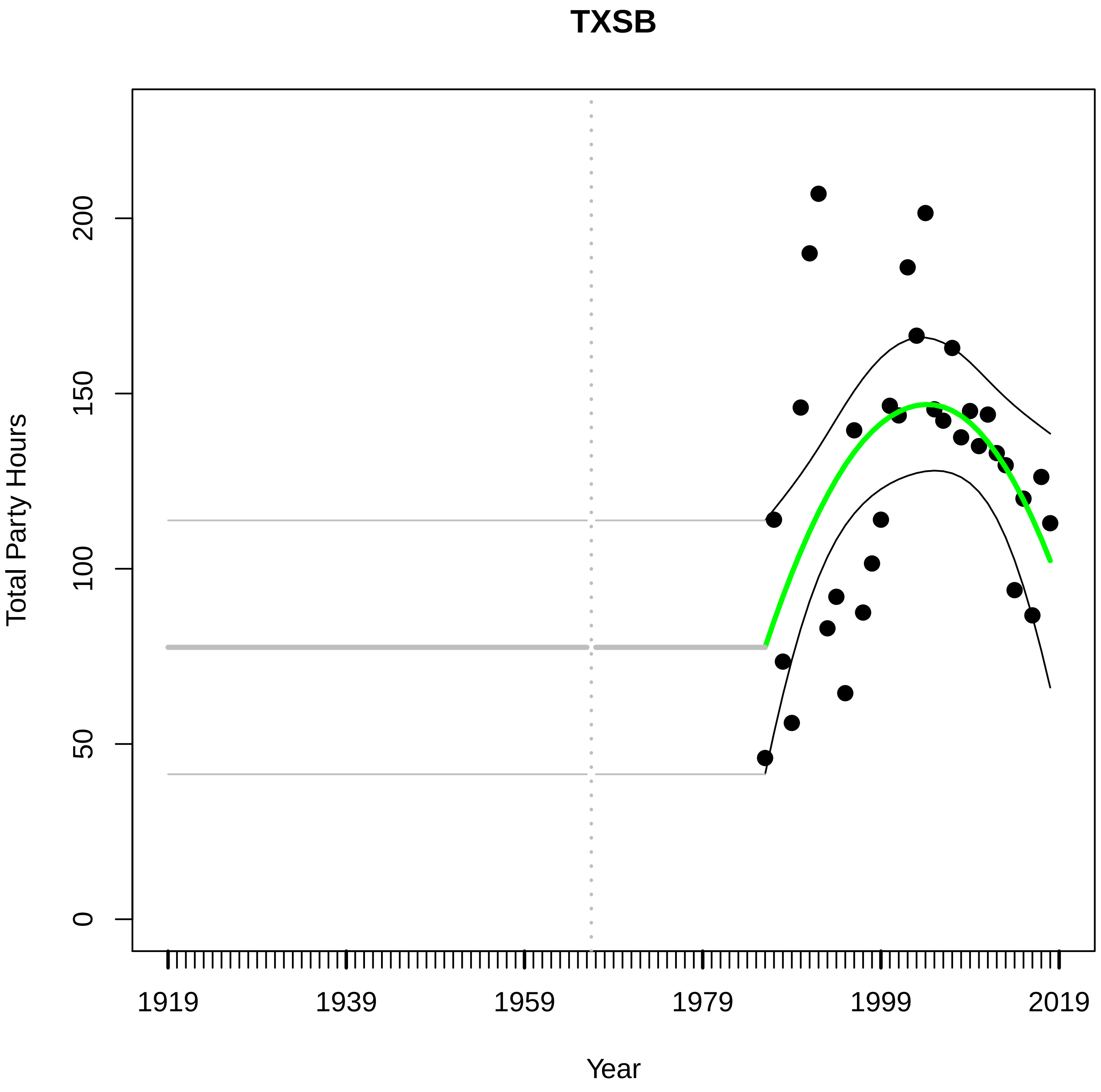
TXSB
Total Party Hours
0
50
100
150
200
1919
1939
1959
1979
1999
2019
Year

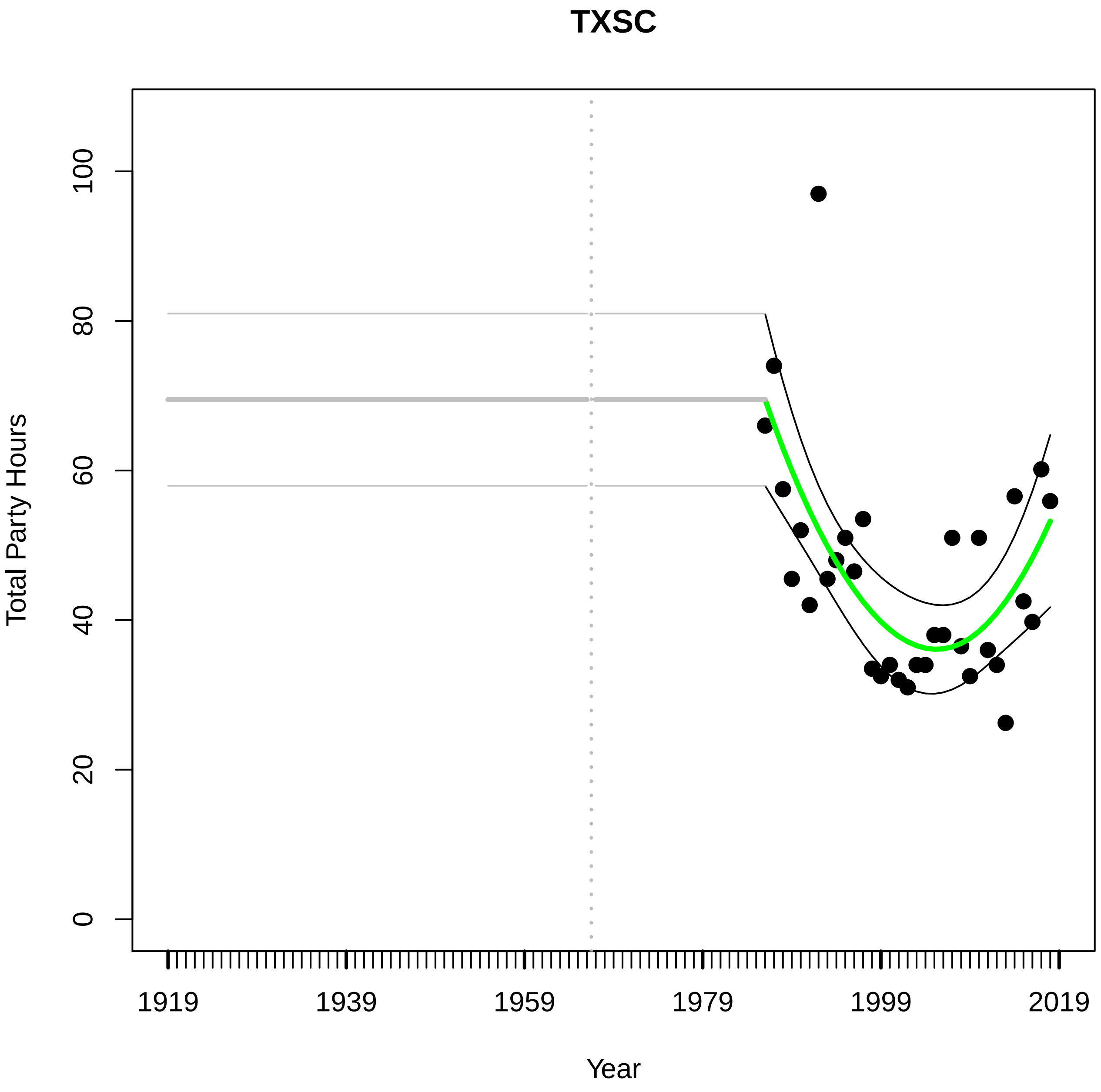

TXSC
Total Party Hours
Year
0
20
40
60
80
100
1919
1939
1959
1979
1999
2019

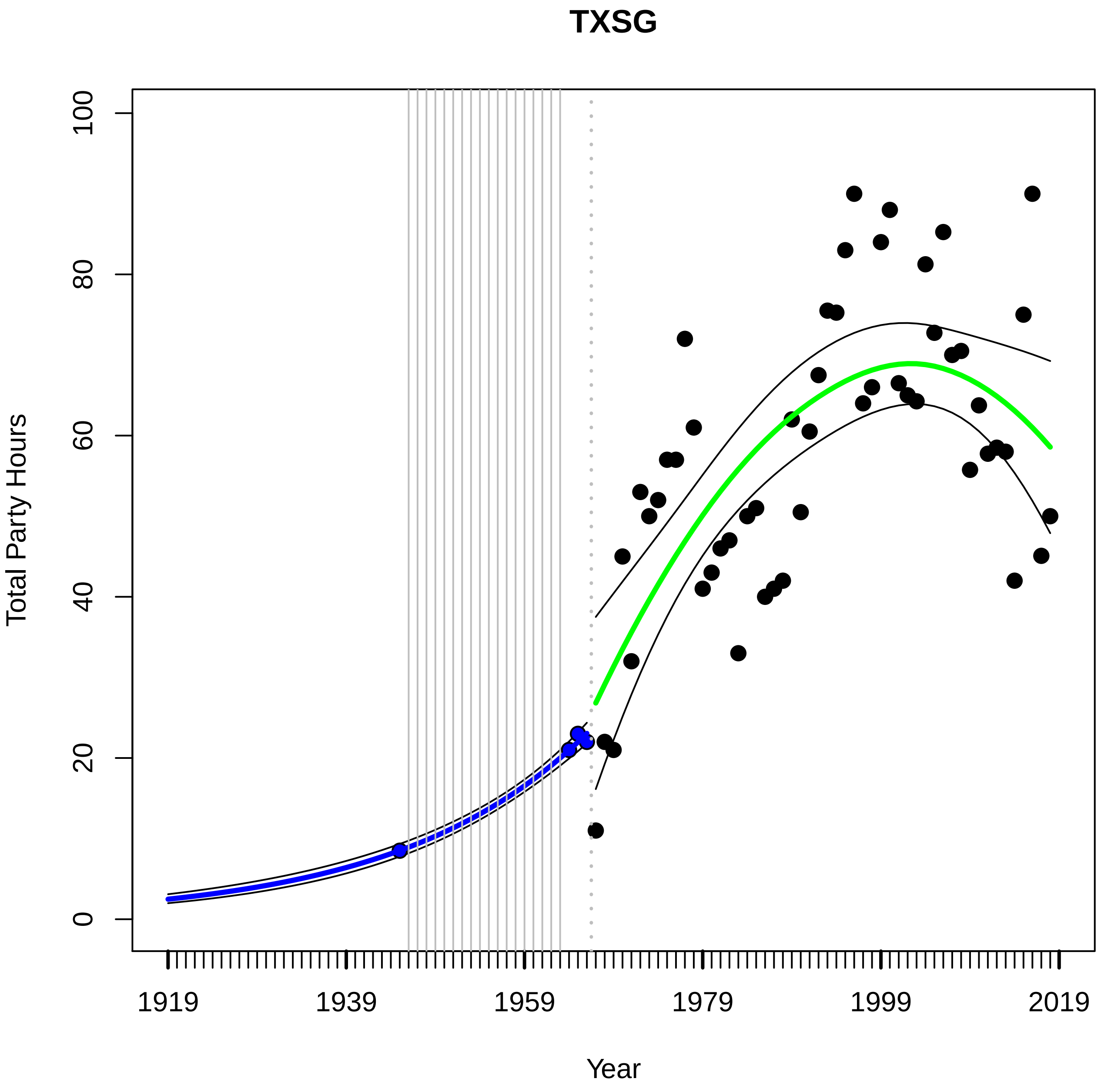

TXSG
Total Party Hours
0
20
40
60
80
100
1919
1939
1959
1979
1999
2019
Year

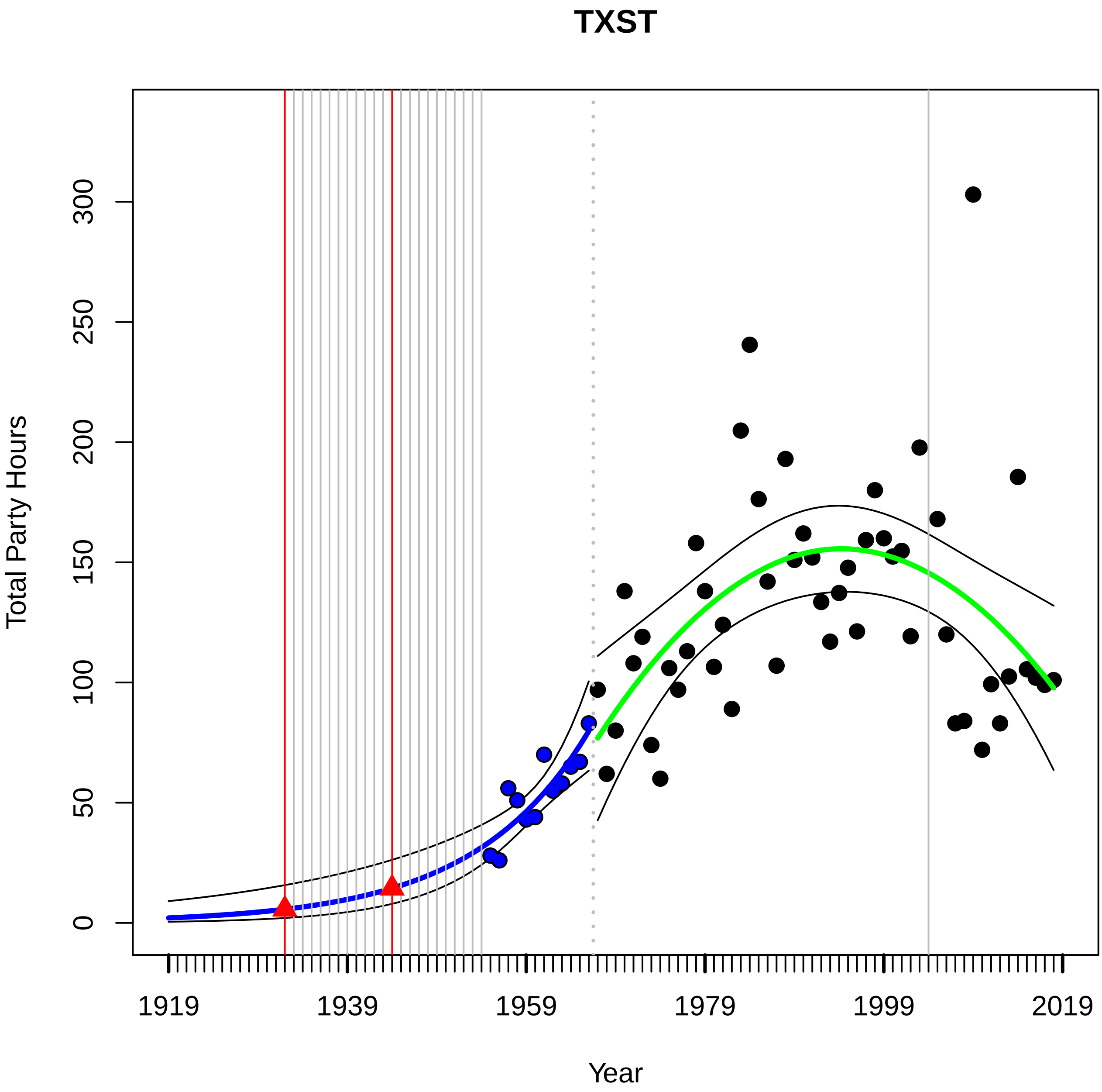
TXST
Total Party Hours
0
50
100
150
200
250
300
1919
1939
1959
1979
1999
2019
Year

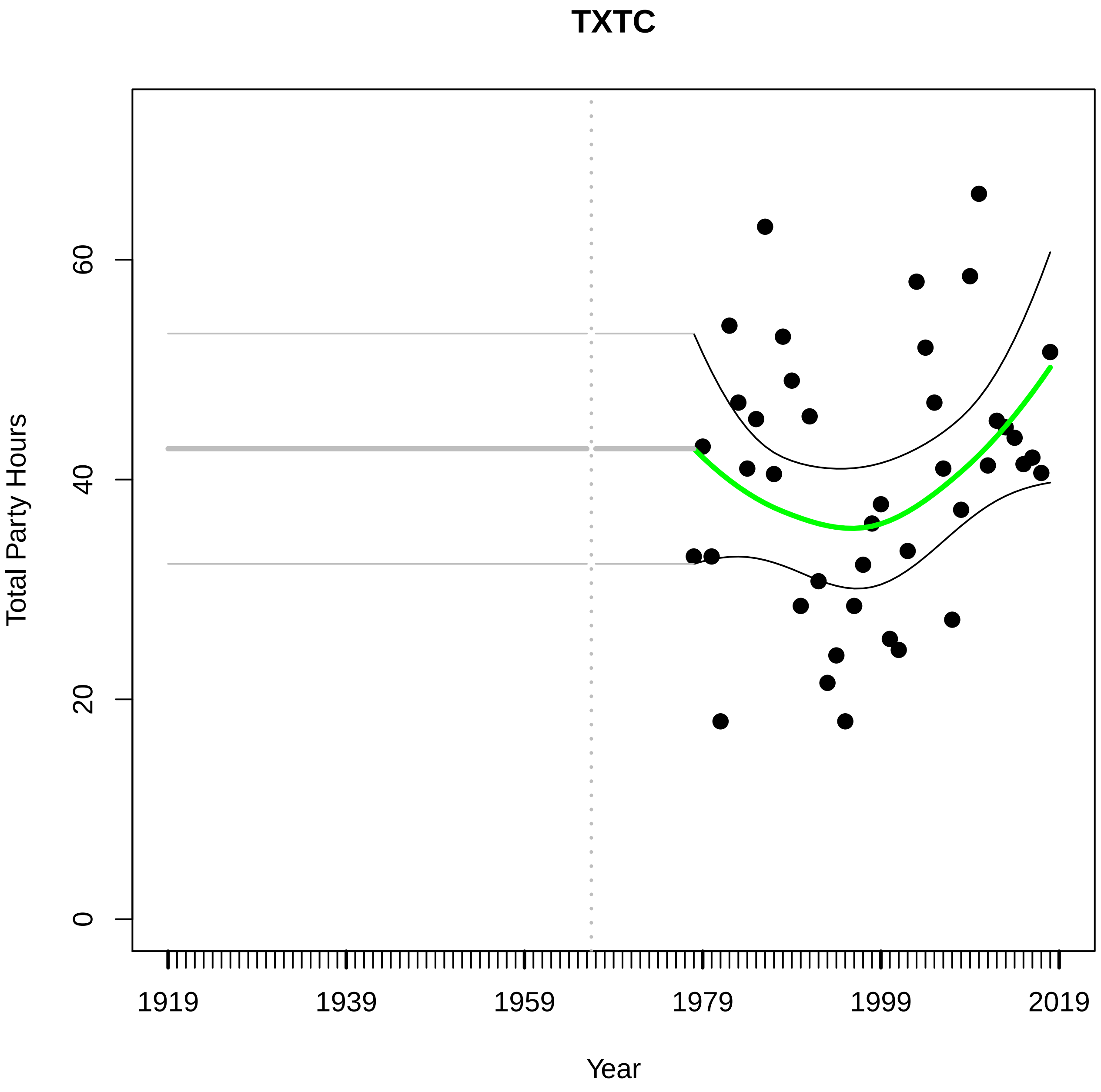
TXTC
Total Party Hours
0
20
40
60
1919
1939
1959
1979
1999
2019
Year

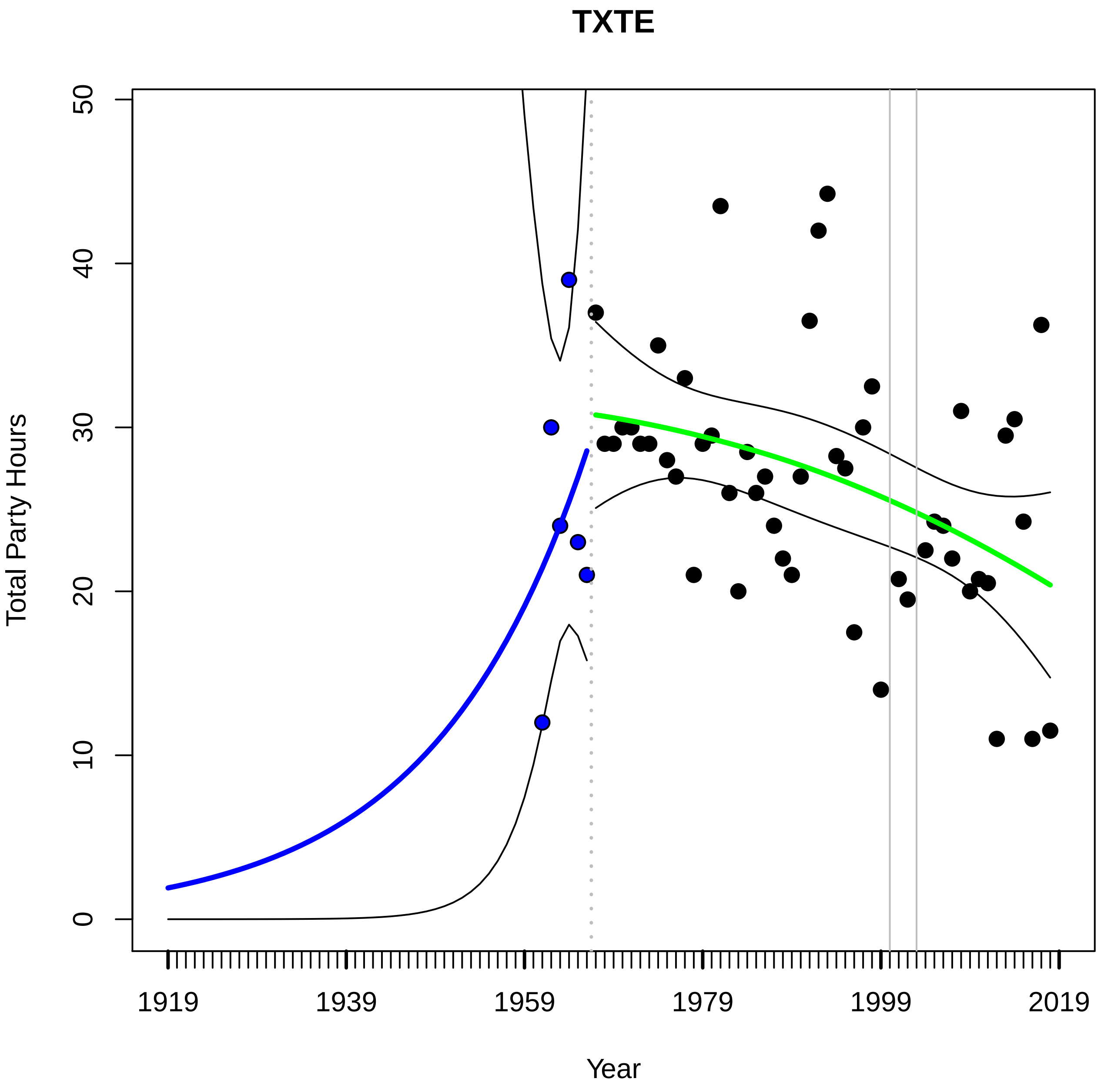

TXTE
Total Party Hours
Year
0
10
20
30
40
50
1919
1939
1959
1979
1999
2019

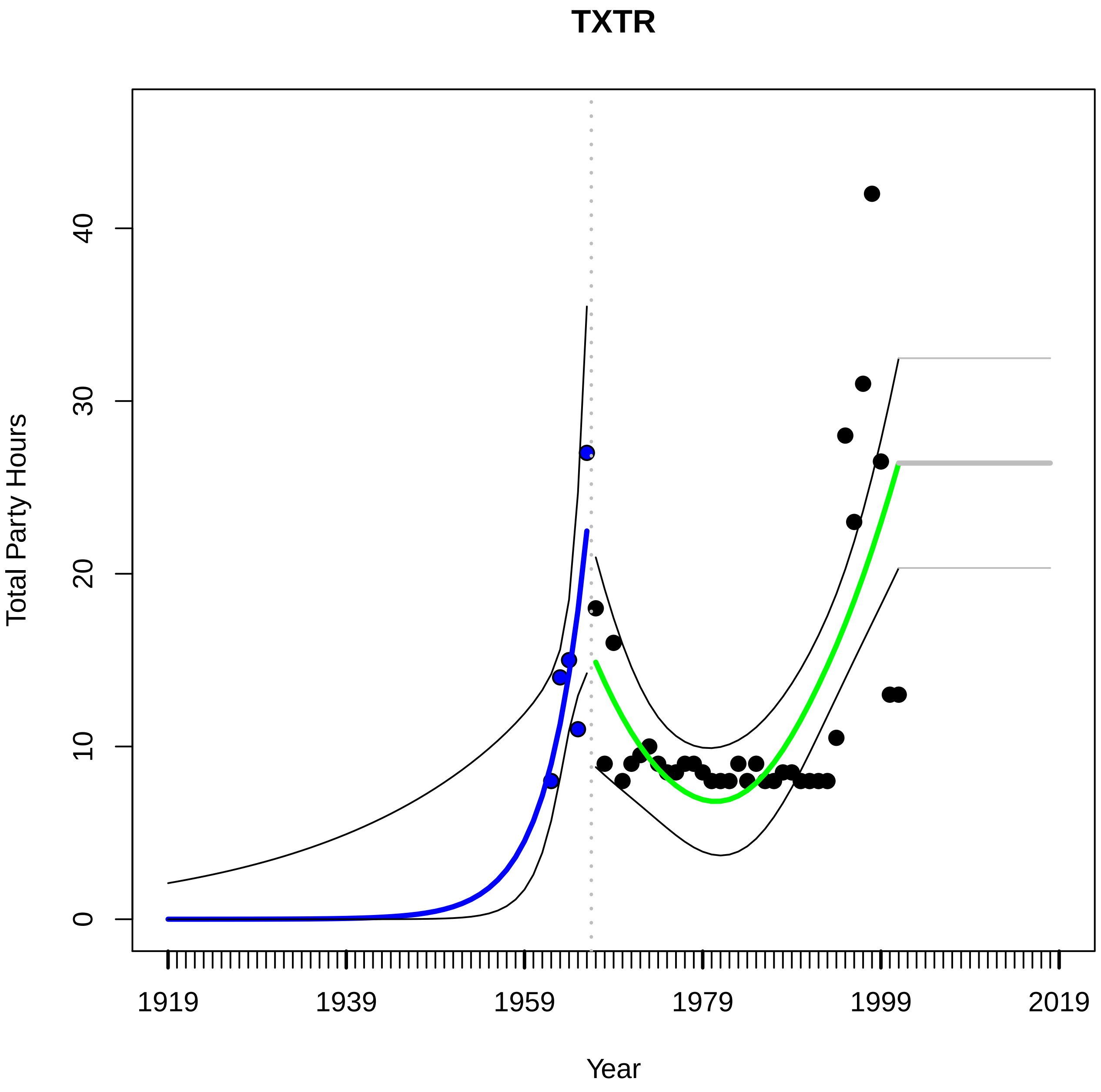

TXTR
Total Party Hours
0
10
20
30
40
Year
1919
1939
1959
1979
1999
2019

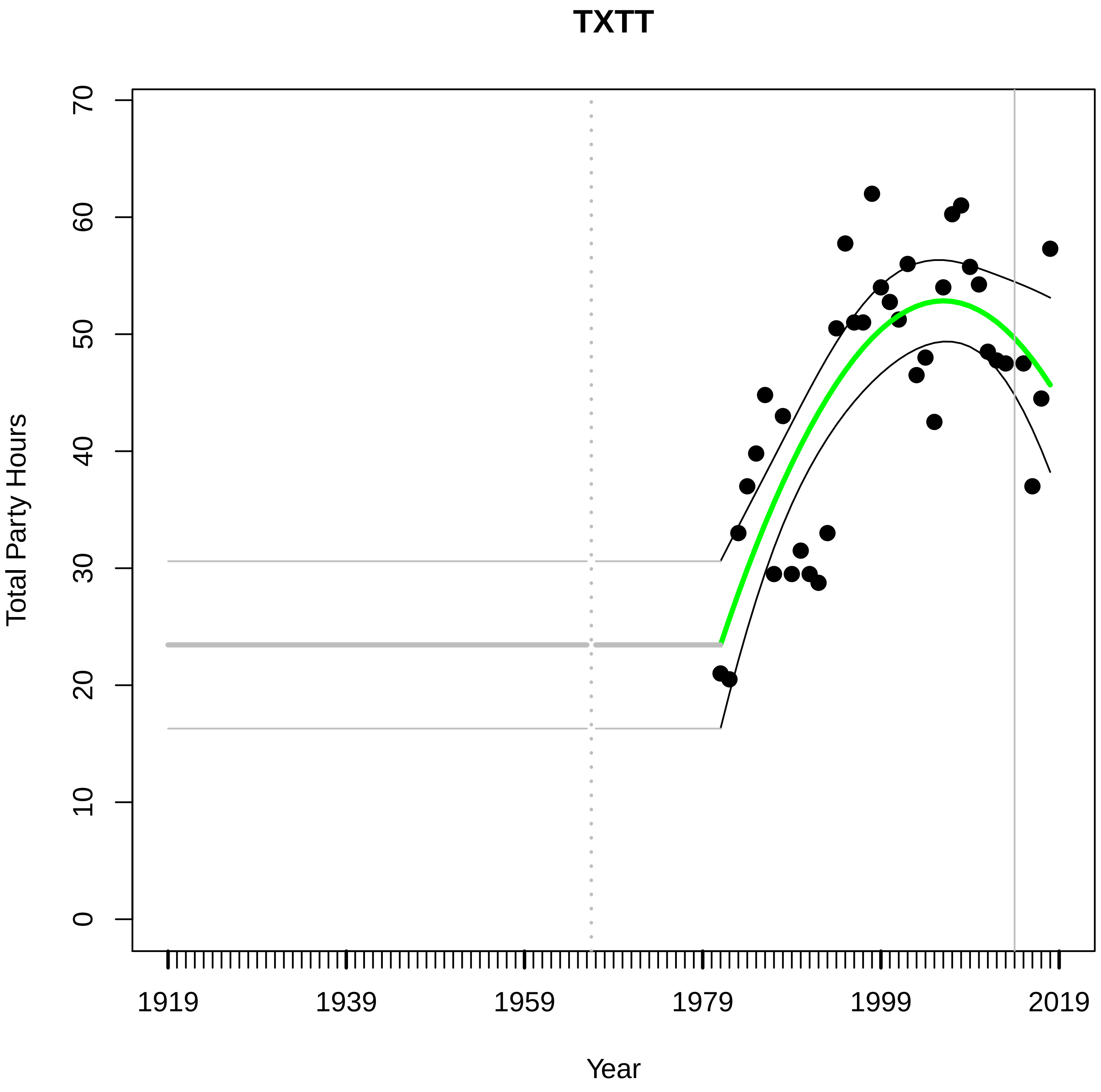

TXTT
Total Party Hours
Year
0
10
20
30
40
50
60
70
1919
1939
1959
1979
1999
2019

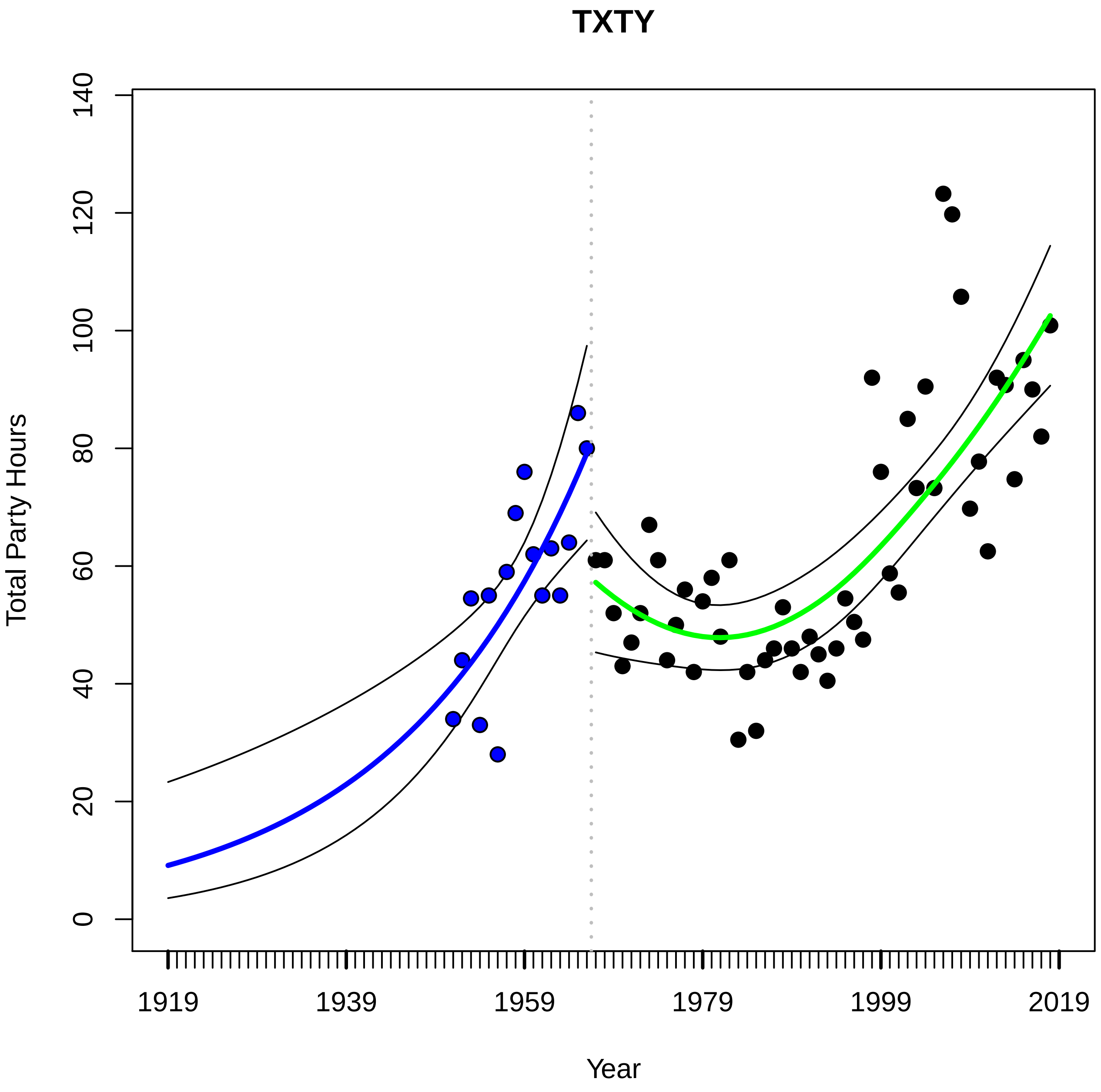

TXTY
Total Party Hours
0
20
40
60
80
100
120
140
1919
1939
1959
1979
1999
2019
Year

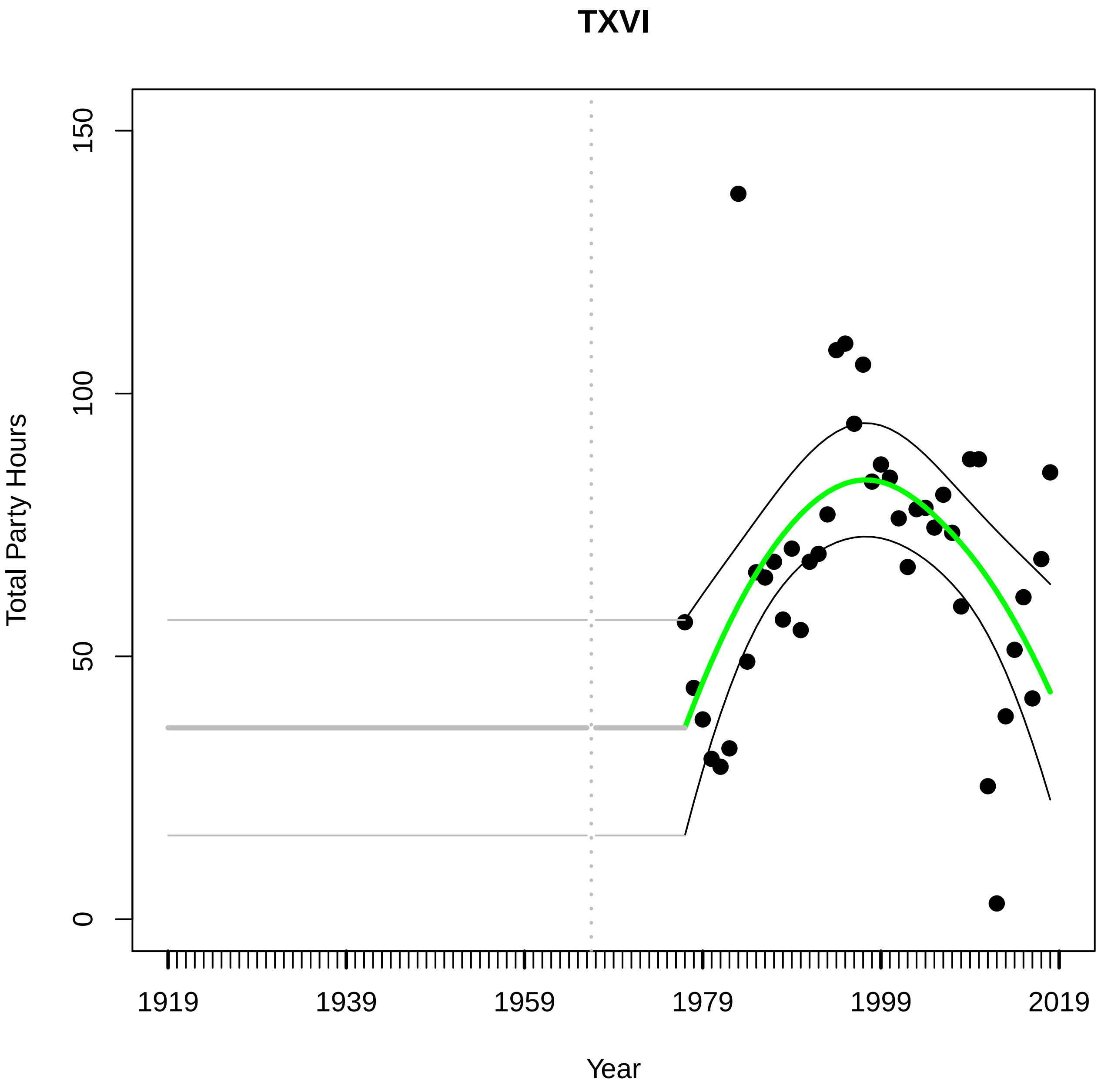
TXVI
Total Party Hours
0
50
100
150
1919
1939
1959
1979
1999
2019
Year

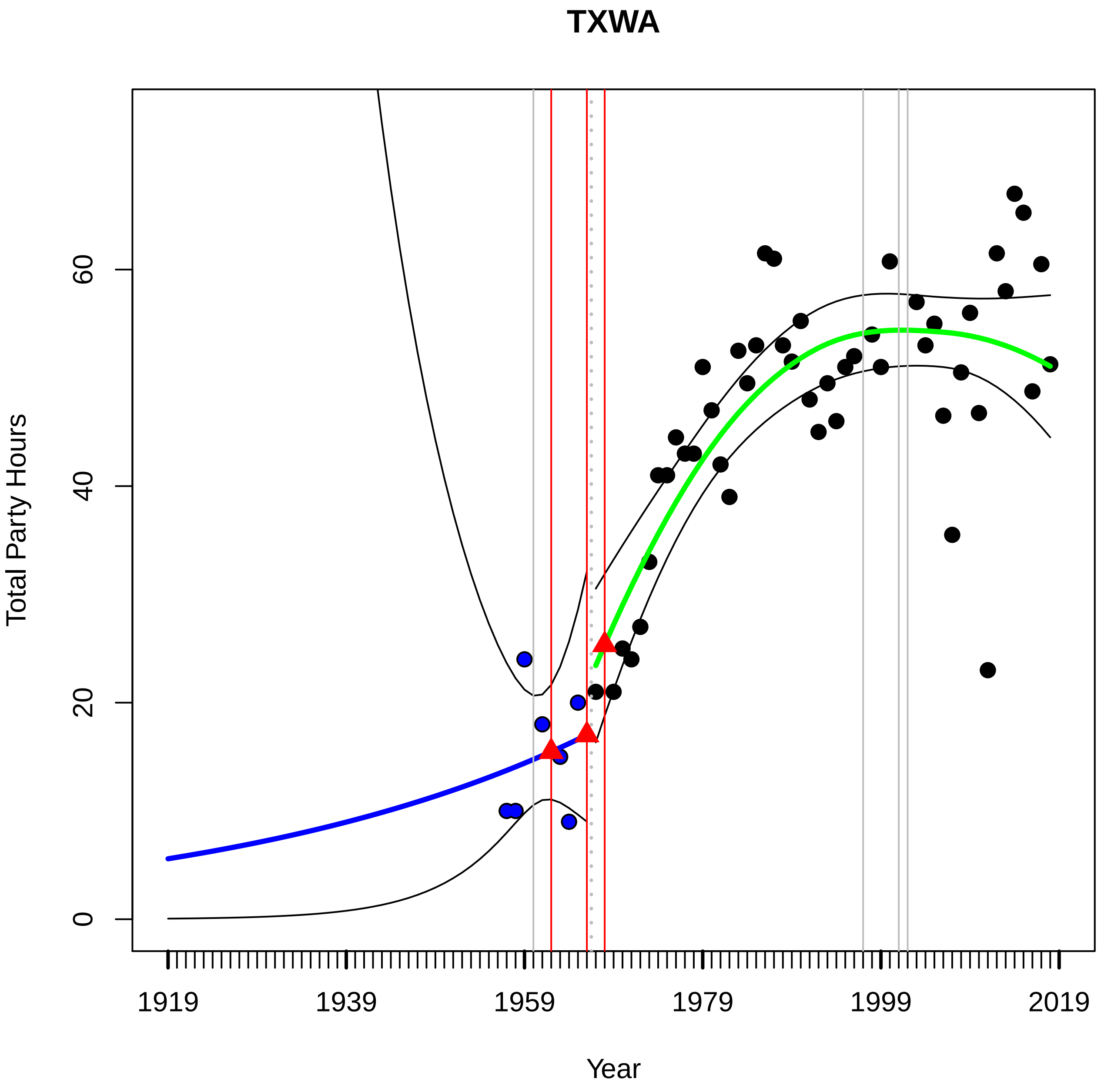

TXWA
Total Party Hours
Year
0
20
40
60
1919
1939
1959
1979
1999
2019

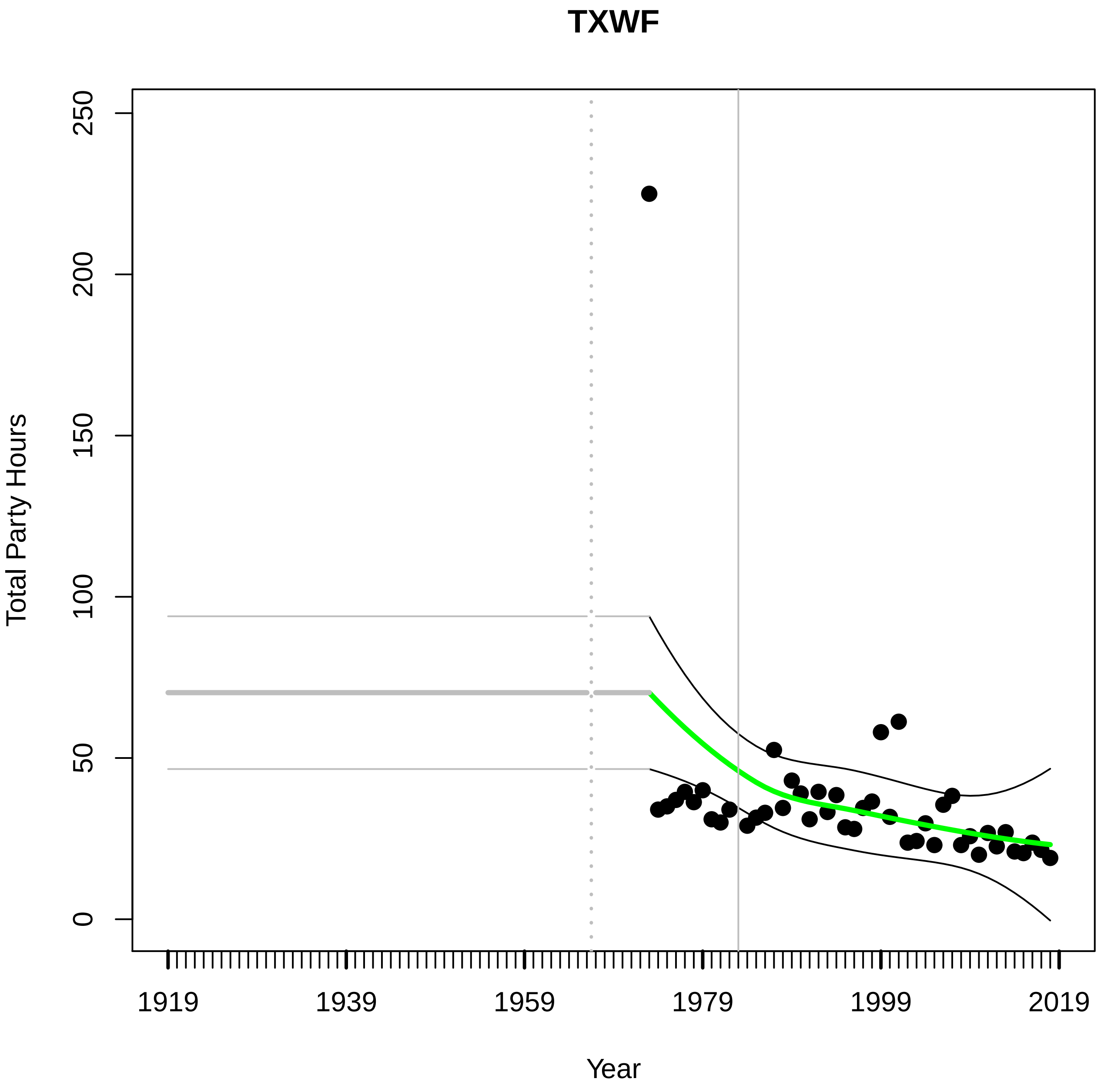
TXWF
Total Party Hours
0
50
100
150
200
250
1919
1939
1959
1979
1999
2019
Year

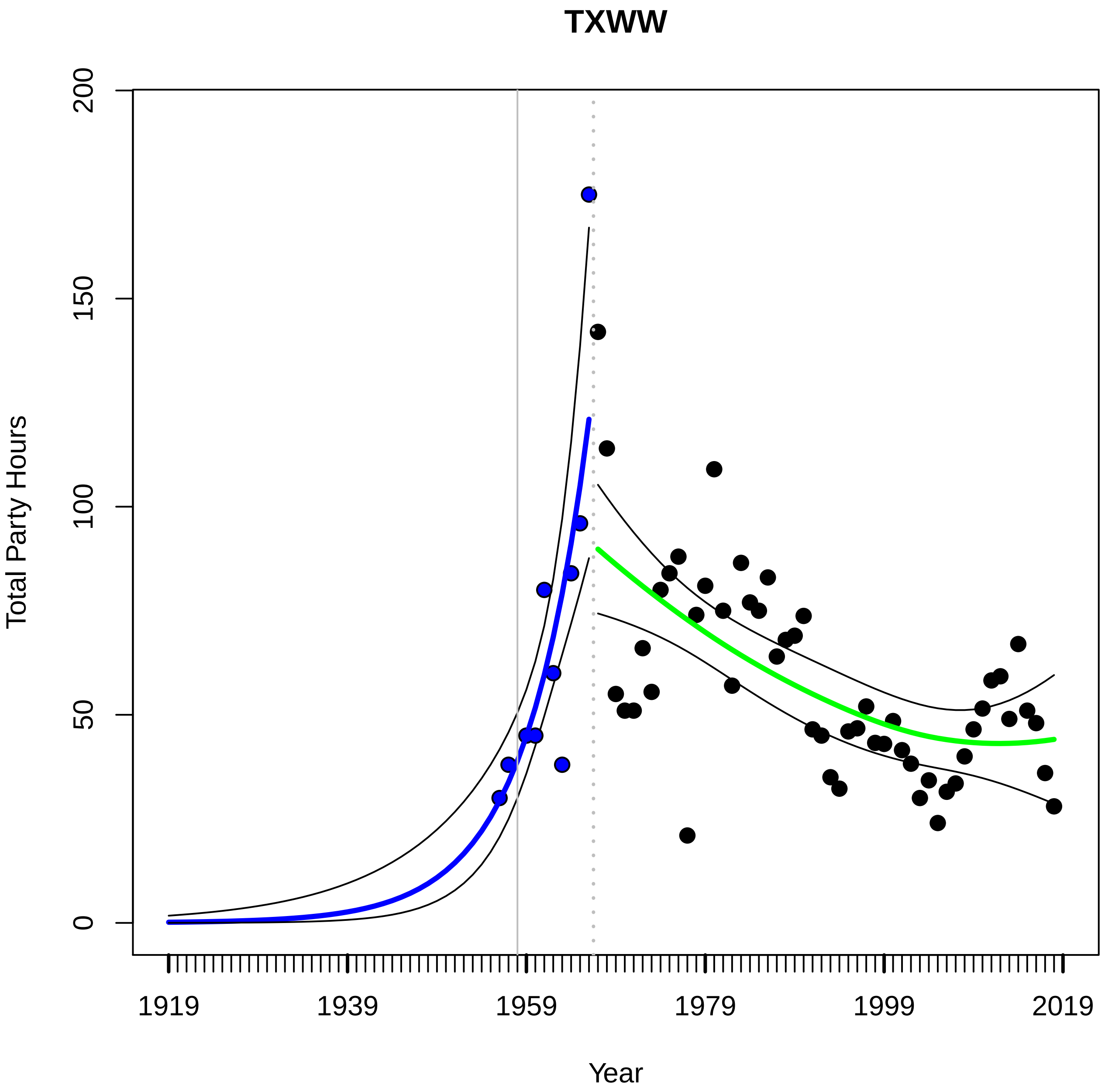
TXWW
Total Party Hours
0
50
100
150
200
1919
1939
1959
1979
1999
2019
Year

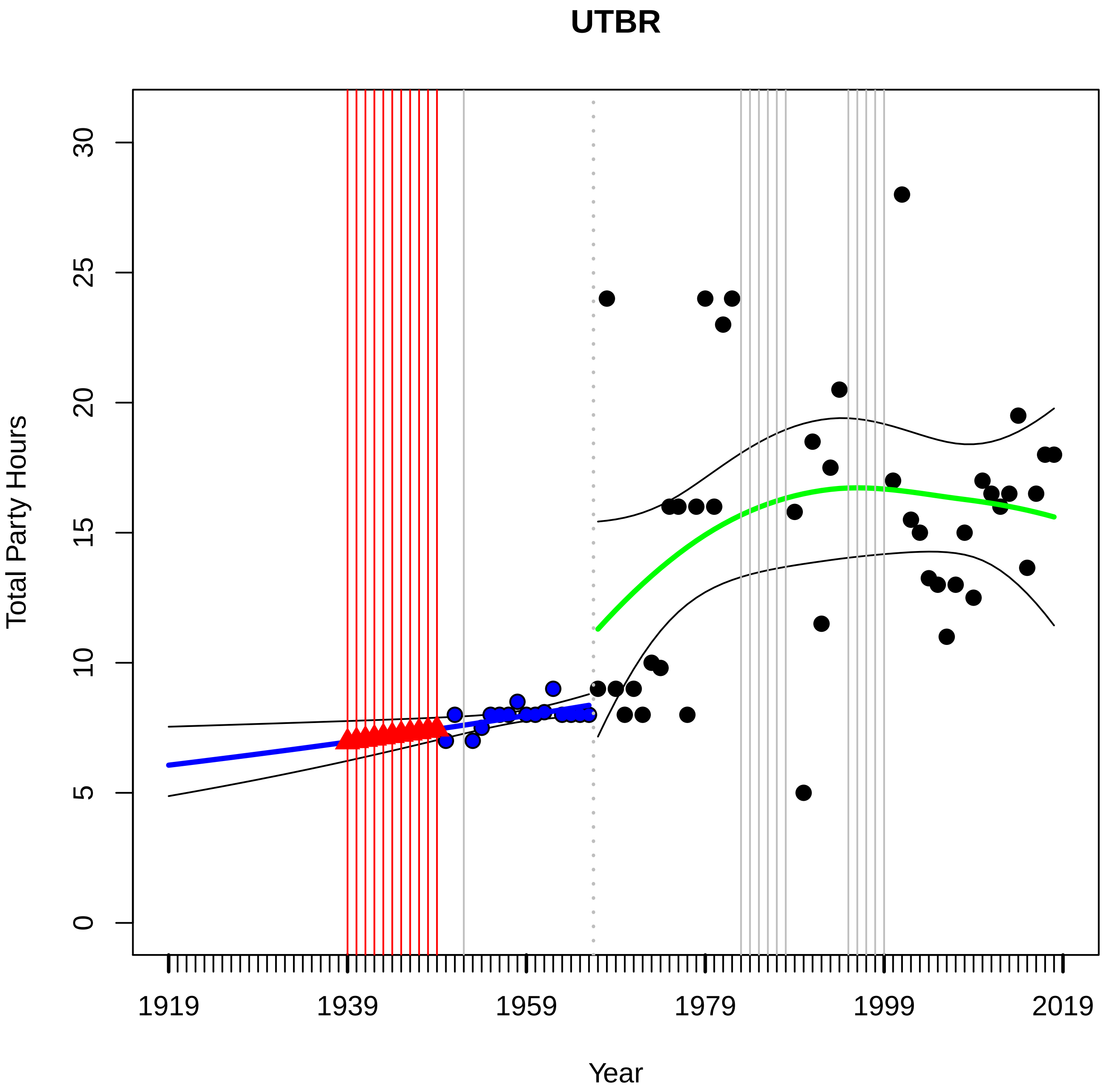

UTBR
Total Party Hours
Year
0
5
10
15
20
25
30
1919
1939
1959
1979
1999
2019

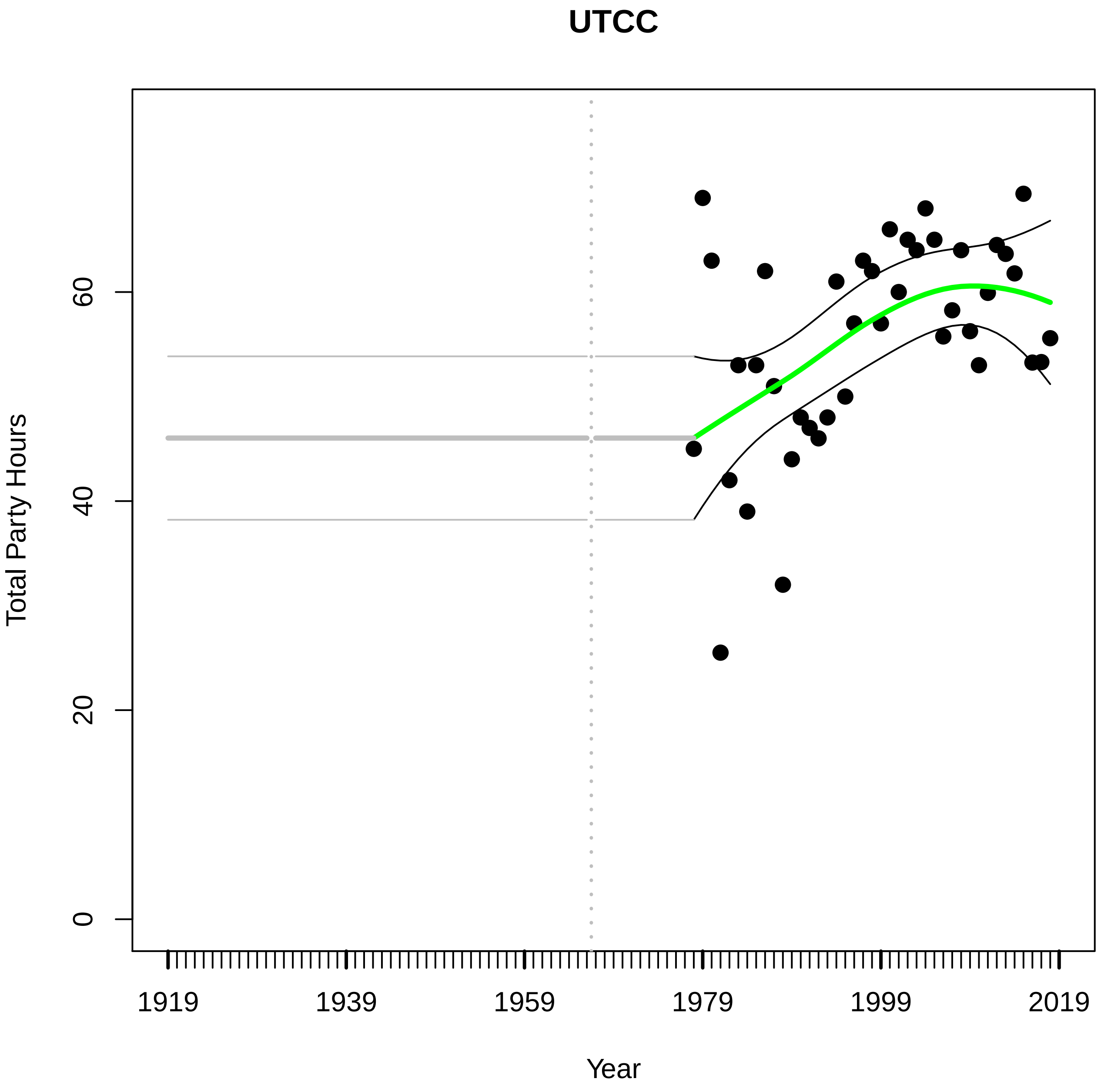

UTCC
Total Party Hours
0
20
40
60
1919
1939
1959
1979
1999
2019
Year

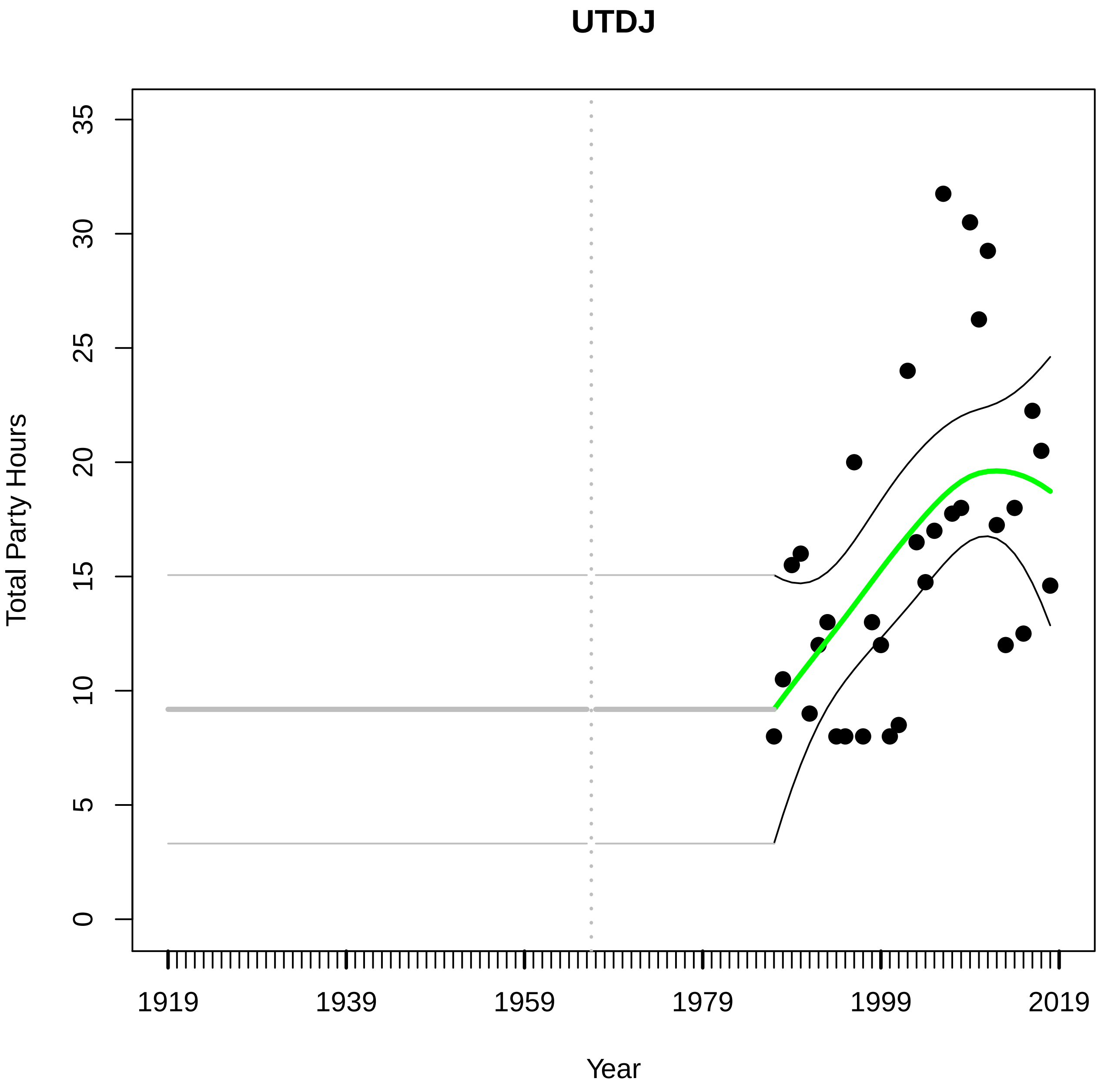
UTDJ
Total Party Hours
0
5
10
15
20
25
30
35
1919
1939
1959
1979
1999
2019
Year

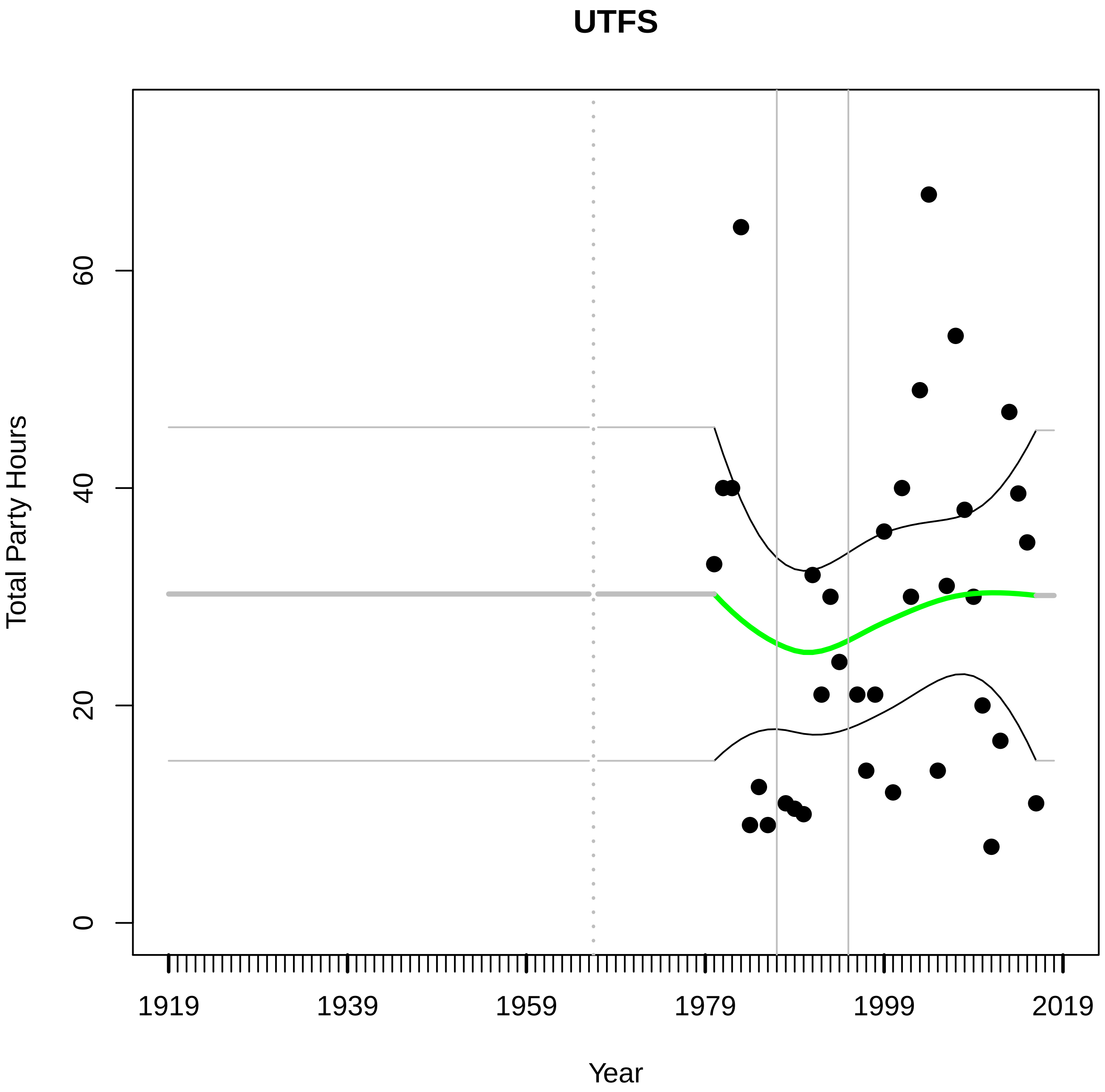

UTFS
Total Party Hours
0
20
40
60
1919
1939
1959
1979
1999
2019
Year

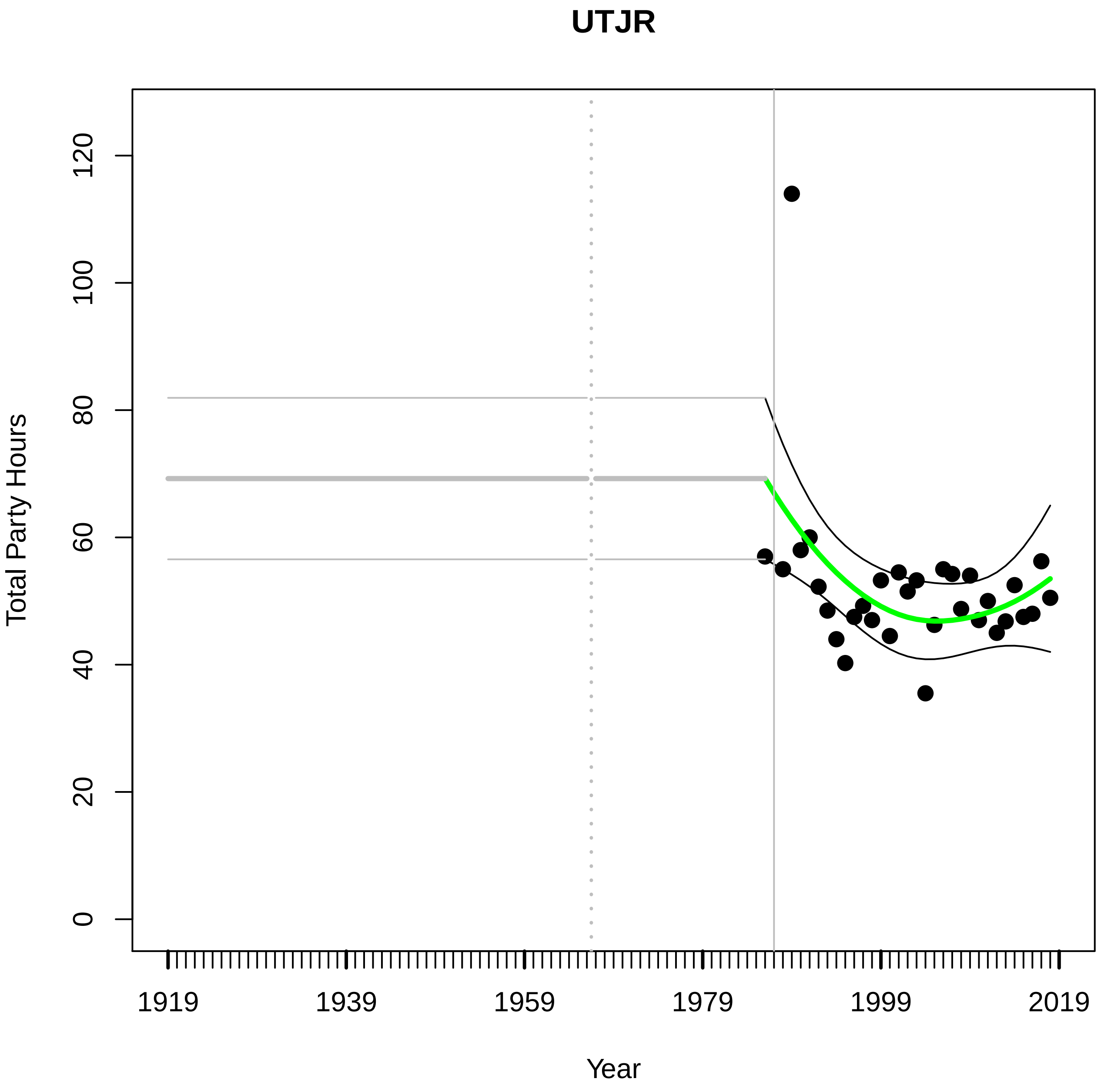
UTJR
Total Party Hours
0
20
40
60
80
100
120
1919
1939
1959
1979
1999
2019
Year

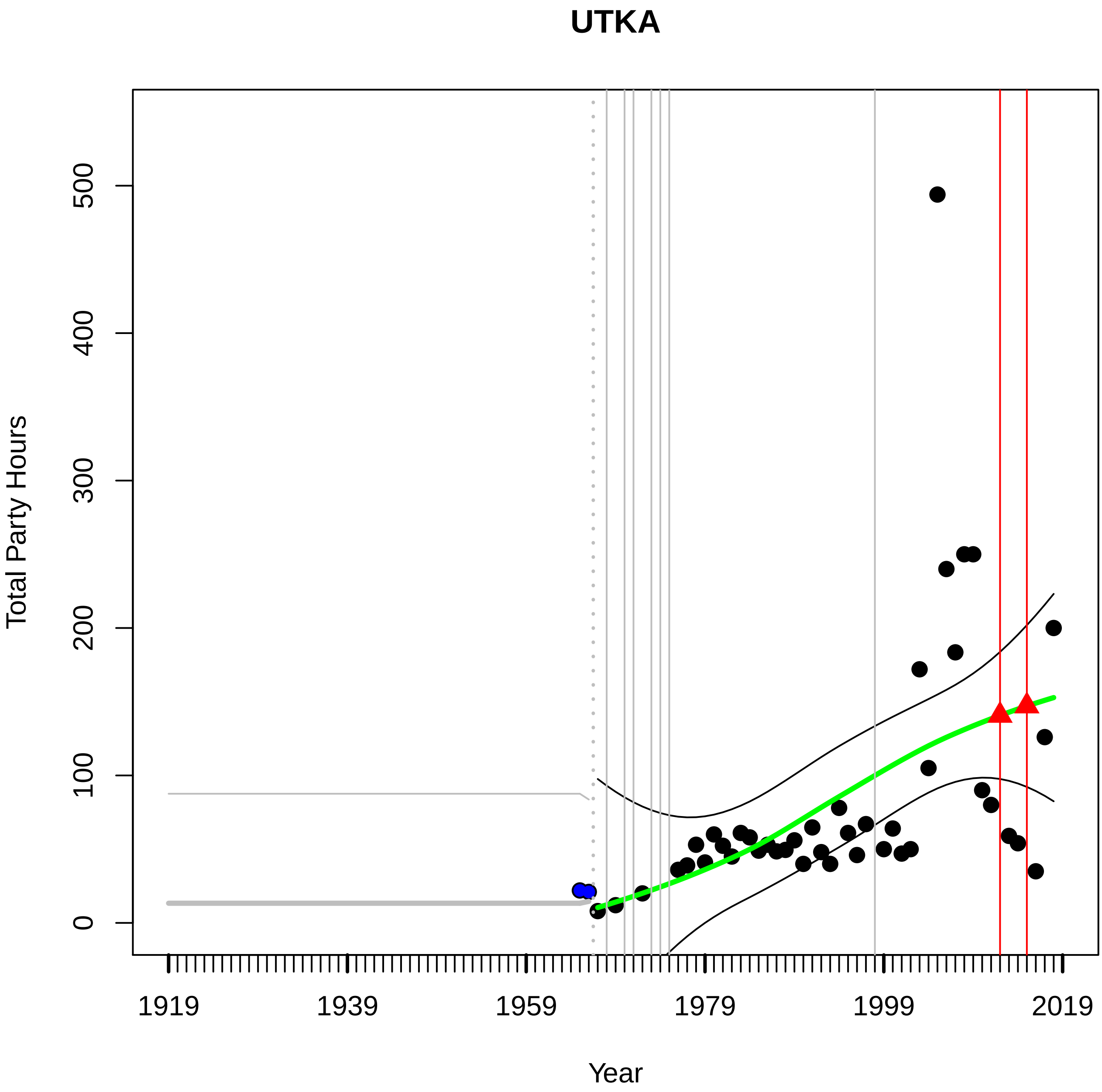
UTKA
Total Party Hours
0
100
200
300
400
500
1919
1939
1959
1979
1999
2019
Year

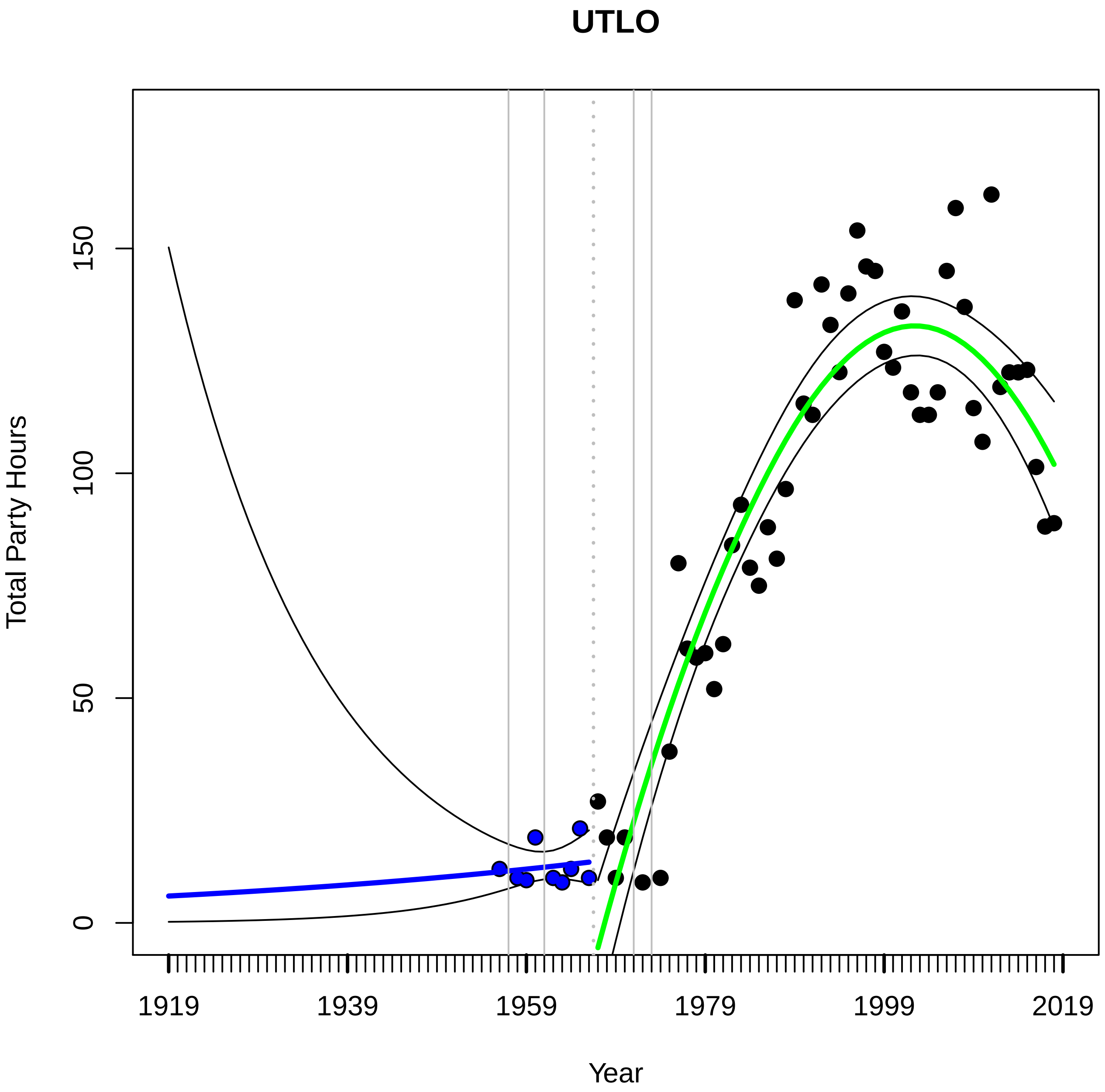

UTLO
Total Party Hours
0
50
100
150
1919
1939
1959
1979
1999
2019
Year

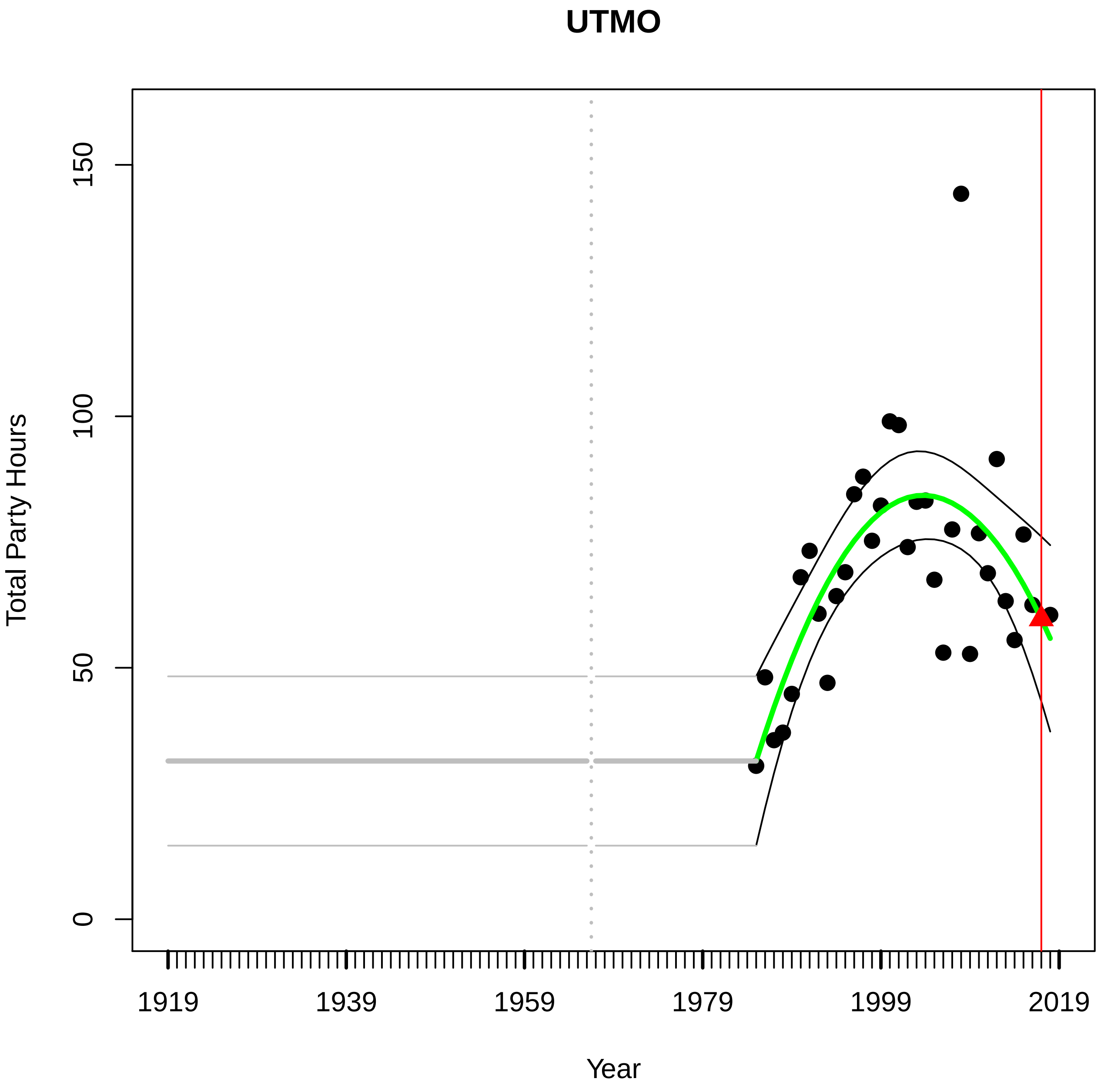

UTMO
Total Party Hours
150
100
50
0
1919
1939
1959
1979
1999
2019
Year

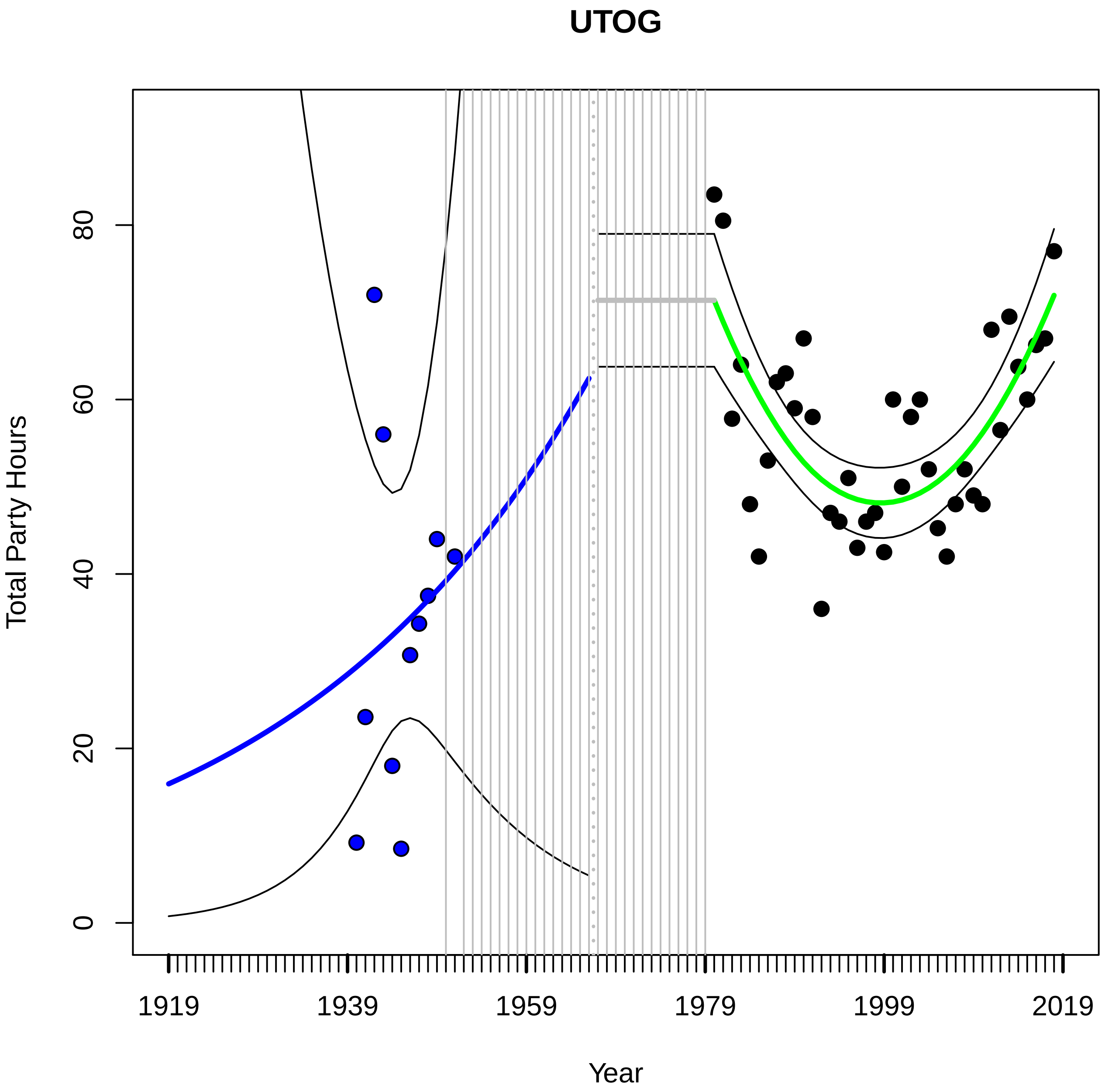

UTOG
Total Party Hours
0
20
40
60
80
1919
1939
1959
1979
1999
2019
Year

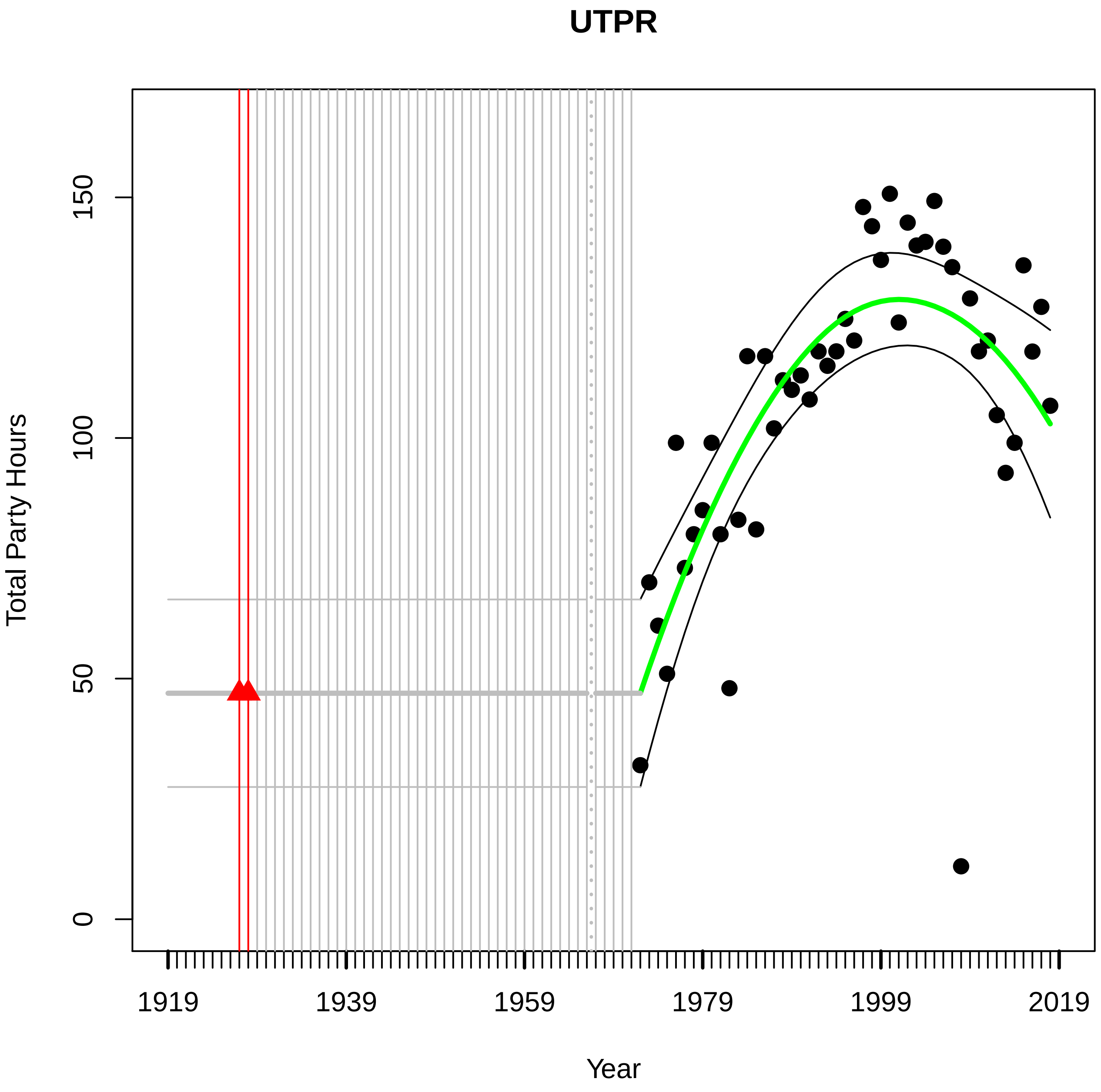

UTPR
Total Party Hours
150
100
50
0
1919
1939
1959
1979
1999
2019
Year

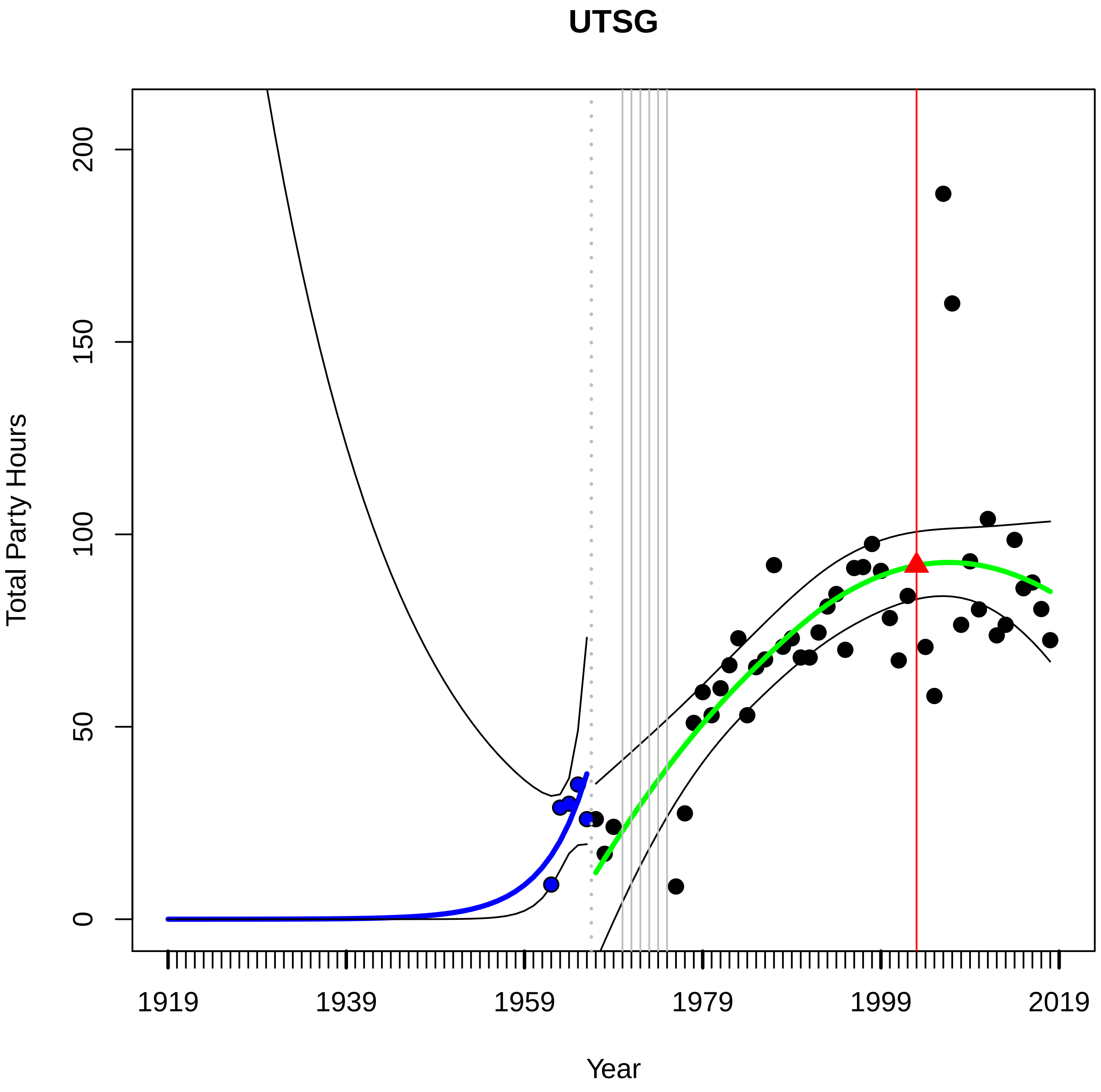
UTSG
Total Party Hours
Year
0
50
100
150
200
1919
1939
1959
1979
1999
2019

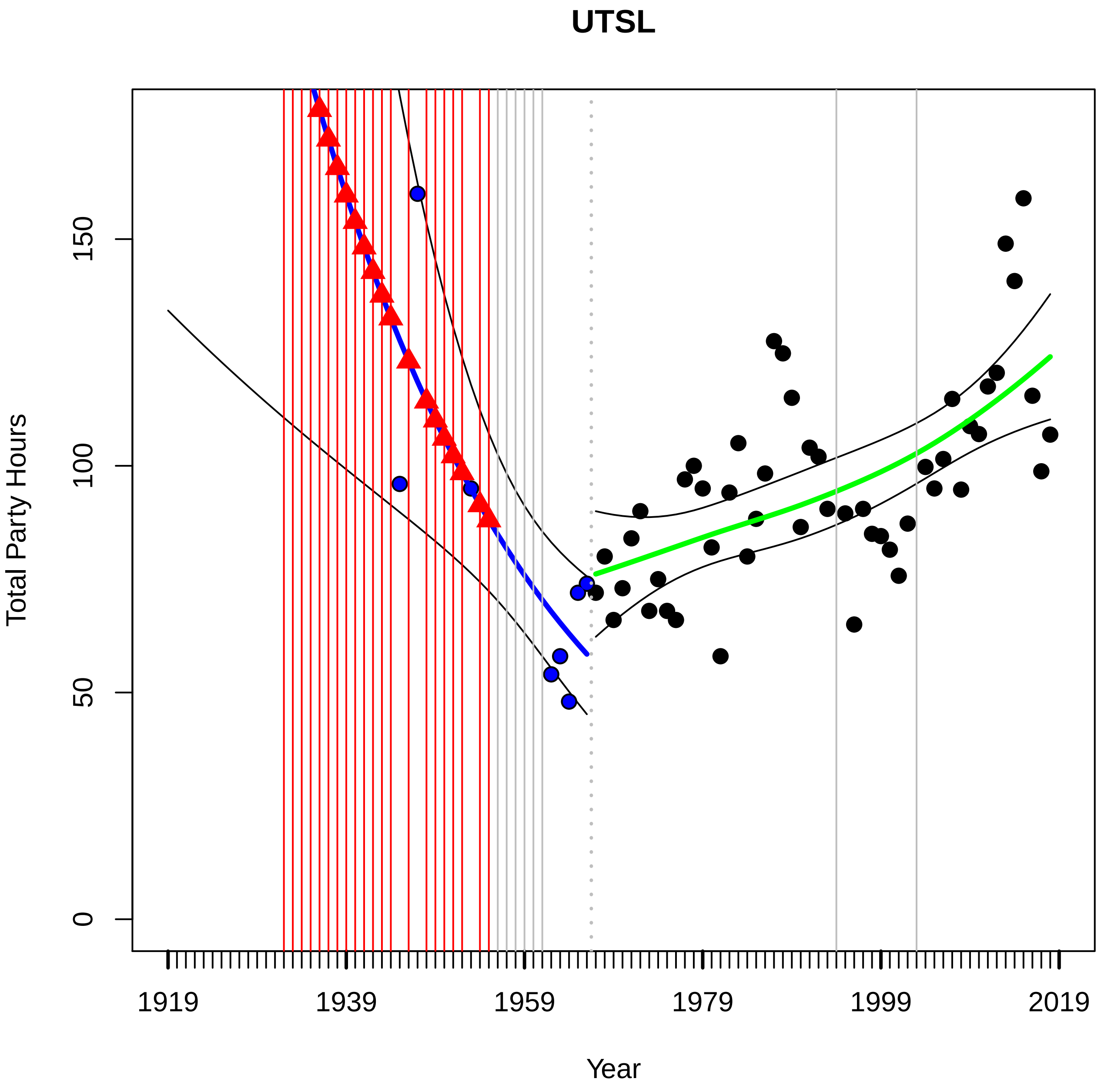

UTSL
Total Party Hours
0
50
100
150
Year
1919
1939
1959
1979
1999
2019

UTZI

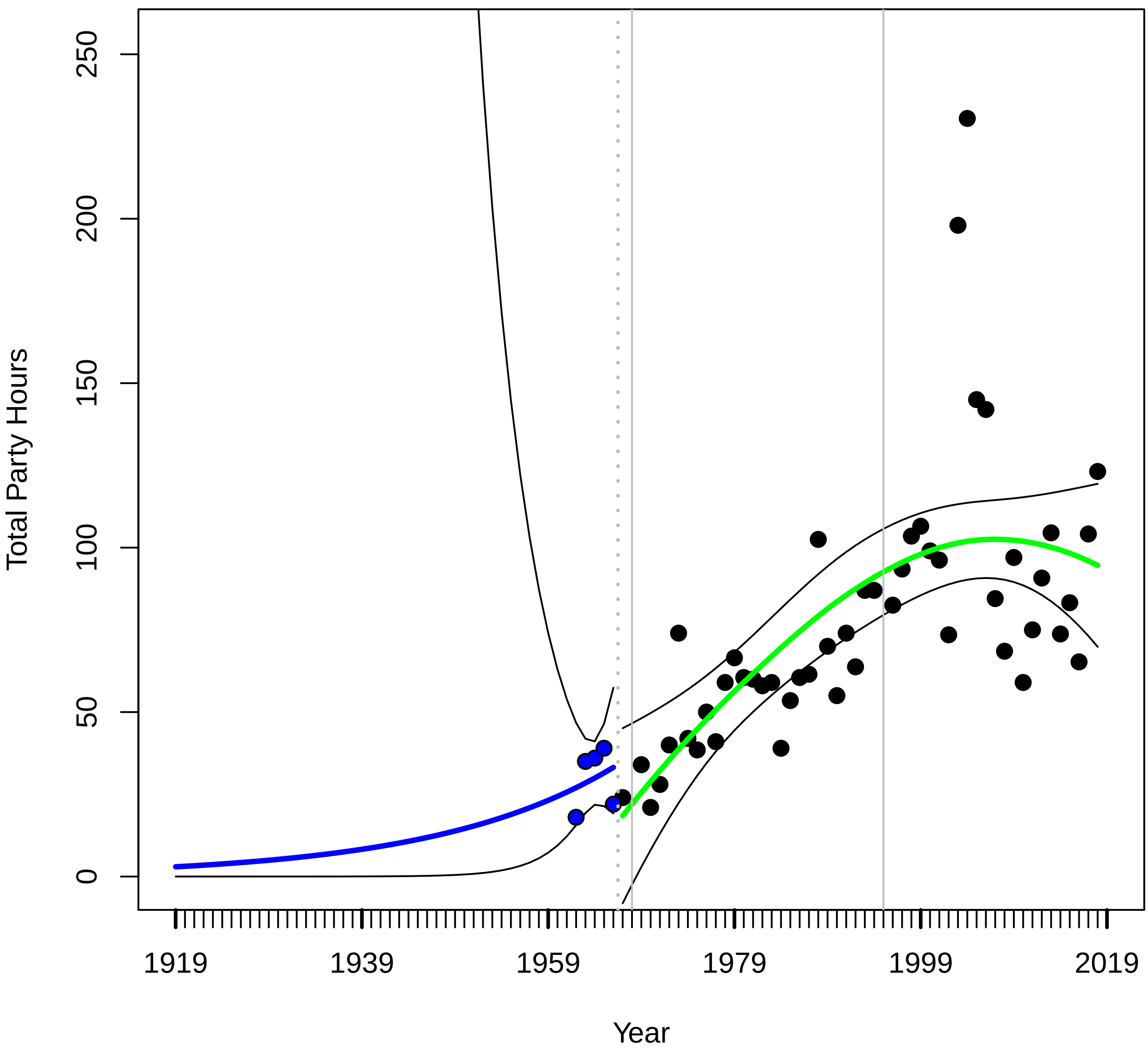

Total Party Hours
250
200
150
100
50
0
1919
1939
1959
1979
1999
2019
Year

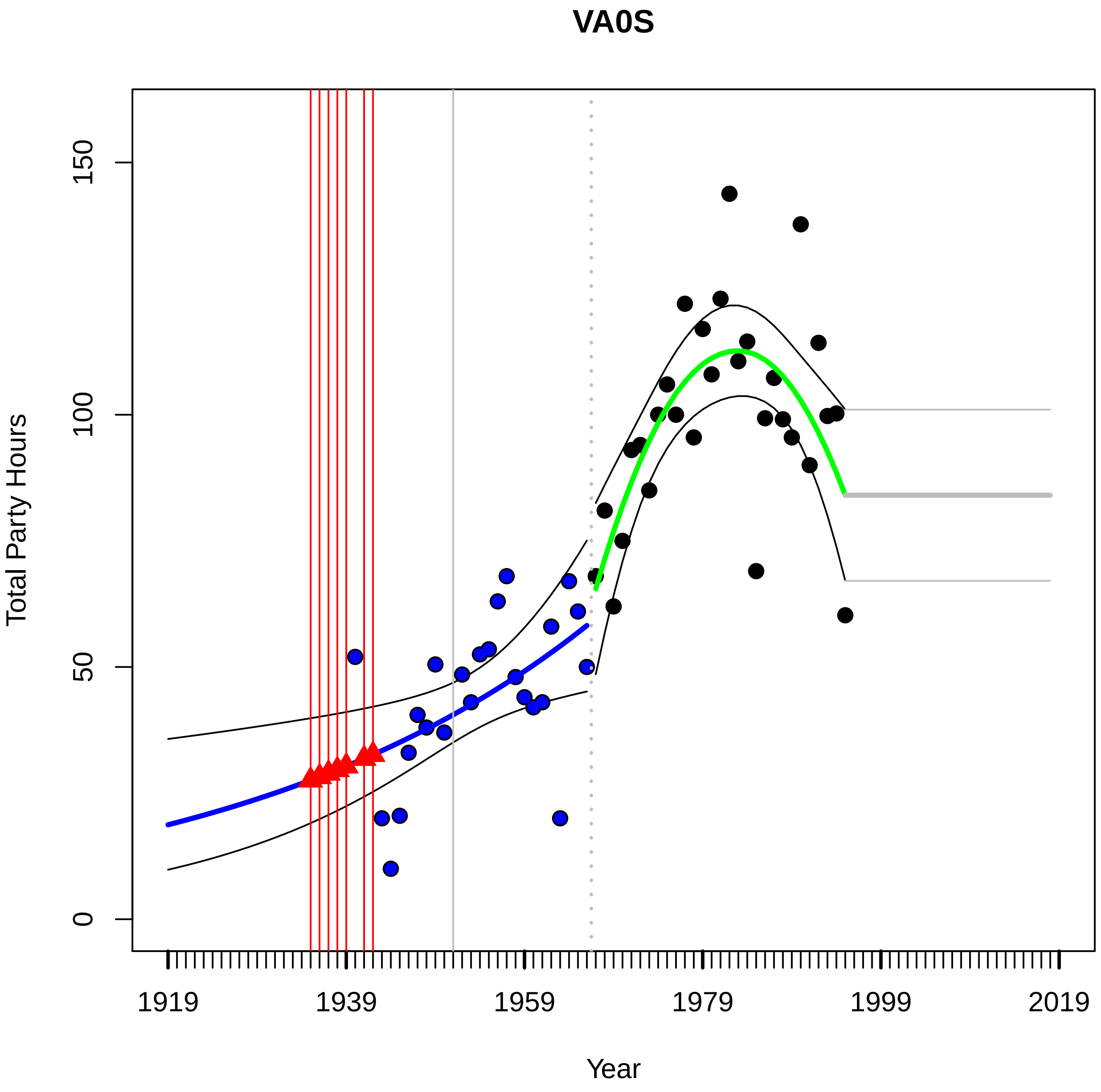
VA0S
Total Party Hours
150
100
50
0
1919
1939
1959
1979
1999
2019
Year

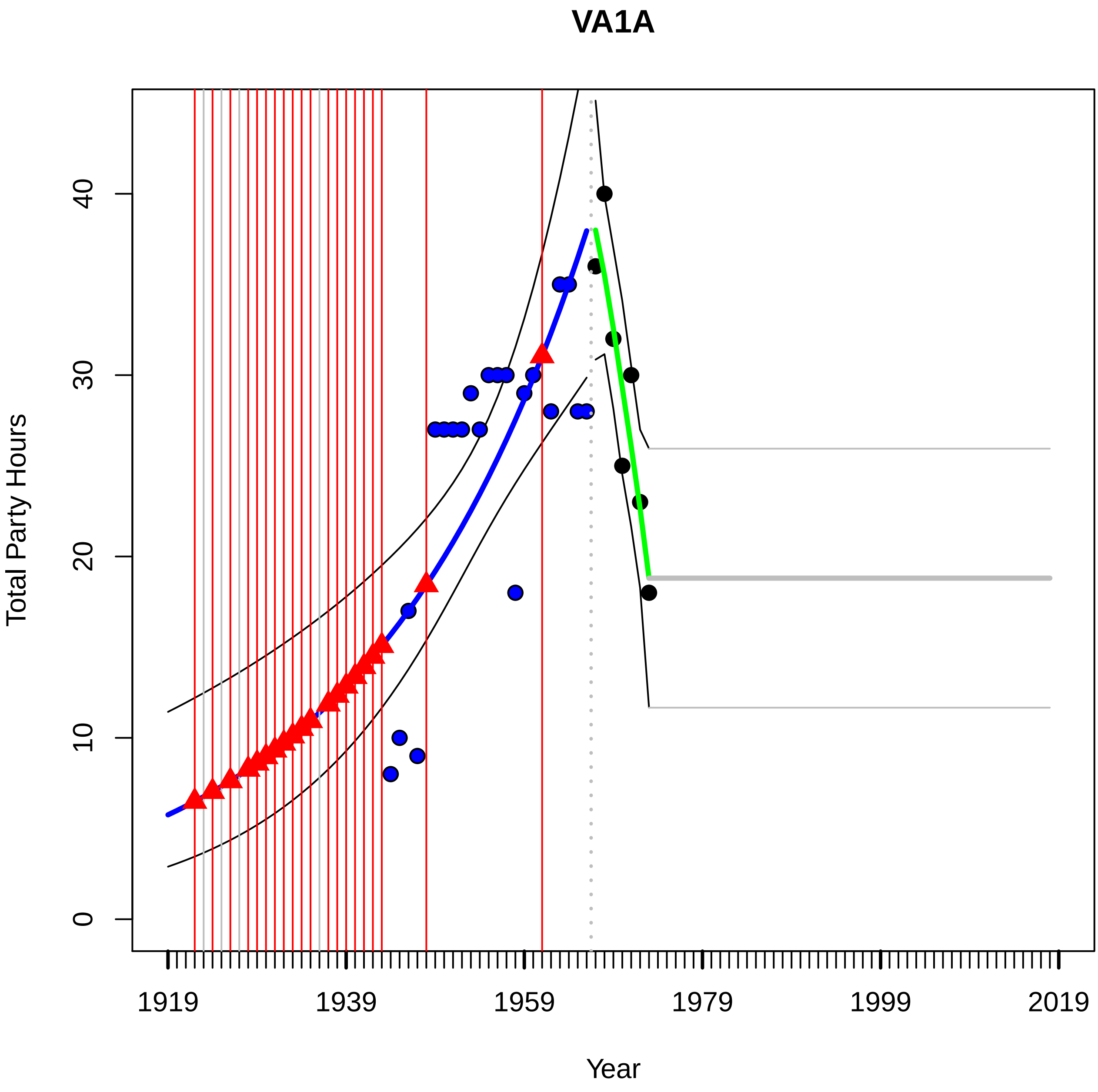

VA1A
Total Party Hours
Year
0
10
20
30
40
1919
1939
1959
1979
1999
2019

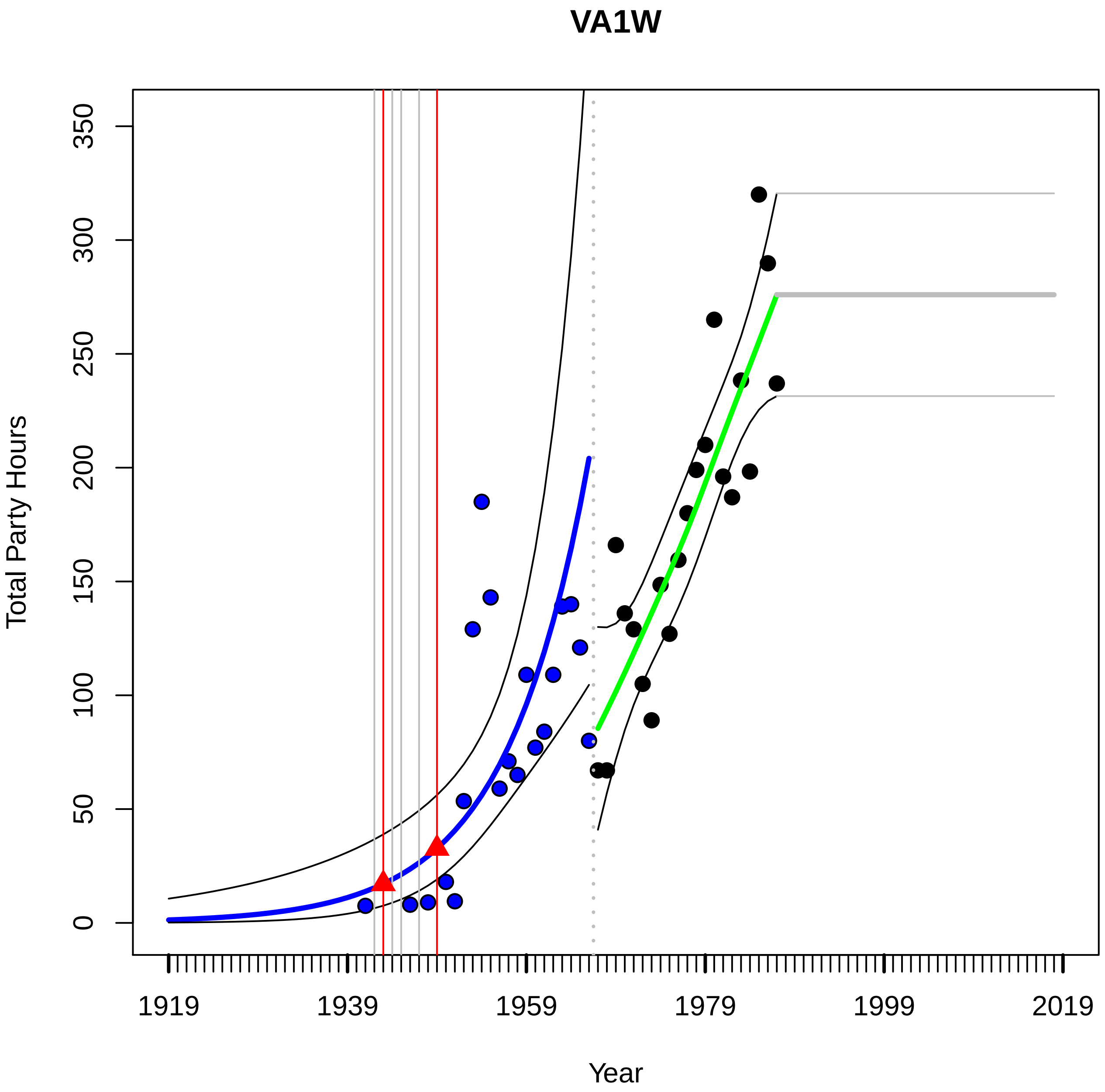
VA1W
Total Party Hours
Year
0
50
100
150
200
250
300
350
1919
1939
1959
1979
1999
2019

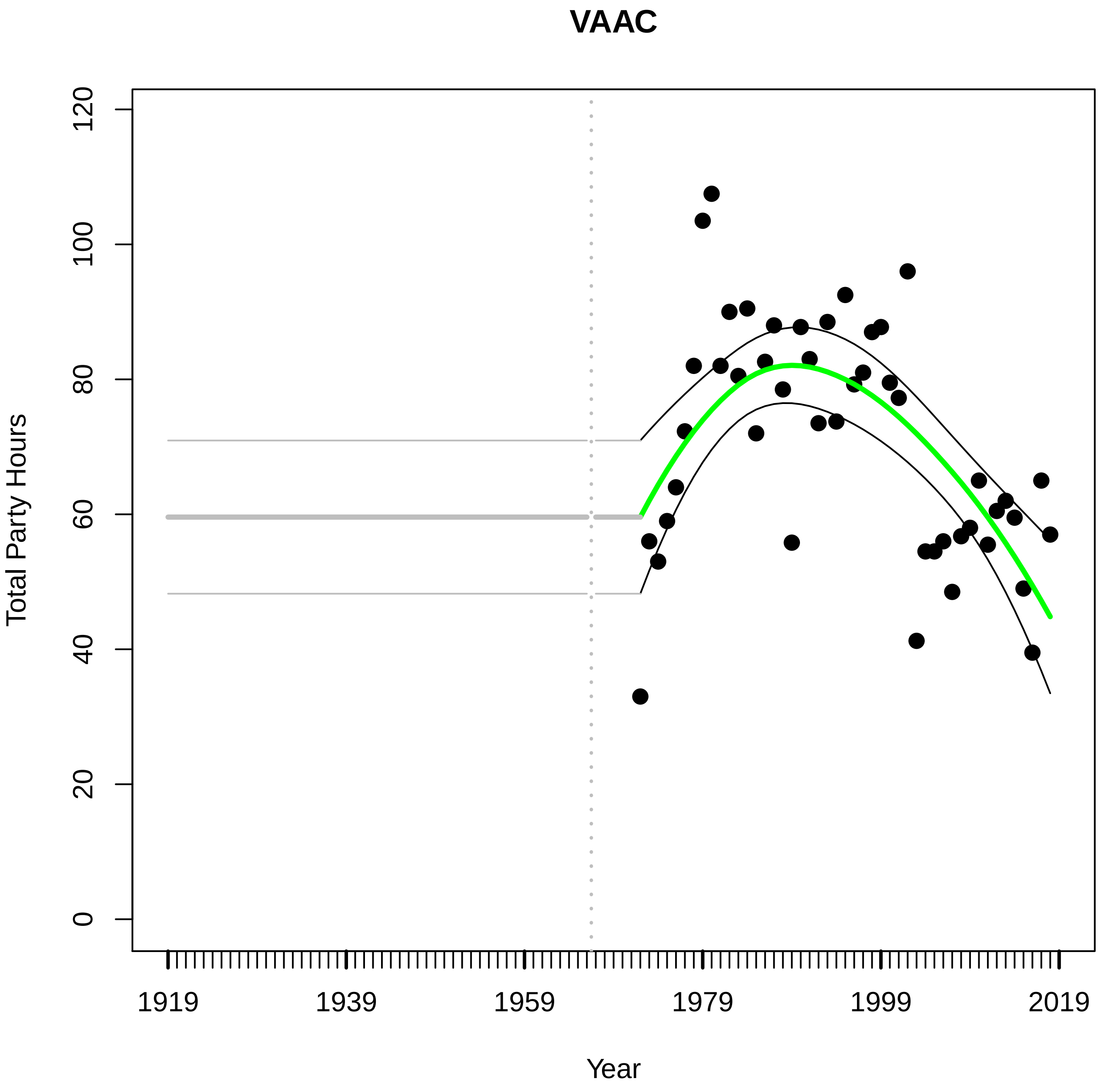

VAAC
Total Party Hours
0
20
40
60
80
100
120
1919
1939
1959
1979
1999
2019
Year

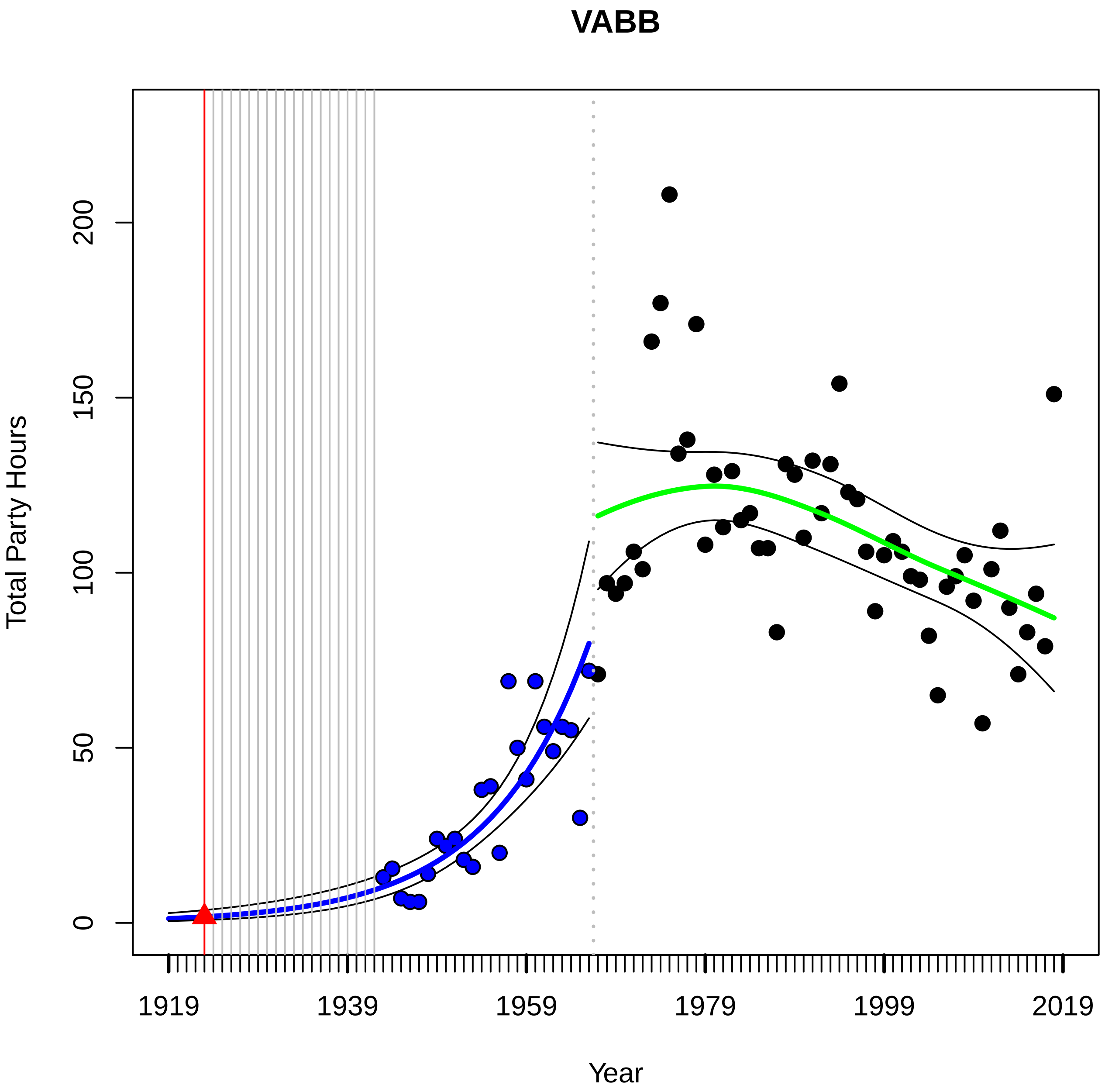
VABB
Total Party Hours
Year
0
50
100
150
200
1919
1939
1959
1979
1999
2019

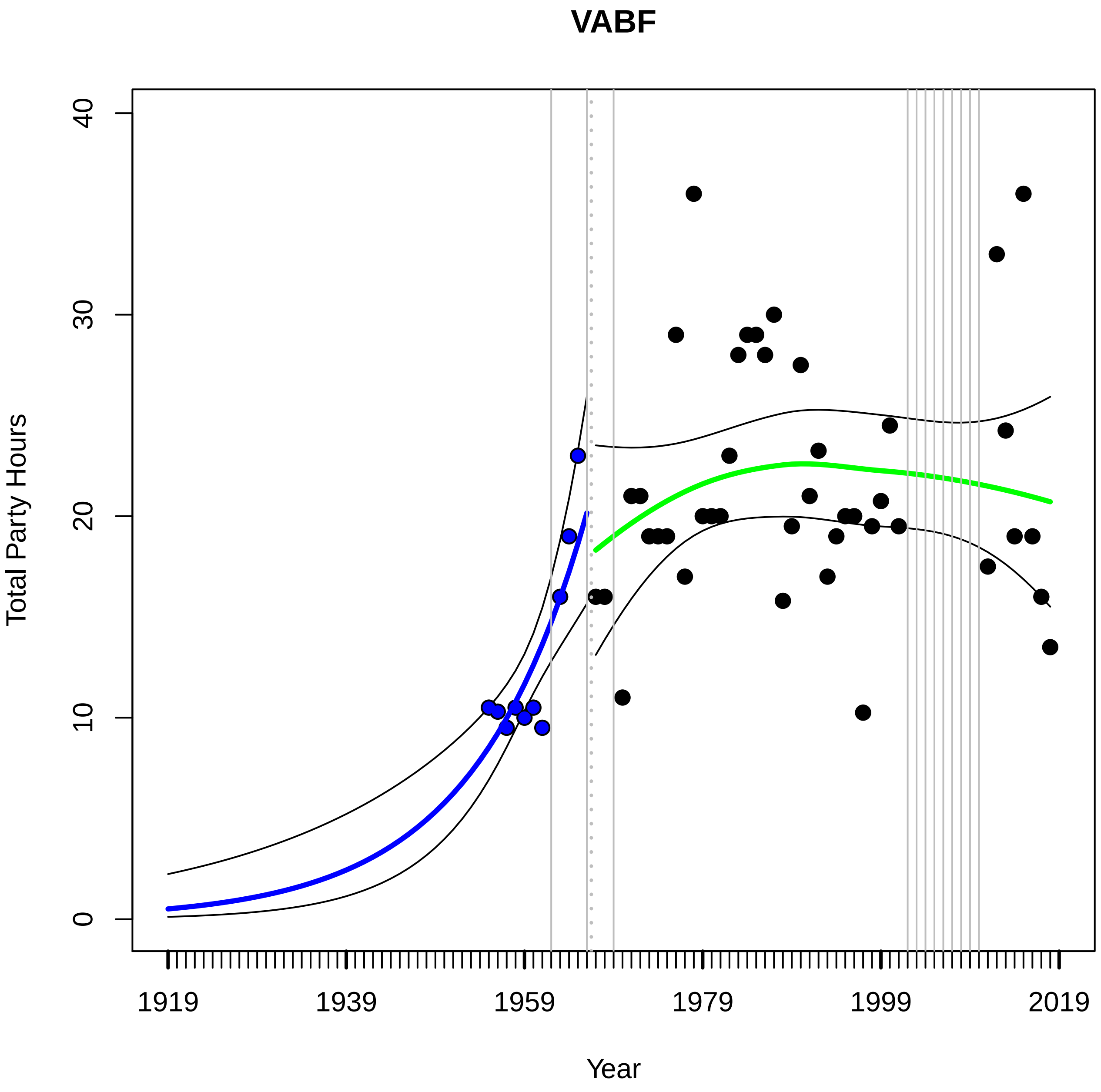

VABF
Total Party Hours
0
10
20
30
40
1919
1939
1959
1979
1999
2019
Year

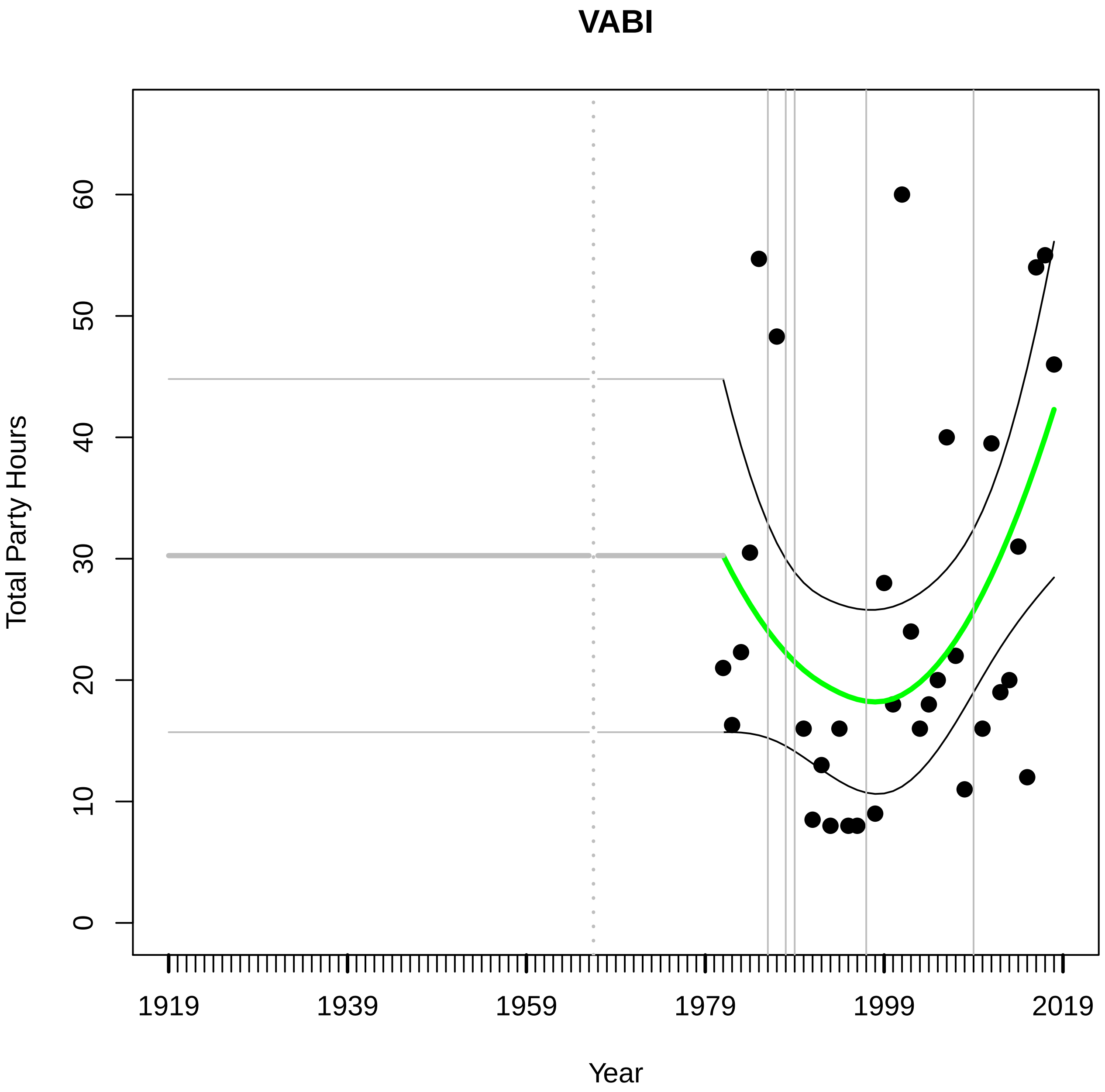
VABI
Total Party Hours
0
10
20
30
40
50
60
1919
1939
1959
1979
1999
2019
Year

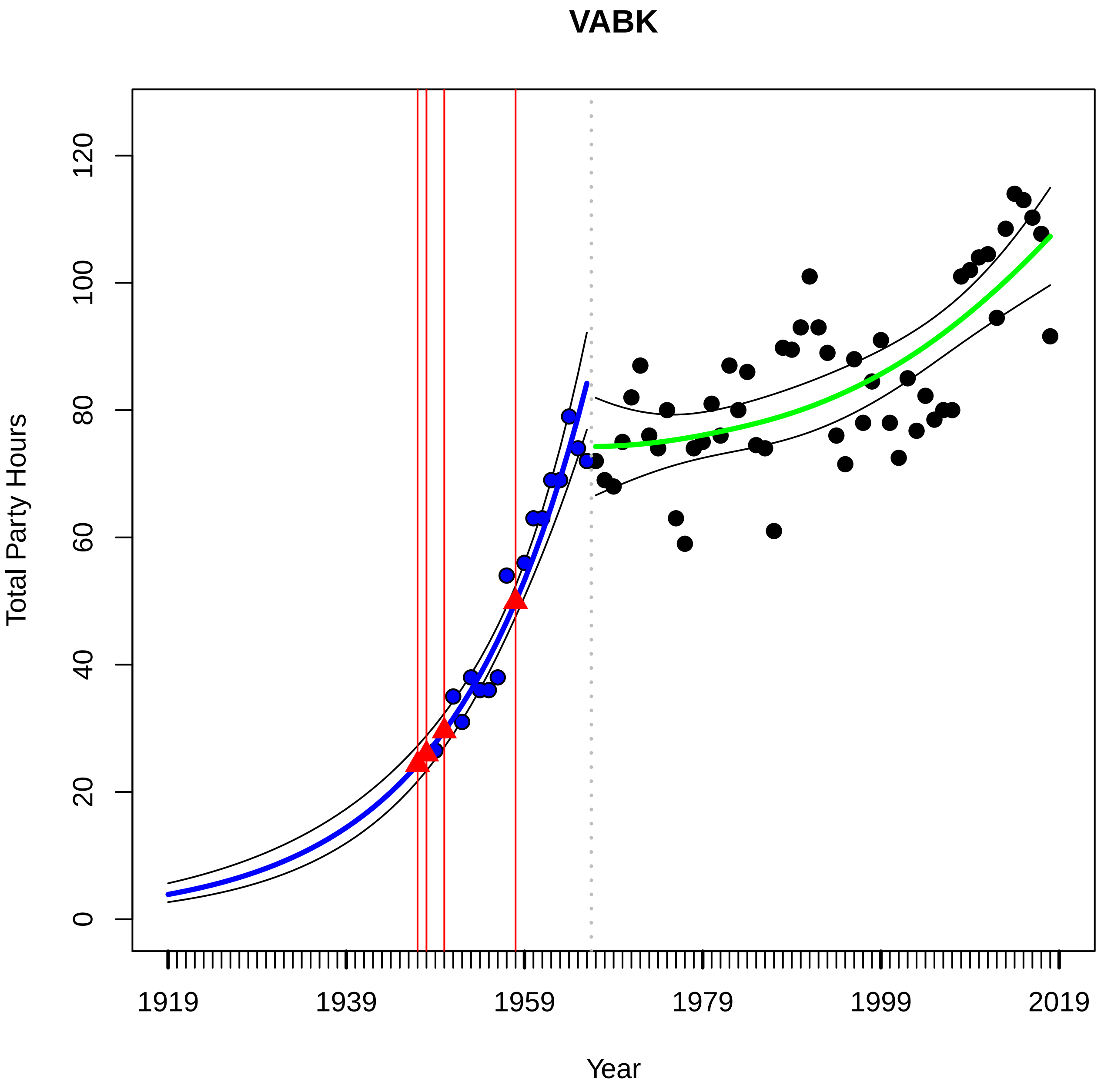

VABK
Total Party Hours
0
20
40
60
80
100
120
1919
1939
1959
1979
1999
2019
Year

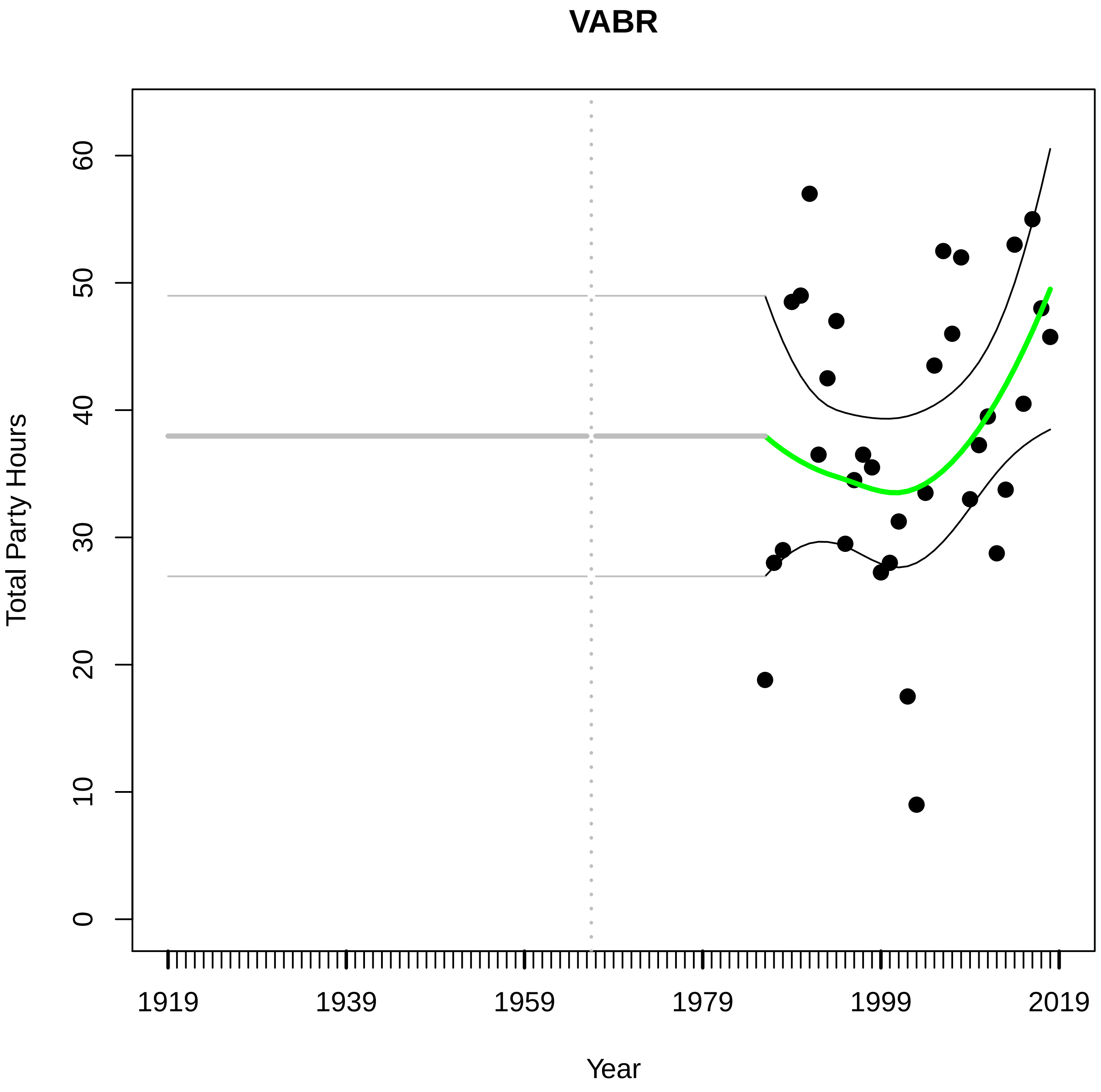
VABR
Total Party Hours
0
10
20
30
40
50
60
1919
1939
1959
1979
1999
2019
Year

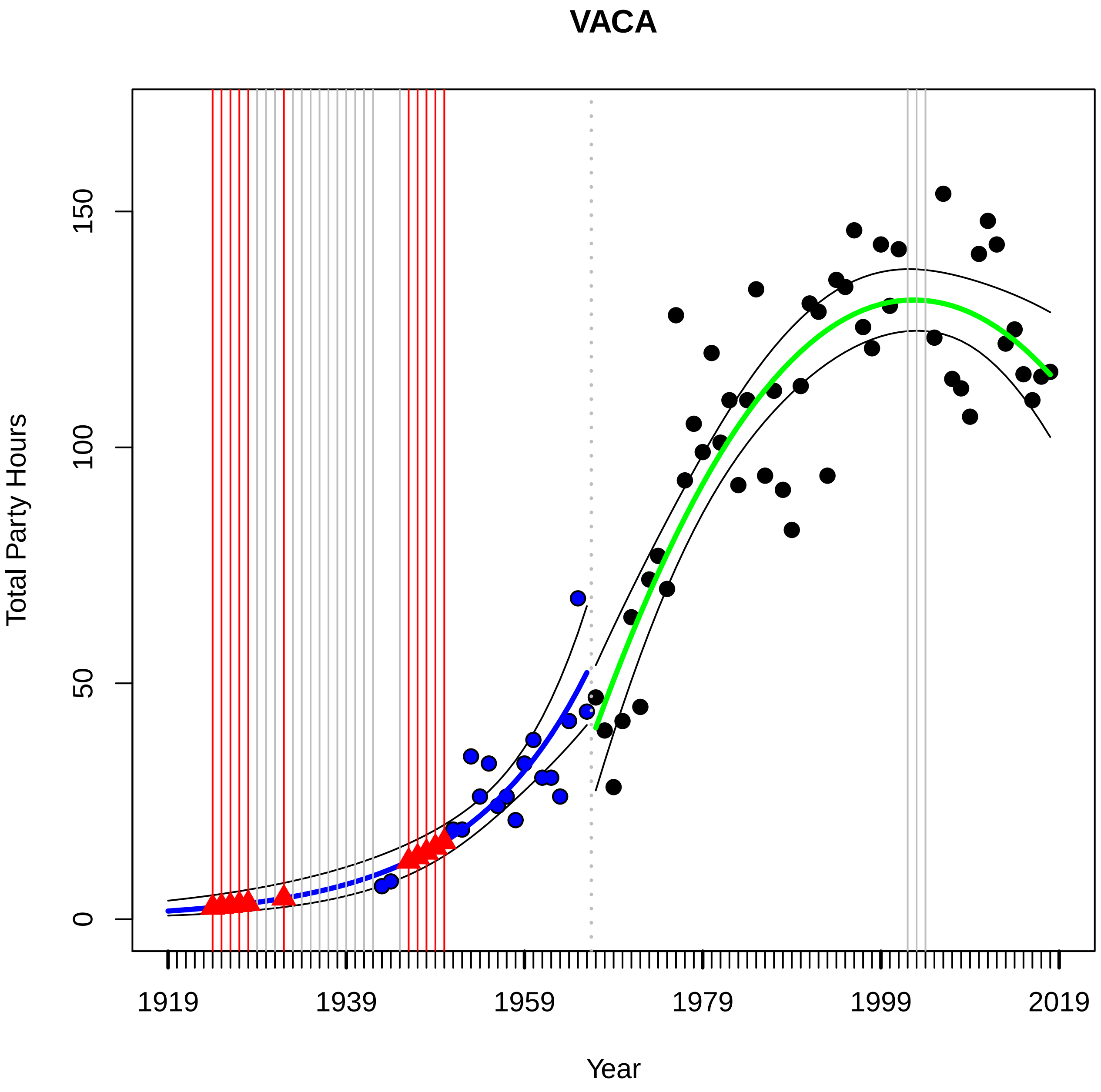

VACA
Total Party Hours
0
50
100
150
1919
1939
1959
1979
1999
2019
Year

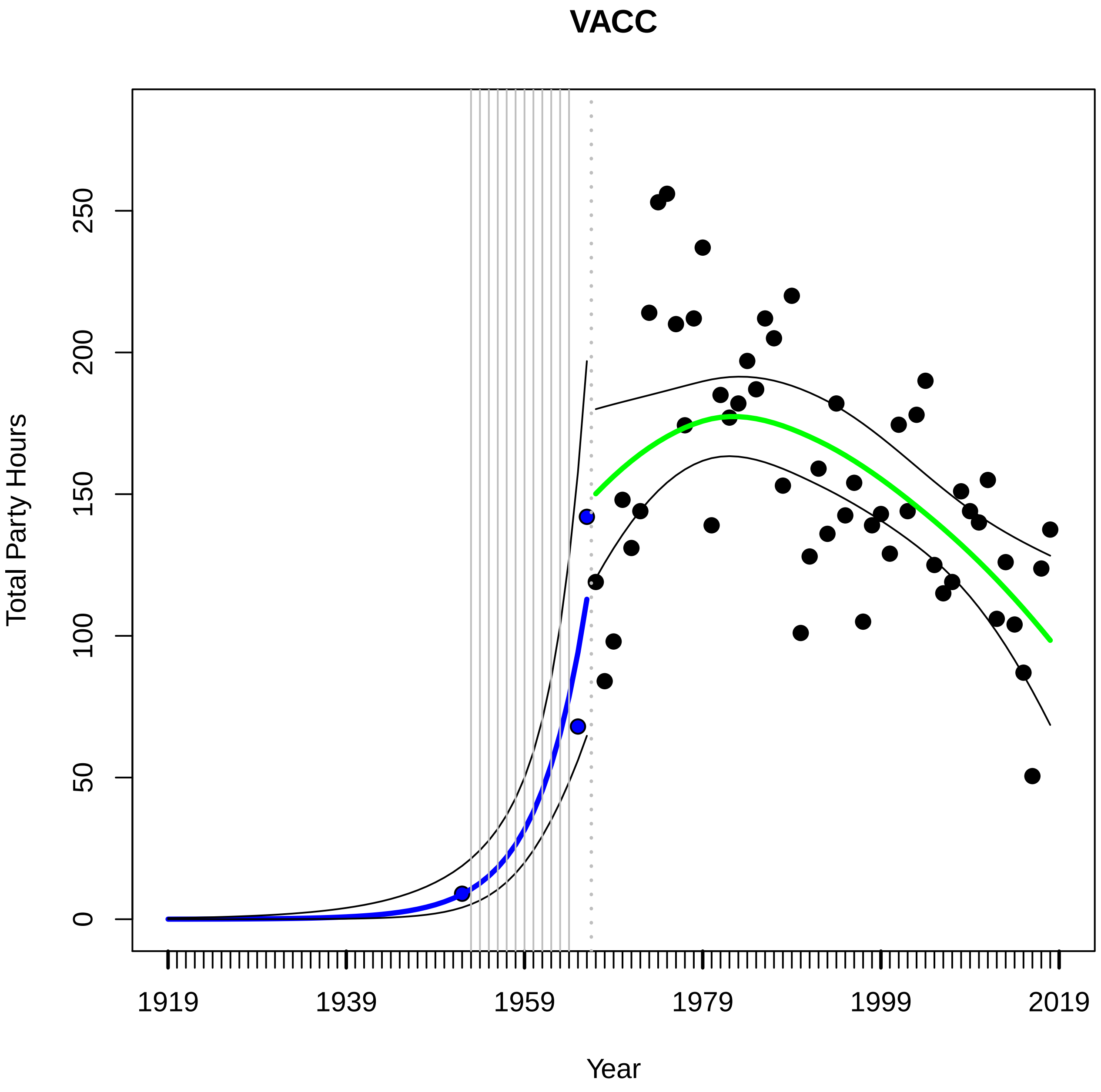
VACC
Total Party Hours
0
50
100
150
200
250
1919
1939
1959
1979
1999
2019
Year

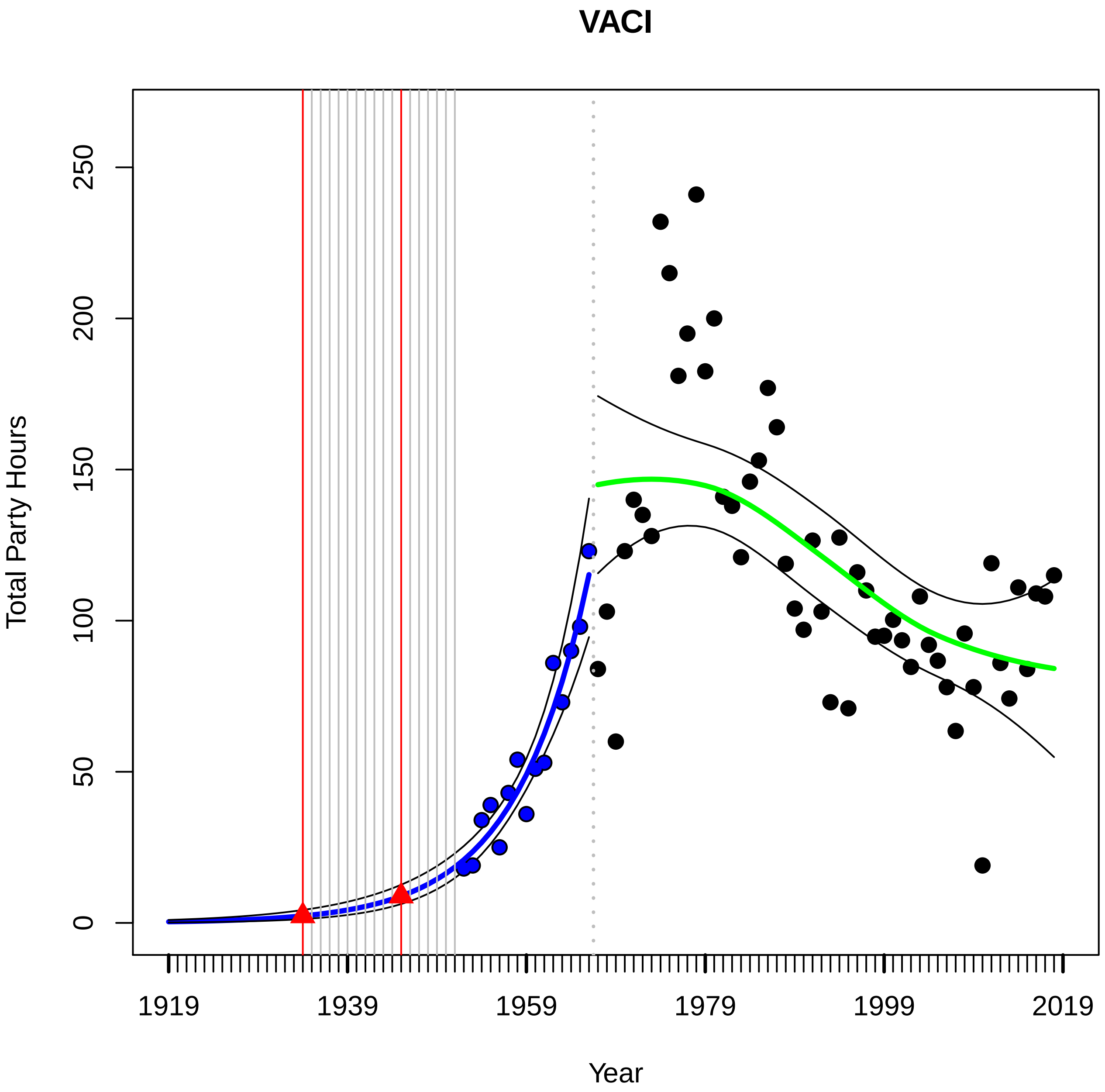
VACI
Total Party Hours
0
50
100
150
200
250
1919
1939
1959
1979
1999
2019
Year

VACN

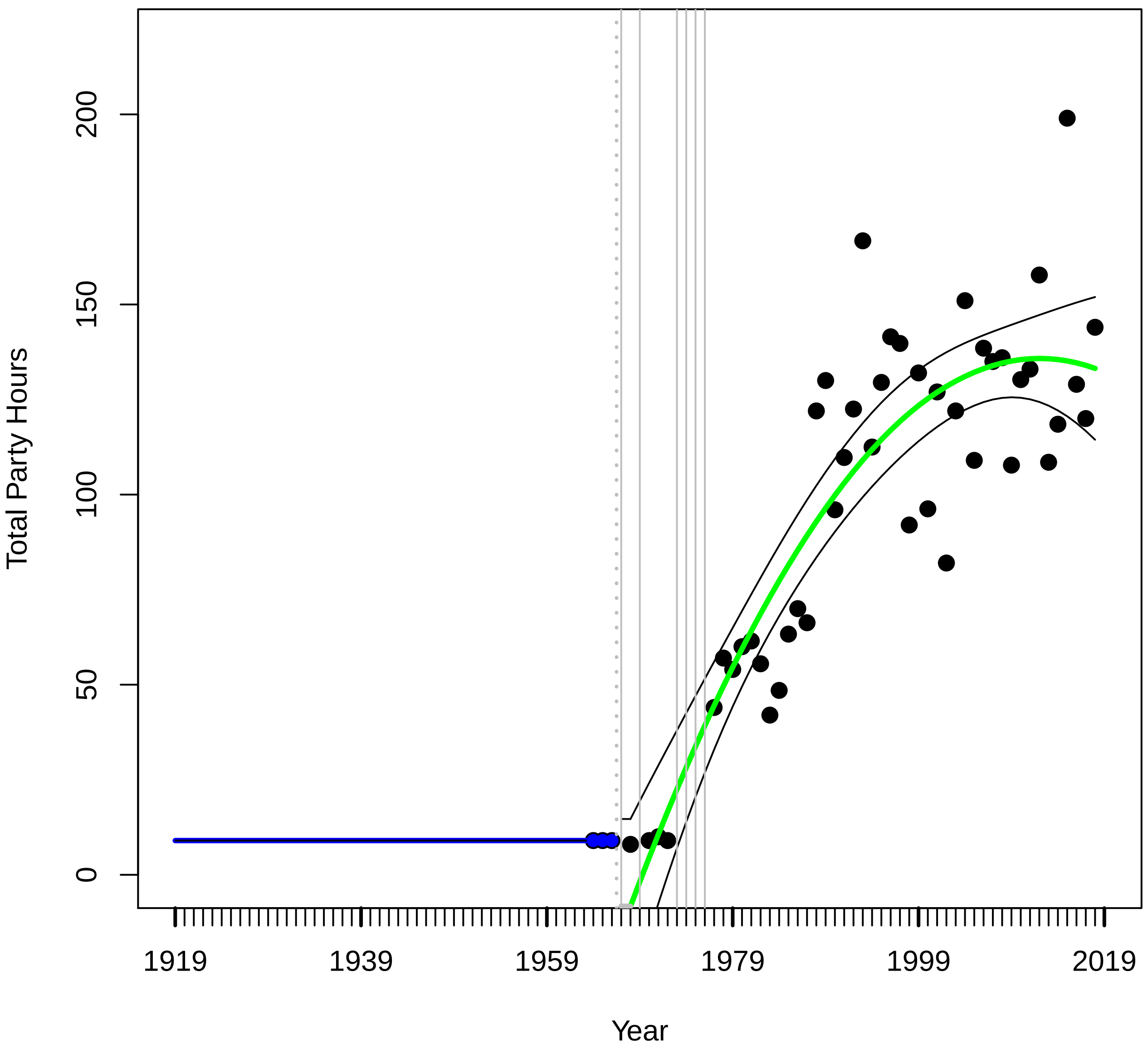

Total Party Hours
0
50
100
150
200
1919
1939
1959
1979
1999
2019
Year

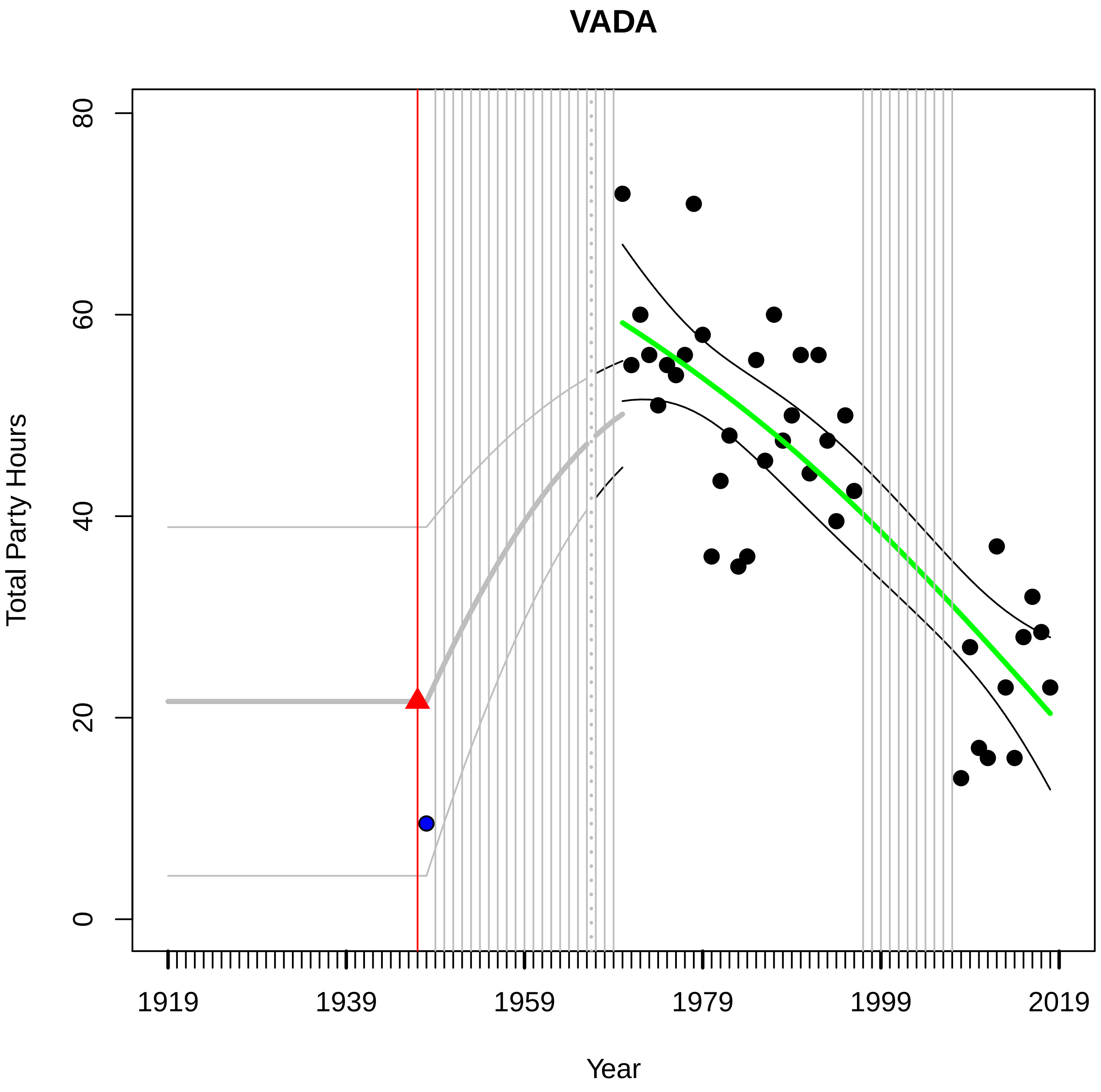

VADA
Total Party Hours
0
20
40
60
80
1919
1939
1959
1979
1999
2019
Year

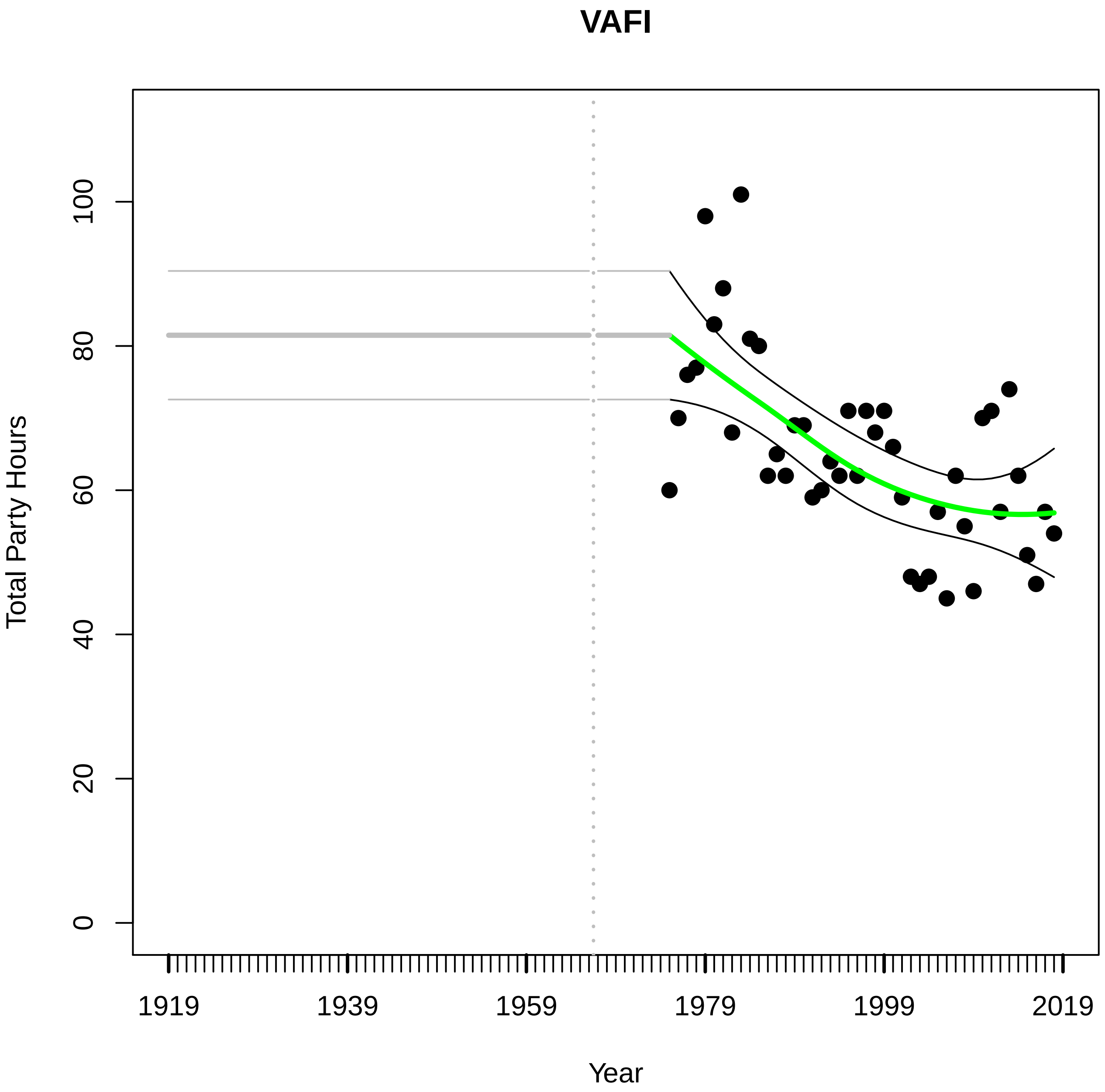
VAFI
Total Party Hours
0
20
40
60
80
100
1919
1939
1959
1979
1999
2019
Year

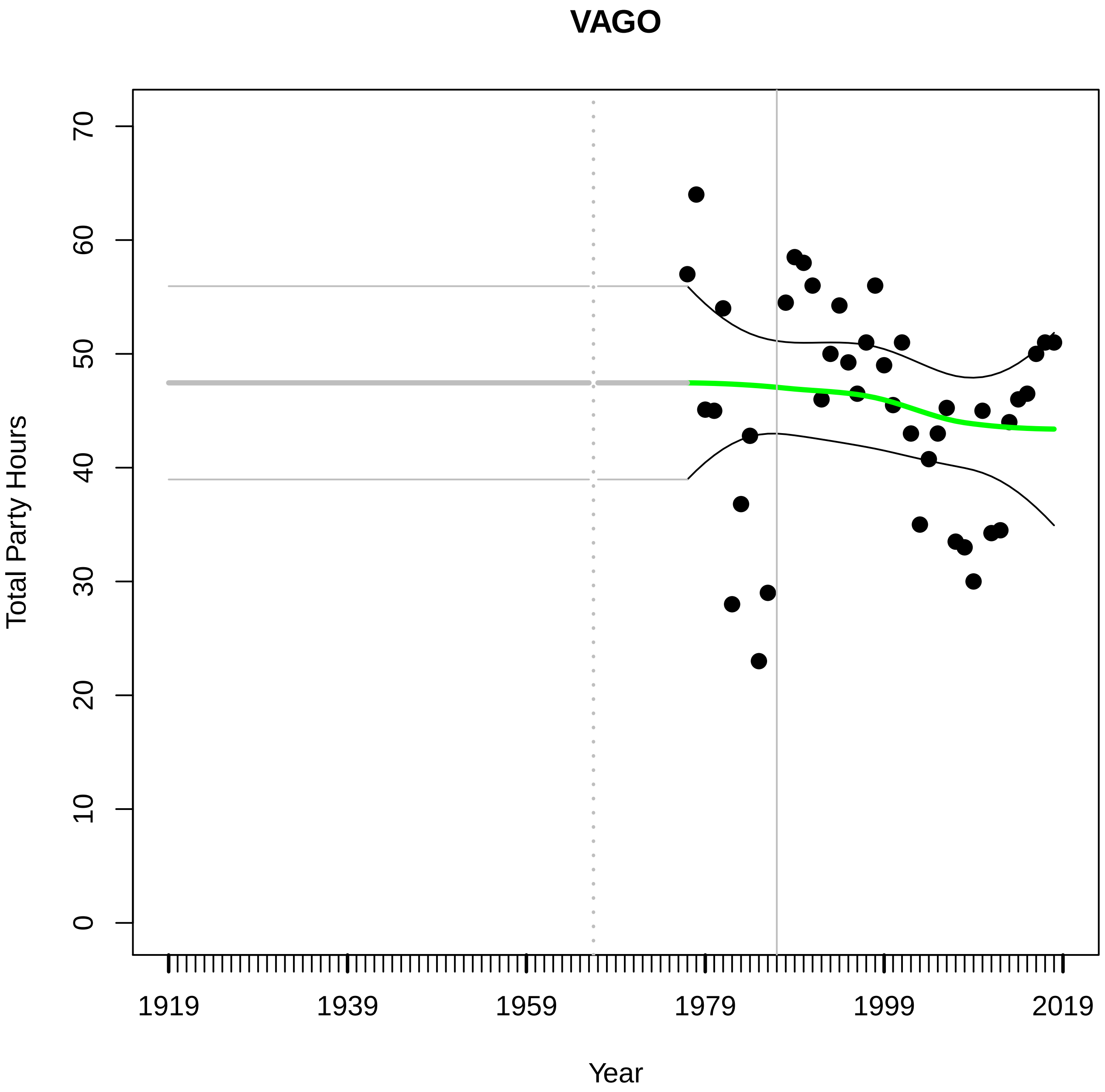
VAGO
Total Party Hours
Year
0
10
20
30
40
50
60
70
1919
1939
1959
1979
1999
2019

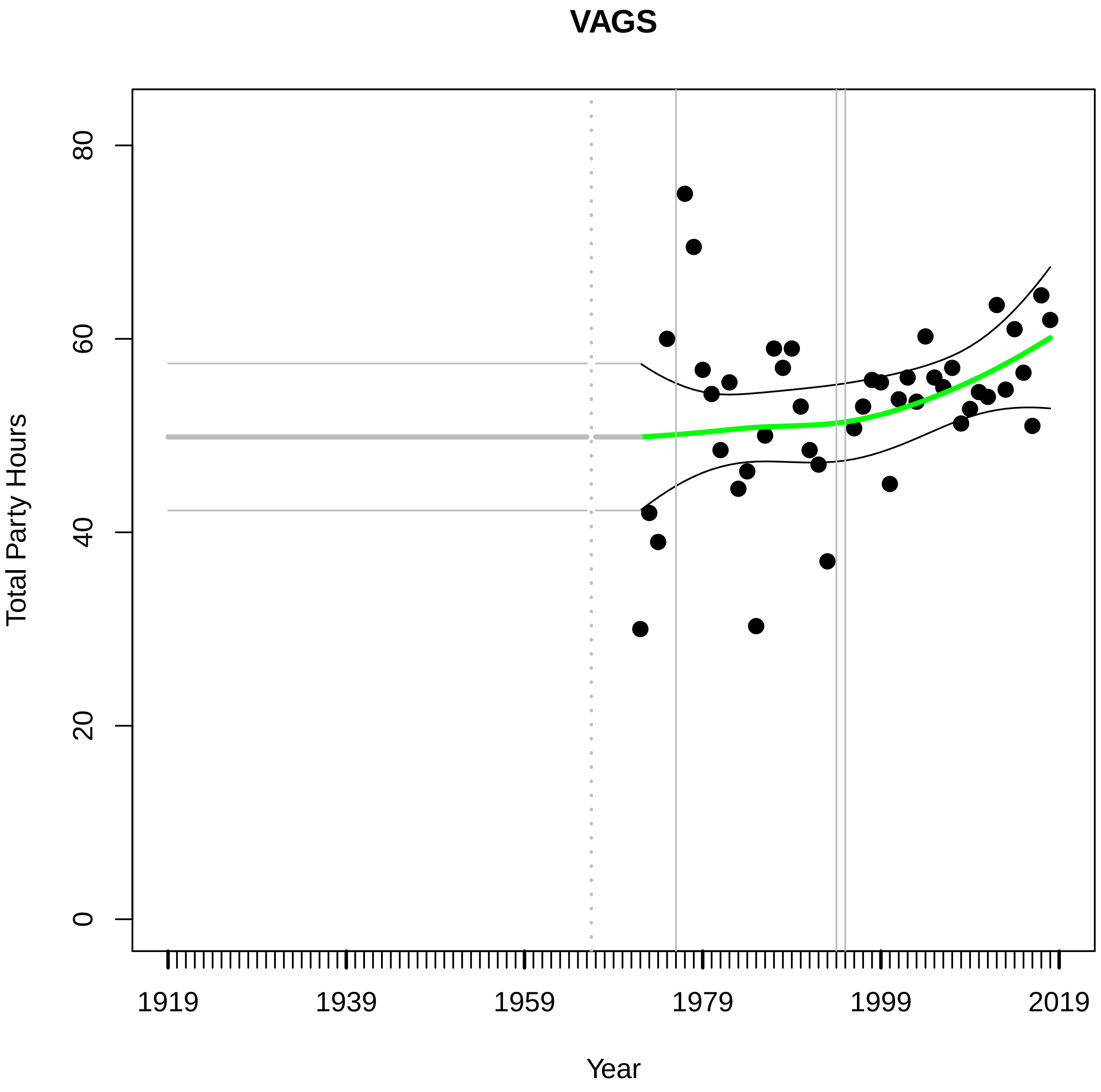
VAGS
80
60
40
20
0
Total Party Hours
1919
1939
1959
1979
1999
2019
Year

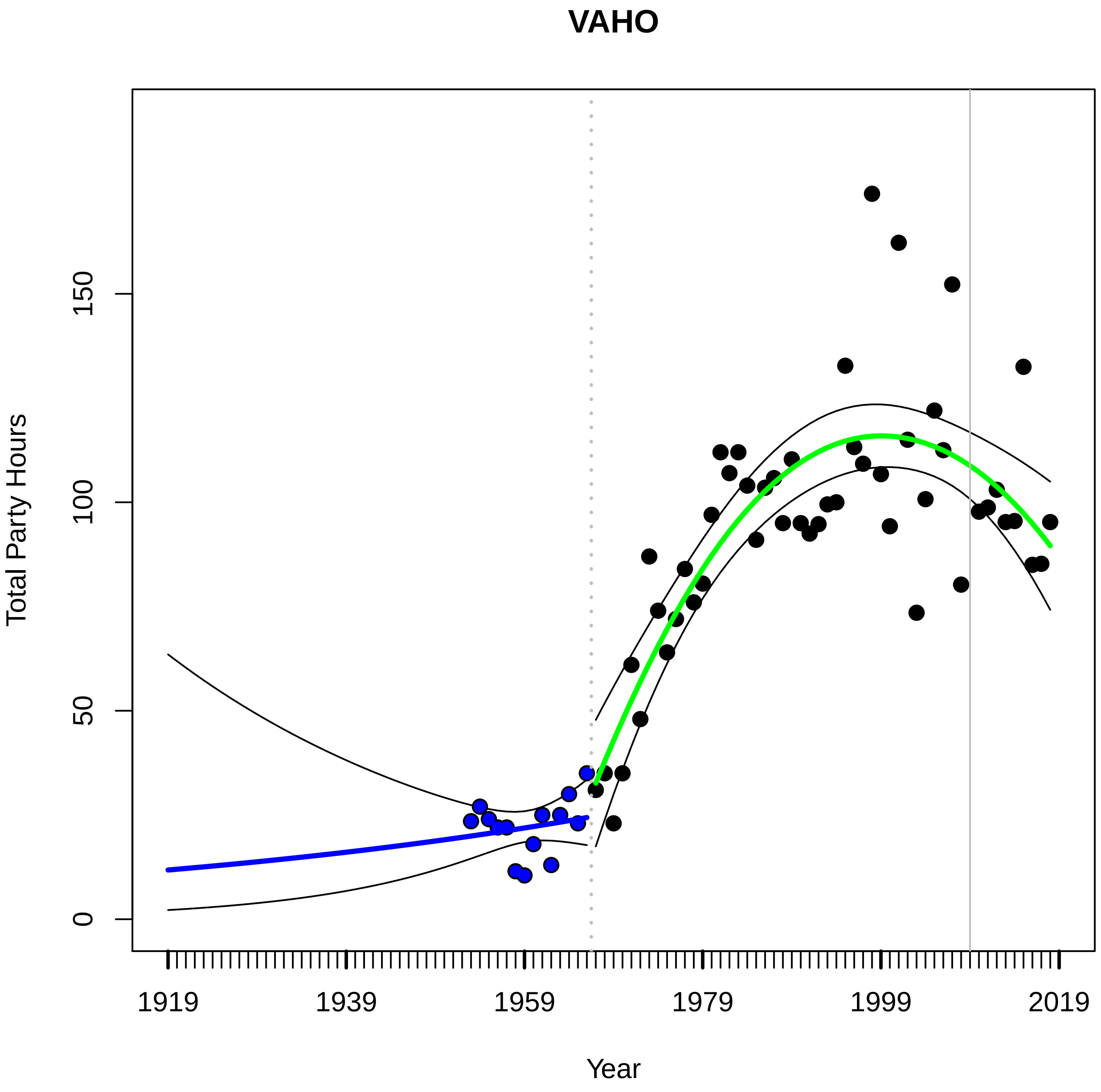
VAHO
Total Party Hours
0
50
100
150
1919
1939
1959
1979
1999
2019
Year

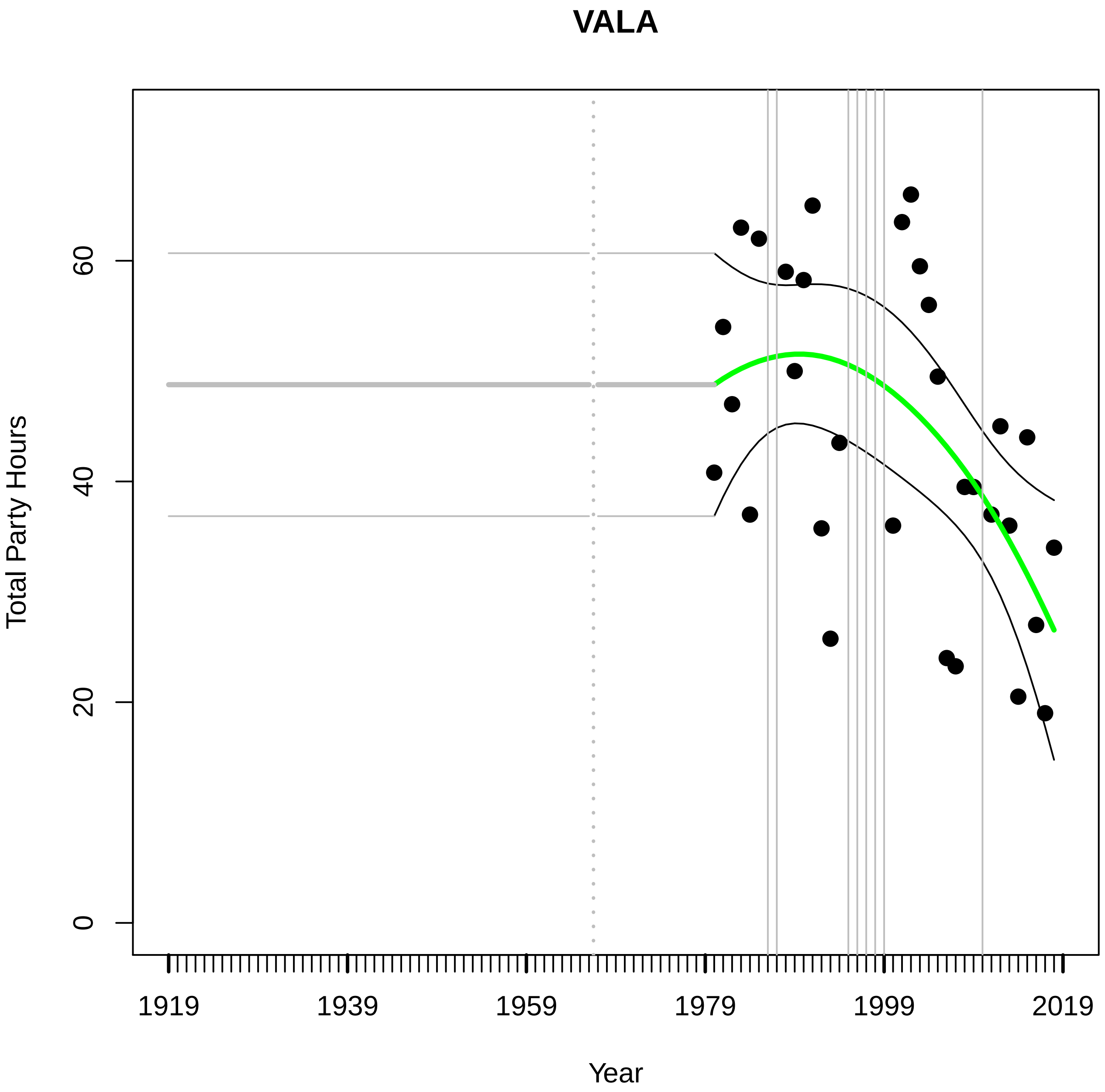

VALA
Total Party Hours
0
20
40
60
1919
1939
1959
1979
1999
2019
Year

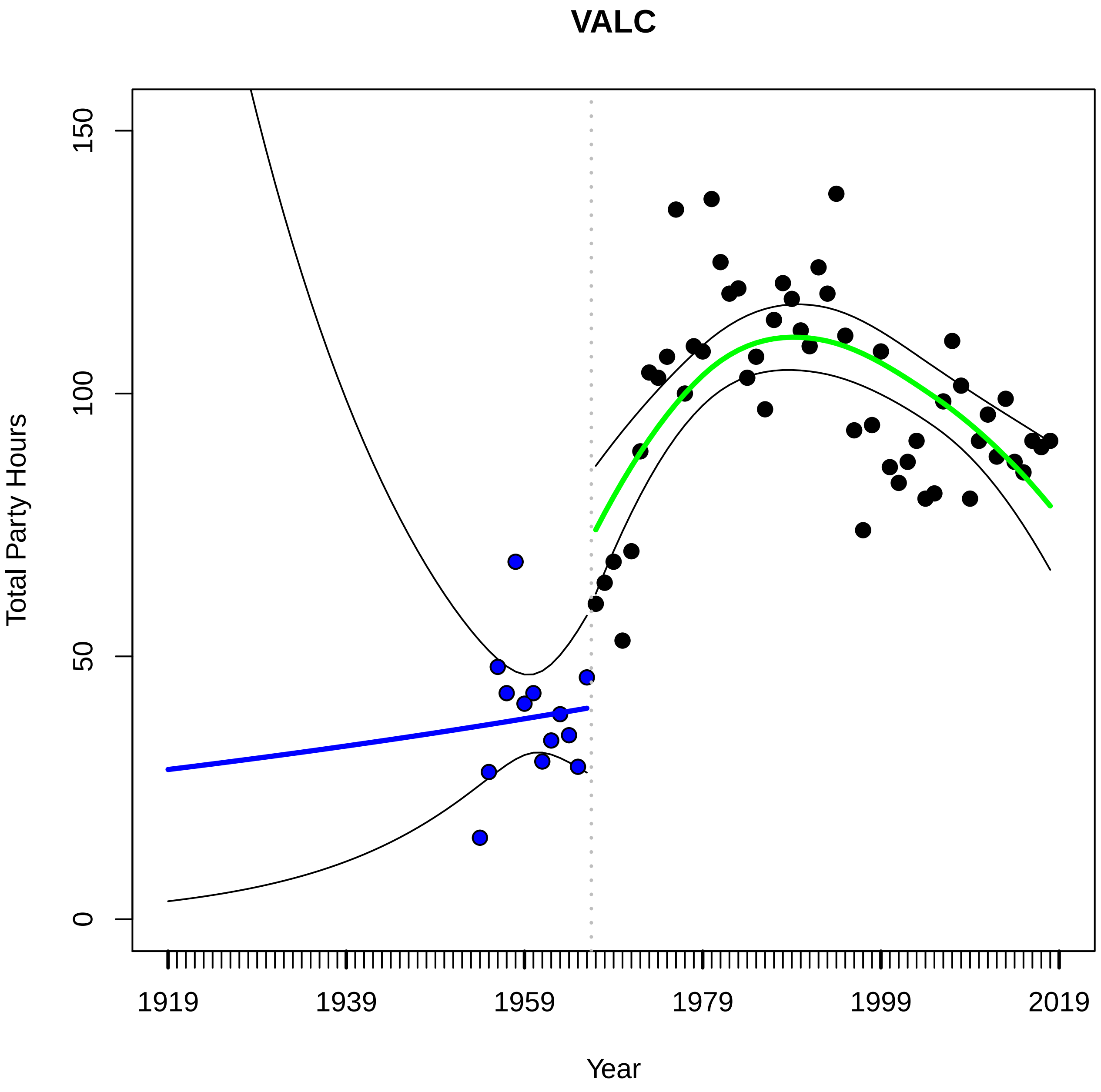

VALC
Total Party Hours
Year
0
50
100
150
1919
1939
1959
1979
1999
2019

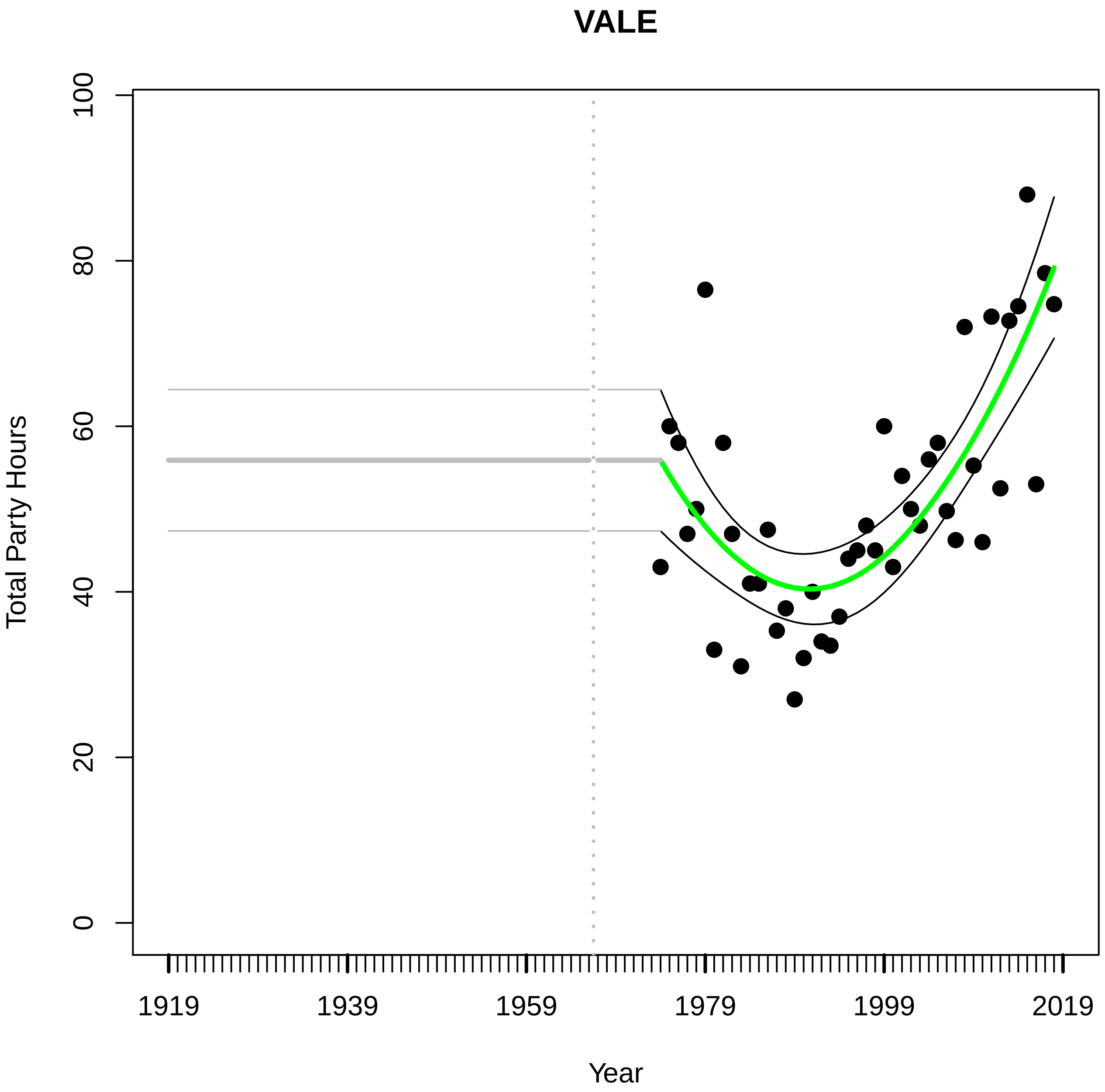
VALE
Total Party Hours
0
20
40
60
80
100
1919
1939
1959
1979
1999
2019
Year

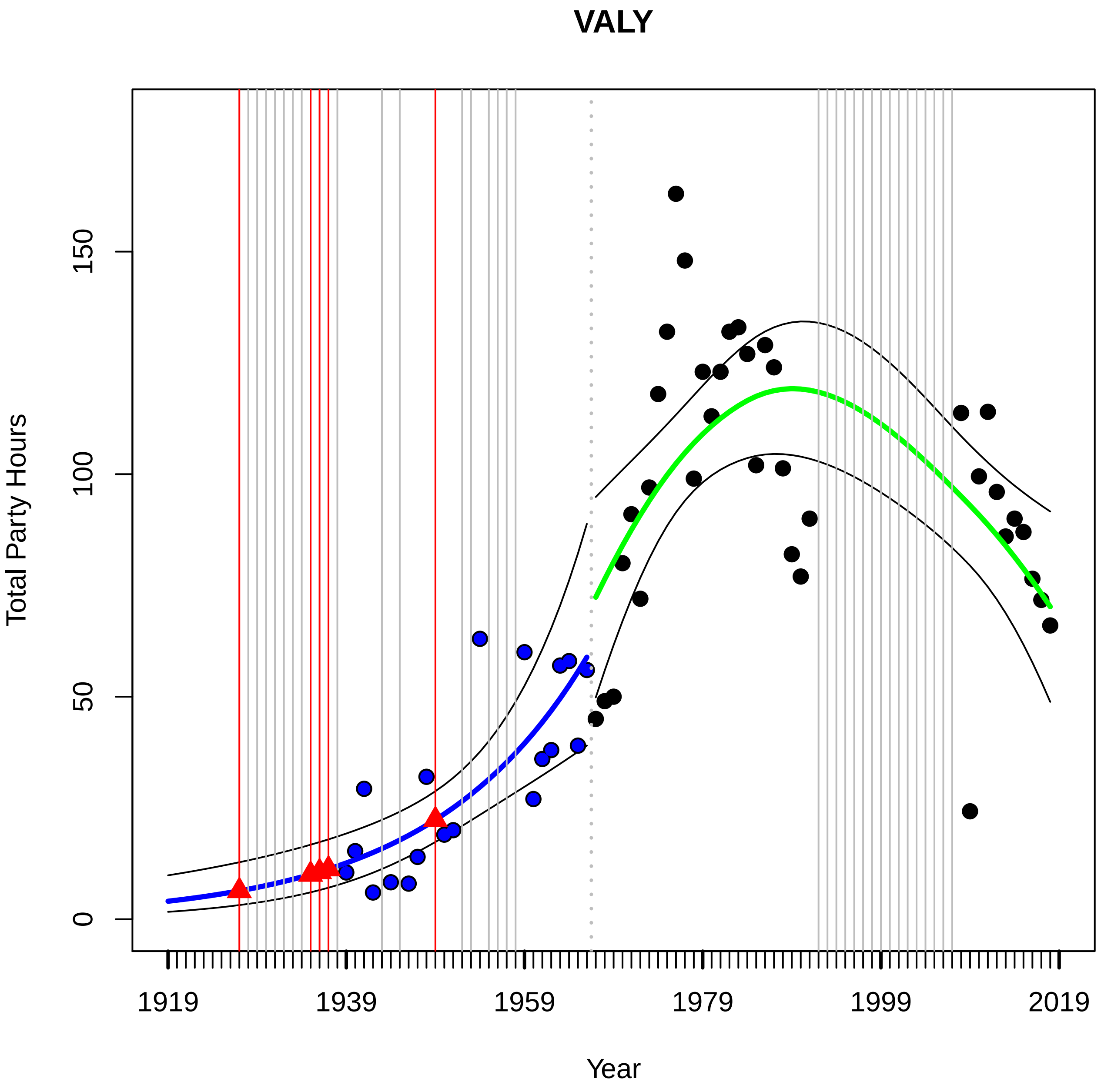
VALY
Total Party Hours
150
100
50
0
1919
1939
1959
1979
1999
2019
Year

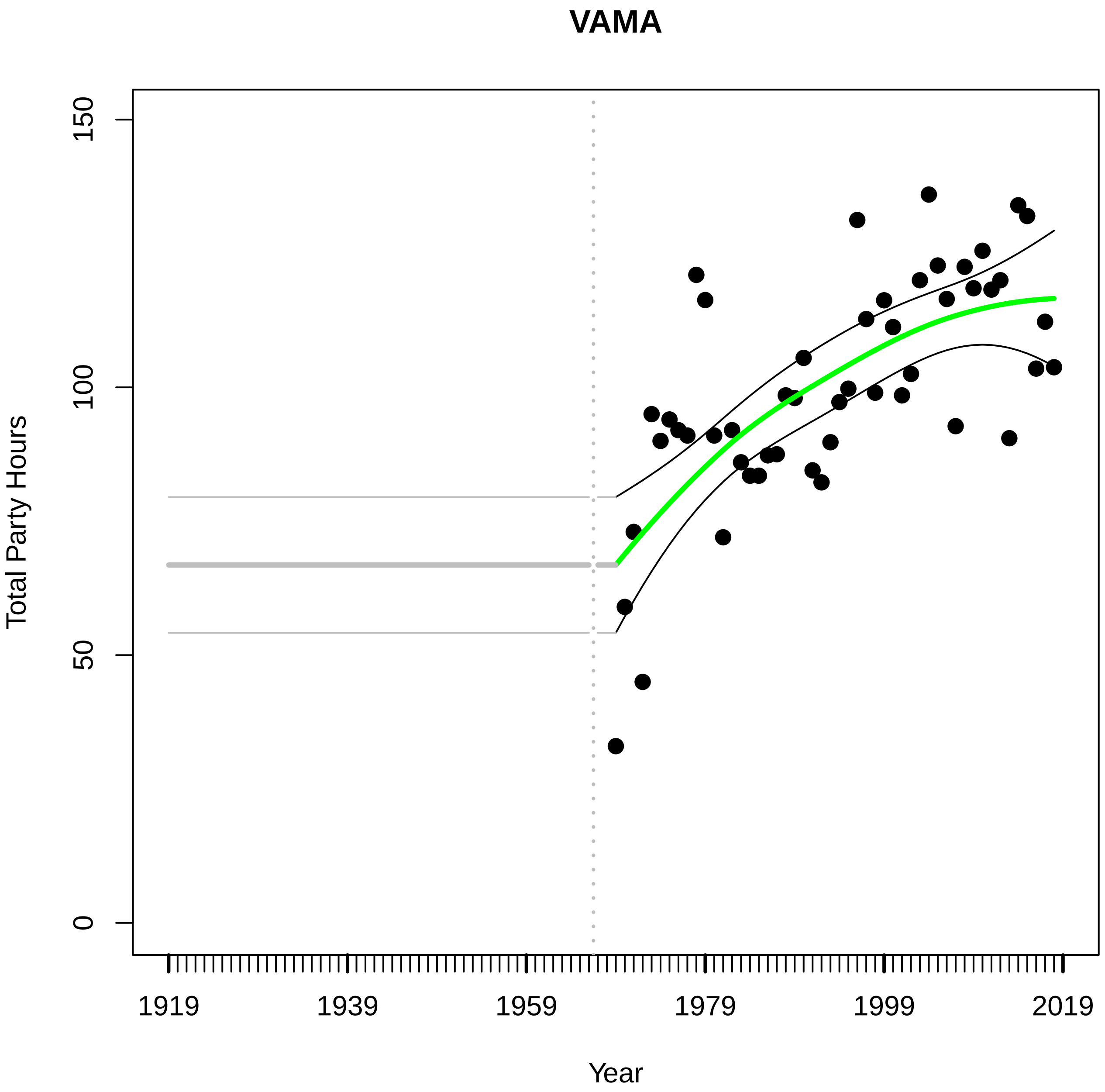
VAMA
Total Party Hours
0
50
100
150
1919
1939
1959
1979
1999
2019
Year

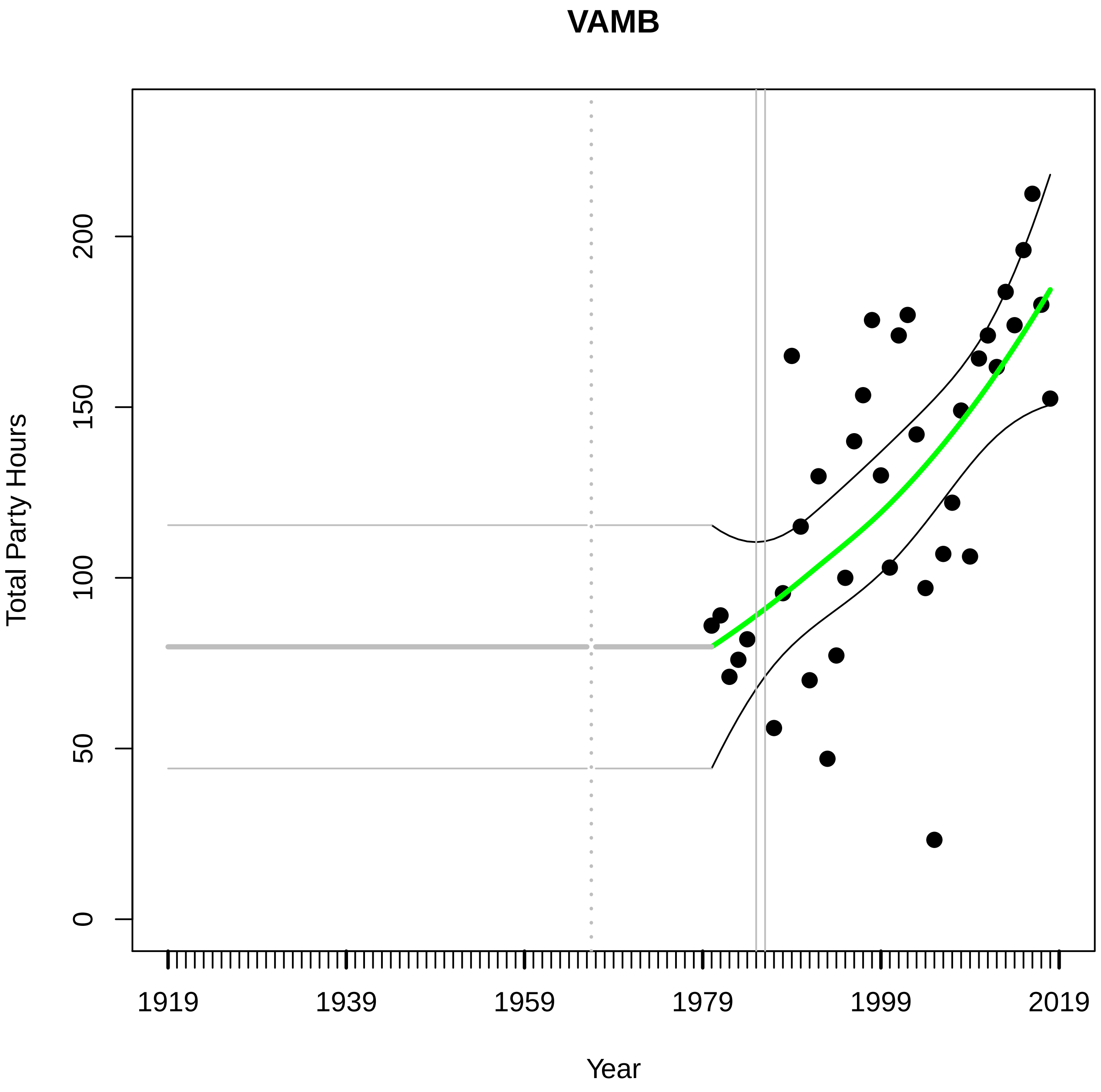
VAMB
Total Party Hours
0
50
100
150
200
1919
1939
1959
1979
1999
2019
Year

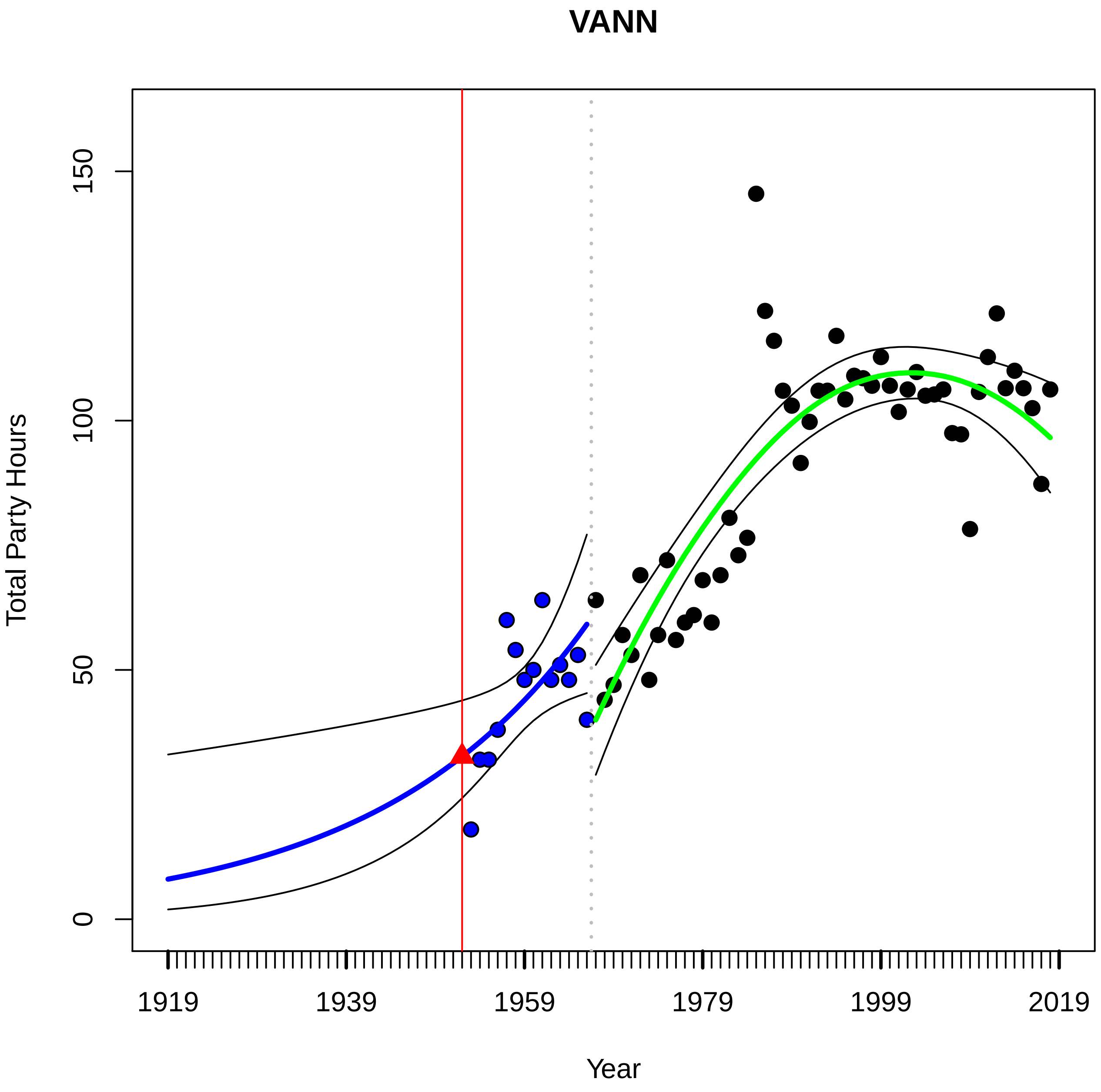
VANN
Total Party Hours
150
100
50
0
1919
1939
1959
1979
1999
2019
Year

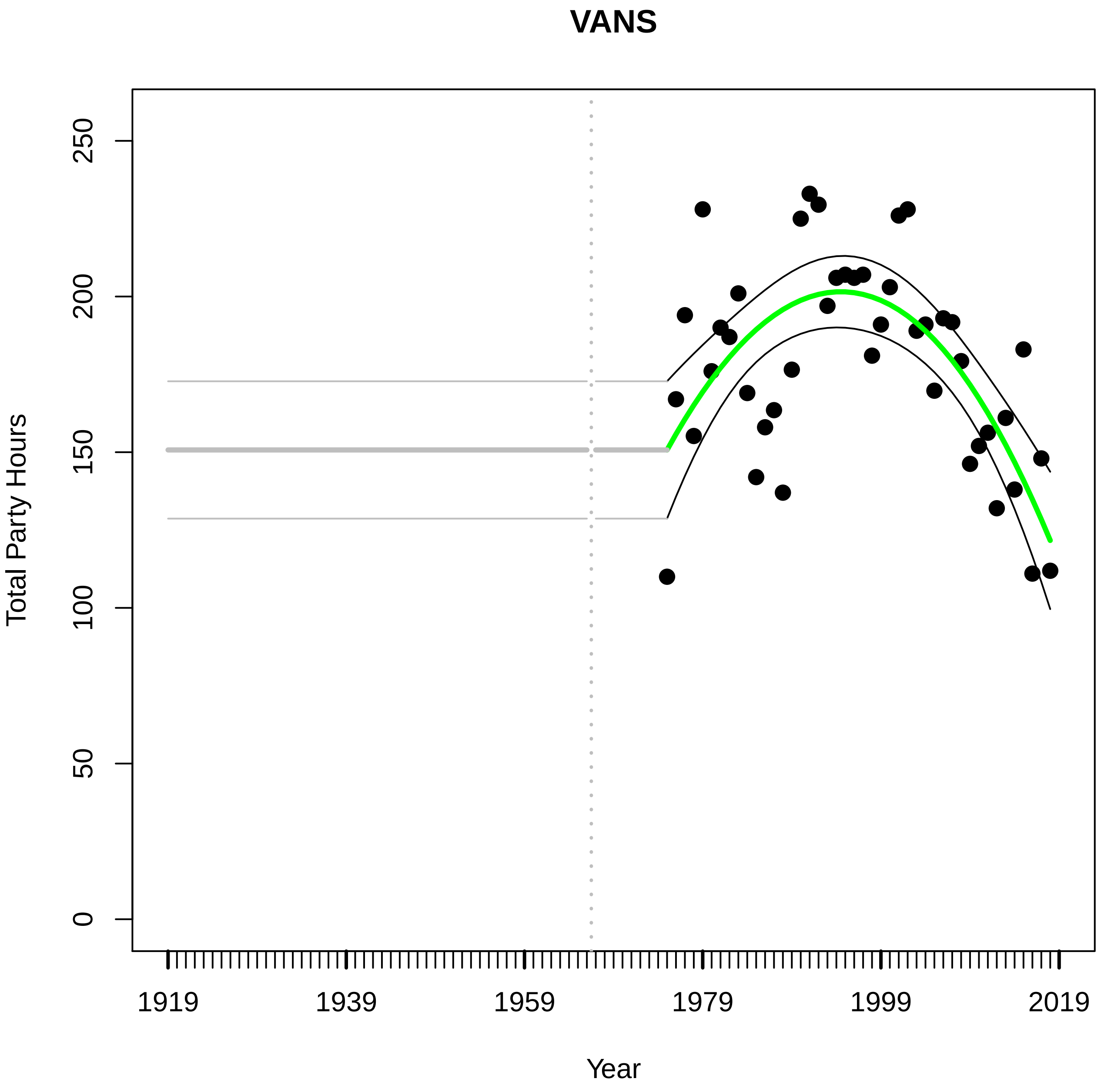

VANS
Total Party Hours
Year
0
50
100
150
200
250
1919
1939
1959
1979
1999
2019

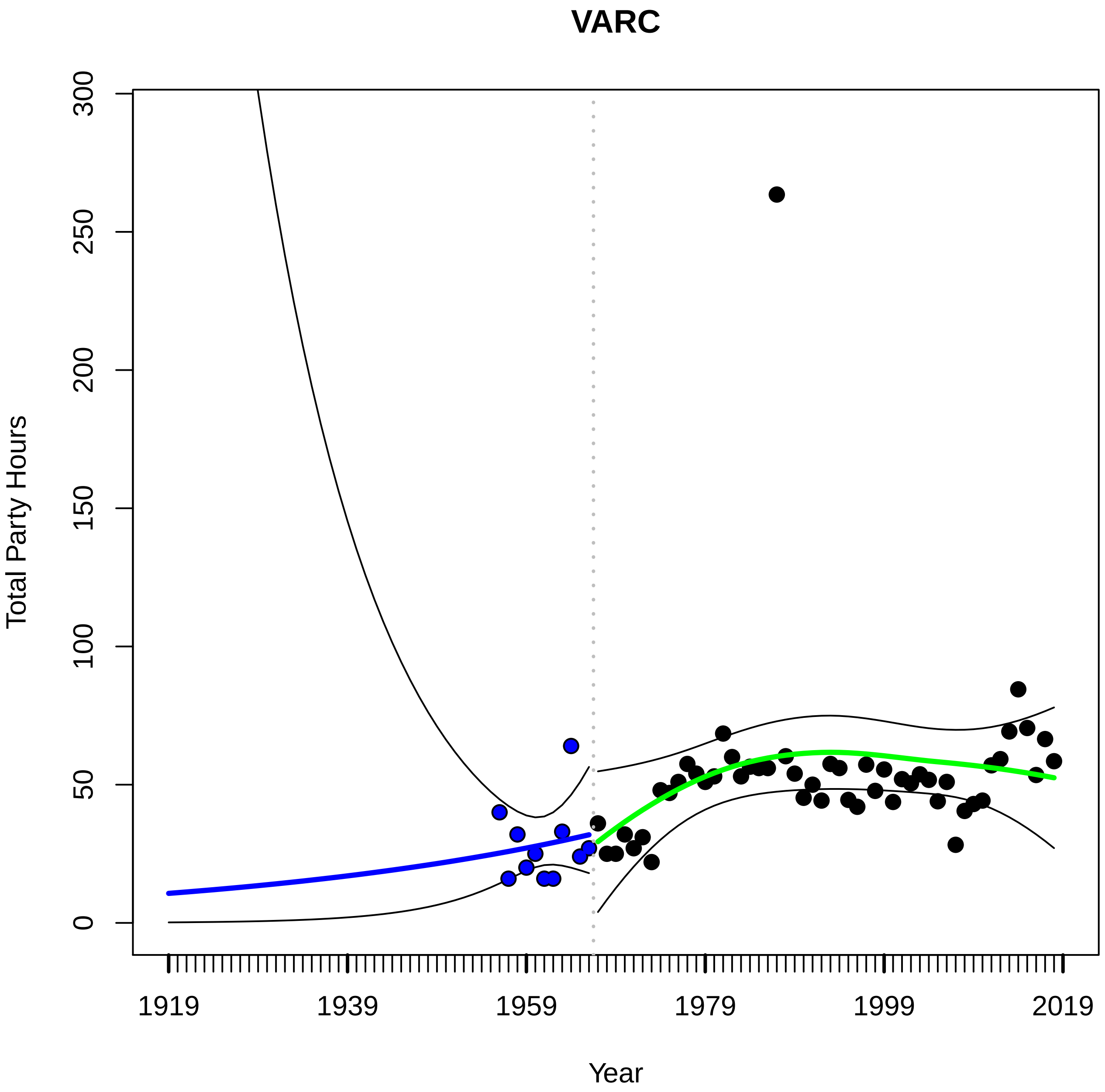
VARC
Total Party Hours
Year
0
50
100
150
200
250
300
1919
1939
1959
1979
1999
2019

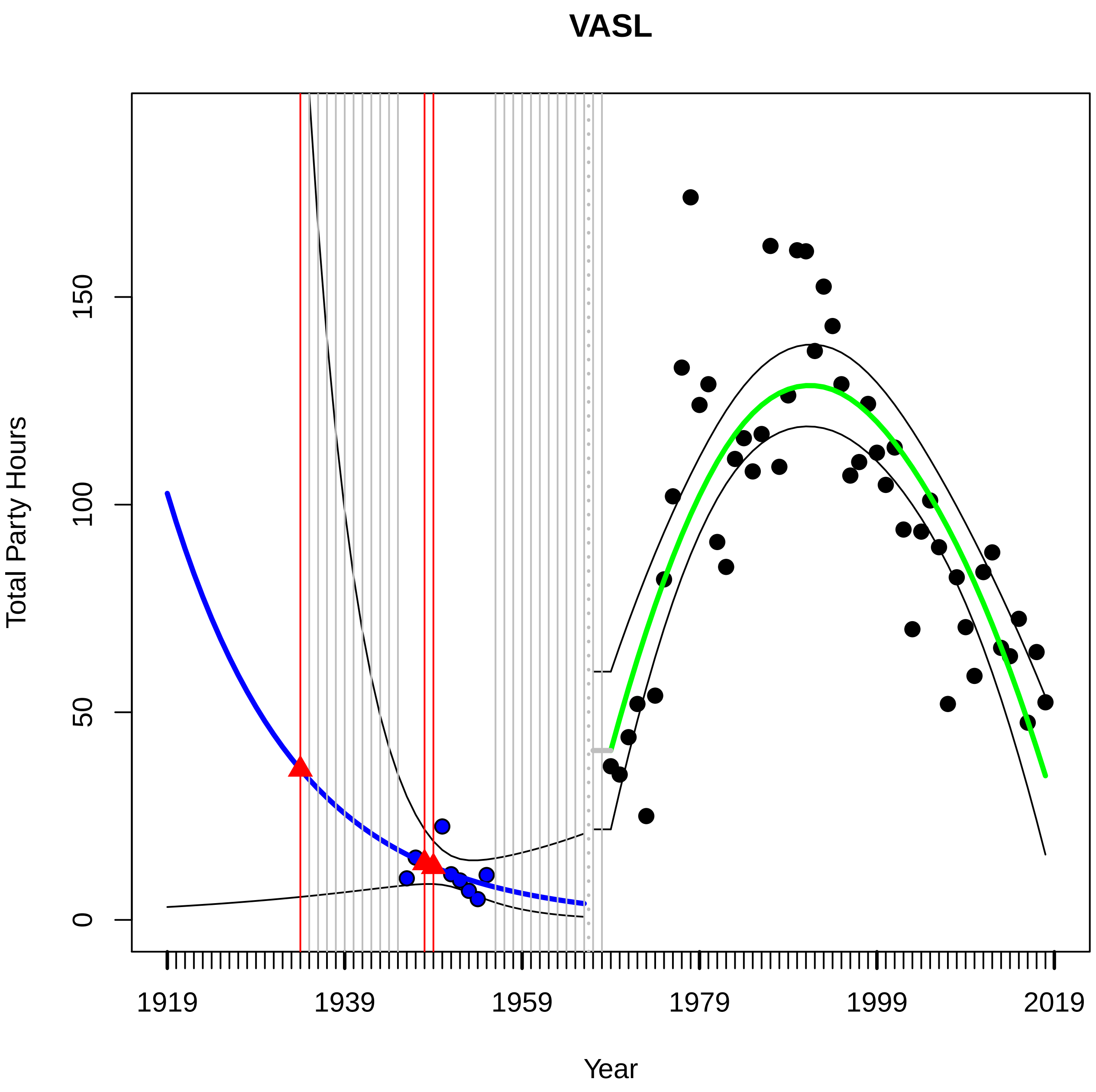

VASL
Total Party Hours
0
50
100
150
1919
1939
1959
1979
1999
2019
Year

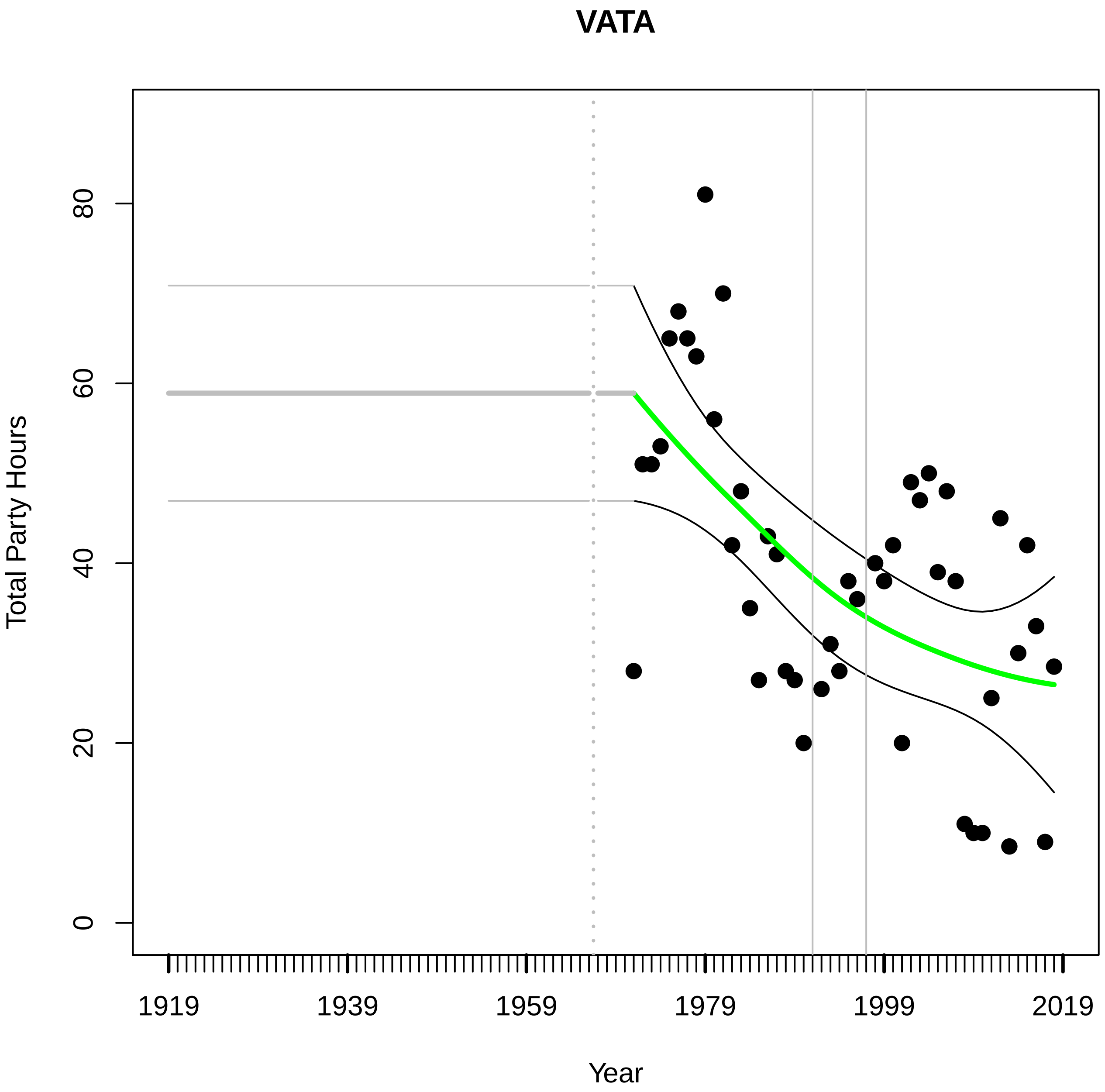

VATA
Total Party Hours
0
20
40
60
80
1919
1939
1959
1979
1999
2019
Year

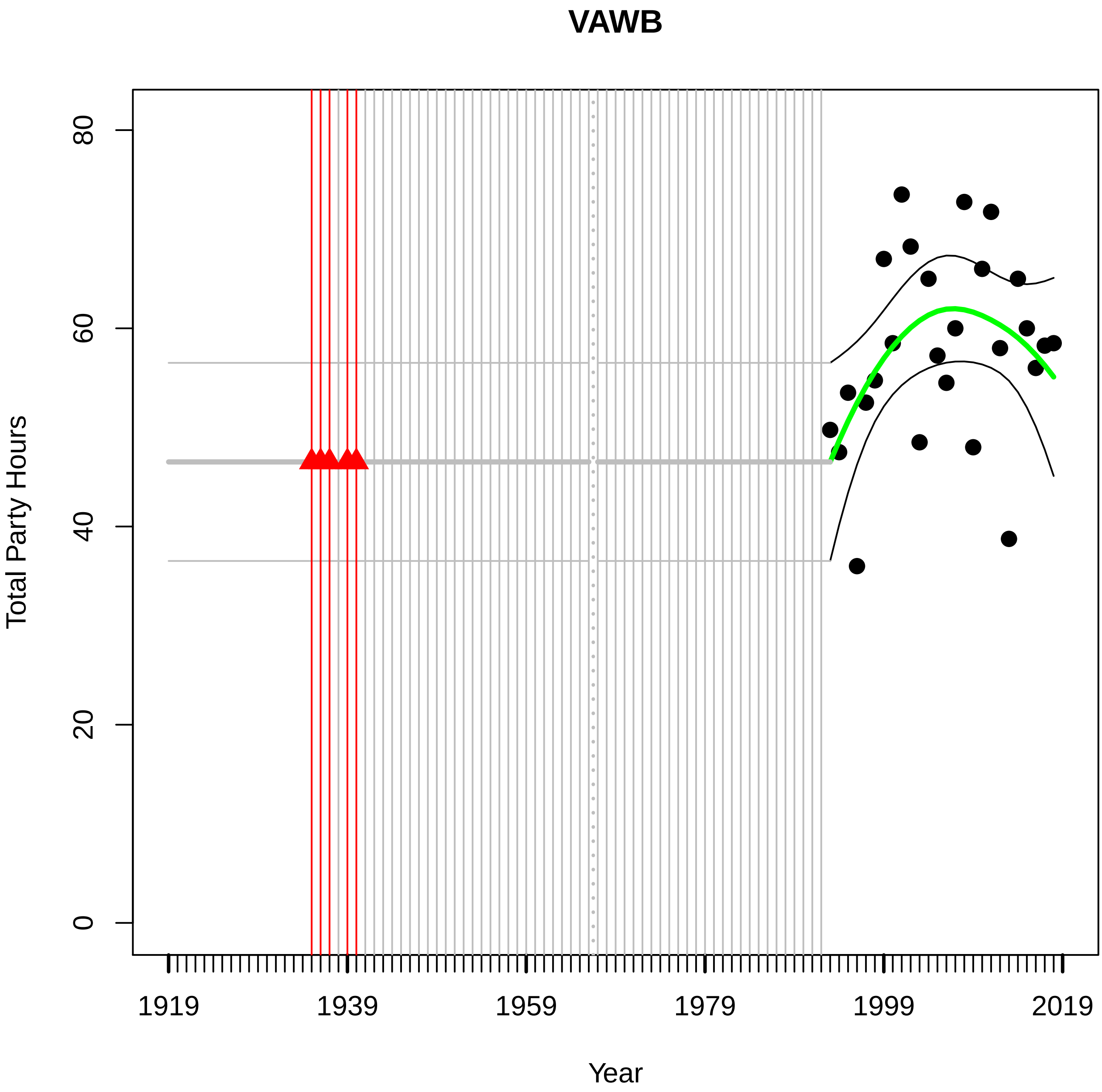

VAWB
Total Party Hours
0
20
40
60
80
Year
1919
1939
1959
1979
1999
2019

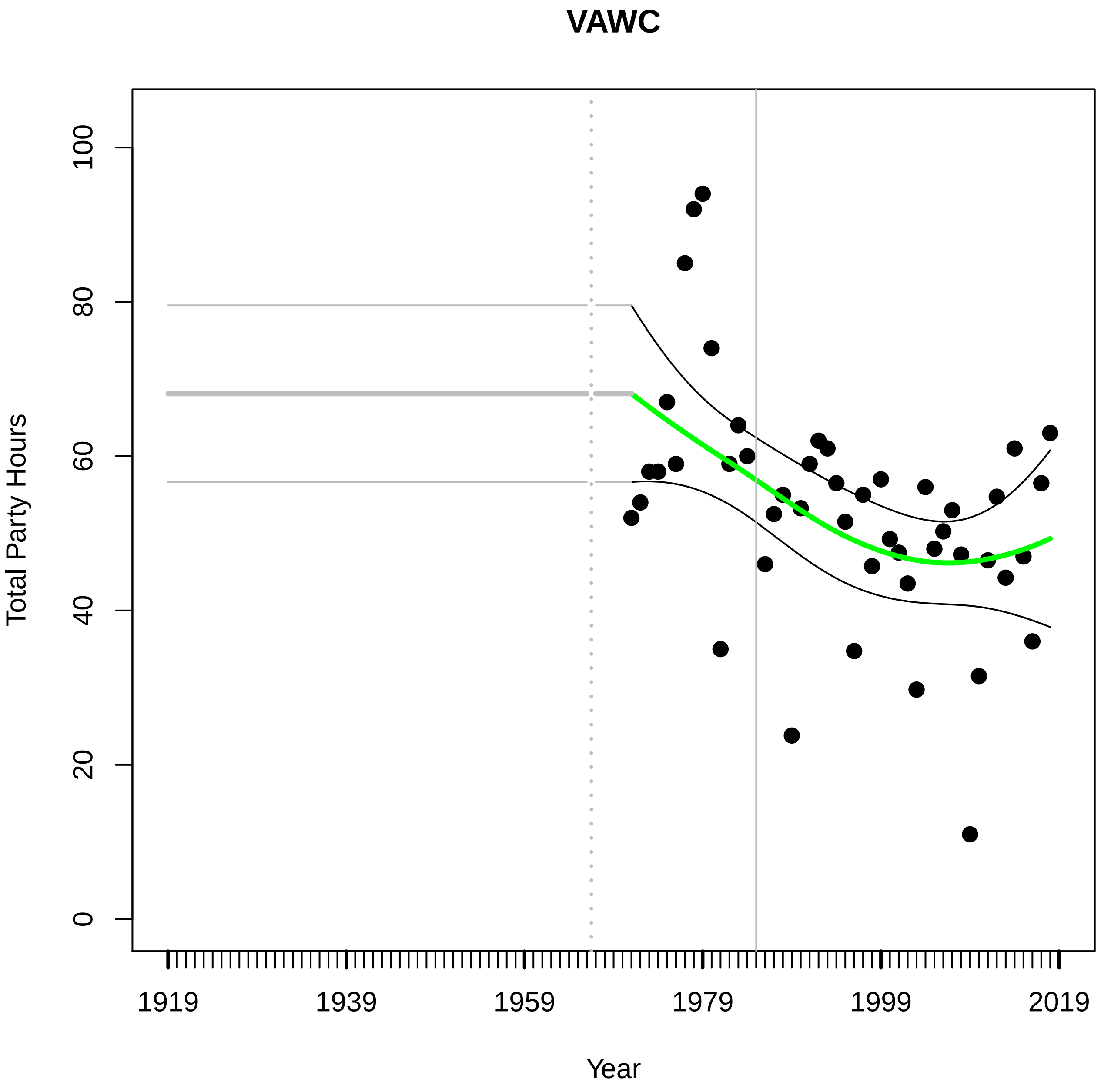

VAWC
Total Party Hours
100
80
60
40
20
0
1919
1939
1959
1979
1999
2019
Year

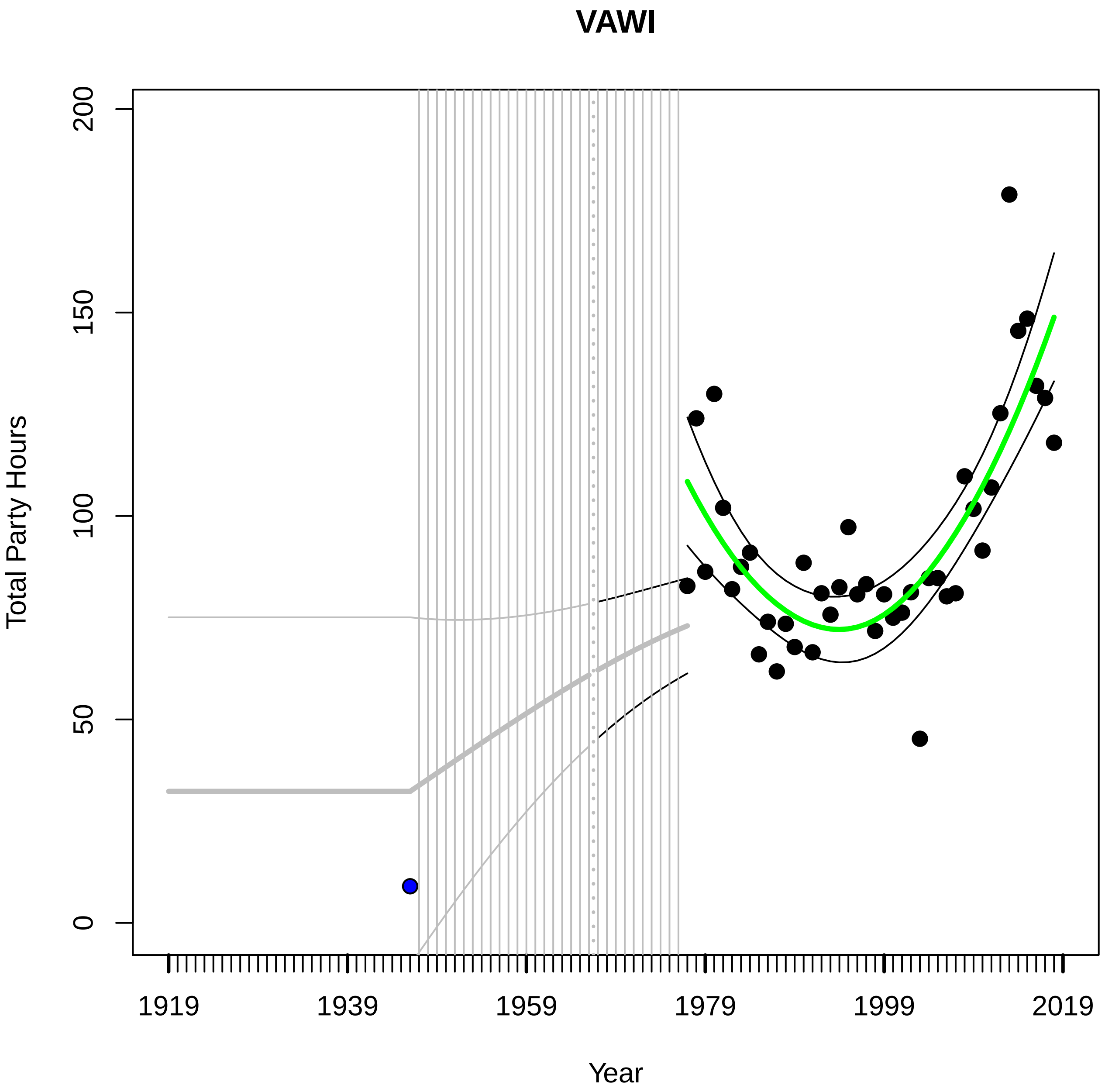
VAWI
Total Party Hours
0
50
100
150
200
1919
1939
1959
1979
1999
2019
Year

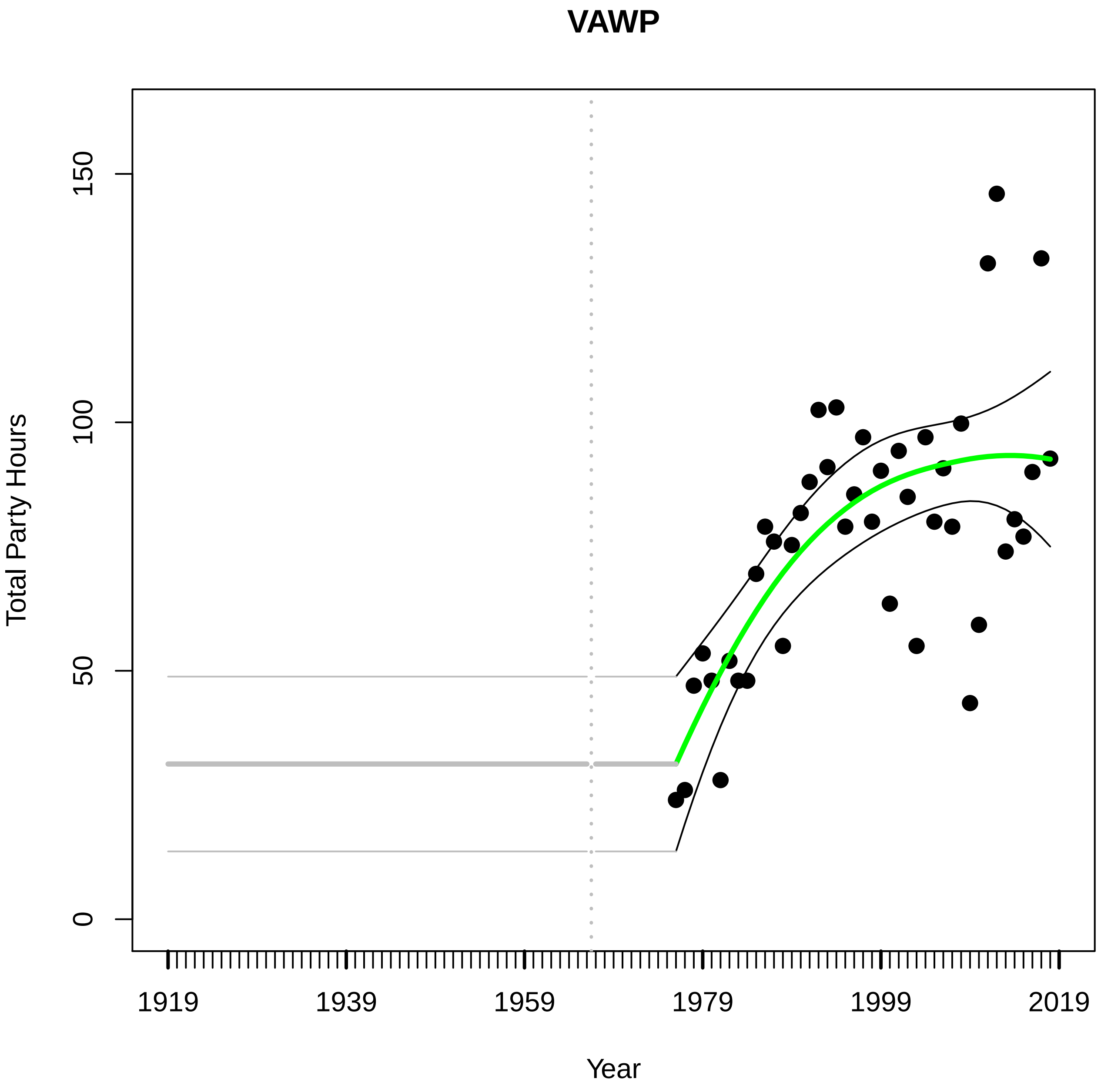

VAWP
Total Party Hours
0
50
100
150
1919
1939
1959
1979
1999
2019
Year

VAWR

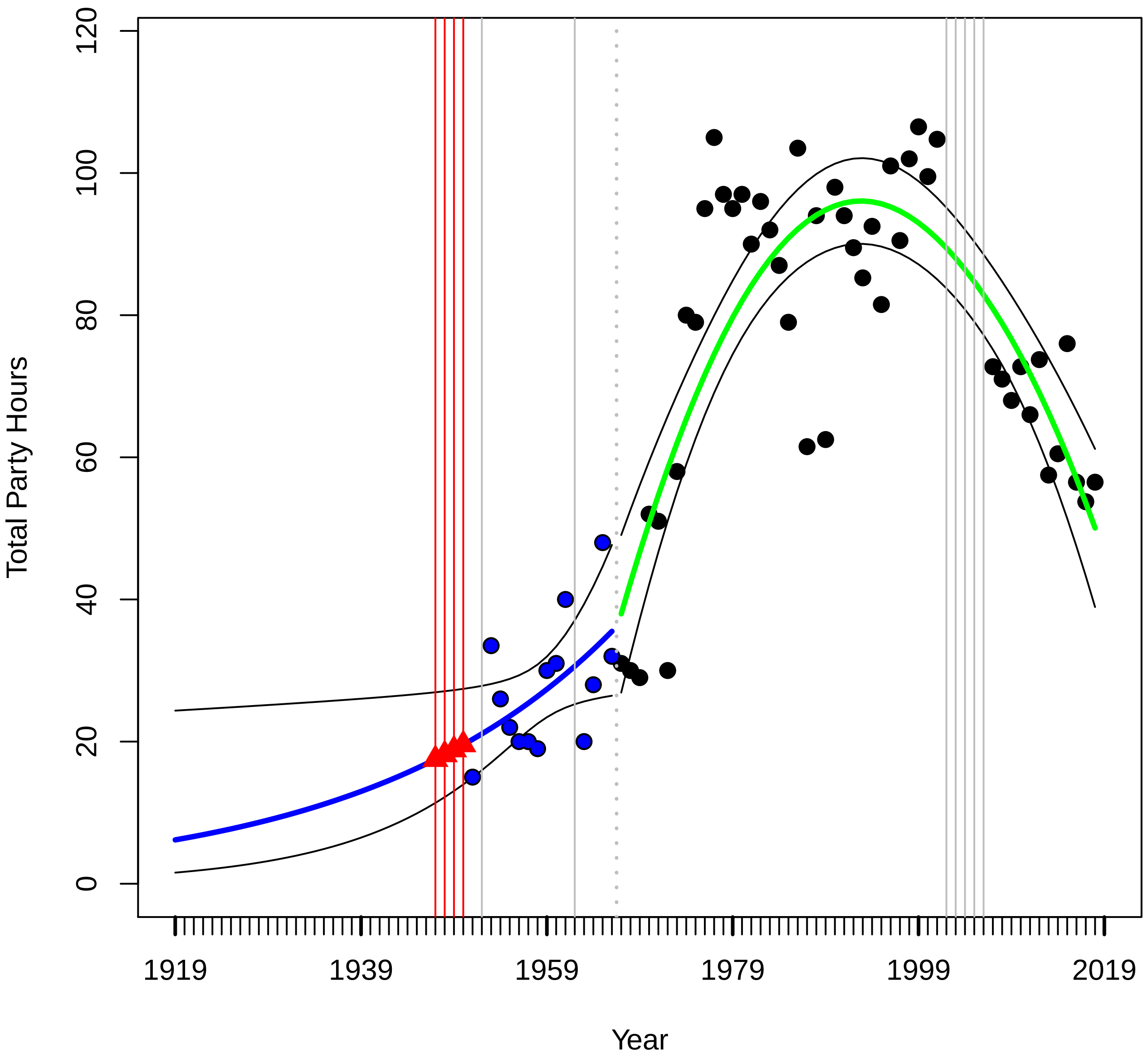

VAWR
120
100
80
60
40
20
0
Total Party Hours
1919
1939
1959
1979
1999
2019
Year

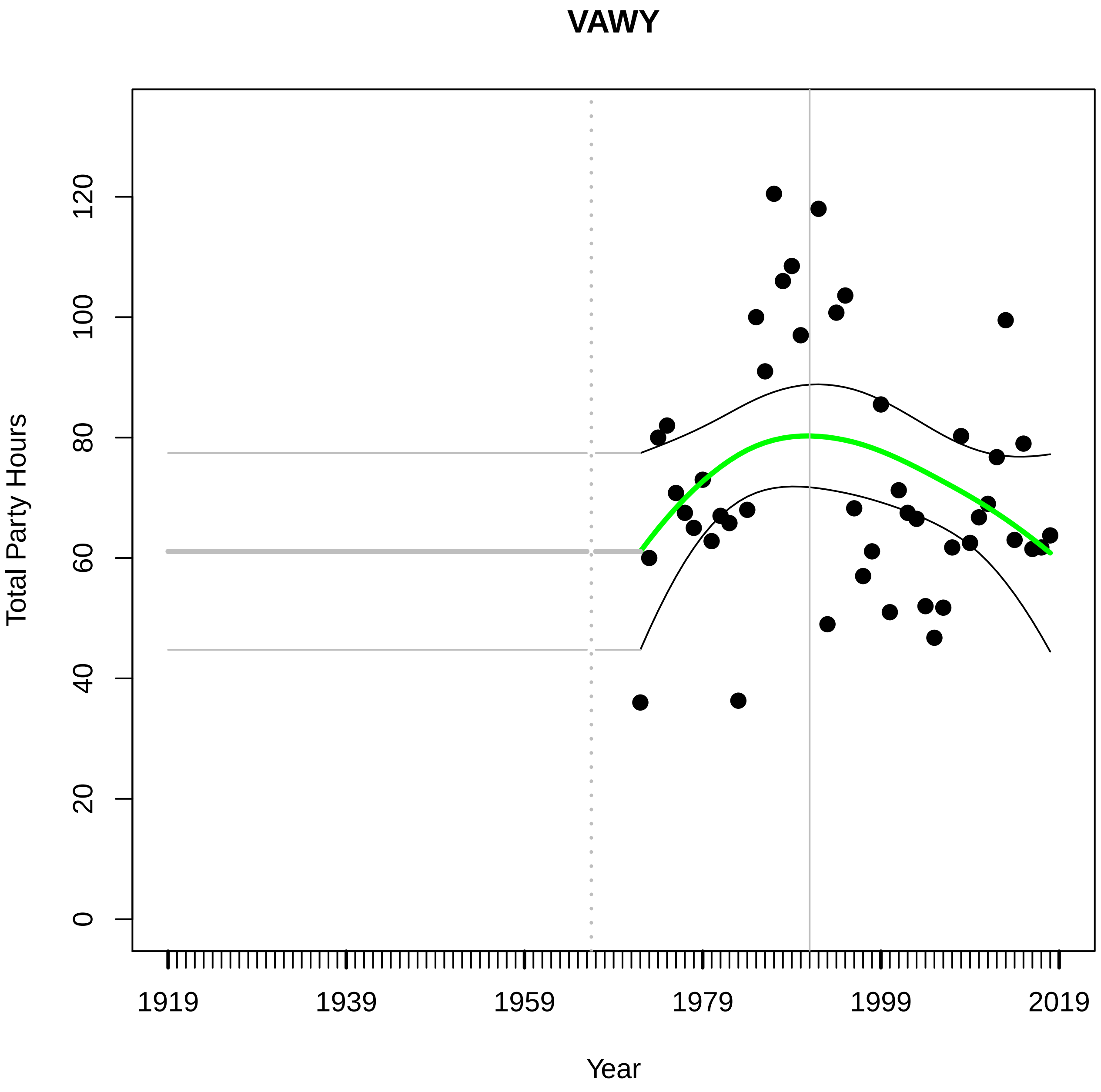
VAWY
Total Party Hours
0
20
40
60
80
100
120
1919
1939
1959
1979
1999
2019
Year

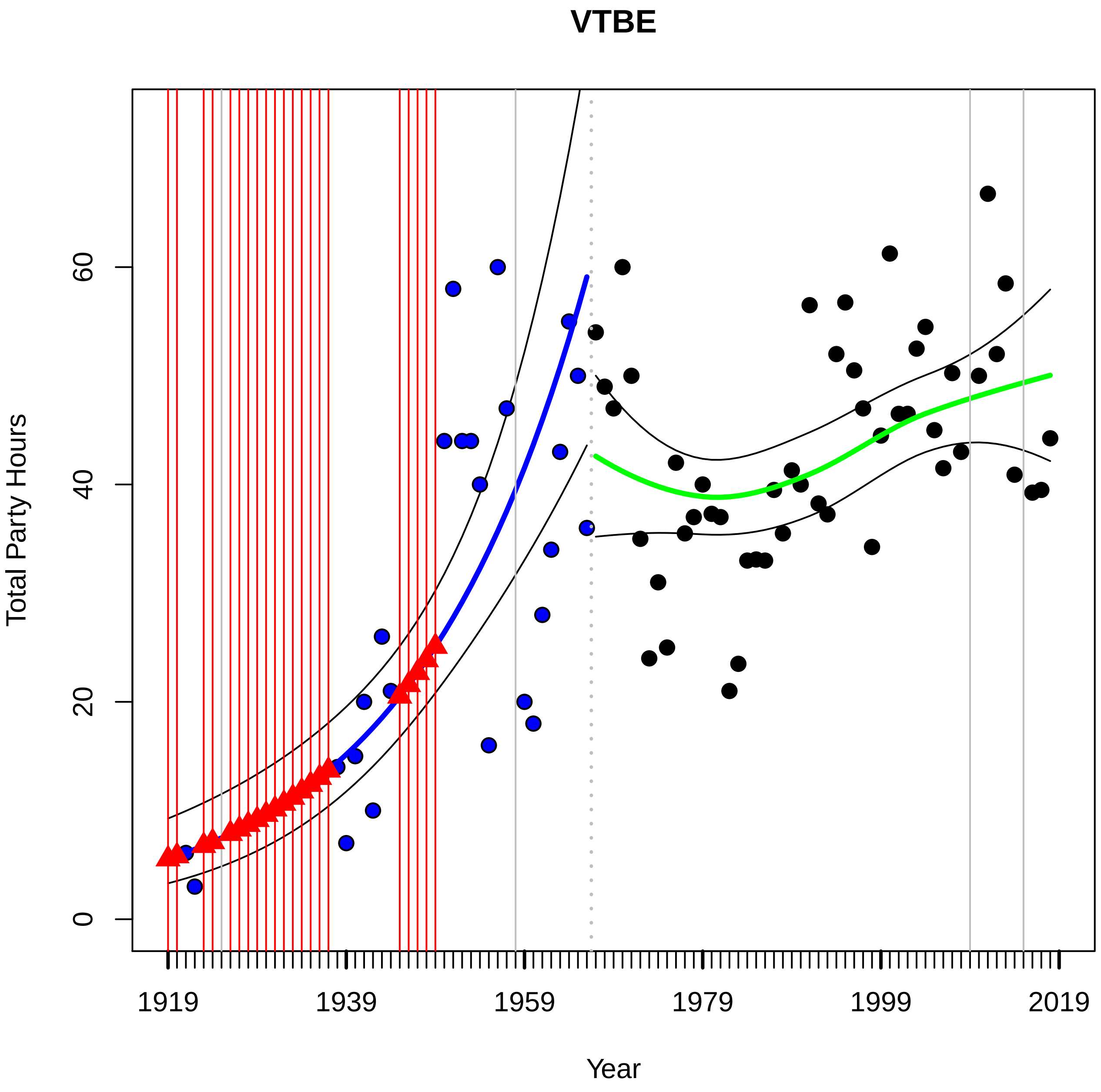

VTBE
Total Party Hours
0
20
40
60
1919
1939
1959
1979
1999
2019
Year

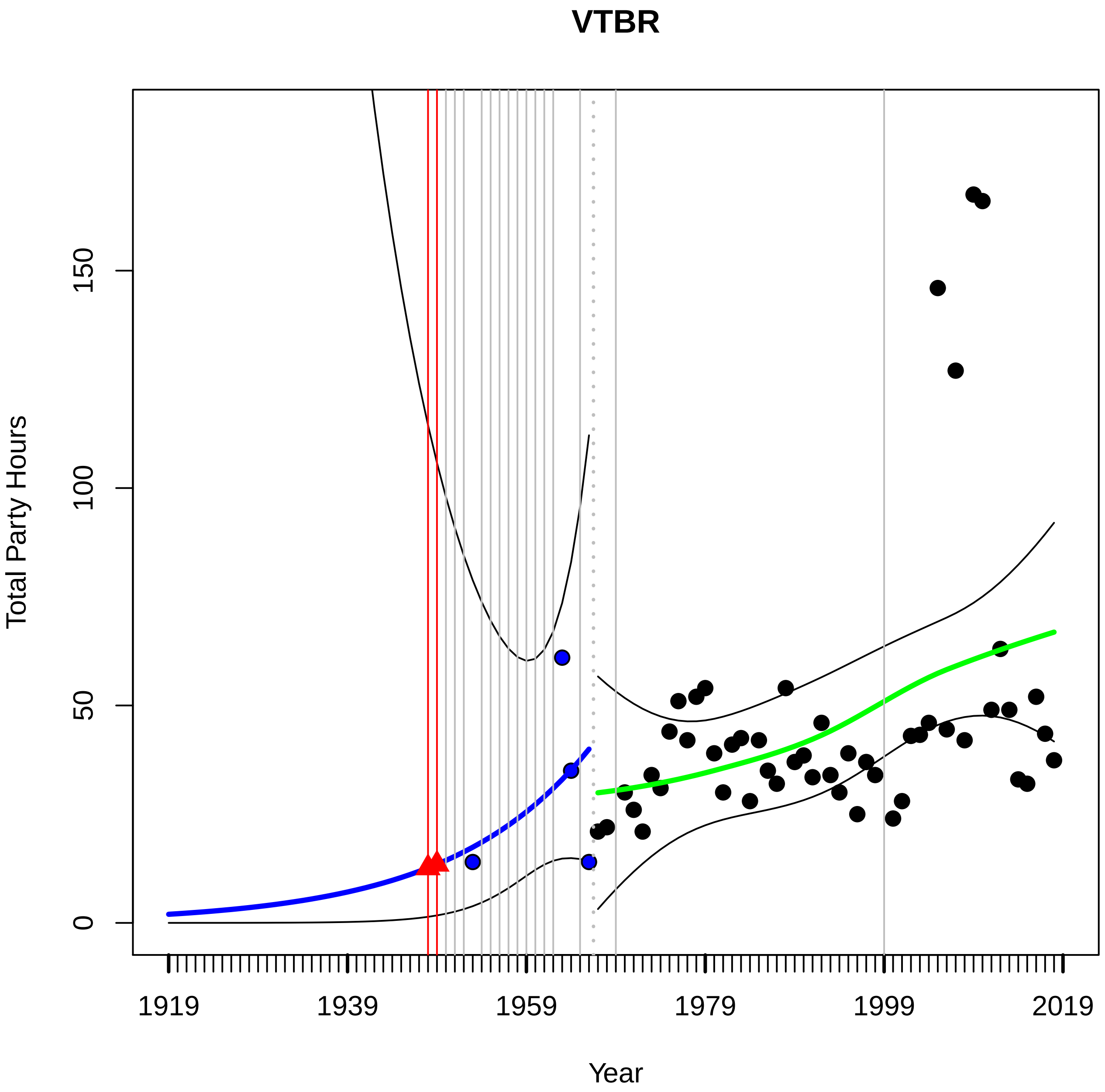
VTBR
Total Party Hours
Year
0
50
100
150
1919
1939
1959
1979
1999
2019

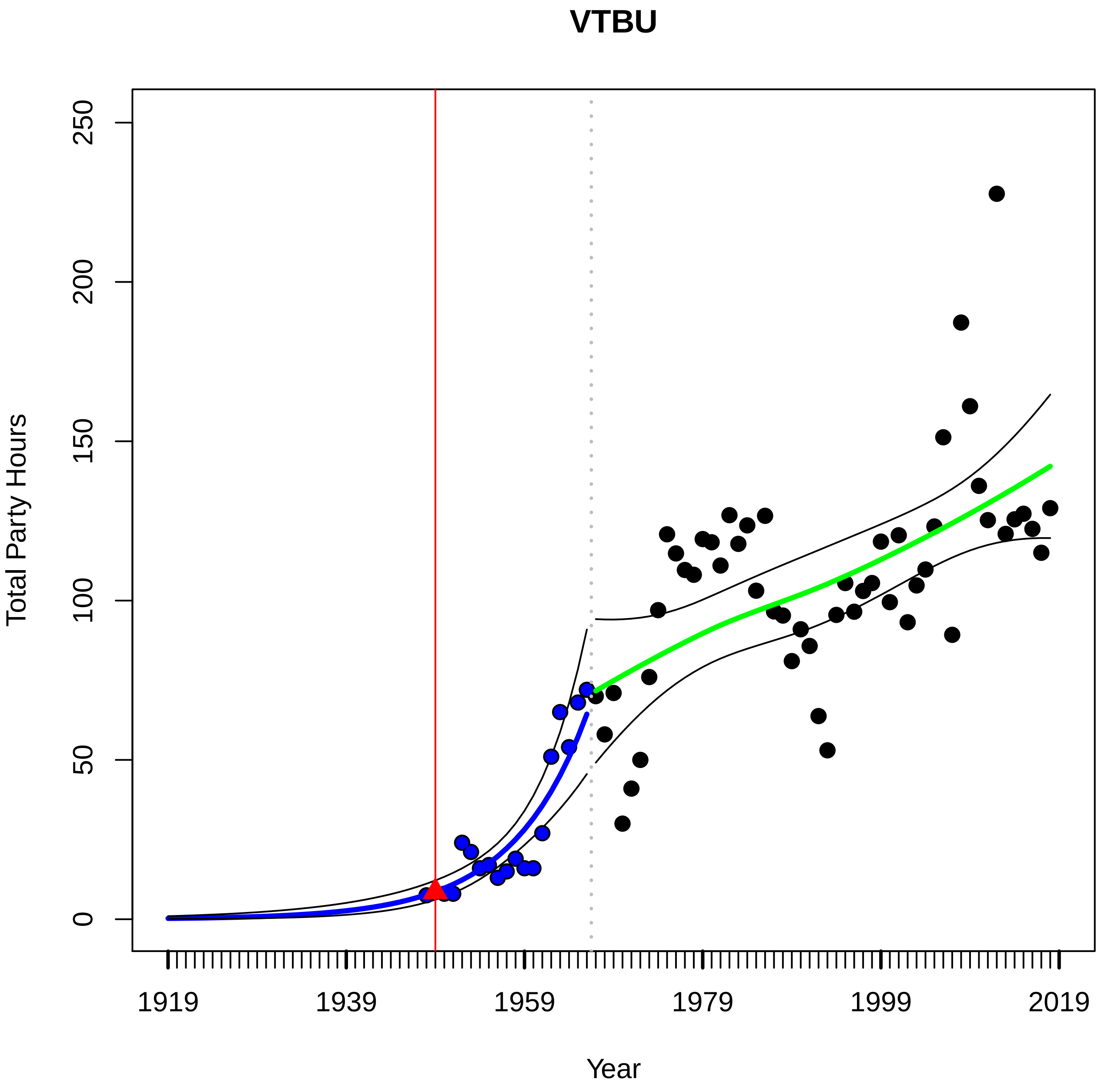

VTBU
Total Party Hours
0
50
100
150
200
250
1919
1939
1959
1979
1999
2019
Year

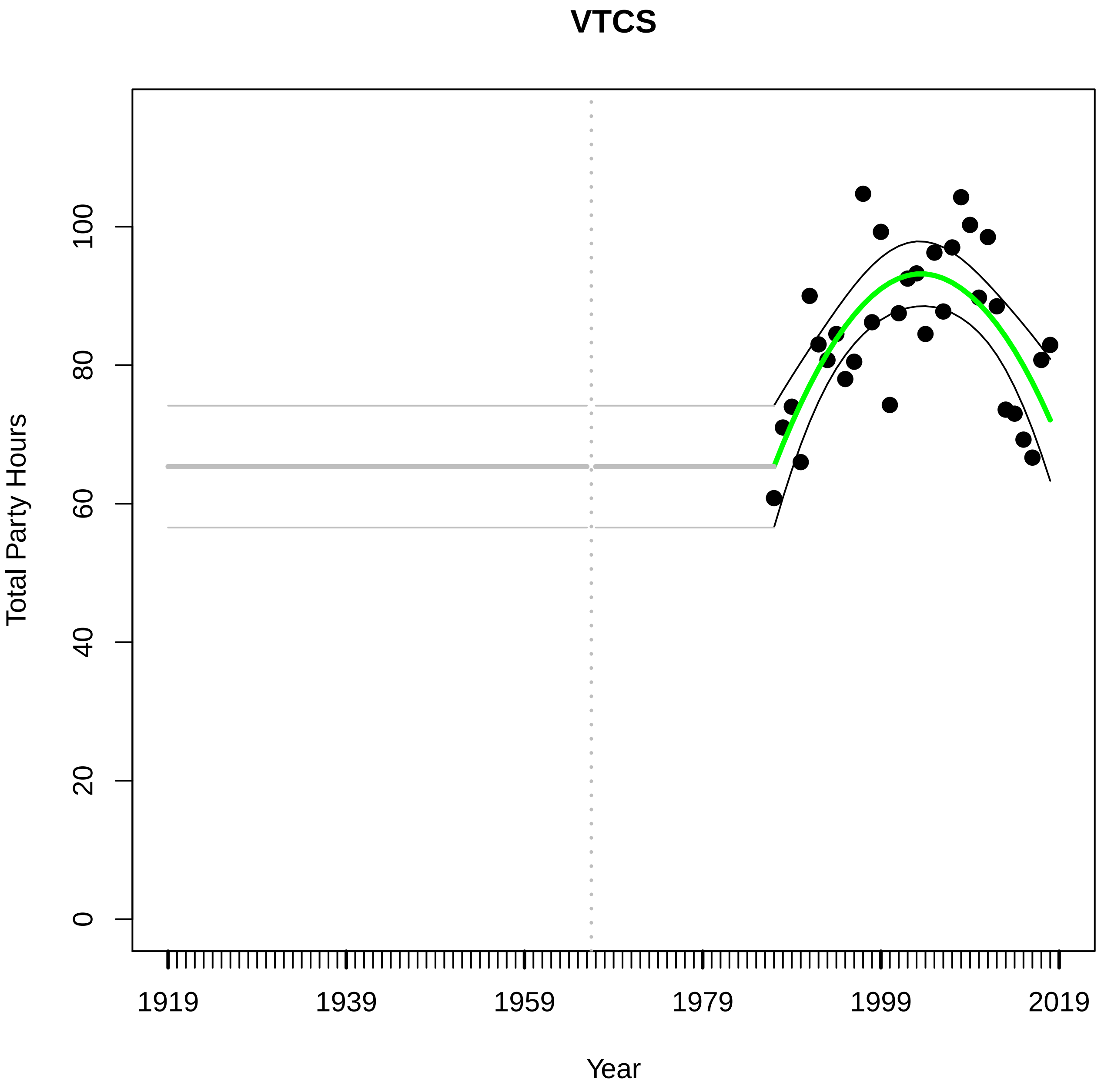

VTCS
Total Party Hours
0
20
40
60
80
100
1919
1939
1959
1979
1999
2019
Year

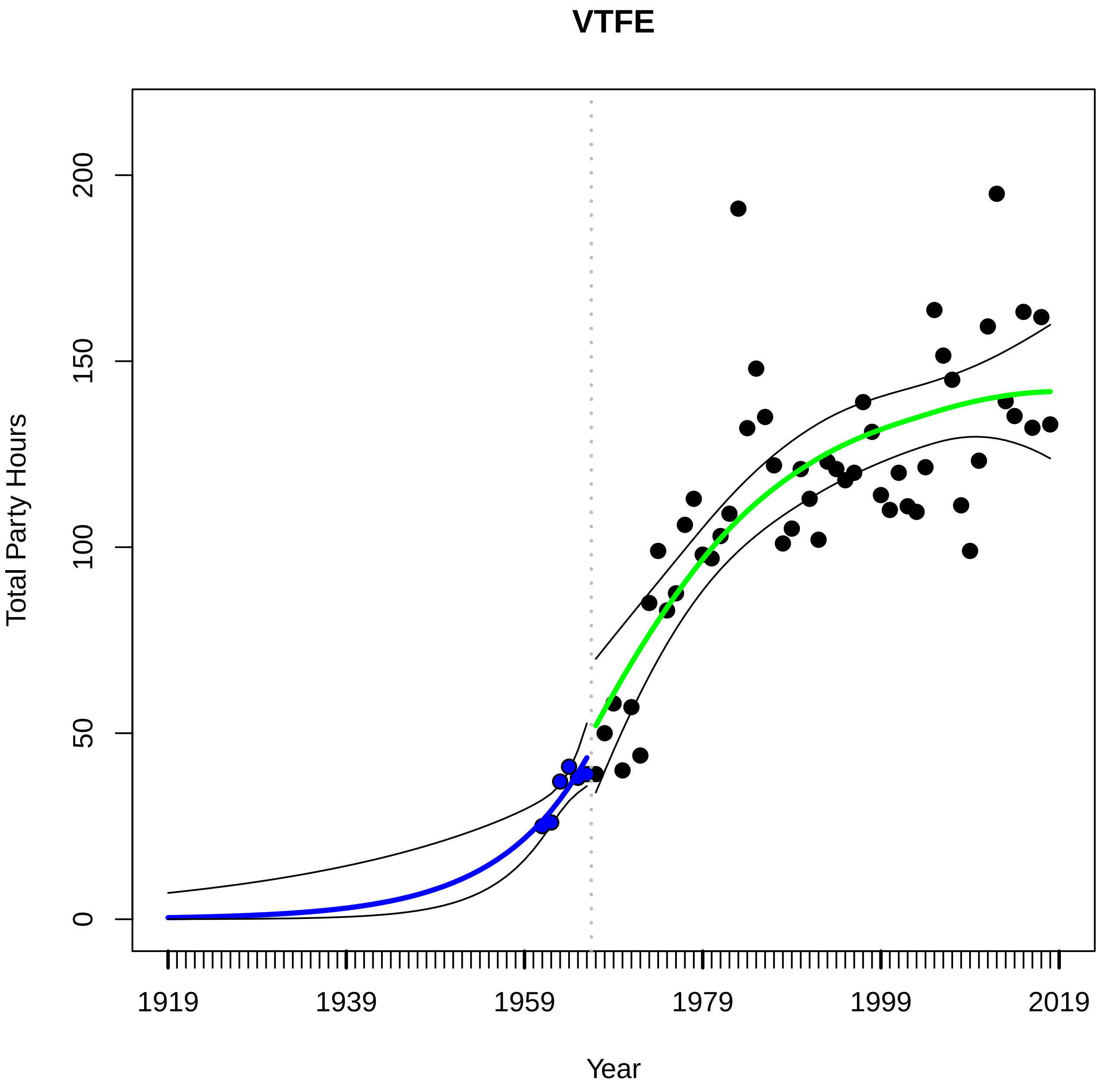
VTFE
Total Party Hours
0
50
100
150
200
1919
1939
1959
1979
1999
2019
Year

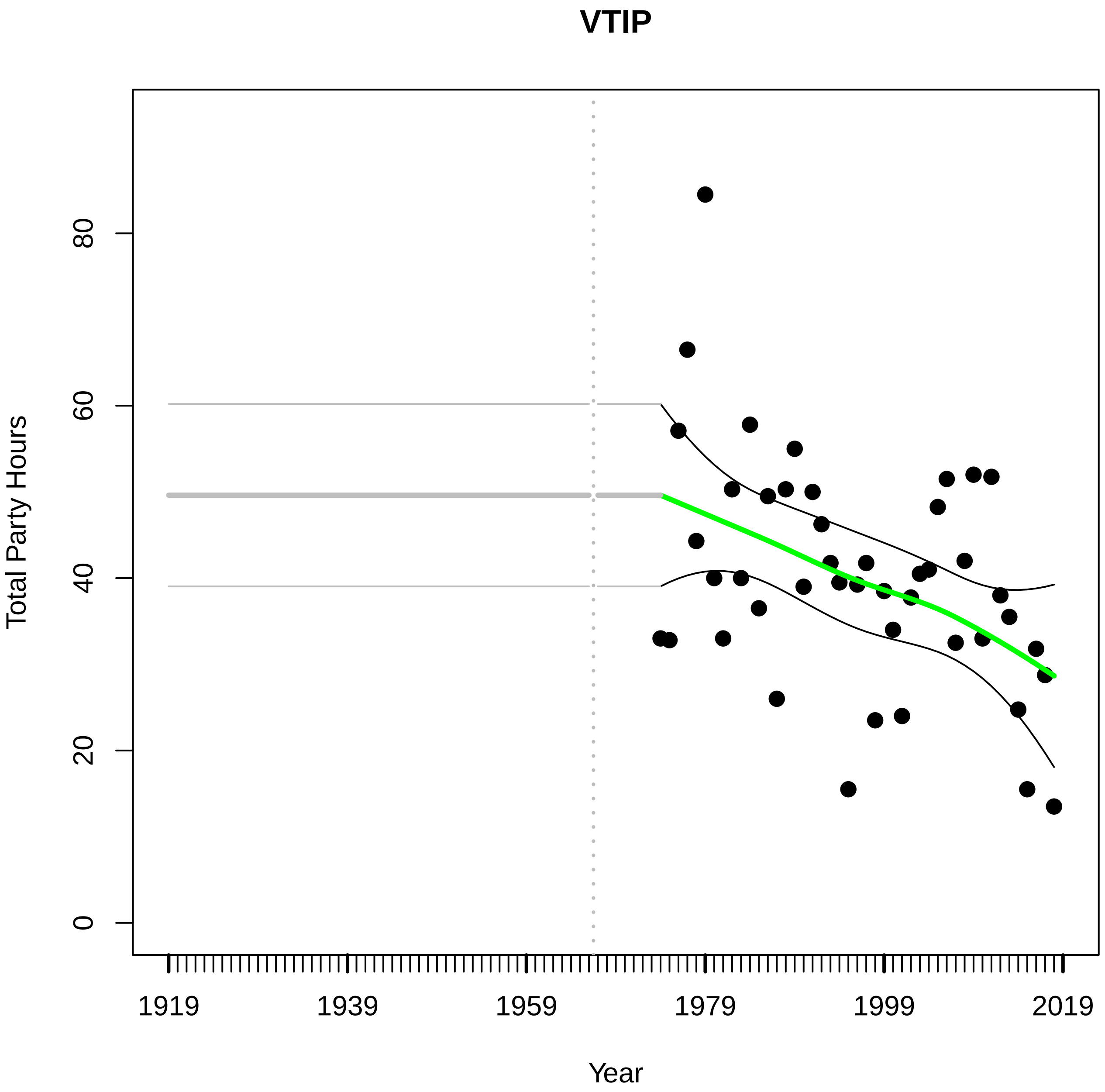

VTIP
Total Party Hours
0
20
40
60
80
1919
1939
1959
1979
1999
2019
Year

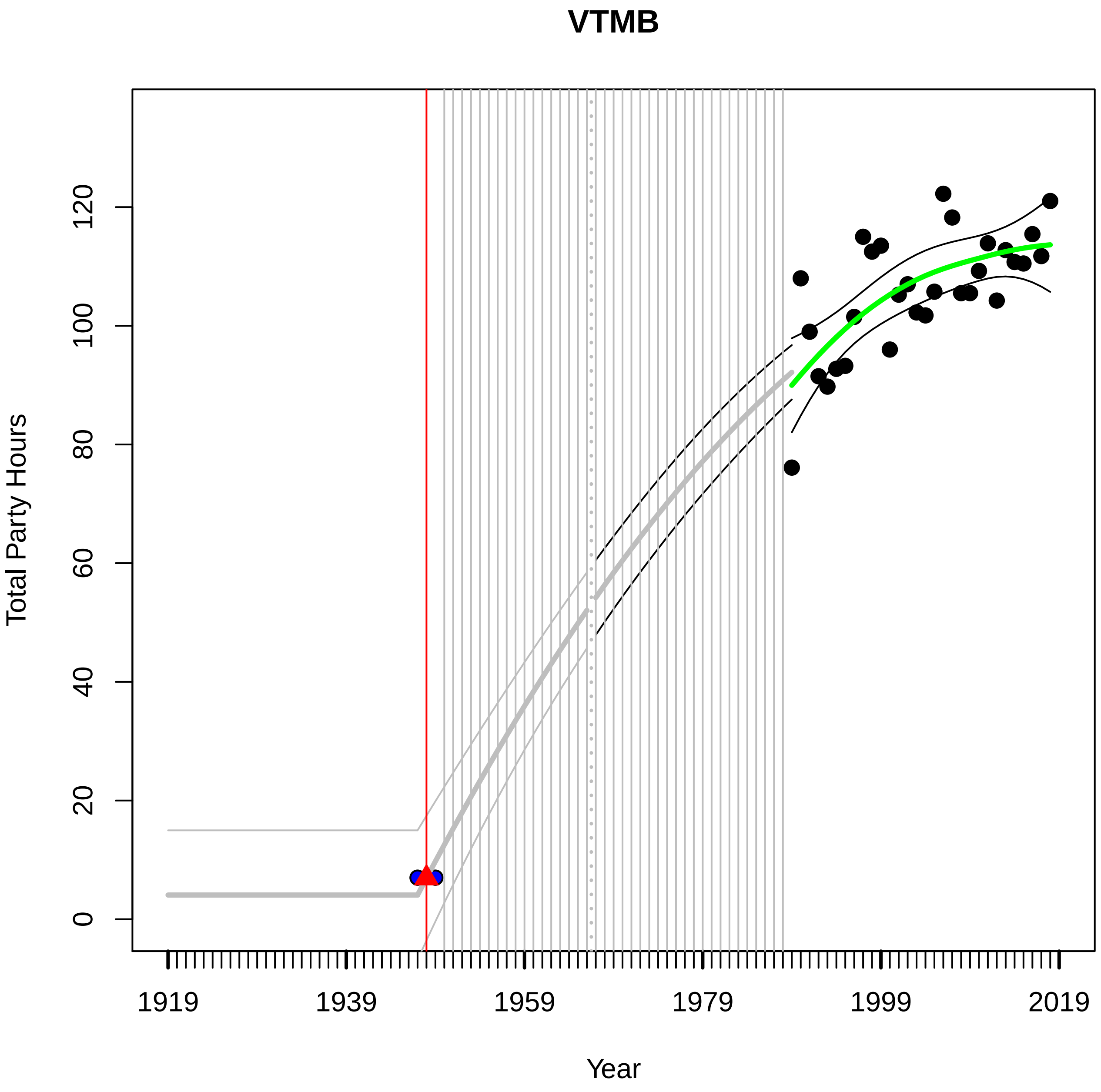
VTMB
Total Party Hours
0
20
40
60
80
100
120
1919
1939
1959
1979
1999
2019
Year

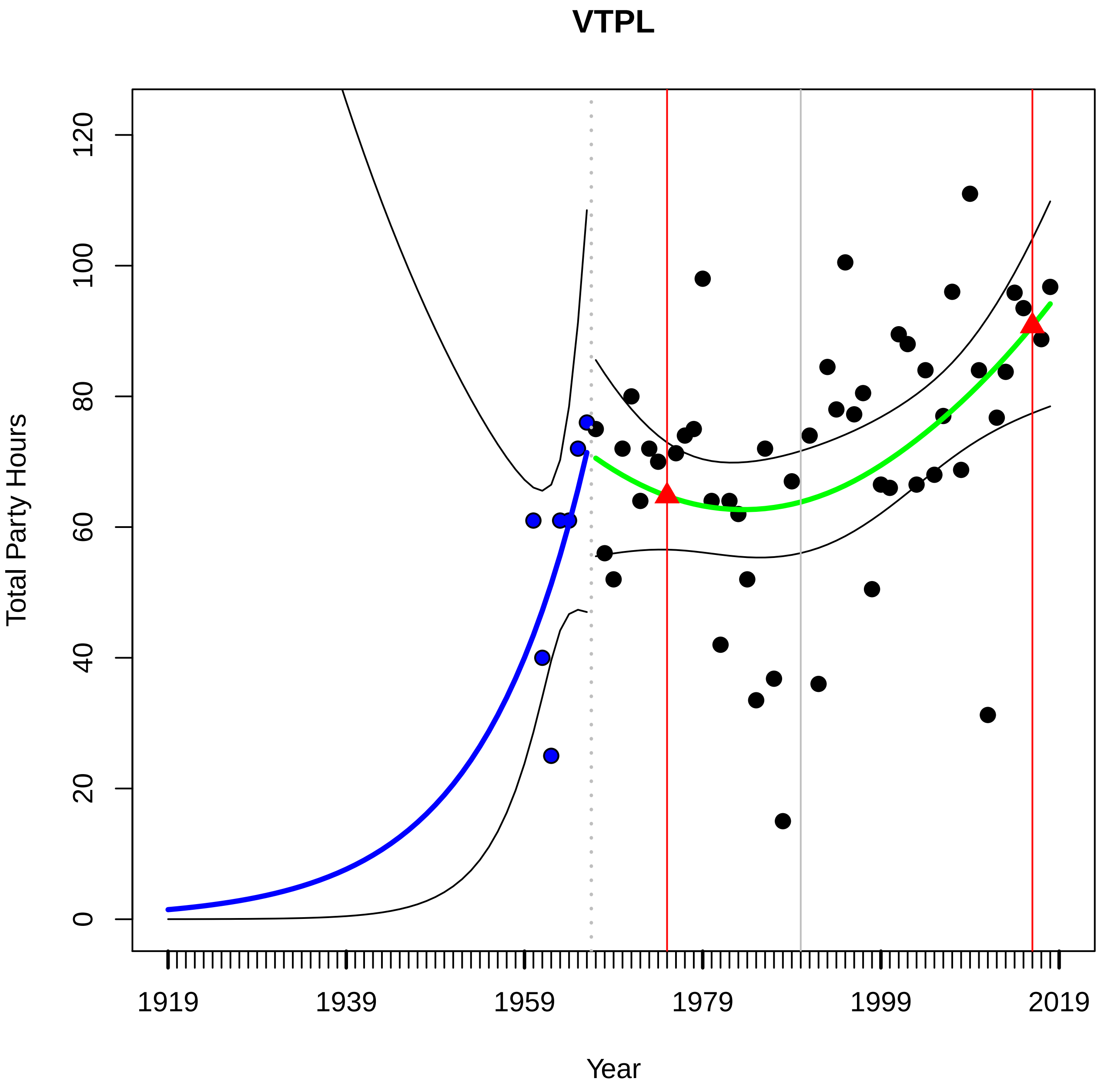

VTPL
Total Party Hours
0
20
40
60
80
100
120
1919
1939
1959
1979
1999
2019
Year

VTRU

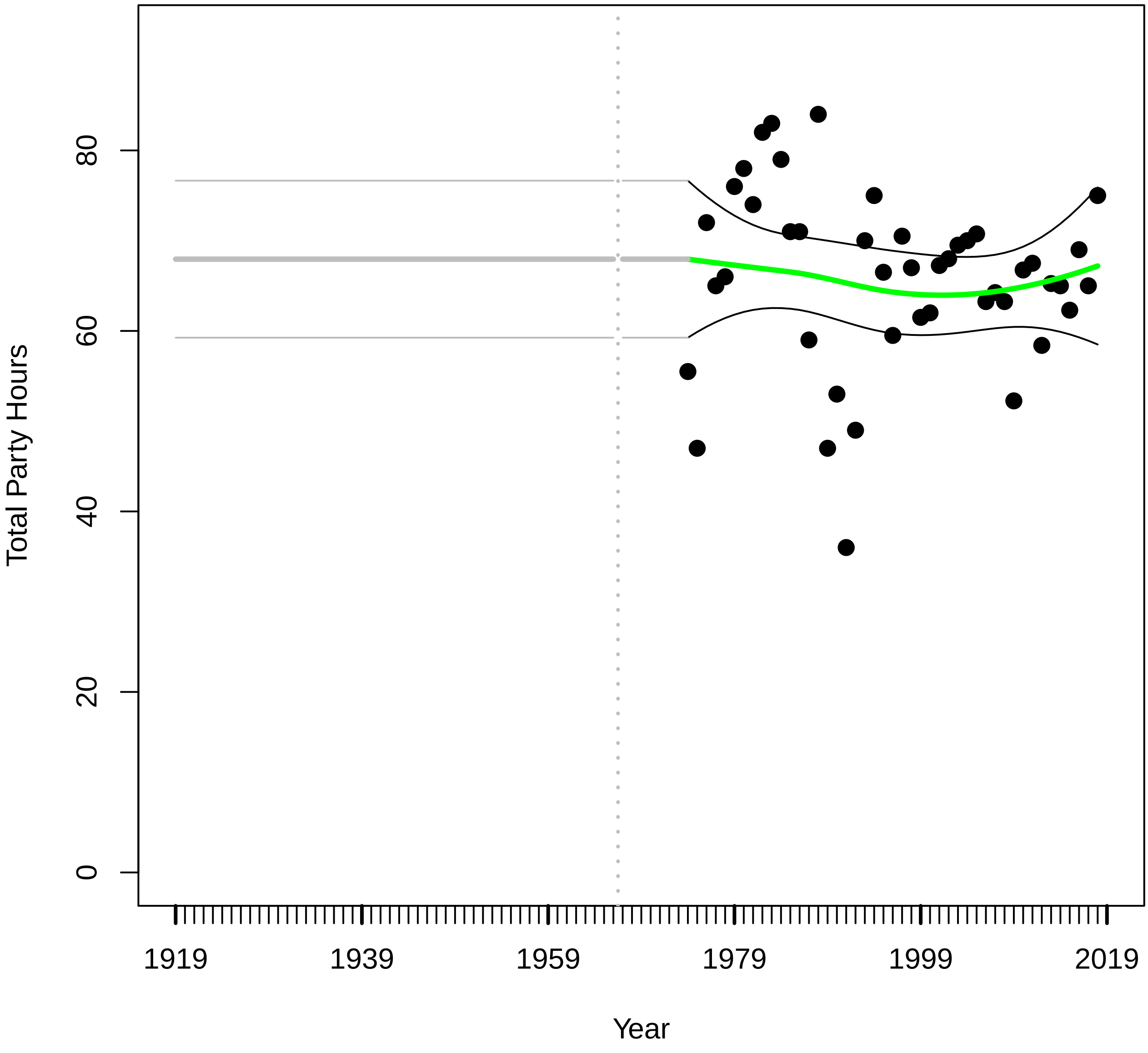

Total Party Hours
0
20
40
60
80
1919
1939
1959
1979
1999
2019
Year

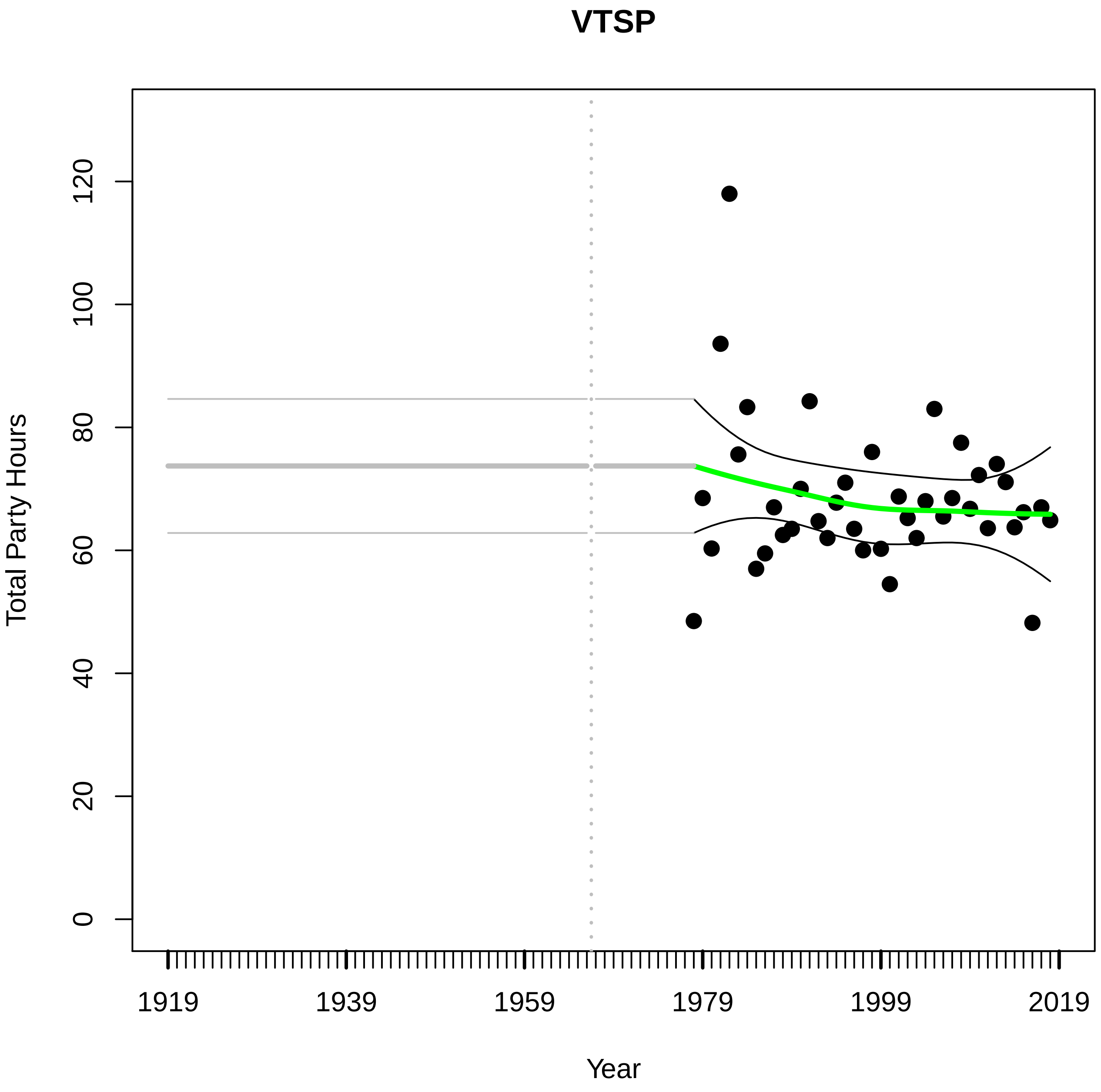

VTSP
Total Party Hours
0
20
40
60
80
100
120
1919
1939
1959
1979
1999
2019
Year

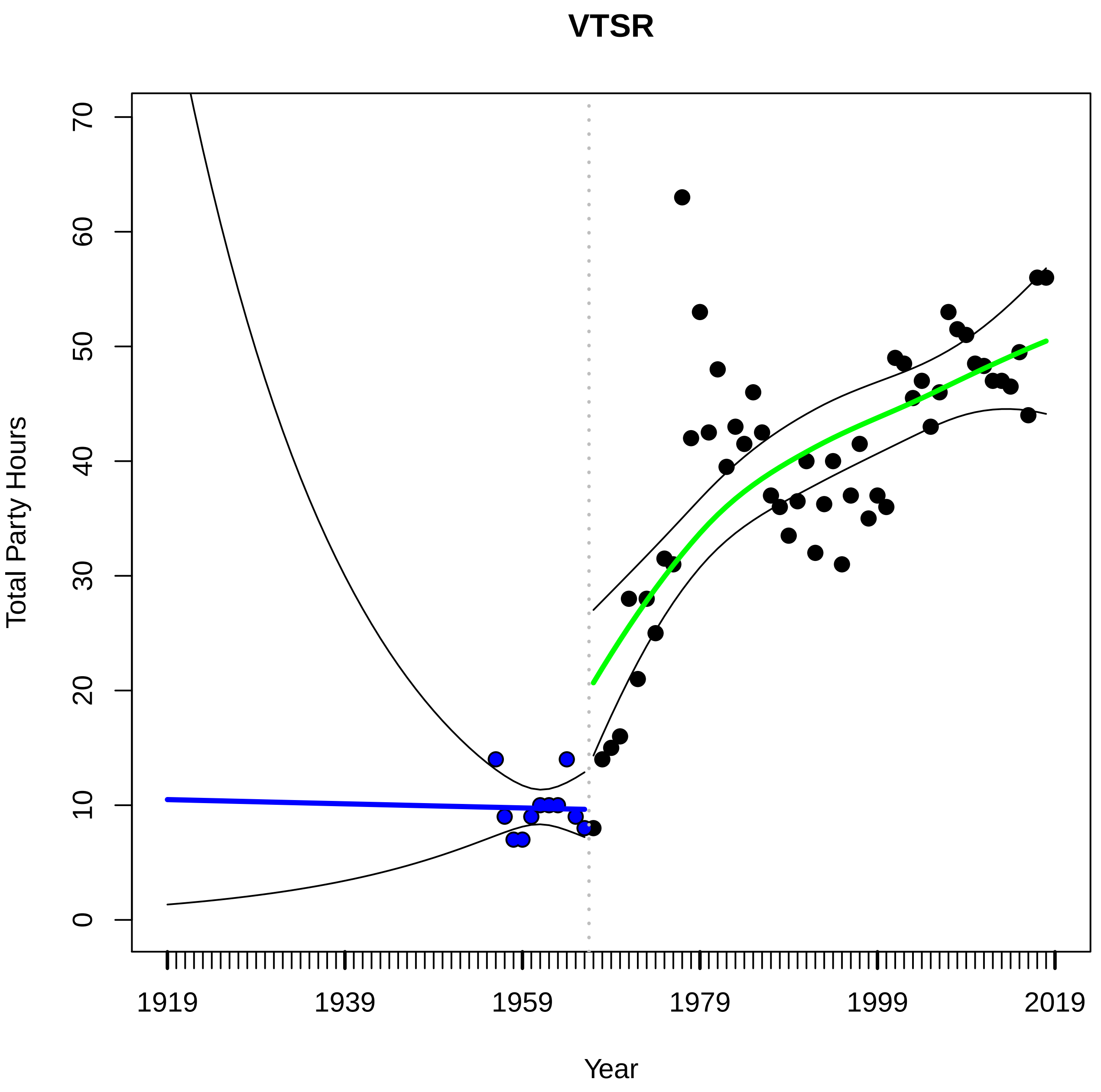

VTSR
Total Party Hours
0
10
20
30
40
50
60
70
1919
1939
1959
1979
1999
2019
Year

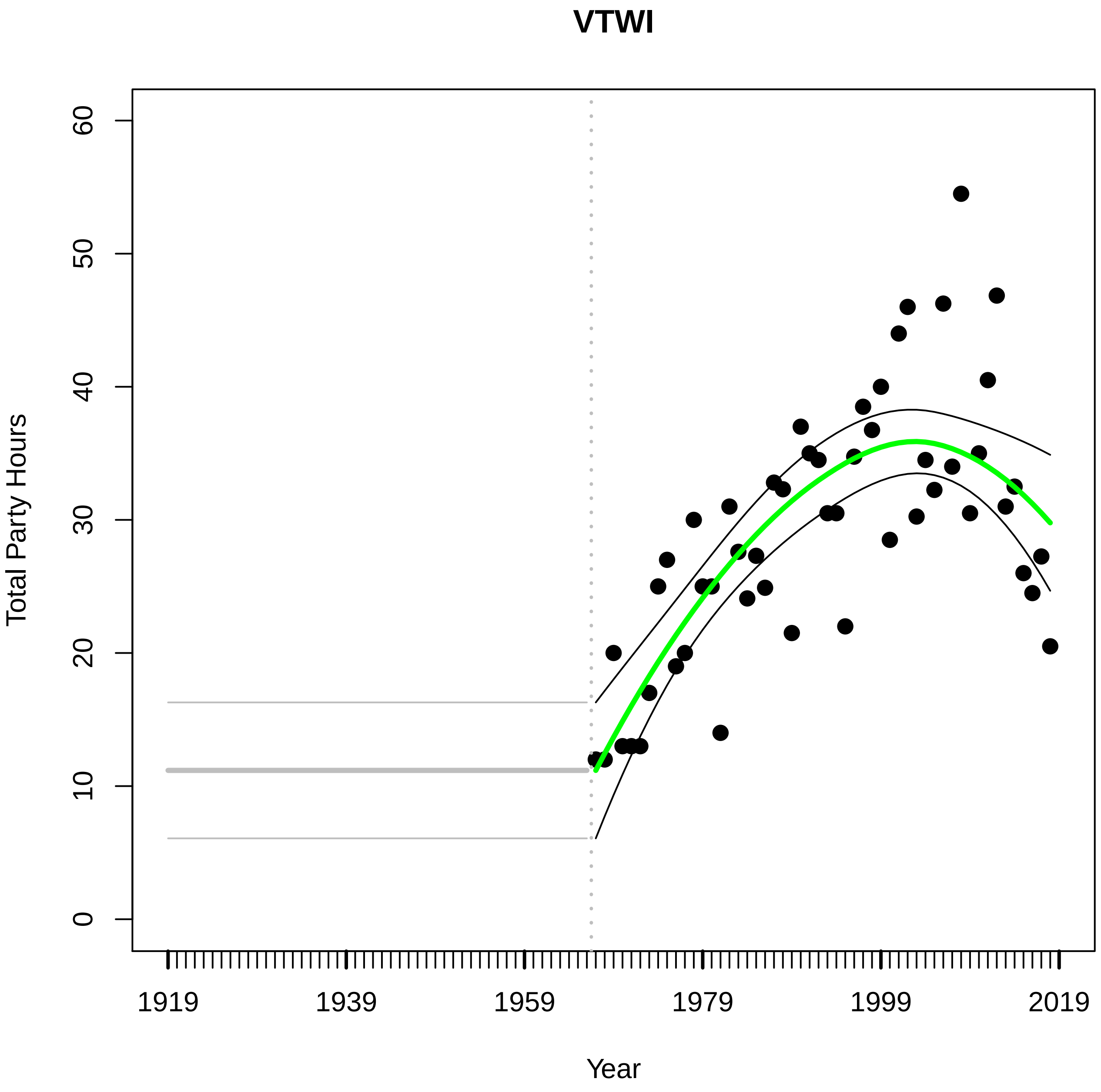

VTWI
Total Party Hours
0
10
20
30
40
50
60
1919
1939
1959
1979
1999
2019
Year

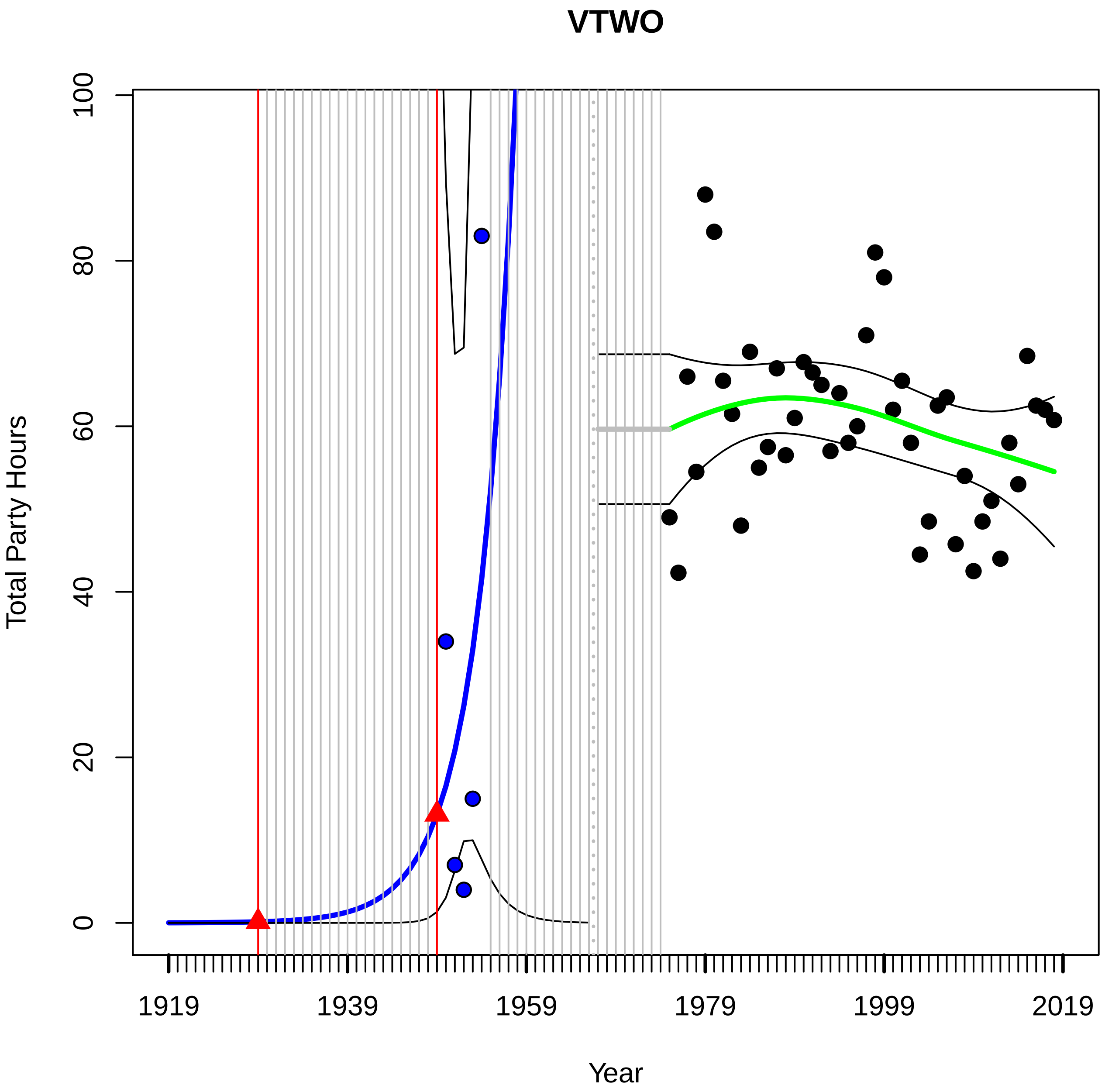

VTWO
Total Party Hours
100
80
60
40
20
0
1919
1939
1959
1979
1999
2019
Year

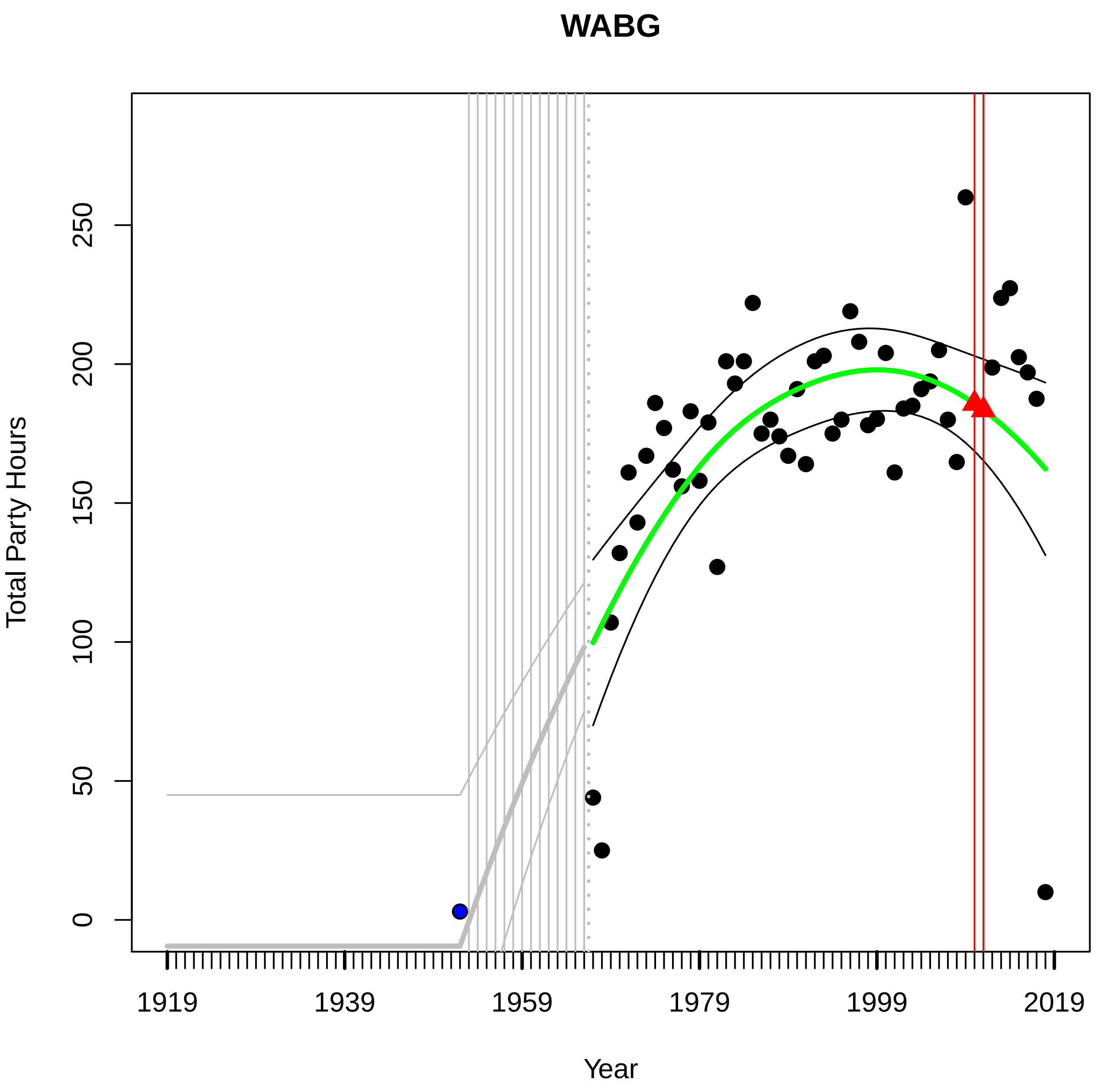

WABG
Total Party Hours
Year
0
50
100
150
200
250
1919
1939
1959
1979
1999
2019

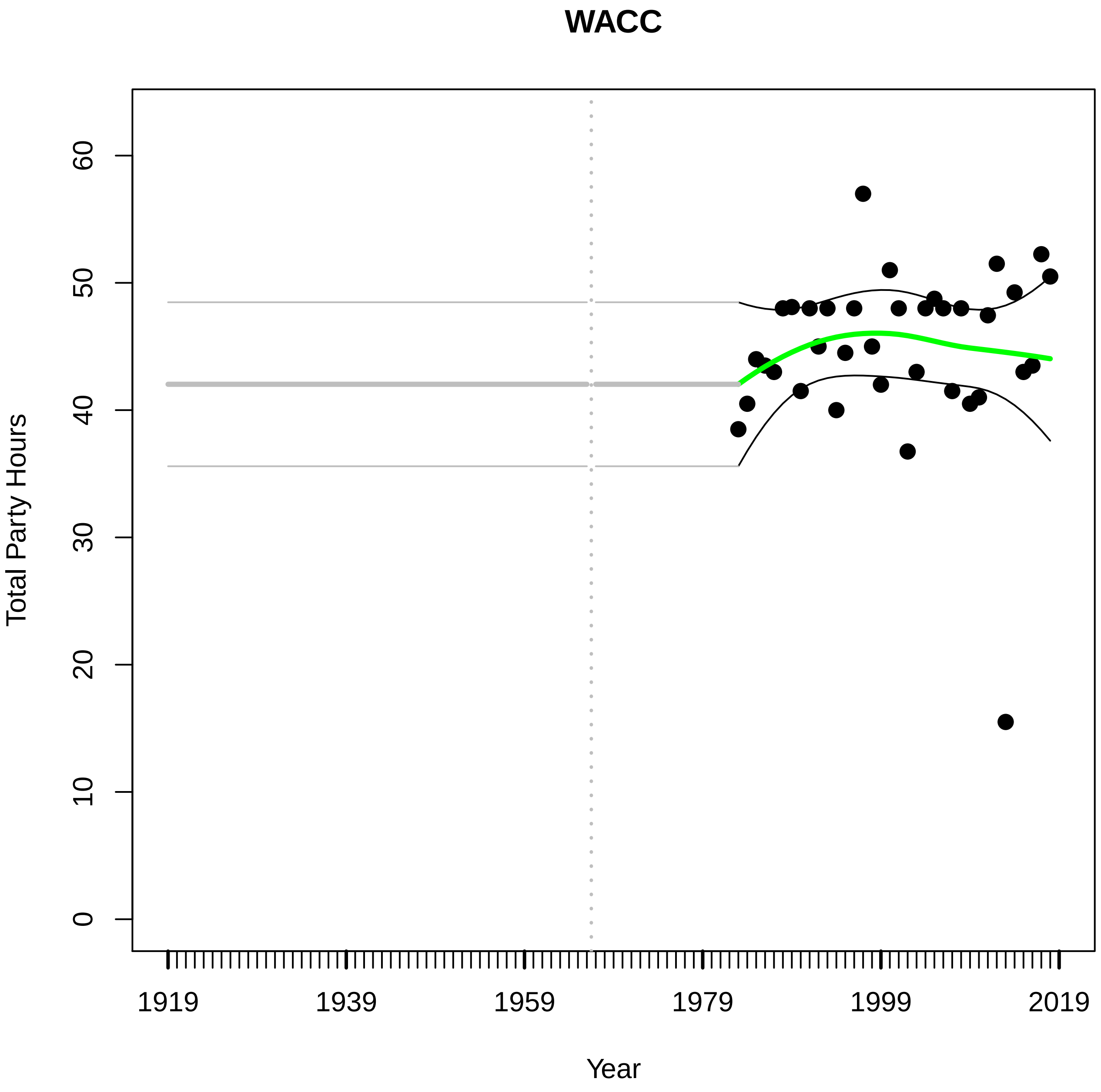
WACC
Total Party Hours
0
10
20
30
40
50
60
1919
1939
1959
1979
1999
2019
Year

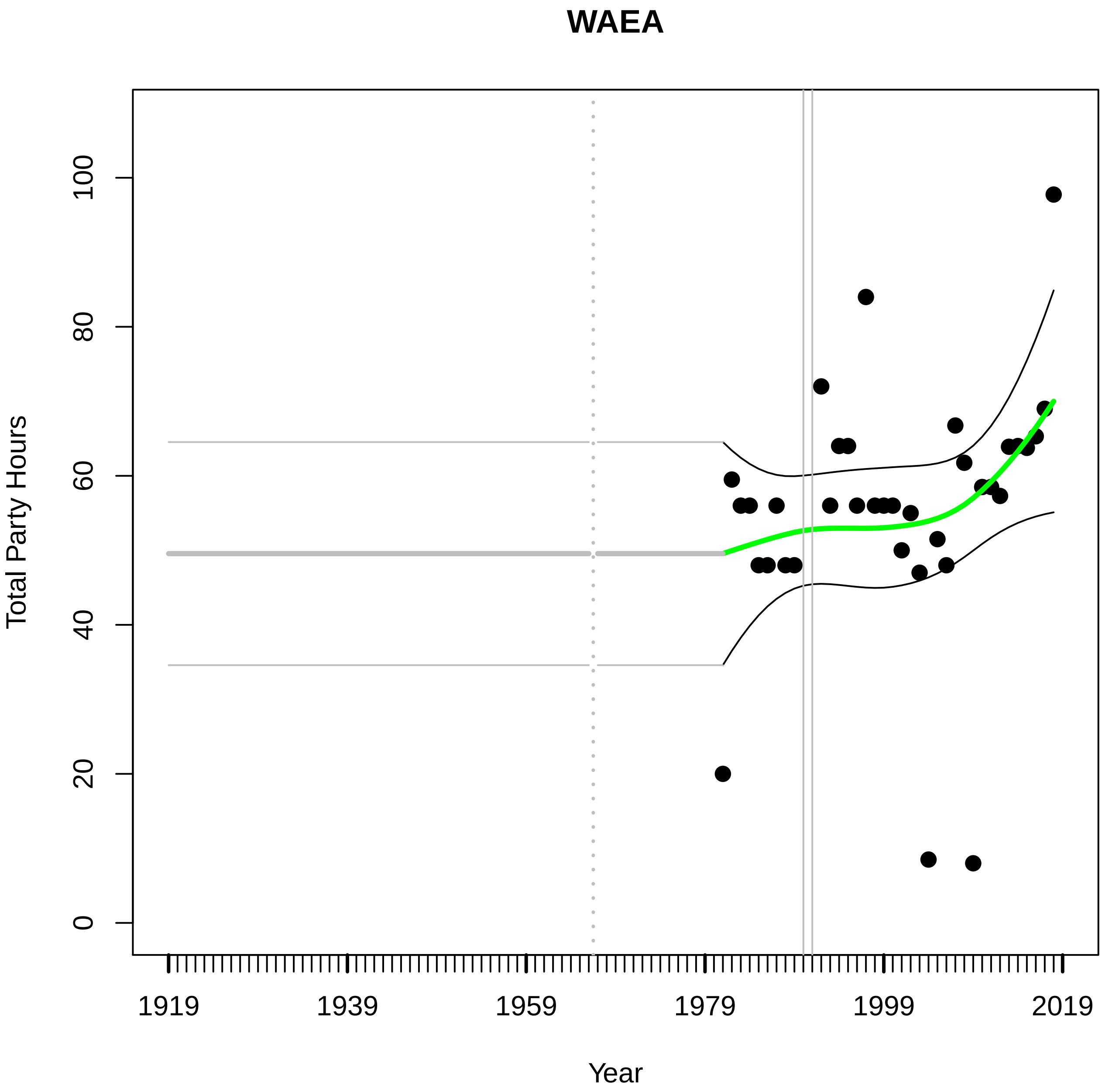

WAEA
Total Party Hours
0
20
40
60
80
100
1919
1939
1959
1979
1999
2019
Year

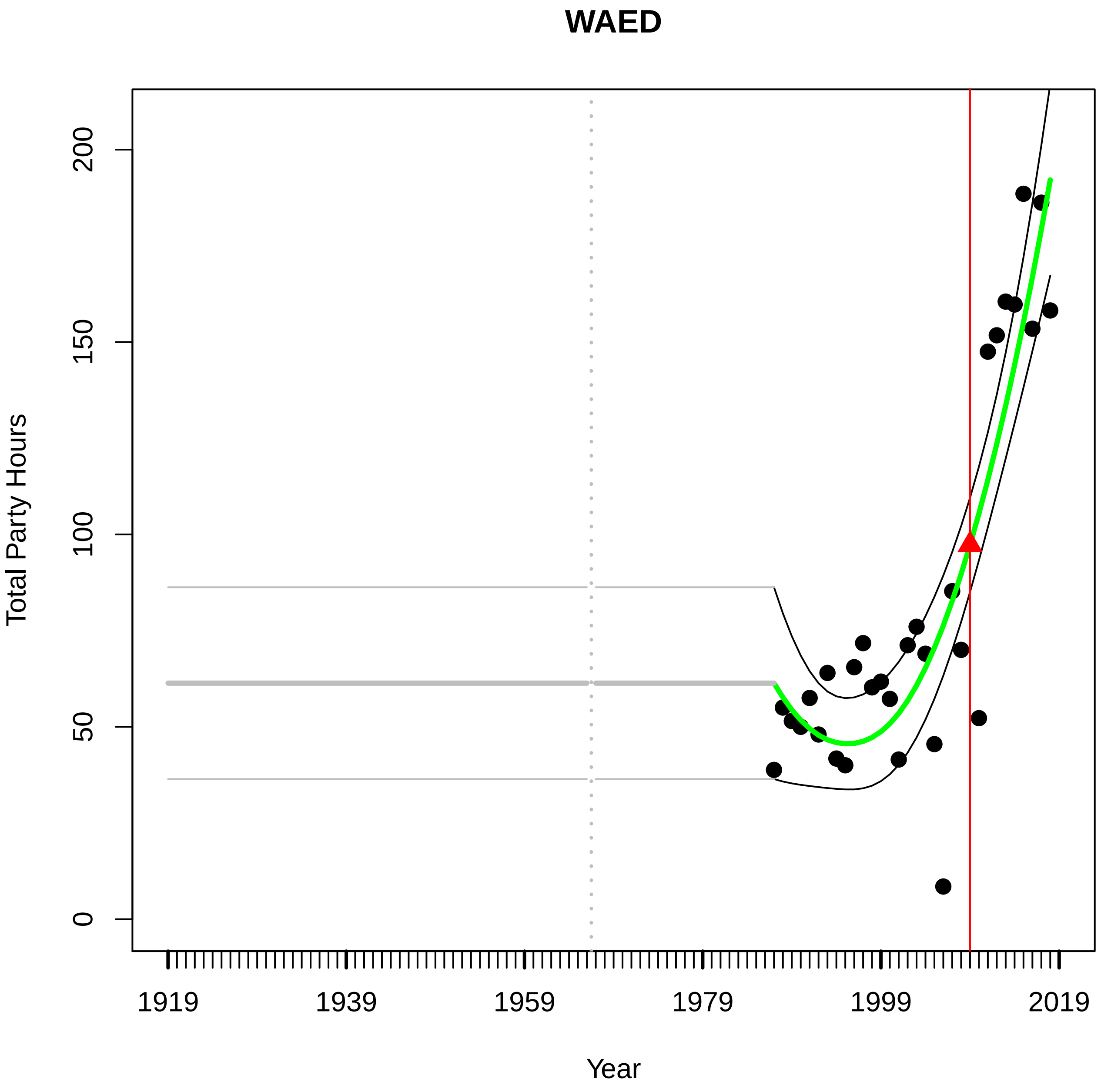

WAED
Total Party Hours
0
50
100
150
200
1919
1939
1959
1979
1999
2019
Year

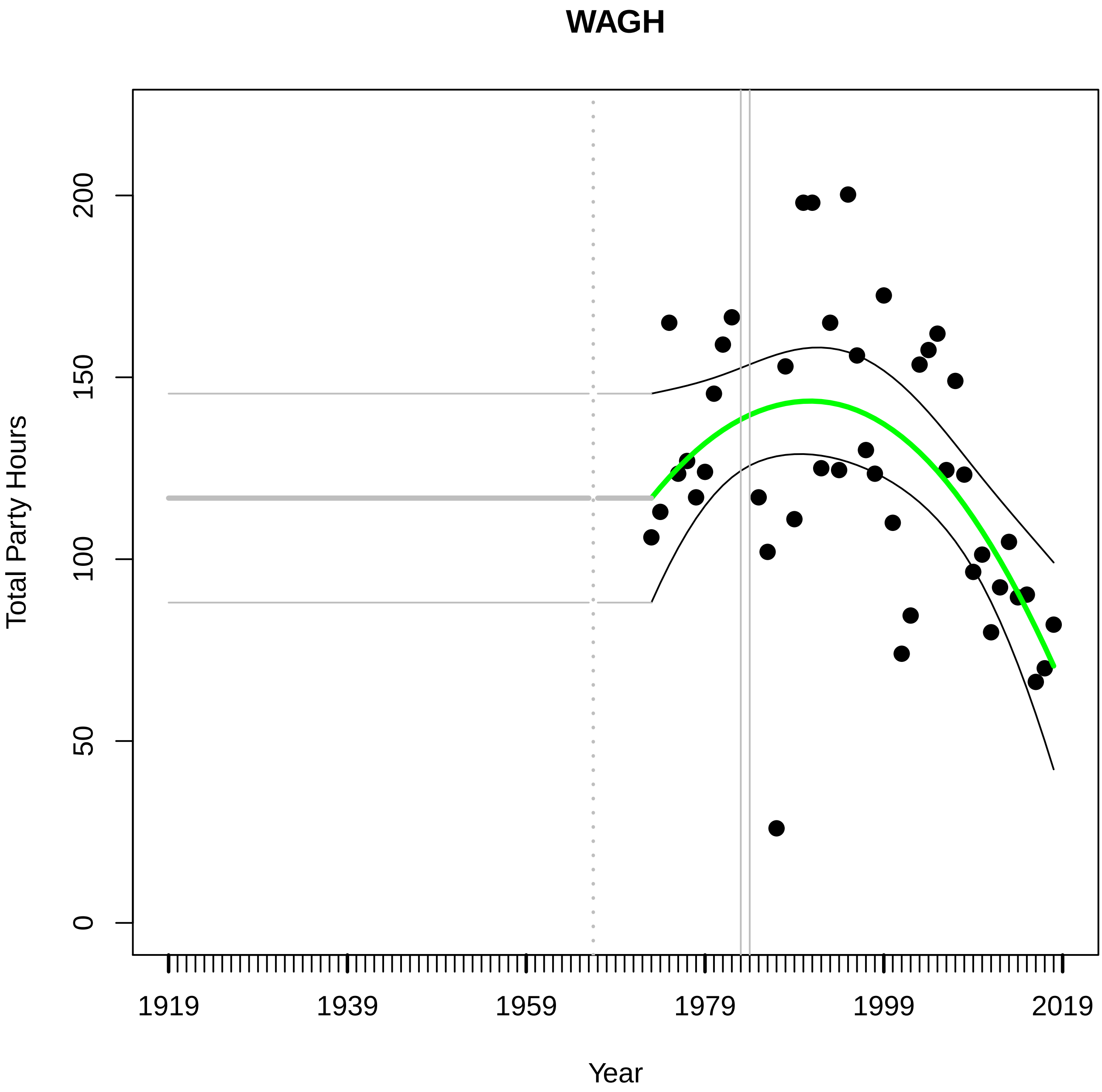
WAGH
Total Party Hours
Year
0
50
100
150
200
1919
1939
1959
1979
1999
2019

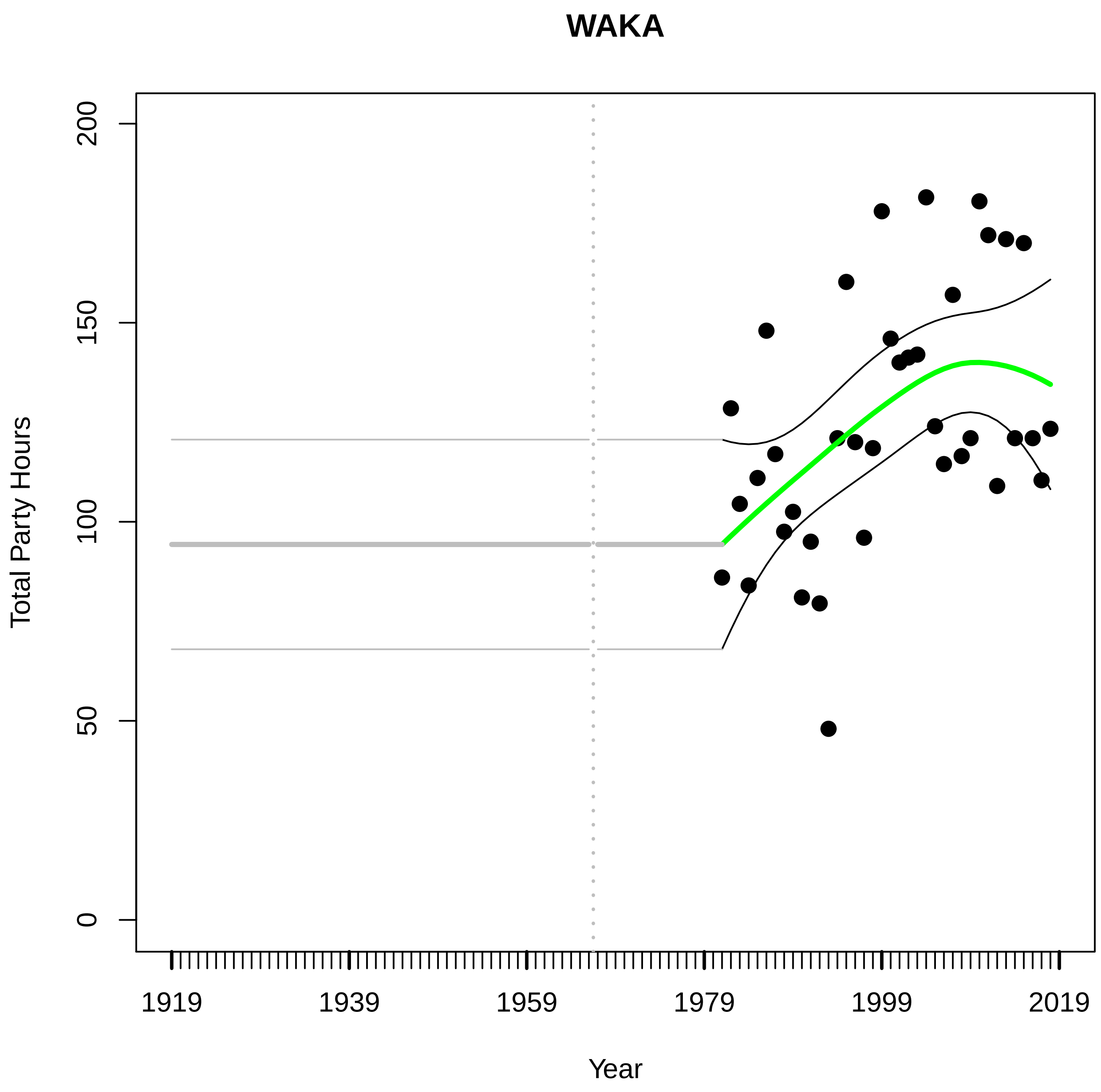
WAKA
Total Party Hours
0
50
100
150
200
1919
1939
1959
1979
1999
2019
Year

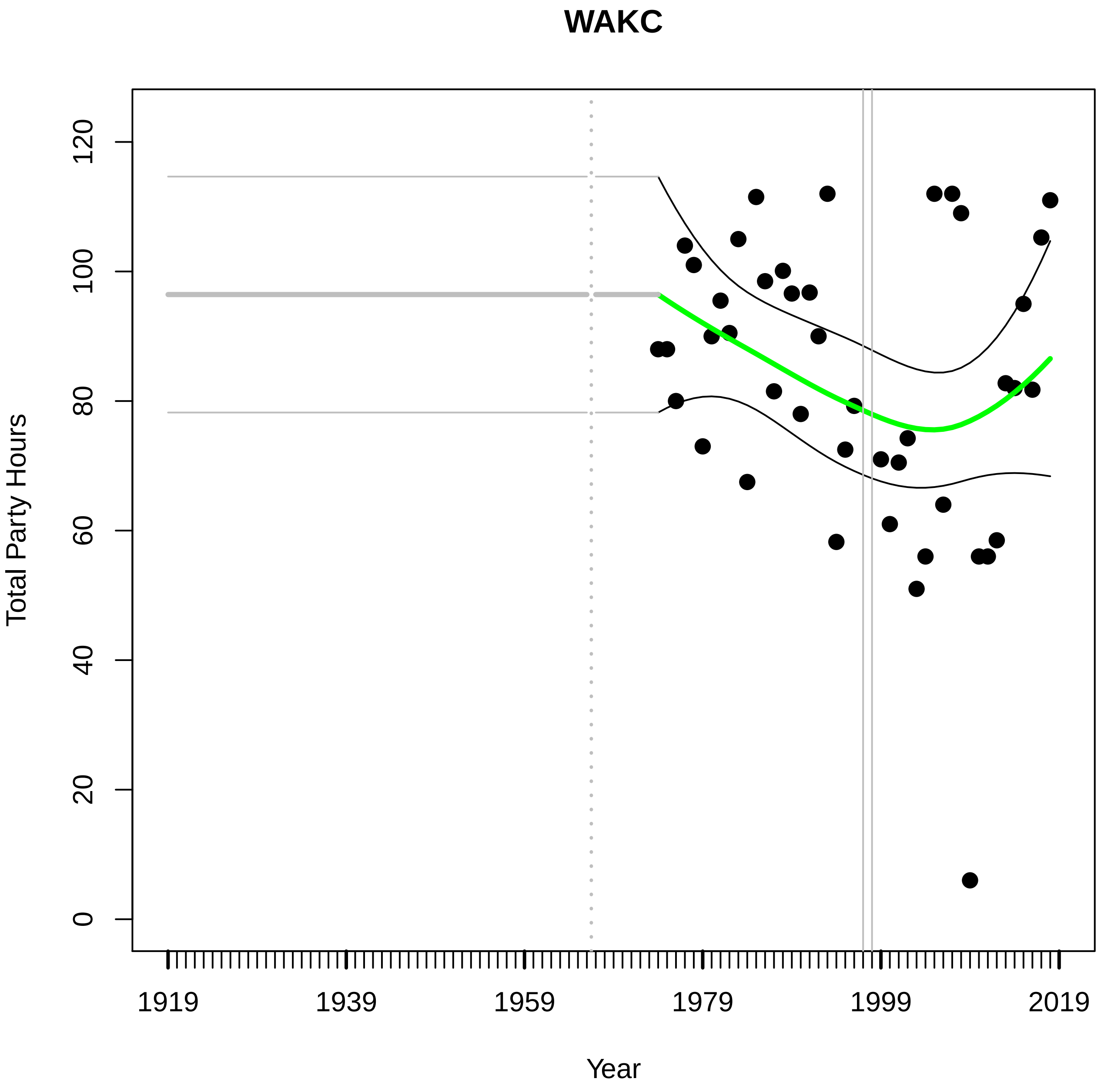

WAKC
Total Party Hours
Year
0
20
40
60
80
100
120
1919
1939
1959
1979
1999
2019

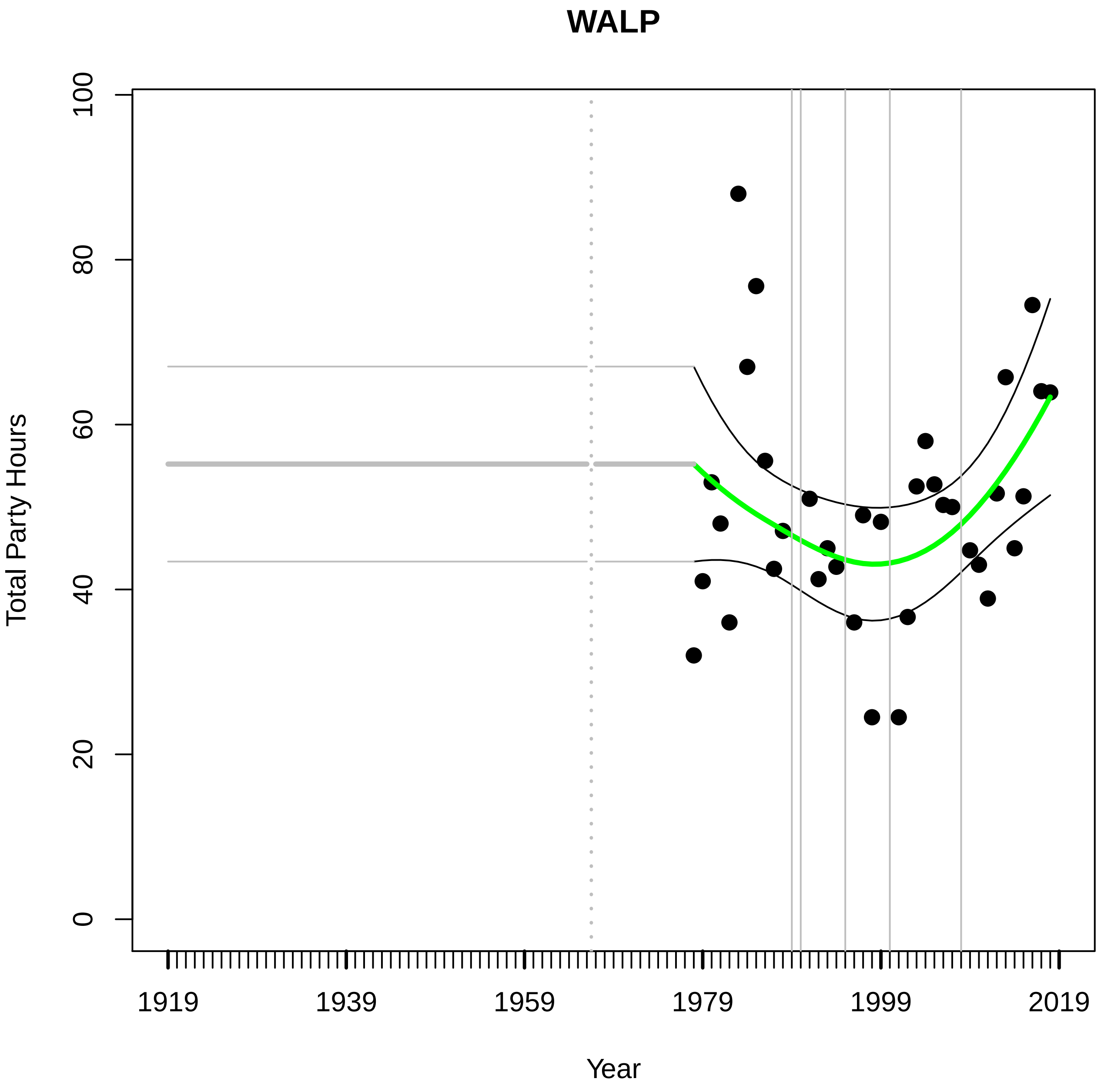

WALP
Total Party Hours
Year
0
20
40
60
80
100
1919
1939
1959
1979
1999
2019

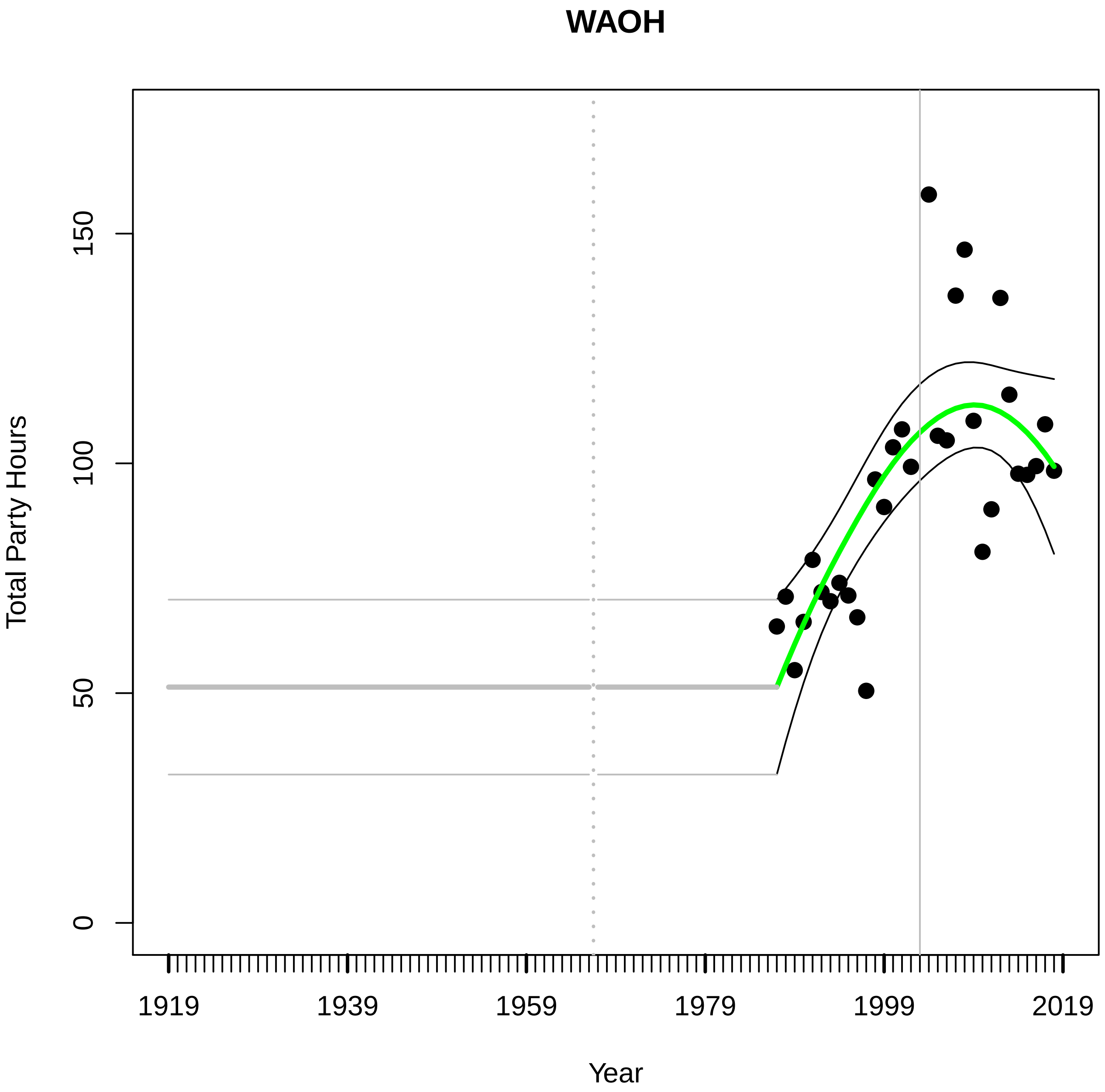
WAOH
Total Party Hours
Year
0
50
100
150
1919
1939
1959
1979
1999
2019

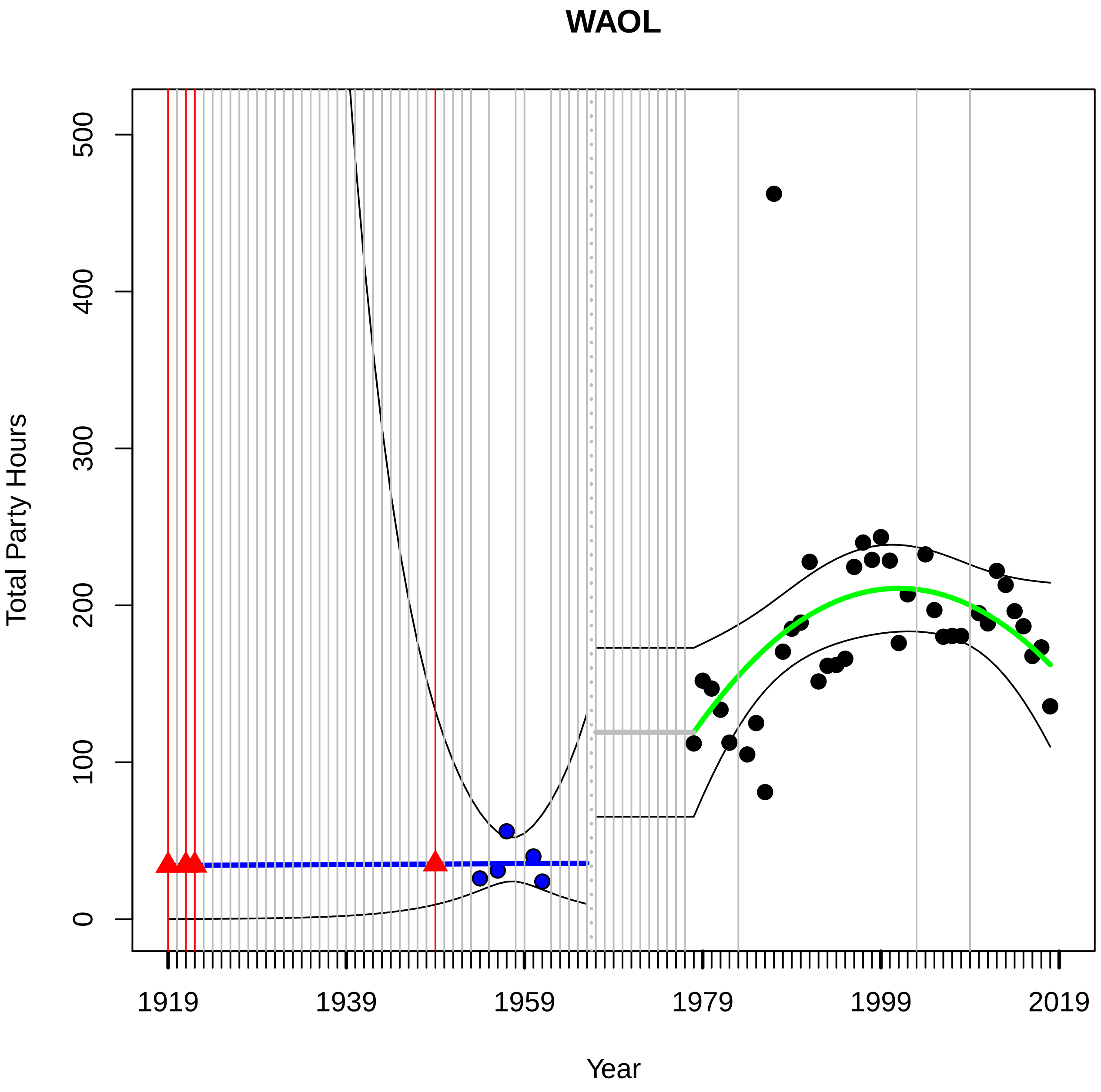
WAOL
Total Party Hours
0
100
200
300
400
500
1919
1939
1959
1979
1999
2019
Year

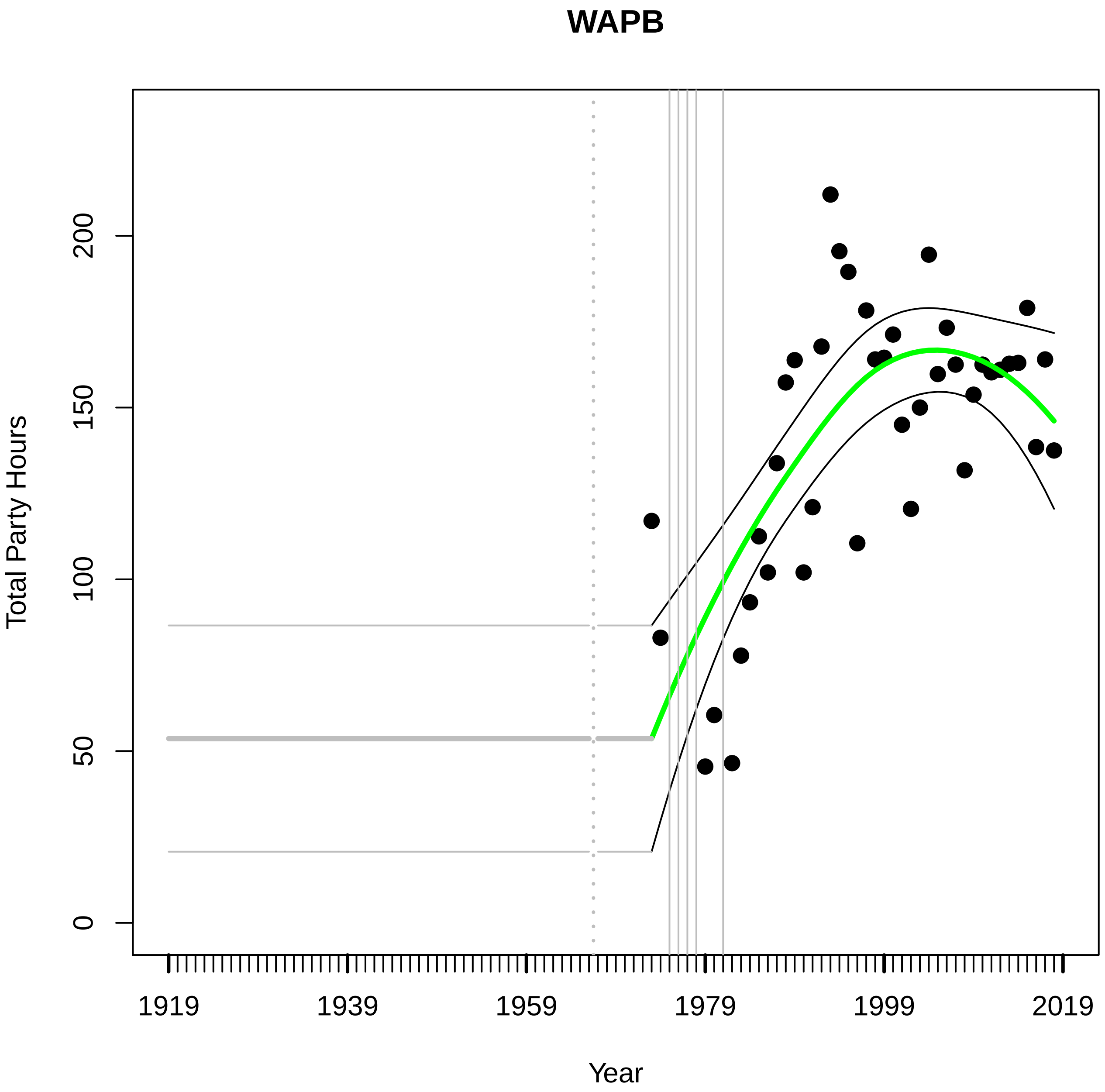
WAPB
Total Party Hours
0
50
100
150
200
1919
1939
1959
1979
1999
2019
Year

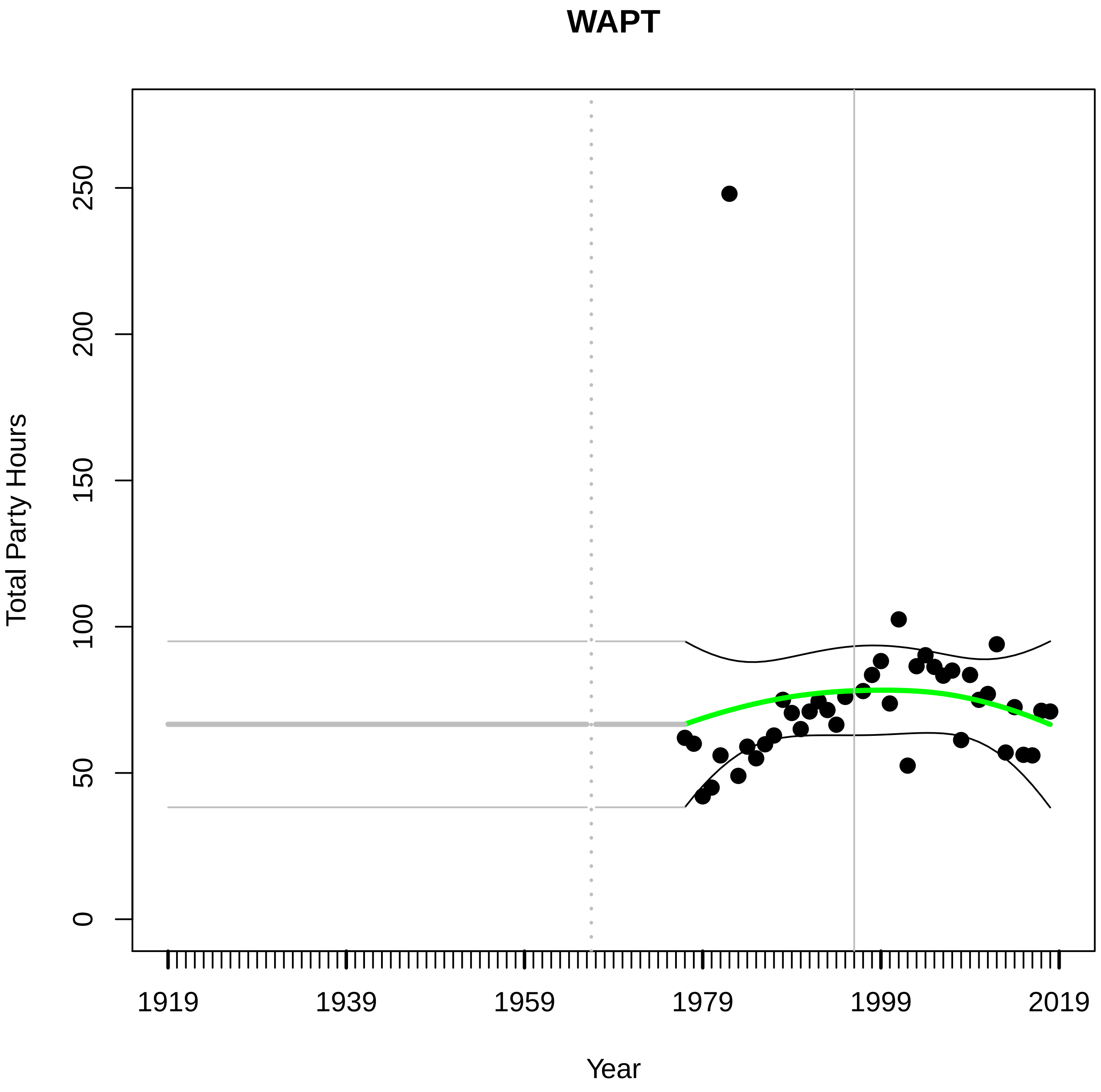
WAPT
Total Party Hours
0
50
100
150
200
250
1919
1939
1959
1979
1999
2019
Year

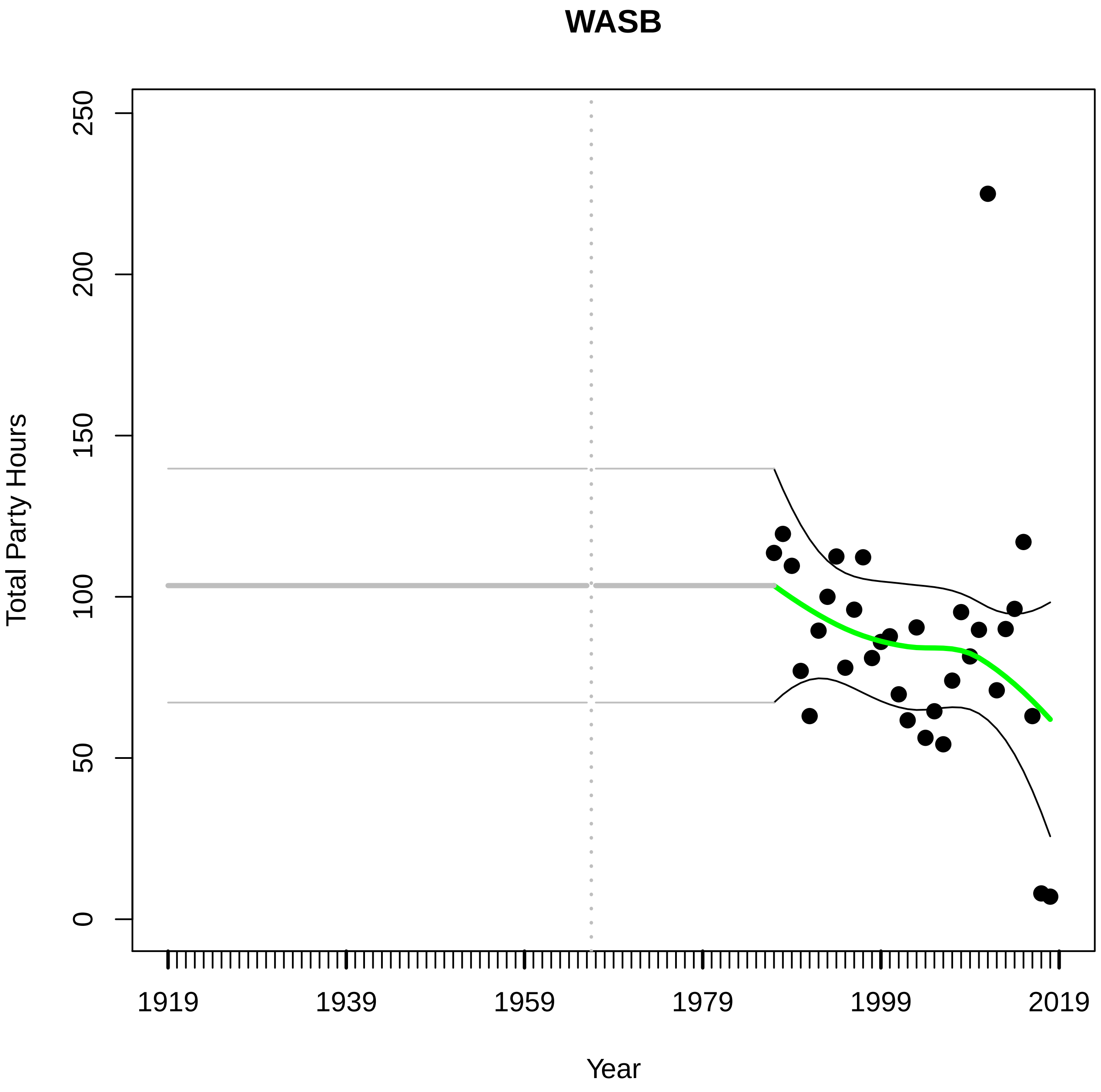

WASB
Total Party Hours
0
50
100
150
200
250
1919
1939
1959
1979
1999
2019
Year

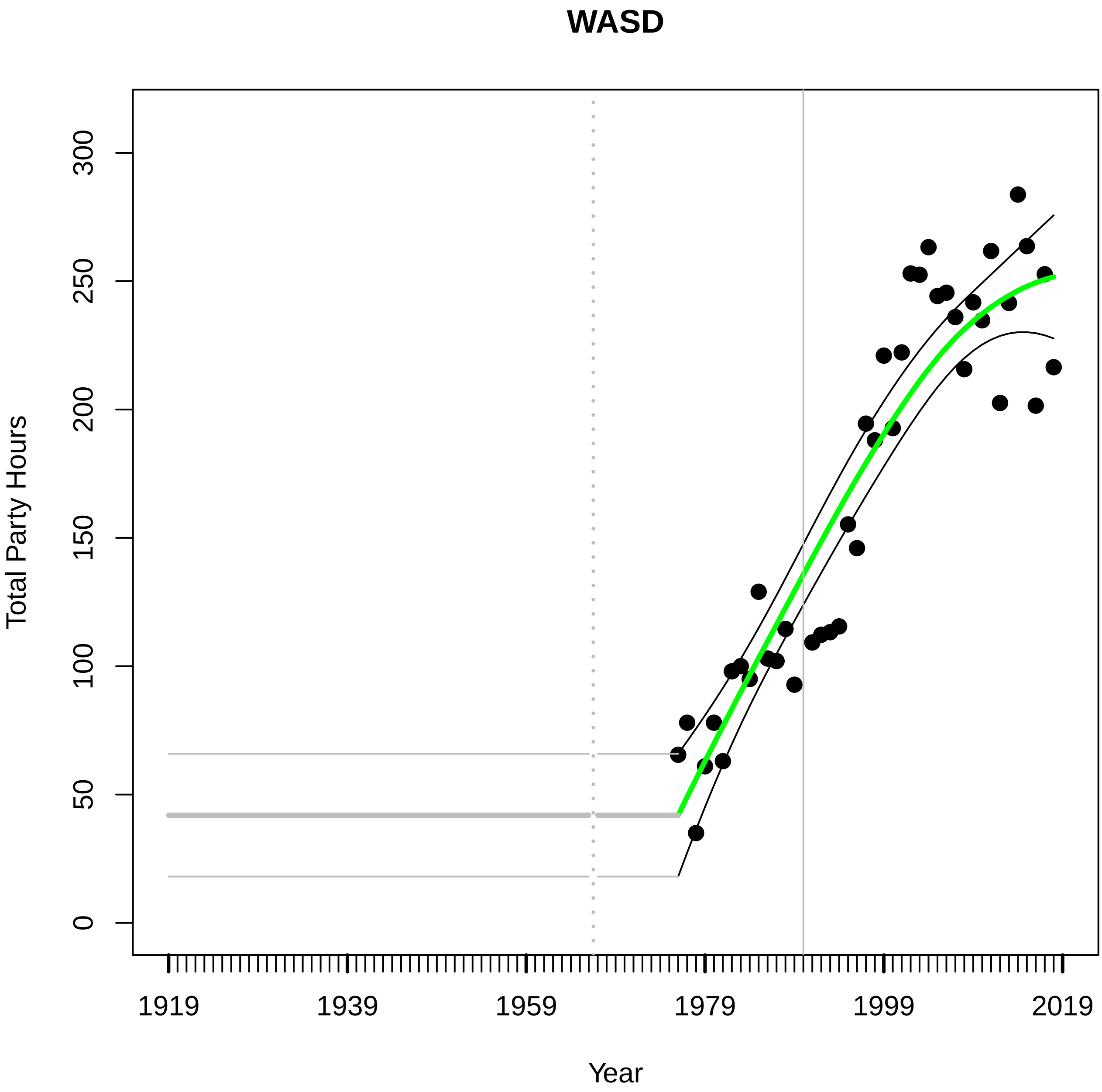

WASD
Total Party Hours
0
50
100
150
200
250
300
1919
1939
1959
1979
1999
2019
Year

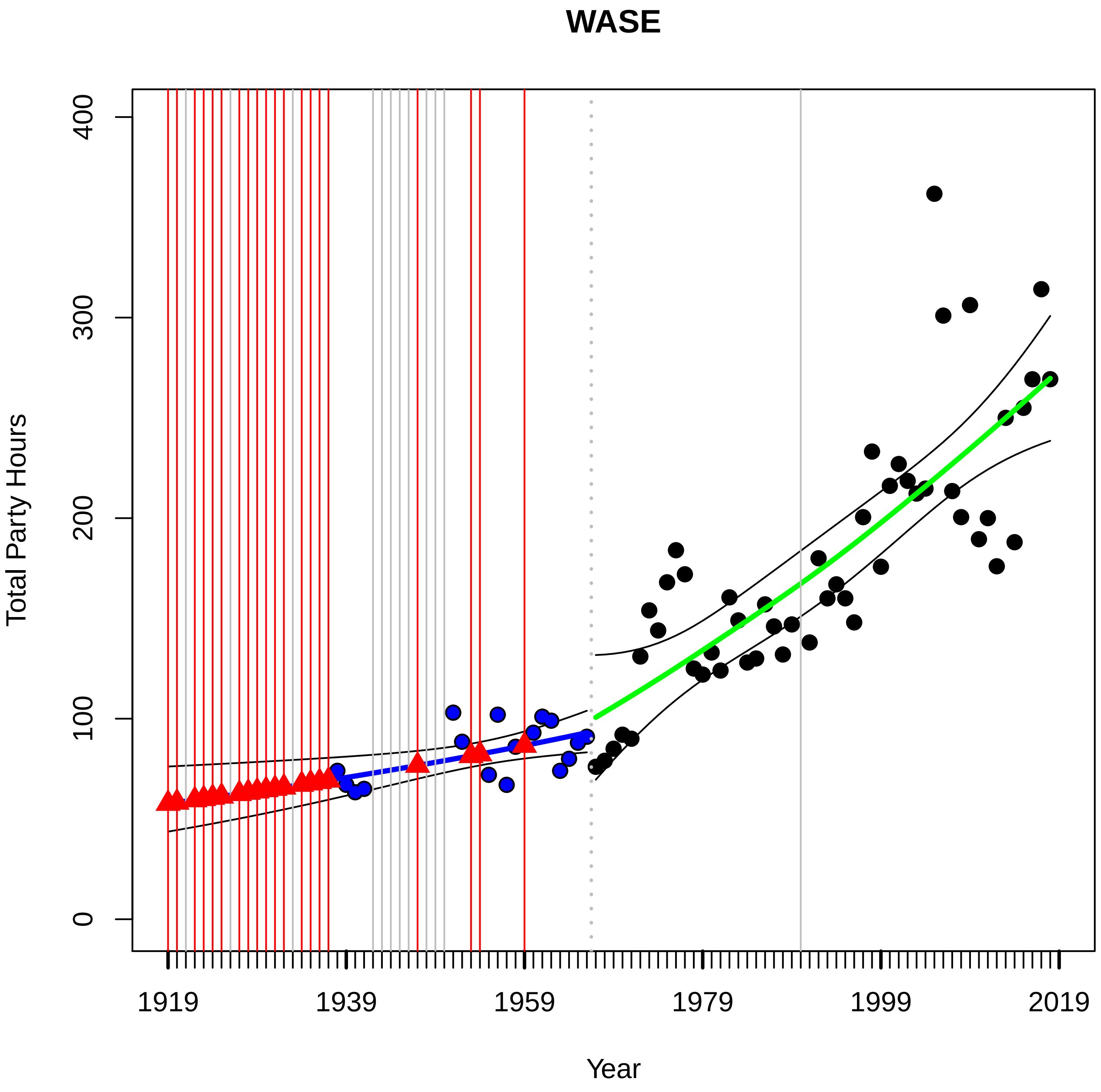
WASE
Total Party Hours
Year
0
100
200
300
400
1919
1939
1959
1979
1999
2019

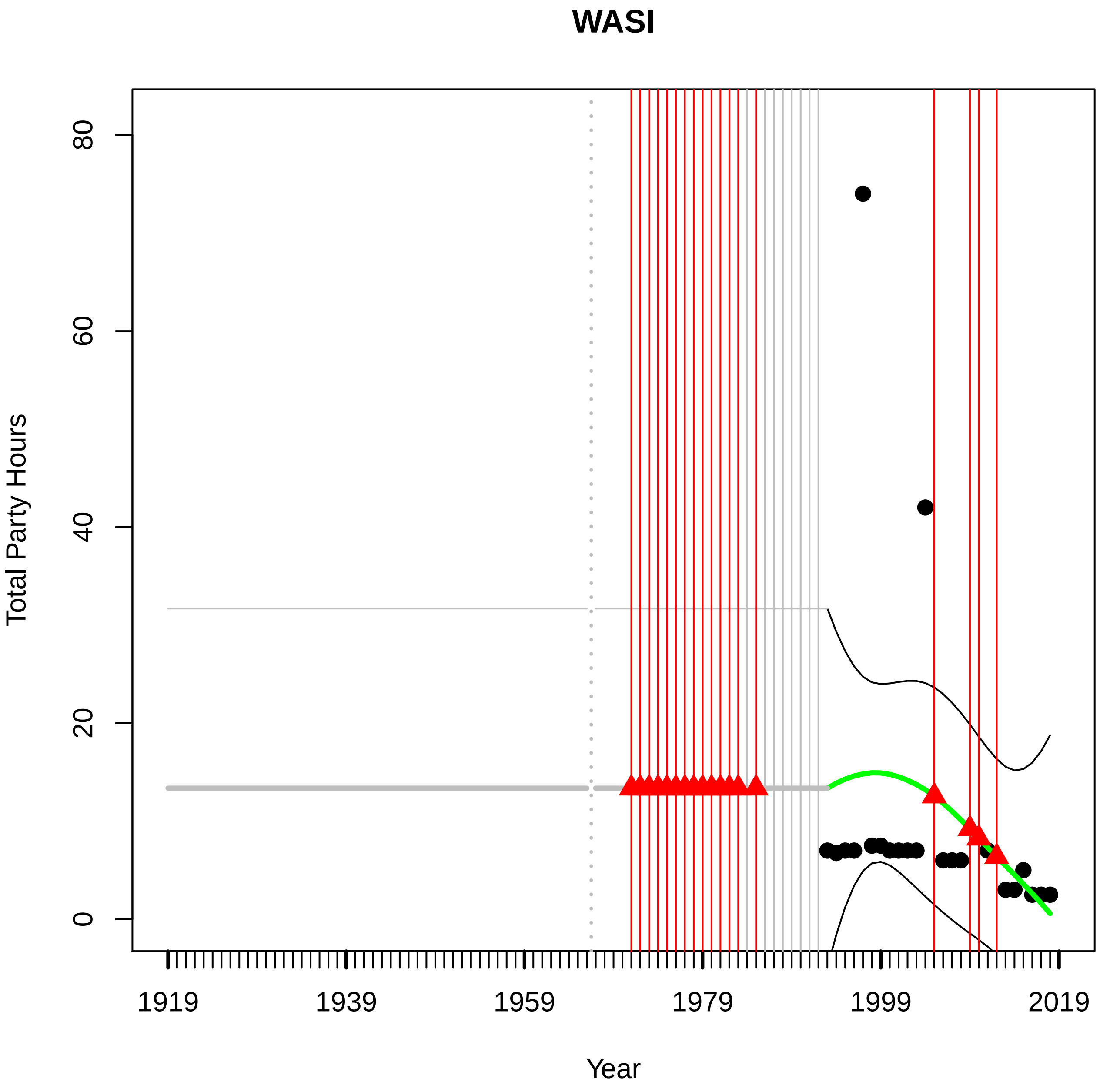

WASI
Total Party Hours
0
20
40
60
80
1919
1939
1959
1979
1999
2019
Year

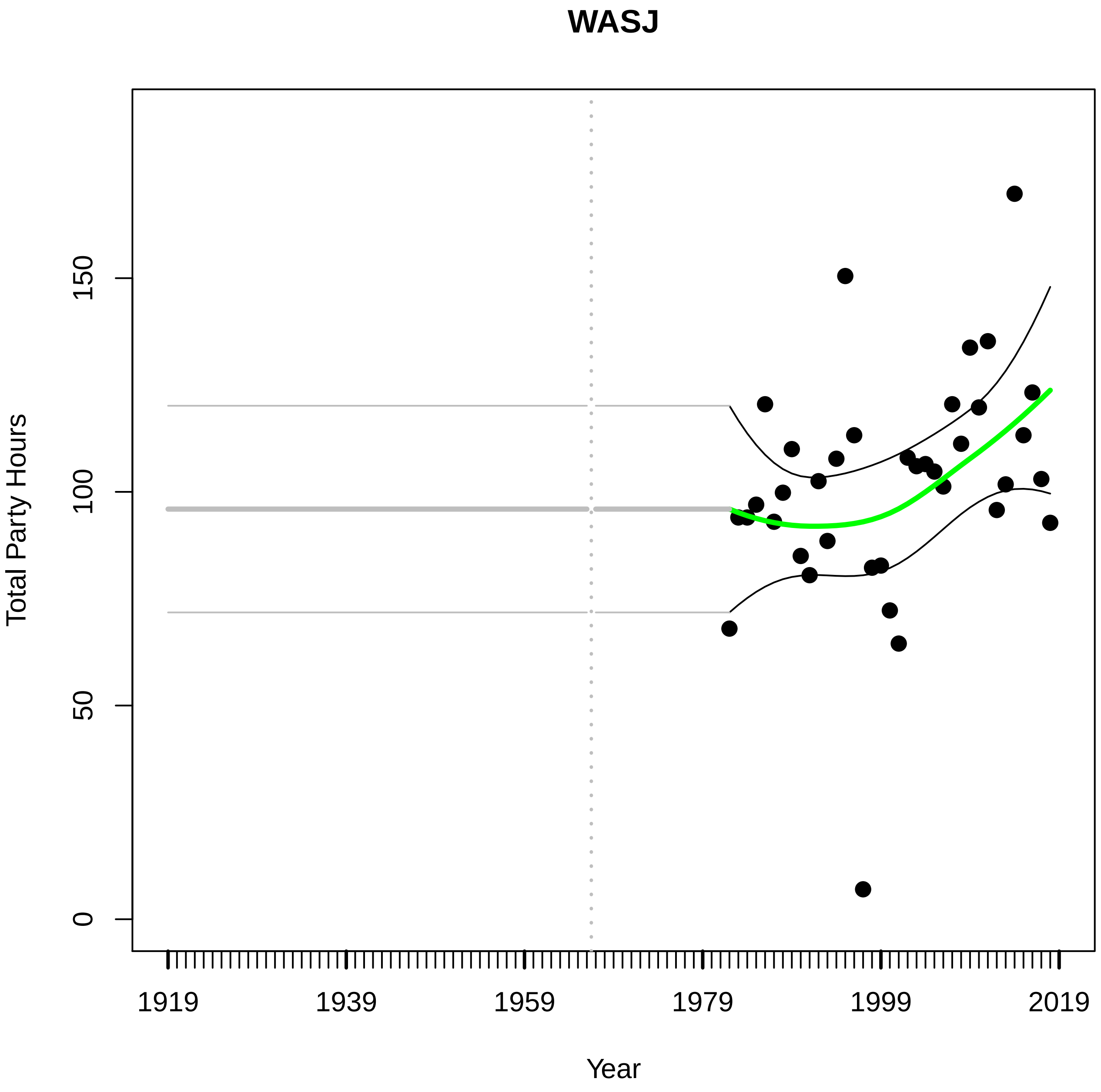

WASJ
Total Party Hours
0
50
100
150
1919
1939
1959
1979
1999
2019
Year

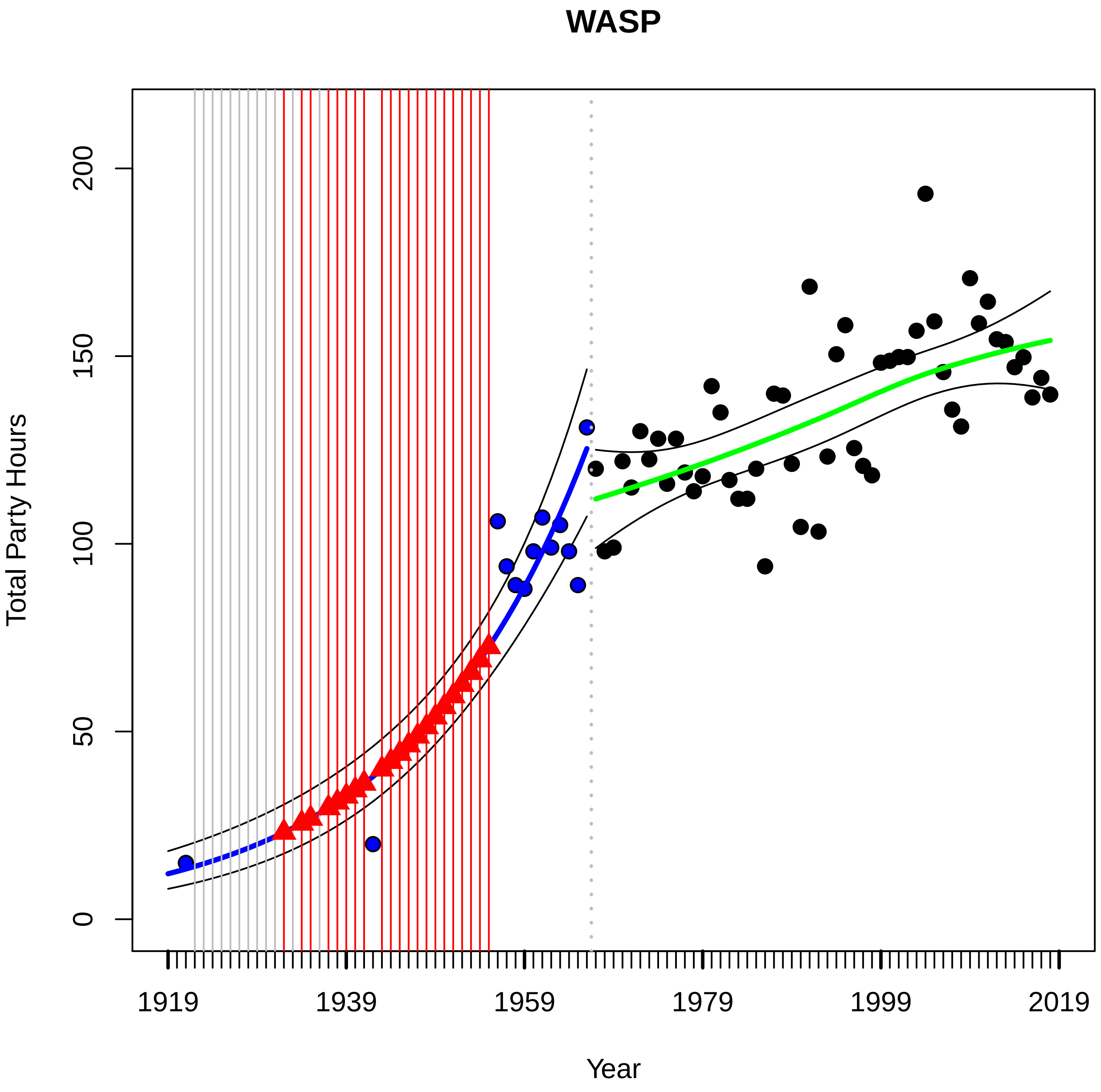

WASP
Total Party Hours
Year
0
50
100
150
200
1919
1939
1959
1979
1999
2019

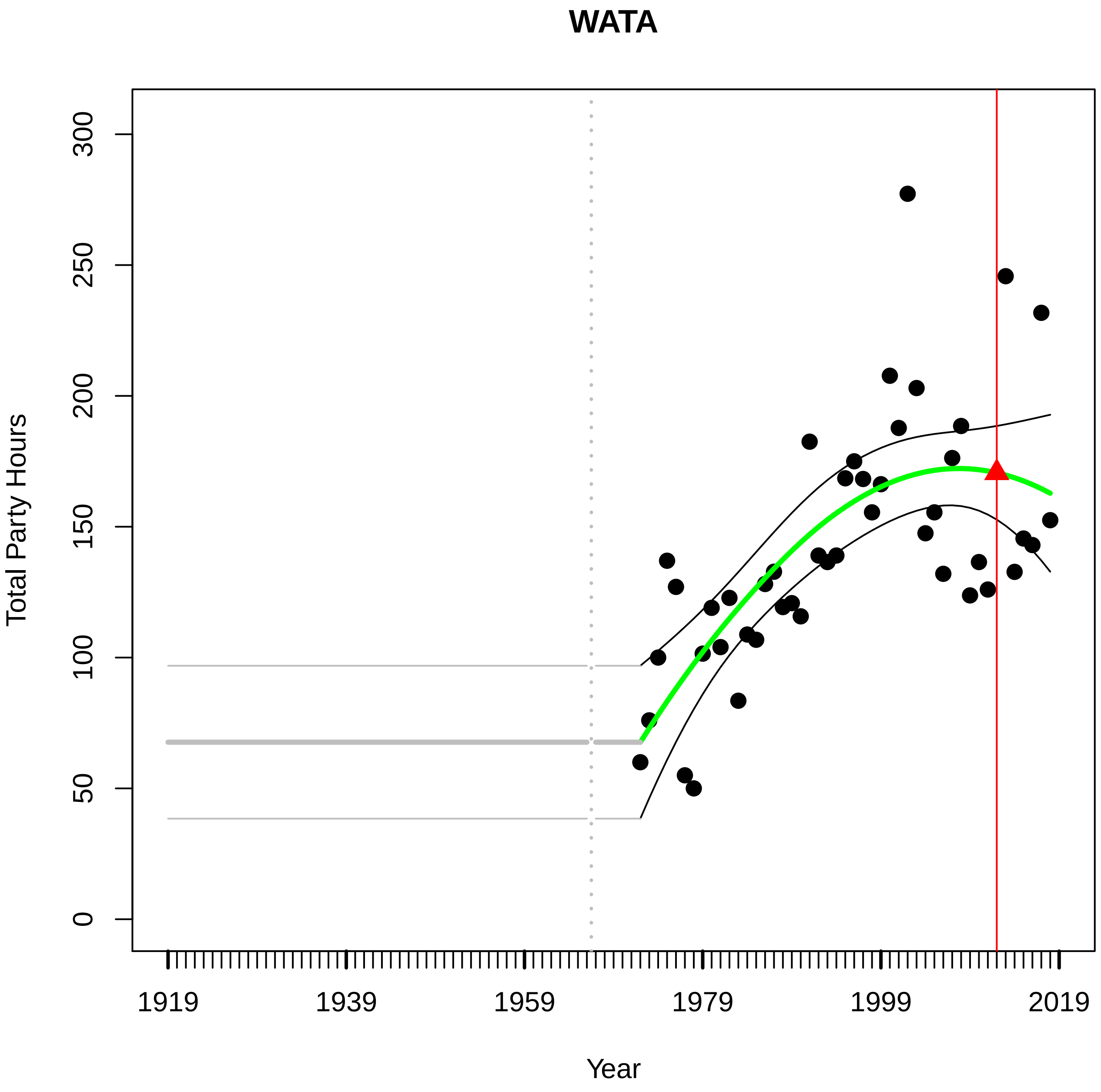
WATA
Total Party Hours
0
50
100
150
200
250
300
1919
1939
1959
1979
1999
2019
Year

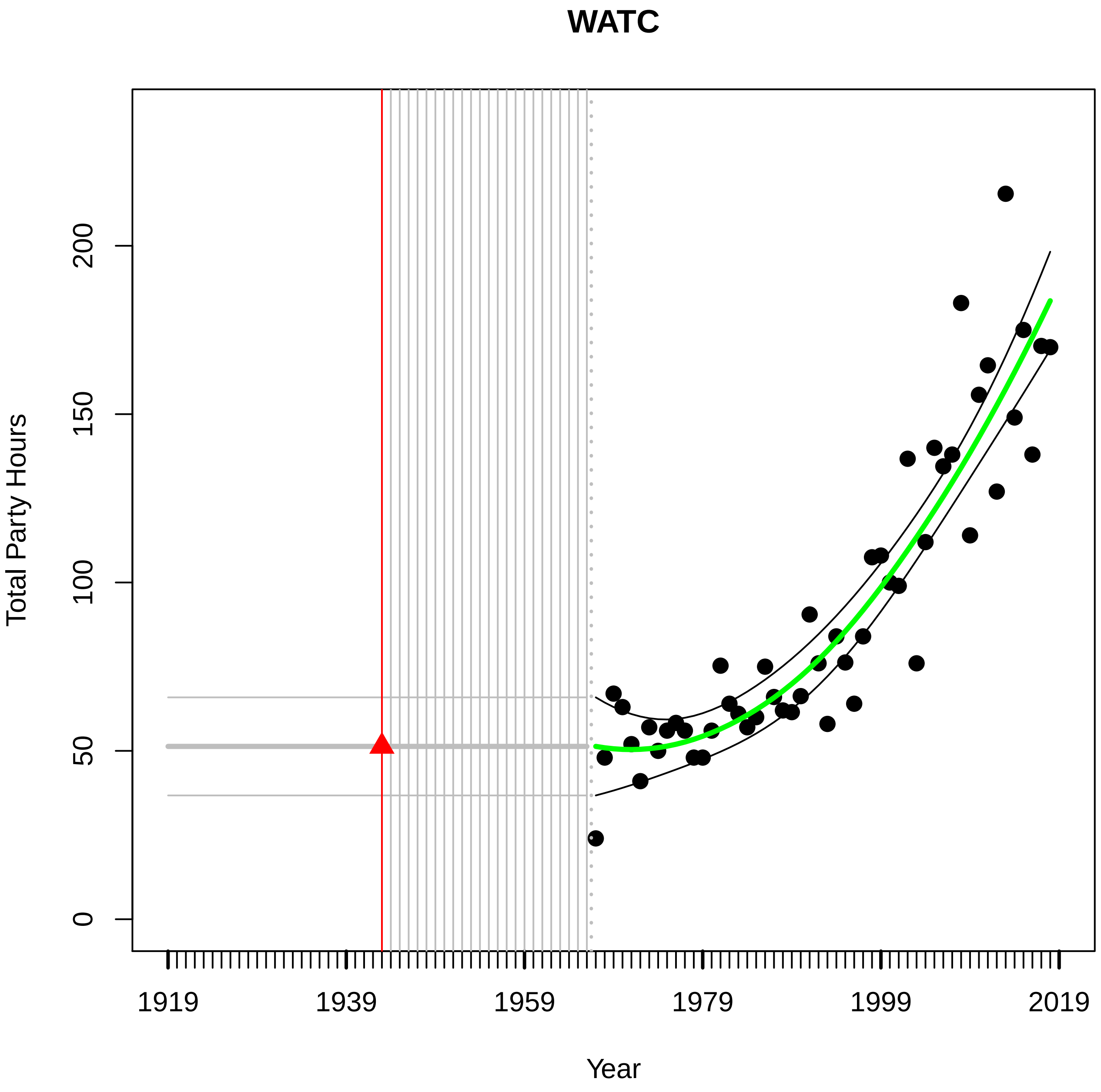

WATC
Total Party Hours
Year
0
50
100
150
200
1919
1939
1959
1979
1999
2019

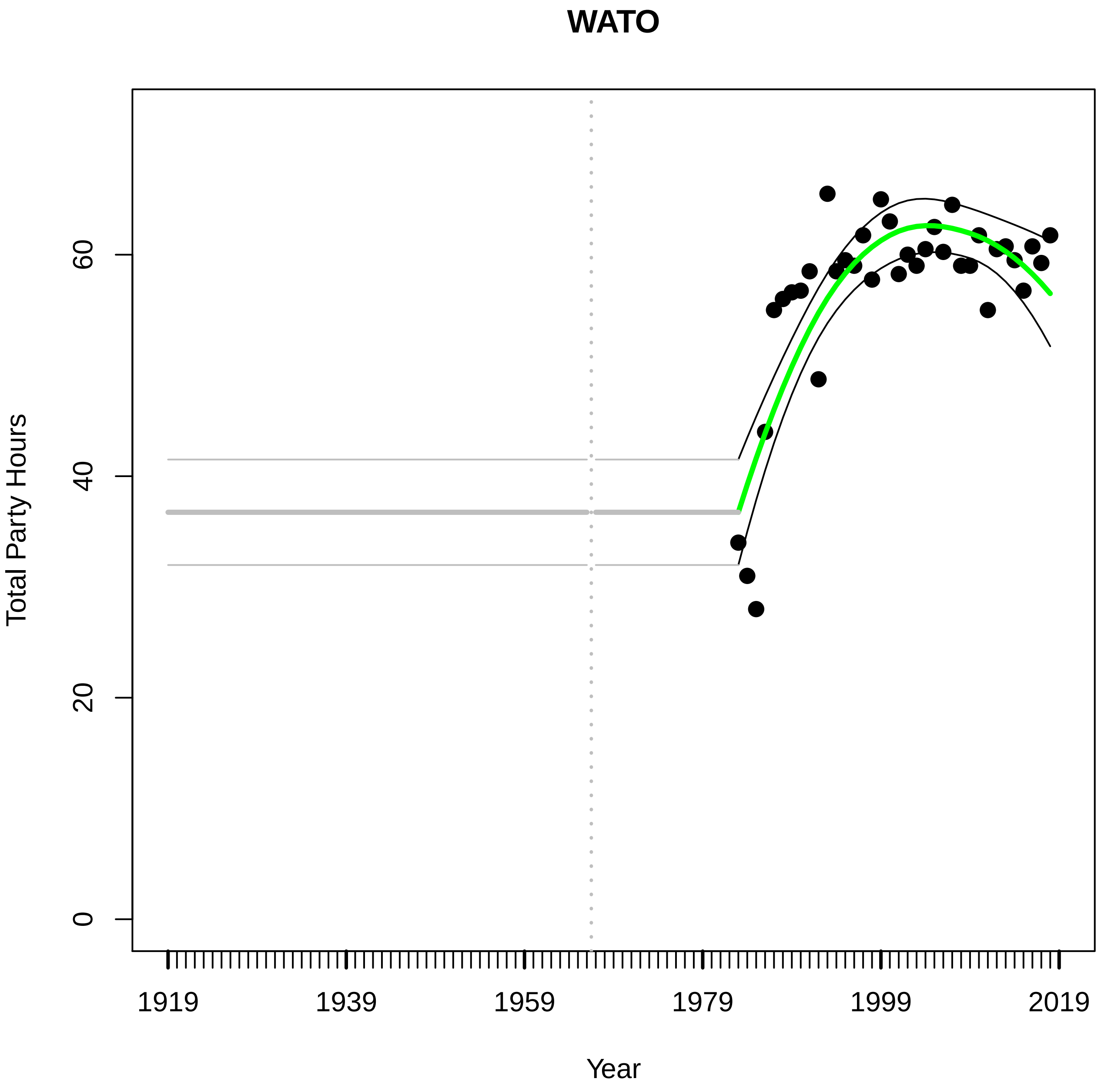

WATO
Total Party Hours
0
20
40
60
1919
1939
1959
1979
1999
2019
Year

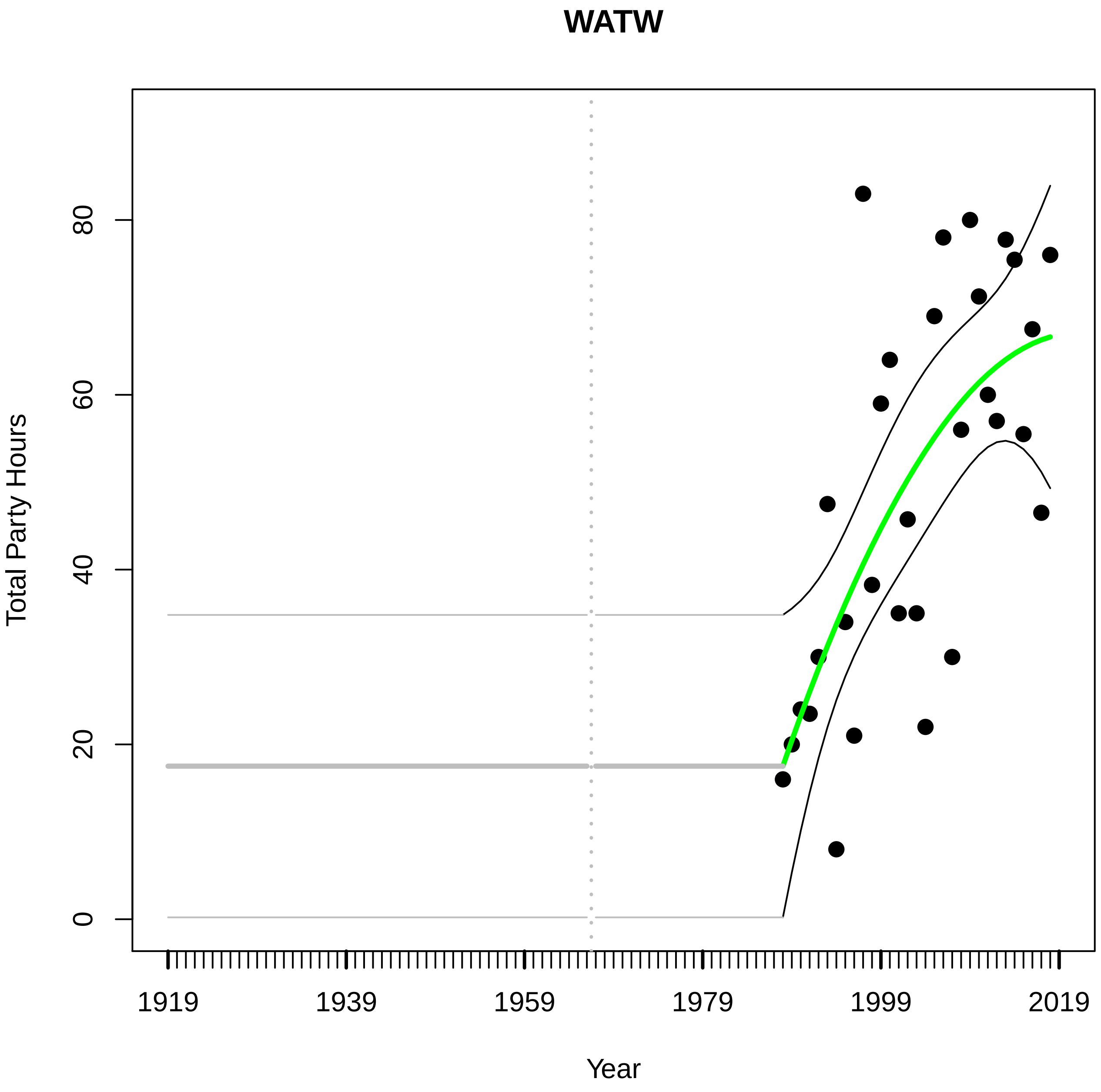
WATW
Total Party Hours
0
20
40
60
80
1919
1939
1959
1979
1999
2019
Year

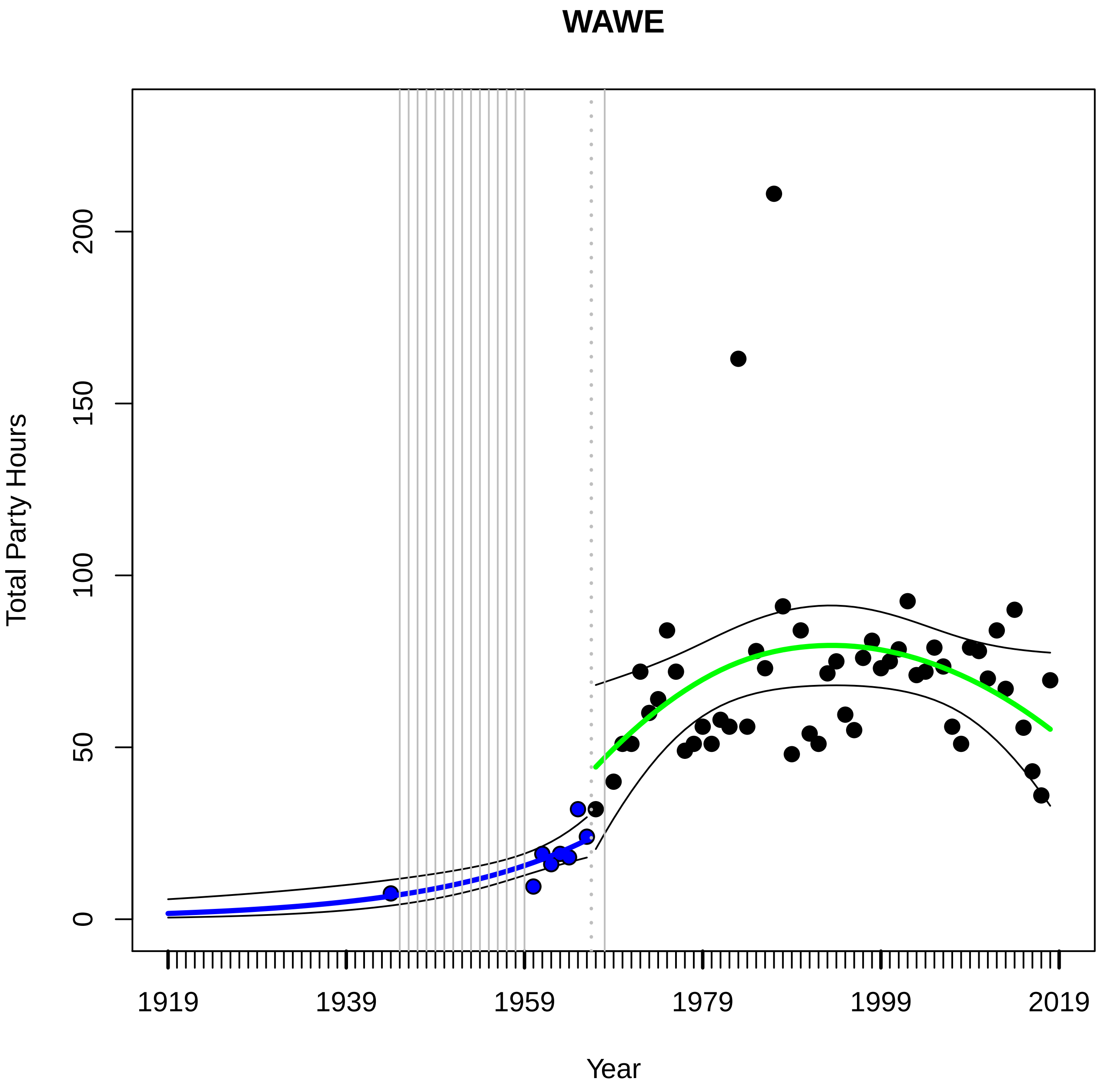

WAWE
Total Party Hours
0
50
100
150
200
Year
1919
1939
1959
1979
1999
2019

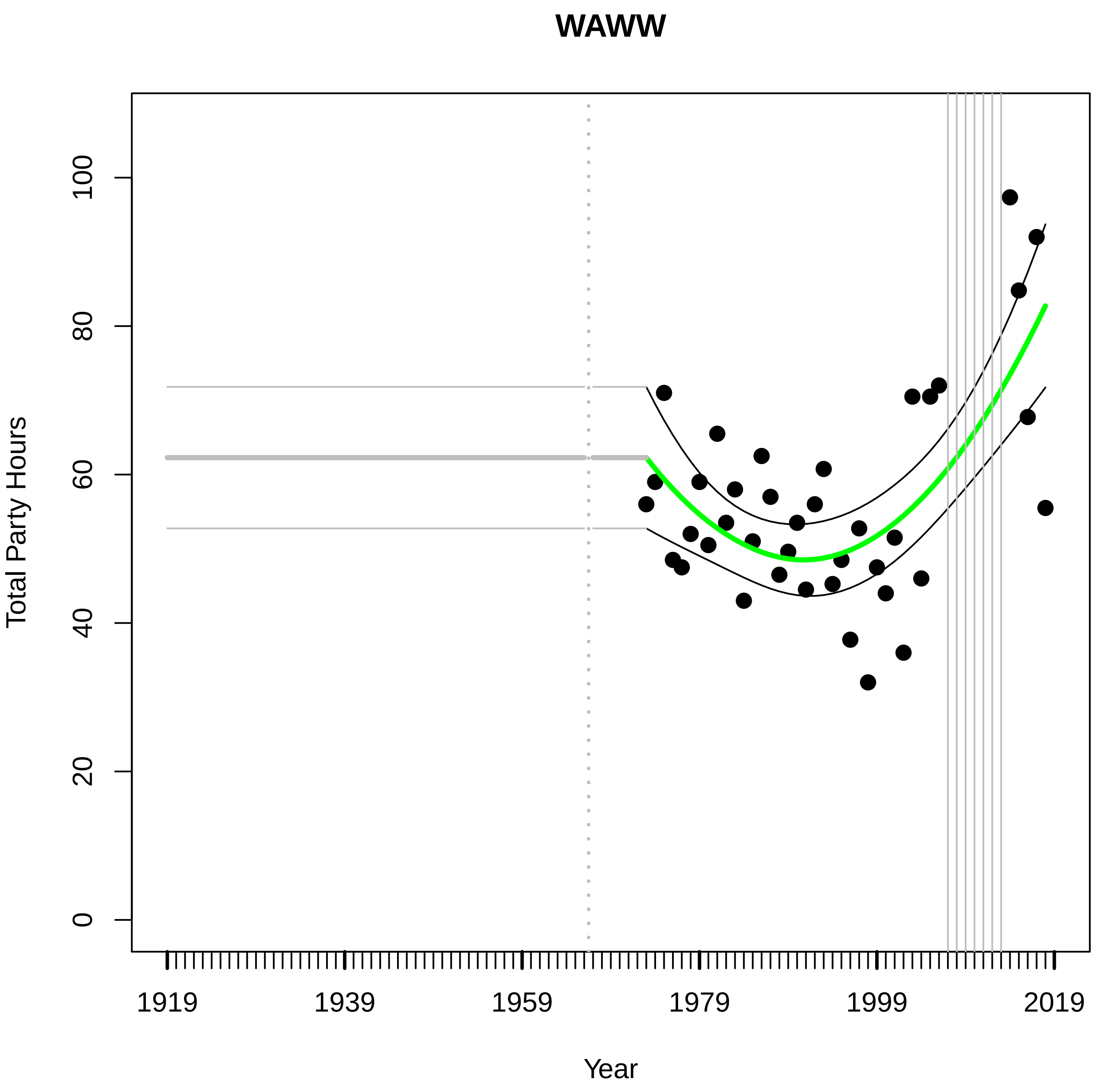

WAWW
Total Party Hours
0
20
40
60
80
100
1919
1939
1959
1979
1999
2019
Year

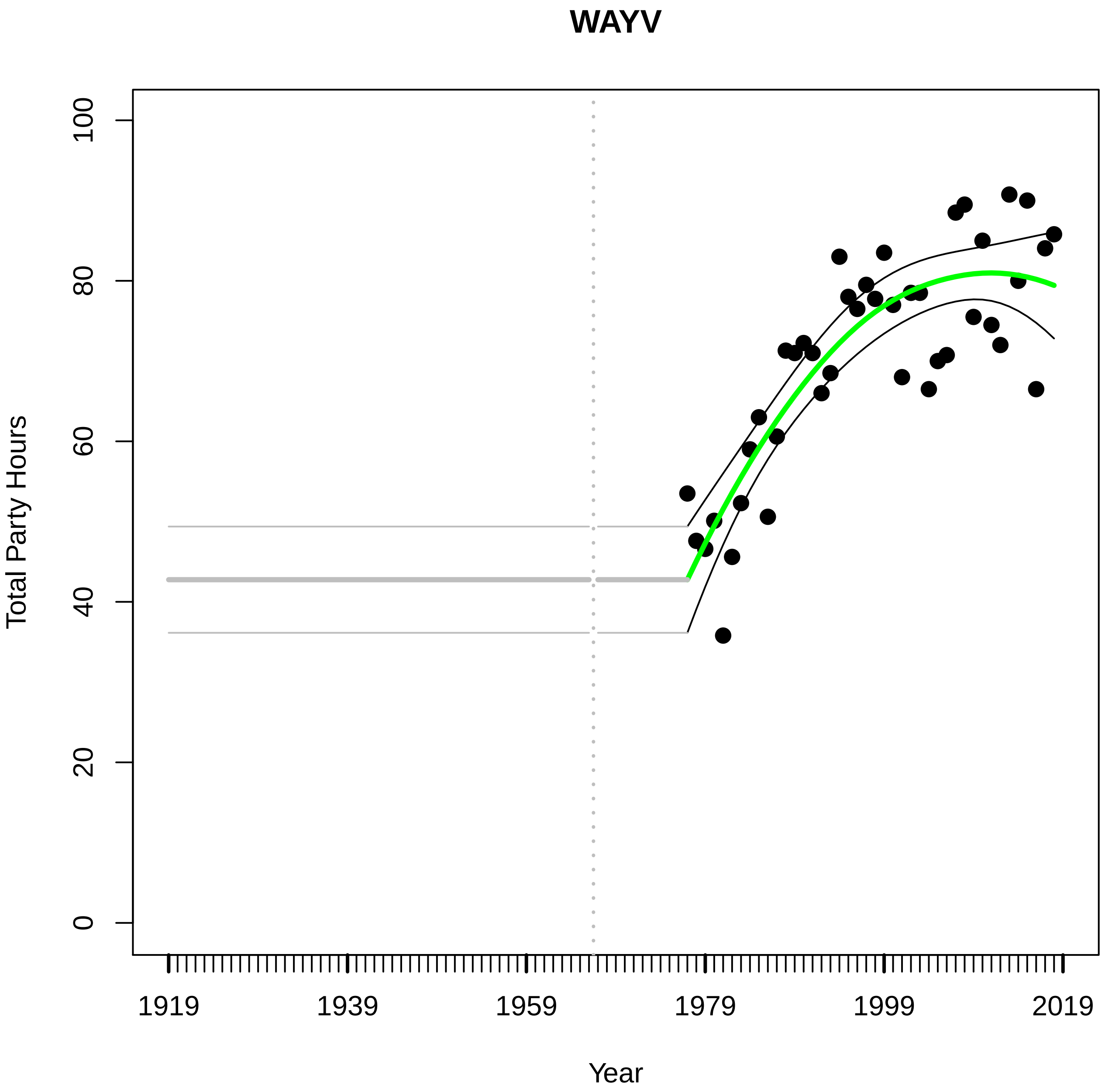

WAYV
Total Party Hours
0
20
40
60
80
100
1919
1939
1959
1979
1999
2019
Year

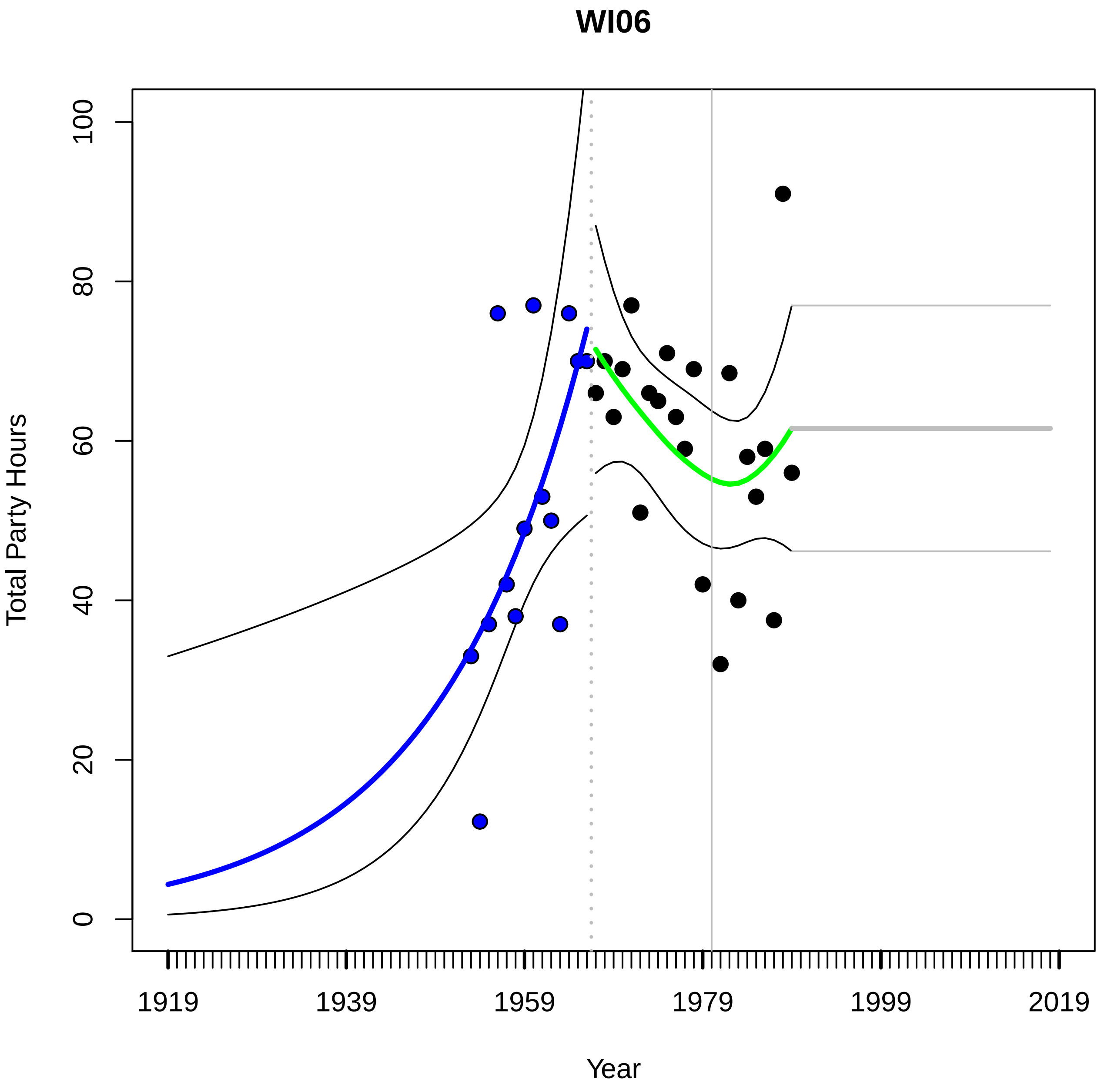
WI06
Total Party Hours
Year
0
20
40
60
80
100
1919
1939
1959
1979
1999
2019

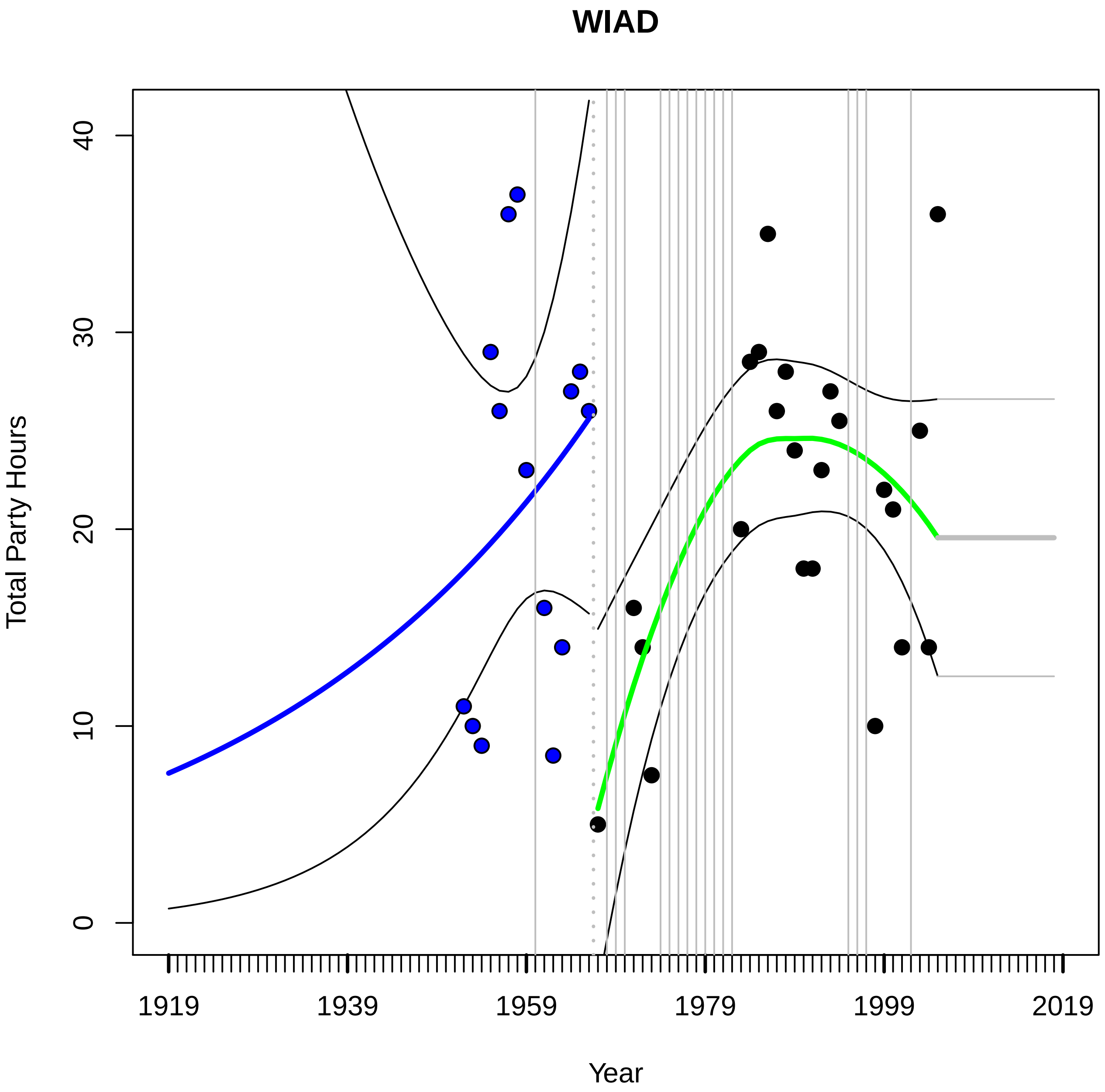

WIAD
Total Party Hours
40
30
20
10
0
1919
1939
1959
1979
1999
2019
Year

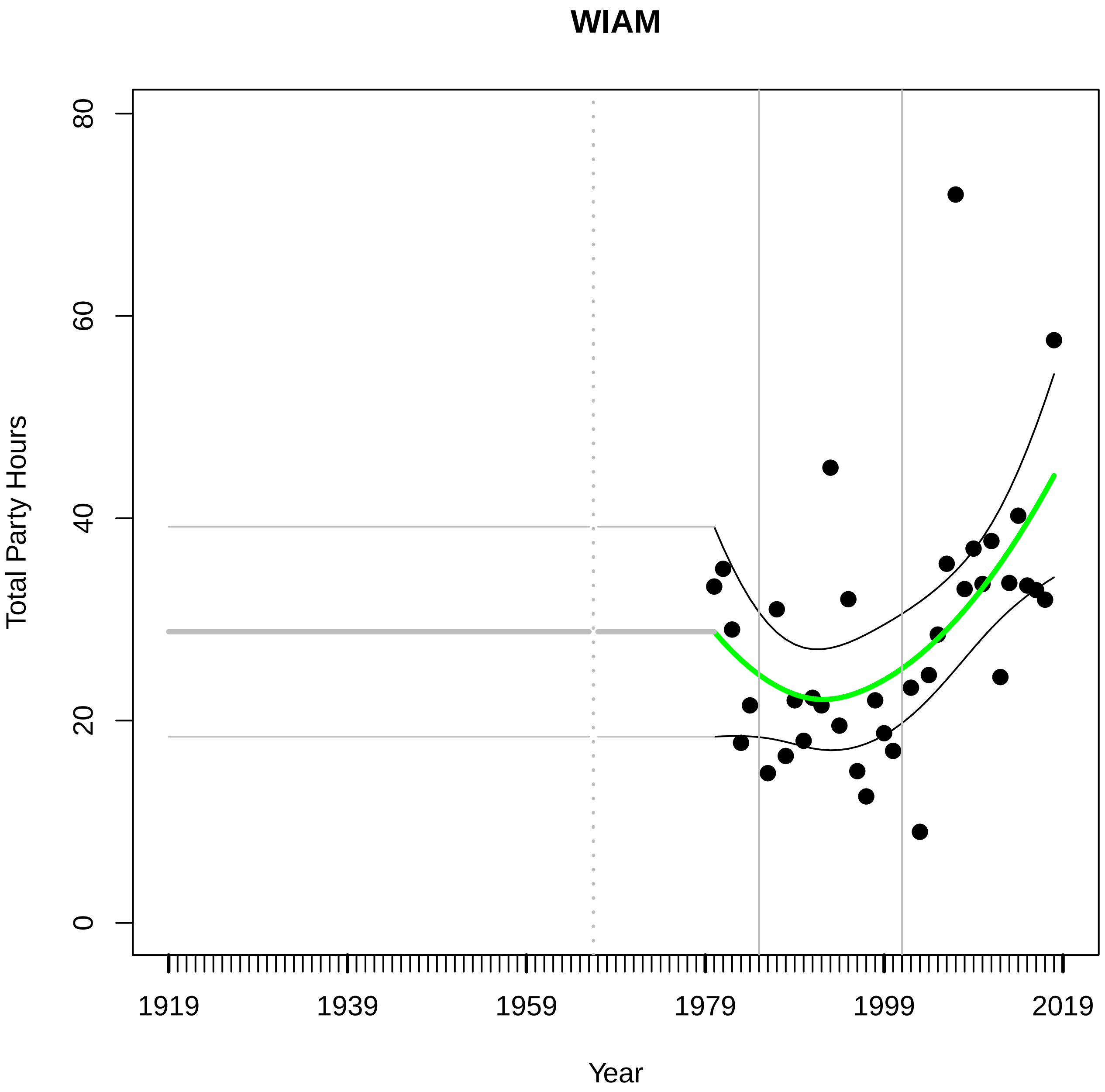

WIAM
Total Party Hours
Year
0
20
40
60
80
1919
1939
1959
1979
1999
2019

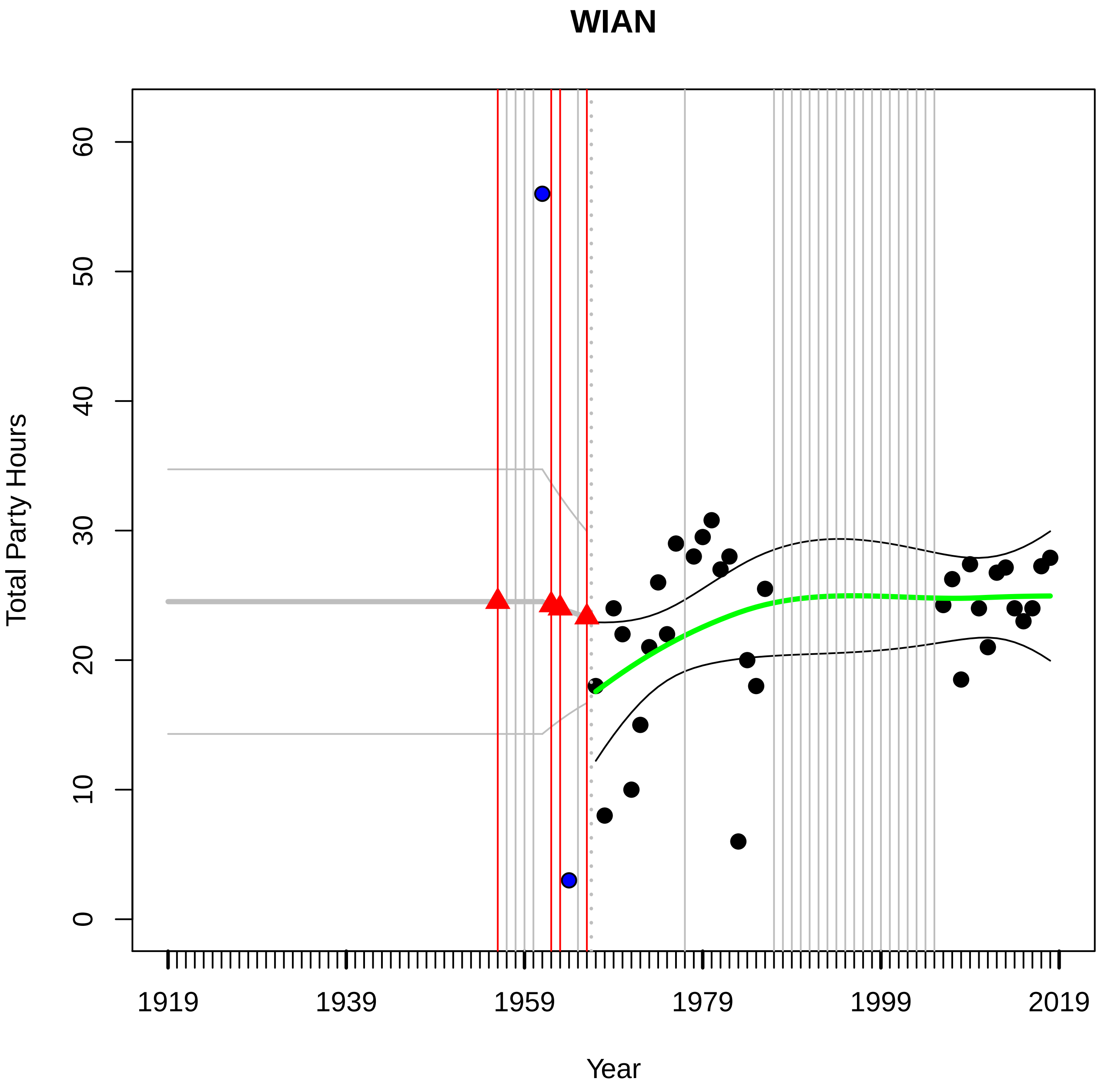

WIAN
Total Party Hours
0
10
20
30
40
50
60
1919
1939
1959
1979
1999
2019
Year

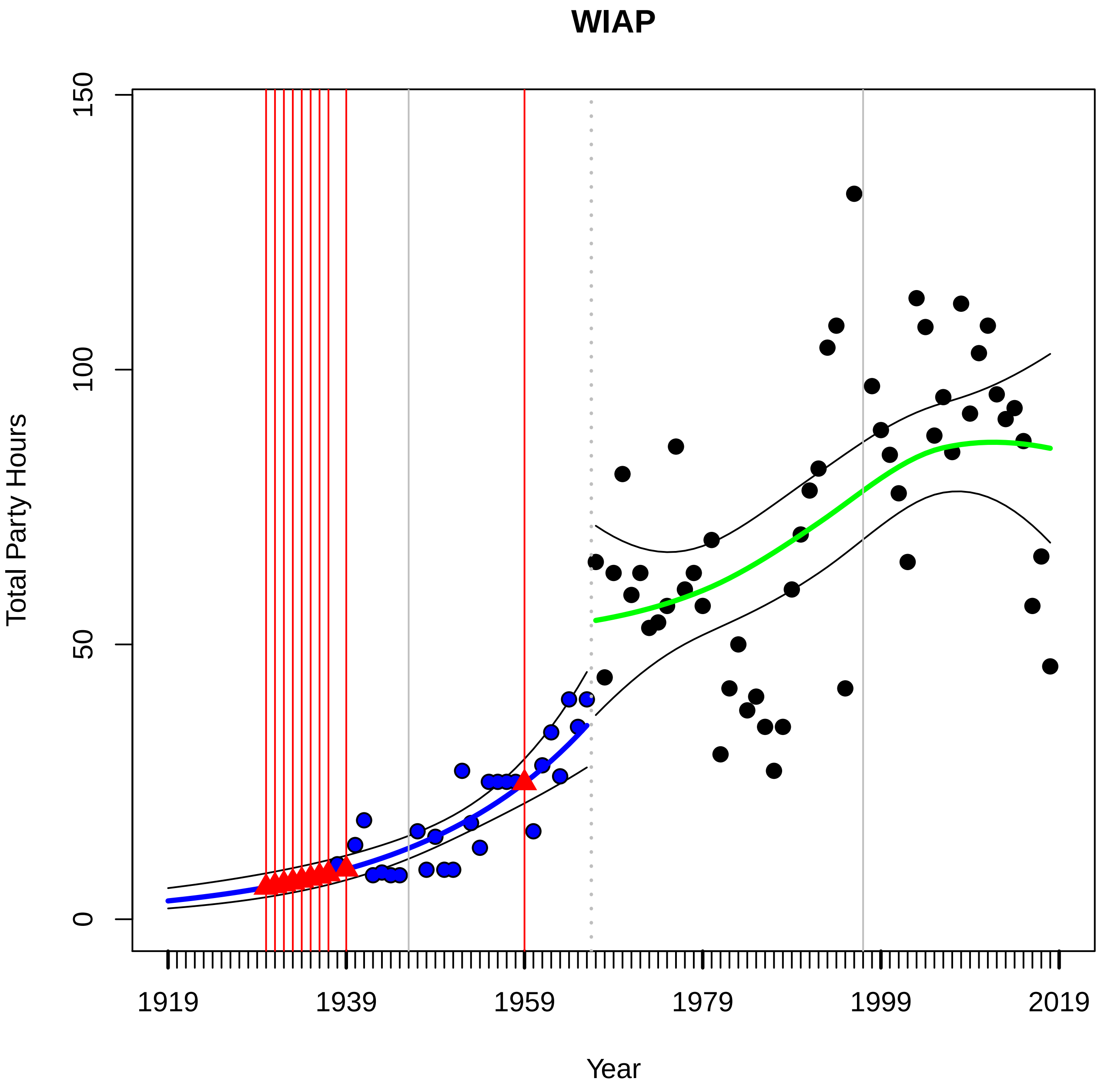

WIAP
Total Party Hours
Year
0
50
100
150
1919
1939
1959
1979
1999
2019

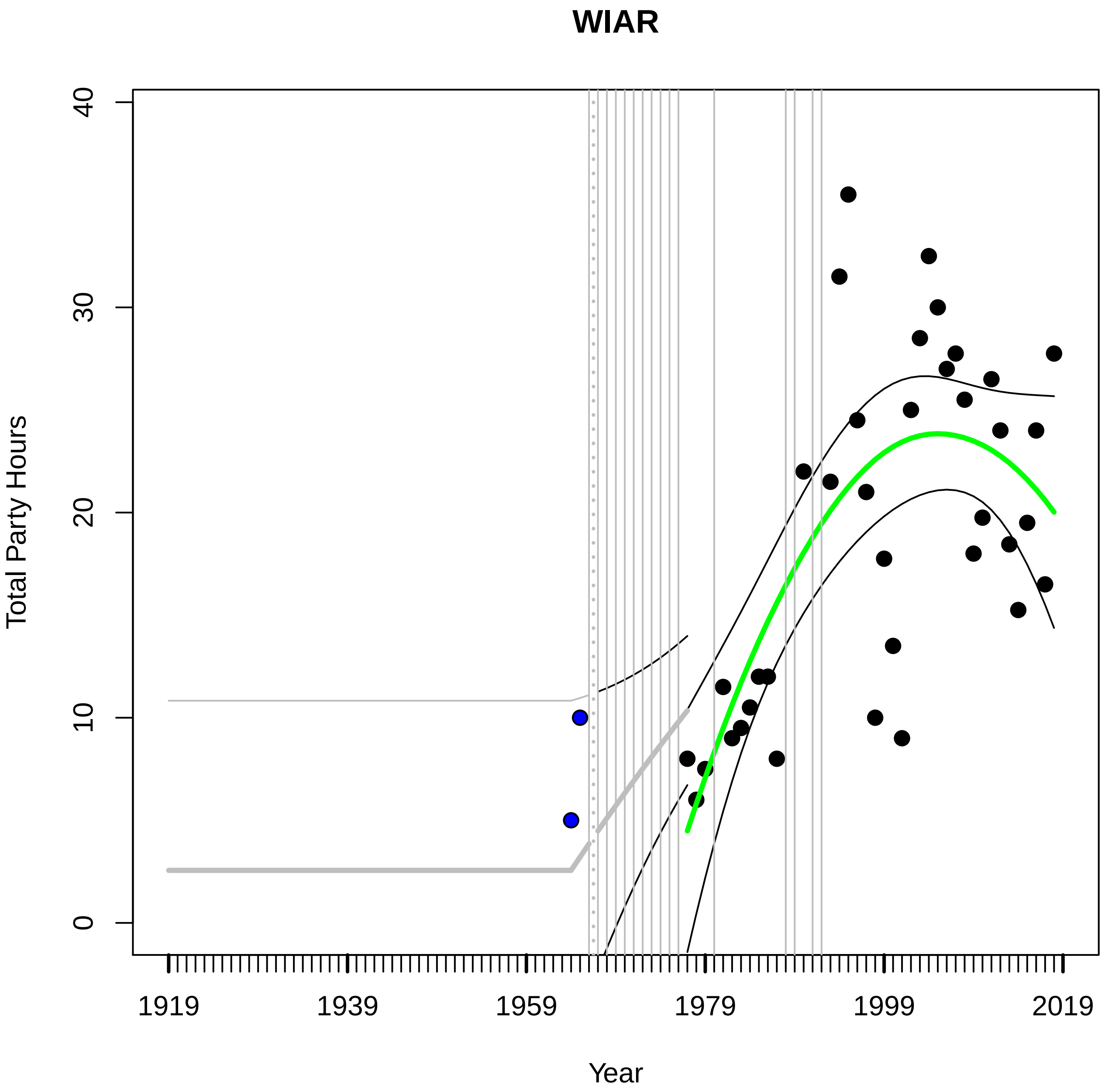
WIAR
Total Party Hours
0
10
20
30
40
1919
1939
1959
1979
1999
2019
Year

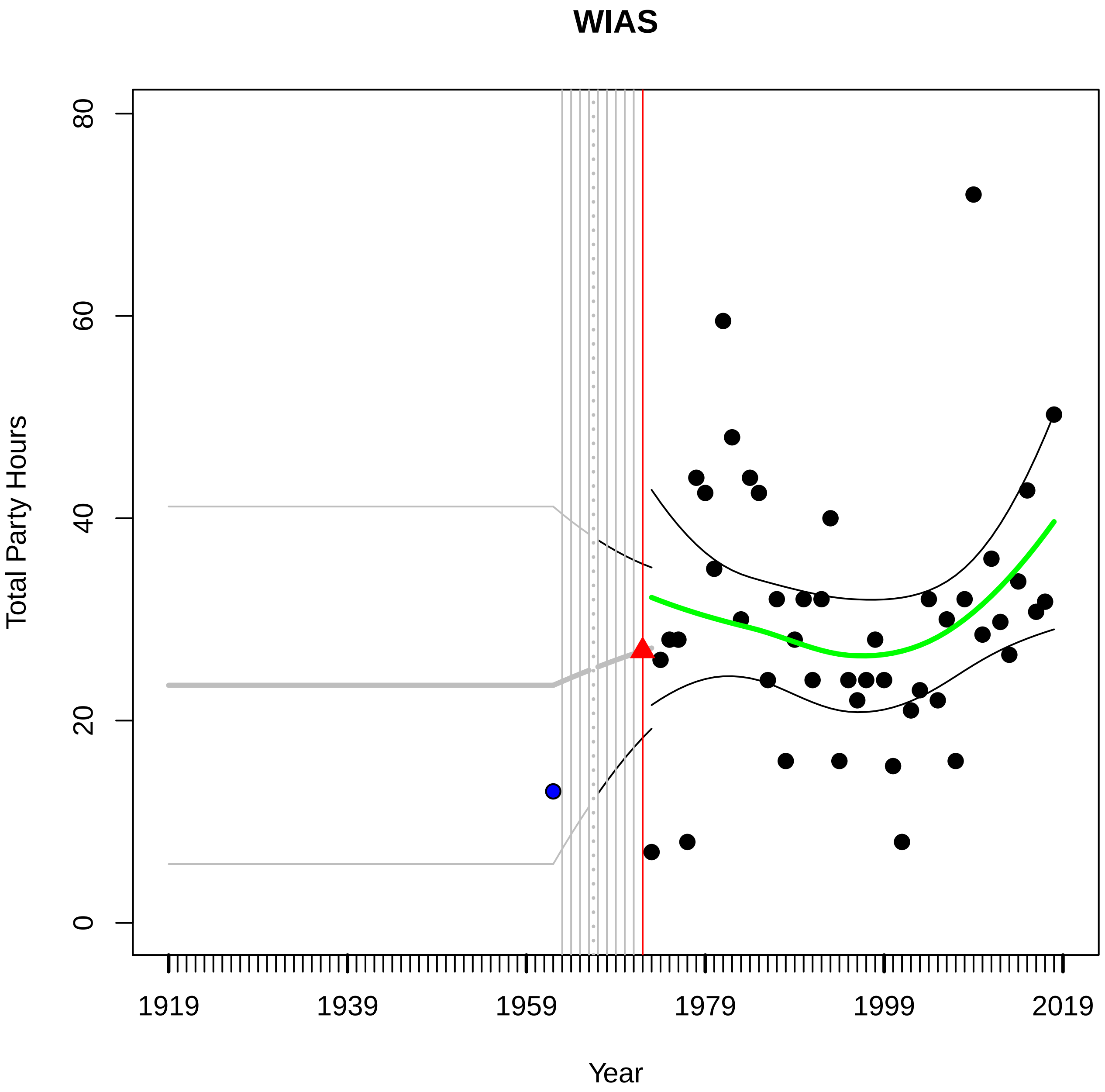
WIAS
Total Party Hours
Year
0
20
40
60
80
1919
1939
1959
1979
1999
2019

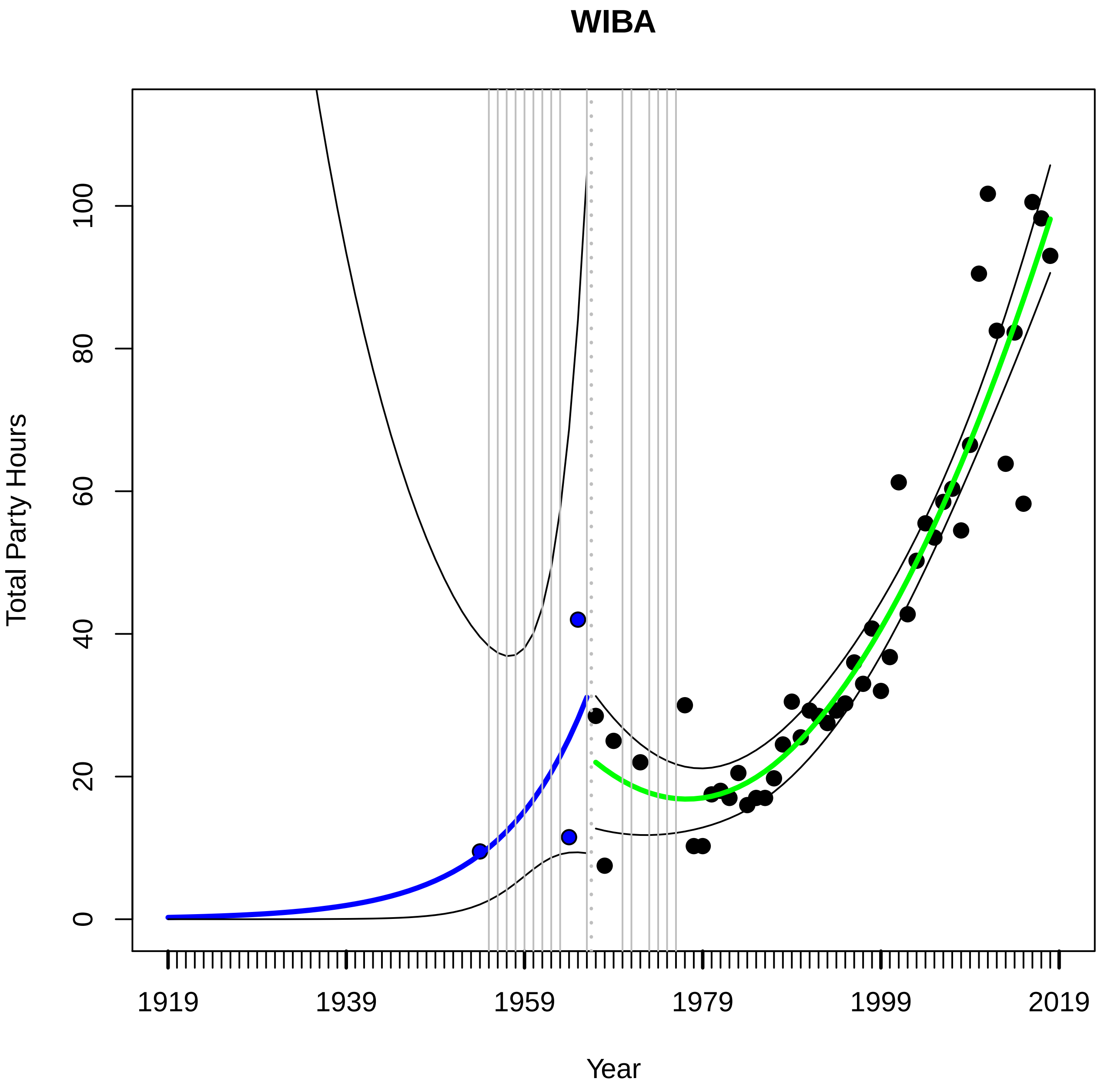

WIBA
Total Party Hours
0
20
40
60
80
100
1919
1939
1959
1979
1999
2019
Year

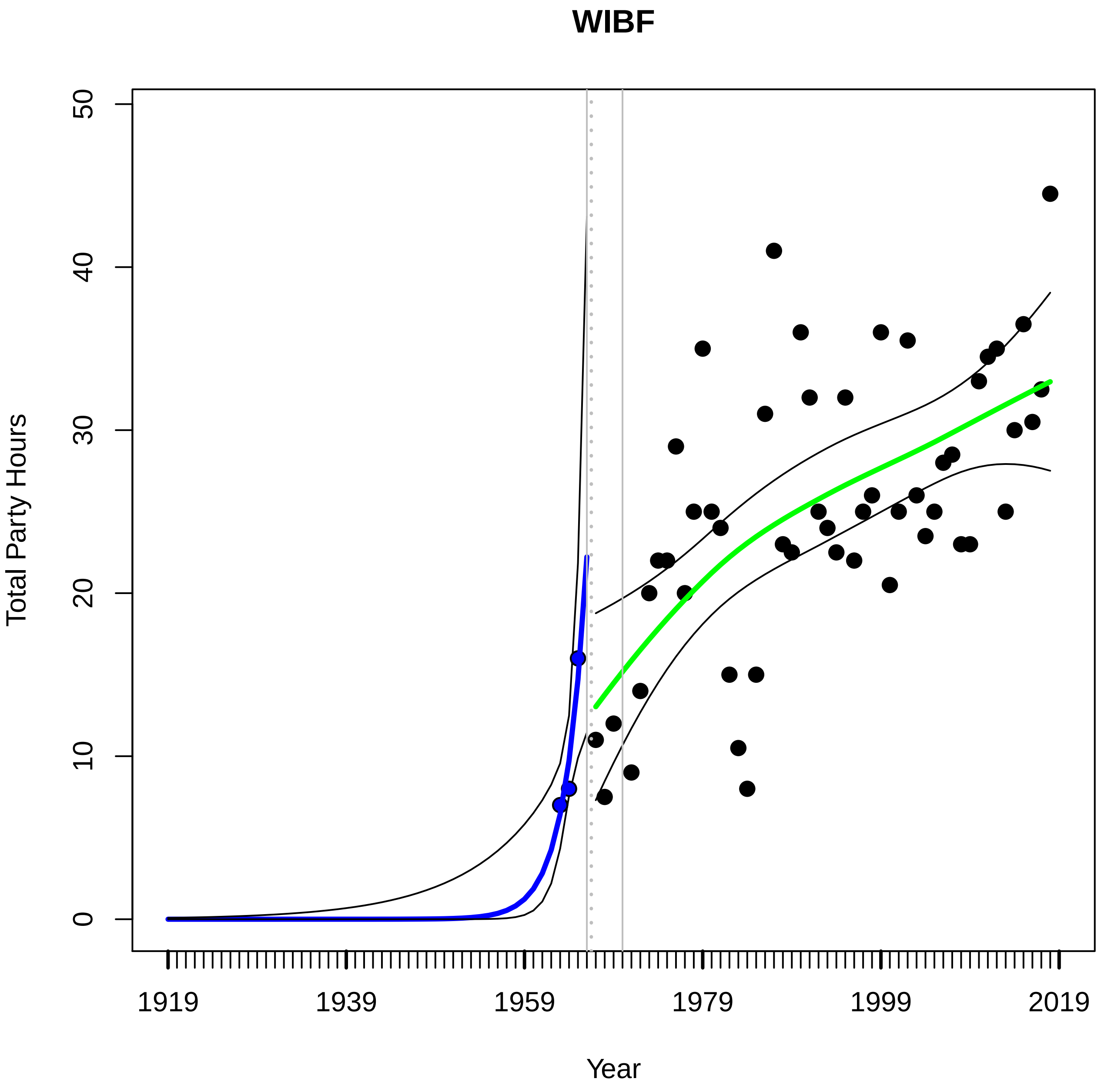

WIBF
Total Party Hours
Year
0
10
20
30
40
50
1919
1939
1959
1979
1999
2019

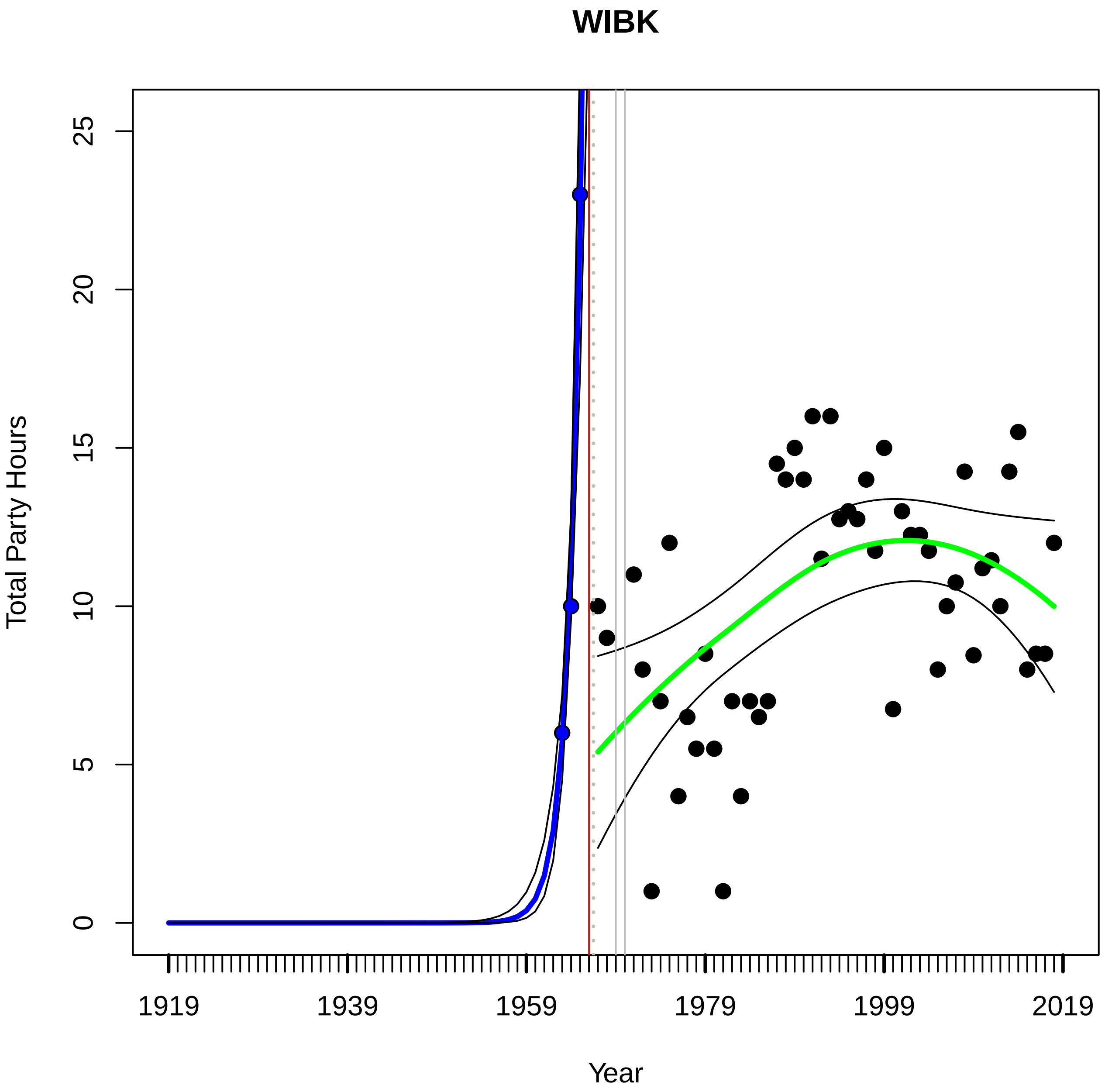

WIBK
Total Party Hours
Year
0
5
10
15
20
25
1919
1939
1959
1979
1999
2019

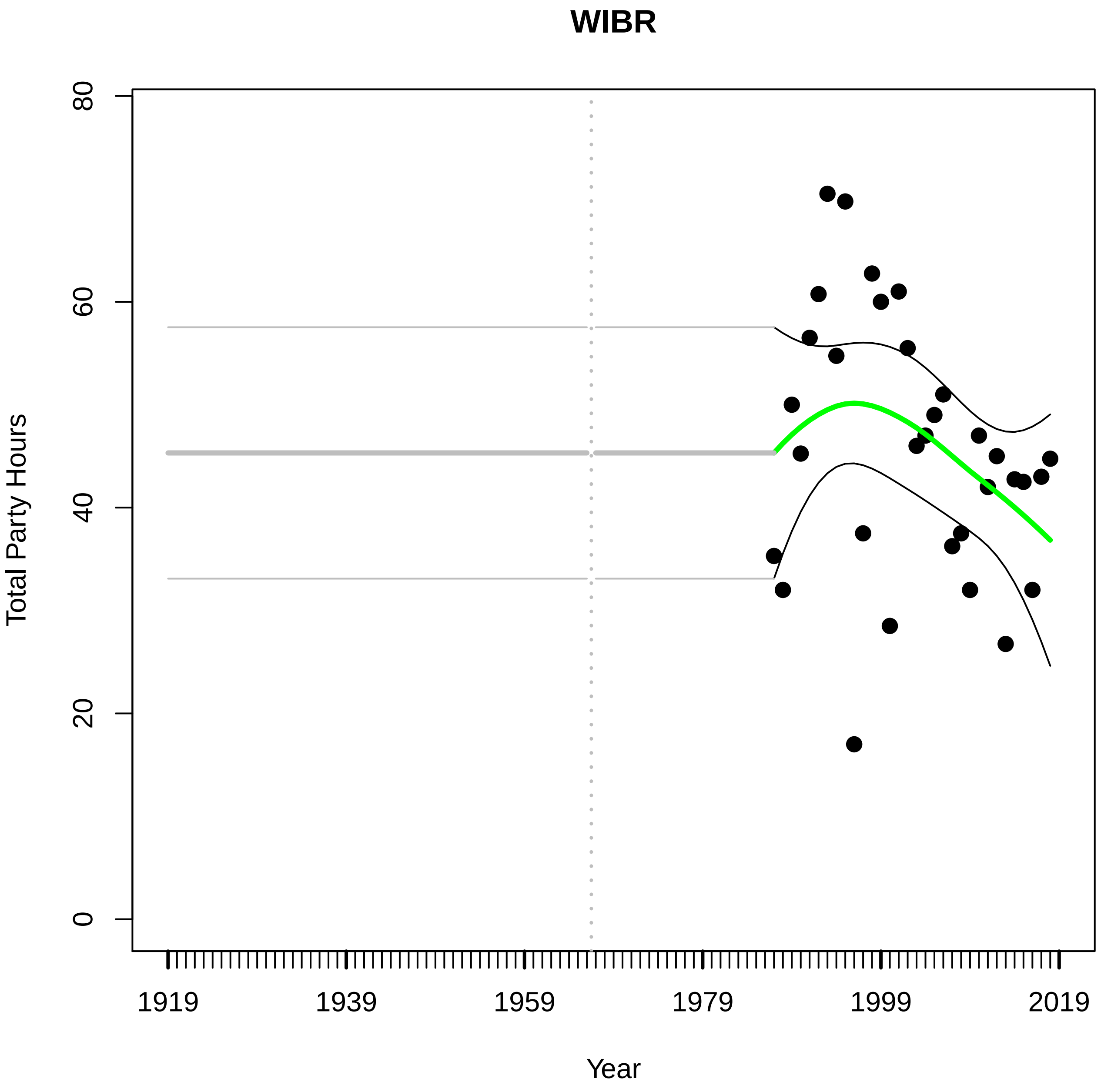
WIBR
Total Party Hours
0
20
40
60
80
1919
1939
1959
1979
1999
2019
Year

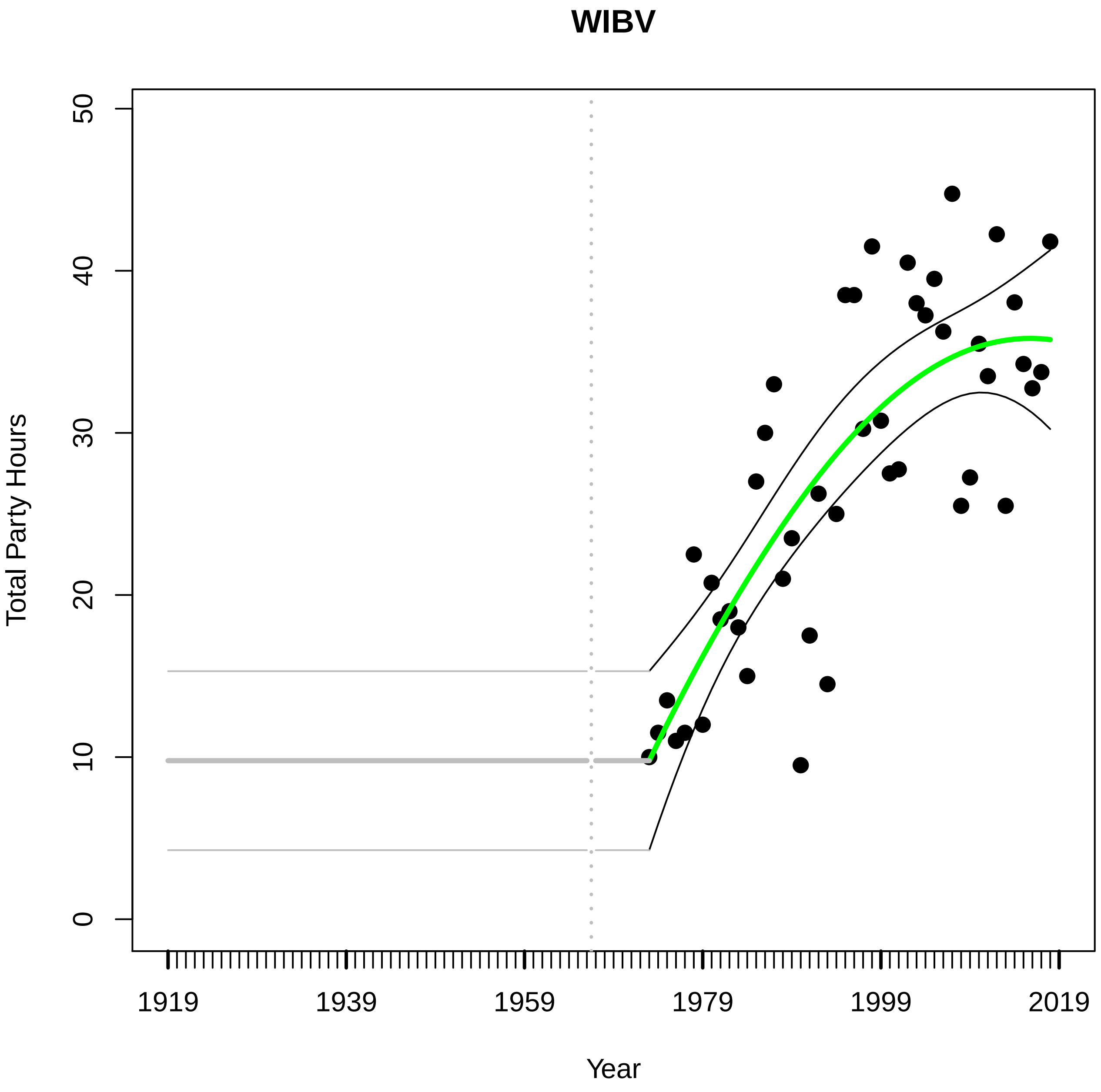

WIBV
Total Party Hours
Year
0
10
20
30
40
50
1919
1939
1959
1979
1999
2019

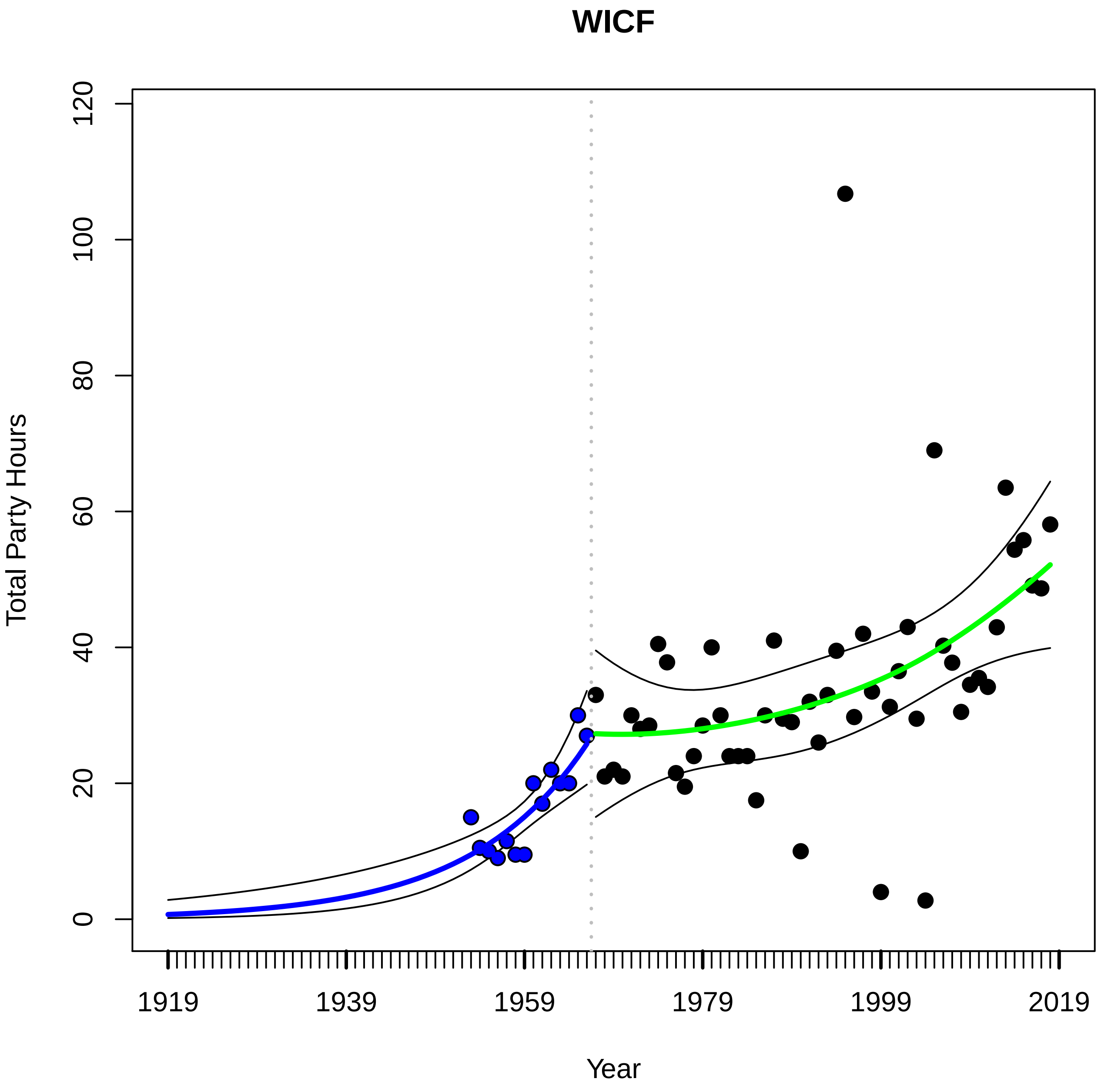
WICF
Total Party Hours
Year
0
20
40
60
80
100
120
1919
1939
1959
1979
1999
2019

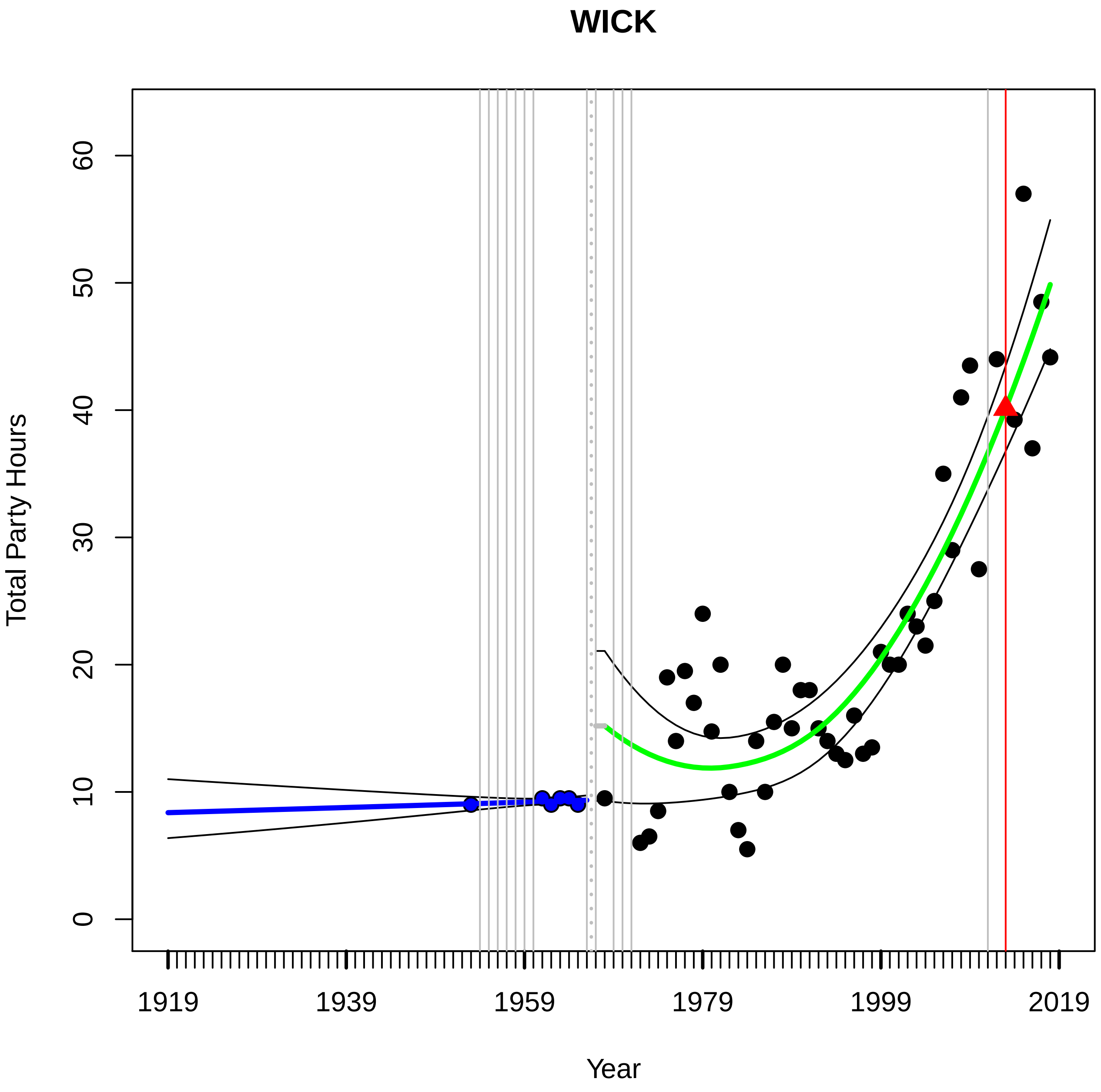

WICK
Total Party Hours
Year
0
10
20
30
40
50
60
1919
1939
1959
1979
1999
2019

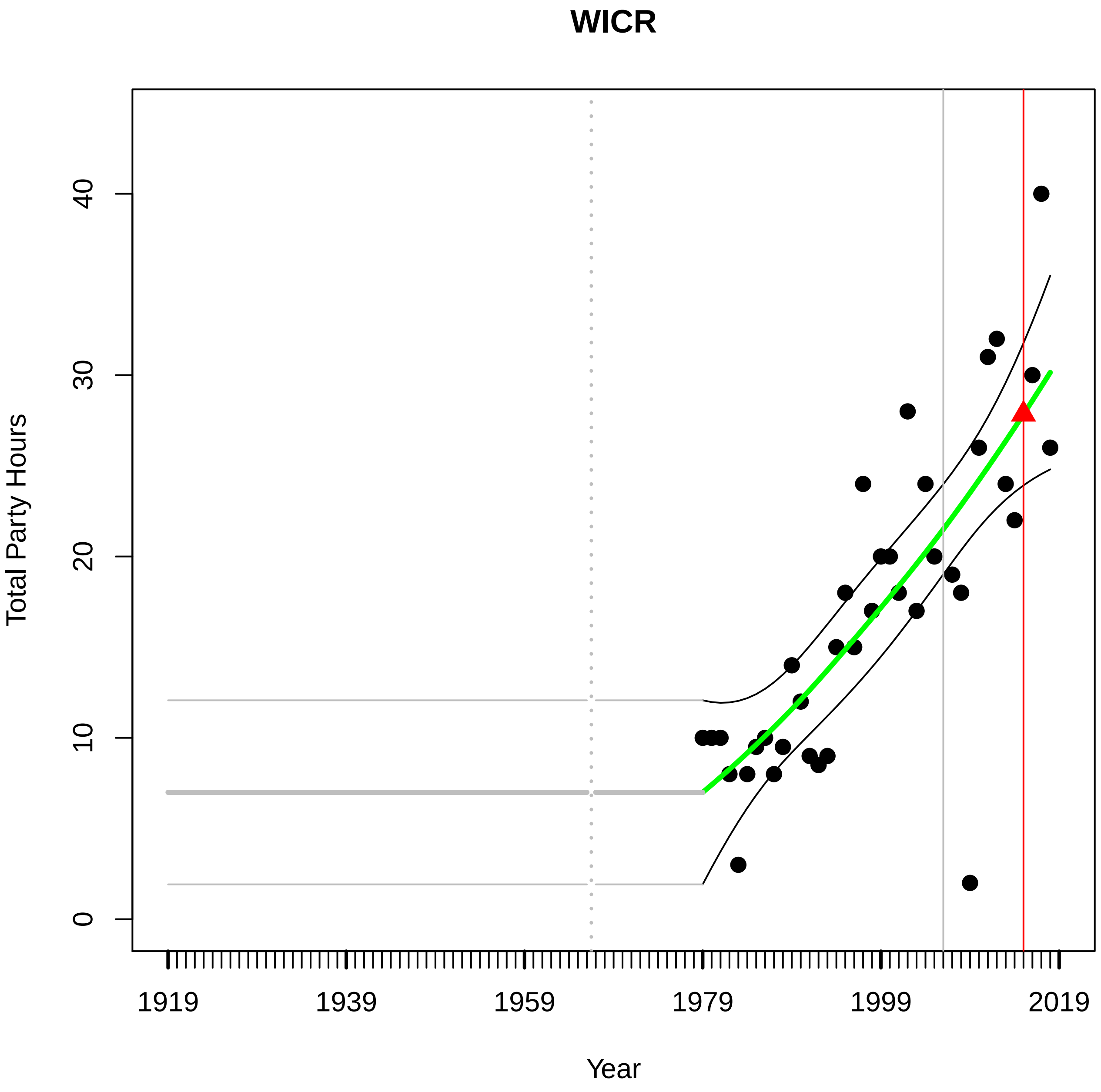
WICR
Total Party Hours
0
10
20
30
40
1919
1939
1959
1979
1999
2019
Year

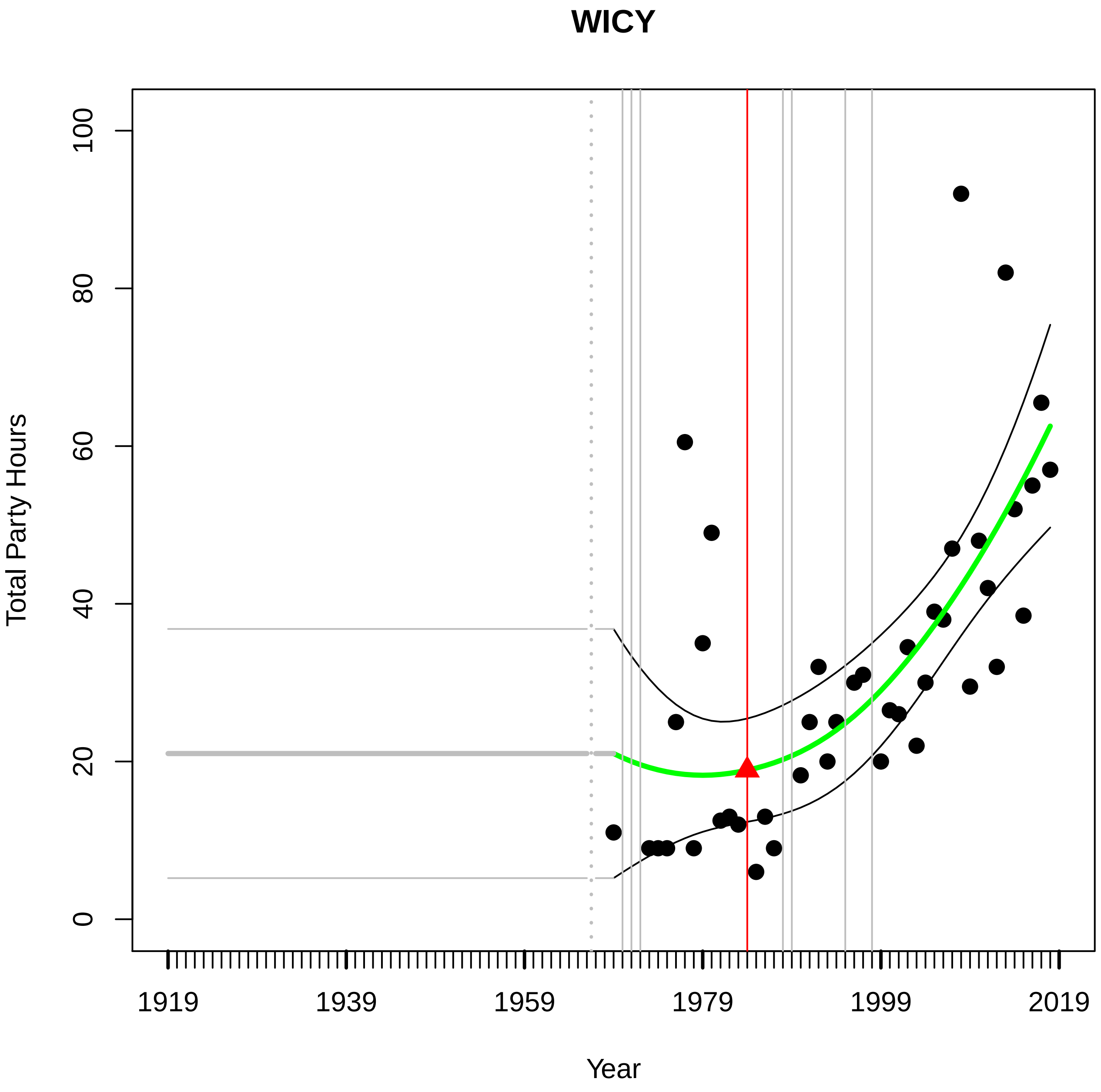

WICY
Total Party Hours
0
20
40
60
80
100
1919
1939
1959
1979
1999
2019
Year

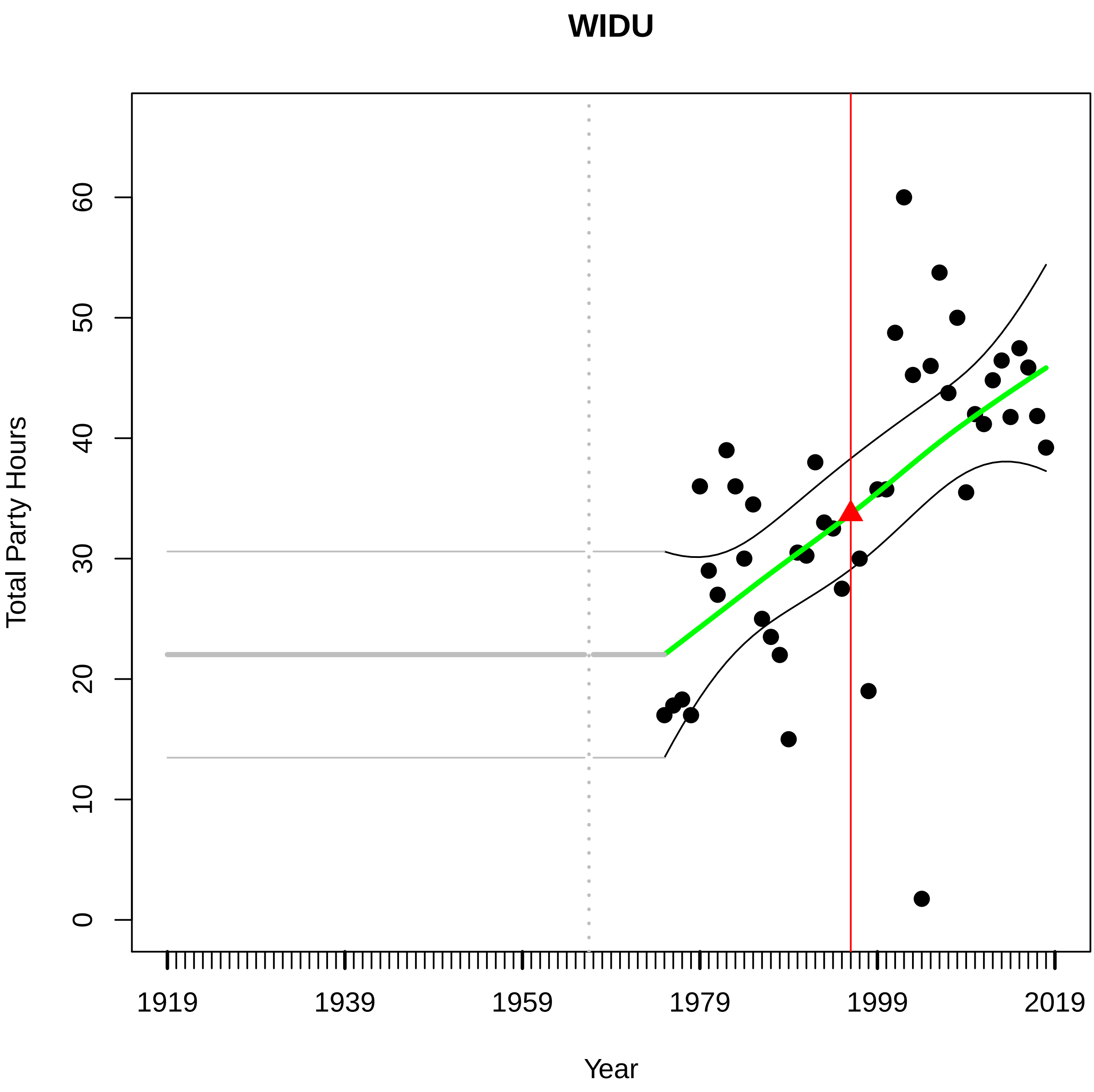
WIDU
Total Party Hours
Year
0
10
20
30
40
50
60
1919
1939
1959
1979
1999
2019

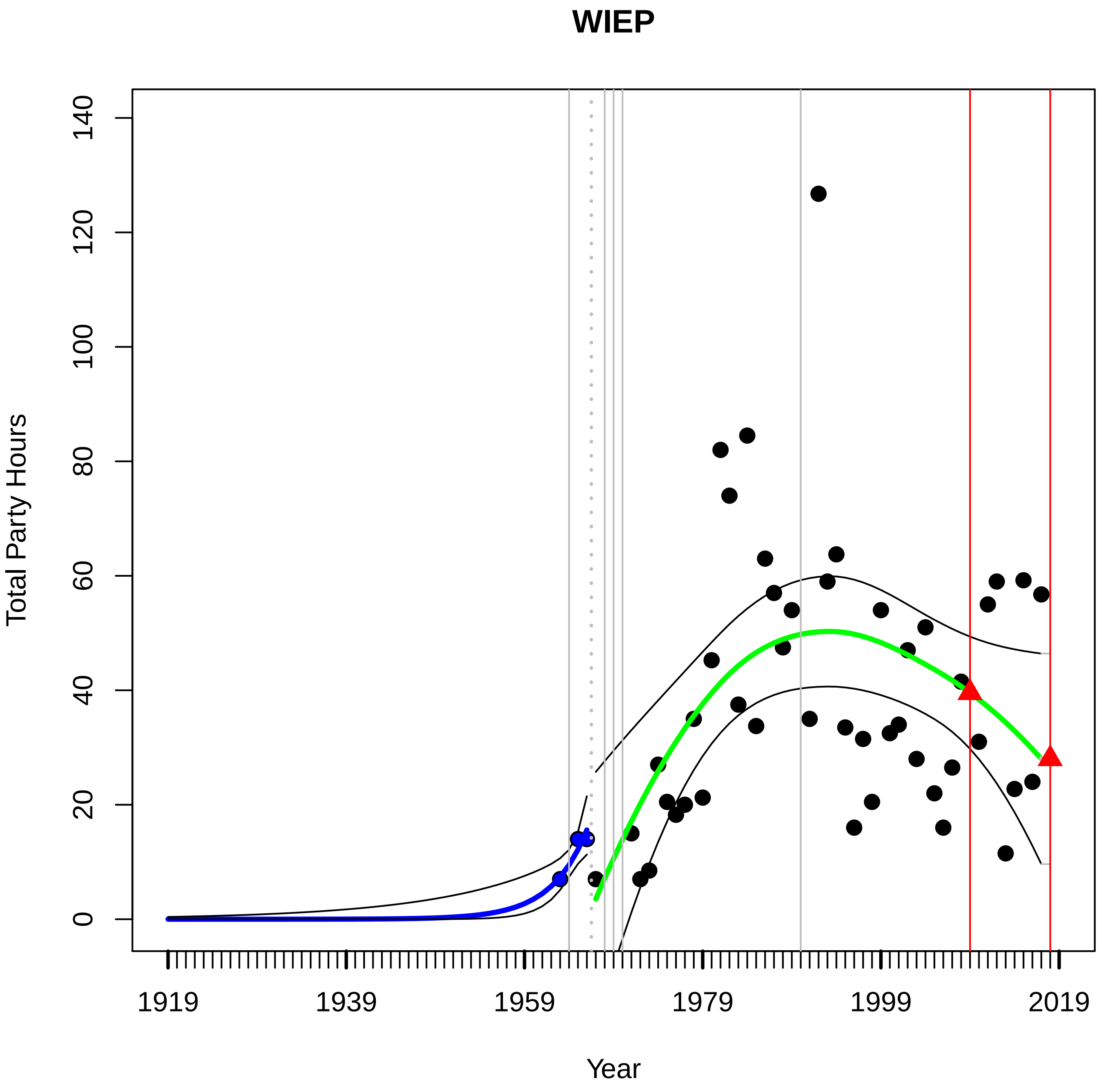

WIEP
Total Party Hours
0
20
40
60
80
100
120
140
1919
1939
1959
1979
1999
2019
Year

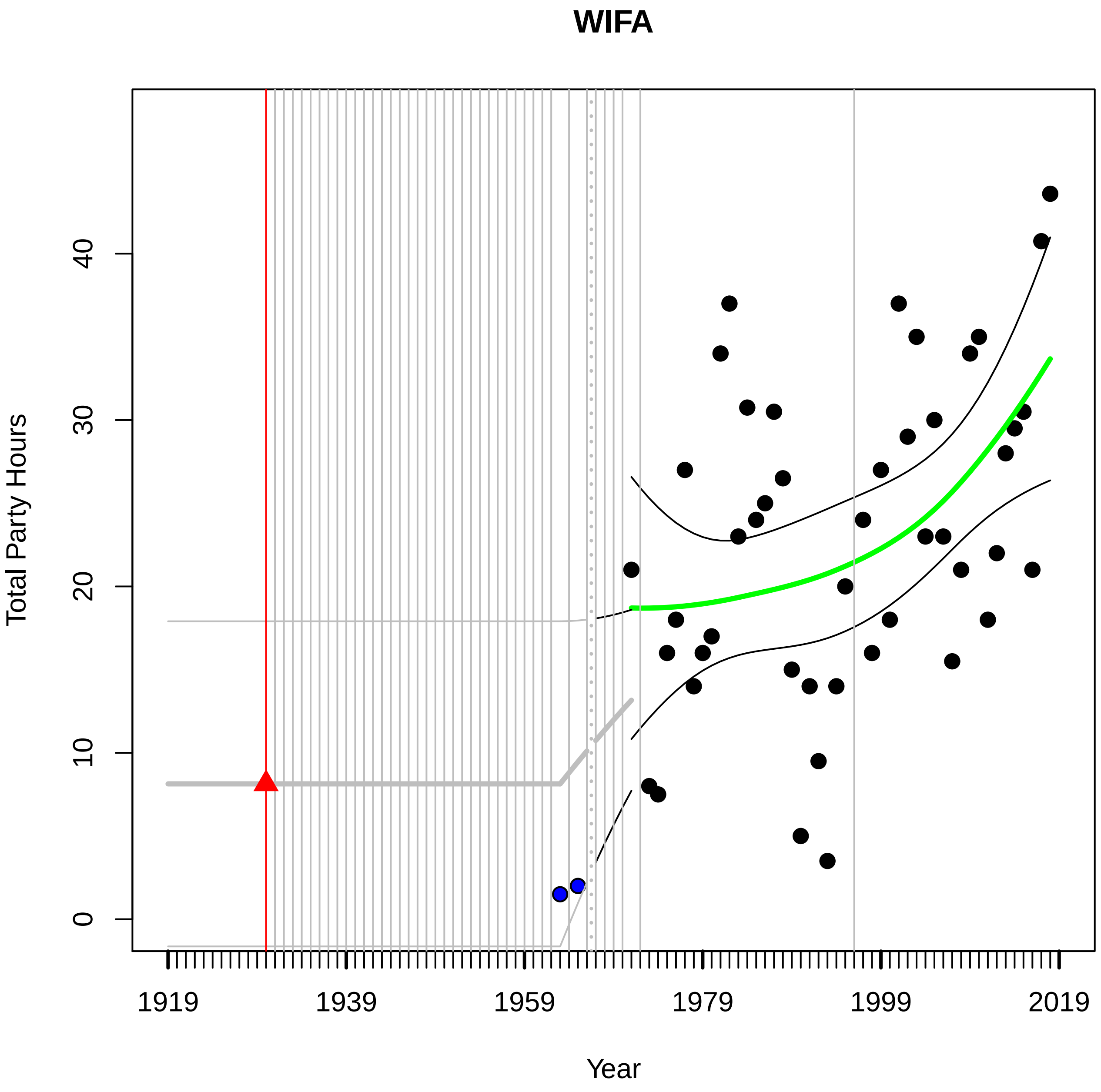

WIFA
Total Party Hours
0
10
20
30
40
1919
1939
1959
1979
1999
2019
Year

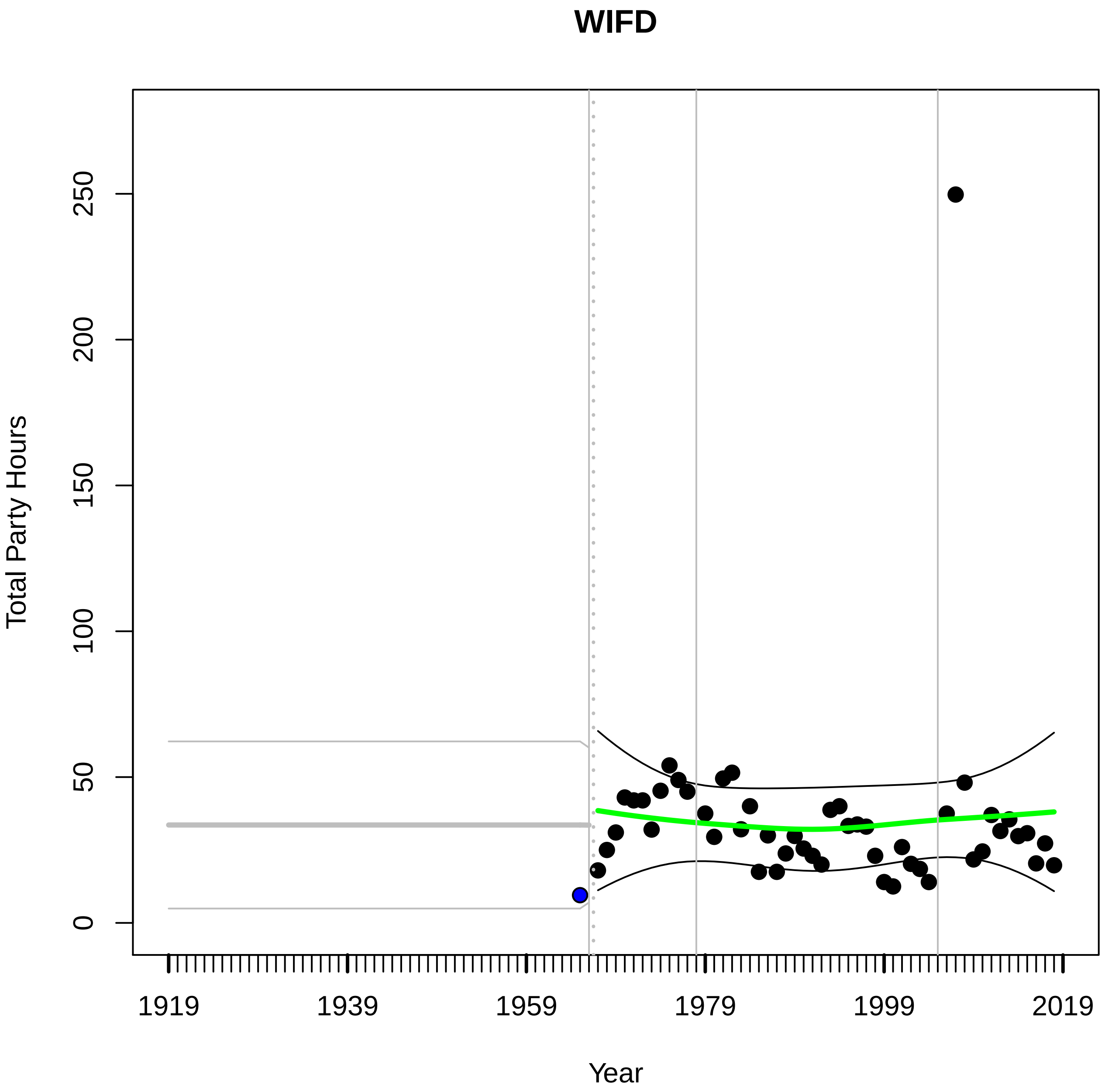
WIFD
Total Party Hours
0
50
100
150
200
250
1919
1939
1959
1979
1999
2019
Year

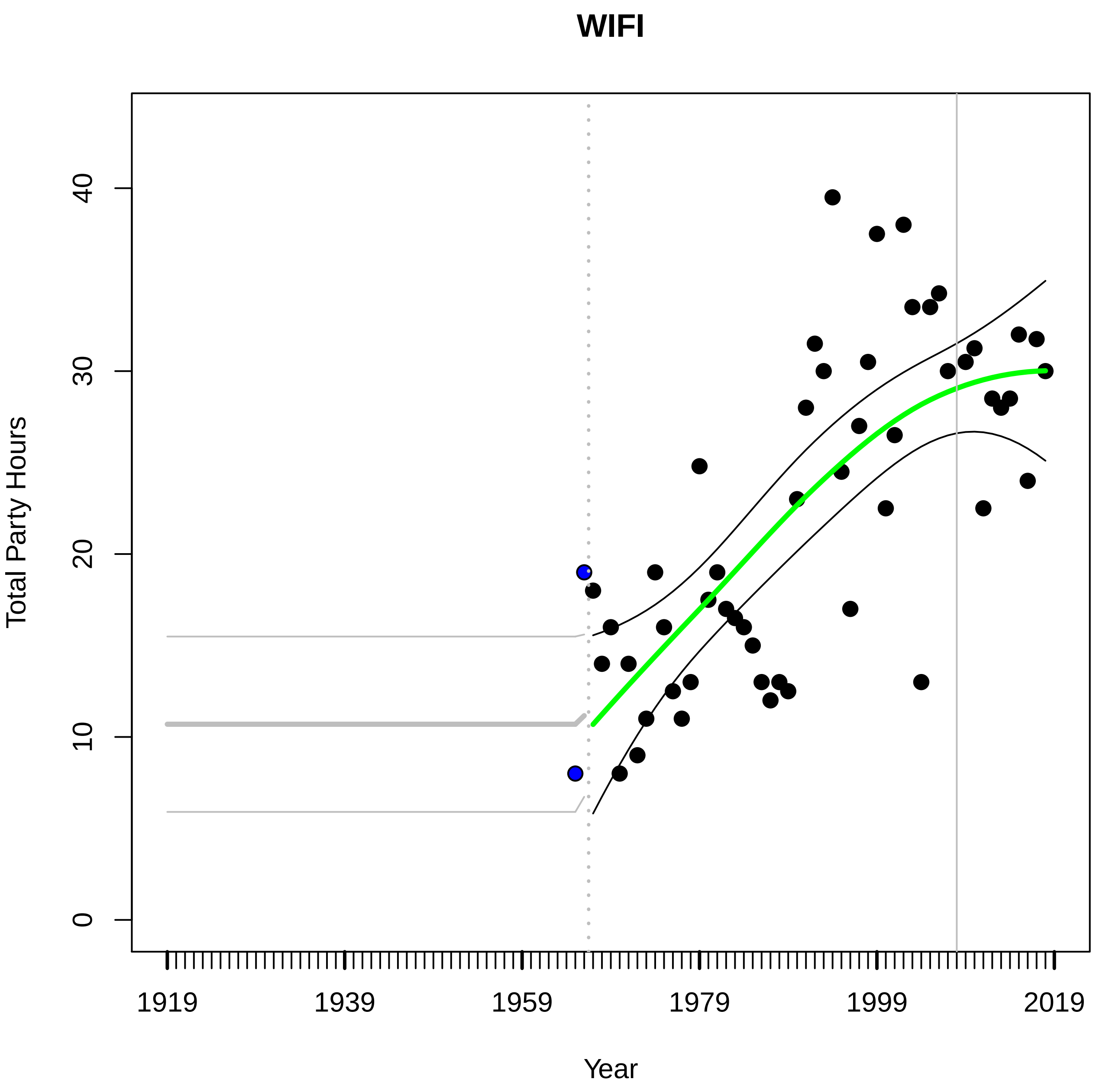

WIFI
Total Party Hours
0
10
20
30
40
1919
1939
1959
1979
1999
2019
Year

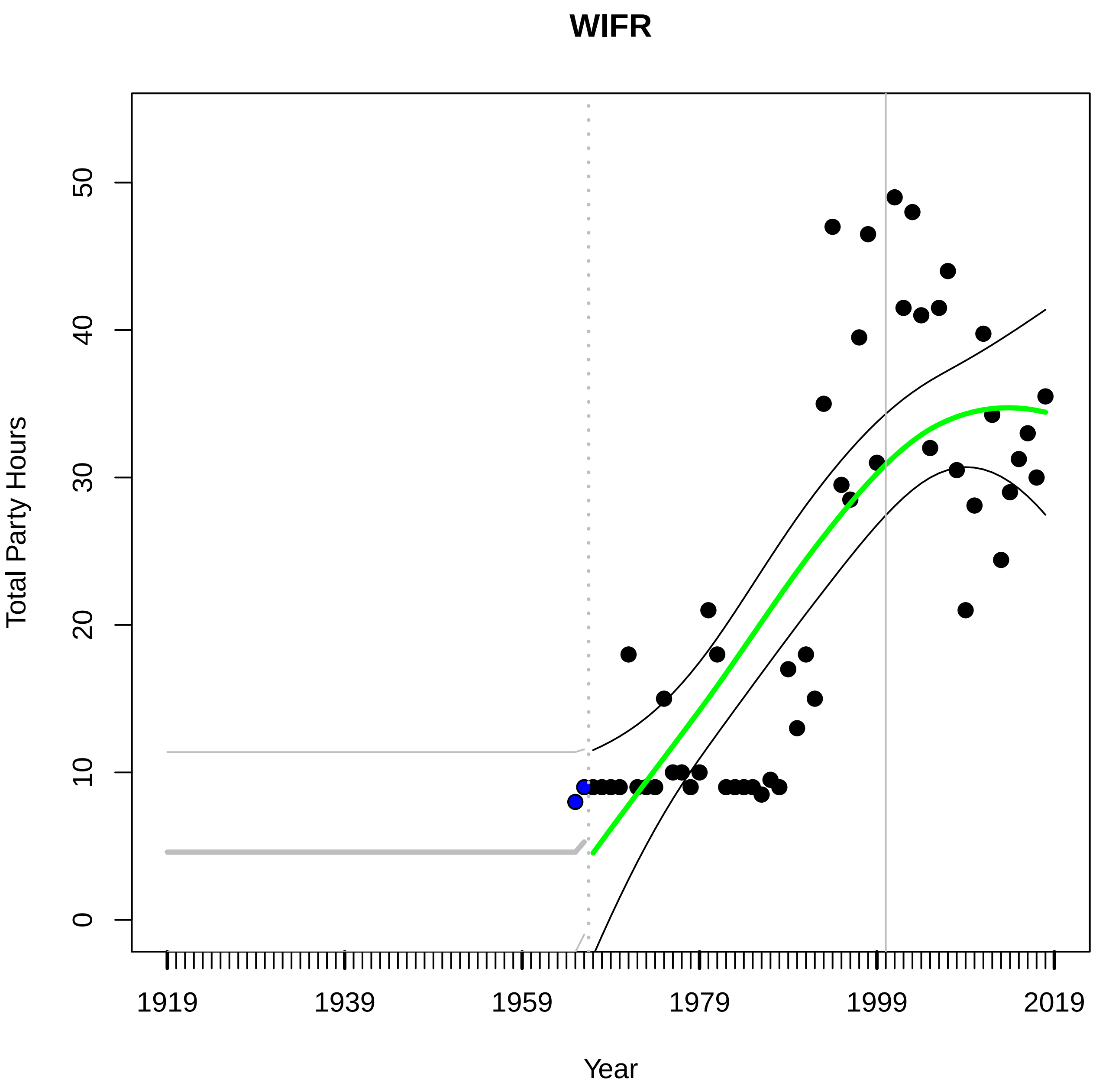
WIFR
Total Party Hours
0
10
20
30
40
50
1919
1939
1959
1979
1999
2019
Year

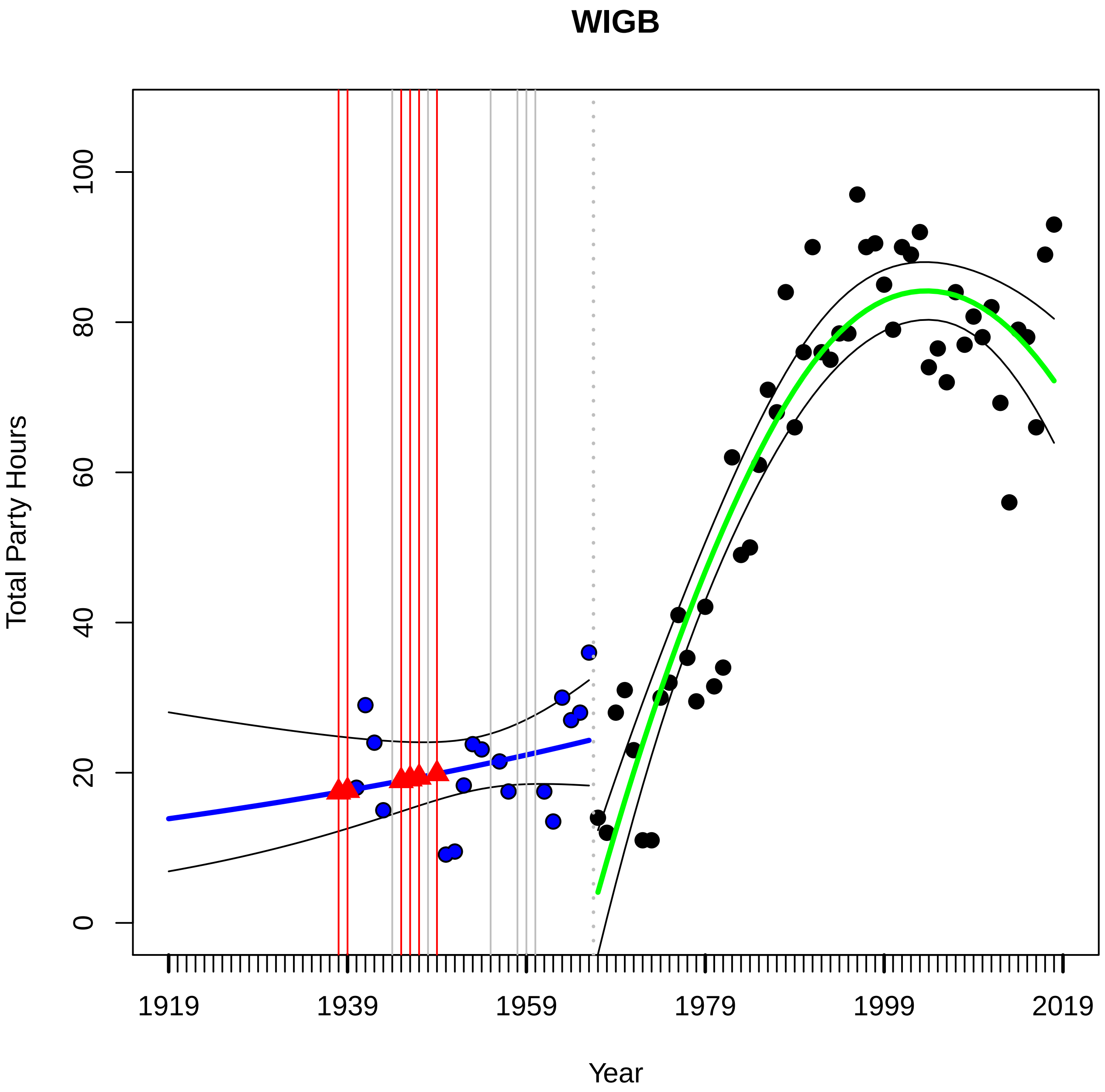
WIGB
Total Party Hours
Year
0
20
40
60
80
100
1919
1939
1959
1979
1999
2019

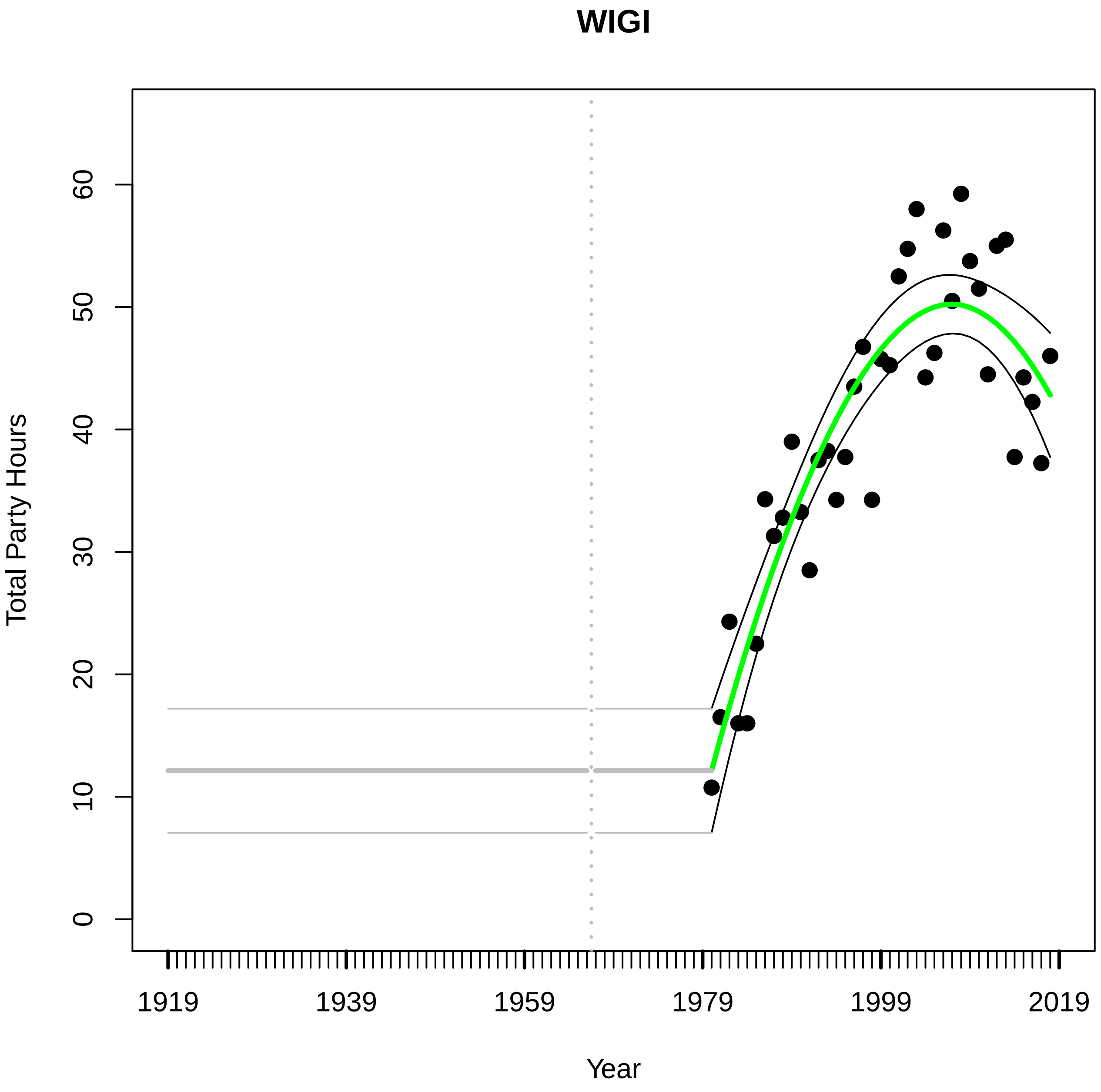
WIGI
Total Party Hours
0
10
20
30
40
50
60
1919
1939
1959
1979
1999
2019
Year

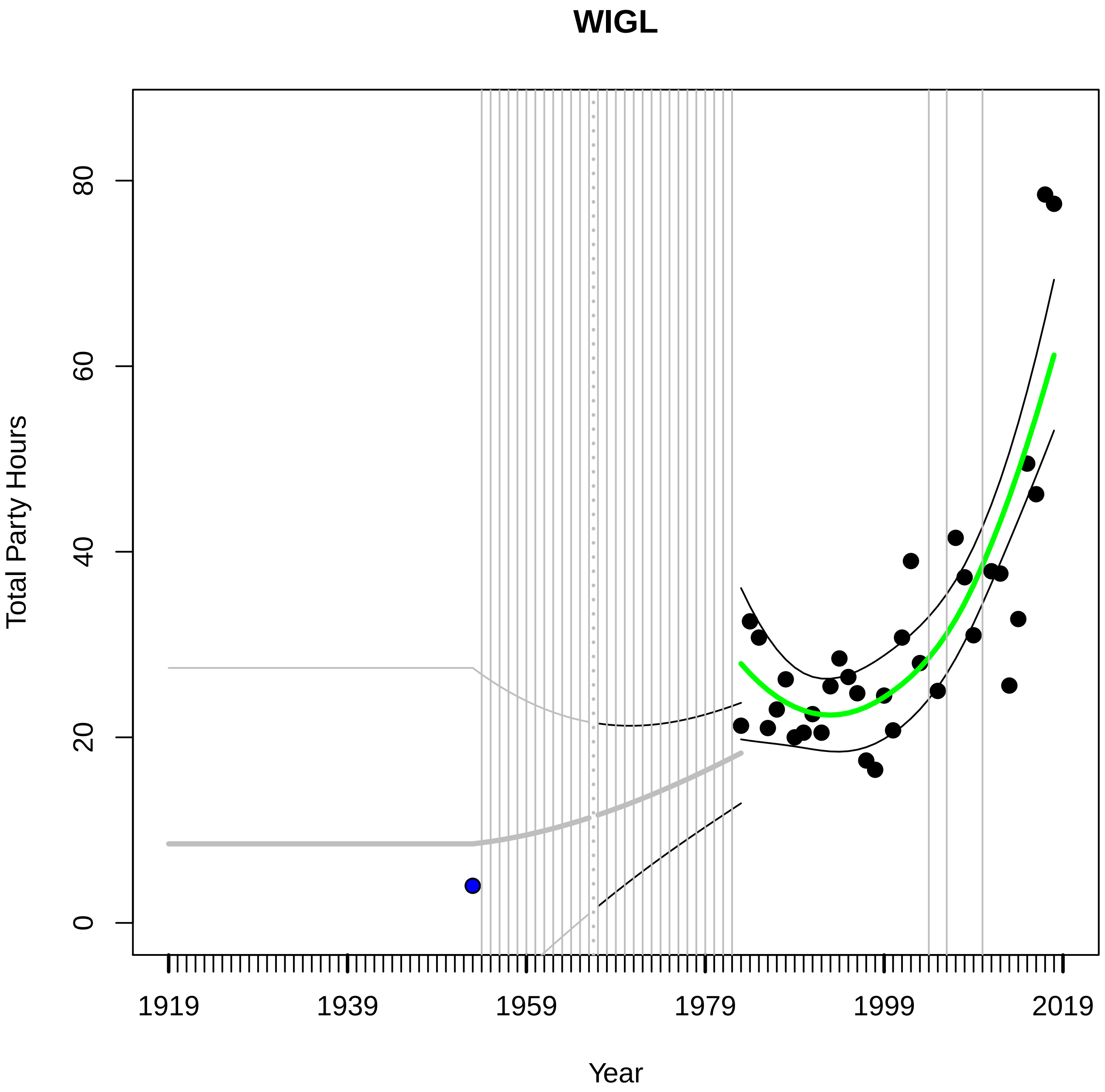
WIGL
Total Party Hours
0
20
40
60
80
1919
1939
1959
1979
1999
2019
Year

WIGR

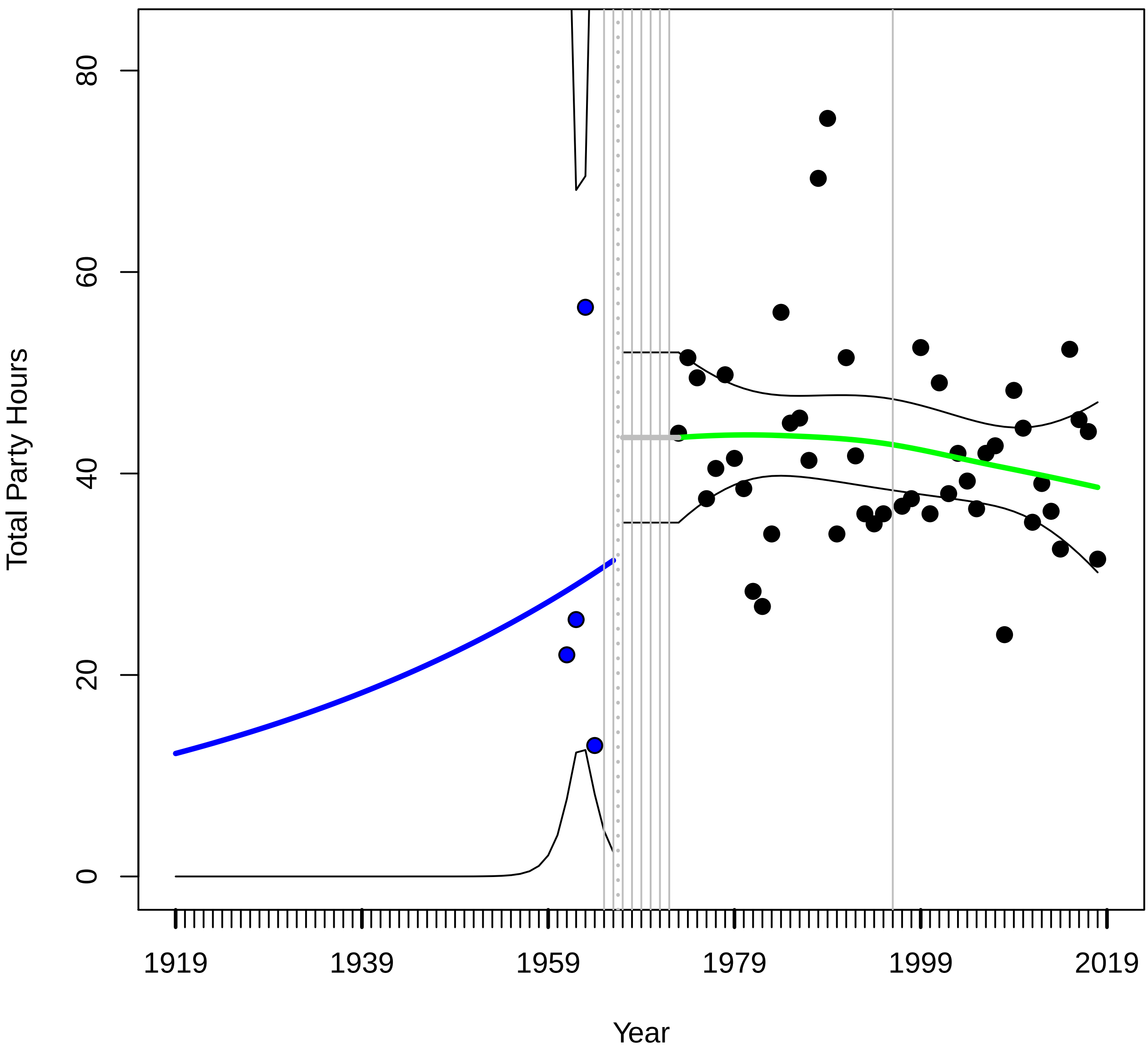

Total Party Hours
0
20
40
60
80
1919
1939
1959
1979
1999
2019
Year

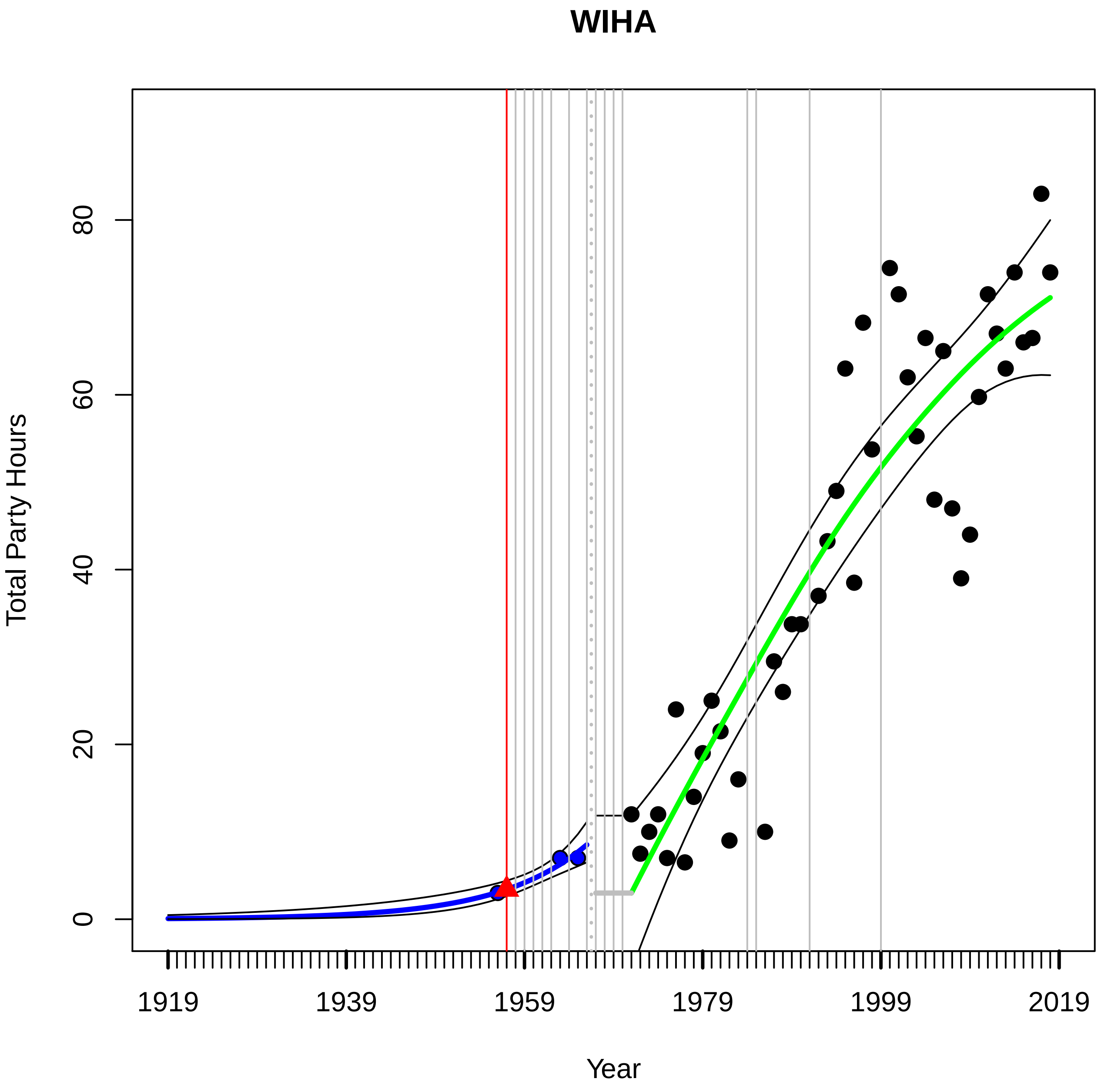

WIHA
Total Party Hours
Year
0
20
40
60
80
1919
1939
1959
1979
1999
2019

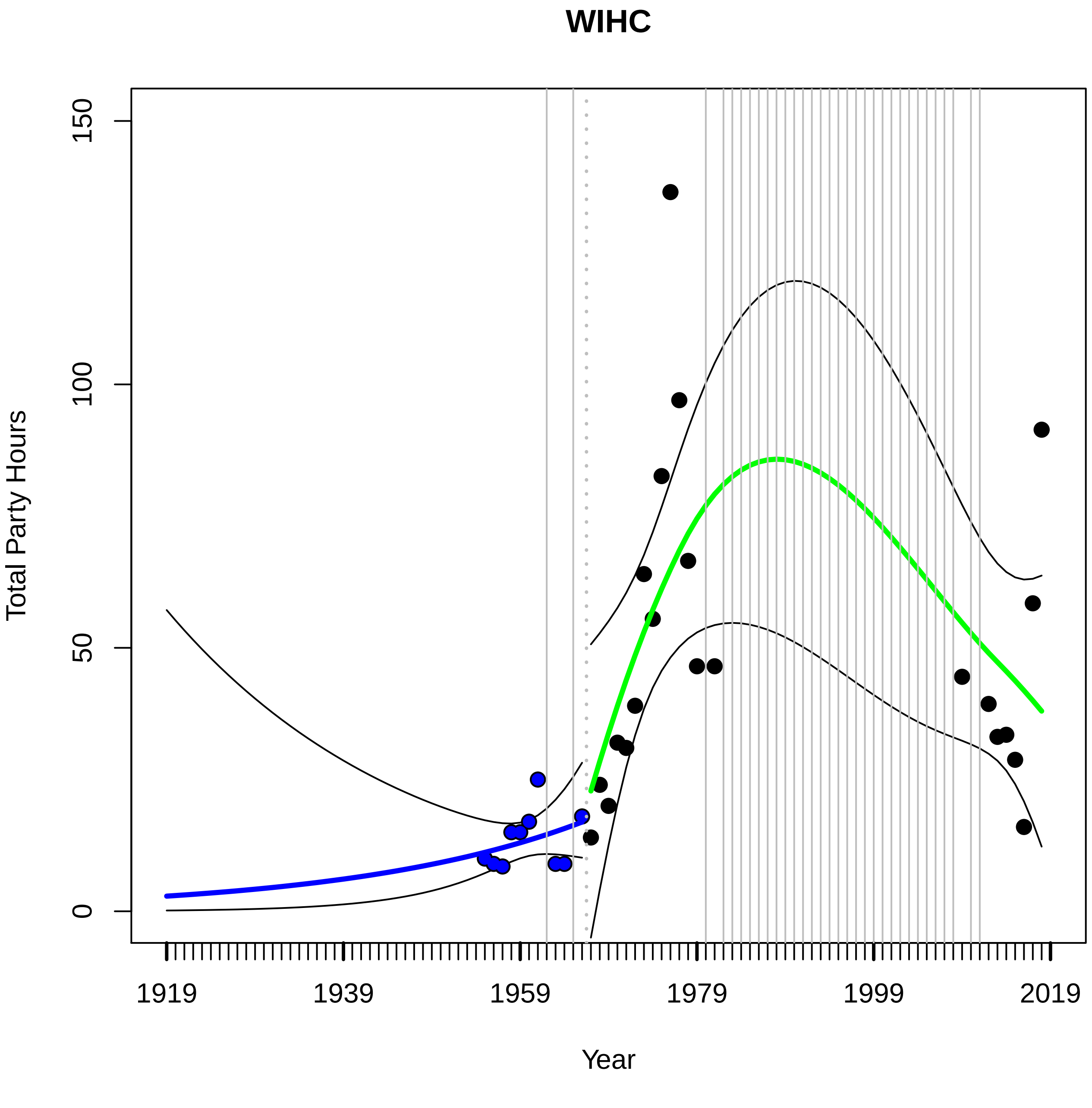
WIHC
Total Party Hours
0
50
100
150
1919
1939
1959
1979
1999
2019
Year

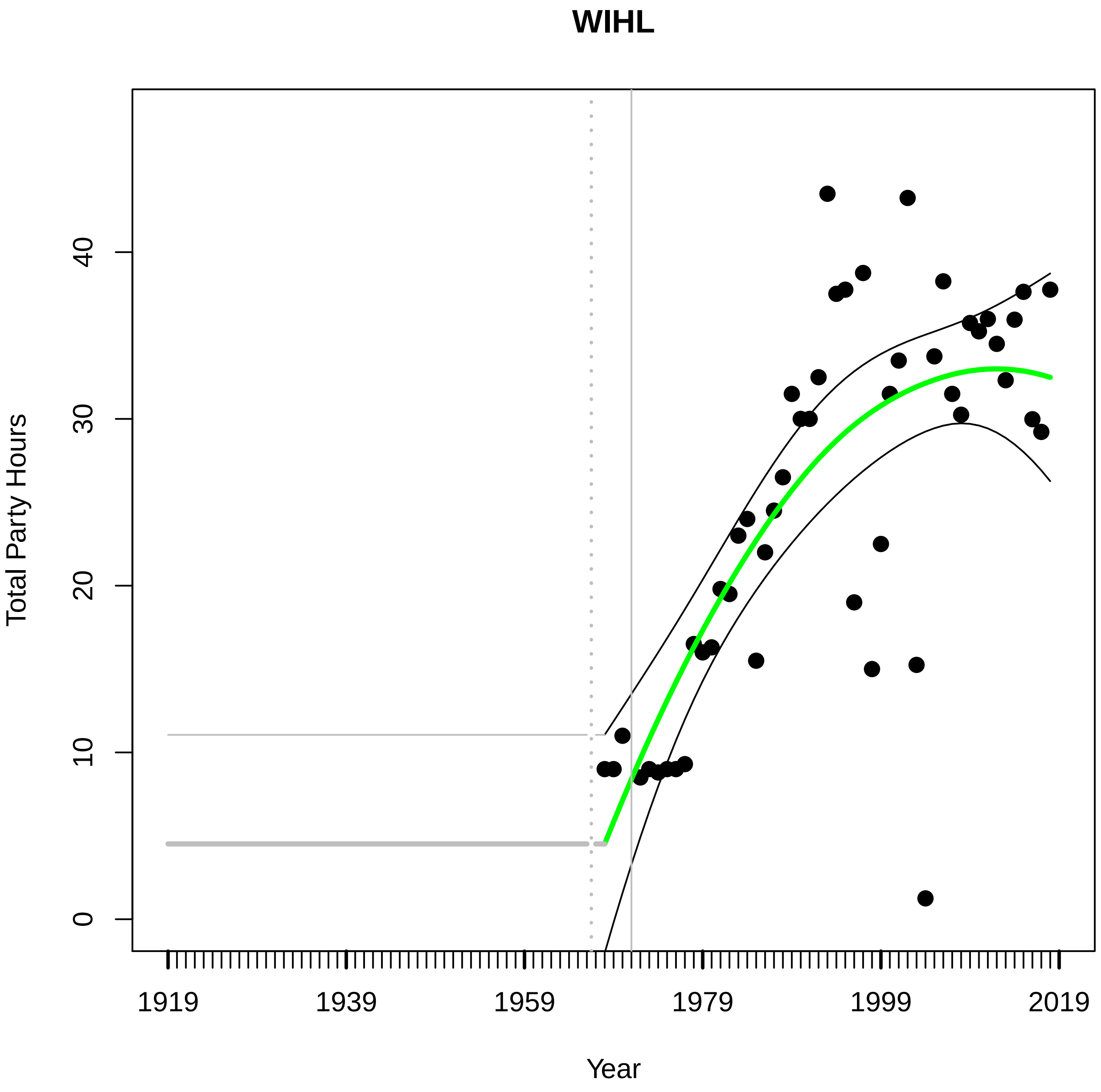

WIHL
Total Party Hours
0
10
20
30
40
1919
1939
1959
1979
1999
2019
Year

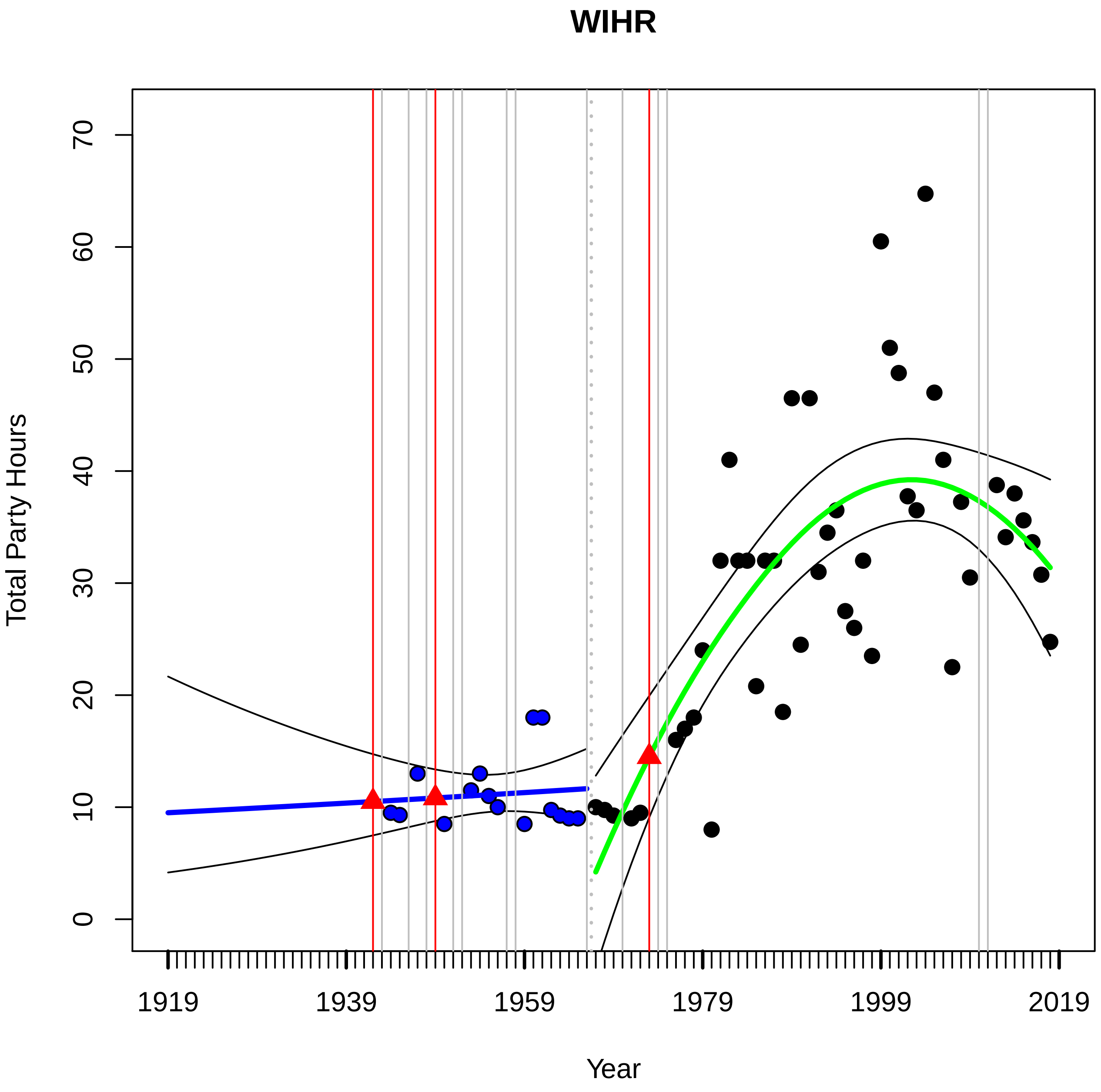

WIHR
Total Party Hours
0
10
20
30
40
50
60
70
1919
1939
1959
1979
1999
2019
Year

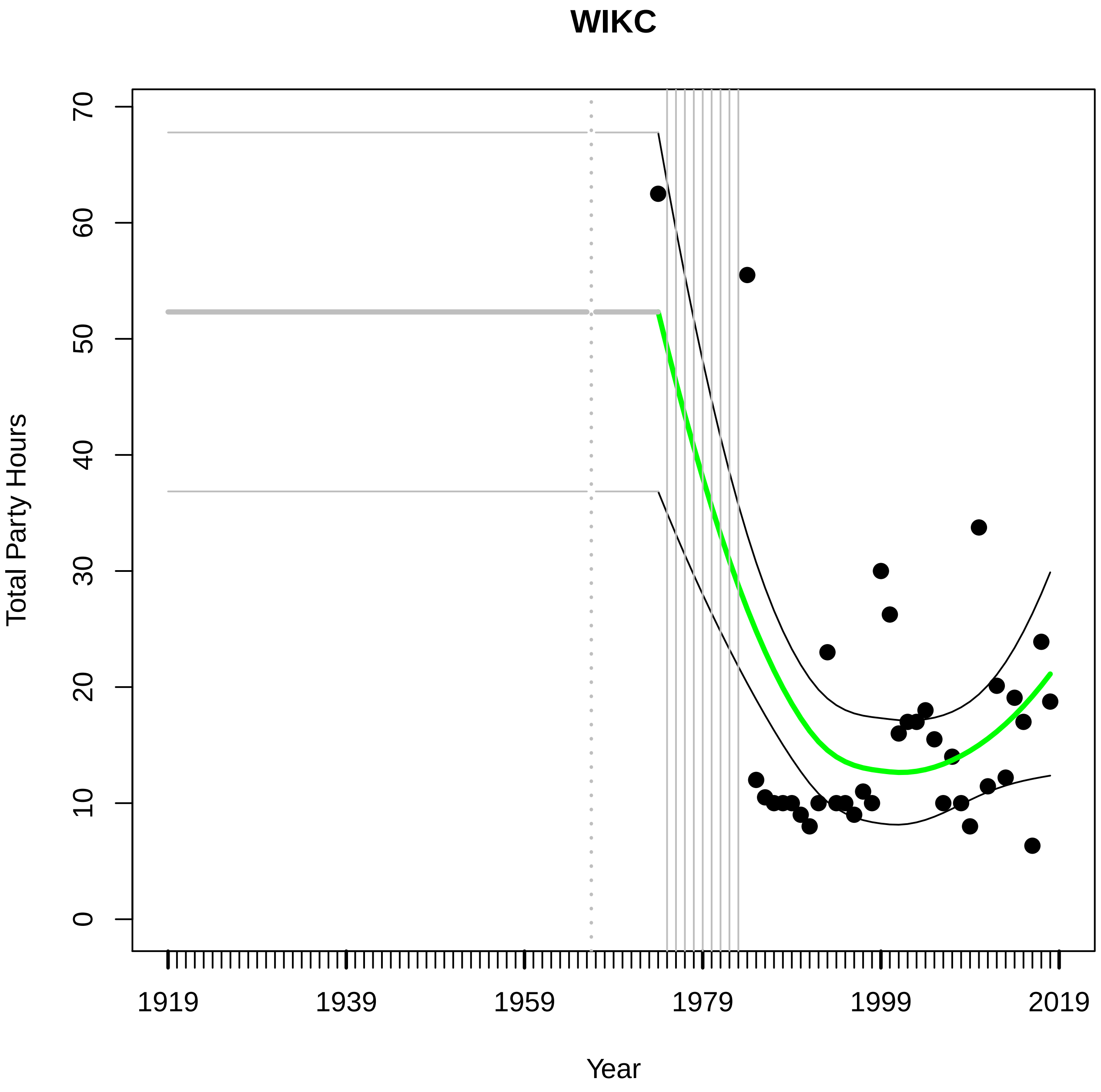

WIKC
Total Party Hours
0
10
20
30
40
50
60
70
1919
1939
1959
1979
1999
2019
Year

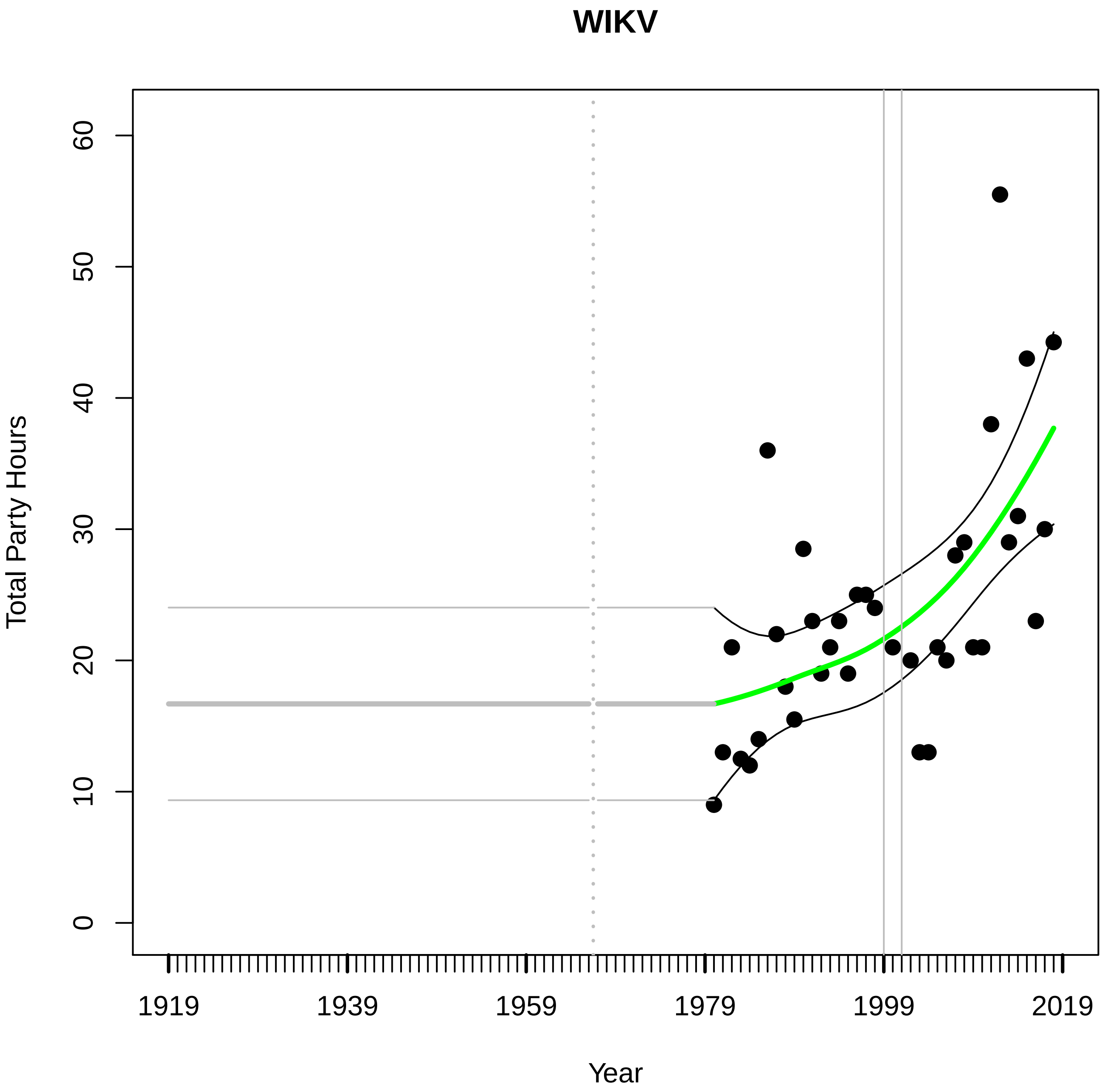

WIKV
Total Party Hours
0
10
20
30
40
50
60
1919
1939
1959
1979
1999
2019
Year

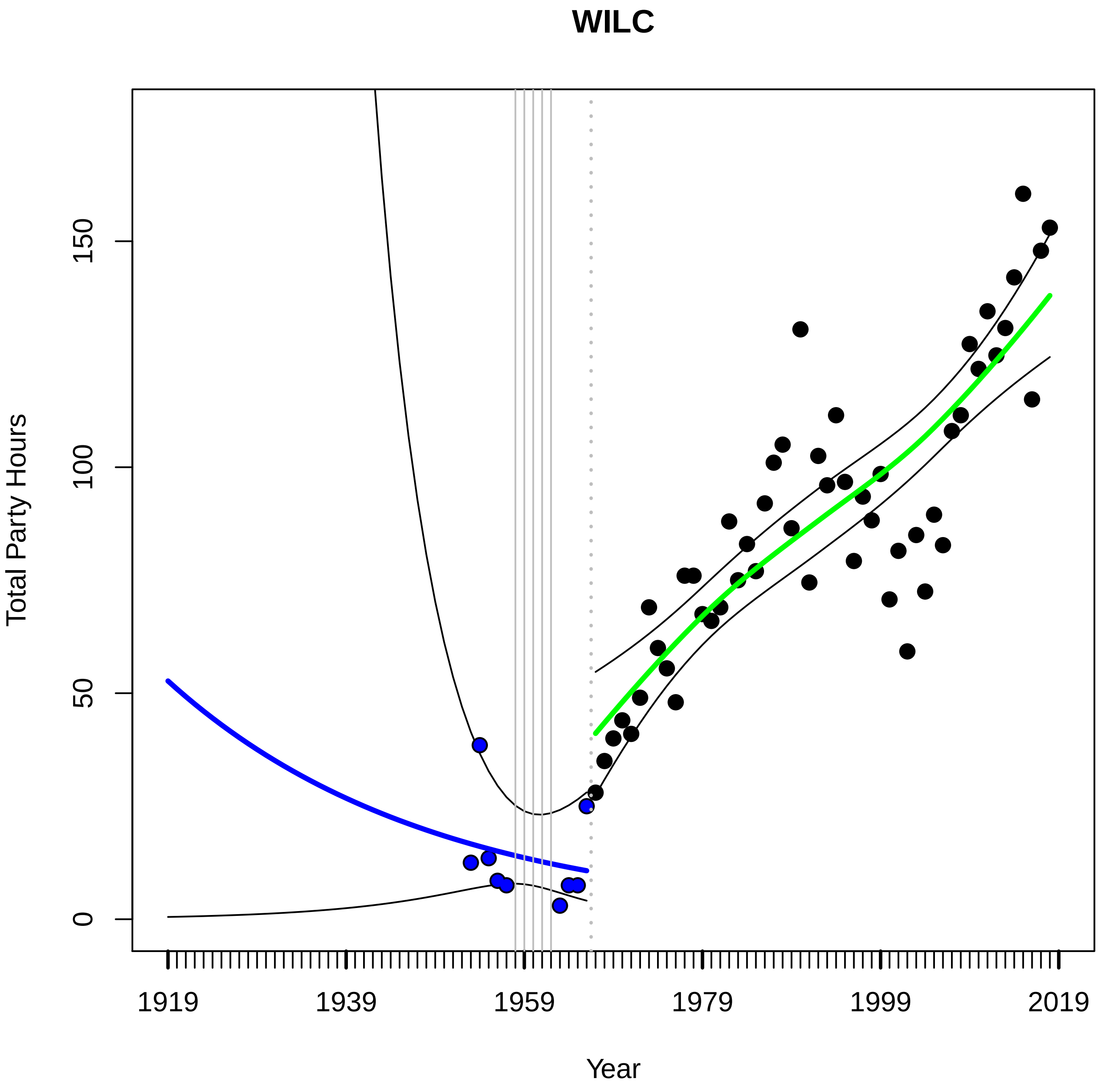

WILC
Total Party Hours
0
50
100
150
1919
1939
1959
1979
1999
2019
Year

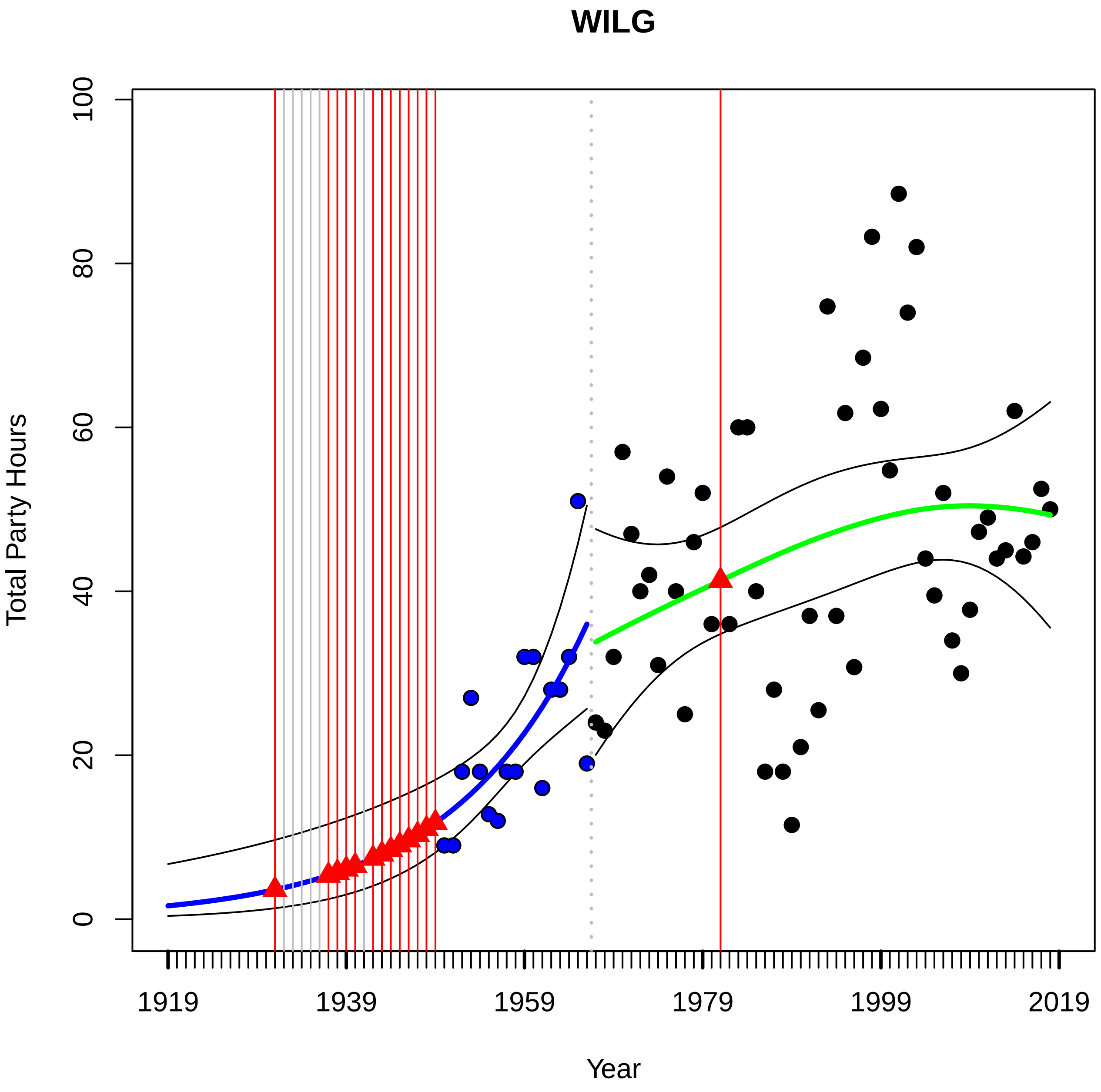

WILG
Total Party Hours
Year
0
20
40
60
80
100
1919
1939
1959
1979
1999
2019

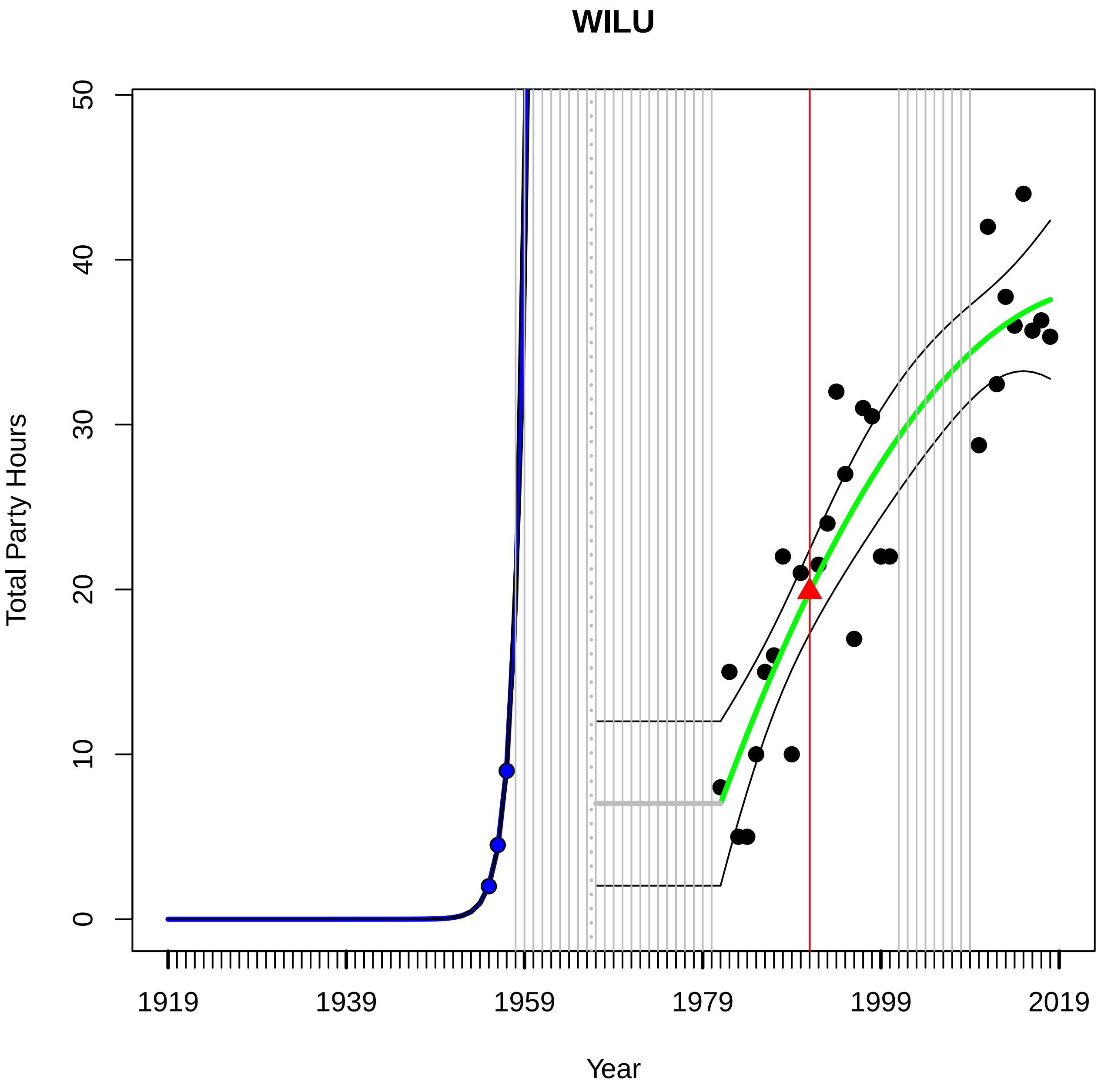

WILU
Total Party Hours
0
10
20
30
40
50
1919
1939
1959
1979
1999
2019
Year

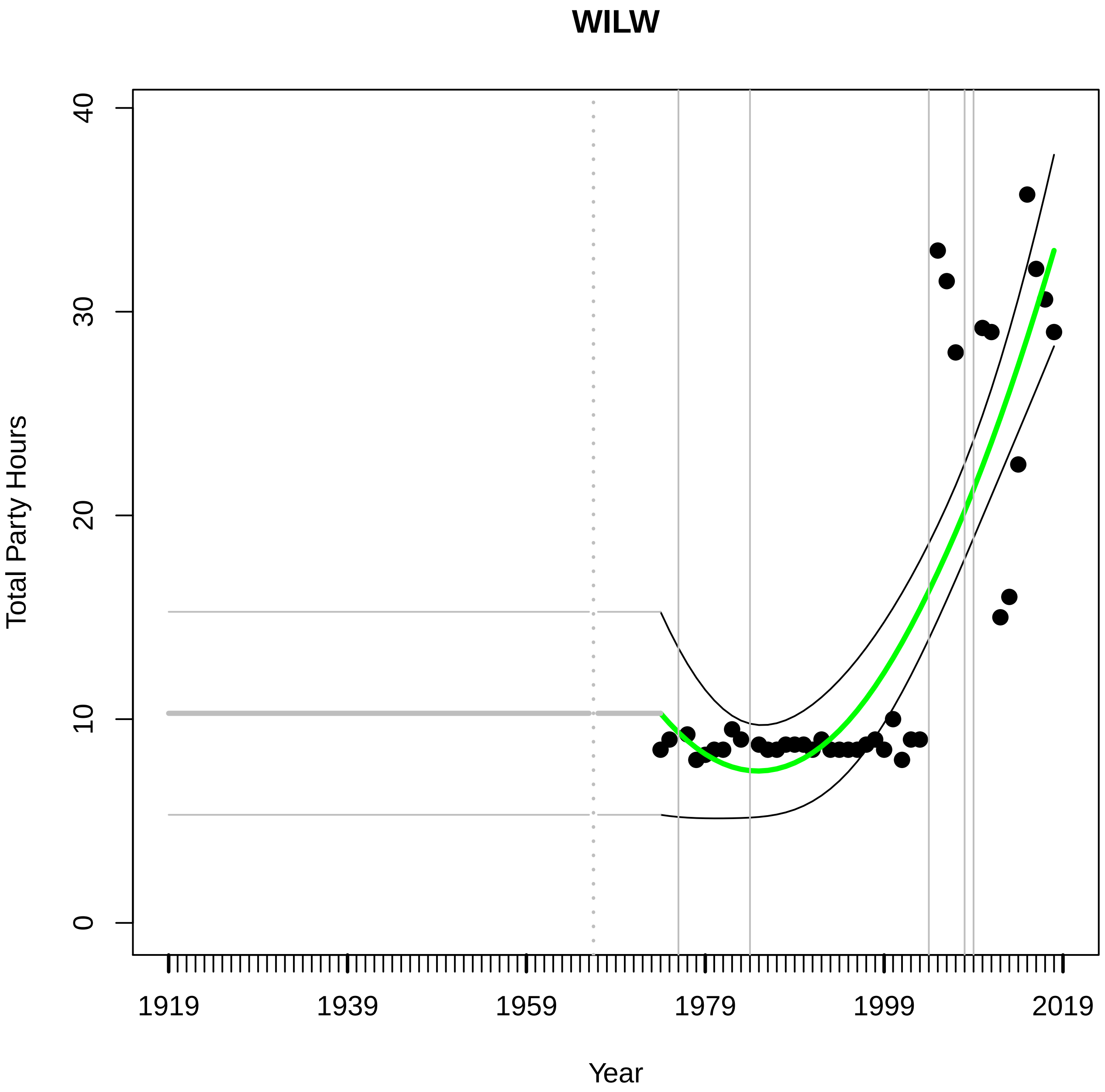
WILW
Total Party Hours
Year
0
10
20
30
40
1919
1939
1959
1979
1999
2019

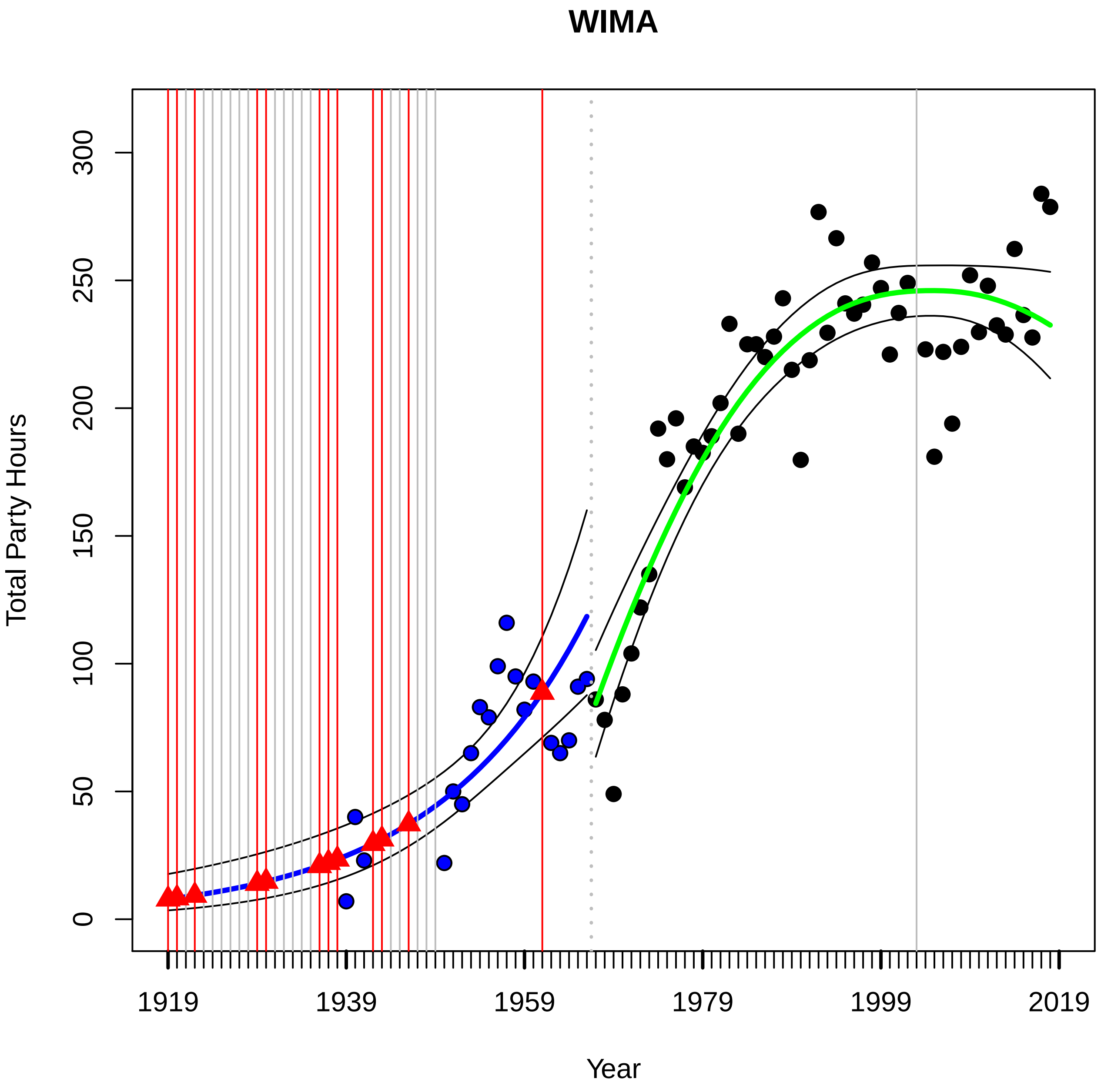

WIMA
Total Party Hours
0
50
100
150
200
250
300
1919
1939
1959
1979
1999
2019
Year

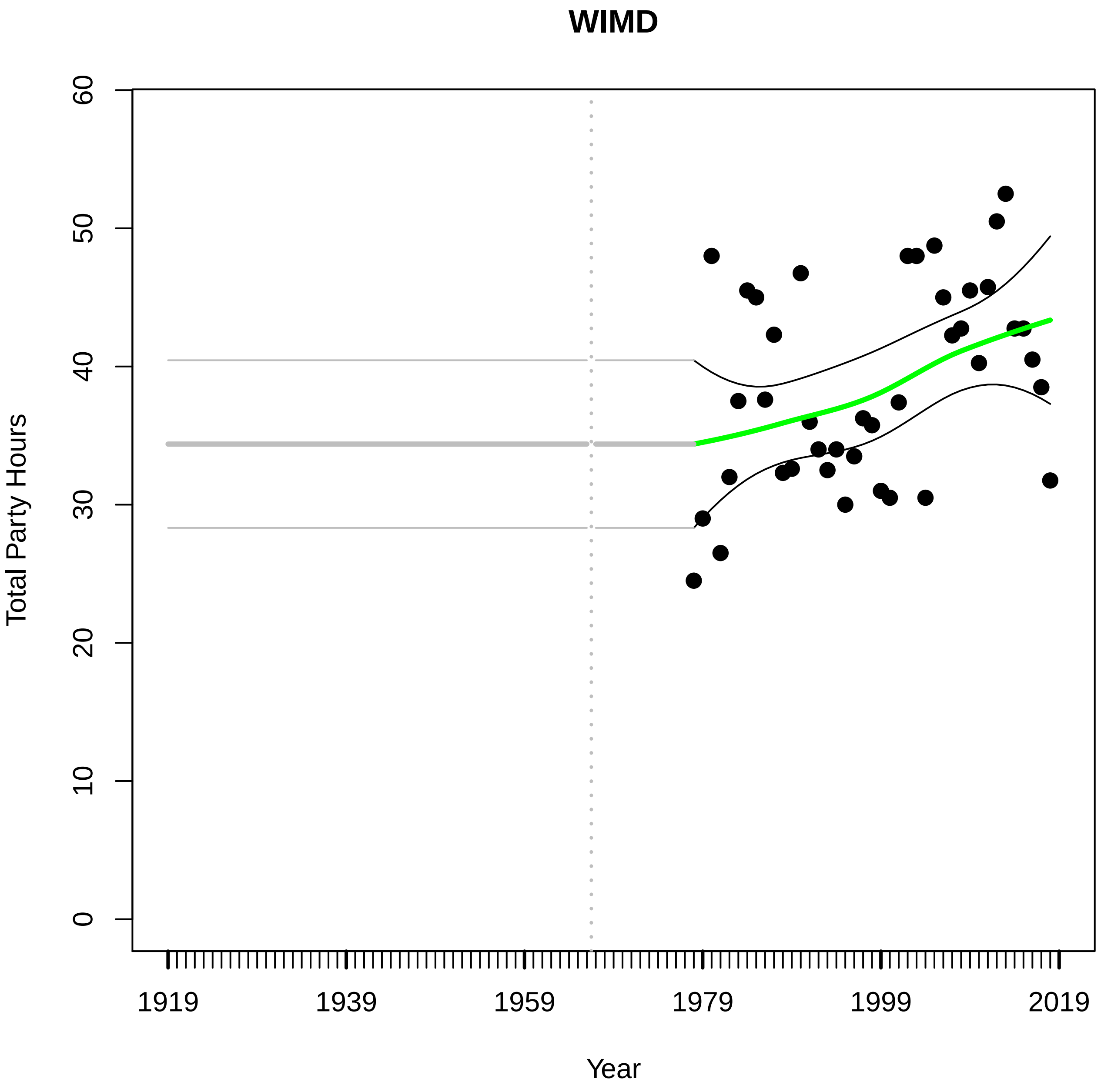
WIMD
Total Party Hours
Year
0
10
20
30
40
50
60
1919
1939
1959
1979
1999
2019

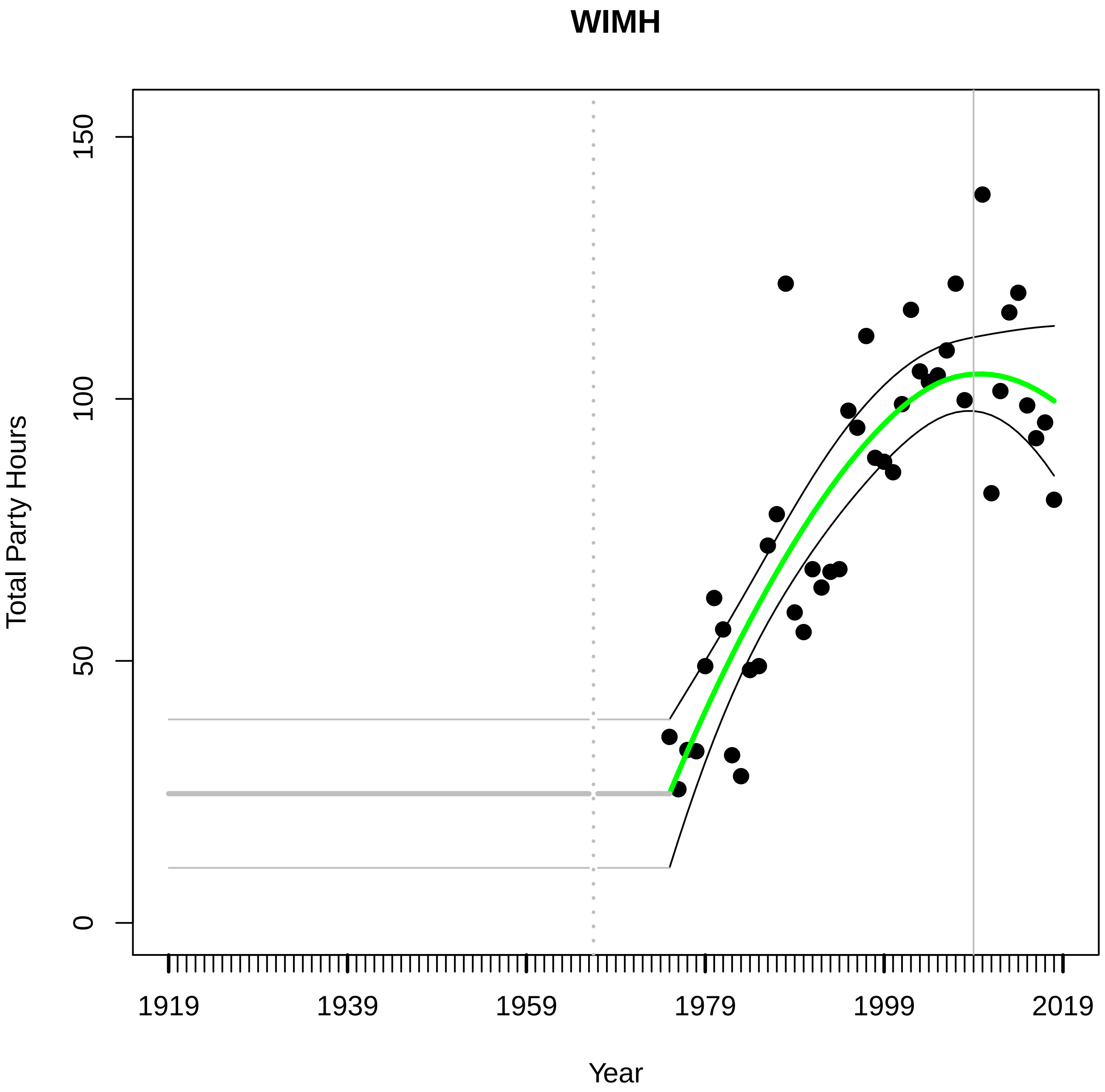
WIMH
Total Party Hours
150
100
50
0
1919
1939
1959
1979
1999
2019
Year

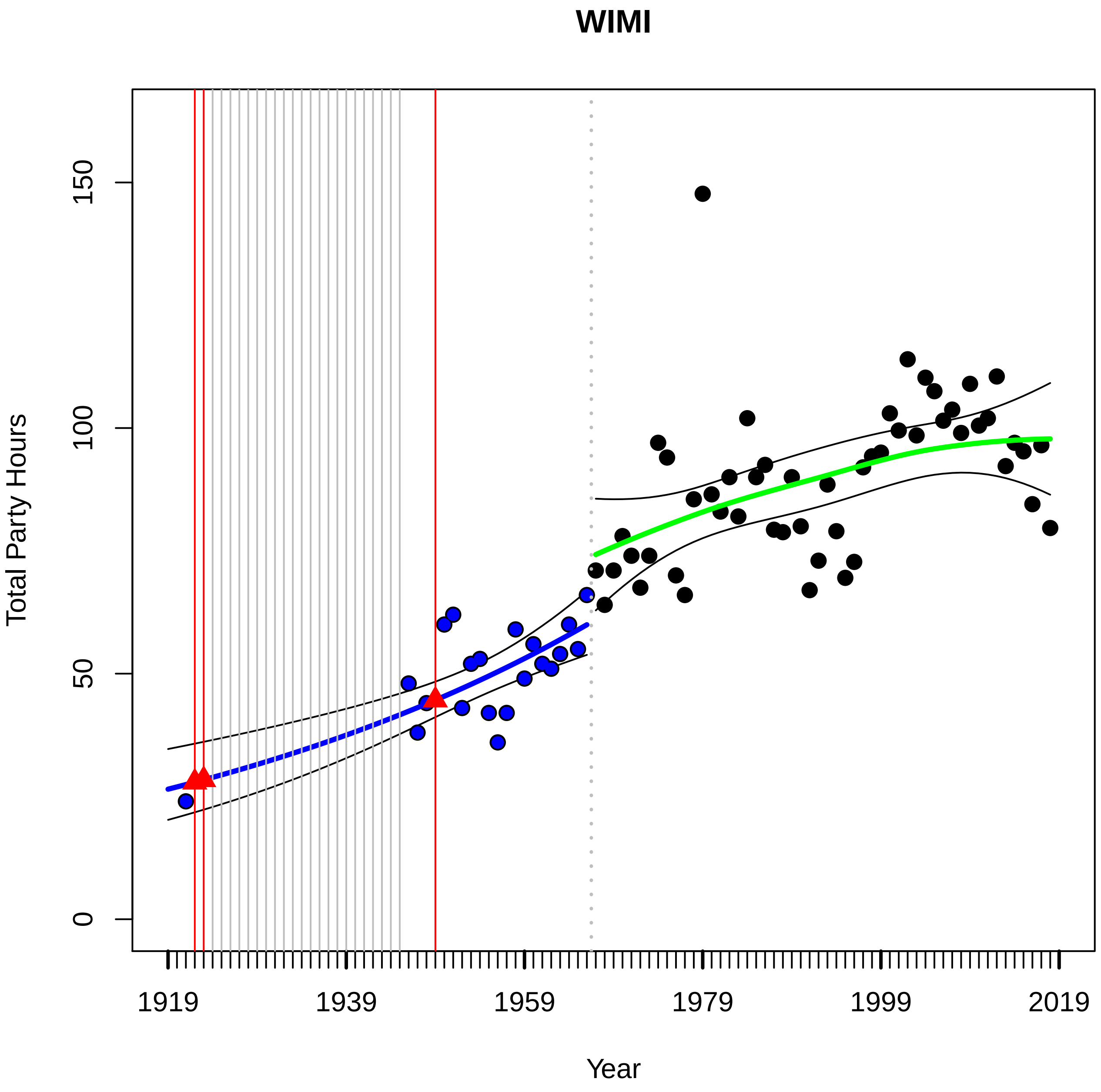

WIMI
Total Party Hours
150
100
50
0
1919
1939
1959
1979
1999
2019
Year

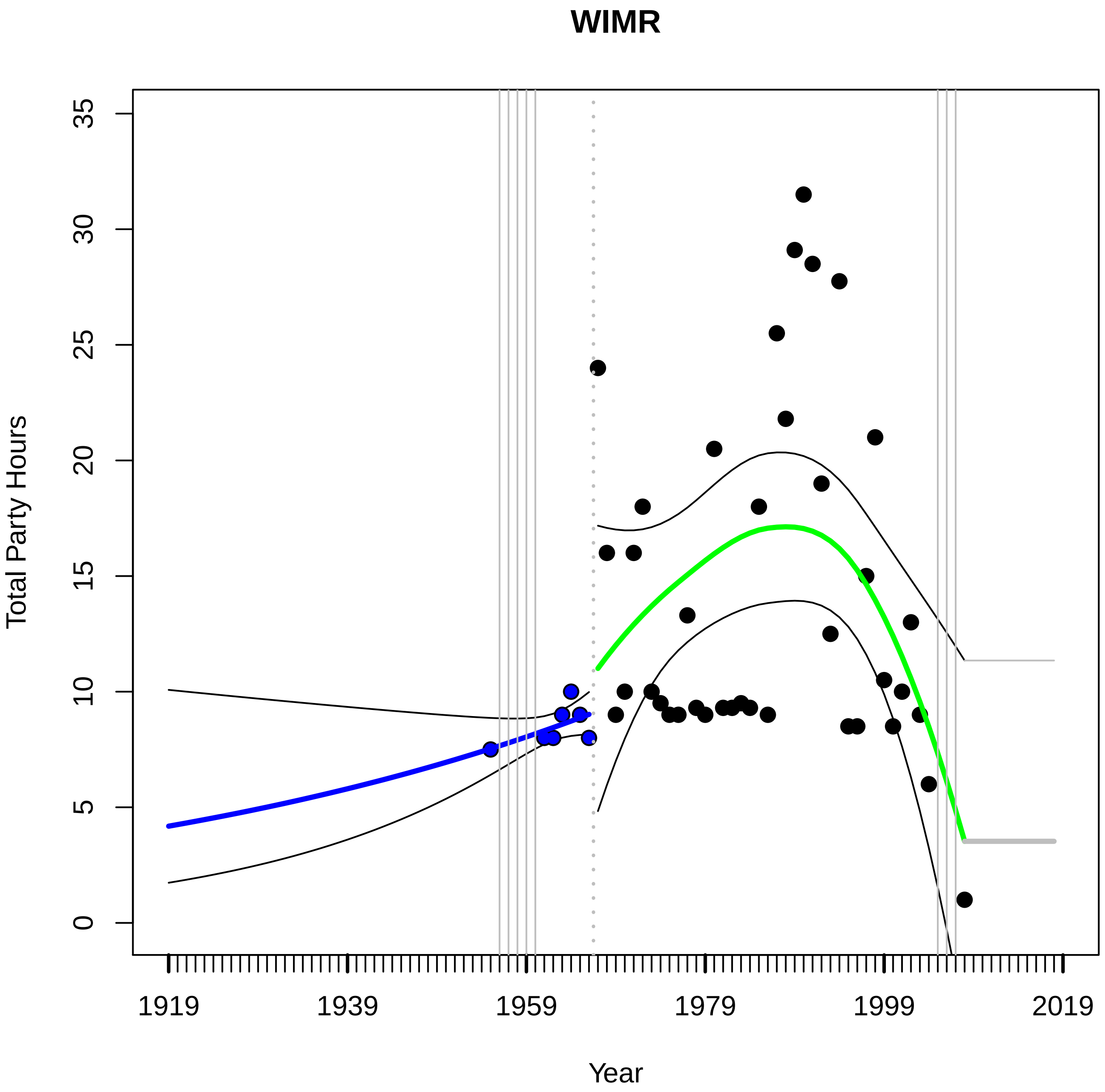

WIMR
Total Party Hours
Year
0
5
10
15
20
25
30
35
1919
1939
1959
1979
1999
2019

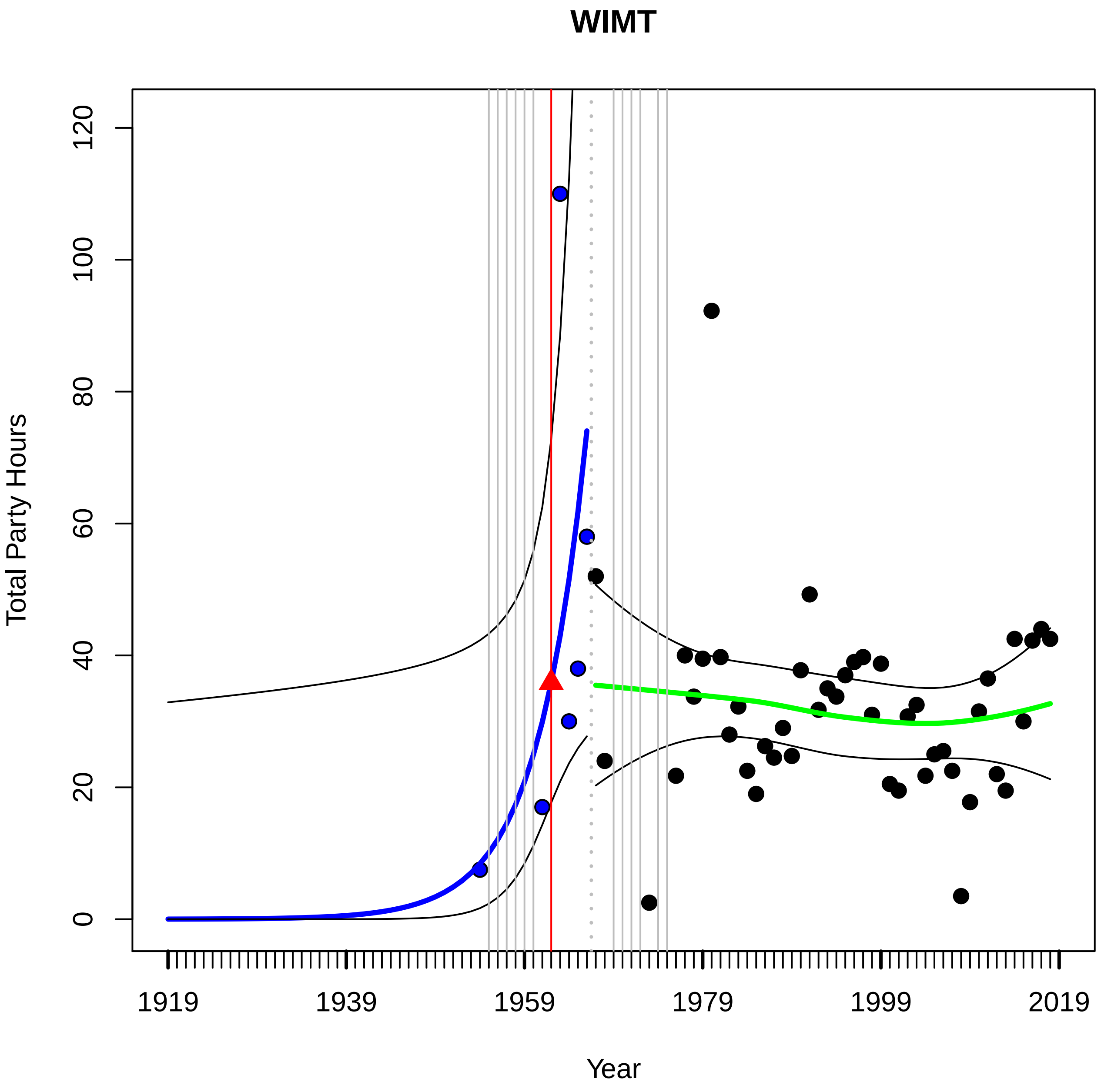
WIMT
Total Party Hours
Year
0
20
40
60
80
100
120
1919
1939
1959
1979
1999
2019

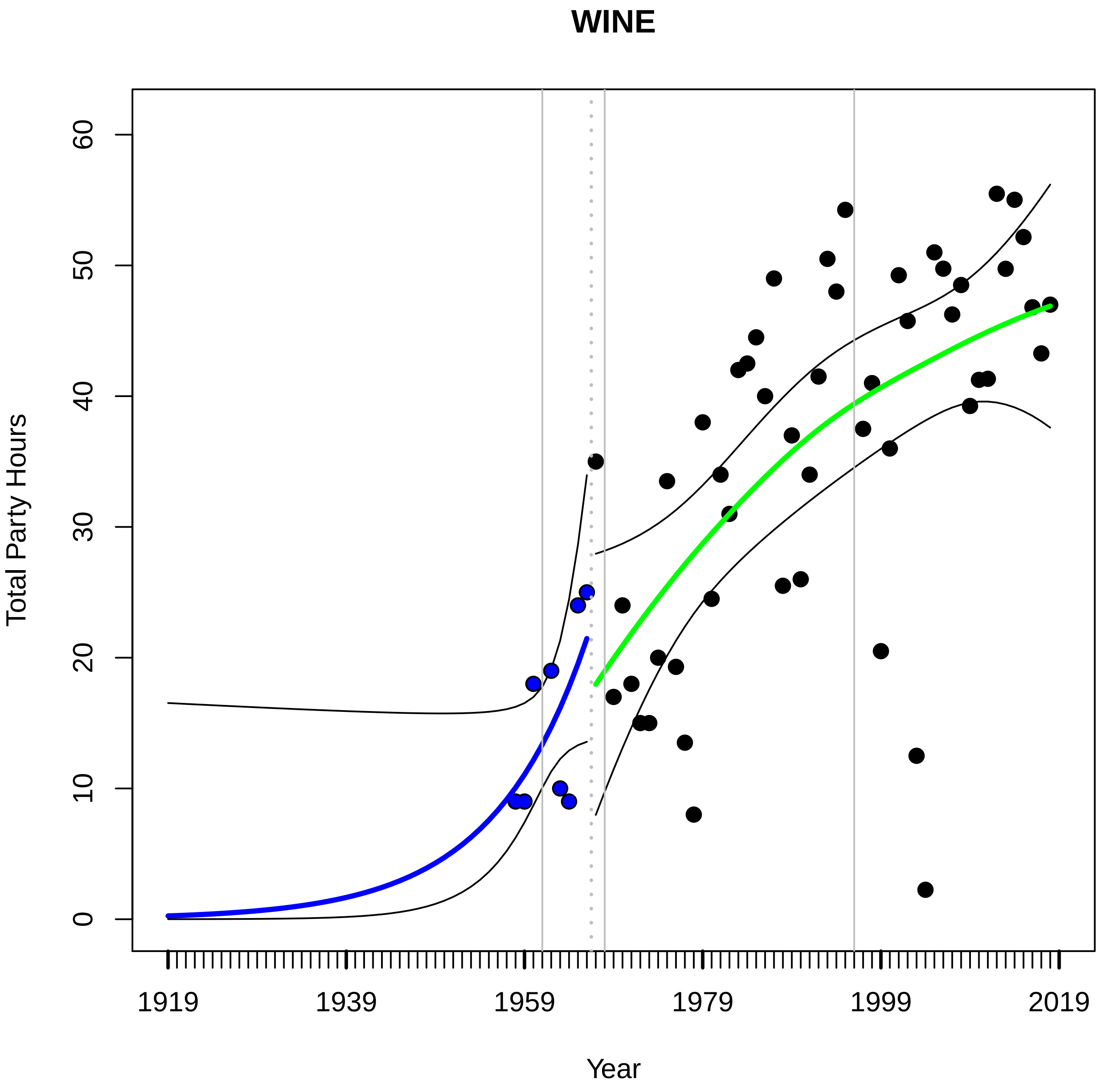

WINE
Total Party Hours
0
10
20
30
40
50
60
1919
1939
1959
1979
1999
2019
Year

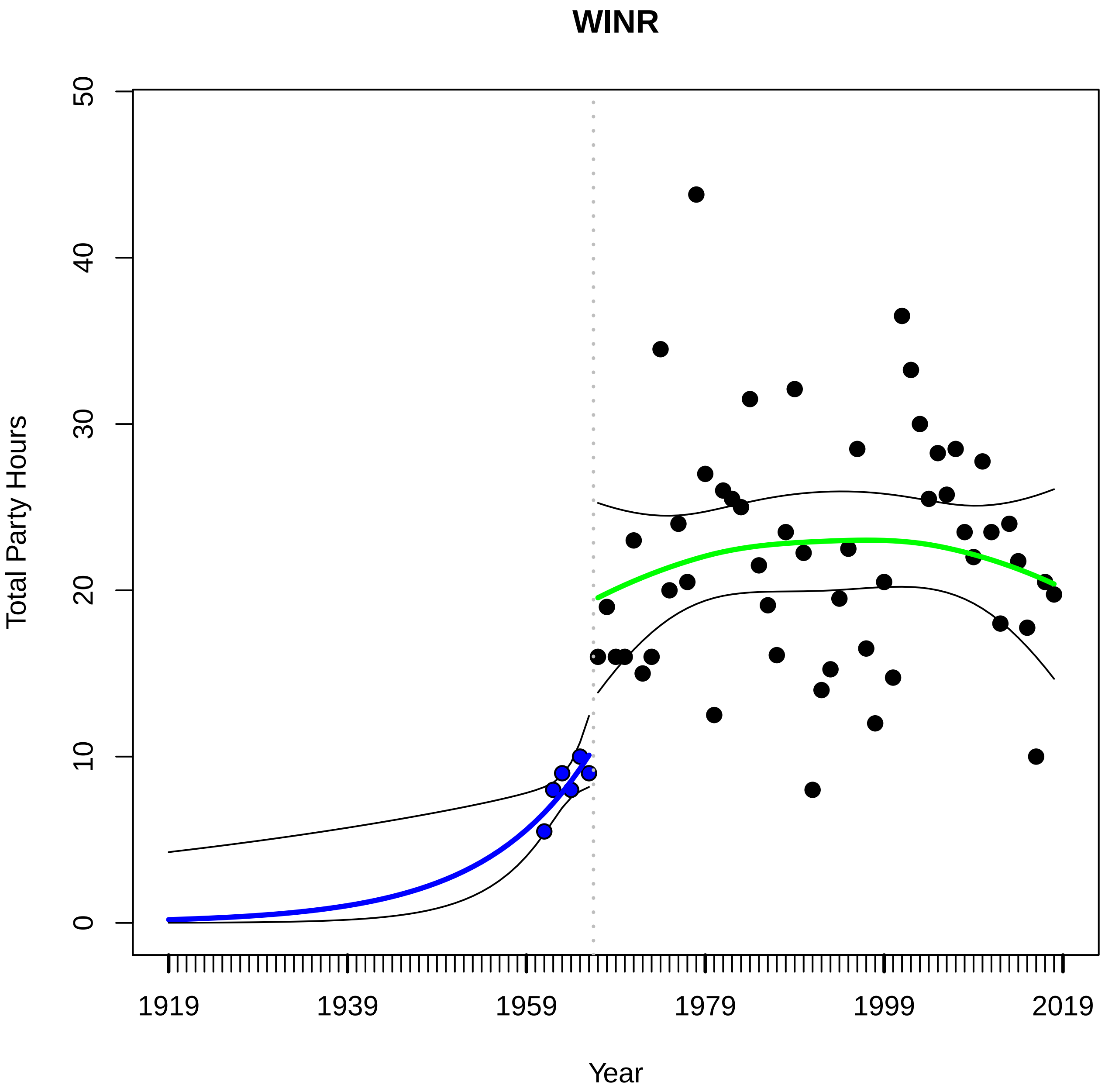

WINR
Total Party Hours
0
10
20
30
40
50
1919
1939
1959
1979
1999
2019
Year

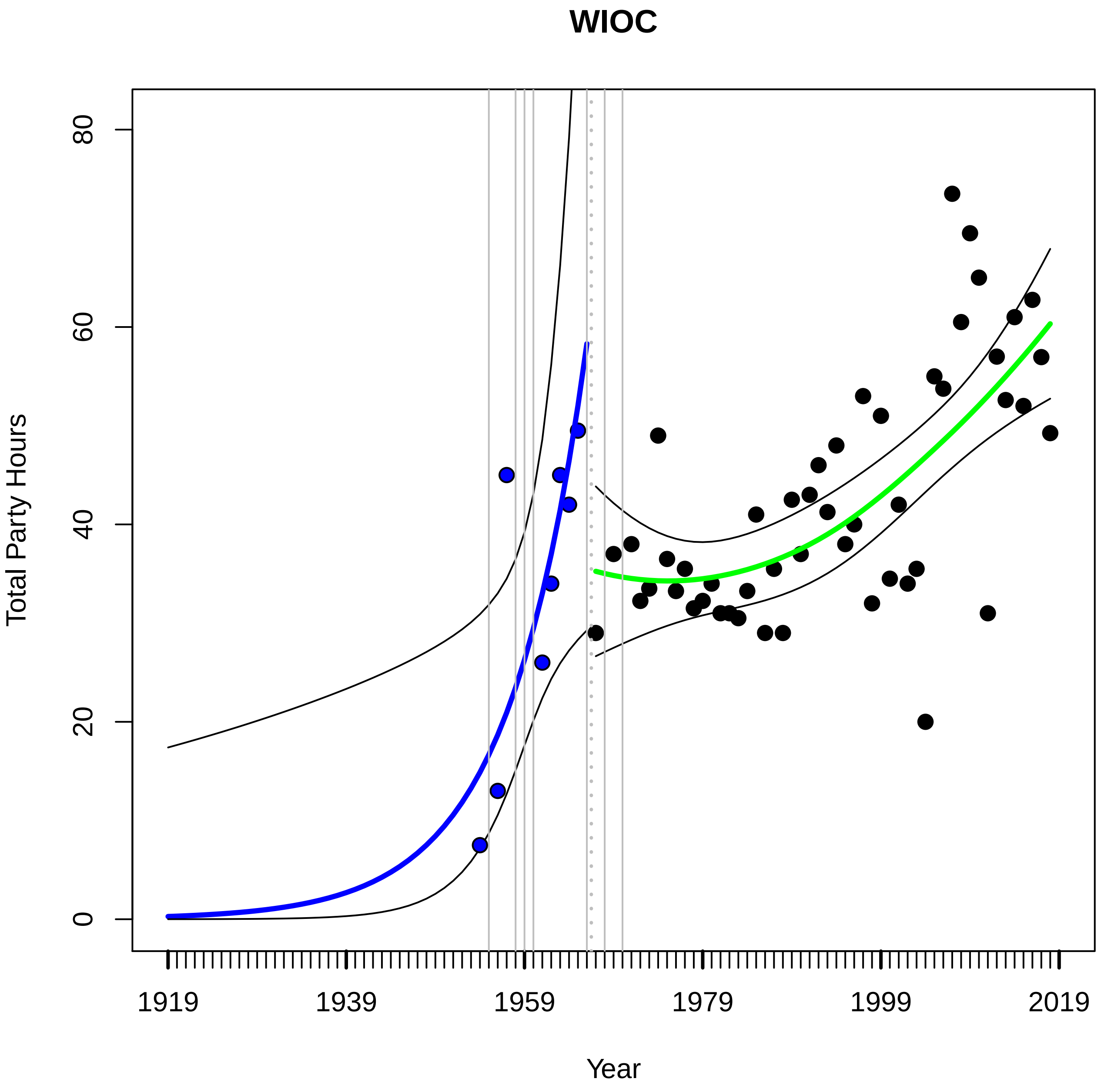

WIOC
Total Party Hours
0
20
40
60
80
1919
1939
1959
1979
1999
2019
Year

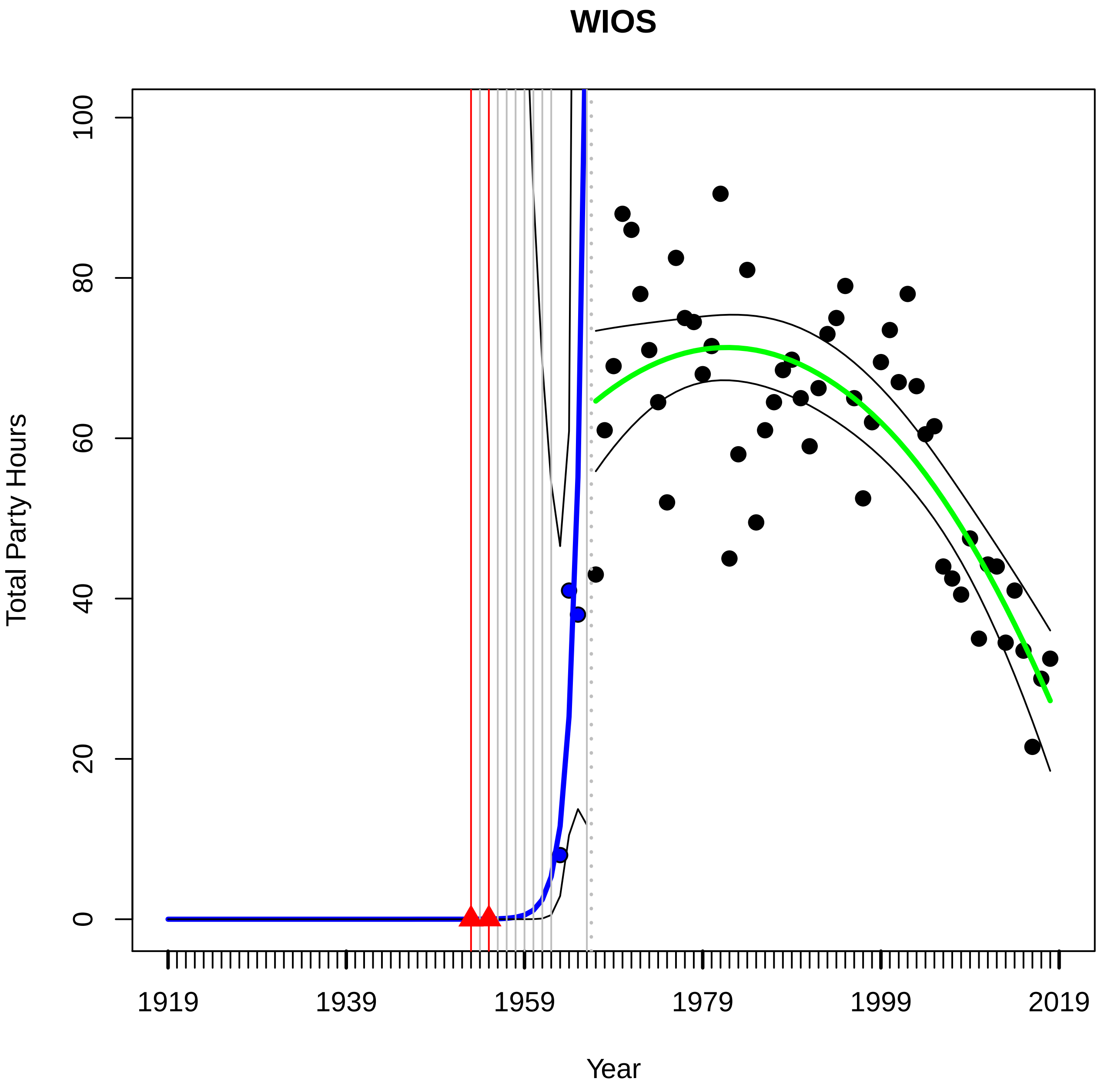

WIOS
Total Party Hours
0
20
40
60
80
100
1919
1939
1959
1979
1999
2019
Year

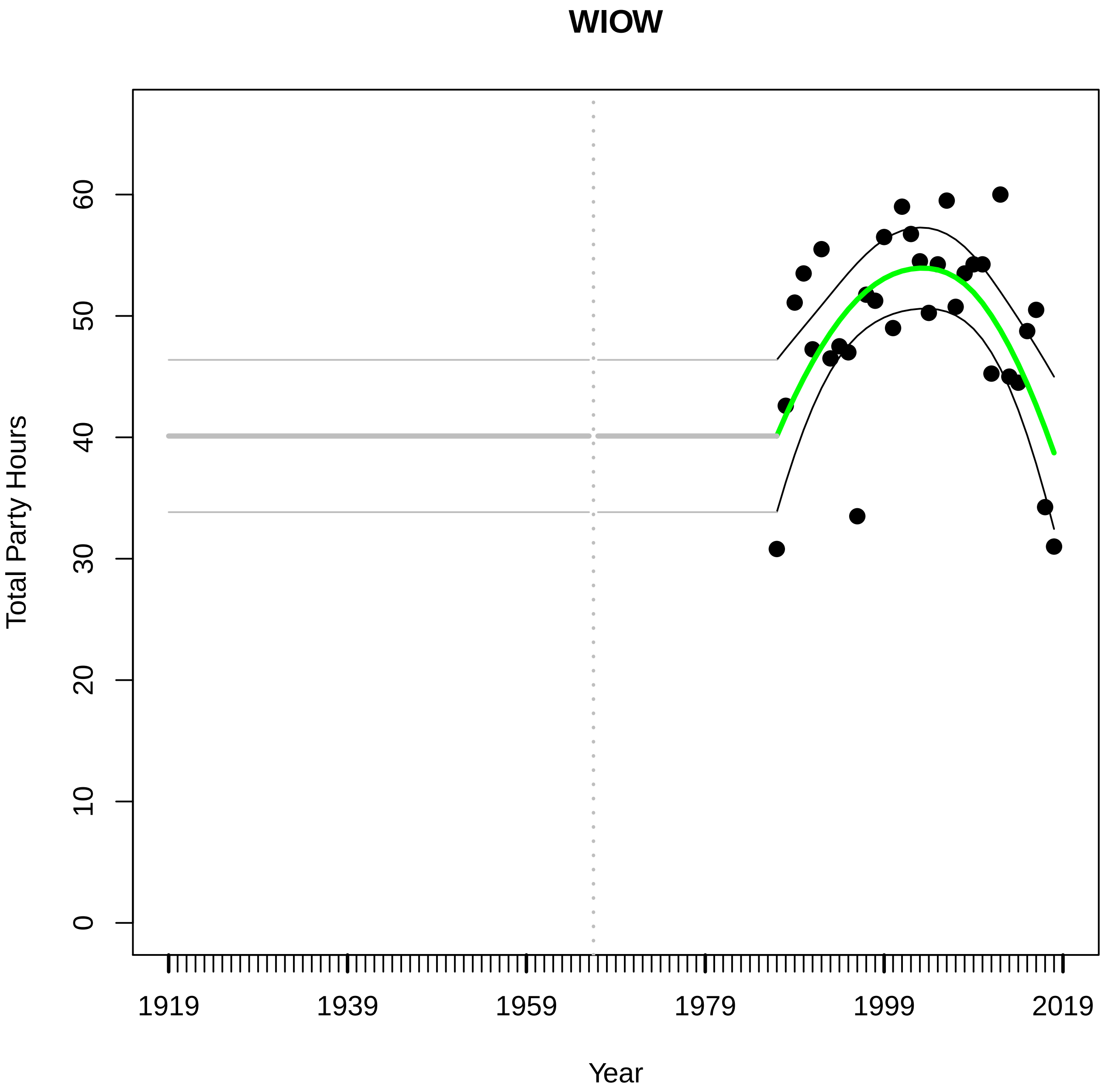
WIOW
Total Party Hours
0
10
20
30
40
50
60
1919
1939
1959
1979
1999
2019
Year

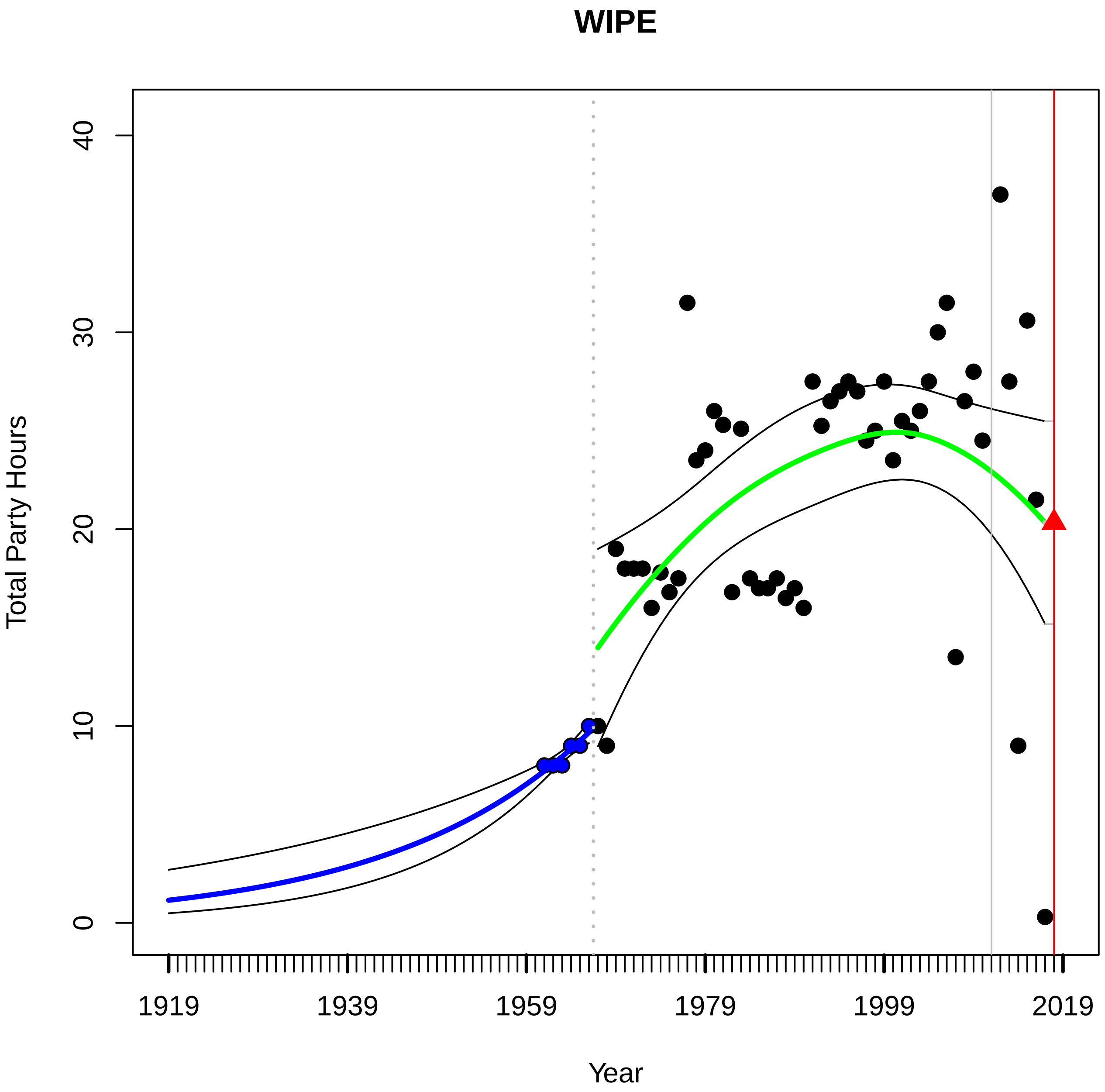

WIPE
Total Party Hours
0
10
20
30
40
Year
1919
1939
1959
1979
1999
2019

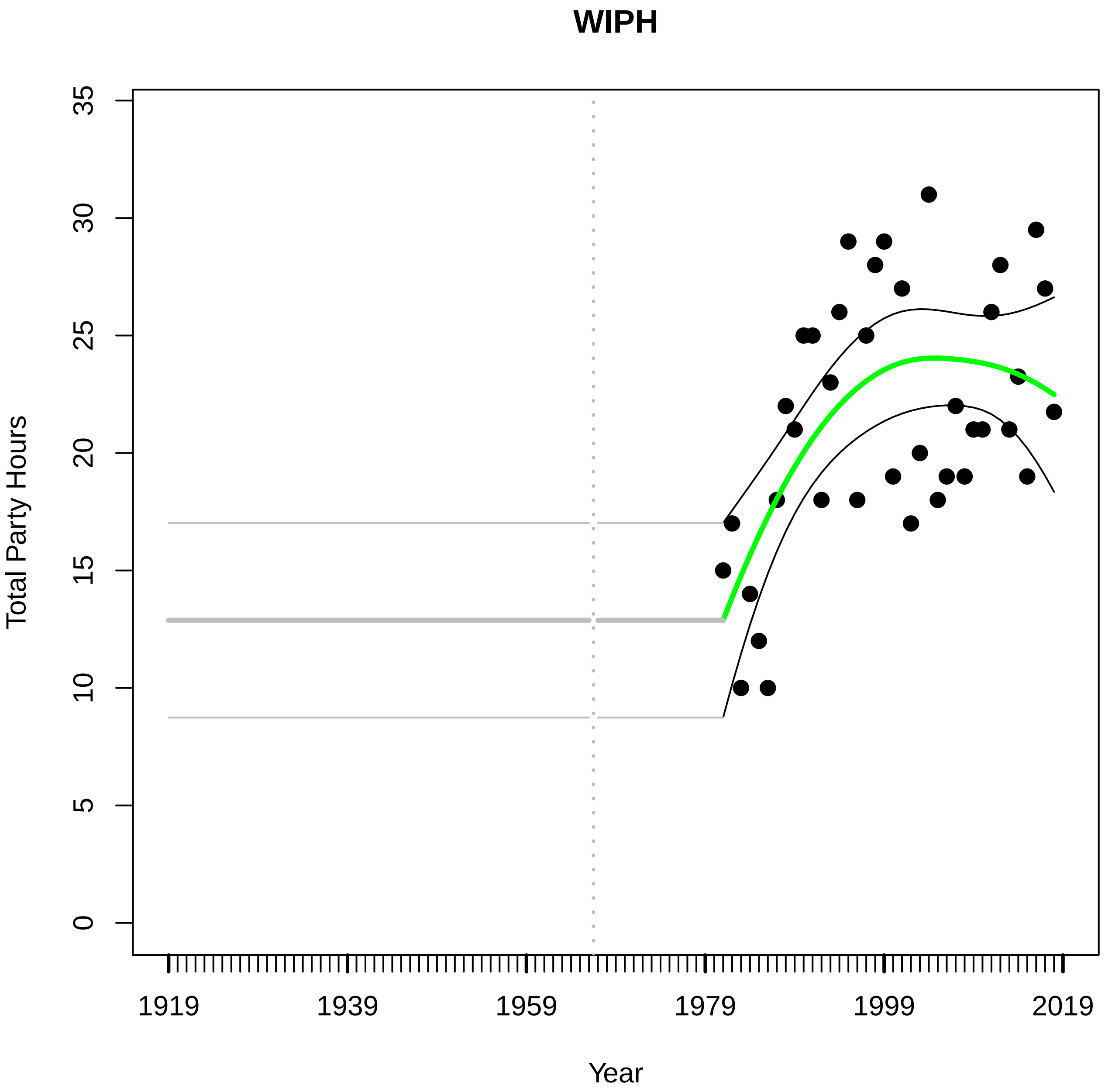

WIPH
Total Party Hours
0
5
10
15
20
25
30
35
1919
1939
1959
1979
1999
2019
Year

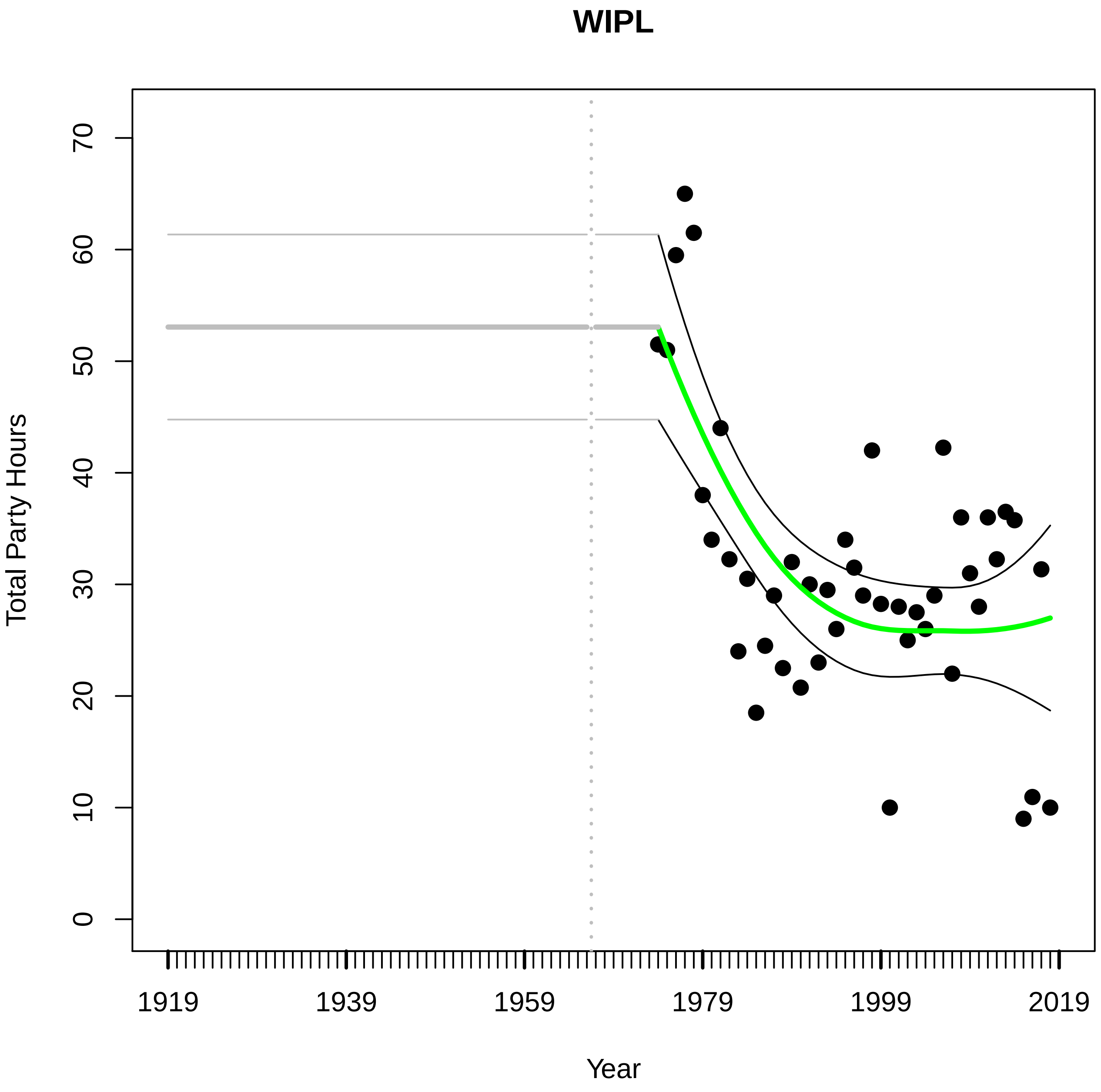

WIPL
Total Party Hours
Year
0
10
20
30
40
50
60
70
1919
1939
1959
1979
1999
2019

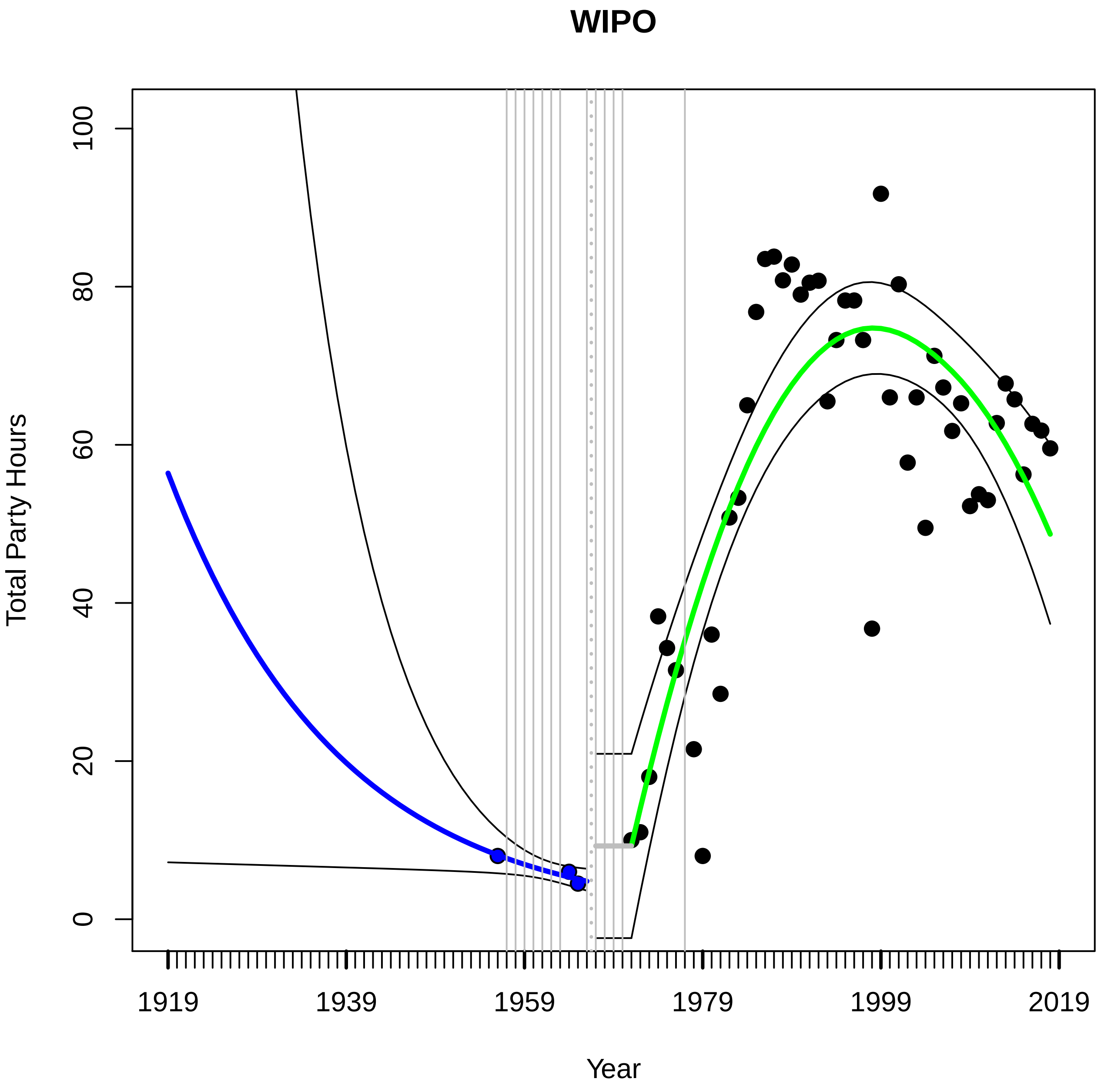

WIPO
Total Party Hours
Year
0
20
40
60
80
100
1919
1939
1959
1979
1999
2019

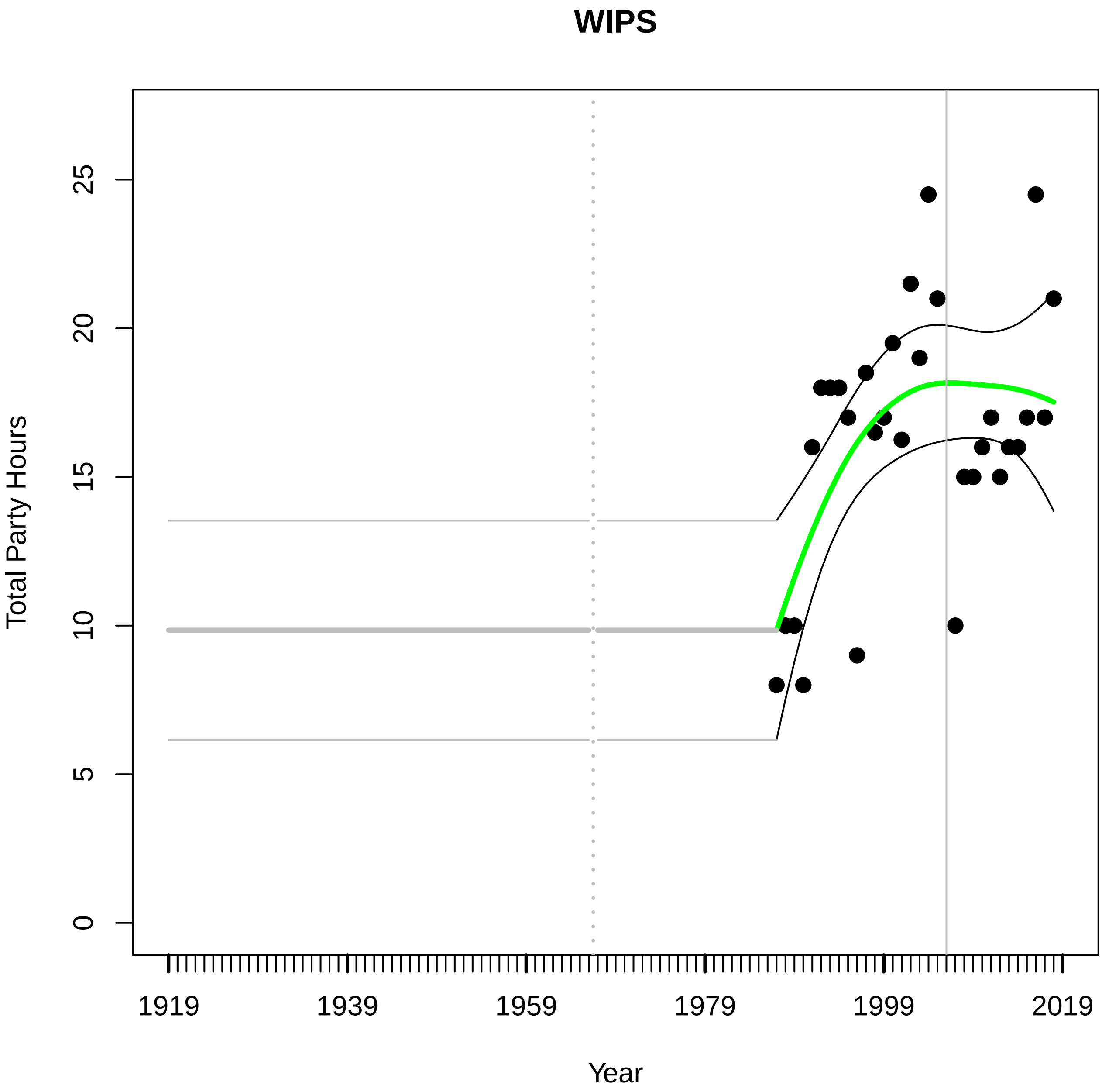
WIPS
Total Party Hours
0
5
10
15
20
25
1919
1939
1959
1979
1999
2019
Year

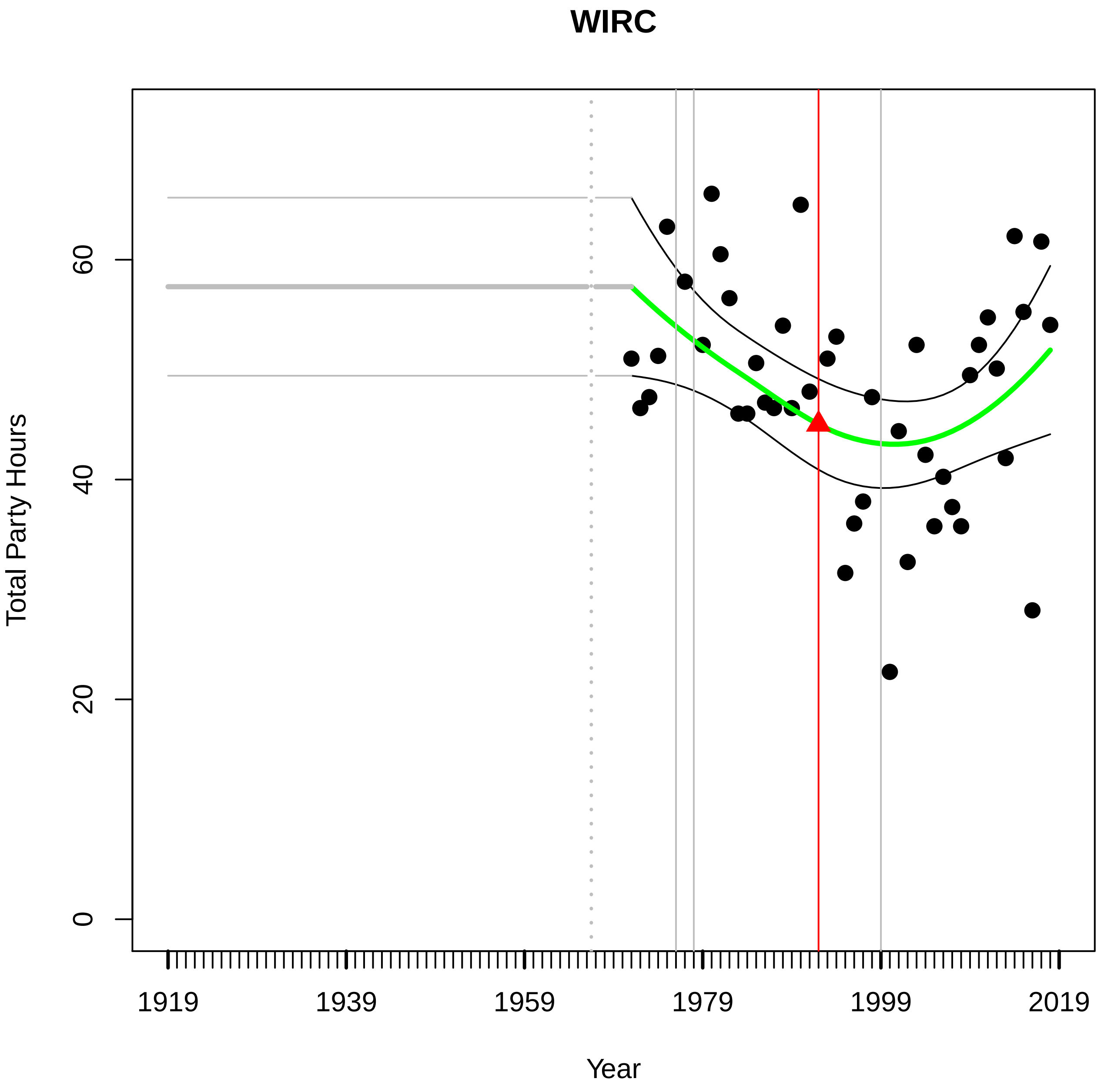

WIRC
Total Party Hours
0
20
40
60
1919
1939
1959
1979
1999
2019
Year

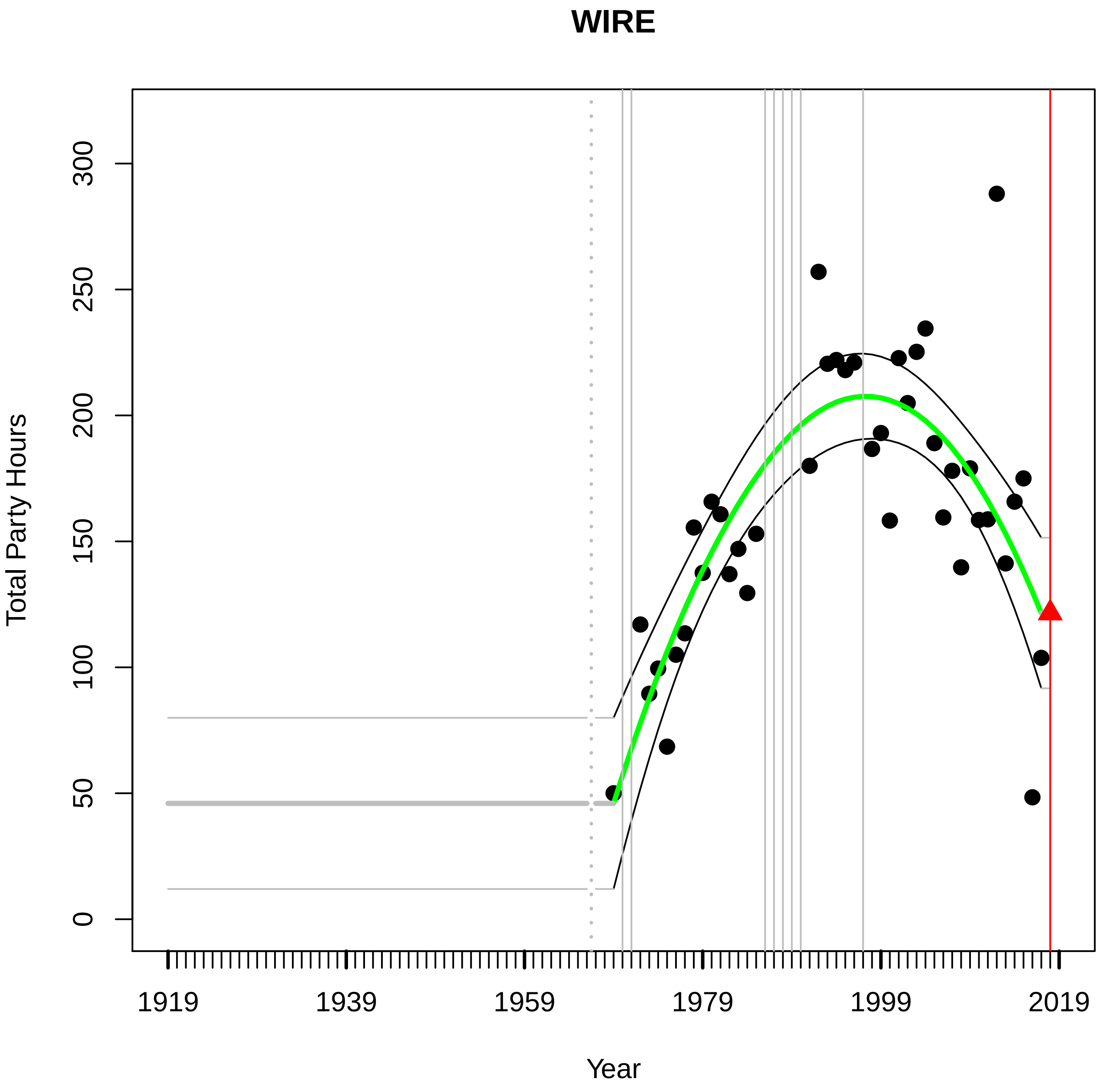

WIRE
Total Party Hours
0
50
100
150
200
250
300
1919
1939
1959
1979
1999
2019
Year

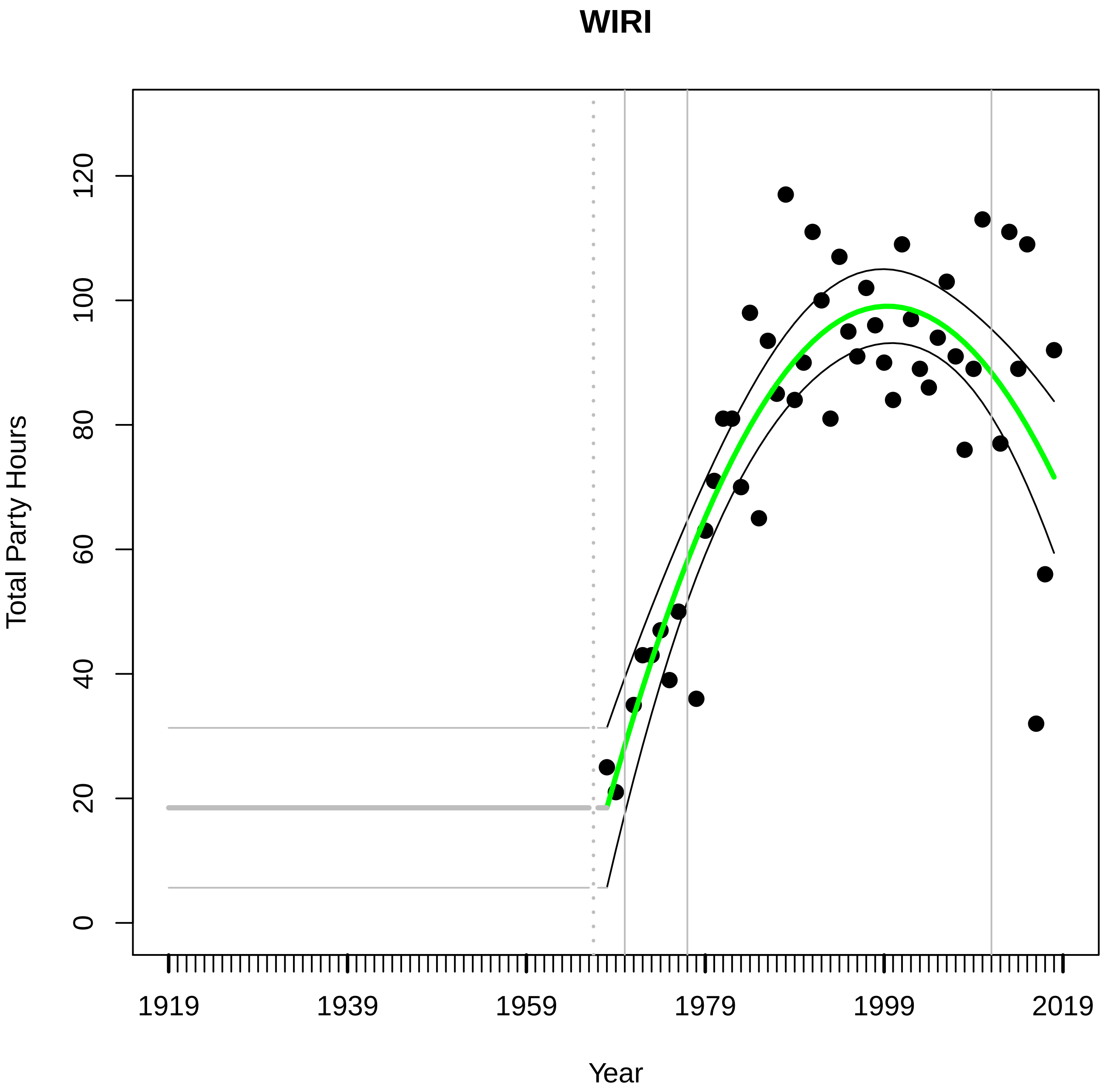

WIRI
Total Party Hours
0
20
40
60
80
100
120
1919
1939
1959
1979
1999
2019
Year

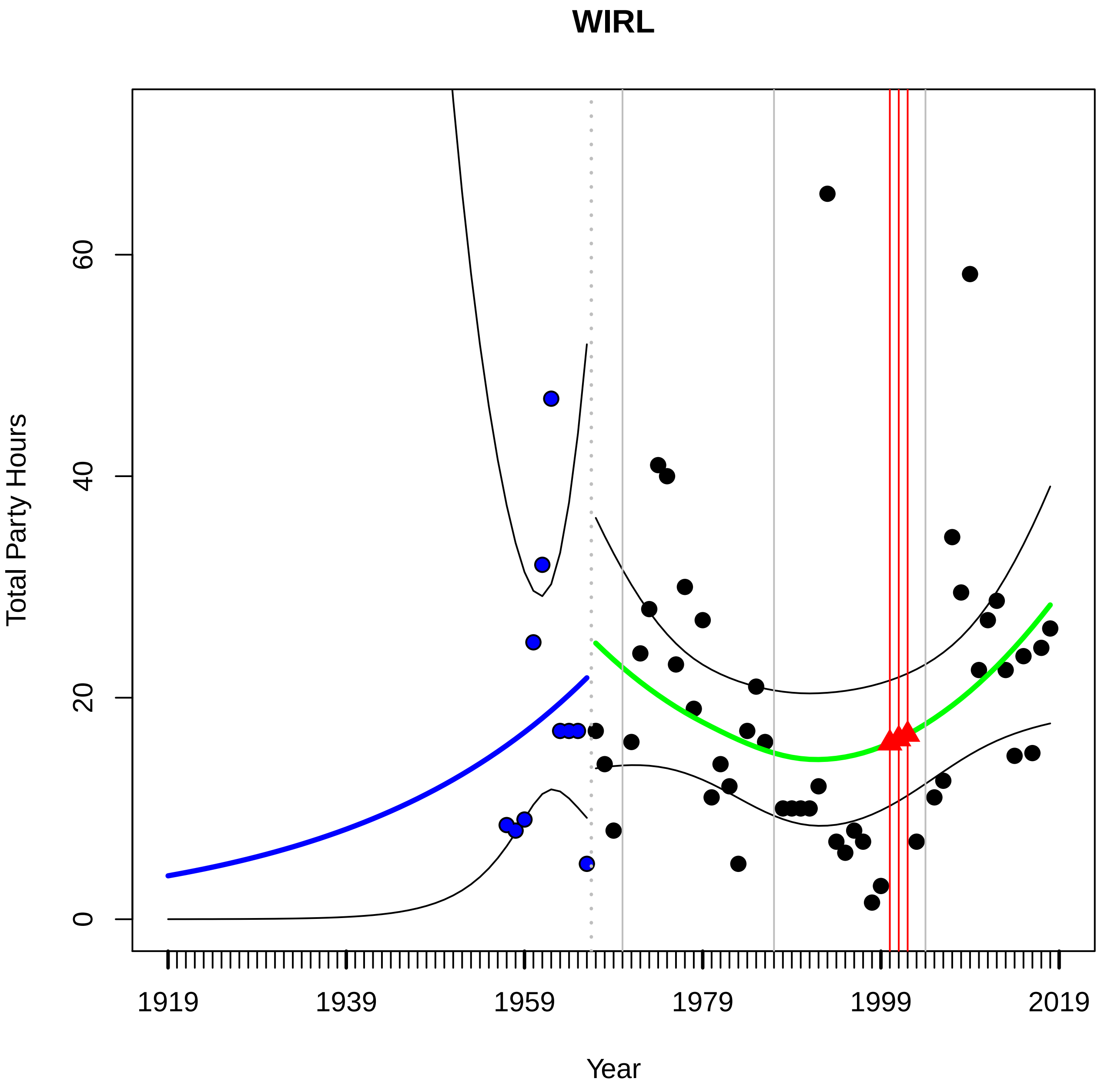

WIRL
Total Party Hours
0
20
40
60
Year
1919
1939
1959
1979
1999
2019

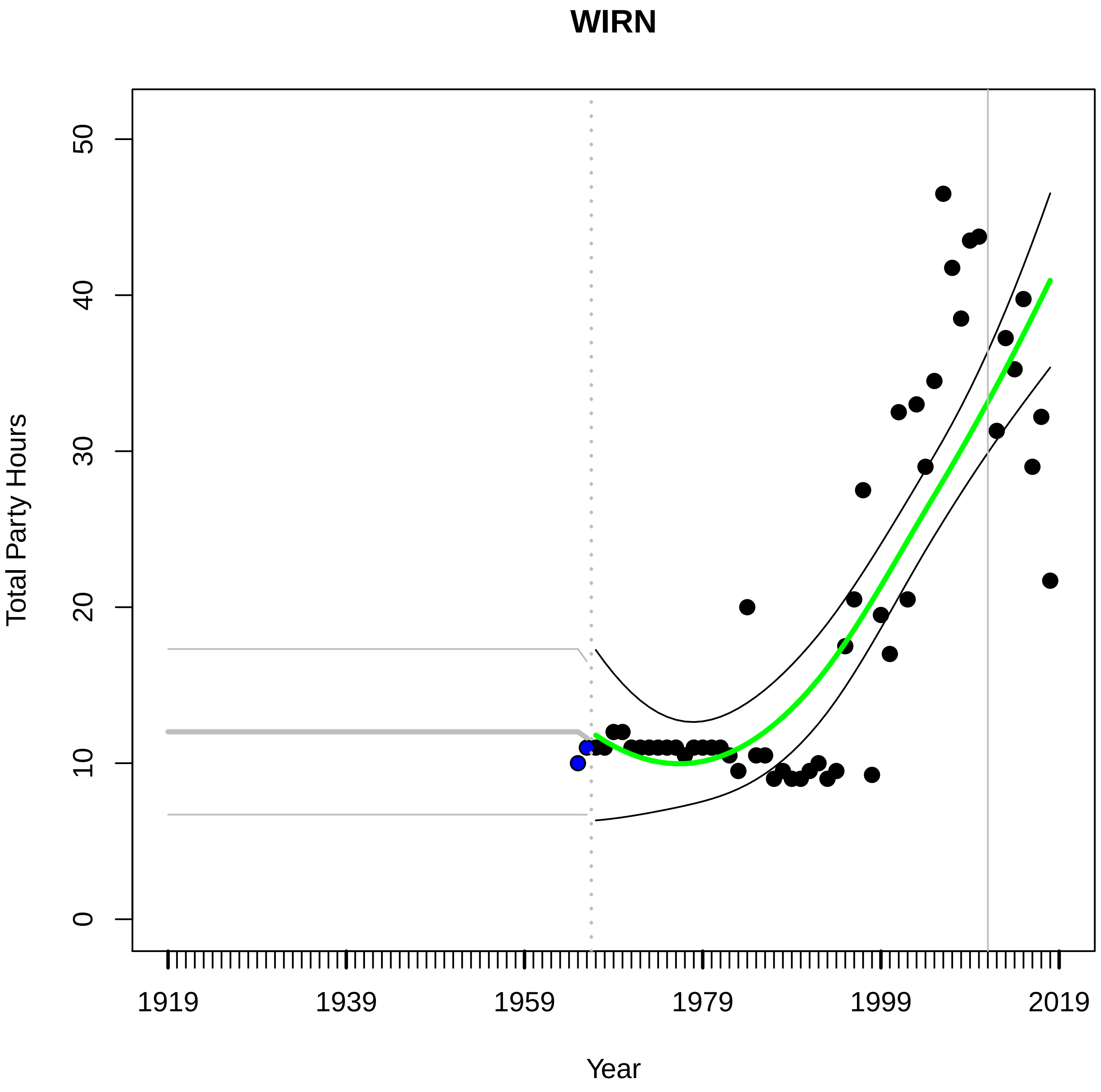

WIRN
Total Party Hours
50
40
30
20
10
0
1919
1939
1959
1979
1999
2019
Year

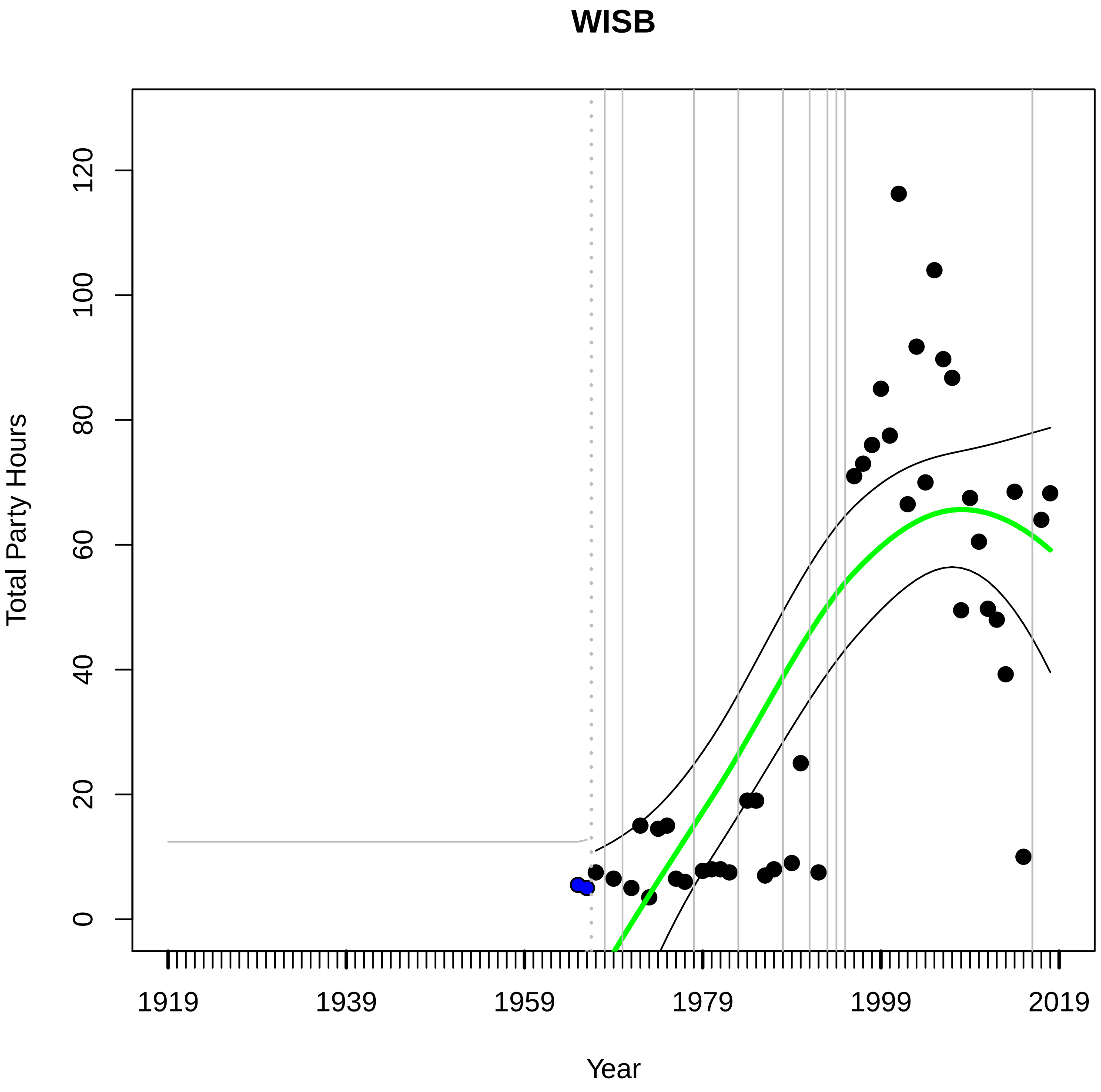

WISB
Total Party Hours
0
20
40
60
80
100
120
1919
1939
1959
1979
1999
2019
Year

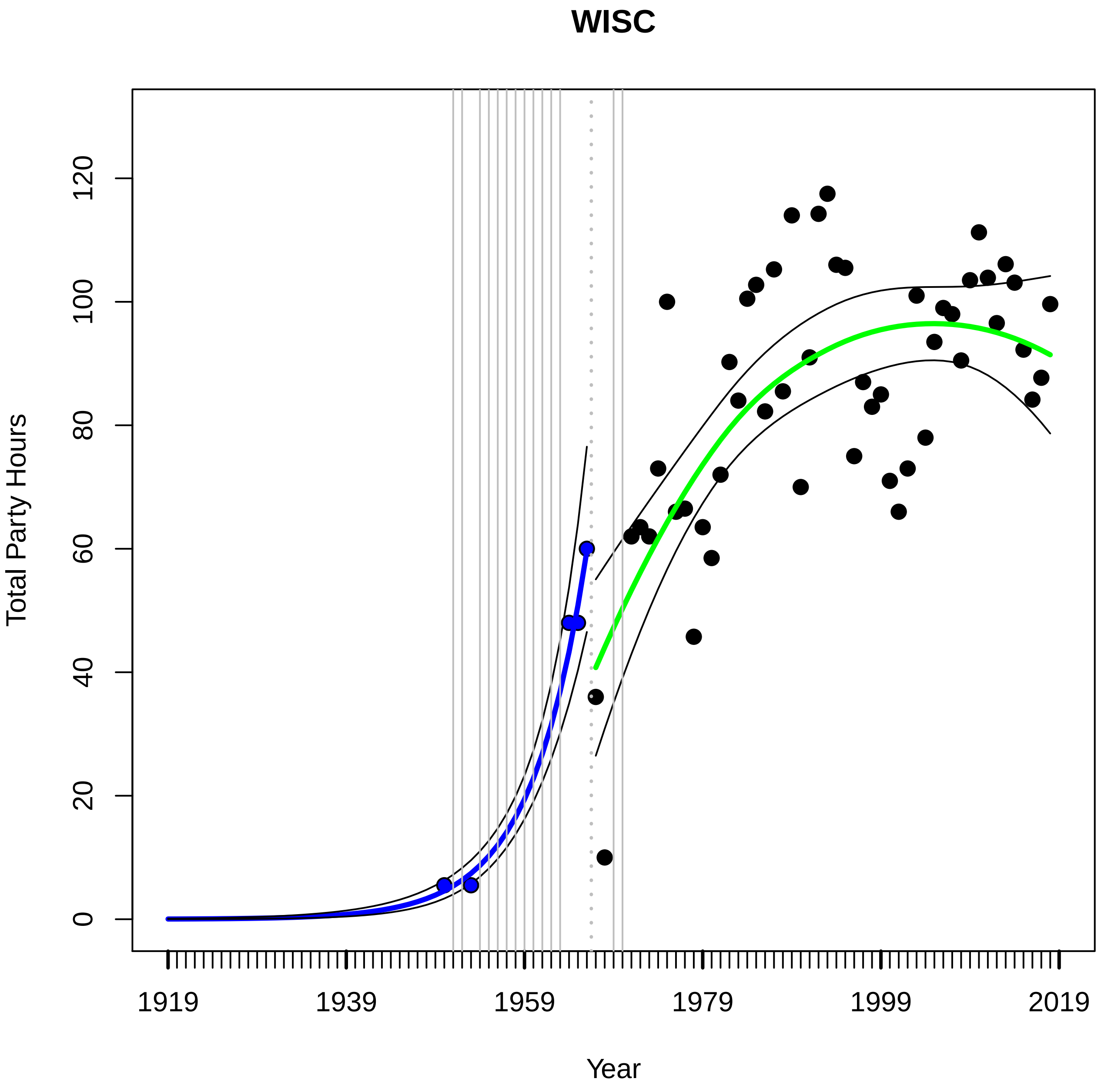

WISC
Total Party Hours
0
20
40
60
80
100
120
1919
1939
1959
1979
1999
2019
Year

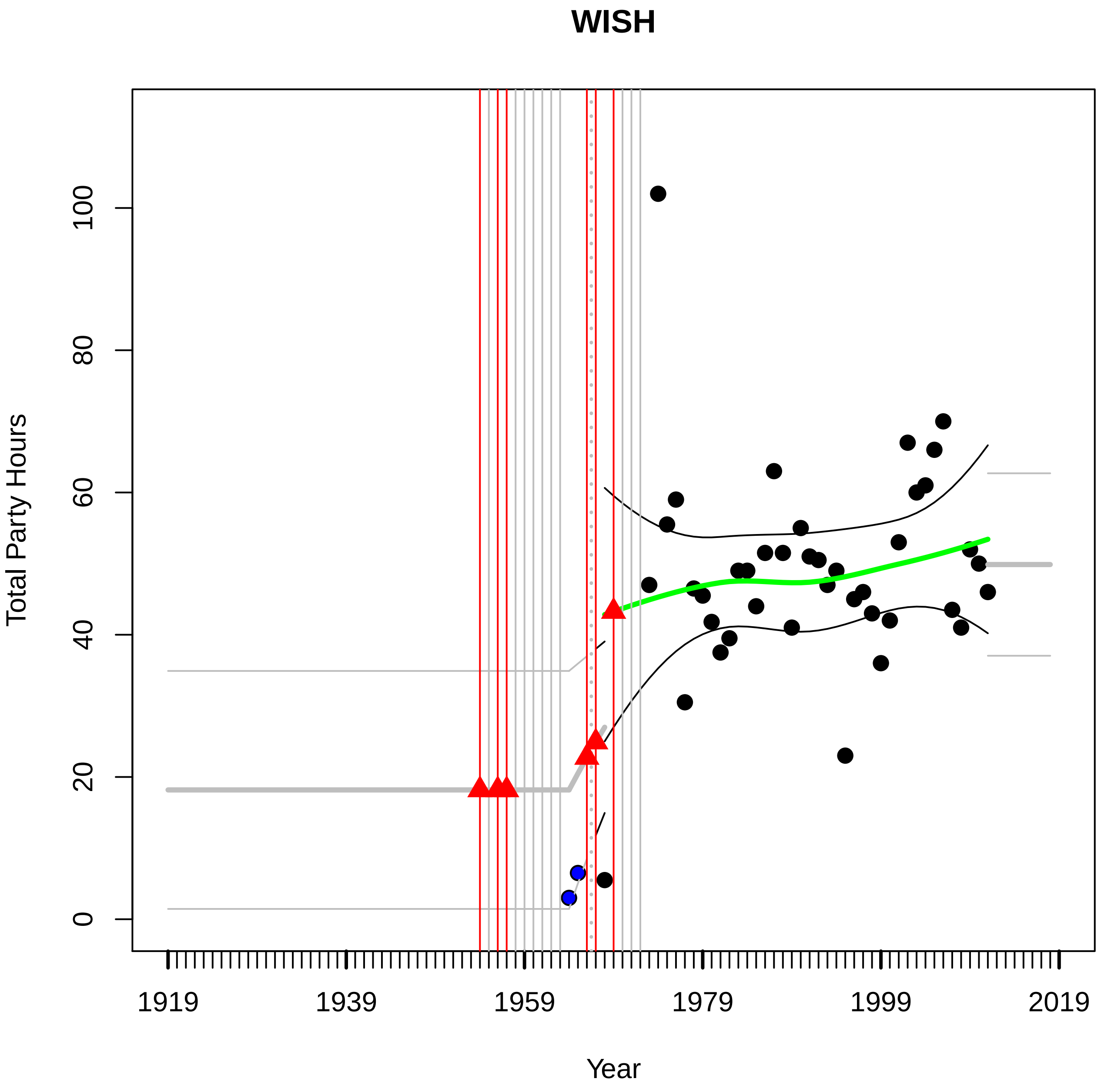

WISH
Total Party Hours
0
20
40
60
80
100
1919
1939
1959
1979
1999
2019
Year

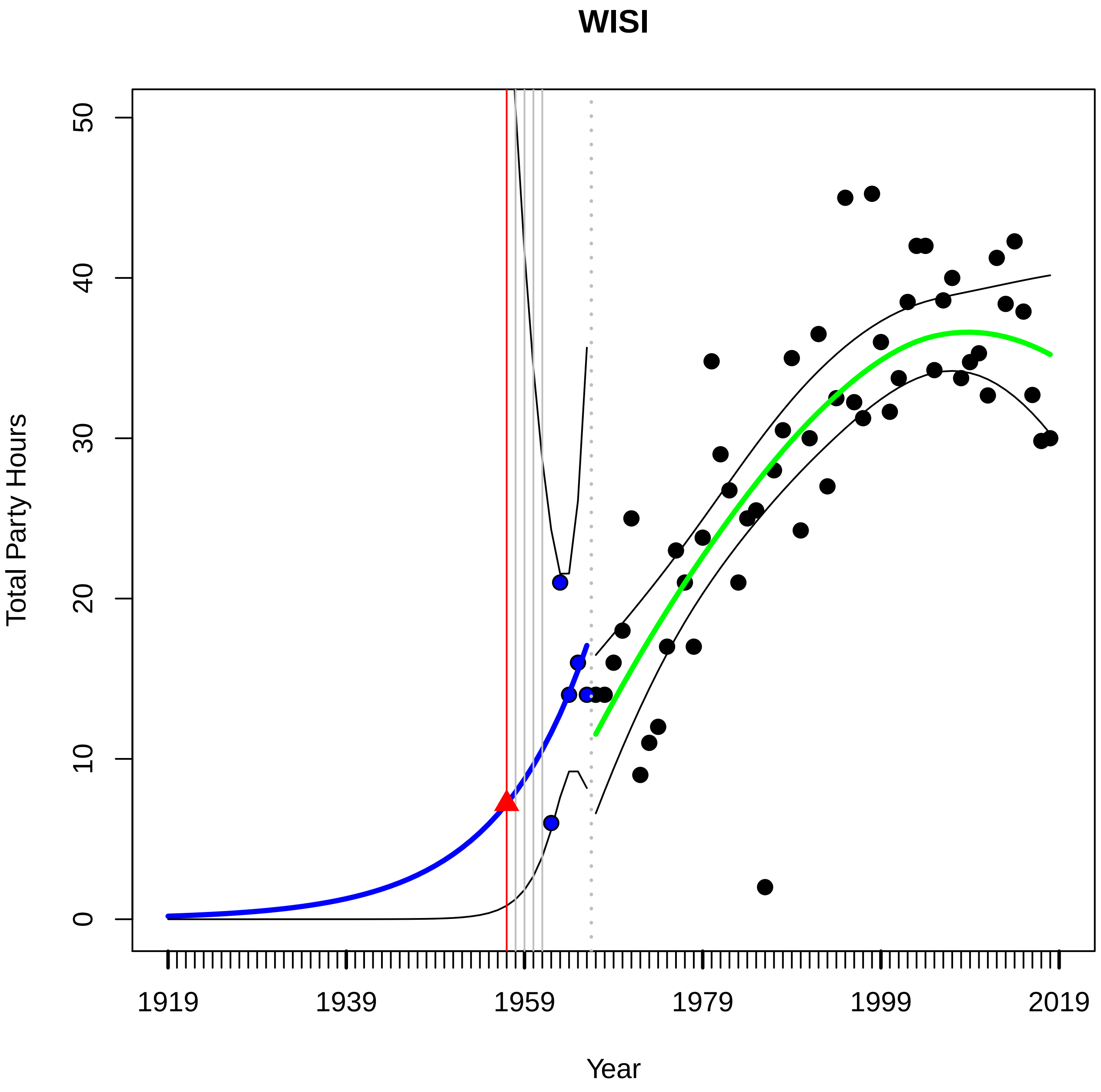

WISI
Total Party Hours
Year
0
10
20
30
40
50
1919
1939
1959
1979
1999
2019

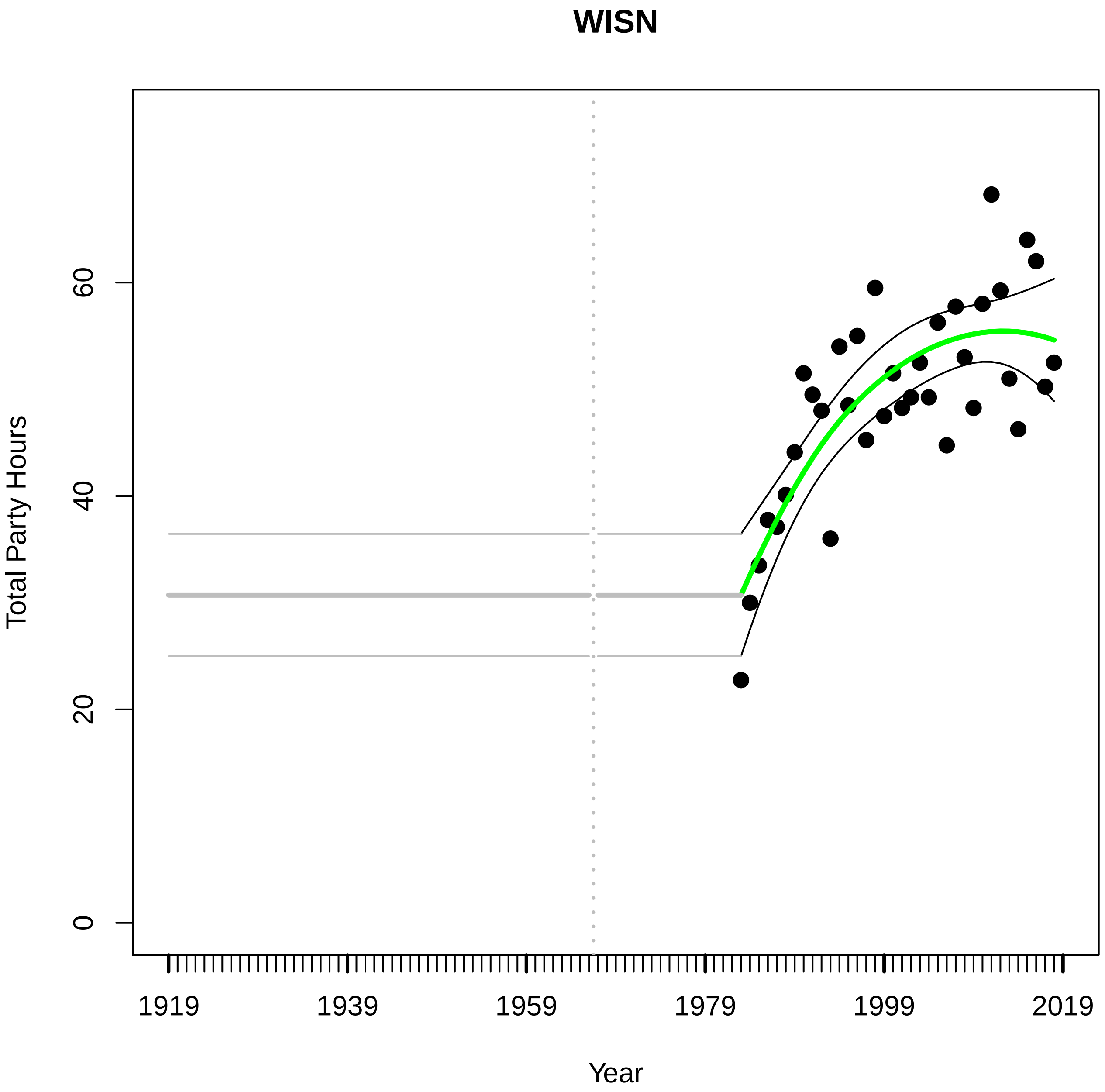

WISN
Total Party Hours
0
20
40
60
1919
1939
1959
1979
1999
2019
Year

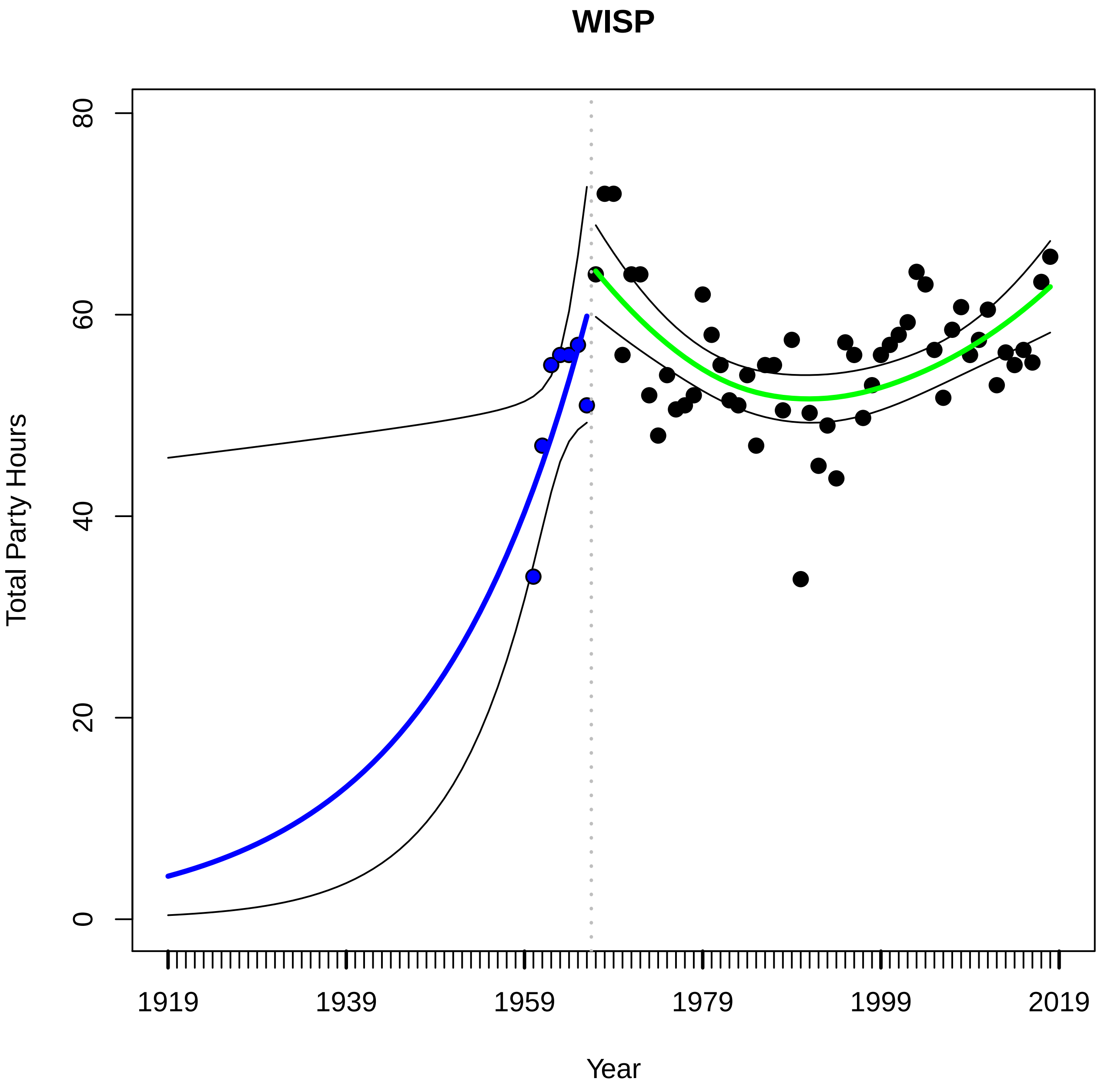

WISP
Total Party Hours
0
20
40
60
80
1919
1939
1959
1979
1999
2019
Year

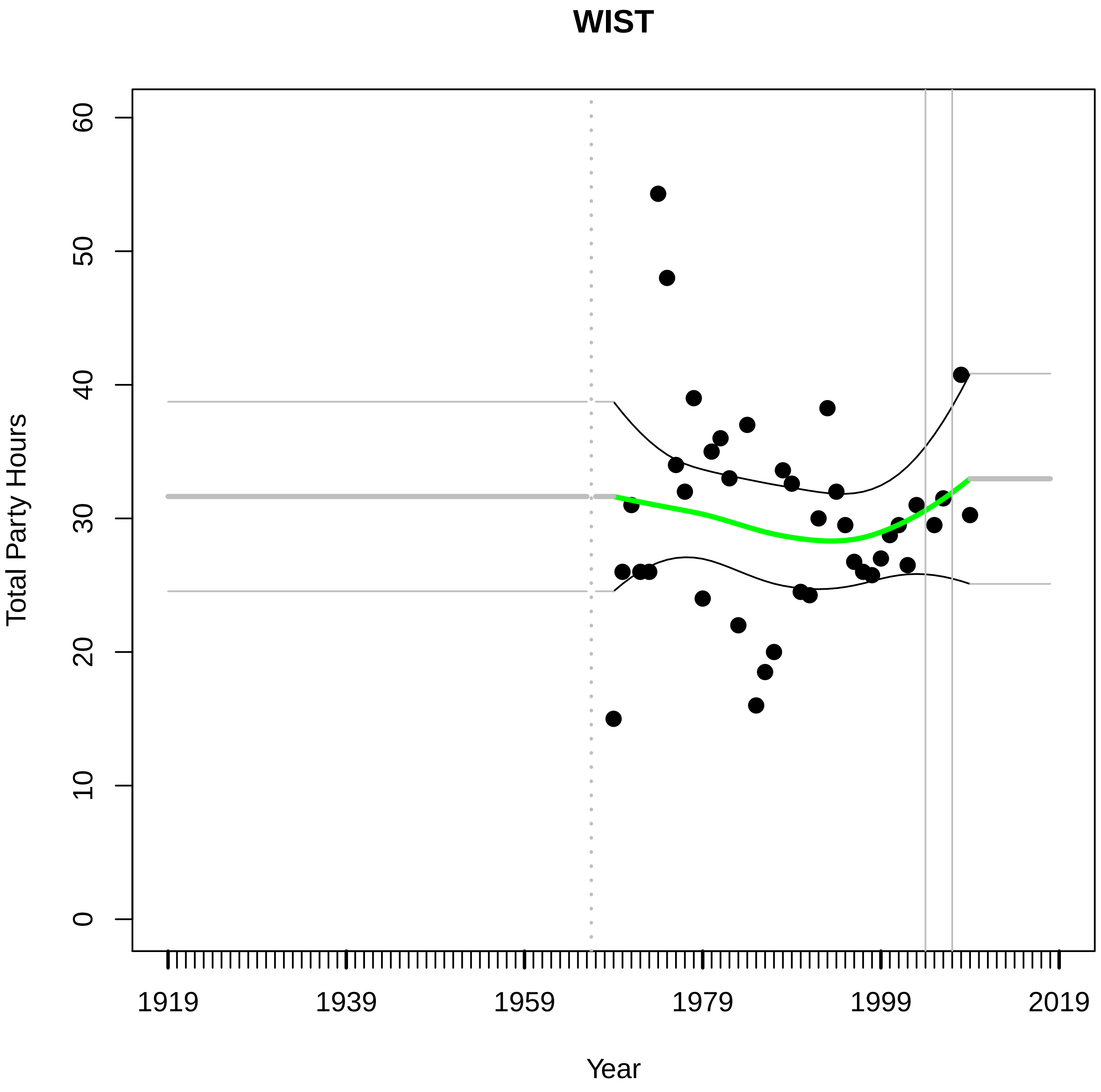

WIST
Total Party Hours
Year
0
10
20
30
40
50
60
1919
1939
1959
1979
1999
2019

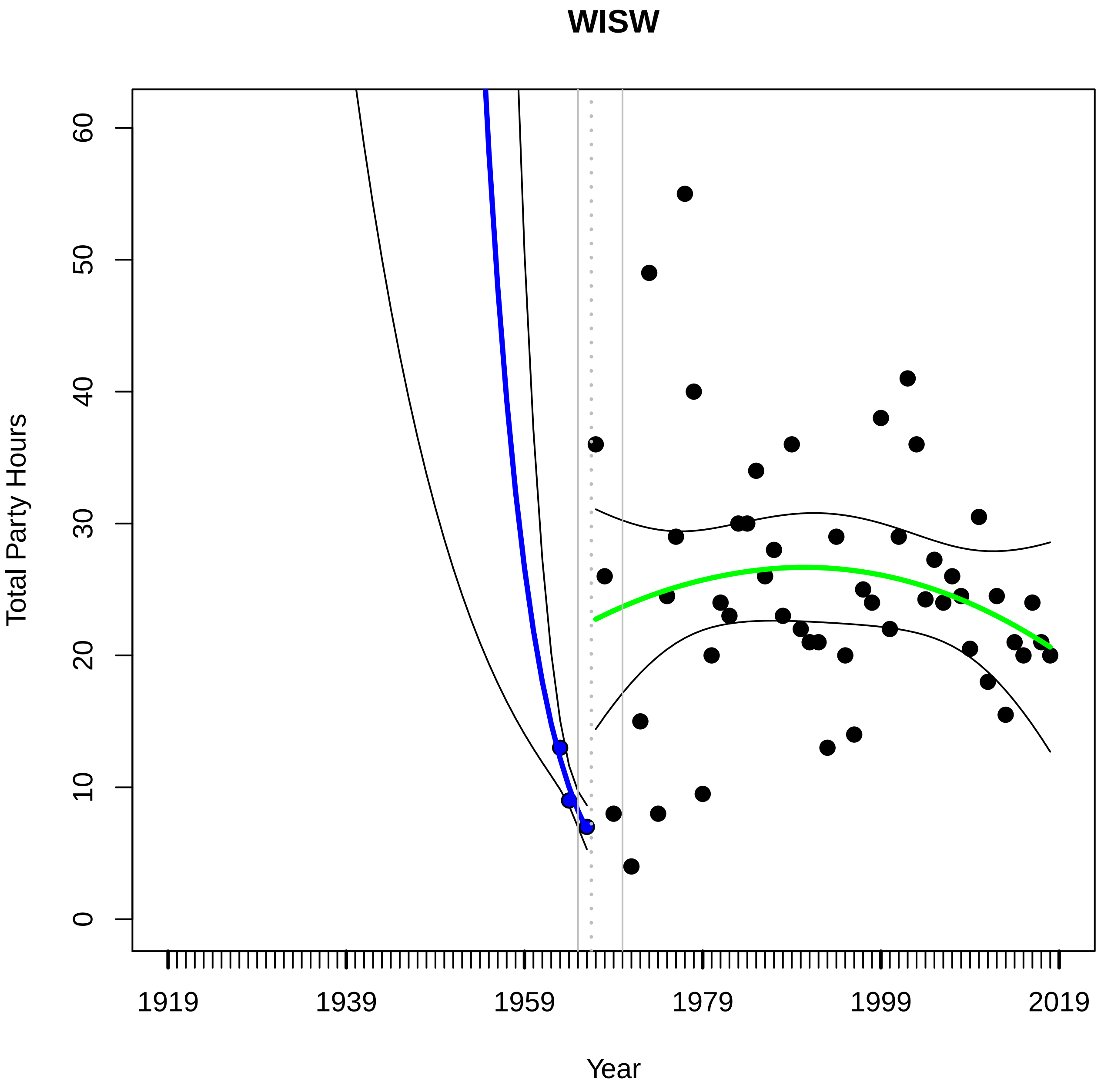
WISW
Total Party Hours
Year
0
10
20
30
40
50
60
1919
1939
1959
1979
1999
2019

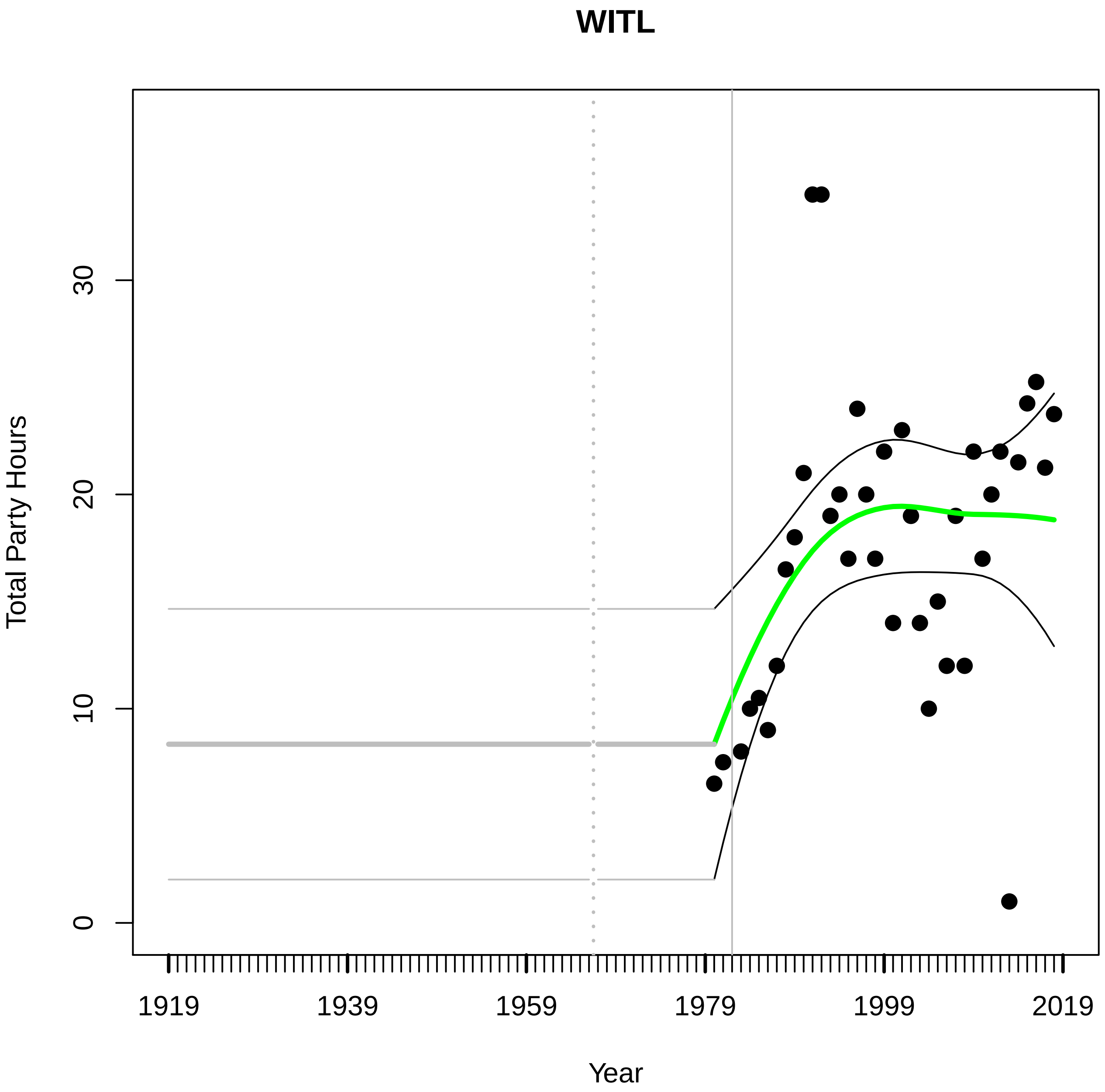
WITL
Total Party Hours
0
10
20
30
1919
1939
1959
1979
1999
2019
Year

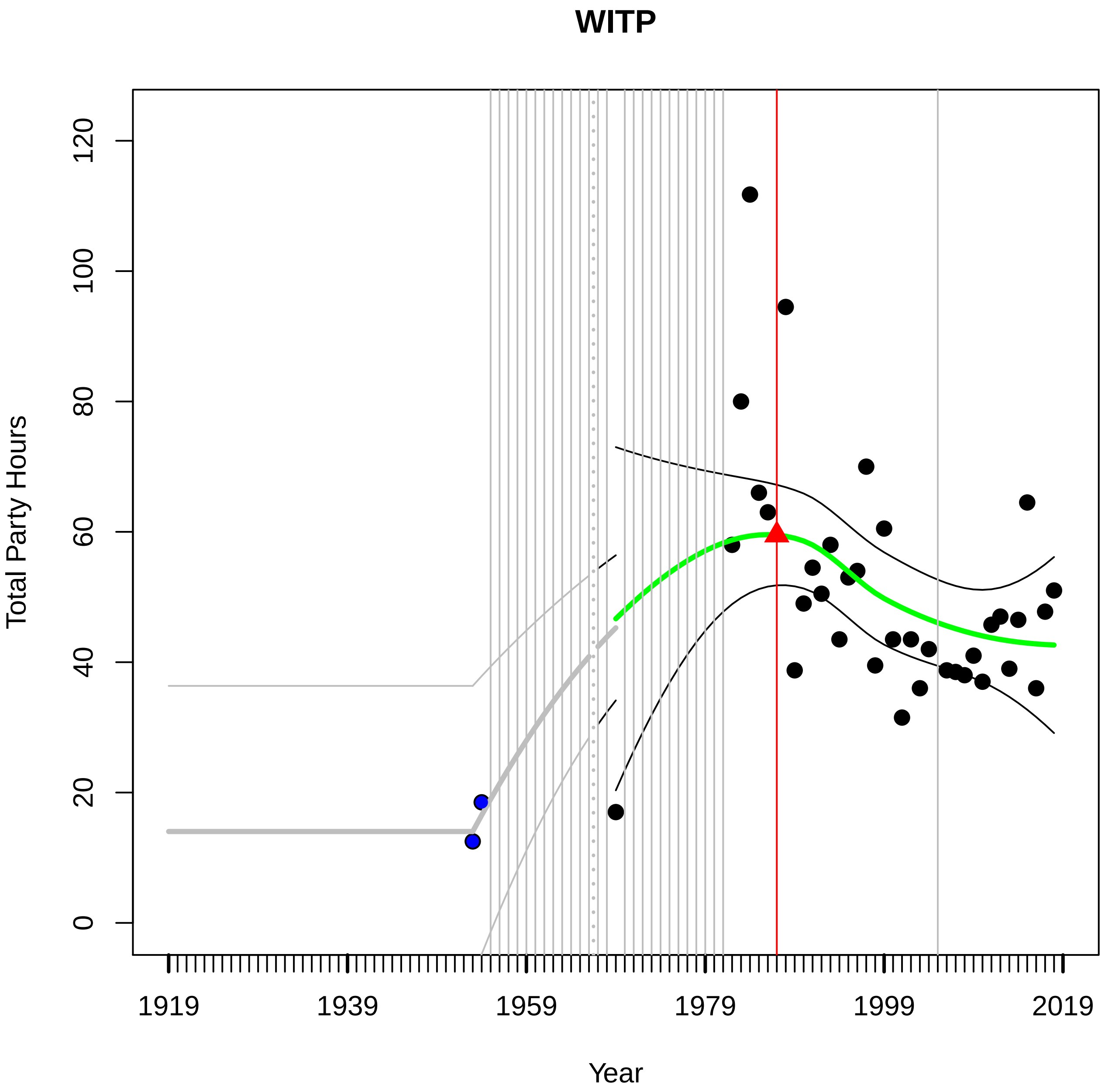
WITP
Total Party Hours
0
20
40
60
80
100
120
1919
1939
1959
1979
1999
2019
Year

WIWI

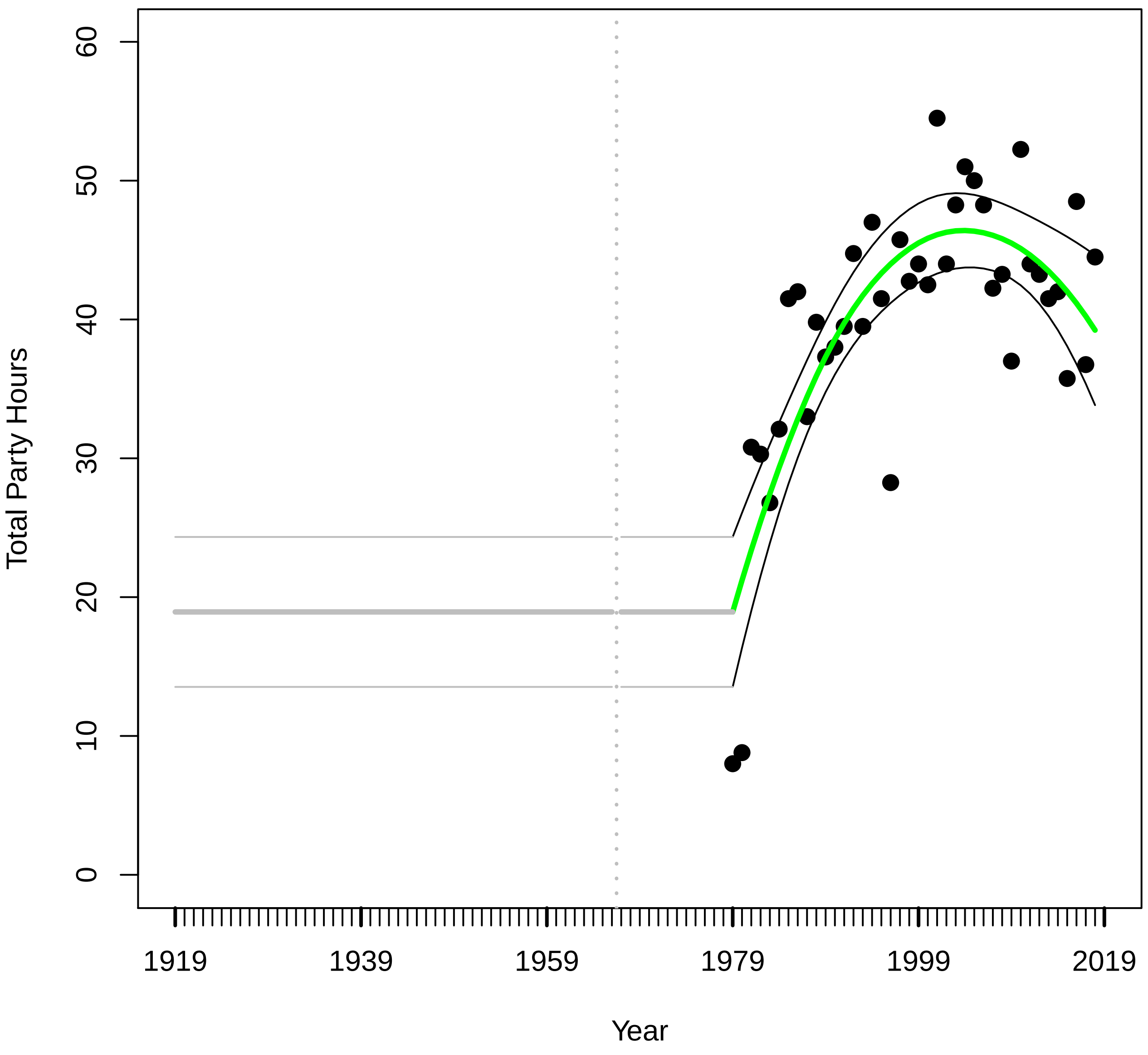

WIWI
Total Party Hours
0
10
20
30
40
50
60
1919
1939
1959
1979
1999
2019
Year

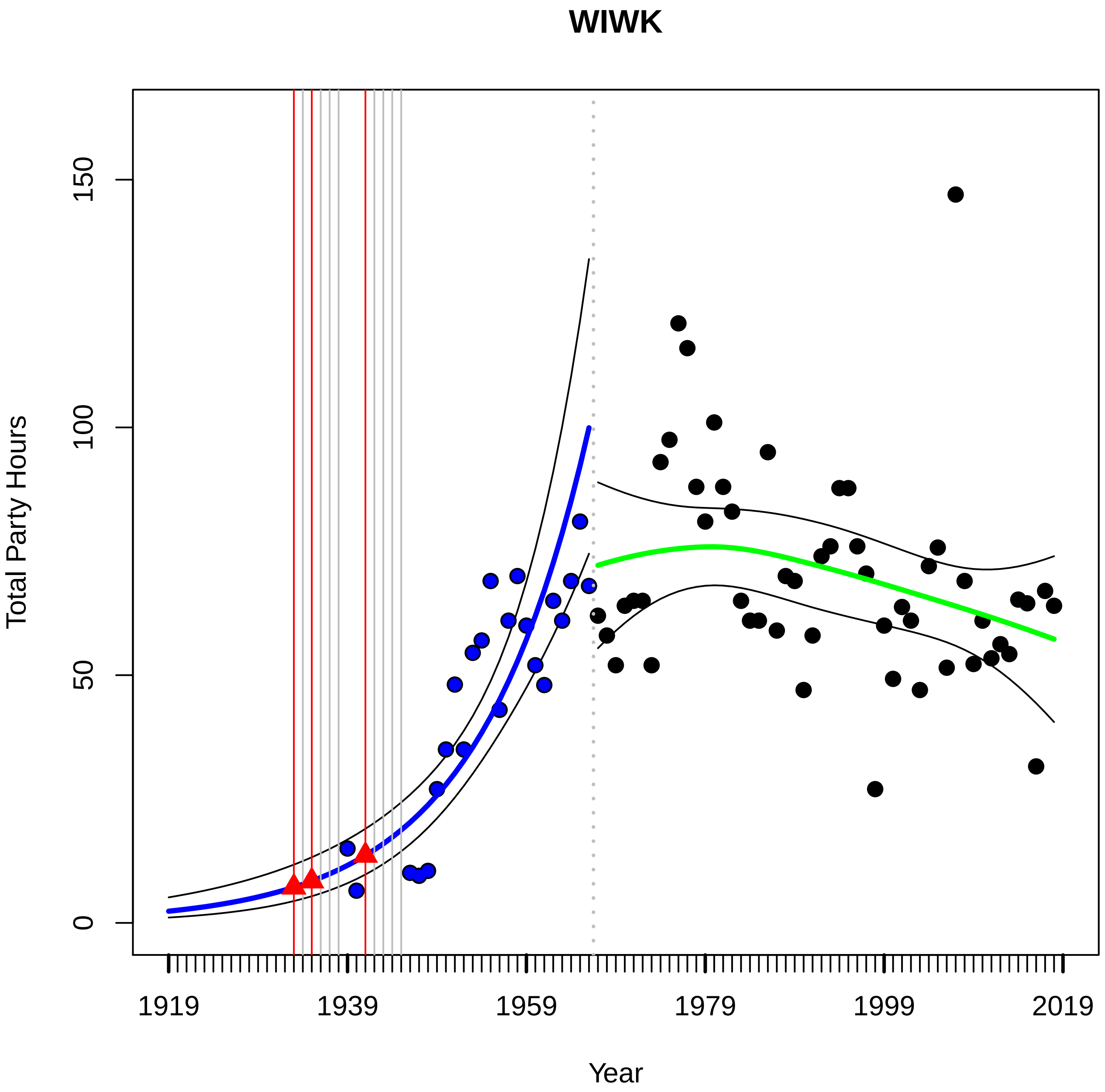

WIWK
Total Party Hours
150
100
50
0
1919
1939
1959
1979
1999
2019
Year

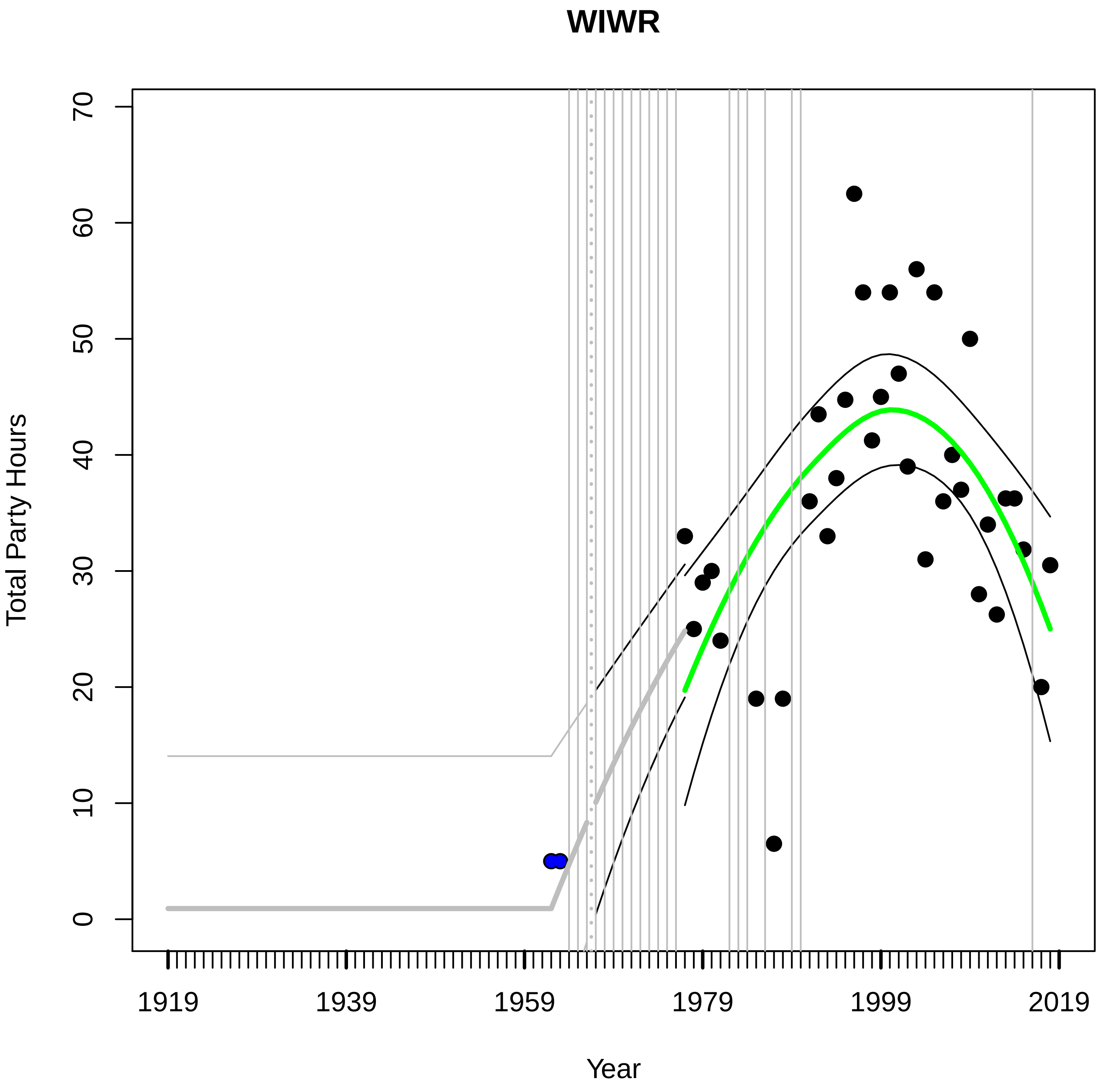
WIWR
Total Party Hours
0
10
20
30
40
50
60
70
1919
1939
1959
1979
1999
2019
Year

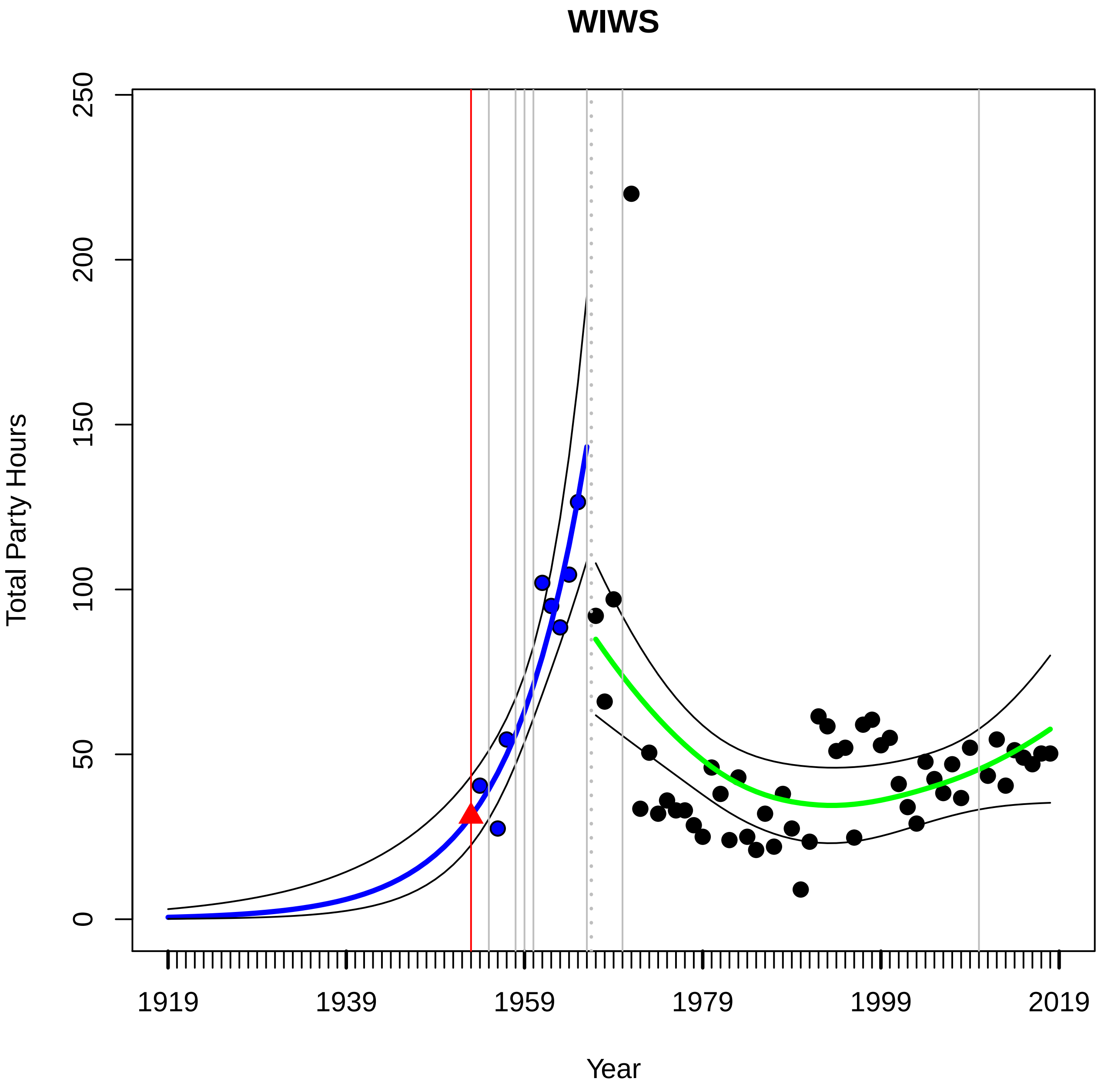
WIWS
Total Party Hours
0
50
100
150
200
250
1919
1939
1959
1979
1999
2019
Year

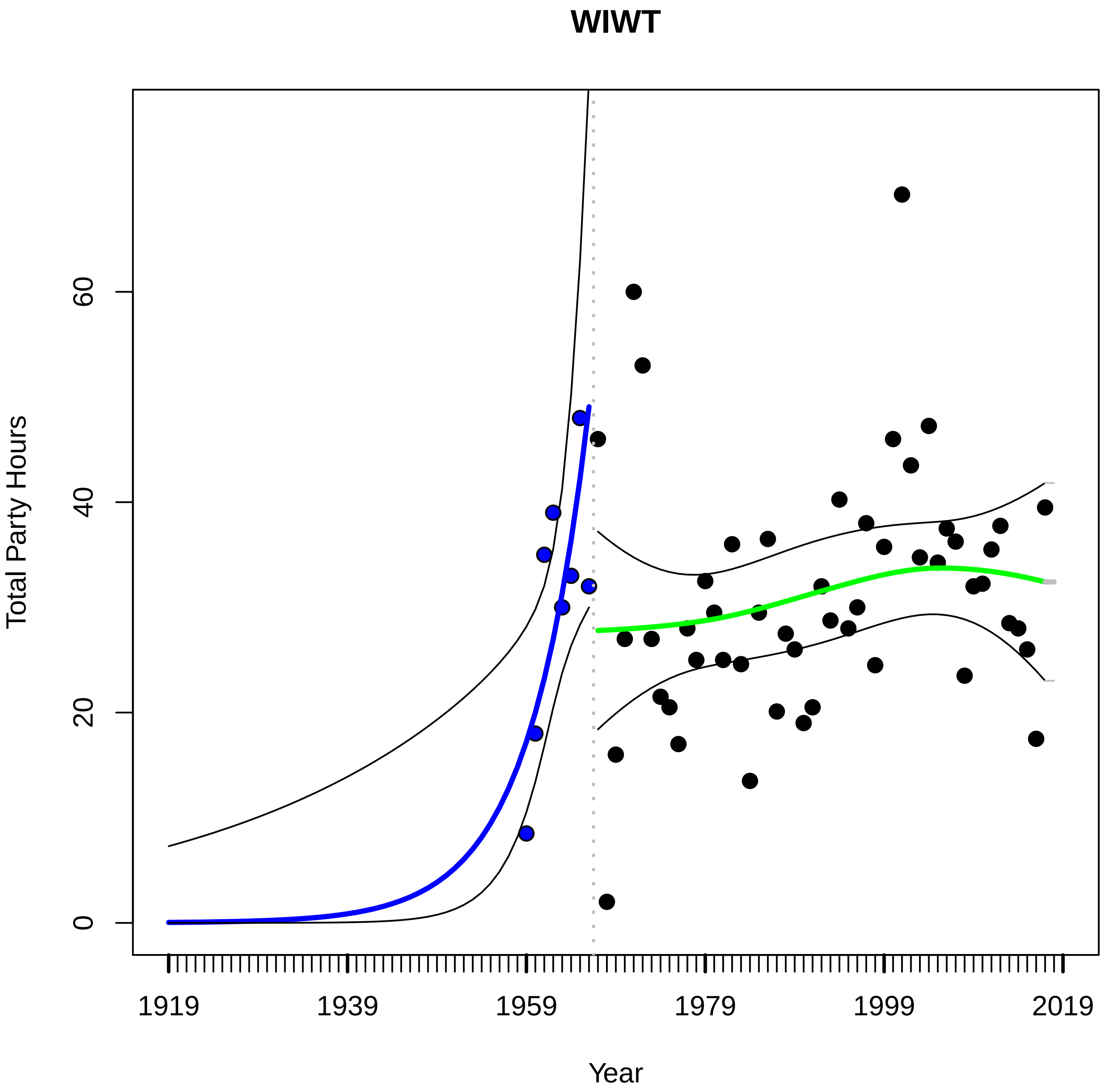

WIWT
Total Party Hours
0
20
40
60
1919
1939
1959
1979
1999
2019
Year

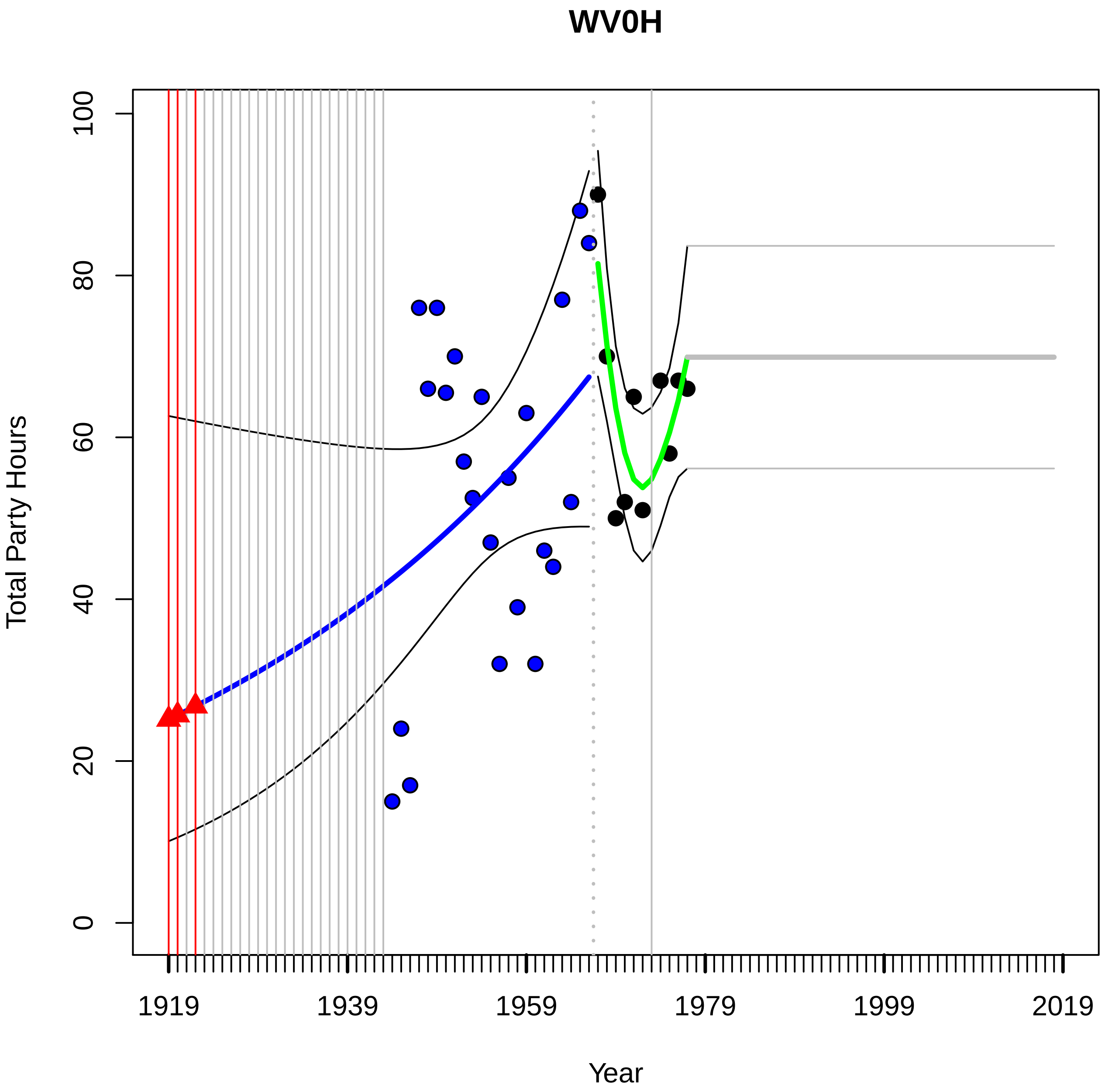
WV0H
Total Party Hours
Year
0
20
40
60
80
100
1919
1939
1959
1979
1999
2019

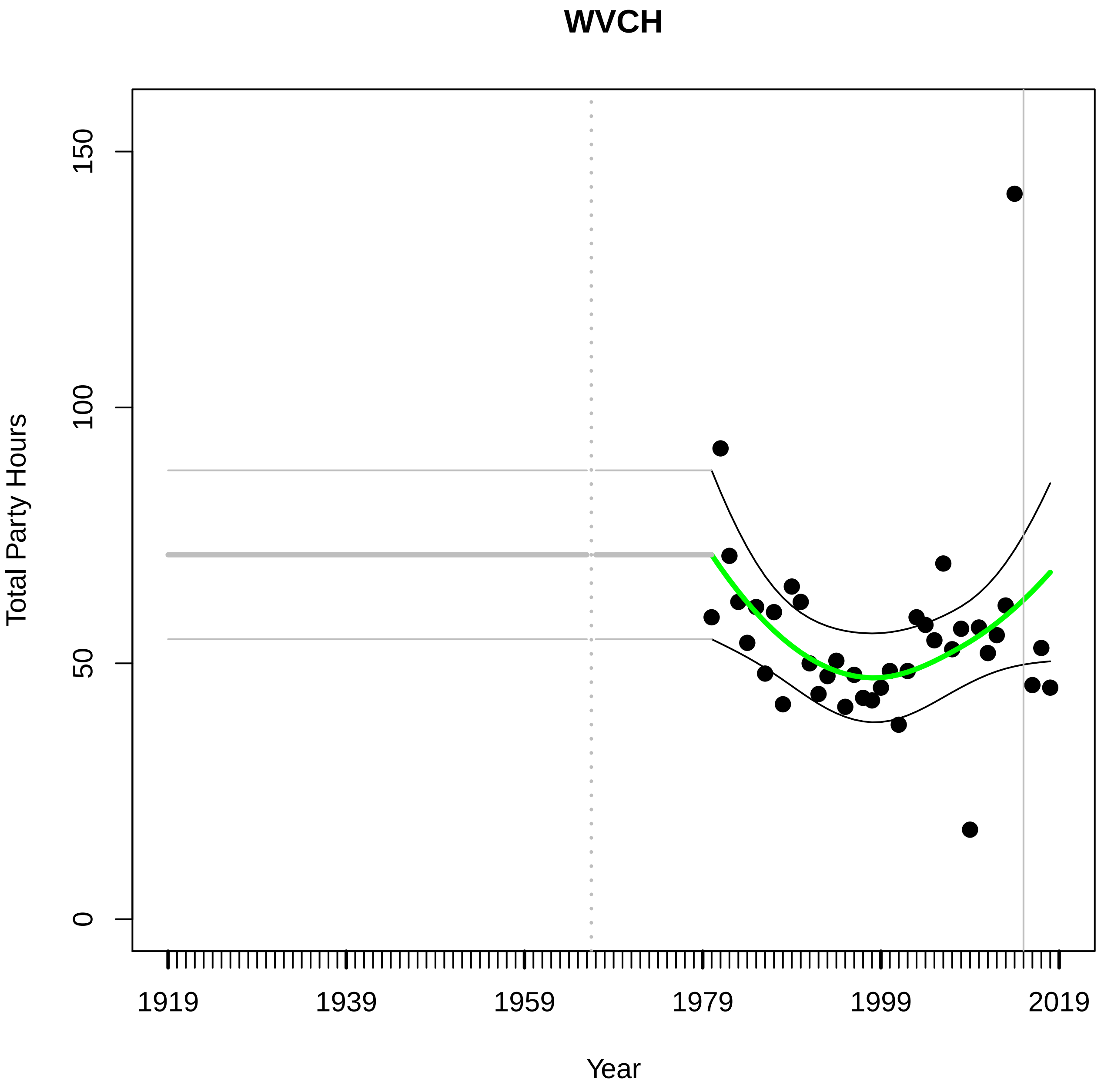

WVCH
Total Party Hours
150
100
50
0
1919
1939
1959
1979
1999
2019
Year

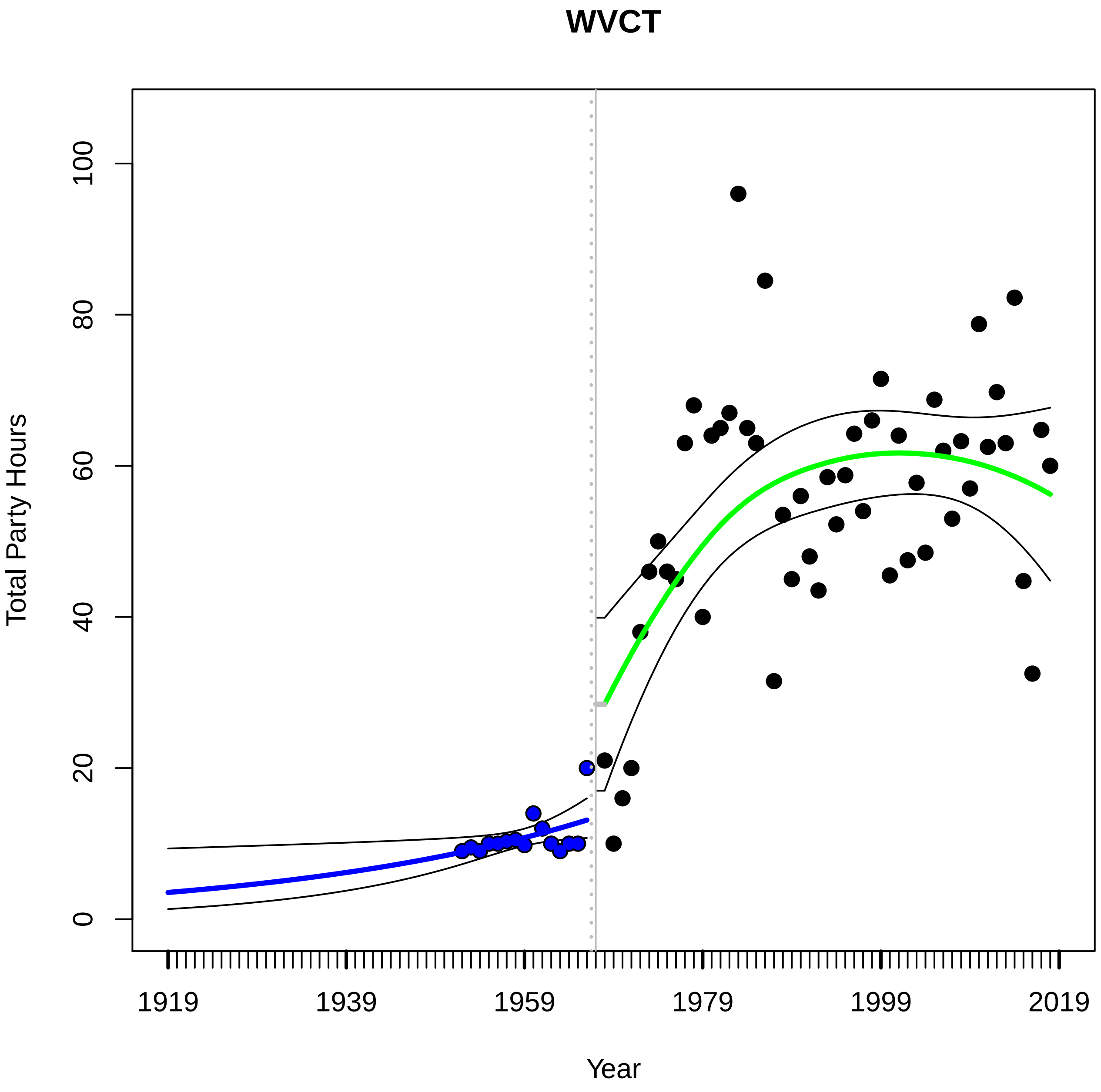
WVCT
Total Party Hours
0
20
40
60
80
100
1919
1939
1959
1979
1999
2019
Year

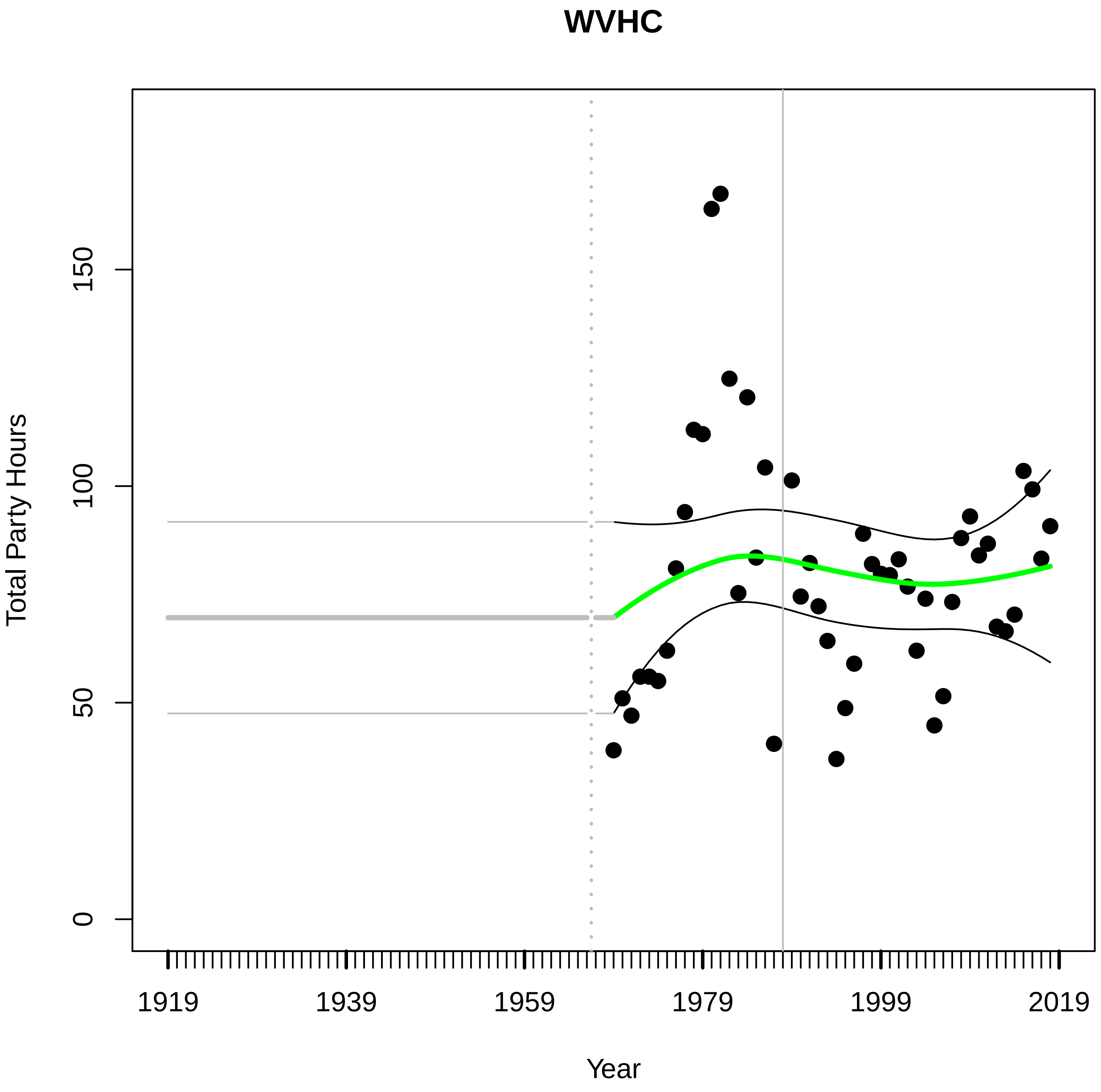

WVHC
Total Party Hours
0
50
100
150
1919
1939
1959
1979
1999
2019
Year

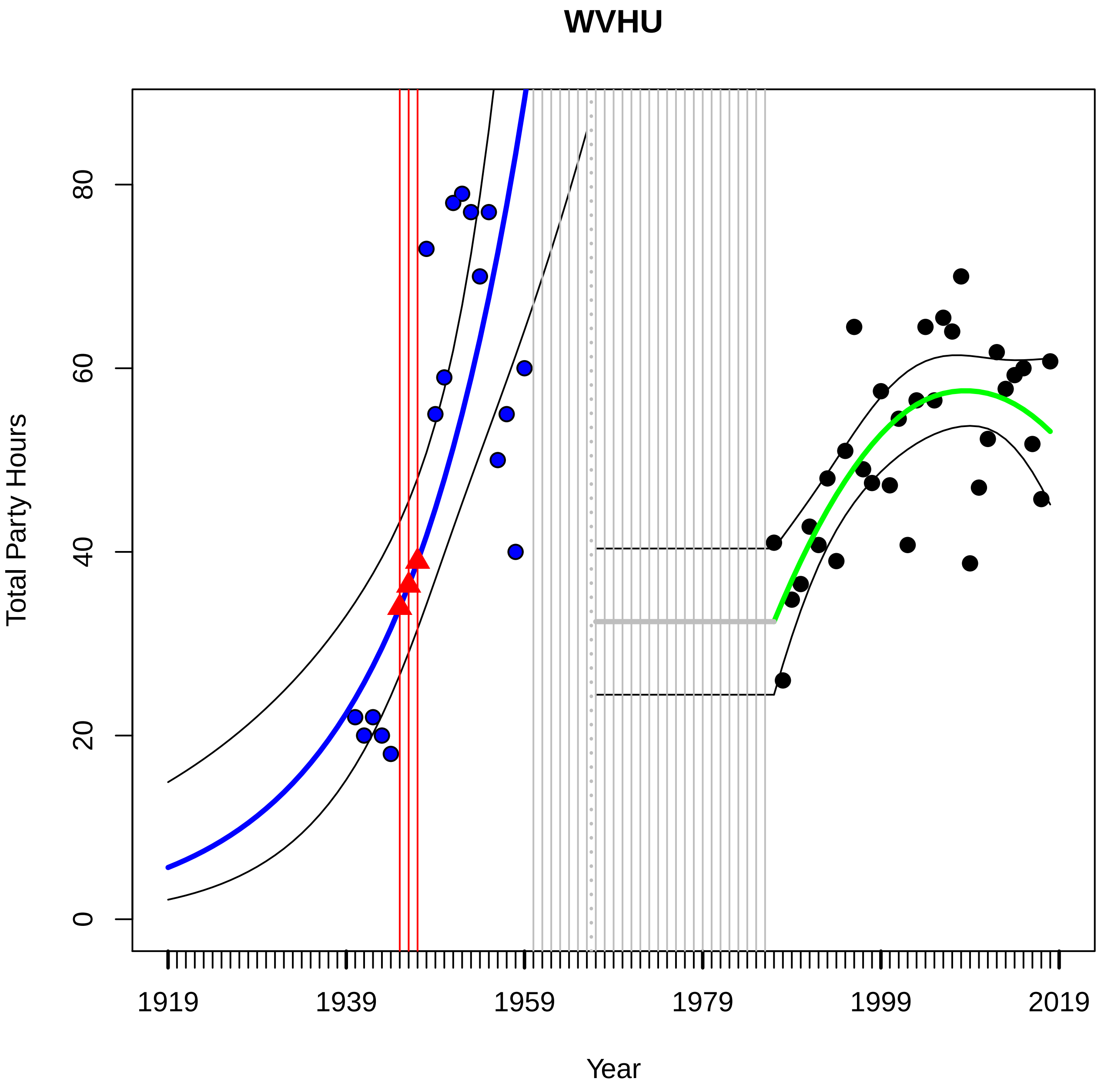
WVHU
Total Party Hours
0
20
40
60
80
1919
1939
1959
1979
1999
2019
Year

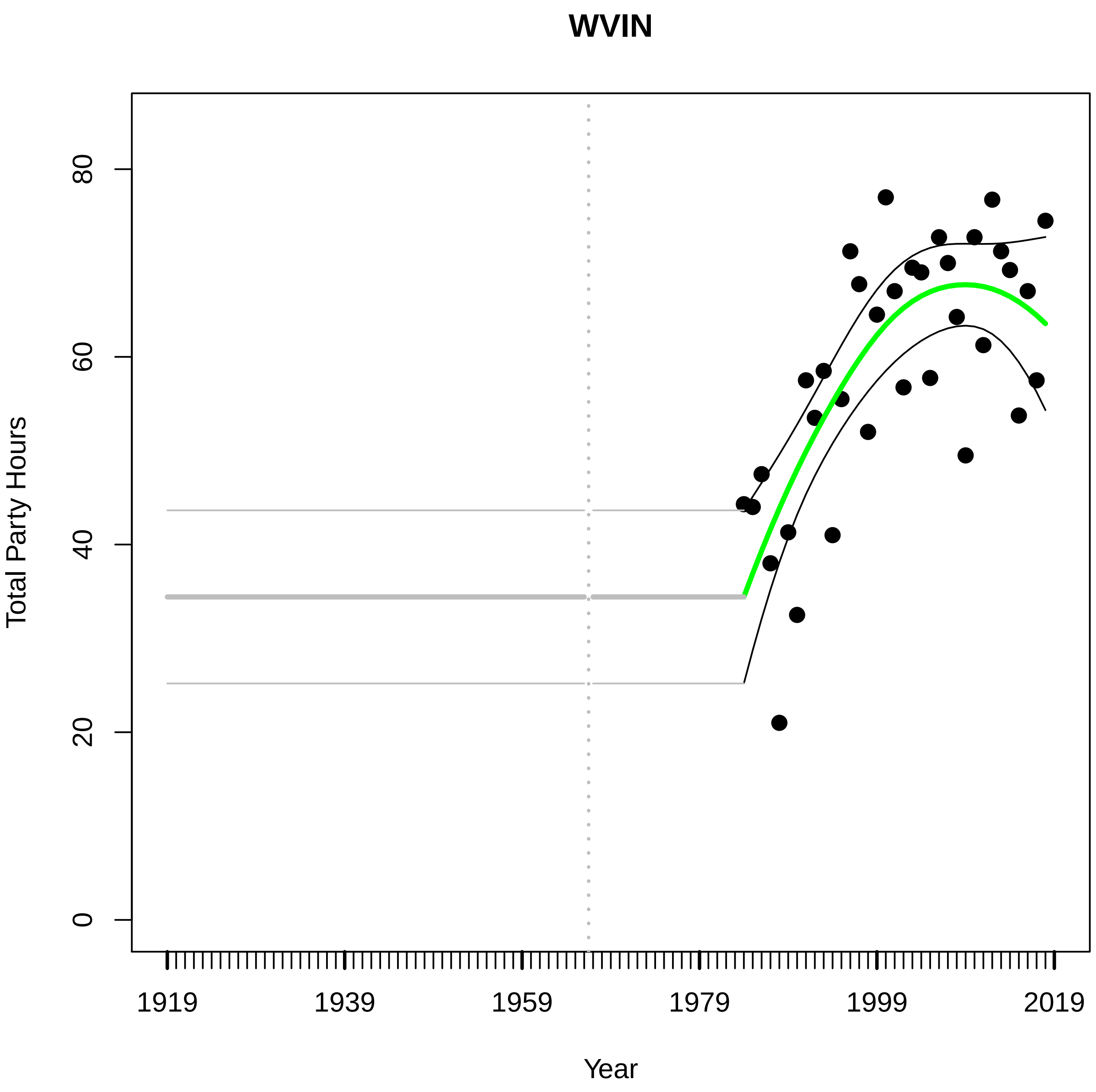
WVIN
Total Party Hours
0
20
40
60
80
1919
1939
1959
1979
1999
2019
Year

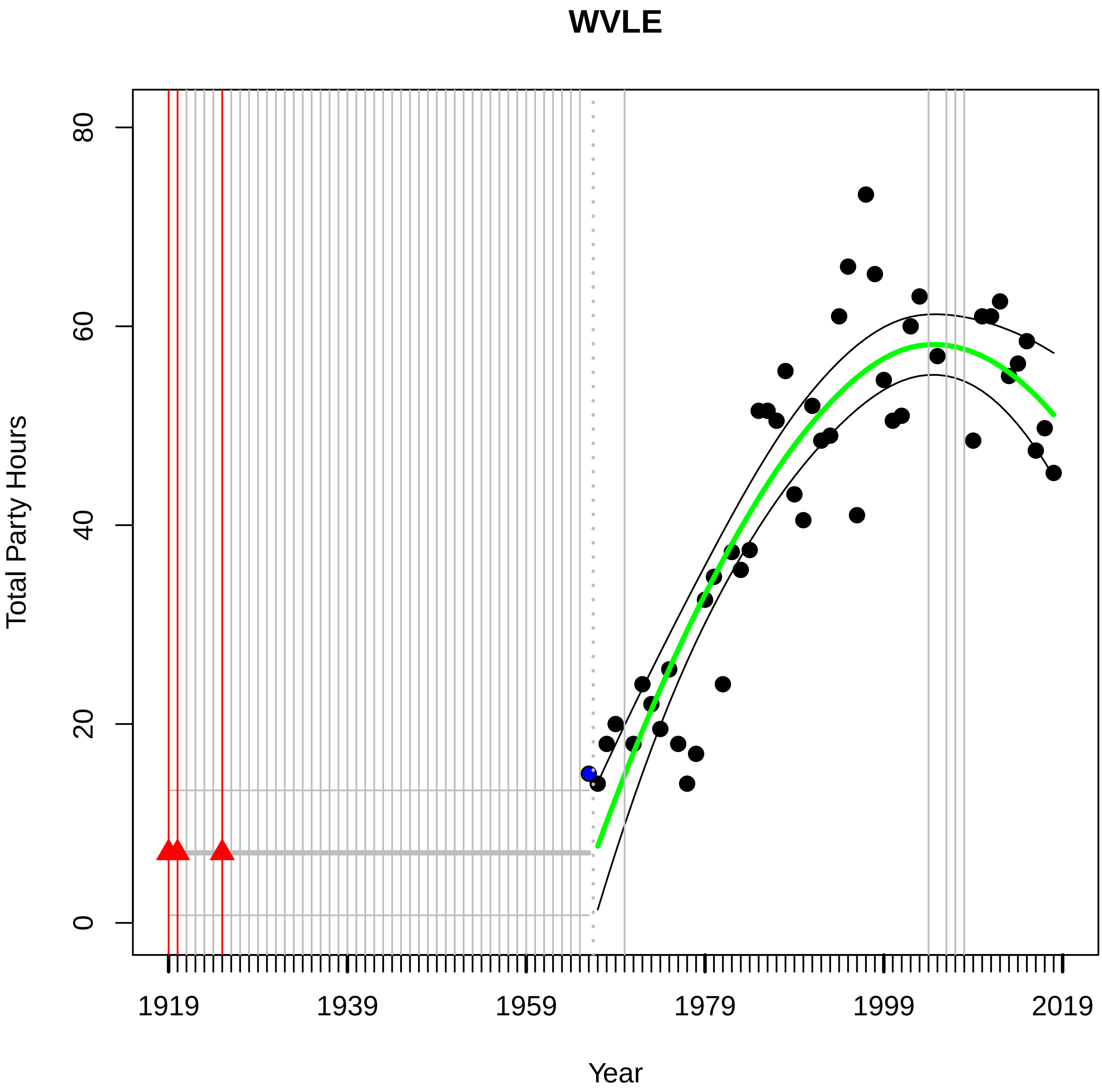

WVLE
Total Party Hours
0
20
40
60
80
Year
1919
1939
1959
1979
1999
2019

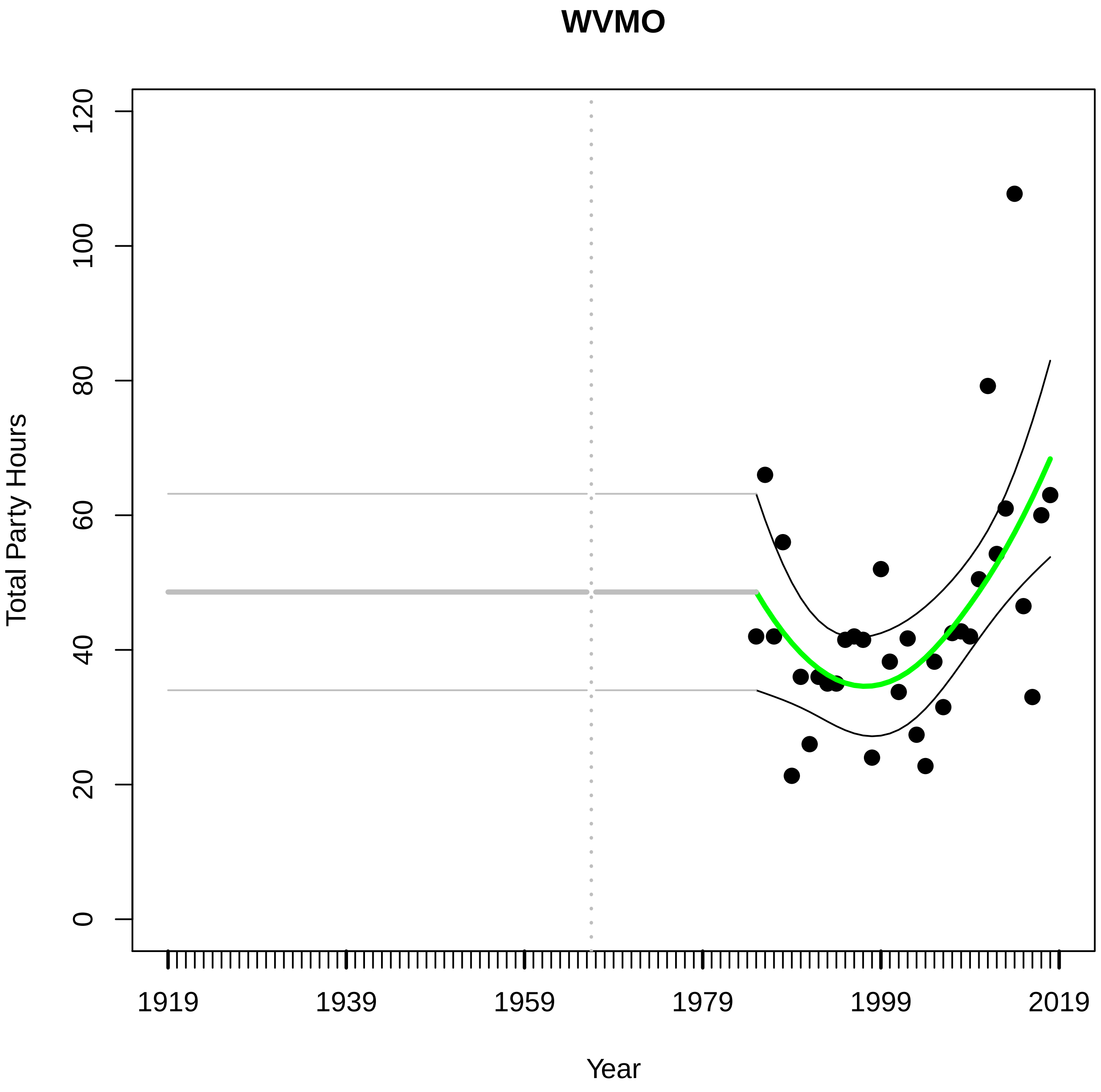

WVMO
Total Party Hours
Year
0
20
40
60
80
100
120
1919
1939
1959
1979
1999
2019

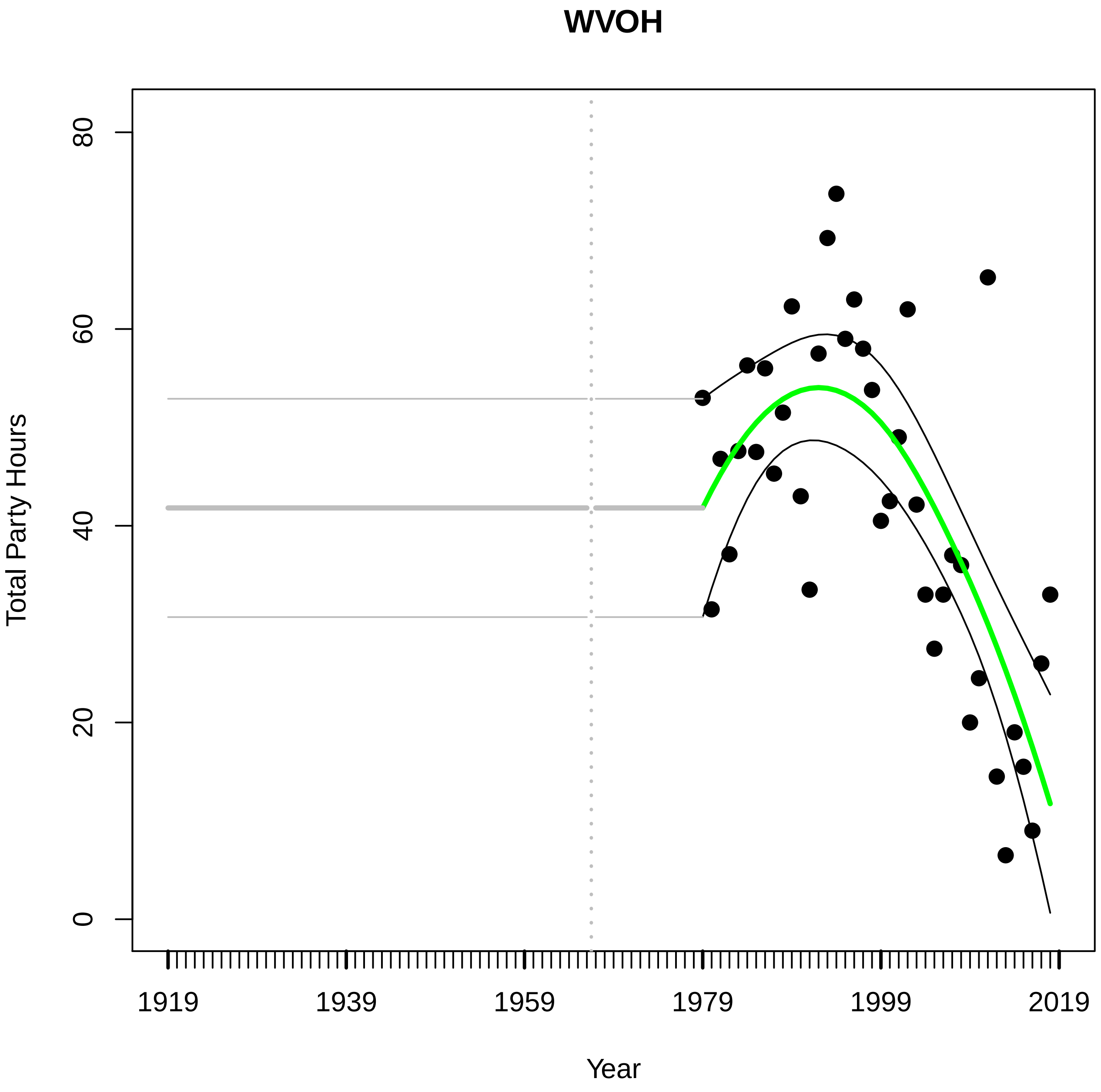
WVOH
Total Party Hours
80
60
40
20
0
1919
1939
1959
1979
1999
2019
Year

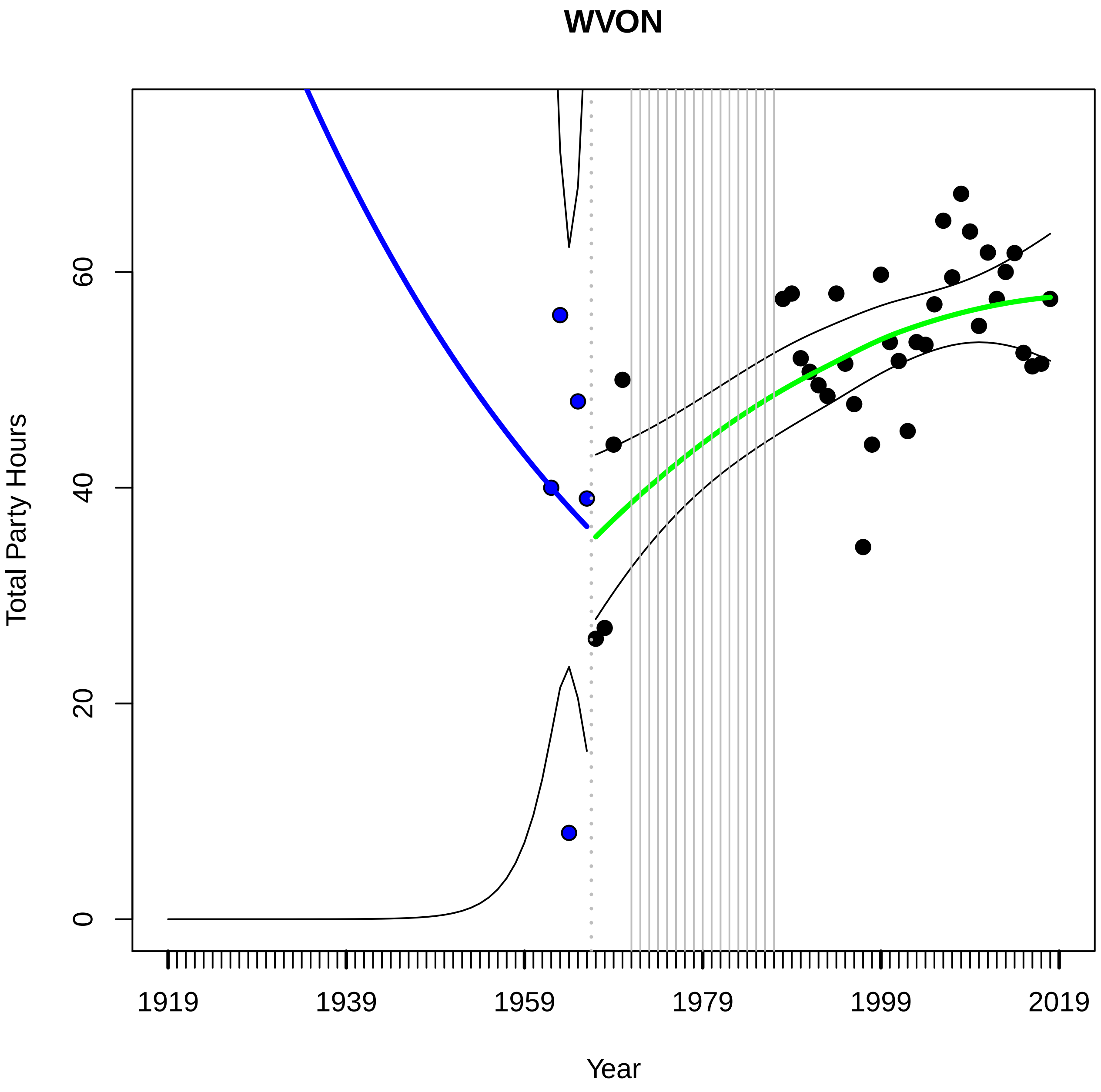

WVON
Total Party Hours
Year
0
20
40
60
1919
1939
1959
1979
1999
2019

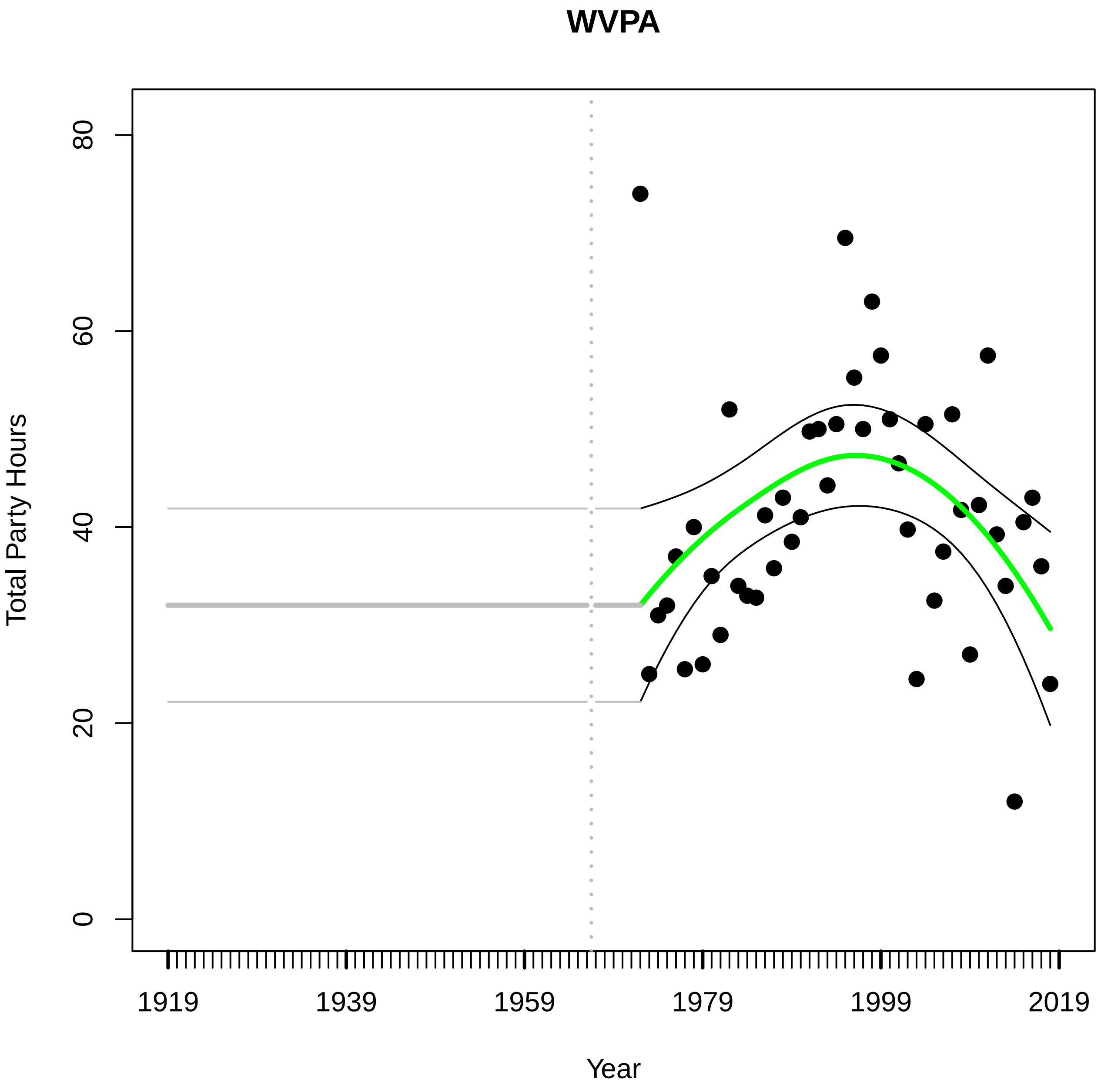

WVPA
Total Party Hours
0
20
40
60
80
1919
1939
1959
1979
1999
2019
Year

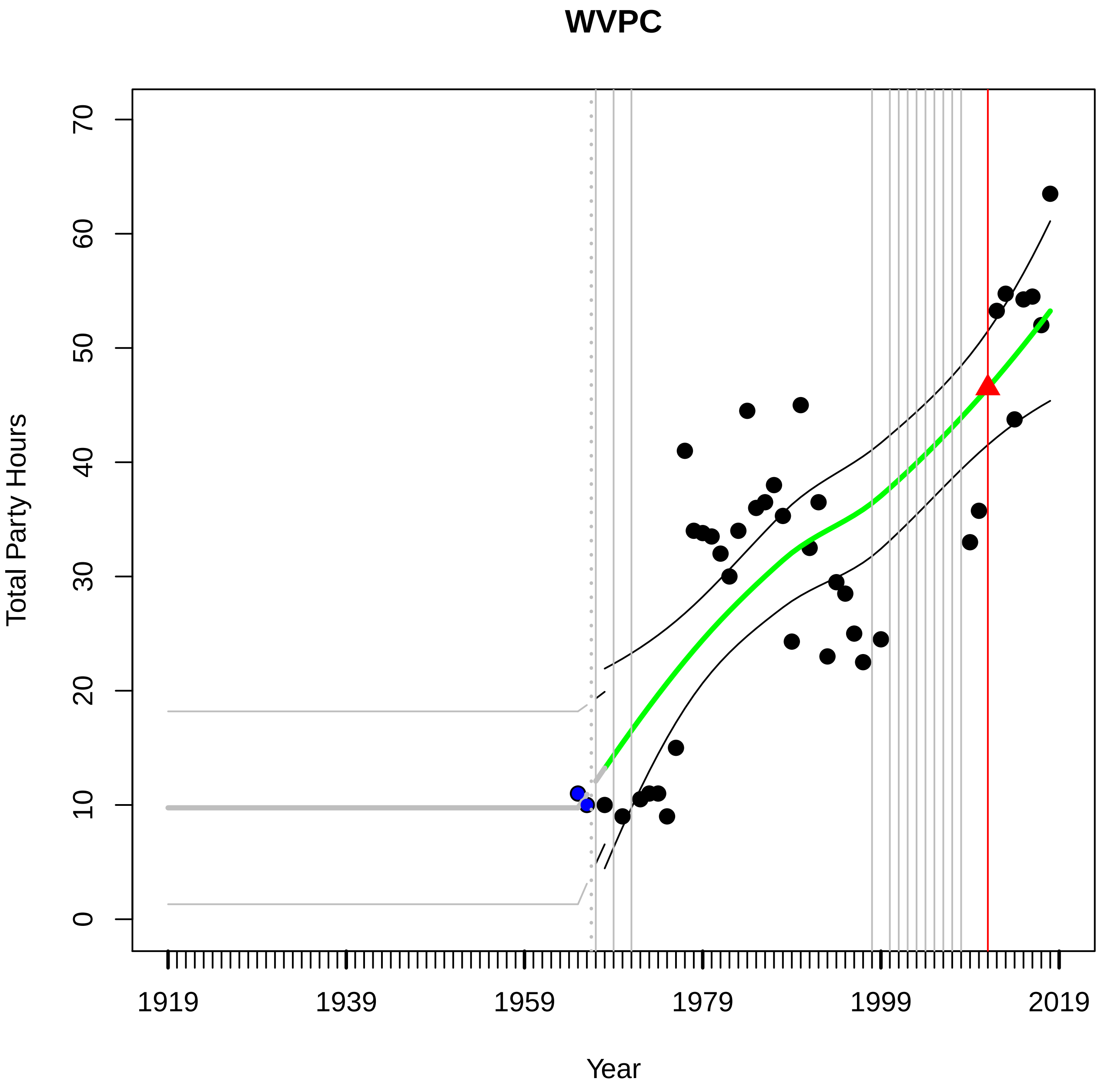

WVPC
Total Party Hours
0
10
20
30
40
50
60
70
1919
1939
1959
1979
1999
2019
Year

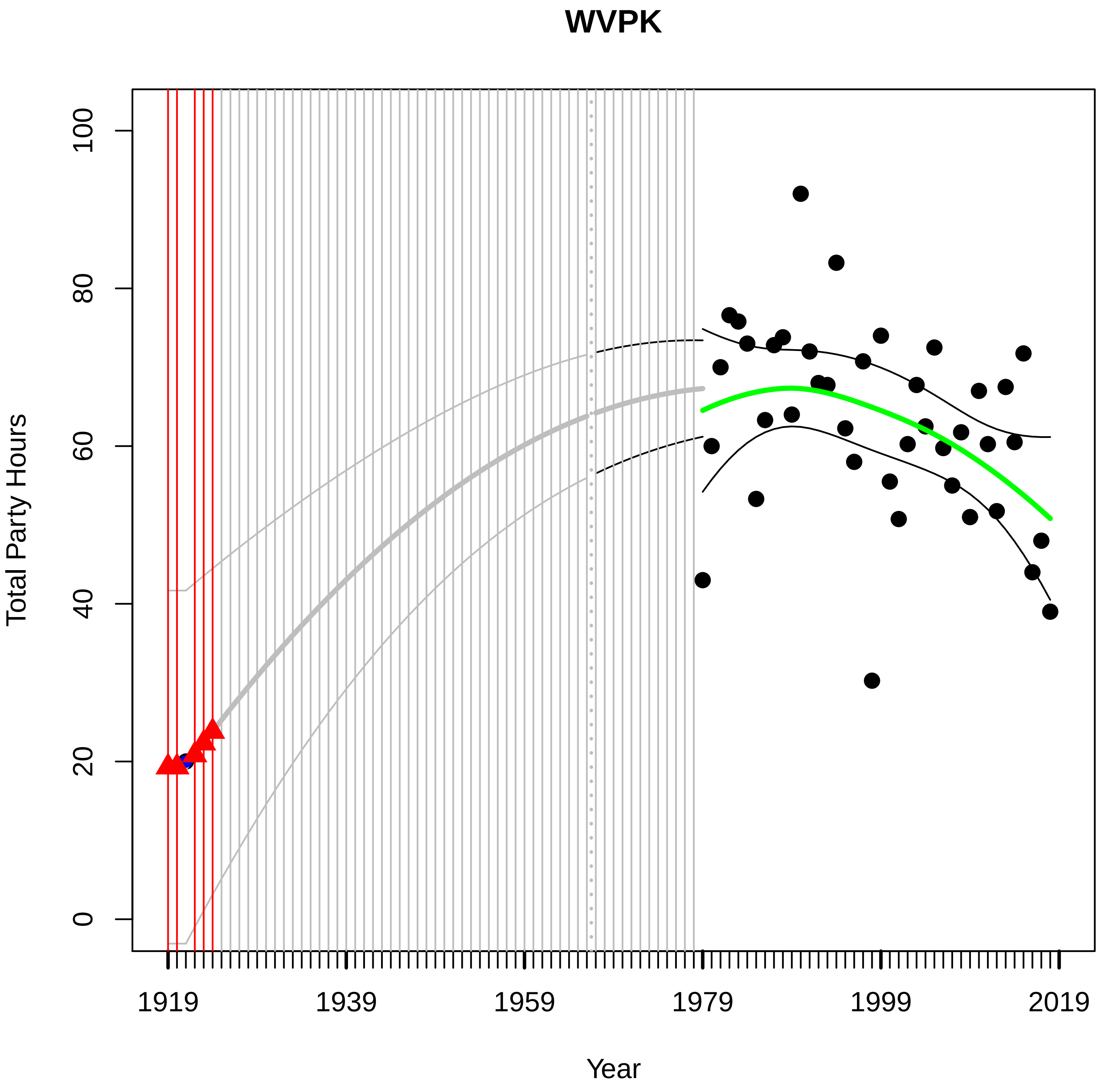

WVPK
Total Party Hours
0
20
40
60
80
100
1919
1939
1959
1979
1999
2019
Year

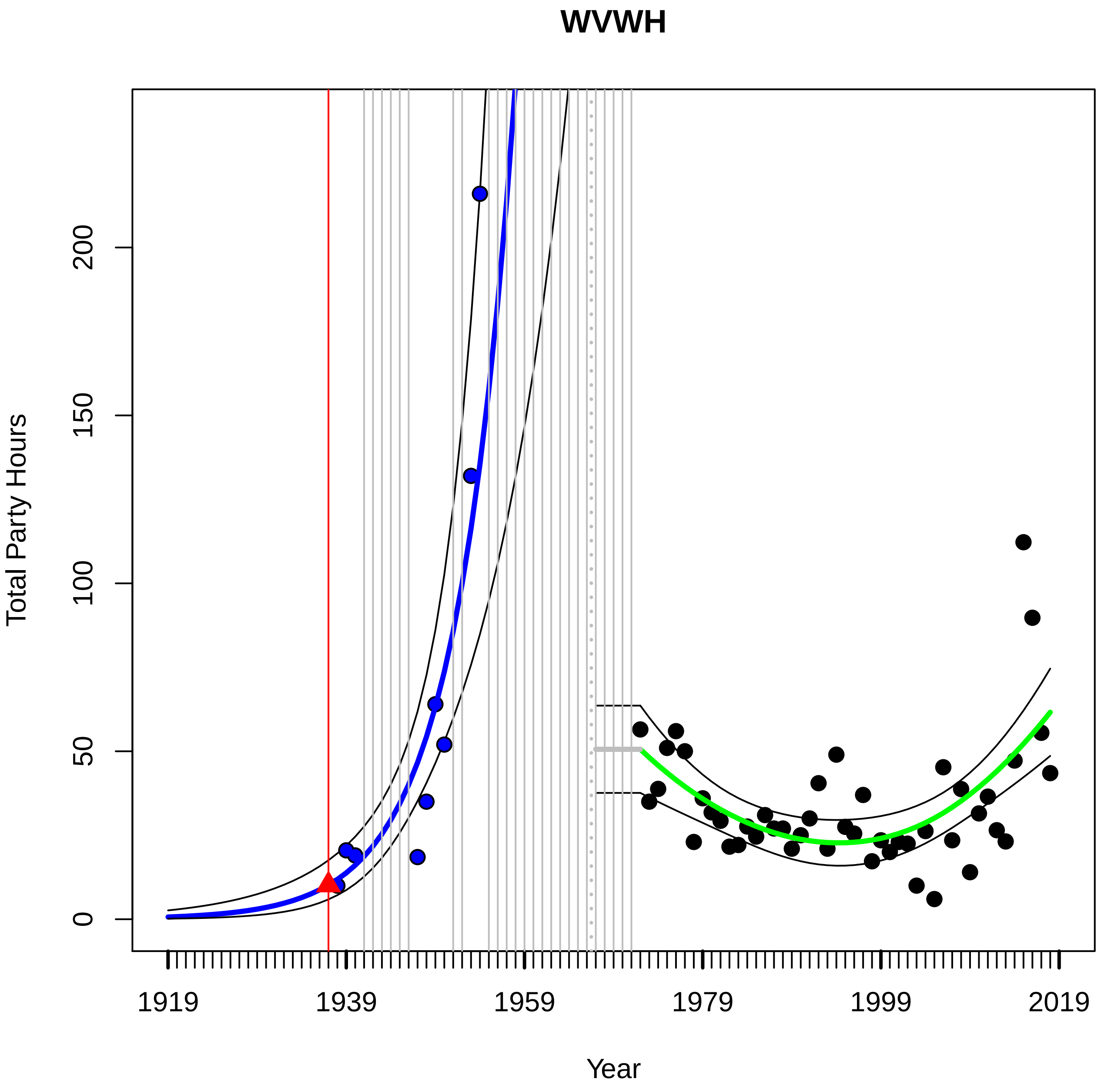

WVWH
Total Party Hours
0
50
100
150
200
1919
1939
1959
1979
1999
2019
Year

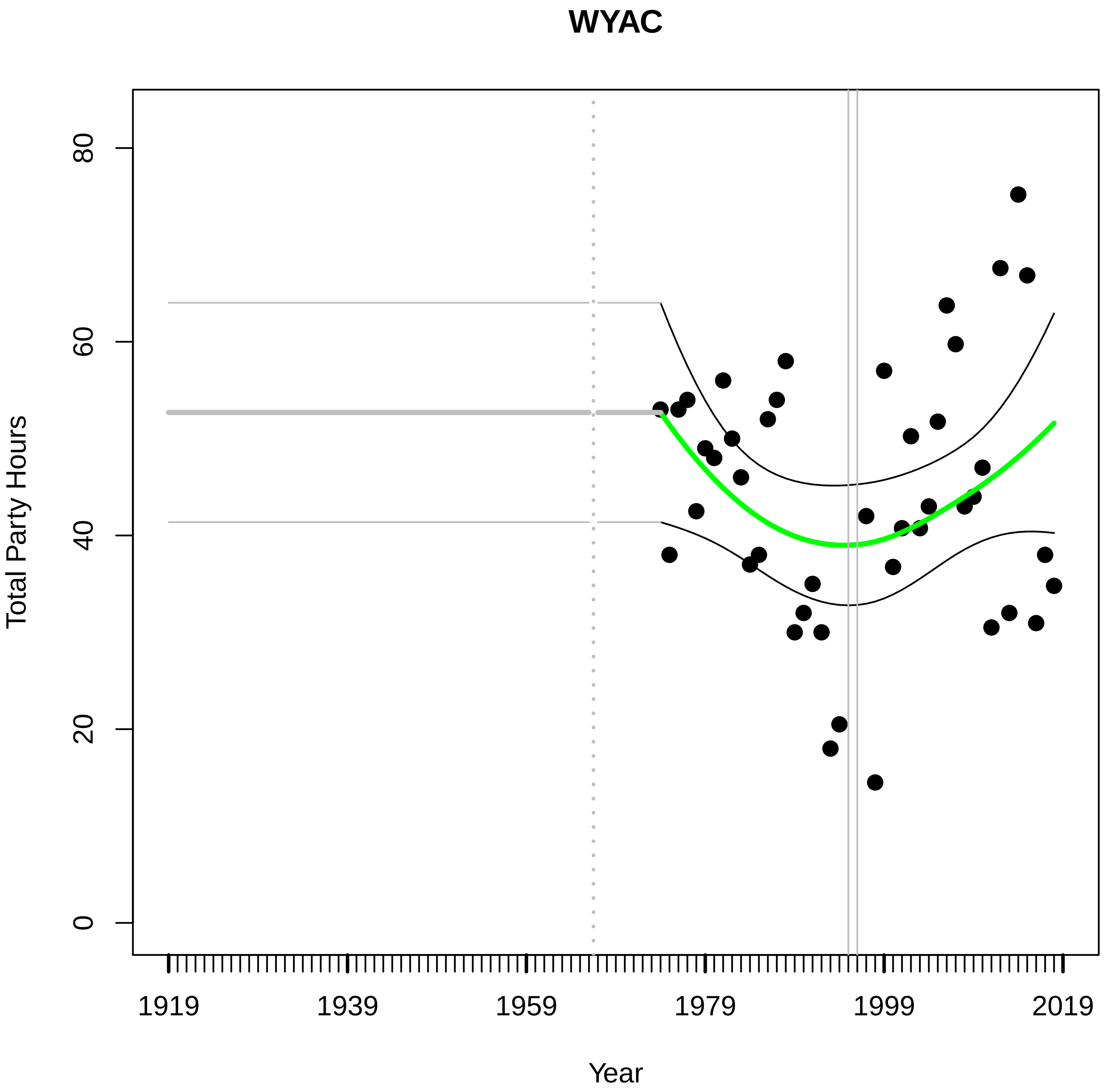

WYAC
Total Party Hours
80
60
40
20
0
1919
1939
1959
1979
1999
2019
Year

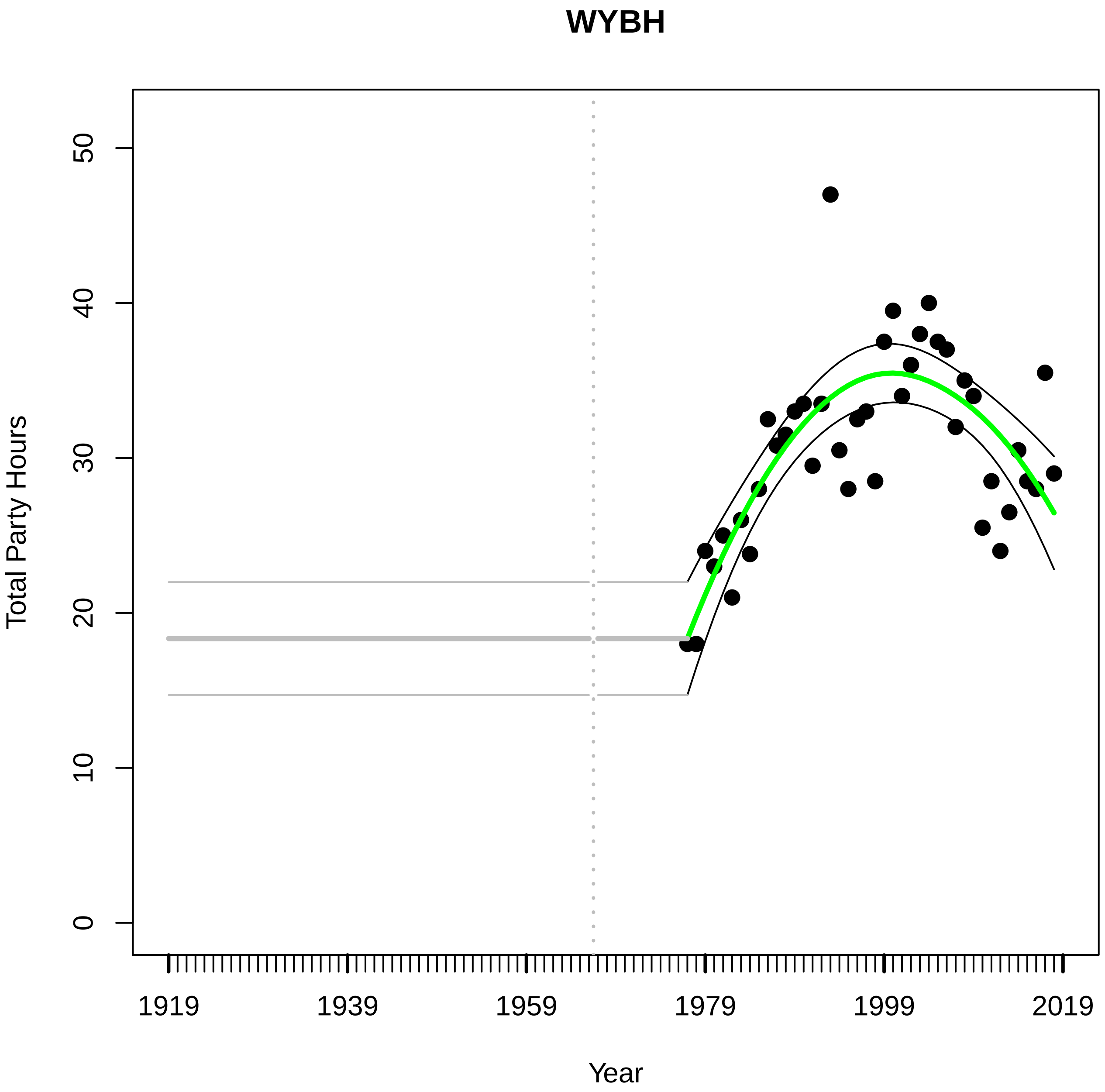
WYBH
Total Party Hours
0
10
20
30
40
50
1919
1939
1959
1979
1999
2019
Year

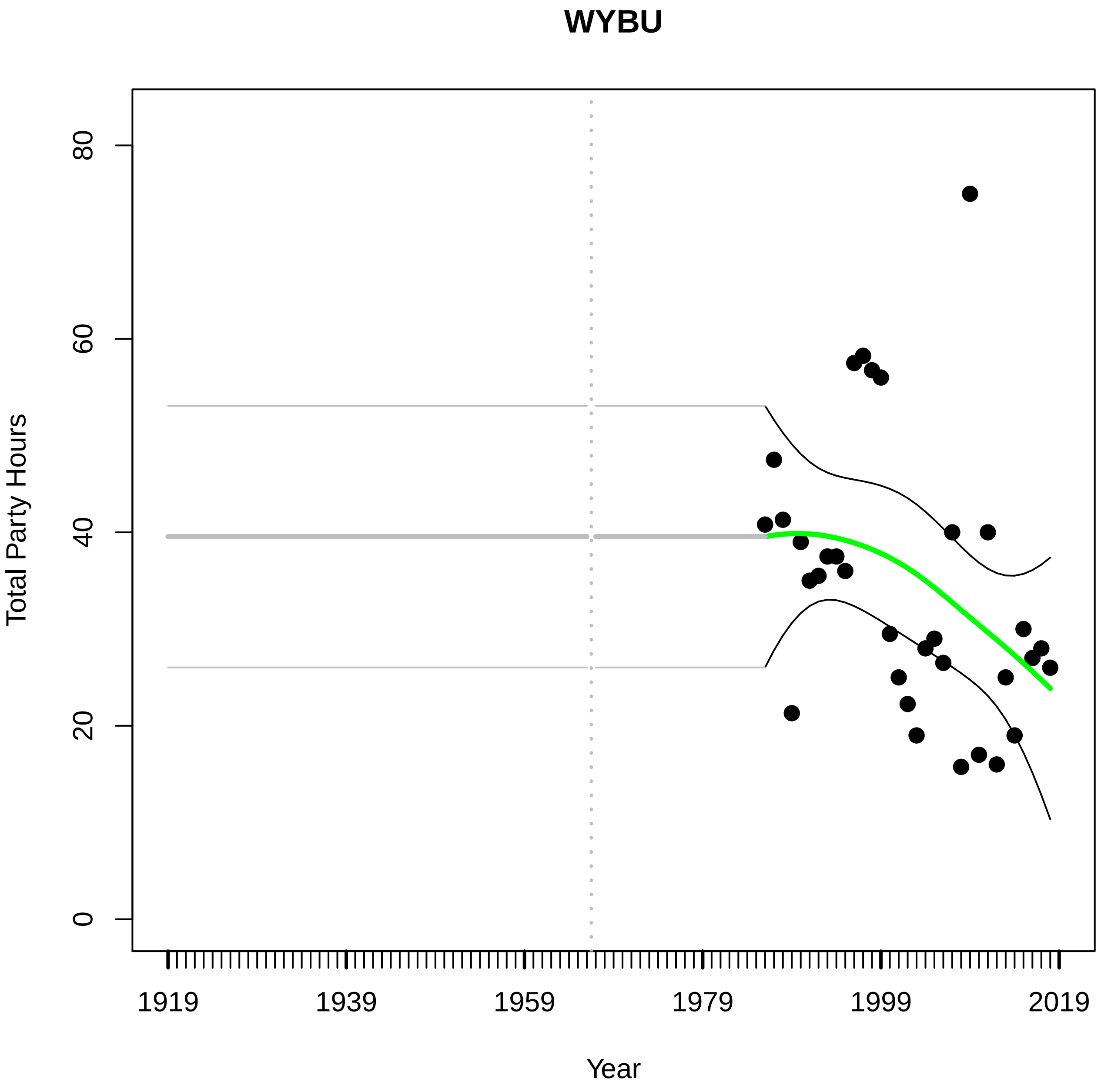
WYBU
Total Party Hours
0
20
40
60
80
1919
1939
1959
1979
1999
2019
Year

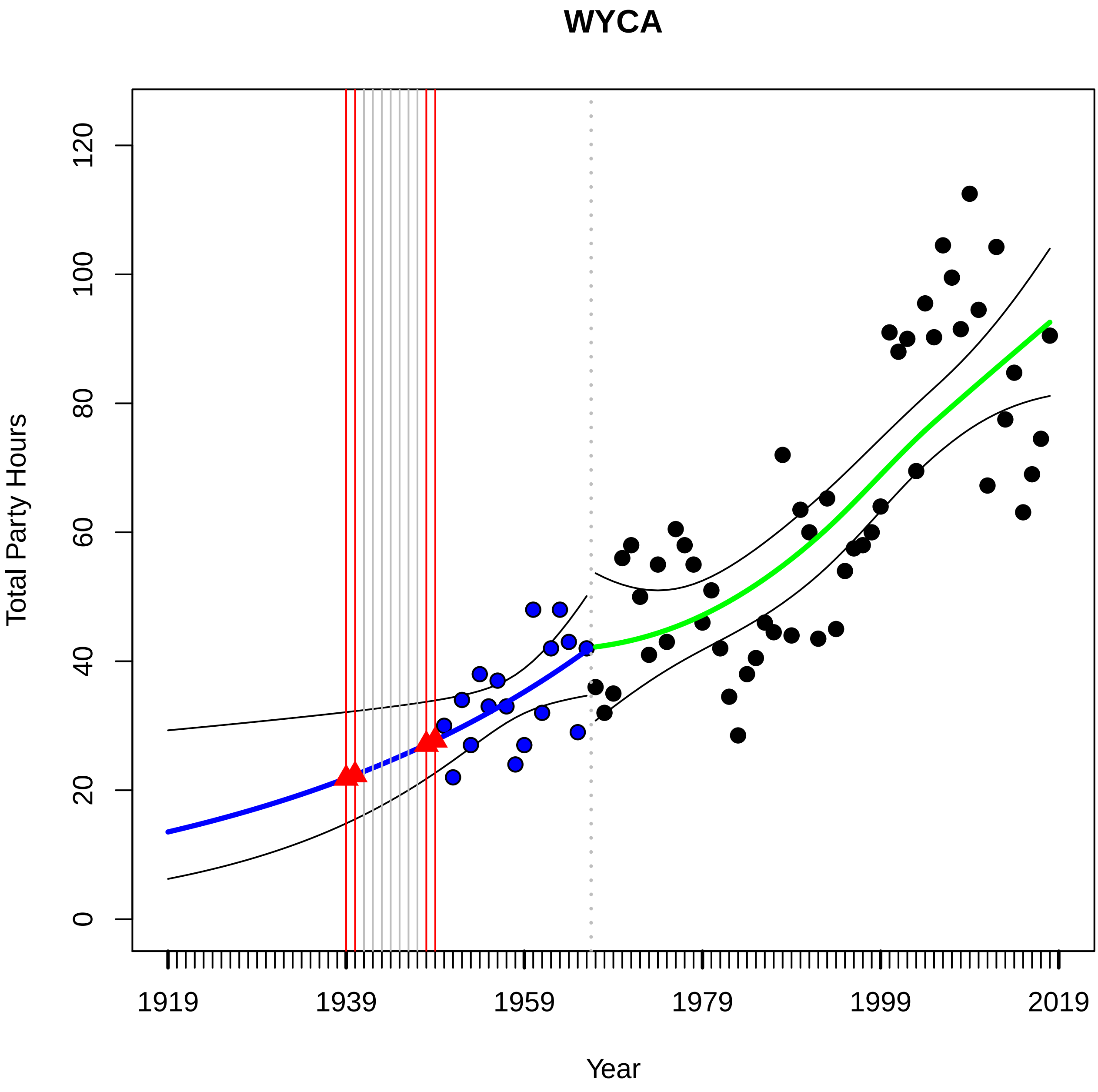

WYCA
Total Party Hours
Year
0
20
40
60
80
100
120
1919
1939
1959
1979
1999
2019

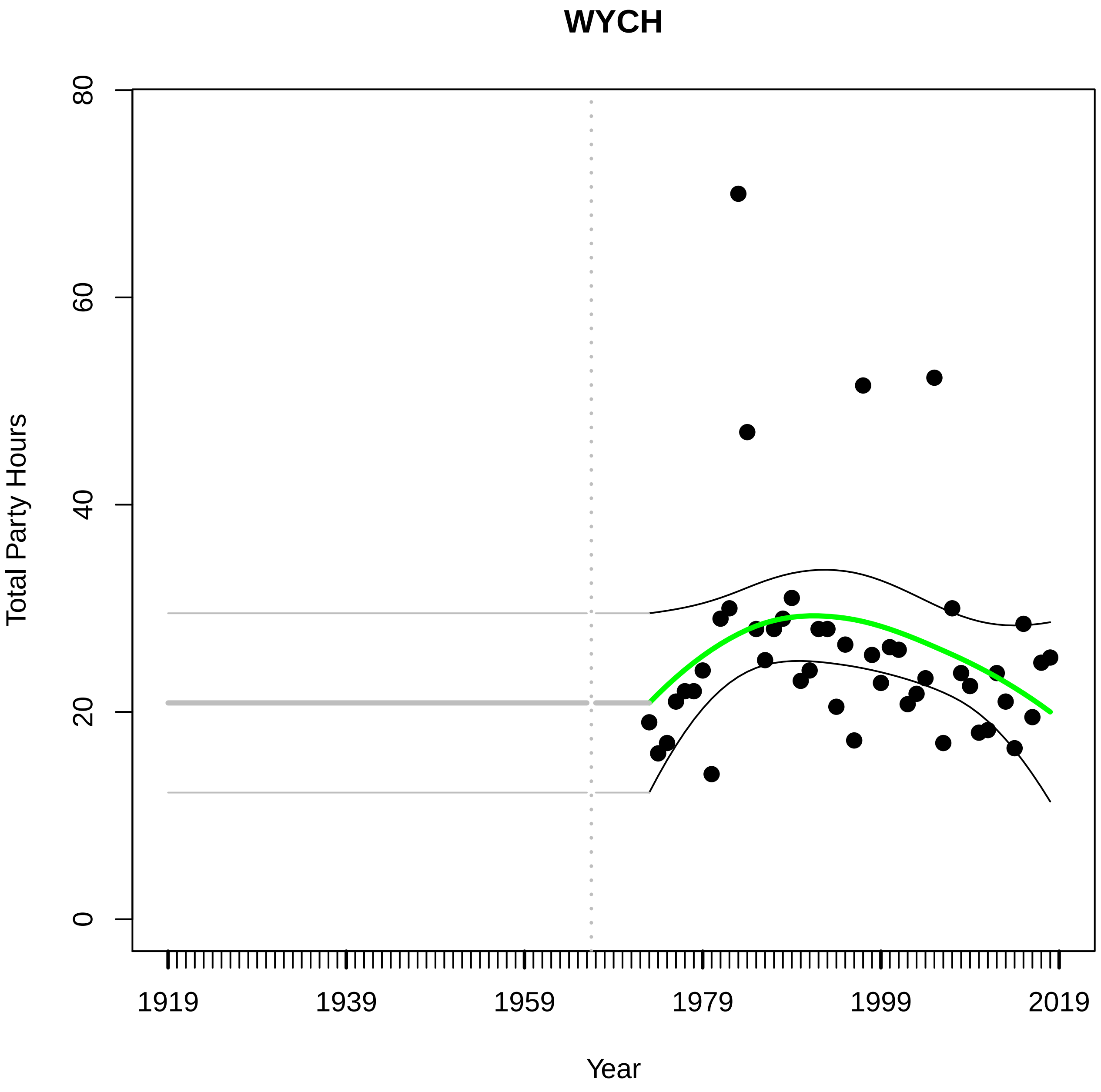

WYCH
Total Party Hours
0
20
40
60
80
1919
1939
1959
1979
1999
2019
Year

WYCO

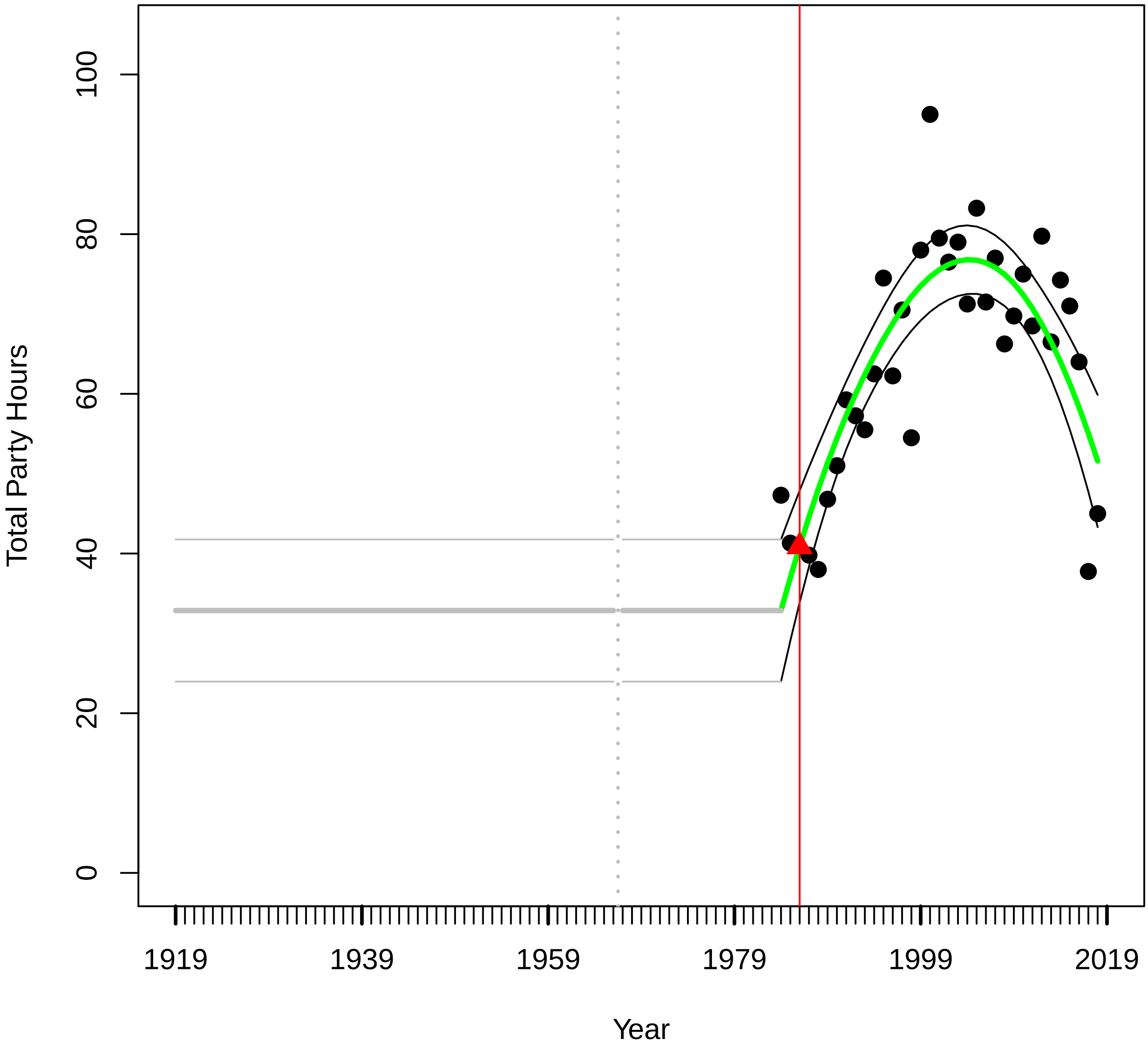

Total Party Hours
0
20
40
60
80
100
1919
1939
1959
1979
1999
2019
Year

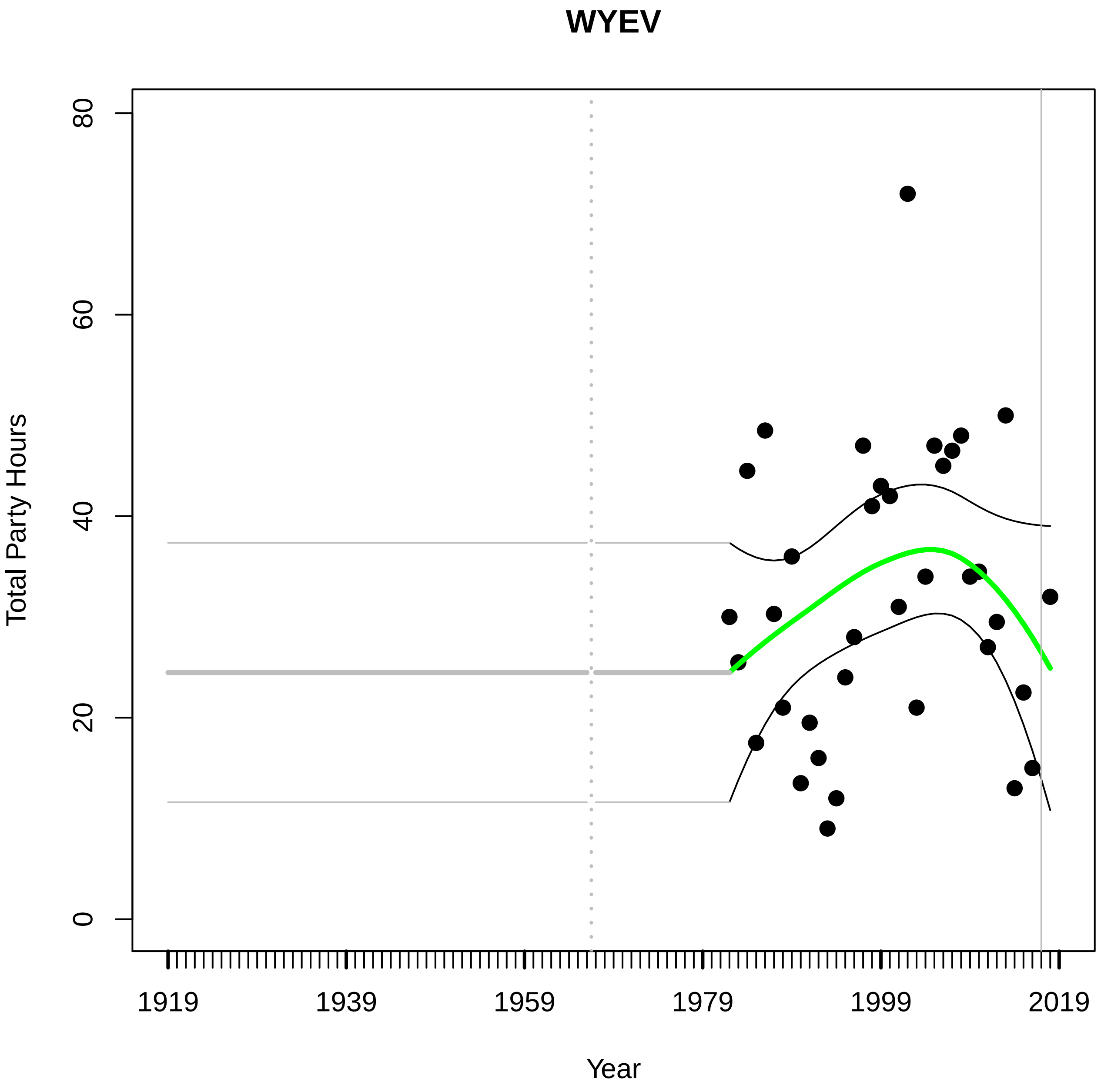

WYEV
Total Party Hours
0
20
40
60
80
1919
1939
1959
1979
1999
2019
Year

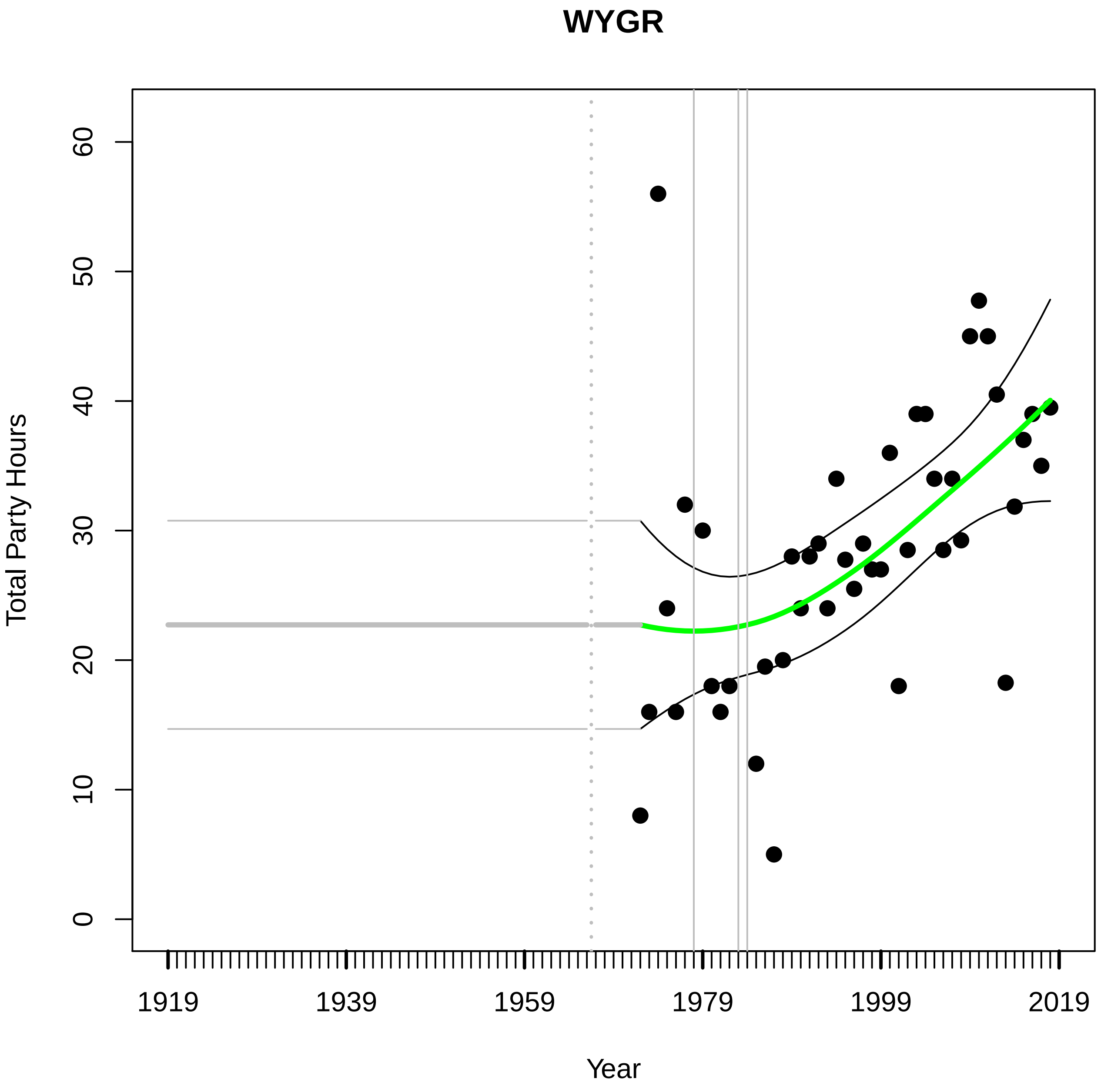
WYGR
Total Party Hours
0
10
20
30
40
50
60
1919
1939
1959
1979
1999
2019
Year

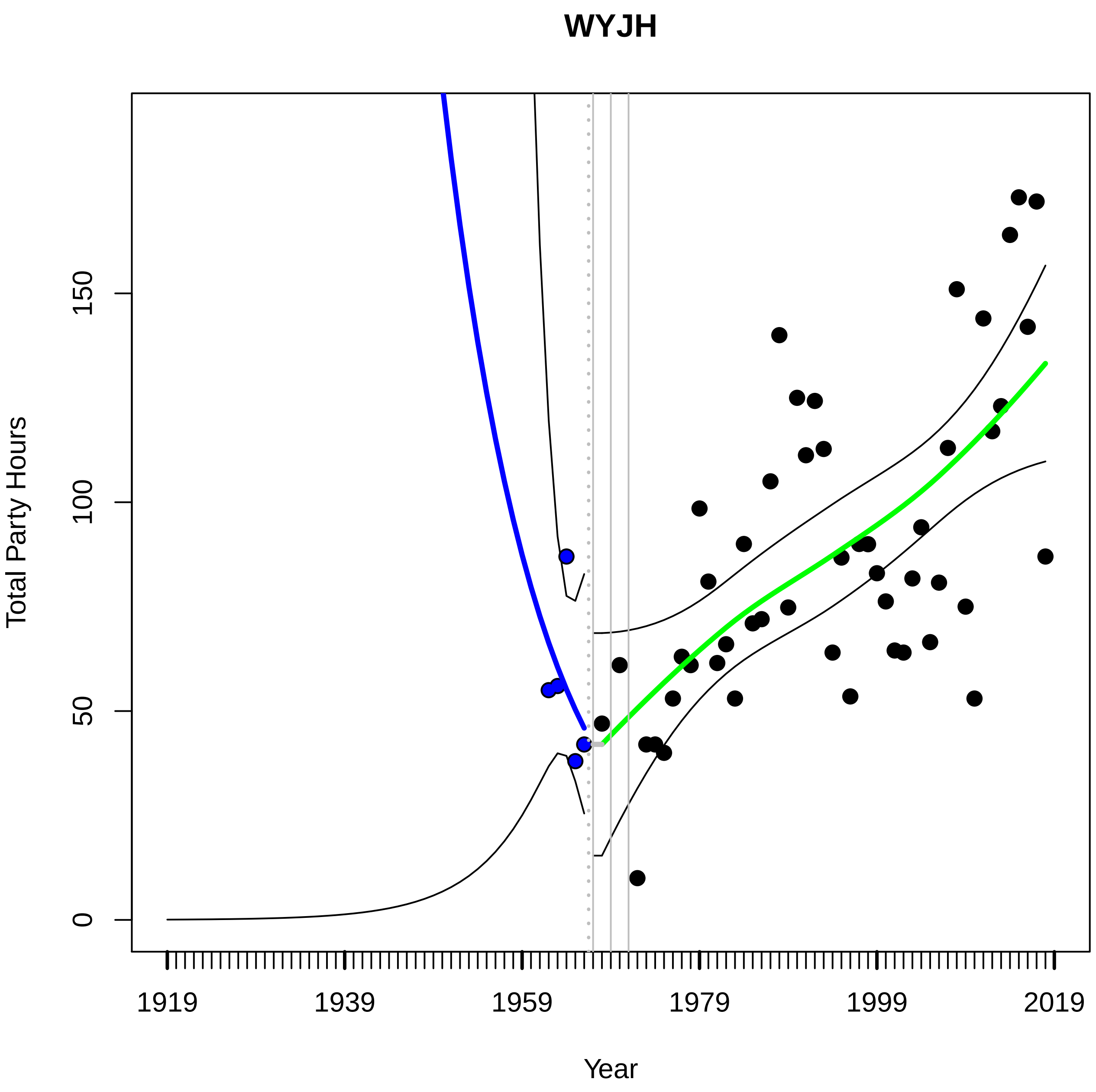

WYJH
Total Party Hours
0
50
100
150
1919
1939
1959
1979
1999
2019
Year

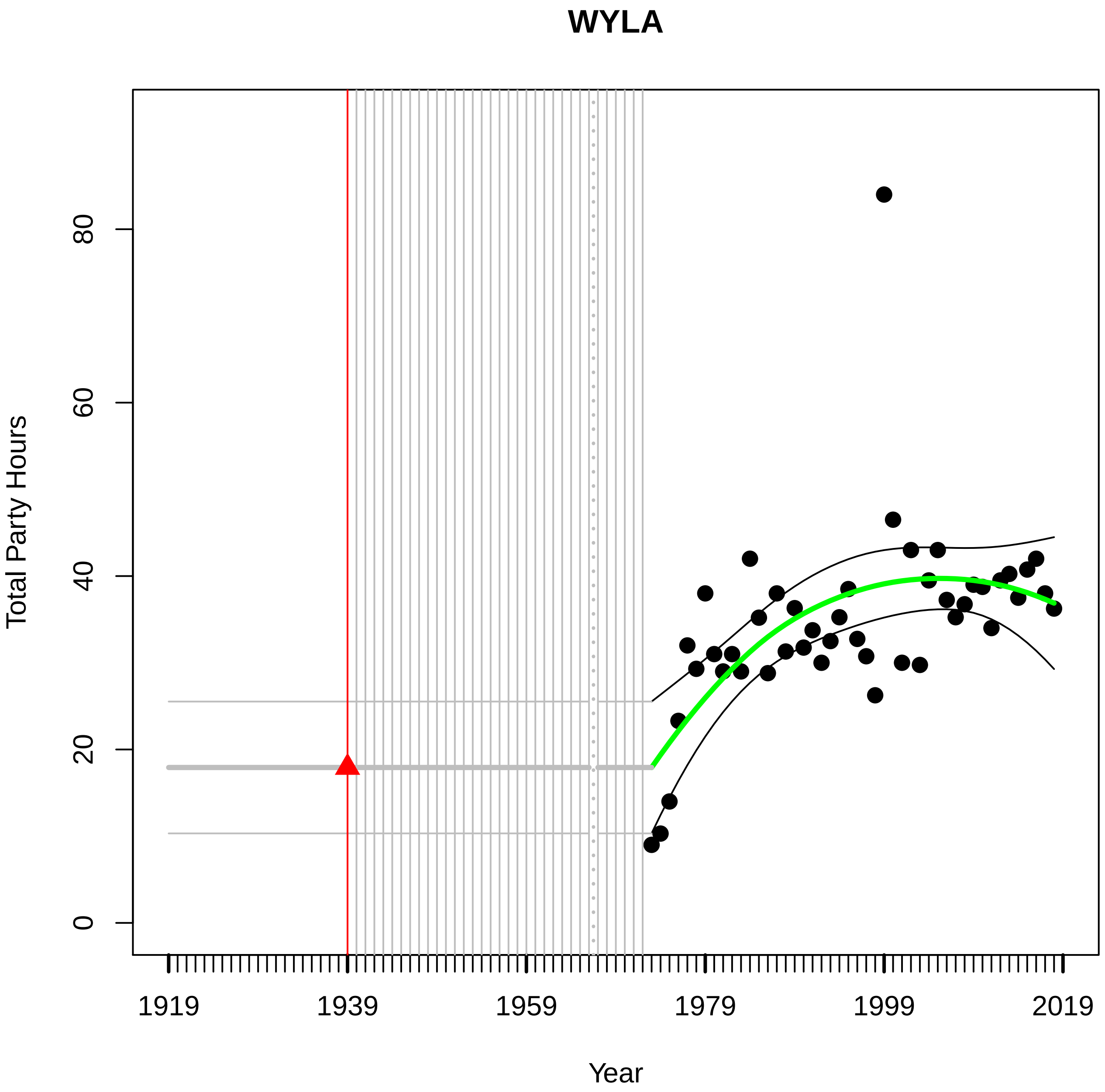
WYLA
Total Party Hours
0
20
40
60
80
1919
1939
1959
1979
1999
2019
Year

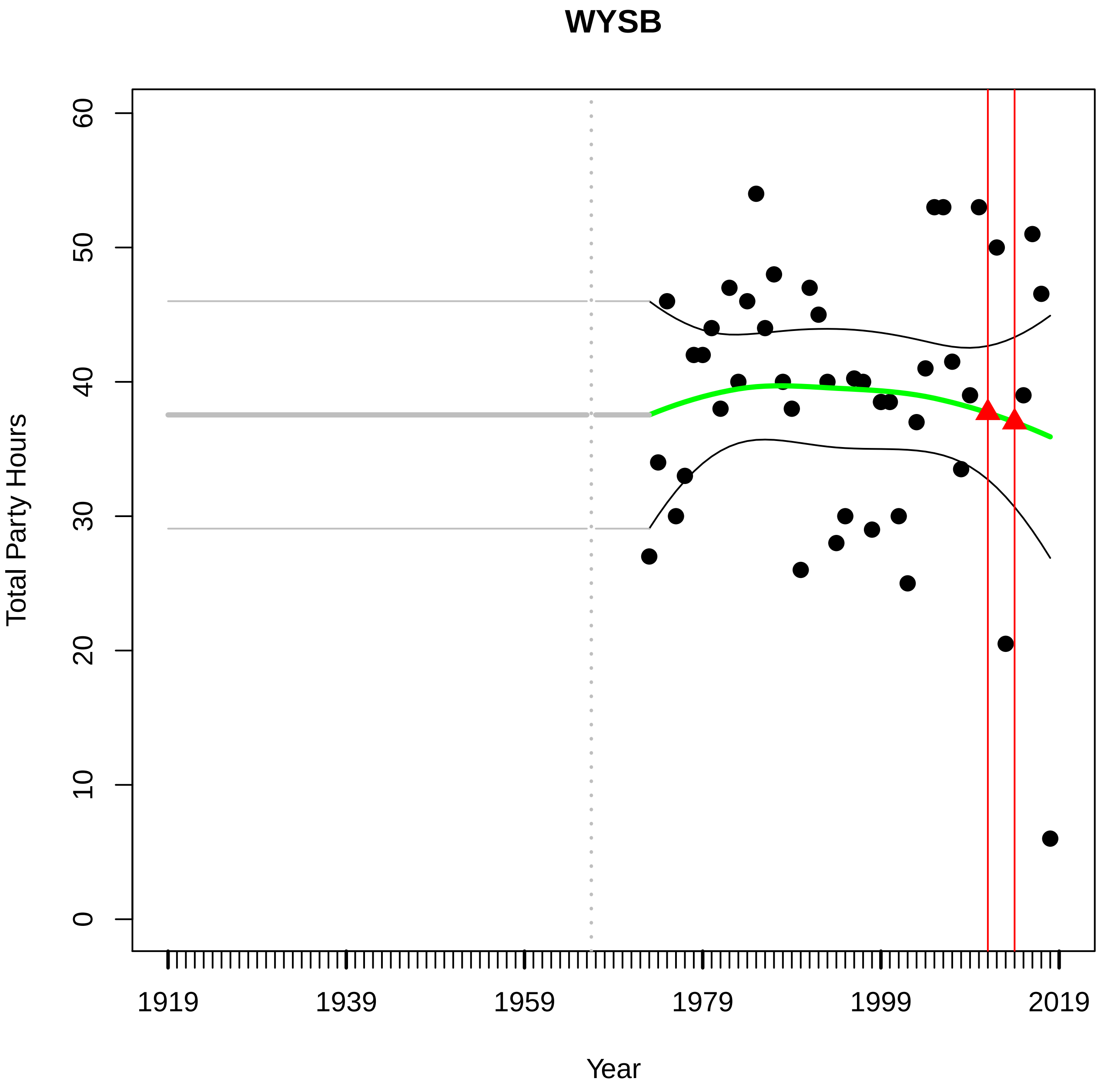

WYSB
Total Party Hours
Year
0
10
20
30
40
50
60
1919
1939
1959
1979
1999
2019

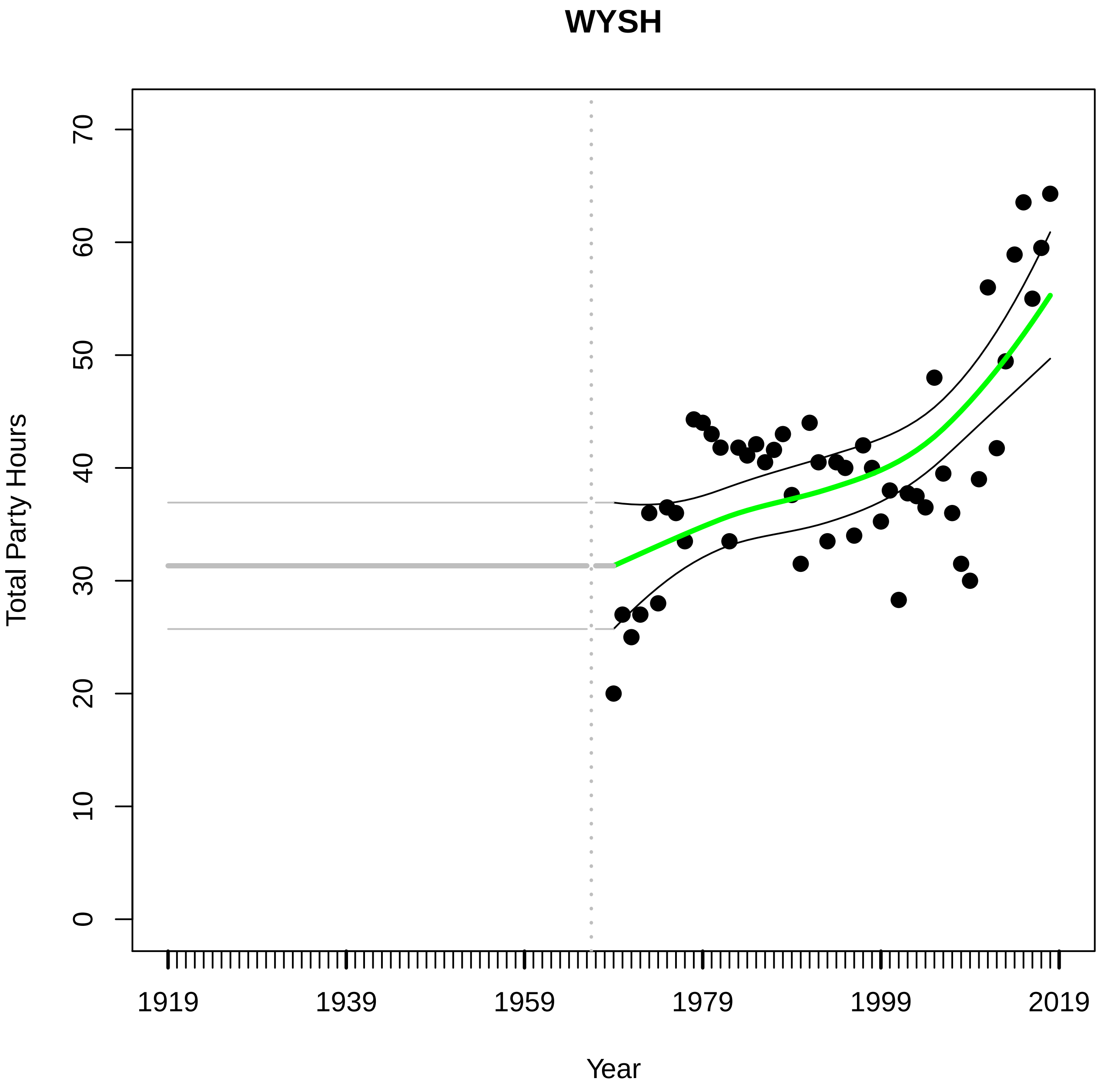
WYSH
Total Party Hours
Year
0
10
20
30
40
50
60
70
1919
1939
1959
1979
1999
2019
